# Mendelian Randomization Mediation Analysis Reveals the Impact of Dietary Preferences on Preeclampsia and Fetal Growth Restriction via Immune Modulation

**DOI:** 10.1002/fsn3.71280

**Published:** 2025-11-26

**Authors:** Yuxiu Wang, Shijun Ni, Xiaoli Gao, Lingyi Fang, Yang Li, Feng Liu, Lining Guo, Cha Han

**Affiliations:** ^1^ Department of Gynecology and Obstetrics Tianjin Medical University General Hospital Tianjin China; ^2^ Tianjin Key Laboratory of Female Reproductive Health and Eugenics Tianjin Medical University General Hospital Tianjin China; ^3^ Department of Radiology and Tianjin Key Laboratory of Functional Imaging Tianjin Medical University General Hospital Tianjin China

**Keywords:** dietary preferences, fetal growth restriction, immune cells, mendelian randomization, preeclampsia

## Abstract

Preeclampsia (PE) and fetal growth restriction (FGR) are major causes of maternal and neonatal complications. Emerging evidence suggests that dietary preferences may influence their development, but the mechanisms, especially involving immune cells, are not well understood. We performed a two‐sample Mendelian randomization (MR) analysis using genome‐wide association study (GWAS) data for dietary preferences, PE, and FGR, sourced from the GWAS Catalog (*n* = 159,579) and FinnGen (*n* = 242,332 for PE; *n* = 254,618 for FGR). Genetic instruments for 731 immune cell traits were extracted from the IEU GWAS database. The primary analysis employed the inverse‐variance weighted (IVW) method, with sensitivity analyses (Cochran's *Q* test, MR‐Egger intercept, and MR‐PRESSO) to assess heterogeneity and pleiotropy. Reverse MR was performed to investigate potential bidirectional causality. We identified 21 dietary preferences significantly associated with PE risk and 11 with FGR risk. Notably, a preference for chili peppers was linked to a lower risk of PE (OR = 0.762, 95% CI: [0.649, 0.895], *p* = 0.001), while a preference for hard cheese was associated with a decreased risk of FGR (OR = 0.337, 95% CI: [0.176, 0.646], *p* = 0.001). Immune cell trait analysis revealed that elevated HLA‐DR expression on HLA‐DR^+^ CD8^+^ T cells was positively associated with PE risk (OR = 1.068, 95% CI: [1.013, 1.126], *p* = 0.015), whereas higher levels of IgD^+^ CD24^+^ B cells were inversely associated with FGR risk (OR = 0.883, 95% CI: [0.813, 0.960], *p* = 0.003). Mediation analysis indicated that 10.9% of the chili pepper's protective effect on PE was mediated by HLA‐DR^+^ CD8^+^ T cells (*p* = 0.042), and 13.9% of the protective effect of hard cheese on FGR was mediated by IgD^+^ CD24^+^ B cells (*p* = 0.043). Reverse MR analyses provided no evidence of reverse causality. Specific dietary preferences—such as the consumption of chili peppers and hard cheese—may reduce the risk of PE and FGR through immune modulation. These findings highlight the potential of targeted dietary interventions during pregnancy to prevent adverse outcomes and warrant further validation in prospective studies.

## Graphical abstract

Wang et al. showed that specific dietary preferences influence the risk of preeclampsia (PE) and fetal growth restriction (FGR) via immune modulation. Genetic analyses revealed that chili pepper consumption lowers PE risk partly through HLA‐DR^+^ CD8^+^ T cells, while hard cheese intake reduces FGR risk via IgD^+^ CD24^+^ B cells. These findings suggest that targeted dietary interventions during pregnancy may help prevent adverse maternal and neonatal outcomes.

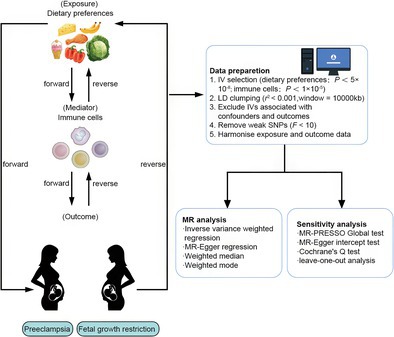

## 1 Introduction

Preeclampsia (PE) and fetal growth restriction (FGR) are severe pregnancy complications that threaten both maternal and fetal health (Aplin et al. 2020). PE typically arises after the 20th week of gestation and is characterized by new‐onset hypertension accompanied by at least one of the following complications: proteinuria, maternal organ dysfunction, or uteroplacental insufficiency. Affecting 5%–7% of pregnancies worldwide each year, PE contributes to over 500,000 perinatal deaths and 70,000 maternal fatalities annually (Dimitriadis et al. 2023; Ives et al. 2020). FGR is defined as the inability of a fetus to reach its genetic growth potential, typically indicated by an ultrasound‐estimated fetal weight below the 10th percentile for gestational age (Blencowe et al. 2019; Fetal Growth Restriction: ACOG Practice Bulletin Summary, Number 227 2021). These conditions heighten the risk of adverse maternal and neonatal outcomes, such as fetal distress, preterm birth, low birth weight, and other complications, along with long‐term cardiometabolic consequences affecting both generations (Sehgal et al. 2020).

Maternal diet and nutrition are pivotal modifiable factors that significantly influence maternal and fetal health by supporting fetal and placental development while maintaining maternal physiological functions (Francis et al. 2022; Kinshella et al. 2021a; Mate et al. 2021; Reijnders et al. 2019). Numerous studies suggest that adherence to healthy dietary patterns—especially the Mediterranean diet, known for its emphasis on nutrient‐rich plant foods, healthy fats, and lean protein sources like fish—may help reduce the risk of PE and FGR (Crovetto et al. 2021; Makarem et al. 2022; Minhas et al. 2022). These diets play crucial roles in optimizing nutritional status during pregnancy, reducing systemic inflammation, and preventing adverse pregnancy outcomes (Abdollahi et al. 2021; Amati et al. 2019; Arvizu et al. 2020; Kinshella et al. 2021b; Li et al. 2021; Maldonado et al. 2024; Xu et al. 2024). Mendelian randomization (MR) studies further strengthen the causal relationship between dietary patterns and adverse pregnancy outcomes, particularly PE and FGR (Mu et al. 2024).

Emerging evidence highlights that the immune system may serve as a key mediator of the effects of dietary patterns, underscoring the importance of understanding the intricate relationship between diet and immune function (Alexander and Turnbaugh 2020; Collins 2020; Harris 2024; Koelman et al. 2022; Lee and Dixit 2020). Specific dietary components, such as polyphenols, dietary fiber, omega‐3 fatty acids, and vitamins, exert their protective effects through immune modulation. They regulate immune cell function, thereby alleviating chronic inflammation and oxidative stress (García‐Montero et al. 2023; Nacka‐Aleksić et al. 2022; Zheng et al. 2020). These mechanisms improve placental function and reduce the risk of adverse pregnancy outcomes. Conversely, unhealthy dietary patterns, such as Western diets and high‐fat diets, can disrupt immune homeostasis, impair immune cell function, and contribute to systemic inflammation and placental dysfunction, ultimately increasing the risk of PE and FGR (Christ et al. 2019; García‐Montero et al. 2023, 2021). Despite established links between dietary factors and PE and FGR, the specific mechanisms by which dietary preferences influence these conditions, particularly immune cell characteristics, remain underexplored.

To explore the causal impact of dietary preferences on PE and FGR risk, we employed an MR framework integrating genetic data with causal inference (Emdin et al. 2017). By leveraging genetic variants as instrumental variables, MR minimized confounding and reverse causation, thereby enhancing the robustness of causal inference. This study aimed to elucidate the mechanisms linking dietary preferences to PE and FGR, identify modifiable dietary factors, and clarify the mediating role of immune cells. Our results may provide valuable insights into the onset and progression of PE and FGR, inform evidence‐based dietary interventions, and support the development of personalized treatments to improve maternal and infant health.

## 2 Methods

### 2.1 Study Design and Data Sources

**FIGURE 1:**
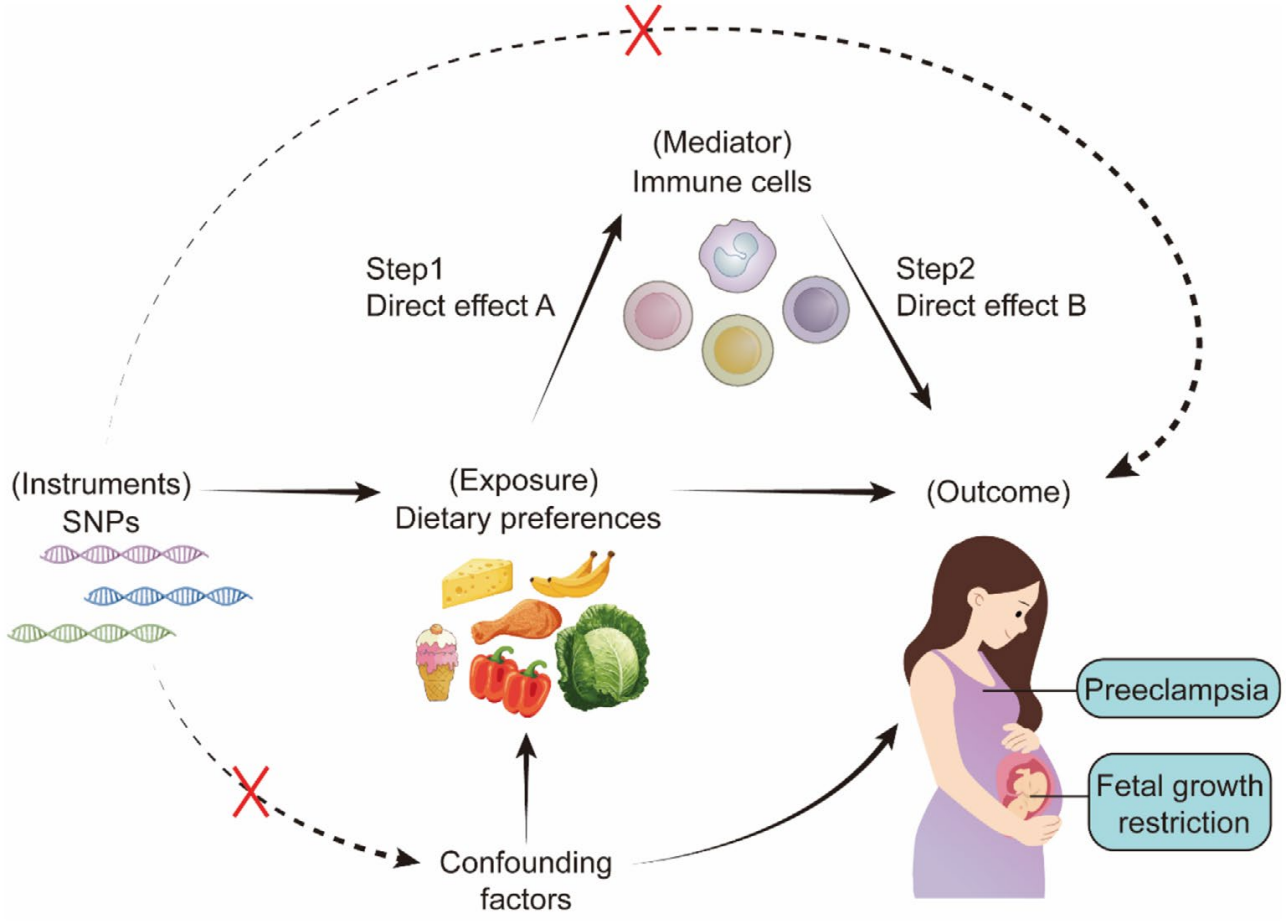
Conceptual framework of the two‐step Mendelian randomization (MR) analysis. Direct effect A: The causal effect of dietary preferences on immune cells; Direct effect B: The causal effect of immune cells on PE and FGR.

All data used in this study were obtained from publicly available genome‐wide association study (GWAS) databases, with prior approval from the respective institutional review boards. We applied a two‐step MR framework to investigate the causal effects of dietary preferences on the risks of PE and FGR, and to explore the potential mediating role of immune cell traits in these associations. The study design is illustrated in the schematic overview presented in Figure 1.

Data for PE were obtained from the FinnGen database (version R11, https://r11.finngen.fi/), which includes 8185 cases and 234,147 controls, providing a robust representation of the European population. For FGR, the data included 4617 cases and 250,001 controls (version R11, https://r11.finngen.fi/). Dietary preference data were sourced from the GWAS catalog compiled by May‐Wilson et al. (2022) (May‐Wilson et al. 2022), which includes 187 dietary traits across 159,579 individuals, identifying 1,076,976 associated SNPs (https://www.ebi.ac.uk/gwas/studies). These traits were assessed using a 9‐point food‐liking questionnaire, with the scores subsequently inverse rank‐normalized as part of the GWAS pipeline. Immune cell trait data were sourced from the IEU database, covering 731 traits from 3658 individuals, with a total of 15,048,965 SNPs (https://gwas.mrcieu.ac.uk/datasets/) (Orrù et al. 2020). These traits, including absolute cell counts, relative proportions, median fluorescence intensities (MFIs) of surface antigens, and morphological parameters, were measured by flow cytometry, and all traits were inverse‐normalized prior to association analyses. Therefore, in MR analyses, effect estimates are interpreted per one standard deviation (SD) increase in the standardized trait value. These large‐scale datasets offer strong statistical power, enhancing the reliability of our analysis and allowing for a robust investigation of the causal relationships between dietary preferences, immune traits, and the risks of PE and FGR. The specific data sources and sample characteristics are summarized in Table 1.

**TABLE 1:** Data sources and sample characteristics for preeclampsia, fetal growth restriction, dietary preferences, and immune cell traits.

Trait	Population	Sources	Cases	Controls	Sample size	SNP	PMID
Preeclampsia	European	FinnGen (finngen_R11_O15_PREECLAMPS)	8185	234,147	242,332	20,088,387	36,653,562
Poor fetal growth	European	FinnGen (finngen_R11_O15_POOR_FETGRO)	4617	250,001	254,618	20,089,073	36,653,562
187 food habits	European	GWAS catalog (GCST90094687~GCST90094873)	—	—	159,579	10,769,765	35585065
731 immune cells	European	IEU (ebi‐a‐GCST90001391~GCST90002121)	—	—	3658	15,048,965	32929287

### 2.2 Selection of Genetic Instruments

Lead single‐nucleotide polymorphisms (SNPs) linked to dietary preferences were identified using a genome‐wide significance threshold of *p* < 5 × 10^−8^. For immune cell traits, a relaxed threshold of *p* < 1 × 10^−5^ was applied to retain a sufficient number of variants for mediation analysis. To ensure the independence of instrumental variables, linkage disequilibrium (LD) clumping was performed using an *R* ^2^ threshold < 0.001 and a clumping window of 10,000 kb. Instrument strength was assessed using *F*‐statistics, with values > 10 indicating adequate strength.

### 2.3 Two‐Step Mendelian Randomization Framework

Firstly, we performed two‐sample MR analyses to estimate the causal effects of dietary preferences on the risks of PE and FGR, as well as the effects of immune cell traits on these outcomes. Secondly, we evaluated the effects of dietary preferences on immune cell traits and calculated the mediation proportion of each trait contributing to PE and FGR pathogenesis. All exposure and outcome datasets were obtained from independent and non‐overlapping populations to avoid bias due to sample overlap.

### 2.4 Statistical Analysis

We used R version 4.3.1 with the TwoSampleMR, ieugwasr, and VariantAnnotation packages to process the data, employing the inverse‐variance weighted (IVW) method as the primary approach due to its efficiency and statistical power. Sensitivity analyses were conducted using complementary methods, including MR‐Egger, weighted median, weighted mode, and simple mode. Cochran's Q test was used to assess heterogeneity. A fixed‐effects IVW model was applied when *p* > 0.05, while a random‐effects IVW model was used in cases of significant heterogeneity (*p* ≤ 0.05). Horizontal pleiotropy was evaluated using the MR‐Egger intercept test, with *p* < 0.05 indicating its presence. Mediation analysis was performed via the product‐of‐coefficients method, with standard errors estimated using the delta method. Considering the large number of exposures (187 dietary traits and 731 immune traits), multiple testing correction was applied using the Benjamini–Hochberg false discovery rate (FDR), with associations at FDR‐adjusted *p* < 0.05 considered statistically significant. Statistical power was calculated using an online MR power calculator. Furthermore, for the key associations, we performed the formal pleiotropy test using MR‐PRESSO.

## 3 Results

### 3.1 Effects of Dietary Preferences on PE and FGR

**FIGURE 2:**
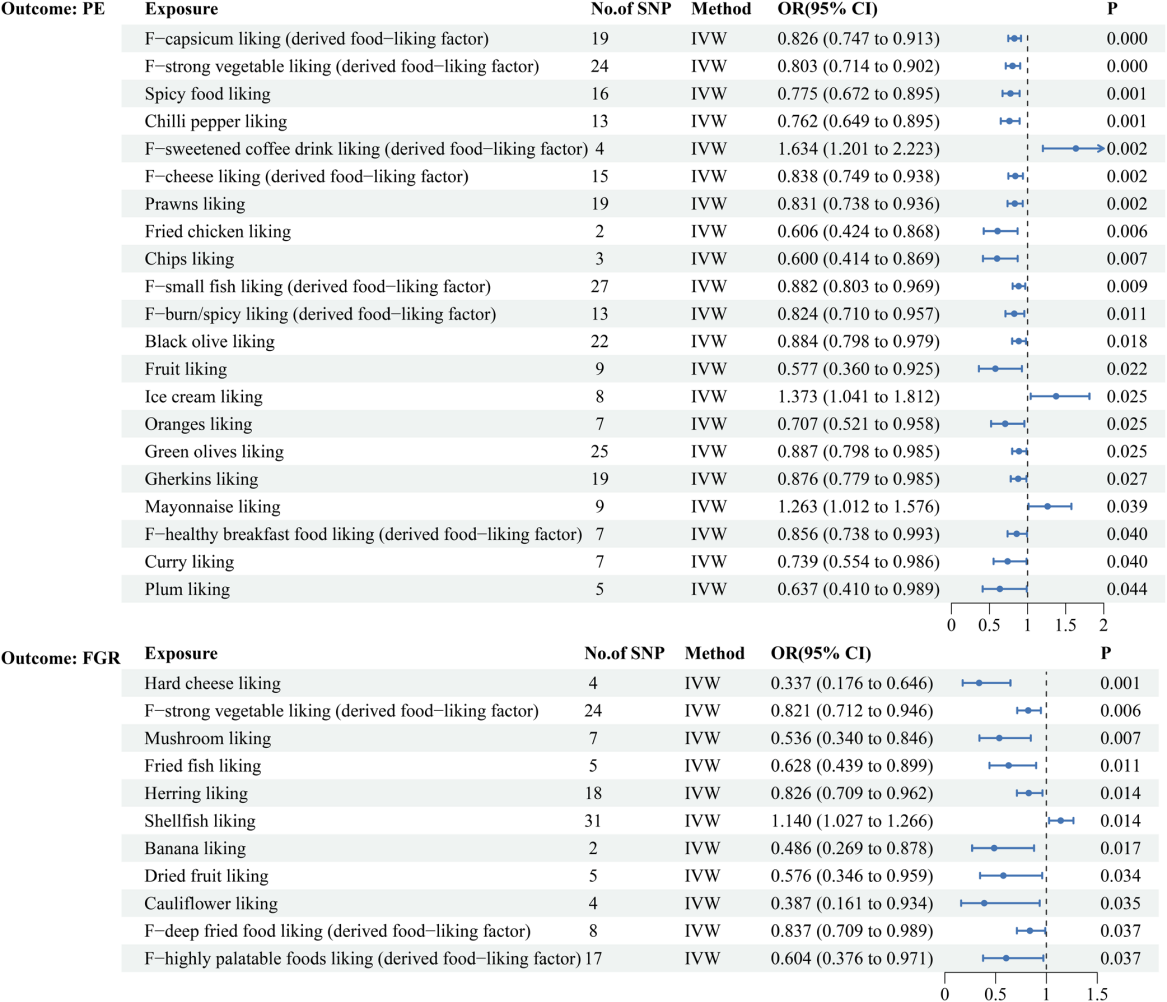
Forest plot showing the associations between dietary preferences and the risks of PE and FGR. ORs and 95% CIs are provided, with IVW as the primary analytical method. Estimates are per 1‐SD increase in inverse‐normalized dietary preference scores.

**FIGURE 3:**
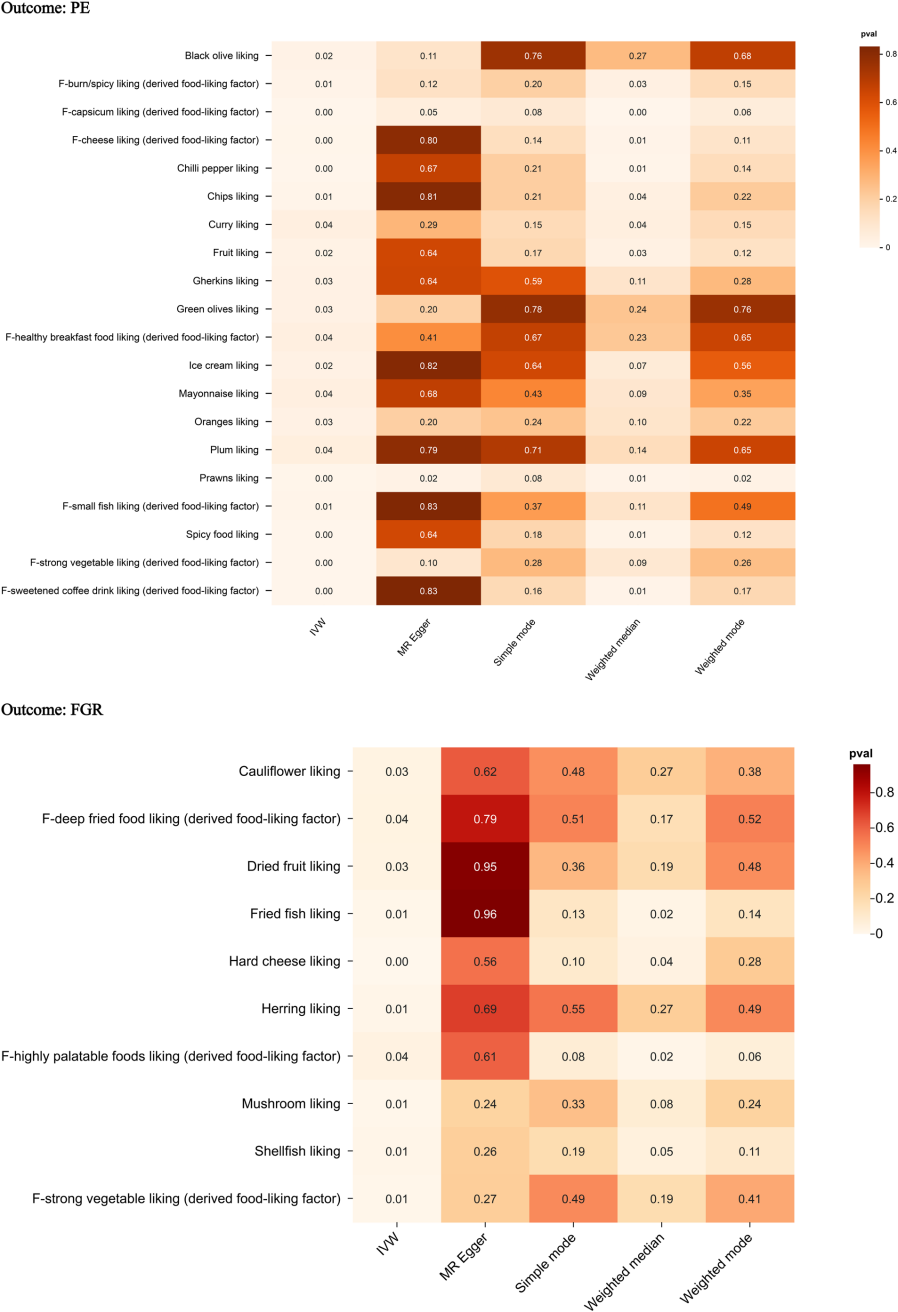
Heatmap showing the associations between dietary preferences and the risks of PE and FGR. The color intensity reflects the strength of the associations. Estimates are per 1‐SD increase in inverse‐normalized dietary preference scores.

We employed 1505 and 1549 SNPs as instrumental variables to assess the effects of dietary preferences on PE and FGR, respectively. MR analysis revealed significant causal associations between 21 dietary preferences and PE and between 11 dietary preferences and FGR. Specifically, 18 dietary preferences were found to be protective against PE, whereas 3 increased its risk. Similarly, 10 preferences were protective against FGR, and 1 was associated with increased risk, as shown in Figures 2 and 3. Notably, a preference for chili peppers was protective against PE (OR = 0.762, 95% CI [0.694, 0.895], *p* = 0.001, FDR‐adjusted *p =* 0.038), and a preference for hard cheese was protective against FGR (OR = 0.337, 95% CI [0.176, 0.646], *p* = 0.001, FDR‐adjusted *p =* 0.177) (Supporting Information S1; Figure S1). No significant heterogeneity or horizontal pleiotropy was detected (*p* > 0.05 for Cochran's *Q* test and MR‐Egger test), reinforcing the robustness of these findings (Supporting Information S7 and S8; Figure S2). The MR‐PRESSO global test for the key associations between chili pepper preference and PE, and hard cheese preference and FGR, also showed *p* > 0.05, further indicating no evidence of horizontal pleiotropy (Supporting Information S9 and S10).

### 3.2 Effects of Immune Cells on PE and FGR

**FIGURE 4:**
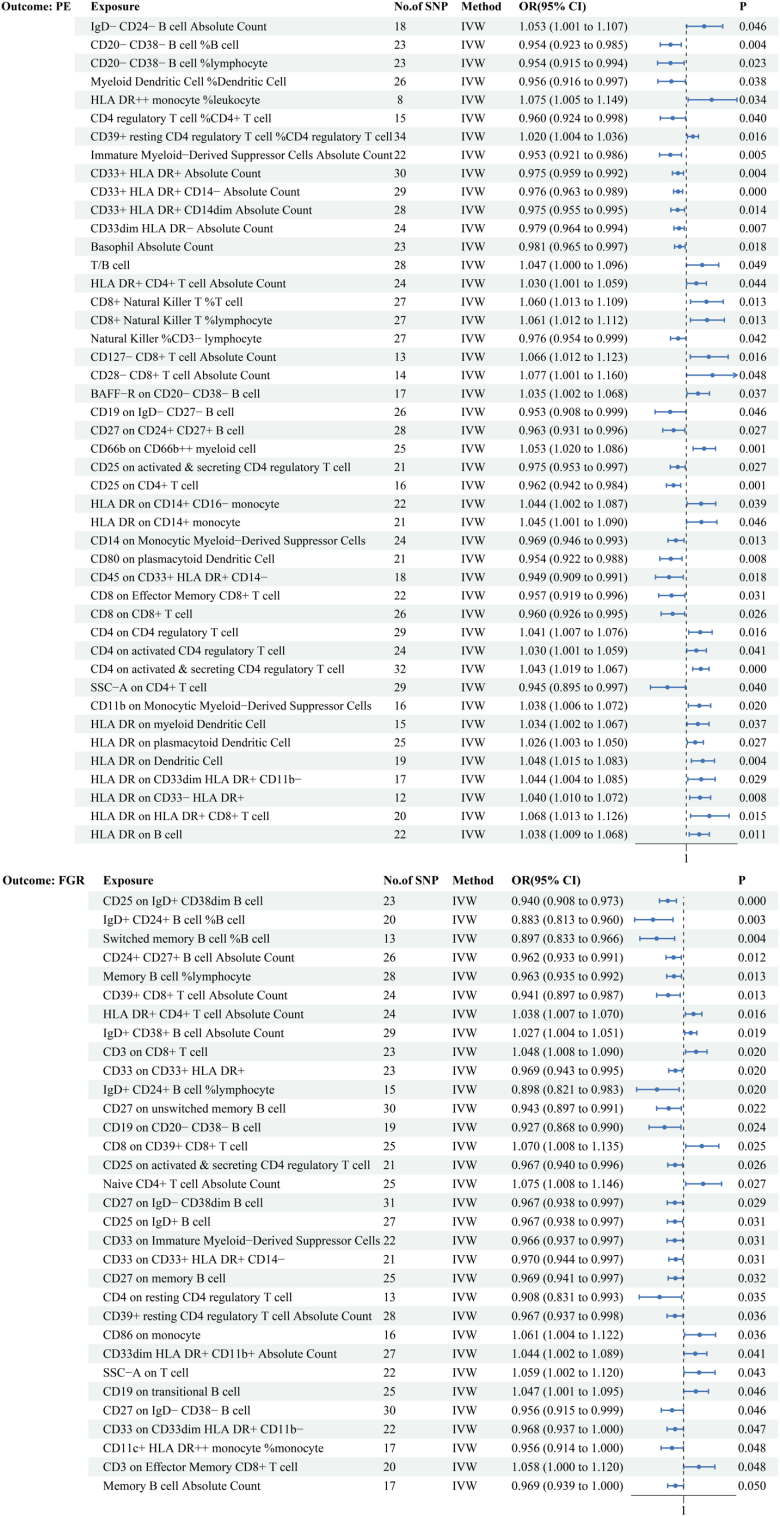
Forest plot showing immune cell traits significantly associated with PE and FGR. ORs and 95% CIs are provided, with IVW as the primary analytical method. Estimates are per 1‐SD increase in inverse‐normalized immune traits.

**FIGURE 5:**
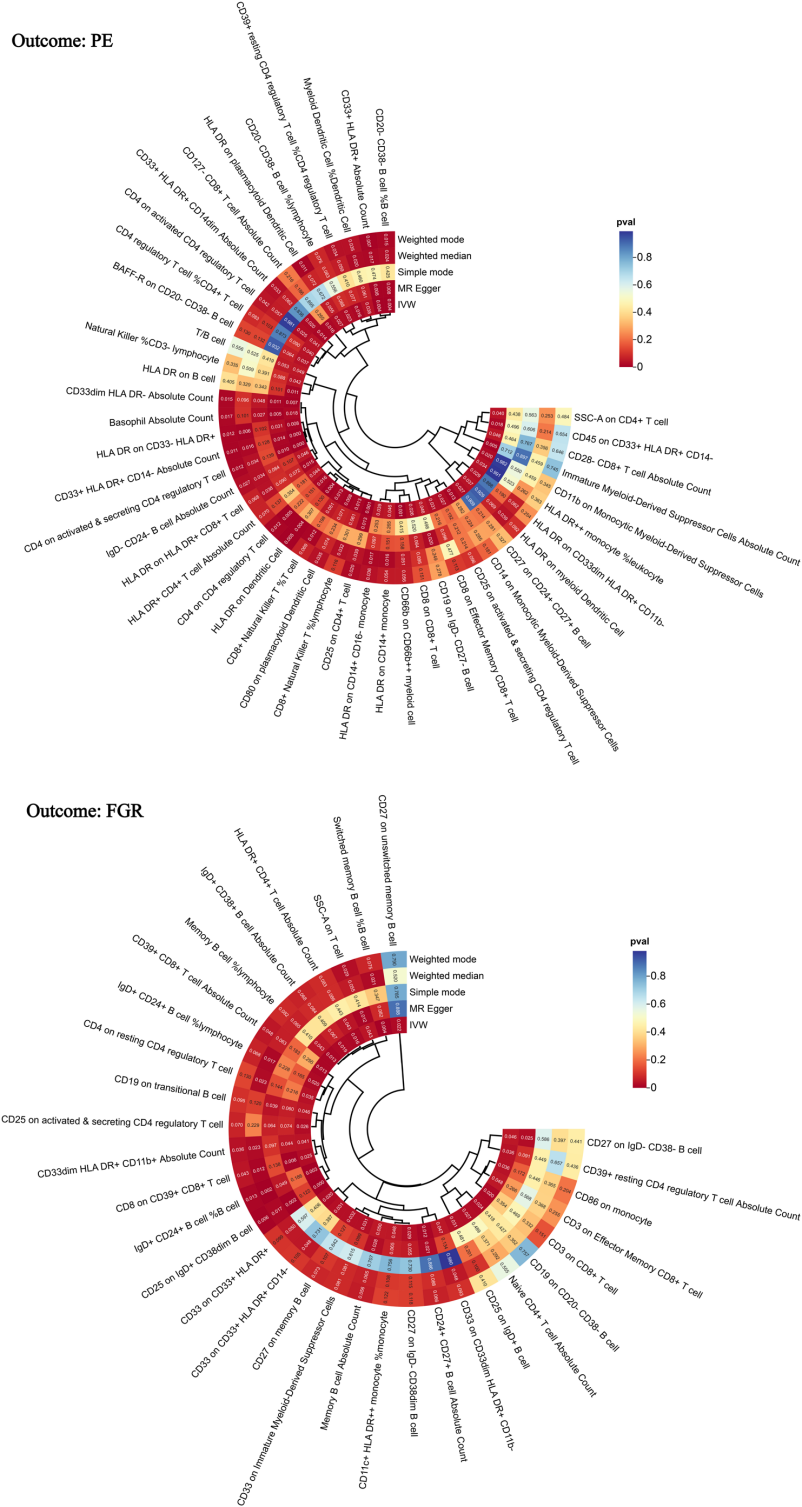
Heatmap showing immune cell traits significantly associated with PE and FGR. The color intensity reflects the strength of the associations. Estimates are per 1‐SD increase in inverse‐normalized immune traits.

As illustrated in Figures 4 and 5, in our IVW analysis, we observed associations between altered levels of certain immune cell traits and the risks of PE and FGR. Specifically, 45 immune cell traits were linked to PE, and 32 were linked to FGR. Increased HLA‐DR expression on HLA‐DR^+^ CD8^+^ T cells was associated with a greater risk of PE (OR = 1.068, 95% CI [1.013, 1.126], *p* = 0.015, FDR‐adjusted *p =* 0.589), whereas elevated IgD^+^ CD24^+^ B cells were inversely associated with FGR risk (OR = 0.883, 95% CI [0.813, 0.960], *p* = 0.003, FDR‐adjusted *p =* 0.956) (Supporting Information S2; Figure S1). No significant heterogeneity or horizontal pleiotropy was found (*p* > 0.05, Cochran's Q test and MR–Egger test), further supporting the robustness of these findings (Supporting Information S7 and S8; Figure S2). The MR‐PRESSO global test for the key associations (HLA‐DR expression on HLA‐DR^+^ CD8^+^ T cells with PE, and IgD^+^ CD24^+^ B‐cell percentage with FGR) also yielded *p* > 0.05, indicating no evidence of horizontal pleiotropy (Supporting Information S11 and S12). These results suggest that immune cell traits may mediate the development of PE and FGR.

### 3.3 Effect of Dietary Preferences on Immune Cells

**FIGURE 6:**
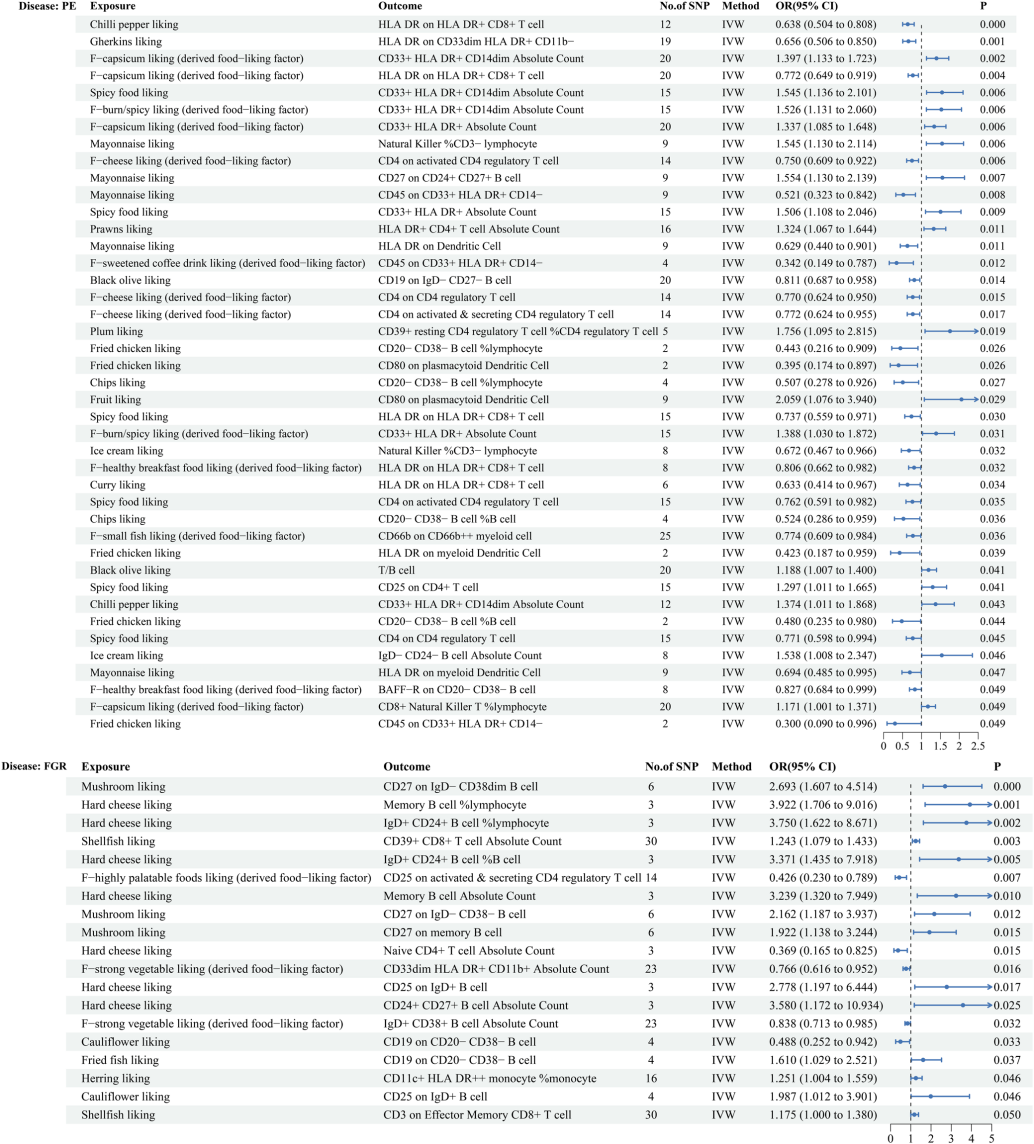
Forest plot showing dietary preferences significantly associated with immune cell traits. ORs and 95% CIs are provided, with IVW as the primary analytical method. Estimates are per 1‐SD increase in inverse‐normalized traits.

We identified 21 dietary preferences and 45 immune cell traits significantly associated with PE and 11 dietary preferences and 32 immune cell traits associated with FGR. MR analysis revealed several key associations between dietary preferences and immune cell traits. For example, chili pepper preference was negatively associated with HLA‐DR expression on HLA‐DR^+^ CD8^+^ T cells (OR = 0.638, 95% CI: [0.504, 0.808], *p* < 0.001, FDR‐adjusted *p =* 0.999), suggesting a potential role in modulating immune responses related to PE. Similarly, hard cheese preference was positively associated with a greater proportion of IgD^+^ CD24^+^ B cells (OR = 3.371, 95% CI: [1.435, 7.918], *p* = 0.005, FDR‐adjusted *p =* 0.878), indicating that an immune‐related mechanism influences FGR risk. Additionally, 20 other significant associations between dietary preferences and immune cell traits were identified, highlighting the broader influence of diet on immune regulation in both PE and FGR (Figure 6) (Supporting Information S3 and S4). Furthermore, no evidence of horizontal pleiotropy or heterogeneity was observed, supporting the validity of the results (Supporting Information S7 and S8; Figure S2). For the key association between chili pepper preference and HLA‐DR expression on HLA‐DR^+^ CD8^+^ T cells, the MR‐PRESSO global test showed *p* > 0.05, indicating no horizontal pleiotropy. In contrast, MR‐PRESSO could not be performed for the association between hard cheese preference and the proportion of IgD^+^ CD24^+^ B cells due to insufficient instruments (Supporting Information S13 and S14).

### 3.4 Reverse MR Analysis

**FIGURE 7:**
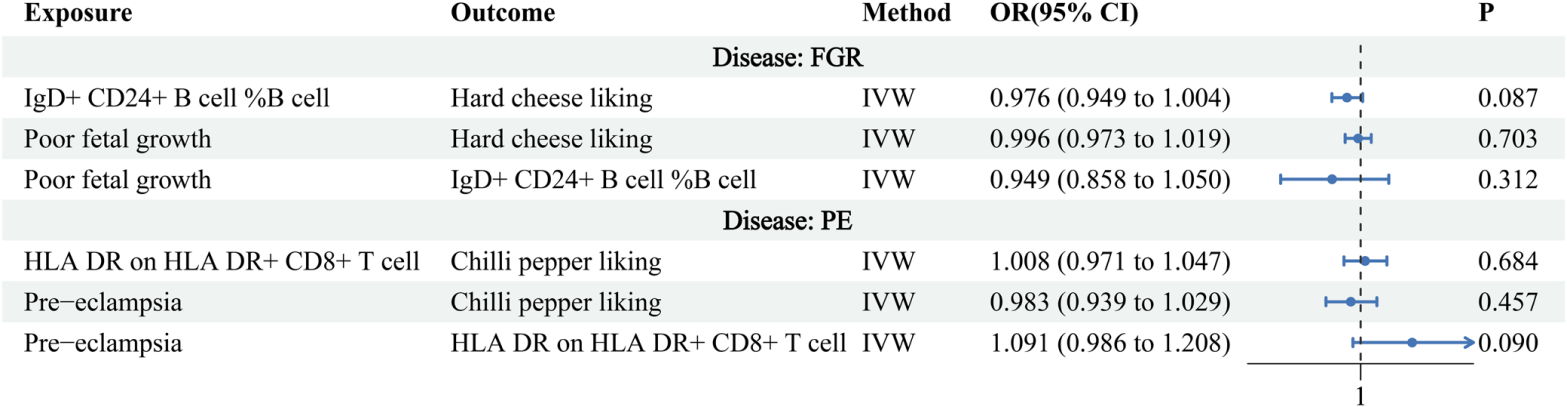
Reverse MR analysis across dietary preferences, immune cell traits, and pregnancy outcomes, with ORs and 95% CIs estimated using the IVW method.

To validate the robustness of our findings, we performed a reverse MR analysis for both PE and FGR. No significant causal effects were detected, indicating that reverse causality is unlikely. Details are provided in Figure 7 (Supporting Information S5). We further evaluated the statistical power of the reverse MR analyses. For the association from PE to chili pepper preference, the power was 71.1%, indicating adequate sensitivity to detect modest effects; thus, the null finding (*p* > 0.05) suggests that reverse causality is unlikely. In contrast, the power for the association from FGR to hard cheese preference was only 9.9%, indicating limited ability to detect potential effects and warranting cautious interpretation of the null result.

### 3.5 Mediation Analysis of Immune Cells on PE and FGR

Mediation analysis revealed that chili pepper preference reduced the risk of PE, which was partially mediated by HLA‐DR^+^ CD8^+^ T cells, accounting for 10.9% of the total effect (mediation effect = −0.030, 95% CI [−0.059, −0.000]; *p* = 0.042). Similarly, hard cheese preference reduced the risk of FGR via an increase in the proportion of IgD^+^ CD24^+^ B cells, contributing to 13.9% of the total effect (mediation effect = −0.151, 95% CI [−0.302, −0.000]; *p* = 0.043). These findings underscore the role of immune cells in mediating the effects of dietary preferences on pregnancy outcomes (Table 2; Supporting Information S6).

**TABLE 2:** Mediating effects of liking chili pepper and liking hard cheese on PE and FGR via immune cells.

Dietary preference	Mediating immune cell	Outcome	Total effect (*β*, 95% CI)	Direct effect (*β*, 95% CI)	Mediation effect (*β*, 95% CI)	*p*	Mediation proportion (%) (*β*, 95% CI)	Direct effect: A & B (*β*, 95% CI)
Chili pepper liking	HLA DR on HLA DR^+^ CD8^+^ T‐cell	PE	−0.272 (−0.433, −0.111)	−0.242 (−0.406, −0.079)	−0.030 (−0.059, −0.000)	0.042	10.9% (0.1%, 21.6%)	A: −0.450 (−0.686, −0.214); B: 0.066 (0.013, 0.119)
Hard cheese liking	IgD^+^ CD24^+^ B‐cell % B‐cell	FGR	−1.087 (−1.738, −0.437)	−0.937 (−1.604, −0.269)	−0.151 (−0.302, −0.000)	0.043	13.9% (0.00%, 27.7%)	A: 1.215 (0.361, 2.069); B: −0.124 (−0.207, −0.041)

*Note:* Total effect: The overall impact of dietary preferences on PE and FGR. Direct effect A: Dietary preferences directly influence immune cell traits, which in turn affect PE and FGR risk. Direct effect B: Immune cell traits directly influence the development of PE and FGR. Mediation effect: Calculated as *β* (Direct effect A) × *β* (Direct effect B). Mediation proportion: (Mediation effect/*β* (Total effect)) × 100%.

## 4 Discussion

Although previous studies have highlighted significant changes in dietary preferences and immune cell traits in patients with PE and FGR, the underlying mechanisms remain poorly understood. Using an MR approach, this study systematically analyzed the genetic and immune‐mediated pathways linking dietary habits to PE and FGR by integrating large‐scale GWAS data. We identified 21 dietary preferences associated with PE risk and 11 with FGR risk, alongside 45 immune cell traits linked to PE and 32 to FGR, underscoring the interplay between dietary factors, immune modulation, and pregnancy outcomes. Mediation analysis revealed specific immune‐mediated mechanisms. Notably, a preference for chili peppers was associated with reduced PE risk, mediated in part by HLA‐DR^+^ CD8^+^ T cells (10.9% of the total effect; mediation effect = −0.030, 95% CI [−0.059, −0.000]; *p* = 0.042). Similarly, a preference for hard cheese was linked to reduced FGR risk through an increase in IgD^+^ CD24^+^ B cells (13.9% of the total effect; mediation effect = −0.151, 95% CI [−0.302, −0.000]; *p* = 0.043). These findings highlight immune cells as critical mediators linking dietary preferences to pregnancy outcomes, providing novel insights into immune‐mediated mechanisms underlying PE and FGR. This work paves the way for targeted dietary interventions to improve maternal and fetal health and supports future research into actionable strategies for preventing pregnancy complications.

### 4.1 Dietary Preferences and Increased Risk of PE and FGR

Our results suggest a significant association between specific dietary preferences that are high in sugar and fat and an increased risk of PE. In particular, preferences for sugar‐sweetened coffee drinks (OR = 1.634, 95% CI: [1.201, 2.223], *p* = 0.002), ice cream (OR = 1.373, 95% CI: [1.041, 1.812], *p* = 0.025), and mayonnaise (OR = 1.263, 95% CI: [1.012, 1.576], *p* = 0.039) were positively correlated with an elevated risk of PE. An analysis from the Norwegian Mother and Child Cohort Study (MoBa) found that diets high in processed meats, salty snacks, and sweetened beverages increased the risk of PE (OR= 1.21) (Brantsaeter et al. 2009). These foods, which are rich in sugars and saturated fats, are likely to disrupt metabolic processes and exacerbate inflammatory responses, leading to oxidative stress and endothelial dysfunction, all of which contribute to the pathogenesis of PE (Casas et al. 2020).

Similarly, a preference for shellfish was linked to a higher risk of FGR (OR = 1.140, 95% CI: [1.027, 1.266], *p* = 0.014). Shellfish are often associated with microbial and heavy metal contaminants, which can impair placental function through inflammation and oxidative stress (Fehrenbach et al. 2022). Guldner et al. (2007) reported that women who consumed shellfish more than twice per week prior to pregnancy had a higher risk of delivering a small‐for‐gestational‐age infant compared to those who consumed shellfish less than once per month. These findings underscore the potential risks of high‐sugar, high‐fat, and shellfish consumption for pregnant women, particularly those at elevated risk for PE and FGR. Reducing the intake of these dietary components may offer protective benefits.

### 4.2 Dietary Preferences and Reduced Risk of PE and FGR

Certain dietary preferences were inversely associated with the risks of PE and FGR. For PE, a preference for spicy foods—including F. capsicum (OR = 0.826, 95% CI: [0.747, 0.913], *p* = 0.000), general spicy food (OR = 0.775, 95% CI: [0.672, 0.895], *p* = 0.001), and chili peppers (OR = 0.762, 95% CI: [0.649, 0.895], *p* = 0.001)—was significantly associated with a reduced risk. Capsaicin, the primary bioactive compound in chili peppers, has been reported to exert anti‐inflammatory and antioxidant effects, potentially enhancing vascular function and attenuating systemic inflammation during pregnancy. A previous study found that women who regularly consumed spicy foods had a significantly lower adjusted odds ratio for hypertension compared to non‐consumers (OR = 0.740) (He et al. 2019). Additionally, preferences for nutrient‐dense plant foods such as dark green vegetables (OR = 0.803, 95% CI: [0.714, 0.902], *p* = 0.000) and fruits (OR = 0.577, 95% CI: [0.360, 0.925], *p* = 0.022) were also inversely associated with PE risk. Higher intake of fresh fruits (≥ 330 g/day) has been linked to a lower risk of PE (OR = 0.79) in large prospective cohorts of nulliparous women (Borgen et al. 2012). Similarly, higher vegetable consumption has been linked to a reduced risk of PE, with women in the highest intake quartile exhibiting an adjusted relative risk (RR) of 0.44 (Mi et al. 2019). The rich content of antioxidants, polyphenols, and dietary fiber in these plant‐based foods likely supports vascular function, alleviates oxidative stress, and modulates immune responses, underscoring the protective role of a plant‐rich diet in preventing hypertensive disorders during pregnancy (Perry et al. 2022).

For FGR, protein‐rich and anti‐inflammatory foods, including hard cheese (OR = 0.337, 95% CI: [0.176, 0.646], *p* = 0.001), mushrooms (OR = 0.536, 95% CI: [0.340, 0.846], *p* = 0.007), fried fish (OR = 0.628, 95% CI: [0.439, 0.899], *p* = 0.011), and cauliflower (OR = 0.387, 95% CI: [0.161, 0.934], *p* = 0.035), among others, were associated with a lower risk. These foods provide essential nutrients, such as high‐quality proteins, calcium, omega‐3 fatty acids, and antioxidant compounds, which increase placental blood flow and reduce inflammation. Similarly, nutrient‐rich vegetables such as cauliflower (OR = 0.387, 95% CI: [0.161, 0.934], *p* = 0.035) demonstrated protective effects, further highlighting the role of anti‐inflammatory and protein‐rich diets in reducing FGR risk and supporting fetal growth.

### 4.3 Immune Modulation as a Mechanism in PE and FGR

This study uses mediation analysis to explore the role of specific immune cells in mediating the relationships between dietary preferences and the risks of PE and FGR. Our findings suggest that HLA‐DR^+^ CD8^+^ T cells and IgD^+^ CD24^+^ B cells may mediate these associations. Specifically, we estimated that 10.9% of the protective effect of a preference for F‐chili on PE risk is mediated by HLA‐DR^+^ CD8^+^ T cells. These cells, known for their cytotoxic functions, may also possess unique immunomodulatory properties during pregnancy, helping to balance pro‐ and anti‐inflammatory responses. However, further studies are needed to clarify their exact role in PE regulation.

Similarly, 13.9% of the protective effect of a preference for hard cheese on FGR risk may be mediated by IgD^+^ CD24^+^ B cells, which are implicated in the production of IL‐10, an anti‐inflammatory cytokine. IL‐10 likely plays a critical role in suppressing excessive inflammatory responses, maintaining placental immune tolerance, and preserving placental blood flow and function, thereby supporting normal fetal growth. Nevertheless, the exact mechanisms by which IgD^+^ CD24^+^ B cells influence pregnancy outcomes remain unclear, and further studies are warranted to elucidate whether their quantity and activity impact maternal immune tolerance and the fetal growth environment.

In conclusion, the immunomodulatory potential of HLA‐DR^+^ CD8^+^ T cells and IgD^+^ CD24^+^ B cells positions these cells as promising therapeutic targets for PE and FGR. Dietary intake of capsaicin or hard cheese may enhance the anti‐inflammatory capacity of immune cells, thereby potentially reducing the risk of PE and FGR. However, experimental studies are necessary to validate these mechanisms and establish causality. Future research should investigate whether targeting these immune cells could improve pregnancy outcomes, with a particular focus on their activity dynamics throughout different pregnancy stages and their distribution within placental tissues. Furthermore, studies should elucidate how specific dietary components influence the anti‐inflammatory and immune tolerance functions of these cells, providing evidence to refine intervention strategies for PE and FGR and ultimately support healthier pregnancy outcomes.

### 4.4 Advantages and Limitations

This study applied MR mediation analysis to investigate causal relationships between dietary preferences, immune cell traits, and the risks of PE and FGR. Using high‐quality, large‐scale GWAS data and robust MR methods, we minimized confounding factors and strengthened causal inferences. Reverse MR analyses confirmed the direction and specificity of these associations, with no evidence of reverse causality or horizontal pleiotropy. Our findings highlight that dietary preferences, such as chili peppers and hard cheese, may modulate immune pathways and reduce the risks of PE and FGR, providing a scientific basis for targeted dietary interventions, especially in high‐risk populations.

Despite these strengths, the study has several limitations. First, some associations did not remain statistically significant after FDR correction, likely due to the large number of traits tested and limitations in sample size and effect magnitude; nevertheless, we reported them at nominal significance (*p* < 0.05) as biologically plausible exploratory findings requiring validation in independent cohorts. Second, although peripheral blood immune traits provide valuable insights into systemic immune modulation, they may not fully capture the complex immune milieu at the maternal–fetal interface, where placental and decidual tissues involve distinct cell populations and spatial regulation that are not reflected in peripheral measurements. This limitation should be considered when interpreting immune‐related findings. Third, the generalizability of our results may be limited, as the GWAS summary statistics were predominantly derived from individuals of European ancestry. Fourth, while MR approaches reduce confounding, residual pleiotropy and weak instrument bias cannot be entirely ruled out. Taken together, these limitations highlight the need for further experimental and population‐based studies to confirm and extend our findings.

## 5 Conclusion

This study suggests that dietary preferences may influence PE and FGR risks via immune modulation. Identifying protective dietary components, such as chili peppers and hard cheese, highlights the potential for targeted dietary interventions to reduce pregnancy complications and support personalized nutrition strategies for long‐term maternal and child health.

## Author Contributions

**Yuxiu Wang:** conceptualization (equal), data curation (equal), formal analysis (equal), visualization (equal), writing – original draft (equal). **Shijun Ni:** formal analysis (equal), methodology (equal), writing – original draft (equal). **Xiaoli Gao:** data curation (equal), formal analysis (equal), writing – review and editing (equal). **Lingyi Fang:** visualization (equal), writing – review and editing (equal). **Yang Li:** visualization (equal), writing – review and editing (equal). **Feng Liu:** formal analysis (equal), methodology (equal), supervision (equal), writing – review and editing (equal). **Lining Guo:** formal analysis (equal), methodology (equal), software (equal), supervision (equal), writing – review and editing (equal). **Cha Han:** funding acquisition (equal), project administration (equal), resources (equal), supervision (equal), validation (equal), writing – review and editing (equal).

## Funding

This work was supported by the Beijing‐Tianjin‐Hebei Basic Research Collaboration Project (22JCZXJC00160), the Tianjin Public Health Science and Technology Major Special Project (24ZXGZSY00070), and the Tianjin Key Medical Discipline (Specialty) Construction Project (TJYXZDXK‐031A).

## Conflicts of Interest

The authors declare no conflicts of interest.

## Supporting information


**Data S1:** fsn371280‐sup‐0001‐Supinfo1.zip.

## Data Availability

Summary‐level data used in this study are publicly available. No new individual‐level data were generated. Data for preeclampsia (PE) and fetal growth restriction (FGR) were obtained from the FinnGen database (version R11, https://r11.finngen.fi/). Dietary preference data were sourced from the GWAS Catalog (https://www.ebi.ac.uk/gwas/studies). Immune cell trait data were obtained from the IEU GWAS database (https://gwas.mrcieu.ac.uk/datasets/).
