# Supplementary material for: Mendelian Randomization Mediation Analysis Reveals the Impact of Dietary Preferences on Preeclampsia and Fetal Growth Restriction via Immune Modulation
Source: Food Sci Nutr. 2025 Nov 26;13(12):e71280. doi: 10.1002/fsn3.71280 (PMC12648301; doi:10.1002/fsn3.71280)
Supplement: Supplementary file 1 — Data S1: fsn371280‐sup‐0001‐Supinfo1.zip. [file FSN3-13-e71280-s001.zip › fsn371280-sup-0011-Supinfo11@Supplementary Material 3_PE - MR (Dietary Preferences to Immune Cells) MR Analysis - 4.docx]

| id.exposure | id.outcome | outcome | exposure | method | nsnp | b | se | pval | lo_ci | up_ci | or | or_lci95 | or_uci95 | p_fdr |
| --- | --- | --- | --- | --- | --- | --- | --- | --- | --- | --- | --- | --- | --- | --- |
| ebi-fl187-GCST90094798 | prot-a-3192 | Guanine nucleotide exchange factor VAV3 \|\| id:prot-a-3192 | Mayonnaise liking \|\| id:ebi-fl187-GCST90094798 | MR Egger | 12 | 0.120933168174173 | 1.01122172707458 | 0.907175511468005 | -1.861061417 | 2.10292775324034 | 1.12854948682422 | 0.155507484468331 | 8.19011347631038 | 0.999950574162623 |
| ebi-fl187-GCST90094798 | prot-a-3233 | Serine/threonine-protein kinase WNK3 \|\| id:prot-a-3233 | Mayonnaise liking \|\| id:ebi-fl187-GCST90094798 | MR Egger | 12 | 0.905817384614384 | 0.845957451886258 | 0.309439877187046 | -0.752259221 | 2.56389399031145 | 2.47395326817484 | 0.471300576857306 | 12.9862874642015 | 0.999950574162623 |
| ebi-fl187-GCST90094798 | prot-a-3250 | 14-3-3 protein theta \|\| id:prot-a-3250 | Mayonnaise liking \|\| id:ebi-fl187-GCST90094798 | MR Egger | 12 | 1.18675823290972 | 0.797380329651869 | 0.167507569040108 | -0.376107213 | 2.74962367902738 | 3.27644250910979 | 0.686528724149585 | 15.6367463412392 | 0.999950574162623 |
| ebi-fl187-GCST90094798 | prot-a-3278 | E3 ubiquitin-protein ligase ZNRF3 \|\| id:prot-a-3278 | Mayonnaise liking \|\| id:ebi-fl187-GCST90094798 | MR Egger | 12 | 0.209309092088635 | 0.797433432897837 | 0.798280648136674 | -1.353660436 | 1.7722786205684 | 1.23282599640766 | 0.258293062798123 | 5.88424606125188 | 0.999950574162623 |
| ebi-fl187-GCST90094798 | prot-a-355 | Calpain-3 \|\| id:prot-a-355 | Mayonnaise liking \|\| id:ebi-fl187-GCST90094798 | MR Egger | 12 | -0.108040078 | 0.797778339584894 | 0.894962171976426 | -1.671685624 | 1.45560546740201 | 0.897591621867022 | 0.187930019195707 | 4.28707836615957 | 0.999950574162623 |
| ebi-fl187-GCST90094798 | prot-a-385 | Coiled-coil domain-containing protein 90B, mitochondrial \|\| id:prot-a-385 | Mayonnaise liking \|\| id:ebi-fl187-GCST90094798 | MR Egger | 12 | 0.230643420742578 | 0.809233200162995 | 0.781448414867496 | -1.355453652 | 1.81674049306205 | 1.25941007986243 | 0.257830302793674 | 6.15177398495458 | 0.999950574162623 |
| ebi-fl187-GCST90094798 | prot-a-390 | C-C motif chemokine 14 \|\| id:prot-a-390 | Mayonnaise liking \|\| id:ebi-fl187-GCST90094798 | MR Egger | 12 | -0.2692809 | 0.797852950600121 | 0.742712210242434 | -1.833072683 | 1.29451088307221 | 0.763928637872686 | 0.159921424175458 | 3.64921064685952 | 0.999950574162623 |
| ebi-fl187-GCST90094798 | prot-a-40 | Pituitary adenylate cyclase-activating polypeptide 27 \|\| id:prot-a-40 | Mayonnaise liking \|\| id:ebi-fl187-GCST90094798 | MR Egger | 12 | 0.394213661052586 | 0.797778339584894 | 0.631881526019918 | -1.169431885 | 1.95785920663898 | 1.48321742061725 | 0.310543315620769 | 7.08414512939969 | 0.999950574162623 |
| ebi-fl187-GCST90094798 | prot-a-401 | C-C motif chemokine 24 \|\| id:prot-a-401 | Mayonnaise liking \|\| id:ebi-fl187-GCST90094798 | MR Egger | 12 | 0.751446404980971 | 0.797433432897837 | 0.368230901404028 | -0.811523123 | 2.31441593346073 | 2.12006427152398 | 0.444181008201035 | 10.1190110166939 | 0.999950574162623 |
| ebi-fl187-GCST90094798 | prot-a-412 | Cerebral cavernous malformations 2 protein \|\| id:prot-a-412 | Mayonnaise liking \|\| id:ebi-fl187-GCST90094798 | MR Egger | 12 | 1.16146717846613 | 1.48271469511371 | 0.451581934842577 | -1.744653624 | 4.067587981 | 3.19461691255239 | 0.17470549228491 | 58.4158922795769 | 0.999950574162623 |
| ebi-fl187-GCST90094798 | prot-a-44 | Adhesion G protein-coupled receptor F5 \|\| id:prot-a-44 | Mayonnaise liking \|\| id:ebi-fl187-GCST90094798 | MR Egger | 12 | 0.398904244271208 | 0.797472356463271 | 0.627750433412572 | -1.164141574 | 1.96195006293922 | 1.49019091745795 | 0.312190539391895 | 7.11318470700527 | 0.999950574162623 |
| ebi-fl187-GCST90094798 | prot-a-48 | Adrenomedullin \|\| id:prot-a-48 | Mayonnaise liking \|\| id:ebi-fl187-GCST90094798 | MR Egger | 12 | -0.013423616 | 0.820771069578263 | 0.98727297860863 | -1.622134913 | 1.59528768017461 | 0.986666078745084 | 0.197476653338608 | 4.92974705864065 | 0.999950574162623 |
| ebi-fl187-GCST90094798 | prot-a-502 | Carcinoembryonic antigen-related cell adhesion molecule 1 \|\| id:prot-a-502 | Mayonnaise liking \|\| id:ebi-fl187-GCST90094798 | MR Egger | 12 | 0.470434252 | 0.797778339584894 | 0.56848811279292 | -1.093211294 | 2.03407979710139 | 1.60068914400055 | 0.335138535420677 | 7.64521373976003 | 0.999950574162623 |
| ebi-fl187-GCST90094798 | prot-a-518 | Complement factor B \|\| id:prot-a-518 | Mayonnaise liking \|\| id:ebi-fl187-GCST90094798 | MR Egger | 12 | 0.765159260581345 | 0.940595155423611 | 0.434881951780086 | -1.078407244 | 2.60872576521162 | 2.14933665200356 | 0.340136849423262 | 13.5817335036735 | 0.999950574162623 |
| ebi-fl187-GCST90094798 | prot-a-525 | Properdin \|\| id:prot-a-525 | Mayonnaise liking \|\| id:ebi-fl187-GCST90094798 | MR Egger | 12 | -0.617272091 | 0.834837665984774 | 0.476667069824154 | -2.253553916 | 1.01900973469026 | 0.539413904636403 | 0.105025309399552 | 2.77044992467627 | 0.999950574162623 |
| ebi-fl187-GCST90094798 | prot-a-533 | Chondroadherin \|\| id:prot-a-533 | Mayonnaise liking \|\| id:ebi-fl187-GCST90094798 | MR Egger | 12 | -0.171077702 | 0.797430847030283 | 0.834440650291819 | -1.734042163 | 1.39188675768637 | 0.842756086678499 | 0.176569243928013 | 4.02243225282894 | 0.999950574162623 |
| ebi-fl187-GCST90094798 | prot-a-535 | Coiled-coil-helix-coiled-coil-helix domain-containing protein 2 \|\| id:prot-a-535 | Mayonnaise liking \|\| id:ebi-fl187-GCST90094798 | MR Egger | 12 | -0.349859834 | 1.17510720484812 | 0.772005889160521 | -2.653069955 | 1.95335028755285 | 0.704786869987768 | 0.0704346495739339 | 7.05227519568692 | 0.999950574162623 |
| ebi-fl187-GCST90094798 | prot-a-591 | Caseinolytic peptidase B protein homolog \|\| id:prot-a-591 | Mayonnaise liking \|\| id:ebi-fl187-GCST90094798 | MR Egger | 11 | 0.134713907794633 | 0.845120069974748 | 0.876871991309526 | -1.521721429 | 1.79114924494514 | 1.14420938813354 | 0.218335713760218 | 5.99633977119633 | 0.999950574162623 |
| ebi-fl187-GCST90094798 | prot-a-605 | Ciliary neurotrophic factor receptor subunit alpha \|\| id:prot-a-605 | Mayonnaise liking \|\| id:ebi-fl187-GCST90094798 | MR Egger | 12 | -0.051139132 | 1.01540639515066 | 0.960824717326553 | -2.041335666 | 1.93905740274768 | 0.950146465797416 | 0.129855151852963 | 6.95219476151053 | 0.999950574162623 |
| ebi-fl187-GCST90094798 | prot-a-606 | Ciliary neurotrophic factor receptor subunit alpha \|\| id:prot-a-606 | Mayonnaise liking \|\| id:ebi-fl187-GCST90094798 | MR Egger | 12 | -0.383343966 | 0.828792615270189 | 0.653599704606309 | -2.007777492 | 1.24108955983294 | 0.68157841909332 | 0.134286796733272 | 3.45938061428677 | 0.999950574162623 |
| ebi-fl187-GCST90094798 | prot-a-707 | Cancer/testis antigen 55 \|\| id:prot-a-707 | Mayonnaise liking \|\| id:ebi-fl187-GCST90094798 | MR Egger | 12 | 0.964888691071681 | 0.797109969907265 | 0.253926882918442 | -0.59744685 | 2.52722423208992 | 2.62449551 | 0.550214624810284 | 12.5187088341354 | 0.999950574162623 |
| ebi-fl187-GCST90094798 | prot-a-719 | Dipeptidyl peptidase 1 \|\| id:prot-a-719 | Mayonnaise liking \|\| id:ebi-fl187-GCST90094798 | MR Egger | 12 | -0.341421261 | 1.0886332459525 | 0.760250946244147 | -2.475142423 | 1.79229990124569 | 0.71075943003045 | 0.0841510043747185 | 6.00324346846395 | 0.999950574162623 |
| ebi-fl187-GCST90094798 | prot-a-752 | Cytochrome b5 \|\| id:prot-a-752 | Mayonnaise liking \|\| id:ebi-fl187-GCST90094798 | MR Egger | 12 | -0.361605541 | 0.79703569679686 | 0.659739988945609 | -1.923795507 | 1.20058442440619 | 0.696557076645597 | 0.146051569039773 | 3.32205784720417 | 0.999950574162623 |
| ebi-fl187-GCST90094798 | prot-a-755 | Cytoglobin \|\| id:prot-a-755 | Mayonnaise liking \|\| id:ebi-fl187-GCST90094798 | MR Egger | 12 | 0.351491568450664 | 0.97661401731086 | 0.72640053223186 | -1.562671905 | 2.26565504237995 | 1.42118576431726 | 0.209575356902252 | 9.63743546260579 | 0.999950574162623 |
| ebi-fl187-GCST90094798 | prot-a-807 | Desmin \|\| id:prot-a-807 | Mayonnaise liking \|\| id:ebi-fl187-GCST90094798 | MR Egger | 12 | -0.1393125 | 1.06343159116552 | 0.898371128635271 | -2.223638418 | 1.94501341888909 | 0.86995612486458 | 0.108214661453784 | 6.99372570243281 | 0.999950574162623 |
| ebi-fl187-GCST90094798 | prot-a-82 | Ameloblastin \|\| id:prot-a-82 | Mayonnaise liking \|\| id:ebi-fl187-GCST90094798 | MR Egger | 12 | 0.616843713226431 | 0.978341655512872 | 0.542507309863787 | -1.300705932 | 2.53439335803166 | 1.85306998157716 | 0.272339472125771 | 12.6087795126391 | 0.999950574162623 |
| ebi-fl187-GCST90094798 | prot-a-84 | Muellerian-inhibiting factor \|\| id:prot-a-84 | Mayonnaise liking \|\| id:ebi-fl187-GCST90094798 | MR Egger | 12 | -0.899768311 | 0.93999241634806 | 0.361031203263024 | -2.742153447 | 0.942616825059514 | 0.406663868378667 | 0.0644314476814798 | 2.56668921459354 | 0.999950574162623 |
| ebi-fl187-GCST90094798 | prot-a-858 | Inactive dipeptidyl peptidase 10 \|\| id:prot-a-858 | Mayonnaise liking \|\| id:ebi-fl187-GCST90094798 | MR Egger | 12 | 0.90220750433691 | 1.02010048948644 | 0.39723044737754 | -1.097189455 | 2.90160446373033 | 2.46503869301984 | 0.333807948773198 | 18.2033285319204 | 0.999950574162623 |
| ebi-fl187-GCST90094798 | prot-a-863 | Dihydropyrimidinase-related protein 3 \|\| id:prot-a-863 | Mayonnaise liking \|\| id:ebi-fl187-GCST90094798 | MR Egger | 12 | 0.0696955907376528 | 0.845559472704804 | 0.935934950030438 | -1.587600976 | 1.72699215723907 | 1.07218174951684 | 0.204415421517006 | 5.62371319867058 | 0.999950574162623 |
| ebi-fl187-GCST90094798 | prot-a-864 | Dihydropyrimidinase-related protein 5 \|\| id:prot-a-864 | Mayonnaise liking \|\| id:ebi-fl187-GCST90094798 | MR Egger | 12 | -0.776725841 | 0.797778339584894 | 0.353211791005341 | -2.340371387 | 0.786919704255571 | 0.459909365087542 | 0.0962918700482167 | 2.19661975605326 | 0.999950574162623 |
| ebi-fl187-GCST90094798 | prot-a-884 | Endothelin-converting enzyme 1 \|\| id:prot-a-884 | Mayonnaise liking \|\| id:ebi-fl187-GCST90094798 | MR Egger | 12 | 0.587764046457151 | 1.33765349360249 | 0.669715361683311 | -2.034036801 | 3.20956489391803 | 1.79995928725949 | 0.130806414452632 | 24.7683070386804 | 0.999950574162623 |
| ebi-fl187-GCST90094798 | prot-a-893 | Early endosome antigen 1 \|\| id:prot-a-893 | Mayonnaise liking \|\| id:ebi-fl187-GCST90094798 | MR Egger | 11 | 1.02909533340457 | 0.887621589124399 | 0.276133062288481 | -0.710642981 | 2.76883364808839 | 2.79853294988772 | 0.491328181053151 | 15.9400314771687 | 0.999950574162623 |
| ebi-fl187-GCST90094798 | prot-a-897 | Ephrin-A2 \|\| id:prot-a-897 | Mayonnaise liking \|\| id:ebi-fl187-GCST90094798 | MR Egger | 12 | 0.4869651999883 | 0.797778339584894 | 0.555213508316504 | -1.076680346 | 2.05061074557469 | 1.62736997589897 | 0.340724738688035 | 7.77264676658009 | 0.999950574162623 |
| ebi-fl187-GCST90094798 | prot-a-90 | Anaphase-promoting complex subunit 10 \|\| id:prot-a-90 | Mayonnaise liking \|\| id:ebi-fl187-GCST90094798 | MR Egger | 12 | 0.570756944291756 | 0.924717484122443 | 0.550882054719964 | -1.241689325 | 2.38320321317174 | 1.76960603778973 | 0.288895766756699 | 10.839568762595 | 0.999950574162623 |
| ebi-fl187-GCST90094798 | prot-a-914 | Histone-lysine N-methyltransferase EHMT2 \|\| id:prot-a-914 | Mayonnaise liking \|\| id:ebi-fl187-GCST90094798 | MR Egger | 12 | 1.16328135902635 | 0.916519352510384 | 0.233100165166777 | -0.633096572 | 2.95965928994671 | 3.2004177847763 | 0.530945143018132 | 19.2913978624768 | 0.999950574162623 |
| ebi-fl187-GCST90094798 | prot-a-932 | RNA polymerase II elongation factor ELL \|\| id:prot-a-932 | Mayonnaise liking \|\| id:ebi-fl187-GCST90094798 | MR Egger | 12 | 0.0843239127902911 | 0.817469789854531 | 0.919881398993096 | -1.517916875 | 1.68656470090517 | 1.08798124778152 | 0.219167965950512 | 5.40089510978767 | 0.999950574162623 |
| ebi-fl187-GCST90094798 | prot-a-991 | Estrogen receptor \|\| id:prot-a-991 | Mayonnaise liking \|\| id:ebi-fl187-GCST90094798 | MR Egger | 12 | 0.940791830442118 | 0.797074628529803 | 0.265192669759308 | -0.621474441 | 2.50305810236053 | 2.56200929230778 | 0.537151854456257 | 12.219806297636 | 0.999950574162623 |
| ebi-fl187-GCST90094798 | prot-a-992 | Persulfide dioxygenase ETHE1, mitochondrial \|\| id:prot-a-992 | Mayonnaise liking \|\| id:ebi-fl187-GCST90094798 | MR Egger | 12 | 1.73531929731909 | 0.825701162046394 | 0.0619072713554627 | 0.116945019708158 | 3.35369357493002 | 5.67073816626142 | 1.12405762697521 | 28.6082052899972 | 0.999950574162623 |
| ebi-fl187-GCST90094798 | prot-a-993 | Ethanolamine kinase 1 \|\| id:prot-a-993 | Mayonnaise liking \|\| id:ebi-fl187-GCST90094798 | MR Egger | 12 | 0.537904849925676 | 0.819297711175186 | 0.526293435954447 | -1.067918664 | 2.14372836382904 | 1.71241533398909 | 0.343723176867208 | 8.53118577224675 | 0.999950574162623 |
| ebi-fl187-GCST90094798 | prot-b-10 | coagulation factor III, tissue factor \|\| id:prot-b-10 | Mayonnaise liking \|\| id:ebi-fl187-GCST90094798 | MR Egger | 8 | 1.38176060598494 | 1.74432162004072 | 0.458439881786435 | -2.037109769 | 4.80063098126475 | 3.98190602727445 | 0.130405067467852 | 121.587112509668 | 0.999950574162623 |
| ebi-fl187-GCST90094798 | prot-b-28 | prolactin \|\| id:prot-b-28 | Mayonnaise liking \|\| id:ebi-fl187-GCST90094798 | MR Egger | 8 | 0.644485929545185 | 1.07809108635865 | 0.571828822139249 | -1.4685726 | 2.75754445880813 | 1.90500746945587 | 0.23025391523398 | 15.7610933781293 | 0.999950574162623 |
| ebi-fl187-GCST90094798 | prot-b-54 | matrix metallopeptidase 7 \|\| id:prot-b-54 | Mayonnaise liking \|\| id:ebi-fl187-GCST90094798 | MR Egger | 8 | 0.13933912013583 | 1.01698153820513 | 0.895502914784795 | -1.853944695 | 2.13262293501788 | 1.14951385720801 | 0.156618135436393 | 8.4369674318456 | 0.999950574162623 |
| ebi-fl187-GCST90094798 | ukb-rsfMRI191-edge-pheno716 | Net100_Pair10_33 (OccipitalPrecuneus)&(Temporal\|Frontal\|Supp_Motor_Area) (Default_mode\|Central_executive)&(Default_mode\|Salience) \|\| id:ukb-rsfMRI191-edge-pheno716 | Mayonnaise liking \|\| id:ebi-fl187-GCST90094798 | MR Egger | 10 | 0.71660078576511 | 0.361340440512996 | 0.0826417975940916 | 0.00837352235963795 | 1.42482804917058 | 2.04746160772844 | 1.00840867835599 | 4.1571429571157 | 0.999950574162623 |
| ebi-fl187-GCST90094798 | ukb-rsfMRI191-edge-pheno903 | Net100_Pair27_38 (Precentral\|Frontal\|Supp_Motor_Area)&(Subcortical) (Attention\|Salience\|Motor)&(Subcortical-cerebellum) \|\| id:ukb-rsfMRI191-edge-pheno903 | Mayonnaise liking \|\| id:ebi-fl187-GCST90094798 | MR Egger | 10 | 0.333073750923515 | 0.4079100666336 | 0.437829699488616 | -0.46642998 | 1.13257748152537 | 1.39525019566584 | 0.627237526666442 | 3.10364578926224 | 0.999950574162623 |
| ebi-fl187-GCST90094798 | ukb-rsfMRI191-node-pheno23 | Net100_Node2 RolandicOper\|SupraMarginal\|Insula Salience\|Motor \|\| id:ukb-rsfMRI191-node-pheno23 | Mayonnaise liking \|\| id:ebi-fl187-GCST90094798 | MR Egger | 10 | 0.495413783936325 | 0.334950170783646 | 0.177384308688515 | -0.161088551 | 1.15191611867227 | 1.64117719133602 | 0.851216691851039 | 3.16425018346908 | 0.999950574162623 |
| ebi-fl187-GCST90094798 | ukb-rsfMRI191-node-pheno55 | Net100_Node34 Parietal\|Postcentral\|Precuneus Attention \|\| id:ukb-rsfMRI191-node-pheno55 | Mayonnaise liking \|\| id:ebi-fl187-GCST90094798 | MR Egger | 9 | 0.523271301821713 | 0.353061334751434 | 0.181872147446927 | -0.168728914 | 1.21527151793452 | 1.68753907967397 | 0.844737868713995 | 3.37120928384832 | 0.999950574162623 |
| ebi-fl187-GCST90094798 | ebi-a-GCST90001414 | IgD- CD24- B cell Absolute Count \|\| id:ebi-a-GCST90001414 | Mayonnaise liking \|\| id:ebi-fl187-GCST90094798 | Weighted median | 9 | -0.056242458 | 0.220590417466341 | 0.798751456418482 | -0.488599676 | 0.376114760711102 | 0.9453099106035 | 0.613484870708585 | 1.45661428627093 | 0.999332001504091 |
| ebi-fl187-GCST90094798 | ebi-a-GCST90001422 | CD20- CD38- B cell %B cell \|\| id:ebi-a-GCST90001422 | Mayonnaise liking \|\| id:ebi-fl187-GCST90094798 | Weighted median | 9 | 0.0495801835995485 | 0.206552490616339 | 0.810301785496048 | -0.355262698 | 0.454423065207573 | 1.05082984815655 | 0.700989270533402 | 1.5752642960376 | 0.999332001504091 |
| ebi-fl187-GCST90094798 | ebi-a-GCST90001444 | CD20- CD38- B cell %lymphocyte \|\| id:ebi-a-GCST90001444 | Mayonnaise liking \|\| id:ebi-fl187-GCST90094798 | Weighted median | 9 | 0.0112882824490431 | 0.211289935600672 | 0.957392842930178 | -0.402839991 | 0.42541655622636 | 1.01135223552295 | 0.66841904361065 | 1.53022771280147 | 0.999332001504091 |
| ebi-fl187-GCST90094798 | ebi-a-GCST90001459 | Myeloid Dendritic Cell %Dendritic Cell \|\| id:ebi-a-GCST90001459 | Mayonnaise liking \|\| id:ebi-fl187-GCST90094798 | Weighted median | 9 | 0.0482102725863232 | 0.213547493694723 | 0.821388852664075 | -0.370342815 | 0.466763360227979 | 1.0493912903476 | 0.690497577093235 | 1.59482395998139 | 0.999332001504091 |
| ebi-fl187-GCST90094798 | ebi-a-GCST90001476 | HLA DR++ monocyte %leukocyte \|\| id:ebi-a-GCST90001476 | Mayonnaise liking \|\| id:ebi-fl187-GCST90094798 | Weighted median | 9 | -0.211671554 | 0.22329447905756 | 0.343155928383723 | -0.649328733 | 0.225985624641968 | 0.809230442173036 | 0.52239632636878 | 1.2535576448087 | 0.999332001504091 |
| ebi-fl187-GCST90094798 | ebi-a-GCST90001478 | CD4 regulatory T cell %CD4+ T cell \|\| id:ebi-a-GCST90001478 | Mayonnaise liking \|\| id:ebi-fl187-GCST90094798 | Weighted median | 9 | -0.310398078 | 0.240004156041966 | 0.195906308095863 | -0.780806224 | 0.160010067905065 | 0.733155045278427 | 0.458036582419458 | 1.17352268584733 | 0.999332001504091 |
| ebi-fl187-GCST90094798 | ebi-a-GCST90001485 | CD39+ resting CD4 regulatory T cell %CD4 regulatory T cell \|\| id:ebi-a-GCST90001485 | Mayonnaise liking \|\| id:ebi-fl187-GCST90094798 | Weighted median | 9 | 0.0930481914115028 | 0.228703888616092 | 0.684118114969755 | -0.35521143 | 0.541307813099044 | 1.09751462477257 | 0.701025209585014 | 1.71825254658493 | 0.999332001504091 |
| ebi-fl187-GCST90094798 | ebi-a-GCST90001515 | Immature Myeloid-Derived Suppressor Cells Absolute Count \|\| id:ebi-a-GCST90001515 | Mayonnaise liking \|\| id:ebi-fl187-GCST90094798 | Weighted median | 9 | 0.355379509855406 | 0.299271434224179 | 0.235037462092482 | -0.231192501 | 0.941951520934797 | 1.42672200661314 | 0.793586684922253 | 2.56498215359253 | 0.999332001504091 |
| ebi-fl187-GCST90094798 | ebi-a-GCST90001517 | CD33+ HLA DR+ Absolute Count \|\| id:ebi-a-GCST90001517 | Mayonnaise liking \|\| id:ebi-fl187-GCST90094798 | Weighted median | 9 | 0.157313388178096 | 0.28218716801977 | 0.57720028071955 | -0.395773461 | 0.710400237496846 | 1.17036233415463 | 0.673159175377224 | 2.03480550115104 | 0.999332001504091 |
| ebi-fl187-GCST90094798 | ebi-a-GCST90001518 | CD33+ HLA DR+ CD14- Absolute Count \|\| id:ebi-a-GCST90001518 | Mayonnaise liking \|\| id:ebi-fl187-GCST90094798 | Weighted median | 9 | 0.0379468168351763 | 0.287176299729179 | 0.894875374039372 | -0.524918731 | 0.600812364304368 | 1.03867599133311 | 0.591603441649801 | 1.82359962606579 | 0.999332001504091 |
| ebi-fl187-GCST90094798 | ebi-a-GCST90001520 | CD33+ HLA DR+ CD14dim Absolute Count \|\| id:ebi-a-GCST90001520 | Mayonnaise liking \|\| id:ebi-fl187-GCST90094798 | Weighted median | 9 | -0.066155184 | 0.292574258031889 | 0.821112628039976 | -0.63960073 | 0.507290361777838 | 0.93598560306405 | 0.527502998278825 | 1.66078496615503 | 0.999332001504091 |
| ebi-fl187-GCST90094798 | ebi-a-GCST90001531 | CD33dim HLA DR- Absolute Count \|\| id:ebi-a-GCST90001531 | Mayonnaise liking \|\| id:ebi-fl187-GCST90094798 | Weighted median | 9 | -0.182461974 | 0.284498707313296 | 0.521298214407612 | -0.74007944 | 0.375155492229061 | 0.833216327122504 | 0.477076014887546 | 1.45521767206671 | 0.999332001504091 |
| ebi-fl187-GCST90094798 | ebi-a-GCST90001532 | Basophil Absolute Count \|\| id:ebi-a-GCST90001532 | Mayonnaise liking \|\| id:ebi-fl187-GCST90094798 | Weighted median | 9 | -0.194174233 | 0.303984695267707 | 0.52297699981418 | -0.789984236 | 0.401635769902299 | 0.823514408528415 | 0.453851949953643 | 1.49426697653976 | 0.999332001504091 |
| ebi-fl187-GCST90094798 | ebi-a-GCST90001588 | T/B cell \|\| id:ebi-a-GCST90001588 | Mayonnaise liking \|\| id:ebi-fl187-GCST90094798 | Weighted median | 9 | 0.129545028316244 | 0.217812491948997 | 0.552007375299568 | -0.297367456 | 0.556457512536279 | 1.13831036650798 | 0.742771026611006 | 1.74448173673597 | 0.999332001504091 |
| ebi-fl187-GCST90094798 | ebi-a-GCST90001624 | HLA DR+ CD4+ T cell Absolute Count \|\| id:ebi-a-GCST90001624 | Mayonnaise liking \|\| id:ebi-fl187-GCST90094798 | Weighted median | 9 | 0.000853903753943123 | 0.214102910374506 | 0.99681781618813 | -0.418787801 | 0.420495608087975 | 1.00085426843355 | 0.657843774525321 | 1.52271603902378 | 0.999364451047625 |
| ebi-fl187-GCST90094798 | ebi-a-GCST90001631 | CD8+ Natural Killer T %T cell \|\| id:ebi-a-GCST90001631 | Mayonnaise liking \|\| id:ebi-fl187-GCST90094798 | Weighted median | 9 | -0.150513735 | 0.208392654207596 | 0.470134474586032 | -0.558963337 | 0.257935867556333 | 0.860265914440142 | 0.571801522223103 | 1.2942558121746 | 0.999332001504091 |
| ebi-fl187-GCST90094798 | ebi-a-GCST90001632 | CD8+ Natural Killer T %lymphocyte \|\| id:ebi-a-GCST90001632 | Mayonnaise liking \|\| id:ebi-fl187-GCST90094798 | Weighted median | 9 | -0.180860899 | 0.202883485012333 | 0.37268670311612 | -0.57851253 | 0.216790731395125 | 0.834551437370623 | 0.560731818384576 | 1.24208414572204 | 0.999332001504091 |
| ebi-fl187-GCST90094798 | ebi-a-GCST90001646 | Natural Killer %CD3- lymphocyte \|\| id:ebi-a-GCST90001646 | Mayonnaise liking \|\| id:ebi-fl187-GCST90094798 | Weighted median | 9 | 0.520531650948757 | 0.232436848398909 | 0.0251263852775838 | 0.0649554280868965 | 0.976107873810618 | 1.68292213905167 | 1.06711146012491 | 2.65410599730484 | 0.999332001504091 |
| ebi-fl187-GCST90094798 | ebi-a-GCST90001684 | CD127- CD8+ T cell Absolute Count \|\| id:ebi-a-GCST90001684 | Mayonnaise liking \|\| id:ebi-fl187-GCST90094798 | Weighted median | 9 | -0.131878214 | 0.226065170429422 | 0.559648489942829 | -0.574965948 | 0.31120952010064 | 0.87644772769462 | 0.562724030369129 | 1.36507520191945 | 0.999332001504091 |
| ebi-fl187-GCST90094798 | ebi-a-GCST90001687 | CD28- CD8+ T cell Absolute Count \|\| id:ebi-a-GCST90001687 | Mayonnaise liking \|\| id:ebi-fl187-GCST90094798 | Weighted median | 9 | -0.1125311 | 0.213467170521169 | 0.598083309920484 | -0.530926754 | 0.305864554680573 | 0.893569557073385 | 0.588059730498387 | 1.35779838665643 | 0.999332001504091 |
| ebi-fl187-GCST90094798 | ebi-a-GCST90001701 | BAFF-R on CD20- CD38- B cell \|\| id:ebi-a-GCST90001701 | Mayonnaise liking \|\| id:ebi-fl187-GCST90094798 | Weighted median | 9 | -0.471784746 | 0.227873722068419 | 0.0384169582858575 | -0.918417241 | -0.025152251 | 0.623887792764608 | 0.399150299987923 | 0.97516143160226 | 0.999332001504091 |
| ebi-fl187-GCST90094798 | ebi-a-GCST90001732 | CD19 on IgD- CD27- B cell \|\| id:ebi-a-GCST90001732 | Mayonnaise liking \|\| id:ebi-fl187-GCST90094798 | Weighted median | 9 | 0.237258679479852 | 0.231444680210973 | 0.305306347707647 | -0.216372894 | 0.690890252693359 | 1.26776902119349 | 0.805434904240105 | 1.99549123416033 | 0.999332001504091 |
| ebi-fl187-GCST90094798 | ebi-a-GCST90001798 | CD27 on CD24+ CD27+ B cell \|\| id:ebi-a-GCST90001798 | Mayonnaise liking \|\| id:ebi-fl187-GCST90094798 | Weighted median | 9 | 0.465232942939948 | 0.208166487460694 | 0.0254233908951212 | 0.0572266275169872 | 0.873239258362909 | 1.59238508054932 | 1.05889575812151 | 2.39465521068326 | 0.999332001504091 |
| ebi-fl187-GCST90094798 | ebi-a-GCST90001836 | CD66b on CD66b++ myeloid cell \|\| id:ebi-a-GCST90001836 | Mayonnaise liking \|\| id:ebi-fl187-GCST90094798 | Weighted median | 9 | 0.165167440492769 | 0.373775399222508 | 0.658569137731969 | -0.567432342 | 0.897767222968884 | 1.1795906134102 | 0.566979380439615 | 2.45411749218567 | 0.999332001504091 |
| ebi-fl187-GCST90094798 | ebi-a-GCST90001943 | CD25 on activated & secreting CD4 regulatory T cell \|\| id:ebi-a-GCST90001943 | Mayonnaise liking \|\| id:ebi-fl187-GCST90094798 | Weighted median | 9 | -0.203875019 | 0.2219830067604 | 0.358395755611604 | -0.638961713 | 0.231211673825726 | 0.815564294512699 | 0.527840189403185 | 1.26012594690082 | 0.999332001504091 |
| ebi-fl187-GCST90094798 | ebi-a-GCST90001960 | CD25 on CD4+ T cell \|\| id:ebi-a-GCST90001960 | Mayonnaise liking \|\| id:ebi-fl187-GCST90094798 | Weighted median | 9 | -0.463245558 | 0.218349292634292 | 0.0338729980406653 | -0.891210172 | -0.035280944 | 0.629238099055851 | 0.410159089402752 | 0.965334172844896 | 0.999332001504091 |
| ebi-fl187-GCST90094798 | ebi-a-GCST90001988 | HLA DR on CD14+ CD16- monocyte \|\| id:ebi-a-GCST90001988 | Mayonnaise liking \|\| id:ebi-fl187-GCST90094798 | Weighted median | 9 | -0.326427261 | 0.221618618659732 | 0.140771677776837 | -0.760799753 | 0.107945231905645 | 0.721496854339572 | 0.467292558790521 | 1.11398673278522 | 0.999332001504091 |
| ebi-fl187-GCST90094798 | ebi-a-GCST90001991 | HLA DR on CD14+ monocyte \|\| id:ebi-a-GCST90001991 | Mayonnaise liking \|\| id:ebi-fl187-GCST90094798 | Weighted median | 9 | -0.327672675 | 0.215465265190102 | 0.128318074239713 | -0.749984594 | 0.0946392452362638 | 0.720598851459497 | 0.472373829930251 | 1.09926221950403 | 0.999332001504091 |
| ebi-fl187-GCST90094798 | ebi-a-GCST90002019 | CD14 on Monocytic Myeloid-Derived Suppressor Cells \|\| id:ebi-a-GCST90002019 | Mayonnaise liking \|\| id:ebi-fl187-GCST90094798 | Weighted median | 9 | 0.019533624820987 | 0.33735217189048 | 0.953826077622735 | -0.641676632 | 0.680743881726327 | 1.01972565437678 | 0.526409089367619 | 1.97534661007342 | 0.999332001504091 |
| ebi-fl187-GCST90094798 | ebi-a-GCST90002037 | CD80 on plasmacytoid Dendritic Cell \|\| id:ebi-a-GCST90002037 | Mayonnaise liking \|\| id:ebi-fl187-GCST90094798 | Weighted median | 9 | -0.07202207 | 0.24738388829095 | 0.770948190721151 | -0.556894491 | 0.412850350941768 | 0.930510359120003 | 0.572985715923296 | 1.51111887149652 | 0.999332001504091 |
| ebi-fl187-GCST90094798 | ebi-a-GCST90002042 | CD45 on CD33+ HLA DR+ CD14- \|\| id:ebi-a-GCST90002042 | Mayonnaise liking \|\| id:ebi-fl187-GCST90094798 | Weighted median | 9 | -0.544579267 | 0.310646780491829 | 0.0795934645187539 | -1.153446957 | 0.0642884228559418 | 0.580085793294129 | 0.3155472150563 | 1.06639992852303 | 0.999332001504091 |
| ebi-fl187-GCST90094798 | ebi-a-GCST90002056 | CD8 on Effector Memory CD8+ T cell \|\| id:ebi-a-GCST90002056 | Mayonnaise liking \|\| id:ebi-fl187-GCST90094798 | Weighted median | 9 | -0.248531678 | 0.233624422913551 | 0.287415399931484 | -0.706435547 | 0.209372191063154 | 0.77994515346024 | 0.493399767178862 | 1.23290378891808 | 0.999332001504091 |
| ebi-fl187-GCST90094798 | ebi-a-GCST90002058 | CD8 on CD8+ T cell \|\| id:ebi-a-GCST90002058 | Mayonnaise liking \|\| id:ebi-fl187-GCST90094798 | Weighted median | 9 | -0.148341011 | 0.246515573929087 | 0.54733984809022 | -0.631511536 | 0.334829513914097 | 0.862137066596102 | 0.531787377496721 | 1.39770207615263 | 0.999332001504091 |
| ebi-fl187-GCST90094798 | ebi-a-GCST90002063 | CD4 on CD4 regulatory T cell \|\| id:ebi-a-GCST90002063 | Mayonnaise liking \|\| id:ebi-fl187-GCST90094798 | Weighted median | 9 | -0.226582911 | 0.22956741957564 | 0.323642920773485 | -0.676535053 | 0.223369231384988 | 0.797253238522742 | 0.508375437916055 | 1.25028213192305 | 0.999332001504091 |
| ebi-fl187-GCST90094798 | ebi-a-GCST90002066 | CD4 on activated CD4 regulatory T cell \|\| id:ebi-a-GCST90002066 | Mayonnaise liking \|\| id:ebi-fl187-GCST90094798 | Weighted median | 9 | -0.16665784 | 0.232826411280875 | 0.474113010421424 | -0.622997606 | 0.28968192646167 | 0.846489196832863 | 0.536334306724781 | 1.33600247340217 | 0.999332001504091 |
| ebi-fl187-GCST90094798 | ebi-a-GCST90002070 | CD4 on activated & secreting CD4 regulatory T cell \|\| id:ebi-a-GCST90002070 | Mayonnaise liking \|\| id:ebi-fl187-GCST90094798 | Weighted median | 9 | -0.170433718 | 0.222845577289358 | 0.444386850659249 | -0.60721105 | 0.266343613174401 | 0.8432989830557 | 0.544868362713014 | 1.30518345987606 | 0.999332001504091 |
| ebi-fl187-GCST90094798 | ebi-a-GCST90002081 | SSC-A on CD4+ T cell \|\| id:ebi-a-GCST90002081 | Mayonnaise liking \|\| id:ebi-fl187-GCST90094798 | Weighted median | 9 | 0.0402978838773322 | 0.220015354414679 | 0.854672936786705 | -0.390932211 | 0.471527978530103 | 1.04112086112403 | 0.676426008879723 | 1.60244081871248 | 0.999332001504091 |
| ebi-fl187-GCST90094798 | ebi-a-GCST90002094 | CD11b on Monocytic Myeloid-Derived Suppressor Cells \|\| id:ebi-a-GCST90002094 | Mayonnaise liking \|\| id:ebi-fl187-GCST90094798 | Weighted median | 9 | -0.457095334 | 0.339423998431163 | 0.178083483445866 | -1.122366371 | 0.208175702777428 | 0.633119979 | 0.325508608371714 | 1.23142951612815 | 0.999332001504091 |
| ebi-fl187-GCST90094798 | ebi-a-GCST90002104 | HLA DR on myeloid Dendritic Cell \|\| id:ebi-a-GCST90002104 | Mayonnaise liking \|\| id:ebi-fl187-GCST90094798 | Weighted median | 9 | -0.380601595 | 0.233947373049082 | 0.103765044541442 | -0.839138446 | 0.0779352560117815 | 0.683450125221075 | 0.432082625480257 | 1.08105266474328 | 0.999332001504091 |
| ebi-fl187-GCST90094798 | ebi-a-GCST90002105 | HLA DR on plasmacytoid Dendritic Cell \|\| id:ebi-a-GCST90002105 | Mayonnaise liking \|\| id:ebi-fl187-GCST90094798 | Weighted median | 9 | -0.186845026 | 0.240876079937651 | 0.437932390643058 | -0.658962142 | 0.285272090907774 | 0.829572288750655 | 0.51738803101165 | 1.33012389350674 | 0.999332001504091 |
| ebi-fl187-GCST90094798 | ebi-a-GCST90002106 | HLA DR on Dendritic Cell \|\| id:ebi-a-GCST90002106 | Mayonnaise liking \|\| id:ebi-fl187-GCST90094798 | Weighted median | 9 | -0.505559699 | 0.229568844613528 | 0.0276502032472293 | -0.955514634 | -0.055604763 | 0.603167888659575 | 0.384614158039913 | 0.945912921573456 | 0.999332001504091 |
| ebi-fl187-GCST90094798 | ebi-a-GCST90002111 | HLA DR on CD33dim HLA DR+ CD11b- \|\| id:ebi-a-GCST90002111 | Mayonnaise liking \|\| id:ebi-fl187-GCST90094798 | Weighted median | 9 | -0.36509402 | 0.350231650923352 | 0.297209664019516 | -1.051548056 | 0.321360015647713 | 0.694131385464544 | 0.349396444981523 | 1.37900195381898 | 0.999332001504091 |
| ebi-fl187-GCST90094798 | ebi-a-GCST90002112 | HLA DR on CD33- HLA DR+ \|\| id:ebi-a-GCST90002112 | Mayonnaise liking \|\| id:ebi-fl187-GCST90094798 | Weighted median | 9 | -0.234997632 | 0.306710172747132 | 0.443564490741817 | -0.83614957 | 0.366154307042099 | 0.790572722064585 | 0.433375998865646 | 1.44217776367069 | 0.999332001504091 |
| ebi-fl187-GCST90094798 | ebi-a-GCST90002115 | HLA DR on HLA DR+ CD8+ T cell \|\| id:ebi-a-GCST90002115 | Mayonnaise liking \|\| id:ebi-fl187-GCST90094798 | Weighted median | 9 | -0.065242932 | 0.219422881441824 | 0.766207875976002 | -0.49531178 | 0.3648259153125 | 0.936839847059442 | 0.60938088493986 | 1.44026325854474 | 0.999332001504091 |
| ebi-fl187-GCST90094798 | ebi-a-GCST90002116 | HLA DR on B cell \|\| id:ebi-a-GCST90002116 | Mayonnaise liking \|\| id:ebi-fl187-GCST90094798 | Weighted median | 9 | -0.087550674 | 0.246309767374816 | 0.722252901476476 | -0.570317818 | 0.39521647039206 | 0.91617244466125 | 0.565345733253881 | 1.4847055509299 | 0.999332001504091 |
| ebi-fl187-GCST90094798 | ebi-cfmet338-GCST90026023 | Sphingomyelin (d18:1/24:1, d18:2/24:0) levels \|\| id:ebi-cfmet338-GCST90026023 | Mayonnaise liking \|\| id:ebi-fl187-GCST90094798 | Weighted median | 9 | -0.053959302 | 0.124392944711106 | 0.664447446131522 | -0.297769473 | 0.189850869861999 | 0.947470666094551 | 0.742472479673087 | 1.20906927554394 | 0.999332001504091 |
| ebi-fl187-GCST90094798 | ebi-cfmet338-GCST90026046 | 1-arachidonoyl-gpc (20:4n6) levels \|\| id:ebi-cfmet338-GCST90026046 | Mayonnaise liking \|\| id:ebi-fl187-GCST90094798 | Weighted median | 9 | 0.0533847670706926 | 0.178671257808019 | 0.765102066814796 | -0.296810898 | 0.40358043237441 | 1.05483543298706 | 0.743184536583889 | 1.49717564872853 | 0.999332001504091 |
| ebi-fl187-GCST90094798 | ebi-cfmet338-GCST90026047 | 1-linoleoyl-gpc (18:2) levels \|\| id:ebi-cfmet338-GCST90026047 | Mayonnaise liking \|\| id:ebi-fl187-GCST90094798 | Weighted median | 9 | -0.087210987 | 0.193486738796817 | 0.652181515024622 | -0.466444995 | 0.292023020896695 | 0.916483708951566 | 0.627228108446746 | 1.33913384534637 | 0.999332001504091 |
| ebi-fl187-GCST90094798 | ebi-cfmet338-GCST90026050 | 1-myristoyl-2-palmitoyl-gpc (14:0/16:0) levels \|\| id:ebi-cfmet338-GCST90026050 | Mayonnaise liking \|\| id:ebi-fl187-GCST90094798 | Weighted median | 9 | -0.10029019 | 0.0965915379531071 | 0.299134280224544 | -0.289609605 | 0.0890292240174169 | 0.904574881024919 | 0.748555743142803 | 1.09311260100124 | 0.999332001504091 |
| ebi-fl187-GCST90094798 | ebi-cfmet338-GCST90026068 | 2,3-dihydroxyisovalerate levels \|\| id:ebi-cfmet338-GCST90026068 | Mayonnaise liking \|\| id:ebi-fl187-GCST90094798 | Weighted median | 9 | -0.146318089 | 0.332038278640205 | 0.659454576830502 | -0.797113115 | 0.5044769371121 | 0.86388286781875 | 0.450627999273286 | 1.65611903946109 | 0.999332001504091 |
| ebi-fl187-GCST90094798 | ebi-cfmet338-GCST90026082 | 3-hydroxyhexanoate levels \|\| id:ebi-cfmet338-GCST90026082 | Mayonnaise liking \|\| id:ebi-fl187-GCST90094798 | Weighted median | 9 | -0.052599269 | 0.0726735864562771 | 0.469204352873703 | -0.195039499 | 0.0898409603979528 | 0.948760133857751 | 0.822802157954368 | 1.09400028049963 | 0.999332001504091 |
| ebi-fl187-GCST90094798 | ebi-cfmet338-GCST90026094 | 3-ureidopropionate levels \|\| id:ebi-cfmet338-GCST90026094 | Mayonnaise liking \|\| id:ebi-fl187-GCST90094798 | Weighted median | 9 | -0.070554067 | 0.172336323449665 | 0.682247942702842 | -0.408333261 | 0.26722512662757 | 0.931877354039772 | 0.664757304023224 | 1.30633450391066 | 0.999332001504091 |
| ebi-fl187-GCST90094798 | ebi-cfmet338-GCST90026173 | Glycerol levels \|\| id:ebi-cfmet338-GCST90026173 | Mayonnaise liking \|\| id:ebi-fl187-GCST90094798 | Weighted median | 8 | -0.034619681 | 0.0949875648461887 | 0.715510507182607 | -0.220795308 | 0.151555946377762 | 0.965972724456268 | 0.801880802222455 | 1.16364340162194 | 0.999332001504091 |
| ebi-fl187-GCST90094798 | ebi-cfmet338-GCST90026179 | Histidine levels \|\| id:ebi-cfmet338-GCST90026179 | Mayonnaise liking \|\| id:ebi-fl187-GCST90094798 | Weighted median | 9 | 0.0142552036849952 | 0.0600027610851069 | 0.812210125613166 | -0.103350208 | 0.131860615411805 | 1.01435729362854 | 0.901811096675193 | 1.14094927743832 | 0.999332001504091 |
| ebi-fl187-GCST90094798 | ebi-cfmet338-GCST90026188 | Isocitrate levels \|\| id:ebi-cfmet338-GCST90026188 | Mayonnaise liking \|\| id:ebi-fl187-GCST90094798 | Weighted median | 9 | 0.0245615266766682 | 0.113278704058198 | 0.828345611116681 | -0.197464733 | 0.246587786630737 | 1.02486564574462 | 0.820809087363685 | 1.27965151458192 | 0.999332001504091 |
| ebi-fl187-GCST90094798 | ebi-cfmet338-GCST90026207 | N-acetyl-isoputreanine levels \|\| id:ebi-cfmet338-GCST90026207 | Mayonnaise liking \|\| id:ebi-fl187-GCST90094798 | Weighted median | 9 | 0.00970922706485532 | 0.0800667146275436 | 0.903481765391365 | -0.147221534 | 0.166639987734841 | 1.00975651452762 | 0.86310274997165 | 1.18132889585215 | 0.999332001504091 |
| ebi-fl187-GCST90094798 | ebi-cfmet338-GCST90026213 | N-acetylglutamate levels \|\| id:ebi-cfmet338-GCST90026213 | Mayonnaise liking \|\| id:ebi-fl187-GCST90094798 | Weighted median | 9 | 0.126795884690726 | 0.137732331280858 | 0.357261196275204 | -0.143159485 | 0.396751254001207 | 1.13518528543661 | 0.866615845974898 | 1.48698600222587 | 0.999332001504091 |
| ebi-fl187-GCST90094798 | ebi-cfmet338-GCST90026224 | N-acetyltaurine levels \|\| id:ebi-cfmet338-GCST90026224 | Mayonnaise liking \|\| id:ebi-fl187-GCST90094798 | Weighted median | 9 | -0.005683812 | 0.100897125276119 | 0.955076736859829 | -0.203442177 | 0.192074553936746 | 0.9943323106929 | 0.815917381630623 | 1.21176085513947 | 0.999332001504091 |
| ebi-fl187-GCST90094798 | ebi-cfmet338-GCST90026250 | Phenyllactate (pla) levels \|\| id:ebi-cfmet338-GCST90026250 | Mayonnaise liking \|\| id:ebi-fl187-GCST90094798 | Weighted median | 9 | -0.069984232 | 0.163867362836497 | 0.669322134382548 | -0.391164263 | 0.251195799639855 | 0.932408522455467 | 0.676269061147314 | 1.2855617722222 | 0.999332001504091 |
| ebi-fl187-GCST90094798 | ebi-cfmet338-GCST90026251 | Pipecolate levels \|\| id:ebi-cfmet338-GCST90026251 | Mayonnaise liking \|\| id:ebi-fl187-GCST90094798 | Weighted median | 9 | -0.148767604 | 0.259129367042529 | 0.565896387999558 | -0.656661163 | 0.359125955482475 | 0.861769363450737 | 0.518579900777658 | 1.432077168 | 0.999332001504091 |
| ebi-fl187-GCST90094798 | ebi-cfmet338-GCST90026278 | Trigonelline (n'-methylnicotinate) levels \|\| id:ebi-cfmet338-GCST90026278 | Mayonnaise liking \|\| id:ebi-fl187-GCST90094798 | Weighted median | 9 | 0.0409609425471799 | 0.303373445807868 | 0.892597469026475 | -0.553651011 | 0.635572896330601 | 1.04181141425068 | 0.574847200801268 | 1.88810351924845 | 0.999332001504091 |
| ebi-fl187-GCST90094798 | ebi-cfmet338-GCST90026299 | 1-carboxyethylvaline levels \|\| id:ebi-cfmet338-GCST90026299 | Mayonnaise liking \|\| id:ebi-fl187-GCST90094798 | Weighted median | 9 | 0.0265223174479966 | 0.170900589649008 | 0.876670355628572 | -0.308442838 | 0.361487473160053 | 1.02687716428367 | 0.734589941435327 | 1.43546304005594 | 0.999332001504091 |
| ebi-fl187-GCST90094798 | ebi-gm430-GCST90011306 | C_Erysipelotrichia abundance \|\| id:ebi-gm430-GCST90011306 | Mayonnaise liking \|\| id:ebi-fl187-GCST90094798 | Weighted median | 8 | 0.00234263067223977 | 0.131055513918934 | 0.985738491607526 | -0.254526177 | 0.25921143795335 | 1.00234537677542 | 0.775283758525439 | 1.29590777995138 | 0.999332001504091 |
| ebi-fl187-GCST90094798 | ebi-gm430-GCST90011312 | F_Erysipelotrichaceae abundance \|\| id:ebi-gm430-GCST90011312 | Mayonnaise liking \|\| id:ebi-fl187-GCST90094798 | Weighted median | 8 | 0.00234263067223977 | 0.133237716445907 | 0.985972046091671 | -0.258803294 | 0.263488554906218 | 1.00234537677542 | 0.771974860534751 | 1.30146239949719 | 0.999332001504091 |
| ebi-fl187-GCST90094798 | ebi-gm430-GCST90011352 | O_Erysipelotrichales abundance \|\| id:ebi-gm430-GCST90011352 | Mayonnaise liking \|\| id:ebi-fl187-GCST90094798 | Weighted median | 8 | 0.00234263067223977 | 0.137916337079823 | 0.986447878421741 | -0.26797339 | 0.272658651348694 | 1.00234537677542 | 0.764928135566511 | 1.31345182328661 | 0.999332001504091 |
| ebi-fl187-GCST90094798 | ebi-gm430-GCST90011362 | OTU97_11 (Parabacteroides) abundance \|\| id:ebi-gm430-GCST90011362 | Mayonnaise liking \|\| id:ebi-fl187-GCST90094798 | Weighted median | 8 | -0.06851657 | 0.185738346327934 | 0.712211517821925 | -0.432563729 | 0.295530588862997 | 0.933777987330338 | 0.64884350183638 | 1.34383919566875 | 0.999332001504091 |
| ebi-fl187-GCST90094798 | ebi-gm430-GCST90011384 | OTU97_235 (Phascolarctobacterium) abundance \|\| id:ebi-gm430-GCST90011384 | Mayonnaise liking \|\| id:ebi-fl187-GCST90094798 | Weighted median | 8 | -0.406422453 | 0.550103156035474 | 0.460021560962328 | -1.484624639 | 0.671779732436642 | 0.666028741882716 | 0.226587376729791 | 1.95771843699337 | 0.999332001504091 |
| ebi-fl187-GCST90094798 | ebi-gm430-GCST90011390 | OTU97_3 (Bacteroides) abundance \|\| id:ebi-gm430-GCST90011390 | Mayonnaise liking \|\| id:ebi-fl187-GCST90094798 | Weighted median | 8 | 0.0683648096389349 | 0.166184475734169 | 0.680794622621821 | -0.257356763 | 0.394086382077905 | 1.07075585929451 | 0.773092353967231 | 1.48302865023823 | 0.999332001504091 |
| ebi-fl187-GCST90094798 | ebi-gm430-GCST90011398 | OTU97_4 (Alistipes) abundance \|\| id:ebi-gm430-GCST90011398 | Mayonnaise liking \|\| id:ebi-fl187-GCST90094798 | Weighted median | 8 | -0.09843262 | 0.170186681844015 | 0.563007409056842 | -0.431998516 | 0.23513327680576 | 0.90625675449197 | 0.649210340097789 | 1.26507736296777 | 0.999332001504091 |
| ebi-fl187-GCST90094798 | ebi-gm430-GCST90011421 | OTU99_108 (Cytophagales) abundance \|\| id:ebi-gm430-GCST90011421 | Mayonnaise liking \|\| id:ebi-fl187-GCST90094798 | Weighted median | 8 | 0.0317533364254458 | 0.521484447077757 | 0.951446586812548 | -0.99035618 | 1.05386285269785 | 1.03226285225458 | 0.371444366460174 | 2.86871115128086 | 0.999332001504091 |
| ebi-fl187-GCST90094798 | ebi-gm430-GCST90011428 | OTU99_123 (Phascolarctobacterium) abundance \|\| id:ebi-gm430-GCST90011428 | Mayonnaise liking \|\| id:ebi-fl187-GCST90094798 | Weighted median | 8 | -0.36501345 | 0.33575550603667 | 0.276974667158795 | -1.023094242 | 0.293067341802892 | 0.694187313975697 | 0.359480896663265 | 1.34053306130533 | 0.999332001504091 |
| ebi-fl187-GCST90094798 | ebi-gm430-GCST90011450 | OTU99_298 (Phascolarctobacterium) abundance \|\| id:ebi-gm430-GCST90011450 | Mayonnaise liking \|\| id:ebi-fl187-GCST90094798 | Weighted median | 8 | -0.399779517 | 0.539953827663018 | 0.459059898983589 | -1.45808902 | 0.658529984897309 | 0.67046785628857 | 0.232680498028306 | 1.93195024991525 | 0.999332001504091 |
| ebi-fl187-GCST90094798 | ebi-gm430-GCST90011451 | OTU99_3 (Bacteroides) abundance \|\| id:ebi-gm430-GCST90011451 | Mayonnaise liking \|\| id:ebi-fl187-GCST90094798 | Weighted median | 8 | 0.0646019494009795 | 0.156755953106945 | 0.680252938235128 | -0.242639719 | 0.371843617490592 | 1.06673432562669 | 0.784554123047369 | 1.45040614540436 | 0.999332001504091 |
| ebi-fl187-GCST90094798 | ebi-gm430-GCST90011472 | OTU99_72 (Parabacteroides) abundance \|\| id:ebi-gm430-GCST90011472 | Mayonnaise liking \|\| id:ebi-fl187-GCST90094798 | Weighted median | 8 | -0.20120933 | 0.4334620594996 | 0.642510671342335 | -1.050794967 | 0.648376306127046 | 0.817741235460916 | 0.349659670655837 | 1.91243310078884 | 0.999332001504091 |
| ebi-fl187-GCST90094798 | ebi-gm430-GCST90011507 | TestASV_3 (Bacteroides) abundance \|\| id:ebi-gm430-GCST90011507 | Mayonnaise liking \|\| id:ebi-fl187-GCST90094798 | Weighted median | 8 | -0.182776541 | 0.189418249184278 | 0.334576727373438 | -0.55403631 | 0.188483227298052 | 0.832954265983738 | 0.57462575583431 | 1.20741683117414 | 0.999332001504091 |
| ebi-fl187-GCST90094798 | ebi-gm430-GCST90011512 | TestASV_34 (Phascolarctobacterium) abundance \|\| id:ebi-gm430-GCST90011512 | Mayonnaise liking \|\| id:ebi-fl187-GCST90094798 | Weighted median | 8 | -0.551827919 | 0.482789628387205 | 0.253039130007739 | -1.49809559 | 0.394439753088365 | 0.575896156409552 | 0.223555496293053 | 1.48355280217551 | 0.999332001504091 |
| ebi-fl187-GCST90094798 | ebi-gm430-GCST90011519 | TestASV_41 (Barnesiella) abundance \|\| id:ebi-gm430-GCST90011519 | Mayonnaise liking \|\| id:ebi-fl187-GCST90094798 | Weighted median | 8 | 0.417188232069552 | 0.583707205518833 | 0.474780998862982 | -0.726877891 | 1.56125435488647 | 1.51768816363391 | 0.483415913773211 | 4.76479424116572 | 0.999332001504091 |
| ebi-fl187-GCST90094798 | ebi-gm430-GCST90011524 | TestASV_46 (Sutterella) abundance \|\| id:ebi-gm430-GCST90011524 | Mayonnaise liking \|\| id:ebi-fl187-GCST90094798 | Weighted median | 8 | -0.329314464 | 0.580036467983707 | 0.57020618821981 | -1.466185941 | 0.807557013564073 | 0.719416750730957 | 0.230804109065022 | 2.24242308045947 | 0.999332001504091 |
| ebi-fl187-GCST90094798 | ebi-gm430-GCST90011581 | OTU97_19 (Bacteria) prevalence \|\| id:ebi-gm430-GCST90011581 | Mayonnaise liking \|\| id:ebi-fl187-GCST90094798 | Weighted median | 8 | -0.706780635 | 0.629521612204223 | 0.261553643396399 | -1.940642995 | 0.527081724683017 | 0.49322952997866 | 0.143611578534191 | 1.69398158369975 | 0.999332001504091 |
| ebi-fl187-GCST90094798 | ebi-gm430-GCST90011614 | OTU97_80 (Ruminococcus) prevalence \|\| id:ebi-gm430-GCST90011614 | Mayonnaise liking \|\| id:ebi-fl187-GCST90094798 | Weighted median | 8 | -0.275710168 | 0.332462984026181 | 0.406937027719818 | -0.927337616 | 0.375917281105179 | 0.759032891218931 | 0.395605563344563 | 1.45632666305649 | 0.999332001504091 |
| ebi-fl187-GCST90094798 | ebi-gm430-GCST90011623 | OTU99_11 (Parabacteroides) prevalence \|\| id:ebi-gm430-GCST90011623 | Mayonnaise liking \|\| id:ebi-fl187-GCST90094798 | Weighted median | 8 | -0.308131827 | 0.291053868324537 | 0.289747232692304 | -0.878597409 | 0.262333754452441 | 0.734818442372272 | 0.415365090486772 | 1.29996033758549 | 0.999332001504091 |
| ebi-fl187-GCST90094798 | ebi-gm430-GCST90011642 | OTU99_19 (Bacteria) prevalence \|\| id:ebi-gm430-GCST90011642 | Mayonnaise liking \|\| id:ebi-fl187-GCST90094798 | Weighted median | 8 | -0.706780635 | 0.652251566945696 | 0.278541621422668 | -1.985193706 | 0.571632435976304 | 0.49322952997866 | 0.137354005183026 | 1.77115599154755 | 0.999332001504091 |
| ebi-fl187-GCST90094798 | ebi-gm430-GCST90011653 | OTU99_30 (Parasutterella) prevalence \|\| id:ebi-gm430-GCST90011653 | Mayonnaise liking \|\| id:ebi-fl187-GCST90094798 | Weighted median | 8 | 0.38911630744522 | 0.301212447759033 | 0.196414837933927 | -0.20126009 | 0.979492705052924 | 1.4756761734781 | 0.817699728238857 | 2.66310491952967 | 0.999332001504091 |
| ebi-fl187-GCST90094798 | ebi-gm430-GCST90011675 | OTU99_8 (Ruminococcaceae) prevalence \|\| id:ebi-gm430-GCST90011675 | Mayonnaise liking \|\| id:ebi-fl187-GCST90094798 | Weighted median | 8 | -0.111227466 | 0.293641005109856 | 0.704845862676371 | -0.686763836 | 0.464308903790422 | 0.894735203741005 | 0.503201880640899 | 1.59091433401211 | 0.999332001504091 |
| ebi-fl187-GCST90094798 | ebi-gm430-GCST90011679 | OTU99_92 (Ruminococcus) prevalence \|\| id:ebi-gm430-GCST90011679 | Mayonnaise liking \|\| id:ebi-fl187-GCST90094798 | Weighted median | 8 | -0.323452505 | 0.330933138319429 | 0.32837347225828 | -0.972081456 | 0.325176445945559 | 0.723646326572329 | 0.378294813949807 | 1.38427487412266 | 0.999332001504091 |
| ebi-fl187-GCST90094798 | ebi-gm430-GCST90011684 | TestASV_11 (Lachnospiraceae) prevalence \|\| id:ebi-gm430-GCST90011684 | Mayonnaise liking \|\| id:ebi-fl187-GCST90094798 | Weighted median | 8 | -0.287973157 | 0.350353720911788 | 0.411105490631465 | -0.97466645 | 0.39872013596639 | 0.749781718344052 | 0.377318187054452 | 1.48991658618838 | 0.999332001504091 |
| ebi-fl187-GCST90094798 | ebi-gm430-GCST90011688 | TestASV_15 (Bacteroides) prevalence \|\| id:ebi-gm430-GCST90011688 | Mayonnaise liking \|\| id:ebi-fl187-GCST90094798 | Weighted median | 8 | -0.185536659 | 0.306226858734822 | 0.54459459320432 | -0.785741302 | 0.414667984045335 | 0.830658383854836 | 0.455781704543973 | 1.51386802890365 | 0.999332001504091 |
| ebi-fl187-GCST90094798 | ebi-gm430-GCST90011692 | TestASV_19 (Prevotella) prevalence \|\| id:ebi-gm430-GCST90011692 | Mayonnaise liking \|\| id:ebi-fl187-GCST90094798 | Weighted median | 8 | 0.0222380002434107 | 0.544660049196962 | 0.967432110056151 | -1.045295696 | 1.08977169666946 | 1.02248710769431 | 0.351587840823401 | 2.97359511339362 | 0.999332001504091 |
| ebi-fl187-GCST90094798 | ebi-gm430-GCST90011693 | TestASV_2 (Bacteroides) prevalence \|\| id:ebi-gm430-GCST90011693 | Mayonnaise liking \|\| id:ebi-fl187-GCST90094798 | Weighted median | 8 | 0.316507290659497 | 0.325333301140946 | 0.330617485965622 | -0.32114598 | 0.954160560895751 | 1.37232624736313 | 0.725317361740014 | 2.59649007254404 | 0.999332001504091 |
| ebi-fl187-GCST90094798 | ebi-gm430-GCST90011716 | TestASV_40 (Barnesiella) prevalence \|\| id:ebi-gm430-GCST90011716 | Mayonnaise liking \|\| id:ebi-fl187-GCST90094798 | Weighted median | 8 | -0.114997169 | 0.579627213530796 | 0.842733291959515 | -1.251066507 | 1.02107216954764 | 0.891368667392273 | 0.28619940023038 | 2.77616969346932 | 0.999332001504091 |
| ebi-fl187-GCST90094798 | ebi-gm430-GCST90011718 | TestASV_42 (Alphaproteobacteria) prevalence \|\| id:ebi-gm430-GCST90011718 | Mayonnaise liking \|\| id:ebi-fl187-GCST90094798 | Weighted median | 8 | 0.489566840208527 | 0.473387331502655 | 0.301052881484577 | -0.43827233 | 1.41740600995373 | 1.63160931931345 | 0.645150065513441 | 4.12640269787746 | 0.999332001504091 |
| ebi-fl187-GCST90094798 | ebi-hbsm44-GCST90104929 | Sulcal depth of the right inferior frontal sulcus \|\| id:ebi-hbsm44-GCST90104929 | Mayonnaise liking \|\| id:ebi-fl187-GCST90094798 | Weighted median | 8 | -0.38650398 | 0.303242883317957 | 0.202461834284177 | -0.980860031 | 0.207852071531404 | 0.679428021400782 | 0.374988458403892 | 1.23103105154073 | 0.999332001504091 |
| ebi-fl187-GCST90094798 | ebi-hbsm44-GCST90104935 | Sulcal opening distance of the left calloso-marginal posterior fissure \|\| id:ebi-hbsm44-GCST90104935 | Mayonnaise liking \|\| id:ebi-fl187-GCST90094798 | Weighted median | 8 | -0.026504735 | 0.0706664931908484 | 0.707609951948964 | -0.165011062 | 0.112001591796692 | 0.973843432814863 | 0.847884325153887 | 1.11851464109153 | 0.999332001504091 |
| ebi-fl187-GCST90094798 | ebi-hbsm44-GCST90104936 | Sulcal opening distance of the left anterior inferior temporal sulcus \|\| id:ebi-hbsm44-GCST90104936 | Mayonnaise liking \|\| id:ebi-fl187-GCST90094798 | Weighted median | 8 | -0.009321022 | 0.0370048653177469 | 0.801128887061647 | -0.081850558 | 0.0632085144381899 | 0.990722284480392 | 0.921409646070021 | 1.06524893585872 | 0.999332001504091 |
| ebi-fl187-GCST90094798 | ebi-hbsm44-GCST90104941 | Sulcal opening distance of the right posterior inferior temporal sulcus \|\| id:ebi-hbsm44-GCST90104941 | Mayonnaise liking \|\| id:ebi-fl187-GCST90094798 | Weighted median | 8 | -0.018441173 | 0.02939989138335 | 0.530493345633766 | -0.07606496 | 0.0391826143054504 | 0.981727825186432 | 0.926756002610712 | 1.03996037795304 | 0.999332001504091 |
| ebi-fl187-GCST90094798 | ebi-IPs91-GCST90274819 | Leukemia inhibitory factor levels \|\| id:ebi-IPs91-GCST90274819 | Mayonnaise liking \|\| id:ebi-fl187-GCST90094798 | Weighted median | 12 | 0.106872178067705 | 0.110069015306605 | 0.331570157040569 | -0.108863092 | 0.322607448068651 | 1.11279200616382 | 0.896853195530559 | 1.38072323893494 | 0.999332001504091 |
| ebi-fl187-GCST90094798 | ebi-IPs91-GCST90274837 | STAM binding protein levels \|\| id:ebi-IPs91-GCST90274837 | Mayonnaise liking \|\| id:ebi-fl187-GCST90094798 | Weighted median | 12 | -0.327176187 | 0.10099937133155 | 0.00119786351763335 | -0.525134955 | -0.12921742 | 0.72095670837785 | 0.591475536299685 | 0.878782880196193 | 0.999332001504091 |
| ebi-fl187-GCST90094798 | ebi-IPs91-GCST90274840 | TNF-beta levels \|\| id:ebi-IPs91-GCST90274840 | Mayonnaise liking \|\| id:ebi-fl187-GCST90094798 | Weighted median | 12 | -0.128148243 | 0.115177533634464 | 0.265873847770983 | -0.353896209 | 0.097599723011009 | 0.879722956784445 | 0.701947819555817 | 1.1025213828334 | 0.999332001504091 |
| ebi-fl187-GCST90094798 | ebi-IPs91-GCST90274842 | Tumor necrosis factor ligand superfamily member 14 levels \|\| id:ebi-IPs91-GCST90274842 | Mayonnaise liking \|\| id:ebi-fl187-GCST90094798 | Weighted median | 12 | 0.0564448932607268 | 0.109658506847674 | 0.606739348317301 | -0.15848578 | 0.271375566682168 | 1.05806830649935 | 0.853435099414605 | 1.31176763410165 | 0.999332001504091 |
| ebi-fl187-GCST90094798 | ebi-met1400-GCST90199628 | 3-methylhistidine levels \|\| id:ebi-met1400-GCST90199628 | Mayonnaise liking \|\| id:ebi-fl187-GCST90094798 | Weighted median | 9 | 0.0958707626368226 | 0.156108699298175 | 0.539130580311574 | -0.210102288 | 0.401843813261245 | 1.10061681398726 | 0.810501337179246 | 1.49457788120042 | 0.999332001504091 |
| ebi-fl187-GCST90094798 | ebi-met1400-GCST90199651 | Docosahexaenoate DHA; 22:6n3 levels \|\| id:ebi-met1400-GCST90199651 | Mayonnaise liking \|\| id:ebi-fl187-GCST90094798 | Weighted median | 9 | -0.131847639 | 0.139629742414555 | 0.345033659933665 | -0.405521934 | 0.141826656056913 | 0.876474525375601 | 0.666628783676396 | 1.15237687367142 | 0.999332001504091 |
| ebi-fl187-GCST90094798 | ebi-met1400-GCST90199652 | 1-stearoyl-2-oleoyl-GPS (18:0/18:1) levels \|\| id:ebi-met1400-GCST90199652 | Mayonnaise liking \|\| id:ebi-fl187-GCST90094798 | Weighted median | 9 | -0.166016133 | 0.144997917735287 | 0.252227679138723 | -0.450212052 | 0.118179785989325 | 0.847032569095997 | 0.637492955929472 | 1.12544643267986 | 0.999332001504091 |
| ebi-fl187-GCST90094798 | ebi-met1400-GCST90199653 | 1-myristoyl-2-palmitoyl-gpc (14:0/16:0) levels \|\| id:ebi-met1400-GCST90199653 | Mayonnaise liking \|\| id:ebi-fl187-GCST90094798 | Weighted median | 9 | 0.139583774380334 | 0.139950846423286 | 0.318581485104107 | -0.134719885 | 0.413887433369975 | 1.14979512505757 | 0.873960687243092 | 1.51268683924045 | 0.999332001504091 |
| ebi-fl187-GCST90094798 | ebi-met1400-GCST90199667 | Erythritol levels in elite athletes \|\| id:ebi-met1400-GCST90199667 | Mayonnaise liking \|\| id:ebi-fl187-GCST90094798 | Weighted median | 9 | 0.0863422084467992 | 0.142593501763466 | 0.544838238865052 | -0.193141055 | 0.365825471903192 | 1.0901793330551 | 0.82436568502811 | 1.44170360291007 | 0.999332001504091 |
| ebi-fl187-GCST90094798 | ebi-met1400-GCST90199674 | 3-hydroxymyristate levels \|\| id:ebi-met1400-GCST90199674 | Mayonnaise liking \|\| id:ebi-fl187-GCST90094798 | Weighted median | 9 | -0.129037935 | 0.147035635735958 | 0.380162819828923 | -0.417227781 | 0.15915191125283 | 0.878940622485529 | 0.658870824705645 | 1.17251605153466 | 0.999332001504091 |
| ebi-fl187-GCST90094798 | ebi-met1400-GCST90199685 | 2-linoleoylglycerol (18:2) levels \|\| id:ebi-met1400-GCST90199685 | Mayonnaise liking \|\| id:ebi-fl187-GCST90094798 | Weighted median | 9 | -0.042905666 | 0.158299631637397 | 0.786359616224438 | -0.353172944 | 0.267361611754806 | 0.958001757698611 | 0.702455697188113 | 1.30651281130952 | 0.999332001504091 |
| ebi-fl187-GCST90094798 | ebi-met1400-GCST90199703 | Gamma-glutamylvaline levels \|\| id:ebi-met1400-GCST90199703 | Mayonnaise liking \|\| id:ebi-fl187-GCST90094798 | Weighted median | 9 | -0.039885129 | 0.139375720427417 | 0.774748034599634 | -0.313061541 | 0.233291283004293 | 0.960899812303069 | 0.731204912050665 | 1.2627492431562 | 0.999332001504091 |
| ebi-fl187-GCST90094798 | ebi-met1400-GCST90199779 | Gamma-glutamyl-2-aminobutyrate levels \|\| id:ebi-met1400-GCST90199779 | Mayonnaise liking \|\| id:ebi-fl187-GCST90094798 | Weighted median | 9 | -0.083827574 | 0.159553024133075 | 0.599312056332875 | -0.396551502 | 0.228896353065881 | 0.919589803401299 | 0.672635634040999 | 1.25721172611574 | 0.999332001504091 |
| ebi-fl187-GCST90094798 | ebi-met1400-GCST90199824 | 4-vinylphenol sulfate levels \|\| id:ebi-met1400-GCST90199824 | Mayonnaise liking \|\| id:ebi-fl187-GCST90094798 | Weighted median | 9 | 0.282156670438229 | 0.156745651805369 | 0.0718460354595208 | -0.025064807 | 0.589378147976752 | 1.32598644659173 | 0.975246707069134 | 1.80286695028064 | 0.999332001504091 |
| ebi-fl187-GCST90094798 | ebi-met1400-GCST90199830 | 4-ethylphenylsulfate levels \|\| id:ebi-met1400-GCST90199830 | Mayonnaise liking \|\| id:ebi-fl187-GCST90094798 | Weighted median | 9 | -0.1180827 | 0.153832673219331 | 0.442722003168976 | -0.41959474 | 0.18342933942164 | 0.888622560411587 | 0.657313148835954 | 1.2013300757346 | 0.999332001504091 |
| ebi-fl187-GCST90094798 | ebi-met1400-GCST90199833 | Hexanoylglutamine levels \|\| id:ebi-met1400-GCST90199833 | Mayonnaise liking \|\| id:ebi-fl187-GCST90094798 | Weighted median | 9 | -0.038988111 | 0.155696252030081 | 0.802269240641798 | -0.344152765 | 0.266176542801681 | 0.961762143298019 | 0.70882063668284 | 1.30496542060341 | 0.999332001504091 |
| ebi-fl187-GCST90094798 | ebi-met1400-GCST90199837 | Carotenoid levels (cryptoxanthin) \|\| id:ebi-met1400-GCST90199837 | Mayonnaise liking \|\| id:ebi-fl187-GCST90094798 | Weighted median | 9 | -0.329025535 | 0.149148130196283 | 0.0273817654242746 | -0.62135587 | -0.0366952 | 0.719624641078557 | 0.537215549156858 | 0.963969909024796 | 0.999332001504091 |
| ebi-fl187-GCST90094798 | ebi-met1400-GCST90199869 | 2-hydroxyhippurate levels \|\| id:ebi-met1400-GCST90199869 | Mayonnaise liking \|\| id:ebi-fl187-GCST90094798 | Weighted median | 9 | -0.030041011 | 0.146801048234116 | 0.837855295908067 | -0.317771065 | 0.257689044005433 | 0.970405735875576 | 0.727769381175078 | 1.29393639878026 | 0.999332001504091 |
| ebi-fl187-GCST90094798 | ebi-met1400-GCST90199902 | Hydantoin-5-propionate levels \|\| id:ebi-met1400-GCST90199902 | Mayonnaise liking \|\| id:ebi-fl187-GCST90094798 | Weighted median | 9 | 0.244114267929015 | 0.147284561583469 | 0.0974319982730964 | -0.044563473 | 0.532792008632615 | 1.27649018404584 | 0.95641489186014 | 1.70368237031138 | 0.999332001504091 |
| ebi-fl187-GCST90094798 | ebi-met1400-GCST90199904 | Pantoate levels \|\| id:ebi-met1400-GCST90199904 | Mayonnaise liking \|\| id:ebi-fl187-GCST90094798 | Weighted median | 9 | 0.142448681769696 | 0.145811415925257 | 0.32859995386058 | -0.143341693 | 0.428239056983199 | 1.15309390470041 | 0.866457955305671 | 1.53455288270527 | 0.999332001504091 |
| ebi-fl187-GCST90094798 | ebi-met1400-GCST90199936 | 3-methyl catechol sulfate (2) levels \|\| id:ebi-met1400-GCST90199936 | Mayonnaise liking \|\| id:ebi-fl187-GCST90094798 | Weighted median | 9 | -0.161797605 | 0.18036628698684 | 0.369692155509328 | -0.515315528 | 0.191720317112453 | 0.850613346682901 | 0.597312096264539 | 1.2113316808415 | 0.999332001504091 |
| ebi-fl187-GCST90094798 | ebi-met1400-GCST90199977 | 4-hydroxychlorothalonil levels \|\| id:ebi-met1400-GCST90199977 | Mayonnaise liking \|\| id:ebi-fl187-GCST90094798 | Weighted median | 9 | 0.0271525681117401 | 0.166023627519877 | 0.870088235803401 | -0.298253742 | 0.352558878050699 | 1.02752455828686 | 0.742113010743963 | 1.42270341928674 | 0.999332001504091 |
| ebi-fl187-GCST90094798 | ebi-met1400-GCST90200040 | Sphingomyelin (d18:2/24:1, d18:1/24:2) levels \|\| id:ebi-met1400-GCST90200040 | Mayonnaise liking \|\| id:ebi-fl187-GCST90094798 | Weighted median | 9 | -0.088522949 | 0.13013821935719 | 0.496363566022084 | -0.343593859 | 0.166547960562816 | 0.915282105340618 | 0.709216911405205 | 1.18122018649682 | 0.999332001504091 |
| ebi-fl187-GCST90094798 | ebi-met1400-GCST90200077 | 1-stearoyl-2-oleoyl-GPI (18:0/18:1) levels \|\| id:ebi-met1400-GCST90200077 | Mayonnaise liking \|\| id:ebi-fl187-GCST90094798 | Weighted median | 9 | -0.031958383 | 0.139507102474602 | 0.818806202339515 | -0.305392304 | 0.241475537693483 | 0.968546889104543 | 0.736834254653357 | 1.27312631093055 | 0.999332001504091 |
| ebi-fl187-GCST90094798 | ebi-met1400-GCST90200080 | 1-palmitoyl-2-oleoyl-GPI (16:0/18:1) levels \|\| id:ebi-met1400-GCST90200080 | Mayonnaise liking \|\| id:ebi-fl187-GCST90094798 | Weighted median | 9 | 0.0278544992790227 | 0.139106636743375 | 0.841294086330946 | -0.244794509 | 0.300503507296038 | 1.02824606299317 | 0.782865393711841 | 1.35053864247079 | 0.999332001504091 |
| ebi-fl187-GCST90094798 | ebi-met1400-GCST90200082 | 1-oleoyl-2-linoleoyl-GPE (18:1/18:2) levels \|\| id:ebi-met1400-GCST90200082 | Mayonnaise liking \|\| id:ebi-fl187-GCST90094798 | Weighted median | 9 | 0.156288722974741 | 0.14051903339616 | 0.266041601778075 | -0.119128582 | 0.431706028431215 | 1.1691637187901 | 0.887693651570577 | 1.53988236698168 | 0.999332001504091 |
| ebi-fl187-GCST90094798 | ebi-met1400-GCST90200100 | Perfluorooctanesulfonate (PFOS) levels \|\| id:ebi-met1400-GCST90200100 | Mayonnaise liking \|\| id:ebi-fl187-GCST90094798 | Weighted median | 9 | -0.097354117 | 0.131586048865543 | 0.45939019068905 | -0.355262773 | 0.160554538530075 | 0.907234681796039 | 0.70098921794913 | 1.17416180845351 | 0.999332001504091 |
| ebi-fl187-GCST90094798 | ebi-met1400-GCST90200102 | 1-palmitoleoyl-2-linolenoyl-GPC (16:1/18:3) levels \|\| id:ebi-met1400-GCST90200102 | Mayonnaise liking \|\| id:ebi-fl187-GCST90094798 | Weighted median | 9 | 0.0403454451724277 | 0.13292676117493 | 0.761496390411339 | -0.220191007 | 0.300881897075291 | 1.0411703793581 | 0.80236552610921 | 1.35104976918605 | 0.999332001504091 |
| ebi-fl187-GCST90094798 | ebi-met1400-GCST90200114 | Glycosyl-N-tricosanoyl-sphingadienine (d18:2/23:0) levels \|\| id:ebi-met1400-GCST90200114 | Mayonnaise liking \|\| id:ebi-fl187-GCST90094798 | Weighted median | 9 | -0.05360688 | 0.149358475 | 0.719659265711852 | -0.34634949 | 0.23913573079692 | 0.947804634621595 | 0.707265261790952 | 1.27015092348152 | 0.999332001504091 |
| ebi-fl187-GCST90094798 | ebi-met1400-GCST90200135 | Arachidoylcarnitine (C20) levels \|\| id:ebi-met1400-GCST90200135 | Mayonnaise liking \|\| id:ebi-fl187-GCST90094798 | Weighted median | 9 | 0.321454670453017 | 0.164578531864307 | 0.0507959860016492 | -0.001119252 | 0.644028592907059 | 1.37913248915824 | 0.998881374127876 | 1.90413643893645 | 0.999332001504091 |
| ebi-fl187-GCST90094798 | ebi-met1400-GCST90200146 | Docosahexaenoylcarnitine (C22:6) levels \|\| id:ebi-met1400-GCST90200146 | Mayonnaise liking \|\| id:ebi-fl187-GCST90094798 | Weighted median | 9 | -0.056799807 | 0.155230513273205 | 0.714435071421261 | -0.361051613 | 0.247451999118621 | 0.944783189514121 | 0.696943026279244 | 1.28075788340097 | 0.999332001504091 |
| ebi-fl187-GCST90094798 | ebi-met1400-GCST90200162 | 3-hydroxyphenylacetoylglutamine levels \|\| id:ebi-met1400-GCST90200162 | Mayonnaise liking \|\| id:ebi-fl187-GCST90094798 | Weighted median | 9 | 0.139542447696558 | 0.176370624329919 | 0.428833875579141 | -0.206143976 | 0.4852288713832 | 1.14974760881988 | 0.813715912294602 | 1.62454677856716 | 0.999332001504091 |
| ebi-fl187-GCST90094798 | ebi-met1400-GCST90200172 | 3-carboxy-4-methyl-5-pentyl-2-furanpropionate (3-CMPFP) levels \|\| id:ebi-met1400-GCST90200172 | Mayonnaise liking \|\| id:ebi-fl187-GCST90094798 | Weighted median | 9 | 0.362546003968954 | 0.15217357235839 | 0.0171978437948491 | 0.0642858021465091 | 0.660806205791399 | 1.43698332640189 | 1.06639713380234 | 1.93635280413253 | 0.999332001504091 |
| ebi-fl187-GCST90094798 | ebi-met1400-GCST90200222 | Dihydrocaffeate sulfate (2) levels \|\| id:ebi-met1400-GCST90200222 | Mayonnaise liking \|\| id:ebi-fl187-GCST90094798 | Weighted median | 9 | 0.126318363111479 | 0.159658159414536 | 0.428838885586659 | -0.186611629 | 0.43924835556397 | 1.13464333937214 | 0.82976593055723 | 1.55154057327588 | 0.999332001504091 |
| ebi-fl187-GCST90094798 | ebi-met1400-GCST90200223 | 4-methylhexanoylglutamine levels \|\| id:ebi-met1400-GCST90200223 | Mayonnaise liking \|\| id:ebi-fl187-GCST90094798 | Weighted median | 9 | 0.115673824284584 | 0.167944591678332 | 0.490973159613218 | -0.213497575 | 0.444845223974115 | 1.12262963788286 | 0.807754118624829 | 1.56024868805201 | 0.999332001504091 |
| ebi-fl187-GCST90094798 | ebi-met1400-GCST90200224 | 2-ketocaprylate levels \|\| id:ebi-met1400-GCST90200224 | Mayonnaise liking \|\| id:ebi-fl187-GCST90094798 | Weighted median | 9 | 0.144266264124355 | 0.150041928368893 | 0.336297643486376 | -0.149815915 | 0.438348443727384 | 1.1551916536731 | 0.860866434025248 | 1.55014495161146 | 0.999332001504091 |
| ebi-fl187-GCST90094798 | ebi-met1400-GCST90200243 | Hydroxypalmitoyl sphingomyelin (d18:1/16:0(OH)) levels \|\| id:ebi-met1400-GCST90200243 | Mayonnaise liking \|\| id:ebi-fl187-GCST90094798 | Weighted median | 9 | -0.032278205 | 0.145933364431518 | 0.824948742540557 | -0.318307599 | 0.253751189469322 | 0.968237176360019 | 0.727379012868443 | 1.28885108464791 | 0.999332001504091 |
| ebi-fl187-GCST90094798 | ebi-met1400-GCST90200273 | 2-methoxyhydroquinone sulfate (2) levels \|\| id:ebi-met1400-GCST90200273 | Mayonnaise liking \|\| id:ebi-fl187-GCST90094798 | Weighted median | 9 | 0.191405292978765 | 0.18578038637386 | 0.302879907202109 | -0.172724264 | 0.55553485027153 | 1.21095014222823 | 0.841369578480728 | 1.74287291158121 | 0.999332001504091 |
| ebi-fl187-GCST90094798 | ebi-met1400-GCST90200276 | 2,4-di-tert-butylphenol levels \|\| id:ebi-met1400-GCST90200276 | Mayonnaise liking \|\| id:ebi-fl187-GCST90094798 | Weighted median | 9 | 0.0127210978951015 | 0.135691168650721 | 0.925307453287886 | -0.253233593 | 0.278675788450515 | 1.0128023552563 | 0.776286525808118 | 1.32137886812461 | 0.999332001504091 |
| ebi-fl187-GCST90094798 | ebi-met1400-GCST90200289 | N-acetylmethionine levels \|\| id:ebi-met1400-GCST90200289 | Mayonnaise liking \|\| id:ebi-fl187-GCST90094798 | Weighted median | 9 | 0.0846612095294555 | 0.141296123660544 | 0.549055698360535 | -0.192279193 | 0.361601611904122 | 1.08834828220495 | 0.825076480881679 | 1.4356268913552 | 0.999332001504091 |
| ebi-fl187-GCST90094798 | ebi-met1400-GCST90200308 | 3-hydroxyisobutyrate levels \|\| id:ebi-met1400-GCST90200308 | Mayonnaise liking \|\| id:ebi-fl187-GCST90094798 | Weighted median | 9 | -0.211353133 | 0.151575368087215 | 0.163203937389575 | -0.508440854 | 0.0857345885497933 | 0.809488159500387 | 0.601432569142945 | 1.08951711960844 | 0.999332001504091 |
| ebi-fl187-GCST90094798 | ebi-met1400-GCST90200383 | Cystathionine levels \|\| id:ebi-met1400-GCST90200383 | Mayonnaise liking \|\| id:ebi-fl187-GCST90094798 | Weighted median | 9 | 0.0168988680099246 | 0.155308195674121 | 0.913354299086383 | -0.287505196 | 0.321302931531201 | 1.01704246159594 | 0.750132669438058 | 1.37892323695754 | 0.999332001504091 |
| ebi-fl187-GCST90094798 | ebi-met1400-GCST90200384 | Guanidinoacetate levels \|\| id:ebi-met1400-GCST90200384 | Mayonnaise liking \|\| id:ebi-fl187-GCST90094798 | Weighted median | 9 | -0.004084812 | 0.134558887887085 | 0.975782289885302 | -0.267820233 | 0.259650607970011 | 0.995923519208979 | 0.765045298986425 | 1.29647702878205 | 0.999332001504091 |
| ebi-fl187-GCST90094798 | ebi-met1400-GCST90200403 | Citrulline levels \|\| id:ebi-met1400-GCST90200403 | Mayonnaise liking \|\| id:ebi-fl187-GCST90094798 | Weighted median | 9 | 0.0385141365451309 | 0.146450313546543 | 0.792562662457616 | -0.248528478 | 0.325556751096355 | 1.03926541987665 | 0.779947649164957 | 1.38480142110533 | 0.999332001504091 |
| ebi-fl187-GCST90094798 | ebi-met1400-GCST90200434 | 12,13-DiHOME levels \|\| id:ebi-met1400-GCST90200434 | Mayonnaise liking \|\| id:ebi-fl187-GCST90094798 | Weighted median | 9 | -0.045467051 | 0.149340034960642 | 0.760782643746936 | -0.33817352 | 0.247239417259458 | 0.955551086254327 | 0.713071545140585 | 1.28048564644633 | 0.999332001504091 |
| ebi-fl187-GCST90094798 | ebi-met1400-GCST90200437 | Fructose levels \|\| id:ebi-met1400-GCST90200437 | Mayonnaise liking \|\| id:ebi-fl187-GCST90094798 | Weighted median | 9 | -0.079054743 | 0.155403219866826 | 0.610957446161645 | -0.383645054 | 0.225535567725422 | 0.923989340914733 | 0.68137323486298 | 1.25299359945615 | 0.999332001504091 |
| ebi-fl187-GCST90094798 | ebi-met1400-GCST90200448 | N-stearoyl-sphinganine (d18:0/18:0) levels \|\| id:ebi-met1400-GCST90200448 | Mayonnaise liking \|\| id:ebi-fl187-GCST90094798 | Weighted median | 9 | 0.00847761419753644 | 0.162240373056144 | 0.958326768801928 | -0.309513517 | 0.326468745387579 | 1.00851365093197 | 0.733803852510341 | 1.38606492816389 | 0.999332001504091 |
| ebi-fl187-GCST90094798 | ebi-met1400-GCST90200468 | X-11847 levels \|\| id:ebi-met1400-GCST90200468 | Mayonnaise liking \|\| id:ebi-fl187-GCST90094798 | Weighted median | 9 | -0.068882623 | 0.151454583807158 | 0.649248008872388 | -0.365733607 | 0.227968361478912 | 0.933436237796015 | 0.693687570079637 | 1.25604558537895 | 0.999332001504091 |
| ebi-fl187-GCST90094798 | ebi-met1400-GCST90200476 | X-11843 levels \|\| id:ebi-met1400-GCST90200476 | Mayonnaise liking \|\| id:ebi-fl187-GCST90094798 | Weighted median | 9 | -0.039242093 | 0.160100506293441 | 0.806371948848709 | -0.353039085 | 0.274554899636059 | 0.961517904502631 | 0.702549733660225 | 1.31594481697755 | 0.999332001504091 |
| ebi-fl187-GCST90094798 | ebi-met1400-GCST90200511 | X-12906 levels \|\| id:ebi-met1400-GCST90200511 | Mayonnaise liking \|\| id:ebi-fl187-GCST90094798 | Weighted median | 9 | 0.00237434790473814 | 0.132696915272865 | 0.98572420128998 | -0.257711606 | 0.262460301839555 | 1.00237716890096 | 0.772818076044938 | 1.30012485457894 | 0.999332001504091 |
| ebi-fl187-GCST90094798 | ebi-met1400-GCST90200515 | X-12849 levels \|\| id:ebi-met1400-GCST90200515 | Mayonnaise liking \|\| id:ebi-fl187-GCST90094798 | Weighted median | 9 | -0.02286086 | 0.160778566220326 | 0.886931130951558 | -0.33798685 | 0.292265129841756 | 0.97739846958437 | 0.713204666662046 | 1.33945810087996 | 0.999332001504091 |
| ebi-fl187-GCST90094798 | ebi-met1400-GCST90200551 | X-18886 levels \|\| id:ebi-met1400-GCST90200551 | Mayonnaise liking \|\| id:ebi-fl187-GCST90094798 | Weighted median | 9 | 0.0160141308891893 | 0.145823321994452 | 0.912553142678723 | -0.26979958 | 0.301827841998315 | 1.0161430443094 | 0.763532506019985 | 1.35232839251425 | 0.999332001504091 |
| ebi-fl187-GCST90094798 | ebi-met1400-GCST90200564 | X-21319 levels \|\| id:ebi-met1400-GCST90200564 | Mayonnaise liking \|\| id:ebi-fl187-GCST90094798 | Weighted median | 9 | 0.104453733700706 | 0.13755902996836 | 0.447650674052179 | -0.165161965 | 0.374069432438693 | 1.11010403227267 | 0.847756386073183 | 1.45363807658967 | 0.999332001504091 |
| ebi-fl187-GCST90094798 | ebi-met1400-GCST90200579 | X-21442 levels \|\| id:ebi-met1400-GCST90200579 | Mayonnaise liking \|\| id:ebi-fl187-GCST90094798 | Weighted median | 9 | -0.041718492 | 0.165251745211729 | 0.800690032912234 | -0.365611913 | 0.282174928192023 | 0.959139747678214 | 0.693771992836548 | 1.32601065634681 | 0.999332001504091 |
| ebi-fl187-GCST90094798 | ebi-met1400-GCST90200581 | X-21441 levels \|\| id:ebi-met1400-GCST90200581 | Mayonnaise liking \|\| id:ebi-fl187-GCST90094798 | Weighted median | 9 | 0.0917652607914076 | 0.149862743679178 | 0.540320296986377 | -0.201965717 | 0.385496238402597 | 1.09610749247401 | 0.817122941035093 | 1.47034378148252 | 0.999332001504091 |
| ebi-fl187-GCST90094798 | ebi-met1400-GCST90200648 | X-24585 levels \|\| id:ebi-met1400-GCST90200648 | Mayonnaise liking \|\| id:ebi-fl187-GCST90094798 | Weighted median | 9 | 0.195972762325364 | 0.149864076777795 | 0.190985251530394 | -0.097760828 | 0.489706352809843 | 1.21649377042154 | 0.906865774574932 | 1.63183696525328 | 0.999332001504091 |
| ebi-fl187-GCST90094798 | ebi-met1400-GCST90200720 | Adenosine 5'-diphosphate (ADP) to pantothenate ratio \|\| id:ebi-met1400-GCST90200720 | Mayonnaise liking \|\| id:ebi-fl187-GCST90094798 | Weighted median | 9 | -0.083565164 | 0.20810018219295 | 0.688006262689686 | -0.491441521 | 0.324311192729524 | 0.919831144502409 | 0.611743916294274 | 1.38307764386431 | 0.999332001504091 |
| ebi-fl187-GCST90094798 | ebi-met1400-GCST90200724 | Cholate to taurocholate ratio \|\| id:ebi-met1400-GCST90200724 | Mayonnaise liking \|\| id:ebi-fl187-GCST90094798 | Weighted median | 9 | 0.0206793378291388 | 0.159493964436478 | 0.896838653203391 | -0.291928832 | 0.333287508124636 | 1.02089463685509 | 0.746821683539432 | 1.39554847232079 | 0.999332001504091 |
| ebi-fl187-GCST90094798 | ebi-met1400-GCST90200726 | N-acetylputrescine to (N(1) + N(8))-acetylspermidine ratio \|\| id:ebi-met1400-GCST90200726 | Mayonnaise liking \|\| id:ebi-fl187-GCST90094798 | Weighted median | 9 | 0.256892140067505 | 0.13755924010402 | 0.0618315339604483 | -0.012723971 | 0.526508250671384 | 1.29290566652049 | 0.987356636932179 | 1.693010408 | 0.999332001504091 |
| ebi-fl187-GCST90094798 | ebi-met1400-GCST90200744 | Arginine to glutamate ratio \|\| id:ebi-met1400-GCST90200744 | Mayonnaise liking \|\| id:ebi-fl187-GCST90094798 | Weighted median | 9 | -0.335021868 | 0.131635675044474 | 0.0109256165188285 | -0.593027791 | -0.077015945 | 0.71532244344659 | 0.552651435813591 | 0.925875090408688 | 0.999332001504091 |
| ebi-fl187-GCST90094798 | ebi-met1400-GCST90200759 | Histidine to trans-urocanate ratio \|\| id:ebi-met1400-GCST90200759 | Mayonnaise liking \|\| id:ebi-fl187-GCST90094798 | Weighted median | 9 | 0.0314980278646499 | 0.141300258095411 | 0.823601216562783 | -0.245450478 | 0.308446533731655 | 1.03199934035126 | 0.782352026470407 | 1.3613087235043 | 0.999332001504091 |
| ebi-fl187-GCST90094798 | ebi-met1400-GCST90200795 | Oleoyl-linoleoyl-glycerol (18:1 to 18:2) [2] to linoleoyl-arachidonoyl-glycerol (18:2 to 20:4) [2] ratio \|\| id:ebi-met1400-GCST90200795 | Mayonnaise liking \|\| id:ebi-fl187-GCST90094798 | Weighted median | 9 | -0.138594264 | 0.147970492235099 | 0.348946613896676 | -0.428616429 | 0.151427900990817 | 0.870581183119346 | 0.651409743433467 | 1.16349441199123 | 0.999332001504091 |
| ebi-fl187-GCST90094798 | ebi-met1400-GCST90200811 | Choline to taurocholate ratio \|\| id:ebi-met1400-GCST90200811 | Mayonnaise liking \|\| id:ebi-fl187-GCST90094798 | Weighted median | 9 | -0.034409316 | 0.148216071749345 | 0.816416505874521 | -0.324912817 | 0.256094184400665 | 0.966175952193627 | 0.722590348593844 | 1.29187439662575 | 0.999332001504091 |
| ebi-fl187-GCST90094798 | ebi-met1400-GCST90200831 | Cholate to phosphate ratio \|\| id:ebi-met1400-GCST90200831 | Mayonnaise liking \|\| id:ebi-fl187-GCST90094798 | Weighted median | 9 | -0.139459737 | 0.140813907822879 | 0.321986823774422 | -0.415454997 | 0.136535522049685 | 0.869828044139617 | 0.660039896453944 | 1.14629559582168 | 0.999332001504091 |
| ebi-fl187-GCST90094798 | ebi-met1400-GCST90200841 | Adenosine 5'-diphosphate (ADP) to oxalate (ethanedioate) ratio \|\| id:ebi-met1400-GCST90200841 | Mayonnaise liking \|\| id:ebi-fl187-GCST90094798 | Weighted median | 9 | -0.339775372 | 0.215413675167875 | 0.114722901931169 | -0.761986175 | 0.082435431759081 | 0.711930224571339 | 0.466738481527549 | 1.08592855467882 | 0.999332001504091 |
| ebi-fl187-GCST90094798 | ebi-met1400-GCST90200844 | Aspartate to phosphate ratio \|\| id:ebi-met1400-GCST90200844 | Mayonnaise liking \|\| id:ebi-fl187-GCST90094798 | Weighted median | 9 | 0.32121767301488 | 0.135454781077342 | 0.0177207686637999 | 0.0557263021032894 | 0.586709043926471 | 1.37880567701981 | 1.057308261 | 1.79806132700878 | 0.999332001504091 |
| ebi-fl187-GCST90094798 | ebi-met1400-GCST90200870 | Adenosine 5'-monophosphate (AMP) to acetoacetate ratio \|\| id:ebi-met1400-GCST90200870 | Mayonnaise liking \|\| id:ebi-fl187-GCST90094798 | Weighted median | 9 | 0.0662350203202362 | 0.172829254707483 | 0.701542093517382 | -0.272510319 | 0.404980359546904 | 1.06847780165497 | 0.761465571642681 | 1.49927305336551 | 0.999332001504091 |
| ebi-fl187-GCST90094798 | ebi-met1400-GCST90200887 | Cholesterol to cortisol ratio \|\| id:ebi-met1400-GCST90200887 | Mayonnaise liking \|\| id:ebi-fl187-GCST90094798 | Weighted median | 9 | 0.164313339920957 | 0.138539311802765 | 0.235606005831129 | -0.107223711 | 0.435850391054377 | 1.17858355451879 | 0.89832468520502 | 1.54627744050596 | 0.999332001504091 |
| ebi-fl187-GCST90094798 | ebi-met1400-GCST90200890 | Cortisol to taurocholate ratio \|\| id:ebi-met1400-GCST90200890 | Mayonnaise liking \|\| id:ebi-fl187-GCST90094798 | Weighted median | 9 | -0.086046479 | 0.144804634243708 | 0.552361885807215 | -0.369863562 | 0.197770603885608 | 0.917551583136074 | 0.690828579235772 | 1.21868280065493 | 0.999332001504091 |
| ebi-fl187-GCST90094798 | ebi-met1400-GCST90200948 | Adenosine 5'-diphosphate (ADP) to mannitol to sorbitol ratio \|\| id:ebi-met1400-GCST90200948 | Mayonnaise liking \|\| id:ebi-fl187-GCST90094798 | Weighted median | 9 | -0.330656507 | 0.192335636251901 | 0.0855845085279586 | -0.707634354 | 0.0463213396146859 | 0.718451909547718 | 0.492808630122253 | 1.0474109315105 | 0.999332001504091 |
| ebi-fl187-GCST90094798 | ebi-met1400-GCST90200972 | Leucine to N-palmitoyl-sphingosine (d18:1 to 16:0) ratio \|\| id:ebi-met1400-GCST90200972 | Mayonnaise liking \|\| id:ebi-fl187-GCST90094798 | Weighted median | 9 | 0.111656931403367 | 0.130697927540802 | 0.392931616450315 | -0.144511007 | 0.367824869383338 | 1.11812919982238 | 0.865445386759739 | 1.4445890250525 | 0.999332001504091 |
| ebi-fl187-GCST90094798 | ebi-met1400-GCST90200973 | Proline to glutamate ratio \|\| id:ebi-met1400-GCST90200973 | Mayonnaise liking \|\| id:ebi-fl187-GCST90094798 | Weighted median | 9 | -0.274050451 | 0.138789783956593 | 0.0483167152607171 | -0.546078427 | -0.002022474 | 0.760293717076494 | 0.579216803317174 | 0.997979569852809 | 0.999332001504091 |
| ebi-fl187-GCST90094798 | ebi-met1400-GCST90200983 | Cholesterol to linoleoyl-arachidonoyl-glycerol (18:2 to 20:4) [1] ratio \|\| id:ebi-met1400-GCST90200983 | Mayonnaise liking \|\| id:ebi-fl187-GCST90094798 | Weighted median | 9 | 0.00278231633949083 | 0.158256857301508 | 0.985973101378959 | -0.307401124 | 0.312965756650446 | 1.00278619057388 | 0.735355572991948 | 1.36747470331157 | 0.999332001504091 |
| ebi-fl187-GCST90094798 | ebi-met1400-GCST90201001 | Serine to alpha-ketobutyrate ratio \|\| id:ebi-met1400-GCST90201001 | Mayonnaise liking \|\| id:ebi-fl187-GCST90094798 | Weighted median | 9 | -0.030176848 | 0.161531336536738 | 0.851803883910131 | -0.346778267 | 0.286424571791353 | 0.970273927545357 | 0.706962067394236 | 1.33165771954962 | 0.999332001504091 |
| ebi-fl187-GCST90094798 | ebi-met1400-GCST90201005 | Fructose to maltose ratio \|\| id:ebi-met1400-GCST90201005 | Mayonnaise liking \|\| id:ebi-fl187-GCST90094798 | Weighted median | 9 | -0.251318032 | 0.157066961208244 | 0.10958319715846 | -0.559169276 | 0.0565332117599057 | 0.777774974728227 | 0.571683777977273 | 1.05816175763088 | 0.999332001504091 |
| ebi-fl187-GCST90094798 | ebi-met1400-GCST90201020 | Paraxanthine to 5-acetylamino-6-formylamino-3-methyluracil ratio \|\| id:ebi-met1400-GCST90201020 | Mayonnaise liking \|\| id:ebi-fl187-GCST90094798 | Weighted median | 9 | 0.00249137445381933 | 0.149054180635058 | 0.986664334750391 | -0.28965482 | 0.294637568498533 | 1.00249448050606 | 0.748521898085691 | 1.34263965558703 | 0.999332001504091 |
| ebi-fl187-GCST90094798 | ebi-pw11-GCST90275197 | Placental weight (fetal genotype, adjusted for sex) \|\| id:ebi-pw11-GCST90275197 | Mayonnaise liking \|\| id:ebi-fl187-GCST90094798 | Weighted median | 9 | 0.101349073222998 | 0.0536731980338209 | 0.0589907536682268 | -0.003850395 | 0.206548541369286 | 1.10666288072521 | 0.996157008342361 | 1.22942741085862 | 0.999332001504091 |
| ebi-fl187-GCST90094798 | prot-a-1017 | FAS-associated factor 2 \|\| id:prot-a-1017 | Mayonnaise liking \|\| id:ebi-fl187-GCST90094798 | Weighted median | 12 | 0.0974202729262839 | 0.19434774291307 | 0.616182636930776 | -0.283501303 | 0.478341849035901 | 1.10232355302865 | 0.75314214065714 | 1.61339692730709 | 0.999332001504091 |
| ebi-fl187-GCST90094798 | prot-a-1019 | Fas apoptotic inhibitory molecule 3 \|\| id:prot-a-1019 | Mayonnaise liking \|\| id:ebi-fl187-GCST90094798 | Weighted median | 12 | -0.168726624 | 0.205569849037983 | 0.411774355559081 | -0.571643528 | 0.234190280363093 | 0.844739803621873 | 0.564596745258038 | 1.26388496181817 | 0.999332001504091 |
| ebi-fl187-GCST90094798 | prot-a-1052 | MIP18 family protein FAM96A \|\| id:prot-a-1052 | Mayonnaise liking \|\| id:ebi-fl187-GCST90094798 | Weighted median | 12 | 0.0684874921413609 | 0.186525646451253 | 0.713488734358058 | -0.297102775 | 0.434077759185817 | 1.07088723036112 | 0.742967650009708 | 1.5435388877773 | 0.999332001504091 |
| ebi-fl187-GCST90094798 | prot-a-1066 | F-box/LRR-repeat protein 4 \|\| id:prot-a-1066 | Mayonnaise liking \|\| id:ebi-fl187-GCST90094798 | Weighted median | 12 | -0.078325348 | 0.191767247803516 | 0.682950927568265 | -0.454189154 | 0.29753845779297 | 0.924663540257026 | 0.634962616423284 | 1.34654015931969 | 0.999332001504091 |
| ebi-fl187-GCST90094798 | prot-a-1076 | Low affinity immunoglobulin gamma Fc region receptor III-B \|\| id:prot-a-1076 | Mayonnaise liking \|\| id:ebi-fl187-GCST90094798 | Weighted median | 12 | 0.156711900918865 | 0.213667065930096 | 0.46329031534379 | -0.262075548 | 0.575499350141853 | 1.1696585877904 | 0.769452890650685 | 1.77801815889588 | 0.999332001504091 |
| ebi-fl187-GCST90094798 | prot-a-1081 | Fc receptor-like protein 3 \|\| id:prot-a-1081 | Mayonnaise liking \|\| id:ebi-fl187-GCST90094798 | Weighted median | 12 | -0.257631325 | 0.18520230203649 | 0.164200993892677 | -0.620627837 | 0.105365186759605 | 0.772880120987393 | 0.537606802053784 | 1.11111630123632 | 0.999332001504091 |
| ebi-fl187-GCST90094798 | prot-a-1105 | Fibroblast growth factor receptor 4 \|\| id:prot-a-1105 | Mayonnaise liking \|\| id:ebi-fl187-GCST90094798 | Weighted median | 12 | -0.246555795 | 0.192112548184045 | 0.199354616344315 | -0.623096389 | 0.12998479983422 | 0.78148775751866 | 0.536281328475288 | 1.13881107307596 | 0.999332001504091 |
| ebi-fl187-GCST90094798 | prot-a-1143 | Follistatin-related protein 1 \|\| id:prot-a-1143 | Mayonnaise liking \|\| id:ebi-fl187-GCST90094798 | Weighted median | 12 | -0.088127022 | 0.185983921581968 | 0.635612470270691 | -0.452655509 | 0.276401463864478 | 0.915644561933257 | 0.635937170695295 | 1.3183770385387 | 0.999332001504091 |
| ebi-fl187-GCST90094798 | prot-a-1151 | Alpha-(1,3)-fucosyltransferase 10 \|\| id:prot-a-1151 | Mayonnaise liking \|\| id:ebi-fl187-GCST90094798 | Weighted median | 12 | 0.124510994312201 | 0.198645655408839 | 0.53079068179548 | -0.26483449 | 0.513856478913525 | 1.13259447248902 | 0.767332940519613 | 1.67172575472133 | 0.999332001504091 |
| ebi-fl187-GCST90094798 | prot-a-1165 | Growth arrest and DNA damage-inducible proteins-interacting protein 1 \|\| id:prot-a-1165 | Mayonnaise liking \|\| id:ebi-fl187-GCST90094798 | Weighted median | 12 | 0.262701165432324 | 0.195418663858457 | 0.178851634418433 | -0.120319416 | 0.645721746594899 | 1.30043804503904 | 0.886637185618026 | 1.90736316547129 | 0.999332001504091 |
| ebi-fl187-GCST90094798 | prot-a-1194 | Growth/differentiation factor 11/8 \|\| id:prot-a-1194 | Mayonnaise liking \|\| id:ebi-fl187-GCST90094798 | Weighted median | 12 | 0.0960583212932295 | 0.199573399780592 | 0.63029032201026 | -0.295105542 | 0.487222184863189 | 1.10082326355821 | 0.744453012053658 | 1.62778823910998 | 0.999332001504091 |
| ebi-fl187-GCST90094798 | prot-a-1216 | D-glucuronyl C5-epimerase \|\| id:prot-a-1216 | Mayonnaise liking \|\| id:ebi-fl187-GCST90094798 | Weighted median | 12 | 0.0344373047990725 | 0.225534392285991 | 0.878641144080839 | -0.407610104 | 0.476484713679616 | 1.03503713448075 | 0.665238201924387 | 1.61040341137218 | 0.999332001504091 |
| ebi-fl187-GCST90094798 | prot-a-1246 | Glypican-3 \|\| id:prot-a-1246 | Mayonnaise liking \|\| id:ebi-fl187-GCST90094798 | Weighted median | 12 | -0.152008183 | 0.196373958045181 | 0.438886407265079 | -0.53690114 | 0.232884775157142 | 0.858981252002572 | 0.58455690834002 | 1.26223602999953 | 0.999332001504091 |
| ebi-fl187-GCST90094798 | prot-a-1275 | Glutamate receptor 4 \|\| id:prot-a-1275 | Mayonnaise liking \|\| id:ebi-fl187-GCST90094798 | Weighted median | 12 | -0.251617389 | 0.213006431054331 | 0.237496819955406 | -0.669109994 | 0.165875216213291 | 0.777542177623219 | 0.512164204462075 | 1.18042579453212 | 0.999332001504091 |
| ebi-fl187-GCST90094798 | prot-a-1289 | Glutathione S-transferase P \|\| id:prot-a-1289 | Mayonnaise liking \|\| id:ebi-fl187-GCST90094798 | Weighted median | 12 | 0.143116121080408 | 0.182857825359487 | 0.43382482029621 | -0.215285217 | 0.501517458785003 | 1.15386378179589 | 0.806311433952851 | 1.65122503647661 | 0.999332001504091 |
| ebi-fl187-GCST90094798 | prot-a-130 | Apolipoprotein D \|\| id:prot-a-130 | Mayonnaise liking \|\| id:ebi-fl187-GCST90094798 | Weighted median | 12 | 0.404090638736749 | 0.210262238067019 | 0.0546257383286691 | -0.008023348 | 0.816204625348106 | 1.49793971216849 | 0.992008753270694 | 2.26189877245885 | 0.999332001504091 |
| ebi-fl187-GCST90094798 | prot-a-1313 | Heparin-binding EGF-like growth factor \|\| id:prot-a-1313 | Mayonnaise liking \|\| id:ebi-fl187-GCST90094798 | Weighted median | 12 | 0.0957370109335609 | 0.192759288777929 | 0.619424507302643 | -0.282071195 | 0.473545216938302 | 1.10046961445806 | 0.754219985875992 | 1.60567658643906 | 0.999332001504091 |
| ebi-fl187-GCST90094798 | prot-a-1347 | HLA class II histocompatibility antigen, DQ alpha 2 chain \|\| id:prot-a-1347 | Mayonnaise liking \|\| id:ebi-fl187-GCST90094798 | Weighted median | 12 | -0.154402932 | 0.213669331701582 | 0.469909926506705 | -0.573194822 | 0.264388957833738 | 0.856926668004751 | 0.563721568396573 | 1.30263476777446 | 0.999332001504091 |
| ebi-fl187-GCST90094798 | prot-a-1357 | Non-histone chromosomal protein HMG-14 \|\| id:prot-a-1357 | Mayonnaise liking \|\| id:ebi-fl187-GCST90094798 | Weighted median | 12 | 0.111035311624866 | 0.188372664074007 | 0.555562844595414 | -0.25817511 | 0.48024573320992 | 1.11743436458089 | 0.772459954831137 | 1.61647157414038 | 0.999332001504091 |
| ebi-fl187-GCST90094798 | prot-a-137 | Apolipoprotein M \|\| id:prot-a-137 | Mayonnaise liking \|\| id:ebi-fl187-GCST90094798 | Weighted median | 12 | -0.059147085 | 0.189168217394122 | 0.754531958109056 | -0.429916791 | 0.311622620977424 | 0.942568121220269 | 0.650563225051605 | 1.36563923217491 | 0.999332001504091 |
| ebi-fl187-GCST90094798 | prot-a-1381 | 17-beta-hydroxysteroid dehydrogenase 14 \|\| id:prot-a-1381 | Mayonnaise liking \|\| id:ebi-fl187-GCST90094798 | Weighted median | 12 | 0.232692872786434 | 0.182919784235681 | 0.203336396267464 | -0.125829904 | 0.591215649888368 | 1.26199382715337 | 0.881764818418647 | 1.80618276722519 | 0.999332001504091 |
| ebi-fl187-GCST90094798 | prot-a-1392 | Serine protease HTRA2, mitochondrial \|\| id:prot-a-1392 | Mayonnaise liking \|\| id:ebi-fl187-GCST90094798 | Weighted median | 12 | -0.29434675 | 0.197337582821665 | 0.135806681916569 | -0.681128412 | 0.0924349126522869 | 0.745018111858836 | 0.506045642087507 | 1.09684174871666 | 0.999332001504091 |
| ebi-fl187-GCST90094798 | prot-a-1416 | Interferon-induced protein with tetratricopeptide repeats 2 \|\| id:prot-a-1416 | Mayonnaise liking \|\| id:ebi-fl187-GCST90094798 | Weighted median | 12 | -0.113386353 | 0.19332798020512 | 0.557541325651301 | -0.492309194 | 0.265536488477962 | 0.892805655576485 | 0.611213353156776 | 1.30413043908892 | 0.999332001504091 |
| ebi-fl187-GCST90094798 | prot-a-1428 | Interferon gamma \|\| id:prot-a-1428 | Mayonnaise liking \|\| id:ebi-fl187-GCST90094798 | Weighted median | 12 | 0.0660983598419038 | 0.190862365425972 | 0.72910654906436 | -0.307991876 | 0.44018859607681 | 1.06833179294455 | 0.734921288196539 | 1.55300008061664 | 0.999332001504091 |
| ebi-fl187-GCST90094798 | prot-a-1483 | Interleukin-17C \|\| id:prot-a-1483 | Mayonnaise liking \|\| id:ebi-fl187-GCST90094798 | Weighted median | 12 | -0.375107461 | 0.200673808738181 | 0.0615894659590773 | -0.768428126 | 0.0182132045869629 | 0.687215426282211 | 0.463741438972889 | 1.01838007654919 | 0.999332001504091 |
| ebi-fl187-GCST90094798 | prot-a-1503 | Interleukin-1 receptor-like 2 \|\| id:prot-a-1503 | Mayonnaise liking \|\| id:ebi-fl187-GCST90094798 | Weighted median | 12 | -0.083632181 | 0.191322872130693 | 0.662020050594196 | -0.458625011 | 0.291360648193597 | 0.919769502415326 | 0.632152250878711 | 1.33824713334075 | 0.999332001504091 |
| ebi-fl187-GCST90094798 | prot-a-1522 | Interleukin-31 receptor subunit alpha \|\| id:prot-a-1522 | Mayonnaise liking \|\| id:ebi-fl187-GCST90094798 | Weighted median | 12 | -0.080042395 | 0.192807810697846 | 0.678038808485156 | -0.457945704 | 0.297860913671687 | 0.923077211425379 | 0.632581821770623 | 1.34697442912266 | 0.999332001504091 |
| ebi-fl187-GCST90094798 | prot-a-1540 | Interleukin-6 receptor subunit alpha \|\| id:prot-a-1540 | Mayonnaise liking \|\| id:ebi-fl187-GCST90094798 | Weighted median | 12 | -0.247695163 | 0.19477766328801 | 0.203486283925167 | -0.629459383 | 0.134069056761434 | 0.780597861902495 | 0.532879806857492 | 1.14347178137621 | 0.999332001504091 |
| ebi-fl187-GCST90094798 | prot-a-1541 | Interleukin-6 receptor subunit alpha \|\| id:prot-a-1541 | Mayonnaise liking \|\| id:ebi-fl187-GCST90094798 | Weighted median | 12 | -0.326894348 | 0.195640757107751 | 0.0947432423288391 | -0.710350232 | 0.05656153612855 | 0.721159931133187 | 0.491472038210836 | 1.05819172981906 | 0.999332001504091 |
| ebi-fl187-GCST90094798 | prot-a-1545 | Interleukin-9 \|\| id:prot-a-1545 | Mayonnaise liking \|\| id:ebi-fl187-GCST90094798 | Weighted median | 12 | 0.0445646440486497 | 0.198175331000332 | 0.822076649874917 | -0.343859005 | 0.432988292809301 | 1.04557256456959 | 0.70902889073486 | 1.54185816976735 | 0.999332001504091 |
| ebi-fl187-GCST90094798 | prot-a-1548 | Inositol monophosphatase 3 \|\| id:prot-a-1548 | Mayonnaise liking \|\| id:ebi-fl187-GCST90094798 | Weighted median | 12 | 0.0205506410366397 | 0.192761748876942 | 0.915097104991226 | -0.357262387 | 0.398363668835447 | 1.02076325944396 | 0.699588910781123 | 1.48938557454728 | 0.999332001504091 |
| ebi-fl187-GCST90094798 | prot-a-1555 | Inhibin beta A chain:Inhibin beta B chain heterodimer \|\| id:prot-a-1555 | Mayonnaise liking \|\| id:ebi-fl187-GCST90094798 | Weighted median | 12 | -0.06259942 | 0.190180064548037 | 0.742035748423098 | -0.435352347 | 0.310153506396567 | 0.939319670898904 | 0.647036645726383 | 1.36363442467328 | 0.999332001504091 |
| ebi-fl187-GCST90094798 | prot-a-1603 | Junctophilin-3 \|\| id:prot-a-1603 | Mayonnaise liking \|\| id:ebi-fl187-GCST90094798 | Weighted median | 12 | 0.222545111170394 | 0.19781285963703 | 0.260576943762612 | -0.165168094 | 0.610258316058973 | 1.24925217360722 | 0.847751190460729 | 1.84090687317607 | 0.999332001504091 |
| ebi-fl187-GCST90094798 | prot-a-1627 | UPF0258 protein KIAA1024 \|\| id:prot-a-1627 | Mayonnaise liking \|\| id:ebi-fl187-GCST90094798 | Weighted median | 12 | -0.061755057 | 0.182343191921868 | 0.734854674895398 | -0.419147713 | 0.295637599599883 | 0.940113133129531 | 0.657607051157963 | 1.34398300858596 | 0.999332001504091 |
| ebi-fl187-GCST90094798 | prot-a-1666 | Kallikrein-7 \|\| id:prot-a-1666 | Mayonnaise liking \|\| id:ebi-fl187-GCST90094798 | Weighted median | 12 | -0.048073666 | 0.198913072550998 | 0.809026745486587 | -0.437943288 | 0.341795955971569 | 0.953063575892391 | 0.645362381334441 | 1.40747308173526 | 0.999332001504091 |
| ebi-fl187-GCST90094798 | prot-a-1678 | Importin subunit alpha-3 \|\| id:prot-a-1678 | Mayonnaise liking \|\| id:ebi-fl187-GCST90094798 | Weighted median | 12 | 0.290653212521835 | 0.198981919780802 | 0.144097344365347 | -0.09935135 | 0.680657775292207 | 1.33730074437512 | 0.905424530997035 | 1.97517652734338 | 0.999332001504091 |
| ebi-fl187-GCST90094798 | prot-a-1718 | Protein LDOC1 \|\| id:prot-a-1718 | Mayonnaise liking \|\| id:ebi-fl187-GCST90094798 | Weighted median | 12 | -0.078280478 | 0.20327144706948 | 0.700160980009276 | -0.476692515 | 0.32013155776336 | 0.924705030294505 | 0.620833397060346 | 1.3773089481023 | 0.999332001504091 |
| ebi-fl187-GCST90094798 | prot-a-1723 | Leptin \|\| id:prot-a-1723 | Mayonnaise liking \|\| id:ebi-fl187-GCST90094798 | Weighted median | 12 | 0.0695701372598107 | 0.199641005179323 | 0.727482590672381 | -0.321726233 | 0.460866507411283 | 1.07204724902444 | 0.724896616017862 | 1.58544719170347 | 0.999332001504091 |
| ebi-fl187-GCST90094798 | prot-a-1730 | Galectin-7 \|\| id:prot-a-1730 | Mayonnaise liking \|\| id:ebi-fl187-GCST90094798 | Weighted median | 12 | 0.199864916545316 | 0.216525746343674 | 0.355979160506814 | -0.224525546 | 0.624255379378916 | 1.22123777799933 | 0.798895167632973 | 1.86685534077224 | 0.999332001504091 |
| ebi-fl187-GCST90094798 | prot-a-1753 | Lipase member N \|\| id:prot-a-1753 | Mayonnaise liking \|\| id:ebi-fl187-GCST90094798 | Weighted median | 12 | 0.0672498683748153 | 0.20849823570941 | 0.747040561270388 | -0.341406674 | 0.475906410365259 | 1.06956269468102 | 0.710769798099996 | 1.60947237897744 | 0.999332001504091 |
| ebi-fl187-GCST90094798 | prot-a-1756 | VIP36-like protein \|\| id:prot-a-1756 | Mayonnaise liking \|\| id:ebi-fl187-GCST90094798 | Weighted median | 12 | 0.143753334630881 | 0.199310330399069 | 0.470753371797366 | -0.246894913 | 0.534401582213055 | 1.15459927374088 | 0.781222785614816 | 1.70642678051668 | 0.999332001504091 |
| ebi-fl187-GCST90094798 | prot-a-1760 | Ig Kappa chain V-I region HK102- like \|\| id:prot-a-1760 | Mayonnaise liking \|\| id:ebi-fl187-GCST90094798 | Weighted median | 12 | -0.005855774 | 0.1874556384976 | 0.975079587528508 | -0.373268825 | 0.361557277538581 | 0.994161337710467 | 0.688480127038228 | 1.43556324515869 | 0.999332001504091 |
| ebi-fl187-GCST90094798 | prot-a-179 | Neutral ceramidase \|\| id:prot-a-179 | Mayonnaise liking \|\| id:ebi-fl187-GCST90094798 | Weighted median | 12 | 0.300576118364831 | 0.224059014191998 | 0.179756792845945 | -0.138579549 | 0.739731786181148 | 1.35063671008542 | 0.870593993239805 | 2.0953734310086 | 0.999332001504091 |
| ebi-fl187-GCST90094798 | prot-a-1809 | Lactotransferrin \|\| id:prot-a-1809 | Mayonnaise liking \|\| id:ebi-fl187-GCST90094798 | Weighted median | 12 | -0.274494443 | 0.181805127385486 | 0.13108735902427 | -0.630832493 | 0.0818436067336761 | 0.759956227358116 | 0.532148606768018 | 1.08528606512381 | 0.999332001504091 |
| ebi-fl187-GCST90094798 | prot-a-1818 | Lysophospholipase-like protein 1 \|\| id:prot-a-1818 | Mayonnaise liking \|\| id:ebi-fl187-GCST90094798 | Weighted median | 12 | -0.24954158 | 0.183956243427017 | 0.174931605109178 | -0.610095817 | 0.111012657584623 | 0.779157883135338 | 0.543298809508967 | 1.11740905046456 | 0.999332001504091 |
| ebi-fl187-GCST90094798 | prot-a-1852 | MAP kinase-activated protein kinase 3 \|\| id:prot-a-1852 | Mayonnaise liking \|\| id:ebi-fl187-GCST90094798 | Weighted median | 12 | 0.122637755374064 | 0.188462109681729 | 0.515221455935651 | -0.24674798 | 0.492023490350254 | 1.13047483833248 | 0.781337581728635 | 1.63562254010033 | 0.999332001504091 |
| ebi-fl187-GCST90094798 | prot-a-1856 | Megakaryocyte-associated tyrosine-protein kinase \|\| id:prot-a-1856 | Mayonnaise liking \|\| id:ebi-fl187-GCST90094798 | Weighted median | 12 | 0.124288433657361 | 0.191553203941251 | 0.516438246971819 | -0.251155846 | 0.499732713382213 | 1.13234242957002 | 0.777901129279742 | 1.64828064845685 | 0.999332001504091 |
| ebi-fl187-GCST90094798 | prot-a-1890 | Beta-1,3-N-acetylglucosaminyltransferase manic fringe \|\| id:prot-a-1890 | Mayonnaise liking \|\| id:ebi-fl187-GCST90094798 | Weighted median | 12 | 0.14341220437271 | 0.193797413202239 | 0.45929336102264 | -0.236430726 | 0.523255134249099 | 1.15420547216518 | 0.789440568505526 | 1.68751179648391 | 0.999332001504091 |
| ebi-fl187-GCST90094798 | prot-a-1919 | Matrilysin \|\| id:prot-a-1919 | Mayonnaise liking \|\| id:ebi-fl187-GCST90094798 | Weighted median | 12 | 0.0585742444611302 | 0.203961589755344 | 0.773972415466053 | -0.341190471 | 0.458338960381605 | 1.06032370593446 | 0.710923484676052 | 1.58144495940923 | 0.999332001504091 |
| ebi-fl187-GCST90094798 | prot-a-1923 | Multimerin-2 \|\| id:prot-a-1923 | Mayonnaise liking \|\| id:ebi-fl187-GCST90094798 | Weighted median | 12 | -0.244980021 | 0.215033497421967 | 0.254592868353065 | -0.666445676 | 0.176485634 | 0.782720176332223 | 0.513530592172778 | 1.193017288 | 0.999332001504091 |
| ebi-fl187-GCST90094798 | prot-a-1948 | Mesothelin \|\| id:prot-a-1948 | Mayonnaise liking \|\| id:ebi-fl187-GCST90094798 | Weighted median | 12 | 0.0964916016056244 | 0.185787529611347 | 0.603506003631614 | -0.267651956 | 0.460635159643864 | 1.10130033195044 | 0.765174048669388 | 1.58508044446003 | 0.999332001504091 |
| ebi-fl187-GCST90094798 | prot-a-195 | ATPase family AAA domain-containing protein 1 \|\| id:prot-a-195 | Mayonnaise liking \|\| id:ebi-fl187-GCST90094798 | Weighted median | 12 | -0.055530901 | 0.205925102380921 | 0.787417584802741 | -0.459144102 | 0.348082299687731 | 0.945982791424531 | 0.631824191433537 | 1.4163488100083 | 0.999332001504091 |
| ebi-fl187-GCST90094798 | prot-a-1966 | Mucin-1 \|\| id:prot-a-1966 | Mayonnaise liking \|\| id:ebi-fl187-GCST90094798 | Weighted median | 12 | 0.232263815030575 | 0.183943287688728 | 0.206699700184388 | -0.128265029 | 0.592792658900482 | 1.26145247505795 | 0.879620223522634 | 1.80903338085755 | 0.999332001504091 |
| ebi-fl187-GCST90094798 | prot-a-1986 | Myocardial zonula adherens protein \|\| id:prot-a-1986 | Mayonnaise liking \|\| id:ebi-fl187-GCST90094798 | Weighted median | 12 | 0.0164959971925117 | 0.203737034949501 | 0.935468115759411 | -0.382828591 | 0.415820585693533 | 1.0166328073925 | 0.681929777959622 | 1.51561392165506 | 0.999332001504091 |
| ebi-fl187-GCST90094798 | prot-a-2071 | Epididymal secretory protein E1 \|\| id:prot-a-2071 | Mayonnaise liking \|\| id:ebi-fl187-GCST90094798 | Weighted median | 12 | -0.142308578 | 0.200285062416922 | 0.477375434554596 | -0.5348673 | 0.25025014476734 | 0.867353569330308 | 0.585747013670913 | 1.28434664910249 | 0.999332001504091 |
| ebi-fl187-GCST90094798 | prot-a-2098 | Neuritin \|\| id:prot-a-2098 | Mayonnaise liking \|\| id:ebi-fl187-GCST90094798 | Weighted median | 12 | -0.001753988 | 0.205860588619881 | 0.99320188905206 | -0.405240742 | 0.401732765558885 | 0.998247549202153 | 0.666816260966074 | 1.49441192097564 | 0.999332001504091 |
| ebi-fl187-GCST90094798 | prot-a-2115 | Neurotrophin-4 \|\| id:prot-a-2115 | Mayonnaise liking \|\| id:ebi-fl187-GCST90094798 | Weighted median | 12 | 0.0385464140127899 | 0.182306935609238 | 0.832546362506531 | -0.31877518 | 0.395868007806896 | 1.03929896527401 | 0.727038983997312 | 1.48567320734429 | 0.999332001504091 |
| ebi-fl187-GCST90094798 | prot-a-2118 | Netrin-1 \|\| id:prot-a-2118 | Mayonnaise liking \|\| id:ebi-fl187-GCST90094798 | Weighted median | 12 | 0.0522819218959441 | 0.199045313553838 | 0.792810017086155 | -0.337846893 | 0.442410736461467 | 1.0536727540649 | 0.713304491684618 | 1.55645490194051 | 0.999332001504091 |
| ebi-fl187-GCST90094798 | prot-a-2120 | Netrin-G1 \|\| id:prot-a-2120 | Mayonnaise liking \|\| id:ebi-fl187-GCST90094798 | Weighted median | 12 | 0.0510514327808935 | 0.195287074646018 | 0.793770665953135 | -0.331711234 | 0.43381409908709 | 1.0523770185679 | 0.71769453905641 | 1.54313197180787 | 0.999332001504091 |
| ebi-fl187-GCST90094798 | prot-a-2145 | Olfactomedin-like protein 3 \|\| id:prot-a-2145 | Mayonnaise liking \|\| id:ebi-fl187-GCST90094798 | Weighted median | 12 | -0.140153879 | 0.203497491540637 | 0.490996125333803 | -0.539008963 | 0.258701203943412 | 0.869224469301073 | 0.583326063783432 | 1.29524673238712 | 0.999332001504091 |
| ebi-fl187-GCST90094798 | prot-a-2162 | Prolyl 4-hydroxylase subunit alpha-2 \|\| id:prot-a-2162 | Mayonnaise liking \|\| id:ebi-fl187-GCST90094798 | Weighted median | 12 | 0.090774745925139 | 0.214064246468965 | 0.671526616032032 | -0.328791177 | 0.510340669004311 | 1.09502231923652 | 0.719793310341735 | 1.66585860468312 | 0.999332001504091 |
| ebi-fl187-GCST90094798 | prot-a-2168 | Polyadenylate-binding protein-interacting protein 1 \|\| id:prot-a-2168 | Mayonnaise liking \|\| id:ebi-fl187-GCST90094798 | Weighted median | 12 | -0.049169682 | 0.19150715000557 | 0.797370880169987 | -0.424523696 | 0.326184332375076 | 0.952019575753557 | 0.654081252695313 | 1.38567076931676 | 0.999332001504091 |
| ebi-fl187-GCST90094798 | prot-a-219 | Beta-1,4-galactosyltransferase 5 \|\| id:prot-a-219 | Mayonnaise liking \|\| id:ebi-fl187-GCST90094798 | Weighted median | 12 | 0.107232854014846 | 0.186924340360656 | 0.566191249973761 | -0.259138853 | 0.473604561121731 | 1.11319343586328 | 0.771715860470546 | 1.60577187683237 | 0.999332001504091 |
| ebi-fl187-GCST90094798 | prot-a-2190 | Pyruvate carboxylase, mitochondrial \|\| id:prot-a-2190 | Mayonnaise liking \|\| id:ebi-fl187-GCST90094798 | Weighted median | 12 | 0.252444636901732 | 0.205061631662747 | 0.218297753378429 | -0.149476161 | 0.654365434960716 | 1.28716823256078 | 0.861158966808199 | 1.92392127675848 | 0.999332001504091 |
| ebi-fl187-GCST90094798 | prot-a-2197 | Protocadherin-9 \|\| id:prot-a-2197 | Mayonnaise liking \|\| id:ebi-fl187-GCST90094798 | Weighted median | 12 | -0.207589988 | 0.187704501231997 | 0.268752325084151 | -0.57549081 | 0.160310834782654 | 0.812540119921771 | 0.562428755369145 | 1.1738756956855 | 0.999332001504091 |
| ebi-fl187-GCST90094798 | prot-a-2198 | Protocadherin alpha-4 \|\| id:prot-a-2198 | Mayonnaise liking \|\| id:ebi-fl187-GCST90094798 | Weighted median | 12 | -0.092884242 | 0.220160524837823 | 0.673102927746638 | -0.524398871 | 0.338630386500443 | 0.911298984172967 | 0.591911072434529 | 1.40302467250524 | 0.999332001504091 |
| ebi-fl187-GCST90094798 | prot-a-2254 | Placenta growth factor \|\| id:prot-a-2254 | Mayonnaise liking \|\| id:ebi-fl187-GCST90094798 | Weighted median | 12 | -0.107842167 | 0.190172477902481 | 0.570662838274374 | -0.480580223 | 0.264895889907532 | 0.897769283064281 | 0.618424463298692 | 1.3032952825226 | 0.999332001504091 |
| ebi-fl187-GCST90094798 | prot-a-2261 | Membrane-associated progesterone receptor component 2 \|\| id:prot-a-2261 | Mayonnaise liking \|\| id:ebi-fl187-GCST90094798 | Weighted median | 12 | 0.01319458689812 | 0.200443661804885 | 0.94751563156472 | -0.37967499 | 0.406064164035695 | 1.01328201958268 | 0.684083706967723 | 1.50089885309605 | 0.999332001504091 |
| ebi-fl187-GCST90094798 | prot-a-2268 | Polymeric immunoglobulin receptor \|\| id:prot-a-2268 | Mayonnaise liking \|\| id:ebi-fl187-GCST90094798 | Weighted median | 12 | 0.122532115601935 | 0.192776347266418 | 0.525025436784061 | -0.255309525 | 0.500373756244114 | 1.13035542153585 | 0.774676679017763 | 1.64933760574222 | 0.999332001504091 |
| ebi-fl187-GCST90094798 | prot-a-2283 | Group 10 secretory phospholipase A2 \|\| id:prot-a-2283 | Mayonnaise liking \|\| id:ebi-fl187-GCST90094798 | Weighted median | 12 | 0.218947078805241 | 0.191005976173386 | 0.251677694765446 | -0.155424634 | 0.593318792105078 | 1.24476540047224 | 0.856051591258649 | 1.80998542381618 | 0.999332001504091 |
| ebi-fl187-GCST90094798 | prot-a-2298 | Inactive phospholipase D5 \|\| id:prot-a-2298 | Mayonnaise liking \|\| id:ebi-fl187-GCST90094798 | Weighted median | 12 | 0.182170260835671 | 0.190810627222111 | 0.33972053560456 | -0.191818569 | 0.556159090191009 | 1.19981845858365 | 0.825456618722855 | 1.74396122207528 | 0.999332001504091 |
| ebi-fl187-GCST90094798 | prot-a-2304 | Procollagen-lysine,2-oxoglutarate 5-dioxygenase 2 \|\| id:prot-a-2304 | Mayonnaise liking \|\| id:ebi-fl187-GCST90094798 | Weighted median | 12 | 0.0794403619291282 | 0.190261211626539 | 0.67628848635997 | -0.293471613 | 0.452352336717144 | 1.08268098859849 | 0.745670390014246 | 1.57200572635075 | 0.999332001504091 |
| ebi-fl187-GCST90094798 | prot-a-2331 | Periostin \|\| id:prot-a-2331 | Mayonnaise liking \|\| id:ebi-fl187-GCST90094798 | Weighted median | 12 | 0.0257274436595575 | 0.183424622281493 | 0.888453248578019 | -0.333784816 | 0.385239703331284 | 1.02606125085889 | 0.716207882114889 | 1.46996663511339 | 0.999332001504091 |
| ebi-fl187-GCST90094798 | prot-a-2333 | Periostin \|\| id:prot-a-2333 | Mayonnaise liking \|\| id:ebi-fl187-GCST90094798 | Weighted median | 11 | 0.0913718645965014 | 0.204288960560234 | 0.654681746308152 | -0.309034498 | 0.491778227294559 | 1.09567637276325 | 0.734155442620374 | 1.63522143150875 | 0.999332001504091 |
| ebi-fl187-GCST90094798 | prot-a-2367 | cAMP-dependent protein kinase catalytic subunit alpha \|\| id:prot-a-2367 | Mayonnaise liking \|\| id:ebi-fl187-GCST90094798 | Weighted median | 12 | -0.175600767 | 0.193288066470857 | 0.363618051043023 | -0.554445378 | 0.203243842971985 | 0.838952853890882 | 0.574390742845654 | 1.22537122998305 | 0.999332001504091 |
| ebi-fl187-GCST90094798 | prot-a-2385 | Phosphoribosyl pyrophosphate synthase-associated protein 1 \|\| id:prot-a-2385 | Mayonnaise liking \|\| id:ebi-fl187-GCST90094798 | Weighted median | 12 | 0.25120695143127 | 0.202384253721908 | 0.214517977505776 | -0.145466186 | 0.64788008872621 | 1.28557610861888 | 0.864619125928656 | 1.91148435361818 | 0.999332001504091 |
| ebi-fl187-GCST90094798 | prot-a-2393 | Trypsin-3 \|\| id:prot-a-2393 | Mayonnaise liking \|\| id:ebi-fl187-GCST90094798 | Weighted median | 12 | 0.116849078922567 | 0.207465299730109 | 0.573283533974129 | -0.289782909 | 0.52348106639358 | 1.12394978917653 | 0.748426026836216 | 1.68789310271597 | 0.999332001504091 |
| ebi-fl187-GCST90094798 | prot-a-2405 | Pregnancy-specific beta-1-glycoprotein 6 \|\| id:prot-a-2405 | Mayonnaise liking \|\| id:ebi-fl187-GCST90094798 | Weighted median | 12 | 0.22295707404433 | 0.202309960530795 | 0.270436965202753 | -0.173570449 | 0.619484596684687 | 1.24976692514491 | 0.840657925905119 | 1.85797019103159 | 0.999332001504091 |
| ebi-fl187-GCST90094798 | prot-a-244 | Beclin-1 \|\| id:prot-a-244 | Mayonnaise liking \|\| id:ebi-fl187-GCST90094798 | Weighted median | 12 | 0.0343801562887756 | 0.186603609656468 | 0.853823889465311 | -0.331362919 | 0.400123231215452 | 1.03497798534058 | 0.717944566290547 | 1.49200854833984 | 0.999332001504091 |
| ebi-fl187-GCST90094798 | prot-a-2454 | tRNA pseudouridine synthase A, mitochondrial \|\| id:prot-a-2454 | Mayonnaise liking \|\| id:ebi-fl187-GCST90094798 | Weighted median | 12 | 0.184211284515183 | 0.199922048723642 | 0.356833513207837 | -0.207635931 | 0.576058500013521 | 1.2022698172583 | 0.8125027899633 | 1.77901261552049 | 0.999332001504091 |
| ebi-fl187-GCST90094798 | prot-a-2466 | Glutaminyl-peptide cyclotransferase-like protein \|\| id:prot-a-2466 | Mayonnaise liking \|\| id:ebi-fl187-GCST90094798 | Weighted median | 12 | -0.067568434 | 0.192966582565615 | 0.726221518809328 | -0.445782936 | 0.310646067617711 | 0.934663755450793 | 0.640322747969442 | 1.364306264 | 0.999332001504091 |
| ebi-fl187-GCST90094798 | prot-a-2474 | Ras-related protein Rab-27A \|\| id:prot-a-2474 | Mayonnaise liking \|\| id:ebi-fl187-GCST90094798 | Weighted median | 12 | -0.18679632 | 0.196209101713907 | 0.341083397742608 | -0.57136616 | 0.197773518978105 | 0.829612694375552 | 0.564753368118963 | 1.2186863532332 | 0.999332001504091 |
| ebi-fl187-GCST90094798 | prot-a-2490 | Rap1 GTPase-GDP dissociation stimulator 1 \|\| id:prot-a-2490 | Mayonnaise liking \|\| id:ebi-fl187-GCST90094798 | Weighted median | 12 | -0.08848155 | 0.202412363678031 | 0.662013370878661 | -0.485209782 | 0.308246683300783 | 0.91531999868437 | 0.615568047926761 | 1.36103669255302 | 0.999332001504091 |
| ebi-fl187-GCST90094798 | prot-a-2545 | Receptor-interacting serine/threonine-protein kinase 2 \|\| id:prot-a-2545 | Mayonnaise liking \|\| id:ebi-fl187-GCST90094798 | Weighted median | 12 | 0.0817409187988268 | 0.193327216360382 | 0.672432991080011 | -0.297180425 | 0.460662262865175 | 1.08517462506093 | 0.742909960540761 | 1.5851234058283 | 0.999332001504091 |
| ebi-fl187-GCST90094798 | prot-a-2610 | RUN and FYVE domain-containing protein 1 \|\| id:prot-a-2610 | Mayonnaise liking \|\| id:ebi-fl187-GCST90094798 | Weighted median | 12 | -0.062399979 | 0.217703879529411 | 0.774397345163944 | -0.489099582 | 0.364299625327952 | 0.939507028969692 | 0.613178262173748 | 1.43950546184455 | 0.999332001504091 |
| ebi-fl187-GCST90094798 | prot-a-2627 | Serine--tRNA ligase, mitochondrial \|\| id:prot-a-2627 | Mayonnaise liking \|\| id:ebi-fl187-GCST90094798 | Weighted median | 12 | -0.11621786 | 0.20435240374215 | 0.569550954197459 | -0.516748571 | 0.284312851809889 | 0.890281245919223 | 0.596456735386933 | 1.3288485984173 | 0.999332001504091 |
| ebi-fl187-GCST90094798 | prot-a-264 | BolA-like protein 2 \|\| id:prot-a-264 | Mayonnaise liking \|\| id:ebi-fl187-GCST90094798 | Weighted median | 12 | -0.012452597 | 0.188617055625643 | 0.947361483314677 | -0.382142026 | 0.357236831705296 | 0.987624615436948 | 0.682398127809127 | 1.42937435093581 | 0.999332001504091 |
| ebi-fl187-GCST90094798 | prot-a-2646 | Secretoglobin family 1C member 1 \|\| id:prot-a-2646 | Mayonnaise liking \|\| id:ebi-fl187-GCST90094798 | Weighted median | 12 | 0.0337581435112715 | 0.19353333858015 | 0.861527038604224 | -0.3455672 | 0.413083487128365 | 1.03433441598419 | 0.707818764709728 | 1.51147120905744 | 0.999332001504091 |
| ebi-fl187-GCST90094798 | prot-a-2672 | Semaphorin-3E \|\| id:prot-a-2672 | Mayonnaise liking \|\| id:ebi-fl187-GCST90094798 | Weighted median | 12 | 0.0822982311062678 | 0.193741441196781 | 0.670994267578511 | -0.297434994 | 0.462031455851958 | 1.08577957479251 | 0.742720863231755 | 1.58729523216415 | 0.999332001504091 |
| ebi-fl187-GCST90094798 | prot-a-2701 | Plasma protease C1 inhibitor \|\| id:prot-a-2701 | Mayonnaise liking \|\| id:ebi-fl187-GCST90094798 | Weighted median | 12 | -0.281374898 | 0.198666377056955 | 0.156682225029948 | -0.670760997 | 0.108011200830743 | 0.754745329768097 | 0.511319317106926 | 1.11406022371659 | 0.999332001504091 |
| ebi-fl187-GCST90094798 | prot-a-2738 | Signaling threshold-regulating transmembrane adapter 1 \|\| id:prot-a-2738 | Mayonnaise liking \|\| id:ebi-fl187-GCST90094798 | Weighted median | 12 | -0.149455568 | 0.189225440718538 | 0.429628174516471 | -0.520337431 | 0.221426296239056 | 0.861176701343749 | 0.594319971924901 | 1.24785527320461 | 0.999332001504091 |
| ebi-fl187-GCST90094798 | prot-a-274 | Serine/threonine-protein kinase BRSK2 \|\| id:prot-a-274 | Mayonnaise liking \|\| id:ebi-fl187-GCST90094798 | Weighted median | 12 | 0.040242640533569 | 0.183211834012558 | 0.826143062563888 | -0.318852554 | 0.399337835198183 | 1.04106334771503 | 0.726982732004957 | 1.49083719081822 | 0.999332001504091 |
| ebi-fl187-GCST90094798 | prot-a-2749 | Mitochondrial glutamate carrier 2 \|\| id:prot-a-2749 | Mayonnaise liking \|\| id:ebi-fl187-GCST90094798 | Weighted median | 12 | -0.081258599 | 0.18156554113527 | 0.654482205849614 | -0.437127059 | 0.274609861812183 | 0.921955244083125 | 0.645889359857629 | 1.31601714615602 | 0.999332001504091 |
| ebi-fl187-GCST90094798 | prot-a-2760 | Sodium-coupled monocarboxylate transporter 1 \|\| id:prot-a-2760 | Mayonnaise liking \|\| id:ebi-fl187-GCST90094798 | Weighted median | 12 | -0.121677222 | 0.224129124122822 | 0.587206272715745 | -0.560970305 | 0.317615861093358 | 0.885434120871643 | 0.570655085378364 | 1.37384841122361 | 0.999332001504091 |
| ebi-fl187-GCST90094798 | prot-a-2777 | Small integral membrane protein 10 \|\| id:prot-a-2777 | Mayonnaise liking \|\| id:ebi-fl187-GCST90094798 | Weighted median | 12 | 0.0874291598419133 | 0.210472187828375 | 0.677852538965998 | -0.325096328 | 0.499954647985528 | 1.09136494923894 | 0.722457757161272 | 1.64864649956472 | 0.999332001504091 |
| ebi-fl187-GCST90094798 | prot-a-2799 | Superoxide dismutase [Mn], mitochondrial \|\| id:prot-a-2799 | Mayonnaise liking \|\| id:ebi-fl187-GCST90094798 | Weighted median | 12 | -0.311974098 | 0.198941678532299 | 0.116841784564564 | -0.701899788 | 0.0779515915146917 | 0.732000487960301 | 0.495642792395016 | 1.08107032442647 | 0.999332001504091 |
| ebi-fl187-GCST90094798 | prot-a-2805 | Protein SPACA7 \|\| id:prot-a-2805 | Mayonnaise liking \|\| id:ebi-fl187-GCST90094798 | Weighted median | 12 | -0.439167615 | 0.208543531259401 | 0.0352147925128939 | -0.847912937 | -0.030422294 | 0.644572730347755 | 0.428307905561801 | 0.97003580674747 | 0.999332001504091 |
| ebi-fl187-GCST90094798 | prot-a-2811 | SPARC-like protein 1 \|\| id:prot-a-2811 | Mayonnaise liking \|\| id:ebi-fl187-GCST90094798 | Weighted median | 12 | -0.038586959 | 0.192829418921075 | 0.841395104582816 | -0.41653262 | 0.339358702145139 | 0.962148033728602 | 0.659329005115708 | 1.40404688952726 | 0.999332001504091 |
| ebi-fl187-GCST90094798 | prot-a-2831 | Sepiapterin reductase \|\| id:prot-a-2831 | Mayonnaise liking \|\| id:ebi-fl187-GCST90094798 | Weighted median | 12 | -0.061394196 | 0.192824682395429 | 0.750186190699715 | -0.439330574 | 0.316542181432133 | 0.940452444046907 | 0.644467700431852 | 1.37237412972153 | 0.999332001504091 |
| ebi-fl187-GCST90094798 | prot-a-2834 | SPRY domain-containing SOCS box protein 1 \|\| id:prot-a-2834 | Mayonnaise liking \|\| id:ebi-fl187-GCST90094798 | Weighted median | 12 | -0.089012005 | 0.18964252642981 | 0.63880709928541 | -0.460711357 | 0.282687346326126 | 0.914834590483528 | 0.630834736976858 | 1.32669030236974 | 0.999332001504091 |
| ebi-fl187-GCST90094798 | prot-a-2835 | Spectrin alpha chain, non-erythrocytic 1 \|\| id:prot-a-2835 | Mayonnaise liking \|\| id:ebi-fl187-GCST90094798 | Weighted median | 12 | 0.336064493547032 | 0.207374603464387 | 0.105110477908544 | -0.070389729 | 0.74251871633723 | 1.39942927605842 | 0.932030509569113 | 2.10122123533787 | 0.999332001504091 |
| ebi-fl187-GCST90094798 | prot-a-2859 | Sia-alpha-2,3-Gal-beta-1,4-GlcNAc-R:alpha 2,8-sialyltransferase \|\| id:prot-a-2859 | Mayonnaise liking \|\| id:ebi-fl187-GCST90094798 | Weighted median | 12 | -0.15160366 | 0.19995497532551 | 0.448337854839241 | -0.543515411 | 0.240308091989383 | 0.859328799934438 | 0.580703249209973 | 1.27164087234124 | 0.999332001504091 |
| ebi-fl187-GCST90094798 | prot-a-2861 | Alpha-2,8-sialyltransferase 8F \|\| id:prot-a-2861 | Mayonnaise liking \|\| id:ebi-fl187-GCST90094798 | Weighted median | 12 | 0.290535884665753 | 0.189331095849734 | 0.124897191845761 | -0.080553063 | 0.661624832531232 | 1.33714385095001 | 0.922605945861268 | 1.93793860331599 | 0.999332001504091 |
| ebi-fl187-GCST90094798 | prot-a-289 | Uncharacterized protein C17orf89 \|\| id:prot-a-289 | Mayonnaise liking \|\| id:ebi-fl187-GCST90094798 | Weighted median | 12 | 0.171481149432393 | 0.19015243268129 | 0.367158409937934 | -0.201217619 | 0.544179917487722 | 1.18706176573331 | 0.817734457942711 | 1.72319464097305 | 0.999332001504091 |
| ebi-fl187-GCST90094798 | prot-a-2921 | Tachykinin-3 \|\| id:prot-a-2921 | Mayonnaise liking \|\| id:ebi-fl187-GCST90094798 | Weighted median | 12 | -0.087703502 | 0.191374352613393 | 0.646749549263377 | -0.462797233 | 0.287390228785852 | 0.916032437940336 | 0.629520265214793 | 1.33294426522172 | 0.999332001504091 |
| ebi-fl187-GCST90094798 | prot-a-2928 | Tax1-binding protein 1 \|\| id:prot-a-2928 | Mayonnaise liking \|\| id:ebi-fl187-GCST90094798 | Weighted median | 12 | -0.020518033 | 0.192775560341881 | 0.915237365762598 | -0.398358131 | 0.357322065756702 | 0.979691030023622 | 0.671421527881573 | 1.42949618749495 | 0.999332001504091 |
| ebi-fl187-GCST90094798 | prot-a-296 | Uncharacterized protein C1orf185 \|\| id:prot-a-296 | Mayonnaise liking \|\| id:ebi-fl187-GCST90094798 | Weighted median | 12 | 0.274238155519833 | 0.198969639389281 | 0.168113317669965 | -0.115742338 | 0.664218648722824 | 1.31552806520482 | 0.890704694768404 | 1.94297178459524 | 0.999332001504091 |
| ebi-fl187-GCST90094798 | prot-a-2964 | Transforming growth factor beta-2 \|\| id:prot-a-2964 | Mayonnaise liking \|\| id:ebi-fl187-GCST90094798 | Weighted median | 12 | -0.059150059 | 0.188405245317391 | 0.753558078165622 | -0.42842434 | 0.310124221843461 | 0.942565318155439 | 0.651534883948547 | 1.36359449183326 | 0.999332001504091 |
| ebi-fl187-GCST90094798 | prot-a-2977 | Thymocyte nuclear protein 1 \|\| id:prot-a-2977 | Mayonnaise liking \|\| id:ebi-fl187-GCST90094798 | Weighted median | 12 | -0.172522492 | 0.18869817104033 | 0.360571050814961 | -0.542370907 | 0.197325923375873 | 0.841539360810752 | 0.581368246982362 | 1.2181409966397 | 0.999332001504091 |
| ebi-fl187-GCST90094798 | prot-a-3015 | Transmembrane protein 70, mitochondrial \|\| id:prot-a-3015 | Mayonnaise liking \|\| id:ebi-fl187-GCST90094798 | Weighted median | 12 | -0.190007679 | 0.191543349868205 | 0.32120599190409 | -0.565432644 | 0.185417287073049 | 0.826952784022579 | 0.568114302102492 | 1.2037206324007 | 0.999332001504091 |
| ebi-fl187-GCST90094798 | prot-a-3030 | Tumor necrosis factor alpha-induced protein 3 \|\| id:prot-a-3030 | Mayonnaise liking \|\| id:ebi-fl187-GCST90094798 | Weighted median | 12 | -0.010289909 | 0.193410849109889 | 0.957570694315934 | -0.389375173 | 0.368795355255923 | 0.989762850993902 | 0.677480049933241 | 1.44599165880104 | 0.999332001504091 |
| ebi-fl187-GCST90094798 | prot-a-3032 | Tumor necrosis factor receptor superfamily member 10A \|\| id:prot-a-3032 | Mayonnaise liking \|\| id:ebi-fl187-GCST90094798 | Weighted median | 12 | 0.10561169209294 | 0.190671620852517 | 0.579652018576961 | -0.268104685 | 0.479328068963874 | 1.11139023109173 | 0.764827711092742 | 1.61498887638545 | 0.999332001504091 |
| ebi-fl187-GCST90094798 | prot-a-3081 | Tumor protein p53-inducible protein 11 \|\| id:prot-a-3081 | Mayonnaise liking \|\| id:ebi-fl187-GCST90094798 | Weighted median | 12 | 0.263594889123845 | 0.190404675613421 | 0.166238168789904 | -0.109598275 | 0.63678805332615 | 1.30160079684127 | 0.896194086490085 | 1.89039925600598 | 0.999332001504091 |
| ebi-fl187-GCST90094798 | prot-a-315 | Complement C4 \|\| id:prot-a-315 | Mayonnaise liking \|\| id:ebi-fl187-GCST90094798 | Weighted median | 12 | -0.240443632 | 0.190250540813544 | 0.206292389181798 | -0.613334692 | 0.132447428202915 | 0.786278965335544 | 0.541541979115892 | 1.14161899754927 | 0.999332001504091 |
| ebi-fl187-GCST90094798 | prot-a-3157 | UDP-glucuronosyltransferase 1-8 \|\| id:prot-a-3157 | Mayonnaise liking \|\| id:ebi-fl187-GCST90094798 | Weighted median | 12 | -0.020915389 | 0.200609106991402 | 0.916963482002316 | -0.414109238 | 0.372278460940416 | 0.979301821002851 | 0.660928748479783 | 1.45103698216395 | 0.999332001504091 |
| ebi-fl187-GCST90094798 | prot-a-317 | Glutaredoxin-like protein C5orf63 \|\| id:prot-a-317 | Mayonnaise liking \|\| id:ebi-fl187-GCST90094798 | Weighted median | 12 | -0.050853299 | 0.224323675787282 | 0.820660071615115 | -0.490527704 | 0.388821105476667 | 0.950418087526619 | 0.612303194309313 | 1.47524061525873 | 0.999332001504091 |
| ebi-fl187-GCST90094798 | prot-a-3192 | Guanine nucleotide exchange factor VAV3 \|\| id:prot-a-3192 | Mayonnaise liking \|\| id:ebi-fl187-GCST90094798 | Weighted median | 12 | 0.151567526494937 | 0.191799416851746 | 0.429387755814586 | -0.224359331 | 0.52749438352436 | 1.1636568768269 | 0.799027967631889 | 1.69468076442897 | 0.999332001504091 |
| ebi-fl187-GCST90094798 | prot-a-3233 | Serine/threonine-protein kinase WNK3 \|\| id:prot-a-3233 | Mayonnaise liking \|\| id:ebi-fl187-GCST90094798 | Weighted median | 12 | 0.327172639293574 | 0.203289112875514 | 0.107529828623257 | -0.071274022 | 0.725619300529583 | 1.38704091427518 | 0.931206686098231 | 2.0660101850583 | 0.999332001504091 |
| ebi-fl187-GCST90094798 | prot-a-3250 | 14-3-3 protein theta \|\| id:prot-a-3250 | Mayonnaise liking \|\| id:ebi-fl187-GCST90094798 | Weighted median | 12 | 0.358211370935591 | 0.199404827396311 | 0.0724304942826676 | -0.032622091 | 0.749044832632361 | 1.43076801129362 | 0.96790427044802 | 2.11497889268899 | 0.999332001504091 |
| ebi-fl187-GCST90094798 | prot-a-3278 | E3 ubiquitin-protein ligase ZNRF3 \|\| id:prot-a-3278 | Mayonnaise liking \|\| id:ebi-fl187-GCST90094798 | Weighted median | 12 | -0.089036288 | 0.193058322428051 | 0.644663335790852 | -0.4674306 | 0.2893580241807 | 0.914812376463445 | 0.626610214118652 | 1.33556980922789 | 0.999332001504091 |
| ebi-fl187-GCST90094798 | prot-a-355 | Calpain-3 \|\| id:prot-a-355 | Mayonnaise liking \|\| id:ebi-fl187-GCST90094798 | Weighted median | 12 | 0.0302483101926181 | 0.196798275217928 | 0.877844620825168 | -0.355476309 | 0.415972929619758 | 1.03071043808781 | 0.700839547347725 | 1.51584483381911 | 0.999332001504091 |
| ebi-fl187-GCST90094798 | prot-a-385 | Coiled-coil domain-containing protein 90B, mitochondrial \|\| id:prot-a-385 | Mayonnaise liking \|\| id:ebi-fl187-GCST90094798 | Weighted median | 12 | 0.109166349037247 | 0.197821891905558 | 0.581056884030519 | -0.278564559 | 0.496897257172141 | 1.11534787195504 | 0.756869403366885 | 1.64361364053136 | 0.999332001504091 |
| ebi-fl187-GCST90094798 | prot-a-390 | C-C motif chemokine 14 \|\| id:prot-a-390 | Mayonnaise liking \|\| id:ebi-fl187-GCST90094798 | Weighted median | 12 | -0.283804709 | 0.181793990043813 | 0.11849222839212 | -0.640120929 | 0.0725115117587313 | 0.752913667818104 | 0.527228662840554 | 1.07520518352157 | 0.999332001504091 |
| ebi-fl187-GCST90094798 | prot-a-40 | Pituitary adenylate cyclase-activating polypeptide 27 \|\| id:prot-a-40 | Mayonnaise liking \|\| id:ebi-fl187-GCST90094798 | Weighted median | 12 | 0.201150295833068 | 0.192463270313858 | 0.295960109653527 | -0.176077714 | 0.578378305648231 | 1.22280854104147 | 0.838552813526469 | 1.78314436959046 | 0.999332001504091 |
| ebi-fl187-GCST90094798 | prot-a-401 | C-C motif chemokine 24 \|\| id:prot-a-401 | Mayonnaise liking \|\| id:ebi-fl187-GCST90094798 | Weighted median | 12 | 0.143874033065872 | 0.196829306473071 | 0.46480456874891 | -0.241911408 | 0.529659473753092 | 1.15473864047676 | 0.785125730626865 | 1.69835387606707 | 0.999332001504091 |
| ebi-fl187-GCST90094798 | prot-a-412 | Cerebral cavernous malformations 2 protein \|\| id:prot-a-412 | Mayonnaise liking \|\| id:ebi-fl187-GCST90094798 | Weighted median | 12 | 0.22518060122606 | 0.218015785320745 | 0.30166766580096 | -0.202130338 | 0.652491540454721 | 1.25254890763444 | 0.816988436361494 | 1.92031942704517 | 0.999332001504091 |
| ebi-fl187-GCST90094798 | prot-a-44 | Adhesion G protein-coupled receptor F5 \|\| id:prot-a-44 | Mayonnaise liking \|\| id:ebi-fl187-GCST90094798 | Weighted median | 12 | 0.0504324342267717 | 0.197073658054636 | 0.798023019170209 | -0.335831936 | 0.436696804013858 | 1.05172580028739 | 0.714743218640987 | 1.54758678381496 | 0.999332001504091 |
| ebi-fl187-GCST90094798 | prot-a-48 | Adrenomedullin \|\| id:prot-a-48 | Mayonnaise liking \|\| id:ebi-fl187-GCST90094798 | Weighted median | 12 | 0.235896573861639 | 0.200371606705209 | 0.239077616335074 | -0.156831775 | 0.628624923003848 | 1.26604336141937 | 0.854847853262726 | 1.87503049446324 | 0.999332001504091 |
| ebi-fl187-GCST90094798 | prot-a-502 | Carcinoembryonic antigen-related cell adhesion molecule 1 \|\| id:prot-a-502 | Mayonnaise liking \|\| id:ebi-fl187-GCST90094798 | Weighted median | 12 | -0.161166392 | 0.191330948100846 | 0.399595675334492 | -0.53617505 | 0.213842266375918 | 0.851150434784399 | 0.584981503511383 | 1.23842729776083 | 0.999332001504091 |
| ebi-fl187-GCST90094798 | prot-a-518 | Complement factor B \|\| id:prot-a-518 | Mayonnaise liking \|\| id:ebi-fl187-GCST90094798 | Weighted median | 12 | -0.268902532 | 0.20337469868649 | 0.186100733906882 | -0.667516941 | 0.12971187748774 | 0.764217738840393 | 0.512980758986703 | 1.13850030849493 | 0.999332001504091 |
| ebi-fl187-GCST90094798 | prot-a-525 | Properdin \|\| id:prot-a-525 | Mayonnaise liking \|\| id:ebi-fl187-GCST90094798 | Weighted median | 12 | -0.049837592 | 0.196134572790233 | 0.799419100941802 | -0.434261355 | 0.334586170213105 | 0.951383923881156 | 0.647742942423464 | 1.39736199553645 | 0.999332001504091 |
| ebi-fl187-GCST90094798 | prot-a-533 | Chondroadherin \|\| id:prot-a-533 | Mayonnaise liking \|\| id:ebi-fl187-GCST90094798 | Weighted median | 12 | -0.205419248 | 0.187728194997715 | 0.273850789195228 | -0.57336651 | 0.16252801454715 | 0.814305849024458 | 0.563624792809795 | 1.17648127657723 | 0.999332001504091 |
| ebi-fl187-GCST90094798 | prot-a-535 | Coiled-coil-helix-coiled-coil-helix domain-containing protein 2 \|\| id:prot-a-535 | Mayonnaise liking \|\| id:ebi-fl187-GCST90094798 | Weighted median | 12 | -0.099169402 | 0.214247099991215 | 0.643454717603189 | -0.519093718 | 0.320754913576923 | 0.905589286025115 | 0.595059595237813 | 1.37816776928995 | 0.999332001504091 |
| ebi-fl187-GCST90094798 | prot-a-591 | Caseinolytic peptidase B protein homolog \|\| id:prot-a-591 | Mayonnaise liking \|\| id:ebi-fl187-GCST90094798 | Weighted median | 11 | 0.0231869772317296 | 0.189305613759298 | 0.90251543044221 | -0.347852026 | 0.394225980199953 | 1.02345788498017 | 0.706203368406442 | 1.48323569270378 | 0.999332001504091 |
| ebi-fl187-GCST90094798 | prot-a-605 | Ciliary neurotrophic factor receptor subunit alpha \|\| id:prot-a-605 | Mayonnaise liking \|\| id:ebi-fl187-GCST90094798 | Weighted median | 12 | -0.004251306 | 0.195986420009347 | 0.982693771917777 | -0.38838469 | 0.379882076892454 | 0.995757717684398 | 0.678151415320346 | 1.46211216245808 | 0.999332001504091 |
| ebi-fl187-GCST90094798 | prot-a-606 | Ciliary neurotrophic factor receptor subunit alpha \|\| id:prot-a-606 | Mayonnaise liking \|\| id:ebi-fl187-GCST90094798 | Weighted median | 12 | -0.285279789 | 0.193264812188608 | 0.139914826686563 | -0.664078821 | 0.0935192425476586 | 0.751803878177855 | 0.514747483827104 | 1.09803173206595 | 0.999332001504091 |
| ebi-fl187-GCST90094798 | prot-a-707 | Cancer/testis antigen 55 \|\| id:prot-a-707 | Mayonnaise liking \|\| id:ebi-fl187-GCST90094798 | Weighted median | 12 | -0.101990745 | 0.189915591780712 | 0.591245527920379 | -0.474225305 | 0.270243814674051 | 0.903037909050865 | 0.62236701430374 | 1.31028387822779 | 0.999332001504091 |
| ebi-fl187-GCST90094798 | prot-a-719 | Dipeptidyl peptidase 1 \|\| id:prot-a-719 | Mayonnaise liking \|\| id:ebi-fl187-GCST90094798 | Weighted median | 12 | -0.022447861 | 0.198626085097172 | 0.910018128067157 | -0.411754988 | 0.366859265732295 | 0.97780221743724 | 0.662486573429512 | 1.44319479785942 | 0.999332001504091 |
| ebi-fl187-GCST90094798 | prot-a-752 | Cytochrome b5 \|\| id:prot-a-752 | Mayonnaise liking \|\| id:ebi-fl187-GCST90094798 | Weighted median | 12 | -0.04208208 | 0.182666868968778 | 0.817799533243606 | -0.400109144 | 0.315944982796191 | 0.958791079403974 | 0.670246888910913 | 1.37155479444057 | 0.999332001504091 |
| ebi-fl187-GCST90094798 | prot-a-755 | Cytoglobin \|\| id:prot-a-755 | Mayonnaise liking \|\| id:ebi-fl187-GCST90094798 | Weighted median | 12 | 0.0274861235727205 | 0.202847226666704 | 0.892215300072593 | -0.370094441 | 0.42506668783946 | 1.02786735188173 | 0.690669100288035 | 1.52969242814505 | 0.999332001504091 |
| ebi-fl187-GCST90094798 | prot-a-807 | Desmin \|\| id:prot-a-807 | Mayonnaise liking \|\| id:ebi-fl187-GCST90094798 | Weighted median | 12 | 0.0795511447854151 | 0.2006669515545 | 0.691785099264301 | -0.31375608 | 0.472858369832236 | 1.08280093773488 | 0.730697237903365 | 1.60457411078184 | 0.999332001504091 |
| ebi-fl187-GCST90094798 | prot-a-82 | Ameloblastin \|\| id:prot-a-82 | Mayonnaise liking \|\| id:ebi-fl187-GCST90094798 | Weighted median | 12 | 0.070055719089425 | 0.203664250108758 | 0.730863947540907 | -0.329126211 | 0.469237649302592 | 1.07256794209837 | 0.719552195524684 | 1.59877490135137 | 0.999332001504091 |
| ebi-fl187-GCST90094798 | prot-a-84 | Muellerian-inhibiting factor \|\| id:prot-a-84 | Mayonnaise liking \|\| id:ebi-fl187-GCST90094798 | Weighted median | 12 | 0.115086504161952 | 0.204986494816193 | 0.574501324227453 | -0.286687026 | 0.516860034001689 | 1.12197048849107 | 0.75074665649796 | 1.67675442327901 | 0.999332001504091 |
| ebi-fl187-GCST90094798 | prot-a-858 | Inactive dipeptidyl peptidase 10 \|\| id:prot-a-858 | Mayonnaise liking \|\| id:ebi-fl187-GCST90094798 | Weighted median | 12 | 0.256238441650072 | 0.205748965093326 | 0.212987109475643 | -0.14702953 | 0.659506413232991 | 1.29206077231548 | 0.863268484779539 | 1.93383758215478 | 0.999332001504091 |
| ebi-fl187-GCST90094798 | prot-a-863 | Dihydropyrimidinase-related protein 3 \|\| id:prot-a-863 | Mayonnaise liking \|\| id:ebi-fl187-GCST90094798 | Weighted median | 12 | 0.112921432175499 | 0.191365343627254 | 0.555135019099182 | -0.262154641 | 0.487997505684917 | 1.11954396935897 | 0.769392034696952 | 1.62905078660156 | 0.999332001504091 |
| ebi-fl187-GCST90094798 | prot-a-864 | Dihydropyrimidinase-related protein 5 \|\| id:prot-a-864 | Mayonnaise liking \|\| id:ebi-fl187-GCST90094798 | Weighted median | 12 | 0.051218635258093 | 0.19356887179957 | 0.791316379196086 | -0.328176353 | 0.430613623985249 | 1.05255299332365 | 0.720235992388975 | 1.53820111111061 | 0.999332001504091 |
| ebi-fl187-GCST90094798 | prot-a-884 | Endothelin-converting enzyme 1 \|\| id:prot-a-884 | Mayonnaise liking \|\| id:ebi-fl187-GCST90094798 | Weighted median | 12 | -0.077442949 | 0.22327557548749 | 0.728704632637085 | -0.515063077 | 0.360177179170591 | 0.925479822638962 | 0.597462907416965 | 1.43358339317639 | 0.999332001504091 |
| ebi-fl187-GCST90094798 | prot-a-893 | Early endosome antigen 1 \|\| id:prot-a-893 | Mayonnaise liking \|\| id:ebi-fl187-GCST90094798 | Weighted median | 11 | -0.00025513 | 0.211096579989017 | 0.99903568100844 | -0.414004427 | 0.413494166524431 | 0.999744902288913 | 0.660998024999685 | 1.51209206661872 | 0.999758154020451 |
| ebi-fl187-GCST90094798 | prot-a-897 | Ephrin-A2 \|\| id:prot-a-897 | Mayonnaise liking \|\| id:ebi-fl187-GCST90094798 | Weighted median | 12 | -0.178251104 | 0.19047334285296 | 0.349359545940913 | -0.551578856 | 0.195076648281049 | 0.836732290524231 | 0.576039608609985 | 1.21540414155783 | 0.999332001504091 |
| ebi-fl187-GCST90094798 | prot-a-90 | Anaphase-promoting complex subunit 10 \|\| id:prot-a-90 | Mayonnaise liking \|\| id:ebi-fl187-GCST90094798 | Weighted median | 12 | -0.091504809 | 0.193647023784244 | 0.636545657673466 | -0.471052975 | 0.288043358035797 | 0.912556928037263 | 0.624344502761397 | 1.33381513447402 | 0.999332001504091 |
| ebi-fl187-GCST90094798 | prot-a-914 | Histone-lysine N-methyltransferase EHMT2 \|\| id:prot-a-914 | Mayonnaise liking \|\| id:ebi-fl187-GCST90094798 | Weighted median | 12 | 0.213644249452258 | 0.204808358131722 | 0.296882419996452 | -0.187780132 | 0.615068631390432 | 1.23818209247535 | 0.828796912718293 | 1.84978354841887 | 0.999332001504091 |
| ebi-fl187-GCST90094798 | prot-a-932 | RNA polymerase II elongation factor ELL \|\| id:prot-a-932 | Mayonnaise liking \|\| id:ebi-fl187-GCST90094798 | Weighted median | 12 | 0.0490261486618428 | 0.199840537246845 | 0.806203754249717 | -0.342661304 | 0.440713601665658 | 1.05024781295589 | 0.709878603648815 | 1.55381562840328 | 0.999332001504091 |
| ebi-fl187-GCST90094798 | prot-a-991 | Estrogen receptor \|\| id:prot-a-991 | Mayonnaise liking \|\| id:ebi-fl187-GCST90094798 | Weighted median | 12 | 0.114326421555583 | 0.191051049380814 | 0.549567920427066 | -0.260133635 | 0.488786478341978 | 1.12111802225133 | 0.770948553031488 | 1.63033657028654 | 0.999332001504091 |
| ebi-fl187-GCST90094798 | prot-a-992 | Persulfide dioxygenase ETHE1, mitochondrial \|\| id:prot-a-992 | Mayonnaise liking \|\| id:ebi-fl187-GCST90094798 | Weighted median | 12 | 0.31847553176259 | 0.211257982253264 | 0.131677544548543 | -0.095590113 | 0.732541176978987 | 1.37502997621233 | 0.908836459571169 | 2.08036046042276 | 0.999332001504091 |
| ebi-fl187-GCST90094798 | prot-a-993 | Ethanolamine kinase 1 \|\| id:prot-a-993 | Mayonnaise liking \|\| id:ebi-fl187-GCST90094798 | Weighted median | 12 | -0.069287442 | 0.191908045172932 | 0.718065799887358 | -0.445427211 | 0.30685232641064 | 0.933058441224295 | 0.640550567535521 | 1.35914024413324 | 0.999332001504091 |
| ebi-fl187-GCST90094798 | prot-b-10 | coagulation factor III, tissue factor \|\| id:prot-b-10 | Mayonnaise liking \|\| id:ebi-fl187-GCST90094798 | Weighted median | 8 | 0.00221918158907737 | 0.28034682054047 | 0.993684136554015 | -0.547260587 | 0.551698949848399 | 1.00222164579504 | 0.578532481186647 | 1.73620023069382 | 0.999332001504091 |
| ebi-fl187-GCST90094798 | prot-b-28 | prolactin \|\| id:prot-b-28 | Mayonnaise liking \|\| id:ebi-fl187-GCST90094798 | Weighted median | 8 | -0.35927321 | 0.260124191796293 | 0.16722967419965 | -0.869116626 | 0.150570205603015 | 0.698183574335465 | 0.419321803566001 | 1.16249691603532 | 0.999332001504091 |
| ebi-fl187-GCST90094798 | prot-b-54 | matrix metallopeptidase 7 \|\| id:prot-b-54 | Mayonnaise liking \|\| id:ebi-fl187-GCST90094798 | Weighted median | 8 | -0.1325193 | 0.233921794202378 | 0.571046369518232 | -0.591006016 | 0.325967416839491 | 0.875886029520363 | 0.553769902791051 | 1.38537022839686 | 0.999332001504091 |
| ebi-fl187-GCST90094798 | ukb-rsfMRI191-edge-pheno716 | Net100_Pair10_33 (OccipitalPrecuneus)&(Temporal\|Frontal\|Supp_Motor_Area) (Default_mode\|Central_executive)&(Default_mode\|Salience) \|\| id:ukb-rsfMRI191-edge-pheno716 | Mayonnaise liking \|\| id:ebi-fl187-GCST90094798 | Weighted median | 10 | 0.0319452194385472 | 0.0701097545459152 | 0.648644659350962 | -0.105469899 | 0.169360338348541 | 1.03246094496569 | 0.899901559951714 | 1.18454689970382 | 0.999332001504091 |
| ebi-fl187-GCST90094798 | ukb-rsfMRI191-edge-pheno903 | Net100_Pair27_38 (Precentral\|Frontal\|Supp_Motor_Area)&(Subcortical) (Attention\|Salience\|Motor)&(Subcortical-cerebellum) \|\| id:ukb-rsfMRI191-edge-pheno903 | Mayonnaise liking \|\| id:ebi-fl187-GCST90094798 | Weighted median | 10 | -0.031156501 | 0.0677679227587087 | 0.645693573805042 | -0.16398163 | 0.101668627670106 | 0.969323861112318 | 0.848757613801362 | 1.10701657627968 | 0.999332001504091 |
| ebi-fl187-GCST90094798 | ukb-rsfMRI191-node-pheno23 | Net100_Node2 RolandicOper\|SupraMarginal\|Insula Salience\|Motor \|\| id:ukb-rsfMRI191-node-pheno23 | Mayonnaise liking \|\| id:ebi-fl187-GCST90094798 | Weighted median | 10 | 0.00362174018950217 | 0.064880517010626 | 0.955483854755924 | -0.123544073 | 0.130787553530329 | 1.00362830661541 | 0.883782689297514 | 1.13972562490486 | 0.999332001504091 |
| ebi-fl187-GCST90094798 | ukb-rsfMRI191-node-pheno55 | Net100_Node34 Parietal\|Postcentral\|Precuneus Attention \|\| id:ukb-rsfMRI191-node-pheno55 | Mayonnaise liking \|\| id:ebi-fl187-GCST90094798 | Weighted median | 9 | -0.015380008 | 0.0695165916039534 | 0.824903806496424 | -0.151632527 | 0.120872511779259 | 0.984737660536102 | 0.859303993481191 | 1.1284810351569 | 0.999332001504091 |
| ebi-fl187-GCST90094798 | ebi-a-GCST90001414 | IgD- CD24- B cell Absolute Count \|\| id:ebi-a-GCST90001414 | Mayonnaise liking \|\| id:ebi-fl187-GCST90094798 | Weighted mode | 9 | -0.024876237 | 0.298969231382693 | 0.93573153619781 | -0.61085593 | 0.561103457005112 | 0.975430627261702 | 0.542885997734007 | 1.75260535834696 | 0.999714303421225 |
| ebi-fl187-GCST90094798 | ebi-a-GCST90001422 | CD20- CD38- B cell %B cell \|\| id:ebi-a-GCST90001422 | Mayonnaise liking \|\| id:ebi-fl187-GCST90094798 | Weighted mode | 9 | 0.0954980699868893 | 0.260528357576084 | 0.723455942471133 | -0.415137511 | 0.606133650836013 | 1.10020669861853 | 0.660249482986594 | 1.83332938665803 | 0.999714303421225 |
| ebi-fl187-GCST90094798 | ebi-a-GCST90001444 | CD20- CD38- B cell %lymphocyte \|\| id:ebi-a-GCST90001444 | Mayonnaise liking \|\| id:ebi-fl187-GCST90094798 | Weighted mode | 9 | 0.0161640124821827 | 0.267916015659921 | 0.953370872953063 | -0.508951378 | 0.541279403175627 | 1.01629535686173 | 0.60112560183045 | 1.71820373185509 | 0.999714303421225 |
| ebi-fl187-GCST90094798 | ebi-a-GCST90001459 | Myeloid Dendritic Cell %Dendritic Cell \|\| id:ebi-a-GCST90001459 | Mayonnaise liking \|\| id:ebi-fl187-GCST90094798 | Weighted mode | 9 | 0.0631537543067565 | 0.290868991393764 | 0.83354903334844 | -0.506949469 | 0.633256977438535 | 1.06519060428412 | 0.602330206167126 | 1.88373588413453 | 0.999714303421225 |
| ebi-fl187-GCST90094798 | ebi-a-GCST90001476 | HLA DR++ monocyte %leukocyte \|\| id:ebi-a-GCST90001476 | Mayonnaise liking \|\| id:ebi-fl187-GCST90094798 | Weighted mode | 9 | -0.280247899 | 0.269085325172345 | 0.328100010213108 | -0.807655136 | 0.247159338355204 | 0.755596406656171 | 0.445902421702103 | 1.28038311066438 | 0.999714303421225 |
| ebi-fl187-GCST90094798 | ebi-a-GCST90001478 | CD4 regulatory T cell %CD4+ T cell \|\| id:ebi-a-GCST90001478 | Mayonnaise liking \|\| id:ebi-fl187-GCST90094798 | Weighted mode | 9 | -0.321208479 | 0.312964278837597 | 0.334759705877194 | -0.934618466 | 0.292201507172311 | 0.725272030986422 | 0.392735679015416 | 1.33937288369087 | 0.999714303421225 |
| ebi-fl187-GCST90094798 | ebi-a-GCST90001485 | CD39+ resting CD4 regulatory T cell %CD4 regulatory T cell \|\| id:ebi-a-GCST90001485 | Mayonnaise liking \|\| id:ebi-fl187-GCST90094798 | Weighted mode | 9 | 0.165979623868896 | 0.269157029362277 | 0.554594778800122 | -0.361568154 | 0.69352740141896 | 1.18054904645606 | 0.696583119753821 | 2.00076058630426 | 0.999714303421225 |
| ebi-fl187-GCST90094798 | ebi-a-GCST90001515 | Immature Myeloid-Derived Suppressor Cells Absolute Count \|\| id:ebi-a-GCST90001515 | Mayonnaise liking \|\| id:ebi-fl187-GCST90094798 | Weighted mode | 9 | 0.361963355595186 | 0.351175119628758 | 0.332823403077141 | -0.326339879 | 1.05026659006755 | 1.43614631426919 | 0.721559902780966 | 2.8584130410238 | 0.999714303421225 |
| ebi-fl187-GCST90094798 | ebi-a-GCST90001517 | CD33+ HLA DR+ Absolute Count \|\| id:ebi-a-GCST90001517 | Mayonnaise liking \|\| id:ebi-fl187-GCST90094798 | Weighted mode | 9 | 0.286677992391893 | 0.422539932249461 | 0.516619134050054 | -0.541500275 | 1.11485625960084 | 1.3319952318131 | 0.581874625350449 | 3.04912986453785 | 0.999714303421225 |
| ebi-fl187-GCST90094798 | ebi-a-GCST90001518 | CD33+ HLA DR+ CD14- Absolute Count \|\| id:ebi-a-GCST90001518 | Mayonnaise liking \|\| id:ebi-fl187-GCST90094798 | Weighted mode | 9 | 0.296021432594499 | 0.414456095715046 | 0.495387078245271 | -0.516312515 | 1.10835538019599 | 1.34449897262454 | 0.59671688055186 | 3.02937212990631 | 0.999714303421225 |
| ebi-fl187-GCST90094798 | ebi-a-GCST90001520 | CD33+ HLA DR+ CD14dim Absolute Count \|\| id:ebi-a-GCST90001520 | Mayonnaise liking \|\| id:ebi-fl187-GCST90094798 | Weighted mode | 9 | 0.071728730524816 | 0.413463339972219 | 0.866581292236107 | -0.738659416 | 0.882116876870364 | 1.07436386240945 | 0.477753955805976 | 2.4160086898791 | 0.999714303421225 |
| ebi-fl187-GCST90094798 | ebi-a-GCST90001531 | CD33dim HLA DR- Absolute Count \|\| id:ebi-a-GCST90001531 | Mayonnaise liking \|\| id:ebi-fl187-GCST90094798 | Weighted mode | 9 | -0.1737244 | 0.386689608994508 | 0.665165173271885 | -0.931636034 | 0.584187233253869 | 0.840528515083378 | 0.393908734829613 | 1.79353266937293 | 0.999714303421225 |
| ebi-fl187-GCST90094798 | ebi-a-GCST90001532 | Basophil Absolute Count \|\| id:ebi-a-GCST90001532 | Mayonnaise liking \|\| id:ebi-fl187-GCST90094798 | Weighted mode | 9 | -0.149051682 | 0.392845410849028 | 0.714252375 | -0.919028688 | 0.620925322990379 | 0.861524588198768 | 0.398906315647397 | 1.86064894677458 | 0.999714303421225 |
| ebi-fl187-GCST90094798 | ebi-a-GCST90001588 | T/B cell \|\| id:ebi-a-GCST90001588 | Mayonnaise liking \|\| id:ebi-fl187-GCST90094798 | Weighted mode | 9 | 0.137335774185285 | 0.276486126392923 | 0.632747771800818 | -0.404577034 | 0.679248581915414 | 1.14721328846404 | 0.66725897934553 | 1.97239508192058 | 0.999714303421225 |
| ebi-fl187-GCST90094798 | ebi-a-GCST90001624 | HLA DR+ CD4+ T cell Absolute Count \|\| id:ebi-a-GCST90001624 | Mayonnaise liking \|\| id:ebi-fl187-GCST90094798 | Weighted mode | 9 | -0.055476348 | 0.288928214074251 | 0.852520618756224 | -0.621775648 | 0.510822951132518 | 0.946034398582861 | 0.536990085156979 | 1.6666622122835 | 0.999714303421225 |
| ebi-fl187-GCST90094798 | ebi-a-GCST90001631 | CD8+ Natural Killer T %T cell \|\| id:ebi-a-GCST90001631 | Mayonnaise liking \|\| id:ebi-fl187-GCST90094798 | Weighted mode | 9 | -0.152210892 | 0.278163379613555 | 0.599169892789885 | -0.697411116 | 0.392989332229511 | 0.858807146245767 | 0.497872571184825 | 1.48140258598218 | 0.999714303421225 |
| ebi-fl187-GCST90094798 | ebi-a-GCST90001632 | CD8+ Natural Killer T %lymphocyte \|\| id:ebi-a-GCST90001632 | Mayonnaise liking \|\| id:ebi-fl187-GCST90094798 | Weighted mode | 9 | -0.174795739 | 0.290933316413599 | 0.564596105558448 | -0.745025039 | 0.395433561124983 | 0.839628506574718 | 0.474722413113898 | 1.48502790173455 | 0.999714303421225 |
| ebi-fl187-GCST90094798 | ebi-a-GCST90001646 | Natural Killer %CD3- lymphocyte \|\| id:ebi-a-GCST90001646 | Mayonnaise liking \|\| id:ebi-fl187-GCST90094798 | Weighted mode | 9 | 0.720176878767203 | 0.37991481260626 | 0.094602810005628 | -0.024456154 | 1.46480991147547 | 2.05479662839698 | 0.975840474739371 | 4.3267207021714 | 0.999714303421225 |
| ebi-fl187-GCST90094798 | ebi-a-GCST90001684 | CD127- CD8+ T cell Absolute Count \|\| id:ebi-a-GCST90001684 | Mayonnaise liking \|\| id:ebi-fl187-GCST90094798 | Weighted mode | 9 | -0.243029654 | 0.29138654171567 | 0.428454841010775 | -0.814147276 | 0.328087967907153 | 0.784248257442189 | 0.443016937683986 | 1.38831109373934 | 0.999714303421225 |
| ebi-fl187-GCST90094798 | ebi-a-GCST90001687 | CD28- CD8+ T cell Absolute Count \|\| id:ebi-a-GCST90001687 | Mayonnaise liking \|\| id:ebi-fl187-GCST90094798 | Weighted mode | 9 | -0.075600902 | 0.306501940115719 | 0.811384971664946 | -0.676344704 | 0.525142901058857 | 0.927186171275475 | 0.508472215962496 | 1.69070043399949 | 0.999714303421225 |
| ebi-fl187-GCST90094798 | ebi-a-GCST90001701 | BAFF-R on CD20- CD38- B cell \|\| id:ebi-a-GCST90001701 | Mayonnaise liking \|\| id:ebi-fl187-GCST90094798 | Weighted mode | 9 | -0.633974753 | 0.377821353318498 | 0.13187127223404 | -1.374504606 | 0.106555099163809 | 0.530479081517685 | 0.252964882093221 | 1.11243921922785 | 0.999714303421225 |
| ebi-fl187-GCST90094798 | ebi-a-GCST90001732 | CD19 on IgD- CD27- B cell \|\| id:ebi-a-GCST90001732 | Mayonnaise liking \|\| id:ebi-fl187-GCST90094798 | Weighted mode | 9 | 0.476107790121795 | 0.30715118297876 | 0.159718900375697 | -0.125908529 | 1.07812410876017 | 1.60979652677058 | 0.881695493089623 | 2.93916083036982 | 0.999714303421225 |
| ebi-fl187-GCST90094798 | ebi-a-GCST90001798 | CD27 on CD24+ CD27+ B cell \|\| id:ebi-a-GCST90001798 | Mayonnaise liking \|\| id:ebi-fl187-GCST90094798 | Weighted mode | 9 | 0.497642754 | 0.274530940491025 | 0.107445571201096 | -0.040437889 | 1.03572339731241 | 1.64483940604978 | 0.960368811730233 | 2.81714341266442 | 0.999714303421225 |
| ebi-fl187-GCST90094798 | ebi-a-GCST90001836 | CD66b on CD66b++ myeloid cell \|\| id:ebi-a-GCST90001836 | Mayonnaise liking \|\| id:ebi-fl187-GCST90094798 | Weighted mode | 9 | 0.902356059738494 | 0.772930119906389 | 0.276644830198052 | -0.612586975 | 2.41729909475502 | 2.46540491503426 | 0.541947050413908 | 11.2155262962182 | 0.999714303421225 |
| ebi-fl187-GCST90094798 | ebi-a-GCST90001943 | CD25 on activated & secreting CD4 regulatory T cell \|\| id:ebi-a-GCST90001943 | Mayonnaise liking \|\| id:ebi-fl187-GCST90094798 | Weighted mode | 9 | -0.207981287 | 0.293481871485343 | 0.498657129402899 | -0.783205755 | 0.367243181446146 | 0.812222235956419 | 0.456938827017975 | 1.44374896939124 | 0.999714303421225 |
| ebi-fl187-GCST90094798 | ebi-a-GCST90001960 | CD25 on CD4+ T cell \|\| id:ebi-a-GCST90001960 | Mayonnaise liking \|\| id:ebi-fl187-GCST90094798 | Weighted mode | 9 | -0.526425746 | 0.343613300455378 | 0.164051994617234 | -1.199907815 | 0.147056322708427 | 0.590712557520468 | 0.301221978757362 | 1.1584192064997 | 0.999714303421225 |
| ebi-fl187-GCST90094798 | ebi-a-GCST90001988 | HLA DR on CD14+ CD16- monocyte \|\| id:ebi-a-GCST90001988 | Mayonnaise liking \|\| id:ebi-fl187-GCST90094798 | Weighted mode | 9 | -0.413877647 | 0.283888646236488 | 0.182980676876085 | -0.970299393 | 0.142544099932582 | 0.6610818318675 | 0.378969560164285 | 1.15320393605184 | 0.999714303421225 |
| ebi-fl187-GCST90094798 | ebi-a-GCST90001991 | HLA DR on CD14+ monocyte \|\| id:ebi-a-GCST90001991 | Mayonnaise liking \|\| id:ebi-fl187-GCST90094798 | Weighted mode | 9 | -0.377151051 | 0.275633740842785 | 0.208408174117854 | -0.917393183 | 0.163091081443027 | 0.685812473680086 | 0.39955926267563 | 1.17714390077106 | 0.999714303421225 |
| ebi-fl187-GCST90094798 | ebi-a-GCST90002019 | CD14 on Monocytic Myeloid-Derived Suppressor Cells \|\| id:ebi-a-GCST90002019 | Mayonnaise liking \|\| id:ebi-fl187-GCST90094798 | Weighted mode | 9 | 0.103709172010427 | 0.454308185876777 | 0.825155910856688 | -0.786734872 | 0.994153216328909 | 1.10927779896701 | 0.455329078359497 | 2.70243499430007 | 0.999714303421225 |
| ebi-fl187-GCST90094798 | ebi-a-GCST90002037 | CD80 on plasmacytoid Dendritic Cell \|\| id:ebi-a-GCST90002037 | Mayonnaise liking \|\| id:ebi-fl187-GCST90094798 | Weighted mode | 9 | 0.267782553053943 | 0.341851979999514 | 0.455995842788655 | -0.402247328 | 0.937812433852991 | 1.30706289227743 | 0.668815308650425 | 2.55438741050345 | 0.999714303421225 |
| ebi-fl187-GCST90094798 | ebi-a-GCST90002042 | CD45 on CD33+ HLA DR+ CD14- \|\| id:ebi-a-GCST90002042 | Mayonnaise liking \|\| id:ebi-fl187-GCST90094798 | Weighted mode | 9 | -0.277488478 | 0.532703420617125 | 0.616539145318251 | -1.321587182 | 0.766610226478967 | 0.757684294643708 | 0.266711645830842 | 2.1524575297842 | 0.999714303421225 |
| ebi-fl187-GCST90094798 | ebi-a-GCST90002056 | CD8 on Effector Memory CD8+ T cell \|\| id:ebi-a-GCST90002056 | Mayonnaise liking \|\| id:ebi-fl187-GCST90094798 | Weighted mode | 9 | -0.268273047 | 0.327926679492496 | 0.43699339417461 | -0.911009339 | 0.374463245089378 | 0.764698954056324 | 0.402118145798278 | 1.45421065038975 | 0.999714303421225 |
| ebi-fl187-GCST90094798 | ebi-a-GCST90002058 | CD8 on CD8+ T cell \|\| id:ebi-a-GCST90002058 | Mayonnaise liking \|\| id:ebi-fl187-GCST90094798 | Weighted mode | 9 | -0.25640386 | 0.340322897416257 | 0.472789322795367 | -0.923436739 | 0.410629018825238 | 0.773829386855029 | 0.397151785930578 | 1.5077659000257 | 0.999714303421225 |
| ebi-fl187-GCST90094798 | ebi-a-GCST90002063 | CD4 on CD4 regulatory T cell \|\| id:ebi-a-GCST90002063 | Mayonnaise liking \|\| id:ebi-fl187-GCST90094798 | Weighted mode | 9 | -0.216260488 | 0.286965667068739 | 0.472677111899725 | -0.778713195 | 0.346192219677713 | 0.805525445009592 | 0.458996270038795 | 1.41367432572613 | 0.999714303421225 |
| ebi-fl187-GCST90094798 | ebi-a-GCST90002066 | CD4 on activated CD4 regulatory T cell \|\| id:ebi-a-GCST90002066 | Mayonnaise liking \|\| id:ebi-fl187-GCST90094798 | Weighted mode | 9 | -0.235129652 | 0.285957604865374 | 0.434753453758305 | -0.795606558 | 0.325347253110896 | 0.790468356845122 | 0.451307407783756 | 1.38451133838428 | 0.999714303421225 |
| ebi-fl187-GCST90094798 | ebi-a-GCST90002070 | CD4 on activated & secreting CD4 regulatory T cell \|\| id:ebi-a-GCST90002070 | Mayonnaise liking \|\| id:ebi-fl187-GCST90094798 | Weighted mode | 9 | -0.180125792 | 0.303021968671854 | 0.568649215421717 | -0.774048851 | 0.413797266225465 | 0.835165147398909 | 0.461142187416076 | 1.51255045073659 | 0.999714303421225 |
| ebi-fl187-GCST90094798 | ebi-a-GCST90002081 | SSC-A on CD4+ T cell \|\| id:ebi-a-GCST90002081 | Mayonnaise liking \|\| id:ebi-fl187-GCST90094798 | Weighted mode | 9 | 0.0263691997400699 | 0.296788441409549 | 0.931386307623391 | -0.555336145 | 0.608074544902785 | 1.02671994324292 | 0.573879321863221 | 1.83689114016204 | 0.999714303421225 |
| ebi-fl187-GCST90094798 | ebi-a-GCST90002094 | CD11b on Monocytic Myeloid-Derived Suppressor Cells \|\| id:ebi-a-GCST90002094 | Mayonnaise liking \|\| id:ebi-fl187-GCST90094798 | Weighted mode | 9 | -0.471370526 | 0.438044641423969 | 0.313270104444777 | -1.329938023 | 0.387196971010266 | 0.624146273026956 | 0.264493653216696 | 1.47284657078058 | 0.999714303421225 |
| ebi-fl187-GCST90094798 | ebi-a-GCST90002104 | HLA DR on myeloid Dendritic Cell \|\| id:ebi-a-GCST90002104 | Mayonnaise liking \|\| id:ebi-fl187-GCST90094798 | Weighted mode | 9 | -0.648231418 | 0.394125288339205 | 0.13864508262124 | -1.420716983 | 0.124254147621647 | 0.522969874704728 | 0.241540774249709 | 1.13230360670258 | 0.999714303421225 |
| ebi-fl187-GCST90094798 | ebi-a-GCST90002105 | HLA DR on plasmacytoid Dendritic Cell \|\| id:ebi-a-GCST90002105 | Mayonnaise liking \|\| id:ebi-fl187-GCST90094798 | Weighted mode | 9 | -0.065134087 | 0.366458008896815 | 0.863345543820387 | -0.783391784 | 0.653123610622882 | 0.936941823409436 | 0.456853830833457 | 1.92153358734518 | 0.999714303421225 |
| ebi-fl187-GCST90094798 | ebi-a-GCST90002106 | HLA DR on Dendritic Cell \|\| id:ebi-a-GCST90002106 | Mayonnaise liking \|\| id:ebi-fl187-GCST90094798 | Weighted mode | 9 | -0.532566772 | 0.400655822430327 | 0.220425582472662 | -1.317852184 | 0.252718640318746 | 0.587096092432336 | 0.267709676150598 | 1.28752096937811 | 0.999714303421225 |
| ebi-fl187-GCST90094798 | ebi-a-GCST90002111 | HLA DR on CD33dim HLA DR+ CD11b- \|\| id:ebi-a-GCST90002111 | Mayonnaise liking \|\| id:ebi-fl187-GCST90094798 | Weighted mode | 9 | -0.1781794 | 0.416621795011145 | 0.68016605997177 | -0.994758118 | 0.638399318210275 | 0.836792289475736 | 0.369812884720512 | 1.89344764516585 | 0.999714303421225 |
| ebi-fl187-GCST90094798 | ebi-a-GCST90002112 | HLA DR on CD33- HLA DR+ \|\| id:ebi-a-GCST90002112 | Mayonnaise liking \|\| id:ebi-fl187-GCST90094798 | Weighted mode | 9 | -0.191006464 | 0.409537198525556 | 0.653368897275193 | -0.993699373 | 0.61168644494514 | 0.826127247911134 | 0.370204629590111 | 1.84353780366515 | 0.999714303421225 |
| ebi-fl187-GCST90094798 | ebi-a-GCST90002115 | HLA DR on HLA DR+ CD8+ T cell \|\| id:ebi-a-GCST90002115 | Mayonnaise liking \|\| id:ebi-fl187-GCST90094798 | Weighted mode | 9 | -0.126561644 | 0.296116035803097 | 0.680355420600681 | -0.706949075 | 0.453825785838484 | 0.881119831822739 | 0.493146457752016 | 1.57432370409875 | 0.999714303421225 |
| ebi-fl187-GCST90094798 | ebi-a-GCST90002116 | HLA DR on B cell \|\| id:ebi-a-GCST90002116 | Mayonnaise liking \|\| id:ebi-fl187-GCST90094798 | Weighted mode | 9 | -0.105812348 | 0.396692533804704 | 0.796418755406665 | -0.883329714 | 0.671705018628562 | 0.899593443080874 | 0.413404100058939 | 1.9575721738578 | 0.999714303421225 |
| ebi-fl187-GCST90094798 | ebi-cfmet338-GCST90026023 | Sphingomyelin (d18:1/24:1, d18:2/24:0) levels \|\| id:ebi-cfmet338-GCST90026023 | Mayonnaise liking \|\| id:ebi-fl187-GCST90094798 | Weighted mode | 9 | -0.06805684 | 0.17435878178441 | 0.706482688462879 | -0.409800052 | 0.273686372124748 | 0.934207371559731 | 0.663782958631451 | 1.31480237889191 | 0.999714303421225 |
| ebi-fl187-GCST90094798 | ebi-cfmet338-GCST90026046 | 1-arachidonoyl-gpc (20:4n6) levels \|\| id:ebi-cfmet338-GCST90026046 | Mayonnaise liking \|\| id:ebi-fl187-GCST90094798 | Weighted mode | 9 | 0.19444294529366 | 0.277835802646192 | 0.503860776818108 | -0.350115228 | 0.739001118480196 | 1.21463418031157 | 0.704606894673057 | 2.09384296851898 | 0.999714303421225 |
| ebi-fl187-GCST90094798 | ebi-cfmet338-GCST90026047 | 1-linoleoyl-gpc (18:2) levels \|\| id:ebi-cfmet338-GCST90026047 | Mayonnaise liking \|\| id:ebi-fl187-GCST90094798 | Weighted mode | 9 | -0.213577266 | 0.293061806949301 | 0.48692053853562 | -0.787978408 | 0.36082387529774 | 0.807689750522454 | 0.454763212334898 | 1.43451078584301 | 0.999714303421225 |
| ebi-fl187-GCST90094798 | ebi-cfmet338-GCST90026050 | 1-myristoyl-2-palmitoyl-gpc (14:0/16:0) levels \|\| id:ebi-cfmet338-GCST90026050 | Mayonnaise liking \|\| id:ebi-fl187-GCST90094798 | Weighted mode | 9 | -0.105790781 | 0.136982785678086 | 0.46214420775689 | -0.374277041 | 0.162695479324688 | 0.899612844843737 | 0.687786340737529 | 1.1766783122503 | 0.999714303421225 |
| ebi-fl187-GCST90094798 | ebi-cfmet338-GCST90026068 | 2,3-dihydroxyisovalerate levels \|\| id:ebi-cfmet338-GCST90026068 | Mayonnaise liking \|\| id:ebi-fl187-GCST90094798 | Weighted mode | 9 | -0.03797638 | 0.497350574180565 | 0.941009844178153 | -1.012783505 | 0.936830745585011 | 0.962735680623399 | 0.36320658380503 | 2.55188102879473 | 0.999714303421225 |
| ebi-fl187-GCST90094798 | ebi-cfmet338-GCST90026082 | 3-hydroxyhexanoate levels \|\| id:ebi-cfmet338-GCST90026082 | Mayonnaise liking \|\| id:ebi-fl187-GCST90094798 | Weighted mode | 9 | -0.052506037 | 0.103084664851182 | 0.624256913815585 | -0.25455198 | 0.149539906412013 | 0.948848593127718 | 0.775263753984948 | 1.16129981319613 | 0.999714303421225 |
| ebi-fl187-GCST90094798 | ebi-cfmet338-GCST90026094 | 3-ureidopropionate levels \|\| id:ebi-cfmet338-GCST90026094 | Mayonnaise liking \|\| id:ebi-fl187-GCST90094798 | Weighted mode | 9 | 0.0655164555632437 | 0.336855648907433 | 0.850637236845988 | -0.594720616 | 0.725753527421813 | 1.06771030694342 | 0.551716684989025 | 2.06628751779709 | 0.999714303421225 |
| ebi-fl187-GCST90094798 | ebi-cfmet338-GCST90026173 | Glycerol levels \|\| id:ebi-cfmet338-GCST90026173 | Mayonnaise liking \|\| id:ebi-fl187-GCST90094798 | Weighted mode | 8 | -0.008652389 | 0.156240040040682 | 0.95738414872401 | -0.314882867 | 0.29757808958689 | 0.99138493529855 | 0.729874361363924 | 1.3465935261793 | 0.999714303421225 |
| ebi-fl187-GCST90094798 | ebi-cfmet338-GCST90026179 | Histidine levels \|\| id:ebi-cfmet338-GCST90026179 | Mayonnaise liking \|\| id:ebi-fl187-GCST90094798 | Weighted mode | 9 | 0.0206388223202681 | 0.0844257336130714 | 0.813026845843998 | -0.144835616 | 0.186113260201888 | 1.02085327562727 | 0.865164501002874 | 1.20455868121138 | 0.999714303421225 |
| ebi-fl187-GCST90094798 | ebi-cfmet338-GCST90026188 | Isocitrate levels \|\| id:ebi-cfmet338-GCST90026188 | Mayonnaise liking \|\| id:ebi-fl187-GCST90094798 | Weighted mode | 9 | -0.029904163 | 0.172864228677398 | 0.866954293396773 | -0.368718052 | 0.308909724751917 | 0.970538542151534 | 0.691620384179577 | 1.361939416 | 0.999714303421225 |
| ebi-fl187-GCST90094798 | ebi-cfmet338-GCST90026207 | N-acetyl-isoputreanine levels \|\| id:ebi-cfmet338-GCST90026207 | Mayonnaise liking \|\| id:ebi-fl187-GCST90094798 | Weighted mode | 9 | -0.000112407 | 0.125192937335992 | 0.999305590539056 | -0.245490564 | 0.245265750411837 | 0.999887599550697 | 0.782320665780315 | 1.27796088671394 | 0.999714303421225 |
| ebi-fl187-GCST90094798 | ebi-cfmet338-GCST90026213 | N-acetylglutamate levels \|\| id:ebi-cfmet338-GCST90026213 | Mayonnaise liking \|\| id:ebi-fl187-GCST90094798 | Weighted mode | 9 | 0.123074046336143 | 0.194243042847221 | 0.544021643403223 | -0.257642318 | 0.503790410316696 | 1.13096816189578 | 0.772871625216988 | 1.65498245955504 | 0.999714303421225 |
| ebi-fl187-GCST90094798 | ebi-cfmet338-GCST90026224 | N-acetyltaurine levels \|\| id:ebi-cfmet338-GCST90026224 | Mayonnaise liking \|\| id:ebi-fl187-GCST90094798 | Weighted mode | 9 | 0.0252978941627772 | 0.148604832966281 | 0.86905096515261 | -0.265967578 | 0.316563366776689 | 1.02562060141207 | 0.766463977047472 | 1.37240320424831 | 0.999714303421225 |
| ebi-fl187-GCST90094798 | ebi-cfmet338-GCST90026250 | Phenyllactate (pla) levels \|\| id:ebi-cfmet338-GCST90026250 | Mayonnaise liking \|\| id:ebi-fl187-GCST90094798 | Weighted mode | 9 | -0.076972996 | 0.213408608474787 | 0.727674190822521 | -0.495253869 | 0.34130787612027 | 0.925914856219704 | 0.609416175719756 | 1.40678629010103 | 0.999714303421225 |
| ebi-fl187-GCST90094798 | ebi-cfmet338-GCST90026251 | Pipecolate levels \|\| id:ebi-cfmet338-GCST90026251 | Mayonnaise liking \|\| id:ebi-fl187-GCST90094798 | Weighted mode | 9 | -0.22446958 | 0.484467025204359 | 0.655469951720398 | -1.174024949 | 0.725085789431654 | 0.798939880106273 | 0.309120240677387 | 2.06490823967232 | 0.999714303421225 |
| ebi-fl187-GCST90094798 | ebi-cfmet338-GCST90026278 | Trigonelline (n'-methylnicotinate) levels \|\| id:ebi-cfmet338-GCST90026278 | Mayonnaise liking \|\| id:ebi-fl187-GCST90094798 | Weighted mode | 9 | 0.465557098259041 | 0.570639016266228 | 0.438199344628028 | -0.652895374 | 1.58400957014085 | 1.59290134431353 | 0.520536445282799 | 4.87446117502368 | 0.999714303421225 |
| ebi-fl187-GCST90094798 | ebi-cfmet338-GCST90026299 | 1-carboxyethylvaline levels \|\| id:ebi-cfmet338-GCST90026299 | Mayonnaise liking \|\| id:ebi-fl187-GCST90094798 | Weighted mode | 9 | 0.10877360814899 | 0.263036767014011 | 0.690081343940129 | -0.406778455 | 0.624325671496453 | 1.11490991524848 | 0.665791676648363 | 1.86698657059943 | 0.999714303421225 |
| ebi-fl187-GCST90094798 | ebi-gm430-GCST90011306 | C_Erysipelotrichia abundance \|\| id:ebi-gm430-GCST90011306 | Mayonnaise liking \|\| id:ebi-fl187-GCST90094798 | Weighted mode | 8 | 0.0685819459326424 | 0.204532194660273 | 0.747215095051732 | -0.332301156 | 0.469465047466777 | 1.07098838449718 | 0.717271280061048 | 1.59913850116828 | 0.999714303421225 |
| ebi-fl187-GCST90094798 | ebi-gm430-GCST90011312 | F_Erysipelotrichaceae abundance \|\| id:ebi-gm430-GCST90011312 | Mayonnaise liking \|\| id:ebi-fl187-GCST90094798 | Weighted mode | 8 | 0.0685819459326424 | 0.199573118955867 | 0.741205302333718 | -0.322581367 | 0.459745259086142 | 1.07098838449718 | 0.724276997002891 | 1.58367050793317 | 0.999714303421225 |
| ebi-fl187-GCST90094798 | ebi-gm430-GCST90011352 | O_Erysipelotrichales abundance \|\| id:ebi-gm430-GCST90011352 | Mayonnaise liking \|\| id:ebi-fl187-GCST90094798 | Weighted mode | 8 | 0.0685819459326424 | 0.206135813861808 | 0.749100520516466 | -0.335444249 | 0.472608141101786 | 1.07098838449718 | 0.715020368532033 | 1.60417265046973 | 0.999714303421225 |
| ebi-fl187-GCST90094798 | ebi-gm430-GCST90011362 | OTU97_11 (Parabacteroides) abundance \|\| id:ebi-gm430-GCST90011362 | Mayonnaise liking \|\| id:ebi-fl187-GCST90094798 | Weighted mode | 8 | -0.155014984 | 0.310276876592561 | 0.632673408592881 | -0.763157662 | 0.453127694492062 | 0.856402345372155 | 0.466192023679804 | 1.57322506586399 | 0.999714303421225 |
| ebi-fl187-GCST90094798 | ebi-gm430-GCST90011384 | OTU97_235 (Phascolarctobacterium) abundance \|\| id:ebi-gm430-GCST90011384 | Mayonnaise liking \|\| id:ebi-fl187-GCST90094798 | Weighted mode | 8 | -0.337570538 | 0.840153100673335 | 0.699818493920346 | -1.984270615 | 1.3091295397877 | 0.713501644286342 | 0.137480854048861 | 3.70294903913228 | 0.999714303421225 |
| ebi-fl187-GCST90094798 | ebi-gm430-GCST90011390 | OTU97_3 (Bacteroides) abundance \|\| id:ebi-gm430-GCST90011390 | Mayonnaise liking \|\| id:ebi-fl187-GCST90094798 | Weighted mode | 8 | 0.106702204043344 | 0.226459193405375 | 0.651845807972579 | -0.337157815 | 0.550562223117879 | 1.11260287650228 | 0.713796183246574 | 1.73422776677068 | 0.999714303421225 |
| ebi-fl187-GCST90094798 | ebi-gm430-GCST90011398 | OTU97_4 (Alistipes) abundance \|\| id:ebi-gm430-GCST90011398 | Mayonnaise liking \|\| id:ebi-fl187-GCST90094798 | Weighted mode | 8 | -0.081860988 | 0.234175512385837 | 0.736941286179545 | -0.540844992 | 0.377123016450719 | 0.921400035616533 | 0.582256042689353 | 1.45808366661657 | 0.999714303421225 |
| ebi-fl187-GCST90094798 | ebi-gm430-GCST90011421 | OTU99_108 (Cytophagales) abundance \|\| id:ebi-gm430-GCST90011421 | Mayonnaise liking \|\| id:ebi-fl187-GCST90094798 | Weighted mode | 8 | 0.0131919901629782 | 0.852525433199362 | 0.98808581748002 | -1.657757859 | 1.68414183923373 | 1.01327938836107 | 0.190565776812463 | 5.38782532756547 | 0.999714303421225 |
| ebi-fl187-GCST90094798 | ebi-gm430-GCST90011428 | OTU99_123 (Phascolarctobacterium) abundance \|\| id:ebi-gm430-GCST90011428 | Mayonnaise liking \|\| id:ebi-fl187-GCST90094798 | Weighted mode | 8 | -0.409865079 | 0.467471117216607 | 0.409689671934998 | -1.326108469 | 0.506378310950067 | 0.663739796668837 | 0.265508488107256 | 1.6592709363929 | 0.999714303421225 |
| ebi-fl187-GCST90094798 | ebi-gm430-GCST90011450 | OTU99_298 (Phascolarctobacterium) abundance \|\| id:ebi-gm430-GCST90011450 | Mayonnaise liking \|\| id:ebi-fl187-GCST90094798 | Weighted mode | 8 | -0.341338668 | 0.932306686238882 | 0.725088353988557 | -2.168659773 | 1.48598243691364 | 0.71081813599986 | 0.114330743408717 | 4.41930496909367 | 0.999714303421225 |
| ebi-fl187-GCST90094798 | ebi-gm430-GCST90011451 | OTU99_3 (Bacteroides) abundance \|\| id:ebi-gm430-GCST90011451 | Mayonnaise liking \|\| id:ebi-fl187-GCST90094798 | Weighted mode | 8 | 0.0985708202345117 | 0.234529236252524 | 0.686873050387323 | -0.361106483 | 0.558248123289458 | 1.1035925583107 | 0.696904786128572 | 1.74760822281693 | 0.999714303421225 |
| ebi-fl187-GCST90094798 | ebi-gm430-GCST90011472 | OTU99_72 (Parabacteroides) abundance \|\| id:ebi-gm430-GCST90011472 | Mayonnaise liking \|\| id:ebi-fl187-GCST90094798 | Weighted mode | 8 | -0.290113793 | 0.588222226994581 | 0.636962884028675 | -1.443029358 | 0.862801771977845 | 0.748178425318037 | 0.236211105765755 | 2.36979101510362 | 0.999714303421225 |
| ebi-fl187-GCST90094798 | ebi-gm430-GCST90011507 | TestASV_3 (Bacteroides) abundance \|\| id:ebi-gm430-GCST90011507 | Mayonnaise liking \|\| id:ebi-fl187-GCST90094798 | Weighted mode | 8 | -0.234048737 | 0.258299671940722 | 0.39499443781909 | -0.740316094 | 0.272218619814927 | 0.791323248085135 | 0.476963126416116 | 1.31287399 | 0.999714303421225 |
| ebi-fl187-GCST90094798 | ebi-gm430-GCST90011512 | TestASV_34 (Phascolarctobacterium) abundance \|\| id:ebi-gm430-GCST90011512 | Mayonnaise liking \|\| id:ebi-fl187-GCST90094798 | Weighted mode | 8 | -0.720967077 | 0.613054935042328 | 0.278027096717852 | -1.922554749 | 0.480620596070072 | 0.486281756778007 | 0.146232896122967 | 1.61707764288726 | 0.999714303421225 |
| ebi-fl187-GCST90094798 | ebi-gm430-GCST90011519 | TestASV_41 (Barnesiella) abundance \|\| id:ebi-gm430-GCST90011519 | Mayonnaise liking \|\| id:ebi-fl187-GCST90094798 | Weighted mode | 8 | 0.239094336098088 | 0.872224225061116 | 0.791905136395131 | -1.470465145 | 1.94865381721787 | 1.27009834705477 | 0.229818561361398 | 7.01923204825272 | 0.999714303421225 |
| ebi-fl187-GCST90094798 | ebi-gm430-GCST90011524 | TestASV_46 (Sutterella) abundance \|\| id:ebi-gm430-GCST90011524 | Mayonnaise liking \|\| id:ebi-fl187-GCST90094798 | Weighted mode | 8 | -1.105005606 | 1.02079753897688 | 0.314907307821914 | -3.105768782 | 0.895757570740536 | 0.331209025593532 | 0.0447900715168823 | 2.44919052190548 | 0.999714303421225 |
| ebi-fl187-GCST90094798 | ebi-gm430-GCST90011581 | OTU97_19 (Bacteria) prevalence \|\| id:ebi-gm430-GCST90011581 | Mayonnaise liking \|\| id:ebi-fl187-GCST90094798 | Weighted mode | 8 | -0.039541735 | 1.06289692621802 | 0.971362758700634 | -2.12281971 | 2.04373624083053 | 0.961229836652258 | 0.119693650858342 | 7.71939691240636 | 0.999714303421225 |
| ebi-fl187-GCST90094798 | ebi-gm430-GCST90011614 | OTU97_80 (Ruminococcus) prevalence \|\| id:ebi-gm430-GCST90011614 | Mayonnaise liking \|\| id:ebi-fl187-GCST90094798 | Weighted mode | 8 | -0.620837917 | 0.469880500157754 | 0.227960443595287 | -1.541803697 | 0.300127863233728 | 0.537493873558772 | 0.21399477131106 | 1.35003141592311 | 0.999714303421225 |
| ebi-fl187-GCST90094798 | ebi-gm430-GCST90011623 | OTU99_11 (Parabacteroides) prevalence \|\| id:ebi-gm430-GCST90011623 | Mayonnaise liking \|\| id:ebi-fl187-GCST90094798 | Weighted mode | 8 | -0.492323186 | 0.406433581719087 | 0.265072310233529 | -1.288933006 | 0.30428663426058 | 0.61120480112993 | 0.275564652092487 | 1.35565757831268 | 0.999714303421225 |
| ebi-fl187-GCST90094798 | ebi-gm430-GCST90011642 | OTU99_19 (Bacteria) prevalence \|\| id:ebi-gm430-GCST90011642 | Mayonnaise liking \|\| id:ebi-fl187-GCST90094798 | Weighted mode | 8 | -0.039541735 | 1.08319461204998 | 0.971899109152368 | -2.162603174 | 2.08351970506117 | 0.961229836652258 | 0.115025300073684 | 8.0326919232434 | 0.999714303421225 |
| ebi-fl187-GCST90094798 | ebi-gm430-GCST90011653 | OTU99_30 (Parasutterella) prevalence \|\| id:ebi-gm430-GCST90011653 | Mayonnaise liking \|\| id:ebi-fl187-GCST90094798 | Weighted mode | 8 | 0.351939652624786 | 0.430622691451865 | 0.440693657483743 | -0.492080823 | 1.19596012787044 | 1.42182271786048 | 0.611352952687673 | 3.30673113156117 | 0.999714303421225 |
| ebi-fl187-GCST90094798 | ebi-gm430-GCST90011675 | OTU99_8 (Ruminococcaceae) prevalence \|\| id:ebi-gm430-GCST90011675 | Mayonnaise liking \|\| id:ebi-fl187-GCST90094798 | Weighted mode | 8 | -0.179938405 | 0.424486287038167 | 0.684363025167372 | -1.011931527 | 0.652054717928057 | 0.835321661742832 | 0.363516159659721 | 1.91948077144621 | 0.999714303421225 |
| ebi-fl187-GCST90094798 | ebi-gm430-GCST90011679 | OTU99_92 (Ruminococcus) prevalence \|\| id:ebi-gm430-GCST90011679 | Mayonnaise liking \|\| id:ebi-fl187-GCST90094798 | Weighted mode | 8 | -0.647758296 | 0.568577802411261 | 0.292074821427088 | -1.762170788 | 0.466654197144743 | 0.52321736176855 | 0.171671795916541 | 1.59464987358278 | 0.999714303421225 |
| ebi-fl187-GCST90094798 | ebi-gm430-GCST90011684 | TestASV_11 (Lachnospiraceae) prevalence \|\| id:ebi-gm430-GCST90011684 | Mayonnaise liking \|\| id:ebi-fl187-GCST90094798 | Weighted mode | 8 | -0.339755778 | 0.56437172161546 | 0.566149795797501 | -1.445924352 | 0.766412796542314 | 0.711944174087966 | 0.235528264836526 | 2.15203261217756 | 0.999714303421225 |
| ebi-fl187-GCST90094798 | ebi-gm430-GCST90011688 | TestASV_15 (Bacteroides) prevalence \|\| id:ebi-gm430-GCST90011688 | Mayonnaise liking \|\| id:ebi-fl187-GCST90094798 | Weighted mode | 8 | -0.275209636 | 0.427309797317039 | 0.540062956567272 | -1.112736839 | 0.562317566786425 | 0.759412906286927 | 0.328658244478021 | 1.75473450590321 | 0.999714303421225 |
| ebi-fl187-GCST90094798 | ebi-gm430-GCST90011692 | TestASV_19 (Prevotella) prevalence \|\| id:ebi-gm430-GCST90011692 | Mayonnaise liking \|\| id:ebi-fl187-GCST90094798 | Weighted mode | 8 | 0.073875972042881 | 0.788168593872021 | 0.927949141535845 | -1.470934472 | 1.61868641603204 | 1.0766732596298 | 0.229710726629634 | 5.04645701578797 | 0.999714303421225 |
| ebi-fl187-GCST90094798 | ebi-gm430-GCST90011693 | TestASV_2 (Bacteroides) prevalence \|\| id:ebi-gm430-GCST90011693 | Mayonnaise liking \|\| id:ebi-fl187-GCST90094798 | Weighted mode | 8 | 0.44718780507482 | 0.412341797157436 | 0.314072764511013 | -0.361002117 | 1.2553777275034 | 1.56390798156353 | 0.696977522717336 | 3.50916363165135 | 0.999714303421225 |
| ebi-fl187-GCST90094798 | ebi-gm430-GCST90011716 | TestASV_40 (Barnesiella) prevalence \|\| id:ebi-gm430-GCST90011716 | Mayonnaise liking \|\| id:ebi-fl187-GCST90094798 | Weighted mode | 8 | -0.209355222 | 0.844726128783327 | 0.811372640612451 | -1.865018434 | 1.44630799076778 | 0.811107061676847 | 0.154893354544755 | 4.24740407640894 | 0.999714303421225 |
| ebi-fl187-GCST90094798 | ebi-gm430-GCST90011718 | TestASV_42 (Alphaproteobacteria) prevalence \|\| id:ebi-gm430-GCST90011718 | Mayonnaise liking \|\| id:ebi-fl187-GCST90094798 | Weighted mode | 8 | 0.481230950497339 | 0.629570056710567 | 0.469618424471312 | -0.752726361 | 1.71518826165005 | 1.61806493465413 | 0.471080465122677 | 5.55772171973944 | 0.999714303421225 |
| ebi-fl187-GCST90094798 | ebi-hbsm44-GCST90104929 | Sulcal depth of the right inferior frontal sulcus \|\| id:ebi-hbsm44-GCST90104929 | Mayonnaise liking \|\| id:ebi-fl187-GCST90094798 | Weighted mode | 8 | -0.610433873 | 0.476287145380372 | 0.240781941241913 | -1.543956678 | 0.323088932163641 | 0.5431151750557 | 0.213534540386819 | 1.38138819528418 | 0.999714303421225 |
| ebi-fl187-GCST90094798 | ebi-hbsm44-GCST90104935 | Sulcal opening distance of the left calloso-marginal posterior fissure \|\| id:ebi-hbsm44-GCST90104935 | Mayonnaise liking \|\| id:ebi-fl187-GCST90094798 | Weighted mode | 8 | -0.078276702 | 0.103619326577259 | 0.474639842824703 | -0.281370582 | 0.124817178024405 | 0.924708522381031 | 0.754748587288002 | 1.13294130756395 | 0.999714303421225 |
| ebi-fl187-GCST90094798 | ebi-hbsm44-GCST90104936 | Sulcal opening distance of the left anterior inferior temporal sulcus \|\| id:ebi-hbsm44-GCST90104936 | Mayonnaise liking \|\| id:ebi-fl187-GCST90094798 | Weighted mode | 8 | -0.022466227 | 0.055631876129078 | 0.698384706827466 | -0.131504704 | 0.0865722499999917 | 0.977784259135226 | 0.876775150404286 | 1.09043014845001 | 0.999714303421225 |
| ebi-fl187-GCST90094798 | ebi-hbsm44-GCST90104941 | Sulcal opening distance of the right posterior inferior temporal sulcus \|\| id:ebi-hbsm44-GCST90104941 | Mayonnaise liking \|\| id:ebi-fl187-GCST90094798 | Weighted mode | 8 | -0.028871994 | 0.0487903715538104 | 0.572621422354915 | -0.124501122 | 0.0667571346261342 | 0.971540819931317 | 0.882937270829585 | 1.06903581486141 | 0.999714303421225 |
| ebi-fl187-GCST90094798 | ebi-IPs91-GCST90274819 | Leukemia inhibitory factor levels \|\| id:ebi-IPs91-GCST90274819 | Mayonnaise liking \|\| id:ebi-fl187-GCST90094798 | Weighted mode | 12 | 0.151016599706133 | 0.157414292306921 | 0.357991144814117 | -0.157515413 | 0.459548612627698 | 1.16301596364481 | 0.854263646557386 | 1.58335911535457 | 0.999714303421225 |
| ebi-fl187-GCST90094798 | ebi-IPs91-GCST90274837 | STAM binding protein levels \|\| id:ebi-IPs91-GCST90274837 | Mayonnaise liking \|\| id:ebi-fl187-GCST90094798 | Weighted mode | 12 | -0.351377863 | 0.151176685093645 | 0.0402725667490536 | -0.647684165 | -0.05507156 | 0.703717794896177 | 0.523256149353989 | 0.946417419967132 | 0.999714303421225 |
| ebi-fl187-GCST90094798 | ebi-IPs91-GCST90274840 | TNF-beta levels \|\| id:ebi-IPs91-GCST90274840 | Mayonnaise liking \|\| id:ebi-fl187-GCST90094798 | Weighted mode | 12 | -0.12590075 | 0.165637124711236 | 0.463175338955238 | -0.450549515 | 0.198748014173788 | 0.881702351169855 | 0.637277861836492 | 1.21987453607467 | 0.999714303421225 |
| ebi-fl187-GCST90094798 | ebi-IPs91-GCST90274842 | Tumor necrosis factor ligand superfamily member 14 levels \|\| id:ebi-IPs91-GCST90274842 | Mayonnaise liking \|\| id:ebi-fl187-GCST90094798 | Weighted mode | 12 | 0.0558391509369787 | 0.162787646059321 | 0.738050355102156 | -0.263224635 | 0.374902937213248 | 1.0574275838207 | 0.768569230108535 | 1.45485019595045 | 0.999714303421225 |
| ebi-fl187-GCST90094798 | ebi-met1400-GCST90199628 | 3-methylhistidine levels \|\| id:ebi-met1400-GCST90199628 | Mayonnaise liking \|\| id:ebi-fl187-GCST90094798 | Weighted mode | 9 | 0.100997877765132 | 0.226256611982515 | 0.667153685010945 | -0.342465082 | 0.544460837250862 | 1.10627429398705 | 0.710017911556515 | 1.72367878840355 | 0.999714303421225 |
| ebi-fl187-GCST90094798 | ebi-met1400-GCST90199651 | Docosahexaenoate DHA; 22:6n3 levels \|\| id:ebi-met1400-GCST90199651 | Mayonnaise liking \|\| id:ebi-fl187-GCST90094798 | Weighted mode | 9 | -0.126579119 | 0.20990822205157 | 0.563195174868671 | -0.537999234 | 0.28484099652709 | 0.881104434953529 | 0.583915362481221 | 1.32955060814956 | 0.999714303421225 |
| ebi-fl187-GCST90094798 | ebi-met1400-GCST90199652 | 1-stearoyl-2-oleoyl-GPS (18:0/18:1) levels \|\| id:ebi-met1400-GCST90199652 | Mayonnaise liking \|\| id:ebi-fl187-GCST90094798 | Weighted mode | 9 | -0.247693853 | 0.24170899934136 | 0.335461392467684 | -0.721443491 | 0.226055786003291 | 0.780598884936997 | 0.486050140128343 | 1.25364559920502 | 0.999714303421225 |
| ebi-fl187-GCST90094798 | ebi-met1400-GCST90199653 | 1-myristoyl-2-palmitoyl-gpc (14:0/16:0) levels \|\| id:ebi-met1400-GCST90199653 | Mayonnaise liking \|\| id:ebi-fl187-GCST90094798 | Weighted mode | 9 | 0.201076076031126 | 0.214255907157991 | 0.375460478461796 | -0.218865502 | 0.621017654060789 | 1.22271778780162 | 0.803429770585506 | 1.86082075041478 | 0.999714303421225 |
| ebi-fl187-GCST90094798 | ebi-met1400-GCST90199667 | Erythritol levels in elite athletes \|\| id:ebi-met1400-GCST90199667 | Mayonnaise liking \|\| id:ebi-fl187-GCST90094798 | Weighted mode | 9 | 0.141653080002084 | 0.193497477490458 | 0.485018810223612 | -0.237601976 | 0.520908135883382 | 1.15217686599879 | 0.788516477219976 | 1.68355585316759 | 0.999714303421225 |
| ebi-fl187-GCST90094798 | ebi-met1400-GCST90199674 | 3-hydroxymyristate levels \|\| id:ebi-met1400-GCST90199674 | Mayonnaise liking \|\| id:ebi-fl187-GCST90094798 | Weighted mode | 9 | -0.090285131 | 0.207684879129665 | 0.675252584096039 | -0.497347494 | 0.316777231831038 | 0.913670632065678 | 0.608141621307853 | 1.37269674472208 | 0.999714303421225 |
| ebi-fl187-GCST90094798 | ebi-met1400-GCST90199685 | 2-linoleoylglycerol (18:2) levels \|\| id:ebi-met1400-GCST90199685 | Mayonnaise liking \|\| id:ebi-fl187-GCST90094798 | Weighted mode | 9 | -0.121444004 | 0.245154028173389 | 0.633652137510338 | -0.601945899 | 0.359057891656045 | 0.885640644680244 | 0.547744742568962 | 1.43197969884829 | 0.999714303421225 |
| ebi-fl187-GCST90094798 | ebi-met1400-GCST90199703 | Gamma-glutamylvaline levels \|\| id:ebi-met1400-GCST90199703 | Mayonnaise liking \|\| id:ebi-fl187-GCST90094798 | Weighted mode | 9 | -0.082374755 | 0.228066618829758 | 0.727312014682598 | -0.529385328 | 0.364635817471684 | 0.920926771707435 | 0.588966879688101 | 1.43998949363096 | 0.999714303421225 |
| ebi-fl187-GCST90094798 | ebi-met1400-GCST90199779 | Gamma-glutamyl-2-aminobutyrate levels \|\| id:ebi-met1400-GCST90199779 | Mayonnaise liking \|\| id:ebi-fl187-GCST90094798 | Weighted mode | 9 | -0.149622262 | 0.217195566497269 | 0.510378107934958 | -0.575325573 | 0.276081047955698 | 0.86103315962159 | 0.562521697275309 | 1.31795467723104 | 0.999714303421225 |
| ebi-fl187-GCST90094798 | ebi-met1400-GCST90199824 | 4-vinylphenol sulfate levels \|\| id:ebi-met1400-GCST90199824 | Mayonnaise liking \|\| id:ebi-fl187-GCST90094798 | Weighted mode | 9 | 0.328975420733431 | 0.222448823414337 | 0.177432720891104 | -0.107024273 | 0.764975114625531 | 1.38954370120283 | 0.898503863198774 | 2.14894089679089 | 0.999714303421225 |
| ebi-fl187-GCST90094798 | ebi-met1400-GCST90199830 | 4-ethylphenylsulfate levels \|\| id:ebi-met1400-GCST90199830 | Mayonnaise liking \|\| id:ebi-fl187-GCST90094798 | Weighted mode | 9 | -0.247364952 | 0.272199185283566 | 0.390030094899287 | -0.780875356 | 0.286145450809852 | 0.780855666416586 | 0.458004918656143 | 1.33128607780883 | 0.999714303421225 |
| ebi-fl187-GCST90094798 | ebi-met1400-GCST90199833 | Hexanoylglutamine levels \|\| id:ebi-met1400-GCST90199833 | Mayonnaise liking \|\| id:ebi-fl187-GCST90094798 | Weighted mode | 9 | 0.13507264228586 | 0.241493779207222 | 0.591252409029084 | -0.338255165 | 0.608400449532014 | 1.14461992913946 | 0.71301332866676 | 1.83748988905022 | 0.999714303421225 |
| ebi-fl187-GCST90094798 | ebi-met1400-GCST90199837 | Carotenoid levels (cryptoxanthin) \|\| id:ebi-met1400-GCST90199837 | Mayonnaise liking \|\| id:ebi-fl187-GCST90094798 | Weighted mode | 9 | -0.333883903 | 0.221988598556731 | 0.170976963240841 | -0.768981556 | 0.101213749901324 | 0.716136918555758 | 0.463484861194787 | 1.10651313356059 | 0.999714303421225 |
| ebi-fl187-GCST90094798 | ebi-met1400-GCST90199869 | 2-hydroxyhippurate levels \|\| id:ebi-met1400-GCST90199869 | Mayonnaise liking \|\| id:ebi-fl187-GCST90094798 | Weighted mode | 9 | -0.086586204 | 0.30746409425141 | 0.78538675787881 | -0.689215829 | 0.516043420904592 | 0.917056491597014 | 0.501969544950048 | 1.67538572258186 | 0.999714303421225 |
| ebi-fl187-GCST90094798 | ebi-met1400-GCST90199902 | Hydantoin-5-propionate levels \|\| id:ebi-met1400-GCST90199902 | Mayonnaise liking \|\| id:ebi-fl187-GCST90094798 | Weighted mode | 9 | 0.298312774030804 | 0.213530884541531 | 0.199932773002671 | -0.12020776 | 0.716833307732204 | 1.34758321100376 | 0.886736189559608 | 2.04793774288282 | 0.999714303421225 |
| ebi-fl187-GCST90094798 | ebi-met1400-GCST90199904 | Pantoate levels \|\| id:ebi-met1400-GCST90199904 | Mayonnaise liking \|\| id:ebi-fl187-GCST90094798 | Weighted mode | 9 | 0.134851420325075 | 0.221833171755072 | 0.56011366853275 | -0.299941596 | 0.569644436965016 | 1.14436674208068 | 0.740861488459307 | 1.76763843279766 | 0.999714303421225 |
| ebi-fl187-GCST90094798 | ebi-met1400-GCST90199936 | 3-methyl catechol sulfate (2) levels \|\| id:ebi-met1400-GCST90199936 | Mayonnaise liking \|\| id:ebi-fl187-GCST90094798 | Weighted mode | 9 | -0.197921888 | 0.272892117231682 | 0.488952395617496 | -0.732790437 | 0.336946662205048 | 0.820433936724168 | 0.480566127706282 | 1.40066435339803 | 0.999714303421225 |
| ebi-fl187-GCST90094798 | ebi-met1400-GCST90199977 | 4-hydroxychlorothalonil levels \|\| id:ebi-met1400-GCST90199977 | Mayonnaise liking \|\| id:ebi-fl187-GCST90094798 | Weighted mode | 9 | 0.451794149758082 | 0.399833863625928 | 0.291233327693648 | -0.331880223 | 1.2354685224649 | 1.57112849810619 | 0.71757326651723 | 3.43998985572876 | 0.999714303421225 |
| ebi-fl187-GCST90094798 | ebi-met1400-GCST90200040 | Sphingomyelin (d18:2/24:1, d18:1/24:2) levels \|\| id:ebi-met1400-GCST90200040 | Mayonnaise liking \|\| id:ebi-fl187-GCST90094798 | Weighted mode | 9 | -0.067914301 | 0.176751329905322 | 0.7108149831191 | -0.414346907 | 0.278518305735159 | 0.934340542309192 | 0.660771684851993 | 1.32117079017721 | 0.999714303421225 |
| ebi-fl187-GCST90094798 | ebi-met1400-GCST90200077 | 1-stearoyl-2-oleoyl-GPI (18:0/18:1) levels \|\| id:ebi-met1400-GCST90200077 | Mayonnaise liking \|\| id:ebi-fl187-GCST90094798 | Weighted mode | 9 | -0.063571245 | 0.205801052354059 | 0.765291478460438 | -0.466941308 | 0.339798817421632 | 0.938407259913276 | 0.626916884459834 | 1.40466496801517 | 0.999714303421225 |
| ebi-fl187-GCST90094798 | ebi-met1400-GCST90200080 | 1-palmitoyl-2-oleoyl-GPI (16:0/18:1) levels \|\| id:ebi-met1400-GCST90200080 | Mayonnaise liking \|\| id:ebi-fl187-GCST90094798 | Weighted mode | 9 | -0.028880446 | 0.244811413304536 | 0.90899949382764 | -0.508710816 | 0.450949924412175 | 0.971532608458919 | 0.601270227484855 | 1.56980267133344 | 0.999714303421225 |
| ebi-fl187-GCST90094798 | ebi-met1400-GCST90200082 | 1-oleoyl-2-linoleoyl-GPE (18:1/18:2) levels \|\| id:ebi-met1400-GCST90200082 | Mayonnaise liking \|\| id:ebi-fl187-GCST90094798 | Weighted mode | 9 | 0.182113329982093 | 0.185104581230955 | 0.354009194547774 | -0.180691649 | 0.544918309194765 | 1.199750154 | 0.834692697153892 | 1.7244675034833 | 0.999714303421225 |
| ebi-fl187-GCST90094798 | ebi-met1400-GCST90200100 | Perfluorooctanesulfonate (PFOS) levels \|\| id:ebi-met1400-GCST90200100 | Mayonnaise liking \|\| id:ebi-fl187-GCST90094798 | Weighted mode | 9 | -0.209161928 | 0.210756816724624 | 0.350051360197708 | -0.622245289 | 0.203921432839948 | 0.811263858721207 | 0.536737951977776 | 1.22620181047766 | 0.999714303421225 |
| ebi-fl187-GCST90094798 | ebi-met1400-GCST90200102 | 1-palmitoleoyl-2-linolenoyl-GPC (16:1/18:3) levels \|\| id:ebi-met1400-GCST90200102 | Mayonnaise liking \|\| id:ebi-fl187-GCST90094798 | Weighted mode | 9 | 0.204835133508959 | 0.209390215078682 | 0.356604150495536 | -0.205569688 | 0.615239955063175 | 1.22732270389479 | 0.814183353743695 | 1.85010048727895 | 0.999714303421225 |
| ebi-fl187-GCST90094798 | ebi-met1400-GCST90200114 | Glycosyl-N-tricosanoyl-sphingadienine (d18:2/23:0) levels \|\| id:ebi-met1400-GCST90200114 | Mayonnaise liking \|\| id:ebi-fl187-GCST90094798 | Weighted mode | 9 | -0.048297336 | 0.205935223604962 | 0.820467012225295 | -0.451930374 | 0.355335702039059 | 0.952850428002293 | 0.636398477749638 | 1.42665950640651 | 0.999714303421225 |
| ebi-fl187-GCST90094798 | ebi-met1400-GCST90200135 | Arachidoylcarnitine (C20) levels \|\| id:ebi-met1400-GCST90200135 | Mayonnaise liking \|\| id:ebi-fl187-GCST90094798 | Weighted mode | 9 | 0.293496630802665 | 0.257977825888005 | 0.288177122788405 | -0.212139908 | 0.799133169543154 | 1.34110866094284 | 0.808851524900953 | 2.22361259772136 | 0.999714303421225 |
| ebi-fl187-GCST90094798 | ebi-met1400-GCST90200146 | Docosahexaenoylcarnitine (C22:6) levels \|\| id:ebi-met1400-GCST90200146 | Mayonnaise liking \|\| id:ebi-fl187-GCST90094798 | Weighted mode | 9 | -0.043657936 | 0.222045451225138 | 0.849030206260205 | -0.47886702 | 0.391551148681284 | 0.957281353231521 | 0.619484858236998 | 1.47927358846629 | 0.999714303421225 |
| ebi-fl187-GCST90094798 | ebi-met1400-GCST90200162 | 3-hydroxyphenylacetoylglutamine levels \|\| id:ebi-met1400-GCST90200162 | Mayonnaise liking \|\| id:ebi-fl187-GCST90094798 | Weighted mode | 9 | 0.194079661186112 | 0.248191821937332 | 0.456747463860352 | -0.29237631 | 0.680535632183283 | 1.21419300315856 | 0.746487572514649 | 1.97493528787483 | 0.999714303421225 |
| ebi-fl187-GCST90094798 | ebi-met1400-GCST90200172 | 3-carboxy-4-methyl-5-pentyl-2-furanpropionate (3-CMPFP) levels \|\| id:ebi-met1400-GCST90200172 | Mayonnaise liking \|\| id:ebi-fl187-GCST90094798 | Weighted mode | 9 | 0.427986873210219 | 0.252659066914937 | 0.128729648773544 | -0.067224898 | 0.923198644363497 | 1.53416594216159 | 0.934984901508524 | 2.51732956787977 | 0.999714303421225 |
| ebi-fl187-GCST90094798 | ebi-met1400-GCST90200222 | Dihydrocaffeate sulfate (2) levels \|\| id:ebi-met1400-GCST90200222 | Mayonnaise liking \|\| id:ebi-fl187-GCST90094798 | Weighted mode | 9 | 0.230966824018519 | 0.258083272178041 | 0.396949950435989 | -0.274876389 | 0.73681003748748 | 1.25981744307576 | 0.759666020155755 | 2.08926021141833 | 0.999714303421225 |
| ebi-fl187-GCST90094798 | ebi-met1400-GCST90200223 | 4-methylhexanoylglutamine levels \|\| id:ebi-met1400-GCST90200223 | Mayonnaise liking \|\| id:ebi-fl187-GCST90094798 | Weighted mode | 9 | 0.197353678770378 | 0.270234963496766 | 0.486037816486376 | -0.33230685 | 0.727014207224039 | 1.21817480709283 | 0.717267195871341 | 2.06889408741599 | 0.999714303421225 |
| ebi-fl187-GCST90094798 | ebi-met1400-GCST90200224 | 2-ketocaprylate levels \|\| id:ebi-met1400-GCST90200224 | Mayonnaise liking \|\| id:ebi-fl187-GCST90094798 | Weighted mode | 9 | 0.203220437467607 | 0.241580505962229 | 0.424658072510702 | -0.270277354 | 0.676718229153576 | 1.22534255 | 0.763167797172796 | 1.96741053556746 | 0.999714303421225 |
| ebi-fl187-GCST90094798 | ebi-met1400-GCST90200243 | Hydroxypalmitoyl sphingomyelin (d18:1/16:0(OH)) levels \|\| id:ebi-met1400-GCST90200243 | Mayonnaise liking \|\| id:ebi-fl187-GCST90094798 | Weighted mode | 9 | -0.062598633 | 0.204223604815394 | 0.76703469915874 | -0.462876898 | 0.337679632855493 | 0.939320410646223 | 0.629470116756019 | 1.40169137560278 | 0.999714303421225 |
| ebi-fl187-GCST90094798 | ebi-met1400-GCST90200273 | 2-methoxyhydroquinone sulfate (2) levels \|\| id:ebi-met1400-GCST90200273 | Mayonnaise liking \|\| id:ebi-fl187-GCST90094798 | Weighted mode | 9 | 0.153150805295819 | 0.297805077197157 | 0.620967631253676 | -0.430547146 | 0.736848756602246 | 1.16550072937263 | 0.650153268620568 | 2.08934110729033 | 0.999714303421225 |
| ebi-fl187-GCST90094798 | ebi-met1400-GCST90200276 | 2,4-di-tert-butylphenol levels \|\| id:ebi-met1400-GCST90200276 | Mayonnaise liking \|\| id:ebi-fl187-GCST90094798 | Weighted mode | 9 | 0.021867254961442 | 0.199782947687138 | 0.915537357264589 | -0.369707323 | 0.413441832428233 | 1.02210809568606 | 0.690936522617793 | 1.51201293471771 | 0.999714303421225 |
| ebi-fl187-GCST90094798 | ebi-met1400-GCST90200289 | N-acetylmethionine levels \|\| id:ebi-met1400-GCST90200289 | Mayonnaise liking \|\| id:ebi-fl187-GCST90094798 | Weighted mode | 9 | 0.111880229246936 | 0.199193622808759 | 0.589727862433065 | -0.278539271 | 0.502299729952104 | 1.11837890353965 | 0.756888543049439 | 1.65251724757689 | 0.999714303421225 |
| ebi-fl187-GCST90094798 | ebi-met1400-GCST90200308 | 3-hydroxyisobutyrate levels \|\| id:ebi-met1400-GCST90200308 | Mayonnaise liking \|\| id:ebi-fl187-GCST90094798 | Weighted mode | 9 | -0.196618973 | 0.210992466230854 | 0.378665053376809 | -0.610164206 | 0.216926261208155 | 0.821503589059597 | 0.543261654700215 | 1.2422524965621 | 0.999714303421225 |
| ebi-fl187-GCST90094798 | ebi-met1400-GCST90200383 | Cystathionine levels \|\| id:ebi-met1400-GCST90200383 | Mayonnaise liking \|\| id:ebi-fl187-GCST90094798 | Weighted mode | 9 | -0.101955083 | 0.257610945636497 | 0.702618751257066 | -0.606872536 | 0.402962370893131 | 0.903070114360616 | 0.545052839394045 | 1.49625058803098 | 0.999714303421225 |
| ebi-fl187-GCST90094798 | ebi-met1400-GCST90200384 | Guanidinoacetate levels \|\| id:ebi-met1400-GCST90200384 | Mayonnaise liking \|\| id:ebi-fl187-GCST90094798 | Weighted mode | 9 | 0.0577826080353274 | 0.208069208012722 | 0.788277830545027 | -0.35003304 | 0.465598255740263 | 1.05948464722424 | 0.704664807441674 | 1.59296690546985 | 0.999714303421225 |
| ebi-fl187-GCST90094798 | ebi-met1400-GCST90200403 | Citrulline levels \|\| id:ebi-met1400-GCST90200403 | Mayonnaise liking \|\| id:ebi-fl187-GCST90094798 | Weighted mode | 9 | 0.114994372934992 | 0.205338996494656 | 0.590796522395927 | -0.28747006 | 0.517458806064518 | 1.12186712473494 | 0.750159026050076 | 1.67775871762558 | 0.999714303421225 |
| ebi-fl187-GCST90094798 | ebi-met1400-GCST90200434 | 12,13-DiHOME levels \|\| id:ebi-met1400-GCST90200434 | Mayonnaise liking \|\| id:ebi-fl187-GCST90094798 | Weighted mode | 9 | -0.14973812 | 0.220294504793479 | 0.515865338614454 | -0.58151535 | 0.282039109133217 | 0.860933407921099 | 0.559050567285955 | 1.32583057105729 | 0.999714303421225 |
| ebi-fl187-GCST90094798 | ebi-met1400-GCST90200437 | Fructose levels \|\| id:ebi-met1400-GCST90200437 | Mayonnaise liking \|\| id:ebi-fl187-GCST90094798 | Weighted mode | 9 | -0.191786732 | 0.269332658462549 | 0.496652697161165 | -0.719678743 | 0.336105278442287 | 0.825482898687936 | 0.48690865378107 | 1.39948635279879 | 0.999714303421225 |
| ebi-fl187-GCST90094798 | ebi-met1400-GCST90200448 | N-stearoyl-sphinganine (d18:0/18:0) levels \|\| id:ebi-met1400-GCST90200448 | Mayonnaise liking \|\| id:ebi-fl187-GCST90094798 | Weighted mode | 9 | -0.012713225 | 0.208550042583627 | 0.95288642592626 | -0.421471309 | 0.396044858122555 | 0.987367246328798 | 0.656080811865859 | 1.48593597235433 | 0.999714303421225 |
| ebi-fl187-GCST90094798 | ebi-met1400-GCST90200468 | X-11847 levels \|\| id:ebi-met1400-GCST90200468 | Mayonnaise liking \|\| id:ebi-fl187-GCST90094798 | Weighted mode | 9 | -0.06317946 | 0.252096633466278 | 0.808427435711746 | -0.557288862 | 0.430929941159316 | 0.938774985604215 | 0.572759791600074 | 1.53868774750089 | 0.999714303421225 |
| ebi-fl187-GCST90094798 | ebi-met1400-GCST90200476 | X-11843 levels \|\| id:ebi-met1400-GCST90200476 | Mayonnaise liking \|\| id:ebi-fl187-GCST90094798 | Weighted mode | 9 | -0.121078911 | 0.316507772616871 | 0.71201922832562 | -0.741434145 | 0.499276323311682 | 0.885964044510258 | 0.476430155242368 | 1.64752856117108 | 0.999714303421225 |
| ebi-fl187-GCST90094798 | ebi-met1400-GCST90200511 | X-12906 levels \|\| id:ebi-met1400-GCST90200511 | Mayonnaise liking \|\| id:ebi-fl187-GCST90094798 | Weighted mode | 9 | -0.05341117 | 0.204628327765555 | 0.800674057829167 | -0.454482692 | 0.347660352606356 | 0.947990147404707 | 0.634776257714878 | 1.41575131182688 | 0.999714303421225 |
| ebi-fl187-GCST90094798 | ebi-met1400-GCST90200515 | X-12849 levels \|\| id:ebi-met1400-GCST90200515 | Mayonnaise liking \|\| id:ebi-fl187-GCST90094798 | Weighted mode | 9 | -0.060097226 | 0.247036597700434 | 0.813916057172893 | -0.544288957 | 0.424094505546826 | 0.94167297408759 | 0.580254222139913 | 1.52820601090456 | 0.999714303421225 |
| ebi-fl187-GCST90094798 | ebi-met1400-GCST90200551 | X-18886 levels \|\| id:ebi-met1400-GCST90200551 | Mayonnaise liking \|\| id:ebi-fl187-GCST90094798 | Weighted mode | 9 | 0.0959842794447254 | 0.242073360348304 | 0.702096108224881 | -0.378479507 | 0.570448065727402 | 1.10074175958629 | 0.68490200671907 | 1.7690595288241 | 0.999714303421225 |
| ebi-fl187-GCST90094798 | ebi-met1400-GCST90200564 | X-21319 levels \|\| id:ebi-met1400-GCST90200564 | Mayonnaise liking \|\| id:ebi-fl187-GCST90094798 | Weighted mode | 9 | 0.175051443253129 | 0.199682932343745 | 0.406231803353157 | -0.216327104 | 0.566429990646868 | 1.19130749976931 | 0.805471785620979 | 1.7619655763764 | 0.999714303421225 |
| ebi-fl187-GCST90094798 | ebi-met1400-GCST90200579 | X-21442 levels \|\| id:ebi-met1400-GCST90200579 | Mayonnaise liking \|\| id:ebi-fl187-GCST90094798 | Weighted mode | 9 | -0.025788034 | 0.228640316460138 | 0.912976996148996 | -0.473923054 | 0.422346986460826 | 0.974541637604027 | 0.622555153814458 | 1.52553777380982 | 0.999714303421225 |
| ebi-fl187-GCST90094798 | ebi-met1400-GCST90200581 | X-21441 levels \|\| id:ebi-met1400-GCST90200581 | Mayonnaise liking \|\| id:ebi-fl187-GCST90094798 | Weighted mode | 9 | 0.0110909184566079 | 0.226561671769558 | 0.96215669553978 | -0.432969958 | 0.455151795124941 | 1.01115265070407 | 0.648579976014623 | 1.57641265662942 | 0.999714303421225 |
| ebi-fl187-GCST90094798 | ebi-met1400-GCST90200648 | X-24585 levels \|\| id:ebi-met1400-GCST90200648 | Mayonnaise liking \|\| id:ebi-fl187-GCST90094798 | Weighted mode | 9 | 0.269339586630087 | 0.236907293501126 | 0.288485619535585 | -0.194998709 | 0.733677881892295 | 1.30909961830076 | 0.822835720638952 | 2.08272656090383 | 0.999714303421225 |
| ebi-fl187-GCST90094798 | ebi-met1400-GCST90200720 | Adenosine 5'-diphosphate (ADP) to pantothenate ratio \|\| id:ebi-met1400-GCST90200720 | Mayonnaise liking \|\| id:ebi-fl187-GCST90094798 | Weighted mode | 9 | -0.332479482 | 0.373150520029193 | 0.398929472555384 | -1.063854501 | 0.398895537238513 | 0.717143383047551 | 0.345122966341707 | 1.49017794237339 | 0.999714303421225 |
| ebi-fl187-GCST90094798 | ebi-met1400-GCST90200724 | Cholate to taurocholate ratio \|\| id:ebi-met1400-GCST90200724 | Mayonnaise liking \|\| id:ebi-fl187-GCST90094798 | Weighted mode | 9 | 0.0731702877913103 | 0.234359120733971 | 0.762860220585539 | -0.386173589 | 0.532514164429894 | 1.07591373628975 | 0.679652535339558 | 1.70320907779535 | 0.999714303421225 |
| ebi-fl187-GCST90094798 | ebi-met1400-GCST90200726 | N-acetylputrescine to (N(1) + N(8))-acetylspermidine ratio \|\| id:ebi-met1400-GCST90200726 | Mayonnaise liking \|\| id:ebi-fl187-GCST90094798 | Weighted mode | 9 | 0.27660650003078 | 0.220502112789755 | 0.245091577308074 | -0.155577641 | 0.7087906410987 | 1.31864738122649 | 0.855920619784432 | 2.03153292001941 | 0.999714303421225 |
| ebi-fl187-GCST90094798 | ebi-met1400-GCST90200744 | Arginine to glutamate ratio \|\| id:ebi-met1400-GCST90200744 | Mayonnaise liking \|\| id:ebi-fl187-GCST90094798 | Weighted mode | 9 | -0.364410597 | 0.176628054286346 | 0.0730016345864354 | -0.710601584 | -0.018219611 | 0.694605932865082 | 0.491348521272737 | 0.98194536277755 | 0.999714303421225 |
| ebi-fl187-GCST90094798 | ebi-met1400-GCST90200759 | Histidine to trans-urocanate ratio \|\| id:ebi-met1400-GCST90200759 | Mayonnaise liking \|\| id:ebi-fl187-GCST90094798 | Weighted mode | 9 | 0.0197700379744054 | 0.210177329674612 | 0.927371859225591 | -0.392177528 | 0.431717604136645 | 1.01996675943344 | 0.675584168080965 | 1.53990019230952 | 0.999714303421225 |
| ebi-fl187-GCST90094798 | ebi-met1400-GCST90200795 | Oleoyl-linoleoyl-glycerol (18:1 to 18:2) [2] to linoleoyl-arachidonoyl-glycerol (18:2 to 20:4) [2] ratio \|\| id:ebi-met1400-GCST90200795 | Mayonnaise liking \|\| id:ebi-fl187-GCST90094798 | Weighted mode | 9 | -0.112825615 | 0.25814415755133 | 0.673622405399645 | -0.618788164 | 0.393136933654187 | 0.893306425644285 | 0.538596733216497 | 1.48162125925224 | 0.999714303421225 |
| ebi-fl187-GCST90094798 | ebi-met1400-GCST90200811 | Choline to taurocholate ratio \|\| id:ebi-met1400-GCST90200811 | Mayonnaise liking \|\| id:ebi-fl187-GCST90094798 | Weighted mode | 9 | 0.0240737018642726 | 0.217487981363467 | 0.91458889216118 | -0.402202742 | 0.450350145336668 | 1.02436581277863 | 0.668845129206179 | 1.56886141883849 | 0.999714303421225 |
| ebi-fl187-GCST90094798 | ebi-met1400-GCST90200831 | Cholate to phosphate ratio \|\| id:ebi-met1400-GCST90200831 | Mayonnaise liking \|\| id:ebi-fl187-GCST90094798 | Weighted mode | 9 | -0.14253817 | 0.207059821829615 | 0.51067140943232 | -0.54837542 | 0.263299081196625 | 0.867154454731059 | 0.577887873059251 | 1.30121582994842 | 0.999714303421225 |
| ebi-fl187-GCST90094798 | ebi-met1400-GCST90200841 | Adenosine 5'-diphosphate (ADP) to oxalate (ethanedioate) ratio \|\| id:ebi-met1400-GCST90200841 | Mayonnaise liking \|\| id:ebi-fl187-GCST90094798 | Weighted mode | 9 | -0.448477448 | 0.386349096451372 | 0.279184118404629 | -1.205721677 | 0.308766781188472 | 0.638599713169833 | 0.299475796743467 | 1.36174474897524 | 0.999714303421225 |
| ebi-fl187-GCST90094798 | ebi-met1400-GCST90200844 | Aspartate to phosphate ratio \|\| id:ebi-met1400-GCST90200844 | Mayonnaise liking \|\| id:ebi-fl187-GCST90094798 | Weighted mode | 9 | 0.409224641252674 | 0.209169121406803 | 0.08613212576297 | -0.000746837 | 0.819196119210008 | 1.50564991358075 | 0.999253442108458 | 2.2686753597589 | 0.999714303421225 |
| ebi-fl187-GCST90094798 | ebi-met1400-GCST90200870 | Adenosine 5'-monophosphate (AMP) to acetoacetate ratio \|\| id:ebi-met1400-GCST90200870 | Mayonnaise liking \|\| id:ebi-fl187-GCST90094798 | Weighted mode | 9 | 0.0128213817432734 | 0.238571945572874 | 0.958458381348405 | -0.454779632 | 0.480422395066106 | 1.0129039280669 | 0.634587795651016 | 1.61675716823519 | 0.999714303421225 |
| ebi-fl187-GCST90094798 | ebi-met1400-GCST90200887 | Cholesterol to cortisol ratio \|\| id:ebi-met1400-GCST90200887 | Mayonnaise liking \|\| id:ebi-fl187-GCST90094798 | Weighted mode | 9 | 0.215295698108045 | 0.195032109767069 | 0.301731314711471 | -0.166967237 | 0.5975586332515 | 1.24022857599371 | 0.846227335799256 | 1.81767576588458 | 0.999714303421225 |
| ebi-fl187-GCST90094798 | ebi-met1400-GCST90200890 | Cortisol to taurocholate ratio \|\| id:ebi-met1400-GCST90200890 | Mayonnaise liking \|\| id:ebi-fl187-GCST90094798 | Weighted mode | 9 | -0.129605196 | 0.215843394715884 | 0.564818643972016 | -0.55265825 | 0.293447857452773 | 0.878442174785313 | 0.575418170286042 | 1.34104325217599 | 0.999714303421225 |
| ebi-fl187-GCST90094798 | ebi-met1400-GCST90200948 | Adenosine 5'-diphosphate (ADP) to mannitol to sorbitol ratio \|\| id:ebi-met1400-GCST90200948 | Mayonnaise liking \|\| id:ebi-fl187-GCST90094798 | Weighted mode | 9 | -0.432978677 | 0.287867974310461 | 0.170969674718738 | -0.997199907 | 0.131242552279534 | 0.64857432096848 | 0.368910981343296 | 1.14024431663172 | 0.999714303421225 |
| ebi-fl187-GCST90094798 | ebi-met1400-GCST90200972 | Leucine to N-palmitoyl-sphingosine (d18:1 to 16:0) ratio \|\| id:ebi-met1400-GCST90200972 | Mayonnaise liking \|\| id:ebi-fl187-GCST90094798 | Weighted mode | 9 | 0.142444330421398 | 0.195035022511497 | 0.486009728420578 | -0.239824314 | 0.524712974543932 | 1.15308888719813 | 0.786766072944659 | 1.68997371328356 | 0.999714303421225 |
| ebi-fl187-GCST90094798 | ebi-met1400-GCST90200973 | Proline to glutamate ratio \|\| id:ebi-met1400-GCST90200973 | Mayonnaise liking \|\| id:ebi-fl187-GCST90094798 | Weighted mode | 9 | -0.293750854 | 0.193672793533266 | 0.167806070780193 | -0.673349529 | 0.0858478213762586 | 0.745462197271053 | 0.50999746224102 | 1.08964049569635 | 0.999714303421225 |
| ebi-fl187-GCST90094798 | ebi-met1400-GCST90200983 | Cholesterol to linoleoyl-arachidonoyl-glycerol (18:2 to 20:4) [1] ratio \|\| id:ebi-met1400-GCST90200983 | Mayonnaise liking \|\| id:ebi-fl187-GCST90094798 | Weighted mode | 9 | 0.00225202026374438 | 0.281050094201144 | 0.993802930044426 | -0.548606164 | 0.553110204897987 | 1.00225455796601 | 0.577754544285739 | 1.73865218179372 | 0.999714303421225 |
| ebi-fl187-GCST90094798 | ebi-met1400-GCST90201001 | Serine to alpha-ketobutyrate ratio \|\| id:ebi-met1400-GCST90201001 | Mayonnaise liking \|\| id:ebi-fl187-GCST90094798 | Weighted mode | 9 | -0.117002348 | 0.239820910167188 | 0.638728541548657 | -0.587051332 | 0.353046635487176 | 0.889583104027957 | 0.555964223865091 | 1.42339752272269 | 0.999714303421225 |
| ebi-fl187-GCST90094798 | ebi-met1400-GCST90201005 | Fructose to maltose ratio \|\| id:ebi-met1400-GCST90201005 | Mayonnaise liking \|\| id:ebi-fl187-GCST90094798 | Weighted mode | 9 | -0.284324283 | 0.263652393285678 | 0.312291744435449 | -0.801082974 | 0.232434407928004 | 0.75252257492262 | 0.448842616041769 | 1.26166768824704 | 0.999714303421225 |
| ebi-fl187-GCST90094798 | ebi-met1400-GCST90201020 | Paraxanthine to 5-acetylamino-6-formylamino-3-methyluracil ratio \|\| id:ebi-met1400-GCST90201020 | Mayonnaise liking \|\| id:ebi-fl187-GCST90094798 | Weighted mode | 9 | -0.038591259 | 0.213766487519272 | 0.861225974099717 | -0.457573574 | 0.380391056780843 | 0.962143896677253 | 0.632817268230078 | 1.46285653756325 | 0.999714303421225 |
| ebi-fl187-GCST90094798 | ebi-pw11-GCST90275197 | Placental weight (fetal genotype, adjusted for sex) \|\| id:ebi-pw11-GCST90275197 | Mayonnaise liking \|\| id:ebi-fl187-GCST90094798 | Weighted mode | 9 | 0.122519636880283 | 0.0660145186612689 | 0.100554058747723 | -0.00686882 | 0.25190809345637 | 1.13034131623318 | 0.993154716726224 | 1.28647779612366 | 0.999714303421225 |
| ebi-fl187-GCST90094798 | prot-a-1017 | FAS-associated factor 2 \|\| id:prot-a-1017 | Mayonnaise liking \|\| id:ebi-fl187-GCST90094798 | Weighted mode | 12 | 0.0731757442742129 | 0.272112252477935 | 0.792970156559252 | -0.460164271 | 0.606515759130966 | 1.07591960701067 | 0.631179952691803 | 1.83403005088033 | 0.999714303421225 |
| ebi-fl187-GCST90094798 | prot-a-1019 | Fas apoptotic inhibitory molecule 3 \|\| id:prot-a-1019 | Mayonnaise liking \|\| id:ebi-fl187-GCST90094798 | Weighted mode | 12 | -0.291613733 | 0.327169391759377 | 0.39183520216611 | -0.932865741 | 0.349638274470866 | 0.747057043450345 | 0.393424640122246 | 1.41855432846137 | 0.999714303421225 |
| ebi-fl187-GCST90094798 | prot-a-1052 | MIP18 family protein FAM96A \|\| id:prot-a-1052 | Mayonnaise liking \|\| id:ebi-fl187-GCST90094798 | Weighted mode | 12 | 0.0508553138625869 | 0.242938124375417 | 0.838013527505473 | -0.42530341 | 0.527014037638405 | 1.05217064776264 | 0.653571454985256 | 1.69386692697315 | 0.999714303421225 |
| ebi-fl187-GCST90094798 | prot-a-1066 | F-box/LRR-repeat protein 4 \|\| id:prot-a-1066 | Mayonnaise liking \|\| id:ebi-fl187-GCST90094798 | Weighted mode | 12 | -0.063650964 | 0.276641303514934 | 0.822248622948136 | -0.605867919 | 0.478565991058517 | 0.938332454345963 | 0.545600684037078 | 1.61375859788893 | 0.999714303421225 |
| ebi-fl187-GCST90094798 | prot-a-1076 | Low affinity immunoglobulin gamma Fc region receptor III-B \|\| id:prot-a-1076 | Mayonnaise liking \|\| id:ebi-fl187-GCST90094798 | Weighted mode | 12 | 0.4917852521178 | 0.325181742255136 | 0.158633331143374 | -0.145570963 | 1.12914146693787 | 1.63523291869061 | 0.864528538615859 | 3.09299991721565 | 0.999714303421225 |
| ebi-fl187-GCST90094798 | prot-a-1081 | Fc receptor-like protein 3 \|\| id:prot-a-1081 | Mayonnaise liking \|\| id:ebi-fl187-GCST90094798 | Weighted mode | 12 | -0.230567333 | 0.277952828601987 | 0.424446273332837 | -0.775354877 | 0.314220211102181 | 0.794082965247393 | 0.460540316821719 | 1.3691912144582 | 0.999714303421225 |
| ebi-fl187-GCST90094798 | prot-a-1105 | Fibroblast growth factor receptor 4 \|\| id:prot-a-1105 | Mayonnaise liking \|\| id:ebi-fl187-GCST90094798 | Weighted mode | 12 | -0.273313964 | 0.260662420344453 | 0.316883721234876 | -0.784212308 | 0.237584380181258 | 0.760853869633703 | 0.456479125361397 | 1.26818200170328 | 0.999714303421225 |
| ebi-fl187-GCST90094798 | prot-a-1143 | Follistatin-related protein 1 \|\| id:prot-a-1143 | Mayonnaise liking \|\| id:ebi-fl187-GCST90094798 | Weighted mode | 12 | -0.056375128 | 0.259523697452024 | 0.832009306434177 | -0.565041575 | 0.452291319016822 | 0.945184504215984 | 0.568336517678409 | 1.57190980910279 | 0.999714303421225 |
| ebi-fl187-GCST90094798 | prot-a-1151 | Alpha-(1,3)-fucosyltransferase 10 \|\| id:prot-a-1151 | Mayonnaise liking \|\| id:ebi-fl187-GCST90094798 | Weighted mode | 12 | 0.173639232465604 | 0.313884801559497 | 0.591196012773575 | -0.441574979 | 0.788853443522217 | 1.18962630984355 | 0.643022875873621 | 2.20087155554032 | 0.999714303421225 |
| ebi-fl187-GCST90094798 | prot-a-1165 | Growth arrest and DNA damage-inducible proteins-interacting protein 1 \|\| id:prot-a-1165 | Mayonnaise liking \|\| id:ebi-fl187-GCST90094798 | Weighted mode | 12 | 0.270430456128709 | 0.293873602334321 | 0.377193446714887 | -0.305561804 | 0.846422716703977 | 1.31052845434015 | 0.736709371507451 | 2.33129222466775 | 0.999714303421225 |
| ebi-fl187-GCST90094798 | prot-a-1194 | Growth/differentiation factor 11/8 \|\| id:prot-a-1194 | Mayonnaise liking \|\| id:ebi-fl187-GCST90094798 | Weighted mode | 12 | 0.080560014707642 | 0.278682040377233 | 0.777899262719192 | -0.465656784 | 0.626776813847019 | 1.0838938942655 | 0.627722691279869 | 1.87156843355571 | 0.999714303421225 |
| ebi-fl187-GCST90094798 | prot-a-1216 | D-glucuronyl C5-epimerase \|\| id:prot-a-1216 | Mayonnaise liking \|\| id:ebi-fl187-GCST90094798 | Weighted mode | 12 | 0.717210205223321 | 0.466845064870769 | 0.152716655856826 | -0.197806122 | 1.63222653237003 | 2.04870975095489 | 0.820528920286388 | 5.11525132129736 | 0.999714303421225 |
| ebi-fl187-GCST90094798 | prot-a-1246 | Glypican-3 \|\| id:prot-a-1246 | Mayonnaise liking \|\| id:ebi-fl187-GCST90094798 | Weighted mode | 12 | -0.197165316 | 0.27733925287162 | 0.491934658627564 | -0.740750252 | 0.346419619711742 | 0.821054888651068 | 0.476756094313512 | 1.41399583186977 | 0.999714303421225 |
| ebi-fl187-GCST90094798 | prot-a-1275 | Glutamate receptor 4 \|\| id:prot-a-1275 | Mayonnaise liking \|\| id:ebi-fl187-GCST90094798 | Weighted mode | 12 | -0.380262996 | 0.281872298672494 | 0.204432027454185 | -0.932732702 | 0.17220670916598 | 0.683681579886703 | 0.393476984659175 | 1.18792336248396 | 0.999714303421225 |
| ebi-fl187-GCST90094798 | prot-a-1289 | Glutathione S-transferase P \|\| id:prot-a-1289 | Mayonnaise liking \|\| id:ebi-fl187-GCST90094798 | Weighted mode | 12 | 0.0696976186147676 | 0.291761630546587 | 0.815586617817335 | -0.502155177 | 0.641550414486078 | 1.07218392377188 | 0.60522488622151 | 1.89942349127746 | 0.999714303421225 |
| ebi-fl187-GCST90094798 | prot-a-130 | Apolipoprotein D \|\| id:prot-a-130 | Mayonnaise liking \|\| id:ebi-fl187-GCST90094798 | Weighted mode | 12 | 0.153543199849683 | 0.3760488107845 | 0.690884024128255 | -0.583512469 | 0.890598868987303 | 1.1659581552512 | 0.55793519056456 | 2.43658841167767 | 0.999714303421225 |
| ebi-fl187-GCST90094798 | prot-a-1313 | Heparin-binding EGF-like growth factor \|\| id:prot-a-1313 | Mayonnaise liking \|\| id:ebi-fl187-GCST90094798 | Weighted mode | 12 | 0.0360464860584664 | 0.274790900393002 | 0.898003009473667 | -0.502543679 | 0.574636650828751 | 1.03670403765531 | 0.604989801140849 | 1.77648492530639 | 0.999714303421225 |
| ebi-fl187-GCST90094798 | prot-a-1347 | HLA class II histocompatibility antigen, DQ alpha 2 chain \|\| id:prot-a-1347 | Mayonnaise liking \|\| id:ebi-fl187-GCST90094798 | Weighted mode | 12 | -0.299099515 | 0.430219138879595 | 0.501336314002201 | -1.142329027 | 0.544129997360819 | 0.741485616938452 | 0.319075021399145 | 1.72310862102498 | 0.999714303421225 |
| ebi-fl187-GCST90094798 | prot-a-1357 | Non-histone chromosomal protein HMG-14 \|\| id:prot-a-1357 | Mayonnaise liking \|\| id:ebi-fl187-GCST90094798 | Weighted mode | 12 | 0.111508558841387 | 0.277960229224785 | 0.695979870076811 | -0.43329349 | 0.656310608121965 | 1.11796331243525 | 0.648370173431072 | 1.92766727892067 | 0.999714303421225 |
| ebi-fl187-GCST90094798 | prot-a-137 | Apolipoprotein M \|\| id:prot-a-137 | Mayonnaise liking \|\| id:ebi-fl187-GCST90094798 | Weighted mode | 12 | -0.197611177 | 0.323045085346589 | 0.553159795231422 | -0.830779544 | 0.435557190210438 | 0.820688893769872 | 0.435709499021693 | 1.54582413711316 | 0.999714303421225 |
| ebi-fl187-GCST90094798 | prot-a-1381 | 17-beta-hydroxysteroid dehydrogenase 14 \|\| id:prot-a-1381 | Mayonnaise liking \|\| id:ebi-fl187-GCST90094798 | Weighted mode | 12 | 0.212482099368342 | 0.281479899181191 | 0.466179132081296 | -0.339218503 | 0.764182701763476 | 1.23674397486879 | 0.712326786524519 | 2.14723872288579 | 0.999714303421225 |
| ebi-fl187-GCST90094798 | prot-a-1392 | Serine protease HTRA2, mitochondrial \|\| id:prot-a-1392 | Mayonnaise liking \|\| id:ebi-fl187-GCST90094798 | Weighted mode | 12 | -0.307048093 | 0.254065200883721 | 0.252177787365048 | -0.805015887 | 0.190919700780329 | 0.73561522214913 | 0.44708082387123 | 1.21036225703425 | 0.999714303421225 |
| ebi-fl187-GCST90094798 | prot-a-1416 | Interferon-induced protein with tetratricopeptide repeats 2 \|\| id:prot-a-1416 | Mayonnaise liking \|\| id:ebi-fl187-GCST90094798 | Weighted mode | 12 | 0.00689755969672201 | 0.32439398065002 | 0.983416729728369 | -0.628914642 | 0.642709761770761 | 1.00692140264948 | 0.533170167388303 | 1.90162685973236 | 0.999714303421225 |
| ebi-fl187-GCST90094798 | prot-a-1428 | Interferon gamma \|\| id:prot-a-1428 | Mayonnaise liking \|\| id:ebi-fl187-GCST90094798 | Weighted mode | 12 | 0.0938291121125809 | 0.261050173883936 | 0.726078650801603 | -0.417829229 | 0.605487452925095 | 1.09837203140252 | 0.658474667398892 | 1.83214507572918 | 0.999714303421225 |
| ebi-fl187-GCST90094798 | prot-a-1483 | Interleukin-17C \|\| id:prot-a-1483 | Mayonnaise liking \|\| id:ebi-fl187-GCST90094798 | Weighted mode | 12 | -0.500277763 | 0.345557424884071 | 0.175583744745533 | -1.177570316 | 0.177014789570713 | 0.606362211209921 | 0.308026236567785 | 1.19364874654914 | 0.999714303421225 |
| ebi-fl187-GCST90094798 | prot-a-1503 | Interleukin-1 receptor-like 2 \|\| id:prot-a-1503 | Mayonnaise liking \|\| id:ebi-fl187-GCST90094798 | Weighted mode | 12 | -0.051204432 | 0.308487628062144 | 0.871179948847198 | -0.655840183 | 0.553431318799434 | 0.950084422826867 | 0.519005819378295 | 1.73921057682848 | 0.999714303421225 |
| ebi-fl187-GCST90094798 | prot-a-1522 | Interleukin-31 receptor subunit alpha \|\| id:prot-a-1522 | Mayonnaise liking \|\| id:ebi-fl187-GCST90094798 | Weighted mode | 12 | -0.099046273 | 0.289725008392331 | 0.738895928467899 | -0.66690729 | 0.468814743265114 | 0.905700797394371 | 0.513293593944617 | 1.59809891274292 | 0.999714303421225 |
| ebi-fl187-GCST90094798 | prot-a-1540 | Interleukin-6 receptor subunit alpha \|\| id:prot-a-1540 | Mayonnaise liking \|\| id:ebi-fl187-GCST90094798 | Weighted mode | 12 | -0.292845143 | 0.280138280225102 | 0.3182851360482 | -0.841916173 | 0.256225885829642 | 0.74613767608631 | 0.430884083583764 | 1.29204454953429 | 0.999714303421225 |
| ebi-fl187-GCST90094798 | prot-a-1541 | Interleukin-6 receptor subunit alpha \|\| id:prot-a-1541 | Mayonnaise liking \|\| id:ebi-fl187-GCST90094798 | Weighted mode | 12 | -0.409026741 | 0.285778214688395 | 0.180144079673319 | -0.969152041 | 0.151098560282057 | 0.664296468460215 | 0.379404621190246 | 1.16311128900941 | 0.999714303421225 |
| ebi-fl187-GCST90094798 | prot-a-1545 | Interleukin-9 \|\| id:prot-a-1545 | Mayonnaise liking \|\| id:ebi-fl187-GCST90094798 | Weighted mode | 12 | -0.183110286 | 0.327294608812578 | 0.587054193440185 | -0.824607719 | 0.458387147050699 | 0.832676317947747 | 0.438406937232361 | 1.58152116581023 | 0.999714303421225 |
| ebi-fl187-GCST90094798 | prot-a-1548 | Inositol monophosphatase 3 \|\| id:prot-a-1548 | Mayonnaise liking \|\| id:ebi-fl187-GCST90094798 | Weighted mode | 12 | -0.016218874 | 0.306359388267352 | 0.958728386975819 | -0.616683275 | 0.584245527449068 | 0.98391194418113 | 0.539731613744673 | 1.79363722496392 | 0.999714303421225 |
| ebi-fl187-GCST90094798 | prot-a-1555 | Inhibin beta A chain:Inhibin beta B chain heterodimer \|\| id:prot-a-1555 | Mayonnaise liking \|\| id:ebi-fl187-GCST90094798 | Weighted mode | 12 | -0.036545017 | 0.289891762691842 | 0.901955647291825 | -0.604732871 | 0.531642838349906 | 0.964114691825233 | 0.546220318220677 | 1.7017256736644 | 0.999714303421225 |
| ebi-fl187-GCST90094798 | prot-a-1603 | Junctophilin-3 \|\| id:prot-a-1603 | Mayonnaise liking \|\| id:ebi-fl187-GCST90094798 | Weighted mode | 12 | 0.290127582604384 | 0.293359025637488 | 0.34392618952335 | -0.284856108 | 0.865111272853861 | 1.33659800380216 | 0.752122471206965 | 2.37527037438603 | 0.999714303421225 |
| ebi-fl187-GCST90094798 | prot-a-1627 | UPF0258 protein KIAA1024 \|\| id:prot-a-1627 | Mayonnaise liking \|\| id:ebi-fl187-GCST90094798 | Weighted mode | 12 | -0.07581829 | 0.254777914315189 | 0.771564235782929 | -0.575183002 | 0.423546422411555 | 0.926984633962207 | 0.562601902278935 | 1.52736865645366 | 0.999714303421225 |
| ebi-fl187-GCST90094798 | prot-a-1666 | Kallikrein-7 \|\| id:prot-a-1666 | Mayonnaise liking \|\| id:ebi-fl187-GCST90094798 | Weighted mode | 12 | -0.018869086 | 0.314742019933844 | 0.953270014814188 | -0.635763446 | 0.598025272638808 | 0.981307820342701 | 0.529531065849636 | 1.81852416292264 | 0.999714303421225 |
| ebi-fl187-GCST90094798 | prot-a-1678 | Importin subunit alpha-3 \|\| id:prot-a-1678 | Mayonnaise liking \|\| id:ebi-fl187-GCST90094798 | Weighted mode | 12 | 0.322740101395841 | 0.286349210427137 | 0.283692280250754 | -0.238504351 | 0.88398455383303 | 1.38090640861528 | 0.787805260476421 | 2.42052523005692 | 0.999714303421225 |
| ebi-fl187-GCST90094798 | prot-a-1718 | Protein LDOC1 \|\| id:prot-a-1718 | Mayonnaise liking \|\| id:ebi-fl187-GCST90094798 | Weighted mode | 12 | -0.013007011 | 0.374329886766678 | 0.972903645750094 | -0.746693589 | 0.720679567438403 | 0.987077214968536 | 0.473930975742613 | 2.05582981104655 | 0.999714303421225 |
| ebi-fl187-GCST90094798 | prot-a-1723 | Leptin \|\| id:prot-a-1723 | Mayonnaise liking \|\| id:ebi-fl187-GCST90094798 | Weighted mode | 12 | -0.176626297 | 0.361689852021258 | 0.634900666682481 | -0.885538407 | 0.532285812852758 | 0.838092923757443 | 0.412492024858537 | 1.70282019171931 | 0.999714303421225 |
| ebi-fl187-GCST90094798 | prot-a-1730 | Galectin-7 \|\| id:prot-a-1730 | Mayonnaise liking \|\| id:ebi-fl187-GCST90094798 | Weighted mode | 12 | 0.240755339012397 | 0.298172399948939 | 0.436526923731404 | -0.343662565 | 0.825173242912318 | 1.2722097371378 | 0.709168185926819 | 2.2822761192438 | 0.999714303421225 |
| ebi-fl187-GCST90094798 | prot-a-1753 | Lipase member N \|\| id:prot-a-1753 | Mayonnaise liking \|\| id:ebi-fl187-GCST90094798 | Weighted mode | 12 | 0.101012324445165 | 0.301360020079334 | 0.743788265254056 | -0.489653315 | 0.691677963800659 | 1.10629027609325 | 0.612838819441025 | 1.99706372402255 | 0.999714303421225 |
| ebi-fl187-GCST90094798 | prot-a-1756 | VIP36-like protein \|\| id:prot-a-1756 | Mayonnaise liking \|\| id:ebi-fl187-GCST90094798 | Weighted mode | 12 | 0.284273530841668 | 0.418799077266388 | 0.511304092223511 | -0.536572661 | 1.10511972228379 | 1.32879634783107 | 0.584748955004381 | 3.01958595889402 | 0.999714303421225 |
| ebi-fl187-GCST90094798 | prot-a-1760 | Ig Kappa chain V-I region HK102- like \|\| id:prot-a-1760 | Mayonnaise liking \|\| id:ebi-fl187-GCST90094798 | Weighted mode | 12 | -0.056181953 | 0.284673922860586 | 0.847148836498087 | -0.614142842 | 0.50177893569088 | 0.945367107749449 | 0.541104508686285 | 1.65165685014225 | 0.999714303421225 |
| ebi-fl187-GCST90094798 | prot-a-179 | Neutral ceramidase \|\| id:prot-a-179 | Mayonnaise liking \|\| id:ebi-fl187-GCST90094798 | Weighted mode | 12 | 0.799819021295372 | 0.456013063477377 | 0.107220890304271 | -0.093966583 | 1.69360462571103 | 2.22513818942283 | 0.910313181558594 | 5.43905115550522 | 0.999714303421225 |
| ebi-fl187-GCST90094798 | prot-a-1809 | Lactotransferrin \|\| id:prot-a-1809 | Mayonnaise liking \|\| id:ebi-fl187-GCST90094798 | Weighted mode | 12 | -0.464045918 | 0.299733773278262 | 0.149848435445512 | -1.051524113 | 0.123432277951767 | 0.628734683770259 | 0.349404810566432 | 1.13137338302481 | 0.999714303421225 |
| ebi-fl187-GCST90094798 | prot-a-1818 | Lysophospholipase-like protein 1 \|\| id:prot-a-1818 | Mayonnaise liking \|\| id:ebi-fl187-GCST90094798 | Weighted mode | 12 | -0.249660655 | 0.280314489473776 | 0.392183609439567 | -0.799077055 | 0.299755744127833 | 0.779065109882049 | 0.449743861647961 | 1.3495291368993 | 0.999714303421225 |
| ebi-fl187-GCST90094798 | prot-a-1852 | MAP kinase-activated protein kinase 3 \|\| id:prot-a-1852 | Mayonnaise liking \|\| id:ebi-fl187-GCST90094798 | Weighted mode | 12 | 0.195494753892302 | 0.319947690341603 | 0.553602084319819 | -0.431602719 | 0.822592226961843 | 1.21591241509796 | 0.64946734636027 | 2.27639312349544 | 0.999714303421225 |
| ebi-fl187-GCST90094798 | prot-a-1856 | Megakaryocyte-associated tyrosine-protein kinase \|\| id:prot-a-1856 | Mayonnaise liking \|\| id:ebi-fl187-GCST90094798 | Weighted mode | 12 | 0.149569299602551 | 0.319804062381159 | 0.649135016421827 | -0.477246663 | 0.776385261869623 | 1.16133394800447 | 0.620489458832607 | 2.1736010492831 | 0.999714303421225 |
| ebi-fl187-GCST90094798 | prot-a-1890 | Beta-1,3-N-acetylglucosaminyltransferase manic fringe \|\| id:prot-a-1890 | Mayonnaise liking \|\| id:ebi-fl187-GCST90094798 | Weighted mode | 12 | 0.14163059112826 | 0.28940460355147 | 0.634180936700173 | -0.425602432 | 0.70886361408914 | 1.15215095512998 | 0.65337605201067 | 2.03168117246091 | 0.999714303421225 |
| ebi-fl187-GCST90094798 | prot-a-1919 | Matrilysin \|\| id:prot-a-1919 | Mayonnaise liking \|\| id:ebi-fl187-GCST90094798 | Weighted mode | 12 | 0.141056808821264 | 0.317975319850327 | 0.665924147835521 | -0.482174818 | 0.764288435727906 | 1.15149006091977 | 0.617439112805703 | 2.14746577095167 | 0.999714303421225 |
| ebi-fl187-GCST90094798 | prot-a-1923 | Multimerin-2 \|\| id:prot-a-1923 | Mayonnaise liking \|\| id:ebi-fl187-GCST90094798 | Weighted mode | 12 | -0.619139534 | 0.46700530040966 | 0.21179021636833 | -1.534469922 | 0.296190855158578 | 0.538407519889225 | 0.215569929670192 | 1.34472678038522 | 0.999714303421225 |
| ebi-fl187-GCST90094798 | prot-a-1948 | Mesothelin \|\| id:prot-a-1948 | Mayonnaise liking \|\| id:ebi-fl187-GCST90094798 | Weighted mode | 12 | 0.207848626167332 | 0.310342511742897 | 0.516835968397285 | -0.400422697 | 0.816119949183411 | 1.23102681019788 | 0.670036763740013 | 2.26170725165461 | 0.999714303421225 |
| ebi-fl187-GCST90094798 | prot-a-195 | ATPase family AAA domain-containing protein 1 \|\| id:prot-a-195 | Mayonnaise liking \|\| id:ebi-fl187-GCST90094798 | Weighted mode | 12 | -0.114881121 | 0.314932327498307 | 0.722191475075005 | -0.732148483 | 0.502386240775388 | 0.891472114813263 | 0.480874728253625 | 1.65266021438847 | 0.999714303421225 |
| ebi-fl187-GCST90094798 | prot-a-1966 | Mucin-1 \|\| id:prot-a-1966 | Mayonnaise liking \|\| id:ebi-fl187-GCST90094798 | Weighted mode | 12 | 0.39825019110856 | 0.302521925974457 | 0.214798273711293 | -0.194692784 | 0.991193166018496 | 1.48921657204702 | 0.823087485026045 | 2.69444747831306 | 0.999714303421225 |
| ebi-fl187-GCST90094798 | prot-a-1986 | Myocardial zonula adherens protein \|\| id:prot-a-1986 | Mayonnaise liking \|\| id:ebi-fl187-GCST90094798 | Weighted mode | 12 | 0.122808501080739 | 0.274494855324417 | 0.663269554185583 | -0.415201415 | 0.660818417516596 | 1.13066787853755 | 0.660207291426337 | 1.93637645048524 | 0.999714303421225 |
| ebi-fl187-GCST90094798 | prot-a-2071 | Epididymal secretory protein E1 \|\| id:prot-a-2071 | Mayonnaise liking \|\| id:ebi-fl187-GCST90094798 | Weighted mode | 12 | -0.219949242 | 0.314977714198266 | 0.499485383712667 | -0.837305562 | 0.397407077876076 | 0.80255953328354 | 0.432875309279959 | 1.48796152299766 | 0.999714303421225 |
| ebi-fl187-GCST90094798 | prot-a-2098 | Neuritin \|\| id:prot-a-2098 | Mayonnaise liking \|\| id:ebi-fl187-GCST90094798 | Weighted mode | 12 | 0.0770713332654571 | 0.319156308865929 | 0.813621998897371 | -0.548475032 | 0.702617698642678 | 1.08011912206867 | 0.577830311511781 | 2.01903101068903 | 0.999714303421225 |
| ebi-fl187-GCST90094798 | prot-a-2115 | Neurotrophin-4 \|\| id:prot-a-2115 | Mayonnaise liking \|\| id:ebi-fl187-GCST90094798 | Weighted mode | 12 | -0.011856684 | 0.277536042895396 | 0.966689360433103 | -0.555827328 | 0.532113959795494 | 0.988213329219066 | 0.573597511351324 | 1.70252758200705 | 0.999714303421225 |
| ebi-fl187-GCST90094798 | prot-a-2118 | Netrin-1 \|\| id:prot-a-2118 | Mayonnaise liking \|\| id:ebi-fl187-GCST90094798 | Weighted mode | 12 | 0.0378960902922658 | 0.394379402324477 | 0.925177027161426 | -0.735087538 | 0.810879718848242 | 1.03862330422719 | 0.479463485736735 | 2.24988638379049 | 0.999714303421225 |
| ebi-fl187-GCST90094798 | prot-a-2120 | Netrin-G1 \|\| id:prot-a-2120 | Mayonnaise liking \|\| id:ebi-fl187-GCST90094798 | Weighted mode | 12 | 0.0996258098501872 | 0.290558671694233 | 0.738153985982249 | -0.469869187 | 0.669120806370884 | 1.10475745136665 | 0.625084032258146 | 1.95251992270714 | 0.999714303421225 |
| ebi-fl187-GCST90094798 | prot-a-2145 | Olfactomedin-like protein 3 \|\| id:prot-a-2145 | Mayonnaise liking \|\| id:ebi-fl187-GCST90094798 | Weighted mode | 12 | -0.173883204 | 0.34130121326707 | 0.6204869756371 | -0.842833582 | 0.495067174454259 | 0.840395047085338 | 0.430488967959667 | 1.64060844233235 | 0.999714303421225 |
| ebi-fl187-GCST90094798 | prot-a-2162 | Prolyl 4-hydroxylase subunit alpha-2 \|\| id:prot-a-2162 | Mayonnaise liking \|\| id:ebi-fl187-GCST90094798 | Weighted mode | 12 | 0.0243635866700784 | 0.366084708025876 | 0.948132746736567 | -0.693162441 | 0.741889614400794 | 1.02466280390787 | 0.499992369807874 | 2.09989976870203 | 0.999714303421225 |
| ebi-fl187-GCST90094798 | prot-a-2168 | Polyadenylate-binding protein-interacting protein 1 \|\| id:prot-a-2168 | Mayonnaise liking \|\| id:ebi-fl187-GCST90094798 | Weighted mode | 12 | 0.0344694684385722 | 0.325432251142061 | 0.917553482584329 | -0.603377744 | 0.672316680677012 | 1.03507042557739 | 0.546961018208956 | 1.95876991273201 | 0.999714303421225 |
| ebi-fl187-GCST90094798 | prot-a-219 | Beta-1,4-galactosyltransferase 5 \|\| id:prot-a-219 | Mayonnaise liking \|\| id:ebi-fl187-GCST90094798 | Weighted mode | 12 | 0.12972051747126 | 0.269978643109099 | 0.640296505466696 | -0.399437623 | 0.658878657965094 | 1.13851014516132 | 0.670697124617255 | 1.93262398638575 | 0.999714303421225 |
| ebi-fl187-GCST90094798 | prot-a-2190 | Pyruvate carboxylase, mitochondrial \|\| id:prot-a-2190 | Mayonnaise liking \|\| id:ebi-fl187-GCST90094798 | Weighted mode | 12 | 0.445117672321825 | 0.374265751952223 | 0.259356928513704 | -0.288443202 | 1.17867854614818 | 1.5606738331415 | 0.749429370399369 | 3.25007653777246 | 0.999714303421225 |
| ebi-fl187-GCST90094798 | prot-a-2197 | Protocadherin-9 \|\| id:prot-a-2197 | Mayonnaise liking \|\| id:ebi-fl187-GCST90094798 | Weighted mode | 12 | -0.195174531 | 0.273676545150866 | 0.490601261215182 | -0.73158056 | 0.341231497249327 | 0.822691060229109 | 0.481147905769187 | 1.40667884545584 | 0.999714303421225 |
| ebi-fl187-GCST90094798 | prot-a-2198 | Protocadherin alpha-4 \|\| id:prot-a-2198 | Mayonnaise liking \|\| id:ebi-fl187-GCST90094798 | Weighted mode | 12 | -0.197265514 | 0.410835351569469 | 0.640521872295379 | -1.002502803 | 0.607971775341277 | 0.820972624863956 | 0.36695986271375 | 1.83670237336493 | 0.999714303421225 |
| ebi-fl187-GCST90094798 | prot-a-2254 | Placenta growth factor \|\| id:prot-a-2254 | Mayonnaise liking \|\| id:ebi-fl187-GCST90094798 | Weighted mode | 12 | -0.144683138 | 0.295840217430485 | 0.634405582873615 | -0.724529964 | 0.435163688075659 | 0.865296429157722 | 0.484552272326652 | 1.54521597168026 | 0.999714303421225 |
| ebi-fl187-GCST90094798 | prot-a-2261 | Membrane-associated progesterone receptor component 2 \|\| id:prot-a-2261 | Mayonnaise liking \|\| id:ebi-fl187-GCST90094798 | Weighted mode | 12 | 0.261359552189749 | 0.309521317791445 | 0.416433154182348 | -0.345302231 | 0.868021335060981 | 1.29869452995964 | 0.708006339890095 | 2.38219262614076 | 0.999714303421225 |
| ebi-fl187-GCST90094798 | prot-a-2268 | Polymeric immunoglobulin receptor \|\| id:prot-a-2268 | Mayonnaise liking \|\| id:ebi-fl187-GCST90094798 | Weighted mode | 12 | 0.284168222869611 | 0.317237940842254 | 0.389563819401019 | -0.337618141 | 0.905954586920428 | 1.32865642235016 | 0.713467679812806 | 2.47429272355478 | 0.999714303421225 |
| ebi-fl187-GCST90094798 | prot-a-2283 | Group 10 secretory phospholipase A2 \|\| id:prot-a-2283 | Mayonnaise liking \|\| id:ebi-fl187-GCST90094798 | Weighted mode | 12 | 0.234988408982675 | 0.246652005825739 | 0.361201892353734 | -0.248449522 | 0.718426340401124 | 1.26489410723841 | 0.780009232807569 | 2.05120277457173 | 0.999714303421225 |
| ebi-fl187-GCST90094798 | prot-a-2298 | Inactive phospholipase D5 \|\| id:prot-a-2298 | Mayonnaise liking \|\| id:ebi-fl187-GCST90094798 | Weighted mode | 12 | 0.0597266425223505 | 0.293319896026016 | 0.842366206431735 | -0.515180354 | 0.634633638733341 | 1.06154632525421 | 0.597392842899036 | 1.88633093625992 | 0.999714303421225 |
| ebi-fl187-GCST90094798 | prot-a-2304 | Procollagen-lysine,2-oxoglutarate 5-dioxygenase 2 \|\| id:prot-a-2304 | Mayonnaise liking \|\| id:ebi-fl187-GCST90094798 | Weighted mode | 12 | -0.10753141 | 0.315608179442453 | 0.739738930834876 | -0.726123441 | 0.511060621988292 | 0.898048314562671 | 0.483780764194503 | 1.66705837639425 | 0.999714303421225 |
| ebi-fl187-GCST90094798 | prot-a-2331 | Periostin \|\| id:prot-a-2331 | Mayonnaise liking \|\| id:ebi-fl187-GCST90094798 | Weighted mode | 12 | 0.0105160817315104 | 0.265692965353712 | 0.969137335867686 | -0.51024213 | 0.531274293824786 | 1.01057157005501 | 0.60035019820177 | 1.70109862753842 | 0.999714303421225 |
| ebi-fl187-GCST90094798 | prot-a-2333 | Periostin \|\| id:prot-a-2333 | Mayonnaise liking \|\| id:ebi-fl187-GCST90094798 | Weighted mode | 11 | 0.410923106398639 | 0.376182422038131 | 0.300296054396624 | -0.326394441 | 1.14824065359338 | 1.50820938044834 | 0.72152053416208 | 3.15264143925441 | 0.999714303421225 |
| ebi-fl187-GCST90094798 | prot-a-2367 | cAMP-dependent protein kinase catalytic subunit alpha \|\| id:prot-a-2367 | Mayonnaise liking \|\| id:ebi-fl187-GCST90094798 | Weighted mode | 12 | -0.309136126 | 0.26444204094883 | 0.267107698905029 | -0.827442526 | 0.209170274608686 | 0.734080835992924 | 0.437165898317457 | 1.23265487048753 | 0.999714303421225 |
| ebi-fl187-GCST90094798 | prot-a-2385 | Phosphoribosyl pyrophosphate synthase-associated protein 1 \|\| id:prot-a-2385 | Mayonnaise liking \|\| id:ebi-fl187-GCST90094798 | Weighted mode | 12 | 0.324464916083878 | 0.321398069401954 | 0.334406041625951 | -0.3054753 | 0.954405132111707 | 1.38329027154156 | 0.736773102941688 | 2.59712517693926 | 0.999714303421225 |
| ebi-fl187-GCST90094798 | prot-a-2393 | Trypsin-3 \|\| id:prot-a-2393 | Mayonnaise liking \|\| id:ebi-fl187-GCST90094798 | Weighted mode | 12 | 0.0755906550353858 | 0.298384034897723 | 0.804682840161113 | -0.509242053 | 0.660423363434922 | 1.07852099664548 | 0.600950894946938 | 1.93561162814785 | 0.999714303421225 |
| ebi-fl187-GCST90094798 | prot-a-2405 | Pregnancy-specific beta-1-glycoprotein 6 \|\| id:prot-a-2405 | Mayonnaise liking \|\| id:ebi-fl187-GCST90094798 | Weighted mode | 12 | 0.180560716923706 | 0.293618224998276 | 0.551096475856257 | -0.394931004 | 0.756052437920326 | 1.19788885139849 | 0.673726522031278 | 2.12985188111414 | 0.999714303421225 |
| ebi-fl187-GCST90094798 | prot-a-244 | Beclin-1 \|\| id:prot-a-244 | Mayonnaise liking \|\| id:ebi-fl187-GCST90094798 | Weighted mode | 12 | 0.047903982835827 | 0.280635042015766 | 0.867560115168281 | -0.5021407 | 0.597948665186728 | 1.04906992176957 | 0.605233648574373 | 1.81838485575602 | 0.999714303421225 |
| ebi-fl187-GCST90094798 | prot-a-2454 | tRNA pseudouridine synthase A, mitochondrial \|\| id:prot-a-2454 | Mayonnaise liking \|\| id:ebi-fl187-GCST90094798 | Weighted mode | 12 | 0.416646771484584 | 0.30513820621087 | 0.199387688803538 | -0.181424113 | 1.01471765565789 | 1.51686661775045 | 0.83408153910724 | 2.75858441670877 | 0.999714303421225 |
| ebi-fl187-GCST90094798 | prot-a-2466 | Glutaminyl-peptide cyclotransferase-like protein \|\| id:prot-a-2466 | Mayonnaise liking \|\| id:ebi-fl187-GCST90094798 | Weighted mode | 12 | -0.002714686 | 0.299405276291846 | 0.992928088527852 | -0.589549027 | 0.584119655801892 | 0.997288995697121 | 0.554577327604793 | 1.79341147110028 | 0.999714303421225 |
| ebi-fl187-GCST90094798 | prot-a-2474 | Ras-related protein Rab-27A \|\| id:prot-a-2474 | Mayonnaise liking \|\| id:ebi-fl187-GCST90094798 | Weighted mode | 12 | -0.173816735 | 0.282471909468148 | 0.550847504385573 | -0.727461678 | 0.379828207500339 | 0.840450908733275 | 0.483133784 | 1.46203340148611 | 0.999714303421225 |
| ebi-fl187-GCST90094798 | prot-a-2490 | Rap1 GTPase-GDP dissociation stimulator 1 \|\| id:prot-a-2490 | Mayonnaise liking \|\| id:ebi-fl187-GCST90094798 | Weighted mode | 12 | -0.322185608 | 0.372933458606107 | 0.406074707120481 | -1.053135187 | 0.408763970955997 | 0.724563693094247 | 0.34884234694879 | 1.50495646512619 | 0.999714303421225 |
| ebi-fl187-GCST90094798 | prot-a-2545 | Receptor-interacting serine/threonine-protein kinase 2 \|\| id:prot-a-2545 | Mayonnaise liking \|\| id:ebi-fl187-GCST90094798 | Weighted mode | 12 | -0.008994912 | 0.320683925546695 | 0.978125366674744 | -0.637535406 | 0.619545582541029 | 0.991045421664461 | 0.528593588760451 | 1.85808350438997 | 0.999714303421225 |
| ebi-fl187-GCST90094798 | prot-a-2610 | RUN and FYVE domain-containing protein 1 \|\| id:prot-a-2610 | Mayonnaise liking \|\| id:ebi-fl187-GCST90094798 | Weighted mode | 12 | 0.381994131346036 | 0.389214914185634 | 0.347465535542183 | -0.3808671 | 1.14485536314988 | 1.46520348633548 | 0.683268689682176 | 3.14198687688769 | 0.999714303421225 |
| ebi-fl187-GCST90094798 | prot-a-2627 | Serine--tRNA ligase, mitochondrial \|\| id:prot-a-2627 | Mayonnaise liking \|\| id:ebi-fl187-GCST90094798 | Weighted mode | 12 | -0.101724667 | 0.32014365697621 | 0.756627224396516 | -0.729206235 | 0.525756900353302 | 0.903278219447005 | 0.482291664439543 | 1.69173884163124 | 0.999714303421225 |
| ebi-fl187-GCST90094798 | prot-a-264 | BolA-like protein 2 \|\| id:prot-a-264 | Mayonnaise liking \|\| id:ebi-fl187-GCST90094798 | Weighted mode | 12 | -0.100821789 | 0.292740067497615 | 0.737034336441737 | -0.674592321 | 0.472948743620407 | 0.904094138344423 | 0.509364035320304 | 1.60471912877545 | 0.999714303421225 |
| ebi-fl187-GCST90094798 | prot-a-2646 | Secretoglobin family 1C member 1 \|\| id:prot-a-2646 | Mayonnaise liking \|\| id:ebi-fl187-GCST90094798 | Weighted mode | 12 | -0.029845442 | 0.303050721864893 | 0.923320235338686 | -0.623824857 | 0.564133973092923 | 0.970595535491708 | 0.535890807177298 | 1.75792471320516 | 0.999714303421225 |
| ebi-fl187-GCST90094798 | prot-a-2672 | Semaphorin-3E \|\| id:prot-a-2672 | Mayonnaise liking \|\| id:ebi-fl187-GCST90094798 | Weighted mode | 12 | 0.227338509656967 | 0.28228858936777 | 0.437685173396365 | -0.325947126 | 0.780624144817795 | 1.25525471187126 | 0.721843353526309 | 2.18283424509992 | 0.999714303421225 |
| ebi-fl187-GCST90094798 | prot-a-2701 | Plasma protease C1 inhibitor \|\| id:prot-a-2701 | Mayonnaise liking \|\| id:ebi-fl187-GCST90094798 | Weighted mode | 12 | -0.291127558 | 0.263606670621169 | 0.292991402789259 | -0.807796632 | 0.225541516367652 | 0.747420332457155 | 0.445839332691051 | 1.25300105308895 | 0.999714303421225 |
| ebi-fl187-GCST90094798 | prot-a-2738 | Signaling threshold-regulating transmembrane adapter 1 \|\| id:prot-a-2738 | Mayonnaise liking \|\| id:ebi-fl187-GCST90094798 | Weighted mode | 12 | -0.194796752 | 0.291910490998303 | 0.518323998134448 | -0.766941314 | 0.377347810479224 | 0.823001914652155 | 0.464431447875566 | 1.45841147196085 | 0.999714303421225 |
| ebi-fl187-GCST90094798 | prot-a-274 | Serine/threonine-protein kinase BRSK2 \|\| id:prot-a-274 | Mayonnaise liking \|\| id:ebi-fl187-GCST90094798 | Weighted mode | 12 | 0.107587513282515 | 0.312334062748214 | 0.7369935659452 | -0.50458725 | 0.719762276269015 | 1.11358831025081 | 0.60375472394647 | 2.05394488116205 | 0.999714303421225 |
| ebi-fl187-GCST90094798 | prot-a-2749 | Mitochondrial glutamate carrier 2 \|\| id:prot-a-2749 | Mayonnaise liking \|\| id:ebi-fl187-GCST90094798 | Weighted mode | 12 | -0.106415484 | 0.25891097632573 | 0.688957193264192 | -0.613880997 | 0.401050029930564 | 0.899051029445767 | 0.541246212561787 | 1.49339198092813 | 0.999714303421225 |
| ebi-fl187-GCST90094798 | prot-a-2760 | Sodium-coupled monocarboxylate transporter 1 \|\| id:prot-a-2760 | Mayonnaise liking \|\| id:ebi-fl187-GCST90094798 | Weighted mode | 12 | 0.0281427770064151 | 0.354919158684638 | 0.938223323493085 | -0.667498774 | 0.723784328028305 | 1.02854252616122 | 0.512990078571234 | 2.06222258931115 | 0.999714303421225 |
| ebi-fl187-GCST90094798 | prot-a-2777 | Small integral membrane protein 10 \|\| id:prot-a-2777 | Mayonnaise liking \|\| id:ebi-fl187-GCST90094798 | Weighted mode | 12 | 0.271966445528423 | 0.345097056881702 | 0.44729649877066 | -0.404423786 | 0.948356677016559 | 1.31254295888679 | 0.667361243008401 | 2.58146399266044 | 0.999714303421225 |
| ebi-fl187-GCST90094798 | prot-a-2799 | Superoxide dismutase [Mn], mitochondrial \|\| id:prot-a-2799 | Mayonnaise liking \|\| id:ebi-fl187-GCST90094798 | Weighted mode | 12 | -0.324265936 | 0.311196702116829 | 0.319773501476353 | -0.934211472 | 0.28567959987006 | 0.723057929474276 | 0.392895552393172 | 1.33066604137185 | 0.999714303421225 |
| ebi-fl187-GCST90094798 | prot-a-2805 | Protein SPACA7 \|\| id:prot-a-2805 | Mayonnaise liking \|\| id:ebi-fl187-GCST90094798 | Weighted mode | 12 | -0.555751257 | 0.284309418755027 | 0.0764971994729586 | -1.112997718 | 0.00149520358022759 | 0.573641147247589 | 0.328572515546895 | 1.00149632195443 | 0.999714303421225 |
| ebi-fl187-GCST90094798 | prot-a-2811 | SPARC-like protein 1 \|\| id:prot-a-2811 | Mayonnaise liking \|\| id:ebi-fl187-GCST90094798 | Weighted mode | 12 | 0.0882018455113309 | 0.284987345025977 | 0.762729609885478 | -0.470373351 | 0.646777041762246 | 1.09220855717523 | 0.624768966777951 | 1.90937705904137 | 0.999714303421225 |
| ebi-fl187-GCST90094798 | prot-a-2831 | Sepiapterin reductase \|\| id:prot-a-2831 | Mayonnaise liking \|\| id:ebi-fl187-GCST90094798 | Weighted mode | 12 | -0.161932137 | 0.370748267668151 | 0.670727243083379 | -0.888598742 | 0.564734467193258 | 0.850498919618959 | 0.411231590738628 | 1.75898065363555 | 0.999714303421225 |
| ebi-fl187-GCST90094798 | prot-a-2834 | SPRY domain-containing SOCS box protein 1 \|\| id:prot-a-2834 | Mayonnaise liking \|\| id:ebi-fl187-GCST90094798 | Weighted mode | 12 | -0.039283065 | 0.383101457743796 | 0.9201738135313 | -0.790161922 | 0.711595792638253 | 0.961478510151444 | 0.453771313902934 | 2.03723967813617 | 0.999714303421225 |
| ebi-fl187-GCST90094798 | prot-a-2835 | Spectrin alpha chain, non-erythrocytic 1 \|\| id:prot-a-2835 | Mayonnaise liking \|\| id:ebi-fl187-GCST90094798 | Weighted mode | 12 | 0.375018038631604 | 0.282356763442519 | 0.211020475613363 | -0.178401218 | 0.92843729497894 | 1.45501766090904 | 0.836606694716086 | 2.53055158048391 | 0.999714303421225 |
| ebi-fl187-GCST90094798 | prot-a-2859 | Sia-alpha-2,3-Gal-beta-1,4-GlcNAc-R:alpha 2,8-sialyltransferase \|\| id:prot-a-2859 | Mayonnaise liking \|\| id:ebi-fl187-GCST90094798 | Weighted mode | 12 | -0.289242768 | 0.293954497705752 | 0.346277599301258 | -0.865393583 | 0.286908047525548 | 0.748830391294486 | 0.420885866299398 | 1.33230169940505 | 0.999714303421225 |
| ebi-fl187-GCST90094798 | prot-a-2861 | Alpha-2,8-sialyltransferase 8F \|\| id:prot-a-2861 | Mayonnaise liking \|\| id:ebi-fl187-GCST90094798 | Weighted mode | 12 | 0.339195025728398 | 0.307868868657649 | 0.294092681975057 | -0.264227957 | 0.942618008297389 | 1.40381709896961 | 0.767798494787043 | 2.56669225159923 | 0.999714303421225 |
| ebi-fl187-GCST90094798 | prot-a-289 | Uncharacterized protein C17orf89 \|\| id:prot-a-289 | Mayonnaise liking \|\| id:ebi-fl187-GCST90094798 | Weighted mode | 12 | 0.222305722652219 | 0.295385546523269 | 0.467493592258757 | -0.356649949 | 0.801261393837827 | 1.24895315277305 | 0.700017497002413 | 2.22834998339531 | 0.999714303421225 |
| ebi-fl187-GCST90094798 | prot-a-2921 | Tachykinin-3 \|\| id:prot-a-2921 | Mayonnaise liking \|\| id:ebi-fl187-GCST90094798 | Weighted mode | 12 | -0.127837352 | 0.279538105367455 | 0.656343505047466 | -0.675732039 | 0.420057334359565 | 0.879996497034302 | 0.508783834803306 | 1.52204881881127 | 0.999714303421225 |
| ebi-fl187-GCST90094798 | prot-a-2928 | Tax1-binding protein 1 \|\| id:prot-a-2928 | Mayonnaise liking \|\| id:ebi-fl187-GCST90094798 | Weighted mode | 12 | -0.006242267 | 0.286944807921702 | 0.983033595939979 | -0.568654091 | 0.556169556432089 | 0.99377717537872 | 0.566287097138197 | 1.74397947488939 | 0.999714303421225 |
| ebi-fl187-GCST90094798 | prot-a-296 | Uncharacterized protein C1orf185 \|\| id:prot-a-296 | Mayonnaise liking \|\| id:ebi-fl187-GCST90094798 | Weighted mode | 12 | 0.396102601357544 | 0.307424079405214 | 0.224027884869685 | -0.206448594 | 0.998653796991763 | 1.48602177758167 | 0.81346807729708 | 2.71462493129959 | 0.999714303421225 |
| ebi-fl187-GCST90094798 | prot-a-2964 | Transforming growth factor beta-2 \|\| id:prot-a-2964 | Mayonnaise liking \|\| id:ebi-fl187-GCST90094798 | Weighted mode | 12 | 0.0537267433682631 | 0.348498029626412 | 0.880270517416501 | -0.629329395 | 0.736782881436031 | 1.05519622339034 | 0.532949079674748 | 2.0892034761309 | 0.999714303421225 |
| ebi-fl187-GCST90094798 | prot-a-2977 | Thymocyte nuclear protein 1 \|\| id:prot-a-2977 | Mayonnaise liking \|\| id:ebi-fl187-GCST90094798 | Weighted mode | 12 | -0.219257673 | 0.256647413426608 | 0.411150188262448 | -0.722286603 | 0.283771257422737 | 0.803114750588277 | 0.485640518225164 | 1.32812909633175 | 0.999714303421225 |
| ebi-fl187-GCST90094798 | prot-a-3015 | Transmembrane protein 70, mitochondrial \|\| id:prot-a-3015 | Mayonnaise liking \|\| id:ebi-fl187-GCST90094798 | Weighted mode | 12 | -0.216673751 | 0.287873386441934 | 0.467450205607873 | -0.780905588 | 0.347558086701611 | 0.805192619966715 | 0.457991072163486 | 1.41560653614102 | 0.999714303421225 |
| ebi-fl187-GCST90094798 | prot-a-3030 | Tumor necrosis factor alpha-induced protein 3 \|\| id:prot-a-3030 | Mayonnaise liking \|\| id:ebi-fl187-GCST90094798 | Weighted mode | 12 | 0.0342148942066691 | 0.257312609944962 | 0.896618304775558 | -0.470117821 | 0.538547609698795 | 1.03480695685643 | 0.624928634049963 | 1.7135163594902 | 0.999714303421225 |
| ebi-fl187-GCST90094798 | prot-a-3032 | Tumor necrosis factor receptor superfamily member 10A \|\| id:prot-a-3032 | Mayonnaise liking \|\| id:ebi-fl187-GCST90094798 | Weighted mode | 12 | 0.216335836053264 | 0.279205688824668 | 0.454775841981807 | -0.330907314 | 0.763578986149614 | 1.24151925592256 | 0.718271739658956 | 2.14594279256814 | 0.999714303421225 |
| ebi-fl187-GCST90094798 | prot-a-3081 | Tumor protein p53-inducible protein 11 \|\| id:prot-a-3081 | Mayonnaise liking \|\| id:ebi-fl187-GCST90094798 | Weighted mode | 12 | 0.338596013664229 | 0.284850494160819 | 0.259594578568317 | -0.219710955 | 0.896902982219435 | 1.40297644739679 | 0.802750795623208 | 2.45199746008601 | 0.999714303421225 |
| ebi-fl187-GCST90094798 | prot-a-315 | Complement C4 \|\| id:prot-a-315 | Mayonnaise liking \|\| id:ebi-fl187-GCST90094798 | Weighted mode | 12 | -0.272219968 | 0.295739937956462 | 0.377071670850619 | -0.851870247 | 0.307430310085927 | 0.76168669572673 | 0.426616307626304 | 1.3599260320712 | 0.999714303421225 |
| ebi-fl187-GCST90094798 | prot-a-3157 | UDP-glucuronosyltransferase 1-8 \|\| id:prot-a-3157 | Mayonnaise liking \|\| id:ebi-fl187-GCST90094798 | Weighted mode | 12 | -0.040579439 | 0.389637996140848 | 0.918927719028313 | -0.804269911 | 0.723111033610084 | 0.960232881708756 | 0.44741445960396 | 2.06083457367666 | 0.999714303421225 |
| ebi-fl187-GCST90094798 | prot-a-317 | Glutaredoxin-like protein C5orf63 \|\| id:prot-a-317 | Mayonnaise liking \|\| id:ebi-fl187-GCST90094798 | Weighted mode | 12 | -0.204920542 | 0.450778202688264 | 0.658242080908023 | -1.088445819 | 0.678604735349584 | 0.814712049295274 | 0.33673944120201 | 1.97112557084965 | 0.999714303421225 |
| ebi-fl187-GCST90094798 | prot-a-3192 | Guanine nucleotide exchange factor VAV3 \|\| id:prot-a-3192 | Mayonnaise liking \|\| id:ebi-fl187-GCST90094798 | Weighted mode | 12 | 0.135598989514065 | 0.267330279840961 | 0.622005174007879 | -0.388368359 | 0.65956633800235 | 1.14522255524789 | 0.678162490009911 | 1.93395347039812 | 0.999714303421225 |
| ebi-fl187-GCST90094798 | prot-a-3233 | Serine/threonine-protein kinase WNK3 \|\| id:prot-a-3233 | Mayonnaise liking \|\| id:ebi-fl187-GCST90094798 | Weighted mode | 12 | 0.438262850773898 | 0.328646390908195 | 0.209310884847203 | -0.205884075 | 1.08240977695396 | 1.55001227580486 | 0.813927425020302 | 2.95178412877025 | 0.999714303421225 |
| ebi-fl187-GCST90094798 | prot-a-3250 | 14-3-3 protein theta \|\| id:prot-a-3250 | Mayonnaise liking \|\| id:ebi-fl187-GCST90094798 | Weighted mode | 12 | 0.461497827292589 | 0.299711219667101 | 0.151864278465292 | -0.125936163 | 1.04893181784011 | 1.58644843205457 | 0.881671128002027 | 2.85460025584803 | 0.999714303421225 |
| ebi-fl187-GCST90094798 | prot-a-3278 | E3 ubiquitin-protein ligase ZNRF3 \|\| id:prot-a-3278 | Mayonnaise liking \|\| id:ebi-fl187-GCST90094798 | Weighted mode | 12 | -0.448954948 | 0.325036802906483 | 0.194617051414703 | -1.086027082 | 0.188117185617899 | 0.638294854455493 | 0.33755491129329 | 1.20697494716753 | 0.999714303421225 |
| ebi-fl187-GCST90094798 | prot-a-355 | Calpain-3 \|\| id:prot-a-355 | Mayonnaise liking \|\| id:ebi-fl187-GCST90094798 | Weighted mode | 12 | 0.19370678266456 | 0.343819872368166 | 0.584466913875743 | -0.480180167 | 0.867593732506166 | 1.21374034106589 | 0.618671917391553 | 2.38117421224147 | 0.999714303421225 |
| ebi-fl187-GCST90094798 | prot-a-385 | Coiled-coil domain-containing protein 90B, mitochondrial \|\| id:prot-a-385 | Mayonnaise liking \|\| id:ebi-fl187-GCST90094798 | Weighted mode | 12 | -0.295257783 | 0.327530178676471 | 0.386652229133706 | -0.937216933 | 0.34670136695219 | 0.744339684426573 | 0.39171649281726 | 1.41439427742124 | 0.999714303421225 |
| ebi-fl187-GCST90094798 | prot-a-390 | C-C motif chemokine 14 \|\| id:prot-a-390 | Mayonnaise liking \|\| id:ebi-fl187-GCST90094798 | Weighted mode | 12 | -0.306412346 | 0.284221789954129 | 0.304074070705942 | -0.863487055 | 0.250662361979271 | 0.73608303573062 | 0.421689062757195 | 1.28487618803236 | 0.999714303421225 |
| ebi-fl187-GCST90094798 | prot-a-40 | Pituitary adenylate cyclase-activating polypeptide 27 \|\| id:prot-a-40 | Mayonnaise liking \|\| id:ebi-fl187-GCST90094798 | Weighted mode | 12 | 0.387214159367814 | 0.312575394881717 | 0.241207738425242 | -0.225433615 | 0.999861933335979 | 1.47287188681163 | 0.798170045526614 | 2.71790655026242 | 0.999714303421225 |
| ebi-fl187-GCST90094798 | prot-a-401 | C-C motif chemokine 24 \|\| id:prot-a-401 | Mayonnaise liking \|\| id:ebi-fl187-GCST90094798 | Weighted mode | 12 | 0.11000967696423 | 0.302068171505645 | 0.722621202074418 | -0.482043939 | 0.702063293115294 | 1.1162888726941 | 0.617519927845081 | 2.01791195896964 | 0.999714303421225 |
| ebi-fl187-GCST90094798 | prot-a-412 | Cerebral cavernous malformations 2 protein \|\| id:prot-a-412 | Mayonnaise liking \|\| id:ebi-fl187-GCST90094798 | Weighted mode | 12 | 0.462984496272081 | 0.258811975680253 | 0.101173725204594 | -0.044286976 | 0.970255968605378 | 1.58880870976718 | 0.956679373996893 | 2.63861977674482 | 0.999714303421225 |
| ebi-fl187-GCST90094798 | prot-a-44 | Adhesion G protein-coupled receptor F5 \|\| id:prot-a-44 | Mayonnaise liking \|\| id:ebi-fl187-GCST90094798 | Weighted mode | 12 | 0.0724013357485827 | 0.280118523472923 | 0.800819021156441 | -0.47663097 | 0.621433641755511 | 1.07508672822995 | 0.620871607111346 | 1.86159498997493 | 0.999714303421225 |
| ebi-fl187-GCST90094798 | prot-a-48 | Adrenomedullin \|\| id:prot-a-48 | Mayonnaise liking \|\| id:ebi-fl187-GCST90094798 | Weighted mode | 12 | -0.188492562 | 0.328559260702768 | 0.577715858975855 | -0.832468712 | 0.455483589468179 | 0.828206664023342 | 0.434974133671023 | 1.57693578821275 | 0.999714303421225 |
| ebi-fl187-GCST90094798 | prot-a-502 | Carcinoembryonic antigen-related cell adhesion molecule 1 \|\| id:prot-a-502 | Mayonnaise liking \|\| id:ebi-fl187-GCST90094798 | Weighted mode | 12 | -0.160578685 | 0.267314672449644 | 0.56020647357154 | -0.684515443 | 0.363358073033681 | 0.851650808819143 | 0.504334549228486 | 1.43815073004983 | 0.999714303421225 |
| ebi-fl187-GCST90094798 | prot-a-518 | Complement factor B \|\| id:prot-a-518 | Mayonnaise liking \|\| id:ebi-fl187-GCST90094798 | Weighted mode | 12 | -0.392108336 | 0.272178931610213 | 0.177541661924188 | -0.925579042 | 0.141362369644205 | 0.675630914634187 | 0.396301877086496 | 1.15184196493169 | 0.999714303421225 |
| ebi-fl187-GCST90094798 | prot-a-525 | Properdin \|\| id:prot-a-525 | Mayonnaise liking \|\| id:ebi-fl187-GCST90094798 | Weighted mode | 12 | -0.106964776 | 0.272759457314472 | 0.702432999498171 | -0.641573312 | 0.427643760543857 | 0.898557323402474 | 0.526463480741104 | 1.53363964069005 | 0.999714303421225 |
| ebi-fl187-GCST90094798 | prot-a-533 | Chondroadherin \|\| id:prot-a-533 | Mayonnaise liking \|\| id:ebi-fl187-GCST90094798 | Weighted mode | 12 | -0.231485423 | 0.31412016902147 | 0.476587507119645 | -0.847160955 | 0.384190107818832 | 0.793354259776364 | 0.428630106434518 | 1.46842457414143 | 0.999714303421225 |
| ebi-fl187-GCST90094798 | prot-a-535 | Coiled-coil-helix-coiled-coil-helix domain-containing protein 2 \|\| id:prot-a-535 | Mayonnaise liking \|\| id:ebi-fl187-GCST90094798 | Weighted mode | 12 | -0.202392329 | 0.317939354862642 | 0.537422981823163 | -0.825553464 | 0.420768807026047 | 0.816774421187012 | 0.437992512265742 | 1.52313210025978 | 0.999714303421225 |
| ebi-fl187-GCST90094798 | prot-a-591 | Caseinolytic peptidase B protein homolog \|\| id:prot-a-591 | Mayonnaise liking \|\| id:ebi-fl187-GCST90094798 | Weighted mode | 11 | 0.0352476107918542 | 0.25847520790047 | 0.894237043550021 | -0.471363797 | 0.541859018276776 | 1.03587617116589 | 0.624150473225722 | 1.71919991735911 | 0.999714303421225 |
| ebi-fl187-GCST90094798 | prot-a-605 | Ciliary neurotrophic factor receptor subunit alpha \|\| id:prot-a-605 | Mayonnaise liking \|\| id:ebi-fl187-GCST90094798 | Weighted mode | 12 | 0.0421961558279143 | 0.286762332816085 | 0.885678487793423 | -0.519858016 | 0.604250328147441 | 1.04309906864576 | 0.594604966076251 | 1.82987988511036 | 0.999714303421225 |
| ebi-fl187-GCST90094798 | prot-a-606 | Ciliary neurotrophic factor receptor subunit alpha \|\| id:prot-a-606 | Mayonnaise liking \|\| id:ebi-fl187-GCST90094798 | Weighted mode | 12 | -0.338564106 | 0.28777479093569 | 0.264231464222067 | -0.902602696 | 0.225474484075427 | 0.712793083497803 | 0.405512858217224 | 1.25291706437122 | 0.999714303421225 |
| ebi-fl187-GCST90094798 | prot-a-707 | Cancer/testis antigen 55 \|\| id:prot-a-707 | Mayonnaise liking \|\| id:ebi-fl187-GCST90094798 | Weighted mode | 12 | -0.110745795 | 0.291240846068592 | 0.710996219074143 | -0.681577854 | 0.460086263003127 | 0.895166275490931 | 0.505818255238414 | 1.58421063786756 | 0.999714303421225 |
| ebi-fl187-GCST90094798 | prot-a-719 | Dipeptidyl peptidase 1 \|\| id:prot-a-719 | Mayonnaise liking \|\| id:ebi-fl187-GCST90094798 | Weighted mode | 12 | 0.132805346521438 | 0.378160661823457 | 0.732081647929493 | -0.608389551 | 0.874000243695414 | 1.14202767703384 | 0.544226613108474 | 2.39647820172174 | 0.999714303421225 |
| ebi-fl187-GCST90094798 | prot-a-752 | Cytochrome b5 \|\| id:prot-a-752 | Mayonnaise liking \|\| id:ebi-fl187-GCST90094798 | Weighted mode | 12 | -0.129116842 | 0.272939905415136 | 0.64541882642767 | -0.664079057 | 0.405845372352003 | 0.878871270239199 | 0.514747362530146 | 1.50057050483018 | 0.999714303421225 |
| ebi-fl187-GCST90094798 | prot-a-755 | Cytoglobin \|\| id:prot-a-755 | Mayonnaise liking \|\| id:ebi-fl187-GCST90094798 | Weighted mode | 12 | 0.0748688512814342 | 0.280692048107841 | 0.794611467492898 | -0.475287563 | 0.625025265572802 | 1.07774279702887 | 0.621706251036496 | 1.86829316033277 | 0.999714303421225 |
| ebi-fl187-GCST90094798 | prot-a-807 | Desmin \|\| id:prot-a-807 | Mayonnaise liking \|\| id:ebi-fl187-GCST90094798 | Weighted mode | 12 | 0.201217142553434 | 0.271516245202044 | 0.474164744538564 | -0.330954698 | 0.73338898314944 | 1.22289028451418 | 0.718237705877548 | 2.08212495072507 | 0.999714303421225 |
| ebi-fl187-GCST90094798 | prot-a-82 | Ameloblastin \|\| id:prot-a-82 | Mayonnaise liking \|\| id:ebi-fl187-GCST90094798 | Weighted mode | 12 | 0.0591633555069859 | 0.310205992600663 | 0.852217182439242 | -0.54884039 | 0.667167101004286 | 1.06094853837158 | 0.577619235216546 | 1.94870899797998 | 0.999714303421225 |
| ebi-fl187-GCST90094798 | prot-a-84 | Muellerian-inhibiting factor \|\| id:prot-a-84 | Mayonnaise liking \|\| id:ebi-fl187-GCST90094798 | Weighted mode | 12 | 0.208864802277472 | 0.350552290912964 | 0.563356430225148 | -0.478217688 | 0.895947292466882 | 1.23227838603638 | 0.619887240334326 | 2.44965523064073 | 0.999714303421225 |
| ebi-fl187-GCST90094798 | prot-a-858 | Inactive dipeptidyl peptidase 10 \|\| id:prot-a-858 | Mayonnaise liking \|\| id:ebi-fl187-GCST90094798 | Weighted mode | 12 | 0.165807230072476 | 0.292483939827338 | 0.582169507143167 | -0.407461292 | 0.739075752134059 | 1.1803455446658 | 0.665337204779364 | 2.09399924550202 | 0.999714303421225 |
| ebi-fl187-GCST90094798 | prot-a-863 | Dihydropyrimidinase-related protein 3 \|\| id:prot-a-863 | Mayonnaise liking \|\| id:ebi-fl187-GCST90094798 | Weighted mode | 12 | 0.125341051807089 | 0.28875076827265 | 0.672621419240631 | -0.440610454 | 0.691292557621482 | 1.13353498130377 | 0.643643386446031 | 1.9962941916239 | 0.999714303421225 |
| ebi-fl187-GCST90094798 | prot-a-864 | Dihydropyrimidinase-related protein 5 \|\| id:prot-a-864 | Mayonnaise liking \|\| id:ebi-fl187-GCST90094798 | Weighted mode | 12 | 0.160765698011263 | 0.288869889871808 | 0.588989105517605 | -0.405419286 | 0.726950682160007 | 1.17440977003061 | 0.666697215347018 | 2.06876266496096 | 0.999714303421225 |
| ebi-fl187-GCST90094798 | prot-a-884 | Endothelin-converting enzyme 1 \|\| id:prot-a-884 | Mayonnaise liking \|\| id:ebi-fl187-GCST90094798 | Weighted mode | 12 | 0.207313668676427 | 0.463737131596802 | 0.663513327806893 | -0.701611109 | 1.11623844660616 | 1.23036843930018 | 0.495785894753982 | 3.05334724614776 | 0.999714303421225 |
| ebi-fl187-GCST90094798 | prot-a-893 | Early endosome antigen 1 \|\| id:prot-a-893 | Mayonnaise liking \|\| id:ebi-fl187-GCST90094798 | Weighted mode | 11 | -0.029866015 | 0.388504755288836 | 0.940239904137299 | -0.791335336 | 0.731603305053492 | 0.970575567100992 | 0.453239164584735 | 2.07841026341248 | 0.999714303421225 |
| ebi-fl187-GCST90094798 | prot-a-897 | Ephrin-A2 \|\| id:prot-a-897 | Mayonnaise liking \|\| id:ebi-fl187-GCST90094798 | Weighted mode | 12 | -0.222239398 | 0.304784898748135 | 0.481136587270125 | -0.8196178 | 0.375139003461447 | 0.800723649679248 | 0.440600019819356 | 1.4551936775185 | 0.999714303421225 |
| ebi-fl187-GCST90094798 | prot-a-90 | Anaphase-promoting complex subunit 10 \|\| id:prot-a-90 | Mayonnaise liking \|\| id:ebi-fl187-GCST90094798 | Weighted mode | 12 | -0.081623965 | 0.259834173663682 | 0.759293105874519 | -0.590898946 | 0.427651015031443 | 0.92161845401851 | 0.553829198498558 | 1.53365076650014 | 0.999714303421225 |
| ebi-fl187-GCST90094798 | prot-a-914 | Histone-lysine N-methyltransferase EHMT2 \|\| id:prot-a-914 | Mayonnaise liking \|\| id:ebi-fl187-GCST90094798 | Weighted mode | 12 | 0.454825457690291 | 0.323667229763723 | 0.187561845274902 | -0.179562313 | 1.08921322802719 | 1.57589829809889 | 0.835635878637597 | 2.97193491739484 | 0.999714303421225 |
| ebi-fl187-GCST90094798 | prot-a-932 | RNA polymerase II elongation factor ELL \|\| id:prot-a-932 | Mayonnaise liking \|\| id:ebi-fl187-GCST90094798 | Weighted mode | 12 | 0.212507035167891 | 0.316819762803928 | 0.516215701376094 | -0.4084597 | 0.83347377026359 | 1.23677481445314 | 0.664673258332054 | 2.30129905557513 | 0.999714303421225 |
| ebi-fl187-GCST90094798 | prot-a-991 | Estrogen receptor \|\| id:prot-a-991 | Mayonnaise liking \|\| id:ebi-fl187-GCST90094798 | Weighted mode | 12 | 0.221360813483161 | 0.299455407220174 | 0.475257677776334 | -0.365571785 | 0.808293411634701 | 1.2477735628777 | 0.693799833334071 | 2.24407500465169 | 0.999714303421225 |
| ebi-fl187-GCST90094798 | prot-a-992 | Persulfide dioxygenase ETHE1, mitochondrial \|\| id:prot-a-992 | Mayonnaise liking \|\| id:ebi-fl187-GCST90094798 | Weighted mode | 12 | 0.58498113324372 | 0.40077512027732 | 0.172354363799702 | -0.200538102 | 1.37050036898727 | 1.79495712030192 | 0.818290310525313 | 3.93732031563984 | 0.999714303421225 |
| ebi-fl187-GCST90094798 | prot-a-993 | Ethanolamine kinase 1 \|\| id:prot-a-993 | Mayonnaise liking \|\| id:ebi-fl187-GCST90094798 | Weighted mode | 12 | -0.168167476 | 0.296042983576475 | 0.581410252639206 | -0.748411724 | 0.412076771747566 | 0.845212270007832 | 0.47311739737676 | 1.50995035340648 | 0.999714303421225 |
| ebi-fl187-GCST90094798 | prot-b-10 | coagulation factor III, tissue factor \|\| id:prot-b-10 | Mayonnaise liking \|\| id:ebi-fl187-GCST90094798 | Weighted mode | 8 | -0.179727503 | 0.513929640885504 | 0.736839847578271 | -1.187029599 | 0.82757459336985 | 0.835497851247948 | 0.305126266688695 | 2.28776324967175 | 0.999714303421225 |
| ebi-fl187-GCST90094798 | prot-b-28 | prolactin \|\| id:prot-b-28 | Mayonnaise liking \|\| id:ebi-fl187-GCST90094798 | Weighted mode | 8 | -0.848219908 | 0.50685853864172 | 0.138146993996624 | -1.841662644 | 0.145222827354467 | 0.428176447268372 | 0.158553588641764 | 1.15629719620911 | 0.999714303421225 |
| ebi-fl187-GCST90094798 | prot-b-54 | matrix metallopeptidase 7 \|\| id:prot-b-54 | Mayonnaise liking \|\| id:ebi-fl187-GCST90094798 | Weighted mode | 8 | -0.156011787 | 0.330776118047711 | 0.651521399150909 | -0.804332978 | 0.492309404322983 | 0.855549105911044 | 0.447386243333524 | 1.63609025429848 | 0.999714303421225 |
| ebi-fl187-GCST90094798 | ukb-rsfMRI191-edge-pheno716 | Net100_Pair10_33 (OccipitalPrecuneus)&(Temporal\|Frontal\|Supp_Motor_Area) (Default_mode\|Central_executive)&(Default_mode\|Salience) \|\| id:ukb-rsfMRI191-edge-pheno716 | Mayonnaise liking \|\| id:ebi-fl187-GCST90094798 | Weighted mode | 10 | -0.007650584 | 0.104006538575243 | 0.942970462952777 | -0.2115034 | 0.196202231177855 | 0.992378606800674 | 0.809366529171822 | 1.21677294988137 | 0.999714303421225 |
| ebi-fl187-GCST90094798 | ukb-rsfMRI191-edge-pheno903 | Net100_Pair27_38 (Precentral\|Frontal\|Supp_Motor_Area)&(Subcortical) (Attention\|Salience\|Motor)&(Subcortical-cerebellum) \|\| id:ukb-rsfMRI191-edge-pheno903 | Mayonnaise liking \|\| id:ebi-fl187-GCST90094798 | Weighted mode | 10 | -0.052578637 | 0.0941235063305027 | 0.590051256880949 | -0.237060709 | 0.131903435569752 | 0.948779709085903 | 0.788943390405486 | 1.1409981341126 | 0.999714303421225 |
| ebi-fl187-GCST90094798 | ukb-rsfMRI191-node-pheno23 | Net100_Node2 RolandicOper\|SupraMarginal\|Insula Salience\|Motor \|\| id:ukb-rsfMRI191-node-pheno23 | Mayonnaise liking \|\| id:ebi-fl187-GCST90094798 | Weighted mode | 10 | 0.00424674810305631 | 0.0864885061210033 | 0.96191051984721 | -0.165270724 | 0.173764220100223 | 1.00425577831627 | 0.84766419007142 | 1.1897750077146 | 0.999714303421225 |
| ebi-fl187-GCST90094798 | ukb-rsfMRI191-node-pheno55 | Net100_Node34 Parietal\|Postcentral\|Precuneus Attention \|\| id:ukb-rsfMRI191-node-pheno55 | Mayonnaise liking \|\| id:ebi-fl187-GCST90094798 | Weighted mode | 9 | -0.009358757 | 0.101048294152889 | 0.92848545032547 | -0.207413413 | 0.188695900013149 | 0.990684900337877 | 0.812683606508377 | 1.20767364309736 | 0.999714303421225 |
| ebi-fl187-GCST90094807 | ebi-a-GCST90001414 | IgD- CD24- B cell Absolute Count \|\| id:ebi-a-GCST90001414 | Oranges liking \|\| id:ebi-fl187-GCST90094807 | Inverse variance weighted | 7 | 0.140152041559473 | 0.229178105274604 | 0.540840808569094 | -0.309037045 | 0.589341127897697 | 1.15044870157533 | 0.734153572965832 | 1.802800209 | 0.998735809224318 |
| ebi-fl187-GCST90094807 | ebi-a-GCST90001422 | CD20- CD38- B cell %B cell \|\| id:ebi-a-GCST90001422 | Oranges liking \|\| id:ebi-fl187-GCST90094807 | Inverse variance weighted | 7 | -0.154054596 | 0.217987989833421 | 0.479745849119593 | -0.581311056 | 0.273201864503775 | 0.85722521903466 | 0.559164789637442 | 1.31416550141772 | 0.998735809224318 |
| ebi-fl187-GCST90094807 | ebi-a-GCST90001444 | CD20- CD38- B cell %lymphocyte \|\| id:ebi-a-GCST90001444 | Oranges liking \|\| id:ebi-fl187-GCST90094807 | Inverse variance weighted | 7 | -0.176984353 | 0.218237588447439 | 0.417382331423553 | -0.604730026 | 0.250761320518279 | 0.837792893500975 | 0.546221872332453 | 1.28500334379421 | 0.998735809224318 |
| ebi-fl187-GCST90094807 | ebi-a-GCST90001459 | Myeloid Dendritic Cell %Dendritic Cell \|\| id:ebi-a-GCST90001459 | Oranges liking \|\| id:ebi-fl187-GCST90094807 | Inverse variance weighted | 7 | 0.000396871276139642 | 0.233493442274782 | 0.99864382781674 | -0.457250276 | 0.458044018134712 | 1.00039695003996 | 0.633021890313245 | 1.58097859325849 | 0.999882738470636 |
| ebi-fl187-GCST90094807 | ebi-a-GCST90001476 | HLA DR++ monocyte %leukocyte \|\| id:ebi-a-GCST90001476 | Oranges liking \|\| id:ebi-fl187-GCST90094807 | Inverse variance weighted (multiplicative random effects) | 7 | 0.192757953672268 | 0.334475410485692 | 0.564412878271019 | -0.462813851 | 0.848329758224225 | 1.21258925521935 | 0.629509804298409 | 2.33574233766247 | 0.998735809224318 |
| ebi-fl187-GCST90094807 | ebi-a-GCST90001478 | CD4 regulatory T cell %CD4+ T cell \|\| id:ebi-a-GCST90001478 | Oranges liking \|\| id:ebi-fl187-GCST90094807 | Inverse variance weighted | 7 | -0.306934286 | 0.324472718069609 | 0.344175161197272 | -0.942900813 | 0.329032241650933 | 0.735698945211788 | 0.389496338907827 | 1.38962265859403 | 0.998735809224318 |
| ebi-fl187-GCST90094807 | ebi-a-GCST90001485 | CD39+ resting CD4 regulatory T cell %CD4 regulatory T cell \|\| id:ebi-a-GCST90001485 | Oranges liking \|\| id:ebi-fl187-GCST90094807 | Inverse variance weighted | 7 | 0.332928612759808 | 0.247971870450949 | 0.179399466754351 | -0.153096253 | 0.818953478843667 | 1.39504770630935 | 0.858047127948233 | 2.26812495431649 | 0.998735809224318 |
| ebi-fl187-GCST90094807 | ebi-a-GCST90001515 | Immature Myeloid-Derived Suppressor Cells Absolute Count \|\| id:ebi-a-GCST90001515 | Oranges liking \|\| id:ebi-fl187-GCST90094807 | Inverse variance weighted | 7 | 0.172518130862461 | 0.303197831629888 | 0.569359352597935 | -0.421749619 | 0.766785880857041 | 1.18829336520324 | 0.655898243207213 | 2.15283565158133 | 0.998735809224318 |
| ebi-fl187-GCST90094807 | ebi-a-GCST90001517 | CD33+ HLA DR+ Absolute Count \|\| id:ebi-a-GCST90001517 | Oranges liking \|\| id:ebi-fl187-GCST90094807 | Inverse variance weighted | 7 | 0.091673390090706 | 0.296600393600184 | 0.757260312356941 | -0.489663381 | 0.673010161547066 | 1.09600679693619 | 0.612832650357479 | 1.96012875330584 | 0.998735809224318 |
| ebi-fl187-GCST90094807 | ebi-a-GCST90001518 | CD33+ HLA DR+ CD14- Absolute Count \|\| id:ebi-a-GCST90001518 | Oranges liking \|\| id:ebi-fl187-GCST90094807 | Inverse variance weighted | 7 | -0.107125901 | 0.304324158723883 | 0.724828905375429 | -0.703601252 | 0.489349450198327 | 0.898412554919952 | 0.494800191222677 | 1.63125466229772 | 0.998735809224318 |
| ebi-fl187-GCST90094807 | ebi-a-GCST90001520 | CD33+ HLA DR+ CD14dim Absolute Count \|\| id:ebi-a-GCST90001520 | Oranges liking \|\| id:ebi-fl187-GCST90094807 | Inverse variance weighted | 7 | 0.289430373881472 | 0.297746175432628 | 0.331015309339306 | -0.29415213 | 0.873012877729422 | 1.33566644079954 | 0.745163121179354 | 2.39411316847593 | 0.998735809224318 |
| ebi-fl187-GCST90094807 | ebi-a-GCST90001531 | CD33dim HLA DR- Absolute Count \|\| id:ebi-a-GCST90001531 | Oranges liking \|\| id:ebi-fl187-GCST90094807 | Inverse variance weighted | 7 | -0.308873447 | 0.368003160144331 | 0.401288186487846 | -1.03015964 | 0.412412747377743 | 0.734273689048055 | 0.35694997238836 | 1.51045774515886 | 0.998735809224318 |
| ebi-fl187-GCST90094807 | ebi-a-GCST90001532 | Basophil Absolute Count \|\| id:ebi-a-GCST90001532 | Oranges liking \|\| id:ebi-fl187-GCST90094807 | Inverse variance weighted | 7 | -0.351394584 | 0.35683740419458 | 0.324748331299455 | -1.050795896 | 0.348006728068622 | 0.703706027819302 | 0.349659345730283 | 1.41624177827984 | 0.998735809224318 |
| ebi-fl187-GCST90094807 | ebi-a-GCST90001588 | T/B cell \|\| id:ebi-a-GCST90001588 | Oranges liking \|\| id:ebi-fl187-GCST90094807 | Inverse variance weighted | 7 | 0.00449600777134053 | 0.293778819368032 | 0.987789605914801 | -0.571310478 | 0.580302493732684 | 1.00450612997844 | 0.564784815337604 | 1.7865787779034 | 0.998955228396052 |
| ebi-fl187-GCST90094807 | ebi-a-GCST90001624 | HLA DR+ CD4+ T cell Absolute Count \|\| id:ebi-a-GCST90001624 | Oranges liking \|\| id:ebi-fl187-GCST90094807 | Inverse variance weighted | 7 | -0.008414397 | 0.218799025614781 | 0.96932315954987 | -0.437260487 | 0.420431693381028 | 0.991620905128742 | 0.645803186145498 | 1.52261871818453 | 0.998735809224318 |
| ebi-fl187-GCST90094807 | ebi-a-GCST90001631 | CD8+ Natural Killer T %T cell \|\| id:ebi-a-GCST90001631 | Oranges liking \|\| id:ebi-fl187-GCST90094807 | Inverse variance weighted | 7 | 0.28805962072278 | 0.234042133699537 | 0.218396776331674 | -0.170662961 | 0.746782202773873 | 1.33383682606842 | 0.843105684810777 | 2.11019888802622 | 0.998735809224318 |
| ebi-fl187-GCST90094807 | ebi-a-GCST90001632 | CD8+ Natural Killer T %lymphocyte \|\| id:ebi-a-GCST90001632 | Oranges liking \|\| id:ebi-fl187-GCST90094807 | Inverse variance weighted | 7 | 0.284096757020021 | 0.228425146924091 | 0.213602901223044 | -0.163616531 | 0.73181004499124 | 1.32856147218301 | 0.849067550587119 | 2.0788399982411 | 0.998735809224318 |
| ebi-fl187-GCST90094807 | ebi-a-GCST90001646 | Natural Killer %CD3- lymphocyte \|\| id:ebi-a-GCST90001646 | Oranges liking \|\| id:ebi-fl187-GCST90094807 | Inverse variance weighted | 7 | -0.022848436 | 0.217393166802432 | 0.916294948715415 | -0.448939043 | 0.403242171244193 | 0.97741061311399 | 0.638305006907892 | 1.49666929804566 | 0.998735809224318 |
| ebi-fl187-GCST90094807 | ebi-a-GCST90001684 | CD127- CD8+ T cell Absolute Count \|\| id:ebi-a-GCST90001684 | Oranges liking \|\| id:ebi-fl187-GCST90094807 | Inverse variance weighted | 7 | -0.092681094 | 0.234958791258394 | 0.693243812642803 | -0.553200325 | 0.367838136989998 | 0.911484131822744 | 0.575106335060169 | 1.44460819141862 | 0.998735809224318 |
| ebi-fl187-GCST90094807 | ebi-a-GCST90001687 | CD28- CD8+ T cell Absolute Count \|\| id:ebi-a-GCST90001687 | Oranges liking \|\| id:ebi-fl187-GCST90094807 | Inverse variance weighted | 7 | -0.05791296 | 0.228553445839512 | 0.799967522899986 | -0.505877714 | 0.390051793938651 | 0.943732086390817 | 0.602976102680683 | 1.47705729451639 | 0.998735809224318 |
| ebi-fl187-GCST90094807 | ebi-a-GCST90001701 | BAFF-R on CD20- CD38- B cell \|\| id:ebi-a-GCST90001701 | Oranges liking \|\| id:ebi-fl187-GCST90094807 | Inverse variance weighted | 7 | -0.157756829 | 0.220381478643174 | 0.474093088011392 | -0.589704527 | 0.274190868837136 | 0.854057438461645 | 0.554491097434015 | 1.31546585971737 | 0.998735809224318 |
| ebi-fl187-GCST90094807 | ebi-a-GCST90001732 | CD19 on IgD- CD27- B cell \|\| id:ebi-a-GCST90001732 | Oranges liking \|\| id:ebi-fl187-GCST90094807 | Inverse variance weighted | 7 | 0.146138160092552 | 0.222168732494964 | 0.510679475166849 | -0.289312556 | 0.581588875782682 | 1.15735607742815 | 0.748778134027282 | 1.78887847960457 | 0.998735809224318 |
| ebi-fl187-GCST90094807 | ebi-a-GCST90001798 | CD27 on CD24+ CD27+ B cell \|\| id:ebi-a-GCST90001798 | Oranges liking \|\| id:ebi-fl187-GCST90094807 | Inverse variance weighted | 7 | -0.067727138 | 0.282648391962574 | 0.810627791606616 | -0.621717987 | 0.486263709813323 | 0.934515432136334 | 0.537021049627552 | 1.62622879216121 | 0.998735809224318 |
| ebi-fl187-GCST90094807 | ebi-a-GCST90001836 | CD66b on CD66b++ myeloid cell \|\| id:ebi-a-GCST90001836 | Oranges liking \|\| id:ebi-fl187-GCST90094807 | Inverse variance weighted (multiplicative random effects) | 7 | 0.129136829389198 | 0.600122324461547 | 0.829623725727094 | -1.047102927 | 1.30537658533383 | 1.13784580426108 | 0.350953014410329 | 3.68907808485386 | 0.998735809224318 |
| ebi-fl187-GCST90094807 | ebi-a-GCST90001943 | CD25 on activated & secreting CD4 regulatory T cell \|\| id:ebi-a-GCST90001943 | Oranges liking \|\| id:ebi-fl187-GCST90094807 | Inverse variance weighted | 7 | -0.010789167 | 0.226665292860054 | 0.962035385829191 | -0.455053141 | 0.433474806687436 | 0.989268826989954 | 0.634414253439044 | 1.54260848767029 | 0.998735809224318 |
| ebi-fl187-GCST90094807 | ebi-a-GCST90001960 | CD25 on CD4+ T cell \|\| id:ebi-a-GCST90001960 | Oranges liking \|\| id:ebi-fl187-GCST90094807 | Inverse variance weighted | 7 | -0.230877123 | 0.251696569182071 | 0.35899407435017 | -0.724202398 | 0.262448152726015 | 0.793837004454625 | 0.484711021070603 | 1.30010905931041 | 0.998735809224318 |
| ebi-fl187-GCST90094807 | ebi-a-GCST90001988 | HLA DR on CD14+ CD16- monocyte \|\| id:ebi-a-GCST90001988 | Oranges liking \|\| id:ebi-fl187-GCST90094807 | Inverse variance weighted | 7 | -0.146526827 | 0.227444002579158 | 0.519424653767211 | -0.592317072 | 0.299263417814095 | 0.863702561267145 | 0.553044355234206 | 1.34886489172015 | 0.998735809224318 |
| ebi-fl187-GCST90094807 | ebi-a-GCST90001991 | HLA DR on CD14+ monocyte \|\| id:ebi-a-GCST90001991 | Oranges liking \|\| id:ebi-fl187-GCST90094807 | Inverse variance weighted | 7 | -0.137151084 | 0.227826757178069 | 0.547175960963575 | -0.583691528 | 0.309389360386771 | 0.871838495610954 | 0.55783529649041 | 1.36259280689357 | 0.998735809224318 |
| ebi-fl187-GCST90094807 | ebi-a-GCST90002019 | CD14 on Monocytic Myeloid-Derived Suppressor Cells \|\| id:ebi-a-GCST90002019 | Oranges liking \|\| id:ebi-fl187-GCST90094807 | Inverse variance weighted | 7 | -0.121559205 | 0.327719273997721 | 0.710693544751961 | -0.763888982 | 0.520770571641297 | 0.885538623133513 | 0.465851212448845 | 1.68332427201165 | 0.998735809224318 |
| ebi-fl187-GCST90094807 | ebi-a-GCST90002037 | CD80 on plasmacytoid Dendritic Cell \|\| id:ebi-a-GCST90002037 | Oranges liking \|\| id:ebi-fl187-GCST90094807 | Inverse variance weighted | 7 | 0.0938529281496804 | 0.332024453120026 | 0.777430794281256 | -0.556915 | 0.744620856264932 | 1.09839819058307 | 0.572973964790441 | 2.10564294228868 | 0.998735809224318 |
| ebi-fl187-GCST90094807 | ebi-a-GCST90002042 | CD45 on CD33+ HLA DR+ CD14- \|\| id:ebi-a-GCST90002042 | Oranges liking \|\| id:ebi-fl187-GCST90094807 | Inverse variance weighted | 7 | 0.0472075677642969 | 0.333247238649608 | 0.887349131159603 | -0.60595702 | 0.700372155517529 | 1.04833958800219 | 0.545552072489519 | 2.0145022761209 | 0.998735809224318 |
| ebi-fl187-GCST90094807 | ebi-a-GCST90002056 | CD8 on Effector Memory CD8+ T cell \|\| id:ebi-a-GCST90002056 | Oranges liking \|\| id:ebi-fl187-GCST90094807 | Inverse variance weighted | 7 | 0.21818992151971 | 0.247457678988657 | 0.377924883534577 | -0.266827129 | 0.703206972337478 | 1.24382327399446 | 0.765805445348149 | 2.02022112316917 | 0.998735809224318 |
| ebi-fl187-GCST90094807 | ebi-a-GCST90002058 | CD8 on CD8+ T cell \|\| id:ebi-a-GCST90002058 | Oranges liking \|\| id:ebi-fl187-GCST90094807 | Inverse variance weighted | 7 | 0.0759709035043477 | 0.239362852677975 | 0.750949191159878 | -0.393180288 | 0.545122094753178 | 1.07893118058416 | 0.674907059145567 | 1.72481896086621 | 0.998735809224318 |
| ebi-fl187-GCST90094807 | ebi-a-GCST90002063 | CD4 on CD4 regulatory T cell \|\| id:ebi-a-GCST90002063 | Oranges liking \|\| id:ebi-fl187-GCST90094807 | Inverse variance weighted | 7 | 0.301030853944085 | 0.246267493163669 | 0.221566421358523 | -0.181653433 | 0.783715140544877 | 1.35125103231865 | 0.833890289484912 | 2.18959181485377 | 0.998735809224318 |
| ebi-fl187-GCST90094807 | ebi-a-GCST90002066 | CD4 on activated CD4 regulatory T cell \|\| id:ebi-a-GCST90002066 | Oranges liking \|\| id:ebi-fl187-GCST90094807 | Inverse variance weighted | 7 | 0.268672764198828 | 0.246282008088943 | 0.275310237286106 | -0.214039972 | 0.751385500053156 | 1.30822697229282 | 0.807316114616123 | 2.11993515309457 | 0.998735809224318 |
| ebi-fl187-GCST90094807 | ebi-a-GCST90002070 | CD4 on activated & secreting CD4 regulatory T cell \|\| id:ebi-a-GCST90002070 | Oranges liking \|\| id:ebi-fl187-GCST90094807 | Inverse variance weighted | 7 | 0.228608076827538 | 0.247435523040567 | 0.35553333874074 | -0.256365548 | 0.713581701987049 | 1.25684935408267 | 0.77385903420313 | 2.04128947138888 | 0.998735809224318 |
| ebi-fl187-GCST90094807 | ebi-a-GCST90002081 | SSC-A on CD4+ T cell \|\| id:ebi-a-GCST90002081 | Oranges liking \|\| id:ebi-fl187-GCST90094807 | Inverse variance weighted | 7 | -0.094028186 | 0.297062962700526 | 0.751603206405904 | -0.676271592 | 0.488215221392138 | 0.91025710582673 | 0.508509392640892 | 1.62940549515707 | 0.998735809224318 |
| ebi-fl187-GCST90094807 | ebi-a-GCST90002094 | CD11b on Monocytic Myeloid-Derived Suppressor Cells \|\| id:ebi-a-GCST90002094 | Oranges liking \|\| id:ebi-fl187-GCST90094807 | Inverse variance weighted | 7 | -0.072034815 | 0.331457411804151 | 0.827953116324794 | -0.721691342 | 0.577621711802084 | 0.930498499631167 | 0.485929687015991 | 1.7817957637714 | 0.998735809224318 |
| ebi-fl187-GCST90094807 | ebi-a-GCST90002104 | HLA DR on myeloid Dendritic Cell \|\| id:ebi-a-GCST90002104 | Oranges liking \|\| id:ebi-fl187-GCST90094807 | Inverse variance weighted | 7 | -0.113412744 | 0.250814666429672 | 0.651141129539546 | -0.60500949 | 0.378184002124766 | 0.892782093537903 | 0.546069244265337 | 1.45963149346427 | 0.998735809224318 |
| ebi-fl187-GCST90094807 | ebi-a-GCST90002105 | HLA DR on plasmacytoid Dendritic Cell \|\| id:ebi-a-GCST90002105 | Oranges liking \|\| id:ebi-fl187-GCST90094807 | Inverse variance weighted | 7 | 0.224050391830224 | 0.245797597329131 | 0.362019383019725 | -0.257712899 | 0.70581368259532 | 1.25113406477524 | 0.772817076865388 | 2.02549412390078 | 0.998735809224318 |
| ebi-fl187-GCST90094807 | ebi-a-GCST90002106 | HLA DR on Dendritic Cell \|\| id:ebi-a-GCST90002106 | Oranges liking \|\| id:ebi-fl187-GCST90094807 | Inverse variance weighted | 7 | -0.045980974 | 0.250491867657547 | 0.85435649519216 | -0.536945034 | 0.44498308692632 | 0.955060133295203 | 0.584531250414053 | 1.5604638033702 | 0.998735809224318 |
| ebi-fl187-GCST90094807 | ebi-a-GCST90002111 | HLA DR on CD33dim HLA DR+ CD11b- \|\| id:ebi-a-GCST90002111 | Oranges liking \|\| id:ebi-fl187-GCST90094807 | Inverse variance weighted | 7 | -0.061785579 | 0.37665738320224 | 0.869702368664771 | -0.80003405 | 0.676462892396095 | 0.940084439327851 | 0.449313664835792 | 1.96690824746971 | 0.998735809224318 |
| ebi-fl187-GCST90094807 | ebi-a-GCST90002112 | HLA DR on CD33- HLA DR+ \|\| id:ebi-a-GCST90002112 | Oranges liking \|\| id:ebi-fl187-GCST90094807 | Inverse variance weighted | 7 | -0.182989333 | 0.456542556970128 | 0.68855596668573 | -1.077812745 | 0.711834078717995 | 0.832777038969476 | 0.340339120710263 | 2.03772518183466 | 0.998735809224318 |
| ebi-fl187-GCST90094807 | ebi-a-GCST90002115 | HLA DR on HLA DR+ CD8+ T cell \|\| id:ebi-a-GCST90002115 | Oranges liking \|\| id:ebi-fl187-GCST90094807 | Inverse variance weighted | 7 | -0.008956387 | 0.281127269814244 | 0.974584627317049 | -0.559965836 | 0.542053061689436 | 0.991083601814286 | 0.571228578978681 | 1.7195335491466 | 0.998955228396052 |
| ebi-fl187-GCST90094807 | ebi-a-GCST90002116 | HLA DR on B cell \|\| id:ebi-a-GCST90002116 | Oranges liking \|\| id:ebi-fl187-GCST90094807 | Inverse variance weighted | 7 | 0.421214405676948 | 0.243593998430381 | 0.083779429392745 | -0.056229831 | 0.898658642600496 | 1.52381095710642 | 0.945321846422997 | 2.45630611604269 | 0.998735809224318 |
| ebi-fl187-GCST90094807 | ebi-cfmet338-GCST90026023 | Sphingomyelin (d18:1/24:1, d18:2/24:0) levels \|\| id:ebi-cfmet338-GCST90026023 | Oranges liking \|\| id:ebi-fl187-GCST90094807 | Inverse variance weighted | 7 | 0.0137681436682343 | 0.132279253872855 | 0.917102749181139 | -0.245499194 | 0.27303548125903 | 1.01386336104527 | 0.782313914399853 | 1.31394686448672 | 0.998735809224318 |
| ebi-fl187-GCST90094807 | ebi-cfmet338-GCST90026046 | 1-arachidonoyl-gpc (20:4n6) levels \|\| id:ebi-cfmet338-GCST90026046 | Oranges liking \|\| id:ebi-fl187-GCST90094807 | Inverse variance weighted | 7 | -0.366332649 | 0.157033381732873 | 0.0196569138996043 | -0.674118077 | -0.058547221 | 0.693272146441336 | 0.509605655242766 | 0.943133703652517 | 0.998735809224318 |
| ebi-fl187-GCST90094807 | ebi-cfmet338-GCST90026047 | 1-linoleoyl-gpc (18:2) levels \|\| id:ebi-cfmet338-GCST90026047 | Oranges liking \|\| id:ebi-fl187-GCST90094807 | Inverse variance weighted | 7 | -0.240618007 | 0.200081930599157 | 0.229132159281553 | -0.632778591 | 0.151542577016568 | 0.786141869763702 | 0.531114000310689 | 1.16362784455699 | 0.998735809224318 |
| ebi-fl187-GCST90094807 | ebi-cfmet338-GCST90026050 | 1-myristoyl-2-palmitoyl-gpc (14:0/16:0) levels \|\| id:ebi-cfmet338-GCST90026050 | Oranges liking \|\| id:ebi-fl187-GCST90094807 | Inverse variance weighted | 7 | -0.175317569 | 0.101134603220303 | 0.0830055476186184 | -0.373541392 | 0.022906253040226 | 0.839190477339851 | 0.688292496190489 | 1.02317061591644 | 0.998735809224318 |
| ebi-fl187-GCST90094807 | ebi-cfmet338-GCST90026068 | 2,3-dihydroxyisovalerate levels \|\| id:ebi-cfmet338-GCST90026068 | Oranges liking \|\| id:ebi-fl187-GCST90094807 | Inverse variance weighted | 7 | -0.181020281 | 0.365358147016359 | 0.620275578987991 | -0.897122249 | 0.535081687057071 | 0.834418435604626 | 0.407741350994128 | 1.70758772437309 | 0.998735809224318 |
| ebi-fl187-GCST90094807 | ebi-cfmet338-GCST90026082 | 3-hydroxyhexanoate levels \|\| id:ebi-cfmet338-GCST90026082 | Oranges liking \|\| id:ebi-fl187-GCST90094807 | Inverse variance weighted (multiplicative random effects) | 7 | 0.0426200638918023 | 0.118934502067757 | 0.720082127578077 | -0.19049156 | 0.275731687944606 | 1.04354134048707 | 0.826552733671614 | 1.3174943169908 | 0.998735809224318 |
| ebi-fl187-GCST90094807 | ebi-cfmet338-GCST90026094 | 3-ureidopropionate levels \|\| id:ebi-cfmet338-GCST90026094 | Oranges liking \|\| id:ebi-fl187-GCST90094807 | Inverse variance weighted | 7 | -0.140622793 | 0.159055769577795 | 0.376636847240193 | -0.452372101 | 0.171126515386841 | 0.868816973752267 | 0.636117425523441 | 1.18664086785378 | 0.998735809224318 |
| ebi-fl187-GCST90094807 | ebi-cfmet338-GCST90026173 | Glycerol levels \|\| id:ebi-cfmet338-GCST90026173 | Oranges liking \|\| id:ebi-fl187-GCST90094807 | Inverse variance weighted | 7 | -0.10729331 | 0.089878665708296 | 0.232572933694751 | -0.283455495 | 0.068868874833137 | 0.898262165112115 | 0.753176641713878 | 1.07129572610727 | 0.998735809224318 |
| ebi-fl187-GCST90094807 | ebi-cfmet338-GCST90026179 | Histidine levels \|\| id:ebi-cfmet338-GCST90026179 | Oranges liking \|\| id:ebi-fl187-GCST90094807 | Inverse variance weighted | 7 | 0.0672332103433758 | 0.0819181020634072 | 0.411796139159735 | -0.09332627 | 0.227792690387654 | 1.06954487802042 | 0.910896253959208 | 1.25582495386015 | 0.998735809224318 |
| ebi-fl187-GCST90094807 | ebi-cfmet338-GCST90026188 | Isocitrate levels \|\| id:ebi-cfmet338-GCST90026188 | Oranges liking \|\| id:ebi-fl187-GCST90094807 | Inverse variance weighted | 7 | -0.052871178 | 0.167930692210423 | 0.752883685480531 | -0.382015335 | 0.276272978371432 | 0.948502192219432 | 0.682484587160235 | 1.31820765709665 | 0.998735809224318 |
| ebi-fl187-GCST90094807 | ebi-cfmet338-GCST90026207 | N-acetyl-isoputreanine levels \|\| id:ebi-cfmet338-GCST90026207 | Oranges liking \|\| id:ebi-fl187-GCST90094807 | Inverse variance weighted | 7 | -0.193633596 | 0.0779895967705883 | 0.013034944060881 | -0.346493206 | -0.040773987 | 0.82395975073867 | 0.707163623650824 | 0.960046088530929 | 0.998735809224318 |
| ebi-fl187-GCST90094807 | ebi-cfmet338-GCST90026213 | N-acetylglutamate levels \|\| id:ebi-cfmet338-GCST90026213 | Oranges liking \|\| id:ebi-fl187-GCST90094807 | Inverse variance weighted | 7 | -0.142703173 | 0.13231007276826 | 0.280787897003777 | -0.402030915 | 0.116624570078506 | 0.867011383485018 | 0.668960064354495 | 1.12369748083237 | 0.998735809224318 |
| ebi-fl187-GCST90094807 | ebi-cfmet338-GCST90026224 | N-acetyltaurine levels \|\| id:ebi-cfmet338-GCST90026224 | Oranges liking \|\| id:ebi-fl187-GCST90094807 | Inverse variance weighted | 7 | -0.194590539 | 0.153410912832282 | 0.204645516877501 | -0.495275928 | 0.10609485047924 | 0.823171646014864 | 0.609402732925769 | 1.11192733834582 | 0.998735809224318 |
| ebi-fl187-GCST90094807 | ebi-cfmet338-GCST90026250 | Phenyllactate (pla) levels \|\| id:ebi-cfmet338-GCST90026250 | Oranges liking \|\| id:ebi-fl187-GCST90094807 | Inverse variance weighted | 7 | 0.238551507733504 | 0.179405102589682 | 0.183623486614006 | -0.113082493 | 0.590185508809281 | 1.26940908873759 | 0.893076984171838 | 1.80432310218347 | 0.998735809224318 |
| ebi-fl187-GCST90094807 | ebi-cfmet338-GCST90026251 | Pipecolate levels \|\| id:ebi-cfmet338-GCST90026251 | Oranges liking \|\| id:ebi-fl187-GCST90094807 | Inverse variance weighted | 7 | -0.001131269 | 0.299071733060544 | 0.996981929465664 | -0.587311865 | 0.58504932821421 | 0.998869371058623 | 0.555819395696738 | 1.79507953152363 | 0.999604360968858 |
| ebi-fl187-GCST90094807 | ebi-cfmet338-GCST90026278 | Trigonelline (n'-methylnicotinate) levels \|\| id:ebi-cfmet338-GCST90026278 | Oranges liking \|\| id:ebi-fl187-GCST90094807 | Inverse variance weighted | 7 | 0.454901352256251 | 0.28387265627103 | 0.109048652018762 | -0.101489054 | 1.01129175854747 | 1.57601790475492 | 0.903491068869743 | 2.74914996029271 | 0.998735809224318 |
| ebi-fl187-GCST90094807 | ebi-cfmet338-GCST90026299 | 1-carboxyethylvaline levels \|\| id:ebi-cfmet338-GCST90026299 | Oranges liking \|\| id:ebi-fl187-GCST90094807 | Inverse variance weighted | 7 | 0.127243716286159 | 0.17059329320006 | 0.455734274404452 | -0.207119138 | 0.461606570958277 | 1.13569377112358 | 0.812922793908471 | 1.58662095765286 | 0.998735809224318 |
| ebi-fl187-GCST90094807 | ebi-gm430-GCST90011306 | C_Erysipelotrichia abundance \|\| id:ebi-gm430-GCST90011306 | Oranges liking \|\| id:ebi-fl187-GCST90094807 | Inverse variance weighted | 5 | -0.208723408 | 0.163791168599503 | 0.202547769924922 | -0.529754098 | 0.112307282917243 | 0.811619692489575 | 0.588749726619124 | 1.1188566133518 | 0.998735809224318 |
| ebi-fl187-GCST90094807 | ebi-gm430-GCST90011312 | F_Erysipelotrichaceae abundance \|\| id:ebi-gm430-GCST90011312 | Oranges liking \|\| id:ebi-fl187-GCST90094807 | Inverse variance weighted | 5 | -0.208723408 | 0.163791168599503 | 0.202547769924922 | -0.529754098 | 0.112307282917243 | 0.811619692489575 | 0.588749726619124 | 1.1188566133518 | 0.998735809224318 |
| ebi-fl187-GCST90094807 | ebi-gm430-GCST90011352 | O_Erysipelotrichales abundance \|\| id:ebi-gm430-GCST90011352 | Oranges liking \|\| id:ebi-fl187-GCST90094807 | Inverse variance weighted | 5 | -0.208723408 | 0.163791168599503 | 0.202547769924922 | -0.529754098 | 0.112307282917243 | 0.811619692489575 | 0.588749726619124 | 1.1188566133518 | 0.998735809224318 |
| ebi-fl187-GCST90094807 | ebi-gm430-GCST90011362 | OTU97_11 (Parabacteroides) abundance \|\| id:ebi-gm430-GCST90011362 | Oranges liking \|\| id:ebi-fl187-GCST90094807 | Inverse variance weighted | 5 | -0.010509569 | 0.218979101916602 | 0.961721438890246 | -0.439708609 | 0.418689470625629 | 0.989545463432511 | 0.644224114917232 | 1.51996828669735 | 0.998735809224318 |
| ebi-fl187-GCST90094807 | ebi-gm430-GCST90011384 | OTU97_235 (Phascolarctobacterium) abundance \|\| id:ebi-gm430-GCST90011384 | Oranges liking \|\| id:ebi-fl187-GCST90094807 | Inverse variance weighted | 5 | -0.097105119 | 0.662937346631662 | 0.883544791908444 | -1.396462318 | 1.20225208073797 | 0.907460610075998 | 0.247470890493504 | 3.32760252002697 | 0.998735809224318 |
| ebi-fl187-GCST90094807 | ebi-gm430-GCST90011390 | OTU97_3 (Bacteroides) abundance \|\| id:ebi-gm430-GCST90011390 | Oranges liking \|\| id:ebi-fl187-GCST90094807 | Inverse variance weighted | 5 | -0.041898843 | 0.169012105850904 | 0.804208382356496 | -0.373162571 | 0.289364884282716 | 0.958966781691512 | 0.68855328518742 | 1.33557897140446 | 0.998735809224318 |
| ebi-fl187-GCST90094807 | ebi-gm430-GCST90011398 | OTU97_4 (Alistipes) abundance \|\| id:ebi-gm430-GCST90011398 | Oranges liking \|\| id:ebi-fl187-GCST90094807 | Inverse variance weighted | 5 | 0.325770669474587 | 0.278854095769798 | 0.242706845367677 | -0.220783358 | 0.87232469718339 | 1.38509768726676 | 0.801890384422577 | 2.39246615315533 | 0.998735809224318 |
| ebi-fl187-GCST90094807 | ebi-gm430-GCST90011421 | OTU99_108 (Cytophagales) abundance \|\| id:ebi-gm430-GCST90011421 | Oranges liking \|\| id:ebi-fl187-GCST90094807 | Inverse variance weighted | 5 | -0.023649535 | 0.631907667169833 | 0.970145647181063 | -1.262188562 | 1.21488949301932 | 0.97662792405056 | 0.28303391082339 | 3.36992164387845 | 0.998735809224318 |
| ebi-fl187-GCST90094807 | ebi-gm430-GCST90011428 | OTU99_123 (Phascolarctobacterium) abundance \|\| id:ebi-gm430-GCST90011428 | Oranges liking \|\| id:ebi-fl187-GCST90094807 | Inverse variance weighted | 5 | 0.293307882678784 | 0.37364165898848 | 0.43245485474015 | -0.439029769 | 1.0256455342962 | 1.34085555308673 | 0.644661588452884 | 2.788895207 | 0.998735809224318 |
| ebi-fl187-GCST90094807 | ebi-gm430-GCST90011450 | OTU99_298 (Phascolarctobacterium) abundance \|\| id:ebi-gm430-GCST90011450 | Oranges liking \|\| id:ebi-fl187-GCST90094807 | Inverse variance weighted | 5 | -0.102310214 | 0.663001505225361 | 0.877362397356532 | -1.401793164 | 1.19717273628851 | 0.902749462747789 | 0.246155171317031 | 3.31074333369097 | 0.998735809224318 |
| ebi-fl187-GCST90094807 | ebi-gm430-GCST90011451 | OTU99_3 (Bacteroides) abundance \|\| id:ebi-gm430-GCST90011451 | Oranges liking \|\| id:ebi-fl187-GCST90094807 | Inverse variance weighted | 5 | -0.034126674 | 0.169007407568515 | 0.839976034108295 | -0.365381193 | 0.297127844341529 | 0.966449072437122 | 0.693932078176882 | 1.34598736531746 | 0.998735809224318 |
| ebi-fl187-GCST90094807 | ebi-gm430-GCST90011472 | OTU99_72 (Parabacteroides) abundance \|\| id:ebi-gm430-GCST90011472 | Oranges liking \|\| id:ebi-fl187-GCST90094807 | Inverse variance weighted | 5 | 0.68136295315458 | 0.507620727687901 | 0.179509182669731 | -0.313573673 | 1.67629957942287 | 1.97656986932397 | 0.730830534459116 | 5.34573784770883 | 0.998735809224318 |
| ebi-fl187-GCST90094807 | ebi-gm430-GCST90011507 | TestASV_3 (Bacteroides) abundance \|\| id:ebi-gm430-GCST90011507 | Oranges liking \|\| id:ebi-fl187-GCST90094807 | Inverse variance weighted | 5 | 0.118983532023191 | 0.215413067688381 | 0.580708185556689 | -0.303226081 | 0.541193144692418 | 1.12635136940739 | 0.738432132283999 | 1.71805552859932 | 0.998735809224318 |
| ebi-fl187-GCST90094807 | ebi-gm430-GCST90011512 | TestASV_34 (Phascolarctobacterium) abundance \|\| id:ebi-gm430-GCST90011512 | Oranges liking \|\| id:ebi-fl187-GCST90094807 | Inverse variance weighted | 5 | 0.142770297216421 | 0.542532455214198 | 0.792430873431721 | -0.920593315 | 1.20613390943625 | 1.15346481715407 | 0.398282663888298 | 3.34054480660356 | 0.998735809224318 |
| ebi-fl187-GCST90094807 | ebi-gm430-GCST90011519 | TestASV_41 (Barnesiella) abundance \|\| id:ebi-gm430-GCST90011519 | Oranges liking \|\| id:ebi-fl187-GCST90094807 | Inverse variance weighted | 5 | -0.484518369 | 0.70963627991617 | 0.494752183570447 | -1.875405478 | 0.906368739422429 | 0.615993806912109 | 0.153292797407862 | 2.4753176703044 | 0.998735809224318 |
| ebi-fl187-GCST90094807 | ebi-gm430-GCST90011524 | TestASV_46 (Sutterella) abundance \|\| id:ebi-gm430-GCST90011524 | Oranges liking \|\| id:ebi-fl187-GCST90094807 | Inverse variance weighted | 5 | -0.413085828 | 0.692692624698219 | 0.550942802995441 | -1.770763373 | 0.944591716154719 | 0.66160549594651 | 0.170203010895364 | 2.57176315485821 | 0.998735809224318 |
| ebi-fl187-GCST90094807 | ebi-gm430-GCST90011581 | OTU97_19 (Bacteria) prevalence \|\| id:ebi-gm430-GCST90011581 | Oranges liking \|\| id:ebi-fl187-GCST90094807 | Inverse variance weighted | 5 | 1.50787620536818 | 0.791887213163889 | 0.0568899660645161 | -0.044222732 | 3.0599751431694 | 4.51712714944806 | 0.956740836525142 | 21.3270270331401 | 0.998735809224318 |
| ebi-fl187-GCST90094807 | ebi-gm430-GCST90011614 | OTU97_80 (Ruminococcus) prevalence \|\| id:ebi-gm430-GCST90011614 | Oranges liking \|\| id:ebi-fl187-GCST90094807 | Inverse variance weighted | 5 | -0.438481165 | 0.387199283458291 | 0.257448184350695 | -1.197391761 | 0.320429430309427 | 0.645015349194445 | 0.301980823838421 | 1.37771927173443 | 0.998735809224318 |
| ebi-fl187-GCST90094807 | ebi-gm430-GCST90011623 | OTU99_11 (Parabacteroides) prevalence \|\| id:ebi-gm430-GCST90011623 | Oranges liking \|\| id:ebi-fl187-GCST90094807 | Inverse variance weighted | 5 | -0.10205477 | 0.342418780708049 | 0.765672018188374 | -0.77319558 | 0.569086040265187 | 0.902980094164641 | 0.461535834525839 | 1.76665166485992 | 0.998735809224318 |
| ebi-fl187-GCST90094807 | ebi-gm430-GCST90011642 | OTU99_19 (Bacteria) prevalence \|\| id:ebi-gm430-GCST90011642 | Oranges liking \|\| id:ebi-fl187-GCST90094807 | Inverse variance weighted | 5 | 1.50787620536818 | 0.791887213163889 | 0.0568899660645161 | -0.044222732 | 3.0599751431694 | 4.51712714944806 | 0.956740836525142 | 21.3270270331401 | 0.998735809224318 |
| ebi-fl187-GCST90094807 | ebi-gm430-GCST90011653 | OTU99_30 (Parasutterella) prevalence \|\| id:ebi-gm430-GCST90011653 | Oranges liking \|\| id:ebi-fl187-GCST90094807 | Inverse variance weighted | 5 | -0.042050342 | 0.479338756760572 | 0.930094668754924 | -0.981554306 | 0.897453620823487 | 0.958821509955369 | 0.374728203795352 | 2.45334799633914 | 0.998735809224318 |
| ebi-fl187-GCST90094807 | ebi-gm430-GCST90011675 | OTU99_8 (Ruminococcaceae) prevalence \|\| id:ebi-gm430-GCST90011675 | Oranges liking \|\| id:ebi-fl187-GCST90094807 | Inverse variance weighted | 5 | 0.140762753824419 | 0.376015082852671 | 0.708140954679363 | -0.596226809 | 0.877752316215655 | 1.15115150929238 | 0.550886319084377 | 2.40548685171313 | 0.998735809224318 |
| ebi-fl187-GCST90094807 | ebi-gm430-GCST90011679 | OTU99_92 (Ruminococcus) prevalence \|\| id:ebi-gm430-GCST90011679 | Oranges liking \|\| id:ebi-fl187-GCST90094807 | Inverse variance weighted | 5 | -0.468360535 | 0.390946580659338 | 0.230910513407668 | -1.234615833 | 0.297894763389702 | 0.626027778226628 | 0.290946513009748 | 1.34702002459895 | 0.998735809224318 |
| ebi-fl187-GCST90094807 | ebi-gm430-GCST90011684 | TestASV_11 (Lachnospiraceae) prevalence \|\| id:ebi-gm430-GCST90011684 | Oranges liking \|\| id:ebi-fl187-GCST90094807 | Inverse variance weighted | 5 | 0.915780366344342 | 0.442653379028784 | 0.0385608172534085 | 0.0481797434479263 | 1.78338098924076 | 2.4987244117113 | 1.04935925382469 | 5.94993912992652 | 0.998735809224318 |
| ebi-fl187-GCST90094807 | ebi-gm430-GCST90011688 | TestASV_15 (Bacteroides) prevalence \|\| id:ebi-gm430-GCST90011688 | Oranges liking \|\| id:ebi-fl187-GCST90094807 | Inverse variance weighted | 5 | 0.818329676117852 | 0.371617313039932 | 0.0276599195798725 | 0.0899597425595858 | 1.54669960967612 | 2.2667105329933 | 1.09413023593581 | 4.69594612380697 | 0.998735809224318 |
| ebi-fl187-GCST90094807 | ebi-gm430-GCST90011692 | TestASV_19 (Prevotella) prevalence \|\| id:ebi-gm430-GCST90011692 | Oranges liking \|\| id:ebi-fl187-GCST90094807 | Inverse variance weighted | 5 | 0.619275647301279 | 0.745217529252765 | 0.405973719951784 | -0.84135071 | 2.0799020046367 | 1.85758200986231 | 0.43112780132613 | 8.00368455188962 | 0.998735809224318 |
| ebi-fl187-GCST90094807 | ebi-gm430-GCST90011693 | TestASV_2 (Bacteroides) prevalence \|\| id:ebi-gm430-GCST90011693 | Oranges liking \|\| id:ebi-fl187-GCST90094807 | Inverse variance weighted | 5 | 0.281632301289794 | 0.366943415774942 | 0.44277906215971 | -0.437576794 | 1.00084139620868 | 1.32529132247464 | 0.645598946638159 | 2.72056994295408 | 0.998735809224318 |
| ebi-fl187-GCST90094807 | ebi-gm430-GCST90011716 | TestASV_40 (Barnesiella) prevalence \|\| id:ebi-gm430-GCST90011716 | Oranges liking \|\| id:ebi-fl187-GCST90094807 | Inverse variance weighted | 5 | -0.583501641 | 0.741967175336175 | 0.43161845857404 | -2.037757305 | 0.870754022173152 | 0.557941231809143 | 0.13032065284537 | 2.38871131594216 | 0.998735809224318 |
| ebi-fl187-GCST90094807 | ebi-gm430-GCST90011718 | TestASV_42 (Alphaproteobacteria) prevalence \|\| id:ebi-gm430-GCST90011718 | Oranges liking \|\| id:ebi-fl187-GCST90094807 | Inverse variance weighted | 5 | 0.662888265762322 | 0.589511371698519 | 0.260813392974485 | -0.492554023 | 1.81833055429142 | 1.94038860672919 | 0.611063728817047 | 6.1615634631322 | 0.998735809224318 |
| ebi-fl187-GCST90094807 | ebi-hbsm44-GCST90104929 | Sulcal depth of the right inferior frontal sulcus \|\| id:ebi-hbsm44-GCST90104929 | Oranges liking \|\| id:ebi-fl187-GCST90094807 | Inverse variance weighted | 5 | -0.167652911 | 0.312186247179478 | 0.591247929135535 | -0.779537956 | 0.444232133031711 | 0.845647298255752 | 0.458617864031604 | 1.55929240688708 | 0.998735809224318 |
| ebi-fl187-GCST90094807 | ebi-hbsm44-GCST90104935 | Sulcal opening distance of the left calloso-marginal posterior fissure \|\| id:ebi-hbsm44-GCST90104935 | Oranges liking \|\| id:ebi-fl187-GCST90094807 | Inverse variance weighted (multiplicative random effects) | 5 | -0.083317341 | 0.112975767549939 | 0.460830651852816 | -0.304749845 | 0.138115163324033 | 0.920059128335993 | 0.737307792207142 | 1.14810776256731 | 0.998735809224318 |
| ebi-fl187-GCST90094807 | ebi-hbsm44-GCST90104936 | Sulcal opening distance of the left anterior inferior temporal sulcus \|\| id:ebi-hbsm44-GCST90104936 | Oranges liking \|\| id:ebi-fl187-GCST90094807 | Inverse variance weighted | 5 | -0.00454922 | 0.0402842035275748 | 0.910087531313986 | -0.083506259 | 0.0744078189811381 | 0.995461112094597 | 0.919885329231727 | 1.07724603730797 | 0.998735809224318 |
| ebi-fl187-GCST90094807 | ebi-hbsm44-GCST90104941 | Sulcal opening distance of the right posterior inferior temporal sulcus \|\| id:ebi-hbsm44-GCST90104941 | Oranges liking \|\| id:ebi-fl187-GCST90094807 | Inverse variance weighted | 5 | 0.00102226365864136 | 0.0328572472692853 | 0.975180001689079 | -0.063377941 | 0.0654224683064406 | 1.00102278634823 | 0.938588675514534 | 1.06760996049633 | 0.998955228396052 |
| ebi-fl187-GCST90094807 | ebi-IPs91-GCST90274819 | Leukemia inhibitory factor levels \|\| id:ebi-IPs91-GCST90274819 | Oranges liking \|\| id:ebi-fl187-GCST90094807 | Inverse variance weighted (multiplicative random effects) | 7 | 0.0317363987062095 | 0.194395700134296 | 0.870316332090121 | -0.349279174 | 0.41275197096943 | 1.03224536822428 | 0.705196230646549 | 1.51097021497628 | 0.998735809224318 |
| ebi-fl187-GCST90094807 | ebi-IPs91-GCST90274837 | STAM binding protein levels \|\| id:ebi-IPs91-GCST90274837 | Oranges liking \|\| id:ebi-fl187-GCST90094807 | Inverse variance weighted | 7 | 0.0441835795871794 | 0.146192447252443 | 0.762477590017959 | -0.242353617 | 0.330720776201968 | 1.04517420992756 | 0.784778617397708 | 1.39197106659202 | 0.998735809224318 |
| ebi-fl187-GCST90094807 | ebi-IPs91-GCST90274840 | TNF-beta levels \|\| id:ebi-IPs91-GCST90274840 | Oranges liking \|\| id:ebi-fl187-GCST90094807 | Inverse variance weighted | 7 | -0.078648414 | 0.156016962040462 | 0.614189919560939 | -0.38444166 | 0.227144831401113 | 0.924364860880997 | 0.680830665233469 | 1.25501161986959 | 0.998735809224318 |
| ebi-fl187-GCST90094807 | ebi-IPs91-GCST90274842 | Tumor necrosis factor ligand superfamily member 14 levels \|\| id:ebi-IPs91-GCST90274842 | Oranges liking \|\| id:ebi-fl187-GCST90094807 | Inverse variance weighted | 7 | -0.049075853 | 0.154808270395535 | 0.751235607302878 | -0.352500063 | 0.254348357118742 | 0.952108906779097 | 0.702928525644483 | 1.28962097467441 | 0.998735809224318 |
| ebi-fl187-GCST90094807 | ebi-met1400-GCST90199628 | 3-methylhistidine levels \|\| id:ebi-met1400-GCST90199628 | Oranges liking \|\| id:ebi-fl187-GCST90094807 | Inverse variance weighted (multiplicative random effects) | 7 | 0.154498793905683 | 0.211673034284975 | 0.465455222074444 | -0.260380353 | 0.569377941104234 | 1.16707287045669 | 0.770758369560351 | 1.76716742723527 | 0.998735809224318 |
| ebi-fl187-GCST90094807 | ebi-met1400-GCST90199651 | Docosahexaenoate DHA; 22:6n3 levels \|\| id:ebi-met1400-GCST90199651 | Oranges liking \|\| id:ebi-fl187-GCST90094807 | Inverse variance weighted | 7 | 0.050543577825328 | 0.13556573740104 | 0.709271657366838 | -0.215165267 | 0.316252423131366 | 1.0518426993737 | 0.806408156119484 | 1.3719765305323 | 0.998735809224318 |
| ebi-fl187-GCST90094807 | ebi-met1400-GCST90199652 | 1-stearoyl-2-oleoyl-GPS (18:0/18:1) levels \|\| id:ebi-met1400-GCST90199652 | Oranges liking \|\| id:ebi-fl187-GCST90094807 | Inverse variance weighted | 7 | -0.012383753 | 0.143779624860277 | 0.931362997415048 | -0.294191818 | 0.269424311481273 | 0.987692609881607 | 0.745133547728881 | 1.30921053626979 | 0.998735809224318 |
| ebi-fl187-GCST90094807 | ebi-met1400-GCST90199653 | 1-myristoyl-2-palmitoyl-gpc (14:0/16:0) levels \|\| id:ebi-met1400-GCST90199653 | Oranges liking \|\| id:ebi-fl187-GCST90094807 | Inverse variance weighted | 7 | -0.047979251 | 0.169552936617445 | 0.777195962642921 | -0.380303007 | 0.284344505003895 | 0.95315356407838 | 0.683654226125811 | 1.3288906613855 | 0.998735809224318 |
| ebi-fl187-GCST90094807 | ebi-met1400-GCST90199667 | Erythritol levels in elite athletes \|\| id:ebi-met1400-GCST90199667 | Oranges liking \|\| id:ebi-fl187-GCST90094807 | Inverse variance weighted | 7 | -0.0134998 | 0.164734894048958 | 0.934687557322476 | -0.336380192 | 0.309380592216104 | 0.986590913517649 | 0.714351463143997 | 1.36258085949966 | 0.998735809224318 |
| ebi-fl187-GCST90094807 | ebi-met1400-GCST90199674 | 3-hydroxymyristate levels \|\| id:ebi-met1400-GCST90199674 | Oranges liking \|\| id:ebi-fl187-GCST90094807 | Inverse variance weighted | 7 | 0.0510704900472304 | 0.138173458989505 | 0.711672031884659 | -0.21974949 | 0.32189046966666 | 1.05239707418813 | 0.802719862473201 | 1.37973364499463 | 0.998735809224318 |
| ebi-fl187-GCST90094807 | ebi-met1400-GCST90199685 | 2-linoleoylglycerol (18:2) levels \|\| id:ebi-met1400-GCST90199685 | Oranges liking \|\| id:ebi-fl187-GCST90094807 | Inverse variance weighted | 7 | -0.250964993 | 0.179340429370042 | 0.161699758414174 | -0.602472234 | 0.10054224875649 | 0.778049608413622 | 0.547456520873795 | 1.10577035813981 | 0.998735809224318 |
| ebi-fl187-GCST90094807 | ebi-met1400-GCST90199703 | Gamma-glutamylvaline levels \|\| id:ebi-met1400-GCST90199703 | Oranges liking \|\| id:ebi-fl187-GCST90094807 | Inverse variance weighted | 7 | 0.100850426323228 | 0.138325467499153 | 0.465952310058819 | -0.17026749 | 0.371968342621568 | 1.10611118427294 | 0.843439174895382 | 1.4505870587828 | 0.998735809224318 |
| ebi-fl187-GCST90094807 | ebi-met1400-GCST90199779 | Gamma-glutamyl-2-aminobutyrate levels \|\| id:ebi-met1400-GCST90199779 | Oranges liking \|\| id:ebi-fl187-GCST90094807 | Inverse variance weighted | 7 | 0.102572371229736 | 0.184060246198846 | 0.577338820732407 | -0.258185711 | 0.463330453779475 | 1.10801748759635 | 0.772451765748621 | 1.58935846515866 | 0.998735809224318 |
| ebi-fl187-GCST90094807 | ebi-met1400-GCST90199824 | 4-vinylphenol sulfate levels \|\| id:ebi-met1400-GCST90199824 | Oranges liking \|\| id:ebi-fl187-GCST90094807 | Inverse variance weighted | 7 | 0.0652484225744602 | 0.189170511248976 | 0.730155588123927 | -0.305525779 | 0.436022624622453 | 1.06742416370832 | 0.736735911920744 | 1.54654378432279 | 0.998735809224318 |
| ebi-fl187-GCST90094807 | ebi-met1400-GCST90199830 | 4-ethylphenylsulfate levels \|\| id:ebi-met1400-GCST90199830 | Oranges liking \|\| id:ebi-fl187-GCST90094807 | Inverse variance weighted | 7 | -0.02956079 | 0.14203829961167 | 0.835136485228613 | -0.307955857 | 0.248834277153934 | 0.970871856464642 | 0.734947759854007 | 1.282529471 | 0.998735809224318 |
| ebi-fl187-GCST90094807 | ebi-met1400-GCST90199833 | Hexanoylglutamine levels \|\| id:ebi-met1400-GCST90199833 | Oranges liking \|\| id:ebi-fl187-GCST90094807 | Inverse variance weighted | 7 | 0.0722250868683399 | 0.139879758063794 | 0.605619097343792 | -0.201939239 | 0.346389412673376 | 1.07489726209502 | 0.817144577007228 | 1.41395311988853 | 0.998735809224318 |
| ebi-fl187-GCST90094807 | ebi-met1400-GCST90199837 | Carotenoid levels (cryptoxanthin) \|\| id:ebi-met1400-GCST90199837 | Oranges liking \|\| id:ebi-fl187-GCST90094807 | Inverse variance weighted | 7 | 0.0446298254694631 | 0.140490961361903 | 0.750734936274093 | -0.230732459 | 0.319992109738793 | 1.04564071869608 | 0.793951852454371 | 1.37711689848105 | 0.998735809224318 |
| ebi-fl187-GCST90094807 | ebi-met1400-GCST90199869 | 2-hydroxyhippurate levels \|\| id:ebi-met1400-GCST90199869 | Oranges liking \|\| id:ebi-fl187-GCST90094807 | Inverse variance weighted (multiplicative random effects) | 7 | 0.211661810502429 | 0.221742060066553 | 0.339810042214514 | -0.222952627 | 0.646276248232874 | 1.23572990352879 | 0.800152753850686 | 1.90842109475541 | 0.998735809224318 |
| ebi-fl187-GCST90094807 | ebi-met1400-GCST90199902 | Hydantoin-5-propionate levels \|\| id:ebi-met1400-GCST90199902 | Oranges liking \|\| id:ebi-fl187-GCST90094807 | Inverse variance weighted | 7 | -0.183825616 | 0.141590573007428 | 0.194187722080781 | -0.46134314 | 0.093691906597008 | 0.832080892356111 | 0.630436312619352 | 1.0982213390398 | 0.998735809224318 |
| ebi-fl187-GCST90094807 | ebi-met1400-GCST90199904 | Pantoate levels \|\| id:ebi-met1400-GCST90199904 | Oranges liking \|\| id:ebi-fl187-GCST90094807 | Inverse variance weighted | 7 | 0.0806015382628245 | 0.143714901425621 | 0.574904371961601 | -0.201079669 | 0.362282745057042 | 1.08393890232787 | 0.817847272267236 | 1.43660507752583 | 0.998735809224318 |
| ebi-fl187-GCST90094807 | ebi-met1400-GCST90199936 | 3-methyl catechol sulfate (2) levels \|\| id:ebi-met1400-GCST90199936 | Oranges liking \|\| id:ebi-fl187-GCST90094807 | Inverse variance weighted | 7 | 0.188008689482541 | 0.161493996101645 | 0.244349545394709 | -0.128519543 | 0.504536921841766 | 1.20684400215393 | 0.879396376315546 | 1.65621838429353 | 0.998735809224318 |
| ebi-fl187-GCST90094807 | ebi-met1400-GCST90199977 | 4-hydroxychlorothalonil levels \|\| id:ebi-met1400-GCST90199977 | Oranges liking \|\| id:ebi-fl187-GCST90094807 | Inverse variance weighted | 7 | -0.034880682 | 0.1406676200156 | 0.804161291160419 | -0.310589217 | 0.240827853197758 | 0.965720637206762 | 0.733014923908846 | 1.27230199373541 | 0.998735809224318 |
| ebi-fl187-GCST90094807 | ebi-met1400-GCST90200040 | Sphingomyelin (d18:2/24:1, d18:1/24:2) levels \|\| id:ebi-met1400-GCST90200040 | Oranges liking \|\| id:ebi-fl187-GCST90094807 | Inverse variance weighted | 7 | 0.031579867994868 | 0.129245814425462 | 0.806967831936128 | -0.221741928 | 0.284901664268774 | 1.03208380276782 | 0.80112208461426 | 1.32963127142921 | 0.998735809224318 |
| ebi-fl187-GCST90094807 | ebi-met1400-GCST90200077 | 1-stearoyl-2-oleoyl-GPI (18:0/18:1) levels \|\| id:ebi-met1400-GCST90200077 | Oranges liking \|\| id:ebi-fl187-GCST90094807 | Inverse variance weighted | 6 | 0.248040264782783 | 0.14866204692988 | 0.0952196614093673 | -0.043337347 | 0.539417876765347 | 1.28151153093864 | 0.957588295842483 | 1.71500822541259 | 0.998735809224318 |
| ebi-fl187-GCST90094807 | ebi-met1400-GCST90200080 | 1-palmitoyl-2-oleoyl-GPI (16:0/18:1) levels \|\| id:ebi-met1400-GCST90200080 | Oranges liking \|\| id:ebi-fl187-GCST90094807 | Inverse variance weighted | 7 | 0.271161093762418 | 0.141607427945301 | 0.0555079331510317 | -0.006389465 | 0.548711652535209 | 1.31148632563474 | 0.993630904215279 | 1.73102142357908 | 0.998735809224318 |
| ebi-fl187-GCST90094807 | ebi-met1400-GCST90200082 | 1-oleoyl-2-linoleoyl-GPE (18:1/18:2) levels \|\| id:ebi-met1400-GCST90200082 | Oranges liking \|\| id:ebi-fl187-GCST90094807 | Inverse variance weighted | 7 | 0.00347565726884547 | 0.142319068862481 | 0.980516330942415 | -0.275469718 | 0.282421032239309 | 1.00348170436942 | 0.759215422533907 | 1.32633703309576 | 0.998955228396052 |
| ebi-fl187-GCST90094807 | ebi-met1400-GCST90200100 | Perfluorooctanesulfonate (PFOS) levels \|\| id:ebi-met1400-GCST90200100 | Oranges liking \|\| id:ebi-fl187-GCST90094807 | Inverse variance weighted | 7 | -0.197881653 | 0.130649690422362 | 0.129874446313869 | -0.453955046 | 0.0581917403013296 | 0.820466947254426 | 0.635111283298818 | 1.059918205 | 0.998735809224318 |
| ebi-fl187-GCST90094807 | ebi-met1400-GCST90200102 | 1-palmitoleoyl-2-linolenoyl-GPC (16:1/18:3) levels \|\| id:ebi-met1400-GCST90200102 | Oranges liking \|\| id:ebi-fl187-GCST90094807 | Inverse variance weighted | 7 | 0.174711867197915 | 0.153372765671817 | 0.254647988470874 | -0.125898754 | 0.475322487914677 | 1.19090302894618 | 0.881704111703189 | 1.60853284625559 | 0.998735809224318 |
| ebi-fl187-GCST90094807 | ebi-met1400-GCST90200114 | Glycosyl-N-tricosanoyl-sphingadienine (d18:2/23:0) levels \|\| id:ebi-met1400-GCST90200114 | Oranges liking \|\| id:ebi-fl187-GCST90094807 | Inverse variance weighted | 7 | -0.174410415 | 0.148376390337273 | 0.239810992637443 | -0.46522814 | 0.116407310308913 | 0.83995209817569 | 0.627991818909354 | 1.12345337309494 | 0.998735809224318 |
| ebi-fl187-GCST90094807 | ebi-met1400-GCST90200135 | Arachidoylcarnitine (C20) levels \|\| id:ebi-met1400-GCST90200135 | Oranges liking \|\| id:ebi-fl187-GCST90094807 | Inverse variance weighted | 7 | -0.07642424 | 0.154730083521203 | 0.621362876733195 | -0.379695203 | 0.226846724107011 | 0.926423097819734 | 0.684069879684747 | 1.25463754751114 | 0.998735809224318 |
| ebi-fl187-GCST90094807 | ebi-met1400-GCST90200146 | Docosahexaenoylcarnitine (C22:6) levels \|\| id:ebi-met1400-GCST90200146 | Oranges liking \|\| id:ebi-fl187-GCST90094807 | Inverse variance weighted | 7 | 0.230383395862473 | 0.180209235636628 | 0.201100779711504 | -0.122826706 | 0.583593497710264 | 1.25908264447993 | 0.884416913439291 | 1.79246810134572 | 0.998735809224318 |
| ebi-fl187-GCST90094807 | ebi-met1400-GCST90200162 | 3-hydroxyphenylacetoylglutamine levels \|\| id:ebi-met1400-GCST90200162 | Oranges liking \|\| id:ebi-fl187-GCST90094807 | Inverse variance weighted | 7 | -0.24579098 | 0.227323674633195 | 0.279591288624094 | -0.691345382 | 0.199763422295246 | 0.782085679402056 | 0.500901711253484 | 1.22111383567672 | 0.998735809224318 |
| ebi-fl187-GCST90094807 | ebi-met1400-GCST90200172 | 3-carboxy-4-methyl-5-pentyl-2-furanpropionate (3-CMPFP) levels \|\| id:ebi-met1400-GCST90200172 | Oranges liking \|\| id:ebi-fl187-GCST90094807 | Inverse variance weighted | 7 | -0.18538418 | 0.140787772720357 | 0.1879179605854 | -0.461328214 | 0.0905598549540823 | 0.830785051884361 | 0.630445722255658 | 1.09478703410824 | 0.998735809224318 |
| ebi-fl187-GCST90094807 | ebi-met1400-GCST90200222 | Dihydrocaffeate sulfate (2) levels \|\| id:ebi-met1400-GCST90200222 | Oranges liking \|\| id:ebi-fl187-GCST90094807 | Inverse variance weighted | 7 | 0.0318129967624083 | 0.160417885352789 | 0.842799963323669 | -0.282606059 | 0.346232052053875 | 1.03232443924131 | 0.753816689030333 | 1.41373063685514 | 0.998735809224318 |
| ebi-fl187-GCST90094807 | ebi-met1400-GCST90200223 | 4-methylhexanoylglutamine levels \|\| id:ebi-met1400-GCST90200223 | Oranges liking \|\| id:ebi-fl187-GCST90094807 | Inverse variance weighted | 7 | 0.195961375305494 | 0.170811951731356 | 0.25128468923724 | -0.13883005 | 0.530752800698952 | 1.21647991826168 | 0.870375936203238 | 1.7002117475688 | 0.998735809224318 |
| ebi-fl187-GCST90094807 | ebi-met1400-GCST90200224 | 2-ketocaprylate levels \|\| id:ebi-met1400-GCST90200224 | Oranges liking \|\| id:ebi-fl187-GCST90094807 | Inverse variance weighted | 7 | -0.278233333 | 0.143534234447501 | 0.0525688581850426 | -0.559560432 | 0.00309376664233074 | 0.757120139883534 | 0.571460204043254 | 1.00309855727744 | 0.998735809224318 |
| ebi-fl187-GCST90094807 | ebi-met1400-GCST90200243 | Hydroxypalmitoyl sphingomyelin (d18:1/16:0(OH)) levels \|\| id:ebi-met1400-GCST90200243 | Oranges liking \|\| id:ebi-fl187-GCST90094807 | Inverse variance weighted | 7 | -0.217585384 | 0.138493124315509 | 0.116161374382067 | -0.489031907 | 0.0538611399287479 | 0.804458914284886 | 0.613219760440915 | 1.05533804766354 | 0.998735809224318 |
| ebi-fl187-GCST90094807 | ebi-met1400-GCST90200273 | 2-methoxyhydroquinone sulfate (2) levels \|\| id:ebi-met1400-GCST90200273 | Oranges liking \|\| id:ebi-fl187-GCST90094807 | Inverse variance weighted | 7 | -0.19097225 | 0.177435874934495 | 0.281798103214092 | -0.538746565 | 0.156802064860356 | 0.826155513639307 | 0.583479147467061 | 1.16976405357341 | 0.998735809224318 |
| ebi-fl187-GCST90094807 | ebi-met1400-GCST90200276 | 2,4-di-tert-butylphenol levels \|\| id:ebi-met1400-GCST90200276 | Oranges liking \|\| id:ebi-fl187-GCST90094807 | Inverse variance weighted | 7 | 0.0306084674883349 | 0.145990421532866 | 0.833932428337031 | -0.255532759 | 0.316749693692753 | 1.0310817228283 | 0.77450376439602 | 1.37265894372979 | 0.998735809224318 |
| ebi-fl187-GCST90094807 | ebi-met1400-GCST90200289 | N-acetylmethionine levels \|\| id:ebi-met1400-GCST90200289 | Oranges liking \|\| id:ebi-fl187-GCST90094807 | Inverse variance weighted | 7 | -0.032379113 | 0.151470563991981 | 0.830730279740662 | -0.329261418 | 0.264503192493045 | 0.968139478301234 | 0.719454913440975 | 1.30278358231309 | 0.998735809224318 |
| ebi-fl187-GCST90094807 | ebi-met1400-GCST90200308 | 3-hydroxyisobutyrate levels \|\| id:ebi-met1400-GCST90200308 | Oranges liking \|\| id:ebi-fl187-GCST90094807 | Inverse variance weighted | 7 | -0.084556106 | 0.151583818105034 | 0.576969011419402 | -0.38166039 | 0.212548177017422 | 0.918920096569052 | 0.682726874743825 | 1.23682569870319 | 0.998735809224318 |
| ebi-fl187-GCST90094807 | ebi-met1400-GCST90200383 | Cystathionine levels \|\| id:ebi-met1400-GCST90200383 | Oranges liking \|\| id:ebi-fl187-GCST90094807 | Inverse variance weighted | 7 | -0.05655058 | 0.163168641691013 | 0.728908753667111 | -0.376361117 | 0.263259958203743 | 0.945018684703691 | 0.686354433876118 | 1.30116492348658 | 0.998735809224318 |
| ebi-fl187-GCST90094807 | ebi-met1400-GCST90200384 | Guanidinoacetate levels \|\| id:ebi-met1400-GCST90200384 | Oranges liking \|\| id:ebi-fl187-GCST90094807 | Inverse variance weighted | 7 | -0.059156558 | 0.153744805789709 | 0.700407011144467 | -0.360496378 | 0.242183261003343 | 0.942559192098496 | 0.697330101042612 | 1.27402765100926 | 0.998735809224318 |
| ebi-fl187-GCST90094807 | ebi-met1400-GCST90200403 | Citrulline levels \|\| id:ebi-met1400-GCST90200403 | Oranges liking \|\| id:ebi-fl187-GCST90094807 | Inverse variance weighted | 7 | 0.00590877209270454 | 0.141935298833682 | 0.966793625958582 | -0.272284414 | 0.284101957806722 | 1.00592626332012 | 0.761637610171225 | 1.32856838176582 | 0.998735809224318 |
| ebi-fl187-GCST90094807 | ebi-met1400-GCST90200434 | 12,13-DiHOME levels \|\| id:ebi-met1400-GCST90200434 | Oranges liking \|\| id:ebi-fl187-GCST90094807 | Inverse variance weighted | 7 | -0.074357827 | 0.14305956188743 | 0.603225069641139 | -0.354754568 | 0.20603891466167 | 0.928339449817985 | 0.701345554773205 | 1.22880102144091 | 0.998735809224318 |
| ebi-fl187-GCST90094807 | ebi-met1400-GCST90200437 | Fructose levels \|\| id:ebi-met1400-GCST90200437 | Oranges liking \|\| id:ebi-fl187-GCST90094807 | Inverse variance weighted | 7 | 0.0013030787971493 | 0.17912409313991 | 0.994195659192985 | -0.349780144 | 0.352386301351372 | 1.00130392817322 | 0.704843036826975 | 1.42245791501128 | 0.999107451094609 |
| ebi-fl187-GCST90094807 | ebi-met1400-GCST90200448 | N-stearoyl-sphinganine (d18:0/18:0) levels \|\| id:ebi-met1400-GCST90200448 | Oranges liking \|\| id:ebi-fl187-GCST90094807 | Inverse variance weighted | 7 | 0.0322247859154975 | 0.158182788352896 | 0.838573358233797 | -0.277813479 | 0.342263051087174 | 1.03274962678566 | 0.75743808625477 | 1.40813065910072 | 0.998735809224318 |
| ebi-fl187-GCST90094807 | ebi-met1400-GCST90200468 | X-11847 levels \|\| id:ebi-met1400-GCST90200468 | Oranges liking \|\| id:ebi-fl187-GCST90094807 | Inverse variance weighted | 7 | -0.023901058 | 0.150495647416492 | 0.873814149264495 | -0.318872527 | 0.271070410767422 | 0.976382310032532 | 0.726968212142623 | 1.31136740151911 | 0.998735809224318 |
| ebi-fl187-GCST90094807 | ebi-met1400-GCST90200476 | X-11843 levels \|\| id:ebi-met1400-GCST90200476 | Oranges liking \|\| id:ebi-fl187-GCST90094807 | Inverse variance weighted | 7 | -0.198023769 | 0.158512802865868 | 0.211569843589912 | -0.508708863 | 0.112661324728218 | 0.820350354089685 | 0.601271401908428 | 1.11925280550357 | 0.998735809224318 |
| ebi-fl187-GCST90094807 | ebi-met1400-GCST90200511 | X-12906 levels \|\| id:ebi-met1400-GCST90200511 | Oranges liking \|\| id:ebi-fl187-GCST90094807 | Inverse variance weighted | 7 | -0.05844572 | 0.142070770625132 | 0.680790583070646 | -0.33690443 | 0.220012990786835 | 0.943229437845081 | 0.713977071385473 | 1.24609291820971 | 0.998735809224318 |
| ebi-fl187-GCST90094807 | ebi-met1400-GCST90200515 | X-12849 levels \|\| id:ebi-met1400-GCST90200515 | Oranges liking \|\| id:ebi-fl187-GCST90094807 | Inverse variance weighted | 7 | 0.0593898500128731 | 0.152767683662063 | 0.697454248498694 | -0.24003481 | 0.358814509990517 | 1.06118886460181 | 0.786600479055008 | 1.43163122365214 | 0.998735809224318 |
| ebi-fl187-GCST90094807 | ebi-met1400-GCST90200551 | X-18886 levels \|\| id:ebi-met1400-GCST90200551 | Oranges liking \|\| id:ebi-fl187-GCST90094807 | Inverse variance weighted | 7 | -0.101504488 | 0.139906041404687 | 0.468133709397868 | -0.375720329 | 0.172711353636479 | 0.90347712496443 | 0.686794382884202 | 1.18852299272754 | 0.998735809224318 |
| ebi-fl187-GCST90094807 | ebi-met1400-GCST90200564 | X-21319 levels \|\| id:ebi-met1400-GCST90200564 | Oranges liking \|\| id:ebi-fl187-GCST90094807 | Inverse variance weighted | 7 | -0.268990368 | 0.143260557852182 | 0.0604317541587017 | -0.549781061 | 0.0118003254617909 | 0.764150615966131 | 0.577076140839918 | 1.01187022397344 | 0.998735809224318 |
| ebi-fl187-GCST90094807 | ebi-met1400-GCST90200579 | X-21442 levels \|\| id:ebi-met1400-GCST90200579 | Oranges liking \|\| id:ebi-fl187-GCST90094807 | Inverse variance weighted | 7 | 0.062127786475094 | 0.154555689722701 | 0.687701172299335 | -0.240801365 | 0.365056938331588 | 1.06409831341299 | 0.78599773724408 | 1.44059603094857 | 0.998735809224318 |
| ebi-fl187-GCST90094807 | ebi-met1400-GCST90200581 | X-21441 levels \|\| id:ebi-met1400-GCST90200581 | Oranges liking \|\| id:ebi-fl187-GCST90094807 | Inverse variance weighted | 7 | 0.13604171426419 | 0.14213264420603 | 0.338493488900546 | -0.142538268 | 0.41462169690801 | 1.14572968586885 | 0.867154369064694 | 1.51379795790801 | 0.998735809224318 |
| ebi-fl187-GCST90094807 | ebi-met1400-GCST90200648 | X-24585 levels \|\| id:ebi-met1400-GCST90200648 | Oranges liking \|\| id:ebi-fl187-GCST90094807 | Inverse variance weighted | 7 | -0.061981831 | 0.138590779592688 | 0.654709647931114 | -0.333619759 | 0.209656097500293 | 0.93989996414705 | 0.716326107360639 | 1.23325386793262 | 0.998735809224318 |
| ebi-fl187-GCST90094807 | ebi-met1400-GCST90200720 | Adenosine 5'-diphosphate (ADP) to pantothenate ratio \|\| id:ebi-met1400-GCST90200720 | Oranges liking \|\| id:ebi-fl187-GCST90094807 | Inverse variance weighted | 7 | -0.195152048 | 0.18942091426627 | 0.302889811486674 | -0.56641704 | 0.176112943786862 | 0.822709557058842 | 0.567555327982288 | 1.19257274472645 | 0.998735809224318 |
| ebi-fl187-GCST90094807 | ebi-met1400-GCST90200724 | Cholate to taurocholate ratio \|\| id:ebi-met1400-GCST90200724 | Oranges liking \|\| id:ebi-fl187-GCST90094807 | Inverse variance weighted | 7 | 0.207193605673425 | 0.17389168353229 | 0.233453600615878 | -0.133634094 | 0.548021305396713 | 1.23022072643817 | 0.874910140867238 | 1.72982683028211 | 0.998735809224318 |
| ebi-fl187-GCST90094807 | ebi-met1400-GCST90200726 | N-acetylputrescine to (N(1) + N(8))-acetylspermidine ratio \|\| id:ebi-met1400-GCST90200726 | Oranges liking \|\| id:ebi-fl187-GCST90094807 | Inverse variance weighted | 7 | 0.0840432202512784 | 0.14118184338247 | 0.551653990961984 | -0.192673193 | 0.36075963328092 | 1.08767590241877 | 0.824751464835721 | 1.43441863293705 | 0.998735809224318 |
| ebi-fl187-GCST90094807 | ebi-met1400-GCST90200744 | Arginine to glutamate ratio \|\| id:ebi-met1400-GCST90200744 | Oranges liking \|\| id:ebi-fl187-GCST90094807 | Inverse variance weighted | 7 | 0.0828033102978416 | 0.136743692937207 | 0.544822516528309 | -0.185214328 | 0.350820948454767 | 1.08632811797959 | 0.830926174137911 | 1.42023300823017 | 0.998735809224318 |
| ebi-fl187-GCST90094807 | ebi-met1400-GCST90200759 | Histidine to trans-urocanate ratio \|\| id:ebi-met1400-GCST90200759 | Oranges liking \|\| id:ebi-fl187-GCST90094807 | Inverse variance weighted | 7 | 0.261675025129705 | 0.143049296070385 | 0.0673599667531715 | -0.018701595 | 0.542051645427659 | 1.29910429757301 | 0.981472194594435 | 1.71953111383868 | 0.998735809224318 |
| ebi-fl187-GCST90094807 | ebi-met1400-GCST90200795 | Oleoyl-linoleoyl-glycerol (18:1 to 18:2) [2] to linoleoyl-arachidonoyl-glycerol (18:2 to 20:4) [2] ratio \|\| id:ebi-met1400-GCST90200795 | Oranges liking \|\| id:ebi-fl187-GCST90094807 | Inverse variance weighted | 7 | -0.041866397 | 0.181735469683321 | 0.817804283266579 | -0.398067918 | 0.314335123343628 | 0.95899789678394 | 0.671616411393775 | 1.36934856032993 | 0.998735809224318 |
| ebi-fl187-GCST90094807 | ebi-met1400-GCST90200811 | Choline to taurocholate ratio \|\| id:ebi-met1400-GCST90200811 | Oranges liking \|\| id:ebi-fl187-GCST90094807 | Inverse variance weighted | 7 | 0.0126355834958679 | 0.148379414698401 | 0.932136373506108 | -0.278188069 | 0.303459236304734 | 1.01271574977441 | 0.757154410613358 | 1.35453637390864 | 0.998735809224318 |
| ebi-fl187-GCST90094807 | ebi-met1400-GCST90200831 | Cholate to phosphate ratio \|\| id:ebi-met1400-GCST90200831 | Oranges liking \|\| id:ebi-fl187-GCST90094807 | Inverse variance weighted | 7 | 0.19742276913958 | 0.170122010715631 | 0.245854441015846 | -0.136016372 | 0.530861910142218 | 1.21825897414754 | 0.872828342545647 | 1.70039726684678 | 0.998735809224318 |
| ebi-fl187-GCST90094807 | ebi-met1400-GCST90200841 | Adenosine 5'-diphosphate (ADP) to oxalate (ethanedioate) ratio \|\| id:ebi-met1400-GCST90200841 | Oranges liking \|\| id:ebi-fl187-GCST90094807 | Inverse variance weighted | 7 | -0.152335068 | 0.190858310900169 | 0.424778867081271 | -0.526417357 | 0.221747221428748 | 0.858700509525352 | 0.590717512960494 | 1.24825580566191 | 0.998735809224318 |
| ebi-fl187-GCST90094807 | ebi-met1400-GCST90200844 | Aspartate to phosphate ratio \|\| id:ebi-met1400-GCST90200844 | Oranges liking \|\| id:ebi-fl187-GCST90094807 | Inverse variance weighted | 7 | -0.0647789 | 0.13328497249498 | 0.626954382188859 | -0.326017446 | 0.196459646253216 | 0.937274672052442 | 0.721792594980876 | 1.21708620589865 | 0.998735809224318 |
| ebi-fl187-GCST90094807 | ebi-met1400-GCST90200870 | Adenosine 5'-monophosphate (AMP) to acetoacetate ratio \|\| id:ebi-met1400-GCST90200870 | Oranges liking \|\| id:ebi-fl187-GCST90094807 | Inverse variance weighted | 7 | -0.069262479 | 0.15653901980289 | 0.658155332618066 | -0.376078958 | 0.23755399978367 | 0.933081733544618 | 0.686548122542833 | 1.2681434744151 | 0.998735809224318 |
| ebi-fl187-GCST90094807 | ebi-met1400-GCST90200887 | Cholesterol to cortisol ratio \|\| id:ebi-met1400-GCST90200887 | Oranges liking \|\| id:ebi-fl187-GCST90094807 | Inverse variance weighted | 7 | 0.154593663331706 | 0.137857273065784 | 0.262116039561043 | -0.115606592 | 0.424793918540642 | 1.16718359524215 | 0.890825612401884 | 1.52927523191577 | 0.998735809224318 |
| ebi-fl187-GCST90094807 | ebi-met1400-GCST90200890 | Cortisol to taurocholate ratio \|\| id:ebi-met1400-GCST90200890 | Oranges liking \|\| id:ebi-fl187-GCST90094807 | Inverse variance weighted | 7 | -0.007492116 | 0.147981976007812 | 0.959621494410817 | -0.297536789 | 0.282552556766454 | 0.992535879733871 | 0.742645261404919 | 1.32651149041933 | 0.998735809224318 |
| ebi-fl187-GCST90094807 | ebi-met1400-GCST90200948 | Adenosine 5'-diphosphate (ADP) to mannitol to sorbitol ratio \|\| id:ebi-met1400-GCST90200948 | Oranges liking \|\| id:ebi-fl187-GCST90094807 | Inverse variance weighted | 7 | -0.281123554 | 0.224104469186601 | 0.209685454800175 | -0.720368313 | 0.158121205725891 | 0.754935054562693 | 0.486573011551056 | 1.17130815535949 | 0.998735809224318 |
| ebi-fl187-GCST90094807 | ebi-met1400-GCST90200972 | Leucine to N-palmitoyl-sphingosine (d18:1 to 16:0) ratio \|\| id:ebi-met1400-GCST90200972 | Oranges liking \|\| id:ebi-fl187-GCST90094807 | Inverse variance weighted | 7 | 0.121943116579857 | 0.153685803651613 | 0.427511461458907 | -0.179281059 | 0.423167291737018 | 1.12968983933084 | 0.835870937683025 | 1.52678969390283 | 0.998735809224318 |
| ebi-fl187-GCST90094807 | ebi-met1400-GCST90200973 | Proline to glutamate ratio \|\| id:ebi-met1400-GCST90200973 | Oranges liking \|\| id:ebi-fl187-GCST90094807 | Inverse variance weighted | 7 | -0.024617362 | 0.138954661138634 | 0.85938184030551 | -0.296968498 | 0.247733773500258 | 0.975683173746614 | 0.743067419981853 | 1.28111881901041 | 0.998735809224318 |
| ebi-fl187-GCST90094807 | ebi-met1400-GCST90200983 | Cholesterol to linoleoyl-arachidonoyl-glycerol (18:2 to 20:4) [1] ratio \|\| id:ebi-met1400-GCST90200983 | Oranges liking \|\| id:ebi-fl187-GCST90094807 | Inverse variance weighted | 7 | 0.0414804937568636 | 0.146048946966387 | 0.776396775646913 | -0.244775442 | 0.327736429810982 | 1.04235282926545 | 0.782880320310774 | 1.38782313527358 | 0.998735809224318 |
| ebi-fl187-GCST90094807 | ebi-met1400-GCST90201001 | Serine to alpha-ketobutyrate ratio \|\| id:ebi-met1400-GCST90201001 | Oranges liking \|\| id:ebi-fl187-GCST90094807 | Inverse variance weighted | 7 | 0.24092961390916 | 0.144303107245143 | 0.0949969580510968 | -0.041904476 | 0.52376370410964 | 1.27243147067913 | 0.958961379744942 | 1.68837023239172 | 0.998735809224318 |
| ebi-fl187-GCST90094807 | ebi-met1400-GCST90201005 | Fructose to maltose ratio \|\| id:ebi-met1400-GCST90201005 | Oranges liking \|\| id:ebi-fl187-GCST90094807 | Inverse variance weighted | 7 | 0.0629028179514063 | 0.161934148479328 | 0.697685342616354 | -0.254488113 | 0.38029374897089 | 1.06492334277034 | 0.775313269132044 | 1.46271419712284 | 0.998735809224318 |
| ebi-fl187-GCST90094807 | ebi-met1400-GCST90201020 | Paraxanthine to 5-acetylamino-6-formylamino-3-methyluracil ratio \|\| id:ebi-met1400-GCST90201020 | Oranges liking \|\| id:ebi-fl187-GCST90094807 | Inverse variance weighted | 7 | 0.185379765199434 | 0.202538966595715 | 0.360044915351832 | -0.211596609 | 0.582356139727034 | 1.20367546739461 | 0.809291092207191 | 1.79025154824847 | 0.998735809224318 |
| ebi-fl187-GCST90094807 | ebi-pw11-GCST90275197 | Placental weight (fetal genotype, adjusted for sex) \|\| id:ebi-pw11-GCST90275197 | Oranges liking \|\| id:ebi-fl187-GCST90094807 | Inverse variance weighted | 7 | 0.0517823177370586 | 0.062095866489964 | 0.404332121777762 | -0.069925581 | 0.173490216057388 | 1.0531464662536 | 0.932463210692166 | 1.18944904921144 | 0.998735809224318 |
| ebi-fl187-GCST90094807 | prot-a-1017 | FAS-associated factor 2 \|\| id:prot-a-1017 | Oranges liking \|\| id:ebi-fl187-GCST90094807 | Inverse variance weighted | 7 | -0.153973649 | 0.253428565535894 | 0.543477794134944 | -0.650693637 | 0.342746339762409 | 0.857294611551644 | 0.521683791979921 | 1.4088113571751 | 0.998735809224318 |
| ebi-fl187-GCST90094807 | prot-a-1019 | Fas apoptotic inhibitory molecule 3 \|\| id:prot-a-1019 | Oranges liking \|\| id:ebi-fl187-GCST90094807 | Inverse variance weighted | 7 | -0.020361837 | 0.228424214362953 | 0.928970291067039 | -0.468073297 | 0.427349622945972 | 0.97984406511714 | 0.626207622611816 | 1.5331886059465 | 0.998735809224318 |
| ebi-fl187-GCST90094807 | prot-a-1052 | MIP18 family protein FAM96A \|\| id:prot-a-1052 | Oranges liking \|\| id:ebi-fl187-GCST90094807 | Inverse variance weighted | 7 | -0.026807926 | 0.292713550770107 | 0.927028432467021 | -0.600526486 | 0.546910633156419 | 0.973548216523764 | 0.54852277057498 | 1.72790662619728 | 0.998735809224318 |
| ebi-fl187-GCST90094807 | prot-a-1066 | F-box/LRR-repeat protein 4 \|\| id:prot-a-1066 | Oranges liking \|\| id:ebi-fl187-GCST90094807 | Inverse variance weighted | 7 | -0.102806001 | 0.228497832556868 | 0.652767318004809 | -0.550661753 | 0.345049750970392 | 0.902302002332797 | 0.576568138613171 | 1.41206016928382 | 0.998735809224318 |
| ebi-fl187-GCST90094807 | prot-a-1076 | Low affinity immunoglobulin gamma Fc region receptor III-B \|\| id:prot-a-1076 | Oranges liking \|\| id:ebi-fl187-GCST90094807 | Inverse variance weighted | 7 | -0.14340623 | 0.236563424463682 | 0.54437779242396 | -0.607070542 | 0.320258081735131 | 0.866402038712357 | 0.544944926258095 | 1.37748322172543 | 0.998735809224318 |
| ebi-fl187-GCST90094807 | prot-a-1081 | Fc receptor-like protein 3 \|\| id:prot-a-1081 | Oranges liking \|\| id:ebi-fl187-GCST90094807 | Inverse variance weighted | 7 | -0.051998585 | 0.228497832556868 | 0.819982814845369 | -0.499854336 | 0.395857167277475 | 0.949330210586588 | 0.606619015620092 | 1.48565710194747 | 0.998735809224318 |
| ebi-fl187-GCST90094807 | prot-a-1105 | Fibroblast growth factor receptor 4 \|\| id:prot-a-1105 | Oranges liking \|\| id:ebi-fl187-GCST90094807 | Inverse variance weighted | 7 | -0.14234777 | 0.228497832556868 | 0.53330286996105 | -0.590203522 | 0.305507981986102 | 0.867319576453715 | 0.554214478617816 | 1.35731431913491 | 0.998735809224318 |
| ebi-fl187-GCST90094807 | prot-a-1143 | Follistatin-related protein 1 \|\| id:prot-a-1143 | Oranges liking \|\| id:ebi-fl187-GCST90094807 | Inverse variance weighted | 7 | 0.266289222606755 | 0.228497832556868 | 0.243860926853216 | -0.181566529 | 0.714144974418216 | 1.30511247213735 | 0.833962760578607 | 2.04243959735886 | 0.998735809224318 |
| ebi-fl187-GCST90094807 | prot-a-1151 | Alpha-(1,3)-fucosyltransferase 10 \|\| id:prot-a-1151 | Oranges liking \|\| id:ebi-fl187-GCST90094807 | Inverse variance weighted | 7 | -0.084771991 | 0.228497832556868 | 0.71063982882528 | -0.532627743 | 0.363083760646838 | 0.91872173719534 | 0.587060297493172 | 1.43775628159395 | 0.998735809224318 |
| ebi-fl187-GCST90094807 | prot-a-1165 | Growth arrest and DNA damage-inducible proteins-interacting protein 1 \|\| id:prot-a-1165 | Oranges liking \|\| id:ebi-fl187-GCST90094807 | Inverse variance weighted | 7 | -0.193786399 | 0.278801169409183 | 0.487011276527801 | -0.740236691 | 0.352663893004644 | 0.823833857181005 | 0.477001000276937 | 1.42285283226595 | 0.998735809224318 |
| ebi-fl187-GCST90094807 | prot-a-1194 | Growth/differentiation factor 11/8 \|\| id:prot-a-1194 | Oranges liking \|\| id:ebi-fl187-GCST90094807 | Inverse variance weighted | 7 | -0.282938512 | 0.228497832556868 | 0.215621640489911 | -0.730794263 | 0.164917240135395 | 0.753566121953012 | 0.481526379342051 | 1.17929551633541 | 0.998735809224318 |
| ebi-fl187-GCST90094807 | prot-a-1216 | D-glucuronyl C5-epimerase \|\| id:prot-a-1216 | Oranges liking \|\| id:ebi-fl187-GCST90094807 | Inverse variance weighted | 7 | -0.173475085 | 0.297359848695284 | 0.55963458410565 | -0.756300388 | 0.409350218456917 | 0.84073809790255 | 0.469399815589964 | 1.50583900075974 | 0.998735809224318 |
| ebi-fl187-GCST90094807 | prot-a-1246 | Glypican-3 \|\| id:prot-a-1246 | Oranges liking \|\| id:ebi-fl187-GCST90094807 | Inverse variance weighted | 7 | -0.029155683 | 0.228424214362953 | 0.89843520389949 | -0.476867143 | 0.418555777105558 | 0.971265243164249 | 0.620724991353251 | 1.51976509037003 | 0.998735809224318 |
| ebi-fl187-GCST90094807 | prot-a-1275 | Glutamate receptor 4 \|\| id:prot-a-1275 | Oranges liking \|\| id:ebi-fl187-GCST90094807 | Inverse variance weighted | 7 | -0.292070122 | 0.228497832556868 | 0.201172448053603 | -0.739925874 | 0.155785629498982 | 0.746716172672668 | 0.477149283318821 | 1.16857566808573 | 0.998735809224318 |
| ebi-fl187-GCST90094807 | prot-a-1289 | Glutathione S-transferase P \|\| id:prot-a-1289 | Oranges liking \|\| id:ebi-fl187-GCST90094807 | Inverse variance weighted | 7 | 0.103987168290319 | 0.228279043682083 | 0.648730461599562 | -0.343439757 | 0.551414093907201 | 1.10958621693603 | 0.709326211564636 | 1.73570573417648 | 0.998735809224318 |
| ebi-fl187-GCST90094807 | prot-a-130 | Apolipoprotein D \|\| id:prot-a-130 | Oranges liking \|\| id:ebi-fl187-GCST90094807 | Inverse variance weighted | 7 | 0.106548670048389 | 0.251132400802884 | 0.671366806262693 | -0.385670836 | 0.598768175622041 | 1.1124320672507 | 0.679994318818703 | 1.81987565189879 | 0.998735809224318 |
| ebi-fl187-GCST90094807 | prot-a-1313 | Heparin-binding EGF-like growth factor \|\| id:prot-a-1313 | Oranges liking \|\| id:ebi-fl187-GCST90094807 | Inverse variance weighted | 7 | -0.04129444 | 0.22921287388663 | 0.857028847214485 | -0.490551673 | 0.407962792499027 | 0.959546559154974 | 0.612288517867209 | 1.50375120930462 | 0.998735809224318 |
| ebi-fl187-GCST90094807 | prot-a-1347 | HLA class II histocompatibility antigen, DQ alpha 2 chain \|\| id:prot-a-1347 | Oranges liking \|\| id:ebi-fl187-GCST90094807 | Inverse variance weighted | 7 | -0.085525337 | 0.240642349270094 | 0.722286693127977 | -0.557184341 | 0.386133667693871 | 0.918029882750631 | 0.572819659916433 | 1.47128132045274 | 0.998735809224318 |
| ebi-fl187-GCST90094807 | prot-a-1357 | Non-histone chromosomal protein HMG-14 \|\| id:prot-a-1357 | Oranges liking \|\| id:ebi-fl187-GCST90094807 | Inverse variance weighted | 7 | -0.108971904 | 0.228497832556868 | 0.633429320457634 | -0.556827655 | 0.338883848284843 | 0.896755612814489 | 0.573024013173655 | 1.40338033071327 | 0.998735809224318 |
| ebi-fl187-GCST90094807 | prot-a-137 | Apolipoprotein M \|\| id:prot-a-137 | Oranges liking \|\| id:ebi-fl187-GCST90094807 | Inverse variance weighted | 7 | -0.174824411 | 0.228424214362953 | 0.444063319308722 | -0.622535871 | 0.272887049606398 | 0.839604433511671 | 0.536582008261517 | 1.31375184765585 | 0.998735809224318 |
| ebi-fl187-GCST90094807 | prot-a-1381 | 17-beta-hydroxysteroid dehydrogenase 14 \|\| id:prot-a-1381 | Oranges liking \|\| id:ebi-fl187-GCST90094807 | Inverse variance weighted | 7 | -0.234435832 | 0.234960362869093 | 0.318392076121437 | -0.694958143 | 0.226086479430182 | 0.791016990404675 | 0.499095338173187 | 1.2536840784751 | 0.998735809224318 |
| ebi-fl187-GCST90094807 | prot-a-1392 | Serine protease HTRA2, mitochondrial \|\| id:prot-a-1392 | Oranges liking \|\| id:ebi-fl187-GCST90094807 | Inverse variance weighted | 7 | -0.287434796 | 0.228337872706176 | 0.208097703867373 | -0.734977027 | 0.160107434318829 | 0.750185480131351 | 0.479516474929283 | 1.17363695310553 | 0.998735809224318 |
| ebi-fl187-GCST90094807 | prot-a-1416 | Interferon-induced protein with tetratricopeptide repeats 2 \|\| id:prot-a-1416 | Oranges liking \|\| id:ebi-fl187-GCST90094807 | Inverse variance weighted | 7 | -0.22498465 | 0.252374740586425 | 0.372676780458853 | -0.719639142 | 0.269669841100576 | 0.798528475719018 | 0.486927936102311 | 1.30953202570032 | 0.998735809224318 |
| ebi-fl187-GCST90094807 | prot-a-1428 | Interferon gamma \|\| id:prot-a-1428 | Oranges liking \|\| id:ebi-fl187-GCST90094807 | Inverse variance weighted | 7 | -0.233415177 | 0.228424214362953 | 0.306852119910395 | -0.681126638 | 0.214296282668841 | 0.791824757461103 | 0.506046540002889 | 1.23898969158994 | 0.998735809224318 |
| ebi-fl187-GCST90094807 | prot-a-1483 | Interleukin-17C \|\| id:prot-a-1483 | Oranges liking \|\| id:ebi-fl187-GCST90094807 | Inverse variance weighted | 7 | -0.47495241 | 0.228205636688239 | 0.0374112581884409 | -0.922235458 | -0.027669362 | 0.621914652697073 | 0.397629163566211 | 0.972709928442954 | 0.998735809224318 |
| ebi-fl187-GCST90094807 | prot-a-1503 | Interleukin-1 receptor-like 2 \|\| id:prot-a-1503 | Oranges liking \|\| id:ebi-fl187-GCST90094807 | Inverse variance weighted | 7 | -0.108270329 | 0.228205636688239 | 0.635184780433862 | -0.555553376 | 0.339012719382616 | 0.897384974880337 | 0.573754671016506 | 1.40356119753109 | 0.998735809224318 |
| ebi-fl187-GCST90094807 | prot-a-1522 | Interleukin-31 receptor subunit alpha \|\| id:prot-a-1522 | Oranges liking \|\| id:ebi-fl187-GCST90094807 | Inverse variance weighted | 7 | -0.202992359 | 0.228205636688239 | 0.373726312522092 | -0.650275407 | 0.244290688544747 | 0.816284478334189 | 0.521902021353729 | 1.27671540309615 | 0.998735809224318 |
| ebi-fl187-GCST90094807 | prot-a-1540 | Interleukin-6 receptor subunit alpha \|\| id:prot-a-1540 | Oranges liking \|\| id:ebi-fl187-GCST90094807 | Inverse variance weighted | 7 | -0.192066847 | 0.228279043682083 | 0.400141403263899 | -0.639493772 | 0.25536007877858 | 0.825251701180796 | 0.527559421567321 | 1.2909263723857 | 0.998735809224318 |
| ebi-fl187-GCST90094807 | prot-a-1541 | Interleukin-6 receptor subunit alpha \|\| id:prot-a-1541 | Oranges liking \|\| id:ebi-fl187-GCST90094807 | Inverse variance weighted | 7 | -0.153408684 | 0.228279043682083 | 0.501569288854647 | -0.600835609 | 0.294018241992669 | 0.85777908990028 | 0.548353235567817 | 1.34180838070236 | 0.998735809224318 |
| ebi-fl187-GCST90094807 | prot-a-1545 | Interleukin-9 \|\| id:prot-a-1545 | Oranges liking \|\| id:ebi-fl187-GCST90094807 | Inverse variance weighted | 7 | -0.04535937 | 0.228192866477041 | 0.842437610231343 | -0.492617389 | 0.401898647953917 | 0.955653986416708 | 0.611025009459424 | 1.49465983816624 | 0.998735809224318 |
| ebi-fl187-GCST90094807 | prot-a-1548 | Inositol monophosphatase 3 \|\| id:prot-a-1548 | Oranges liking \|\| id:ebi-fl187-GCST90094807 | Inverse variance weighted | 7 | -0.501368085 | 0.228424214362953 | 0.0281708417887534 | -0.949079546 | -0.053656625 | 0.605701441329533 | 0.387097164835276 | 0.947757486637216 | 0.998735809224318 |
| ebi-fl187-GCST90094807 | prot-a-1555 | Inhibin beta A chain:Inhibin beta B chain heterodimer \|\| id:prot-a-1555 | Oranges liking \|\| id:ebi-fl187-GCST90094807 | Inverse variance weighted | 7 | -0.434305202 | 0.228337872706176 | 0.0571673941779329 | -0.881847432 | 0.013237028533135 | 0.647714541560835 | 0.414017335653186 | 1.01332502584094 | 0.998735809224318 |
| ebi-fl187-GCST90094807 | prot-a-1603 | Junctophilin-3 \|\| id:prot-a-1603 | Oranges liking \|\| id:ebi-fl187-GCST90094807 | Inverse variance weighted | 7 | 0.04337427553324 | 0.228497832556868 | 0.849447381317811 | -0.404481476 | 0.491230027344701 | 1.04432868839055 | 0.667322743836258 | 1.63432524886807 | 0.998735809224318 |
| ebi-fl187-GCST90094807 | prot-a-1627 | UPF0258 protein KIAA1024 \|\| id:prot-a-1627 | Oranges liking \|\| id:ebi-fl187-GCST90094807 | Inverse variance weighted | 7 | 0.2510200042761 | 0.238680201122469 | 0.292937140336808 | -0.21679319 | 0.718833198476139 | 1.28533579628617 | 0.805096454147956 | 2.05203749277846 | 0.998735809224318 |
| ebi-fl187-GCST90094807 | prot-a-1666 | Kallikrein-7 \|\| id:prot-a-1666 | Oranges liking \|\| id:ebi-fl187-GCST90094807 | Inverse variance weighted | 7 | 0.0687392275743968 | 0.228424214362953 | 0.763469446553077 | -0.378972233 | 0.516450687725785 | 1.07115684455608 | 0.684564620997779 | 1.67606819056322 | 0.998735809224318 |
| ebi-fl187-GCST90094807 | prot-a-1678 | Importin subunit alpha-3 \|\| id:prot-a-1678 | Oranges liking \|\| id:ebi-fl187-GCST90094807 | Inverse variance weighted | 7 | -0.002678562 | 0.228497832556868 | 0.990647027555675 | -0.450534313 | 0.445177190296597 | 0.997325022630199 | 0.637287549405377 | 1.56076672405164 | 0.999107451094609 |
| ebi-fl187-GCST90094807 | prot-a-1718 | Protein LDOC1 \|\| id:prot-a-1718 | Oranges liking \|\| id:ebi-fl187-GCST90094807 | Inverse variance weighted | 7 | -0.391176767 | 0.228411407424344 | 0.0867866444484289 | -0.838863126 | 0.056509591564862 | 0.676260604923796 | 0.432201603193027 | 1.05813676393896 | 0.998735809224318 |
| ebi-fl187-GCST90094807 | prot-a-1723 | Leptin \|\| id:prot-a-1723 | Oranges liking \|\| id:ebi-fl187-GCST90094807 | Inverse variance weighted | 7 | -0.325935772 | 0.228497832556868 | 0.153744752939912 | -0.773791524 | 0.121919979572614 | 0.721851548851722 | 0.461260866982944 | 1.12966370199122 | 0.998735809224318 |
| ebi-fl187-GCST90094807 | prot-a-1730 | Galectin-7 \|\| id:prot-a-1730 | Oranges liking \|\| id:ebi-fl187-GCST90094807 | Inverse variance weighted | 7 | -0.117194138 | 0.228497832556868 | 0.608028620498704 | -0.56504989 | 0.330661613312068 | 0.889412507191969 | 0.568331791800467 | 1.39188871599714 | 0.998735809224318 |
| ebi-fl187-GCST90094807 | prot-a-1753 | Lipase member N \|\| id:prot-a-1753 | Oranges liking \|\| id:ebi-fl187-GCST90094807 | Inverse variance weighted | 7 | 0.0388107933826825 | 0.228424214362953 | 0.865083550046984 | -0.408900667 | 0.486522253534071 | 1.03957377080443 | 0.664380224079041 | 1.62664929776112 | 0.998735809224318 |
| ebi-fl187-GCST90094807 | prot-a-1756 | VIP36-like protein \|\| id:prot-a-1756 | Oranges liking \|\| id:ebi-fl187-GCST90094807 | Inverse variance weighted | 7 | 0.156201777480512 | 0.228394970352444 | 0.494031519836564 | -0.291452364 | 0.603855919371302 | 1.16906206969177 | 0.747177605001096 | 1.82915830673217 | 0.998735809224318 |
| ebi-fl187-GCST90094807 | prot-a-1760 | Ig Kappa chain V-I region HK102- like \|\| id:prot-a-1760 | Oranges liking \|\| id:ebi-fl187-GCST90094807 | Inverse variance weighted | 7 | -0.404323101 | 0.271590871543277 | 0.136560787569143 | -0.936641209 | 0.127995007172161 | 0.667428439595907 | 0.391942078406007 | 1.13654732809775 | 0.998735809224318 |
| ebi-fl187-GCST90094807 | prot-a-179 | Neutral ceramidase \|\| id:prot-a-179 | Oranges liking \|\| id:ebi-fl187-GCST90094807 | Inverse variance weighted | 7 | -0.574988005 | 0.254461367347486 | 0.0238443545560807 | -1.073732285 | -0.076243725 | 0.562711618516753 | 0.341730697965279 | 0.92659034584571 | 0.998735809224318 |
| ebi-fl187-GCST90094807 | prot-a-1809 | Lactotransferrin \|\| id:prot-a-1809 | Oranges liking \|\| id:ebi-fl187-GCST90094807 | Inverse variance weighted | 7 | -0.1375678 | 0.305571010931202 | 0.652566872081557 | -0.736486981 | 0.461351381770095 | 0.871475262271942 | 0.47879297328719 | 1.58621612079592 | 0.998735809224318 |
| ebi-fl187-GCST90094807 | prot-a-1818 | Lysophospholipase-like protein 1 \|\| id:prot-a-1818 | Oranges liking \|\| id:ebi-fl187-GCST90094807 | Inverse variance weighted | 7 | 0.182795590500316 | 0.228497832556868 | 0.423717581357775 | -0.265060161 | 0.630651342311777 | 1.20056897529371 | 0.767159795248365 | 1.87883394485129 | 0.998735809224318 |
| ebi-fl187-GCST90094807 | prot-a-1852 | MAP kinase-activated protein kinase 3 \|\| id:prot-a-1852 | Oranges liking \|\| id:ebi-fl187-GCST90094807 | Inverse variance weighted | 7 | 0.286346485584414 | 0.239458519054064 | 0.231771374585041 | -0.182992212 | 0.75568518293038 | 1.33155373950912 | 0.832774641559316 | 2.12906982599857 | 0.998735809224318 |
| ebi-fl187-GCST90094807 | prot-a-1856 | Megakaryocyte-associated tyrosine-protein kinase \|\| id:prot-a-1856 | Oranges liking \|\| id:ebi-fl187-GCST90094807 | Inverse variance weighted | 7 | -0.150025688 | 0.228424214362953 | 0.511318915841804 | -0.597737148 | 0.297685772068857 | 0.860685866771502 | 0.550054921628921 | 1.3467385385199 | 0.998735809224318 |
| ebi-fl187-GCST90094807 | prot-a-1890 | Beta-1,3-N-acetylglucosaminyltransferase manic fringe \|\| id:prot-a-1890 | Oranges liking \|\| id:ebi-fl187-GCST90094807 | Inverse variance weighted | 7 | -0.232312435 | 0.249340799596515 | 0.351488245253433 | -0.721020402 | 0.256395532619937 | 0.792698418208201 | 0.486255826404495 | 1.29226375933863 | 0.998735809224318 |
| ebi-fl187-GCST90094807 | prot-a-1919 | Matrilysin \|\| id:prot-a-1919 | Oranges liking \|\| id:ebi-fl187-GCST90094807 | Inverse variance weighted | 7 | 0.101775572379229 | 0.228497832556868 | 0.656022371302669 | -0.346080179 | 0.54963132419069 | 1.10713497217622 | 0.707455761431135 | 1.7326141271873 | 0.998735809224318 |
| ebi-fl187-GCST90094807 | prot-a-1923 | Multimerin-2 \|\| id:prot-a-1923 | Oranges liking \|\| id:ebi-fl187-GCST90094807 | Inverse variance weighted | 7 | -0.23894782 | 0.267712970836953 | 0.372096988757848 | -0.763665243 | 0.285769602764594 | 0.787455970693521 | 0.465955453433463 | 1.33078581055693 | 0.998735809224318 |
| ebi-fl187-GCST90094807 | prot-a-1948 | Mesothelin \|\| id:prot-a-1948 | Oranges liking \|\| id:ebi-fl187-GCST90094807 | Inverse variance weighted | 7 | -0.104302104 | 0.228424214362953 | 0.647947224982642 | -0.552013564 | 0.343409356155311 | 0.900953074779332 | 0.575789253747149 | 1.40974573191807 | 0.998735809224318 |
| ebi-fl187-GCST90094807 | prot-a-195 | ATPase family AAA domain-containing protein 1 \|\| id:prot-a-195 | Oranges liking \|\| id:ebi-fl187-GCST90094807 | Inverse variance weighted | 7 | 0.140683961241639 | 0.228337872706176 | 0.537814077026008 | -0.306858269 | 0.588226191745744 | 1.15106081066502 | 0.735754872598565 | 1.80079132220946 | 0.998735809224318 |
| ebi-fl187-GCST90094807 | prot-a-1966 | Mucin-1 \|\| id:prot-a-1966 | Oranges liking \|\| id:ebi-fl187-GCST90094807 | Inverse variance weighted | 7 | -0.046070708 | 0.228497832556868 | 0.840210473401079 | -0.49392646 | 0.401785044073214 | 0.954974435720987 | 0.610225657710258 | 1.49449004865285 | 0.998735809224318 |
| ebi-fl187-GCST90094807 | prot-a-1986 | Myocardial zonula adherens protein \|\| id:prot-a-1986 | Oranges liking \|\| id:ebi-fl187-GCST90094807 | Inverse variance weighted | 7 | -0.630740871 | 0.228497832556868 | 0.0057734144852335 | -1.078596623 | -0.18288512 | 0.532197365092871 | 0.340072440682955 | 0.832863829962189 | 0.998735809224318 |
| ebi-fl187-GCST90094807 | prot-a-2071 | Epididymal secretory protein E1 \|\| id:prot-a-2071 | Oranges liking \|\| id:ebi-fl187-GCST90094807 | Inverse variance weighted | 7 | -0.338074134 | 0.228434007517966 | 0.138882823335214 | -0.785804789 | 0.10965652075968 | 0.713142417856017 | 0.455752769470191 | 1.11589471795583 | 0.998735809224318 |
| ebi-fl187-GCST90094807 | prot-a-2098 | Neuritin \|\| id:prot-a-2098 | Oranges liking \|\| id:ebi-fl187-GCST90094807 | Inverse variance weighted | 7 | 0.126530646096139 | 0.27829323305559 | 0.649349092379657 | -0.418924091 | 0.671985382885095 | 1.13488423041438 | 0.657754123032578 | 1.95812108406874 | 0.998735809224318 |
| ebi-fl187-GCST90094807 | prot-a-2115 | Neurotrophin-4 \|\| id:prot-a-2115 | Oranges liking \|\| id:ebi-fl187-GCST90094807 | Inverse variance weighted | 7 | -0.049098176 | 0.228497832556868 | 0.82986578559099 | -0.496953928 | 0.398757575703775 | 0.952087652850051 | 0.6083810125447 | 1.48997236931834 | 0.998735809224318 |
| ebi-fl187-GCST90094807 | prot-a-2118 | Netrin-1 \|\| id:prot-a-2118 | Oranges liking \|\| id:ebi-fl187-GCST90094807 | Inverse variance weighted | 7 | 0.0857514528034106 | 0.228497832556868 | 0.707449680094427 | -0.362104299 | 0.533607204614872 | 1.08953549365639 | 0.696209750068529 | 1.70507177157492 | 0.998735809224318 |
| ebi-fl187-GCST90094807 | prot-a-2120 | Netrin-G1 \|\| id:prot-a-2120 | Oranges liking \|\| id:ebi-fl187-GCST90094807 | Inverse variance weighted | 7 | -0.215159848 | 0.228497832556868 | 0.346383372978649 | -0.6630156 | 0.23269590352111 | 0.80641252621063 | 0.515295065276958 | 1.26199765192762 | 0.998735809224318 |
| ebi-fl187-GCST90094807 | prot-a-2145 | Olfactomedin-like protein 3 \|\| id:prot-a-2145 | Oranges liking \|\| id:ebi-fl187-GCST90094807 | Inverse variance weighted | 7 | 0.0635040539392683 | 0.255345365019587 | 0.803593873868198 | -0.436972861 | 0.563980969377658 | 1.06556380552366 | 0.645988962344764 | 1.75765576476847 | 0.998735809224318 |
| ebi-fl187-GCST90094807 | prot-a-2162 | Prolyl 4-hydroxylase subunit alpha-2 \|\| id:prot-a-2162 | Oranges liking \|\| id:ebi-fl187-GCST90094807 | Inverse variance weighted | 7 | -0.198203034 | 0.228497832556868 | 0.385713260950727 | -0.646058785 | 0.249652718202761 | 0.820203307393844 | 0.524107331033068 | 1.28357957545409 | 0.998735809224318 |
| ebi-fl187-GCST90094807 | prot-a-2168 | Polyadenylate-binding protein-interacting protein 1 \|\| id:prot-a-2168 | Oranges liking \|\| id:ebi-fl187-GCST90094807 | Inverse variance weighted | 7 | 0.0177991446408442 | 0.269059218902149 | 0.947255807805255 | -0.509556924 | 0.545155213689056 | 1.01795849343593 | 0.600761702698867 | 1.72487608598074 | 0.998735809224318 |
| ebi-fl187-GCST90094807 | prot-a-219 | Beta-1,4-galactosyltransferase 5 \|\| id:prot-a-219 | Oranges liking \|\| id:ebi-fl187-GCST90094807 | Inverse variance weighted | 7 | 0.0774775084547864 | 0.228497832556868 | 0.73455453046082 | -0.370378243 | 0.525333260266247 | 1.08055792876774 | 0.690473114370337 | 1.69102230502862 | 0.998735809224318 |
| ebi-fl187-GCST90094807 | prot-a-2190 | Pyruvate carboxylase, mitochondrial \|\| id:prot-a-2190 | Oranges liking \|\| id:ebi-fl187-GCST90094807 | Inverse variance weighted | 7 | 0.00986628925092733 | 0.228394970352444 | 0.965543408563138 | -0.437787853 | 0.457520431141717 | 1.00991512154845 | 0.645462701541512 | 1.58015103010041 | 0.998735809224318 |
| ebi-fl187-GCST90094807 | prot-a-2197 | Protocadherin-9 \|\| id:prot-a-2197 | Oranges liking \|\| id:ebi-fl187-GCST90094807 | Inverse variance weighted | 7 | -0.544949139 | 0.228424214362953 | 0.0170471597164583 | -0.992660599 | -0.097237679 | 0.579871275555468 | 0.370589388468725 | 0.907340325106753 | 0.998735809224318 |
| ebi-fl187-GCST90094807 | prot-a-2198 | Protocadherin alpha-4 \|\| id:prot-a-2198 | Oranges liking \|\| id:ebi-fl187-GCST90094807 | Inverse variance weighted | 7 | 0.221300471818578 | 0.282249810410945 | 0.433005558682621 | -0.331909157 | 0.774510100224031 | 1.24769827241549 | 0.717552504812356 | 2.16952901501708 | 0.998735809224318 |
| ebi-fl187-GCST90094807 | prot-a-2254 | Placenta growth factor \|\| id:prot-a-2254 | Oranges liking \|\| id:ebi-fl187-GCST90094807 | Inverse variance weighted | 7 | -0.085894936 | 0.271899937584259 | 0.752073538396265 | -0.618818814 | 0.44702894162964 | 0.917690642372458 | 0.538580225612297 | 1.56365955348723 | 0.998735809224318 |
| ebi-fl187-GCST90094807 | prot-a-2261 | Membrane-associated progesterone receptor component 2 \|\| id:prot-a-2261 | Oranges liking \|\| id:ebi-fl187-GCST90094807 | Inverse variance weighted | 7 | -0.43566695 | 0.228424214362953 | 0.0564854635020475 | -0.883378411 | 0.0120445097827429 | 0.646833117596755 | 0.413383969160878 | 1.01211733698631 | 0.998735809224318 |
| ebi-fl187-GCST90094807 | prot-a-2268 | Polymeric immunoglobulin receptor \|\| id:prot-a-2268 | Oranges liking \|\| id:ebi-fl187-GCST90094807 | Inverse variance weighted | 7 | -0.30252806 | 0.240328615570486 | 0.208098974225014 | -0.773572147 | 0.16851602642254 | 0.738947753023426 | 0.461362068309758 | 1.1835471947203 | 0.998735809224318 |
| ebi-fl187-GCST90094807 | prot-a-2283 | Group 10 secretory phospholipase A2 \|\| id:prot-a-2283 | Oranges liking \|\| id:ebi-fl187-GCST90094807 | Inverse variance weighted | 7 | 0.00270652215802417 | 0.228497832556868 | 0.99054939942329 | -0.44514923 | 0.450562273969485 | 1.00271018809569 | 0.640728653182753 | 1.5691942545671 | 0.999107451094609 |
| ebi-fl187-GCST90094807 | prot-a-2298 | Inactive phospholipase D5 \|\| id:prot-a-2298 | Oranges liking \|\| id:ebi-fl187-GCST90094807 | Inverse variance weighted (multiplicative random effects) | 7 | 0.37817631915823 | 0.389965382953965 | 0.332161674160538 | -0.386155831 | 1.14250847 | 1.45962027920743 | 0.679664604319337 | 3.13462161474071 | 0.998735809224318 |
| ebi-fl187-GCST90094807 | prot-a-2304 | Procollagen-lysine,2-oxoglutarate 5-dioxygenase 2 \|\| id:prot-a-2304 | Oranges liking \|\| id:ebi-fl187-GCST90094807 | Inverse variance weighted | 7 | -0.065123623 | 0.228337872706176 | 0.775485345197119 | -0.512665854 | 0.382418607485832 | 0.936951627429398 | 0.598896877456938 | 1.46582556227423 | 0.998735809224318 |
| ebi-fl187-GCST90094807 | prot-a-2331 | Periostin \|\| id:prot-a-2331 | Oranges liking \|\| id:ebi-fl187-GCST90094807 | Inverse variance weighted | 7 | 0.018805055340178 | 0.228424214362953 | 0.934388163743542 | -0.428906405 | 0.466516515491566 | 1.01898298396256 | 0.651220877469725 | 1.59443033466554 | 0.998735809224318 |
| ebi-fl187-GCST90094807 | prot-a-2333 | Periostin \|\| id:prot-a-2333 | Oranges liking \|\| id:ebi-fl187-GCST90094807 | Inverse variance weighted | 7 | -0.053382071 | 0.228497832556868 | 0.815278614617981 | -0.501237823 | 0.394473680565261 | 0.948017732961759 | 0.605780346550132 | 1.48360313623277 | 0.998735809224318 |
| ebi-fl187-GCST90094807 | prot-a-2367 | cAMP-dependent protein kinase catalytic subunit alpha \|\| id:prot-a-2367 | Oranges liking \|\| id:ebi-fl187-GCST90094807 | Inverse variance weighted | 7 | -0.429387856 | 0.228424214362953 | 0.0601377175669941 | -0.877099316 | 0.0183236045590978 | 0.650907422132325 | 0.415987812617041 | 1.01849251188758 | 0.998735809224318 |
| ebi-fl187-GCST90094807 | prot-a-2385 | Phosphoribosyl pyrophosphate synthase-associated protein 1 \|\| id:prot-a-2385 | Oranges liking \|\| id:ebi-fl187-GCST90094807 | Inverse variance weighted | 7 | 0.440719579950711 | 0.292456114510715 | 0.131820986148038 | -0.132494404 | 1.01393356439171 | 1.55382491758379 | 0.875907835243288 | 2.75642228252666 | 0.998735809224318 |
| ebi-fl187-GCST90094807 | prot-a-2393 | Trypsin-3 \|\| id:prot-a-2393 | Oranges liking \|\| id:ebi-fl187-GCST90094807 | Inverse variance weighted | 7 | -0.282853831 | 0.29653123413425 | 0.340146748407783 | -0.86405505 | 0.29834738771947 | 0.753629937005186 | 0.421449613299511 | 1.34762985663676 | 0.998735809224318 |
| ebi-fl187-GCST90094807 | prot-a-2405 | Pregnancy-specific beta-1-glycoprotein 6 \|\| id:prot-a-2405 | Oranges liking \|\| id:ebi-fl187-GCST90094807 | Inverse variance weighted | 7 | 0.212546063926363 | 0.230655809476261 | 0.356796827120763 | -0.239539323 | 0.664631450499835 | 1.23682308518063 | 0.786990326190626 | 1.94377401236974 | 0.998735809224318 |
| ebi-fl187-GCST90094807 | prot-a-244 | Beclin-1 \|\| id:prot-a-244 | Oranges liking \|\| id:ebi-fl187-GCST90094807 | Inverse variance weighted | 7 | -0.463653873 | 0.31712827553377 | 0.143730552050828 | -1.085225293 | 0.157917546880168 | 0.628981224074001 | 0.33782566749076 | 1.17106963238206 | 0.998735809224318 |
| ebi-fl187-GCST90094807 | prot-a-2454 | tRNA pseudouridine synthase A, mitochondrial \|\| id:prot-a-2454 | Oranges liking \|\| id:ebi-fl187-GCST90094807 | Inverse variance weighted | 7 | 0.266925826296001 | 0.228411407424344 | 0.242557318348375 | -0.180760532 | 0.714612184847715 | 1.30594357606584 | 0.834635202976064 | 2.04339406939265 | 0.998735809224318 |
| ebi-fl187-GCST90094807 | prot-a-2466 | Glutaminyl-peptide cyclotransferase-like protein \|\| id:prot-a-2466 | Oranges liking \|\| id:ebi-fl187-GCST90094807 | Inverse variance weighted | 7 | 0.270536904349323 | 0.325329473740318 | 0.405647060612708 | -0.367108864 | 0.908182672880346 | 1.31066796518739 | 0.692734226994546 | 2.4798118066454 | 0.998735809224318 |
| ebi-fl187-GCST90094807 | prot-a-2474 | Ras-related protein Rab-27A \|\| id:prot-a-2474 | Oranges liking \|\| id:ebi-fl187-GCST90094807 | Inverse variance weighted | 7 | 0.0700283435752939 | 0.233351435644029 | 0.764102259444156 | -0.38734047 | 0.527397157437591 | 1.07253858040141 | 0.678859923942304 | 1.6945160052595 | 0.998735809224318 |
| ebi-fl187-GCST90094807 | prot-a-2490 | Rap1 GTPase-GDP dissociation stimulator 1 \|\| id:prot-a-2490 | Oranges liking \|\| id:ebi-fl187-GCST90094807 | Inverse variance weighted | 7 | 0.00786989377431728 | 0.288975291174217 | 0.978273261364768 | -0.558521677 | 0.574261464475783 | 1.00790094278569 | 0.572054119866327 | 1.7758185374238 | 0.998955228396052 |
| ebi-fl187-GCST90094807 | prot-a-2545 | Receptor-interacting serine/threonine-protein kinase 2 \|\| id:prot-a-2545 | Oranges liking \|\| id:ebi-fl187-GCST90094807 | Inverse variance weighted | 7 | -0.041226464 | 0.228497832556868 | 0.856819814455897 | -0.489082216 | 0.406629288049004 | 0.959611788042677 | 0.613188911243218 | 1.50174728679203 | 0.998735809224318 |
| ebi-fl187-GCST90094807 | prot-a-2610 | RUN and FYVE domain-containing protein 1 \|\| id:prot-a-2610 | Oranges liking \|\| id:ebi-fl187-GCST90094807 | Inverse variance weighted | 7 | -0.009191946 | 0.228497832556868 | 0.967911582729243 | -0.457047697 | 0.438663806295834 | 0.990850171271695 | 0.633150139773217 | 1.55063388639669 | 0.998735809224318 |
| ebi-fl187-GCST90094807 | prot-a-2627 | Serine--tRNA ligase, mitochondrial \|\| id:prot-a-2627 | Oranges liking \|\| id:ebi-fl187-GCST90094807 | Inverse variance weighted | 7 | -0.154001566 | 0.228497832556868 | 0.500327933095804 | -0.601857318 | 0.293854185753398 | 0.857270678474748 | 0.547793264447985 | 1.34158826672163 | 0.998735809224318 |
| ebi-fl187-GCST90094807 | prot-a-264 | BolA-like protein 2 \|\| id:prot-a-264 | Oranges liking \|\| id:ebi-fl187-GCST90094807 | Inverse variance weighted | 7 | -0.14675083 | 0.228424214362953 | 0.520581915098993 | -0.59446229 | 0.300960630183076 | 0.863509111205362 | 0.551859226260564 | 1.35115614572077 | 0.998735809224318 |
| ebi-fl187-GCST90094807 | prot-a-2646 | Secretoglobin family 1C member 1 \|\| id:prot-a-2646 | Oranges liking \|\| id:ebi-fl187-GCST90094807 | Inverse variance weighted | 7 | -0.024748815 | 0.228497832556868 | 0.913749046526401 | -0.472604566 | 0.423106937275075 | 0.975554926471498 | 0.623376526502623 | 1.52669754811309 | 0.998735809224318 |
| ebi-fl187-GCST90094807 | prot-a-2672 | Semaphorin-3E \|\| id:prot-a-2672 | Oranges liking \|\| id:ebi-fl187-GCST90094807 | Inverse variance weighted | 7 | 0.0311607694615139 | 0.27518993493266 | 0.909845293354762 | -0.508211503 | 0.570533041929528 | 1.03165134858657 | 0.601570524331459 | 1.76920986317152 | 0.998735809224318 |
| ebi-fl187-GCST90094807 | prot-a-2701 | Plasma protease C1 inhibitor \|\| id:prot-a-2701 | Oranges liking \|\| id:ebi-fl187-GCST90094807 | Inverse variance weighted | 7 | -0.386975973 | 0.228205636688239 | 0.0899363366824271 | -0.834259021 | 0.0603070752883982 | 0.679107411902081 | 0.434196092655448 | 1.06216266037731 | 0.998735809224318 |
| ebi-fl187-GCST90094807 | prot-a-2738 | Signaling threshold-regulating transmembrane adapter 1 \|\| id:prot-a-2738 | Oranges liking \|\| id:ebi-fl187-GCST90094807 | Inverse variance weighted | 7 | 0.0895108014994474 | 0.249286821584391 | 0.719544576728708 | -0.399091369 | 0.578112971804854 | 1.09363914619119 | 0.67092939653497 | 1.78267130380453 | 0.998735809224318 |
| ebi-fl187-GCST90094807 | prot-a-274 | Serine/threonine-protein kinase BRSK2 \|\| id:prot-a-274 | Oranges liking \|\| id:ebi-fl187-GCST90094807 | Inverse variance weighted | 7 | -0.100877425 | 0.244948029658717 | 0.680462600251027 | -0.580975563 | 0.379220713004168 | 0.904043839153538 | 0.559352416638409 | 1.46114549396826 | 0.998735809224318 |
| ebi-fl187-GCST90094807 | prot-a-2749 | Mitochondrial glutamate carrier 2 \|\| id:prot-a-2749 | Oranges liking \|\| id:ebi-fl187-GCST90094807 | Inverse variance weighted | 7 | 0.0917818666564277 | 0.228424214362953 | 0.687827944217412 | -0.355929593 | 0.539493326807816 | 1.09612569443821 | 0.700521939800508 | 1.71513762773768 | 0.998735809224318 |
| ebi-fl187-GCST90094807 | prot-a-2760 | Sodium-coupled monocarboxylate transporter 1 \|\| id:prot-a-2760 | Oranges liking \|\| id:ebi-fl187-GCST90094807 | Inverse variance weighted | 7 | -0.495045489 | 0.283461008584119 | 0.0807358446486722 | -1.050629065 | 0.0605380882698817 | 0.609543179427227 | 0.349717684612818 | 1.0624080620847 | 0.998735809224318 |
| ebi-fl187-GCST90094807 | prot-a-2777 | Small integral membrane protein 10 \|\| id:prot-a-2777 | Oranges liking \|\| id:ebi-fl187-GCST90094807 | Inverse variance weighted | 7 | 0.279950059677757 | 0.228497832556868 | 0.220508916396243 | -0.167905692 | 0.727805811489218 | 1.32306373645818 | 0.845433561960469 | 2.07053248119398 | 0.998735809224318 |
| ebi-fl187-GCST90094807 | prot-a-2799 | Superoxide dismutase [Mn], mitochondrial \|\| id:prot-a-2799 | Oranges liking \|\| id:ebi-fl187-GCST90094807 | Inverse variance weighted | 7 | 0.0787355582459424 | 0.228497832556868 | 0.730410883674527 | -0.369120194 | 0.526591310057403 | 1.08191817989677 | 0.691342310559073 | 1.69315103402908 | 0.998735809224318 |
| ebi-fl187-GCST90094807 | prot-a-2805 | Protein SPACA7 \|\| id:prot-a-2805 | Oranges liking \|\| id:ebi-fl187-GCST90094807 | Inverse variance weighted | 7 | -0.354836492 | 0.228424214362953 | 0.120325181036244 | -0.802547952 | 0.0928749684573961 | 0.701288100263924 | 0.44818555285093 | 1.09732452651226 | 0.998735809224318 |
| ebi-fl187-GCST90094807 | prot-a-2811 | SPARC-like protein 1 \|\| id:prot-a-2811 | Oranges liking \|\| id:ebi-fl187-GCST90094807 | Inverse variance weighted | 7 | -0.342598761 | 0.256553616953033 | 0.181749212101007 | -0.84544385 | 0.160246328107689 | 0.709923003132144 | 0.429366741333539 | 1.17379997530981 | 0.998735809224318 |
| ebi-fl187-GCST90094807 | prot-a-2831 | Sepiapterin reductase \|\| id:prot-a-2831 | Oranges liking \|\| id:ebi-fl187-GCST90094807 | Inverse variance weighted | 7 | -0.355302332 | 0.228424214362953 | 0.119839051164368 | -0.803013792 | 0.0924091284826578 | 0.700961488313371 | 0.447976818726436 | 1.09681346792755 | 0.998735809224318 |
| ebi-fl187-GCST90094807 | prot-a-2834 | SPRY domain-containing SOCS box protein 1 \|\| id:prot-a-2834 | Oranges liking \|\| id:ebi-fl187-GCST90094807 | Inverse variance weighted | 7 | -0.031588518 | 0.26160089461734 | 0.903888419937089 | -0.544326271 | 0.48114923587233 | 0.968905187517589 | 0.580232571176428 | 1.61793272048674 | 0.998735809224318 |
| ebi-fl187-GCST90094807 | prot-a-2835 | Spectrin alpha chain, non-erythrocytic 1 \|\| id:prot-a-2835 | Oranges liking \|\| id:ebi-fl187-GCST90094807 | Inverse variance weighted | 7 | 0.119719948700105 | 0.228497832556868 | 0.600317854495518 | -0.328135803 | 0.567575700511566 | 1.12718113883041 | 0.720265198808261 | 1.7639854345833 | 0.998735809224318 |
| ebi-fl187-GCST90094807 | prot-a-2859 | Sia-alpha-2,3-Gal-beta-1,4-GlcNAc-R:alpha 2,8-sialyltransferase \|\| id:prot-a-2859 | Oranges liking \|\| id:ebi-fl187-GCST90094807 | Inverse variance weighted | 7 | -0.146264315 | 0.228411407424344 | 0.521942035480574 | -0.593950674 | 0.30142204346765 | 0.863929323452115 | 0.552141638774813 | 1.35177973097014 | 0.998735809224318 |
| ebi-fl187-GCST90094807 | prot-a-2861 | Alpha-2,8-sialyltransferase 8F \|\| id:prot-a-2861 | Oranges liking \|\| id:ebi-fl187-GCST90094807 | Inverse variance weighted | 7 | 0.0281289501183805 | 0.243936438718485 | 0.908197334943931 | -0.44998647 | 0.506244370006611 | 1.02852830471719 | 0.637636778935779 | 1.65904870696137 | 0.998735809224318 |
| ebi-fl187-GCST90094807 | prot-a-289 | Uncharacterized protein C17orf89 \|\| id:prot-a-289 | Oranges liking \|\| id:ebi-fl187-GCST90094807 | Inverse variance weighted | 7 | 0.359439134717344 | 0.251871829213294 | 0.153559257110078 | -0.134229651 | 0.8531079199754 | 1.43252573525208 | 0.874389237582995 | 2.34692959834691 | 0.998735809224318 |
| ebi-fl187-GCST90094807 | prot-a-2921 | Tachykinin-3 \|\| id:prot-a-2921 | Oranges liking \|\| id:ebi-fl187-GCST90094807 | Inverse variance weighted | 7 | -0.109102289 | 0.254950690569687 | 0.668698543967886 | -0.608805642 | 0.390601064613238 | 0.896638696618325 | 0.544000212000247 | 1.47786882162657 | 0.998735809224318 |
| ebi-fl187-GCST90094807 | prot-a-2928 | Tax1-binding protein 1 \|\| id:prot-a-2928 | Oranges liking \|\| id:ebi-fl187-GCST90094807 | Inverse variance weighted | 7 | -0.156884167 | 0.228497832556868 | 0.492341572132188 | -0.604739919 | 0.29097158454939 | 0.854803067263036 | 0.546216468652919 | 1.33772657130665 | 0.998735809224318 |
| ebi-fl187-GCST90094807 | prot-a-296 | Uncharacterized protein C1orf185 \|\| id:prot-a-296 | Oranges liking \|\| id:ebi-fl187-GCST90094807 | Inverse variance weighted | 7 | 0.271814963683845 | 0.263555909545325 | 0.302382808488059 | -0.244754619 | 0.788384546392682 | 1.31234414751683 | 0.782896622610566 | 2.19983981509443 | 0.998735809224318 |
| ebi-fl187-GCST90094807 | prot-a-2964 | Transforming growth factor beta-2 \|\| id:prot-a-2964 | Oranges liking \|\| id:ebi-fl187-GCST90094807 | Inverse variance weighted | 7 | 0.00835110137359696 | 0.228497832556868 | 0.970845536334418 | -0.43950465 | 0.456206853185058 | 1.00838606909255 | 0.644355523269364 | 1.57807674120743 | 0.998955228396052 |
| ebi-fl187-GCST90094807 | prot-a-2977 | Thymocyte nuclear protein 1 \|\| id:prot-a-2977 | Oranges liking \|\| id:ebi-fl187-GCST90094807 | Inverse variance weighted | 7 | -0.219478673 | 0.228192866477041 | 0.336143971515949 | -0.666736691 | 0.227779345780353 | 0.802937282143543 | 0.51338116869755 | 1.25580819548112 | 0.998735809224318 |
| ebi-fl187-GCST90094807 | prot-a-3015 | Transmembrane protein 70, mitochondrial \|\| id:prot-a-3015 | Oranges liking \|\| id:ebi-fl187-GCST90094807 | Inverse variance weighted | 7 | -0.284125892 | 0.228497832556868 | 0.213701632605843 | -0.731981644 | 0.163729859822046 | 0.7526718833807 | 0.480954963709833 | 1.17789607505462 | 0.998735809224318 |
| ebi-fl187-GCST90094807 | prot-a-3030 | Tumor necrosis factor alpha-induced protein 3 \|\| id:prot-a-3030 | Oranges liking \|\| id:ebi-fl187-GCST90094807 | Inverse variance weighted | 7 | -0.274551321 | 0.228411407424344 | 0.229362154830193 | -0.72223768 | 0.173135037236038 | 0.759913003513007 | 0.48566427796361 | 1.18902665711689 | 0.998735809224318 |
| ebi-fl187-GCST90094807 | prot-a-3032 | Tumor necrosis factor receptor superfamily member 10A \|\| id:prot-a-3032 | Oranges liking \|\| id:ebi-fl187-GCST90094807 | Inverse variance weighted | 7 | 0.144139207063662 | 0.228497832556868 | 0.528163363723534 | -0.303716545 | 0.591994958875123 | 1.15504488774107 | 0.73807004663379 | 1.80759089029763 | 0.998735809224318 |
| ebi-fl187-GCST90094807 | prot-a-3081 | Tumor protein p53-inducible protein 11 \|\| id:prot-a-3081 | Oranges liking \|\| id:ebi-fl187-GCST90094807 | Inverse variance weighted | 7 | -0.12829593 | 0.228497832556868 | 0.574473440929018 | -0.576151682 | 0.319559821489447 | 0.879593042373501 | 0.562057184697829 | 1.37652171568239 | 0.998735809224318 |
| ebi-fl187-GCST90094807 | prot-a-315 | Complement C4 \|\| id:prot-a-315 | Oranges liking \|\| id:ebi-fl187-GCST90094807 | Inverse variance weighted | 7 | -0.23626872 | 0.228497832556868 | 0.301132150065498 | -0.684124472 | 0.211587031414938 | 0.789568472269672 | 0.504531767841809 | 1.23563750022918 | 0.998735809224318 |
| ebi-fl187-GCST90094807 | prot-a-3157 | UDP-glucuronosyltransferase 1-8 \|\| id:prot-a-3157 | Oranges liking \|\| id:ebi-fl187-GCST90094807 | Inverse variance weighted | 7 | 0.123260689653953 | 0.295554389522649 | 0.676642500407183 | -0.456025914 | 0.702547293118346 | 1.13117926924613 | 0.63379741277973 | 2.01888886475607 | 0.998735809224318 |
| ebi-fl187-GCST90094807 | prot-a-317 | Glutaredoxin-like protein C5orf63 \|\| id:prot-a-317 | Oranges liking \|\| id:ebi-fl187-GCST90094807 | Inverse variance weighted | 7 | -0.224676045 | 0.228279043682083 | 0.325008981202235 | -0.67210297 | 0.222750880808296 | 0.798774944139324 | 0.510633600499892 | 1.24950925822382 | 0.998735809224318 |
| ebi-fl187-GCST90094807 | prot-a-3192 | Guanine nucleotide exchange factor VAV3 \|\| id:prot-a-3192 | Oranges liking \|\| id:ebi-fl187-GCST90094807 | Inverse variance weighted | 7 | -0.132166452 | 0.228497832556868 | 0.562984329470914 | -0.580022204 | 0.315689299946079 | 0.876195138625408 | 0.559885934901021 | 1.37120415622964 | 0.998735809224318 |
| ebi-fl187-GCST90094807 | prot-a-3233 | Serine/threonine-protein kinase WNK3 \|\| id:prot-a-3233 | Oranges liking \|\| id:ebi-fl187-GCST90094807 | Inverse variance weighted | 7 | 0.246178342561788 | 0.228279043682083 | 0.280850945346189 | -0.201248583 | 0.69360526817867 | 1.27912767610755 | 0.817709137651581 | 2.00091638511376 | 0.998735809224318 |
| ebi-fl187-GCST90094807 | prot-a-3250 | 14-3-3 protein theta \|\| id:prot-a-3250 | Oranges liking \|\| id:ebi-fl187-GCST90094807 | Inverse variance weighted | 7 | -0.125426073 | 0.255315282247824 | 0.623242879028087 | -0.625844026 | 0.374991880271216 | 0.882120974631657 | 0.534809844482624 | 1.4549796005305 | 0.998735809224318 |
| ebi-fl187-GCST90094807 | prot-a-3278 | E3 ubiquitin-protein ligase ZNRF3 \|\| id:prot-a-3278 | Oranges liking \|\| id:ebi-fl187-GCST90094807 | Inverse variance weighted | 7 | 0.0321153864499762 | 0.228337872706176 | 0.888147606799776 | -0.415426844 | 0.479657616954081 | 1.03263665070834 | 0.660058478529533 | 1.61552118042892 | 0.998735809224318 |
| ebi-fl187-GCST90094807 | prot-a-355 | Calpain-3 \|\| id:prot-a-355 | Oranges liking \|\| id:ebi-fl187-GCST90094807 | Inverse variance weighted | 7 | 0.0356176349755286 | 0.228497832556868 | 0.876129704807511 | -0.412238117 | 0.48347338678699 | 1.03625954132426 | 0.662166584266427 | 1.62169741346159 | 0.998735809224318 |
| ebi-fl187-GCST90094807 | prot-a-385 | Coiled-coil domain-containing protein 90B, mitochondrial \|\| id:prot-a-385 | Oranges liking \|\| id:ebi-fl187-GCST90094807 | Inverse variance weighted | 7 | 0.285851884513698 | 0.23481621890866 | 0.223473733603801 | -0.174387905 | 0.746091673574671 | 1.33089531444613 | 0.839971005882306 | 2.10874223706581 | 0.998735809224318 |
| ebi-fl187-GCST90094807 | prot-a-390 | C-C motif chemokine 14 \|\| id:prot-a-390 | Oranges liking \|\| id:ebi-fl187-GCST90094807 | Inverse variance weighted | 7 | 0.419376550168077 | 0.270634637513298 | 0.121236562720649 | -0.111067339 | 0.949820439694141 | 1.52101298466651 | 0.894878486357284 | 2.58524541018016 | 0.998735809224318 |
| ebi-fl187-GCST90094807 | prot-a-40 | Pituitary adenylate cyclase-activating polypeptide 27 \|\| id:prot-a-40 | Oranges liking \|\| id:ebi-fl187-GCST90094807 | Inverse variance weighted | 7 | 0.160419961427129 | 0.228497832556868 | 0.482639636344873 | -0.28743579 | 0.60827571323859 | 1.17400380379108 | 0.750184734298026 | 1.83726070166655 | 0.998735809224318 |
| ebi-fl187-GCST90094807 | prot-a-401 | C-C motif chemokine 24 \|\| id:prot-a-401 | Oranges liking \|\| id:ebi-fl187-GCST90094807 | Inverse variance weighted | 7 | -0.012406605 | 0.250036957390143 | 0.960425938762668 | -0.502479041 | 0.477665831765666 | 0.987670039907349 | 0.605028907437887 | 1.6123066116981 | 0.998735809224318 |
| ebi-fl187-GCST90094807 | prot-a-412 | Cerebral cavernous malformations 2 protein \|\| id:prot-a-412 | Oranges liking \|\| id:ebi-fl187-GCST90094807 | Inverse variance weighted | 7 | -0.031158132 | 0.235737249342969 | 0.894847345749066 | -0.493203141 | 0.430886876212628 | 0.969322279601022 | 0.610667204789033 | 1.53862148542187 | 0.998735809224318 |
| ebi-fl187-GCST90094807 | prot-a-44 | Adhesion G protein-coupled receptor F5 \|\| id:prot-a-44 | Oranges liking \|\| id:ebi-fl187-GCST90094807 | Inverse variance weighted | 7 | -0.153055221 | 0.247967538253636 | 0.537077134083203 | -0.639071596 | 0.332961154260961 | 0.858082336581928 | 0.527782191916548 | 1.39509310399455 | 0.998735809224318 |
| ebi-fl187-GCST90094807 | prot-a-48 | Adrenomedullin \|\| id:prot-a-48 | Oranges liking \|\| id:ebi-fl187-GCST90094807 | Inverse variance weighted | 7 | -0.182225667 | 0.228497832556868 | 0.425164144294901 | -0.630081419 | 0.26563008488055 | 0.833413245383853 | 0.532548439817492 | 1.30425250671898 | 0.998735809224318 |
| ebi-fl187-GCST90094807 | prot-a-502 | Carcinoembryonic antigen-related cell adhesion molecule 1 \|\| id:prot-a-502 | Oranges liking \|\| id:ebi-fl187-GCST90094807 | Inverse variance weighted | 7 | -0.083576016 | 0.228424214362953 | 0.714454082510155 | -0.531287476 | 0.364135443812884 | 0.919821162576725 | 0.587847642243306 | 1.43926914105716 | 0.998735809224318 |
| ebi-fl187-GCST90094807 | prot-a-518 | Complement factor B \|\| id:prot-a-518 | Oranges liking \|\| id:ebi-fl187-GCST90094807 | Inverse variance weighted | 7 | -0.021288308 | 0.235368330268804 | 0.927932183144426 | -0.482610235 | 0.440033619505856 | 0.978936688776618 | 0.617170327801981 | 1.55275942063826 | 0.998735809224318 |
| ebi-fl187-GCST90094807 | prot-a-525 | Properdin \|\| id:prot-a-525 | Oranges liking \|\| id:ebi-fl187-GCST90094807 | Inverse variance weighted | 7 | -0.219806449 | 0.283182546303086 | 0.437630580697385 | -0.774844239 | 0.335231342224895 | 0.802674141689225 | 0.460775546139076 | 1.39826382527268 | 0.998735809224318 |
| ebi-fl187-GCST90094807 | prot-a-533 | Chondroadherin \|\| id:prot-a-533 | Oranges liking \|\| id:ebi-fl187-GCST90094807 | Inverse variance weighted | 7 | 0.350034890672196 | 0.228337872706176 | 0.1252834821088 | -0.09750734 | 0.797577121176301 | 1.41911706167769 | 0.907095683601525 | 2.22015523957601 | 0.998735809224318 |
| ebi-fl187-GCST90094807 | prot-a-535 | Coiled-coil-helix-coiled-coil-helix domain-containing protein 2 \|\| id:prot-a-535 | Oranges liking \|\| id:ebi-fl187-GCST90094807 | Inverse variance weighted | 7 | -0.07913073 | 0.301134845154133 | 0.792723950758208 | -0.669355027 | 0.511093566118644 | 0.923919132247212 | 0.512038722517438 | 1.66711329708735 | 0.998735809224318 |
| ebi-fl187-GCST90094807 | prot-a-591 | Caseinolytic peptidase B protein homolog \|\| id:prot-a-591 | Oranges liking \|\| id:ebi-fl187-GCST90094807 | Inverse variance weighted | 7 | -0.088063025 | 0.315772455551011 | 0.780335939548119 | -0.706977038 | 0.530850988071004 | 0.915703162887722 | 0.493132668001997 | 1.70037869508816 | 0.998735809224318 |
| ebi-fl187-GCST90094807 | prot-a-605 | Ciliary neurotrophic factor receptor subunit alpha \|\| id:prot-a-605 | Oranges liking \|\| id:ebi-fl187-GCST90094807 | Inverse variance weighted | 7 | -0.004618141 | 0.228424214362953 | 0.98386995562289 | -0.452329602 | 0.4430933187574 | 0.995392505824553 | 0.636144460969403 | 1.55751767317414 | 0.998955228396052 |
| ebi-fl187-GCST90094807 | prot-a-606 | Ciliary neurotrophic factor receptor subunit alpha \|\| id:prot-a-606 | Oranges liking \|\| id:ebi-fl187-GCST90094807 | Inverse variance weighted | 7 | -0.183436555 | 0.228424214362953 | 0.421944597192505 | -0.631148015 | 0.26427490537948 | 0.83240468616777 | 0.531980728498608 | 1.30248620755418 | 0.998735809224318 |
| ebi-fl187-GCST90094807 | prot-a-707 | Cancer/testis antigen 55 \|\| id:prot-a-707 | Oranges liking \|\| id:ebi-fl187-GCST90094807 | Inverse variance weighted | 7 | -0.086463336 | 0.228337872706176 | 0.704937377210021 | -0.534005567 | 0.36107889440174 | 0.917169175165017 | 0.586251988924022 | 1.43487666014878 | 0.998735809224318 |
| ebi-fl187-GCST90094807 | prot-a-719 | Dipeptidyl peptidase 1 \|\| id:prot-a-719 | Oranges liking \|\| id:ebi-fl187-GCST90094807 | Inverse variance weighted | 7 | -0.417107524 | 0.228205636688239 | 0.0675840064295788 | -0.864390572 | 0.030175523436377 | 0.658950062876792 | 0.421308231277881 | 1.03063541874864 | 0.998735809224318 |
| ebi-fl187-GCST90094807 | prot-a-752 | Cytochrome b5 \|\| id:prot-a-752 | Oranges liking \|\| id:ebi-fl187-GCST90094807 | Inverse variance weighted | 7 | -0.311911172 | 0.228424214362953 | 0.172098830113081 | -0.759622633 | 0.135800287781053 | 0.732046551300304 | 0.467842942413636 | 1.14545310976791 | 0.998735809224318 |
| ebi-fl187-GCST90094807 | prot-a-755 | Cytoglobin \|\| id:prot-a-755 | Oranges liking \|\| id:ebi-fl187-GCST90094807 | Inverse variance weighted | 7 | 0.00188150371391733 | 0.228497832556868 | 0.993430109234086 | -0.445974248 | 0.449737255525378 | 1.00188327485266 | 0.640200258223974 | 1.56790017425816 | 0.999107451094609 |
| ebi-fl187-GCST90094807 | prot-a-807 | Desmin \|\| id:prot-a-807 | Oranges liking \|\| id:ebi-fl187-GCST90094807 | Inverse variance weighted | 7 | -0.186359197 | 0.276202945165736 | 0.499854721078247 | -0.727716969 | 0.354998575880955 | 0.82997541704857 | 0.483010460038945 | 1.42617862323189 | 0.998735809224318 |
| ebi-fl187-GCST90094807 | prot-a-82 | Ameloblastin \|\| id:prot-a-82 | Oranges liking \|\| id:ebi-fl187-GCST90094807 | Inverse variance weighted | 7 | -0.018214268 | 0.228424214362953 | 0.936445014893305 | -0.465925729 | 0.429497191756421 | 0.981950608834659 | 0.627553891656286 | 1.53648477208212 | 0.998735809224318 |
| ebi-fl187-GCST90094807 | prot-a-84 | Muellerian-inhibiting factor \|\| id:prot-a-84 | Oranges liking \|\| id:ebi-fl187-GCST90094807 | Inverse variance weighted | 7 | -0.12673169 | 0.329393732566237 | 0.700428380798465 | -0.772343405 | 0.518880026264829 | 0.880970014337035 | 0.461929311326018 | 1.68014487743394 | 0.998735809224318 |
| ebi-fl187-GCST90094807 | prot-a-858 | Inactive dipeptidyl peptidase 10 \|\| id:prot-a-858 | Oranges liking \|\| id:ebi-fl187-GCST90094807 | Inverse variance weighted | 7 | 0.0495902513064 | 0.228411407424344 | 0.828123130549731 | -0.398096107 | 0.497276609858114 | 1.05084042765666 | 0.671597479176587 | 1.64423726806051 | 0.998735809224318 |
| ebi-fl187-GCST90094807 | prot-a-863 | Dihydropyrimidinase-related protein 3 \|\| id:prot-a-863 | Oranges liking \|\| id:ebi-fl187-GCST90094807 | Inverse variance weighted | 7 | 0.0640373271531938 | 0.228411407424344 | 0.779201641456286 | -0.383649031 | 0.511723685704908 | 1.0661321936985 | 0.681370524879406 | 1.66816410886214 | 0.998735809224318 |
| ebi-fl187-GCST90094807 | prot-a-864 | Dihydropyrimidinase-related protein 5 \|\| id:prot-a-864 | Oranges liking \|\| id:ebi-fl187-GCST90094807 | Inverse variance weighted | 7 | -0.40192868 | 0.228497832556868 | 0.0785767707484031 | -0.849784432 | 0.0459270717714553 | 0.669028459071804 | 0.427507078925882 | 1.04699805245937 | 0.998735809224318 |
| ebi-fl187-GCST90094807 | prot-a-884 | Endothelin-converting enzyme 1 \|\| id:prot-a-884 | Oranges liking \|\| id:ebi-fl187-GCST90094807 | Inverse variance weighted | 7 | -0.149237698 | 0.25212532532669 | 0.553905023774194 | -0.643403335 | 0.344927940121847 | 0.861364346395808 | 0.525500921468398 | 1.41188817551198 | 0.998735809224318 |
| ebi-fl187-GCST90094807 | prot-a-893 | Early endosome antigen 1 \|\| id:prot-a-893 | Oranges liking \|\| id:ebi-fl187-GCST90094807 | Inverse variance weighted | 7 | -0.438500304 | 0.228321451503266 | 0.0547898229440681 | -0.886010349 | 0.00900974048592484 | 0.645003004240194 | 0.412297398338478 | 1.00905045036771 | 0.998735809224318 |
| ebi-fl187-GCST90094807 | prot-a-897 | Ephrin-A2 \|\| id:prot-a-897 | Oranges liking \|\| id:ebi-fl187-GCST90094807 | Inverse variance weighted | 7 | 0.149742013557696 | 0.228497832556868 | 0.512253819473878 | -0.298113738 | 0.597597765369157 | 1.16153454390623 | 0.742216916490792 | 1.81774689677825 | 0.998735809224318 |
| ebi-fl187-GCST90094807 | prot-a-90 | Anaphase-promoting complex subunit 10 \|\| id:prot-a-90 | Oranges liking \|\| id:ebi-fl187-GCST90094807 | Inverse variance weighted | 7 | -0.141370477 | 0.228497832556868 | 0.536117274838704 | -0.589226229 | 0.306485275010644 | 0.868167616149974 | 0.554756373313692 | 1.3586414613489 | 0.998735809224318 |
| ebi-fl187-GCST90094807 | prot-a-914 | Histone-lysine N-methyltransferase EHMT2 \|\| id:prot-a-914 | Oranges liking \|\| id:ebi-fl187-GCST90094807 | Inverse variance weighted | 7 | -0.28218654 | 0.228424214362953 | 0.216695896429116 | -0.729898001 | 0.165524919764753 | 0.754132995150881 | 0.481958147054863 | 1.18001236798369 | 0.998735809224318 |
| ebi-fl187-GCST90094807 | prot-a-932 | RNA polymerase II elongation factor ELL \|\| id:prot-a-932 | Oranges liking \|\| id:ebi-fl187-GCST90094807 | Inverse variance weighted | 7 | -0.071504434 | 0.228497832556868 | 0.754331825616149 | -0.519360186 | 0.376351317828829 | 0.930992149582689 | 0.594901052375571 | 1.45695889950688 | 0.998735809224318 |
| ebi-fl187-GCST90094807 | prot-a-991 | Estrogen receptor \|\| id:prot-a-991 | Oranges liking \|\| id:ebi-fl187-GCST90094807 | Inverse variance weighted | 7 | 0.103261847209817 | 0.228497832556868 | 0.651329432207846 | -0.344593905 | 0.551117599021278 | 1.10878170246318 | 0.70850801689981 | 1.73519118258755 | 0.998735809224318 |
| ebi-fl187-GCST90094807 | prot-a-992 | Persulfide dioxygenase ETHE1, mitochondrial \|\| id:prot-a-992 | Oranges liking \|\| id:ebi-fl187-GCST90094807 | Inverse variance weighted | 7 | 0.0676879408810162 | 0.228497832556868 | 0.767054346350583 | -0.380167811 | 0.515543692692477 | 1.07003134333476 | 0.683746659421385 | 1.67454869423087 | 0.998735809224318 |
| ebi-fl187-GCST90094807 | prot-a-993 | Ethanolamine kinase 1 \|\| id:prot-a-993 | Oranges liking \|\| id:ebi-fl187-GCST90094807 | Inverse variance weighted | 7 | 0.139686021783867 | 0.228337872706176 | 0.540702224313867 | -0.307856209 | 0.587228252287972 | 1.14991269463419 | 0.735021000021056 | 1.79899513788449 | 0.998735809224318 |
| ebi-fl187-GCST90094807 | prot-b-10 | coagulation factor III, tissue factor \|\| id:prot-b-10 | Oranges liking \|\| id:ebi-fl187-GCST90094807 | Inverse variance weighted | 6 | -0.05200979 | 0.295335157438248 | 0.860212005517328 | -0.630866699 | 0.526847118430405 | 0.949319572817744 | 0.532130404345505 | 1.69358421164321 | 0.998735809224318 |
| ebi-fl187-GCST90094807 | prot-b-28 | prolactin \|\| id:prot-b-28 | Oranges liking \|\| id:ebi-fl187-GCST90094807 | Inverse variance weighted (multiplicative random effects) | 6 | 0.101598531164019 | 0.478041931987002 | 0.83169311984983 | -0.835363656 | 1.03856071785854 | 1.10693898100511 | 0.433716729263736 | 2.82514790183143 | 0.998735809224318 |
| ebi-fl187-GCST90094807 | prot-b-54 | matrix metallopeptidase 7 \|\| id:prot-b-54 | Oranges liking \|\| id:ebi-fl187-GCST90094807 | Inverse variance weighted | 6 | -0.050021735 | 0.356752113494814 | 0.888490701825356 | -0.749255878 | 0.64921240725828 | 0.951208749571648 | 0.472718182065765 | 1.91403275691177 | 0.998735809224318 |
| ebi-fl187-GCST90094807 | ukb-rsfMRI191-edge-pheno716 | Net100_Pair10_33 (OccipitalPrecuneus)&(Temporal\|Frontal\|Supp_Motor_Area) (Default_mode\|Central_executive)&(Default_mode\|Salience) \|\| id:ukb-rsfMRI191-edge-pheno716 | Oranges liking \|\| id:ebi-fl187-GCST90094807 | Inverse variance weighted | 7 | 0.00522938277821207 | 0.0852209636185084 | 0.951070410204952 | -0.161803706 | 0.172262471470488 | 1.0052430798657 | 0.850608157504524 | 1.18798960567515 | 0.998735809224318 |
| ebi-fl187-GCST90094807 | ukb-rsfMRI191-edge-pheno903 | Net100_Pair27_38 (Precentral\|Frontal\|Supp_Motor_Area)&(Subcortical) (Attention\|Salience\|Motor)&(Subcortical-cerebellum) \|\| id:ukb-rsfMRI191-edge-pheno903 | Oranges liking \|\| id:ebi-fl187-GCST90094807 | Inverse variance weighted | 7 | -0.142165073 | 0.0788645729903328 | 0.0714434977795481 | -0.296739636 | 0.0124094903714423 | 0.867478047731795 | 0.743237499645936 | 1.01248680758849 | 0.998735809224318 |
| ebi-fl187-GCST90094807 | ukb-rsfMRI191-node-pheno23 | Net100_Node2 RolandicOper\|SupraMarginal\|Insula Salience\|Motor \|\| id:ukb-rsfMRI191-node-pheno23 | Oranges liking \|\| id:ebi-fl187-GCST90094807 | Inverse variance weighted | 7 | -0.077957465 | 0.064816626460588 | 0.229077455777729 | -0.204998052 | 0.0490831232894935 | 0.925003771136906 | 0.814648902990736 | 1.05030765213861 | 0.998735809224318 |
| ebi-fl187-GCST90094807 | ukb-rsfMRI191-node-pheno55 | Net100_Node34 Parietal\|Postcentral\|Precuneus Attention \|\| id:ukb-rsfMRI191-node-pheno55 | Oranges liking \|\| id:ebi-fl187-GCST90094807 | Inverse variance weighted | 7 | 0.0269430896878059 | 0.0672013488897686 | 0.688471082476671 | -0.104771554 | 0.158657733511752 | 1.02730933660478 | 0.900530221494219 | 1.17193676334839 | 0.998735809224318 |
| ebi-fl187-GCST90094807 | ebi-a-GCST90001414 | IgD- CD24- B cell Absolute Count \|\| id:ebi-a-GCST90001414 | Oranges liking \|\| id:ebi-fl187-GCST90094807 | MR Egger | 7 | 0.440270481927025 | 1.80194382531167 | 0.816684226918679 | -3.091539416 | 3.97208037953789 | 1.55312725455542 | 0.0454319618699909 | 53.0948735109807 | 0.999950574162623 |
| ebi-fl187-GCST90094807 | ebi-a-GCST90001422 | CD20- CD38- B cell %B cell \|\| id:ebi-a-GCST90001422 | Oranges liking \|\| id:ebi-fl187-GCST90094807 | MR Egger | 7 | 0.653441594865054 | 1.71875185122815 | 0.719423571973892 | -2.715312034 | 4.02219522327223 | 1.92214470190401 | 0.0661842980637654 | 55.8235165008177 | 0.999950574162623 |
| ebi-fl187-GCST90094807 | ebi-a-GCST90001444 | CD20- CD38- B cell %lymphocyte \|\| id:ebi-a-GCST90001444 | Oranges liking \|\| id:ebi-fl187-GCST90094807 | MR Egger | 7 | 0.615046538786727 | 1.72140920412573 | 0.735458580631105 | -2.758915501 | 3.98900857887315 | 1.84974268233541 | 0.0633604454335343 | 54.0013247609289 | 0.999950574162623 |
| ebi-fl187-GCST90094807 | ebi-a-GCST90001459 | Myeloid Dendritic Cell %Dendritic Cell \|\| id:ebi-a-GCST90001459 | Oranges liking \|\| id:ebi-fl187-GCST90094807 | MR Egger | 7 | 0.319681157839126 | 1.83610123345021 | 0.868609984295435 | -3.27907726 | 3.91843957540153 | 1.37668874793596 | 0.0376629939394858 | 50.3218600130059 | 0.999950574162623 |
| ebi-fl187-GCST90094807 | ebi-a-GCST90001476 | HLA DR++ monocyte %leukocyte \|\| id:ebi-a-GCST90001476 | Oranges liking \|\| id:ebi-fl187-GCST90094807 | MR Egger | 7 | -2.971921414 | 2.50211135891649 | 0.288268014837514 | -7.876059678 | 1.93221684936566 | 0.0512048300685055 | 0.000379726357083837 | 6.90480018948393 | 0.999950574162623 |
| ebi-fl187-GCST90094807 | ebi-a-GCST90001478 | CD4 regulatory T cell %CD4+ T cell \|\| id:ebi-a-GCST90001478 | Oranges liking \|\| id:ebi-fl187-GCST90094807 | MR Egger | 7 | 0.803928746040235 | 2.75305609845472 | 0.782004780493788 | -4.592061207 | 6.19991869901148 | 2.2343017117804 | 0.0101319527840953 | 492.708981737577 | 0.999950574162623 |
| ebi-fl187-GCST90094807 | ebi-a-GCST90001485 | CD39+ resting CD4 regulatory T cell %CD4 regulatory T cell \|\| id:ebi-a-GCST90001485 | Oranges liking \|\| id:ebi-fl187-GCST90094807 | MR Egger | 7 | 2.2787665570086 | 1.94762098034468 | 0.294721065093593 | -1.538570564 | 6.09610367848416 | 9.76462886557274 | 0.21468776451631 | 444.123944823751 | 0.999950574162623 |
| ebi-fl187-GCST90094807 | ebi-a-GCST90001515 | Immature Myeloid-Derived Suppressor Cells Absolute Count \|\| id:ebi-a-GCST90001515 | Oranges liking \|\| id:ebi-fl187-GCST90094807 | MR Egger | 7 | -0.310496183 | 2.3828178068353 | 0.901404308589668 | -4.980819084 | 4.35982671873785 | 0.733083122834442 | 0.00686843442772223 | 78.2435751028809 | 0.999950574162623 |
| ebi-fl187-GCST90094807 | ebi-a-GCST90001517 | CD33+ HLA DR+ Absolute Count \|\| id:ebi-a-GCST90001517 | Oranges liking \|\| id:ebi-fl187-GCST90094807 | MR Egger | 7 | -2.693198383 | 2.33087717810246 | 0.300120619644582 | -7.261717652 | 1.87532088585651 | 0.0676641769434649 | 0.000701901377074933 | 6.52291189471158 | 0.999950574162623 |
| ebi-fl187-GCST90094807 | ebi-a-GCST90001518 | CD33+ HLA DR+ CD14- Absolute Count \|\| id:ebi-a-GCST90001518 | Oranges liking \|\| id:ebi-fl187-GCST90094807 | MR Egger | 7 | -2.775314494 | 2.36039014676236 | 0.292611653017481 | -7.401679182 | 1.85105019358496 | 0.062329871232094 | 0.000610227217942306 | 6.36650207263735 | 0.999950574162623 |
| ebi-fl187-GCST90094807 | ebi-a-GCST90001520 | CD33+ HLA DR+ CD14dim Absolute Count \|\| id:ebi-a-GCST90001520 | Oranges liking \|\| id:ebi-fl187-GCST90094807 | MR Egger | 7 | -2.644272613 | 2.33991587152683 | 0.309723550072737 | -7.230507721 | 1.94196249536717 | 0.071057020912016 | 0.000724153102047582 | 6.97242089637408 | 0.999950574162623 |
| ebi-fl187-GCST90094807 | ebi-a-GCST90001531 | CD33dim HLA DR- Absolute Count \|\| id:ebi-a-GCST90001531 | Oranges liking \|\| id:ebi-fl187-GCST90094807 | MR Egger | 7 | 1.81184937654167 | 3.02070496693169 | 0.574777321033865 | -4.108732359 | 7.73243111172779 | 6.1217584062721 | 0.0164285868883531 | 2281.14117418895 | 0.999950574162623 |
| ebi-fl187-GCST90094807 | ebi-a-GCST90001532 | Basophil Absolute Count \|\| id:ebi-a-GCST90001532 | Oranges liking \|\| id:ebi-fl187-GCST90094807 | MR Egger | 7 | 1.56426525059905 | 2.94830941171201 | 0.618431413660936 | -4.214421196 | 7.34295169755458 | 4.77916215901457 | 0.0147808745156918 | 1545.26654819434 | 0.999950574162623 |
| ebi-fl187-GCST90094807 | ebi-a-GCST90001588 | T/B cell \|\| id:ebi-a-GCST90001588 | Oranges liking \|\| id:ebi-fl187-GCST90094807 | MR Egger | 7 | 1.07889006812419 | 2.48684845507947 | 0.682486876555223 | -3.795332904 | 5.95311304007995 | 2.94141297054404 | 0.0224754224169398 | 384.949839997834 | 0.999950574162623 |
| ebi-fl187-GCST90094807 | ebi-a-GCST90001624 | HLA DR+ CD4+ T cell Absolute Count \|\| id:ebi-a-GCST90001624 | Oranges liking \|\| id:ebi-fl187-GCST90094807 | MR Egger | 7 | 2.76167419115542 | 1.72196199251556 | 0.169663689414599 | -0.613371314 | 6.13671969648591 | 15.8263170607601 | 0.541522146918083 | 462.533828271286 | 0.999950574162623 |
| ebi-fl187-GCST90094807 | ebi-a-GCST90001631 | CD8+ Natural Killer T %T cell \|\| id:ebi-a-GCST90001631 | Oranges liking \|\| id:ebi-fl187-GCST90094807 | MR Egger | 7 | -3.746536685 | 1.74816439415301 | 0.0849805843672557 | -7.172938898 | -0.320134473 | 0.0235993364212317 | 0.000767065089589228 | 0.726051396525837 | 0.999950574162623 |
| ebi-fl187-GCST90094807 | ebi-a-GCST90001632 | CD8+ Natural Killer T %lymphocyte \|\| id:ebi-a-GCST90001632 | Oranges liking \|\| id:ebi-fl187-GCST90094807 | MR Egger | 7 | -3.516968181 | 1.75923706803421 | 0.102050980612886 | -6.965072834 | -0.068863528 | 0.0296893114694477 | 0.000944294155950912 | 0.933454061930782 | 0.999950574162623 |
| ebi-fl187-GCST90094807 | ebi-a-GCST90001646 | Natural Killer %CD3- lymphocyte \|\| id:ebi-a-GCST90001646 | Oranges liking \|\| id:ebi-fl187-GCST90094807 | MR Egger | 7 | 0.103518279275487 | 1.71156095903496 | 0.954114976920643 | -3.2511412 | 3.45817775898401 | 1.10906606610394 | 0.0387299839279226 | 31.7590511081124 | 0.999950574162623 |
| ebi-fl187-GCST90094807 | ebi-a-GCST90001684 | CD127- CD8+ T cell Absolute Count \|\| id:ebi-a-GCST90001684 | Oranges liking \|\| id:ebi-fl187-GCST90094807 | MR Egger | 7 | 0.729153490278432 | 1.84927433165012 | 0.709621384954775 | -2.8954242 | 4.35373118031266 | 2.07332477503624 | 0.0552755722364505 | 77.7680890283824 | 0.999950574162623 |
| ebi-fl187-GCST90094807 | ebi-a-GCST90001687 | CD28- CD8+ T cell Absolute Count \|\| id:ebi-a-GCST90001687 | Oranges liking \|\| id:ebi-fl187-GCST90094807 | MR Egger | 7 | -0.410536672 | 1.80099792050651 | 0.828713993424149 | -3.940492596 | 3.11941925253964 | 0.663294183413485 | 0.0194386369947655 | 22.6332316339173 | 0.999950574162623 |
| ebi-fl187-GCST90094807 | ebi-a-GCST90001701 | BAFF-R on CD20- CD38- B cell \|\| id:ebi-a-GCST90001701 | Oranges liking \|\| id:ebi-fl187-GCST90094807 | MR Egger | 7 | 0.602650583317898 | 1.79909435993373 | 0.751240520883598 | -2.923574362 | 4.12887552878801 | 1.82695488447659 | 0.0537412528897568 | 62.1080449456559 | 0.999950574162623 |
| ebi-fl187-GCST90094807 | ebi-a-GCST90001732 | CD19 on IgD- CD27- B cell \|\| id:ebi-a-GCST90001732 | Oranges liking \|\| id:ebi-fl187-GCST90094807 | MR Egger | 7 | 1.64428648044601 | 1.74842509113339 | 0.39017039261097 | -1.782626698 | 5.07119965906746 | 5.17731447360924 | 0.168195767008918 | 159.365396854622 | 0.999950574162623 |
| ebi-fl187-GCST90094807 | ebi-a-GCST90001798 | CD27 on CD24+ CD27+ B cell \|\| id:ebi-a-GCST90001798 | Oranges liking \|\| id:ebi-fl187-GCST90094807 | MR Egger | 7 | -3.096694781 | 2.00581022458536 | 0.183273538996366 | -7.028082821 | 0.834693258990149 | 0.0451983462050507 | 0.000886629980237649 | 2.30410717571727 | 0.999950574162623 |
| ebi-fl187-GCST90094807 | ebi-a-GCST90001836 | CD66b on CD66b++ myeloid cell \|\| id:ebi-a-GCST90001836 | Oranges liking \|\| id:ebi-fl187-GCST90094807 | MR Egger | 7 | 0.871297751141226 | 5.18295581655772 | 0.873086464959717 | -9.287295649 | 11.0298911515944 | 2.39001048064574 | 9.25931262793528e-05 | 61690.865479182 | 0.999950574162623 |
| ebi-fl187-GCST90094807 | ebi-a-GCST90001943 | CD25 on activated & secreting CD4 regulatory T cell \|\| id:ebi-a-GCST90001943 | Oranges liking \|\| id:ebi-fl187-GCST90094807 | MR Egger | 7 | 1.66288991231882 | 1.78548981910661 | 0.394428812285068 | -1.836670133 | 5.16244995776777 | 5.27453177462835 | 0.159347148455458 | 174.591674286162 | 0.999950574162623 |
| ebi-fl187-GCST90094807 | ebi-a-GCST90001960 | CD25 on CD4+ T cell \|\| id:ebi-a-GCST90001960 | Oranges liking \|\| id:ebi-fl187-GCST90094807 | MR Egger | 7 | 2.55286840713322 | 1.90954380636807 | 0.238854304177821 | -1.189837453 | 6.29557426761464 | 12.8438925078059 | 0.304270718233779 | 542.167106022092 | 0.999950574162623 |
| ebi-fl187-GCST90094807 | ebi-a-GCST90001988 | HLA DR on CD14+ CD16- monocyte \|\| id:ebi-a-GCST90001988 | Oranges liking \|\| id:ebi-fl187-GCST90094807 | MR Egger | 7 | -0.068476774 | 1.78410148917142 | 0.970868713657453 | -3.565315693 | 3.42836214438008 | 0.933815148272589 | 0.0282880537290054 | 30.8261126586179 | 0.999950574162623 |
| ebi-fl187-GCST90094807 | ebi-a-GCST90001991 | HLA DR on CD14+ monocyte \|\| id:ebi-a-GCST90001991 | Oranges liking \|\| id:ebi-fl187-GCST90094807 | MR Egger | 7 | -0.041591373 | 1.78720094830755 | 0.982333655951727 | -3.544505231 | 3.46132248598005 | 0.959261681004489 | 0.0288829093320894 | 31.8590818557609 | 0.999950574162623 |
| ebi-fl187-GCST90094807 | ebi-a-GCST90002019 | CD14 on Monocytic Myeloid-Derived Suppressor Cells \|\| id:ebi-a-GCST90002019 | Oranges liking \|\| id:ebi-fl187-GCST90094807 | MR Egger | 7 | -0.159123635 | 2.56770048364057 | 0.952986694805208 | -5.191816583 | 4.87356931288014 | 0.852890905236072 | 0.00556189399028841 | 130.78690415613 | 0.999950574162623 |
| ebi-fl187-GCST90094807 | ebi-a-GCST90002037 | CD80 on plasmacytoid Dendritic Cell \|\| id:ebi-a-GCST90002037 | Oranges liking \|\| id:ebi-fl187-GCST90094807 | MR Egger | 7 | 1.18950156022259 | 2.82977130207865 | 0.691681851875084 | -4.356850192 | 6.73585331229674 | 3.28544320361482 | 0.0128187005747387 | 842.061719223739 | 0.999950574162623 |
| ebi-fl187-GCST90094807 | ebi-a-GCST90002042 | CD45 on CD33+ HLA DR+ CD14- \|\| id:ebi-a-GCST90002042 | Oranges liking \|\| id:ebi-fl187-GCST90094807 | MR Egger | 7 | 2.08177970696357 | 2.60852175665799 | 0.461036490782321 | -3.030922936 | 7.19448235001323 | 8.0187272073911 | 0.0482710664516855 | 1332.06060593072 | 0.999950574162623 |
| ebi-fl187-GCST90094807 | ebi-a-GCST90002056 | CD8 on Effector Memory CD8+ T cell \|\| id:ebi-a-GCST90002056 | Oranges liking \|\| id:ebi-fl187-GCST90094807 | MR Egger | 7 | 0.510451695954761 | 1.95135165409152 | 0.804072388321749 | -3.314197546 | 4.33510093797415 | 1.66604357015177 | 0.0363632166790729 | 76.3326633653253 | 0.999950574162623 |
| ebi-fl187-GCST90094807 | ebi-a-GCST90002058 | CD8 on CD8+ T cell \|\| id:ebi-a-GCST90002058 | Oranges liking \|\| id:ebi-fl187-GCST90094807 | MR Egger | 7 | 1.44530827306105 | 1.89070854022645 | 0.479115842402917 | -2.260480466 | 5.1510970119049 | 4.24315999314217 | 0.10430035996001 | 172.620753507517 | 0.999950574162623 |
| ebi-fl187-GCST90094807 | ebi-a-GCST90002063 | CD4 on CD4 regulatory T cell \|\| id:ebi-a-GCST90002063 | Oranges liking \|\| id:ebi-fl187-GCST90094807 | MR Egger | 7 | 0.306076566935011 | 1.94201702233396 | 0.880933170807667 | -3.500276797 | 4.11242993070958 | 1.35808628707159 | 0.0301890260387264 | 61.0949939479963 | 0.999950574162623 |
| ebi-fl187-GCST90094807 | ebi-a-GCST90002066 | CD4 on activated CD4 regulatory T cell \|\| id:ebi-a-GCST90002066 | Oranges liking \|\| id:ebi-fl187-GCST90094807 | MR Egger | 7 | 0.615483691550057 | 1.94216149893803 | 0.76411922315201 | -3.191152846 | 4.42212022946859 | 1.85055147923146 | 0.0411244334464965 | 83.2726554577615 | 0.999950574162623 |
| ebi-fl187-GCST90094807 | ebi-a-GCST90002070 | CD4 on activated & secreting CD4 regulatory T cell \|\| id:ebi-a-GCST90002070 | Oranges liking \|\| id:ebi-fl187-GCST90094807 | MR Egger | 7 | 0.260819695518186 | 1.95124937777956 | 0.89887852186045 | -3.563629085 | 4.08526847596613 | 1.29799361026843 | 0.0283358048509773 | 59.4578986253699 | 0.999950574162623 |
| ebi-fl187-GCST90094807 | ebi-a-GCST90002081 | SSC-A on CD4+ T cell \|\| id:ebi-a-GCST90002081 | Oranges liking \|\| id:ebi-fl187-GCST90094807 | MR Egger | 7 | -0.579641682 | 2.55773062311333 | 0.829690232588442 | -5.592793704 | 4.43351033897343 | 0.560099023991683 | 0.00372460786575287 | 84.2265623613573 | 0.999950574162623 |
| ebi-fl187-GCST90094807 | ebi-a-GCST90002094 | CD11b on Monocytic Myeloid-Derived Suppressor Cells \|\| id:ebi-a-GCST90002094 | Oranges liking \|\| id:ebi-fl187-GCST90094807 | MR Egger | 7 | -0.898083128 | 2.59881394709935 | 0.743727183990487 | -5.991758464 | 4.19559220850129 | 0.407349749240327 | 0.00249926531542302 | 66.3930384590129 | 0.999950574162623 |
| ebi-fl187-GCST90094807 | ebi-a-GCST90002104 | HLA DR on myeloid Dendritic Cell \|\| id:ebi-a-GCST90002104 | Oranges liking \|\| id:ebi-fl187-GCST90094807 | MR Egger | 7 | -1.183796971 | 1.97731100656919 | 0.575467770902976 | -5.059326544 | 2.6917326021223 | 0.306114222433534 | 0.00634983440970791 | 14.7572221777666 | 0.999950574162623 |
| ebi-fl187-GCST90094807 | ebi-a-GCST90002105 | HLA DR on plasmacytoid Dendritic Cell \|\| id:ebi-a-GCST90002105 | Oranges liking \|\| id:ebi-fl187-GCST90094807 | MR Egger | 7 | -0.554232653 | 1.93439443455553 | 0.785976482044909 | -4.345645745 | 3.23718043876242 | 0.574512942899401 | 0.0129631346690154 | 25.4618292555314 | 0.999950574162623 |
| ebi-fl187-GCST90094807 | ebi-a-GCST90002106 | HLA DR on Dendritic Cell \|\| id:ebi-a-GCST90002106 | Oranges liking \|\| id:ebi-fl187-GCST90094807 | MR Egger | 7 | -1.166892276 | 2.06511333774413 | 0.596449790443524 | -5.214514418 | 2.88072986562043 | 0.311332976253926 | 0.00543707297852333 | 17.8272799511793 | 0.999950574162623 |
| ebi-fl187-GCST90094807 | ebi-a-GCST90002111 | HLA DR on CD33dim HLA DR+ CD11b- \|\| id:ebi-a-GCST90002111 | Oranges liking \|\| id:ebi-fl187-GCST90094807 | MR Egger | 7 | 1.18981360088346 | 3.1842587872904 | 0.72398084795177 | -5.051333622 | 7.43096082397265 | 3.28646855545072 | 0.00640079151396981 | 1687.42811609991 | 0.999950574162623 |
| ebi-fl187-GCST90094807 | ebi-a-GCST90002112 | HLA DR on CD33- HLA DR+ \|\| id:ebi-a-GCST90002112 | Oranges liking \|\| id:ebi-fl187-GCST90094807 | MR Egger | 7 | -5.192453783 | 3.19751842846947 | 0.165324611957196 | -11.4595899 | 1.07468233726622 | 0.0055583510828688 | 1.05478353168293e-05 | 2.92906229879554 | 0.999950574162623 |
| ebi-fl187-GCST90094807 | ebi-a-GCST90002115 | HLA DR on HLA DR+ CD8+ T cell \|\| id:ebi-a-GCST90002115 | Oranges liking \|\| id:ebi-fl187-GCST90094807 | MR Egger | 7 | 0.42536120781703 | 2.42298106956494 | 0.867533421649655 | -4.323681689 | 5.17440410416432 | 1.53014301947549 | 0.0132510075420656 | 176.691293293506 | 0.999950574162623 |
| ebi-fl187-GCST90094807 | ebi-a-GCST90002116 | HLA DR on B cell \|\| id:ebi-a-GCST90002116 | Oranges liking \|\| id:ebi-fl187-GCST90094807 | MR Egger | 7 | -0.89443492 | 1.91615964354445 | 0.660286200819385 | -4.650107821 | 2.86123798133338 | 0.408838559866706 | 0.00956057104118808 | 17.4831573672519 | 0.999950574162623 |
| ebi-fl187-GCST90094807 | ebi-cfmet338-GCST90026023 | Sphingomyelin (d18:1/24:1, d18:2/24:0) levels \|\| id:ebi-cfmet338-GCST90026023 | Oranges liking \|\| id:ebi-fl187-GCST90094807 | MR Egger | 7 | -1.272489445 | 1.0225066989372 | 0.268475528327774 | -3.276602575 | 0.731623684649208 | 0.280133376309064 | 0.0377563133887415 | 2.07845262100499 | 0.999950574162623 |
| ebi-fl187-GCST90094807 | ebi-cfmet338-GCST90026046 | 1-arachidonoyl-gpc (20:4n6) levels \|\| id:ebi-cfmet338-GCST90026046 | Oranges liking \|\| id:ebi-fl187-GCST90094807 | MR Egger | 7 | -0.294476447 | 1.20813303001873 | 0.817113459090498 | -2.662417185 | 2.07346429226088 | 0.744921491586839 | 0.0697793481050938 | 7.95232463037948 | 0.999950574162623 |
| ebi-fl187-GCST90094807 | ebi-cfmet338-GCST90026047 | 1-linoleoyl-gpc (18:2) levels \|\| id:ebi-cfmet338-GCST90026047 | Oranges liking \|\| id:ebi-fl187-GCST90094807 | MR Egger | 7 | -1.54955637 | 1.57899856956524 | 0.371486352283989 | -4.644393566 | 1.54528082661685 | 0.212342154341579 | 0.00961535897178867 | 4.68928831910635 | 0.999950574162623 |
| ebi-fl187-GCST90094807 | ebi-cfmet338-GCST90026050 | 1-myristoyl-2-palmitoyl-gpc (14:0/16:0) levels \|\| id:ebi-cfmet338-GCST90026050 | Oranges liking \|\| id:ebi-fl187-GCST90094807 | MR Egger | 7 | -0.5508237 | 0.770542380669173 | 0.506679399847545 | -2.061086766 | 0.959439366072755 | 0.576474772470508 | 0.127315532472782 | 2.61023267813744 | 0.999950574162623 |
| ebi-fl187-GCST90094807 | ebi-cfmet338-GCST90026068 | 2,3-dihydroxyisovalerate levels \|\| id:ebi-cfmet338-GCST90026068 | Oranges liking \|\| id:ebi-fl187-GCST90094807 | MR Egger | 7 | -2.913477566 | 2.81905924146806 | 0.348747536354808 | -8.438833679 | 2.61187854717189 | 0.0542866159361128 | 0.000216302281030144 | 13.6246213204951 | 0.999950574162623 |
| ebi-fl187-GCST90094807 | ebi-cfmet338-GCST90026082 | 3-hydroxyhexanoate levels \|\| id:ebi-cfmet338-GCST90026082 | Oranges liking \|\| id:ebi-fl187-GCST90094807 | MR Egger | 7 | 1.35590170977224 | 0.804994629823588 | 0.152929180706079 | -0.221887765 | 2.93369118422647 | 3.88025824640562 | 0.801005260369931 | 18.7968853685746 | 0.999950574162623 |
| ebi-fl187-GCST90094807 | ebi-cfmet338-GCST90026094 | 3-ureidopropionate levels \|\| id:ebi-cfmet338-GCST90026094 | Oranges liking \|\| id:ebi-fl187-GCST90094807 | MR Egger | 7 | -2.062407413 | 1.21399455604623 | 0.150097116294684 | -4.441836743 | 0.317021916897779 | 0.127147504599081 | 0.0117742922999203 | 1.37303266421224 | 0.999950574162623 |
| ebi-fl187-GCST90094807 | ebi-cfmet338-GCST90026173 | Glycerol levels \|\| id:ebi-cfmet338-GCST90026173 | Oranges liking \|\| id:ebi-fl187-GCST90094807 | MR Egger | 7 | -0.30979049 | 0.694276202032505 | 0.674108401497478 | -1.670571846 | 1.05099086607882 | 0.733600636864107 | 0.188139448101476 | 2.86048407092782 | 0.999950574162623 |
| ebi-fl187-GCST90094807 | ebi-cfmet338-GCST90026179 | Histidine levels \|\| id:ebi-cfmet338-GCST90026179 | Oranges liking \|\| id:ebi-fl187-GCST90094807 | MR Egger | 7 | 0.626670891110942 | 0.638707978592958 | 0.371575819695259 | -0.625196747 | 1.87853852915314 | 1.87137020240525 | 0.535156127834643 | 6.54393410128784 | 0.999950574162623 |
| ebi-fl187-GCST90094807 | ebi-cfmet338-GCST90026188 | Isocitrate levels \|\| id:ebi-cfmet338-GCST90026188 | Oranges liking \|\| id:ebi-fl187-GCST90094807 | MR Egger | 7 | -1.316578563 | 1.29327167653689 | 0.355370685165832 | -3.851391049 | 1.21823392310279 | 0.268050853954805 | 0.0212501558627321 | 3.38121097887619 | 0.999950574162623 |
| ebi-fl187-GCST90094807 | ebi-cfmet338-GCST90026207 | N-acetyl-isoputreanine levels \|\| id:ebi-cfmet338-GCST90026207 | Oranges liking \|\| id:ebi-fl187-GCST90094807 | MR Egger | 7 | 0.0841694156617113 | 0.599033977323994 | 0.893743230559523 | -1.08993718 | 1.25827601121674 | 1.08781317078683 | 0.336237615526167 | 3.51934893627392 | 0.999950574162623 |
| ebi-fl187-GCST90094807 | ebi-cfmet338-GCST90026213 | N-acetylglutamate levels \|\| id:ebi-cfmet338-GCST90026213 | Oranges liking \|\| id:ebi-fl187-GCST90094807 | MR Egger | 7 | -0.707309351 | 1.06990549062227 | 0.537792238710493 | -2.804324112 | 1.38970541085528 | 0.4929688207943 | 0.0605476807734874 | 4.01366749594371 | 0.999950574162623 |
| ebi-fl187-GCST90094807 | ebi-cfmet338-GCST90026224 | N-acetyltaurine levels \|\| id:ebi-cfmet338-GCST90026224 | Oranges liking \|\| id:ebi-fl187-GCST90094807 | MR Egger | 7 | -0.205352285 | 1.29706351711653 | 0.880399610057535 | -2.747596779 | 2.33689220847186 | 0.814360378864075 | 0.0640816787640113 | 10.3490239246992 | 0.999950574162623 |
| ebi-fl187-GCST90094807 | ebi-cfmet338-GCST90026250 | Phenyllactate (pla) levels \|\| id:ebi-cfmet338-GCST90026250 | Oranges liking \|\| id:ebi-fl187-GCST90094807 | MR Egger | 7 | -1.251784505 | 1.33004017245545 | 0.389833155809068 | -3.858663243 | 1.35509423295716 | 0.285993983511818 | 0.0210961811492384 | 3.87712629249538 | 0.999950574162623 |
| ebi-fl187-GCST90094807 | ebi-cfmet338-GCST90026251 | Pipecolate levels \|\| id:ebi-cfmet338-GCST90026251 | Oranges liking \|\| id:ebi-fl187-GCST90094807 | MR Egger | 7 | 0.86475557650042 | 2.4865840467893 | 0.742175848347387 | -4.008949155 | 5.73846030820744 | 2.37442564961709 | 0.0181524606364058 | 310.585836184236 | 0.999950574162623 |
| ebi-fl187-GCST90094807 | ebi-cfmet338-GCST90026278 | Trigonelline (n'-methylnicotinate) levels \|\| id:ebi-cfmet338-GCST90026278 | Oranges liking \|\| id:ebi-fl187-GCST90094807 | MR Egger | 7 | -0.493922078 | 2.16260916439903 | 0.828388548903686 | -4.73263604 | 3.74479188452166 | 0.610228331632972 | 0.00880323474904111 | 42.3002029757438 | 0.999950574162623 |
| ebi-fl187-GCST90094807 | ebi-cfmet338-GCST90026299 | 1-carboxyethylvaline levels \|\| id:ebi-cfmet338-GCST90026299 | Oranges liking \|\| id:ebi-fl187-GCST90094807 | MR Egger | 7 | -1.651626882 | 1.30507769507428 | 0.261444520558039 | -4.209579164 | 0.90632540069241 | 0.191737720163619 | 0.0148526175392235 | 2.47521039550477 | 0.999950574162623 |
| ebi-fl187-GCST90094807 | ebi-gm430-GCST90011306 | C_Erysipelotrichia abundance \|\| id:ebi-gm430-GCST90011306 | Oranges liking \|\| id:ebi-fl187-GCST90094807 | MR Egger | 5 | -0.432243748 | 1.05187471069527 | 0.708715619189954 | -2.493918181 | 1.62943068507711 | 0.649051152556444 | 0.0825857459243645 | 5.10096983347057 | 0.999950574162623 |
| ebi-fl187-GCST90094807 | ebi-gm430-GCST90011312 | F_Erysipelotrichaceae abundance \|\| id:ebi-gm430-GCST90011312 | Oranges liking \|\| id:ebi-fl187-GCST90094807 | MR Egger | 5 | -0.432243748 | 1.05187471069527 | 0.708715619189954 | -2.493918181 | 1.62943068507711 | 0.649051152556444 | 0.0825857459243645 | 5.10096983347057 | 0.999950574162623 |
| ebi-fl187-GCST90094807 | ebi-gm430-GCST90011352 | O_Erysipelotrichales abundance \|\| id:ebi-gm430-GCST90011352 | Oranges liking \|\| id:ebi-fl187-GCST90094807 | MR Egger | 5 | -0.432243748 | 1.05187471069527 | 0.708715619189954 | -2.493918181 | 1.62943068507711 | 0.649051152556444 | 0.0825857459243645 | 5.10096983347057 | 0.999950574162623 |
| ebi-fl187-GCST90094807 | ebi-gm430-GCST90011362 | OTU97_11 (Parabacteroides) abundance \|\| id:ebi-gm430-GCST90011362 | Oranges liking \|\| id:ebi-fl187-GCST90094807 | MR Egger | 5 | -0.611218121 | 1.58927110912335 | 0.726184356597106 | -3.726189495 | 2.50375325288819 | 0.542689404927476 | 0.0240844350694582 | 12.2283038556305 | 0.999950574162623 |
| ebi-fl187-GCST90094807 | ebi-gm430-GCST90011384 | OTU97_235 (Phascolarctobacterium) abundance \|\| id:ebi-gm430-GCST90011384 | Oranges liking \|\| id:ebi-fl187-GCST90094807 | MR Egger | 5 | 5.80237762922542 | 4.02723015599392 | 0.245288150276448 | -2.090993477 | 13.6957487349735 | 331.085824148446 | 0.123564316570985 | 887131.705932938 | 0.999950574162623 |
| ebi-fl187-GCST90094807 | ebi-gm430-GCST90011390 | OTU97_3 (Bacteroides) abundance \|\| id:ebi-gm430-GCST90011390 | Oranges liking \|\| id:ebi-fl187-GCST90094807 | MR Egger | 5 | 0.918385086135523 | 1.08661799307366 | 0.460087699870387 | -1.21138618 | 3.0481563525599 | 2.50524137238285 | 0.297784210599029 | 21.0764510357124 | 0.999950574162623 |
| ebi-fl187-GCST90094807 | ebi-gm430-GCST90011398 | OTU97_4 (Alistipes) abundance \|\| id:ebi-gm430-GCST90011398 | Oranges liking \|\| id:ebi-fl187-GCST90094807 | MR Egger | 5 | -0.853364661 | 1.94811109858868 | 0.690975037507238 | -4.671662414 | 2.96493309232582 | 0.42597924229458 | 0.00935670187712942 | 19.3934056303966 | 0.999950574162623 |
| ebi-fl187-GCST90094807 | ebi-gm430-GCST90011421 | OTU99_108 (Cytophagales) abundance \|\| id:ebi-gm430-GCST90011421 | Oranges liking \|\| id:ebi-fl187-GCST90094807 | MR Egger | 5 | 0.129542725918702 | 3.97611477528513 | 0.976055752744243 | -7.663642234 | 7.92272768547756 | 1.13830774566801 | 0.000469593921447826 | 2759.28725792022 | 0.999950574162623 |
| ebi-fl187-GCST90094807 | ebi-gm430-GCST90011428 | OTU99_123 (Phascolarctobacterium) abundance \|\| id:ebi-gm430-GCST90011428 | Oranges liking \|\| id:ebi-fl187-GCST90094807 | MR Egger | 5 | -2.517751319 | 2.40889861641178 | 0.372736118461826 | -7.239192608 | 2.20368996868158 | 0.0806407382765761 | 0.000717891145999519 | 9.05837703394047 | 0.999950574162623 |
| ebi-fl187-GCST90094807 | ebi-gm430-GCST90011450 | OTU99_298 (Phascolarctobacterium) abundance \|\| id:ebi-gm430-GCST90011450 | Oranges liking \|\| id:ebi-fl187-GCST90094807 | MR Egger | 5 | 5.649037373 | 4.0276764338827 | 0.255321242713395 | -2.245208437 | 13.5432831836451 | 284.017930926988 | 0.105905463437173 | 761681.054687998 | 0.999950574162623 |
| ebi-fl187-GCST90094807 | ebi-gm430-GCST90011451 | OTU99_3 (Bacteroides) abundance \|\| id:ebi-gm430-GCST90011451 | Oranges liking \|\| id:ebi-fl187-GCST90094807 | MR Egger | 5 | 0.901868564795656 | 1.08624573125896 | 0.467288991670843 | -1.227173068 | 3.03091019806322 | 2.4642033355119 | 0.293120037819653 | 20.7160797464281 | 0.999950574162623 |
| ebi-fl187-GCST90094807 | ebi-gm430-GCST90011472 | OTU99_72 (Parabacteroides) abundance \|\| id:ebi-gm430-GCST90011472 | Oranges liking \|\| id:ebi-fl187-GCST90094807 | MR Egger | 5 | 3.67858769591148 | 3.2237645664537 | 0.336673661103837 | -2.639990854 | 9.99716624616073 | 39.5904408289264 | 0.0713619222054377 | 21964.1365673484 | 0.999950574162623 |
| ebi-fl187-GCST90094807 | ebi-gm430-GCST90011507 | TestASV_3 (Bacteroides) abundance \|\| id:ebi-gm430-GCST90011507 | Oranges liking \|\| id:ebi-fl187-GCST90094807 | MR Egger | 5 | 0.232358009426739 | 1.49386918421168 | 0.886271237736974 | -2.695625592 | 3.16034161048163 | 1.26157130240847 | 0.067500141039744 | 23.5786492671697 | 0.999950574162623 |
| ebi-fl187-GCST90094807 | ebi-gm430-GCST90011512 | TestASV_34 (Phascolarctobacterium) abundance \|\| id:ebi-gm430-GCST90011512 | Oranges liking \|\| id:ebi-fl187-GCST90094807 | MR Egger | 5 | -0.023891821 | 3.5155081603232 | 0.99500418693904 | -6.914287815 | 6.8665041730487 | 0.976391328902084 | 0.000993488754194192 | 959.588141416278 | 0.999950574162623 |
| ebi-fl187-GCST90094807 | ebi-gm430-GCST90011519 | TestASV_41 (Barnesiella) abundance \|\| id:ebi-gm430-GCST90011519 | Oranges liking \|\| id:ebi-fl187-GCST90094807 | MR Egger | 5 | -1.195048836 | 5.22228992761595 | 0.833708633177507 | -11.43073709 | 9.04063942233936 | 0.302689171757001 | 1.08566029754487e-05 | 8439.17152594894 | 0.999950574162623 |
| ebi-fl187-GCST90094807 | ebi-gm430-GCST90011524 | TestASV_46 (Sutterella) abundance \|\| id:ebi-gm430-GCST90011524 | Oranges liking \|\| id:ebi-fl187-GCST90094807 | MR Egger | 5 | 1.08233096573257 | 4.52394208218707 | 0.826328959422962 | -7.784595515 | 9.94925744681923 | 2.9515515042246 | 0.000416095600440373 | 20936.6700173484 | 0.999950574162623 |
| ebi-fl187-GCST90094807 | ebi-gm430-GCST90011581 | OTU97_19 (Bacteria) prevalence \|\| id:ebi-gm430-GCST90011581 | Oranges liking \|\| id:ebi-fl187-GCST90094807 | MR Egger | 5 | 6.24050251699579 | 5.12557536952522 | 0.310437002475207 | -3.805625207 | 16.2866302412652 | 513.116295826231 | 0.0222452849035604 | 11835691.6615751 | 0.999950574162623 |
| ebi-fl187-GCST90094807 | ebi-gm430-GCST90011614 | OTU97_80 (Ruminococcus) prevalence \|\| id:ebi-gm430-GCST90011614 | Oranges liking \|\| id:ebi-fl187-GCST90094807 | MR Egger | 5 | 3.56308519266765 | 2.39793223919115 | 0.233995989487656 | -1.136861996 | 8.2630323814823 | 35.2718499042743 | 0.320824191427249 | 3877.83536564065 | 0.999950574162623 |
| ebi-fl187-GCST90094807 | ebi-gm430-GCST90011623 | OTU99_11 (Parabacteroides) prevalence \|\| id:ebi-gm430-GCST90011623 | Oranges liking \|\| id:ebi-fl187-GCST90094807 | MR Egger | 5 | -1.795583669 | 2.3397022176742 | 0.498689433017803 | -6.381400015 | 2.79023267791582 | 0.166030517240179 | 0.0016927514189169 | 16.2848084762967 | 0.999950574162623 |
| ebi-fl187-GCST90094807 | ebi-gm430-GCST90011642 | OTU99_19 (Bacteria) prevalence \|\| id:ebi-gm430-GCST90011642 | Oranges liking \|\| id:ebi-fl187-GCST90094807 | MR Egger | 5 | 6.24050251699579 | 5.12557536952522 | 0.310437002475207 | -3.805625207 | 16.2866302412652 | 513.116295826231 | 0.0222452849035604 | 11835691.6615751 | 0.999950574162623 |
| ebi-fl187-GCST90094807 | ebi-gm430-GCST90011653 | OTU99_30 (Parasutterella) prevalence \|\| id:ebi-gm430-GCST90011653 | Oranges liking \|\| id:ebi-fl187-GCST90094807 | MR Egger | 5 | -3.907715086 | 2.75178718995438 | 0.250672252019591 | -9.301217979 | 1.48578780597506 | 0.0200863442302992 | 9.13129465016168e-05 | 4.41844491931851 | 0.999950574162623 |
| ebi-fl187-GCST90094807 | ebi-gm430-GCST90011675 | OTU99_8 (Ruminococcaceae) prevalence \|\| id:ebi-gm430-GCST90011675 | Oranges liking \|\| id:ebi-fl187-GCST90094807 | MR Egger | 5 | 1.2215783377928 | 2.57847883210794 | 0.668015557798244 | -3.832240173 | 6.27539684872437 | 3.39253808124984 | 0.0216610367649113 | 531.337200413891 | 0.999950574162623 |
| ebi-fl187-GCST90094807 | ebi-gm430-GCST90011679 | OTU99_92 (Ruminococcus) prevalence \|\| id:ebi-gm430-GCST90011679 | Oranges liking \|\| id:ebi-fl187-GCST90094807 | MR Egger | 5 | 3.58727993301356 | 2.50642523763515 | 0.247754481017322 | -1.325313533 | 8.49987339877846 | 36.1356507350071 | 0.26571963421907 | 4914.14666394533 | 0.999950574162623 |
| ebi-fl187-GCST90094807 | ebi-gm430-GCST90011684 | TestASV_11 (Lachnospiraceae) prevalence \|\| id:ebi-gm430-GCST90011684 | Oranges liking \|\| id:ebi-fl187-GCST90094807 | MR Egger | 5 | -2.552638792 | 2.5762798019097 | 0.394814071575699 | -7.602147204 | 2.49686961964927 | 0.0778758963293368 | 0.000499378015039095 | 12.1444177485924 | 0.999950574162623 |
| ebi-fl187-GCST90094807 | ebi-gm430-GCST90011688 | TestASV_15 (Bacteroides) prevalence \|\| id:ebi-gm430-GCST90011688 | Oranges liking \|\| id:ebi-fl187-GCST90094807 | MR Egger | 5 | -0.601764608 | 2.39762626819371 | 0.8180355733545 | -5.301112093 | 4.09758287782036 | 0.547844052732344 | 0.00498604587331473 | 60.1946138764003 | 0.999950574162623 |
| ebi-fl187-GCST90094807 | ebi-gm430-GCST90011692 | TestASV_19 (Prevotella) prevalence \|\| id:ebi-gm430-GCST90011692 | Oranges liking \|\| id:ebi-fl187-GCST90094807 | MR Egger | 5 | -4.513806605 | 4.68936285696984 | 0.406773865870318 | -13.70495781 | 4.67734459427628 | 0.0109566729622543 | 1.11689526698338e-06 | 107.484278920828 | 0.999950574162623 |
| ebi-fl187-GCST90094807 | ebi-gm430-GCST90011693 | TestASV_2 (Bacteroides) prevalence \|\| id:ebi-gm430-GCST90011693 | Oranges liking \|\| id:ebi-fl187-GCST90094807 | MR Egger | 5 | 4.09319703717953 | 2.35752955828023 | 0.180925208126172 | -0.527560897 | 8.71395497140879 | 59.9311879867512 | 0.590042390092005 | 6087.26992130728 | 0.999950574162623 |
| ebi-fl187-GCST90094807 | ebi-gm430-GCST90011716 | TestASV_40 (Barnesiella) prevalence \|\| id:ebi-gm430-GCST90011716 | Oranges liking \|\| id:ebi-fl187-GCST90094807 | MR Egger | 5 | -4.165121032 | 5.07521397832981 | 0.471967121526207 | -14.11254043 | 5.78229836553252 | 0.015527835421487 | 7.43021816812567e-07 | 324.504163163232 | 0.999950574162623 |
| ebi-fl187-GCST90094807 | ebi-gm430-GCST90011718 | TestASV_42 (Alphaproteobacteria) prevalence \|\| id:ebi-gm430-GCST90011718 | Oranges liking \|\| id:ebi-fl187-GCST90094807 | MR Egger | 5 | -1.382200921 | 4.23386675935938 | 0.765523354770044 | -9.680579769 | 6.91617792775524 | 0.251025457525486 | 6.24852662549642e-05 | 1008.45821907454 | 0.999950574162623 |
| ebi-fl187-GCST90094807 | ebi-hbsm44-GCST90104929 | Sulcal depth of the right inferior frontal sulcus \|\| id:ebi-hbsm44-GCST90104929 | Oranges liking \|\| id:ebi-fl187-GCST90094807 | MR Egger | 5 | 0.421680370324695 | 2.02938643936909 | 0.84870140438795 | -3.555917051 | 4.39927779148811 | 1.52452116459522 | 0.0285551763569839 | 81.392065390986 | 0.999950574162623 |
| ebi-fl187-GCST90094807 | ebi-hbsm44-GCST90104935 | Sulcal opening distance of the left calloso-marginal posterior fissure \|\| id:ebi-hbsm44-GCST90104935 | Oranges liking \|\| id:ebi-fl187-GCST90094807 | MR Egger | 5 | -0.228933306 | 0.843254198450587 | 0.803626012938595 | -1.881711535 | 1.42384492324677 | 0.795381579140776 | 0.152329165866662 | 4.1530579704627 | 0.999950574162623 |
| ebi-fl187-GCST90094807 | ebi-hbsm44-GCST90104936 | Sulcal opening distance of the left anterior inferior temporal sulcus \|\| id:ebi-hbsm44-GCST90104936 | Oranges liking \|\| id:ebi-fl187-GCST90094807 | MR Egger | 5 | -0.041077861 | 0.261750894900922 | 0.885263069092464 | -0.554109615 | 0.471953893038077 | 0.959754399632211 | 0.574583634167547 | 1.6031234668699 | 0.999950574162623 |
| ebi-fl187-GCST90094807 | ebi-hbsm44-GCST90104941 | Sulcal opening distance of the right posterior inferior temporal sulcus \|\| id:ebi-hbsm44-GCST90104941 | Oranges liking \|\| id:ebi-fl187-GCST90094807 | MR Egger | 5 | -0.086729467 | 0.213549687690601 | 0.711878410153801 | -0.505286854 | 0.331827921261318 | 0.916925120941348 | 0.603332481973005 | 1.3935130339144 | 0.999950574162623 |
| ebi-fl187-GCST90094807 | ebi-IPs91-GCST90274819 | Leukemia inhibitory factor levels \|\| id:ebi-IPs91-GCST90274819 | Oranges liking \|\| id:ebi-fl187-GCST90094807 | MR Egger | 7 | -0.748243697 | 1.54095238297062 | 0.647799903307058 | -3.768510368 | 2.27202297329254 | 0.473196900336276 | 0.0230864279684744 | 9.69900180286126 | 0.999950574162623 |
| ebi-fl187-GCST90094807 | ebi-IPs91-GCST90274837 | STAM binding protein levels \|\| id:ebi-IPs91-GCST90274837 | Oranges liking \|\| id:ebi-fl187-GCST90094807 | MR Egger | 7 | -1.892911621 | 0.802596557961582 | 0.0648729479100279 | -3.466000875 | -0.319822368 | 0.150632584671383 | 0.0312417207174017 | 0.72627803602837 | 0.999950574162623 |
| ebi-fl187-GCST90094807 | ebi-IPs91-GCST90274840 | TNF-beta levels \|\| id:ebi-IPs91-GCST90274840 | Oranges liking \|\| id:ebi-fl187-GCST90094807 | MR Egger | 7 | -1.777404297 | 1.00673349320221 | 0.137740938778098 | -3.750601944 | 0.195793349557947 | 0.169076450399666 | 0.0235035937546375 | 1.2162755354853 | 0.999950574162623 |
| ebi-fl187-GCST90094807 | ebi-IPs91-GCST90274842 | Tumor necrosis factor ligand superfamily member 14 levels \|\| id:ebi-IPs91-GCST90274842 | Oranges liking \|\| id:ebi-fl187-GCST90094807 | MR Egger | 7 | -1.880223075 | 0.943138641014988 | 0.102778721833323 | -3.728774811 | -0.031671339 | 0.152556070530982 | 0.0240222496033934 | 0.968824945211064 | 0.999950574162623 |
| ebi-fl187-GCST90094807 | ebi-met1400-GCST90199628 | 3-methylhistidine levels \|\| id:ebi-met1400-GCST90199628 | Oranges liking \|\| id:ebi-fl187-GCST90094807 | MR Egger | 7 | 0.851890867585688 | 1.68358618427291 | 0.634372064182276 | -2.447938054 | 4.15171978876059 | 2.34407499952127 | 0.0864717028203745 | 63.5431872412022 | 0.999950574162623 |
| ebi-fl187-GCST90094807 | ebi-met1400-GCST90199651 | Docosahexaenoate DHA; 22:6n3 levels \|\| id:ebi-met1400-GCST90199651 | Oranges liking \|\| id:ebi-fl187-GCST90094807 | MR Egger | 7 | -0.093199323 | 1.00158231405667 | 0.929475699335115 | -2.056300658 | 1.86990201285092 | 0.911011896846746 | 0.127926338875142 | 6.48766066076777 | 0.999950574162623 |
| ebi-fl187-GCST90094807 | ebi-met1400-GCST90199652 | 1-stearoyl-2-oleoyl-GPS (18:0/18:1) levels \|\| id:ebi-met1400-GCST90199652 | Oranges liking \|\| id:ebi-fl187-GCST90094807 | MR Egger | 7 | 0.104563469217715 | 1.06114131743892 | 0.925333057502221 | -1.975273513 | 2.18440045139801 | 1.1102258567967 | 0.138723364415201 | 8.88531977505079 | 0.999950574162623 |
| ebi-fl187-GCST90094807 | ebi-met1400-GCST90199653 | 1-myristoyl-2-palmitoyl-gpc (14:0/16:0) levels \|\| id:ebi-met1400-GCST90199653 | Oranges liking \|\| id:ebi-fl187-GCST90094807 | MR Egger | 7 | -1.638736221 | 1.16904955558602 | 0.219906764328383 | -3.93007335 | 0.652600908032252 | 0.194225345182668 | 0.0196422317451786 | 1.92052945921414 | 0.999950574162623 |
| ebi-fl187-GCST90094807 | ebi-met1400-GCST90199667 | Erythritol levels in elite athletes \|\| id:ebi-met1400-GCST90199667 | Oranges liking \|\| id:ebi-fl187-GCST90094807 | MR Egger | 7 | -0.316944433 | 1.3269502836844 | 0.820701674257852 | -2.917766989 | 2.28387812258505 | 0.728371227087384 | 0.0540542563585673 | 9.81466918959279 | 0.999950574162623 |
| ebi-fl187-GCST90094807 | ebi-met1400-GCST90199674 | 3-hydroxymyristate levels \|\| id:ebi-met1400-GCST90199674 | Oranges liking \|\| id:ebi-fl187-GCST90094807 | MR Egger | 7 | -0.181452477 | 1.02076062721237 | 0.865887196534467 | -2.182143306 | 1.81923835251823 | 0.834057881446142 | 0.112799507489646 | 6.16715945915172 | 0.999950574162623 |
| ebi-fl187-GCST90094807 | ebi-met1400-GCST90199685 | 2-linoleoylglycerol (18:2) levels \|\| id:ebi-met1400-GCST90199685 | Oranges liking \|\| id:ebi-fl187-GCST90094807 | MR Egger | 7 | -2.468530325 | 1.19579731684426 | 0.0939134288044389 | -4.812293066 | -0.124767584 | 0.0847092626148958 | 0.00812919751928678 | 0.88270203248659 | 0.999950574162623 |
| ebi-fl187-GCST90094807 | ebi-met1400-GCST90199703 | Gamma-glutamylvaline levels \|\| id:ebi-met1400-GCST90199703 | Oranges liking \|\| id:ebi-fl187-GCST90094807 | MR Egger | 7 | -0.751752582 | 1.02172803203327 | 0.494918044411819 | -2.754339524 | 1.25083436125031 | 0.47153941686622 | 0.0636510457563607 | 3.49325637963011 | 0.999950574162623 |
| ebi-fl187-GCST90094807 | ebi-met1400-GCST90199779 | Gamma-glutamyl-2-aminobutyrate levels \|\| id:ebi-met1400-GCST90199779 | Oranges liking \|\| id:ebi-fl187-GCST90094807 | MR Egger | 7 | 2.90587967155276 | 1.08497221183615 | 0.0439095317215112 | 0.779334136353904 | 5.03242520675163 | 18.2813181365511 | 2.18002018591879 | 153.304356981877 | 0.999950574162623 |
| ebi-fl187-GCST90094807 | ebi-met1400-GCST90199824 | 4-vinylphenol sulfate levels \|\| id:ebi-met1400-GCST90199824 | Oranges liking \|\| id:ebi-fl187-GCST90094807 | MR Egger | 7 | 0.576085171111616 | 1.51363051688006 | 0.719134698878046 | -2.390630642 | 3.54280098419654 | 1.77906006437322 | 0.0915719165700097 | 34.563595818461 | 0.999950574162623 |
| ebi-fl187-GCST90094807 | ebi-met1400-GCST90199830 | 4-ethylphenylsulfate levels \|\| id:ebi-met1400-GCST90199830 | Oranges liking \|\| id:ebi-fl187-GCST90094807 | MR Egger | 7 | -0.039610538 | 1.07857599285651 | 0.972125523799054 | -2.153619484 | 2.07439840835282 | 0.961163703345228 | 0.116063307359616 | 7.95975649535698 | 0.999950574162623 |
| ebi-fl187-GCST90094807 | ebi-met1400-GCST90199833 | Hexanoylglutamine levels \|\| id:ebi-met1400-GCST90199833 | Oranges liking \|\| id:ebi-fl187-GCST90094807 | MR Egger | 7 | 1.10915391931822 | 1.0331049153155 | 0.33205502707737 | -0.915731715 | 3.13403955333659 | 3.03179216818687 | 0.400223669381291 | 22.9665670830735 | 0.999950574162623 |
| ebi-fl187-GCST90094807 | ebi-met1400-GCST90199837 | Carotenoid levels (cryptoxanthin) \|\| id:ebi-met1400-GCST90199837 | Oranges liking \|\| id:ebi-fl187-GCST90094807 | MR Egger | 7 | 0.00428807300258857 | 1.03827824215406 | 0.996864471238582 | -2.030737282 | 2.03931342762454 | 1.00429727994293 | 0.131238725569966 | 7.68533085124379 | 0.999950574162623 |
| ebi-fl187-GCST90094807 | ebi-met1400-GCST90199869 | 2-hydroxyhippurate levels \|\| id:ebi-met1400-GCST90199869 | Oranges liking \|\| id:ebi-fl187-GCST90094807 | MR Egger | 7 | 1.69962876726585 | 1.66342050408676 | 0.353757600972338 | -1.560675421 | 4.9599329552759 | 5.4719156604163 | 0.209994188859705 | 142.584236055756 | 0.999950574162623 |
| ebi-fl187-GCST90094807 | ebi-met1400-GCST90199902 | Hydantoin-5-propionate levels \|\| id:ebi-met1400-GCST90199902 | Oranges liking \|\| id:ebi-fl187-GCST90094807 | MR Egger | 7 | 0.499428196808259 | 1.04652962013692 | 0.653333032922383 | -1.551769859 | 2.55062625227662 | 1.64777879609592 | 0.211872657138892 | 12.8151267725095 | 0.999950574162623 |
| ebi-fl187-GCST90094807 | ebi-met1400-GCST90199904 | Pantoate levels \|\| id:ebi-met1400-GCST90199904 | Oranges liking \|\| id:ebi-fl187-GCST90094807 | MR Egger | 7 | -0.901240934 | 1.06172681209536 | 0.434714239905779 | -2.982225486 | 1.17974361773912 | 0.406065446551893 | 0.0506799208305276 | 3.25353994602268 | 0.999950574162623 |
| ebi-fl187-GCST90094807 | ebi-met1400-GCST90199936 | 3-methyl catechol sulfate (2) levels \|\| id:ebi-met1400-GCST90199936 | Oranges liking \|\| id:ebi-fl187-GCST90094807 | MR Egger | 7 | -0.689511898 | 1.19115602028217 | 0.587780819802296 | -3.024177698 | 1.64515390171017 | 0.501820949085361 | 0.0485977668949024 | 5.18180733459476 | 0.999950574162623 |
| ebi-fl187-GCST90094807 | ebi-met1400-GCST90199977 | 4-hydroxychlorothalonil levels \|\| id:ebi-met1400-GCST90199977 | Oranges liking \|\| id:ebi-fl187-GCST90094807 | MR Egger | 7 | 0.0521655667031107 | 1.03863195520304 | 0.961887532097852 | -1.983553065 | 2.08788419890107 | 1.05355016090073 | 0.13757953874861 | 8.06782717568292 | 0.999950574162623 |
| ebi-fl187-GCST90094807 | ebi-met1400-GCST90200040 | Sphingomyelin (d18:2/24:1, d18:1/24:2) levels \|\| id:ebi-met1400-GCST90200040 | Oranges liking \|\| id:ebi-fl187-GCST90094807 | MR Egger | 7 | 0.0507357523640268 | 0.954522908727423 | 0.95966832102431 | -1.820129149 | 1.92160065346978 | 1.05204485618331 | 0.162004826863209 | 6.8318852027558 | 0.999950574162623 |
| ebi-fl187-GCST90094807 | ebi-met1400-GCST90200077 | 1-stearoyl-2-oleoyl-GPI (18:0/18:1) levels \|\| id:ebi-met1400-GCST90200077 | Oranges liking \|\| id:ebi-fl187-GCST90094807 | MR Egger | 6 | 0.232950528374748 | 2.51313193027014 | 0.9306041756146 | -4.692788055 | 5.15868911170422 | 1.26231902880857 | 0.00916110884243575 | 173.936295037898 | 0.999950574162623 |
| ebi-fl187-GCST90094807 | ebi-met1400-GCST90200080 | 1-palmitoyl-2-oleoyl-GPI (16:0/18:1) levels \|\| id:ebi-met1400-GCST90200080 | Oranges liking \|\| id:ebi-fl187-GCST90094807 | MR Egger | 7 | -1.102856445 | 1.04470321077796 | 0.339436768252156 | -3.150474739 | 0.944761847664286 | 0.331921612304322 | 0.0428317881371252 | 2.5722007300276 | 0.999950574162623 |
| ebi-fl187-GCST90094807 | ebi-met1400-GCST90200082 | 1-oleoyl-2-linoleoyl-GPE (18:1/18:2) levels \|\| id:ebi-met1400-GCST90200082 | Oranges liking \|\| id:ebi-fl187-GCST90094807 | MR Egger | 7 | -1.57988379 | 1.05128326401868 | 0.193203981800817 | -3.640398988 | 0.480631407041294 | 0.205999035872246 | 0.0262418716870806 | 1.61709512516162 | 0.999950574162623 |
| ebi-fl187-GCST90094807 | ebi-met1400-GCST90200100 | Perfluorooctanesulfonate (PFOS) levels \|\| id:ebi-met1400-GCST90200100 | Oranges liking \|\| id:ebi-fl187-GCST90094807 | MR Egger | 7 | 0.594473032591706 | 0.96590431704924 | 0.56518376597478 | -1.298699429 | 2.48764549400822 | 1.81207578843222 | 0.272886470620085 | 12.0329111793663 | 0.999950574162623 |
| ebi-fl187-GCST90094807 | ebi-met1400-GCST90200102 | 1-palmitoleoyl-2-linolenoyl-GPC (16:1/18:3) levels \|\| id:ebi-met1400-GCST90200102 | Oranges liking \|\| id:ebi-fl187-GCST90094807 | MR Egger | 7 | -1.814551992 | 1.025419507 | 0.137023418701973 | -3.824374225 | 0.195270240908617 | 0.16291087741681 | 0.0218320932365585 | 1.21563945761614 | 0.999950574162623 |
| ebi-fl187-GCST90094807 | ebi-met1400-GCST90200114 | Glycosyl-N-tricosanoyl-sphingadienine (d18:2/23:0) levels \|\| id:ebi-met1400-GCST90200114 | Oranges liking \|\| id:ebi-fl187-GCST90094807 | MR Egger | 7 | -1.079266412 | 1.09383871200148 | 0.369109734398431 | -3.223190287 | 1.064657464 | 0.33984474040199 | 0.0398277932689408 | 2.89984551237899 | 0.999950574162623 |
| ebi-fl187-GCST90094807 | ebi-met1400-GCST90200135 | Arachidoylcarnitine (C20) levels \|\| id:ebi-met1400-GCST90200135 | Oranges liking \|\| id:ebi-fl187-GCST90094807 | MR Egger | 7 | -0.614558581 | 1.22826984337188 | 0.638072680403405 | -3.021967474 | 1.79285031234159 | 0.540879597332524 | 0.0487052976412084 | 6.00654862979571 | 0.999950574162623 |
| ebi-fl187-GCST90094807 | ebi-met1400-GCST90200146 | Docosahexaenoylcarnitine (C22:6) levels \|\| id:ebi-met1400-GCST90200146 | Oranges liking \|\| id:ebi-fl187-GCST90094807 | MR Egger | 7 | -0.152727392 | 1.44203366884794 | 0.919770773744257 | -2.979113382 | 2.67365859941196 | 0.85836368713092 | 0.0508378876514876 | 14.4928960155847 | 0.999950574162623 |
| ebi-fl187-GCST90094807 | ebi-met1400-GCST90200162 | 3-hydroxyphenylacetoylglutamine levels \|\| id:ebi-met1400-GCST90200162 | Oranges liking \|\| id:ebi-fl187-GCST90094807 | MR Egger | 7 | 1.4705993777926 | 1.662980227 | 0.417016050759427 | -1.788841866 | 4.73004062207365 | 4.35184275741669 | 0.167153643847467 | 113.300164742816 | 0.999950574162623 |
| ebi-fl187-GCST90094807 | ebi-met1400-GCST90200172 | 3-carboxy-4-methyl-5-pentyl-2-furanpropionate (3-CMPFP) levels \|\| id:ebi-met1400-GCST90200172 | Oranges liking \|\| id:ebi-fl187-GCST90094807 | MR Egger | 7 | -0.407731364 | 1.04024788436401 | 0.711239923268099 | -2.446617217 | 1.63115448983079 | 0.665157540402339 | 0.0865859932830047 | 5.10977049264769 | 0.999950574162623 |
| ebi-fl187-GCST90094807 | ebi-met1400-GCST90200222 | Dihydrocaffeate sulfate (2) levels \|\| id:ebi-met1400-GCST90200222 | Oranges liking \|\| id:ebi-fl187-GCST90094807 | MR Egger | 7 | 0.0512233128598543 | 1.1858220788397 | 0.967216904927203 | -2.272987962 | 2.37543458738567 | 1.0525579167589 | 0.103003947971574 | 10.7556864561888 | 0.999950574162623 |
| ebi-fl187-GCST90094807 | ebi-met1400-GCST90200223 | 4-methylhexanoylglutamine levels \|\| id:ebi-met1400-GCST90200223 | Oranges liking \|\| id:ebi-fl187-GCST90094807 | MR Egger | 7 | -0.556843691 | 1.25731656298437 | 0.676354813927604 | -3.021184155 | 1.90749677209738 | 0.573014824226286 | 0.0487434643665617 | 6.73620541851204 | 0.999950574162623 |
| ebi-fl187-GCST90094807 | ebi-met1400-GCST90200224 | 2-ketocaprylate levels \|\| id:ebi-met1400-GCST90200224 | Oranges liking \|\| id:ebi-fl187-GCST90094807 | MR Egger | 7 | -0.868137037 | 1.06060240169712 | 0.45028840996946 | -2.946917744 | 1.21064367026428 | 0.419732767921448 | 0.0525012791778803 | 3.35564388574413 | 0.999950574162623 |
| ebi-fl187-GCST90094807 | ebi-met1400-GCST90200243 | Hydroxypalmitoyl sphingomyelin (d18:1/16:0(OH)) levels \|\| id:ebi-met1400-GCST90200243 | Oranges liking \|\| id:ebi-fl187-GCST90094807 | MR Egger | 7 | -0.174449889 | 1.06009224642912 | 0.875735348996249 | -2.252230692 | 1.9033309135139 | 0.839918941943589 | 0.105164373341177 | 6.70820170959376 | 0.999950574162623 |
| ebi-fl187-GCST90094807 | ebi-met1400-GCST90200273 | 2-methoxyhydroquinone sulfate (2) levels \|\| id:ebi-met1400-GCST90200273 | Oranges liking \|\| id:ebi-fl187-GCST90094807 | MR Egger | 7 | -2.3979068 | 1.24808860904756 | 0.11274590975032 | -4.844160474 | 0.0483468734765 | 0.0909080429643724 | 0.00787422529781713 | 1.04953464792316 | 0.999950574162623 |
| ebi-fl187-GCST90094807 | ebi-met1400-GCST90200276 | 2,4-di-tert-butylphenol levels \|\| id:ebi-met1400-GCST90200276 | Oranges liking \|\| id:ebi-fl187-GCST90094807 | MR Egger | 7 | -1.066253109 | 1.07229101711382 | 0.365698404444221 | -3.167943503 | 1.0354372844221 | 0.344296143695185 | 0.0420900670719657 | 2.81633750691572 | 0.999950574162623 |
| ebi-fl187-GCST90094807 | ebi-met1400-GCST90200289 | N-acetylmethionine levels \|\| id:ebi-met1400-GCST90200289 | Oranges liking \|\| id:ebi-fl187-GCST90094807 | MR Egger | 7 | 1.48138492261968 | 1.04178052175659 | 0.214300108978122 | -0.5605049 | 3.5232747452626 | 4.39903378556311 | 0.570920733174409 | 33.8952452101858 | 0.999950574162623 |
| ebi-fl187-GCST90094807 | ebi-met1400-GCST90200308 | 3-hydroxyisobutyrate levels \|\| id:ebi-met1400-GCST90200308 | Oranges liking \|\| id:ebi-fl187-GCST90094807 | MR Egger | 7 | 0.0926367583896139 | 1.22387850229506 | 0.942600029729266 | -2.306165106 | 2.49143862288793 | 1.09706316389326 | 0.0996426387491934 | 12.0786402355411 | 0.999950574162623 |
| ebi-fl187-GCST90094807 | ebi-met1400-GCST90200383 | Cystathionine levels \|\| id:ebi-met1400-GCST90200383 | Oranges liking \|\| id:ebi-fl187-GCST90094807 | MR Egger | 7 | -0.102739954 | 1.32076047865123 | 0.94101331217019 | -2.691430492 | 2.48595058457726 | 0.902361598877554 | 0.0677839056684912 | 12.0125337585461 | 0.999950574162623 |
| ebi-fl187-GCST90094807 | ebi-met1400-GCST90200384 | Guanidinoacetate levels \|\| id:ebi-met1400-GCST90200384 | Oranges liking \|\| id:ebi-fl187-GCST90094807 | MR Egger | 7 | -0.125691714 | 1.24336096138583 | 0.923407382936007 | -2.562679198 | 2.31129577049836 | 0.881886678357447 | 0.0770979029300122 | 10.0874872585099 | 0.999950574162623 |
| ebi-fl187-GCST90094807 | ebi-met1400-GCST90200403 | Citrulline levels \|\| id:ebi-met1400-GCST90200403 | Oranges liking \|\| id:ebi-fl187-GCST90094807 | MR Egger | 7 | -0.376564015 | 1.04815873627509 | 0.734072502642307 | -2.430955138 | 1.67782710802127 | 0.686215188161979 | 0.0879527853873162 | 5.3539098550492 | 0.999950574162623 |
| ebi-fl187-GCST90094807 | ebi-met1400-GCST90200434 | 12,13-DiHOME levels \|\| id:ebi-met1400-GCST90200434 | Oranges liking \|\| id:ebi-fl187-GCST90094807 | MR Egger | 7 | 0.58423317080109 | 1.05670276467041 | 0.604151134994889 | -1.486904248 | 2.6553705895551 | 1.79361506175706 | 0.226071434464252 | 14.230258667512 | 0.999950574162623 |
| ebi-fl187-GCST90094807 | ebi-met1400-GCST90200437 | Fructose levels \|\| id:ebi-met1400-GCST90200437 | Oranges liking \|\| id:ebi-fl187-GCST90094807 | MR Egger | 7 | 1.74406942353582 | 1.21951329345344 | 0.212074196266593 | -0.646176632 | 4.13431547870457 | 5.72057556473996 | 0.524045570608209 | 62.4468302516501 | 0.999950574162623 |
| ebi-fl187-GCST90094807 | ebi-met1400-GCST90200448 | N-stearoyl-sphinganine (d18:0/18:0) levels \|\| id:ebi-met1400-GCST90200448 | Oranges liking \|\| id:ebi-fl187-GCST90094807 | MR Egger | 7 | 0.0738509157313892 | 1.16882427409439 | 0.952068176633456 | -2.217044661 | 2.36474649295639 | 1.07664628250721 | 0.108930560271368 | 10.6413408206924 | 0.999950574162623 |
| ebi-fl187-GCST90094807 | ebi-met1400-GCST90200468 | X-11847 levels \|\| id:ebi-met1400-GCST90200468 | Oranges liking \|\| id:ebi-fl187-GCST90094807 | MR Egger | 7 | -0.078471054 | 1.11032893445927 | 0.946397117309679 | -2.254715766 | 2.09777365723355 | 0.924528820672009 | 0.104903356615903 | 8.14800944246999 | 0.999950574162623 |
| ebi-fl187-GCST90094807 | ebi-met1400-GCST90200476 | X-11843 levels \|\| id:ebi-met1400-GCST90200476 | Oranges liking \|\| id:ebi-fl187-GCST90094807 | MR Egger | 7 | 2.15150490792521 | 1.10128964233453 | 0.10816724282431 | -0.007022791 | 4.31003260690089 | 8.59778754476487 | 0.993001811120946 | 74.442916253564 | 0.999950574162623 |
| ebi-fl187-GCST90094807 | ebi-met1400-GCST90200511 | X-12906 levels \|\| id:ebi-met1400-GCST90200511 | Oranges liking \|\| id:ebi-fl187-GCST90094807 | MR Egger | 7 | -0.62922439 | 1.04819008414542 | 0.574477841989544 | -2.683676955 | 1.425228175 | 0.533005044938836 | 0.068311513467736 | 4.15880667121261 | 0.999950574162623 |
| ebi-fl187-GCST90094807 | ebi-met1400-GCST90200515 | X-12849 levels \|\| id:ebi-met1400-GCST90200515 | Oranges liking \|\| id:ebi-fl187-GCST90094807 | MR Egger | 7 | -0.325437769 | 1.22020941483026 | 0.800345183474397 | -2.717048222 | 2.06617268437464 | 0.722211123010032 | 0.0660694893588593 | 7.89455028729493 | 0.999950574162623 |
| ebi-fl187-GCST90094807 | ebi-met1400-GCST90200551 | X-18886 levels \|\| id:ebi-met1400-GCST90200551 | Oranges liking \|\| id:ebi-fl187-GCST90094807 | MR Egger | 7 | 1.31170457230189 | 1.03334048831414 | 0.260179471293749 | -0.713642785 | 3.33705192939759 | 3.71249654059413 | 0.489856501527142 | 28.1360572350385 | 0.999950574162623 |
| ebi-fl187-GCST90094807 | ebi-met1400-GCST90200564 | X-21319 levels \|\| id:ebi-met1400-GCST90200564 | Oranges liking \|\| id:ebi-fl187-GCST90094807 | MR Egger | 7 | 1.27201585567284 | 1.05673852322929 | 0.282572505033732 | -0.79919165 | 3.34322336120224 | 3.56803796712972 | 0.449692326091883 | 28.3102339004067 | 0.999950574162623 |
| ebi-fl187-GCST90094807 | ebi-met1400-GCST90200579 | X-21442 levels \|\| id:ebi-met1400-GCST90200579 | Oranges liking \|\| id:ebi-fl187-GCST90094807 | MR Egger | 7 | 1.39612694618493 | 1.1389841497722 | 0.274867399106515 | -0.836281987 | 3.62853587973844 | 4.0395243350203 | 0.433318616210474 | 37.6576409200366 | 0.999950574162623 |
| ebi-fl187-GCST90094807 | ebi-met1400-GCST90200581 | X-21441 levels \|\| id:ebi-met1400-GCST90200581 | Oranges liking \|\| id:ebi-fl187-GCST90094807 | MR Egger | 7 | 0.444444027919505 | 1.05019169561604 | 0.68973338826337 | -1.613931695 | 2.50281975132694 | 1.55962284798483 | 0.199103259776314 | 12.2168940412581 | 0.999950574162623 |
| ebi-fl187-GCST90094807 | ebi-met1400-GCST90200648 | X-24585 levels \|\| id:ebi-met1400-GCST90200648 | Oranges liking \|\| id:ebi-fl187-GCST90094807 | MR Egger | 7 | -1.519084402 | 1.02340728285961 | 0.19784165721593 | -3.524962676 | 0.486793872670293 | 0.218912230879825 | 0.0294529066447756 | 1.62709118684836 | 0.999950574162623 |
| ebi-fl187-GCST90094807 | ebi-met1400-GCST90200720 | Adenosine 5'-diphosphate (ADP) to pantothenate ratio \|\| id:ebi-met1400-GCST90200720 | Oranges liking \|\| id:ebi-fl187-GCST90094807 | MR Egger | 7 | -2.673339869 | 1.3903359834223 | 0.112523806130154 | -5.398398396 | 0.0517186589517094 | 0.069021317794619 | 0.00452382051234313 | 1.05307942636262 | 0.999950574162623 |
| ebi-fl187-GCST90094807 | ebi-met1400-GCST90200724 | Cholate to taurocholate ratio \|\| id:ebi-met1400-GCST90200724 | Oranges liking \|\| id:ebi-fl187-GCST90094807 | MR Egger | 7 | -1.970577619 | 1.11947728502173 | 0.138675823055171 | -4.164753098 | 0.223597859223649 | 0.139376326486804 | 0.0155335496902074 | 1.25056801390363 | 0.999950574162623 |
| ebi-fl187-GCST90094807 | ebi-met1400-GCST90200726 | N-acetylputrescine to (N(1) + N(8))-acetylspermidine ratio \|\| id:ebi-met1400-GCST90200726 | Oranges liking \|\| id:ebi-fl187-GCST90094807 | MR Egger | 7 | 0.517766522829842 | 1.04246666699398 | 0.640481982509944 | -1.525468144 | 2.56100119013804 | 1.67827507155247 | 0.2175192026122 | 12.9487750137444 | 0.999950574162623 |
| ebi-fl187-GCST90094807 | ebi-met1400-GCST90200744 | Arginine to glutamate ratio \|\| id:ebi-met1400-GCST90200744 | Oranges liking \|\| id:ebi-fl187-GCST90094807 | MR Egger | 7 | 0.953477504715176 | 1.00984087622907 | 0.388428201482877 | -1.025810613 | 2.93276562212416 | 2.59471712953096 | 0.358505738286067 | 18.7794957326713 | 0.999950574162623 |
| ebi-fl187-GCST90094807 | ebi-met1400-GCST90200759 | Histidine to trans-urocanate ratio \|\| id:ebi-met1400-GCST90200759 | Oranges liking \|\| id:ebi-fl187-GCST90094807 | MR Egger | 7 | -0.221354018 | 1.05617436471656 | 0.842266575756453 | -2.291455772 | 1.84874773732466 | 0.801432908772465 | 0.101119148190886 | 6.35186034252396 | 0.999950574162623 |
| ebi-fl187-GCST90094807 | ebi-met1400-GCST90200795 | Oleoyl-linoleoyl-glycerol (18:1 to 18:2) [2] to linoleoyl-arachidonoyl-glycerol (18:2 to 20:4) [2] ratio \|\| id:ebi-met1400-GCST90200795 | Oranges liking \|\| id:ebi-fl187-GCST90094807 | MR Egger | 7 | 0.151650452438695 | 1.4658109272918 | 0.921620715817258 | -2.721338965 | 3.02463986993063 | 1.1637533781728 | 0.0657866094570212 | 20.5865895261464 | 0.999950574162623 |
| ebi-fl187-GCST90094807 | ebi-met1400-GCST90200811 | Choline to taurocholate ratio \|\| id:ebi-met1400-GCST90200811 | Oranges liking \|\| id:ebi-fl187-GCST90094807 | MR Egger | 7 | -0.248713924 | 1.1633885970858 | 0.839159801847574 | -2.528955574 | 2.03152772629124 | 0.779803024410811 | 0.0797422616772665 | 7.62572748865997 | 0.999950574162623 |
| ebi-fl187-GCST90094807 | ebi-met1400-GCST90200831 | Cholate to phosphate ratio \|\| id:ebi-met1400-GCST90200831 | Oranges liking \|\| id:ebi-fl187-GCST90094807 | MR Egger | 7 | -2.350610942 | 1.09129109038297 | 0.0838230248201618 | -4.489541479 | -0.211680405 | 0.0953109149661627 | 0.0112257898863795 | 0.809223279932323 | 0.999950574162623 |
| ebi-fl187-GCST90094807 | ebi-met1400-GCST90200841 | Adenosine 5'-diphosphate (ADP) to oxalate (ethanedioate) ratio \|\| id:ebi-met1400-GCST90200841 | Oranges liking \|\| id:ebi-fl187-GCST90094807 | MR Egger | 7 | -1.855544015 | 1.40920034695419 | 0.24504780591173 | -4.617576695 | 0.906488664592728 | 0.156367853006045 | 0.00987670133972463 | 2.47561454099834 | 0.999950574162623 |
| ebi-fl187-GCST90094807 | ebi-met1400-GCST90200844 | Aspartate to phosphate ratio \|\| id:ebi-met1400-GCST90200844 | Oranges liking \|\| id:ebi-fl187-GCST90094807 | MR Egger | 7 | -1.373079139 | 0.984638600877413 | 0.221956986044697 | -3.302970797 | 0.55681251878135 | 0.253325732290011 | 0.036773757608375 | 1.74510114858796 | 0.999950574162623 |
| ebi-fl187-GCST90094807 | ebi-met1400-GCST90200870 | Adenosine 5'-monophosphate (AMP) to acetoacetate ratio \|\| id:ebi-met1400-GCST90200870 | Oranges liking \|\| id:ebi-fl187-GCST90094807 | MR Egger | 7 | -0.242192808 | 1.15427705826079 | 0.842088250104454 | -2.504575842 | 2.02019022623706 | 0.784904827067656 | 0.0817102486798854 | 7.53975905724744 | 0.999950574162623 |
| ebi-fl187-GCST90094807 | ebi-met1400-GCST90200887 | Cholesterol to cortisol ratio \|\| id:ebi-met1400-GCST90200887 | Oranges liking \|\| id:ebi-fl187-GCST90094807 | MR Egger | 7 | -0.284527553 | 1.0177989305264 | 0.791016699711945 | -2.279413457 | 1.71035835044703 | 0.752369624848673 | 0.102344218376839 | 5.53094313848447 | 0.999950574162623 |
| ebi-fl187-GCST90094807 | ebi-met1400-GCST90200890 | Cortisol to taurocholate ratio \|\| id:ebi-met1400-GCST90200890 | Oranges liking \|\| id:ebi-fl187-GCST90094807 | MR Egger | 7 | -0.162896461 | 1.09212589939858 | 0.887260419230933 | -2.303463224 | 1.97767030147061 | 0.84967915849286 | 0.0999122254266596 | 7.22588921720179 | 0.999950574162623 |
| ebi-fl187-GCST90094807 | ebi-met1400-GCST90200948 | Adenosine 5'-diphosphate (ADP) to mannitol to sorbitol ratio \|\| id:ebi-met1400-GCST90200948 | Oranges liking \|\| id:ebi-fl187-GCST90094807 | MR Egger | 7 | -0.744733095 | 1.80187693899459 | 0.696508096836278 | -4.276411896 | 2.78694570495605 | 0.474861025584596 | 0.0138924202143159 | 16.2313686269644 | 0.999950574162623 |
| ebi-fl187-GCST90094807 | ebi-met1400-GCST90200972 | Leucine to N-palmitoyl-sphingosine (d18:1 to 16:0) ratio \|\| id:ebi-met1400-GCST90200972 | Oranges liking \|\| id:ebi-fl187-GCST90094807 | MR Egger | 7 | 1.04823686024384 | 1.17111583371843 | 0.411759660124686 | -1.247150174 | 3.34362389433197 | 2.85261711889379 | 0.287322450255654 | 28.3215753581573 | 0.999950574162623 |
| ebi-fl187-GCST90094807 | ebi-met1400-GCST90200973 | Proline to glutamate ratio \|\| id:ebi-met1400-GCST90200973 | Oranges liking \|\| id:ebi-fl187-GCST90094807 | MR Egger | 7 | 0.711402211109787 | 1.02632212959955 | 0.519083972010078 | -1.300189163 | 2.7229935851249 | 2.03684534433452 | 0.272480245003889 | 15.2258339193655 | 0.999950574162623 |
| ebi-fl187-GCST90094807 | ebi-met1400-GCST90200983 | Cholesterol to linoleoyl-arachidonoyl-glycerol (18:2 to 20:4) [1] ratio \|\| id:ebi-met1400-GCST90200983 | Oranges liking \|\| id:ebi-fl187-GCST90094807 | MR Egger | 7 | 0.337399455751373 | 1.07637735318536 | 0.766587109763485 | -1.772300156 | 2.44709906799468 | 1.40129870878299 | 0.169941646542159 | 11.5547784265451 | 0.999950574162623 |
| ebi-fl187-GCST90094807 | ebi-met1400-GCST90201001 | Serine to alpha-ketobutyrate ratio \|\| id:ebi-met1400-GCST90201001 | Oranges liking \|\| id:ebi-fl187-GCST90094807 | MR Egger | 7 | 0.937060108897721 | 1.0662501712193 | 0.419712529431873 | -1.152790227 | 3.02691044448755 | 2.55246640381035 | 0.315754512434259 | 20.6333860198973 | 0.999950574162623 |
| ebi-fl187-GCST90094807 | ebi-met1400-GCST90201005 | Fructose to maltose ratio \|\| id:ebi-met1400-GCST90201005 | Oranges liking \|\| id:ebi-fl187-GCST90094807 | MR Egger | 7 | 0.576380611867973 | 1.2006460460013 | 0.651450988427174 | -1.776885638 | 2.92964686203052 | 1.77958574887473 | 0.169164166137868 | 18.7210182268567 | 0.999950574162623 |
| ebi-fl187-GCST90094807 | ebi-met1400-GCST90201020 | Paraxanthine to 5-acetylamino-6-formylamino-3-methyluracil ratio \|\| id:ebi-met1400-GCST90201020 | Oranges liking \|\| id:ebi-fl187-GCST90094807 | MR Egger | 7 | 3.05697338217024 | 1.10685209340149 | 0.0397467834166874 | 0.887543279103318 | 5.22640348523716 | 21.2631043834207 | 2.4291545599605 | 186.122207072569 | 0.999950574162623 |
| ebi-fl187-GCST90094807 | ebi-pw11-GCST90275197 | Placental weight (fetal genotype, adjusted for sex) \|\| id:ebi-pw11-GCST90275197 | Oranges liking \|\| id:ebi-fl187-GCST90094807 | MR Egger | 7 | 0.482448600965646 | 0.461933686611496 | 0.344136110240815 | -0.422941425 | 1.38783862672418 | 1.6200363721969 | 0.655117005602245 | 4.00618183438574 | 0.999950574162623 |
| ebi-fl187-GCST90094807 | prot-a-1017 | FAS-associated factor 2 \|\| id:prot-a-1017 | Oranges liking \|\| id:ebi-fl187-GCST90094807 | MR Egger | 7 | 0.475116064003147 | 2.0168667884648 | 0.823109978563465 | -3.477942841 | 4.42817496939415 | 1.60820084088174 | 0.0308708519953631 | 83.7783791973479 | 0.999950574162623 |
| ebi-fl187-GCST90094807 | prot-a-1019 | Fas apoptotic inhibitory molecule 3 \|\| id:prot-a-1019 | Oranges liking \|\| id:ebi-fl187-GCST90094807 | MR Egger | 7 | 0.502651888782257 | 1.67585447727309 | 0.776293897767032 | -2.782022887 | 3.78732666423752 | 1.65309929859879 | 0.0619131373549972 | 44.1382460617181 | 0.999950574162623 |
| ebi-fl187-GCST90094807 | prot-a-1052 | MIP18 family protein FAM96A \|\| id:prot-a-1052 | Oranges liking \|\| id:ebi-fl187-GCST90094807 | MR Egger | 7 | -1.797198086 | 2.21257362392519 | 0.453559577209001 | -6.133842389 | 2.53944621661613 | 0.165762690908977 | 0.00216823373651449 | 12.6726511236541 | 0.999950574162623 |
| ebi-fl187-GCST90094807 | prot-a-1066 | F-box/LRR-repeat protein 4 \|\| id:prot-a-1066 | Oranges liking \|\| id:ebi-fl187-GCST90094807 | MR Egger | 7 | -0.024881574 | 1.67585552407569 | 0.988728389276514 | -3.309558401 | 3.25979525320067 | 0.975425420931483 | 0.0365323028141506 | 26.0442041291418 | 0.999950574162623 |
| ebi-fl187-GCST90094807 | prot-a-1076 | Low affinity immunoglobulin gamma Fc region receptor III-B \|\| id:prot-a-1076 | Oranges liking \|\| id:ebi-fl187-GCST90094807 | MR Egger | 7 | -1.867754041 | 1.73456197914672 | 0.330763454201491 | -5.26749552 | 1.53198743835296 | 0.154470206291944 | 0.00515650878826117 | 4.62736429077665 | 0.999950574162623 |
| ebi-fl187-GCST90094807 | prot-a-1081 | Fc receptor-like protein 3 \|\| id:prot-a-1081 | Oranges liking \|\| id:ebi-fl187-GCST90094807 | MR Egger | 7 | 0.831327788832424 | 1.67585552407569 | 0.640884637276391 | -2.453349038 | 4.11600461602078 | 2.29636580575879 | 0.0860050693654558 | 61.3137801383631 | 0.999950574162623 |
| ebi-fl187-GCST90094807 | prot-a-1105 | Fibroblast growth factor receptor 4 \|\| id:prot-a-1105 | Oranges liking \|\| id:ebi-fl187-GCST90094807 | MR Egger | 7 | -0.193584018 | 1.67585552407569 | 0.912533862853499 | -3.478260845 | 3.09109280918881 | 0.824000602404467 | 0.0308610365078819 | 22.0011078561641 | 0.999950574162623 |
| ebi-fl187-GCST90094807 | prot-a-1143 | Follistatin-related protein 1 \|\| id:prot-a-1143 | Oranges liking \|\| id:ebi-fl187-GCST90094807 | MR Egger | 7 | -1.25381951 | 1.67585552407569 | 0.4880358020064 | -4.538496338 | 2.0308573168033 | 0.285412576015482 | 0.0106894678262612 | 7.62061684190364 | 0.999950574162623 |
| ebi-fl187-GCST90094807 | prot-a-1151 | Alpha-(1,3)-fucosyltransferase 10 \|\| id:prot-a-1151 | Oranges liking \|\| id:ebi-fl187-GCST90094807 | MR Egger | 7 | -0.751944517 | 1.67585552407569 | 0.672430361332597 | -4.036621344 | 2.53273231022773 | 0.471448920432511 | 0.0176570287723255 | 12.5878531118068 | 0.999950574162623 |
| ebi-fl187-GCST90094807 | prot-a-1165 | Growth arrest and DNA damage-inducible proteins-interacting protein 1 \|\| id:prot-a-1165 | Oranges liking \|\| id:ebi-fl187-GCST90094807 | MR Egger | 7 | -0.970224117 | 2.21320621482367 | 0.679404465148696 | -5.308108298 | 3.36766006387492 | 0.378998088601792 | 0.00495128421718848 | 29.0105647066602 | 0.999950574162623 |
| ebi-fl187-GCST90094807 | prot-a-1194 | Growth/differentiation factor 11/8 \|\| id:prot-a-1194 | Oranges liking \|\| id:ebi-fl187-GCST90094807 | MR Egger | 7 | 1.81092321407417 | 1.67585552407569 | 0.329219622110495 | -1.473753613 | 5.09560004126252 | 6.11609128814223 | 0.229064051625834 | 163.301803051974 | 0.999950574162623 |
| ebi-fl187-GCST90094807 | prot-a-1216 | D-glucuronyl C5-epimerase \|\| id:prot-a-1216 | Oranges liking \|\| id:ebi-fl187-GCST90094807 | MR Egger | 7 | -1.829094059 | 2.27008062162158 | 0.456985321020382 | -6.278452078 | 2.6202639590241 | 0.16055895879423 | 0.00187630271826989 | 13.7393497318268 | 0.999950574162623 |
| ebi-fl187-GCST90094807 | prot-a-1246 | Glypican-3 \|\| id:prot-a-1246 | Oranges liking \|\| id:ebi-fl187-GCST90094807 | MR Egger | 7 | 0.854364144011264 | 1.67585447727309 | 0.631884471189208 | -2.430310631 | 4.13903891946653 | 2.34987972033794 | 0.0880094898209232 | 62.7424918754928 | 0.999950574162623 |
| ebi-fl187-GCST90094807 | prot-a-1275 | Glutamate receptor 4 \|\| id:prot-a-1275 | Oranges liking \|\| id:ebi-fl187-GCST90094807 | MR Egger | 7 | -2.594339857 | 1.67585552407569 | 0.182284363888922 | -5.879016684 | 0.690336970198934 | 0.0746951692962925 | 0.00279753478321341 | 1.99438746916777 | 0.999950574162623 |
| ebi-fl187-GCST90094807 | prot-a-1289 | Glutathione S-transferase P \|\| id:prot-a-1289 | Oranges liking \|\| id:ebi-fl187-GCST90094807 | MR Egger | 7 | -0.892854421 | 1.67433500133837 | 0.616696351419476 | -4.174551023 | 2.38884218204675 | 0.409485239884604 | 0.0153820963092739 | 10.9008654160005 | 0.999950574162623 |
| ebi-fl187-GCST90094807 | prot-a-130 | Apolipoprotein D \|\| id:prot-a-130 | Oranges liking \|\| id:ebi-fl187-GCST90094807 | MR Egger | 7 | 2.88071961092718 | 1.67585552407569 | 0.146259001253992 | -0.403957216 | 6.16539643811553 | 17.8270971388291 | 0.667672686191691 | 475.989806037995 | 0.999950574162623 |
| ebi-fl187-GCST90094807 | prot-a-1313 | Heparin-binding EGF-like growth factor \|\| id:prot-a-1313 | Oranges liking \|\| id:ebi-fl187-GCST90094807 | MR Egger | 7 | -2.256641328 | 1.67585447727309 | 0.2359384256826 | -5.541316103 | 1.02803344784747 | 0.104701553078744 | 0.00392136252343733 | 2.79556280542302 | 0.999950574162623 |
| ebi-fl187-GCST90094807 | prot-a-1347 | HLA class II histocompatibility antigen, DQ alpha 2 chain \|\| id:prot-a-1347 | Oranges liking \|\| id:ebi-fl187-GCST90094807 | MR Egger | 7 | 2.73953266811893 | 1.67585447727309 | 0.16303794782767 | -0.545142107 | 6.0242074435742 | 15.4797492253711 | 0.579759389423053 | 413.313937560955 | 0.999950574162623 |
| ebi-fl187-GCST90094807 | prot-a-1357 | Non-histone chromosomal protein HMG-14 \|\| id:prot-a-1357 | Oranges liking \|\| id:ebi-fl187-GCST90094807 | MR Egger | 7 | 0.331287709723697 | 1.67585552407569 | 0.851078725471258 | -2.953389117 | 3.61596453691205 | 1.39276044539246 | 0.0521626207876148 | 37.1871970572147 | 0.999950574162623 |
| ebi-fl187-GCST90094807 | prot-a-137 | Apolipoprotein M \|\| id:prot-a-137 | Oranges liking \|\| id:ebi-fl187-GCST90094807 | MR Egger | 7 | -0.264491455 | 1.67585447727309 | 0.880770651892158 | -3.549166231 | 3.02018332029833 | 0.767596207828682 | 0.0287485993665081 | 20.4950484982372 | 0.999950574162623 |
| ebi-fl187-GCST90094807 | prot-a-1381 | 17-beta-hydroxysteroid dehydrogenase 14 \|\| id:prot-a-1381 | Oranges liking \|\| id:ebi-fl187-GCST90094807 | MR Egger | 7 | -1.589846393 | 1.7865572292773 | 0.414284400730302 | -5.091498562 | 1.91180577681476 | 0.203956938629558 | 0.00614879862390756 | 6.76529438667901 | 0.999950574162623 |
| ebi-fl187-GCST90094807 | prot-a-1392 | Serine protease HTRA2, mitochondrial \|\| id:prot-a-1392 | Oranges liking \|\| id:ebi-fl187-GCST90094807 | MR Egger | 7 | -0.955817122 | 1.8025805726917 | 0.618633795470328 | -4.488875044 | 2.57724080057668 | 0.384497834554922 | 0.0112332736386279 | 13.1607748135906 | 0.999950574162623 |
| ebi-fl187-GCST90094807 | prot-a-1416 | Interferon-induced protein with tetratricopeptide repeats 2 \|\| id:prot-a-1416 | Oranges liking \|\| id:ebi-fl187-GCST90094807 | MR Egger | 7 | -2.711775977 | 1.68863476456284 | 0.169204543413588 | -6.021500115 | 0.597948161985741 | 0.0664187437756226 | 0.00242602754340692 | 1.8183839407432 | 0.999950574162623 |
| ebi-fl187-GCST90094807 | prot-a-1428 | Interferon gamma \|\| id:prot-a-1428 | Oranges liking \|\| id:ebi-fl187-GCST90094807 | MR Egger | 7 | -0.490171667 | 1.67585447727309 | 0.781660302181925 | -3.774846442 | 2.79450310849109 | 0.612521235497547 | 0.0229406130765144 | 16.3545003215078 | 0.999950574162623 |
| ebi-fl187-GCST90094807 | prot-a-1483 | Interleukin-17C \|\| id:prot-a-1483 | Oranges liking \|\| id:ebi-fl187-GCST90094807 | MR Egger | 7 | -2.073540116 | 1.67433403361957 | 0.270528147671418 | -5.355234822 | 1.20815458978312 | 0.125739859160716 | 0.00472336021834958 | 3.34730180440928 | 0.999950574162623 |
| ebi-fl187-GCST90094807 | prot-a-1503 | Interleukin-1 receptor-like 2 \|\| id:prot-a-1503 | Oranges liking \|\| id:ebi-fl187-GCST90094807 | MR Egger | 7 | -2.295937176 | 1.67433403361957 | 0.228636849947917 | -5.577631882 | 0.985757530132265 | 0.100667006368244 | 0.00378150998699913 | 2.6798411761398 | 0.999950574162623 |
| ebi-fl187-GCST90094807 | prot-a-1522 | Interleukin-31 receptor subunit alpha \|\| id:prot-a-1522 | Oranges liking \|\| id:ebi-fl187-GCST90094807 | MR Egger | 7 | -1.654547964 | 1.67433403361957 | 0.368440219295597 | -4.93624267 | 1.62714674178854 | 0.191178455700216 | 0.00718153112535082 | 5.08933280187293 | 0.999950574162623 |
| ebi-fl187-GCST90094807 | prot-a-1540 | Interleukin-6 receptor subunit alpha \|\| id:prot-a-1540 | Oranges liking \|\| id:ebi-fl187-GCST90094807 | MR Egger | 7 | -1.738127791 | 1.67433500133837 | 0.346795181312287 | -5.019824394 | 1.54356881169466 | 0.17584931930946 | 0.00660568660863243 | 4.68126705575008 | 0.999950574162623 |
| ebi-fl187-GCST90094807 | prot-a-1541 | Interleukin-6 receptor subunit alpha \|\| id:prot-a-1541 | Oranges liking \|\| id:ebi-fl187-GCST90094807 | MR Egger | 7 | -1.540938018 | 1.67433500133837 | 0.399627366186403 | -4.822634621 | 1.74075858450415 | 0.214180102354578 | 0.00804556218650685 | 5.70166697879126 | 0.999950574162623 |
| ebi-fl187-GCST90094807 | prot-a-1545 | Interleukin-9 \|\| id:prot-a-1545 | Oranges liking \|\| id:ebi-fl187-GCST90094807 | MR Egger | 7 | 0.519336570080858 | 1.67432648734628 | 0.768938911261251 | -2.762343345 | 3.80101648527956 | 1.68091211231261 | 0.0631436275435746 | 44.7466456907217 | 0.999950574162623 |
| ebi-fl187-GCST90094807 | prot-a-1548 | Inositol monophosphatase 3 \|\| id:prot-a-1548 | Oranges liking \|\| id:ebi-fl187-GCST90094807 | MR Egger | 7 | -1.154446716 | 1.67585447727309 | 0.521559191291582 | -4.439121491 | 2.13022805974054 | 0.315231901519695 | 0.0118063059091543 | 8.41678612263235 | 0.999950574162623 |
| ebi-fl187-GCST90094807 | prot-a-1555 | Inhibin beta A chain:Inhibin beta B chain heterodimer \|\| id:prot-a-1555 | Oranges liking \|\| id:ebi-fl187-GCST90094807 | MR Egger | 7 | -0.48813147 | 1.6758461803312 | 0.782537852453585 | -3.772789983 | 2.79652704376396 | 0.613772175303611 | 0.0229878380547607 | 16.3876342907727 | 0.999950574162623 |
| ebi-fl187-GCST90094807 | prot-a-1603 | Junctophilin-3 \|\| id:prot-a-1603 | Oranges liking \|\| id:ebi-fl187-GCST90094807 | MR Egger | 7 | 0.593621688095084 | 1.67585552407569 | 0.737622643021517 | -2.691055139 | 3.87829851528344 | 1.81053374418085 | 0.0678093533122088 | 48.3418920650192 | 0.999950574162623 |
| ebi-fl187-GCST90094807 | prot-a-1627 | UPF0258 protein KIAA1024 \|\| id:prot-a-1627 | Oranges liking \|\| id:ebi-fl187-GCST90094807 | MR Egger | 7 | -2.05685747 | 1.67152702564712 | 0.273228476408931 | -5.33305044 | 1.21933550053137 | 0.127855127847725 | 0.00482931598187797 | 3.3849376968291 | 0.999950574162623 |
| ebi-fl187-GCST90094807 | prot-a-1666 | Kallikrein-7 \|\| id:prot-a-1666 | Oranges liking \|\| id:ebi-fl187-GCST90094807 | MR Egger | 7 | -1.00168827 | 1.67585447727309 | 0.57606776307466 | -4.286363045 | 2.28298650578169 | 0.367258885447484 | 0.013754860242713 | 9.80592216570021 | 0.999950574162623 |
| ebi-fl187-GCST90094807 | prot-a-1678 | Importin subunit alpha-3 \|\| id:prot-a-1678 | Oranges liking \|\| id:ebi-fl187-GCST90094807 | MR Egger | 7 | -1.704816518 | 1.67585552407569 | 0.355690231535976 | -4.989493345 | 1.57986030918962 | 0.181805741196488 | 0.00680911348855212 | 4.85427766589226 | 0.999950574162623 |
| ebi-fl187-GCST90094807 | prot-a-1718 | Protein LDOC1 \|\| id:prot-a-1718 | Oranges liking \|\| id:ebi-fl187-GCST90094807 | MR Egger | 7 | -1.239974431 | 1.67584723135401 | 0.492611084893982 | -4.524635004 | 2.04468614250824 | 0.289391617314451 | 0.010838669781336 | 7.72673306424422 | 0.999950574162623 |
| ebi-fl187-GCST90094807 | prot-a-1723 | Leptin \|\| id:prot-a-1723 | Oranges liking \|\| id:ebi-fl187-GCST90094807 | MR Egger | 7 | -0.244454304 | 1.67585552407569 | 0.889723669745056 | -3.529131131 | 3.04022252324713 | 0.783131773583294 | 0.0293303890610181 | 20.9098956553162 | 0.999950574162623 |
| ebi-fl187-GCST90094807 | prot-a-1730 | Galectin-7 \|\| id:prot-a-1730 | Oranges liking \|\| id:ebi-fl187-GCST90094807 | MR Egger | 7 | -0.078208708 | 1.67585552407569 | 0.964584508186822 | -3.362885535 | 3.20646811920455 | 0.924771399226859 | 0.0346351736026721 | 24.691723813448 | 0.999950574162623 |
| ebi-fl187-GCST90094807 | prot-a-1753 | Lipase member N \|\| id:prot-a-1753 | Oranges liking \|\| id:ebi-fl187-GCST90094807 | MR Egger | 7 | -1.425919123 | 1.67585447727309 | 0.433692910203496 | -4.710593898 | 1.85875565258031 | 0.240287507936266 | 0.00899943124781317 | 6.41574838234928 | 0.999950574162623 |
| ebi-fl187-GCST90094807 | prot-a-1756 | VIP36-like protein \|\| id:prot-a-1756 | Oranges liking \|\| id:ebi-fl187-GCST90094807 | MR Egger | 7 | 0.546603174483829 | 1.67153601806872 | 0.756909010204457 | -2.729607421 | 3.82281376989851 | 1.72737544798149 | 0.0652448984225574 | 45.732708770034 | 0.999950574162623 |
| ebi-fl187-GCST90094807 | prot-a-1760 | Ig Kappa chain V-I region HK102- like \|\| id:prot-a-1760 | Oranges liking \|\| id:ebi-fl187-GCST90094807 | MR Egger | 7 | -3.634585141 | 1.67585447727309 | 0.0822680714561471 | -6.919259916 | -0.349910365 | 0.0263948822771508 | 0.000988561288048093 | 0.704751257051811 | 0.999950574162623 |
| ebi-fl187-GCST90094807 | prot-a-179 | Neutral ceramidase \|\| id:prot-a-179 | Oranges liking \|\| id:ebi-fl187-GCST90094807 | MR Egger | 7 | -2.168425728 | 1.91530650896118 | 0.308924670750809 | -5.922426485 | 1.58557502963336 | 0.114357505103422 | 0.00267869247628839 | 4.88209792249081 | 0.999950574162623 |
| ebi-fl187-GCST90094807 | prot-a-1809 | Lactotransferrin \|\| id:prot-a-1809 | Oranges liking \|\| id:ebi-fl187-GCST90094807 | MR Egger | 7 | 3.49169054421808 | 1.82236827552499 | 0.113506761612318 | -0.080151276 | 7.06353236424705 | 32.8414206771043 | 0.922976711774718 | 1168.56568354434 | 0.999950574162623 |
| ebi-fl187-GCST90094807 | prot-a-1818 | Lysophospholipase-like protein 1 \|\| id:prot-a-1818 | Oranges liking \|\| id:ebi-fl187-GCST90094807 | MR Egger | 7 | 0.631938412659087 | 1.67585552407569 | 0.721585570545481 | -2.652738415 | 3.91661523984744 | 1.88125369316835 | 0.0704580054141247 | 50.2301397443485 | 0.999950574162623 |
| ebi-fl187-GCST90094807 | prot-a-1852 | MAP kinase-activated protein kinase 3 \|\| id:prot-a-1852 | Oranges liking \|\| id:ebi-fl187-GCST90094807 | MR Egger | 7 | 2.59753555942831 | 1.67433500133837 | 0.181508642515594 | -0.684161043 | 5.87923216205153 | 13.430598304962 | 0.504513316954514 | 357.534607645475 | 0.999950574162623 |
| ebi-fl187-GCST90094807 | prot-a-1856 | Megakaryocyte-associated tyrosine-protein kinase \|\| id:prot-a-1856 | Oranges liking \|\| id:ebi-fl187-GCST90094807 | MR Egger | 7 | -0.581715398 | 1.79768699268609 | 0.759344180671612 | -4.105181903 | 2.9417511080862 | 0.55893874156768 | 0.0164870195232543 | 18.9489990222075 | 0.999950574162623 |
| ebi-fl187-GCST90094807 | prot-a-1890 | Beta-1,3-N-acetylglucosaminyltransferase manic fringe \|\| id:prot-a-1890 | Oranges liking \|\| id:ebi-fl187-GCST90094807 | MR Egger | 7 | -2.042711743 | 1.8296818789479 | 0.314996672008682 | -5.628888226 | 1.54346473985156 | 0.129676584101107 | 0.00359256723206399 | 4.68077989300995 | 0.999950574162623 |
| ebi-fl187-GCST90094807 | prot-a-1919 | Matrilysin \|\| id:prot-a-1919 | Oranges liking \|\| id:ebi-fl187-GCST90094807 | MR Egger | 7 | 0.176518408893091 | 1.67585552407569 | 0.920208776963754 | -3.108158418 | 3.46119523608145 | 1.19305638940497 | 0.0446831673204476 | 31.8550280487538 | 0.999950574162623 |
| ebi-fl187-GCST90094807 | prot-a-1923 | Multimerin-2 \|\| id:prot-a-1923 | Oranges liking \|\| id:ebi-fl187-GCST90094807 | MR Egger | 7 | 1.22761918284513 | 2.04717349767251 | 0.574866497889134 | -2.784840873 | 5.24007923828325 | 3.41309390288965 | 0.0617389126024131 | 188.685052892998 | 0.999950574162623 |
| ebi-fl187-GCST90094807 | prot-a-1948 | Mesothelin \|\| id:prot-a-1948 | Oranges liking \|\| id:ebi-fl187-GCST90094807 | MR Egger | 7 | -0.167522333 | 1.67585447727309 | 0.924258563381437 | -3.452197108 | 3.11715244274975 | 0.845757729019633 | 0.031675964347833 | 22.5819845085595 | 0.999950574162623 |
| ebi-fl187-GCST90094807 | prot-a-195 | ATPase family AAA domain-containing protein 1 \|\| id:prot-a-195 | Oranges liking \|\| id:ebi-fl187-GCST90094807 | MR Egger | 7 | -0.074827947 | 1.6758461803312 | 0.966113992345532 | -3.35948646 | 3.20983056687992 | 0.927903121510742 | 0.0347531014700112 | 24.7748881823497 | 0.999950574162623 |
| ebi-fl187-GCST90094807 | prot-a-1966 | Mucin-1 \|\| id:prot-a-1966 | Oranges liking \|\| id:ebi-fl187-GCST90094807 | MR Egger | 7 | 1.07119284532681 | 1.67585552407569 | 0.550828132171259 | -2.213483982 | 4.35586967251517 | 2.91885917173193 | 0.109319118453713 | 77.9345734297242 | 0.999950574162623 |
| ebi-fl187-GCST90094807 | prot-a-1986 | Myocardial zonula adherens protein \|\| id:prot-a-1986 | Oranges liking \|\| id:ebi-fl187-GCST90094807 | MR Egger | 7 | -0.994153415 | 1.67585552407569 | 0.57884728961021 | -4.278830242 | 2.29052341264162 | 0.370036579563016 | 0.0138588641292528 | 9.88010770129967 | 0.999950574162623 |
| ebi-fl187-GCST90094807 | prot-a-2071 | Epididymal secretory protein E1 \|\| id:prot-a-2071 | Oranges liking \|\| id:ebi-fl187-GCST90094807 | MR Egger | 7 | 0.676937232458581 | 1.77727143475772 | 0.718934432463461 | -2.80651478 | 4.16038924458372 | 1.96784145216141 | 0.0604151861290124 | 64.0964669474903 | 0.999950574162623 |
| ebi-fl187-GCST90094807 | prot-a-2098 | Neuritin \|\| id:prot-a-2098 | Oranges liking \|\| id:ebi-fl187-GCST90094807 | MR Egger | 7 | -2.129478307 | 1.99127458204754 | 0.333773178655936 | -6.032376488 | 1.77341987346501 | 0.118899306569758 | 0.00239978413802867 | 5.89096530756404 | 0.999950574162623 |
| ebi-fl187-GCST90094807 | prot-a-2115 | Neurotrophin-4 \|\| id:prot-a-2115 | Oranges liking \|\| id:ebi-fl187-GCST90094807 | MR Egger | 7 | 1.30746024731214 | 1.67585552407569 | 0.470589532871395 | -1.97721658 | 4.5921370745005 | 3.69677289049727 | 0.138454077341783 | 98.7051451737297 | 0.999950574162623 |
| ebi-fl187-GCST90094807 | prot-a-2118 | Netrin-1 \|\| id:prot-a-2118 | Oranges liking \|\| id:ebi-fl187-GCST90094807 | MR Egger | 7 | 0.225183852317513 | 1.67585552407569 | 0.89835167566705 | -3.059492975 | 3.50986067950587 | 1.25255297979211 | 0.046911474487547 | 33.4436080793531 | 0.999950574162623 |
| ebi-fl187-GCST90094807 | prot-a-2120 | Netrin-G1 \|\| id:prot-a-2120 | Oranges liking \|\| id:ebi-fl187-GCST90094807 | MR Egger | 7 | 1.14506080831187 | 1.71984721747138 | 0.535023641152557 | -2.225839738 | 4.51596135455577 | 3.14263244920318 | 0.107976708415922 | 91.4654545010049 | 0.999950574162623 |
| ebi-fl187-GCST90094807 | prot-a-2145 | Olfactomedin-like protein 3 \|\| id:prot-a-2145 | Oranges liking \|\| id:ebi-fl187-GCST90094807 | MR Egger | 7 | 0.237881899609126 | 2.05065697642248 | 0.912165405805459 | -3.781405774 | 4.25716957339718 | 1.26855936662077 | 0.0227906304153436 | 70.6098443664766 | 0.999950574162623 |
| ebi-fl187-GCST90094807 | prot-a-2162 | Prolyl 4-hydroxylase subunit alpha-2 \|\| id:prot-a-2162 | Oranges liking \|\| id:ebi-fl187-GCST90094807 | MR Egger | 7 | -1.686291468 | 1.67585552407569 | 0.360489985626492 | -4.970968295 | 1.59838535894508 | 0.185205090945131 | 0.00693642827010666 | 4.94504150786335 | 0.999950574162623 |
| ebi-fl187-GCST90094807 | prot-a-2168 | Polyadenylate-binding protein-interacting protein 1 \|\| id:prot-a-2168 | Oranges liking \|\| id:ebi-fl187-GCST90094807 | MR Egger | 7 | -2.540885806 | 1.82734505523945 | 0.223099146318689 | -6.122482115 | 1.04071050185634 | 0.0787965703602152 | 0.00219300590984485 | 2.83122789257399 | 0.999950574162623 |
| ebi-fl187-GCST90094807 | prot-a-219 | Beta-1,4-galactosyltransferase 5 \|\| id:prot-a-219 | Oranges liking \|\| id:ebi-fl187-GCST90094807 | MR Egger | 7 | -2.541666945 | 1.67585552407569 | 0.189802683234752 | -5.826343773 | 0.743009881733383 | 0.0787350433164495 | 0.00294883891971465 | 2.10225353599276 | 0.999950574162623 |
| ebi-fl187-GCST90094807 | prot-a-2190 | Pyruvate carboxylase, mitochondrial \|\| id:prot-a-2190 | Oranges liking \|\| id:ebi-fl187-GCST90094807 | MR Egger | 7 | -2.041718237 | 1.67153601806872 | 0.276356189153735 | -5.317928832 | 1.23449235860575 | 0.129805482595623 | 0.00490289794065268 | 3.43663350039865 | 0.999950574162623 |
| ebi-fl187-GCST90094807 | prot-a-2197 | Protocadherin-9 \|\| id:prot-a-2197 | Oranges liking \|\| id:ebi-fl187-GCST90094807 | MR Egger | 7 | -1.585729249 | 1.67585447727309 | 0.387484505191934 | -4.870404025 | 1.69894552603545 | 0.204798389540431 | 0.00767026568364441 | 5.46817829893293 | 0.999950574162623 |
| ebi-fl187-GCST90094807 | prot-a-2198 | Protocadherin alpha-4 \|\| id:prot-a-2198 | Oranges liking \|\| id:ebi-fl187-GCST90094807 | MR Egger | 7 | 0.48310423769831 | 2.26543045003634 | 0.839553977411848 | -3.957139444 | 4.92334791976953 | 1.62109887582091 | 0.0191177234595117 | 137.462055602667 | 0.999950574162623 |
| ebi-fl187-GCST90094807 | prot-a-2254 | Placenta growth factor \|\| id:prot-a-2254 | Oranges liking \|\| id:ebi-fl187-GCST90094807 | MR Egger | 7 | -2.411050234 | 1.91581221590394 | 0.263775757580372 | -6.166042177 | 1.34394170915831 | 0.0897210170103808 | 0.00209952914263724 | 3.83412677152247 | 0.999950574162623 |
| ebi-fl187-GCST90094807 | prot-a-2261 | Membrane-associated progesterone receptor component 2 \|\| id:prot-a-2261 | Oranges liking \|\| id:ebi-fl187-GCST90094807 | MR Egger | 7 | -1.785156973 | 1.67585447727309 | 0.335488734857349 | -5.069831749 | 1.49951780235328 | 0.167770723442215 | 0.00628347725598186 | 4.47952853136026 | 0.999950574162623 |
| ebi-fl187-GCST90094807 | prot-a-2268 | Polymeric immunoglobulin receptor \|\| id:prot-a-2268 | Oranges liking \|\| id:ebi-fl187-GCST90094807 | MR Egger | 7 | -1.335759108 | 1.87377544374066 | 0.507803760947418 | -5.008358977 | 2.33684076196569 | 0.262958485843911 | 0.00668185939582766 | 10.3484915172714 | 0.999950574162623 |
| ebi-fl187-GCST90094807 | prot-a-2283 | Group 10 secretory phospholipase A2 \|\| id:prot-a-2283 | Oranges liking \|\| id:ebi-fl187-GCST90094807 | MR Egger | 7 | 1.23815240799947 | 1.67585552407569 | 0.493217761056714 | -2.046524419 | 4.52282923518782 | 3.44923479537203 | 0.129183110587075 | 92.0957903826436 | 0.999950574162623 |
| ebi-fl187-GCST90094807 | prot-a-2298 | Inactive phospholipase D5 \|\| id:prot-a-2298 | Oranges liking \|\| id:ebi-fl187-GCST90094807 | MR Egger | 7 | 0.168153160080248 | 3.13397439027776 | 0.959287901359287 | -5.974436645 | 6.31074296502465 | 1.1831178031894 | 0.00254293425839495 | 550.453764820184 | 0.999950574162623 |
| ebi-fl187-GCST90094807 | prot-a-2304 | Procollagen-lysine,2-oxoglutarate 5-dioxygenase 2 \|\| id:prot-a-2304 | Oranges liking \|\| id:ebi-fl187-GCST90094807 | MR Egger | 7 | -0.893473233 | 1.6758461803312 | 0.616768131528565 | -4.178131747 | 2.39118528018251 | 0.40923192360747 | 0.0153271157690934 | 10.9264371602888 | 0.999950574162623 |
| ebi-fl187-GCST90094807 | prot-a-2331 | Periostin \|\| id:prot-a-2331 | Oranges liking \|\| id:ebi-fl187-GCST90094807 | MR Egger | 7 | 0.259429649842836 | 1.67585447727309 | 0.883030510696266 | -3.025245126 | 3.5441044252981 | 1.29619059329214 | 0.0485459199630503 | 34.6086768036903 | 0.999950574162623 |
| ebi-fl187-GCST90094807 | prot-a-2333 | Periostin \|\| id:prot-a-2333 | Oranges liking \|\| id:ebi-fl187-GCST90094807 | MR Egger | 7 | 1.06244119351906 | 1.67585552407569 | 0.553966352364133 | -2.222235634 | 4.34711802070742 | 2.89342578698026 | 0.108366569859634 | 77.2554930510981 | 0.999950574162623 |
| ebi-fl187-GCST90094807 | prot-a-2367 | cAMP-dependent protein kinase catalytic subunit alpha \|\| id:prot-a-2367 | Oranges liking \|\| id:ebi-fl187-GCST90094807 | MR Egger | 7 | 0.0634100208976577 | 1.67585447727309 | 0.971281542023856 | -3.221264755 | 3.34808479635292 | 1.06546361202882 | 0.0399045568612888 | 28.44819735 | 0.999950574162623 |
| ebi-fl187-GCST90094807 | prot-a-2385 | Phosphoribosyl pyrophosphate synthase-associated protein 1 \|\| id:prot-a-2385 | Oranges liking \|\| id:ebi-fl187-GCST90094807 | MR Egger | 7 | 2.32269628736319 | 2.19163137301056 | 0.337722714686097 | -1.972901204 | 6.61829377846388 | 10.2031478697837 | 0.139052849798419 | 748.66661563276 | 0.999950574162623 |
| ebi-fl187-GCST90094807 | prot-a-2393 | Trypsin-3 \|\| id:prot-a-2393 | Oranges liking \|\| id:ebi-fl187-GCST90094807 | MR Egger | 7 | -3.510947989 | 1.88586896615772 | 0.121702461673603 | -7.207251163 | 0.185355184226849 | 0.0298685859073783 | 0.000741191773053015 | 1.20364588024458 | 0.999950574162623 |
| ebi-fl187-GCST90094807 | prot-a-2405 | Pregnancy-specific beta-1-glycoprotein 6 \|\| id:prot-a-2405 | Oranges liking \|\| id:ebi-fl187-GCST90094807 | MR Egger | 7 | 3.81589183250409 | 1.67433500133837 | 0.0716232055807477 | 0.534195229880873 | 7.0975884351273 | 45.4172429001321 | 1.70607469169924 | 1209.04785862284 | 0.999950574162623 |
| ebi-fl187-GCST90094807 | prot-a-244 | Beclin-1 \|\| id:prot-a-244 | Oranges liking \|\| id:ebi-fl187-GCST90094807 | MR Egger | 7 | 1.31565147993944 | 2.4198324405596 | 0.610005882980731 | -3.427220104 | 6.05852306343626 | 3.72717837640997 | 0.0324770983706812 | 427.743220500233 | 0.999950574162623 |
| ebi-fl187-GCST90094807 | prot-a-2454 | tRNA pseudouridine synthase A, mitochondrial \|\| id:prot-a-2454 | Oranges liking \|\| id:ebi-fl187-GCST90094807 | MR Egger | 7 | 0.0796303144849839 | 1.67584723135401 | 0.963941155264548 | -3.205030259 | 3.36429088793885 | 1.08288666615332 | 0.0405576743858972 | 28.9129874798837 | 0.999950574162623 |
| ebi-fl187-GCST90094807 | prot-a-2466 | Glutaminyl-peptide cyclotransferase-like protein \|\| id:prot-a-2466 | Oranges liking \|\| id:ebi-fl187-GCST90094807 | MR Egger | 7 | 0.495235054741585 | 2.61278477266998 | 0.857121573039728 | -4.6258231 | 5.61629320917474 | 1.64088389126961 | 0.00979558897042895 | 274.868612061638 | 0.999950574162623 |
| ebi-fl187-GCST90094807 | prot-a-2474 | Ras-related protein Rab-27A \|\| id:prot-a-2474 | Oranges liking \|\| id:ebi-fl187-GCST90094807 | MR Egger | 7 | 0.75261559667781 | 1.85072112706266 | 0.701079334068034 | -2.874797812 | 4.38002900572062 | 2.12254448270563 | 0.0564275473028187 | 79.8403491983616 | 0.999950574162623 |
| ebi-fl187-GCST90094807 | prot-a-2490 | Rap1 GTPase-GDP dissociation stimulator 1 \|\| id:prot-a-2490 | Oranges liking \|\| id:ebi-fl187-GCST90094807 | MR Egger | 7 | -1.313298497 | 2.24468382886643 | 0.583906961841269 | -5.712878801 | 3.08628180771107 | 0.268931521993576 | 0.00330314973211003 | 21.8955147018362 | 0.999950574162623 |
| ebi-fl187-GCST90094807 | prot-a-2545 | Receptor-interacting serine/threonine-protein kinase 2 \|\| id:prot-a-2545 | Oranges liking \|\| id:ebi-fl187-GCST90094807 | MR Egger | 7 | -1.835031305 | 1.67585552407569 | 0.323444724897535 | -5.119708133 | 1.4496455218025 | 0.159608505084069 | 0.00597776735598803 | 4.26160360182847 | 0.999950574162623 |
| ebi-fl187-GCST90094807 | prot-a-2610 | RUN and FYVE domain-containing protein 1 \|\| id:prot-a-2610 | Oranges liking \|\| id:ebi-fl187-GCST90094807 | MR Egger | 7 | -0.478206292 | 1.67585552407569 | 0.786818424694475 | -3.76288312 | 2.8064705347196 | 0.619894304264402 | 0.0232167072440335 | 16.551397423431 | 0.999950574162623 |
| ebi-fl187-GCST90094807 | prot-a-2627 | Serine--tRNA ligase, mitochondrial \|\| id:prot-a-2627 | Oranges liking \|\| id:ebi-fl187-GCST90094807 | MR Egger | 7 | 1.29150119546624 | 1.67585552407569 | 0.475732166350222 | -1.993175632 | 4.57617802265459 | 3.63824417391099 | 0.136262019649037 | 97.1424077163298 | 0.999950574162623 |
| ebi-fl187-GCST90094807 | prot-a-264 | BolA-like protein 2 \|\| id:prot-a-264 | Oranges liking \|\| id:ebi-fl187-GCST90094807 | MR Egger | 7 | 0.0382710331463564 | 1.67585447727309 | 0.982663858605489 | -3.246403742 | 3.32294580860162 | 1.03901280162827 | 0.0389139009104523 | 27.7419527903835 | 0.999950574162623 |
| ebi-fl187-GCST90094807 | prot-a-2646 | Secretoglobin family 1C member 1 \|\| id:prot-a-2646 | Oranges liking \|\| id:ebi-fl187-GCST90094807 | MR Egger | 7 | -1.783779055 | 1.67585552407569 | 0.335827125781951 | -5.068455882 | 1.50089777243611 | 0.16800205714347 | 0.00629212843264271 | 4.48571441390353 | 0.999950574162623 |
| ebi-fl187-GCST90094807 | prot-a-2672 | Semaphorin-3E \|\| id:prot-a-2672 | Oranges liking \|\| id:ebi-fl187-GCST90094807 | MR Egger | 7 | -1.967836744 | 2.01852102807656 | 0.374388497428689 | -5.924137959 | 1.98846447138585 | 0.139758863687635 | 0.00267411188685437 | 7.30430917093587 | 0.999950574162623 |
| ebi-fl187-GCST90094807 | prot-a-2701 | Plasma protease C1 inhibitor \|\| id:prot-a-2701 | Oranges liking \|\| id:ebi-fl187-GCST90094807 | MR Egger | 7 | -0.233464381 | 1.75453425718501 | 0.899332676877402 | -3.672351525 | 3.20542276281754 | 0.791785797646422 | 0.0254166317649056 | 24.6659256487407 | 0.999950574162623 |
| ebi-fl187-GCST90094807 | prot-a-2738 | Signaling threshold-regulating transmembrane adapter 1 \|\| id:prot-a-2738 | Oranges liking \|\| id:ebi-fl187-GCST90094807 | MR Egger | 7 | 1.15731457037699 | 1.94462272791425 | 0.577662907330481 | -2.654145976 | 4.96877511708892 | 3.18137842666527 | 0.0703589011808288 | 143.850579298259 | 0.999950574162623 |
| ebi-fl187-GCST90094807 | prot-a-274 | Serine/threonine-protein kinase BRSK2 \|\| id:prot-a-274 | Oranges liking \|\| id:ebi-fl187-GCST90094807 | MR Egger | 7 | 1.90059772852725 | 1.74841361451635 | 0.326621515998211 | -1.526292956 | 5.3274884129793 | 6.68989198672178 | 0.2173398642544 | 205.9201378 | 0.999950574162623 |
| ebi-fl187-GCST90094807 | prot-a-2749 | Mitochondrial glutamate carrier 2 \|\| id:prot-a-2749 | Oranges liking \|\| id:ebi-fl187-GCST90094807 | MR Egger | 7 | -1.092202795 | 1.67585447727309 | 0.543341433910371 | -4.37687757 | 2.19247198074089 | 0.335476692905303 | 0.0125645292964868 | 8.95732811209634 | 0.999950574162623 |
| ebi-fl187-GCST90094807 | prot-a-2760 | Sodium-coupled monocarboxylate transporter 1 \|\| id:prot-a-2760 | Oranges liking \|\| id:ebi-fl187-GCST90094807 | MR Egger | 7 | -2.986687633 | 1.98037297519858 | 0.191885764671955 | -6.868218664 | 0.894843398332266 | 0.0504542833427088 | 0.00104032858933619 | 2.44695256260394 | 0.999950574162623 |
| ebi-fl187-GCST90094807 | prot-a-2777 | Small integral membrane protein 10 \|\| id:prot-a-2777 | Oranges liking \|\| id:ebi-fl187-GCST90094807 | MR Egger | 7 | -2.03463562 | 1.67585552407569 | 0.278924742958797 | -5.319312447 | 1.2500412070514 | 0.130728108516795 | 0.00489611890788745 | 3.49048678716686 | 0.999950574162623 |
| ebi-fl187-GCST90094807 | prot-a-2799 | Superoxide dismutase [Mn], mitochondrial \|\| id:prot-a-2799 | Oranges liking \|\| id:ebi-fl187-GCST90094807 | MR Egger | 7 | 0.760537142725166 | 1.67585552407569 | 0.668976992548241 | -2.524139684 | 4.04521396991352 | 2.13942508853849 | 0.0801272178328527 | 57.1234074171393 | 0.999950574162623 |
| ebi-fl187-GCST90094807 | prot-a-2805 | Protein SPACA7 \|\| id:prot-a-2805 | Oranges liking \|\| id:ebi-fl187-GCST90094807 | MR Egger | 7 | -1.119326331 | 1.67585447727309 | 0.533776190148206 | -4.404001106 | 2.16534844441174 | 0.326499673246188 | 0.0122283151007246 | 8.71763900028632 | 0.999950574162623 |
| ebi-fl187-GCST90094807 | prot-a-2811 | SPARC-like protein 1 \|\| id:prot-a-2811 | Oranges liking \|\| id:ebi-fl187-GCST90094807 | MR Egger | 7 | -3.189008322 | 1.67433500133837 | 0.115175268780123 | -6.470704925 | 0.0926882802521103 | 0.0412127204138253 | 0.00154813402981579 | 1.09711968808685 | 0.999950574162623 |
| ebi-fl187-GCST90094807 | prot-a-2831 | Sepiapterin reductase \|\| id:prot-a-2831 | Oranges liking \|\| id:ebi-fl187-GCST90094807 | MR Egger | 7 | 0.784417101463908 | 1.67585447727309 | 0.659428251292797 | -2.500257674 | 4.06909187691917 | 2.19112936232241 | 0.0820638501794921 | 58.5038098009104 | 0.999950574162623 |
| ebi-fl187-GCST90094807 | prot-a-2834 | SPRY domain-containing SOCS box protein 1 \|\| id:prot-a-2834 | Oranges liking \|\| id:ebi-fl187-GCST90094807 | MR Egger | 7 | 4.32943948820133 | 1.67153601806872 | 0.0488311539048082 | 1.05322889278665 | 7.60565008361601 | 75.9017308281354 | 2.86689307976725 | 2009.51782379497 | 0.999950574162623 |
| ebi-fl187-GCST90094807 | prot-a-2835 | Spectrin alpha chain, non-erythrocytic 1 \|\| id:prot-a-2835 | Oranges liking \|\| id:ebi-fl187-GCST90094807 | MR Egger | 7 | 0.643438392855029 | 1.67585552407569 | 0.716803343327725 | -2.641238434 | 3.92811522004338 | 1.90301294908561 | 0.0712729480116028 | 50.8111195821164 | 0.999950574162623 |
| ebi-fl187-GCST90094807 | prot-a-2859 | Sia-alpha-2,3-Gal-beta-1,4-GlcNAc-R:alpha 2,8-sialyltransferase \|\| id:prot-a-2859 | Oranges liking \|\| id:ebi-fl187-GCST90094807 | MR Egger | 7 | -2.202089943 | 1.67584723135401 | 0.245894403424289 | -5.486750516 | 1.08257063044859 | 0.1105718278945 | 0.00414127935283272 | 2.95225897175181 | 0.999950574162623 |
| ebi-fl187-GCST90094807 | prot-a-2861 | Alpha-2,8-sialyltransferase 8F \|\| id:prot-a-2861 | Oranges liking \|\| id:ebi-fl187-GCST90094807 | MR Egger | 7 | -3.044180045 | 1.67153483802822 | 0.12821295053361 | -6.320388328 | 0.232028237082346 | 0.0476353548044469 | 0.00179924467348675 | 1.26115533967273 | 0.999950574162623 |
| ebi-fl187-GCST90094807 | prot-a-289 | Uncharacterized protein C17orf89 \|\| id:prot-a-289 | Oranges liking \|\| id:ebi-fl187-GCST90094807 | MR Egger | 7 | -1.965858391 | 1.73170262729642 | 0.307754677504237 | -5.359995541 | 1.42827875812612 | 0.140035629632772 | 0.00470092706955404 | 4.17151282640663 | 0.999950574162623 |
| ebi-fl187-GCST90094807 | prot-a-2921 | Tachykinin-3 \|\| id:prot-a-2921 | Oranges liking \|\| id:ebi-fl187-GCST90094807 | MR Egger | 7 | 2.66389452766557 | 1.67432551552151 | 0.172477653623045 | -0.617783483 | 5.94557253808772 | 14.3520749513504 | 0.539138123140192 | 382.058041470793 | 0.999950574162623 |
| ebi-fl187-GCST90094807 | prot-a-2928 | Tax1-binding protein 1 \|\| id:prot-a-2928 | Oranges liking \|\| id:ebi-fl187-GCST90094807 | MR Egger | 7 | 0.696443109857978 | 1.67585552407569 | 0.694954158028877 | -2.588233717 | 3.98111993704633 | 2.00660273369057 | 0.0751526637729852 | 53.5770035113757 | 0.999950574162623 |
| ebi-fl187-GCST90094807 | prot-a-296 | Uncharacterized protein C1orf185 \|\| id:prot-a-296 | Oranges liking \|\| id:ebi-fl187-GCST90094807 | MR Egger | 7 | -2.022776148 | 1.84759651095002 | 0.323511233139728 | -5.64406531 | 1.5985130130056 | 0.132287704530637 | 0.00353845421398151 | 4.94567280278411 | 0.999950574162623 |
| ebi-fl187-GCST90094807 | prot-a-2964 | Transforming growth factor beta-2 \|\| id:prot-a-2964 | Oranges liking \|\| id:ebi-fl187-GCST90094807 | MR Egger | 7 | -0.667893908 | 1.67585552407569 | 0.706682601134581 | -3.952570736 | 2.61678291879859 | 0.51278741859912 | 0.0192052665980965 | 13.6916056504839 | 0.999950574162623 |
| ebi-fl187-GCST90094807 | prot-a-2977 | Thymocyte nuclear protein 1 \|\| id:prot-a-2977 | Oranges liking \|\| id:ebi-fl187-GCST90094807 | MR Egger | 7 | 1.09825096655791 | 1.67432648734628 | 0.54084422642604 | -2.183428949 | 4.37993088175661 | 2.99891622947632 | 0.112654580832253 | 79.8325153311619 | 0.999950574162623 |
| ebi-fl187-GCST90094807 | prot-a-3015 | Transmembrane protein 70, mitochondrial \|\| id:prot-a-3015 | Oranges liking \|\| id:ebi-fl187-GCST90094807 | MR Egger | 7 | 2.09055275737129 | 1.67585552407569 | 0.267472369049081 | -1.19412407 | 5.37522958455965 | 8.08938539612137 | 0.302969217871381 | 215.989454462539 | 0.999950574162623 |
| ebi-fl187-GCST90094807 | prot-a-3030 | Tumor necrosis factor alpha-induced protein 3 \|\| id:prot-a-3030 | Oranges liking \|\| id:ebi-fl187-GCST90094807 | MR Egger | 7 | 0.427755439702223 | 1.67584723135401 | 0.808699065612046 | -2.856905134 | 3.71241601315609 | 1.53381092583867 | 0.0574462739675923 | 40.9526291913945 | 0.999950574162623 |
| ebi-fl187-GCST90094807 | prot-a-3032 | Tumor necrosis factor receptor superfamily member 10A \|\| id:prot-a-3032 | Oranges liking \|\| id:ebi-fl187-GCST90094807 | MR Egger | 7 | -0.713553305 | 1.67585552407569 | 0.68797081460024 | -3.998230133 | 2.57112352176441 | 0.489900335539319 | 0.018348083843853 | 13.0805124286558 | 0.999950574162623 |
| ebi-fl187-GCST90094807 | prot-a-3081 | Tumor protein p53-inducible protein 11 \|\| id:prot-a-3081 | Oranges liking \|\| id:ebi-fl187-GCST90094807 | MR Egger | 7 | -0.824529248 | 1.67585552407569 | 0.64355424577157 | -4.109206076 | 2.46014757869314 | 0.43844134081269 | 0.0164208062298763 | 11.70653905 | 0.999950574162623 |
| ebi-fl187-GCST90094807 | prot-a-315 | Complement C4 \|\| id:prot-a-315 | Oranges liking \|\| id:ebi-fl187-GCST90094807 | MR Egger | 7 | -2.141738841 | 1.67585552407569 | 0.257364429324883 | -5.426415669 | 1.14293798575542 | 0.117450437671696 | 0.00439883445992157 | 3.13596827408659 | 0.999950574162623 |
| ebi-fl187-GCST90094807 | prot-a-3157 | UDP-glucuronosyltransferase 1-8 \|\| id:prot-a-3157 | Oranges liking \|\| id:ebi-fl187-GCST90094807 | MR Egger | 7 | -2.793358982 | 1.97105548129131 | 0.215615347898451 | -6.656627725 | 1.06990976153578 | 0.0612152472883517 | 0.00128547404104501 | 2.91511643247787 | 0.999950574162623 |
| ebi-fl187-GCST90094807 | prot-a-317 | Glutaredoxin-like protein C5orf63 \|\| id:prot-a-317 | Oranges liking \|\| id:ebi-fl187-GCST90094807 | MR Egger | 7 | -1.317157214 | 1.67433500133837 | 0.467102713879986 | -4.598853817 | 1.96453938821131 | 0.267895790793536 | 0.0100633635927794 | 7.13162692207493 | 0.999950574162623 |
| ebi-fl187-GCST90094807 | prot-a-3192 | Guanine nucleotide exchange factor VAV3 \|\| id:prot-a-3192 | Oranges liking \|\| id:ebi-fl187-GCST90094807 | MR Egger | 7 | -1.499582795 | 1.67585552407569 | 0.41188559077248 | -4.784259622 | 1.78509403221277 | 0.223223270594065 | 0.00836031124626623 | 5.96014036642054 | 0.999950574162623 |
| ebi-fl187-GCST90094807 | prot-a-3233 | Serine/threonine-protein kinase WNK3 \|\| id:prot-a-3233 | Oranges liking \|\| id:ebi-fl187-GCST90094807 | MR Egger | 7 | 2.38973789423648 | 1.67433500133837 | 0.212851641202472 | -0.891958708 | 5.67143449685969 | 10.910633828222 | 0.40985218511961 | 290.450886577076 | 0.999950574162623 |
| ebi-fl187-GCST90094807 | prot-a-3250 | 14-3-3 protein theta \|\| id:prot-a-3250 | Oranges liking \|\| id:ebi-fl187-GCST90094807 | MR Egger | 7 | 2.26476856037324 | 1.74532591650658 | 0.251056259318184 | -1.156070236 | 5.68560735672615 | 9.6288958351497 | 0.314720531361651 | 294.596716023023 | 0.999950574162623 |
| ebi-fl187-GCST90094807 | prot-a-3278 | E3 ubiquitin-protein ligase ZNRF3 \|\| id:prot-a-3278 | Oranges liking \|\| id:ebi-fl187-GCST90094807 | MR Egger | 7 | 0.693650749104786 | 1.69272222039191 | 0.698930119682294 | -2.624084803 | 4.01138630107292 | 2.00100739071353 | 0.0725060840389817 | 55.2233737452635 | 0.999950574162623 |
| ebi-fl187-GCST90094807 | prot-a-355 | Calpain-3 \|\| id:prot-a-355 | Oranges liking \|\| id:ebi-fl187-GCST90094807 | MR Egger | 7 | 1.87044243184125 | 1.67585552407569 | 0.315120525403295 | -1.414234395 | 5.1551192590296 | 6.4911676633317 | 0.243111669642362 | 173.316475081051 | 0.999950574162623 |
| ebi-fl187-GCST90094807 | prot-a-385 | Coiled-coil domain-containing protein 90B, mitochondrial \|\| id:prot-a-385 | Oranges liking \|\| id:ebi-fl187-GCST90094807 | MR Egger | 7 | -2.542286062 | 1.67585447727309 | 0.189712355863476 | -5.826960838 | 0.742388713040558 | 0.0786863122024932 | 0.00294701985505683 | 2.10094808740569 | 0.999950574162623 |
| ebi-fl187-GCST90094807 | prot-a-390 | C-C motif chemokine 14 \|\| id:prot-a-390 | Oranges liking \|\| id:ebi-fl187-GCST90094807 | MR Egger | 7 | -3.198297892 | 1.67585447727309 | 0.114612750678889 | -6.482972667 | 0.0863768835783123 | 0.0408316447379466 | 0.0015292579406656 | 1.09021713582225 | 0.999950574162623 |
| ebi-fl187-GCST90094807 | prot-a-40 | Pituitary adenylate cyclase-activating polypeptide 27 \|\| id:prot-a-40 | Oranges liking \|\| id:ebi-fl187-GCST90094807 | MR Egger | 7 | -0.735608641 | 1.67585552407569 | 0.679020982735842 | -4.020285468 | 2.54906818605696 | 0.479213701026786 | 0.0179478406682105 | 12.7951755031201 | 0.999950574162623 |
| ebi-fl187-GCST90094807 | prot-a-401 | C-C motif chemokine 24 \|\| id:prot-a-401 | Oranges liking \|\| id:ebi-fl187-GCST90094807 | MR Egger | 7 | 3.19414849563891 | 1.67585552407569 | 0.11497756289663 | -0.090528332 | 6.47882532282726 | 24.3893971698462 | 0.913448454124301 | 651.205540523641 | 0.999950574162623 |
| ebi-fl187-GCST90094807 | prot-a-412 | Cerebral cavernous malformations 2 protein \|\| id:prot-a-412 | Oranges liking \|\| id:ebi-fl187-GCST90094807 | MR Egger | 7 | 1.99716955931697 | 1.67585552407569 | 0.286842656552235 | -1.287507268 | 5.28184638650533 | 7.36817138435491 | 0.275957815352775 | 196.732784972312 | 0.999950574162623 |
| ebi-fl187-GCST90094807 | prot-a-44 | Adhesion G protein-coupled receptor F5 \|\| id:prot-a-44 | Oranges liking \|\| id:ebi-fl187-GCST90094807 | MR Egger | 7 | -1.94472536 | 1.82077213631131 | 0.334316794955741 | -5.513438747 | 1.62398802724172 | 0.143026498752395 | 0.00403221773837245 | 5.07328241495857 | 0.999950574162623 |
| ebi-fl187-GCST90094807 | prot-a-48 | Adrenomedullin \|\| id:prot-a-48 | Oranges liking \|\| id:ebi-fl187-GCST90094807 | MR Egger | 7 | -2.734394282 | 1.67585552407569 | 0.16368373372622 | -6.019071109 | 0.550282545429786 | 0.0649333264947656 | 0.00243192754190432 | 1.73374280977723 | 0.999950574162623 |
| ebi-fl187-GCST90094807 | prot-a-502 | Carcinoembryonic antigen-related cell adhesion molecule 1 \|\| id:prot-a-502 | Oranges liking \|\| id:ebi-fl187-GCST90094807 | MR Egger | 7 | 0.128119985055695 | 1.67585447727309 | 0.942025477476822 | -3.15655479 | 3.41279476051096 | 1.13668938025386 | 0.0425721587259014 | 30.3499466752622 | 0.999950574162623 |
| ebi-fl187-GCST90094807 | prot-a-518 | Complement factor B \|\| id:prot-a-518 | Oranges liking \|\| id:ebi-fl187-GCST90094807 | MR Egger | 7 | -0.58780797 | 1.87363355957283 | 0.766395274684666 | -4.260129747 | 3.084513807 | 0.555543719446292 | 0.0141204702042319 | 21.8568376089718 | 0.999950574162623 |
| ebi-fl187-GCST90094807 | prot-a-525 | Properdin \|\| id:prot-a-525 | Oranges liking \|\| id:ebi-fl187-GCST90094807 | MR Egger | 7 | 0.825993818241108 | 2.22725496108632 | 0.725937496711937 | -3.539425905 | 5.1914135419703 | 2.28414966730926 | 0.029029988256134 | 179.722418646401 | 0.999950574162623 |
| ebi-fl187-GCST90094807 | prot-a-533 | Chondroadherin \|\| id:prot-a-533 | Oranges liking \|\| id:ebi-fl187-GCST90094807 | MR Egger | 7 | 0.612836481186252 | 1.6758461803312 | 0.729559470997263 | -2.671822032 | 3.89749499463541 | 1.84565915853491 | 0.069126160402594 | 49.2788505776168 | 0.999950574162623 |
| ebi-fl187-GCST90094807 | prot-a-535 | Coiled-coil-helix-coiled-coil-helix domain-containing protein 2 \|\| id:prot-a-535 | Oranges liking \|\| id:ebi-fl187-GCST90094807 | MR Egger | 7 | -3.933819598 | 1.68108029271731 | 0.0663756735638116 | -7.228736971 | -0.638902224 | 0.0195687847369852 | 0.000725436531790965 | 0.527871590829682 | 0.999950574162623 |
| ebi-fl187-GCST90094807 | prot-a-591 | Caseinolytic peptidase B protein homolog \|\| id:prot-a-591 | Oranges liking \|\| id:ebi-fl187-GCST90094807 | MR Egger | 7 | 0.241384704518443 | 2.53357967030531 | 0.927797605182529 | -4.724431449 | 5.20720085831684 | 1.27301067407741 | 0.00887575879470957 | 182.582268603442 | 0.999950574162623 |
| ebi-fl187-GCST90094807 | prot-a-605 | Ciliary neurotrophic factor receptor subunit alpha \|\| id:prot-a-605 | Oranges liking \|\| id:ebi-fl187-GCST90094807 | MR Egger | 7 | -0.559800245 | 1.67585447727309 | 0.751905295149828 | -3.84447502 | 2.72487453088906 | 0.57132317736017 | 0.0213976319413941 | 15.2544998382496 | 0.999950574162623 |
| ebi-fl187-GCST90094807 | prot-a-606 | Ciliary neurotrophic factor receptor subunit alpha \|\| id:prot-a-606 | Oranges liking \|\| id:ebi-fl187-GCST90094807 | MR Egger | 7 | -1.579668134 | 1.67585447727309 | 0.389162615692366 | -4.86434291 | 1.70500664135607 | 0.206043465660179 | 0.00771689722530964 | 5.50142220399391 | 0.999950574162623 |
| ebi-fl187-GCST90094807 | prot-a-707 | Cancer/testis antigen 55 \|\| id:prot-a-707 | Oranges liking \|\| id:ebi-fl187-GCST90094807 | MR Egger | 7 | 0.190122599710559 | 1.77638527900513 | 0.918928714357139 | -3.291592547 | 3.67183774656062 | 1.20939786039622 | 0.0371945681015818 | 39.324107238894 | 0.999950574162623 |
| ebi-fl187-GCST90094807 | prot-a-719 | Dipeptidyl peptidase 1 \|\| id:prot-a-719 | Oranges liking \|\| id:ebi-fl187-GCST90094807 | MR Egger | 7 | -1.916663905 | 1.67433403361957 | 0.304142595329387 | -5.198358611 | 1.36503080076457 | 0.147096873605858 | 0.00552562668410064 | 3.91584366111399 | 0.999950574162623 |
| ebi-fl187-GCST90094807 | prot-a-752 | Cytochrome b5 \|\| id:prot-a-752 | Oranges liking \|\| id:ebi-fl187-GCST90094807 | MR Egger | 7 | 0.0265583437798253 | 1.67585447727309 | 0.987968861573524 | -3.258116432 | 3.31123311923509 | 1.02691415956754 | 0.0384607733286124 | 27.4189154261174 | 0.999950574162623 |
| ebi-fl187-GCST90094807 | prot-a-755 | Cytoglobin \|\| id:prot-a-755 | Oranges liking \|\| id:ebi-fl187-GCST90094807 | MR Egger | 7 | -1.108904149 | 1.67585552407569 | 0.537438522075313 | -4.393580976 | 2.17577267815959 | 0.329920306536989 | 0.0123564019189052 | 8.80898900665617 | 0.999950574162623 |
| ebi-fl187-GCST90094807 | prot-a-807 | Desmin \|\| id:prot-a-807 | Oranges liking \|\| id:ebi-fl187-GCST90094807 | MR Egger | 7 | 2.24977866934502 | 1.92918829830511 | 0.296137142174231 | -1.531430395 | 6.03098773402304 | 9.48563614194353 | 0.216226157121791 | 416.125848116818 | 0.999950574162623 |
| ebi-fl187-GCST90094807 | prot-a-82 | Ameloblastin \|\| id:prot-a-82 | Oranges liking \|\| id:ebi-fl187-GCST90094807 | MR Egger | 7 | -1.80400872 | 1.67585447727309 | 0.330891871174646 | -5.088683495 | 1.48066605587922 | 0.164637577722243 | 0.00616613228978518 | 4.3958726028552 | 0.999950574162623 |
| ebi-fl187-GCST90094807 | prot-a-84 | Muellerian-inhibiting factor \|\| id:prot-a-84 | Oranges liking \|\| id:ebi-fl187-GCST90094807 | MR Egger | 7 | -2.73073292 | 2.37197431358681 | 0.301690161056572 | -7.379802575 | 1.91833673423844 | 0.0651715066335167 | 0.000623724012544471 | 6.80962283230948 | 0.999950574162623 |
| ebi-fl187-GCST90094807 | prot-a-858 | Inactive dipeptidyl peptidase 10 \|\| id:prot-a-858 | Oranges liking \|\| id:ebi-fl187-GCST90094807 | MR Egger | 7 | -1.273207931 | 1.67584723135401 | 0.481674904486555 | -4.557868505 | 2.01145264223206 | 0.279932176701114 | 0.0104843825560336 | 7.47416675553561 | 0.999950574162623 |
| ebi-fl187-GCST90094807 | prot-a-863 | Dihydropyrimidinase-related protein 3 \|\| id:prot-a-863 | Oranges liking \|\| id:ebi-fl187-GCST90094807 | MR Egger | 7 | 1.67799802241311 | 1.67584723135401 | 0.362653953047782 | -1.606662551 | 4.96265859586698 | 5.35482499349901 | 0.200555843254836 | 142.973399556187 | 0.999950574162623 |
| ebi-fl187-GCST90094807 | prot-a-864 | Dihydropyrimidinase-related protein 5 \|\| id:prot-a-864 | Oranges liking \|\| id:ebi-fl187-GCST90094807 | MR Egger | 7 | -0.726753567 | 1.67585552407569 | 0.682607199518005 | -4.011430394 | 2.55792326061433 | 0.483476017793183 | 0.0181074758832238 | 12.9089808700349 | 0.999950574162623 |
| ebi-fl187-GCST90094807 | prot-a-884 | Endothelin-converting enzyme 1 \|\| id:prot-a-884 | Oranges liking \|\| id:ebi-fl187-GCST90094807 | MR Egger | 7 | 1.43856752549066 | 1.89527689346108 | 0.48206463844564 | -2.276175186 | 5.15331023667438 | 4.21465410615853 | 0.102676173935826 | 173.003225126614 | 0.999950574162623 |
| ebi-fl187-GCST90094807 | prot-a-893 | Early endosome antigen 1 \|\| id:prot-a-893 | Oranges liking \|\| id:ebi-fl187-GCST90094807 | MR Egger | 7 | 1.24734999444169 | 1.67153483802822 | 0.489105998095569 | -2.028858288 | 4.523558277 | 3.48110577392373 | 0.13148555410763 | 92.1629565429342 | 0.999950574162623 |
| ebi-fl187-GCST90094807 | prot-a-897 | Ephrin-A2 \|\| id:prot-a-897 | Oranges liking \|\| id:ebi-fl187-GCST90094807 | MR Egger | 7 | 1.20091870272476 | 1.67585552407569 | 0.505688706555913 | -2.083758124 | 4.48559552991311 | 3.32316852474289 | 0.124461590033636 | 88.7297763177964 | 0.999950574162623 |
| ebi-fl187-GCST90094807 | prot-a-90 | Anaphase-promoting complex subunit 10 \|\| id:prot-a-90 | Oranges liking \|\| id:ebi-fl187-GCST90094807 | MR Egger | 7 | 0.484260541848255 | 1.67585552407569 | 0.784207010948102 | -2.800416285 | 3.76893736903661 | 1.62297444333276 | 0.0607847535558793 | 43.3339923191399 | 0.999950574162623 |
| ebi-fl187-GCST90094807 | prot-a-914 | Histone-lysine N-methyltransferase EHMT2 \|\| id:prot-a-914 | Oranges liking \|\| id:ebi-fl187-GCST90094807 | MR Egger | 7 | 0.935888137467972 | 1.67585447727309 | 0.600617384939392 | -2.348786638 | 4.22056291292323 | 2.54947673835314 | 0.095484949766068 | 68.0717919978794 | 0.999950574162623 |
| ebi-fl187-GCST90094807 | prot-a-932 | RNA polymerase II elongation factor ELL \|\| id:prot-a-932 | Oranges liking \|\| id:ebi-fl187-GCST90094807 | MR Egger | 7 | 0.614896614944679 | 1.67585552407569 | 0.728698874977666 | -2.669780212 | 3.89957344213303 | 1.84946538259312 | 0.0692674477733615 | 49.3813805960055 | 0.999950574162623 |
| ebi-fl187-GCST90094807 | prot-a-991 | Estrogen receptor \|\| id:prot-a-991 | Oranges liking \|\| id:ebi-fl187-GCST90094807 | MR Egger | 7 | -1.830367296 | 1.67585552407569 | 0.32455511410031 | -5.115044123 | 1.45430953112636 | 0.160354659322522 | 0.00600571283700616 | 4.28152618420237 | 0.999950574162623 |
| ebi-fl187-GCST90094807 | prot-a-992 | Persulfide dioxygenase ETHE1, mitochondrial \|\| id:prot-a-992 | Oranges liking \|\| id:ebi-fl187-GCST90094807 | MR Egger | 7 | -1.345093597 | 1.67585552407569 | 0.458623886947299 | -4.629770424 | 1.93958323035456 | 0.260515323309418 | 0.00975699881778509 | 6.95585137873544 | 0.999950574162623 |
| ebi-fl187-GCST90094807 | prot-a-993 | Ethanolamine kinase 1 \|\| id:prot-a-993 | Oranges liking \|\| id:ebi-fl187-GCST90094807 | MR Egger | 7 | -2.182606228 | 1.6758461803312 | 0.24954399638396 | -5.467264742 | 1.10205228535014 | 0.112747302206688 | 0.00422276673418185 | 3.0103377607831 | 0.999950574162623 |
| ebi-fl187-GCST90094807 | prot-b-10 | coagulation factor III, tissue factor \|\| id:prot-b-10 | Oranges liking \|\| id:ebi-fl187-GCST90094807 | MR Egger | 6 | 1.88827803391511 | 1.89030693283544 | 0.374362015585657 | -1.816723554 | 5.59327962227258 | 6.6079801608078 | 0.162557490117305 | 268.615132862347 | 0.999950574162623 |
| ebi-fl187-GCST90094807 | prot-b-28 | prolactin \|\| id:prot-b-28 | Oranges liking \|\| id:ebi-fl187-GCST90094807 | MR Egger | 6 | -0.58804815 | 3.43060933392036 | 0.872221805129785 | -7.312042444 | 6.13594614468085 | 0.555410305157366 | 0.000667452424566967 | 462.176172743902 | 0.999950574162623 |
| ebi-fl187-GCST90094807 | prot-b-54 | matrix metallopeptidase 7 \|\| id:prot-b-54 | Oranges liking \|\| id:ebi-fl187-GCST90094807 | MR Egger | 6 | 2.45859449028844 | 2.23809657634971 | 0.333657282369329 | -1.928074799 | 6.84526377993388 | 11.6883718694541 | 0.145427907049839 | 939.42104875253 | 0.999950574162623 |
| ebi-fl187-GCST90094807 | ukb-rsfMRI191-edge-pheno716 | Net100_Pair10_33 (OccipitalPrecuneus)&(Temporal\|Frontal\|Supp_Motor_Area) (Default_mode\|Central_executive)&(Default_mode\|Salience) \|\| id:ukb-rsfMRI191-edge-pheno716 | Oranges liking \|\| id:ebi-fl187-GCST90094807 | MR Egger | 7 | -0.541587749 | 0.645351695143988 | 0.439617658495422 | -1.806477071 | 0.723301573610556 | 0.581823728643798 | 0.164231695400827 | 2.06122728250949 | 0.999950574162623 |
| ebi-fl187-GCST90094807 | ukb-rsfMRI191-edge-pheno903 | Net100_Pair27_38 (Precentral\|Frontal\|Supp_Motor_Area)&(Subcortical) (Attention\|Salience\|Motor)&(Subcortical-cerebellum) \|\| id:ukb-rsfMRI191-edge-pheno903 | Oranges liking \|\| id:ebi-fl187-GCST90094807 | MR Egger | 7 | 0.705254394724707 | 0.518447966595591 | 0.231843992749512 | -0.31090362 | 1.72141240925207 | 2.02436160633648 | 0.73278449838054 | 5.59242167685848 | 0.999950574162623 |
| ebi-fl187-GCST90094807 | ukb-rsfMRI191-node-pheno23 | Net100_Node2 RolandicOper\|SupraMarginal\|Insula Salience\|Motor \|\| id:ukb-rsfMRI191-node-pheno23 | Oranges liking \|\| id:ebi-fl187-GCST90094807 | MR Egger | 7 | -0.088032344 | 0.479726135489809 | 0.861611007721298 | -1.028295569 | 0.852230881641435 | 0.915731257907076 | 0.357615972991332 | 2.34487215348293 | 0.999950574162623 |
| ebi-fl187-GCST90094807 | ukb-rsfMRI191-node-pheno55 | Net100_Node34 Parietal\|Postcentral\|Precuneus Attention \|\| id:ukb-rsfMRI191-node-pheno55 | Oranges liking \|\| id:ebi-fl187-GCST90094807 | MR Egger | 7 | 0.0283969244588819 | 0.501572321108486 | 0.957044051934293 | -0.954684825 | 1.01147867383151 | 1.02880396084407 | 0.384933446868558 | 2.74966386646538 | 0.999950574162623 |
| ebi-fl187-GCST90094807 | ebi-a-GCST90001414 | IgD- CD24- B cell Absolute Count \|\| id:ebi-a-GCST90001414 | Oranges liking \|\| id:ebi-fl187-GCST90094807 | Weighted median | 7 | 0.134720249560309 | 0.2937377064889 | 0.646491758842953 | -0.441005655 | 0.710446154278554 | 1.14421664446437 | 0.643389068095874 | 2.03489893501613 | 0.999332001504091 |
| ebi-fl187-GCST90094807 | ebi-a-GCST90001422 | CD20- CD38- B cell %B cell \|\| id:ebi-a-GCST90001422 | Oranges liking \|\| id:ebi-fl187-GCST90094807 | Weighted median | 7 | -0.360087628 | 0.278239230747883 | 0.195608242571897 | -0.90543652 | 0.185261264411555 | 0.697615192870109 | 0.404365332967812 | 1.2035328393543 | 0.999332001504091 |
| ebi-fl187-GCST90094807 | ebi-a-GCST90001444 | CD20- CD38- B cell %lymphocyte \|\| id:ebi-a-GCST90001444 | Oranges liking \|\| id:ebi-fl187-GCST90094807 | Weighted median | 7 | -0.217332074 | 0.262011713369014 | 0.406835875978316 | -0.730875032 | 0.296210884181196 | 0.80466271734878 | 0.481487488634765 | 1.34475371421805 | 0.999332001504091 |
| ebi-fl187-GCST90094807 | ebi-a-GCST90001459 | Myeloid Dendritic Cell %Dendritic Cell \|\| id:ebi-a-GCST90001459 | Oranges liking \|\| id:ebi-fl187-GCST90094807 | Weighted median | 7 | 0.0446558775400725 | 0.305626188502657 | 0.883832395171445 | -0.554371452 | 0.64368320700528 | 1.04566796015676 | 0.574433206634942 | 1.90347889061587 | 0.999332001504091 |
| ebi-fl187-GCST90094807 | ebi-a-GCST90001476 | HLA DR++ monocyte %leukocyte \|\| id:ebi-a-GCST90001476 | Oranges liking \|\| id:ebi-fl187-GCST90094807 | Weighted median | 7 | 0.0912025675574525 | 0.313881830490221 | 0.771385265914273 | -0.52400582 | 0.706410955318285 | 1.09549089369856 | 0.592143769200285 | 2.02670425764548 | 0.999332001504091 |
| ebi-fl187-GCST90094807 | ebi-a-GCST90001478 | CD4 regulatory T cell %CD4+ T cell \|\| id:ebi-a-GCST90001478 | Oranges liking \|\| id:ebi-fl187-GCST90094807 | Weighted median | 7 | -0.073716342 | 0.346253048416733 | 0.831407061924302 | -0.752372317 | 0.604939632898575 | 0.928935156363131 | 0.471247277749722 | 1.83114166483459 | 0.999332001504091 |
| ebi-fl187-GCST90094807 | ebi-a-GCST90001485 | CD39+ resting CD4 regulatory T cell %CD4 regulatory T cell \|\| id:ebi-a-GCST90001485 | Oranges liking \|\| id:ebi-fl187-GCST90094807 | Weighted median | 7 | 0.476840734421465 | 0.314988790161781 | 0.130067951541907 | -0.140537294 | 1.09421876313856 | 1.61097685046142 | 0.86889125964101 | 2.98684833565348 | 0.999332001504091 |
| ebi-fl187-GCST90094807 | ebi-a-GCST90001515 | Immature Myeloid-Derived Suppressor Cells Absolute Count \|\| id:ebi-a-GCST90001515 | Oranges liking \|\| id:ebi-fl187-GCST90094807 | Weighted median | 7 | 0.134857468057794 | 0.371765217854863 | 0.716792378379345 | -0.593802359 | 0.863517295053326 | 1.1443736629258 | 0.552223535568342 | 2.37148726203854 | 0.999332001504091 |
| ebi-fl187-GCST90094807 | ebi-a-GCST90001517 | CD33+ HLA DR+ Absolute Count \|\| id:ebi-a-GCST90001517 | Oranges liking \|\| id:ebi-fl187-GCST90094807 | Weighted median | 7 | 0.0253368964242497 | 0.380075948215387 | 0.946850213583394 | -0.719611962 | 0.770285754926408 | 1.02566060371502 | 0.486941170944666 | 2.16038350581903 | 0.999332001504091 |
| ebi-fl187-GCST90094807 | ebi-a-GCST90001518 | CD33+ HLA DR+ CD14- Absolute Count \|\| id:ebi-a-GCST90001518 | Oranges liking \|\| id:ebi-fl187-GCST90094807 | Weighted median | 7 | -0.119872647 | 0.387781443198317 | 0.75722701535245 | -0.879924275 | 0.640178982004786 | 0.887033396186345 | 0.414814322168864 | 1.89682034563304 | 0.999332001504091 |
| ebi-fl187-GCST90094807 | ebi-a-GCST90001520 | CD33+ HLA DR+ CD14dim Absolute Count \|\| id:ebi-a-GCST90001520 | Oranges liking \|\| id:ebi-fl187-GCST90094807 | Weighted median | 7 | 0.156599251981501 | 0.369147559381967 | 0.671406414660405 | -0.566929964 | 0.880128468370156 | 1.16952683441449 | 0.567264289726537 | 2.41120945066885 | 0.999332001504091 |
| ebi-fl187-GCST90094807 | ebi-a-GCST90001531 | CD33dim HLA DR- Absolute Count \|\| id:ebi-a-GCST90001531 | Oranges liking \|\| id:ebi-fl187-GCST90094807 | Weighted median | 7 | -0.552605851 | 0.426952185952801 | 0.19556049324533 | -1.389432135 | 0.284220433945115 | 0.575448322591741 | 0.249216785949401 | 1.32872579474194 | 0.999332001504091 |
| ebi-fl187-GCST90094807 | ebi-a-GCST90001532 | Basophil Absolute Count \|\| id:ebi-a-GCST90001532 | Oranges liking \|\| id:ebi-fl187-GCST90094807 | Weighted median | 7 | -0.598889986 | 0.417075971673131 | 0.151023566717367 | -1.416358891 | 0.218578918245058 | 0.549421162793808 | 0.242595728278238 | 1.24430721129387 | 0.999332001504091 |
| ebi-fl187-GCST90094807 | ebi-a-GCST90001588 | T/B cell \|\| id:ebi-a-GCST90001588 | Oranges liking \|\| id:ebi-fl187-GCST90094807 | Weighted median | 7 | -0.054766925 | 0.319907838015886 | 0.864069696059665 | -0.681786287 | 0.572252437954703 | 0.946705776109676 | 0.505712836765061 | 1.77225445225508 | 0.999332001504091 |
| ebi-fl187-GCST90094807 | ebi-a-GCST90001624 | HLA DR+ CD4+ T cell Absolute Count \|\| id:ebi-a-GCST90001624 | Oranges liking \|\| id:ebi-fl187-GCST90094807 | Weighted median | 7 | 0.173053231307948 | 0.27417976516439 | 0.527931309297673 | -0.364339108 | 0.710445571030154 | 1.18892939166618 | 0.694655591241603 | 2.03489774816493 | 0.999332001504091 |
| ebi-fl187-GCST90094807 | ebi-a-GCST90001631 | CD8+ Natural Killer T %T cell \|\| id:ebi-a-GCST90001631 | Oranges liking \|\| id:ebi-fl187-GCST90094807 | Weighted median | 7 | 0.179117894257198 | 0.29760000727067 | 0.547257592926835 | -0.40417812 | 0.762413908507711 | 1.19616175645722 | 0.667525211092967 | 2.14344405849197 | 0.999332001504091 |
| ebi-fl187-GCST90094807 | ebi-a-GCST90001632 | CD8+ Natural Killer T %lymphocyte \|\| id:ebi-a-GCST90001632 | Oranges liking \|\| id:ebi-fl187-GCST90094807 | Weighted median | 7 | 0.105518884266877 | 0.295031191927558 | 0.720602773124556 | -0.472742252 | 0.683780020444891 | 1.11128709016669 | 0.623290702462931 | 1.98135315012917 | 0.999332001504091 |
| ebi-fl187-GCST90094807 | ebi-a-GCST90001646 | Natural Killer %CD3- lymphocyte \|\| id:ebi-a-GCST90001646 | Oranges liking \|\| id:ebi-fl187-GCST90094807 | Weighted median | 7 | 0.137044163382573 | 0.287634008608328 | 0.63375149289517 | -0.426718493 | 0.700806820254895 | 1.14687879744907 | 0.652647250820515 | 2.01537809955467 | 0.999332001504091 |
| ebi-fl187-GCST90094807 | ebi-a-GCST90001684 | CD127- CD8+ T cell Absolute Count \|\| id:ebi-a-GCST90001684 | Oranges liking \|\| id:ebi-fl187-GCST90094807 | Weighted median | 7 | -0.245065904 | 0.304391828405841 | 0.420761894913196 | -0.841673887 | 0.351542079950137 | 0.782652956796704 | 0.430988493090468 | 1.42125755235429 | 0.999332001504091 |
| ebi-fl187-GCST90094807 | ebi-a-GCST90001687 | CD28- CD8+ T cell Absolute Count \|\| id:ebi-a-GCST90001687 | Oranges liking \|\| id:ebi-fl187-GCST90094807 | Weighted median | 7 | -0.102474336 | 0.289472826776485 | 0.7233364104496 | -0.669841076 | 0.46489240466402 | 0.902601313980174 | 0.511789906869537 | 1.59184290480041 | 0.999332001504091 |
| ebi-fl187-GCST90094807 | ebi-a-GCST90001701 | BAFF-R on CD20- CD38- B cell \|\| id:ebi-a-GCST90001701 | Oranges liking \|\| id:ebi-fl187-GCST90094807 | Weighted median | 7 | -0.219748617 | 0.290117226541912 | 0.448781706856371 | -0.788378381 | 0.348881147221002 | 0.802720563064167 | 0.454581355754519 | 1.41748070880852 | 0.999332001504091 |
| ebi-fl187-GCST90094807 | ebi-a-GCST90001732 | CD19 on IgD- CD27- B cell \|\| id:ebi-a-GCST90001732 | Oranges liking \|\| id:ebi-fl187-GCST90094807 | Weighted median | 7 | 0.234093696883159 | 0.273892081543893 | 0.392721021589538 | -0.302734783 | 0.770922176709191 | 1.26376289730513 | 0.738795011428079 | 2.16175885854709 | 0.999332001504091 |
| ebi-fl187-GCST90094807 | ebi-a-GCST90001798 | CD27 on CD24+ CD27+ B cell \|\| id:ebi-a-GCST90001798 | Oranges liking \|\| id:ebi-fl187-GCST90094807 | Weighted median | 7 | 0.14428563568386 | 0.300208603427828 | 0.630788074199252 | -0.444123227 | 0.732694498402402 | 1.15521403175371 | 0.641386379815755 | 2.08067944870301 | 0.999332001504091 |
| ebi-fl187-GCST90094807 | ebi-a-GCST90001836 | CD66b on CD66b++ myeloid cell \|\| id:ebi-a-GCST90001836 | Oranges liking \|\| id:ebi-fl187-GCST90094807 | Weighted median | 7 | -0.294872162 | 0.453643963618339 | 0.515687116589166 | -1.18401433 | 0.594270007097279 | 0.744626773280844 | 0.306047692819692 | 1.8117079281931 | 0.999332001504091 |
| ebi-fl187-GCST90094807 | ebi-a-GCST90001943 | CD25 on activated & secreting CD4 regulatory T cell \|\| id:ebi-a-GCST90001943 | Oranges liking \|\| id:ebi-fl187-GCST90094807 | Weighted median | 7 | -0.021786249 | 0.303463848530041 | 0.942767512585508 | -0.616575392 | 0.573002894385998 | 0.978449357490294 | 0.539789844593314 | 1.77358495118496 | 0.999332001504091 |
| ebi-fl187-GCST90094807 | ebi-a-GCST90001960 | CD25 on CD4+ T cell \|\| id:ebi-a-GCST90001960 | Oranges liking \|\| id:ebi-fl187-GCST90094807 | Weighted median | 7 | -0.37253335 | 0.330200207129322 | 0.259233576132788 | -1.019725756 | 0.274659055941385 | 0.688986673447057 | 0.360693844731219 | 1.31608188806599 | 0.999332001504091 |
| ebi-fl187-GCST90094807 | ebi-a-GCST90001988 | HLA DR on CD14+ CD16- monocyte \|\| id:ebi-a-GCST90001988 | Oranges liking \|\| id:ebi-fl187-GCST90094807 | Weighted median | 7 | -0.206339 | 0.284731116478554 | 0.468648249798527 | -0.764411988 | 0.351733988176821 | 0.813557233529475 | 0.465607633176854 | 1.42153032954408 | 0.999332001504091 |
| ebi-fl187-GCST90094807 | ebi-a-GCST90001991 | HLA DR on CD14+ monocyte \|\| id:ebi-a-GCST90001991 | Oranges liking \|\| id:ebi-fl187-GCST90094807 | Weighted median | 7 | -0.247922969 | 0.269299574699097 | 0.357248091360111 | -0.775750135 | 0.279904197359839 | 0.780420057460799 | 0.46035832034334 | 1.32300305907989 | 0.999332001504091 |
| ebi-fl187-GCST90094807 | ebi-a-GCST90002019 | CD14 on Monocytic Myeloid-Derived Suppressor Cells \|\| id:ebi-a-GCST90002019 | Oranges liking \|\| id:ebi-fl187-GCST90094807 | Weighted median | 7 | 0.148777265556824 | 0.394323099139075 | 0.705952254617627 | -0.624096009 | 0.92165053986941 | 1.16041449614585 | 0.535745518937533 | 2.51343549365726 | 0.999332001504091 |
| ebi-fl187-GCST90094807 | ebi-a-GCST90002037 | CD80 on plasmacytoid Dendritic Cell \|\| id:ebi-a-GCST90002037 | Oranges liking \|\| id:ebi-fl187-GCST90094807 | Weighted median | 7 | 0.129384528693292 | 0.341617929564414 | 0.70488061173033 | -0.540186613 | 0.798955670639542 | 1.13812768278408 | 0.582639513973287 | 2.22321794394955 | 0.999332001504091 |
| ebi-fl187-GCST90094807 | ebi-a-GCST90002042 | CD45 on CD33+ HLA DR+ CD14- \|\| id:ebi-a-GCST90002042 | Oranges liking \|\| id:ebi-fl187-GCST90094807 | Weighted median | 7 | 0.332315445202675 | 0.413566069932016 | 0.421664690891279 | -0.478274052 | 1.14290494226943 | 1.39419257051275 | 0.619852302024221 | 3.13586465247488 | 0.999332001504091 |
| ebi-fl187-GCST90094807 | ebi-a-GCST90002056 | CD8 on Effector Memory CD8+ T cell \|\| id:ebi-a-GCST90002056 | Oranges liking \|\| id:ebi-fl187-GCST90094807 | Weighted median | 7 | 0.224820485098503 | 0.311138488286295 | 0.469941838135294 | -0.385010952 | 0.834651922139641 | 1.25209792578004 | 0.680443183989062 | 2.30401193315192 | 0.999332001504091 |
| ebi-fl187-GCST90094807 | ebi-a-GCST90002058 | CD8 on CD8+ T cell \|\| id:ebi-a-GCST90002058 | Oranges liking \|\| id:ebi-fl187-GCST90094807 | Weighted median | 7 | 0.0810229612907412 | 0.314562723759712 | 0.796736231996255 | -0.535519977 | 0.697565899859776 | 1.08439579540804 | 0.585364834583171 | 2.00885699247033 | 0.999332001504091 |
| ebi-fl187-GCST90094807 | ebi-a-GCST90002063 | CD4 on CD4 regulatory T cell \|\| id:ebi-a-GCST90002063 | Oranges liking \|\| id:ebi-fl187-GCST90094807 | Weighted median | 7 | 0.157544553360927 | 0.303322455804369 | 0.603484429117455 | -0.43696746 | 0.75205656673749 | 1.17063291245052 | 0.6459924516529 | 2.12135824839128 | 0.999332001504091 |
| ebi-fl187-GCST90094807 | ebi-a-GCST90002066 | CD4 on activated CD4 regulatory T cell \|\| id:ebi-a-GCST90002066 | Oranges liking \|\| id:ebi-fl187-GCST90094807 | Weighted median | 7 | 0.0399418265053487 | 0.31256159936486 | 0.898316228151282 | -0.572678908 | 0.652562561260474 | 1.04075022835348 | 0.564012475386119 | 1.9204558145213 | 0.999332001504091 |
| ebi-fl187-GCST90094807 | ebi-a-GCST90002070 | CD4 on activated & secreting CD4 regulatory T cell \|\| id:ebi-a-GCST90002070 | Oranges liking \|\| id:ebi-fl187-GCST90094807 | Weighted median | 7 | 0.020421098954049 | 0.323316580931379 | 0.949638052994289 | -0.6132794 | 0.654121597579553 | 1.02063103620992 | 0.541571922944949 | 1.92345221002311 | 0.999332001504091 |
| ebi-fl187-GCST90094807 | ebi-a-GCST90002081 | SSC-A on CD4+ T cell \|\| id:ebi-a-GCST90002081 | Oranges liking \|\| id:ebi-fl187-GCST90094807 | Weighted median | 7 | 0.0578637841535257 | 0.319727460216577 | 0.856384481327178 | -0.568802038 | 0.684529606178017 | 1.05957065556606 | 0.566203322716725 | 1.98283890096203 | 0.999332001504091 |
| ebi-fl187-GCST90094807 | ebi-a-GCST90002094 | CD11b on Monocytic Myeloid-Derived Suppressor Cells \|\| id:ebi-a-GCST90002094 | Oranges liking \|\| id:ebi-fl187-GCST90094807 | Weighted median | 7 | -0.039489881 | 0.405263729344867 | 0.922375053759284 | -0.833806791 | 0.754827028467888 | 0.961279681084287 | 0.434392493545291 | 2.12724353895669 | 0.999332001504091 |
| ebi-fl187-GCST90094807 | ebi-a-GCST90002104 | HLA DR on myeloid Dendritic Cell \|\| id:ebi-a-GCST90002104 | Oranges liking \|\| id:ebi-fl187-GCST90094807 | Weighted median | 7 | -0.235309932 | 0.346576356710884 | 0.497166042092627 | -0.914599592 | 0.443979726731652 | 0.790325864057247 | 0.400677028433335 | 1.55889888132621 | 0.999332001504091 |
| ebi-fl187-GCST90094807 | ebi-a-GCST90002105 | HLA DR on plasmacytoid Dendritic Cell \|\| id:ebi-a-GCST90002105 | Oranges liking \|\| id:ebi-fl187-GCST90094807 | Weighted median | 7 | 0.370345404488127 | 0.299474075925871 | 0.216216070842048 | -0.216623784 | 0.957314593302834 | 1.44823475506724 | 0.805232853546787 | 2.60469241480448 | 0.999332001504091 |
| ebi-fl187-GCST90094807 | ebi-a-GCST90002106 | HLA DR on Dendritic Cell \|\| id:ebi-a-GCST90002106 | Oranges liking \|\| id:ebi-fl187-GCST90094807 | Weighted median | 7 | -0.186576281 | 0.330372608031497 | 0.572247217243245 | -0.834106592 | 0.460954031139502 | 0.829795262254773 | 0.434262281422406 | 1.58558596202535 | 0.999332001504091 |
| ebi-fl187-GCST90094807 | ebi-a-GCST90002111 | HLA DR on CD33dim HLA DR+ CD11b- \|\| id:ebi-a-GCST90002111 | Oranges liking \|\| id:ebi-fl187-GCST90094807 | Weighted median | 7 | 0.194675291572826 | 0.42411131454471 | 0.646220284852173 | -0.636582885 | 1.02593346808046 | 1.21491642883235 | 0.529097324949779 | 2.78969833987885 | 0.999332001504091 |
| ebi-fl187-GCST90094807 | ebi-a-GCST90002112 | HLA DR on CD33- HLA DR+ \|\| id:ebi-a-GCST90002112 | Oranges liking \|\| id:ebi-fl187-GCST90094807 | Weighted median | 7 | -0.205977947 | 0.423770491226991 | 0.626924522097809 | -1.03656811 | 0.624612215663897 | 0.813851023826895 | 0.354669783421071 | 1.86752162136611 | 0.999332001504091 |
| ebi-fl187-GCST90094807 | ebi-a-GCST90002115 | HLA DR on HLA DR+ CD8+ T cell \|\| id:ebi-a-GCST90002115 | Oranges liking \|\| id:ebi-fl187-GCST90094807 | Weighted median | 7 | -0.232631297 | 0.338876498229861 | 0.49241166580417 | -0.896829233 | 0.431566639772097 | 0.792445696964814 | 0.407860843222455 | 1.53966773980199 | 0.999332001504091 |
| ebi-fl187-GCST90094807 | ebi-a-GCST90002116 | HLA DR on B cell \|\| id:ebi-a-GCST90002116 | Oranges liking \|\| id:ebi-fl187-GCST90094807 | Weighted median | 7 | 0.382461857348813 | 0.303314994342918 | 0.20733064535073 | -0.212035532 | 0.976959246260931 | 1.46588896039992 | 0.808935954296786 | 2.65636659219872 | 0.999332001504091 |
| ebi-fl187-GCST90094807 | ebi-cfmet338-GCST90026023 | Sphingomyelin (d18:1/24:1, d18:2/24:0) levels \|\| id:ebi-cfmet338-GCST90026023 | Oranges liking \|\| id:ebi-fl187-GCST90094807 | Weighted median | 7 | 0.0781111852471119 | 0.173196771173685 | 0.651991873306437 | -0.261354486 | 0.417576856747534 | 1.0812428702428 | 0.770007914009115 | 1.51827808933019 | 0.999332001504091 |
| ebi-fl187-GCST90094807 | ebi-cfmet338-GCST90026046 | 1-arachidonoyl-gpc (20:4n6) levels \|\| id:ebi-cfmet338-GCST90026046 | Oranges liking \|\| id:ebi-fl187-GCST90094807 | Weighted median | 7 | -0.427276475 | 0.210275465331777 | 0.0421552304090109 | -0.839416387 | -0.015136563 | 0.652283187099226 | 0.431962548682522 | 0.984977418690603 | 0.999332001504091 |
| ebi-fl187-GCST90094807 | ebi-cfmet338-GCST90026047 | 1-linoleoyl-gpc (18:2) levels \|\| id:ebi-cfmet338-GCST90026047 | Oranges liking \|\| id:ebi-fl187-GCST90094807 | Weighted median | 7 | -0.192846856 | 0.264723553166888 | 0.466317397761888 | -0.711705021 | 0.326011307853115 | 0.824608247983473 | 0.490806648217799 | 1.38543103503485 | 0.999332001504091 |
| ebi-fl187-GCST90094807 | ebi-cfmet338-GCST90026050 | 1-myristoyl-2-palmitoyl-gpc (14:0/16:0) levels \|\| id:ebi-cfmet338-GCST90026050 | Oranges liking \|\| id:ebi-fl187-GCST90094807 | Weighted median | 7 | -0.242506926 | 0.129756860019476 | 0.0616320848676323 | -0.496830371 | 0.0118165198803541 | 0.784658313206457 | 0.608456186631393 | 1.01188661075607 | 0.999332001504091 |
| ebi-fl187-GCST90094807 | ebi-cfmet338-GCST90026068 | 2,3-dihydroxyisovalerate levels \|\| id:ebi-cfmet338-GCST90026068 | Oranges liking \|\| id:ebi-fl187-GCST90094807 | Weighted median | 7 | -0.230561791 | 0.433584784586465 | 0.594894233952424 | -1.080387968 | 0.619264387103141 | 0.794087366282884 | 0.339463798841042 | 1.85756109323858 | 0.999332001504091 |
| ebi-fl187-GCST90094807 | ebi-cfmet338-GCST90026082 | 3-hydroxyhexanoate levels \|\| id:ebi-cfmet338-GCST90026082 | Oranges liking \|\| id:ebi-fl187-GCST90094807 | Weighted median | 7 | -0.072805349 | 0.105681986715016 | 0.490880077422729 | -0.279942043 | 0.134331345270311 | 0.92978179565612 | 0.755827545946009 | 1.14377174022085 | 0.999332001504091 |
| ebi-fl187-GCST90094807 | ebi-cfmet338-GCST90026094 | 3-ureidopropionate levels \|\| id:ebi-cfmet338-GCST90026094 | Oranges liking \|\| id:ebi-fl187-GCST90094807 | Weighted median | 7 | -0.204585983 | 0.205849727089966 | 0.320290620984691 | -0.608051448 | 0.198879482200953 | 0.814984664163692 | 0.544410648684163 | 1.22003492111584 | 0.999332001504091 |
| ebi-fl187-GCST90094807 | ebi-cfmet338-GCST90026173 | Glycerol levels \|\| id:ebi-cfmet338-GCST90026173 | Oranges liking \|\| id:ebi-fl187-GCST90094807 | Weighted median | 7 | -0.094813547 | 0.117020240537219 | 0.417806833200946 | -0.324173218 | 0.134546124683436 | 0.909542505797649 | 0.723124973108234 | 1.14401742522706 | 0.999332001504091 |
| ebi-fl187-GCST90094807 | ebi-cfmet338-GCST90026179 | Histidine levels \|\| id:ebi-cfmet338-GCST90026179 | Oranges liking \|\| id:ebi-fl187-GCST90094807 | Weighted median | 7 | 0.116971648157347 | 0.0876848957945729 | 0.182203867117815 | -0.054890748 | 0.28883404391471 | 1.12408755928514 | 0.946588559376318 | 1.33487018031588 | 0.999332001504091 |
| ebi-fl187-GCST90094807 | ebi-cfmet338-GCST90026188 | Isocitrate levels \|\| id:ebi-cfmet338-GCST90026188 | Oranges liking \|\| id:ebi-fl187-GCST90094807 | Weighted median | 7 | 0.0365845364688867 | 0.170567351140666 | 0.830167042893562 | -0.297727472 | 0.370896544704592 | 1.03726198677717 | 0.742503665388857 | 1.44903315547872 | 0.999332001504091 |
| ebi-fl187-GCST90094807 | ebi-cfmet338-GCST90026207 | N-acetyl-isoputreanine levels \|\| id:ebi-cfmet338-GCST90026207 | Oranges liking \|\| id:ebi-fl187-GCST90094807 | Weighted median | 7 | -0.222007057 | 0.100673893434458 | 0.0274393755175591 | -0.419327888 | -0.024686226 | 0.800909712421118 | 0.657488577322992 | 0.975615987219443 | 0.999332001504091 |
| ebi-fl187-GCST90094807 | ebi-cfmet338-GCST90026213 | N-acetylglutamate levels \|\| id:ebi-cfmet338-GCST90026213 | Oranges liking \|\| id:ebi-fl187-GCST90094807 | Weighted median | 7 | -0.072091533 | 0.183511350400636 | 0.694433898595253 | -0.43177378 | 0.287590713611014 | 0.930445725262616 | 0.649356257469711 | 1.33321152710977 | 0.999332001504091 |
| ebi-fl187-GCST90094807 | ebi-cfmet338-GCST90026224 | N-acetyltaurine levels \|\| id:ebi-cfmet338-GCST90026224 | Oranges liking \|\| id:ebi-fl187-GCST90094807 | Weighted median | 7 | -0.156733393 | 0.160487914347292 | 0.328764455253121 | -0.471289705 | 0.157822919350461 | 0.854931959477711 | 0.624196719372302 | 1.17095882219856 | 0.999332001504091 |
| ebi-fl187-GCST90094807 | ebi-cfmet338-GCST90026250 | Phenyllactate (pla) levels \|\| id:ebi-cfmet338-GCST90026250 | Oranges liking \|\| id:ebi-fl187-GCST90094807 | Weighted median | 7 | 0.205595730115067 | 0.213071743906537 | 0.3345883084368 | -0.212024888 | 0.623216348171879 | 1.2282565564755 | 0.808944564350771 | 1.86491662718053 | 0.999332001504091 |
| ebi-fl187-GCST90094807 | ebi-cfmet338-GCST90026251 | Pipecolate levels \|\| id:ebi-cfmet338-GCST90026251 | Oranges liking \|\| id:ebi-fl187-GCST90094807 | Weighted median | 7 | 0.112992654921773 | 0.317832855927446 | 0.72220779903397 | -0.509959743 | 0.735945052539568 | 1.11962370919466 | 0.600519753631924 | 2.08745381414904 | 0.999332001504091 |
| ebi-fl187-GCST90094807 | ebi-cfmet338-GCST90026278 | Trigonelline (n'-methylnicotinate) levels \|\| id:ebi-cfmet338-GCST90026278 | Oranges liking \|\| id:ebi-fl187-GCST90094807 | Weighted median | 7 | 0.724167665514216 | 0.364142676466633 | 0.0467351980381147 | 0.0104480196396158 | 1.43788731138882 | 2.06301326807265 | 1.0105027907806 | 4.21178821382182 | 0.999332001504091 |
| ebi-fl187-GCST90094807 | ebi-cfmet338-GCST90026299 | 1-carboxyethylvaline levels \|\| id:ebi-cfmet338-GCST90026299 | Oranges liking \|\| id:ebi-fl187-GCST90094807 | Weighted median | 7 | -0.062361994 | 0.22129315288506 | 0.778091383875568 | -0.496096574 | 0.371372585695094 | 0.939542716436838 | 0.608902834285861 | 1.44972311886972 | 0.999332001504091 |
| ebi-fl187-GCST90094807 | ebi-gm430-GCST90011306 | C_Erysipelotrichia abundance \|\| id:ebi-gm430-GCST90011306 | Oranges liking \|\| id:ebi-fl187-GCST90094807 | Weighted median | 5 | -0.255197302 | 0.196858477505492 | 0.194855253096543 | -0.641039918 | 0.130645313421826 | 0.774763620089403 | 0.526744367965453 | 1.13956352173737 | 0.999332001504091 |
| ebi-fl187-GCST90094807 | ebi-gm430-GCST90011312 | F_Erysipelotrichaceae abundance \|\| id:ebi-gm430-GCST90011312 | Oranges liking \|\| id:ebi-fl187-GCST90094807 | Weighted median | 5 | -0.255197302 | 0.206069693928466 | 0.215566713212822 | -0.659093903 | 0.148699297610855 | 0.774763620089403 | 0.517319864382699 | 1.1603240245381 | 0.999332001504091 |
| ebi-fl187-GCST90094807 | ebi-gm430-GCST90011352 | O_Erysipelotrichales abundance \|\| id:ebi-gm430-GCST90011352 | Oranges liking \|\| id:ebi-fl187-GCST90094807 | Weighted median | 5 | -0.255197302 | 0.200623465370927 | 0.203365584602884 | -0.648419295 | 0.138024689638079 | 0.774763620089403 | 0.522871629874333 | 1.14800389372493 | 0.999332001504091 |
| ebi-fl187-GCST90094807 | ebi-gm430-GCST90011362 | OTU97_11 (Parabacteroides) abundance \|\| id:ebi-gm430-GCST90011362 | Oranges liking \|\| id:ebi-fl187-GCST90094807 | Weighted median | 5 | 0.185683666903815 | 0.259246363760415 | 0.47384066442999 | -0.322439206 | 0.693806539874229 | 1.20404132200977 | 0.724379968375878 | 2.00131915347884 | 0.999332001504091 |
| ebi-fl187-GCST90094807 | ebi-gm430-GCST90011384 | OTU97_235 (Phascolarctobacterium) abundance \|\| id:ebi-gm430-GCST90011384 | Oranges liking \|\| id:ebi-fl187-GCST90094807 | Weighted median | 5 | 0.163393344011793 | 0.820135406581461 | 0.842084962951049 | -1.444072053 | 1.77085874091146 | 1.17749976108923 | 0.235964937977006 | 5.87589706865813 | 0.999332001504091 |
| ebi-fl187-GCST90094807 | ebi-gm430-GCST90011390 | OTU97_3 (Bacteroides) abundance \|\| id:ebi-gm430-GCST90011390 | Oranges liking \|\| id:ebi-fl187-GCST90094807 | Weighted median | 5 | -0.176402561 | 0.212881871580601 | 0.407307810024297 | -0.593651029 | 0.240845907661994 | 0.838280456690383 | 0.552307109881555 | 1.27232496467362 | 0.999332001504091 |
| ebi-fl187-GCST90094807 | ebi-gm430-GCST90011398 | OTU97_4 (Alistipes) abundance \|\| id:ebi-gm430-GCST90011398 | Oranges liking \|\| id:ebi-fl187-GCST90094807 | Weighted median | 5 | 0.436057107616805 | 0.280106159571362 | 0.119528289130779 | -0.112950965 | 0.985065180376674 | 1.54659711470286 | 0.893194456704626 | 2.67798643089678 | 0.999332001504091 |
| ebi-fl187-GCST90094807 | ebi-gm430-GCST90011421 | OTU99_108 (Cytophagales) abundance \|\| id:ebi-gm430-GCST90011421 | Oranges liking \|\| id:ebi-fl187-GCST90094807 | Weighted median | 5 | -0.253154851 | 0.764422647368264 | 0.740515088602619 | -1.75142324 | 1.24511353797877 | 0.776347654411021 | 0.173526797390223 | 3.47332913171976 | 0.999332001504091 |
| ebi-fl187-GCST90094807 | ebi-gm430-GCST90011428 | OTU99_123 (Phascolarctobacterium) abundance \|\| id:ebi-gm430-GCST90011428 | Oranges liking \|\| id:ebi-fl187-GCST90094807 | Weighted median | 5 | 0.283735525985994 | 0.452352424058208 | 0.530499041360155 | -0.602875225 | 1.17034627714008 | 1.32808164121879 | 0.547235945383689 | 3.22310853411815 | 0.999332001504091 |
| ebi-fl187-GCST90094807 | ebi-gm430-GCST90011450 | OTU99_298 (Phascolarctobacterium) abundance \|\| id:ebi-gm430-GCST90011450 | Oranges liking \|\| id:ebi-fl187-GCST90094807 | Weighted median | 5 | 0.157013599295475 | 0.791493413671573 | 0.842750595147696 | -1.394313492 | 1.70834069009176 | 1.17001152512516 | 0.248003234267544 | 5.51979482432444 | 0.999332001504091 |
| ebi-fl187-GCST90094807 | ebi-gm430-GCST90011451 | OTU99_3 (Bacteroides) abundance \|\| id:ebi-gm430-GCST90011451 | Oranges liking \|\| id:ebi-fl187-GCST90094807 | Weighted median | 5 | -0.165481809 | 0.22902816160003 | 0.469963076020942 | -0.614377006 | 0.283413387869894 | 0.847485279782848 | 0.540977816497412 | 1.32765388440294 | 0.999332001504091 |
| ebi-fl187-GCST90094807 | ebi-gm430-GCST90011472 | OTU99_72 (Parabacteroides) abundance \|\| id:ebi-gm430-GCST90011472 | Oranges liking \|\| id:ebi-fl187-GCST90094807 | Weighted median | 5 | 0.765600924637689 | 0.626321820146548 | 0.221565372224115 | -0.461989843 | 1.99319169212492 | 2.15028614641107 | 0.630028739206107 | 7.33891999478259 | 0.999332001504091 |
| ebi-fl187-GCST90094807 | ebi-gm430-GCST90011507 | TestASV_3 (Bacteroides) abundance \|\| id:ebi-gm430-GCST90011507 | Oranges liking \|\| id:ebi-fl187-GCST90094807 | Weighted median | 5 | 0.259644055805954 | 0.262412414626408 | 0.322442849853002 | -0.254684277 | 0.773972388473713 | 1.29646853407969 | 0.775161195655986 | 2.16836274735907 | 0.999332001504091 |
| ebi-fl187-GCST90094807 | ebi-gm430-GCST90011512 | TestASV_34 (Phascolarctobacterium) abundance \|\| id:ebi-gm430-GCST90011512 | Oranges liking \|\| id:ebi-fl187-GCST90094807 | Weighted median | 5 | -0.023331286 | 0.623091296611518 | 0.970130663865018 | -1.244590227 | 1.19792765572753 | 0.976938784376081 | 0.288058922721451 | 3.31324362183051 | 0.999332001504091 |
| ebi-fl187-GCST90094807 | ebi-gm430-GCST90011519 | TestASV_41 (Barnesiella) abundance \|\| id:ebi-gm430-GCST90011519 | Oranges liking \|\| id:ebi-fl187-GCST90094807 | Weighted median | 5 | -0.92608753 | 0.852499183113997 | 0.277337121175355 | -2.596985929 | 0.744810868747512 | 0.39610041360718 | 0.074497781761051 | 2.10604307874583 | 0.999332001504091 |
| ebi-fl187-GCST90094807 | ebi-gm430-GCST90011524 | TestASV_46 (Sutterella) abundance \|\| id:ebi-gm430-GCST90011524 | Oranges liking \|\| id:ebi-fl187-GCST90094807 | Weighted median | 5 | -0.523967164 | 0.864553469660191 | 0.54447806912859 | -2.218491965 | 1.17055763607916 | 0.592166659403355 | 0.108773018723471 | 3.22378983891584 | 0.999332001504091 |
| ebi-fl187-GCST90094807 | ebi-gm430-GCST90011581 | OTU97_19 (Bacteria) prevalence \|\| id:ebi-gm430-GCST90011581 | Oranges liking \|\| id:ebi-fl187-GCST90094807 | Weighted median | 5 | 1.75756581737011 | 0.940166388676599 | 0.0615644090619665 | -0.085160304 | 3.60029193917624 | 5.79830606635405 | 0.918365054608832 | 36.6089204618511 | 0.999332001504091 |
| ebi-fl187-GCST90094807 | ebi-gm430-GCST90011614 | OTU97_80 (Ruminococcus) prevalence \|\| id:ebi-gm430-GCST90011614 | Oranges liking \|\| id:ebi-fl187-GCST90094807 | Weighted median | 5 | -0.262566895 | 0.482223724856967 | 0.586103045784733 | -1.207725395 | 0.682591606021276 | 0.769074915613254 | 0.298876332323645 | 1.97899988007428 | 0.999332001504091 |
| ebi-fl187-GCST90094807 | ebi-gm430-GCST90011623 | OTU99_11 (Parabacteroides) prevalence \|\| id:ebi-gm430-GCST90011623 | Oranges liking \|\| id:ebi-fl187-GCST90094807 | Weighted median | 5 | -0.168496494 | 0.398506158458563 | 0.672426374592074 | -0.949568565 | 0.612575576583025 | 0.844934225756722 | 0.386907913230989 | 1.84517768037713 | 0.999332001504091 |
| ebi-fl187-GCST90094807 | ebi-gm430-GCST90011642 | OTU99_19 (Bacteria) prevalence \|\| id:ebi-gm430-GCST90011642 | Oranges liking \|\| id:ebi-fl187-GCST90094807 | Weighted median | 5 | 1.75756581737011 | 0.974136024477868 | 0.071195197397077 | -0.151740791 | 3.66687242534673 | 5.79830606635405 | 0.85921096743238 | 39.1293343700995 | 0.999332001504091 |
| ebi-fl187-GCST90094807 | ebi-gm430-GCST90011653 | OTU99_30 (Parasutterella) prevalence \|\| id:ebi-gm430-GCST90011653 | Oranges liking \|\| id:ebi-fl187-GCST90094807 | Weighted median | 5 | 0.356217654205478 | 0.514471854355631 | 0.488689409144847 | -0.65214718 | 1.36458248874252 | 1.42791830686823 | 0.520926052891712 | 3.91408853477574 | 0.999332001504091 |
| ebi-fl187-GCST90094807 | ebi-gm430-GCST90011675 | OTU99_8 (Ruminococcaceae) prevalence \|\| id:ebi-gm430-GCST90011675 | Oranges liking \|\| id:ebi-fl187-GCST90094807 | Weighted median | 5 | 0.0624381698788977 | 0.484011890870396 | 0.897356609153209 | -0.886225136 | 1.01110147598487 | 1.06442864313127 | 0.412208851800967 | 2.74862689476001 | 0.999332001504091 |
| ebi-fl187-GCST90094807 | ebi-gm430-GCST90011679 | OTU99_92 (Ruminococcus) prevalence \|\| id:ebi-gm430-GCST90011679 | Oranges liking \|\| id:ebi-fl187-GCST90094807 | Weighted median | 5 | -0.280692022 | 0.505724195862304 | 0.578874247305594 | -1.271911446 | 0.710527402246603 | 0.755260903677217 | 0.280295340129092 | 2.03506427313639 | 0.999332001504091 |
| ebi-fl187-GCST90094807 | ebi-gm430-GCST90011684 | TestASV_11 (Lachnospiraceae) prevalence \|\| id:ebi-gm430-GCST90011684 | Oranges liking \|\| id:ebi-fl187-GCST90094807 | Weighted median | 5 | 0.727128616897589 | 0.548764903547791 | 0.185162013132455 | -0.348450594 | 1.80270782785126 | 2.06913080245412 | 0.705780783928385 | 6.06605106735078 | 0.999332001504091 |
| ebi-fl187-GCST90094807 | ebi-gm430-GCST90011688 | TestASV_15 (Bacteroides) prevalence \|\| id:ebi-gm430-GCST90011688 | Oranges liking \|\| id:ebi-fl187-GCST90094807 | Weighted median | 5 | 0.668083504301571 | 0.433406319663635 | 0.123202065861027 | -0.181392882 | 1.51755989084229 | 1.95049561983923 | 0.834107588255445 | 4.56108206732549 | 0.999332001504091 |
| ebi-fl187-GCST90094807 | ebi-gm430-GCST90011692 | TestASV_19 (Prevotella) prevalence \|\| id:ebi-gm430-GCST90011692 | Oranges liking \|\| id:ebi-fl187-GCST90094807 | Weighted median | 5 | 0.667196901741936 | 0.989382325882253 | 0.500084370622059 | -1.271992457 | 2.60638626047115 | 1.94876707181091 | 0.280272633915881 | 13.5499961131213 | 0.999332001504091 |
| ebi-fl187-GCST90094807 | ebi-gm430-GCST90011693 | TestASV_2 (Bacteroides) prevalence \|\| id:ebi-gm430-GCST90011693 | Oranges liking \|\| id:ebi-fl187-GCST90094807 | Weighted median | 5 | 0.188064526 | 0.477219670762923 | 0.693519195261166 | -0.747286029 | 1.12341508029133 | 1.20691138951387 | 0.473650283034978 | 3.07533881919105 | 0.999332001504091 |
| ebi-fl187-GCST90094807 | ebi-gm430-GCST90011716 | TestASV_40 (Barnesiella) prevalence \|\| id:ebi-gm430-GCST90011716 | Oranges liking \|\| id:ebi-fl187-GCST90094807 | Weighted median | 5 | -0.153769524 | 0.977967576508711 | 0.875060618846649 | -2.070585974 | 1.76304692612715 | 0.857469624554153 | 0.126111861804345 | 5.83017446981904 | 0.999332001504091 |
| ebi-fl187-GCST90094807 | ebi-gm430-GCST90011718 | TestASV_42 (Alphaproteobacteria) prevalence \|\| id:ebi-gm430-GCST90011718 | Oranges liking \|\| id:ebi-fl187-GCST90094807 | Weighted median | 5 | 0.467700168115926 | 0.744528088715244 | 0.529883819364239 | -0.991574886 | 1.9269752219978 | 1.59631870368152 | 0.37099196074295 | 6.86870248783921 | 0.999332001504091 |
| ebi-fl187-GCST90094807 | ebi-hbsm44-GCST90104929 | Sulcal depth of the right inferior frontal sulcus \|\| id:ebi-hbsm44-GCST90104929 | Oranges liking \|\| id:ebi-fl187-GCST90094807 | Weighted median | 5 | -0.197408489 | 0.382311595472269 | 0.605606517013024 | -0.946739216 | 0.551922238559335 | 0.820855254831987 | 0.388004160805068 | 1.73658794789008 | 0.999332001504091 |
| ebi-fl187-GCST90094807 | ebi-hbsm44-GCST90104935 | Sulcal opening distance of the left calloso-marginal posterior fissure \|\| id:ebi-hbsm44-GCST90104935 | Oranges liking \|\| id:ebi-fl187-GCST90094807 | Weighted median | 5 | -0.032805835 | 0.0979963403971031 | 0.737801673084758 | -0.224878662 | 0.159266992464389 | 0.967726440229594 | 0.798613115084854 | 1.17265099386696 | 0.999332001504091 |
| ebi-fl187-GCST90094807 | ebi-hbsm44-GCST90104936 | Sulcal opening distance of the left anterior inferior temporal sulcus \|\| id:ebi-hbsm44-GCST90104936 | Oranges liking \|\| id:ebi-fl187-GCST90094807 | Weighted median | 5 | 0.0140056566253711 | 0.049312445284621 | 0.776396049640572 | -0.082646736 | 0.110658049383228 | 1.01410419532979 | 0.920676331460402 | 1.11701287829805 | 0.999332001504091 |
| ebi-fl187-GCST90094807 | ebi-hbsm44-GCST90104941 | Sulcal opening distance of the right posterior inferior temporal sulcus \|\| id:ebi-hbsm44-GCST90104941 | Oranges liking \|\| id:ebi-fl187-GCST90094807 | Weighted median | 5 | 0.00387399433179766 | 0.0387596469328264 | 0.920384684017833 | -0.072094914 | 0.0798429023201375 | 1.00388150794727 | 0.930442579912618 | 1.08311689915689 | 0.999332001504091 |
| ebi-fl187-GCST90094807 | ebi-IPs91-GCST90274819 | Leukemia inhibitory factor levels \|\| id:ebi-IPs91-GCST90274819 | Oranges liking \|\| id:ebi-fl187-GCST90094807 | Weighted median | 7 | -0.019242553 | 0.173302605602276 | 0.911589059823816 | -0.35891566 | 0.320430554334814 | 0.980941403452574 | 0.698433254989426 | 1.37772082032674 | 0.999332001504091 |
| ebi-fl187-GCST90094807 | ebi-IPs91-GCST90274837 | STAM binding protein levels \|\| id:ebi-IPs91-GCST90274837 | Oranges liking \|\| id:ebi-fl187-GCST90094807 | Weighted median | 7 | -0.076774318 | 0.137814536098546 | 0.577469815901165 | -0.346890809 | 0.193342172770331 | 0.926098833877002 | 0.706882509438644 | 1.21329787999626 | 0.999332001504091 |
| ebi-fl187-GCST90094807 | ebi-IPs91-GCST90274840 | TNF-beta levels \|\| id:ebi-IPs91-GCST90274840 | Oranges liking \|\| id:ebi-fl187-GCST90094807 | Weighted median | 7 | -0.168455167 | 0.163894137905231 | 0.304030218092055 | -0.489687677 | 0.152777343563189 | 0.844969145298679 | 0.612817761364845 | 1.16506554071907 | 0.999332001504091 |
| ebi-fl187-GCST90094807 | ebi-IPs91-GCST90274842 | Tumor necrosis factor ligand superfamily member 14 levels \|\| id:ebi-IPs91-GCST90274842 | Oranges liking \|\| id:ebi-fl187-GCST90094807 | Weighted median | 7 | -0.02396182 | 0.168806311002422 | 0.887120601271277 | -0.35482219 | 0.306898549099739 | 0.976322984603891 | 0.701298129922588 | 1.35920306870213 | 0.999332001504091 |
| ebi-fl187-GCST90094807 | ebi-met1400-GCST90199628 | 3-methylhistidine levels \|\| id:ebi-met1400-GCST90199628 | Oranges liking \|\| id:ebi-fl187-GCST90094807 | Weighted median | 7 | 0.19605072950718 | 0.212757502862351 | 0.35680246422474 | -0.220953976 | 0.613055435117388 | 1.21658862071008 | 0.801753579265201 | 1.84606331710765 | 0.999332001504091 |
| ebi-fl187-GCST90094807 | ebi-met1400-GCST90199651 | Docosahexaenoate DHA; 22:6n3 levels \|\| id:ebi-met1400-GCST90199651 | Oranges liking \|\| id:ebi-fl187-GCST90094807 | Weighted median | 7 | 0.0829460371424385 | 0.171011074976173 | 0.627652844126213 | -0.25223567 | 0.418127744095738 | 1.08648317722936 | 0.777061586531025 | 1.51911471994411 | 0.999332001504091 |
| ebi-fl187-GCST90094807 | ebi-met1400-GCST90199652 | 1-stearoyl-2-oleoyl-GPS (18:0/18:1) levels \|\| id:ebi-met1400-GCST90199652 | Oranges liking \|\| id:ebi-fl187-GCST90094807 | Weighted median | 7 | -0.027215713 | 0.179201575891285 | 0.879287843643783 | -0.378450802 | 0.324019375833753 | 0.973151297579989 | 0.684921667235171 | 1.38267409732338 | 0.999332001504091 |
| ebi-fl187-GCST90094807 | ebi-met1400-GCST90199653 | 1-myristoyl-2-palmitoyl-gpc (14:0/16:0) levels \|\| id:ebi-met1400-GCST90199653 | Oranges liking \|\| id:ebi-fl187-GCST90094807 | Weighted median | 7 | -0.094549053 | 0.209387508889769 | 0.651592773758539 | -0.50494857 | 0.31585046475746 | 0.909783106244181 | 0.603536614462367 | 1.37142516389769 | 0.999332001504091 |
| ebi-fl187-GCST90094807 | ebi-met1400-GCST90199667 | Erythritol levels in elite athletes \|\| id:ebi-met1400-GCST90199667 | Oranges liking \|\| id:ebi-fl187-GCST90094807 | Weighted median | 7 | 0.143044065993161 | 0.166068598014162 | 0.389042390678366 | -0.182450386 | 0.468538518100919 | 1.15378064303574 | 0.833225982481249 | 1.59765753857048 | 0.999332001504091 |
| ebi-fl187-GCST90094807 | ebi-met1400-GCST90199674 | 3-hydroxymyristate levels \|\| id:ebi-met1400-GCST90199674 | Oranges liking \|\| id:ebi-fl187-GCST90094807 | Weighted median | 7 | 0.106799067303327 | 0.170214856279372 | 0.530372941767612 | -0.226822051 | 0.440420185610897 | 1.11271065206362 | 0.79706260616138 | 1.55335978083154 | 0.999332001504091 |
| ebi-fl187-GCST90094807 | ebi-met1400-GCST90199685 | 2-linoleoylglycerol (18:2) levels \|\| id:ebi-met1400-GCST90199685 | Oranges liking \|\| id:ebi-fl187-GCST90094807 | Weighted median | 7 | -0.169584927 | 0.221962291036153 | 0.444851839682317 | -0.604631018 | 0.265461162953543 | 0.844015071365644 | 0.546275955501926 | 1.30403220847937 | 0.999332001504091 |
| ebi-fl187-GCST90094807 | ebi-met1400-GCST90199703 | Gamma-glutamylvaline levels \|\| id:ebi-met1400-GCST90199703 | Oranges liking \|\| id:ebi-fl187-GCST90094807 | Weighted median | 7 | 0.0382864828919833 | 0.162741289152054 | 0.814007095645562 | -0.280686444 | 0.357259409630009 | 1.03902885423576 | 0.755265116381325 | 1.42940662360661 | 0.999332001504091 |
| ebi-fl187-GCST90094807 | ebi-met1400-GCST90199779 | Gamma-glutamyl-2-aminobutyrate levels \|\| id:ebi-met1400-GCST90199779 | Oranges liking \|\| id:ebi-fl187-GCST90094807 | Weighted median | 7 | 0.164367702901938 | 0.203318897408779 | 0.418847031359921 | -0.234137336 | 0.562872741823145 | 1.17864762757573 | 0.791253140876655 | 1.75570896116855 | 0.999332001504091 |
| ebi-fl187-GCST90094807 | ebi-met1400-GCST90199824 | 4-vinylphenol sulfate levels \|\| id:ebi-met1400-GCST90199824 | Oranges liking \|\| id:ebi-fl187-GCST90094807 | Weighted median | 7 | 0.0587457544381765 | 0.195285497389288 | 0.763551869403416 | -0.32401382 | 0.441505329321182 | 1.06050557762489 | 0.723240246808842 | 1.55504631432763 | 0.999332001504091 |
| ebi-fl187-GCST90094807 | ebi-met1400-GCST90199830 | 4-ethylphenylsulfate levels \|\| id:ebi-met1400-GCST90199830 | Oranges liking \|\| id:ebi-fl187-GCST90094807 | Weighted median | 7 | 0.0530026966306077 | 0.183644577565518 | 0.77287552835885 | -0.306940675 | 0.412946068659022 | 1.05443248853044 | 0.735694244381059 | 1.51126351926797 | 0.999332001504091 |
| ebi-fl187-GCST90094807 | ebi-met1400-GCST90199833 | Hexanoylglutamine levels \|\| id:ebi-met1400-GCST90199833 | Oranges liking \|\| id:ebi-fl187-GCST90094807 | Weighted median | 7 | 0.114840688988769 | 0.181320025123454 | 0.526499163588811 | -0.24054656 | 0.470227938230739 | 1.12169472501594 | 0.786198039016158 | 1.60035893463063 | 0.999332001504091 |
| ebi-fl187-GCST90094807 | ebi-met1400-GCST90199837 | Carotenoid levels (cryptoxanthin) \|\| id:ebi-met1400-GCST90199837 | Oranges liking \|\| id:ebi-fl187-GCST90094807 | Weighted median | 7 | 0.130509929705901 | 0.187129174386298 | 0.48553229611552 | -0.236263252 | 0.497283111503046 | 1.13940925383618 | 0.789572789882922 | 1.64424795834216 | 0.999332001504091 |
| ebi-fl187-GCST90094807 | ebi-met1400-GCST90199869 | 2-hydroxyhippurate levels \|\| id:ebi-met1400-GCST90199869 | Oranges liking \|\| id:ebi-fl187-GCST90094807 | Weighted median | 7 | 0.0699989981475269 | 0.209403211033023 | 0.738169387992542 | -0.340431295 | 0.480429291772253 | 1.07250710675978 | 0.711463405632341 | 1.61676831857274 | 0.999332001504091 |
| ebi-fl187-GCST90094807 | ebi-met1400-GCST90199902 | Hydantoin-5-propionate levels \|\| id:ebi-met1400-GCST90199902 | Oranges liking \|\| id:ebi-fl187-GCST90094807 | Weighted median | 7 | -0.158064277 | 0.1723806760161 | 0.359169400561306 | -0.495930402 | 0.179801848036363 | 0.853794900868118 | 0.609004025092525 | 1.19698015565275 | 0.999332001504091 |
| ebi-fl187-GCST90094807 | ebi-met1400-GCST90199904 | Pantoate levels \|\| id:ebi-met1400-GCST90199904 | Oranges liking \|\| id:ebi-fl187-GCST90094807 | Weighted median | 7 | -0.003827488 | 0.171134770961552 | 0.982156523547757 | -0.339251639 | 0.331596662768525 | 0.996179827180986 | 0.712303182988806 | 1.3931908094505 | 0.999332001504091 |
| ebi-fl187-GCST90094807 | ebi-met1400-GCST90199936 | 3-methyl catechol sulfate (2) levels \|\| id:ebi-met1400-GCST90199936 | Oranges liking \|\| id:ebi-fl187-GCST90094807 | Weighted median | 7 | 0.340411219367625 | 0.214996068146545 | 0.113344684725502 | -0.080981074 | 0.761803512934853 | 1.4055254510289 | 0.922211144863498 | 2.14213610895191 | 0.999332001504091 |
| ebi-fl187-GCST90094807 | ebi-met1400-GCST90199977 | 4-hydroxychlorothalonil levels \|\| id:ebi-met1400-GCST90199977 | Oranges liking \|\| id:ebi-fl187-GCST90094807 | Weighted median | 7 | -0.04923758 | 0.184111579318888 | 0.789135511711205 | -0.410096275 | 0.311621115946185 | 0.951954937834208 | 0.663586360294748 | 1.36563717684675 | 0.999332001504091 |
| ebi-fl187-GCST90094807 | ebi-met1400-GCST90200040 | Sphingomyelin (d18:2/24:1, d18:1/24:2) levels \|\| id:ebi-met1400-GCST90200040 | Oranges liking \|\| id:ebi-fl187-GCST90094807 | Weighted median | 7 | 0.0318755626533866 | 0.168632669571788 | 0.850074281167806 | -0.29864447 | 0.362395595014091 | 1.03238902956018 | 0.741823103141661 | 1.43676720749513 | 0.999332001504091 |
| ebi-fl187-GCST90094807 | ebi-met1400-GCST90200077 | 1-stearoyl-2-oleoyl-GPI (18:0/18:1) levels \|\| id:ebi-met1400-GCST90200077 | Oranges liking \|\| id:ebi-fl187-GCST90094807 | Weighted median | 6 | 0.198488386438025 | 0.19599970633432 | 0.311204736152248 | -0.185671038 | 0.582647810853292 | 1.21955786392117 | 0.830546768392556 | 1.79077378909142 | 0.999332001504091 |
| ebi-fl187-GCST90094807 | ebi-met1400-GCST90200080 | 1-palmitoyl-2-oleoyl-GPI (16:0/18:1) levels \|\| id:ebi-met1400-GCST90200080 | Oranges liking \|\| id:ebi-fl187-GCST90094807 | Weighted median | 7 | 0.176376069414972 | 0.182811473149738 | 0.334646201364713 | -0.181934418 | 0.534686556788458 | 1.19288658246654 | 0.833656011486126 | 1.70691313806039 | 0.999332001504091 |
| ebi-fl187-GCST90094807 | ebi-met1400-GCST90200082 | 1-oleoyl-2-linoleoyl-GPE (18:1/18:2) levels \|\| id:ebi-met1400-GCST90200082 | Oranges liking \|\| id:ebi-fl187-GCST90094807 | Weighted median | 7 | 0.00383495539306636 | 0.183589019397623 | 0.983334356268394 | -0.355999523 | 0.363669433412406 | 1.00384231824356 | 0.700472954622557 | 1.43859858292401 | 0.999332001504091 |
| ebi-fl187-GCST90094807 | ebi-met1400-GCST90200100 | Perfluorooctanesulfonate (PFOS) levels \|\| id:ebi-met1400-GCST90200100 | Oranges liking \|\| id:ebi-fl187-GCST90094807 | Weighted median | 7 | -0.203590836 | 0.161153218171165 | 0.206468066726803 | -0.519451143 | 0.112269471954406 | 0.815796097579128 | 0.594846944134341 | 1.11881430910576 | 0.999332001504091 |
| ebi-fl187-GCST90094807 | ebi-met1400-GCST90200102 | 1-palmitoleoyl-2-linolenoyl-GPC (16:1/18:3) levels \|\| id:ebi-met1400-GCST90200102 | Oranges liking \|\| id:ebi-fl187-GCST90094807 | Weighted median | 7 | 0.20454816135687 | 0.195901220565274 | 0.296420950168228 | -0.179418231 | 0.588514553664807 | 1.22697054698918 | 0.835756287145965 | 1.8013106767284 | 0.999332001504091 |
| ebi-fl187-GCST90094807 | ebi-met1400-GCST90200114 | Glycosyl-N-tricosanoyl-sphingadienine (d18:2/23:0) levels \|\| id:ebi-met1400-GCST90200114 | Oranges liking \|\| id:ebi-fl187-GCST90094807 | Weighted median | 7 | 0.0140281496393501 | 0.203963083862631 | 0.945166421166168 | -0.385739495 | 0.413795794010108 | 1.01412700584617 | 0.67994763255133 | 1.51254822393822 | 0.999332001504091 |
| ebi-fl187-GCST90094807 | ebi-met1400-GCST90200135 | Arachidoylcarnitine (C20) levels \|\| id:ebi-met1400-GCST90200135 | Oranges liking \|\| id:ebi-fl187-GCST90094807 | Weighted median | 7 | -0.017126136 | 0.204592258362355 | 0.933288103781005 | -0.418126962 | 0.383874690869324 | 0.983019683114799 | 0.658278646804058 | 1.46796148117919 | 0.999332001504091 |
| ebi-fl187-GCST90094807 | ebi-met1400-GCST90200146 | Docosahexaenoylcarnitine (C22:6) levels \|\| id:ebi-met1400-GCST90200146 | Oranges liking \|\| id:ebi-fl187-GCST90094807 | Weighted median | 7 | 0.102567550287957 | 0.224571783278166 | 0.647868711050612 | -0.337593145 | 0.542728245513161 | 1.10801214592143 | 0.71348551404788 | 1.72069494241619 | 0.999332001504091 |
| ebi-fl187-GCST90094807 | ebi-met1400-GCST90200162 | 3-hydroxyphenylacetoylglutamine levels \|\| id:ebi-met1400-GCST90200162 | Oranges liking \|\| id:ebi-fl187-GCST90094807 | Weighted median | 7 | -0.264562607 | 0.243312477641557 | 0.276887998351306 | -0.741455063 | 0.212329849520037 | 0.767541594149679 | 0.476420189624344 | 1.23655569511937 | 0.999332001504091 |
| ebi-fl187-GCST90094807 | ebi-met1400-GCST90200172 | 3-carboxy-4-methyl-5-pentyl-2-furanpropionate (3-CMPFP) levels \|\| id:ebi-met1400-GCST90200172 | Oranges liking \|\| id:ebi-fl187-GCST90094807 | Weighted median | 7 | -0.120080744 | 0.176728350088381 | 0.496843222300541 | -0.46646831 | 0.226306822538596 | 0.886848826428545 | 0.627213485031569 | 1.25396034955802 | 0.999332001504091 |
| ebi-fl187-GCST90094807 | ebi-met1400-GCST90200222 | Dihydrocaffeate sulfate (2) levels \|\| id:ebi-met1400-GCST90200222 | Oranges liking \|\| id:ebi-fl187-GCST90094807 | Weighted median | 7 | 0.0347936489615818 | 0.203990679621672 | 0.86456590266607 | -0.365028083 | 0.434615381020058 | 1.03540602964452 | 0.694177155959908 | 1.54436895109546 | 0.999332001504091 |
| ebi-fl187-GCST90094807 | ebi-met1400-GCST90200223 | 4-methylhexanoylglutamine levels \|\| id:ebi-met1400-GCST90200223 | Oranges liking \|\| id:ebi-fl187-GCST90094807 | Weighted median | 7 | 0.266446053133787 | 0.224441328172166 | 0.235167527066467 | -0.17345895 | 0.706351056351232 | 1.30531716966518 | 0.840751663238957 | 2.02658286378964 | 0.999332001504091 |
| ebi-fl187-GCST90094807 | ebi-met1400-GCST90200224 | 2-ketocaprylate levels \|\| id:ebi-met1400-GCST90200224 | Oranges liking \|\| id:ebi-fl187-GCST90094807 | Weighted median | 7 | -0.248384007 | 0.187320009239135 | 0.184843809859361 | -0.615531225 | 0.118763211532069 | 0.780060337456657 | 0.540353769790658 | 1.12610323845568 | 0.999332001504091 |
| ebi-fl187-GCST90094807 | ebi-met1400-GCST90200243 | Hydroxypalmitoyl sphingomyelin (d18:1/16:0(OH)) levels \|\| id:ebi-met1400-GCST90200243 | Oranges liking \|\| id:ebi-fl187-GCST90094807 | Weighted median | 7 | -0.254094187 | 0.190120686492798 | 0.181389526048202 | -0.626730733 | 0.118542358283413 | 0.775618745215198 | 0.534335835233377 | 1.12585456235862 | 0.999332001504091 |
| ebi-fl187-GCST90094807 | ebi-met1400-GCST90200273 | 2-methoxyhydroquinone sulfate (2) levels \|\| id:ebi-met1400-GCST90200273 | Oranges liking \|\| id:ebi-fl187-GCST90094807 | Weighted median | 7 | -0.085535577 | 0.228124530216778 | 0.707696725716601 | -0.532659656 | 0.361588502341392 | 0.918020482165439 | 0.587041562859176 | 1.43560807103778 | 0.999332001504091 |
| ebi-fl187-GCST90094807 | ebi-met1400-GCST90200276 | 2,4-di-tert-butylphenol levels \|\| id:ebi-met1400-GCST90200276 | Oranges liking \|\| id:ebi-fl187-GCST90094807 | Weighted median | 7 | 0.252695647123815 | 0.191592699948167 | 0.187195504278657 | -0.122826045 | 0.628217339022223 | 1.28749136549783 | 0.884417498225431 | 1.87426641779192 | 0.999332001504091 |
| ebi-fl187-GCST90094807 | ebi-met1400-GCST90200289 | N-acetylmethionine levels \|\| id:ebi-met1400-GCST90200289 | Oranges liking \|\| id:ebi-fl187-GCST90094807 | Weighted median | 7 | 0.130675879269193 | 0.195558950238719 | 0.503994875817601 | -0.252619663 | 0.513971421737082 | 1.13959835399438 | 0.776763257301747 | 1.67191791864351 | 0.999332001504091 |
| ebi-fl187-GCST90094807 | ebi-met1400-GCST90200308 | 3-hydroxyisobutyrate levels \|\| id:ebi-met1400-GCST90200308 | Oranges liking \|\| id:ebi-fl187-GCST90094807 | Weighted median | 7 | -0.148764622 | 0.192958432093089 | 0.440726373589309 | -0.526963149 | 0.229433904834198 | 0.861771933123809 | 0.590395192230113 | 1.25788772417796 | 0.999332001504091 |
| ebi-fl187-GCST90094807 | ebi-met1400-GCST90200383 | Cystathionine levels \|\| id:ebi-met1400-GCST90200383 | Oranges liking \|\| id:ebi-fl187-GCST90094807 | Weighted median | 7 | 0.0237997955246068 | 0.195462566897487 | 0.903087985849435 | -0.359306836 | 0.406906426643682 | 1.02408527091122 | 0.698160098114256 | 1.50216353660144 | 0.999332001504091 |
| ebi-fl187-GCST90094807 | ebi-met1400-GCST90200384 | Guanidinoacetate levels \|\| id:ebi-met1400-GCST90200384 | Oranges liking \|\| id:ebi-fl187-GCST90094807 | Weighted median | 7 | -0.246510588 | 0.179617288753352 | 0.169932332168388 | -0.598560474 | 0.105539298271878 | 0.781523086973175 | 0.54960223381677 | 1.11130977621846 | 0.999332001504091 |
| ebi-fl187-GCST90094807 | ebi-met1400-GCST90200403 | Citrulline levels \|\| id:ebi-met1400-GCST90200403 | Oranges liking \|\| id:ebi-fl187-GCST90094807 | Weighted median | 7 | 0.0590433413070118 | 0.172795040797423 | 0.732579826196528 | -0.279634939 | 0.397721621269962 | 1.06082121712187 | 0.756059699251899 | 1.48842962508043 | 0.999332001504091 |
| ebi-fl187-GCST90094807 | ebi-met1400-GCST90200434 | 12,13-DiHOME levels \|\| id:ebi-met1400-GCST90200434 | Oranges liking \|\| id:ebi-fl187-GCST90094807 | Weighted median | 7 | -0.028663435 | 0.172591454477768 | 0.868096516635999 | -0.366942686 | 0.309615815432412 | 0.971743463939934 | 0.692849353790867 | 1.36290140785064 | 0.999332001504091 |
| ebi-fl187-GCST90094807 | ebi-met1400-GCST90200437 | Fructose levels \|\| id:ebi-met1400-GCST90200437 | Oranges liking \|\| id:ebi-fl187-GCST90094807 | Weighted median | 7 | 0.00931655071058641 | 0.205806895540027 | 0.963893367625368 | -0.394064965 | 0.41269806596904 | 1.00936008486017 | 0.674310248557067 | 1.51088876832146 | 0.999332001504091 |
| ebi-fl187-GCST90094807 | ebi-met1400-GCST90200448 | N-stearoyl-sphinganine (d18:0/18:0) levels \|\| id:ebi-met1400-GCST90200448 | Oranges liking \|\| id:ebi-fl187-GCST90094807 | Weighted median | 7 | 0.0101253652976124 | 0.191999373709974 | 0.957941896828185 | -0.366193407 | 0.386444137769161 | 1.01017680026151 | 0.69336868576272 | 1.47173818019204 | 0.999332001504091 |
| ebi-fl187-GCST90094807 | ebi-met1400-GCST90200468 | X-11847 levels \|\| id:ebi-met1400-GCST90200468 | Oranges liking \|\| id:ebi-fl187-GCST90094807 | Weighted median | 7 | -0.008571483 | 0.189062240384628 | 0.963838829642605 | -0.379133474 | 0.361990508058709 | 0.99146514733232 | 0.684454249552147 | 1.43618530970901 | 0.999332001504091 |
| ebi-fl187-GCST90094807 | ebi-met1400-GCST90200476 | X-11843 levels \|\| id:ebi-met1400-GCST90200476 | Oranges liking \|\| id:ebi-fl187-GCST90094807 | Weighted median | 7 | -0.023775178 | 0.201200483489014 | 0.905935649321953 | -0.418128126 | 0.370577769438585 | 0.976505224743559 | 0.658277880616056 | 1.448571313 | 0.999332001504091 |
| ebi-fl187-GCST90094807 | ebi-met1400-GCST90200511 | X-12906 levels \|\| id:ebi-met1400-GCST90200511 | Oranges liking \|\| id:ebi-fl187-GCST90094807 | Weighted median | 7 | 0.0486608519752115 | 0.172199523081554 | 0.777495649082739 | -0.288850213 | 0.386171917215058 | 1.04986423097455 | 0.749124405898529 | 1.47133759733505 | 0.999332001504091 |
| ebi-fl187-GCST90094807 | ebi-met1400-GCST90200515 | X-12849 levels \|\| id:ebi-met1400-GCST90200515 | Oranges liking \|\| id:ebi-fl187-GCST90094807 | Weighted median | 7 | 0.122150977917509 | 0.196477723772961 | 0.53413655061159 | -0.262945361 | 0.507247316512513 | 1.12992468257855 | 0.768783901995154 | 1.66071347876412 | 0.999332001504091 |
| ebi-fl187-GCST90094807 | ebi-met1400-GCST90200551 | X-18886 levels \|\| id:ebi-met1400-GCST90200551 | Oranges liking \|\| id:ebi-fl187-GCST90094807 | Weighted median | 7 | -0.2440508 | 0.18570057391602 | 0.188773281206893 | -0.608023925 | 0.119922324516183 | 0.783447833820548 | 0.544425632572407 | 1.1274092761172 | 0.999332001504091 |
| ebi-fl187-GCST90094807 | ebi-met1400-GCST90200564 | X-21319 levels \|\| id:ebi-met1400-GCST90200564 | Oranges liking \|\| id:ebi-fl187-GCST90094807 | Weighted median | 7 | -0.194456786 | 0.178871435103775 | 0.276978663176572 | -0.545044799 | 0.156131226981556 | 0.823281754932241 | 0.579815807806934 | 1.1689795946888 | 0.999332001504091 |
| ebi-fl187-GCST90094807 | ebi-met1400-GCST90200579 | X-21442 levels \|\| id:ebi-met1400-GCST90200579 | Oranges liking \|\| id:ebi-fl187-GCST90094807 | Weighted median | 7 | 0.205525508857692 | 0.193324090343139 | 0.287730345706392 | -0.173389708 | 0.584440725930245 | 1.22817030978394 | 0.840809880470827 | 1.79398737439922 | 0.999332001504091 |
| ebi-fl187-GCST90094807 | ebi-met1400-GCST90200581 | X-21441 levels \|\| id:ebi-met1400-GCST90200581 | Oranges liking \|\| id:ebi-fl187-GCST90094807 | Weighted median | 7 | 0.0789398230503941 | 0.180673228893746 | 0.662169062304279 | -0.275179706 | 0.433059351682137 | 1.0821392002748 | 0.759435636139101 | 1.54196773636376 | 0.999332001504091 |
| ebi-fl187-GCST90094807 | ebi-met1400-GCST90200648 | X-24585 levels \|\| id:ebi-met1400-GCST90200648 | Oranges liking \|\| id:ebi-fl187-GCST90094807 | Weighted median | 7 | -0.051861733 | 0.181413155634393 | 0.774972819016563 | -0.407431518 | 0.303708052112716 | 0.949460136838065 | 0.665357014834192 | 1.35487344590363 | 0.999332001504091 |
| ebi-fl187-GCST90094807 | ebi-met1400-GCST90200720 | Adenosine 5'-diphosphate (ADP) to pantothenate ratio \|\| id:ebi-met1400-GCST90200720 | Oranges liking \|\| id:ebi-fl187-GCST90094807 | Weighted median | 7 | -0.055194977 | 0.250596282372464 | 0.82567289741009 | -0.54636369 | 0.435973736447607 | 0.946300623106134 | 0.579051597542842 | 1.54646817846799 | 0.999332001504091 |
| ebi-fl187-GCST90094807 | ebi-met1400-GCST90200724 | Cholate to taurocholate ratio \|\| id:ebi-met1400-GCST90200724 | Oranges liking \|\| id:ebi-fl187-GCST90094807 | Weighted median | 7 | 0.0684462924617376 | 0.201188395051566 | 0.733698430705635 | -0.325882962 | 0.462775546762807 | 1.07084311105917 | 0.721889671127025 | 1.58847676364814 | 0.999332001504091 |
| ebi-fl187-GCST90094807 | ebi-met1400-GCST90200726 | N-acetylputrescine to (N(1) + N(8))-acetylspermidine ratio \|\| id:ebi-met1400-GCST90200726 | Oranges liking \|\| id:ebi-fl187-GCST90094807 | Weighted median | 7 | 0.0279035391506639 | 0.169769529456604 | 0.869446733876003 | -0.304844739 | 0.360651816885607 | 1.02829648928455 | 0.737237830095344 | 1.43426398742749 | 0.999332001504091 |
| ebi-fl187-GCST90094807 | ebi-met1400-GCST90200744 | Arginine to glutamate ratio \|\| id:ebi-met1400-GCST90200744 | Oranges liking \|\| id:ebi-fl187-GCST90094807 | Weighted median | 7 | 0.0244219661994127 | 0.177389817400293 | 0.890498066556335 | -0.323262076 | 0.372106008303987 | 1.02472262498623 | 0.723784143125178 | 1.45078676858643 | 0.999332001504091 |
| ebi-fl187-GCST90094807 | ebi-met1400-GCST90200759 | Histidine to trans-urocanate ratio \|\| id:ebi-met1400-GCST90200759 | Oranges liking \|\| id:ebi-fl187-GCST90094807 | Weighted median | 7 | 0.312666520915073 | 0.205957746227107 | 0.128986662333159 | -0.091010662 | 0.716343703520204 | 1.36706556723024 | 0.913007976639308 | 2.04693530935585 | 0.999332001504091 |
| ebi-fl187-GCST90094807 | ebi-met1400-GCST90200795 | Oleoyl-linoleoyl-glycerol (18:1 to 18:2) [2] to linoleoyl-arachidonoyl-glycerol (18:2 to 20:4) [2] ratio \|\| id:ebi-met1400-GCST90200795 | Oranges liking \|\| id:ebi-fl187-GCST90094807 | Weighted median | 7 | -0.030349069 | 0.191630547709674 | 0.874163035326071 | -0.405944942 | 0.345246804576309 | 0.970106840277064 | 0.666346853843092 | 1.41233844824899 | 0.999332001504091 |
| ebi-fl187-GCST90094807 | ebi-met1400-GCST90200811 | Choline to taurocholate ratio \|\| id:ebi-met1400-GCST90200811 | Oranges liking \|\| id:ebi-fl187-GCST90094807 | Weighted median | 7 | -0.087973781 | 0.201023351771068 | 0.66165476558238 | -0.481979551 | 0.306031988027207 | 0.915784886965831 | 0.617559690165923 | 1.35802574641763 | 0.999332001504091 |
| ebi-fl187-GCST90094807 | ebi-met1400-GCST90200831 | Cholate to phosphate ratio \|\| id:ebi-met1400-GCST90200831 | Oranges liking \|\| id:ebi-fl187-GCST90094807 | Weighted median | 7 | 0.0829822284026583 | 0.200075338451692 | 0.678321333008567 | -0.309165435 | 0.475129891767975 | 1.0865224991363 | 0.734059320904232 | 1.60822307885851 | 0.999332001504091 |
| ebi-fl187-GCST90094807 | ebi-met1400-GCST90200841 | Adenosine 5'-diphosphate (ADP) to oxalate (ethanedioate) ratio \|\| id:ebi-met1400-GCST90200841 | Oranges liking \|\| id:ebi-fl187-GCST90094807 | Weighted median | 7 | -0.194437531 | 0.248076084952078 | 0.433168128717634 | -0.680666658 | 0.291791595056184 | 0.823297606857981 | 0.506279364670602 | 1.3388239710285 | 0.999332001504091 |
| ebi-fl187-GCST90094807 | ebi-met1400-GCST90200844 | Aspartate to phosphate ratio \|\| id:ebi-met1400-GCST90200844 | Oranges liking \|\| id:ebi-fl187-GCST90094807 | Weighted median | 7 | -0.085717527 | 0.170106010825131 | 0.614327071157224 | -0.419125309 | 0.247690253884025 | 0.917853463120895 | 0.657621784471955 | 1.28106306642423 | 0.999332001504091 |
| ebi-fl187-GCST90094807 | ebi-met1400-GCST90200870 | Adenosine 5'-monophosphate (AMP) to acetoacetate ratio \|\| id:ebi-met1400-GCST90200870 | Oranges liking \|\| id:ebi-fl187-GCST90094807 | Weighted median | 7 | -0.094009532 | 0.201868464747751 | 0.641432421150889 | -0.489671723 | 0.301652658431496 | 0.910274085035274 | 0.612827538120233 | 1.35209150755272 | 0.999332001504091 |
| ebi-fl187-GCST90094807 | ebi-met1400-GCST90200887 | Cholesterol to cortisol ratio \|\| id:ebi-met1400-GCST90200887 | Oranges liking \|\| id:ebi-fl187-GCST90094807 | Weighted median | 7 | 0.222927771676579 | 0.165341686828452 | 0.177566626910331 | -0.101141935 | 0.546997477860346 | 1.2497303045514 | 0.903804742700929 | 1.72805669225278 | 0.999332001504091 |
| ebi-fl187-GCST90094807 | ebi-met1400-GCST90200890 | Cortisol to taurocholate ratio \|\| id:ebi-met1400-GCST90200890 | Oranges liking \|\| id:ebi-fl187-GCST90094807 | Weighted median | 7 | -0.096051947 | 0.18644775433415 | 0.606435514519434 | -0.461489546 | 0.269385651448553 | 0.908416825272049 | 0.630344019748867 | 1.30915992312598 | 0.999332001504091 |
| ebi-fl187-GCST90094807 | ebi-met1400-GCST90200948 | Adenosine 5'-diphosphate (ADP) to mannitol to sorbitol ratio \|\| id:ebi-met1400-GCST90200948 | Oranges liking \|\| id:ebi-fl187-GCST90094807 | Weighted median | 7 | -0.351644931 | 0.262558806919695 | 0.180473127226441 | -0.866260192 | 0.16297033090628 | 0.703529879525673 | 0.420521280929244 | 1.17700176859465 | 0.999332001504091 |
| ebi-fl187-GCST90094807 | ebi-met1400-GCST90200972 | Leucine to N-palmitoyl-sphingosine (d18:1 to 16:0) ratio \|\| id:ebi-met1400-GCST90200972 | Oranges liking \|\| id:ebi-fl187-GCST90094807 | Weighted median | 7 | 0.120617514117702 | 0.188254228918582 | 0.5217071562097 | -0.248360775 | 0.489595802798124 | 1.12819331181777 | 0.780078460039784 | 1.63165657562887 | 0.999332001504091 |
| ebi-fl187-GCST90094807 | ebi-met1400-GCST90200973 | Proline to glutamate ratio \|\| id:ebi-met1400-GCST90200973 | Oranges liking \|\| id:ebi-fl187-GCST90094807 | Weighted median | 7 | -0.042722017 | 0.187525299573121 | 0.819786164676579 | -0.410271604 | 0.324827570259081 | 0.958177710255248 | 0.663470024505064 | 1.38379201850887 | 0.999332001504091 |
| ebi-fl187-GCST90094807 | ebi-met1400-GCST90200983 | Cholesterol to linoleoyl-arachidonoyl-glycerol (18:2 to 20:4) [1] ratio \|\| id:ebi-met1400-GCST90200983 | Oranges liking \|\| id:ebi-fl187-GCST90094807 | Weighted median | 7 | -0.055198459 | 0.197615205965174 | 0.779997008667012 | -0.442524263 | 0.332127344821463 | 0.946297328218148 | 0.642412754199741 | 1.39393034702169 | 0.999332001504091 |
| ebi-fl187-GCST90094807 | ebi-met1400-GCST90201001 | Serine to alpha-ketobutyrate ratio \|\| id:ebi-met1400-GCST90201001 | Oranges liking \|\| id:ebi-fl187-GCST90094807 | Weighted median | 7 | 0.232254052302008 | 0.183256191620178 | 0.205021748559821 | -0.126928083 | 0.591436187877558 | 1.26144015989995 | 0.880797014357411 | 1.80658114306768 | 0.999332001504091 |
| ebi-fl187-GCST90094807 | ebi-met1400-GCST90201005 | Fructose to maltose ratio \|\| id:ebi-met1400-GCST90201005 | Oranges liking \|\| id:ebi-fl187-GCST90094807 | Weighted median | 7 | -0.029995912 | 0.207397110306771 | 0.885002751467535 | -0.436494248 | 0.376502424310814 | 0.970449500844263 | 0.646298215323577 | 1.45717907207491 | 0.999332001504091 |
| ebi-fl187-GCST90094807 | ebi-met1400-GCST90201020 | Paraxanthine to 5-acetylamino-6-formylamino-3-methyluracil ratio \|\| id:ebi-met1400-GCST90201020 | Oranges liking \|\| id:ebi-fl187-GCST90094807 | Weighted median | 7 | 0.285528317395296 | 0.204051804915593 | 0.161725056898669 | -0.11441322 | 0.685469855029858 | 1.33046475014632 | 0.891889333002855 | 1.98470413971893 | 0.999332001504091 |
| ebi-fl187-GCST90094807 | ebi-pw11-GCST90275197 | Placental weight (fetal genotype, adjusted for sex) \|\| id:ebi-pw11-GCST90275197 | Oranges liking \|\| id:ebi-fl187-GCST90094807 | Weighted median | 7 | 0.104191177242337 | 0.067958125225677 | 0.125234632189318 | -0.029006748 | 0.237389102684664 | 1.10981260554919 | 0.971409909176148 | 1.26793437847517 | 0.999332001504091 |
| ebi-fl187-GCST90094807 | prot-a-1017 | FAS-associated factor 2 \|\| id:prot-a-1017 | Oranges liking \|\| id:ebi-fl187-GCST90094807 | Weighted median | 7 | -0.131173522 | 0.310859786890871 | 0.673046873288603 | -0.740458704 | 0.478111660309108 | 0.877065571016727 | 0.476895111501923 | 1.61302558426371 | 0.999332001504091 |
| ebi-fl187-GCST90094807 | prot-a-1019 | Fas apoptotic inhibitory molecule 3 \|\| id:prot-a-1019 | Oranges liking \|\| id:ebi-fl187-GCST90094807 | Weighted median | 7 | 0.151956525832001 | 0.320311922252838 | 0.635213469024908 | -0.475854842 | 0.779767893447563 | 1.16410962663453 | 0.621353670292093 | 2.18096599024859 | 0.999332001504091 |
| ebi-fl187-GCST90094807 | prot-a-1052 | MIP18 family protein FAM96A \|\| id:prot-a-1052 | Oranges liking \|\| id:ebi-fl187-GCST90094807 | Weighted median | 7 | -0.330255412 | 0.297201787681073 | 0.266475341542245 | -0.912770916 | 0.252260091562276 | 0.718740134920602 | 0.401410407020916 | 1.28693071357955 | 0.999332001504091 |
| ebi-fl187-GCST90094807 | prot-a-1066 | F-box/LRR-repeat protein 4 \|\| id:prot-a-1066 | Oranges liking \|\| id:ebi-fl187-GCST90094807 | Weighted median | 7 | -0.195978868 | 0.298979956468015 | 0.512151254718082 | -0.781979582 | 0.390021846904361 | 0.822029605810915 | 0.457499456406283 | 1.47701306169326 | 0.999332001504091 |
| ebi-fl187-GCST90094807 | prot-a-1076 | Low affinity immunoglobulin gamma Fc region receptor III-B \|\| id:prot-a-1076 | Oranges liking \|\| id:ebi-fl187-GCST90094807 | Weighted median | 7 | -0.366801231 | 0.312379736982507 | 0.24030858868647 | -0.979065515 | 0.245463053690498 | 0.692947367953687 | 0.375661985261633 | 1.27821305746313 | 0.999332001504091 |
| ebi-fl187-GCST90094807 | prot-a-1081 | Fc receptor-like protein 3 \|\| id:prot-a-1081 | Oranges liking \|\| id:ebi-fl187-GCST90094807 | Weighted median | 7 | -0.195798133 | 0.296082169099854 | 0.50842211879539 | -0.776119184 | 0.384522918449625 | 0.822178188583146 | 0.460188456929332 | 1.46891336278274 | 0.999332001504091 |
| ebi-fl187-GCST90094807 | prot-a-1105 | Fibroblast growth factor receptor 4 \|\| id:prot-a-1105 | Oranges liking \|\| id:ebi-fl187-GCST90094807 | Weighted median | 7 | -0.165509507 | 0.300607435394734 | 0.581919192385995 | -0.75470008 | 0.423681066338653 | 0.847461806317549 | 0.470151601263268 | 1.52757432121313 | 0.999332001504091 |
| ebi-fl187-GCST90094807 | prot-a-1143 | Follistatin-related protein 1 \|\| id:prot-a-1143 | Oranges liking \|\| id:ebi-fl187-GCST90094807 | Weighted median | 7 | 0.0854045871232237 | 0.297154922706631 | 0.773800232413931 | -0.497019061 | 0.66782823562822 | 1.08915763672289 | 0.608341387873217 | 1.94999778295376 | 0.999332001504091 |
| ebi-fl187-GCST90094807 | prot-a-1151 | Alpha-(1,3)-fucosyltransferase 10 \|\| id:prot-a-1151 | Oranges liking \|\| id:ebi-fl187-GCST90094807 | Weighted median | 7 | -0.095532288 | 0.27903330218463 | 0.732073389720064 | -0.64243756 | 0.451372984505924 | 0.908889015175021 | 0.526008682325808 | 1.57046693270007 | 0.999332001504091 |
| ebi-fl187-GCST90094807 | prot-a-1165 | Growth arrest and DNA damage-inducible proteins-interacting protein 1 \|\| id:prot-a-1165 | Oranges liking \|\| id:ebi-fl187-GCST90094807 | Weighted median | 7 | -0.369921879 | 0.318378343357369 | 0.245278534168376 | -0.993943432 | 0.254099673583474 | 0.69078829332757 | 0.370114288805213 | 1.28930030704531 | 0.999332001504091 |
| ebi-fl187-GCST90094807 | prot-a-1194 | Growth/differentiation factor 11/8 \|\| id:prot-a-1194 | Oranges liking \|\| id:ebi-fl187-GCST90094807 | Weighted median | 7 | -0.082894251 | 0.282596767003028 | 0.769269542774159 | -0.636783914 | 0.470995412349125 | 0.920448478601194 | 0.528990971539404 | 1.60158764013261 | 0.999332001504091 |
| ebi-fl187-GCST90094807 | prot-a-1216 | D-glucuronyl C5-epimerase \|\| id:prot-a-1216 | Oranges liking \|\| id:ebi-fl187-GCST90094807 | Weighted median | 7 | 0.052057448783513 | 0.324286698736061 | 0.872464307923435 | -0.583544481 | 0.687659378306192 | 1.05343625940665 | 0.557917330535283 | 1.9890544564514 | 0.999332001504091 |
| ebi-fl187-GCST90094807 | prot-a-1246 | Glypican-3 \|\| id:prot-a-1246 | Oranges liking \|\| id:ebi-fl187-GCST90094807 | Weighted median | 7 | -0.113137897 | 0.280730200146203 | 0.686938722661626 | -0.663369089 | 0.437093295658419 | 0.89302750614302 | 0.515112946426433 | 1.54820051070473 | 0.999332001504091 |
| ebi-fl187-GCST90094807 | prot-a-1275 | Glutamate receptor 4 \|\| id:prot-a-1275 | Oranges liking \|\| id:ebi-fl187-GCST90094807 | Weighted median | 7 | -0.438945413 | 0.292125309817977 | 0.132944058501504 | -1.01151102 | 0.133620194318191 | 0.644715971828352 | 0.363669052927478 | 1.14295863501332 | 0.999332001504091 |
| ebi-fl187-GCST90094807 | prot-a-1289 | Glutathione S-transferase P \|\| id:prot-a-1289 | Oranges liking \|\| id:ebi-fl187-GCST90094807 | Weighted median | 7 | 0.0866859759185198 | 0.290097298280584 | 0.765079753989637 | -0.481904729 | 0.655276680548464 | 1.09055416567208 | 0.617605899071719 | 1.92567524055761 | 0.999332001504091 |
| ebi-fl187-GCST90094807 | prot-a-130 | Apolipoprotein D \|\| id:prot-a-130 | Oranges liking \|\| id:ebi-fl187-GCST90094807 | Weighted median | 7 | 0.182086124383861 | 0.300236591015026 | 0.544199073708475 | -0.406377594 | 0.770549842773312 | 1.19971751436233 | 0.666058620194154 | 2.16095411218936 | 0.999332001504091 |
| ebi-fl187-GCST90094807 | prot-a-1313 | Heparin-binding EGF-like growth factor \|\| id:prot-a-1313 | Oranges liking \|\| id:ebi-fl187-GCST90094807 | Weighted median | 7 | -0.087883441 | 0.301247277776893 | 0.77049161624275 | -0.678328105 | 0.502561223441095 | 0.915867623114882 | 0.507464711007565 | 1.65294942658111 | 0.999332001504091 |
| ebi-fl187-GCST90094807 | prot-a-1347 | HLA class II histocompatibility antigen, DQ alpha 2 chain \|\| id:prot-a-1347 | Oranges liking \|\| id:ebi-fl187-GCST90094807 | Weighted median | 7 | 0.0206142357736001 | 0.314676162640565 | 0.947768449711171 | -0.596151043 | 0.637379514549107 | 1.02082817667912 | 0.550928058878743 | 1.89151768458278 | 0.999332001504091 |
| ebi-fl187-GCST90094807 | prot-a-1357 | Non-histone chromosomal protein HMG-14 \|\| id:prot-a-1357 | Oranges liking \|\| id:ebi-fl187-GCST90094807 | Weighted median | 7 | -0.209690079 | 0.288890065267143 | 0.467932375938908 | -0.775914607 | 0.356534449052387 | 0.810835502087454 | 0.46028261082238 | 1.42837073568943 | 0.999332001504091 |
| ebi-fl187-GCST90094807 | prot-a-137 | Apolipoprotein M \|\| id:prot-a-137 | Oranges liking \|\| id:ebi-fl187-GCST90094807 | Weighted median | 7 | -0.148317322 | 0.273623658426063 | 0.58778502460816 | -0.684619692 | 0.38798504888244 | 0.862157490308414 | 0.504281975506318 | 1.4740077460603 | 0.999332001504091 |
| ebi-fl187-GCST90094807 | prot-a-1381 | 17-beta-hydroxysteroid dehydrogenase 14 \|\| id:prot-a-1381 | Oranges liking \|\| id:ebi-fl187-GCST90094807 | Weighted median | 7 | -0.31449016 | 0.302492981129847 | 0.298497418940171 | -0.907376403 | 0.278396083298478 | 0.730161044901687 | 0.403581672041004 | 1.3210093233316 | 0.999332001504091 |
| ebi-fl187-GCST90094807 | prot-a-1392 | Serine protease HTRA2, mitochondrial \|\| id:prot-a-1392 | Oranges liking \|\| id:ebi-fl187-GCST90094807 | Weighted median | 7 | -0.320776207 | 0.28200638620931 | 0.255338298237285 | -0.873508724 | 0.231956309560177 | 0.725585613505361 | 0.417484139606072 | 1.26106463115634 | 0.999332001504091 |
| ebi-fl187-GCST90094807 | prot-a-1416 | Interferon-induced protein with tetratricopeptide repeats 2 \|\| id:prot-a-1416 | Oranges liking \|\| id:ebi-fl187-GCST90094807 | Weighted median | 7 | -0.242078523 | 0.32164883843389 | 0.45167983982479 | -0.872510246 | 0.38835320079947 | 0.784994535374009 | 0.417901196728107 | 1.47455050474042 | 0.999332001504091 |
| ebi-fl187-GCST90094807 | prot-a-1428 | Interferon gamma \|\| id:prot-a-1428 | Oranges liking \|\| id:ebi-fl187-GCST90094807 | Weighted median | 7 | -0.097282664 | 0.286275409486037 | 0.733990627644476 | -0.658382466 | 0.463817138799932 | 0.907299509163401 | 0.517688035412528 | 1.59013217037588 | 0.999332001504091 |
| ebi-fl187-GCST90094807 | prot-a-1483 | Interleukin-17C \|\| id:prot-a-1483 | Oranges liking \|\| id:ebi-fl187-GCST90094807 | Weighted median | 7 | -0.458062798 | 0.294591345050818 | 0.119967691302123 | -1.035461835 | 0.11933623786641 | 0.632507754464182 | 0.355062362921756 | 1.12674871018499 | 0.999332001504091 |
| ebi-fl187-GCST90094807 | prot-a-1503 | Interleukin-1 receptor-like 2 \|\| id:prot-a-1503 | Oranges liking \|\| id:ebi-fl187-GCST90094807 | Weighted median | 7 | -0.129296027 | 0.305212188795703 | 0.671838091824494 | -0.727511917 | 0.46891986264693 | 0.878713803683777 | 0.483109512327601 | 1.59826691274259 | 0.999332001504091 |
| ebi-fl187-GCST90094807 | prot-a-1522 | Interleukin-31 receptor subunit alpha \|\| id:prot-a-1522 | Oranges liking \|\| id:ebi-fl187-GCST90094807 | Weighted median | 7 | -0.12458145 | 0.29935638788122 | 0.677289760611519 | -0.71131997 | 0.462157070227005 | 0.88286634895585 | 0.490995669850931 | 1.58749463178622 | 0.999332001504091 |
| ebi-fl187-GCST90094807 | prot-a-1540 | Interleukin-6 receptor subunit alpha \|\| id:prot-a-1540 | Oranges liking \|\| id:ebi-fl187-GCST90094807 | Weighted median | 7 | -0.26773531 | 0.275504146717113 | 0.331149369079683 | -0.807723438 | 0.272252817516752 | 0.765110271303504 | 0.445871967029788 | 1.31291888824895 | 0.999332001504091 |
| ebi-fl187-GCST90094807 | prot-a-1541 | Interleukin-6 receptor subunit alpha \|\| id:prot-a-1541 | Oranges liking \|\| id:ebi-fl187-GCST90094807 | Weighted median | 7 | -0.141360942 | 0.303899152587487 | 0.641818737759556 | -0.737003281 | 0.454281397160315 | 0.868175894071863 | 0.478545836325686 | 1.57504114722776 | 0.999332001504091 |
| ebi-fl187-GCST90094807 | prot-a-1545 | Interleukin-9 \|\| id:prot-a-1545 | Oranges liking \|\| id:ebi-fl187-GCST90094807 | Weighted median | 7 | -0.067159144 | 0.283025015495427 | 0.812431440697181 | -0.621888174 | 0.487569886260464 | 0.935046382370393 | 0.536929662972348 | 1.62835432176335 | 0.999332001504091 |
| ebi-fl187-GCST90094807 | prot-a-1548 | Inositol monophosphatase 3 \|\| id:prot-a-1548 | Oranges liking \|\| id:ebi-fl187-GCST90094807 | Weighted median | 7 | -0.609948388 | 0.29249443640782 | 0.0370390568569363 | -1.183237483 | -0.036659293 | 0.543378913298082 | 0.306285537444093 | 0.964004522971966 | 0.999332001504091 |
| ebi-fl187-GCST90094807 | prot-a-1555 | Inhibin beta A chain:Inhibin beta B chain heterodimer \|\| id:prot-a-1555 | Oranges liking \|\| id:ebi-fl187-GCST90094807 | Weighted median | 7 | -0.296360977 | 0.286204295202259 | 0.300441290219136 | -0.857321395 | 0.264599441805288 | 0.743518986474621 | 0.424297085657212 | 1.30290898037153 | 0.999332001504091 |
| ebi-fl187-GCST90094807 | prot-a-1603 | Junctophilin-3 \|\| id:prot-a-1603 | Oranges liking \|\| id:ebi-fl187-GCST90094807 | Weighted median | 7 | 0.18325601801246 | 0.286427038609456 | 0.522302635540527 | -0.378140978 | 0.744653013686994 | 1.2011218775561 | 0.68513390528096 | 2.10571065542622 | 0.999332001504091 |
| ebi-fl187-GCST90094807 | prot-a-1627 | UPF0258 protein KIAA1024 \|\| id:prot-a-1627 | Oranges liking \|\| id:ebi-fl187-GCST90094807 | Weighted median | 7 | -0.127791793 | 0.30369989065027 | 0.673913446725302 | -0.723043578 | 0.467459993113584 | 0.880036590235754 | 0.485273039584923 | 1.59593535387049 | 0.999332001504091 |
| ebi-fl187-GCST90094807 | prot-a-1666 | Kallikrein-7 \|\| id:prot-a-1666 | Oranges liking \|\| id:ebi-fl187-GCST90094807 | Weighted median | 7 | 0.105396933709462 | 0.291536867603801 | 0.717709409909026 | -0.466015327 | 0.676809194212912 | 1.11115157634976 | 0.627497666446176 | 1.96758950932362 | 0.999332001504091 |
| ebi-fl187-GCST90094807 | prot-a-1678 | Importin subunit alpha-3 \|\| id:prot-a-1678 | Oranges liking \|\| id:ebi-fl187-GCST90094807 | Weighted median | 7 | -0.073592903 | 0.294829594161214 | 0.802887685505101 | -0.651458907 | 0.50427310201082 | 0.929049830688284 | 0.521284715763814 | 1.65578149867145 | 0.999332001504091 |
| ebi-fl187-GCST90094807 | prot-a-1718 | Protein LDOC1 \|\| id:prot-a-1718 | Oranges liking \|\| id:ebi-fl187-GCST90094807 | Weighted median | 7 | -0.24227887 | 0.290975657656262 | 0.405046050056189 | -0.812591159 | 0.328033418623324 | 0.784837279158517 | 0.443706860191045 | 1.38823536442896 | 0.999332001504091 |
| ebi-fl187-GCST90094807 | prot-a-1723 | Leptin \|\| id:prot-a-1723 | Oranges liking \|\| id:ebi-fl187-GCST90094807 | Weighted median | 7 | -0.353290482 | 0.279012193447006 | 0.205434761546351 | -0.900154382 | 0.193573416715123 | 0.702373136677992 | 0.406506897711956 | 1.21357848022654 | 0.999332001504091 |
| ebi-fl187-GCST90094807 | prot-a-1730 | Galectin-7 \|\| id:prot-a-1730 | Oranges liking \|\| id:ebi-fl187-GCST90094807 | Weighted median | 7 | -0.129741242 | 0.298633615877243 | 0.663962017092281 | -0.715063129 | 0.455580644819859 | 0.87832267427427 | 0.489161230357342 | 1.57708884569766 | 0.999332001504091 |
| ebi-fl187-GCST90094807 | prot-a-1753 | Lipase member N \|\| id:prot-a-1753 | Oranges liking \|\| id:ebi-fl187-GCST90094807 | Weighted median | 7 | 0.0393501621019109 | 0.292080700690298 | 0.892830416949986 | -0.533128011 | 0.611828335454895 | 1.04013463562061 | 0.586766683299887 | 1.84379940274264 | 0.999332001504091 |
| ebi-fl187-GCST90094807 | prot-a-1756 | VIP36-like protein \|\| id:prot-a-1756 | Oranges liking \|\| id:ebi-fl187-GCST90094807 | Weighted median | 7 | 0.18856396377628 | 0.276456824834797 | 0.495192271143528 | -0.353291413 | 0.730419340452483 | 1.20751431769215 | 0.702372483148949 | 2.07595095538833 | 0.999332001504091 |
| ebi-fl187-GCST90094807 | prot-a-1760 | Ig Kappa chain V-I region HK102- like \|\| id:prot-a-1760 | Oranges liking \|\| id:ebi-fl187-GCST90094807 | Weighted median | 7 | -0.6900341 | 0.316083389756068 | 0.0290302047880145 | -1.309557544 | -0.070510656 | 0.501558965473102 | 0.26993946624783 | 0.931917808622662 | 0.999332001504091 |
| ebi-fl187-GCST90094807 | prot-a-179 | Neutral ceramidase \|\| id:prot-a-179 | Oranges liking \|\| id:ebi-fl187-GCST90094807 | Weighted median | 7 | -0.672800968 | 0.339432322925903 | 0.0474640406409627 | -1.338088321 | -0.007513615 | 0.510277303745211 | 0.262346712175761 | 0.992514541379257 | 0.999332001504091 |
| ebi-fl187-GCST90094807 | prot-a-1809 | Lactotransferrin \|\| id:prot-a-1809 | Oranges liking \|\| id:ebi-fl187-GCST90094807 | Weighted median | 7 | -0.143804293 | 0.320100272594496 | 0.65325302164926 | -0.771200828 | 0.483592240993854 | 0.866057224683745 | 0.462457403146286 | 1.6218901704762 | 0.999332001504091 |
| ebi-fl187-GCST90094807 | prot-a-1818 | Lysophospholipase-like protein 1 \|\| id:prot-a-1818 | Oranges liking \|\| id:ebi-fl187-GCST90094807 | Weighted median | 7 | 0.365095449982924 | 0.279297983237501 | 0.191148289053739 | -0.182328597 | 0.912519497128426 | 1.44065151174895 | 0.833327466380133 | 2.49058966857307 | 0.999332001504091 |
| ebi-fl187-GCST90094807 | prot-a-1852 | MAP kinase-activated protein kinase 3 \|\| id:prot-a-1852 | Oranges liking \|\| id:ebi-fl187-GCST90094807 | Weighted median | 7 | 0.133902250486395 | 0.314164406601211 | 0.669949639307694 | -0.481859986 | 0.749664487424769 | 1.14328105901503 | 0.617633532773283 | 2.11628985562597 | 0.999332001504091 |
| ebi-fl187-GCST90094807 | prot-a-1856 | Megakaryocyte-associated tyrosine-protein kinase \|\| id:prot-a-1856 | Oranges liking \|\| id:ebi-fl187-GCST90094807 | Weighted median | 7 | -0.114034191 | 0.288478545622936 | 0.692625024021528 | -0.679452141 | 0.45138375823125 | 0.892227449042909 | 0.506894623286067 | 1.57048385257059 | 0.999332001504091 |
| ebi-fl187-GCST90094807 | prot-a-1890 | Beta-1,3-N-acetylglucosaminyltransferase manic fringe \|\| id:prot-a-1890 | Oranges liking \|\| id:ebi-fl187-GCST90094807 | Weighted median | 7 | -0.191595741 | 0.318601355843118 | 0.547596829295701 | -0.816054398 | 0.432862916856838 | 0.825640574001976 | 0.442172855285372 | 1.54166486994858 | 0.999332001504091 |
| ebi-fl187-GCST90094807 | prot-a-1919 | Matrilysin \|\| id:prot-a-1919 | Oranges liking \|\| id:ebi-fl187-GCST90094807 | Weighted median | 7 | 0.175525600017365 | 0.299127600256646 | 0.557343596259219 | -0.410764496 | 0.76181569652039 | 1.19187250021741 | 0.663143085739783 | 2.14216220800941 | 0.999332001504091 |
| ebi-fl187-GCST90094807 | prot-a-1923 | Multimerin-2 \|\| id:prot-a-1923 | Oranges liking \|\| id:ebi-fl187-GCST90094807 | Weighted median | 7 | -0.149337809 | 0.319488898306098 | 0.64019424754561 | -0.77553605 | 0.4768604312844 | 0.861278117910555 | 0.460456886882094 | 1.61100858196414 | 0.999332001504091 |
| ebi-fl187-GCST90094807 | prot-a-1948 | Mesothelin \|\| id:prot-a-1948 | Oranges liking \|\| id:ebi-fl187-GCST90094807 | Weighted median | 7 | -0.202079546 | 0.275686496222302 | 0.463555456099821 | -0.742425078 | 0.338265986908825 | 0.817029934149964 | 0.475958278747359 | 1.402513504 | 0.999332001504091 |
| ebi-fl187-GCST90094807 | prot-a-195 | ATPase family AAA domain-containing protein 1 \|\| id:prot-a-195 | Oranges liking \|\| id:ebi-fl187-GCST90094807 | Weighted median | 7 | 0.0490163367071459 | 0.295183270414875 | 0.868114523686192 | -0.529542873 | 0.627575546720301 | 1.05023750802248 | 0.588874098230447 | 1.87306391395336 | 0.999332001504091 |
| ebi-fl187-GCST90094807 | prot-a-1966 | Mucin-1 \|\| id:prot-a-1966 | Oranges liking \|\| id:ebi-fl187-GCST90094807 | Weighted median | 7 | 0.0439485442159996 | 0.297253809942195 | 0.882462294485869 | -0.538668923 | 0.626566011702701 | 1.04492858588548 | 0.583524451487449 | 1.87117394449771 | 0.999332001504091 |
| ebi-fl187-GCST90094807 | prot-a-1986 | Myocardial zonula adherens protein \|\| id:prot-a-1986 | Oranges liking \|\| id:ebi-fl187-GCST90094807 | Weighted median | 7 | -0.700749401 | 0.275395345223944 | 0.0109427547264647 | -1.240524278 | -0.160974525 | 0.496213301527931 | 0.289232539942973 | 0.851313758340596 | 0.999332001504091 |
| ebi-fl187-GCST90094807 | prot-a-2071 | Epididymal secretory protein E1 \|\| id:prot-a-2071 | Oranges liking \|\| id:ebi-fl187-GCST90094807 | Weighted median | 7 | 0.0458926825039259 | 0.324636448085765 | 0.88758050502699 | -0.590394756 | 0.682180120752026 | 1.04696204758234 | 0.554108504040015 | 1.97818571829506 | 0.999332001504091 |
| ebi-fl187-GCST90094807 | prot-a-2098 | Neuritin \|\| id:prot-a-2098 | Oranges liking \|\| id:ebi-fl187-GCST90094807 | Weighted median | 7 | -0.051576963 | 0.322594656345044 | 0.872974311507739 | -0.683862489 | 0.580708563679324 | 0.949730553267623 | 0.504663963911184 | 1.78730440116934 | 0.999332001504091 |
| ebi-fl187-GCST90094807 | prot-a-2115 | Neurotrophin-4 \|\| id:prot-a-2115 | Oranges liking \|\| id:ebi-fl187-GCST90094807 | Weighted median | 7 | -0.121698116 | 0.27338090068647 | 0.656204463021009 | -0.657524682 | 0.414128449135191 | 0.885415620784102 | 0.518132290866304 | 1.513051465 | 0.999332001504091 |
| ebi-fl187-GCST90094807 | prot-a-2118 | Netrin-1 \|\| id:prot-a-2118 | Oranges liking \|\| id:ebi-fl187-GCST90094807 | Weighted median | 7 | 0.0294378643127938 | 0.309004309164325 | 0.924102880077954 | -0.576210582 | 0.63508631027487 | 1.02987544146546 | 0.562024080776706 | 1.88718501788731 | 0.999332001504091 |
| ebi-fl187-GCST90094807 | prot-a-2120 | Netrin-G1 \|\| id:prot-a-2120 | Oranges liking \|\| id:ebi-fl187-GCST90094807 | Weighted median | 7 | -0.257497347 | 0.307804308589454 | 0.402838887784899 | -0.860793792 | 0.345799097835323 | 0.772983677036453 | 0.422826312994885 | 1.41311868869434 | 0.999332001504091 |
| ebi-fl187-GCST90094807 | prot-a-2145 | Olfactomedin-like protein 3 \|\| id:prot-a-2145 | Oranges liking \|\| id:ebi-fl187-GCST90094807 | Weighted median | 7 | -0.096089623 | 0.308366536993571 | 0.755338101663676 | -0.700488035 | 0.508308789845584 | 0.908382600753806 | 0.496343011826954 | 1.66247721775105 | 0.999332001504091 |
| ebi-fl187-GCST90094807 | prot-a-2162 | Prolyl 4-hydroxylase subunit alpha-2 \|\| id:prot-a-2162 | Oranges liking \|\| id:ebi-fl187-GCST90094807 | Weighted median | 7 | -0.246242567 | 0.29725537929944 | 0.407451223132977 | -0.828863111 | 0.336377976311788 | 0.781732579308959 | 0.436545308065836 | 1.39986804178611 | 0.999332001504091 |
| ebi-fl187-GCST90094807 | prot-a-2168 | Polyadenylate-binding protein-interacting protein 1 \|\| id:prot-a-2168 | Oranges liking \|\| id:ebi-fl187-GCST90094807 | Weighted median | 7 | 0.176736274173082 | 0.333550624794552 | 0.59620663126823 | -0.47702295 | 0.830495498770404 | 1.19331634328572 | 0.620628285447541 | 2.29445535845333 | 0.999332001504091 |
| ebi-fl187-GCST90094807 | prot-a-219 | Beta-1,4-galactosyltransferase 5 \|\| id:prot-a-219 | Oranges liking \|\| id:ebi-fl187-GCST90094807 | Weighted median | 7 | 0.179280376127022 | 0.317366556962204 | 0.572141766331559 | -0.442758076 | 0.801318827772942 | 1.19635612684642 | 0.642262567332647 | 2.22847796997902 | 0.999332001504091 |
| ebi-fl187-GCST90094807 | prot-a-2190 | Pyruvate carboxylase, mitochondrial \|\| id:prot-a-2190 | Oranges liking \|\| id:ebi-fl187-GCST90094807 | Weighted median | 7 | -0.124058018 | 0.299192295079257 | 0.678403198499333 | -0.710474916 | 0.462358880367491 | 0.883328590448611 | 0.491410763031854 | 1.58781503663027 | 0.999332001504091 |
| ebi-fl187-GCST90094807 | prot-a-2197 | Protocadherin-9 \|\| id:prot-a-2197 | Oranges liking \|\| id:ebi-fl187-GCST90094807 | Weighted median | 7 | -0.288323836 | 0.304377188129551 | 0.343507143468164 | -0.884903125 | 0.308255452628591 | 0.749518831674524 | 0.412754156967611 | 1.36104862798226 | 0.999332001504091 |
| ebi-fl187-GCST90094807 | prot-a-2198 | Protocadherin alpha-4 \|\| id:prot-a-2198 | Oranges liking \|\| id:ebi-fl187-GCST90094807 | Weighted median | 7 | -0.115416801 | 0.33795494421567 | 0.732715651316742 | -0.777808492 | 0.546974889695608 | 0.89099469905067 | 0.459411713515838 | 1.72801765906438 | 0.999332001504091 |
| ebi-fl187-GCST90094807 | prot-a-2254 | Placenta growth factor \|\| id:prot-a-2254 | Oranges liking \|\| id:ebi-fl187-GCST90094807 | Weighted median | 7 | -0.252788208 | 0.302649490968301 | 0.40357655274503 | -0.84598121 | 0.340404794414193 | 0.776632349015499 | 0.429136078872892 | 1.40551642062234 | 0.999332001504091 |
| ebi-fl187-GCST90094807 | prot-a-2261 | Membrane-associated progesterone receptor component 2 \|\| id:prot-a-2261 | Oranges liking \|\| id:ebi-fl187-GCST90094807 | Weighted median | 7 | -0.43918096 | 0.289073938240312 | 0.128694898009877 | -1.005765879 | 0.127403959137056 | 0.644564128870726 | 0.365764396319239 | 1.13587577251303 | 0.999332001504091 |
| ebi-fl187-GCST90094807 | prot-a-2268 | Polymeric immunoglobulin receptor \|\| id:prot-a-2268 | Oranges liking \|\| id:ebi-fl187-GCST90094807 | Weighted median | 7 | -0.294555288 | 0.294662702681155 | 0.317486953615769 | -0.872094185 | 0.28298360944361 | 0.744862763371136 | 0.418075105207932 | 1.32708341000343 | 0.999332001504091 |
| ebi-fl187-GCST90094807 | prot-a-2283 | Group 10 secretory phospholipase A2 \|\| id:prot-a-2283 | Oranges liking \|\| id:ebi-fl187-GCST90094807 | Weighted median | 7 | -0.073518072 | 0.277192395602224 | 0.790836919175434 | -0.616815167 | 0.46977902367877 | 0.929119354872077 | 0.539660431874677 | 1.59964067144796 | 0.999332001504091 |
| ebi-fl187-GCST90094807 | prot-a-2298 | Inactive phospholipase D5 \|\| id:prot-a-2298 | Oranges liking \|\| id:ebi-fl187-GCST90094807 | Weighted median | 7 | 0.747719166370058 | 0.358465565922675 | 0.0369887331402952 | 0.0451266571616157 | 1.4503116755785 | 2.112176994 | 1.04616035521899 | 4.26444343093609 | 0.999332001504091 |
| ebi-fl187-GCST90094807 | prot-a-2304 | Procollagen-lysine,2-oxoglutarate 5-dioxygenase 2 \|\| id:prot-a-2304 | Oranges liking \|\| id:ebi-fl187-GCST90094807 | Weighted median | 7 | -0.047081553 | 0.279337271577237 | 0.866152707455827 | -0.594582605 | 0.500419499292985 | 0.954009592075043 | 0.551792833218021 | 1.64941305319851 | 0.999332001504091 |
| ebi-fl187-GCST90094807 | prot-a-2331 | Periostin \|\| id:prot-a-2331 | Oranges liking \|\| id:ebi-fl187-GCST90094807 | Weighted median | 7 | -0.058846349 | 0.280897607420363 | 0.834062658661411 | -0.60940566 | 0.491712961526339 | 0.942851628106896 | 0.54367390045409 | 1.63511471100845 | 0.999332001504091 |
| ebi-fl187-GCST90094807 | prot-a-2333 | Periostin \|\| id:prot-a-2333 | Oranges liking \|\| id:ebi-fl187-GCST90094807 | Weighted median | 7 | 0.00644900973774898 | 0.294159345180643 | 0.982508959102155 | -0.570103307 | 0.583001326291809 | 1.0064698493753 | 0.565467019084546 | 1.79140696718528 | 0.999332001504091 |
| ebi-fl187-GCST90094807 | prot-a-2367 | cAMP-dependent protein kinase catalytic subunit alpha \|\| id:prot-a-2367 | Oranges liking \|\| id:ebi-fl187-GCST90094807 | Weighted median | 7 | -0.548480748 | 0.284595848678995 | 0.0539513709468966 | -1.106288611 | 0.00932711554537213 | 0.577827008785482 | 0.330784355037199 | 1.00937074863903 | 0.999332001504091 |
| ebi-fl187-GCST90094807 | prot-a-2385 | Phosphoribosyl pyrophosphate synthase-associated protein 1 \|\| id:prot-a-2385 | Oranges liking \|\| id:ebi-fl187-GCST90094807 | Weighted median | 7 | 0.492463484554788 | 0.320379675844001 | 0.12426279725057 | -0.13548068 | 1.12040764920903 | 1.63634236288609 | 0.873296034758037 | 3.06610384337485 | 0.999332001504091 |
| ebi-fl187-GCST90094807 | prot-a-2393 | Trypsin-3 \|\| id:prot-a-2393 | Oranges liking \|\| id:ebi-fl187-GCST90094807 | Weighted median | 7 | -0.397684955 | 0.316425447670346 | 0.208824403990088 | -1.017878832 | 0.222508922767834 | 0.671873664985745 | 0.361360634380997 | 1.24920696598466 | 0.999332001504091 |
| ebi-fl187-GCST90094807 | prot-a-2405 | Pregnancy-specific beta-1-glycoprotein 6 \|\| id:prot-a-2405 | Oranges liking \|\| id:ebi-fl187-GCST90094807 | Weighted median | 7 | 0.357438951040785 | 0.295558182749369 | 0.226521051612334 | -0.221855087 | 0.936732989229549 | 1.42966328432831 | 0.801031435674244 | 2.55163157839866 | 0.999332001504091 |
| ebi-fl187-GCST90094807 | prot-a-244 | Beclin-1 \|\| id:prot-a-244 | Oranges liking \|\| id:ebi-fl187-GCST90094807 | Weighted median | 7 | -0.396993977 | 0.309165892369105 | 0.199113695731472 | -1.002959126 | 0.208971171863203 | 0.672338074991505 | 0.366792448537302 | 1.23240946994936 | 0.999332001504091 |
| ebi-fl187-GCST90094807 | prot-a-2454 | tRNA pseudouridine synthase A, mitochondrial \|\| id:prot-a-2454 | Oranges liking \|\| id:ebi-fl187-GCST90094807 | Weighted median | 7 | 0.332282946496334 | 0.270857436906248 | 0.219904651293214 | -0.19859763 | 0.863163522832579 | 1.39414726179406 | 0.819879722106885 | 2.37064844410736 | 0.999332001504091 |
| ebi-fl187-GCST90094807 | prot-a-2466 | Glutaminyl-peptide cyclotransferase-like protein \|\| id:prot-a-2466 | Oranges liking \|\| id:ebi-fl187-GCST90094807 | Weighted median | 7 | 0.179436744623157 | 0.309642940992418 | 0.562254765115926 | -0.42746342 | 0.786336908968296 | 1.1965432138817 | 0.652161257799934 | 2.19533994938037 | 0.999332001504091 |
| ebi-fl187-GCST90094807 | prot-a-2474 | Ras-related protein Rab-27A \|\| id:prot-a-2474 | Oranges liking \|\| id:ebi-fl187-GCST90094807 | Weighted median | 7 | 0.315853873982145 | 0.295556044714463 | 0.285215350491272 | -0.263435974 | 0.895143721622493 | 1.37142983940219 | 0.768406819141911 | 2.44768754980994 | 0.999332001504091 |
| ebi-fl187-GCST90094807 | prot-a-2490 | Rap1 GTPase-GDP dissociation stimulator 1 \|\| id:prot-a-2490 | Oranges liking \|\| id:ebi-fl187-GCST90094807 | Weighted median | 7 | -0.150341437 | 0.319118654306549 | 0.637558944708084 | -0.775813999 | 0.475131125431765 | 0.860414149032532 | 0.460328920963293 | 1.60822506286632 | 0.999332001504091 |
| ebi-fl187-GCST90094807 | prot-a-2545 | Receptor-interacting serine/threonine-protein kinase 2 \|\| id:prot-a-2545 | Oranges liking \|\| id:ebi-fl187-GCST90094807 | Weighted median | 7 | -0.14370851 | 0.283034744150471 | 0.611634579343834 | -0.698456609 | 0.411039588321188 | 0.866140182283028 | 0.497352320956361 | 1.5083850698087 | 0.999332001504091 |
| ebi-fl187-GCST90094807 | prot-a-2610 | RUN and FYVE domain-containing protein 1 \|\| id:prot-a-2610 | Oranges liking \|\| id:ebi-fl187-GCST90094807 | Weighted median | 7 | -0.194136578 | 0.292965050116619 | 0.507547550203946 | -0.768348077 | 0.380074919920822 | 0.823545418147603 | 0.463778562557636 | 1.46239414778388 | 0.999332001504091 |
| ebi-fl187-GCST90094807 | prot-a-2627 | Serine--tRNA ligase, mitochondrial \|\| id:prot-a-2627 | Oranges liking \|\| id:ebi-fl187-GCST90094807 | Weighted median | 7 | -0.11926779 | 0.275590775713622 | 0.665180893304556 | -0.659425711 | 0.42089012993785 | 0.887570086132114 | 0.5171482418475 | 1.52331690229138 | 0.999332001504091 |
| ebi-fl187-GCST90094807 | prot-a-264 | BolA-like protein 2 \|\| id:prot-a-264 | Oranges liking \|\| id:ebi-fl187-GCST90094807 | Weighted median | 7 | -0.292184922 | 0.270399359698217 | 0.279889278881603 | -0.822167667 | 0.237797822660777 | 0.74663045455004 | 0.439477979184732 | 1.26845271450398 | 0.999332001504091 |
| ebi-fl187-GCST90094807 | prot-a-2646 | Secretoglobin family 1C member 1 \|\| id:prot-a-2646 | Oranges liking \|\| id:ebi-fl187-GCST90094807 | Weighted median | 7 | -0.158677712 | 0.29453585953887 | 0.590067528315066 | -0.735967996 | 0.418612573097054 | 0.853271314106657 | 0.479041524046895 | 1.51985140938644 | 0.999332001504091 |
| ebi-fl187-GCST90094807 | prot-a-2672 | Semaphorin-3E \|\| id:prot-a-2672 | Oranges liking \|\| id:ebi-fl187-GCST90094807 | Weighted median | 7 | -0.311502369 | 0.29895771326026 | 0.297429619504874 | -0.897459487 | 0.274454749019515 | 0.732345875597471 | 0.407603868416616 | 1.31581303089213 | 0.999332001504091 |
| ebi-fl187-GCST90094807 | prot-a-2701 | Plasma protease C1 inhibitor \|\| id:prot-a-2701 | Oranges liking \|\| id:ebi-fl187-GCST90094807 | Weighted median | 7 | -0.584199138 | 0.308216349460829 | 0.0580369079479057 | -1.188303183 | 0.0199049071641025 | 0.557552205555994 | 0.304737910166856 | 1.02010433080066 | 0.999332001504091 |
| ebi-fl187-GCST90094807 | prot-a-2738 | Signaling threshold-regulating transmembrane adapter 1 \|\| id:prot-a-2738 | Oranges liking \|\| id:ebi-fl187-GCST90094807 | Weighted median | 7 | 0.379139446048237 | 0.300406458233851 | 0.206917050923747 | -0.209657212 | 0.967936104186585 | 1.4610267559466 | 0.810862152078358 | 2.63250563134628 | 0.999332001504091 |
| ebi-fl187-GCST90094807 | prot-a-274 | Serine/threonine-protein kinase BRSK2 \|\| id:prot-a-274 | Oranges liking \|\| id:ebi-fl187-GCST90094807 | Weighted median | 7 | -0.190292797 | 0.327041281556252 | 0.560659837592691 | -0.831293709 | 0.450708114485773 | 0.826717037932678 | 0.435485530088689 | 1.56942312335611 | 0.999332001504091 |
| ebi-fl187-GCST90094807 | prot-a-2749 | Mitochondrial glutamate carrier 2 \|\| id:prot-a-2749 | Oranges liking \|\| id:ebi-fl187-GCST90094807 | Weighted median | 7 | -0.014345851 | 0.282525223648527 | 0.959503026810148 | -0.56809529 | 0.539403587081347 | 0.98575656014336 | 0.566603627364891 | 1.71498371866209 | 0.999332001504091 |
| ebi-fl187-GCST90094807 | prot-a-2760 | Sodium-coupled monocarboxylate transporter 1 \|\| id:prot-a-2760 | Oranges liking \|\| id:ebi-fl187-GCST90094807 | Weighted median | 7 | -0.636689442 | 0.322776345889213 | 0.048547927926107 | -1.26933108 | -0.004047804 | 0.529040948690265 | 0.28101953841758 | 0.995960376873162 | 0.999332001504091 |
| ebi-fl187-GCST90094807 | prot-a-2777 | Small integral membrane protein 10 \|\| id:prot-a-2777 | Oranges liking \|\| id:ebi-fl187-GCST90094807 | Weighted median | 7 | 0.277585487916967 | 0.299007947887733 | 0.353223514579298 | -0.30847009 | 0.863641065776923 | 1.31993895315235 | 0.734569922898868 | 2.37178080089834 | 0.999332001504091 |
| ebi-fl187-GCST90094807 | prot-a-2799 | Superoxide dismutase [Mn], mitochondrial \|\| id:prot-a-2799 | Oranges liking \|\| id:ebi-fl187-GCST90094807 | Weighted median | 7 | 0.125454941507718 | 0.290231011663675 | 0.665553245895736 | -0.443397841 | 0.694307724368522 | 1.13366408661519 | 0.641851801096603 | 2.00232243500027 | 0.999332001504091 |
| ebi-fl187-GCST90094807 | prot-a-2805 | Protein SPACA7 \|\| id:prot-a-2805 | Oranges liking \|\| id:ebi-fl187-GCST90094807 | Weighted median | 7 | -0.162235591 | 0.294302449227254 | 0.581459127691354 | -0.739068392 | 0.414597209443915 | 0.850240871810344 | 0.477558605993902 | 1.51376088928872 | 0.999332001504091 |
| ebi-fl187-GCST90094807 | prot-a-2811 | SPARC-like protein 1 \|\| id:prot-a-2811 | Oranges liking \|\| id:ebi-fl187-GCST90094807 | Weighted median | 7 | -0.34028071 | 0.327000095227381 | 0.298054968348164 | -0.981200897 | 0.300639476700904 | 0.711570549795065 | 0.374860659552082 | 1.35072228689099 | 0.999332001504091 |
| ebi-fl187-GCST90094807 | prot-a-2831 | Sepiapterin reductase \|\| id:prot-a-2831 | Oranges liking \|\| id:ebi-fl187-GCST90094807 | Weighted median | 7 | -0.273072013 | 0.293009012761812 | 0.351358372839076 | -0.847369678 | 0.301225651703711 | 0.76103798079194 | 0.428540650561482 | 1.3515142786315 | 0.999332001504091 |
| ebi-fl187-GCST90094807 | prot-a-2834 | SPRY domain-containing SOCS box protein 1 \|\| id:prot-a-2834 | Oranges liking \|\| id:ebi-fl187-GCST90094807 | Weighted median | 7 | 0.220431636989719 | 0.282969477263738 | 0.435983015979373 | -0.334188538 | 0.775051812426645 | 1.24661469949162 | 0.715918791284882 | 2.17070459374237 | 0.999332001504091 |
| ebi-fl187-GCST90094807 | prot-a-2835 | Spectrin alpha chain, non-erythrocytic 1 \|\| id:prot-a-2835 | Oranges liking \|\| id:ebi-fl187-GCST90094807 | Weighted median | 7 | 0.296048137425718 | 0.301990094879137 | 0.326926212062818 | -0.295852449 | 0.887948723388826 | 1.34453487772209 | 0.743897183040113 | 2.4301396464808 | 0.999332001504091 |
| ebi-fl187-GCST90094807 | prot-a-2859 | Sia-alpha-2,3-Gal-beta-1,4-GlcNAc-R:alpha 2,8-sialyltransferase \|\| id:prot-a-2859 | Oranges liking \|\| id:ebi-fl187-GCST90094807 | Weighted median | 7 | -0.307066895 | 0.308245745627267 | 0.31916482545812 | -0.911228557 | 0.297094766297311 | 0.735601391109069 | 0.402030003907858 | 1.34594284342423 | 0.999332001504091 |
| ebi-fl187-GCST90094807 | prot-a-2861 | Alpha-2,8-sialyltransferase 8F \|\| id:prot-a-2861 | Oranges liking \|\| id:ebi-fl187-GCST90094807 | Weighted median | 7 | -0.161694469 | 0.296748147039855 | 0.58583063285435 | -0.743320837 | 0.419931899614606 | 0.850701080744249 | 0.475532125967471 | 1.52185791297923 | 0.999332001504091 |
| ebi-fl187-GCST90094807 | prot-a-289 | Uncharacterized protein C17orf89 \|\| id:prot-a-289 | Oranges liking \|\| id:ebi-fl187-GCST90094807 | Weighted median | 7 | 0.0696554195630118 | 0.305366655863429 | 0.819565227854057 | -0.528863226 | 0.668174065055333 | 1.07213867958163 | 0.589274461003859 | 1.95067226619127 | 0.999332001504091 |
| ebi-fl187-GCST90094807 | prot-a-2921 | Tachykinin-3 \|\| id:prot-a-2921 | Oranges liking \|\| id:ebi-fl187-GCST90094807 | Weighted median | 7 | -0.115977015 | 0.310914284482145 | 0.709134113174571 | -0.725369012 | 0.493414982826248 | 0.890495691320533 | 0.484145880182242 | 1.63790008078131 | 0.999332001504091 |
| ebi-fl187-GCST90094807 | prot-a-2928 | Tax1-binding protein 1 \|\| id:prot-a-2928 | Oranges liking \|\| id:ebi-fl187-GCST90094807 | Weighted median | 7 | -0.037219124 | 0.301251480125033 | 0.901672886904975 | -0.627672025 | 0.553233776651728 | 0.963464993534527 | 0.533833105473367 | 1.7388670433681 | 0.999332001504091 |
| ebi-fl187-GCST90094807 | prot-a-296 | Uncharacterized protein C1orf185 \|\| id:prot-a-296 | Oranges liking \|\| id:ebi-fl187-GCST90094807 | Weighted median | 7 | 0.0983844793320312 | 0.330491114623637 | 0.765938438212161 | -0.549378105 | 0.74614706399436 | 1.10338693303621 | 0.577308723983909 | 2.10885904441831 | 0.999332001504091 |
| ebi-fl187-GCST90094807 | prot-a-2964 | Transforming growth factor beta-2 \|\| id:prot-a-2964 | Oranges liking \|\| id:ebi-fl187-GCST90094807 | Weighted median | 7 | -0.081216695 | 0.29568077342527 | 0.78356443655782 | -0.660751011 | 0.498317621000884 | 0.921993878413213 | 0.516463319264028 | 1.64594982862057 | 0.999332001504091 |
| ebi-fl187-GCST90094807 | prot-a-2977 | Thymocyte nuclear protein 1 \|\| id:prot-a-2977 | Oranges liking \|\| id:ebi-fl187-GCST90094807 | Weighted median | 7 | -0.023385096 | 0.284056269180803 | 0.93438781694665 | -0.580135383 | 0.533365191691071 | 0.976886216448477 | 0.559822570697063 | 1.70465917209941 | 0.999332001504091 |
| ebi-fl187-GCST90094807 | prot-a-3015 | Transmembrane protein 70, mitochondrial \|\| id:prot-a-3015 | Oranges liking \|\| id:ebi-fl187-GCST90094807 | Weighted median | 7 | -0.202965198 | 0.286834731251181 | 0.479191651562569 | -0.765161272 | 0.359230874827441 | 0.816306649688476 | 0.465258891841818 | 1.4322274286639 | 0.999332001504091 |
| ebi-fl187-GCST90094807 | prot-a-3030 | Tumor necrosis factor alpha-induced protein 3 \|\| id:prot-a-3030 | Oranges liking \|\| id:ebi-fl187-GCST90094807 | Weighted median | 7 | -0.108680347 | 0.29078882404901 | 0.708595379306883 | -0.678626442 | 0.461265748589057 | 0.897017106290687 | 0.50731333847547 | 1.58608029387941 | 0.999332001504091 |
| ebi-fl187-GCST90094807 | prot-a-3032 | Tumor necrosis factor receptor superfamily member 10A \|\| id:prot-a-3032 | Oranges liking \|\| id:ebi-fl187-GCST90094807 | Weighted median | 7 | 0.125375346252312 | 0.281132168632236 | 0.655621915027066 | -0.425643704 | 0.676394396771496 | 1.13357385592369 | 0.653349086 | 1.96677352747469 | 0.999332001504091 |
| ebi-fl187-GCST90094807 | prot-a-3081 | Tumor protein p53-inducible protein 11 \|\| id:prot-a-3081 | Oranges liking \|\| id:ebi-fl187-GCST90094807 | Weighted median | 7 | 0.0286742413956844 | 0.295417860117074 | 0.922676101329372 | -0.550344764 | 0.607693247225149 | 1.02908930517064 | 0.576750932890704 | 1.83619087135014 | 0.999332001504091 |
| ebi-fl187-GCST90094807 | prot-a-315 | Complement C4 \|\| id:prot-a-315 | Oranges liking \|\| id:ebi-fl187-GCST90094807 | Weighted median | 7 | -0.07690816 | 0.304332113 | 0.800491324159691 | -0.673399101 | 0.519582780447099 | 0.925974891005043 | 0.509972181441347 | 1.68132602125164 | 0.999332001504091 |
| ebi-fl187-GCST90094807 | prot-a-3157 | UDP-glucuronosyltransferase 1-8 \|\| id:prot-a-3157 | Oranges liking \|\| id:ebi-fl187-GCST90094807 | Weighted median | 7 | 0.0678281654880546 | 0.318069223461453 | 0.831132207124798 | -0.555587512 | 0.691243843472503 | 1.07018139857987 | 0.573735085626279 | 1.99619694621988 | 0.999332001504091 |
| ebi-fl187-GCST90094807 | prot-a-317 | Glutaredoxin-like protein C5orf63 \|\| id:prot-a-317 | Oranges liking \|\| id:ebi-fl187-GCST90094807 | Weighted median | 7 | -0.258572874 | 0.281995022755847 | 0.359173417107875 | -0.811283118 | 0.294137370723536 | 0.772152759232922 | 0.444287626666499 | 1.34196823815344 | 0.999332001504091 |
| ebi-fl187-GCST90094807 | prot-a-3192 | Guanine nucleotide exchange factor VAV3 \|\| id:prot-a-3192 | Oranges liking \|\| id:ebi-fl187-GCST90094807 | Weighted median | 7 | -0.111075969 | 0.29338532439583 | 0.704984199625757 | -0.686111205 | 0.463959266581473 | 0.894870763699906 | 0.503530393070157 | 1.59035818839495 | 0.999332001504091 |
| ebi-fl187-GCST90094807 | prot-a-3233 | Serine/threonine-protein kinase WNK3 \|\| id:prot-a-3233 | Oranges liking \|\| id:ebi-fl187-GCST90094807 | Weighted median | 7 | 0.143026456803661 | 0.304981488939504 | 0.639092748646359 | -0.454737262 | 0.740790175125089 | 1.15376032607264 | 0.634614683744751 | 2.09759232510077 | 0.999332001504091 |
| ebi-fl187-GCST90094807 | prot-a-3250 | 14-3-3 protein theta \|\| id:prot-a-3250 | Oranges liking \|\| id:ebi-fl187-GCST90094807 | Weighted median | 7 | -0.119751133 | 0.308197004765029 | 0.697606211531464 | -0.723817262 | 0.484314996570869 | 0.887141189618669 | 0.484897736861957 | 1.62306282436224 | 0.999332001504091 |
| ebi-fl187-GCST90094807 | prot-a-3278 | E3 ubiquitin-protein ligase ZNRF3 \|\| id:prot-a-3278 | Oranges liking \|\| id:ebi-fl187-GCST90094807 | Weighted median | 7 | 0.102160942655984 | 0.294743667570221 | 0.728884740000702 | -0.475536646 | 0.679858531093617 | 1.10756171130786 | 0.621551414004714 | 1.97359850965746 | 0.999332001504091 |
| ebi-fl187-GCST90094807 | prot-a-355 | Calpain-3 \|\| id:prot-a-355 | Oranges liking \|\| id:ebi-fl187-GCST90094807 | Weighted median | 7 | 0.0556499220454291 | 0.281561682814286 | 0.843320981182321 | -0.496210976 | 0.607510820361429 | 1.05722750690189 | 0.608833178168731 | 1.83585593136025 | 0.999332001504091 |
| ebi-fl187-GCST90094807 | prot-a-385 | Coiled-coil domain-containing protein 90B, mitochondrial \|\| id:prot-a-385 | Oranges liking \|\| id:ebi-fl187-GCST90094807 | Weighted median | 7 | 0.287854541246427 | 0.3135459609175 | 0.358586559154383 | -0.326695542 | 0.902404624644727 | 1.33356331155766 | 0.721303316054999 | 2.46552465010022 | 0.999332001504091 |
| ebi-fl187-GCST90094807 | prot-a-390 | C-C motif chemokine 14 \|\| id:prot-a-390 | Oranges liking \|\| id:ebi-fl187-GCST90094807 | Weighted median | 7 | 0.367553465723465 | 0.321099105272885 | 0.252344653446025 | -0.261800781 | 0.99690771205832 | 1.44419701150345 | 0.769664340494579 | 2.70988910139094 | 0.999332001504091 |
| ebi-fl187-GCST90094807 | prot-a-40 | Pituitary adenylate cyclase-activating polypeptide 27 \|\| id:prot-a-40 | Oranges liking \|\| id:ebi-fl187-GCST90094807 | Weighted median | 7 | 0.141507719598775 | 0.294469427763675 | 0.630835306134534 | -0.435652359 | 0.718667798015578 | 1.15200939727682 | 0.646842555963792 | 2.05169811289964 | 0.999332001504091 |
| ebi-fl187-GCST90094807 | prot-a-401 | C-C motif chemokine 24 \|\| id:prot-a-401 | Oranges liking \|\| id:ebi-fl187-GCST90094807 | Weighted median | 7 | 0.00102472954201649 | 0.316499488372981 | 0.99741669581739 | -0.619314268 | 0.621363726753059 | 1.00102525475672 | 0.538313449995208 | 1.86146484110638 | 0.999364451047625 |
| ebi-fl187-GCST90094807 | prot-a-412 | Cerebral cavernous malformations 2 protein \|\| id:prot-a-412 | Oranges liking \|\| id:ebi-fl187-GCST90094807 | Weighted median | 7 | -0.224853181 | 0.312633235287396 | 0.472003200120281 | -0.837614322 | 0.387907960264083 | 0.798633464799347 | 0.43274167520917 | 1.47389412121935 | 0.999332001504091 |
| ebi-fl187-GCST90094807 | prot-a-44 | Adhesion G protein-coupled receptor F5 \|\| id:prot-a-44 | Oranges liking \|\| id:ebi-fl187-GCST90094807 | Weighted median | 7 | -0.164112881 | 0.323362214152925 | 0.611789812158409 | -0.79790282 | 0.469677059015456 | 0.848646220673208 | 0.450272276427595 | 1.59947757294076 | 0.999332001504091 |
| ebi-fl187-GCST90094807 | prot-a-48 | Adrenomedullin \|\| id:prot-a-48 | Oranges liking \|\| id:ebi-fl187-GCST90094807 | Weighted median | 7 | -0.227855737 | 0.28550667257856 | 0.424826968908791 | -0.787448816 | 0.331737340891012 | 0.796239119105336 | 0.455004115227091 | 1.39338681470434 | 0.999332001504091 |
| ebi-fl187-GCST90094807 | prot-a-502 | Carcinoembryonic antigen-related cell adhesion molecule 1 \|\| id:prot-a-502 | Oranges liking \|\| id:ebi-fl187-GCST90094807 | Weighted median | 7 | 0.0247335127220436 | 0.298737016539047 | 0.934015658996718 | -0.56079104 | 0.610258065138576 | 1.0250419234923 | 0.570757393473609 | 1.84090641125505 | 0.999332001504091 |
| ebi-fl187-GCST90094807 | prot-a-518 | Complement factor B \|\| id:prot-a-518 | Oranges liking \|\| id:ebi-fl187-GCST90094807 | Weighted median | 7 | -0.211350268 | 0.296151292027397 | 0.475439667276486 | -0.7918068 | 0.369106264722573 | 0.809490478889677 | 0.453025528843162 | 1.44644130119187 | 0.999332001504091 |
| ebi-fl187-GCST90094807 | prot-a-525 | Properdin \|\| id:prot-a-525 | Oranges liking \|\| id:ebi-fl187-GCST90094807 | Weighted median | 7 | -0.286989809 | 0.320699747693397 | 0.370847873759155 | -0.915561314 | 0.341581696513527 | 0.75051937736694 | 0.400291873407662 | 1.40717154961977 | 0.999332001504091 |
| ebi-fl187-GCST90094807 | prot-a-533 | Chondroadherin \|\| id:prot-a-533 | Oranges liking \|\| id:ebi-fl187-GCST90094807 | Weighted median | 7 | 0.159149684055411 | 0.293170980239426 | 0.587228815520036 | -0.415465437 | 0.733764805324686 | 1.17251344011285 | 0.660033005278785 | 2.08290760651367 | 0.999332001504091 |
| ebi-fl187-GCST90094807 | prot-a-535 | Coiled-coil-helix-coiled-coil-helix domain-containing protein 2 \|\| id:prot-a-535 | Oranges liking \|\| id:ebi-fl187-GCST90094807 | Weighted median | 7 | -0.025591944 | 0.324403183959152 | 0.937120664725263 | -0.661422184 | 0.610238296644607 | 0.974732754099761 | 0.516116798994061 | 1.84087001966746 | 0.999332001504091 |
| ebi-fl187-GCST90094807 | prot-a-591 | Caseinolytic peptidase B protein homolog \|\| id:prot-a-591 | Oranges liking \|\| id:ebi-fl187-GCST90094807 | Weighted median | 7 | -0.180730772 | 0.310467480160287 | 0.560482725248193 | -0.789247033 | 0.427785489119045 | 0.834660042306817 | 0.454186654074753 | 1.533857017 | 0.999332001504091 |
| ebi-fl187-GCST90094807 | prot-a-605 | Ciliary neurotrophic factor receptor subunit alpha \|\| id:prot-a-605 | Oranges liking \|\| id:ebi-fl187-GCST90094807 | Weighted median | 7 | 0.0761950988605186 | 0.29151855221042 | 0.793804822679987 | -0.495181263 | 0.647571461192942 | 1.07917309906196 | 0.609460424370843 | 1.91089450794324 | 0.999332001504091 |
| ebi-fl187-GCST90094807 | prot-a-606 | Ciliary neurotrophic factor receptor subunit alpha \|\| id:prot-a-606 | Oranges liking \|\| id:ebi-fl187-GCST90094807 | Weighted median | 7 | 0.0164604759668933 | 0.305205954854426 | 0.956989058446851 | -0.581743196 | 0.614664147481569 | 1.01659669599054 | 0.558923204421686 | 1.84903549203723 | 0.999332001504091 |
| ebi-fl187-GCST90094807 | prot-a-707 | Cancer/testis antigen 55 \|\| id:prot-a-707 | Oranges liking \|\| id:ebi-fl187-GCST90094807 | Weighted median | 7 | -0.037475255 | 0.31542936233285 | 0.905428165041604 | -0.655716805 | 0.580766295013025 | 0.963218252108079 | 0.519069857162405 | 1.78740758761467 | 0.999332001504091 |
| ebi-fl187-GCST90094807 | prot-a-719 | Dipeptidyl peptidase 1 \|\| id:prot-a-719 | Oranges liking \|\| id:ebi-fl187-GCST90094807 | Weighted median | 7 | -0.469782407 | 0.301226195437758 | 0.118862261422209 | -1.06018575 | 0.120620936443174 | 0.625138279439022 | 0.346391462253138 | 1.12819717286909 | 0.999332001504091 |
| ebi-fl187-GCST90094807 | prot-a-752 | Cytochrome b5 \|\| id:prot-a-752 | Oranges liking \|\| id:ebi-fl187-GCST90094807 | Weighted median | 7 | -0.316490296 | 0.289918931258457 | 0.274986523032217 | -0.884731401 | 0.251750809206375 | 0.728702082806618 | 0.412825042647429 | 1.28627546934018 | 0.999332001504091 |
| ebi-fl187-GCST90094807 | prot-a-755 | Cytoglobin \|\| id:prot-a-755 | Oranges liking \|\| id:ebi-fl187-GCST90094807 | Weighted median | 7 | 0.0148772602344725 | 0.284749323947927 | 0.958331988939872 | -0.543231415 | 0.572985935172409 | 1.01498847752224 | 0.580868190368827 | 1.77355487283401 | 0.999332001504091 |
| ebi-fl187-GCST90094807 | prot-a-807 | Desmin \|\| id:prot-a-807 | Oranges liking \|\| id:ebi-fl187-GCST90094807 | Weighted median | 7 | -0.229903681 | 0.307323933078228 | 0.454410348364243 | -0.83225859 | 0.372451227928412 | 0.794610134946651 | 0.435065541234554 | 1.45128769510966 | 0.999332001504091 |
| ebi-fl187-GCST90094807 | prot-a-82 | Ameloblastin \|\| id:prot-a-82 | Oranges liking \|\| id:ebi-fl187-GCST90094807 | Weighted median | 7 | -0.11386346 | 0.291418085107341 | 0.696002727874038 | -0.685042907 | 0.45731598681106 | 0.892379793101941 | 0.504068601135272 | 1.57982801020165 | 0.999332001504091 |
| ebi-fl187-GCST90094807 | prot-a-84 | Muellerian-inhibiting factor \|\| id:prot-a-84 | Oranges liking \|\| id:ebi-fl187-GCST90094807 | Weighted median | 7 | -0.361054672 | 0.310060693548744 | 0.244235819319632 | -0.968773632 | 0.246664286959518 | 0.696940893996691 | 0.379548218687899 | 1.27974941208806 | 0.999332001504091 |
| ebi-fl187-GCST90094807 | prot-a-858 | Inactive dipeptidyl peptidase 10 \|\| id:prot-a-858 | Oranges liking \|\| id:ebi-fl187-GCST90094807 | Weighted median | 7 | 0.108123295298002 | 0.305073642110382 | 0.723026370929821 | -0.489821043 | 0.70606763383435 | 1.1141851107033 | 0.612736037630448 | 2.02600856596203 | 0.999332001504091 |
| ebi-fl187-GCST90094807 | prot-a-863 | Dihydropyrimidinase-related protein 3 \|\| id:prot-a-863 | Oranges liking \|\| id:ebi-fl187-GCST90094807 | Weighted median | 7 | 0.171330411988915 | 0.286732320788462 | 0.550155333391799 | -0.390664937 | 0.7333257607343 | 1.18688284456289 | 0.676606824140185 | 2.08199331791817 | 0.999332001504091 |
| ebi-fl187-GCST90094807 | prot-a-864 | Dihydropyrimidinase-related protein 5 \|\| id:prot-a-864 | Oranges liking \|\| id:ebi-fl187-GCST90094807 | Weighted median | 7 | -0.419756861 | 0.278771755290974 | 0.132134780784779 | -0.966149501 | 0.126635779727914 | 0.657206593179553 | 0.380545510775562 | 1.13500355118737 | 0.999332001504091 |
| ebi-fl187-GCST90094807 | prot-a-884 | Endothelin-converting enzyme 1 \|\| id:prot-a-884 | Oranges liking \|\| id:ebi-fl187-GCST90094807 | Weighted median | 7 | -0.239173754 | 0.310201531020042 | 0.440690931363982 | -0.847168755 | 0.368821246713059 | 0.787278077704851 | 0.428626763072645 | 1.44602909811677 | 0.999332001504091 |
| ebi-fl187-GCST90094807 | prot-a-893 | Early endosome antigen 1 \|\| id:prot-a-893 | Oranges liking \|\| id:ebi-fl187-GCST90094807 | Weighted median | 7 | -0.477047334 | 0.295460699664174 | 0.106399921034829 | -1.056150305 | 0.102055637382883 | 0.620613152520731 | 0.347792129987615 | 1.10744508536014 | 0.999332001504091 |
| ebi-fl187-GCST90094807 | prot-a-897 | Ephrin-A2 \|\| id:prot-a-897 | Oranges liking \|\| id:ebi-fl187-GCST90094807 | Weighted median | 7 | 0.363364460698765 | 0.291527965963625 | 0.212612541603787 | -0.208030353 | 0.93475927398747 | 1.43815991650438 | 0.812182384498939 | 2.54660035099859 | 0.999332001504091 |
| ebi-fl187-GCST90094807 | prot-a-90 | Anaphase-promoting complex subunit 10 \|\| id:prot-a-90 | Oranges liking \|\| id:ebi-fl187-GCST90094807 | Weighted median | 7 | -0.068629753 | 0.293255220847459 | 0.81496371864993 | -0.643409985 | 0.506150480347263 | 0.933672305914944 | 0.525497426785381 | 1.65889294675568 | 0.999332001504091 |
| ebi-fl187-GCST90094807 | prot-a-914 | Histone-lysine N-methyltransferase EHMT2 \|\| id:prot-a-914 | Oranges liking \|\| id:ebi-fl187-GCST90094807 | Weighted median | 7 | -0.202995594 | 0.28508106629414 | 0.476425950000889 | -0.761754484 | 0.355763295886361 | 0.816281837914526 | 0.466846633120536 | 1.42726966767495 | 0.999332001504091 |
| ebi-fl187-GCST90094807 | prot-a-932 | RNA polymerase II elongation factor ELL \|\| id:prot-a-932 | Oranges liking \|\| id:ebi-fl187-GCST90094807 | Weighted median | 7 | -0.126727848 | 0.286393777881006 | 0.658130964861514 | -0.688059652 | 0.434603957126812 | 0.880973399070007 | 0.502550245920218 | 1.5443513084902 | 0.999332001504091 |
| ebi-fl187-GCST90094807 | prot-a-991 | Estrogen receptor \|\| id:prot-a-991 | Oranges liking \|\| id:ebi-fl187-GCST90094807 | Weighted median | 7 | -0.055971905 | 0.277636101209312 | 0.840228131423925 | -0.600138663 | 0.488194853823366 | 0.945565701614043 | 0.548735541547475 | 1.62937230846656 | 0.999332001504091 |
| ebi-fl187-GCST90094807 | prot-a-992 | Persulfide dioxygenase ETHE1, mitochondrial \|\| id:prot-a-992 | Oranges liking \|\| id:ebi-fl187-GCST90094807 | Weighted median | 7 | -0.026849285 | 0.290807010938754 | 0.926438385376931 | -0.596831026 | 0.543132456820307 | 0.973507953089637 | 0.55055356447205 | 1.72139060735641 | 0.999332001504091 |
| ebi-fl187-GCST90094807 | prot-a-993 | Ethanolamine kinase 1 \|\| id:prot-a-993 | Oranges liking \|\| id:ebi-fl187-GCST90094807 | Weighted median | 7 | 0.160345503806979 | 0.316956054845614 | 0.612932532236336 | -0.460888364 | 0.781579371304384 | 1.17391639351602 | 0.630723085065536 | 2.18492034237573 | 0.999332001504091 |
| ebi-fl187-GCST90094807 | prot-b-10 | coagulation factor III, tissue factor \|\| id:prot-b-10 | Oranges liking \|\| id:ebi-fl187-GCST90094807 | Weighted median | 6 | -0.08304477 | 0.360102716047781 | 0.817614599319342 | -0.788846093 | 0.622756553512752 | 0.920309944075996 | 0.454368792053008 | 1.86405934557749 | 0.999332001504091 |
| ebi-fl187-GCST90094807 | prot-b-28 | prolactin \|\| id:prot-b-28 | Oranges liking \|\| id:ebi-fl187-GCST90094807 | Weighted median | 6 | -0.050730058 | 0.373880947796787 | 0.89207026090494 | -0.783536716 | 0.68207659980559 | 0.950535225400961 | 0.456787623209371 | 1.97798094523661 | 0.999332001504091 |
| ebi-fl187-GCST90094807 | prot-b-54 | matrix metallopeptidase 7 \|\| id:prot-b-54 | Oranges liking \|\| id:ebi-fl187-GCST90094807 | Weighted median | 6 | 0.220799306620079 | 0.401137297609099 | 0.582022247794255 | -0.565429797 | 1.00702840993391 | 1.24707312612699 | 0.568115919933303 | 2.73745432462222 | 0.999332001504091 |
| ebi-fl187-GCST90094807 | ukb-rsfMRI191-edge-pheno716 | Net100_Pair10_33 (OccipitalPrecuneus)&(Temporal\|Frontal\|Supp_Motor_Area) (Default_mode\|Central_executive)&(Default_mode\|Salience) \|\| id:ukb-rsfMRI191-edge-pheno716 | Oranges liking \|\| id:ebi-fl187-GCST90094807 | Weighted median | 7 | 0.0894794970582964 | 0.102523751074185 | 0.382789290287132 | -0.111467055 | 0.290426049163699 | 1.09360491096475 | 0.894520860865454 | 1.33699699314907 | 0.999332001504091 |
| ebi-fl187-GCST90094807 | ukb-rsfMRI191-edge-pheno903 | Net100_Pair27_38 (Precentral\|Frontal\|Supp_Motor_Area)&(Subcortical) (Attention\|Salience\|Motor)&(Subcortical-cerebellum) \|\| id:ukb-rsfMRI191-edge-pheno903 | Oranges liking \|\| id:ebi-fl187-GCST90094807 | Weighted median | 7 | -0.16820435 | 0.0991839076487465 | 0.0899079493725085 | -0.362604809 | 0.0261961088235585 | 0.845181104135894 | 0.69586137721041 | 1.02654224272656 | 0.999332001504091 |
| ebi-fl187-GCST90094807 | ukb-rsfMRI191-node-pheno23 | Net100_Node2 RolandicOper\|SupraMarginal\|Insula Salience\|Motor \|\| id:ukb-rsfMRI191-node-pheno23 | Oranges liking \|\| id:ebi-fl187-GCST90094807 | Weighted median | 7 | -0.009926808 | 0.0884362343108064 | 0.910626609850672 | -0.183261828 | 0.16340821083205 | 0.990122299715537 | 0.832550142536485 | 1.17751726689666 | 0.999332001504091 |
| ebi-fl187-GCST90094807 | ukb-rsfMRI191-node-pheno55 | Net100_Node34 Parietal\|Postcentral\|Precuneus Attention \|\| id:ukb-rsfMRI191-node-pheno55 | Oranges liking \|\| id:ebi-fl187-GCST90094807 | Weighted median | 7 | 0.0648747028776332 | 0.0875422082510026 | 0.458652393147877 | -0.106708025 | 0.236457431049598 | 1.06702532080584 | 0.89878805806225 | 1.26675363009992 | 0.999332001504091 |
| ebi-fl187-GCST90094807 | ebi-a-GCST90001414 | IgD- CD24- B cell Absolute Count \|\| id:ebi-a-GCST90001414 | Oranges liking \|\| id:ebi-fl187-GCST90094807 | Weighted mode | 7 | -0.16625725 | 0.442704159648819 | 0.720170790983297 | -1.033957403 | 0.701442902930307 | 0.846828359586749 | 0.355596932056214 | 2.01666045444683 | 0.999714303421225 |
| ebi-fl187-GCST90094807 | ebi-a-GCST90001422 | CD20- CD38- B cell %B cell \|\| id:ebi-a-GCST90001422 | Oranges liking \|\| id:ebi-fl187-GCST90094807 | Weighted mode | 7 | -0.501923331 | 0.388067304058594 | 0.243435515325308 | -1.262535247 | 0.258688585351134 | 0.605365221853136 | 0.282935804424984 | 1.29523038829983 | 0.999714303421225 |
| ebi-fl187-GCST90094807 | ebi-a-GCST90001444 | CD20- CD38- B cell %lymphocyte \|\| id:ebi-a-GCST90001444 | Oranges liking \|\| id:ebi-fl187-GCST90094807 | Weighted mode | 7 | -0.235748787 | 0.359712636507467 | 0.536526198592607 | -0.940785555 | 0.469287980131744 | 0.789979101693982 | 0.390321096214099 | 1.59885537104281 | 0.999714303421225 |
| ebi-fl187-GCST90094807 | ebi-a-GCST90001459 | Myeloid Dendritic Cell %Dendritic Cell \|\| id:ebi-a-GCST90001459 | Oranges liking \|\| id:ebi-fl187-GCST90094807 | Weighted mode | 7 | 0.176271975573691 | 0.437819817849618 | 0.701185120545211 | -0.681854867 | 1.03439881855894 | 1.19276241678251 | 0.505678155993979 | 2.81341435461529 | 0.999714303421225 |
| ebi-fl187-GCST90094807 | ebi-a-GCST90001476 | HLA DR++ monocyte %leukocyte \|\| id:ebi-a-GCST90001476 | Oranges liking \|\| id:ebi-fl187-GCST90094807 | Weighted mode | 7 | 0.132669445458905 | 0.408931434090438 | 0.756625247410098 | -0.668836165 | 0.934175056276163 | 1.14187248480472 | 0.51230446864775 | 2.54511301647581 | 0.999714303421225 |
| ebi-fl187-GCST90094807 | ebi-a-GCST90001478 | CD4 regulatory T cell %CD4+ T cell \|\| id:ebi-a-GCST90001478 | Oranges liking \|\| id:ebi-fl187-GCST90094807 | Weighted mode | 7 | 0.289020606404768 | 0.600957879287399 | 0.647598788881688 | -0.888856837 | 1.46689804980807 | 1.33511924025253 | 0.411125467644421 | 4.33576493304032 | 0.999714303421225 |
| ebi-fl187-GCST90094807 | ebi-a-GCST90001485 | CD39+ resting CD4 regulatory T cell %CD4 regulatory T cell \|\| id:ebi-a-GCST90001485 | Oranges liking \|\| id:ebi-fl187-GCST90094807 | Weighted mode | 7 | 0.64604144012645 | 0.453943421113503 | 0.204538585714141 | -0.243687665 | 1.53577054550892 | 1.90797303461803 | 0.783732382892285 | 4.64490326072165 | 0.999714303421225 |
| ebi-fl187-GCST90094807 | ebi-a-GCST90001515 | Immature Myeloid-Derived Suppressor Cells Absolute Count \|\| id:ebi-a-GCST90001515 | Oranges liking \|\| id:ebi-fl187-GCST90094807 | Weighted mode | 7 | 0.149663801278669 | 0.482101179604347 | 0.766726856447208 | -0.795254511 | 1.09458211330319 | 1.16144370119494 | 0.451466317270188 | 2.98793380467871 | 0.999714303421225 |
| ebi-fl187-GCST90094807 | ebi-a-GCST90001517 | CD33+ HLA DR+ Absolute Count \|\| id:ebi-a-GCST90001517 | Oranges liking \|\| id:ebi-fl187-GCST90094807 | Weighted mode | 7 | 0.0109286488159159 | 0.458649647587599 | 0.98176259213557 | -0.88802466 | 0.90988195808761 | 1.01098858463858 | 0.411467739010073 | 2.48402929650944 | 0.999714303421225 |
| ebi-fl187-GCST90094807 | ebi-a-GCST90001518 | CD33+ HLA DR+ CD14- Absolute Count \|\| id:ebi-a-GCST90001518 | Oranges liking \|\| id:ebi-fl187-GCST90094807 | Weighted mode | 7 | -0.170166778 | 0.462550775596864 | 0.72558622446601 | -1.076766299 | 0.736432741817504 | 0.843524123300962 | 0.340695453659014 | 2.08847209127362 | 0.999714303421225 |
| ebi-fl187-GCST90094807 | ebi-a-GCST90001520 | CD33+ HLA DR+ CD14dim Absolute Count \|\| id:ebi-a-GCST90001520 | Oranges liking \|\| id:ebi-fl187-GCST90094807 | Weighted mode | 7 | 0.0933646734833316 | 0.47248085699738 | 0.849878485461904 | -0.832697806 | 1.0194271531982 | 1.09786202344471 | 0.434874495231529 | 2.77160660314284 | 0.999714303421225 |
| ebi-fl187-GCST90094807 | ebi-a-GCST90001531 | CD33dim HLA DR- Absolute Count \|\| id:ebi-a-GCST90001531 | Oranges liking \|\| id:ebi-fl187-GCST90094807 | Weighted mode | 7 | -0.611760926 | 0.62546311190943 | 0.365801865935183 | -1.837668625 | 0.614146773310139 | 0.542394910317804 | 0.159188120970577 | 1.84807909626017 | 0.999714303421225 |
| ebi-fl187-GCST90094807 | ebi-a-GCST90001532 | Basophil Absolute Count \|\| id:ebi-a-GCST90001532 | Oranges liking \|\| id:ebi-fl187-GCST90094807 | Weighted mode | 7 | -0.613831606 | 0.684514611607064 | 0.404399651372661 | -1.955480245 | 0.727817032427255 | 0.541272945883189 | 0.141496507374041 | 2.07055571464101 | 0.999714303421225 |
| ebi-fl187-GCST90094807 | ebi-a-GCST90001588 | T/B cell \|\| id:ebi-a-GCST90001588 | Oranges liking \|\| id:ebi-fl187-GCST90094807 | Weighted mode | 7 | -0.143571083 | 0.559418157336651 | 0.806027429108066 | -1.240030671 | 0.952888505817578 | 0.86625922207362 | 0.28937534238857 | 2.59318929399298 | 0.999714303421225 |
| ebi-fl187-GCST90094807 | ebi-a-GCST90001624 | HLA DR+ CD4+ T cell Absolute Count \|\| id:ebi-a-GCST90001624 | Oranges liking \|\| id:ebi-fl187-GCST90094807 | Weighted mode | 7 | 0.233115584789414 | 0.36894594391886 | 0.550789731113361 | -0.490018465 | 0.95624963487038 | 1.26252739985764 | 0.612615081963877 | 2.60192000216751 | 0.999714303421225 |
| ebi-fl187-GCST90094807 | ebi-a-GCST90001631 | CD8+ Natural Killer T %T cell \|\| id:ebi-a-GCST90001631 | Oranges liking \|\| id:ebi-fl187-GCST90094807 | Weighted mode | 7 | 0.169399574161421 | 0.416401990330551 | 0.698256358510247 | -0.646748327 | 0.985547475209301 | 1.18459337727611 | 0.52374606186488 | 2.67927832142527 | 0.999714303421225 |
| ebi-fl187-GCST90094807 | ebi-a-GCST90001632 | CD8+ Natural Killer T %lymphocyte \|\| id:ebi-a-GCST90001632 | Oranges liking \|\| id:ebi-fl187-GCST90094807 | Weighted mode | 7 | 0.149299134034892 | 0.424395651887187 | 0.737019482071107 | -0.682516344 | 0.981114611733778 | 1.16102023793791 | 0.505343772507974 | 2.66742773184193 | 0.999714303421225 |
| ebi-fl187-GCST90094807 | ebi-a-GCST90001646 | Natural Killer %CD3- lymphocyte \|\| id:ebi-a-GCST90001646 | Oranges liking \|\| id:ebi-fl187-GCST90094807 | Weighted mode | 7 | 0.338022204673827 | 0.416888254584247 | 0.448426871925467 | -0.479078774 | 1.15512318365895 | 1.40217163782352 | 0.619353693610419 | 3.17441442942841 | 0.999714303421225 |
| ebi-fl187-GCST90094807 | ebi-a-GCST90001684 | CD127- CD8+ T cell Absolute Count \|\| id:ebi-a-GCST90001684 | Oranges liking \|\| id:ebi-fl187-GCST90094807 | Weighted mode | 7 | -0.376760352 | 0.417374331682481 | 0.401474155769645 | -1.194814042 | 0.441293337873032 | 0.686080471855236 | 0.30276024954061 | 1.55471669274723 | 0.999714303421225 |
| ebi-fl187-GCST90094807 | ebi-a-GCST90001687 | CD28- CD8+ T cell Absolute Count \|\| id:ebi-a-GCST90001687 | Oranges liking \|\| id:ebi-fl187-GCST90094807 | Weighted mode | 7 | 0.265460570728564 | 0.419998312800461 | 0.550661803945017 | -0.557736122 | 1.08865726381747 | 1.30403143619915 | 0.572503676145025 | 2.97028308716894 | 0.999714303421225 |
| ebi-fl187-GCST90094807 | ebi-a-GCST90001701 | BAFF-R on CD20- CD38- B cell \|\| id:ebi-a-GCST90001701 | Oranges liking \|\| id:ebi-fl187-GCST90094807 | Weighted mode | 7 | -0.321552837 | 0.388298973507621 | 0.439300090685836 | -1.082618825 | 0.439513151331633 | 0.725022321197119 | 0.33870734795272 | 1.55195146905238 | 0.999714303421225 |
| ebi-fl187-GCST90094807 | ebi-a-GCST90001732 | CD19 on IgD- CD27- B cell \|\| id:ebi-a-GCST90001732 | Oranges liking \|\| id:ebi-fl187-GCST90094807 | Weighted mode | 7 | 0.299286957321745 | 0.349455092102177 | 0.424633028104708 | -0.385645023 | 0.984218937842011 | 1.3488966437093 | 0.680011871280777 | 2.67572116348971 | 0.999714303421225 |
| ebi-fl187-GCST90094807 | ebi-a-GCST90001798 | CD27 on CD24+ CD27+ B cell \|\| id:ebi-a-GCST90001798 | Oranges liking \|\| id:ebi-fl187-GCST90094807 | Weighted mode | 7 | 0.142948202295956 | 0.402135349722719 | 0.734398403191909 | -0.645237083 | 0.931133487752485 | 1.1536700426589 | 0.524538168196939 | 2.53738364150629 | 0.999714303421225 |
| ebi-fl187-GCST90094807 | ebi-a-GCST90001836 | CD66b on CD66b++ myeloid cell \|\| id:ebi-a-GCST90001836 | Oranges liking \|\| id:ebi-fl187-GCST90094807 | Weighted mode | 7 | -0.556318094 | 0.578225280421267 | 0.373147427520911 | -1.689639644 | 0.577003455183606 | 0.573316078209379 | 0.184586028680034 | 1.78069449721542 | 0.999714303421225 |
| ebi-fl187-GCST90094807 | ebi-a-GCST90001943 | CD25 on activated & secreting CD4 regulatory T cell \|\| id:ebi-a-GCST90001943 | Oranges liking \|\| id:ebi-fl187-GCST90094807 | Weighted mode | 7 | -0.321775846 | 0.452613193854036 | 0.503813717842194 | -1.208897706 | 0.565346014376566 | 0.72486065284202 | 0.298526161870944 | 1.76005668228738 | 0.999714303421225 |
| ebi-fl187-GCST90094807 | ebi-a-GCST90001960 | CD25 on CD4+ T cell \|\| id:ebi-a-GCST90001960 | Oranges liking \|\| id:ebi-fl187-GCST90094807 | Weighted mode | 7 | -0.603236744 | 0.538035096171846 | 0.305067061299929 | -1.657785532 | 0.451312044939775 | 0.54703814528268 | 0.190560503330849 | 1.57037123204255 | 0.999714303421225 |
| ebi-fl187-GCST90094807 | ebi-a-GCST90001988 | HLA DR on CD14+ CD16- monocyte \|\| id:ebi-a-GCST90001988 | Oranges liking \|\| id:ebi-fl187-GCST90094807 | Weighted mode | 7 | -0.308598101 | 0.389600431821225 | 0.458471841919792 | -1.072214947 | 0.455018745484581 | 0.73447589592942 | 0.342249612422273 | 1.57620292944477 | 0.999714303421225 |
| ebi-fl187-GCST90094807 | ebi-a-GCST90001991 | HLA DR on CD14+ monocyte \|\| id:ebi-a-GCST90001991 | Oranges liking \|\| id:ebi-fl187-GCST90094807 | Weighted mode | 7 | -0.282578844 | 0.359320999818679 | 0.461540282607613 | -0.986848004 | 0.421690315564867 | 0.753837204015559 | 0.372749747131307 | 1.52453632639974 | 0.999714303421225 |
| ebi-fl187-GCST90094807 | ebi-a-GCST90002019 | CD14 on Monocytic Myeloid-Derived Suppressor Cells \|\| id:ebi-a-GCST90002019 | Oranges liking \|\| id:ebi-fl187-GCST90094807 | Weighted mode | 7 | 0.203045036339858 | 0.507105464924984 | 0.702729002485811 | -0.790881675 | 1.19697174759283 | 1.22512764226994 | 0.453444828058353 | 3.31007797857328 | 0.999714303421225 |
| ebi-fl187-GCST90094807 | ebi-a-GCST90002037 | CD80 on plasmacytoid Dendritic Cell \|\| id:ebi-a-GCST90002037 | Oranges liking \|\| id:ebi-fl187-GCST90094807 | Weighted mode | 7 | 0.0116023090993416 | 0.470603028954877 | 0.981130341711704 | -0.910779628 | 0.9339842458509 | 1.01166987694907 | 0.402210527317099 | 2.54462742870787 | 0.999714303421225 |
| ebi-fl187-GCST90094807 | ebi-a-GCST90002042 | CD45 on CD33+ HLA DR+ CD14- \|\| id:ebi-a-GCST90002042 | Oranges liking \|\| id:ebi-fl187-GCST90094807 | Weighted mode | 7 | 0.460937984762199 | 0.594772368021694 | 0.467783089073043 | -0.704815857 | 1.62669182608472 | 1.58556051931888 | 0.49419956948641 | 5.08701811099393 | 0.999714303421225 |
| ebi-fl187-GCST90094807 | ebi-a-GCST90002056 | CD8 on Effector Memory CD8+ T cell \|\| id:ebi-a-GCST90002056 | Oranges liking \|\| id:ebi-fl187-GCST90094807 | Weighted mode | 7 | 0.263514537724101 | 0.416041767284708 | 0.549847712183432 | -0.551927326 | 1.07895640160213 | 1.30149621559701 | 0.575838910798224 | 2.94160809116786 | 0.999714303421225 |
| ebi-fl187-GCST90094807 | ebi-a-GCST90002058 | CD8 on CD8+ T cell \|\| id:ebi-a-GCST90002058 | Oranges liking \|\| id:ebi-fl187-GCST90094807 | Weighted mode | 7 | 0.155612047605946 | 0.45509518949981 | 0.744059832733886 | -0.736374524 | 1.04759861902557 | 1.16837284211307 | 0.478846820063875 | 2.85079704195443 | 0.999714303421225 |
| ebi-fl187-GCST90094807 | ebi-a-GCST90002063 | CD4 on CD4 regulatory T cell \|\| id:ebi-a-GCST90002063 | Oranges liking \|\| id:ebi-fl187-GCST90094807 | Weighted mode | 7 | -0.040202085 | 0.507068364766132 | 0.939385388965801 | -1.03405608 | 0.953651909507226 | 0.960595297218443 | 0.355561844377226 | 2.59516970009646 | 0.999714303421225 |
| ebi-fl187-GCST90094807 | ebi-a-GCST90002066 | CD4 on activated CD4 regulatory T cell \|\| id:ebi-a-GCST90002066 | Oranges liking \|\| id:ebi-fl187-GCST90094807 | Weighted mode | 7 | -0.048068213 | 0.466194695619584 | 0.921237453039965 | -0.961809816 | 0.865673390452143 | 0.953068773215895 | 0.382200546851898 | 2.37660593099892 | 0.999714303421225 |
| ebi-fl187-GCST90094807 | ebi-a-GCST90002070 | CD4 on activated & secreting CD4 regulatory T cell \|\| id:ebi-a-GCST90002070 | Oranges liking \|\| id:ebi-fl187-GCST90094807 | Weighted mode | 7 | -0.005681577 | 0.517849921839486 | 0.991601911740295 | -1.020667423 | 1.00930427018816 | 0.994334533015382 | 0.360354350960294 | 2.74369148287558 | 0.999714303421225 |
| ebi-fl187-GCST90094807 | ebi-a-GCST90002081 | SSC-A on CD4+ T cell \|\| id:ebi-a-GCST90002081 | Oranges liking \|\| id:ebi-fl187-GCST90094807 | Weighted mode | 7 | 0.214987277860784 | 0.389298063023169 | 0.600731780037082 | -0.548036926 | 0.978011481386195 | 1.23984612337074 | 0.578083518158288 | 2.65916318551143 | 0.999714303421225 |
| ebi-fl187-GCST90094807 | ebi-a-GCST90002094 | CD11b on Monocytic Myeloid-Derived Suppressor Cells \|\| id:ebi-a-GCST90002094 | Oranges liking \|\| id:ebi-fl187-GCST90094807 | Weighted mode | 7 | -0.000279489 | 0.544775636570082 | 0.999607288942123 | -1.068039737 | 1.06748075837964 | 0.999720549755778 | 0.343681563791072 | 2.90804419817982 | 0.999714303421225 |
| ebi-fl187-GCST90094807 | ebi-a-GCST90002104 | HLA DR on myeloid Dendritic Cell \|\| id:ebi-a-GCST90002104 | Oranges liking \|\| id:ebi-fl187-GCST90094807 | Weighted mode | 7 | -0.308997384 | 0.438781528064171 | 0.507694861902629 | -1.169009179 | 0.551014410699134 | 0.73418269042054 | 0.310674611649553 | 1.73501214035852 | 0.999714303421225 |
| ebi-fl187-GCST90094807 | ebi-a-GCST90002105 | HLA DR on plasmacytoid Dendritic Cell \|\| id:ebi-a-GCST90002105 | Oranges liking \|\| id:ebi-fl187-GCST90094807 | Weighted mode | 7 | 0.383338207171277 | 0.401116906050157 | 0.376138433722402 | -0.402850929 | 1.16952734302958 | 1.4671741549879 | 0.66841173291175 | 3.22047010109658 | 0.999714303421225 |
| ebi-fl187-GCST90094807 | ebi-a-GCST90002106 | HLA DR on Dendritic Cell \|\| id:ebi-a-GCST90002106 | Oranges liking \|\| id:ebi-fl187-GCST90094807 | Weighted mode | 7 | -0.249576151 | 0.474031404465686 | 0.61742970048075 | -1.178677704 | 0.679525401667494 | 0.779130946902964 | 0.307685320849682 | 1.97294115541664 | 0.999714303421225 |
| ebi-fl187-GCST90094807 | ebi-a-GCST90002111 | HLA DR on CD33dim HLA DR+ CD11b- \|\| id:ebi-a-GCST90002111 | Oranges liking \|\| id:ebi-fl187-GCST90094807 | Weighted mode | 7 | 0.355280056425213 | 0.509526692610952 | 0.51172996865317 | -0.643392261 | 1.35395237394268 | 1.42658012127126 | 0.525506740932782 | 3.87270168750632 | 0.999714303421225 |
| ebi-fl187-GCST90094807 | ebi-a-GCST90002112 | HLA DR on CD33- HLA DR+ \|\| id:ebi-a-GCST90002112 | Oranges liking \|\| id:ebi-fl187-GCST90094807 | Weighted mode | 7 | -0.156047906 | 0.472808321899445 | 0.752585070116233 | -1.082752217 | 0.770656405273984 | 0.855518205234524 | 0.338662170198856 | 2.16118440113325 | 0.999714303421225 |
| ebi-fl187-GCST90094807 | ebi-a-GCST90002115 | HLA DR on HLA DR+ CD8+ T cell \|\| id:ebi-a-GCST90002115 | Oranges liking \|\| id:ebi-fl187-GCST90094807 | Weighted mode | 7 | -0.436681438 | 0.619964026639913 | 0.50760858849238 | -1.65181093 | 0.77844805451005 | 0.646177246333546 | 0.191702434416083 | 2.17808936517175 | 0.999714303421225 |
| ebi-fl187-GCST90094807 | ebi-a-GCST90002116 | HLA DR on B cell \|\| id:ebi-a-GCST90002116 | Oranges liking \|\| id:ebi-fl187-GCST90094807 | Weighted mode | 7 | 0.2192561373406 | 0.417187716333676 | 0.618043920633027 | -0.598431787 | 1.03694406135461 | 1.24515016529814 | 0.549672965012763 | 2.82058429798521 | 0.999714303421225 |
| ebi-fl187-GCST90094807 | ebi-cfmet338-GCST90026023 | Sphingomyelin (d18:1/24:1, d18:2/24:0) levels \|\| id:ebi-cfmet338-GCST90026023 | Oranges liking \|\| id:ebi-fl187-GCST90094807 | Weighted mode | 7 | 0.0952619620933845 | 0.260470450867896 | 0.727114292228851 | -0.415260122 | 0.605784045794461 | 1.09994696179667 | 0.660168534267991 | 1.83268855748671 | 0.999714303421225 |
| ebi-fl187-GCST90094807 | ebi-cfmet338-GCST90026046 | 1-arachidonoyl-gpc (20:4n6) levels \|\| id:ebi-cfmet338-GCST90026046 | Oranges liking \|\| id:ebi-fl187-GCST90094807 | Weighted mode | 7 | -0.485728236 | 0.280890451318575 | 0.134495287358112 | -1.036273521 | 0.0648170485013005 | 0.615248987070378 | 0.354774280727774 | 1.06696380389982 | 0.999714303421225 |
| ebi-fl187-GCST90094807 | ebi-cfmet338-GCST90026047 | 1-linoleoyl-gpc (18:2) levels \|\| id:ebi-cfmet338-GCST90026047 | Oranges liking \|\| id:ebi-fl187-GCST90094807 | Weighted mode | 7 | -0.199255395 | 0.359076381999855 | 0.599016581394424 | -0.903045104 | 0.504534313818933 | 0.819340611195164 | 0.405333496076173 | 1.6562140648438 | 0.999714303421225 |
| ebi-fl187-GCST90094807 | ebi-cfmet338-GCST90026050 | 1-myristoyl-2-palmitoyl-gpc (14:0/16:0) levels \|\| id:ebi-cfmet338-GCST90026050 | Oranges liking \|\| id:ebi-fl187-GCST90094807 | Weighted mode | 7 | -0.266611292 | 0.174795043936118 | 0.178034061454143 | -0.609209578 | 0.0759869943111977 | 0.765970752715844 | 0.543780515378076 | 1.07894854159707 | 0.999714303421225 |
| ebi-fl187-GCST90094807 | ebi-cfmet338-GCST90026068 | 2,3-dihydroxyisovalerate levels \|\| id:ebi-cfmet338-GCST90026068 | Oranges liking \|\| id:ebi-fl187-GCST90094807 | Weighted mode | 7 | -0.182387347 | 0.601841086639586 | 0.772084980947422 | -1.361995877 | 0.997221182750986 | 0.833278509912903 | 0.256149024511123 | 2.71073870536062 | 0.999714303421225 |
| ebi-fl187-GCST90094807 | ebi-cfmet338-GCST90026082 | 3-hydroxyhexanoate levels \|\| id:ebi-cfmet338-GCST90026082 | Oranges liking \|\| id:ebi-fl187-GCST90094807 | Weighted mode | 7 | -0.168024806 | 0.180307032624365 | 0.387359238147725 | -0.52142659 | 0.185376977617833 | 0.845332864822009 | 0.593673015425915 | 1.2036721120557 | 0.999714303421225 |
| ebi-fl187-GCST90094807 | ebi-cfmet338-GCST90026094 | 3-ureidopropionate levels \|\| id:ebi-cfmet338-GCST90026094 | Oranges liking \|\| id:ebi-fl187-GCST90094807 | Weighted mode | 7 | -0.319366163 | 0.271526326271626 | 0.284068018936443 | -0.851557762 | 0.212825436846226 | 0.726609443353778 | 0.42674963946854 | 1.23716866832833 | 0.999714303421225 |
| ebi-fl187-GCST90094807 | ebi-cfmet338-GCST90026173 | Glycerol levels \|\| id:ebi-cfmet338-GCST90026173 | Oranges liking \|\| id:ebi-fl187-GCST90094807 | Weighted mode | 7 | -0.101143359 | 0.151579614454233 | 0.529416564001606 | -0.398239404 | 0.195952684852072 | 0.903803454806266 | 0.671501248460894 | 1.21646934654554 | 0.999714303421225 |
| ebi-fl187-GCST90094807 | ebi-cfmet338-GCST90026179 | Histidine levels \|\| id:ebi-cfmet338-GCST90026179 | Oranges liking \|\| id:ebi-fl187-GCST90094807 | Weighted mode | 7 | 0.147319845558812 | 0.116696500755999 | 0.253645424327627 | -0.081405296 | 0.37604498704057 | 1.15872451665727 | 0.92182000583304 | 1.45651265649121 | 0.999714303421225 |
| ebi-fl187-GCST90094807 | ebi-cfmet338-GCST90026188 | Isocitrate levels \|\| id:ebi-cfmet338-GCST90026188 | Oranges liking \|\| id:ebi-fl187-GCST90094807 | Weighted mode | 7 | 0.0965201737737396 | 0.25481735576067 | 0.717891284538332 | -0.402921844 | 0.595962191064653 | 1.10133179893821 | 0.668364334287898 | 1.81477626666716 | 0.999714303421225 |
| ebi-fl187-GCST90094807 | ebi-cfmet338-GCST90026207 | N-acetyl-isoputreanine levels \|\| id:ebi-cfmet338-GCST90026207 | Oranges liking \|\| id:ebi-fl187-GCST90094807 | Weighted mode | 7 | -0.245792322 | 0.152897757312399 | 0.159055773618443 | -0.545471926 | 0.0538872826269746 | 0.782084630063144 | 0.579568205463923 | 1.05536563740828 | 0.999714303421225 |
| ebi-fl187-GCST90094807 | ebi-cfmet338-GCST90026213 | N-acetylglutamate levels \|\| id:ebi-cfmet338-GCST90026213 | Oranges liking \|\| id:ebi-fl187-GCST90094807 | Weighted mode | 7 | 0.126651052783353 | 0.319003528350591 | 0.705089403271072 | -0.498595863 | 0.751897968350512 | 1.13502088629191 | 0.607382910182976 | 2.12102183107321 | 0.999714303421225 |
| ebi-fl187-GCST90094807 | ebi-cfmet338-GCST90026224 | N-acetyltaurine levels \|\| id:ebi-cfmet338-GCST90026224 | Oranges liking \|\| id:ebi-fl187-GCST90094807 | Weighted mode | 7 | -0.432614654 | 0.284596150179878 | 0.179297500586417 | -0.990423108 | 0.125193800361707 | 0.648810460161244 | 0.37141950707912 | 1.133368079 | 0.999714303421225 |
| ebi-fl187-GCST90094807 | ebi-cfmet338-GCST90026250 | Phenyllactate (pla) levels \|\| id:ebi-cfmet338-GCST90026250 | Oranges liking \|\| id:ebi-fl187-GCST90094807 | Weighted mode | 7 | 0.144204233669795 | 0.311859260501641 | 0.660086785635642 | -0.467039917 | 0.755448384253012 | 1.15511999883214 | 0.626855067793499 | 2.12856572476736 | 0.999714303421225 |
| ebi-fl187-GCST90094807 | ebi-cfmet338-GCST90026251 | Pipecolate levels \|\| id:ebi-cfmet338-GCST90026251 | Oranges liking \|\| id:ebi-fl187-GCST90094807 | Weighted mode | 7 | 0.125142431714784 | 0.383196432403552 | 0.755080475325913 | -0.625922576 | 0.876207439225746 | 1.13330986083862 | 0.534767837003214 | 2.40177353947025 | 0.999714303421225 |
| ebi-fl187-GCST90094807 | ebi-cfmet338-GCST90026278 | Trigonelline (n'-methylnicotinate) levels \|\| id:ebi-cfmet338-GCST90026278 | Oranges liking \|\| id:ebi-fl187-GCST90094807 | Weighted mode | 7 | 0.773594641437641 | 0.475789846948682 | 0.155092376137881 | -0.158953459 | 1.70614274145706 | 2.16754380944354 | 0.853036059553271 | 5.50767592206767 | 0.999714303421225 |
| ebi-fl187-GCST90094807 | ebi-cfmet338-GCST90026299 | 1-carboxyethylvaline levels \|\| id:ebi-cfmet338-GCST90026299 | Oranges liking \|\| id:ebi-fl187-GCST90094807 | Weighted mode | 7 | -0.14125073 | 0.302343330350792 | 0.656850629935167 | -0.733843657 | 0.451342197719767 | 0.868271582870911 | 0.480060252337226 | 1.57041858381471 | 0.999714303421225 |
| ebi-fl187-GCST90094807 | ebi-gm430-GCST90011306 | C_Erysipelotrichia abundance \|\| id:ebi-gm430-GCST90011306 | Oranges liking \|\| id:ebi-fl187-GCST90094807 | Weighted mode | 5 | -0.301676941 | 0.254194283335504 | 0.300975803908606 | -0.799897737 | 0.196543853946496 | 0.739576953001641 | 0.449374916316563 | 1.21718869823583 | 0.999714303421225 |
| ebi-fl187-GCST90094807 | ebi-gm430-GCST90011312 | F_Erysipelotrichaceae abundance \|\| id:ebi-gm430-GCST90011312 | Oranges liking \|\| id:ebi-fl187-GCST90094807 | Weighted mode | 5 | -0.301676941 | 0.256660008499912 | 0.305023423301465 | -0.804730558 | 0.201376675268735 | 0.739576953001641 | 0.447208407032218 | 1.22308539108431 | 0.999714303421225 |
| ebi-fl187-GCST90094807 | ebi-gm430-GCST90011352 | O_Erysipelotrichales abundance \|\| id:ebi-gm430-GCST90011352 | Oranges liking \|\| id:ebi-fl187-GCST90094807 | Weighted mode | 5 | -0.301676941 | 0.250197668262467 | 0.294356883581681 | -0.792064371 | 0.188710488403345 | 0.739576953001641 | 0.452908857558093 | 1.2076912612402 | 0.999714303421225 |
| ebi-fl187-GCST90094807 | ebi-gm430-GCST90011362 | OTU97_11 (Parabacteroides) abundance \|\| id:ebi-gm430-GCST90011362 | Oranges liking \|\| id:ebi-fl187-GCST90094807 | Weighted mode | 5 | 0.292580590542715 | 0.339691140752872 | 0.437635559144053 | -0.373214045 | 0.958375226418345 | 1.33988071392677 | 0.688517843039538 | 2.60745650341818 | 0.999714303421225 |
| ebi-fl187-GCST90094807 | ebi-gm430-GCST90011384 | OTU97_235 (Phascolarctobacterium) abundance \|\| id:ebi-gm430-GCST90011384 | Oranges liking \|\| id:ebi-fl187-GCST90094807 | Weighted mode | 5 | 0.185789270871546 | 0.96480605565408 | 0.856680150624355 | -1.705230598 | 2.07680913995354 | 1.20416848026478 | 0.181730474620956 | 7.97896848003928 | 0.999714303421225 |
| ebi-fl187-GCST90094807 | ebi-gm430-GCST90011390 | OTU97_3 (Bacteroides) abundance \|\| id:ebi-gm430-GCST90011390 | Oranges liking \|\| id:ebi-fl187-GCST90094807 | Weighted mode | 5 | -0.216930589 | 0.341387599065727 | 0.559677438785777 | -0.886050283 | 0.452189105118341 | 0.804985842197537 | 0.41228093406025 | 1.57174914628427 | 0.999714303421225 |
| ebi-fl187-GCST90094807 | ebi-gm430-GCST90011398 | OTU97_4 (Alistipes) abundance \|\| id:ebi-gm430-GCST90011398 | Oranges liking \|\| id:ebi-fl187-GCST90094807 | Weighted mode | 5 | 0.462638216939806 | 0.400599052173662 | 0.312439560609955 | -0.322535925 | 1.24781235920018 | 1.58825863339359 | 0.724309910273783 | 3.48271568670895 | 0.999714303421225 |
| ebi-fl187-GCST90094807 | ebi-gm430-GCST90011421 | OTU99_108 (Cytophagales) abundance \|\| id:ebi-gm430-GCST90011421 | Oranges liking \|\| id:ebi-fl187-GCST90094807 | Weighted mode | 5 | -0.259608203 | 0.931129466729828 | 0.7942112637234 | -2.084621957 | 1.56540555228556 | 0.771353741071447 | 0.124354122449567 | 4.7846149540094 | 0.999714303421225 |
| ebi-fl187-GCST90094807 | ebi-gm430-GCST90011428 | OTU99_123 (Phascolarctobacterium) abundance \|\| id:ebi-gm430-GCST90011428 | Oranges liking \|\| id:ebi-fl187-GCST90094807 | Weighted mode | 5 | 0.30442798153755 | 0.558324159035206 | 0.614558083055702 | -0.78988737 | 1.39874333324655 | 1.3558492103629 | 0.453895914622562 | 4.05010713253577 | 0.999714303421225 |
| ebi-fl187-GCST90094807 | ebi-gm430-GCST90011450 | OTU99_298 (Phascolarctobacterium) abundance \|\| id:ebi-gm430-GCST90011450 | Oranges liking \|\| id:ebi-fl187-GCST90094807 | Weighted mode | 5 | 0.177908541787776 | 0.946775971416738 | 0.860094821738676 | -1.677772362 | 2.03358944576458 | 1.19471604964397 | 0.186789612527316 | 7.64146581795686 | 0.999714303421225 |
| ebi-fl187-GCST90094807 | ebi-gm430-GCST90011451 | OTU99_3 (Bacteroides) abundance \|\| id:ebi-gm430-GCST90011451 | Oranges liking \|\| id:ebi-fl187-GCST90094807 | Weighted mode | 5 | -0.207240478 | 0.329133238767308 | 0.563094633746823 | -0.852341626 | 0.437860669909293 | 0.812824160093964 | 0.426415256895623 | 1.54938901586764 | 0.999714303421225 |
| ebi-fl187-GCST90094807 | ebi-gm430-GCST90011472 | OTU99_72 (Parabacteroides) abundance \|\| id:ebi-gm430-GCST90011472 | Oranges liking \|\| id:ebi-fl187-GCST90094807 | Weighted mode | 5 | 0.662551397485643 | 0.752964192587259 | 0.428590393080112 | -0.81325842 | 2.13836121495667 | 1.93973506144867 | 0.443410890842317 | 8.48552028450582 | 0.999714303421225 |
| ebi-fl187-GCST90094807 | ebi-gm430-GCST90011507 | TestASV_3 (Bacteroides) abundance \|\| id:ebi-gm430-GCST90011507 | Oranges liking \|\| id:ebi-fl187-GCST90094807 | Weighted mode | 5 | 0.311025841895162 | 0.310553648593555 | 0.373248678601166 | -0.297659309 | 0.91971099313853 | 1.36482449038184 | 0.742554277959436 | 2.50856529258029 | 0.999714303421225 |
| ebi-fl187-GCST90094807 | ebi-gm430-GCST90011512 | TestASV_34 (Phascolarctobacterium) abundance \|\| id:ebi-gm430-GCST90011512 | Oranges liking \|\| id:ebi-fl187-GCST90094807 | Weighted mode | 5 | -0.084109182 | 0.782893951191343 | 0.919617900727294 | -1.618581327 | 1.45036296199035 | 0.919330875914779 | 0.198179651931157 | 4.26466214454665 | 0.999714303421225 |
| ebi-fl187-GCST90094807 | ebi-gm430-GCST90011519 | TestASV_41 (Barnesiella) abundance \|\| id:ebi-gm430-GCST90011519 | Oranges liking \|\| id:ebi-fl187-GCST90094807 | Weighted mode | 5 | -1.165318956 | 1.02424162710343 | 0.318754324247602 | -3.172832545 | 0.842194633519879 | 0.311823188416613 | 0.0418847891786987 | 2.3214561357695 | 0.999714303421225 |
| ebi-fl187-GCST90094807 | ebi-gm430-GCST90011524 | TestASV_46 (Sutterella) abundance \|\| id:ebi-gm430-GCST90011524 | Oranges liking \|\| id:ebi-fl187-GCST90094807 | Weighted mode | 5 | -0.493962585 | 1.16958636909782 | 0.694490114371615 | -2.786351868 | 1.79842669860776 | 0.610203613539185 | 0.0616456958160641 | 6.04013702898701 | 0.999714303421225 |
| ebi-fl187-GCST90094807 | ebi-gm430-GCST90011581 | OTU97_19 (Bacteria) prevalence \|\| id:ebi-gm430-GCST90011581 | Oranges liking \|\| id:ebi-fl187-GCST90094807 | Weighted mode | 5 | 1.90779407548226 | 1.11427013998818 | 0.162032875146682 | -0.276175399 | 4.0917635498591 | 6.738208413 | 0.758679847483669 | 59.845338935138 | 0.999714303421225 |
| ebi-fl187-GCST90094807 | ebi-gm430-GCST90011614 | OTU97_80 (Ruminococcus) prevalence \|\| id:ebi-gm430-GCST90011614 | Oranges liking \|\| id:ebi-fl187-GCST90094807 | Weighted mode | 5 | 0.145686210691703 | 0.614063009910886 | 0.824119006171137 | -1.057877289 | 1.34924971011704 | 1.15683312922435 | 0.347192017082688 | 3.85453242881528 | 0.999714303421225 |
| ebi-fl187-GCST90094807 | ebi-gm430-GCST90011623 | OTU99_11 (Parabacteroides) prevalence \|\| id:ebi-gm430-GCST90011623 | Oranges liking \|\| id:ebi-fl187-GCST90094807 | Weighted mode | 5 | -0.027954087 | 0.458647693715593 | 0.954323644120916 | -0.926903566 | 0.870995393077595 | 0.97243301347739 | 0.395777313206663 | 2.3892879509416 | 0.999714303421225 |
| ebi-fl187-GCST90094807 | ebi-gm430-GCST90011642 | OTU99_19 (Bacteria) prevalence \|\| id:ebi-gm430-GCST90011642 | Oranges liking \|\| id:ebi-fl187-GCST90094807 | Weighted mode | 5 | 1.90779407548226 | 1.1449016626127 | 0.170974533898062 | -0.336213183 | 4.15180133420316 | 6.738208413 | 0.714470776385573 | 63.5483691098045 | 0.999714303421225 |
| ebi-fl187-GCST90094807 | ebi-gm430-GCST90011653 | OTU99_30 (Parasutterella) prevalence \|\| id:ebi-gm430-GCST90011653 | Oranges liking \|\| id:ebi-fl187-GCST90094807 | Weighted mode | 5 | 0.579172270799905 | 0.875927340537881 | 0.544620749167733 | -1.137645317 | 2.29598985825415 | 1.78456068625895 | 0.320572981660581 | 9.93426466087185 | 0.999714303421225 |
| ebi-fl187-GCST90094807 | ebi-gm430-GCST90011675 | OTU99_8 (Ruminococcaceae) prevalence \|\| id:ebi-gm430-GCST90011675 | Oranges liking \|\| id:ebi-fl187-GCST90094807 | Weighted mode | 5 | 0.0103318604903997 | 0.641873856992856 | 0.987928349094953 | -1.247740899 | 1.2684046201964 | 1.01038541845321 | 0.287152771716184 | 3.55517617929136 | 0.999714303421225 |
| ebi-fl187-GCST90094807 | ebi-gm430-GCST90011679 | OTU99_92 (Ruminococcus) prevalence \|\| id:ebi-gm430-GCST90011679 | Oranges liking \|\| id:ebi-fl187-GCST90094807 | Weighted mode | 5 | -0.124225062 | 0.577891332921535 | 0.840311466018737 | -1.256892075 | 1.00844195042039 | 0.883181047926745 | 0.284536973400279 | 2.74132656327826 | 0.999714303421225 |
| ebi-fl187-GCST90094807 | ebi-gm430-GCST90011684 | TestASV_11 (Lachnospiraceae) prevalence \|\| id:ebi-gm430-GCST90011684 | Oranges liking \|\| id:ebi-fl187-GCST90094807 | Weighted mode | 5 | 1.52890670743982 | 0.831313693438004 | 0.139736880795731 | -0.100468132 | 3.15828154657831 | 4.6131305629599 | 0.904413934089299 | 23.5301257408685 | 0.999714303421225 |
| ebi-fl187-GCST90094807 | ebi-gm430-GCST90011688 | TestASV_15 (Bacteroides) prevalence \|\| id:ebi-gm430-GCST90011688 | Oranges liking \|\| id:ebi-fl187-GCST90094807 | Weighted mode | 5 | 0.557190117626437 | 0.542763959780342 | 0.362641003868205 | -0.506627244 | 1.62100747879591 | 1.74576022119092 | 0.602524323460729 | 5.05818376325055 | 0.999714303421225 |
| ebi-fl187-GCST90094807 | ebi-gm430-GCST90011692 | TestASV_19 (Prevotella) prevalence \|\| id:ebi-gm430-GCST90011692 | Oranges liking \|\| id:ebi-fl187-GCST90094807 | Weighted mode | 5 | 1.41401663096519 | 1.43703143842321 | 0.380832006619113 | -1.402564988 | 4.23059825027468 | 4.11244043034955 | 0.245965256111311 | 68.7583545763877 | 0.999714303421225 |
| ebi-fl187-GCST90094807 | ebi-gm430-GCST90011693 | TestASV_2 (Bacteroides) prevalence \|\| id:ebi-gm430-GCST90011693 | Oranges liking \|\| id:ebi-fl187-GCST90094807 | Weighted mode | 5 | 0.143334374267648 | 0.644275159701273 | 0.83484306160009 | -1.119444939 | 1.40611368728214 | 1.15411564372774 | 0.326460950166282 | 4.08006813194244 | 0.999714303421225 |
| ebi-fl187-GCST90094807 | ebi-gm430-GCST90011716 | TestASV_40 (Barnesiella) prevalence \|\| id:ebi-gm430-GCST90011716 | Oranges liking \|\| id:ebi-fl187-GCST90094807 | Weighted mode | 5 | 0.622034347373574 | 1.53869261384984 | 0.706702552251986 | -2.393803176 | 3.63787187051926 | 1.86271359648694 | 0.0912818619170477 | 38.0108585612594 | 0.999714303421225 |
| ebi-fl187-GCST90094807 | ebi-gm430-GCST90011718 | TestASV_42 (Alphaproteobacteria) prevalence \|\| id:ebi-gm430-GCST90011718 | Oranges liking \|\| id:ebi-fl187-GCST90094807 | Weighted mode | 5 | 0.267593161855846 | 0.924424556930398 | 0.786605905297307 | -1.54427897 | 2.07946529343943 | 1.30681536951033 | 0.213465731001614 | 8.00019001633333 | 0.999714303421225 |
| ebi-fl187-GCST90094807 | ebi-hbsm44-GCST90104929 | Sulcal depth of the right inferior frontal sulcus \|\| id:ebi-hbsm44-GCST90104929 | Oranges liking \|\| id:ebi-fl187-GCST90094807 | Weighted mode | 5 | -0.223728294 | 0.487142298604158 | 0.669892789960323 | -1.178527199 | 0.731070611036532 | 0.79953234241233 | 0.307731632312691 | 2.0773034015359 | 0.999714303421225 |
| ebi-fl187-GCST90094807 | ebi-hbsm44-GCST90104935 | Sulcal opening distance of the left calloso-marginal posterior fissure \|\| id:ebi-hbsm44-GCST90104935 | Oranges liking \|\| id:ebi-fl187-GCST90094807 | Weighted mode | 5 | -0.062667063 | 0.117995493907668 | 0.623472643823702 | -0.293938231 | 0.168604105039811 | 0.939256134739713 | 0.745322527790366 | 1.18365144451171 | 0.999714303421225 |
| ebi-fl187-GCST90094807 | ebi-hbsm44-GCST90104936 | Sulcal opening distance of the left anterior inferior temporal sulcus \|\| id:ebi-hbsm44-GCST90104936 | Oranges liking \|\| id:ebi-fl187-GCST90094807 | Weighted mode | 5 | 0.0228992021359189 | 0.062524449291136 | 0.732732067071758 | -0.099648718 | 0.145447122746545 | 1.02316340166377 | 0.905155326538732 | 1.15655657743001 | 0.999714303421225 |
| ebi-fl187-GCST90094807 | ebi-hbsm44-GCST90104941 | Sulcal opening distance of the right posterior inferior temporal sulcus \|\| id:ebi-hbsm44-GCST90104941 | Oranges liking \|\| id:ebi-fl187-GCST90094807 | Weighted mode | 5 | 0.00617018312994389 | 0.046543339483007 | 0.9009359480053 | -0.085054762 | 0.0973951285166376 | 1.00618925792134 | 0.918461985973151 | 1.10229583610214 | 0.999714303421225 |
| ebi-fl187-GCST90094807 | ebi-IPs91-GCST90274819 | Leukemia inhibitory factor levels \|\| id:ebi-IPs91-GCST90274819 | Oranges liking \|\| id:ebi-fl187-GCST90094807 | Weighted mode | 7 | 0.0124757528623336 | 0.1923714765129 | 0.950398277788878 | -0.364572341 | 0.389523846827617 | 1.01255389970918 | 0.694493593742632 | 1.47627769219742 | 0.999714303421225 |
| ebi-fl187-GCST90094807 | ebi-IPs91-GCST90274837 | STAM binding protein levels \|\| id:ebi-IPs91-GCST90274837 | Oranges liking \|\| id:ebi-fl187-GCST90094807 | Weighted mode | 7 | -0.099215629 | 0.161053771396921 | 0.560496396143489 | -0.41488102 | 0.216449763389837 | 0.905547425093484 | 0.660418852344709 | 1.24166070696211 | 0.999714303421225 |
| ebi-fl187-GCST90094807 | ebi-IPs91-GCST90274840 | TNF-beta levels \|\| id:ebi-IPs91-GCST90274840 | Oranges liking \|\| id:ebi-fl187-GCST90094807 | Weighted mode | 7 | -0.121359391 | 0.183374545584782 | 0.532670656726662 | -0.4807735 | 0.238054718507365 | 0.885715584318931 | 0.618304947800235 | 1.26877861659759 | 0.999714303421225 |
| ebi-fl187-GCST90094807 | ebi-IPs91-GCST90274842 | Tumor necrosis factor ligand superfamily member 14 levels \|\| id:ebi-IPs91-GCST90274842 | Oranges liking \|\| id:ebi-fl187-GCST90094807 | Weighted mode | 7 | -0.120202482 | 0.224202419118294 | 0.611149607355228 | -0.559639224 | 0.319234259422768 | 0.886740869429943 | 0.571415179822337 | 1.37607364536911 | 0.999714303421225 |
| ebi-fl187-GCST90094807 | ebi-met1400-GCST90199628 | 3-methylhistidine levels \|\| id:ebi-met1400-GCST90199628 | Oranges liking \|\| id:ebi-fl187-GCST90094807 | Weighted mode | 7 | 0.0218659633092315 | 0.348793017820742 | 0.95204932053364 | -0.661768352 | 0.705500278237886 | 1.02210677547873 | 0.515938167235709 | 2.02485942468034 | 0.999714303421225 |
| ebi-fl187-GCST90094807 | ebi-met1400-GCST90199651 | Docosahexaenoate DHA; 22:6n3 levels \|\| id:ebi-met1400-GCST90199651 | Oranges liking \|\| id:ebi-fl187-GCST90094807 | Weighted mode | 7 | 0.17161882905513 | 0.226322603392062 | 0.476994259963851 | -0.271973474 | 0.615211131703572 | 1.18722521120068 | 0.761874470613847 | 1.85004716193581 | 0.999714303421225 |
| ebi-fl187-GCST90094807 | ebi-met1400-GCST90199652 | 1-stearoyl-2-oleoyl-GPS (18:0/18:1) levels \|\| id:ebi-met1400-GCST90199652 | Oranges liking \|\| id:ebi-fl187-GCST90094807 | Weighted mode | 7 | -0.029308708 | 0.22365630338019 | 0.900024355873333 | -0.467675063 | 0.409057646526814 | 0.971116626620713 | 0.626457049836744 | 1.50539849897923 | 0.999714303421225 |
| ebi-fl187-GCST90094807 | ebi-met1400-GCST90199653 | 1-myristoyl-2-palmitoyl-gpc (14:0/16:0) levels \|\| id:ebi-met1400-GCST90199653 | Oranges liking \|\| id:ebi-fl187-GCST90094807 | Weighted mode | 7 | -0.35894859 | 0.347487821642724 | 0.341440353938962 | -1.04002472 | 0.322127540767566 | 0.698410255942815 | 0.353445944641618 | 1.38006077874425 | 0.999714303421225 |
| ebi-fl187-GCST90094807 | ebi-met1400-GCST90199667 | Erythritol levels in elite athletes \|\| id:ebi-met1400-GCST90199667 | Oranges liking \|\| id:ebi-fl187-GCST90094807 | Weighted mode | 7 | 0.165881627548225 | 0.196499170578544 | 0.430931834705649 | -0.219256747 | 0.551020001882172 | 1.18043336266152 | 0.803115494359372 | 1.73502184115608 | 0.999714303421225 |
| ebi-fl187-GCST90094807 | ebi-met1400-GCST90199674 | 3-hydroxymyristate levels \|\| id:ebi-met1400-GCST90199674 | Oranges liking \|\| id:ebi-fl187-GCST90094807 | Weighted mode | 7 | 0.168398272415626 | 0.214339099451417 | 0.461953992294229 | -0.251706363 | 0.588502907340404 | 1.18340783550097 | 0.777472999775065 | 1.80128969820207 | 0.999714303421225 |
| ebi-fl187-GCST90094807 | ebi-met1400-GCST90199685 | 2-linoleoylglycerol (18:2) levels \|\| id:ebi-met1400-GCST90199685 | Oranges liking \|\| id:ebi-fl187-GCST90094807 | Weighted mode | 7 | -0.177608403 | 0.31613152588179 | 0.594588395950944 | -0.797226194 | 0.442009387698756 | 0.837270231786424 | 0.450577045770776 | 1.55583034603216 | 0.999714303421225 |
| ebi-fl187-GCST90094807 | ebi-met1400-GCST90199703 | Gamma-glutamylvaline levels \|\| id:ebi-met1400-GCST90199703 | Oranges liking \|\| id:ebi-fl187-GCST90094807 | Weighted mode | 7 | 0.010334260957497 | 0.215644437594839 | 0.963333206743166 | -0.412328837 | 0.432997358643381 | 1.01038784385307 | 0.662106515309881 | 1.54187214806105 | 0.999714303421225 |
| ebi-fl187-GCST90094807 | ebi-met1400-GCST90199779 | Gamma-glutamyl-2-aminobutyrate levels \|\| id:ebi-met1400-GCST90199779 | Oranges liking \|\| id:ebi-fl187-GCST90094807 | Weighted mode | 7 | 0.338729242847468 | 0.27772380644341 | 0.268356559021634 | -0.205609418 | 0.88306790347655 | 1.40316337725496 | 0.81415100709627 | 2.41830747135186 | 0.999714303421225 |
| ebi-fl187-GCST90094807 | ebi-met1400-GCST90199824 | 4-vinylphenol sulfate levels \|\| id:ebi-met1400-GCST90199824 | Oranges liking \|\| id:ebi-fl187-GCST90094807 | Weighted mode | 7 | 0.0123110823391162 | 0.276935715069457 | 0.965984561582122 | -0.530482919 | 0.555105083875251 | 1.01238717565637 | 0.588320789662552 | 1.74212404430135 | 0.999714303421225 |
| ebi-fl187-GCST90094807 | ebi-met1400-GCST90199830 | 4-ethylphenylsulfate levels \|\| id:ebi-met1400-GCST90199830 | Oranges liking \|\| id:ebi-fl187-GCST90094807 | Weighted mode | 7 | 0.0554132394481871 | 0.229628688462438 | 0.817344934951582 | -0.39465899 | 0.505485468834566 | 1.05697730915957 | 0.67390981009555 | 1.65779013058113 | 0.999714303421225 |
| ebi-fl187-GCST90094807 | ebi-met1400-GCST90199833 | Hexanoylglutamine levels \|\| id:ebi-met1400-GCST90199833 | Oranges liking \|\| id:ebi-fl187-GCST90094807 | Weighted mode | 7 | 0.166304081018611 | 0.224993583304254 | 0.487713289751751 | -0.274683342 | 0.607291504294949 | 1.18093214618113 | 0.759812685704579 | 1.83545334280744 | 0.999714303421225 |
| ebi-fl187-GCST90094807 | ebi-met1400-GCST90199837 | Carotenoid levels (cryptoxanthin) \|\| id:ebi-met1400-GCST90199837 | Oranges liking \|\| id:ebi-fl187-GCST90094807 | Weighted mode | 7 | 0.228815854396544 | 0.23858218592847 | 0.374561557882549 | -0.23880523 | 0.696436938816346 | 1.25711052631799 | 0.787568262087412 | 2.00659035089977 | 0.999714303421225 |
| ebi-fl187-GCST90094807 | ebi-met1400-GCST90199869 | 2-hydroxyhippurate levels \|\| id:ebi-met1400-GCST90199869 | Oranges liking \|\| id:ebi-fl187-GCST90094807 | Weighted mode | 7 | 0.0410731189894611 | 0.281462733671594 | 0.888757934660589 | -0.510593839 | 0.592740076985785 | 1.04192828750375 | 0.600139086973975 | 1.80893826091945 | 0.999714303421225 |
| ebi-fl187-GCST90094807 | ebi-met1400-GCST90199902 | Hydantoin-5-propionate levels \|\| id:ebi-met1400-GCST90199902 | Oranges liking \|\| id:ebi-fl187-GCST90094807 | Weighted mode | 7 | -0.144181988 | 0.222367740506271 | 0.540735116882163 | -0.580022759 | 0.291658783705567 | 0.865730181488995 | 0.559885623939242 | 1.3386461718158 | 0.999714303421225 |
| ebi-fl187-GCST90094807 | ebi-met1400-GCST90199904 | Pantoate levels \|\| id:ebi-met1400-GCST90199904 | Oranges liking \|\| id:ebi-fl187-GCST90094807 | Weighted mode | 7 | -0.023079099 | 0.238973297157265 | 0.926208142522108 | -0.491466762 | 0.44530856303062 | 0.97718518595664 | 0.61172847585306 | 1.56097177971242 | 0.999714303421225 |
| ebi-fl187-GCST90094807 | ebi-met1400-GCST90199936 | 3-methyl catechol sulfate (2) levels \|\| id:ebi-met1400-GCST90199936 | Oranges liking \|\| id:ebi-fl187-GCST90094807 | Weighted mode | 7 | 0.444816729186028 | 0.347643923072236 | 0.247963098008875 | -0.23656536 | 1.12619881840761 | 1.5602042297297 | 0.789334289698701 | 3.0839116838515 | 0.999714303421225 |
| ebi-fl187-GCST90094807 | ebi-met1400-GCST90199977 | 4-hydroxychlorothalonil levels \|\| id:ebi-met1400-GCST90199977 | Oranges liking \|\| id:ebi-fl187-GCST90094807 | Weighted mode | 7 | -0.067372101 | 0.244059403759474 | 0.791772933198478 | -0.545728533 | 0.410984330102097 | 0.934847278753192 | 0.579419503518516 | 1.5083017214389 | 0.999714303421225 |
| ebi-fl187-GCST90094807 | ebi-met1400-GCST90200040 | Sphingomyelin (d18:2/24:1, d18:1/24:2) levels \|\| id:ebi-met1400-GCST90200040 | Oranges liking \|\| id:ebi-fl187-GCST90094807 | Weighted mode | 7 | -0.107828989 | 0.278424967370468 | 0.711912192795023 | -0.653541925 | 0.437883946822778 | 0.897781113549019 | 0.520200000363167 | 1.54942508128146 | 0.999714303421225 |
| ebi-fl187-GCST90094807 | ebi-met1400-GCST90200077 | 1-stearoyl-2-oleoyl-GPI (18:0/18:1) levels \|\| id:ebi-met1400-GCST90200077 | Oranges liking \|\| id:ebi-fl187-GCST90094807 | Weighted mode | 6 | 0.52332274481846 | 0.319665678946404 | 0.16253733197438 | -0.103221986 | 1.14986747555341 | 1.68762589397433 | 0.901926736224264 | 3.15777439965421 | 0.999714303421225 |
| ebi-fl187-GCST90094807 | ebi-met1400-GCST90200080 | 1-palmitoyl-2-oleoyl-GPI (16:0/18:1) levels \|\| id:ebi-met1400-GCST90200080 | Oranges liking \|\| id:ebi-fl187-GCST90094807 | Weighted mode | 7 | 0.0795864694740116 | 0.279735286159873 | 0.785586494197326 | -0.468694691 | 0.627867630347362 | 1.0828391880164 | 0.625818621799646 | 1.87361108516105 | 0.999714303421225 |
| ebi-fl187-GCST90094807 | ebi-met1400-GCST90200082 | 1-oleoyl-2-linoleoyl-GPE (18:1/18:2) levels \|\| id:ebi-met1400-GCST90200082 | Oranges liking \|\| id:ebi-fl187-GCST90094807 | Weighted mode | 7 | 0.0173878611191027 | 0.277466050126397 | 0.952067454286173 | -0.526445597 | 0.56122131936684 | 1.0175399099659 | 0.590700831434648 | 1.75281193672735 | 0.999714303421225 |
| ebi-fl187-GCST90094807 | ebi-met1400-GCST90200100 | Perfluorooctanesulfonate (PFOS) levels \|\| id:ebi-met1400-GCST90200100 | Oranges liking \|\| id:ebi-fl187-GCST90094807 | Weighted mode | 7 | -0.248124212 | 0.231329348998401 | 0.324683875278533 | -0.701529736 | 0.205281312369324 | 0.780263019487853 | 0.495826240253236 | 1.22787043152327 | 0.999714303421225 |
| ebi-fl187-GCST90094807 | ebi-met1400-GCST90200102 | 1-palmitoleoyl-2-linolenoyl-GPC (16:1/18:3) levels \|\| id:ebi-met1400-GCST90200102 | Oranges liking \|\| id:ebi-fl187-GCST90094807 | Weighted mode | 7 | 0.275071881795811 | 0.34447688429457 | 0.455005111211759 | -0.400102811 | 0.950246575013169 | 1.31662531285811 | 0.670251133021399 | 2.58634730932019 | 0.999714303421225 |
| ebi-fl187-GCST90094807 | ebi-met1400-GCST90200114 | Glycosyl-N-tricosanoyl-sphingadienine (d18:2/23:0) levels \|\| id:ebi-met1400-GCST90200114 | Oranges liking \|\| id:ebi-fl187-GCST90094807 | Weighted mode | 7 | 0.0559413637267832 | 0.354587491726101 | 0.879817811429202 | -0.63905012 | 0.750932847509941 | 1.05753567196796 | 0.52779352649698 | 2.11897577620465 | 0.999714303421225 |
| ebi-fl187-GCST90094807 | ebi-met1400-GCST90200135 | Arachidoylcarnitine (C20) levels \|\| id:ebi-met1400-GCST90200135 | Oranges liking \|\| id:ebi-fl187-GCST90094807 | Weighted mode | 7 | 0.164874052319349 | 0.321869776843176 | 0.626787171947775 | -0.46599071 | 0.795738814931974 | 1.17924458623737 | 0.627513113433054 | 2.2160776634009 | 0.999714303421225 |
| ebi-fl187-GCST90094807 | ebi-met1400-GCST90200146 | Docosahexaenoylcarnitine (C22:6) levels \|\| id:ebi-met1400-GCST90200146 | Oranges liking \|\| id:ebi-fl187-GCST90094807 | Weighted mode | 7 | 0.0356728173699845 | 0.333947282514174 | 0.918412628492714 | -0.618863856 | 0.690209491097765 | 1.03631672618481 | 0.538555967074191 | 1.99413324265045 | 0.999714303421225 |
| ebi-fl187-GCST90094807 | ebi-met1400-GCST90200162 | 3-hydroxyphenylacetoylglutamine levels \|\| id:ebi-met1400-GCST90200162 | Oranges liking \|\| id:ebi-fl187-GCST90094807 | Weighted mode | 7 | -0.74378077 | 0.553176231104601 | 0.227366438351324 | -1.828006182 | 0.340444643452156 | 0.475313463466859 | 0.160733722216097 | 1.4055724302155 | 0.999714303421225 |
| ebi-fl187-GCST90094807 | ebi-met1400-GCST90200172 | 3-carboxy-4-methyl-5-pentyl-2-furanpropionate (3-CMPFP) levels \|\| id:ebi-met1400-GCST90200172 | Oranges liking \|\| id:ebi-fl187-GCST90094807 | Weighted mode | 7 | -0.091969209 | 0.217479232928254 | 0.687114726815922 | -0.518228506 | 0.334290087211306 | 0.912133234307887 | 0.595574671043354 | 1.39694832164614 | 0.999714303421225 |
| ebi-fl187-GCST90094807 | ebi-met1400-GCST90200222 | Dihydrocaffeate sulfate (2) levels \|\| id:ebi-met1400-GCST90200222 | Oranges liking \|\| id:ebi-fl187-GCST90094807 | Weighted mode | 7 | 0.0609002331835197 | 0.255961754075865 | 0.819854510140804 | -0.440784805 | 0.562585271172215 | 1.06279287743585 | 0.643531176490292 | 1.75520431890903 | 0.999714303421225 |
| ebi-fl187-GCST90094807 | ebi-met1400-GCST90200223 | 4-methylhexanoylglutamine levels \|\| id:ebi-met1400-GCST90200223 | Oranges liking \|\| id:ebi-fl187-GCST90094807 | Weighted mode | 7 | 0.33289706469948 | 0.337052694925474 | 0.36145402813035 | -0.327726217 | 0.993520346753408 | 1.39500369595438 | 0.72056026959916 | 2.70072524649316 | 0.999714303421225 |
| ebi-fl187-GCST90094807 | ebi-met1400-GCST90200224 | 2-ketocaprylate levels \|\| id:ebi-met1400-GCST90200224 | Oranges liking \|\| id:ebi-fl187-GCST90094807 | Weighted mode | 7 | -0.271163223 | 0.2347092041007 | 0.291887563935073 | -0.731193263 | 0.188866817274321 | 0.76249203019151 | 0.481334288972063 | 1.20788007300954 | 0.999714303421225 |
| ebi-fl187-GCST90094807 | ebi-met1400-GCST90200243 | Hydroxypalmitoyl sphingomyelin (d18:1/16:0(OH)) levels \|\| id:ebi-met1400-GCST90200243 | Oranges liking \|\| id:ebi-fl187-GCST90094807 | Weighted mode | 7 | -0.35356468 | 0.292484595301945 | 0.272198819054871 | -0.926834487 | 0.219705126887296 | 0.702180574146763 | 0.395804654286009 | 1.24570935023112 | 0.999714303421225 |
| ebi-fl187-GCST90094807 | ebi-met1400-GCST90200273 | 2-methoxyhydroquinone sulfate (2) levels \|\| id:ebi-met1400-GCST90200273 | Oranges liking \|\| id:ebi-fl187-GCST90094807 | Weighted mode | 7 | -0.130449214 | 0.33999171778464 | 0.71444070101843 | -0.796832981 | 0.535934552830764 | 0.87770106671943 | 0.450754253303236 | 1.70904468870798 | 0.999714303421225 |
| ebi-fl187-GCST90094807 | ebi-met1400-GCST90200276 | 2,4-di-tert-butylphenol levels \|\| id:ebi-met1400-GCST90200276 | Oranges liking \|\| id:ebi-fl187-GCST90094807 | Weighted mode | 7 | 0.306216301712766 | 0.318840078894724 | 0.373938377257741 | -0.318710253 | 0.931142856346425 | 1.35827607221657 | 0.727086189888398 | 2.53740741333466 | 0.999714303421225 |
| ebi-fl187-GCST90094807 | ebi-met1400-GCST90200289 | N-acetylmethionine levels \|\| id:ebi-met1400-GCST90200289 | Oranges liking \|\| id:ebi-fl187-GCST90094807 | Weighted mode | 7 | 0.149707106054956 | 0.217178464546784 | 0.5163750543611 | -0.275962684 | 0.575376896566653 | 1.16149399834364 | 0.758841246806295 | 1.77780044754559 | 0.999714303421225 |
| ebi-fl187-GCST90094807 | ebi-met1400-GCST90200308 | 3-hydroxyisobutyrate levels \|\| id:ebi-met1400-GCST90200308 | Oranges liking \|\| id:ebi-fl187-GCST90094807 | Weighted mode | 7 | -0.183266639 | 0.301677177993357 | 0.565790675503396 | -0.774553908 | 0.408020629464575 | 0.832546136534553 | 0.460909342992277 | 1.50383818422666 | 0.999714303421225 |
| ebi-fl187-GCST90094807 | ebi-met1400-GCST90200383 | Cystathionine levels \|\| id:ebi-met1400-GCST90200383 | Oranges liking \|\| id:ebi-fl187-GCST90094807 | Weighted mode | 7 | 0.266227645937747 | 0.343186938868179 | 0.467361722481144 | -0.406418754 | 0.938874046119378 | 1.30503211013287 | 0.66603120562683 | 2.55710061944467 | 0.999714303421225 |
| ebi-fl187-GCST90094807 | ebi-met1400-GCST90200384 | Guanidinoacetate levels \|\| id:ebi-met1400-GCST90200384 | Oranges liking \|\| id:ebi-fl187-GCST90094807 | Weighted mode | 7 | -0.278880333 | 0.228054478719058 | 0.267228626793247 | -0.725867112 | 0.168106445052091 | 0.75663044131324 | 0.483904787563942 | 1.18306253509885 | 0.999714303421225 |
| ebi-fl187-GCST90094807 | ebi-met1400-GCST90200403 | Citrulline levels \|\| id:ebi-met1400-GCST90200403 | Oranges liking \|\| id:ebi-fl187-GCST90094807 | Weighted mode | 7 | 0.113295388007283 | 0.268576505992486 | 0.687840658783293 | -0.413114564 | 0.639705339752556 | 1.11996270764518 | 0.661586484665384 | 1.89592214410235 | 0.999714303421225 |
| ebi-fl187-GCST90094807 | ebi-met1400-GCST90200434 | 12,13-DiHOME levels \|\| id:ebi-met1400-GCST90200434 | Oranges liking \|\| id:ebi-fl187-GCST90094807 | Weighted mode | 7 | 0.0172059300747587 | 0.218015321896873 | 0.939661955519752 | -0.410104101 | 0.444515960992631 | 1.01735480470613 | 0.663581167181607 | 1.55973504048433 | 0.999714303421225 |
| ebi-fl187-GCST90094807 | ebi-met1400-GCST90200437 | Fructose levels \|\| id:ebi-met1400-GCST90200437 | Oranges liking \|\| id:ebi-fl187-GCST90094807 | Weighted mode | 7 | 0.0484405398250061 | 0.262299535653485 | 0.859566887159859 | -0.46566655 | 0.562547629705836 | 1.0496329586054 | 0.627716561205966 | 1.75513825168812 | 0.999714303421225 |
| ebi-fl187-GCST90094807 | ebi-met1400-GCST90200448 | N-stearoyl-sphinganine (d18:0/18:0) levels \|\| id:ebi-met1400-GCST90200448 | Oranges liking \|\| id:ebi-fl187-GCST90094807 | Weighted mode | 7 | -0.003802709 | 0.268400147631143 | 0.98915526166701 | -0.529866999 | 0.522261580012682 | 0.996204511798618 | 0.588683260109935 | 1.68583599462738 | 0.999714303421225 |
| ebi-fl187-GCST90094807 | ebi-met1400-GCST90200468 | X-11847 levels \|\| id:ebi-met1400-GCST90200468 | Oranges liking \|\| id:ebi-fl187-GCST90094807 | Weighted mode | 7 | 0.00568813463393492 | 0.240850772327203 | 0.981924081525078 | -0.466379379 | 0.477755648395253 | 1.00570434278856 | 0.62726926603388 | 1.61245143014728 | 0.999714303421225 |
| ebi-fl187-GCST90094807 | ebi-met1400-GCST90200476 | X-11843 levels \|\| id:ebi-met1400-GCST90200476 | Oranges liking \|\| id:ebi-fl187-GCST90094807 | Weighted mode | 7 | 0.0528093773055629 | 0.254472256487642 | 0.842464174987557 | -0.445956245 | 0.55157500002134 | 1.0542286660555 | 0.640211783652794 | 1.73598504231204 | 0.999714303421225 |
| ebi-fl187-GCST90094807 | ebi-met1400-GCST90200511 | X-12906 levels \|\| id:ebi-met1400-GCST90200511 | Oranges liking \|\| id:ebi-fl187-GCST90094807 | Weighted mode | 7 | 0.126970437482056 | 0.230689609033129 | 0.601921843245743 | -0.325181196 | 0.579122071186988 | 1.13538345249166 | 0.722396446274986 | 1.78447110425179 | 0.999714303421225 |
| ebi-fl187-GCST90094807 | ebi-met1400-GCST90200515 | X-12849 levels \|\| id:ebi-met1400-GCST90200515 | Oranges liking \|\| id:ebi-fl187-GCST90094807 | Weighted mode | 7 | 0.140439016182171 | 0.258088201046838 | 0.605952132725153 | -0.365413858 | 0.646291890233973 | 1.15077889853421 | 0.693909411573165 | 1.90845094651374 | 0.999714303421225 |
| ebi-fl187-GCST90094807 | ebi-met1400-GCST90200551 | X-18886 levels \|\| id:ebi-met1400-GCST90200551 | Oranges liking \|\| id:ebi-fl187-GCST90094807 | Weighted mode | 7 | -0.207000355 | 0.298128136027549 | 0.513445859277967 | -0.791331502 | 0.377330791720156 | 0.813019361451957 | 0.453240902384486 | 1.45838665181859 | 0.999714303421225 |
| ebi-fl187-GCST90094807 | ebi-met1400-GCST90200564 | X-21319 levels \|\| id:ebi-met1400-GCST90200564 | Oranges liking \|\| id:ebi-fl187-GCST90094807 | Weighted mode | 7 | -0.197461549 | 0.237267695583923 | 0.437142111654203 | -0.662506232 | 0.267583134190557 | 0.820811700925325 | 0.515557606748782 | 1.30680226526891 | 0.999714303421225 |
| ebi-fl187-GCST90094807 | ebi-met1400-GCST90200579 | X-21442 levels \|\| id:ebi-met1400-GCST90200579 | Oranges liking \|\| id:ebi-fl187-GCST90094807 | Weighted mode | 7 | 0.241324595440244 | 0.253823790765109 | 0.378437776615327 | -0.256170034 | 0.738819225339858 | 1.27293415687897 | 0.774010349171362 | 2.09346214748147 | 0.999714303421225 |
| ebi-fl187-GCST90094807 | ebi-met1400-GCST90200581 | X-21441 levels \|\| id:ebi-met1400-GCST90200581 | Oranges liking \|\| id:ebi-fl187-GCST90094807 | Weighted mode | 7 | 0.0639401345641308 | 0.235590720350443 | 0.795177025190049 | -0.397817677 | 0.525697946450999 | 1.06602857858571 | 0.671784498045334 | 1.69163910996466 | 0.999714303421225 |
| ebi-fl187-GCST90094807 | ebi-met1400-GCST90200648 | X-24585 levels \|\| id:ebi-met1400-GCST90200648 | Oranges liking \|\| id:ebi-fl187-GCST90094807 | Weighted mode | 7 | -0.057515066 | 0.249328027097554 | 0.825227609319957 | -0.546197999 | 0.431167867471381 | 0.944107666693215 | 0.579147549536254 | 1.53905388535726 | 0.999714303421225 |
| ebi-fl187-GCST90094807 | ebi-met1400-GCST90200720 | Adenosine 5'-diphosphate (ADP) to pantothenate ratio \|\| id:ebi-met1400-GCST90200720 | Oranges liking \|\| id:ebi-fl187-GCST90094807 | Weighted mode | 7 | -0.171389933 | 0.377770368337237 | 0.666000687343217 | -0.911819855 | 0.569039989162172 | 0.842492993779593 | 0.401792354574729 | 1.76657031047535 | 0.999714303421225 |
| ebi-fl187-GCST90094807 | ebi-met1400-GCST90200724 | Cholate to taurocholate ratio \|\| id:ebi-met1400-GCST90200724 | Oranges liking \|\| id:ebi-fl187-GCST90094807 | Weighted mode | 7 | 0.0614227536264935 | 0.23307769413224 | 0.800959628477837 | -0.395409527 | 0.518255034125685 | 1.06334835355213 | 0.673404205653685 | 1.67909512816961 | 0.999714303421225 |
| ebi-fl187-GCST90094807 | ebi-met1400-GCST90200726 | N-acetylputrescine to (N(1) + N(8))-acetylspermidine ratio \|\| id:ebi-met1400-GCST90200726 | Oranges liking \|\| id:ebi-fl187-GCST90094807 | Weighted mode | 7 | -0.016975929 | 0.226899054798824 | 0.942792342896581 | -0.461698077 | 0.427746218037214 | 0.983167349809089 | 0.630212587037692 | 1.53379678161334 | 0.999714303421225 |
| ebi-fl187-GCST90094807 | ebi-met1400-GCST90200744 | Arginine to glutamate ratio \|\| id:ebi-met1400-GCST90200744 | Oranges liking \|\| id:ebi-fl187-GCST90094807 | Weighted mode | 7 | -0.089084378 | 0.288050394339445 | 0.767576945645001 | -0.653663151 | 0.475494394614522 | 0.914768383725236 | 0.520136942458285 | 1.60880938759775 | 0.999714303421225 |
| ebi-fl187-GCST90094807 | ebi-met1400-GCST90200759 | Histidine to trans-urocanate ratio \|\| id:ebi-met1400-GCST90200759 | Oranges liking \|\| id:ebi-fl187-GCST90094807 | Weighted mode | 7 | 0.507925376518511 | 0.321725203523195 | 0.165470313728168 | -0.122656022 | 1.13850677542397 | 1.66183992401107 | 0.884567881784076 | 3.12210288199369 | 0.999714303421225 |
| ebi-fl187-GCST90094807 | ebi-met1400-GCST90200795 | Oleoyl-linoleoyl-glycerol (18:1 to 18:2) [2] to linoleoyl-arachidonoyl-glycerol (18:2 to 20:4) [2] ratio \|\| id:ebi-met1400-GCST90200795 | Oranges liking \|\| id:ebi-fl187-GCST90094807 | Weighted mode | 7 | -0.035127402 | 0.230084500366843 | 0.883661936176618 | -0.486093023 | 0.415838218642407 | 0.965482403958532 | 0.615024593345423 | 1.51564064663347 | 0.999714303421225 |
| ebi-fl187-GCST90094807 | ebi-met1400-GCST90200811 | Choline to taurocholate ratio \|\| id:ebi-met1400-GCST90200811 | Oranges liking \|\| id:ebi-fl187-GCST90094807 | Weighted mode | 7 | -0.174977395 | 0.349015149175196 | 0.633987108108827 | -0.859047088 | 0.509092297242096 | 0.839475996791031 | 0.423565510930626 | 1.66378029136493 | 0.999714303421225 |
| ebi-fl187-GCST90094807 | ebi-met1400-GCST90200831 | Cholate to phosphate ratio \|\| id:ebi-met1400-GCST90200831 | Oranges liking \|\| id:ebi-fl187-GCST90094807 | Weighted mode | 7 | 0.00875087390932872 | 0.253749102618097 | 0.973608009822873 | -0.488597367 | 0.506099115040798 | 1.00878927473829 | 0.6134862869614 | 1.65880773939945 | 0.999714303421225 |
| ebi-fl187-GCST90094807 | ebi-met1400-GCST90200841 | Adenosine 5'-diphosphate (ADP) to oxalate (ethanedioate) ratio \|\| id:ebi-met1400-GCST90200841 | Oranges liking \|\| id:ebi-fl187-GCST90094807 | Weighted mode | 7 | -0.224612136 | 0.356448904453024 | 0.551831246900082 | -0.923251989 | 0.474027716787645 | 0.798825994573304 | 0.397225166651611 | 1.60645151208583 | 0.999714303421225 |
| ebi-fl187-GCST90094807 | ebi-met1400-GCST90200844 | Aspartate to phosphate ratio \|\| id:ebi-met1400-GCST90200844 | Oranges liking \|\| id:ebi-fl187-GCST90094807 | Weighted mode | 7 | -0.279181155 | 0.289360995033196 | 0.37189520717466 | -0.846328705 | 0.287966395045035 | 0.756402864475436 | 0.428986982007632 | 1.33371248402234 | 0.999714303421225 |
| ebi-fl187-GCST90094807 | ebi-met1400-GCST90200870 | Adenosine 5'-monophosphate (AMP) to acetoacetate ratio \|\| id:ebi-met1400-GCST90200870 | Oranges liking \|\| id:ebi-fl187-GCST90094807 | Weighted mode | 7 | -0.122339754 | 0.284787043545687 | 0.682497344171494 | -0.68052236 | 0.435842851135129 | 0.884847686695891 | 0.50635242523996 | 1.54626578174288 | 0.999714303421225 |
| ebi-fl187-GCST90094807 | ebi-met1400-GCST90200887 | Cholesterol to cortisol ratio \|\| id:ebi-met1400-GCST90200887 | Oranges liking \|\| id:ebi-fl187-GCST90094807 | Weighted mode | 7 | 0.290169817913463 | 0.226194441666819 | 0.246874479635011 | -0.153171288 | 0.733510923580429 | 1.3366544566241 | 0.857982747286958 | 2.0823788614196 | 0.999714303421225 |
| ebi-fl187-GCST90094807 | ebi-met1400-GCST90200890 | Cortisol to taurocholate ratio \|\| id:ebi-met1400-GCST90200890 | Oranges liking \|\| id:ebi-fl187-GCST90094807 | Weighted mode | 7 | -0.090240444 | 0.264161958108579 | 0.744290709982258 | -0.607997882 | 0.42751699427099 | 0.91371146276344 | 0.544439811625767 | 1.53344523923091 | 0.999714303421225 |
| ebi-fl187-GCST90094807 | ebi-met1400-GCST90200948 | Adenosine 5'-diphosphate (ADP) to mannitol to sorbitol ratio \|\| id:ebi-met1400-GCST90200948 | Oranges liking \|\| id:ebi-fl187-GCST90094807 | Weighted mode | 7 | -0.433986332 | 0.388461554748464 | 0.306640859682552 | -1.195370979 | 0.327398315192363 | 0.647921111136309 | 0.30259167807165 | 1.38735397130355 | 0.999714303421225 |
| ebi-fl187-GCST90094807 | ebi-met1400-GCST90200972 | Leucine to N-palmitoyl-sphingosine (d18:1 to 16:0) ratio \|\| id:ebi-met1400-GCST90200972 | Oranges liking \|\| id:ebi-fl187-GCST90094807 | Weighted mode | 7 | 0.0535287577579755 | 0.236307064825614 | 0.828315458775353 | -0.409633089 | 0.516690604816179 | 1.05498733040167 | 0.663893795190912 | 1.67647035620808 | 0.999714303421225 |
| ebi-fl187-GCST90094807 | ebi-met1400-GCST90200973 | Proline to glutamate ratio \|\| id:ebi-met1400-GCST90200973 | Oranges liking \|\| id:ebi-fl187-GCST90094807 | Weighted mode | 7 | -0.076253512 | 0.26147942748216 | 0.780394387738142 | -0.588753189 | 0.436246166330002 | 0.926581277740006 | 0.555018856909116 | 1.54688954000511 | 0.999714303421225 |
| ebi-fl187-GCST90094807 | ebi-met1400-GCST90200983 | Cholesterol to linoleoyl-arachidonoyl-glycerol (18:2 to 20:4) [1] ratio \|\| id:ebi-met1400-GCST90200983 | Oranges liking \|\| id:ebi-fl187-GCST90094807 | Weighted mode | 7 | -0.139126612 | 0.318499987123836 | 0.677525147249174 | -0.763386587 | 0.48513336237853 | 0.870117853787668 | 0.466085312701003 | 1.6243916271306 | 0.999714303421225 |
| ebi-fl187-GCST90094807 | ebi-met1400-GCST90201001 | Serine to alpha-ketobutyrate ratio \|\| id:ebi-met1400-GCST90201001 | Oranges liking \|\| id:ebi-fl187-GCST90094807 | Weighted mode | 7 | 0.211276766131527 | 0.226918228670052 | 0.387746445946717 | -0.233482962 | 0.656036494324829 | 1.23525418427787 | 0.791771085772174 | 1.92713895113751 | 0.999714303421225 |
| ebi-fl187-GCST90094807 | ebi-met1400-GCST90201005 | Fructose to maltose ratio \|\| id:ebi-met1400-GCST90201005 | Oranges liking \|\| id:ebi-fl187-GCST90094807 | Weighted mode | 7 | -0.059321637 | 0.271606985028868 | 0.834349058470547 | -0.591671328 | 0.473028053457897 | 0.942403608349198 | 0.553401595882858 | 1.60484640383578 | 0.999714303421225 |
| ebi-fl187-GCST90094807 | ebi-met1400-GCST90201020 | Paraxanthine to 5-acetylamino-6-formylamino-3-methyluracil ratio \|\| id:ebi-met1400-GCST90201020 | Oranges liking \|\| id:ebi-fl187-GCST90094807 | Weighted mode | 7 | 0.344780767126109 | 0.27068443045607 | 0.249871564017594 | -0.185760717 | 0.875322250820005 | 1.41168039898946 | 0.830472289468616 | 2.39964845806736 | 0.999714303421225 |
| ebi-fl187-GCST90094807 | ebi-pw11-GCST90275197 | Placental weight (fetal genotype, adjusted for sex) \|\| id:ebi-pw11-GCST90275197 | Oranges liking \|\| id:ebi-fl187-GCST90094807 | Weighted mode | 7 | 0.133786710302608 | 0.0767996338543088 | 0.132140294161399 | -0.016740572 | 0.284313992657053 | 1.14314897174219 | 0.983398772670816 | 1.32885011443132 | 0.999714303421225 |
| ebi-fl187-GCST90094807 | prot-a-1017 | FAS-associated factor 2 \|\| id:prot-a-1017 | Oranges liking \|\| id:ebi-fl187-GCST90094807 | Weighted mode | 7 | 0.0889337816321043 | 0.393950349948689 | 0.828890092683454 | -0.683208904 | 0.861076467531535 | 1.09300827670575 | 0.504993912483614 | 2.36570592915025 | 0.999714303421225 |
| ebi-fl187-GCST90094807 | prot-a-1019 | Fas apoptotic inhibitory molecule 3 \|\| id:prot-a-1019 | Oranges liking \|\| id:ebi-fl187-GCST90094807 | Weighted mode | 7 | 0.136797725309018 | 0.442440501190084 | 0.767633196281099 | -0.730385657 | 1.00398110764158 | 1.14659619767073 | 0.481723174336342 | 2.72912517095359 | 0.999714303421225 |
| ebi-fl187-GCST90094807 | prot-a-1052 | MIP18 family protein FAM96A \|\| id:prot-a-1052 | Oranges liking \|\| id:ebi-fl187-GCST90094807 | Weighted mode | 7 | -0.348352883 | 0.341109300686866 | 0.346541079030645 | -1.016927112 | 0.320221346358036 | 0.705849749891772 | 0.361704712146025 | 1.37743262028928 | 0.999714303421225 |
| ebi-fl187-GCST90094807 | prot-a-1066 | F-box/LRR-repeat protein 4 \|\| id:prot-a-1066 | Oranges liking \|\| id:ebi-fl187-GCST90094807 | Weighted mode | 7 | -0.233580305 | 0.385958251730742 | 0.567218403366379 | -0.990058478 | 0.522897868356107 | 0.791694016170816 | 0.371554962436457 | 1.68690901375827 | 0.999714303421225 |
| ebi-fl187-GCST90094807 | prot-a-1076 | Low affinity immunoglobulin gamma Fc region receptor III-B \|\| id:prot-a-1076 | Oranges liking \|\| id:ebi-fl187-GCST90094807 | Weighted mode | 7 | -0.480862713 | 0.393265049633322 | 0.267271688022842 | -1.25166221 | 0.289936784452776 | 0.618249789641757 | 0.286028961269254 | 1.33634300770075 | 0.999714303421225 |
| ebi-fl187-GCST90094807 | prot-a-1081 | Fc receptor-like protein 3 \|\| id:prot-a-1081 | Oranges liking \|\| id:ebi-fl187-GCST90094807 | Weighted mode | 7 | -0.220654993 | 0.444898355891504 | 0.63755898700426 | -1.092655771 | 0.651345784385183 | 0.801993325746445 | 0.335324764429063 | 1.91812046938129 | 0.999714303421225 |
| ebi-fl187-GCST90094807 | prot-a-1105 | Fibroblast growth factor receptor 4 \|\| id:prot-a-1105 | Oranges liking \|\| id:ebi-fl187-GCST90094807 | Weighted mode | 7 | -0.187637914 | 0.426409713939207 | 0.67531578860262 | -1.023400953 | 0.648125125730031 | 0.828914791681593 | 0.359370656806461 | 1.91195279540749 | 0.999714303421225 |
| ebi-fl187-GCST90094807 | prot-a-1143 | Follistatin-related protein 1 \|\| id:prot-a-1143 | Oranges liking \|\| id:ebi-fl187-GCST90094807 | Weighted mode | 7 | -0.064270903 | 0.389033699759007 | 0.874207129678955 | -0.826776954 | 0.698235148591992 | 0.937750925638927 | 0.437456960307689 | 2.01020186744348 | 0.999714303421225 |
| ebi-fl187-GCST90094807 | prot-a-1151 | Alpha-(1,3)-fucosyltransferase 10 \|\| id:prot-a-1151 | Oranges liking \|\| id:ebi-fl187-GCST90094807 | Weighted mode | 7 | -0.154359785 | 0.404634239243605 | 0.715991105567936 | -0.947442894 | 0.638723323794201 | 0.856963642769985 | 0.387731226718043 | 1.8940612321732 | 0.999714303421225 |
| ebi-fl187-GCST90094807 | prot-a-1165 | Growth arrest and DNA damage-inducible proteins-interacting protein 1 \|\| id:prot-a-1165 | Oranges liking \|\| id:ebi-fl187-GCST90094807 | Weighted mode | 7 | -0.357968161 | 0.382719738521622 | 0.38571862348259 | -1.108098848 | 0.392162526721433 | 0.699095333301638 | 0.330186098612694 | 1.48017826037374 | 0.999714303421225 |
| ebi-fl187-GCST90094807 | prot-a-1194 | Growth/differentiation factor 11/8 \|\| id:prot-a-1194 | Oranges liking \|\| id:ebi-fl187-GCST90094807 | Weighted mode | 7 | -0.057465164 | 0.347934621739035 | 0.874241565954914 | -0.739417023 | 0.624486694464945 | 0.944154780253924 | 0.477392143170768 | 1.86728722252445 | 0.999714303421225 |
| ebi-fl187-GCST90094807 | prot-a-1216 | D-glucuronyl C5-epimerase \|\| id:prot-a-1216 | Oranges liking \|\| id:ebi-fl187-GCST90094807 | Weighted mode | 7 | 0.109000478924211 | 0.495796177726849 | 0.833277460163509 | -0.862760029 | 1.08076098726884 | 1.11516288441982 | 0.421995752813223 | 2.94692126756536 | 0.999714303421225 |
| ebi-fl187-GCST90094807 | prot-a-1246 | Glypican-3 \|\| id:prot-a-1246 | Oranges liking \|\| id:ebi-fl187-GCST90094807 | Weighted mode | 7 | -0.161427549 | 0.380676091413241 | 0.686308539418307 | -0.907552688 | 0.584697590013543 | 0.850928179696567 | 0.403510532676085 | 1.79444824450955 | 0.999714303421225 |
| ebi-fl187-GCST90094807 | prot-a-1275 | Glutamate receptor 4 \|\| id:prot-a-1275 | Oranges liking \|\| id:ebi-fl187-GCST90094807 | Weighted mode | 7 | -0.563873065 | 0.431830440843381 | 0.239457083539742 | -1.410260729 | 0.282514599212479 | 0.569001012829584 | 0.244079636243002 | 1.32646114024342 | 0.999714303421225 |
| ebi-fl187-GCST90094807 | prot-a-1289 | Glutathione S-transferase P \|\| id:prot-a-1289 | Oranges liking \|\| id:ebi-fl187-GCST90094807 | Weighted mode | 7 | 0.154902729488008 | 0.364518673110836 | 0.685689777024665 | -0.55955387 | 0.869359328785246 | 1.16754438794117 | 0.571463954310376 | 2.38538211820889 | 0.999714303421225 |
| ebi-fl187-GCST90094807 | prot-a-130 | Apolipoprotein D \|\| id:prot-a-130 | Oranges liking \|\| id:ebi-fl187-GCST90094807 | Weighted mode | 7 | 0.495070808737685 | 0.375055490478632 | 0.234959766112849 | -0.240037953 | 1.2301795700758 | 1.64061440477925 | 0.786598007060174 | 3.42184394189962 | 0.999714303421225 |
| ebi-fl187-GCST90094807 | prot-a-1313 | Heparin-binding EGF-like growth factor \|\| id:prot-a-1313 | Oranges liking \|\| id:ebi-fl187-GCST90094807 | Weighted mode | 7 | -0.279596804 | 0.394561349138076 | 0.505142607080182 | -1.052937049 | 0.493743439897081 | 0.756088531565477 | 0.348911472741182 | 1.63843814900548 | 0.999714303421225 |
| ebi-fl187-GCST90094807 | prot-a-1347 | HLA class II histocompatibility antigen, DQ alpha 2 chain \|\| id:prot-a-1347 | Oranges liking \|\| id:ebi-fl187-GCST90094807 | Weighted mode | 7 | 0.147018713130334 | 0.394278459402017 | 0.722055273213064 | -0.625767067 | 0.919804493558288 | 1.15837563966136 | 0.534851004413075 | 2.50879985545383 | 0.999714303421225 |
| ebi-fl187-GCST90094807 | prot-a-1357 | Non-histone chromosomal protein HMG-14 \|\| id:prot-a-1357 | Oranges liking \|\| id:ebi-fl187-GCST90094807 | Weighted mode | 7 | -0.380358645 | 0.422440780967999 | 0.402606887973079 | -1.208342575 | 0.447625285897176 | 0.683616189849904 | 0.298691928716116 | 1.56459231099298 | 0.999714303421225 |
| ebi-fl187-GCST90094807 | prot-a-137 | Apolipoprotein M \|\| id:prot-a-137 | Oranges liking \|\| id:ebi-fl187-GCST90094807 | Weighted mode | 7 | -0.139948808 | 0.400376238567145 | 0.738623074925475 | -0.924686236 | 0.644788619564549 | 0.86940274070122 | 0.396655856031303 | 1.90558418348207 | 0.999714303421225 |
| ebi-fl187-GCST90094807 | prot-a-1381 | 17-beta-hydroxysteroid dehydrogenase 14 \|\| id:prot-a-1381 | Oranges liking \|\| id:ebi-fl187-GCST90094807 | Weighted mode | 7 | -0.489620994 | 0.395018350200241 | 0.26144459263064 | -1.26385696 | 0.284614972851059 | 0.612858627550705 | 0.282562091413063 | 1.32925013219226 | 0.999714303421225 |
| ebi-fl187-GCST90094807 | prot-a-1392 | Serine protease HTRA2, mitochondrial \|\| id:prot-a-1392 | Oranges liking \|\| id:ebi-fl187-GCST90094807 | Weighted mode | 7 | -0.286151298 | 0.38458929192578 | 0.48495753917446 | -1.039946311 | 0.467643713804318 | 0.75114895973571 | 0.35347365925755 | 1.59622858715175 | 0.999714303421225 |
| ebi-fl187-GCST90094807 | prot-a-1416 | Interferon-induced protein with tetratricopeptide repeats 2 \|\| id:prot-a-1416 | Oranges liking \|\| id:ebi-fl187-GCST90094807 | Weighted mode | 7 | -0.225268376 | 0.473813349261809 | 0.6512897606209 | -1.15394254 | 0.703405788995094 | 0.798301945277722 | 0.315390873825503 | 2.02062281671088 | 0.999714303421225 |
| ebi-fl187-GCST90094807 | prot-a-1428 | Interferon gamma \|\| id:prot-a-1428 | Oranges liking \|\| id:ebi-fl187-GCST90094807 | Weighted mode | 7 | -0.082650198 | 0.391544675059874 | 0.839805807864326 | -0.850077761 | 0.684777365042374 | 0.920673144137404 | 0.427381696946211 | 1.98333022773911 | 0.999714303421225 |
| ebi-fl187-GCST90094807 | prot-a-1483 | Interleukin-17C \|\| id:prot-a-1483 | Oranges liking \|\| id:ebi-fl187-GCST90094807 | Weighted mode | 7 | -0.593880553 | 0.405781402885355 | 0.193649081966872 | -1.389212102 | 0.201450997009216 | 0.552180356850437 | 0.249271627822111 | 1.22317629629741 | 0.999714303421225 |
| ebi-fl187-GCST90094807 | prot-a-1503 | Interleukin-1 receptor-like 2 \|\| id:prot-a-1503 | Oranges liking \|\| id:ebi-fl187-GCST90094807 | Weighted mode | 7 | -0.074804845 | 0.503047257330786 | 0.88665965930845 | -1.060777469 | 0.911167779710553 | 0.9279245580941 | 0.346186556350481 | 2.48722536944041 | 0.999714303421225 |
| ebi-fl187-GCST90094807 | prot-a-1522 | Interleukin-31 receptor subunit alpha \|\| id:prot-a-1522 | Oranges liking \|\| id:ebi-fl187-GCST90094807 | Weighted mode | 7 | -0.566100764 | 0.420308592298545 | 0.226665377771199 | -1.389905605 | 0.257704076740448 | 0.567734860484845 | 0.249098817187367 | 1.29395585032947 | 0.999714303421225 |
| ebi-fl187-GCST90094807 | prot-a-1540 | Interleukin-6 receptor subunit alpha \|\| id:prot-a-1540 | Oranges liking \|\| id:ebi-fl187-GCST90094807 | Weighted mode | 7 | -0.365501189 | 0.404034632531509 | 0.400525697642576 | -1.157409069 | 0.426406690418706 | 0.693848814087904 | 0.314299455027469 | 1.53174359392101 | 0.999714303421225 |
| ebi-fl187-GCST90094807 | prot-a-1541 | Interleukin-6 receptor subunit alpha \|\| id:prot-a-1541 | Oranges liking \|\| id:ebi-fl187-GCST90094807 | Weighted mode | 7 | -0.408226684 | 0.421938716816081 | 0.370657252252453 | -1.235226569 | 0.418773201118403 | 0.664828155939755 | 0.290768875748767 | 1.52009555971924 | 0.999714303421225 |
| ebi-fl187-GCST90094807 | prot-a-1545 | Interleukin-9 \|\| id:prot-a-1545 | Oranges liking \|\| id:ebi-fl187-GCST90094807 | Weighted mode | 7 | -0.156706958 | 0.439223266169302 | 0.733467040100574 | -1.01758456 | 0.704170643614927 | 0.854954559640586 | 0.36146698846504 | 2.02216889059269 | 0.999714303421225 |
| ebi-fl187-GCST90094807 | prot-a-1548 | Inositol monophosphatase 3 \|\| id:prot-a-1548 | Oranges liking \|\| id:ebi-fl187-GCST90094807 | Weighted mode | 7 | -0.6339742 | 0.387191434335083 | 0.152668327796257 | -1.392869412 | 0.124921010904945 | 0.530479374845447 | 0.248361629444854 | 1.13305895023088 | 0.999714303421225 |
| ebi-fl187-GCST90094807 | prot-a-1555 | Inhibin beta A chain:Inhibin beta B chain heterodimer \|\| id:prot-a-1555 | Oranges liking \|\| id:ebi-fl187-GCST90094807 | Weighted mode | 7 | -0.133515843 | 0.417742044154845 | 0.760097442367208 | -0.95229025 | 0.685258563128391 | 0.875013605664018 | 0.385856303343358 | 1.98428483210711 | 0.999714303421225 |
| ebi-fl187-GCST90094807 | prot-a-1603 | Junctophilin-3 \|\| id:prot-a-1603 | Oranges liking \|\| id:ebi-fl187-GCST90094807 | Weighted mode | 7 | 0.233311253444911 | 0.368483706140701 | 0.549981432752209 | -0.488916811 | 0.955539317480684 | 1.26277446106678 | 0.613290344133214 | 2.60007246938839 | 0.999714303421225 |
| ebi-fl187-GCST90094807 | prot-a-1627 | UPF0258 protein KIAA1024 \|\| id:prot-a-1627 | Oranges liking \|\| id:ebi-fl187-GCST90094807 | Weighted mode | 7 | -0.13527304 | 0.371472173980412 | 0.728231794921983 | -0.863358501 | 0.592812420634027 | 0.873477384539599 | 0.421743275750419 | 1.80906913084643 | 0.999714303421225 |
| ebi-fl187-GCST90094807 | prot-a-1666 | Kallikrein-7 \|\| id:prot-a-1666 | Oranges liking \|\| id:ebi-fl187-GCST90094807 | Weighted mode | 7 | 0.0993827481176239 | 0.392476538216025 | 0.808551391242962 | -0.669871267 | 0.868636763021033 | 1.1044889597378 | 0.511774455916763 | 2.38365914531128 | 0.999714303421225 |
| ebi-fl187-GCST90094807 | prot-a-1678 | Importin subunit alpha-3 \|\| id:prot-a-1678 | Oranges liking \|\| id:ebi-fl187-GCST90094807 | Weighted mode | 7 | -0.105773109 | 0.379747976888895 | 0.789952162902547 | -0.850079144 | 0.638532925510798 | 0.899628742414257 | 0.427381106005375 | 1.89370064049504 | 0.999714303421225 |
| ebi-fl187-GCST90094807 | prot-a-1718 | Protein LDOC1 \|\| id:prot-a-1718 | Oranges liking \|\| id:ebi-fl187-GCST90094807 | Weighted mode | 7 | -0.222438619 | 0.35475187372877 | 0.553737102037067 | -0.917752292 | 0.472875053175015 | 0.800564144403023 | 0.399415803036399 | 1.60460088066505 | 0.999714303421225 |
| ebi-fl187-GCST90094807 | prot-a-1723 | Leptin \|\| id:prot-a-1723 | Oranges liking \|\| id:ebi-fl187-GCST90094807 | Weighted mode | 7 | -0.415314361 | 0.338252289442232 | 0.265492151240025 | -1.078288849 | 0.247660125809968 | 0.660132727770974 | 0.340177122393019 | 1.2810244710427 | 0.999714303421225 |
| ebi-fl187-GCST90094807 | prot-a-1730 | Galectin-7 \|\| id:prot-a-1730 | Oranges liking \|\| id:ebi-fl187-GCST90094807 | Weighted mode | 7 | -0.342360621 | 0.421339995269007 | 0.447507344462327 | -1.168187012 | 0.48346576926061 | 0.710092084081841 | 0.310930143130206 | 1.62168506018581 | 0.999714303421225 |
| ebi-fl187-GCST90094807 | prot-a-1753 | Lipase member N \|\| id:prot-a-1753 | Oranges liking \|\| id:ebi-fl187-GCST90094807 | Weighted mode | 7 | 0.0921728895830458 | 0.367553681548202 | 0.810355715236868 | -0.628232326 | 0.812578105417522 | 1.09655438852433 | 0.533534082129683 | 2.25371080736264 | 0.999714303421225 |
| ebi-fl187-GCST90094807 | prot-a-1756 | VIP36-like protein \|\| id:prot-a-1756 | Oranges liking \|\| id:ebi-fl187-GCST90094807 | Weighted mode | 7 | 0.184567241595332 | 0.357848166404652 | 0.624462495100281 | -0.516815165 | 0.885949647748449 | 1.2026978498881 | 0.596417016449491 | 2.42528646606442 | 0.999714303421225 |
| ebi-fl187-GCST90094807 | prot-a-1760 | Ig Kappa chain V-I region HK102- like \|\| id:prot-a-1760 | Oranges liking \|\| id:ebi-fl187-GCST90094807 | Weighted mode | 7 | -0.848294251 | 0.410404887524323 | 0.0842400119756654 | -1.65268783 | -0.043900671 | 0.428144616756067 | 0.191534404142151 | 0.9570490151793 | 0.999714303421225 |
| ebi-fl187-GCST90094807 | prot-a-179 | Neutral ceramidase \|\| id:prot-a-179 | Oranges liking \|\| id:ebi-fl187-GCST90094807 | Weighted mode | 7 | -1.055114803 | 0.572295782899979 | 0.114795526866958 | -2.176814537 | 0.0665849319296668 | 0.348152456220593 | 0.113402194384968 | 1.06885173986103 | 0.999714303421225 |
| ebi-fl187-GCST90094807 | prot-a-1809 | Lactotransferrin \|\| id:prot-a-1809 | Oranges liking \|\| id:ebi-fl187-GCST90094807 | Weighted mode | 7 | -0.17433906 | 0.376708531982879 | 0.659820017118221 | -0.912687783 | 0.564009662455577 | 0.840012034693897 | 0.401443778951837 | 1.75770619804582 | 0.999714303421225 |
| ebi-fl187-GCST90094807 | prot-a-1818 | Lysophospholipase-like protein 1 \|\| id:prot-a-1818 | Oranges liking \|\| id:ebi-fl187-GCST90094807 | Weighted mode | 7 | 0.37670527711827 | 0.338593723002393 | 0.308476214152629 | -0.28693842 | 1.04034897420296 | 1.4574746949236 | 0.75055794679757 | 2.83020451039944 | 0.999714303421225 |
| ebi-fl187-GCST90094807 | prot-a-1852 | MAP kinase-activated protein kinase 3 \|\| id:prot-a-1852 | Oranges liking \|\| id:ebi-fl187-GCST90094807 | Weighted mode | 7 | -0.012850165 | 0.518554991659848 | 0.981033479144296 | -1.029217948 | 1.0035176188307 | 0.987232046027628 | 0.357286267608787 | 2.72786054506598 | 0.999714303421225 |
| ebi-fl187-GCST90094807 | prot-a-1856 | Megakaryocyte-associated tyrosine-protein kinase \|\| id:prot-a-1856 | Oranges liking \|\| id:ebi-fl187-GCST90094807 | Weighted mode | 7 | -0.10996863 | 0.35516137546582 | 0.76731377617485 | -0.806084925 | 0.58614766636612 | 0.895862238460069 | 0.446603132515275 | 1.79705221899946 | 0.999714303421225 |
| ebi-fl187-GCST90094807 | prot-a-1890 | Beta-1,3-N-acetylglucosaminyltransferase manic fringe \|\| id:prot-a-1890 | Oranges liking \|\| id:ebi-fl187-GCST90094807 | Weighted mode | 7 | -0.16181996 | 0.472768550160749 | 0.743810435927477 | -1.088446319 | 0.764806398044673 | 0.8505943315288 | 0.336739273035367 | 2.1485783654137 | 0.999714303421225 |
| ebi-fl187-GCST90094807 | prot-a-1919 | Matrilysin \|\| id:prot-a-1919 | Oranges liking \|\| id:ebi-fl187-GCST90094807 | Weighted mode | 7 | 0.17827062800113 | 0.42380962499934 | 0.688668822258074 | -0.652396237 | 1.00893749299984 | 1.19514871818135 | 0.520796328940715 | 2.74268534395356 | 0.999714303421225 |
| ebi-fl187-GCST90094807 | prot-a-1923 | Multimerin-2 \|\| id:prot-a-1923 | Oranges liking \|\| id:ebi-fl187-GCST90094807 | Weighted mode | 7 | -0.037060517 | 0.433861088272113 | 0.93470636834504 | -0.88742825 | 0.813307215748209 | 0.963617818069487 | 0.411713215752388 | 2.25535461037873 | 0.999714303421225 |
| ebi-fl187-GCST90094807 | prot-a-1948 | Mesothelin \|\| id:prot-a-1948 | Oranges liking \|\| id:ebi-fl187-GCST90094807 | Weighted mode | 7 | -0.290250146 | 0.364005463766282 | 0.455619294648182 | -1.003700855 | 0.423200563168906 | 0.748076415988679 | 0.366520488971068 | 1.52684049322722 | 0.999714303421225 |
| ebi-fl187-GCST90094807 | prot-a-195 | ATPase family AAA domain-containing protein 1 \|\| id:prot-a-195 | Oranges liking \|\| id:ebi-fl187-GCST90094807 | Weighted mode | 7 | -0.102805374 | 0.400424083091033 | 0.805955485781949 | -0.887636577 | 0.682025828928159 | 0.902302567995847 | 0.411627453908483 | 1.97788052395774 | 0.999714303421225 |
| ebi-fl187-GCST90094807 | prot-a-1966 | Mucin-1 \|\| id:prot-a-1966 | Oranges liking \|\| id:ebi-fl187-GCST90094807 | Weighted mode | 7 | 0.264813489865304 | 0.394494300149432 | 0.527030178120553 | -0.508395338 | 1.03802231815819 | 1.30318789536069 | 0.601459944525361 | 2.82362725244295 | 0.999714303421225 |
| ebi-fl187-GCST90094807 | prot-a-1986 | Myocardial zonula adherens protein \|\| id:prot-a-1986 | Oranges liking \|\| id:ebi-fl187-GCST90094807 | Weighted mode | 7 | -0.798286847 | 0.373439963287761 | 0.076397066831462 | -1.530229175 | -0.066344519 | 0.450099393125458 | 0.216486048432005 | 0.935808405018468 | 0.999714303421225 |
| ebi-fl187-GCST90094807 | prot-a-2071 | Epididymal secretory protein E1 \|\| id:prot-a-2071 | Oranges liking \|\| id:ebi-fl187-GCST90094807 | Weighted mode | 7 | 0.142587363250393 | 0.544307604296443 | 0.802112839948721 | -0.924255541 | 1.20943026767142 | 1.15325382855948 | 0.396826730300929 | 3.35157460808778 | 0.999714303421225 |
| ebi-fl187-GCST90094807 | prot-a-2098 | Neuritin \|\| id:prot-a-2098 | Oranges liking \|\| id:ebi-fl187-GCST90094807 | Weighted mode | 7 | -0.281497934 | 0.420185443492868 | 0.527824221700616 | -1.105061404 | 0.542065534860043 | 0.754652474494406 | 0.331190545315241 | 1.71955499731568 | 0.999714303421225 |
| ebi-fl187-GCST90094807 | prot-a-2115 | Neurotrophin-4 \|\| id:prot-a-2115 | Oranges liking \|\| id:ebi-fl187-GCST90094807 | Weighted mode | 7 | -0.310462041 | 0.404550622192295 | 0.471938848721936 | -1.103381261 | 0.482457178062823 | 0.733108151617728 | 0.331747460410008 | 1.62005026746588 | 0.999714303421225 |
| ebi-fl187-GCST90094807 | prot-a-2118 | Netrin-1 \|\| id:prot-a-2118 | Oranges liking \|\| id:ebi-fl187-GCST90094807 | Weighted mode | 7 | 0.492128756912545 | 0.492671564659793 | 0.356409654890626 | -0.47350751 | 1.45776502364574 | 1.63579472552485 | 0.622813906782047 | 4.29634655700027 | 0.999714303421225 |
| ebi-fl187-GCST90094807 | prot-a-2120 | Netrin-G1 \|\| id:prot-a-2120 | Oranges liking \|\| id:ebi-fl187-GCST90094807 | Weighted mode | 7 | -0.277106351 | 0.380265601263046 | 0.493623859064838 | -1.02242693 | 0.468214227128302 | 0.757973881280315 | 0.359720862650237 | 1.59713951665285 | 0.999714303421225 |
| ebi-fl187-GCST90094807 | prot-a-2145 | Olfactomedin-like protein 3 \|\| id:prot-a-2145 | Oranges liking \|\| id:ebi-fl187-GCST90094807 | Weighted mode | 7 | -0.309776929 | 0.392386969221046 | 0.459889781275405 | -1.078855389 | 0.459301530288949 | 0.733610584938097 | 0.339984452942357 | 1.58296794360907 | 0.999714303421225 |
| ebi-fl187-GCST90094807 | prot-a-2162 | Prolyl 4-hydroxylase subunit alpha-2 \|\| id:prot-a-2162 | Oranges liking \|\| id:ebi-fl187-GCST90094807 | Weighted mode | 7 | -0.368761399 | 0.379330086479125 | 0.368525475977282 | -1.112248369 | 0.374725570398327 | 0.691590404858125 | 0.328818823418237 | 1.45459217668771 | 0.999714303421225 |
| ebi-fl187-GCST90094807 | prot-a-2168 | Polyadenylate-binding protein-interacting protein 1 \|\| id:prot-a-2168 | Oranges liking \|\| id:ebi-fl187-GCST90094807 | Weighted mode | 7 | 0.470163401210995 | 0.62594268235928 | 0.480986819283768 | -0.756684256 | 1.69701105863518 | 1.60025565556717 | 0.46921966270232 | 5.45761051109088 | 0.999714303421225 |
| ebi-fl187-GCST90094807 | prot-a-219 | Beta-1,4-galactosyltransferase 5 \|\| id:prot-a-219 | Oranges liking \|\| id:ebi-fl187-GCST90094807 | Weighted mode | 7 | 0.302554553590229 | 0.508396401484541 | 0.573510337868447 | -0.693902393 | 1.29901150049993 | 1.35331150240857 | 0.499622536170928 | 3.66567136179939 | 0.999714303421225 |
| ebi-fl187-GCST90094807 | prot-a-2190 | Pyruvate carboxylase, mitochondrial \|\| id:prot-a-2190 | Oranges liking \|\| id:ebi-fl187-GCST90094807 | Weighted mode | 7 | -0.041568258 | 0.367619433307404 | 0.913660194871996 | -0.762102347 | 0.678965831147578 | 0.959283854179928 | 0.466684262492131 | 1.97183746453378 | 0.999714303421225 |
| ebi-fl187-GCST90094807 | prot-a-2197 | Protocadherin-9 \|\| id:prot-a-2197 | Oranges liking \|\| id:ebi-fl187-GCST90094807 | Weighted mode | 7 | -0.227960803 | 0.4074427150336 | 0.596077734737428 | -1.026548524 | 0.570626918503086 | 0.7961554661593 | 0.35824129027643 | 1.76937595832748 | 0.999714303421225 |
| ebi-fl187-GCST90094807 | prot-a-2198 | Protocadherin alpha-4 \|\| id:prot-a-2198 | Oranges liking \|\| id:ebi-fl187-GCST90094807 | Weighted mode | 7 | -0.363339241 | 0.720809058522236 | 0.632181490823803 | -1.776124995 | 1.049446514 | 0.695350502279948 | 0.169292888605154 | 2.85606988577462 | 0.999714303421225 |
| ebi-fl187-GCST90094807 | prot-a-2254 | Placenta growth factor \|\| id:prot-a-2254 | Oranges liking \|\| id:ebi-fl187-GCST90094807 | Weighted mode | 7 | -0.300624192 | 0.343913814466419 | 0.415661846417411 | -0.974695269 | 0.37344688399077 | 0.740355951893026 | 0.37730731338781 | 1.45273339809357 | 0.999714303421225 |
| ebi-fl187-GCST90094807 | prot-a-2261 | Membrane-associated progesterone receptor component 2 \|\| id:prot-a-2261 | Oranges liking \|\| id:ebi-fl187-GCST90094807 | Weighted mode | 7 | -0.433217319 | 0.356093404340929 | 0.2694440196056 | -1.131160392 | 0.264725753134431 | 0.648419562358936 | 0.322658628653428 | 1.30307356293069 | 0.999714303421225 |
| ebi-fl187-GCST90094807 | prot-a-2268 | Polymeric immunoglobulin receptor \|\| id:prot-a-2268 | Oranges liking \|\| id:ebi-fl187-GCST90094807 | Weighted mode | 7 | -0.19972064 | 0.494663370202347 | 0.700392135051132 | -1.169260845 | 0.769819565759405 | 0.818959505785165 | 0.310596435212622 | 2.15937659315617 | 0.999714303421225 |
| ebi-fl187-GCST90094807 | prot-a-2283 | Group 10 secretory phospholipase A2 \|\| id:prot-a-2283 | Oranges liking \|\| id:ebi-fl187-GCST90094807 | Weighted mode | 7 | -0.110286337 | 0.390207324181526 | 0.786953574951679 | -0.875092693 | 0.654520018031552 | 0.895577661232238 | 0.416823381378126 | 1.92421870540561 | 0.999714303421225 |
| ebi-fl187-GCST90094807 | prot-a-2298 | Inactive phospholipase D5 \|\| id:prot-a-2298 | Oranges liking \|\| id:ebi-fl187-GCST90094807 | Weighted mode | 7 | 1.06269087404897 | 0.583537110463314 | 0.118436468118013 | -0.081041862 | 2.20642361055707 | 2.89414830926013 | 0.922155086956944 | 9.08317326929665 | 0.999714303421225 |
| ebi-fl187-GCST90094807 | prot-a-2304 | Procollagen-lysine,2-oxoglutarate 5-dioxygenase 2 \|\| id:prot-a-2304 | Oranges liking \|\| id:ebi-fl187-GCST90094807 | Weighted mode | 7 | -0.049795361 | 0.360543666102368 | 0.894670560699745 | -0.756460946 | 0.656870225017114 | 0.951424103491948 | 0.469324455896611 | 1.92874633599931 | 0.999714303421225 |
| ebi-fl187-GCST90094807 | prot-a-2331 | Periostin \|\| id:prot-a-2331 | Oranges liking \|\| id:ebi-fl187-GCST90094807 | Weighted mode | 7 | -0.074890467 | 0.367418872182541 | 0.845224991864645 | -0.795031456 | 0.645250522459774 | 0.927845110404628 | 0.451567029982798 | 1.90646458164709 | 0.999714303421225 |
| ebi-fl187-GCST90094807 | prot-a-2333 | Periostin \|\| id:prot-a-2333 | Oranges liking \|\| id:ebi-fl187-GCST90094807 | Weighted mode | 7 | -0.032673839 | 0.409071595138809 | 0.93893558809908 | -0.834454165 | 0.769106487803826 | 0.967854184723717 | 0.434111369894799 | 2.15783733817941 | 0.999714303421225 |
| ebi-fl187-GCST90094807 | prot-a-2367 | cAMP-dependent protein kinase catalytic subunit alpha \|\| id:prot-a-2367 | Oranges liking \|\| id:ebi-fl187-GCST90094807 | Weighted mode | 7 | -0.088608995 | 0.432725085032611 | 0.844521849878679 | -0.936750162 | 0.759532171711913 | 0.915203352754029 | 0.391899377726244 | 2.13727610835174 | 0.999714303421225 |
| ebi-fl187-GCST90094807 | prot-a-2385 | Phosphoribosyl pyrophosphate synthase-associated protein 1 \|\| id:prot-a-2385 | Oranges liking \|\| id:ebi-fl187-GCST90094807 | Weighted mode | 7 | -0.085932899 | 0.613454913007966 | 0.893180740857468 | -1.288304528 | 1.11643873070242 | 0.917655804966233 | 0.275737892789141 | 3.05395884428616 | 0.999714303421225 |
| ebi-fl187-GCST90094807 | prot-a-2393 | Trypsin-3 \|\| id:prot-a-2393 | Oranges liking \|\| id:ebi-fl187-GCST90094807 | Weighted mode | 7 | -0.614995737 | 0.420353095596679 | 0.1937812394128 | -1.438887804 | 0.208896330361478 | 0.540643200062035 | 0.237191415289861 | 1.23231723802532 | 0.999714303421225 |
| ebi-fl187-GCST90094807 | prot-a-2405 | Pregnancy-specific beta-1-glycoprotein 6 \|\| id:prot-a-2405 | Oranges liking \|\| id:ebi-fl187-GCST90094807 | Weighted mode | 7 | 0.365578202862215 | 0.362544849898325 | 0.352199302483033 | -0.345009703 | 1.07616610866293 | 1.44134715831348 | 0.708213481682602 | 2.93341158352787 | 0.999714303421225 |
| ebi-fl187-GCST90094807 | prot-a-244 | Beclin-1 \|\| id:prot-a-244 | Oranges liking \|\| id:ebi-fl187-GCST90094807 | Weighted mode | 7 | -0.307801634 | 0.362059644176161 | 0.427861913009362 | -1.017438537 | 0.40183526835918 | 0.73506111451492 | 0.3615197747976 | 1.49456511023337 | 0.999714303421225 |
| ebi-fl187-GCST90094807 | prot-a-2454 | tRNA pseudouridine synthase A, mitochondrial \|\| id:prot-a-2454 | Oranges liking \|\| id:ebi-fl187-GCST90094807 | Weighted mode | 7 | 0.337801056475897 | 0.381055970183801 | 0.409477257439476 | -0.409068645 | 1.08467075803615 | 1.40186158437777 | 0.66426863198084 | 2.9584656073461 | 0.999714303421225 |
| ebi-fl187-GCST90094807 | prot-a-2466 | Glutaminyl-peptide cyclotransferase-like protein \|\| id:prot-a-2466 | Oranges liking \|\| id:ebi-fl187-GCST90094807 | Weighted mode | 7 | -0.022600106 | 0.383101997559568 | 0.954873883746846 | -0.773480021 | 0.728279809108269 | 0.97765336322089 | 0.461404573381284 | 2.07151414129413 | 0.999714303421225 |
| ebi-fl187-GCST90094807 | prot-a-2474 | Ras-related protein Rab-27A \|\| id:prot-a-2474 | Oranges liking \|\| id:ebi-fl187-GCST90094807 | Weighted mode | 7 | 0.362192709833135 | 0.387728750246729 | 0.386283558548278 | -0.397755641 | 1.12214106031672 | 1.43647573828864 | 0.671826174612809 | 3.07142327087972 | 0.999714303421225 |
| ebi-fl187-GCST90094807 | prot-a-2490 | Rap1 GTPase-GDP dissociation stimulator 1 \|\| id:prot-a-2490 | Oranges liking \|\| id:ebi-fl187-GCST90094807 | Weighted mode | 7 | -0.39542967 | 0.497547921090974 | 0.457031404947094 | -1.370623595 | 0.579764255441062 | 0.673390641392853 | 0.25394854906065 | 1.78561743153406 | 0.999714303421225 |
| ebi-fl187-GCST90094807 | prot-a-2545 | Receptor-interacting serine/threonine-protein kinase 2 \|\| id:prot-a-2545 | Oranges liking \|\| id:ebi-fl187-GCST90094807 | Weighted mode | 7 | -0.321834897 | 0.41900310363056 | 0.471568167197138 | -1.14308098 | 0.499411186494301 | 0.724817850327313 | 0.318835182263539 | 1.64775076709966 | 0.999714303421225 |
| ebi-fl187-GCST90094807 | prot-a-2610 | RUN and FYVE domain-containing protein 1 \|\| id:prot-a-2610 | Oranges liking \|\| id:ebi-fl187-GCST90094807 | Weighted mode | 7 | -0.286394374 | 0.421847130738772 | 0.522508527280936 | -1.113214751 | 0.540426001818157 | 0.75096639559577 | 0.328501212291949 | 1.71673803995859 | 0.999714303421225 |
| ebi-fl187-GCST90094807 | prot-a-2627 | Serine--tRNA ligase, mitochondrial \|\| id:prot-a-2627 | Oranges liking \|\| id:ebi-fl187-GCST90094807 | Weighted mode | 7 | -0.090361933 | 0.411507628934001 | 0.833472368447402 | -0.896916885 | 0.716193019956519 | 0.913600463493397 | 0.407825094898852 | 2.04662689308602 | 0.999714303421225 |
| ebi-fl187-GCST90094807 | prot-a-264 | BolA-like protein 2 \|\| id:prot-a-264 | Oranges liking \|\| id:ebi-fl187-GCST90094807 | Weighted mode | 7 | -0.269480209 | 0.386108816235961 | 0.511343209902209 | -1.026253488 | 0.487293071183372 | 0.763776395547063 | 0.358346999935247 | 1.6279036311181 | 0.999714303421225 |
| ebi-fl187-GCST90094807 | prot-a-2646 | Secretoglobin family 1C member 1 \|\| id:prot-a-2646 | Oranges liking \|\| id:ebi-fl187-GCST90094807 | Weighted mode | 7 | -0.228948307 | 0.388015742835573 | 0.576688478452704 | -0.989459163 | 0.531562549030487 | 0.795369647543494 | 0.371777707841413 | 1.70158904875306 | 0.999714303421225 |
| ebi-fl187-GCST90094807 | prot-a-2672 | Semaphorin-3E \|\| id:prot-a-2672 | Oranges liking \|\| id:ebi-fl187-GCST90094807 | Weighted mode | 7 | -0.360881546 | 0.352209998208177 | 0.345064913960595 | -1.051213143 | 0.329450050348104 | 0.697061563209594 | 0.349513482110805 | 1.39020337633259 | 0.999714303421225 |
| ebi-fl187-GCST90094807 | prot-a-2701 | Plasma protease C1 inhibitor \|\| id:prot-a-2701 | Oranges liking \|\| id:ebi-fl187-GCST90094807 | Weighted mode | 7 | -0.708014705 | 0.397001585372828 | 0.12478702847183 | -1.486137812 | 0.070108402775428 | 0.492621225970811 | 0.2262447701822 | 1.07262445041957 | 0.999714303421225 |
| ebi-fl187-GCST90094807 | prot-a-2738 | Signaling threshold-regulating transmembrane adapter 1 \|\| id:prot-a-2738 | Oranges liking \|\| id:ebi-fl187-GCST90094807 | Weighted mode | 7 | 0.535781055567061 | 0.425769932351075 | 0.255003600867484 | -0.298728012 | 1.37029012297517 | 1.70878237515743 | 0.741761132245402 | 3.93649259676046 | 0.999714303421225 |
| ebi-fl187-GCST90094807 | prot-a-274 | Serine/threonine-protein kinase BRSK2 \|\| id:prot-a-274 | Oranges liking \|\| id:ebi-fl187-GCST90094807 | Weighted mode | 7 | -0.482868401 | 0.466714736583652 | 0.340737520104984 | -1.397629285 | 0.431892482363076 | 0.617011015853367 | 0.24718226857301 | 1.54016951087232 | 0.999714303421225 |
| ebi-fl187-GCST90094807 | prot-a-2749 | Mitochondrial glutamate carrier 2 \|\| id:prot-a-2749 | Oranges liking \|\| id:ebi-fl187-GCST90094807 | Weighted mode | 7 | -0.001386088 | 0.380962820841679 | 0.997214950893228 | -0.748073217 | 0.745301040471981 | 0.998614871799104 | 0.473277577869102 | 2.10707565456261 | 0.999714303421225 |
| ebi-fl187-GCST90094807 | prot-a-2760 | Sodium-coupled monocarboxylate transporter 1 \|\| id:prot-a-2760 | Oranges liking \|\| id:ebi-fl187-GCST90094807 | Weighted mode | 7 | -0.938467664 | 0.392477041123575 | 0.0539423185073557 | -1.707722665 | -0.169212664 | 0.391226867200204 | 0.181278154009344 | 0.844329326143716 | 0.999714303421225 |
| ebi-fl187-GCST90094807 | prot-a-2777 | Small integral membrane protein 10 \|\| id:prot-a-2777 | Oranges liking \|\| id:ebi-fl187-GCST90094807 | Weighted mode | 7 | 0.150430018944633 | 0.386318343514209 | 0.710430136719403 | -0.606753934 | 0.907613972232483 | 1.16233396089962 | 0.545117487398492 | 2.47840193699927 | 0.999714303421225 |
| ebi-fl187-GCST90094807 | prot-a-2799 | Superoxide dismutase [Mn], mitochondrial \|\| id:prot-a-2799 | Oranges liking \|\| id:ebi-fl187-GCST90094807 | Weighted mode | 7 | -0.312347214 | 0.436508687929683 | 0.50114874096867 | -1.167904243 | 0.543209813985857 | 0.731727417851088 | 0.311018077121822 | 1.72152377440524 | 0.999714303421225 |
| ebi-fl187-GCST90094807 | prot-a-2805 | Protein SPACA7 \|\| id:prot-a-2805 | Oranges liking \|\| id:ebi-fl187-GCST90094807 | Weighted mode | 7 | -0.176259895 | 0.373239143201436 | 0.653439048137089 | -0.907808615 | 0.555288825923397 | 0.838400059244651 | 0.403407276609267 | 1.74244417515122 | 0.999714303421225 |
| ebi-fl187-GCST90094807 | prot-a-2811 | SPARC-like protein 1 \|\| id:prot-a-2811 | Oranges liking \|\| id:ebi-fl187-GCST90094807 | Weighted mode | 7 | -0.135016548 | 0.468291442827754 | 0.782804721582596 | -1.052867776 | 0.782834679952728 | 0.873701453565781 | 0.348935643650282 | 2.18766481399655 | 0.999714303421225 |
| ebi-fl187-GCST90094807 | prot-a-2831 | Sepiapterin reductase \|\| id:prot-a-2831 | Oranges liking \|\| id:ebi-fl187-GCST90094807 | Weighted mode | 7 | -0.134484458 | 0.415166202487485 | 0.756985668331723 | -0.948210215 | 0.679241298511996 | 0.874166464748457 | 0.387433826443932 | 1.97238071622381 | 0.999714303421225 |
| ebi-fl187-GCST90094807 | prot-a-2834 | SPRY domain-containing SOCS box protein 1 \|\| id:prot-a-2834 | Oranges liking \|\| id:ebi-fl187-GCST90094807 | Weighted mode | 7 | 0.275619483718906 | 0.363125189633656 | 0.47659005478624 | -0.436105888 | 0.98734485540087 | 1.31734649685427 | 0.646549260526458 | 2.68409833360749 | 0.999714303421225 |
| ebi-fl187-GCST90094807 | prot-a-2835 | Spectrin alpha chain, non-erythrocytic 1 \|\| id:prot-a-2835 | Oranges liking \|\| id:ebi-fl187-GCST90094807 | Weighted mode | 7 | 0.302226337056345 | 0.445731317683708 | 0.523016516061283 | -0.571407046 | 1.17585971971641 | 1.35286739608349 | 0.564730278161978 | 3.2409280361992 | 0.999714303421225 |
| ebi-fl187-GCST90094807 | prot-a-2859 | Sia-alpha-2,3-Gal-beta-1,4-GlcNAc-R:alpha 2,8-sialyltransferase \|\| id:prot-a-2859 | Oranges liking \|\| id:ebi-fl187-GCST90094807 | Weighted mode | 7 | -0.331657884 | 0.374147633251956 | 0.409502555595859 | -1.064987246 | 0.401671476834277 | 0.717732828497029 | 0.344732251622067 | 1.49432033318164 | 0.999714303421225 |
| ebi-fl187-GCST90094807 | prot-a-2861 | Alpha-2,8-sialyltransferase 8F \|\| id:prot-a-2861 | Oranges liking \|\| id:ebi-fl187-GCST90094807 | Weighted mode | 7 | -0.248320199 | 0.373572031387911 | 0.530933675232404 | -0.98052138 | 0.483880982990471 | 0.780110113171206 | 0.375115470134749 | 1.6223585458989 | 0.999714303421225 |
| ebi-fl187-GCST90094807 | prot-a-289 | Uncharacterized protein C17orf89 \|\| id:prot-a-289 | Oranges liking \|\| id:ebi-fl187-GCST90094807 | Weighted mode | 7 | -0.001845365 | 0.439621680611742 | 0.996786877002318 | -0.863503859 | 0.859813128937495 | 0.998156336577709 | 0.421681976576776 | 2.36271912861525 | 0.999714303421225 |
| ebi-fl187-GCST90094807 | prot-a-2921 | Tachykinin-3 \|\| id:prot-a-2921 | Oranges liking \|\| id:ebi-fl187-GCST90094807 | Weighted mode | 7 | -0.180887762 | 0.509800752272286 | 0.73486268850483 | -1.180097236 | 0.818321712867403 | 0.834529019652883 | 0.307248861486016 | 2.2666924826815 | 0.999714303421225 |
| ebi-fl187-GCST90094807 | prot-a-2928 | Tax1-binding protein 1 \|\| id:prot-a-2928 | Oranges liking \|\| id:ebi-fl187-GCST90094807 | Weighted mode | 7 | 0.0369336240962836 | 0.399633518895655 | 0.929373907753187 | -0.746348073 | 0.820215321131767 | 1.03762414531198 | 0.474094754650557 | 2.27098877676468 | 0.999714303421225 |
| ebi-fl187-GCST90094807 | prot-a-296 | Uncharacterized protein C1orf185 \|\| id:prot-a-296 | Oranges liking \|\| id:ebi-fl187-GCST90094807 | Weighted mode | 7 | -0.23100072 | 0.410939552328559 | 0.594390000994953 | -1.036442243 | 0.574440802517108 | 0.793738894505832 | 0.354714427571097 | 1.77613703780081 | 0.999714303421225 |
| ebi-fl187-GCST90094807 | prot-a-2964 | Transforming growth factor beta-2 \|\| id:prot-a-2964 | Oranges liking \|\| id:ebi-fl187-GCST90094807 | Weighted mode | 7 | -0.108128536 | 0.376273833640414 | 0.783498373206348 | -0.84562525 | 0.629368178011404 | 0.897512226452845 | 0.429288861480596 | 1.87642463830558 | 0.999714303421225 |
| ebi-fl187-GCST90094807 | prot-a-2977 | Thymocyte nuclear protein 1 \|\| id:prot-a-2977 | Oranges liking \|\| id:ebi-fl187-GCST90094807 | Weighted mode | 7 | 0.0129042901984588 | 0.368918899153028 | 0.973231406069913 | -0.710176752 | 0.735985332538393 | 1.01298790984818 | 0.491557305975574 | 2.08753789847967 | 0.999714303421225 |
| ebi-fl187-GCST90094807 | prot-a-3015 | Transmembrane protein 70, mitochondrial \|\| id:prot-a-3015 | Oranges liking \|\| id:ebi-fl187-GCST90094807 | Weighted mode | 7 | -0.181028102 | 0.414931747603191 | 0.677891683520734 | -0.994294327 | 0.632238123641177 | 0.834411910005624 | 0.369984440487967 | 1.88181761006211 | 0.999714303421225 |
| ebi-fl187-GCST90094807 | prot-a-3030 | Tumor necrosis factor alpha-induced protein 3 \|\| id:prot-a-3030 | Oranges liking \|\| id:ebi-fl187-GCST90094807 | Weighted mode | 7 | -0.068137753 | 0.351891784881437 | 0.852852386062935 | -0.757845651 | 0.621570145315496 | 0.934131785209314 | 0.468675029563619 | 1.86184912166286 | 0.999714303421225 |
| ebi-fl187-GCST90094807 | prot-a-3032 | Tumor necrosis factor receptor superfamily member 10A \|\| id:prot-a-3032 | Oranges liking \|\| id:ebi-fl187-GCST90094807 | Weighted mode | 7 | 0.118895553588708 | 0.375629671088731 | 0.762328139443154 | -0.617338602 | 0.855129708922621 | 1.1262522791362 | 0.539378028814468 | 2.35167939459358 | 0.999714303421225 |
| ebi-fl187-GCST90094807 | prot-a-3081 | Tumor protein p53-inducible protein 11 \|\| id:prot-a-3081 | Oranges liking \|\| id:ebi-fl187-GCST90094807 | Weighted mode | 7 | 0.20432208708829 | 0.489866943260072 | 0.691119483621093 | -0.755817122 | 1.16446129587803 | 1.2266931918728 | 0.469626715724794 | 3.20419630443022 | 0.999714303421225 |
| ebi-fl187-GCST90094807 | prot-a-315 | Complement C4 \|\| id:prot-a-315 | Oranges liking \|\| id:ebi-fl187-GCST90094807 | Weighted mode | 7 | 0.103461722270154 | 0.39285674208373 | 0.801085884148701 | -0.666537492 | 0.873460936754264 | 1.10900334242227 | 0.513483443691588 | 2.39518611284081 | 0.999714303421225 |
| ebi-fl187-GCST90094807 | prot-a-3157 | UDP-glucuronosyltransferase 1-8 \|\| id:prot-a-3157 | Oranges liking \|\| id:ebi-fl187-GCST90094807 | Weighted mode | 7 | 0.0359015506284797 | 0.379720899885878 | 0.927752914103599 | -0.708351413 | 0.7801545144048 | 1.03655379339797 | 0.492455384092925 | 2.18180936042923 | 0.999714303421225 |
| ebi-fl187-GCST90094807 | prot-a-317 | Glutaredoxin-like protein C5orf63 \|\| id:prot-a-317 | Oranges liking \|\| id:ebi-fl187-GCST90094807 | Weighted mode | 7 | -0.26409454 | 0.404789310777319 | 0.538304561948182 | -1.057481589 | 0.529292509141844 | 0.767900938884212 | 0.347329428019762 | 1.69773075463593 | 0.999714303421225 |
| ebi-fl187-GCST90094807 | prot-a-3192 | Guanine nucleotide exchange factor VAV3 \|\| id:prot-a-3192 | Oranges liking \|\| id:ebi-fl187-GCST90094807 | Weighted mode | 7 | -0.290355493 | 0.385350639372135 | 0.479671696084165 | -1.045642746 | 0.464931759914484 | 0.747997612202875 | 0.351465843349133 | 1.5919055534094 | 0.999714303421225 |
| ebi-fl187-GCST90094807 | prot-a-3233 | Serine/threonine-protein kinase WNK3 \|\| id:prot-a-3233 | Oranges liking \|\| id:ebi-fl187-GCST90094807 | Weighted mode | 7 | -0.036452126 | 0.492801162496838 | 0.943439261792536 | -1.002342405 | 0.929438152242953 | 0.964204252863945 | 0.367018727086912 | 2.53308556928422 | 0.999714303421225 |
| ebi-fl187-GCST90094807 | prot-a-3250 | 14-3-3 protein theta \|\| id:prot-a-3250 | Oranges liking \|\| id:ebi-fl187-GCST90094807 | Weighted mode | 7 | -0.14496732 | 0.410431284494559 | 0.736011362920023 | -0.949412637 | 0.659477997805136 | 0.865050562670577 | 0.386968247387216 | 1.9337826321132 | 0.999714303421225 |
| ebi-fl187-GCST90094807 | prot-a-3278 | E3 ubiquitin-protein ligase ZNRF3 \|\| id:prot-a-3278 | Oranges liking \|\| id:ebi-fl187-GCST90094807 | Weighted mode | 7 | 0.249301430262681 | 0.366715240963742 | 0.521966761058253 | -0.469460442 | 0.968063302551615 | 1.28312874861875 | 0.625339584232734 | 2.63284050305564 | 0.999714303421225 |
| ebi-fl187-GCST90094807 | prot-a-355 | Calpain-3 \|\| id:prot-a-355 | Oranges liking \|\| id:ebi-fl187-GCST90094807 | Weighted mode | 7 | 0.104060704364167 | 0.379961495526443 | 0.793367348919358 | -0.640663827 | 0.848785235595994 | 1.10966781455017 | 0.526942509319102 | 2.33680645776647 | 0.999714303421225 |
| ebi-fl187-GCST90094807 | prot-a-385 | Coiled-coil domain-containing protein 90B, mitochondrial \|\| id:prot-a-385 | Oranges liking \|\| id:ebi-fl187-GCST90094807 | Weighted mode | 7 | 0.110011313759656 | 0.40202513221532 | 0.793535065270692 | -0.677957945 | 0.897980572901682 | 1.11629069983211 | 0.507652588946741 | 2.45464113384518 | 0.999714303421225 |
| ebi-fl187-GCST90094807 | prot-a-390 | C-C motif chemokine 14 \|\| id:prot-a-390 | Oranges liking \|\| id:ebi-fl187-GCST90094807 | Weighted mode | 7 | 0.514029114541821 | 0.558660114346502 | 0.393004627412198 | -0.58094471 | 1.60900293866097 | 1.67201437906004 | 0.5593696749855 | 4.99782560407121 | 0.999714303421225 |
| ebi-fl187-GCST90094807 | prot-a-40 | Pituitary adenylate cyclase-activating polypeptide 27 \|\| id:prot-a-40 | Oranges liking \|\| id:ebi-fl187-GCST90094807 | Weighted mode | 7 | 0.0324040168847828 | 0.41365451698508 | 0.940107984803817 | -0.778358836 | 0.84316687017554 | 1.03293474409118 | 0.459158948239605 | 2.32371423804624 | 0.999714303421225 |
| ebi-fl187-GCST90094807 | prot-a-401 | C-C motif chemokine 24 \|\| id:prot-a-401 | Oranges liking \|\| id:ebi-fl187-GCST90094807 | Weighted mode | 7 | 0.0939652388516521 | 0.475050045044455 | 0.849732186708476 | -0.837132849 | 1.02506332713878 | 1.09852155938258 | 0.432950078646608 | 2.78727196493558 | 0.999714303421225 |
| ebi-fl187-GCST90094807 | prot-a-412 | Cerebral cavernous malformations 2 protein \|\| id:prot-a-412 | Oranges liking \|\| id:ebi-fl187-GCST90094807 | Weighted mode | 7 | -0.356668944 | 0.45508339060287 | 0.462997363172969 | -1.248632389 | 0.535294501825731 | 0.700004200140587 | 0.286896891930814 | 1.70795116293079 | 0.999714303421225 |
| ebi-fl187-GCST90094807 | prot-a-44 | Adhesion G protein-coupled receptor F5 \|\| id:prot-a-44 | Oranges liking \|\| id:ebi-fl187-GCST90094807 | Weighted mode | 7 | -0.230019268 | 0.50467364460052 | 0.664579694604108 | -1.219179611 | 0.759141075519548 | 0.79451829365883 | 0.295472469790255 | 2.13644039123728 | 0.999714303421225 |
| ebi-fl187-GCST90094807 | prot-a-48 | Adrenomedullin \|\| id:prot-a-48 | Oranges liking \|\| id:ebi-fl187-GCST90094807 | Weighted mode | 7 | -0.464311048 | 0.385743169287356 | 0.274043411173386 | -1.22036766 | 0.291745563979189 | 0.628568009345172 | 0.295121642662108 | 1.33876234493757 | 0.999714303421225 |
| ebi-fl187-GCST90094807 | prot-a-502 | Carcinoembryonic antigen-related cell adhesion molecule 1 \|\| id:prot-a-502 | Oranges liking \|\| id:ebi-fl187-GCST90094807 | Weighted mode | 7 | 0.0837382244883629 | 0.40865061412913 | 0.844414084172116 | -0.717216979 | 0.884693428181457 | 1.08734421646108 | 0.488108784358274 | 2.42224168660636 | 0.999714303421225 |
| ebi-fl187-GCST90094807 | prot-a-518 | Complement factor B \|\| id:prot-a-518 | Oranges liking \|\| id:ebi-fl187-GCST90094807 | Weighted mode | 7 | -0.21685507 | 0.330938493581742 | 0.536591016777386 | -0.865494517 | 0.431784377616818 | 0.805046636417794 | 0.42084338685754 | 1.54000302023753 | 0.999714303421225 |
| ebi-fl187-GCST90094807 | prot-a-525 | Properdin \|\| id:prot-a-525 | Oranges liking \|\| id:ebi-fl187-GCST90094807 | Weighted mode | 7 | -0.47014793 | 0.423956447929801 | 0.309910320277116 | -1.301102568 | 0.360806707476035 | 0.624909818243925 | 0.272231473680363 | 1.434486159 | 0.999714303421225 |
| ebi-fl187-GCST90094807 | prot-a-533 | Chondroadherin \|\| id:prot-a-533 | Oranges liking \|\| id:ebi-fl187-GCST90094807 | Weighted mode | 7 | 0.088493922238697 | 0.349000420645003 | 0.808297441521967 | -0.595546902 | 0.772534746702902 | 1.09252761246826 | 0.55126099754476 | 2.16524765822685 | 0.999714303421225 |
| ebi-fl187-GCST90094807 | prot-a-535 | Coiled-coil-helix-coiled-coil-helix domain-containing protein 2 \|\| id:prot-a-535 | Oranges liking \|\| id:ebi-fl187-GCST90094807 | Weighted mode | 7 | 0.118417680913884 | 0.488104736885193 | 0.816390181613637 | -0.838267603 | 1.07510296520886 | 1.12571420252334 | 0.432459065478862 | 2.93029460339685 | 0.999714303421225 |
| ebi-fl187-GCST90094807 | prot-a-591 | Caseinolytic peptidase B protein homolog \|\| id:prot-a-591 | Oranges liking \|\| id:ebi-fl187-GCST90094807 | Weighted mode | 7 | -0.172350968 | 0.347811725901848 | 0.637850121067343 | -0.85406195 | 0.50936001516137 | 0.841683717604278 | 0.425682314990416 | 1.66422577479195 | 0.999714303421225 |
| ebi-fl187-GCST90094807 | prot-a-605 | Ciliary neurotrophic factor receptor subunit alpha \|\| id:prot-a-605 | Oranges liking \|\| id:ebi-fl187-GCST90094807 | Weighted mode | 7 | 0.239814087493911 | 0.421725523851699 | 0.590227343891042 | -0.586767939 | 1.06639611424324 | 1.27101283117403 | 0.556121802624401 | 2.90489171506209 | 0.999714303421225 |
| ebi-fl187-GCST90094807 | prot-a-606 | Ciliary neurotrophic factor receptor subunit alpha \|\| id:prot-a-606 | Oranges liking \|\| id:ebi-fl187-GCST90094807 | Weighted mode | 7 | 0.178961167743838 | 0.482700025816541 | 0.723560862377784 | -0.767130883 | 1.12505321834426 | 1.19597430088573 | 0.464343414590205 | 3.08038077732065 | 0.999714303421225 |
| ebi-fl187-GCST90094807 | prot-a-707 | Cancer/testis antigen 55 \|\| id:prot-a-707 | Oranges liking \|\| id:ebi-fl187-GCST90094807 | Weighted mode | 7 | 0.0581456869575479 | 0.44960773474963 | 0.90132661571208 | -0.823085473 | 0.939376847066822 | 1.05986939361049 | 0.439074808792807 | 2.55838665534178 | 0.999714303421225 |
| ebi-fl187-GCST90094807 | prot-a-719 | Dipeptidyl peptidase 1 \|\| id:prot-a-719 | Oranges liking \|\| id:ebi-fl187-GCST90094807 | Weighted mode | 7 | -0.719068084 | 0.475483649936592 | 0.18121918329328 | -1.651016038 | 0.212879869674675 | 0.4872060795073 | 0.191854877697156 | 1.23723601275107 | 0.999714303421225 |
| ebi-fl187-GCST90094807 | prot-a-752 | Cytochrome b5 \|\| id:prot-a-752 | Oranges liking \|\| id:ebi-fl187-GCST90094807 | Weighted mode | 7 | -0.356712257 | 0.384228193145026 | 0.389028956814542 | -1.109799515 | 0.396375001684843 | 0.699973881428811 | 0.329625039180888 | 1.48642662553811 | 0.999714303421225 |
| ebi-fl187-GCST90094807 | prot-a-755 | Cytoglobin \|\| id:prot-a-755 | Oranges liking \|\| id:ebi-fl187-GCST90094807 | Weighted mode | 7 | 0.0710844762232437 | 0.379083517210008 | 0.857436138203525 | -0.671919218 | 0.81408816995486 | 1.07367192178643 | 0.510727439534886 | 2.25711662698678 | 0.999714303421225 |
| ebi-fl187-GCST90094807 | prot-a-807 | Desmin \|\| id:prot-a-807 | Oranges liking \|\| id:ebi-fl187-GCST90094807 | Weighted mode | 7 | -0.379288112 | 0.506891431973615 | 0.48259013528544 | -1.372795319 | 0.614219094657054 | 0.684348415261868 | 0.253397641469084 | 1.8482127566628 | 0.999714303421225 |
| ebi-fl187-GCST90094807 | prot-a-82 | Ameloblastin \|\| id:prot-a-82 | Oranges liking \|\| id:ebi-fl187-GCST90094807 | Weighted mode | 7 | -0.246025705 | 0.38996707208633 | 0.5513725943026 | -1.010361166 | 0.518309756150603 | 0.781902125764636 | 0.36408745965545 | 1.67918701416912 | 0.999714303421225 |
| ebi-fl187-GCST90094807 | prot-a-84 | Muellerian-inhibiting factor \|\| id:prot-a-84 | Oranges liking \|\| id:ebi-fl187-GCST90094807 | Weighted mode | 7 | -0.451919219 | 0.387035098230275 | 0.287247164217151 | -1.210508012 | 0.306669573377637 | 0.636405577029797 | 0.298045830199736 | 1.3588918798267 | 0.999714303421225 |
| ebi-fl187-GCST90094807 | prot-a-858 | Inactive dipeptidyl peptidase 10 \|\| id:prot-a-858 | Oranges liking \|\| id:ebi-fl187-GCST90094807 | Weighted mode | 7 | 0.152383918150307 | 0.364995734231601 | 0.690843888402767 | -0.563007721 | 0.867775557244244 | 1.16460726448234 | 0.569493607484526 | 2.38160720798241 | 0.999714303421225 |
| ebi-fl187-GCST90094807 | prot-a-863 | Dihydropyrimidinase-related protein 3 \|\| id:prot-a-863 | Oranges liking \|\| id:ebi-fl187-GCST90094807 | Weighted mode | 7 | 0.174292726130975 | 0.389117143867177 | 0.669931203891365 | -0.588376876 | 0.936962328110642 | 1.19040397717386 | 0.555227757329804 | 2.55221683383817 | 0.999714303421225 |
| ebi-fl187-GCST90094807 | prot-a-864 | Dihydropyrimidinase-related protein 5 \|\| id:prot-a-864 | Oranges liking \|\| id:ebi-fl187-GCST90094807 | Weighted mode | 7 | -0.515692402 | 0.403956138385711 | 0.248923192741056 | -1.307446433 | 0.276061629240752 | 0.597087027208339 | 0.270509940371752 | 1.31792908449334 | 0.999714303421225 |
| ebi-fl187-GCST90094807 | prot-a-884 | Endothelin-converting enzyme 1 \|\| id:prot-a-884 | Oranges liking \|\| id:ebi-fl187-GCST90094807 | Weighted mode | 7 | -0.316157491 | 0.47945624658408 | 0.53410937892148 | -1.255891735 | 0.623576751946826 | 0.728944638645924 | 0.284821749520223 | 1.86558887130531 | 0.999714303421225 |
| ebi-fl187-GCST90094807 | prot-a-893 | Early endosome antigen 1 \|\| id:prot-a-893 | Oranges liking \|\| id:ebi-fl187-GCST90094807 | Weighted mode | 7 | -0.430840625 | 0.365331710190257 | 0.282912866860819 | -1.146890777 | 0.285209526869858 | 0.649962490225452 | 0.317622795774592 | 1.3300406781882 | 0.999714303421225 |
| ebi-fl187-GCST90094807 | prot-a-897 | Ephrin-A2 \|\| id:prot-a-897 | Oranges liking \|\| id:ebi-fl187-GCST90094807 | Weighted mode | 7 | 0.502309379113553 | 0.439390325082161 | 0.296511218641966 | -0.358895658 | 1.36351441627459 | 1.65253319305954 | 0.698447224896799 | 3.90991023633469 | 0.999714303421225 |
| ebi-fl187-GCST90094807 | prot-a-90 | Anaphase-promoting complex subunit 10 \|\| id:prot-a-90 | Oranges liking \|\| id:ebi-fl187-GCST90094807 | Weighted mode | 7 | 0.0592894163378437 | 0.375951378915642 | 0.879862790023229 | -0.677575286 | 0.796154119012502 | 1.06108229085613 | 0.507846883973905 | 2.21699820063548 | 0.999714303421225 |
| ebi-fl187-GCST90094807 | prot-a-914 | Histone-lysine N-methyltransferase EHMT2 \|\| id:prot-a-914 | Oranges liking \|\| id:ebi-fl187-GCST90094807 | Weighted mode | 7 | -0.131752545 | 0.378865376478418 | 0.739898708413906 | -0.874328683 | 0.610823592722547 | 0.876557876719916 | 0.417141960162484 | 1.8419477890463 | 0.999714303421225 |
| ebi-fl187-GCST90094807 | prot-a-932 | RNA polymerase II elongation factor ELL \|\| id:prot-a-932 | Oranges liking \|\| id:ebi-fl187-GCST90094807 | Weighted mode | 7 | -0.165750183 | 0.366923800980976 | 0.667333897626939 | -0.884920833 | 0.553420466608495 | 0.847257866905921 | 0.412746847817573 | 1.73919170268563 | 0.999714303421225 |
| ebi-fl187-GCST90094807 | prot-a-991 | Estrogen receptor \|\| id:prot-a-991 | Oranges liking \|\| id:ebi-fl187-GCST90094807 | Weighted mode | 7 | -0.063864848 | 0.37936695301812 | 0.871842675715917 | -0.807424075 | 0.679694380355445 | 0.938131781762353 | 0.446005464196446 | 1.97327456859355 | 0.999714303421225 |
| ebi-fl187-GCST90094807 | prot-a-992 | Persulfide dioxygenase ETHE1, mitochondrial \|\| id:prot-a-992 | Oranges liking \|\| id:ebi-fl187-GCST90094807 | Weighted mode | 7 | -0.094117819 | 0.416627836725943 | 0.828774825174192 | -0.910708379 | 0.722472740950712 | 0.910175519924466 | 0.402239185289959 | 2.05951957781686 | 0.999714303421225 |
| ebi-fl187-GCST90094807 | prot-a-993 | Ethanolamine kinase 1 \|\| id:prot-a-993 | Oranges liking \|\| id:ebi-fl187-GCST90094807 | Weighted mode | 7 | -0.203675773 | 0.446238756780654 | 0.664138396958764 | -1.078303737 | 0.67095219005997 | 0.815726808784426 | 0.340172057970122 | 1.95609901218891 | 0.999714303421225 |
| ebi-fl187-GCST90094807 | prot-b-10 | coagulation factor III, tissue factor \|\| id:prot-b-10 | Oranges liking \|\| id:ebi-fl187-GCST90094807 | Weighted mode | 6 | -0.145482242 | 0.367235410006319 | 0.708331255546039 | -0.865263646 | 0.574299161117762 | 0.864605243169892 | 0.420940558656829 | 1.77588548108119 | 0.999714303421225 |
| ebi-fl187-GCST90094807 | prot-b-28 | prolactin \|\| id:prot-b-28 | Oranges liking \|\| id:ebi-fl187-GCST90094807 | Weighted mode | 6 | 0.00356641570899896 | 0.446425041596866 | 0.993934847487526 | -0.871426666 | 0.878559497238856 | 1.00357278293665 | 0.418354271550877 | 2.40742929889919 | 0.999714303421225 |
| ebi-fl187-GCST90094807 | prot-b-54 | matrix metallopeptidase 7 \|\| id:prot-b-54 | Oranges liking \|\| id:ebi-fl187-GCST90094807 | Weighted mode | 6 | 0.507335645553196 | 0.543429877128055 | 0.393375042060915 | -0.557786914 | 1.57245820472418 | 1.6608601744712 | 0.572474598701865 | 4.81847845371575 | 0.999714303421225 |
| ebi-fl187-GCST90094807 | ukb-rsfMRI191-edge-pheno716 | Net100_Pair10_33 (OccipitalPrecuneus)&(Temporal\|Frontal\|Supp_Motor_Area) (Default_mode\|Central_executive)&(Default_mode\|Salience) \|\| id:ukb-rsfMRI191-edge-pheno716 | Oranges liking \|\| id:ebi-fl187-GCST90094807 | Weighted mode | 7 | 0.143254393255986 | 0.159351700584952 | 0.403295465098133 | -0.16907494 | 0.455583726402493 | 1.1540233400823 | 0.844445618352587 | 1.57709370563474 | 0.999714303421225 |
| ebi-fl187-GCST90094807 | ukb-rsfMRI191-edge-pheno903 | Net100_Pair27_38 (Precentral\|Frontal\|Supp_Motor_Area)&(Subcortical) (Attention\|Salience\|Motor)&(Subcortical-cerebellum) \|\| id:ukb-rsfMRI191-edge-pheno903 | Oranges liking \|\| id:ebi-fl187-GCST90094807 | Weighted mode | 7 | -0.249428713 | 0.161005753591665 | 0.172308777132876 | -0.56499999 | 0.0661425641895528 | 0.779245829063521 | 0.56836015250379 | 1.06837901889831 | 0.999714303421225 |
| ebi-fl187-GCST90094807 | ukb-rsfMRI191-node-pheno23 | Net100_Node2 RolandicOper\|SupraMarginal\|Insula Salience\|Motor \|\| id:ukb-rsfMRI191-node-pheno23 | Oranges liking \|\| id:ebi-fl187-GCST90094807 | Weighted mode | 7 | 0.0263718718992004 | 0.142508235938591 | 0.859282748614802 | -0.252944271 | 0.305688014338839 | 1.02672268680566 | 0.776511155164948 | 1.35755870162291 | 0.999714303421225 |
| ebi-fl187-GCST90094807 | ukb-rsfMRI191-node-pheno55 | Net100_Node34 Parietal\|Postcentral\|Precuneus Attention \|\| id:ukb-rsfMRI191-node-pheno55 | Oranges liking \|\| id:ebi-fl187-GCST90094807 | Weighted mode | 7 | 0.0921373051987233 | 0.10533055331553 | 0.415351070869017 | -0.114310579 | 0.298585189697162 | 1.09651536900579 | 0.891980882060229 | 1.34795036378898 | 0.999714303421225 |
| ebi-fl187-GCST90094812 | ebi-a-GCST90001414 | IgD- CD24- B cell Absolute Count \|\| id:ebi-a-GCST90001414 | Plum liking \|\| id:ebi-fl187-GCST90094812 | Inverse variance weighted | 5 | -0.219240641 | 0.294624096960999 | 0.456793701792696 | -0.796703871 | 0.35822258942991 | 0.803128429579886 | 0.450812454044645 | 1.43078406244646 | 0.998735809224318 |
| ebi-fl187-GCST90094812 | ebi-a-GCST90001422 | CD20- CD38- B cell %B cell \|\| id:ebi-a-GCST90001422 | Plum liking \|\| id:ebi-fl187-GCST90094812 | Inverse variance weighted | 5 | -0.205398646 | 0.231593828233834 | 0.375137205860538 | -0.659322549 | 0.248525257224759 | 0.814322625147564 | 0.517201594340019 | 1.28213320508689 | 0.998735809224318 |
| ebi-fl187-GCST90094812 | ebi-a-GCST90001444 | CD20- CD38- B cell %lymphocyte \|\| id:ebi-a-GCST90001444 | Plum liking \|\| id:ebi-fl187-GCST90094812 | Inverse variance weighted | 5 | -0.237451479 | 0.231740490690023 | 0.305531245863333 | -0.69166284 | 0.216759883242947 | 0.788635155805958 | 0.500742721238166 | 1.24204583031228 | 0.998735809224318 |
| ebi-fl187-GCST90094812 | ebi-a-GCST90001459 | Myeloid Dendritic Cell %Dendritic Cell \|\| id:ebi-a-GCST90001459 | Plum liking \|\| id:ebi-fl187-GCST90094812 | Inverse variance weighted | 5 | -0.126140011 | 0.309087851972913 | 0.683197271153274 | -0.731952201 | 0.479672179083952 | 0.881491419839204 | 0.48096912474793 | 1.61554470602945 | 0.998735809224318 |
| ebi-fl187-GCST90094812 | ebi-a-GCST90001476 | HLA DR++ monocyte %leukocyte \|\| id:ebi-a-GCST90001476 | Plum liking \|\| id:ebi-fl187-GCST90094812 | Inverse variance weighted | 5 | 0.127322222541414 | 0.24353892367243 | 0.601113230299055 | -0.350014068 | 0.604658512939376 | 1.13578293368853 | 0.704678176337486 | 1.83062696671383 | 0.998735809224318 |
| ebi-fl187-GCST90094812 | ebi-a-GCST90001478 | CD4 regulatory T cell %CD4+ T cell \|\| id:ebi-a-GCST90001478 | Plum liking \|\| id:ebi-fl187-GCST90094812 | Inverse variance weighted | 5 | -0.051188181 | 0.245825316623522 | 0.835049463185428 | -0.533005802 | 0.430629439412495 | 0.950099862805405 | 0.586838396144402 | 1.53822543861074 | 0.998735809224318 |
| ebi-fl187-GCST90094812 | ebi-a-GCST90001485 | CD39+ resting CD4 regulatory T cell %CD4 regulatory T cell \|\| id:ebi-a-GCST90001485 | Plum liking \|\| id:ebi-fl187-GCST90094812 | Inverse variance weighted | 5 | 0.562878295221526 | 0.240806230073914 | 0.0194145527395497 | 0.0908980842766541 | 1.0348585061664 | 1.75571871134693 | 1.09515738581352 | 2.81470794362938 | 0.998735809224318 |
| ebi-fl187-GCST90094812 | ebi-a-GCST90001515 | Immature Myeloid-Derived Suppressor Cells Absolute Count \|\| id:ebi-a-GCST90001515 | Plum liking \|\| id:ebi-fl187-GCST90094812 | Inverse variance weighted | 5 | -0.343929199 | 0.375471666179029 | 0.359670872752925 | -1.079853664 | 0.391995267113152 | 0.708979122988632 | 0.339645224227217 | 1.47993070704113 | 0.998735809224318 |
| ebi-fl187-GCST90094812 | ebi-a-GCST90001517 | CD33+ HLA DR+ Absolute Count \|\| id:ebi-a-GCST90001517 | Plum liking \|\| id:ebi-fl187-GCST90094812 | Inverse variance weighted | 5 | 0.0663609527342008 | 0.314652057395663 | 0.83296323623287 | -0.55035708 | 0.683078985229699 | 1.06861236611664 | 0.576743830057806 | 1.97996463855182 | 0.998735809224318 |
| ebi-fl187-GCST90094812 | ebi-a-GCST90001518 | CD33+ HLA DR+ CD14- Absolute Count \|\| id:ebi-a-GCST90001518 | Plum liking \|\| id:ebi-fl187-GCST90094812 | Inverse variance weighted | 5 | 0.0445580976553441 | 0.346621398766855 | 0.897713921980695 | -0.634819844 | 0.72393603923838 | 1.04556571986275 | 0.530030968014086 | 2.06253547532918 | 0.998735809224318 |
| ebi-fl187-GCST90094812 | ebi-a-GCST90001520 | CD33+ HLA DR+ CD14dim Absolute Count \|\| id:ebi-a-GCST90001520 | Plum liking \|\| id:ebi-fl187-GCST90094812 | Inverse variance weighted | 5 | -0.000450236 | 0.31579287821436 | 0.998862430043301 | -0.619404278 | 0.618503804975057 | 0.999549865016076 | 0.538264998605734 | 1.85614880261882 | 0.999882738470636 |
| ebi-fl187-GCST90094812 | ebi-a-GCST90001531 | CD33dim HLA DR- Absolute Count \|\| id:ebi-a-GCST90001531 | Plum liking \|\| id:ebi-fl187-GCST90094812 | Inverse variance weighted | 5 | -0.384441415 | 0.321257312625912 | 0.231432259052579 | -1.014105748 | 0.245222917622348 | 0.68083083181441 | 0.362726653924276 | 1.2779061492566 | 0.998735809224318 |
| ebi-fl187-GCST90094812 | ebi-a-GCST90001532 | Basophil Absolute Count \|\| id:ebi-a-GCST90001532 | Plum liking \|\| id:ebi-fl187-GCST90094812 | Inverse variance weighted | 5 | -0.311156438 | 0.320272312768968 | 0.331280880670374 | -0.938890171 | 0.316577294562331 | 0.732599260200804 | 0.39106160592716 | 1.37242231891906 | 0.998735809224318 |
| ebi-fl187-GCST90094812 | ebi-a-GCST90001588 | T/B cell \|\| id:ebi-a-GCST90001588 | Plum liking \|\| id:ebi-fl187-GCST90094812 | Inverse variance weighted | 5 | 0.0945356954886789 | 0.247822769164446 | 0.70285828866107 | -0.391196932 | 0.580268323050994 | 1.09914839707191 | 0.676246968207502 | 1.78651773033169 | 0.998735809224318 |
| ebi-fl187-GCST90094812 | ebi-a-GCST90001624 | HLA DR+ CD4+ T cell Absolute Count \|\| id:ebi-a-GCST90001624 | Plum liking \|\| id:ebi-fl187-GCST90094812 | Inverse variance weighted | 5 | -0.196545534 | 0.232883950202957 | 0.398689848519529 | -0.652998077 | 0.25990700799149 | 0.821563921018147 | 0.520482987279068 | 1.29680948813955 | 0.998735809224318 |
| ebi-fl187-GCST90094812 | ebi-a-GCST90001631 | CD8+ Natural Killer T %T cell \|\| id:ebi-a-GCST90001631 | Plum liking \|\| id:ebi-fl187-GCST90094812 | Inverse variance weighted | 5 | -0.089574596 | 0.236228839624612 | 0.704549974299257 | -0.552583122 | 0.373433929175104 | 0.914320057514076 | 0.575461401742191 | 1.45271457832209 | 0.998735809224318 |
| ebi-fl187-GCST90094812 | ebi-a-GCST90001632 | CD8+ Natural Killer T %lymphocyte \|\| id:ebi-a-GCST90001632 | Plum liking \|\| id:ebi-fl187-GCST90094812 | Inverse variance weighted | 5 | -0.068025963 | 0.237680702078258 | 0.774719317300445 | -0.533880139 | 0.397828213015391 | 0.934236217633237 | 0.586325525642426 | 1.48858828784803 | 0.998735809224318 |
| ebi-fl187-GCST90094812 | ebi-a-GCST90001646 | Natural Killer %CD3- lymphocyte \|\| id:ebi-a-GCST90001646 | Plum liking \|\| id:ebi-fl187-GCST90094812 | Inverse variance weighted | 5 | -0.137545584 | 0.231745832754132 | 0.552833787529938 | -0.591767416 | 0.316676248268711 | 0.871494622942353 | 0.553348423034349 | 1.37255813191373 | 0.998735809224318 |
| ebi-fl187-GCST90094812 | ebi-a-GCST90001684 | CD127- CD8+ T cell Absolute Count \|\| id:ebi-a-GCST90001684 | Plum liking \|\| id:ebi-fl187-GCST90094812 | Inverse variance weighted | 5 | 0.274735093764431 | 0.281732623589813 | 0.329479652844083 | -0.277460848 | 0.826931036000465 | 1.31618196387243 | 0.757705229340064 | 2.28629141642838 | 0.998735809224318 |
| ebi-fl187-GCST90094812 | ebi-a-GCST90001687 | CD28- CD8+ T cell Absolute Count \|\| id:ebi-a-GCST90001687 | Plum liking \|\| id:ebi-fl187-GCST90094812 | Inverse variance weighted | 5 | 0.133932241202469 | 0.258770830670946 | 0.604757717848909 | -0.373258587 | 0.641123069317523 | 1.14331534734683 | 0.68848717605016 | 1.89861195524078 | 0.998735809224318 |
| ebi-fl187-GCST90094812 | ebi-a-GCST90001701 | BAFF-R on CD20- CD38- B cell \|\| id:ebi-a-GCST90001701 | Plum liking \|\| id:ebi-fl187-GCST90094812 | Inverse variance weighted | 5 | -0.060795519 | 0.233240152470027 | 0.794357556819272 | -0.517946218 | 0.396355180053878 | 0.941015640123404 | 0.595742818500239 | 1.48639716243009 | 0.998735809224318 |
| ebi-fl187-GCST90094812 | ebi-a-GCST90001732 | CD19 on IgD- CD27- B cell \|\| id:ebi-a-GCST90001732 | Plum liking \|\| id:ebi-fl187-GCST90094812 | Inverse variance weighted | 5 | 0.043583960121371 | 0.344470498408952 | 0.899316750839335 | -0.631578217 | 0.718746137002917 | 1.04454769098138 | 0.531751918632765 | 2.05185884714793 | 0.998735809224318 |
| ebi-fl187-GCST90094812 | ebi-a-GCST90001798 | CD27 on CD24+ CD27+ B cell \|\| id:ebi-a-GCST90001798 | Plum liking \|\| id:ebi-fl187-GCST90094812 | Inverse variance weighted | 5 | -0.173374537 | 0.296361804165899 | 0.558541177913286 | -0.754243674 | 0.407494598701155 | 0.840822636284797 | 0.470366230616915 | 1.50304732710438 | 0.998735809224318 |
| ebi-fl187-GCST90094812 | ebi-a-GCST90001836 | CD66b on CD66b++ myeloid cell \|\| id:ebi-a-GCST90001836 | Plum liking \|\| id:ebi-fl187-GCST90094812 | Inverse variance weighted | 5 | -0.017429939 | 0.357616702423754 | 0.961127165388497 | -0.718358676 | 0.683498798000346 | 0.982721083920581 | 0.487551830363763 | 1.98079602749415 | 0.998735809224318 |
| ebi-fl187-GCST90094812 | ebi-a-GCST90001943 | CD25 on activated & secreting CD4 regulatory T cell \|\| id:ebi-a-GCST90001943 | Plum liking \|\| id:ebi-fl187-GCST90094812 | Inverse variance weighted | 5 | 0.0331688192165776 | 0.299104789733445 | 0.911700688135262 | -0.553076569 | 0.619414207094129 | 1.03372503716252 | 0.575177512371112 | 1.85783941387331 | 0.998735809224318 |
| ebi-fl187-GCST90094812 | ebi-a-GCST90001960 | CD25 on CD4+ T cell \|\| id:ebi-a-GCST90001960 | Plum liking \|\| id:ebi-fl187-GCST90094812 | Inverse variance weighted | 5 | -0.087016298 | 0.256578386313313 | 0.734503375070494 | -0.589909935 | 0.415877339624534 | 0.916662156164495 | 0.554377212622945 | 1.51569994114394 | 0.998735809224318 |
| ebi-fl187-GCST90094812 | ebi-a-GCST90001988 | HLA DR on CD14+ CD16- monocyte \|\| id:ebi-a-GCST90001988 | Plum liking \|\| id:ebi-fl187-GCST90094812 | Inverse variance weighted | 5 | -0.054470401 | 0.24010202809265 | 0.820529836452268 | -0.525070376 | 0.416129573667391 | 0.946986537924245 | 0.591513734261771 | 1.51608230048785 | 0.998735809224318 |
| ebi-fl187-GCST90094812 | ebi-a-GCST90001991 | HLA DR on CD14+ monocyte \|\| id:ebi-a-GCST90001991 | Plum liking \|\| id:ebi-fl187-GCST90094812 | Inverse variance weighted | 5 | -0.059164283 | 0.240551625996174 | 0.805718973333972 | -0.53064547 | 0.41231690405135 | 0.942551911274728 | 0.588225165503978 | 1.51031298480145 | 0.998735809224318 |
| ebi-fl187-GCST90094812 | ebi-a-GCST90002019 | CD14 on Monocytic Myeloid-Derived Suppressor Cells \|\| id:ebi-a-GCST90002019 | Plum liking \|\| id:ebi-fl187-GCST90094812 | Inverse variance weighted | 5 | -0.039258494 | 0.502638305841487 | 0.937744641385884 | -1.024429574 | 0.945912585004072 | 0.961502134059367 | 0.359001190661123 | 2.57516236115601 | 0.998735809224318 |
| ebi-fl187-GCST90094812 | ebi-a-GCST90002037 | CD80 on plasmacytoid Dendritic Cell \|\| id:ebi-a-GCST90002037 | Plum liking \|\| id:ebi-fl187-GCST90094812 | Inverse variance weighted (multiplicative random effects) | 5 | 0.341110678976797 | 0.424037710600794 | 0.421145926240493 | -0.490003234 | 1.17222459175435 | 1.406508903 | 0.612624413075921 | 3.22916823521214 | 0.998735809224318 |
| ebi-fl187-GCST90094812 | ebi-a-GCST90002042 | CD45 on CD33+ HLA DR+ CD14- \|\| id:ebi-a-GCST90002042 | Plum liking \|\| id:ebi-fl187-GCST90094812 | Inverse variance weighted | 5 | -0.362839278 | 0.52797332989846 | 0.491937603156674 | -1.397667005 | 0.671988448257159 | 0.695698238287187 | 0.247172945058409 | 1.9581270864476 | 0.998735809224318 |
| ebi-fl187-GCST90094812 | ebi-a-GCST90002056 | CD8 on Effector Memory CD8+ T cell \|\| id:ebi-a-GCST90002056 | Plum liking \|\| id:ebi-fl187-GCST90094812 | Inverse variance weighted | 5 | 0.187081860248039 | 0.263552741178102 | 0.47779963730055 | -0.329481512 | 0.70364523295712 | 1.20572598203815 | 0.719296583079768 | 2.0211067005731 | 0.998735809224318 |
| ebi-fl187-GCST90094812 | ebi-a-GCST90002058 | CD8 on CD8+ T cell \|\| id:ebi-a-GCST90002058 | Plum liking \|\| id:ebi-fl187-GCST90094812 | Inverse variance weighted | 5 | -0.325249438 | 0.360281488926962 | 0.366650749495192 | -1.031401156 | 0.380902280728166 | 0.722347150651307 | 0.356507088453466 | 1.46360457604805 | 0.998735809224318 |
| ebi-fl187-GCST90094812 | ebi-a-GCST90002063 | CD4 on CD4 regulatory T cell \|\| id:ebi-a-GCST90002063 | Plum liking \|\| id:ebi-fl187-GCST90094812 | Inverse variance weighted | 5 | -0.266911616 | 0.260850720641273 | 0.306196696424185 | -0.778179028 | 0.24435579676347 | 0.765740747939583 | 0.459241516232136 | 1.27679853046797 | 0.998735809224318 |
| ebi-fl187-GCST90094812 | ebi-a-GCST90002066 | CD4 on activated CD4 regulatory T cell \|\| id:ebi-a-GCST90002066 | Plum liking \|\| id:ebi-fl187-GCST90094812 | Inverse variance weighted | 5 | -0.229013823 | 0.260867152104282 | 0.380001004989316 | -0.740313441 | 0.282285794846075 | 0.795317539533408 | 0.476964391700803 | 1.3261576748611 | 0.998735809224318 |
| ebi-fl187-GCST90094812 | ebi-a-GCST90002070 | CD4 on activated & secreting CD4 regulatory T cell \|\| id:ebi-a-GCST90002070 | Plum liking \|\| id:ebi-fl187-GCST90094812 | Inverse variance weighted | 5 | -0.217898665 | 0.267342712561288 | 0.415041576851372 | -0.741890382 | 0.306093051628133 | 0.804206931853466 | 0.476212840104784 | 1.35810867489179 | 0.998735809224318 |
| ebi-fl187-GCST90094812 | ebi-a-GCST90002081 | SSC-A on CD4+ T cell \|\| id:ebi-a-GCST90002081 | Plum liking \|\| id:ebi-fl187-GCST90094812 | Inverse variance weighted | 5 | -0.085003627 | 0.247586400462918 | 0.731350856473637 | -0.570272972 | 0.400265717450129 | 0.918508952543511 | 0.565371086951185 | 1.49222115416634 | 0.998735809224318 |
| ebi-fl187-GCST90094812 | ebi-a-GCST90002094 | CD11b on Monocytic Myeloid-Derived Suppressor Cells \|\| id:ebi-a-GCST90002094 | Plum liking \|\| id:ebi-fl187-GCST90094812 | Inverse variance weighted | 5 | -0.093919655 | 0.350180628002782 | 0.788542714721109 | -0.780273686 | 0.592434376140961 | 0.910355902080064 | 0.458280569333865 | 1.8083853514816 | 0.998735809224318 |
| ebi-fl187-GCST90094812 | ebi-a-GCST90002104 | HLA DR on myeloid Dendritic Cell \|\| id:ebi-a-GCST90002104 | Plum liking \|\| id:ebi-fl187-GCST90094812 | Inverse variance weighted | 5 | -0.079287983 | 0.265726194730702 | 0.765411425754114 | -0.600111325 | 0.441535358637397 | 0.923773854936978 | 0.548750543200101 | 1.55509301200628 | 0.998735809224318 |
| ebi-fl187-GCST90094812 | ebi-a-GCST90002105 | HLA DR on plasmacytoid Dendritic Cell \|\| id:ebi-a-GCST90002105 | Plum liking \|\| id:ebi-fl187-GCST90094812 | Inverse variance weighted | 5 | -0.058431557 | 0.260277440586682 | 0.822370445626525 | -0.56857534 | 0.451712226991909 | 0.943242796974082 | 0.566331694293659 | 1.57099979218566 | 0.998735809224318 |
| ebi-fl187-GCST90094812 | ebi-a-GCST90002106 | HLA DR on Dendritic Cell \|\| id:ebi-a-GCST90002106 | Plum liking \|\| id:ebi-fl187-GCST90094812 | Inverse variance weighted | 5 | -0.140700884 | 0.26525507569641 | 0.595809559338148 | -0.660600833 | 0.37919906412346 | 0.868749129392729 | 0.516540886630179 | 1.46111386214616 | 0.998735809224318 |
| ebi-fl187-GCST90094812 | ebi-a-GCST90002111 | HLA DR on CD33dim HLA DR+ CD11b- \|\| id:ebi-a-GCST90002111 | Plum liking \|\| id:ebi-fl187-GCST90094812 | Inverse variance weighted | 5 | -0.140659785 | 0.341132910802476 | 0.680096591655848 | -0.80928029 | 0.527960720066374 | 0.868784834964229 | 0.445178350139419 | 1.69547124029604 | 0.998735809224318 |
| ebi-fl187-GCST90094812 | ebi-a-GCST90002112 | HLA DR on CD33- HLA DR+ \|\| id:ebi-a-GCST90002112 | Plum liking \|\| id:ebi-fl187-GCST90094812 | Inverse variance weighted | 5 | -0.346023278 | 0.344613102563786 | 0.31533424484485 | -1.021464959 | 0.329418402863768 | 0.707496017708429 | 0.360067070051446 | 1.3901593805892 | 0.998735809224318 |
| ebi-fl187-GCST90094812 | ebi-a-GCST90002115 | HLA DR on HLA DR+ CD8+ T cell \|\| id:ebi-a-GCST90002115 | Plum liking \|\| id:ebi-fl187-GCST90094812 | Inverse variance weighted | 5 | -0.324632959 | 0.299788407513047 | 0.278864521405131 | -0.912218238 | 0.262952319921926 | 0.722792599621474 | 0.401632319287478 | 1.30076469691082 | 0.998735809224318 |
| ebi-fl187-GCST90094812 | ebi-a-GCST90002116 | HLA DR on B cell \|\| id:ebi-a-GCST90002116 | Plum liking \|\| id:ebi-fl187-GCST90094812 | Inverse variance weighted | 5 | -0.000911954 | 0.257178612367006 | 0.997170711981844 | -0.504982034 | 0.503158126396475 | 0.999088461860672 | 0.603516418056006 | 1.6539363715048 | 0.999604360968858 |
| ebi-fl187-GCST90094812 | ebi-cfmet338-GCST90026023 | Sphingomyelin (d18:1/24:1, d18:2/24:0) levels \|\| id:ebi-cfmet338-GCST90026023 | Plum liking \|\| id:ebi-fl187-GCST90094812 | Inverse variance weighted | 5 | -0.103640662 | 0.137530596611053 | 0.451099909088893 | -0.373200631 | 0.16591930785736 | 0.901549200559416 | 0.688527079206841 | 1.18047784259354 | 0.998735809224318 |
| ebi-fl187-GCST90094812 | ebi-cfmet338-GCST90026046 | 1-arachidonoyl-gpc (20:4n6) levels \|\| id:ebi-cfmet338-GCST90026046 | Plum liking \|\| id:ebi-fl187-GCST90094812 | Inverse variance weighted (multiplicative random effects) | 5 | -0.206236069 | 0.281402071172028 | 0.463627030028592 | -0.757784128 | 0.34531199093197 | 0.813640978551296 | 0.468703864911961 | 1.41243051644615 | 0.998735809224318 |
| ebi-fl187-GCST90094812 | ebi-cfmet338-GCST90026047 | 1-linoleoyl-gpc (18:2) levels \|\| id:ebi-cfmet338-GCST90026047 | Plum liking \|\| id:ebi-fl187-GCST90094812 | Inverse variance weighted | 5 | -0.145592381 | 0.199002202963747 | 0.464405479513303 | -0.535636699 | 0.244451936910002 | 0.864510022171883 | 0.585296513950164 | 1.27692128796664 | 0.998735809224318 |
| ebi-fl187-GCST90094812 | ebi-cfmet338-GCST90026050 | 1-myristoyl-2-palmitoyl-gpc (14:0/16:0) levels \|\| id:ebi-cfmet338-GCST90026050 | Plum liking \|\| id:ebi-fl187-GCST90094812 | Inverse variance weighted | 5 | 0.0147975487614721 | 0.106356561300585 | 0.889346230558602 | -0.193661311 | 0.223256408910618 | 1.01490757452011 | 0.823936915067428 | 1.25014107995633 | 0.998735809224318 |
| ebi-fl187-GCST90094812 | ebi-cfmet338-GCST90026068 | 2,3-dihydroxyisovalerate levels \|\| id:ebi-cfmet338-GCST90026068 | Plum liking \|\| id:ebi-fl187-GCST90094812 | Inverse variance weighted | 5 | 0.209961016813779 | 0.341215292853406 | 0.538334853614156 | -0.458820957 | 0.878742990806456 | 1.23362996819259 | 0.632028394916688 | 2.4078710872214 | 0.998735809224318 |
| ebi-fl187-GCST90094812 | ebi-cfmet338-GCST90026082 | 3-hydroxyhexanoate levels \|\| id:ebi-cfmet338-GCST90026082 | Plum liking \|\| id:ebi-fl187-GCST90094812 | Inverse variance weighted | 5 | -0.067778998 | 0.0908167958466744 | 0.455470095092947 | -0.245779918 | 0.110221921633908 | 0.934466969616806 | 0.782094330804078 | 1.1165258242021 | 0.998735809224318 |
| ebi-fl187-GCST90094812 | ebi-cfmet338-GCST90026094 | 3-ureidopropionate levels \|\| id:ebi-cfmet338-GCST90026094 | Plum liking \|\| id:ebi-fl187-GCST90094812 | Inverse variance weighted | 5 | 0.200737340277068 | 0.16643766609528 | 0.227786182426463 | -0.125480485 | 0.526955165823816 | 1.22230367971034 | 0.882072977675359 | 1.69376720888881 | 0.998735809224318 |
| ebi-fl187-GCST90094812 | ebi-cfmet338-GCST90026173 | Glycerol levels \|\| id:ebi-cfmet338-GCST90026173 | Plum liking \|\| id:ebi-fl187-GCST90094812 | Inverse variance weighted | 5 | -0.045153861 | 0.0939325892970117 | 0.63072620766053 | -0.229261736 | 0.138954013733238 | 0.955850402143581 | 0.795120394388634 | 1.14907125729123 | 0.998735809224318 |
| ebi-fl187-GCST90094812 | ebi-cfmet338-GCST90026179 | Histidine levels \|\| id:ebi-cfmet338-GCST90026179 | Plum liking \|\| id:ebi-fl187-GCST90094812 | Inverse variance weighted | 5 | -0.085154318 | 0.0652472376422192 | 0.191858023826785 | -0.213038904 | 0.0427302674209198 | 0.918370552030317 | 0.808124697211326 | 1.04365634876262 | 0.998735809224318 |
| ebi-fl187-GCST90094812 | ebi-cfmet338-GCST90026188 | Isocitrate levels \|\| id:ebi-cfmet338-GCST90026188 | Plum liking \|\| id:ebi-fl187-GCST90094812 | Inverse variance weighted | 5 | 0.00088663394400124 | 0.146208062468695 | 0.995161503495896 | -0.285681168 | 0.287454436382644 | 1.00088702712007 | 0.751502180325954 | 1.33302985311732 | 0.999107451094609 |
| ebi-fl187-GCST90094812 | ebi-cfmet338-GCST90026207 | N-acetyl-isoputreanine levels \|\| id:ebi-cfmet338-GCST90026207 | Plum liking \|\| id:ebi-fl187-GCST90094812 | Inverse variance weighted | 5 | -0.01366866 | 0.100380418533199 | 0.891688007575418 | -0.21041428 | 0.18307696054621 | 0.986424332177244 | 0.810248506594396 | 1.20090682696983 | 0.998735809224318 |
| ebi-fl187-GCST90094812 | ebi-cfmet338-GCST90026213 | N-acetylglutamate levels \|\| id:ebi-cfmet338-GCST90026213 | Plum liking \|\| id:ebi-fl187-GCST90094812 | Inverse variance weighted | 5 | -0.016180567 | 0.138721089733549 | 0.907144545149318 | -0.288073903 | 0.255712769073295 | 0.98394963537238 | 0.749706184893752 | 1.29138174988732 | 0.998735809224318 |
| ebi-fl187-GCST90094812 | ebi-cfmet338-GCST90026224 | N-acetyltaurine levels \|\| id:ebi-cfmet338-GCST90026224 | Plum liking \|\| id:ebi-fl187-GCST90094812 | Inverse variance weighted | 5 | -0.13440989 | 0.126040698238085 | 0.286242539622914 | -0.381449659 | 0.112629878105543 | 0.874231651955945 | 0.682870761598212 | 1.11921760933632 | 0.998735809224318 |
| ebi-fl187-GCST90094812 | ebi-cfmet338-GCST90026250 | Phenyllactate (pla) levels \|\| id:ebi-cfmet338-GCST90026250 | Plum liking \|\| id:ebi-fl187-GCST90094812 | Inverse variance weighted | 5 | 0.114482000995213 | 0.179499212784032 | 0.523613298577387 | -0.237336456 | 0.466300458051916 | 1.121292459 | 0.788725871769251 | 1.5940858833417 | 0.998735809224318 |
| ebi-fl187-GCST90094812 | ebi-cfmet338-GCST90026251 | Pipecolate levels \|\| id:ebi-cfmet338-GCST90026251 | Plum liking \|\| id:ebi-fl187-GCST90094812 | Inverse variance weighted | 5 | 0.0914426320771911 | 0.262637164238995 | 0.727711799916385 | -0.42332621 | 0.606211473985622 | 1.09575391376346 | 0.654864974872054 | 1.83347206767704 | 0.998735809224318 |
| ebi-fl187-GCST90094812 | ebi-cfmet338-GCST90026278 | Trigonelline (n'-methylnicotinate) levels \|\| id:ebi-cfmet338-GCST90026278 | Plum liking \|\| id:ebi-fl187-GCST90094812 | Inverse variance weighted | 5 | -0.139932542 | 0.314454645726753 | 0.656319238322906 | -0.756263647 | 0.47639856383521 | 0.869416882727987 | 0.469417062132424 | 1.61026468134473 | 0.998735809224318 |
| ebi-fl187-GCST90094812 | ebi-cfmet338-GCST90026299 | 1-carboxyethylvaline levels \|\| id:ebi-cfmet338-GCST90026299 | Plum liking \|\| id:ebi-fl187-GCST90094812 | Inverse variance weighted | 5 | 0.149655994593078 | 0.206496427665574 | 0.468612223579144 | -0.255077004 | 0.554388992817603 | 1.16143463420453 | 0.774856828874068 | 1.74087697141407 | 0.998735809224318 |
| ebi-fl187-GCST90094812 | ebi-gm430-GCST90011306 | C_Erysipelotrichia abundance \|\| id:ebi-gm430-GCST90011306 | Plum liking \|\| id:ebi-fl187-GCST90094812 | Inverse variance weighted | 5 | -0.111518368 | 0.154154168218398 | 0.469421250348308 | -0.413660538 | 0.190623801429697 | 0.894474961287294 | 0.661225374069159 | 1.21000416461063 | 0.998735809224318 |
| ebi-fl187-GCST90094812 | ebi-gm430-GCST90011312 | F_Erysipelotrichaceae abundance \|\| id:ebi-gm430-GCST90011312 | Plum liking \|\| id:ebi-fl187-GCST90094812 | Inverse variance weighted | 5 | -0.111518368 | 0.154154168218398 | 0.469421250348308 | -0.413660538 | 0.190623801429697 | 0.894474961287294 | 0.661225374069159 | 1.21000416461063 | 0.998735809224318 |
| ebi-fl187-GCST90094812 | ebi-gm430-GCST90011352 | O_Erysipelotrichales abundance \|\| id:ebi-gm430-GCST90011352 | Plum liking \|\| id:ebi-fl187-GCST90094812 | Inverse variance weighted | 5 | -0.111518368 | 0.154154168218398 | 0.469421250348308 | -0.413660538 | 0.190623801429697 | 0.894474961287294 | 0.661225374069159 | 1.21000416461063 | 0.998735809224318 |
| ebi-fl187-GCST90094812 | ebi-gm430-GCST90011362 | OTU97_11 (Parabacteroides) abundance \|\| id:ebi-gm430-GCST90011362 | Plum liking \|\| id:ebi-fl187-GCST90094812 | Inverse variance weighted | 5 | -0.256637055 | 0.202695297621439 | 0.205469266523507 | -0.653919839 | 0.140645728000384 | 0.773648954574284 | 0.520003446951547 | 1.15101680272064 | 0.998735809224318 |
| ebi-fl187-GCST90094812 | ebi-gm430-GCST90011384 | OTU97_235 (Phascolarctobacterium) abundance \|\| id:ebi-gm430-GCST90011384 | Plum liking \|\| id:ebi-fl187-GCST90094812 | Inverse variance weighted (multiplicative random effects) | 5 | 0.458304107609555 | 0.957318629772921 | 0.632125547934218 | -1.418040407 | 2.33464862196448 | 1.58138984262904 | 0.242188142448993 | 10.3258310216281 | 0.998735809224318 |
| ebi-fl187-GCST90094812 | ebi-gm430-GCST90011390 | OTU97_3 (Bacteroides) abundance \|\| id:ebi-gm430-GCST90011390 | Plum liking \|\| id:ebi-fl187-GCST90094812 | Inverse variance weighted | 5 | 0.0658692764435936 | 0.181318916905544 | 0.716396801762239 | -0.289515801 | 0.421254353578459 | 1.06808708389735 | 0.748625964009704 | 1.52387183137235 | 0.998735809224318 |
| ebi-fl187-GCST90094812 | ebi-gm430-GCST90011398 | OTU97_4 (Alistipes) abundance \|\| id:ebi-gm430-GCST90011398 | Plum liking \|\| id:ebi-fl187-GCST90094812 | Inverse variance weighted | 5 | 0.123455632596588 | 0.213011932932493 | 0.562203657731992 | -0.294047756 | 0.540959021144275 | 1.13139980615688 | 0.745240900905445 | 1.71765333842599 | 0.998735809224318 |
| ebi-fl187-GCST90094812 | ebi-gm430-GCST90011421 | OTU99_108 (Cytophagales) abundance \|\| id:ebi-gm430-GCST90011421 | Plum liking \|\| id:ebi-fl187-GCST90094812 | Inverse variance weighted | 5 | 0.611132825375955 | 0.577259040456197 | 0.289745362184646 | -0.520294894 | 1.7425605446701 | 1.8425174675256 | 0.594345253324291 | 5.71195041795785 | 0.998735809224318 |
| ebi-fl187-GCST90094812 | ebi-gm430-GCST90011428 | OTU99_123 (Phascolarctobacterium) abundance \|\| id:ebi-gm430-GCST90011428 | Plum liking \|\| id:ebi-fl187-GCST90094812 | Inverse variance weighted | 5 | -0.121205205 | 0.333570270279571 | 0.716337967461226 | -0.775002935 | 0.532592524795639 | 0.885852159690202 | 0.460702428973436 | 1.70334254711091 | 0.998735809224318 |
| ebi-fl187-GCST90094812 | ebi-gm430-GCST90011450 | OTU99_298 (Phascolarctobacterium) abundance \|\| id:ebi-gm430-GCST90011450 | Plum liking \|\| id:ebi-fl187-GCST90094812 | Inverse variance weighted (multiplicative random effects) | 5 | 0.458263158380208 | 0.958154552966537 | 0.632453158429624 | -1.419719765 | 2.33624608219462 | 1.58132508725954 | 0.241781763011567 | 10.3423393081834 | 0.998735809224318 |
| ebi-fl187-GCST90094812 | ebi-gm430-GCST90011451 | OTU99_3 (Bacteroides) abundance \|\| id:ebi-gm430-GCST90011451 | Plum liking \|\| id:ebi-fl187-GCST90094812 | Inverse variance weighted | 5 | 0.063950621207224 | 0.18301478376485 | 0.726767476843707 | -0.294758355 | 0.42265959738633 | 1.06603975770555 | 0.744711521561511 | 1.52601474813499 | 0.998735809224318 |
| ebi-fl187-GCST90094812 | ebi-gm430-GCST90011472 | OTU99_72 (Parabacteroides) abundance \|\| id:ebi-gm430-GCST90011472 | Plum liking \|\| id:ebi-fl187-GCST90094812 | Inverse variance weighted | 5 | -0.316852004 | 0.457518302407059 | 0.488595148323767 | -1.213587877 | 0.579883868697455 | 0.728438553125932 | 0.297129301380083 | 1.78583102782393 | 0.998735809224318 |
| ebi-fl187-GCST90094812 | ebi-gm430-GCST90011507 | TestASV_3 (Bacteroides) abundance \|\| id:ebi-gm430-GCST90011507 | Plum liking \|\| id:ebi-fl187-GCST90094812 | Inverse variance weighted | 5 | 0.403376718890458 | 0.268054164778435 | 0.132367049917375 | -0.122009444 | 0.92876288185619 | 1.49687068492471 | 0.885140009134279 | 2.53137562901314 | 0.998735809224318 |
| ebi-fl187-GCST90094812 | ebi-gm430-GCST90011512 | TestASV_34 (Phascolarctobacterium) abundance \|\| id:ebi-gm430-GCST90011512 | Plum liking \|\| id:ebi-fl187-GCST90094812 | Inverse variance weighted | 5 | -0.253065892 | 0.491405932373362 | 0.606564628946082 | -1.216221519 | 0.710089735922175 | 0.776416720852863 | 0.296347798651292 | 2.03417378891768 | 0.998735809224318 |
| ebi-fl187-GCST90094812 | ebi-gm430-GCST90011519 | TestASV_41 (Barnesiella) abundance \|\| id:ebi-gm430-GCST90011519 | Plum liking \|\| id:ebi-fl187-GCST90094812 | Inverse variance weighted | 5 | -1.274288572 | 0.61514542558631 | 0.0383098449330734 | -2.479973606 | -0.068603538 | 0.279629833886854 | 0.0837454359035047 | 0.933696781872233 | 0.998735809224318 |
| ebi-fl187-GCST90094812 | ebi-gm430-GCST90011524 | TestASV_46 (Sutterella) abundance \|\| id:ebi-gm430-GCST90011524 | Plum liking \|\| id:ebi-fl187-GCST90094812 | Inverse variance weighted | 5 | 0.0691302760277853 | 0.634178975510595 | 0.913196527446123 | -1.173860516 | 1.31212106802855 | 1.07157580069422 | 0.309171074547751 | 3.71404310158421 | 0.998735809224318 |
| ebi-fl187-GCST90094812 | ebi-gm430-GCST90011581 | OTU97_19 (Bacteria) prevalence \|\| id:ebi-gm430-GCST90011581 | Plum liking \|\| id:ebi-fl187-GCST90094812 | Inverse variance weighted | 5 | 0.357435705186193 | 0.716037909644213 | 0.61764874476552 | -1.045998598 | 1.76087000808885 | 1.42965864385671 | 0.351340796025066 | 5.81749646234756 | 0.998735809224318 |
| ebi-fl187-GCST90094812 | ebi-gm430-GCST90011614 | OTU97_80 (Ruminococcus) prevalence \|\| id:ebi-gm430-GCST90011614 | Plum liking \|\| id:ebi-fl187-GCST90094812 | Inverse variance weighted | 5 | 0.152668238633752 | 0.333749917359197 | 0.647359822197318 | -0.501481599 | 0.806818076657777 | 1.16493843325961 | 0.605632689637243 | 2.24076668334763 | 0.998735809224318 |
| ebi-fl187-GCST90094812 | ebi-gm430-GCST90011623 | OTU99_11 (Parabacteroides) prevalence \|\| id:ebi-gm430-GCST90011623 | Plum liking \|\| id:ebi-fl187-GCST90094812 | Inverse variance weighted | 5 | 0.174315599460999 | 0.303406641350166 | 0.565610583959577 | -0.420361418 | 0.768992616507324 | 1.19043120598829 | 0.656809394447429 | 2.15759163643353 | 0.998735809224318 |
| ebi-fl187-GCST90094812 | ebi-gm430-GCST90011642 | OTU99_19 (Bacteria) prevalence \|\| id:ebi-gm430-GCST90011642 | Plum liking \|\| id:ebi-fl187-GCST90094812 | Inverse variance weighted | 5 | 0.357435705186193 | 0.716037909644213 | 0.61764874476552 | -1.045998598 | 1.76087000808885 | 1.42965864385671 | 0.351340796025066 | 5.81749646234756 | 0.998735809224318 |
| ebi-fl187-GCST90094812 | ebi-gm430-GCST90011653 | OTU99_30 (Parasutterella) prevalence \|\| id:ebi-gm430-GCST90011653 | Plum liking \|\| id:ebi-fl187-GCST90094812 | Inverse variance weighted | 5 | -0.500434523 | 0.325612373953724 | 0.124317148829059 | -1.138634776 | 0.137765730340041 | 0.606267165678984 | 0.320255944742793 | 1.14770664593167 | 0.998735809224318 |
| ebi-fl187-GCST90094812 | ebi-gm430-GCST90011675 | OTU99_8 (Ruminococcaceae) prevalence \|\| id:ebi-gm430-GCST90011675 | Plum liking \|\| id:ebi-fl187-GCST90094812 | Inverse variance weighted | 5 | 0.309662265819928 | 0.357652813310706 | 0.386589942349657 | -0.391337248 | 1.01066177990891 | 1.36296471661953 | 0.676152086462638 | 2.74741859996081 | 0.998735809224318 |
| ebi-fl187-GCST90094812 | ebi-gm430-GCST90011679 | OTU99_92 (Ruminococcus) prevalence \|\| id:ebi-gm430-GCST90011679 | Plum liking \|\| id:ebi-fl187-GCST90094812 | Inverse variance weighted | 5 | 0.0363979905149818 | 0.348210667152186 | 0.916749834394942 | -0.646094917 | 0.718890898133267 | 1.03706850779732 | 0.524088394495157 | 2.05215589805415 | 0.998735809224318 |
| ebi-fl187-GCST90094812 | ebi-gm430-GCST90011684 | TestASV_11 (Lachnospiraceae) prevalence \|\| id:ebi-gm430-GCST90011684 | Plum liking \|\| id:ebi-fl187-GCST90094812 | Inverse variance weighted | 5 | 0.073985564446718 | 0.431729442805092 | 0.8639326787408 | -0.772204143 | 0.920175272344698 | 1.0767912613064 | 0.461993644979209 | 2.50973023769192 | 0.998735809224318 |
| ebi-fl187-GCST90094812 | ebi-gm430-GCST90011688 | TestASV_15 (Bacteroides) prevalence \|\| id:ebi-gm430-GCST90011688 | Plum liking \|\| id:ebi-fl187-GCST90094812 | Inverse variance weighted | 5 | -0.057522643 | 0.334098602618098 | 0.863301796117729 | -0.712355904 | 0.597310618167817 | 0.944100512910797 | 0.490487294194488 | 1.81722501077674 | 0.998735809224318 |
| ebi-fl187-GCST90094812 | ebi-gm430-GCST90011692 | TestASV_19 (Prevotella) prevalence \|\| id:ebi-gm430-GCST90011692 | Plum liking \|\| id:ebi-fl187-GCST90094812 | Inverse variance weighted | 5 | 0.103597245736847 | 0.65126932253978 | 0.873613923858995 | -1.172890626 | 1.38008511791482 | 1.10915364858459 | 0.309471081799938 | 3.97523997723248 | 0.998735809224318 |
| ebi-fl187-GCST90094812 | ebi-gm430-GCST90011693 | TestASV_2 (Bacteroides) prevalence \|\| id:ebi-gm430-GCST90011693 | Plum liking \|\| id:ebi-fl187-GCST90094812 | Inverse variance weighted | 5 | 0.303069214423613 | 0.327185920027535 | 0.354295023710684 | -0.338215189 | 0.944353617677582 | 1.3540081780947 | 0.713041832749974 | 2.57115089485946 | 0.998735809224318 |
| ebi-fl187-GCST90094812 | ebi-gm430-GCST90011716 | TestASV_40 (Barnesiella) prevalence \|\| id:ebi-gm430-GCST90011716 | Plum liking \|\| id:ebi-fl187-GCST90094812 | Inverse variance weighted | 5 | -0.604165534 | 0.705293835452738 | 0.391657493030918 | -1.986541452 | 0.77821038343401 | 0.546530297330904 | 0.137169011687003 | 2.1775717578412 | 0.998735809224318 |
| ebi-fl187-GCST90094812 | ebi-gm430-GCST90011718 | TestASV_42 (Alphaproteobacteria) prevalence \|\| id:ebi-gm430-GCST90011718 | Plum liking \|\| id:ebi-fl187-GCST90094812 | Inverse variance weighted | 5 | -0.097541193 | 0.52199630215384 | 0.851768953762496 | -1.120653945 | 0.925571558967557 | 0.907064975828283 | 0.326066495178305 | 2.52331006877737 | 0.998735809224318 |
| ebi-fl187-GCST90094812 | ebi-hbsm44-GCST90104929 | Sulcal depth of the right inferior frontal sulcus \|\| id:ebi-hbsm44-GCST90104929 | Plum liking \|\| id:ebi-fl187-GCST90094812 | Inverse variance weighted | 4 | -0.102348998 | 0.295939796923963 | 0.72945996868085 | -0.682391 | 0.4776930038482 | 0.902714451038502 | 0.505407118070523 | 1.61235042202163 | 0.998735809224318 |
| ebi-fl187-GCST90094812 | ebi-hbsm44-GCST90104935 | Sulcal opening distance of the left calloso-marginal posterior fissure \|\| id:ebi-hbsm44-GCST90104935 | Plum liking \|\| id:ebi-fl187-GCST90094812 | Inverse variance weighted | 5 | 0.0768845411148066 | 0.0637149322859821 | 0.227549317598105 | -0.047996726 | 0.201765808395332 | 1.07991738313711 | 0.95313690748468 | 1.22356142674125 | 0.998735809224318 |
| ebi-fl187-GCST90094812 | ebi-hbsm44-GCST90104936 | Sulcal opening distance of the left anterior inferior temporal sulcus \|\| id:ebi-hbsm44-GCST90104936 | Plum liking \|\| id:ebi-fl187-GCST90094812 | Inverse variance weighted | 5 | -0.021010385 | 0.0363393548696954 | 0.563148233183144 | -0.092235521 | 0.050214750574926 | 0.979208795463009 | 0.911890355366403 | 1.05149688169127 | 0.998735809224318 |
| ebi-fl187-GCST90094812 | ebi-hbsm44-GCST90104941 | Sulcal opening distance of the right posterior inferior temporal sulcus \|\| id:ebi-hbsm44-GCST90104941 | Plum liking \|\| id:ebi-fl187-GCST90094812 | Inverse variance weighted | 5 | 0.0498904418002755 | 0.0296427162269115 | 0.0923636608445191 | -0.008209282 | 0.107990165605022 | 1.05115592731626 | 0.991824322132891 | 1.11403678945479 | 0.998735809224318 |
| ebi-fl187-GCST90094812 | ebi-IPs91-GCST90274819 | Leukemia inhibitory factor levels \|\| id:ebi-IPs91-GCST90274819 | Plum liking \|\| id:ebi-fl187-GCST90094812 | Inverse variance weighted | 5 | 0.204868007900832 | 0.164834850613075 | 0.21391637477716 | -0.118208299 | 0.52794431510246 | 1.22736305204552 | 0.888510957126538 | 1.69544342637967 | 0.998735809224318 |
| ebi-fl187-GCST90094812 | ebi-IPs91-GCST90274837 | STAM binding protein levels \|\| id:ebi-IPs91-GCST90274837 | Plum liking \|\| id:ebi-fl187-GCST90094812 | Inverse variance weighted | 5 | -0.177570133 | 0.111068952651136 | 0.109878875539242 | -0.39526528 | 0.0401250145500239 | 0.837302275052312 | 0.673501349216687 | 1.04094089881655 | 0.998735809224318 |
| ebi-fl187-GCST90094812 | ebi-IPs91-GCST90274840 | TNF-beta levels \|\| id:ebi-IPs91-GCST90274840 | Plum liking \|\| id:ebi-fl187-GCST90094812 | Inverse variance weighted | 5 | -0.039289454 | 0.194135705659199 | 0.839618575693815 | -0.419795437 | 0.341216529053285 | 0.961472366804939 | 0.65718124094605 | 1.40665779016869 | 0.998735809224318 |
| ebi-fl187-GCST90094812 | ebi-IPs91-GCST90274842 | Tumor necrosis factor ligand superfamily member 14 levels \|\| id:ebi-IPs91-GCST90274842 | Plum liking \|\| id:ebi-fl187-GCST90094812 | Inverse variance weighted | 5 | -0.226231496 | 0.127049678125136 | 0.0749690194968107 | -0.475248865 | 0.0227858727517247 | 0.797533454191466 | 0.621730309986571 | 1.02304745375581 | 0.998735809224318 |
| ebi-fl187-GCST90094812 | ebi-met1400-GCST90199628 | 3-methylhistidine levels \|\| id:ebi-met1400-GCST90199628 | Plum liking \|\| id:ebi-fl187-GCST90094812 | Inverse variance weighted | 5 | 0.00998794216950527 | 0.146415287356298 | 0.945613272244174 | -0.276986021 | 0.296961905387849 | 1.01003798814389 | 0.758065093991368 | 1.34576403211275 | 0.998735809224318 |
| ebi-fl187-GCST90094812 | ebi-met1400-GCST90199651 | Docosahexaenoate DHA; 22:6n3 levels \|\| id:ebi-met1400-GCST90199651 | Plum liking \|\| id:ebi-fl187-GCST90094812 | Inverse variance weighted | 5 | -0.102102704 | 0.140453853054601 | 0.46725756573085 | -0.377392256 | 0.173186848151697 | 0.902936811832962 | 0.685647072084611 | 1.18908826327266 | 0.998735809224318 |
| ebi-fl187-GCST90094812 | ebi-met1400-GCST90199652 | 1-stearoyl-2-oleoyl-GPS (18:0/18:1) levels \|\| id:ebi-met1400-GCST90199652 | Plum liking \|\| id:ebi-fl187-GCST90094812 | Inverse variance weighted (multiplicative random effects) | 5 | 0.0875507097810724 | 0.294177108123315 | 0.76599912717163 | -0.489036422 | 0.66413784170277 | 1.09149761264457 | 0.613216991911671 | 1.94281478517869 | 0.998735809224318 |
| ebi-fl187-GCST90094812 | ebi-met1400-GCST90199653 | 1-myristoyl-2-palmitoyl-gpc (14:0/16:0) levels \|\| id:ebi-met1400-GCST90199653 | Plum liking \|\| id:ebi-fl187-GCST90094812 | Inverse variance weighted | 5 | -0.056824015 | 0.145869187363021 | 0.69686591934706 | -0.342727622 | 0.22907959240295 | 0.944760318544018 | 0.709831527680683 | 1.25744211786676 | 0.998735809224318 |
| ebi-fl187-GCST90094812 | ebi-met1400-GCST90199667 | Erythritol levels in elite athletes \|\| id:ebi-met1400-GCST90199667 | Plum liking \|\| id:ebi-fl187-GCST90094812 | Inverse variance weighted | 5 | 0.236388349582008 | 0.137875903538266 | 0.0864367867262279 | -0.033848421 | 0.506625120517009 | 1.26666612392263 | 0.966718027342991 | 1.65968051087552 | 0.998735809224318 |
| ebi-fl187-GCST90094812 | ebi-met1400-GCST90199674 | 3-hydroxymyristate levels \|\| id:ebi-met1400-GCST90199674 | Plum liking \|\| id:ebi-fl187-GCST90094812 | Inverse variance weighted | 5 | -0.118168533 | 0.143159414063524 | 0.409125695452385 | -0.398760984 | 0.162423919007401 | 0.888546291016587 | 0.671151097953223 | 1.17635881649762 | 0.998735809224318 |
| ebi-fl187-GCST90094812 | ebi-met1400-GCST90199685 | 2-linoleoylglycerol (18:2) levels \|\| id:ebi-met1400-GCST90199685 | Plum liking \|\| id:ebi-fl187-GCST90094812 | Inverse variance weighted | 5 | -0.329580183 | 0.167743742442307 | 0.0494392693622574 | -0.658357918 | -0.000802448 | 0.719225613344759 | 0.517700743981592 | 0.999197874263704 | 0.998735809224318 |
| ebi-fl187-GCST90094812 | ebi-met1400-GCST90199703 | Gamma-glutamylvaline levels \|\| id:ebi-met1400-GCST90199703 | Plum liking \|\| id:ebi-fl187-GCST90094812 | Inverse variance weighted | 5 | -0.026967219 | 0.1432897683038 | 0.850719410353246 | -0.307815165 | 0.253880727004348 | 0.973393149927655 | 0.735051168822761 | 1.28901805005434 | 0.998735809224318 |
| ebi-fl187-GCST90094812 | ebi-met1400-GCST90199779 | Gamma-glutamyl-2-aminobutyrate levels \|\| id:ebi-met1400-GCST90199779 | Plum liking \|\| id:ebi-fl187-GCST90094812 | Inverse variance weighted | 5 | -0.026801417 | 0.152105011620875 | 0.860134164681339 | -0.32492724 | 0.271324405460016 | 0.973554553404866 | 0.722579926577118 | 1.31170052418304 | 0.998735809224318 |
| ebi-fl187-GCST90094812 | ebi-met1400-GCST90199824 | 4-vinylphenol sulfate levels \|\| id:ebi-met1400-GCST90199824 | Plum liking \|\| id:ebi-fl187-GCST90094812 | Inverse variance weighted | 5 | 0.100983649779392 | 0.146984522566881 | 0.492060079973345 | -0.187106014 | 0.389073314010478 | 1.10625855404415 | 0.829355808023401 | 1.47561273045467 | 0.998735809224318 |
| ebi-fl187-GCST90094812 | ebi-met1400-GCST90199830 | 4-ethylphenylsulfate levels \|\| id:ebi-met1400-GCST90199830 | Plum liking \|\| id:ebi-fl187-GCST90094812 | Inverse variance weighted | 5 | 0.160657960855917 | 0.14723670643523 | 0.275205006410978 | -0.127925984 | 0.449241905468968 | 1.17428324927839 | 0.879918504996198 | 1.56712370714578 | 0.998735809224318 |
| ebi-fl187-GCST90094812 | ebi-met1400-GCST90199833 | Hexanoylglutamine levels \|\| id:ebi-met1400-GCST90199833 | Plum liking \|\| id:ebi-fl187-GCST90094812 | Inverse variance weighted | 5 | -0.055963659 | 0.182269578020825 | 0.758814565233581 | -0.413212032 | 0.301284714195137 | 0.945573498611903 | 0.661522004356859 | 1.35159410478933 | 0.998735809224318 |
| ebi-fl187-GCST90094812 | ebi-met1400-GCST90199837 | Carotenoid levels (cryptoxanthin) \|\| id:ebi-met1400-GCST90199837 | Plum liking \|\| id:ebi-fl187-GCST90094812 | Inverse variance weighted (multiplicative random effects) | 5 | 0.170931055539032 | 0.267231831733521 | 0.522409380277573 | -0.352843335 | 0.694705445736733 | 1.18640894987641 | 0.702687271495542 | 2.00311895980568 | 0.998735809224318 |
| ebi-fl187-GCST90094812 | ebi-met1400-GCST90199869 | 2-hydroxyhippurate levels \|\| id:ebi-met1400-GCST90199869 | Plum liking \|\| id:ebi-fl187-GCST90094812 | Inverse variance weighted | 5 | -0.104193806 | 0.149138946991451 | 0.484779671385217 | -0.396506142 | 0.18811853014965 | 0.90105065151729 | 0.672666145134852 | 1.20697656998476 | 0.998735809224318 |
| ebi-fl187-GCST90094812 | ebi-met1400-GCST90199902 | Hydantoin-5-propionate levels \|\| id:ebi-met1400-GCST90199902 | Plum liking \|\| id:ebi-fl187-GCST90094812 | Inverse variance weighted | 5 | -0.137967351 | 0.146781665539442 | 0.347243470711713 | -0.425659416 | 0.149724712989377 | 0.871127132303641 | 0.653338821030007 | 1.16151444887233 | 0.998735809224318 |
| ebi-fl187-GCST90094812 | ebi-met1400-GCST90199904 | Pantoate levels \|\| id:ebi-met1400-GCST90199904 | Plum liking \|\| id:ebi-fl187-GCST90094812 | Inverse variance weighted | 5 | -0.077255565 | 0.148918062922848 | 0.603914860247799 | -0.369134968 | 0.21462383880095 | 0.925653259237086 | 0.691332096541982 | 1.23939559673577 | 0.998735809224318 |
| ebi-fl187-GCST90094812 | ebi-met1400-GCST90199936 | 3-methyl catechol sulfate (2) levels \|\| id:ebi-met1400-GCST90199936 | Plum liking \|\| id:ebi-fl187-GCST90094812 | Inverse variance weighted | 5 | 0.0128680172108048 | 0.167730924263157 | 0.938847713292626 | -0.315884594 | 0.341620628766593 | 1.01295116641663 | 0.72914359260618 | 1.4072263350451 | 0.998735809224318 |
| ebi-fl187-GCST90094812 | ebi-met1400-GCST90199977 | 4-hydroxychlorothalonil levels \|\| id:ebi-met1400-GCST90199977 | Plum liking \|\| id:ebi-fl187-GCST90094812 | Inverse variance weighted | 5 | -0.205559389 | 0.145711063214937 | 0.158323389080031 | -0.491153073 | 0.0800342945598462 | 0.814191738820094 | 0.611920398192274 | 1.08332421916517 | 0.998735809224318 |
| ebi-fl187-GCST90094812 | ebi-met1400-GCST90200040 | Sphingomyelin (d18:2/24:1, d18:1/24:2) levels \|\| id:ebi-met1400-GCST90200040 | Plum liking \|\| id:ebi-fl187-GCST90094812 | Inverse variance weighted | 5 | 0.126476882933857 | 0.146014174542738 | 0.386382727854917 | -0.159710899 | 0.412664665037624 | 1.13482321708948 | 0.852390180057176 | 1.51083830407211 | 0.998735809224318 |
| ebi-fl187-GCST90094812 | ebi-met1400-GCST90200077 | 1-stearoyl-2-oleoyl-GPI (18:0/18:1) levels \|\| id:ebi-met1400-GCST90200077 | Plum liking \|\| id:ebi-fl187-GCST90094812 | Inverse variance weighted | 5 | 0.142069026539867 | 0.164454348900012 | 0.387653134452021 | -0.180261497 | 0.464399550383891 | 1.15265620966087 | 0.835051819058496 | 1.5910585515133 | 0.998735809224318 |
| ebi-fl187-GCST90094812 | ebi-met1400-GCST90200080 | 1-palmitoyl-2-oleoyl-GPI (16:0/18:1) levels \|\| id:ebi-met1400-GCST90200080 | Plum liking \|\| id:ebi-fl187-GCST90094812 | Inverse variance weighted | 5 | 0.134445632567859 | 0.146470262362977 | 0.358669152845057 | -0.152636082 | 0.421527346799294 | 1.14390246627207 | 0.858442067782958 | 1.5242878948404 | 0.998735809224318 |
| ebi-fl187-GCST90094812 | ebi-met1400-GCST90200082 | 1-oleoyl-2-linoleoyl-GPE (18:1/18:2) levels \|\| id:ebi-met1400-GCST90200082 | Plum liking \|\| id:ebi-fl187-GCST90094812 | Inverse variance weighted | 5 | 0.0481328431833376 | 0.147466583964781 | 0.744123019102912 | -0.240901661 | 0.337167347754308 | 1.04931003975212 | 0.785918908763284 | 1.40097349389036 | 0.998735809224318 |
| ebi-fl187-GCST90094812 | ebi-met1400-GCST90200100 | Perfluorooctanesulfonate (PFOS) levels \|\| id:ebi-met1400-GCST90200100 | Plum liking \|\| id:ebi-fl187-GCST90094812 | Inverse variance weighted | 5 | 0.0224421699579019 | 0.13531842887749 | 0.86827727336947 | -0.242781951 | 0.287666290557783 | 1.02269588990794 | 0.784442542317303 | 1.33331229097405 | 0.998735809224318 |
| ebi-fl187-GCST90094812 | ebi-met1400-GCST90200102 | 1-palmitoleoyl-2-linolenoyl-GPC (16:1/18:3) levels \|\| id:ebi-met1400-GCST90200102 | Plum liking \|\| id:ebi-fl187-GCST90094812 | Inverse variance weighted | 5 | -0.013619069 | 0.143960078232468 | 0.924630092957514 | -0.295780822 | 0.2685426846589 | 0.986473251260003 | 0.743950467718579 | 1.30805680979771 | 0.998735809224318 |
| ebi-fl187-GCST90094812 | ebi-met1400-GCST90200114 | Glycosyl-N-tricosanoyl-sphingadienine (d18:2/23:0) levels \|\| id:ebi-met1400-GCST90200114 | Plum liking \|\| id:ebi-fl187-GCST90094812 | Inverse variance weighted | 5 | 0.0533693183854409 | 0.153856099398346 | 0.728682866049705 | -0.248188636 | 0.354927273206199 | 1.05481913729234 | 0.780212752843246 | 1.42607693650669 | 0.998735809224318 |
| ebi-fl187-GCST90094812 | ebi-met1400-GCST90200135 | Arachidoylcarnitine (C20) levels \|\| id:ebi-met1400-GCST90200135 | Plum liking \|\| id:ebi-fl187-GCST90094812 | Inverse variance weighted | 5 | 0.184796147847015 | 0.150660163591758 | 0.219981976711417 | -0.110497773 | 0.480090068486861 | 1.20297318645676 | 0.895388324402561 | 1.61621996612422 | 0.998735809224318 |
| ebi-fl187-GCST90094812 | ebi-met1400-GCST90200146 | Docosahexaenoylcarnitine (C22:6) levels \|\| id:ebi-met1400-GCST90200146 | Plum liking \|\| id:ebi-fl187-GCST90094812 | Inverse variance weighted | 5 | -0.053948261 | 0.164508492154942 | 0.742960033625083 | -0.376384906 | 0.268488383427409 | 0.947481126773711 | 0.68633810666271 | 1.30798578263053 | 0.998735809224318 |
| ebi-fl187-GCST90094812 | ebi-met1400-GCST90200162 | 3-hydroxyphenylacetoylglutamine levels \|\| id:ebi-met1400-GCST90200162 | Plum liking \|\| id:ebi-fl187-GCST90094812 | Inverse variance weighted | 5 | -0.335530312 | 0.17852585418799 | 0.0601831549385903 | -0.685440986 | 0.0143803626731455 | 0.71495883488332 | 0.50386798197762 | 1.0144842575062 | 0.998735809224318 |
| ebi-fl187-GCST90094812 | ebi-met1400-GCST90200172 | 3-carboxy-4-methyl-5-pentyl-2-furanpropionate (3-CMPFP) levels \|\| id:ebi-met1400-GCST90200172 | Plum liking \|\| id:ebi-fl187-GCST90094812 | Inverse variance weighted | 5 | 0.112837327999258 | 0.148076892409218 | 0.446048976638109 | -0.177393381 | 0.403068037121325 | 1.11944981499511 | 0.837450282584862 | 1.49640869954043 | 0.998735809224318 |
| ebi-fl187-GCST90094812 | ebi-met1400-GCST90200222 | Dihydrocaffeate sulfate (2) levels \|\| id:ebi-met1400-GCST90200222 | Plum liking \|\| id:ebi-fl187-GCST90094812 | Inverse variance weighted | 5 | 0.236105833745955 | 0.166502458372644 | 0.156181387870109 | -0.090238985 | 0.562450652156338 | 1.26630832122848 | 0.91371279583051 | 1.7549680509344 | 0.998735809224318 |
| ebi-fl187-GCST90094812 | ebi-met1400-GCST90200223 | 4-methylhexanoylglutamine levels \|\| id:ebi-met1400-GCST90200223 | Plum liking \|\| id:ebi-fl187-GCST90094812 | Inverse variance weighted | 5 | -0.301641179 | 0.175289294029859 | 0.0852836415296666 | -0.645208195 | 0.0419258377277338 | 0.739603402832268 | 0.524553321427192 | 1.0428171381944 | 0.998735809224318 |
| ebi-fl187-GCST90094812 | ebi-met1400-GCST90200224 | 2-ketocaprylate levels \|\| id:ebi-met1400-GCST90200224 | Plum liking \|\| id:ebi-fl187-GCST90094812 | Inverse variance weighted | 5 | -0.246115488 | 0.148668254311977 | 0.0978296706143762 | -0.537505266 | 0.0452742909110398 | 0.781831927865103 | 0.584203869190787 | 1.0463148151962 | 0.998735809224318 |
| ebi-fl187-GCST90094812 | ebi-met1400-GCST90200243 | Hydroxypalmitoyl sphingomyelin (d18:1/16:0(OH)) levels \|\| id:ebi-met1400-GCST90200243 | Plum liking \|\| id:ebi-fl187-GCST90094812 | Inverse variance weighted | 5 | 0.0759191871164553 | 0.143501003228057 | 0.59677085532119 | -0.205342779 | 0.357181153443446 | 1.07887538360354 | 0.814368120101509 | 1.42929476807195 | 0.998735809224318 |
| ebi-fl187-GCST90094812 | ebi-met1400-GCST90200273 | 2-methoxyhydroquinone sulfate (2) levels \|\| id:ebi-met1400-GCST90200273 | Plum liking \|\| id:ebi-fl187-GCST90094812 | Inverse variance weighted | 5 | -0.056933716 | 0.256989218342034 | 0.824671011927487 | -0.560632584 | 0.446765152099068 | 0.944656683055386 | 0.5708478405117 | 1.56324713086642 | 0.998735809224318 |
| ebi-fl187-GCST90094812 | ebi-met1400-GCST90200276 | 2,4-di-tert-butylphenol levels \|\| id:ebi-met1400-GCST90200276 | Plum liking \|\| id:ebi-fl187-GCST90094812 | Inverse variance weighted (multiplicative random effects) | 5 | 0.102280942337844 | 0.231402875152496 | 0.65848648055517 | -0.351268693 | 0.555830577636735 | 1.10769462633558 | 0.703794623786365 | 1.74338840301394 | 0.998735809224318 |
| ebi-fl187-GCST90094812 | ebi-met1400-GCST90200289 | N-acetylmethionine levels \|\| id:ebi-met1400-GCST90200289 | Plum liking \|\| id:ebi-fl187-GCST90094812 | Inverse variance weighted | 5 | 0.095594888308486 | 0.146295796561746 | 0.513475464812388 | -0.191144873 | 0.382334649569507 | 1.10031322394122 | 0.826012912553051 | 1.46570249978044 | 0.998735809224318 |
| ebi-fl187-GCST90094812 | ebi-met1400-GCST90200308 | 3-hydroxyisobutyrate levels \|\| id:ebi-met1400-GCST90200308 | Plum liking \|\| id:ebi-fl187-GCST90094812 | Inverse variance weighted | 5 | 0.11204002310382 | 0.148787867040838 | 0.451438770261104 | -0.179584196 | 0.403664242503863 | 1.11855762789725 | 0.835617592074909 | 1.49730113247179 | 0.998735809224318 |
| ebi-fl187-GCST90094812 | ebi-met1400-GCST90200383 | Cystathionine levels \|\| id:ebi-met1400-GCST90200383 | Plum liking \|\| id:ebi-fl187-GCST90094812 | Inverse variance weighted | 5 | -0.058290195 | 0.197663991617882 | 0.768073813105117 | -0.445711618 | 0.329131228699932 | 0.943376144791907 | 0.640368415877792 | 1.38976022004852 | 0.998735809224318 |
| ebi-fl187-GCST90094812 | ebi-met1400-GCST90200384 | Guanidinoacetate levels \|\| id:ebi-met1400-GCST90200384 | Plum liking \|\| id:ebi-fl187-GCST90094812 | Inverse variance weighted | 5 | 0.0277161176292827 | 0.140421167865889 | 0.843531363502706 | -0.247509371 | 0.302941606646426 | 1.02810378245137 | 0.780742904132068 | 1.35383540714449 | 0.998735809224318 |
| ebi-fl187-GCST90094812 | ebi-met1400-GCST90200403 | Citrulline levels \|\| id:ebi-met1400-GCST90200403 | Plum liking \|\| id:ebi-fl187-GCST90094812 | Inverse variance weighted | 5 | -0.08754147 | 0.177619389519143 | 0.622111600666166 | -0.435675474 | 0.260592533363278 | 0.916180876755756 | 0.646827604543143 | 1.29769878873028 | 0.998735809224318 |
| ebi-fl187-GCST90094812 | ebi-met1400-GCST90200434 | 12,13-DiHOME levels \|\| id:ebi-met1400-GCST90200434 | Plum liking \|\| id:ebi-fl187-GCST90094812 | Inverse variance weighted | 5 | -0.132145941 | 0.226029366547621 | 0.558789414014266 | -0.575163499 | 0.310871617911891 | 0.876213110749566 | 0.562612874669731 | 1.36461401794284 | 0.998735809224318 |
| ebi-fl187-GCST90094812 | ebi-met1400-GCST90200437 | Fructose levels \|\| id:ebi-met1400-GCST90200437 | Plum liking \|\| id:ebi-fl187-GCST90094812 | Inverse variance weighted | 5 | 0.194449254831923 | 0.14776863784138 | 0.18820646317163 | -0.095177275 | 0.484075785001028 | 1.21464184411659 | 0.909211739363029 | 1.62267461538997 | 0.998735809224318 |
| ebi-fl187-GCST90094812 | ebi-met1400-GCST90200448 | N-stearoyl-sphinganine (d18:0/18:0) levels \|\| id:ebi-met1400-GCST90200448 | Plum liking \|\| id:ebi-fl187-GCST90094812 | Inverse variance weighted | 5 | 0.0468210602779029 | 0.163314493871912 | 0.774347688200318 | -0.273275348 | 0.36691746826685 | 1.04793447519776 | 0.760883251320963 | 1.443278798 | 0.998735809224318 |
| ebi-fl187-GCST90094812 | ebi-met1400-GCST90200468 | X-11847 levels \|\| id:ebi-met1400-GCST90200468 | Plum liking \|\| id:ebi-fl187-GCST90094812 | Inverse variance weighted | 5 | 0.0377140171999315 | 0.19286990818908 | 0.844969271341551 | -0.340311003 | 0.415739037250528 | 1.03843421608487 | 0.711548994581843 | 1.51549033073895 | 0.998735809224318 |
| ebi-fl187-GCST90094812 | ebi-met1400-GCST90200476 | X-11843 levels \|\| id:ebi-met1400-GCST90200476 | Plum liking \|\| id:ebi-fl187-GCST90094812 | Inverse variance weighted | 5 | -0.06314769 | 0.15459352744049 | 0.682924512134076 | -0.366151004 | 0.239855623917034 | 0.938804811492768 | 0.693398087661967 | 1.27106562559722 | 0.998735809224318 |
| ebi-fl187-GCST90094812 | ebi-met1400-GCST90200511 | X-12906 levels \|\| id:ebi-met1400-GCST90200511 | Plum liking \|\| id:ebi-fl187-GCST90094812 | Inverse variance weighted | 5 | -0.068085991 | 0.147153509694577 | 0.643588857864911 | -0.35650687 | 0.220334887697186 | 0.934180138754739 | 0.700117661430838 | 1.24649409623561 | 0.998735809224318 |
| ebi-fl187-GCST90094812 | ebi-met1400-GCST90200515 | X-12849 levels \|\| id:ebi-met1400-GCST90200515 | Plum liking \|\| id:ebi-fl187-GCST90094812 | Inverse variance weighted | 5 | 0.322690324676624 | 0.224155202124289 | 0.149985025226592 | -0.116653871 | 0.762034520840229 | 1.38083767333543 | 0.889893157258026 | 2.14263101648905 | 0.998735809224318 |
| ebi-fl187-GCST90094812 | ebi-met1400-GCST90200551 | X-18886 levels \|\| id:ebi-met1400-GCST90200551 | Plum liking \|\| id:ebi-fl187-GCST90094812 | Inverse variance weighted | 5 | -0.191049328 | 0.156547969394423 | 0.222317666447277 | -0.497883348 | 0.115784691556761 | 0.826091837311011 | 0.607815833414504 | 1.12275410766816 | 0.998735809224318 |
| ebi-fl187-GCST90094812 | ebi-met1400-GCST90200564 | X-21319 levels \|\| id:ebi-met1400-GCST90200564 | Plum liking \|\| id:ebi-fl187-GCST90094812 | Inverse variance weighted | 5 | -0.35913569 | 0.148403229889098 | 0.0155205434063136 | -0.650006021 | -0.06826536 | 0.698279595295831 | 0.52204263369925 | 0.934012591560508 | 0.998735809224318 |
| ebi-fl187-GCST90094812 | ebi-met1400-GCST90200579 | X-21442 levels \|\| id:ebi-met1400-GCST90200579 | Plum liking \|\| id:ebi-fl187-GCST90094812 | Inverse variance weighted | 5 | -0.025349313 | 0.160263060613644 | 0.87432036076263 | -0.339464912 | 0.288766285712388 | 0.974969283005461 | 0.712151284512128 | 1.33477973497637 | 0.998735809224318 |
| ebi-fl187-GCST90094812 | ebi-met1400-GCST90200581 | X-21441 levels \|\| id:ebi-met1400-GCST90200581 | Plum liking \|\| id:ebi-fl187-GCST90094812 | Inverse variance weighted | 5 | -0.064143707 | 0.147240720997662 | 0.663099128855171 | -0.352735521 | 0.224448105796668 | 0.937870210995038 | 0.702763035206697 | 1.25163175722976 | 0.998735809224318 |
| ebi-fl187-GCST90094812 | ebi-met1400-GCST90200648 | X-24585 levels \|\| id:ebi-met1400-GCST90200648 | Plum liking \|\| id:ebi-fl187-GCST90094812 | Inverse variance weighted | 5 | 0.0541127198536254 | 0.211030726836866 | 0.797625738106897 | -0.359507505 | 0.467732944453883 | 1.05560358293056 | 0.698020012975146 | 1.5963710260203 | 0.998735809224318 |
| ebi-fl187-GCST90094812 | ebi-met1400-GCST90200720 | Adenosine 5'-diphosphate (ADP) to pantothenate ratio \|\| id:ebi-met1400-GCST90200720 | Plum liking \|\| id:ebi-fl187-GCST90094812 | Inverse variance weighted | 5 | -0.008344047 | 0.194793747861673 | 0.965832829848912 | -0.390139793 | 0.373451698494858 | 0.991690667627297 | 0.676962233218552 | 1.45274039230129 | 0.998735809224318 |
| ebi-fl187-GCST90094812 | ebi-met1400-GCST90200724 | Cholate to taurocholate ratio \|\| id:ebi-met1400-GCST90200724 | Plum liking \|\| id:ebi-fl187-GCST90094812 | Inverse variance weighted | 5 | 0.306382150650022 | 0.15748113048215 | 0.0517128359964576 | -0.002280865 | 0.615045166395036 | 1.35850135954096 | 0.997721734101285 | 1.84974014376566 | 0.998735809224318 |
| ebi-fl187-GCST90094812 | ebi-met1400-GCST90200726 | N-acetylputrescine to (N(1) + N(8))-acetylspermidine ratio \|\| id:ebi-met1400-GCST90200726 | Plum liking \|\| id:ebi-fl187-GCST90094812 | Inverse variance weighted | 5 | -0.160777407 | 0.146285917474639 | 0.271740682936371 | -0.447497805 | 0.125942991016268 | 0.851481583655191 | 0.639225619039437 | 1.13421750585253 | 0.998735809224318 |
| ebi-fl187-GCST90094812 | ebi-met1400-GCST90200744 | Arginine to glutamate ratio \|\| id:ebi-met1400-GCST90200744 | Plum liking \|\| id:ebi-fl187-GCST90094812 | Inverse variance weighted | 5 | 0.250857044577575 | 0.14160590922486 | 0.076475039190776 | -0.026690538 | 0.528404626658301 | 1.28512635541798 | 0.973662506937243 | 1.69622403822965 | 0.998735809224318 |
| ebi-fl187-GCST90094812 | ebi-met1400-GCST90200759 | Histidine to trans-urocanate ratio \|\| id:ebi-met1400-GCST90200759 | Plum liking \|\| id:ebi-fl187-GCST90094812 | Inverse variance weighted | 5 | 0.0627580881009314 | 0.177719702337941 | 0.723991280007008 | -0.285572528 | 0.411088704683296 | 1.06476922772697 | 0.751583827967771 | 1.50845915801544 | 0.998735809224318 |
| ebi-fl187-GCST90094812 | ebi-met1400-GCST90200795 | Oleoyl-linoleoyl-glycerol (18:1 to 18:2) [2] to linoleoyl-arachidonoyl-glycerol (18:2 to 20:4) [2] ratio \|\| id:ebi-met1400-GCST90200795 | Plum liking \|\| id:ebi-fl187-GCST90094812 | Inverse variance weighted | 5 | 0.100704415933327 | 0.15241294801004 | 0.508782919002206 | -0.198024962 | 0.399433794033007 | 1.10594969233769 | 0.820349375184675 | 1.49098025668207 | 0.998735809224318 |
| ebi-fl187-GCST90094812 | ebi-met1400-GCST90200811 | Choline to taurocholate ratio \|\| id:ebi-met1400-GCST90200811 | Plum liking \|\| id:ebi-fl187-GCST90094812 | Inverse variance weighted | 5 | 0.0889879958379705 | 0.185280315124082 | 0.631022298708751 | -0.274161422 | 0.452137413481171 | 1.09306753488778 | 0.760209350990173 | 1.57166790209752 | 0.998735809224318 |
| ebi-fl187-GCST90094812 | ebi-met1400-GCST90200831 | Cholate to phosphate ratio \|\| id:ebi-met1400-GCST90200831 | Plum liking \|\| id:ebi-fl187-GCST90094812 | Inverse variance weighted | 5 | 0.174369321949787 | 0.173963451111542 | 0.316182750329357 | -0.166599042 | 0.51533768612841 | 1.1904951606333 | 0.846538969676946 | 1.67420376173841 | 0.998735809224318 |
| ebi-fl187-GCST90094812 | ebi-met1400-GCST90200841 | Adenosine 5'-diphosphate (ADP) to oxalate (ethanedioate) ratio \|\| id:ebi-met1400-GCST90200841 | Plum liking \|\| id:ebi-fl187-GCST90094812 | Inverse variance weighted | 5 | -0.012539824 | 0.256410061549647 | 0.960994726127933 | -0.515103544 | 0.490023897105701 | 0.987538472441226 | 0.597438730118652 | 1.63235522805471 | 0.998735809224318 |
| ebi-fl187-GCST90094812 | ebi-met1400-GCST90200844 | Aspartate to phosphate ratio \|\| id:ebi-met1400-GCST90200844 | Plum liking \|\| id:ebi-fl187-GCST90094812 | Inverse variance weighted | 5 | -0.039705106 | 0.154588865395013 | 0.797299786203579 | -0.342699282 | 0.263289070604585 | 0.961072812387219 | 0.709851644815706 | 1.30120280407279 | 0.998735809224318 |
| ebi-fl187-GCST90094812 | ebi-met1400-GCST90200870 | Adenosine 5'-monophosphate (AMP) to acetoacetate ratio \|\| id:ebi-met1400-GCST90200870 | Plum liking \|\| id:ebi-fl187-GCST90094812 | Inverse variance weighted | 5 | 0.23459277714026 | 0.161867863427664 | 0.147257817409204 | -0.082668235 | 0.551853789458481 | 1.26439377383274 | 0.920656538010855 | 1.7364690840746 | 0.998735809224318 |
| ebi-fl187-GCST90094812 | ebi-met1400-GCST90200887 | Cholesterol to cortisol ratio \|\| id:ebi-met1400-GCST90200887 | Plum liking \|\| id:ebi-fl187-GCST90094812 | Inverse variance weighted | 5 | 0.0765196639883716 | 0.142807576342945 | 0.592080497545965 | -0.203383186 | 0.356422513620544 | 1.07952341786439 | 0.815965515242067 | 1.42821085934238 | 0.998735809224318 |
| ebi-fl187-GCST90094812 | ebi-met1400-GCST90200890 | Cortisol to taurocholate ratio \|\| id:ebi-met1400-GCST90200890 | Plum liking \|\| id:ebi-fl187-GCST90094812 | Inverse variance weighted | 5 | 0.0797912405666102 | 0.163294905909744 | 0.62510171554993 | -0.240266775 | 0.399849256149708 | 1.08306094488397 | 0.786418036395182 | 1.49159983119143 | 0.998735809224318 |
| ebi-fl187-GCST90094812 | ebi-met1400-GCST90200948 | Adenosine 5'-diphosphate (ADP) to mannitol to sorbitol ratio \|\| id:ebi-met1400-GCST90200948 | Plum liking \|\| id:ebi-fl187-GCST90094812 | Inverse variance weighted | 5 | -0.033642086 | 0.196888076494034 | 0.864326702490431 | -0.419542716 | 0.352258543639424 | 0.96691751574916 | 0.657347345377803 | 1.42227619665093 | 0.998735809224318 |
| ebi-fl187-GCST90094812 | ebi-met1400-GCST90200972 | Leucine to N-palmitoyl-sphingosine (d18:1 to 16:0) ratio \|\| id:ebi-met1400-GCST90200972 | Plum liking \|\| id:ebi-fl187-GCST90094812 | Inverse variance weighted | 5 | -0.060075253 | 0.141676371622786 | 0.671542794380193 | -0.337760942 | 0.217610435007488 | 0.941693665292935 | 0.713365803493749 | 1.24310270398406 | 0.998735809224318 |
| ebi-fl187-GCST90094812 | ebi-met1400-GCST90200973 | Proline to glutamate ratio \|\| id:ebi-met1400-GCST90200973 | Plum liking \|\| id:ebi-fl187-GCST90094812 | Inverse variance weighted | 5 | 0.125198930689899 | 0.143971216308842 | 0.384513110523499 | -0.156984653 | 0.407382514655229 | 1.13337389349312 | 0.854717175826116 | 1.50287886891965 | 0.998735809224318 |
| ebi-fl187-GCST90094812 | ebi-met1400-GCST90200983 | Cholesterol to linoleoyl-arachidonoyl-glycerol (18:2 to 20:4) [1] ratio \|\| id:ebi-met1400-GCST90200983 | Plum liking \|\| id:ebi-fl187-GCST90094812 | Inverse variance weighted | 5 | 0.275617257616459 | 0.151172145469512 | 0.0682729027817016 | -0.020680148 | 0.571914662736703 | 1.31734356430928 | 0.979532220294761 | 1.77165592970985 | 0.998735809224318 |
| ebi-fl187-GCST90094812 | ebi-met1400-GCST90201001 | Serine to alpha-ketobutyrate ratio \|\| id:ebi-met1400-GCST90201001 | Plum liking \|\| id:ebi-fl187-GCST90094812 | Inverse variance weighted (multiplicative random effects) | 5 | 0.13855357942405 | 0.235251862976344 | 0.555889127071258 | -0.322540072 | 0.599647230857684 | 1.14861122184924 | 0.724306906792064 | 1.82147612646853 | 0.998735809224318 |
| ebi-fl187-GCST90094812 | ebi-met1400-GCST90201005 | Fructose to maltose ratio \|\| id:ebi-met1400-GCST90201005 | Plum liking \|\| id:ebi-fl187-GCST90094812 | Inverse variance weighted | 5 | -0.011541973 | 0.181083169514852 | 0.949178431360289 | -0.366464986 | 0.343381038753275 | 0.988524379553195 | 0.69318040725322 | 1.40970581214662 | 0.998735809224318 |
| ebi-fl187-GCST90094812 | ebi-met1400-GCST90201020 | Paraxanthine to 5-acetylamino-6-formylamino-3-methyluracil ratio \|\| id:ebi-met1400-GCST90201020 | Plum liking \|\| id:ebi-fl187-GCST90094812 | Inverse variance weighted | 5 | 0.398364099997306 | 0.155377684817222 | 0.0103520147111777 | 0.0938238377555504 | 0.702904362239062 | 1.48938621671367 | 1.09836623821155 | 2.01960987634566 | 0.998735809224318 |
| ebi-fl187-GCST90094812 | ebi-pw11-GCST90275197 | Placental weight (fetal genotype, adjusted for sex) \|\| id:ebi-pw11-GCST90275197 | Plum liking \|\| id:ebi-fl187-GCST90094812 | Inverse variance weighted | 5 | -0.057288029 | 0.0804424701544282 | 0.476364781146977 | -0.214955271 | 0.100379212143016 | 0.944322037720013 | 0.806577516887265 | 1.10559009178075 | 0.998735809224318 |
| ebi-fl187-GCST90094812 | prot-a-1017 | FAS-associated factor 2 \|\| id:prot-a-1017 | Plum liking \|\| id:ebi-fl187-GCST90094812 | Inverse variance weighted | 5 | 0.146811536255094 | 0.236935286095222 | 0.535503287237725 | -0.317581624 | 0.611204697001729 | 1.15813567587431 | 0.727907263289129 | 1.84264989701041 | 0.998735809224318 |
| ebi-fl187-GCST90094812 | prot-a-1019 | Fas apoptotic inhibitory molecule 3 \|\| id:prot-a-1019 | Plum liking \|\| id:ebi-fl187-GCST90094812 | Inverse variance weighted | 5 | 0.129681195503175 | 0.237021661795527 | 0.584290774591847 | -0.334881262 | 0.594243652622409 | 1.13846537758191 | 0.715423029483811 | 1.8116601822112 | 0.998735809224318 |
| ebi-fl187-GCST90094812 | prot-a-1052 | MIP18 family protein FAM96A \|\| id:prot-a-1052 | Plum liking \|\| id:ebi-fl187-GCST90094812 | Inverse variance weighted | 5 | -0.67546435 | 0.236640300120678 | 0.00431191920496533 | -1.139279338 | -0.211649362 | 0.508920048715846 | 0.320049586231284 | 0.809248401270461 | 0.998735809224318 |
| ebi-fl187-GCST90094812 | prot-a-1066 | F-box/LRR-repeat protein 4 \|\| id:prot-a-1066 | Plum liking \|\| id:ebi-fl187-GCST90094812 | Inverse variance weighted | 5 | -0.213893802 | 0.237021661795527 | 0.366832201114516 | -0.678456259 | 0.250668655049744 | 0.807434128303171 | 0.507399681671665 | 1.28488427387421 | 0.998735809224318 |
| ebi-fl187-GCST90094812 | prot-a-1076 | Low affinity immunoglobulin gamma Fc region receptor III-B \|\| id:prot-a-1076 | Plum liking \|\| id:ebi-fl187-GCST90094812 | Inverse variance weighted | 5 | -0.419306017 | 0.236640300120678 | 0.0764090054070524 | -0.883121005 | 0.044508971682191 | 0.657502957688793 | 0.413490390258998 | 1.04551435669091 | 0.998735809224318 |
| ebi-fl187-GCST90094812 | prot-a-1081 | Fc receptor-like protein 3 \|\| id:prot-a-1081 | Plum liking \|\| id:ebi-fl187-GCST90094812 | Inverse variance weighted | 5 | -0.177162579 | 0.270217887489315 | 0.512063110344435 | -0.706789638 | 0.352464480536856 | 0.837643590243289 | 0.493225089362437 | 1.4225691259596 | 0.998735809224318 |
| ebi-fl187-GCST90094812 | prot-a-1105 | Fibroblast growth factor receptor 4 \|\| id:prot-a-1105 | Plum liking \|\| id:ebi-fl187-GCST90094812 | Inverse variance weighted | 5 | 0.171674190288172 | 0.237021661795527 | 0.468883129079123 | -0.292888267 | 0.636236647407406 | 1.18729093927165 | 0.746105500772056 | 1.88935716600113 | 0.998735809224318 |
| ebi-fl187-GCST90094812 | prot-a-1143 | Follistatin-related protein 1 \|\| id:prot-a-1143 | Plum liking \|\| id:ebi-fl187-GCST90094812 | Inverse variance weighted | 5 | -0.04429236 | 0.237021661795527 | 0.851762157207973 | -0.508854817 | 0.420270097006409 | 0.956674223199636 | 0.601183649909732 | 1.52237268839904 | 0.998735809224318 |
| ebi-fl187-GCST90094812 | prot-a-1151 | Alpha-(1,3)-fucosyltransferase 10 \|\| id:prot-a-1151 | Plum liking \|\| id:ebi-fl187-GCST90094812 | Inverse variance weighted | 5 | 0.094219990161995 | 0.237021661795527 | 0.690986718781411 | -0.370342467 | 0.558782447281228 | 1.09880144483836 | 0.690497817454168 | 1.74854226133597 | 0.998735809224318 |
| ebi-fl187-GCST90094812 | prot-a-1165 | Growth arrest and DNA damage-inducible proteins-interacting protein 1 \|\| id:prot-a-1165 | Plum liking \|\| id:ebi-fl187-GCST90094812 | Inverse variance weighted | 5 | -0.34216764 | 0.324107604321043 | 0.291095085704688 | -0.977418544 | 0.293083264673211 | 0.710229132061964 | 0.376281199436973 | 1.34055440660936 | 0.998735809224318 |
| ebi-fl187-GCST90094812 | prot-a-1194 | Growth/differentiation factor 11/8 \|\| id:prot-a-1194 | Plum liking \|\| id:ebi-fl187-GCST90094812 | Inverse variance weighted | 5 | -0.049883155 | 0.236640300120678 | 0.833045385492859 | -0.513698143 | 0.413931833704311 | 0.951340577841543 | 0.598278961639453 | 1.51275400453292 | 0.998735809224318 |
| ebi-fl187-GCST90094812 | prot-a-1216 | D-glucuronyl C5-epimerase \|\| id:prot-a-1216 | Plum liking \|\| id:ebi-fl187-GCST90094812 | Inverse variance weighted | 5 | 0.346235552393458 | 0.236554340527589 | 0.143286377172679 | -0.117410955 | 0.809882059827533 | 1.4137355854011 | 0.889219688752307 | 2.24764288365429 | 0.998735809224318 |
| ebi-fl187-GCST90094812 | prot-a-1246 | Glypican-3 \|\| id:prot-a-1246 | Plum liking \|\| id:ebi-fl187-GCST90094812 | Inverse variance weighted | 5 | 0.101481036178284 | 0.259180637006594 | 0.69539399482409 | -0.406513012 | 0.609475084711208 | 1.10680892886572 | 0.665968429742297 | 1.83946558171069 | 0.998735809224318 |
| ebi-fl187-GCST90094812 | prot-a-1275 | Glutamate receptor 4 \|\| id:prot-a-1275 | Plum liking \|\| id:ebi-fl187-GCST90094812 | Inverse variance weighted | 5 | -0.216807046 | 0.256983203654811 | 0.398858127683211 | -0.720494126 | 0.286880032813184 | 0.805085298465557 | 0.486511798664397 | 1.33226437587896 | 0.998735809224318 |
| ebi-fl187-GCST90094812 | prot-a-1289 | Glutathione S-transferase P \|\| id:prot-a-1289 | Plum liking \|\| id:ebi-fl187-GCST90094812 | Inverse variance weighted | 5 | 0.0164462538294689 | 0.236935286095222 | 0.944661335515618 | -0.447946907 | 0.480839414576103 | 1.01658223791544 | 0.638938606351741 | 1.61743152811811 | 0.998735809224318 |
| ebi-fl187-GCST90094812 | prot-a-130 | Apolipoprotein D \|\| id:prot-a-130 | Plum liking \|\| id:ebi-fl187-GCST90094812 | Inverse variance weighted (multiplicative random effects) | 5 | 0.265932075928469 | 0.395757913661535 | 0.501611432429484 | -0.509753435 | 1.04161758670508 | 1.30464643877922 | 0.600643658350724 | 2.83379722162224 | 0.998735809224318 |
| ebi-fl187-GCST90094812 | prot-a-1313 | Heparin-binding EGF-like growth factor \|\| id:prot-a-1313 | Plum liking \|\| id:ebi-fl187-GCST90094812 | Inverse variance weighted | 5 | 0.00132377870024153 | 0.28746169975026 | 0.996325705808371 | -0.562101153 | 0.564748710210751 | 1.00132465528202 | 0.570010126335615 | 1.75900570700618 | 0.999391239686106 |
| ebi-fl187-GCST90094812 | prot-a-1347 | HLA class II histocompatibility antigen, DQ alpha 2 chain \|\| id:prot-a-1347 | Plum liking \|\| id:ebi-fl187-GCST90094812 | Inverse variance weighted | 5 | 0.0253707975142502 | 0.244281677343443 | 0.917281417219129 | -0.45342129 | 0.504162885107399 | 1.02569537531685 | 0.635450368291142 | 1.65559901361858 | 0.998735809224318 |
| ebi-fl187-GCST90094812 | prot-a-1357 | Non-histone chromosomal protein HMG-14 \|\| id:prot-a-1357 | Plum liking \|\| id:ebi-fl187-GCST90094812 | Inverse variance weighted | 5 | 0.156375864533143 | 0.236554340527589 | 0.508575863362393 | -0.307270643 | 0.620022371967218 | 1.16926560597783 | 0.735451529234435 | 1.85896963018876 | 0.998735809224318 |
| ebi-fl187-GCST90094812 | prot-a-137 | Apolipoprotein M \|\| id:prot-a-137 | Plum liking \|\| id:ebi-fl187-GCST90094812 | Inverse variance weighted | 5 | 0.234097016742058 | 0.295156637901038 | 0.427703205814745 | -0.344409994 | 0.812604027028092 | 1.26376709282659 | 0.70863833134133 | 2.2537692279337 | 0.998735809224318 |
| ebi-fl187-GCST90094812 | prot-a-1381 | 17-beta-hydroxysteroid dehydrogenase 14 \|\| id:prot-a-1381 | Plum liking \|\| id:ebi-fl187-GCST90094812 | Inverse variance weighted | 5 | 0.0214207407428621 | 0.237021661795527 | 0.927989530733712 | -0.443141716 | 0.485983197862096 | 1.02165181176462 | 0.64201621642875 | 1.62577267952509 | 0.998735809224318 |
| ebi-fl187-GCST90094812 | prot-a-1392 | Serine protease HTRA2, mitochondrial \|\| id:prot-a-1392 | Plum liking \|\| id:ebi-fl187-GCST90094812 | Inverse variance weighted | 5 | 0.109563902782006 | 0.236935286095222 | 0.64377928880141 | -0.354829258 | 0.57395706352864 | 1.11579137082973 | 0.701293173210596 | 1.77527805884432 | 0.998735809224318 |
| ebi-fl187-GCST90094812 | prot-a-1416 | Interferon-induced protein with tetratricopeptide repeats 2 \|\| id:prot-a-1416 | Plum liking \|\| id:ebi-fl187-GCST90094812 | Inverse variance weighted | 5 | -0.080960113 | 0.278110676770977 | 0.770969079363773 | -0.626057039 | 0.464136813656761 | 0.922230475889191 | 0.53469593508809 | 1.59064057690785 | 0.998735809224318 |
| ebi-fl187-GCST90094812 | prot-a-1428 | Interferon gamma \|\| id:prot-a-1428 | Plum liking \|\| id:ebi-fl187-GCST90094812 | Inverse variance weighted | 5 | -0.006182978 | 0.284394295270981 | 0.982654666741482 | -0.563595796 | 0.551229841046796 | 0.993836097587953 | 0.569158800718079 | 1.73538595489116 | 0.998955228396052 |
| ebi-fl187-GCST90094812 | prot-a-1483 | Interleukin-17C \|\| id:prot-a-1483 | Plum liking \|\| id:ebi-fl187-GCST90094812 | Inverse variance weighted | 5 | -0.460680364 | 0.236554340527589 | 0.0514794853910951 | -0.924326871 | 0.00296614375867138 | 0.630854289121453 | 0.396798425684021 | 1.00297054711566 | 0.998735809224318 |
| ebi-fl187-GCST90094812 | prot-a-1503 | Interleukin-1 receptor-like 2 \|\| id:prot-a-1503 | Plum liking \|\| id:ebi-fl187-GCST90094812 | Inverse variance weighted | 5 | 0.767489690151258 | 0.236542900623836 | 0.00117611181616111 | 0.30386560492854 | 1.23111377537398 | 2.15435137064785 | 1.35508692684694 | 3.42504214029401 | 0.998735809224318 |
| ebi-fl187-GCST90094812 | prot-a-1522 | Interleukin-31 receptor subunit alpha \|\| id:prot-a-1522 | Plum liking \|\| id:ebi-fl187-GCST90094812 | Inverse variance weighted | 5 | 0.0336226726470412 | 0.237021661795527 | 0.887194656831076 | -0.430939784 | 0.498185129766275 | 1.03419430329776 | 0.649898043550251 | 1.64573176914148 | 0.998735809224318 |
| ebi-fl187-GCST90094812 | prot-a-1540 | Interleukin-6 receptor subunit alpha \|\| id:prot-a-1540 | Plum liking \|\| id:ebi-fl187-GCST90094812 | Inverse variance weighted | 5 | -0.214027036 | 0.236640300120678 | 0.365761949434361 | -0.677842025 | 0.249787951866127 | 0.80732655754777 | 0.50771143983946 | 1.28375317035997 | 0.998735809224318 |
| ebi-fl187-GCST90094812 | prot-a-1541 | Interleukin-6 receptor subunit alpha \|\| id:prot-a-1541 | Plum liking \|\| id:ebi-fl187-GCST90094812 | Inverse variance weighted | 5 | -0.172994788 | 0.236640300120678 | 0.464751460973354 | -0.636809776 | 0.290820200209697 | 0.841141998842727 | 0.528977290972635 | 1.33752407578067 | 0.998735809224318 |
| ebi-fl187-GCST90094812 | prot-a-1545 | Interleukin-9 \|\| id:prot-a-1545 | Plum liking \|\| id:ebi-fl187-GCST90094812 | Inverse variance weighted | 5 | -0.299608031 | 0.281118373112687 | 0.286526816840177 | -0.850600043 | 0.251383980061282 | 0.741108655198281 | 0.427158541737343 | 1.28580371254177 | 0.998735809224318 |
| ebi-fl187-GCST90094812 | prot-a-1548 | Inositol monophosphatase 3 \|\| id:prot-a-1548 | Plum liking \|\| id:ebi-fl187-GCST90094812 | Inverse variance weighted | 5 | 0.181837910154819 | 0.236640300120678 | 0.442240787241055 | -0.281977078 | 0.645652898391348 | 1.19941976435886 | 0.754290974131009 | 1.90723185146425 | 0.998735809224318 |
| ebi-fl187-GCST90094812 | prot-a-1555 | Inhibin beta A chain:Inhibin beta B chain heterodimer \|\| id:prot-a-1555 | Plum liking \|\| id:ebi-fl187-GCST90094812 | Inverse variance weighted | 5 | -0.260348323 | 0.237021661795527 | 0.272022993685633 | -0.72491078 | 0.204214133783387 | 0.770783057313174 | 0.484367782100698 | 1.22656077343627 | 0.998735809224318 |
| ebi-fl187-GCST90094812 | prot-a-1603 | Junctophilin-3 \|\| id:prot-a-1603 | Plum liking \|\| id:ebi-fl187-GCST90094812 | Inverse variance weighted | 5 | 0.282388826454938 | 0.236935286095222 | 0.233324194973033 | -0.182004334 | 0.746781987201573 | 1.32629431805911 | 0.833597727352198 | 2.11019843312584 | 0.998735809224318 |
| ebi-fl187-GCST90094812 | prot-a-1627 | UPF0258 protein KIAA1024 \|\| id:prot-a-1627 | Plum liking \|\| id:ebi-fl187-GCST90094812 | Inverse variance weighted | 5 | -0.090174327 | 0.2837618836803 | 0.750650550759887 | -0.646347619 | 0.465998964866891 | 0.913771876141962 | 0.523955973012333 | 1.59360534975397 | 0.998735809224318 |
| ebi-fl187-GCST90094812 | prot-a-1666 | Kallikrein-7 \|\| id:prot-a-1666 | Plum liking \|\| id:ebi-fl187-GCST90094812 | Inverse variance weighted | 5 | 0.176035239330764 | 0.236640300120678 | 0.456940759174851 | -0.287779749 | 0.639850227567293 | 1.19248008011023 | 0.749926746237154 | 1.89619686001974 | 0.998735809224318 |
| ebi-fl187-GCST90094812 | prot-a-1678 | Importin subunit alpha-3 \|\| id:prot-a-1678 | Plum liking \|\| id:ebi-fl187-GCST90094812 | Inverse variance weighted | 5 | 0.0660095592714094 | 0.237021661795527 | 0.780631717596633 | -0.398552898 | 0.530572016390643 | 1.06823692868392 | 0.67129076981574 | 1.69990440374634 | 0.998735809224318 |
| ebi-fl187-GCST90094812 | prot-a-1718 | Protein LDOC1 \|\| id:prot-a-1718 | Plum liking \|\| id:ebi-fl187-GCST90094812 | Inverse variance weighted | 5 | -0.45263808 | 0.236935286095222 | 0.0560834092783052 | -0.91703124 | 0.0117550811377186 | 0.635948254622184 | 0.399703906250939 | 1.01182444362477 | 0.998735809224318 |
| ebi-fl187-GCST90094812 | prot-a-1723 | Leptin \|\| id:prot-a-1723 | Plum liking \|\| id:ebi-fl187-GCST90094812 | Inverse variance weighted (multiplicative random effects) | 5 | -0.097276141 | 0.417440485765776 | 0.815738138303349 | -0.915459493 | 0.720907210746896 | 0.907305426988109 | 0.400332633597443 | 2.05629786021854 | 0.998735809224318 |
| ebi-fl187-GCST90094812 | prot-a-1730 | Galectin-7 \|\| id:prot-a-1730 | Plum liking \|\| id:ebi-fl187-GCST90094812 | Inverse variance weighted | 5 | 0.0953724882418902 | 0.342334458374743 | 0.780556054255707 | -0.57560305 | 0.766348026656386 | 1.10006854141665 | 0.562365631837433 | 2.15189322978469 | 0.998735809224318 |
| ebi-fl187-GCST90094812 | prot-a-1753 | Lipase member N \|\| id:prot-a-1753 | Plum liking \|\| id:ebi-fl187-GCST90094812 | Inverse variance weighted | 5 | -0.117882999 | 0.237021661795527 | 0.618941440032025 | -0.582445456 | 0.346679458569223 | 0.888800037424442 | 0.558530832733882 | 1.41436329066918 | 0.998735809224318 |
| ebi-fl187-GCST90094812 | prot-a-1756 | VIP36-like protein \|\| id:prot-a-1756 | Plum liking \|\| id:ebi-fl187-GCST90094812 | Inverse variance weighted (multiplicative random effects) | 5 | -0.065577205 | 0.411326097609055 | 0.873331090086162 | -0.871776357 | 0.740621946108108 | 0.936526739228915 | 0.418208002529374 | 2.09723947888621 | 0.998735809224318 |
| ebi-fl187-GCST90094812 | prot-a-1760 | Ig Kappa chain V-I region HK102- like \|\| id:prot-a-1760 | Plum liking \|\| id:ebi-fl187-GCST90094812 | Inverse variance weighted | 5 | -0.133479728 | 0.285940005308015 | 0.640635634379818 | -0.693922139 | 0.426962682087458 | 0.875045207437533 | 0.499612671021253 | 1.53259546739322 | 0.998735809224318 |
| ebi-fl187-GCST90094812 | prot-a-179 | Neutral ceramidase \|\| id:prot-a-179 | Plum liking \|\| id:ebi-fl187-GCST90094812 | Inverse variance weighted | 5 | 0.101779426634672 | 0.237021661795527 | 0.667625016284081 | -0.36278303 | 0.566341883753905 | 1.10713923936543 | 0.695737370924335 | 1.76181034190267 | 0.998735809224318 |
| ebi-fl187-GCST90094812 | prot-a-1809 | Lactotransferrin \|\| id:prot-a-1809 | Plum liking \|\| id:ebi-fl187-GCST90094812 | Inverse variance weighted | 5 | -0.12637663 | 0.308157297330742 | 0.681729446029729 | -0.730364932 | 0.477611673208907 | 0.881282867092765 | 0.481733158006114 | 1.61221929386348 | 0.998735809224318 |
| ebi-fl187-GCST90094812 | prot-a-1818 | Lysophospholipase-like protein 1 \|\| id:prot-a-1818 | Plum liking \|\| id:ebi-fl187-GCST90094812 | Inverse variance weighted | 5 | -0.005346525 | 0.237021661795527 | 0.982003550372318 | -0.469908983 | 0.45921593163791 | 0.994667741748012 | 0.625059156952696 | 1.5828324494876 | 0.998955228396052 |
| ebi-fl187-GCST90094812 | prot-a-1852 | MAP kinase-activated protein kinase 3 \|\| id:prot-a-1852 | Plum liking \|\| id:ebi-fl187-GCST90094812 | Inverse variance weighted | 5 | 0.104273285900036 | 0.274725801673926 | 0.70427648821245 | -0.434189285 | 0.642735857180931 | 1.10990373451372 | 0.647789626773585 | 1.90167648411274 | 0.998735809224318 |
| ebi-fl187-GCST90094812 | prot-a-1856 | Megakaryocyte-associated tyrosine-protein kinase \|\| id:prot-a-1856 | Plum liking \|\| id:ebi-fl187-GCST90094812 | Inverse variance weighted | 5 | 0.104932362890211 | 0.236554340527589 | 0.657341412228923 | -0.358714145 | 0.568578870324285 | 1.11063548764078 | 0.698574014006303 | 1.76575589941131 | 0.998735809224318 |
| ebi-fl187-GCST90094812 | prot-a-1890 | Beta-1,3-N-acetylglucosaminyltransferase manic fringe \|\| id:prot-a-1890 | Plum liking \|\| id:ebi-fl187-GCST90094812 | Inverse variance weighted | 5 | 0.142101376384538 | 0.237021661795527 | 0.54882013098722 | -0.322461081 | 0.606663833503771 | 1.15269349851335 | 0.724364122977801 | 1.8343016438373 | 0.998735809224318 |
| ebi-fl187-GCST90094812 | prot-a-1919 | Matrilysin \|\| id:prot-a-1919 | Plum liking \|\| id:ebi-fl187-GCST90094812 | Inverse variance weighted | 5 | -0.399118448 | 0.236554340527589 | 0.0915616799135209 | -0.862764955 | 0.0645280595250527 | 0.67091122861468 | 0.421993674099892 | 1.06665550767168 | 0.998735809224318 |
| ebi-fl187-GCST90094812 | prot-a-1923 | Multimerin-2 \|\| id:prot-a-1923 | Plum liking \|\| id:ebi-fl187-GCST90094812 | Inverse variance weighted | 5 | 0.586330539767212 | 0.239932984567326 | 0.0145364506159305 | 0.116061890015252 | 1.05659918951917 | 1.79738088210165 | 1.1230653765157 | 2.87657166083004 | 0.998735809224318 |
| ebi-fl187-GCST90094812 | prot-a-1948 | Mesothelin \|\| id:prot-a-1948 | Plum liking \|\| id:ebi-fl187-GCST90094812 | Inverse variance weighted | 5 | -0.163327009 | 0.236935286095222 | 0.490614538201967 | -0.62772017 | 0.301066151399933 | 0.849313409575717 | 0.53380740488139 | 1.35129872888408 | 0.998735809224318 |
| ebi-fl187-GCST90094812 | prot-a-195 | ATPase family AAA domain-containing protein 1 \|\| id:prot-a-195 | Plum liking \|\| id:ebi-fl187-GCST90094812 | Inverse variance weighted | 5 | -0.401105335 | 0.236640300120678 | 0.0900753997329786 | -0.864920323 | 0.0627096532746023 | 0.669579527189156 | 0.421085102004856 | 1.06471765706326 | 0.998735809224318 |
| ebi-fl187-GCST90094812 | prot-a-1966 | Mucin-1 \|\| id:prot-a-1966 | Plum liking \|\| id:ebi-fl187-GCST90094812 | Inverse variance weighted | 5 | -0.512519251 | 0.236640300120678 | 0.030325281550286 | -0.976334239 | -0.048704263 | 0.598984683569467 | 0.376689424240628 | 0.952462766572457 | 0.998735809224318 |
| ebi-fl187-GCST90094812 | prot-a-1986 | Myocardial zonula adherens protein \|\| id:prot-a-1986 | Plum liking \|\| id:ebi-fl187-GCST90094812 | Inverse variance weighted | 5 | -0.069377921 | 0.236640300120678 | 0.76938548229187 | -0.533192909 | 0.394437067362641 | 0.93297402308606 | 0.586728604633831 | 1.48354881776493 | 0.998735809224318 |
| ebi-fl187-GCST90094812 | prot-a-2071 | Epididymal secretory protein E1 \|\| id:prot-a-2071 | Plum liking \|\| id:ebi-fl187-GCST90094812 | Inverse variance weighted | 5 | 0.272244369323188 | 0.236640300120678 | 0.249955904486747 | -0.191570619 | 0.736059357559717 | 1.3129077965029 | 0.82566131574273 | 2.0876924342368 | 0.998735809224318 |
| ebi-fl187-GCST90094812 | prot-a-2098 | Neuritin \|\| id:prot-a-2098 | Plum liking \|\| id:ebi-fl187-GCST90094812 | Inverse variance weighted | 5 | -0.1187231 | 0.237021661795527 | 0.616444622448428 | -0.583285557 | 0.345839357417612 | 0.888053669045813 | 0.558061807380047 | 1.4131755814077 | 0.998735809224318 |
| ebi-fl187-GCST90094812 | prot-a-2115 | Neurotrophin-4 \|\| id:prot-a-2115 | Plum liking \|\| id:ebi-fl187-GCST90094812 | Inverse variance weighted | 5 | -0.078779112 | 0.236640300120678 | 0.739204804317549 | -0.542594101 | 0.38503587584526 | 0.924244055958601 | 0.581238503833114 | 1.4696670460429 | 0.998735809224318 |
| ebi-fl187-GCST90094812 | prot-a-2118 | Netrin-1 \|\| id:prot-a-2118 | Plum liking \|\| id:ebi-fl187-GCST90094812 | Inverse variance weighted | 5 | -0.003879322 | 0.236935286095222 | 0.986936884821682 | -0.468272483 | 0.460513838636017 | 0.996128192738754 | 0.626082903554648 | 1.58488815256775 | 0.998955228396052 |
| ebi-fl187-GCST90094812 | prot-a-2120 | Netrin-G1 \|\| id:prot-a-2120 | Plum liking \|\| id:ebi-fl187-GCST90094812 | Inverse variance weighted | 5 | -0.018641751 | 0.261249305532831 | 0.943114289719615 | -0.530690389 | 0.493406888264546 | 0.981530932151536 | 0.588198743275627 | 1.63788682275171 | 0.998735809224318 |
| ebi-fl187-GCST90094812 | prot-a-2145 | Olfactomedin-like protein 3 \|\| id:prot-a-2145 | Plum liking \|\| id:ebi-fl187-GCST90094812 | Inverse variance weighted | 5 | 0.0813272271950268 | 0.237021661795527 | 0.731507060182392 | -0.38323523 | 0.54588968431426 | 1.08472579027588 | 0.681652535351306 | 1.72614342215163 | 0.998735809224318 |
| ebi-fl187-GCST90094812 | prot-a-2162 | Prolyl 4-hydroxylase subunit alpha-2 \|\| id:prot-a-2162 | Plum liking \|\| id:ebi-fl187-GCST90094812 | Inverse variance weighted | 5 | -0.358163195 | 0.236640300120678 | 0.130144012823674 | -0.821978183 | 0.105651793509367 | 0.698958999275316 | 0.439561261292734 | 1.11143480030784 | 0.998735809224318 |
| ebi-fl187-GCST90094812 | prot-a-2168 | Polyadenylate-binding protein-interacting protein 1 \|\| id:prot-a-2168 | Plum liking \|\| id:ebi-fl187-GCST90094812 | Inverse variance weighted | 5 | 0.02717999912419 | 0.237021661795527 | 0.908704268442956 | -0.437382458 | 0.491742456243424 | 1.0275527447124 | 0.64572442171054 | 1.63516293896548 | 0.998735809224318 |
| ebi-fl187-GCST90094812 | prot-a-219 | Beta-1,4-galactosyltransferase 5 \|\| id:prot-a-219 | Plum liking \|\| id:ebi-fl187-GCST90094812 | Inverse variance weighted | 5 | -0.545575203 | 0.265123415453508 | 0.0396078007643128 | -1.065217097 | -0.025933309 | 0.579508352505228 | 0.344653023404287 | 0.974400071428898 | 0.998735809224318 |
| ebi-fl187-GCST90094812 | prot-a-2190 | Pyruvate carboxylase, mitochondrial \|\| id:prot-a-2190 | Plum liking \|\| id:ebi-fl187-GCST90094812 | Inverse variance weighted | 5 | -0.204719578 | 0.264103160631288 | 0.438250999187648 | -0.722361773 | 0.31292261640095 | 0.814875793119087 | 0.485604013968296 | 1.36741571138416 | 0.998735809224318 |
| ebi-fl187-GCST90094812 | prot-a-2197 | Protocadherin-9 \|\| id:prot-a-2197 | Plum liking \|\| id:ebi-fl187-GCST90094812 | Inverse variance weighted | 5 | -0.200828878 | 0.236935286095222 | 0.396654826023498 | -0.665222039 | 0.263564282424027 | 0.818052406077104 | 0.514159351567456 | 1.30156095974604 | 0.998735809224318 |
| ebi-fl187-GCST90094812 | prot-a-2198 | Protocadherin alpha-4 \|\| id:prot-a-2198 | Plum liking \|\| id:ebi-fl187-GCST90094812 | Inverse variance weighted | 5 | 0.353557317580117 | 0.283152395176258 | 0.211794305803078 | -0.201421377 | 0.908536012125583 | 1.42412461198862 | 0.817567854699004 | 2.48068817629627 | 0.998735809224318 |
| ebi-fl187-GCST90094812 | prot-a-2254 | Placenta growth factor \|\| id:prot-a-2254 | Plum liking \|\| id:ebi-fl187-GCST90094812 | Inverse variance weighted | 5 | -0.39127344 | 0.247178178621108 | 0.113430332773638 | -0.87574267 | 0.093195790527389 | 0.676195232224173 | 0.416552543834406 | 1.0976766289164 | 0.998735809224318 |
| ebi-fl187-GCST90094812 | prot-a-2261 | Membrane-associated progesterone receptor component 2 \|\| id:prot-a-2261 | Plum liking \|\| id:ebi-fl187-GCST90094812 | Inverse variance weighted | 5 | -0.292286224 | 0.297268385802918 | 0.325489238471777 | -0.874932261 | 0.290359811800014 | 0.746554823203206 | 0.416890258639956 | 1.33690843692588 | 0.998735809224318 |
| ebi-fl187-GCST90094812 | prot-a-2268 | Polymeric immunoglobulin receptor \|\| id:prot-a-2268 | Plum liking \|\| id:ebi-fl187-GCST90094812 | Inverse variance weighted | 5 | -0.210746225 | 0.237021661795527 | 0.373926064313172 | -0.675308682 | 0.253816231800199 | 0.809979593114327 | 0.508999277217126 | 1.28893491725292 | 0.998735809224318 |
| ebi-fl187-GCST90094812 | prot-a-2283 | Group 10 secretory phospholipase A2 \|\| id:prot-a-2283 | Plum liking \|\| id:ebi-fl187-GCST90094812 | Inverse variance weighted | 5 | -0.036740672 | 0.237021661795527 | 0.876813619152067 | -0.501303129 | 0.427821785069458 | 0.9639260759128 | 0.605740786683132 | 1.53391269046358 | 0.998735809224318 |
| ebi-fl187-GCST90094812 | prot-a-2298 | Inactive phospholipase D5 \|\| id:prot-a-2298 | Plum liking \|\| id:ebi-fl187-GCST90094812 | Inverse variance weighted (multiplicative random effects) | 5 | -0.200824394 | 0.390231228170626 | 0.606812139467132 | -0.965677601 | 0.564028813007268 | 0.81805607432676 | 0.380725132424858 | 1.75773985941154 | 0.998735809224318 |
| ebi-fl187-GCST90094812 | prot-a-2304 | Procollagen-lysine,2-oxoglutarate 5-dioxygenase 2 \|\| id:prot-a-2304 | Plum liking \|\| id:ebi-fl187-GCST90094812 | Inverse variance weighted | 5 | -0.263244062 | 0.236640300120678 | 0.265956412716926 | -0.72705905 | 0.200570926665685 | 0.768554299849726 | 0.483328346532715 | 1.22210028866478 | 0.998735809224318 |
| ebi-fl187-GCST90094812 | prot-a-2331 | Periostin \|\| id:prot-a-2331 | Plum liking \|\| id:ebi-fl187-GCST90094812 | Inverse variance weighted | 5 | 0.401701440006852 | 0.295113638762486 | 0.173458250519465 | -0.176721292 | 0.980124171981325 | 1.49436510843047 | 0.838013313019927 | 2.66478711328218 | 0.998735809224318 |
| ebi-fl187-GCST90094812 | prot-a-2333 | Periostin \|\| id:prot-a-2333 | Plum liking \|\| id:ebi-fl187-GCST90094812 | Inverse variance weighted | 5 | 0.542187075122547 | 0.349513094757966 | 0.12083843465144 | -0.142858591 | 1.22723274084816 | 1.71976400518263 | 0.866876644732144 | 3.41177519488445 | 0.998735809224318 |
| ebi-fl187-GCST90094812 | prot-a-2367 | cAMP-dependent protein kinase catalytic subunit alpha \|\| id:prot-a-2367 | Plum liking \|\| id:ebi-fl187-GCST90094812 | Inverse variance weighted | 5 | -0.190378236 | 0.236935286095222 | 0.421684007366219 | -0.654771397 | 0.274014924550628 | 0.8266464072123 | 0.519560822204566 | 1.31523443137511 | 0.998735809224318 |
| ebi-fl187-GCST90094812 | prot-a-2385 | Phosphoribosyl pyrophosphate synthase-associated protein 1 \|\| id:prot-a-2385 | Plum liking \|\| id:ebi-fl187-GCST90094812 | Inverse variance weighted | 5 | -0.272540709 | 0.236640300120678 | 0.249440766945669 | -0.736355697 | 0.191274279084478 | 0.761442430868556 | 0.478855835122528 | 1.21079150132661 | 0.998735809224318 |
| ebi-fl187-GCST90094812 | prot-a-2393 | Trypsin-3 \|\| id:prot-a-2393 | Plum liking \|\| id:ebi-fl187-GCST90094812 | Inverse variance weighted | 5 | -0.17946266 | 0.237021661795527 | 0.448955681992477 | -0.644025117 | 0.285099796754091 | 0.835719155808686 | 0.525174275845104 | 1.32989474067763 | 0.998735809224318 |
| ebi-fl187-GCST90094812 | prot-a-2405 | Pregnancy-specific beta-1-glycoprotein 6 \|\| id:prot-a-2405 | Plum liking \|\| id:ebi-fl187-GCST90094812 | Inverse variance weighted | 5 | -0.060402229 | 0.236640300120678 | 0.798530691239169 | -0.524217218 | 0.40341275875443 | 0.941385804296795 | 0.592018604709004 | 1.49692463291271 | 0.998735809224318 |
| ebi-fl187-GCST90094812 | prot-a-244 | Beclin-1 \|\| id:prot-a-244 | Plum liking \|\| id:ebi-fl187-GCST90094812 | Inverse variance weighted | 5 | -0.233959381 | 0.339553419705705 | 0.49081015572653 | -0.899484084 | 0.431565321565122 | 0.79139396082808 | 0.40677946978033 | 1.53966571020257 | 0.998735809224318 |
| ebi-fl187-GCST90094812 | prot-a-2454 | tRNA pseudouridine synthase A, mitochondrial \|\| id:prot-a-2454 | Plum liking \|\| id:ebi-fl187-GCST90094812 | Inverse variance weighted | 5 | 0.319184550725397 | 0.236935286095222 | 0.177935759927823 | -0.14520861 | 0.783577711472031 | 1.37600524424079 | 0.864841859612577 | 2.18929092195693 | 0.998735809224318 |
| ebi-fl187-GCST90094812 | prot-a-2466 | Glutaminyl-peptide cyclotransferase-like protein \|\| id:prot-a-2466 | Plum liking \|\| id:ebi-fl187-GCST90094812 | Inverse variance weighted | 5 | 0.380397322856861 | 0.236935286095222 | 0.108385928263806 | -0.083995838 | 0.844790483603496 | 1.46286570396224 | 0.919435082877323 | 2.32749011613958 | 0.998735809224318 |
| ebi-fl187-GCST90094812 | prot-a-2474 | Ras-related protein Rab-27A \|\| id:prot-a-2474 | Plum liking \|\| id:ebi-fl187-GCST90094812 | Inverse variance weighted | 5 | 0.0503896556803747 | 0.31088957819279 | 0.871241151330701 | -0.558953918 | 0.659733228938243 | 1.05168080994877 | 0.571806908252827 | 1.93427625663707 | 0.998735809224318 |
| ebi-fl187-GCST90094812 | prot-a-2490 | Rap1 GTPase-GDP dissociation stimulator 1 \|\| id:prot-a-2490 | Plum liking \|\| id:ebi-fl187-GCST90094812 | Inverse variance weighted | 5 | 0.0125949162038173 | 0.313271111016392 | 0.967930070147858 | -0.601416461 | 0.626606293795946 | 1.01267456620467 | 0.54803481589983 | 1.87124932081917 | 0.998735809224318 |
| ebi-fl187-GCST90094812 | prot-a-2545 | Receptor-interacting serine/threonine-protein kinase 2 \|\| id:prot-a-2545 | Plum liking \|\| id:ebi-fl187-GCST90094812 | Inverse variance weighted | 5 | 0.108188350602826 | 0.27379436139074 | 0.692736209939528 | -0.428448598 | 0.644825298928677 | 1.11425759671308 | 0.651519079257643 | 1.90565408038009 | 0.998735809224318 |
| ebi-fl187-GCST90094812 | prot-a-2610 | RUN and FYVE domain-containing protein 1 \|\| id:prot-a-2610 | Plum liking \|\| id:ebi-fl187-GCST90094812 | Inverse variance weighted | 5 | 0.00704328107674011 | 0.2577978459633 | 0.978203750461752 | -0.498240497 | 0.512327059164809 | 1.00706814331721 | 0.60759879163621 | 1.66917093852879 | 0.998955228396052 |
| ebi-fl187-GCST90094812 | prot-a-2627 | Serine--tRNA ligase, mitochondrial \|\| id:prot-a-2627 | Plum liking \|\| id:ebi-fl187-GCST90094812 | Inverse variance weighted | 5 | 0.177035661426437 | 0.237021661795527 | 0.455113274253555 | -0.287526796 | 0.641598118545671 | 1.19367366047356 | 0.750116466611284 | 1.89951410365015 | 0.998735809224318 |
| ebi-fl187-GCST90094812 | prot-a-264 | BolA-like protein 2 \|\| id:prot-a-264 | Plum liking \|\| id:ebi-fl187-GCST90094812 | Inverse variance weighted | 5 | 0.153524577258803 | 0.282979479734054 | 0.587454127671953 | -0.401115203 | 0.70816435753755 | 1.16593644229167 | 0.669572919772136 | 2.0302610026797 | 0.998735809224318 |
| ebi-fl187-GCST90094812 | prot-a-2646 | Secretoglobin family 1C member 1 \|\| id:prot-a-2646 | Plum liking \|\| id:ebi-fl187-GCST90094812 | Inverse variance weighted | 5 | -0.221094042 | 0.310183947067435 | 0.475979652562976 | -0.829054578 | 0.386866494340589 | 0.801641288866066 | 0.436461731775251 | 1.47235990977041 | 0.998735809224318 |
| ebi-fl187-GCST90094812 | prot-a-2672 | Semaphorin-3E \|\| id:prot-a-2672 | Plum liking \|\| id:ebi-fl187-GCST90094812 | Inverse variance weighted | 5 | -0.115196445 | 0.286253979578989 | 0.687369508527407 | -0.676254245 | 0.445861355300342 | 0.891191056972863 | 0.508518214208493 | 1.56183491138977 | 0.998735809224318 |
| ebi-fl187-GCST90094812 | prot-a-2701 | Plasma protease C1 inhibitor \|\| id:prot-a-2701 | Plum liking \|\| id:ebi-fl187-GCST90094812 | Inverse variance weighted | 5 | 0.103712564217792 | 0.236640300120678 | 0.661189896900869 | -0.360102424 | 0.567527552454321 | 1.10928156187372 | 0.697604870917362 | 1.76390050415625 | 0.998735809224318 |
| ebi-fl187-GCST90094812 | prot-a-2738 | Signaling threshold-regulating transmembrane adapter 1 \|\| id:prot-a-2738 | Plum liking \|\| id:ebi-fl187-GCST90094812 | Inverse variance weighted | 5 | -0.439424264 | 0.265388897303788 | 0.0977674212185831 | -0.959586502 | 0.080737975081773 | 0.644407323032637 | 0.383051244022103 | 1.08408680159287 | 0.998735809224318 |
| ebi-fl187-GCST90094812 | prot-a-274 | Serine/threonine-protein kinase BRSK2 \|\| id:prot-a-274 | Plum liking \|\| id:ebi-fl187-GCST90094812 | Inverse variance weighted | 5 | -0.336991063 | 0.236554340527589 | 0.154278056629074 | -0.800637571 | 0.126655444031675 | 0.713915219847253 | 0.449042576379653 | 1.13502587046141 | 0.998735809224318 |
| ebi-fl187-GCST90094812 | prot-a-2749 | Mitochondrial glutamate carrier 2 \|\| id:prot-a-2749 | Plum liking \|\| id:ebi-fl187-GCST90094812 | Inverse variance weighted | 5 | -0.059449313 | 0.237021661795527 | 0.801954777812625 | -0.524011771 | 0.405113143686748 | 0.9422832934866 | 0.592140245749074 | 1.49947214626619 | 0.998735809224318 |
| ebi-fl187-GCST90094812 | prot-a-2760 | Sodium-coupled monocarboxylate transporter 1 \|\| id:prot-a-2760 | Plum liking \|\| id:ebi-fl187-GCST90094812 | Inverse variance weighted | 5 | -0.078760233 | 0.237021661795527 | 0.739669318417473 | -0.54332269 | 0.38580222406426 | 0.924261505237667 | 0.580815173770906 | 1.4707937544365 | 0.998735809224318 |
| ebi-fl187-GCST90094812 | prot-a-2777 | Small integral membrane protein 10 \|\| id:prot-a-2777 | Plum liking \|\| id:ebi-fl187-GCST90094812 | Inverse variance weighted | 5 | -0.12380965 | 0.236640300120678 | 0.60083671251911 | -0.587624639 | 0.340005337922656 | 0.883548007962961 | 0.555645577488164 | 1.40495509008518 | 0.998735809224318 |
| ebi-fl187-GCST90094812 | prot-a-2799 | Superoxide dismutase [Mn], mitochondrial \|\| id:prot-a-2799 | Plum liking \|\| id:ebi-fl187-GCST90094812 | Inverse variance weighted | 5 | 0.215800903628661 | 0.237021661795527 | 0.362575160385457 | -0.248761553 | 0.680363360747895 | 1.24085530461718 | 0.779765883672123 | 1.97459509224183 | 0.998735809224318 |
| ebi-fl187-GCST90094812 | prot-a-2805 | Protein SPACA7 \|\| id:prot-a-2805 | Plum liking \|\| id:ebi-fl187-GCST90094812 | Inverse variance weighted | 5 | -0.272575287 | 0.237021661795527 | 0.250143217685823 | -0.737137744 | 0.191987169931294 | 0.761416102140055 | 0.478481493787543 | 1.21165497124865 | 0.998735809224318 |
| ebi-fl187-GCST90094812 | prot-a-2811 | SPARC-like protein 1 \|\| id:prot-a-2811 | Plum liking \|\| id:ebi-fl187-GCST90094812 | Inverse variance weighted | 5 | -0.060699558 | 0.236640300120678 | 0.79756047116381 | -0.524514546 | 0.403115430447195 | 0.941105945256217 | 0.591842606985263 | 1.49647962100615 | 0.998735809224318 |
| ebi-fl187-GCST90094812 | prot-a-2831 | Sepiapterin reductase \|\| id:prot-a-2831 | Plum liking \|\| id:ebi-fl187-GCST90094812 | Inverse variance weighted (multiplicative random effects) | 5 | -0.11852742 | 0.423565525955215 | 0.779605979096899 | -0.948715851 | 0.711661010452614 | 0.888227459753229 | 0.387237975452009 | 2.03737254678796 | 0.998735809224318 |
| ebi-fl187-GCST90094812 | prot-a-2834 | SPRY domain-containing SOCS box protein 1 \|\| id:prot-a-2834 | Plum liking \|\| id:ebi-fl187-GCST90094812 | Inverse variance weighted | 5 | 0.164631471613528 | 0.236640300120678 | 0.486614568722394 | -0.299183517 | 0.628446459850057 | 1.17895855894706 | 0.741423333442921 | 1.87469590046498 | 0.998735809224318 |
| ebi-fl187-GCST90094812 | prot-a-2835 | Spectrin alpha chain, non-erythrocytic 1 \|\| id:prot-a-2835 | Plum liking \|\| id:ebi-fl187-GCST90094812 | Inverse variance weighted | 5 | -0.140886583 | 0.237021661795527 | 0.552242047785255 | -0.605449041 | 0.32367587368056 | 0.868587818354987 | 0.545829272120793 | 1.38219922735788 | 0.998735809224318 |
| ebi-fl187-GCST90094812 | prot-a-2859 | Sia-alpha-2,3-Gal-beta-1,4-GlcNAc-R:alpha 2,8-sialyltransferase \|\| id:prot-a-2859 | Plum liking \|\| id:ebi-fl187-GCST90094812 | Inverse variance weighted | 5 | -0.397288062 | 0.237021661795527 | 0.0937053421022481 | -0.861850519 | 0.0672743950501346 | 0.672140379594486 | 0.422379736860542 | 1.06958892781967 | 0.998735809224318 |
| ebi-fl187-GCST90094812 | prot-a-2861 | Alpha-2,8-sialyltransferase 8F \|\| id:prot-a-2861 | Plum liking \|\| id:ebi-fl187-GCST90094812 | Inverse variance weighted | 5 | 0.0737763911718023 | 0.293427228787033 | 0.801481720535902 | -0.501340977 | 0.648893759594386 | 1.0765660489069 | 0.605717860990155 | 1.91342295200678 | 0.998735809224318 |
| ebi-fl187-GCST90094812 | prot-a-289 | Uncharacterized protein C17orf89 \|\| id:prot-a-289 | Plum liking \|\| id:ebi-fl187-GCST90094812 | Inverse variance weighted | 5 | -0.026373336 | 0.275454363044567 | 0.923723221037515 | -0.566263888 | 0.513517215481302 | 0.973971403052776 | 0.56764225714684 | 1.67115869550071 | 0.998735809224318 |
| ebi-fl187-GCST90094812 | prot-a-2921 | Tachykinin-3 \|\| id:prot-a-2921 | Plum liking \|\| id:ebi-fl187-GCST90094812 | Inverse variance weighted | 5 | -0.470962733 | 0.236640300120678 | 0.0465683464759643 | -0.934777721 | -0.007147745 | 0.624400847684221 | 0.392673138832024 | 0.992877739863825 | 0.998735809224318 |
| ebi-fl187-GCST90094812 | prot-a-2928 | Tax1-binding protein 1 \|\| id:prot-a-2928 | Plum liking \|\| id:ebi-fl187-GCST90094812 | Inverse variance weighted | 5 | -0.011138613 | 0.262723412101046 | 0.966182436165505 | -0.526076501 | 0.503799274505232 | 0.988923191453733 | 0.590918897106677 | 1.65499712969661 | 0.998735809224318 |
| ebi-fl187-GCST90094812 | prot-a-296 | Uncharacterized protein C1orf185 \|\| id:prot-a-296 | Plum liking \|\| id:ebi-fl187-GCST90094812 | Inverse variance weighted | 5 | -0.013464223 | 0.270906678905338 | 0.960360983069025 | -0.544441314 | 0.517512867782855 | 0.986626014331888 | 0.580165823611248 | 1.67784942259663 | 0.998735809224318 |
| ebi-fl187-GCST90094812 | prot-a-2964 | Transforming growth factor beta-2 \|\| id:prot-a-2964 | Plum liking \|\| id:ebi-fl187-GCST90094812 | Inverse variance weighted | 5 | -0.114961992 | 0.288852160981696 | 0.690633165717165 | -0.681112228 | 0.451188243187806 | 0.89140002329528 | 0.506053832071446 | 1.57017682936673 | 0.998735809224318 |
| ebi-fl187-GCST90094812 | prot-a-2977 | Thymocyte nuclear protein 1 \|\| id:prot-a-2977 | Plum liking \|\| id:ebi-fl187-GCST90094812 | Inverse variance weighted (multiplicative random effects) | 5 | 0.0257582219764114 | 0.438180906941091 | 0.953123797121637 | -0.833076356 | 0.88459279958095 | 1.02609283178318 | 0.434709904908515 | 2.42199795207889 | 0.998735809224318 |
| ebi-fl187-GCST90094812 | prot-a-3015 | Transmembrane protein 70, mitochondrial \|\| id:prot-a-3015 | Plum liking \|\| id:ebi-fl187-GCST90094812 | Inverse variance weighted | 5 | -0.361295206 | 0.24447281577485 | 0.139446775814722 | -0.840461925 | 0.117871512453631 | 0.69677327612728 | 0.4315111514309 | 1.12509954080036 | 0.998735809224318 |
| ebi-fl187-GCST90094812 | prot-a-3030 | Tumor necrosis factor alpha-induced protein 3 \|\| id:prot-a-3030 | Plum liking \|\| id:ebi-fl187-GCST90094812 | Inverse variance weighted | 5 | -0.146150159 | 0.237021661795527 | 0.537491352220661 | -0.610712616 | 0.318412298587322 | 0.864027952274439 | 0.542963806670877 | 1.37494303144977 | 0.998735809224318 |
| ebi-fl187-GCST90094812 | prot-a-3032 | Tumor necrosis factor receptor superfamily member 10A \|\| id:prot-a-3032 | Plum liking \|\| id:ebi-fl187-GCST90094812 | Inverse variance weighted | 5 | 0.293857192715357 | 0.236935286095222 | 0.214885742469866 | -0.170535968 | 0.758250353461991 | 1.34159230083258 | 0.843212760380236 | 2.13453826391529 | 0.998735809224318 |
| ebi-fl187-GCST90094812 | prot-a-3081 | Tumor protein p53-inducible protein 11 \|\| id:prot-a-3081 | Plum liking \|\| id:ebi-fl187-GCST90094812 | Inverse variance weighted | 5 | 0.253838709250502 | 0.236554340527589 | 0.283240955973197 | -0.209807798 | 0.717485216684577 | 1.28896388954907 | 0.810740056707704 | 2.04927324709759 | 0.998735809224318 |
| ebi-fl187-GCST90094812 | prot-a-315 | Complement C4 \|\| id:prot-a-315 | Plum liking \|\| id:ebi-fl187-GCST90094812 | Inverse variance weighted | 5 | -0.244472369 | 0.237021661795527 | 0.302337011202098 | -0.709034826 | 0.220090088314072 | 0.783117626542139 | 0.492118948766747 | 1.24618899259592 | 0.998735809224318 |
| ebi-fl187-GCST90094812 | prot-a-3157 | UDP-glucuronosyltransferase 1-8 \|\| id:prot-a-3157 | Plum liking \|\| id:ebi-fl187-GCST90094812 | Inverse variance weighted | 5 | -0.314655701 | 0.237021661795527 | 0.184330167262761 | -0.779218158 | 0.149906756075325 | 0.730040183076832 | 0.458764552446097 | 1.16172591379425 | 0.998735809224318 |
| ebi-fl187-GCST90094812 | prot-a-317 | Glutaredoxin-like protein C5orf63 \|\| id:prot-a-317 | Plum liking \|\| id:ebi-fl187-GCST90094812 | Inverse variance weighted | 5 | -0.082619801 | 0.257954055087173 | 0.748749395575712 | -0.588209749 | 0.422970147404517 | 0.920701130732616 | 0.555320558806767 | 1.52648872563583 | 0.998735809224318 |
| ebi-fl187-GCST90094812 | prot-a-3192 | Guanine nucleotide exchange factor VAV3 \|\| id:prot-a-3192 | Plum liking \|\| id:ebi-fl187-GCST90094812 | Inverse variance weighted | 5 | -0.036248456 | 0.236640300120678 | 0.87825667897764 | -0.500063444 | 0.42756653202194 | 0.964400652378757 | 0.606492179928519 | 1.53352120454083 | 0.998735809224318 |
| ebi-fl187-GCST90094812 | prot-a-3233 | Serine/threonine-protein kinase WNK3 \|\| id:prot-a-3233 | Plum liking \|\| id:ebi-fl187-GCST90094812 | Inverse variance weighted | 5 | 0.10562769917525 | 0.236640300120678 | 0.65533436874314 | -0.358187289 | 0.569442687411779 | 1.11140802134902 | 0.698942158526538 | 1.76728184850513 | 0.998735809224318 |
| ebi-fl187-GCST90094812 | prot-a-3250 | 14-3-3 protein theta \|\| id:prot-a-3250 | Plum liking \|\| id:ebi-fl187-GCST90094812 | Inverse variance weighted | 5 | 0.16109612444842 | 0.236554340527589 | 0.49586448614659 | -0.302550383 | 0.624742631882495 | 1.17479789018573 | 0.738931257758101 | 1.86776519235658 | 0.998735809224318 |
| ebi-fl187-GCST90094812 | prot-a-3278 | E3 ubiquitin-protein ligase ZNRF3 \|\| id:prot-a-3278 | Plum liking \|\| id:ebi-fl187-GCST90094812 | Inverse variance weighted | 5 | 0.0222130676666448 | 0.275227996126901 | 0.935674271135624 | -0.517233805 | 0.561659940075372 | 1.02246161477381 | 0.59616738457642 | 1.75358092497571 | 0.998735809224318 |
| ebi-fl187-GCST90094812 | prot-a-355 | Calpain-3 \|\| id:prot-a-355 | Plum liking \|\| id:ebi-fl187-GCST90094812 | Inverse variance weighted | 5 | 0.148546481918086 | 0.314051247545929 | 0.636212629807907 | -0.466993963 | 0.764086927108107 | 1.16014672236607 | 0.626883874728434 | 2.14703308168488 | 0.998735809224318 |
| ebi-fl187-GCST90094812 | prot-a-385 | Coiled-coil domain-containing protein 90B, mitochondrial \|\| id:prot-a-385 | Plum liking \|\| id:ebi-fl187-GCST90094812 | Inverse variance weighted | 5 | -0.091740337 | 0.275467962392181 | 0.73910823655143 | -0.631657543 | 0.44817686955492 | 0.912342020499393 | 0.531709738413636 | 1.56545555259585 | 0.998735809224318 |
| ebi-fl187-GCST90094812 | prot-a-390 | C-C motif chemokine 14 \|\| id:prot-a-390 | Plum liking \|\| id:ebi-fl187-GCST90094812 | Inverse variance weighted | 5 | 0.0623792280141239 | 0.237021661795527 | 0.792412277541725 | -0.402183229 | 0.526941685133357 | 1.06436590557108 | 0.668858180176106 | 1.69374437589126 | 0.998735809224318 |
| ebi-fl187-GCST90094812 | prot-a-40 | Pituitary adenylate cyclase-activating polypeptide 27 \|\| id:prot-a-40 | Plum liking \|\| id:ebi-fl187-GCST90094812 | Inverse variance weighted | 5 | 0.277597739119999 | 0.237021661795527 | 0.241521839106669 | -0.186964718 | 0.742160196239232 | 1.31995512409151 | 0.829473001336206 | 2.1004680403205 | 0.998735809224318 |
| ebi-fl187-GCST90094812 | prot-a-401 | C-C motif chemokine 24 \|\| id:prot-a-401 | Plum liking \|\| id:ebi-fl187-GCST90094812 | Inverse variance weighted | 5 | -0.019689848 | 0.237021661795527 | 0.933794342407112 | -0.484252305 | 0.444872609361048 | 0.980502731272397 | 0.616157722700336 | 1.56029141665111 | 0.998735809224318 |
| ebi-fl187-GCST90094812 | prot-a-412 | Cerebral cavernous malformations 2 protein \|\| id:prot-a-412 | Plum liking \|\| id:ebi-fl187-GCST90094812 | Inverse variance weighted | 5 | 0.493097334026936 | 0.257617756615592 | 0.0556117504054743 | -0.011833469 | 0.998028136993497 | 1.63737988641119 | 0.988236271193656 | 2.71292703027954 | 0.998735809224318 |
| ebi-fl187-GCST90094812 | prot-a-44 | Adhesion G protein-coupled receptor F5 \|\| id:prot-a-44 | Plum liking \|\| id:ebi-fl187-GCST90094812 | Inverse variance weighted | 5 | -0.327352742 | 0.237021661795527 | 0.16724545943585 | -0.791915199 | 0.137209715054679 | 0.720829431314231 | 0.45297642391836 | 1.14706868086902 | 0.998735809224318 |
| ebi-fl187-GCST90094812 | prot-a-48 | Adrenomedullin \|\| id:prot-a-48 | Plum liking \|\| id:ebi-fl187-GCST90094812 | Inverse variance weighted | 5 | -0.050672917 | 0.303918620309708 | 0.867581034509324 | -0.646353413 | 0.545007578469628 | 0.950589541047603 | 0.523952937228319 | 1.72462145231821 | 0.998735809224318 |
| ebi-fl187-GCST90094812 | prot-a-502 | Carcinoembryonic antigen-related cell adhesion molecule 1 \|\| id:prot-a-502 | Plum liking \|\| id:ebi-fl187-GCST90094812 | Inverse variance weighted | 5 | -0.126320905 | 0.236935286095222 | 0.593933087856599 | -0.590714066 | 0.338072255584757 | 0.881331977417843 | 0.553931599807692 | 1.40224181954759 | 0.998735809224318 |
| ebi-fl187-GCST90094812 | prot-a-518 | Complement factor B \|\| id:prot-a-518 | Plum liking \|\| id:ebi-fl187-GCST90094812 | Inverse variance weighted | 5 | -0.200493131 | 0.236640300120678 | 0.396856643849139 | -0.66430812 | 0.263321856751029 | 0.818327110698055 | 0.514629466537264 | 1.30124546619784 | 0.998735809224318 |
| ebi-fl187-GCST90094812 | prot-a-525 | Properdin \|\| id:prot-a-525 | Plum liking \|\| id:ebi-fl187-GCST90094812 | Inverse variance weighted (multiplicative random effects) | 5 | -0.049227521 | 0.38744589947697 | 0.89889567857596 | -0.808621484 | 0.710166442193228 | 0.951964513346916 | 0.445471733171033 | 2.03432982878824 | 0.998735809224318 |
| ebi-fl187-GCST90094812 | prot-a-533 | Chondroadherin \|\| id:prot-a-533 | Plum liking \|\| id:ebi-fl187-GCST90094812 | Inverse variance weighted | 5 | 0.420168384010768 | 0.236554340527589 | 0.0756995651843571 | -0.043478123 | 0.883814891444842 | 1.52221785118704 | 0.957453499666773 | 2.42011459280155 | 0.998735809224318 |
| ebi-fl187-GCST90094812 | prot-a-535 | Coiled-coil-helix-coiled-coil-helix domain-containing protein 2 \|\| id:prot-a-535 | Plum liking \|\| id:ebi-fl187-GCST90094812 | Inverse variance weighted | 5 | -0.501013043 | 0.236640300120678 | 0.0342435227738789 | -0.964828031 | -0.037198055 | 0.605916529068272 | 0.381048722502329 | 0.963485293395513 | 0.998735809224318 |
| ebi-fl187-GCST90094812 | prot-a-591 | Caseinolytic peptidase B protein homolog \|\| id:prot-a-591 | Plum liking \|\| id:ebi-fl187-GCST90094812 | Inverse variance weighted | 5 | 0.0525597251051345 | 0.289717784736147 | 0.85604029313103 | -0.515287133 | 0.620406583187982 | 1.05396550839958 | 0.597329057121534 | 1.85968400440624 | 0.998735809224318 |
| ebi-fl187-GCST90094812 | prot-a-605 | Ciliary neurotrophic factor receptor subunit alpha \|\| id:prot-a-605 | Plum liking \|\| id:ebi-fl187-GCST90094812 | Inverse variance weighted | 5 | -0.062719154 | 0.237021661795527 | 0.791307145112155 | -0.527281611 | 0.401843302805284 | 0.939207208945886 | 0.590207203458635 | 1.49457711828443 | 0.998735809224318 |
| ebi-fl187-GCST90094812 | prot-a-606 | Ciliary neurotrophic factor receptor subunit alpha \|\| id:prot-a-606 | Plum liking \|\| id:ebi-fl187-GCST90094812 | Inverse variance weighted | 5 | 0.168022848397233 | 0.313629842288702 | 0.59214090662348 | -0.446691642 | 0.782737339283089 | 1.18296363916217 | 0.639741146851209 | 2.18745187560256 | 0.998735809224318 |
| ebi-fl187-GCST90094812 | prot-a-707 | Cancer/testis antigen 55 \|\| id:prot-a-707 | Plum liking \|\| id:ebi-fl187-GCST90094812 | Inverse variance weighted | 5 | -0.107837202 | 0.237021661795527 | 0.649132472076738 | -0.572399659 | 0.356725255372925 | 0.897773740531278 | 0.564169997515526 | 1.42864330385691 | 0.998735809224318 |
| ebi-fl187-GCST90094812 | prot-a-719 | Dipeptidyl peptidase 1 \|\| id:prot-a-719 | Plum liking \|\| id:ebi-fl187-GCST90094812 | Inverse variance weighted | 5 | 0.180926302145202 | 0.318845874836184 | 0.570414430274898 | -0.444011613 | 0.805864216824123 | 1.19832686191977 | 0.641457971831741 | 2.23863032506695 | 0.998735809224318 |
| ebi-fl187-GCST90094812 | prot-a-752 | Cytochrome b5 \|\| id:prot-a-752 | Plum liking \|\| id:ebi-fl187-GCST90094812 | Inverse variance weighted | 5 | -0.107233232 | 0.242896445642266 | 0.658867596629384 | -0.583310265 | 0.368843801684707 | 0.898316132690166 | 0.558048018729269 | 1.4460617136299 | 0.998735809224318 |
| ebi-fl187-GCST90094812 | prot-a-755 | Cytoglobin \|\| id:prot-a-755 | Plum liking \|\| id:ebi-fl187-GCST90094812 | Inverse variance weighted (multiplicative random effects) | 5 | 0.122794195989935 | 0.401691525878897 | 0.759838577232316 | -0.664521195 | 0.910109586712573 | 1.13065170434656 | 0.514519823539695 | 2.48459479704219 | 0.998735809224318 |
| ebi-fl187-GCST90094812 | prot-a-807 | Desmin \|\| id:prot-a-807 | Plum liking \|\| id:ebi-fl187-GCST90094812 | Inverse variance weighted | 5 | 0.333229151995996 | 0.272119235461894 | 0.220737138660112 | -0.20012455 | 0.866582853501308 | 1.39546703589079 | 0.818628786914474 | 2.37876834944638 | 0.998735809224318 |
| ebi-fl187-GCST90094812 | prot-a-82 | Ameloblastin \|\| id:prot-a-82 | Plum liking \|\| id:ebi-fl187-GCST90094812 | Inverse variance weighted | 5 | -0.145664022 | 0.237781256740807 | 0.540143015975324 | -0.611715285 | 0.32038724108935 | 0.864448089834483 | 0.542419666164889 | 1.37766114805897 | 0.998735809224318 |
| ebi-fl187-GCST90094812 | prot-a-84 | Muellerian-inhibiting factor \|\| id:prot-a-84 | Plum liking \|\| id:ebi-fl187-GCST90094812 | Inverse variance weighted | 5 | 0.0581747698284903 | 0.33525775759241 | 0.862240730596339 | -0.598930435 | 0.715279974709615 | 1.05990021810351 | 0.549398939806443 | 2.04475908295644 | 0.998735809224318 |
| ebi-fl187-GCST90094812 | prot-a-858 | Inactive dipeptidyl peptidase 10 \|\| id:prot-a-858 | Plum liking \|\| id:ebi-fl187-GCST90094812 | Inverse variance weighted | 5 | -0.350273656 | 0.3092711226206 | 0.257391530341024 | -0.956445056 | 0.255897744539195 | 0.704495274121513 | 0.384256470989666 | 1.29162064592243 | 0.998735809224318 |
| ebi-fl187-GCST90094812 | prot-a-863 | Dihydropyrimidinase-related protein 3 \|\| id:prot-a-863 | Plum liking \|\| id:ebi-fl187-GCST90094812 | Inverse variance weighted | 5 | 0.159569391438673 | 0.241796151039105 | 0.50929647544713 | -0.314351065 | 0.633489847475319 | 1.17300565594672 | 0.730262613802128 | 1.88417460085916 | 0.998735809224318 |
| ebi-fl187-GCST90094812 | prot-a-864 | Dihydropyrimidinase-related protein 5 \|\| id:prot-a-864 | Plum liking \|\| id:ebi-fl187-GCST90094812 | Inverse variance weighted | 5 | -0.033766004 | 0.237021661795527 | 0.886717013234246 | -0.498328461 | 0.430796453448979 | 0.966797705286057 | 0.60754534730151 | 1.53848236530485 | 0.998735809224318 |
| ebi-fl187-GCST90094812 | prot-a-884 | Endothelin-converting enzyme 1 \|\| id:prot-a-884 | Plum liking \|\| id:ebi-fl187-GCST90094812 | Inverse variance weighted | 5 | 0.108039602480683 | 0.302085919196033 | 0.720608789739327 | -0.484048799 | 0.700128004104907 | 1.11409186531441 | 0.616283127089702 | 2.01401049258173 | 0.998735809224318 |
| ebi-fl187-GCST90094812 | prot-a-893 | Early endosome antigen 1 \|\| id:prot-a-893 | Plum liking \|\| id:ebi-fl187-GCST90094812 | Inverse variance weighted | 5 | 0.185768298814415 | 0.236640300120678 | 0.432439537255913 | -0.278046689 | 0.649583287050944 | 1.20414322663943 | 0.757261464588774 | 1.91474276463943 | 0.998735809224318 |
| ebi-fl187-GCST90094812 | prot-a-897 | Ephrin-A2 \|\| id:prot-a-897 | Plum liking \|\| id:ebi-fl187-GCST90094812 | Inverse variance weighted | 5 | 0.134976687460148 | 0.246570839170779 | 0.584093341624047 | -0.348302157 | 0.618255532234876 | 1.14451010260293 | 0.705885555503949 | 1.85568802867059 | 0.998735809224318 |
| ebi-fl187-GCST90094812 | prot-a-90 | Anaphase-promoting complex subunit 10 \|\| id:prot-a-90 | Plum liking \|\| id:ebi-fl187-GCST90094812 | Inverse variance weighted | 5 | -0.384590197 | 0.236640300120678 | 0.104117806754313 | -0.848405186 | 0.079224790926284 | 0.680729543850198 | 0.428097123299444 | 1.08244761912676 | 0.998735809224318 |
| ebi-fl187-GCST90094812 | prot-a-914 | Histone-lysine N-methyltransferase EHMT2 \|\| id:prot-a-914 | Plum liking \|\| id:ebi-fl187-GCST90094812 | Inverse variance weighted (multiplicative random effects) | 5 | -0.24638792 | 0.446710312948096 | 0.581249265824337 | -1.121940133 | 0.62916429362981 | 0.781618960677569 | 0.325647382065559 | 1.87604210362634 | 0.998735809224318 |
| ebi-fl187-GCST90094812 | prot-a-932 | RNA polymerase II elongation factor ELL \|\| id:prot-a-932 | Plum liking \|\| id:ebi-fl187-GCST90094812 | Inverse variance weighted | 5 | 0.086352254153395 | 0.318835650436252 | 0.786516972639865 | -0.538565621 | 0.711270129008449 | 1.09019028473182 | 0.583584734176012 | 2.03657633128764 | 0.998735809224318 |
| ebi-fl187-GCST90094812 | prot-a-991 | Estrogen receptor \|\| id:prot-a-991 | Plum liking \|\| id:ebi-fl187-GCST90094812 | Inverse variance weighted | 5 | -0.011644736 | 0.237021661795527 | 0.960816158953418 | -0.476207194 | 0.452917720649039 | 0.988422801067068 | 0.621134773770841 | 1.57289476442952 | 0.998735809224318 |
| ebi-fl187-GCST90094812 | prot-a-992 | Persulfide dioxygenase ETHE1, mitochondrial \|\| id:prot-a-992 | Plum liking \|\| id:ebi-fl187-GCST90094812 | Inverse variance weighted | 5 | 0.178207020394545 | 0.282336760258167 | 0.52791889157498 | -0.37517303 | 0.731587070500554 | 1.19507270004956 | 0.687170367613252 | 2.07837652162492 | 0.998735809224318 |
| ebi-fl187-GCST90094812 | prot-a-993 | Ethanolamine kinase 1 \|\| id:prot-a-993 | Plum liking \|\| id:ebi-fl187-GCST90094812 | Inverse variance weighted | 5 | 0.0777200960008612 | 0.237021661795527 | 0.742985062680352 | -0.386842361 | 0.542282553120095 | 1.08082009046136 | 0.67919815452552 | 1.71992821264507 | 0.998735809224318 |
| ebi-fl187-GCST90094812 | prot-b-10 | coagulation factor III, tissue factor \|\| id:prot-b-10 | Plum liking \|\| id:ebi-fl187-GCST90094812 | Inverse variance weighted | 4 | -0.275126146 | 0.287029130479098 | 0.337795371676678 | -0.837703242 | 0.287450949613249 | 0.759476312187593 | 0.432703197615696 | 1.33302520515773 | 0.998735809224318 |
| ebi-fl187-GCST90094812 | prot-b-28 | prolactin \|\| id:prot-b-28 | Plum liking \|\| id:ebi-fl187-GCST90094812 | Inverse variance weighted | 4 | 0.356433873680516 | 0.286987311060377 | 0.214242175978496 | -0.206061256 | 0.918929003358853 | 1.42822708399555 | 0.813783225652613 | 2.50660438696384 | 0.998735809224318 |
| ebi-fl187-GCST90094812 | prot-b-54 | matrix metallopeptidase 7 \|\| id:prot-b-54 | Plum liking \|\| id:ebi-fl187-GCST90094812 | Inverse variance weighted | 4 | -0.180482449 | 0.288436174847892 | 0.531493687949834 | -0.745817351 | 0.384852454119715 | 0.834867333673772 | 0.474346433783331 | 1.46939750189822 | 0.998735809224318 |
| ebi-fl187-GCST90094812 | ukb-rsfMRI191-edge-pheno716 | Net100_Pair10_33 (OccipitalPrecuneus)&(Temporal\|Frontal\|Supp_Motor_Area) (Default_mode\|Central_executive)&(Default_mode\|Salience) \|\| id:ukb-rsfMRI191-edge-pheno716 | Plum liking \|\| id:ebi-fl187-GCST90094812 | Inverse variance weighted | 5 | 0.0244569639333538 | 0.0726079225370146 | 0.73624051514814 | -0.117854564 | 0.166768492105902 | 1.0247584885836 | 0.888825310200264 | 1.18148071153321 | 0.998735809224318 |
| ebi-fl187-GCST90094812 | ukb-rsfMRI191-edge-pheno903 | Net100_Pair27_38 (Precentral\|Frontal\|Supp_Motor_Area)&(Subcortical) (Attention\|Salience\|Motor)&(Subcortical-cerebellum) \|\| id:ukb-rsfMRI191-edge-pheno903 | Plum liking \|\| id:ebi-fl187-GCST90094812 | Inverse variance weighted | 5 | -0.085894866 | 0.0720950893565654 | 0.233492365898959 | -0.227201241 | 0.0554115090194717 | 0.917690706533822 | 0.796760425084958 | 1.05697548013726 | 0.998735809224318 |
| ebi-fl187-GCST90094812 | ukb-rsfMRI191-node-pheno23 | Net100_Node2 RolandicOper\|SupraMarginal\|Insula Salience\|Motor \|\| id:ukb-rsfMRI191-node-pheno23 | Plum liking \|\| id:ebi-fl187-GCST90094812 | Inverse variance weighted | 5 | 0.0323978133067901 | 0.0900508172997661 | 0.719017223015708 | -0.144101789 | 0.208897415214332 | 1.03292833621981 | 0.865799615042467 | 1.23231857490891 | 0.998735809224318 |
| ebi-fl187-GCST90094812 | ukb-rsfMRI191-node-pheno55 | Net100_Node34 Parietal\|Postcentral\|Precuneus Attention \|\| id:ukb-rsfMRI191-node-pheno55 | Plum liking \|\| id:ebi-fl187-GCST90094812 | Inverse variance weighted (multiplicative random effects) | 5 | 0.0065018670597472 | 0.110172007632147 | 0.952939683299165 | -0.209435268 | 0.222439002018755 | 1.00652305008222 | 0.81104213819529 | 1.2491196235514 | 0.998735809224318 |
| ebi-fl187-GCST90094812 | ebi-a-GCST90001414 | IgD- CD24- B cell Absolute Count \|\| id:ebi-a-GCST90001414 | Plum liking \|\| id:ebi-fl187-GCST90094812 | MR Egger | 5 | -2.099184575 | 0.984966226111159 | 0.122868617 | -4.029718379 | -0.168650772 | 0.122556322967297 | 0.0177793362917427 | 0.844803880909787 | 0.999950574162623 |
| ebi-fl187-GCST90094812 | ebi-a-GCST90001422 | CD20- CD38- B cell %B cell \|\| id:ebi-a-GCST90001422 | Plum liking \|\| id:ebi-fl187-GCST90094812 | MR Egger | 5 | 1.1121376924613 | 0.941895476863516 | 0.322791998773897 | -0.733977442 | 2.95825282711379 | 3.04085185754113 | 0.479996031803127 | 19.2642842999667 | 0.999950574162623 |
| ebi-fl187-GCST90094812 | ebi-a-GCST90001444 | CD20- CD38- B cell %lymphocyte \|\| id:ebi-a-GCST90001444 | Plum liking \|\| id:ebi-fl187-GCST90094812 | MR Egger | 5 | 1.11375041855249 | 0.943826812418482 | 0.323034484831374 | -0.736150134 | 2.96365097089271 | 3.04575987525156 | 0.478954280570334 | 19.3685568623499 | 0.999950574162623 |
| ebi-fl187-GCST90094812 | ebi-a-GCST90001459 | Myeloid Dendritic Cell %Dendritic Cell \|\| id:ebi-a-GCST90001459 | Plum liking \|\| id:ebi-fl187-GCST90094812 | MR Egger | 5 | 1.83526310484595 | 1.00286481168124 | 0.164666422186764 | -0.130351926 | 3.80087813574118 | 6.26678274945825 | 0.877786460635205 | 44.7404554411653 | 0.999950574162623 |
| ebi-fl187-GCST90094812 | ebi-a-GCST90001476 | HLA DR++ monocyte %leukocyte \|\| id:ebi-a-GCST90001476 | Plum liking \|\| id:ebi-fl187-GCST90094812 | MR Egger | 5 | -0.61875269 | 0.98033578644802 | 0.572742877540688 | -2.540210832 | 1.30270545129083 | 0.538615839628035 | 0.0788497740152523 | 3.67923721179082 | 0.999950574162623 |
| ebi-fl187-GCST90094812 | ebi-a-GCST90001478 | CD4 regulatory T cell %CD4+ T cell \|\| id:ebi-a-GCST90001478 | Plum liking \|\| id:ebi-fl187-GCST90094812 | MR Egger | 5 | -0.443142226 | 0.989595782518789 | 0.684658029584667 | -2.38274996 | 1.49646550774052 | 0.642015889244568 | 0.0922964167749748 | 4.46587653611113 | 0.999950574162623 |
| ebi-fl187-GCST90094812 | ebi-a-GCST90001485 | CD39+ resting CD4 regulatory T cell %CD4 regulatory T cell \|\| id:ebi-a-GCST90001485 | Plum liking \|\| id:ebi-fl187-GCST90094812 | MR Egger | 5 | 0.177130153600005 | 0.966804723861644 | 0.866314564886607 | -1.717807105 | 2.07206741236883 | 1.19378645862146 | 0.179459252002123 | 7.94122394297677 | 0.999950574162623 |
| ebi-fl187-GCST90094812 | ebi-a-GCST90001515 | Immature Myeloid-Derived Suppressor Cells Absolute Count \|\| id:ebi-a-GCST90001515 | Plum liking \|\| id:ebi-fl187-GCST90094812 | MR Egger | 5 | -0.090142458 | 1.75714641659971 | 0.962310786744457 | -3.534149434 | 3.35386451890309 | 0.91380099807175 | 0.0291835689824233 | 28.6130961082879 | 0.999950574162623 |
| ebi-fl187-GCST90094812 | ebi-a-GCST90001517 | CD33+ HLA DR+ Absolute Count \|\| id:ebi-a-GCST90001517 | Plum liking \|\| id:ebi-fl187-GCST90094812 | MR Egger | 5 | 0.583504220447118 | 1.43765109921758 | 0.712049600870898 | -2.234291934 | 3.40130037491358 | 1.79230808184251 | 0.107067914167442 | 30.0030899566624 | 0.999950574162623 |
| ebi-fl187-GCST90094812 | ebi-a-GCST90001518 | CD33+ HLA DR+ CD14- Absolute Count \|\| id:ebi-a-GCST90001518 | Plum liking \|\| id:ebi-fl187-GCST90094812 | MR Egger | 5 | 0.763108703604492 | 1.57091531681463 | 0.660397837739859 | -2.315885317 | 3.84210272456117 | 2.14493383039721 | 0.0986787832800222 | 46.6234076247866 | 0.999950574162623 |
| ebi-fl187-GCST90094812 | ebi-a-GCST90001520 | CD33+ HLA DR+ CD14dim Absolute Count \|\| id:ebi-a-GCST90001520 | Plum liking \|\| id:ebi-fl187-GCST90094812 | MR Egger | 5 | 0.217124670528256 | 1.28652505266082 | 0.876716135648113 | -2.304464433 | 2.73871377374347 | 1.24249899548837 | 0.0998122425160167 | 15.4670781346474 | 0.999950574162623 |
| ebi-fl187-GCST90094812 | ebi-a-GCST90001531 | CD33dim HLA DR- Absolute Count \|\| id:ebi-a-GCST90001531 | Plum liking \|\| id:ebi-fl187-GCST90094812 | MR Egger | 5 | -1.195277138 | 1.30562309899371 | 0.427445462629702 | -3.754298412 | 1.36374413596667 | 0.302620075018811 | 0.0234168738429751 | 3.91080852288334 | 0.999950574162623 |
| ebi-fl187-GCST90094812 | ebi-a-GCST90001532 | Basophil Absolute Count \|\| id:ebi-a-GCST90001532 | Plum liking \|\| id:ebi-fl187-GCST90094812 | MR Egger | 5 | -1.257339309 | 1.3016949559815 | 0.405333630766969 | -3.808661423 | 1.29398280459773 | 0.284409747103077 | 0.0221778458545948 | 3.64728408600051 | 0.999950574162623 |
| ebi-fl187-GCST90094812 | ebi-a-GCST90001588 | T/B cell \|\| id:ebi-a-GCST90001588 | Plum liking \|\| id:ebi-fl187-GCST90094812 | MR Egger | 5 | 1.20623561544809 | 0.965518397132892 | 0.300147588606606 | -0.686180443 | 3.09865167382855 | 3.34088457737127 | 0.503495530899022 | 22.1680413714649 | 0.999950574162623 |
| ebi-fl187-GCST90094812 | ebi-a-GCST90001624 | HLA DR+ CD4+ T cell Absolute Count \|\| id:ebi-a-GCST90001624 | Plum liking \|\| id:ebi-fl187-GCST90094812 | MR Egger | 5 | -0.24978934 | 0.938823115346463 | 0.807426113984361 | -2.089882646 | 1.59030396605124 | 0.77896486250457 | 0.123701651836109 | 4.9052397280894 | 0.999950574162623 |
| ebi-fl187-GCST90094812 | ebi-a-GCST90001631 | CD8+ Natural Killer T %T cell \|\| id:ebi-a-GCST90001631 | Plum liking \|\| id:ebi-fl187-GCST90094812 | MR Egger | 5 | -0.204205584 | 0.952306864025834 | 0.843958303188096 | -2.070727038 | 1.66231586933374 | 0.815294742274749 | 0.126094073232952 | 5.27150483554324 | 0.999950574162623 |
| ebi-fl187-GCST90094812 | ebi-a-GCST90001632 | CD8+ Natural Killer T %lymphocyte \|\| id:ebi-a-GCST90001632 | Plum liking \|\| id:ebi-fl187-GCST90094812 | MR Egger | 5 | -0.0047558 | 0.958141348165184 | 0.996351274806194 | -1.882712843 | 1.87320124194763 | 0.995255490456657 | 0.152176713765278 | 6.50910028726176 | 0.999950574162623 |
| ebi-fl187-GCST90094812 | ebi-a-GCST90001646 | Natural Killer %CD3- lymphocyte \|\| id:ebi-a-GCST90001646 | Plum liking \|\| id:ebi-fl187-GCST90094812 | MR Egger | 5 | 0.268084235331233 | 0.931903887443924 | 0.792324486456327 | -1.558447384 | 2.09461585472132 | 1.30745726947264 | 0.210462585224568 | 8.12232021987589 | 0.999950574162623 |
| ebi-fl187-GCST90094812 | ebi-a-GCST90001684 | CD127- CD8+ T cell Absolute Count \|\| id:ebi-a-GCST90001684 | Plum liking \|\| id:ebi-fl187-GCST90094812 | MR Egger | 5 | 1.56255712242107 | 1.06353942225457 | 0.238107275809384 | -0.521980145 | 3.64709439004002 | 4.77100570558078 | 0.593344475743256 | 38.3630359314811 | 0.999950574162623 |
| ebi-fl187-GCST90094812 | ebi-a-GCST90001687 | CD28- CD8+ T cell Absolute Count \|\| id:ebi-a-GCST90001687 | Plum liking \|\| id:ebi-fl187-GCST90094812 | MR Egger | 5 | 0.920071094147174 | 1.11143434590127 | 0.468475736192738 | -1.258340224 | 3.09848241211367 | 2.50946879213818 | 0.2841252196259 | 22.1642894883007 | 0.999950574162623 |
| ebi-fl187-GCST90094812 | ebi-a-GCST90001701 | BAFF-R on CD20- CD38- B cell \|\| id:ebi-a-GCST90001701 | Plum liking \|\| id:ebi-fl187-GCST90094812 | MR Egger | 5 | 0.101682019507671 | 0.954012316593384 | 0.921846998563028 | -1.768182121 | 1.9715461600307 | 1.10703140136512 | 0.170642915205161 | 7.18177207729364 | 0.999950574162623 |
| ebi-fl187-GCST90094812 | ebi-a-GCST90001732 | CD19 on IgD- CD27- B cell \|\| id:ebi-a-GCST90001732 | Plum liking \|\| id:ebi-fl187-GCST90094812 | MR Egger | 5 | 1.10611358831746 | 1.49357356933095 | 0.512643624614654 | -1.821290608 | 4.03351778420612 | 3.02258851463626 | 0.16181677415551 | 56.459173509608 | 0.999950574162623 |
| ebi-fl187-GCST90094812 | ebi-a-GCST90001798 | CD27 on CD24+ CD27+ B cell \|\| id:ebi-a-GCST90001798 | Plum liking \|\| id:ebi-fl187-GCST90094812 | MR Egger | 5 | 0.168080691288354 | 1.37576359449385 | 0.910486811093945 | -2.528415954 | 2.86457733649629 | 1.18303206717817 | 0.0797853038378283 | 17.541637427572 | 0.999950574162623 |
| ebi-fl187-GCST90094812 | ebi-a-GCST90001836 | CD66b on CD66b++ myeloid cell \|\| id:ebi-a-GCST90001836 | Plum liking \|\| id:ebi-fl187-GCST90094812 | MR Egger | 5 | 0.431708828976977 | 1.46916256637383 | 0.788031901524744 | -2.447849801 | 3.31126745906967 | 1.53988667949875 | 0.0864793344987876 | 27.4198570033044 | 0.999950574162623 |
| ebi-fl187-GCST90094812 | ebi-a-GCST90001943 | CD25 on activated & secreting CD4 regulatory T cell \|\| id:ebi-a-GCST90001943 | Plum liking \|\| id:ebi-fl187-GCST90094812 | MR Egger | 5 | -1.237209461 | 1.17923277126197 | 0.371168263163299 | -3.548505692 | 1.0740867711493 | 0.29019288380514 | 0.028767595193801 | 2.92731836790064 | 0.999950574162623 |
| ebi-fl187-GCST90094812 | ebi-a-GCST90001960 | CD25 on CD4+ T cell \|\| id:ebi-a-GCST90001960 | Plum liking \|\| id:ebi-fl187-GCST90094812 | MR Egger | 5 | -1.065682932 | 1.0427459776834 | 0.382006326609569 | -3.109465048 | 0.978099184631667 | 0.344492509583701 | 0.0446248210985143 | 2.65939641298029 | 0.999950574162623 |
| ebi-fl187-GCST90094812 | ebi-a-GCST90001988 | HLA DR on CD14+ CD16- monocyte \|\| id:ebi-a-GCST90001988 | Plum liking \|\| id:ebi-fl187-GCST90094812 | MR Egger | 5 | -0.34644502 | 1.02608179668385 | 0.757880165658145 | -2.357565342 | 1.66467530148783 | 0.707197699939117 | 0.094650384262168 | 5.28395727812254 | 0.999950574162623 |
| ebi-fl187-GCST90094812 | ebi-a-GCST90001991 | HLA DR on CD14+ monocyte \|\| id:ebi-a-GCST90001991 | Plum liking \|\| id:ebi-fl187-GCST90094812 | MR Egger | 5 | -0.445995978 | 1.00613531573117 | 0.687584253905118 | -2.418021197 | 1.52602924075029 | 0.640186346832949 | 0.0890977500386317 | 4.59987551305859 | 0.999950574162623 |
| ebi-fl187-GCST90094812 | ebi-a-GCST90002019 | CD14 on Monocytic Myeloid-Derived Suppressor Cells \|\| id:ebi-a-GCST90002019 | Plum liking \|\| id:ebi-fl187-GCST90094812 | MR Egger | 5 | 2.61718614753049 | 1.77254882019801 | 0.236298565739965 | -0.85700954 | 6.09138183511859 | 13.6971276125001 | 0.42442942559925 | 442.03180438827 | 0.999950574162623 |
| ebi-fl187-GCST90094812 | ebi-a-GCST90002037 | CD80 on plasmacytoid Dendritic Cell \|\| id:ebi-a-GCST90002037 | Plum liking \|\| id:ebi-fl187-GCST90094812 | MR Egger | 5 | 1.3281257173775 | 1.90217829885866 | 0.535298321995539 | -2.400143748 | 5.05639518314046 | 3.77396328078576 | 0.0907049136673293 | 157.023454065083 | 0.999950574162623 |
| ebi-fl187-GCST90094812 | ebi-a-GCST90002042 | CD45 on CD33+ HLA DR+ CD14- \|\| id:ebi-a-GCST90002042 | Plum liking \|\| id:ebi-fl187-GCST90094812 | MR Egger | 5 | 3.62172668606469 | 1.44024490091092 | 0.0865819980904188 | 0.798846680279289 | 6.4446066918501 | 37.4020937720992 | 2.22297564782913 | 629.299119809536 | 0.999950574162623 |
| ebi-fl187-GCST90094812 | ebi-a-GCST90002056 | CD8 on Effector Memory CD8+ T cell \|\| id:ebi-a-GCST90002056 | Plum liking \|\| id:ebi-fl187-GCST90094812 | MR Egger | 5 | 1.43892011116276 | 1.06567796858927 | 0.269771214019178 | -0.649808707 | 3.52764892959773 | 4.21614039481586 | 0.522145649873875 | 34.0438340012826 | 0.999950574162623 |
| ebi-fl187-GCST90094812 | ebi-a-GCST90002058 | CD8 on CD8+ T cell \|\| id:ebi-a-GCST90002058 | Plum liking \|\| id:ebi-fl187-GCST90094812 | MR Egger | 5 | -0.388451979 | 1.6903178517549 | 0.833017206171124 | -3.701474968 | 2.92457101070095 | 0.678105784593126 | 0.0246870869357244 | 18.6262338807276 | 0.999950574162623 |
| ebi-fl187-GCST90094812 | ebi-a-GCST90002063 | CD4 on CD4 regulatory T cell \|\| id:ebi-a-GCST90002063 | Plum liking \|\| id:ebi-fl187-GCST90094812 | MR Egger | 5 | 0.510290297752999 | 1.06624950928288 | 0.664949624471324 | -1.57955874 | 2.60013933594744 | 1.66577469541402 | 0.206066006741447 | 13.4656141483988 | 0.999950574162623 |
| ebi-fl187-GCST90094812 | ebi-a-GCST90002066 | CD4 on activated CD4 regulatory T cell \|\| id:ebi-a-GCST90002066 | Plum liking \|\| id:ebi-fl187-GCST90094812 | MR Egger | 5 | 0.570802110133687 | 1.06019677343056 | 0.627688137986464 | -1.507183566 | 2.64878778605759 | 1.76968596534129 | 0.221533033364009 | 14.1368913176031 | 0.999950574162623 |
| ebi-fl187-GCST90094812 | ebi-a-GCST90002070 | CD4 on activated & secreting CD4 regulatory T cell \|\| id:ebi-a-GCST90002070 | Plum liking \|\| id:ebi-fl187-GCST90094812 | MR Egger | 5 | 0.931067317213958 | 1.06513866868141 | 0.446387201199325 | -1.156604473 | 3.01873910782953 | 2.53721574701918 | 0.314552440780479 | 20.4654706571318 | 0.999950574162623 |
| ebi-fl187-GCST90094812 | ebi-a-GCST90002081 | SSC-A on CD4+ T cell \|\| id:ebi-a-GCST90002081 | Plum liking \|\| id:ebi-fl187-GCST90094812 | MR Egger | 5 | -1.508787101 | 1.00388262245337 | 0.229876548088974 | -3.476397041 | 0.458822839407184 | 0.221178082097589 | 0.0309186090843524 | 1.58221037262396 | 0.999950574162623 |
| ebi-fl187-GCST90094812 | ebi-a-GCST90002094 | CD11b on Monocytic Myeloid-Derived Suppressor Cells \|\| id:ebi-a-GCST90002094 | Plum liking \|\| id:ebi-fl187-GCST90094812 | MR Egger | 5 | -0.156438597 | 1.43641802368498 | 0.920150831314747 | -2.971817924 | 2.65894072932254 | 0.85518402687005 | 0.0512101295607005 | 14.2811534766145 | 0.999950574162623 |
| ebi-fl187-GCST90094812 | ebi-a-GCST90002104 | HLA DR on myeloid Dendritic Cell \|\| id:ebi-a-GCST90002104 | Plum liking \|\| id:ebi-fl187-GCST90094812 | MR Egger | 5 | -0.133924647 | 1.0780362053387 | 0.908989493477052 | -2.24687561 | 1.97902631510941 | 0.874655969761518 | 0.105729047844252 | 7.23569426792159 | 0.999950574162623 |
| ebi-fl187-GCST90094812 | ebi-a-GCST90002105 | HLA DR on plasmacytoid Dendritic Cell \|\| id:ebi-a-GCST90002105 | Plum liking \|\| id:ebi-fl187-GCST90094812 | MR Egger | 5 | -0.682293185 | 1.05338813612902 | 0.563321004023304 | -2.746933932 | 1.38234756152781 | 0.505456556788958 | 0.0641241691629751 | 3.98424391513934 | 0.999950574162623 |
| ebi-fl187-GCST90094812 | ebi-a-GCST90002106 | HLA DR on Dendritic Cell \|\| id:ebi-a-GCST90002106 | Plum liking \|\| id:ebi-fl187-GCST90094812 | MR Egger | 5 | 0.134880402969976 | 1.07540073490054 | 0.908121412068314 | -1.972905037 | 2.24266584337503 | 1.14439990933624 | 0.139052316712868 | 9.41840584499658 | 0.999950574162623 |
| ebi-fl187-GCST90094812 | ebi-a-GCST90002111 | HLA DR on CD33dim HLA DR+ CD11b- \|\| id:ebi-a-GCST90002111 | Plum liking \|\| id:ebi-fl187-GCST90094812 | MR Egger | 5 | 1.82341854813795 | 1.3909708006514 | 0.281209857068893 | -0.902884221 | 4.54972131741469 | 6.19299334984887 | 0.405398712380882 | 94.6060395851446 | 0.999950574162623 |
| ebi-fl187-GCST90094812 | ebi-a-GCST90002112 | HLA DR on CD33- HLA DR+ \|\| id:ebi-a-GCST90002112 | Plum liking \|\| id:ebi-fl187-GCST90094812 | MR Egger | 5 | -1.771725055 | 1.4054063159699 | 0.296564432347428 | -4.526321434 | 0.982871324269727 | 0.170039408340094 | 0.0108204065283619 | 2.67211775388132 | 0.999950574162623 |
| ebi-fl187-GCST90094812 | ebi-a-GCST90002115 | HLA DR on HLA DR+ CD8+ T cell \|\| id:ebi-a-GCST90002115 | Plum liking \|\| id:ebi-fl187-GCST90094812 | MR Egger | 5 | 0.996594369025539 | 1.17552914005828 | 0.458839417303224 | -1.307442745 | 3.30063148353977 | 2.7090401095412 | 0.270510937944606 | 27.1297655128674 | 0.999950574162623 |
| ebi-fl187-GCST90094812 | ebi-a-GCST90002116 | HLA DR on B cell \|\| id:ebi-a-GCST90002116 | Plum liking \|\| id:ebi-fl187-GCST90094812 | MR Egger | 5 | 0.657730348998964 | 1.04164036736727 | 0.572586477239139 | -1.383884771 | 2.69934546903882 | 1.93040601063846 | 0.250603123868578 | 14.8699956663883 | 0.999950574162623 |
| ebi-fl187-GCST90094812 | ebi-cfmet338-GCST90026023 | Sphingomyelin (d18:1/24:1, d18:2/24:0) levels \|\| id:ebi-cfmet338-GCST90026023 | Plum liking \|\| id:ebi-fl187-GCST90094812 | MR Egger | 5 | -0.321604724 | 0.535760953985168 | 0.590639173902933 | -1.371696194 | 0.728486745574091 | 0.724984702582092 | 0.253676310176216 | 2.07194285746641 | 0.999950574162623 |
| ebi-fl187-GCST90094812 | ebi-cfmet338-GCST90026046 | 1-arachidonoyl-gpc (20:4n6) levels \|\| id:ebi-cfmet338-GCST90026046 | Plum liking \|\| id:ebi-fl187-GCST90094812 | MR Egger | 5 | -0.448186384 | 1.26235620776481 | 0.746053581859262 | -2.922404552 | 2.02603178281757 | 0.638785613261605 | 0.0538041567590063 | 7.58393187979295 | 0.999950574162623 |
| ebi-fl187-GCST90094812 | ebi-cfmet338-GCST90026047 | 1-linoleoyl-gpc (18:2) levels \|\| id:ebi-cfmet338-GCST90026047 | Plum liking \|\| id:ebi-fl187-GCST90094812 | MR Egger | 5 | -0.067422472 | 0.774382121995868 | 0.936104791146366 | -1.585211431 | 1.45036648740944 | 0.934800191274101 | 0.204904465418565 | 4.26467717929452 | 0.999950574162623 |
| ebi-fl187-GCST90094812 | ebi-cfmet338-GCST90026050 | 1-myristoyl-2-palmitoyl-gpc (14:0/16:0) levels \|\| id:ebi-cfmet338-GCST90026050 | Plum liking \|\| id:ebi-fl187-GCST90094812 | MR Egger | 5 | -0.00109425 | 0.417166490272901 | 0.998071782796224 | -0.818740571 | 0.816552070969093 | 0.998906348507388 | 0.440986696392532 | 2.26268479582474 | 0.999950574162623 |
| ebi-fl187-GCST90094812 | ebi-cfmet338-GCST90026068 | 2,3-dihydroxyisovalerate levels \|\| id:ebi-cfmet338-GCST90026068 | Plum liking \|\| id:ebi-fl187-GCST90094812 | MR Egger | 5 | 2.11127988191875 | 1.34067433535484 | 0.213379176792706 | -0.516441815 | 4.73900157921423 | 8.25880481810406 | 0.59663972982649 | 114.320005212148 | 0.999950574162623 |
| ebi-fl187-GCST90094812 | ebi-cfmet338-GCST90026082 | 3-hydroxyhexanoate levels \|\| id:ebi-cfmet338-GCST90026082 | Plum liking \|\| id:ebi-fl187-GCST90094812 | MR Egger | 5 | -0.458395989 | 0.337676081956093 | 0.267714004076622 | -1.12024111 | 0.20344913147728 | 0.632297043853232 | 0.32620113485328 | 1.22562281043369 | 0.999950574162623 |
| ebi-fl187-GCST90094812 | ebi-cfmet338-GCST90026094 | 3-ureidopropionate levels \|\| id:ebi-cfmet338-GCST90026094 | Plum liking \|\| id:ebi-fl187-GCST90094812 | MR Egger | 5 | 0.204222888868904 | 0.650088118037254 | 0.773988372716191 | -1.069949822 | 1.47839560022192 | 1.22657151212774 | 0.343025729165856 | 4.38590329075844 | 0.999950574162623 |
| ebi-fl187-GCST90094812 | ebi-cfmet338-GCST90026173 | Glycerol levels \|\| id:ebi-cfmet338-GCST90026173 | Plum liking \|\| id:ebi-fl187-GCST90094812 | MR Egger | 5 | -0.412696523 | 0.366240501705499 | 0.341802144555184 | -1.130527906 | 0.305134860490074 | 0.661863112682009 | 0.322862770169175 | 1.35680797045624 | 0.999950574162623 |
| ebi-fl187-GCST90094812 | ebi-cfmet338-GCST90026179 | Histidine levels \|\| id:ebi-cfmet338-GCST90026179 | Plum liking \|\| id:ebi-fl187-GCST90094812 | MR Egger | 5 | 0.0408470754209817 | 0.255177404233558 | 0.882994244860524 | -0.459300637 | 0.540994787718755 | 1.04169279293256 | 0.631725296428061 | 1.71771477410071 | 0.999950574162623 |
| ebi-fl187-GCST90094812 | ebi-cfmet338-GCST90026188 | Isocitrate levels \|\| id:ebi-cfmet338-GCST90026188 | Plum liking \|\| id:ebi-fl187-GCST90094812 | MR Egger | 5 | 0.291635507075388 | 0.638265638750301 | 0.678784370031987 | -0.959365145 | 1.54263615902598 | 1.33861501300656 | 0.383136044663125 | 4.67690309488392 | 0.999950574162623 |
| ebi-fl187-GCST90094812 | ebi-cfmet338-GCST90026207 | N-acetyl-isoputreanine levels \|\| id:ebi-cfmet338-GCST90026207 | Plum liking \|\| id:ebi-fl187-GCST90094812 | MR Egger | 5 | 0.219217824575632 | 0.431083090471354 | 0.646120406192527 | -0.625705033 | 1.06414068189949 | 1.24510246106635 | 0.53488418468328 | 2.89834731133702 | 0.999950574162623 |
| ebi-fl187-GCST90094812 | ebi-cfmet338-GCST90026213 | N-acetylglutamate levels \|\| id:ebi-cfmet338-GCST90026213 | Plum liking \|\| id:ebi-fl187-GCST90094812 | MR Egger | 5 | 0.208954228968033 | 0.542030305699566 | 0.725575173513612 | -0.85342517 | 1.27133362813918 | 1.23238858954179 | 0.425953467370712 | 3.56560458354243 | 0.999950574162623 |
| ebi-fl187-GCST90094812 | ebi-cfmet338-GCST90026224 | N-acetyltaurine levels \|\| id:ebi-cfmet338-GCST90026224 | Plum liking \|\| id:ebi-fl187-GCST90094812 | MR Egger | 5 | 0.533401633387873 | 0.435131113629915 | 0.307710047496118 | -0.319455349 | 1.38625861610251 | 1.70472129390402 | 0.726544642359169 | 3.99985702248584 | 0.999950574162623 |
| ebi-fl187-GCST90094812 | ebi-cfmet338-GCST90026250 | Phenyllactate (pla) levels \|\| id:ebi-cfmet338-GCST90026250 | Plum liking \|\| id:ebi-fl187-GCST90094812 | MR Egger | 5 | -0.23089401 | 0.786720440113589 | 0.788280290616597 | -1.772866072 | 1.31107805276361 | 0.793823599051702 | 0.169845501054761 | 3.71017131745059 | 0.999950574162623 |
| ebi-fl187-GCST90094812 | ebi-cfmet338-GCST90026251 | Pipecolate levels \|\| id:ebi-cfmet338-GCST90026251 | Plum liking \|\| id:ebi-fl187-GCST90094812 | MR Egger | 5 | 0.98940250445887 | 1.02676532050959 | 0.406324269331146 | -1.023057524 | 3.00186253265767 | 2.68962695204283 | 0.359494096368631 | 20.1229817519375 | 0.999950574162623 |
| ebi-fl187-GCST90094812 | ebi-cfmet338-GCST90026278 | Trigonelline (n'-methylnicotinate) levels \|\| id:ebi-cfmet338-GCST90026278 | Plum liking \|\| id:ebi-fl187-GCST90094812 | MR Egger | 5 | -0.911547691 | 1.34673980387496 | 0.54701527732373 | -3.551157707 | 1.72806232417256 | 0.401901722589138 | 0.028691404179526 | 5.62973473202748 | 0.999950574162623 |
| ebi-fl187-GCST90094812 | ebi-cfmet338-GCST90026299 | 1-carboxyethylvaline levels \|\| id:ebi-cfmet338-GCST90026299 | Plum liking \|\| id:ebi-fl187-GCST90094812 | MR Egger | 5 | 0.785462203362746 | 0.852503529748769 | 0.424814122071533 | -0.885444715 | 2.45636912167033 | 2.1934205128139 | 0.412530673923721 | 11.6623898540024 | 0.999950574162623 |
| ebi-fl187-GCST90094812 | ebi-gm430-GCST90011306 | C_Erysipelotrichia abundance \|\| id:ebi-gm430-GCST90011306 | Plum liking \|\| id:ebi-fl187-GCST90094812 | MR Egger | 5 | 0.401892986547229 | 0.613740254604427 | 0.559307443035149 | -0.801037912 | 1.60482388557191 | 1.49465137631299 | 0.448862841917754 | 4.97698300703546 | 0.999950574162623 |
| ebi-fl187-GCST90094812 | ebi-gm430-GCST90011312 | F_Erysipelotrichaceae abundance \|\| id:ebi-gm430-GCST90011312 | Plum liking \|\| id:ebi-fl187-GCST90094812 | MR Egger | 5 | 0.401892986547229 | 0.613740254604427 | 0.559307443035149 | -0.801037912 | 1.60482388557191 | 1.49465137631299 | 0.448862841917754 | 4.97698300703546 | 0.999950574162623 |
| ebi-fl187-GCST90094812 | ebi-gm430-GCST90011352 | O_Erysipelotrichales abundance \|\| id:ebi-gm430-GCST90011352 | Plum liking \|\| id:ebi-fl187-GCST90094812 | MR Egger | 5 | 0.401892986547229 | 0.613740254604427 | 0.559307443035149 | -0.801037912 | 1.60482388557191 | 1.49465137631299 | 0.448862841917754 | 4.97698300703546 | 0.999950574162623 |
| ebi-fl187-GCST90094812 | ebi-gm430-GCST90011362 | OTU97_11 (Parabacteroides) abundance \|\| id:ebi-gm430-GCST90011362 | Plum liking \|\| id:ebi-fl187-GCST90094812 | MR Egger | 5 | -0.630602616 | 0.872244621104268 | 0.521967868767651 | -2.340202073 | 1.07899684173716 | 0.532270949549452 | 0.0963081749830299 | 2.94172705260168 | 0.999950574162623 |
| ebi-fl187-GCST90094812 | ebi-gm430-GCST90011384 | OTU97_235 (Phascolarctobacterium) abundance \|\| id:ebi-gm430-GCST90011384 | Plum liking \|\| id:ebi-fl187-GCST90094812 | MR Egger | 5 | 1.85385564441244 | 4.18780387983046 | 0.687970663438501 | -6.35423996 | 10.0619512488801 | 6.38438805854116 | 0.00173935667687473 | 23434.1877223717 | 0.999950574162623 |
| ebi-fl187-GCST90094812 | ebi-gm430-GCST90011390 | OTU97_3 (Bacteroides) abundance \|\| id:ebi-gm430-GCST90011390 | Plum liking \|\| id:ebi-fl187-GCST90094812 | MR Egger | 5 | -0.310487332 | 0.77518146658961 | 0.715580718138463 | -1.829843007 | 1.2088683420588 | 0.733089610797235 | 0.160438753563778 | 3.34969180152103 | 0.999950574162623 |
| ebi-fl187-GCST90094812 | ebi-gm430-GCST90011398 | OTU97_4 (Alistipes) abundance \|\| id:ebi-gm430-GCST90011398 | Plum liking \|\| id:ebi-fl187-GCST90094812 | MR Egger | 5 | -0.384449638 | 0.896424201728813 | 0.696949328573527 | -2.141441074 | 1.37254179704385 | 0.68082523321559 | 0.11748541582579 | 3.94536628163604 | 0.999950574162623 |
| ebi-fl187-GCST90094812 | ebi-gm430-GCST90011421 | OTU99_108 (Cytophagales) abundance \|\| id:ebi-gm430-GCST90011421 | Plum liking \|\| id:ebi-fl187-GCST90094812 | MR Egger | 5 | -0.96562133 | 2.23663091862843 | 0.695084200869462 | -5.349417931 | 3.41817527044085 | 0.380746556945097 | 0.00475091555740173 | 30.5136849674568 | 0.999950574162623 |
| ebi-fl187-GCST90094812 | ebi-gm430-GCST90011428 | OTU99_123 (Phascolarctobacterium) abundance \|\| id:ebi-gm430-GCST90011428 | Plum liking \|\| id:ebi-fl187-GCST90094812 | MR Egger | 5 | -0.373732166 | 1.28703920756923 | 0.790440469688883 | -2.896329013 | 2.14886468034994 | 0.688161199781148 | 0.0552255807687329 | 8.57511736938307 | 0.999950574162623 |
| ebi-fl187-GCST90094812 | ebi-gm430-GCST90011450 | OTU99_298 (Phascolarctobacterium) abundance \|\| id:ebi-gm430-GCST90011450 | Plum liking \|\| id:ebi-fl187-GCST90094812 | MR Egger | 5 | 1.86135986207848 | 4.19047396929758 | 0.686993847140014 | -6.351969118 | 10.0746888419017 | 6.43247810962683 | 0.00174331096969802 | 23734.5920206053 | 0.999950574162623 |
| ebi-fl187-GCST90094812 | ebi-gm430-GCST90011451 | OTU99_3 (Bacteroides) abundance \|\| id:ebi-gm430-GCST90011451 | Plum liking \|\| id:ebi-fl187-GCST90094812 | MR Egger | 5 | -0.334486132 | 0.779121939722104 | 0.696661263619271 | -1.861565134 | 1.19259286979131 | 0.715705770120886 | 0.155429172425737 | 3.29561523998383 | 0.999950574162623 |
| ebi-fl187-GCST90094812 | ebi-gm430-GCST90011472 | OTU99_72 (Parabacteroides) abundance \|\| id:ebi-gm430-GCST90011472 | Plum liking \|\| id:ebi-fl187-GCST90094812 | MR Egger | 5 | -0.266792701 | 1.76272888405547 | 0.889302801007555 | -3.721741313 | 3.18815591206897 | 0.765831811425399 | 0.024191805631525 | 24.2436787201541 | 0.999950574162623 |
| ebi-fl187-GCST90094812 | ebi-gm430-GCST90011507 | TestASV_3 (Bacteroides) abundance \|\| id:ebi-gm430-GCST90011507 | Plum liking \|\| id:ebi-fl187-GCST90094812 | MR Egger | 5 | 0.832551772546793 | 1.16488693068495 | 0.526386170508658 | -1.450626612 | 3.11573015668929 | 2.29917824094359 | 0.234423349672738 | 22.5498892964723 | 0.999950574162623 |
| ebi-fl187-GCST90094812 | ebi-gm430-GCST90011512 | TestASV_34 (Phascolarctobacterium) abundance \|\| id:ebi-gm430-GCST90011512 | Plum liking \|\| id:ebi-fl187-GCST90094812 | MR Egger | 5 | 2.87163442972403 | 1.90475386679939 | 0.228764070171511 | -0.861683149 | 6.60495200865082 | 17.665868236883 | 0.422450436467334 | 738.744415019778 | 0.999950574162623 |
| ebi-fl187-GCST90094812 | ebi-gm430-GCST90011519 | TestASV_41 (Barnesiella) abundance \|\| id:ebi-gm430-GCST90011519 | Plum liking \|\| id:ebi-fl187-GCST90094812 | MR Egger | 5 | -1.408585317 | 2.40178286832147 | 0.598765398793555 | -6.116079739 | 3.29890910530756 | 0.244488913023495 | 0.00220709140081378 | 27.0830780136113 | 0.999950574162623 |
| ebi-fl187-GCST90094812 | ebi-gm430-GCST90011524 | TestASV_46 (Sutterella) abundance \|\| id:ebi-gm430-GCST90011524 | Plum liking \|\| id:ebi-fl187-GCST90094812 | MR Egger | 5 | -1.296975166 | 2.42445307552533 | 0.629791347441579 | -6.048903194 | 3.45495286160855 | 0.273357404391877 | 0.00236044954137523 | 31.6567963966427 | 0.999950574162623 |
| ebi-fl187-GCST90094812 | ebi-gm430-GCST90011581 | OTU97_19 (Bacteria) prevalence \|\| id:ebi-gm430-GCST90011581 | Plum liking \|\| id:ebi-fl187-GCST90094812 | MR Egger | 5 | -2.639255935 | 2.77919370955557 | 0.412350974130161 | -8.086475605 | 2.80796373596422 | 0.0714143867550901 | 0.000307672206758198 | 16.5761304517627 | 0.999950574162623 |
| ebi-fl187-GCST90094812 | ebi-gm430-GCST90011614 | OTU97_80 (Ruminococcus) prevalence \|\| id:ebi-gm430-GCST90011614 | Plum liking \|\| id:ebi-fl187-GCST90094812 | MR Egger | 5 | -0.535683134 | 1.28753713318084 | 0.705343163843527 | -3.059255915 | 1.98788964685484 | 0.585269336061454 | 0.0469225966238407 | 7.30011168136793 | 0.999950574162623 |
| ebi-fl187-GCST90094812 | ebi-gm430-GCST90011623 | OTU99_11 (Parabacteroides) prevalence \|\| id:ebi-gm430-GCST90011623 | Plum liking \|\| id:ebi-fl187-GCST90094812 | MR Egger | 5 | 0.663497965923748 | 1.31918364815603 | 0.64959498050538 | -1.922101984 | 3.24909791630956 | 1.94157202270374 | 0.146299120226717 | 25.767085362537 | 0.999950574162623 |
| ebi-fl187-GCST90094812 | ebi-gm430-GCST90011642 | OTU99_19 (Bacteria) prevalence \|\| id:ebi-gm430-GCST90011642 | Plum liking \|\| id:ebi-fl187-GCST90094812 | MR Egger | 5 | -2.639255935 | 2.77919370955557 | 0.412350974130161 | -8.086475605 | 2.80796373596422 | 0.0714143867550901 | 0.000307672206758198 | 16.5761304517627 | 0.999950574162623 |
| ebi-fl187-GCST90094812 | ebi-gm430-GCST90011653 | OTU99_30 (Parasutterella) prevalence \|\| id:ebi-gm430-GCST90011653 | Plum liking \|\| id:ebi-fl187-GCST90094812 | MR Egger | 5 | -0.683548993 | 1.44402239829809 | 0.668266789206116 | -3.513832893 | 2.14673490805871 | 0.504822199143617 | 0.0297825420833268 | 8.55687375628248 | 0.999950574162623 |
| ebi-fl187-GCST90094812 | ebi-gm430-GCST90011675 | OTU99_8 (Ruminococcaceae) prevalence \|\| id:ebi-gm430-GCST90011675 | Plum liking \|\| id:ebi-fl187-GCST90094812 | MR Egger | 5 | -1.819694823 | 1.29250085033889 | 0.253895815146116 | -4.352996489 | 0.713606844042623 | 0.162075205073541 | 0.0128681953418342 | 2.04134079424739 | 0.999950574162623 |
| ebi-fl187-GCST90094812 | ebi-gm430-GCST90011679 | OTU99_92 (Ruminococcus) prevalence \|\| id:ebi-gm430-GCST90011679 | Plum liking \|\| id:ebi-fl187-GCST90094812 | MR Egger | 5 | -0.525061976 | 1.34286019494158 | 0.721909803789682 | -3.157067958 | 2.10694400608034 | 0.591518703264567 | 0.0425503176740435 | 8.22307318577876 | 0.999950574162623 |
| ebi-fl187-GCST90094812 | ebi-gm430-GCST90011684 | TestASV_11 (Lachnospiraceae) prevalence \|\| id:ebi-gm430-GCST90011684 | Plum liking \|\| id:ebi-fl187-GCST90094812 | MR Egger | 5 | -2.472977254 | 1.36605324084013 | 0.167938837082696 | -5.150441606 | 0.204487098089763 | 0.0843334029054801 | 0.00579684424030241 | 1.2268956264464 | 0.999950574162623 |
| ebi-fl187-GCST90094812 | ebi-gm430-GCST90011688 | TestASV_15 (Bacteroides) prevalence \|\| id:ebi-gm430-GCST90011688 | Plum liking \|\| id:ebi-fl187-GCST90094812 | MR Egger | 5 | -0.03680001 | 1.28776914951317 | 0.978997035013997 | -2.560827543 | 2.48722752331665 | 0.963868880473299 | 0.0772407939267634 | 12.0278828260845 | 0.999950574162623 |
| ebi-fl187-GCST90094812 | ebi-gm430-GCST90011692 | TestASV_19 (Prevotella) prevalence \|\| id:ebi-gm430-GCST90011692 | Plum liking \|\| id:ebi-fl187-GCST90094812 | MR Egger | 5 | -0.380385847 | 2.5253112954467 | 0.889826150779326 | -5.329995986 | 4.569224293 | 0.683597594559508 | 0.00484408945829334 | 96.4692488260089 | 0.999950574162623 |
| ebi-fl187-GCST90094812 | ebi-gm430-GCST90011693 | TestASV_2 (Bacteroides) prevalence \|\| id:ebi-gm430-GCST90011693 | Plum liking \|\| id:ebi-fl187-GCST90094812 | MR Egger | 5 | 0.406433553843844 | 1.26100202128079 | 0.768372860553905 | -2.065130408 | 2.8779975155542 | 1.501453372 | 0.12680175354359 | 17.7786360679462 | 0.999950574162623 |
| ebi-fl187-GCST90094812 | ebi-gm430-GCST90011716 | TestASV_40 (Barnesiella) prevalence \|\| id:ebi-gm430-GCST90011716 | Plum liking \|\| id:ebi-fl187-GCST90094812 | MR Egger | 5 | 3.70934043929723 | 2.53755227971522 | 0.2399612963447 | -1.264262029 | 8.68294290753906 | 40.8268698458111 | 0.282447657444619 | 5901.38830141873 | 0.999950574162623 |
| ebi-fl187-GCST90094812 | ebi-gm430-GCST90011718 | TestASV_42 (Alphaproteobacteria) prevalence \|\| id:ebi-gm430-GCST90011718 | Plum liking \|\| id:ebi-fl187-GCST90094812 | MR Egger | 5 | 2.42043880850198 | 2.00659152159266 | 0.314173698525573 | -1.512480574 | 6.3533581908236 | 11.2507951764311 | 0.220362673546695 | 574.418480519947 | 0.999950574162623 |
| ebi-fl187-GCST90094812 | ebi-hbsm44-GCST90104929 | Sulcal depth of the right inferior frontal sulcus \|\| id:ebi-hbsm44-GCST90104929 | Plum liking \|\| id:ebi-fl187-GCST90094812 | MR Egger | 4 | 0.258785264949455 | 1.48156143013747 | 0.877420644683782 | -2.645075138 | 3.16264566801891 | 1.29535561670692 | 0.0710000187312812 | 23.6330384655926 | 0.999950574162623 |
| ebi-fl187-GCST90094812 | ebi-hbsm44-GCST90104935 | Sulcal opening distance of the left calloso-marginal posterior fissure \|\| id:ebi-hbsm44-GCST90104935 | Plum liking \|\| id:ebi-fl187-GCST90094812 | MR Egger | 5 | -0.150107011 | 0.246293949985615 | 0.585274554972934 | -0.632843153 | 0.332629130633235 | 0.860615875840374 | 0.531079711434606 | 1.39462997700995 | 0.999950574162623 |
| ebi-fl187-GCST90094812 | ebi-hbsm44-GCST90104936 | Sulcal opening distance of the left anterior inferior temporal sulcus \|\| id:ebi-hbsm44-GCST90104936 | Plum liking \|\| id:ebi-fl187-GCST90094812 | MR Egger | 5 | -0.016917206 | 0.140527458861878 | 0.911789130399566 | -0.292351025 | 0.258516613588516 | 0.983225086618435 | 0.746506447438492 | 1.29500766439862 | 0.999950574162623 |
| ebi-fl187-GCST90094812 | ebi-hbsm44-GCST90104941 | Sulcal opening distance of the right posterior inferior temporal sulcus \|\| id:ebi-hbsm44-GCST90104941 | Plum liking \|\| id:ebi-fl187-GCST90094812 | MR Egger | 5 | 0.00264742622407818 | 0.1282729087076 | 0.984829590362386 | -0.248767475 | 0.254062327290975 | 1.00265093375151 | 0.779761266417326 | 1.28925215735804 | 0.999950574162623 |
| ebi-fl187-GCST90094812 | ebi-IPs91-GCST90274819 | Leukemia inhibitory factor levels \|\| id:ebi-IPs91-GCST90274819 | Plum liking \|\| id:ebi-fl187-GCST90094812 | MR Egger | 5 | 0.704853658613762 | 0.658924032268274 | 0.363172126305815 | -0.586637445 | 1.99634476185958 | 2.02355053406304 | 0.556194378264842 | 7.36209664089235 | 0.999950574162623 |
| ebi-fl187-GCST90094812 | ebi-IPs91-GCST90274837 | STAM binding protein levels \|\| id:ebi-IPs91-GCST90274837 | Plum liking \|\| id:ebi-fl187-GCST90094812 | MR Egger | 5 | -0.35517863 | 0.424993809277617 | 0.464639370073765 | -1.188166496 | 0.477809236585178 | 0.701048204063659 | 0.304779566705963 | 1.61253784081604 | 0.999950574162623 |
| ebi-fl187-GCST90094812 | ebi-IPs91-GCST90274840 | TNF-beta levels \|\| id:ebi-IPs91-GCST90274840 | Plum liking \|\| id:ebi-fl187-GCST90094812 | MR Egger | 5 | 0.864051261179669 | 0.659369715741313 | 0.281351449043123 | -0.428313382 | 2.15641590403264 | 2.37275389404471 | 0.651607181050112 | 8.6401150960787 | 0.999950574162623 |
| ebi-fl187-GCST90094812 | ebi-IPs91-GCST90274842 | Tumor necrosis factor ligand superfamily member 14 levels \|\| id:ebi-IPs91-GCST90274842 | Plum liking \|\| id:ebi-fl187-GCST90094812 | MR Egger | 5 | -0.136104979 | 0.483235194035013 | 0.796520094034576 | -1.083245959 | 0.811036001380458 | 0.872751007214148 | 0.338494999509471 | 2.25023802920905 | 0.999950574162623 |
| ebi-fl187-GCST90094812 | ebi-met1400-GCST90199628 | 3-methylhistidine levels \|\| id:ebi-met1400-GCST90199628 | Plum liking \|\| id:ebi-fl187-GCST90094812 | MR Egger | 5 | 0.29015609149187 | 0.563480329919756 | 0.642136083465681 | -0.814265355 | 1.39457753813459 | 1.33663610926743 | 0.442964629539382 | 4.03327030976576 | 0.999950574162623 |
| ebi-fl187-GCST90094812 | ebi-met1400-GCST90199651 | Docosahexaenoate DHA; 22:6n3 levels \|\| id:ebi-met1400-GCST90199651 | Plum liking \|\| id:ebi-fl187-GCST90094812 | MR Egger | 5 | 0.862553654999202 | 0.540359772998216 | 0.208707313665347 | -0.1965515 | 1.92165881007571 | 2.36920310265548 | 0.821559019852712 | 6.83228253356488 | 0.999950574162623 |
| ebi-fl187-GCST90094812 | ebi-met1400-GCST90199652 | 1-stearoyl-2-oleoyl-GPS (18:0/18:1) levels \|\| id:ebi-met1400-GCST90199652 | Plum liking \|\| id:ebi-fl187-GCST90094812 | MR Egger | 5 | -0.018180787 | 1.30530342718689 | 0.989761602907432 | -2.576575504 | 2.54021393028718 | 0.981983486462071 | 0.0760339365898543 | 12.6823838266566 | 0.999950574162623 |
| ebi-fl187-GCST90094812 | ebi-met1400-GCST90199653 | 1-myristoyl-2-palmitoyl-gpc (14:0/16:0) levels \|\| id:ebi-met1400-GCST90199653 | Plum liking \|\| id:ebi-fl187-GCST90094812 | MR Egger | 5 | -0.100781748 | 0.561300760984455 | 0.868948127727305 | -1.200931239 | 0.999367743897049 | 0.904130339940951 | 0.300913858624938 | 2.71656372138254 | 0.999950574162623 |
| ebi-fl187-GCST90094812 | ebi-met1400-GCST90199667 | Erythritol levels in elite athletes \|\| id:ebi-met1400-GCST90199667 | Plum liking \|\| id:ebi-fl187-GCST90094812 | MR Egger | 5 | 0.657889719903452 | 0.530648651348247 | 0.30319457476692 | -0.382181637 | 1.69796107654602 | 1.93071368570699 | 0.68237109828719 | 5.46279780244648 | 0.999950574162623 |
| ebi-fl187-GCST90094812 | ebi-met1400-GCST90199674 | 3-hydroxymyristate levels \|\| id:ebi-met1400-GCST90199674 | Plum liking \|\| id:ebi-fl187-GCST90094812 | MR Egger | 5 | 0.132554442708482 | 0.618168739951329 | 0.843959518128561 | -1.079056288 | 1.34416517301309 | 1.14174117387904 | 0.339916157423088 | 3.83498365600831 | 0.999950574162623 |
| ebi-fl187-GCST90094812 | ebi-met1400-GCST90199685 | 2-linoleoylglycerol (18:2) levels \|\| id:ebi-met1400-GCST90199685 | Plum liking \|\| id:ebi-fl187-GCST90094812 | MR Egger | 5 | -0.002111407 | 0.64638510322236 | 0.997598796397074 | -1.269026209 | 1.26480339576912 | 0.997890820904134 | 0.281105226318005 | 3.54239621755814 | 0.999950574162623 |
| ebi-fl187-GCST90094812 | ebi-met1400-GCST90199703 | Gamma-glutamylvaline levels \|\| id:ebi-met1400-GCST90199703 | Plum liking \|\| id:ebi-fl187-GCST90094812 | MR Egger | 5 | 0.100171250233991 | 0.616348592804731 | 0.881223750292662 | -1.107871992 | 1.30821449213126 | 1.10536019506037 | 0.330261012011989 | 3.69956221408159 | 0.999950574162623 |
| ebi-fl187-GCST90094812 | ebi-met1400-GCST90199779 | Gamma-glutamyl-2-aminobutyrate levels \|\| id:ebi-met1400-GCST90199779 | Plum liking \|\| id:ebi-fl187-GCST90094812 | MR Egger | 5 | -0.003742761 | 0.585435586730307 | 0.995300425972915 | -1.151196511 | 1.14371098924122 | 0.99626423464874 | 0.316258136647546 | 3.13839332565972 | 0.999950574162623 |
| ebi-fl187-GCST90094812 | ebi-met1400-GCST90199824 | 4-vinylphenol sulfate levels \|\| id:ebi-met1400-GCST90199824 | Plum liking \|\| id:ebi-fl187-GCST90094812 | MR Egger | 5 | -0.203317949 | 0.584324774152541 | 0.750858525918079 | -1.348594506 | 0.941958608659145 | 0.816018748091338 | 0.259604877445246 | 2.56500033354342 | 0.999950574162623 |
| ebi-fl187-GCST90094812 | ebi-met1400-GCST90199830 | 4-ethylphenylsulfate levels \|\| id:ebi-met1400-GCST90199830 | Plum liking \|\| id:ebi-fl187-GCST90094812 | MR Egger | 5 | -0.473223318 | 0.566383574624186 | 0.464740574935717 | -1.583335124 | 0.636888488668678 | 0.622990930805786 | 0.205289290004016 | 1.89058912843757 | 0.999950574162623 |
| ebi-fl187-GCST90094812 | ebi-met1400-GCST90199833 | Hexanoylglutamine levels \|\| id:ebi-met1400-GCST90199833 | Plum liking \|\| id:ebi-fl187-GCST90094812 | MR Egger | 5 | 0.763241194491346 | 0.645350538159046 | 0.322130476589081 | -0.50164586 | 2.02812824928308 | 2.14521803340938 | 0.605533216030486 | 7.5998480166497 | 0.999950574162623 |
| ebi-fl187-GCST90094812 | ebi-met1400-GCST90199837 | Carotenoid levels (cryptoxanthin) \|\| id:ebi-met1400-GCST90199837 | Plum liking \|\| id:ebi-fl187-GCST90094812 | MR Egger | 5 | 1.32842049310125 | 0.964814247437197 | 0.262310304418145 | -0.562615432 | 3.21945641807816 | 3.77507591752435 | 0.569717057426642 | 25.0145190446704 | 0.999950574162623 |
| ebi-fl187-GCST90094812 | ebi-met1400-GCST90199869 | 2-hydroxyhippurate levels \|\| id:ebi-met1400-GCST90199869 | Plum liking \|\| id:ebi-fl187-GCST90094812 | MR Egger | 5 | -0.053093824 | 0.661857863398343 | 0.941114486515055 | -1.350335237 | 1.24414758777147 | 0.948291035386162 | 0.259153368348994 | 3.46997568861524 | 0.999950574162623 |
| ebi-fl187-GCST90094812 | ebi-met1400-GCST90199902 | Hydantoin-5-propionate levels \|\| id:ebi-met1400-GCST90199902 | Plum liking \|\| id:ebi-fl187-GCST90094812 | MR Egger | 5 | 0.0470846843998901 | 0.564898487034294 | 0.938822864944873 | -1.06011635 | 1.15428571898711 | 1.04821077242139 | 0.346415502476584 | 3.17175708236243 | 0.999950574162623 |
| ebi-fl187-GCST90094812 | ebi-met1400-GCST90199904 | Pantoate levels \|\| id:ebi-met1400-GCST90199904 | Plum liking \|\| id:ebi-fl187-GCST90094812 | MR Egger | 5 | 0.170065698703163 | 0.572846734348133 | 0.785927727539324 | -0.952713901 | 1.2928452980255 | 1.18538272687008 | 0.385692869687392 | 3.64313763513654 | 0.999950574162623 |
| ebi-fl187-GCST90094812 | ebi-met1400-GCST90199936 | 3-methyl catechol sulfate (2) levels \|\| id:ebi-met1400-GCST90199936 | Plum liking \|\| id:ebi-fl187-GCST90094812 | MR Egger | 5 | -0.639849234 | 0.645475877573481 | 0.394622421848792 | -1.904981954 | 0.625283486236472 | 0.527371927907225 | 0.148825328321871 | 1.86877565452487 | 0.999950574162623 |
| ebi-fl187-GCST90094812 | ebi-met1400-GCST90199977 | 4-hydroxychlorothalonil levels \|\| id:ebi-met1400-GCST90199977 | Plum liking \|\| id:ebi-fl187-GCST90094812 | MR Egger | 5 | -0.274182011 | 0.5607464563777 | 0.658387042604145 | -1.373245066 | 0.824881043010332 | 0.760193698680445 | 0.253283702185151 | 2.28160933580722 | 0.999950574162623 |
| ebi-fl187-GCST90094812 | ebi-met1400-GCST90200040 | Sphingomyelin (d18:2/24:1, d18:1/24:2) levels \|\| id:ebi-met1400-GCST90200040 | Plum liking \|\| id:ebi-fl187-GCST90094812 | MR Egger | 5 | 0.0683303688168273 | 0.647977294993466 | 0.922672758453035 | -1.201705129 | 1.33836586700402 | 1.07071898221748 | 0.300681074422516 | 3.81280777675373 | 0.999950574162623 |
| ebi-fl187-GCST90094812 | ebi-met1400-GCST90200077 | 1-stearoyl-2-oleoyl-GPI (18:0/18:1) levels \|\| id:ebi-met1400-GCST90200077 | Plum liking \|\| id:ebi-fl187-GCST90094812 | MR Egger | 5 | 0.730985713029415 | 0.640353925908185 | 0.336513045234396 | -0.524107982 | 1.98607940780946 | 2.07712705010298 | 0.592083277966622 | 7.28690868805911 | 0.999950574162623 |
| ebi-fl187-GCST90094812 | ebi-met1400-GCST90200080 | 1-palmitoyl-2-oleoyl-GPI (16:0/18:1) levels \|\| id:ebi-met1400-GCST90200080 | Plum liking \|\| id:ebi-fl187-GCST90094812 | MR Egger | 5 | -0.015963653 | 0.563525051186118 | 0.979179502661178 | -1.120472753 | 1.08854544760666 | 0.984163091058465 | 0.326125581312565 | 2.96995097993693 | 0.999950574162623 |
| ebi-fl187-GCST90094812 | ebi-met1400-GCST90200082 | 1-oleoyl-2-linoleoyl-GPE (18:1/18:2) levels \|\| id:ebi-met1400-GCST90200082 | Plum liking \|\| id:ebi-fl187-GCST90094812 | MR Egger | 5 | 0.00764951155653495 | 0.567497611324282 | 0.99009164350272 | -1.104645807 | 1.11994482975213 | 1.00767884381484 | 0.331328215715685 | 3.06468511919116 | 0.999950574162623 |
| ebi-fl187-GCST90094812 | ebi-met1400-GCST90200100 | Perfluorooctanesulfonate (PFOS) levels \|\| id:ebi-met1400-GCST90200100 | Plum liking \|\| id:ebi-fl187-GCST90094812 | MR Egger | 5 | -0.582631412 | 0.520667562953801 | 0.344658010400615 | -1.603139835 | 0.437877011696189 | 0.558426980217235 | 0.20126359038851 | 1.54941433585964 | 0.999950574162623 |
| ebi-fl187-GCST90094812 | ebi-met1400-GCST90200102 | 1-palmitoleoyl-2-linolenoyl-GPC (16:1/18:3) levels \|\| id:ebi-met1400-GCST90200102 | Plum liking \|\| id:ebi-fl187-GCST90094812 | MR Egger | 5 | 0.18450819548656 | 0.553933802559984 | 0.760988703938929 | -0.901202058 | 1.27021844850413 | 1.20262683735657 | 0.406081233236419 | 3.56163051023887 | 0.999950574162623 |
| ebi-fl187-GCST90094812 | ebi-met1400-GCST90200114 | Glycosyl-N-tricosanoyl-sphingadienine (d18:2/23:0) levels \|\| id:ebi-met1400-GCST90200114 | Plum liking \|\| id:ebi-fl187-GCST90094812 | MR Egger | 5 | 0.253977139083404 | 0.591778204884989 | 0.696749629949534 | -0.905908142 | 1.41386242065798 | 1.28914233295558 | 0.404174670194666 | 4.11180629854347 | 0.999950574162623 |
| ebi-fl187-GCST90094812 | ebi-met1400-GCST90200135 | Arachidoylcarnitine (C20) levels \|\| id:ebi-met1400-GCST90200135 | Plum liking \|\| id:ebi-fl187-GCST90094812 | MR Egger | 5 | 0.577843410567112 | 0.579456980514898 | 0.392155255702534 | -0.557892271 | 1.71357909237631 | 1.78219082948293 | 0.572414287315363 | 5.5487855965118 | 0.999950574162623 |
| ebi-fl187-GCST90094812 | ebi-met1400-GCST90200146 | Docosahexaenoylcarnitine (C22:6) levels \|\| id:ebi-met1400-GCST90200146 | Plum liking \|\| id:ebi-fl187-GCST90094812 | MR Egger | 5 | -0.08626097 | 0.632086412207578 | 0.900092922000692 | -1.325150338 | 1.15262839800843 | 0.917354797972282 | 0.265763001848408 | 3.16650481635809 | 0.999950574162623 |
| ebi-fl187-GCST90094812 | ebi-met1400-GCST90200162 | 3-hydroxyphenylacetoylglutamine levels \|\| id:ebi-met1400-GCST90200162 | Plum liking \|\| id:ebi-fl187-GCST90094812 | MR Egger | 5 | -0.316501442 | 0.686576338181287 | 0.676174515653402 | -1.662191065 | 1.02918818042056 | 0.7286939604801 | 0.189722829262841 | 2.7987927973841 | 0.999950574162623 |
| ebi-fl187-GCST90094812 | ebi-met1400-GCST90200172 | 3-carboxy-4-methyl-5-pentyl-2-furanpropionate (3-CMPFP) levels \|\| id:ebi-met1400-GCST90200172 | Plum liking \|\| id:ebi-fl187-GCST90094812 | MR Egger | 5 | -0.166726008 | 0.636379336330451 | 0.81028709132849 | -1.414029507 | 1.08057749125319 | 0.846431495065307 | 0.243161485454252 | 2.9463805688639 | 0.999950574162623 |
| ebi-fl187-GCST90094812 | ebi-met1400-GCST90200222 | Dihydrocaffeate sulfate (2) levels \|\| id:ebi-met1400-GCST90200222 | Plum liking \|\| id:ebi-fl187-GCST90094812 | MR Egger | 5 | 0.957127601460805 | 0.640300941940576 | 0.231833589793087 | -0.297862245 | 2.21211744766433 | 2.60420540410685 | 0.742403602703275 | 9.13503889539973 | 0.999950574162623 |
| ebi-fl187-GCST90094812 | ebi-met1400-GCST90200223 | 4-methylhexanoylglutamine levels \|\| id:ebi-met1400-GCST90200223 | Plum liking \|\| id:ebi-fl187-GCST90094812 | MR Egger | 5 | 0.614636743137404 | 0.674967539756989 | 0.429636523597106 | -0.708299635 | 1.9375731210611 | 1.84898482112654 | 0.492480883285995 | 6.94188340051973 | 0.999950574162623 |
| ebi-fl187-GCST90094812 | ebi-met1400-GCST90200224 | 2-ketocaprylate levels \|\| id:ebi-met1400-GCST90200224 | Plum liking \|\| id:ebi-fl187-GCST90094812 | MR Egger | 5 | -0.927902045 | 0.572137189808847 | 0.203293696001158 | -2.049290937 | 0.193486846854592 | 0.395382335138414 | 0.128826217089181 | 1.21347342545412 | 0.999950574162623 |
| ebi-fl187-GCST90094812 | ebi-met1400-GCST90200243 | Hydroxypalmitoyl sphingomyelin (d18:1/16:0(OH)) levels \|\| id:ebi-met1400-GCST90200243 | Plum liking \|\| id:ebi-fl187-GCST90094812 | MR Egger | 5 | 0.0970210993332021 | 0.612346416090455 | 0.88417370479445 | -1.103177876 | 1.29722007487049 | 1.10188362238544 | 0.331814939638527 | 3.65911046260886 | 0.999950574162623 |
| ebi-fl187-GCST90094812 | ebi-met1400-GCST90200273 | 2-methoxyhydroquinone sulfate (2) levels \|\| id:ebi-met1400-GCST90200273 | Plum liking \|\| id:ebi-fl187-GCST90094812 | MR Egger | 5 | 1.30041841204066 | 0.797272583638516 | 0.201370046541492 | -0.262235852 | 2.86307267597215 | 3.67083226675991 | 0.769329554478391 | 17.5152630654126 | 0.999950574162623 |
| ebi-fl187-GCST90094812 | ebi-met1400-GCST90200276 | 2,4-di-tert-butylphenol levels \|\| id:ebi-met1400-GCST90200276 | Plum liking \|\| id:ebi-fl187-GCST90094812 | MR Egger | 5 | -0.010422588 | 1.02599092297493 | 0.992532563045469 | -2.021364797 | 2.00051962149731 | 0.989631539421032 | 0.132474540847597 | 7.39289660904388 | 0.999950574162623 |
| ebi-fl187-GCST90094812 | ebi-met1400-GCST90200289 | N-acetylmethionine levels \|\| id:ebi-met1400-GCST90200289 | Plum liking \|\| id:ebi-fl187-GCST90094812 | MR Egger | 5 | 0.222463196867313 | 0.562837197700363 | 0.719083917101455 | -0.880697711 | 1.32562410436003 | 1.24914984617714 | 0.414493614171577 | 3.764534084 | 0.999950574162623 |
| ebi-fl187-GCST90094812 | ebi-met1400-GCST90200308 | 3-hydroxyisobutyrate levels \|\| id:ebi-met1400-GCST90200308 | Plum liking \|\| id:ebi-fl187-GCST90094812 | MR Egger | 5 | 0.794283559288474 | 0.570545509917916 | 0.25812840929637 | -0.32398564 | 1.91255275872759 | 2.21285504930721 | 0.723260628218942 | 6.77034982714708 | 0.999950574162623 |
| ebi-fl187-GCST90094812 | ebi-met1400-GCST90200383 | Cystathionine levels \|\| id:ebi-met1400-GCST90200383 | Plum liking \|\| id:ebi-fl187-GCST90094812 | MR Egger | 5 | -0.331932512 | 0.862867143526333 | 0.726120716553649 | -2.023152114 | 1.35928708898479 | 0.71753574603836 | 0.132237978307976 | 3.89341665254248 | 0.999950574162623 |
| ebi-fl187-GCST90094812 | ebi-met1400-GCST90200384 | Guanidinoacetate levels \|\| id:ebi-met1400-GCST90200384 | Plum liking \|\| id:ebi-fl187-GCST90094812 | MR Egger | 5 | -0.61101067 | 0.540175064303682 | 0.340249257281408 | -1.669753796 | 0.447732456526638 | 0.542801998328851 | 0.188293418611095 | 1.56475999832122 | 0.999950574162623 |
| ebi-fl187-GCST90094812 | ebi-met1400-GCST90200403 | Citrulline levels \|\| id:ebi-met1400-GCST90200403 | Plum liking \|\| id:ebi-fl187-GCST90094812 | MR Egger | 5 | -0.359956956 | 0.772240683913626 | 0.67288743538562 | -1.873548697 | 1.15363478428552 | 0.697706357367936 | 0.153577693003662 | 3.16969314742882 | 0.999950574162623 |
| ebi-fl187-GCST90094812 | ebi-met1400-GCST90200434 | 12,13-DiHOME levels \|\| id:ebi-met1400-GCST90200434 | Plum liking \|\| id:ebi-fl187-GCST90094812 | MR Egger | 5 | -1.252247771 | 0.748428395249889 | 0.192887351681207 | -2.719167425 | 0.214671883957037 | 0.285861522999938 | 0.0659296229101185 | 1.23945514512116 | 0.999950574162623 |
| ebi-fl187-GCST90094812 | ebi-met1400-GCST90200437 | Fructose levels \|\| id:ebi-met1400-GCST90200437 | Plum liking \|\| id:ebi-fl187-GCST90094812 | MR Egger | 5 | 0.494028492018834 | 0.568604846128703 | 0.448860868345781 | -0.620437006 | 1.60849399043109 | 1.63890525584795 | 0.537709403795516 | 4.99528261675614 | 0.999950574162623 |
| ebi-fl187-GCST90094812 | ebi-met1400-GCST90200448 | N-stearoyl-sphinganine (d18:0/18:0) levels \|\| id:ebi-met1400-GCST90200448 | Plum liking \|\| id:ebi-fl187-GCST90094812 | MR Egger | 5 | 0.679347923597386 | 0.627760308303247 | 0.35839816715036 | -0.551062281 | 1.90975812787175 | 1.97259103269841 | 0.576337253156788 | 6.75145561209043 | 0.999950574162623 |
| ebi-fl187-GCST90094812 | ebi-met1400-GCST90200468 | X-11847 levels \|\| id:ebi-met1400-GCST90200468 | Plum liking \|\| id:ebi-fl187-GCST90094812 | MR Egger | 5 | -0.662765425 | 0.747916833574213 | 0.440805418967685 | -2.128682419 | 0.803151568687093 | 0.51542399533825 | 0.118993974819206 | 2.23256593767945 | 0.999950574162623 |
| ebi-fl187-GCST90094812 | ebi-met1400-GCST90200476 | X-11843 levels \|\| id:ebi-met1400-GCST90200476 | Plum liking \|\| id:ebi-fl187-GCST90094812 | MR Egger | 5 | -0.075592111 | 0.596206451171304 | 0.907128479298052 | -1.244156755 | 1.09297253379023 | 0.927194322262819 | 0.288183815319834 | 2.98312835605394 | 0.999950574162623 |
| ebi-fl187-GCST90094812 | ebi-met1400-GCST90200511 | X-12906 levels \|\| id:ebi-met1400-GCST90200511 | Plum liking \|\| id:ebi-fl187-GCST90094812 | MR Egger | 5 | -0.036094891 | 0.566213025253415 | 0.953180855383129 | -1.145872421 | 1.07368263847328 | 0.964548762121272 | 0.317946413782247 | 2.92613558191241 | 0.999950574162623 |
| ebi-fl187-GCST90094812 | ebi-met1400-GCST90200515 | X-12849 levels \|\| id:ebi-met1400-GCST90200515 | Plum liking \|\| id:ebi-fl187-GCST90094812 | MR Egger | 5 | -0.225552524 | 0.941354454504304 | 0.826074755780634 | -2.070607254 | 1.61950220730584 | 0.798075141629303 | 0.12610917810135 | 5.05057555108921 | 0.999950574162623 |
| ebi-fl187-GCST90094812 | ebi-met1400-GCST90200551 | X-18886 levels \|\| id:ebi-met1400-GCST90200551 | Plum liking \|\| id:ebi-fl187-GCST90094812 | MR Egger | 5 | -0.313847722 | 0.691652156357289 | 0.680812460441483 | -1.669485949 | 1.04179050404138 | 0.730630278300208 | 0.188343859130096 | 2.8342872766577 | 0.999950574162623 |
| ebi-fl187-GCST90094812 | ebi-met1400-GCST90200564 | X-21319 levels \|\| id:ebi-met1400-GCST90200564 | Plum liking \|\| id:ebi-fl187-GCST90094812 | MR Egger | 5 | -0.75224341 | 0.570923709926604 | 0.279228121329709 | -1.871253882 | 0.366767061167808 | 0.47130802855271 | 0.153930530076673 | 1.44306173484622 | 0.999950574162623 |
| ebi-fl187-GCST90094812 | ebi-met1400-GCST90200579 | X-21442 levels \|\| id:ebi-met1400-GCST90200579 | Plum liking \|\| id:ebi-fl187-GCST90094812 | MR Egger | 5 | 0.197711322721041 | 0.615927415211733 | 0.769274243743995 | -1.009506411 | 1.40492905653604 | 1.21861055786061 | 0.364398798393848 | 4.0752376195388 | 0.999950574162623 |
| ebi-fl187-GCST90094812 | ebi-met1400-GCST90200581 | X-21441 levels \|\| id:ebi-met1400-GCST90200581 | Plum liking \|\| id:ebi-fl187-GCST90094812 | MR Egger | 5 | -0.204122 | 0.566750019619292 | 0.742589436594018 | -1.314952039 | 0.906708038367519 | 0.815362890776072 | 0.268487199968515 | 2.47615768547877 | 0.999950574162623 |
| ebi-fl187-GCST90094812 | ebi-met1400-GCST90200648 | X-24585 levels \|\| id:ebi-met1400-GCST90200648 | Plum liking \|\| id:ebi-fl187-GCST90094812 | MR Egger | 5 | 0.480592042469271 | 0.902624413857069 | 0.631334771741219 | -1.288551809 | 2.24973589362913 | 1.61703147015698 | 0.275669716641889 | 9.48523039574495 | 0.999950574162623 |
| ebi-fl187-GCST90094812 | ebi-met1400-GCST90200720 | Adenosine 5'-diphosphate (ADP) to pantothenate ratio \|\| id:ebi-met1400-GCST90200720 | Plum liking \|\| id:ebi-fl187-GCST90094812 | MR Egger | 5 | -0.225193789 | 0.748109582685116 | 0.783058664485094 | -1.691488571 | 1.24110099310929 | 0.798361490129791 | 0.184245057909429 | 3.45942016656742 | 0.999950574162623 |
| ebi-fl187-GCST90094812 | ebi-met1400-GCST90200724 | Cholate to taurocholate ratio \|\| id:ebi-met1400-GCST90200724 | Plum liking \|\| id:ebi-fl187-GCST90094812 | MR Egger | 5 | 0.364033544595285 | 0.606338491768363 | 0.590578510635511 | -0.824389899 | 1.55245698846128 | 1.43912248812981 | 0.438502441530593 | 4.72306044320264 | 0.999950574162623 |
| ebi-fl187-GCST90094812 | ebi-met1400-GCST90200726 | N-acetylputrescine to (N(1) + N(8))-acetylspermidine ratio \|\| id:ebi-met1400-GCST90200726 | Plum liking \|\| id:ebi-fl187-GCST90094812 | MR Egger | 5 | -0.325534612 | 0.56294672102573 | 0.603634459994207 | -1.428910185 | 0.777840961271993 | 0.722141185127314 | 0.239569866826038 | 2.17676746314576 | 0.999950574162623 |
| ebi-fl187-GCST90094812 | ebi-met1400-GCST90200744 | Arginine to glutamate ratio \|\| id:ebi-met1400-GCST90200744 | Plum liking \|\| id:ebi-fl187-GCST90094812 | MR Egger | 5 | -0.16420281 | 0.544921820541149 | 0.782840034852111 | -1.232249578 | 0.903843958296405 | 0.848569905995093 | 0.291635781696763 | 2.46907591781463 | 0.999950574162623 |
| ebi-fl187-GCST90094812 | ebi-met1400-GCST90200759 | Histidine to trans-urocanate ratio \|\| id:ebi-met1400-GCST90200759 | Plum liking \|\| id:ebi-fl187-GCST90094812 | MR Egger | 5 | -0.348499982 | 0.750495392243668 | 0.674013107159613 | -1.819470951 | 1.12247098688542 | 0.705745927789342 | 0.162111493221362 | 3.07243678220346 | 0.999950574162623 |
| ebi-fl187-GCST90094812 | ebi-met1400-GCST90200795 | Oleoyl-linoleoyl-glycerol (18:1 to 18:2) [2] to linoleoyl-arachidonoyl-glycerol (18:2 to 20:4) [2] ratio \|\| id:ebi-met1400-GCST90200795 | Plum liking \|\| id:ebi-fl187-GCST90094812 | MR Egger | 5 | 0.261941804462955 | 0.586554359800792 | 0.685448882537257 | -0.887704741 | 1.41158834967251 | 1.29945091798486 | 0.411599396708297 | 4.1024663829826 | 0.999950574162623 |
| ebi-fl187-GCST90094812 | ebi-met1400-GCST90200811 | Choline to taurocholate ratio \|\| id:ebi-met1400-GCST90200811 | Plum liking \|\| id:ebi-fl187-GCST90094812 | MR Egger | 5 | 0.927744505301487 | 0.653527402861826 | 0.250795582405603 | -0.353169204 | 2.20865821491067 | 2.52879904760913 | 0.702458324346495 | 9.10349326294625 | 0.999950574162623 |
| ebi-fl187-GCST90094812 | ebi-met1400-GCST90200831 | Cholate to phosphate ratio \|\| id:ebi-met1400-GCST90200831 | Plum liking \|\| id:ebi-fl187-GCST90094812 | MR Egger | 5 | -0.267210851 | 0.726659696220687 | 0.737490372841285 | -1.691463855 | 1.15704215406855 | 0.765511645915974 | 0.184249611748099 | 3.18051188533389 | 0.999950574162623 |
| ebi-fl187-GCST90094812 | ebi-met1400-GCST90200841 | Adenosine 5'-diphosphate (ADP) to oxalate (ethanedioate) ratio \|\| id:ebi-met1400-GCST90200841 | Plum liking \|\| id:ebi-fl187-GCST90094812 | MR Egger | 5 | -0.970002319 | 0.982054846320701 | 0.396108169672725 | -2.894829818 | 0.954825179739666 | 0.379082158992313 | 0.0553084368031969 | 2.59821632235983 | 0.999950574162623 |
| ebi-fl187-GCST90094812 | ebi-met1400-GCST90200844 | Aspartate to phosphate ratio \|\| id:ebi-met1400-GCST90200844 | Plum liking \|\| id:ebi-fl187-GCST90094812 | MR Egger | 5 | 0.154856481780268 | 0.676982629335312 | 0.833773280498313 | -1.172029472 | 1.48174243527748 | 1.16749039293812 | 0.309737699066909 | 4.40060677698896 | 0.999950574162623 |
| ebi-fl187-GCST90094812 | ebi-met1400-GCST90200870 | Adenosine 5'-monophosphate (AMP) to acetoacetate ratio \|\| id:ebi-met1400-GCST90200870 | Plum liking \|\| id:ebi-fl187-GCST90094812 | MR Egger | 5 | -0.618079443 | 0.623098672177109 | 0.394346479988553 | -1.839352841 | 0.603193953990663 | 0.538978583043114 | 0.158920239507263 | 1.82794786793589 | 0.999950574162623 |
| ebi-fl187-GCST90094812 | ebi-met1400-GCST90200887 | Cholesterol to cortisol ratio \|\| id:ebi-met1400-GCST90200887 | Plum liking \|\| id:ebi-fl187-GCST90094812 | MR Egger | 5 | 0.523476642307934 | 0.549515614438122 | 0.411063647366755 | -0.553573962 | 1.60052724660665 | 1.68788563534884 | 0.574891494050701 | 4.95564458249876 | 0.999950574162623 |
| ebi-fl187-GCST90094812 | ebi-met1400-GCST90200890 | Cortisol to taurocholate ratio \|\| id:ebi-met1400-GCST90200890 | Plum liking \|\| id:ebi-fl187-GCST90094812 | MR Egger | 5 | 0.916041476719231 | 0.590085235635353 | 0.218378454745701 | -0.240525585 | 2.07260853856452 | 2.4993769397664 | 0.786214529792932 | 7.94552231015285 | 0.999950574162623 |
| ebi-fl187-GCST90094812 | ebi-met1400-GCST90200948 | Adenosine 5'-diphosphate (ADP) to mannitol to sorbitol ratio \|\| id:ebi-met1400-GCST90200948 | Plum liking \|\| id:ebi-fl187-GCST90094812 | MR Egger | 5 | -0.350487533 | 0.756055566478677 | 0.674516216416062 | -1.832356443 | 1.13138137735075 | 0.704344614791771 | 0.160036007331466 | 3.09993572483127 | 0.999950574162623 |
| ebi-fl187-GCST90094812 | ebi-met1400-GCST90200972 | Leucine to N-palmitoyl-sphingosine (d18:1 to 16:0) ratio \|\| id:ebi-met1400-GCST90200972 | Plum liking \|\| id:ebi-fl187-GCST90094812 | MR Egger | 5 | -0.672144225 | 0.545046544032691 | 0.305322549781108 | -1.740435452 | 0.39614700102053 | 0.510612534817709 | 0.175443986618395 | 1.48608775791253 | 0.999950574162623 |
| ebi-fl187-GCST90094812 | ebi-met1400-GCST90200973 | Proline to glutamate ratio \|\| id:ebi-met1400-GCST90200973 | Plum liking \|\| id:ebi-fl187-GCST90094812 | MR Egger | 5 | -0.285242751 | 0.554086525530516 | 0.642221357588027 | -1.371252341 | 0.800766839457382 | 0.751831724576649 | 0.253788930267021 | 2.22724821561436 | 0.999950574162623 |
| ebi-fl187-GCST90094812 | ebi-met1400-GCST90200983 | Cholesterol to linoleoyl-arachidonoyl-glycerol (18:2 to 20:4) [1] ratio \|\| id:ebi-met1400-GCST90200983 | Plum liking \|\| id:ebi-fl187-GCST90094812 | MR Egger | 5 | 0.228944953758901 | 0.607874550658274 | 0.731507983683493 | -0.962489166 | 1.42037907304912 | 1.25727282896171 | 0.381940987409199 | 4.13868900839342 | 0.999950574162623 |
| ebi-fl187-GCST90094812 | ebi-met1400-GCST90201001 | Serine to alpha-ketobutyrate ratio \|\| id:ebi-met1400-GCST90201001 | Plum liking \|\| id:ebi-fl187-GCST90094812 | MR Egger | 5 | -0.819317002 | 0.874147897729289 | 0.417760301402098 | -2.532646881 | 0.894012877606459 | 0.44073257122136 | 0.0794484510981841 | 2.44492116146277 | 0.999950574162623 |
| ebi-fl187-GCST90094812 | ebi-met1400-GCST90201005 | Fructose to maltose ratio \|\| id:ebi-met1400-GCST90201005 | Plum liking \|\| id:ebi-fl187-GCST90094812 | MR Egger | 5 | 0.0507484900129373 | 0.803015694523046 | 0.953584458517314 | -1.523162271 | 1.62465925127811 | 1.05205825684668 | 0.218021353042792 | 5.07668886717727 | 0.999950574162623 |
| ebi-fl187-GCST90094812 | ebi-met1400-GCST90201020 | Paraxanthine to 5-acetylamino-6-formylamino-3-methyluracil ratio \|\| id:ebi-met1400-GCST90201020 | Plum liking \|\| id:ebi-fl187-GCST90094812 | MR Egger | 5 | 0.857938810324195 | 0.597652542614538 | 0.24664594381314 | -0.313460173 | 2.02933779384869 | 2.35829478778192 | 0.730913488369034 | 7.60904593304123 | 0.999950574162623 |
| ebi-fl187-GCST90094812 | ebi-pw11-GCST90275197 | Placental weight (fetal genotype, adjusted for sex) \|\| id:ebi-pw11-GCST90275197 | Plum liking \|\| id:ebi-fl187-GCST90094812 | MR Egger | 5 | 0.472448472986327 | 0.202553183156828 | 0.101918451762067 | 0.0754442339989451 | 0.869452711973709 | 1.60391653569299 | 1.07836309004403 | 2.38560488319788 | 0.999950574162623 |
| ebi-fl187-GCST90094812 | prot-a-1017 | FAS-associated factor 2 \|\| id:prot-a-1017 | Plum liking \|\| id:ebi-fl187-GCST90094812 | MR Egger | 5 | -1.205196164 | 0.909037144151468 | 0.27681926673138 | -2.986908966 | 0.576516638773479 | 0.299633216568492 | 0.0504431173682828 | 1.7798278368821 | 0.999950574162623 |
| ebi-fl187-GCST90094812 | prot-a-1019 | Fas apoptotic inhibitory molecule 3 \|\| id:prot-a-1019 | Plum liking \|\| id:ebi-fl187-GCST90094812 | MR Egger | 5 | 0.178323378552077 | 0.974411343318073 | 0.866462423866852 | -1.731522854 | 2.0881696114555 | 1.19521176459755 | 0.17701463709926 | 8.07013016348101 | 0.999950574162623 |
| ebi-fl187-GCST90094812 | prot-a-1052 | MIP18 family protein FAM96A \|\| id:prot-a-1052 | Plum liking \|\| id:ebi-fl187-GCST90094812 | MR Egger | 5 | -1.485984923 | 0.908953058710118 | 0.200597907352591 | -3.267532918 | 0.295563072186683 | 0.22627936316345 | 0.0381003078070477 | 1.34388284874132 | 0.999950574162623 |
| ebi-fl187-GCST90094812 | prot-a-1066 | F-box/LRR-repeat protein 4 \|\| id:prot-a-1066 | Plum liking \|\| id:ebi-fl187-GCST90094812 | MR Egger | 5 | 0.323658935428782 | 0.953447290973273 | 0.756636878421709 | -1.545097755 | 2.1924156257364 | 1.38217581551764 | 0.213291019965963 | 8.95682333605383 | 0.999950574162623 |
| ebi-fl187-GCST90094812 | prot-a-1076 | Low affinity immunoglobulin gamma Fc region receptor III-B \|\| id:prot-a-1076 | Plum liking \|\| id:ebi-fl187-GCST90094812 | MR Egger | 5 | -0.168793348 | 0.924866666945114 | 0.866822402404251 | -1.981532015 | 1.64394531955199 | 0.844683441160401 | 0.137857875132189 | 5.1755484776363 | 0.999950574162623 |
| ebi-fl187-GCST90094812 | prot-a-1081 | Fc receptor-like protein 3 \|\| id:prot-a-1081 | Plum liking \|\| id:ebi-fl187-GCST90094812 | MR Egger | 5 | -0.166153566 | 1.19847687650261 | 0.898519814010773 | -2.515168244 | 2.18286111177949 | 0.846916166534357 | 0.0808493086423389 | 8.87165277207619 | 0.999950574162623 |
| ebi-fl187-GCST90094812 | prot-a-1105 | Fibroblast growth factor receptor 4 \|\| id:prot-a-1105 | Plum liking \|\| id:ebi-fl187-GCST90094812 | MR Egger | 5 | 0.140569618099747 | 0.910272093037976 | 0.887078008307817 | -1.643563684 | 1.92470292045418 | 1.15092920227983 | 0.193289988964841 | 6.8531124439219 | 0.999950574162623 |
| ebi-fl187-GCST90094812 | prot-a-1143 | Follistatin-related protein 1 \|\| id:prot-a-1143 | Plum liking \|\| id:ebi-fl187-GCST90094812 | MR Egger | 5 | -0.310765073 | 0.910272093037976 | 0.755317477126436 | -2.094898375 | 1.4733682298307 | 0.732886030712177 | 0.123082751318009 | 4.36390906330398 | 0.999950574162623 |
| ebi-fl187-GCST90094812 | prot-a-1151 | Alpha-(1,3)-fucosyltransferase 10 \|\| id:prot-a-1151 | Plum liking \|\| id:ebi-fl187-GCST90094812 | MR Egger | 5 | -1.430042222 | 0.910272093037976 | 0.214214712154984 | -3.214175524 | 0.354091080408637 | 0.239298818368715 | 0.0401884545723279 | 1.42488495967429 | 0.999950574162623 |
| ebi-fl187-GCST90094812 | prot-a-1165 | Growth arrest and DNA damage-inducible proteins-interacting protein 1 \|\| id:prot-a-1165 | Plum liking \|\| id:ebi-fl187-GCST90094812 | MR Egger | 5 | 0.24045080064778 | 1.3946557544185 | 0.874091237362934 | -2.493074478 | 2.97397607930803 | 1.27182235945368 | 0.0826554531543609 | 19.5695752945125 | 0.999950574162623 |
| ebi-fl187-GCST90094812 | prot-a-1194 | Growth/differentiation factor 11/8 \|\| id:prot-a-1194 | Plum liking \|\| id:ebi-fl187-GCST90094812 | MR Egger | 5 | 0.691882963204879 | 0.908953058710118 | 0.501910509336524 | -1.089665032 | 2.47343095827671 | 1.99747316286209 | 0.336329134382395 | 11.863078836988 | 0.999950574162623 |
| ebi-fl187-GCST90094812 | prot-a-1216 | D-glucuronyl C5-epimerase \|\| id:prot-a-1216 | Plum liking \|\| id:ebi-fl187-GCST90094812 | MR Egger | 5 | -0.055003689 | 0.971733550014968 | 0.958419942535938 | -1.959601447 | 1.84959406879404 | 0.946481656153511 | 0.140914571618766 | 6.35723839730845 | 0.999950574162623 |
| ebi-fl187-GCST90094812 | prot-a-1246 | Glypican-3 \|\| id:prot-a-1246 | Plum liking \|\| id:ebi-fl187-GCST90094812 | MR Egger | 5 | -1.142777703 | 0.910272093037976 | 0.298210613801368 | -2.926911006 | 0.641355598917834 | 0.318931892082368 | 0.0535622363035225 | 1.89905349005284 | 0.999950574162623 |
| ebi-fl187-GCST90094812 | prot-a-1275 | Glutamate receptor 4 \|\| id:prot-a-1275 | Plum liking \|\| id:ebi-fl187-GCST90094812 | MR Egger | 5 | -1.438801954 | 0.910272093037976 | 0.212097103460145 | -3.222935256 | 0.34533134865097 | 0.237211779206855 | 0.0398379518865275 | 1.41245785814383 | 0.999950574162623 |
| ebi-fl187-GCST90094812 | prot-a-1289 | Glutathione S-transferase P \|\| id:prot-a-1289 | Plum liking \|\| id:ebi-fl187-GCST90094812 | MR Egger | 5 | -0.973614751 | 0.909037144151468 | 0.362657999211018 | -2.755327553 | 0.80809805178477 | 0.377715221054529 | 0.0635881877371404 | 2.24363664531585 | 0.999950574162623 |
| ebi-fl187-GCST90094812 | prot-a-130 | Apolipoprotein D \|\| id:prot-a-130 | Plum liking \|\| id:ebi-fl187-GCST90094812 | MR Egger | 5 | -1.794088884 | 1.25046904229433 | 0.246847946752286 | -4.245008207 | 0.656830438534397 | 0.166278882641266 | 0.0143356160224099 | 1.92866959949309 | 0.999950574162623 |
| ebi-fl187-GCST90094812 | prot-a-1313 | Heparin-binding EGF-like growth factor \|\| id:prot-a-1313 | Plum liking \|\| id:ebi-fl187-GCST90094812 | MR Egger | 5 | -1.116351634 | 1.08400637544346 | 0.378847188858341 | -3.24100413 | 1.00830086180832 | 0.327472356841254 | 0.0391245891971604 | 2.74093982060134 | 0.999950574162623 |
| ebi-fl187-GCST90094812 | prot-a-1347 | HLA class II histocompatibility antigen, DQ alpha 2 chain \|\| id:prot-a-1347 | Plum liking \|\| id:ebi-fl187-GCST90094812 | MR Egger | 5 | 0.532942524106226 | 1.039898068 | 0.643651818113867 | -1.505257688 | 2.5711427364964 | 1.70393882016959 | 0.221960089947198 | 13.0807637696111 | 0.999950574162623 |
| ebi-fl187-GCST90094812 | prot-a-1357 | Non-histone chromosomal protein HMG-14 \|\| id:prot-a-1357 | Plum liking \|\| id:ebi-fl187-GCST90094812 | MR Egger | 5 | -0.52370611 | 0.907718741447522 | 0.604421126178148 | -2.302834843 | 1.25542262310368 | 0.5923212672484 | 0.0999750280808468 | 3.5093211811957 | 0.999950574162623 |
| ebi-fl187-GCST90094812 | prot-a-137 | Apolipoprotein M \|\| id:prot-a-137 | Plum liking \|\| id:ebi-fl187-GCST90094812 | MR Egger | 5 | 0.239099207218683 | 1.30910215356105 | 0.866723134149908 | -2.326741014 | 2.80493942819835 | 1.27010453387206 | 0.0976133498408058 | 16.5260748616168 | 0.999950574162623 |
| ebi-fl187-GCST90094812 | prot-a-1381 | 17-beta-hydroxysteroid dehydrogenase 14 \|\| id:prot-a-1381 | Plum liking \|\| id:ebi-fl187-GCST90094812 | MR Egger | 5 | 0.615463626801845 | 0.972880880205117 | 0.57190942131083 | -1.291382898 | 2.52231015200388 | 1.85051434875449 | 0.27489037465793 | 12.4573418010999 | 0.999950574162623 |
| ebi-fl187-GCST90094812 | prot-a-1392 | Serine protease HTRA2, mitochondrial \|\| id:prot-a-1392 | Plum liking \|\| id:ebi-fl187-GCST90094812 | MR Egger | 5 | -0.894587845 | 0.909037144151468 | 0.397627191676192 | -2.676300647 | 0.887124957942392 | 0.408776043181642 | 0.0688172631849727 | 2.4281386057173 | 0.999950574162623 |
| ebi-fl187-GCST90094812 | prot-a-1416 | Interferon-induced protein with tetratricopeptide repeats 2 \|\| id:prot-a-1416 | Plum liking \|\| id:ebi-fl187-GCST90094812 | MR Egger | 5 | 1.12370179184005 | 1.0010591708551 | 0.343378098093478 | -0.838374183 | 3.08577776671604 | 3.07622068076065 | 0.432412976597041 | 21.8844812457098 | 0.999950574162623 |
| ebi-fl187-GCST90094812 | prot-a-1428 | Interferon gamma \|\| id:prot-a-1428 | Plum liking \|\| id:ebi-fl187-GCST90094812 | MR Egger | 5 | -1.373082424 | 0.958745648147959 | 0.247512697639166 | -3.252223894 | 0.506059046758416 | 0.253324900199133 | 0.038688073916132 | 1.65874127515409 | 0.999950574162623 |
| ebi-fl187-GCST90094812 | prot-a-1483 | Interleukin-17C \|\| id:prot-a-1483 | Plum liking \|\| id:ebi-fl187-GCST90094812 | MR Egger | 5 | 0.645620168631751 | 0.907718741447522 | 0.528240767974215 | -1.133508565 | 2.42474890186889 | 1.90716942924579 | 0.321901859322941 | 11.2993918068696 | 0.999950574162623 |
| ebi-fl187-GCST90094812 | prot-a-1503 | Interleukin-1 receptor-like 2 \|\| id:prot-a-1503 | Plum liking \|\| id:ebi-fl187-GCST90094812 | MR Egger | 5 | 2.1977372005261 | 0.908549843830509 | 0.0942574864816119 | 0.416979506618306 | 3.9784948944339 | 9.00461479143245 | 1.51737141654484 | 53.436546028207 | 0.999950574162623 |
| ebi-fl187-GCST90094812 | prot-a-1522 | Interleukin-31 receptor subunit alpha \|\| id:prot-a-1522 | Plum liking \|\| id:ebi-fl187-GCST90094812 | MR Egger | 5 | 1.41556575691338 | 0.910272093037976 | 0.217766038454648 | -0.368567545 | 3.19969905926781 | 4.11881605557878 | 0.691724485184682 | 24.5251484702962 | 0.999950574162623 |
| ebi-fl187-GCST90094812 | prot-a-1540 | Interleukin-6 receptor subunit alpha \|\| id:prot-a-1540 | Plum liking \|\| id:ebi-fl187-GCST90094812 | MR Egger | 5 | 0.05306426871198 | 0.908953058710118 | 0.957117360537711 | -1.728483726 | 1.83461226378381 | 1.05449741413221 | 0.177553425546597 | 6.26270539691556 | 0.999950574162623 |
| ebi-fl187-GCST90094812 | prot-a-1541 | Interleukin-6 receptor subunit alpha \|\| id:prot-a-1541 | Plum liking \|\| id:ebi-fl187-GCST90094812 | MR Egger | 5 | -0.137342096 | 0.908953058710118 | 0.889485887212042 | -1.918890091 | 1.64420589955637 | 0.871671980045369 | 0.146769772913484 | 5.17689729781145 | 0.999950574162623 |
| ebi-fl187-GCST90094812 | prot-a-1545 | Interleukin-9 \|\| id:prot-a-1545 | Plum liking \|\| id:ebi-fl187-GCST90094812 | MR Egger | 5 | -1.464301039 | 1.03261935651912 | 0.251203665048719 | -3.488234978 | 0.559632899473562 | 0.231239562370377 | 0.0305547544168203 | 1.75002994544795 | 0.999950574162623 |
| ebi-fl187-GCST90094812 | prot-a-1548 | Inositol monophosphatase 3 \|\| id:prot-a-1548 | Plum liking \|\| id:ebi-fl187-GCST90094812 | MR Egger | 5 | 0.192484455159359 | 1.04042232005365 | 0.86502497756001 | -1.846743292 | 2.23171220246452 | 1.21225765920885 | 0.157750076574238 | 9.31580297280512 | 0.999950574162623 |
| ebi-fl187-GCST90094812 | prot-a-1555 | Inhibin beta A chain:Inhibin beta B chain heterodimer \|\| id:prot-a-1555 | Plum liking \|\| id:ebi-fl187-GCST90094812 | MR Egger | 5 | -0.314902718 | 0.910272093037976 | 0.752224399042859 | -2.09903602 | 1.46923058478905 | 0.729859873360932 | 0.122574530725579 | 4.34589006043221 | 0.999950574162623 |
| ebi-fl187-GCST90094812 | prot-a-1603 | Junctophilin-3 \|\| id:prot-a-1603 | Plum liking \|\| id:ebi-fl187-GCST90094812 | MR Egger | 5 | -0.164293705 | 0.909037144151468 | 0.868096712673955 | -1.946006507 | 1.6174190979004 | 0.84849277901689 | 0.142843378074893 | 5.0400656001452 | 0.999950574162623 |
| ebi-fl187-GCST90094812 | prot-a-1627 | UPF0258 protein KIAA1024 \|\| id:prot-a-1627 | Plum liking \|\| id:ebi-fl187-GCST90094812 | MR Egger | 5 | -1.247087761 | 1.04960576197986 | 0.320264994372938 | -3.304315054 | 0.810139532847378 | 0.28734038353204 | 0.0367243574212653 | 2.24822166556224 | 0.999950574162623 |
| ebi-fl187-GCST90094812 | prot-a-1666 | Kallikrein-7 \|\| id:prot-a-1666 | Plum liking \|\| id:ebi-fl187-GCST90094812 | MR Egger | 5 | -0.669613579 | 0.908953058710118 | 0.514693634236945 | -2.451161574 | 1.11193441600619 | 0.511906350902663 | 0.0861934082945325 | 3.04023378677675 | 0.999950574162623 |
| ebi-fl187-GCST90094812 | prot-a-1678 | Importin subunit alpha-3 \|\| id:prot-a-1678 | Plum liking \|\| id:ebi-fl187-GCST90094812 | MR Egger | 5 | 0.18937198995027 | 0.910272093037976 | 0.848521554493578 | -1.594761312 | 1.9735052923047 | 1.20849041516936 | 0.202956966031876 | 7.19585591029595 | 0.999950574162623 |
| ebi-fl187-GCST90094812 | prot-a-1718 | Protein LDOC1 \|\| id:prot-a-1718 | Plum liking \|\| id:ebi-fl187-GCST90094812 | MR Egger | 5 | -0.217142626 | 0.909037144151468 | 0.826593763906701 | -1.998855429 | 1.56457017628563 | 0.804815173347614 | 0.135490272787125 | 4.78061967052223 | 0.999950574162623 |
| ebi-fl187-GCST90094812 | prot-a-1723 | Leptin \|\| id:prot-a-1723 | Plum liking \|\| id:ebi-fl187-GCST90094812 | MR Egger | 5 | 1.05323038645304 | 1.71858392030336 | 0.58330675230635 | -2.315194097 | 4.42165487024762 | 2.86689736195227 | 0.0987470156087091 | 83.2339127750207 | 0.999950574162623 |
| ebi-fl187-GCST90094812 | prot-a-1730 | Galectin-7 \|\| id:prot-a-1730 | Plum liking \|\| id:ebi-fl187-GCST90094812 | MR Egger | 5 | 0.444131177646618 | 1.50243519356331 | 0.786809441565892 | -2.500641802 | 3.3889041570307 | 1.55913499586755 | 0.0820323332313401 | 29.633460851144 | 0.999950574162623 |
| ebi-fl187-GCST90094812 | prot-a-1753 | Lipase member N \|\| id:prot-a-1753 | Plum liking \|\| id:ebi-fl187-GCST90094812 | MR Egger | 5 | -1.11205467 | 0.910272093037976 | 0.309073371096075 | -2.896187973 | 0.672078632096526 | 0.328882521248014 | 0.0552333703731169 | 1.95830368582931 | 0.999950574162623 |
| ebi-fl187-GCST90094812 | prot-a-1756 | VIP36-like protein \|\| id:prot-a-1756 | Plum liking \|\| id:ebi-fl187-GCST90094812 | MR Egger | 5 | -0.113878138 | 1.82412228249483 | 0.95414781435133 | -3.689157812 | 3.46140153543084 | 0.892366694615721 | 0.0249930420225749 | 31.8616003982274 | 0.999950574162623 |
| ebi-fl187-GCST90094812 | prot-a-1760 | Ig Kappa chain V-I region HK102- like \|\| id:prot-a-1760 | Plum liking \|\| id:ebi-fl187-GCST90094812 | MR Egger | 5 | 0.872515645496527 | 1.11648139314461 | 0.491518354868245 | -1.315787885 | 3.06081917605997 | 2.39292303415045 | 0.268262879636673 | 21.3450353441484 | 0.999950574162623 |
| ebi-fl187-GCST90094812 | prot-a-179 | Neutral ceramidase \|\| id:prot-a-179 | Plum liking \|\| id:ebi-fl187-GCST90094812 | MR Egger | 5 | -0.232782839 | 0.910272093037976 | 0.814691853650025 | -2.016916141 | 1.5513504634755 | 0.792325617162318 | 0.133065187236973 | 4.71783714919852 | 0.999950574162623 |
| ebi-fl187-GCST90094812 | prot-a-1809 | Lactotransferrin \|\| id:prot-a-1809 | Plum liking \|\| id:ebi-fl187-GCST90094812 | MR Egger | 5 | -0.479570921 | 1.3489718797649 | 0.745736155330912 | -3.123555806 | 2.16441396300282 | 0.61904895552675 | 0.044000432935214 | 8.70949633388866 | 0.999950574162623 |
| ebi-fl187-GCST90094812 | prot-a-1818 | Lysophospholipase-like protein 1 \|\| id:prot-a-1818 | Plum liking \|\| id:ebi-fl187-GCST90094812 | MR Egger | 5 | 0.220479360272703 | 1.02198350074732 | 0.843028500106525 | -1.782608301 | 2.22356702173744 | 1.24667419345731 | 0.168198861332115 | 9.24023226033383 | 0.999950574162623 |
| ebi-fl187-GCST90094812 | prot-a-1852 | MAP kinase-activated protein kinase 3 \|\| id:prot-a-1852 | Plum liking \|\| id:ebi-fl187-GCST90094812 | MR Egger | 5 | -1.408358178 | 0.908953058710118 | 0.21904858933342 | -3.189906173 | 0.373189816720107 | 0.244544452114827 | 0.0411757341359962 | 1.45235999588072 | 0.999950574162623 |
| ebi-fl187-GCST90094812 | prot-a-1856 | Megakaryocyte-associated tyrosine-protein kinase \|\| id:prot-a-1856 | Plum liking \|\| id:ebi-fl187-GCST90094812 | MR Egger | 5 | -0.338281953 | 0.907718741447523 | 0.734164089121474 | -2.117410686 | 1.44084678025057 | 0.712994228702802 | 0.120342830787048 | 4.22427133248236 | 0.999950574162623 |
| ebi-fl187-GCST90094812 | prot-a-1890 | Beta-1,3-N-acetylglucosaminyltransferase manic fringe \|\| id:prot-a-1890 | Plum liking \|\| id:ebi-fl187-GCST90094812 | MR Egger | 5 | 0.0429224272508404 | 0.910272093037976 | 0.965354403594631 | -1.741210875 | 1.82705572960527 | 1.04385691685904 | 0.175307995957427 | 6.21555940402785 | 0.999950574162623 |
| ebi-fl187-GCST90094812 | prot-a-1919 | Matrilysin \|\| id:prot-a-1919 | Plum liking \|\| id:ebi-fl187-GCST90094812 | MR Egger | 5 | -1.350172138 | 0.907718741447522 | 0.233621777981227 | -3.129300872 | 0.42895659493173 | 0.25919563930736 | 0.043748372295603 | 1.53565437776764 | 0.999950574162623 |
| ebi-fl187-GCST90094812 | prot-a-1923 | Multimerin-2 \|\| id:prot-a-1923 | Plum liking \|\| id:ebi-fl187-GCST90094812 | MR Egger | 5 | 2.18505347635202 | 0.907718741447522 | 0.0952568416893804 | 0.405924743114874 | 3.96418220958916 | 8.89112400553569 | 1.5006896109826 | 52.6771728832404 | 0.999950574162623 |
| ebi-fl187-GCST90094812 | prot-a-1948 | Mesothelin \|\| id:prot-a-1948 | Plum liking \|\| id:ebi-fl187-GCST90094812 | MR Egger | 5 | -0.788858322 | 0.909037144151468 | 0.449352774777795 | -2.570571125 | 0.992854480212007 | 0.454363235642684 | 0.0764918465510568 | 2.69892752250255 | 0.999950574162623 |
| ebi-fl187-GCST90094812 | prot-a-195 | ATPase family AAA domain-containing protein 1 \|\| id:prot-a-195 | Plum liking \|\| id:ebi-fl187-GCST90094812 | MR Egger | 5 | -1.437216439 | 0.908953058710118 | 0.211976909903117 | -3.218764434 | 0.344331556293381 | 0.237588180339131 | 0.0400044558888851 | 1.41104639927231 | 0.999950574162623 |
| ebi-fl187-GCST90094812 | prot-a-1966 | Mucin-1 \|\| id:prot-a-1966 | Plum liking \|\| id:ebi-fl187-GCST90094812 | MR Egger | 5 | -1.704257117 | 0.908953058710118 | 0.157477168563377 | -3.485805112 | 0.077290877882096 | 0.181907471926667 | 0.0306290886447368 | 1.08035628243994 | 0.999950574162623 |
| ebi-fl187-GCST90094812 | prot-a-1986 | Myocardial zonula adherens protein \|\| id:prot-a-1986 | Plum liking \|\| id:ebi-fl187-GCST90094812 | MR Egger | 5 | 0.24971684867346 | 0.908953058710118 | 0.801356822882189 | -1.531831146 | 2.03126484374529 | 1.283661895 | 0.216139521619758 | 7.62372308147644 | 0.999950574162623 |
| ebi-fl187-GCST90094812 | prot-a-2071 | Epididymal secretory protein E1 \|\| id:prot-a-2071 | Plum liking \|\| id:ebi-fl187-GCST90094812 | MR Egger | 5 | 1.25703106590775 | 0.908953058710118 | 0.260638530043945 | -0.524516929 | 3.03857906097958 | 3.51497026549337 | 0.591841196544208 | 20.8755592538068 | 0.999950574162623 |
| ebi-fl187-GCST90094812 | prot-a-2098 | Neuritin \|\| id:prot-a-2098 | Plum liking \|\| id:ebi-fl187-GCST90094812 | MR Egger | 5 | -0.230305642 | 0.910272093037976 | 0.816608775123726 | -2.014438944 | 1.55382766042139 | 0.79429079682489 | 0.133395224527312 | 4.72953864845099 | 0.999950574162623 |
| ebi-fl187-GCST90094812 | prot-a-2115 | Neurotrophin-4 \|\| id:prot-a-2115 | Plum liking \|\| id:ebi-fl187-GCST90094812 | MR Egger | 5 | 0.0690571030040067 | 0.908953058710118 | 0.944222385916604 | -1.712490892 | 1.85060509807584 | 1.07149739312137 | 0.180415836078186 | 6.36366900169638 | 0.999950574162623 |
| ebi-fl187-GCST90094812 | prot-a-2118 | Netrin-1 \|\| id:prot-a-2118 | Plum liking \|\| id:ebi-fl187-GCST90094812 | MR Egger | 5 | 1.24444551435142 | 0.909037144151468 | 0.264499392516765 | -0.537267288 | 3.02615831688829 | 3.47100964061789 | 0.584342913290145 | 20.617872915454 | 0.999950574162623 |
| ebi-fl187-GCST90094812 | prot-a-2120 | Netrin-G1 \|\| id:prot-a-2120 | Plum liking \|\| id:ebi-fl187-GCST90094812 | MR Egger | 5 | 0.177133435838277 | 1.1527877002642 | 0.887634710014425 | -2.082330457 | 2.43659732835612 | 1.19379037691949 | 0.124639406737464 | 11.4340680955537 | 0.999950574162623 |
| ebi-fl187-GCST90094812 | prot-a-2145 | Olfactomedin-like protein 3 \|\| id:prot-a-2145 | Plum liking \|\| id:ebi-fl187-GCST90094812 | MR Egger | 5 | 0.511032267980683 | 0.910272093037976 | 0.613727286648865 | -1.273101034 | 2.29516557033512 | 1.66701110927846 | 0.279962102167915 | 9.92607934052118 | 0.999950574162623 |
| ebi-fl187-GCST90094812 | prot-a-2162 | Prolyl 4-hydroxylase subunit alpha-2 \|\| id:prot-a-2162 | Plum liking \|\| id:ebi-fl187-GCST90094812 | MR Egger | 5 | -0.127870839 | 0.908953058710118 | 0.897038072732251 | -1.909418834 | 1.6536771561433 | 0.879967029289261 | 0.148166470893576 | 5.2261619512579 | 0.999950574162623 |
| ebi-fl187-GCST90094812 | prot-a-2168 | Polyadenylate-binding protein-interacting protein 1 \|\| id:prot-a-2168 | Plum liking \|\| id:ebi-fl187-GCST90094812 | MR Egger | 5 | 0.0810281726806765 | 0.910272093037976 | 0.9346793147596 | -1.70310513 | 1.86516147503511 | 1.0844014466321 | 0.182117147812203 | 6.45697844263625 | 0.999950574162623 |
| ebi-fl187-GCST90094812 | prot-a-219 | Beta-1,4-galactosyltransferase 5 \|\| id:prot-a-219 | Plum liking \|\| id:ebi-fl187-GCST90094812 | MR Egger | 5 | 1.06642111873425 | 0.907718741447522 | 0.324822684903067 | -0.712707615 | 2.8455498519714 | 2.90496435130898 | 0.490314815041378 | 17.2110195806827 | 0.999950574162623 |
| ebi-fl187-GCST90094812 | prot-a-2190 | Pyruvate carboxylase, mitochondrial \|\| id:prot-a-2190 | Plum liking \|\| id:ebi-fl187-GCST90094812 | MR Egger | 5 | -1.912025523 | 0.907718741447522 | 0.125795956890533 | -3.691154256 | -0.132896789 | 0.147780749984279 | 0.0249431945912066 | 0.875555454056202 | 0.999950574162623 |
| ebi-fl187-GCST90094812 | prot-a-2197 | Protocadherin-9 \|\| id:prot-a-2197 | Plum liking \|\| id:ebi-fl187-GCST90094812 | MR Egger | 5 | -1.229525145 | 0.909037144151468 | 0.269112684073647 | -3.011237948 | 0.552187657409493 | 0.292431407194928 | 0.0492306959963964 | 1.73704893224067 | 0.999950574162623 |
| ebi-fl187-GCST90094812 | prot-a-2198 | Protocadherin alpha-4 \|\| id:prot-a-2198 | Plum liking \|\| id:ebi-fl187-GCST90094812 | MR Egger | 5 | 0.56424038777139 | 1.24932601164195 | 0.682184027499609 | -1.884438595 | 3.01291937058961 | 1.75811179215209 | 0.151914320946309 | 20.3467129000738 | 0.999950574162623 |
| ebi-fl187-GCST90094812 | prot-a-2254 | Placenta growth factor \|\| id:prot-a-2254 | Plum liking \|\| id:ebi-fl187-GCST90094812 | MR Egger | 5 | -1.641973461 | 0.908953058710118 | 0.168588956507728 | -3.423521456 | 0.139574533806436 | 0.193597607675239 | 0.0325974421176296 | 1.14978450033984 | 0.999950574162623 |
| ebi-fl187-GCST90094812 | prot-a-2261 | Membrane-associated progesterone receptor component 2 \|\| id:prot-a-2261 | Plum liking \|\| id:ebi-fl187-GCST90094812 | MR Egger | 5 | 1.72127962669593 | 0.909037144151468 | 0.154616487724434 | -0.060433176 | 3.50299242923281 | 5.59167915011162 | 0.941356672284647 | 33.2146960215507 | 0.999950574162623 |
| ebi-fl187-GCST90094812 | prot-a-2268 | Polymeric immunoglobulin receptor \|\| id:prot-a-2268 | Plum liking \|\| id:ebi-fl187-GCST90094812 | MR Egger | 5 | -0.563418795 | 0.910272093037976 | 0.57976779913507 | -2.347552097 | 1.22071450724904 | 0.569259551487682 | 0.0956029026001132 | 3.3896087686314 | 0.999950574162623 |
| ebi-fl187-GCST90094812 | prot-a-2283 | Group 10 secretory phospholipase A2 \|\| id:prot-a-2283 | Plum liking \|\| id:ebi-fl187-GCST90094812 | MR Egger | 5 | -0.773722937 | 0.910272093037976 | 0.457784531930706 | -2.557856239 | 1.01041036578631 | 0.461292504789408 | 0.0774706410991125 | 2.74672794694768 | 0.999950574162623 |
| ebi-fl187-GCST90094812 | prot-a-2298 | Inactive phospholipase D5 \|\| id:prot-a-2298 | Plum liking \|\| id:ebi-fl187-GCST90094812 | MR Egger | 5 | -0.714964488 | 1.70151064685389 | 0.702624865575343 | -4.049925356 | 2.61999637985142 | 0.489209484303778 | 0.0174236751669727 | 13.7356738598078 | 0.999950574162623 |
| ebi-fl187-GCST90094812 | prot-a-2304 | Procollagen-lysine,2-oxoglutarate 5-dioxygenase 2 \|\| id:prot-a-2304 | Plum liking \|\| id:ebi-fl187-GCST90094812 | MR Egger | 5 | -1.156541086 | 0.908953058710118 | 0.292900726907419 | -2.938089082 | 0.625006908586992 | 0.314572379921797 | 0.0529668474184214 | 1.86825886441653 | 0.999950574162623 |
| ebi-fl187-GCST90094812 | prot-a-2331 | Periostin \|\| id:prot-a-2331 | Plum liking \|\| id:ebi-fl187-GCST90094812 | MR Egger | 5 | 2.27002574698347 | 0.908953058710118 | 0.08790742547165 | 0.488477751911634 | 4.0515737420553 | 9.67965003265386 | 1.62983331998427 | 57.4878569518755 | 0.999950574162623 |
| ebi-fl187-GCST90094812 | prot-a-2333 | Periostin \|\| id:prot-a-2333 | Plum liking \|\| id:ebi-fl187-GCST90094812 | MR Egger | 5 | 2.75528065182152 | 0.908953058710118 | 0.0562544791590274 | 0.973732656749693 | 4.53682864689336 | 15.7254536607645 | 2.64780940031111 | 93.3941441584871 | 0.999950574162623 |
| ebi-fl187-GCST90094812 | prot-a-2367 | cAMP-dependent protein kinase catalytic subunit alpha \|\| id:prot-a-2367 | Plum liking \|\| id:ebi-fl187-GCST90094812 | MR Egger | 5 | -1.325545461 | 0.909037144151468 | 0.24086393395586 | -3.107258264 | 0.456167341284905 | 0.265658013408804 | 0.0447234071832018 | 1.57801438962862 | 0.999950574162623 |
| ebi-fl187-GCST90094812 | prot-a-2385 | Phosphoribosyl pyrophosphate synthase-associated protein 1 \|\| id:prot-a-2385 | Plum liking \|\| id:ebi-fl187-GCST90094812 | MR Egger | 5 | 0.906791345297419 | 0.908953058710118 | 0.391986785346486 | -0.87475665 | 2.68833934036925 | 2.47636397516606 | 0.416963475489183 | 14.7072319231456 | 0.999950574162623 |
| ebi-fl187-GCST90094812 | prot-a-2393 | Trypsin-3 \|\| id:prot-a-2393 | Plum liking \|\| id:ebi-fl187-GCST90094812 | MR Egger | 5 | 0.640351756855918 | 0.910272093037976 | 0.532443223946471 | -1.143781545 | 2.42448505921035 | 1.89714809679865 | 0.318611895474108 | 11.2964109385527 | 0.999950574162623 |
| ebi-fl187-GCST90094812 | prot-a-2405 | Pregnancy-specific beta-1-glycoprotein 6 \|\| id:prot-a-2405 | Plum liking \|\| id:ebi-fl187-GCST90094812 | MR Egger | 5 | -0.841690889 | 0.908953058710118 | 0.422746093841829 | -2.623238884 | 0.939857106131097 | 0.430981165684662 | 0.072567444251488 | 2.55961563881453 | 0.999950574162623 |
| ebi-fl187-GCST90094812 | prot-a-244 | Beclin-1 \|\| id:prot-a-244 | Plum liking \|\| id:ebi-fl187-GCST90094812 | MR Egger | 5 | 1.06478133095451 | 1.29005000709955 | 0.469667244042266 | -1.463716683 | 3.59327934486963 | 2.90020472971628 | 0.231374728164118 | 36.353095003109 | 0.999950574162623 |
| ebi-fl187-GCST90094812 | prot-a-2454 | tRNA pseudouridine synthase A, mitochondrial \|\| id:prot-a-2454 | Plum liking \|\| id:ebi-fl187-GCST90094812 | MR Egger | 5 | -0.08362243 | 0.909037144151468 | 0.932504431118161 | -1.865335232 | 1.69809037295765 | 0.919778471686404 | 0.154844292403381 | 5.46350416826411 | 0.999950574162623 |
| ebi-fl187-GCST90094812 | prot-a-2466 | Glutaminyl-peptide cyclotransferase-like protein \|\| id:prot-a-2466 | Plum liking \|\| id:ebi-fl187-GCST90094812 | MR Egger | 5 | -0.285148383 | 0.909037144151468 | 0.774308593345569 | -2.066861185 | 1.49656441965714 | 0.751902676557045 | 0.126582477728811 | 4.46631828636557 | 0.999950574162623 |
| ebi-fl187-GCST90094812 | prot-a-2474 | Ras-related protein Rab-27A \|\| id:prot-a-2474 | Plum liking \|\| id:ebi-fl187-GCST90094812 | MR Egger | 5 | -2.032865625 | 0.907718741447522 | 0.111009704915512 | -3.811994358 | -0.253736892 | 0.130959701526618 | 0.0221040515705344 | 0.775895919768984 | 0.999950574162623 |
| ebi-fl187-GCST90094812 | prot-a-2490 | Rap1 GTPase-GDP dissociation stimulator 1 \|\| id:prot-a-2490 | Plum liking \|\| id:ebi-fl187-GCST90094812 | MR Egger | 5 | -1.844101528 | 0.907718741447522 | 0.135138274487527 | -3.623230261 | -0.064972795 | 0.158167366027172 | 0.0266963010352433 | 0.937092956921152 | 0.999950574162623 |
| ebi-fl187-GCST90094812 | prot-a-2545 | Receptor-interacting serine/threonine-protein kinase 2 \|\| id:prot-a-2545 | Plum liking \|\| id:ebi-fl187-GCST90094812 | MR Egger | 5 | -1.194421038 | 0.929755497218939 | 0.289120421074927 | -3.016741812 | 0.627899736679281 | 0.302879259050807 | 0.0489604811988266 | 1.87367124090612 | 0.999950574162623 |
| ebi-fl187-GCST90094812 | prot-a-2610 | RUN and FYVE domain-containing protein 1 \|\| id:prot-a-2610 | Plum liking \|\| id:ebi-fl187-GCST90094812 | MR Egger | 5 | 1.46748904809775 | 0.909037144151468 | 0.204862380536368 | -0.314223754 | 3.24920185063462 | 4.33832812004514 | 0.730355589569741 | 25.7697635863403 | 0.999950574162623 |
| ebi-fl187-GCST90094812 | prot-a-2627 | Serine--tRNA ligase, mitochondrial \|\| id:prot-a-2627 | Plum liking \|\| id:ebi-fl187-GCST90094812 | MR Egger | 5 | -0.33553337 | 0.910272093037976 | 0.736896253489448 | -2.119666673 | 1.44859993193943 | 0.714956647913609 | 0.12007164499069 | 4.25715004100677 | 0.999950574162623 |
| ebi-fl187-GCST90094812 | prot-a-264 | BolA-like protein 2 \|\| id:prot-a-264 | Plum liking \|\| id:ebi-fl187-GCST90094812 | MR Egger | 5 | -0.730784442 | 1.13664892897675 | 0.566032722928634 | -2.958616342 | 1.49704745912223 | 0.481531108828453 | 0.0518906664346 | 4.46847621550202 | 0.999950574162623 |
| ebi-fl187-GCST90094812 | prot-a-2646 | Secretoglobin family 1C member 1 \|\| id:prot-a-2646 | Plum liking \|\| id:ebi-fl187-GCST90094812 | MR Egger | 5 | -1.578646783 | 1.11073093608381 | 0.250355952046794 | -3.755679417 | 0.598385852092289 | 0.206254015960767 | 0.0233845573366172 | 1.81918000360568 | 0.999950574162623 |
| ebi-fl187-GCST90094812 | prot-a-2672 | Semaphorin-3E \|\| id:prot-a-2672 | Plum liking \|\| id:ebi-fl187-GCST90094812 | MR Egger | 5 | -0.179558257 | 1.26903622883638 | 0.89644859352808 | -2.666869266 | 2.30775275128523 | 0.835639267492674 | 0.069469375364967 | 10.0518103366716 | 0.999950574162623 |
| ebi-fl187-GCST90094812 | prot-a-2701 | Plasma protease C1 inhibitor \|\| id:prot-a-2701 | Plum liking \|\| id:ebi-fl187-GCST90094812 | MR Egger | 5 | 1.27000552542038 | 0.908953058710118 | 0.256756664408204 | -0.51154247 | 3.05155352049221 | 3.56087223761719 | 0.5995700465923 | 21.1481730361607 | 0.999950574162623 |
| ebi-fl187-GCST90094812 | prot-a-2738 | Signaling threshold-regulating transmembrane adapter 1 \|\| id:prot-a-2738 | Plum liking \|\| id:ebi-fl187-GCST90094812 | MR Egger | 5 | -0.448310642 | 1.17706498538903 | 0.728669324353172 | -2.755358013 | 1.85873672977113 | 0.63870624448752 | 0.0635862508917494 | 6.41562697951554 | 0.999950574162623 |
| ebi-fl187-GCST90094812 | prot-a-274 | Serine/threonine-protein kinase BRSK2 \|\| id:prot-a-274 | Plum liking \|\| id:ebi-fl187-GCST90094812 | MR Egger | 5 | -0.994407125 | 0.907718741447522 | 0.353366368219808 | -2.773535858 | 0.784721608291331 | 0.369942709343169 | 0.0624408320280312 | 2.1917966777689 | 0.999950574162623 |
| ebi-fl187-GCST90094812 | prot-a-2749 | Mitochondrial glutamate carrier 2 \|\| id:prot-a-2749 | Plum liking \|\| id:ebi-fl187-GCST90094812 | MR Egger | 5 | 0.751032695145424 | 0.910272093037976 | 0.469821504679624 | -1.033100607 | 2.53516599749986 | 2.11918736148852 | 0.355901736531796 | 12.6185253178483 | 0.999950574162623 |
| ebi-fl187-GCST90094812 | prot-a-2760 | Sodium-coupled monocarboxylate transporter 1 \|\| id:prot-a-2760 | Plum liking \|\| id:ebi-fl187-GCST90094812 | MR Egger | 5 | -0.149297047 | 1.0031415720391 | 0.891129638815088 | -2.115454529 | 1.81686043377158 | 0.861313226019324 | 0.120578470729635 | 6.15251187734202 | 0.999950574162623 |
| ebi-fl187-GCST90094812 | prot-a-2777 | Small integral membrane protein 10 \|\| id:prot-a-2777 | Plum liking \|\| id:ebi-fl187-GCST90094812 | MR Egger | 5 | 1.18315367052482 | 0.908953058710118 | 0.283966749881915 | -0.598394325 | 2.96470166559665 | 3.26465362732936 | 0.549693557316564 | 19.3889179972632 | 0.999950574162623 |
| ebi-fl187-GCST90094812 | prot-a-2799 | Superoxide dismutase [Mn], mitochondrial \|\| id:prot-a-2799 | Plum liking \|\| id:ebi-fl187-GCST90094812 | MR Egger | 5 | 0.233461007591716 | 0.910272093037976 | 0.814167371198341 | -1.550672295 | 2.01759430994615 | 1.2629635809392 | 0.212105328580741 | 7.52021185630688 | 0.999950574162623 |
| ebi-fl187-GCST90094812 | prot-a-2805 | Protein SPACA7 \|\| id:prot-a-2805 | Plum liking \|\| id:ebi-fl187-GCST90094812 | MR Egger | 5 | 0.587828067809582 | 0.910272093037976 | 0.564420116776891 | -1.196305235 | 2.37196137016401 | 1.80007452677624 | 0.302309112260995 | 10.7183944199183 | 0.999950574162623 |
| ebi-fl187-GCST90094812 | prot-a-2811 | SPARC-like protein 1 \|\| id:prot-a-2811 | Plum liking \|\| id:ebi-fl187-GCST90094812 | MR Egger | 5 | 0.165786052612563 | 0.908953058710118 | 0.866903862364337 | -1.615761942 | 1.94733404768439 | 1.18032054821003 | 0.198739184913215 | 7.00997438997843 | 0.999950574162623 |
| ebi-fl187-GCST90094812 | prot-a-2831 | Sepiapterin reductase \|\| id:prot-a-2831 | Plum liking \|\| id:ebi-fl187-GCST90094812 | MR Egger | 5 | 2.31598509022391 | 1.18386213281022 | 0.145376818401366 | -0.00438469 | 4.63635487053194 | 10.1349017955684 | 0.99562490863518 | 103.167602090853 | 0.999950574162623 |
| ebi-fl187-GCST90094812 | prot-a-2834 | SPRY domain-containing SOCS box protein 1 \|\| id:prot-a-2834 | Plum liking \|\| id:ebi-fl187-GCST90094812 | MR Egger | 5 | 0.856524036239902 | 0.908953058710118 | 0.41554716189171 | -0.925023959 | 2.63807203131173 | 2.35496069248498 | 0.396521918759177 | 13.9862126172085 | 0.999950574162623 |
| ebi-fl187-GCST90094812 | prot-a-2835 | Spectrin alpha chain, non-erythrocytic 1 \|\| id:prot-a-2835 | Plum liking \|\| id:ebi-fl187-GCST90094812 | MR Egger | 5 | 0.413928216673547 | 0.914776312635683 | 0.681632895262302 | -1.379033356 | 2.20688978943949 | 1.51274853286505 | 0.251821857510794 | 9.08740864000361 | 0.999950574162623 |
| ebi-fl187-GCST90094812 | prot-a-2859 | Sia-alpha-2,3-Gal-beta-1,4-GlcNAc-R:alpha 2,8-sialyltransferase \|\| id:prot-a-2859 | Plum liking \|\| id:ebi-fl187-GCST90094812 | MR Egger | 5 | 0.065615510041785 | 0.910272093037976 | 0.947072205605733 | -1.718517792 | 1.84974881239622 | 1.06781607366936 | 0.179331757928449 | 6.35822221539574 | 0.999950574162623 |
| ebi-fl187-GCST90094812 | prot-a-2861 | Alpha-2,8-sialyltransferase 8F \|\| id:prot-a-2861 | Plum liking \|\| id:ebi-fl187-GCST90094812 | MR Egger | 5 | -1.714737391 | 0.908953058710118 | 0.15569212376412 | -3.496285386 | 0.0668106040416545 | 0.180010987006419 | 0.0303097636377953 | 1.0690929771098 | 0.999950574162623 |
| ebi-fl187-GCST90094812 | prot-a-289 | Uncharacterized protein C17orf89 \|\| id:prot-a-289 | Plum liking \|\| id:ebi-fl187-GCST90094812 | MR Egger | 5 | -1.502660537 | 0.907718741447522 | 0.196413730804191 | -3.28178927 | 0.276468196200367 | 0.222537303102107 | 0.0375609898831117 | 1.31846501985363 | 0.999950574162623 |
| ebi-fl187-GCST90094812 | prot-a-2921 | Tachykinin-3 \|\| id:prot-a-2921 | Plum liking \|\| id:ebi-fl187-GCST90094812 | MR Egger | 5 | -1.310775861 | 0.908953058710118 | 0.244958185183682 | -3.092323856 | 0.470772133796525 | 0.269610794641176 | 0.0453963371662465 | 1.60123007988172 | 0.999950574162623 |
| ebi-fl187-GCST90094812 | prot-a-2928 | Tax1-binding protein 1 \|\| id:prot-a-2928 | Plum liking \|\| id:ebi-fl187-GCST90094812 | MR Egger | 5 | -0.424168954 | 1.1374009994766 | 0.733992474915667 | -2.653474913 | 1.80513700452309 | 0.654313323421077 | 0.0704061322755283 | 6.08080448917292 | 0.999950574162623 |
| ebi-fl187-GCST90094812 | prot-a-296 | Uncharacterized protein C1orf185 \|\| id:prot-a-296 | Plum liking \|\| id:ebi-fl187-GCST90094812 | MR Egger | 5 | 0.979732659502949 | 1.04428507718303 | 0.41736023629548 | -1.067066092 | 3.0265314107817 | 2.66374402008044 | 0.3440163506511 | 20.6255667531064 | 0.999950574162623 |
| ebi-fl187-GCST90094812 | prot-a-2964 | Transforming growth factor beta-2 \|\| id:prot-a-2964 | Plum liking \|\| id:ebi-fl187-GCST90094812 | MR Egger | 5 | -0.024102046 | 1.27999079766571 | 0.986159163273815 | -2.53288401 | 2.48467991702527 | 0.9761860883994 | 0.0794296138530075 | 11.9972795152704 | 0.999950574162623 |
| ebi-fl187-GCST90094812 | prot-a-2977 | Thymocyte nuclear protein 1 \|\| id:prot-a-2977 | Plum liking \|\| id:ebi-fl187-GCST90094812 | MR Egger | 5 | -2.785542323 | 0.971132859043478 | 0.0641372030261567 | -4.688962727 | -0.88212192 | 0.0616956209839448 | 0.00919622020139864 | 0.41390370883199 | 0.999950574162623 |
| ebi-fl187-GCST90094812 | prot-a-3015 | Transmembrane protein 70, mitochondrial \|\| id:prot-a-3015 | Plum liking \|\| id:ebi-fl187-GCST90094812 | MR Egger | 5 | -0.549587701 | 1.07827170545973 | 0.645394716752084 | -2.663000244 | 1.56382484155955 | 0.577187735173399 | 0.0697386745266278 | 4.7770578362139 | 0.999950574162623 |
| ebi-fl187-GCST90094812 | prot-a-3030 | Tumor necrosis factor alpha-induced protein 3 \|\| id:prot-a-3030 | Plum liking \|\| id:ebi-fl187-GCST90094812 | MR Egger | 5 | -0.365814967 | 0.910272093037976 | 0.714693924076358 | -2.149948269 | 1.41831833572639 | 0.693631134244104 | 0.116490183773368 | 4.13016903921098 | 0.999950574162623 |
| ebi-fl187-GCST90094812 | prot-a-3032 | Tumor necrosis factor receptor superfamily member 10A \|\| id:prot-a-3032 | Plum liking \|\| id:ebi-fl187-GCST90094812 | MR Egger | 5 | -1.002710127 | 0.909037144151468 | 0.35054787113459 | -2.784422929 | 0.779002675786593 | 0.366883791035481 | 0.0617647213605669 | 2.17929771493315 | 0.999950574162623 |
| ebi-fl187-GCST90094812 | prot-a-3081 | Tumor protein p53-inducible protein 11 \|\| id:prot-a-3081 | Plum liking \|\| id:ebi-fl187-GCST90094812 | MR Egger | 5 | 0.834806764977942 | 0.945358323520783 | 0.442234268541642 | -1.018095549 | 2.68770907907868 | 2.3043687205214 | 0.361282329865399 | 14.697965444631 | 0.999950574162623 |
| ebi-fl187-GCST90094812 | prot-a-315 | Complement C4 \|\| id:prot-a-315 | Plum liking \|\| id:ebi-fl187-GCST90094812 | MR Egger | 5 | -0.481528367 | 0.910272093037976 | 0.633450955743414 | -2.26566167 | 1.30260493493301 | 0.617838385771361 | 0.103761356068856 | 3.67886740685279 | 0.999950574162623 |
| ebi-fl187-GCST90094812 | prot-a-3157 | UDP-glucuronosyltransferase 1-8 \|\| id:prot-a-3157 | Plum liking \|\| id:ebi-fl187-GCST90094812 | MR Egger | 5 | -0.243149874 | 0.910272093037976 | 0.806688626999033 | -2.027283176 | 1.54098342839373 | 0.784153980701318 | 0.131692821744602 | 4.66917981788123 | 0.999950574162623 |
| ebi-fl187-GCST90094812 | prot-a-317 | Glutaredoxin-like protein C5orf63 \|\| id:prot-a-317 | Plum liking \|\| id:ebi-fl187-GCST90094812 | MR Egger | 5 | -0.605937799 | 1.09909559971115 | 0.619828125928868 | -2.760165174 | 1.54828957665655 | 0.545562558762137 | 0.0632813150550606 | 4.70341846189695 | 0.999950574162623 |
| ebi-fl187-GCST90094812 | prot-a-3192 | Guanine nucleotide exchange factor VAV3 \|\| id:prot-a-3192 | Plum liking \|\| id:ebi-fl187-GCST90094812 | MR Egger | 5 | 0.523650883003813 | 0.908953058710118 | 0.604924096316547 | -1.257897112 | 2.30519887807564 | 1.68817975933995 | 0.28425114674739 | 10.0261720399595 | 0.999950574162623 |
| ebi-fl187-GCST90094812 | prot-a-3233 | Serine/threonine-protein kinase WNK3 \|\| id:prot-a-3233 | Plum liking \|\| id:ebi-fl187-GCST90094812 | MR Egger | 5 | 1.27796966567262 | 0.908953058710118 | 0.254404630653671 | -0.503578329 | 3.05951766074445 | 3.58934475259424 | 0.604364171736991 | 21.3172725245243 | 0.999950574162623 |
| ebi-fl187-GCST90094812 | prot-a-3250 | 14-3-3 protein theta \|\| id:prot-a-3250 | Plum liking \|\| id:ebi-fl187-GCST90094812 | MR Egger | 5 | -0.091130193 | 0.907718741447522 | 0.926364101177085 | -1.870258926 | 1.68799854042486 | 0.912898850293386 | 0.154083760350242 | 5.40864468113092 | 0.999950574162623 |
| ebi-fl187-GCST90094812 | prot-a-3278 | E3 ubiquitin-protein ligase ZNRF3 \|\| id:prot-a-3278 | Plum liking \|\| id:ebi-fl187-GCST90094812 | MR Egger | 5 | -0.577976634 | 1.16548664946736 | 0.654012923273468 | -2.862330467 | 1.70637719897306 | 0.561032392781468 | 0.057135452707794 | 5.50896738947449 | 0.999950574162623 |
| ebi-fl187-GCST90094812 | prot-a-355 | Calpain-3 \|\| id:prot-a-355 | Plum liking \|\| id:ebi-fl187-GCST90094812 | MR Egger | 5 | 1.06278387363974 | 1.28114311133065 | 0.467630781172275 | -1.448256625 | 3.57382437184782 | 2.89441747638452 | 0.234979588849831 | 35.6526818716755 | 0.999950574162623 |
| ebi-fl187-GCST90094812 | prot-a-385 | Coiled-coil domain-containing protein 90B, mitochondrial \|\| id:prot-a-385 | Plum liking \|\| id:ebi-fl187-GCST90094812 | MR Egger | 5 | -1.535097849 | 0.909037144151468 | 0.18985827176039 | -3.316810652 | 0.24661495352468 | 0.215434610074588 | 0.0362683197999131 | 1.27968627921112 | 0.999950574162623 |
| ebi-fl187-GCST90094812 | prot-a-390 | C-C motif chemokine 14 \|\| id:prot-a-390 | Plum liking \|\| id:ebi-fl187-GCST90094812 | MR Egger | 5 | -1.165927912 | 0.910272093037976 | 0.290286915964204 | -2.950061214 | 0.618205390812372 | 0.31163335963859 | 0.0523365021291501 | 1.85559498416582 | 0.999950574162623 |
| ebi-fl187-GCST90094812 | prot-a-40 | Pituitary adenylate cyclase-activating polypeptide 27 \|\| id:prot-a-40 | Plum liking \|\| id:ebi-fl187-GCST90094812 | MR Egger | 5 | -0.417305798 | 0.910272093037976 | 0.67780680357109 | -2.2014391 | 1.3668275044132 | 0.658819423513742 | 0.110643816186561 | 3.92288559594803 | 0.999950574162623 |
| ebi-fl187-GCST90094812 | prot-a-401 | C-C motif chemokine 24 \|\| id:prot-a-401 | Plum liking \|\| id:ebi-fl187-GCST90094812 | MR Egger | 5 | 0.856345299076126 | 0.910272093037976 | 0.41623124547502 | -0.927788003 | 2.64047860143056 | 2.35453981110469 | 0.395427427859331 | 14.0199119522106 | 0.999950574162623 |
| ebi-fl187-GCST90094812 | prot-a-412 | Cerebral cavernous malformations 2 protein \|\| id:prot-a-412 | Plum liking \|\| id:ebi-fl187-GCST90094812 | MR Egger | 5 | 0.972762188300767 | 1.10583362578321 | 0.443808651977044 | -1.194671718 | 3.14019609483585 | 2.64524103129146 | 0.302803342683525 | 23.1083978519391 | 0.999950574162623 |
| ebi-fl187-GCST90094812 | prot-a-44 | Adhesion G protein-coupled receptor F5 \|\| id:prot-a-44 | Plum liking \|\| id:ebi-fl187-GCST90094812 | MR Egger | 5 | 0.944893267116384 | 0.910272093037976 | 0.375572467457096 | -0.839240035 | 2.72902656947082 | 2.57253878945153 | 0.432038732912274 | 15.3179687816938 | 0.999950574162623 |
| ebi-fl187-GCST90094812 | prot-a-48 | Adrenomedullin \|\| id:prot-a-48 | Plum liking \|\| id:ebi-fl187-GCST90094812 | MR Egger | 5 | 1.72672707181156 | 0.907718741447522 | 0.153288106789083 | -0.052401661 | 3.5058558050487 | 5.62222263182562 | 0.948947634625146 | 33.3099384712609 | 0.999950574162623 |
| ebi-fl187-GCST90094812 | prot-a-502 | Carcinoembryonic antigen-related cell adhesion molecule 1 \|\| id:prot-a-502 | Plum liking \|\| id:ebi-fl187-GCST90094812 | MR Egger | 5 | -0.446729658 | 0.96546831515851 | 0.675070718261913 | -2.339047555 | 1.4455882400379 | 0.639716827435956 | 0.0964194286766691 | 4.24434810412592 | 0.999950574162623 |
| ebi-fl187-GCST90094812 | prot-a-518 | Complement factor B \|\| id:prot-a-518 | Plum liking \|\| id:ebi-fl187-GCST90094812 | MR Egger | 5 | -0.199174516 | 0.969255718145402 | 0.850341377653746 | -2.098915723 | 1.70056669187259 | 0.819406881495105 | 0.122589276919465 | 5.477050312 | 0.999950574162623 |
| ebi-fl187-GCST90094812 | prot-a-525 | Properdin \|\| id:prot-a-525 | Plum liking \|\| id:ebi-fl187-GCST90094812 | MR Egger | 5 | 1.75432117573615 | 1.33788036802129 | 0.281099115687808 | -0.867924346 | 4.37656669705787 | 5.7795231295126 | 0.419822050998131 | 79.564395260219 | 0.999950574162623 |
| ebi-fl187-GCST90094812 | prot-a-533 | Chondroadherin \|\| id:prot-a-533 | Plum liking \|\| id:ebi-fl187-GCST90094812 | MR Egger | 5 | 0.312643922850235 | 0.907718741447522 | 0.753254835678326 | -1.46648481 | 2.09177265608738 | 1.36703467454297 | 0.230735139073793 | 8.09925964854943 | 0.999950574162623 |
| ebi-fl187-GCST90094812 | prot-a-535 | Coiled-coil-helix-coiled-coil-helix domain-containing protein 2 \|\| id:prot-a-535 | Plum liking \|\| id:ebi-fl187-GCST90094812 | MR Egger | 5 | -1.327220197 | 0.908953058710118 | 0.240366672371985 | -3.108768192 | 0.454327798317654 | 0.265213478845111 | 0.0446559290131891 | 1.57511423265546 | 0.999950574162623 |
| ebi-fl187-GCST90094812 | prot-a-591 | Caseinolytic peptidase B protein homolog \|\| id:prot-a-591 | Plum liking \|\| id:ebi-fl187-GCST90094812 | MR Egger | 5 | 1.82892764142338 | 0.907718741447522 | 0.137336303258789 | 0.0497989081862333 | 3.60805637466052 | 6.22720527975794 | 1.05105971561868 | 36.8942744356058 | 0.999950574162623 |
| ebi-fl187-GCST90094812 | prot-a-605 | Ciliary neurotrophic factor receptor subunit alpha \|\| id:prot-a-605 | Plum liking \|\| id:ebi-fl187-GCST90094812 | MR Egger | 5 | 1.03216828526233 | 0.910272093037976 | 0.339247423040225 | -0.751965017 | 2.81630158761677 | 2.80714593377979 | 0.471439255766726 | 16.7149175575561 | 0.999950574162623 |
| ebi-fl187-GCST90094812 | prot-a-606 | Ciliary neurotrophic factor receptor subunit alpha \|\| id:prot-a-606 | Plum liking \|\| id:ebi-fl187-GCST90094812 | MR Egger | 5 | 2.0922212715913 | 0.908953058710118 | 0.104815977901688 | 0.310673276519471 | 3.87376926666313 | 8.10289391713174 | 1.36434338533811 | 48.1234347143621 | 0.999950574162623 |
| ebi-fl187-GCST90094812 | prot-a-707 | Cancer/testis antigen 55 \|\| id:prot-a-707 | Plum liking \|\| id:ebi-fl187-GCST90094812 | MR Egger | 5 | 0.358933375044502 | 0.910272093037976 | 0.719707559317004 | -1.425199927 | 2.14306667739893 | 1.43180140468892 | 0.240460383804631 | 8.52554266957667 | 0.999950574162623 |
| ebi-fl187-GCST90094812 | prot-a-719 | Dipeptidyl peptidase 1 \|\| id:prot-a-719 | Plum liking \|\| id:ebi-fl187-GCST90094812 | MR Egger | 5 | 1.01395481151192 | 1.32202290860664 | 0.498930332362848 | -1.577210089 | 3.60511971238092 | 2.75648084918441 | 0.206550552683568 | 36.7860873437636 | 0.999950574162623 |
| ebi-fl187-GCST90094812 | prot-a-752 | Cytochrome b5 \|\| id:prot-a-752 | Plum liking \|\| id:ebi-fl187-GCST90094812 | MR Egger | 5 | 0.284678570240793 | 1.0513405256862 | 0.804124070158179 | -1.77594886 | 2.34530600058574 | 1.32933467171918 | 0.169322709687782 | 10.4364658036314 | 0.999950574162623 |
| ebi-fl187-GCST90094812 | prot-a-755 | Cytoglobin \|\| id:prot-a-755 | Plum liking \|\| id:ebi-fl187-GCST90094812 | MR Egger | 5 | -1.041563563 | 1.63993869683979 | 0.570477996925633 | -4.255843408 | 2.17271628318796 | 0.352902465256102 | 0.014181125219902 | 8.78210635987141 | 0.999950574162623 |
| ebi-fl187-GCST90094812 | prot-a-807 | Desmin \|\| id:prot-a-807 | Plum liking \|\| id:ebi-fl187-GCST90094812 | MR Egger | 5 | 1.58129873912739 | 0.948524100282057 | 0.194082519147393 | -0.277808497 | 3.44040597568022 | 4.86126522856797 | 0.75744185969251 | 31.1996218852726 | 0.999950574162623 |
| ebi-fl187-GCST90094812 | prot-a-82 | Ameloblastin \|\| id:prot-a-82 | Plum liking \|\| id:ebi-fl187-GCST90094812 | MR Egger | 5 | -1.071651254 | 0.908953058710118 | 0.323391884446216 | -2.853199249 | 0.709896741519595 | 0.342442590758665 | 0.0576595582192953 | 2.03378124264338 | 0.999950574162623 |
| ebi-fl187-GCST90094812 | prot-a-84 | Muellerian-inhibiting factor \|\| id:prot-a-84 | Plum liking \|\| id:ebi-fl187-GCST90094812 | MR Egger | 5 | 1.1540496984777 | 1.33451463681715 | 0.450775303768075 | -1.46159899 | 3.76969838663931 | 3.17100857097562 | 0.231865228051198 | 43.3669828016668 | 0.999950574162623 |
| ebi-fl187-GCST90094812 | prot-a-858 | Inactive dipeptidyl peptidase 10 \|\| id:prot-a-858 | Plum liking \|\| id:ebi-fl187-GCST90094812 | MR Egger | 5 | -2.219004383 | 0.908953058710118 | 0.0923950898966034 | -4.000552378 | -0.437456388 | 0.108717295727622 | 0.0183055245218283 | 0.645676684993822 | 0.999950574162623 |
| ebi-fl187-GCST90094812 | prot-a-863 | Dihydropyrimidinase-related protein 3 \|\| id:prot-a-863 | Plum liking \|\| id:ebi-fl187-GCST90094812 | MR Egger | 5 | -1.317130234 | 0.907718741447522 | 0.242672057731805 | -3.096258967 | 0.461998499609145 | 0.267903018929488 | 0.0452180485850881 | 1.58724292173904 | 0.999950574162623 |
| ebi-fl187-GCST90094812 | prot-a-864 | Dihydropyrimidinase-related protein 5 \|\| id:prot-a-864 | Plum liking \|\| id:ebi-fl187-GCST90094812 | MR Egger | 5 | 0.51297241581758 | 0.910272093037976 | 0.61244516738371 | -1.271160887 | 2.29710571817201 | 1.67024849676587 | 0.280505797288758 | 9.94535609571326 | 0.999950574162623 |
| ebi-fl187-GCST90094812 | prot-a-884 | Endothelin-converting enzyme 1 \|\| id:prot-a-884 | Plum liking \|\| id:ebi-fl187-GCST90094812 | MR Egger | 5 | 1.54641559489221 | 1.02524375215232 | 0.228591622744782 | -0.463062159 | 3.55589334911076 | 4.6946125950633 | 0.629353511102019 | 35.0190902711184 | 0.999950574162623 |
| ebi-fl187-GCST90094812 | prot-a-893 | Early endosome antigen 1 \|\| id:prot-a-893 | Plum liking \|\| id:ebi-fl187-GCST90094812 | MR Egger | 5 | 0.646721317750115 | 0.908953058710118 | 0.528108586041426 | -1.134826677 | 2.42826931282195 | 1.90927066385538 | 0.321477835905481 | 11.3392404101244 | 0.999950574162623 |
| ebi-fl187-GCST90094812 | prot-a-897 | Ephrin-A2 \|\| id:prot-a-897 | Plum liking \|\| id:ebi-fl187-GCST90094812 | MR Egger | 5 | 0.207293762944681 | 1.09166892359249 | 0.861519566946335 | -1.932377327 | 2.34696485318596 | 1.23034394815984 | 0.14480354355043 | 10.4537927295015 | 0.999950574162623 |
| ebi-fl187-GCST90094812 | prot-a-90 | Anaphase-promoting complex subunit 10 \|\| id:prot-a-90 | Plum liking \|\| id:ebi-fl187-GCST90094812 | MR Egger | 5 | -1.333781059 | 0.908953058710118 | 0.23856098744133 | -3.115329055 | 0.447766935615137 | 0.263479145207174 | 0.0443639066010622 | 1.56481395074979 | 0.999950574162623 |
| ebi-fl187-GCST90094812 | prot-a-914 | Histone-lysine N-methyltransferase EHMT2 \|\| id:prot-a-914 | Plum liking \|\| id:ebi-fl187-GCST90094812 | MR Egger | 5 | -1.609665468 | 1.80638470754205 | 0.438525425546201 | -5.150179495 | 1.93084855831064 | 0.199954493970248 | 0.00579836385463519 | 6.89535887385481 | 0.999950574162623 |
| ebi-fl187-GCST90094812 | prot-a-932 | RNA polymerase II elongation factor ELL \|\| id:prot-a-932 | Plum liking \|\| id:ebi-fl187-GCST90094812 | MR Egger | 5 | 1.84675446310694 | 0.942077578768478 | 0.144809844903609 | 0.000282408720726046 | 3.69322651749316 | 6.33921195311073 | 1.00028244860182 | 40.1742610226069 | 0.999950574162623 |
| ebi-fl187-GCST90094812 | prot-a-991 | Estrogen receptor \|\| id:prot-a-991 | Plum liking \|\| id:ebi-fl187-GCST90094812 | MR Egger | 5 | 0.589571171345426 | 0.910272093037976 | 0.563335855213038 | -1.194562131 | 2.37370447369986 | 1.80321497932044 | 0.302836527879968 | 10.7370939840336 | 0.999950574162623 |
| ebi-fl187-GCST90094812 | prot-a-992 | Persulfide dioxygenase ETHE1, mitochondrial \|\| id:prot-a-992 | Plum liking \|\| id:ebi-fl187-GCST90094812 | MR Egger | 5 | 1.81697260872894 | 0.907718741447522 | 0.13909784560668 | 0.0378438754917978 | 3.59610134196609 | 6.15320207381016 | 1.03856907413442 | 36.4558282198969 | 0.999950574162623 |
| ebi-fl187-GCST90094812 | prot-a-993 | Ethanolamine kinase 1 \|\| id:prot-a-993 | Plum liking \|\| id:ebi-fl187-GCST90094812 | MR Egger | 5 | -0.015814929 | 0.910272093037976 | 0.987229250765643 | -1.799948231 | 1.76831837343741 | 0.98430947041906 | 0.165307445756364 | 5.86098907477281 | 0.999950574162623 |
| ebi-fl187-GCST90094812 | prot-b-10 | coagulation factor III, tissue factor \|\| id:prot-b-10 | Plum liking \|\| id:ebi-fl187-GCST90094812 | MR Egger | 4 | -0.69325499 | 1.10250854631914 | 0.5937220795265 | -2.854171741 | 1.46766176065772 | 0.499946098121698 | 0.0576035120002012 | 4.33907746850986 | 0.999950574162623 |
| ebi-fl187-GCST90094812 | prot-b-28 | prolactin \|\| id:prot-b-28 | Plum liking \|\| id:ebi-fl187-GCST90094812 | MR Egger | 4 | 0.435779448888642 | 1.10178966673453 | 0.730660663789178 | -1.723728298 | 2.59528719568831 | 1.54616774812646 | 0.178399778949192 | 13.4004353560734 | 0.999950574162623 |
| ebi-fl187-GCST90094812 | prot-b-54 | matrix metallopeptidase 7 \|\| id:prot-b-54 | Plum liking \|\| id:ebi-fl187-GCST90094812 | MR Egger | 4 | 1.56219290625962 | 1.10178966673453 | 0.291981243248238 | -0.597314841 | 3.72170065305929 | 4.76926834460311 | 0.55028726311072 | 41.3346302334035 | 0.999950574162623 |
| ebi-fl187-GCST90094812 | ukb-rsfMRI191-edge-pheno716 | Net100_Pair10_33 (OccipitalPrecuneus)&(Temporal\|Frontal\|Supp_Motor_Area) (Default_mode\|Central_executive)&(Default_mode\|Salience) \|\| id:ukb-rsfMRI191-edge-pheno716 | Plum liking \|\| id:ebi-fl187-GCST90094812 | MR Egger | 5 | -0.115669575 | 0.280435621788516 | 0.707703446554624 | -0.665323393 | 0.433984244126626 | 0.890769507564965 | 0.51410724199074 | 1.54339455039581 | 0.999950574162623 |
| ebi-fl187-GCST90094812 | ukb-rsfMRI191-edge-pheno903 | Net100_Pair27_38 (Precentral\|Frontal\|Supp_Motor_Area)&(Subcortical) (Attention\|Salience\|Motor)&(Subcortical-cerebellum) \|\| id:ukb-rsfMRI191-edge-pheno903 | Plum liking \|\| id:ebi-fl187-GCST90094812 | MR Egger | 5 | -0.29607015 | 0.295139672709465 | 0.389700655338289 | -0.874543908 | 0.282403608580152 | 0.743735253213923 | 0.417052190291281 | 1.32631392365269 | 0.999950574162623 |
| ebi-fl187-GCST90094812 | ukb-rsfMRI191-node-pheno23 | Net100_Node2 RolandicOper\|SupraMarginal\|Insula Salience\|Motor \|\| id:ukb-rsfMRI191-node-pheno23 | Plum liking \|\| id:ebi-fl187-GCST90094812 | MR Egger | 5 | -0.360998306 | 0.325581808820867 | 0.348421188471709 | -0.999138651 | 0.27714203954474 | 0.696980179328612 | 0.368196450256178 | 1.31935375813361 | 0.999950574162623 |
| ebi-fl187-GCST90094812 | ukb-rsfMRI191-node-pheno55 | Net100_Node34 Parietal\|Postcentral\|Precuneus Attention \|\| id:ukb-rsfMRI191-node-pheno55 | Plum liking \|\| id:ebi-fl187-GCST90094812 | MR Egger | 5 | -0.643124543 | 0.301083864424523 | 0.122311124963849 | -1.233248918 | -0.053000169 | 0.525647447235828 | 0.291344484142235 | 0.948379852115717 | 0.999950574162623 |
| ebi-fl187-GCST90094812 | ebi-a-GCST90001414 | IgD- CD24- B cell Absolute Count \|\| id:ebi-a-GCST90001414 | Plum liking \|\| id:ebi-fl187-GCST90094812 | Weighted median | 5 | -0.427610654 | 0.291445283011376 | 0.142319664868018 | -0.998843409 | 0.143622100591467 | 0.652065244304128 | 0.368305173442571 | 1.15444776095631 | 0.999332001504091 |
| ebi-fl187-GCST90094812 | ebi-a-GCST90001422 | CD20- CD38- B cell %B cell \|\| id:ebi-a-GCST90001422 | Plum liking \|\| id:ebi-fl187-GCST90094812 | Weighted median | 5 | -0.139072614 | 0.276547066544947 | 0.615041968552655 | -0.681104865 | 0.402959636282875 | 0.870164839888038 | 0.506057558304831 | 1.49624649637437 | 0.999332001504091 |
| ebi-fl187-GCST90094812 | ebi-a-GCST90001444 | CD20- CD38- B cell %lymphocyte \|\| id:ebi-a-GCST90001444 | Plum liking \|\| id:ebi-fl187-GCST90094812 | Weighted median | 5 | -0.171309273 | 0.282270652550107 | 0.543919126433709 | -0.724559752 | 0.381941206070535 | 0.842560951879763 | 0.484537838856479 | 1.46512594208934 | 0.999332001504091 |
| ebi-fl187-GCST90094812 | ebi-a-GCST90001459 | Myeloid Dendritic Cell %Dendritic Cell \|\| id:ebi-a-GCST90001459 | Plum liking \|\| id:ebi-fl187-GCST90094812 | Weighted median | 5 | 0.107364489463678 | 0.293661763796414 | 0.714659131218618 | -0.468212568 | 0.682941546504649 | 1.11333998122592 | 0.626120416610884 | 1.97969253343557 | 0.999332001504091 |
| ebi-fl187-GCST90094812 | ebi-a-GCST90001476 | HLA DR++ monocyte %leukocyte \|\| id:ebi-a-GCST90001476 | Plum liking \|\| id:ebi-fl187-GCST90094812 | Weighted median | 5 | 0.112755882515018 | 0.298685312833248 | 0.70579664907371 | -0.472667331 | 0.698179095668184 | 1.1193586445756 | 0.623337401945172 | 2.01008919290926 | 0.999332001504091 |
| ebi-fl187-GCST90094812 | ebi-a-GCST90001478 | CD4 regulatory T cell %CD4+ T cell \|\| id:ebi-a-GCST90001478 | Plum liking \|\| id:ebi-fl187-GCST90094812 | Weighted median | 5 | 0.00391129438809892 | 0.281389176371688 | 0.988909804722304 | -0.547611491 | 0.555434080076607 | 1.0039189534824 | 0.578329507074658 | 1.742697291 | 0.999332001504091 |
| ebi-fl187-GCST90094812 | ebi-a-GCST90001485 | CD39+ resting CD4 regulatory T cell %CD4 regulatory T cell \|\| id:ebi-a-GCST90001485 | Plum liking \|\| id:ebi-fl187-GCST90094812 | Weighted median | 5 | 0.299276568908596 | 0.297990893251797 | 0.315227056516745 | -0.284785582 | 0.883338719682118 | 1.34888263088645 | 0.752175517101557 | 2.41896247689421 | 0.999332001504091 |
| ebi-fl187-GCST90094812 | ebi-a-GCST90001515 | Immature Myeloid-Derived Suppressor Cells Absolute Count \|\| id:ebi-a-GCST90001515 | Plum liking \|\| id:ebi-fl187-GCST90094812 | Weighted median | 5 | -0.321895277 | 0.413225353729699 | 0.435990083315459 | -1.13181697 | 0.488026416137199 | 0.724774086747083 | 0.322446847435475 | 1.62909788387739 | 0.999332001504091 |
| ebi-fl187-GCST90094812 | ebi-a-GCST90001517 | CD33+ HLA DR+ Absolute Count \|\| id:ebi-a-GCST90001517 | Plum liking \|\| id:ebi-fl187-GCST90094812 | Weighted median | 5 | 0.039227599678599 | 0.408648535765542 | 0.923525741850596 | -0.76172353 | 0.840178729779061 | 1.04000716201099 | 0.466861083911699 | 2.31678101753868 | 0.999332001504091 |
| ebi-fl187-GCST90094812 | ebi-a-GCST90001518 | CD33+ HLA DR+ CD14- Absolute Count \|\| id:ebi-a-GCST90001518 | Plum liking \|\| id:ebi-fl187-GCST90094812 | Weighted median | 5 | -0.013077408 | 0.428770081613799 | 0.975668441946778 | -0.853466768 | 0.827311952347063 | 0.98700773014782 | 0.425935749192726 | 2.28716246809036 | 0.999332001504091 |
| ebi-fl187-GCST90094812 | ebi-a-GCST90001520 | CD33+ HLA DR+ CD14dim Absolute Count \|\| id:ebi-a-GCST90001520 | Plum liking \|\| id:ebi-fl187-GCST90094812 | Weighted median | 5 | -0.071726764 | 0.384139714938494 | 0.851879773550232 | -0.824640605 | 0.681187077448646 | 0.93078518524745 | 0.438392520187384 | 1.97622226927097 | 0.999332001504091 |
| ebi-fl187-GCST90094812 | ebi-a-GCST90001531 | CD33dim HLA DR- Absolute Count \|\| id:ebi-a-GCST90001531 | Plum liking \|\| id:ebi-fl187-GCST90094812 | Weighted median | 5 | -0.582453665 | 0.371314876371084 | 0.116735427335946 | -1.310230822 | 0.145323493012334 | 0.558526247769885 | 0.269757783069186 | 1.15641360148597 | 0.999332001504091 |
| ebi-fl187-GCST90094812 | ebi-a-GCST90001532 | Basophil Absolute Count \|\| id:ebi-a-GCST90001532 | Plum liking \|\| id:ebi-fl187-GCST90094812 | Weighted median | 5 | -0.413972152 | 0.377435740314622 | 0.272728097690567 | -1.153746203 | 0.325801898710181 | 0.661019358874124 | 0.315452802735917 | 1.38514094348419 | 0.999332001504091 |
| ebi-fl187-GCST90094812 | ebi-a-GCST90001588 | T/B cell \|\| id:ebi-a-GCST90001588 | Plum liking \|\| id:ebi-fl187-GCST90094812 | Weighted median | 5 | 0.156806249246725 | 0.279671603384818 | 0.575015765537778 | -0.391350093 | 0.704962591880969 | 1.16976894832841 | 0.67614340126477 | 2.02377097804069 | 0.999332001504091 |
| ebi-fl187-GCST90094812 | ebi-a-GCST90001624 | HLA DR+ CD4+ T cell Absolute Count \|\| id:ebi-a-GCST90001624 | Plum liking \|\| id:ebi-fl187-GCST90094812 | Weighted median | 5 | -0.141467987 | 0.279351088679059 | 0.612564327465206 | -0.688996121 | 0.406060146601849 | 0.868082964898516 | 0.502079843560636 | 1.50089282334631 | 0.999332001504091 |
| ebi-fl187-GCST90094812 | ebi-a-GCST90001631 | CD8+ Natural Killer T %T cell \|\| id:ebi-a-GCST90001631 | Plum liking \|\| id:ebi-fl187-GCST90094812 | Weighted median | 5 | -0.236498209 | 0.283391233858727 | 0.403982939890208 | -0.791945028 | 0.318948608984217 | 0.789387295794261 | 0.452962912486247 | 1.37568062546466 | 0.999332001504091 |
| ebi-fl187-GCST90094812 | ebi-a-GCST90001632 | CD8+ Natural Killer T %lymphocyte \|\| id:ebi-a-GCST90001632 | Plum liking \|\| id:ebi-fl187-GCST90094812 | Weighted median | 5 | -0.168511652 | 0.290626222705204 | 0.562034255613091 | -0.738139048 | 0.401115744518923 | 0.844921418351343 | 0.478002628053713 | 1.49349012179202 | 0.999332001504091 |
| ebi-fl187-GCST90094812 | ebi-a-GCST90001646 | Natural Killer %CD3- lymphocyte \|\| id:ebi-a-GCST90001646 | Plum liking \|\| id:ebi-fl187-GCST90094812 | Weighted median | 5 | -0.21367472 | 0.273399848656068 | 0.43448108365762 | -0.749538424 | 0.322188982866772 | 0.807611041618484 | 0.472584636195912 | 1.38014557518054 | 0.999332001504091 |
| ebi-fl187-GCST90094812 | ebi-a-GCST90001684 | CD127- CD8+ T cell Absolute Count \|\| id:ebi-a-GCST90001684 | Plum liking \|\| id:ebi-fl187-GCST90094812 | Weighted median | 5 | 0.323276821801421 | 0.294580856814704 | 0.272460952466933 | -0.254101658 | 0.90065530115824 | 1.38164776819659 | 0.775612951122102 | 2.46121542014078 | 0.999332001504091 |
| ebi-fl187-GCST90094812 | ebi-a-GCST90001687 | CD28- CD8+ T cell Absolute Count \|\| id:ebi-a-GCST90001687 | Plum liking \|\| id:ebi-fl187-GCST90094812 | Weighted median | 5 | 0.215377327852654 | 0.291395322305904 | 0.459831596987342 | -0.355757504 | 0.786512159572226 | 1.24032981966781 | 0.700642502734081 | 2.19572471774679 | 0.999332001504091 |
| ebi-fl187-GCST90094812 | ebi-a-GCST90001701 | BAFF-R on CD20- CD38- B cell \|\| id:ebi-a-GCST90001701 | Plum liking \|\| id:ebi-fl187-GCST90094812 | Weighted median | 5 | -0.014497871 | 0.268811473031833 | 0.956988362841582 | -0.541368358 | 0.512372616602408 | 0.985606717540244 | 0.581951389646916 | 1.66924698341186 | 0.999332001504091 |
| ebi-fl187-GCST90094812 | ebi-a-GCST90001732 | CD19 on IgD- CD27- B cell \|\| id:ebi-a-GCST90001732 | Plum liking \|\| id:ebi-fl187-GCST90094812 | Weighted median | 5 | 0.152294339640033 | 0.317038885327154 | 0.630967976837966 | -0.469101876 | 0.773690554881255 | 1.16450294537098 | 0.625563850216627 | 2.16775171600485 | 0.999332001504091 |
| ebi-fl187-GCST90094812 | ebi-a-GCST90001798 | CD27 on CD24+ CD27+ B cell \|\| id:ebi-a-GCST90001798 | Plum liking \|\| id:ebi-fl187-GCST90094812 | Weighted median | 5 | 0.160242248124133 | 0.297389154944406 | 0.590004045838491 | -0.422640496 | 0.743124991815169 | 1.17379518623498 | 0.655314179121817 | 2.10249554049753 | 0.999332001504091 |
| ebi-fl187-GCST90094812 | ebi-a-GCST90001836 | CD66b on CD66b++ myeloid cell \|\| id:ebi-a-GCST90001836 | Plum liking \|\| id:ebi-fl187-GCST90094812 | Weighted median | 5 | -0.113688213 | 0.425585803806882 | 0.789366397145045 | -0.947836389 | 0.720459962254274 | 0.892536193501964 | 0.387578686577063 | 2.0553783897314 | 0.999332001504091 |
| ebi-fl187-GCST90094812 | ebi-a-GCST90001943 | CD25 on activated & secreting CD4 regulatory T cell \|\| id:ebi-a-GCST90001943 | Plum liking \|\| id:ebi-fl187-GCST90094812 | Weighted median | 5 | -0.047821209 | 0.303427961370236 | 0.874769503293295 | -0.642540014 | 0.546897594858913 | 0.953304213648558 | 0.525954793574556 | 1.7278840973835 | 0.999332001504091 |
| ebi-fl187-GCST90094812 | ebi-a-GCST90001960 | CD25 on CD4+ T cell \|\| id:ebi-a-GCST90001960 | Plum liking \|\| id:ebi-fl187-GCST90094812 | Weighted median | 5 | -0.064189506 | 0.308132175534404 | 0.83498060963427 | -0.66812857 | 0.539749558535894 | 0.937827259255381 | 0.512667101421354 | 1.71557715672415 | 0.999332001504091 |
| ebi-fl187-GCST90094812 | ebi-a-GCST90001988 | HLA DR on CD14+ CD16- monocyte \|\| id:ebi-a-GCST90001988 | Plum liking \|\| id:ebi-fl187-GCST90094812 | Weighted median | 5 | -0.083240006 | 0.298768669308274 | 0.780543966020448 | -0.668826598 | 0.502346586101229 | 0.920130284164487 | 0.512309370283113 | 1.65259467998555 | 0.999332001504091 |
| ebi-fl187-GCST90094812 | ebi-a-GCST90001991 | HLA DR on CD14+ monocyte \|\| id:ebi-a-GCST90001991 | Plum liking \|\| id:ebi-fl187-GCST90094812 | Weighted median | 5 | -0.107131213 | 0.295632571347016 | 0.717068382596846 | -0.686571053 | 0.47230862716895 | 0.898407782771124 | 0.503298899127311 | 1.6036922503571 | 0.999332001504091 |
| ebi-fl187-GCST90094812 | ebi-a-GCST90002019 | CD14 on Monocytic Myeloid-Derived Suppressor Cells \|\| id:ebi-a-GCST90002019 | Plum liking \|\| id:ebi-fl187-GCST90094812 | Weighted median | 5 | 0.2355497396388 | 0.427938794171599 | 0.582025455543861 | -0.603210297 | 1.07430977621513 | 1.26560433039392 | 0.547052612783672 | 2.92797124752103 | 0.999332001504091 |
| ebi-fl187-GCST90094812 | ebi-a-GCST90002037 | CD80 on plasmacytoid Dendritic Cell \|\| id:ebi-a-GCST90002037 | Plum liking \|\| id:ebi-fl187-GCST90094812 | Weighted median | 5 | 0.381704650761377 | 0.351192878850226 | 0.27708967683509 | -0.306633392 | 1.07004269330782 | 1.46477939975899 | 0.735920345903212 | 2.9155039698284 | 0.999332001504091 |
| ebi-fl187-GCST90094812 | ebi-a-GCST90002042 | CD45 on CD33+ HLA DR+ CD14- \|\| id:ebi-a-GCST90002042 | Plum liking \|\| id:ebi-fl187-GCST90094812 | Weighted median | 5 | 0.0893385333808804 | 0.437766427737456 | 0.838292481134033 | -0.768683665 | 0.947360731746294 | 1.09345076325973 | 0.463622949941874 | 2.57889427566774 | 0.999332001504091 |
| ebi-fl187-GCST90094812 | ebi-a-GCST90002056 | CD8 on Effector Memory CD8+ T cell \|\| id:ebi-a-GCST90002056 | Plum liking \|\| id:ebi-fl187-GCST90094812 | Weighted median | 5 | 0.366500374244154 | 0.325999121292009 | 0.260912366555959 | -0.272457903 | 1.00545865197649 | 1.4426769404636 | 0.761505485225134 | 2.73316055488438 | 0.999332001504091 |
| ebi-fl187-GCST90094812 | ebi-a-GCST90002058 | CD8 on CD8+ T cell \|\| id:ebi-a-GCST90002058 | Plum liking \|\| id:ebi-fl187-GCST90094812 | Weighted median | 5 | -0.324041095 | 0.323223592134855 | 0.316088061938127 | -0.957559336 | 0.309477145272864 | 0.723220520796587 | 0.383828540242107 | 1.36271242719826 | 0.999332001504091 |
| ebi-fl187-GCST90094812 | ebi-a-GCST90002063 | CD4 on CD4 regulatory T cell \|\| id:ebi-a-GCST90002063 | Plum liking \|\| id:ebi-fl187-GCST90094812 | Weighted median | 5 | 0.0237639285492974 | 0.299978713533359 | 0.936858658064473 | -0.56419435 | 0.611722207074682 | 1.0240485407288 | 0.56881823062623 | 1.84360373368179 | 0.999332001504091 |
| ebi-fl187-GCST90094812 | ebi-a-GCST90002066 | CD4 on activated CD4 regulatory T cell \|\| id:ebi-a-GCST90002066 | Plum liking \|\| id:ebi-fl187-GCST90094812 | Weighted median | 5 | -0.076981782 | 0.312545648309514 | 0.805445580534702 | -0.689571252 | 0.535607688993062 | 0.925906721905209 | 0.501791164719379 | 1.70848615508941 | 0.999332001504091 |
| ebi-fl187-GCST90094812 | ebi-a-GCST90002070 | CD4 on activated & secreting CD4 regulatory T cell \|\| id:ebi-a-GCST90002070 | Plum liking \|\| id:ebi-fl187-GCST90094812 | Weighted median | 5 | -0.020231475 | 0.311933428709598 | 0.948286815267539 | -0.631620995 | 0.591158045565453 | 0.979971808365419 | 0.531729171720874 | 1.80607872628646 | 0.999332001504091 |
| ebi-fl187-GCST90094812 | ebi-a-GCST90002081 | SSC-A on CD4+ T cell \|\| id:ebi-a-GCST90002081 | Plum liking \|\| id:ebi-fl187-GCST90094812 | Weighted median | 5 | -0.149600903 | 0.30290206752535 | 0.621382465402946 | -0.743288956 | 0.444087148989224 | 0.86105155064117 | 0.475547286682837 | 1.55906635075818 | 0.999332001504091 |
| ebi-fl187-GCST90094812 | ebi-a-GCST90002094 | CD11b on Monocytic Myeloid-Derived Suppressor Cells \|\| id:ebi-a-GCST90002094 | Plum liking \|\| id:ebi-fl187-GCST90094812 | Weighted median | 5 | -0.179028951 | 0.415289143369139 | 0.666399510491241 | -0.992995672 | 0.634937770219865 | 0.836081693826422 | 0.370465234822164 | 1.88690471613948 | 0.999332001504091 |
| ebi-fl187-GCST90094812 | ebi-a-GCST90002104 | HLA DR on myeloid Dendritic Cell \|\| id:ebi-a-GCST90002104 | Plum liking \|\| id:ebi-fl187-GCST90094812 | Weighted median | 5 | -0.087122651 | 0.321296471443078 | 0.786268216927174 | -0.716863735 | 0.542618433412818 | 0.916564671517638 | 0.488281236588484 | 1.7205059996648 | 0.999332001504091 |
| ebi-fl187-GCST90094812 | ebi-a-GCST90002105 | HLA DR on plasmacytoid Dendritic Cell \|\| id:ebi-a-GCST90002105 | Plum liking \|\| id:ebi-fl187-GCST90094812 | Weighted median | 5 | -0.123193686 | 0.31362920600036 | 0.694466888540651 | -0.73790693 | 0.491519557415843 | 0.884092409349365 | 0.478113594127463 | 1.63479850368109 | 0.999332001504091 |
| ebi-fl187-GCST90094812 | ebi-a-GCST90002106 | HLA DR on Dendritic Cell \|\| id:ebi-a-GCST90002106 | Plum liking \|\| id:ebi-fl187-GCST90094812 | Weighted median | 5 | -0.114191372 | 0.305970317979375 | 0.708991693280889 | -0.713893195 | 0.485510451416864 | 0.892087219188679 | 0.489733851786102 | 1.62500428291278 | 0.999332001504091 |
| ebi-fl187-GCST90094812 | ebi-a-GCST90002111 | HLA DR on CD33dim HLA DR+ CD11b- \|\| id:ebi-a-GCST90002111 | Plum liking \|\| id:ebi-fl187-GCST90094812 | Weighted median | 5 | 0.0403381639744656 | 0.410461922058564 | 0.921713893229294 | -0.764167203 | 0.844843531209252 | 1.04116279841806 | 0.465721620965965 | 2.32761358719255 | 0.999332001504091 |
| ebi-fl187-GCST90094812 | ebi-a-GCST90002112 | HLA DR on CD33- HLA DR+ \|\| id:ebi-a-GCST90002112 | Plum liking \|\| id:ebi-fl187-GCST90094812 | Weighted median | 5 | -0.439241146 | 0.428341821405541 | 0.305153080994018 | -1.278791116 | 0.400308824384424 | 0.644525336458418 | 0.27837361867308 | 1.49228548063204 | 0.999332001504091 |
| ebi-fl187-GCST90094812 | ebi-a-GCST90002115 | HLA DR on HLA DR+ CD8+ T cell \|\| id:ebi-a-GCST90002115 | Plum liking \|\| id:ebi-fl187-GCST90094812 | Weighted median | 5 | -0.178891049 | 0.299020891341142 | 0.549669116655033 | -0.764971996 | 0.407189898387429 | 0.836196999233503 | 0.465346962521817 | 1.50258941787835 | 0.999332001504091 |
| ebi-fl187-GCST90094812 | ebi-a-GCST90002116 | HLA DR on B cell \|\| id:ebi-a-GCST90002116 | Plum liking \|\| id:ebi-fl187-GCST90094812 | Weighted median | 5 | 0.061775828590137 | 0.299424405123923 | 0.836544647202893 | -0.525096005 | 0.648647662633026 | 1.06372386152073 | 0.591498574552341 | 1.91295212236974 | 0.999332001504091 |
| ebi-fl187-GCST90094812 | ebi-cfmet338-GCST90026023 | Sphingomyelin (d18:1/24:1, d18:2/24:0) levels \|\| id:ebi-cfmet338-GCST90026023 | Plum liking \|\| id:ebi-fl187-GCST90094812 | Weighted median | 5 | -0.117110366 | 0.176756324692745 | 0.507617123884531 | -0.463552762 | 0.22933203048113 | 0.889487018695787 | 0.629044824116474 | 1.25775958420703 | 0.999332001504091 |
| ebi-fl187-GCST90094812 | ebi-cfmet338-GCST90026046 | 1-arachidonoyl-gpc (20:4n6) levels \|\| id:ebi-cfmet338-GCST90026046 | Plum liking \|\| id:ebi-fl187-GCST90094812 | Weighted median | 5 | -0.298207236 | 0.228101206676674 | 0.191095175095482 | -0.745285601 | 0.148871129317883 | 0.742147524297831 | 0.474598734777789 | 1.16052342212686 | 0.999332001504091 |
| ebi-fl187-GCST90094812 | ebi-cfmet338-GCST90026047 | 1-linoleoyl-gpc (18:2) levels \|\| id:ebi-cfmet338-GCST90026047 | Plum liking \|\| id:ebi-fl187-GCST90094812 | Weighted median | 5 | -0.190984548 | 0.242469274664387 | 0.430892715466884 | -0.666224326 | 0.284255230466555 | 0.826145353753308 | 0.513644274427764 | 1.32877203058196 | 0.999332001504091 |
| ebi-fl187-GCST90094812 | ebi-cfmet338-GCST90026050 | 1-myristoyl-2-palmitoyl-gpc (14:0/16:0) levels \|\| id:ebi-cfmet338-GCST90026050 | Plum liking \|\| id:ebi-fl187-GCST90094812 | Weighted median | 5 | -0.074059797 | 0.126221705513427 | 0.557375818314271 | -0.32145434 | 0.173334745665616 | 0.928616163589637 | 0.725093737090053 | 1.18926413947613 | 0.999332001504091 |
| ebi-fl187-GCST90094812 | ebi-cfmet338-GCST90026068 | 2,3-dihydroxyisovalerate levels \|\| id:ebi-cfmet338-GCST90026068 | Plum liking \|\| id:ebi-fl187-GCST90094812 | Weighted median | 5 | 0.269377918229388 | 0.414803063389937 | 0.516072372595701 | -0.543636086 | 1.08239192247367 | 1.30914979914452 | 0.58063317723121 | 2.95173142666918 | 0.999332001504091 |
| ebi-fl187-GCST90094812 | ebi-cfmet338-GCST90026082 | 3-hydroxyhexanoate levels \|\| id:ebi-cfmet338-GCST90026082 | Plum liking \|\| id:ebi-fl187-GCST90094812 | Weighted median | 5 | -0.15456002 | 0.0967007317176994 | 0.109968774395434 | -0.344093454 | 0.0349734146245889 | 0.856792066331338 | 0.708862679107567 | 1.03559217682686 | 0.999332001504091 |
| ebi-fl187-GCST90094812 | ebi-cfmet338-GCST90026094 | 3-ureidopropionate levels \|\| id:ebi-cfmet338-GCST90026094 | Plum liking \|\| id:ebi-fl187-GCST90094812 | Weighted median | 5 | 0.223036274542123 | 0.192854811156062 | 0.247477366347643 | -0.154959155 | 0.601031704408005 | 1.24986591122733 | 0.856450158198649 | 1.82399965846673 | 0.999332001504091 |
| ebi-fl187-GCST90094812 | ebi-cfmet338-GCST90026173 | Glycerol levels \|\| id:ebi-cfmet338-GCST90026173 | Plum liking \|\| id:ebi-fl187-GCST90094812 | Weighted median | 5 | -0.071759342 | 0.10846501284103 | 0.508234170228841 | -0.284350767 | 0.14083208306873 | 0.930754862371345 | 0.752502645110624 | 1.1512313205232 | 0.999332001504091 |
| ebi-fl187-GCST90094812 | ebi-cfmet338-GCST90026179 | Histidine levels \|\| id:ebi-cfmet338-GCST90026179 | Plum liking \|\| id:ebi-fl187-GCST90094812 | Weighted median | 5 | -0.080260518 | 0.0804146564129053 | 0.318239015351309 | -0.237873244 | 0.0773522088851197 | 0.922875889576837 | 0.788302606646599 | 1.08042254380628 | 0.999332001504091 |
| ebi-fl187-GCST90094812 | ebi-cfmet338-GCST90026188 | Isocitrate levels \|\| id:ebi-cfmet338-GCST90026188 | Plum liking \|\| id:ebi-fl187-GCST90094812 | Weighted median | 5 | -0.008648623 | 0.166495029070799 | 0.958572334116811 | -0.33497888 | 0.317681634304652 | 0.991388669078097 | 0.715353194700798 | 1.37393877661723 | 0.999332001504091 |
| ebi-fl187-GCST90094812 | ebi-cfmet338-GCST90026207 | N-acetyl-isoputreanine levels \|\| id:ebi-cfmet338-GCST90026207 | Plum liking \|\| id:ebi-fl187-GCST90094812 | Weighted median | 5 | -0.062831732 | 0.10521319894841 | 0.550383952301291 | -0.269049602 | 0.143386138261973 | 0.939101481426402 | 0.764105353848023 | 1.15417538690963 | 0.999332001504091 |
| ebi-fl187-GCST90094812 | ebi-cfmet338-GCST90026213 | N-acetylglutamate levels \|\| id:ebi-cfmet338-GCST90026213 | Plum liking \|\| id:ebi-fl187-GCST90094812 | Weighted median | 5 | -0.02015062 | 0.159458674537081 | 0.899439904295262 | -0.332689622 | 0.292388381839084 | 0.980051046652071 | 0.716992698135169 | 1.33962320194054 | 0.999332001504091 |
| ebi-fl187-GCST90094812 | ebi-cfmet338-GCST90026224 | N-acetyltaurine levels \|\| id:ebi-cfmet338-GCST90026224 | Plum liking \|\| id:ebi-fl187-GCST90094812 | Weighted median | 5 | -0.193209364 | 0.136003158078617 | 0.155426068649244 | -0.459775554 | 0.073356825906062 | 0.824309375423018 | 0.631425350648196 | 1.07611445392991 | 0.999332001504091 |
| ebi-fl187-GCST90094812 | ebi-cfmet338-GCST90026250 | Phenyllactate (pla) levels \|\| id:ebi-cfmet338-GCST90026250 | Plum liking \|\| id:ebi-fl187-GCST90094812 | Weighted median | 5 | 0.117365712214349 | 0.217691918183523 | 0.589792502764224 | -0.309310447 | 0.544041871854054 | 1.12453060907844 | 0.733952880872093 | 1.72295677789527 | 0.999332001504091 |
| ebi-fl187-GCST90094812 | ebi-cfmet338-GCST90026251 | Pipecolate levels \|\| id:ebi-cfmet338-GCST90026251 | Plum liking \|\| id:ebi-fl187-GCST90094812 | Weighted median | 5 | 0.188123669375074 | 0.316384058638954 | 0.552107270404019 | -0.431989086 | 0.808236424307424 | 1.20698277292537 | 0.649216462482374 | 2.24394712445877 | 0.999332001504091 |
| ebi-fl187-GCST90094812 | ebi-cfmet338-GCST90026278 | Trigonelline (n'-methylnicotinate) levels \|\| id:ebi-cfmet338-GCST90026278 | Plum liking \|\| id:ebi-fl187-GCST90094812 | Weighted median | 5 | -0.498087956 | 0.374423767606321 | 0.183426468788488 | -1.23195854 | 0.23578262894956 | 0.607691482707859 | 0.291720671189824 | 1.26589911043835 | 0.999332001504091 |
| ebi-fl187-GCST90094812 | ebi-cfmet338-GCST90026299 | 1-carboxyethylvaline levels \|\| id:ebi-cfmet338-GCST90026299 | Plum liking \|\| id:ebi-fl187-GCST90094812 | Weighted median | 5 | 0.276759732983866 | 0.222155837380986 | 0.212841150807759 | -0.158665708 | 0.712185174250599 | 1.31884945694073 | 0.853281556253569 | 2.0384407436506 | 0.999332001504091 |
| ebi-fl187-GCST90094812 | ebi-gm430-GCST90011306 | C_Erysipelotrichia abundance \|\| id:ebi-gm430-GCST90011306 | Plum liking \|\| id:ebi-fl187-GCST90094812 | Weighted median | 5 | -0.234612581 | 0.186820212676008 | 0.209181197557123 | -0.600780197 | 0.13155503619233 | 0.790877191408871 | 0.548383621618503 | 1.14060068031339 | 0.999332001504091 |
| ebi-fl187-GCST90094812 | ebi-gm430-GCST90011312 | F_Erysipelotrichaceae abundance \|\| id:ebi-gm430-GCST90011312 | Plum liking \|\| id:ebi-fl187-GCST90094812 | Weighted median | 5 | -0.234612581 | 0.184713862033681 | 0.204034541986522 | -0.59665175 | 0.127426588933369 | 0.790877191408871 | 0.550652274262171 | 1.13590147744125 | 0.999332001504091 |
| ebi-fl187-GCST90094812 | ebi-gm430-GCST90011352 | O_Erysipelotrichales abundance \|\| id:ebi-gm430-GCST90011352 | Plum liking \|\| id:ebi-fl187-GCST90094812 | Weighted median | 5 | -0.234612581 | 0.178989665243077 | 0.189938696141389 | -0.585432325 | 0.116207163223784 | 0.790877191408871 | 0.556865063338114 | 1.12322853967767 | 0.999332001504091 |
| ebi-fl187-GCST90094812 | ebi-gm430-GCST90011362 | OTU97_11 (Parabacteroides) abundance \|\| id:ebi-gm430-GCST90011362 | Plum liking \|\| id:ebi-fl187-GCST90094812 | Weighted median | 5 | -0.36608582 | 0.243888520195798 | 0.133345856510627 | -0.84410732 | 0.111935679220617 | 0.69344328710131 | 0.429940986699345 | 1.11844091933978 | 0.999332001504091 |
| ebi-fl187-GCST90094812 | ebi-gm430-GCST90011384 | OTU97_235 (Phascolarctobacterium) abundance \|\| id:ebi-gm430-GCST90011384 | Plum liking \|\| id:ebi-fl187-GCST90094812 | Weighted median | 5 | 1.37076931846767 | 0.818654470941423 | 0.0940485888428725 | -0.233793445 | 2.97533208151286 | 3.93837939830641 | 0.79152529285275 | 19.5961296815943 | 0.999332001504091 |
| ebi-fl187-GCST90094812 | ebi-gm430-GCST90011390 | OTU97_3 (Bacteroides) abundance \|\| id:ebi-gm430-GCST90011390 | Plum liking \|\| id:ebi-fl187-GCST90094812 | Weighted median | 5 | 0.0037598671968178 | 0.1903862556846 | 0.984243899396033 | -0.369397194 | 0.376916928338634 | 1.00376694436444 | 0.691150834997323 | 1.45778320386842 | 0.999332001504091 |
| ebi-fl187-GCST90094812 | ebi-gm430-GCST90011398 | OTU97_4 (Alistipes) abundance \|\| id:ebi-gm430-GCST90011398 | Plum liking \|\| id:ebi-fl187-GCST90094812 | Weighted median | 5 | 0.00414832795408539 | 0.22545287602832 | 0.985319770011066 | -0.437739309 | 0.446035964969593 | 1.00415694417668 | 0.645494035371316 | 1.56210764667749 | 0.999332001504091 |
| ebi-fl187-GCST90094812 | ebi-gm430-GCST90011421 | OTU99_108 (Cytophagales) abundance \|\| id:ebi-gm430-GCST90011421 | Plum liking \|\| id:ebi-fl187-GCST90094812 | Weighted median | 5 | 0.484383506307287 | 0.713431160616327 | 0.497170058599131 | -0.913941569 | 1.88270858111529 | 1.62317402377761 | 0.400940769927863 | 6.57127962302368 | 0.999332001504091 |
| ebi-fl187-GCST90094812 | ebi-gm430-GCST90011428 | OTU99_123 (Phascolarctobacterium) abundance \|\| id:ebi-gm430-GCST90011428 | Plum liking \|\| id:ebi-fl187-GCST90094812 | Weighted median | 5 | -0.010439143 | 0.404233853358366 | 0.979397308206625 | -0.802737496 | 0.781859209363494 | 0.989615155528282 | 0.448100610038708 | 2.18553185180147 | 0.999332001504091 |
| ebi-fl187-GCST90094812 | ebi-gm430-GCST90011450 | OTU99_298 (Phascolarctobacterium) abundance \|\| id:ebi-gm430-GCST90011450 | Plum liking \|\| id:ebi-fl187-GCST90094812 | Weighted median | 5 | 1.3548166816347 | 0.849317945894156 | 0.110671547440137 | -0.309846492 | 3.01947985558725 | 3.87605034028846 | 0.73355955460866 | 20.4806360247944 | 0.999332001504091 |
| ebi-fl187-GCST90094812 | ebi-gm430-GCST90011451 | OTU99_3 (Bacteroides) abundance \|\| id:ebi-gm430-GCST90011451 | Plum liking \|\| id:ebi-fl187-GCST90094812 | Weighted median | 5 | 0.00600311908958066 | 0.189176731656087 | 0.974685088115091 | -0.364783275 | 0.376789513135511 | 1.00602117391933 | 0.69434711698174 | 1.45759747195818 | 0.999332001504091 |
| ebi-fl187-GCST90094812 | ebi-gm430-GCST90011472 | OTU99_72 (Parabacteroides) abundance \|\| id:ebi-gm430-GCST90011472 | Plum liking \|\| id:ebi-fl187-GCST90094812 | Weighted median | 5 | -0.370188761 | 0.546347248997653 | 0.498044076415272 | -1.441029369 | 0.700651847174726 | 0.690603959335494 | 0.236683998098024 | 2.01506579440295 | 0.999332001504091 |
| ebi-fl187-GCST90094812 | ebi-gm430-GCST90011507 | TestASV_3 (Bacteroides) abundance \|\| id:ebi-gm430-GCST90011507 | Plum liking \|\| id:ebi-fl187-GCST90094812 | Weighted median | 5 | 0.637206785679446 | 0.26133352251467 | 0.0147569502365518 | 0.124993081550692 | 1.1494204898082 | 1.89119099308652 | 1.13314061346381 | 3.15636323491973 | 0.999332001504091 |
| ebi-fl187-GCST90094812 | ebi-gm430-GCST90011512 | TestASV_34 (Phascolarctobacterium) abundance \|\| id:ebi-gm430-GCST90011512 | Plum liking \|\| id:ebi-fl187-GCST90094812 | Weighted median | 5 | -0.0161477 | 0.580115507484185 | 0.97779349711442 | -1.153174094 | 1.12087869499538 | 0.983981975505247 | 0.315633327752358 | 3.06754845888411 | 0.999332001504091 |
| ebi-fl187-GCST90094812 | ebi-gm430-GCST90011519 | TestASV_41 (Barnesiella) abundance \|\| id:ebi-gm430-GCST90011519 | Plum liking \|\| id:ebi-fl187-GCST90094812 | Weighted median | 5 | -0.736063653 | 0.71419297733272 | 0.302717682893018 | -2.135881888 | 0.663754582771934 | 0.478995702800597 | 0.118140357814789 | 1.9420703267306 | 0.999332001504091 |
| ebi-fl187-GCST90094812 | ebi-gm430-GCST90011524 | TestASV_46 (Sutterella) abundance \|\| id:ebi-gm430-GCST90011524 | Plum liking \|\| id:ebi-fl187-GCST90094812 | Weighted median | 5 | 0.0373213049898318 | 0.786211069163366 | 0.962138776209069 | -1.503652391 | 1.57829500055003 | 1.03802649035351 | 0.222316688118632 | 4.84668516697518 | 0.999332001504091 |
| ebi-fl187-GCST90094812 | ebi-gm430-GCST90011581 | OTU97_19 (Bacteria) prevalence \|\| id:ebi-gm430-GCST90011581 | Plum liking \|\| id:ebi-fl187-GCST90094812 | Weighted median | 5 | 0.617772289655786 | 0.930583617318177 | 0.506783324698891 | -1.2061716 | 2.44171617959941 | 1.85479149784001 | 0.299341085885747 | 11.4927474465455 | 0.999332001504091 |
| ebi-fl187-GCST90094812 | ebi-gm430-GCST90011614 | OTU97_80 (Ruminococcus) prevalence \|\| id:ebi-gm430-GCST90011614 | Plum liking \|\| id:ebi-fl187-GCST90094812 | Weighted median | 5 | -0.079237867 | 0.404441755314359 | 0.844673483604219 | -0.871943707 | 0.713467973783837 | 0.923820152319407 | 0.418138021054775 | 2.04105733240569 | 0.999332001504091 |
| ebi-fl187-GCST90094812 | ebi-gm430-GCST90011623 | OTU99_11 (Parabacteroides) prevalence \|\| id:ebi-gm430-GCST90011623 | Plum liking \|\| id:ebi-fl187-GCST90094812 | Weighted median | 5 | 0.0481023280005914 | 0.384482808515575 | 0.900437117659162 | -0.705483977 | 0.801688632691118 | 1.04927802035304 | 0.493869495082945 | 2.22930222448966 | 0.999332001504091 |
| ebi-fl187-GCST90094812 | ebi-gm430-GCST90011642 | OTU99_19 (Bacteria) prevalence \|\| id:ebi-gm430-GCST90011642 | Plum liking \|\| id:ebi-fl187-GCST90094812 | Weighted median | 5 | 0.617772289655786 | 0.87611558026887 | 0.480731532804674 | -1.099414248 | 2.33495882698277 | 1.85479149784001 | 0.333066120826696 | 10.3290346430931 | 0.999332001504091 |
| ebi-fl187-GCST90094812 | ebi-gm430-GCST90011653 | OTU99_30 (Parasutterella) prevalence \|\| id:ebi-gm430-GCST90011653 | Plum liking \|\| id:ebi-fl187-GCST90094812 | Weighted median | 5 | -0.702213273 | 0.399359320833876 | 0.0786880553902176 | -1.484957542 | 0.0805309957332537 | 0.495487440280158 | 0.226511957731609 | 1.08386244123272 | 0.999332001504091 |
| ebi-fl187-GCST90094812 | ebi-gm430-GCST90011675 | OTU99_8 (Ruminococcaceae) prevalence \|\| id:ebi-gm430-GCST90011675 | Plum liking \|\| id:ebi-fl187-GCST90094812 | Weighted median | 5 | 0.0811998168613907 | 0.420905578179361 | 0.847024016752551 | -0.743775116 | 0.906174750092938 | 1.08458759380506 | 0.475316150489293 | 2.47483753166165 | 0.999332001504091 |
| ebi-fl187-GCST90094812 | ebi-gm430-GCST90011679 | OTU99_92 (Ruminococcus) prevalence \|\| id:ebi-gm430-GCST90011679 | Plum liking \|\| id:ebi-fl187-GCST90094812 | Weighted median | 5 | -0.132305783 | 0.432457106768572 | 0.759650584386927 | -0.979921712 | 0.715310146729973 | 0.876073066273185 | 0.375340482430885 | 2.04482077839985 | 0.999332001504091 |
| ebi-fl187-GCST90094812 | ebi-gm430-GCST90011684 | TestASV_11 (Lachnospiraceae) prevalence \|\| id:ebi-gm430-GCST90011684 | Plum liking \|\| id:ebi-fl187-GCST90094812 | Weighted median | 5 | -0.123608589 | 0.454333324120211 | 0.785571376040077 | -1.014101904 | 0.76688472668523 | 0.8837256735085 | 0.362728048250111 | 2.15304846092175 | 0.999332001504091 |
| ebi-fl187-GCST90094812 | ebi-gm430-GCST90011688 | TestASV_15 (Bacteroides) prevalence \|\| id:ebi-gm430-GCST90011688 | Plum liking \|\| id:ebi-fl187-GCST90094812 | Weighted median | 5 | -0.368845578 | 0.410323865359567 | 0.368698845675435 | -1.173080354 | 0.435389198166256 | 0.691532190031916 | 0.309412372163256 | 1.5455644727678 | 0.999332001504091 |
| ebi-fl187-GCST90094812 | ebi-gm430-GCST90011692 | TestASV_19 (Prevotella) prevalence \|\| id:ebi-gm430-GCST90011692 | Plum liking \|\| id:ebi-fl187-GCST90094812 | Weighted median | 5 | 0.259332528675918 | 0.752352191384218 | 0.730323187708183 | -1.215277766 | 1.73394282378899 | 1.29606471186214 | 0.29662760965566 | 5.66293791493072 | 0.999332001504091 |
| ebi-fl187-GCST90094812 | ebi-gm430-GCST90011693 | TestASV_2 (Bacteroides) prevalence \|\| id:ebi-gm430-GCST90011693 | Plum liking \|\| id:ebi-fl187-GCST90094812 | Weighted median | 5 | 0.317492858025301 | 0.35821621055209 | 0.375447120442814 | -0.384610915 | 1.0195966307074 | 1.37367943404655 | 0.680715441086417 | 2.77207636793258 | 0.999332001504091 |
| ebi-fl187-GCST90094812 | ebi-gm430-GCST90011716 | TestASV_40 (Barnesiella) prevalence \|\| id:ebi-gm430-GCST90011716 | Plum liking \|\| id:ebi-fl187-GCST90094812 | Weighted median | 5 | -0.66230112 | 0.81231858763401 | 0.414888086186897 | -2.254445552 | 0.929843311623307 | 0.515663364831511 | 0.10493170679585 | 2.53411208060016 | 0.999332001504091 |
| ebi-fl187-GCST90094812 | ebi-gm430-GCST90011718 | TestASV_42 (Alphaproteobacteria) prevalence \|\| id:ebi-gm430-GCST90011718 | Plum liking \|\| id:ebi-fl187-GCST90094812 | Weighted median | 5 | -0.219267059 | 0.649411766877664 | 0.73563487760224 | -1.492114122 | 1.05358000386442 | 0.803107212329694 | 0.224896693481725 | 2.86789985442086 | 0.999332001504091 |
| ebi-fl187-GCST90094812 | ebi-hbsm44-GCST90104929 | Sulcal depth of the right inferior frontal sulcus \|\| id:ebi-hbsm44-GCST90104929 | Plum liking \|\| id:ebi-fl187-GCST90094812 | Weighted median | 4 | -0.129582791 | 0.346345903420499 | 0.708298153479718 | -0.808420761 | 0.549255180037731 | 0.878461856962959 | 0.445561158294663 | 1.73196253706761 | 0.999332001504091 |
| ebi-fl187-GCST90094812 | ebi-hbsm44-GCST90104935 | Sulcal opening distance of the left calloso-marginal posterior fissure \|\| id:ebi-hbsm44-GCST90104935 | Plum liking \|\| id:ebi-fl187-GCST90094812 | Weighted median | 5 | 0.085197935504607 | 0.0809787780801513 | 0.292752748261022 | -0.07352047 | 0.243916340541704 | 1.08893258378891 | 0.929117127003649 | 1.27623755668055 | 0.999332001504091 |
| ebi-fl187-GCST90094812 | ebi-hbsm44-GCST90104936 | Sulcal opening distance of the left anterior inferior temporal sulcus \|\| id:ebi-hbsm44-GCST90104936 | Plum liking \|\| id:ebi-fl187-GCST90094812 | Weighted median | 5 | -0.032231838 | 0.0428300585511043 | 0.451719283678655 | -0.116178753 | 0.0517150765699179 | 0.968282071292059 | 0.8903160624497 | 1.05307565383682 | 0.999332001504091 |
| ebi-fl187-GCST90094812 | ebi-hbsm44-GCST90104941 | Sulcal opening distance of the right posterior inferior temporal sulcus \|\| id:ebi-hbsm44-GCST90104941 | Plum liking \|\| id:ebi-fl187-GCST90094812 | Weighted median | 5 | 0.0624790286529929 | 0.0361887267862084 | 0.0842615134396225 | -0.008450876 | 0.133408933153961 | 1.06447213526925 | 0.99158473242569 | 1.14271719774548 | 0.999332001504091 |
| ebi-fl187-GCST90094812 | ebi-IPs91-GCST90274819 | Leukemia inhibitory factor levels \|\| id:ebi-IPs91-GCST90274819 | Plum liking \|\| id:ebi-fl187-GCST90094812 | Weighted median | 5 | 0.254222223359439 | 0.160047706204327 | 0.112192462689538 | -0.059471281 | 0.56791572751992 | 1.28945832019113 | 0.942262594229562 | 1.76458533925952 | 0.999332001504091 |
| ebi-fl187-GCST90094812 | ebi-IPs91-GCST90274837 | STAM binding protein levels \|\| id:ebi-IPs91-GCST90274837 | Plum liking \|\| id:ebi-fl187-GCST90094812 | Weighted median | 5 | -0.225874861 | 0.126585060939388 | 0.0743632082382721 | -0.473981581 | 0.0222318580712469 | 0.797817933262345 | 0.622518718751929 | 1.02248082742175 | 0.999332001504091 |
| ebi-fl187-GCST90094812 | ebi-IPs91-GCST90274840 | TNF-beta levels \|\| id:ebi-IPs91-GCST90274840 | Plum liking \|\| id:ebi-fl187-GCST90094812 | Weighted median | 5 | 0.100127050047196 | 0.157759878499097 | 0.525637097390373 | -0.209082312 | 0.409336411905426 | 1.105311339 | 0.811328450980722 | 1.50581821045956 | 0.999332001504091 |
| ebi-fl187-GCST90094812 | ebi-IPs91-GCST90274842 | Tumor necrosis factor ligand superfamily member 14 levels \|\| id:ebi-IPs91-GCST90274842 | Plum liking \|\| id:ebi-fl187-GCST90094812 | Weighted median | 5 | -0.265714918 | 0.145210677733695 | 0.0672711778962881 | -0.550327846 | 0.0188980106229584 | 0.766657656851348 | 0.576760691 | 1.01907770821647 | 0.999332001504091 |
| ebi-fl187-GCST90094812 | ebi-met1400-GCST90199628 | 3-methylhistidine levels \|\| id:ebi-met1400-GCST90199628 | Plum liking \|\| id:ebi-fl187-GCST90094812 | Weighted median | 5 | 0.101791344651232 | 0.172288570615713 | 0.554641766583778 | -0.235894254 | 0.439476943058029 | 1.10715243434785 | 0.789864194688924 | 1.55189527658629 | 0.999332001504091 |
| ebi-fl187-GCST90094812 | ebi-met1400-GCST90199651 | Docosahexaenoate DHA; 22:6n3 levels \|\| id:ebi-met1400-GCST90199651 | Plum liking \|\| id:ebi-fl187-GCST90094812 | Weighted median | 5 | -0.031839464 | 0.170757214993746 | 0.852083703996805 | -0.366523606 | 0.302844677015053 | 0.9686620743716 | 0.69313977419798 | 1.35370418673721 | 0.999332001504091 |
| ebi-fl187-GCST90094812 | ebi-met1400-GCST90199652 | 1-stearoyl-2-oleoyl-GPS (18:0/18:1) levels \|\| id:ebi-met1400-GCST90199652 | Plum liking \|\| id:ebi-fl187-GCST90094812 | Weighted median | 5 | -0.042027944 | 0.177737214425424 | 0.813074860859169 | -0.390392884 | 0.306336996559586 | 0.958842986563702 | 0.67679092194083 | 1.35844001903261 | 0.999332001504091 |
| ebi-fl187-GCST90094812 | ebi-met1400-GCST90199653 | 1-myristoyl-2-palmitoyl-gpc (14:0/16:0) levels \|\| id:ebi-met1400-GCST90199653 | Plum liking \|\| id:ebi-fl187-GCST90094812 | Weighted median | 5 | -0.04755357 | 0.169249156979217 | 0.77873506908089 | -0.379281917 | 0.284174778172873 | 0.95355939005855 | 0.684352654694064 | 1.32866513212449 | 0.999332001504091 |
| ebi-fl187-GCST90094812 | ebi-met1400-GCST90199667 | Erythritol levels in elite athletes \|\| id:ebi-met1400-GCST90199667 | Plum liking \|\| id:ebi-fl187-GCST90094812 | Weighted median | 5 | 0.322843139290734 | 0.172381905378281 | 0.0610909540376739 | -0.015025395 | 0.660711673832165 | 1.38104870163533 | 0.985086922756048 | 1.93616976555982 | 0.999332001504091 |
| ebi-fl187-GCST90094812 | ebi-met1400-GCST90199674 | 3-hydroxymyristate levels \|\| id:ebi-met1400-GCST90199674 | Plum liking \|\| id:ebi-fl187-GCST90094812 | Weighted median | 5 | -0.003851184 | 0.182329895899065 | 0.983148285062061 | -0.36121778 | 0.35351541240759 | 0.996156222742088 | 0.696827227244337 | 1.42406493505144 | 0.999332001504091 |
| ebi-fl187-GCST90094812 | ebi-met1400-GCST90199685 | 2-linoleoylglycerol (18:2) levels \|\| id:ebi-met1400-GCST90199685 | Plum liking \|\| id:ebi-fl187-GCST90094812 | Weighted median | 5 | -0.187712553 | 0.199617612254261 | 0.347032586531426 | -0.578963073 | 0.203537967440525 | 0.828852924630097 | 0.56047924163564 | 1.22573169465297 | 0.999332001504091 |
| ebi-fl187-GCST90094812 | ebi-met1400-GCST90199703 | Gamma-glutamylvaline levels \|\| id:ebi-met1400-GCST90199703 | Plum liking \|\| id:ebi-fl187-GCST90094812 | Weighted median | 5 | 0.0393488338603221 | 0.172894054683643 | 0.819965552618011 | -0.299523513 | 0.378221181040262 | 1.04013325407144 | 0.741171294807336 | 1.45968576198904 | 0.999332001504091 |
| ebi-fl187-GCST90094812 | ebi-met1400-GCST90199779 | Gamma-glutamyl-2-aminobutyrate levels \|\| id:ebi-met1400-GCST90199779 | Plum liking \|\| id:ebi-fl187-GCST90094812 | Weighted median | 5 | 0.121193574198686 | 0.183191513977773 | 0.508248363930976 | -0.237861793 | 0.480248941595122 | 1.12884340617707 | 0.788311633595356 | 1.61647676041218 | 0.999332001504091 |
| ebi-fl187-GCST90094812 | ebi-met1400-GCST90199824 | 4-vinylphenol sulfate levels \|\| id:ebi-met1400-GCST90199824 | Plum liking \|\| id:ebi-fl187-GCST90094812 | Weighted median | 5 | 0.072649996498573 | 0.182043436726747 | 0.689833846531512 | -0.284155139 | 0.429455132482998 | 1.07535409334232 | 0.752649869934394 | 1.53642015000797 | 0.999332001504091 |
| ebi-fl187-GCST90094812 | ebi-met1400-GCST90199830 | 4-ethylphenylsulfate levels \|\| id:ebi-met1400-GCST90199830 | Plum liking \|\| id:ebi-fl187-GCST90094812 | Weighted median | 5 | 0.231360106522171 | 0.181025797389782 | 0.201231629224236 | -0.123450456 | 0.586170669406143 | 1.26031300467538 | 0.883865430068424 | 1.79709355713903 | 0.999332001504091 |
| ebi-fl187-GCST90094812 | ebi-met1400-GCST90199833 | Hexanoylglutamine levels \|\| id:ebi-met1400-GCST90199833 | Plum liking \|\| id:ebi-fl187-GCST90094812 | Weighted median | 5 | 0.133578483263396 | 0.186861301782697 | 0.47469948110775 | -0.232669668 | 0.499826634757481 | 1.14291096199732 | 0.792415290240115 | 1.64843546451233 | 0.999332001504091 |
| ebi-fl187-GCST90094812 | ebi-met1400-GCST90199837 | Carotenoid levels (cryptoxanthin) \|\| id:ebi-met1400-GCST90199837 | Plum liking \|\| id:ebi-fl187-GCST90094812 | Weighted median | 5 | 0.131037540915322 | 0.192485644460698 | 0.496020031555186 | -0.246234322 | 0.508309404058289 | 1.14001057754924 | 0.781739024632676 | 1.66247823886599 | 0.999332001504091 |
| ebi-fl187-GCST90094812 | ebi-met1400-GCST90199869 | 2-hydroxyhippurate levels \|\| id:ebi-met1400-GCST90199869 | Plum liking \|\| id:ebi-fl187-GCST90094812 | Weighted median | 5 | -0.092277309 | 0.190512798492822 | 0.628128425849914 | -0.465682394 | 0.281127775653465 | 0.911852249287634 | 0.627706615503415 | 1.32462284767237 | 0.999332001504091 |
| ebi-fl187-GCST90094812 | ebi-met1400-GCST90199902 | Hydantoin-5-propionate levels \|\| id:ebi-met1400-GCST90199902 | Plum liking \|\| id:ebi-fl187-GCST90094812 | Weighted median | 5 | -0.116117327 | 0.178636943318018 | 0.515680238980314 | -0.466245736 | 0.234011081912633 | 0.890370752647979 | 0.627353101928721 | 1.26365849588323 | 0.999332001504091 |
| ebi-fl187-GCST90094812 | ebi-met1400-GCST90199904 | Pantoate levels \|\| id:ebi-met1400-GCST90199904 | Plum liking \|\| id:ebi-fl187-GCST90094812 | Weighted median | 5 | -0.099599143 | 0.175612025302746 | 0.570609244761129 | -0.443798713 | 0.2446004261247 | 0.905200200731809 | 0.641594552433487 | 1.27711091108407 | 0.999332001504091 |
| ebi-fl187-GCST90094812 | ebi-met1400-GCST90199936 | 3-methyl catechol sulfate (2) levels \|\| id:ebi-met1400-GCST90199936 | Plum liking \|\| id:ebi-fl187-GCST90094812 | Weighted median | 5 | -0.123593371 | 0.205405522871125 | 0.547371038455397 | -0.526188195 | 0.279001454283036 | 0.883739122188795 | 0.590852898436756 | 1.32180926615293 | 0.999332001504091 |
| ebi-fl187-GCST90094812 | ebi-met1400-GCST90199977 | 4-hydroxychlorothalonil levels \|\| id:ebi-met1400-GCST90199977 | Plum liking \|\| id:ebi-fl187-GCST90094812 | Weighted median | 5 | -0.291367635 | 0.178014137113458 | 0.10167919925317 | -0.640275343 | 0.0575400740777426 | 0.747240915851637 | 0.527147257536758 | 1.05922771737811 | 0.999332001504091 |
| ebi-fl187-GCST90094812 | ebi-met1400-GCST90200040 | Sphingomyelin (d18:2/24:1, d18:1/24:2) levels \|\| id:ebi-met1400-GCST90200040 | Plum liking \|\| id:ebi-fl187-GCST90094812 | Weighted median | 5 | 0.00408650577708157 | 0.163571919725028 | 0.98006857945784 | -0.316514457 | 0.324687468438136 | 1.00409486692723 | 0.7286844769767 | 1.38359816030753 | 0.999332001504091 |
| ebi-fl187-GCST90094812 | ebi-met1400-GCST90200077 | 1-stearoyl-2-oleoyl-GPI (18:0/18:1) levels \|\| id:ebi-met1400-GCST90200077 | Plum liking \|\| id:ebi-fl187-GCST90094812 | Weighted median | 5 | 0.307986014906408 | 0.178693435809372 | 0.0847901795217872 | -0.042253119 | 0.658225149092777 | 1.36068195953953 | 0.958627102858548 | 1.93136141206049 | 0.999332001504091 |
| ebi-fl187-GCST90094812 | ebi-met1400-GCST90200080 | 1-palmitoyl-2-oleoyl-GPI (16:0/18:1) levels \|\| id:ebi-met1400-GCST90200080 | Plum liking \|\| id:ebi-fl187-GCST90094812 | Weighted median | 5 | 0.218084414897073 | 0.181006965081983 | 0.228265110972081 | -0.136689237 | 0.57285806645776 | 1.24369204932432 | 0.872241244617935 | 1.77332810515061 | 0.999332001504091 |
| ebi-fl187-GCST90094812 | ebi-met1400-GCST90200082 | 1-oleoyl-2-linoleoyl-GPE (18:1/18:2) levels \|\| id:ebi-met1400-GCST90200082 | Plum liking \|\| id:ebi-fl187-GCST90094812 | Weighted median | 5 | 0.0964515427690539 | 0.18061276357904 | 0.593324958948377 | -0.257549474 | 0.450452559383972 | 1.10125621602405 | 0.772943384885631 | 1.56902210051395 | 0.999332001504091 |
| ebi-fl187-GCST90094812 | ebi-met1400-GCST90200100 | Perfluorooctanesulfonate (PFOS) levels \|\| id:ebi-met1400-GCST90200100 | Plum liking \|\| id:ebi-fl187-GCST90094812 | Weighted median | 5 | 0.0225061240409284 | 0.166591750020872 | 0.892534810060233 | -0.304013706 | 0.349025954081838 | 1.02276129757731 | 0.737850753398875 | 1.41768598460257 | 0.999332001504091 |
| ebi-fl187-GCST90094812 | ebi-met1400-GCST90200102 | 1-palmitoleoyl-2-linolenoyl-GPC (16:1/18:3) levels \|\| id:ebi-met1400-GCST90200102 | Plum liking \|\| id:ebi-fl187-GCST90094812 | Weighted median | 5 | 0.0491373021515737 | 0.177130434666959 | 0.781467261956861 | -0.29803835 | 0.396312954098813 | 1.05036455815358 | 0.742272873188919 | 1.48633439921543 | 0.999332001504091 |
| ebi-fl187-GCST90094812 | ebi-met1400-GCST90200114 | Glycosyl-N-tricosanoyl-sphingadienine (d18:2/23:0) levels \|\| id:ebi-met1400-GCST90200114 | Plum liking \|\| id:ebi-fl187-GCST90094812 | Weighted median | 5 | -0.034406296 | 0.193802941300263 | 0.859090241260171 | -0.41426006 | 0.345447469417466 | 0.966178870722836 | 0.660829073391823 | 1.41262188335615 | 0.999332001504091 |
| ebi-fl187-GCST90094812 | ebi-met1400-GCST90200135 | Arachidoylcarnitine (C20) levels \|\| id:ebi-met1400-GCST90200135 | Plum liking \|\| id:ebi-fl187-GCST90094812 | Weighted median | 5 | 0.194154104349535 | 0.185828665824629 | 0.296114633049911 | -0.170070081 | 0.558378289365808 | 1.2142833948912 | 0.84360569407523 | 1.74783571692798 | 0.999332001504091 |
| ebi-fl187-GCST90094812 | ebi-met1400-GCST90200146 | Docosahexaenoylcarnitine (C22:6) levels \|\| id:ebi-met1400-GCST90200146 | Plum liking \|\| id:ebi-fl187-GCST90094812 | Weighted median | 5 | -0.168677233 | 0.207044095450305 | 0.41524845446466 | -0.57448366 | 0.237129194032877 | 0.844781526943834 | 0.562995490787859 | 1.26760487418267 | 0.999332001504091 |
| ebi-fl187-GCST90094812 | ebi-met1400-GCST90200162 | 3-hydroxyphenylacetoylglutamine levels \|\| id:ebi-met1400-GCST90200162 | Plum liking \|\| id:ebi-fl187-GCST90094812 | Weighted median | 5 | -0.305923924 | 0.207567938958459 | 0.140522090309105 | -0.712757084 | 0.100909236580139 | 0.736442643098721 | 0.49029055994682 | 1.10617623686872 | 0.999332001504091 |
| ebi-fl187-GCST90094812 | ebi-met1400-GCST90200172 | 3-carboxy-4-methyl-5-pentyl-2-furanpropionate (3-CMPFP) levels \|\| id:ebi-met1400-GCST90200172 | Plum liking \|\| id:ebi-fl187-GCST90094812 | Weighted median | 5 | 0.00345676041934231 | 0.180402330920898 | 0.984712353839589 | -0.350131808 | 0.357045329024302 | 1.00346274190584 | 0.704595212181326 | 1.42910064812371 | 0.999332001504091 |
| ebi-fl187-GCST90094812 | ebi-met1400-GCST90200222 | Dihydrocaffeate sulfate (2) levels \|\| id:ebi-met1400-GCST90200222 | Plum liking \|\| id:ebi-fl187-GCST90094812 | Weighted median | 5 | 0.209896515635621 | 0.206683085801543 | 0.30984485954254 | -0.195202333 | 0.614995363806646 | 1.23355040017237 | 0.822668188675089 | 1.84964802421257 | 0.999332001504091 |
| ebi-fl187-GCST90094812 | ebi-met1400-GCST90200223 | 4-methylhexanoylglutamine levels \|\| id:ebi-met1400-GCST90200223 | Plum liking \|\| id:ebi-fl187-GCST90094812 | Weighted median | 5 | -0.244200201 | 0.21776859587797 | 0.262128386938251 | -0.671026649 | 0.182626247003562 | 0.783330795020026 | 0.511183502349885 | 1.20036568395886 | 0.999332001504091 |
| ebi-fl187-GCST90094812 | ebi-met1400-GCST90200224 | 2-ketocaprylate levels \|\| id:ebi-met1400-GCST90200224 | Plum liking \|\| id:ebi-fl187-GCST90094812 | Weighted median | 5 | -0.246908079 | 0.18095009714508 | 0.172407348369582 | -0.60157027 | 0.107754111029723 | 0.781212499772612 | 0.547950530028864 | 1.11377384700919 | 0.999332001504091 |
| ebi-fl187-GCST90094812 | ebi-met1400-GCST90200243 | Hydroxypalmitoyl sphingomyelin (d18:1/16:0(OH)) levels \|\| id:ebi-met1400-GCST90200243 | Plum liking \|\| id:ebi-fl187-GCST90094812 | Weighted median | 5 | 0.0123877905515088 | 0.178709130609664 | 0.944736360037462 | -0.337882105 | 0.362657686546451 | 1.01246483704546 | 0.713279374697085 | 1.43714382136568 | 0.999332001504091 |
| ebi-fl187-GCST90094812 | ebi-met1400-GCST90200273 | 2-methoxyhydroquinone sulfate (2) levels \|\| id:ebi-met1400-GCST90200273 | Plum liking \|\| id:ebi-fl187-GCST90094812 | Weighted median | 5 | 0.190043982042562 | 0.225259084590011 | 0.398854704203419 | -0.251463824 | 0.631551787838983 | 1.20930278409414 | 0.777661589977997 | 1.88052649438326 | 0.999332001504091 |
| ebi-fl187-GCST90094812 | ebi-met1400-GCST90200276 | 2,4-di-tert-butylphenol levels \|\| id:ebi-met1400-GCST90200276 | Plum liking \|\| id:ebi-fl187-GCST90094812 | Weighted median | 5 | 0.302535669973259 | 0.18414428073605 | 0.100397971337295 | -0.05838712 | 0.663458460215918 | 1.35328594723381 | 0.943284712114488 | 1.94149532104176 | 0.999332001504091 |
| ebi-fl187-GCST90094812 | ebi-met1400-GCST90200289 | N-acetylmethionine levels \|\| id:ebi-met1400-GCST90200289 | Plum liking \|\| id:ebi-fl187-GCST90094812 | Weighted median | 5 | 0.163772632431257 | 0.172997795736244 | 0.343804577925729 | -0.175303047 | 0.502848312074294 | 1.17794645782062 | 0.839202664202624 | 1.6534240376972 | 0.999332001504091 |
| ebi-fl187-GCST90094812 | ebi-met1400-GCST90200308 | 3-hydroxyisobutyrate levels \|\| id:ebi-met1400-GCST90200308 | Plum liking \|\| id:ebi-fl187-GCST90094812 | Weighted median | 5 | 0.167794865163362 | 0.179291628852013 | 0.349336654217959 | -0.183616727 | 0.519206457713308 | 1.1826939740269 | 0.832254723149016 | 1.68069341908569 | 0.999332001504091 |
| ebi-fl187-GCST90094812 | ebi-met1400-GCST90200383 | Cystathionine levels \|\| id:ebi-met1400-GCST90200383 | Plum liking \|\| id:ebi-fl187-GCST90094812 | Weighted median | 5 | -0.19499747 | 0.194782269269668 | 0.316776130397844 | -0.576770718 | 0.186775777286817 | 0.82283673943395 | 0.561709358670334 | 1.20535698633367 | 0.999332001504091 |
| ebi-fl187-GCST90094812 | ebi-met1400-GCST90200384 | Guanidinoacetate levels \|\| id:ebi-met1400-GCST90200384 | Plum liking \|\| id:ebi-fl187-GCST90094812 | Weighted median | 5 | -0.035452436 | 0.172419292059897 | 0.83708965094566 | -0.373394249 | 0.302489376370793 | 0.965168640355179 | 0.688393781119383 | 1.35322330020223 | 0.999332001504091 |
| ebi-fl187-GCST90094812 | ebi-met1400-GCST90200403 | Citrulline levels \|\| id:ebi-met1400-GCST90200403 | Plum liking \|\| id:ebi-fl187-GCST90094812 | Weighted median | 5 | 0.0340053458890468 | 0.186939404823592 | 0.855656720276466 | -0.332395888 | 0.400406579343287 | 1.03459013751742 | 0.717203334762528 | 1.4924313660682 | 0.999332001504091 |
| ebi-fl187-GCST90094812 | ebi-met1400-GCST90200434 | 12,13-DiHOME levels \|\| id:ebi-met1400-GCST90200434 | Plum liking \|\| id:ebi-fl187-GCST90094812 | Weighted median | 5 | -0.34152437 | 0.195176094217778 | 0.0801481964648359 | -0.724069514 | 0.0410207749501471 | 0.710686148188731 | 0.484775435730466 | 1.04187375019587 | 0.999332001504091 |
| ebi-fl187-GCST90094812 | ebi-met1400-GCST90200437 | Fructose levels \|\| id:ebi-met1400-GCST90200437 | Plum liking \|\| id:ebi-fl187-GCST90094812 | Weighted median | 5 | 0.26597324514739 | 0.179224747521864 | 0.137803414491172 | -0.08530726 | 0.617253750290243 | 1.30470015115971 | 0.918230105674457 | 1.85382996475143 | 0.999332001504091 |
| ebi-fl187-GCST90094812 | ebi-met1400-GCST90200448 | N-stearoyl-sphinganine (d18:0/18:0) levels \|\| id:ebi-met1400-GCST90200448 | Plum liking \|\| id:ebi-fl187-GCST90094812 | Weighted median | 5 | 0.104281212981749 | 0.195575359483788 | 0.59389361510447 | -0.279046492 | 0.487608917569974 | 1.10991253284619 | 0.75650473127719 | 1.62841787980524 | 0.999332001504091 |
| ebi-fl187-GCST90094812 | ebi-met1400-GCST90200468 | X-11847 levels \|\| id:ebi-met1400-GCST90200468 | Plum liking \|\| id:ebi-fl187-GCST90094812 | Weighted median | 5 | -0.284215784 | 0.210436154610639 | 0.176822514270211 | -0.696670647 | 0.128239079219664 | 0.752604227370296 | 0.498241366914577 | 1.13682476138668 | 0.999332001504091 |
| ebi-fl187-GCST90094812 | ebi-met1400-GCST90200476 | X-11843 levels \|\| id:ebi-met1400-GCST90200476 | Plum liking \|\| id:ebi-fl187-GCST90094812 | Weighted median | 5 | -0.080151829 | 0.187762344916551 | 0.669467356428502 | -0.448166025 | 0.287862366817611 | 0.922976200992228 | 0.638798618523678 | 1.33357374749312 | 0.999332001504091 |
| ebi-fl187-GCST90094812 | ebi-met1400-GCST90200511 | X-12906 levels \|\| id:ebi-met1400-GCST90200511 | Plum liking \|\| id:ebi-fl187-GCST90094812 | Weighted median | 5 | -0.049821635 | 0.170457072667494 | 0.770070567452853 | -0.383917497 | 0.284274227380639 | 0.951399105623822 | 0.681187624559688 | 1.32879727338983 | 0.999332001504091 |
| ebi-fl187-GCST90094812 | ebi-met1400-GCST90200515 | X-12849 levels \|\| id:ebi-met1400-GCST90200515 | Plum liking \|\| id:ebi-fl187-GCST90094812 | Weighted median | 5 | 0.172902865724272 | 0.1921084466075 | 0.368105669384758 | -0.20362969 | 0.549435421074971 | 1.18875063104431 | 0.815764401281589 | 1.73227473592643 | 0.999332001504091 |
| ebi-fl187-GCST90094812 | ebi-met1400-GCST90200551 | X-18886 levels \|\| id:ebi-met1400-GCST90200551 | Plum liking \|\| id:ebi-fl187-GCST90094812 | Weighted median | 5 | -0.083645767 | 0.179865880936625 | 0.641899079449193 | -0.436182893 | 0.268891359866632 | 0.919757006891985 | 0.646499474631872 | 1.30851297629995 | 0.999332001504091 |
| ebi-fl187-GCST90094812 | ebi-met1400-GCST90200564 | X-21319 levels \|\| id:ebi-met1400-GCST90200564 | Plum liking \|\| id:ebi-fl187-GCST90094812 | Weighted median | 5 | -0.367966488 | 0.181025399566779 | 0.0420849864953218 | -0.722776272 | -0.013156705 | 0.69214037604265 | 0.485402773658683 | 0.986929465889943 | 0.999332001504091 |
| ebi-fl187-GCST90094812 | ebi-met1400-GCST90200579 | X-21442 levels \|\| id:ebi-met1400-GCST90200579 | Plum liking \|\| id:ebi-fl187-GCST90094812 | Weighted median | 5 | -0.107088525 | 0.191377935093264 | 0.575775717106092 | -0.482189278 | 0.268012227658794 | 0.898446134414317 | 0.61743018481094 | 1.30736312590742 | 0.999332001504091 |
| ebi-fl187-GCST90094812 | ebi-met1400-GCST90200581 | X-21441 levels \|\| id:ebi-met1400-GCST90200581 | Plum liking \|\| id:ebi-fl187-GCST90094812 | Weighted median | 5 | 0.0121630893072998 | 0.184017176003325 | 0.947300140570226 | -0.348510576 | 0.372836754273816 | 1.01223736049495 | 0.705738451335104 | 1.45184731261761 | 0.999332001504091 |
| ebi-fl187-GCST90094812 | ebi-met1400-GCST90200648 | X-24585 levels \|\| id:ebi-met1400-GCST90200648 | Plum liking \|\| id:ebi-fl187-GCST90094812 | Weighted median | 5 | -0.144074108 | 0.190063687820876 | 0.44843261040159 | -0.516598937 | 0.228450719741564 | 0.865823580892259 | 0.596545992476498 | 1.25665159549057 | 0.999332001504091 |
| ebi-fl187-GCST90094812 | ebi-met1400-GCST90200720 | Adenosine 5'-diphosphate (ADP) to pantothenate ratio \|\| id:ebi-met1400-GCST90200720 | Plum liking \|\| id:ebi-fl187-GCST90094812 | Weighted median | 5 | -0.001575991 | 0.230544543377065 | 0.99454574242505 | -0.453443296 | 0.450291313732409 | 0.998425249935492 | 0.63543638458009 | 1.56876912291935 | 0.999332001504091 |
| ebi-fl187-GCST90094812 | ebi-met1400-GCST90200724 | Cholate to taurocholate ratio \|\| id:ebi-met1400-GCST90200724 | Plum liking \|\| id:ebi-fl187-GCST90094812 | Weighted median | 5 | 0.357964298109372 | 0.187523983958214 | 0.0562751546415908 | -0.00958271 | 0.725511306667471 | 1.43041455106441 | 0.990463057410994 | 2.06578708068642 | 0.999332001504091 |
| ebi-fl187-GCST90094812 | ebi-met1400-GCST90200726 | N-acetylputrescine to (N(1) + N(8))-acetylspermidine ratio \|\| id:ebi-met1400-GCST90200726 | Plum liking \|\| id:ebi-fl187-GCST90094812 | Weighted median | 5 | -0.164691518 | 0.169296547861421 | 0.330653192159498 | -0.496512752 | 0.167129715335365 | 0.848155303979763 | 0.608649474641014 | 1.18190756690177 | 0.999332001504091 |
| ebi-fl187-GCST90094812 | ebi-met1400-GCST90200744 | Arginine to glutamate ratio \|\| id:ebi-met1400-GCST90200744 | Plum liking \|\| id:ebi-fl187-GCST90094812 | Weighted median | 5 | 0.184638666873373 | 0.176374944161401 | 0.295167309882385 | -0.161056224 | 0.53033355742972 | 1.20278375598433 | 0.851244209677113 | 1.69949909463527 | 0.999332001504091 |
| ebi-fl187-GCST90094812 | ebi-met1400-GCST90200759 | Histidine to trans-urocanate ratio \|\| id:ebi-met1400-GCST90200759 | Plum liking \|\| id:ebi-fl187-GCST90094812 | Weighted median | 5 | -0.030918548 | 0.183583822334993 | 0.866255615408789 | -0.39074284 | 0.328905743934841 | 0.969554542169887 | 0.676554116585292 | 1.38944688561916 | 0.999332001504091 |
| ebi-fl187-GCST90094812 | ebi-met1400-GCST90200795 | Oleoyl-linoleoyl-glycerol (18:1 to 18:2) [2] to linoleoyl-arachidonoyl-glycerol (18:2 to 20:4) [2] ratio \|\| id:ebi-met1400-GCST90200795 | Plum liking \|\| id:ebi-fl187-GCST90094812 | Weighted median | 5 | 0.204121295543251 | 0.19378827589685 | 0.29219381471132 | -0.175703725 | 0.583946316301076 | 1.22644690697831 | 0.838866481510194 | 1.79310062899251 | 0.999332001504091 |
| ebi-fl187-GCST90094812 | ebi-met1400-GCST90200811 | Choline to taurocholate ratio \|\| id:ebi-met1400-GCST90200811 | Plum liking \|\| id:ebi-fl187-GCST90094812 | Weighted median | 5 | 0.148910490779677 | 0.198408747604944 | 0.452939284307996 | -0.239970655 | 0.537791636085368 | 1.16056910292423 | 0.786650945372696 | 1.71222147584685 | 0.999332001504091 |
| ebi-fl187-GCST90094812 | ebi-met1400-GCST90200831 | Cholate to phosphate ratio \|\| id:ebi-met1400-GCST90200831 | Plum liking \|\| id:ebi-fl187-GCST90094812 | Weighted median | 5 | 0.247097756166809 | 0.191936944976959 | 0.197958292637465 | -0.129098656 | 0.623294168321649 | 1.28030426429823 | 0.878887253777946 | 1.86506176091886 | 0.999332001504091 |
| ebi-fl187-GCST90094812 | ebi-met1400-GCST90200841 | Adenosine 5'-diphosphate (ADP) to oxalate (ethanedioate) ratio \|\| id:ebi-met1400-GCST90200841 | Plum liking \|\| id:ebi-fl187-GCST90094812 | Weighted median | 5 | -0.102722236 | 0.267087158457074 | 0.700532367027692 | -0.626213066 | 0.420768595050787 | 0.902377587110788 | 0.534612514692168 | 1.52313177739349 | 0.999332001504091 |
| ebi-fl187-GCST90094812 | ebi-met1400-GCST90200844 | Aspartate to phosphate ratio \|\| id:ebi-met1400-GCST90200844 | Plum liking \|\| id:ebi-fl187-GCST90094812 | Weighted median | 5 | -0.003690074 | 0.172914149801089 | 0.982974039833021 | -0.342601807 | 0.33522165984104 | 0.996316726186433 | 0.70992084052612 | 1.39825028681114 | 0.999332001504091 |
| ebi-fl187-GCST90094812 | ebi-met1400-GCST90200870 | Adenosine 5'-monophosphate (AMP) to acetoacetate ratio \|\| id:ebi-met1400-GCST90200870 | Plum liking \|\| id:ebi-fl187-GCST90094812 | Weighted median | 5 | 0.208147418948916 | 0.198681165683344 | 0.29480198965839 | -0.181267666 | 0.59756250368827 | 1.23139468707945 | 0.834212038784819 | 1.81768280109731 | 0.999332001504091 |
| ebi-fl187-GCST90094812 | ebi-met1400-GCST90200887 | Cholesterol to cortisol ratio \|\| id:ebi-met1400-GCST90200887 | Plum liking \|\| id:ebi-fl187-GCST90094812 | Weighted median | 5 | 0.105569108548355 | 0.176218958638532 | 0.549120087352214 | -0.23982005 | 0.450958267479879 | 1.11134290516393 | 0.786769427185744 | 1.56981576835805 | 0.999332001504091 |
| ebi-fl187-GCST90094812 | ebi-met1400-GCST90200890 | Cortisol to taurocholate ratio \|\| id:ebi-met1400-GCST90200890 | Plum liking \|\| id:ebi-fl187-GCST90094812 | Weighted median | 5 | 0.244226914112041 | 0.19130276877064 | 0.201725408405968 | -0.130726513 | 0.619180340902496 | 1.27663398389185 | 0.87745771513949 | 1.85740497884673 | 0.999332001504091 |
| ebi-fl187-GCST90094812 | ebi-met1400-GCST90200948 | Adenosine 5'-diphosphate (ADP) to mannitol to sorbitol ratio \|\| id:ebi-met1400-GCST90200948 | Plum liking \|\| id:ebi-fl187-GCST90094812 | Weighted median | 5 | -0.127684377 | 0.243131482440287 | 0.59946782943162 | -0.604222083 | 0.348853328356961 | 0.880131124737993 | 0.546499392596509 | 1.41744127665388 | 0.999332001504091 |
| ebi-fl187-GCST90094812 | ebi-met1400-GCST90200972 | Leucine to N-palmitoyl-sphingosine (d18:1 to 16:0) ratio \|\| id:ebi-met1400-GCST90200972 | Plum liking \|\| id:ebi-fl187-GCST90094812 | Weighted median | 5 | -0.125408965 | 0.176122358159043 | 0.476430156970317 | -0.470608787 | 0.219790856531545 | 0.88213606562268 | 0.624621890540706 | 1.2458161490284 | 0.999332001504091 |
| ebi-fl187-GCST90094812 | ebi-met1400-GCST90200973 | Proline to glutamate ratio \|\| id:ebi-met1400-GCST90200973 | Plum liking \|\| id:ebi-fl187-GCST90094812 | Weighted median | 5 | -0.013345171 | 0.183308047737423 | 0.941963772337757 | -0.372628945 | 0.345938602479523 | 0.986743480912957 | 0.688920813176444 | 1.4133158390656 | 0.999332001504091 |
| ebi-fl187-GCST90094812 | ebi-met1400-GCST90200983 | Cholesterol to linoleoyl-arachidonoyl-glycerol (18:2 to 20:4) [1] ratio \|\| id:ebi-met1400-GCST90200983 | Plum liking \|\| id:ebi-fl187-GCST90094812 | Weighted median | 5 | 0.309895959623746 | 0.182650184151313 | 0.0897602360628203 | -0.048098401 | 0.667890320560318 | 1.36328327024918 | 0.953040002075916 | 1.95011885219196 | 0.999332001504091 |
| ebi-fl187-GCST90094812 | ebi-met1400-GCST90201001 | Serine to alpha-ketobutyrate ratio \|\| id:ebi-met1400-GCST90201001 | Plum liking \|\| id:ebi-fl187-GCST90094812 | Weighted median | 5 | -0.060639349 | 0.204208516660881 | 0.766506066573248 | -0.460888041 | 0.339609343871334 | 0.941162610014949 | 0.630723288316779 | 1.40439884636901 | 0.999332001504091 |
| ebi-fl187-GCST90094812 | ebi-met1400-GCST90201005 | Fructose to maltose ratio \|\| id:ebi-met1400-GCST90201005 | Plum liking \|\| id:ebi-fl187-GCST90094812 | Weighted median | 5 | 0.00268773736759813 | 0.200680092421302 | 0.989314136680197 | -0.390645244 | 0.39602071851335 | 1.00269135257186 | 0.676620148674901 | 1.48590010287359 | 0.999332001504091 |
| ebi-fl187-GCST90094812 | ebi-met1400-GCST90201020 | Paraxanthine to 5-acetylamino-6-formylamino-3-methyluracil ratio \|\| id:ebi-met1400-GCST90201020 | Plum liking \|\| id:ebi-fl187-GCST90094812 | Weighted median | 5 | 0.492022607548023 | 0.196991374504464 | 0.0125006849795273 | 0.105919513519274 | 0.878125701576771 | 1.63562109616974 | 1.11173239347763 | 2.40638519299323 | 0.999332001504091 |
| ebi-fl187-GCST90094812 | ebi-pw11-GCST90275197 | Placental weight (fetal genotype, adjusted for sex) \|\| id:ebi-pw11-GCST90275197 | Plum liking \|\| id:ebi-fl187-GCST90094812 | Weighted median | 5 | -0.005429425 | 0.068813510411051 | 0.937111714624339 | -0.140303905 | 0.129445055622511 | 0.994585287904403 | 0.86909407306232 | 1.13819657224259 | 0.999332001504091 |
| ebi-fl187-GCST90094812 | prot-a-1017 | FAS-associated factor 2 \|\| id:prot-a-1017 | Plum liking \|\| id:ebi-fl187-GCST90094812 | Weighted median | 5 | -0.046555853 | 0.286725468827203 | 0.87101383744137 | -0.608537772 | 0.515426065510214 | 0.954511246391137 | 0.54414595292535 | 1.67435173337061 | 0.999332001504091 |
| ebi-fl187-GCST90094812 | prot-a-1019 | Fas apoptotic inhibitory molecule 3 \|\| id:prot-a-1019 | Plum liking \|\| id:ebi-fl187-GCST90094812 | Weighted median | 5 | 0.0243119497888822 | 0.289548492095337 | 0.933084230176848 | -0.543203095 | 0.591826994295743 | 1.02460989488244 | 0.580884640780484 | 1.80728730455059 | 0.999332001504091 |
| ebi-fl187-GCST90094812 | prot-a-1052 | MIP18 family protein FAM96A \|\| id:prot-a-1052 | Plum liking \|\| id:ebi-fl187-GCST90094812 | Weighted median | 5 | -0.761721024 | 0.2725838214093 | 0.00519885723592804 | -1.295985314 | -0.227456734 | 0.466862253968439 | 0.273628121797829 | 0.796556884388995 | 0.999332001504091 |
| ebi-fl187-GCST90094812 | prot-a-1066 | F-box/LRR-repeat protein 4 \|\| id:prot-a-1066 | Plum liking \|\| id:ebi-fl187-GCST90094812 | Weighted median | 5 | -0.103312801 | 0.281301538007795 | 0.713419790773222 | -0.654663815 | 0.448038213819904 | 0.901844831684311 | 0.519616720485238 | 1.56523850825314 | 0.999332001504091 |
| ebi-fl187-GCST90094812 | prot-a-1076 | Low affinity immunoglobulin gamma Fc region receptor III-B \|\| id:prot-a-1076 | Plum liking \|\| id:ebi-fl187-GCST90094812 | Weighted median | 5 | -0.641504461 | 0.299295563935303 | 0.0320825078443385 | -1.228123766 | -0.054885156 | 0.526499729558184 | 0.292841501635419 | 0.946593852567904 | 0.999332001504091 |
| ebi-fl187-GCST90094812 | prot-a-1081 | Fc receptor-like protein 3 \|\| id:prot-a-1081 | Plum liking \|\| id:ebi-fl187-GCST90094812 | Weighted median | 5 | -0.430203251 | 0.298321165115679 | 0.14927955597424 | -1.014912734 | 0.154506232927261 | 0.650376891730433 | 0.362434056504658 | 1.16708155236925 | 0.999332001504091 |
| ebi-fl187-GCST90094812 | prot-a-1105 | Fibroblast growth factor receptor 4 \|\| id:prot-a-1105 | Plum liking \|\| id:ebi-fl187-GCST90094812 | Weighted median | 5 | 0.185403610709193 | 0.28920689516604 | 0.521473281213379 | -0.381441904 | 0.752249125234631 | 1.20370416999192 | 0.682876057398619 | 2.12176677327869 | 0.999332001504091 |
| ebi-fl187-GCST90094812 | prot-a-1143 | Follistatin-related protein 1 \|\| id:prot-a-1143 | Plum liking \|\| id:ebi-fl187-GCST90094812 | Weighted median | 5 | -0.064262526 | 0.283161506178609 | 0.820465306030567 | -0.619259079 | 0.490734025708238 | 0.937758780774175 | 0.538343159880602 | 1.63351482187329 | 0.999332001504091 |
| ebi-fl187-GCST90094812 | prot-a-1151 | Alpha-(1,3)-fucosyltransferase 10 \|\| id:prot-a-1151 | Plum liking \|\| id:ebi-fl187-GCST90094812 | Weighted median | 5 | 0.0282297279391531 | 0.297635696260883 | 0.924436687953502 | -0.555136237 | 0.611595692610483 | 1.02863196278147 | 0.573994056794814 | 1.84337050589689 | 0.999332001504091 |
| ebi-fl187-GCST90094812 | prot-a-1165 | Growth arrest and DNA damage-inducible proteins-interacting protein 1 \|\| id:prot-a-1165 | Plum liking \|\| id:ebi-fl187-GCST90094812 | Weighted median | 5 | -0.364126926 | 0.307174927130222 | 0.235857057397166 | -0.966189784 | 0.237935930757425 | 0.694803000290015 | 0.380530181729199 | 1.26862791019176 | 0.999332001504091 |
| ebi-fl187-GCST90094812 | prot-a-1194 | Growth/differentiation factor 11/8 \|\| id:prot-a-1194 | Plum liking \|\| id:ebi-fl187-GCST90094812 | Weighted median | 5 | -0.164454519 | 0.286293780784673 | 0.565679734977734 | -0.725590329 | 0.39668129182168 | 0.848356340572006 | 0.484038742561576 | 1.48688197308332 | 0.999332001504091 |
| ebi-fl187-GCST90094812 | prot-a-1216 | D-glucuronyl C5-epimerase \|\| id:prot-a-1216 | Plum liking \|\| id:ebi-fl187-GCST90094812 | Weighted median | 5 | 0.357355017758946 | 0.282213468701203 | 0.205420785126756 | -0.195783381 | 0.910493416413303 | 1.42954329303264 | 0.8221903175198 | 2.48554864136475 | 0.999332001504091 |
| ebi-fl187-GCST90094812 | prot-a-1246 | Glypican-3 \|\| id:prot-a-1246 | Plum liking \|\| id:ebi-fl187-GCST90094812 | Weighted median | 5 | 0.138127469546073 | 0.303834634431496 | 0.64938698222779 | -0.457388414 | 0.733643353031805 | 1.1481218915233 | 0.632934451748677 | 2.08265464797051 | 0.999332001504091 |
| ebi-fl187-GCST90094812 | prot-a-1275 | Glutamate receptor 4 \|\| id:prot-a-1275 | Plum liking \|\| id:ebi-fl187-GCST90094812 | Weighted median | 5 | -0.230436751 | 0.298589292945896 | 0.440261590674012 | -0.815671765 | 0.354798263628368 | 0.794186665286978 | 0.442342077729834 | 1.42589297079008 | 0.999332001504091 |
| ebi-fl187-GCST90094812 | prot-a-1289 | Glutathione S-transferase P \|\| id:prot-a-1289 | Plum liking \|\| id:ebi-fl187-GCST90094812 | Weighted median | 5 | 0.0471760179730487 | 0.290467614208211 | 0.870979817310829 | -0.522140506 | 0.616492541821142 | 1.04830651362878 | 0.593249334250014 | 1.8524193506358 | 0.999332001504091 |
| ebi-fl187-GCST90094812 | prot-a-130 | Apolipoprotein D \|\| id:prot-a-130 | Plum liking \|\| id:ebi-fl187-GCST90094812 | Weighted median | 5 | 0.247225683578567 | 0.325500469864856 | 0.447538676589757 | -0.390755237 | 0.885206604513685 | 1.28046806078583 | 0.676545728896457 | 2.42348504271404 | 0.999332001504091 |
| ebi-fl187-GCST90094812 | prot-a-1313 | Heparin-binding EGF-like growth factor \|\| id:prot-a-1313 | Plum liking \|\| id:ebi-fl187-GCST90094812 | Weighted median | 5 | -0.042188678 | 0.282822466938557 | 0.881419350198587 | -0.596520713 | 0.512143357109755 | 0.958688879920434 | 0.550724434784043 | 1.66886433655972 | 0.999332001504091 |
| ebi-fl187-GCST90094812 | prot-a-1347 | HLA class II histocompatibility antigen, DQ alpha 2 chain \|\| id:prot-a-1347 | Plum liking \|\| id:ebi-fl187-GCST90094812 | Weighted median | 5 | -0.078313964 | 0.282937674062587 | 0.781942322470203 | -0.632871805 | 0.476243876934633 | 0.924674066385135 | 0.531064495114043 | 1.61001561375631 | 0.999332001504091 |
| ebi-fl187-GCST90094812 | prot-a-1357 | Non-histone chromosomal protein HMG-14 \|\| id:prot-a-1357 | Plum liking \|\| id:ebi-fl187-GCST90094812 | Weighted median | 5 | 0.173636446862193 | 0.283459259481446 | 0.540165813647164 | -0.381943702 | 0.729216595445827 | 1.18962299602106 | 0.682533477583614 | 2.07345561667158 | 0.999332001504091 |
| ebi-fl187-GCST90094812 | prot-a-137 | Apolipoprotein M \|\| id:prot-a-137 | Plum liking \|\| id:ebi-fl187-GCST90094812 | Weighted median | 5 | 0.200974624174395 | 0.29248799378834 | 0.492006521883981 | -0.372301844 | 0.774251091999542 | 1.22259374710398 | 0.68914619672323 | 2.16896716192435 | 0.999332001504091 |
| ebi-fl187-GCST90094812 | prot-a-1381 | 17-beta-hydroxysteroid dehydrogenase 14 \|\| id:prot-a-1381 | Plum liking \|\| id:ebi-fl187-GCST90094812 | Weighted median | 5 | 0.234891782026438 | 0.2973722784038 | 0.429591239998323 | -0.347957884 | 0.817741447697887 | 1.26477189027569 | 0.706128615151556 | 2.26537758151637 | 0.999332001504091 |
| ebi-fl187-GCST90094812 | prot-a-1392 | Serine protease HTRA2, mitochondrial \|\| id:prot-a-1392 | Plum liking \|\| id:ebi-fl187-GCST90094812 | Weighted median | 5 | -0.031660906 | 0.284585331059995 | 0.911415907390188 | -0.589448155 | 0.526126342670027 | 0.968835052337079 | 0.554633271848699 | 1.69236395701311 | 0.999332001504091 |
| ebi-fl187-GCST90094812 | prot-a-1416 | Interferon-induced protein with tetratricopeptide repeats 2 \|\| id:prot-a-1416 | Plum liking \|\| id:ebi-fl187-GCST90094812 | Weighted median | 5 | 0.205825011409436 | 0.322248906032865 | 0.523008753801094 | -0.425782844 | 0.837432867233851 | 1.22853820501573 | 0.653258185382506 | 2.31042818131626 | 0.999332001504091 |
| ebi-fl187-GCST90094812 | prot-a-1428 | Interferon gamma \|\| id:prot-a-1428 | Plum liking \|\| id:ebi-fl187-GCST90094812 | Weighted median | 5 | -0.101061582 | 0.302122083203803 | 0.737997931659541 | -0.693220866 | 0.491097700626719 | 0.903877368186594 | 0.499963158871215 | 1.63410899827996 | 0.999332001504091 |
| ebi-fl187-GCST90094812 | prot-a-1483 | Interleukin-17C \|\| id:prot-a-1483 | Plum liking \|\| id:ebi-fl187-GCST90094812 | Weighted median | 5 | -0.381931249 | 0.29154104420143 | 0.190181353903633 | -0.953351695 | 0.189489198076645 | 0.682541977337381 | 0.385446955297727 | 1.20863206836795 | 0.999332001504091 |
| ebi-fl187-GCST90094812 | prot-a-1503 | Interleukin-1 receptor-like 2 \|\| id:prot-a-1503 | Plum liking \|\| id:ebi-fl187-GCST90094812 | Weighted median | 5 | 0.745014308250926 | 0.310880547117543 | 0.0165540804995834 | 0.135688435900541 | 1.35434018060131 | 2.10647157468896 | 1.14532499584857 | 3.87420383826084 | 0.999332001504091 |
| ebi-fl187-GCST90094812 | prot-a-1522 | Interleukin-31 receptor subunit alpha \|\| id:prot-a-1522 | Plum liking \|\| id:ebi-fl187-GCST90094812 | Weighted median | 5 | 0.104240760666779 | 0.3080720384705 | 0.735088230894898 | -0.499580435 | 0.708061956068959 | 1.10986763522293 | 0.606785192302146 | 2.03005311161576 | 0.999332001504091 |
| ebi-fl187-GCST90094812 | prot-a-1540 | Interleukin-6 receptor subunit alpha \|\| id:prot-a-1540 | Plum liking \|\| id:ebi-fl187-GCST90094812 | Weighted median | 5 | -0.27226053 | 0.27455418870694 | 0.321370301013968 | -0.810386739 | 0.26586568033385 | 0.761655801409358 | 0.444686055346336 | 1.30455981887877 | 0.999332001504091 |
| ebi-fl187-GCST90094812 | prot-a-1541 | Interleukin-6 receptor subunit alpha \|\| id:prot-a-1541 | Plum liking \|\| id:ebi-fl187-GCST90094812 | Weighted median | 5 | -0.257424656 | 0.294508697012456 | 0.382073774237982 | -0.834661702 | 0.319812389868324 | 0.773039867821764 | 0.434021284950103 | 1.37686942544901 | 0.999332001504091 |
| ebi-fl187-GCST90094812 | prot-a-1545 | Interleukin-9 \|\| id:prot-a-1545 | Plum liking \|\| id:ebi-fl187-GCST90094812 | Weighted median | 5 | -0.513616617 | 0.281657519724575 | 0.0682201063156845 | -1.065665356 | 0.0384321217087855 | 0.598327738809086 | 0.344498564442854 | 1.0391801881885 | 0.999332001504091 |
| ebi-fl187-GCST90094812 | prot-a-1548 | Inositol monophosphatase 3 \|\| id:prot-a-1548 | Plum liking \|\| id:ebi-fl187-GCST90094812 | Weighted median | 5 | 0.00218335639101741 | 0.302116020384662 | 0.994233833780234 | -0.589964044 | 0.594330756344954 | 1.00218574164922 | 0.554347216726982 | 1.81181799142984 | 0.999332001504091 |
| ebi-fl187-GCST90094812 | prot-a-1555 | Inhibin beta A chain:Inhibin beta B chain heterodimer \|\| id:prot-a-1555 | Plum liking \|\| id:ebi-fl187-GCST90094812 | Weighted median | 5 | -0.168177969 | 0.286168419522633 | 0.55674120912906 | -0.729068071 | 0.392712133412666 | 0.845203401420038 | 0.48235830432944 | 1.4809919998477 | 0.999332001504091 |
| ebi-fl187-GCST90094812 | prot-a-1603 | Junctophilin-3 \|\| id:prot-a-1603 | Plum liking \|\| id:ebi-fl187-GCST90094812 | Weighted median | 5 | 0.228307382347554 | 0.293981822036971 | 0.437392594593836 | -0.347896989 | 0.804511753540016 | 1.25647148323441 | 0.706171616021673 | 2.235604706 | 0.999332001504091 |
| ebi-fl187-GCST90094812 | prot-a-1627 | UPF0258 protein KIAA1024 \|\| id:prot-a-1627 | Plum liking \|\| id:ebi-fl187-GCST90094812 | Weighted median | 5 | -0.168535197 | 0.279405463412089 | 0.546380834170142 | -0.716169905 | 0.3790995113445 | 0.844901524944609 | 0.48862013802862 | 1.46096841144094 | 0.999332001504091 |
| ebi-fl187-GCST90094812 | prot-a-1666 | Kallikrein-7 \|\| id:prot-a-1666 | Plum liking \|\| id:ebi-fl187-GCST90094812 | Weighted median | 5 | 0.291699772773872 | 0.308618442181446 | 0.344567329645901 | -0.313192374 | 0.896591919449507 | 1.33870104279972 | 0.731109252700116 | 2.45123485357961 | 0.999332001504091 |
| ebi-fl187-GCST90094812 | prot-a-1678 | Importin subunit alpha-3 \|\| id:prot-a-1678 | Plum liking \|\| id:ebi-fl187-GCST90094812 | Weighted median | 5 | 0.0614906779122866 | 0.280921551586927 | 0.826736403380292 | -0.489115563 | 0.612096919022664 | 1.06342058318264 | 0.613168463190808 | 1.84429468347364 | 0.999332001504091 |
| ebi-fl187-GCST90094812 | prot-a-1718 | Protein LDOC1 \|\| id:prot-a-1718 | Plum liking \|\| id:ebi-fl187-GCST90094812 | Weighted median | 5 | -0.306861411 | 0.285104356772342 | 0.281787476292124 | -0.86566595 | 0.251943128329793 | 0.735752561094701 | 0.420771246599439 | 1.28652286849995 | 0.999332001504091 |
| ebi-fl187-GCST90094812 | prot-a-1723 | Leptin \|\| id:prot-a-1723 | Plum liking \|\| id:ebi-fl187-GCST90094812 | Weighted median | 5 | -0.192756056 | 0.303715973780729 | 0.525650881693359 | -0.788039365 | 0.402527252139022 | 0.824683125715121 | 0.454735492115925 | 1.49559968296005 | 0.999332001504091 |
| ebi-fl187-GCST90094812 | prot-a-1730 | Galectin-7 \|\| id:prot-a-1730 | Plum liking \|\| id:ebi-fl187-GCST90094812 | Weighted median | 5 | -0.071056857 | 0.309482737977813 | 0.818403560572143 | -0.677643023 | 0.535529309596233 | 0.931408933653104 | 0.507812485145056 | 1.70835225022285 | 0.999332001504091 |
| ebi-fl187-GCST90094812 | prot-a-1753 | Lipase member N \|\| id:prot-a-1753 | Plum liking \|\| id:ebi-fl187-GCST90094812 | Weighted median | 5 | -0.115946496 | 0.285089422199438 | 0.684226721259232 | -0.674721764 | 0.442828771053275 | 0.890522868150892 | 0.509298105979496 | 1.55710568994658 | 0.999332001504091 |
| ebi-fl187-GCST90094812 | prot-a-1756 | VIP36-like protein \|\| id:prot-a-1756 | Plum liking \|\| id:ebi-fl187-GCST90094812 | Weighted median | 5 | -0.29355561 | 0.358684780439312 | 0.413116114013344 | -0.996577779 | 0.40946656015956 | 0.745607758835436 | 0.369140562547805 | 1.50601420282445 | 0.999332001504091 |
| ebi-fl187-GCST90094812 | prot-a-1760 | Ig Kappa chain V-I region HK102- like \|\| id:prot-a-1760 | Plum liking \|\| id:ebi-fl187-GCST90094812 | Weighted median | 5 | 0.0582223324744659 | 0.300330982098605 | 0.846285066084748 | -0.530426392 | 0.646871057387731 | 1.05995063096123 | 0.588354046469526 | 1.90955657875856 | 0.999332001504091 |
| ebi-fl187-GCST90094812 | prot-a-179 | Neutral ceramidase \|\| id:prot-a-179 | Plum liking \|\| id:ebi-fl187-GCST90094812 | Weighted median | 5 | 0.130660143449047 | 0.27141903202251 | 0.630234798619341 | -0.401321159 | 0.662641446213167 | 1.13958042162073 | 0.669435031214257 | 1.93990973998736 | 0.999332001504091 |
| ebi-fl187-GCST90094812 | prot-a-1809 | Lactotransferrin \|\| id:prot-a-1809 | Plum liking \|\| id:ebi-fl187-GCST90094812 | Weighted median | 5 | -0.382666484 | 0.309164660292159 | 0.215811261833451 | -0.988629218 | 0.223296250226664 | 0.682040332757998 | 0.372086390881503 | 1.25019088821441 | 0.999332001504091 |
| ebi-fl187-GCST90094812 | prot-a-1818 | Lysophospholipase-like protein 1 \|\| id:prot-a-1818 | Plum liking \|\| id:ebi-fl187-GCST90094812 | Weighted median | 5 | 0.107121515244094 | 0.287381458848374 | 0.709334321274465 | -0.456146144 | 0.670389174586907 | 1.11306950117402 | 0.63372121571477 | 1.95499800814844 | 0.999332001504091 |
| ebi-fl187-GCST90094812 | prot-a-1852 | MAP kinase-activated protein kinase 3 \|\| id:prot-a-1852 | Plum liking \|\| id:ebi-fl187-GCST90094812 | Weighted median | 5 | 0.307572887555521 | 0.339578429478399 | 0.365068619823392 | -0.358000834 | 0.973146609333184 | 1.36011994070686 | 0.699072491824531 | 2.64625811306099 | 0.999332001504091 |
| ebi-fl187-GCST90094812 | prot-a-1856 | Megakaryocyte-associated tyrosine-protein kinase \|\| id:prot-a-1856 | Plum liking \|\| id:ebi-fl187-GCST90094812 | Weighted median | 5 | -0.014158169 | 0.28043106708936 | 0.95973417815838 | -0.563803061 | 0.535486722274376 | 0.985941586316881 | 0.56904084664165 | 1.7082794976249 | 0.999332001504091 |
| ebi-fl187-GCST90094812 | prot-a-1890 | Beta-1,3-N-acetylglucosaminyltransferase manic fringe \|\| id:prot-a-1890 | Plum liking \|\| id:ebi-fl187-GCST90094812 | Weighted median | 5 | 0.0397165683014552 | 0.281670234886248 | 0.887866954856665 | -0.512357092 | 0.591790228678502 | 1.04051581722885 | 0.599081822273742 | 1.80722085973876 | 0.999332001504091 |
| ebi-fl187-GCST90094812 | prot-a-1919 | Matrilysin \|\| id:prot-a-1919 | Plum liking \|\| id:ebi-fl187-GCST90094812 | Weighted median | 5 | -0.488937674 | 0.285727018703582 | 0.087043165862445 | -1.04896263 | 0.0710872830793804 | 0.613277549197117 | 0.350300952305869 | 1.07367493543329 | 0.999332001504091 |
| ebi-fl187-GCST90094812 | prot-a-1923 | Multimerin-2 \|\| id:prot-a-1923 | Plum liking \|\| id:ebi-fl187-GCST90094812 | Weighted median | 5 | 0.774835974483688 | 0.283703488229872 | 0.00631142837507136 | 0.218777137553139 | 1.33089481141424 | 2.17023612388669 | 1.24455388145492 | 3.78442822251833 | 0.999332001504091 |
| ebi-fl187-GCST90094812 | prot-a-1948 | Mesothelin \|\| id:prot-a-1948 | Plum liking \|\| id:ebi-fl187-GCST90094812 | Weighted median | 5 | -0.209349071 | 0.287344236321747 | 0.466267544119251 | -0.772543774 | 0.353845632049764 | 0.811112050411589 | 0.461836764313176 | 1.42453526691687 | 0.999332001504091 |
| ebi-fl187-GCST90094812 | prot-a-195 | ATPase family AAA domain-containing protein 1 \|\| id:prot-a-195 | Plum liking \|\| id:ebi-fl187-GCST90094812 | Weighted median | 5 | -0.448646654 | 0.277975683287433 | 0.106532185669158 | -0.993478993 | 0.0961856856801153 | 0.638491667595075 | 0.370286224450416 | 1.10096347816722 | 0.999332001504091 |
| ebi-fl187-GCST90094812 | prot-a-1966 | Mucin-1 \|\| id:prot-a-1966 | Plum liking \|\| id:ebi-fl187-GCST90094812 | Weighted median | 5 | -0.507277955 | 0.295583795856158 | 0.0861272724470087 | -1.086622195 | 0.0720662845647673 | 0.60213238132609 | 0.337354087599356 | 1.07472657948645 | 0.999332001504091 |
| ebi-fl187-GCST90094812 | prot-a-1986 | Myocardial zonula adherens protein \|\| id:prot-a-1986 | Plum liking \|\| id:ebi-fl187-GCST90094812 | Weighted median | 5 | 0.0280022367632221 | 0.261796202015547 | 0.914819152412715 | -0.485118319 | 0.541122792713694 | 1.02839798470164 | 0.615624352281907 | 1.7179346642449 | 0.999332001504091 |
| ebi-fl187-GCST90094812 | prot-a-2071 | Epididymal secretory protein E1 \|\| id:prot-a-2071 | Plum liking \|\| id:ebi-fl187-GCST90094812 | Weighted median | 5 | 0.331592289947573 | 0.278672710781919 | 0.234086218438348 | -0.214606223 | 0.877790803080133 | 1.39318471728985 | 0.806859100036254 | 2.40557943314117 | 0.999332001504091 |
| ebi-fl187-GCST90094812 | prot-a-2098 | Neuritin \|\| id:prot-a-2098 | Plum liking \|\| id:ebi-fl187-GCST90094812 | Weighted median | 5 | -0.081100676 | 0.280653655587364 | 0.772603818208608 | -0.631181841 | 0.468980488501723 | 0.922100852931398 | 0.531962733768845 | 1.59836381197763 | 0.999332001504091 |
| ebi-fl187-GCST90094812 | prot-a-2115 | Neurotrophin-4 \|\| id:prot-a-2115 | Plum liking \|\| id:ebi-fl187-GCST90094812 | Weighted median | 5 | -0.079788927 | 0.284848615030164 | 0.779393161986532 | -0.638092213 | 0.478514358163962 | 0.923311211615392 | 0.528299345995428 | 1.61367527701248 | 0.999332001504091 |
| ebi-fl187-GCST90094812 | prot-a-2118 | Netrin-1 \|\| id:prot-a-2118 | Plum liking \|\| id:ebi-fl187-GCST90094812 | Weighted median | 5 | 0.163512060714247 | 0.265336268703808 | 0.537733051967903 | -0.356547026 | 0.683571147373712 | 1.17763955827597 | 0.70008954832276 | 1.9809393420298 | 0.999332001504091 |
| ebi-fl187-GCST90094812 | prot-a-2120 | Netrin-G1 \|\| id:prot-a-2120 | Plum liking \|\| id:ebi-fl187-GCST90094812 | Weighted median | 5 | -0.08197848 | 0.298528101292089 | 0.783616712460227 | -0.667093558 | 0.503136599011829 | 0.921291785123834 | 0.51319799246175 | 1.65390076696359 | 0.999332001504091 |
| ebi-fl187-GCST90094812 | prot-a-2145 | Olfactomedin-like protein 3 \|\| id:prot-a-2145 | Plum liking \|\| id:ebi-fl187-GCST90094812 | Weighted median | 5 | 0.0579062795802563 | 0.273513469695552 | 0.832331166900487 | -0.478180121 | 0.593992680183538 | 1.05961568342995 | 0.619910528006842 | 1.81120556248776 | 0.999332001504091 |
| ebi-fl187-GCST90094812 | prot-a-2162 | Prolyl 4-hydroxylase subunit alpha-2 \|\| id:prot-a-2162 | Plum liking \|\| id:ebi-fl187-GCST90094812 | Weighted median | 5 | -0.42719812 | 0.293884617540513 | 0.146050214448416 | -1.00321197 | 0.148815730593703 | 0.652334299092979 | 0.366699719012474 | 1.1604591323907 | 0.999332001504091 |
| ebi-fl187-GCST90094812 | prot-a-2168 | Polyadenylate-binding protein-interacting protein 1 \|\| id:prot-a-2168 | Plum liking \|\| id:ebi-fl187-GCST90094812 | Weighted median | 5 | -0.121569048 | 0.284799739441058 | 0.669482755281909 | -0.679776538 | 0.436638440921795 | 0.885529906829977 | 0.506730214820435 | 1.54749646450071 | 0.999332001504091 |
| ebi-fl187-GCST90094812 | prot-a-219 | Beta-1,4-galactosyltransferase 5 \|\| id:prot-a-219 | Plum liking \|\| id:ebi-fl187-GCST90094812 | Weighted median | 5 | -0.504013897 | 0.29934228187103 | 0.0922323645591612 | -1.090724769 | 0.0826969757467168 | 0.60410098777495 | 0.335972902636005 | 1.08621260990815 | 0.999332001504091 |
| ebi-fl187-GCST90094812 | prot-a-2190 | Pyruvate carboxylase, mitochondrial \|\| id:prot-a-2190 | Plum liking \|\| id:ebi-fl187-GCST90094812 | Weighted median | 5 | -0.333338008 | 0.299614908416369 | 0.265899677457931 | -0.920583228 | 0.253907212699385 | 0.716527961182257 | 0.398286681270537 | 1.28905219104544 | 0.999332001504091 |
| ebi-fl187-GCST90094812 | prot-a-2197 | Protocadherin-9 \|\| id:prot-a-2197 | Plum liking \|\| id:ebi-fl187-GCST90094812 | Weighted median | 5 | -0.25954291 | 0.285486010749444 | 0.363283333559215 | -0.819095491 | 0.300009671249265 | 0.771404106472716 | 0.44083020917146 | 1.34987186246013 | 0.999332001504091 |
| ebi-fl187-GCST90094812 | prot-a-2198 | Protocadherin alpha-4 \|\| id:prot-a-2198 | Plum liking \|\| id:ebi-fl187-GCST90094812 | Weighted median | 5 | 0.0584597635529571 | 0.30563336909547 | 0.848310810778516 | -0.54058164 | 0.657501166980079 | 1.06020232606162 | 0.582409401308008 | 1.92996364698451 | 0.999332001504091 |
| ebi-fl187-GCST90094812 | prot-a-2254 | Placenta growth factor \|\| id:prot-a-2254 | Plum liking \|\| id:ebi-fl187-GCST90094812 | Weighted median | 5 | -0.556965676 | 0.300949554012066 | 0.0642135200128169 | -1.146826801 | 0.0328954503559381 | 0.572944929759833 | 0.317643116566757 | 1.03344248754879 | 0.999332001504091 |
| ebi-fl187-GCST90094812 | prot-a-2261 | Membrane-associated progesterone receptor component 2 \|\| id:prot-a-2261 | Plum liking \|\| id:ebi-fl187-GCST90094812 | Weighted median | 5 | -0.084151872 | 0.321629310480208 | 0.793597133505592 | -0.714545321 | 0.546241576166697 | 0.919291630489967 | 0.489414587791519 | 1.72675094484289 | 0.999332001504091 |
| ebi-fl187-GCST90094812 | prot-a-2268 | Polymeric immunoglobulin receptor \|\| id:prot-a-2268 | Plum liking \|\| id:ebi-fl187-GCST90094812 | Weighted median | 5 | -0.267586943 | 0.279388595041729 | 0.338184295731014 | -0.815188589 | 0.280014703613861 | 0.765223797132084 | 0.442555858346415 | 1.32314926727031 | 0.999332001504091 |
| ebi-fl187-GCST90094812 | prot-a-2283 | Group 10 secretory phospholipase A2 \|\| id:prot-a-2283 | Plum liking \|\| id:ebi-fl187-GCST90094812 | Weighted median | 5 | -0.218290525 | 0.29403777168761 | 0.457851440617891 | -0.794604557 | 0.358023507901473 | 0.803891857371886 | 0.451759844822093 | 1.43049924812002 | 0.999332001504091 |
| ebi-fl187-GCST90094812 | prot-a-2298 | Inactive phospholipase D5 \|\| id:prot-a-2298 | Plum liking \|\| id:ebi-fl187-GCST90094812 | Weighted median | 5 | -0.461160702 | 0.299964398964877 | 0.124199110873259 | -1.049090924 | 0.126769519990774 | 0.630551338406767 | 0.350256013778797 | 1.1351553570117 | 0.999332001504091 |
| ebi-fl187-GCST90094812 | prot-a-2304 | Procollagen-lysine,2-oxoglutarate 5-dioxygenase 2 \|\| id:prot-a-2304 | Plum liking \|\| id:ebi-fl187-GCST90094812 | Weighted median | 5 | -0.210541465 | 0.288376652502945 | 0.465333997912742 | -0.775759704 | 0.354676774038415 | 0.810145461882766 | 0.460353915512139 | 1.42571975016021 | 0.999332001504091 |
| ebi-fl187-GCST90094812 | prot-a-2331 | Periostin \|\| id:prot-a-2331 | Plum liking \|\| id:ebi-fl187-GCST90094812 | Weighted median | 5 | 0.553742403635266 | 0.309117184616991 | 0.0732343794035406 | -0.052127278 | 1.15961208548457 | 1.73975170302896 | 0.9492080456493 | 3.18869609467096 | 0.999332001504091 |
| ebi-fl187-GCST90094812 | prot-a-2333 | Periostin \|\| id:prot-a-2333 | Plum liking \|\| id:ebi-fl187-GCST90094812 | Weighted median | 5 | 0.350318636220766 | 0.325805274680795 | 0.282267603482119 | -0.288259702 | 0.988896974595125 | 1.41951978695989 | 0.749566902820581 | 2.68826760891946 | 0.999332001504091 |
| ebi-fl187-GCST90094812 | prot-a-2367 | cAMP-dependent protein kinase catalytic subunit alpha \|\| id:prot-a-2367 | Plum liking \|\| id:ebi-fl187-GCST90094812 | Weighted median | 5 | -0.255155688 | 0.279678959970391 | 0.36160220950934 | -0.80332645 | 0.293015073425467 | 0.774795862062101 | 0.447836777156352 | 1.34046299564846 | 0.999332001504091 |
| ebi-fl187-GCST90094812 | prot-a-2385 | Phosphoribosyl pyrophosphate synthase-associated protein 1 \|\| id:prot-a-2385 | Plum liking \|\| id:ebi-fl187-GCST90094812 | Weighted median | 5 | -0.147626777 | 0.287774886990198 | 0.607955551079589 | -0.711665555 | 0.416412001833172 | 0.862753054430642 | 0.490826018477126 | 1.5165105453025 | 0.999332001504091 |
| ebi-fl187-GCST90094812 | prot-a-2393 | Trypsin-3 \|\| id:prot-a-2393 | Plum liking \|\| id:ebi-fl187-GCST90094812 | Weighted median | 5 | -0.037225544 | 0.280119762875748 | 0.894279164389408 | -0.58626028 | 0.511809190946151 | 0.963458808208381 | 0.55640419494135 | 1.66830675173497 | 0.999332001504091 |
| ebi-fl187-GCST90094812 | prot-a-2405 | Pregnancy-specific beta-1-glycoprotein 6 \|\| id:prot-a-2405 | Plum liking \|\| id:ebi-fl187-GCST90094812 | Weighted median | 5 | -0.15444067 | 0.299975971733878 | 0.606662406486072 | -0.742393575 | 0.433512234543651 | 0.856894330127666 | 0.475973273396797 | 1.54266622527945 | 0.999332001504091 |
| ebi-fl187-GCST90094812 | prot-a-244 | Beclin-1 \|\| id:prot-a-244 | Plum liking \|\| id:ebi-fl187-GCST90094812 | Weighted median | 5 | -0.352787425 | 0.308689896457139 | 0.253099292543866 | -0.957819622 | 0.252244772480237 | 0.702726559897451 | 0.383728648148828 | 1.28691099913337 | 0.999332001504091 |
| ebi-fl187-GCST90094812 | prot-a-2454 | tRNA pseudouridine synthase A, mitochondrial \|\| id:prot-a-2454 | Plum liking \|\| id:ebi-fl187-GCST90094812 | Weighted median | 5 | 0.255542997070127 | 0.287411785162869 | 0.373939655852564 | -0.307784102 | 0.818870095989351 | 1.29116252803018 | 0.735074001996471 | 2.26793583946843 | 0.999332001504091 |
| ebi-fl187-GCST90094812 | prot-a-2466 | Glutaminyl-peptide cyclotransferase-like protein \|\| id:prot-a-2466 | Plum liking \|\| id:ebi-fl187-GCST90094812 | Weighted median | 5 | 0.270674775786467 | 0.288448656686626 | 0.348048580927447 | -0.294684591 | 0.836034142892255 | 1.31084868132088 | 0.744766456229513 | 2.30719878822141 | 0.999332001504091 |
| ebi-fl187-GCST90094812 | prot-a-2474 | Ras-related protein Rab-27A \|\| id:prot-a-2474 | Plum liking \|\| id:ebi-fl187-GCST90094812 | Weighted median | 5 | -0.082211546 | 0.347671594969845 | 0.813073240835099 | -0.763647873 | 0.599224779753659 | 0.921077087554694 | 0.465963547330838 | 1.82070680437987 | 0.999332001504091 |
| ebi-fl187-GCST90094812 | prot-a-2490 | Rap1 GTPase-GDP dissociation stimulator 1 \|\| id:prot-a-2490 | Plum liking \|\| id:ebi-fl187-GCST90094812 | Weighted median | 5 | -0.154193392 | 0.306918479216904 | 0.615391796612428 | -0.755753612 | 0.447366826849219 | 0.857106247134442 | 0.469656542674239 | 1.56418798020757 | 0.999332001504091 |
| ebi-fl187-GCST90094812 | prot-a-2545 | Receptor-interacting serine/threonine-protein kinase 2 \|\| id:prot-a-2545 | Plum liking \|\| id:ebi-fl187-GCST90094812 | Weighted median | 5 | -0.109146335 | 0.299120879326493 | 0.715193226663482 | -0.695423258 | 0.477130588576959 | 0.896599204140384 | 0.498863255239041 | 1.61144386647593 | 0.999332001504091 |
| ebi-fl187-GCST90094812 | prot-a-2610 | RUN and FYVE domain-containing protein 1 \|\| id:prot-a-2610 | Plum liking \|\| id:ebi-fl187-GCST90094812 | Weighted median | 5 | -0.005854069 | 0.289801856708403 | 0.983883631909836 | -0.573865708 | 0.562157570447254 | 0.994163032971235 | 0.563343502652793 | 1.75445377726442 | 0.999332001504091 |
| ebi-fl187-GCST90094812 | prot-a-2627 | Serine--tRNA ligase, mitochondrial \|\| id:prot-a-2627 | Plum liking \|\| id:ebi-fl187-GCST90094812 | Weighted median | 5 | 0.048780253311743 | 0.28337770539093 | 0.863328534426823 | -0.506640049 | 0.604200555877965 | 1.04998959365099 | 0.602516607757506 | 1.82978881010213 | 0.999332001504091 |
| ebi-fl187-GCST90094812 | prot-a-264 | BolA-like protein 2 \|\| id:prot-a-264 | Plum liking \|\| id:ebi-fl187-GCST90094812 | Weighted median | 5 | 0.0107448233993748 | 0.297996499577517 | 0.971237006403095 | -0.573328316 | 0.594817962571308 | 1.0108027563214 | 0.563646320346447 | 1.81270093550675 | 0.999332001504091 |
| ebi-fl187-GCST90094812 | prot-a-2646 | Secretoglobin family 1C member 1 \|\| id:prot-a-2646 | Plum liking \|\| id:ebi-fl187-GCST90094812 | Weighted median | 5 | -0.225444298 | 0.350786190045071 | 0.520429855875571 | -0.912985231 | 0.462096634100675 | 0.79816151809316 | 0.401324388076422 | 1.58739869265923 | 0.999332001504091 |
| ebi-fl187-GCST90094812 | prot-a-2672 | Semaphorin-3E \|\| id:prot-a-2672 | Plum liking \|\| id:ebi-fl187-GCST90094812 | Weighted median | 5 | -0.220920748 | 0.304497027235333 | 0.468128920971174 | -0.817734922 | 0.375893425213561 | 0.801780220323899 | 0.441430397444326 | 1.45629192149985 | 0.999332001504091 |
| ebi-fl187-GCST90094812 | prot-a-2701 | Plasma protease C1 inhibitor \|\| id:prot-a-2701 | Plum liking \|\| id:ebi-fl187-GCST90094812 | Weighted median | 5 | 0.0472371547518811 | 0.305134073608647 | 0.876972789874163 | -0.55082563 | 0.645299939024829 | 1.04837060567143 | 0.576473660173744 | 1.90655879490595 | 0.999332001504091 |
| ebi-fl187-GCST90094812 | prot-a-2738 | Signaling threshold-regulating transmembrane adapter 1 \|\| id:prot-a-2738 | Plum liking \|\| id:ebi-fl187-GCST90094812 | Weighted median | 5 | -0.241883417 | 0.305809836186319 | 0.428967201299304 | -0.841270696 | 0.357503861512105 | 0.785147706767139 | 0.431162298835027 | 1.42975608745784 | 0.999332001504091 |
| ebi-fl187-GCST90094812 | prot-a-274 | Serine/threonine-protein kinase BRSK2 \|\| id:prot-a-274 | Plum liking \|\| id:ebi-fl187-GCST90094812 | Weighted median | 5 | -0.358687056 | 0.287018980533623 | 0.211409875782659 | -0.921244258 | 0.203870146154192 | 0.698592937830799 | 0.398023489124828 | 1.22613892426336 | 0.999332001504091 |
| ebi-fl187-GCST90094812 | prot-a-2749 | Mitochondrial glutamate carrier 2 \|\| id:prot-a-2749 | Plum liking \|\| id:ebi-fl187-GCST90094812 | Weighted median | 5 | -0.1413061 | 0.281334400625484 | 0.615476933513222 | -0.692721525 | 0.41010932563649 | 0.868223508159161 | 0.5002128731744 | 1.50698252793143 | 0.999332001504091 |
| ebi-fl187-GCST90094812 | prot-a-2760 | Sodium-coupled monocarboxylate transporter 1 \|\| id:prot-a-2760 | Plum liking \|\| id:ebi-fl187-GCST90094812 | Weighted median | 5 | 0.0590701020296728 | 0.291125973325127 | 0.839211470497061 | -0.511536806 | 0.629677009746922 | 1.0608496058441 | 0.599573442545003 | 1.87700422727633 | 0.999332001504091 |
| ebi-fl187-GCST90094812 | prot-a-2777 | Small integral membrane protein 10 \|\| id:prot-a-2777 | Plum liking \|\| id:ebi-fl187-GCST90094812 | Weighted median | 5 | 0.0399006074609192 | 0.289864432656059 | 0.890514942421359 | -0.528233681 | 0.608034895466795 | 1.04070733050769 | 0.589645552818214 | 1.8368183099082 | 0.999332001504091 |
| ebi-fl187-GCST90094812 | prot-a-2799 | Superoxide dismutase [Mn], mitochondrial \|\| id:prot-a-2799 | Plum liking \|\| id:ebi-fl187-GCST90094812 | Weighted median | 5 | 0.225722912016049 | 0.28586319705986 | 0.429750512605978 | -0.334568954 | 0.786014778253375 | 1.25322836264328 | 0.715646496279666 | 2.19463287684398 | 0.999332001504091 |
| ebi-fl187-GCST90094812 | prot-a-2805 | Protein SPACA7 \|\| id:prot-a-2805 | Plum liking \|\| id:ebi-fl187-GCST90094812 | Weighted median | 5 | -0.269593466 | 0.294633735695361 | 0.360185218683246 | -0.847075588 | 0.307888655465189 | 0.763689896766474 | 0.428666698556063 | 1.36054949075292 | 0.999332001504091 |
| ebi-fl187-GCST90094812 | prot-a-2811 | SPARC-like protein 1 \|\| id:prot-a-2811 | Plum liking \|\| id:ebi-fl187-GCST90094812 | Weighted median | 5 | 0.00276460063881276 | 0.274583111615016 | 0.99196674957214 | -0.535418298 | 0.540947499404244 | 1.00276842567124 | 0.585424357009016 | 1.71763354818477 | 0.999332001504091 |
| ebi-fl187-GCST90094812 | prot-a-2831 | Sepiapterin reductase \|\| id:prot-a-2831 | Plum liking \|\| id:ebi-fl187-GCST90094812 | Weighted median | 5 | -0.245461071 | 0.286099238811183 | 0.39091577091153 | -0.806215579 | 0.315293437088191 | 0.782343739075423 | 0.446544786023596 | 1.37066145485835 | 0.999332001504091 |
| ebi-fl187-GCST90094812 | prot-a-2834 | SPRY domain-containing SOCS box protein 1 \|\| id:prot-a-2834 | Plum liking \|\| id:ebi-fl187-GCST90094812 | Weighted median | 5 | 0.187804569268613 | 0.294567725411671 | 0.523760206640257 | -0.389548173 | 0.765157311075488 | 1.20659768603794 | 0.677362856507553 | 2.14933246186325 | 0.999332001504091 |
| ebi-fl187-GCST90094812 | prot-a-2835 | Spectrin alpha chain, non-erythrocytic 1 \|\| id:prot-a-2835 | Plum liking \|\| id:ebi-fl187-GCST90094812 | Weighted median | 5 | 0.0526268889082604 | 0.288454589025079 | 0.855234080917883 | -0.512744106 | 0.617997883397416 | 1.05403629910875 | 0.598850014377027 | 1.85520997439502 | 0.999332001504091 |
| ebi-fl187-GCST90094812 | prot-a-2859 | Sia-alpha-2,3-Gal-beta-1,4-GlcNAc-R:alpha 2,8-sialyltransferase \|\| id:prot-a-2859 | Plum liking \|\| id:ebi-fl187-GCST90094812 | Weighted median | 5 | -0.367508777 | 0.289467029322172 | 0.204225380326543 | -0.934864155 | 0.19984660014618 | 0.692457248859456 | 0.392639200060759 | 1.22121540952561 | 0.999332001504091 |
| ebi-fl187-GCST90094812 | prot-a-2861 | Alpha-2,8-sialyltransferase 8F \|\| id:prot-a-2861 | Plum liking \|\| id:ebi-fl187-GCST90094812 | Weighted median | 5 | -0.220995729 | 0.302580739931837 | 0.465164385668994 | -0.81405398 | 0.372062521020733 | 0.801720104232492 | 0.443058271367361 | 1.45072367918309 | 0.999332001504091 |
| ebi-fl187-GCST90094812 | prot-a-289 | Uncharacterized protein C17orf89 \|\| id:prot-a-289 | Plum liking \|\| id:ebi-fl187-GCST90094812 | Weighted median | 5 | -0.140326766 | 0.305550573219083 | 0.646048385293111 | -0.73920589 | 0.458552357389399 | 0.869074204989615 | 0.477492947105841 | 1.58178247104227 | 0.999332001504091 |
| ebi-fl187-GCST90094812 | prot-a-2921 | Tachykinin-3 \|\| id:prot-a-2921 | Plum liking \|\| id:ebi-fl187-GCST90094812 | Weighted median | 5 | -0.5259832 | 0.291879940783327 | 0.0715370256776519 | -1.098067884 | 0.0461014837204242 | 0.59097403283492 | 0.333514850910976 | 1.04718067735579 | 0.999332001504091 |
| ebi-fl187-GCST90094812 | prot-a-2928 | Tax1-binding protein 1 \|\| id:prot-a-2928 | Plum liking \|\| id:ebi-fl187-GCST90094812 | Weighted median | 5 | 0.0251347828554416 | 0.296793370147624 | 0.9325095811593 | -0.556580223 | 0.606849788344786 | 1.02545332473767 | 0.573165815597348 | 1.83464277282416 | 0.999332001504091 |
| ebi-fl187-GCST90094812 | prot-a-296 | Uncharacterized protein C1orf185 \|\| id:prot-a-296 | Plum liking \|\| id:ebi-fl187-GCST90094812 | Weighted median | 5 | -0.128838116 | 0.287247597762815 | 0.653773375759065 | -0.691843407 | 0.434167175861333 | 0.879116269101479 | 0.500652311736397 | 1.54367691206392 | 0.999332001504091 |
| ebi-fl187-GCST90094812 | prot-a-2964 | Transforming growth factor beta-2 \|\| id:prot-a-2964 | Plum liking \|\| id:ebi-fl187-GCST90094812 | Weighted median | 5 | -0.267612329 | 0.296208766274547 | 0.366282602045266 | -0.848181511 | 0.312956853060384 | 0.765204371277409 | 0.428192888552297 | 1.36746252793159 | 0.999332001504091 |
| ebi-fl187-GCST90094812 | prot-a-2977 | Thymocyte nuclear protein 1 \|\| id:prot-a-2977 | Plum liking \|\| id:ebi-fl187-GCST90094812 | Weighted median | 5 | -0.21419849 | 0.303679957420566 | 0.480596892744123 | -0.809411207 | 0.381014226260039 | 0.807188150115296 | 0.44512007274084 | 1.46376842921192 | 0.999332001504091 |
| ebi-fl187-GCST90094812 | prot-a-3015 | Transmembrane protein 70, mitochondrial \|\| id:prot-a-3015 | Plum liking \|\| id:ebi-fl187-GCST90094812 | Weighted median | 5 | -0.208961196 | 0.315481545116218 | 0.507742887804974 | -0.827305025 | 0.409382631931389 | 0.811426721232231 | 0.437226013192589 | 1.5058878110248 | 0.999332001504091 |
| ebi-fl187-GCST90094812 | prot-a-3030 | Tumor necrosis factor alpha-induced protein 3 \|\| id:prot-a-3030 | Plum liking \|\| id:ebi-fl187-GCST90094812 | Weighted median | 5 | -0.121100339 | 0.294460932834887 | 0.680881060354123 | -0.698243767 | 0.456043089224731 | 0.88594506017488 | 0.497458189317509 | 1.57781833027036 | 0.999332001504091 |
| ebi-fl187-GCST90094812 | prot-a-3032 | Tumor necrosis factor receptor superfamily member 10A \|\| id:prot-a-3032 | Plum liking \|\| id:ebi-fl187-GCST90094812 | Weighted median | 5 | 0.20264402610937 | 0.294446442707348 | 0.491314047340254 | -0.374471002 | 0.779759053815772 | 1.22463645204447 | 0.687652949904068 | 2.1809467113975 | 0.999332001504091 |
| ebi-fl187-GCST90094812 | prot-a-3081 | Tumor protein p53-inducible protein 11 \|\| id:prot-a-3081 | Plum liking \|\| id:ebi-fl187-GCST90094812 | Weighted median | 5 | 0.228704070727758 | 0.301572588248185 | 0.448228585492586 | -0.362378202 | 0.819786343694202 | 1.25697000974517 | 0.696019082082267 | 2.27001478274415 | 0.999332001504091 |
| ebi-fl187-GCST90094812 | prot-a-315 | Complement C4 \|\| id:prot-a-315 | Plum liking \|\| id:ebi-fl187-GCST90094812 | Weighted median | 5 | -0.229587868 | 0.2837916063302 | 0.418514217841232 | -0.785819416 | 0.326643680819584 | 0.794861123039939 | 0.455746103093729 | 1.38630742124058 | 0.999332001504091 |
| ebi-fl187-GCST90094812 | prot-a-3157 | UDP-glucuronosyltransferase 1-8 \|\| id:prot-a-3157 | Plum liking \|\| id:ebi-fl187-GCST90094812 | Weighted median | 5 | -0.261587011 | 0.288838840375463 | 0.365120924755486 | -0.827711138 | 0.304537116621828 | 0.769828889302724 | 0.437048486195008 | 1.35599718915543 | 0.999332001504091 |
| ebi-fl187-GCST90094812 | prot-a-317 | Glutaredoxin-like protein C5orf63 \|\| id:prot-a-317 | Plum liking \|\| id:ebi-fl187-GCST90094812 | Weighted median | 5 | 0.0906490144770998 | 0.294774931835293 | 0.758448047784873 | -0.487109852 | 0.668407880874273 | 1.0948846491496 | 0.614399536268628 | 1.95112841755029 | 0.999332001504091 |
| ebi-fl187-GCST90094812 | prot-a-3192 | Guanine nucleotide exchange factor VAV3 \|\| id:prot-a-3192 | Plum liking \|\| id:ebi-fl187-GCST90094812 | Weighted median | 5 | -0.015469291 | 0.283705938106866 | 0.956516260342178 | -0.57153293 | 0.540594347802869 | 0.984649744008109 | 0.564659192145659 | 1.71702707024228 | 0.999332001504091 |
| ebi-fl187-GCST90094812 | prot-a-3233 | Serine/threonine-protein kinase WNK3 \|\| id:prot-a-3233 | Plum liking \|\| id:ebi-fl187-GCST90094812 | Weighted median | 5 | 0.343559363706633 | 0.291617727834198 | 0.238750565623991 | -0.228011383 | 0.915130110261661 | 1.40995722028533 | 0.796115197725276 | 2.49710012912069 | 0.999332001504091 |
| ebi-fl187-GCST90094812 | prot-a-3250 | 14-3-3 protein theta \|\| id:prot-a-3250 | Plum liking \|\| id:ebi-fl187-GCST90094812 | Weighted median | 5 | 0.143524634200019 | 0.287874031250398 | 0.618084110173665 | -0.420708467 | 0.707757735450799 | 1.15433524658221 | 0.656581488647774 | 2.02943562153462 | 0.999332001504091 |
| ebi-fl187-GCST90094812 | prot-a-3278 | E3 ubiquitin-protein ligase ZNRF3 \|\| id:prot-a-3278 | Plum liking \|\| id:ebi-fl187-GCST90094812 | Weighted median | 5 | -0.057605031 | 0.277057944751574 | 0.835293848332863 | -0.600638602 | 0.4854285409821 | 0.944022733781472 | 0.548461275524347 | 1.62487118355667 | 0.999332001504091 |
| ebi-fl187-GCST90094812 | prot-a-355 | Calpain-3 \|\| id:prot-a-355 | Plum liking \|\| id:ebi-fl187-GCST90094812 | Weighted median | 5 | -0.015910055 | 0.303539353165425 | 0.958197918218745 | -0.610847187 | 0.579027077367617 | 0.984215841528858 | 0.542890744193025 | 1.78430159857716 | 0.999332001504091 |
| ebi-fl187-GCST90094812 | prot-a-385 | Coiled-coil domain-containing protein 90B, mitochondrial \|\| id:prot-a-385 | Plum liking \|\| id:ebi-fl187-GCST90094812 | Weighted median | 5 | -0.065679993 | 0.277327660789319 | 0.812787324689903 | -0.609242208 | 0.4778822218783 | 0.936430480406538 | 0.543762771838848 | 1.612655537 | 0.999332001504091 |
| ebi-fl187-GCST90094812 | prot-a-390 | C-C motif chemokine 14 \|\| id:prot-a-390 | Plum liking \|\| id:ebi-fl187-GCST90094812 | Weighted median | 5 | -0.160198376 | 0.30207672724672 | 0.595887682707992 | -0.752268761 | 0.431872009777656 | 0.851974761174757 | 0.47129608069632 | 1.54013797994321 | 0.999332001504091 |
| ebi-fl187-GCST90094812 | prot-a-40 | Pituitary adenylate cyclase-activating polypeptide 27 \|\| id:prot-a-40 | Plum liking \|\| id:ebi-fl187-GCST90094812 | Weighted median | 5 | 0.210554043271541 | 0.279512486191646 | 0.451275489234842 | -0.33729043 | 0.758398516207167 | 1.23436176036743 | 0.713701529704133 | 2.13485454639421 | 0.999332001504091 |
| ebi-fl187-GCST90094812 | prot-a-401 | C-C motif chemokine 24 \|\| id:prot-a-401 | Plum liking \|\| id:ebi-fl187-GCST90094812 | Weighted median | 5 | -0.167429476 | 0.272364784487086 | 0.538736263948652 | -0.701264454 | 0.366405501278896 | 0.845836266675234 | 0.495957791376014 | 1.44254007591683 | 0.999332001504091 |
| ebi-fl187-GCST90094812 | prot-a-412 | Cerebral cavernous malformations 2 protein \|\| id:prot-a-412 | Plum liking \|\| id:ebi-fl187-GCST90094812 | Weighted median | 5 | 0.460520982073809 | 0.303295559219023 | 0.128916079622493 | -0.133938314 | 1.05498027814309 | 1.58489947415811 | 0.874644016234028 | 2.87191851377685 | 0.999332001504091 |
| ebi-fl187-GCST90094812 | prot-a-44 | Adhesion G protein-coupled receptor F5 \|\| id:prot-a-44 | Plum liking \|\| id:ebi-fl187-GCST90094812 | Weighted median | 5 | -0.259148695 | 0.286402771724481 | 0.365550136783482 | -0.820498128 | 0.30220073755147 | 0.771708265329448 | 0.440212317972562 | 1.35283276379128 | 0.999332001504091 |
| ebi-fl187-GCST90094812 | prot-a-48 | Adrenomedullin \|\| id:prot-a-48 | Plum liking \|\| id:ebi-fl187-GCST90094812 | Weighted median | 5 | 0.191423131321778 | 0.30670742354921 | 0.53254686577793 | -0.409723419 | 0.79256968147823 | 1.2109717437649 | 0.663833828681885 | 2.20906573427994 | 0.999332001504091 |
| ebi-fl187-GCST90094812 | prot-a-502 | Carcinoembryonic antigen-related cell adhesion molecule 1 \|\| id:prot-a-502 | Plum liking \|\| id:ebi-fl187-GCST90094812 | Weighted median | 5 | -0.216749305 | 0.293123408213537 | 0.459635444973705 | -0.791271185 | 0.35777257483972 | 0.805131786311506 | 0.453268240955434 | 1.4301403335975 | 0.999332001504091 |
| ebi-fl187-GCST90094812 | prot-a-518 | Complement factor B \|\| id:prot-a-518 | Plum liking \|\| id:ebi-fl187-GCST90094812 | Weighted median | 5 | -0.103404665 | 0.284697060776435 | 0.716448423338518 | -0.661410904 | 0.454601574141123 | 0.901761988140587 | 0.516122621016782 | 1.57554551988688 | 0.999332001504091 |
| ebi-fl187-GCST90094812 | prot-a-525 | Properdin \|\| id:prot-a-525 | Plum liking \|\| id:ebi-fl187-GCST90094812 | Weighted median | 5 | 0.187282727165682 | 0.338774585617404 | 0.580383921295685 | -0.476715461 | 0.851280914975794 | 1.20596819682534 | 0.620819151645576 | 2.34264566081628 | 0.999332001504091 |
| ebi-fl187-GCST90094812 | prot-a-533 | Chondroadherin \|\| id:prot-a-533 | Plum liking \|\| id:ebi-fl187-GCST90094812 | Weighted median | 5 | 0.358480836075923 | 0.291331419086613 | 0.218513106515109 | -0.212528745 | 0.929490417485685 | 1.43115360534631 | 0.808537074319282 | 2.53321796507618 | 0.999332001504091 |
| ebi-fl187-GCST90094812 | prot-a-535 | Coiled-coil-helix-coiled-coil-helix domain-containing protein 2 \|\| id:prot-a-535 | Plum liking \|\| id:ebi-fl187-GCST90094812 | Weighted median | 5 | -0.60475648 | 0.289299954938093 | 0.0365806871809794 | -1.171784392 | -0.037728568 | 0.546207422905506 | 0.309813618913032 | 0.962974287198208 | 0.999332001504091 |
| ebi-fl187-GCST90094812 | prot-a-591 | Caseinolytic peptidase B protein homolog \|\| id:prot-a-591 | Plum liking \|\| id:ebi-fl187-GCST90094812 | Weighted median | 5 | 0.242674606623889 | 0.29489118666951 | 0.410548182554874 | -0.335312119 | 0.820661332496129 | 1.27465379272944 | 0.715114850406661 | 2.27200188948056 | 0.999332001504091 |
| ebi-fl187-GCST90094812 | prot-a-605 | Ciliary neurotrophic factor receptor subunit alpha \|\| id:prot-a-605 | Plum liking \|\| id:ebi-fl187-GCST90094812 | Weighted median | 5 | 0.0524531427789626 | 0.286728148736904 | 0.854847572904414 | -0.509534029 | 0.614440314303295 | 1.05385318029019 | 0.600775457693217 | 1.84862166286239 | 0.999332001504091 |
| ebi-fl187-GCST90094812 | prot-a-606 | Ciliary neurotrophic factor receptor subunit alpha \|\| id:prot-a-606 | Plum liking \|\| id:ebi-fl187-GCST90094812 | Weighted median | 5 | 0.108585906038671 | 0.33321633376663 | 0.744521126344397 | -0.544518108 | 0.761689920221265 | 1.11470066394364 | 0.580121271709227 | 2.14189279171821 | 0.999332001504091 |
| ebi-fl187-GCST90094812 | prot-a-707 | Cancer/testis antigen 55 \|\| id:prot-a-707 | Plum liking \|\| id:ebi-fl187-GCST90094812 | Weighted median | 5 | -0.114283002 | 0.277456810193143 | 0.68041657324444 | -0.65809835 | 0.429532346113441 | 0.892005480943851 | 0.517835140043245 | 1.53653878716578 | 0.999332001504091 |
| ebi-fl187-GCST90094812 | prot-a-719 | Dipeptidyl peptidase 1 \|\| id:prot-a-719 | Plum liking \|\| id:ebi-fl187-GCST90094812 | Weighted median | 5 | 0.137984559089182 | 0.280649065287533 | 0.622958108417248 | -0.412087609 | 0.688056727052746 | 1.14795782462294 | 0.662266253109521 | 1.98984496178925 | 0.999332001504091 |
| ebi-fl187-GCST90094812 | prot-a-752 | Cytochrome b5 \|\| id:prot-a-752 | Plum liking \|\| id:ebi-fl187-GCST90094812 | Weighted median | 5 | 0.0284018908043598 | 0.302460340225805 | 0.925186322398582 | -0.564420376 | 0.621224157646937 | 1.02880907025266 | 0.568689677410229 | 1.86120505625182 | 0.999332001504091 |
| ebi-fl187-GCST90094812 | prot-a-755 | Cytoglobin \|\| id:prot-a-755 | Plum liking \|\| id:ebi-fl187-GCST90094812 | Weighted median | 5 | -0.278824449 | 0.30501646243419 | 0.360648970008994 | -0.876656716 | 0.319007816908195 | 0.756672725859659 | 0.416171969536315 | 1.37576207906988 | 0.999332001504091 |
| ebi-fl187-GCST90094812 | prot-a-807 | Desmin \|\| id:prot-a-807 | Plum liking \|\| id:ebi-fl187-GCST90094812 | Weighted median | 5 | 0.301466982548679 | 0.300662643514295 | 0.316017589244935 | -0.287831799 | 0.890765763836697 | 1.35184048 | 0.749887713690647 | 2.4369950996698 | 0.999332001504091 |
| ebi-fl187-GCST90094812 | prot-a-82 | Ameloblastin \|\| id:prot-a-82 | Plum liking \|\| id:ebi-fl187-GCST90094812 | Weighted median | 5 | -0.384495999 | 0.308641256319662 | 0.212848868559579 | -0.989432862 | 0.220440863182761 | 0.680793670304494 | 0.371787486205041 | 1.24662620105254 | 0.999332001504091 |
| ebi-fl187-GCST90094812 | prot-a-84 | Muellerian-inhibiting factor \|\| id:prot-a-84 | Plum liking \|\| id:ebi-fl187-GCST90094812 | Weighted median | 5 | 0.288174313014674 | 0.321040384939899 | 0.369384779461694 | -0.341064841 | 0.917413467496875 | 1.33398981564421 | 0.711012803598065 | 2.50280841531575 | 0.999332001504091 |
| ebi-fl187-GCST90094812 | prot-a-858 | Inactive dipeptidyl peptidase 10 \|\| id:prot-a-858 | Plum liking \|\| id:ebi-fl187-GCST90094812 | Weighted median | 5 | -0.210163869 | 0.3412105132637 | 0.53793670232165 | -0.878936475 | 0.45860873724258 | 0.81045142742227 | 0.415224278441702 | 1.58187165421979 | 0.999332001504091 |
| ebi-fl187-GCST90094812 | prot-a-863 | Dihydropyrimidinase-related protein 3 \|\| id:prot-a-863 | Plum liking \|\| id:ebi-fl187-GCST90094812 | Weighted median | 5 | 0.106689463373732 | 0.303455489930121 | 0.725151827338193 | -0.488083297 | 0.701462223636769 | 1.11258870128692 | 0.613801743137512 | 2.01669941812791 | 0.999332001504091 |
| ebi-fl187-GCST90094812 | prot-a-864 | Dihydropyrimidinase-related protein 5 \|\| id:prot-a-864 | Plum liking \|\| id:ebi-fl187-GCST90094812 | Weighted median | 5 | -0.001049558 | 0.286114661232866 | 0.997073117717605 | -0.561834294 | 0.559735178408751 | 0.998950992985277 | 0.570162259072152 | 1.75020894580115 | 0.999364451047625 |
| ebi-fl187-GCST90094812 | prot-a-884 | Endothelin-converting enzyme 1 \|\| id:prot-a-884 | Plum liking \|\| id:ebi-fl187-GCST90094812 | Weighted median | 5 | 0.1372654037887 | 0.295197643516893 | 0.641935096545651 | -0.441321978 | 0.715852785081811 | 1.14713256145039 | 0.643185581941463 | 2.04593067768657 | 0.999332001504091 |
| ebi-fl187-GCST90094812 | prot-a-893 | Early endosome antigen 1 \|\| id:prot-a-893 | Plum liking \|\| id:ebi-fl187-GCST90094812 | Weighted median | 5 | 0.248621499919375 | 0.278717375718622 | 0.372382161686413 | -0.297664556 | 0.794907556327875 | 1.28225660697958 | 0.742550381682722 | 2.21423629520845 | 0.999332001504091 |
| ebi-fl187-GCST90094812 | prot-a-897 | Ephrin-A2 \|\| id:prot-a-897 | Plum liking \|\| id:ebi-fl187-GCST90094812 | Weighted median | 5 | 0.107824746447496 | 0.300655825048912 | 0.719870537501498 | -0.481460671 | 0.697110163543364 | 1.11385252166875 | 0.617880212852105 | 2.00794169196806 | 0.999332001504091 |
| ebi-fl187-GCST90094812 | prot-a-90 | Anaphase-promoting complex subunit 10 \|\| id:prot-a-90 | Plum liking \|\| id:ebi-fl187-GCST90094812 | Weighted median | 5 | -0.450444298 | 0.284152961526178 | 0.112916885349045 | -1.007384102 | 0.106495506744736 | 0.637344917731857 | 0.365172986360263 | 1.11237292825894 | 0.999332001504091 |
| ebi-fl187-GCST90094812 | prot-a-914 | Histone-lysine N-methyltransferase EHMT2 \|\| id:prot-a-914 | Plum liking \|\| id:ebi-fl187-GCST90094812 | Weighted median | 5 | -0.556107141 | 0.316700217134294 | 0.0790983509204555 | -1.176839567 | 0.0646252841232523 | 0.573437033703284 | 0.308251408673199 | 1.06675921786634 | 0.999332001504091 |
| ebi-fl187-GCST90094812 | prot-a-932 | RNA polymerase II elongation factor ELL \|\| id:prot-a-932 | Plum liking \|\| id:ebi-fl187-GCST90094812 | Weighted median | 5 | 0.0632275078014096 | 0.2917881017994 | 0.828450091218176 | -0.508677172 | 0.635132187328233 | 1.06526916871082 | 0.601290456970423 | 1.88727159836109 | 0.999332001504091 |
| ebi-fl187-GCST90094812 | prot-a-991 | Estrogen receptor \|\| id:prot-a-991 | Plum liking \|\| id:ebi-fl187-GCST90094812 | Weighted median | 5 | 0.131427594986813 | 0.288095251887535 | 0.648249806624913 | -0.433239099 | 0.696094288686382 | 1.14045533004968 | 0.648405440363281 | 2.00590291023779 | 0.999332001504091 |
| ebi-fl187-GCST90094812 | prot-a-992 | Persulfide dioxygenase ETHE1, mitochondrial \|\| id:prot-a-992 | Plum liking \|\| id:ebi-fl187-GCST90094812 | Weighted median | 5 | 0.128107363694293 | 0.308454191628285 | 0.677907261447431 | -0.476462852 | 0.732677579285731 | 1.13667503377693 | 0.620975995803032 | 2.08064424574247 | 0.999332001504091 |
| ebi-fl187-GCST90094812 | prot-a-993 | Ethanolamine kinase 1 \|\| id:prot-a-993 | Plum liking \|\| id:ebi-fl187-GCST90094812 | Weighted median | 5 | 0.0571664360701686 | 0.291303375710845 | 0.844419468865297 | -0.51378818 | 0.628121052463424 | 1.05883202357195 | 0.598225096489914 | 1.87408595981626 | 0.999332001504091 |
| ebi-fl187-GCST90094812 | prot-b-10 | coagulation factor III, tissue factor \|\| id:prot-b-10 | Plum liking \|\| id:ebi-fl187-GCST90094812 | Weighted median | 4 | -0.305519347 | 0.332489620656531 | 0.358156320697702 | -0.957199004 | 0.346160309075732 | 0.736740650667389 | 0.383966870867841 | 1.41362921524714 | 0.999332001504091 |
| ebi-fl187-GCST90094812 | prot-b-28 | prolactin \|\| id:prot-b-28 | Plum liking \|\| id:ebi-fl187-GCST90094812 | Weighted median | 4 | 0.363430068252716 | 0.336200577074992 | 0.279700759530877 | -0.295523063 | 1.0223831993197 | 1.43825427375393 | 0.744142252510654 | 2.77981172147166 | 0.999332001504091 |
| ebi-fl187-GCST90094812 | prot-b-54 | matrix metallopeptidase 7 \|\| id:prot-b-54 | Plum liking \|\| id:ebi-fl187-GCST90094812 | Weighted median | 4 | 0.0177614856837768 | 0.331769644924069 | 0.95730516977678 | -0.632507018 | 0.668029989734951 | 1.01792015890255 | 0.53125825588896 | 1.95039124270431 | 0.999332001504091 |
| ebi-fl187-GCST90094812 | ukb-rsfMRI191-edge-pheno716 | Net100_Pair10_33 (OccipitalPrecuneus)&(Temporal\|Frontal\|Supp_Motor_Area) (Default_mode\|Central_executive)&(Default_mode\|Salience) \|\| id:ukb-rsfMRI191-edge-pheno716 | Plum liking \|\| id:ebi-fl187-GCST90094812 | Weighted median | 5 | 0.0175465021700425 | 0.0873530401658537 | 0.840801148199346 | -0.153665457 | 0.188758460895116 | 1.01770134637148 | 0.857558863724642 | 1.20774919858898 | 0.999332001504091 |
| ebi-fl187-GCST90094812 | ukb-rsfMRI191-edge-pheno903 | Net100_Pair27_38 (Precentral\|Frontal\|Supp_Motor_Area)&(Subcortical) (Attention\|Salience\|Motor)&(Subcortical-cerebellum) \|\| id:ukb-rsfMRI191-edge-pheno903 | Plum liking \|\| id:ebi-fl187-GCST90094812 | Weighted median | 5 | -0.139013324 | 0.0901071542432587 | 0.122890067349473 | -0.315623346 | 0.0375966985923282 | 0.870216433857026 | 0.729334105017368 | 1.0383123955746 | 0.999332001504091 |
| ebi-fl187-GCST90094812 | ukb-rsfMRI191-node-pheno23 | Net100_Node2 RolandicOper\|SupraMarginal\|Insula Salience\|Motor \|\| id:ukb-rsfMRI191-node-pheno23 | Plum liking \|\| id:ebi-fl187-GCST90094812 | Weighted median | 5 | -0.047981386 | 0.0823275776438065 | 0.560020802926757 | -0.209343438 | 0.11338066658878 | 0.953151529262795 | 0.81111661971544 | 1.12005822054877 | 0.999332001504091 |
| ebi-fl187-GCST90094812 | ukb-rsfMRI191-node-pheno55 | Net100_Node34 Parietal\|Postcentral\|Precuneus Attention \|\| id:ukb-rsfMRI191-node-pheno55 | Plum liking \|\| id:ebi-fl187-GCST90094812 | Weighted median | 5 | -0.025122576 | 0.105527227790727 | 0.81182894174141 | -0.231955943 | 0.181710790431576 | 0.975190369729912 | 0.792981059293701 | 1.19926730414091 | 0.999332001504091 |
| ebi-fl187-GCST90094812 | ebi-a-GCST90001414 | IgD- CD24- B cell Absolute Count \|\| id:ebi-a-GCST90001414 | Plum liking \|\| id:ebi-fl187-GCST90094812 | Weighted mode | 5 | -0.482351435 | 0.321410979173411 | 0.20782041702506 | -1.112316954 | 0.147614084346303 | 0.617330072346853 | 0.328796272016583 | 1.15906550851814 | 0.999714303421225 |
| ebi-fl187-GCST90094812 | ebi-a-GCST90001422 | CD20- CD38- B cell %B cell \|\| id:ebi-a-GCST90001422 | Plum liking \|\| id:ebi-fl187-GCST90094812 | Weighted mode | 5 | -0.148845595 | 0.282561809404392 | 0.626212159764235 | -0.702666741 | 0.404975551881202 | 0.86170215613552 | 0.495262803576239 | 1.4992658453792 | 0.999714303421225 |
| ebi-fl187-GCST90094812 | ebi-a-GCST90001444 | CD20- CD38- B cell %lymphocyte \|\| id:ebi-a-GCST90001444 | Plum liking \|\| id:ebi-fl187-GCST90094812 | Weighted mode | 5 | -0.154762996 | 0.319408717929434 | 0.653346057673688 | -0.780804083 | 0.471278091121877 | 0.85661817534407 | 0.458037562901831 | 1.60204043895647 | 0.999714303421225 |
| ebi-fl187-GCST90094812 | ebi-a-GCST90001459 | Myeloid Dendritic Cell %Dendritic Cell \|\| id:ebi-a-GCST90001459 | Plum liking \|\| id:ebi-fl187-GCST90094812 | Weighted mode | 5 | 0.117690649932021 | 0.328800769877931 | 0.73847820277329 | -0.526758859 | 0.762140158892765 | 1.12489607086095 | 0.59051581635041 | 2.14285737181259 | 0.999714303421225 |
| ebi-fl187-GCST90094812 | ebi-a-GCST90001476 | HLA DR++ monocyte %leukocyte \|\| id:ebi-a-GCST90001476 | Plum liking \|\| id:ebi-fl187-GCST90094812 | Weighted mode | 5 | -0.099753575 | 0.348698914144326 | 0.789025938325016 | -0.783203447 | 0.583696296454271 | 0.905060419829103 | 0.456939881535722 | 1.79265237428656 | 0.999714303421225 |
| ebi-fl187-GCST90094812 | ebi-a-GCST90001478 | CD4 regulatory T cell %CD4+ T cell \|\| id:ebi-a-GCST90001478 | Plum liking \|\| id:ebi-fl187-GCST90094812 | Weighted mode | 5 | 0.0145959653199752 | 0.309178315980831 | 0.964609761983881 | -0.591393534 | 0.620585464642404 | 1.01470300657788 | 0.55355534879915 | 1.86001669714111 | 0.999714303421225 |
| ebi-fl187-GCST90094812 | ebi-a-GCST90001485 | CD39+ resting CD4 regulatory T cell %CD4 regulatory T cell \|\| id:ebi-a-GCST90001485 | Plum liking \|\| id:ebi-fl187-GCST90094812 | Weighted mode | 5 | 0.368651725042825 | 0.338813896002951 | 0.337735791821625 | -0.295423511 | 1.03272696120861 | 1.44578398562538 | 0.744216336818014 | 2.80871465685379 | 0.999714303421225 |
| ebi-fl187-GCST90094812 | ebi-a-GCST90001515 | Immature Myeloid-Derived Suppressor Cells Absolute Count \|\| id:ebi-a-GCST90001515 | Plum liking \|\| id:ebi-fl187-GCST90094812 | Weighted mode | 5 | -0.520085078 | 0.480059663462574 | 0.339579867881283 | -1.461002019 | 0.420831862197665 | 0.59446996939026 | 0.232003686217012 | 1.52322814464378 | 0.999714303421225 |
| ebi-fl187-GCST90094812 | ebi-a-GCST90001517 | CD33+ HLA DR+ Absolute Count \|\| id:ebi-a-GCST90001517 | Plum liking \|\| id:ebi-fl187-GCST90094812 | Weighted mode | 5 | 0.589151729938917 | 0.466756367031524 | 0.275446501754704 | -0.325690749 | 1.5039942093207 | 1.8024587948919 | 0.722028440606874 | 4.49962567201984 | 0.999714303421225 |
| ebi-fl187-GCST90094812 | ebi-a-GCST90001518 | CD33+ HLA DR+ CD14- Absolute Count \|\| id:ebi-a-GCST90001518 | Plum liking \|\| id:ebi-fl187-GCST90094812 | Weighted mode | 5 | 0.624875398686313 | 0.535360851902464 | 0.30796258878631 | -0.424431871 | 1.67418266841514 | 1.86801318603375 | 0.654141316205095 | 5.33443336592714 | 0.999714303421225 |
| ebi-fl187-GCST90094812 | ebi-a-GCST90001520 | CD33+ HLA DR+ CD14dim Absolute Count \|\| id:ebi-a-GCST90001520 | Plum liking \|\| id:ebi-fl187-GCST90094812 | Weighted mode | 5 | 0.378965148185823 | 0.435758539890889 | 0.433556194349338 | -0.47512159 | 1.23305188637196 | 1.46077212429763 | 0.621809446057634 | 3.43168668899125 | 0.999714303421225 |
| ebi-fl187-GCST90094812 | ebi-a-GCST90001531 | CD33dim HLA DR- Absolute Count \|\| id:ebi-a-GCST90001531 | Plum liking \|\| id:ebi-fl187-GCST90094812 | Weighted mode | 5 | -0.544128865 | 0.470747611869798 | 0.312068488797049 | -1.466794184 | 0.378536454142143 | 0.580347123818553 | 0.230663766661703 | 1.4601460341989 | 0.999714303421225 |
| ebi-fl187-GCST90094812 | ebi-a-GCST90001532 | Basophil Absolute Count \|\| id:ebi-a-GCST90001532 | Plum liking \|\| id:ebi-fl187-GCST90094812 | Weighted mode | 5 | -0.490269256 | 0.428891112626734 | 0.316761687568605 | -1.330895837 | 0.350357324540552 | 0.612461462930069 | 0.264240438888037 | 1.41957470685772 | 0.999714303421225 |
| ebi-fl187-GCST90094812 | ebi-a-GCST90001588 | T/B cell \|\| id:ebi-a-GCST90001588 | Plum liking \|\| id:ebi-fl187-GCST90094812 | Weighted mode | 5 | 0.196434716457976 | 0.318757903356715 | 0.571070636958994 | -0.428330774 | 0.821200207037138 | 1.21705586456695 | 0.65159584810473 | 2.2732265433937 | 0.999714303421225 |
| ebi-fl187-GCST90094812 | ebi-a-GCST90001624 | HLA DR+ CD4+ T cell Absolute Count \|\| id:ebi-a-GCST90001624 | Plum liking \|\| id:ebi-fl187-GCST90094812 | Weighted mode | 5 | -0.06593239 | 0.328146916997518 | 0.850561565105618 | -0.709100348 | 0.577235566827444 | 0.936194157782408 | 0.492086705265152 | 1.78110786511424 | 0.999714303421225 |
| ebi-fl187-GCST90094812 | ebi-a-GCST90001631 | CD8+ Natural Killer T %T cell \|\| id:ebi-a-GCST90001631 | Plum liking \|\| id:ebi-fl187-GCST90094812 | Weighted mode | 5 | -0.2685917 | 0.338958279073864 | 0.47247128985258 | -0.932949927 | 0.395766527036312 | 0.764455319082286 | 0.393391520784738 | 1.48552244773211 | 0.999714303421225 |
| ebi-fl187-GCST90094812 | ebi-a-GCST90001632 | CD8+ Natural Killer T %lymphocyte \|\| id:ebi-a-GCST90001632 | Plum liking \|\| id:ebi-fl187-GCST90094812 | Weighted mode | 5 | -0.182312178 | 0.350503797791367 | 0.630424686512057 | -0.869299621 | 0.504675266126769 | 0.833341149411359 | 0.41924507680275 | 1.65644752849174 | 0.999714303421225 |
| ebi-fl187-GCST90094812 | ebi-a-GCST90001646 | Natural Killer %CD3- lymphocyte \|\| id:ebi-a-GCST90001646 | Plum liking \|\| id:ebi-fl187-GCST90094812 | Weighted mode | 5 | -0.207619578 | 0.321496750716671 | 0.55359502447019 | -0.83775321 | 0.422514053007875 | 0.812516076593971 | 0.432681576869901 | 1.52579266142909 | 0.999714303421225 |
| ebi-fl187-GCST90094812 | ebi-a-GCST90001684 | CD127- CD8+ T cell Absolute Count \|\| id:ebi-a-GCST90001684 | Plum liking \|\| id:ebi-fl187-GCST90094812 | Weighted mode | 5 | 0.292716423426592 | 0.334672703288472 | 0.431145440930682 | -0.363242075 | 0.948674921871998 | 1.34006272614953 | 0.695418069779267 | 2.58228566103454 | 0.999714303421225 |
| ebi-fl187-GCST90094812 | ebi-a-GCST90001687 | CD28- CD8+ T cell Absolute Count \|\| id:ebi-a-GCST90001687 | Plum liking \|\| id:ebi-fl187-GCST90094812 | Weighted mode | 5 | 0.196081751258211 | 0.304459329536344 | 0.554624613950768 | -0.400658535 | 0.792822037149445 | 1.21662636200477 | 0.669878762386345 | 2.20962327489238 | 0.999714303421225 |
| ebi-fl187-GCST90094812 | ebi-a-GCST90001701 | BAFF-R on CD20- CD38- B cell \|\| id:ebi-a-GCST90001701 | Plum liking \|\| id:ebi-fl187-GCST90094812 | Weighted mode | 5 | -0.004825355 | 0.318402023581876 | 0.988634358828106 | -0.628893321 | 0.619242611151889 | 0.995186268254112 | 0.533181535277682 | 1.85752064351918 | 0.999714303421225 |
| ebi-fl187-GCST90094812 | ebi-a-GCST90001732 | CD19 on IgD- CD27- B cell \|\| id:ebi-a-GCST90001732 | Plum liking \|\| id:ebi-fl187-GCST90094812 | Weighted mode | 5 | 0.330054631118344 | 0.298699571868739 | 0.33116277726769 | -0.25539653 | 0.915505791981072 | 1.39104412068397 | 0.774609281434496 | 2.49803842022912 | 0.999714303421225 |
| ebi-fl187-GCST90094812 | ebi-a-GCST90001798 | CD27 on CD24+ CD27+ B cell \|\| id:ebi-a-GCST90001798 | Plum liking \|\| id:ebi-fl187-GCST90094812 | Weighted mode | 5 | 0.101720158678825 | 0.309544089739434 | 0.758932269539536 | -0.504986257 | 0.708426574568114 | 1.10707362343036 | 0.603513869334137 | 2.03079344149531 | 0.999714303421225 |
| ebi-fl187-GCST90094812 | ebi-a-GCST90001836 | CD66b on CD66b++ myeloid cell \|\| id:ebi-a-GCST90001836 | Plum liking \|\| id:ebi-fl187-GCST90094812 | Weighted mode | 5 | -0.091109961 | 0.497017794173712 | 0.863469129111847 | -1.065264838 | 0.883044915334503 | 0.912917319853843 | 0.344636569879826 | 2.41825187959518 | 0.999714303421225 |
| ebi-fl187-GCST90094812 | ebi-a-GCST90001943 | CD25 on activated & secreting CD4 regulatory T cell \|\| id:ebi-a-GCST90001943 | Plum liking \|\| id:ebi-fl187-GCST90094812 | Weighted mode | 5 | -0.096496982 | 0.365801315336599 | 0.804969748986997 | -0.813467561 | 0.620473595611986 | 0.908012637571196 | 0.443318165353769 | 1.85980863051496 | 0.999714303421225 |
| ebi-fl187-GCST90094812 | ebi-a-GCST90001960 | CD25 on CD4+ T cell \|\| id:ebi-a-GCST90001960 | Plum liking \|\| id:ebi-fl187-GCST90094812 | Weighted mode | 5 | -0.056285798 | 0.371507688008038 | 0.886910325772792 | -0.784440867 | 0.671869270236529 | 0.945268941063774 | 0.456374804786254 | 1.95789373464278 | 0.999714303421225 |
| ebi-fl187-GCST90094812 | ebi-a-GCST90001988 | HLA DR on CD14+ CD16- monocyte \|\| id:ebi-a-GCST90001988 | Plum liking \|\| id:ebi-fl187-GCST90094812 | Weighted mode | 5 | -0.21266586 | 0.336227983544571 | 0.561408846380132 | -0.871672708 | 0.446340987863986 | 0.808426219722618 | 0.418251351570564 | 1.56258419794907 | 0.999714303421225 |
| ebi-fl187-GCST90094812 | ebi-a-GCST90001991 | HLA DR on CD14+ monocyte \|\| id:ebi-a-GCST90001991 | Plum liking \|\| id:ebi-fl187-GCST90094812 | Weighted mode | 5 | -0.242034813 | 0.346992864088519 | 0.523894251779904 | -0.922140826 | 0.438071200918483 | 0.785028848106261 | 0.397666793619313 | 1.54971524464021 | 0.999714303421225 |
| ebi-fl187-GCST90094812 | ebi-a-GCST90002019 | CD14 on Monocytic Myeloid-Derived Suppressor Cells \|\| id:ebi-a-GCST90002019 | Plum liking \|\| id:ebi-fl187-GCST90094812 | Weighted mode | 5 | 0.277022642893243 | 0.482877796167394 | 0.596891630973392 | -0.669417838 | 1.22346312338134 | 1.31919624111644 | 0.512006562012114 | 3.39893831777603 | 0.999714303421225 |
| ebi-fl187-GCST90094812 | ebi-a-GCST90002037 | CD80 on plasmacytoid Dendritic Cell \|\| id:ebi-a-GCST90002037 | Plum liking \|\| id:ebi-fl187-GCST90094812 | Weighted mode | 5 | 0.409617484380694 | 0.38428659206969 | 0.346525257881312 | -0.343584236 | 1.16281920483729 | 1.50624151399798 | 0.709223736403638 | 3.19893904002066 | 0.999714303421225 |
| ebi-fl187-GCST90094812 | ebi-a-GCST90002042 | CD45 on CD33+ HLA DR+ CD14- \|\| id:ebi-a-GCST90002042 | Plum liking \|\| id:ebi-fl187-GCST90094812 | Weighted mode | 5 | 0.217558911154688 | 0.457539777203968 | 0.659235255221784 | -0.679219052 | 1.11433687447447 | 1.24303865619333 | 0.507012788336794 | 3.04754660303466 | 0.999714303421225 |
| ebi-fl187-GCST90094812 | ebi-a-GCST90002056 | CD8 on Effector Memory CD8+ T cell \|\| id:ebi-a-GCST90002056 | Plum liking \|\| id:ebi-fl187-GCST90094812 | Weighted mode | 5 | 0.447433708795141 | 0.35927029812915 | 0.280957800209974 | -0.256736076 | 1.15160349312828 | 1.56429259964201 | 0.773572351492486 | 3.16326111264659 | 0.999714303421225 |
| ebi-fl187-GCST90094812 | ebi-a-GCST90002058 | CD8 on CD8+ T cell \|\| id:ebi-a-GCST90002058 | Plum liking \|\| id:ebi-fl187-GCST90094812 | Weighted mode | 5 | 0.0183608892265892 | 0.402315066571975 | 0.965786280686213 | -0.770176641 | 0.80689841970766 | 1.01853048675016 | 0.462931288325073 | 2.24094672060932 | 0.999714303421225 |
| ebi-fl187-GCST90094812 | ebi-a-GCST90002063 | CD4 on CD4 regulatory T cell \|\| id:ebi-a-GCST90002063 | Plum liking \|\| id:ebi-fl187-GCST90094812 | Weighted mode | 5 | -0.025834787 | 0.340439280533801 | 0.943153213545203 | -0.693095777 | 0.641426202678319 | 0.974496075566395 | 0.500025702433475 | 1.899187575 | 0.999714303421225 |
| ebi-fl187-GCST90094812 | ebi-a-GCST90002066 | CD4 on activated CD4 regulatory T cell \|\| id:ebi-a-GCST90002066 | Plum liking \|\| id:ebi-fl187-GCST90094812 | Weighted mode | 5 | 0.00899119802909532 | 0.340952377436237 | 0.980224740558366 | -0.659275462 | 0.67725785780412 | 1.00903174026676 | 0.517225948749998 | 1.96847249316544 | 0.999714303421225 |
| ebi-fl187-GCST90094812 | ebi-a-GCST90002070 | CD4 on activated & secreting CD4 regulatory T cell \|\| id:ebi-a-GCST90002070 | Plum liking \|\| id:ebi-fl187-GCST90094812 | Weighted mode | 5 | 0.0152353795183058 | 0.316513231753074 | 0.963916129870923 | -0.605130555 | 0.63560131375433 | 1.01535202956252 | 0.546003138700676 | 1.88815717504858 | 0.999714303421225 |
| ebi-fl187-GCST90094812 | ebi-a-GCST90002081 | SSC-A on CD4+ T cell \|\| id:ebi-a-GCST90002081 | Plum liking \|\| id:ebi-fl187-GCST90094812 | Weighted mode | 5 | -0.073744248 | 0.326194169254195 | 0.832225492692158 | -0.71308482 | 0.565596323776586 | 0.928909233894339 | 0.490129900622527 | 1.76049729616212 | 0.999714303421225 |
| ebi-fl187-GCST90094812 | ebi-a-GCST90002094 | CD11b on Monocytic Myeloid-Derived Suppressor Cells \|\| id:ebi-a-GCST90002094 | Plum liking \|\| id:ebi-fl187-GCST90094812 | Weighted mode | 5 | -0.249229023 | 0.481283355723331 | 0.631890750599949 | -1.192544401 | 0.694086353732287 | 0.779401451705624 | 0.303448187194235 | 2.00187922866713 | 0.999714303421225 |
| ebi-fl187-GCST90094812 | ebi-a-GCST90002104 | HLA DR on myeloid Dendritic Cell \|\| id:ebi-a-GCST90002104 | Plum liking \|\| id:ebi-fl187-GCST90094812 | Weighted mode | 5 | -0.087684322 | 0.371842713437086 | 0.825161687097534 | -0.816496041 | 0.641127396095635 | 0.916050007698329 | 0.441977616063207 | 1.8986201701312 | 0.999714303421225 |
| ebi-fl187-GCST90094812 | ebi-a-GCST90002105 | HLA DR on plasmacytoid Dendritic Cell \|\| id:ebi-a-GCST90002105 | Plum liking \|\| id:ebi-fl187-GCST90094812 | Weighted mode | 5 | -0.195915001 | 0.334167857325052 | 0.58917751167023 | -0.850884001 | 0.45905399970315 | 0.822082108150135 | 0.427037263670908 | 1.58257615911801 | 0.999714303421225 |
| ebi-fl187-GCST90094812 | ebi-a-GCST90002106 | HLA DR on Dendritic Cell \|\| id:ebi-a-GCST90002106 | Plum liking \|\| id:ebi-fl187-GCST90094812 | Weighted mode | 5 | -0.097746832 | 0.358357238456809 | 0.798537415886737 | -0.800127019 | 0.604633355399285 | 0.906878467323142 | 0.449271894268195 | 1.83058091322182 | 0.999714303421225 |
| ebi-fl187-GCST90094812 | ebi-a-GCST90002111 | HLA DR on CD33dim HLA DR+ CD11b- \|\| id:ebi-a-GCST90002111 | Plum liking \|\| id:ebi-fl187-GCST90094812 | Weighted mode | 5 | 0.0615434031199262 | 0.433454704822223 | 0.893957275995927 | -0.788027818 | 0.911114624571482 | 1.06347665373185 | 0.454740742863195 | 2.48709316414373 | 0.999714303421225 |
| ebi-fl187-GCST90094812 | ebi-a-GCST90002112 | HLA DR on CD33- HLA DR+ \|\| id:ebi-a-GCST90002112 | Plum liking \|\| id:ebi-fl187-GCST90094812 | Weighted mode | 5 | -0.734529199 | 0.518900985870016 | 0.229836766526475 | -1.751575132 | 0.28251673283954 | 0.47973126355147 | 0.173500442048079 | 1.32646397041983 | 0.999714303421225 |
| ebi-fl187-GCST90094812 | ebi-a-GCST90002115 | HLA DR on HLA DR+ CD8+ T cell \|\| id:ebi-a-GCST90002115 | Plum liking \|\| id:ebi-fl187-GCST90094812 | Weighted mode | 5 | -0.096546153 | 0.376295807475744 | 0.810166797145089 | -0.834085936 | 0.640993629532375 | 0.907967991076975 | 0.434271251884889 | 1.89836621522182 | 0.999714303421225 |
| ebi-fl187-GCST90094812 | ebi-a-GCST90002116 | HLA DR on B cell \|\| id:ebi-a-GCST90002116 | Plum liking \|\| id:ebi-fl187-GCST90094812 | Weighted mode | 5 | 0.117759612133697 | 0.339963546291279 | 0.746504125882305 | -0.548568939 | 0.784088162864603 | 1.1249736488456 | 0.57777605204574 | 2.19040873382684 | 0.999714303421225 |
| ebi-fl187-GCST90094812 | ebi-cfmet338-GCST90026023 | Sphingomyelin (d18:1/24:1, d18:2/24:0) levels \|\| id:ebi-cfmet338-GCST90026023 | Plum liking \|\| id:ebi-fl187-GCST90094812 | Weighted mode | 5 | -0.232422252 | 0.220929352662328 | 0.352143845000783 | -0.665443783 | 0.200599279398341 | 0.792611371042947 | 0.514045352472045 | 1.22213493903876 | 0.999714303421225 |
| ebi-fl187-GCST90094812 | ebi-cfmet338-GCST90026046 | 1-arachidonoyl-gpc (20:4n6) levels \|\| id:ebi-cfmet338-GCST90026046 | Plum liking \|\| id:ebi-fl187-GCST90094812 | Weighted mode | 5 | -0.630108569 | 0.226353150338138 | 0.0496283341615599 | -1.073760743 | -0.186456394 | 0.532533981370146 | 0.341720973008404 | 0.82989474955921 | 0.999714303421225 |
| ebi-fl187-GCST90094812 | ebi-cfmet338-GCST90026047 | 1-linoleoyl-gpc (18:2) levels \|\| id:ebi-cfmet338-GCST90026047 | Plum liking \|\| id:ebi-fl187-GCST90094812 | Weighted mode | 5 | -0.345499697 | 0.319687511152734 | 0.340619596811891 | -0.972087219 | 0.281087824700638 | 0.707866546175035 | 0.37829263393709 | 1.32456992878456 | 0.999714303421225 |
| ebi-fl187-GCST90094812 | ebi-cfmet338-GCST90026050 | 1-myristoyl-2-palmitoyl-gpc (14:0/16:0) levels \|\| id:ebi-cfmet338-GCST90026050 | Plum liking \|\| id:ebi-fl187-GCST90094812 | Weighted mode | 5 | -0.075579694 | 0.169908502415184 | 0.679455006474826 | -0.408600359 | 0.257440970893964 | 0.927205834996255 | 0.664579772866594 | 1.29361544776308 | 0.999714303421225 |
| ebi-fl187-GCST90094812 | ebi-cfmet338-GCST90026068 | 2,3-dihydroxyisovalerate levels \|\| id:ebi-cfmet338-GCST90026068 | Plum liking \|\| id:ebi-fl187-GCST90094812 | Weighted mode | 5 | 0.437691559443294 | 0.458063467900743 | 0.393421748267827 | -0.460112838 | 1.33549595652875 | 1.54912702012289 | 0.631212416967538 | 3.80188104664336 | 0.999714303421225 |
| ebi-fl187-GCST90094812 | ebi-cfmet338-GCST90026082 | 3-hydroxyhexanoate levels \|\| id:ebi-cfmet338-GCST90026082 | Plum liking \|\| id:ebi-fl187-GCST90094812 | Weighted mode | 5 | -0.182688632 | 0.11073245858317 | 0.174324650328885 | -0.399724251 | 0.0343469866432413 | 0.83302749331509 | 0.670504911603222 | 1.03494365605701 | 0.999714303421225 |
| ebi-fl187-GCST90094812 | ebi-cfmet338-GCST90026094 | 3-ureidopropionate levels \|\| id:ebi-cfmet338-GCST90026094 | Plum liking \|\| id:ebi-fl187-GCST90094812 | Weighted mode | 5 | 0.221394656756312 | 0.233587035312906 | 0.396898822439315 | -0.236435932 | 0.679225245969608 | 1.2478157923338 | 0.789436457936051 | 1.97234905475288 | 0.999714303421225 |
| ebi-fl187-GCST90094812 | ebi-cfmet338-GCST90026173 | Glycerol levels \|\| id:ebi-cfmet338-GCST90026173 | Plum liking \|\| id:ebi-fl187-GCST90094812 | Weighted mode | 5 | -0.099248931 | 0.133014455709785 | 0.49704001812472 | -0.359957264 | 0.161459401916294 | 0.905517268397175 | 0.697706142278443 | 1.17522474531727 | 0.999714303421225 |
| ebi-fl187-GCST90094812 | ebi-cfmet338-GCST90026179 | Histidine levels \|\| id:ebi-cfmet338-GCST90026179 | Plum liking \|\| id:ebi-fl187-GCST90094812 | Weighted mode | 5 | -0.047164173 | 0.0966624815363976 | 0.651139208315065 | -0.236622637 | 0.142294290485043 | 0.95393077474573 | 0.78928908021276 | 1.15291589079339 | 0.999714303421225 |
| ebi-fl187-GCST90094812 | ebi-cfmet338-GCST90026188 | Isocitrate levels \|\| id:ebi-cfmet338-GCST90026188 | Plum liking \|\| id:ebi-fl187-GCST90094812 | Weighted mode | 5 | -0.006652573 | 0.188540146963911 | 0.973543374190504 | -0.376191261 | 0.362886114935075 | 0.993369506261644 | 0.686471025238694 | 1.43747214331062 | 0.999714303421225 |
| ebi-fl187-GCST90094812 | ebi-cfmet338-GCST90026207 | N-acetyl-isoputreanine levels \|\| id:ebi-cfmet338-GCST90026207 | Plum liking \|\| id:ebi-fl187-GCST90094812 | Weighted mode | 5 | -0.117303476 | 0.128761911413321 | 0.413824423278206 | -0.369676822 | 0.135069870293092 | 0.889315266299063 | 0.690957596543339 | 1.14461675626569 | 0.999714303421225 |
| ebi-fl187-GCST90094812 | ebi-cfmet338-GCST90026213 | N-acetylglutamate levels \|\| id:ebi-cfmet338-GCST90026213 | Plum liking \|\| id:ebi-fl187-GCST90094812 | Weighted mode | 5 | 0.127782865344255 | 0.206573032262531 | 0.569676455522778 | -0.277100278 | 0.532666008578816 | 1.13630624444294 | 0.75797848481604 | 1.70346771976434 | 0.999714303421225 |
| ebi-fl187-GCST90094812 | ebi-cfmet338-GCST90026224 | N-acetyltaurine levels \|\| id:ebi-cfmet338-GCST90026224 | Plum liking \|\| id:ebi-fl187-GCST90094812 | Weighted mode | 5 | -0.217396052 | 0.174961003585831 | 0.281903370778882 | -0.560319619 | 0.125527515161474 | 0.8046112384092 | 0.571026523812199 | 1.13374636374563 | 0.999714303421225 |
| ebi-fl187-GCST90094812 | ebi-cfmet338-GCST90026250 | Phenyllactate (pla) levels \|\| id:ebi-cfmet338-GCST90026250 | Plum liking \|\| id:ebi-fl187-GCST90094812 | Weighted mode | 5 | 0.143793602430409 | 0.272516105321619 | 0.625654227824438 | -0.390337964 | 0.677925168860783 | 1.15464576784907 | 0.676828092310905 | 1.96978651500675 | 0.999714303421225 |
| ebi-fl187-GCST90094812 | ebi-cfmet338-GCST90026251 | Pipecolate levels \|\| id:ebi-cfmet338-GCST90026251 | Plum liking \|\| id:ebi-fl187-GCST90094812 | Weighted mode | 5 | 0.271507823696087 | 0.390820354115216 | 0.525476839244615 | -0.49450007 | 1.03751571776191 | 1.31194113604507 | 0.609875726042226 | 2.82219716403018 | 0.999714303421225 |
| ebi-fl187-GCST90094812 | ebi-cfmet338-GCST90026278 | Trigonelline (n'-methylnicotinate) levels \|\| id:ebi-cfmet338-GCST90026278 | Plum liking \|\| id:ebi-fl187-GCST90094812 | Weighted mode | 5 | -0.542379072 | 0.430938811336619 | 0.27662530876947 | -1.387019142 | 0.30226099863423 | 0.581363500430381 | 0.249818870476162 | 1.3529142894148 | 0.999714303421225 |
| ebi-fl187-GCST90094812 | ebi-cfmet338-GCST90026299 | 1-carboxyethylvaline levels \|\| id:ebi-cfmet338-GCST90026299 | Plum liking \|\| id:ebi-fl187-GCST90094812 | Weighted mode | 5 | 0.30988123061825 | 0.269541493803098 | 0.314346399255153 | -0.218420097 | 0.838182558472322 | 1.36326319059028 | 0.803787701738034 | 2.3121609385162 | 0.999714303421225 |
| ebi-fl187-GCST90094812 | ebi-gm430-GCST90011306 | C_Erysipelotrichia abundance \|\| id:ebi-gm430-GCST90011306 | Plum liking \|\| id:ebi-fl187-GCST90094812 | Weighted mode | 5 | -0.280724998 | 0.215526073887303 | 0.262672507477205 | -0.703156103 | 0.141706106808366 | 0.755235998326945 | 0.495020500148363 | 1.15223796387816 | 0.999714303421225 |
| ebi-fl187-GCST90094812 | ebi-gm430-GCST90011312 | F_Erysipelotrichaceae abundance \|\| id:ebi-gm430-GCST90011312 | Plum liking \|\| id:ebi-fl187-GCST90094812 | Weighted mode | 5 | -0.280724998 | 0.217506706790966 | 0.26637284454317 | -0.707038143 | 0.145588147299545 | 0.755235998326945 | 0.493102535739253 | 1.15671969180566 | 0.999714303421225 |
| ebi-fl187-GCST90094812 | ebi-gm430-GCST90011352 | O_Erysipelotrichales abundance \|\| id:ebi-gm430-GCST90011352 | Plum liking \|\| id:ebi-fl187-GCST90094812 | Weighted mode | 5 | -0.280724998 | 0.209512108322671 | 0.251331957797925 | -0.69136873 | 0.129918734301687 | 0.755235998326945 | 0.500890016308653 | 1.13873583940117 | 0.999714303421225 |
| ebi-fl187-GCST90094812 | ebi-gm430-GCST90011362 | OTU97_11 (Parabacteroides) abundance \|\| id:ebi-gm430-GCST90011362 | Plum liking \|\| id:ebi-fl187-GCST90094812 | Weighted mode | 5 | -0.368238094 | 0.306491665207526 | 0.295843448857576 | -0.968961758 | 0.232485569551108 | 0.691952412179653 | 0.379476822397704 | 1.26173223886503 | 0.999714303421225 |
| ebi-fl187-GCST90094812 | ebi-gm430-GCST90011384 | OTU97_235 (Phascolarctobacterium) abundance \|\| id:ebi-gm430-GCST90011384 | Plum liking \|\| id:ebi-fl187-GCST90094812 | Weighted mode | 5 | 1.93067308390613 | 0.920582850647409 | 0.10397807710034 | 0.126330696637205 | 3.73501547117505 | 6.89414902101476 | 1.13465733361126 | 41.8886736250918 | 0.999714303421225 |
| ebi-fl187-GCST90094812 | ebi-gm430-GCST90011390 | OTU97_3 (Bacteroides) abundance \|\| id:ebi-gm430-GCST90011390 | Plum liking \|\| id:ebi-fl187-GCST90094812 | Weighted mode | 5 | -0.026299129 | 0.231097493909691 | 0.914878739649049 | -0.479250217 | 0.42665195865251 | 0.974043680914409 | 0.619247518756415 | 1.53211932804287 | 0.999714303421225 |
| ebi-fl187-GCST90094812 | ebi-gm430-GCST90011398 | OTU97_4 (Alistipes) abundance \|\| id:ebi-gm430-GCST90011398 | Plum liking \|\| id:ebi-fl187-GCST90094812 | Weighted mode | 5 | -0.003125156 | 0.278882743531086 | 0.991595731070074 | -0.549735333 | 0.543485021463507 | 0.996879722359092 | 0.577102530062076 | 1.72199761582027 | 0.999714303421225 |
| ebi-fl187-GCST90094812 | ebi-gm430-GCST90011421 | OTU99_108 (Cytophagales) abundance \|\| id:ebi-gm430-GCST90011421 | Plum liking \|\| id:ebi-fl187-GCST90094812 | Weighted mode | 5 | -0.260852569 | 0.976199755514217 | 0.802517144232811 | -2.17420409 | 1.65249895166266 | 0.770394491161258 | 0.113698611535069 | 5.2200080897958 | 0.999714303421225 |
| ebi-fl187-GCST90094812 | ebi-gm430-GCST90011428 | OTU99_123 (Phascolarctobacterium) abundance \|\| id:ebi-gm430-GCST90011428 | Plum liking \|\| id:ebi-fl187-GCST90094812 | Weighted mode | 5 | 0.00176599012078771 | 0.499354399022683 | 0.997347596933123 | -0.976968632 | 0.980500612205247 | 1.00176755039969 | 0.376450531066797 | 2.66579043517332 | 0.999714303421225 |
| ebi-fl187-GCST90094812 | ebi-gm430-GCST90011450 | OTU99_298 (Phascolarctobacterium) abundance \|\| id:ebi-gm430-GCST90011450 | Plum liking \|\| id:ebi-fl187-GCST90094812 | Weighted mode | 5 | 1.92675005406326 | 0.904183567215801 | 0.100103380226072 | 0.154550262320293 | 3.69894984580623 | 6.86715605039765 | 1.16713293939288 | 40.4048507490874 | 0.999714303421225 |
| ebi-fl187-GCST90094812 | ebi-gm430-GCST90011451 | OTU99_3 (Bacteroides) abundance \|\| id:ebi-gm430-GCST90011451 | Plum liking \|\| id:ebi-fl187-GCST90094812 | Weighted mode | 5 | -0.036388853 | 0.232871168851648 | 0.883396083873169 | -0.492816344 | 0.420038638341046 | 0.96426526350946 | 0.610903455119055 | 1.52202036282438 | 0.999714303421225 |
| ebi-fl187-GCST90094812 | ebi-gm430-GCST90011472 | OTU99_72 (Parabacteroides) abundance \|\| id:ebi-gm430-GCST90011472 | Plum liking \|\| id:ebi-fl187-GCST90094812 | Weighted mode | 5 | -0.514942751 | 0.707753904645839 | 0.507176034546706 | -1.902140405 | 0.872254901649448 | 0.597534801636224 | 0.149248824235099 | 2.39229917552995 | 0.999714303421225 |
| ebi-fl187-GCST90094812 | ebi-gm430-GCST90011507 | TestASV_3 (Bacteroides) abundance \|\| id:ebi-gm430-GCST90011507 | Plum liking \|\| id:ebi-fl187-GCST90094812 | Weighted mode | 5 | 0.775584187204268 | 0.326813780660022 | 0.0765563076081051 | 0.135029177110625 | 1.41613919729791 | 2.17186052978604 | 1.14457017911486 | 4.12117862837411 | 0.999714303421225 |
| ebi-fl187-GCST90094812 | ebi-gm430-GCST90011512 | TestASV_34 (Phascolarctobacterium) abundance \|\| id:ebi-gm430-GCST90011512 | Plum liking \|\| id:ebi-fl187-GCST90094812 | Weighted mode | 5 | -0.045440465 | 0.698958531913028 | 0.951284129423678 | -1.415399187 | 1.32451825760652 | 0.955576491179358 | 0.242828659934836 | 3.76037338730814 | 0.999714303421225 |
| ebi-fl187-GCST90094812 | ebi-gm430-GCST90011519 | TestASV_41 (Barnesiella) abundance \|\| id:ebi-gm430-GCST90011519 | Plum liking \|\| id:ebi-fl187-GCST90094812 | Weighted mode | 5 | -0.678540451 | 0.905403220864046 | 0.495264716452282 | -2.453130764 | 1.09604986177622 | 0.507356964512147 | 0.0860238441145948 | 2.9923225599646 | 0.999714303421225 |
| ebi-fl187-GCST90094812 | ebi-gm430-GCST90011524 | TestASV_46 (Sutterella) abundance \|\| id:ebi-gm430-GCST90011524 | Plum liking \|\| id:ebi-fl187-GCST90094812 | Weighted mode | 5 | -0.840383544 | 1.13880468407055 | 0.501494517835521 | -3.072440725 | 1.39167363629688 | 0.431544974989883 | 0.0463079917284189 | 4.02157507782248 | 0.999714303421225 |
| ebi-fl187-GCST90094812 | ebi-gm430-GCST90011581 | OTU97_19 (Bacteria) prevalence \|\| id:ebi-gm430-GCST90011581 | Plum liking \|\| id:ebi-fl187-GCST90094812 | Weighted mode | 5 | -1.188988499 | 1.21284045216458 | 0.382427391038741 | -3.566155785 | 1.18817878729875 | 0.304529139878721 | 0.0282642991403789 | 3.28110018135163 | 0.999714303421225 |
| ebi-fl187-GCST90094812 | ebi-gm430-GCST90011614 | OTU97_80 (Ruminococcus) prevalence \|\| id:ebi-gm430-GCST90011614 | Plum liking \|\| id:ebi-fl187-GCST90094812 | Weighted mode | 5 | -0.190845672 | 0.516472477260381 | 0.730472813796958 | -1.203131728 | 0.82144038319329 | 0.826260093183949 | 0.300252429138808 | 2.27377258377727 | 0.999714303421225 |
| ebi-fl187-GCST90094812 | ebi-gm430-GCST90011623 | OTU99_11 (Parabacteroides) prevalence \|\| id:ebi-gm430-GCST90011623 | Plum liking \|\| id:ebi-fl187-GCST90094812 | Weighted mode | 5 | 0.0903965927727075 | 0.45525877375018 | 0.852290006837361 | -0.801910604 | 0.982703789323061 | 1.09460831137864 | 0.448471294095669 | 2.67167011827429 | 0.999714303421225 |
| ebi-fl187-GCST90094812 | ebi-gm430-GCST90011642 | OTU99_19 (Bacteria) prevalence \|\| id:ebi-gm430-GCST90011642 | Plum liking \|\| id:ebi-fl187-GCST90094812 | Weighted mode | 5 | -1.188988499 | 1.31206281296632 | 0.416082517041934 | -3.760631612 | 1.38265461447015 | 0.304529139878721 | 0.0232690387201023 | 3.98546747679597 | 0.999714303421225 |
| ebi-fl187-GCST90094812 | ebi-gm430-GCST90011653 | OTU99_30 (Parasutterella) prevalence \|\| id:ebi-gm430-GCST90011653 | Plum liking \|\| id:ebi-fl187-GCST90094812 | Weighted mode | 5 | -0.757898283 | 0.485180837842338 | 0.19329635254358 | -1.708852725 | 0.193056159301144 | 0.468650363166307 | 0.181073414488878 | 1.21295091008184 | 0.999714303421225 |
| ebi-fl187-GCST90094812 | ebi-gm430-GCST90011675 | OTU99_8 (Ruminococcaceae) prevalence \|\| id:ebi-gm430-GCST90011675 | Plum liking \|\| id:ebi-fl187-GCST90094812 | Weighted mode | 5 | 0.0354603915004061 | 0.491535301018439 | 0.945952006901656 | -0.927948798 | 0.998869581496547 | 1.0360966090832 | 0.395363850131726 | 2.71521076850108 | 0.999714303421225 |
| ebi-fl187-GCST90094812 | ebi-gm430-GCST90011679 | OTU99_92 (Ruminococcus) prevalence \|\| id:ebi-gm430-GCST90011679 | Plum liking \|\| id:ebi-fl187-GCST90094812 | Weighted mode | 5 | -0.281472272 | 0.526493496913916 | 0.621248761956048 | -1.313399526 | 0.750454981779895 | 0.754671840796643 | 0.268904353414556 | 2.11796343219992 | 0.999714303421225 |
| ebi-fl187-GCST90094812 | ebi-gm430-GCST90011684 | TestASV_11 (Lachnospiraceae) prevalence \|\| id:ebi-gm430-GCST90011684 | Plum liking \|\| id:ebi-fl187-GCST90094812 | Weighted mode | 5 | -0.428409149 | 0.53636156341299 | 0.469183688872953 | -1.479677813 | 0.622859515571179 | 0.651544781543853 | 0.22771104210213 | 1.8642512828457 | 0.999714303421225 |
| ebi-fl187-GCST90094812 | ebi-gm430-GCST90011688 | TestASV_15 (Bacteroides) prevalence \|\| id:ebi-gm430-GCST90011688 | Plum liking \|\| id:ebi-fl187-GCST90094812 | Weighted mode | 5 | -0.379241568 | 0.493695756464193 | 0.485225982324018 | -1.346885251 | 0.588402114230151 | 0.684380268022573 | 0.260048987797281 | 1.80110814976049 | 0.999714303421225 |
| ebi-fl187-GCST90094812 | ebi-gm430-GCST90011692 | TestASV_19 (Prevotella) prevalence \|\| id:ebi-gm430-GCST90011692 | Plum liking \|\| id:ebi-fl187-GCST90094812 | Weighted mode | 5 | 0.285173264921909 | 0.972448232897756 | 0.783913834974569 | -1.620825272 | 2.19117180140151 | 1.3299924491968 | 0.197735446289166 | 8.94568954689953 | 0.999714303421225 |
| ebi-fl187-GCST90094812 | ebi-gm430-GCST90011693 | TestASV_2 (Bacteroides) prevalence \|\| id:ebi-gm430-GCST90011693 | Plum liking \|\| id:ebi-fl187-GCST90094812 | Weighted mode | 5 | 0.332371384210615 | 0.480566718104992 | 0.527221405864821 | -0.609539383 | 1.2742821516964 | 1.39427056244339 | 0.543601203221868 | 3.57613336720814 | 0.999714303421225 |
| ebi-fl187-GCST90094812 | ebi-gm430-GCST90011716 | TestASV_40 (Barnesiella) prevalence \|\| id:ebi-gm430-GCST90011716 | Plum liking \|\| id:ebi-fl187-GCST90094812 | Weighted mode | 5 | -0.669713357 | 0.991597979969824 | 0.536459886712862 | -2.613245398 | 1.27381868333476 | 0.511855276284619 | 0.0732962817277026 | 3.57447632655807 | 0.999714303421225 |
| ebi-fl187-GCST90094812 | ebi-gm430-GCST90011718 | TestASV_42 (Alphaproteobacteria) prevalence \|\| id:ebi-gm430-GCST90011718 | Plum liking \|\| id:ebi-fl187-GCST90094812 | Weighted mode | 5 | -0.957399197 | 0.817865676678819 | 0.306738403846423 | -2.560415923 | 0.645617529354837 | 0.383890011067211 | 0.077272594291888 | 1.90716439570419 | 0.999714303421225 |
| ebi-fl187-GCST90094812 | ebi-hbsm44-GCST90104929 | Sulcal depth of the right inferior frontal sulcus \|\| id:ebi-hbsm44-GCST90104929 | Plum liking \|\| id:ebi-fl187-GCST90094812 | Weighted mode | 4 | -0.166679318 | 0.391110440003102 | 0.698713205735698 | -0.93325578 | 0.599897144593496 | 0.846471015994537 | 0.393271219093918 | 1.82193139525858 | 0.999714303421225 |
| ebi-fl187-GCST90094812 | ebi-hbsm44-GCST90104935 | Sulcal opening distance of the left calloso-marginal posterior fissure \|\| id:ebi-hbsm44-GCST90104935 | Plum liking \|\| id:ebi-fl187-GCST90094812 | Weighted mode | 5 | -0.019328075 | 0.0976453163329556 | 0.852743229575115 | -0.210712895 | 0.172056744878541 | 0.980857514490019 | 0.810006590323706 | 1.18774522976048 | 0.999714303421225 |
| ebi-fl187-GCST90094812 | ebi-hbsm44-GCST90104936 | Sulcal opening distance of the left anterior inferior temporal sulcus \|\| id:ebi-hbsm44-GCST90104936 | Plum liking \|\| id:ebi-fl187-GCST90094812 | Weighted mode | 5 | -0.042693089 | 0.0508182360498555 | 0.448127101385012 | -0.142296831 | 0.0569106539669098 | 0.95820542902548 | 0.867363757517117 | 1.05856122792378 | 0.999714303421225 |
| ebi-fl187-GCST90094812 | ebi-hbsm44-GCST90104941 | Sulcal opening distance of the right posterior inferior temporal sulcus \|\| id:ebi-hbsm44-GCST90104941 | Plum liking \|\| id:ebi-fl187-GCST90094812 | Weighted mode | 5 | 0.0555914892625612 | 0.0450691935850502 | 0.284926779521848 | -0.03274413 | 0.143927108689259 | 1.05716573196139 | 0.967786155196238 | 1.15479993057645 | 0.999714303421225 |
| ebi-fl187-GCST90094812 | ebi-IPs91-GCST90274819 | Leukemia inhibitory factor levels \|\| id:ebi-IPs91-GCST90274819 | Plum liking \|\| id:ebi-fl187-GCST90094812 | Weighted mode | 5 | 0.253618774316767 | 0.179506520669686 | 0.230566518600859 | -0.098214006 | 0.605451554829352 | 1.28868043253363 | 0.906454896031254 | 1.83207930639034 | 0.999714303421225 |
| ebi-fl187-GCST90094812 | ebi-IPs91-GCST90274837 | STAM binding protein levels \|\| id:ebi-IPs91-GCST90274837 | Plum liking \|\| id:ebi-fl187-GCST90094812 | Weighted mode | 5 | -0.250548784 | 0.154948681814475 | 0.181190039382333 | -0.5542482 | 0.0531506324116194 | 0.778373506957355 | 0.574504010823822 | 1.05458848836285 | 0.999714303421225 |
| ebi-fl187-GCST90094812 | ebi-IPs91-GCST90274840 | TNF-beta levels \|\| id:ebi-IPs91-GCST90274840 | Plum liking \|\| id:ebi-fl187-GCST90094812 | Weighted mode | 5 | 0.12013630273556 | 0.180583595579711 | 0.542276838862726 | -0.233807545 | 0.474080150071793 | 1.12765054295862 | 0.791514132406471 | 1.60653574582274 | 0.999714303421225 |
| ebi-fl187-GCST90094812 | ebi-IPs91-GCST90274842 | Tumor necrosis factor ligand superfamily member 14 levels \|\| id:ebi-IPs91-GCST90274842 | Plum liking \|\| id:ebi-fl187-GCST90094812 | Weighted mode | 5 | -0.279973832 | 0.177336378989602 | 0.189531608646649 | -0.627553135 | 0.067605470394795 | 0.755803518742356 | 0.533896576767799 | 1.06994310096835 | 0.999714303421225 |
| ebi-fl187-GCST90094812 | ebi-met1400-GCST90199628 | 3-methylhistidine levels \|\| id:ebi-met1400-GCST90199628 | Plum liking \|\| id:ebi-fl187-GCST90094812 | Weighted mode | 5 | 0.152402261673122 | 0.215323859620281 | 0.518141606994864 | -0.269632503 | 0.574437026528872 | 1.16462862767821 | 0.763660085426477 | 1.77613033114091 | 0.999714303421225 |
| ebi-fl187-GCST90094812 | ebi-met1400-GCST90199651 | Docosahexaenoate DHA; 22:6n3 levels \|\| id:ebi-met1400-GCST90199651 | Plum liking \|\| id:ebi-fl187-GCST90094812 | Weighted mode | 5 | -0.001977648 | 0.186142086803352 | 0.992031889259884 | -0.366816138 | 0.36286084261444 | 0.998024306736239 | 0.692937038361635 | 1.43743581551275 | 0.999714303421225 |
| ebi-fl187-GCST90094812 | ebi-met1400-GCST90199652 | 1-stearoyl-2-oleoyl-GPS (18:0/18:1) levels \|\| id:ebi-met1400-GCST90199652 | Plum liking \|\| id:ebi-fl187-GCST90094812 | Weighted mode | 5 | -0.034000864 | 0.21003159622335 | 0.879244964135515 | -0.445662793 | 0.377661064692466 | 0.966570669611621 | 0.640399683230388 | 1.4588683970622 | 0.999714303421225 |
| ebi-fl187-GCST90094812 | ebi-met1400-GCST90199653 | 1-myristoyl-2-palmitoyl-gpc (14:0/16:0) levels \|\| id:ebi-met1400-GCST90199653 | Plum liking \|\| id:ebi-fl187-GCST90094812 | Weighted mode | 5 | 0.0220843931606389 | 0.207767524456122 | 0.920466766507231 | -0.385139955 | 0.429308741094638 | 1.02233005849477 | 0.680355410553813 | 1.53619524779138 | 0.999714303421225 |
| ebi-fl187-GCST90094812 | ebi-met1400-GCST90199667 | Erythritol levels in elite athletes \|\| id:ebi-met1400-GCST90199667 | Plum liking \|\| id:ebi-fl187-GCST90094812 | Weighted mode | 5 | 0.353084289538182 | 0.217301559193008 | 0.179517596831418 | -0.072826766 | 0.778995345556477 | 1.42345112041469 | 0.929761881999065 | 2.17928174023796 | 0.999714303421225 |
| ebi-fl187-GCST90094812 | ebi-met1400-GCST90199674 | 3-hydroxymyristate levels \|\| id:ebi-met1400-GCST90199674 | Plum liking \|\| id:ebi-fl187-GCST90094812 | Weighted mode | 5 | 0.0298600326060432 | 0.219205172477543 | 0.898228360872355 | -0.399782105 | 0.459502170662028 | 1.03031031401109 | 0.670466121034361 | 1.5832855827524 | 0.999714303421225 |
| ebi-fl187-GCST90094812 | ebi-met1400-GCST90199685 | 2-linoleoylglycerol (18:2) levels \|\| id:ebi-met1400-GCST90199685 | Plum liking \|\| id:ebi-fl187-GCST90094812 | Weighted mode | 5 | -0.18738328 | 0.242904324275606 | 0.483495157478254 | -0.663475756 | 0.288709195102963 | 0.829125887710777 | 0.515058003730603 | 1.33470353376306 | 0.999714303421225 |
| ebi-fl187-GCST90094812 | ebi-met1400-GCST90199703 | Gamma-glutamylvaline levels \|\| id:ebi-met1400-GCST90199703 | Plum liking \|\| id:ebi-fl187-GCST90094812 | Weighted mode | 5 | 0.132749780615722 | 0.195412275807361 | 0.534205214439014 | -0.25025828 | 0.51575784119815 | 1.14196422099463 | 0.778599660405179 | 1.67490733473121 | 0.999714303421225 |
| ebi-fl187-GCST90094812 | ebi-met1400-GCST90199779 | Gamma-glutamyl-2-aminobutyrate levels \|\| id:ebi-met1400-GCST90199779 | Plum liking \|\| id:ebi-fl187-GCST90094812 | Weighted mode | 5 | 0.127544572593913 | 0.219982048995006 | 0.593142141127385 | -0.303620243 | 0.558709388624126 | 1.13603550316184 | 0.738141127169779 | 1.74841451985283 | 0.999714303421225 |
| ebi-fl187-GCST90094812 | ebi-met1400-GCST90199824 | 4-vinylphenol sulfate levels \|\| id:ebi-met1400-GCST90199824 | Plum liking \|\| id:ebi-fl187-GCST90094812 | Weighted mode | 5 | -0.086364622 | 0.215724455458731 | 0.709361389179543 | -0.509184555 | 0.336455310383725 | 0.91725971687638 | 0.600985449625026 | 1.39997630346806 | 0.999714303421225 |
| ebi-fl187-GCST90094812 | ebi-met1400-GCST90199830 | 4-ethylphenylsulfate levels \|\| id:ebi-met1400-GCST90199830 | Plum liking \|\| id:ebi-fl187-GCST90094812 | Weighted mode | 5 | 0.247331131308418 | 0.219236526199002 | 0.32234150669221 | -0.18237246 | 0.677034722658461 | 1.28060309035514 | 0.833290915039898 | 1.96803330676973 | 0.999714303421225 |
| ebi-fl187-GCST90094812 | ebi-met1400-GCST90199833 | Hexanoylglutamine levels \|\| id:ebi-met1400-GCST90199833 | Plum liking \|\| id:ebi-fl187-GCST90094812 | Weighted mode | 5 | 0.173512998531128 | 0.206307657744005 | 0.447664415910578 | -0.230850011 | 0.577876007709377 | 1.18947614811186 | 0.793858527432855 | 1.78224892475781 | 0.999714303421225 |
| ebi-fl187-GCST90094812 | ebi-met1400-GCST90199837 | Carotenoid levels (cryptoxanthin) \|\| id:ebi-met1400-GCST90199837 | Plum liking \|\| id:ebi-fl187-GCST90094812 | Weighted mode | 5 | 0.150673909331156 | 0.213943816335406 | 0.520105989483377 | -0.268655971 | 0.570003789348552 | 1.1626174775507 | 0.764406188553775 | 1.76827375202662 | 0.999714303421225 |
| ebi-fl187-GCST90094812 | ebi-met1400-GCST90199869 | 2-hydroxyhippurate levels \|\| id:ebi-met1400-GCST90199869 | Plum liking \|\| id:ebi-fl187-GCST90094812 | Weighted mode | 5 | -0.13180703 | 0.22128096797343 | 0.583472464177274 | -0.565517727 | 0.30190366752641 | 0.87651011918021 | 0.568065967562764 | 1.35243093741648 | 0.999714303421225 |
| ebi-fl187-GCST90094812 | ebi-met1400-GCST90199902 | Hydantoin-5-propionate levels \|\| id:ebi-met1400-GCST90199902 | Plum liking \|\| id:ebi-fl187-GCST90094812 | Weighted mode | 5 | -0.122175084 | 0.204394956392745 | 0.582208574656263 | -0.522789198 | 0.27843903091608 | 0.884993407093607 | 0.592864622786799 | 1.32106605875319 | 0.999714303421225 |
| ebi-fl187-GCST90094812 | ebi-met1400-GCST90199904 | Pantoate levels \|\| id:ebi-met1400-GCST90199904 | Plum liking \|\| id:ebi-fl187-GCST90094812 | Weighted mode | 5 | -0.097180668 | 0.207799554086316 | 0.664365973981553 | -0.504467794 | 0.310106458377309 | 0.907392054949637 | 0.603826850416658 | 1.3635702698838 | 0.999714303421225 |
| ebi-fl187-GCST90094812 | ebi-met1400-GCST90199936 | 3-methyl catechol sulfate (2) levels \|\| id:ebi-met1400-GCST90199936 | Plum liking \|\| id:ebi-fl187-GCST90094812 | Weighted mode | 5 | -0.139149542 | 0.251889374479413 | 0.610073135137178 | -0.632852716 | 0.354553632150352 | 0.870097902696834 | 0.531074633009904 | 1.42554419514763 | 0.999714303421225 |
| ebi-fl187-GCST90094812 | ebi-met1400-GCST90199977 | 4-hydroxychlorothalonil levels \|\| id:ebi-met1400-GCST90199977 | Plum liking \|\| id:ebi-fl187-GCST90094812 | Weighted mode | 5 | -0.344116425 | 0.229103329533283 | 0.207505619645254 | -0.793158951 | 0.104926101202528 | 0.708846396028477 | 0.452413384077495 | 1.110628533 | 0.999714303421225 |
| ebi-fl187-GCST90094812 | ebi-met1400-GCST90200040 | Sphingomyelin (d18:2/24:1, d18:1/24:2) levels \|\| id:ebi-met1400-GCST90200040 | Plum liking \|\| id:ebi-fl187-GCST90094812 | Weighted mode | 5 | 0.000709342390380591 | 0.197863067705544 | 0.997311244718516 | -0.38710227 | 0.388520955093246 | 1.00070959403319 | 0.679021647619412 | 1.47479788766818 | 0.999714303421225 |
| ebi-fl187-GCST90094812 | ebi-met1400-GCST90200077 | 1-stearoyl-2-oleoyl-GPI (18:0/18:1) levels \|\| id:ebi-met1400-GCST90200077 | Plum liking \|\| id:ebi-fl187-GCST90094812 | Weighted mode | 5 | 0.333124555937898 | 0.218243908868419 | 0.201617006551335 | -0.094633505 | 0.760882617319999 | 1.3953210831728 | 0.90970627577806 | 2.1401643332419 | 0.999714303421225 |
| ebi-fl187-GCST90094812 | ebi-met1400-GCST90200080 | 1-palmitoyl-2-oleoyl-GPI (16:0/18:1) levels \|\| id:ebi-met1400-GCST90200080 | Plum liking \|\| id:ebi-fl187-GCST90094812 | Weighted mode | 5 | 0.247490762894697 | 0.211108856679722 | 0.306117647345565 | -0.166282596 | 0.661264121986952 | 1.28080753137504 | 0.846806895964091 | 1.93723969448708 | 0.999714303421225 |
| ebi-fl187-GCST90094812 | ebi-met1400-GCST90200082 | 1-oleoyl-2-linoleoyl-GPE (18:1/18:2) levels \|\| id:ebi-met1400-GCST90200082 | Plum liking \|\| id:ebi-fl187-GCST90094812 | Weighted mode | 5 | 0.153136460218572 | 0.220358533030463 | 0.52534728313041 | -0.278766265 | 0.585039184958278 | 1.16548401029455 | 0.756716754098964 | 1.79506132366487 | 0.999714303421225 |
| ebi-fl187-GCST90094812 | ebi-met1400-GCST90200100 | Perfluorooctanesulfonate (PFOS) levels \|\| id:ebi-met1400-GCST90200100 | Plum liking \|\| id:ebi-fl187-GCST90094812 | Weighted mode | 5 | -0.101158375 | 0.187534212731258 | 0.618226272631012 | -0.468725432 | 0.26640868218213 | 0.903789884034541 | 0.625799384227467 | 1.30526838963182 | 0.999714303421225 |
| ebi-fl187-GCST90094812 | ebi-met1400-GCST90200102 | 1-palmitoleoyl-2-linolenoyl-GPC (16:1/18:3) levels \|\| id:ebi-met1400-GCST90200102 | Plum liking \|\| id:ebi-fl187-GCST90094812 | Weighted mode | 5 | 0.0764191537343475 | 0.222822622445621 | 0.748894669850627 | -0.360313186 | 0.513151493727765 | 1.0794149201441 | 0.697457857644867 | 1.67054762815928 | 0.999714303421225 |
| ebi-fl187-GCST90094812 | ebi-met1400-GCST90200114 | Glycosyl-N-tricosanoyl-sphingadienine (d18:2/23:0) levels \|\| id:ebi-met1400-GCST90200114 | Plum liking \|\| id:ebi-fl187-GCST90094812 | Weighted mode | 5 | -0.089477868 | 0.225804044673138 | 0.712140275709269 | -0.532053796 | 0.353098059265555 | 0.914408502320686 | 0.587397335773732 | 1.4234707210835 | 0.999714303421225 |
| ebi-fl187-GCST90094812 | ebi-met1400-GCST90200135 | Arachidoylcarnitine (C20) levels \|\| id:ebi-met1400-GCST90200135 | Plum liking \|\| id:ebi-fl187-GCST90094812 | Weighted mode | 5 | 0.184664075140515 | 0.232865579250733 | 0.472156878496153 | -0.27175246 | 0.641080610471952 | 1.20281431702356 | 0.762042873691773 | 1.89853134408031 | 0.999714303421225 |
| ebi-fl187-GCST90094812 | ebi-met1400-GCST90200146 | Docosahexaenoylcarnitine (C22:6) levels \|\| id:ebi-met1400-GCST90200146 | Plum liking \|\| id:ebi-fl187-GCST90094812 | Weighted mode | 5 | -0.236413044 | 0.251509379572375 | 0.400449483542741 | -0.729371428 | 0.256545339587287 | 0.789454526829543 | 0.482211999647431 | 1.29245736395473 | 0.999714303421225 |
| ebi-fl187-GCST90094812 | ebi-met1400-GCST90200162 | 3-hydroxyphenylacetoylglutamine levels \|\| id:ebi-met1400-GCST90200162 | Plum liking \|\| id:ebi-fl187-GCST90094812 | Weighted mode | 5 | -0.376603681 | 0.255504802315978 | 0.214500634212535 | -0.877393094 | 0.124185731191767 | 0.686187969105133 | 0.415865622439141 | 1.13222614118226 | 0.999714303421225 |
| ebi-fl187-GCST90094812 | ebi-met1400-GCST90200172 | 3-carboxy-4-methyl-5-pentyl-2-furanpropionate (3-CMPFP) levels \|\| id:ebi-met1400-GCST90200172 | Plum liking \|\| id:ebi-fl187-GCST90094812 | Weighted mode | 5 | -0.033580156 | 0.205653139071378 | 0.878211459375099 | -0.436660309 | 0.369499996558316 | 0.966977399063639 | 0.646190899623182 | 1.44701092331251 | 0.999714303421225 |
| ebi-fl187-GCST90094812 | ebi-met1400-GCST90200222 | Dihydrocaffeate sulfate (2) levels \|\| id:ebi-met1400-GCST90200222 | Plum liking \|\| id:ebi-fl187-GCST90094812 | Weighted mode | 5 | 0.214431424085807 | 0.233617803127103 | 0.410622524046939 | -0.24345947 | 0.672322318214929 | 1.23915714172693 | 0.783911247277323 | 1.95878095540279 | 0.999714303421225 |
| ebi-fl187-GCST90094812 | ebi-met1400-GCST90200223 | 4-methylhexanoylglutamine levels \|\| id:ebi-met1400-GCST90200223 | Plum liking \|\| id:ebi-fl187-GCST90094812 | Weighted mode | 5 | -0.116528227 | 0.262346241604792 | 0.679886229494964 | -0.630726861 | 0.397670406219863 | 0.890004974161975 | 0.532204821523731 | 1.48835339703464 | 0.999714303421225 |
| ebi-fl187-GCST90094812 | ebi-met1400-GCST90200224 | 2-ketocaprylate levels \|\| id:ebi-met1400-GCST90200224 | Plum liking \|\| id:ebi-fl187-GCST90094812 | Weighted mode | 5 | -0.23636648 | 0.210220722463538 | 0.323763412307918 | -0.648399096 | 0.175666136427031 | 0.789491288456321 | 0.522882191457688 | 1.19204001346996 | 0.999714303421225 |
| ebi-fl187-GCST90094812 | ebi-met1400-GCST90200243 | Hydroxypalmitoyl sphingomyelin (d18:1/16:0(OH)) levels \|\| id:ebi-met1400-GCST90200243 | Plum liking \|\| id:ebi-fl187-GCST90094812 | Weighted mode | 5 | -0.015066103 | 0.204291337028956 | 0.944751487244424 | -0.415477124 | 0.385344917086192 | 0.985046822417522 | 0.660025291614856 | 1.47012130395916 | 0.999714303421225 |
| ebi-fl187-GCST90094812 | ebi-met1400-GCST90200273 | 2-methoxyhydroquinone sulfate (2) levels \|\| id:ebi-met1400-GCST90200273 | Plum liking \|\| id:ebi-fl187-GCST90094812 | Weighted mode | 5 | 0.196923186920678 | 0.238951719535225 | 0.456178280011456 | -0.271422183 | 0.665268557209719 | 1.21765050562872 | 0.762294600358203 | 1.94501279841306 | 0.999714303421225 |
| ebi-fl187-GCST90094812 | ebi-met1400-GCST90200276 | 2,4-di-tert-butylphenol levels \|\| id:ebi-met1400-GCST90200276 | Plum liking \|\| id:ebi-fl187-GCST90094812 | Weighted mode | 5 | 0.360646155888037 | 0.211959904904251 | 0.164069589290237 | -0.054795258 | 0.776087569500368 | 1.43425586808552 | 0.946678953315996 | 2.17295408114044 | 0.999714303421225 |
| ebi-fl187-GCST90094812 | ebi-met1400-GCST90200289 | N-acetylmethionine levels \|\| id:ebi-met1400-GCST90200289 | Plum liking \|\| id:ebi-fl187-GCST90094812 | Weighted mode | 5 | 0.185410069480869 | 0.211512098335104 | 0.430198003883174 | -0.229153643 | 0.599973782217673 | 1.20371194446743 | 0.79520634602655 | 1.82207102910266 | 0.999714303421225 |
| ebi-fl187-GCST90094812 | ebi-met1400-GCST90200308 | 3-hydroxyisobutyrate levels \|\| id:ebi-met1400-GCST90200308 | Plum liking \|\| id:ebi-fl187-GCST90094812 | Weighted mode | 5 | 0.210115095166311 | 0.193322737157455 | 0.338208273853822 | -0.16879747 | 0.589027659994923 | 1.23382005850974 | 0.844679959380856 | 1.80223517780253 | 0.999714303421225 |
| ebi-fl187-GCST90094812 | ebi-met1400-GCST90200383 | Cystathionine levels \|\| id:ebi-met1400-GCST90200383 | Plum liking \|\| id:ebi-fl187-GCST90094812 | Weighted mode | 5 | -0.261588012 | 0.229658109940147 | 0.318273160434812 | -0.711717908 | 0.188541883208451 | 0.7698281181192 | 0.49080032313716 | 1.20748765538471 | 0.999714303421225 |
| ebi-fl187-GCST90094812 | ebi-met1400-GCST90200384 | Guanidinoacetate levels \|\| id:ebi-met1400-GCST90200384 | Plum liking \|\| id:ebi-fl187-GCST90094812 | Weighted mode | 5 | -0.057311498 | 0.203634833724132 | 0.792330482667521 | -0.456435773 | 0.341812775686419 | 0.944299875635919 | 0.633537698621653 | 1.40749675523024 | 0.999714303421225 |
| ebi-fl187-GCST90094812 | ebi-met1400-GCST90200403 | Citrulline levels \|\| id:ebi-met1400-GCST90200403 | Plum liking \|\| id:ebi-fl187-GCST90094812 | Weighted mode | 5 | 0.124400214391587 | 0.230003267677848 | 0.617314334285401 | -0.32640619 | 0.57520661904017 | 1.13246901071272 | 0.72151205673458 | 1.7774977538545 | 0.999714303421225 |
| ebi-fl187-GCST90094812 | ebi-met1400-GCST90200434 | 12,13-DiHOME levels \|\| id:ebi-met1400-GCST90200434 | Plum liking \|\| id:ebi-fl187-GCST90094812 | Weighted mode | 5 | -0.378185875 | 0.206993866893525 | 0.14171384495079 | -0.783893854 | 0.0275221038023316 | 0.685103145071335 | 0.456624515724971 | 1.0279043354504 | 0.999714303421225 |
| ebi-fl187-GCST90094812 | ebi-met1400-GCST90200437 | Fructose levels \|\| id:ebi-met1400-GCST90200437 | Plum liking \|\| id:ebi-fl187-GCST90094812 | Weighted mode | 5 | 0.274637674771336 | 0.216753299565123 | 0.273885125273923 | -0.150198792 | 0.699474141918978 | 1.31605374899616 | 0.860536891246897 | 2.01269403771549 | 0.999714303421225 |
| ebi-fl187-GCST90094812 | ebi-met1400-GCST90200448 | N-stearoyl-sphinganine (d18:0/18:0) levels \|\| id:ebi-met1400-GCST90200448 | Plum liking \|\| id:ebi-fl187-GCST90094812 | Weighted mode | 5 | 0.151688750128382 | 0.230003073532898 | 0.545606816495057 | -0.299117274 | 0.602494774252862 | 1.163797948 | 0.741472448898927 | 1.82667025051903 | 0.999714303421225 |
| ebi-fl187-GCST90094812 | ebi-met1400-GCST90200468 | X-11847 levels \|\| id:ebi-met1400-GCST90200468 | Plum liking \|\| id:ebi-fl187-GCST90094812 | Weighted mode | 5 | -0.285921361 | 0.245498075303927 | 0.308881467647003 | -0.767097589 | 0.1952548661254 | 0.751321696457396 | 0.464358874599971 | 1.21562076756669 | 0.999714303421225 |
| ebi-fl187-GCST90094812 | ebi-met1400-GCST90200476 | X-11843 levels \|\| id:ebi-met1400-GCST90200476 | Plum liking \|\| id:ebi-fl187-GCST90094812 | Weighted mode | 5 | -0.046347244 | 0.239667364142727 | 0.856082827712848 | -0.516095278 | 0.423400789711958 | 0.954710387164212 | 0.596846523784761 | 1.52714623782904 | 0.999714303421225 |
| ebi-fl187-GCST90094812 | ebi-met1400-GCST90200511 | X-12906 levels \|\| id:ebi-met1400-GCST90200511 | Plum liking \|\| id:ebi-fl187-GCST90094812 | Weighted mode | 5 | -0.037602958 | 0.193288122923378 | 0.855231478199664 | -0.416447679 | 0.341241762867678 | 0.963095254195037 | 0.659385011581416 | 1.40669328595813 | 0.999714303421225 |
| ebi-fl187-GCST90094812 | ebi-met1400-GCST90200515 | X-12849 levels \|\| id:ebi-met1400-GCST90200515 | Plum liking \|\| id:ebi-fl187-GCST90094812 | Weighted mode | 5 | 0.144730795403969 | 0.193976698459048 | 0.497054541320459 | -0.235463534 | 0.524925124383704 | 1.15572840098849 | 0.79020447841515 | 1.69033227896954 | 0.999714303421225 |
| ebi-fl187-GCST90094812 | ebi-met1400-GCST90200551 | X-18886 levels \|\| id:ebi-met1400-GCST90200551 | Plum liking \|\| id:ebi-fl187-GCST90094812 | Weighted mode | 5 | 0.000482251344341877 | 0.213736923443335 | 0.99830778829026 | -0.418442119 | 0.419406621293279 | 1.00048236764622 | 0.658071218570253 | 1.52105872392612 | 0.999714303421225 |
| ebi-fl187-GCST90094812 | ebi-met1400-GCST90200564 | X-21319 levels \|\| id:ebi-met1400-GCST90200564 | Plum liking \|\| id:ebi-fl187-GCST90094812 | Weighted mode | 5 | -0.362156305 | 0.207657636821806 | 0.156105381725592 | -0.769165273 | 0.0448526629749939 | 0.696173543795075 | 0.463399719002378 | 1.0458737526289 | 0.999714303421225 |
| ebi-fl187-GCST90094812 | ebi-met1400-GCST90200579 | X-21442 levels \|\| id:ebi-met1400-GCST90200579 | Plum liking \|\| id:ebi-fl187-GCST90094812 | Weighted mode | 5 | -0.145251852 | 0.262704861096305 | 0.609770098823397 | -0.660153379 | 0.369649676126338 | 0.864804463274342 | 0.516772066238235 | 1.44722752749264 | 0.999714303421225 |
| ebi-fl187-GCST90094812 | ebi-met1400-GCST90200581 | X-21441 levels \|\| id:ebi-met1400-GCST90200581 | Plum liking \|\| id:ebi-fl187-GCST90094812 | Weighted mode | 5 | 0.0497602956195749 | 0.224152182706648 | 0.835192542232097 | -0.389577982 | 0.489098573724605 | 1.05101913228886 | 0.677342664657531 | 1.63084547021078 | 0.999714303421225 |
| ebi-fl187-GCST90094812 | ebi-met1400-GCST90200648 | X-24585 levels \|\| id:ebi-met1400-GCST90200648 | Plum liking \|\| id:ebi-fl187-GCST90094812 | Weighted mode | 5 | -0.189330141 | 0.206524078045172 | 0.411146823120749 | -0.594117334 | 0.215457052333662 | 0.827513265838309 | 0.552049626534756 | 1.24042870826083 | 0.999714303421225 |
| ebi-fl187-GCST90094812 | ebi-met1400-GCST90200720 | Adenosine 5'-diphosphate (ADP) to pantothenate ratio \|\| id:ebi-met1400-GCST90200720 | Plum liking \|\| id:ebi-fl187-GCST90094812 | Weighted mode | 5 | 0.0505480701172748 | 0.306590241119134 | 0.877041484785124 | -0.550368802 | 0.651464942710777 | 1.05184742456881 | 0.576737069093928 | 1.91834904302266 | 0.999714303421225 |
| ebi-fl187-GCST90094812 | ebi-met1400-GCST90200724 | Cholate to taurocholate ratio \|\| id:ebi-met1400-GCST90200724 | Plum liking \|\| id:ebi-fl187-GCST90094812 | Weighted mode | 5 | 0.414314408413318 | 0.22561779168649 | 0.140189870168981 | -0.027896463 | 0.856525280118838 | 1.51333285667604 | 0.972489049903555 | 2.3549636217728 | 0.999714303421225 |
| ebi-fl187-GCST90094812 | ebi-met1400-GCST90200726 | N-acetylputrescine to (N(1) + N(8))-acetylspermidine ratio \|\| id:ebi-met1400-GCST90200726 | Plum liking \|\| id:ebi-fl187-GCST90094812 | Weighted mode | 5 | -0.265708818 | 0.216903482580058 | 0.287774636438547 | -0.690839644 | 0.159422007387715 | 0.766662332914501 | 0.501155100322291 | 1.17283278636077 | 0.999714303421225 |
| ebi-fl187-GCST90094812 | ebi-met1400-GCST90200744 | Arginine to glutamate ratio \|\| id:ebi-met1400-GCST90200744 | Plum liking \|\| id:ebi-fl187-GCST90094812 | Weighted mode | 5 | 0.140998756079772 | 0.213705118797346 | 0.54544787249597 | -0.277863277 | 0.559860788922569 | 1.15142321570522 | 0.757400368665613 | 1.75042880425409 | 0.999714303421225 |
| ebi-fl187-GCST90094812 | ebi-met1400-GCST90200759 | Histidine to trans-urocanate ratio \|\| id:ebi-met1400-GCST90200759 | Plum liking \|\| id:ebi-fl187-GCST90094812 | Weighted mode | 5 | -0.094423576 | 0.230511842544441 | 0.70306748251608 | -0.546226787 | 0.357379635631415 | 0.90989727018017 | 0.5791308770497 | 1.4295784857803 | 0.999714303421225 |
| ebi-fl187-GCST90094812 | ebi-met1400-GCST90200795 | Oleoyl-linoleoyl-glycerol (18:1 to 18:2) [2] to linoleoyl-arachidonoyl-glycerol (18:2 to 20:4) [2] ratio \|\| id:ebi-met1400-GCST90200795 | Plum liking \|\| id:ebi-fl187-GCST90094812 | Weighted mode | 5 | 0.226878124249703 | 0.226385544155213 | 0.372967837060119 | -0.216837542 | 0.67059379079392 | 1.25467694392768 | 0.805060747003526 | 1.9553980733541 | 0.999714303421225 |
| ebi-fl187-GCST90094812 | ebi-met1400-GCST90200811 | Choline to taurocholate ratio \|\| id:ebi-met1400-GCST90200811 | Plum liking \|\| id:ebi-fl187-GCST90094812 | Weighted mode | 5 | 0.278480740993552 | 0.234629533634472 | 0.300940951269389 | -0.181393145 | 0.738354626917117 | 1.32112116167001 | 0.834107369143035 | 2.09248975417338 | 0.999714303421225 |
| ebi-fl187-GCST90094812 | ebi-met1400-GCST90200831 | Cholate to phosphate ratio \|\| id:ebi-met1400-GCST90200831 | Plum liking \|\| id:ebi-fl187-GCST90094812 | Weighted mode | 5 | 0.261453480582493 | 0.226783365744485 | 0.313166646777378 | -0.183041916 | 0.705948877441683 | 1.29881651997858 | 0.832733249928217 | 2.02576797877914 | 0.999714303421225 |
| ebi-fl187-GCST90094812 | ebi-met1400-GCST90200841 | Adenosine 5'-diphosphate (ADP) to oxalate (ethanedioate) ratio \|\| id:ebi-met1400-GCST90200841 | Plum liking \|\| id:ebi-fl187-GCST90094812 | Weighted mode | 5 | -0.18311698 | 0.338422387635736 | 0.617171499313475 | -0.846424859 | 0.480190900263031 | 0.832670744629771 | 0.428945735269088 | 1.61638294067045 | 0.999714303421225 |
| ebi-fl187-GCST90094812 | ebi-met1400-GCST90200844 | Aspartate to phosphate ratio \|\| id:ebi-met1400-GCST90200844 | Plum liking \|\| id:ebi-fl187-GCST90094812 | Weighted mode | 5 | 0.0854682175143871 | 0.214845301190004 | 0.711085210987569 | -0.335628573 | 0.506565007846794 | 1.0892269424543 | 0.714888585562573 | 1.6595807460469 | 0.999714303421225 |
| ebi-fl187-GCST90094812 | ebi-met1400-GCST90200870 | Adenosine 5'-monophosphate (AMP) to acetoacetate ratio \|\| id:ebi-met1400-GCST90200870 | Plum liking \|\| id:ebi-fl187-GCST90094812 | Weighted mode | 5 | 0.088712426827544 | 0.230039658133727 | 0.719394971300292 | -0.362165303 | 0.539590156769649 | 1.09276636084788 | 0.696167279710228 | 1.71530371248957 | 0.999714303421225 |
| ebi-fl187-GCST90094812 | ebi-met1400-GCST90200887 | Cholesterol to cortisol ratio \|\| id:ebi-met1400-GCST90200887 | Plum liking \|\| id:ebi-fl187-GCST90094812 | Weighted mode | 5 | 0.242817029878696 | 0.201816399016123 | 0.295255218549845 | -0.152743112 | 0.638377171950297 | 1.27483534599975 | 0.858350193190825 | 1.89340571284637 | 0.999714303421225 |
| ebi-fl187-GCST90094812 | ebi-met1400-GCST90200890 | Cortisol to taurocholate ratio \|\| id:ebi-met1400-GCST90200890 | Plum liking \|\| id:ebi-fl187-GCST90094812 | Weighted mode | 5 | 0.24927722714301 | 0.212384769466809 | 0.305627489994105 | -0.166996921 | 0.665551375297956 | 1.28309769327591 | 0.846202216779702 | 1.94556296100861 | 0.999714303421225 |
| ebi-fl187-GCST90094812 | ebi-met1400-GCST90200948 | Adenosine 5'-diphosphate (ADP) to mannitol to sorbitol ratio \|\| id:ebi-met1400-GCST90200948 | Plum liking \|\| id:ebi-fl187-GCST90094812 | Weighted mode | 5 | -0.188602707 | 0.288628808213073 | 0.549128117326815 | -0.754315171 | 0.377109757461721 | 0.828115446119133 | 0.470332601995627 | 1.45806433402944 | 0.999714303421225 |
| ebi-fl187-GCST90094812 | ebi-met1400-GCST90200972 | Leucine to N-palmitoyl-sphingosine (d18:1 to 16:0) ratio \|\| id:ebi-met1400-GCST90200972 | Plum liking \|\| id:ebi-fl187-GCST90094812 | Weighted mode | 5 | -0.155834489 | 0.194456199582828 | 0.467810264469026 | -0.53696864 | 0.225299662130952 | 0.855700806503395 | 0.584517452165847 | 1.25269804611891 | 0.999714303421225 |
| ebi-fl187-GCST90094812 | ebi-met1400-GCST90200973 | Proline to glutamate ratio \|\| id:ebi-met1400-GCST90200973 | Plum liking \|\| id:ebi-fl187-GCST90094812 | Weighted mode | 5 | -0.024369685 | 0.198554862838212 | 0.908236295703357 | -0.413537216 | 0.364797846152361 | 0.975924858269816 | 0.661306922609339 | 1.44022283213206 | 0.999714303421225 |
| ebi-fl187-GCST90094812 | ebi-met1400-GCST90200983 | Cholesterol to linoleoyl-arachidonoyl-glycerol (18:2 to 20:4) [1] ratio \|\| id:ebi-met1400-GCST90200983 | Plum liking \|\| id:ebi-fl187-GCST90094812 | Weighted mode | 5 | 0.28788434875453 | 0.214975240211146 | 0.251553936264088 | -0.133467122 | 0.709235819568376 | 1.33360306234931 | 0.875056238551779 | 2.03243751607424 | 0.999714303421225 |
| ebi-fl187-GCST90094812 | ebi-met1400-GCST90201001 | Serine to alpha-ketobutyrate ratio \|\| id:ebi-met1400-GCST90201001 | Plum liking \|\| id:ebi-fl187-GCST90094812 | Weighted mode | 5 | -0.131170614 | 0.215503747509953 | 0.575614763877459 | -0.553557959 | 0.291216730988301 | 0.877068121409406 | 0.57490069396346 | 1.33805455041165 | 0.999714303421225 |
| ebi-fl187-GCST90094812 | ebi-met1400-GCST90201005 | Fructose to maltose ratio \|\| id:ebi-met1400-GCST90201005 | Plum liking \|\| id:ebi-fl187-GCST90094812 | Weighted mode | 5 | 0.0645247879888742 | 0.25331474114078 | 0.811497784984794 | -0.431972105 | 0.561021680624803 | 1.06665201807531 | 0.649227486862439 | 1.75246204248465 | 0.999714303421225 |
| ebi-fl187-GCST90094812 | ebi-met1400-GCST90201020 | Paraxanthine to 5-acetylamino-6-formylamino-3-methyluracil ratio \|\| id:ebi-met1400-GCST90201020 | Plum liking \|\| id:ebi-fl187-GCST90094812 | Weighted mode | 5 | 0.533799378371155 | 0.234878312603352 | 0.0854758419108463 | 0.0734378856685846 | 0.994160871073725 | 1.70539947310881 | 1.07620168704749 | 2.70245568082951 | 0.999714303421225 |
| ebi-fl187-GCST90094812 | ebi-pw11-GCST90275197 | Placental weight (fetal genotype, adjusted for sex) \|\| id:ebi-pw11-GCST90275197 | Plum liking \|\| id:ebi-fl187-GCST90094812 | Weighted mode | 5 | 0.015575977358816 | 0.074893617726945 | 0.845408769018049 | -0.131215513 | 0.162367468103628 | 1.01569791517175 | 0.877028742588402 | 1.17629241185359 | 0.999714303421225 |
| ebi-fl187-GCST90094812 | prot-a-1017 | FAS-associated factor 2 \|\| id:prot-a-1017 | Plum liking \|\| id:ebi-fl187-GCST90094812 | Weighted mode | 5 | -0.014481865 | 0.321836327876477 | 0.966266022193289 | -0.645281068 | 0.616317337606332 | 0.985622492803107 | 0.524515097150607 | 1.85209482738773 | 0.999714303421225 |
| ebi-fl187-GCST90094812 | prot-a-1019 | Fas apoptotic inhibitory molecule 3 \|\| id:prot-a-1019 | Plum liking \|\| id:ebi-fl187-GCST90094812 | Weighted mode | 5 | -0.010876253 | 0.328115825741201 | 0.975144988204704 | -0.653983271 | 0.632230765872545 | 0.989182680006147 | 0.519970462953238 | 1.88180376413446 | 0.999714303421225 |
| ebi-fl187-GCST90094812 | prot-a-1052 | MIP18 family protein FAM96A \|\| id:prot-a-1052 | Plum liking \|\| id:ebi-fl187-GCST90094812 | Weighted mode | 5 | -0.794204829 | 0.351876906662694 | 0.0869635198666158 | -1.483883566 | -0.104526092 | 0.45194046206893 | 0.226755356808831 | 0.900751294829494 | 0.999714303421225 |
| ebi-fl187-GCST90094812 | prot-a-1066 | F-box/LRR-repeat protein 4 \|\| id:prot-a-1066 | Plum liking \|\| id:ebi-fl187-GCST90094812 | Weighted mode | 5 | -0.09772 | 0.330653358272444 | 0.782290794103628 | -0.745800582 | 0.550360581936437 | 0.906902800739212 | 0.474354388066933 | 1.73387811028866 | 0.999714303421225 |
| ebi-fl187-GCST90094812 | prot-a-1076 | Low affinity immunoglobulin gamma Fc region receptor III-B \|\| id:prot-a-1076 | Plum liking \|\| id:ebi-fl187-GCST90094812 | Weighted mode | 5 | -0.700843097 | 0.386534197147952 | 0.14401953228056 | -1.458450124 | 0.0567639291610534 | 0.496166810527972 | 0.232596491310975 | 1.05840592212701 | 0.999714303421225 |
| ebi-fl187-GCST90094812 | prot-a-1081 | Fc receptor-like protein 3 \|\| id:prot-a-1081 | Plum liking \|\| id:ebi-fl187-GCST90094812 | Weighted mode | 5 | -0.587590224 | 0.39954697688989 | 0.215343593533376 | -1.370702298 | 0.195521851193673 | 0.555664700381934 | 0.253928563339768 | 1.21594536348951 | 0.999714303421225 |
| ebi-fl187-GCST90094812 | prot-a-1105 | Fibroblast growth factor receptor 4 \|\| id:prot-a-1105 | Plum liking \|\| id:ebi-fl187-GCST90094812 | Weighted mode | 5 | 0.239225103084651 | 0.344850960735424 | 0.526045041013034 | -0.43668278 | 0.915132986126082 | 1.27026444484807 | 0.646176379001039 | 2.49710731045244 | 0.999714303421225 |
| ebi-fl187-GCST90094812 | prot-a-1143 | Follistatin-related protein 1 \|\| id:prot-a-1143 | Plum liking \|\| id:ebi-fl187-GCST90094812 | Weighted mode | 5 | -0.069829434 | 0.340272327995102 | 0.847423380142178 | -0.736763197 | 0.597104329203189 | 0.932552868464303 | 0.478660741530143 | 1.81685017597424 | 0.999714303421225 |
| ebi-fl187-GCST90094812 | prot-a-1151 | Alpha-(1,3)-fucosyltransferase 10 \|\| id:prot-a-1151 | Plum liking \|\| id:ebi-fl187-GCST90094812 | Weighted mode | 5 | -0.187982434 | 0.374022170334239 | 0.641657392884413 | -0.921065887 | 0.545101020348184 | 0.828629263215046 | 0.398094490976585 | 1.72478261171591 | 0.999714303421225 |
| ebi-fl187-GCST90094812 | prot-a-1165 | Growth arrest and DNA damage-inducible proteins-interacting protein 1 \|\| id:prot-a-1165 | Plum liking \|\| id:ebi-fl187-GCST90094812 | Weighted mode | 5 | -0.515497327 | 0.398292489826203 | 0.265238655469786 | -1.296150607 | 0.265155952796282 | 0.59720351516181 | 0.273582896679939 | 1.30363426533518 | 0.999714303421225 |
| ebi-fl187-GCST90094812 | prot-a-1194 | Growth/differentiation factor 11/8 \|\| id:prot-a-1194 | Plum liking \|\| id:ebi-fl187-GCST90094812 | Weighted mode | 5 | -0.129236248 | 0.333294566469605 | 0.717948384611848 | -0.782493598 | 0.52402110255857 | 0.878766334475802 | 0.457264354998713 | 1.68880487220617 | 0.999714303421225 |
| ebi-fl187-GCST90094812 | prot-a-1216 | D-glucuronyl C5-epimerase \|\| id:prot-a-1216 | Plum liking \|\| id:ebi-fl187-GCST90094812 | Weighted mode | 5 | 0.271567868054036 | 0.365790523764432 | 0.499067048114993 | -0.445381559 | 0.988517294632323 | 1.31201991307328 | 0.640579810709132 | 2.68724712131531 | 0.999714303421225 |
| ebi-fl187-GCST90094812 | prot-a-1246 | Glypican-3 \|\| id:prot-a-1246 | Plum liking \|\| id:ebi-fl187-GCST90094812 | Weighted mode | 5 | 0.212179508413382 | 0.362114575202358 | 0.589379812993485 | -0.497565059 | 0.921924075810002 | 1.23636980394161 | 0.608009325595418 | 2.51412310263773 | 0.999714303421225 |
| ebi-fl187-GCST90094812 | prot-a-1275 | Glutamate receptor 4 \|\| id:prot-a-1275 | Plum liking \|\| id:ebi-fl187-GCST90094812 | Weighted mode | 5 | -0.238137372 | 0.3490768141948 | 0.532574373065318 | -0.922327928 | 0.446053183857034 | 0.788094421578224 | 0.397592396534567 | 1.56213454466481 | 0.999714303421225 |
| ebi-fl187-GCST90094812 | prot-a-1289 | Glutathione S-transferase P \|\| id:prot-a-1289 | Plum liking \|\| id:ebi-fl187-GCST90094812 | Weighted mode | 5 | 0.0713047762753978 | 0.380657592270495 | 0.860527640875223 | -0.674784105 | 0.817393657125568 | 1.0739084778225 | 0.509266357016441 | 2.26458984154298 | 0.999714303421225 |
| ebi-fl187-GCST90094812 | prot-a-130 | Apolipoprotein D \|\| id:prot-a-130 | Plum liking \|\| id:ebi-fl187-GCST90094812 | Weighted mode | 5 | 0.130286194939668 | 0.390017925659546 | 0.75512001571486 | -0.634148939 | 0.894721329232378 | 1.13915435688885 | 0.530386687528815 | 2.44665388353727 | 0.999714303421225 |
| ebi-fl187-GCST90094812 | prot-a-1313 | Heparin-binding EGF-like growth factor \|\| id:prot-a-1313 | Plum liking \|\| id:ebi-fl187-GCST90094812 | Weighted mode | 5 | -0.091331722 | 0.322602015872425 | 0.791140334534466 | -0.723631673 | 0.540968228923509 | 0.912714892896446 | 0.484987736807945 | 1.71766915427154 | 0.999714303421225 |
| ebi-fl187-GCST90094812 | prot-a-1347 | HLA class II histocompatibility antigen, DQ alpha 2 chain \|\| id:prot-a-1347 | Plum liking \|\| id:ebi-fl187-GCST90094812 | Weighted mode | 5 | 0.0134665154560221 | 0.346043906611708 | 0.970822484960855 | -0.664779542 | 0.691712572414969 | 1.01355759736805 | 0.514386916173902 | 1.99713284082674 | 0.999714303421225 |
| ebi-fl187-GCST90094812 | prot-a-1357 | Non-histone chromosomal protein HMG-14 \|\| id:prot-a-1357 | Plum liking \|\| id:ebi-fl187-GCST90094812 | Weighted mode | 5 | 0.181474530277943 | 0.341406153965029 | 0.623186039351237 | -0.487681531 | 0.8506305920494 | 1.19898399853162 | 0.614048396982954 | 2.34112267990301 | 0.999714303421225 |
| ebi-fl187-GCST90094812 | prot-a-137 | Apolipoprotein M \|\| id:prot-a-137 | Plum liking \|\| id:ebi-fl187-GCST90094812 | Weighted mode | 5 | 0.154395723239587 | 0.335628113632232 | 0.669395955643942 | -0.503435379 | 0.812226825958761 | 1.16695258567757 | 0.604450571722052 | 2.25291926408461 | 0.999714303421225 |
| ebi-fl187-GCST90094812 | prot-a-1381 | 17-beta-hydroxysteroid dehydrogenase 14 \|\| id:prot-a-1381 | Plum liking \|\| id:ebi-fl187-GCST90094812 | Weighted mode | 5 | 0.275353973397086 | 0.344951694704286 | 0.469439040700768 | -0.400751348 | 0.951459295017486 | 1.31699677419149 | 0.66981659141855 | 2.58948572706667 | 0.999714303421225 |
| ebi-fl187-GCST90094812 | prot-a-1392 | Serine protease HTRA2, mitochondrial \|\| id:prot-a-1392 | Plum liking \|\| id:ebi-fl187-GCST90094812 | Weighted mode | 5 | -0.139125587 | 0.315432650807703 | 0.681958913084228 | -0.757373583 | 0.479122408105257 | 0.870118745577436 | 0.468896328445625 | 1.61465677053824 | 0.999714303421225 |
| ebi-fl187-GCST90094812 | prot-a-1416 | Interferon-induced protein with tetratricopeptide repeats 2 \|\| id:prot-a-1416 | Plum liking \|\| id:ebi-fl187-GCST90094812 | Weighted mode | 5 | 0.264613077664687 | 0.346980748101082 | 0.488185088674084 | -0.415469189 | 0.944695343942808 | 1.30292674677632 | 0.660030529235898 | 2.57202967479463 | 0.999714303421225 |
| ebi-fl187-GCST90094812 | prot-a-1428 | Interferon gamma \|\| id:prot-a-1428 | Plum liking \|\| id:ebi-fl187-GCST90094812 | Weighted mode | 5 | -0.068674423 | 0.342909248898624 | 0.851039522947392 | -0.74077655 | 0.603427705216264 | 0.933630599600659 | 0.476743556307361 | 1.82837520293345 | 0.999714303421225 |
| ebi-fl187-GCST90094812 | prot-a-1483 | Interleukin-17C \|\| id:prot-a-1483 | Plum liking \|\| id:ebi-fl187-GCST90094812 | Weighted mode | 5 | -0.287249401 | 0.35629820591435 | 0.465324584539161 | -0.985593885 | 0.411095082459235 | 0.75032457370097 | 0.37321751292686 | 1.50846877866064 | 0.999714303421225 |
| ebi-fl187-GCST90094812 | prot-a-1503 | Interleukin-1 receptor-like 2 \|\| id:prot-a-1503 | Plum liking \|\| id:ebi-fl187-GCST90094812 | Weighted mode | 5 | 0.75672453662035 | 0.340702086619333 | 0.0905041133021692 | 0.0889484468464584 | 1.42450062639424 | 2.13128383294726 | 1.09302430602395 | 4.15578203663737 | 0.999714303421225 |
| ebi-fl187-GCST90094812 | prot-a-1522 | Interleukin-31 receptor subunit alpha \|\| id:prot-a-1522 | Plum liking \|\| id:ebi-fl187-GCST90094812 | Weighted mode | 5 | 0.212107416228983 | 0.317158535628781 | 0.540256129060254 | -0.409523314 | 0.833738146061394 | 1.23628067455453 | 0.66396667859522 | 2.30190754378017 | 0.999714303421225 |
| ebi-fl187-GCST90094812 | prot-a-1540 | Interleukin-6 receptor subunit alpha \|\| id:prot-a-1540 | Plum liking \|\| id:ebi-fl187-GCST90094812 | Weighted mode | 5 | -0.35211574 | 0.375860383172026 | 0.401886621509262 | -1.088802091 | 0.384570611171351 | 0.703198729153742 | 0.336619491845942 | 1.4689834209296 | 0.999714303421225 |
| ebi-fl187-GCST90094812 | prot-a-1541 | Interleukin-6 receptor subunit alpha \|\| id:prot-a-1541 | Plum liking \|\| id:ebi-fl187-GCST90094812 | Weighted mode | 5 | -0.310009197 | 0.326121618287364 | 0.395639525691929 | -0.949207569 | 0.329189174541041 | 0.733440210522012 | 0.387047610432664 | 1.38984075320667 | 0.999714303421225 |
| ebi-fl187-GCST90094812 | prot-a-1545 | Interleukin-9 \|\| id:prot-a-1545 | Plum liking \|\| id:ebi-fl187-GCST90094812 | Weighted mode | 5 | -0.552557045 | 0.311371041501093 | 0.150630572223751 | -1.162844286 | 0.0577301962690606 | 0.575476408291032 | 0.312595803241735 | 1.05942911921772 | 0.999714303421225 |
| ebi-fl187-GCST90094812 | prot-a-1548 | Inositol monophosphatase 3 \|\| id:prot-a-1548 | Plum liking \|\| id:ebi-fl187-GCST90094812 | Weighted mode | 5 | -0.127151353 | 0.358706834443219 | 0.740883579311356 | -0.830216748 | 0.575914042638209 | 0.880600381114941 | 0.435954783587496 | 1.77875564258866 | 0.999714303421225 |
| ebi-fl187-GCST90094812 | prot-a-1555 | Inhibin beta A chain:Inhibin beta B chain heterodimer \|\| id:prot-a-1555 | Plum liking \|\| id:ebi-fl187-GCST90094812 | Weighted mode | 5 | -0.169481647 | 0.353940842876024 | 0.657051113553269 | -0.863205699 | 0.524242404758452 | 0.844102245911597 | 0.421807723912836 | 1.68917864979693 | 0.999714303421225 |
| ebi-fl187-GCST90094812 | prot-a-1603 | Junctophilin-3 \|\| id:prot-a-1603 | Plum liking \|\| id:ebi-fl187-GCST90094812 | Weighted mode | 5 | 0.209586931457346 | 0.331717263898962 | 0.561811352367871 | -0.440578906 | 0.859752768699311 | 1.23316857159246 | 0.643663692571226 | 2.36257651862991 | 0.999714303421225 |
| ebi-fl187-GCST90094812 | prot-a-1627 | UPF0258 protein KIAA1024 \|\| id:prot-a-1627 | Plum liking \|\| id:ebi-fl187-GCST90094812 | Weighted mode | 5 | -0.169154433 | 0.310364784190383 | 0.614705411012805 | -0.77746941 | 0.439160544155108 | 0.844378493532484 | 0.459567518061337 | 1.55140433629391 | 0.999714303421225 |
| ebi-fl187-GCST90094812 | prot-a-1666 | Kallikrein-7 \|\| id:prot-a-1666 | Plum liking \|\| id:ebi-fl187-GCST90094812 | Weighted mode | 5 | 0.548668328155066 | 0.329822572087788 | 0.171540257943984 | -0.097783913 | 1.19512056944713 | 1.73094642977343 | 0.906844839840223 | 3.30395610264848 | 0.999714303421225 |
| ebi-fl187-GCST90094812 | prot-a-1678 | Importin subunit alpha-3 \|\| id:prot-a-1678 | Plum liking \|\| id:ebi-fl187-GCST90094812 | Weighted mode | 5 | 0.0682435947964644 | 0.349507293110124 | 0.854709203849005 | -0.6167907 | 0.753277889292308 | 1.07062607565771 | 0.539673636114457 | 2.1239506938507 | 0.999714303421225 |
| ebi-fl187-GCST90094812 | prot-a-1718 | Protein LDOC1 \|\| id:prot-a-1718 | Plum liking \|\| id:ebi-fl187-GCST90094812 | Weighted mode | 5 | -0.260725728 | 0.348922046413263 | 0.496455185076818 | -0.944612939 | 0.423161482886869 | 0.770492215014306 | 0.388830042703499 | 1.52678082503605 | 0.999714303421225 |
| ebi-fl187-GCST90094812 | prot-a-1723 | Leptin \|\| id:prot-a-1723 | Plum liking \|\| id:ebi-fl187-GCST90094812 | Weighted mode | 5 | -0.297217037 | 0.389575131374962 | 0.488019475867193 | -1.060784295 | 0.466350220174685 | 0.742882761580027 | 0.346184193362144 | 1.59416521041286 | 0.999714303421225 |
| ebi-fl187-GCST90094812 | prot-a-1730 | Galectin-7 \|\| id:prot-a-1730 | Plum liking \|\| id:ebi-fl187-GCST90094812 | Weighted mode | 5 | -0.034464325 | 0.332320650905776 | 0.922392758734609 | -0.685812801 | 0.616884150392828 | 0.966122805133256 | 0.503680670920007 | 1.8531449159914 | 0.999714303421225 |
| ebi-fl187-GCST90094812 | prot-a-1753 | Lipase member N \|\| id:prot-a-1753 | Plum liking \|\| id:ebi-fl187-GCST90094812 | Weighted mode | 5 | -0.257719407 | 0.36376586913194 | 0.517753915699454 | -0.970700511 | 0.455261696350906 | 0.772812047223744 | 0.378817579388559 | 1.57658591583354 | 0.999714303421225 |
| ebi-fl187-GCST90094812 | prot-a-1756 | VIP36-like protein \|\| id:prot-a-1756 | Plum liking \|\| id:ebi-fl187-GCST90094812 | Weighted mode | 5 | -0.587665028 | 0.326041516260823 | 0.145826540441955 | -1.2267064 | 0.0513763433743372 | 0.55562313544625 | 0.293256859514571 | 1.05271900256363 | 0.999714303421225 |
| ebi-fl187-GCST90094812 | prot-a-1760 | Ig Kappa chain V-I region HK102- like \|\| id:prot-a-1760 | Plum liking \|\| id:ebi-fl187-GCST90094812 | Weighted mode | 5 | 0.216815190133573 | 0.369555668048412 | 0.588924666597776 | -0.507513919 | 0.94114429950846 | 1.24211452590482 | 0.601990316565947 | 2.5629124804946 | 0.999714303421225 |
| ebi-fl187-GCST90094812 | prot-a-179 | Neutral ceramidase \|\| id:prot-a-179 | Plum liking \|\| id:ebi-fl187-GCST90094812 | Weighted mode | 5 | 0.137366948551941 | 0.364424682681891 | 0.725361284688431 | -0.57690543 | 0.851639326608447 | 1.14724905266918 | 0.56163369519456 | 2.34348544275712 | 0.999714303421225 |
| ebi-fl187-GCST90094812 | prot-a-1809 | Lactotransferrin \|\| id:prot-a-1809 | Plum liking \|\| id:ebi-fl187-GCST90094812 | Weighted mode | 5 | -0.445152355 | 0.344682881609861 | 0.266111030475815 | -1.120730803 | 0.23042609318117 | 0.640726650831491 | 0.326041435565887 | 1.25913640508057 | 0.999714303421225 |
| ebi-fl187-GCST90094812 | prot-a-1818 | Lysophospholipase-like protein 1 \|\| id:prot-a-1818 | Plum liking \|\| id:ebi-fl187-GCST90094812 | Weighted mode | 5 | 0.111173050732989 | 0.330463986680651 | 0.753466224523856 | -0.536536363 | 0.758882464627065 | 1.11758828959416 | 0.584770180279329 | 2.13588795591694 | 0.999714303421225 |
| ebi-fl187-GCST90094812 | prot-a-1852 | MAP kinase-activated protein kinase 3 \|\| id:prot-a-1852 | Plum liking \|\| id:ebi-fl187-GCST90094812 | Weighted mode | 5 | 0.522040831526755 | 0.373821878705737 | 0.235072320328753 | -0.210650051 | 1.254731714 | 1.68546388995633 | 0.810057496309659 | 3.50689739591125 | 0.999714303421225 |
| ebi-fl187-GCST90094812 | prot-a-1856 | Megakaryocyte-associated tyrosine-protein kinase \|\| id:prot-a-1856 | Plum liking \|\| id:ebi-fl187-GCST90094812 | Weighted mode | 5 | -0.102235127 | 0.343201087652672 | 0.780621042420431 | -0.774909259 | 0.570439004674613 | 0.902817249886874 | 0.460745587652596 | 1.76904349935494 | 0.999714303421225 |
| ebi-fl187-GCST90094812 | prot-a-1890 | Beta-1,3-N-acetylglucosaminyltransferase manic fringe \|\| id:prot-a-1890 | Plum liking \|\| id:ebi-fl187-GCST90094812 | Weighted mode | 5 | -0.017670142 | 0.344940414528153 | 0.961601002857763 | -0.693753354 | 0.65841307083424 | 0.982485059823414 | 0.49969700506495 | 1.93172439096519 | 0.999714303421225 |
| ebi-fl187-GCST90094812 | prot-a-1919 | Matrilysin \|\| id:prot-a-1919 | Plum liking \|\| id:ebi-fl187-GCST90094812 | Weighted mode | 5 | -0.567419981 | 0.343846413391154 | 0.174244074010246 | -1.241358951 | 0.106518989162555 | 0.566986388857922 | 0.288991225959946 | 1.11239904977151 | 0.999714303421225 |
| ebi-fl187-GCST90094812 | prot-a-1923 | Multimerin-2 \|\| id:prot-a-1923 | Plum liking \|\| id:ebi-fl187-GCST90094812 | Weighted mode | 5 | 0.81103264751478 | 0.328966999295558 | 0.0692887634885955 | 0.166257328895487 | 1.45580796613407 | 2.25023048222561 | 1.18087693638663 | 4.28794658199631 | 0.999714303421225 |
| ebi-fl187-GCST90094812 | prot-a-1948 | Mesothelin \|\| id:prot-a-1948 | Plum liking \|\| id:ebi-fl187-GCST90094812 | Weighted mode | 5 | -0.24155244 | 0.334497895064132 | 0.510172197747037 | -0.897168314 | 0.414063434450592 | 0.785407616031761 | 0.407722568840254 | 1.51295309718894 | 0.999714303421225 |
| ebi-fl187-GCST90094812 | prot-a-195 | ATPase family AAA domain-containing protein 1 \|\| id:prot-a-195 | Plum liking \|\| id:ebi-fl187-GCST90094812 | Weighted mode | 5 | -0.608775823 | 0.335991661677743 | 0.144232077063433 | -1.26731948 | 0.0497678339341698 | 0.544016434037789 | 0.281585406417516 | 1.05102705523159 | 0.999714303421225 |
| ebi-fl187-GCST90094812 | prot-a-1966 | Mucin-1 \|\| id:prot-a-1966 | Plum liking \|\| id:ebi-fl187-GCST90094812 | Weighted mode | 5 | -0.441613823 | 0.35240098082903 | 0.2784031497942 | -1.132319746 | 0.249092099336331 | 0.64299789845821 | 0.322284769959671 | 1.2828601782002 | 0.999714303421225 |
| ebi-fl187-GCST90094812 | prot-a-1986 | Myocardial zonula adherens protein \|\| id:prot-a-1986 | Plum liking \|\| id:ebi-fl187-GCST90094812 | Weighted mode | 5 | 0.0514280980666644 | 0.324609798537238 | 0.88179439629347 | -0.584807107 | 0.687663303199651 | 1.05277348712162 | 0.557213333961204 | 1.98906226329355 | 0.999714303421225 |
| ebi-fl187-GCST90094812 | prot-a-2071 | Epididymal secretory protein E1 \|\| id:prot-a-2071 | Plum liking \|\| id:ebi-fl187-GCST90094812 | Weighted mode | 5 | 0.368760169061456 | 0.350902737536425 | 0.352604588729517 | -0.319009197 | 1.05652953463285 | 1.44594078075244 | 0.726868864618863 | 2.87637130053613 | 0.999714303421225 |
| ebi-fl187-GCST90094812 | prot-a-2098 | Neuritin \|\| id:prot-a-2098 | Plum liking \|\| id:ebi-fl187-GCST90094812 | Weighted mode | 5 | -0.11267618 | 0.361819694636504 | 0.771040344181681 | -0.821842782 | 0.596490421303892 | 0.893439926831362 | 0.439620782485173 | 1.81573513959921 | 0.999714303421225 |
| ebi-fl187-GCST90094812 | prot-a-2115 | Neurotrophin-4 \|\| id:prot-a-2115 | Plum liking \|\| id:ebi-fl187-GCST90094812 | Weighted mode | 5 | 0.143964014837051 | 0.37046773427504 | 0.717369334186302 | -0.582152744 | 0.870080774016129 | 1.15484255057982 | 0.558694344964997 | 2.3871036616869 | 0.999714303421225 |
| ebi-fl187-GCST90094812 | prot-a-2118 | Netrin-1 \|\| id:prot-a-2118 | Plum liking \|\| id:ebi-fl187-GCST90094812 | Weighted mode | 5 | 0.163485781200796 | 0.325543279049602 | 0.641916989665396 | -0.474579046 | 0.801550608138016 | 1.177608611 | 0.622146896738516 | 2.22899454728039 | 0.999714303421225 |
| ebi-fl187-GCST90094812 | prot-a-2120 | Netrin-G1 \|\| id:prot-a-2120 | Plum liking \|\| id:ebi-fl187-GCST90094812 | Weighted mode | 5 | -0.161449096 | 0.355269073995945 | 0.673079491057801 | -0.857776481 | 0.534878288608333 | 0.850909844717153 | 0.424104037894755 | 1.70724043899872 | 0.999714303421225 |
| ebi-fl187-GCST90094812 | prot-a-2145 | Olfactomedin-like protein 3 \|\| id:prot-a-2145 | Plum liking \|\| id:ebi-fl187-GCST90094812 | Weighted mode | 5 | 0.0549541579781396 | 0.335789458942193 | 0.877937665717091 | -0.603193182 | 0.713101497504838 | 1.05649218182768 | 0.547061975882057 | 2.04030947035451 | 0.999714303421225 |
| ebi-fl187-GCST90094812 | prot-a-2162 | Prolyl 4-hydroxylase subunit alpha-2 \|\| id:prot-a-2162 | Plum liking \|\| id:ebi-fl187-GCST90094812 | Weighted mode | 5 | -0.456254114 | 0.343010634614152 | 0.254243363652412 | -1.128554958 | 0.216046730152567 | 0.63365279678703 | 0.323500390628516 | 1.24116037725932 | 0.999714303421225 |
| ebi-fl187-GCST90094812 | prot-a-2168 | Polyadenylate-binding protein-interacting protein 1 \|\| id:prot-a-2168 | Plum liking \|\| id:ebi-fl187-GCST90094812 | Weighted mode | 5 | -0.166639929 | 0.35455670971727 | 0.66283741546777 | -0.86157108 | 0.528291222501418 | 0.846504358525033 | 0.422497782977047 | 1.69603169027944 | 0.999714303421225 |
| ebi-fl187-GCST90094812 | prot-a-219 | Beta-1,4-galactosyltransferase 5 \|\| id:prot-a-219 | Plum liking \|\| id:ebi-fl187-GCST90094812 | Weighted mode | 5 | -0.503021938 | 0.336652781960531 | 0.209433795598296 | -1.162861391 | 0.156817514728571 | 0.604700528380378 | 0.312590456604613 | 1.16978212641349 | 0.999714303421225 |
| ebi-fl187-GCST90094812 | prot-a-2190 | Pyruvate carboxylase, mitochondrial \|\| id:prot-a-2190 | Plum liking \|\| id:ebi-fl187-GCST90094812 | Weighted mode | 5 | -0.3707369 | 0.347246272723654 | 0.345830744061511 | -1.051339594 | 0.309865794967964 | 0.690225516301407 | 0.349469288407668 | 1.36324214789883 | 0.999714303421225 |
| ebi-fl187-GCST90094812 | prot-a-2197 | Protocadherin-9 \|\| id:prot-a-2197 | Plum liking \|\| id:ebi-fl187-GCST90094812 | Weighted mode | 5 | -0.302242105 | 0.320383448950942 | 0.398904142922751 | -0.930193665 | 0.325709454821316 | 0.739159089023101 | 0.394477306499712 | 1.38501290158718 | 0.999714303421225 |
| ebi-fl187-GCST90094812 | prot-a-2198 | Protocadherin alpha-4 \|\| id:prot-a-2198 | Plum liking \|\| id:ebi-fl187-GCST90094812 | Weighted mode | 5 | -0.051113375 | 0.375596273383668 | 0.898327419174298 | -0.787282071 | 0.685055320604474 | 0.950170938579136 | 0.455079991012679 | 1.98388158202983 | 0.999714303421225 |
| ebi-fl187-GCST90094812 | prot-a-2254 | Placenta growth factor \|\| id:prot-a-2254 | Plum liking \|\| id:ebi-fl187-GCST90094812 | Weighted mode | 5 | -0.647724792 | 0.3231348488212 | 0.115521174019681 | -1.281069096 | -0.014380489 | 0.523234891600463 | 0.277740210748472 | 0.985722416823849 | 0.999714303421225 |
| ebi-fl187-GCST90094812 | prot-a-2261 | Membrane-associated progesterone receptor component 2 \|\| id:prot-a-2261 | Plum liking \|\| id:ebi-fl187-GCST90094812 | Weighted mode | 5 | 0.049601084967609 | 0.340287462383358 | 0.89115948759722 | -0.617362341 | 0.71656451123899 | 1.05085181216751 | 0.539365224370151 | 2.04738733837593 | 0.999714303421225 |
| ebi-fl187-GCST90094812 | prot-a-2268 | Polymeric immunoglobulin receptor \|\| id:prot-a-2268 | Plum liking \|\| id:ebi-fl187-GCST90094812 | Weighted mode | 5 | -0.276910788 | 0.348622602676985 | 0.471484388706336 | -0.96021109 | 0.406389512833648 | 0.758122127371701 | 0.382812069775977 | 1.50138724817882 | 0.999714303421225 |
| ebi-fl187-GCST90094812 | prot-a-2283 | Group 10 secretory phospholipase A2 \|\| id:prot-a-2283 | Plum liking \|\| id:ebi-fl187-GCST90094812 | Weighted mode | 5 | -0.224263852 | 0.331601406620466 | 0.535934750877472 | -0.874202609 | 0.425674905024431 | 0.799104261332006 | 0.417194554293788 | 1.53062309636308 | 0.999714303421225 |
| ebi-fl187-GCST90094812 | prot-a-2298 | Inactive phospholipase D5 \|\| id:prot-a-2298 | Plum liking \|\| id:ebi-fl187-GCST90094812 | Weighted mode | 5 | -0.591471422 | 0.359976876378701 | 0.175710864889973 | -1.2970261 | 0.114083255761768 | 0.553512235193482 | 0.273343481773177 | 1.12084543784043 | 0.999714303421225 |
| ebi-fl187-GCST90094812 | prot-a-2304 | Procollagen-lysine,2-oxoglutarate 5-dioxygenase 2 \|\| id:prot-a-2304 | Plum liking \|\| id:ebi-fl187-GCST90094812 | Weighted mode | 5 | -0.083617396 | 0.337912029926867 | 0.816740246833304 | -0.745924974 | 0.57869018307744 | 0.919783101862891 | 0.474295385966932 | 1.7837005788024 | 0.999714303421225 |
| ebi-fl187-GCST90094812 | prot-a-2331 | Periostin \|\| id:prot-a-2331 | Plum liking \|\| id:ebi-fl187-GCST90094812 | Weighted mode | 5 | 0.718976527498185 | 0.358359685838521 | 0.115284721166047 | 0.0165915432546844 | 1.42136151174169 | 2.05233163038423 | 1.01672994729451 | 4.1427570145679 | 0.999714303421225 |
| ebi-fl187-GCST90094812 | prot-a-2333 | Periostin \|\| id:prot-a-2333 | Plum liking \|\| id:ebi-fl187-GCST90094812 | Weighted mode | 5 | 1.01125312099099 | 0.379662539269419 | 0.0561816472753305 | 0.267114544022928 | 1.75539169795905 | 2.74904374190787 | 1.30619005402565 | 5.78571355036087 | 0.999714303421225 |
| ebi-fl187-GCST90094812 | prot-a-2367 | cAMP-dependent protein kinase catalytic subunit alpha \|\| id:prot-a-2367 | Plum liking \|\| id:ebi-fl187-GCST90094812 | Weighted mode | 5 | -0.307336399 | 0.31784241287154 | 0.388327477315367 | -0.930307528 | 0.315634730045001 | 0.735403170266042 | 0.39443239255112 | 1.37112933179608 | 0.999714303421225 |
| ebi-fl187-GCST90094812 | prot-a-2385 | Phosphoribosyl pyrophosphate synthase-associated protein 1 \|\| id:prot-a-2385 | Plum liking \|\| id:ebi-fl187-GCST90094812 | Weighted mode | 5 | -0.081962091 | 0.337591529180334 | 0.820113407305928 | -0.743641488 | 0.579717306492843 | 0.921306884132844 | 0.47537967050609 | 1.78553360064163 | 0.999714303421225 |
| ebi-fl187-GCST90094812 | prot-a-2393 | Trypsin-3 \|\| id:prot-a-2393 | Plum liking \|\| id:ebi-fl187-GCST90094812 | Weighted mode | 5 | 0.0595961719395646 | 0.369324613267652 | 0.879628102573969 | -0.66428007 | 0.783472413944163 | 1.06140783372123 | 0.514643901919733 | 2.1890604071716 | 0.999714303421225 |
| ebi-fl187-GCST90094812 | prot-a-2405 | Pregnancy-specific beta-1-glycoprotein 6 \|\| id:prot-a-2405 | Plum liking \|\| id:ebi-fl187-GCST90094812 | Weighted mode | 5 | -0.233957815 | 0.334779441718705 | 0.523151245690073 | -0.89012552 | 0.42220989121008 | 0.791395200547277 | 0.410604210342754 | 1.52532864416186 | 0.999714303421225 |
| ebi-fl187-GCST90094812 | prot-a-244 | Beclin-1 \|\| id:prot-a-244 | Plum liking \|\| id:ebi-fl187-GCST90094812 | Weighted mode | 5 | -0.315148713 | 0.378979202706982 | 0.452410510220627 | -1.057947951 | 0.427650523879578 | 0.72968035293472 | 0.347167484667781 | 1.53365001324489 | 0.999714303421225 |
| ebi-fl187-GCST90094812 | prot-a-2454 | tRNA pseudouridine synthase A, mitochondrial \|\| id:prot-a-2454 | Plum liking \|\| id:ebi-fl187-GCST90094812 | Weighted mode | 5 | 0.273697418793972 | 0.33313513847871 | 0.45746285057171 | -0.379247453 | 0.926642290212245 | 1.31481690315911 | 0.684376241014565 | 2.52601330266827 | 0.999714303421225 |
| ebi-fl187-GCST90094812 | prot-a-2466 | Glutaminyl-peptide cyclotransferase-like protein \|\| id:prot-a-2466 | Plum liking \|\| id:ebi-fl187-GCST90094812 | Weighted mode | 5 | 0.21003661920398 | 0.355954608474214 | 0.586868264194451 | -0.487634413 | 0.907707651813439 | 1.23372323709244 | 0.614077330451019 | 2.47863412352957 | 0.999714303421225 |
| ebi-fl187-GCST90094812 | prot-a-2474 | Ras-related protein Rab-27A \|\| id:prot-a-2474 | Plum liking \|\| id:ebi-fl187-GCST90094812 | Weighted mode | 5 | -0.437382478 | 0.383915505892749 | 0.318185768337485 | -1.189856869 | 0.315091913939104 | 0.645724409044242 | 0.304264810627985 | 1.37038526267613 | 0.999714303421225 |
| ebi-fl187-GCST90094812 | prot-a-2490 | Rap1 GTPase-GDP dissociation stimulator 1 \|\| id:prot-a-2490 | Plum liking \|\| id:ebi-fl187-GCST90094812 | Weighted mode | 5 | -0.255630361 | 0.321985287767512 | 0.471682145127483 | -0.886721525 | 0.375460803346734 | 0.774428174998318 | 0.41200428685375 | 1.4556620340315 | 0.999714303421225 |
| ebi-fl187-GCST90094812 | prot-a-2545 | Receptor-interacting serine/threonine-protein kinase 2 \|\| id:prot-a-2545 | Plum liking \|\| id:ebi-fl187-GCST90094812 | Weighted mode | 5 | -0.129855856 | 0.329522600879298 | 0.713633270562313 | -0.775720153 | 0.516008442066836 | 0.878222012532696 | 0.460372123050524 | 1.67532712056141 | 0.999714303421225 |
| ebi-fl187-GCST90094812 | prot-a-2610 | RUN and FYVE domain-containing protein 1 \|\| id:prot-a-2610 | Plum liking \|\| id:ebi-fl187-GCST90094812 | Weighted mode | 5 | -0.016680051 | 0.375131596252953 | 0.966665330323153 | -0.751937979 | 0.718577877999861 | 0.983458291138327 | 0.47145200261011 | 2.05151363246745 | 0.999714303421225 |
| ebi-fl187-GCST90094812 | prot-a-2627 | Serine--tRNA ligase, mitochondrial \|\| id:prot-a-2627 | Plum liking \|\| id:ebi-fl187-GCST90094812 | Weighted mode | 5 | -0.051633728 | 0.338794615930573 | 0.886246594577914 | -0.715671176 | 0.612403718837664 | 0.949676642745251 | 0.488863888142377 | 1.84486059954837 | 0.999714303421225 |
| ebi-fl187-GCST90094812 | prot-a-264 | BolA-like protein 2 \|\| id:prot-a-264 | Plum liking \|\| id:ebi-fl187-GCST90094812 | Weighted mode | 5 | 0.0286149193878875 | 0.32938855117741 | 0.934947606965727 | -0.616986641 | 0.67421647969561 | 1.02902825933755 | 0.539567902162707 | 1.96249471896118 | 0.999714303421225 |
| ebi-fl187-GCST90094812 | prot-a-2646 | Secretoglobin family 1C member 1 \|\| id:prot-a-2646 | Plum liking \|\| id:ebi-fl187-GCST90094812 | Weighted mode | 5 | -0.883188256 | 0.408321124834598 | 0.0965667295772738 | -1.68349766 | -0.082878851 | 0.413462583661063 | 0.185723241887532 | 0.920462653732933 | 0.999714303421225 |
| ebi-fl187-GCST90094812 | prot-a-2672 | Semaphorin-3E \|\| id:prot-a-2672 | Plum liking \|\| id:ebi-fl187-GCST90094812 | Weighted mode | 5 | 0.0257856446181686 | 0.358147218068553 | 0.94606023433219 | -0.676182903 | 0.727754192032533 | 1.02612097034513 | 0.508554494134353 | 2.07042560419075 | 0.999714303421225 |
| ebi-fl187-GCST90094812 | prot-a-2701 | Plasma protease C1 inhibitor \|\| id:prot-a-2701 | Plum liking \|\| id:ebi-fl187-GCST90094812 | Weighted mode | 5 | 0.460050076713567 | 0.369042933048558 | 0.280558935337557 | -0.263274072 | 1.18337422548874 | 1.5841533121999 | 0.768531235504097 | 3.26537374230191 | 0.999714303421225 |
| ebi-fl187-GCST90094812 | prot-a-2738 | Signaling threshold-regulating transmembrane adapter 1 \|\| id:prot-a-2738 | Plum liking \|\| id:ebi-fl187-GCST90094812 | Weighted mode | 5 | -0.142694499 | 0.365281753168067 | 0.715974283914485 | -0.858646735 | 0.573257737313639 | 0.867018903672228 | 0.423735120357246 | 1.7740369943639 | 0.999714303421225 |
| ebi-fl187-GCST90094812 | prot-a-274 | Serine/threonine-protein kinase BRSK2 \|\| id:prot-a-274 | Plum liking \|\| id:ebi-fl187-GCST90094812 | Weighted mode | 5 | -0.55665628 | 0.322405083560294 | 0.159319735915981 | -1.188570244 | 0.0752576837434488 | 0.573122223752934 | 0.304656537394467 | 1.07816193989695 | 0.999714303421225 |
| ebi-fl187-GCST90094812 | prot-a-2749 | Mitochondrial glutamate carrier 2 \|\| id:prot-a-2749 | Plum liking \|\| id:ebi-fl187-GCST90094812 | Weighted mode | 5 | -0.130109864 | 0.340480660863291 | 0.721795609470729 | -0.797451959 | 0.53723223115593 | 0.877998965023781 | 0.450475332424135 | 1.71126391856907 | 0.999714303421225 |
| ebi-fl187-GCST90094812 | prot-a-2760 | Sodium-coupled monocarboxylate transporter 1 \|\| id:prot-a-2760 | Plum liking \|\| id:ebi-fl187-GCST90094812 | Weighted mode | 5 | 0.15251568298281 | 0.366706920177409 | 0.698823565678929 | -0.566229881 | 0.871261246530531 | 1.16476072887386 | 0.567661561335547 | 2.38992323583601 | 0.999714303421225 |
| ebi-fl187-GCST90094812 | prot-a-2777 | Small integral membrane protein 10 \|\| id:prot-a-2777 | Plum liking \|\| id:ebi-fl187-GCST90094812 | Weighted mode | 5 | 0.0951813323547148 | 0.337586012868298 | 0.791970312623637 | -0.566487253 | 0.75684991757658 | 1.09985827693595 | 0.567515479772094 | 2.13155107210523 | 0.999714303421225 |
| ebi-fl187-GCST90094812 | prot-a-2799 | Superoxide dismutase [Mn], mitochondrial \|\| id:prot-a-2799 | Plum liking \|\| id:ebi-fl187-GCST90094812 | Weighted mode | 5 | 0.303572998188138 | 0.336906832727355 | 0.418504784521829 | -0.356764394 | 0.963910390333754 | 1.35469047728305 | 0.699937387787251 | 2.62192922004503 | 0.999714303421225 |
| ebi-fl187-GCST90094812 | prot-a-2805 | Protein SPACA7 \|\| id:prot-a-2805 | Plum liking \|\| id:ebi-fl187-GCST90094812 | Weighted mode | 5 | -0.3086807 | 0.347095732564909 | 0.424080170774517 | -0.988988335 | 0.37162693620328 | 0.734415231652078 | 0.371952792199563 | 1.45009190358004 | 0.999714303421225 |
| ebi-fl187-GCST90094812 | prot-a-2811 | SPARC-like protein 1 \|\| id:prot-a-2811 | Plum liking \|\| id:ebi-fl187-GCST90094812 | Weighted mode | 5 | 0.160469173517825 | 0.342135639927025 | 0.663475604477592 | -0.510116681 | 0.831055027774794 | 1.17406158039439 | 0.600425516631811 | 2.29573953200833 | 0.999714303421225 |
| ebi-fl187-GCST90094812 | prot-a-2831 | Sepiapterin reductase \|\| id:prot-a-2831 | Plum liking \|\| id:ebi-fl187-GCST90094812 | Weighted mode | 5 | -0.252603976 | 0.346379823272258 | 0.506244631962466 | -0.931508429 | 0.426300477828068 | 0.776775442803454 | 0.393959002605423 | 1.53158091210529 | 0.999714303421225 |
| ebi-fl187-GCST90094812 | prot-a-2834 | SPRY domain-containing SOCS box protein 1 \|\| id:prot-a-2834 | Plum liking \|\| id:ebi-fl187-GCST90094812 | Weighted mode | 5 | 0.400069976743852 | 0.33990163 | 0.304444608116979 | -0.266137217 | 1.06627717072849 | 1.49192909432864 | 0.766333966053858 | 2.90454621757928 | 0.999714303421225 |
| ebi-fl187-GCST90094812 | prot-a-2835 | Spectrin alpha chain, non-erythrocytic 1 \|\| id:prot-a-2835 | Plum liking \|\| id:ebi-fl187-GCST90094812 | Weighted mode | 5 | 0.0992348948310768 | 0.338397948344561 | 0.78391653558065 | -0.564025084 | 0.762494873586417 | 1.10432566948695 | 0.568914520391397 | 2.14361760963456 | 0.999714303421225 |
| ebi-fl187-GCST90094812 | prot-a-2859 | Sia-alpha-2,3-Gal-beta-1,4-GlcNAc-R:alpha 2,8-sialyltransferase \|\| id:prot-a-2859 | Plum liking \|\| id:ebi-fl187-GCST90094812 | Weighted mode | 5 | -0.067723861 | 0.342811530054433 | 0.853026828217127 | -0.73963446 | 0.604186737880164 | 0.934518494928582 | 0.477288351653334 | 1.82976352625907 | 0.999714303421225 |
| ebi-fl187-GCST90094812 | prot-a-2861 | Alpha-2,8-sialyltransferase 8F \|\| id:prot-a-2861 | Plum liking \|\| id:ebi-fl187-GCST90094812 | Weighted mode | 5 | -0.254725136 | 0.311201902355869 | 0.459019174800839 | -0.864680864 | 0.355230592916789 | 0.775129524116598 | 0.421185946645456 | 1.42650955935855 | 0.999714303421225 |
| ebi-fl187-GCST90094812 | prot-a-289 | Uncharacterized protein C17orf89 \|\| id:prot-a-289 | Plum liking \|\| id:ebi-fl187-GCST90094812 | Weighted mode | 5 | -0.217266854 | 0.325958847697775 | 0.541538775922624 | -0.856146196 | 0.421612487122807 | 0.804715198886795 | 0.424796012506897 | 1.52441767872974 | 0.999714303421225 |
| ebi-fl187-GCST90094812 | prot-a-2921 | Tachykinin-3 \|\| id:prot-a-2921 | Plum liking \|\| id:ebi-fl187-GCST90094812 | Weighted mode | 5 | -0.587831571 | 0.381206375190218 | 0.197927563067195 | -1.334996066 | 0.159332924332978 | 0.555530608261409 | 0.263159210613728 | 1.17272831148701 | 0.999714303421225 |
| ebi-fl187-GCST90094812 | prot-a-2928 | Tax1-binding protein 1 \|\| id:prot-a-2928 | Plum liking \|\| id:ebi-fl187-GCST90094812 | Weighted mode | 5 | 0.0185526667770766 | 0.329648274967781 | 0.95781769005509 | -0.627557952 | 0.664663285713927 | 1.01872583676328 | 0.533894005039389 | 1.94383589381657 | 0.999714303421225 |
| ebi-fl187-GCST90094812 | prot-a-296 | Uncharacterized protein C1orf185 \|\| id:prot-a-296 | Plum liking \|\| id:ebi-fl187-GCST90094812 | Weighted mode | 5 | -0.150403754 | 0.344175461847382 | 0.684669536122258 | -0.82498766 | 0.524180150922165 | 0.860360532025453 | 0.438240400528666 | 1.68907349521898 | 0.999714303421225 |
| ebi-fl187-GCST90094812 | prot-a-2964 | Transforming growth factor beta-2 \|\| id:prot-a-2964 | Plum liking \|\| id:ebi-fl187-GCST90094812 | Weighted mode | 5 | -0.361793208 | 0.329597761827794 | 0.333983033422769 | -1.007804821 | 0.284218405197176 | 0.696426368363983 | 0.365019383559302 | 1.32872309909495 | 0.999714303421225 |
| ebi-fl187-GCST90094812 | prot-a-2977 | Thymocyte nuclear protein 1 \|\| id:prot-a-2977 | Plum liking \|\| id:ebi-fl187-GCST90094812 | Weighted mode | 5 | -0.456931414 | 0.325319722400047 | 0.232841706783182 | -1.09455807 | 0.180695241424529 | 0.633223768555179 | 0.334687482581662 | 1.19805000763776 | 0.999714303421225 |
| ebi-fl187-GCST90094812 | prot-a-3015 | Transmembrane protein 70, mitochondrial \|\| id:prot-a-3015 | Plum liking \|\| id:ebi-fl187-GCST90094812 | Weighted mode | 5 | -0.251496693 | 0.348071218492339 | 0.509946227187351 | -0.933716281 | 0.430722895647373 | 0.777636029560775 | 0.393090159144459 | 1.53836920208632 | 0.999714303421225 |
| ebi-fl187-GCST90094812 | prot-a-3030 | Tumor necrosis factor alpha-induced protein 3 \|\| id:prot-a-3030 | Plum liking \|\| id:ebi-fl187-GCST90094812 | Weighted mode | 5 | -0.101774155 | 0.347741951245799 | 0.78432735231846 | -0.78334838 | 0.579800069197103 | 0.903233519188667 | 0.456873660806156 | 1.78568138234629 | 0.999714303421225 |
| ebi-fl187-GCST90094812 | prot-a-3032 | Tumor necrosis factor receptor superfamily member 10A \|\| id:prot-a-3032 | Plum liking \|\| id:ebi-fl187-GCST90094812 | Weighted mode | 5 | 0.168539688624272 | 0.359944199012727 | 0.663989868034296 | -0.536950941 | 0.874030318689217 | 1.18357520038412 | 0.584527797510822 | 2.39655027687263 | 0.999714303421225 |
| ebi-fl187-GCST90094812 | prot-a-3081 | Tumor protein p53-inducible protein 11 \|\| id:prot-a-3081 | Plum liking \|\| id:ebi-fl187-GCST90094812 | Weighted mode | 5 | 0.689358031335354 | 0.372216971847306 | 0.137664861882534 | -0.040187233 | 1.41890329615607 | 1.9924360410854 | 0.960609564036775 | 4.1325857314326 | 0.999714303421225 |
| ebi-fl187-GCST90094812 | prot-a-315 | Complement C4 \|\| id:prot-a-315 | Plum liking \|\| id:ebi-fl187-GCST90094812 | Weighted mode | 5 | -0.230257376 | 0.355536051137617 | 0.552516567379784 | -0.927108037 | 0.466593283774434 | 0.794329134574852 | 0.395696396734904 | 1.59455274104288 | 0.999714303421225 |
| ebi-fl187-GCST90094812 | prot-a-3157 | UDP-glucuronosyltransferase 1-8 \|\| id:prot-a-3157 | Plum liking \|\| id:ebi-fl187-GCST90094812 | Weighted mode | 5 | -0.234464989 | 0.337001129718359 | 0.524897775983742 | -0.894987204 | 0.426057224782987 | 0.790993926527161 | 0.408612827334413 | 1.53120839569434 | 0.999714303421225 |
| ebi-fl187-GCST90094812 | prot-a-317 | Glutaredoxin-like protein C5orf63 \|\| id:prot-a-317 | Plum liking \|\| id:ebi-fl187-GCST90094812 | Weighted mode | 5 | 0.191788720638766 | 0.351218709945407 | 0.614048360994894 | -0.496599951 | 0.880177392131763 | 1.21141454303401 | 0.608596403589367 | 2.4113274189909 | 0.999714303421225 |
| ebi-fl187-GCST90094812 | prot-a-3192 | Guanine nucleotide exchange factor VAV3 \|\| id:prot-a-3192 | Plum liking \|\| id:ebi-fl187-GCST90094812 | Weighted mode | 5 | -0.032626306 | 0.34732510056592 | 0.929677266598354 | -0.713383504 | 0.648130890691819 | 0.967900190104977 | 0.489983528608779 | 1.91196381777407 | 0.999714303421225 |
| ebi-fl187-GCST90094812 | prot-a-3233 | Serine/threonine-protein kinase WNK3 \|\| id:prot-a-3233 | Plum liking \|\| id:ebi-fl187-GCST90094812 | Weighted mode | 5 | 0.389806124243307 | 0.322988277761328 | 0.293970330680477 | -0.2432509 | 1.02286314865551 | 1.47669447087005 | 0.784074764599523 | 2.78114621 | 0.999714303421225 |
| ebi-fl187-GCST90094812 | prot-a-3250 | 14-3-3 protein theta \|\| id:prot-a-3250 | Plum liking \|\| id:ebi-fl187-GCST90094812 | Weighted mode | 5 | 0.189190356977612 | 0.347936844541857 | 0.61550142514146 | -0.492765858 | 0.871146572279652 | 1.20827093339599 | 0.610934297500853 | 2.38964918889265 | 0.999714303421225 |
| ebi-fl187-GCST90094812 | prot-a-3278 | E3 ubiquitin-protein ligase ZNRF3 \|\| id:prot-a-3278 | Plum liking \|\| id:ebi-fl187-GCST90094812 | Weighted mode | 5 | -0.04640586 | 0.350566699634443 | 0.901080393131445 | -0.733516591 | 0.640704871286472 | 0.954654427510503 | 0.480217289390956 | 1.89781812545996 | 0.999714303421225 |
| ebi-fl187-GCST90094812 | prot-a-355 | Calpain-3 \|\| id:prot-a-355 | Plum liking \|\| id:ebi-fl187-GCST90094812 | Weighted mode | 5 | -0.018577812 | 0.345615469137304 | 0.95970962110552 | -0.695984132 | 0.65882850743685 | 0.981593691780797 | 0.498583534660687 | 1.93252706670224 | 0.999714303421225 |
| ebi-fl187-GCST90094812 | prot-a-385 | Coiled-coil domain-containing protein 90B, mitochondrial \|\| id:prot-a-385 | Plum liking \|\| id:ebi-fl187-GCST90094812 | Weighted mode | 5 | -0.094683848 | 0.32985942095558 | 0.788334924597234 | -0.741208313 | 0.5518406174102 | 0.909660480298655 | 0.476537760858151 | 1.73644621137063 | 0.999714303421225 |
| ebi-fl187-GCST90094812 | prot-a-390 | C-C motif chemokine 14 \|\| id:prot-a-390 | Plum liking \|\| id:ebi-fl187-GCST90094812 | Weighted mode | 5 | -0.200191179 | 0.3165228621101 | 0.561429560228726 | -0.820575989 | 0.420193630670921 | 0.818574243859343 | 0.440178043851002 | 1.52225628258893 | 0.999714303421225 |
| ebi-fl187-GCST90094812 | prot-a-40 | Pituitary adenylate cyclase-activating polypeptide 27 \|\| id:prot-a-40 | Plum liking \|\| id:ebi-fl187-GCST90094812 | Weighted mode | 5 | 0.171376436747808 | 0.328400973619314 | 0.629337630463439 | -0.472289472 | 0.815042345041663 | 1.18693747181674 | 0.623572980154825 | 2.25927133926317 | 0.999714303421225 |
| ebi-fl187-GCST90094812 | prot-a-401 | C-C motif chemokine 24 \|\| id:prot-a-401 | Plum liking \|\| id:ebi-fl187-GCST90094812 | Weighted mode | 5 | -0.182587555 | 0.33277664244663 | 0.612412611488484 | -0.834829774 | 0.469654664665262 | 0.833111698031736 | 0.433948344556098 | 1.59944175408093 | 0.999714303421225 |
| ebi-fl187-GCST90094812 | prot-a-412 | Cerebral cavernous malformations 2 protein \|\| id:prot-a-412 | Plum liking \|\| id:ebi-fl187-GCST90094812 | Weighted mode | 5 | 0.420424846259833 | 0.349938604950168 | 0.295856440505753 | -0.265454819 | 1.10630451196216 | 1.52260829266533 | 0.766857089133768 | 3.02316565334486 | 0.999714303421225 |
| ebi-fl187-GCST90094812 | prot-a-44 | Adhesion G protein-coupled receptor F5 \|\| id:prot-a-44 | Plum liking \|\| id:ebi-fl187-GCST90094812 | Weighted mode | 5 | -0.010868518 | 0.349207053085471 | 0.97666214405711 | -0.695314342 | 0.673577306077466 | 0.989190330978134 | 0.498917592570649 | 1.96124074490733 | 0.999714303421225 |
| ebi-fl187-GCST90094812 | prot-a-48 | Adrenomedullin \|\| id:prot-a-48 | Plum liking \|\| id:ebi-fl187-GCST90094812 | Weighted mode | 5 | 0.314966620794879 | 0.331403554262412 | 0.395725969001357 | -0.334584346 | 0.964517587149205 | 1.37021357355373 | 0.715635481607196 | 2.6235217305525 | 0.999714303421225 |
| ebi-fl187-GCST90094812 | prot-a-502 | Carcinoembryonic antigen-related cell adhesion molecule 1 \|\| id:prot-a-502 | Plum liking \|\| id:ebi-fl187-GCST90094812 | Weighted mode | 5 | -0.211010315 | 0.336333981944565 | 0.56444115280821 | -0.870224919 | 0.448204290026409 | 0.809765714440958 | 0.4188573295982 | 1.56549847871372 | 0.999714303421225 |
| ebi-fl187-GCST90094812 | prot-a-518 | Complement factor B \|\| id:prot-a-518 | Plum liking \|\| id:ebi-fl187-GCST90094812 | Weighted mode | 5 | -0.105770029 | 0.33932473831074 | 0.770834004609148 | -0.770846516 | 0.559306457868643 | 0.899631513248987 | 0.462621286045844 | 1.74945875609896 | 0.999714303421225 |
| ebi-fl187-GCST90094812 | prot-a-525 | Properdin \|\| id:prot-a-525 | Plum liking \|\| id:ebi-fl187-GCST90094812 | Weighted mode | 5 | 0.382293447600404 | 0.361954462229364 | 0.350448726405311 | -0.327137298 | 1.09172419356996 | 1.4656421111954 | 0.720984746200876 | 2.97940672036189 | 0.999714303421225 |
| ebi-fl187-GCST90094812 | prot-a-533 | Chondroadherin \|\| id:prot-a-533 | Plum liking \|\| id:ebi-fl187-GCST90094812 | Weighted mode | 5 | 0.300066084157214 | 0.355383133295469 | 0.446016868233752 | -0.396484857 | 0.996617025416334 | 1.34994801480522 | 0.672680462955812 | 2.7091014873079 | 0.999714303421225 |
| ebi-fl187-GCST90094812 | prot-a-535 | Coiled-coil-helix-coiled-coil-helix domain-containing protein 2 \|\| id:prot-a-535 | Plum liking \|\| id:ebi-fl187-GCST90094812 | Weighted mode | 5 | -0.609333266 | 0.353740083776897 | 0.16007254769651 | -1.30266383 | 0.0839972980765831 | 0.543713260300134 | 0.271806780661848 | 1.08762595512282 | 0.999714303421225 |
| ebi-fl187-GCST90094812 | prot-a-591 | Caseinolytic peptidase B protein homolog \|\| id:prot-a-591 | Plum liking \|\| id:ebi-fl187-GCST90094812 | Weighted mode | 5 | 0.297315351480953 | 0.341707189528484 | 0.433351767061345 | -0.37243074 | 0.967061442956781 | 1.34623977122125 | 0.689057374022488 | 2.63020408741565 | 0.999714303421225 |
| ebi-fl187-GCST90094812 | prot-a-605 | Ciliary neurotrophic factor receptor subunit alpha \|\| id:prot-a-605 | Plum liking \|\| id:ebi-fl187-GCST90094812 | Weighted mode | 5 | 0.151046942144793 | 0.330828441985232 | 0.671671664772528 | -0.497376804 | 0.799470688435848 | 1.16305125292073 | 0.608123797066455 | 2.22436323565324 | 0.999714303421225 |
| ebi-fl187-GCST90094812 | prot-a-606 | Ciliary neurotrophic factor receptor subunit alpha \|\| id:prot-a-606 | Plum liking \|\| id:ebi-fl187-GCST90094812 | Weighted mode | 5 | 0.687312581518258 | 0.364242351391016 | 0.132213080914868 | -0.026602427 | 1.40122759024465 | 1.98836477835086 | 0.973748300407567 | 4.06018114756296 | 0.999714303421225 |
| ebi-fl187-GCST90094812 | prot-a-707 | Cancer/testis antigen 55 \|\| id:prot-a-707 | Plum liking \|\| id:ebi-fl187-GCST90094812 | Weighted mode | 5 | -0.029400952 | 0.335681199015387 | 0.934415322151672 | -0.687336102 | 0.628534198559225 | 0.971027051640478 | 0.502913998025621 | 1.87486039107936 | 0.999714303421225 |
| ebi-fl187-GCST90094812 | prot-a-719 | Dipeptidyl peptidase 1 \|\| id:prot-a-719 | Plum liking \|\| id:ebi-fl187-GCST90094812 | Weighted mode | 5 | 0.190157740339725 | 0.340821893119368 | 0.606637151706564 | -0.47785317 | 0.858168650853687 | 1.20944036014467 | 0.620113241417002 | 2.35883688179985 | 0.999714303421225 |
| ebi-fl187-GCST90094812 | prot-a-752 | Cytochrome b5 \|\| id:prot-a-752 | Plum liking \|\| id:ebi-fl187-GCST90094812 | Weighted mode | 5 | 0.115083215996501 | 0.348302700877369 | 0.75767063667492 | -0.567590078 | 0.797756509716145 | 1.12196679927253 | 0.566889954580604 | 2.22055354570738 | 0.999714303421225 |
| ebi-fl187-GCST90094812 | prot-a-755 | Cytoglobin \|\| id:prot-a-755 | Plum liking \|\| id:ebi-fl187-GCST90094812 | Weighted mode | 5 | -0.291967933 | 0.321676593372987 | 0.415402782629109 | -0.922454056 | 0.338518189940081 | 0.74679248293095 | 0.397542252045695 | 1.40286726679324 | 0.999714303421225 |
| ebi-fl187-GCST90094812 | prot-a-807 | Desmin \|\| id:prot-a-807 | Plum liking \|\| id:ebi-fl187-GCST90094812 | Weighted mode | 5 | 0.269876068893647 | 0.351836546161649 | 0.485821233340389 | -0.419723562 | 0.959475699370479 | 1.30980211544899 | 0.657228477905181 | 2.61032751822138 | 0.999714303421225 |
| ebi-fl187-GCST90094812 | prot-a-82 | Ameloblastin \|\| id:prot-a-82 | Plum liking \|\| id:ebi-fl187-GCST90094812 | Weighted mode | 5 | -0.509099657 | 0.326444547 | 0.193882288956209 | -1.14893097 | 0.130731655137283 | 0.601036473992374 | 0.316975444568034 | 1.13966191785449 | 0.999714303421225 |
| ebi-fl187-GCST90094812 | prot-a-84 | Muellerian-inhibiting factor \|\| id:prot-a-84 | Plum liking \|\| id:ebi-fl187-GCST90094812 | Weighted mode | 5 | 0.476192082566439 | 0.377865814996864 | 0.276099446160194 | -0.264424915 | 1.21680907996029 | 1.60993222617434 | 0.767647285632639 | 3.37639671420008 | 0.999714303421225 |
| ebi-fl187-GCST90094812 | prot-a-858 | Inactive dipeptidyl peptidase 10 \|\| id:prot-a-858 | Plum liking \|\| id:ebi-fl187-GCST90094812 | Weighted mode | 5 | -0.114644305 | 0.399834310567596 | 0.78855837615044 | -0.898319553 | 0.66903094415642 | 0.891683255177268 | 0.407253452774385 | 1.95234447282638 | 0.999714303421225 |
| ebi-fl187-GCST90094812 | prot-a-863 | Dihydropyrimidinase-related protein 3 \|\| id:prot-a-863 | Plum liking \|\| id:ebi-fl187-GCST90094812 | Weighted mode | 5 | -0.058848679 | 0.357992735240157 | 0.877400348831539 | -0.76051444 | 0.642817082330459 | 0.942849431526638 | 0.467425902654565 | 1.90183095434286 | 0.999714303421225 |
| ebi-fl187-GCST90094812 | prot-a-864 | Dihydropyrimidinase-related protein 5 \|\| id:prot-a-864 | Plum liking \|\| id:ebi-fl187-GCST90094812 | Weighted mode | 5 | -0.008208553 | 0.340009946164852 | 0.981895625280026 | -0.674628048 | 0.658210941278684 | 0.991825044974765 | 0.509345837740487 | 1.93133397183153 | 0.999714303421225 |
| ebi-fl187-GCST90094812 | prot-a-884 | Endothelin-converting enzyme 1 \|\| id:prot-a-884 | Plum liking \|\| id:ebi-fl187-GCST90094812 | Weighted mode | 5 | 0.190796439655164 | 0.33647517783969 | 0.600991311919394 | -0.468694909 | 0.850287788220956 | 1.21021307561491 | 0.625818485677046 | 2.34032027162765 | 0.999714303421225 |
| ebi-fl187-GCST90094812 | prot-a-893 | Early endosome antigen 1 \|\| id:prot-a-893 | Plum liking \|\| id:ebi-fl187-GCST90094812 | Weighted mode | 5 | 0.239686604634124 | 0.310941246909472 | 0.483806082806701 | -0.369758239 | 0.849131448576688 | 1.27085080915122 | 0.690901343234541 | 2.3376156305605 | 0.999714303421225 |
| ebi-fl187-GCST90094812 | prot-a-897 | Ephrin-A2 \|\| id:prot-a-897 | Plum liking \|\| id:ebi-fl187-GCST90094812 | Weighted mode | 5 | -0.204155046 | 0.379372395798915 | 0.61902825876151 | -0.947724942 | 0.53941484969135 | 0.815335946748779 | 0.387621883399432 | 1.71500303396364 | 0.999714303421225 |
| ebi-fl187-GCST90094812 | prot-a-90 | Anaphase-promoting complex subunit 10 \|\| id:prot-a-90 | Plum liking \|\| id:ebi-fl187-GCST90094812 | Weighted mode | 5 | -0.523357576 | 0.355272345428625 | 0.214714412145496 | -1.219691373 | 0.172976220671553 | 0.592527747190237 | 0.295321296865932 | 1.18883783498256 | 0.999714303421225 |
| ebi-fl187-GCST90094812 | prot-a-914 | Histone-lysine N-methyltransferase EHMT2 \|\| id:prot-a-914 | Plum liking \|\| id:ebi-fl187-GCST90094812 | Weighted mode | 5 | -0.762540851 | 0.350318482349171 | 0.0950938997291725 | -1.449165077 | -0.075916626 | 0.466479664437354 | 0.23476621804571 | 0.926893482141531 | 0.999714303421225 |
| ebi-fl187-GCST90094812 | prot-a-932 | RNA polymerase II elongation factor ELL \|\| id:prot-a-932 | Plum liking \|\| id:ebi-fl187-GCST90094812 | Weighted mode | 5 | 0.0391769019145309 | 0.339680266524773 | 0.913737901751169 | -0.62659642 | 0.704950224303087 | 1.03995443730979 | 0.53440760792522 | 2.02374594905028 | 0.999714303421225 |
| ebi-fl187-GCST90094812 | prot-a-991 | Estrogen receptor \|\| id:prot-a-991 | Plum liking \|\| id:ebi-fl187-GCST90094812 | Weighted mode | 5 | 0.213824683039366 | 0.354532819153802 | 0.578958673911509 | -0.481059643 | 0.908709008580818 | 1.2384055222683 | 0.618128049899987 | 2.48111736368019 | 0.999714303421225 |
| ebi-fl187-GCST90094812 | prot-a-992 | Persulfide dioxygenase ETHE1, mitochondrial \|\| id:prot-a-992 | Plum liking \|\| id:ebi-fl187-GCST90094812 | Weighted mode | 5 | 0.72208622171917 | 0.370638666325323 | 0.123208464284143 | -0.004365564 | 1.4485380077168 | 2.05872368771562 | 0.995643950945795 | 4.25688642846195 | 0.999714303421225 |
| ebi-fl187-GCST90094812 | prot-a-993 | Ethanolamine kinase 1 \|\| id:prot-a-993 | Plum liking \|\| id:ebi-fl187-GCST90094812 | Weighted mode | 5 | 0.0996828367764675 | 0.358128686334369 | 0.794544635997166 | -0.602249388 | 0.801615061991831 | 1.1048204540848 | 0.5475785329286 | 2.22913821919915 | 0.999714303421225 |
| ebi-fl187-GCST90094812 | prot-b-10 | coagulation factor III, tissue factor \|\| id:prot-b-10 | Plum liking \|\| id:ebi-fl187-GCST90094812 | Weighted mode | 4 | -0.310653827 | 0.347339005949978 | 0.43701823066173 | -0.991438279 | 0.370130624264782 | 0.732967565246491 | 0.371042644194735 | 1.44792373628463 | 0.999714303421225 |
| ebi-fl187-GCST90094812 | prot-b-28 | prolactin \|\| id:prot-b-28 | Plum liking \|\| id:ebi-fl187-GCST90094812 | Weighted mode | 4 | 0.352396978737699 | 0.340548048913471 | 0.376863937703078 | -0.315077197 | 1.0198711546081 | 1.42247310322506 | 0.729732538834979 | 2.77283747361623 | 0.999714303421225 |
| ebi-fl187-GCST90094812 | prot-b-54 | matrix metallopeptidase 7 \|\| id:prot-b-54 | Plum liking \|\| id:ebi-fl187-GCST90094812 | Weighted mode | 4 | 0.134586384307293 | 0.363762097480846 | 0.735970229100383 | -0.578387327 | 0.847560095369752 | 1.14406348386543 | 0.560802028140564 | 2.33394529519468 | 0.999714303421225 |
| ebi-fl187-GCST90094812 | ukb-rsfMRI191-edge-pheno716 | Net100_Pair10_33 (OccipitalPrecuneus)&(Temporal\|Frontal\|Supp_Motor_Area) (Default_mode\|Central_executive)&(Default_mode\|Salience) \|\| id:ukb-rsfMRI191-edge-pheno716 | Plum liking \|\| id:ebi-fl187-GCST90094812 | Weighted mode | 5 | 0.0229291761967164 | 0.111873428639631 | 0.847613279108817 | -0.196342744 | 0.242201096330394 | 1.02319407048541 | 0.821730543245416 | 1.27405037391172 | 0.999714303421225 |
| ebi-fl187-GCST90094812 | ukb-rsfMRI191-edge-pheno903 | Net100_Pair27_38 (Precentral\|Frontal\|Supp_Motor_Area)&(Subcortical) (Attention\|Salience\|Motor)&(Subcortical-cerebellum) \|\| id:ukb-rsfMRI191-edge-pheno903 | Plum liking \|\| id:ebi-fl187-GCST90094812 | Weighted mode | 5 | -0.16012335 | 0.10759171429987 | 0.210908162293558 | -0.37100311 | 0.0507564099074298 | 0.852038683409841 | 0.6900417954233 | 1.05206658907007 | 0.999714303421225 |
| ebi-fl187-GCST90094812 | ukb-rsfMRI191-node-pheno23 | Net100_Node2 RolandicOper\|SupraMarginal\|Insula Salience\|Motor \|\| id:ukb-rsfMRI191-node-pheno23 | Plum liking \|\| id:ebi-fl187-GCST90094812 | Weighted mode | 5 | -0.057404615 | 0.0917672150484453 | 0.565531624238446 | -0.237268356 | 0.122459126527643 | 0.944211949778891 | 0.78877958551394 | 1.13027292095087 | 0.999714303421225 |
| ebi-fl187-GCST90094812 | ukb-rsfMRI191-node-pheno55 | Net100_Node34 Parietal\|Postcentral\|Precuneus Attention \|\| id:ukb-rsfMRI191-node-pheno55 | Plum liking \|\| id:ebi-fl187-GCST90094812 | Weighted mode | 5 | -0.16832738 | 0.105430308223351 | 0.185596517153952 | -0.374970784 | 0.0383160241868923 | 0.845077128101256 | 0.687309358894437 | 1.03905954894694 | 0.999714303421225 |
| ebi-fl187-GCST90094817 | ebi-a-GCST90001414 | IgD- CD24- B cell Absolute Count \|\| id:ebi-a-GCST90001414 | Prawns liking \|\| id:ebi-fl187-GCST90094817 | Inverse variance weighted | 16 | -0.091370276 | 0.115324556613101 | 0.428192695394641 | -0.317406407 | 0.134665854470044 | 0.912679704486251 | 0.728034816218306 | 1.14415440638945 | 0.998735809224318 |
| ebi-fl187-GCST90094817 | ebi-a-GCST90001422 | CD20- CD38- B cell %B cell \|\| id:ebi-a-GCST90001422 | Prawns liking \|\| id:ebi-fl187-GCST90094817 | Inverse variance weighted | 16 | -0.066530042 | 0.131886214133274 | 0.613944971209821 | -0.325027021 | 0.191966937971593 | 0.935634807346209 | 0.722507830182932 | 1.21163045734208 | 0.998735809224318 |
| ebi-fl187-GCST90094817 | ebi-a-GCST90001444 | CD20- CD38- B cell %lymphocyte \|\| id:ebi-a-GCST90001444 | Prawns liking \|\| id:ebi-fl187-GCST90094817 | Inverse variance weighted | 16 | -0.107851003 | 0.129762551364284 | 0.405893866071213 | -0.362185604 | 0.146483597715158 | 0.897761350250582 | 0.696153147297093 | 1.157755941 | 0.998735809224318 |
| ebi-fl187-GCST90094817 | ebi-a-GCST90001459 | Myeloid Dendritic Cell %Dendritic Cell \|\| id:ebi-a-GCST90001459 | Prawns liking \|\| id:ebi-fl187-GCST90094817 | Inverse variance weighted | 16 | 0.154367375916627 | 0.117948534396263 | 0.19061263804986 | -0.076811752 | 0.385546503333303 | 1.16691950616461 | 0.926064167389196 | 1.4704176900683 | 0.998735809224318 |
| ebi-fl187-GCST90094817 | ebi-a-GCST90001476 | HLA DR++ monocyte %leukocyte \|\| id:ebi-a-GCST90001476 | Prawns liking \|\| id:ebi-fl187-GCST90094817 | Inverse variance weighted | 16 | 0.00602954040112703 | 0.120957022015344 | 0.960243023145382 | -0.231046223 | 0.243105303551202 | 1.00604775466934 | 0.79370277806309 | 1.27520290044237 | 0.998735809224318 |
| ebi-fl187-GCST90094817 | ebi-a-GCST90001478 | CD4 regulatory T cell %CD4+ T cell \|\| id:ebi-a-GCST90001478 | Prawns liking \|\| id:ebi-fl187-GCST90094817 | Inverse variance weighted | 16 | -0.042140636 | 0.122848770697519 | 0.731576979088868 | -0.282924227 | 0.198642954409411 | 0.958734938292858 | 0.75357688668518 | 1.21974638307532 | 0.998735809224318 |
| ebi-fl187-GCST90094817 | ebi-a-GCST90001485 | CD39+ resting CD4 regulatory T cell %CD4 regulatory T cell \|\| id:ebi-a-GCST90001485 | Prawns liking \|\| id:ebi-fl187-GCST90094817 | Inverse variance weighted | 16 | 0.088550091807551 | 0.113790018860979 | 0.436457855402146 | -0.134478345 | 0.31157852877507 | 1.09258898099671 | 0.874171808722288 | 1.36557901946101 | 0.998735809224318 |
| ebi-fl187-GCST90094817 | ebi-a-GCST90001515 | Immature Myeloid-Derived Suppressor Cells Absolute Count \|\| id:ebi-a-GCST90001515 | Prawns liking \|\| id:ebi-fl187-GCST90094817 | Inverse variance weighted | 16 | -0.237635745 | 0.152964499300935 | 0.120295308814688 | -0.537446164 | 0.0621746732052534 | 0.788489849826627 | 0.584238397791694 | 1.06414820667313 | 0.998735809224318 |
| ebi-fl187-GCST90094817 | ebi-a-GCST90001517 | CD33+ HLA DR+ Absolute Count \|\| id:ebi-a-GCST90001517 | Prawns liking \|\| id:ebi-fl187-GCST90094817 | Inverse variance weighted | 16 | 0.0763075701662541 | 0.149664789652957 | 0.610151978922983 | -0.217035418 | 0.369650557886049 | 1.07929448189544 | 0.804901461159564 | 1.44722880360013 | 0.998735809224318 |
| ebi-fl187-GCST90094817 | ebi-a-GCST90001518 | CD33+ HLA DR+ CD14- Absolute Count \|\| id:ebi-a-GCST90001518 | Prawns liking \|\| id:ebi-fl187-GCST90094817 | Inverse variance weighted | 16 | -0.012293142 | 0.151557447796793 | 0.935352805069787 | -0.30934574 | 0.28475945521695 | 0.987782109534248 | 0.733926978134843 | 1.32944219927103 | 0.998735809224318 |
| ebi-fl187-GCST90094817 | ebi-a-GCST90001520 | CD33+ HLA DR+ CD14dim Absolute Count \|\| id:ebi-a-GCST90001520 | Prawns liking \|\| id:ebi-fl187-GCST90094817 | Inverse variance weighted | 16 | 0.034690817836653 | 0.15022716336178 | 0.817375084544215 | -0.259754422 | 0.329136058025741 | 1.03529956315186 | 0.771240962090499 | 1.38976693166962 | 0.998735809224318 |
| ebi-fl187-GCST90094817 | ebi-a-GCST90001531 | CD33dim HLA DR- Absolute Count \|\| id:ebi-a-GCST90001531 | Prawns liking \|\| id:ebi-fl187-GCST90094817 | Inverse variance weighted | 16 | -0.109671582 | 0.152619443649199 | 0.472390444475104 | -0.408805691 | 0.189462527929085 | 0.896128392006004 | 0.664443326981324 | 1.20859983440219 | 0.998735809224318 |
| ebi-fl187-GCST90094817 | ebi-a-GCST90001532 | Basophil Absolute Count \|\| id:ebi-a-GCST90001532 | Prawns liking \|\| id:ebi-fl187-GCST90094817 | Inverse variance weighted | 16 | -0.114281739 | 0.152146478443052 | 0.452574601871133 | -0.412488837 | 0.183925358718216 | 0.892006607400262 | 0.662000586708737 | 1.20192610644287 | 0.998735809224318 |
| ebi-fl187-GCST90094817 | ebi-a-GCST90001588 | T/B cell \|\| id:ebi-a-GCST90001588 | Prawns liking \|\| id:ebi-fl187-GCST90094817 | Inverse variance weighted | 16 | 0.0669864064790349 | 0.11346513577693 | 0.554942652665292 | -0.15540526 | 0.289378072601817 | 1.06928094278289 | 0.856068177291223 | 1.33559658556227 | 0.998735809224318 |
| ebi-fl187-GCST90094817 | ebi-a-GCST90001624 | HLA DR+ CD4+ T cell Absolute Count \|\| id:ebi-a-GCST90001624 | Prawns liking \|\| id:ebi-fl187-GCST90094817 | Inverse variance weighted | 16 | 0.280974700060837 | 0.110255084870057 | 0.010821650198128 | 0.0648747337155249 | 0.49707466640615 | 1.32442009576353 | 1.06702535371065 | 1.64390525863544 | 0.998735809224318 |
| ebi-fl187-GCST90094817 | ebi-a-GCST90001631 | CD8+ Natural Killer T %T cell \|\| id:ebi-a-GCST90001631 | Prawns liking \|\| id:ebi-fl187-GCST90094817 | Inverse variance weighted | 16 | -0.069722469 | 0.111888992603992 | 0.533192746591918 | -0.289024895 | 0.149579956208893 | 0.932652623731531 | 0.748993559126649 | 1.16134632394913 | 0.998735809224318 |
| ebi-fl187-GCST90094817 | ebi-a-GCST90001632 | CD8+ Natural Killer T %lymphocyte \|\| id:ebi-a-GCST90001632 | Prawns liking \|\| id:ebi-fl187-GCST90094817 | Inverse variance weighted | 16 | -0.054949859 | 0.112563356108729 | 0.625430658283088 | -0.275574037 | 0.165674319289964 | 0.946532607155009 | 0.759136226105601 | 1.18018867444093 | 0.998735809224318 |
| ebi-fl187-GCST90094817 | ebi-a-GCST90001646 | Natural Killer %CD3- lymphocyte \|\| id:ebi-a-GCST90001646 | Prawns liking \|\| id:ebi-fl187-GCST90094817 | Inverse variance weighted | 16 | -0.060587034 | 0.109501014130571 | 0.580057235187997 | -0.275209021 | 0.154034954084865 | 0.941211848387601 | 0.759413373058694 | 1.1665316611125 | 0.998735809224318 |
| ebi-fl187-GCST90094817 | ebi-a-GCST90001684 | CD127- CD8+ T cell Absolute Count \|\| id:ebi-a-GCST90001684 | Prawns liking \|\| id:ebi-fl187-GCST90094817 | Inverse variance weighted | 16 | 0.124053995473356 | 0.118755542554819 | 0.296200265121919 | -0.108706868 | 0.356814858880801 | 1.13207699638221 | 0.896993316468259 | 1.42877132104374 | 0.998735809224318 |
| ebi-fl187-GCST90094817 | ebi-a-GCST90001687 | CD28- CD8+ T cell Absolute Count \|\| id:ebi-a-GCST90001687 | Prawns liking \|\| id:ebi-fl187-GCST90094817 | Inverse variance weighted | 16 | 0.114362415245103 | 0.115597438050505 | 0.322508472651601 | -0.112208563 | 0.340933393824093 | 1.12115837615157 | 0.893857812092884 | 1.40625957217031 | 0.998735809224318 |
| ebi-fl187-GCST90094817 | ebi-a-GCST90001701 | BAFF-R on CD20- CD38- B cell \|\| id:ebi-a-GCST90001701 | Prawns liking \|\| id:ebi-fl187-GCST90094817 | Inverse variance weighted | 16 | -0.093723652 | 0.124671445697736 | 0.452192284225243 | -0.338079686 | 0.150632381554571 | 0.910534351811254 | 0.713138458781977 | 1.16256919763432 | 0.998735809224318 |
| ebi-fl187-GCST90094817 | ebi-a-GCST90001732 | CD19 on IgD- CD27- B cell \|\| id:ebi-a-GCST90001732 | Prawns liking \|\| id:ebi-fl187-GCST90094817 | Inverse variance weighted (multiplicative random effects) | 16 | 0.00749526891588382 | 0.162286804971399 | 0.96316253852901 | -0.310586869 | 0.325577406659826 | 1.00752342875517 | 0.733016645349017 | 1.38483002525439 | 0.998735809224318 |
| ebi-fl187-GCST90094817 | ebi-a-GCST90001798 | CD27 on CD24+ CD27+ B cell \|\| id:ebi-a-GCST90001798 | Prawns liking \|\| id:ebi-fl187-GCST90094817 | Inverse variance weighted (multiplicative random effects) | 16 | -0.022348667 | 0.160625835547975 | 0.889343511553691 | -0.337175305 | 0.292477970248165 | 0.977899214001497 | 0.713783699011463 | 1.33974322202809 | 0.998735809224318 |
| ebi-fl187-GCST90094817 | ebi-a-GCST90001836 | CD66b on CD66b++ myeloid cell \|\| id:ebi-a-GCST90001836 | Prawns liking \|\| id:ebi-fl187-GCST90094817 | Inverse variance weighted (multiplicative random effects) | 16 | 0.00165950122187155 | 0.235419173385895 | 0.9943756516563 | -0.459762079 | 0.463081081058225 | 1.00166087895604 | 0.631433859255343 | 1.58896217192757 | 0.999107451094609 |
| ebi-fl187-GCST90094817 | ebi-a-GCST90001943 | CD25 on activated & secreting CD4 regulatory T cell \|\| id:ebi-a-GCST90001943 | Prawns liking \|\| id:ebi-fl187-GCST90094817 | Inverse variance weighted | 16 | 0.0187203266129952 | 0.126584992498713 | 0.882431624765106 | -0.229386259 | 0.266826911910474 | 1.01889665048882 | 0.795021390274199 | 1.30581440584798 | 0.998735809224318 |
| ebi-fl187-GCST90094817 | ebi-a-GCST90001960 | CD25 on CD4+ T cell \|\| id:ebi-a-GCST90001960 | Prawns liking \|\| id:ebi-fl187-GCST90094817 | Inverse variance weighted | 16 | -0.154974194 | 0.133958658828928 | 0.24732108259506 | -0.417533166 | 0.107584776978387 | 0.856437278139385 | 0.658669646291329 | 1.11358526313869 | 0.998735809224318 |
| ebi-fl187-GCST90094817 | ebi-a-GCST90001988 | HLA DR on CD14+ CD16- monocyte \|\| id:ebi-a-GCST90001988 | Prawns liking \|\| id:ebi-fl187-GCST90094817 | Inverse variance weighted | 16 | 0.0312966573810279 | 0.114597702608885 | 0.78477618038788 | -0.19331484 | 0.255908154494443 | 1.03179154706741 | 0.824222435313723 | 1.29163409170554 | 0.998735809224318 |
| ebi-fl187-GCST90094817 | ebi-a-GCST90001991 | HLA DR on CD14+ monocyte \|\| id:ebi-a-GCST90001991 | Prawns liking \|\| id:ebi-fl187-GCST90094817 | Inverse variance weighted | 16 | 0.0195363994886089 | 0.114799968986442 | 0.864870355798238 | -0.20547154 | 0.244544338702035 | 1.01972848378047 | 0.814263268394075 | 1.27703928323334 | 0.998735809224318 |
| ebi-fl187-GCST90094817 | ebi-a-GCST90002019 | CD14 on Monocytic Myeloid-Derived Suppressor Cells \|\| id:ebi-a-GCST90002019 | Prawns liking \|\| id:ebi-fl187-GCST90094817 | Inverse variance weighted | 16 | -0.205202156 | 0.164744120285073 | 0.212918239994754 | -0.528100631 | 0.117696320132765 | 0.814482647518146 | 0.58972400988306 | 1.12490244926557 | 0.998735809224318 |
| ebi-fl187-GCST90094817 | ebi-a-GCST90002037 | CD80 on plasmacytoid Dendritic Cell \|\| id:ebi-a-GCST90002037 | Prawns liking \|\| id:ebi-fl187-GCST90094817 | Inverse variance weighted | 16 | -0.129161862 | 0.127733306421573 | 0.311928422984187 | -0.379519143 | 0.121195418417548 | 0.878831704426915 | 0.684190328000692 | 1.12884548801329 | 0.998735809224318 |
| ebi-fl187-GCST90094817 | ebi-a-GCST90002042 | CD45 on CD33+ HLA DR+ CD14- \|\| id:ebi-a-GCST90002042 | Prawns liking \|\| id:ebi-fl187-GCST90094817 | Inverse variance weighted | 16 | -0.026474896 | 0.167760915345088 | 0.874603955340049 | -0.35528629 | 0.302336498341443 | 0.973872491881839 | 0.700972733127822 | 1.35301643790358 | 0.998735809224318 |
| ebi-fl187-GCST90094817 | ebi-a-GCST90002056 | CD8 on Effector Memory CD8+ T cell \|\| id:ebi-a-GCST90002056 | Prawns liking \|\| id:ebi-fl187-GCST90094817 | Inverse variance weighted | 16 | -0.119981152 | 0.125191406822179 | 0.337870323410927 | -0.36535631 | 0.125394005010207 | 0.886937153230669 | 0.693949345916038 | 1.13359500720115 | 0.998735809224318 |
| ebi-fl187-GCST90094817 | ebi-a-GCST90002058 | CD8 on CD8+ T cell \|\| id:ebi-a-GCST90002058 | Prawns liking \|\| id:ebi-fl187-GCST90094817 | Inverse variance weighted | 16 | -0.028124137 | 0.12910379506008 | 0.827552770965828 | -0.281167576 | 0.224919300844164 | 0.972267664458037 | 0.754901821610025 | 1.25222165888351 | 0.998735809224318 |
| ebi-fl187-GCST90094817 | ebi-a-GCST90002063 | CD4 on CD4 regulatory T cell \|\| id:ebi-a-GCST90002063 | Prawns liking \|\| id:ebi-fl187-GCST90094817 | Inverse variance weighted | 16 | -0.117723967 | 0.124573053300917 | 0.344648912084373 | -0.361887151 | 0.126439217808375 | 0.88894139621295 | 0.696360946953033 | 1.13478047463556 | 0.998735809224318 |
| ebi-fl187-GCST90094817 | ebi-a-GCST90002066 | CD4 on activated CD4 regulatory T cell \|\| id:ebi-a-GCST90002066 | Prawns liking \|\| id:ebi-fl187-GCST90094817 | Inverse variance weighted | 16 | -0.075931832 | 0.124580896754709 | 0.542194265313713 | -0.32011039 | 0.168246725280672 | 0.926879387587661 | 0.726068881907341 | 1.18322850702272 | 0.998735809224318 |
| ebi-fl187-GCST90094817 | ebi-a-GCST90002070 | CD4 on activated & secreting CD4 regulatory T cell \|\| id:ebi-a-GCST90002070 | Prawns liking \|\| id:ebi-fl187-GCST90094817 | Inverse variance weighted | 16 | -0.096637197 | 0.125171516792562 | 0.440091782889291 | -0.34197337 | 0.148698976166892 | 0.907885330141303 | 0.710367121775304 | 1.16032365155901 | 0.998735809224318 |
| ebi-fl187-GCST90094817 | ebi-a-GCST90002081 | SSC-A on CD4+ T cell \|\| id:ebi-a-GCST90002081 | Prawns liking \|\| id:ebi-fl187-GCST90094817 | Inverse variance weighted | 16 | 0.0353557296600098 | 0.118094384383089 | 0.764646032024865 | -0.196109264 | 0.266820723050864 | 1.03598817497982 | 0.82192242346127 | 1.30580632437095 | 0.998735809224318 |
| ebi-fl187-GCST90094817 | ebi-a-GCST90002094 | CD11b on Monocytic Myeloid-Derived Suppressor Cells \|\| id:ebi-a-GCST90002094 | Prawns liking \|\| id:ebi-fl187-GCST90094817 | Inverse variance weighted | 16 | -0.203821446 | 0.166770225212161 | 0.22164361901915 | -0.530691087 | 0.123048195619432 | 0.81560798842144 | 0.588198332837694 | 1.1309389259361 | 0.998735809224318 |
| ebi-fl187-GCST90094817 | ebi-a-GCST90002104 | HLA DR on myeloid Dendritic Cell \|\| id:ebi-a-GCST90002104 | Prawns liking \|\| id:ebi-fl187-GCST90094817 | Inverse variance weighted | 16 | -0.006990104 | 0.148066463168005 | 0.962346471033255 | -0.297200372 | 0.283220164054109 | 0.993034270194861 | 0.742895142385866 | 1.32739737483603 | 0.998735809224318 |
| ebi-fl187-GCST90094817 | ebi-a-GCST90002105 | HLA DR on plasmacytoid Dendritic Cell \|\| id:ebi-a-GCST90002105 | Prawns liking \|\| id:ebi-fl187-GCST90094817 | Inverse variance weighted | 15 | 0.12817310278858 | 0.132179600260998 | 0.33220154117823 | -0.130898914 | 0.387245119300136 | 1.13674976022034 | 0.877306453552043 | 1.47291748753145 | 0.998735809224318 |
| ebi-fl187-GCST90094817 | ebi-a-GCST90002106 | HLA DR on Dendritic Cell \|\| id:ebi-a-GCST90002106 | Prawns liking \|\| id:ebi-fl187-GCST90094817 | Inverse variance weighted | 16 | 0.0915879614612148 | 0.126501663012142 | 0.469062059136003 | -0.156355298 | 0.339531220965014 | 1.09591317057685 | 0.855255265860454 | 1.40428913493504 | 0.998735809224318 |
| ebi-fl187-GCST90094817 | ebi-a-GCST90002111 | HLA DR on CD33dim HLA DR+ CD11b- \|\| id:ebi-a-GCST90002111 | Prawns liking \|\| id:ebi-fl187-GCST90094817 | Inverse variance weighted | 16 | 0.141197440931783 | 0.18044749725502 | 0.433929659052449 | -0.212479654 | 0.494874535551622 | 1.15165200878455 | 0.808576767709236 | 1.64029242776169 | 0.998735809224318 |
| ebi-fl187-GCST90094817 | ebi-a-GCST90002112 | HLA DR on CD33- HLA DR+ \|\| id:ebi-a-GCST90002112 | Prawns liking \|\| id:ebi-fl187-GCST90094817 | Inverse variance weighted | 16 | 0.250485337844819 | 0.174300002156342 | 0.150691529467624 | -0.091142666 | 0.59211334207125 | 1.28464875406854 | 0.912887463257305 | 1.80780489135133 | 0.998735809224318 |
| ebi-fl187-GCST90094817 | ebi-a-GCST90002115 | HLA DR on HLA DR+ CD8+ T cell \|\| id:ebi-a-GCST90002115 | Prawns liking \|\| id:ebi-fl187-GCST90094817 | Inverse variance weighted | 16 | -0.052651142 | 0.119574615209097 | 0.659705067560947 | -0.287017388 | 0.181715103396591 | 0.948710919761186 | 0.750498678885169 | 1.199272477 | 0.998735809224318 |
| ebi-fl187-GCST90094817 | ebi-a-GCST90002116 | HLA DR on B cell \|\| id:ebi-a-GCST90002116 | Prawns liking \|\| id:ebi-fl187-GCST90094817 | Inverse variance weighted (multiplicative random effects) | 16 | 0.0731448618235573 | 0.168707814978308 | 0.664608410710866 | -0.257522456 | 0.403812179181041 | 1.07588638048956 | 0.772964268793236 | 1.49752265461129 | 0.998735809224318 |
| ebi-fl187-GCST90094817 | ebi-cfmet338-GCST90026023 | Sphingomyelin (d18:1/24:1, d18:2/24:0) levels \|\| id:ebi-cfmet338-GCST90026023 | Prawns liking \|\| id:ebi-fl187-GCST90094817 | Inverse variance weighted | 13 | -0.089080985 | 0.0710079089401567 | 0.209652329255568 | -0.228256486 | 0.0500945165681437 | 0.914771487847199 | 0.795920090912982 | 1.05137046360809 | 0.998735809224318 |
| ebi-fl187-GCST90094817 | ebi-cfmet338-GCST90026046 | 1-arachidonoyl-gpc (20:4n6) levels \|\| id:ebi-cfmet338-GCST90026046 | Prawns liking \|\| id:ebi-fl187-GCST90094817 | Inverse variance weighted | 13 | 0.0338158497628863 | 0.0853149342019934 | 0.69183581787989 | -0.133401421 | 0.201033120798793 | 1.03439410526846 | 0.875113732323376 | 1.222665267 | 0.998735809224318 |
| ebi-fl187-GCST90094817 | ebi-cfmet338-GCST90026047 | 1-linoleoyl-gpc (18:2) levels \|\| id:ebi-cfmet338-GCST90026047 | Prawns liking \|\| id:ebi-fl187-GCST90094817 | Inverse variance weighted | 13 | -0.001886982 | 0.102860870627987 | 0.98536363503034 | -0.203494288 | 0.19972032457691 | 0.998114797377013 | 0.81587486435423 | 1.22106120959061 | 0.998955228396052 |
| ebi-fl187-GCST90094817 | ebi-cfmet338-GCST90026050 | 1-myristoyl-2-palmitoyl-gpc (14:0/16:0) levels \|\| id:ebi-cfmet338-GCST90026050 | Prawns liking \|\| id:ebi-fl187-GCST90094817 | Inverse variance weighted | 13 | -0.003273443 | 0.0631500559749466 | 0.958659406581388 | -0.127047553 | 0.120500666289058 | 0.996731908452802 | 0.880691791947708 | 1.12806149258054 | 0.998735809224318 |
| ebi-fl187-GCST90094817 | ebi-cfmet338-GCST90026068 | 2,3-dihydroxyisovalerate levels \|\| id:ebi-cfmet338-GCST90026068 | Prawns liking \|\| id:ebi-fl187-GCST90094817 | Inverse variance weighted | 13 | -0.032779516 | 0.17626108758209 | 0.852467212192471 | -0.378251248 | 0.312692215527081 | 0.967751909750602 | 0.68505835963964 | 1.36710069390086 | 0.998735809224318 |
| ebi-fl187-GCST90094817 | ebi-cfmet338-GCST90026082 | 3-hydroxyhexanoate levels \|\| id:ebi-cfmet338-GCST90026082 | Prawns liking \|\| id:ebi-fl187-GCST90094817 | Inverse variance weighted | 13 | 0.00419943290468704 | 0.0375853658597326 | 0.911037070247392 | -0.069467884 | 0.077866749989763 | 1.00420826287902 | 0.932890093433415 | 1.08097860866229 | 0.998735809224318 |
| ebi-fl187-GCST90094817 | ebi-cfmet338-GCST90026094 | 3-ureidopropionate levels \|\| id:ebi-cfmet338-GCST90026094 | Prawns liking \|\| id:ebi-fl187-GCST90094817 | Inverse variance weighted | 13 | 0.115585314636816 | 0.0856898812454696 | 0.177375709553217 | -0.052366853 | 0.283537481877936 | 1.12253027872622 | 0.948980666948671 | 1.32781864851766 | 0.998735809224318 |
| ebi-fl187-GCST90094817 | ebi-cfmet338-GCST90026173 | Glycerol levels \|\| id:ebi-cfmet338-GCST90026173 | Prawns liking \|\| id:ebi-fl187-GCST90094817 | Inverse variance weighted | 13 | -0.058881133 | 0.0596407968813416 | 0.323513881064853 | -0.175777095 | 0.0580148289213219 | 0.942818832574771 | 0.838804936437106 | 1.0597307102571 | 0.998735809224318 |
| ebi-fl187-GCST90094817 | ebi-cfmet338-GCST90026179 | Histidine levels \|\| id:ebi-cfmet338-GCST90026179 | Prawns liking \|\| id:ebi-fl187-GCST90094817 | Inverse variance weighted | 13 | 0.0512515413721421 | 0.0380626056211502 | 0.178139263684648 | -0.023351166 | 0.125854248389596 | 1.05258762932235 | 0.976919363012161 | 1.13411685687784 | 0.998735809224318 |
| ebi-fl187-GCST90094817 | ebi-cfmet338-GCST90026188 | Isocitrate levels \|\| id:ebi-cfmet338-GCST90026188 | Prawns liking \|\| id:ebi-fl187-GCST90094817 | Inverse variance weighted | 13 | -0.03543472 | 0.0658392209911361 | 0.590438440666872 | -0.164479593 | 0.0936101529600182 | 0.965185739322309 | 0.848335068465537 | 1.09813155912109 | 0.998735809224318 |
| ebi-fl187-GCST90094817 | ebi-cfmet338-GCST90026207 | N-acetyl-isoputreanine levels \|\| id:ebi-cfmet338-GCST90026207 | Prawns liking \|\| id:ebi-fl187-GCST90094817 | Inverse variance weighted | 13 | 0.00615384510047514 | 0.0461616179319081 | 0.893947537253787 | -0.084322926 | 0.096630616247015 | 1.00617281890589 | 0.919134395729517 | 1.101453439 | 0.998735809224318 |
| ebi-fl187-GCST90094817 | ebi-cfmet338-GCST90026213 | N-acetylglutamate levels \|\| id:ebi-cfmet338-GCST90026213 | Prawns liking \|\| id:ebi-fl187-GCST90094817 | Inverse variance weighted | 13 | -0.009468295 | 0.0711831380395391 | 0.894182962356337 | -0.148987246 | 0.130050655487473 | 0.990576388100061 | 0.861580103742444 | 1.13888607269266 | 0.998735809224318 |
| ebi-fl187-GCST90094817 | ebi-cfmet338-GCST90026224 | N-acetyltaurine levels \|\| id:ebi-cfmet338-GCST90026224 | Prawns liking \|\| id:ebi-fl187-GCST90094817 | Inverse variance weighted | 13 | 0.0475959813561663 | 0.0734489321268189 | 0.516975498621878 | -0.096363926 | 0.191555888324731 | 1.04874685643625 | 0.908133462897347 | 1.21113251941608 | 0.998735809224318 |
| ebi-fl187-GCST90094817 | ebi-cfmet338-GCST90026250 | Phenyllactate (pla) levels \|\| id:ebi-cfmet338-GCST90026250 | Prawns liking \|\| id:ebi-fl187-GCST90094817 | Inverse variance weighted | 13 | -0.039995338 | 0.108384953975521 | 0.71211885405619 | -0.252429848 | 0.17243917187636 | 0.960793918444161 | 0.776910712995078 | 1.18819954246806 | 0.998735809224318 |
| ebi-fl187-GCST90094817 | ebi-cfmet338-GCST90026251 | Pipecolate levels \|\| id:ebi-cfmet338-GCST90026251 | Prawns liking \|\| id:ebi-fl187-GCST90094817 | Inverse variance weighted | 13 | -0.145452615 | 0.156711292019898 | 0.353326359256458 | -0.452606748 | 0.161701517183841 | 0.86463085948511 | 0.635968180512597 | 1.17550931961941 | 0.998735809224318 |
| ebi-fl187-GCST90094817 | ebi-cfmet338-GCST90026278 | Trigonelline (n'-methylnicotinate) levels \|\| id:ebi-cfmet338-GCST90026278 | Prawns liking \|\| id:ebi-fl187-GCST90094817 | Inverse variance weighted | 12 | 0.0941753637259351 | 0.159667516250713 | 0.55531020182361 | -0.218772968 | 0.407123695577332 | 1.09875241034006 | 0.803504118493718 | 1.50248994552918 | 0.998735809224318 |
| ebi-fl187-GCST90094817 | ebi-cfmet338-GCST90026299 | 1-carboxyethylvaline levels \|\| id:ebi-cfmet338-GCST90026299 | Prawns liking \|\| id:ebi-fl187-GCST90094817 | Inverse variance weighted | 13 | 0.109373049910208 | 0.0917816576979678 | 0.233392367163508 | -0.070518999 | 0.289265098998225 | 1.11557843916223 | 0.931910033833169 | 1.33544570692587 | 0.998735809224318 |
| ebi-fl187-GCST90094817 | ebi-gm430-GCST90011306 | C_Erysipelotrichia abundance \|\| id:ebi-gm430-GCST90011306 | Prawns liking \|\| id:ebi-fl187-GCST90094817 | Inverse variance weighted | 13 | 0.0209651389045604 | 0.0889450501027164 | 0.813658572125313 | -0.153367159 | 0.195297437105884 | 1.02118645133866 | 0.857814709339619 | 1.2156725188362 | 0.998735809224318 |
| ebi-fl187-GCST90094817 | ebi-gm430-GCST90011312 | F_Erysipelotrichaceae abundance \|\| id:ebi-gm430-GCST90011312 | Prawns liking \|\| id:ebi-fl187-GCST90094817 | Inverse variance weighted | 13 | 0.0209651389045604 | 0.0889450501027164 | 0.813658572125313 | -0.153367159 | 0.195297437105884 | 1.02118645133866 | 0.857814709339619 | 1.2156725188362 | 0.998735809224318 |
| ebi-fl187-GCST90094817 | ebi-gm430-GCST90011352 | O_Erysipelotrichales abundance \|\| id:ebi-gm430-GCST90011352 | Prawns liking \|\| id:ebi-fl187-GCST90094817 | Inverse variance weighted | 13 | 0.0209651389045604 | 0.0889450501027164 | 0.813658572125313 | -0.153367159 | 0.195297437105884 | 1.02118645133866 | 0.857814709339619 | 1.2156725188362 | 0.998735809224318 |
| ebi-fl187-GCST90094817 | ebi-gm430-GCST90011362 | OTU97_11 (Parabacteroides) abundance \|\| id:ebi-gm430-GCST90011362 | Prawns liking \|\| id:ebi-fl187-GCST90094817 | Inverse variance weighted | 13 | 0.0700814570723641 | 0.0976636455976944 | 0.473016430312142 | -0.121339288 | 0.261502202443845 | 1.07259554818903 | 0.885733389630585 | 1.29887980227859 | 0.998735809224318 |
| ebi-fl187-GCST90094817 | ebi-gm430-GCST90011384 | OTU97_235 (Phascolarctobacterium) abundance \|\| id:ebi-gm430-GCST90011384 | Prawns liking \|\| id:ebi-fl187-GCST90094817 | Inverse variance weighted | 13 | 0.191901825689438 | 0.384438976538545 | 0.617657021948897 | -0.561598568 | 0.945402219704986 | 1.21155156788624 | 0.570296676582747 | 2.57384842297011 | 0.998735809224318 |
| ebi-fl187-GCST90094817 | ebi-gm430-GCST90011390 | OTU97_3 (Bacteroides) abundance \|\| id:ebi-gm430-GCST90011390 | Prawns liking \|\| id:ebi-fl187-GCST90094817 | Inverse variance weighted | 13 | -0.100726624 | 0.0776810366372183 | 0.194744958334327 | -0.252981456 | 0.051528207733606 | 0.904180180195081 | 0.776482280867492 | 1.05287888520038 | 0.998735809224318 |
| ebi-fl187-GCST90094817 | ebi-gm430-GCST90011398 | OTU97_4 (Alistipes) abundance \|\| id:ebi-gm430-GCST90011398 | Prawns liking \|\| id:ebi-fl187-GCST90094817 | Inverse variance weighted | 13 | -0.222021565 | 0.0910093006973641 | 0.014705645576708 | -0.400399794 | -0.043643336 | 0.800898092872093 | 0.670052109496764 | 0.95729532983323 | 0.998735809224318 |
| ebi-fl187-GCST90094817 | ebi-gm430-GCST90011421 | OTU99_108 (Cytophagales) abundance \|\| id:ebi-gm430-GCST90011421 | Prawns liking \|\| id:ebi-fl187-GCST90094817 | Inverse variance weighted | 13 | 0.143767686765708 | 0.358374835442072 | 0.688298142290594 | -0.558646991 | 0.84618236423217 | 1.15461584482424 | 0.571982438097311 | 2.33073196015223 | 0.998735809224318 |
| ebi-fl187-GCST90094817 | ebi-gm430-GCST90011428 | OTU99_123 (Phascolarctobacterium) abundance \|\| id:ebi-gm430-GCST90011428 | Prawns liking \|\| id:ebi-fl187-GCST90094817 | Inverse variance weighted | 13 | -0.11891489 | 0.189674151810988 | 0.530696570057281 | -0.490676228 | 0.252846447309784 | 0.887883365086604 | 0.612212259232779 | 1.28768553407514 | 0.998735809224318 |
| ebi-fl187-GCST90094817 | ebi-gm430-GCST90011450 | OTU99_298 (Phascolarctobacterium) abundance \|\| id:ebi-gm430-GCST90011450 | Prawns liking \|\| id:ebi-fl187-GCST90094817 | Inverse variance weighted | 13 | 0.199836041532143 | 0.381757937522887 | 0.600652299916769 | -0.548409516 | 0.948081599077001 | 1.22120251525151 | 0.577868169939857 | 2.58075398652229 | 0.998735809224318 |
| ebi-fl187-GCST90094817 | ebi-gm430-GCST90011451 | OTU99_3 (Bacteroides) abundance \|\| id:ebi-gm430-GCST90011451 | Prawns liking \|\| id:ebi-fl187-GCST90094817 | Inverse variance weighted | 13 | -0.103212575 | 0.077681532877688 | 0.183959192244393 | -0.255468379 | 0.0490432294952499 | 0.90193522427099 | 0.774553628035148 | 1.05026575221703 | 0.998735809224318 |
| ebi-fl187-GCST90094817 | ebi-gm430-GCST90011472 | OTU99_72 (Parabacteroides) abundance \|\| id:ebi-gm430-GCST90011472 | Prawns liking \|\| id:ebi-fl187-GCST90094817 | Inverse variance weighted | 13 | 0.0267990351146573 | 0.231081221535887 | 0.907674445762551 | -0.426120159 | 0.479718229324995 | 1.02716135865551 | 0.653037868966358 | 1.61561910396559 | 0.998735809224318 |
| ebi-fl187-GCST90094817 | ebi-gm430-GCST90011507 | TestASV_3 (Bacteroides) abundance \|\| id:ebi-gm430-GCST90011507 | Prawns liking \|\| id:ebi-fl187-GCST90094817 | Inverse variance weighted | 13 | 0.0148878388877777 | 0.100020568302169 | 0.881673752763294 | -0.181152475 | 0.21092815276003 | 1.01499921479024 | 0.83430813787667 | 1.23482363320433 | 0.998735809224318 |
| ebi-fl187-GCST90094817 | ebi-gm430-GCST90011512 | TestASV_34 (Phascolarctobacterium) abundance \|\| id:ebi-gm430-GCST90011512 | Prawns liking \|\| id:ebi-fl187-GCST90094817 | Inverse variance weighted (multiplicative random effects) | 13 | 0.224957898236009 | 0.345315989709454 | 0.51475268555167 | -0.451861442 | 0.901777238066539 | 1.25226999230632 | 0.636442348052947 | 2.46397829815751 | 0.998735809224318 |
| ebi-fl187-GCST90094817 | ebi-gm430-GCST90011519 | TestASV_41 (Barnesiella) abundance \|\| id:ebi-gm430-GCST90011519 | Prawns liking \|\| id:ebi-fl187-GCST90094817 | Inverse variance weighted | 13 | -0.171512841 | 0.356243872654452 | 0.630198245893651 | -0.869750832 | 0.52672514904517 | 0.842389450526432 | 0.419055951674036 | 1.69337765881489 | 0.998735809224318 |
| ebi-fl187-GCST90094817 | ebi-gm430-GCST90011524 | TestASV_46 (Sutterella) abundance \|\| id:ebi-gm430-GCST90011524 | Prawns liking \|\| id:ebi-fl187-GCST90094817 | Inverse variance weighted (multiplicative random effects) | 13 | -0.299043143 | 0.456298737250185 | 0.5122314815307 | -1.193388668 | 0.595302382412524 | 0.741527417325752 | 0.303192104048869 | 1.81357925652698 | 0.998735809224318 |
| ebi-fl187-GCST90094817 | ebi-gm430-GCST90011581 | OTU97_19 (Bacteria) prevalence \|\| id:ebi-gm430-GCST90011581 | Prawns liking \|\| id:ebi-fl187-GCST90094817 | Inverse variance weighted | 13 | -0.358646057 | 0.367277280983283 | 0.328817028861697 | -1.078509527 | 0.361217414188413 | 0.698621580136614 | 0.340102060911211 | 1.43507543272429 | 0.998735809224318 |
| ebi-fl187-GCST90094817 | ebi-gm430-GCST90011614 | OTU97_80 (Ruminococcus) prevalence \|\| id:ebi-gm430-GCST90011614 | Prawns liking \|\| id:ebi-fl187-GCST90094817 | Inverse variance weighted | 13 | -0.020704722 | 0.171760419487462 | 0.904052071261404 | -0.357355144 | 0.315945700467224 | 0.979508149345919 | 0.699524021909071 | 1.37155577876607 | 0.998735809224318 |
| ebi-fl187-GCST90094817 | ebi-gm430-GCST90011623 | OTU99_11 (Parabacteroides) prevalence \|\| id:ebi-gm430-GCST90011623 | Prawns liking \|\| id:ebi-fl187-GCST90094817 | Inverse variance weighted | 13 | 0.269461794799731 | 0.181978776211584 | 0.13867800677786 | -0.087216607 | 0.626140196174436 | 1.30925961074498 | 0.916478558850072 | 1.87037733919155 | 0.998735809224318 |
| ebi-fl187-GCST90094817 | ebi-gm430-GCST90011642 | OTU99_19 (Bacteria) prevalence \|\| id:ebi-gm430-GCST90011642 | Prawns liking \|\| id:ebi-fl187-GCST90094817 | Inverse variance weighted | 13 | -0.358646057 | 0.367277280983283 | 0.328817028861697 | -1.078509527 | 0.361217414188413 | 0.698621580136614 | 0.340102060911211 | 1.43507543272429 | 0.998735809224318 |
| ebi-fl187-GCST90094817 | ebi-gm430-GCST90011653 | OTU99_30 (Parasutterella) prevalence \|\| id:ebi-gm430-GCST90011653 | Prawns liking \|\| id:ebi-fl187-GCST90094817 | Inverse variance weighted | 13 | -0.379699972 | 0.162902795103754 | 0.0197619206739868 | -0.698989451 | -0.060410494 | 0.684066617403362 | 0.497087381396291 | 0.94137802438525 | 0.998735809224318 |
| ebi-fl187-GCST90094817 | ebi-gm430-GCST90011675 | OTU99_8 (Ruminococcaceae) prevalence \|\| id:ebi-gm430-GCST90011675 | Prawns liking \|\| id:ebi-fl187-GCST90094817 | Inverse variance weighted | 13 | 0.216276241283033 | 0.17273929468499 | 0.210555848386454 | -0.122292776 | 0.554845258865613 | 1.24144527007238 | 0.884889255971602 | 1.74167145570421 | 0.998735809224318 |
| ebi-fl187-GCST90094817 | ebi-gm430-GCST90011679 | OTU99_92 (Ruminococcus) prevalence \|\| id:ebi-gm430-GCST90011679 | Prawns liking \|\| id:ebi-fl187-GCST90094817 | Inverse variance weighted | 13 | -0.022158732 | 0.179784892836898 | 0.901908057133003 | -0.374537122 | 0.330219657630007 | 0.97808496902229 | 0.687607483317222 | 1.39127369878549 | 0.998735809224318 |
| ebi-fl187-GCST90094817 | ebi-gm430-GCST90011684 | TestASV_11 (Lachnospiraceae) prevalence \|\| id:ebi-gm430-GCST90011684 | Prawns liking \|\| id:ebi-fl187-GCST90094817 | Inverse variance weighted | 13 | -0.140418143 | 0.182057932637777 | 0.440539353357935 | -0.497251691 | 0.21641540461044 | 0.868994795015945 | 0.608199885907302 | 1.24161804574869 | 0.998735809224318 |
| ebi-fl187-GCST90094817 | ebi-gm430-GCST90011688 | TestASV_15 (Bacteroides) prevalence \|\| id:ebi-gm430-GCST90011688 | Prawns liking \|\| id:ebi-fl187-GCST90094817 | Inverse variance weighted | 13 | -0.079103634 | 0.171109381734341 | 0.643867174843174 | -0.414478023 | 0.256270753747569 | 0.923944167036101 | 0.660685053348039 | 1.29210252218364 | 0.998735809224318 |
| ebi-fl187-GCST90094817 | ebi-gm430-GCST90011692 | TestASV_19 (Prevotella) prevalence \|\| id:ebi-gm430-GCST90011692 | Prawns liking \|\| id:ebi-fl187-GCST90094817 | Inverse variance weighted | 13 | 0.0105025418413399 | 0.334099718401351 | 0.974922349233082 | -0.644332906 | 0.665337989907988 | 1.01055788711958 | 0.525012657989325 | 1.94514785058829 | 0.998955228396052 |
| ebi-fl187-GCST90094817 | ebi-gm430-GCST90011693 | TestASV_2 (Bacteroides) prevalence \|\| id:ebi-gm430-GCST90011693 | Prawns liking \|\| id:ebi-fl187-GCST90094817 | Inverse variance weighted | 13 | -0.220451096 | 0.214534485625856 | 0.304147986059634 | -0.640938688 | 0.20003649581217 | 0.802156866570192 | 0.526797693291048 | 1.22144733505925 | 0.998735809224318 |
| ebi-fl187-GCST90094817 | ebi-gm430-GCST90011716 | TestASV_40 (Barnesiella) prevalence \|\| id:ebi-gm430-GCST90011716 | Prawns liking \|\| id:ebi-fl187-GCST90094817 | Inverse variance weighted | 13 | 0.0478770075477483 | 0.404179514297127 | 0.905707299373383 | -0.74431484 | 0.840068855570117 | 1.04904162318789 | 0.475059680123478 | 2.31652647704106 | 0.998735809224318 |
| ebi-fl187-GCST90094817 | ebi-gm430-GCST90011718 | TestASV_42 (Alphaproteobacteria) prevalence \|\| id:ebi-gm430-GCST90011718 | Prawns liking \|\| id:ebi-fl187-GCST90094817 | Inverse variance weighted | 13 | -0.206076469 | 0.26800689161424 | 0.441939452132216 | -0.731369977 | 0.319217038330059 | 0.813770845470496 | 0.481249237980874 | 1.37604994808134 | 0.998735809224318 |
| ebi-fl187-GCST90094817 | ebi-hbsm44-GCST90104929 | Sulcal depth of the right inferior frontal sulcus \|\| id:ebi-hbsm44-GCST90104929 | Prawns liking \|\| id:ebi-fl187-GCST90094817 | Inverse variance weighted | 17 | 0.00774856550820837 | 0.118901633655394 | 0.948040347421139 | -0.225298636 | 0.240795767472781 | 1.00777866333002 | 0.798277788309242 | 1.27226117165846 | 0.998735809224318 |
| ebi-fl187-GCST90094817 | ebi-hbsm44-GCST90104935 | Sulcal opening distance of the left calloso-marginal posterior fissure \|\| id:ebi-hbsm44-GCST90104935 | Prawns liking \|\| id:ebi-fl187-GCST90094817 | Inverse variance weighted (multiplicative random effects) | 18 | -0.021467029 | 0.0333478380903741 | 0.519750052114428 | -0.086828791 | 0.043894734135563 | 0.978761748159006 | 0.916834052274002 | 1.04487235970701 | 0.998735809224318 |
| ebi-fl187-GCST90094817 | ebi-hbsm44-GCST90104936 | Sulcal opening distance of the left anterior inferior temporal sulcus \|\| id:ebi-hbsm44-GCST90104936 | Prawns liking \|\| id:ebi-fl187-GCST90094817 | Inverse variance weighted | 18 | -0.016761564 | 0.0153506994448731 | 0.274873179411021 | -0.046848935 | 0.01332580675479 | 0.983378129276879 | 0.954231537624127 | 1.01341499102811 | 0.998735809224318 |
| ebi-fl187-GCST90094817 | ebi-hbsm44-GCST90104941 | Sulcal opening distance of the right posterior inferior temporal sulcus \|\| id:ebi-hbsm44-GCST90104941 | Prawns liking \|\| id:ebi-fl187-GCST90094817 | Inverse variance weighted | 17 | -0.018575583 | 0.0125641038941616 | 0.139283459227257 | -0.043201227 | 0.00605006049734556 | 0.981595879693788 | 0.957718652046874 | 1.00606839907788 | 0.998735809224318 |
| ebi-fl187-GCST90094817 | ebi-IPs91-GCST90274819 | Leukemia inhibitory factor levels \|\| id:ebi-IPs91-GCST90274819 | Prawns liking \|\| id:ebi-fl187-GCST90094817 | Inverse variance weighted | 21 | -0.021429871 | 0.0488762758203414 | 0.661059096626033 | -0.117227372 | 0.0743676292760786 | 0.978798116870707 | 0.889382949445694 | 1.07720274397743 | 0.998735809224318 |
| ebi-fl187-GCST90094817 | ebi-IPs91-GCST90274837 | STAM binding protein levels \|\| id:ebi-IPs91-GCST90274837 | Prawns liking \|\| id:ebi-fl187-GCST90094817 | Inverse variance weighted | 21 | 0.0444894692217944 | 0.0433168939937211 | 0.304387646104718 | -0.040411643 | 0.129390581449488 | 1.0454939667874 | 0.960394018291254 | 1.13813457161431 | 0.998735809224318 |
| ebi-fl187-GCST90094817 | ebi-IPs91-GCST90274840 | TNF-beta levels \|\| id:ebi-IPs91-GCST90274840 | Prawns liking \|\| id:ebi-fl187-GCST90094817 | Inverse variance weighted (multiplicative random effects) | 21 | 0.0092171711694605 | 0.0652763463800756 | 0.887710117950289 | -0.118724468 | 0.137158810074409 | 1.00925978010231 | 0.88805245415915 | 1.14701029084663 | 0.998735809224318 |
| ebi-fl187-GCST90094817 | ebi-IPs91-GCST90274842 | Tumor necrosis factor ligand superfamily member 14 levels \|\| id:ebi-IPs91-GCST90274842 | Prawns liking \|\| id:ebi-fl187-GCST90094817 | Inverse variance weighted | 21 | 0.077636180646516 | 0.0491576015589367 | 0.114259890839225 | -0.018712718 | 0.173985079702032 | 1.08072939686583 | 0.981461277503641 | 1.19003780996918 | 0.998735809224318 |
| ebi-fl187-GCST90094817 | ebi-met1400-GCST90199628 | 3-methylhistidine levels \|\| id:ebi-met1400-GCST90199628 | Prawns liking \|\| id:ebi-fl187-GCST90094817 | Inverse variance weighted | 18 | 0.0491481521255981 | 0.0698971057812516 | 0.48196225558548 | -0.087850175 | 0.186146479456851 | 1.05037595464358 | 0.91589809068712 | 1.20459869641796 | 0.998735809224318 |
| ebi-fl187-GCST90094817 | ebi-met1400-GCST90199651 | Docosahexaenoate DHA; 22:6n3 levels \|\| id:ebi-met1400-GCST90199651 | Prawns liking \|\| id:ebi-fl187-GCST90094817 | Inverse variance weighted | 18 | 0.0432357075812117 | 0.0617729368769217 | 0.483981375285749 | -0.077839249 | 0.164310663859978 | 1.04418398792861 | 0.925113127731456 | 1.17858040056155 | 0.998735809224318 |
| ebi-fl187-GCST90094817 | ebi-met1400-GCST90199652 | 1-stearoyl-2-oleoyl-GPS (18:0/18:1) levels \|\| id:ebi-met1400-GCST90199652 | Prawns liking \|\| id:ebi-fl187-GCST90094817 | Inverse variance weighted | 18 | -0.020007784 | 0.0635257355598189 | 0.752795393079018 | -0.144518226 | 0.10450265784717 | 0.980191043616933 | 0.865439139157386 | 1.11015834449351 | 0.998735809224318 |
| ebi-fl187-GCST90094817 | ebi-met1400-GCST90199653 | 1-myristoyl-2-palmitoyl-gpc (14:0/16:0) levels \|\| id:ebi-met1400-GCST90199653 | Prawns liking \|\| id:ebi-fl187-GCST90094817 | Inverse variance weighted | 17 | -0.006101854 | 0.0678117131722139 | 0.928301277209247 | -0.139012812 | 0.126809103634175 | 0.993916724321889 | 0.870216879167387 | 1.13520029148588 | 0.998735809224318 |
| ebi-fl187-GCST90094817 | ebi-met1400-GCST90199667 | Erythritol levels in elite athletes \|\| id:ebi-met1400-GCST90199667 | Prawns liking \|\| id:ebi-fl187-GCST90094817 | Inverse variance weighted (multiplicative random effects) | 18 | -0.01306715 | 0.0776160055658515 | 0.866302921150141 | -0.165194521 | 0.139060221103498 | 0.987017854737918 | 0.84772878723905 | 1.1491933036087 | 0.998735809224318 |
| ebi-fl187-GCST90094817 | ebi-met1400-GCST90199674 | 3-hydroxymyristate levels \|\| id:ebi-met1400-GCST90199674 | Prawns liking \|\| id:ebi-fl187-GCST90094817 | Inverse variance weighted | 18 | 0.0689502667945695 | 0.0611146705477434 | 0.259230659029585 | -0.050834487 | 0.188735021068147 | 1.07138292451627 | 0.950435966567705 | 1.20772088948853 | 0.998735809224318 |
| ebi-fl187-GCST90094817 | ebi-met1400-GCST90199685 | 2-linoleoylglycerol (18:2) levels \|\| id:ebi-met1400-GCST90199685 | Prawns liking \|\| id:ebi-fl187-GCST90094817 | Inverse variance weighted | 18 | 0.0450322847403081 | 0.0717014297325118 | 0.5299693423978 | -0.095502518 | 0.185567087016031 | 1.04606163119167 | 0.908916073422387 | 1.20390096318923 | 0.998735809224318 |
| ebi-fl187-GCST90094817 | ebi-met1400-GCST90199703 | Gamma-glutamylvaline levels \|\| id:ebi-met1400-GCST90199703 | Prawns liking \|\| id:ebi-fl187-GCST90094817 | Inverse variance weighted (multiplicative random effects) | 18 | 0.0568008970916344 | 0.0813868450805341 | 0.485231912207186 | -0.102717319 | 0.216319113449481 | 1.0584450499269 | 0.902382023443507 | 1.24149849466155 | 0.998735809224318 |
| ebi-fl187-GCST90094817 | ebi-met1400-GCST90199779 | Gamma-glutamyl-2-aminobutyrate levels \|\| id:ebi-met1400-GCST90199779 | Prawns liking \|\| id:ebi-fl187-GCST90094817 | Inverse variance weighted | 18 | 0.109530538531077 | 0.0738648825661612 | 0.138114277563314 | -0.035244631 | 0.254305708360753 | 1.11575414390747 | 0.965369227841371 | 1.28956597511441 | 0.998735809224318 |
| ebi-fl187-GCST90094817 | ebi-met1400-GCST90199824 | 4-vinylphenol sulfate levels \|\| id:ebi-met1400-GCST90199824 | Prawns liking \|\| id:ebi-fl187-GCST90094817 | Inverse variance weighted | 18 | -0.017782075 | 0.0627408838597159 | 0.776854242180717 | -0.140754207 | 0.105190057307375 | 0.982375093068374 | 0.86870280616054 | 1.11092172908527 | 0.998735809224318 |
| ebi-fl187-GCST90094817 | ebi-met1400-GCST90199830 | 4-ethylphenylsulfate levels \|\| id:ebi-met1400-GCST90199830 | Prawns liking \|\| id:ebi-fl187-GCST90094817 | Inverse variance weighted | 18 | -0.011149614 | 0.0627899170107158 | 0.859060577669053 | -0.134217852 | 0.111918623068531 | 0.988912312310553 | 0.874399554498852 | 1.11842184320405 | 0.998735809224318 |
| ebi-fl187-GCST90094817 | ebi-met1400-GCST90199833 | Hexanoylglutamine levels \|\| id:ebi-met1400-GCST90199833 | Prawns liking \|\| id:ebi-fl187-GCST90094817 | Inverse variance weighted | 18 | 0.167270092003845 | 0.0618528344605832 | 0.00684435589513401 | 0.0460385364611025 | 0.288501647546589 | 1.18207349079412 | 1.04711476227674 | 1.334426548 | 0.998735809224318 |
| ebi-fl187-GCST90094817 | ebi-met1400-GCST90199837 | Carotenoid levels (cryptoxanthin) \|\| id:ebi-met1400-GCST90199837 | Prawns liking \|\| id:ebi-fl187-GCST90094817 | Inverse variance weighted | 18 | -0.052179323 | 0.0726173805296346 | 0.472417348761585 | -0.194509388 | 0.0901507432211097 | 0.949158645968801 | 0.823238449283059 | 1.09433923549356 | 0.998735809224318 |
| ebi-fl187-GCST90094817 | ebi-met1400-GCST90199869 | 2-hydroxyhippurate levels \|\| id:ebi-met1400-GCST90199869 | Prawns liking \|\| id:ebi-fl187-GCST90094817 | Inverse variance weighted | 18 | 0.129686522732995 | 0.0631464944780942 | 0.0400007997541063 | 0.00591939355592998 | 0.253453651910059 | 1.13847144246477 | 1.00593694778567 | 1.28846766008637 | 0.998735809224318 |
| ebi-fl187-GCST90094817 | ebi-met1400-GCST90199902 | Hydantoin-5-propionate levels \|\| id:ebi-met1400-GCST90199902 | Prawns liking \|\| id:ebi-fl187-GCST90094817 | Inverse variance weighted | 18 | 0.0975750364107001 | 0.0626724751544304 | 0.119493244955003 | -0.025263015 | 0.220413087713384 | 1.10249416566464 | 0.975053424728638 | 1.24659157590554 | 0.998735809224318 |
| ebi-fl187-GCST90094817 | ebi-met1400-GCST90199904 | Pantoate levels \|\| id:ebi-met1400-GCST90199904 | Prawns liking \|\| id:ebi-fl187-GCST90094817 | Inverse variance weighted | 18 | -0.02652503 | 0.0635585949463527 | 0.676435654232304 | -0.151099876 | 0.0980498165289221 | 0.973823669146762 | 0.859761825090542 | 1.10301773225462 | 0.998735809224318 |
| ebi-fl187-GCST90094817 | ebi-met1400-GCST90199936 | 3-methyl catechol sulfate (2) levels \|\| id:ebi-met1400-GCST90199936 | Prawns liking \|\| id:ebi-fl187-GCST90094817 | Inverse variance weighted | 18 | 0.0127705856372616 | 0.0737504017904796 | 0.862526025690913 | -0.131780202 | 0.157321373146602 | 1.01285247779833 | 0.876533634359614 | 1.17037167949832 | 0.998735809224318 |
| ebi-fl187-GCST90094817 | ebi-met1400-GCST90199977 | 4-hydroxychlorothalonil levels \|\| id:ebi-met1400-GCST90199977 | Prawns liking \|\| id:ebi-fl187-GCST90094817 | Inverse variance weighted | 18 | 0.0324304908767244 | 0.0661130274155487 | 0.623758183739725 | -0.097151043 | 0.1620120246112 | 1.03296209035925 | 0.907418936632482 | 1.17587438066824 | 0.998735809224318 |
| ebi-fl187-GCST90094817 | ebi-met1400-GCST90200040 | Sphingomyelin (d18:2/24:1, d18:1/24:2) levels \|\| id:ebi-met1400-GCST90200040 | Prawns liking \|\| id:ebi-fl187-GCST90094817 | Inverse variance weighted | 18 | -0.005121122 | 0.059878826359396 | 0.931844221219363 | -0.122483621 | 0.112241378109187 | 0.994891969032066 | 0.884720395465847 | 1.11878287775125 | 0.998735809224318 |
| ebi-fl187-GCST90094817 | ebi-met1400-GCST90200077 | 1-stearoyl-2-oleoyl-GPI (18:0/18:1) levels \|\| id:ebi-met1400-GCST90200077 | Prawns liking \|\| id:ebi-fl187-GCST90094817 | Inverse variance weighted | 18 | -0.013995271 | 0.062052751621511 | 0.821560553697156 | -0.135618664 | 0.107628122663092 | 0.986102208007366 | 0.873175542545895 | 1.11363353330054 | 0.998735809224318 |
| ebi-fl187-GCST90094817 | ebi-met1400-GCST90200080 | 1-palmitoyl-2-oleoyl-GPI (16:0/18:1) levels \|\| id:ebi-met1400-GCST90200080 | Prawns liking \|\| id:ebi-fl187-GCST90094817 | Inverse variance weighted | 18 | -0.002261246 | 0.062585311995571 | 0.971178209987644 | -0.124928457 | 0.120405965618029 | 0.997741308797248 | 0.882560040961945 | 1.12795466945842 | 0.998955228396052 |
| ebi-fl187-GCST90094817 | ebi-met1400-GCST90200082 | 1-oleoyl-2-linoleoyl-GPE (18:1/18:2) levels \|\| id:ebi-met1400-GCST90200082 | Prawns liking \|\| id:ebi-fl187-GCST90094817 | Inverse variance weighted | 18 | -0.108677134 | 0.0794868041188379 | 0.171551683821238 | -0.26447127 | 0.0471170019693568 | 0.897019987912031 | 0.767611701899503 | 1.04824464859324 | 0.998735809224318 |
| ebi-fl187-GCST90094817 | ebi-met1400-GCST90200100 | Perfluorooctanesulfonate (PFOS) levels \|\| id:ebi-met1400-GCST90200100 | Prawns liking \|\| id:ebi-fl187-GCST90094817 | Inverse variance weighted | 18 | 0.0711362221567102 | 0.0577894842410676 | 0.21833990502809 | -0.042131167 | 0.184403611269203 | 1.07372748137974 | 0.958744016790582 | 1.20250106814686 | 0.998735809224318 |
| ebi-fl187-GCST90094817 | ebi-met1400-GCST90200102 | 1-palmitoleoyl-2-linolenoyl-GPC (16:1/18:3) levels \|\| id:ebi-met1400-GCST90200102 | Prawns liking \|\| id:ebi-fl187-GCST90094817 | Inverse variance weighted | 18 | -0.095600497 | 0.0613596646407009 | 0.119224982023807 | -0.21586544 | 0.0246644454962529 | 0.908827022493474 | 0.805843728994883 | 1.02497112913513 | 0.998735809224318 |
| ebi-fl187-GCST90094817 | ebi-met1400-GCST90200114 | Glycosyl-N-tricosanoyl-sphingadienine (d18:2/23:0) levels \|\| id:ebi-met1400-GCST90200114 | Prawns liking \|\| id:ebi-fl187-GCST90094817 | Inverse variance weighted | 18 | -0.110086477 | 0.0655657349100588 | 0.0931472651438296 | -0.238595317 | 0.0184223638472265 | 0.895756669976922 | 0.787733600274879 | 1.01859310245006 | 0.998735809224318 |
| ebi-fl187-GCST90094817 | ebi-met1400-GCST90200135 | Arachidoylcarnitine (C20) levels \|\| id:ebi-met1400-GCST90200135 | Prawns liking \|\| id:ebi-fl187-GCST90094817 | Inverse variance weighted (multiplicative random effects) | 18 | 0.0399564081262278 | 0.0937155049139917 | 0.669846589633496 | -0.143725982 | 0.223638797757652 | 1.04076540428939 | 0.86612504982765 | 1.25061921137276 | 0.998735809224318 |
| ebi-fl187-GCST90094817 | ebi-met1400-GCST90200146 | Docosahexaenoylcarnitine (C22:6) levels \|\| id:ebi-met1400-GCST90200146 | Prawns liking \|\| id:ebi-fl187-GCST90094817 | Inverse variance weighted | 18 | 0.0191105982157067 | 0.07036961846384 | 0.7859491621979 | -0.118813854 | 0.157035050404833 | 1.01929437452275 | 0.887973078038806 | 1.17003662343953 | 0.998735809224318 |
| ebi-fl187-GCST90094817 | ebi-met1400-GCST90200162 | 3-hydroxyphenylacetoylglutamine levels \|\| id:ebi-met1400-GCST90200162 | Prawns liking \|\| id:ebi-fl187-GCST90094817 | Inverse variance weighted | 18 | 0.123120024804972 | 0.081720610212048 | 0.131913991178921 | -0.037052371 | 0.283292420820586 | 1.13102016327562 | 0.963625667784713 | 1.32749329174345 | 0.998735809224318 |
| ebi-fl187-GCST90094817 | ebi-met1400-GCST90200172 | 3-carboxy-4-methyl-5-pentyl-2-furanpropionate (3-CMPFP) levels \|\| id:ebi-met1400-GCST90200172 | Prawns liking \|\| id:ebi-fl187-GCST90094817 | Inverse variance weighted (multiplicative random effects) | 18 | 0.0291389005354826 | 0.0793428509520592 | 0.713430304154199 | -0.126373087 | 0.184650888401519 | 1.02956759203353 | 0.881285988803841 | 1.20279845592968 | 0.998735809224318 |
| ebi-fl187-GCST90094817 | ebi-met1400-GCST90200222 | Dihydrocaffeate sulfate (2) levels \|\| id:ebi-met1400-GCST90200222 | Prawns liking \|\| id:ebi-fl187-GCST90094817 | Inverse variance weighted | 18 | 0.0268450820333295 | 0.0711144366853207 | 0.705808519393015 | -0.112539214 | 0.166229377936558 | 1.02720865736002 | 0.89356230638545 | 1.18084393020516 | 0.998735809224318 |
| ebi-fl187-GCST90094817 | ebi-met1400-GCST90200223 | 4-methylhexanoylglutamine levels \|\| id:ebi-met1400-GCST90200223 | Prawns liking \|\| id:ebi-fl187-GCST90094817 | Inverse variance weighted | 18 | 0.139645540201424 | 0.0754595372902795 | 0.0642268898373316 | -0.008255153 | 0.287546233290372 | 1.14986614529085 | 0.991778827318853 | 1.33315222675242 | 0.998735809224318 |
| ebi-fl187-GCST90094817 | ebi-met1400-GCST90200224 | 2-ketocaprylate levels \|\| id:ebi-met1400-GCST90200224 | Prawns liking \|\| id:ebi-fl187-GCST90094817 | Inverse variance weighted | 18 | 0.0248148217468114 | 0.0638941573756744 | 0.697739371652527 | -0.100417727 | 0.150047370203133 | 1.02512527203989 | 0.904459522212766 | 1.16188928035593 | 0.998735809224318 |
| ebi-fl187-GCST90094817 | ebi-met1400-GCST90200243 | Hydroxypalmitoyl sphingomyelin (d18:1/16:0(OH)) levels \|\| id:ebi-met1400-GCST90200243 | Prawns liking \|\| id:ebi-fl187-GCST90094817 | Inverse variance weighted | 18 | 0.067644096336781 | 0.0612573850645275 | 0.269480253565273 | -0.052420378 | 0.187708571063255 | 1.06998442932667 | 0.948929873372549 | 1.2064818603851 | 0.998735809224318 |
| ebi-fl187-GCST90094817 | ebi-met1400-GCST90200273 | 2-methoxyhydroquinone sulfate (2) levels \|\| id:ebi-met1400-GCST90200273 | Prawns liking \|\| id:ebi-fl187-GCST90094817 | Inverse variance weighted | 18 | -0.130228604 | 0.078228648932066 | 0.0959691219359175 | -0.283556756 | 0.0230995478968374 | 0.877894717726674 | 0.753100378024642 | 1.02336840864948 | 0.998735809224318 |
| ebi-fl187-GCST90094817 | ebi-met1400-GCST90200276 | 2,4-di-tert-butylphenol levels \|\| id:ebi-met1400-GCST90200276 | Prawns liking \|\| id:ebi-fl187-GCST90094817 | Inverse variance weighted | 18 | 0.00700238064765574 | 0.0725078531059861 | 0.923064625635567 | -0.135113011 | 0.149117772735389 | 1.00702695464035 | 0.873617177373795 | 1.16080969289174 | 0.998735809224318 |
| ebi-fl187-GCST90094817 | ebi-met1400-GCST90200289 | N-acetylmethionine levels \|\| id:ebi-met1400-GCST90200289 | Prawns liking \|\| id:ebi-fl187-GCST90094817 | Inverse variance weighted | 18 | 0.0621373346353665 | 0.0624447112958074 | 0.319698514943794 | -0.0602543 | 0.184528968775149 | 1.06410847364274 | 0.941525073778627 | 1.20265182013041 | 0.998735809224318 |
| ebi-fl187-GCST90094817 | ebi-met1400-GCST90200308 | 3-hydroxyisobutyrate levels \|\| id:ebi-met1400-GCST90200308 | Prawns liking \|\| id:ebi-fl187-GCST90094817 | Inverse variance weighted | 18 | 0.12073210900316 | 0.0752539933501924 | 0.108641712728991 | -0.026765718 | 0.268229935969537 | 1.12832260440911 | 0.973589309293574 | 1.30764778070983 | 0.998735809224318 |
| ebi-fl187-GCST90094817 | ebi-met1400-GCST90200383 | Cystathionine levels \|\| id:ebi-met1400-GCST90200383 | Prawns liking \|\| id:ebi-fl187-GCST90094817 | Inverse variance weighted (multiplicative random effects) | 18 | -0.023483864 | 0.082937201893808 | 0.7770600484614 | -0.18604078 | 0.13907305156412 | 0.976789735876546 | 0.830239737231655 | 1.14920804838272 | 0.998735809224318 |
| ebi-fl187-GCST90094817 | ebi-met1400-GCST90200384 | Guanidinoacetate levels \|\| id:ebi-met1400-GCST90200384 | Prawns liking \|\| id:ebi-fl187-GCST90094817 | Inverse variance weighted | 18 | -0.064370636 | 0.070928595476285 | 0.364120572670622 | -0.203390683 | 0.0746494110741144 | 0.937657405473427 | 0.815959397523469 | 1.07750632287297 | 0.998735809224318 |
| ebi-fl187-GCST90094817 | ebi-met1400-GCST90200403 | Citrulline levels \|\| id:ebi-met1400-GCST90200403 | Prawns liking \|\| id:ebi-fl187-GCST90094817 | Inverse variance weighted | 17 | -0.00579168 | 0.0645440610872473 | 0.92850003436836 | -0.13229804 | 0.120714679710908 | 0.994225059426533 | 0.876079849545128 | 1.12830293871606 | 0.998735809224318 |
| ebi-fl187-GCST90094817 | ebi-met1400-GCST90200434 | 12,13-DiHOME levels \|\| id:ebi-met1400-GCST90200434 | Prawns liking \|\| id:ebi-fl187-GCST90094817 | Inverse variance weighted | 18 | -0.080783195 | 0.0632994083607732 | 0.201882094910403 | -0.204850035 | 0.0432836457894168 | 0.922393649694113 | 0.814769494169557 | 1.04423404543785 | 0.998735809224318 |
| ebi-fl187-GCST90094817 | ebi-met1400-GCST90200437 | Fructose levels \|\| id:ebi-met1400-GCST90200437 | Prawns liking \|\| id:ebi-fl187-GCST90094817 | Inverse variance weighted | 18 | 0.0213607093012185 | 0.0685888559707478 | 0.755472842869975 | -0.113073448 | 0.155794867003884 | 1.02159048237437 | 0.893085062036845 | 1.16858646285905 | 0.998735809224318 |
| ebi-fl187-GCST90094817 | ebi-met1400-GCST90200448 | N-stearoyl-sphinganine (d18:0/18:0) levels \|\| id:ebi-met1400-GCST90200448 | Prawns liking \|\| id:ebi-fl187-GCST90094817 | Inverse variance weighted | 18 | -0.012567939 | 0.0850122994681759 | 0.882471630402903 | -0.179192046 | 0.154056167556567 | 0.987510707328779 | 0.835945343721143 | 1.16655640756136 | 0.998735809224318 |
| ebi-fl187-GCST90094817 | ebi-met1400-GCST90200468 | X-11847 levels \|\| id:ebi-met1400-GCST90200468 | Prawns liking \|\| id:ebi-fl187-GCST90094817 | Inverse variance weighted | 18 | 0.0505835021859476 | 0.070952996156296 | 0.475897947265559 | -0.08848437 | 0.189651374652288 | 1.05188469435926 | 0.915317416778774 | 1.20882809607313 | 0.998735809224318 |
| ebi-fl187-GCST90094817 | ebi-met1400-GCST90200476 | X-11843 levels \|\| id:ebi-met1400-GCST90200476 | Prawns liking \|\| id:ebi-fl187-GCST90094817 | Inverse variance weighted (multiplicative random effects) | 18 | -0.034353037 | 0.0866809085750467 | 0.691871873582079 | -0.204247618 | 0.135541543771994 | 0.966230329326604 | 0.815260473152363 | 1.14515676897795 | 0.998735809224318 |
| ebi-fl187-GCST90094817 | ebi-met1400-GCST90200511 | X-12906 levels \|\| id:ebi-met1400-GCST90200511 | Prawns liking \|\| id:ebi-fl187-GCST90094817 | Inverse variance weighted | 18 | -0.139821825 | 0.0692748913996718 | 0.0435535493884726 | -0.275600612 | -0.004043038 | 0.869513147368655 | 0.759116052190887 | 0.995965124521999 | 0.998735809224318 |
| ebi-fl187-GCST90094817 | ebi-met1400-GCST90200515 | X-12849 levels \|\| id:ebi-met1400-GCST90200515 | Prawns liking \|\| id:ebi-fl187-GCST90094817 | Inverse variance weighted | 18 | 0.0617429530978856 | 0.0659549066662776 | 0.34920168210718 | -0.067528664 | 0.19101457016379 | 1.06368889164999 | 0.934700927994531 | 1.21047708880226 | 0.998735809224318 |
| ebi-fl187-GCST90094817 | ebi-met1400-GCST90200551 | X-18886 levels \|\| id:ebi-met1400-GCST90200551 | Prawns liking \|\| id:ebi-fl187-GCST90094817 | Inverse variance weighted | 18 | 0.135660242364668 | 0.0732891170589232 | 0.0641654571352312 | -0.007986427 | 0.279306911800157 | 1.1452927055424 | 0.992045379707362 | 1.322213084 | 0.998735809224318 |
| ebi-fl187-GCST90094817 | ebi-met1400-GCST90200564 | X-21319 levels \|\| id:ebi-met1400-GCST90200564 | Prawns liking \|\| id:ebi-fl187-GCST90094817 | Inverse variance weighted | 18 | 0.00656346834291602 | 0.0660621629414458 | 0.920858067510368 | -0.122918371 | 0.13604530770815 | 1.00658505510342 | 0.88433584704573 | 1.14573380299167 | 0.998735809224318 |
| ebi-fl187-GCST90094817 | ebi-met1400-GCST90200579 | X-21442 levels \|\| id:ebi-met1400-GCST90200579 | Prawns liking \|\| id:ebi-fl187-GCST90094817 | Inverse variance weighted | 18 | -0.082472382 | 0.0685186157160751 | 0.228725164846387 | -0.216768869 | 0.0518241045319275 | 0.920836868928194 | 0.805116035015239 | 1.05319047478843 | 0.998735809224318 |
| ebi-fl187-GCST90094817 | ebi-met1400-GCST90200581 | X-21441 levels \|\| id:ebi-met1400-GCST90200581 | Prawns liking \|\| id:ebi-fl187-GCST90094817 | Inverse variance weighted | 18 | 0.0421001229040361 | 0.0688949752233323 | 0.541148719645259 | -0.092934029 | 0.177134274341767 | 1.04299890160205 | 0.91125361505035 | 1.19379137791731 | 0.998735809224318 |
| ebi-fl187-GCST90094817 | ebi-met1400-GCST90200648 | X-24585 levels \|\| id:ebi-met1400-GCST90200648 | Prawns liking \|\| id:ebi-fl187-GCST90094817 | Inverse variance weighted | 18 | -0.005183562 | 0.0612890624317363 | 0.93259875625285 | -0.125310125 | 0.114942999924867 | 0.994829849035373 | 0.882223260836268 | 1.12180949252416 | 0.998735809224318 |
| ebi-fl187-GCST90094817 | ebi-met1400-GCST90200720 | Adenosine 5'-diphosphate (ADP) to pantothenate ratio \|\| id:ebi-met1400-GCST90200720 | Prawns liking \|\| id:ebi-fl187-GCST90094817 | Inverse variance weighted | 18 | 0.0356210540697211 | 0.0831529069603265 | 0.668374380106701 | -0.127358644 | 0.198600751711961 | 1.03626308439929 | 0.880417859761886 | 1.21969490757396 | 0.998735809224318 |
| ebi-fl187-GCST90094817 | ebi-met1400-GCST90200724 | Cholate to taurocholate ratio \|\| id:ebi-met1400-GCST90200724 | Prawns liking \|\| id:ebi-fl187-GCST90094817 | Inverse variance weighted | 18 | -0.05673558 | 0.0672389807510695 | 0.398786759430463 | -0.188523982 | 0.0750528220933147 | 0.94484387178637 | 0.828180641398923 | 1.0779410884856 | 0.998735809224318 |
| ebi-fl187-GCST90094817 | ebi-met1400-GCST90200726 | N-acetylputrescine to (N(1) + N(8))-acetylspermidine ratio \|\| id:ebi-met1400-GCST90200726 | Prawns liking \|\| id:ebi-fl187-GCST90094817 | Inverse variance weighted | 18 | 0.0444514434260967 | 0.0648913205314184 | 0.493335277368433 | -0.082735545 | 0.171638431667677 | 1.04545421180328 | 0.92059457103851 | 1.1872484841446 | 0.998735809224318 |
| ebi-fl187-GCST90094817 | ebi-met1400-GCST90200744 | Arginine to glutamate ratio \|\| id:ebi-met1400-GCST90200744 | Prawns liking \|\| id:ebi-fl187-GCST90094817 | Inverse variance weighted | 18 | -0.037725353 | 0.0722921015657357 | 0.601777470765305 | -0.179417872 | 0.10396716589506 | 0.96297738325744 | 0.835756586938857 | 1.10956402277592 | 0.998735809224318 |
| ebi-fl187-GCST90094817 | ebi-met1400-GCST90200759 | Histidine to trans-urocanate ratio \|\| id:ebi-met1400-GCST90200759 | Prawns liking \|\| id:ebi-fl187-GCST90094817 | Inverse variance weighted | 18 | 0.0308412773141423 | 0.063248835761213 | 0.625820370425272 | -0.093126441 | 0.15480899540612 | 1.03132179672929 | 0.911078295564646 | 1.16743495436881 | 0.998735809224318 |
| ebi-fl187-GCST90094817 | ebi-met1400-GCST90200795 | Oleoyl-linoleoyl-glycerol (18:1 to 18:2) [2] to linoleoyl-arachidonoyl-glycerol (18:2 to 20:4) [2] ratio \|\| id:ebi-met1400-GCST90200795 | Prawns liking \|\| id:ebi-fl187-GCST90094817 | Inverse variance weighted | 18 | 0.127812728021618 | 0.07133645053534 | 0.0731828103651837 | -0.012006715 | 0.267632171070885 | 1.13634017809637 | 0.988065077955337 | 1.30686634834641 | 0.998735809224318 |
| ebi-fl187-GCST90094817 | ebi-met1400-GCST90200811 | Choline to taurocholate ratio \|\| id:ebi-met1400-GCST90200811 | Prawns liking \|\| id:ebi-fl187-GCST90094817 | Inverse variance weighted | 18 | -0.022012522 | 0.0656464126460331 | 0.737384090179257 | -0.150679491 | 0.106654446927742 | 0.978227985742117 | 0.860123332059935 | 1.11254974306685 | 0.998735809224318 |
| ebi-fl187-GCST90094817 | ebi-met1400-GCST90200831 | Cholate to phosphate ratio \|\| id:ebi-met1400-GCST90200831 | Prawns liking \|\| id:ebi-fl187-GCST90094817 | Inverse variance weighted | 18 | -0.023733111 | 0.0654492317556372 | 0.716889783029643 | -0.152013606 | 0.104547382746184 | 0.976546303964824 | 0.858976593652928 | 1.11020799732371 | 0.998735809224318 |
| ebi-fl187-GCST90094817 | ebi-met1400-GCST90200841 | Adenosine 5'-diphosphate (ADP) to oxalate (ethanedioate) ratio \|\| id:ebi-met1400-GCST90200841 | Prawns liking \|\| id:ebi-fl187-GCST90094817 | Inverse variance weighted | 18 | 0.0847671804833785 | 0.0843407170207489 | 0.314869675327467 | -0.080540625 | 0.250074985844046 | 1.08846362162182 | 0.922617421602874 | 1.28412170402744 | 0.998735809224318 |
| ebi-fl187-GCST90094817 | ebi-met1400-GCST90200844 | Aspartate to phosphate ratio \|\| id:ebi-met1400-GCST90200844 | Prawns liking \|\| id:ebi-fl187-GCST90094817 | Inverse variance weighted (multiplicative random effects) | 18 | 0.0645186961249088 | 0.0753892721212974 | 0.392104327513792 | -0.083244277 | 0.212281669482652 | 1.06664552019611 | 0.920126353845716 | 1.23649611925494 | 0.998735809224318 |
| ebi-fl187-GCST90094817 | ebi-met1400-GCST90200870 | Adenosine 5'-monophosphate (AMP) to acetoacetate ratio \|\| id:ebi-met1400-GCST90200870 | Prawns liking \|\| id:ebi-fl187-GCST90094817 | Inverse variance weighted | 18 | -0.044766284 | 0.0809168604845996 | 0.580100429373587 | -0.203363331 | 0.113830762112979 | 0.956220939434692 | 0.815981716118449 | 1.12056246721175 | 0.998735809224318 |
| ebi-fl187-GCST90094817 | ebi-met1400-GCST90200887 | Cholesterol to cortisol ratio \|\| id:ebi-met1400-GCST90200887 | Prawns liking \|\| id:ebi-fl187-GCST90094817 | Inverse variance weighted | 18 | -0.057182634 | 0.0682091810204379 | 0.401838550678855 | -0.190872629 | 0.0765073610348036 | 0.94442157034755 | 0.826237820545847 | 1.07951013661961 | 0.998735809224318 |
| ebi-fl187-GCST90094817 | ebi-met1400-GCST90200890 | Cortisol to taurocholate ratio \|\| id:ebi-met1400-GCST90200890 | Prawns liking \|\| id:ebi-fl187-GCST90094817 | Inverse variance weighted | 18 | -0.00219873 | 0.0654334949916051 | 0.973194115724885 | -0.13044838 | 0.126050920104741 | 0.997803685357553 | 0.877701798515971 | 1.13433992752038 | 0.998955228396052 |
| ebi-fl187-GCST90094817 | ebi-met1400-GCST90200948 | Adenosine 5'-diphosphate (ADP) to mannitol to sorbitol ratio \|\| id:ebi-met1400-GCST90200948 | Prawns liking \|\| id:ebi-fl187-GCST90094817 | Inverse variance weighted | 18 | -0.088050753 | 0.0996724889862488 | 0.377019778388847 | -0.283408831 | 0.107307325538047 | 0.915714400405432 | 0.753211788358483 | 1.11327634016103 | 0.998735809224318 |
| ebi-fl187-GCST90094817 | ebi-met1400-GCST90200972 | Leucine to N-palmitoyl-sphingosine (d18:1 to 16:0) ratio \|\| id:ebi-met1400-GCST90200972 | Prawns liking \|\| id:ebi-fl187-GCST90094817 | Inverse variance weighted | 18 | -0.042066667 | 0.0719025587952052 | 0.558513394733286 | -0.182995682 | 0.0988623480823256 | 0.95880585758178 | 0.832771751308907 | 1.10391433317498 | 0.998735809224318 |
| ebi-fl187-GCST90094817 | ebi-met1400-GCST90200973 | Proline to glutamate ratio \|\| id:ebi-met1400-GCST90200973 | Prawns liking \|\| id:ebi-fl187-GCST90094817 | Inverse variance weighted | 18 | -0.029271515 | 0.0614586695520993 | 0.633875146129648 | -0.149730507 | 0.0911874773931007 | 0.971152746197556 | 0.860939962241444 | 1.09547436268565 | 0.998735809224318 |
| ebi-fl187-GCST90094817 | ebi-met1400-GCST90200983 | Cholesterol to linoleoyl-arachidonoyl-glycerol (18:2 to 20:4) [1] ratio \|\| id:ebi-met1400-GCST90200983 | Prawns liking \|\| id:ebi-fl187-GCST90094817 | Inverse variance weighted (multiplicative random effects) | 18 | 0.0860696038158552 | 0.0844652532258118 | 0.308205716265401 | -0.079482293 | 0.251621500138446 | 1.08988218562407 | 0.923594374364971 | 1.28610915301148 | 0.998735809224318 |
| ebi-fl187-GCST90094817 | ebi-met1400-GCST90201001 | Serine to alpha-ketobutyrate ratio \|\| id:ebi-met1400-GCST90201001 | Prawns liking \|\| id:ebi-fl187-GCST90094817 | Inverse variance weighted | 18 | -0.136063292 | 0.0746874606069713 | 0.0684900594758664 | -0.282450715 | 0.0103241303094269 | 0.872787389861893 | 0.75393379846746 | 1.01037760802126 | 0.998735809224318 |
| ebi-fl187-GCST90094817 | ebi-met1400-GCST90201005 | Fructose to maltose ratio \|\| id:ebi-met1400-GCST90201005 | Prawns liking \|\| id:ebi-fl187-GCST90094817 | Inverse variance weighted (multiplicative random effects) | 18 | -0.084072981 | 0.0919580892351855 | 0.360583795701267 | -0.264310836 | 0.0961648740498507 | 0.919364157667995 | 0.767734863120454 | 1.10094056556081 | 0.998735809224318 |
| ebi-fl187-GCST90094817 | ebi-met1400-GCST90201020 | Paraxanthine to 5-acetylamino-6-formylamino-3-methyluracil ratio \|\| id:ebi-met1400-GCST90201020 | Prawns liking \|\| id:ebi-fl187-GCST90094817 | Inverse variance weighted | 18 | 0.00661238831940118 | 0.0663352605179212 | 0.920597307350252 | -0.123404722 | 0.136629498934527 | 1.00663429842513 | 0.883905853752769 | 1.14640332617288 | 0.998735809224318 |
| ebi-fl187-GCST90094817 | ebi-pw11-GCST90275197 | Placental weight (fetal genotype, adjusted for sex) \|\| id:ebi-pw11-GCST90275197 | Prawns liking \|\| id:ebi-fl187-GCST90094817 | Inverse variance weighted | 17 | 0.0492330297044985 | 0.0247917366742664 | 0.0470483717121488 | 0.000641225822936444 | 0.0978248335860606 | 1.05046511179521 | 1.00064143145216 | 1.1027696 | 0.998735809224318 |
| ebi-fl187-GCST90094817 | prot-a-1017 | FAS-associated factor 2 \|\| id:prot-a-1017 | Prawns liking \|\| id:ebi-fl187-GCST90094817 | Inverse variance weighted | 20 | -0.0363622 | 0.10220151692303 | 0.721998404476807 | -0.236677174 | 0.16395277268824 | 0.964290963572479 | 0.789246036312791 | 1.17815867251188 | 0.998735809224318 |
| ebi-fl187-GCST90094817 | prot-a-1019 | Fas apoptotic inhibitory molecule 3 \|\| id:prot-a-1019 | Prawns liking \|\| id:ebi-fl187-GCST90094817 | Inverse variance weighted | 20 | -0.00777834 | 0.0951138210216711 | 0.934822230864416 | -0.194201429 | 0.178644749141081 | 0.992251832943015 | 0.823492012171562 | 1.1955959322331 | 0.998735809224318 |
| ebi-fl187-GCST90094817 | prot-a-1052 | MIP18 family protein FAM96A \|\| id:prot-a-1052 | Prawns liking \|\| id:ebi-fl187-GCST90094817 | Inverse variance weighted | 20 | 0.0321207552989136 | 0.0883980979235761 | 0.716332408725875 | -0.141139517 | 0.205381027229123 | 1.03264219479341 | 0.868368151446715 | 1.2279928745558 | 0.998735809224318 |
| ebi-fl187-GCST90094817 | prot-a-1066 | F-box/LRR-repeat protein 4 \|\| id:prot-a-1066 | Prawns liking \|\| id:ebi-fl187-GCST90094817 | Inverse variance weighted | 20 | -0.034223819 | 0.0883973596212184 | 0.698638622638687 | -0.207482644 | 0.139035006022019 | 0.966355191937176 | 0.812627345860263 | 1.14916432697124 | 0.998735809224318 |
| ebi-fl187-GCST90094817 | prot-a-1076 | Low affinity immunoglobulin gamma Fc region receptor III-B \|\| id:prot-a-1076 | Prawns liking \|\| id:ebi-fl187-GCST90094817 | Inverse variance weighted | 20 | 0.0750157706107455 | 0.102221102345059 | 0.463035305572935 | -0.12533759 | 0.275369131207061 | 1.07790114991005 | 0.882199030750089 | 1.31701673712962 | 0.998735809224318 |
| ebi-fl187-GCST90094817 | prot-a-1081 | Fc receptor-like protein 3 \|\| id:prot-a-1081 | Prawns liking \|\| id:ebi-fl187-GCST90094817 | Inverse variance weighted | 20 | -0.027316474 | 0.0883980979235761 | 0.757308949251657 | -0.200576746 | 0.145943798053006 | 0.973053246857016 | 0.818258689692119 | 1.15713115319953 | 0.998735809224318 |
| ebi-fl187-GCST90094817 | prot-a-1105 | Fibroblast growth factor receptor 4 \|\| id:prot-a-1105 | Prawns liking \|\| id:ebi-fl187-GCST90094817 | Inverse variance weighted | 20 | -0.081784942 | 0.107317392861087 | 0.446009462391717 | -0.292127032 | 0.128557148408549 | 0.9214701072765 | 0.7466736787913 | 1.13718640782769 | 0.998735809224318 |
| ebi-fl187-GCST90094817 | prot-a-1143 | Follistatin-related protein 1 \|\| id:prot-a-1143 | Prawns liking \|\| id:ebi-fl187-GCST90094817 | Inverse variance weighted | 20 | -0.162555954 | 0.0939885530782322 | 0.0837144121816618 | -0.346773518 | 0.0216616102352996 | 0.849968529927361 | 0.706965425190158 | 1.0218979261575 | 0.998735809224318 |
| ebi-fl187-GCST90094817 | prot-a-1151 | Alpha-(1,3)-fucosyltransferase 10 \|\| id:prot-a-1151 | Prawns liking \|\| id:ebi-fl187-GCST90094817 | Inverse variance weighted | 20 | 0.0196740902049651 | 0.0883980979235761 | 0.823876125997882 | -0.153586182 | 0.192934362135174 | 1.01986890059271 | 0.857626849252331 | 1.21280318509496 | 0.998735809224318 |
| ebi-fl187-GCST90094817 | prot-a-1165 | Growth arrest and DNA damage-inducible proteins-interacting protein 1 \|\| id:prot-a-1165 | Prawns liking \|\| id:ebi-fl187-GCST90094817 | Inverse variance weighted | 20 | -0.07528364 | 0.104751744382078 | 0.47233424777194 | -0.280597059 | 0.130029779036701 | 0.927480378526168 | 0.755332628699246 | 1.1388622970418 | 0.998735809224318 |
| ebi-fl187-GCST90094817 | prot-a-1194 | Growth/differentiation factor 11/8 \|\| id:prot-a-1194 | Prawns liking \|\| id:ebi-fl187-GCST90094817 | Inverse variance weighted | 20 | -0.122809816 | 0.0883807882319759 | 0.164664566402379 | -0.296036161 | 0.0504165293666162 | 0.884431851736148 | 0.743760532779134 | 1.05170907286864 | 0.998735809224318 |
| ebi-fl187-GCST90094817 | prot-a-1216 | D-glucuronyl C5-epimerase \|\| id:prot-a-1216 | Prawns liking \|\| id:ebi-fl187-GCST90094817 | Inverse variance weighted | 20 | -0.150457793 | 0.100927323054355 | 0.136026138561693 | -0.348275346 | 0.0473597601869937 | 0.86031404051623 | 0.705904481345995 | 1.04849914948562 | 0.998735809224318 |
| ebi-fl187-GCST90094817 | prot-a-1246 | Glypican-3 \|\| id:prot-a-1246 | Prawns liking \|\| id:ebi-fl187-GCST90094817 | Inverse variance weighted | 20 | -0.086617855 | 0.0949541437709193 | 0.361659649181928 | -0.272727976 | 0.0994922672222242 | 0.917027466539211 | 0.761299851021363 | 1.10460992900382 | 0.998735809224318 |
| ebi-fl187-GCST90094817 | prot-a-1275 | Glutamate receptor 4 \|\| id:prot-a-1275 | Prawns liking \|\| id:ebi-fl187-GCST90094817 | Inverse variance weighted | 19 | -0.207408179 | 0.100976807031702 | 0.0399739495694576 | -0.405322721 | -0.009493637 | 0.812687859960129 | 0.666761598145661 | 0.990551284842127 | 0.998735809224318 |
| ebi-fl187-GCST90094817 | prot-a-1289 | Glutathione S-transferase P \|\| id:prot-a-1289 | Prawns liking \|\| id:ebi-fl187-GCST90094817 | Inverse variance weighted | 20 | 0.0301486903421079 | 0.0883980979235761 | 0.733061520935842 | -0.143111582 | 0.203408962272317 | 1.03060776398233 | 0.866657360495527 | 1.2255735791286 | 0.998735809224318 |
| ebi-fl187-GCST90094817 | prot-a-130 | Apolipoprotein D \|\| id:prot-a-130 | Prawns liking \|\| id:ebi-fl187-GCST90094817 | Inverse variance weighted | 20 | -0.034925591 | 0.0955278433319286 | 0.714658797039509 | -0.222160164 | 0.152308981808918 | 0.965677268546698 | 0.800787096756606 | 1.16451999634461 | 0.998735809224318 |
| ebi-fl187-GCST90094817 | prot-a-1313 | Heparin-binding EGF-like growth factor \|\| id:prot-a-1313 | Prawns liking \|\| id:ebi-fl187-GCST90094817 | Inverse variance weighted | 20 | 0.0109258270801417 | 0.104196208533398 | 0.91648832604739 | -0.193298742 | 0.215150395805601 | 1.01098573189995 | 0.82423570382505 | 1.24004838101775 | 0.998735809224318 |
| ebi-fl187-GCST90094817 | prot-a-1347 | HLA class II histocompatibility antigen, DQ alpha 2 chain \|\| id:prot-a-1347 | Prawns liking \|\| id:ebi-fl187-GCST90094817 | Inverse variance weighted (multiplicative random effects) | 19 | 0.104081009235207 | 0.131045895749776 | 0.427059648116169 | -0.152768946 | 0.360930964904768 | 1.10969034644079 | 0.858328018651118 | 1.43466441526525 | 0.998735809224318 |
| ebi-fl187-GCST90094817 | prot-a-1357 | Non-histone chromosomal protein HMG-14 \|\| id:prot-a-1357 | Prawns liking \|\| id:ebi-fl187-GCST90094817 | Inverse variance weighted | 20 | 0.0597803472512982 | 0.107616657590823 | 0.578557196377751 | -0.151148302 | 0.270708996129312 | 1.06160333684275 | 0.859720191301775 | 1.3108935397797 | 0.998735809224318 |
| ebi-fl187-GCST90094817 | prot-a-137 | Apolipoprotein M \|\| id:prot-a-137 | Prawns liking \|\| id:ebi-fl187-GCST90094817 | Inverse variance weighted | 20 | -0.127250955 | 0.0884112153109565 | 0.150063324462729 | -0.300536937 | 0.0460350272701899 | 0.880512676039198 | 0.740420554925116 | 1.04711108775758 | 0.998735809224318 |
| ebi-fl187-GCST90094817 | prot-a-1381 | 17-beta-hydroxysteroid dehydrogenase 14 \|\| id:prot-a-1381 | Prawns liking \|\| id:ebi-fl187-GCST90094817 | Inverse variance weighted | 20 | -0.056904288 | 0.0902554160901997 | 0.528380201325034 | -0.233804903 | 0.119996327985656 | 0.944684483104877 | 0.791516223203903 | 1.12749271140237 | 0.998735809224318 |
| ebi-fl187-GCST90094817 | prot-a-1392 | Serine protease HTRA2, mitochondrial \|\| id:prot-a-1392 | Prawns liking \|\| id:ebi-fl187-GCST90094817 | Inverse variance weighted | 20 | -0.045834294 | 0.0921272763703246 | 0.618829050071907 | -0.226403756 | 0.134735167786859 | 0.955200231583323 | 0.797396083539703 | 1.14423371427482 | 0.998735809224318 |
| ebi-fl187-GCST90094817 | prot-a-1416 | Interferon-induced protein with tetratricopeptide repeats 2 \|\| id:prot-a-1416 | Prawns liking \|\| id:ebi-fl187-GCST90094817 | Inverse variance weighted | 20 | -0.107499264 | 0.101457834295326 | 0.289351095811085 | -0.306356619 | 0.0913580914032876 | 0.898077183601015 | 0.736124056791136 | 1.09566128190479 | 0.998735809224318 |
| ebi-fl187-GCST90094817 | prot-a-1428 | Interferon gamma \|\| id:prot-a-1428 | Prawns liking \|\| id:ebi-fl187-GCST90094817 | Inverse variance weighted | 20 | -0.082772002 | 0.0883807882319759 | 0.348996048456456 | -0.255998347 | 0.0904543424717172 | 0.920561008937975 | 0.774143248142159 | 1.09467152650447 | 0.998735809224318 |
| ebi-fl187-GCST90094817 | prot-a-1483 | Interleukin-17C \|\| id:prot-a-1483 | Prawns liking \|\| id:ebi-fl187-GCST90094817 | Inverse variance weighted | 20 | -0.1149755 | 0.0973151975544743 | 0.237413968339846 | -0.305713287 | 0.0757622870150866 | 0.891387982474017 | 0.736597781049408 | 1.07870612122547 | 0.998735809224318 |
| ebi-fl187-GCST90094817 | prot-a-1503 | Interleukin-1 receptor-like 2 \|\| id:prot-a-1503 | Prawns liking \|\| id:ebi-fl187-GCST90094817 | Inverse variance weighted | 20 | -0.102067749 | 0.0959329061655126 | 0.287351666405362 | -0.290096245 | 0.0859607474252375 | 0.902968374699915 | 0.74819155460887 | 1.08976355144031 | 0.998735809224318 |
| ebi-fl187-GCST90094817 | prot-a-1522 | Interleukin-31 receptor subunit alpha \|\| id:prot-a-1522 | Prawns liking \|\| id:ebi-fl187-GCST90094817 | Inverse variance weighted | 20 | -0.112738357 | 0.0883980979235761 | 0.202186016054767 | -0.285998629 | 0.0605219147550773 | 0.893384377151621 | 0.7512636458495 | 1.06239087935113 | 0.998735809224318 |
| ebi-fl187-GCST90094817 | prot-a-1540 | Interleukin-6 receptor subunit alpha \|\| id:prot-a-1540 | Prawns liking \|\| id:ebi-fl187-GCST90094817 | Inverse variance weighted | 20 | -0.031002908 | 0.0883980979235761 | 0.725799039977293 | -0.20426318 | 0.142257364109582 | 0.969472754019056 | 0.815247786242072 | 1.15287331857435 | 0.998735809224318 |
| ebi-fl187-GCST90094817 | prot-a-1541 | Interleukin-6 receptor subunit alpha \|\| id:prot-a-1541 | Prawns liking \|\| id:ebi-fl187-GCST90094817 | Inverse variance weighted | 20 | -0.057114832 | 0.0883980979235761 | 0.518208578276527 | -0.230375104 | 0.116145439491335 | 0.944485605553493 | 0.794235625367408 | 1.12315921195941 | 0.998735809224318 |
| ebi-fl187-GCST90094817 | prot-a-1545 | Interleukin-9 \|\| id:prot-a-1545 | Prawns liking \|\| id:ebi-fl187-GCST90094817 | Inverse variance weighted | 20 | -0.001824586 | 0.108129072384381 | 0.98653701366523 | -0.213757568 | 0.210108395486333 | 0.998177077158774 | 0.807544135623214 | 1.23381179233789 | 0.998955228396052 |
| ebi-fl187-GCST90094817 | prot-a-1548 | Inositol monophosphatase 3 \|\| id:prot-a-1548 | Prawns liking \|\| id:ebi-fl187-GCST90094817 | Inverse variance weighted | 20 | -0.012380262 | 0.0907475563847629 | 0.891485082897085 | -0.190245472 | 0.165484948914566 | 0.987696058559888 | 0.826756163449623 | 1.17996520282873 | 0.998735809224318 |
| ebi-fl187-GCST90094817 | prot-a-1555 | Inhibin beta A chain:Inhibin beta B chain heterodimer \|\| id:prot-a-1555 | Prawns liking \|\| id:ebi-fl187-GCST90094817 | Inverse variance weighted | 20 | -0.162612466 | 0.0883980979235761 | 0.0658347543001448 | -0.335872738 | 0.0106478056734713 | 0.849920497473144 | 0.714714055835263 | 1.01070469529355 | 0.998735809224318 |
| ebi-fl187-GCST90094817 | prot-a-1603 | Junctophilin-3 \|\| id:prot-a-1603 | Prawns liking \|\| id:ebi-fl187-GCST90094817 | Inverse variance weighted | 20 | 0.0211575885012246 | 0.0888128353699489 | 0.811705271177051 | -0.152915569 | 0.195230745826325 | 1.02138299717131 | 0.858202177771802 | 1.21559144678383 | 0.998735809224318 |
| ebi-fl187-GCST90094817 | prot-a-1627 | UPF0258 protein KIAA1024 \|\| id:prot-a-1627 | Prawns liking \|\| id:ebi-fl187-GCST90094817 | Inverse variance weighted | 20 | 0.0582857416877341 | 0.0995416022558071 | 0.55818365307188 | -0.136815799 | 0.253387282109116 | 1.06001784372777 | 0.872130858945914 | 1.288382148 | 0.998735809224318 |
| ebi-fl187-GCST90094817 | prot-a-1666 | Kallikrein-7 \|\| id:prot-a-1666 | Prawns liking \|\| id:ebi-fl187-GCST90094817 | Inverse variance weighted | 20 | -0.031392424 | 0.0883980979235761 | 0.722495530139691 | -0.204652696 | 0.141867848047499 | 0.969095202345608 | 0.814930295972547 | 1.15242434334643 | 0.998735809224318 |
| ebi-fl187-GCST90094817 | prot-a-1678 | Importin subunit alpha-3 \|\| id:prot-a-1678 | Prawns liking \|\| id:ebi-fl187-GCST90094817 | Inverse variance weighted (multiplicative random effects) | 20 | -0.081365903 | 0.12065701039022 | 0.500084352964657 | -0.317853644 | 0.155121836895406 | 0.921856319300009 | 0.727709285361943 | 1.16780023359285 | 0.998735809224318 |
| ebi-fl187-GCST90094817 | prot-a-1718 | Protein LDOC1 \|\| id:prot-a-1718 | Prawns liking \|\| id:ebi-fl187-GCST90094817 | Inverse variance weighted | 20 | 0.0789693143580933 | 0.103341635637133 | 0.444773226067465 | -0.123580291 | 0.281518920206874 | 1.08217111444552 | 0.883750680735802 | 1.32514106802749 | 0.998735809224318 |
| ebi-fl187-GCST90094817 | prot-a-1723 | Leptin \|\| id:prot-a-1723 | Prawns liking \|\| id:ebi-fl187-GCST90094817 | Inverse variance weighted | 20 | 0.0157134051278372 | 0.0888554172661025 | 0.85963218451298 | -0.158443213 | 0.189870022969398 | 1.0158375098621 | 0.853471428741177 | 1.2090924331994 | 0.998735809224318 |
| ebi-fl187-GCST90094817 | prot-a-1730 | Galectin-7 \|\| id:prot-a-1730 | Prawns liking \|\| id:ebi-fl187-GCST90094817 | Inverse variance weighted | 20 | -0.084865315 | 0.0999656789991057 | 0.395912166029457 | -0.280798046 | 0.111067415752431 | 0.918636002480951 | 0.755180831928127 | 1.11747023941214 | 0.998735809224318 |
| ebi-fl187-GCST90094817 | prot-a-1753 | Lipase member N \|\| id:prot-a-1753 | Prawns liking \|\| id:ebi-fl187-GCST90094817 | Inverse variance weighted | 20 | 0.0376385253892435 | 0.0883886856793765 | 0.670231961082103 | -0.135603299 | 0.210880349320821 | 1.03835582576457 | 0.873188959122939 | 1.23476460579871 | 0.998735809224318 |
| ebi-fl187-GCST90094817 | prot-a-1756 | VIP36-like protein \|\| id:prot-a-1756 | Prawns liking \|\| id:ebi-fl187-GCST90094817 | Inverse variance weighted | 20 | -0.042089758 | 0.0883980979235761 | 0.63397554943683 | -0.21535003 | 0.131170514237279 | 0.958783718495585 | 0.806259176184237 | 1.14016217862203 | 0.998735809224318 |
| ebi-fl187-GCST90094817 | prot-a-1760 | Ig Kappa chain V-I region HK102- like \|\| id:prot-a-1760 | Prawns liking \|\| id:ebi-fl187-GCST90094817 | Inverse variance weighted | 20 | -0.074658687 | 0.0883973596212184 | 0.398345092742611 | -0.247917512 | 0.0986001374771254 | 0.928060190932695 | 0.780424316078218 | 1.10362491307576 | 0.998735809224318 |
| ebi-fl187-GCST90094817 | prot-a-179 | Neutral ceramidase \|\| id:prot-a-179 | Prawns liking \|\| id:ebi-fl187-GCST90094817 | Inverse variance weighted | 20 | 0.148600806423476 | 0.107018580116582 | 0.164969184154621 | -0.061155611 | 0.358357223451978 | 1.16020974847486 | 0.940676849092713 | 1.4309767076275 | 0.998735809224318 |
| ebi-fl187-GCST90094817 | prot-a-1809 | Lactotransferrin \|\| id:prot-a-1809 | Prawns liking \|\| id:ebi-fl187-GCST90094817 | Inverse variance weighted | 20 | -0.095446716 | 0.0883980979235761 | 0.280259219525252 | -0.268706987 | 0.0778135564111815 | 0.908966794187158 | 0.764367192019166 | 1.08092110907105 | 0.998735809224318 |
| ebi-fl187-GCST90094817 | prot-a-1818 | Lysophospholipase-like protein 1 \|\| id:prot-a-1818 | Prawns liking \|\| id:ebi-fl187-GCST90094817 | Inverse variance weighted | 20 | -0.15209181 | 0.0883623252366896 | 0.0852092497809591 | -0.325281968 | 0.0210983473053808 | 0.858909420511033 | 0.722323653041853 | 1.02132249101339 | 0.998735809224318 |
| ebi-fl187-GCST90094817 | prot-a-1852 | MAP kinase-activated protein kinase 3 \|\| id:prot-a-1852 | Prawns liking \|\| id:ebi-fl187-GCST90094817 | Inverse variance weighted | 20 | 0.0291502286883921 | 0.0989840815554077 | 0.768380365936504 | -0.164858571 | 0.223159028536991 | 1.02957925519871 | 0.848013629190987 | 1.25001934667819 | 0.998735809224318 |
| ebi-fl187-GCST90094817 | prot-a-1856 | Megakaryocyte-associated tyrosine-protein kinase \|\| id:prot-a-1856 | Prawns liking \|\| id:ebi-fl187-GCST90094817 | Inverse variance weighted | 20 | -0.078071877 | 0.0884112153109565 | 0.377207096339385 | -0.251357859 | 0.0952141047142035 | 0.924897944991623 | 0.777743998826102 | 1.09989432247744 | 0.998735809224318 |
| ebi-fl187-GCST90094817 | prot-a-1890 | Beta-1,3-N-acetylglucosaminyltransferase manic fringe \|\| id:prot-a-1890 | Prawns liking \|\| id:ebi-fl187-GCST90094817 | Inverse variance weighted | 20 | -0.011394214 | 0.106991297975828 | 0.915188318205864 | -0.221097158 | 0.19830872989305 | 0.988670454070838 | 0.801638790746782 | 1.21933878204928 | 0.998735809224318 |
| ebi-fl187-GCST90094817 | prot-a-1919 | Matrilysin \|\| id:prot-a-1919 | Prawns liking \|\| id:ebi-fl187-GCST90094817 | Inverse variance weighted | 20 | -0.143504427 | 0.0967313795160403 | 0.137932215365066 | -0.333097931 | 0.0460890769631773 | 0.866316965090352 | 0.716700003757345 | 1.04716768531992 | 0.998735809224318 |
| ebi-fl187-GCST90094817 | prot-a-1923 | Multimerin-2 \|\| id:prot-a-1923 | Prawns liking \|\| id:ebi-fl187-GCST90094817 | Inverse variance weighted | 20 | -0.173917485 | 0.100444250023589 | 0.083365700865635 | -0.370788215 | 0.0229532448935589 | 0.840366237489392 | 0.69019009785399 | 1.02321869772968 | 0.998735809224318 |
| ebi-fl187-GCST90094817 | prot-a-1948 | Mesothelin \|\| id:prot-a-1948 | Prawns liking \|\| id:ebi-fl187-GCST90094817 | Inverse variance weighted | 20 | -0.0048465 | 0.0883980979235761 | 0.956277214932178 | -0.178106772 | 0.168413772058826 | 0.995165225459199 | 0.836853066408876 | 1.18342617804233 | 0.998735809224318 |
| ebi-fl187-GCST90094817 | prot-a-195 | ATPase family AAA domain-containing protein 1 \|\| id:prot-a-195 | Prawns liking \|\| id:ebi-fl187-GCST90094817 | Inverse variance weighted | 20 | 0.0847888840490657 | 0.0989645855291536 | 0.39157761577836 | -0.109181704 | 0.278759471686207 | 1.08848724541989 | 0.896567493166256 | 1.32148945001073 | 0.998735809224318 |
| ebi-fl187-GCST90094817 | prot-a-1966 | Mucin-1 \|\| id:prot-a-1966 | Prawns liking \|\| id:ebi-fl187-GCST90094817 | Inverse variance weighted | 20 | -0.048829306 | 0.0885712806149814 | 0.581428359277449 | -0.222429016 | 0.124770404455835 | 0.952343675605636 | 0.800571832880646 | 1.1328883170953 | 0.998735809224318 |
| ebi-fl187-GCST90094817 | prot-a-1986 | Myocardial zonula adherens protein \|\| id:prot-a-1986 | Prawns liking \|\| id:ebi-fl187-GCST90094817 | Inverse variance weighted | 20 | -0.18124184 | 0.0961591588391434 | 0.0594553592404399 | -0.369713792 | 0.00723011093862505 | 0.834233582926219 | 0.690932052821921 | 1.00725631129649 | 0.998735809224318 |
| ebi-fl187-GCST90094817 | prot-a-2071 | Epididymal secretory protein E1 \|\| id:prot-a-2071 | Prawns liking \|\| id:ebi-fl187-GCST90094817 | Inverse variance weighted | 20 | -0.023462965 | 0.0957450153570899 | 0.806412468712775 | -0.211123195 | 0.164197265114943 | 0.976810150177569 | 0.809674312841128 | 1.17844675860076 | 0.998735809224318 |
| ebi-fl187-GCST90094817 | prot-a-2098 | Neuritin \|\| id:prot-a-2098 | Prawns liking \|\| id:ebi-fl187-GCST90094817 | Inverse variance weighted | 20 | -0.028132364 | 0.0883980979235761 | 0.750298148323098 | -0.201392636 | 0.145127908265641 | 0.972259666431448 | 0.817591353057369 | 1.15618744674158 | 0.998735809224318 |
| ebi-fl187-GCST90094817 | prot-a-2115 | Neurotrophin-4 \|\| id:prot-a-2115 | Prawns liking \|\| id:ebi-fl187-GCST90094817 | Inverse variance weighted | 20 | -0.042481518 | 0.0884112153109565 | 0.630872474220706 | -0.2157675 | 0.130804464070794 | 0.958408178715986 | 0.805922657152151 | 1.13974489844412 | 0.998735809224318 |
| ebi-fl187-GCST90094817 | prot-a-2118 | Netrin-1 \|\| id:prot-a-2118 | Prawns liking \|\| id:ebi-fl187-GCST90094817 | Inverse variance weighted | 20 | 0.0188566451151151 | 0.0903348194512846 | 0.834649873724937 | -0.158199601 | 0.195912891239633 | 1.0190355544214 | 0.853679369697919 | 1.21642093979897 | 0.998735809224318 |
| ebi-fl187-GCST90094817 | prot-a-2120 | Netrin-G1 \|\| id:prot-a-2120 | Prawns liking \|\| id:ebi-fl187-GCST90094817 | Inverse variance weighted | 20 | -0.07877087 | 0.088388893607389 | 0.3728298474884 | -0.252013101 | 0.0944713616920972 | 0.924251674175961 | 0.777234555258906 | 1.09907768695707 | 0.998735809224318 |
| ebi-fl187-GCST90094817 | prot-a-2145 | Olfactomedin-like protein 3 \|\| id:prot-a-2145 | Prawns liking \|\| id:ebi-fl187-GCST90094817 | Inverse variance weighted | 20 | -0.089795057 | 0.0883973596212184 | 0.309719143134924 | -0.263053882 | 0.0834637675024592 | 0.914118507940114 | 0.768700476912646 | 1.08704582819404 | 0.998735809224318 |
| ebi-fl187-GCST90094817 | prot-a-2162 | Prolyl 4-hydroxylase subunit alpha-2 \|\| id:prot-a-2162 | Prawns liking \|\| id:ebi-fl187-GCST90094817 | Inverse variance weighted | 20 | -0.16779608 | 0.092871072503638 | 0.0707991351189921 | -0.349823382 | 0.0142312220810697 | 0.845526236794117 | 0.704812561217507 | 1.01433296800537 | 0.998735809224318 |
| ebi-fl187-GCST90094817 | prot-a-2168 | Polyadenylate-binding protein-interacting protein 1 \|\| id:prot-a-2168 | Prawns liking \|\| id:ebi-fl187-GCST90094817 | Inverse variance weighted (multiplicative random effects) | 20 | -0.182355426 | 0.112916725070285 | 0.10632044812031 | -0.403672207 | 0.038961355495974 | 0.833305109771427 | 0.667863006358122 | 1.0397303030119 | 0.998735809224318 |
| ebi-fl187-GCST90094817 | prot-a-219 | Beta-1,4-galactosyltransferase 5 \|\| id:prot-a-219 | Prawns liking \|\| id:ebi-fl187-GCST90094817 | Inverse variance weighted | 20 | -0.008094307 | 0.104375247118472 | 0.9381859678622 | -0.212669791 | 0.196481177523406 | 0.991938363864422 | 0.808423041564782 | 1.21711241159271 | 0.998735809224318 |
| ebi-fl187-GCST90094817 | prot-a-2190 | Pyruvate carboxylase, mitochondrial \|\| id:prot-a-2190 | Prawns liking \|\| id:ebi-fl187-GCST90094817 | Inverse variance weighted | 20 | -0.058826389 | 0.0978813058518495 | 0.547841862924753 | -0.250673749 | 0.133020970437591 | 0.942870447599577 | 0.778276243934291 | 1.14227395206436 | 0.998735809224318 |
| ebi-fl187-GCST90094817 | prot-a-2197 | Protocadherin-9 \|\| id:prot-a-2197 | Prawns liking \|\| id:ebi-fl187-GCST90094817 | Inverse variance weighted | 20 | -0.148622941 | 0.0884112153109565 | 0.0927548131929149 | -0.321908923 | 0.0246630414425274 | 0.861894038914941 | 0.724764196979762 | 1.02496969002161 | 0.998735809224318 |
| ebi-fl187-GCST90094817 | prot-a-2198 | Protocadherin alpha-4 \|\| id:prot-a-2198 | Prawns liking \|\| id:ebi-fl187-GCST90094817 | Inverse variance weighted (multiplicative random effects) | 20 | 0.00617023176411922 | 0.124633224611597 | 0.960515164771781 | -0.238110888 | 0.250451352002849 | 1.00618930685653 | 0.788115293345462 | 1.2846050949409 | 0.998735809224318 |
| ebi-fl187-GCST90094817 | prot-a-2254 | Placenta growth factor \|\| id:prot-a-2254 | Prawns liking \|\| id:ebi-fl187-GCST90094817 | Inverse variance weighted | 20 | 0.00432520003179252 | 0.0883980979235761 | 0.960976152854491 | -0.168935072 | 0.177585471962002 | 1.00433456720955 | 0.844563737526021 | 1.19433013527996 | 0.998735809224318 |
| ebi-fl187-GCST90094817 | prot-a-2261 | Membrane-associated progesterone receptor component 2 \|\| id:prot-a-2261 | Prawns liking \|\| id:ebi-fl187-GCST90094817 | Inverse variance weighted | 20 | -0.059556939 | 0.105044742293032 | 0.570736362729825 | -0.265444634 | 0.146330755460803 | 0.942181884761068 | 0.766864899700889 | 1.15757900031435 | 0.998735809224318 |
| ebi-fl187-GCST90094817 | prot-a-2268 | Polymeric immunoglobulin receptor \|\| id:prot-a-2268 | Prawns liking \|\| id:ebi-fl187-GCST90094817 | Inverse variance weighted | 20 | -0.063289049 | 0.0883797377322458 | 0.473927373640087 | -0.236513335 | 0.109935236537895 | 0.93867211184556 | 0.789375355617364 | 1.11620577876731 | 0.998735809224318 |
| ebi-fl187-GCST90094817 | prot-a-2283 | Group 10 secretory phospholipase A2 \|\| id:prot-a-2283 | Prawns liking \|\| id:ebi-fl187-GCST90094817 | Inverse variance weighted | 20 | 0.0230453286556535 | 0.09480616044428 | 0.807944673038929 | -0.162774746 | 0.208865403126442 | 1.02331292389509 | 0.849782583940717 | 1.2322791264498 | 0.998735809224318 |
| ebi-fl187-GCST90094817 | prot-a-2298 | Inactive phospholipase D5 \|\| id:prot-a-2298 | Prawns liking \|\| id:ebi-fl187-GCST90094817 | Inverse variance weighted | 20 | 0.124073349920074 | 0.0900464763000039 | 0.16823995964854 | -0.052417744 | 0.300564443468082 | 1.13209890731815 | 0.948932373579986 | 1.35062094163333 | 0.998735809224318 |
| ebi-fl187-GCST90094817 | prot-a-2304 | Procollagen-lysine,2-oxoglutarate 5-dioxygenase 2 \|\| id:prot-a-2304 | Prawns liking \|\| id:ebi-fl187-GCST90094817 | Inverse variance weighted | 20 | 0.00244680345895343 | 0.0883980979235761 | 0.977917879565586 | -0.170813468 | 0.175707075389163 | 1.00244979932547 | 0.842978800931662 | 1.19208881534987 | 0.998955228396052 |
| ebi-fl187-GCST90094817 | prot-a-2331 | Periostin \|\| id:prot-a-2331 | Prawns liking \|\| id:ebi-fl187-GCST90094817 | Inverse variance weighted | 20 | -0.073121029 | 0.106281409826312 | 0.491456226111328 | -0.281432592 | 0.135190534677115 | 0.929488328563168 | 0.754701787017972 | 1.14475487907461 | 0.998735809224318 |
| ebi-fl187-GCST90094817 | prot-a-2333 | Periostin \|\| id:prot-a-2333 | Prawns liking \|\| id:ebi-fl187-GCST90094817 | Inverse variance weighted | 20 | -0.124451287 | 0.0883980979235761 | 0.159175409314641 | -0.297711559 | 0.0488089853210818 | 0.882981273330651 | 0.742515481112603 | 1.05001976239521 | 0.998735809224318 |
| ebi-fl187-GCST90094817 | prot-a-2367 | cAMP-dependent protein kinase catalytic subunit alpha \|\| id:prot-a-2367 | Prawns liking \|\| id:ebi-fl187-GCST90094817 | Inverse variance weighted | 20 | -0.052421562 | 0.0917675450770271 | 0.567834974780691 | -0.232285951 | 0.127442826139637 | 0.948928750009492 | 0.792719412332453 | 1.13591992145758 | 0.998735809224318 |
| ebi-fl187-GCST90094817 | prot-a-2385 | Phosphoribosyl pyrophosphate synthase-associated protein 1 \|\| id:prot-a-2385 | Prawns liking \|\| id:ebi-fl187-GCST90094817 | Inverse variance weighted (multiplicative random effects) | 20 | 0.116520367887916 | 0.118413398957571 | 0.325108938606486 | -0.115569894 | 0.348610629844754 | 1.1235803951937 | 0.890858304349301 | 1.41709730750698 | 0.998735809224318 |
| ebi-fl187-GCST90094817 | prot-a-2393 | Trypsin-3 \|\| id:prot-a-2393 | Prawns liking \|\| id:ebi-fl187-GCST90094817 | Inverse variance weighted | 19 | -0.106716095 | 0.0996131116222335 | 0.284031995192611 | -0.301957794 | 0.0885256039427012 | 0.89878080528304 | 0.739369270333999 | 1.092562226 | 0.998735809224318 |
| ebi-fl187-GCST90094817 | prot-a-2405 | Pregnancy-specific beta-1-glycoprotein 6 \|\| id:prot-a-2405 | Prawns liking \|\| id:ebi-fl187-GCST90094817 | Inverse variance weighted | 20 | 0.0397943816055211 | 0.11040989345018 | 0.718530039803573 | -0.17660901 | 0.256197772767874 | 1.04059678635271 | 0.838107412457744 | 1.29200822671663 | 0.998735809224318 |
| ebi-fl187-GCST90094817 | prot-a-244 | Beclin-1 \|\| id:prot-a-244 | Prawns liking \|\| id:ebi-fl187-GCST90094817 | Inverse variance weighted | 20 | -0.078839397 | 0.0883980979235761 | 0.372463915522885 | -0.252099669 | 0.0944208745841633 | 0.924188339626929 | 0.777167274508862 | 1.09902219910399 | 0.998735809224318 |
| ebi-fl187-GCST90094817 | prot-a-2454 | tRNA pseudouridine synthase A, mitochondrial \|\| id:prot-a-2454 | Prawns liking \|\| id:ebi-fl187-GCST90094817 | Inverse variance weighted | 20 | -0.074788772 | 0.0979644908150417 | 0.445208573119257 | -0.266799174 | 0.117221630224136 | 0.927939472638152 | 0.765826854142376 | 1.12436859614208 | 0.998735809224318 |
| ebi-fl187-GCST90094817 | prot-a-2466 | Glutaminyl-peptide cyclotransferase-like protein \|\| id:prot-a-2466 | Prawns liking \|\| id:ebi-fl187-GCST90094817 | Inverse variance weighted | 20 | -0.206683021 | 0.0884112153109565 | 0.0194003955935709 | -0.379969003 | -0.033397039 | 0.813277401170712 | 0.683882607336897 | 0.967154485519991 | 0.998735809224318 |
| ebi-fl187-GCST90094817 | prot-a-2474 | Ras-related protein Rab-27A \|\| id:prot-a-2474 | Prawns liking \|\| id:ebi-fl187-GCST90094817 | Inverse variance weighted (multiplicative random effects) | 20 | -0.096576914 | 0.121932031088655 | 0.428328421474488 | -0.335563695 | 0.142409867191194 | 0.907940061845935 | 0.714934967710097 | 1.15304914871512 | 0.998735809224318 |
| ebi-fl187-GCST90094817 | prot-a-2490 | Rap1 GTPase-GDP dissociation stimulator 1 \|\| id:prot-a-2490 | Prawns liking \|\| id:ebi-fl187-GCST90094817 | Inverse variance weighted | 20 | -0.065038403 | 0.102891290626409 | 0.527316340124507 | -0.266705333 | 0.136628526746943 | 0.937031477978265 | 0.765898723673143 | 1.14640221165434 | 0.998735809224318 |
| ebi-fl187-GCST90094817 | prot-a-2545 | Receptor-interacting serine/threonine-protein kinase 2 \|\| id:prot-a-2545 | Prawns liking \|\| id:ebi-fl187-GCST90094817 | Inverse variance weighted | 20 | 0.0469489380428581 | 0.0988360450305506 | 0.634773872385766 | -0.14676971 | 0.240667586302737 | 1.04806849128492 | 0.863492808092483 | 1.27209810218431 | 0.998735809224318 |
| ebi-fl187-GCST90094817 | prot-a-2610 | RUN and FYVE domain-containing protein 1 \|\| id:prot-a-2610 | Prawns liking \|\| id:ebi-fl187-GCST90094817 | Inverse variance weighted (multiplicative random effects) | 20 | 0.0211508726857254 | 0.115461970749806 | 0.854653056652679 | -0.20515459 | 0.247456335355345 | 1.02137613777458 | 0.814521389829636 | 1.28076343708238 | 0.998735809224318 |
| ebi-fl187-GCST90094817 | prot-a-2627 | Serine--tRNA ligase, mitochondrial \|\| id:prot-a-2627 | Prawns liking \|\| id:ebi-fl187-GCST90094817 | Inverse variance weighted | 20 | -0.062842341 | 0.105036855119584 | 0.549647455095512 | -0.268714578 | 0.143029894560586 | 0.939091517803284 | 0.76436139044914 | 1.15376429242701 | 0.998735809224318 |
| ebi-fl187-GCST90094817 | prot-a-264 | BolA-like protein 2 \|\| id:prot-a-264 | Prawns liking \|\| id:ebi-fl187-GCST90094817 | Inverse variance weighted | 20 | 0.0267337723289436 | 0.0942410212306132 | 0.776659856613508 | -0.157978629 | 0.211446173940945 | 1.02709432543127 | 0.853868029545464 | 1.23546346370959 | 0.998735809224318 |
| ebi-fl187-GCST90094817 | prot-a-2646 | Secretoglobin family 1C member 1 \|\| id:prot-a-2646 | Prawns liking \|\| id:ebi-fl187-GCST90094817 | Inverse variance weighted | 20 | -0.064101844 | 0.0994962595150527 | 0.519404348802364 | -0.259114513 | 0.130910824772427 | 0.937909474329271 | 0.771734644699572 | 1.13986612895817 | 0.998735809224318 |
| ebi-fl187-GCST90094817 | prot-a-2672 | Semaphorin-3E \|\| id:prot-a-2672 | Prawns liking \|\| id:ebi-fl187-GCST90094817 | Inverse variance weighted | 20 | -0.007960045 | 0.0883980979235761 | 0.928249310264896 | -0.181220317 | 0.165300226596763 | 0.992071551933267 | 0.834251538430997 | 1.17974725705188 | 0.998735809224318 |
| ebi-fl187-GCST90094817 | prot-a-2701 | Plasma protease C1 inhibitor \|\| id:prot-a-2701 | Prawns liking \|\| id:ebi-fl187-GCST90094817 | Inverse variance weighted | 20 | -0.092198092 | 0.0883841708955129 | 0.29687806889314 | -0.265431067 | 0.0810348824856308 | 0.911924486278112 | 0.766875303753252 | 1.08440872277871 | 0.998735809224318 |
| ebi-fl187-GCST90094817 | prot-a-2738 | Signaling threshold-regulating transmembrane adapter 1 \|\| id:prot-a-2738 | Prawns liking \|\| id:ebi-fl187-GCST90094817 | Inverse variance weighted | 20 | -0.100712433 | 0.102354979946935 | 0.325138893982765 | -0.301328194 | 0.0999033276088286 | 0.904193011496374 | 0.739834923675599 | 1.10506408372439 | 0.998735809224318 |
| ebi-fl187-GCST90094817 | prot-a-274 | Serine/threonine-protein kinase BRSK2 \|\| id:prot-a-274 | Prawns liking \|\| id:ebi-fl187-GCST90094817 | Inverse variance weighted | 20 | 0.0106649528320818 | 0.0883797377322458 | 0.90395089931465 | -0.162559333 | 0.183889238787284 | 1.01072202615586 | 0.84996565761224 | 1.20188269373895 | 0.998735809224318 |
| ebi-fl187-GCST90094817 | prot-a-2749 | Mitochondrial glutamate carrier 2 \|\| id:prot-a-2749 | Prawns liking \|\| id:ebi-fl187-GCST90094817 | Inverse variance weighted | 20 | -0.083706873 | 0.0883980979235761 | 0.343674093067442 | -0.256967145 | 0.0895533991932715 | 0.919700805967076 | 0.77339362345295 | 1.09368573368894 | 0.998735809224318 |
| ebi-fl187-GCST90094817 | prot-a-2760 | Sodium-coupled monocarboxylate transporter 1 \|\| id:prot-a-2760 | Prawns liking \|\| id:ebi-fl187-GCST90094817 | Inverse variance weighted | 20 | -0.1837309 | 0.0883846207573437 | 0.0376389099611773 | -0.356964756 | -0.010497043 | 0.832159708074568 | 0.699797160638828 | 0.989557858609477 | 0.998735809224318 |
| ebi-fl187-GCST90094817 | prot-a-2777 | Small integral membrane protein 10 \|\| id:prot-a-2777 | Prawns liking \|\| id:ebi-fl187-GCST90094817 | Inverse variance weighted | 20 | 0.0694389960442513 | 0.0978151003872772 | 0.47776579361778 | -0.122278601 | 0.261156592803315 | 1.07190666866321 | 0.884901799883139 | 1.29843097446111 | 0.998735809224318 |
| ebi-fl187-GCST90094817 | prot-a-2799 | Superoxide dismutase [Mn], mitochondrial \|\| id:prot-a-2799 | Prawns liking \|\| id:ebi-fl187-GCST90094817 | Inverse variance weighted | 20 | -0.127830649 | 0.0983207862153924 | 0.193553471279329 | -0.32053939 | 0.0648780919861691 | 0.88000239581548 | 0.725757465174909 | 1.06702893707659 | 0.998735809224318 |
| ebi-fl187-GCST90094817 | prot-a-2805 | Protein SPACA7 \|\| id:prot-a-2805 | Prawns liking \|\| id:ebi-fl187-GCST90094817 | Inverse variance weighted | 20 | -0.109668817 | 0.0883980979235761 | 0.214744619844106 | -0.282929089 | 0.0635914552955002 | 0.896130869794251 | 0.753573222923793 | 1.06565694131546 | 0.998735809224318 |
| ebi-fl187-GCST90094817 | prot-a-2811 | SPARC-like protein 1 \|\| id:prot-a-2811 | Prawns liking \|\| id:ebi-fl187-GCST90094817 | Inverse variance weighted | 20 | -0.1767399 | 0.088367760363759 | 0.045494912704329 | -0.34994071 | -0.00353909 | 0.837997719277636 | 0.70472987160236 | 0.996467165380431 | 0.998735809224318 |
| ebi-fl187-GCST90094817 | prot-a-2831 | Sepiapterin reductase \|\| id:prot-a-2831 | Prawns liking \|\| id:ebi-fl187-GCST90094817 | Inverse variance weighted | 20 | -0.095722004 | 0.102948430985093 | 0.352471864146819 | -0.297500929 | 0.106056920508262 | 0.908716600336219 | 0.742671893312954 | 1.11188516377399 | 0.998735809224318 |
| ebi-fl187-GCST90094817 | prot-a-2834 | SPRY domain-containing SOCS box protein 1 \|\| id:prot-a-2834 | Prawns liking \|\| id:ebi-fl187-GCST90094817 | Inverse variance weighted | 20 | -0.021681158 | 0.0917273796056009 | 0.813149317502461 | -0.201466822 | 0.158104505958278 | 0.978552188786353 | 0.817530701065527 | 1.17128859494882 | 0.998735809224318 |
| ebi-fl187-GCST90094817 | prot-a-2835 | Spectrin alpha chain, non-erythrocytic 1 \|\| id:prot-a-2835 | Prawns liking \|\| id:ebi-fl187-GCST90094817 | Inverse variance weighted | 20 | -0.044828191 | 0.0939371612578764 | 0.633209272481005 | -0.228945027 | 0.139288645529663 | 0.956161745358865 | 0.795372256619527 | 1.14945583741306 | 0.998735809224318 |
| ebi-fl187-GCST90094817 | prot-a-2859 | Sia-alpha-2,3-Gal-beta-1,4-GlcNAc-R:alpha 2,8-sialyltransferase \|\| id:prot-a-2859 | Prawns liking \|\| id:ebi-fl187-GCST90094817 | Inverse variance weighted | 20 | -0.219238168 | 0.104925584450061 | 0.0366660296289861 | -0.424892314 | -0.013584022 | 0.803130415394146 | 0.653840191074356 | 0.986507823985726 | 0.998735809224318 |
| ebi-fl187-GCST90094817 | prot-a-2861 | Alpha-2,8-sialyltransferase 8F \|\| id:prot-a-2861 | Prawns liking \|\| id:ebi-fl187-GCST90094817 | Inverse variance weighted | 20 | -0.095996956 | 0.0980904508257995 | 0.327749232686952 | -0.28825424 | 0.0962603271733427 | 0.908466781032909 | 0.749570997033497 | 1.10104565879223 | 0.998735809224318 |
| ebi-fl187-GCST90094817 | prot-a-289 | Uncharacterized protein C17orf89 \|\| id:prot-a-289 | Prawns liking \|\| id:ebi-fl187-GCST90094817 | Inverse variance weighted | 20 | 0.0187711249990383 | 0.0884112153109565 | 0.831860430752967 | -0.154514857 | 0.192057107008513 | 1.01894841010885 | 0.856830762103957 | 1.21173971381922 | 0.998735809224318 |
| ebi-fl187-GCST90094817 | prot-a-2921 | Tachykinin-3 \|\| id:prot-a-2921 | Prawns liking \|\| id:ebi-fl187-GCST90094817 | Inverse variance weighted | 20 | 0.0635261830388662 | 0.0883499666922559 | 0.47212308764243 | -0.109639752 | 0.236692117755688 | 1.06558738575215 | 0.896156916177744 | 1.26705095522455 | 0.998735809224318 |
| ebi-fl187-GCST90094817 | prot-a-2928 | Tax1-binding protein 1 \|\| id:prot-a-2928 | Prawns liking \|\| id:ebi-fl187-GCST90094817 | Inverse variance weighted | 20 | -0.138349328 | 0.0931941502284965 | 0.137668894189871 | -0.321009862 | 0.0443112067403691 | 0.87079444598065 | 0.725416096788633 | 1.04530761104918 | 0.998735809224318 |
| ebi-fl187-GCST90094817 | prot-a-296 | Uncharacterized protein C1orf185 \|\| id:prot-a-296 | Prawns liking \|\| id:ebi-fl187-GCST90094817 | Inverse variance weighted | 20 | 0.147008199928927 | 0.0957430802831023 | 0.124674644383347 | -0.040648237 | 0.334664637283807 | 1.15836346148897 | 0.960166821303302 | 1.3974716467409 | 0.998735809224318 |
| ebi-fl187-GCST90094817 | prot-a-2964 | Transforming growth factor beta-2 \|\| id:prot-a-2964 | Prawns liking \|\| id:ebi-fl187-GCST90094817 | Inverse variance weighted | 20 | -0.108017985 | 0.0883887601260192 | 0.221678019912025 | -0.281259955 | 0.0652239845003821 | 0.89761145243216 | 0.754832087452027 | 1.06739807823628 | 0.998735809224318 |
| ebi-fl187-GCST90094817 | prot-a-2977 | Thymocyte nuclear protein 1 \|\| id:prot-a-2977 | Prawns liking \|\| id:ebi-fl187-GCST90094817 | Inverse variance weighted | 20 | 0.053293826903304 | 0.0956447123390602 | 0.577386552783608 | -0.134169809 | 0.240757463087862 | 1.05473951043789 | 0.874441563701845 | 1.27221243941016 | 0.998735809224318 |
| ebi-fl187-GCST90094817 | prot-a-3015 | Transmembrane protein 70, mitochondrial \|\| id:prot-a-3015 | Prawns liking \|\| id:ebi-fl187-GCST90094817 | Inverse variance weighted | 20 | -0.026434134 | 0.0884112153109565 | 0.764947120461529 | -0.199720116 | 0.146851848417819 | 0.973912189820966 | 0.818959935113385 | 1.15818236376917 | 0.998735809224318 |
| ebi-fl187-GCST90094817 | prot-a-3030 | Tumor necrosis factor alpha-induced protein 3 \|\| id:prot-a-3030 | Prawns liking \|\| id:ebi-fl187-GCST90094817 | Inverse variance weighted | 20 | -0.021898206 | 0.10158491108067 | 0.829326418573298 | -0.221004632 | 0.177208219405458 | 0.978339818800258 | 0.801712966722465 | 1.19387965616061 | 0.998735809224318 |
| ebi-fl187-GCST90094817 | prot-a-3032 | Tumor necrosis factor receptor superfamily member 10A \|\| id:prot-a-3032 | Prawns liking \|\| id:ebi-fl187-GCST90094817 | Inverse variance weighted | 20 | 0.0733603071985971 | 0.0883980979235761 | 0.406603475767386 | -0.099899965 | 0.246620579128806 | 1.07611820020565 | 0.90492793821745 | 1.27969347823978 | 0.998735809224318 |
| ebi-fl187-GCST90094817 | prot-a-3081 | Tumor protein p53-inducible protein 11 \|\| id:prot-a-3081 | Prawns liking \|\| id:ebi-fl187-GCST90094817 | Inverse variance weighted | 20 | 0.0203419968964217 | 0.0983615734269125 | 0.836159517360322 | -0.172446687 | 0.21313068081317 | 1.0205503053879 | 0.841603155987709 | 1.23754636424219 | 0.998735809224318 |
| ebi-fl187-GCST90094817 | prot-a-315 | Complement C4 \|\| id:prot-a-315 | Prawns liking \|\| id:ebi-fl187-GCST90094817 | Inverse variance weighted | 19 | -0.125822564 | 0.0951445377884789 | 0.186023000994817 | -0.312305858 | 0.0606607301620332 | 0.881771290959558 | 0.731757680079502 | 1.06253836580986 | 0.998735809224318 |
| ebi-fl187-GCST90094817 | prot-a-3157 | UDP-glucuronosyltransferase 1-8 \|\| id:prot-a-3157 | Prawns liking \|\| id:ebi-fl187-GCST90094817 | Inverse variance weighted | 20 | -0.061514903 | 0.10442254162634 | 0.555796865814902 | -0.266183085 | 0.1431532783356 | 0.940338931526914 | 0.766298816961016 | 1.15390665700344 | 0.998735809224318 |
| ebi-fl187-GCST90094817 | prot-a-317 | Glutaredoxin-like protein C5orf63 \|\| id:prot-a-317 | Prawns liking \|\| id:ebi-fl187-GCST90094817 | Inverse variance weighted | 20 | -0.083807684 | 0.101856529900054 | 0.410621006129067 | -0.283446482 | 0.115831114970954 | 0.919608094777804 | 0.753183429753562 | 1.122806231 | 0.998735809224318 |
| ebi-fl187-GCST90094817 | prot-a-3192 | Guanine nucleotide exchange factor VAV3 \|\| id:prot-a-3192 | Prawns liking \|\| id:ebi-fl187-GCST90094817 | Inverse variance weighted | 19 | -0.035957471 | 0.0979058134318544 | 0.713420652249358 | -0.227852866 | 0.155937923034413 | 0.964681319260849 | 0.796241405703938 | 1.1687536481579 | 0.998735809224318 |
| ebi-fl187-GCST90094817 | prot-a-3233 | Serine/threonine-protein kinase WNK3 \|\| id:prot-a-3233 | Prawns liking \|\| id:ebi-fl187-GCST90094817 | Inverse variance weighted | 20 | 0.102990838634818 | 0.0983422485491995 | 0.2949752934165 | -0.089759969 | 0.295741645791249 | 1.10848125382793 | 0.914150583855003 | 1.34412285217424 | 0.998735809224318 |
| ebi-fl187-GCST90094817 | prot-a-3250 | 14-3-3 protein theta \|\| id:prot-a-3250 | Prawns liking \|\| id:ebi-fl187-GCST90094817 | Inverse variance weighted | 20 | 0.0727831182142985 | 0.0883980979235761 | 0.410305487346021 | -0.100477154 | 0.246043390144508 | 1.07549725585304 | 0.904405774 | 1.27895506638296 | 0.998735809224318 |
| ebi-fl187-GCST90094817 | prot-a-3278 | E3 ubiquitin-protein ligase ZNRF3 \|\| id:prot-a-3278 | Prawns liking \|\| id:ebi-fl187-GCST90094817 | Inverse variance weighted | 20 | -0.04706137 | 0.0883815261191909 | 0.594393782970051 | -0.220289162 | 0.126166420771702 | 0.954028846640906 | 0.802286773878215 | 1.13447095210514 | 0.998735809224318 |
| ebi-fl187-GCST90094817 | prot-a-355 | Calpain-3 \|\| id:prot-a-355 | Prawns liking \|\| id:ebi-fl187-GCST90094817 | Inverse variance weighted | 20 | -0.083925017 | 0.0884112153109565 | 0.342489617383024 | -0.257210999 | 0.0893609647948429 | 0.919500200196439 | 0.773205050886622 | 1.09347529118155 | 0.998735809224318 |
| ebi-fl187-GCST90094817 | prot-a-385 | Coiled-coil domain-containing protein 90B, mitochondrial \|\| id:prot-a-385 | Prawns liking \|\| id:ebi-fl187-GCST90094817 | Inverse variance weighted | 20 | 0.0263702922638169 | 0.108203957569934 | 0.807456102530545 | -0.185709465 | 0.238450049100888 | 1.02672106495945 | 0.830514853920604 | 1.26928030276055 | 0.998735809224318 |
| ebi-fl187-GCST90094817 | prot-a-390 | C-C motif chemokine 14 \|\| id:prot-a-390 | Prawns liking \|\| id:ebi-fl187-GCST90094817 | Inverse variance weighted | 20 | -0.030128755 | 0.0891555215028188 | 0.735412309324109 | -0.204873577 | 0.144616067310138 | 0.970320592037169 | 0.814750313095086 | 1.15559581407793 | 0.998735809224318 |
| ebi-fl187-GCST90094817 | prot-a-40 | Pituitary adenylate cyclase-activating polypeptide 27 \|\| id:prot-a-40 | Prawns liking \|\| id:ebi-fl187-GCST90094817 | Inverse variance weighted | 20 | 0.0445636326954324 | 0.0954001631392455 | 0.640411712420903 | -0.142420687 | 0.231547952448354 | 1.04557150712694 | 0.867256336216507 | 1.26054977157618 | 0.998735809224318 |
| ebi-fl187-GCST90094817 | prot-a-401 | C-C motif chemokine 24 \|\| id:prot-a-401 | Prawns liking \|\| id:ebi-fl187-GCST90094817 | Inverse variance weighted | 20 | -0.016434537 | 0.0949414405567779 | 0.862571387948031 | -0.20251976 | 0.169650686558707 | 0.983699773287521 | 0.816670344686499 | 1.18489088071073 | 0.998735809224318 |
| ebi-fl187-GCST90094817 | prot-a-412 | Cerebral cavernous malformations 2 protein \|\| id:prot-a-412 | Prawns liking \|\| id:ebi-fl187-GCST90094817 | Inverse variance weighted (multiplicative random effects) | 19 | -0.132652186 | 0.125447260926595 | 0.290313609287356 | -0.378528817 | 0.113224445717306 | 0.875769644348733 | 0.684868234844062 | 1.11988325774423 | 0.998735809224318 |
| ebi-fl187-GCST90094817 | prot-a-44 | Adhesion G protein-coupled receptor F5 \|\| id:prot-a-44 | Prawns liking \|\| id:ebi-fl187-GCST90094817 | Inverse variance weighted | 20 | 0.169688544349329 | 0.0997127798141187 | 0.0887978772719694 | -0.025748504 | 0.365125592785002 | 1.18493573891071 | 0.974580161718344 | 1.44069493767682 | 0.998735809224318 |
| ebi-fl187-GCST90094817 | prot-a-48 | Adrenomedullin \|\| id:prot-a-48 | Prawns liking \|\| id:ebi-fl187-GCST90094817 | Inverse variance weighted | 20 | 0.0903126789273602 | 0.0883815261191909 | 0.306851812145607 | -0.082915112 | 0.263540470120974 | 1.09451646243984 | 0.920429277059348 | 1.30152996695103 | 0.998735809224318 |
| ebi-fl187-GCST90094817 | prot-a-502 | Carcinoembryonic antigen-related cell adhesion molecule 1 \|\| id:prot-a-502 | Prawns liking \|\| id:ebi-fl187-GCST90094817 | Inverse variance weighted | 20 | 0.00731581729017987 | 0.0884112153109565 | 0.934052222615022 | -0.165970165 | 0.180601799299655 | 1.00734264325955 | 0.847071506428567 | 1.19793806452952 | 0.998735809224318 |
| ebi-fl187-GCST90094817 | prot-a-518 | Complement factor B \|\| id:prot-a-518 | Prawns liking \|\| id:ebi-fl187-GCST90094817 | Inverse variance weighted | 20 | 0.08127695155266 | 0.088349601510954 | 0.357600452161831 | -0.091888267 | 0.25444217051413 | 1.08467125636086 | 0.912207067110538 | 1.28974196407194 | 0.998735809224318 |
| ebi-fl187-GCST90094817 | prot-a-525 | Properdin \|\| id:prot-a-525 | Prawns liking \|\| id:ebi-fl187-GCST90094817 | Inverse variance weighted | 20 | 0.0476284020189608 | 0.101682014132898 | 0.639494724577874 | -0.151668346 | 0.24692514971944 | 1.04878085805561 | 0.85927321516121 | 1.28008329459857 | 0.998735809224318 |
| ebi-fl187-GCST90094817 | prot-a-533 | Chondroadherin \|\| id:prot-a-533 | Prawns liking \|\| id:ebi-fl187-GCST90094817 | Inverse variance weighted | 20 | -0.101143539 | 0.0883499666922559 | 0.252289682344136 | -0.274309474 | 0.0720223953740834 | 0.90380329224411 | 0.760096808613278 | 1.07467941164175 | 0.998735809224318 |
| ebi-fl187-GCST90094817 | prot-a-535 | Coiled-coil-helix-coiled-coil-helix domain-containing protein 2 \|\| id:prot-a-535 | Prawns liking \|\| id:ebi-fl187-GCST90094817 | Inverse variance weighted (multiplicative random effects) | 20 | -0.039144457 | 0.120333315472692 | 0.74495384548027 | -0.274997755 | 0.196708841325014 | 0.961611787557108 | 0.759573828217205 | 1.21738953557567 | 0.998735809224318 |
| ebi-fl187-GCST90094817 | prot-a-591 | Caseinolytic peptidase B protein homolog \|\| id:prot-a-591 | Prawns liking \|\| id:ebi-fl187-GCST90094817 | Inverse variance weighted | 19 | -0.133259734 | 0.0907120715964309 | 0.141821724850249 | -0.311055394 | 0.0445359266562202 | 0.875237733872576 | 0.732673289039726 | 1.04554253888307 | 0.998735809224318 |
| ebi-fl187-GCST90094817 | prot-a-605 | Ciliary neurotrophic factor receptor subunit alpha \|\| id:prot-a-605 | Prawns liking \|\| id:ebi-fl187-GCST90094817 | Inverse variance weighted | 20 | -0.021347125 | 0.0890749592240303 | 0.810598892370854 | -0.195934045 | 0.153239795061149 | 0.978879112157856 | 0.822066452203285 | 1.16560445162407 | 0.998735809224318 |
| ebi-fl187-GCST90094817 | prot-a-606 | Ciliary neurotrophic factor receptor subunit alpha \|\| id:prot-a-606 | Prawns liking \|\| id:ebi-fl187-GCST90094817 | Inverse variance weighted | 20 | -0.109381775 | 0.0883980979235761 | 0.215947145899859 | -0.282642047 | 0.0638784969080956 | 0.896388133565151 | 0.753789560844417 | 1.06596287310784 | 0.998735809224318 |
| ebi-fl187-GCST90094817 | prot-a-707 | Cancer/testis antigen 55 \|\| id:prot-a-707 | Prawns liking \|\| id:ebi-fl187-GCST90094817 | Inverse variance weighted | 20 | -0.092866895 | 0.100545035608924 | 0.355676483561098 | -0.289935165 | 0.104201374915189 | 0.911314792890042 | 0.748312083065297 | 1.10982392311277 | 0.998735809224318 |
| ebi-fl187-GCST90094817 | prot-a-719 | Dipeptidyl peptidase 1 \|\| id:prot-a-719 | Prawns liking \|\| id:ebi-fl187-GCST90094817 | Inverse variance weighted | 20 | -0.174844501 | 0.0969956346390605 | 0.0714507820542062 | -0.364955945 | 0.0152669431843681 | 0.839587565891016 | 0.694227234662277 | 1.0153840783007 | 0.998735809224318 |
| ebi-fl187-GCST90094817 | prot-a-752 | Cytochrome b5 \|\| id:prot-a-752 | Prawns liking \|\| id:ebi-fl187-GCST90094817 | Inverse variance weighted | 20 | -0.068014441 | 0.0900325217206597 | 0.44998320047689 | -0.244478184 | 0.108449301202522 | 0.934246981673487 | 0.783113072618858 | 1.11454840084226 | 0.998735809224318 |
| ebi-fl187-GCST90094817 | prot-a-755 | Cytoglobin \|\| id:prot-a-755 | Prawns liking \|\| id:ebi-fl187-GCST90094817 | Inverse variance weighted | 20 | -0.092274254 | 0.0883973596212184 | 0.296551284237916 | -0.265533079 | 0.0809845704760636 | 0.911855035010488 | 0.766797077402223 | 1.08435416536913 | 0.998735809224318 |
| ebi-fl187-GCST90094817 | prot-a-807 | Desmin \|\| id:prot-a-807 | Prawns liking \|\| id:ebi-fl187-GCST90094817 | Inverse variance weighted | 20 | -0.041423949 | 0.0883973596212184 | 0.6393479268074 | -0.214682774 | 0.131834876180692 | 0.959422297902158 | 0.806797337054171 | 1.14091991065912 | 0.998735809224318 |
| ebi-fl187-GCST90094817 | prot-a-82 | Ameloblastin \|\| id:prot-a-82 | Prawns liking \|\| id:ebi-fl187-GCST90094817 | Inverse variance weighted | 20 | -0.086718094 | 0.0978666588817707 | 0.375572192369597 | -0.278536745 | 0.105100557794813 | 0.916935549188951 | 0.756890455282699 | 1.11082230658123 | 0.998735809224318 |
| ebi-fl187-GCST90094817 | prot-a-84 | Muellerian-inhibiting factor \|\| id:prot-a-84 | Prawns liking \|\| id:ebi-fl187-GCST90094817 | Inverse variance weighted | 20 | -0.061389355 | 0.0883966197153722 | 0.48738357880401 | -0.234646729 | 0.11186802009233 | 0.940456997270766 | 0.790850184569156 | 1.11836524916206 | 0.998735809224318 |
| ebi-fl187-GCST90094817 | prot-a-858 | Inactive dipeptidyl peptidase 10 \|\| id:prot-a-858 | Prawns liking \|\| id:ebi-fl187-GCST90094817 | Inverse variance weighted | 20 | 0.106823833075896 | 0.0998348112283099 | 0.284616670884204 | -0.088852397 | 0.302500063083383 | 1.11273820954381 | 0.914980617554439 | 1.35323776178799 | 0.998735809224318 |
| ebi-fl187-GCST90094817 | prot-a-863 | Dihydropyrimidinase-related protein 3 \|\| id:prot-a-863 | Prawns liking \|\| id:ebi-fl187-GCST90094817 | Inverse variance weighted | 20 | -0.074659741 | 0.0883980979235761 | 0.398342374739763 | -0.247920013 | 0.0986005308158462 | 0.928059213004726 | 0.780422364390439 | 1.10362534717425 | 0.998735809224318 |
| ebi-fl187-GCST90094817 | prot-a-864 | Dihydropyrimidinase-related protein 5 \|\| id:prot-a-864 | Prawns liking \|\| id:ebi-fl187-GCST90094817 | Inverse variance weighted | 20 | -0.139034554 | 0.0883980979235761 | 0.115759827578671 | -0.312294826 | 0.0342257176734157 | 0.870197958895006 | 0.731765752715088 | 1.03481815711573 | 0.998735809224318 |
| ebi-fl187-GCST90094817 | prot-a-884 | Endothelin-converting enzyme 1 \|\| id:prot-a-884 | Prawns liking \|\| id:ebi-fl187-GCST90094817 | Inverse variance weighted | 20 | -0.192352673 | 0.0907044506098661 | 0.0339510071177682 | -0.370133396 | -0.01457195 | 0.825015856187522 | 0.690642195305372 | 0.985533707015794 | 0.998735809224318 |
| ebi-fl187-GCST90094817 | prot-a-893 | Early endosome antigen 1 \|\| id:prot-a-893 | Prawns liking \|\| id:ebi-fl187-GCST90094817 | Inverse variance weighted | 19 | -0.176963057 | 0.102802683210344 | 0.0851808787324142 | -0.378456316 | 0.0245302023732721 | 0.837810735428698 | 0.684917890483956 | 1.02483354304499 | 0.998735809224318 |
| ebi-fl187-GCST90094817 | prot-a-897 | Ephrin-A2 \|\| id:prot-a-897 | Prawns liking \|\| id:ebi-fl187-GCST90094817 | Inverse variance weighted | 20 | 0.0899364319382071 | 0.0956905427987299 | 0.347285526026824 | -0.097617032 | 0.277489895823718 | 1.09410473137741 | 0.906996187814176 | 1.31981278345536 | 0.998735809224318 |
| ebi-fl187-GCST90094817 | prot-a-90 | Anaphase-promoting complex subunit 10 \|\| id:prot-a-90 | Prawns liking \|\| id:ebi-fl187-GCST90094817 | Inverse variance weighted | 20 | -0.026655744 | 0.0884112153109565 | 0.76303529851377 | -0.199941726 | 0.146630238137509 | 0.973696384780773 | 0.818778465281135 | 1.1579257270886 | 0.998735809224318 |
| ebi-fl187-GCST90094817 | prot-a-914 | Histone-lysine N-methyltransferase EHMT2 \|\| id:prot-a-914 | Prawns liking \|\| id:ebi-fl187-GCST90094817 | Inverse variance weighted | 20 | -0.048967242 | 0.107103157812655 | 0.647529454483623 | -0.258889432 | 0.160954946872406 | 0.952212321339491 | 0.771908366880272 | 1.17463204677425 | 0.998735809224318 |
| ebi-fl187-GCST90094817 | prot-a-932 | RNA polymerase II elongation factor ELL \|\| id:prot-a-932 | Prawns liking \|\| id:ebi-fl187-GCST90094817 | Inverse variance weighted | 20 | -0.168922654 | 0.104848812992872 | 0.107156407852341 | -0.374426328 | 0.0365810192820621 | 0.844574225142463 | 0.687683670761736 | 1.0372583385394 | 0.998735809224318 |
| ebi-fl187-GCST90094817 | prot-a-991 | Estrogen receptor \|\| id:prot-a-991 | Prawns liking \|\| id:ebi-fl187-GCST90094817 | Inverse variance weighted | 20 | 0.0293297821877134 | 0.0883815261191909 | 0.739999342261528 | -0.143898009 | 0.202557573381328 | 1.02976413635434 | 0.865976065315114 | 1.22453058345871 | 0.998735809224318 |
| ebi-fl187-GCST90094817 | prot-a-992 | Persulfide dioxygenase ETHE1, mitochondrial \|\| id:prot-a-992 | Prawns liking \|\| id:ebi-fl187-GCST90094817 | Inverse variance weighted | 20 | 0.0879562736293063 | 0.0981475169325554 | 0.370165167321807 | -0.10441286 | 0.280325406817115 | 1.0919403743947 | 0.900853294740516 | 1.32356043785874 | 0.998735809224318 |
| ebi-fl187-GCST90094817 | prot-a-993 | Ethanolamine kinase 1 \|\| id:prot-a-993 | Prawns liking \|\| id:ebi-fl187-GCST90094817 | Inverse variance weighted | 20 | -0.061510995 | 0.109002718232214 | 0.572545232073831 | -0.275156322 | 0.152134333020047 | 0.940342606883541 | 0.759453394329777 | 1.16431663209681 | 0.998735809224318 |
| ebi-fl187-GCST90094817 | prot-b-10 | coagulation factor III, tissue factor \|\| id:prot-b-10 | Prawns liking \|\| id:ebi-fl187-GCST90094817 | Inverse variance weighted | 12 | -0.323243861 | 0.126298431980465 | 0.01048633969653 | -0.570788788 | -0.075698935 | 0.723797326697656 | 0.565079534922858 | 0.927095280854919 | 0.998735809224318 |
| ebi-fl187-GCST90094817 | prot-b-28 | prolactin \|\| id:prot-b-28 | Prawns liking \|\| id:ebi-fl187-GCST90094817 | Inverse variance weighted (multiplicative random effects) | 12 | -0.226506931 | 0.178932645069962 | 0.205556700484459 | -0.577214915 | 0.124201053752143 | 0.797313816442676 | 0.561459904650104 | 1.13224348991858 | 0.998735809224318 |
| ebi-fl187-GCST90094817 | prot-b-54 | matrix metallopeptidase 7 \|\| id:prot-b-54 | Prawns liking \|\| id:ebi-fl187-GCST90094817 | Inverse variance weighted | 12 | -0.048229271 | 0.126298431980465 | 0.70255962264268 | -0.295774197 | 0.199315655893224 | 0.952915286391478 | 0.743955396066153 | 1.22056718432325 | 0.998735809224318 |
| ebi-fl187-GCST90094817 | ukb-rsfMRI191-edge-pheno716 | Net100_Pair10_33 (OccipitalPrecuneus)&(Temporal\|Frontal\|Supp_Motor_Area) (Default_mode\|Central_executive)&(Default_mode\|Salience) \|\| id:ukb-rsfMRI191-edge-pheno716 | Prawns liking \|\| id:ebi-fl187-GCST90094817 | Inverse variance weighted | 19 | -0.018397107 | 0.0281033442020179 | 0.512710256744919 | -0.073479661 | 0.0366854480057772 | 0.981771087130491 | 0.929155043436491 | 1.03736666375989 | 0.998735809224318 |
| ebi-fl187-GCST90094817 | ukb-rsfMRI191-edge-pheno903 | Net100_Pair27_38 (Precentral\|Frontal\|Supp_Motor_Area)&(Subcortical) (Attention\|Salience\|Motor)&(Subcortical-cerebellum) \|\| id:ukb-rsfMRI191-edge-pheno903 | Prawns liking \|\| id:ebi-fl187-GCST90094817 | Inverse variance weighted | 19 | 0.0472402885753858 | 0.0338401785109861 | 0.162719840016228 | -0.019086461 | 0.113567038456919 | 1.04837389108502 | 0.981094531860908 | 1.12026698734528 | 0.998735809224318 |
| ebi-fl187-GCST90094817 | ukb-rsfMRI191-node-pheno23 | Net100_Node2 RolandicOper\|SupraMarginal\|Insula Salience\|Motor \|\| id:ukb-rsfMRI191-node-pheno23 | Prawns liking \|\| id:ebi-fl187-GCST90094817 | Inverse variance weighted (multiplicative random effects) | 19 | 0.0442905756388447 | 0.0441726976270605 | 0.316020798534809 | -0.042287912 | 0.130869062987883 | 1.0452860454242 | 0.958593750472163 | 1.13981852710846 | 0.998735809224318 |
| ebi-fl187-GCST90094817 | ukb-rsfMRI191-node-pheno55 | Net100_Node34 Parietal\|Postcentral\|Precuneus Attention \|\| id:ukb-rsfMRI191-node-pheno55 | Prawns liking \|\| id:ebi-fl187-GCST90094817 | Inverse variance weighted (multiplicative random effects) | 18 | -0.014883833 | 0.0352454246520491 | 0.672812479923452 | -0.083964865 | 0.0541971996158225 | 0.985226384042345 | 0.919463560861236 | 1.05569276383716 | 0.998735809224318 |
| ebi-fl187-GCST90094817 | ebi-a-GCST90001414 | IgD- CD24- B cell Absolute Count \|\| id:ebi-a-GCST90001414 | Prawns liking \|\| id:ebi-fl187-GCST90094817 | MR Egger | 16 | 0.460371435951332 | 0.744706599026988 | 0.546375155558394 | -0.999253498 | 1.91999637004423 | 1.58466247630873 | 0.368154166386641 | 6.82093370955813 | 0.999950574162623 |
| ebi-fl187-GCST90094817 | ebi-a-GCST90001422 | CD20- CD38- B cell %B cell \|\| id:ebi-a-GCST90001422 | Prawns liking \|\| id:ebi-fl187-GCST90094817 | MR Egger | 16 | -1.781634918 | 0.744845004417115 | 0.0313481325224786 | -3.241531126 | -0.321738709 | 0.168362663055647 | 0.0391039761151821 | 0.724887572243174 | 0.999950574162623 |
| ebi-fl187-GCST90094817 | ebi-a-GCST90001444 | CD20- CD38- B cell %lymphocyte \|\| id:ebi-a-GCST90001444 | Prawns liking \|\| id:ebi-fl187-GCST90094817 | MR Egger | 16 | -1.651202284 | 0.754766677315645 | 0.0461514566578968 | -3.130544971 | -0.171859596 | 0.191819148885603 | 0.0436939787996842 | 0.842097398542772 | 0.999950574162623 |
| ebi-fl187-GCST90094817 | ebi-a-GCST90001459 | Myeloid Dendritic Cell %Dendritic Cell \|\| id:ebi-a-GCST90001459 | Prawns liking \|\| id:ebi-fl187-GCST90094817 | MR Egger | 16 | 0.0883949397662546 | 0.773561261736585 | 0.910646283677337 | -1.427785133 | 1.60457501276996 | 1.09241947673582 | 0.239839547036301 | 4.97574452544785 | 0.999950574162623 |
| ebi-fl187-GCST90094817 | ebi-a-GCST90001476 | HLA DR++ monocyte %leukocyte \|\| id:ebi-a-GCST90001476 | Prawns liking \|\| id:ebi-fl187-GCST90094817 | MR Egger | 16 | -0.259760858 | 0.814912208515939 | 0.75461497430595 | -1.856988787 | 1.33746707083039 | 0.771235998778766 | 0.156142100368482 | 3.80938237931084 | 0.999950574162623 |
| ebi-fl187-GCST90094817 | ebi-a-GCST90001478 | CD4 regulatory T cell %CD4+ T cell \|\| id:ebi-a-GCST90001478 | Prawns liking \|\| id:ebi-fl187-GCST90094817 | MR Egger | 16 | -0.183399482 | 0.831557064557851 | 0.828627134451086 | -1.813251329 | 1.44645236417683 | 0.832435545992053 | 0.163122907448406 | 4.24801733288294 | 0.999950574162623 |
| ebi-fl187-GCST90094817 | ebi-a-GCST90001485 | CD39+ resting CD4 regulatory T cell %CD4 regulatory T cell \|\| id:ebi-a-GCST90001485 | Prawns liking \|\| id:ebi-fl187-GCST90094817 | MR Egger | 16 | -0.142186401 | 0.746483242480834 | 0.851671315926027 | -1.605293556 | 1.32092075409292 | 0.867459545940995 | 0.200830591144297 | 3.74686973511668 | 0.999950574162623 |
| ebi-fl187-GCST90094817 | ebi-a-GCST90001515 | Immature Myeloid-Derived Suppressor Cells Absolute Count \|\| id:ebi-a-GCST90001515 | Prawns liking \|\| id:ebi-fl187-GCST90094817 | MR Egger | 16 | 0.803144488040313 | 1.02334622312163 | 0.445636137933698 | -1.202614109 | 2.8089030853587 | 2.2325501297246 | 0.300407885547647 | 16.5917085453563 | 0.999950574162623 |
| ebi-fl187-GCST90094817 | ebi-a-GCST90001517 | CD33+ HLA DR+ Absolute Count \|\| id:ebi-a-GCST90001517 | Prawns liking \|\| id:ebi-fl187-GCST90094817 | MR Egger | 16 | -0.084009482 | 1.00072622240341 | 0.934285954506487 | -2.045432878 | 1.87741391343627 | 0.919422537653104 | 0.129324196209791 | 6.53657882685122 | 0.999950574162623 |
| ebi-fl187-GCST90094817 | ebi-a-GCST90001518 | CD33+ HLA DR+ CD14- Absolute Count \|\| id:ebi-a-GCST90001518 | Prawns liking \|\| id:ebi-fl187-GCST90094817 | MR Egger | 16 | 0.0423035047871492 | 1.0143100087954 | 0.967321499545338 | -1.945744112 | 2.03035112202613 | 1.04321105025561 | 0.142880864341189 | 7.61676030162205 | 0.999950574162623 |
| ebi-fl187-GCST90094817 | ebi-a-GCST90001520 | CD33+ HLA DR+ CD14dim Absolute Count \|\| id:ebi-a-GCST90001520 | Prawns liking \|\| id:ebi-fl187-GCST90094817 | MR Egger | 16 | 0.0326859440351452 | 1.00350068511458 | 0.974475781872357 | -1.934175399 | 1.99954728685971 | 1.03322599749428 | 0.144543410365829 | 7.38571173321663 | 0.999950574162623 |
| ebi-fl187-GCST90094817 | ebi-a-GCST90001531 | CD33dim HLA DR- Absolute Count \|\| id:ebi-a-GCST90001531 | Prawns liking \|\| id:ebi-fl187-GCST90094817 | MR Egger | 16 | 0.770400295661573 | 1.02195607472859 | 0.463431115994571 | -1.232633611 | 2.7734342021296 | 2.16063097190623 | 0.291523805557262 | 16.0135333985391 | 0.999950574162623 |
| ebi-fl187-GCST90094817 | ebi-a-GCST90001532 | Basophil Absolute Count \|\| id:ebi-a-GCST90001532 | Prawns liking \|\| id:ebi-fl187-GCST90094817 | MR Egger | 16 | 0.730993137586928 | 1.01872966859611 | 0.484834213087872 | -1.265717013 | 2.7277032880353 | 2.07714247190947 | 0.282036999468512 | 15.2977122035079 | 0.999950574162623 |
| ebi-fl187-GCST90094817 | ebi-a-GCST90001588 | T/B cell \|\| id:ebi-a-GCST90001588 | Prawns liking \|\| id:ebi-fl187-GCST90094817 | MR Egger | 16 | -0.034734272 | 0.741093849384195 | 0.963279716477616 | -1.487278217 | 1.41780967272677 | 0.965862038684011 | 0.225986906583532 | 4.12806870926395 | 0.999950574162623 |
| ebi-fl187-GCST90094817 | ebi-a-GCST90001624 | HLA DR+ CD4+ T cell Absolute Count \|\| id:ebi-a-GCST90001624 | Prawns liking \|\| id:ebi-fl187-GCST90094817 | MR Egger | 16 | 0.763997728096052 | 0.7202381806722 | 0.306757985390554 | -0.647669106 | 2.17566456221356 | 2.14684157699628 | 0.523264029375862 | 8.80803666595868 | 0.999950574162623 |
| ebi-fl187-GCST90094817 | ebi-a-GCST90001631 | CD8+ Natural Killer T %T cell \|\| id:ebi-a-GCST90001631 | Prawns liking \|\| id:ebi-fl187-GCST90094817 | MR Egger | 16 | -1.068300945 | 0.730498616983747 | 0.165708639564683 | -2.500078235 | 0.363476344074468 | 0.343591803058788 | 0.0820785769961242 | 1.43832083169233 | 0.999950574162623 |
| ebi-fl187-GCST90094817 | ebi-a-GCST90001632 | CD8+ Natural Killer T %lymphocyte \|\| id:ebi-a-GCST90001632 | Prawns liking \|\| id:ebi-fl187-GCST90094817 | MR Egger | 16 | -1.129836346 | 0.734801375297639 | 0.146434481649273 | -2.570047042 | 0.310374349573994 | 0.323086126429846 | 0.0765319451546346 | 1.36393560728831 | 0.999950574162623 |
| ebi-fl187-GCST90094817 | ebi-a-GCST90001646 | Natural Killer %CD3- lymphocyte \|\| id:ebi-a-GCST90001646 | Prawns liking \|\| id:ebi-fl187-GCST90094817 | MR Egger | 16 | 0.0529663892418866 | 0.715473952225586 | 0.942034031783457 | -1.349362557 | 1.45529533560403 | 1.05439420553519 | 0.259405564184366 | 4.28574901298581 | 0.999950574162623 |
| ebi-fl187-GCST90094817 | ebi-a-GCST90001684 | CD127- CD8+ T cell Absolute Count \|\| id:ebi-a-GCST90001684 | Prawns liking \|\| id:ebi-fl187-GCST90094817 | MR Egger | 16 | -0.502785097 | 0.775603222179717 | 0.527312867937418 | -2.022967412 | 1.01739721850048 | 0.604843763184594 | 0.132262405076275 | 2.76598612925816 | 0.999950574162623 |
| ebi-fl187-GCST90094817 | ebi-a-GCST90001687 | CD28- CD8+ T cell Absolute Count \|\| id:ebi-a-GCST90001687 | Prawns liking \|\| id:ebi-fl187-GCST90094817 | MR Egger | 16 | -0.199657504 | 0.755536581112895 | 0.795430589644961 | -1.680509203 | 1.28119419539155 | 0.819011213447483 | 0.186279098089741 | 3.60093738176448 | 0.999950574162623 |
| ebi-fl187-GCST90094817 | ebi-a-GCST90001701 | BAFF-R on CD20- CD38- B cell \|\| id:ebi-a-GCST90001701 | Prawns liking \|\| id:ebi-fl187-GCST90094817 | MR Egger | 16 | -0.758389706 | 0.805244139817731 | 0.36225692951961 | -2.33666822 | 0.819888808348775 | 0.468420114260723 | 0.0966491160036193 | 2.2702473909416 | 0.999950574162623 |
| ebi-fl187-GCST90094817 | ebi-a-GCST90001732 | CD19 on IgD- CD27- B cell \|\| id:ebi-a-GCST90001732 | Prawns liking \|\| id:ebi-fl187-GCST90094817 | MR Egger | 16 | 0.594186187786314 | 1.06721329448411 | 0.586479211100535 | -1.497551869 | 2.68592424497518 | 1.81155607844695 | 0.223677081114522 | 14.6717554118915 | 0.999950574162623 |
| ebi-fl187-GCST90094817 | ebi-a-GCST90001798 | CD27 on CD24+ CD27+ B cell \|\| id:ebi-a-GCST90001798 | Prawns liking \|\| id:ebi-fl187-GCST90094817 | MR Egger | 16 | 2.0046194308865 | 0.921899399598733 | 0.0473096248583517 | 0.197696607672983 | 3.81154225410002 | 7.42326829248973 | 1.21859262607962 | 45.2201260396294 | 0.999950574162623 |
| ebi-fl187-GCST90094817 | ebi-a-GCST90001836 | CD66b on CD66b++ myeloid cell \|\| id:ebi-a-GCST90001836 | Prawns liking \|\| id:ebi-fl187-GCST90094817 | MR Egger | 16 | -1.498501324 | 1.58912187699552 | 0.3616831926548 | -4.613180203 | 1.6161775552939 | 0.223464810753552 | 0.00992021978159033 | 5.03381202680524 | 0.999950574162623 |
| ebi-fl187-GCST90094817 | ebi-a-GCST90001943 | CD25 on activated & secreting CD4 regulatory T cell \|\| id:ebi-a-GCST90001943 | Prawns liking \|\| id:ebi-fl187-GCST90094817 | MR Egger | 16 | -0.085063918 | 0.852759030897999 | 0.921956013009381 | -1.756471618 | 1.58634378299177 | 0.918453577206009 | 0.172652975996763 | 4.88585249464989 | 0.999950574162623 |
| ebi-fl187-GCST90094817 | ebi-a-GCST90001960 | CD25 on CD4+ T cell \|\| id:ebi-a-GCST90001960 | Prawns liking \|\| id:ebi-fl187-GCST90094817 | MR Egger | 16 | 0.0298701192217995 | 0.91068924383213 | 0.974297540500548 | -1.755080799 | 1.81482103713277 | 1.03032070640775 | 0.172893272177581 | 6.13997725118081 | 0.999950574162623 |
| ebi-fl187-GCST90094817 | ebi-a-GCST90001988 | HLA DR on CD14+ CD16- monocyte \|\| id:ebi-a-GCST90001988 | Prawns liking \|\| id:ebi-fl187-GCST90094817 | MR Egger | 16 | -0.320383782 | 0.742038556585062 | 0.672489883230782 | -1.774779353 | 1.13401178865344 | 0.725870407 | 0.16952084961299 | 3.10810056453503 | 0.999950574162623 |
| ebi-fl187-GCST90094817 | ebi-a-GCST90001991 | HLA DR on CD14+ monocyte \|\| id:ebi-a-GCST90001991 | Prawns liking \|\| id:ebi-fl187-GCST90094817 | MR Egger | 16 | -0.263192951 | 0.743291256973504 | 0.728546742029752 | -1.720043814 | 1.19365791293323 | 0.768593582306376 | 0.179058302407016 | 3.29912708219333 | 0.999950574162623 |
| ebi-fl187-GCST90094817 | ebi-a-GCST90002019 | CD14 on Monocytic Myeloid-Derived Suppressor Cells \|\| id:ebi-a-GCST90002019 | Prawns liking \|\| id:ebi-fl187-GCST90094817 | MR Egger | 16 | 0.297847762519661 | 1.11628450941175 | 0.79349723556365 | -1.890069876 | 2.48576540096669 | 1.34695671497365 | 0.151061252922436 | 12.0103094401326 | 0.999950574162623 |
| ebi-fl187-GCST90094817 | ebi-a-GCST90002037 | CD80 on plasmacytoid Dendritic Cell \|\| id:ebi-a-GCST90002037 | Prawns liking \|\| id:ebi-fl187-GCST90094817 | MR Egger | 16 | -1.273718477 | 0.840135247947957 | 0.151747786509433 | -2.920383563 | 0.372946608512873 | 0.279789294856915 | 0.0539130042764346 | 1.45200681296009 | 0.999950574162623 |
| ebi-fl187-GCST90094817 | ebi-a-GCST90002042 | CD45 on CD33+ HLA DR+ CD14- \|\| id:ebi-a-GCST90002042 | Prawns liking \|\| id:ebi-fl187-GCST90094817 | MR Egger | 16 | 0.873200762483035 | 1.137394022 | 0.455407468670531 | -1.35609152 | 3.10249304499151 | 2.39456302809826 | 0.257665893419793 | 22.2533608132347 | 0.999950574162623 |
| ebi-fl187-GCST90094817 | ebi-a-GCST90002056 | CD8 on Effector Memory CD8+ T cell \|\| id:ebi-a-GCST90002056 | Prawns liking \|\| id:ebi-fl187-GCST90094817 | MR Egger | 16 | 0.99300513968661 | 0.823095142001987 | 0.247644222971016 | -0.620261339 | 2.6062716180105 | 2.69933417213713 | 0.537803870297052 | 13.5484427972648 | 0.999950574162623 |
| ebi-fl187-GCST90094817 | ebi-a-GCST90002058 | CD8 on CD8+ T cell \|\| id:ebi-a-GCST90002058 | Prawns liking \|\| id:ebi-fl187-GCST90094817 | MR Egger | 16 | 0.0887046545185953 | 0.876156987223803 | 0.920793345449979 | -1.62856304 | 1.80597234947725 | 1.09275786756312 | 0.196211319381314 | 6.08588618070736 | 0.999950574162623 |
| ebi-fl187-GCST90094817 | ebi-a-GCST90002063 | CD4 on CD4 regulatory T cell \|\| id:ebi-a-GCST90002063 | Prawns liking \|\| id:ebi-fl187-GCST90094817 | MR Egger | 16 | 0.633035758091898 | 0.819263353541856 | 0.452554890263308 | -0.972720415 | 2.23879193103393 | 1.88331921140274 | 0.378053176437729 | 9.38199034712475 | 0.999950574162623 |
| ebi-fl187-GCST90094817 | ebi-a-GCST90002066 | CD4 on activated CD4 regulatory T cell \|\| id:ebi-a-GCST90002066 | Prawns liking \|\| id:ebi-fl187-GCST90094817 | MR Egger | 16 | 0.61708818214745 | 0.819455019522223 | 0.46389748461312 | -0.989043656 | 2.22322002041101 | 1.85352305497505 | 0.371932216092918 | 9.23702644372639 | 0.999950574162623 |
| ebi-fl187-GCST90094817 | ebi-a-GCST90002070 | CD4 on activated & secreting CD4 regulatory T cell \|\| id:ebi-a-GCST90002070 | Prawns liking \|\| id:ebi-fl187-GCST90094817 | MR Egger | 16 | 0.480298822407654 | 0.82321688311321 | 0.568876900244939 | -1.133206268 | 2.09380391330955 | 1.61655739359744 | 0.321999183712839 | 8.11572804832034 | 0.999950574162623 |
| ebi-fl187-GCST90094817 | ebi-a-GCST90002081 | SSC-A on CD4+ T cell \|\| id:ebi-a-GCST90002081 | Prawns liking \|\| id:ebi-fl187-GCST90094817 | MR Egger | 16 | 1.44891755912787 | 0.776447807345667 | 0.083117946312775 | -0.072920143 | 2.97075526152538 | 4.2585024423734 | 0.929675067872778 | 19.5066466536477 | 0.999950574162623 |
| ebi-fl187-GCST90094817 | ebi-a-GCST90002094 | CD11b on Monocytic Myeloid-Derived Suppressor Cells \|\| id:ebi-a-GCST90002094 | Prawns liking \|\| id:ebi-fl187-GCST90094817 | MR Egger | 16 | -0.961656175 | 1.12851019133925 | 0.408474014295696 | -3.17353615 | 1.25022379987239 | 0.382259273123036 | 0.0418553291780054 | 3.49112418318616 | 0.999950574162623 |
| ebi-fl187-GCST90094817 | ebi-a-GCST90002104 | HLA DR on myeloid Dendritic Cell \|\| id:ebi-a-GCST90002104 | Prawns liking \|\| id:ebi-fl187-GCST90094817 | MR Egger | 16 | -2.482706846 | 0.836347509307808 | 0.0101663880436706 | -4.121947964 | -0.843465728 | 0.0835168521120117 | 0.0162129015125346 | 0.430216922079434 | 0.999950574162623 |
| ebi-fl187-GCST90094817 | ebi-a-GCST90002105 | HLA DR on plasmacytoid Dendritic Cell \|\| id:ebi-a-GCST90002105 | Prawns liking \|\| id:ebi-fl187-GCST90094817 | MR Egger | 15 | -0.878392822 | 1.02429079875693 | 0.40666685973818 | -2.886002787 | 1.12921714378126 | 0.415450077726059 | 0.0557988071342429 | 3.093233995 | 0.999950574162623 |
| ebi-fl187-GCST90094817 | ebi-a-GCST90002106 | HLA DR on Dendritic Cell \|\| id:ebi-a-GCST90002106 | Prawns liking \|\| id:ebi-fl187-GCST90094817 | MR Egger | 16 | -0.746009353 | 0.835633710436132 | 0.387081020127586 | -2.383851425 | 0.89183271995251 | 0.47425536743286 | 0.0921948114492373 | 2.43959665412101 | 0.999950574162623 |
| ebi-fl187-GCST90094817 | ebi-a-GCST90002111 | HLA DR on CD33dim HLA DR+ CD11b- \|\| id:ebi-a-GCST90002111 | Prawns liking \|\| id:ebi-fl187-GCST90094817 | MR Egger | 16 | -1.025598407 | 1.22460956842457 | 0.416385607147891 | -3.425833161 | 1.37463634683154 | 0.358581823216927 | 0.0325221734788549 | 3.95363870822395 | 0.999950574162623 |
| ebi-fl187-GCST90094817 | ebi-a-GCST90002112 | HLA DR on CD33- HLA DR+ \|\| id:ebi-a-GCST90002112 | Prawns liking \|\| id:ebi-fl187-GCST90094817 | MR Egger | 16 | 0.841491876405878 | 1.21093904089801 | 0.498484417085468 | -1.531948644 | 3.21493239656597 | 2.31982528906693 | 0.216114127289387 | 24.901608420019 | 0.999950574162623 |
| ebi-fl187-GCST90094817 | ebi-a-GCST90002115 | HLA DR on HLA DR+ CD8+ T cell \|\| id:ebi-a-GCST90002115 | Prawns liking \|\| id:ebi-fl187-GCST90094817 | MR Egger | 16 | -0.313876821 | 0.808252318096607 | 0.70360567504438 | -1.898051365 | 1.27029772214472 | 0.730609018067955 | 0.149860358053732 | 3.56191286484736 | 0.999950574162623 |
| ebi-fl187-GCST90094817 | ebi-a-GCST90002116 | HLA DR on B cell \|\| id:ebi-a-GCST90002116 | Prawns liking \|\| id:ebi-fl187-GCST90094817 | MR Egger | 16 | -2.859488031 | 0.835627495455132 | 0.004128520144687 | -4.497317922 | -1.22165814 | 0.0572980875896148 | 0.011138831722273 | 0.29474103957082 | 0.999950574162623 |
| ebi-fl187-GCST90094817 | ebi-cfmet338-GCST90026023 | Sphingomyelin (d18:1/24:1, d18:2/24:0) levels \|\| id:ebi-cfmet338-GCST90026023 | Prawns liking \|\| id:ebi-fl187-GCST90094817 | MR Egger | 13 | -0.283271186 | 0.495420821546092 | 0.578968300897951 | -1.254295996 | 0.68775362454547 | 0.753315471785033 | 0.285276613447555 | 1.98924192618766 | 0.999950574162623 |
| ebi-fl187-GCST90094817 | ebi-cfmet338-GCST90026046 | 1-arachidonoyl-gpc (20:4n6) levels \|\| id:ebi-cfmet338-GCST90026046 | Prawns liking \|\| id:ebi-fl187-GCST90094817 | MR Egger | 13 | -0.436176147 | 0.604605029171561 | 0.485700748313959 | -1.621202004 | 0.748849709943683 | 0.646503836043471 | 0.197660966881015 | 2.11456625257998 | 0.999950574162623 |
| ebi-fl187-GCST90094817 | ebi-cfmet338-GCST90026047 | 1-linoleoyl-gpc (18:2) levels \|\| id:ebi-cfmet338-GCST90026047 | Prawns liking \|\| id:ebi-fl187-GCST90094817 | MR Egger | 13 | 0.0800149742916342 | 0.71746064195508 | 0.913208843212927 | -1.326207884 | 1.48623783252359 | 1.08330328925289 | 0.265482093786388 | 4.42043378432288 | 0.999950574162623 |
| ebi-fl187-GCST90094817 | ebi-cfmet338-GCST90026050 | 1-myristoyl-2-palmitoyl-gpc (14:0/16:0) levels \|\| id:ebi-cfmet338-GCST90026050 | Prawns liking \|\| id:ebi-fl187-GCST90094817 | MR Egger | 13 | -0.44365431 | 0.437339284458646 | 0.332166868685644 | -1.300839307 | 0.413530687735489 | 0.641687207467246 | 0.272303151062945 | 1.51214729106065 | 0.999950574162623 |
| ebi-fl187-GCST90094817 | ebi-cfmet338-GCST90026068 | 2,3-dihydroxyisovalerate levels \|\| id:ebi-cfmet338-GCST90026068 | Prawns liking \|\| id:ebi-fl187-GCST90094817 | MR Egger | 13 | 0.374695542990466 | 1.22340704011038 | 0.76511638802066 | -2.023182256 | 2.77257334160682 | 1.4545484997109 | 0.132233992452565 | 15.9997539117651 | 0.999950574162623 |
| ebi-fl187-GCST90094817 | ebi-cfmet338-GCST90026082 | 3-hydroxyhexanoate levels \|\| id:ebi-cfmet338-GCST90026082 | Prawns liking \|\| id:ebi-fl187-GCST90094817 | MR Egger | 13 | -0.513611326 | 0.26133024622652 | 0.0751227621587964 | -1.025818608 | -0.001404043 | 0.598330904685308 | 0.35850287180465 | 0.998596942053548 | 0.999950574162623 |
| ebi-fl187-GCST90094817 | ebi-cfmet338-GCST90026094 | 3-ureidopropionate levels \|\| id:ebi-cfmet338-GCST90026094 | Prawns liking \|\| id:ebi-fl187-GCST90094817 | MR Egger | 13 | 0.323616118099652 | 0.618796739848629 | 0.61136262208264 | -0.889225492 | 1.53645772820296 | 1.3821166357078 | 0.410973932116921 | 4.64809625481744 | 0.999950574162623 |
| ebi-fl187-GCST90094817 | ebi-cfmet338-GCST90026173 | Glycerol levels \|\| id:ebi-cfmet338-GCST90026173 | Prawns liking \|\| id:ebi-fl187-GCST90094817 | MR Egger | 13 | -0.148070415 | 0.43227762667787 | 0.738403744227942 | -0.995334564 | 0.699193733072787 | 0.862370388806988 | 0.369599769262541 | 2.01212973962343 | 0.999950574162623 |
| ebi-fl187-GCST90094817 | ebi-cfmet338-GCST90026179 | Histidine levels \|\| id:ebi-cfmet338-GCST90026179 | Prawns liking \|\| id:ebi-fl187-GCST90094817 | MR Egger | 13 | -0.544662601 | 0.231574005652501 | 0.0383515241313244 | -0.998547653 | -0.09077755 | 0.580037454125024 | 0.368414118122244 | 0.913220833942643 | 0.999950574162623 |
| ebi-fl187-GCST90094817 | ebi-cfmet338-GCST90026188 | Isocitrate levels \|\| id:ebi-cfmet338-GCST90026188 | Prawns liking \|\| id:ebi-fl187-GCST90094817 | MR Egger | 13 | -0.022724098 | 0.45777176757915 | 0.961298633010863 | -0.919956762 | 0.874508566896489 | 0.977532150077479 | 0.398536272617847 | 2.39769669686098 | 0.999950574162623 |
| ebi-fl187-GCST90094817 | ebi-cfmet338-GCST90026207 | N-acetyl-isoputreanine levels \|\| id:ebi-cfmet338-GCST90026207 | Prawns liking \|\| id:ebi-fl187-GCST90094817 | MR Egger | 13 | -0.17701359 | 0.330128875293088 | 0.602497724634645 | -0.824066186 | 0.470039005510998 | 0.837768399119925 | 0.43864441372686 | 1.60005660302562 | 0.999950574162623 |
| ebi-fl187-GCST90094817 | ebi-cfmet338-GCST90026213 | N-acetylglutamate levels \|\| id:ebi-cfmet338-GCST90026213 | Prawns liking \|\| id:ebi-fl187-GCST90094817 | MR Egger | 13 | 0.0508719833848571 | 0.496003177001155 | 0.920155014493238 | -0.921294244 | 1.02303821030712 | 1.05218818709087 | 0.398003594020024 | 2.78163312514677 | 0.999950574162623 |
| ebi-fl187-GCST90094817 | ebi-cfmet338-GCST90026224 | N-acetyltaurine levels \|\| id:ebi-cfmet338-GCST90026224 | Prawns liking \|\| id:ebi-fl187-GCST90094817 | MR Egger | 13 | -0.675731607 | 0.486432469721763 | 0.192264472026459 | -1.629139248 | 0.277676033405003 | 0.508784054308577 | 0.196098293520816 | 1.32005847307995 | 0.999950574162623 |
| ebi-fl187-GCST90094817 | ebi-cfmet338-GCST90026250 | Phenyllactate (pla) levels \|\| id:ebi-cfmet338-GCST90026250 | Prawns liking \|\| id:ebi-fl187-GCST90094817 | MR Egger | 13 | -0.050381711 | 0.783617677255273 | 0.949889860846328 | -1.586272358 | 1.4855089368399 | 0.950866399454623 | 0.204687191976616 | 4.41721292319596 | 0.999950574162623 |
| ebi-fl187-GCST90094817 | ebi-cfmet338-GCST90026251 | Pipecolate levels \|\| id:ebi-cfmet338-GCST90026251 | Prawns liking \|\| id:ebi-fl187-GCST90094817 | MR Egger | 13 | -0.899349185 | 1.1158494986866 | 0.437333291780119 | -3.086414202 | 1.28771583288279 | 0.406834347681688 | 0.045665408103984 | 3.62449813383239 | 0.999950574162623 |
| ebi-fl187-GCST90094817 | ebi-cfmet338-GCST90026278 | Trigonelline (n'-methylnicotinate) levels \|\| id:ebi-cfmet338-GCST90026278 | Prawns liking \|\| id:ebi-fl187-GCST90094817 | MR Egger | 12 | 1.20995703198045 | 1.12335891451632 | 0.306737876920798 | -0.99182644 | 3.41174050443243 | 3.35334056305055 | 0.370898647706652 | 30.3179669198846 | 0.999950574162623 |
| ebi-fl187-GCST90094817 | ebi-cfmet338-GCST90026299 | 1-carboxyethylvaline levels \|\| id:ebi-cfmet338-GCST90026299 | Prawns liking \|\| id:ebi-fl187-GCST90094817 | MR Egger | 13 | -0.152614499 | 0.638693495009882 | 0.815539389245169 | -1.404453749 | 1.09922475150882 | 0.858460595697722 | 0.245501125084309 | 3.00183795130311 | 0.999950574162623 |
| ebi-fl187-GCST90094817 | ebi-gm430-GCST90011306 | C_Erysipelotrichia abundance \|\| id:ebi-gm430-GCST90011306 | Prawns liking \|\| id:ebi-fl187-GCST90094817 | MR Egger | 13 | -0.005843151 | 0.683921400522942 | 0.993336254912982 | -1.346329096 | 1.33464279366703 | 0.994173886649586 | 0.26019365549607 | 3.79863880620591 | 0.999950574162623 |
| ebi-fl187-GCST90094817 | ebi-gm430-GCST90011312 | F_Erysipelotrichaceae abundance \|\| id:ebi-gm430-GCST90011312 | Prawns liking \|\| id:ebi-fl187-GCST90094817 | MR Egger | 13 | -0.005843151 | 0.683921400522942 | 0.993336254912982 | -1.346329096 | 1.33464279366703 | 0.994173886649586 | 0.26019365549607 | 3.79863880620591 | 0.999950574162623 |
| ebi-fl187-GCST90094817 | ebi-gm430-GCST90011352 | O_Erysipelotrichales abundance \|\| id:ebi-gm430-GCST90011352 | Prawns liking \|\| id:ebi-fl187-GCST90094817 | MR Egger | 13 | -0.005843151 | 0.683921400522942 | 0.993336254912982 | -1.346329096 | 1.33464279366703 | 0.994173886649586 | 0.26019365549607 | 3.79863880620591 | 0.999950574162623 |
| ebi-fl187-GCST90094817 | ebi-gm430-GCST90011362 | OTU97_11 (Parabacteroides) abundance \|\| id:ebi-gm430-GCST90011362 | Prawns liking \|\| id:ebi-fl187-GCST90094817 | MR Egger | 13 | 0.78654772071834 | 0.719209053853539 | 0.297488444758596 | -0.623102025 | 2.19619746627128 | 2.19580280162267 | 0.536278306116272 | 8.99076074609772 | 0.999950574162623 |
| ebi-fl187-GCST90094817 | ebi-gm430-GCST90011384 | OTU97_235 (Phascolarctobacterium) abundance \|\| id:ebi-gm430-GCST90011384 | Prawns liking \|\| id:ebi-fl187-GCST90094817 | MR Egger | 13 | 0.439891061404315 | 3.00561605642263 | 0.886287802435178 | -5.451116409 | 6.33089853199268 | 1.55253807798057 | 0.00429151093313353 | 561.661037600954 | 0.999950574162623 |
| ebi-fl187-GCST90094817 | ebi-gm430-GCST90011390 | OTU97_3 (Bacteroides) abundance \|\| id:ebi-gm430-GCST90011390 | Prawns liking \|\| id:ebi-fl187-GCST90094817 | MR Egger | 13 | -0.139148932 | 0.572212963062081 | 0.81234359631031 | -1.26068634 | 0.98238847562103 | 0.870098433325027 | 0.283459410307735 | 2.6708278368771 | 0.999950574162623 |
| ebi-fl187-GCST90094817 | ebi-gm430-GCST90011398 | OTU97_4 (Alistipes) abundance \|\| id:ebi-gm430-GCST90011398 | Prawns liking \|\| id:ebi-fl187-GCST90094817 | MR Egger | 13 | 0.148352583331579 | 0.668841261220211 | 0.828529464936379 | -1.162576289 | 1.45928145532319 | 1.15992179336388 | 0.31267958944204 | 4.30286661537871 | 0.999950574162623 |
| ebi-fl187-GCST90094817 | ebi-gm430-GCST90011421 | OTU99_108 (Cytophagales) abundance \|\| id:ebi-gm430-GCST90011421 | Prawns liking \|\| id:ebi-fl187-GCST90094817 | MR Egger | 13 | -0.183617706 | 2.74637832605903 | 0.947894377935781 | -5.566519225 | 5.19928381283386 | 0.832253908492507 | 0.00382376696671089 | 181.142463500293 | 0.999950574162623 |
| ebi-fl187-GCST90094817 | ebi-gm430-GCST90011428 | OTU99_123 (Phascolarctobacterium) abundance \|\| id:ebi-gm430-GCST90011428 | Prawns liking \|\| id:ebi-fl187-GCST90094817 | MR Egger | 13 | 3.09208127201284 | 1.26033278034112 | 0.0320527591186093 | 0.621829022544246 | 5.56233352148143 | 22.0228658850972 | 1.86233117439737 | 260.429846453018 | 0.999950574162623 |
| ebi-fl187-GCST90094817 | ebi-gm430-GCST90011450 | OTU99_298 (Phascolarctobacterium) abundance \|\| id:ebi-gm430-GCST90011450 | Prawns liking \|\| id:ebi-fl187-GCST90094817 | MR Egger | 13 | 0.494276066016727 | 2.98407105647877 | 0.871446730402437 | -5.354503205 | 6.34305533671511 | 1.63931105640507 | 0.00472681717494295 | 568.530713203299 | 0.999950574162623 |
| ebi-fl187-GCST90094817 | ebi-gm430-GCST90011451 | OTU99_3 (Bacteroides) abundance \|\| id:ebi-gm430-GCST90011451 | Prawns liking \|\| id:ebi-fl187-GCST90094817 | MR Egger | 13 | -0.145862241 | 0.572219295285073 | 0.803498464139141 | -1.26741206 | 0.975687577376339 | 0.864276756555345 | 0.281559338363232 | 2.65299072040784 | 0.999950574162623 |
| ebi-fl187-GCST90094817 | ebi-gm430-GCST90011472 | OTU99_72 (Parabacteroides) abundance \|\| id:ebi-gm430-GCST90011472 | Prawns liking \|\| id:ebi-fl187-GCST90094817 | MR Egger | 13 | 2.07074012804908 | 1.71210077500245 | 0.251833645684503 | -1.284977391 | 5.42645764705388 | 7.93069067282615 | 0.276656838507976 | 227.342490022123 | 0.999950574162623 |
| ebi-fl187-GCST90094817 | ebi-gm430-GCST90011507 | TestASV_3 (Bacteroides) abundance \|\| id:ebi-gm430-GCST90011507 | Prawns liking \|\| id:ebi-fl187-GCST90094817 | MR Egger | 13 | 0.852379135726188 | 0.736804431285932 | 0.271835130526369 | -0.59175755 | 2.29651582104661 | 2.34521981612847 | 0.553353882691898 | 9.93949108878676 | 0.999950574162623 |
| ebi-fl187-GCST90094817 | ebi-gm430-GCST90011512 | TestASV_34 (Phascolarctobacterium) abundance \|\| id:ebi-gm430-GCST90011512 | Prawns liking \|\| id:ebi-fl187-GCST90094817 | MR Egger | 13 | 2.28918694058371 | 2.55575860856655 | 0.389594357963584 | -2.720099932 | 7.29847381337415 | 9.86691203321266 | 0.0658681717457417 | 1478.04243674717 | 0.999950574162623 |
| ebi-fl187-GCST90094817 | ebi-gm430-GCST90011519 | TestASV_41 (Barnesiella) abundance \|\| id:ebi-gm430-GCST90011519 | Prawns liking \|\| id:ebi-fl187-GCST90094817 | MR Egger | 13 | -1.514641725 | 2.70458827029479 | 0.586684822377541 | -6.815634735 | 3.78635128440246 | 0.219886950651741 | 0.00109649699006034 | 44.0952154955395 | 0.999950574162623 |
| ebi-fl187-GCST90094817 | ebi-gm430-GCST90011524 | TestASV_46 (Sutterella) abundance \|\| id:ebi-gm430-GCST90011524 | Prawns liking \|\| id:ebi-fl187-GCST90094817 | MR Egger | 13 | 2.40578067735591 | 3.44651967213611 | 0.499647175939719 | -4.34939788 | 9.16095923474268 | 11.0870823386396 | 0.0129145863702146 | 9518.1828716773 | 0.999950574162623 |
| ebi-fl187-GCST90094817 | ebi-gm430-GCST90011581 | OTU97_19 (Bacteria) prevalence \|\| id:ebi-gm430-GCST90011581 | Prawns liking \|\| id:ebi-fl187-GCST90094817 | MR Egger | 13 | -3.223066831 | 2.68025634380238 | 0.254407417666075 | -8.476369265 | 2.03023560277596 | 0.0398327105520806 | 0.000208333735826765 | 7.61588047000303 | 0.999950574162623 |
| ebi-fl187-GCST90094817 | ebi-gm430-GCST90011614 | OTU97_80 (Ruminococcus) prevalence \|\| id:ebi-gm430-GCST90011614 | Prawns liking \|\| id:ebi-fl187-GCST90094817 | MR Egger | 13 | 1.64417013042862 | 1.26630766715075 | 0.220712170752341 | -0.837792897 | 4.12613315804408 | 5.17671212802232 | 0.432664405210168 | 61.9379549916892 | 0.999950574162623 |
| ebi-fl187-GCST90094817 | ebi-gm430-GCST90011623 | OTU99_11 (Parabacteroides) prevalence \|\| id:ebi-gm430-GCST90011623 | Prawns liking \|\| id:ebi-fl187-GCST90094817 | MR Egger | 13 | -1.005865768 | 1.34420800961224 | 0.469978649019733 | -3.640513467 | 1.62878193064922 | 0.365727862148431 | 0.0262388677126833 | 5.09766162992646 | 0.999950574162623 |
| ebi-fl187-GCST90094817 | ebi-gm430-GCST90011642 | OTU99_19 (Bacteria) prevalence \|\| id:ebi-gm430-GCST90011642 | Prawns liking \|\| id:ebi-fl187-GCST90094817 | MR Egger | 13 | -3.223066831 | 2.68025634380238 | 0.254407417666075 | -8.476369265 | 2.03023560277596 | 0.0398327105520806 | 0.000208333735826765 | 7.61588047000303 | 0.999950574162623 |
| ebi-fl187-GCST90094817 | ebi-gm430-GCST90011653 | OTU99_30 (Parasutterella) prevalence \|\| id:ebi-gm430-GCST90011653 | Prawns liking \|\| id:ebi-fl187-GCST90094817 | MR Egger | 13 | 0.186754635060656 | 1.19406404079362 | 0.87854927386567 | -2.153610885 | 2.52712015501614 | 1.20533150267305 | 0.116064305363255 | 12.5174059913517 | 0.999950574162623 |
| ebi-fl187-GCST90094817 | ebi-gm430-GCST90011675 | OTU99_8 (Ruminococcaceae) prevalence \|\| id:ebi-gm430-GCST90011675 | Prawns liking \|\| id:ebi-fl187-GCST90094817 | MR Egger | 13 | -0.657120598 | 1.27425057534556 | 0.616275882553486 | -3.154651725 | 1.84041053004621 | 0.518341702102908 | 0.0426532534548274 | 6.29912370983582 | 0.999950574162623 |
| ebi-fl187-GCST90094817 | ebi-gm430-GCST90011679 | OTU99_92 (Ruminococcus) prevalence \|\| id:ebi-gm430-GCST90011679 | Prawns liking \|\| id:ebi-fl187-GCST90094817 | MR Egger | 13 | 2.54045606301054 | 1.32807936384736 | 0.0821387561084485 | -0.06257949 | 5.14349161615136 | 12.6854550185943 | 0.939338391714561 | 171.312884098193 | 0.999950574162623 |
| ebi-fl187-GCST90094817 | ebi-gm430-GCST90011684 | TestASV_11 (Lachnospiraceae) prevalence \|\| id:ebi-gm430-GCST90011684 | Prawns liking \|\| id:ebi-fl187-GCST90094817 | MR Egger | 13 | 0.0104459107417472 | 1.34097430672488 | 0.993924188962068 | -2.61786373 | 2.63875555192251 | 1.01050065973567 | 0.0729585556089105 | 13.9957757497259 | 0.999950574162623 |
| ebi-fl187-GCST90094817 | ebi-gm430-GCST90011688 | TestASV_15 (Bacteroides) prevalence \|\| id:ebi-gm430-GCST90011688 | Prawns liking \|\| id:ebi-fl187-GCST90094817 | MR Egger | 13 | -0.140431733 | 1.30262286609863 | 0.9160901220175 | -2.69357255 | 2.41270908467716 | 0.868982985877041 | 0.0676388639634289 | 11.164164882368 | 0.999950574162623 |
| ebi-fl187-GCST90094817 | ebi-gm430-GCST90011692 | TestASV_19 (Prevotella) prevalence \|\| id:ebi-gm430-GCST90011692 | Prawns liking \|\| id:ebi-fl187-GCST90094817 | MR Egger | 13 | 2.48241352934436 | 2.43661595366412 | 0.330184589495102 | -2.29335374 | 7.25818079852603 | 11.9701198176001 | 0.100927409351315 | 1419.67151806058 | 0.999950574162623 |
| ebi-fl187-GCST90094817 | ebi-gm430-GCST90011693 | TestASV_2 (Bacteroides) prevalence \|\| id:ebi-gm430-GCST90011693 | Prawns liking \|\| id:ebi-fl187-GCST90094817 | MR Egger | 13 | 3.37126903219633 | 1.23573870591826 | 0.0196435237040013 | 0.949221168596544 | 5.79331689579612 | 29.1154520694142 | 2.58369661144716 | 328.099493357907 | 0.999950574162623 |
| ebi-fl187-GCST90094817 | ebi-gm430-GCST90011716 | TestASV_40 (Barnesiella) prevalence \|\| id:ebi-gm430-GCST90011716 | Prawns liking \|\| id:ebi-fl187-GCST90094817 | MR Egger | 13 | -3.993757542 | 2.83346543218432 | 0.186328429484922 | -9.547349789 | 1.55983470526336 | 0.0184303311068165 | 7.13902117109793e-05 | 4.75803470204091 | 0.999950574162623 |
| ebi-fl187-GCST90094817 | ebi-gm430-GCST90011718 | TestASV_42 (Alphaproteobacteria) prevalence \|\| id:ebi-gm430-GCST90011718 | Prawns liking \|\| id:ebi-fl187-GCST90094817 | MR Egger | 13 | -1.550261688 | 1.95970045866325 | 0.445624569806563 | -5.391274587 | 2.29075121132638 | 0.212192438419275 | 0.0045561624095498 | 9.88235863320918 | 0.999950574162623 |
| ebi-fl187-GCST90094817 | ebi-hbsm44-GCST90104929 | Sulcal depth of the right inferior frontal sulcus \|\| id:ebi-hbsm44-GCST90104929 | Prawns liking \|\| id:ebi-fl187-GCST90094817 | MR Egger | 17 | 0.527446919546419 | 0.508267246684818 | 0.315833330855104 | -0.468756884 | 1.52365072304866 | 1.69460033004743 | 0.625779701749941 | 4.58894762896029 | 0.999950574162623 |
| ebi-fl187-GCST90094817 | ebi-hbsm44-GCST90104935 | Sulcal opening distance of the left calloso-marginal posterior fissure \|\| id:ebi-hbsm44-GCST90104935 | Prawns liking \|\| id:ebi-fl187-GCST90094817 | MR Egger | 18 | 0.147791322643886 | 0.140834167182394 | 0.309587603167065 | -0.128243645 | 0.423826290321378 | 1.15927095752184 | 0.879639033351718 | 1.52779617774902 | 0.999950574162623 |
| ebi-fl187-GCST90094817 | ebi-hbsm44-GCST90104936 | Sulcal opening distance of the left anterior inferior temporal sulcus \|\| id:ebi-hbsm44-GCST90104936 | Prawns liking \|\| id:ebi-fl187-GCST90094817 | MR Egger | 18 | 0.121012707950316 | 0.0640859498634785 | 0.0772571224561757 | -0.004595754 | 0.246621169682734 | 1.12863925496779 | 0.995414790535099 | 1.27969423396801 | 0.999950574162623 |
| ebi-fl187-GCST90094817 | ebi-hbsm44-GCST90104941 | Sulcal opening distance of the right posterior inferior temporal sulcus \|\| id:ebi-hbsm44-GCST90104941 | Prawns liking \|\| id:ebi-fl187-GCST90094817 | MR Egger | 17 | 0.0907391356067895 | 0.052419453427603 | 0.103959138110887 | -0.012002993 | 0.193481264324891 | 1.09498332583742 | 0.988068755457735 | 1.21346665122159 | 0.999950574162623 |
| ebi-fl187-GCST90094817 | ebi-IPs91-GCST90274819 | Leukemia inhibitory factor levels \|\| id:ebi-IPs91-GCST90274819 | Prawns liking \|\| id:ebi-fl187-GCST90094817 | MR Egger | 21 | -0.046960569 | 0.172974764556327 | 0.788943610731622 | -0.385991107 | 0.292069969903803 | 0.954125019308537 | 0.679776570799762 | 1.33919671782668 | 0.999950574162623 |
| ebi-fl187-GCST90094817 | ebi-IPs91-GCST90274837 | STAM binding protein levels \|\| id:ebi-IPs91-GCST90274837 | Prawns liking \|\| id:ebi-fl187-GCST90094817 | MR Egger | 21 | -0.03831396 | 0.150307250570596 | 0.801535761575799 | -0.332916171 | 0.25628825128536 | 0.962410735139758 | 0.716830282837709 | 1.29212513099415 | 0.999950574162623 |
| ebi-fl187-GCST90094817 | ebi-IPs91-GCST90274840 | TNF-beta levels \|\| id:ebi-IPs91-GCST90274840 | Prawns liking \|\| id:ebi-fl187-GCST90094817 | MR Egger | 21 | 0.0972789806190941 | 0.235031899082863 | 0.683584346641662 | -0.363383542 | 0.557941502821506 | 1.10216781419319 | 0.695319698332341 | 1.74707245250912 | 0.999950574162623 |
| ebi-fl187-GCST90094817 | ebi-IPs91-GCST90274842 | Tumor necrosis factor ligand superfamily member 14 levels \|\| id:ebi-IPs91-GCST90274842 | Prawns liking \|\| id:ebi-fl187-GCST90094817 | MR Egger | 21 | -0.001809875 | 0.173204262927796 | 0.991771700868551 | -0.341290231 | 0.337670479935941 | 0.998191761434308 | 0.710852566997303 | 1.4016785460931 | 0.999950574162623 |
| ebi-fl187-GCST90094817 | ebi-met1400-GCST90199628 | 3-methylhistidine levels \|\| id:ebi-met1400-GCST90199628 | Prawns liking \|\| id:ebi-fl187-GCST90094817 | MR Egger | 18 | -0.354849123 | 0.461645996973385 | 0.453292076206246 | -1.259675277 | 0.549977031252878 | 0.701279242265042 | 0.283746150476047 | 1.73321320767433 | 0.999950574162623 |
| ebi-fl187-GCST90094817 | ebi-met1400-GCST90199651 | Docosahexaenoate DHA; 22:6n3 levels \|\| id:ebi-met1400-GCST90199651 | Prawns liking \|\| id:ebi-fl187-GCST90094817 | MR Egger | 18 | -0.70922544 | 0.393313642680608 | 0.0902193157024973 | -1.48012018 | 0.0616692994935375 | 0.492025152828982 | 0.227610332572503 | 1.06361055001433 | 0.999950574162623 |
| ebi-fl187-GCST90094817 | ebi-met1400-GCST90199652 | 1-stearoyl-2-oleoyl-GPS (18:0/18:1) levels \|\| id:ebi-met1400-GCST90199652 | Prawns liking \|\| id:ebi-fl187-GCST90094817 | MR Egger | 18 | -0.497637473 | 0.415851929854617 | 0.248866702920743 | -1.312707256 | 0.317432309155442 | 0.607965298573394 | 0.269090572347459 | 1.37359626182729 | 0.999950574162623 |
| ebi-fl187-GCST90094817 | ebi-met1400-GCST90199653 | 1-myristoyl-2-palmitoyl-gpc (14:0/16:0) levels \|\| id:ebi-met1400-GCST90199653 | Prawns liking \|\| id:ebi-fl187-GCST90094817 | MR Egger | 17 | -0.159673618 | 0.454449633281521 | 0.730207827291509 | -1.050394899 | 0.7310476633858 | 0.852421958883967 | 0.349799586298736 | 2.07725573284992 | 0.999950574162623 |
| ebi-fl187-GCST90094817 | ebi-met1400-GCST90199667 | Erythritol levels in elite athletes \|\| id:ebi-met1400-GCST90199667 | Prawns liking \|\| id:ebi-fl187-GCST90094817 | MR Egger | 18 | 0.410119909575934 | 0.515542155464608 | 0.437959055721605 | -0.600342715 | 1.42058253428657 | 1.50699847782765 | 0.548623582266525 | 4.13953115684986 | 0.999950574162623 |
| ebi-fl187-GCST90094817 | ebi-met1400-GCST90199674 | 3-hydroxymyristate levels \|\| id:ebi-met1400-GCST90199674 | Prawns liking \|\| id:ebi-fl187-GCST90094817 | MR Egger | 18 | -0.209771698 | 0.401328806025345 | 0.608350489810299 | -0.996376157 | 0.576832762273931 | 0.810769325477278 | 0.369214996842337 | 1.78039057123019 | 0.999950574162623 |
| ebi-fl187-GCST90094817 | ebi-met1400-GCST90199685 | 2-linoleoylglycerol (18:2) levels \|\| id:ebi-met1400-GCST90199685 | Prawns liking \|\| id:ebi-fl187-GCST90094817 | MR Egger | 18 | -0.238248443 | 0.470757172161211 | 0.619690790902921 | -1.160932501 | 0.684435613950131 | 0.788006891593207 | 0.313193990984803 | 1.98265253827465 | 0.999950574162623 |
| ebi-fl187-GCST90094817 | ebi-met1400-GCST90199703 | Gamma-glutamylvaline levels \|\| id:ebi-met1400-GCST90199703 | Prawns liking \|\| id:ebi-fl187-GCST90094817 | MR Egger | 18 | -0.163633581 | 0.546937508434652 | 0.76865276530024 | -1.235631098 | 0.908363935267914 | 0.849053073843205 | 0.290651275095641 | 2.48026134399507 | 0.999950574162623 |
| ebi-fl187-GCST90094817 | ebi-met1400-GCST90199779 | Gamma-glutamyl-2-aminobutyrate levels \|\| id:ebi-met1400-GCST90199779 | Prawns liking \|\| id:ebi-fl187-GCST90094817 | MR Egger | 18 | 0.196070834344189 | 0.500351471698852 | 0.700327182014261 | -0.78461805 | 1.17675971887394 | 1.21661308027188 | 0.456293949895997 | 3.24384618166865 | 0.999950574162623 |
| ebi-fl187-GCST90094817 | ebi-met1400-GCST90199824 | 4-vinylphenol sulfate levels \|\| id:ebi-met1400-GCST90199824 | Prawns liking \|\| id:ebi-fl187-GCST90094817 | MR Egger | 18 | 0.227308136729629 | 0.41167867037215 | 0.588476386579408 | -0.579582057 | 1.03419833065904 | 1.25521658669009 | 0.560132420963829 | 2.81285035561916 | 0.999950574162623 |
| ebi-fl187-GCST90094817 | ebi-met1400-GCST90199830 | 4-ethylphenylsulfate levels \|\| id:ebi-met1400-GCST90199830 | Prawns liking \|\| id:ebi-fl187-GCST90094817 | MR Egger | 18 | -0.050894853 | 0.411978248757563 | 0.90321989806834 | -0.85837222 | 0.756582514738907 | 0.950378594902516 | 0.423851457850258 | 2.13098116550062 | 0.999950574162623 |
| ebi-fl187-GCST90094817 | ebi-met1400-GCST90199833 | Hexanoylglutamine levels \|\| id:ebi-met1400-GCST90199833 | Prawns liking \|\| id:ebi-fl187-GCST90094817 | MR Egger | 18 | -0.474536381 | 0.405463911719275 | 0.258996074844647 | -1.269245648 | 0.32017288642741 | 0.622173441322858 | 0.281043547734322 | 1.37736587161739 | 0.999950574162623 |
| ebi-fl187-GCST90094817 | ebi-met1400-GCST90199837 | Carotenoid levels (cryptoxanthin) \|\| id:ebi-met1400-GCST90199837 | Prawns liking \|\| id:ebi-fl187-GCST90094817 | MR Egger | 18 | -0.644023056 | 0.468343131719396 | 0.188048112147977 | -1.561975595 | 0.273929481821664 | 0.525175358301838 | 0.209721337338253 | 1.31512205895683 | 0.999950574162623 |
| ebi-fl187-GCST90094817 | ebi-met1400-GCST90199869 | 2-hydroxyhippurate levels \|\| id:ebi-met1400-GCST90199869 | Prawns liking \|\| id:ebi-fl187-GCST90094817 | MR Egger | 18 | 0.479843744250233 | 0.414674541243122 | 0.264192193042219 | -0.332918357 | 1.29260584508675 | 1.61582190100339 | 0.716828716110132 | 3.64226537955979 | 0.999950574162623 |
| ebi-fl187-GCST90094817 | ebi-met1400-GCST90199902 | Hydantoin-5-propionate levels \|\| id:ebi-met1400-GCST90199902 | Prawns liking \|\| id:ebi-fl187-GCST90094817 | MR Egger | 18 | 0.0483729122703011 | 0.410780872779163 | 0.907724695115171 | -0.756757598 | 0.85350342291746 | 1.04956197689525 | 0.469185250378977 | 2.34785799948845 | 0.999950574162623 |
| ebi-fl187-GCST90094817 | ebi-met1400-GCST90199904 | Pantoate levels \|\| id:ebi-met1400-GCST90199904 | Prawns liking \|\| id:ebi-fl187-GCST90094817 | MR Egger | 18 | 0.239453101271965 | 0.417577761643638 | 0.574323445720166 | -0.578999312 | 1.0579055140935 | 1.2705540958576 | 0.560458930822552 | 2.8803318525611 | 0.999950574162623 |
| ebi-fl187-GCST90094817 | ebi-met1400-GCST90199936 | 3-methyl catechol sulfate (2) levels \|\| id:ebi-met1400-GCST90199936 | Prawns liking \|\| id:ebi-fl187-GCST90094817 | MR Egger | 18 | 1.08059126048509 | 0.47473880835096 | 0.0369325755429468 | 0.150103196117207 | 2.01107932485297 | 2.94642113854051 | 1.16195414569762 | 7.47137703994863 | 0.999950574162623 |
| ebi-fl187-GCST90094817 | ebi-met1400-GCST90199977 | 4-hydroxychlorothalonil levels \|\| id:ebi-met1400-GCST90199977 | Prawns liking \|\| id:ebi-fl187-GCST90094817 | MR Egger | 18 | -0.130316236 | 0.445117488978335 | 0.773462094965483 | -1.002746514 | 0.742114042593208 | 0.877817789608063 | 0.366870441312182 | 2.10037109829922 | 0.999950574162623 |
| ebi-fl187-GCST90094817 | ebi-met1400-GCST90200040 | Sphingomyelin (d18:2/24:1, d18:1/24:2) levels \|\| id:ebi-met1400-GCST90200040 | Prawns liking \|\| id:ebi-fl187-GCST90094817 | MR Egger | 18 | 0.18348806753501 | 0.402107891499082 | 0.654296171502811 | -0.6046434 | 0.97161953487321 | 1.20140062965521 | 0.546269191612272 | 2.64222016379133 | 0.999950574162623 |
| ebi-fl187-GCST90094817 | ebi-met1400-GCST90200077 | 1-stearoyl-2-oleoyl-GPI (18:0/18:1) levels \|\| id:ebi-met1400-GCST90200077 | Prawns liking \|\| id:ebi-fl187-GCST90094817 | MR Egger | 18 | -0.933159154 | 0.406399106282077 | 0.0355122212369705 | -1.729701402 | -0.136616906 | 0.393309221305494 | 0.17733735461805 | 0.872304337104329 | 0.999950574162623 |
| ebi-fl187-GCST90094817 | ebi-met1400-GCST90200080 | 1-palmitoyl-2-oleoyl-GPI (16:0/18:1) levels \|\| id:ebi-met1400-GCST90200080 | Prawns liking \|\| id:ebi-fl187-GCST90094817 | MR Egger | 18 | -0.6226886 | 0.410397973059427 | 0.148704988089564 | -1.427068627 | 0.181691427480645 | 0.536500062875417 | 0.240011455168049 | 1.19924408301177 | 0.999950574162623 |
| ebi-fl187-GCST90094817 | ebi-met1400-GCST90200082 | 1-oleoyl-2-linoleoyl-GPE (18:1/18:2) levels \|\| id:ebi-met1400-GCST90200082 | Prawns liking \|\| id:ebi-fl187-GCST90094817 | MR Egger | 18 | -0.598191646 | 0.522320916987569 | 0.268945160441792 | -1.621940643 | 0.425557351195761 | 0.549804979644235 | 0.19751502069214 | 1.5304431763332 | 0.999950574162623 |
| ebi-fl187-GCST90094817 | ebi-met1400-GCST90200100 | Perfluorooctanesulfonate (PFOS) levels \|\| id:ebi-met1400-GCST90200100 | Prawns liking \|\| id:ebi-fl187-GCST90094817 | MR Egger | 18 | -0.201596404 | 0.379904590385957 | 0.602949528680603 | -0.946209401 | 0.543016592954303 | 0.817424770578655 | 0.388209785437477 | 1.72119117193964 | 0.999950574162623 |
| ebi-fl187-GCST90094817 | ebi-met1400-GCST90200102 | 1-palmitoleoyl-2-linolenoyl-GPC (16:1/18:3) levels \|\| id:ebi-met1400-GCST90200102 | Prawns liking \|\| id:ebi-fl187-GCST90094817 | MR Egger | 18 | -0.528704307 | 0.402696385458036 | 0.207733051928757 | -1.317989222 | 0.260580608616091 | 0.589368115381541 | 0.267672992059149 | 1.29768331409256 | 0.999950574162623 |
| ebi-fl187-GCST90094817 | ebi-met1400-GCST90200114 | Glycosyl-N-tricosanoyl-sphingadienine (d18:2/23:0) levels \|\| id:ebi-met1400-GCST90200114 | Prawns liking \|\| id:ebi-fl187-GCST90094817 | MR Egger | 18 | 0.233156740465397 | 0.426983810325964 | 0.592561053203782 | -0.603731528 | 1.07004500870429 | 1.26257936109548 | 0.546767546392149 | 2.9155107203838 | 0.999950574162623 |
| ebi-fl187-GCST90094817 | ebi-met1400-GCST90200135 | Arachidoylcarnitine (C20) levels \|\| id:ebi-met1400-GCST90200135 | Prawns liking \|\| id:ebi-fl187-GCST90094817 | MR Egger | 18 | -0.004189089 | 0.631699693185583 | 0.99479088243016 | -1.242320488 | 1.23394230952197 | 0.995819672872871 | 0.288713483917809 | 3.43474370307878 | 0.999950574162623 |
| ebi-fl187-GCST90094817 | ebi-met1400-GCST90200146 | Docosahexaenoylcarnitine (C22:6) levels \|\| id:ebi-met1400-GCST90200146 | Prawns liking \|\| id:ebi-fl187-GCST90094817 | MR Egger | 18 | -0.337668052 | 0.45856497307415 | 0.472171897194098 | -1.236455399 | 0.561119295353116 | 0.713432071036528 | 0.290411789588926 | 1.75263311694037 | 0.999950574162623 |
| ebi-fl187-GCST90094817 | ebi-met1400-GCST90200162 | 3-hydroxyphenylacetoylglutamine levels \|\| id:ebi-met1400-GCST90200162 | Prawns liking \|\| id:ebi-fl187-GCST90094817 | MR Egger | 18 | 0.102023922569405 | 0.556605531127465 | 0.856867916792778 | -0.988922918 | 1.19297076357924 | 1.10740996350277 | 0.371977125035246 | 3.2968608678531 | 0.999950574162623 |
| ebi-fl187-GCST90094817 | ebi-met1400-GCST90200172 | 3-carboxy-4-methyl-5-pentyl-2-furanpropionate (3-CMPFP) levels \|\| id:ebi-met1400-GCST90200172 | Prawns liking \|\| id:ebi-fl187-GCST90094817 | MR Egger | 18 | 0.754095288810848 | 0.503795839281941 | 0.153903964463711 | -0.233344556 | 1.74153513380345 | 2.12568751986892 | 0.791880679130081 | 5.70609632386831 | 0.999950574162623 |
| ebi-fl187-GCST90094817 | ebi-met1400-GCST90200222 | Dihydrocaffeate sulfate (2) levels \|\| id:ebi-met1400-GCST90200222 | Prawns liking \|\| id:ebi-fl187-GCST90094817 | MR Egger | 18 | 0.755669964145446 | 0.468269868178048 | 0.12612800058753 | -0.162138977 | 1.67347890577442 | 2.12903742438933 | 0.850323020574383 | 5.33068051172896 | 0.999950574162623 |
| ebi-fl187-GCST90094817 | ebi-met1400-GCST90200223 | 4-methylhexanoylglutamine levels \|\| id:ebi-met1400-GCST90200223 | Prawns liking \|\| id:ebi-fl187-GCST90094817 | MR Egger | 18 | -0.206821129 | 0.496026150374895 | 0.682251048242259 | -1.179032384 | 0.76539012552283 | 0.813165088460012 | 0.307576210337425 | 2.1498329157667 | 0.999950574162623 |
| ebi-fl187-GCST90094817 | ebi-met1400-GCST90200224 | 2-ketocaprylate levels \|\| id:ebi-met1400-GCST90200224 | Prawns liking \|\| id:ebi-fl187-GCST90094817 | MR Egger | 18 | 0.151438254566965 | 0.430708939899667 | 0.729724203596204 | -0.692751268 | 0.995627776770312 | 1.16350645838159 | 0.500197995653715 | 2.70642283747345 | 0.999950574162623 |
| ebi-fl187-GCST90094817 | ebi-met1400-GCST90200243 | Hydroxypalmitoyl sphingomyelin (d18:1/16:0(OH)) levels \|\| id:ebi-met1400-GCST90200243 | Prawns liking \|\| id:ebi-fl187-GCST90094817 | MR Egger | 18 | 0.125460025682672 | 0.402426218836686 | 0.759249327008321 | -0.663295363 | 0.914215414602576 | 1.1336698503764 | 0.515150924877355 | 2.49481708677593 | 0.999950574162623 |
| ebi-fl187-GCST90094817 | ebi-met1400-GCST90200273 | 2-methoxyhydroquinone sulfate (2) levels \|\| id:ebi-met1400-GCST90200273 | Prawns liking \|\| id:ebi-fl187-GCST90094817 | MR Egger | 18 | -0.110331185 | 0.529763068736148 | 0.837649972815503 | -1.148666799 | 0.928004430144363 | 0.895537497969601 | 0.31705919126522 | 2.52945643073565 | 0.999950574162623 |
| ebi-fl187-GCST90094817 | ebi-met1400-GCST90200276 | 2,4-di-tert-butylphenol levels \|\| id:ebi-met1400-GCST90200276 | Prawns liking \|\| id:ebi-fl187-GCST90094817 | MR Egger | 18 | -0.469216175 | 0.475501673606671 | 0.338438262002089 | -1.401199456 | 0.462767104782702 | 0.625492352426545 | 0.246301359111828 | 1.58846335381551 | 0.999950574162623 |
| ebi-fl187-GCST90094817 | ebi-met1400-GCST90200289 | N-acetylmethionine levels \|\| id:ebi-met1400-GCST90200289 | Prawns liking \|\| id:ebi-fl187-GCST90094817 | MR Egger | 18 | 0.00738096126320204 | 0.40983896916253 | 0.98585401021452 | -0.795903418 | 0.810665340821761 | 1.00740826769903 | 0.451173452399954 | 2.2494041092841 | 0.999950574162623 |
| ebi-fl187-GCST90094817 | ebi-met1400-GCST90200308 | 3-hydroxyisobutyrate levels \|\| id:ebi-met1400-GCST90200308 | Prawns liking \|\| id:ebi-fl187-GCST90094817 | MR Egger | 18 | -0.081724539 | 0.50601942013336 | 0.873717673568697 | -1.073522603 | 0.910073524251621 | 0.921525767953756 | 0.341802360352311 | 2.48450519805493 | 0.999950574162623 |
| ebi-fl187-GCST90094817 | ebi-met1400-GCST90200383 | Cystathionine levels \|\| id:ebi-met1400-GCST90200383 | Prawns liking \|\| id:ebi-fl187-GCST90094817 | MR Egger | 18 | 0.0693757271329952 | 0.561227682839729 | 0.903160282788533 | -1.030630531 | 1.16938198549886 | 1.07183885244067 | 0.356781927482805 | 3.22000201553563 | 0.999950574162623 |
| ebi-fl187-GCST90094817 | ebi-met1400-GCST90200384 | Guanidinoacetate levels \|\| id:ebi-met1400-GCST90200384 | Prawns liking \|\| id:ebi-fl187-GCST90094817 | MR Egger | 18 | 0.26331841531058 | 0.472474073188098 | 0.585023163663722 | -0.662730768 | 1.18936759875925 | 1.30124098804676 | 0.515441858687031 | 3.28500311031397 | 0.999950574162623 |
| ebi-fl187-GCST90094817 | ebi-met1400-GCST90200403 | Citrulline levels \|\| id:ebi-met1400-GCST90200403 | Prawns liking \|\| id:ebi-fl187-GCST90094817 | MR Egger | 17 | -0.109979611 | 0.420759580216516 | 0.797351668330422 | -0.934668388 | 0.714709166511297 | 0.895852400901962 | 0.392716073328162 | 2.04359225075868 | 0.999950574162623 |
| ebi-fl187-GCST90094817 | ebi-met1400-GCST90200434 | 12,13-DiHOME levels \|\| id:ebi-met1400-GCST90200434 | Prawns liking \|\| id:ebi-fl187-GCST90094817 | MR Egger | 18 | 0.0265204602950547 | 0.414805933091294 | 0.94981424575324 | -0.786499169 | 0.83954008915399 | 1.02687525721749 | 0.455436413777281 | 2.31530189942422 | 0.999950574162623 |
| ebi-fl187-GCST90094817 | ebi-met1400-GCST90200437 | Fructose levels \|\| id:ebi-met1400-GCST90200437 | Prawns liking \|\| id:ebi-fl187-GCST90094817 | MR Egger | 18 | 0.491748411615209 | 0.45009916597234 | 0.290776547867831 | -0.390445954 | 1.373942777 | 1.63517267699771 | 0.676755005800984 | 3.95089753408658 | 0.999950574162623 |
| ebi-fl187-GCST90094817 | ebi-met1400-GCST90200448 | N-stearoyl-sphinganine (d18:0/18:0) levels \|\| id:ebi-met1400-GCST90200448 | Prawns liking \|\| id:ebi-fl187-GCST90094817 | MR Egger | 18 | -0.157701668 | 0.573330998500043 | 0.786788800145407 | -1.281430425 | 0.966027089377523 | 0.854104550953703 | 0.277639873331807 | 2.62748493292967 | 0.999950574162623 |
| ebi-fl187-GCST90094817 | ebi-met1400-GCST90200468 | X-11847 levels \|\| id:ebi-met1400-GCST90200468 | Prawns liking \|\| id:ebi-fl187-GCST90094817 | MR Egger | 18 | -0.380737317 | 0.466815748640919 | 0.426701501904732 | -1.295696184 | 0.534221550281278 | 0.683357372372454 | 0.273707247273545 | 1.70611959685921 | 0.999950574162623 |
| ebi-fl187-GCST90094817 | ebi-met1400-GCST90200476 | X-11843 levels \|\| id:ebi-met1400-GCST90200476 | Prawns liking \|\| id:ebi-fl187-GCST90094817 | MR Egger | 18 | -0.282445707 | 0.577743625174756 | 0.631562107328903 | -1.414823212 | 0.84993179826586 | 0.753937574323067 | 0.242968563476667 | 2.3394872893947 | 0.999950574162623 |
| ebi-fl187-GCST90094817 | ebi-met1400-GCST90200511 | X-12906 levels \|\| id:ebi-met1400-GCST90200511 | Prawns liking \|\| id:ebi-fl187-GCST90094817 | MR Egger | 18 | -0.347433198 | 0.467389805886923 | 0.468046775297895 | -1.263517218 | 0.568650821547241 | 0.706499207919574 | 0.282658106046586 | 1.76588295227847 | 0.999950574162623 |
| ebi-fl187-GCST90094817 | ebi-met1400-GCST90200515 | X-12849 levels \|\| id:ebi-met1400-GCST90200515 | Prawns liking \|\| id:ebi-fl187-GCST90094817 | MR Egger | 18 | -0.226797476 | 0.430210862510542 | 0.605303651225393 | -1.070010766 | 0.616415814580757 | 0.797082194266895 | 0.343004824450909 | 1.85227722506351 | 0.999950574162623 |
| ebi-fl187-GCST90094817 | ebi-met1400-GCST90200551 | X-18886 levels \|\| id:ebi-met1400-GCST90200551 | Prawns liking \|\| id:ebi-fl187-GCST90094817 | MR Egger | 18 | 0.443918422953495 | 0.489415595632712 | 0.377845194976281 | -0.515336144 | 1.40317299039361 | 1.55880331786425 | 0.597299781840548 | 4.06808751260057 | 0.999950574162623 |
| ebi-fl187-GCST90094817 | ebi-met1400-GCST90200564 | X-21319 levels \|\| id:ebi-met1400-GCST90200564 | Prawns liking \|\| id:ebi-fl187-GCST90094817 | MR Egger | 18 | -0.190772908 | 0.444015010695514 | 0.673177390976073 | -1.061042329 | 0.679496512566078 | 0.826320217228508 | 0.346094877405061 | 1.97288415974279 | 0.999950574162623 |
| ebi-fl187-GCST90094817 | ebi-met1400-GCST90200579 | X-21442 levels \|\| id:ebi-met1400-GCST90200579 | Prawns liking \|\| id:ebi-fl187-GCST90094817 | MR Egger | 18 | 0.20082392791949 | 0.454376822962461 | 0.664421730143225 | -0.689754645 | 1.09140250092591 | 1.22240952068648 | 0.50169914831731 | 2.97844842128348 | 0.999950574162623 |
| ebi-fl187-GCST90094817 | ebi-met1400-GCST90200581 | X-21441 levels \|\| id:ebi-met1400-GCST90200581 | Prawns liking \|\| id:ebi-fl187-GCST90094817 | MR Egger | 18 | 0.894962096610079 | 0.415451180896186 | 0.046812675953188 | 0.0806777820535536 | 1.7092464111666 | 2.4472430288976 | 1.08402154908932 | 5.52479648353782 | 0.999950574162623 |
| ebi-fl187-GCST90094817 | ebi-met1400-GCST90200648 | X-24585 levels \|\| id:ebi-met1400-GCST90200648 | Prawns liking \|\| id:ebi-fl187-GCST90094817 | MR Egger | 18 | 0.00535343197193817 | 0.40213190915318 | 0.989542960931061 | -0.78282511 | 0.79353197391217 | 1.00536778719401 | 0.457112791517342 | 2.21119252465514 | 0.999950574162623 |
| ebi-fl187-GCST90094817 | ebi-met1400-GCST90200720 | Adenosine 5'-diphosphate (ADP) to pantothenate ratio \|\| id:ebi-met1400-GCST90200720 | Prawns liking \|\| id:ebi-fl187-GCST90094817 | MR Egger | 18 | -0.751715255 | 0.540593203130533 | 0.18340579160536 | -1.811277933 | 0.307847423451715 | 0.471557018276168 | 0.163445131386044 | 1.36049339371449 | 0.999950574162623 |
| ebi-fl187-GCST90094817 | ebi-met1400-GCST90200724 | Cholate to taurocholate ratio \|\| id:ebi-met1400-GCST90200724 | Prawns liking \|\| id:ebi-fl187-GCST90094817 | MR Egger | 18 | -0.036470016 | 0.444467813316316 | 0.935622105792254 | -0.90762693 | 0.834686898159837 | 0.964187003703739 | 0.403480576474633 | 2.30409251972912 | 0.999950574162623 |
| ebi-fl187-GCST90094817 | ebi-met1400-GCST90200726 | N-acetylputrescine to (N(1) + N(8))-acetylspermidine ratio \|\| id:ebi-met1400-GCST90200726 | Prawns liking \|\| id:ebi-fl187-GCST90094817 | MR Egger | 18 | 0.602117756868898 | 0.416104186174651 | 0.167195060242391 | -0.213446448 | 1.41768196177121 | 1.82598169388688 | 0.807795418025512 | 4.12754154332762 | 0.999950574162623 |
| ebi-fl187-GCST90094817 | ebi-met1400-GCST90200744 | Arginine to glutamate ratio \|\| id:ebi-met1400-GCST90200744 | Prawns liking \|\| id:ebi-fl187-GCST90094817 | MR Egger | 18 | -0.033084185 | 0.488926220923992 | 0.946889092667915 | -0.991379578 | 0.925211208176263 | 0.967457110943687 | 0.37106442548744 | 2.52240095580703 | 0.999950574162623 |
| ebi-fl187-GCST90094817 | ebi-met1400-GCST90200759 | Histidine to trans-urocanate ratio \|\| id:ebi-met1400-GCST90200759 | Prawns liking \|\| id:ebi-fl187-GCST90094817 | MR Egger | 18 | 0.136214440075155 | 0.416235924376271 | 0.747719119904556 | -0.679607972 | 0.952036851852647 | 1.14592760004994 | 0.506815639497779 | 2.59098173422088 | 0.999950574162623 |
| ebi-fl187-GCST90094817 | ebi-met1400-GCST90200795 | Oleoyl-linoleoyl-glycerol (18:1 to 18:2) [2] to linoleoyl-arachidonoyl-glycerol (18:2 to 20:4) [2] ratio \|\| id:ebi-met1400-GCST90200795 | Prawns liking \|\| id:ebi-fl187-GCST90094817 | MR Egger | 18 | 0.00701216659874903 | 0.481583544789114 | 0.988562716415541 | -0.936891581 | 0.950915914385412 | 1.0070368094051 | 0.391843959402693 | 2.58807903289535 | 0.999950574162623 |
| ebi-fl187-GCST90094817 | ebi-met1400-GCST90200811 | Choline to taurocholate ratio \|\| id:ebi-met1400-GCST90200811 | Prawns liking \|\| id:ebi-fl187-GCST90094817 | MR Egger | 18 | -0.007574589 | 0.431335506782436 | 0.986206364116616 | -0.852992182 | 0.837843004243422 | 0.992454025855189 | 0.426137939985067 | 2.31137596776924 | 0.999950574162623 |
| ebi-fl187-GCST90094817 | ebi-met1400-GCST90200831 | Cholate to phosphate ratio \|\| id:ebi-met1400-GCST90200831 | Prawns liking \|\| id:ebi-fl187-GCST90094817 | MR Egger | 18 | -0.306433021 | 0.432420164058103 | 0.488741082356302 | -1.153976543 | 0.541110500323842 | 0.736067817445359 | 0.315380149875164 | 1.71791354685205 | 0.999950574162623 |
| ebi-fl187-GCST90094817 | ebi-met1400-GCST90200841 | Adenosine 5'-diphosphate (ADP) to oxalate (ethanedioate) ratio \|\| id:ebi-met1400-GCST90200841 | Prawns liking \|\| id:ebi-fl187-GCST90094817 | MR Egger | 18 | -0.242810826 | 0.548801714210738 | 0.664094732874632 | -1.318462186 | 0.832840533458498 | 0.784419891275581 | 0.267546422339228 | 2.29984224961388 | 0.999950574162623 |
| ebi-fl187-GCST90094817 | ebi-met1400-GCST90200844 | Aspartate to phosphate ratio \|\| id:ebi-met1400-GCST90200844 | Prawns liking \|\| id:ebi-fl187-GCST90094817 | MR Egger | 18 | -0.448957165 | 0.493220125549979 | 0.376196275667208 | -1.415668611 | 0.517754281147983 | 0.638293439452368 | 0.242763244996198 | 1.67825452676873 | 0.999950574162623 |
| ebi-fl187-GCST90094817 | ebi-met1400-GCST90200870 | Adenosine 5'-monophosphate (AMP) to acetoacetate ratio \|\| id:ebi-met1400-GCST90200870 | Prawns liking \|\| id:ebi-fl187-GCST90094817 | MR Egger | 18 | -0.387208689 | 0.543867941320152 | 0.486745856777009 | -1.453189854 | 0.678772476459954 | 0.678949391192454 | 0.233823235363634 | 1.97145623737399 | 0.999950574162623 |
| ebi-fl187-GCST90094817 | ebi-met1400-GCST90200887 | Cholesterol to cortisol ratio \|\| id:ebi-met1400-GCST90200887 | Prawns liking \|\| id:ebi-fl187-GCST90094817 | MR Egger | 18 | -0.265121741 | 0.458789573988206 | 0.571394341463854 | -1.164349306 | 0.634105824245582 | 0.767112555416837 | 0.31212569435218 | 1.88533556617154 | 0.999950574162623 |
| ebi-fl187-GCST90094817 | ebi-met1400-GCST90200890 | Cortisol to taurocholate ratio \|\| id:ebi-met1400-GCST90200890 | Prawns liking \|\| id:ebi-fl187-GCST90094817 | MR Egger | 18 | 0.0222458496886372 | 0.429489911454871 | 0.959332416907744 | -0.819554377 | 0.864046076140185 | 1.02249513368236 | 0.440627964822568 | 2.37274159125398 | 0.999950574162623 |
| ebi-fl187-GCST90094817 | ebi-met1400-GCST90200948 | Adenosine 5'-diphosphate (ADP) to mannitol to sorbitol ratio \|\| id:ebi-met1400-GCST90200948 | Prawns liking \|\| id:ebi-fl187-GCST90094817 | MR Egger | 18 | -1.367430549 | 0.584144697398474 | 0.0325148831944203 | -2.512354156 | -0.222506942 | 0.254760714538788 | 0.0810771461775001 | 0.800509449731039 | 0.999950574162623 |
| ebi-fl187-GCST90094817 | ebi-met1400-GCST90200972 | Leucine to N-palmitoyl-sphingosine (d18:1 to 16:0) ratio \|\| id:ebi-met1400-GCST90200972 | Prawns liking \|\| id:ebi-fl187-GCST90094817 | MR Egger | 18 | 0.315861860259006 | 0.477368059999863 | 0.517597024053131 | -0.619779537 | 1.25150325785874 | 1.37144079206432 | 0.538063047329957 | 3.49559378863013 | 0.999950574162623 |
| ebi-fl187-GCST90094817 | ebi-met1400-GCST90200973 | Proline to glutamate ratio \|\| id:ebi-met1400-GCST90200973 | Prawns liking \|\| id:ebi-fl187-GCST90094817 | MR Egger | 18 | 0.163266354685985 | 0.403201109829759 | 0.690894572909142 | -0.627007821 | 0.953540529952311 | 1.17735024068238 | 0.534187797796558 | 2.5948806673468 | 0.999950574162623 |
| ebi-fl187-GCST90094817 | ebi-met1400-GCST90200983 | Cholesterol to linoleoyl-arachidonoyl-glycerol (18:2 to 20:4) [1] ratio \|\| id:ebi-met1400-GCST90200983 | Prawns liking \|\| id:ebi-fl187-GCST90094817 | MR Egger | 18 | 0.42181651241008 | 0.564667012713604 | 0.465889433631782 | -0.684930833 | 1.52856385732874 | 1.52472873021467 | 0.5041250974373 | 4.61154922173099 | 0.999950574162623 |
| ebi-fl187-GCST90094817 | ebi-met1400-GCST90201001 | Serine to alpha-ketobutyrate ratio \|\| id:ebi-met1400-GCST90201001 | Prawns liking \|\| id:ebi-fl187-GCST90094817 | MR Egger | 18 | -0.056869766 | 0.505915923447206 | 0.911896934357057 | -1.048464976 | 0.934725443707255 | 0.944717095405994 | 0.350475324372384 | 2.54651420025238 | 0.999950574162623 |
| ebi-fl187-GCST90094817 | ebi-met1400-GCST90201005 | Fructose to maltose ratio \|\| id:ebi-met1400-GCST90201005 | Prawns liking \|\| id:ebi-fl187-GCST90094817 | MR Egger | 18 | 0.256209585042465 | 0.615765719436965 | 0.682876885959337 | -0.950691225 | 1.46311039513892 | 1.29202348836272 | 0.386473790739299 | 4.31937361467038 | 0.999950574162623 |
| ebi-fl187-GCST90094817 | ebi-met1400-GCST90201020 | Paraxanthine to 5-acetylamino-6-formylamino-3-methyluracil ratio \|\| id:ebi-met1400-GCST90201020 | Prawns liking \|\| id:ebi-fl187-GCST90094817 | MR Egger | 18 | 0.547968334231966 | 0.433969201515887 | 0.224794113603631 | -0.302611301 | 1.3985479692031 | 1.72973520176696 | 0.738886245096923 | 4.04931596451535 | 0.999950574162623 |
| ebi-fl187-GCST90094817 | ebi-pw11-GCST90275197 | Placental weight (fetal genotype, adjusted for sex) \|\| id:ebi-pw11-GCST90275197 | Prawns liking \|\| id:ebi-fl187-GCST90094817 | MR Egger | 17 | 0.149857071512213 | 0.0823193506473111 | 0.0887013122530915 | -0.011488856 | 0.311202998780943 | 1.16166819538364 | 0.98857688912808 | 1.36506629985667 | 0.999950574162623 |
| ebi-fl187-GCST90094817 | prot-a-1017 | FAS-associated factor 2 \|\| id:prot-a-1017 | Prawns liking \|\| id:ebi-fl187-GCST90094817 | MR Egger | 20 | -0.117767112 | 0.360431059496865 | 0.747633581960953 | -0.824211989 | 0.588677764440755 | 0.888903043208944 | 0.438580462652274 | 1.80160469403442 | 0.999950574162623 |
| ebi-fl187-GCST90094817 | prot-a-1019 | Fas apoptotic inhibitory molecule 3 \|\| id:prot-a-1019 | Prawns liking \|\| id:ebi-fl187-GCST90094817 | MR Egger | 20 | -0.283738105 | 0.329601616509449 | 0.400641679296849 | -0.929757273 | 0.362281063732762 | 0.752963816626408 | 0.394649490840045 | 1.43660266212886 | 0.999950574162623 |
| ebi-fl187-GCST90094817 | prot-a-1052 | MIP18 family protein FAM96A \|\| id:prot-a-1052 | Prawns liking \|\| id:ebi-fl187-GCST90094817 | MR Egger | 20 | -0.458780013 | 0.304417294615684 | 0.149140822720048 | -1.055437911 | 0.137877884374953 | 0.632054273284765 | 0.348039983560604 | 1.1478353730814 | 0.999950574162623 |
| ebi-fl187-GCST90094817 | prot-a-1066 | F-box/LRR-repeat protein 4 \|\| id:prot-a-1066 | Prawns liking \|\| id:ebi-fl187-GCST90094817 | MR Egger | 20 | -0.371769316 | 0.304407249817619 | 0.237732200507801 | -0.968407525 | 0.224868893811855 | 0.689513283978495 | 0.379687199112947 | 1.25215853969673 | 0.999950574162623 |
| ebi-fl187-GCST90094817 | prot-a-1076 | Low affinity immunoglobulin gamma Fc region receptor III-B \|\| id:prot-a-1076 | Prawns liking \|\| id:ebi-fl187-GCST90094817 | MR Egger | 20 | 0.0399485193448497 | 0.361562695279279 | 0.913244576584886 | -0.668714363 | 0.748611402092236 | 1.04075719395103 | 0.512366872134361 | 2.11406239487859 | 0.999950574162623 |
| ebi-fl187-GCST90094817 | prot-a-1081 | Fc receptor-like protein 3 \|\| id:prot-a-1081 | Prawns liking \|\| id:ebi-fl187-GCST90094817 | MR Egger | 20 | 0.166415008397266 | 0.304417294615684 | 0.591320261572648 | -0.430242889 | 0.763072905844006 | 1.18106315115436 | 0.650351112374493 | 2.14485704794402 | 0.999950574162623 |
| ebi-fl187-GCST90094817 | prot-a-1105 | Fibroblast growth factor receptor 4 \|\| id:prot-a-1105 | Prawns liking \|\| id:ebi-fl187-GCST90094817 | MR Egger | 20 | -0.742190091 | 0.342360461569233 | 0.0438200879665361 | -1.413216596 | -0.071163586 | 0.476070136079493 | 0.243359234608546 | 0.931309530256008 | 0.999950574162623 |
| ebi-fl187-GCST90094817 | prot-a-1143 | Follistatin-related protein 1 \|\| id:prot-a-1143 | Prawns liking \|\| id:ebi-fl187-GCST90094817 | MR Egger | 20 | 0.032970732112125 | 0.329032411463791 | 0.921289133894688 | -0.611932794 | 0.677874258581156 | 1.03352028984268 | 0.542301697823685 | 1.96968623517713 | 0.999950574162623 |
| ebi-fl187-GCST90094817 | prot-a-1151 | Alpha-(1,3)-fucosyltransferase 10 \|\| id:prot-a-1151 | Prawns liking \|\| id:ebi-fl187-GCST90094817 | MR Egger | 20 | -0.197468487 | 0.304417294615684 | 0.524737487266901 | -0.794126385 | 0.399189410273285 | 0.820806006137474 | 0.451975915609109 | 1.49061592984087 | 0.999950574162623 |
| ebi-fl187-GCST90094817 | prot-a-1165 | Growth arrest and DNA damage-inducible proteins-interacting protein 1 \|\| id:prot-a-1165 | Prawns liking \|\| id:ebi-fl187-GCST90094817 | MR Egger | 20 | -0.026043221 | 0.370420943226928 | 0.944724467651588 | -0.752068269 | 0.699981828128267 | 0.974292979174999 | 0.471390581125564 | 2.01371611414706 | 0.999950574162623 |
| ebi-fl187-GCST90094817 | prot-a-1194 | Growth/differentiation factor 11/8 \|\| id:prot-a-1194 | Prawns liking \|\| id:ebi-fl187-GCST90094817 | MR Egger | 20 | -0.184457212 | 0.304393027940003 | 0.552091398778269 | -0.781067547 | 0.412153122472811 | 0.831555519494967 | 0.45791690243905 | 1.51006564361223 | 0.999950574162623 |
| ebi-fl187-GCST90094817 | prot-a-1216 | D-glucuronyl C5-epimerase \|\| id:prot-a-1216 | Prawns liking \|\| id:ebi-fl187-GCST90094817 | MR Egger | 20 | 0.0640994645412928 | 0.353230188518837 | 0.858029181172999 | -0.628231705 | 0.756430634038213 | 1.06619844242663 | 0.533534413612272 | 2.13065753516527 | 0.999950574162623 |
| ebi-fl187-GCST90094817 | prot-a-1246 | Glypican-3 \|\| id:prot-a-1246 | Prawns liking \|\| id:ebi-fl187-GCST90094817 | MR Egger | 20 | -0.418046819 | 0.325885193635249 | 0.215833773366525 | -1.056781799 | 0.220688160433567 | 0.658331405224964 | 0.347572570914534 | 1.24693452640728 | 0.999950574162623 |
| ebi-fl187-GCST90094817 | prot-a-1275 | Glutamate receptor 4 \|\| id:prot-a-1275 | Prawns liking \|\| id:ebi-fl187-GCST90094817 | MR Egger | 19 | -0.914616659 | 0.694353988242201 | 0.205244313627402 | -2.275550476 | 0.446317158220756 | 0.40067019007316 | 0.102740336808403 | 1.56254696256877 | 0.999950574162623 |
| ebi-fl187-GCST90094817 | prot-a-1289 | Glutathione S-transferase P \|\| id:prot-a-1289 | Prawns liking \|\| id:ebi-fl187-GCST90094817 | MR Egger | 20 | 0.237170473414141 | 0.304417294615684 | 0.446045554515413 | -0.359487424 | 0.833828370860881 | 1.26765720120757 | 0.69803402985615 | 2.30211524229638 | 0.999950574162623 |
| ebi-fl187-GCST90094817 | prot-a-130 | Apolipoprotein D \|\| id:prot-a-130 | Prawns liking \|\| id:ebi-fl187-GCST90094817 | MR Egger | 20 | -0.326887418 | 0.329679247821432 | 0.334568743377774 | -0.973058744 | 0.319283907531165 | 0.721164928503101 | 0.377925291689626 | 1.37614196651861 | 0.999950574162623 |
| ebi-fl187-GCST90094817 | prot-a-1313 | Heparin-binding EGF-like growth factor \|\| id:prot-a-1313 | Prawns liking \|\| id:ebi-fl187-GCST90094817 | MR Egger | 20 | 0.528995972992018 | 0.345935423537122 | 0.143604463006255 | -0.149037457 | 1.20702940312478 | 1.69722739073099 | 0.861536843587763 | 3.3435375832004 | 0.999950574162623 |
| ebi-fl187-GCST90094817 | prot-a-1347 | HLA class II histocompatibility antigen, DQ alpha 2 chain \|\| id:prot-a-1347 | Prawns liking \|\| id:ebi-fl187-GCST90094817 | MR Egger | 19 | 0.540794739428821 | 0.922888496949747 | 0.565588030585339 | -1.268066715 | 2.34965619345032 | 1.71737118256615 | 0.281375074610124 | 10.4819653368207 | 0.999950574162623 |
| ebi-fl187-GCST90094817 | prot-a-1357 | Non-histone chromosomal protein HMG-14 \|\| id:prot-a-1357 | Prawns liking \|\| id:ebi-fl187-GCST90094817 | MR Egger | 20 | -0.089463358 | 0.37897597588864 | 0.816046687834608 | -0.832256271 | 0.653329554509864 | 0.91442177054094 | 0.435066550050315 | 1.92192935619281 | 0.999950574162623 |
| ebi-fl187-GCST90094817 | prot-a-137 | Apolipoprotein M \|\| id:prot-a-137 | Prawns liking \|\| id:ebi-fl187-GCST90094817 | MR Egger | 20 | -0.073243548 | 0.304435712283202 | 0.812593429313643 | -0.669937544 | 0.523450448089455 | 0.929374455183896 | 0.511740538024461 | 1.6878414230828 | 0.999950574162623 |
| ebi-fl187-GCST90094817 | prot-a-1381 | 17-beta-hydroxysteroid dehydrogenase 14 \|\| id:prot-a-1381 | Prawns liking \|\| id:ebi-fl187-GCST90094817 | MR Egger | 20 | -0.342759521 | 0.311516019280257 | 0.285701355668223 | -0.953330919 | 0.267811876540111 | 0.709808884991694 | 0.385454963486397 | 1.30710122048002 | 0.999950574162623 |
| ebi-fl187-GCST90094817 | prot-a-1392 | Serine protease HTRA2, mitochondrial \|\| id:prot-a-1392 | Prawns liking \|\| id:ebi-fl187-GCST90094817 | MR Egger | 20 | 0.175319456916321 | 0.321400243012078 | 0.592115271630414 | -0.454625019 | 0.805263933219994 | 1.19162682924673 | 0.634685918246529 | 2.2372869152409 | 0.999950574162623 |
| ebi-fl187-GCST90094817 | prot-a-1416 | Interferon-induced protein with tetratricopeptide repeats 2 \|\| id:prot-a-1416 | Prawns liking \|\| id:ebi-fl187-GCST90094817 | MR Egger | 20 | -0.043060685 | 0.358102563773263 | 0.905619681345467 | -0.74494171 | 0.658820339582401 | 0.957853260582345 | 0.474761972814702 | 1.93251128216691 | 0.999950574162623 |
| ebi-fl187-GCST90094817 | prot-a-1428 | Interferon gamma \|\| id:prot-a-1428 | Prawns liking \|\| id:ebi-fl187-GCST90094817 | MR Egger | 20 | -0.26727334 | 0.304393027940003 | 0.39148472363627 | -0.863883675 | 0.329336994806329 | 0.765463811022574 | 0.421521845571453 | 1.39004621502085 | 0.999950574162623 |
| ebi-fl187-GCST90094817 | prot-a-1483 | Interleukin-17C \|\| id:prot-a-1483 | Prawns liking \|\| id:ebi-fl187-GCST90094817 | MR Egger | 20 | 0.402710773021632 | 0.319273342403235 | 0.223290615321018 | -0.223064978 | 1.02848652413197 | 1.49587418192128 | 0.800062861049966 | 2.79682969560926 | 0.999950574162623 |
| ebi-fl187-GCST90094817 | prot-a-1503 | Interleukin-1 receptor-like 2 \|\| id:prot-a-1503 | Prawns liking \|\| id:ebi-fl187-GCST90094817 | MR Egger | 20 | 0.780807768737687 | 0.304416552290854 | 0.0194814394470357 | 0.184151326247614 | 1.37746421122776 | 2.18323510248285 | 1.20219773340394 | 3.96483488553689 | 0.999950574162623 |
| ebi-fl187-GCST90094817 | prot-a-1522 | Interleukin-31 receptor subunit alpha \|\| id:prot-a-1522 | Prawns liking \|\| id:ebi-fl187-GCST90094817 | MR Egger | 20 | -0.222348467 | 0.304417294615684 | 0.474541742951128 | -0.819006365 | 0.374309429957695 | 0.800636319990477 | 0.440869500334767 | 1.45398698798884 | 0.999950574162623 |
| ebi-fl187-GCST90094817 | prot-a-1540 | Interleukin-6 receptor subunit alpha \|\| id:prot-a-1540 | Prawns liking \|\| id:ebi-fl187-GCST90094817 | MR Egger | 20 | -0.521840376 | 0.304417294615684 | 0.103653980402825 | -1.118498274 | 0.0748175209989124 | 0.593427412554833 | 0.326770145602581 | 1.07768747760641 | 0.999950574162623 |
| ebi-fl187-GCST90094817 | prot-a-1541 | Interleukin-6 receptor subunit alpha \|\| id:prot-a-1541 | Prawns liking \|\| id:ebi-fl187-GCST90094817 | MR Egger | 20 | -0.53710844 | 0.304417294615684 | 0.0946305768597913 | -1.133766337 | 0.0595494576802035 | 0.584435742610731 | 0.321818892534923 | 1.06135825199849 | 0.999950574162623 |
| ebi-fl187-GCST90094817 | prot-a-1545 | Interleukin-9 \|\| id:prot-a-1545 | Prawns liking \|\| id:ebi-fl187-GCST90094817 | MR Egger | 20 | -0.437475039 | 0.367269990990191 | 0.249063960564317 | -1.157324222 | 0.282374143164991 | 0.645664642548401 | 0.314326123709588 | 1.32627484383805 | 0.999950574162623 |
| ebi-fl187-GCST90094817 | prot-a-1548 | Inositol monophosphatase 3 \|\| id:prot-a-1548 | Prawns liking \|\| id:ebi-fl187-GCST90094817 | MR Egger | 20 | -0.027292601 | 0.321050585958301 | 0.933191620857021 | -0.656551749 | 0.601966547865233 | 0.973076477091516 | 0.518636643903913 | 1.82570560988796 | 0.999950574162623 |
| ebi-fl187-GCST90094817 | prot-a-1555 | Inhibin beta A chain:Inhibin beta B chain heterodimer \|\| id:prot-a-1555 | Prawns liking \|\| id:ebi-fl187-GCST90094817 | MR Egger | 20 | 0.112458758330739 | 0.304417294615684 | 0.716122579540812 | -0.484199139 | 0.709116655777479 | 1.11902610545657 | 0.616190482065648 | 2.03219533754481 | 0.999950574162623 |
| ebi-fl187-GCST90094817 | prot-a-1603 | Junctophilin-3 \|\| id:prot-a-1603 | Prawns liking \|\| id:ebi-fl187-GCST90094817 | MR Egger | 20 | 0.216038484905946 | 0.310538271163581 | 0.495507619354405 | -0.392616527 | 0.824693496386565 | 1.2411501436281 | 0.675287652810628 | 2.28118146780339 | 0.999950574162623 |
| ebi-fl187-GCST90094817 | prot-a-1627 | UPF0258 protein KIAA1024 \|\| id:prot-a-1627 | Prawns liking \|\| id:ebi-fl187-GCST90094817 | MR Egger | 20 | 0.497861158953274 | 0.335128821250236 | 0.154694030636398 | -0.158991331 | 1.15471364860374 | 1.64519868643853 | 0.853003753884919 | 3.17311466160819 | 0.999950574162623 |
| ebi-fl187-GCST90094817 | prot-a-1666 | Kallikrein-7 \|\| id:prot-a-1666 | Prawns liking \|\| id:ebi-fl187-GCST90094817 | MR Egger | 20 | -0.222063088 | 0.304417294615684 | 0.475100872004413 | -0.818720985 | 0.374594809813617 | 0.800864838073799 | 0.440995333563533 | 1.45440198579918 | 0.999950574162623 |
| ebi-fl187-GCST90094817 | prot-a-1678 | Importin subunit alpha-3 \|\| id:prot-a-1678 | Prawns liking \|\| id:ebi-fl187-GCST90094817 | MR Egger | 20 | -0.538454759 | 0.411778953940147 | 0.207454744714598 | -1.345541508 | 0.26863199118489 | 0.583649435228822 | 0.260398661648276 | 1.30817363302366 | 0.999950574162623 |
| ebi-fl187-GCST90094817 | prot-a-1718 | Protein LDOC1 \|\| id:prot-a-1718 | Prawns liking \|\| id:ebi-fl187-GCST90094817 | MR Egger | 20 | -0.046328649 | 0.36430737015952 | 0.900216414819645 | -0.760371095 | 0.667713796294431 | 0.954728139968005 | 0.467492910660526 | 1.94977463927504 | 0.999950574162623 |
| ebi-fl187-GCST90094817 | prot-a-1723 | Leptin \|\| id:prot-a-1723 | Prawns liking \|\| id:ebi-fl187-GCST90094817 | MR Egger | 20 | 0.492941205133964 | 0.304417294615684 | 0.122771514931442 | -0.103716692 | 1.0895991025807 | 1.63712426405769 | 0.901480657646935 | 2.97308193274195 | 0.999950574162623 |
| ebi-fl187-GCST90094817 | prot-a-1730 | Galectin-7 \|\| id:prot-a-1730 | Prawns liking \|\| id:ebi-fl187-GCST90094817 | MR Egger | 20 | -0.16356784 | 0.353187669168662 | 0.648829516948895 | -0.855815672 | 0.52867999150244 | 0.849108893442436 | 0.424936441081181 | 1.69669118301223 | 0.999950574162623 |
| ebi-fl187-GCST90094817 | prot-a-1753 | Lipase member N \|\| id:prot-a-1753 | Prawns liking \|\| id:ebi-fl187-GCST90094817 | MR Egger | 20 | -0.399067938 | 0.304413521307054 | 0.206357318563285 | -0.99571844 | 0.197582563423228 | 0.670945116908464 | 0.369457915790105 | 1.21845366052208 | 0.999950574162623 |
| ebi-fl187-GCST90094817 | prot-a-1756 | VIP36-like protein \|\| id:prot-a-1756 | Prawns liking \|\| id:ebi-fl187-GCST90094817 | MR Egger | 20 | -0.249397746 | 0.304417294615684 | 0.423351804502457 | -0.846055643 | 0.347260151422801 | 0.779269960207286 | 0.429104138051801 | 1.41518483983521 | 0.999950574162623 |
| ebi-fl187-GCST90094817 | prot-a-1760 | Ig Kappa chain V-I region HK102- like \|\| id:prot-a-1760 | Prawns liking \|\| id:ebi-fl187-GCST90094817 | MR Egger | 20 | 0.118371257612543 | 0.304407249817619 | 0.701942578920692 | -0.478266952 | 0.715009467255077 | 1.1256619443667 | 0.619856702888346 | 2.04420603518691 | 0.999950574162623 |
| ebi-fl187-GCST90094817 | prot-a-179 | Neutral ceramidase \|\| id:prot-a-179 | Prawns liking \|\| id:ebi-fl187-GCST90094817 | MR Egger | 20 | 0.409110543875927 | 0.372560086738404 | 0.286630480664822 | -0.321107226 | 1.1393283138832 | 1.50547813267538 | 0.725345470831457 | 3.12466886346682 | 0.999950574162623 |
| ebi-fl187-GCST90094817 | prot-a-1809 | Lactotransferrin \|\| id:prot-a-1809 | Prawns liking \|\| id:ebi-fl187-GCST90094817 | MR Egger | 20 | -0.415365003 | 0.304417294615684 | 0.189240044396414 | -1.012022901 | 0.181292894099449 | 0.660099298274547 | 0.363482945421576 | 1.19876623943712 | 0.999950574162623 |
| ebi-fl187-GCST90094817 | prot-a-1818 | Lysophospholipase-like protein 1 \|\| id:prot-a-1818 | Prawns liking \|\| id:ebi-fl187-GCST90094817 | MR Egger | 20 | -0.785526975 | 0.303748225235326 | 0.0186352057205487 | -1.380873496 | -0.190180453 | 0.455879401567342 | 0.251358896109829 | 0.826809919958394 | 0.999950574162623 |
| ebi-fl187-GCST90094817 | prot-a-1852 | MAP kinase-activated protein kinase 3 \|\| id:prot-a-1852 | Prawns liking \|\| id:ebi-fl187-GCST90094817 | MR Egger | 20 | -0.462615826 | 0.328642457979593 | 0.176268113053655 | -1.106755043 | 0.181523392040216 | 0.629634475497224 | 0.33063010261791 | 1.19904258443402 | 0.999950574162623 |
| ebi-fl187-GCST90094817 | prot-a-1856 | Megakaryocyte-associated tyrosine-protein kinase \|\| id:prot-a-1856 | Prawns liking \|\| id:ebi-fl187-GCST90094817 | MR Egger | 20 | -0.452889083 | 0.304435712283202 | 0.154156343657247 | -1.049583079 | 0.14380491351548 | 0.635788649765277 | 0.350083676053211 | 1.15465882821944 | 0.999950574162623 |
| ebi-fl187-GCST90094817 | prot-a-1890 | Beta-1,3-N-acetylglucosaminyltransferase manic fringe \|\| id:prot-a-1890 | Prawns liking \|\| id:ebi-fl187-GCST90094817 | MR Egger | 20 | -0.584001395 | 0.351286194211128 | 0.113734338007144 | -1.272522335 | 0.104519546085455 | 0.557662468620817 | 0.280124162886592 | 1.11017709327048 | 0.999950574162623 |
| ebi-fl187-GCST90094817 | prot-a-1919 | Matrilysin \|\| id:prot-a-1919 | Prawns liking \|\| id:ebi-fl187-GCST90094817 | MR Egger | 20 | -0.446744057 | 0.333992708030683 | 0.19768793568408 | -1.101369764 | 0.207881651080221 | 0.6397076162279 | 0.332415440870725 | 1.23106746542237 | 0.999950574162623 |
| ebi-fl187-GCST90094817 | prot-a-1923 | Multimerin-2 \|\| id:prot-a-1923 | Prawns liking \|\| id:ebi-fl187-GCST90094817 | MR Egger | 20 | -0.328368471 | 0.353305596196507 | 0.364970084485128 | -1.02084744 | 0.364110497112821 | 0.720097635407899 | 0.36028948704997 | 1.43923323648944 | 0.999950574162623 |
| ebi-fl187-GCST90094817 | prot-a-1948 | Mesothelin \|\| id:prot-a-1948 | Prawns liking \|\| id:ebi-fl187-GCST90094817 | MR Egger | 20 | -0.57080839 | 0.304417294615684 | 0.0771003591426069 | -1.167466288 | 0.0258495072898841 | 0.565068458235581 | 0.311154318905019 | 1.02618650326433 | 0.999950574162623 |
| ebi-fl187-GCST90094817 | prot-a-195 | ATPase family AAA domain-containing protein 1 \|\| id:prot-a-195 | Prawns liking \|\| id:ebi-fl187-GCST90094817 | MR Egger | 20 | 0.236597177452902 | 0.34814077337401 | 0.505403063670009 | -0.445758738 | 0.918953093265961 | 1.26693066673342 | 0.64033824248144 | 2.506664772 | 0.999950574162623 |
| ebi-fl187-GCST90094817 | prot-a-1966 | Mucin-1 \|\| id:prot-a-1966 | Prawns liking \|\| id:ebi-fl187-GCST90094817 | MR Egger | 20 | -0.293344829 | 0.307529583782055 | 0.352782182001232 | -0.896102813 | 0.309413155580996 | 0.745764935251578 | 0.408157229314826 | 1.36262523043982 | 0.999950574162623 |
| ebi-fl187-GCST90094817 | prot-a-1986 | Myocardial zonula adherens protein \|\| id:prot-a-1986 | Prawns liking \|\| id:ebi-fl187-GCST90094817 | MR Egger | 20 | -0.443285853 | 0.334039395651282 | 0.201081894272954 | -1.098003069 | 0.211431362194744 | 0.641923684866866 | 0.333536468507322 | 1.23544516447385 | 0.999950574162623 |
| ebi-fl187-GCST90094817 | prot-a-2071 | Epididymal secretory protein E1 \|\| id:prot-a-2071 | Prawns liking \|\| id:ebi-fl187-GCST90094817 | MR Egger | 20 | 0.144867492869834 | 0.33627612328816 | 0.671728891464585 | -0.514233709 | 0.803968694514628 | 1.15588639693071 | 0.597958629552512 | 2.23439097050798 | 0.999950574162623 |
| ebi-fl187-GCST90094817 | prot-a-2098 | Neuritin \|\| id:prot-a-2098 | Prawns liking \|\| id:ebi-fl187-GCST90094817 | MR Egger | 20 | 0.0456236885207385 | 0.304417294615684 | 0.882532006027724 | -0.551034209 | 0.642281585967479 | 1.04668045896543 | 0.576353432179722 | 1.9008128034162 | 0.999950574162623 |
| ebi-fl187-GCST90094817 | prot-a-2115 | Neurotrophin-4 \|\| id:prot-a-2115 | Prawns liking \|\| id:ebi-fl187-GCST90094817 | MR Egger | 20 | -0.267626867 | 0.304435712283202 | 0.390936533284339 | -0.864320863 | 0.329067129144421 | 0.765193246746016 | 0.421337601435374 | 1.38967113989117 | 0.999950574162623 |
| ebi-fl187-GCST90094817 | prot-a-2118 | Netrin-1 \|\| id:prot-a-2118 | Prawns liking \|\| id:ebi-fl187-GCST90094817 | MR Egger | 20 | 0.0699594096220987 | 0.319355454516273 | 0.829064685893539 | -0.555977281 | 0.695896100473994 | 1.07246464862535 | 0.573511505203805 | 2.00550540331768 | 0.999950574162623 |
| ebi-fl187-GCST90094817 | prot-a-2120 | Netrin-G1 \|\| id:prot-a-2120 | Prawns liking \|\| id:ebi-fl187-GCST90094817 | MR Egger | 20 | 0.000607717718172894 | 0.304404738995403 | 0.998429053229392 | -0.596025571 | 0.597241006149163 | 1.000607902 | 0.550997189420308 | 1.81709851447827 | 0.999974376584515 |
| ebi-fl187-GCST90094817 | prot-a-2145 | Olfactomedin-like protein 3 \|\| id:prot-a-2145 | Prawns liking \|\| id:ebi-fl187-GCST90094817 | MR Egger | 20 | -0.098077238 | 0.304407249817619 | 0.75102002047616 | -0.694715448 | 0.498560971194796 | 0.906578878304316 | 0.4992164807793 | 1.6463504195705 | 0.999950574162623 |
| ebi-fl187-GCST90094817 | prot-a-2162 | Prolyl 4-hydroxylase subunit alpha-2 \|\| id:prot-a-2162 | Prawns liking \|\| id:ebi-fl187-GCST90094817 | MR Egger | 20 | -0.148322683 | 0.327997166062769 | 0.656521960171855 | -0.791197128 | 0.494551762495764 | 0.86215286798876 | 0.453301809833347 | 1.63976307099792 | 0.999950574162623 |
| ebi-fl187-GCST90094817 | prot-a-2168 | Polyadenylate-binding protein-interacting protein 1 \|\| id:prot-a-2168 | Prawns liking \|\| id:ebi-fl187-GCST90094817 | MR Egger | 20 | 0.549981171947041 | 0.356558596618809 | 0.140356868303659 | -0.148873677 | 1.24883602131991 | 1.733220384 | 0.861677957401871 | 3.48628263619526 | 0.999950574162623 |
| ebi-fl187-GCST90094817 | prot-a-219 | Beta-1,4-galactosyltransferase 5 \|\| id:prot-a-219 | Prawns liking \|\| id:ebi-fl187-GCST90094817 | MR Egger | 20 | 0.065625872500184 | 0.368875465543011 | 0.86078213094802 | -0.65737004 | 0.788621784964486 | 1.06782713892633 | 0.518212421864084 | 2.2003617638611 | 0.999950574162623 |
| ebi-fl187-GCST90094817 | prot-a-2190 | Pyruvate carboxylase, mitochondrial \|\| id:prot-a-2190 | Prawns liking \|\| id:ebi-fl187-GCST90094817 | MR Egger | 20 | -0.192569615 | 0.344789920012525 | 0.583375898284049 | -0.868357858 | 0.483218628002063 | 0.824836895000902 | 0.419640092183091 | 1.62128432442028 | 0.999950574162623 |
| ebi-fl187-GCST90094817 | prot-a-2197 | Protocadherin-9 \|\| id:prot-a-2197 | Prawns liking \|\| id:ebi-fl187-GCST90094817 | MR Egger | 20 | -0.286093038 | 0.304435712283202 | 0.359787424809927 | -0.882787034 | 0.310600958186416 | 0.751192723310651 | 0.413628507048849 | 1.3642447218664 | 0.999950574162623 |
| ebi-fl187-GCST90094817 | prot-a-2198 | Protocadherin alpha-4 \|\| id:prot-a-2198 | Prawns liking \|\| id:ebi-fl187-GCST90094817 | MR Egger | 20 | 0.462323698573859 | 0.42567857139551 | 0.291772242075102 | -0.372006301 | 1.29665369850906 | 1.58775917543198 | 0.689349898667734 | 3.6570386157169 | 0.999950574162623 |
| ebi-fl187-GCST90094817 | prot-a-2254 | Placenta growth factor \|\| id:prot-a-2254 | Prawns liking \|\| id:ebi-fl187-GCST90094817 | MR Egger | 20 | 0.240810854550152 | 0.304417294615684 | 0.439211474832743 | -0.355847043 | 0.837468751996892 | 1.27228036650599 | 0.700579770692766 | 2.31051109197167 | 0.999950574162623 |
| ebi-fl187-GCST90094817 | prot-a-2261 | Membrane-associated progesterone receptor component 2 \|\| id:prot-a-2261 | Prawns liking \|\| id:ebi-fl187-GCST90094817 | MR Egger | 20 | 0.574702317450674 | 0.337215192698533 | 0.105533053853847 | -0.08623946 | 1.2356440951398 | 1.77660158490061 | 0.917374530192623 | 3.44059387697261 | 0.999950574162623 |
| ebi-fl187-GCST90094817 | prot-a-2268 | Polymeric immunoglobulin receptor \|\| id:prot-a-2268 | Prawns liking \|\| id:ebi-fl187-GCST90094817 | MR Egger | 20 | -0.12624314 | 0.304416552290854 | 0.683258337665231 | -0.722899583 | 0.470413302237607 | 0.881400516784172 | 0.485342921746704 | 1.6006556110709 | 0.999950574162623 |
| ebi-fl187-GCST90094817 | prot-a-2283 | Group 10 secretory phospholipase A2 \|\| id:prot-a-2283 | Prawns liking \|\| id:ebi-fl187-GCST90094817 | MR Egger | 20 | 0.146309600842094 | 0.33408600402315 | 0.666639921408492 | -0.508498967 | 0.801118168727469 | 1.15755451243099 | 0.601397619293307 | 2.22803085057749 | 0.999950574162623 |
| ebi-fl187-GCST90094817 | prot-a-2298 | Inactive phospholipase D5 \|\| id:prot-a-2298 | Prawns liking \|\| id:ebi-fl187-GCST90094817 | MR Egger | 20 | -0.232917518 | 0.306284412139455 | 0.456825233526043 | -0.833234966 | 0.367399929383063 | 0.792218914305039 | 0.434640960788112 | 1.44397529180094 | 0.999950574162623 |
| ebi-fl187-GCST90094817 | prot-a-2304 | Procollagen-lysine,2-oxoglutarate 5-dioxygenase 2 \|\| id:prot-a-2304 | Prawns liking \|\| id:ebi-fl187-GCST90094817 | MR Egger | 20 | 0.287141332435954 | 0.304417294615684 | 0.35804279065388 | -0.309516565 | 0.883799229882694 | 1.33261254154361 | 0.733801615866193 | 2.42007669032326 | 0.999950574162623 |
| ebi-fl187-GCST90094817 | prot-a-2331 | Periostin \|\| id:prot-a-2331 | Prawns liking \|\| id:ebi-fl187-GCST90094817 | MR Egger | 20 | -0.033303588 | 0.375903601420038 | 0.930381201268489 | -0.770074647 | 0.70346747042024 | 0.96724487072407 | 0.462978506996943 | 2.02074745544981 | 0.999950574162623 |
| ebi-fl187-GCST90094817 | prot-a-2333 | Periostin \|\| id:prot-a-2333 | Prawns liking \|\| id:ebi-fl187-GCST90094817 | MR Egger | 20 | -0.065969303 | 0.304417294615684 | 0.830873983888535 | -0.6626272 | 0.530688594501801 | 0.936159601193371 | 0.515495244603224 | 1.70010258694257 | 0.999950574162623 |
| ebi-fl187-GCST90094817 | prot-a-2367 | cAMP-dependent protein kinase catalytic subunit alpha \|\| id:prot-a-2367 | Prawns liking \|\| id:ebi-fl187-GCST90094817 | MR Egger | 20 | 0.104552739311229 | 0.322341128351951 | 0.749408731068557 | -0.527235872 | 0.736341350881054 | 1.11021394424099 | 0.590234199666344 | 2.08828123257496 | 0.999950574162623 |
| ebi-fl187-GCST90094817 | prot-a-2385 | Phosphoribosyl pyrophosphate synthase-associated protein 1 \|\| id:prot-a-2385 | Prawns liking \|\| id:ebi-fl187-GCST90094817 | MR Egger | 20 | -0.618765195 | 0.377851851163082 | 0.118870505742906 | -1.359354823 | 0.121824433444177 | 0.538609104447027 | 0.256826421983532 | 1.12955577215428 | 0.999950574162623 |
| ebi-fl187-GCST90094817 | prot-a-2393 | Trypsin-3 \|\| id:prot-a-2393 | Prawns liking \|\| id:ebi-fl187-GCST90094817 | MR Egger | 19 | 0.0304480224007036 | 0.70496671591086 | 0.966052751495417 | -1.351286741 | 1.41218278558599 | 1.0309163041016 | 0.258906900149781 | 4.1049057612897 | 0.999950574162623 |
| ebi-fl187-GCST90094817 | prot-a-2405 | Pregnancy-specific beta-1-glycoprotein 6 \|\| id:prot-a-2405 | Prawns liking \|\| id:ebi-fl187-GCST90094817 | MR Egger | 20 | 0.0397728327285599 | 0.390675162440514 | 0.920036674328907 | -0.725950486 | 0.805496151111968 | 1.0405743629022 | 0.483864444105955 | 2.23780651361978 | 0.999950574162623 |
| ebi-fl187-GCST90094817 | prot-a-244 | Beclin-1 \|\| id:prot-a-244 | Prawns liking \|\| id:ebi-fl187-GCST90094817 | MR Egger | 20 | -0.111817846 | 0.304417294615684 | 0.717665182402141 | -0.708475743 | 0.484840051645766 | 0.894207126249139 | 0.492394160871861 | 1.62391524549949 | 0.999950574162623 |
| ebi-fl187-GCST90094817 | prot-a-2454 | tRNA pseudouridine synthase A, mitochondrial \|\| id:prot-a-2454 | Prawns liking \|\| id:ebi-fl187-GCST90094817 | MR Egger | 20 | -0.413697794 | 0.33645324429735 | 0.234683212165825 | -1.073146152 | 0.245750565162267 | 0.661200740130986 | 0.341931056137315 | 1.27858061121595 | 0.999950574162623 |
| ebi-fl187-GCST90094817 | prot-a-2466 | Glutaminyl-peptide cyclotransferase-like protein \|\| id:prot-a-2466 | Prawns liking \|\| id:ebi-fl187-GCST90094817 | MR Egger | 20 | -0.147428828 | 0.304435712283202 | 0.634034698566664 | -0.744122824 | 0.449265168018674 | 0.862923852103612 | 0.475150908104381 | 1.56716016286294 | 0.999950574162623 |
| ebi-fl187-GCST90094817 | prot-a-2474 | Ras-related protein Rab-27A \|\| id:prot-a-2474 | Prawns liking \|\| id:ebi-fl187-GCST90094817 | MR Egger | 20 | 0.0723689016555321 | 0.429382121149693 | 0.868036352051796 | -0.769220056 | 0.913957859108931 | 1.07505185933244 | 0.463374333534414 | 2.49417461566912 | 0.999950574162623 |
| ebi-fl187-GCST90094817 | prot-a-2490 | Rap1 GTPase-GDP dissociation stimulator 1 \|\| id:prot-a-2490 | Prawns liking \|\| id:ebi-fl187-GCST90094817 | MR Egger | 20 | 0.016246628718895 | 0.363560893419333 | 0.964848421717887 | -0.696332722 | 0.728825979820788 | 1.01637932282793 | 0.498409763315974 | 2.0726458506738 | 0.999950574162623 |
| ebi-fl187-GCST90094817 | prot-a-2545 | Receptor-interacting serine/threonine-protein kinase 2 \|\| id:prot-a-2545 | Prawns liking \|\| id:ebi-fl187-GCST90094817 | MR Egger | 20 | 0.4572599164168 | 0.334785448839764 | 0.188817078599127 | -0.198919563 | 1.11343939614274 | 1.57973943110561 | 0.819615817865682 | 3.04481272297607 | 0.999950574162623 |
| ebi-fl187-GCST90094817 | prot-a-2610 | RUN and FYVE domain-containing protein 1 \|\| id:prot-a-2610 | Prawns liking \|\| id:ebi-fl187-GCST90094817 | MR Egger | 20 | 0.598122460785979 | 0.382995507207475 | 0.135769334127043 | -0.152548733 | 1.34879365491263 | 1.81870091050536 | 0.858517054532823 | 3.85277495002469 | 0.999950574162623 |
| ebi-fl187-GCST90094817 | prot-a-2627 | Serine--tRNA ligase, mitochondrial \|\| id:prot-a-2627 | Prawns liking \|\| id:ebi-fl187-GCST90094817 | MR Egger | 20 | -0.15604664 | 0.370885671383805 | 0.678925156477038 | -0.882982556 | 0.570889276090527 | 0.855519288173423 | 0.413547641576662 | 1.76984022843491 | 0.999950574162623 |
| ebi-fl187-GCST90094817 | prot-a-264 | BolA-like protein 2 \|\| id:prot-a-264 | Prawns liking \|\| id:ebi-fl187-GCST90094817 | MR Egger | 20 | -0.267095272 | 0.325451974253497 | 0.422558944296852 | -0.904981142 | 0.370790597410371 | 0.765600127638476 | 0.404549514162017 | 1.44887964246895 | 0.999950574162623 |
| ebi-fl187-GCST90094817 | prot-a-2646 | Secretoglobin family 1C member 1 \|\| id:prot-a-2646 | Prawns liking \|\| id:ebi-fl187-GCST90094817 | MR Egger | 20 | -0.481229641 | 0.336075795346623 | 0.169306587766259 | -1.1399382 | 0.177478917721045 | 0.618022977893417 | 0.319838787230534 | 1.1942028811188 | 0.999950574162623 |
| ebi-fl187-GCST90094817 | prot-a-2672 | Semaphorin-3E \|\| id:prot-a-2672 | Prawns liking \|\| id:ebi-fl187-GCST90094817 | MR Egger | 20 | -0.04070627 | 0.304417294615684 | 0.895108754453892 | -0.637364167 | 0.555951627793915 | 0.960111102301258 | 0.528684112085313 | 1.74359945322773 | 0.999950574162623 |
| ebi-fl187-GCST90094817 | prot-a-2701 | Plasma protease C1 inhibitor \|\| id:prot-a-2701 | Prawns liking \|\| id:ebi-fl187-GCST90094817 | MR Egger | 20 | -0.50142027 | 0.304418460611437 | 0.116875117609519 | -1.098080453 | 0.0952399125156998 | 0.6056698336886 | 0.3335106590122 | 1.09992270869807 | 0.999950574162623 |
| ebi-fl187-GCST90094817 | prot-a-2738 | Signaling threshold-regulating transmembrane adapter 1 \|\| id:prot-a-2738 | Prawns liking \|\| id:ebi-fl187-GCST90094817 | MR Egger | 20 | 0.200785053384192 | 0.354487415782222 | 0.578110038220036 | -0.494010282 | 0.895580388317348 | 1.22236200100808 | 0.610174509519333 | 2.44875660683616 | 0.999950574162623 |
| ebi-fl187-GCST90094817 | prot-a-274 | Serine/threonine-protein kinase BRSK2 \|\| id:prot-a-274 | Prawns liking \|\| id:ebi-fl187-GCST90094817 | MR Egger | 20 | 0.0970275697932911 | 0.304416552290854 | 0.75359789177648 | -0.499628873 | 0.693684012283364 | 1.10189075210251 | 0.606755801576254 | 2.00107395168671 | 0.999950574162623 |
| ebi-fl187-GCST90094817 | prot-a-2749 | Mitochondrial glutamate carrier 2 \|\| id:prot-a-2749 | Prawns liking \|\| id:ebi-fl187-GCST90094817 | MR Egger | 20 | -0.11035803 | 0.304417294615684 | 0.721183448138478 | -0.707015928 | 0.48629986699946 | 0.895513456810136 | 0.49311349034433 | 1.62628759308138 | 0.999950574162623 |
| ebi-fl187-GCST90094817 | prot-a-2760 | Sodium-coupled monocarboxylate transporter 1 \|\| id:prot-a-2760 | Prawns liking \|\| id:ebi-fl187-GCST90094817 | MR Egger | 20 | -0.057975024 | 0.304370089298493 | 0.851068355334993 | -0.654540399 | 0.538590351459321 | 0.943673516742027 | 0.519680853761203 | 1.71358959976129 | 0.999950574162623 |
| ebi-fl187-GCST90094817 | prot-a-2777 | Small integral membrane protein 10 \|\| id:prot-a-2777 | Prawns liking \|\| id:ebi-fl187-GCST90094817 | MR Egger | 20 | 0.270852309541054 | 0.342543342080671 | 0.439408003746874 | -0.400532641 | 0.942237260019169 | 1.31108142186801 | 0.669963101208341 | 2.56571517396583 | 0.999950574162623 |
| ebi-fl187-GCST90094817 | prot-a-2799 | Superoxide dismutase [Mn], mitochondrial \|\| id:prot-a-2799 | Prawns liking \|\| id:ebi-fl187-GCST90094817 | MR Egger | 20 | 0.0528198501854753 | 0.345010965536514 | 0.880025646890744 | -0.623401642 | 0.729041342637043 | 1.05423970692354 | 0.536117651856236 | 2.0730922695906 | 0.999950574162623 |
| ebi-fl187-GCST90094817 | prot-a-2805 | Protein SPACA7 \|\| id:prot-a-2805 | Prawns liking \|\| id:ebi-fl187-GCST90094817 | MR Egger | 20 | -0.09977654 | 0.304417294615684 | 0.746872887863553 | -0.696434438 | 0.496881356968459 | 0.905039635165457 | 0.498359069874948 | 1.64358750694747 | 0.999950574162623 |
| ebi-fl187-GCST90094817 | prot-a-2811 | SPARC-like protein 1 \|\| id:prot-a-2811 | Prawns liking \|\| id:ebi-fl187-GCST90094817 | MR Egger | 20 | -0.155812062 | 0.304391438157504 | 0.614957464018202 | -0.75241928 | 0.440795157130845 | 0.855719997857329 | 0.471225146823575 | 1.55394235572725 | 0.999950574162623 |
| ebi-fl187-GCST90094817 | prot-a-2831 | Sepiapterin reductase \|\| id:prot-a-2831 | Prawns liking \|\| id:ebi-fl187-GCST90094817 | MR Egger | 20 | -0.088783032 | 0.364222536759584 | 0.810172999848855 | -0.802659204 | 0.625093139849277 | 0.915044087141088 | 0.44813569390474 | 1.86841997368291 | 0.999950574162623 |
| ebi-fl187-GCST90094817 | prot-a-2834 | SPRY domain-containing SOCS box protein 1 \|\| id:prot-a-2834 | Prawns liking \|\| id:ebi-fl187-GCST90094817 | MR Egger | 20 | 0.258764834566972 | 0.317101374116942 | 0.425149706899201 | -0.362753859 | 0.880283527836177 | 1.29532915236656 | 0.695757667119513 | 2.41158336050711 | 0.999950574162623 |
| ebi-fl187-GCST90094817 | prot-a-2835 | Spectrin alpha chain, non-erythrocytic 1 \|\| id:prot-a-2835 | Prawns liking \|\| id:ebi-fl187-GCST90094817 | MR Egger | 20 | -0.058877529 | 0.33233863993999 | 0.86136000102363 | -0.710261264 | 0.592506205028962 | 0.942822230229083 | 0.491515765537601 | 1.80851525045566 | 0.999950574162623 |
| ebi-fl187-GCST90094817 | prot-a-2859 | Sia-alpha-2,3-Gal-beta-1,4-GlcNAc-R:alpha 2,8-sialyltransferase \|\| id:prot-a-2859 | Prawns liking \|\| id:ebi-fl187-GCST90094817 | MR Egger | 20 | -0.766378465 | 0.345229709240585 | 0.0395070743136673 | -1.443028695 | -0.089728235 | 0.464692926450533 | 0.236211262366089 | 0.914179593852257 | 0.999950574162623 |
| ebi-fl187-GCST90094817 | prot-a-2861 | Alpha-2,8-sialyltransferase 8F \|\| id:prot-a-2861 | Prawns liking \|\| id:ebi-fl187-GCST90094817 | MR Egger | 20 | -0.196485407 | 0.346167517442456 | 0.577316899022125 | -0.874973741 | 0.48200292706547 | 0.821613320910937 | 0.416872966073209 | 1.61931452513462 | 0.999950574162623 |
| ebi-fl187-GCST90094817 | prot-a-289 | Uncharacterized protein C17orf89 \|\| id:prot-a-289 | Prawns liking \|\| id:ebi-fl187-GCST90094817 | MR Egger | 20 | 0.0729506356350677 | 0.312026276540184 | 0.817781400070089 | -0.538620866 | 0.684522137653828 | 1.07567743547058 | 0.583552494529906 | 1.98282409213704 | 0.999950574162623 |
| ebi-fl187-GCST90094817 | prot-a-2921 | Tachykinin-3 \|\| id:prot-a-2921 | Prawns liking \|\| id:ebi-fl187-GCST90094817 | MR Egger | 20 | -0.06487169 | 0.303739952763489 | 0.833277880682448 | -0.660201997 | 0.53045861770539 | 0.937187706488445 | 0.516746942550585 | 1.69971164775125 | 0.999950574162623 |
| ebi-fl187-GCST90094817 | prot-a-2928 | Tax1-binding protein 1 \|\| id:prot-a-2928 | Prawns liking \|\| id:ebi-fl187-GCST90094817 | MR Egger | 20 | 0.0850593442534589 | 0.325095159549445 | 0.796566065356651 | -0.552127168 | 0.722245856970372 | 1.08878167771708 | 0.575723845318234 | 2.05905235882171 | 0.999950574162623 |
| ebi-fl187-GCST90094817 | prot-a-296 | Uncharacterized protein C1orf185 \|\| id:prot-a-296 | Prawns liking \|\| id:ebi-fl187-GCST90094817 | MR Egger | 20 | 0.359820159976359 | 0.334665790390357 | 0.296503163651393 | -0.296124789 | 1.01576510914146 | 1.43307166774183 | 0.743694617181457 | 2.76147541939686 | 0.999950574162623 |
| ebi-fl187-GCST90094817 | prot-a-2964 | Transforming growth factor beta-2 \|\| id:prot-a-2964 | Prawns liking \|\| id:ebi-fl187-GCST90094817 | MR Egger | 20 | -0.557523306 | 0.3043987052644 | 0.0836199197112591 | -1.154144768 | 0.0390981565491158 | 0.572625527391374 | 0.315327099416101 | 1.03987254894181 | 0.999950574162623 |
| ebi-fl187-GCST90094817 | prot-a-2977 | Thymocyte nuclear protein 1 \|\| id:prot-a-2977 | Prawns liking \|\| id:ebi-fl187-GCST90094817 | MR Egger | 20 | -0.063142197 | 0.33729815631895 | 0.853597774617625 | -0.724246584 | 0.597962189196891 | 0.938809968059536 | 0.484689604536057 | 1.81840944777758 | 0.999950574162623 |
| ebi-fl187-GCST90094817 | prot-a-3015 | Transmembrane protein 70, mitochondrial \|\| id:prot-a-3015 | Prawns liking \|\| id:ebi-fl187-GCST90094817 | MR Egger | 20 | -0.288889994 | 0.304435712283202 | 0.355220779353133 | -0.88558399 | 0.3078040018285 | 0.749094605586813 | 0.412473222559538 | 1.36043432016551 | 0.999950574162623 |
| ebi-fl187-GCST90094817 | prot-a-3030 | Tumor necrosis factor alpha-induced protein 3 \|\| id:prot-a-3030 | Prawns liking \|\| id:ebi-fl187-GCST90094817 | MR Egger | 20 | -0.263903372 | 0.354405183764067 | 0.466102492200183 | -0.958537532 | 0.430730788547515 | 0.768047751273419 | 0.383453264309803 | 1.53838134432873 | 0.999950574162623 |
| ebi-fl187-GCST90094817 | prot-a-3032 | Tumor necrosis factor receptor superfamily member 10A \|\| id:prot-a-3032 | Prawns liking \|\| id:ebi-fl187-GCST90094817 | MR Egger | 20 | 0.418403443000879 | 0.304417294615684 | 0.186176785550449 | -0.178254454 | 1.01506134044762 | 1.51953359594836 | 0.836729486860666 | 2.75953266315359 | 0.999950574162623 |
| ebi-fl187-GCST90094817 | prot-a-3081 | Tumor protein p53-inducible protein 11 \|\| id:prot-a-3081 | Prawns liking \|\| id:ebi-fl187-GCST90094817 | MR Egger | 20 | 0.540431057389921 | 0.322936658950112 | 0.111519953276027 | -0.092524794 | 1.17338690893214 | 1.71674671907289 | 0.911626607675298 | 3.23292373504008 | 0.999950574162623 |
| ebi-fl187-GCST90094817 | prot-a-315 | Complement C4 \|\| id:prot-a-315 | Prawns liking \|\| id:ebi-fl187-GCST90094817 | MR Egger | 19 | 0.0591815814154911 | 0.339850421183825 | 0.863812292942147 | -0.606925244 | 0.725288406935787 | 1.06096787529879 | 0.545024111450022 | 2.06532666861519 | 0.999950574162623 |
| ebi-fl187-GCST90094817 | prot-a-3157 | UDP-glucuronosyltransferase 1-8 \|\| id:prot-a-3157 | Prawns liking \|\| id:ebi-fl187-GCST90094817 | MR Egger | 20 | -0.642298918 | 0.340007979711654 | 0.0751032278472033 | -1.308714558 | 0.0241167224394013 | 0.526081614415179 | 0.27016711742506 | 1.02440988253298 | 0.999950574162623 |
| ebi-fl187-GCST90094817 | prot-a-317 | Glutaredoxin-like protein C5orf63 \|\| id:prot-a-317 | Prawns liking \|\| id:ebi-fl187-GCST90094817 | MR Egger | 20 | 0.378784303167928 | 0.341888355930916 | 0.282482667140757 | -0.291316874 | 1.04888548079252 | 1.46050797482245 | 0.747278846918614 | 2.85446798516469 | 0.999950574162623 |
| ebi-fl187-GCST90094817 | prot-a-3192 | Guanine nucleotide exchange factor VAV3 \|\| id:prot-a-3192 | Prawns liking \|\| id:ebi-fl187-GCST90094817 | MR Egger | 19 | 0.166611314858845 | 0.337846570953984 | 0.628211605027556 | -0.495567964 | 0.828790593928653 | 1.18129502424081 | 0.60922479113702 | 2.29054686315654 | 0.999950574162623 |
| ebi-fl187-GCST90094817 | prot-a-3233 | Serine/threonine-protein kinase WNK3 \|\| id:prot-a-3233 | Prawns liking \|\| id:ebi-fl187-GCST90094817 | MR Egger | 20 | -0.542014297 | 0.309688929363154 | 0.097112139044192 | -1.149004598 | 0.0649760047755623 | 0.581575605873409 | 0.316952107066503 | 1.06713341797111 | 0.999950574162623 |
| ebi-fl187-GCST90094817 | prot-a-3250 | 14-3-3 protein theta \|\| id:prot-a-3250 | Prawns liking \|\| id:ebi-fl187-GCST90094817 | MR Egger | 20 | 0.0428590585319917 | 0.304417294615684 | 0.889599070778227 | -0.553798839 | 0.639516955978732 | 1.04379077107937 | 0.574762228754842 | 1.89556501677352 | 0.999950574162623 |
| ebi-fl187-GCST90094817 | prot-a-3278 | E3 ubiquitin-protein ligase ZNRF3 \|\| id:prot-a-3278 | Prawns liking \|\| id:ebi-fl187-GCST90094817 | MR Egger | 20 | -0.397840683 | 0.304403072330218 | 0.207679798126042 | -0.994470705 | 0.198789338270745 | 0.671769043032233 | 0.369919189015828 | 1.21992494732987 | 0.999950574162623 |
| ebi-fl187-GCST90094817 | prot-a-355 | Calpain-3 \|\| id:prot-a-355 | Prawns liking \|\| id:ebi-fl187-GCST90094817 | MR Egger | 20 | -0.475995577 | 0.304435712283202 | 0.135337348714994 | -1.072689573 | 0.120698419250475 | 0.62126623021089 | 0.342087210522308 | 1.12828459213993 | 0.999950574162623 |
| ebi-fl187-GCST90094817 | prot-a-385 | Coiled-coil domain-containing protein 90B, mitochondrial \|\| id:prot-a-385 | Prawns liking \|\| id:ebi-fl187-GCST90094817 | MR Egger | 20 | -0.164627651 | 0.379930940701889 | 0.669938079396527 | -0.909292295 | 0.580036992996695 | 0.848209475433077 | 0.402809193444154 | 1.78610450288593 | 0.999950574162623 |
| ebi-fl187-GCST90094817 | prot-a-390 | C-C motif chemokine 14 \|\| id:prot-a-390 | Prawns liking \|\| id:ebi-fl187-GCST90094817 | MR Egger | 20 | 0.190470221014319 | 0.310723604149775 | 0.547551225477246 | -0.418548043 | 0.799488485147877 | 1.20981834593784 | 0.658001516387476 | 2.22440282235745 | 0.999950574162623 |
| ebi-fl187-GCST90094817 | prot-a-40 | Pituitary adenylate cyclase-activating polypeptide 27 \|\| id:prot-a-40 | Prawns liking \|\| id:ebi-fl187-GCST90094817 | MR Egger | 20 | 0.225141520420123 | 0.33461920416518 | 0.509602485334642 | -0.43071212 | 0.880995160583876 | 1.25249995797016 | 0.650046019255714 | 2.41330013298356 | 0.999950574162623 |
| ebi-fl187-GCST90094817 | prot-a-401 | C-C motif chemokine 24 \|\| id:prot-a-401 | Prawns liking \|\| id:ebi-fl187-GCST90094817 | MR Egger | 20 | -0.172172592 | 0.333744142215644 | 0.612215657979287 | -0.826311111 | 0.481965926413363 | 0.841833866561545 | 0.437660794215303 | 1.61925461054967 | 0.999950574162623 |
| ebi-fl187-GCST90094817 | prot-a-412 | Cerebral cavernous malformations 2 protein \|\| id:prot-a-412 | Prawns liking \|\| id:ebi-fl187-GCST90094817 | MR Egger | 19 | 0.406029183542189 | 0.416038560912134 | 0.342777754159015 | -0.409406396 | 1.22146476292997 | 1.50084635183173 | 0.664044312628869 | 3.39215279608239 | 0.999950574162623 |
| ebi-fl187-GCST90094817 | prot-a-44 | Adhesion G protein-coupled receptor F5 \|\| id:prot-a-44 | Prawns liking \|\| id:ebi-fl187-GCST90094817 | MR Egger | 20 | 0.558129875645606 | 0.338948470424007 | 0.116976824349772 | -0.106209126 | 1.22246887767666 | 1.74740158447969 | 0.899236574316568 | 3.39556061736343 | 0.999950574162623 |
| ebi-fl187-GCST90094817 | prot-a-48 | Adrenomedullin \|\| id:prot-a-48 | Prawns liking \|\| id:ebi-fl187-GCST90094817 | MR Egger | 20 | 0.355277770796927 | 0.304403072330218 | 0.258387671738218 | -0.241352251 | 0.951907792564155 | 1.42657686064311 | 0.785564861661131 | 2.59064736553893 | 0.999950574162623 |
| ebi-fl187-GCST90094817 | prot-a-502 | Carcinoembryonic antigen-related cell adhesion molecule 1 \|\| id:prot-a-502 | Prawns liking \|\| id:ebi-fl187-GCST90094817 | MR Egger | 20 | -0.10842876 | 0.304435712283202 | 0.725858827605499 | -0.705122756 | 0.488265236106176 | 0.897242812146049 | 0.494047923164992 | 1.62948699144496 | 0.999950574162623 |
| ebi-fl187-GCST90094817 | prot-a-518 | Complement factor B \|\| id:prot-a-518 | Prawns liking \|\| id:ebi-fl187-GCST90094817 | MR Egger | 20 | 0.494984118924016 | 0.303711202861479 | 0.120521120221054 | -0.100289839 | 1.09025807653252 | 1.64047218638673 | 0.904575199151469 | 2.97504176195951 | 0.999950574162623 |
| ebi-fl187-GCST90094817 | prot-a-525 | Properdin \|\| id:prot-a-525 | Prawns liking \|\| id:ebi-fl187-GCST90094817 | MR Egger | 20 | 0.213146057352978 | 0.357440946 | 0.55839277217574 | -0.487438196 | 0.913730310630978 | 1.23756539356943 | 0.614197834979767 | 2.49360713459875 | 0.999950574162623 |
| ebi-fl187-GCST90094817 | prot-a-533 | Chondroadherin \|\| id:prot-a-533 | Prawns liking \|\| id:ebi-fl187-GCST90094817 | MR Egger | 20 | -0.375185717 | 0.303739952763489 | 0.232629140711026 | -0.970516025 | 0.220144590204014 | 0.687161649193859 | 0.378887472382057 | 1.24625691410214 | 0.999950574162623 |
| ebi-fl187-GCST90094817 | prot-a-535 | Coiled-coil-helix-coiled-coil-helix domain-containing protein 2 \|\| id:prot-a-535 | Prawns liking \|\| id:ebi-fl187-GCST90094817 | MR Egger | 20 | 0.47022868595696 | 0.406231108258237 | 0.262181804286483 | -0.325984286 | 1.2664416581431 | 1.60036013126142 | 0.72181652980212 | 3.54820434831713 | 0.999950574162623 |
| ebi-fl187-GCST90094817 | prot-a-591 | Caseinolytic peptidase B protein homolog \|\| id:prot-a-591 | Prawns liking \|\| id:ebi-fl187-GCST90094817 | MR Egger | 19 | -0.066702393 | 0.307713930043246 | 0.830969529000332 | -0.669821696 | 0.536416909742969 | 0.935473563261612 | 0.51179982559389 | 1.70986925707898 | 0.999950574162623 |
| ebi-fl187-GCST90094817 | prot-a-605 | Ciliary neurotrophic factor receptor subunit alpha \|\| id:prot-a-605 | Prawns liking \|\| id:ebi-fl187-GCST90094817 | MR Egger | 20 | -0.008587901 | 0.315138153689385 | 0.978559220124706 | -0.626258682 | 0.609082880510502 | 0.991448869962421 | 0.534588128443589 | 1.8387442770411 | 0.999950574162623 |
| ebi-fl187-GCST90094817 | prot-a-606 | Ciliary neurotrophic factor receptor subunit alpha \|\| id:prot-a-606 | Prawns liking \|\| id:ebi-fl187-GCST90094817 | MR Egger | 20 | 0.0302428790749566 | 0.304417294615684 | 0.921961007160788 | -0.566415018 | 0.626900776521697 | 1.03070484019335 | 0.567556475447021 | 1.87180045256513 | 0.999950574162623 |
| ebi-fl187-GCST90094817 | prot-a-707 | Cancer/testis antigen 55 \|\| id:prot-a-707 | Prawns liking \|\| id:ebi-fl187-GCST90094817 | MR Egger | 20 | -0.065465075 | 0.35569647955697 | 0.856033667104212 | -0.762630175 | 0.631700024540357 | 0.936631757686045 | 0.466437998513537 | 1.88080527809012 | 0.999950574162623 |
| ebi-fl187-GCST90094817 | prot-a-719 | Dipeptidyl peptidase 1 \|\| id:prot-a-719 | Prawns liking \|\| id:ebi-fl187-GCST90094817 | MR Egger | 20 | -0.228235791 | 0.342996200529341 | 0.514220246492017 | -0.900508344 | 0.444036761726976 | 0.795936562782417 | 0.406363034874726 | 1.5589877956522 | 0.999950574162623 |
| ebi-fl187-GCST90094817 | prot-a-752 | Cytochrome b5 \|\| id:prot-a-752 | Prawns liking \|\| id:ebi-fl187-GCST90094817 | MR Egger | 20 | -0.528822621 | 0.304403072330218 | 0.0994245095227632 | -1.125452643 | 0.0678074003953816 | 0.589298388718356 | 0.324505548899741 | 1.07015917639468 | 0.999950574162623 |
| ebi-fl187-GCST90094817 | prot-a-755 | Cytoglobin \|\| id:prot-a-755 | Prawns liking \|\| id:ebi-fl187-GCST90094817 | MR Egger | 20 | -0.160953379 | 0.304407249817619 | 0.603442787761765 | -0.757591588 | 0.435684830893542 | 0.851331760333654 | 0.468794117688182 | 1.54602145975491 | 0.999950574162623 |
| ebi-fl187-GCST90094817 | prot-a-807 | Desmin \|\| id:prot-a-807 | Prawns liking \|\| id:ebi-fl187-GCST90094817 | MR Egger | 20 | -0.070524896 | 0.304407249817619 | 0.819400376095803 | -0.667163106 | 0.526113313520258 | 0.931904538427656 | 0.513162301956862 | 1.6923419070933 | 0.999950574162623 |
| ebi-fl187-GCST90094817 | prot-a-82 | Ameloblastin \|\| id:prot-a-82 | Prawns liking \|\| id:ebi-fl187-GCST90094817 | MR Egger | 20 | -0.655797356 | 0.316007829743533 | 0.0525726179111848 | -1.275172702 | -0.036422009 | 0.519028047528463 | 0.279382714075235 | 0.96423329200197 | 0.999950574162623 |
| ebi-fl187-GCST90094817 | prot-a-84 | Muellerian-inhibiting factor \|\| id:prot-a-84 | Prawns liking \|\| id:ebi-fl187-GCST90094817 | MR Egger | 20 | -0.376602378 | 0.304397184014435 | 0.231908198606091 | -0.973220858 | 0.220016102968311 | 0.686188863652994 | 0.377864029508695 | 1.24609679628304 | 0.999950574162623 |
| ebi-fl187-GCST90094817 | prot-a-858 | Inactive dipeptidyl peptidase 10 \|\| id:prot-a-858 | Prawns liking \|\| id:ebi-fl187-GCST90094817 | MR Egger | 20 | -0.121776193 | 0.348725070650416 | 0.730988019993771 | -0.805277332 | 0.56172494537547 | 0.885346492985588 | 0.446963952152699 | 1.75369492073511 | 0.999950574162623 |
| ebi-fl187-GCST90094817 | prot-a-863 | Dihydropyrimidinase-related protein 3 \|\| id:prot-a-863 | Prawns liking \|\| id:ebi-fl187-GCST90094817 | MR Egger | 20 | 0.339207200638255 | 0.304417294615684 | 0.27981547742693 | -0.257450697 | 0.935865098084995 | 1.40383419042029 | 0.773019737714142 | 2.54941800065881 | 0.999950574162623 |
| ebi-fl187-GCST90094817 | prot-a-864 | Dihydropyrimidinase-related protein 5 \|\| id:prot-a-864 | Prawns liking \|\| id:ebi-fl187-GCST90094817 | MR Egger | 20 | -0.548942952 | 0.304417294615684 | 0.0881136171940345 | -1.145600849 | 0.0477149458964139 | 0.577559996724537 | 0.318032770699592 | 1.04887162754538 | 0.999950574162623 |
| ebi-fl187-GCST90094817 | prot-a-884 | Endothelin-converting enzyme 1 \|\| id:prot-a-884 | Prawns liking \|\| id:ebi-fl187-GCST90094817 | MR Egger | 20 | -0.091152627 | 0.319941581932348 | 0.778969786458367 | -0.718238127 | 0.535932873850715 | 0.91287837061931 | 0.48761060739061 | 1.70904181925845 | 0.999950574162623 |
| ebi-fl187-GCST90094817 | prot-a-893 | Early endosome antigen 1 \|\| id:prot-a-893 | Prawns liking \|\| id:ebi-fl187-GCST90094817 | MR Egger | 19 | -0.393155301 | 0.353988534859613 | 0.282184207807287 | -1.086972829 | 0.30066222762605 | 0.674923923289782 | 0.337235820578212 | 1.3507530174222 | 0.999950574162623 |
| ebi-fl187-GCST90094817 | prot-a-897 | Ephrin-A2 \|\| id:prot-a-897 | Prawns liking \|\| id:ebi-fl187-GCST90094817 | MR Egger | 20 | -0.04812845 | 0.336909457310883 | 0.887993413796248 | -0.708470986 | 0.612214086309724 | 0.953011364886602 | 0.492396503146614 | 1.84451078713813 | 0.999950574162623 |
| ebi-fl187-GCST90094817 | prot-a-90 | Anaphase-promoting complex subunit 10 \|\| id:prot-a-90 | Prawns liking \|\| id:ebi-fl187-GCST90094817 | MR Egger | 20 | -0.160886234 | 0.304435712283202 | 0.603626316074213 | -0.75758023 | 0.435807762541742 | 0.851388925107446 | 0.468799442649133 | 1.54621152640347 | 0.999950574162623 |
| ebi-fl187-GCST90094817 | prot-a-914 | Histone-lysine N-methyltransferase EHMT2 \|\| id:prot-a-914 | Prawns liking \|\| id:ebi-fl187-GCST90094817 | MR Egger | 20 | -0.677198257 | 0.345194543595465 | 0.0654404319080373 | -1.353779563 | -0.000616952 | 0.508038393219465 | 0.258262295182501 | 0.999383238666826 | 0.999950574162623 |
| ebi-fl187-GCST90094817 | prot-a-932 | RNA polymerase II elongation factor ELL \|\| id:prot-a-932 | Prawns liking \|\| id:ebi-fl187-GCST90094817 | MR Egger | 20 | -0.759333707 | 0.340570800893945 | 0.0387491134631875 | -1.426852476 | -0.091814937 | 0.467978133855936 | 0.240063339365069 | 0.91227396214064 | 0.999950574162623 |
| ebi-fl187-GCST90094817 | prot-a-991 | Estrogen receptor \|\| id:prot-a-991 | Prawns liking \|\| id:ebi-fl187-GCST90094817 | MR Egger | 20 | 0.0852182894612172 | 0.304403072330218 | 0.782706023201545 | -0.511411732 | 0.681848311228445 | 1.08895474810104 | 0.599648437912823 | 1.97752944631898 | 0.999950574162623 |
| ebi-fl187-GCST90094817 | prot-a-992 | Persulfide dioxygenase ETHE1, mitochondrial \|\| id:prot-a-992 | Prawns liking \|\| id:ebi-fl187-GCST90094817 | MR Egger | 20 | 0.0679917371463146 | 0.347218386194012 | 0.846945968422902 | -0.6125563 | 0.748539774086578 | 1.07035646424338 | 0.54196367515701 | 2.11391097422846 | 0.999950574162623 |
| ebi-fl187-GCST90094817 | prot-a-993 | Ethanolamine kinase 1 \|\| id:prot-a-993 | Prawns liking \|\| id:ebi-fl187-GCST90094817 | MR Egger | 20 | 0.218214663502136 | 0.379454979899888 | 0.572360260324427 | -0.525517097 | 0.961946424105916 | 1.24385404902876 | 0.591249551876629 | 2.61678489290101 | 0.999950574162623 |
| ebi-fl187-GCST90094817 | prot-b-10 | coagulation factor III, tissue factor \|\| id:prot-b-10 | Prawns liking \|\| id:ebi-fl187-GCST90094817 | MR Egger | 12 | -1.169831283 | 0.982960315316953 | 0.261489576869836 | -3.096433501 | 0.756770934695916 | 0.310419309761344 | 0.0452101571651111 | 2.13138272270972 | 0.999950574162623 |
| ebi-fl187-GCST90094817 | prot-b-28 | prolactin \|\| id:prot-b-28 | Prawns liking \|\| id:ebi-fl187-GCST90094817 | MR Egger | 12 | -0.262339759 | 1.46053060111155 | 0.86103873423578 | -3.124979737 | 2.60030021916205 | 0.769249619808578 | 0.0439378239179347 | 13.467780713967 | 0.999950574162623 |
| ebi-fl187-GCST90094817 | prot-b-54 | matrix metallopeptidase 7 \|\| id:prot-b-54 | Prawns liking \|\| id:ebi-fl187-GCST90094817 | MR Egger | 12 | 0.0790472811535985 | 0.989298897149246 | 0.937891362424227 | -1.859978557 | 2.01807311956612 | 1.08225549114875 | 0.155675968451692 | 7.52381346826232 | 0.999950574162623 |
| ebi-fl187-GCST90094817 | ukb-rsfMRI191-edge-pheno716 | Net100_Pair10_33 (OccipitalPrecuneus)&(Temporal\|Frontal\|Supp_Motor_Area) (Default_mode\|Central_executive)&(Default_mode\|Salience) \|\| id:ukb-rsfMRI191-edge-pheno716 | Prawns liking \|\| id:ebi-fl187-GCST90094817 | MR Egger | 19 | -0.095243867 | 0.0946215594467352 | 0.328251492145012 | -0.280702124 | 0.0902143893627268 | 0.909151195318348 | 0.755253274051253 | 1.09440888818006 | 0.999950574162623 |
| ebi-fl187-GCST90094817 | ukb-rsfMRI191-edge-pheno903 | Net100_Pair27_38 (Precentral\|Frontal\|Supp_Motor_Area)&(Subcortical) (Attention\|Salience\|Motor)&(Subcortical-cerebellum) \|\| id:ukb-rsfMRI191-edge-pheno903 | Prawns liking \|\| id:ebi-fl187-GCST90094817 | MR Egger | 19 | 0.171218753215495 | 0.112927309212957 | 0.147846364567387 | -0.050118773 | 0.39255627927289 | 1.18675032607884 | 0.951116450987893 | 1.48076119909964 | 0.999950574162623 |
| ebi-fl187-GCST90094817 | ukb-rsfMRI191-node-pheno23 | Net100_Node2 RolandicOper\|SupraMarginal\|Insula Salience\|Motor \|\| id:ukb-rsfMRI191-node-pheno23 | Prawns liking \|\| id:ebi-fl187-GCST90094817 | MR Egger | 19 | 0.0736164209260247 | 0.152855528729517 | 0.636225771889688 | -0.225980415 | 0.373213257235878 | 1.07639384414565 | 0.797733724821495 | 1.45239404034711 | 0.999950574162623 |
| ebi-fl187-GCST90094817 | ukb-rsfMRI191-node-pheno55 | Net100_Node34 Parietal\|Postcentral\|Precuneus Attention \|\| id:ukb-rsfMRI191-node-pheno55 | Prawns liking \|\| id:ebi-fl187-GCST90094817 | MR Egger | 18 | -0.043152405 | 0.120220828956733 | 0.724330608377568 | -0.278785229 | 0.192480420082612 | 0.957765411019489 | 0.756702403172446 | 1.21225276766603 | 0.999950574162623 |
| ebi-fl187-GCST90094817 | ebi-a-GCST90001414 | IgD- CD24- B cell Absolute Count \|\| id:ebi-a-GCST90001414 | Prawns liking \|\| id:ebi-fl187-GCST90094817 | Weighted median | 16 | -0.161790071 | 0.155089639689854 | 0.296854005083582 | -0.465765765 | 0.142185622863555 | 0.850619755613493 | 0.627654285607104 | 1.1527906129727 | 0.999332001504091 |
| ebi-fl187-GCST90094817 | ebi-a-GCST90001422 | CD20- CD38- B cell %B cell \|\| id:ebi-a-GCST90001422 | Prawns liking \|\| id:ebi-fl187-GCST90094817 | Weighted median | 16 | -0.169123729 | 0.152324957235739 | 0.266877326356672 | -0.467680645 | 0.129433187029024 | 0.844404419478682 | 0.626453552580147 | 1.13818306353033 | 0.999332001504091 |
| ebi-fl187-GCST90094817 | ebi-a-GCST90001444 | CD20- CD38- B cell %lymphocyte \|\| id:ebi-a-GCST90001444 | Prawns liking \|\| id:ebi-fl187-GCST90094817 | Weighted median | 16 | -0.201367032 | 0.157808812391688 | 0.201948828329417 | -0.510672304 | 0.107938240738147 | 0.817612286971336 | 0.600091999009694 | 1.1139789447446 | 0.999332001504091 |
| ebi-fl187-GCST90094817 | ebi-a-GCST90001459 | Myeloid Dendritic Cell %Dendritic Cell \|\| id:ebi-a-GCST90001459 | Prawns liking \|\| id:ebi-fl187-GCST90094817 | Weighted median | 16 | 0.220908211656863 | 0.153473825539002 | 0.150040938134451 | -0.079900486 | 0.521716909713307 | 1.24720894606727 | 0.923208213588813 | 1.68491801985099 | 0.999332001504091 |
| ebi-fl187-GCST90094817 | ebi-a-GCST90001476 | HLA DR++ monocyte %leukocyte \|\| id:ebi-a-GCST90001476 | Prawns liking \|\| id:ebi-fl187-GCST90094817 | Weighted median | 16 | -0.025967427 | 0.154927861148418 | 0.866890263351502 | -0.329626035 | 0.277691180494256 | 0.974366826795013 | 0.719192635873582 | 1.3200784682749 | 0.999332001504091 |
| ebi-fl187-GCST90094817 | ebi-a-GCST90001478 | CD4 regulatory T cell %CD4+ T cell \|\| id:ebi-a-GCST90001478 | Prawns liking \|\| id:ebi-fl187-GCST90094817 | Weighted median | 16 | -0.046390344 | 0.14777493829984 | 0.753577507322039 | -0.336029223 | 0.243248535266186 | 0.954669240230187 | 0.714602222783652 | 1.27538556302198 | 0.999332001504091 |
| ebi-fl187-GCST90094817 | ebi-a-GCST90001485 | CD39+ resting CD4 regulatory T cell %CD4 regulatory T cell \|\| id:ebi-a-GCST90001485 | Prawns liking \|\| id:ebi-fl187-GCST90094817 | Weighted median | 16 | 0.119455851212722 | 0.147775745267663 | 0.418883956701031 | -0.17018461 | 0.409096311937341 | 1.12688349242938 | 0.843509082421834 | 1.50545670695551 | 0.999332001504091 |
| ebi-fl187-GCST90094817 | ebi-a-GCST90001515 | Immature Myeloid-Derived Suppressor Cells Absolute Count \|\| id:ebi-a-GCST90001515 | Prawns liking \|\| id:ebi-fl187-GCST90094817 | Weighted median | 16 | -0.130352737 | 0.205467914620868 | 0.525807449997553 | -0.53306985 | 0.272364375336037 | 0.877785748512278 | 0.586800811389779 | 1.31306536278706 | 0.999332001504091 |
| ebi-fl187-GCST90094817 | ebi-a-GCST90001517 | CD33+ HLA DR+ Absolute Count \|\| id:ebi-a-GCST90001517 | Prawns liking \|\| id:ebi-fl187-GCST90094817 | Weighted median | 16 | 0.0149027864909003 | 0.192296030527781 | 0.938226436100379 | -0.361997433 | 0.391803006325351 | 1.01501438670907 | 0.696284154961695 | 1.47964620174799 | 0.999332001504091 |
| ebi-fl187-GCST90094817 | ebi-a-GCST90001518 | CD33+ HLA DR+ CD14- Absolute Count \|\| id:ebi-a-GCST90001518 | Prawns liking \|\| id:ebi-fl187-GCST90094817 | Weighted median | 16 | -0.050826331 | 0.202533066853788 | 0.801850255406472 | -0.447791142 | 0.346138480091147 | 0.950443718865191 | 0.639038138337954 | 1.41359835749359 | 0.999332001504091 |
| ebi-fl187-GCST90094817 | ebi-a-GCST90001520 | CD33+ HLA DR+ CD14dim Absolute Count \|\| id:ebi-a-GCST90001520 | Prawns liking \|\| id:ebi-fl187-GCST90094817 | Weighted median | 16 | 0.138078697872087 | 0.200728517104214 | 0.491523440344943 | -0.255349196 | 0.531506591396346 | 1.1480658970622 | 0.774645947729428 | 1.70149383451962 | 0.999332001504091 |
| ebi-fl187-GCST90094817 | ebi-a-GCST90001531 | CD33dim HLA DR- Absolute Count \|\| id:ebi-a-GCST90001531 | Prawns liking \|\| id:ebi-fl187-GCST90094817 | Weighted median | 16 | -0.154921217 | 0.209040938881949 | 0.458629974665434 | -0.564641457 | 0.254799022936693 | 0.856482650865493 | 0.568563964573009 | 1.29020229374629 | 0.999332001504091 |
| ebi-fl187-GCST90094817 | ebi-a-GCST90001532 | Basophil Absolute Count \|\| id:ebi-a-GCST90001532 | Prawns liking \|\| id:ebi-fl187-GCST90094817 | Weighted median | 16 | -0.165440245 | 0.202066382621698 | 0.412933597695267 | -0.561490355 | 0.230609864797483 | 0.847520505160103 | 0.570358393576846 | 1.25936781987598 | 0.999332001504091 |
| ebi-fl187-GCST90094817 | ebi-a-GCST90001588 | T/B cell \|\| id:ebi-a-GCST90001588 | Prawns liking \|\| id:ebi-fl187-GCST90094817 | Weighted median | 16 | 0.0235466378835425 | 0.152159378278933 | 0.877018544672611 | -0.274685744 | 0.321779019310251 | 1.02382604871322 | 0.75981086117963 | 1.37957988175679 | 0.999332001504091 |
| ebi-fl187-GCST90094817 | ebi-a-GCST90001624 | HLA DR+ CD4+ T cell Absolute Count \|\| id:ebi-a-GCST90001624 | Prawns liking \|\| id:ebi-fl187-GCST90094817 | Weighted median | 16 | 0.324147005721208 | 0.152059277879205 | 0.0330303034015905 | 0.0261108210779667 | 0.622183190364449 | 1.38285057912475 | 1.02645469498635 | 1.86299086898431 | 0.999332001504091 |
| ebi-fl187-GCST90094817 | ebi-a-GCST90001631 | CD8+ Natural Killer T %T cell \|\| id:ebi-a-GCST90001631 | Prawns liking \|\| id:ebi-fl187-GCST90094817 | Weighted median | 16 | -0.040200687 | 0.151455131911319 | 0.790678475266559 | -0.337052746 | 0.256651371491461 | 0.960596640496315 | 0.713871185345031 | 1.29259441293575 | 0.999332001504091 |
| ebi-fl187-GCST90094817 | ebi-a-GCST90001632 | CD8+ Natural Killer T %lymphocyte \|\| id:ebi-a-GCST90001632 | Prawns liking \|\| id:ebi-fl187-GCST90094817 | Weighted median | 16 | -0.08358575 | 0.150427033707387 | 0.578445742427685 | -0.378422736 | 0.211251236524143 | 0.919812209813431 | 0.68494089055156 | 1.23522264912607 | 0.999332001504091 |
| ebi-fl187-GCST90094817 | ebi-a-GCST90001646 | Natural Killer %CD3- lymphocyte \|\| id:ebi-a-GCST90001646 | Prawns liking \|\| id:ebi-fl187-GCST90094817 | Weighted median | 16 | -0.097422881 | 0.147184186999792 | 0.508028051387887 | -0.385903887 | 0.191058125827212 | 0.907172299357836 | 0.67983586346061 | 1.21052981308313 | 0.999332001504091 |
| ebi-fl187-GCST90094817 | ebi-a-GCST90001684 | CD127- CD8+ T cell Absolute Count \|\| id:ebi-a-GCST90001684 | Prawns liking \|\| id:ebi-fl187-GCST90094817 | Weighted median | 16 | 0.202648790333713 | 0.169048458772821 | 0.230620682238944 | -0.128686189 | 0.533983769528442 | 1.22464228650116 | 0.879249840650924 | 1.70571396268495 | 0.999332001504091 |
| ebi-fl187-GCST90094817 | ebi-a-GCST90001687 | CD28- CD8+ T cell Absolute Count \|\| id:ebi-a-GCST90001687 | Prawns liking \|\| id:ebi-fl187-GCST90094817 | Weighted median | 16 | 0.173321452437659 | 0.164331185951928 | 0.291558791720678 | -0.148767672 | 0.495410576903437 | 1.1892483304219 | 0.86176930475801 | 1.64117192803523 | 0.999332001504091 |
| ebi-fl187-GCST90094817 | ebi-a-GCST90001701 | BAFF-R on CD20- CD38- B cell \|\| id:ebi-a-GCST90001701 | Prawns liking \|\| id:ebi-fl187-GCST90094817 | Weighted median | 16 | -0.217037278 | 0.156914790025919 | 0.166617731938692 | -0.524590266 | 0.0905157104181982 | 0.804899963658643 | 0.591797794088919 | 1.09473870630944 | 0.999332001504091 |
| ebi-fl187-GCST90094817 | ebi-a-GCST90001732 | CD19 on IgD- CD27- B cell \|\| id:ebi-a-GCST90001732 | Prawns liking \|\| id:ebi-fl187-GCST90094817 | Weighted median | 16 | 0.176569851165365 | 0.171189924525376 | 0.302340786645659 | -0.158962401 | 0.512102103235102 | 1.19311776451521 | 0.853028431463748 | 1.66879549085965 | 0.999332001504091 |
| ebi-fl187-GCST90094817 | ebi-a-GCST90001798 | CD27 on CD24+ CD27+ B cell \|\| id:ebi-a-GCST90001798 | Prawns liking \|\| id:ebi-fl187-GCST90094817 | Weighted median | 16 | -0.143554937 | 0.168902981199688 | 0.395366552138551 | -0.47460478 | 0.187494906023314 | 0.866273208317783 | 0.622130886278437 | 1.20622410492786 | 0.999332001504091 |
| ebi-fl187-GCST90094817 | ebi-a-GCST90001836 | CD66b on CD66b++ myeloid cell \|\| id:ebi-a-GCST90001836 | Prawns liking \|\| id:ebi-fl187-GCST90094817 | Weighted median | 16 | 0.221832777687992 | 0.253624978763735 | 0.381765038594692 | -0.275272181 | 0.718937736064913 | 1.24836260632789 | 0.759365410494061 | 2.05225201904287 | 0.999332001504091 |
| ebi-fl187-GCST90094817 | ebi-a-GCST90001943 | CD25 on activated & secreting CD4 regulatory T cell \|\| id:ebi-a-GCST90001943 | Prawns liking \|\| id:ebi-fl187-GCST90094817 | Weighted median | 16 | 0.151639110082854 | 0.156160231706506 | 0.331524282726061 | -0.154434944 | 0.457713164227606 | 1.16374017854273 | 0.856899236712523 | 1.58045560683468 | 0.999332001504091 |
| ebi-fl187-GCST90094817 | ebi-a-GCST90001960 | CD25 on CD4+ T cell \|\| id:ebi-a-GCST90001960 | Prawns liking \|\| id:ebi-fl187-GCST90094817 | Weighted median | 16 | -0.14897222 | 0.172011400984709 | 0.386457177057046 | -0.486114566 | 0.188170126204711 | 0.861593049858086 | 0.615011344099591 | 1.20703884682093 | 0.999332001504091 |
| ebi-fl187-GCST90094817 | ebi-a-GCST90001988 | HLA DR on CD14+ CD16- monocyte \|\| id:ebi-a-GCST90001988 | Prawns liking \|\| id:ebi-fl187-GCST90094817 | Weighted median | 16 | 0.0140500199114651 | 0.165326891513015 | 0.932274690945178 | -0.309990687 | 0.338090727276974 | 1.01414918532228 | 0.733453786514579 | 1.40226772156612 | 0.999332001504091 |
| ebi-fl187-GCST90094817 | ebi-a-GCST90001991 | HLA DR on CD14+ monocyte \|\| id:ebi-a-GCST90001991 | Prawns liking \|\| id:ebi-fl187-GCST90094817 | Weighted median | 16 | 0.0196943415400324 | 0.157465225495744 | 0.900467299797944 | -0.2889375 | 0.32832618351169 | 1.01988955450867 | 0.74905901980514 | 1.38864185049997 | 0.999332001504091 |
| ebi-fl187-GCST90094817 | ebi-a-GCST90002019 | CD14 on Monocytic Myeloid-Derived Suppressor Cells \|\| id:ebi-a-GCST90002019 | Prawns liking \|\| id:ebi-fl187-GCST90094817 | Weighted median | 16 | -0.060291618 | 0.215362128898507 | 0.779512337299447 | -0.482401391 | 0.361818154197398 | 0.941489937717211 | 0.617299233621005 | 1.43593779895565 | 0.999332001504091 |
| ebi-fl187-GCST90094817 | ebi-a-GCST90002037 | CD80 on plasmacytoid Dendritic Cell \|\| id:ebi-a-GCST90002037 | Prawns liking \|\| id:ebi-fl187-GCST90094817 | Weighted median | 16 | -0.042918011 | 0.157502453534596 | 0.78524446326056 | -0.35162282 | 0.265786798180914 | 0.957989931726185 | 0.703545435433885 | 1.30445691645026 | 0.999332001504091 |
| ebi-fl187-GCST90094817 | ebi-a-GCST90002042 | CD45 on CD33+ HLA DR+ CD14- \|\| id:ebi-a-GCST90002042 | Prawns liking \|\| id:ebi-fl187-GCST90094817 | Weighted median | 16 | -0.002213141 | 0.219263508434976 | 0.991946670936749 | -0.431969618 | 0.427543335365451 | 0.99778930602415 | 0.649229101459056 | 1.5334856323888 | 0.999332001504091 |
| ebi-fl187-GCST90094817 | ebi-a-GCST90002056 | CD8 on Effector Memory CD8+ T cell \|\| id:ebi-a-GCST90002056 | Prawns liking \|\| id:ebi-fl187-GCST90094817 | Weighted median | 16 | 0.0176273387251604 | 0.177050552038754 | 0.920692818347512 | -0.329391743 | 0.364646420721118 | 1.01778361716765 | 0.719361156649891 | 1.4400047622797 | 0.999332001504091 |
| ebi-fl187-GCST90094817 | ebi-a-GCST90002058 | CD8 on CD8+ T cell \|\| id:ebi-a-GCST90002058 | Prawns liking \|\| id:ebi-fl187-GCST90094817 | Weighted median | 16 | -0.171226218 | 0.166086210454858 | 0.302565285155354 | -0.496755191 | 0.154302754190496 | 0.842630933371154 | 0.608501932454249 | 1.16684410024832 | 0.999332001504091 |
| ebi-fl187-GCST90094817 | ebi-a-GCST90002063 | CD4 on CD4 regulatory T cell \|\| id:ebi-a-GCST90002063 | Prawns liking \|\| id:ebi-fl187-GCST90094817 | Weighted median | 16 | -0.029049794 | 0.174141151820533 | 0.867513637467357 | -0.370366452 | 0.3122668635056 | 0.971368094898587 | 0.690481256287966 | 1.36651931851051 | 0.999332001504091 |
| ebi-fl187-GCST90094817 | ebi-a-GCST90002066 | CD4 on activated CD4 regulatory T cell \|\| id:ebi-a-GCST90002066 | Prawns liking \|\| id:ebi-fl187-GCST90094817 | Weighted median | 16 | 0.089309553813254 | 0.176035157143853 | 0.611916766437758 | -0.255719354 | 0.434338461815207 | 1.09341907598854 | 0.774359258982507 | 1.54394134488244 | 0.999332001504091 |
| ebi-fl187-GCST90094817 | ebi-a-GCST90002070 | CD4 on activated & secreting CD4 regulatory T cell \|\| id:ebi-a-GCST90002070 | Prawns liking \|\| id:ebi-fl187-GCST90094817 | Weighted median | 16 | -0.017593122 | 0.16607608870582 | 0.915634634178678 | -0.343102256 | 0.307916011992752 | 0.982560733511177 | 0.709565650693913 | 1.36058671117168 | 0.999332001504091 |
| ebi-fl187-GCST90094817 | ebi-a-GCST90002081 | SSC-A on CD4+ T cell \|\| id:ebi-a-GCST90002081 | Prawns liking \|\| id:ebi-fl187-GCST90094817 | Weighted median | 16 | -0.003652711 | 0.148467109697743 | 0.980371763825781 | -0.294648246 | 0.287342824292415 | 0.996353952317444 | 0.744793525702604 | 1.33288107917171 | 0.999332001504091 |
| ebi-fl187-GCST90094817 | ebi-a-GCST90002094 | CD11b on Monocytic Myeloid-Derived Suppressor Cells \|\| id:ebi-a-GCST90002094 | Prawns liking \|\| id:ebi-fl187-GCST90094817 | Weighted median | 16 | -0.144088129 | 0.225424964362986 | 0.522702955100592 | -0.585921059 | 0.297744801171795 | 0.865811441617925 | 0.556592970608979 | 1.34681803763408 | 0.999332001504091 |
| ebi-fl187-GCST90094817 | ebi-a-GCST90002104 | HLA DR on myeloid Dendritic Cell \|\| id:ebi-a-GCST90002104 | Prawns liking \|\| id:ebi-fl187-GCST90094817 | Weighted median | 16 | 0.0367864550875698 | 0.172672826701208 | 0.831294696836297 | -0.301652285 | 0.375225195421938 | 1.03747145043135 | 0.739595188342516 | 1.45531910891998 | 0.999332001504091 |
| ebi-fl187-GCST90094817 | ebi-a-GCST90002105 | HLA DR on plasmacytoid Dendritic Cell \|\| id:ebi-a-GCST90002105 | Prawns liking \|\| id:ebi-fl187-GCST90094817 | Weighted median | 15 | 0.174485797942211 | 0.156821202096398 | 0.265862373523842 | -0.132883758 | 0.481855354051151 | 1.19063383281444 | 0.875566863690986 | 1.6190755756407 | 0.999332001504091 |
| ebi-fl187-GCST90094817 | ebi-a-GCST90002106 | HLA DR on Dendritic Cell \|\| id:ebi-a-GCST90002106 | Prawns liking \|\| id:ebi-fl187-GCST90094817 | Weighted median | 16 | 0.111811299219295 | 0.167777167296191 | 0.505137962035337 | -0.217031949 | 0.440654547119828 | 1.11830181630775 | 0.804904253264792 | 1.55372387123641 | 0.999332001504091 |
| ebi-fl187-GCST90094817 | ebi-a-GCST90002111 | HLA DR on CD33dim HLA DR+ CD11b- \|\| id:ebi-a-GCST90002111 | Prawns liking \|\| id:ebi-fl187-GCST90094817 | Weighted median | 16 | 0.10962000431416 | 0.226217150684295 | 0.627975252504954 | -0.333765611 | 0.553005619655378 | 1.11585397019114 | 0.716221637008683 | 1.73847035394189 | 0.999332001504091 |
| ebi-fl187-GCST90094817 | ebi-a-GCST90002112 | HLA DR on CD33- HLA DR+ \|\| id:ebi-a-GCST90002112 | Prawns liking \|\| id:ebi-fl187-GCST90094817 | Weighted median | 16 | 0.0857343769878969 | 0.231103116779749 | 0.710653257354445 | -0.367227732 | 0.538696485876205 | 1.08951688910815 | 0.692651888151123 | 1.71377148024596 | 0.999332001504091 |
| ebi-fl187-GCST90094817 | ebi-a-GCST90002115 | HLA DR on HLA DR+ CD8+ T cell \|\| id:ebi-a-GCST90002115 | Prawns liking \|\| id:ebi-fl187-GCST90094817 | Weighted median | 16 | -0.087519681 | 0.165887240155002 | 0.597787148174928 | -0.412658672 | 0.237619309429042 | 0.916200839472972 | 0.661888165253512 | 1.26822629912029 | 0.999332001504091 |
| ebi-fl187-GCST90094817 | ebi-a-GCST90002116 | HLA DR on B cell \|\| id:ebi-a-GCST90002116 | Prawns liking \|\| id:ebi-fl187-GCST90094817 | Weighted median | 16 | 0.104635993806179 | 0.176741186680142 | 0.553830844101341 | -0.241776732 | 0.451048719699257 | 1.11030637838993 | 0.785231474974676 | 1.56995776810031 | 0.999332001504091 |
| ebi-fl187-GCST90094817 | ebi-cfmet338-GCST90026023 | Sphingomyelin (d18:1/24:1, d18:2/24:0) levels \|\| id:ebi-cfmet338-GCST90026023 | Prawns liking \|\| id:ebi-fl187-GCST90094817 | Weighted median | 13 | -0.070875631 | 0.0975993460448771 | 0.46772254088347 | -0.262170349 | 0.120419087262933 | 0.931577744329664 | 0.769379949259446 | 1.12796947017617 | 0.999332001504091 |
| ebi-fl187-GCST90094817 | ebi-cfmet338-GCST90026046 | 1-arachidonoyl-gpc (20:4n6) levels \|\| id:ebi-cfmet338-GCST90026046 | Prawns liking \|\| id:ebi-fl187-GCST90094817 | Weighted median | 13 | -0.034372815 | 0.1229113521257 | 0.779741952482857 | -0.275279065 | 0.206533435375307 | 0.966211219647915 | 0.759360182836673 | 1.22940883927582 | 0.999332001504091 |
| ebi-fl187-GCST90094817 | ebi-cfmet338-GCST90026047 | 1-linoleoyl-gpc (18:2) levels \|\| id:ebi-cfmet338-GCST90026047 | Prawns liking \|\| id:ebi-fl187-GCST90094817 | Weighted median | 13 | 0.0248441275263968 | 0.13639677312141 | 0.855468144597884 | -0.242493548 | 0.292181802844361 | 1.02515531457537 | 0.784668810409104 | 1.33934649250833 | 0.999332001504091 |
| ebi-fl187-GCST90094817 | ebi-cfmet338-GCST90026050 | 1-myristoyl-2-palmitoyl-gpc (14:0/16:0) levels \|\| id:ebi-cfmet338-GCST90026050 | Prawns liking \|\| id:ebi-fl187-GCST90094817 | Weighted median | 13 | -0.0475748 | 0.0780484490295132 | 0.542156816870042 | -0.20054976 | 0.105400159921852 | 0.953539145569097 | 0.818280771137399 | 1.11115516116652 | 0.999332001504091 |
| ebi-fl187-GCST90094817 | ebi-cfmet338-GCST90026068 | 2,3-dihydroxyisovalerate levels \|\| id:ebi-cfmet338-GCST90026068 | Prawns liking \|\| id:ebi-fl187-GCST90094817 | Weighted median | 13 | -0.176961968 | 0.229607299382113 | 0.440875419881615 | -0.626992274 | 0.273068339253875 | 0.83781164795919 | 0.534196102481546 | 1.31399003885533 | 0.999332001504091 |
| ebi-fl187-GCST90094817 | ebi-cfmet338-GCST90026082 | 3-hydroxyhexanoate levels \|\| id:ebi-cfmet338-GCST90026082 | Prawns liking \|\| id:ebi-fl187-GCST90094817 | Weighted median | 13 | 0.0738919940225428 | 0.0508929825515169 | 0.146526829857909 | -0.025858252 | 0.173642239823516 | 1.07669051020507 | 0.974473209663819 | 1.18962988748103 | 0.999332001504091 |
| ebi-fl187-GCST90094817 | ebi-cfmet338-GCST90026094 | 3-ureidopropionate levels \|\| id:ebi-cfmet338-GCST90026094 | Prawns liking \|\| id:ebi-fl187-GCST90094817 | Weighted median | 13 | 0.070658202368279 | 0.115971034480527 | 0.542342458835881 | -0.156645025 | 0.297961429950113 | 1.07321434105165 | 0.8550075110642 | 1.34710982878423 | 0.999332001504091 |
| ebi-fl187-GCST90094817 | ebi-cfmet338-GCST90026173 | Glycerol levels \|\| id:ebi-cfmet338-GCST90026173 | Prawns liking \|\| id:ebi-fl187-GCST90094817 | Weighted median | 13 | -0.017394654 | 0.0726133340741824 | 0.81067774244021 | -0.159716789 | 0.124927480618075 | 0.982755759435875 | 0.85238515967893 | 1.13306628082096 | 0.999332001504091 |
| ebi-fl187-GCST90094817 | ebi-cfmet338-GCST90026179 | Histidine levels \|\| id:ebi-cfmet338-GCST90026179 | Prawns liking \|\| id:ebi-fl187-GCST90094817 | Weighted median | 13 | 0.0432163293363015 | 0.050241859527329 | 0.389697653542778 | -0.055257715 | 0.141690374009866 | 1.04416375367161 | 0.946241255643109 | 1.15221983609305 | 0.999332001504091 |
| ebi-fl187-GCST90094817 | ebi-cfmet338-GCST90026188 | Isocitrate levels \|\| id:ebi-cfmet338-GCST90026188 | Prawns liking \|\| id:ebi-fl187-GCST90094817 | Weighted median | 13 | 0.062020472201186 | 0.0906942351943878 | 0.494075324858296 | -0.115740229 | 0.239781173182186 | 1.06398412660217 | 0.890706573180494 | 1.27097099734997 | 0.999332001504091 |
| ebi-fl187-GCST90094817 | ebi-cfmet338-GCST90026207 | N-acetyl-isoputreanine levels \|\| id:ebi-cfmet338-GCST90026207 | Prawns liking \|\| id:ebi-fl187-GCST90094817 | Weighted median | 13 | -0.011583678 | 0.0563478512959204 | 0.837123348144854 | -0.122025466 | 0.0988581107921316 | 0.988483154742955 | 0.885125827346483 | 1.10390965557951 | 0.999332001504091 |
| ebi-fl187-GCST90094817 | ebi-cfmet338-GCST90026213 | N-acetylglutamate levels \|\| id:ebi-cfmet338-GCST90026213 | Prawns liking \|\| id:ebi-fl187-GCST90094817 | Weighted median | 13 | 0.0119870161589147 | 0.0929037182513922 | 0.897336896831878 | -0.170104272 | 0.194078303931643 | 1.01205914836562 | 0.843576850890682 | 1.2141913551908 | 0.999332001504091 |
| ebi-fl187-GCST90094817 | ebi-cfmet338-GCST90026224 | N-acetyltaurine levels \|\| id:ebi-cfmet338-GCST90026224 | Prawns liking \|\| id:ebi-fl187-GCST90094817 | Weighted median | 13 | 0.0360379076155219 | 0.0781003616409568 | 0.644489601793799 | -0.117038801 | 0.189114616431797 | 1.03669514438702 | 0.889550676859385 | 1.20817942176162 | 0.999332001504091 |
| ebi-fl187-GCST90094817 | ebi-cfmet338-GCST90026250 | Phenyllactate (pla) levels \|\| id:ebi-cfmet338-GCST90026250 | Prawns liking \|\| id:ebi-fl187-GCST90094817 | Weighted median | 13 | -0.113804879 | 0.133782735786194 | 0.394953028278003 | -0.376019041 | 0.148409283115764 | 0.892432071110769 | 0.686589259457691 | 1.15998756254377 | 0.999332001504091 |
| ebi-fl187-GCST90094817 | ebi-cfmet338-GCST90026251 | Pipecolate levels \|\| id:ebi-cfmet338-GCST90026251 | Prawns liking \|\| id:ebi-fl187-GCST90094817 | Weighted median | 13 | -0.159622571 | 0.187224611445077 | 0.393896370148032 | -0.52658281 | 0.207337667196636 | 0.852465473246106 | 0.590619785433914 | 1.23039796667635 | 0.999332001504091 |
| ebi-fl187-GCST90094817 | ebi-cfmet338-GCST90026278 | Trigonelline (n'-methylnicotinate) levels \|\| id:ebi-cfmet338-GCST90026278 | Prawns liking \|\| id:ebi-fl187-GCST90094817 | Weighted median | 12 | 0.132089396392331 | 0.221689911156844 | 0.551289111701663 | -0.302422829 | 0.566601622259745 | 1.14121033479408 | 0.739025517045523 | 1.76226801132306 | 0.999332001504091 |
| ebi-fl187-GCST90094817 | ebi-cfmet338-GCST90026299 | 1-carboxyethylvaline levels \|\| id:ebi-cfmet338-GCST90026299 | Prawns liking \|\| id:ebi-fl187-GCST90094817 | Weighted median | 13 | 0.0704500592100298 | 0.122582163168938 | 0.56548267438983 | -0.169810981 | 0.310711099021148 | 1.07299098207535 | 0.843824300685218 | 1.36439498919398 | 0.999332001504091 |
| ebi-fl187-GCST90094817 | ebi-gm430-GCST90011306 | C_Erysipelotrichia abundance \|\| id:ebi-gm430-GCST90011306 | Prawns liking \|\| id:ebi-fl187-GCST90094817 | Weighted median | 13 | 0.0460049120404534 | 0.111084045421434 | 0.678767852236865 | -0.171719817 | 0.263729641066465 | 1.04707955424143 | 0.842215114483166 | 1.30177620189498 | 0.999332001504091 |
| ebi-fl187-GCST90094817 | ebi-gm430-GCST90011312 | F_Erysipelotrichaceae abundance \|\| id:ebi-gm430-GCST90011312 | Prawns liking \|\| id:ebi-fl187-GCST90094817 | Weighted median | 13 | 0.0460049120404534 | 0.110210164679228 | 0.67636469176725 | -0.170007011 | 0.26201683481174 | 1.04707955424143 | 0.843658901910174 | 1.29954841989823 | 0.999332001504091 |
| ebi-fl187-GCST90094817 | ebi-gm430-GCST90011352 | O_Erysipelotrichales abundance \|\| id:ebi-gm430-GCST90011352 | Prawns liking \|\| id:ebi-fl187-GCST90094817 | Weighted median | 13 | 0.0460049120404534 | 0.110115744149432 | 0.676102951863064 | -0.169821946 | 0.26183177057334 | 1.04707955424143 | 0.843815047450358 | 1.29930794221223 | 0.999332001504091 |
| ebi-fl187-GCST90094817 | ebi-gm430-GCST90011362 | OTU97_11 (Parabacteroides) abundance \|\| id:ebi-gm430-GCST90011362 | Prawns liking \|\| id:ebi-fl187-GCST90094817 | Weighted median | 13 | 0.139049693805708 | 0.132796266564452 | 0.295057893719719 | -0.121230989 | 0.399330376272034 | 1.14918120577226 | 0.885829319430976 | 1.49082607081519 | 0.999332001504091 |
| ebi-fl187-GCST90094817 | ebi-gm430-GCST90011384 | OTU97_235 (Phascolarctobacterium) abundance \|\| id:ebi-gm430-GCST90011384 | Prawns liking \|\| id:ebi-fl187-GCST90094817 | Weighted median | 13 | 0.521295257646432 | 0.449316650980779 | 0.245969127122252 | -0.359365378 | 1.40195589356876 | 1.68420772044553 | 0.698119227146406 | 4.06313926806296 | 0.999332001504091 |
| ebi-fl187-GCST90094817 | ebi-gm430-GCST90011390 | OTU97_3 (Bacteroides) abundance \|\| id:ebi-gm430-GCST90011390 | Prawns liking \|\| id:ebi-fl187-GCST90094817 | Weighted median | 13 | -0.118310955 | 0.101350410773997 | 0.243070834028421 | -0.31695776 | 0.0803358499303817 | 0.888419750928647 | 0.728361520245364 | 1.08365095066284 | 0.999332001504091 |
| ebi-fl187-GCST90094817 | ebi-gm430-GCST90011398 | OTU97_4 (Alistipes) abundance \|\| id:ebi-gm430-GCST90011398 | Prawns liking \|\| id:ebi-fl187-GCST90094817 | Weighted median | 13 | -0.294584978 | 0.124594032856492 | 0.0180613186648765 | -0.538789282 | -0.050380674 | 0.744840648606981 | 0.583454223238035 | 0.950867385513685 | 0.999332001504091 |
| ebi-fl187-GCST90094817 | ebi-gm430-GCST90011421 | OTU99_108 (Cytophagales) abundance \|\| id:ebi-gm430-GCST90011421 | Prawns liking \|\| id:ebi-fl187-GCST90094817 | Weighted median | 13 | -0.028621908 | 0.40529187962423 | 0.943699808202293 | -0.822993992 | 0.765750175853176 | 0.971783818498581 | 0.439114977579099 | 2.15060710318307 | 0.999332001504091 |
| ebi-fl187-GCST90094817 | ebi-gm430-GCST90011428 | OTU99_123 (Phascolarctobacterium) abundance \|\| id:ebi-gm430-GCST90011428 | Prawns liking \|\| id:ebi-fl187-GCST90094817 | Weighted median | 13 | -0.164500949 | 0.242776372064361 | 0.498036633545221 | -0.640342638 | 0.311340740297838 | 0.84831695193502 | 0.527111784467699 | 1.36525433910958 | 0.999332001504091 |
| ebi-fl187-GCST90094817 | ebi-gm430-GCST90011450 | OTU99_298 (Phascolarctobacterium) abundance \|\| id:ebi-gm430-GCST90011450 | Prawns liking \|\| id:ebi-fl187-GCST90094817 | Weighted median | 13 | 0.520974502010201 | 0.457940856710179 | 0.255268405047875 | -0.376589577 | 1.41853858116215 | 1.68366758795651 | 0.686197647309718 | 4.13107879027139 | 0.999332001504091 |
| ebi-fl187-GCST90094817 | ebi-gm430-GCST90011451 | OTU99_3 (Bacteroides) abundance \|\| id:ebi-gm430-GCST90011451 | Prawns liking \|\| id:ebi-fl187-GCST90094817 | Weighted median | 13 | -0.116924986 | 0.102126417838039 | 0.252248755757923 | -0.317092765 | 0.0832427925014215 | 0.889651926599797 | 0.728263194348316 | 1.08680564477927 | 0.999332001504091 |
| ebi-fl187-GCST90094817 | ebi-gm430-GCST90011472 | OTU99_72 (Parabacteroides) abundance \|\| id:ebi-gm430-GCST90011472 | Prawns liking \|\| id:ebi-fl187-GCST90094817 | Weighted median | 13 | -0.043387792 | 0.313295392128387 | 0.889854400303182 | -0.657446761 | 0.570671176543937 | 0.957539991684039 | 0.518172665802974 | 1.76945426917386 | 0.999332001504091 |
| ebi-fl187-GCST90094817 | ebi-gm430-GCST90011507 | TestASV_3 (Bacteroides) abundance \|\| id:ebi-gm430-GCST90011507 | Prawns liking \|\| id:ebi-fl187-GCST90094817 | Weighted median | 13 | -0.002298744 | 0.133466989553711 | 0.986258464413215 | -0.263894043 | 0.259296555881227 | 0.997703896443775 | 0.768054916010129 | 1.29601808963093 | 0.999332001504091 |
| ebi-fl187-GCST90094817 | ebi-gm430-GCST90011512 | TestASV_34 (Phascolarctobacterium) abundance \|\| id:ebi-gm430-GCST90011512 | Prawns liking \|\| id:ebi-fl187-GCST90094817 | Weighted median | 13 | 0.240179723992544 | 0.395940869207088 | 0.544113059414859 | -0.53586438 | 1.01622382763844 | 1.27147764482658 | 0.58516326825582 | 2.76274244983363 | 0.999332001504091 |
| ebi-fl187-GCST90094817 | ebi-gm430-GCST90011519 | TestASV_41 (Barnesiella) abundance \|\| id:ebi-gm430-GCST90011519 | Prawns liking \|\| id:ebi-fl187-GCST90094817 | Weighted median | 13 | 0.0633413179140109 | 0.436853140896523 | 0.88471518683723 | -0.792890838 | 0.919573474071196 | 1.0653904140142 | 0.4525346979438 | 2.50822034074018 | 0.999332001504091 |
| ebi-fl187-GCST90094817 | ebi-gm430-GCST90011524 | TestASV_46 (Sutterella) abundance \|\| id:ebi-gm430-GCST90011524 | Prawns liking \|\| id:ebi-fl187-GCST90094817 | Weighted median | 13 | -0.362926587 | 0.48956896421495 | 0.458500276930747 | -1.322481757 | 0.596628582922183 | 0.695637500502755 | 0.266473159092205 | 1.81598602183523 | 0.999332001504091 |
| ebi-fl187-GCST90094817 | ebi-gm430-GCST90011581 | OTU97_19 (Bacteria) prevalence \|\| id:ebi-gm430-GCST90011581 | Prawns liking \|\| id:ebi-fl187-GCST90094817 | Weighted median | 13 | -0.651194358 | 0.486088569827819 | 0.180355275223375 | -1.603927955 | 0.301539238414942 | 0.52142263917641 | 0.201105032971247 | 1.35193816200794 | 0.999332001504091 |
| ebi-fl187-GCST90094817 | ebi-gm430-GCST90011614 | OTU97_80 (Ruminococcus) prevalence \|\| id:ebi-gm430-GCST90011614 | Prawns liking \|\| id:ebi-fl187-GCST90094817 | Weighted median | 13 | -0.197697343 | 0.230736065633205 | 0.391549001142234 | -0.649940031 | 0.254545345783892 | 0.820618181510911 | 0.522077084002796 | 1.28987504041198 | 0.999332001504091 |
| ebi-fl187-GCST90094817 | ebi-gm430-GCST90011623 | OTU99_11 (Parabacteroides) prevalence \|\| id:ebi-gm430-GCST90011623 | Prawns liking \|\| id:ebi-fl187-GCST90094817 | Weighted median | 13 | 0.312273097913393 | 0.202987375099836 | 0.123954068403914 | -0.085582157 | 0.710128353109072 | 1.36652783797575 | 0.917977721401193 | 2.03425234450386 | 0.999332001504091 |
| ebi-fl187-GCST90094817 | ebi-gm430-GCST90011642 | OTU99_19 (Bacteria) prevalence \|\| id:ebi-gm430-GCST90011642 | Prawns liking \|\| id:ebi-fl187-GCST90094817 | Weighted median | 13 | -0.651194358 | 0.491302504961497 | 0.185023660944835 | -1.614147268 | 0.31175855127695 | 0.52142263917641 | 0.199060343178164 | 1.36582487654185 | 0.999332001504091 |
| ebi-fl187-GCST90094817 | ebi-gm430-GCST90011653 | OTU99_30 (Parasutterella) prevalence \|\| id:ebi-gm430-GCST90011653 | Prawns liking \|\| id:ebi-fl187-GCST90094817 | Weighted median | 13 | -0.291752877 | 0.20653949763605 | 0.157780230821604 | -0.696570293 | 0.113064538214669 | 0.746953102345116 | 0.498291370104796 | 1.11970419432641 | 0.999332001504091 |
| ebi-fl187-GCST90094817 | ebi-gm430-GCST90011675 | OTU99_8 (Ruminococcaceae) prevalence \|\| id:ebi-gm430-GCST90011675 | Prawns liking \|\| id:ebi-fl187-GCST90094817 | Weighted median | 13 | 0.364642703737003 | 0.243094722037691 | 0.133613741158588 | -0.111822951 | 0.841108358930878 | 1.43999940981482 | 0.894202560746906 | 2.31893576611433 | 0.999332001504091 |
| ebi-fl187-GCST90094817 | ebi-gm430-GCST90011679 | OTU99_92 (Ruminococcus) prevalence \|\| id:ebi-gm430-GCST90011679 | Prawns liking \|\| id:ebi-fl187-GCST90094817 | Weighted median | 13 | -0.090120858 | 0.241011712180806 | 0.708458407171193 | -0.562503814 | 0.382262097806124 | 0.913820735988135 | 0.569780651616059 | 1.46559616433694 | 0.999332001504091 |
| ebi-fl187-GCST90094817 | ebi-gm430-GCST90011684 | TestASV_11 (Lachnospiraceae) prevalence \|\| id:ebi-gm430-GCST90011684 | Prawns liking \|\| id:ebi-fl187-GCST90094817 | Weighted median | 13 | -0.179760576 | 0.256466451316232 | 0.483357539728162 | -0.68243482 | 0.322913668778631 | 0.835470219254837 | 0.505384971470663 | 1.38114610972804 | 0.999332001504091 |
| ebi-fl187-GCST90094817 | ebi-gm430-GCST90011688 | TestASV_15 (Bacteroides) prevalence \|\| id:ebi-gm430-GCST90011688 | Prawns liking \|\| id:ebi-fl187-GCST90094817 | Weighted median | 13 | -0.347181959 | 0.240424864018221 | 0.148729082521664 | -0.818414693 | 0.124050774087344 | 0.7066767300927 | 0.441130427689482 | 1.13207334953108 | 0.999332001504091 |
| ebi-fl187-GCST90094817 | ebi-gm430-GCST90011692 | TestASV_19 (Prevotella) prevalence \|\| id:ebi-gm430-GCST90011692 | Prawns liking \|\| id:ebi-fl187-GCST90094817 | Weighted median | 13 | 0.212643000041214 | 0.432493983250233 | 0.622954802000479 | -0.635045207 | 1.06033120721167 | 1.23694298381642 | 0.52991153199696 | 2.88732713448752 | 0.999332001504091 |
| ebi-fl187-GCST90094817 | ebi-gm430-GCST90011693 | TestASV_2 (Bacteroides) prevalence \|\| id:ebi-gm430-GCST90011693 | Prawns liking \|\| id:ebi-fl187-GCST90094817 | Weighted median | 13 | -0.120400623 | 0.240266832637327 | 0.61629221535542 | -0.591323615 | 0.350522369365086 | 0.886565187507375 | 0.553594054426299 | 1.41980901965165 | 0.999332001504091 |
| ebi-fl187-GCST90094817 | ebi-gm430-GCST90011716 | TestASV_40 (Barnesiella) prevalence \|\| id:ebi-gm430-GCST90011716 | Prawns liking \|\| id:ebi-fl187-GCST90094817 | Weighted median | 13 | -0.24651647 | 0.47338699125794 | 0.602540652311737 | -1.174354973 | 0.681322032854738 | 0.78151848981302 | 0.309018240563631 | 1.97648898914709 | 0.999332001504091 |
| ebi-fl187-GCST90094817 | ebi-gm430-GCST90011718 | TestASV_42 (Alphaproteobacteria) prevalence \|\| id:ebi-gm430-GCST90011718 | Prawns liking \|\| id:ebi-fl187-GCST90094817 | Weighted median | 13 | -0.44907402 | 0.369365833175722 | 0.224062109382951 | -1.173031053 | 0.274883013285438 | 0.638218856152207 | 0.309427626965244 | 1.31637666727795 | 0.999332001504091 |
| ebi-fl187-GCST90094817 | ebi-hbsm44-GCST90104929 | Sulcal depth of the right inferior frontal sulcus \|\| id:ebi-hbsm44-GCST90104929 | Prawns liking \|\| id:ebi-fl187-GCST90094817 | Weighted median | 17 | -0.017311623 | 0.161972734075195 | 0.914884293122352 | -0.334778182 | 0.300154936068994 | 0.982837362458291 | 0.71549677916959 | 1.35006796559599 | 0.999332001504091 |
| ebi-fl187-GCST90094817 | ebi-hbsm44-GCST90104935 | Sulcal opening distance of the left calloso-marginal posterior fissure \|\| id:ebi-hbsm44-GCST90104935 | Prawns liking \|\| id:ebi-fl187-GCST90094817 | Weighted median | 18 | -0.023065228 | 0.0383653294997805 | 0.547706928229386 | -0.098261274 | 0.0521308180053397 | 0.97719874115645 | 0.906412051243262 | 1.05351355204066 | 0.999332001504091 |
| ebi-fl187-GCST90094817 | ebi-hbsm44-GCST90104936 | Sulcal opening distance of the left anterior inferior temporal sulcus \|\| id:ebi-hbsm44-GCST90104936 | Prawns liking \|\| id:ebi-fl187-GCST90094817 | Weighted median | 18 | -0.017069825 | 0.021314285949299 | 0.423210915028664 | -0.058845825 | 0.0247061757369219 | 0.983075039295367 | 0.942852122004051 | 1.02501390231949 | 0.999332001504091 |
| ebi-fl187-GCST90094817 | ebi-hbsm44-GCST90104941 | Sulcal opening distance of the right posterior inferior temporal sulcus \|\| id:ebi-hbsm44-GCST90104941 | Prawns liking \|\| id:ebi-fl187-GCST90094817 | Weighted median | 17 | -0.019915726 | 0.0168850004427489 | 0.238202246407127 | -0.053010327 | 0.0131788747861697 | 0.980281281970577 | 0.948370218660462 | 1.01326609890722 | 0.999332001504091 |
| ebi-fl187-GCST90094817 | ebi-IPs91-GCST90274819 | Leukemia inhibitory factor levels \|\| id:ebi-IPs91-GCST90274819 | Prawns liking \|\| id:ebi-fl187-GCST90094817 | Weighted median | 21 | -0.011453947 | 0.0678104470895424 | 0.865866451107944 | -0.144362424 | 0.121454528998932 | 0.988611399427164 | 0.865573986771901 | 1.12913802172164 | 0.999332001504091 |
| ebi-fl187-GCST90094817 | ebi-IPs91-GCST90274837 | STAM binding protein levels \|\| id:ebi-IPs91-GCST90274837 | Prawns liking \|\| id:ebi-fl187-GCST90094817 | Weighted median | 21 | 0.0371964584858947 | 0.0610098673250949 | 0.542074212076987 | -0.082382881 | 0.156775798443081 | 1.03789690446453 | 0.920919288253141 | 1.16973332846619 | 0.999332001504091 |
| ebi-fl187-GCST90094817 | ebi-IPs91-GCST90274840 | TNF-beta levels \|\| id:ebi-IPs91-GCST90274840 | Prawns liking \|\| id:ebi-fl187-GCST90094817 | Weighted median | 21 | -0.02209167 | 0.0775764804504547 | 0.775818227796269 | -0.174141572 | 0.129958231708162 | 0.978150563903723 | 0.840177943854179 | 1.13878081740173 | 0.999332001504091 |
| ebi-fl187-GCST90094817 | ebi-IPs91-GCST90274842 | Tumor necrosis factor ligand superfamily member 14 levels \|\| id:ebi-IPs91-GCST90274842 | Prawns liking \|\| id:ebi-fl187-GCST90094817 | Weighted median | 21 | 0.0313022142800195 | 0.0714116486223391 | 0.661143566546617 | -0.108664617 | 0.171269045579804 | 1.03179728064475 | 0.897031216056662 | 1.18681001205944 | 0.999332001504091 |
| ebi-fl187-GCST90094817 | ebi-met1400-GCST90199628 | 3-methylhistidine levels \|\| id:ebi-met1400-GCST90199628 | Prawns liking \|\| id:ebi-fl187-GCST90094817 | Weighted median | 18 | 0.0653537604238579 | 0.0883012287730515 | 0.459225432118857 | -0.107716648 | 0.238424168819039 | 1.06753660979643 | 0.897881977068813 | 1.26924745385364 | 0.999332001504091 |
| ebi-fl187-GCST90094817 | ebi-met1400-GCST90199651 | Docosahexaenoate DHA; 22:6n3 levels \|\| id:ebi-met1400-GCST90199651 | Prawns liking \|\| id:ebi-fl187-GCST90094817 | Weighted median | 18 | 0.0622707287257859 | 0.0828852357283572 | 0.452479017414436 | -0.100184333 | 0.224725790753366 | 1.06425042889248 | 0.904670641738791 | 1.25197936480053 | 0.999332001504091 |
| ebi-fl187-GCST90094817 | ebi-met1400-GCST90199652 | 1-stearoyl-2-oleoyl-GPS (18:0/18:1) levels \|\| id:ebi-met1400-GCST90199652 | Prawns liking \|\| id:ebi-fl187-GCST90094817 | Weighted median | 18 | -0.000826511 | 0.0870408889688712 | 0.992423666616977 | -0.171426654 | 0.169773630956922 | 0.999173830044418 | 0.842462057143646 | 1.18503656536238 | 0.999332001504091 |
| ebi-fl187-GCST90094817 | ebi-met1400-GCST90199653 | 1-myristoyl-2-palmitoyl-gpc (14:0/16:0) levels \|\| id:ebi-met1400-GCST90199653 | Prawns liking \|\| id:ebi-fl187-GCST90094817 | Weighted median | 17 | -0.075000342 | 0.0931256382881912 | 0.42060776459763 | -0.257526594 | 0.107525908581856 | 0.92774316861079 | 0.772961070293863 | 1.11351971008937 | 0.999332001504091 |
| ebi-fl187-GCST90094817 | ebi-met1400-GCST90199667 | Erythritol levels in elite athletes \|\| id:ebi-met1400-GCST90199667 | Prawns liking \|\| id:ebi-fl187-GCST90094817 | Weighted median | 18 | 0.0288516717770452 | 0.0869013773751744 | 0.739885657446421 | -0.141475028 | 0.199178371432387 | 1.02927191307813 | 0.868076853035046 | 1.22039963091694 | 0.999332001504091 |
| ebi-fl187-GCST90094817 | ebi-met1400-GCST90199674 | 3-hydroxymyristate levels \|\| id:ebi-met1400-GCST90199674 | Prawns liking \|\| id:ebi-fl187-GCST90094817 | Weighted median | 18 | 0.0756530997271828 | 0.082705279034432 | 0.360332757126907 | -0.086449247 | 0.23775544663467 | 1.07858834665951 | 0.917182097181071 | 1.26839896365752 | 0.999332001504091 |
| ebi-fl187-GCST90094817 | ebi-met1400-GCST90199685 | 2-linoleoylglycerol (18:2) levels \|\| id:ebi-met1400-GCST90199685 | Prawns liking \|\| id:ebi-fl187-GCST90094817 | Weighted median | 18 | 0.131036615729464 | 0.103234288963341 | 0.204329483700553 | -0.071302591 | 0.333375822097612 | 1.14000952282806 | 0.931180083117278 | 1.39567172419321 | 0.999332001504091 |
| ebi-fl187-GCST90094817 | ebi-met1400-GCST90199703 | Gamma-glutamylvaline levels \|\| id:ebi-met1400-GCST90199703 | Prawns liking \|\| id:ebi-fl187-GCST90094817 | Weighted median | 18 | 0.0175210468018776 | 0.0930506730910949 | 0.850644812361834 | -0.164858272 | 0.199900366060424 | 1.01767544073874 | 0.848013882495697 | 1.22128107105375 | 0.999332001504091 |
| ebi-fl187-GCST90094817 | ebi-met1400-GCST90199779 | Gamma-glutamyl-2-aminobutyrate levels \|\| id:ebi-met1400-GCST90199779 | Prawns liking \|\| id:ebi-fl187-GCST90094817 | Weighted median | 18 | 0.136817075628804 | 0.0934780645824871 | 0.143295726231144 | -0.046399931 | 0.320034082210479 | 1.14661838488849 | 0.954660087715545 | 1.37717470069412 | 0.999332001504091 |
| ebi-fl187-GCST90094817 | ebi-met1400-GCST90199824 | 4-vinylphenol sulfate levels \|\| id:ebi-met1400-GCST90199824 | Prawns liking \|\| id:ebi-fl187-GCST90094817 | Weighted median | 18 | -0.013649579 | 0.0852982946741241 | 0.872863975728195 | -0.180834236 | 0.153535078938892 | 0.986443154473845 | 0.834573689350115 | 1.16594868664748 | 0.999332001504091 |
| ebi-fl187-GCST90094817 | ebi-met1400-GCST90199830 | 4-ethylphenylsulfate levels \|\| id:ebi-met1400-GCST90199830 | Prawns liking \|\| id:ebi-fl187-GCST90094817 | Weighted median | 18 | -0.005492439 | 0.0844613742564273 | 0.948150907723951 | -0.171036733 | 0.160051854546411 | 0.99452261686979 | 0.842790615064078 | 1.17357172444348 | 0.999332001504091 |
| ebi-fl187-GCST90094817 | ebi-met1400-GCST90199833 | Hexanoylglutamine levels \|\| id:ebi-met1400-GCST90199833 | Prawns liking \|\| id:ebi-fl187-GCST90094817 | Weighted median | 18 | 0.0729014489142185 | 0.0851101787741443 | 0.391691515658078 | -0.093914501 | 0.239717399311541 | 1.07562452772603 | 0.910360593394071 | 1.27088994519452 | 0.999332001504091 |
| ebi-fl187-GCST90094817 | ebi-met1400-GCST90199837 | Carotenoid levels (cryptoxanthin) \|\| id:ebi-met1400-GCST90199837 | Prawns liking \|\| id:ebi-fl187-GCST90094817 | Weighted median | 18 | -0.076690293 | 0.0925067968784045 | 0.407091206326787 | -0.258003615 | 0.104623029158235 | 0.926176652841064 | 0.7725924394853 | 1.11029198375193 | 0.999332001504091 |
| ebi-fl187-GCST90094817 | ebi-met1400-GCST90199869 | 2-hydroxyhippurate levels \|\| id:ebi-met1400-GCST90199869 | Prawns liking \|\| id:ebi-fl187-GCST90094817 | Weighted median | 18 | 0.17806522863269 | 0.0859043787857942 | 0.0381880436508166 | 0.00969264621253343 | 0.346437811052847 | 1.19490326059872 | 1.00973977204277 | 1.41402155458423 | 0.999332001504091 |
| ebi-fl187-GCST90094817 | ebi-met1400-GCST90199902 | Hydantoin-5-propionate levels \|\| id:ebi-met1400-GCST90199902 | Prawns liking \|\| id:ebi-fl187-GCST90094817 | Weighted median | 18 | 0.0679000039818358 | 0.085995642530017 | 0.429776050355993 | -0.100651455 | 0.236451463340669 | 1.07025828156117 | 0.904248148796819 | 1.26674607050552 | 0.999332001504091 |
| ebi-fl187-GCST90094817 | ebi-met1400-GCST90199904 | Pantoate levels \|\| id:ebi-met1400-GCST90199904 | Prawns liking \|\| id:ebi-fl187-GCST90094817 | Weighted median | 18 | 0.017772112831223 | 0.0878799579304903 | 0.839735596133457 | -0.154472605 | 0.190016830374984 | 1.01793097654765 | 0.856866965937408 | 1.2092699499527 | 0.999332001504091 |
| ebi-fl187-GCST90094817 | ebi-met1400-GCST90199936 | 3-methyl catechol sulfate (2) levels \|\| id:ebi-met1400-GCST90199936 | Prawns liking \|\| id:ebi-fl187-GCST90094817 | Weighted median | 18 | 0.00329987432678186 | 0.0999647038881364 | 0.97366629842502 | -0.192630945 | 0.199230693947529 | 1.00330532490583 | 0.824786309246403 | 1.22046348696564 | 0.999332001504091 |
| ebi-fl187-GCST90094817 | ebi-met1400-GCST90199977 | 4-hydroxychlorothalonil levels \|\| id:ebi-met1400-GCST90199977 | Prawns liking \|\| id:ebi-fl187-GCST90094817 | Weighted median | 18 | 0.033020701367883 | 0.0861134669658545 | 0.701381896968966 | -0.135761694 | 0.201803096620958 | 1.03357193537271 | 0.873050661011557 | 1.22360705202644 | 0.999332001504091 |
| ebi-fl187-GCST90094817 | ebi-met1400-GCST90200040 | Sphingomyelin (d18:2/24:1, d18:1/24:2) levels \|\| id:ebi-met1400-GCST90200040 | Prawns liking \|\| id:ebi-fl187-GCST90094817 | Weighted median | 18 | -0.053540804 | 0.0828924440457893 | 0.518339631458432 | -0.216009994 | 0.108928386292866 | 0.947867263403933 | 0.805727249099709 | 1.11508249229098 | 0.999332001504091 |
| ebi-fl187-GCST90094817 | ebi-met1400-GCST90200077 | 1-stearoyl-2-oleoyl-GPI (18:0/18:1) levels \|\| id:ebi-met1400-GCST90200077 | Prawns liking \|\| id:ebi-fl187-GCST90094817 | Weighted median | 18 | 0.0134065994158773 | 0.0821269168645152 | 0.870327541349598 | -0.147562158 | 0.174375356470327 | 1.01349687082962 | 0.862808806496628 | 1.19050234472247 | 0.999332001504091 |
| ebi-fl187-GCST90094817 | ebi-met1400-GCST90200080 | 1-palmitoyl-2-oleoyl-GPI (16:0/18:1) levels \|\| id:ebi-met1400-GCST90200080 | Prawns liking \|\| id:ebi-fl187-GCST90094817 | Weighted median | 18 | -0.01902322 | 0.0857958800529012 | 0.824526801494351 | -0.187183145 | 0.14913670492254 | 0.981156579541022 | 0.829291841917601 | 1.16083166976613 | 0.999332001504091 |
| ebi-fl187-GCST90094817 | ebi-met1400-GCST90200082 | 1-oleoyl-2-linoleoyl-GPE (18:1/18:2) levels \|\| id:ebi-met1400-GCST90200082 | Prawns liking \|\| id:ebi-fl187-GCST90094817 | Weighted median | 18 | -0.074073411 | 0.090452024541956 | 0.412829288376873 | -0.251359379 | 0.103212557057138 | 0.928603521584021 | 0.777742816778552 | 1.10872705179066 | 0.999332001504091 |
| ebi-fl187-GCST90094817 | ebi-met1400-GCST90200100 | Perfluorooctanesulfonate (PFOS) levels \|\| id:ebi-met1400-GCST90200100 | Prawns liking \|\| id:ebi-fl187-GCST90094817 | Weighted median | 18 | 0.0705556400514249 | 0.0801145374300183 | 0.378488549588676 | -0.086468853 | 0.227580133414261 | 1.07310427534674 | 0.917164114964906 | 1.25555804787622 | 0.999332001504091 |
| ebi-fl187-GCST90094817 | ebi-met1400-GCST90200102 | 1-palmitoleoyl-2-linolenoyl-GPC (16:1/18:3) levels \|\| id:ebi-met1400-GCST90200102 | Prawns liking \|\| id:ebi-fl187-GCST90094817 | Weighted median | 18 | -0.137774683 | 0.0854699173609269 | 0.106969128336701 | -0.305295721 | 0.0297463546473551 | 0.871294986872102 | 0.736905423457834 | 1.03019319709471 | 0.999332001504091 |
| ebi-fl187-GCST90094817 | ebi-met1400-GCST90200114 | Glycosyl-N-tricosanoyl-sphingadienine (d18:2/23:0) levels \|\| id:ebi-met1400-GCST90200114 | Prawns liking \|\| id:ebi-fl187-GCST90094817 | Weighted median | 18 | -0.091741775 | 0.0933521981260794 | 0.325731011168077 | -0.274712083 | 0.0912285333683485 | 0.912340708347222 | 0.759790848220603 | 1.09551933937725 | 0.999332001504091 |
| ebi-fl187-GCST90094817 | ebi-met1400-GCST90200135 | Arachidoylcarnitine (C20) levels \|\| id:ebi-met1400-GCST90200135 | Prawns liking \|\| id:ebi-fl187-GCST90094817 | Weighted median | 18 | 0.0313337437793177 | 0.0935822183895184 | 0.737756475868954 | -0.152087404 | 0.214754891822774 | 1.03182981320925 | 0.858913204783569 | 1.23955803391766 | 0.999332001504091 |
| ebi-fl187-GCST90094817 | ebi-met1400-GCST90200146 | Docosahexaenoylcarnitine (C22:6) levels \|\| id:ebi-met1400-GCST90200146 | Prawns liking \|\| id:ebi-fl187-GCST90094817 | Weighted median | 18 | 0.0902876417273458 | 0.0942113167877721 | 0.337885088177439 | -0.094366539 | 0.274941822631379 | 1.0944890591553 | 0.909949169087757 | 1.31645408480518 | 0.999332001504091 |
| ebi-fl187-GCST90094817 | ebi-met1400-GCST90200162 | 3-hydroxyphenylacetoylglutamine levels \|\| id:ebi-met1400-GCST90200162 | Prawns liking \|\| id:ebi-fl187-GCST90094817 | Weighted median | 18 | 0.174906942649576 | 0.109299964173614 | 0.109543793488973 | -0.039320987 | 0.38913487242986 | 1.19113536755348 | 0.961442049086386 | 1.4757035696379 | 0.999332001504091 |
| ebi-fl187-GCST90094817 | ebi-met1400-GCST90200172 | 3-carboxy-4-methyl-5-pentyl-2-furanpropionate (3-CMPFP) levels \|\| id:ebi-met1400-GCST90200172 | Prawns liking \|\| id:ebi-fl187-GCST90094817 | Weighted median | 18 | 0.00289407708895258 | 0.091765770461001 | 0.974840763771453 | -0.176966833 | 0.182754987192514 | 1.00289826897295 | 0.837807571613672 | 1.2005202292117 | 0.999332001504091 |
| ebi-fl187-GCST90094817 | ebi-met1400-GCST90200222 | Dihydrocaffeate sulfate (2) levels \|\| id:ebi-met1400-GCST90200222 | Prawns liking \|\| id:ebi-fl187-GCST90094817 | Weighted median | 18 | 0.0193494071914228 | 0.0983123078532709 | 0.843971752635237 | -0.173342716 | 0.212041530583834 | 1.01953782023564 | 0.840849392748767 | 1.23619922408794 | 0.999332001504091 |
| ebi-fl187-GCST90094817 | ebi-met1400-GCST90200223 | 4-methylhexanoylglutamine levels \|\| id:ebi-met1400-GCST90200223 | Prawns liking \|\| id:ebi-fl187-GCST90094817 | Weighted median | 18 | 0.201986903928245 | 0.101545294306355 | 0.0466867333560535 | 0.00295812708779011 | 0.4010156807687 | 1.22383198061498 | 1.00296250666311 | 1.49334068504624 | 0.999332001504091 |
| ebi-fl187-GCST90094817 | ebi-met1400-GCST90200224 | 2-ketocaprylate levels \|\| id:ebi-met1400-GCST90200224 | Prawns liking \|\| id:ebi-fl187-GCST90094817 | Weighted median | 18 | 0.050353981861086 | 0.0922888275767128 | 0.58533200286803 | -0.13053212 | 0.231240083911443 | 1.05164329314679 | 0.877628302908807 | 1.26016174769553 | 0.999332001504091 |
| ebi-fl187-GCST90094817 | ebi-met1400-GCST90200243 | Hydroxypalmitoyl sphingomyelin (d18:1/16:0(OH)) levels \|\| id:ebi-met1400-GCST90200243 | Prawns liking \|\| id:ebi-fl187-GCST90094817 | Weighted median | 18 | 0.0819680315110776 | 0.0864428293866725 | 0.343010332289409 | -0.087459914 | 0.251395977108956 | 1.08542111000217 | 0.916255599857175 | 1.28581913848274 | 0.999332001504091 |
| ebi-fl187-GCST90094817 | ebi-met1400-GCST90200273 | 2-methoxyhydroquinone sulfate (2) levels \|\| id:ebi-met1400-GCST90200273 | Prawns liking \|\| id:ebi-fl187-GCST90094817 | Weighted median | 18 | -0.131710558 | 0.104705685448868 | 0.208423515532966 | -0.336933701 | 0.0735125856473216 | 0.876594681828544 | 0.713956172691019 | 1.07628208229336 | 0.999332001504091 |
| ebi-fl187-GCST90094817 | ebi-met1400-GCST90200276 | 2,4-di-tert-butylphenol levels \|\| id:ebi-met1400-GCST90200276 | Prawns liking \|\| id:ebi-fl187-GCST90094817 | Weighted median | 18 | 0.0284245833158011 | 0.0914119926777418 | 0.755838431559575 | -0.150742922 | 0.207592088964175 | 1.02883241677915 | 0.86006877471556 | 1.23071104652728 | 0.999332001504091 |
| ebi-fl187-GCST90094817 | ebi-met1400-GCST90200289 | N-acetylmethionine levels \|\| id:ebi-met1400-GCST90200289 | Prawns liking \|\| id:ebi-fl187-GCST90094817 | Weighted median | 18 | 0.0407423071911337 | 0.0864423769260214 | 0.63740987231451 | -0.128684752 | 0.210169365966136 | 1.04158366233941 | 0.879251104377536 | 1.23388702072819 | 0.999332001504091 |
| ebi-fl187-GCST90094817 | ebi-met1400-GCST90200308 | 3-hydroxyisobutyrate levels \|\| id:ebi-met1400-GCST90200308 | Prawns liking \|\| id:ebi-fl187-GCST90094817 | Weighted median | 18 | 0.122144635258352 | 0.0963586418776029 | 0.204939271586089 | -0.066718303 | 0.311007573338453 | 1.12991751587414 | 0.935458680295003 | 1.36479955723599 | 0.999332001504091 |
| ebi-fl187-GCST90094817 | ebi-met1400-GCST90200383 | Cystathionine levels \|\| id:ebi-met1400-GCST90200383 | Prawns liking \|\| id:ebi-fl187-GCST90094817 | Weighted median | 18 | -0.063172464 | 0.0945181436850345 | 0.503901675658351 | -0.248428026 | 0.122083097202645 | 0.938781553310662 | 0.780026000372867 | 1.12984798508652 | 0.999332001504091 |
| ebi-fl187-GCST90094817 | ebi-met1400-GCST90200384 | Guanidinoacetate levels \|\| id:ebi-met1400-GCST90200384 | Prawns liking \|\| id:ebi-fl187-GCST90094817 | Weighted median | 18 | -0.059870864 | 0.0870581543396674 | 0.491634689877558 | -0.230504847 | 0.11076311807083 | 0.941886156732236 | 0.794132585879379 | 1.11713024754102 | 0.999332001504091 |
| ebi-fl187-GCST90094817 | ebi-met1400-GCST90200403 | Citrulline levels \|\| id:ebi-met1400-GCST90200403 | Prawns liking \|\| id:ebi-fl187-GCST90094817 | Weighted median | 17 | 0.0539602970353357 | 0.0895231271907785 | 0.54667328851284 | -0.121505032 | 0.229425626329262 | 1.0554426971191 | 0.885586596836895 | 1.25787731079133 | 0.999332001504091 |
| ebi-fl187-GCST90094817 | ebi-met1400-GCST90200434 | 12,13-DiHOME levels \|\| id:ebi-met1400-GCST90200434 | Prawns liking \|\| id:ebi-fl187-GCST90094817 | Weighted median | 18 | -0.077229529 | 0.0803865595219399 | 0.336689506823503 | -0.234787186 | 0.080328127296509 | 0.925677359082754 | 0.790739112053873 | 1.08364258205561 | 0.999332001504091 |
| ebi-fl187-GCST90094817 | ebi-met1400-GCST90200437 | Fructose levels \|\| id:ebi-met1400-GCST90200437 | Prawns liking \|\| id:ebi-fl187-GCST90094817 | Weighted median | 18 | -0.024388583 | 0.0906118062052601 | 0.787810727129098 | -0.201987723 | 0.153210557090429 | 0.975906415356243 | 0.817104959286887 | 1.16557037221345 | 0.999332001504091 |
| ebi-fl187-GCST90094817 | ebi-met1400-GCST90200448 | N-stearoyl-sphinganine (d18:0/18:0) levels \|\| id:ebi-met1400-GCST90200448 | Prawns liking \|\| id:ebi-fl187-GCST90094817 | Weighted median | 18 | 0.0322301866690375 | 0.10181870553308 | 0.751588989980108 | -0.167334476 | 0.231794849513875 | 1.03275520442692 | 0.845916625055947 | 1.26086103603928 | 0.999332001504091 |
| ebi-fl187-GCST90094817 | ebi-met1400-GCST90200468 | X-11847 levels \|\| id:ebi-met1400-GCST90200468 | Prawns liking \|\| id:ebi-fl187-GCST90094817 | Weighted median | 18 | 0.0458969031861615 | 0.0906810924724704 | 0.612761661503794 | -0.131838038 | 0.223631844432203 | 1.04696646648578 | 0.876482940461707 | 1.25061051544061 | 0.999332001504091 |
| ebi-fl187-GCST90094817 | ebi-met1400-GCST90200476 | X-11843 levels \|\| id:ebi-met1400-GCST90200476 | Prawns liking \|\| id:ebi-fl187-GCST90094817 | Weighted median | 18 | -0.056651766 | 0.0961219760283024 | 0.555610602696343 | -0.245050839 | 0.131747306571699 | 0.944923065998998 | 0.782664746977886 | 1.14082000512306 | 0.999332001504091 |
| ebi-fl187-GCST90094817 | ebi-met1400-GCST90200511 | X-12906 levels \|\| id:ebi-met1400-GCST90200511 | Prawns liking \|\| id:ebi-fl187-GCST90094817 | Weighted median | 18 | -0.127623889 | 0.0901332285800037 | 0.156790975186697 | -0.304285017 | 0.0490372388108611 | 0.880184363737262 | 0.737650593362806 | 1.05025946042523 | 0.999332001504091 |
| ebi-fl187-GCST90094817 | ebi-met1400-GCST90200515 | X-12849 levels \|\| id:ebi-met1400-GCST90200515 | Prawns liking \|\| id:ebi-fl187-GCST90094817 | Weighted median | 18 | 0.0688719688168561 | 0.09268524172633 | 0.457437039808286 | -0.112791105 | 0.250535042600463 | 1.07129904068393 | 0.893337254341482 | 1.28471260880788 | 0.999332001504091 |
| ebi-fl187-GCST90094817 | ebi-met1400-GCST90200551 | X-18886 levels \|\| id:ebi-met1400-GCST90200551 | Prawns liking \|\| id:ebi-fl187-GCST90094817 | Weighted median | 18 | 0.163687628481343 | 0.0891265804053782 | 0.0662726954578656 | -0.011000469 | 0.338375726075884 | 1.17784633197453 | 0.989059814794247 | 1.40266742313705 | 0.999332001504091 |
| ebi-fl187-GCST90094817 | ebi-met1400-GCST90200564 | X-21319 levels \|\| id:ebi-met1400-GCST90200564 | Prawns liking \|\| id:ebi-fl187-GCST90094817 | Weighted median | 18 | -0.001222766 | 0.0886211759173541 | 0.98899140338022 | -0.17492027 | 0.172474739274549 | 0.998777981749687 | 0.839523953075816 | 1.18824180438588 | 0.999332001504091 |
| ebi-fl187-GCST90094817 | ebi-met1400-GCST90200579 | X-21442 levels \|\| id:ebi-met1400-GCST90200579 | Prawns liking \|\| id:ebi-fl187-GCST90094817 | Weighted median | 18 | -0.072170415 | 0.0964889837874174 | 0.454481064889787 | -0.261288824 | 0.116947992808165 | 0.930372332513463 | 0.770058476402176 | 1.12406096891591 | 0.999332001504091 |
| ebi-fl187-GCST90094817 | ebi-met1400-GCST90200581 | X-21441 levels \|\| id:ebi-met1400-GCST90200581 | Prawns liking \|\| id:ebi-fl187-GCST90094817 | Weighted median | 18 | -0.025770418 | 0.0882750620354429 | 0.770337724159294 | -0.19878954 | 0.147248703190779 | 0.974558804698287 | 0.819722393964789 | 1.15864208518348 | 0.999332001504091 |
| ebi-fl187-GCST90094817 | ebi-met1400-GCST90200648 | X-24585 levels \|\| id:ebi-met1400-GCST90200648 | Prawns liking \|\| id:ebi-fl187-GCST90094817 | Weighted median | 18 | -0.037295381 | 0.0821687886869899 | 0.649909848960594 | -0.198346206 | 0.123755445237928 | 0.963391526161085 | 0.820085884990576 | 1.13173906497568 | 0.999332001504091 |
| ebi-fl187-GCST90094817 | ebi-met1400-GCST90200720 | Adenosine 5'-diphosphate (ADP) to pantothenate ratio \|\| id:ebi-met1400-GCST90200720 | Prawns liking \|\| id:ebi-fl187-GCST90094817 | Weighted median | 18 | 0.109644786258357 | 0.110848079716578 | 0.32259236911795 | -0.10761745 | 0.32690702250285 | 1.11588162356461 | 0.897971049569706 | 1.38667254184405 | 0.999332001504091 |
| ebi-fl187-GCST90094817 | ebi-met1400-GCST90200724 | Cholate to taurocholate ratio \|\| id:ebi-met1400-GCST90200724 | Prawns liking \|\| id:ebi-fl187-GCST90094817 | Weighted median | 18 | -0.028083331 | 0.0941603094788068 | 0.765512208481805 | -0.212637538 | 0.156470875242103 | 0.972307339755275 | 0.808449116268827 | 1.16937670400968 | 0.999332001504091 |
| ebi-fl187-GCST90094817 | ebi-met1400-GCST90200726 | N-acetylputrescine to (N(1) + N(8))-acetylspermidine ratio \|\| id:ebi-met1400-GCST90200726 | Prawns liking \|\| id:ebi-fl187-GCST90094817 | Weighted median | 18 | 0.00357500357006402 | 0.0857022282612025 | 0.966726499621488 | -0.164401364 | 0.171551370962021 | 1.00358140151728 | 0.848401435892523 | 1.18714512595307 | 0.999332001504091 |
| ebi-fl187-GCST90094817 | ebi-met1400-GCST90200744 | Arginine to glutamate ratio \|\| id:ebi-met1400-GCST90200744 | Prawns liking \|\| id:ebi-fl187-GCST90094817 | Weighted median | 18 | -0.094163048 | 0.090151053899444 | 0.296252722080926 | -0.270859114 | 0.0825330174688309 | 0.91013435439763 | 0.762723946101256 | 1.08603453095837 | 0.999332001504091 |
| ebi-fl187-GCST90094817 | ebi-met1400-GCST90200759 | Histidine to trans-urocanate ratio \|\| id:ebi-met1400-GCST90200759 | Prawns liking \|\| id:ebi-fl187-GCST90094817 | Weighted median | 18 | 0.102582313927891 | 0.0832604394969561 | 0.217924622476162 | -0.060608147 | 0.265772775341925 | 1.10802850433455 | 0.941191975967995 | 1.30443862438921 | 0.999332001504091 |
| ebi-fl187-GCST90094817 | ebi-met1400-GCST90200795 | Oleoyl-linoleoyl-glycerol (18:1 to 18:2) [2] to linoleoyl-arachidonoyl-glycerol (18:2 to 20:4) [2] ratio \|\| id:ebi-met1400-GCST90200795 | Prawns liking \|\| id:ebi-fl187-GCST90094817 | Weighted median | 18 | 0.10909446975315 | 0.0939548114092984 | 0.245585922135878 | -0.075056961 | 0.293245900115375 | 1.11526770442972 | 0.927690642999515 | 1.34077244599801 | 0.999332001504091 |
| ebi-fl187-GCST90094817 | ebi-met1400-GCST90200811 | Choline to taurocholate ratio \|\| id:ebi-met1400-GCST90200811 | Prawns liking \|\| id:ebi-fl187-GCST90094817 | Weighted median | 18 | -0.04307744 | 0.0936567287678895 | 0.645551898840258 | -0.226644628 | 0.140489748444071 | 0.957837212337705 | 0.797204035689679 | 1.15083728163162 | 0.999332001504091 |
| ebi-fl187-GCST90094817 | ebi-met1400-GCST90200831 | Cholate to phosphate ratio \|\| id:ebi-met1400-GCST90200831 | Prawns liking \|\| id:ebi-fl187-GCST90094817 | Weighted median | 18 | -0.071124411 | 0.0857861274559707 | 0.407053542901785 | -0.239265221 | 0.0970163986509552 | 0.931346015078886 | 0.7872060711205 | 1.10187844279283 | 0.999332001504091 |
| ebi-fl187-GCST90094817 | ebi-met1400-GCST90200841 | Adenosine 5'-diphosphate (ADP) to oxalate (ethanedioate) ratio \|\| id:ebi-met1400-GCST90200841 | Prawns liking \|\| id:ebi-fl187-GCST90094817 | Weighted median | 18 | 0.0978962716570836 | 0.118872856415452 | 0.410202321482737 | -0.135094527 | 0.330887070231369 | 1.10284838254001 | 0.873633325919743 | 1.39220256231714 | 0.999332001504091 |
| ebi-fl187-GCST90094817 | ebi-met1400-GCST90200844 | Aspartate to phosphate ratio \|\| id:ebi-met1400-GCST90200844 | Prawns liking \|\| id:ebi-fl187-GCST90094817 | Weighted median | 18 | 0.0478403487803276 | 0.0870185925625178 | 0.58247605658495 | -0.122716093 | 0.218396790202863 | 1.04900316731989 | 0.884514747161547 | 1.24408060869356 | 0.999332001504091 |
| ebi-fl187-GCST90094817 | ebi-met1400-GCST90200870 | Adenosine 5'-monophosphate (AMP) to acetoacetate ratio \|\| id:ebi-met1400-GCST90200870 | Prawns liking \|\| id:ebi-fl187-GCST90094817 | Weighted median | 18 | -0.039659524 | 0.0967139076512429 | 0.681754208353865 | -0.229218783 | 0.149899735401066 | 0.961116620981858 | 0.795154548500335 | 1.16171775772367 | 0.999332001504091 |
| ebi-fl187-GCST90094817 | ebi-met1400-GCST90200887 | Cholesterol to cortisol ratio \|\| id:ebi-met1400-GCST90200887 | Prawns liking \|\| id:ebi-fl187-GCST90094817 | Weighted median | 18 | -0.022619773 | 0.0839763610819064 | 0.787653576725504 | -0.187213441 | 0.141973894676729 | 0.977634135964496 | 0.829266718172453 | 1.15254656054373 | 0.999332001504091 |
| ebi-fl187-GCST90094817 | ebi-met1400-GCST90200890 | Cortisol to taurocholate ratio \|\| id:ebi-met1400-GCST90200890 | Prawns liking \|\| id:ebi-fl187-GCST90094817 | Weighted median | 18 | -0.041195367 | 0.0833442028723098 | 0.621108497749646 | -0.204550004 | 0.12215927093162 | 0.959641629616191 | 0.815013986671969 | 1.12993405309874 | 0.999332001504091 |
| ebi-fl187-GCST90094817 | ebi-met1400-GCST90200948 | Adenosine 5'-diphosphate (ADP) to mannitol to sorbitol ratio \|\| id:ebi-met1400-GCST90200948 | Prawns liking \|\| id:ebi-fl187-GCST90094817 | Weighted median | 18 | -0.215088243 | 0.118052600500121 | 0.0684596060733775 | -0.44647134 | 0.0162948535916921 | 0.806470271367778 | 0.639882098707435 | 1.01642833877337 | 0.999332001504091 |
| ebi-fl187-GCST90094817 | ebi-met1400-GCST90200972 | Leucine to N-palmitoyl-sphingosine (d18:1 to 16:0) ratio \|\| id:ebi-met1400-GCST90200972 | Prawns liking \|\| id:ebi-fl187-GCST90094817 | Weighted median | 18 | -0.057630171 | 0.083904269632714 | 0.492173326450885 | -0.222082539 | 0.106822197484936 | 0.94399900109886 | 0.800849259928874 | 1.11273638956074 | 0.999332001504091 |
| ebi-fl187-GCST90094817 | ebi-met1400-GCST90200973 | Proline to glutamate ratio \|\| id:ebi-met1400-GCST90200973 | Prawns liking \|\| id:ebi-fl187-GCST90094817 | Weighted median | 18 | 0.0185525085336382 | 0.088404243524025 | 0.83377699544847 | -0.154719809 | 0.191824825840727 | 1.01872567555662 | 0.856655171123114 | 1.21145828219034 | 0.999332001504091 |
| ebi-fl187-GCST90094817 | ebi-met1400-GCST90200983 | Cholesterol to linoleoyl-arachidonoyl-glycerol (18:2 to 20:4) [1] ratio \|\| id:ebi-met1400-GCST90200983 | Prawns liking \|\| id:ebi-fl187-GCST90094817 | Weighted median | 18 | 0.062173079511281 | 0.0920472526098688 | 0.499391545066558 | -0.118239536 | 0.242585694626624 | 1.0641465107479 | 0.888483203762284 | 1.27454046575304 | 0.999332001504091 |
| ebi-fl187-GCST90094817 | ebi-met1400-GCST90201001 | Serine to alpha-ketobutyrate ratio \|\| id:ebi-met1400-GCST90201001 | Prawns liking \|\| id:ebi-fl187-GCST90094817 | Weighted median | 18 | -0.242642994 | 0.090178451081276 | 0.00713026711803199 | -0.419392758 | -0.06589323 | 0.784551553405821 | 0.657445927337892 | 0.936230820447598 | 0.999332001504091 |
| ebi-fl187-GCST90094817 | ebi-met1400-GCST90201005 | Fructose to maltose ratio \|\| id:ebi-met1400-GCST90201005 | Prawns liking \|\| id:ebi-fl187-GCST90094817 | Weighted median | 18 | -0.162104811 | 0.106497263421845 | 0.127971496909275 | -0.370839447 | 0.0466298254333132 | 0.850352073725743 | 0.690154738953488 | 1.04773409277192 | 0.999332001504091 |
| ebi-fl187-GCST90094817 | ebi-met1400-GCST90201020 | Paraxanthine to 5-acetylamino-6-formylamino-3-methyluracil ratio \|\| id:ebi-met1400-GCST90201020 | Prawns liking \|\| id:ebi-fl187-GCST90094817 | Weighted median | 18 | -0.016286935 | 0.0898883454794001 | 0.856217867968871 | -0.192468092 | 0.159894221906472 | 0.98384497976188 | 0.824920639043923 | 1.17338674581412 | 0.999332001504091 |
| ebi-fl187-GCST90094817 | ebi-pw11-GCST90275197 | Placental weight (fetal genotype, adjusted for sex) \|\| id:ebi-pw11-GCST90275197 | Prawns liking \|\| id:ebi-fl187-GCST90094817 | Weighted median | 17 | 0.0834708070086234 | 0.0348953202323829 | 0.0167552710772899 | 0.0150759793531529 | 0.151865634664094 | 1.08705348048678 | 1.01519019517987 | 1.16400382415932 | 0.999332001504091 |
| ebi-fl187-GCST90094817 | prot-a-1017 | FAS-associated factor 2 \|\| id:prot-a-1017 | Prawns liking \|\| id:ebi-fl187-GCST90094817 | Weighted median | 20 | -0.039920308 | 0.13002384984712 | 0.758825892611734 | -0.294767054 | 0.214926437850193 | 0.960866009579261 | 0.744705043657955 | 1.23977069341406 | 0.999332001504091 |
| ebi-fl187-GCST90094817 | prot-a-1019 | Fas apoptotic inhibitory molecule 3 \|\| id:prot-a-1019 | Prawns liking \|\| id:ebi-fl187-GCST90094817 | Weighted median | 20 | -0.047694519 | 0.129068248573895 | 0.711733127941458 | -0.300668286 | 0.205279248610268 | 0.953424996203569 | 0.740323307774951 | 1.22786789749716 | 0.999332001504091 |
| ebi-fl187-GCST90094817 | prot-a-1052 | MIP18 family protein FAM96A \|\| id:prot-a-1052 | Prawns liking \|\| id:ebi-fl187-GCST90094817 | Weighted median | 20 | 0.0640000371117338 | 0.127607406834787 | 0.615992135951087 | -0.18611048 | 0.314110554507916 | 1.06609243832604 | 0.830181871185918 | 1.36904108184439 | 0.999332001504091 |
| ebi-fl187-GCST90094817 | prot-a-1066 | F-box/LRR-repeat protein 4 \|\| id:prot-a-1066 | Prawns liking \|\| id:ebi-fl187-GCST90094817 | Weighted median | 20 | -0.084777249 | 0.127880223725459 | 0.507367312392641 | -0.335422487 | 0.165867989593912 | 0.91871690680492 | 0.715035928850627 | 1.18041726407502 | 0.999332001504091 |
| ebi-fl187-GCST90094817 | prot-a-1076 | Low affinity immunoglobulin gamma Fc region receptor III-B \|\| id:prot-a-1076 | Prawns liking \|\| id:ebi-fl187-GCST90094817 | Weighted median | 20 | 0.0358166725801681 | 0.137402828889785 | 0.794347692349038 | -0.233492872 | 0.305126217204147 | 1.03646581646872 | 0.791763239373816 | 1.35679624322768 | 0.999332001504091 |
| ebi-fl187-GCST90094817 | prot-a-1081 | Fc receptor-like protein 3 \|\| id:prot-a-1081 | Prawns liking \|\| id:ebi-fl187-GCST90094817 | Weighted median | 20 | 0.0580967948887959 | 0.125840834794955 | 0.644318804585386 | -0.188551241 | 0.304744831086908 | 1.05981757566998 | 0.828158066447742 | 1.3562788786407 | 0.999332001504091 |
| ebi-fl187-GCST90094817 | prot-a-1105 | Fibroblast growth factor receptor 4 \|\| id:prot-a-1105 | Prawns liking \|\| id:ebi-fl187-GCST90094817 | Weighted median | 20 | -0.040843167 | 0.142090418203024 | 0.773771681871027 | -0.319340386 | 0.237654052894155 | 0.959979674842189 | 0.726628172814206 | 1.26827036246192 | 0.999332001504091 |
| ebi-fl187-GCST90094817 | prot-a-1143 | Follistatin-related protein 1 \|\| id:prot-a-1143 | Prawns liking \|\| id:ebi-fl187-GCST90094817 | Weighted median | 20 | -0.152330875 | 0.132120451544247 | 0.248922830125573 | -0.41128696 | 0.106625210447962 | 0.85870411037054 | 0.662796708426881 | 1.11251721650426 | 0.999332001504091 |
| ebi-fl187-GCST90094817 | prot-a-1151 | Alpha-(1,3)-fucosyltransferase 10 \|\| id:prot-a-1151 | Prawns liking \|\| id:ebi-fl187-GCST90094817 | Weighted median | 20 | -0.061045777 | 0.12325755519743 | 0.620409499245411 | -0.302630585 | 0.180539030926423 | 0.940780172451199 | 0.738871996028592 | 1.19786287426579 | 0.999332001504091 |
| ebi-fl187-GCST90094817 | prot-a-1165 | Growth arrest and DNA damage-inducible proteins-interacting protein 1 \|\| id:prot-a-1165 | Prawns liking \|\| id:ebi-fl187-GCST90094817 | Weighted median | 20 | -0.067265547 | 0.136099988686206 | 0.621139345488925 | -0.334021525 | 0.199490430549448 | 0.93494689576888 | 0.716038369263482 | 1.22078052717621 | 0.999332001504091 |
| ebi-fl187-GCST90094817 | prot-a-1194 | Growth/differentiation factor 11/8 \|\| id:prot-a-1194 | Prawns liking \|\| id:ebi-fl187-GCST90094817 | Weighted median | 20 | -0.263389253 | 0.123192949609779 | 0.0325149466526591 | -0.504847435 | -0.021931072 | 0.768442720212319 | 0.603597656551792 | 0.978307665441773 | 0.999332001504091 |
| ebi-fl187-GCST90094817 | prot-a-1216 | D-glucuronyl C5-epimerase \|\| id:prot-a-1216 | Prawns liking \|\| id:ebi-fl187-GCST90094817 | Weighted median | 20 | -0.153006787 | 0.135267228583505 | 0.257994260274075 | -0.418130555 | 0.112116980981728 | 0.858123897668744 | 0.658276281511423 | 1.11864371303103 | 0.999332001504091 |
| ebi-fl187-GCST90094817 | prot-a-1246 | Glypican-3 \|\| id:prot-a-1246 | Prawns liking \|\| id:ebi-fl187-GCST90094817 | Weighted median | 20 | -0.26201577 | 0.130735054783938 | 0.0450514476898777 | -0.518256477 | -0.005775062 | 0.769498888930798 | 0.595558012398894 | 0.994241581404727 | 0.999332001504091 |
| ebi-fl187-GCST90094817 | prot-a-1275 | Glutamate receptor 4 \|\| id:prot-a-1275 | Prawns liking \|\| id:ebi-fl187-GCST90094817 | Weighted median | 19 | -0.173701387 | 0.13232559370062 | 0.189290221679311 | -0.433059551 | 0.0856567764350176 | 0.840547858520777 | 0.6485218706125 | 1.08943234527555 | 0.999332001504091 |
| ebi-fl187-GCST90094817 | prot-a-1289 | Glutathione S-transferase P \|\| id:prot-a-1289 | Prawns liking \|\| id:ebi-fl187-GCST90094817 | Weighted median | 20 | 0.129210676757943 | 0.124308185126831 | 0.298600983716906 | -0.114433366 | 0.372854719606532 | 1.13792983428242 | 0.891871365313892 | 1.45187339577192 | 0.999332001504091 |
| ebi-fl187-GCST90094817 | prot-a-130 | Apolipoprotein D \|\| id:prot-a-130 | Prawns liking \|\| id:ebi-fl187-GCST90094817 | Weighted median | 20 | -0.043656436 | 0.13044373930186 | 0.737869235406623 | -0.299326165 | 0.212013293065102 | 0.957282788918603 | 0.74131757815217 | 1.23616431738203 | 0.999332001504091 |
| ebi-fl187-GCST90094817 | prot-a-1313 | Heparin-binding EGF-like growth factor \|\| id:prot-a-1313 | Prawns liking \|\| id:ebi-fl187-GCST90094817 | Weighted median | 20 | 0.25272024295274 | 0.131147226867358 | 0.0539800701163112 | -0.004328322 | 0.509768807612761 | 1.28752303280464 | 0.995681031977002 | 1.66490623680049 | 0.999332001504091 |
| ebi-fl187-GCST90094817 | prot-a-1347 | HLA class II histocompatibility antigen, DQ alpha 2 chain \|\| id:prot-a-1347 | Prawns liking \|\| id:ebi-fl187-GCST90094817 | Weighted median | 19 | -0.069358089 | 0.143886687476729 | 0.6297827138298 | -0.351375997 | 0.212659818357219 | 0.93299252580204 | 0.703719108147872 | 1.23696378728934 | 0.999332001504091 |
| ebi-fl187-GCST90094817 | prot-a-1357 | Non-histone chromosomal protein HMG-14 \|\| id:prot-a-1357 | Prawns liking \|\| id:ebi-fl187-GCST90094817 | Weighted median | 20 | -0.034969701 | 0.131681242663862 | 0.790575581094829 | -0.293064936 | 0.223125535029575 | 0.965634673973675 | 0.745973698417612 | 1.24997747984707 | 0.999332001504091 |
| ebi-fl187-GCST90094817 | prot-a-137 | Apolipoprotein M \|\| id:prot-a-137 | Prawns liking \|\| id:ebi-fl187-GCST90094817 | Weighted median | 20 | -0.128376919 | 0.12326681172101 | 0.297664118880128 | -0.36997987 | 0.113226031482746 | 0.879521807749107 | 0.690748234939141 | 1.1198850336178 | 0.999332001504091 |
| ebi-fl187-GCST90094817 | prot-a-1381 | 17-beta-hydroxysteroid dehydrogenase 14 \|\| id:prot-a-1381 | Prawns liking \|\| id:ebi-fl187-GCST90094817 | Weighted median | 20 | -0.14331919 | 0.126967264415211 | 0.258987058848003 | -0.392175028 | 0.105536648443368 | 0.866477453977216 | 0.675585857126982 | 1.11130683144204 | 0.999332001504091 |
| ebi-fl187-GCST90094817 | prot-a-1392 | Serine protease HTRA2, mitochondrial \|\| id:prot-a-1392 | Prawns liking \|\| id:ebi-fl187-GCST90094817 | Weighted median | 20 | -0.101028497 | 0.137682566247621 | 0.463083792117931 | -0.370886326 | 0.168829333291019 | 0.903907274276111 | 0.690122385796458 | 1.18391806628085 | 0.999332001504091 |
| ebi-fl187-GCST90094817 | prot-a-1416 | Interferon-induced protein with tetratricopeptide repeats 2 \|\| id:prot-a-1416 | Prawns liking \|\| id:ebi-fl187-GCST90094817 | Weighted median | 20 | -0.164580676 | 0.134018576061494 | 0.219430540236328 | -0.427257085 | 0.0980957326632418 | 0.848249320467631 | 0.652295834870545 | 1.10306837972773 | 0.999332001504091 |
| ebi-fl187-GCST90094817 | prot-a-1428 | Interferon gamma \|\| id:prot-a-1428 | Prawns liking \|\| id:ebi-fl187-GCST90094817 | Weighted median | 20 | -0.169116603 | 0.123978648846503 | 0.172542875552259 | -0.412114755 | 0.0738815482525981 | 0.844410436444385 | 0.662248275241171 | 1.07667926340244 | 0.999332001504091 |
| ebi-fl187-GCST90094817 | prot-a-1483 | Interleukin-17C \|\| id:prot-a-1483 | Prawns liking \|\| id:ebi-fl187-GCST90094817 | Weighted median | 20 | -0.065845648 | 0.130548292387156 | 0.613995980156069 | -0.321720302 | 0.190029004615122 | 0.936275368680646 | 0.724900915645526 | 1.20928467198507 | 0.999332001504091 |
| ebi-fl187-GCST90094817 | prot-a-1503 | Interleukin-1 receptor-like 2 \|\| id:prot-a-1503 | Prawns liking \|\| id:ebi-fl187-GCST90094817 | Weighted median | 20 | -0.089324147 | 0.128318877244775 | 0.486359490617324 | -0.340829146 | 0.162180852763223 | 0.914549077515524 | 0.711180405818183 | 1.17607291812582 | 0.999332001504091 |
| ebi-fl187-GCST90094817 | prot-a-1522 | Interleukin-31 receptor subunit alpha \|\| id:prot-a-1522 | Prawns liking \|\| id:ebi-fl187-GCST90094817 | Weighted median | 20 | -0.081603236 | 0.122011713069189 | 0.503613641857543 | -0.320746194 | 0.157539721444898 | 0.921637558610111 | 0.725607391285775 | 1.17062725606425 | 0.999332001504091 |
| ebi-fl187-GCST90094817 | prot-a-1540 | Interleukin-6 receptor subunit alpha \|\| id:prot-a-1540 | Prawns liking \|\| id:ebi-fl187-GCST90094817 | Weighted median | 20 | -0.094851877 | 0.12666182937709 | 0.453942069950818 | -0.343109063 | 0.153405308555693 | 0.909507643470625 | 0.70956082079032 | 1.16579739085669 | 0.999332001504091 |
| ebi-fl187-GCST90094817 | prot-a-1541 | Interleukin-6 receptor subunit alpha \|\| id:prot-a-1541 | Prawns liking \|\| id:ebi-fl187-GCST90094817 | Weighted median | 20 | -0.177256498 | 0.129134008437773 | 0.169859491217112 | -0.430359154 | 0.0758461586851822 | 0.837564923363992 | 0.650275503475807 | 1.07879659790357 | 0.999332001504091 |
| ebi-fl187-GCST90094817 | prot-a-1545 | Interleukin-9 \|\| id:prot-a-1545 | Prawns liking \|\| id:ebi-fl187-GCST90094817 | Weighted median | 20 | 0.0122466425476538 | 0.127428267324485 | 0.923436252130352 | -0.237512761 | 0.262006046503644 | 1.01232193973981 | 0.788586827438351 | 1.29953440004511 | 0.999332001504091 |
| ebi-fl187-GCST90094817 | prot-a-1548 | Inositol monophosphatase 3 \|\| id:prot-a-1548 | Prawns liking \|\| id:ebi-fl187-GCST90094817 | Weighted median | 20 | -0.116175459 | 0.116494649788096 | 0.318638303417178 | -0.344504973 | 0.112154054183719 | 0.890318994754517 | 0.70857102846454 | 1.11868518550411 | 0.999332001504091 |
| ebi-fl187-GCST90094817 | prot-a-1555 | Inhibin beta A chain:Inhibin beta B chain heterodimer \|\| id:prot-a-1555 | Prawns liking \|\| id:ebi-fl187-GCST90094817 | Weighted median | 20 | -0.13041147 | 0.119304953112271 | 0.274352746332054 | -0.364249178 | 0.103426238447575 | 0.877734195622538 | 0.694718064887704 | 1.10896399144259 | 0.999332001504091 |
| ebi-fl187-GCST90094817 | prot-a-1603 | Junctophilin-3 \|\| id:prot-a-1603 | Prawns liking \|\| id:ebi-fl187-GCST90094817 | Weighted median | 20 | 0.0862760123494933 | 0.130659924387589 | 0.509055121960741 | -0.169817439 | 0.342369464149168 | 1.09010716982637 | 0.843818851 | 1.40828051056877 | 0.999332001504091 |
| ebi-fl187-GCST90094817 | prot-a-1627 | UPF0258 protein KIAA1024 \|\| id:prot-a-1627 | Prawns liking \|\| id:ebi-fl187-GCST90094817 | Weighted median | 20 | 0.0718905281613208 | 0.128861214621509 | 0.576918731334676 | -0.180677452 | 0.324458508819479 | 1.0745377060065 | 0.834704547147986 | 1.38328140846344 | 0.999332001504091 |
| ebi-fl187-GCST90094817 | prot-a-1666 | Kallikrein-7 \|\| id:prot-a-1666 | Prawns liking \|\| id:ebi-fl187-GCST90094817 | Weighted median | 20 | -0.05447974 | 0.131531783844133 | 0.678731678335789 | -0.312282036 | 0.203322556488545 | 0.94697769457744 | 0.731775112063708 | 1.22546768705784 | 0.999332001504091 |
| ebi-fl187-GCST90094817 | prot-a-1678 | Importin subunit alpha-3 \|\| id:prot-a-1678 | Prawns liking \|\| id:ebi-fl187-GCST90094817 | Weighted median | 20 | -0.299627055 | 0.129185938134555 | 0.020376213420659 | -0.552831494 | -0.046422616 | 0.741094556642266 | 0.57531849121394 | 0.954638431186042 | 0.999332001504091 |
| ebi-fl187-GCST90094817 | prot-a-1718 | Protein LDOC1 \|\| id:prot-a-1718 | Prawns liking \|\| id:ebi-fl187-GCST90094817 | Weighted median | 20 | 0.0616231423973734 | 0.12088868628272 | 0.610225838958934 | -0.175318683 | 0.298564967511504 | 1.06356145797287 | 0.839189542947771 | 1.34792310556207 | 0.999332001504091 |
| ebi-fl187-GCST90094817 | prot-a-1723 | Leptin \|\| id:prot-a-1723 | Prawns liking \|\| id:ebi-fl187-GCST90094817 | Weighted median | 20 | 0.0212426908947206 | 0.133386720307481 | 0.873466858161423 | -0.240195281 | 0.282680662697383 | 1.02146992300779 | 0.786474262661538 | 1.32668143529394 | 0.999332001504091 |
| ebi-fl187-GCST90094817 | prot-a-1730 | Galectin-7 \|\| id:prot-a-1730 | Prawns liking \|\| id:ebi-fl187-GCST90094817 | Weighted median | 20 | 0.0216582886827362 | 0.129272147359597 | 0.866944975302997 | -0.23171512 | 0.275031697507547 | 1.02189453187546 | 0.793172049865069 | 1.31657240627001 | 0.999332001504091 |
| ebi-fl187-GCST90094817 | prot-a-1753 | Lipase member N \|\| id:prot-a-1753 | Prawns liking \|\| id:ebi-fl187-GCST90094817 | Weighted median | 20 | -0.043899395 | 0.12524187110584 | 0.725950793008677 | -0.289373462 | 0.201574672240698 | 0.957050236547636 | 0.748732529663706 | 1.22332758226397 | 0.999332001504091 |
| ebi-fl187-GCST90094817 | prot-a-1756 | VIP36-like protein \|\| id:prot-a-1756 | Prawns liking \|\| id:ebi-fl187-GCST90094817 | Weighted median | 20 | -0.121194895 | 0.124144585812833 | 0.32894560509216 | -0.364518284 | 0.122128492708901 | 0.885861292401832 | 0.694531137293112 | 1.12989927627198 | 0.999332001504091 |
| ebi-fl187-GCST90094817 | prot-a-1760 | Ig Kappa chain V-I region HK102- like \|\| id:prot-a-1760 | Prawns liking \|\| id:ebi-fl187-GCST90094817 | Weighted median | 20 | -0.137179822 | 0.126302105739406 | 0.277423878653856 | -0.384731949 | 0.110372305616846 | 0.871813441119742 | 0.680633056206471 | 1.11669374442854 | 0.999332001504091 |
| ebi-fl187-GCST90094817 | prot-a-179 | Neutral ceramidase \|\| id:prot-a-179 | Prawns liking \|\| id:ebi-fl187-GCST90094817 | Weighted median | 20 | 0.247288742836948 | 0.126718450370223 | 0.0509997655196996 | -0.00107942 | 0.495656905562586 | 1.28054880869805 | 0.998921162475401 | 1.64157624551115 | 0.999332001504091 |
| ebi-fl187-GCST90094817 | prot-a-1809 | Lactotransferrin \|\| id:prot-a-1809 | Prawns liking \|\| id:ebi-fl187-GCST90094817 | Weighted median | 20 | -0.033430537 | 0.12483064557624 | 0.788847783905107 | -0.278098602 | 0.21123752867906 | 0.967122088437964 | 0.75722215422967 | 1.2352057170014 | 0.999332001504091 |
| ebi-fl187-GCST90094817 | prot-a-1818 | Lysophospholipase-like protein 1 \|\| id:prot-a-1818 | Prawns liking \|\| id:ebi-fl187-GCST90094817 | Weighted median | 20 | -0.048060231 | 0.127611192959402 | 0.706460077098416 | -0.29817817 | 0.202057706837431 | 0.953076380259254 | 0.742169096023396 | 1.2239186345472 | 0.999332001504091 |
| ebi-fl187-GCST90094817 | prot-a-1852 | MAP kinase-activated protein kinase 3 \|\| id:prot-a-1852 | Prawns liking \|\| id:ebi-fl187-GCST90094817 | Weighted median | 20 | 0.0328505592623795 | 0.129714039689934 | 0.800072179395347 | -0.221388959 | 0.287090077054649 | 1.03339609622668 | 0.801404906386332 | 1.33254423973009 | 0.999332001504091 |
| ebi-fl187-GCST90094817 | prot-a-1856 | Megakaryocyte-associated tyrosine-protein kinase \|\| id:prot-a-1856 | Prawns liking \|\| id:ebi-fl187-GCST90094817 | Weighted median | 20 | -0.180836759 | 0.123934565887692 | 0.144529072756166 | -0.423748508 | 0.0620749898904201 | 0.834571583668454 | 0.654588484722225 | 1.06404213413934 | 0.999332001504091 |
| ebi-fl187-GCST90094817 | prot-a-1890 | Beta-1,3-N-acetylglucosaminyltransferase manic fringe \|\| id:prot-a-1890 | Prawns liking \|\| id:ebi-fl187-GCST90094817 | Weighted median | 20 | -0.225713203 | 0.126190821123939 | 0.0736686574123534 | -0.473047213 | 0.0216208061032168 | 0.797946917395228 | 0.62310065221739 | 1.02185622935025 | 0.999332001504091 |
| ebi-fl187-GCST90094817 | prot-a-1919 | Matrilysin \|\| id:prot-a-1919 | Prawns liking \|\| id:ebi-fl187-GCST90094817 | Weighted median | 20 | -0.149809958 | 0.130727624313268 | 0.251807495279596 | -0.406036101 | 0.106416185891857 | 0.860871562838706 | 0.666286113118444 | 1.11228469738887 | 0.999332001504091 |
| ebi-fl187-GCST90094817 | prot-a-1923 | Multimerin-2 \|\| id:prot-a-1923 | Prawns liking \|\| id:ebi-fl187-GCST90094817 | Weighted median | 20 | -0.220404179 | 0.124204006674498 | 0.0759749354251821 | -0.463844032 | 0.0230356744994336 | 0.802194502593258 | 0.628861629320137 | 1.02330304471995 | 0.999332001504091 |
| ebi-fl187-GCST90094817 | prot-a-1948 | Mesothelin \|\| id:prot-a-1948 | Prawns liking \|\| id:ebi-fl187-GCST90094817 | Weighted median | 20 | -0.092317926 | 0.127958965300667 | 0.470623415882297 | -0.343117498 | 0.158481646404439 | 0.911815214073354 | 0.709554835690135 | 1.17173041856164 | 0.999332001504091 |
| ebi-fl187-GCST90094817 | prot-a-195 | ATPase family AAA domain-containing protein 1 \|\| id:prot-a-195 | Prawns liking \|\| id:ebi-fl187-GCST90094817 | Weighted median | 20 | 0.0436737624120163 | 0.125912718453671 | 0.728698426482706 | -0.203115166 | 0.290462690581212 | 1.04464149796869 | 0.816184239536871 | 1.33704598351164 | 0.999332001504091 |
| ebi-fl187-GCST90094817 | prot-a-1966 | Mucin-1 \|\| id:prot-a-1966 | Prawns liking \|\| id:ebi-fl187-GCST90094817 | Weighted median | 20 | -0.16892604 | 0.121355273910273 | 0.163923648299299 | -0.406782377 | 0.0689302966589904 | 0.844571365401093 | 0.665789065504543 | 1.07136152906765 | 0.999332001504091 |
| ebi-fl187-GCST90094817 | prot-a-1986 | Myocardial zonula adherens protein \|\| id:prot-a-1986 | Prawns liking \|\| id:ebi-fl187-GCST90094817 | Weighted median | 20 | -0.265328675 | 0.127883225541498 | 0.0380075698454961 | -0.515979797 | -0.014677553 | 0.766953830121167 | 0.596915452096779 | 0.985429637432403 | 0.999332001504091 |
| ebi-fl187-GCST90094817 | prot-a-2071 | Epididymal secretory protein E1 \|\| id:prot-a-2071 | Prawns liking \|\| id:ebi-fl187-GCST90094817 | Weighted median | 20 | -0.006058727 | 0.137075481670145 | 0.964745034737188 | -0.274726671 | 0.262609216843242 | 0.99395958984619 | 0.759779764458997 | 1.30031847709274 | 0.999332001504091 |
| ebi-fl187-GCST90094817 | prot-a-2098 | Neuritin \|\| id:prot-a-2098 | Prawns liking \|\| id:ebi-fl187-GCST90094817 | Weighted median | 20 | 0.014961908780445 | 0.127055484581657 | 0.906258729133659 | -0.234066841 | 0.263990658560492 | 1.01507439845753 | 0.791308922248514 | 1.30211603260593 | 0.999332001504091 |
| ebi-fl187-GCST90094817 | prot-a-2115 | Neurotrophin-4 \|\| id:prot-a-2115 | Prawns liking \|\| id:ebi-fl187-GCST90094817 | Weighted median | 20 | -0.058383219 | 0.124493168197159 | 0.6390930453884 | -0.302389828 | 0.185623390923313 | 0.943288392371761 | 0.739049906077471 | 1.20396874942574 | 0.999332001504091 |
| ebi-fl187-GCST90094817 | prot-a-2118 | Netrin-1 \|\| id:prot-a-2118 | Prawns liking \|\| id:ebi-fl187-GCST90094817 | Weighted median | 20 | 0.0514409162032554 | 0.13247296151709 | 0.697784735837005 | -0.208206088 | 0.311087920776751 | 1.05278698180246 | 0.812039667534459 | 1.3649092197897 | 0.999332001504091 |
| ebi-fl187-GCST90094817 | prot-a-2120 | Netrin-G1 \|\| id:prot-a-2120 | Prawns liking \|\| id:ebi-fl187-GCST90094817 | Weighted median | 20 | -0.082174109 | 0.132973675049401 | 0.536593296235126 | -0.342802512 | 0.178454294383391 | 0.921111571183734 | 0.709778370565399 | 1.19536824698209 | 0.999332001504091 |
| ebi-fl187-GCST90094817 | prot-a-2145 | Olfactomedin-like protein 3 \|\| id:prot-a-2145 | Prawns liking \|\| id:ebi-fl187-GCST90094817 | Weighted median | 20 | -0.152550754 | 0.128165800998228 | 0.233943826703041 | -0.403755724 | 0.0986542162862287 | 0.858515320047136 | 0.667807230874543 | 1.10368459741057 | 0.999332001504091 |
| ebi-fl187-GCST90094817 | prot-a-2162 | Prolyl 4-hydroxylase subunit alpha-2 \|\| id:prot-a-2162 | Prawns liking \|\| id:ebi-fl187-GCST90094817 | Weighted median | 20 | -0.207371862 | 0.135195037338275 | 0.125061182769646 | -0.472354135 | 0.0576104113007771 | 0.81271737517595 | 0.623532659035108 | 1.05930222313455 | 0.999332001504091 |
| ebi-fl187-GCST90094817 | prot-a-2168 | Polyadenylate-binding protein-interacting protein 1 \|\| id:prot-a-2168 | Prawns liking \|\| id:ebi-fl187-GCST90094817 | Weighted median | 20 | -0.059544675 | 0.137639023476705 | 0.665294656585928 | -0.329317161 | 0.210227811371405 | 0.942193440495458 | 0.719414810485814 | 1.23395913786261 | 0.999332001504091 |
| ebi-fl187-GCST90094817 | prot-a-219 | Beta-1,4-galactosyltransferase 5 \|\| id:prot-a-219 | Prawns liking \|\| id:ebi-fl187-GCST90094817 | Weighted median | 20 | 0.104305338509753 | 0.128809480407638 | 0.418075566124851 | -0.148161243 | 0.356771920108723 | 1.10993931039509 | 0.862292065095551 | 1.42870997267476 | 0.999332001504091 |
| ebi-fl187-GCST90094817 | prot-a-2190 | Pyruvate carboxylase, mitochondrial \|\| id:prot-a-2190 | Prawns liking \|\| id:ebi-fl187-GCST90094817 | Weighted median | 20 | -0.116829524 | 0.128137743268901 | 0.361900595389179 | -0.367979501 | 0.134320452364794 | 0.88973685862264 | 0.69213136937171 | 1.14375928129121 | 0.999332001504091 |
| ebi-fl187-GCST90094817 | prot-a-2197 | Protocadherin-9 \|\| id:prot-a-2197 | Prawns liking \|\| id:ebi-fl187-GCST90094817 | Weighted median | 20 | -0.171800999 | 0.115494671206405 | 0.136876689891534 | -0.398170554 | 0.0545685568041746 | 0.842146744740617 | 0.671547482566656 | 1.05608487573598 | 0.999332001504091 |
| ebi-fl187-GCST90094817 | prot-a-2198 | Protocadherin alpha-4 \|\| id:prot-a-2198 | Prawns liking \|\| id:ebi-fl187-GCST90094817 | Weighted median | 20 | 0.157848641937874 | 0.139573438926774 | 0.258081983811797 | -0.115715298 | 0.431412582234351 | 1.17098894267661 | 0.89072877914734 | 1.53943056065113 | 0.999332001504091 |
| ebi-fl187-GCST90094817 | prot-a-2254 | Placenta growth factor \|\| id:prot-a-2254 | Prawns liking \|\| id:ebi-fl187-GCST90094817 | Weighted median | 20 | 0.0815915147785322 | 0.127647642769516 | 0.522697206706972 | -0.168597865 | 0.331780894606783 | 1.08501250772002 | 0.844848578224895 | 1.39344750319922 | 0.999332001504091 |
| ebi-fl187-GCST90094817 | prot-a-2261 | Membrane-associated progesterone receptor component 2 \|\| id:prot-a-2261 | Prawns liking \|\| id:ebi-fl187-GCST90094817 | Weighted median | 20 | -0.016205145 | 0.141528059607118 | 0.908840424324444 | -0.293600142 | 0.26118985151786 | 0.983925451655962 | 0.745574555692439 | 1.29847415932438 | 0.999332001504091 |
| ebi-fl187-GCST90094817 | prot-a-2268 | Polymeric immunoglobulin receptor \|\| id:prot-a-2268 | Prawns liking \|\| id:ebi-fl187-GCST90094817 | Weighted median | 20 | -0.055274678 | 0.126904102641536 | 0.663154128140947 | -0.304006719 | 0.193457363289916 | 0.946225205114409 | 0.737855908732194 | 1.21343764845931 | 0.999332001504091 |
| ebi-fl187-GCST90094817 | prot-a-2283 | Group 10 secretory phospholipase A2 \|\| id:prot-a-2283 | Prawns liking \|\| id:ebi-fl187-GCST90094817 | Weighted median | 20 | -0.025184444 | 0.128016713298165 | 0.844040570970329 | -0.276097202 | 0.225728314479271 | 0.975130038960269 | 0.758739176477258 | 1.25323513318171 | 0.999332001504091 |
| ebi-fl187-GCST90094817 | prot-a-2298 | Inactive phospholipase D5 \|\| id:prot-a-2298 | Prawns liking \|\| id:ebi-fl187-GCST90094817 | Weighted median | 20 | 0.0902797261679282 | 0.127418111067788 | 0.478615351321761 | -0.159459772 | 0.340019223860792 | 1.09448039569641 | 0.852604265675976 | 1.4049745993401 | 0.999332001504091 |
| ebi-fl187-GCST90094817 | prot-a-2304 | Procollagen-lysine,2-oxoglutarate 5-dioxygenase 2 \|\| id:prot-a-2304 | Prawns liking \|\| id:ebi-fl187-GCST90094817 | Weighted median | 20 | 0.0627259693240713 | 0.128432119295734 | 0.625268073343159 | -0.189000984 | 0.31445292314371 | 1.06473502919095 | 0.827785691742989 | 1.36950987881804 | 0.999332001504091 |
| ebi-fl187-GCST90094817 | prot-a-2331 | Periostin \|\| id:prot-a-2331 | Prawns liking \|\| id:ebi-fl187-GCST90094817 | Weighted median | 20 | 0.0423964050142145 | 0.138525053043011 | 0.759562110634453 | -0.229112699 | 0.313905508978517 | 1.04330796930089 | 0.795238905864958 | 1.36876039486878 | 0.999332001504091 |
| ebi-fl187-GCST90094817 | prot-a-2333 | Periostin \|\| id:prot-a-2333 | Prawns liking \|\| id:ebi-fl187-GCST90094817 | Weighted median | 20 | 0.00521163917230452 | 0.122856136399538 | 0.966163353804851 | -0.235586388 | 0.246009666515399 | 1.00522524338689 | 0.790107404127102 | 1.27891193610391 | 0.999332001504091 |
| ebi-fl187-GCST90094817 | prot-a-2367 | cAMP-dependent protein kinase catalytic subunit alpha \|\| id:prot-a-2367 | Prawns liking \|\| id:ebi-fl187-GCST90094817 | Weighted median | 20 | -0.132012618 | 0.130572219970854 | 0.312001387188652 | -0.387934169 | 0.123908932903214 | 0.876329937268439 | 0.678457005037598 | 1.13191278629417 | 0.999332001504091 |
| ebi-fl187-GCST90094817 | prot-a-2385 | Phosphoribosyl pyrophosphate synthase-associated protein 1 \|\| id:prot-a-2385 | Prawns liking \|\| id:ebi-fl187-GCST90094817 | Weighted median | 20 | 0.096625075811049 | 0.139570692801363 | 0.488747667186189 | -0.176933482 | 0.37018363370172 | 1.10144733694765 | 0.83783551374547 | 1.44800049194099 | 0.999332001504091 |
| ebi-fl187-GCST90094817 | prot-a-2393 | Trypsin-3 \|\| id:prot-a-2393 | Prawns liking \|\| id:ebi-fl187-GCST90094817 | Weighted median | 19 | -0.024139034 | 0.137223575000128 | 0.860364446242804 | -0.293097241 | 0.244819172507505 | 0.97614998180513 | 0.745949599917495 | 1.27739030503341 | 0.999332001504091 |
| ebi-fl187-GCST90094817 | prot-a-2405 | Pregnancy-specific beta-1-glycoprotein 6 \|\| id:prot-a-2405 | Prawns liking \|\| id:ebi-fl187-GCST90094817 | Weighted median | 20 | 0.0660253104108752 | 0.13206455692234 | 0.617112229604219 | -0.192821221 | 0.324871841978662 | 1.06825375476528 | 0.824629387249372 | 1.3838532827172 | 0.999332001504091 |
| ebi-fl187-GCST90094817 | prot-a-244 | Beclin-1 \|\| id:prot-a-244 | Prawns liking \|\| id:ebi-fl187-GCST90094817 | Weighted median | 20 | -0.137891317 | 0.115448263748656 | 0.232321837540933 | -0.364169914 | 0.0883872795154204 | 0.871193370133499 | 0.694773132767366 | 1.09241110856066 | 0.999332001504091 |
| ebi-fl187-GCST90094817 | prot-a-2454 | tRNA pseudouridine synthase A, mitochondrial \|\| id:prot-a-2454 | Prawns liking \|\| id:ebi-fl187-GCST90094817 | Weighted median | 20 | -0.201214189 | 0.131469063391687 | 0.125891531524381 | -0.458893553 | 0.0564651750096497 | 0.817737262273704 | 0.631982513654806 | 1.05808976619271 | 0.999332001504091 |
| ebi-fl187-GCST90094817 | prot-a-2466 | Glutaminyl-peptide cyclotransferase-like protein \|\| id:prot-a-2466 | Prawns liking \|\| id:ebi-fl187-GCST90094817 | Weighted median | 20 | -0.185703431 | 0.127786511948773 | 0.146159387997996 | -0.436164994 | 0.0647581324969757 | 0.830519864972197 | 0.646511046470081 | 1.06690094450746 | 0.999332001504091 |
| ebi-fl187-GCST90094817 | prot-a-2474 | Ras-related protein Rab-27A \|\| id:prot-a-2474 | Prawns liking \|\| id:ebi-fl187-GCST90094817 | Weighted median | 20 | -0.185980319 | 0.140331877280942 | 0.185075256294087 | -0.461030798 | 0.0890701608947584 | 0.830289936109493 | 0.63063325482664 | 1.09315735053366 | 0.999332001504091 |
| ebi-fl187-GCST90094817 | prot-a-2490 | Rap1 GTPase-GDP dissociation stimulator 1 \|\| id:prot-a-2490 | Prawns liking \|\| id:ebi-fl187-GCST90094817 | Weighted median | 20 | -0.085798941 | 0.140532727939625 | 0.541513159201024 | -0.361243088 | 0.189645205962692 | 0.917778740531172 | 0.696809592133439 | 1.20882063921083 | 0.999332001504091 |
| ebi-fl187-GCST90094817 | prot-a-2545 | Receptor-interacting serine/threonine-protein kinase 2 \|\| id:prot-a-2545 | Prawns liking \|\| id:ebi-fl187-GCST90094817 | Weighted median | 20 | 0.202623215183422 | 0.138032003453236 | 0.142119506015338 | -0.067919512 | 0.473165941951765 | 1.22461096649114 | 0.934335673748335 | 1.60506770894666 | 0.999332001504091 |
| ebi-fl187-GCST90094817 | prot-a-2610 | RUN and FYVE domain-containing protein 1 \|\| id:prot-a-2610 | Prawns liking \|\| id:ebi-fl187-GCST90094817 | Weighted median | 20 | 0.215691931404604 | 0.137549475908402 | 0.116856781712257 | -0.053905041 | 0.485288904185072 | 1.24072009322218 | 0.947522077622794 | 1.62464430758949 | 0.999332001504091 |
| ebi-fl187-GCST90094817 | prot-a-2627 | Serine--tRNA ligase, mitochondrial \|\| id:prot-a-2627 | Prawns liking \|\| id:ebi-fl187-GCST90094817 | Weighted median | 20 | -0.082817241 | 0.136512068614313 | 0.544072539007424 | -0.350380896 | 0.184746413322055 | 0.92051936489751 | 0.704419728205626 | 1.20291335864456 | 0.999332001504091 |
| ebi-fl187-GCST90094817 | prot-a-264 | BolA-like protein 2 \|\| id:prot-a-264 | Prawns liking \|\| id:ebi-fl187-GCST90094817 | Weighted median | 20 | -0.072104283 | 0.127164680711547 | 0.570703983352681 | -0.321347057 | 0.177138491411317 | 0.93043386251897 | 0.725171531471956 | 1.19379641222919 | 0.999332001504091 |
| ebi-fl187-GCST90094817 | prot-a-2646 | Secretoglobin family 1C member 1 \|\| id:prot-a-2646 | Prawns liking \|\| id:ebi-fl187-GCST90094817 | Weighted median | 20 | -0.15785543 | 0.124265216402224 | 0.203973989719566 | -0.401415254 | 0.0857043941147213 | 0.853973231926103 | 0.669372043777525 | 1.08948422275116 | 0.999332001504091 |
| ebi-fl187-GCST90094817 | prot-a-2672 | Semaphorin-3E \|\| id:prot-a-2672 | Prawns liking \|\| id:ebi-fl187-GCST90094817 | Weighted median | 20 | -0.047698298 | 0.127525284675737 | 0.708381907435527 | -0.297647855 | 0.202251260446031 | 0.953421393289922 | 0.742562783124857 | 1.22415555134286 | 0.999332001504091 |
| ebi-fl187-GCST90094817 | prot-a-2701 | Plasma protease C1 inhibitor \|\| id:prot-a-2701 | Prawns liking \|\| id:ebi-fl187-GCST90094817 | Weighted median | 20 | -0.142388507 | 0.125050435708694 | 0.254849737278414 | -0.387487361 | 0.102710346878916 | 0.867284244928807 | 0.678760212980225 | 1.10817037757581 | 0.999332001504091 |
| ebi-fl187-GCST90094817 | prot-a-2738 | Signaling threshold-regulating transmembrane adapter 1 \|\| id:prot-a-2738 | Prawns liking \|\| id:ebi-fl187-GCST90094817 | Weighted median | 20 | -0.017548364 | 0.138138031601911 | 0.898912701849148 | -0.288298906 | 0.253202177949404 | 0.982604711831069 | 0.749537517543267 | 1.2881436847581 | 0.999332001504091 |
| ebi-fl187-GCST90094817 | prot-a-274 | Serine/threonine-protein kinase BRSK2 \|\| id:prot-a-274 | Prawns liking \|\| id:ebi-fl187-GCST90094817 | Weighted median | 20 | 0.0584104319007485 | 0.126624829250262 | 0.644592459627792 | -0.189774233 | 0.306595097231263 | 1.06015002581924 | 0.827145854747621 | 1.35879067856474 | 0.999332001504091 |
| ebi-fl187-GCST90094817 | prot-a-2749 | Mitochondrial glutamate carrier 2 \|\| id:prot-a-2749 | Prawns liking \|\| id:ebi-fl187-GCST90094817 | Weighted median | 20 | -0.090770205 | 0.126795460100675 | 0.474066075009402 | -0.339289307 | 0.157748896378093 | 0.913227541529425 | 0.712276352589043 | 1.17087214755402 | 0.999332001504091 |
| ebi-fl187-GCST90094817 | prot-a-2760 | Sodium-coupled monocarboxylate transporter 1 \|\| id:prot-a-2760 | Prawns liking \|\| id:ebi-fl187-GCST90094817 | Weighted median | 20 | -0.143653335 | 0.12627712715456 | 0.255285974189358 | -0.391156504 | 0.10384983405944 | 0.866187972929512 | 0.67627430786104 | 1.10943384322964 | 0.999332001504091 |
| ebi-fl187-GCST90094817 | prot-a-2777 | Small integral membrane protein 10 \|\| id:prot-a-2777 | Prawns liking \|\| id:ebi-fl187-GCST90094817 | Weighted median | 20 | 0.199067236032504 | 0.124383422184187 | 0.109502732971301 | -0.044724271 | 0.442858743513509 | 1.22026400885228 | 0.956261113977786 | 1.55715236093437 | 0.999332001504091 |
| ebi-fl187-GCST90094817 | prot-a-2799 | Superoxide dismutase [Mn], mitochondrial \|\| id:prot-a-2799 | Prawns liking \|\| id:ebi-fl187-GCST90094817 | Weighted median | 20 | -0.142236041 | 0.133068502593796 | 0.285117718562282 | -0.403050306 | 0.118578224543833 | 0.867416486863929 | 0.6682784803121 | 1.1258949432757 | 0.999332001504091 |
| ebi-fl187-GCST90094817 | prot-a-2805 | Protein SPACA7 \|\| id:prot-a-2805 | Prawns liking \|\| id:ebi-fl187-GCST90094817 | Weighted median | 20 | -0.02440656 | 0.131331384964611 | 0.852570621982449 | -0.281816074 | 0.233002954684157 | 0.975888871864272 | 0.754412427549147 | 1.26238520927133 | 0.999332001504091 |
| ebi-fl187-GCST90094817 | prot-a-2811 | SPARC-like protein 1 \|\| id:prot-a-2811 | Prawns liking \|\| id:ebi-fl187-GCST90094817 | Weighted median | 20 | -0.174806937 | 0.125749779127421 | 0.164493276038703 | -0.421276504 | 0.0716626299844292 | 0.839619104417266 | 0.656208631882235 | 1.07429284872462 | 0.999332001504091 |
| ebi-fl187-GCST90094817 | prot-a-2831 | Sepiapterin reductase \|\| id:prot-a-2831 | Prawns liking \|\| id:ebi-fl187-GCST90094817 | Weighted median | 20 | -0.171757277 | 0.128858646688443 | 0.182560581668721 | -0.424320224 | 0.080805670818951 | 0.842183565944469 | 0.654214353094868 | 1.08416019213215 | 0.999332001504091 |
| ebi-fl187-GCST90094817 | prot-a-2834 | SPRY domain-containing SOCS box protein 1 \|\| id:prot-a-2834 | Prawns liking \|\| id:ebi-fl187-GCST90094817 | Weighted median | 20 | -0.059085712 | 0.129885827220838 | 0.649177588332299 | -0.313661933 | 0.195490509608902 | 0.942625971578591 | 0.730766034214949 | 1.21590725443203 | 0.999332001504091 |
| ebi-fl187-GCST90094817 | prot-a-2835 | Spectrin alpha chain, non-erythrocytic 1 \|\| id:prot-a-2835 | Prawns liking \|\| id:ebi-fl187-GCST90094817 | Weighted median | 20 | 0.0235846232128842 | 0.132908449705802 | 0.85915482492781 | -0.236915938 | 0.284085184636257 | 1.02386493982151 | 0.789057614824926 | 1.32854609764876 | 0.999332001504091 |
| ebi-fl187-GCST90094817 | prot-a-2859 | Sia-alpha-2,3-Gal-beta-1,4-GlcNAc-R:alpha 2,8-sialyltransferase \|\| id:prot-a-2859 | Prawns liking \|\| id:ebi-fl187-GCST90094817 | Weighted median | 20 | -0.325579661 | 0.132445506260748 | 0.0139629169978049 | -0.585172853 | -0.065986469 | 0.722108654122025 | 0.557009572598277 | 0.936143531475701 | 0.999332001504091 |
| ebi-fl187-GCST90094817 | prot-a-2861 | Alpha-2,8-sialyltransferase 8F \|\| id:prot-a-2861 | Prawns liking \|\| id:ebi-fl187-GCST90094817 | Weighted median | 20 | -0.177580471 | 0.129586088620625 | 0.170572437072473 | -0.431569204 | 0.0764082629025818 | 0.837293618942517 | 0.649489113419838 | 1.07940316448181 | 0.999332001504091 |
| ebi-fl187-GCST90094817 | prot-a-289 | Uncharacterized protein C17orf89 \|\| id:prot-a-289 | Prawns liking \|\| id:ebi-fl187-GCST90094817 | Weighted median | 20 | 0.0458118169608662 | 0.12846566383859 | 0.721385675141656 | -0.205980884 | 0.297604518084502 | 1.04687738785087 | 0.813848633532234 | 1.34662911509337 | 0.999332001504091 |
| ebi-fl187-GCST90094817 | prot-a-2921 | Tachykinin-3 \|\| id:prot-a-2921 | Prawns liking \|\| id:ebi-fl187-GCST90094817 | Weighted median | 20 | 0.00492839981061813 | 0.123285936104941 | 0.968112769605123 | -0.236712035 | 0.246568834576303 | 1.00494056434866 | 0.789218522645561 | 1.27962726278657 | 0.999332001504091 |
| ebi-fl187-GCST90094817 | prot-a-2928 | Tax1-binding protein 1 \|\| id:prot-a-2928 | Prawns liking \|\| id:ebi-fl187-GCST90094817 | Weighted median | 20 | -0.081889644 | 0.121748210247792 | 0.501192404665777 | -0.320516136 | 0.156736848029427 | 0.921373632142837 | 0.725774342016387 | 1.16968776775649 | 0.999332001504091 |
| ebi-fl187-GCST90094817 | prot-a-296 | Uncharacterized protein C1orf185 \|\| id:prot-a-296 | Prawns liking \|\| id:ebi-fl187-GCST90094817 | Weighted median | 20 | 0.165824463260599 | 0.12552908072274 | 0.186499909605667 | -0.080212535 | 0.41186146147717 | 1.1803658859579 | 0.922920172742216 | 1.50962528058462 | 0.999332001504091 |
| ebi-fl187-GCST90094817 | prot-a-2964 | Transforming growth factor beta-2 \|\| id:prot-a-2964 | Prawns liking \|\| id:ebi-fl187-GCST90094817 | Weighted median | 20 | -0.112031409 | 0.119392938981226 | 0.348068682998863 | -0.346041569 | 0.121978751445745 | 0.894016176943301 | 0.707483076876039 | 1.12973009639403 | 0.999332001504091 |
| ebi-fl187-GCST90094817 | prot-a-2977 | Thymocyte nuclear protein 1 \|\| id:prot-a-2977 | Prawns liking \|\| id:ebi-fl187-GCST90094817 | Weighted median | 20 | -0.017994142 | 0.127127586239663 | 0.887440232686809 | -0.267164211 | 0.231175926750889 | 0.982166785600602 | 0.765547349611114 | 1.26008090188941 | 0.999332001504091 |
| ebi-fl187-GCST90094817 | prot-a-3015 | Transmembrane protein 70, mitochondrial \|\| id:prot-a-3015 | Prawns liking \|\| id:ebi-fl187-GCST90094817 | Weighted median | 20 | -0.057686409 | 0.128207157044035 | 0.652748748967817 | -0.308972437 | 0.193599618487419 | 0.943945913670246 | 0.734201006437795 | 1.21361027855013 | 0.999332001504091 |
| ebi-fl187-GCST90094817 | prot-a-3030 | Tumor necrosis factor alpha-induced protein 3 \|\| id:prot-a-3030 | Prawns liking \|\| id:ebi-fl187-GCST90094817 | Weighted median | 20 | -0.219210854 | 0.129592871471498 | 0.0907347036492632 | -0.473212882 | 0.0347911737904004 | 0.803152352176502 | 0.622997431885168 | 1.03540346684053 | 0.999332001504091 |
| ebi-fl187-GCST90094817 | prot-a-3032 | Tumor necrosis factor receptor superfamily member 10A \|\| id:prot-a-3032 | Prawns liking \|\| id:ebi-fl187-GCST90094817 | Weighted median | 20 | 0.0662155199158458 | 0.122344530529137 | 0.588354753365032 | -0.17357976 | 0.306010799752955 | 1.06845696610891 | 0.840650098302201 | 1.35799697250051 | 0.999332001504091 |
| ebi-fl187-GCST90094817 | prot-a-3081 | Tumor protein p53-inducible protein 11 \|\| id:prot-a-3081 | Prawns liking \|\| id:ebi-fl187-GCST90094817 | Weighted median | 20 | 0.163912889174814 | 0.130158405504444 | 0.207909762559387 | -0.091197586 | 0.419023363963525 | 1.17811168434168 | 0.912837329555325 | 1.52047587871818 | 0.999332001504091 |
| ebi-fl187-GCST90094817 | prot-a-315 | Complement C4 \|\| id:prot-a-315 | Prawns liking \|\| id:ebi-fl187-GCST90094817 | Weighted median | 19 | -0.112568917 | 0.123386420718669 | 0.361595947904831 | -0.354406302 | 0.129268467451454 | 0.893535765041782 | 0.701589852242145 | 1.13799559793696 | 0.999332001504091 |
| ebi-fl187-GCST90094817 | prot-a-3157 | UDP-glucuronosyltransferase 1-8 \|\| id:prot-a-3157 | Prawns liking \|\| id:ebi-fl187-GCST90094817 | Weighted median | 20 | -0.03017936 | 0.127466655856979 | 0.812840824983874 | -0.280014006 | 0.219655285205158 | 0.970271489779939 | 0.755773156208543 | 1.24564726352892 | 0.999332001504091 |
| ebi-fl187-GCST90094817 | prot-a-317 | Glutaredoxin-like protein C5orf63 \|\| id:prot-a-317 | Prawns liking \|\| id:ebi-fl187-GCST90094817 | Weighted median | 20 | 0.00323192526772266 | 0.127622747747505 | 0.979796488313375 | -0.24690866 | 0.253372510852832 | 1.00323715356917 | 0.781212045933003 | 1.28836311669966 | 0.999332001504091 |
| ebi-fl187-GCST90094817 | prot-a-3192 | Guanine nucleotide exchange factor VAV3 \|\| id:prot-a-3192 | Prawns liking \|\| id:ebi-fl187-GCST90094817 | Weighted median | 19 | 0.094339022352447 | 0.131698742454972 | 0.473791065308259 | -0.163790513 | 0.352468557564192 | 1.09893224536579 | 0.848919841044328 | 1.42257492582464 | 0.999332001504091 |
| ebi-fl187-GCST90094817 | prot-a-3233 | Serine/threonine-protein kinase WNK3 \|\| id:prot-a-3233 | Prawns liking \|\| id:ebi-fl187-GCST90094817 | Weighted median | 20 | -0.009941489 | 0.13005770620086 | 0.939069793082102 | -0.264854593 | 0.244971615015124 | 0.990107764112569 | 0.767317514978163 | 1.27758504845792 | 0.999332001504091 |
| ebi-fl187-GCST90094817 | prot-a-3250 | 14-3-3 protein theta \|\| id:prot-a-3250 | Prawns liking \|\| id:ebi-fl187-GCST90094817 | Weighted median | 20 | 0.0759303281887156 | 0.125764449471948 | 0.546009645188665 | -0.170567993 | 0.322428649153734 | 1.0788874034991 | 0.843185757138971 | 1.38047638918691 | 0.999332001504091 |
| ebi-fl187-GCST90094817 | prot-a-3278 | E3 ubiquitin-protein ligase ZNRF3 \|\| id:prot-a-3278 | Prawns liking \|\| id:ebi-fl187-GCST90094817 | Weighted median | 20 | -0.080683039 | 0.127342995060467 | 0.526349500396175 | -0.330275309 | 0.16890923115114 | 0.922486037053496 | 0.718725834151505 | 1.18401266257987 | 0.999332001504091 |
| ebi-fl187-GCST90094817 | prot-a-355 | Calpain-3 \|\| id:prot-a-355 | Prawns liking \|\| id:ebi-fl187-GCST90094817 | Weighted median | 20 | -0.066455059 | 0.122587199231863 | 0.587746595190358 | -0.306725969 | 0.173815851726076 | 0.935704966645056 | 0.735852219407295 | 1.18983643931855 | 0.999332001504091 |
| ebi-fl187-GCST90094817 | prot-a-385 | Coiled-coil domain-containing protein 90B, mitochondrial \|\| id:prot-a-385 | Prawns liking \|\| id:ebi-fl187-GCST90094817 | Weighted median | 20 | -0.060742622 | 0.132312922843473 | 0.646174518622585 | -0.320075951 | 0.198590706846236 | 0.941065418212896 | 0.726093887640236 | 1.21968265596392 | 0.999332001504091 |
| ebi-fl187-GCST90094817 | prot-a-390 | C-C motif chemokine 14 \|\| id:prot-a-390 | Prawns liking \|\| id:ebi-fl187-GCST90094817 | Weighted median | 20 | -0.060773667 | 0.127085808447917 | 0.632500954880801 | -0.309861852 | 0.188314517443562 | 0.941036203114144 | 0.733548287693993 | 1.20721314523864 | 0.999332001504091 |
| ebi-fl187-GCST90094817 | prot-a-40 | Pituitary adenylate cyclase-activating polypeptide 27 \|\| id:prot-a-40 | Prawns liking \|\| id:ebi-fl187-GCST90094817 | Weighted median | 20 | 0.100701659194197 | 0.127073311115045 | 0.428087263162082 | -0.148362031 | 0.349765348979684 | 1.1059466435271 | 0.862118945006498 | 1.41873460200974 | 0.999332001504091 |
| ebi-fl187-GCST90094817 | prot-a-401 | C-C motif chemokine 24 \|\| id:prot-a-401 | Prawns liking \|\| id:ebi-fl187-GCST90094817 | Weighted median | 20 | -0.072513955 | 0.13625236195532 | 0.594585205097807 | -0.339568585 | 0.194540674234593 | 0.930052767499349 | 0.712077457666169 | 1.21475289102428 | 0.999332001504091 |
| ebi-fl187-GCST90094817 | prot-a-412 | Cerebral cavernous malformations 2 protein \|\| id:prot-a-412 | Prawns liking \|\| id:ebi-fl187-GCST90094817 | Weighted median | 19 | -0.032840589 | 0.138627779678504 | 0.812736228178762 | -0.304551037 | 0.238869859201525 | 0.967692808203122 | 0.737454389712518 | 1.26981327131715 | 0.999332001504091 |
| ebi-fl187-GCST90094817 | prot-a-44 | Adhesion G protein-coupled receptor F5 \|\| id:prot-a-44 | Prawns liking \|\| id:ebi-fl187-GCST90094817 | Weighted median | 20 | 0.18432163052767 | 0.131420431489198 | 0.160755828832879 | -0.073262415 | 0.441905676246499 | 1.2024024902584 | 0.929356920650443 | 1.55566899697452 | 0.999332001504091 |
| ebi-fl187-GCST90094817 | prot-a-48 | Adrenomedullin \|\| id:prot-a-48 | Prawns liking \|\| id:ebi-fl187-GCST90094817 | Weighted median | 20 | 0.126471371158738 | 0.127586859382203 | 0.321560089858391 | -0.123598873 | 0.376541615547855 | 1.13481696221634 | 0.883734259263271 | 1.45723618184443 | 0.999332001504091 |
| ebi-fl187-GCST90094817 | prot-a-502 | Carcinoembryonic antigen-related cell adhesion molecule 1 \|\| id:prot-a-502 | Prawns liking \|\| id:ebi-fl187-GCST90094817 | Weighted median | 20 | 0.0306120402389775 | 0.121163126902068 | 0.800537564667686 | -0.206867688 | 0.26809176896703 | 1.03108540663277 | 0.813127228962684 | 1.30746711941663 | 0.999332001504091 |
| ebi-fl187-GCST90094817 | prot-a-518 | Complement factor B \|\| id:prot-a-518 | Prawns liking \|\| id:ebi-fl187-GCST90094817 | Weighted median | 20 | -0.073855822 | 0.125188271541475 | 0.555218807867624 | -0.319224835 | 0.17151318993847 | 0.928805597258827 | 0.726712140973263 | 1.18709980040235 | 0.999332001504091 |
| ebi-fl187-GCST90094817 | prot-a-525 | Properdin \|\| id:prot-a-525 | Prawns liking \|\| id:ebi-fl187-GCST90094817 | Weighted median | 20 | 0.0351860533186933 | 0.138090137506595 | 0.798873736592093 | -0.235470616 | 0.30584272283162 | 1.03581240720888 | 0.790198881718138 | 1.35776874373073 | 0.999332001504091 |
| ebi-fl187-GCST90094817 | prot-a-533 | Chondroadherin \|\| id:prot-a-533 | Prawns liking \|\| id:ebi-fl187-GCST90094817 | Weighted median | 20 | -0.115222669 | 0.12768922844816 | 0.366861433992366 | -0.365493557 | 0.135048218864518 | 0.891167686489496 | 0.693854110041562 | 1.14459197394603 | 0.999332001504091 |
| ebi-fl187-GCST90094817 | prot-a-535 | Coiled-coil-helix-coiled-coil-helix domain-containing protein 2 \|\| id:prot-a-535 | Prawns liking \|\| id:ebi-fl187-GCST90094817 | Weighted median | 20 | 0.208319454695189 | 0.130846577428959 | 0.111364608626755 | -0.048139837 | 0.464778746455949 | 1.2316065492068 | 0.953000512964172 | 1.5916619890698 | 0.999332001504091 |
| ebi-fl187-GCST90094817 | prot-a-591 | Caseinolytic peptidase B protein homolog \|\| id:prot-a-591 | Prawns liking \|\| id:ebi-fl187-GCST90094817 | Weighted median | 19 | -0.077150178 | 0.132466458192597 | 0.560288572063526 | -0.336784437 | 0.182484079582608 | 0.925750815320913 | 0.714062749155831 | 1.20019504319544 | 0.999332001504091 |
| ebi-fl187-GCST90094817 | prot-a-605 | Ciliary neurotrophic factor receptor subunit alpha \|\| id:prot-a-605 | Prawns liking \|\| id:ebi-fl187-GCST90094817 | Weighted median | 20 | 0.0217045092605868 | 0.131013381854606 | 0.868419525947136 | -0.235081719 | 0.278490737695615 | 1.0219417655228 | 0.79050624747122 | 1.32113436859066 | 0.999332001504091 |
| ebi-fl187-GCST90094817 | prot-a-606 | Ciliary neurotrophic factor receptor subunit alpha \|\| id:prot-a-606 | Prawns liking \|\| id:ebi-fl187-GCST90094817 | Weighted median | 20 | -0.043537319 | 0.125340530546447 | 0.728326681083557 | -0.289204759 | 0.202130121146949 | 0.957396824596342 | 0.748858854416262 | 1.22400726697911 | 0.999332001504091 |
| ebi-fl187-GCST90094817 | prot-a-707 | Cancer/testis antigen 55 \|\| id:prot-a-707 | Prawns liking \|\| id:ebi-fl187-GCST90094817 | Weighted median | 20 | -0.057069345 | 0.130206362940628 | 0.661169401424994 | -0.312273817 | 0.198135125869108 | 0.944528568294788 | 0.731781126783925 | 1.21912711830354 | 0.999332001504091 |
| ebi-fl187-GCST90094817 | prot-a-719 | Dipeptidyl peptidase 1 \|\| id:prot-a-719 | Prawns liking \|\| id:ebi-fl187-GCST90094817 | Weighted median | 20 | -0.120166468 | 0.135657003283333 | 0.375719372170973 | -0.386054194 | 0.145721258401197 | 0.886772805103935 | 0.67973368687556 | 1.1568736742863 | 0.999332001504091 |
| ebi-fl187-GCST90094817 | prot-a-752 | Cytochrome b5 \|\| id:prot-a-752 | Prawns liking \|\| id:ebi-fl187-GCST90094817 | Weighted median | 20 | -0.081817067 | 0.132146405064667 | 0.535824422628126 | -0.340824021 | 0.177189886545802 | 0.921440504804434 | 0.711184050434088 | 1.19385776913306 | 0.999332001504091 |
| ebi-fl187-GCST90094817 | prot-a-755 | Cytoglobin \|\| id:prot-a-755 | Prawns liking \|\| id:ebi-fl187-GCST90094817 | Weighted median | 20 | -0.191823774 | 0.12555021118092 | 0.126546058221766 | -0.437902188 | 0.0542546397565356 | 0.825452321705366 | 0.645388906502943 | 1.05575340471966 | 0.999332001504091 |
| ebi-fl187-GCST90094817 | prot-a-807 | Desmin \|\| id:prot-a-807 | Prawns liking \|\| id:ebi-fl187-GCST90094817 | Weighted median | 20 | 0.0102028375143917 | 0.127164685996956 | 0.936051736515973 | -0.239039947 | 0.259445622068425 | 1.01025506392915 | 0.787383428107424 | 1.29621129650604 | 0.999332001504091 |
| ebi-fl187-GCST90094817 | prot-a-82 | Ameloblastin \|\| id:prot-a-82 | Prawns liking \|\| id:ebi-fl187-GCST90094817 | Weighted median | 20 | -0.083282955 | 0.128768682908098 | 0.517784662161663 | -0.335669573 | 0.169103663543629 | 0.920090766141331 | 0.714859275275274 | 1.18424289537623 | 0.999332001504091 |
| ebi-fl187-GCST90094817 | prot-a-84 | Muellerian-inhibiting factor \|\| id:prot-a-84 | Prawns liking \|\| id:ebi-fl187-GCST90094817 | Weighted median | 20 | -0.063662893 | 0.125920826143821 | 0.613152407413761 | -0.310467712 | 0.183141926263979 | 0.938321260906797 | 0.733103994330275 | 1.20098484727812 | 0.999332001504091 |
| ebi-fl187-GCST90094817 | prot-a-858 | Inactive dipeptidyl peptidase 10 \|\| id:prot-a-858 | Prawns liking \|\| id:ebi-fl187-GCST90094817 | Weighted median | 20 | 0.0407336774793786 | 0.126767395881495 | 0.747963252130532 | -0.207730418 | 0.289197773407109 | 1.04157467381142 | 0.812426022261061 | 1.33535580028079 | 0.999332001504091 |
| ebi-fl187-GCST90094817 | prot-a-863 | Dihydropyrimidinase-related protein 3 \|\| id:prot-a-863 | Prawns liking \|\| id:ebi-fl187-GCST90094817 | Weighted median | 20 | 0.00112077323862867 | 0.122556998888498 | 0.992703515855574 | -0.239090945 | 0.241332491060084 | 1.00112140153966 | 0.787343274510922 | 1.27294420752283 | 0.999332001504091 |
| ebi-fl187-GCST90094817 | prot-a-864 | Dihydropyrimidinase-related protein 5 \|\| id:prot-a-864 | Prawns liking \|\| id:ebi-fl187-GCST90094817 | Weighted median | 20 | -0.13660974 | 0.127604416358506 | 0.284361830533072 | -0.386714396 | 0.113494915756683 | 0.872310587376064 | 0.679285073508533 | 1.12018619357873 | 0.999332001504091 |
| ebi-fl187-GCST90094817 | prot-a-884 | Endothelin-converting enzyme 1 \|\| id:prot-a-884 | Prawns liking \|\| id:ebi-fl187-GCST90094817 | Weighted median | 20 | -0.178805382 | 0.128228076961798 | 0.163187076718715 | -0.430132413 | 0.0725216486868898 | 0.836268636357908 | 0.650422964562875 | 1.07521608285452 | 0.999332001504091 |
| ebi-fl187-GCST90094817 | prot-a-893 | Early endosome antigen 1 \|\| id:prot-a-893 | Prawns liking \|\| id:ebi-fl187-GCST90094817 | Weighted median | 19 | -0.204238776 | 0.131494764151629 | 0.120373347991663 | -0.461968514 | 0.0534909615372033 | 0.815267681425632 | 0.63004217717718 | 1.05494745662116 | 0.999332001504091 |
| ebi-fl187-GCST90094817 | prot-a-897 | Ephrin-A2 \|\| id:prot-a-897 | Prawns liking \|\| id:ebi-fl187-GCST90094817 | Weighted median | 20 | -0.016775798 | 0.128198081746006 | 0.895887311715301 | -0.268044038 | 0.234492442433308 | 0.983364132334377 | 0.764874096827259 | 1.26426691761812 | 0.999332001504091 |
| ebi-fl187-GCST90094817 | prot-a-90 | Anaphase-promoting complex subunit 10 \|\| id:prot-a-90 | Prawns liking \|\| id:ebi-fl187-GCST90094817 | Weighted median | 20 | -0.004054426 | 0.124557002237942 | 0.974032829191139 | -0.248186151 | 0.240077298107426 | 0.995953781810508 | 0.78021469227512 | 1.27134741927274 | 0.999332001504091 |
| ebi-fl187-GCST90094817 | prot-a-914 | Histone-lysine N-methyltransferase EHMT2 \|\| id:prot-a-914 | Prawns liking \|\| id:ebi-fl187-GCST90094817 | Weighted median | 20 | -0.158661254 | 0.129730605546367 | 0.221327591187763 | -0.412933241 | 0.0956107332322068 | 0.853285357327766 | 0.661706456539189 | 1.10033065845845 | 0.999332001504091 |
| ebi-fl187-GCST90094817 | prot-a-932 | RNA polymerase II elongation factor ELL \|\| id:prot-a-932 | Prawns liking \|\| id:ebi-fl187-GCST90094817 | Weighted median | 20 | -0.308890392 | 0.136127130066678 | 0.0232603154913127 | -0.575699567 | -0.042081217 | 0.734261246501263 | 0.562311356732828 | 0.958791907113748 | 0.999332001504091 |
| ebi-fl187-GCST90094817 | prot-a-991 | Estrogen receptor \|\| id:prot-a-991 | Prawns liking \|\| id:ebi-fl187-GCST90094817 | Weighted median | 20 | 0.131021348527256 | 0.128053293954536 | 0.306223562400056 | -0.119963108 | 0.382005804678148 | 1.13999211820502 | 0.886953157923293 | 1.46522059024222 | 0.999332001504091 |
| ebi-fl187-GCST90094817 | prot-a-992 | Persulfide dioxygenase ETHE1, mitochondrial \|\| id:prot-a-992 | Prawns liking \|\| id:ebi-fl187-GCST90094817 | Weighted median | 20 | 0.0692281032498891 | 0.137513980120523 | 0.614664845160844 | -0.200299298 | 0.338755504286115 | 1.07168063510582 | 0.818485745442931 | 1.40320022682777 | 0.999332001504091 |
| ebi-fl187-GCST90094817 | prot-a-993 | Ethanolamine kinase 1 \|\| id:prot-a-993 | Prawns liking \|\| id:ebi-fl187-GCST90094817 | Weighted median | 20 | 0.0365040737286909 | 0.133516874544445 | 0.784542527727777 | -0.225189 | 0.298197147835803 | 1.03717852919308 | 0.798365313152913 | 1.34742740409248 | 0.999332001504091 |
| ebi-fl187-GCST90094817 | prot-b-10 | coagulation factor III, tissue factor \|\| id:prot-b-10 | Prawns liking \|\| id:ebi-fl187-GCST90094817 | Weighted median | 12 | -0.415957951 | 0.157571050591056 | 0.00829520273989499 | -0.724797211 | -0.107118692 | 0.659708009735075 | 0.484422794849662 | 0.89841903134158 | 0.999332001504091 |
| ebi-fl187-GCST90094817 | prot-b-28 | prolactin \|\| id:prot-b-28 | Prawns liking \|\| id:ebi-fl187-GCST90094817 | Weighted median | 12 | -0.105453799 | 0.186863676477946 | 0.572525854049982 | -0.471706604 | 0.260799007335451 | 0.899916049302495 | 0.623936546266959 | 1.2979667574172 | 0.999332001504091 |
| ebi-fl187-GCST90094817 | prot-b-54 | matrix metallopeptidase 7 \|\| id:prot-b-54 | Prawns liking \|\| id:ebi-fl187-GCST90094817 | Weighted median | 12 | 0.048317200713763 | 0.176288620737255 | 0.784023075480736 | -0.297208496 | 0.393842897358782 | 1.04950350579261 | 0.742889106857763 | 1.48266759938085 | 0.999332001504091 |
| ebi-fl187-GCST90094817 | ukb-rsfMRI191-edge-pheno716 | Net100_Pair10_33 (OccipitalPrecuneus)&(Temporal\|Frontal\|Supp_Motor_Area) (Default_mode\|Central_executive)&(Default_mode\|Salience) \|\| id:ukb-rsfMRI191-edge-pheno716 | Prawns liking \|\| id:ebi-fl187-GCST90094817 | Weighted median | 19 | -0.024436536 | 0.0384416675997645 | 0.52498608048092 | -0.099782205 | 0.0509091320817541 | 0.975859618504283 | 0.905034508645373 | 1.05222727523694 | 0.999332001504091 |
| ebi-fl187-GCST90094817 | ukb-rsfMRI191-edge-pheno903 | Net100_Pair27_38 (Precentral\|Frontal\|Supp_Motor_Area)&(Subcortical) (Attention\|Salience\|Motor)&(Subcortical-cerebellum) \|\| id:ukb-rsfMRI191-edge-pheno903 | Prawns liking \|\| id:ebi-fl187-GCST90094817 | Weighted median | 19 | 0.0829948135190375 | 0.0389403239481103 | 0.0330616843093958 | 0.00667177858074138 | 0.159317848457334 | 1.08653617323445 | 1.00669408447453 | 1.17271063171409 | 0.999332001504091 |
| ebi-fl187-GCST90094817 | ukb-rsfMRI191-node-pheno23 | Net100_Node2 RolandicOper\|SupraMarginal\|Insula Salience\|Motor \|\| id:ukb-rsfMRI191-node-pheno23 | Prawns liking \|\| id:ebi-fl187-GCST90094817 | Weighted median | 19 | 0.0531523929805577 | 0.0394675730164053 | 0.178065342316259 | -0.02420405 | 0.130508836092712 | 1.05459034504022 | 0.976086518853513 | 1.13940800776387 | 0.999332001504091 |
| ebi-fl187-GCST90094817 | ukb-rsfMRI191-node-pheno55 | Net100_Node34 Parietal\|Postcentral\|Precuneus Attention \|\| id:ukb-rsfMRI191-node-pheno55 | Prawns liking \|\| id:ebi-fl187-GCST90094817 | Weighted median | 18 | -0.02486489 | 0.0413044596909679 | 0.547180499796566 | -0.105821631 | 0.0560918510553825 | 0.975441695112534 | 0.899585091919732 | 1.05769482966144 | 0.999332001504091 |
| ebi-fl187-GCST90094817 | ebi-a-GCST90001414 | IgD- CD24- B cell Absolute Count \|\| id:ebi-a-GCST90001414 | Prawns liking \|\| id:ebi-fl187-GCST90094817 | Weighted mode | 16 | -0.207650358 | 0.2445873860253 | 0.409236345588796 | -0.687041634 | 0.271740918945397 | 0.812491068329261 | 0.503062111562657 | 1.31224697893513 | 0.999714303421225 |
| ebi-fl187-GCST90094817 | ebi-a-GCST90001422 | CD20- CD38- B cell %B cell \|\| id:ebi-a-GCST90001422 | Prawns liking \|\| id:ebi-fl187-GCST90094817 | Weighted mode | 16 | -0.239251067 | 0.233446615835771 | 0.321672086696554 | -0.696806434 | 0.21830429964731 | 0.787217212987899 | 0.498173716520808 | 1.24396554830799 | 0.999714303421225 |
| ebi-fl187-GCST90094817 | ebi-a-GCST90001444 | CD20- CD38- B cell %lymphocyte \|\| id:ebi-a-GCST90001444 | Prawns liking \|\| id:ebi-fl187-GCST90094817 | Weighted mode | 16 | -0.215796414 | 0.258676149059853 | 0.417237382780404 | -0.722801667 | 0.291208837717674 | 0.805899354645236 | 0.485390446981596 | 1.33804398882666 | 0.999714303421225 |
| ebi-fl187-GCST90094817 | ebi-a-GCST90001459 | Myeloid Dendritic Cell %Dendritic Cell \|\| id:ebi-a-GCST90001459 | Prawns liking \|\| id:ebi-fl187-GCST90094817 | Weighted mode | 16 | 0.240788585911822 | 0.242107218398483 | 0.335727395062291 | -0.233741562 | 0.715318733972847 | 1.2722520348701 | 0.791566360172339 | 2.04483833784791 | 0.999714303421225 |
| ebi-fl187-GCST90094817 | ebi-a-GCST90001476 | HLA DR++ monocyte %leukocyte \|\| id:ebi-a-GCST90001476 | Prawns liking \|\| id:ebi-fl187-GCST90094817 | Weighted mode | 16 | -0.053597135 | 0.221164061053751 | 0.811798116379923 | -0.487078695 | 0.379884424806227 | 0.947813870765447 | 0.614418679656261 | 1.4621155953754 | 0.999714303421225 |
| ebi-fl187-GCST90094817 | ebi-a-GCST90001478 | CD4 regulatory T cell %CD4+ T cell \|\| id:ebi-a-GCST90001478 | Prawns liking \|\| id:ebi-fl187-GCST90094817 | Weighted mode | 16 | -0.046534739 | 0.218176808066491 | 0.833973275504027 | -0.474161283 | 0.381091804567164 | 0.954531400295575 | 0.622406860793142 | 1.46388199029356 | 0.999714303421225 |
| ebi-fl187-GCST90094817 | ebi-a-GCST90001485 | CD39+ resting CD4 regulatory T cell %CD4 regulatory T cell \|\| id:ebi-a-GCST90001485 | Prawns liking \|\| id:ebi-fl187-GCST90094817 | Weighted mode | 16 | 0.143568352182893 | 0.251577597232424 | 0.57667168817264 | -0.349523738 | 0.636660442758444 | 1.15438571289389 | 0.705023785534166 | 1.89015803647514 | 0.999714303421225 |
| ebi-fl187-GCST90094817 | ebi-a-GCST90001515 | Immature Myeloid-Derived Suppressor Cells Absolute Count \|\| id:ebi-a-GCST90001515 | Prawns liking \|\| id:ebi-fl187-GCST90094817 | Weighted mode | 16 | -0.129486445 | 0.322549716850087 | 0.693752570869509 | -0.76168389 | 0.502711000350346 | 0.878546497318105 | 0.466879590987957 | 1.65319701877871 | 0.999714303421225 |
| ebi-fl187-GCST90094817 | ebi-a-GCST90001517 | CD33+ HLA DR+ Absolute Count \|\| id:ebi-a-GCST90001517 | Prawns liking \|\| id:ebi-fl187-GCST90094817 | Weighted mode | 16 | 0.0259780144314838 | 0.337751689148985 | 0.939708085638202 | -0.636015296 | 0.687971325163494 | 1.02631838403225 | 0.52939771982014 | 1.98967503252644 | 0.999714303421225 |
| ebi-fl187-GCST90094817 | ebi-a-GCST90001518 | CD33+ HLA DR+ CD14- Absolute Count \|\| id:ebi-a-GCST90001518 | Prawns liking \|\| id:ebi-fl187-GCST90094817 | Weighted mode | 16 | -0.176447061 | 0.34563720234647 | 0.617132692252698 | -0.853895977 | 0.501001855700335 | 0.838243153819792 | 0.425752972571976 | 1.65037387920284 | 0.999714303421225 |
| ebi-fl187-GCST90094817 | ebi-a-GCST90001520 | CD33+ HLA DR+ CD14dim Absolute Count \|\| id:ebi-a-GCST90001520 | Prawns liking \|\| id:ebi-fl187-GCST90094817 | Weighted mode | 16 | 0.122886497743254 | 0.313459549261864 | 0.700547512836372 | -0.491494219 | 0.737267214296507 | 1.13075607029778 | 0.611711679864585 | 2.09021559110709 | 0.999714303421225 |
| ebi-fl187-GCST90094817 | ebi-a-GCST90001531 | CD33dim HLA DR- Absolute Count \|\| id:ebi-a-GCST90001531 | Prawns liking \|\| id:ebi-fl187-GCST90094817 | Weighted mode | 16 | -0.271252251 | 0.324843823169989 | 0.416806422657795 | -0.907946145 | 0.365441642218508 | 0.762424149743629 | 0.40335180015156 | 1.44115034045683 | 0.999714303421225 |
| ebi-fl187-GCST90094817 | ebi-a-GCST90001532 | Basophil Absolute Count \|\| id:ebi-a-GCST90001532 | Prawns liking \|\| id:ebi-fl187-GCST90094817 | Weighted mode | 16 | -0.267641878 | 0.353719355536777 | 0.460979356105626 | -0.960931814 | 0.425648059330504 | 0.765181760829418 | 0.382536267057508 | 1.53058200627547 | 0.999714303421225 |
| ebi-fl187-GCST90094817 | ebi-a-GCST90001588 | T/B cell \|\| id:ebi-a-GCST90001588 | Prawns liking \|\| id:ebi-fl187-GCST90094817 | Weighted mode | 16 | -0.059046571 | 0.232076519063073 | 0.802620499110116 | -0.513916548 | 0.395823406730098 | 0.942662867727898 | 0.598148308654355 | 1.48560694619715 | 0.999714303421225 |
| ebi-fl187-GCST90094817 | ebi-a-GCST90001624 | HLA DR+ CD4+ T cell Absolute Count \|\| id:ebi-a-GCST90001624 | Prawns liking \|\| id:ebi-fl187-GCST90094817 | Weighted mode | 16 | 0.378351308007516 | 0.240806836394475 | 0.136991746014229 | -0.093630091 | 0.850332707340687 | 1.45987571882938 | 0.910619546016406 | 2.34042539911524 | 0.999714303421225 |
| ebi-fl187-GCST90094817 | ebi-a-GCST90001631 | CD8+ Natural Killer T %T cell \|\| id:ebi-a-GCST90001631 | Prawns liking \|\| id:ebi-fl187-GCST90094817 | Weighted mode | 16 | -0.092533743 | 0.219452434609867 | 0.679255649145965 | -0.522660514 | 0.337593029221817 | 0.911618450056447 | 0.592940919705846 | 1.40156998929269 | 0.999714303421225 |
| ebi-fl187-GCST90094817 | ebi-a-GCST90001632 | CD8+ Natural Killer T %lymphocyte \|\| id:ebi-a-GCST90001632 | Prawns liking \|\| id:ebi-fl187-GCST90094817 | Weighted mode | 16 | -0.084709629 | 0.217070818794626 | 0.701845099048777 | -0.510168434 | 0.340749175425329 | 0.918779032079403 | 0.600394443307638 | 1.40600053714392 | 0.999714303421225 |
| ebi-fl187-GCST90094817 | ebi-a-GCST90001646 | Natural Killer %CD3- lymphocyte \|\| id:ebi-a-GCST90001646 | Prawns liking \|\| id:ebi-fl187-GCST90094817 | Weighted mode | 16 | -0.236692604 | 0.232093617747293 | 0.323983513655161 | -0.691596095 | 0.218210886863683 | 0.789233858126542 | 0.500776144705047 | 1.2438493514506 | 0.999714303421225 |
| ebi-fl187-GCST90094817 | ebi-a-GCST90001684 | CD127- CD8+ T cell Absolute Count \|\| id:ebi-a-GCST90001684 | Prawns liking \|\| id:ebi-fl187-GCST90094817 | Weighted mode | 16 | 0.318527577800412 | 0.277964446464422 | 0.269777715261146 | -0.226282737 | 0.863337892870679 | 1.37510154293684 | 0.797492588909427 | 2.37106185020865 | 0.999714303421225 |
| ebi-fl187-GCST90094817 | ebi-a-GCST90001687 | CD28- CD8+ T cell Absolute Count \|\| id:ebi-a-GCST90001687 | Prawns liking \|\| id:ebi-fl187-GCST90094817 | Weighted mode | 16 | 0.349430252216768 | 0.306903907508427 | 0.272742653047887 | -0.252101406 | 0.950961910933285 | 1.41825926828301 | 0.777165924396786 | 2.5881980783343 | 0.999714303421225 |
| ebi-fl187-GCST90094817 | ebi-a-GCST90001701 | BAFF-R on CD20- CD38- B cell \|\| id:ebi-a-GCST90001701 | Prawns liking \|\| id:ebi-fl187-GCST90094817 | Weighted mode | 16 | -0.35711237 | 0.270191269147838 | 0.206071727711668 | -0.886687257 | 0.172462517755467 | 0.69969386887482 | 0.412018405410468 | 1.18822728235474 | 0.999714303421225 |
| ebi-fl187-GCST90094817 | ebi-a-GCST90001732 | CD19 on IgD- CD27- B cell \|\| id:ebi-a-GCST90001732 | Prawns liking \|\| id:ebi-fl187-GCST90094817 | Weighted mode | 16 | 0.174691075018922 | 0.299118244577163 | 0.567886400757659 | -0.411580684 | 0.760962834390161 | 1.19087826773466 | 0.662602057219836 | 2.14033601784031 | 0.999714303421225 |
| ebi-fl187-GCST90094817 | ebi-a-GCST90001798 | CD27 on CD24+ CD27+ B cell \|\| id:ebi-a-GCST90001798 | Prawns liking \|\| id:ebi-fl187-GCST90094817 | Weighted mode | 16 | -0.193067645 | 0.333362227887546 | 0.571081825015608 | -0.846457612 | 0.460322321222155 | 0.824426203581644 | 0.428931686313286 | 1.58458464795164 | 0.999714303421225 |
| ebi-fl187-GCST90094817 | ebi-a-GCST90001836 | CD66b on CD66b++ myeloid cell \|\| id:ebi-a-GCST90001836 | Prawns liking \|\| id:ebi-fl187-GCST90094817 | Weighted mode | 16 | 0.244426415665958 | 0.344828368535077 | 0.489297902779292 | -0.431437187 | 0.92029001799471 | 1.27688869976268 | 0.649574863221687 | 2.51001823484229 | 0.999714303421225 |
| ebi-fl187-GCST90094817 | ebi-a-GCST90001943 | CD25 on activated & secreting CD4 regulatory T cell \|\| id:ebi-a-GCST90001943 | Prawns liking \|\| id:ebi-fl187-GCST90094817 | Weighted mode | 16 | 0.209706451567641 | 0.228494011488606 | 0.373257684309832 | -0.238141811 | 0.657554714085309 | 1.23331596884445 | 0.788090923246472 | 1.93006699371794 | 0.999714303421225 |
| ebi-fl187-GCST90094817 | ebi-a-GCST90001960 | CD25 on CD4+ T cell \|\| id:ebi-a-GCST90001960 | Prawns liking \|\| id:ebi-fl187-GCST90094817 | Weighted mode | 16 | -0.187478314 | 0.273689963356086 | 0.503790827718946 | -0.723910642 | 0.348954014087897 | 0.829047096626158 | 0.484852459147799 | 1.41758399994986 | 0.999714303421225 |
| ebi-fl187-GCST90094817 | ebi-a-GCST90001988 | HLA DR on CD14+ CD16- monocyte \|\| id:ebi-a-GCST90001988 | Prawns liking \|\| id:ebi-fl187-GCST90094817 | Weighted mode | 16 | 0.0100564897182254 | 0.302010650860328 | 0.973875636999817 | -0.581884386 | 0.601997365404469 | 1.01010722614511 | 0.558844295390305 | 1.82576187450919 | 0.999714303421225 |
| ebi-fl187-GCST90094817 | ebi-a-GCST90001991 | HLA DR on CD14+ monocyte \|\| id:ebi-a-GCST90001991 | Prawns liking \|\| id:ebi-fl187-GCST90094817 | Weighted mode | 16 | -0.019882881 | 0.295926882106516 | 0.947319043494808 | -0.59989957 | 0.560133807969052 | 0.980313479957549 | 0.548866756075644 | 1.75090676990106 | 0.999714303421225 |
| ebi-fl187-GCST90094817 | ebi-a-GCST90002019 | CD14 on Monocytic Myeloid-Derived Suppressor Cells \|\| id:ebi-a-GCST90002019 | Prawns liking \|\| id:ebi-fl187-GCST90094817 | Weighted mode | 16 | 0.0531360699587882 | 0.347108852930214 | 0.880374034856958 | -0.627197282 | 0.733469421702007 | 1.05457313107956 | 0.534086599520054 | 2.0822924405786 | 0.999714303421225 |
| ebi-fl187-GCST90094817 | ebi-a-GCST90002037 | CD80 on plasmacytoid Dendritic Cell \|\| id:ebi-a-GCST90002037 | Prawns liking \|\| id:ebi-fl187-GCST90094817 | Weighted mode | 16 | 0.0484663628409603 | 0.220936863454857 | 0.829320948103962 | -0.38456989 | 0.48150261521248 | 1.049660064 | 0.68074336809617 | 1.61850456551707 | 0.999714303421225 |
| ebi-fl187-GCST90094817 | ebi-a-GCST90002042 | CD45 on CD33+ HLA DR+ CD14- \|\| id:ebi-a-GCST90002042 | Prawns liking \|\| id:ebi-fl187-GCST90094817 | Weighted mode | 16 | 0.0958168380279731 | 0.351594730816124 | 0.788937456984044 | -0.593308834 | 0.784942510427575 | 1.10055746525627 | 0.55249613871177 | 2.19228090381855 | 0.999714303421225 |
| ebi-fl187-GCST90094817 | ebi-a-GCST90002056 | CD8 on Effector Memory CD8+ T cell \|\| id:ebi-a-GCST90002056 | Prawns liking \|\| id:ebi-fl187-GCST90094817 | Weighted mode | 16 | 0.0821027971314212 | 0.26184095586457 | 0.758173467096303 | -0.431105476 | 0.595311070625978 | 1.08556739730842 | 0.649790369635088 | 1.81359501335913 | 0.999714303421225 |
| ebi-fl187-GCST90094817 | ebi-a-GCST90002058 | CD8 on CD8+ T cell \|\| id:ebi-a-GCST90002058 | Prawns liking \|\| id:ebi-fl187-GCST90094817 | Weighted mode | 16 | -0.469013272 | 0.339234839091275 | 0.187038306577423 | -1.133913557 | 0.195887012578143 | 0.62561927985663 | 0.32177151802304 | 1.21638946086056 | 0.999714303421225 |
| ebi-fl187-GCST90094817 | ebi-a-GCST90002063 | CD4 on CD4 regulatory T cell \|\| id:ebi-a-GCST90002063 | Prawns liking \|\| id:ebi-fl187-GCST90094817 | Weighted mode | 16 | -0.034837014 | 0.291060581870395 | 0.906317154734587 | -0.605315755 | 0.535641726288523 | 0.965762809076643 | 0.545902028323058 | 1.70854430832715 | 0.999714303421225 |
| ebi-fl187-GCST90094817 | ebi-a-GCST90002066 | CD4 on activated CD4 regulatory T cell \|\| id:ebi-a-GCST90002066 | Prawns liking \|\| id:ebi-fl187-GCST90094817 | Weighted mode | 16 | 0.127514384722002 | 0.289696428566363 | 0.666093709094407 | -0.440290615 | 0.695319384712074 | 1.13600120918522 | 0.643849281460115 | 2.00434913019349 | 0.999714303421225 |
| ebi-fl187-GCST90094817 | ebi-a-GCST90002070 | CD4 on activated & secreting CD4 regulatory T cell \|\| id:ebi-a-GCST90002070 | Prawns liking \|\| id:ebi-fl187-GCST90094817 | Weighted mode | 16 | 0.0334763322936347 | 0.280595537874189 | 0.906617232262397 | -0.516490922 | 0.583443586527045 | 1.03404297001128 | 0.596610431619356 | 1.79219941047215 | 0.999714303421225 |
| ebi-fl187-GCST90094817 | ebi-a-GCST90002081 | SSC-A on CD4+ T cell \|\| id:ebi-a-GCST90002081 | Prawns liking \|\| id:ebi-fl187-GCST90094817 | Weighted mode | 16 | 0.0395700405831029 | 0.212052285198921 | 0.854470611006585 | -0.376052438 | 0.455192519572987 | 1.04036336398982 | 0.686566329653852 | 1.576476856 | 0.999714303421225 |
| ebi-fl187-GCST90094817 | ebi-a-GCST90002094 | CD11b on Monocytic Myeloid-Derived Suppressor Cells \|\| id:ebi-a-GCST90002094 | Prawns liking \|\| id:ebi-fl187-GCST90094817 | Weighted mode | 16 | -0.277549512 | 0.344737896405121 | 0.433337320749236 | -0.953235789 | 0.398136764518764 | 0.757638051169322 | 0.385491633426022 | 1.48904766486924 | 0.999714303421225 |
| ebi-fl187-GCST90094817 | ebi-a-GCST90002104 | HLA DR on myeloid Dendritic Cell \|\| id:ebi-a-GCST90002104 | Prawns liking \|\| id:ebi-fl187-GCST90094817 | Weighted mode | 16 | 0.0195692752656047 | 0.224304360948752 | 0.931631364453631 | -0.420067272 | 0.459205822725158 | 1.01976200869777 | 0.657002620320676 | 1.58281644885334 | 0.999714303421225 |
| ebi-fl187-GCST90094817 | ebi-a-GCST90002105 | HLA DR on plasmacytoid Dendritic Cell \|\| id:ebi-a-GCST90002105 | Prawns liking \|\| id:ebi-fl187-GCST90094817 | Weighted mode | 15 | 0.225186559914559 | 0.242850691359904 | 0.369500374400966 | -0.250800795 | 0.70117391497997 | 1.25255637120545 | 0.778177372826107 | 2.01611807003537 | 0.999714303421225 |
| ebi-fl187-GCST90094817 | ebi-a-GCST90002106 | HLA DR on Dendritic Cell \|\| id:ebi-a-GCST90002106 | Prawns liking \|\| id:ebi-fl187-GCST90094817 | Weighted mode | 16 | 0.192168179079729 | 0.242090419358543 | 0.439697074 | -0.282329043 | 0.666665401022472 | 1.21187431173391 | 0.754025536988261 | 1.94773157591795 | 0.999714303421225 |
| ebi-fl187-GCST90094817 | ebi-a-GCST90002111 | HLA DR on CD33dim HLA DR+ CD11b- \|\| id:ebi-a-GCST90002111 | Prawns liking \|\| id:ebi-fl187-GCST90094817 | Weighted mode | 16 | 0.154699437462292 | 0.308813659100334 | 0.623678845064283 | -0.450575334 | 0.759974209298946 | 1.16730705960173 | 0.637261407738382 | 2.13822107356519 | 0.999714303421225 |
| ebi-fl187-GCST90094817 | ebi-a-GCST90002112 | HLA DR on CD33- HLA DR+ \|\| id:ebi-a-GCST90002112 | Prawns liking \|\| id:ebi-fl187-GCST90094817 | Weighted mode | 16 | 0.00599838321387569 | 0.333876990802392 | 0.985902894468477 | -0.648400519 | 0.660397285186564 | 1.00601640953938 | 0.522881447329525 | 1.93556115144527 | 0.999714303421225 |
| ebi-fl187-GCST90094817 | ebi-a-GCST90002115 | HLA DR on HLA DR+ CD8+ T cell \|\| id:ebi-a-GCST90002115 | Prawns liking \|\| id:ebi-fl187-GCST90094817 | Weighted mode | 16 | -0.091145652 | 0.267556343061846 | 0.738084112959906 | -0.615556085 | 0.433264780288655 | 0.91288473762502 | 0.540340336855615 | 1.54228453318558 | 0.999714303421225 |
| ebi-fl187-GCST90094817 | ebi-a-GCST90002116 | HLA DR on B cell \|\| id:ebi-a-GCST90002116 | Prawns liking \|\| id:ebi-fl187-GCST90094817 | Weighted mode | 16 | 0.00815052543911099 | 0.254454562658638 | 0.974869373844202 | -0.490580417 | 0.506881468250042 | 1.0081838313971 | 0.612270918354949 | 1.66010602074896 | 0.999714303421225 |
| ebi-fl187-GCST90094817 | ebi-cfmet338-GCST90026023 | Sphingomyelin (d18:1/24:1, d18:2/24:0) levels \|\| id:ebi-cfmet338-GCST90026023 | Prawns liking \|\| id:ebi-fl187-GCST90094817 | Weighted mode | 13 | 0.0420454468130468 | 0.17561447956346 | 0.814821143517809 | -0.302158933 | 0.386249826757429 | 1.04294187605819 | 0.739220568913013 | 1.47145223303948 | 0.999714303421225 |
| ebi-fl187-GCST90094817 | ebi-cfmet338-GCST90026046 | 1-arachidonoyl-gpc (20:4n6) levels \|\| id:ebi-cfmet338-GCST90026046 | Prawns liking \|\| id:ebi-fl187-GCST90094817 | Weighted mode | 13 | 0.295428722284761 | 0.225957909319047 | 0.215556066880508 | -0.14744878 | 0.738306224550093 | 1.34370231034033 | 0.862906635284106 | 2.09238847516739 | 0.999714303421225 |
| ebi-fl187-GCST90094817 | ebi-cfmet338-GCST90026047 | 1-linoleoyl-gpc (18:2) levels \|\| id:ebi-cfmet338-GCST90026047 | Prawns liking \|\| id:ebi-fl187-GCST90094817 | Weighted mode | 13 | 0.0590172836920448 | 0.208035253302089 | 0.78148401006046 | -0.348731813 | 0.466766380164139 | 1.06079357501119 | 0.705582333062285 | 1.59482877625521 | 0.999714303421225 |
| ebi-fl187-GCST90094817 | ebi-cfmet338-GCST90026050 | 1-myristoyl-2-palmitoyl-gpc (14:0/16:0) levels \|\| id:ebi-cfmet338-GCST90026050 | Prawns liking \|\| id:ebi-fl187-GCST90094817 | Weighted mode | 13 | -0.135827168 | 0.153634925560672 | 0.394015972893449 | -0.436951622 | 0.165297286102637 | 0.872993500666873 | 0.646002682910972 | 1.1797437880171 | 0.999714303421225 |
| ebi-fl187-GCST90094817 | ebi-cfmet338-GCST90026068 | 2,3-dihydroxyisovalerate levels \|\| id:ebi-cfmet338-GCST90026068 | Prawns liking \|\| id:ebi-fl187-GCST90094817 | Weighted mode | 13 | -0.293283016 | 0.331620661450949 | 0.39385906610857 | -0.943259513 | 0.356693480027188 | 0.745811034058935 | 0.389356651750613 | 1.42859790894321 | 0.999714303421225 |
| ebi-fl187-GCST90094817 | ebi-cfmet338-GCST90026082 | 3-hydroxyhexanoate levels \|\| id:ebi-cfmet338-GCST90026082 | Prawns liking \|\| id:ebi-fl187-GCST90094817 | Weighted mode | 13 | 0.102799945970904 | 0.0852206283301518 | 0.250953919574481 | -0.064232486 | 0.269832377498002 | 1.10826967308373 | 0.937786952264104 | 1.30974488961669 | 0.999714303421225 |
| ebi-fl187-GCST90094817 | ebi-cfmet338-GCST90026094 | 3-ureidopropionate levels \|\| id:ebi-cfmet338-GCST90026094 | Prawns liking \|\| id:ebi-fl187-GCST90094817 | Weighted mode | 13 | 0.142415577456904 | 0.223653366366644 | 0.536233657038364 | -0.295945021 | 0.580776175535527 | 1.15305573295094 | 0.743828322114701 | 1.78742524822281 | 0.999714303421225 |
| ebi-fl187-GCST90094817 | ebi-cfmet338-GCST90026173 | Glycerol levels \|\| id:ebi-cfmet338-GCST90026173 | Prawns liking \|\| id:ebi-fl187-GCST90094817 | Weighted mode | 13 | 0.0193507221860231 | 0.144474532148197 | 0.89567118864528 | -0.263819361 | 0.30252080519649 | 1.01953916092325 | 0.768112278294198 | 1.35326583108981 | 0.999714303421225 |
| ebi-fl187-GCST90094817 | ebi-cfmet338-GCST90026179 | Histidine levels \|\| id:ebi-cfmet338-GCST90026179 | Prawns liking \|\| id:ebi-fl187-GCST90094817 | Weighted mode | 13 | 0.0230273943318756 | 0.0864012894963864 | 0.794365687860376 | -0.146319133 | 0.192373921744793 | 1.02329457163436 | 0.863881965875108 | 1.21212367163563 | 0.999714303421225 |
| ebi-fl187-GCST90094817 | ebi-cfmet338-GCST90026188 | Isocitrate levels \|\| id:ebi-cfmet338-GCST90026188 | Prawns liking \|\| id:ebi-fl187-GCST90094817 | Weighted mode | 13 | 0.0994159375783555 | 0.147170723829008 | 0.512156590830549 | -0.189038681 | 0.387870556283212 | 1.10452561773909 | 0.827754487599424 | 1.47383899274286 | 0.999714303421225 |
| ebi-fl187-GCST90094817 | ebi-cfmet338-GCST90026207 | N-acetyl-isoputreanine levels \|\| id:ebi-cfmet338-GCST90026207 | Prawns liking \|\| id:ebi-fl187-GCST90094817 | Weighted mode | 13 | -0.031535296 | 0.0921433164705422 | 0.738088476224857 | -0.212136196 | 0.149065604324845 | 0.968956755593782 | 0.808854527119228 | 1.16074913687469 | 0.999714303421225 |
| ebi-fl187-GCST90094817 | ebi-cfmet338-GCST90026213 | N-acetylglutamate levels \|\| id:ebi-cfmet338-GCST90026213 | Prawns liking \|\| id:ebi-fl187-GCST90094817 | Weighted mode | 13 | 0.094451765892865 | 0.167960310008739 | 0.584232677260151 | -0.234750442 | 0.423653973509994 | 1.0990561498624 | 0.790768167746977 | 1.52753293546441 | 0.999714303421225 |
| ebi-fl187-GCST90094817 | ebi-cfmet338-GCST90026224 | N-acetyltaurine levels \|\| id:ebi-cfmet338-GCST90026224 | Prawns liking \|\| id:ebi-fl187-GCST90094817 | Weighted mode | 13 | 0.0371833139532329 | 0.114002340857053 | 0.74991907798897 | -0.186261274 | 0.260627902033058 | 1.03788326188444 | 0.830056694310072 | 1.29774468742189 | 0.999714303421225 |
| ebi-fl187-GCST90094817 | ebi-cfmet338-GCST90026250 | Phenyllactate (pla) levels \|\| id:ebi-cfmet338-GCST90026250 | Prawns liking \|\| id:ebi-fl187-GCST90094817 | Weighted mode | 13 | -0.210818299 | 0.239350341684727 | 0.395727902898014 | -0.679944969 | 0.258308370626681 | 0.809921216946205 | 0.506644872885187 | 1.29473801624412 | 0.999714303421225 |
| ebi-fl187-GCST90094817 | ebi-cfmet338-GCST90026251 | Pipecolate levels \|\| id:ebi-cfmet338-GCST90026251 | Prawns liking \|\| id:ebi-fl187-GCST90094817 | Weighted mode | 13 | -0.211765225 | 0.28861163112974 | 0.477200105884389 | -0.777444022 | 0.353913571897853 | 0.809154644455685 | 0.459579185590319 | 1.42463205291427 | 0.999714303421225 |
| ebi-fl187-GCST90094817 | ebi-cfmet338-GCST90026278 | Trigonelline (n'-methylnicotinate) levels \|\| id:ebi-cfmet338-GCST90026278 | Prawns liking \|\| id:ebi-fl187-GCST90094817 | Weighted mode | 12 | 0.173044964559016 | 0.318246909030348 | 0.597465038887678 | -0.450718977 | 0.796808906258498 | 1.18891956312603 | 0.637169876321114 | 2.21845033815033 | 0.999714303421225 |
| ebi-fl187-GCST90094817 | ebi-cfmet338-GCST90026299 | 1-carboxyethylvaline levels \|\| id:ebi-cfmet338-GCST90026299 | Prawns liking \|\| id:ebi-fl187-GCST90094817 | Weighted mode | 13 | 0.0049994260757561 | 0.213218129198673 | 0.981678730620566 | -0.412908107 | 0.422906959305155 | 1.00501194405852 | 0.661723087652066 | 1.52639227276193 | 0.999714303421225 |
| ebi-fl187-GCST90094817 | ebi-gm430-GCST90011306 | C_Erysipelotrichia abundance \|\| id:ebi-gm430-GCST90011306 | Prawns liking \|\| id:ebi-fl187-GCST90094817 | Weighted mode | 13 | 0.0731093963052081 | 0.211230937989464 | 0.735251758425232 | -0.340903242 | 0.487122034764558 | 1.07584822429801 | 0.711127712063249 | 1.6276252241204 | 0.999714303421225 |
| ebi-fl187-GCST90094817 | ebi-gm430-GCST90011312 | F_Erysipelotrichaceae abundance \|\| id:ebi-gm430-GCST90011312 | Prawns liking \|\| id:ebi-fl187-GCST90094817 | Weighted mode | 13 | 0.0731093963052081 | 0.207054101661144 | 0.73014347735374 | -0.332716643 | 0.47893543556105 | 1.07584822429801 | 0.716973324820882 | 1.61435490227525 | 0.999714303421225 |
| ebi-fl187-GCST90094817 | ebi-gm430-GCST90011352 | O_Erysipelotrichales abundance \|\| id:ebi-gm430-GCST90011352 | Prawns liking \|\| id:ebi-fl187-GCST90094817 | Weighted mode | 13 | 0.0731093963052081 | 0.211792209031247 | 0.735923827858585 | -0.342003333 | 0.488222126006453 | 1.07584822429801 | 0.710345836841233 | 1.62941674561242 | 0.999714303421225 |
| ebi-fl187-GCST90094817 | ebi-gm430-GCST90011362 | OTU97_11 (Parabacteroides) abundance \|\| id:ebi-gm430-GCST90011362 | Prawns liking \|\| id:ebi-fl187-GCST90094817 | Weighted mode | 13 | 0.174831110388423 | 0.192071938517247 | 0.380617368998494 | -0.201629889 | 0.551292109882227 | 1.19104504448996 | 0.81739739964759 | 1.73549401871807 | 0.999714303421225 |
| ebi-fl187-GCST90094817 | ebi-gm430-GCST90011384 | OTU97_235 (Phascolarctobacterium) abundance \|\| id:ebi-gm430-GCST90011384 | Prawns liking \|\| id:ebi-fl187-GCST90094817 | Weighted mode | 13 | 0.966333103645466 | 0.845704578794287 | 0.275475282279508 | -0.691247871 | 2.62391407808227 | 2.62828910384557 | 0.500950557299936 | 13.7895916328073 | 0.999714303421225 |
| ebi-fl187-GCST90094817 | ebi-gm430-GCST90011390 | OTU97_3 (Bacteroides) abundance \|\| id:ebi-gm430-GCST90011390 | Prawns liking \|\| id:ebi-fl187-GCST90094817 | Weighted mode | 13 | -0.170120447 | 0.17748684149376 | 0.356726753645196 | -0.517994657 | 0.17775376190171 | 0.843563205460291 | 0.595713961938165 | 1.19453114593998 | 0.999714303421225 |
| ebi-fl187-GCST90094817 | ebi-gm430-GCST90011398 | OTU97_4 (Alistipes) abundance \|\| id:ebi-gm430-GCST90011398 | Prawns liking \|\| id:ebi-fl187-GCST90094817 | Weighted mode | 13 | -0.321841744 | 0.19436907866618 | 0.123652903641378 | -0.702805138 | 0.0591216503947989 | 0.724812887393758 | 0.495194265436517 | 1.06090429231642 | 0.999714303421225 |
| ebi-fl187-GCST90094817 | ebi-gm430-GCST90011421 | OTU99_108 (Cytophagales) abundance \|\| id:ebi-gm430-GCST90011421 | Prawns liking \|\| id:ebi-fl187-GCST90094817 | Weighted mode | 13 | -0.129839731 | 0.608668669653667 | 0.83466041842475 | -1.322830324 | 1.06315086136262 | 0.878236173535966 | 0.266380291560926 | 2.89547988699714 | 0.999714303421225 |
| ebi-fl187-GCST90094817 | ebi-gm430-GCST90011428 | OTU99_123 (Phascolarctobacterium) abundance \|\| id:ebi-gm430-GCST90011428 | Prawns liking \|\| id:ebi-fl187-GCST90094817 | Weighted mode | 13 | -0.283180485 | 0.425635831572473 | 0.518434539780383 | -1.117426715 | 0.551065744595146 | 0.753383800896811 | 0.327120486675367 | 1.73510120757737 | 0.999714303421225 |
| ebi-fl187-GCST90094817 | ebi-gm430-GCST90011450 | OTU99_298 (Phascolarctobacterium) abundance \|\| id:ebi-gm430-GCST90011450 | Prawns liking \|\| id:ebi-fl187-GCST90094817 | Weighted mode | 13 | 0.959819314104956 | 0.831774848097653 | 0.270989265768045 | -0.670459388 | 2.59009801637636 | 2.61122461933821 | 0.511473558907818 | 13.331078203139 | 0.999714303421225 |
| ebi-fl187-GCST90094817 | ebi-gm430-GCST90011451 | OTU99_3 (Bacteroides) abundance \|\| id:ebi-gm430-GCST90011451 | Prawns liking \|\| id:ebi-fl187-GCST90094817 | Weighted mode | 13 | -0.174584959 | 0.16563427616648 | 0.312626107374502 | -0.49922814 | 0.150058222181242 | 0.839805502074366 | 0.606998996952314 | 1.16190188922138 | 0.999714303421225 |
| ebi-fl187-GCST90094817 | ebi-gm430-GCST90011472 | OTU99_72 (Parabacteroides) abundance \|\| id:ebi-gm430-GCST90011472 | Prawns liking \|\| id:ebi-fl187-GCST90094817 | Weighted mode | 13 | -0.287908927 | 0.543343451725729 | 0.605857973017438 | -1.352862092 | 0.777044238430729 | 0.749829878422213 | 0.25849935186364 | 2.1750338734747 | 0.999714303421225 |
| ebi-fl187-GCST90094817 | ebi-gm430-GCST90011507 | TestASV_3 (Bacteroides) abundance \|\| id:ebi-gm430-GCST90011507 | Prawns liking \|\| id:ebi-fl187-GCST90094817 | Weighted mode | 13 | -0.016901505 | 0.226092982470577 | 0.94164165439592 | -0.46004375 | 0.426240740979608 | 0.983240524472777 | 0.631256027259017 | 1.5314894230211 | 0.999714303421225 |
| ebi-fl187-GCST90094817 | ebi-gm430-GCST90011512 | TestASV_34 (Phascolarctobacterium) abundance \|\| id:ebi-gm430-GCST90011512 | Prawns liking \|\| id:ebi-fl187-GCST90094817 | Weighted mode | 13 | -0.074594101 | 0.856741461381636 | 0.932054085643142 | -1.753807365 | 1.60461916346705 | 0.928120133064586 | 0.173113580513489 | 4.97596421288682 | 0.999714303421225 |
| ebi-fl187-GCST90094817 | ebi-gm430-GCST90011519 | TestASV_41 (Barnesiella) abundance \|\| id:ebi-gm430-GCST90011519 | Prawns liking \|\| id:ebi-fl187-GCST90094817 | Weighted mode | 13 | 0.444325824413936 | 0.704630560252893 | 0.540138779781124 | -0.936750074 | 1.82540172250961 | 1.55943850599199 | 0.3918994121876 | 6.2052873220601 | 0.999714303421225 |
| ebi-fl187-GCST90094817 | ebi-gm430-GCST90011524 | TestASV_46 (Sutterella) abundance \|\| id:ebi-gm430-GCST90011524 | Prawns liking \|\| id:ebi-fl187-GCST90094817 | Weighted mode | 13 | -0.466880048 | 0.783990196984132 | 0.562560728841797 | -2.003500834 | 1.06974073773499 | 0.626955290221207 | 0.134862325172713 | 2.91462375005743 | 0.999714303421225 |
| ebi-fl187-GCST90094817 | ebi-gm430-GCST90011581 | OTU97_19 (Bacteria) prevalence \|\| id:ebi-gm430-GCST90011581 | Prawns liking \|\| id:ebi-fl187-GCST90094817 | Weighted mode | 13 | -0.963388398 | 0.756487105168922 | 0.226955264409884 | -2.446103124 | 0.519326328140642 | 0.381597688051945 | 0.0866305179587282 | 1.68089489659941 | 0.999714303421225 |
| ebi-fl187-GCST90094817 | ebi-gm430-GCST90011614 | OTU97_80 (Ruminococcus) prevalence \|\| id:ebi-gm430-GCST90011614 | Prawns liking \|\| id:ebi-fl187-GCST90094817 | Weighted mode | 13 | -0.210028894 | 0.378257031739058 | 0.58892201834944 | -0.951412676 | 0.531354888448954 | 0.810560825482203 | 0.386195069423632 | 1.70123573246787 | 0.999714303421225 |
| ebi-fl187-GCST90094817 | ebi-gm430-GCST90011623 | OTU99_11 (Parabacteroides) prevalence \|\| id:ebi-gm430-GCST90011623 | Prawns liking \|\| id:ebi-fl187-GCST90094817 | Weighted mode | 13 | 0.370046888388524 | 0.317796327575493 | 0.266883452178098 | -0.252833914 | 0.992927690436491 | 1.44780249819788 | 0.776596853242689 | 2.69912511882529 | 0.999714303421225 |
| ebi-fl187-GCST90094817 | ebi-gm430-GCST90011642 | OTU99_19 (Bacteria) prevalence \|\| id:ebi-gm430-GCST90011642 | Prawns liking \|\| id:ebi-fl187-GCST90094817 | Weighted mode | 13 | -0.963388398 | 0.793136600342065 | 0.247858343097842 | -2.517936135 | 0.591159338680002 | 0.381597688051945 | 0.0806258360215125 | 1.80608106175465 | 0.999714303421225 |
| ebi-fl187-GCST90094817 | ebi-gm430-GCST90011653 | OTU99_30 (Parasutterella) prevalence \|\| id:ebi-gm430-GCST90011653 | Prawns liking \|\| id:ebi-fl187-GCST90094817 | Weighted mode | 13 | -0.243437812 | 0.29433615414073 | 0.424336653063436 | -0.820336675 | 0.333461049624827 | 0.783928225060015 | 0.440283397310497 | 1.3957906789121 | 0.999714303421225 |
| ebi-fl187-GCST90094817 | ebi-gm430-GCST90011675 | OTU99_8 (Ruminococcaceae) prevalence \|\| id:ebi-gm430-GCST90011675 | Prawns liking \|\| id:ebi-fl187-GCST90094817 | Weighted mode | 13 | 0.411138049738511 | 0.412935795520886 | 0.339075523615284 | -0.398216109 | 1.22049220895945 | 1.50853359485241 | 0.671516890812137 | 3.38885534814489 | 0.999714303421225 |
| ebi-fl187-GCST90094817 | ebi-gm430-GCST90011679 | OTU99_92 (Ruminococcus) prevalence \|\| id:ebi-gm430-GCST90011679 | Prawns liking \|\| id:ebi-fl187-GCST90094817 | Weighted mode | 13 | -0.169057786 | 0.380053622408323 | 0.66436255639244 | -0.913962886 | 0.575847313509888 | 0.844460103657931 | 0.400932222831784 | 1.77863695173525 | 0.999714303421225 |
| ebi-fl187-GCST90094817 | ebi-gm430-GCST90011684 | TestASV_11 (Lachnospiraceae) prevalence \|\| id:ebi-gm430-GCST90011684 | Prawns liking \|\| id:ebi-fl187-GCST90094817 | Weighted mode | 13 | 0.0563686274517949 | 0.489400075447571 | 0.910208101472747 | -0.90285552 | 1.01559277532903 | 1.05798761514109 | 0.405410347780087 | 2.76099956481402 | 0.999714303421225 |
| ebi-fl187-GCST90094817 | ebi-gm430-GCST90011688 | TestASV_15 (Bacteroides) prevalence \|\| id:ebi-gm430-GCST90011688 | Prawns liking \|\| id:ebi-fl187-GCST90094817 | Weighted mode | 13 | -0.446279616 | 0.430403231944675 | 0.320231710786701 | -1.28986995 | 0.397310718990625 | 0.640004791702727 | 0.275306584319013 | 1.48781815159138 | 0.999714303421225 |
| ebi-fl187-GCST90094817 | ebi-gm430-GCST90011692 | TestASV_19 (Prevotella) prevalence \|\| id:ebi-gm430-GCST90011692 | Prawns liking \|\| id:ebi-fl187-GCST90094817 | Weighted mode | 13 | 0.600103625359635 | 0.730533325552581 | 0.42740388107601 | -0.831741693 | 2.03194894344269 | 1.82230762788999 | 0.435290483445038 | 7.62894025245864 | 0.999714303421225 |
| ebi-fl187-GCST90094817 | ebi-gm430-GCST90011693 | TestASV_2 (Bacteroides) prevalence \|\| id:ebi-gm430-GCST90011693 | Prawns liking \|\| id:ebi-fl187-GCST90094817 | Weighted mode | 13 | 0.029570161157952 | 0.356549653263446 | 0.935271232177341 | -0.669267159 | 0.728407481554305 | 1.03001169975035 | 0.51208371613195 | 2.07177863345537 | 0.999714303421225 |
| ebi-fl187-GCST90094817 | ebi-gm430-GCST90011716 | TestASV_40 (Barnesiella) prevalence \|\| id:ebi-gm430-GCST90011716 | Prawns liking \|\| id:ebi-fl187-GCST90094817 | Weighted mode | 13 | -0.17378777 | 0.800340888105152 | 0.831745596190681 | -1.742455911 | 1.39488037041896 | 0.840475252569982 | 0.175089867035071 | 4.03449189918614 | 0.999714303421225 |
| ebi-fl187-GCST90094817 | ebi-gm430-GCST90011718 | TestASV_42 (Alphaproteobacteria) prevalence \|\| id:ebi-gm430-GCST90011718 | Prawns liking \|\| id:ebi-fl187-GCST90094817 | Weighted mode | 13 | -0.644621615 | 0.644367273348676 | 0.33686576426682 | -1.907581471 | 0.618338240612548 | 0.524861104027481 | 0.148438956748862 | 1.85584151596416 | 0.999714303421225 |
| ebi-fl187-GCST90094817 | ebi-hbsm44-GCST90104929 | Sulcal depth of the right inferior frontal sulcus \|\| id:ebi-hbsm44-GCST90104929 | Prawns liking \|\| id:ebi-fl187-GCST90094817 | Weighted mode | 17 | 0.0776134535569684 | 0.268047987024758 | 0.775878563020286 | -0.447760601 | 0.602987508125494 | 1.08070483531116 | 0.639057655476859 | 1.82757053460761 | 0.999714303421225 |
| ebi-fl187-GCST90094817 | ebi-hbsm44-GCST90104935 | Sulcal opening distance of the left calloso-marginal posterior fissure \|\| id:ebi-hbsm44-GCST90104935 | Prawns liking \|\| id:ebi-fl187-GCST90094817 | Weighted mode | 18 | -0.083175637 | 0.0677512305684635 | 0.236302204846071 | -0.215968049 | 0.0496167746816206 | 0.920189513486503 | 0.805761046215138 | 1.05086829986125 | 0.999714303421225 |
| ebi-fl187-GCST90094817 | ebi-hbsm44-GCST90104936 | Sulcal opening distance of the left anterior inferior temporal sulcus \|\| id:ebi-hbsm44-GCST90104936 | Prawns liking \|\| id:ebi-fl187-GCST90094817 | Weighted mode | 18 | -0.02442514 | 0.0394324212438299 | 0.543854228965668 | -0.101712685 | 0.0528624057972683 | 0.975870740023179 | 0.903289042448324 | 1.05428457169386 | 0.999714303421225 |
| ebi-fl187-GCST90094817 | ebi-hbsm44-GCST90104941 | Sulcal opening distance of the right posterior inferior temporal sulcus \|\| id:ebi-hbsm44-GCST90104941 | Prawns liking \|\| id:ebi-fl187-GCST90094817 | Weighted mode | 17 | -0.032646451 | 0.0332027321486726 | 0.34012197790765 | -0.097723806 | 0.032430904250413 | 0.967880692587359 | 0.90689934953186 | 1.03296251735869 | 0.999714303421225 |
| ebi-fl187-GCST90094817 | ebi-IPs91-GCST90274819 | Leukemia inhibitory factor levels \|\| id:ebi-IPs91-GCST90274819 | Prawns liking \|\| id:ebi-fl187-GCST90094817 | Weighted mode | 21 | -0.006452096 | 0.0906436812965848 | 0.943960788680866 | -0.184113711 | 0.171209519254874 | 0.993568673991336 | 0.831841208597013 | 1.18673936772366 | 0.999714303421225 |
| ebi-fl187-GCST90094817 | ebi-IPs91-GCST90274837 | STAM binding protein levels \|\| id:ebi-IPs91-GCST90274837 | Prawns liking \|\| id:ebi-fl187-GCST90094817 | Weighted mode | 21 | 0.044018575163973 | 0.0954417685928362 | 0.649625282693597 | -0.143047291 | 0.231084441605932 | 1.04500176578731 | 0.866713079957089 | 1.25996562847843 | 0.999714303421225 |
| ebi-fl187-GCST90094817 | ebi-IPs91-GCST90274840 | TNF-beta levels \|\| id:ebi-IPs91-GCST90274840 | Prawns liking \|\| id:ebi-fl187-GCST90094817 | Weighted mode | 21 | -0.038882344 | 0.0940984469749177 | 0.683850013053414 | -0.2233153 | 0.145550612060856 | 0.961863871535187 | 0.79986261278509 | 1.15667627487077 | 0.999714303421225 |
| ebi-fl187-GCST90094817 | ebi-IPs91-GCST90274842 | Tumor necrosis factor ligand superfamily member 14 levels \|\| id:ebi-IPs91-GCST90274842 | Prawns liking \|\| id:ebi-fl187-GCST90094817 | Weighted mode | 21 | 0.0275099250004499 | 0.100772386156233 | 0.787659289108734 | -0.170003952 | 0.225023801866667 | 1.02789181688337 | 0.843661482552864 | 1.25235252416492 | 0.999714303421225 |
| ebi-fl187-GCST90094817 | ebi-met1400-GCST90199628 | 3-methylhistidine levels \|\| id:ebi-met1400-GCST90199628 | Prawns liking \|\| id:ebi-fl187-GCST90094817 | Weighted mode | 18 | 0.165166348027083 | 0.172856655109048 | 0.352710891922552 | -0.173632696 | 0.503965392040817 | 1.17958932474863 | 0.840605598771454 | 1.65527207657731 | 0.999714303421225 |
| ebi-fl187-GCST90094817 | ebi-met1400-GCST90199651 | Docosahexaenoate DHA; 22:6n3 levels \|\| id:ebi-met1400-GCST90199651 | Prawns liking \|\| id:ebi-fl187-GCST90094817 | Weighted mode | 18 | 0.109445252515846 | 0.145575717159797 | 0.462448025990104 | -0.175883153 | 0.394773658149048 | 1.11565898974028 | 0.838715978959285 | 1.48404825067572 | 0.999714303421225 |
| ebi-fl187-GCST90094817 | ebi-met1400-GCST90199652 | 1-stearoyl-2-oleoyl-GPS (18:0/18:1) levels \|\| id:ebi-met1400-GCST90199652 | Prawns liking \|\| id:ebi-fl187-GCST90094817 | Weighted mode | 18 | 0.00877298859944165 | 0.149752068129317 | 0.95396704746557 | -0.284741065 | 0.302287042132903 | 1.00881158404717 | 0.752209002392407 | 1.35294952449513 | 0.999714303421225 |
| ebi-fl187-GCST90094817 | ebi-met1400-GCST90199653 | 1-myristoyl-2-palmitoyl-gpc (14:0/16:0) levels \|\| id:ebi-met1400-GCST90199653 | Prawns liking \|\| id:ebi-fl187-GCST90094817 | Weighted mode | 17 | -0.141052195 | 0.174325830834787 | 0.43030917200355 | -0.482730824 | 0.200626433223536 | 0.868443981896372 | 0.617095908644442 | 1.22216812512782 | 0.999714303421225 |
| ebi-fl187-GCST90094817 | ebi-met1400-GCST90199667 | Erythritol levels in elite athletes \|\| id:ebi-met1400-GCST90199667 | Prawns liking \|\| id:ebi-fl187-GCST90094817 | Weighted mode | 18 | 0.105090675948872 | 0.141055475549947 | 0.466429209879783 | -0.171378056 | 0.381559408026768 | 1.11081132966056 | 0.842502999833248 | 1.46456666664271 | 0.999714303421225 |
| ebi-fl187-GCST90094817 | ebi-met1400-GCST90199674 | 3-hydroxymyristate levels \|\| id:ebi-met1400-GCST90199674 | Prawns liking \|\| id:ebi-fl187-GCST90094817 | Weighted mode | 18 | 0.082707369095239 | 0.152170180515687 | 0.593829080774343 | -0.215546185 | 0.380960922905985 | 1.08622389935304 | 0.806101039851191 | 1.46369040752451 | 0.999714303421225 |
| ebi-fl187-GCST90094817 | ebi-met1400-GCST90199685 | 2-linoleoylglycerol (18:2) levels \|\| id:ebi-met1400-GCST90199685 | Prawns liking \|\| id:ebi-fl187-GCST90094817 | Weighted mode | 18 | -0.160910938 | 0.209207152400607 | 0.452361016347722 | -0.570956957 | 0.249135080414708 | 0.851367892010624 | 0.564984514036844 | 1.28291531809905 | 0.999714303421225 |
| ebi-fl187-GCST90094817 | ebi-met1400-GCST90199703 | Gamma-glutamylvaline levels \|\| id:ebi-met1400-GCST90199703 | Prawns liking \|\| id:ebi-fl187-GCST90094817 | Weighted mode | 18 | -0.009604352 | 0.185485320452379 | 0.959307895903775 | -0.37315558 | 0.353946875859481 | 0.990441622260885 | 0.688558098425115 | 1.42467949959557 | 0.999714303421225 |
| ebi-fl187-GCST90094817 | ebi-met1400-GCST90199779 | Gamma-glutamyl-2-aminobutyrate levels \|\| id:ebi-met1400-GCST90199779 | Prawns liking \|\| id:ebi-fl187-GCST90094817 | Weighted mode | 18 | 0.19585035853317 | 0.1825047413687 | 0.298212656424904 | -0.161858935 | 0.553559651615823 | 1.21634487608367 | 0.850561180873949 | 1.73943378894251 | 0.999714303421225 |
| ebi-fl187-GCST90094817 | ebi-met1400-GCST90199824 | 4-vinylphenol sulfate levels \|\| id:ebi-met1400-GCST90199824 | Prawns liking \|\| id:ebi-fl187-GCST90094817 | Weighted mode | 18 | -0.037546345 | 0.158496281166011 | 0.815572364351549 | -0.348199056 | 0.273106366144322 | 0.963149779566687 | 0.705958336965866 | 1.31404000676064 | 0.999714303421225 |
| ebi-fl187-GCST90094817 | ebi-met1400-GCST90199830 | 4-ethylphenylsulfate levels \|\| id:ebi-met1400-GCST90199830 | Prawns liking \|\| id:ebi-fl187-GCST90094817 | Weighted mode | 18 | 0.0304192085777124 | 0.157739272831684 | 0.8493655638109 | -0.278749766 | 0.339588183327813 | 1.03088659988964 | 0.756729238778863 | 1.40436912884052 | 0.999714303421225 |
| ebi-fl187-GCST90094817 | ebi-met1400-GCST90199833 | Hexanoylglutamine levels \|\| id:ebi-met1400-GCST90199833 | Prawns liking \|\| id:ebi-fl187-GCST90094817 | Weighted mode | 18 | 0.0443500713969234 | 0.165205563087796 | 0.79158048834463 | -0.279452832 | 0.368152975049003 | 1.04534823735994 | 0.756197395099773 | 1.44506308066209 | 0.999714303421225 |
| ebi-fl187-GCST90094817 | ebi-met1400-GCST90199837 | Carotenoid levels (cryptoxanthin) \|\| id:ebi-met1400-GCST90199837 | Prawns liking \|\| id:ebi-fl187-GCST90094817 | Weighted mode | 18 | -0.027829887 | 0.17224988274616 | 0.873551540875304 | -0.365439657 | 0.30977988352415 | 0.972553797106282 | 0.693891509655189 | 1.36312503482838 | 0.999714303421225 |
| ebi-fl187-GCST90094817 | ebi-met1400-GCST90199869 | 2-hydroxyhippurate levels \|\| id:ebi-met1400-GCST90199869 | Prawns liking \|\| id:ebi-fl187-GCST90094817 | Weighted mode | 18 | 0.203231720591733 | 0.153406173843343 | 0.202772476184386 | -0.09744438 | 0.503907821324686 | 1.22535637565708 | 0.907152795863056 | 1.65517678412153 | 0.999714303421225 |
| ebi-fl187-GCST90094817 | ebi-met1400-GCST90199902 | Hydantoin-5-propionate levels \|\| id:ebi-met1400-GCST90199902 | Prawns liking \|\| id:ebi-fl187-GCST90094817 | Weighted mode | 18 | 0.0760346205601116 | 0.158141525764414 | 0.636787344939491 | -0.23392277 | 0.385992011058362 | 1.07899992909256 | 0.791422935177672 | 1.47107291845211 | 0.999714303421225 |
| ebi-fl187-GCST90094817 | ebi-met1400-GCST90199904 | Pantoate levels \|\| id:ebi-met1400-GCST90199904 | Prawns liking \|\| id:ebi-fl187-GCST90094817 | Weighted mode | 18 | 0.0258216433063468 | 0.143328801284676 | 0.859159650563062 | -0.255102807 | 0.306746093824312 | 1.02615791001886 | 0.774836835051719 | 1.35899586681885 | 0.999714303421225 |
| ebi-fl187-GCST90094817 | ebi-met1400-GCST90199936 | 3-methyl catechol sulfate (2) levels \|\| id:ebi-met1400-GCST90199936 | Prawns liking \|\| id:ebi-fl187-GCST90094817 | Weighted mode | 18 | -0.015762908 | 0.16913328628651 | 0.926835718301458 | -0.347264149 | 0.315738333393166 | 0.984360676699582 | 0.706618651099512 | 1.37127139274449 | 0.999714303421225 |
| ebi-fl187-GCST90094817 | ebi-met1400-GCST90199977 | 4-hydroxychlorothalonil levels \|\| id:ebi-met1400-GCST90199977 | Prawns liking \|\| id:ebi-fl187-GCST90094817 | Weighted mode | 18 | 0.0176952301620397 | 0.156188407875901 | 0.911124540912121 | -0.288434049 | 0.323824509598805 | 1.01785271830552 | 0.749436229380578 | 1.38240468707821 | 0.999714303421225 |
| ebi-fl187-GCST90094817 | ebi-met1400-GCST90200040 | Sphingomyelin (d18:2/24:1, d18:1/24:2) levels \|\| id:ebi-met1400-GCST90200040 | Prawns liking \|\| id:ebi-fl187-GCST90094817 | Weighted mode | 18 | -0.124128622 | 0.168267117565315 | 0.470764872370656 | -0.453932173 | 0.20567492826008 | 0.883266225959359 | 0.635125810719978 | 1.2283538359685 | 0.999714303421225 |
| ebi-fl187-GCST90094817 | ebi-met1400-GCST90200077 | 1-stearoyl-2-oleoyl-GPI (18:0/18:1) levels \|\| id:ebi-met1400-GCST90200077 | Prawns liking \|\| id:ebi-fl187-GCST90094817 | Weighted mode | 18 | 0.100689533897109 | 0.165963452977547 | 0.55206896556126 | -0.224598834 | 0.425977901733101 | 1.10593323367678 | 0.798836620628475 | 1.53108694039155 | 0.999714303421225 |
| ebi-fl187-GCST90094817 | ebi-met1400-GCST90200080 | 1-palmitoyl-2-oleoyl-GPI (16:0/18:1) levels \|\| id:ebi-met1400-GCST90200080 | Prawns liking \|\| id:ebi-fl187-GCST90094817 | Weighted mode | 18 | -0.060483253 | 0.172704077615302 | 0.730480276554544 | -0.398983246 | 0.27801673874153 | 0.941309532635257 | 0.671001943554316 | 1.32050830067124 | 0.999714303421225 |
| ebi-fl187-GCST90094817 | ebi-met1400-GCST90200082 | 1-oleoyl-2-linoleoyl-GPE (18:1/18:2) levels \|\| id:ebi-met1400-GCST90200082 | Prawns liking \|\| id:ebi-fl187-GCST90094817 | Weighted mode | 18 | -0.006039183 | 0.1643892928191 | 0.971122589042903 | -0.328242196 | 0.316163831383909 | 0.993979016666814 | 0.720188571453225 | 1.37185499011781 | 0.999714303421225 |
| ebi-fl187-GCST90094817 | ebi-met1400-GCST90200100 | Perfluorooctanesulfonate (PFOS) levels \|\| id:ebi-met1400-GCST90200100 | Prawns liking \|\| id:ebi-fl187-GCST90094817 | Weighted mode | 18 | -0.02009233 | 0.135907009226748 | 0.884209310799389 | -0.286470068 | 0.24628540841492 | 0.980108176065053 | 0.750909554604272 | 1.2792646343351 | 0.999714303421225 |
| ebi-fl187-GCST90094817 | ebi-met1400-GCST90200102 | 1-palmitoleoyl-2-linolenoyl-GPC (16:1/18:3) levels \|\| id:ebi-met1400-GCST90200102 | Prawns liking \|\| id:ebi-fl187-GCST90094817 | Weighted mode | 18 | -0.158882239 | 0.154773361868062 | 0.319019452673175 | -0.462238028 | 0.144473550205293 | 0.853096814540255 | 0.629872394630665 | 1.1554311336433 | 0.999714303421225 |
| ebi-fl187-GCST90094817 | ebi-met1400-GCST90200114 | Glycosyl-N-tricosanoyl-sphingadienine (d18:2/23:0) levels \|\| id:ebi-met1400-GCST90200114 | Prawns liking \|\| id:ebi-fl187-GCST90094817 | Weighted mode | 18 | -0.115010201 | 0.17519712782184 | 0.520314941727364 | -0.458396572 | 0.228376169435153 | 0.891357051041913 | 0.632296675559408 | 1.25655791522106 | 0.999714303421225 |
| ebi-fl187-GCST90094817 | ebi-met1400-GCST90200135 | Arachidoylcarnitine (C20) levels \|\| id:ebi-met1400-GCST90200135 | Prawns liking \|\| id:ebi-fl187-GCST90094817 | Weighted mode | 18 | -9.34E-05 | 0.176708159591471 | 0.999584312592344 | -0.346441419 | 0.346254567080494 | 0.999906578645257 | 0.707200246912296 | 1.41376246739639 | 0.999714303421225 |
| ebi-fl187-GCST90094817 | ebi-met1400-GCST90200146 | Docosahexaenoylcarnitine (C22:6) levels \|\| id:ebi-met1400-GCST90200146 | Prawns liking \|\| id:ebi-fl187-GCST90094817 | Weighted mode | 18 | 0.151301146546895 | 0.195838923095079 | 0.450379711855438 | -0.232543143 | 0.535145435813249 | 1.16334694325039 | 0.792515557332858 | 1.70769658443639 | 0.999714303421225 |
| ebi-fl187-GCST90094817 | ebi-met1400-GCST90200162 | 3-hydroxyphenylacetoylglutamine levels \|\| id:ebi-met1400-GCST90200162 | Prawns liking \|\| id:ebi-fl187-GCST90094817 | Weighted mode | 18 | 0.230818995650955 | 0.199188728708988 | 0.262559006281471 | -0.159590913 | 0.621228903920571 | 1.25963122008458 | 0.852492461551278 | 1.86121389006127 | 0.999714303421225 |
| ebi-fl187-GCST90094817 | ebi-met1400-GCST90200172 | 3-carboxy-4-methyl-5-pentyl-2-furanpropionate (3-CMPFP) levels \|\| id:ebi-met1400-GCST90200172 | Prawns liking \|\| id:ebi-fl187-GCST90094817 | Weighted mode | 18 | -0.011853359 | 0.159578022302425 | 0.941655120497018 | -0.324626283 | 0.300919564910513 | 0.988216615505466 | 0.722797425209614 | 1.35110066126463 | 0.999714303421225 |
| ebi-fl187-GCST90094817 | ebi-met1400-GCST90200222 | Dihydrocaffeate sulfate (2) levels \|\| id:ebi-met1400-GCST90200222 | Prawns liking \|\| id:ebi-fl187-GCST90094817 | Weighted mode | 18 | -0.007208655 | 0.158083178681493 | 0.964160143480415 | -0.317051685 | 0.302634375691774 | 0.992817265505827 | 0.728293112513005 | 1.35341953088821 | 0.999714303421225 |
| ebi-fl187-GCST90094817 | ebi-met1400-GCST90200223 | 4-methylhexanoylglutamine levels \|\| id:ebi-met1400-GCST90200223 | Prawns liking \|\| id:ebi-fl187-GCST90094817 | Weighted mode | 18 | 0.131897026494808 | 0.193885318904654 | 0.505484757131512 | -0.248118199 | 0.51191225154793 | 1.14099082139348 | 0.780267711308722 | 1.66847869729301 | 0.999714303421225 |
| ebi-fl187-GCST90094817 | ebi-met1400-GCST90200224 | 2-ketocaprylate levels \|\| id:ebi-met1400-GCST90200224 | Prawns liking \|\| id:ebi-fl187-GCST90094817 | Weighted mode | 18 | 0.153138765654738 | 0.179179776790648 | 0.404620348524055 | -0.198053597 | 0.504331128164407 | 1.16548669724664 | 0.820325885072107 | 1.65587758009059 | 0.999714303421225 |
| ebi-fl187-GCST90094817 | ebi-met1400-GCST90200243 | Hydroxypalmitoyl sphingomyelin (d18:1/16:0(OH)) levels \|\| id:ebi-met1400-GCST90200243 | Prawns liking \|\| id:ebi-fl187-GCST90094817 | Weighted mode | 18 | -0.085317775 | 0.161403777387378 | 0.603917443648961 | -0.401669179 | 0.231033628601031 | 0.918220450459659 | 0.669202095343415 | 1.25990160746533 | 0.999714303421225 |
| ebi-fl187-GCST90094817 | ebi-met1400-GCST90200273 | 2-methoxyhydroquinone sulfate (2) levels \|\| id:ebi-met1400-GCST90200273 | Prawns liking \|\| id:ebi-fl187-GCST90094817 | Weighted mode | 18 | -0.199135639 | 0.215368103900795 | 0.368098436459084 | -0.621257123 | 0.222985844781239 | 0.819438738054798 | 0.537268600427173 | 1.24980288237756 | 0.999714303421225 |
| ebi-fl187-GCST90094817 | ebi-met1400-GCST90200276 | 2,4-di-tert-butylphenol levels \|\| id:ebi-met1400-GCST90200276 | Prawns liking \|\| id:ebi-fl187-GCST90094817 | Weighted mode | 18 | 0.0818763953361497 | 0.195156807600822 | 0.680074348204416 | -0.300630948 | 0.464383738233761 | 1.08532165072057 | 0.740350950658834 | 1.59103339365552 | 0.999714303421225 |
| ebi-fl187-GCST90094817 | ebi-met1400-GCST90200289 | N-acetylmethionine levels \|\| id:ebi-met1400-GCST90200289 | Prawns liking \|\| id:ebi-fl187-GCST90094817 | Weighted mode | 18 | 0.0594531429211782 | 0.143539529265595 | 0.683911424182993 | -0.221884334 | 0.340790620281745 | 1.06125603245692 | 0.801008008017016 | 1.40605880984185 | 0.999714303421225 |
| ebi-fl187-GCST90094817 | ebi-met1400-GCST90200308 | 3-hydroxyisobutyrate levels \|\| id:ebi-met1400-GCST90200308 | Prawns liking \|\| id:ebi-fl187-GCST90094817 | Weighted mode | 18 | 0.0494355299609883 | 0.16976683561768 | 0.774423959971343 | -0.283307468 | 0.382178527771641 | 1.05067785278911 | 0.753288140364768 | 1.46547368953263 | 0.999714303421225 |
| ebi-fl187-GCST90094817 | ebi-met1400-GCST90200383 | Cystathionine levels \|\| id:ebi-met1400-GCST90200383 | Prawns liking \|\| id:ebi-fl187-GCST90094817 | Weighted mode | 18 | -0.17639867 | 0.184070609027443 | 0.351332953768372 | -0.537177063 | 0.184379724017837 | 0.838283718412483 | 0.584395637900314 | 1.20247234404465 | 0.999714303421225 |
| ebi-fl187-GCST90094817 | ebi-met1400-GCST90200384 | Guanidinoacetate levels \|\| id:ebi-met1400-GCST90200384 | Prawns liking \|\| id:ebi-fl187-GCST90094817 | Weighted mode | 18 | -0.023816797 | 0.140909192414541 | 0.86777392934587 | -0.299998815 | 0.252365219681863 | 0.976464584173468 | 0.74081909886065 | 1.28706601329728 | 0.999714303421225 |
| ebi-fl187-GCST90094817 | ebi-met1400-GCST90200403 | Citrulline levels \|\| id:ebi-met1400-GCST90200403 | Prawns liking \|\| id:ebi-fl187-GCST90094817 | Weighted mode | 17 | 0.12506143388182 | 0.154897032371764 | 0.431284847381083 | -0.17853675 | 0.428659617330477 | 1.13321806891334 | 0.836493315545522 | 1.53519839052652 | 0.999714303421225 |
| ebi-fl187-GCST90094817 | ebi-met1400-GCST90200434 | 12,13-DiHOME levels \|\| id:ebi-met1400-GCST90200434 | Prawns liking \|\| id:ebi-fl187-GCST90094817 | Weighted mode | 18 | -0.083099797 | 0.132353463205437 | 0.538437480690988 | -0.342512585 | 0.176312991054095 | 0.920259303677392 | 0.709984184383398 | 1.19281133950933 | 0.999714303421225 |
| ebi-fl187-GCST90094817 | ebi-met1400-GCST90200437 | Fructose levels \|\| id:ebi-met1400-GCST90200437 | Prawns liking \|\| id:ebi-fl187-GCST90094817 | Weighted mode | 18 | -0.074990828 | 0.145788428107144 | 0.613607698766079 | -0.360736147 | 0.210754491382696 | 0.92775199590238 | 0.697162922871281 | 1.23460921065042 | 0.999714303421225 |
| ebi-fl187-GCST90094817 | ebi-met1400-GCST90200448 | N-stearoyl-sphinganine (d18:0/18:0) levels \|\| id:ebi-met1400-GCST90200448 | Prawns liking \|\| id:ebi-fl187-GCST90094817 | Weighted mode | 18 | 0.254922604649511 | 0.201785441544368 | 0.223520618021124 | -0.140576861 | 0.650422070076472 | 1.29036174900831 | 0.868856881350896 | 1.91634949212233 | 0.999714303421225 |
| ebi-fl187-GCST90094817 | ebi-met1400-GCST90200468 | X-11847 levels \|\| id:ebi-met1400-GCST90200468 | Prawns liking \|\| id:ebi-fl187-GCST90094817 | Weighted mode | 18 | 0.0505693533817957 | 0.180234933761082 | 0.782422559733027 | -0.302691117 | 0.403829823553515 | 1.05186981155401 | 0.738827272468397 | 1.4975490776919 | 0.999714303421225 |
| ebi-fl187-GCST90094817 | ebi-met1400-GCST90200476 | X-11843 levels \|\| id:ebi-met1400-GCST90200476 | Prawns liking \|\| id:ebi-fl187-GCST90094817 | Weighted mode | 18 | -0.095599397 | 0.161417194034168 | 0.561478242962833 | -0.411977098 | 0.220778302856968 | 0.908828021976104 | 0.662339444937356 | 1.24704693317355 | 0.999714303421225 |
| ebi-fl187-GCST90094817 | ebi-met1400-GCST90200511 | X-12906 levels \|\| id:ebi-met1400-GCST90200511 | Prawns liking \|\| id:ebi-fl187-GCST90094817 | Weighted mode | 18 | -0.138843875 | 0.141139691626882 | 0.339040957177323 | -0.41547767 | 0.137789920737558 | 0.870363903545228 | 0.660024930995282 | 1.14773440974747 | 0.999714303421225 |
| ebi-fl187-GCST90094817 | ebi-met1400-GCST90200515 | X-12849 levels \|\| id:ebi-met1400-GCST90200515 | Prawns liking \|\| id:ebi-fl187-GCST90094817 | Weighted mode | 18 | 0.0533615850012537 | 0.159150044830308 | 0.741507544379314 | -0.258572503 | 0.365295672868657 | 1.05481098000224 | 0.77215304571074 | 1.44093999203118 | 0.999714303421225 |
| ebi-fl187-GCST90094817 | ebi-met1400-GCST90200551 | X-18886 levels \|\| id:ebi-met1400-GCST90200551 | Prawns liking \|\| id:ebi-fl187-GCST90094817 | Weighted mode | 18 | 0.158206820940241 | 0.17564635098801 | 0.380324589068032 | -0.186060027 | 0.50247366887674 | 1.17140844145122 | 0.830223757647761 | 1.65280470964957 | 0.999714303421225 |
| ebi-fl187-GCST90094817 | ebi-met1400-GCST90200564 | X-21319 levels \|\| id:ebi-met1400-GCST90200564 | Prawns liking \|\| id:ebi-fl187-GCST90094817 | Weighted mode | 18 | -0.017205007 | 0.144335539439008 | 0.906513047149414 | -0.300102664 | 0.26569265028747 | 0.982942153943095 | 0.740742168991671 | 1.30433411036055 | 0.999714303421225 |
| ebi-fl187-GCST90094817 | ebi-met1400-GCST90200579 | X-21442 levels \|\| id:ebi-met1400-GCST90200579 | Prawns liking \|\| id:ebi-fl187-GCST90094817 | Weighted mode | 18 | -0.061521435 | 0.173963238890506 | 0.727951850474551 | -0.402489383 | 0.279446513621553 | 0.940332789862584 | 0.66865343809621 | 1.32239768064054 | 0.999714303421225 |
| ebi-fl187-GCST90094817 | ebi-met1400-GCST90200581 | X-21441 levels \|\| id:ebi-met1400-GCST90200581 | Prawns liking \|\| id:ebi-fl187-GCST90094817 | Weighted mode | 18 | -0.03652641 | 0.142537552285851 | 0.800826785070188 | -0.315900012 | 0.242847192533206 | 0.964132630868344 | 0.72913235069689 | 1.27487379899777 | 0.999714303421225 |
| ebi-fl187-GCST90094817 | ebi-met1400-GCST90200648 | X-24585 levels \|\| id:ebi-met1400-GCST90200648 | Prawns liking \|\| id:ebi-fl187-GCST90094817 | Weighted mode | 18 | -0.099108075 | 0.141560922161841 | 0.493329842466857 | -0.376567483 | 0.178351332183287 | 0.905644824939856 | 0.686212808637242 | 1.19524517557341 | 0.999714303421225 |
| ebi-fl187-GCST90094817 | ebi-met1400-GCST90200720 | Adenosine 5'-diphosphate (ADP) to pantothenate ratio \|\| id:ebi-met1400-GCST90200720 | Prawns liking \|\| id:ebi-fl187-GCST90094817 | Weighted mode | 18 | 0.178339752198086 | 0.212157089632472 | 0.412238303979954 | -0.237488143 | 0.594167647877731 | 1.1952313347321 | 0.788606241050087 | 1.8115224926742 | 0.999714303421225 |
| ebi-fl187-GCST90094817 | ebi-met1400-GCST90200724 | Cholate to taurocholate ratio \|\| id:ebi-met1400-GCST90200724 | Prawns liking \|\| id:ebi-fl187-GCST90094817 | Weighted mode | 18 | 0.117666747160003 | 0.169854821656603 | 0.497822305843592 | -0.215248703 | 0.450582197606946 | 1.12486918304797 | 0.806340875611669 | 1.56922551893598 | 0.999714303421225 |
| ebi-fl187-GCST90094817 | ebi-met1400-GCST90200726 | N-acetylputrescine to (N(1) + N(8))-acetylspermidine ratio \|\| id:ebi-met1400-GCST90200726 | Prawns liking \|\| id:ebi-fl187-GCST90094817 | Weighted mode | 18 | -0.029707794 | 0.143834851016709 | 0.838821841973774 | -0.311624102 | 0.252208513516016 | 0.970729144527769 | 0.732256729997749 | 1.28686433791945 | 0.999714303421225 |
| ebi-fl187-GCST90094817 | ebi-met1400-GCST90200744 | Arginine to glutamate ratio \|\| id:ebi-met1400-GCST90200744 | Prawns liking \|\| id:ebi-fl187-GCST90094817 | Weighted mode | 18 | -0.097352904 | 0.160897374156599 | 0.553129563918264 | -0.412711758 | 0.218005949143122 | 0.907235782311004 | 0.66185302947415 | 1.24359446591852 | 0.999714303421225 |
| ebi-fl187-GCST90094817 | ebi-met1400-GCST90200759 | Histidine to trans-urocanate ratio \|\| id:ebi-met1400-GCST90200759 | Prawns liking \|\| id:ebi-fl187-GCST90094817 | Weighted mode | 18 | 0.139771046487945 | 0.143396288724591 | 0.343366589136243 | -0.141285679 | 0.420827772388143 | 1.15001046977736 | 0.86824123761807 | 1.52322191494355 | 0.999714303421225 |
| ebi-fl187-GCST90094817 | ebi-met1400-GCST90200795 | Oleoyl-linoleoyl-glycerol (18:1 to 18:2) [2] to linoleoyl-arachidonoyl-glycerol (18:2 to 20:4) [2] ratio \|\| id:ebi-met1400-GCST90200795 | Prawns liking \|\| id:ebi-fl187-GCST90094817 | Weighted mode | 18 | -0.09139873 | 0.193099919351114 | 0.642003357499854 | -0.469874572 | 0.287077111779834 | 0.912653735780702 | 0.625080665935899 | 1.33252696303982 | 0.999714303421225 |
| ebi-fl187-GCST90094817 | ebi-met1400-GCST90200811 | Choline to taurocholate ratio \|\| id:ebi-met1400-GCST90200811 | Prawns liking \|\| id:ebi-fl187-GCST90094817 | Weighted mode | 18 | -0.086854558 | 0.164726961346908 | 0.604823909377174 | -0.409719402 | 0.23601028667623 | 0.916810429079168 | 0.663836495328325 | 1.26618733495905 | 0.999714303421225 |
| ebi-fl187-GCST90094817 | ebi-met1400-GCST90200831 | Cholate to phosphate ratio \|\| id:ebi-met1400-GCST90200831 | Prawns liking \|\| id:ebi-fl187-GCST90094817 | Weighted mode | 18 | -0.084380815 | 0.154565881453537 | 0.592212570787268 | -0.387329943 | 0.218568312529241 | 0.919081189430857 | 0.67886707069025 | 1.24429401459518 | 0.999714303421225 |
| ebi-fl187-GCST90094817 | ebi-met1400-GCST90200841 | Adenosine 5'-diphosphate (ADP) to oxalate (ethanedioate) ratio \|\| id:ebi-met1400-GCST90200841 | Prawns liking \|\| id:ebi-fl187-GCST90094817 | Weighted mode | 18 | 0.119448982718702 | 0.203555088172273 | 0.565040805584776 | -0.27951899 | 0.518416955536357 | 1.12687575246343 | 0.756147368365477 | 1.67936703163432 | 0.999714303421225 |
| ebi-fl187-GCST90094817 | ebi-met1400-GCST90200844 | Aspartate to phosphate ratio \|\| id:ebi-met1400-GCST90200844 | Prawns liking \|\| id:ebi-fl187-GCST90094817 | Weighted mode | 18 | -0.004331064 | 0.131529538439162 | 0.974115174915621 | -0.262128959 | 0.253466831589378 | 0.995678301779447 | 0.76941179466295 | 1.28848464178885 | 0.999714303421225 |
| ebi-fl187-GCST90094817 | ebi-met1400-GCST90200870 | Adenosine 5'-monophosphate (AMP) to acetoacetate ratio \|\| id:ebi-met1400-GCST90200870 | Prawns liking \|\| id:ebi-fl187-GCST90094817 | Weighted mode | 18 | -0.050110605 | 0.159811998259636 | 0.757670114829879 | -0.363342122 | 0.263120911187746 | 0.951124219206885 | 0.695348498787901 | 1.30098401296448 | 0.999714303421225 |
| ebi-fl187-GCST90094817 | ebi-met1400-GCST90200887 | Cholesterol to cortisol ratio \|\| id:ebi-met1400-GCST90200887 | Prawns liking \|\| id:ebi-fl187-GCST90094817 | Weighted mode | 18 | -0.048182254 | 0.161544957559274 | 0.769120102261874 | -0.36481037 | 0.268445863215064 | 0.95296009084133 | 0.694328303581431 | 1.30793016797969 | 0.999714303421225 |
| ebi-fl187-GCST90094817 | ebi-met1400-GCST90200890 | Cortisol to taurocholate ratio \|\| id:ebi-met1400-GCST90200890 | Prawns liking \|\| id:ebi-fl187-GCST90094817 | Weighted mode | 18 | -0.084578421 | 0.152218091120149 | 0.585696035869415 | -0.38292588 | 0.213769037465126 | 0.918899591406549 | 0.681863437317923 | 1.23833661239916 | 0.999714303421225 |
| ebi-fl187-GCST90094817 | ebi-met1400-GCST90200948 | Adenosine 5'-diphosphate (ADP) to mannitol to sorbitol ratio \|\| id:ebi-met1400-GCST90200948 | Prawns liking \|\| id:ebi-fl187-GCST90094817 | Weighted mode | 18 | -0.346866085 | 0.235865506720766 | 0.159663644052345 | -0.809162479 | 0.115430307773687 | 0.706899986149153 | 0.445230800450611 | 1.12235629231384 | 0.999714303421225 |
| ebi-fl187-GCST90094817 | ebi-met1400-GCST90200972 | Leucine to N-palmitoyl-sphingosine (d18:1 to 16:0) ratio \|\| id:ebi-met1400-GCST90200972 | Prawns liking \|\| id:ebi-fl187-GCST90094817 | Weighted mode | 18 | -0.108071277 | 0.148748448250443 | 0.477395103246115 | -0.399618235 | 0.183475681759084 | 0.897563618677283 | 0.670575999365367 | 1.20138574946837 | 0.999714303421225 |
| ebi-fl187-GCST90094817 | ebi-met1400-GCST90200973 | Proline to glutamate ratio \|\| id:ebi-met1400-GCST90200973 | Prawns liking \|\| id:ebi-fl187-GCST90094817 | Weighted mode | 18 | 0.0710228857575003 | 0.15327867902602 | 0.64898488744782 | -0.229403325 | 0.3714490966485 | 1.0736057958691 | 0.795007822197955 | 1.44983404281112 | 0.999714303421225 |
| ebi-fl187-GCST90094817 | ebi-met1400-GCST90200983 | Cholesterol to linoleoyl-arachidonoyl-glycerol (18:2 to 20:4) [1] ratio \|\| id:ebi-met1400-GCST90200983 | Prawns liking \|\| id:ebi-fl187-GCST90094817 | Weighted mode | 18 | -0.007246022 | 0.148060460006512 | 0.96153780170026 | -0.297444524 | 0.282952479215529 | 0.992780166729213 | 0.742713784860424 | 1.32704209823722 | 0.999714303421225 |
| ebi-fl187-GCST90094817 | ebi-met1400-GCST90201001 | Serine to alpha-ketobutyrate ratio \|\| id:ebi-met1400-GCST90201001 | Prawns liking \|\| id:ebi-fl187-GCST90094817 | Weighted mode | 18 | -0.317002799 | 0.182269040042243 | 0.100072743683947 | -0.674250118 | 0.0402445194554351 | 0.728328716510843 | 0.509538371283117 | 1.04106530379356 | 0.999714303421225 |
| ebi-fl187-GCST90094817 | ebi-met1400-GCST90201005 | Fructose to maltose ratio \|\| id:ebi-met1400-GCST90201005 | Prawns liking \|\| id:ebi-fl187-GCST90094817 | Weighted mode | 18 | -0.31787199 | 0.197744034496603 | 0.126359318749228 | -0.705450297 | 0.0697063179556726 | 0.727695935058361 | 0.493886128600665 | 1.07219325110592 | 0.999714303421225 |
| ebi-fl187-GCST90094817 | ebi-met1400-GCST90201020 | Paraxanthine to 5-acetylamino-6-formylamino-3-methyluracil ratio \|\| id:ebi-met1400-GCST90201020 | Prawns liking \|\| id:ebi-fl187-GCST90094817 | Weighted mode | 18 | -0.066104011 | 0.169417498944518 | 0.701246072062111 | -0.398162309 | 0.265954286948596 | 0.936033501464015 | 0.671553019774497 | 1.30467541662934 | 0.999714303421225 |
| ebi-fl187-GCST90094817 | ebi-pw11-GCST90275197 | Placental weight (fetal genotype, adjusted for sex) \|\| id:ebi-pw11-GCST90275197 | Prawns liking \|\| id:ebi-fl187-GCST90094817 | Weighted mode | 17 | 0.103712666674971 | 0.0526001369187799 | 0.0661868539481684 | 0.000616398314162214 | 0.206808935035779 | 1.10928167552758 | 1.00061658832664 | 1.22974758765402 | 0.999714303421225 |
| ebi-fl187-GCST90094817 | prot-a-1017 | FAS-associated factor 2 \|\| id:prot-a-1017 | Prawns liking \|\| id:ebi-fl187-GCST90094817 | Weighted mode | 20 | -0.137895241 | 0.191939726018626 | 0.481234412278097 | -0.514097104 | 0.238306622329577 | 0.871189952243895 | 0.598040319337282 | 1.26909826703954 | 0.999714303421225 |
| ebi-fl187-GCST90094817 | prot-a-1019 | Fas apoptotic inhibitory molecule 3 \|\| id:prot-a-1019 | Prawns liking \|\| id:ebi-fl187-GCST90094817 | Weighted mode | 20 | -0.075094655 | 0.191328258314839 | 0.699063633902098 | -0.450098042 | 0.299908730878715 | 0.927655674536712 | 0.637565640528365 | 1.3497356127708 | 0.999714303421225 |
| ebi-fl187-GCST90094817 | prot-a-1052 | MIP18 family protein FAM96A \|\| id:prot-a-1052 | Prawns liking \|\| id:ebi-fl187-GCST90094817 | Weighted mode | 20 | 0.160160167646341 | 0.209100837376145 | 0.453119058867565 | -0.249677474 | 0.569997808903585 | 1.17369884451921 | 0.779052007386853 | 1.76826317699438 | 0.999714303421225 |
| ebi-fl187-GCST90094817 | prot-a-1066 | F-box/LRR-repeat protein 4 \|\| id:prot-a-1066 | Prawns liking \|\| id:ebi-fl187-GCST90094817 | Weighted mode | 20 | -0.118554125 | 0.170607509035843 | 0.495532761036303 | -0.452944842 | 0.215836593185571 | 0.888203740750513 | 0.635753199384944 | 1.24089959098346 | 0.999714303421225 |
| ebi-fl187-GCST90094817 | prot-a-1076 | Low affinity immunoglobulin gamma Fc region receptor III-B \|\| id:prot-a-1076 | Prawns liking \|\| id:ebi-fl187-GCST90094817 | Weighted mode | 20 | -0.049764607 | 0.187009001741437 | 0.793022270252721 | -0.416302251 | 0.316773036263261 | 0.951453363461775 | 0.659480911880765 | 1.37269098549193 | 0.999714303421225 |
| ebi-fl187-GCST90094817 | prot-a-1081 | Fc receptor-like protein 3 \|\| id:prot-a-1081 | Prawns liking \|\| id:ebi-fl187-GCST90094817 | Weighted mode | 20 | 0.102914789152744 | 0.178161993080319 | 0.570285201581295 | -0.246282717 | 0.452112295590169 | 1.10839695760807 | 0.781701193243426 | 1.57162842561025 | 0.999714303421225 |
| ebi-fl187-GCST90094817 | prot-a-1105 | Fibroblast growth factor receptor 4 \|\| id:prot-a-1105 | Prawns liking \|\| id:ebi-fl187-GCST90094817 | Weighted mode | 20 | -0.089623642 | 0.298524473020597 | 0.767267850631853 | -0.674731609 | 0.495484325538098 | 0.914275215701342 | 0.509293092099733 | 1.64129296668733 | 0.999714303421225 |
| ebi-fl187-GCST90094817 | prot-a-1143 | Follistatin-related protein 1 \|\| id:prot-a-1143 | Prawns liking \|\| id:ebi-fl187-GCST90094817 | Weighted mode | 20 | -0.090114129 | 0.186805215975928 | 0.635035263016468 | -0.456252352 | 0.276024094294619 | 0.913826885154299 | 0.633653912878799 | 1.31787961702453 | 0.999714303421225 |
| ebi-fl187-GCST90094817 | prot-a-1151 | Alpha-(1,3)-fucosyltransferase 10 \|\| id:prot-a-1151 | Prawns liking \|\| id:ebi-fl187-GCST90094817 | Weighted mode | 20 | -0.096140222 | 0.165878250925925 | 0.569005160915118 | -0.421261594 | 0.22898114948395 | 0.908336638057697 | 0.656218416058282 | 1.25731833768694 | 0.999714303421225 |
| ebi-fl187-GCST90094817 | prot-a-1165 | Growth arrest and DNA damage-inducible proteins-interacting protein 1 \|\| id:prot-a-1165 | Prawns liking \|\| id:ebi-fl187-GCST90094817 | Weighted mode | 20 | -0.067758748 | 0.176452319854969 | 0.705237684202988 | -0.413605295 | 0.278087798858194 | 0.934485892921557 | 0.661261903160166 | 1.3206021394792 | 0.999714303421225 |
| ebi-fl187-GCST90094817 | prot-a-1194 | Growth/differentiation factor 11/8 \|\| id:prot-a-1194 | Prawns liking \|\| id:ebi-fl187-GCST90094817 | Weighted mode | 20 | -0.281298322 | 0.154029298569166 | 0.0835669814026186 | -0.583195747 | 0.0205991031751402 | 0.75480312749561 | 0.558111928941123 | 1.0208127290131 | 0.999714303421225 |
| ebi-fl187-GCST90094817 | prot-a-1216 | D-glucuronyl C5-epimerase \|\| id:prot-a-1216 | Prawns liking \|\| id:ebi-fl187-GCST90094817 | Weighted mode | 20 | -0.127272834 | 0.186997000244218 | 0.504326016811308 | -0.493786954 | 0.239241286597823 | 0.880493411388462 | 0.61031079329499 | 1.27028500235579 | 0.999714303421225 |
| ebi-fl187-GCST90094817 | prot-a-1246 | Glypican-3 \|\| id:prot-a-1246 | Prawns liking \|\| id:ebi-fl187-GCST90094817 | Weighted mode | 20 | -0.355904363 | 0.188148236366131 | 0.0738858253897026 | -0.724674906 | 0.0128661806683635 | 0.700539614812483 | 0.484482045617554 | 1.01294930609053 | 0.999714303421225 |
| ebi-fl187-GCST90094817 | prot-a-1275 | Glutamate receptor 4 \|\| id:prot-a-1275 | Prawns liking \|\| id:ebi-fl187-GCST90094817 | Weighted mode | 19 | -0.047186894 | 0.231009655110108 | 0.840439184868192 | -0.499965818 | 0.40559203002444 | 0.953909101050314 | 0.60655139249363 | 1.5001903949568 | 0.999714303421225 |
| ebi-fl187-GCST90094817 | prot-a-1289 | Glutathione S-transferase P \|\| id:prot-a-1289 | Prawns liking \|\| id:ebi-fl187-GCST90094817 | Weighted mode | 20 | 0.187733799901003 | 0.181245019273902 | 0.313298962106165 | -0.167506438 | 0.54297403767785 | 1.20651229890417 | 0.845771172301517 | 1.72111792773196 | 0.999714303421225 |
| ebi-fl187-GCST90094817 | prot-a-130 | Apolipoprotein D \|\| id:prot-a-130 | Prawns liking \|\| id:ebi-fl187-GCST90094817 | Weighted mode | 20 | -0.084047851 | 0.227517966395712 | 0.71590572033403 | -0.529983065 | 0.361887362994839 | 0.919387261313281 | 0.588614937625256 | 1.43603718192297 | 0.999714303421225 |
| ebi-fl187-GCST90094817 | prot-a-1313 | Heparin-binding EGF-like growth factor \|\| id:prot-a-1313 | Prawns liking \|\| id:ebi-fl187-GCST90094817 | Weighted mode | 20 | 0.319162843771084 | 0.174994160832451 | 0.0839453385517451 | -0.023825711 | 0.662151399002688 | 1.375975376 | 0.976455879997308 | 1.9389593255237 | 0.999714303421225 |
| ebi-fl187-GCST90094817 | prot-a-1347 | HLA class II histocompatibility antigen, DQ alpha 2 chain \|\| id:prot-a-1347 | Prawns liking \|\| id:ebi-fl187-GCST90094817 | Weighted mode | 19 | -0.126040643 | 0.239471868392719 | 0.605086071070378 | -0.595405505 | 0.3433242189235 | 0.881579015928057 | 0.55133894973432 | 1.40962571517791 | 0.999714303421225 |
| ebi-fl187-GCST90094817 | prot-a-1357 | Non-histone chromosomal protein HMG-14 \|\| id:prot-a-1357 | Prawns liking \|\| id:ebi-fl187-GCST90094817 | Weighted mode | 20 | -0.13553047 | 0.186256221930882 | 0.475696254240919 | -0.500592665 | 0.229531724757101 | 0.873252554319165 | 0.606171296592167 | 1.25801077667656 | 0.999714303421225 |
| ebi-fl187-GCST90094817 | prot-a-137 | Apolipoprotein M \|\| id:prot-a-137 | Prawns liking \|\| id:ebi-fl187-GCST90094817 | Weighted mode | 20 | -0.107585695 | 0.167882882622248 | 0.529282444862487 | -0.436636145 | 0.221464755360703 | 0.897999565458834 | 0.646206514422357 | 1.24790326554522 | 0.999714303421225 |
| ebi-fl187-GCST90094817 | prot-a-1381 | 17-beta-hydroxysteroid dehydrogenase 14 \|\| id:prot-a-1381 | Prawns liking \|\| id:ebi-fl187-GCST90094817 | Weighted mode | 20 | -0.128217331 | 0.166277850894671 | 0.450129686381506 | -0.454121919 | 0.197687256651072 | 0.879662180417198 | 0.63500530940541 | 1.21858123104655 | 0.999714303421225 |
| ebi-fl187-GCST90094817 | prot-a-1392 | Serine protease HTRA2, mitochondrial \|\| id:prot-a-1392 | Prawns liking \|\| id:ebi-fl187-GCST90094817 | Weighted mode | 20 | -0.104206201 | 0.174990065754101 | 0.558538011067075 | -0.44718673 | 0.2387743281704 | 0.901039483285296 | 0.639424497654881 | 1.26969197054009 | 0.999714303421225 |
| ebi-fl187-GCST90094817 | prot-a-1416 | Interferon-induced protein with tetratricopeptide repeats 2 \|\| id:prot-a-1416 | Prawns liking \|\| id:ebi-fl187-GCST90094817 | Weighted mode | 20 | -0.191475054 | 0.169789585513035 | 0.273478540588594 | -0.524262641 | 0.141312533996535 | 0.82574022408803 | 0.591991713765065 | 1.15178456357169 | 0.999714303421225 |
| ebi-fl187-GCST90094817 | prot-a-1428 | Interferon gamma \|\| id:prot-a-1428 | Prawns liking \|\| id:ebi-fl187-GCST90094817 | Weighted mode | 20 | -0.201531136 | 0.16564215785961 | 0.238629700328828 | -0.526189766 | 0.123127493034326 | 0.817478123862085 | 0.590851970559982 | 1.13102861002514 | 0.999714303421225 |
| ebi-fl187-GCST90094817 | prot-a-1483 | Interleukin-17C \|\| id:prot-a-1483 | Prawns liking \|\| id:ebi-fl187-GCST90094817 | Weighted mode | 20 | 0.187606135823928 | 0.188674782035043 | 0.332554814877648 | -0.182196437 | 0.557408708612613 | 1.20635828045657 | 0.833437606380841 | 1.74614187035031 | 0.999714303421225 |
| ebi-fl187-GCST90094817 | prot-a-1503 | Interleukin-1 receptor-like 2 \|\| id:prot-a-1503 | Prawns liking \|\| id:ebi-fl187-GCST90094817 | Weighted mode | 20 | -0.089572294 | 0.252817128788028 | 0.727018185243559 | -0.585093866 | 0.405949278446492 | 0.914322162748573 | 0.557053570743296 | 1.50072643135156 | 0.999714303421225 |
| ebi-fl187-GCST90094817 | prot-a-1522 | Interleukin-31 receptor subunit alpha \|\| id:prot-a-1522 | Prawns liking \|\| id:ebi-fl187-GCST90094817 | Weighted mode | 20 | -0.086940938 | 0.182398587738716 | 0.639044812238698 | -0.44444217 | 0.270560293971663 | 0.916731238018103 | 0.641181846783802 | 1.31069862157463 | 0.999714303421225 |
| ebi-fl187-GCST90094817 | prot-a-1540 | Interleukin-6 receptor subunit alpha \|\| id:prot-a-1540 | Prawns liking \|\| id:ebi-fl187-GCST90094817 | Weighted mode | 20 | -0.25434967 | 0.174481589569755 | 0.161242216491124 | -0.596333585 | 0.0876342459006098 | 0.775420613576759 | 0.550827500431175 | 1.09158879592811 | 0.999714303421225 |
| ebi-fl187-GCST90094817 | prot-a-1541 | Interleukin-6 receptor subunit alpha \|\| id:prot-a-1541 | Prawns liking \|\| id:ebi-fl187-GCST90094817 | Weighted mode | 20 | -0.293323095 | 0.189868500350345 | 0.138869889243676 | -0.665465355 | 0.0788191659150787 | 0.745781143778585 | 0.514034263389263 | 1.08200864033553 | 0.999714303421225 |
| ebi-fl187-GCST90094817 | prot-a-1545 | Interleukin-9 \|\| id:prot-a-1545 | Prawns liking \|\| id:ebi-fl187-GCST90094817 | Weighted mode | 20 | -0.362394116 | 0.184828513472427 | 0.0647410409789037 | -0.724658003 | -0.00013023 | 0.696008005703133 | 0.4844902349905 | 0.999869778618658 | 0.999714303421225 |
| ebi-fl187-GCST90094817 | prot-a-1548 | Inositol monophosphatase 3 \|\| id:prot-a-1548 | Prawns liking \|\| id:ebi-fl187-GCST90094817 | Weighted mode | 20 | -0.148265058 | 0.170186176688421 | 0.394523639269026 | -0.481829964 | 0.185299848532029 | 0.862202551160307 | 0.617652075871238 | 1.20357927750625 | 0.999714303421225 |
| ebi-fl187-GCST90094817 | prot-a-1555 | Inhibin beta A chain:Inhibin beta B chain heterodimer \|\| id:prot-a-1555 | Prawns liking \|\| id:ebi-fl187-GCST90094817 | Weighted mode | 20 | -0.155826377 | 0.161114517004178 | 0.34560606569164 | -0.47161083 | 0.159958076173304 | 0.855707747887932 | 0.623996306011827 | 1.1734616739567 | 0.999714303421225 |
| ebi-fl187-GCST90094817 | prot-a-1603 | Junctophilin-3 \|\| id:prot-a-1603 | Prawns liking \|\| id:ebi-fl187-GCST90094817 | Weighted mode | 20 | 0.112215061022337 | 0.168227934007089 | 0.512765663980868 | -0.21751169 | 0.441941811676231 | 1.11875343503251 | 0.804518200343517 | 1.55572521275793 | 0.999714303421225 |
| ebi-fl187-GCST90094817 | prot-a-1627 | UPF0258 protein KIAA1024 \|\| id:prot-a-1627 | Prawns liking \|\| id:ebi-fl187-GCST90094817 | Weighted mode | 20 | 0.0400138769948324 | 0.20933691309303 | 0.850439116546541 | -0.370286473 | 0.450314226657172 | 1.04082521761834 | 0.690536482471517 | 1.56880506842004 | 0.999714303421225 |
| ebi-fl187-GCST90094817 | prot-a-1666 | Kallikrein-7 \|\| id:prot-a-1666 | Prawns liking \|\| id:ebi-fl187-GCST90094817 | Weighted mode | 20 | -0.235327222 | 0.176079268857244 | 0.197176545775693 | -0.580442589 | 0.109788144697122 | 0.790312199566535 | 0.559650616412049 | 1.11604160607916 | 0.999714303421225 |
| ebi-fl187-GCST90094817 | prot-a-1678 | Importin subunit alpha-3 \|\| id:prot-a-1678 | Prawns liking \|\| id:ebi-fl187-GCST90094817 | Weighted mode | 20 | -0.317839094 | 0.168604987566686 | 0.0748031960198586 | -0.64830487 | 0.0126266815609079 | 0.727719873437669 | 0.5229314628395 | 1.0127067346848 | 0.999714303421225 |
| ebi-fl187-GCST90094817 | prot-a-1718 | Protein LDOC1 \|\| id:prot-a-1718 | Prawns liking \|\| id:ebi-fl187-GCST90094817 | Weighted mode | 20 | 0.130575616545639 | 0.190685988187657 | 0.50175922307891 | -0.24316892 | 0.504320153393447 | 1.13948410048743 | 0.784139045579066 | 1.65585940731313 | 0.999714303421225 |
| ebi-fl187-GCST90094817 | prot-a-1723 | Leptin \|\| id:prot-a-1723 | Prawns liking \|\| id:ebi-fl187-GCST90094817 | Weighted mode | 20 | -0.255280223 | 0.221131842529293 | 0.262639858953669 | -0.688698634 | 0.178138188666576 | 0.77469937919707 | 0.502229227991763 | 1.19499044396152 | 0.999714303421225 |
| ebi-fl187-GCST90094817 | prot-a-1730 | Galectin-7 \|\| id:prot-a-1730 | Prawns liking \|\| id:ebi-fl187-GCST90094817 | Weighted mode | 20 | 0.0714559745791323 | 0.180609685099704 | 0.696779680628014 | -0.282539008 | 0.425450957374553 | 1.0740708632386 | 0.753867234369625 | 1.53028035529726 | 0.999714303421225 |
| ebi-fl187-GCST90094817 | prot-a-1753 | Lipase member N \|\| id:prot-a-1753 | Prawns liking \|\| id:ebi-fl187-GCST90094817 | Weighted mode | 20 | -0.231488015 | 0.194154370526203 | 0.247826986101894 | -0.612030581 | 0.149054551582733 | 0.793352204051072 | 0.54224867061891 | 1.16073630748472 | 0.999714303421225 |
| ebi-fl187-GCST90094817 | prot-a-1756 | VIP36-like protein \|\| id:prot-a-1756 | Prawns liking \|\| id:ebi-fl187-GCST90094817 | Weighted mode | 20 | -0.145343139 | 0.186415118073126 | 0.445186573559521 | -0.510716771 | 0.22003049196589 | 0.864725520750478 | 0.60006531528595 | 1.24611472649583 | 0.999714303421225 |
| ebi-fl187-GCST90094817 | prot-a-1760 | Ig Kappa chain V-I region HK102- like \|\| id:prot-a-1760 | Prawns liking \|\| id:ebi-fl187-GCST90094817 | Weighted mode | 20 | -0.111149438 | 0.189276969258979 | 0.563962004438977 | -0.482132298 | 0.259833421959028 | 0.894805021253717 | 0.617465367214052 | 1.29671406458542 | 0.999714303421225 |
| ebi-fl187-GCST90094817 | prot-a-179 | Neutral ceramidase \|\| id:prot-a-179 | Prawns liking \|\| id:ebi-fl187-GCST90094817 | Weighted mode | 20 | 0.280336534750857 | 0.176946653363811 | 0.129628902686298 | -0.066478906 | 0.627151975343926 | 1.32357516643355 | 0.935682653085683 | 1.8722707056953 | 0.999714303421225 |
| ebi-fl187-GCST90094817 | prot-a-1809 | Lactotransferrin \|\| id:prot-a-1809 | Prawns liking \|\| id:ebi-fl187-GCST90094817 | Weighted mode | 20 | -0.263189872 | 0.190168344286991 | 0.182406674283795 | -0.635919827 | 0.109540082812626 | 0.768595948613639 | 0.529448263572721 | 1.11576479302998 | 0.999714303421225 |
| ebi-fl187-GCST90094817 | prot-a-1818 | Lysophospholipase-like protein 1 \|\| id:prot-a-1818 | Prawns liking \|\| id:ebi-fl187-GCST90094817 | Weighted mode | 20 | -0.018119822 | 0.249021409889508 | 0.942754374782744 | -0.506201785 | 0.469962141487509 | 0.9820433550116 | 0.602780726953832 | 1.59993362096381 | 0.999714303421225 |
| ebi-fl187-GCST90094817 | prot-a-1852 | MAP kinase-activated protein kinase 3 \|\| id:prot-a-1852 | Prawns liking \|\| id:ebi-fl187-GCST90094817 | Weighted mode | 20 | -0.234200284 | 0.19713485743971 | 0.249464412011109 | -0.620584605 | 0.152184036439502 | 0.791203334544195 | 0.537630044641877 | 1.16437450405296 | 0.999714303421225 |
| ebi-fl187-GCST90094817 | prot-a-1856 | Megakaryocyte-associated tyrosine-protein kinase \|\| id:prot-a-1856 | Prawns liking \|\| id:ebi-fl187-GCST90094817 | Weighted mode | 20 | -0.279132893 | 0.196288117585435 | 0.171218841288365 | -0.663857604 | 0.105591817212444 | 0.756439370844951 | 0.514861367579432 | 1.11136814256321 | 0.999714303421225 |
| ebi-fl187-GCST90094817 | prot-a-1890 | Beta-1,3-N-acetylglucosaminyltransferase manic fringe \|\| id:prot-a-1890 | Prawns liking \|\| id:ebi-fl187-GCST90094817 | Weighted mode | 20 | -0.270433594 | 0.181196778632262 | 0.151991063484808 | -0.62557928 | 0.0847120922311988 | 0.763048569402556 | 0.534951452065036 | 1.0884036617149 | 0.999714303421225 |
| ebi-fl187-GCST90094817 | prot-a-1919 | Matrilysin \|\| id:prot-a-1919 | Prawns liking \|\| id:ebi-fl187-GCST90094817 | Weighted mode | 20 | -0.122252961 | 0.168437003510686 | 0.476802427997423 | -0.452389488 | 0.207883566163942 | 0.884924489054224 | 0.636106365929463 | 1.23106982302189 | 0.999714303421225 |
| ebi-fl187-GCST90094817 | prot-a-1923 | Multimerin-2 \|\| id:prot-a-1923 | Prawns liking \|\| id:ebi-fl187-GCST90094817 | Weighted mode | 20 | -0.212122578 | 0.1892885762658 | 0.276410436801475 | -0.583128187 | 0.15888303171724 | 0.808865542560161 | 0.558149636240758 | 1.17220082834368 | 0.999714303421225 |
| ebi-fl187-GCST90094817 | prot-a-1948 | Mesothelin \|\| id:prot-a-1948 | Prawns liking \|\| id:ebi-fl187-GCST90094817 | Weighted mode | 20 | -0.265971486 | 0.19800564592485 | 0.195016060294405 | -0.654062552 | 0.122119579731021 | 0.766460981841978 | 0.519929240673128 | 1.1298892055496 | 0.999714303421225 |
| ebi-fl187-GCST90094817 | prot-a-195 | ATPase family AAA domain-containing protein 1 \|\| id:prot-a-195 | Prawns liking \|\| id:ebi-fl187-GCST90094817 | Weighted mode | 20 | -0.080045182 | 0.183482638774551 | 0.66756867343414 | -0.439671154 | 0.279580789873181 | 0.923074638970762 | 0.644248244631556 | 1.32257525916628 | 0.999714303421225 |
| ebi-fl187-GCST90094817 | prot-a-1966 | Mucin-1 \|\| id:prot-a-1966 | Prawns liking \|\| id:ebi-fl187-GCST90094817 | Weighted mode | 20 | -0.290400495 | 0.19559515860109 | 0.154025014882492 | -0.673767006 | 0.0929660155836374 | 0.747963951557076 | 0.509784594539408 | 1.09742443930527 | 0.999714303421225 |
| ebi-fl187-GCST90094817 | prot-a-1986 | Myocardial zonula adherens protein \|\| id:prot-a-1986 | Prawns liking \|\| id:ebi-fl187-GCST90094817 | Weighted mode | 20 | -0.349819778 | 0.175843378415493 | 0.0612508866729222 | -0.6944728 | -0.005166756 | 0.704815101345511 | 0.499337629473838 | 0.994846568259103 | 0.999714303421225 |
| ebi-fl187-GCST90094817 | prot-a-2071 | Epididymal secretory protein E1 \|\| id:prot-a-2071 | Prawns liking \|\| id:ebi-fl187-GCST90094817 | Weighted mode | 20 | 0.0633627821595302 | 0.183645593480445 | 0.733863843542795 | -0.296582581 | 0.423308145381203 | 1.06541328206104 | 0.743354237746832 | 1.5270047629414 | 0.999714303421225 |
| ebi-fl187-GCST90094817 | prot-a-2098 | Neuritin \|\| id:prot-a-2098 | Prawns liking \|\| id:ebi-fl187-GCST90094817 | Weighted mode | 20 | 0.0259692950302592 | 0.174404062303407 | 0.883199223332301 | -0.315862667 | 0.367801257144937 | 1.02630943518949 | 0.729159580903013 | 1.44455491547476 | 0.999714303421225 |
| ebi-fl187-GCST90094817 | prot-a-2115 | Neurotrophin-4 \|\| id:prot-a-2115 | Prawns liking \|\| id:ebi-fl187-GCST90094817 | Weighted mode | 20 | -0.055786931 | 0.165253629031895 | 0.739377981712821 | -0.379684044 | 0.268110182230799 | 0.945740622743519 | 0.684077513756945 | 1.30749119437522 | 0.999714303421225 |
| ebi-fl187-GCST90094817 | prot-a-2118 | Netrin-1 \|\| id:prot-a-2118 | Prawns liking \|\| id:ebi-fl187-GCST90094817 | Weighted mode | 20 | 0.00222375208102399 | 0.173105926870638 | 0.989884434969863 | -0.337063865 | 0.341511368747474 | 1.00222622645147 | 0.713863247866502 | 1.40707258986808 | 0.999714303421225 |
| ebi-fl187-GCST90094817 | prot-a-2120 | Netrin-G1 \|\| id:prot-a-2120 | Prawns liking \|\| id:ebi-fl187-GCST90094817 | Weighted mode | 20 | -0.224353664 | 0.197862188807056 | 0.270945953207728 | -0.612163554 | 0.1634562259655 | 0.799032495287279 | 0.54217657082937 | 1.17757380690274 | 0.999714303421225 |
| ebi-fl187-GCST90094817 | prot-a-2145 | Olfactomedin-like protein 3 \|\| id:prot-a-2145 | Prawns liking \|\| id:ebi-fl187-GCST90094817 | Weighted mode | 20 | -0.148223207 | 0.169064752719046 | 0.391586828436469 | -0.479590122 | 0.183143708361563 | 0.862238635790114 | 0.619037069306192 | 1.20098698755222 | 0.999714303421225 |
| ebi-fl187-GCST90094817 | prot-a-2162 | Prolyl 4-hydroxylase subunit alpha-2 \|\| id:prot-a-2162 | Prawns liking \|\| id:ebi-fl187-GCST90094817 | Weighted mode | 20 | -0.226598984 | 0.172455542500877 | 0.204505230596944 | -0.564611848 | 0.111413879092859 | 0.797240424194568 | 0.568580799984171 | 1.11785746896066 | 0.999714303421225 |
| ebi-fl187-GCST90094817 | prot-a-2168 | Polyadenylate-binding protein-interacting protein 1 \|\| id:prot-a-2168 | Prawns liking \|\| id:ebi-fl187-GCST90094817 | Weighted mode | 20 | 0.0708796006096586 | 0.209106666224094 | 0.738354503005284 | -0.338969465 | 0.480728666408883 | 1.07345197512429 | 0.712504204937866 | 1.61725241045943 | 0.999714303421225 |
| ebi-fl187-GCST90094817 | prot-a-219 | Beta-1,4-galactosyltransferase 5 \|\| id:prot-a-219 | Prawns liking \|\| id:ebi-fl187-GCST90094817 | Weighted mode | 20 | 0.212955946805866 | 0.177734445103188 | 0.245584031366002 | -0.135403566 | 0.561315459208114 | 1.23733014169801 | 0.87336338114445 | 1.75297695393208 | 0.999714303421225 |
| ebi-fl187-GCST90094817 | prot-a-2190 | Pyruvate carboxylase, mitochondrial \|\| id:prot-a-2190 | Prawns liking \|\| id:ebi-fl187-GCST90094817 | Weighted mode | 20 | -0.169792349 | 0.179992897207584 | 0.357354710095428 | -0.522578427 | 0.182993729660133 | 0.843840022741896 | 0.592989594477648 | 1.20080687858998 | 0.999714303421225 |
| ebi-fl187-GCST90094817 | prot-a-2197 | Protocadherin-9 \|\| id:prot-a-2197 | Prawns liking \|\| id:ebi-fl187-GCST90094817 | Weighted mode | 20 | -0.232097871 | 0.170185719133091 | 0.188573620622268 | -0.56566188 | 0.101466138820763 | 0.792868520927878 | 0.56798408490843 | 1.10679244046021 | 0.999714303421225 |
| ebi-fl187-GCST90094817 | prot-a-2198 | Protocadherin alpha-4 \|\| id:prot-a-2198 | Prawns liking \|\| id:ebi-fl187-GCST90094817 | Weighted mode | 20 | 0.377253741370915 | 0.201712460027552 | 0.0769356940028042 | -0.01810268 | 0.772610163024917 | 1.45827428694667 | 0.982060188962864 | 2.16541095939919 | 0.999714303421225 |
| ebi-fl187-GCST90094817 | prot-a-2254 | Placenta growth factor \|\| id:prot-a-2254 | Prawns liking \|\| id:ebi-fl187-GCST90094817 | Weighted mode | 20 | 0.246039444275079 | 0.191691683200082 | 0.214745181566968 | -0.129676255 | 0.621755143347239 | 1.27895001980322 | 0.878379756126004 | 1.86219359194795 | 0.999714303421225 |
| ebi-fl187-GCST90094817 | prot-a-2261 | Membrane-associated progesterone receptor component 2 \|\| id:prot-a-2261 | Prawns liking \|\| id:ebi-fl187-GCST90094817 | Weighted mode | 20 | -0.448631897 | 0.245746589500685 | 0.0836729167476972 | -0.930295213 | 0.0330314182897488 | 0.63850108952325 | 0.394437250354567 | 1.03358301214174 | 0.999714303421225 |
| ebi-fl187-GCST90094817 | prot-a-2268 | Polymeric immunoglobulin receptor \|\| id:prot-a-2268 | Prawns liking \|\| id:ebi-fl187-GCST90094817 | Weighted mode | 20 | -0.044167756 | 0.170860438556975 | 0.798799144455327 | -0.379054216 | 0.290718703560319 | 0.956793436158677 | 0.684508500632815 | 1.33738832845758 | 0.999714303421225 |
| ebi-fl187-GCST90094817 | prot-a-2283 | Group 10 secretory phospholipase A2 \|\| id:prot-a-2283 | Prawns liking \|\| id:ebi-fl187-GCST90094817 | Weighted mode | 20 | -0.058090199 | 0.172685528713366 | 0.740261383044611 | -0.396553835 | 0.28037343767202 | 0.943564835365374 | 0.672634064549415 | 1.32362401112482 | 0.999714303421225 |
| ebi-fl187-GCST90094817 | prot-a-2298 | Inactive phospholipase D5 \|\| id:prot-a-2298 | Prawns liking \|\| id:ebi-fl187-GCST90094817 | Weighted mode | 20 | -0.127390565 | 0.203886343274379 | 0.539526413487264 | -0.527007798 | 0.272226667555529 | 0.880389755784695 | 0.590368832199108 | 1.31288455591983 | 0.999714303421225 |
| ebi-fl187-GCST90094817 | prot-a-2304 | Procollagen-lysine,2-oxoglutarate 5-dioxygenase 2 \|\| id:prot-a-2304 | Prawns liking \|\| id:ebi-fl187-GCST90094817 | Weighted mode | 20 | 0.0653726940963495 | 0.17860329783989 | 0.718392581410601 | -0.28468977 | 0.415435157862535 | 1.06755682237632 | 0.752247588141531 | 1.51502987442987 | 0.999714303421225 |
| ebi-fl187-GCST90094817 | prot-a-2331 | Periostin \|\| id:prot-a-2331 | Prawns liking \|\| id:ebi-fl187-GCST90094817 | Weighted mode | 20 | 0.0920045222650385 | 0.170332632766612 | 0.595371489574364 | -0.241847438 | 0.425856482487598 | 1.09636978014433 | 0.785175956462363 | 1.53090104825612 | 0.999714303421225 |
| ebi-fl187-GCST90094817 | prot-a-2333 | Periostin \|\| id:prot-a-2333 | Prawns liking \|\| id:ebi-fl187-GCST90094817 | Weighted mode | 20 | 0.0381050107768066 | 0.158425231186595 | 0.8125005352407 | -0.272408442 | 0.348618463902534 | 1.03884031657954 | 0.761543151085479 | 1.41710840917266 | 0.999714303421225 |
| ebi-fl187-GCST90094817 | prot-a-2367 | cAMP-dependent protein kinase catalytic subunit alpha \|\| id:prot-a-2367 | Prawns liking \|\| id:ebi-fl187-GCST90094817 | Weighted mode | 20 | -0.106240549 | 0.183384363080444 | 0.569170654158701 | -0.465673901 | 0.253192802551903 | 0.899208318319119 | 0.627711947087127 | 1.28813160795562 | 0.999714303421225 |
| ebi-fl187-GCST90094817 | prot-a-2385 | Phosphoribosyl pyrophosphate synthase-associated protein 1 \|\| id:prot-a-2385 | Prawns liking \|\| id:ebi-fl187-GCST90094817 | Weighted mode | 20 | -0.15791822 | 0.251091860831376 | 0.536886251979695 | -0.650058267 | 0.334221827148636 | 0.85391961258994 | 0.522015359443896 | 1.39685296912058 | 0.999714303421225 |
| ebi-fl187-GCST90094817 | prot-a-2393 | Trypsin-3 \|\| id:prot-a-2393 | Prawns liking \|\| id:ebi-fl187-GCST90094817 | Weighted mode | 19 | -0.089604798 | 0.247920623871069 | 0.721989083928442 | -0.575529221 | 0.396319624424562 | 0.914292443752265 | 0.562407152275049 | 1.48634431358311 | 0.999714303421225 |
| ebi-fl187-GCST90094817 | prot-a-2405 | Pregnancy-specific beta-1-glycoprotein 6 \|\| id:prot-a-2405 | Prawns liking \|\| id:ebi-fl187-GCST90094817 | Weighted mode | 20 | 0.0652871368921382 | 0.185353789188275 | 0.728542666496549 | -0.29800629 | 0.428580563701157 | 1.06746548910643 | 0.742296670748731 | 1.53507703231899 | 0.999714303421225 |
| ebi-fl187-GCST90094817 | prot-a-244 | Beclin-1 \|\| id:prot-a-244 | Prawns liking \|\| id:ebi-fl187-GCST90094817 | Weighted mode | 20 | -0.160343003 | 0.163292945941991 | 0.338471246349984 | -0.480397177 | 0.159711170809274 | 0.851851551010331 | 0.61853767389963 | 1.17317197574041 | 0.999714303421225 |
| ebi-fl187-GCST90094817 | prot-a-2454 | tRNA pseudouridine synthase A, mitochondrial \|\| id:prot-a-2454 | Prawns liking \|\| id:ebi-fl187-GCST90094817 | Weighted mode | 20 | -0.338673794 | 0.192519302992583 | 0.0946432338226652 | -0.716011628 | 0.0386640394155816 | 0.71271490273022 | 0.488697481437617 | 1.03942122042343 | 0.999714303421225 |
| ebi-fl187-GCST90094817 | prot-a-2466 | Glutaminyl-peptide cyclotransferase-like protein \|\| id:prot-a-2466 | Prawns liking \|\| id:ebi-fl187-GCST90094817 | Weighted mode | 20 | -0.167840473 | 0.174509375351134 | 0.348239154459713 | -0.509878849 | 0.174197902520242 | 0.845488702061038 | 0.600568333945578 | 1.19029110412211 | 0.999714303421225 |
| ebi-fl187-GCST90094817 | prot-a-2474 | Ras-related protein Rab-27A \|\| id:prot-a-2474 | Prawns liking \|\| id:ebi-fl187-GCST90094817 | Weighted mode | 20 | -0.193786922 | 0.188990939006499 | 0.318063790700536 | -0.564209163 | 0.176635318080468 | 0.823833426040098 | 0.568809804870122 | 1.19319587681147 | 0.999714303421225 |
| ebi-fl187-GCST90094817 | prot-a-2490 | Rap1 GTPase-GDP dissociation stimulator 1 \|\| id:prot-a-2490 | Prawns liking \|\| id:ebi-fl187-GCST90094817 | Weighted mode | 20 | -0.09327036 | 0.179586343612181 | 0.609505751179141 | -0.445259594 | 0.258718873286404 | 0.910947183143788 | 0.64065794369488 | 1.29526961874809 | 0.999714303421225 |
| ebi-fl187-GCST90094817 | prot-a-2545 | Receptor-interacting serine/threonine-protein kinase 2 \|\| id:prot-a-2545 | Prawns liking \|\| id:ebi-fl187-GCST90094817 | Weighted mode | 20 | 0.282468936939653 | 0.185495460892475 | 0.144284152833013 | -0.081102166 | 0.646040040288905 | 1.32640057239579 | 0.922099479038953 | 1.90797036376761 | 0.999714303421225 |
| ebi-fl187-GCST90094817 | prot-a-2610 | RUN and FYVE domain-containing protein 1 \|\| id:prot-a-2610 | Prawns liking \|\| id:ebi-fl187-GCST90094817 | Weighted mode | 20 | 0.290874208346378 | 0.191423478250446 | 0.145094716575866 | -0.084315809 | 0.666064225717253 | 1.33759631491451 | 0.919140937252114 | 1.94656099968933 | 0.999714303421225 |
| ebi-fl187-GCST90094817 | prot-a-2627 | Serine--tRNA ligase, mitochondrial \|\| id:prot-a-2627 | Prawns liking \|\| id:ebi-fl187-GCST90094817 | Weighted mode | 20 | -0.036561838 | 0.182036345299592 | 0.842950984260876 | -0.393353075 | 0.320229398703683 | 0.964098474050998 | 0.674790453968569 | 1.3774437118975 | 0.999714303421225 |
| ebi-fl187-GCST90094817 | prot-a-264 | BolA-like protein 2 \|\| id:prot-a-264 | Prawns liking \|\| id:ebi-fl187-GCST90094817 | Weighted mode | 20 | -0.209202184 | 0.180381004230493 | 0.260505960961211 | -0.562748952 | 0.144344584456173 | 0.811231201225601 | 0.56964099373987 | 1.15528213220984 | 0.999714303421225 |
| ebi-fl187-GCST90094817 | prot-a-2646 | Secretoglobin family 1C member 1 \|\| id:prot-a-2646 | Prawns liking \|\| id:ebi-fl187-GCST90094817 | Weighted mode | 20 | -0.302639265 | 0.188954934535684 | 0.125728955208694 | -0.672990937 | 0.0677124067040118 | 0.738865582988542 | 0.510180376349983 | 1.07005752285246 | 0.999714303421225 |
| ebi-fl187-GCST90094817 | prot-a-2672 | Semaphorin-3E \|\| id:prot-a-2672 | Prawns liking \|\| id:ebi-fl187-GCST90094817 | Weighted mode | 20 | -0.019921127 | 0.173783000975467 | 0.909939402327682 | -0.360535809 | 0.320693555027137 | 0.980275987678627 | 0.697302605088713 | 1.37808320950852 | 0.999714303421225 |
| ebi-fl187-GCST90094817 | prot-a-2701 | Plasma protease C1 inhibitor \|\| id:prot-a-2701 | Prawns liking \|\| id:ebi-fl187-GCST90094817 | Weighted mode | 20 | -0.34666305 | 0.181193006937189 | 0.0709098113733898 | -0.701801343 | 0.00847524379541737 | 0.707043526581658 | 0.495691588318597 | 1.00851126035191 | 0.999714303421225 |
| ebi-fl187-GCST90094817 | prot-a-2738 | Signaling threshold-regulating transmembrane adapter 1 \|\| id:prot-a-2738 | Prawns liking \|\| id:ebi-fl187-GCST90094817 | Weighted mode | 20 | 0.00559296894401795 | 0.202376254210319 | 0.978240297431765 | -0.391064489 | 0.402250427196243 | 1.00560863879486 | 0.6763365381574 | 1.49518572096354 | 0.999714303421225 |
| ebi-fl187-GCST90094817 | prot-a-274 | Serine/threonine-protein kinase BRSK2 \|\| id:prot-a-274 | Prawns liking \|\| id:ebi-fl187-GCST90094817 | Weighted mode | 20 | 0.0419150588280403 | 0.170731649971278 | 0.808699625744766 | -0.292718975 | 0.376549092771744 | 1.04280589783364 | 0.746231820944327 | 1.45724707796636 | 0.999714303421225 |
| ebi-fl187-GCST90094817 | prot-a-2749 | Mitochondrial glutamate carrier 2 \|\| id:prot-a-2749 | Prawns liking \|\| id:ebi-fl187-GCST90094817 | Weighted mode | 20 | -0.0727598 | 0.157546767705653 | 0.649447418544996 | -0.381551465 | 0.236031864719297 | 0.929824146979535 | 0.682801245001578 | 1.26621465709867 | 0.999714303421225 |
| ebi-fl187-GCST90094817 | prot-a-2760 | Sodium-coupled monocarboxylate transporter 1 \|\| id:prot-a-2760 | Prawns liking \|\| id:ebi-fl187-GCST90094817 | Weighted mode | 20 | -0.163393229 | 0.178298928879339 | 0.370942593538824 | -0.51285913 | 0.186072671558591 | 0.849257170160147 | 0.598781136173939 | 1.20450979080094 | 0.999714303421225 |
| ebi-fl187-GCST90094817 | prot-a-2777 | Small integral membrane protein 10 \|\| id:prot-a-2777 | Prawns liking \|\| id:ebi-fl187-GCST90094817 | Weighted mode | 20 | 0.202263220176869 | 0.15519127950917 | 0.208040228416077 | -0.101911688 | 0.506438128014842 | 1.2241701920013 | 0.903109303842176 | 1.65937019207854 | 0.999714303421225 |
| ebi-fl187-GCST90094817 | prot-a-2799 | Superoxide dismutase [Mn], mitochondrial \|\| id:prot-a-2799 | Prawns liking \|\| id:ebi-fl187-GCST90094817 | Weighted mode | 20 | -0.157238139 | 0.196619250123861 | 0.433764553506586 | -0.542611869 | 0.228135591671292 | 0.854500545047284 | 0.581228176370758 | 1.25625565168805 | 0.999714303421225 |
| ebi-fl187-GCST90094817 | prot-a-2805 | Protein SPACA7 \|\| id:prot-a-2805 | Prawns liking \|\| id:ebi-fl187-GCST90094817 | Weighted mode | 20 | 0.0357725439714365 | 0.174805324526502 | 0.84002794945106 | -0.306845892 | 0.378390980043381 | 1.0364200796834 | 0.735763979212141 | 1.4599336362201 | 0.999714303421225 |
| ebi-fl187-GCST90094817 | prot-a-2811 | SPARC-like protein 1 \|\| id:prot-a-2811 | Prawns liking \|\| id:ebi-fl187-GCST90094817 | Weighted mode | 20 | -0.130197385 | 0.17122878188837 | 0.456365706207316 | -0.465805798 | 0.205411027369684 | 0.877922125043005 | 0.62762915928123 | 1.22802971506725 | 0.999714303421225 |
| ebi-fl187-GCST90094817 | prot-a-2831 | Sepiapterin reductase \|\| id:prot-a-2831 | Prawns liking \|\| id:ebi-fl187-GCST90094817 | Weighted mode | 20 | -0.17973126 | 0.174540044003911 | 0.316062184961258 | -0.521829746 | 0.162367226717447 | 0.835494712485193 | 0.593433721119324 | 1.17629212791289 | 0.999714303421225 |
| ebi-fl187-GCST90094817 | prot-a-2834 | SPRY domain-containing SOCS box protein 1 \|\| id:prot-a-2834 | Prawns liking \|\| id:ebi-fl187-GCST90094817 | Weighted mode | 20 | 0.0617311171220978 | 0.190612781489947 | 0.749584786428573 | -0.311869935 | 0.435332168842393 | 1.06367630192853 | 0.732076739891631 | 1.54547633278424 | 0.999714303421225 |
| ebi-fl187-GCST90094817 | prot-a-2835 | Spectrin alpha chain, non-erythrocytic 1 \|\| id:prot-a-2835 | Prawns liking \|\| id:ebi-fl187-GCST90094817 | Weighted mode | 20 | 0.103236160594359 | 0.175258471833882 | 0.562765948867089 | -0.240270444 | 0.446742765388768 | 1.10875322197975 | 0.786415150888345 | 1.56321213529749 | 0.999714303421225 |
| ebi-fl187-GCST90094817 | prot-a-2859 | Sia-alpha-2,3-Gal-beta-1,4-GlcNAc-R:alpha 2,8-sialyltransferase \|\| id:prot-a-2859 | Prawns liking \|\| id:ebi-fl187-GCST90094817 | Weighted mode | 20 | -0.302130079 | 0.234818609278038 | 0.213671914293826 | -0.762374553 | 0.158114395091551 | 0.739241898719072 | 0.466557245588842 | 1.17130017803511 | 0.999714303421225 |
| ebi-fl187-GCST90094817 | prot-a-2861 | Alpha-2,8-sialyltransferase 8F \|\| id:prot-a-2861 | Prawns liking \|\| id:ebi-fl187-GCST90094817 | Weighted mode | 20 | -0.240523986 | 0.185280709049714 | 0.209770233635736 | -0.603674176 | 0.122626203538267 | 0.786215786893487 | 0.54679890541452 | 1.1304617793482 | 0.999714303421225 |
| ebi-fl187-GCST90094817 | prot-a-289 | Uncharacterized protein C17orf89 \|\| id:prot-a-289 | Prawns liking \|\| id:ebi-fl187-GCST90094817 | Weighted mode | 20 | 0.0510146788747787 | 0.162985443365588 | 0.757689323980827 | -0.26843679 | 0.370466147871331 | 1.05233834031255 | 0.764573749896087 | 1.44840963038855 | 0.999714303421225 |
| ebi-fl187-GCST90094817 | prot-a-2921 | Tachykinin-3 \|\| id:prot-a-2921 | Prawns liking \|\| id:ebi-fl187-GCST90094817 | Weighted mode | 20 | -0.027742168 | 0.172790223630817 | 0.874139394765327 | -0.366411006 | 0.310926670455543 | 0.972639112097646 | 0.693217825841843 | 1.36468914548359 | 0.999714303421225 |
| ebi-fl187-GCST90094817 | prot-a-2928 | Tax1-binding protein 1 \|\| id:prot-a-2928 | Prawns liking \|\| id:ebi-fl187-GCST90094817 | Weighted mode | 20 | -0.054024932 | 0.174192797027168 | 0.759827294997561 | -0.395442814 | 0.28739295031907 | 0.947408485557132 | 0.673381790316768 | 1.33294789287875 | 0.999714303421225 |
| ebi-fl187-GCST90094817 | prot-a-296 | Uncharacterized protein C1orf185 \|\| id:prot-a-296 | Prawns liking \|\| id:ebi-fl187-GCST90094817 | Weighted mode | 20 | 0.270319497170779 | 0.187696384077808 | 0.166087398182928 | -0.097565416 | 0.638204409963282 | 1.31038304753576 | 0.907043004832956 | 1.89307863256753 | 0.999714303421225 |
| ebi-fl187-GCST90094817 | prot-a-2964 | Transforming growth factor beta-2 \|\| id:prot-a-2964 | Prawns liking \|\| id:ebi-fl187-GCST90094817 | Weighted mode | 20 | -0.16734704 | 0.162314972976934 | 0.315486309827517 | -0.485484387 | 0.150790307158889 | 0.845905997279921 | 0.615399033320287 | 1.16275281157571 | 0.999714303421225 |
| ebi-fl187-GCST90094817 | prot-a-2977 | Thymocyte nuclear protein 1 \|\| id:prot-a-2977 | Prawns liking \|\| id:ebi-fl187-GCST90094817 | Weighted mode | 20 | -0.101939187 | 0.193088272637717 | 0.603652250357029 | -0.480392201 | 0.276513827847359 | 0.903084469706002 | 0.618540751992503 | 1.3185251849567 | 0.999714303421225 |
| ebi-fl187-GCST90094817 | prot-a-3015 | Transmembrane protein 70, mitochondrial \|\| id:prot-a-3015 | Prawns liking \|\| id:ebi-fl187-GCST90094817 | Weighted mode | 20 | -0.091690007 | 0.174016514261698 | 0.604358236944773 | -0.432762375 | 0.24938236049036 | 0.912387939163874 | 0.648714624034445 | 1.28323259672268 | 0.999714303421225 |
| ebi-fl187-GCST90094817 | prot-a-3030 | Tumor necrosis factor alpha-induced protein 3 \|\| id:prot-a-3030 | Prawns liking \|\| id:ebi-fl187-GCST90094817 | Weighted mode | 20 | -0.253778255 | 0.154490449613791 | 0.116896558323403 | -0.556579537 | 0.0490230259390527 | 0.775863826661395 | 0.573166208839036 | 1.05024453332823 | 0.999714303421225 |
| ebi-fl187-GCST90094817 | prot-a-3032 | Tumor necrosis factor receptor superfamily member 10A \|\| id:prot-a-3032 | Prawns liking \|\| id:ebi-fl187-GCST90094817 | Weighted mode | 20 | 0.157608639803657 | 0.17203199772594 | 0.371066079191984 | -0.179574076 | 0.4947913553465 | 1.17070793655361 | 0.835626049033206 | 1.64015599357548 | 0.999714303421225 |
| ebi-fl187-GCST90094817 | prot-a-3081 | Tumor protein p53-inducible protein 11 \|\| id:prot-a-3081 | Prawns liking \|\| id:ebi-fl187-GCST90094817 | Weighted mode | 20 | 0.209424651894231 | 0.17908930870703 | 0.256709454471003 | -0.141590393 | 0.56043969696001 | 1.23296846977208 | 0.867976712870793 | 1.75144243492901 | 0.999714303421225 |
| ebi-fl187-GCST90094817 | prot-a-315 | Complement C4 \|\| id:prot-a-315 | Prawns liking \|\| id:ebi-fl187-GCST90094817 | Weighted mode | 19 | -0.07016032 | 0.202987309682031 | 0.733620740513623 | -0.468015447 | 0.327694807439007 | 0.932244350941436 | 0.626243850298098 | 1.38776537198494 | 0.999714303421225 |
| ebi-fl187-GCST90094817 | prot-a-3157 | UDP-glucuronosyltransferase 1-8 \|\| id:prot-a-3157 | Prawns liking \|\| id:ebi-fl187-GCST90094817 | Weighted mode | 20 | 0.217281577171486 | 0.212638816694424 | 0.319694763922646 | -0.199490504 | 0.634053657892556 | 1.24269396713074 | 0.819147999774351 | 1.88523721765608 | 0.999714303421225 |
| ebi-fl187-GCST90094817 | prot-a-317 | Glutaredoxin-like protein C5orf63 \|\| id:prot-a-317 | Prawns liking \|\| id:ebi-fl187-GCST90094817 | Weighted mode | 20 | 0.0762534675266944 | 0.194010353208553 | 0.698666391766507 | -0.304006825 | 0.456513759815458 | 1.07923609079468 | 0.737855830742921 | 1.57856113775104 | 0.999714303421225 |
| ebi-fl187-GCST90094817 | prot-a-3192 | Guanine nucleotide exchange factor VAV3 \|\| id:prot-a-3192 | Prawns liking \|\| id:ebi-fl187-GCST90094817 | Weighted mode | 19 | 0.118169848967745 | 0.177186798175126 | 0.513280866661574 | -0.229116275 | 0.465455973390992 | 1.12543524914994 | 0.795236061693792 | 1.59274027051969 | 0.999714303421225 |
| ebi-fl187-GCST90094817 | prot-a-3233 | Serine/threonine-protein kinase WNK3 \|\| id:prot-a-3233 | Prawns liking \|\| id:ebi-fl187-GCST90094817 | Weighted mode | 20 | -0.176043242 | 0.218960552250258 | 0.431344677232336 | -0.605205924 | 0.253119440858787 | 0.838581720978201 | 0.545961988407366 | 1.28803711190615 | 0.999714303421225 |
| ebi-fl187-GCST90094817 | prot-a-3250 | 14-3-3 protein theta \|\| id:prot-a-3250 | Prawns liking \|\| id:ebi-fl187-GCST90094817 | Weighted mode | 20 | 0.0490277463900746 | 0.15790830783856 | 0.759574322249822 | -0.260472537 | 0.358528029753653 | 1.05024949096781 | 0.770687321491751 | 1.43122114834213 | 0.999714303421225 |
| ebi-fl187-GCST90094817 | prot-a-3278 | E3 ubiquitin-protein ligase ZNRF3 \|\| id:prot-a-3278 | Prawns liking \|\| id:ebi-fl187-GCST90094817 | Weighted mode | 20 | -0.274232197 | 0.207499148338713 | 0.201992842486249 | -0.680930527 | 0.132466134226693 | 0.760155549296253 | 0.50614579071027 | 1.14164035290112 | 0.999714303421225 |
| ebi-fl187-GCST90094817 | prot-a-355 | Calpain-3 \|\| id:prot-a-355 | Prawns liking \|\| id:ebi-fl187-GCST90094817 | Weighted mode | 20 | -0.172988356 | 0.166321390169326 | 0.311355735837471 | -0.498978281 | 0.153001568418725 | 0.841147408844625 | 0.607150680273874 | 1.16532680666169 | 0.999714303421225 |
| ebi-fl187-GCST90094817 | prot-a-385 | Coiled-coil domain-containing protein 90B, mitochondrial \|\| id:prot-a-385 | Prawns liking \|\| id:ebi-fl187-GCST90094817 | Weighted mode | 20 | -0.073506972 | 0.163783889366453 | 0.658649364220783 | -0.394523396 | 0.247509450759746 | 0.929129667506633 | 0.674001194674874 | 1.28083146715699 | 0.999714303421225 |
| ebi-fl187-GCST90094817 | prot-a-390 | C-C motif chemokine 14 \|\| id:prot-a-390 | Prawns liking \|\| id:ebi-fl187-GCST90094817 | Weighted mode | 20 | -0.200264628 | 0.207642700004765 | 0.346927649184975 | -0.60724432 | 0.206715063812123 | 0.818514122499334 | 0.544850235022563 | 1.22963215516115 | 0.999714303421225 |
| ebi-fl187-GCST90094817 | prot-a-40 | Pituitary adenylate cyclase-activating polypeptide 27 \|\| id:prot-a-40 | Prawns liking \|\| id:ebi-fl187-GCST90094817 | Weighted mode | 20 | 0.101604600894117 | 0.164656076601498 | 0.544512026632518 | -0.221121309 | 0.424330511033053 | 1.10694569984635 | 0.801619430543916 | 1.52856671847003 | 0.999714303421225 |
| ebi-fl187-GCST90094817 | prot-a-401 | C-C motif chemokine 24 \|\| id:prot-a-401 | Prawns liking \|\| id:ebi-fl187-GCST90094817 | Weighted mode | 20 | -0.107491156 | 0.180510800435347 | 0.558547545981737 | -0.461292325 | 0.246310012401037 | 0.898084464668536 | 0.630468348604752 | 1.27929610973161 | 0.999714303421225 |
| ebi-fl187-GCST90094817 | prot-a-412 | Cerebral cavernous malformations 2 protein \|\| id:prot-a-412 | Prawns liking \|\| id:ebi-fl187-GCST90094817 | Weighted mode | 19 | -0.035507079 | 0.167896084141818 | 0.834886805939277 | -0.364583404 | 0.293569246289697 | 0.965115902509114 | 0.69448591098914 | 1.34120604973734 | 0.999714303421225 |
| ebi-fl187-GCST90094817 | prot-a-44 | Adhesion G protein-coupled receptor F5 \|\| id:prot-a-44 | Prawns liking \|\| id:ebi-fl187-GCST90094817 | Weighted mode | 20 | 0.44835945509942 | 0.201541362850509 | 0.0384191198495735 | 0.0533383839124212 | 0.843380526286418 | 1.56574140824607 | 1.05478650752289 | 2.32421076683445 | 0.999714303421225 |
| ebi-fl187-GCST90094817 | prot-a-48 | Adrenomedullin \|\| id:prot-a-48 | Prawns liking \|\| id:ebi-fl187-GCST90094817 | Weighted mode | 20 | 0.114376748288543 | 0.177573713642168 | 0.527204955065964 | -0.23366773 | 0.462421227027194 | 1.12117444587845 | 0.791624805019174 | 1.58791403468009 | 0.999714303421225 |
| ebi-fl187-GCST90094817 | prot-a-502 | Carcinoembryonic antigen-related cell adhesion molecule 1 \|\| id:prot-a-502 | Prawns liking \|\| id:ebi-fl187-GCST90094817 | Weighted mode | 20 | 0.0404154659562412 | 0.159543782880686 | 0.802742480278037 | -0.272290348 | 0.353121280402386 | 1.04124328547659 | 0.761633089965509 | 1.42350377607567 | 0.999714303421225 |
| ebi-fl187-GCST90094817 | prot-a-518 | Complement factor B \|\| id:prot-a-518 | Prawns liking \|\| id:ebi-fl187-GCST90094817 | Weighted mode | 20 | -0.132049751 | 0.262920591445911 | 0.621268281680387 | -0.64737411 | 0.383274608530923 | 0.876297397583258 | 0.523418412985803 | 1.46708084767362 | 0.999714303421225 |
| ebi-fl187-GCST90094817 | prot-a-525 | Properdin \|\| id:prot-a-525 | Prawns liking \|\| id:ebi-fl187-GCST90094817 | Weighted mode | 20 | 0.0366053636337689 | 0.176583946612826 | 0.83798332257615 | -0.309499172 | 0.382709898994908 | 1.03728359022857 | 0.733814379196667 | 1.46625260700854 | 0.999714303421225 |
| ebi-fl187-GCST90094817 | prot-a-533 | Chondroadherin \|\| id:prot-a-533 | Prawns liking \|\| id:ebi-fl187-GCST90094817 | Weighted mode | 20 | -0.134015325 | 0.19316596459212 | 0.496213483569765 | -0.512620616 | 0.244589965650517 | 0.87457666165699 | 0.598923970949804 | 1.27709755196823 | 0.999714303421225 |
| ebi-fl187-GCST90094817 | prot-a-535 | Coiled-coil-helix-coiled-coil-helix domain-containing protein 2 \|\| id:prot-a-535 | Prawns liking \|\| id:ebi-fl187-GCST90094817 | Weighted mode | 20 | 0.322566124483654 | 0.191425594931348 | 0.108329350992321 | -0.052628042 | 0.697760290549096 | 1.38066618367969 | 0.948732836025381 | 2.00924753352338 | 0.999714303421225 |
| ebi-fl187-GCST90094817 | prot-a-591 | Caseinolytic peptidase B protein homolog \|\| id:prot-a-591 | Prawns liking \|\| id:ebi-fl187-GCST90094817 | Weighted mode | 19 | -0.074866412 | 0.159367259220315 | 0.644158321898877 | -0.387226241 | 0.237493415611609 | 0.927867429576901 | 0.678937474374279 | 1.26806664731992 | 0.999714303421225 |
| ebi-fl187-GCST90094817 | prot-a-605 | Ciliary neurotrophic factor receptor subunit alpha \|\| id:prot-a-605 | Prawns liking \|\| id:ebi-fl187-GCST90094817 | Weighted mode | 20 | 0.0119557385051223 | 0.191197936099432 | 0.950793363465438 | -0.362792216 | 0.38670369326001 | 1.012027494 | 0.695730980073555 | 1.4721202274969 | 0.999714303421225 |
| ebi-fl187-GCST90094817 | prot-a-606 | Ciliary neurotrophic factor receptor subunit alpha \|\| id:prot-a-606 | Prawns liking \|\| id:ebi-fl187-GCST90094817 | Weighted mode | 20 | -0.002036037 | 0.192594272259809 | 0.991675422929762 | -0.379520811 | 0.375448736138323 | 0.997966033827431 | 0.684189186522374 | 1.45564446836034 | 0.999714303421225 |
| ebi-fl187-GCST90094817 | prot-a-707 | Cancer/testis antigen 55 \|\| id:prot-a-707 | Prawns liking \|\| id:ebi-fl187-GCST90094817 | Weighted mode | 20 | -0.088743746 | 0.180280924087253 | 0.628180445088045 | -0.442094358 | 0.264606864858223 | 0.915080036128972 | 0.642688990027014 | 1.30291865196975 | 0.999714303421225 |
| ebi-fl187-GCST90094817 | prot-a-719 | Dipeptidyl peptidase 1 \|\| id:prot-a-719 | Prawns liking \|\| id:ebi-fl187-GCST90094817 | Weighted mode | 20 | -0.0889102 | 0.184833568774168 | 0.635989220905496 | -0.451183995 | 0.273363594763137 | 0.914927730364424 | 0.636873649936957 | 1.31437805893313 | 0.999714303421225 |
| ebi-fl187-GCST90094817 | prot-a-752 | Cytochrome b5 \|\| id:prot-a-752 | Prawns liking \|\| id:ebi-fl187-GCST90094817 | Weighted mode | 20 | -0.348429617 | 0.223529342344238 | 0.135553480140643 | -0.786547128 | 0.0896878938876083 | 0.705795589211167 | 0.455414571781093 | 1.09383283850958 | 0.999714303421225 |
| ebi-fl187-GCST90094817 | prot-a-755 | Cytoglobin \|\| id:prot-a-755 | Prawns liking \|\| id:ebi-fl187-GCST90094817 | Weighted mode | 20 | -0.164646279 | 0.174981930706115 | 0.358549376595919 | -0.507610863 | 0.178318305014552 | 0.848193674802975 | 0.601931959977921 | 1.19520570066119 | 0.999714303421225 |
| ebi-fl187-GCST90094817 | prot-a-807 | Desmin \|\| id:prot-a-807 | Prawns liking \|\| id:ebi-fl187-GCST90094817 | Weighted mode | 20 | 0.0395523423909077 | 0.165472301482608 | 0.813644016438438 | -0.284773369 | 0.363878053296819 | 1.04034495160199 | 0.752184703740451 | 1.4388987345018 | 0.999714303421225 |
| ebi-fl187-GCST90094817 | prot-a-82 | Ameloblastin \|\| id:prot-a-82 | Prawns liking \|\| id:ebi-fl187-GCST90094817 | Weighted mode | 20 | -0.084458586 | 0.250347057271555 | 0.739539461817329 | -0.575138818 | 0.406221646587112 | 0.919009714764795 | 0.562626760710493 | 1.50113523708955 | 0.999714303421225 |
| ebi-fl187-GCST90094817 | prot-a-84 | Muellerian-inhibiting factor \|\| id:prot-a-84 | Prawns liking \|\| id:ebi-fl187-GCST90094817 | Weighted mode | 20 | -0.057129024 | 0.175212997237066 | 0.74794682955906 | -0.400546499 | 0.286288450487603 | 0.94447220183174 | 0.669953817095097 | 1.33147646490128 | 0.999714303421225 |
| ebi-fl187-GCST90094817 | prot-a-858 | Inactive dipeptidyl peptidase 10 \|\| id:prot-a-858 | Prawns liking \|\| id:ebi-fl187-GCST90094817 | Weighted mode | 20 | 0.00855504807908747 | 0.166564963054813 | 0.959573500152884 | -0.31791228 | 0.335022375666521 | 1.00859174708216 | 0.727666616888416 | 1.3979716654203 | 0.999714303421225 |
| ebi-fl187-GCST90094817 | prot-a-863 | Dihydropyrimidinase-related protein 3 \|\| id:prot-a-863 | Prawns liking \|\| id:ebi-fl187-GCST90094817 | Weighted mode | 20 | -0.008152803 | 0.177734349084111 | 0.963891986544727 | -0.356512127 | 0.340206520947396 | 0.991880340709773 | 0.700113980812215 | 1.40523777163424 | 0.999714303421225 |
| ebi-fl187-GCST90094817 | prot-a-864 | Dihydropyrimidinase-related protein 5 \|\| id:prot-a-864 | Prawns liking \|\| id:ebi-fl187-GCST90094817 | Weighted mode | 20 | -0.21135135 | 0.180727711009524 | 0.256686030561824 | -0.565577664 | 0.142874963654897 | 0.80948960280075 | 0.568031920655767 | 1.15358555252675 | 0.999714303421225 |
| ebi-fl187-GCST90094817 | prot-a-884 | Endothelin-converting enzyme 1 \|\| id:prot-a-884 | Prawns liking \|\| id:ebi-fl187-GCST90094817 | Weighted mode | 20 | -0.128291744 | 0.171036401305179 | 0.462391856 | -0.46352309 | 0.206939603005248 | 0.879596725034191 | 0.629063489539349 | 1.22990828677314 | 0.999714303421225 |
| ebi-fl187-GCST90094817 | prot-a-893 | Early endosome antigen 1 \|\| id:prot-a-893 | Prawns liking \|\| id:ebi-fl187-GCST90094817 | Weighted mode | 19 | -0.349358823 | 0.182159729224254 | 0.0711340908694604 | -0.706391892 | 0.00767424615255602 | 0.705140064306874 | 0.493421306695844 | 1.00770376865222 | 0.999714303421225 |
| ebi-fl187-GCST90094817 | prot-a-897 | Ephrin-A2 \|\| id:prot-a-897 | Prawns liking \|\| id:ebi-fl187-GCST90094817 | Weighted mode | 20 | -0.082848068 | 0.20392743732405 | 0.689089065767036 | -0.482545845 | 0.316849709583878 | 0.920490989028199 | 0.617210068938959 | 1.37279623830296 | 0.999714303421225 |
| ebi-fl187-GCST90094817 | prot-a-90 | Anaphase-promoting complex subunit 10 \|\| id:prot-a-90 | Prawns liking \|\| id:ebi-fl187-GCST90094817 | Weighted mode | 20 | 0.00662641028654032 | 0.168896658155507 | 0.969113528891503 | -0.32441104 | 0.337663860271333 | 1.00664841351715 | 0.722953018907962 | 1.40166926748195 | 0.999714303421225 |
| ebi-fl187-GCST90094817 | prot-a-914 | Histone-lysine N-methyltransferase EHMT2 \|\| id:prot-a-914 | Prawns liking \|\| id:ebi-fl187-GCST90094817 | Weighted mode | 20 | -0.231933869 | 0.224400955566487 | 0.314314958560797 | -0.671759742 | 0.207892003646271 | 0.792998563151316 | 0.51080889445875 | 1.23108021019559 | 0.999714303421225 |
| ebi-fl187-GCST90094817 | prot-a-932 | RNA polymerase II elongation factor ELL \|\| id:prot-a-932 | Prawns liking \|\| id:ebi-fl187-GCST90094817 | Weighted mode | 20 | -0.518332881 | 0.202289840214221 | 0.0190538323213264 | -0.914820968 | -0.121844794 | 0.595512511110689 | 0.400588337880474 | 0.885285759355215 | 0.999714303421225 |
| ebi-fl187-GCST90094817 | prot-a-991 | Estrogen receptor \|\| id:prot-a-991 | Prawns liking \|\| id:ebi-fl187-GCST90094817 | Weighted mode | 20 | 0.118881434411892 | 0.169828054893937 | 0.492403632607243 | -0.213981553 | 0.451744422004008 | 1.12623637749339 | 0.807363278170202 | 1.57105037135717 | 0.999714303421225 |
| ebi-fl187-GCST90094817 | prot-a-992 | Persulfide dioxygenase ETHE1, mitochondrial \|\| id:prot-a-992 | Prawns liking \|\| id:ebi-fl187-GCST90094817 | Weighted mode | 20 | 0.0200446447289611 | 0.18971387942317 | 0.916961824579618 | -0.351794559 | 0.391883848398374 | 1.02024688765579 | 0.703424619432106 | 1.47976582424948 | 0.999714303421225 |
| ebi-fl187-GCST90094817 | prot-a-993 | Ethanolamine kinase 1 \|\| id:prot-a-993 | Prawns liking \|\| id:ebi-fl187-GCST90094817 | Weighted mode | 20 | 0.0224295103158321 | 0.178631591564259 | 0.901396985338589 | -0.327688409 | 0.372547429781779 | 1.02268294302598 | 0.720587513204081 | 1.45142731839161 | 0.999714303421225 |
| ebi-fl187-GCST90094817 | prot-b-10 | coagulation factor III, tissue factor \|\| id:prot-b-10 | Prawns liking \|\| id:ebi-fl187-GCST90094817 | Weighted mode | 12 | -0.447773166 | 0.250899567685531 | 0.101885969264539 | -0.939536319 | 0.0439899862149109 | 0.639049625488489 | 0.390809004018929 | 1.04497189071211 | 0.999714303421225 |
| ebi-fl187-GCST90094817 | prot-b-28 | prolactin \|\| id:prot-b-28 | Prawns liking \|\| id:ebi-fl187-GCST90094817 | Weighted mode | 12 | -0.141640501 | 0.33736504447552 | 0.682683037103369 | -0.802875988 | 0.519594986412728 | 0.86793322174112 | 0.448038555927869 | 1.68134654358452 | 0.999714303421225 |
| ebi-fl187-GCST90094817 | prot-b-54 | matrix metallopeptidase 7 \|\| id:prot-b-54 | Prawns liking \|\| id:ebi-fl187-GCST90094817 | Weighted mode | 12 | 0.032051955742308 | 0.244258400295782 | 0.897969293150317 | -0.446694509 | 0.51079842032204 | 1.03257115191217 | 0.639739313132567 | 1.6666213282101 | 0.999714303421225 |
| ebi-fl187-GCST90094817 | ukb-rsfMRI191-edge-pheno716 | Net100_Pair10_33 (OccipitalPrecuneus)&(Temporal\|Frontal\|Supp_Motor_Area) (Default_mode\|Central_executive)&(Default_mode\|Salience) \|\| id:ukb-rsfMRI191-edge-pheno716 | Prawns liking \|\| id:ebi-fl187-GCST90094817 | Weighted mode | 19 | -0.048512127 | 0.0662756896914924 | 0.473607497845309 | -0.178412479 | 0.0813882244739173 | 0.952645786194144 | 0.836597273405666 | 1.08479195761542 | 0.999714303421225 |
| ebi-fl187-GCST90094817 | ukb-rsfMRI191-edge-pheno903 | Net100_Pair27_38 (Precentral\|Frontal\|Supp_Motor_Area)&(Subcortical) (Attention\|Salience\|Motor)&(Subcortical-cerebellum) \|\| id:ukb-rsfMRI191-edge-pheno903 | Prawns liking \|\| id:ebi-fl187-GCST90094817 | Weighted mode | 19 | 0.103467659026465 | 0.061874304143025 | 0.111772372123472 | -0.017805977 | 0.224741295146794 | 1.10900992632441 | 0.982351612583959 | 1.25199877613165 | 0.999714303421225 |
| ebi-fl187-GCST90094817 | ukb-rsfMRI191-node-pheno23 | Net100_Node2 RolandicOper\|SupraMarginal\|Insula Salience\|Motor \|\| id:ukb-rsfMRI191-node-pheno23 | Prawns liking \|\| id:ebi-fl187-GCST90094817 | Weighted mode | 19 | 0.05207861320885 | 0.0596639231914289 | 0.394231487689627 | -0.064862676 | 0.169019902664051 | 1.05345855501564 | 0.937196153834823 | 1.18414370630382 | 0.999714303421225 |
| ebi-fl187-GCST90094817 | ukb-rsfMRI191-node-pheno55 | Net100_Node34 Parietal\|Postcentral\|Precuneus Attention \|\| id:ukb-rsfMRI191-node-pheno55 | Prawns liking \|\| id:ebi-fl187-GCST90094817 | Weighted mode | 18 | -0.015305619 | 0.066032600053162 | 0.819469156511989 | -0.144729515 | 0.114118277178435 | 0.984810916751809 | 0.865256300286062 | 1.12088469212289 | 0.999714303421225 |
| ebi-fl187-GCST90094840 | ebi-a-GCST90001414 | IgD- CD24- B cell Absolute Count \|\| id:ebi-a-GCST90001414 | F-small fish liking (derived food-liking factor) \|\| id:ebi-fl187-GCST90094840 | Inverse variance weighted | 25 | 0.0207184741160332 | 0.080627358103887 | 0.797205169236394 | -0.137311148 | 0.178748095999652 | 1.02093459166233 | 0.871698956747468 | 1.19571949970186 | 0.998735809224318 |
| ebi-fl187-GCST90094840 | ebi-a-GCST90001422 | CD20- CD38- B cell %B cell \|\| id:ebi-a-GCST90001422 | F-small fish liking (derived food-liking factor) \|\| id:ebi-fl187-GCST90094840 | Inverse variance weighted | 25 | -0.083105397 | 0.0767415681902713 | 0.278841517640468 | -0.23351887 | 0.0673080769335209 | 0.920254150340168 | 0.791742655120579 | 1.06962495419592 | 0.998735809224318 |
| ebi-fl187-GCST90094840 | ebi-a-GCST90001444 | CD20- CD38- B cell %lymphocyte \|\| id:ebi-a-GCST90001444 | F-small fish liking (derived food-liking factor) \|\| id:ebi-fl187-GCST90094840 | Inverse variance weighted | 25 | -0.079492389 | 0.076829383589607 | 0.300827118155629 | -0.230077981 | 0.0710932023975249 | 0.92358504894304 | 0.794471646176734 | 1.07368129087562 | 0.998735809224318 |
| ebi-fl187-GCST90094840 | ebi-a-GCST90001459 | Myeloid Dendritic Cell %Dendritic Cell \|\| id:ebi-a-GCST90001459 | F-small fish liking (derived food-liking factor) \|\| id:ebi-fl187-GCST90094840 | Inverse variance weighted | 25 | -0.049415652 | 0.0867810563722506 | 0.569065028699192 | -0.219506522 | 0.120675218758543 | 0.951785436204695 | 0.802914920887652 | 1.12825841568601 | 0.998735809224318 |
| ebi-fl187-GCST90094840 | ebi-a-GCST90001476 | HLA DR++ monocyte %leukocyte \|\| id:ebi-a-GCST90001476 | F-small fish liking (derived food-liking factor) \|\| id:ebi-fl187-GCST90094840 | Inverse variance weighted | 25 | -0.004091546 | 0.0803700498057492 | 0.959398164917187 | -0.161616843 | 0.153433751973258 | 0.995916813322623 | 0.850767119249458 | 1.16583055059026 | 0.998735809224318 |
| ebi-fl187-GCST90094840 | ebi-a-GCST90001478 | CD4 regulatory T cell %CD4+ T cell \|\| id:ebi-a-GCST90001478 | F-small fish liking (derived food-liking factor) \|\| id:ebi-fl187-GCST90094840 | Inverse variance weighted | 25 | -0.00965124 | 0.0985544147379245 | 0.921989439603042 | -0.202817893 | 0.183515412876677 | 0.990395183738256 | 0.816426905028163 | 1.20143348281509 | 0.998735809224318 |
| ebi-fl187-GCST90094840 | ebi-a-GCST90001485 | CD39+ resting CD4 regulatory T cell %CD4 regulatory T cell \|\| id:ebi-a-GCST90001485 | F-small fish liking (derived food-liking factor) \|\| id:ebi-fl187-GCST90094840 | Inverse variance weighted | 25 | 0.0668476339483171 | 0.0793389858753061 | 0.399476135401286 | -0.088656778 | 0.222352046263917 | 1.06913256625595 | 0.915159622256966 | 1.24901101 | 0.998735809224318 |
| ebi-fl187-GCST90094840 | ebi-a-GCST90001515 | Immature Myeloid-Derived Suppressor Cells Absolute Count \|\| id:ebi-a-GCST90001515 | F-small fish liking (derived food-liking factor) \|\| id:ebi-fl187-GCST90094840 | Inverse variance weighted | 25 | 0.08374829560611 | 0.106314009905704 | 0.430846065782646 | -0.124627164 | 0.29212375502129 | 1.08735516728786 | 0.882825990712433 | 1.33926874861656 | 0.998735809224318 |
| ebi-fl187-GCST90094840 | ebi-a-GCST90001517 | CD33+ HLA DR+ Absolute Count \|\| id:ebi-a-GCST90001517 | F-small fish liking (derived food-liking factor) \|\| id:ebi-fl187-GCST90094840 | Inverse variance weighted | 25 | -0.037477889 | 0.104029361381711 | 0.718650756078711 | -0.241375438 | 0.166419658859501 | 0.963215714715895 | 0.785546647147565 | 1.18106864365671 | 0.998735809224318 |
| ebi-fl187-GCST90094840 | ebi-a-GCST90001518 | CD33+ HLA DR+ CD14- Absolute Count \|\| id:ebi-a-GCST90001518 | F-small fish liking (derived food-liking factor) \|\| id:ebi-fl187-GCST90094840 | Inverse variance weighted | 25 | -0.071121566 | 0.105376808625792 | 0.499722674235926 | -0.277660111 | 0.135416979283976 | 0.931348665265156 | 0.757554262478552 | 1.14501413199526 | 0.998735809224318 |
| ebi-fl187-GCST90094840 | ebi-a-GCST90001520 | CD33+ HLA DR+ CD14dim Absolute Count \|\| id:ebi-a-GCST90001520 | F-small fish liking (derived food-liking factor) \|\| id:ebi-fl187-GCST90094840 | Inverse variance weighted | 25 | -0.016780482 | 0.104401778296901 | 0.87230617865391 | -0.221407968 | 0.187847003358225 | 0.98335952595797 | 0.801389672596617 | 1.20664888799862 | 0.998735809224318 |
| ebi-fl187-GCST90094840 | ebi-a-GCST90001531 | CD33dim HLA DR- Absolute Count \|\| id:ebi-a-GCST90001531 | F-small fish liking (derived food-liking factor) \|\| id:ebi-fl187-GCST90094840 | Inverse variance weighted | 25 | -0.052328326 | 0.106143008170238 | 0.622013765647402 | -0.260368622 | 0.155711969991278 | 0.949017228634366 | 0.770767411577761 | 1.16848959454741 | 0.998735809224318 |
| ebi-fl187-GCST90094840 | ebi-a-GCST90001532 | Basophil Absolute Count \|\| id:ebi-a-GCST90001532 | F-small fish liking (derived food-liking factor) \|\| id:ebi-fl187-GCST90094840 | Inverse variance weighted | 25 | -0.056869287 | 0.105774126744308 | 0.590819830276619 | -0.264186575 | 0.150448001657971 | 0.944717548386986 | 0.76783026822139 | 1.16235486300598 | 0.998735809224318 |
| ebi-fl187-GCST90094840 | ebi-a-GCST90001588 | T/B cell \|\| id:ebi-a-GCST90001588 | F-small fish liking (derived food-liking factor) \|\| id:ebi-fl187-GCST90094840 | Inverse variance weighted (multiplicative random effects) | 25 | -0.001369609 | 0.0980192922186281 | 0.988851639949524 | -0.193487422 | 0.190748203692664 | 0.99863132843059 | 0.824080201571922 | 1.21015470123027 | 0.999095833221658 |
| ebi-fl187-GCST90094840 | ebi-a-GCST90001624 | HLA DR+ CD4+ T cell Absolute Count \|\| id:ebi-a-GCST90001624 | F-small fish liking (derived food-liking factor) \|\| id:ebi-fl187-GCST90094840 | Inverse variance weighted | 25 | -0.068215102 | 0.076939847046132 | 0.375292658165142 | -0.219017202 | 0.0825869981465703 | 0.934059533833227 | 0.803307899311964 | 1.08609315742073 | 0.998735809224318 |
| ebi-fl187-GCST90094840 | ebi-a-GCST90001631 | CD8+ Natural Killer T %T cell \|\| id:ebi-a-GCST90001631 | F-small fish liking (derived food-liking factor) \|\| id:ebi-fl187-GCST90094840 | Inverse variance weighted | 25 | -0.018758897 | 0.0780219138878945 | 0.809996051643845 | -0.171681848 | 0.134164054706169 | 0.981415956528039 | 0.8422470933675 | 1.14358041400516 | 0.998735809224318 |
| ebi-fl187-GCST90094840 | ebi-a-GCST90001632 | CD8+ Natural Killer T %lymphocyte \|\| id:ebi-a-GCST90001632 | F-small fish liking (derived food-liking factor) \|\| id:ebi-fl187-GCST90094840 | Inverse variance weighted | 25 | -0.017939224 | 0.0784940692532722 | 0.819224588422595 | -0.1717876 | 0.135909151824105 | 0.982220726077287 | 0.84215802883448 | 1.14557781521241 | 0.998735809224318 |
| ebi-fl187-GCST90094840 | ebi-a-GCST90001646 | Natural Killer %CD3- lymphocyte \|\| id:ebi-a-GCST90001646 | F-small fish liking (derived food-liking factor) \|\| id:ebi-fl187-GCST90094840 | Inverse variance weighted | 25 | 0.0131659298876519 | 0.0829924762579404 | 0.873952479606388 | -0.149499324 | 0.175831183353215 | 1.0132529823653 | 0.861139020512959 | 1.19223677224684 | 0.998735809224318 |
| ebi-fl187-GCST90094840 | ebi-a-GCST90001684 | CD127- CD8+ T cell Absolute Count \|\| id:ebi-a-GCST90001684 | F-small fish liking (derived food-liking factor) \|\| id:ebi-fl187-GCST90094840 | Inverse variance weighted | 25 | -0.088655671 | 0.0827192803889623 | 0.283825371728315 | -0.250785461 | 0.0734741184896189 | 0.915160635608776 | 0.778189305890772 | 1.07624068157705 | 0.998735809224318 |
| ebi-fl187-GCST90094840 | ebi-a-GCST90001687 | CD28- CD8+ T cell Absolute Count \|\| id:ebi-a-GCST90001687 | F-small fish liking (derived food-liking factor) \|\| id:ebi-fl187-GCST90094840 | Inverse variance weighted | 25 | -0.092498381 | 0.0866413846496951 | 0.285700780530904 | -0.262315495 | 0.0773187326661146 | 0.911650686700289 | 0.769268284996956 | 1.08038637594997 | 0.998735809224318 |
| ebi-fl187-GCST90094840 | ebi-a-GCST90001701 | BAFF-R on CD20- CD38- B cell \|\| id:ebi-a-GCST90001701 | F-small fish liking (derived food-liking factor) \|\| id:ebi-fl187-GCST90094840 | Inverse variance weighted | 25 | -0.117149244 | 0.0775522398622906 | 0.130894237270735 | -0.269151635 | 0.0348531457262815 | 0.889452437458391 | 0.764027393926339 | 1.03546763478607 | 0.998735809224318 |
| ebi-fl187-GCST90094840 | ebi-a-GCST90001732 | CD19 on IgD- CD27- B cell \|\| id:ebi-a-GCST90001732 | F-small fish liking (derived food-liking factor) \|\| id:ebi-fl187-GCST90094840 | Inverse variance weighted | 25 | 0.0246579735797786 | 0.0813754138650754 | 0.761878406476591 | -0.134837838 | 0.184153784755326 | 1.02496449562906 | 0.873857607049408 | 1.20220068901997 | 0.998735809224318 |
| ebi-fl187-GCST90094840 | ebi-a-GCST90001798 | CD27 on CD24+ CD27+ B cell \|\| id:ebi-a-GCST90001798 | F-small fish liking (derived food-liking factor) \|\| id:ebi-fl187-GCST90094840 | Inverse variance weighted | 25 | 0.0508393549908526 | 0.0920510097173988 | 0.580745938844276 | -0.129580624 | 0.231259334036954 | 1.05215385644021 | 0.878463760250317 | 1.26018600620083 | 0.998735809224318 |
| ebi-fl187-GCST90094840 | ebi-a-GCST90001836 | CD66b on CD66b++ myeloid cell \|\| id:ebi-a-GCST90001836 | F-small fish liking (derived food-liking factor) \|\| id:ebi-fl187-GCST90094840 | Inverse variance weighted | 25 | -0.25604872 | 0.122221417334433 | 0.036174738203753 | -0.495602698 | -0.016494742 | 0.774104253871773 | 0.609203631115412 | 0.983640551789243 | 0.998735809224318 |
| ebi-fl187-GCST90094840 | ebi-a-GCST90001943 | CD25 on activated & secreting CD4 regulatory T cell \|\| id:ebi-a-GCST90001943 | F-small fish liking (derived food-liking factor) \|\| id:ebi-fl187-GCST90094840 | Inverse variance weighted | 25 | 0.0397247886791602 | 0.0917736239947556 | 0.665119190039674 | -0.140151514 | 0.219601091708881 | 1.04052437069703 | 0.869226525128615 | 1.24557975937774 | 0.998735809224318 |
| ebi-fl187-GCST90094840 | ebi-a-GCST90001960 | CD25 on CD4+ T cell \|\| id:ebi-a-GCST90001960 | F-small fish liking (derived food-liking factor) \|\| id:ebi-fl187-GCST90094840 | Inverse variance weighted | 25 | 0.0433846734250922 | 0.0849607705995425 | 0.609600642854019 | -0.123138437 | 0.209907783800195 | 1.04433954726376 | 0.884141256269343 | 1.2335643000996 | 0.998735809224318 |
| ebi-fl187-GCST90094840 | ebi-a-GCST90001988 | HLA DR on CD14+ CD16- monocyte \|\| id:ebi-a-GCST90001988 | F-small fish liking (derived food-liking factor) \|\| id:ebi-fl187-GCST90094840 | Inverse variance weighted | 25 | 0.0934193731340753 | 0.0949333494109328 | 0.325089838378607 | -0.092649992 | 0.279488737979504 | 1.09792207775642 | 0.911512481393563 | 1.32245351921243 | 0.998735809224318 |
| ebi-fl187-GCST90094840 | ebi-a-GCST90001991 | HLA DR on CD14+ monocyte \|\| id:ebi-a-GCST90001991 | F-small fish liking (derived food-liking factor) \|\| id:ebi-fl187-GCST90094840 | Inverse variance weighted | 25 | 0.0906719486347064 | 0.095088936114958 | 0.340312041999362 | -0.095702366 | 0.277046263420024 | 1.09490975969465 | 0.908734445953387 | 1.31922740159459 | 0.998735809224318 |
| ebi-fl187-GCST90094840 | ebi-a-GCST90002019 | CD14 on Monocytic Myeloid-Derived Suppressor Cells \|\| id:ebi-a-GCST90002019 | F-small fish liking (derived food-liking factor) \|\| id:ebi-fl187-GCST90094840 | Inverse variance weighted | 25 | -0.025291414 | 0.114352848628355 | 0.824960106993682 | -0.249422997 | 0.198840169598779 | 0.975025734754329 | 0.779250283109617 | 1.21998695931111 | 0.998735809224318 |
| ebi-fl187-GCST90094840 | ebi-a-GCST90002037 | CD80 on plasmacytoid Dendritic Cell \|\| id:ebi-a-GCST90002037 | F-small fish liking (derived food-liking factor) \|\| id:ebi-fl187-GCST90094840 | Inverse variance weighted | 25 | 0.0516037584838978 | 0.0885918975495676 | 0.560237708468074 | -0.122036361 | 0.22524387768105 | 1.05295843399507 | 0.885116184461733 | 1.25262816699662 | 0.998735809224318 |
| ebi-fl187-GCST90094840 | ebi-a-GCST90002042 | CD45 on CD33+ HLA DR+ CD14- \|\| id:ebi-a-GCST90002042 | F-small fish liking (derived food-liking factor) \|\| id:ebi-fl187-GCST90094840 | Inverse variance weighted | 25 | 0.189404001787213 | 0.116333234218114 | 0.103500288201054 | -0.038609137 | 0.417417140854716 | 1.20852910178669 | 0.96212669511889 | 1.51803561555358 | 0.998735809224318 |
| ebi-fl187-GCST90094840 | ebi-a-GCST90002056 | CD8 on Effector Memory CD8+ T cell \|\| id:ebi-a-GCST90002056 | F-small fish liking (derived food-liking factor) \|\| id:ebi-fl187-GCST90094840 | Inverse variance weighted | 25 | 0.0101084360828898 | 0.0924865885721204 | 0.912967531335869 | -0.171165278 | 0.191382149684246 | 1.0101596989063 | 0.842682285524347 | 1.21092211717673 | 0.998735809224318 |
| ebi-fl187-GCST90094840 | ebi-a-GCST90002058 | CD8 on CD8+ T cell \|\| id:ebi-a-GCST90002058 | F-small fish liking (derived food-liking factor) \|\| id:ebi-fl187-GCST90094840 | Inverse variance weighted | 25 | -0.009850824 | 0.0961259866975426 | 0.91837706489462 | -0.198257758 | 0.178556109693781 | 0.990197536208628 | 0.820158423363532 | 1.19548995996721 | 0.998735809224318 |
| ebi-fl187-GCST90094840 | ebi-a-GCST90002063 | CD4 on CD4 regulatory T cell \|\| id:ebi-a-GCST90002063 | F-small fish liking (derived food-liking factor) \|\| id:ebi-fl187-GCST90094840 | Inverse variance weighted | 25 | 0.0732853681135561 | 0.0902983535923629 | 0.417026121904508 | -0.103699405 | 0.250270141154587 | 1.07603755991392 | 0.901496242025111 | 1.28437233165222 | 0.998735809224318 |
| ebi-fl187-GCST90094840 | ebi-a-GCST90002066 | CD4 on activated CD4 regulatory T cell \|\| id:ebi-a-GCST90002066 | F-small fish liking (derived food-liking factor) \|\| id:ebi-fl187-GCST90094840 | Inverse variance weighted | 25 | 0.08743387606254 | 0.095577756471494 | 0.360300236236477 | -0.099898527 | 0.274766278746668 | 1.09137009636896 | 0.904929239604325 | 1.3162230096236 | 0.998735809224318 |
| ebi-fl187-GCST90094840 | ebi-a-GCST90002070 | CD4 on activated & secreting CD4 regulatory T cell \|\| id:ebi-a-GCST90002070 | F-small fish liking (derived food-liking factor) \|\| id:ebi-fl187-GCST90094840 | Inverse variance weighted | 25 | 0.100205010388252 | 0.0881471497362626 | 0.255625078902053 | -0.072563403 | 0.272973423871327 | 1.10539751282099 | 0.930006779482923 | 1.31386532690676 | 0.998735809224318 |
| ebi-fl187-GCST90094840 | ebi-a-GCST90002081 | SSC-A on CD4+ T cell \|\| id:ebi-a-GCST90002081 | F-small fish liking (derived food-liking factor) \|\| id:ebi-fl187-GCST90094840 | Inverse variance weighted | 25 | 0.0204339343962023 | 0.0820206539791396 | 0.803259008920037 | -0.140326547 | 0.181194416195316 | 1.02064413654462 | 0.86907439507102 | 1.19864819326288 | 0.998735809224318 |
| ebi-fl187-GCST90094840 | ebi-a-GCST90002094 | CD11b on Monocytic Myeloid-Derived Suppressor Cells \|\| id:ebi-a-GCST90002094 | F-small fish liking (derived food-liking factor) \|\| id:ebi-fl187-GCST90094840 | Inverse variance weighted | 25 | -0.103217432 | 0.115763707935433 | 0.372595745798705 | -0.330114299 | 0.123679435827573 | 0.901930843779896 | 0.718841565663254 | 1.13165304542586 | 0.998735809224318 |
| ebi-fl187-GCST90094840 | ebi-a-GCST90002104 | HLA DR on myeloid Dendritic Cell \|\| id:ebi-a-GCST90002104 | F-small fish liking (derived food-liking factor) \|\| id:ebi-fl187-GCST90094840 | Inverse variance weighted | 24 | 0.1251617131138 | 0.0913165758670939 | 0.170488704648913 | -0.053818776 | 0.304142201813304 | 1.13333171284892 | 0.947603819889243 | 1.3554617915103 | 0.998735809224318 |
| ebi-fl187-GCST90094840 | ebi-a-GCST90002105 | HLA DR on plasmacytoid Dendritic Cell \|\| id:ebi-a-GCST90002105 | F-small fish liking (derived food-liking factor) \|\| id:ebi-fl187-GCST90094840 | Inverse variance weighted | 25 | 0.0476240513825206 | 0.104340279766302 | 0.648080676424402 | -0.156882897 | 0.252130999724472 | 1.04877629520132 | 0.854804153122319 | 1.2867645920513 | 0.998735809224318 |
| ebi-fl187-GCST90094840 | ebi-a-GCST90002106 | HLA DR on Dendritic Cell \|\| id:ebi-a-GCST90002106 | F-small fish liking (derived food-liking factor) \|\| id:ebi-fl187-GCST90094840 | Inverse variance weighted | 24 | 0.0559479923516157 | 0.105306532142206 | 0.59521990725789 | -0.150452811 | 0.26234879535034 | 1.05754268199841 | 0.860318326914662 | 1.29997989030324 | 0.998735809224318 |
| ebi-fl187-GCST90094840 | ebi-a-GCST90002111 | HLA DR on CD33dim HLA DR+ CD11b- \|\| id:ebi-a-GCST90002111 | F-small fish liking (derived food-liking factor) \|\| id:ebi-fl187-GCST90094840 | Inverse variance weighted | 25 | -0.092617586 | 0.127474077633788 | 0.467495441477364 | -0.342466778 | 0.157231605944326 | 0.911542019883916 | 0.710016706898886 | 1.1702666232224 | 0.998735809224318 |
| ebi-fl187-GCST90094840 | ebi-a-GCST90002112 | HLA DR on CD33- HLA DR+ \|\| id:ebi-a-GCST90002112 | F-small fish liking (derived food-liking factor) \|\| id:ebi-fl187-GCST90094840 | Inverse variance weighted | 25 | 0.0880647062312023 | 0.114248190331026 | 0.440814065901334 | -0.135861747 | 0.311991159280014 | 1.09205878275016 | 0.872963314102504 | 1.36614261529177 | 0.998735809224318 |
| ebi-fl187-GCST90094840 | ebi-a-GCST90002115 | HLA DR on HLA DR+ CD8+ T cell \|\| id:ebi-a-GCST90002115 | F-small fish liking (derived food-liking factor) \|\| id:ebi-fl187-GCST90094840 | Inverse variance weighted | 25 | 0.0697331842005646 | 0.0809288290863095 | 0.388874307671145 | -0.088887321 | 0.228353689209731 | 1.07222205729933 | 0.914948663441869 | 1.25652966783338 | 0.998735809224318 |
| ebi-fl187-GCST90094840 | ebi-a-GCST90002116 | HLA DR on B cell \|\| id:ebi-a-GCST90002116 | F-small fish liking (derived food-liking factor) \|\| id:ebi-fl187-GCST90094840 | Inverse variance weighted | 25 | 0.0100665910589654 | 0.102439370677614 | 0.921718875258985 | -0.190714575 | 0.210847757587088 | 1.01011742963392 | 0.826368420312121 | 1.23472436333525 | 0.998735809224318 |
| ebi-fl187-GCST90094840 | ebi-cfmet338-GCST90026023 | Sphingomyelin (d18:1/24:1, d18:2/24:0) levels \|\| id:ebi-cfmet338-GCST90026023 | F-small fish liking (derived food-liking factor) \|\| id:ebi-fl187-GCST90094840 | Inverse variance weighted | 23 | -0.0349895 | 0.0505505182970204 | 0.488831220573251 | -0.134068516 | 0.064089515491524 | 0.965615554809775 | 0.87453014303969 | 1.06618783481803 | 0.998735809224318 |
| ebi-fl187-GCST90094840 | ebi-cfmet338-GCST90026046 | 1-arachidonoyl-gpc (20:4n6) levels \|\| id:ebi-cfmet338-GCST90026046 | F-small fish liking (derived food-liking factor) \|\| id:ebi-fl187-GCST90094840 | Inverse variance weighted | 23 | 0.00810434364904137 | 0.0617549268712676 | 0.895590231474975 | -0.112935313 | 0.129144000316726 | 1.00813727273814 | 0.893208437204808 | 1.13785396370014 | 0.998735809224318 |
| ebi-fl187-GCST90094840 | ebi-cfmet338-GCST90026047 | 1-linoleoyl-gpc (18:2) levels \|\| id:ebi-cfmet338-GCST90026047 | F-small fish liking (derived food-liking factor) \|\| id:ebi-fl187-GCST90094840 | Inverse variance weighted | 23 | -0.030818878 | 0.0681915389080091 | 0.651308042203736 | -0.164474295 | 0.102836537922816 | 0.969651182006985 | 0.848339563574783 | 1.10831022757629 | 0.998735809224318 |
| ebi-fl187-GCST90094840 | ebi-cfmet338-GCST90026050 | 1-myristoyl-2-palmitoyl-gpc (14:0/16:0) levels \|\| id:ebi-cfmet338-GCST90026050 | F-small fish liking (derived food-liking factor) \|\| id:ebi-fl187-GCST90094840 | Inverse variance weighted | 23 | -0.006175455 | 0.0359996511888229 | 0.863797545557706 | -0.076734771 | 0.0643838612782796 | 0.993843573879815 | 0.92613545866218 | 1.06650170890659 | 0.998735809224318 |
| ebi-fl187-GCST90094840 | ebi-cfmet338-GCST90026068 | 2,3-dihydroxyisovalerate levels \|\| id:ebi-cfmet338-GCST90026068 | F-small fish liking (derived food-liking factor) \|\| id:ebi-fl187-GCST90094840 | Inverse variance weighted | 23 | -0.042547906 | 0.121102541473931 | 0.725334903744077 | -0.279908888 | 0.194813074957712 | 0.958344553649636 | 0.755852605848252 | 1.21508383566294 | 0.998735809224318 |
| ebi-fl187-GCST90094840 | ebi-cfmet338-GCST90026082 | 3-hydroxyhexanoate levels \|\| id:ebi-cfmet338-GCST90026082 | F-small fish liking (derived food-liking factor) \|\| id:ebi-fl187-GCST90094840 | Inverse variance weighted | 23 | 0.0237306262571764 | 0.0295460410491653 | 0.421873891661348 | -0.034179614 | 0.0816408667135405 | 1.024014438 | 0.966397910261218 | 1.08506605650814 | 0.998735809224318 |
| ebi-fl187-GCST90094840 | ebi-cfmet338-GCST90026094 | 3-ureidopropionate levels \|\| id:ebi-cfmet338-GCST90026094 | F-small fish liking (derived food-liking factor) \|\| id:ebi-fl187-GCST90094840 | Inverse variance weighted | 23 | -0.05107394 | 0.0566593771310172 | 0.367364637192598 | -0.162126319 | 0.0599784392748498 | 0.950208409618283 | 0.850333784375506 | 1.06181365282623 | 0.998735809224318 |
| ebi-fl187-GCST90094840 | ebi-cfmet338-GCST90026173 | Glycerol levels \|\| id:ebi-cfmet338-GCST90026173 | F-small fish liking (derived food-liking factor) \|\| id:ebi-fl187-GCST90094840 | Inverse variance weighted | 22 | -0.039720626 | 0.0370260059504982 | 0.28337144847411 | -0.112291597 | 0.0328503460054844 | 0.961057896568486 | 0.893783594596639 | 1.03339587584786 | 0.998735809224318 |
| ebi-fl187-GCST90094840 | ebi-cfmet338-GCST90026179 | Histidine levels \|\| id:ebi-cfmet338-GCST90026179 | F-small fish liking (derived food-liking factor) \|\| id:ebi-fl187-GCST90094840 | Inverse variance weighted | 23 | 0.0315535076101743 | 0.0221675264265865 | 0.154616958613172 | -0.011894844 | 0.0750018594062838 | 1.03205659700032 | 0.988175619810365 | 1.07788615511106 | 0.998735809224318 |
| ebi-fl187-GCST90094840 | ebi-cfmet338-GCST90026188 | Isocitrate levels \|\| id:ebi-cfmet338-GCST90026188 | F-small fish liking (derived food-liking factor) \|\| id:ebi-fl187-GCST90094840 | Inverse variance weighted | 22 | -0.015951054 | 0.0442555220794874 | 0.718524992203385 | -0.102691877 | 0.0707897697539002 | 0.984175490800493 | 0.9024049825618 | 1.07335555034578 | 0.998735809224318 |
| ebi-fl187-GCST90094840 | ebi-cfmet338-GCST90026207 | N-acetyl-isoputreanine levels \|\| id:ebi-cfmet338-GCST90026207 | F-small fish liking (derived food-liking factor) \|\| id:ebi-fl187-GCST90094840 | Inverse variance weighted | 23 | 0.0273321920903163 | 0.0277266207434606 | 0.324243845560534 | -0.027011985 | 0.0816763687474991 | 1.02770914291352 | 0.973349576281354 | 1.08510457924394 | 0.998735809224318 |
| ebi-fl187-GCST90094840 | ebi-cfmet338-GCST90026213 | N-acetylglutamate levels \|\| id:ebi-cfmet338-GCST90026213 | F-small fish liking (derived food-liking factor) \|\| id:ebi-fl187-GCST90094840 | Inverse variance weighted | 23 | 0.0601050572736938 | 0.0471802509166368 | 0.202682814799115 | -0.032368235 | 0.152578349070302 | 1.06194810605801 | 0.968150010175078 | 1.16483372215868 | 0.998735809224318 |
| ebi-fl187-GCST90094840 | ebi-cfmet338-GCST90026224 | N-acetyltaurine levels \|\| id:ebi-cfmet338-GCST90026224 | F-small fish liking (derived food-liking factor) \|\| id:ebi-fl187-GCST90094840 | Inverse variance weighted | 23 | 0.0770734920436817 | 0.041209135599403 | 0.0614420155344899 | -0.003696414 | 0.157843397818512 | 1.08012145380883 | 0.996310409596218 | 1.17098280188693 | 0.998735809224318 |
| ebi-fl187-GCST90094840 | ebi-cfmet338-GCST90026250 | Phenyllactate (pla) levels \|\| id:ebi-cfmet338-GCST90026250 | F-small fish liking (derived food-liking factor) \|\| id:ebi-fl187-GCST90094840 | Inverse variance weighted | 23 | 0.0533607855760417 | 0.0605443240547261 | 0.378128004028841 | -0.06530609 | 0.172027660723305 | 1.05481013676009 | 0.936780680692166 | 1.18771068569619 | 0.998735809224318 |
| ebi-fl187-GCST90094840 | ebi-cfmet338-GCST90026251 | Pipecolate levels \|\| id:ebi-cfmet338-GCST90026251 | F-small fish liking (derived food-liking factor) \|\| id:ebi-fl187-GCST90094840 | Inverse variance weighted | 23 | 0.101293408041492 | 0.10348489903282 | 0.327667401644383 | -0.101536994 | 0.30412381014582 | 1.10660127984962 | 0.903447756520929 | 1.35543686253699 | 0.998735809224318 |
| ebi-fl187-GCST90094840 | ebi-cfmet338-GCST90026278 | Trigonelline (n'-methylnicotinate) levels \|\| id:ebi-cfmet338-GCST90026278 | F-small fish liking (derived food-liking factor) \|\| id:ebi-fl187-GCST90094840 | Inverse variance weighted | 23 | 0.197789142121676 | 0.113997616811926 | 0.0827357137587322 | -0.025646187 | 0.421224471073051 | 1.21870539309379 | 0.974679883188397 | 1.52382629494448 | 0.998735809224318 |
| ebi-fl187-GCST90094840 | ebi-cfmet338-GCST90026299 | 1-carboxyethylvaline levels \|\| id:ebi-cfmet338-GCST90026299 | F-small fish liking (derived food-liking factor) \|\| id:ebi-fl187-GCST90094840 | Inverse variance weighted | 23 | -0.012627169 | 0.0607698914485557 | 0.835395595281127 | -0.131736156 | 0.106481818063118 | 0.987452219023921 | 0.876572242534241 | 1.1123577014443 | 0.998735809224318 |
| ebi-fl187-GCST90094840 | ebi-gm430-GCST90011306 | C_Erysipelotrichia abundance \|\| id:ebi-gm430-GCST90011306 | F-small fish liking (derived food-liking factor) \|\| id:ebi-fl187-GCST90094840 | Inverse variance weighted | 21 | -0.009691976 | 0.0540029713513509 | 0.857567763253867 | -0.1155378 | 0.0961538481166333 | 0.990354840096745 | 0.890886896449513 | 1.10092842673058 | 0.998735809224318 |
| ebi-fl187-GCST90094840 | ebi-gm430-GCST90011312 | F_Erysipelotrichaceae abundance \|\| id:ebi-gm430-GCST90011312 | F-small fish liking (derived food-liking factor) \|\| id:ebi-fl187-GCST90094840 | Inverse variance weighted | 21 | -0.009691976 | 0.0540029713513509 | 0.857567763253867 | -0.1155378 | 0.0961538481166333 | 0.990354840096745 | 0.890886896449513 | 1.10092842673058 | 0.998735809224318 |
| ebi-fl187-GCST90094840 | ebi-gm430-GCST90011352 | O_Erysipelotrichales abundance \|\| id:ebi-gm430-GCST90011352 | F-small fish liking (derived food-liking factor) \|\| id:ebi-fl187-GCST90094840 | Inverse variance weighted | 21 | -0.009691976 | 0.0540029713513509 | 0.857567763253867 | -0.1155378 | 0.0961538481166333 | 0.990354840096745 | 0.890886896449513 | 1.10092842673058 | 0.998735809224318 |
| ebi-fl187-GCST90094840 | ebi-gm430-GCST90011362 | OTU97_11 (Parabacteroides) abundance \|\| id:ebi-gm430-GCST90011362 | F-small fish liking (derived food-liking factor) \|\| id:ebi-fl187-GCST90094840 | Inverse variance weighted | 21 | -0.051408126 | 0.0650790676465106 | 0.429566691642406 | -0.178963099 | 0.0761468464642083 | 0.949890916114634 | 0.836136753352477 | 1.07912102763019 | 0.998735809224318 |
| ebi-fl187-GCST90094840 | ebi-gm430-GCST90011384 | OTU97_235 (Phascolarctobacterium) abundance \|\| id:ebi-gm430-GCST90011384 | F-small fish liking (derived food-liking factor) \|\| id:ebi-fl187-GCST90094840 | Inverse variance weighted | 21 | -0.065750387 | 0.221245074421631 | 0.766326411841725 | -0.499390733 | 0.367889958441372 | 0.936364563493085 | 0.606900311248081 | 1.44468305505152 | 0.998735809224318 |
| ebi-fl187-GCST90094840 | ebi-gm430-GCST90011390 | OTU97_3 (Bacteroides) abundance \|\| id:ebi-gm430-GCST90011390 | F-small fish liking (derived food-liking factor) \|\| id:ebi-fl187-GCST90094840 | Inverse variance weighted | 21 | -0.00902153 | 0.0515575901485083 | 0.861095617119747 | -0.110074406 | 0.0920313471717878 | 0.991019042379615 | 0.89576748215314 | 1.09639919055591 | 0.998735809224318 |
| ebi-fl187-GCST90094840 | ebi-gm430-GCST90011398 | OTU97_4 (Alistipes) abundance \|\| id:ebi-gm430-GCST90011398 | F-small fish liking (derived food-liking factor) \|\| id:ebi-fl187-GCST90094840 | Inverse variance weighted | 21 | -0.073259842 | 0.060299573768052 | 0.224392279466687 | -0.191447007 | 0.0449273226254515 | 0.92935931210377 | 0.825763384001478 | 1.04595184011265 | 0.998735809224318 |
| ebi-fl187-GCST90094840 | ebi-gm430-GCST90011421 | OTU99_108 (Cytophagales) abundance \|\| id:ebi-gm430-GCST90011421 | F-small fish liking (derived food-liking factor) \|\| id:ebi-fl187-GCST90094840 | Inverse variance weighted | 21 | 0.165272991545145 | 0.190966744988364 | 0.386789485988262 | -0.209021829 | 0.539567811722337 | 1.17971512701197 | 0.811377524188676 | 1.71526538437518 | 0.998735809224318 |
| ebi-fl187-GCST90094840 | ebi-gm430-GCST90011428 | OTU99_123 (Phascolarctobacterium) abundance \|\| id:ebi-gm430-GCST90011428 | F-small fish liking (derived food-liking factor) \|\| id:ebi-fl187-GCST90094840 | Inverse variance weighted | 21 | -0.06914357 | 0.114554166734948 | 0.546117303570701 | -0.293669737 | 0.155382596563701 | 0.933192691764332 | 0.745522669314867 | 1.16810478850048 | 0.998735809224318 |
| ebi-fl187-GCST90094840 | ebi-gm430-GCST90011450 | OTU99_298 (Phascolarctobacterium) abundance \|\| id:ebi-gm430-GCST90011450 | F-small fish liking (derived food-liking factor) \|\| id:ebi-fl187-GCST90094840 | Inverse variance weighted | 21 | -0.063513252 | 0.220698659034448 | 0.773513095358804 | -0.496082623 | 0.369056119916256 | 0.938461682919932 | 0.608911328610012 | 1.44636877149148 | 0.998735809224318 |
| ebi-fl187-GCST90094840 | ebi-gm430-GCST90011451 | OTU99_3 (Bacteroides) abundance \|\| id:ebi-gm430-GCST90011451 | F-small fish liking (derived food-liking factor) \|\| id:ebi-fl187-GCST90094840 | Inverse variance weighted | 21 | -0.006223918 | 0.0515575731671007 | 0.903914548243812 | -0.107276761 | 0.0948289258528996 | 0.993795410899887 | 0.898277030569391 | 1.09947074801595 | 0.998735809224318 |
| ebi-fl187-GCST90094840 | ebi-gm430-GCST90011472 | OTU99_72 (Parabacteroides) abundance \|\| id:ebi-gm430-GCST90011472 | F-small fish liking (derived food-liking factor) \|\| id:ebi-fl187-GCST90094840 | Inverse variance weighted | 21 | 0.0278850700233601 | 0.157369552298466 | 0.859355361678914 | -0.280559252 | 0.336329392528354 | 1.02827749772117 | 0.755361185691431 | 1.39980003255242 | 0.998735809224318 |
| ebi-fl187-GCST90094840 | ebi-gm430-GCST90011507 | TestASV_3 (Bacteroides) abundance \|\| id:ebi-gm430-GCST90011507 | F-small fish liking (derived food-liking factor) \|\| id:ebi-fl187-GCST90094840 | Inverse variance weighted | 21 | -0.017112683 | 0.0663143577142635 | 0.796365254651768 | -0.147088824 | 0.112863457789683 | 0.983032906971903 | 0.863217299208889 | 1.11947906636633 | 0.998735809224318 |
| ebi-fl187-GCST90094840 | ebi-gm430-GCST90011512 | TestASV_34 (Phascolarctobacterium) abundance \|\| id:ebi-gm430-GCST90011512 | F-small fish liking (derived food-liking factor) \|\| id:ebi-fl187-GCST90094840 | Inverse variance weighted | 21 | 0.0186443780809896 | 0.180126207051817 | 0.917560352838407 | -0.334402988 | 0.37169174390255 | 1.01881926972246 | 0.715765279466824 | 1.45018588374533 | 0.998735809224318 |
| ebi-fl187-GCST90094840 | ebi-gm430-GCST90011519 | TestASV_41 (Barnesiella) abundance \|\| id:ebi-gm430-GCST90011519 | F-small fish liking (derived food-liking factor) \|\| id:ebi-fl187-GCST90094840 | Inverse variance weighted | 21 | 0.244220454456984 | 0.21191452561314 | 0.249137058685615 | -0.171132016 | 0.659572924658738 | 1.27662573730331 | 0.84271031509798 | 1.93396620872705 | 0.998735809224318 |
| ebi-fl187-GCST90094840 | ebi-gm430-GCST90011524 | TestASV_46 (Sutterella) abundance \|\| id:ebi-gm430-GCST90011524 | F-small fish liking (derived food-liking factor) \|\| id:ebi-fl187-GCST90094840 | Inverse variance weighted | 21 | -0.143829207 | 0.264578023046032 | 0.586704810820903 | -0.662402132 | 0.374743718664071 | 0.866035648682849 | 0.515611279513097 | 1.45461857525262 | 0.998735809224318 |
| ebi-fl187-GCST90094840 | ebi-gm430-GCST90011581 | OTU97_19 (Bacteria) prevalence \|\| id:ebi-gm430-GCST90011581 | F-small fish liking (derived food-liking factor) \|\| id:ebi-fl187-GCST90094840 | Inverse variance weighted | 21 | -0.134614536 | 0.247229053732433 | 0.586102134815047 | -0.619183481 | 0.349954409700339 | 0.874052762972313 | 0.538383858859649 | 1.4190028543532 | 0.998735809224318 |
| ebi-fl187-GCST90094840 | ebi-gm430-GCST90011614 | OTU97_80 (Ruminococcus) prevalence \|\| id:ebi-gm430-GCST90011614 | F-small fish liking (derived food-liking factor) \|\| id:ebi-fl187-GCST90094840 | Inverse variance weighted | 21 | -0.024098119 | 0.115047393203092 | 0.834087179239451 | -0.249591009 | 0.201394772030369 | 0.976189922623636 | 0.779119370473975 | 1.22310752516937 | 0.998735809224318 |
| ebi-fl187-GCST90094840 | ebi-gm430-GCST90011623 | OTU99_11 (Parabacteroides) prevalence \|\| id:ebi-gm430-GCST90011623 | F-small fish liking (derived food-liking factor) \|\| id:ebi-fl187-GCST90094840 | Inverse variance weighted | 21 | 0.116604518599473 | 0.100413095396524 | 0.245541009474361 | -0.080205148 | 0.313414185576659 | 1.12367494926179 | 0.922926989989477 | 1.36808805603667 | 0.998735809224318 |
| ebi-fl187-GCST90094840 | ebi-gm430-GCST90011642 | OTU99_19 (Bacteria) prevalence \|\| id:ebi-gm430-GCST90011642 | F-small fish liking (derived food-liking factor) \|\| id:ebi-fl187-GCST90094840 | Inverse variance weighted | 21 | -0.134614536 | 0.247229053732433 | 0.586102134815047 | -0.619183481 | 0.349954409700339 | 0.874052762972313 | 0.538383858859649 | 1.4190028543532 | 0.998735809224318 |
| ebi-fl187-GCST90094840 | ebi-gm430-GCST90011653 | OTU99_30 (Parasutterella) prevalence \|\| id:ebi-gm430-GCST90011653 | F-small fish liking (derived food-liking factor) \|\| id:ebi-fl187-GCST90094840 | Inverse variance weighted | 21 | 0.0669340476213105 | 0.115680744476638 | 0.562852285331955 | -0.159800212 | 0.29366830679552 | 1.06922495791981 | 0.852314054458488 | 1.34133891686787 | 0.998735809224318 |
| ebi-fl187-GCST90094840 | ebi-gm430-GCST90011675 | OTU99_8 (Ruminococcaceae) prevalence \|\| id:ebi-gm430-GCST90011675 | F-small fish liking (derived food-liking factor) \|\| id:ebi-fl187-GCST90094840 | Inverse variance weighted | 21 | 0.189496566139063 | 0.140414863221427 | 0.177161086083519 | -0.085716566 | 0.464709698053059 | 1.20864097367728 | 0.917854345690933 | 1.59155209114569 | 0.998735809224318 |
| ebi-fl187-GCST90094840 | ebi-gm430-GCST90011679 | OTU99_92 (Ruminococcus) prevalence \|\| id:ebi-gm430-GCST90011679 | F-small fish liking (derived food-liking factor) \|\| id:ebi-fl187-GCST90094840 | Inverse variance weighted | 21 | 0.00618952514937334 | 0.120270748181786 | 0.958956374890619 | -0.229541141 | 0.241920191585674 | 1.00620871984174 | 0.794898264827516 | 1.27369253737802 | 0.998735809224318 |
| ebi-fl187-GCST90094840 | ebi-gm430-GCST90011684 | TestASV_11 (Lachnospiraceae) prevalence \|\| id:ebi-gm430-GCST90011684 | F-small fish liking (derived food-liking factor) \|\| id:ebi-fl187-GCST90094840 | Inverse variance weighted | 21 | 0.0368699009608715 | 0.145204486763935 | 0.799559610276442 | -0.247730893 | 0.321470695018184 | 1.03755802675473 | 0.780569971784824 | 1.37915458933376 | 0.998735809224318 |
| ebi-fl187-GCST90094840 | ebi-gm430-GCST90011688 | TestASV_15 (Bacteroides) prevalence \|\| id:ebi-gm430-GCST90011688 | F-small fish liking (derived food-liking factor) \|\| id:ebi-fl187-GCST90094840 | Inverse variance weighted | 21 | 0.0255211224842727 | 0.114881005399647 | 0.824195034617485 | -0.199645648 | 0.250687893067581 | 1.0258495745331 | 0.819020923284859 | 1.28490899273856 | 0.998735809224318 |
| ebi-fl187-GCST90094840 | ebi-gm430-GCST90011692 | TestASV_19 (Prevotella) prevalence \|\| id:ebi-gm430-GCST90011692 | F-small fish liking (derived food-liking factor) \|\| id:ebi-fl187-GCST90094840 | Inverse variance weighted | 21 | 0.17179638045927 | 0.225451803552533 | 0.446054428589524 | -0.270089155 | 0.613681915422234 | 1.18743602341841 | 0.763311438650685 | 1.84722020176221 | 0.998735809224318 |
| ebi-fl187-GCST90094840 | ebi-gm430-GCST90011693 | TestASV_2 (Bacteroides) prevalence \|\| id:ebi-gm430-GCST90011693 | F-small fish liking (derived food-liking factor) \|\| id:ebi-fl187-GCST90094840 | Inverse variance weighted | 21 | 0.146203189003176 | 0.113019993126595 | 0.195802014395088 | -0.075315998 | 0.367722375531302 | 1.15743134148021 | 0.927450367997857 | 1.44444097114615 | 0.998735809224318 |
| ebi-fl187-GCST90094840 | ebi-gm430-GCST90011716 | TestASV_40 (Barnesiella) prevalence \|\| id:ebi-gm430-GCST90011716 | F-small fish liking (derived food-liking factor) \|\| id:ebi-fl187-GCST90094840 | Inverse variance weighted | 21 | -0.287272001 | 0.22544246873636 | 0.202571788895911 | -0.72913924 | 0.154595237593104 | 0.750307616559268 | 0.4823239767195 | 1.16718543269568 | 0.998735809224318 |
| ebi-fl187-GCST90094840 | ebi-gm430-GCST90011718 | TestASV_42 (Alphaproteobacteria) prevalence \|\| id:ebi-gm430-GCST90011718 | F-small fish liking (derived food-liking factor) \|\| id:ebi-fl187-GCST90094840 | Inverse variance weighted | 21 | 0.140265259968232 | 0.179855777490674 | 0.435463684232798 | -0.212252064 | 0.492782583849954 | 1.15057896092042 | 0.808760812456075 | 1.63686460189936 | 0.998735809224318 |
| ebi-fl187-GCST90094840 | ebi-hbsm44-GCST90104929 | Sulcal depth of the right inferior frontal sulcus \|\| id:ebi-hbsm44-GCST90104929 | F-small fish liking (derived food-liking factor) \|\| id:ebi-fl187-GCST90094840 | Inverse variance weighted | 24 | 0.0699504774237653 | 0.0976400882490716 | 0.473737325515679 | -0.121424096 | 0.261325050391946 | 1.07245506920118 | 0.885658276206868 | 1.29864972343652 | 0.998735809224318 |
| ebi-fl187-GCST90094840 | ebi-hbsm44-GCST90104935 | Sulcal opening distance of the left calloso-marginal posterior fissure \|\| id:ebi-hbsm44-GCST90104935 | F-small fish liking (derived food-liking factor) \|\| id:ebi-fl187-GCST90094840 | Inverse variance weighted (multiplicative random effects) | 27 | 0.00450779455763731 | 0.0260941891540246 | 0.862847235904729 | -0.046636816 | 0.0556524052995255 | 1.0045179699473 | 0.954433969622853 | 1.05723013226968 | 0.998735809224318 |
| ebi-fl187-GCST90094840 | ebi-hbsm44-GCST90104936 | Sulcal opening distance of the left anterior inferior temporal sulcus \|\| id:ebi-hbsm44-GCST90104936 | F-small fish liking (derived food-liking factor) \|\| id:ebi-fl187-GCST90094840 | Inverse variance weighted | 26 | -0.009456145 | 0.0120004678694765 | 0.430707608499049 | -0.032977062 | 0.0140647720203134 | 0.990588423741833 | 0.967560753215925 | 1.0141641462718 | 0.998735809224318 |
| ebi-fl187-GCST90094840 | ebi-hbsm44-GCST90104941 | Sulcal opening distance of the right posterior inferior temporal sulcus \|\| id:ebi-hbsm44-GCST90104941 | F-small fish liking (derived food-liking factor) \|\| id:ebi-fl187-GCST90094840 | Inverse variance weighted (multiplicative random effects) | 26 | 0.00451495907411886 | 0.0120481570768391 | 0.707851822569205 | -0.019099429 | 0.0281293469447235 | 1.00452516685863 | 0.981081809609532 | 1.0285287128644 | 0.998735809224318 |
| ebi-fl187-GCST90094840 | ebi-IPs91-GCST90274819 | Leukemia inhibitory factor levels \|\| id:ebi-IPs91-GCST90274819 | F-small fish liking (derived food-liking factor) \|\| id:ebi-fl187-GCST90094840 | Inverse variance weighted | 28 | -0.005073532 | 0.036991880294431 | 0.890910198830303 | -0.077577617 | 0.0674305538620858 | 0.994939317107537 | 0.925355198414644 | 1.06975596659786 | 0.998735809224318 |
| ebi-fl187-GCST90094840 | ebi-IPs91-GCST90274837 | STAM binding protein levels \|\| id:ebi-IPs91-GCST90274837 | F-small fish liking (derived food-liking factor) \|\| id:ebi-fl187-GCST90094840 | Inverse variance weighted | 27 | 0.00287408724152643 | 0.0340950872934133 | 0.932820911597812 | -0.063952284 | 0.0697004583366166 | 1.00287822138995 | 0.938049758582501 | 1.07218696848032 | 0.998735809224318 |
| ebi-fl187-GCST90094840 | ebi-IPs91-GCST90274840 | TNF-beta levels \|\| id:ebi-IPs91-GCST90274840 | F-small fish liking (derived food-liking factor) \|\| id:ebi-fl187-GCST90094840 | Inverse variance weighted | 28 | 0.0391876433588005 | 0.0442441343140219 | 0.375771733165845 | -0.04753086 | 0.125906146614283 | 1.03996560798241 | 0.953581045266023 | 1.13417571705666 | 0.998735809224318 |
| ebi-fl187-GCST90094840 | ebi-IPs91-GCST90274842 | Tumor necrosis factor ligand superfamily member 14 levels \|\| id:ebi-IPs91-GCST90274842 | F-small fish liking (derived food-liking factor) \|\| id:ebi-fl187-GCST90094840 | Inverse variance weighted | 28 | -0.014490168 | 0.0374813092059417 | 0.699054453321933 | -0.087953534 | 0.0589731979741495 | 0.985614309176136 | 0.915803429353227 | 1.06074681019573 | 0.998735809224318 |
| ebi-fl187-GCST90094840 | ebi-met1400-GCST90199628 | 3-methylhistidine levels \|\| id:ebi-met1400-GCST90199628 | F-small fish liking (derived food-liking factor) \|\| id:ebi-fl187-GCST90094840 | Inverse variance weighted | 27 | 0.0387940996083637 | 0.0470197368329116 | 0.409337581436345 | -0.053364585 | 0.13095278380087 | 1.03955641653937 | 0.948034310772424 | 1.13991395763691 | 0.998735809224318 |
| ebi-fl187-GCST90094840 | ebi-met1400-GCST90199651 | Docosahexaenoate DHA; 22:6n3 levels \|\| id:ebi-met1400-GCST90199651 | F-small fish liking (derived food-liking factor) \|\| id:ebi-fl187-GCST90094840 | Inverse variance weighted | 27 | 0.0539313945322449 | 0.052566246792349 | 0.304905704416205 | -0.049098449 | 0.156961238245249 | 1.05541219262411 | 0.952087392860585 | 1.1699502636968 | 0.998735809224318 |
| ebi-fl187-GCST90094840 | ebi-met1400-GCST90199652 | 1-stearoyl-2-oleoyl-GPS (18:0/18:1) levels \|\| id:ebi-met1400-GCST90199652 | F-small fish liking (derived food-liking factor) \|\| id:ebi-fl187-GCST90094840 | Inverse variance weighted | 27 | -0.066043907 | 0.0455503373176675 | 0.147083510321276 | -0.155322568 | 0.023234753917351 | 0.936089762285216 | 0.856138969587923 | 1.02350678357388 | 0.998735809224318 |
| ebi-fl187-GCST90094840 | ebi-met1400-GCST90199653 | 1-myristoyl-2-palmitoyl-gpc (14:0/16:0) levels \|\| id:ebi-met1400-GCST90199653 | F-small fish liking (derived food-liking factor) \|\| id:ebi-fl187-GCST90094840 | Inverse variance weighted | 26 | -0.0078125 | 0.0533513665680091 | 0.883578102255722 | -0.112381179 | 0.0967561780824281 | 0.992217937872416 | 0.893703531668636 | 1.10159174866148 | 0.998735809224318 |
| ebi-fl187-GCST90094840 | ebi-met1400-GCST90199667 | Erythritol levels in elite athletes \|\| id:ebi-met1400-GCST90199667 | F-small fish liking (derived food-liking factor) \|\| id:ebi-fl187-GCST90094840 | Inverse variance weighted | 27 | -0.052166828 | 0.0446603091652842 | 0.242774365988916 | -0.139701034 | 0.0353673775416109 | 0.949170505015741 | 0.869618182472856 | 1.0360002421177 | 0.998735809224318 |
| ebi-fl187-GCST90094840 | ebi-met1400-GCST90199674 | 3-hydroxymyristate levels \|\| id:ebi-met1400-GCST90199674 | F-small fish liking (derived food-liking factor) \|\| id:ebi-fl187-GCST90094840 | Inverse variance weighted (multiplicative random effects) | 27 | 0.0309240696271829 | 0.0547268272186247 | 0.572031355575169 | -0.076340512 | 0.138188650975687 | 1.03140718578106 | 0.92650066850279 | 1.14819213741085 | 0.998735809224318 |
| ebi-fl187-GCST90094840 | ebi-met1400-GCST90199685 | 2-linoleoylglycerol (18:2) levels \|\| id:ebi-met1400-GCST90199685 | F-small fish liking (derived food-liking factor) \|\| id:ebi-fl187-GCST90094840 | Inverse variance weighted | 27 | 0.0641327466433842 | 0.0527034222815533 | 0.223657052131408 | -0.039165961 | 0.167431454315229 | 1.06623392834256 | 0.961591109253602 | 1.18226424829494 | 0.998735809224318 |
| ebi-fl187-GCST90094840 | ebi-met1400-GCST90199703 | Gamma-glutamylvaline levels \|\| id:ebi-met1400-GCST90199703 | F-small fish liking (derived food-liking factor) \|\| id:ebi-fl187-GCST90094840 | Inverse variance weighted | 27 | 0.0431679597390204 | 0.0497052092023912 | 0.385131840459405 | -0.05425425 | 0.140590169775707 | 1.0441132491128 | 0.947191252226635 | 1.15095285604691 | 0.998735809224318 |
| ebi-fl187-GCST90094840 | ebi-met1400-GCST90199779 | Gamma-glutamyl-2-aminobutyrate levels \|\| id:ebi-met1400-GCST90199779 | F-small fish liking (derived food-liking factor) \|\| id:ebi-fl187-GCST90094840 | Inverse variance weighted | 27 | 0.0308692876621814 | 0.053286641913401 | 0.562382511589434 | -0.07357253 | 0.135311105812447 | 1.03135068481633 | 0.929068757537249 | 1.14489291179127 | 0.998735809224318 |
| ebi-fl187-GCST90094840 | ebi-met1400-GCST90199824 | 4-vinylphenol sulfate levels \|\| id:ebi-met1400-GCST90199824 | F-small fish liking (derived food-liking factor) \|\| id:ebi-fl187-GCST90094840 | Inverse variance weighted | 27 | -0.045340633 | 0.0449919361738646 | 0.313574397091707 | -0.133524828 | 0.04284356206161 | 0.955671893152875 | 0.875005744292866 | 1.04377459613193 | 0.998735809224318 |
| ebi-fl187-GCST90094840 | ebi-met1400-GCST90199830 | 4-ethylphenylsulfate levels \|\| id:ebi-met1400-GCST90199830 | F-small fish liking (derived food-liking factor) \|\| id:ebi-fl187-GCST90094840 | Inverse variance weighted | 27 | -0.086866362 | 0.0450466995823351 | 0.0538100651526231 | -0.175157893 | 0.00142516943377612 | 0.916799606944144 | 0.839324486904803 | 1.00142618547035 | 0.998735809224318 |
| ebi-fl187-GCST90094840 | ebi-met1400-GCST90199833 | Hexanoylglutamine levels \|\| id:ebi-met1400-GCST90199833 | F-small fish liking (derived food-liking factor) \|\| id:ebi-fl187-GCST90094840 | Inverse variance weighted | 27 | 0.0746155219225416 | 0.0503490948638103 | 0.138350001505982 | -0.024068704 | 0.17329974785561 | 1.07746980771661 | 0.976218637318328 | 1.18922251856406 | 0.998735809224318 |
| ebi-fl187-GCST90094840 | ebi-met1400-GCST90199837 | Carotenoid levels (cryptoxanthin) \|\| id:ebi-met1400-GCST90199837 | F-small fish liking (derived food-liking factor) \|\| id:ebi-fl187-GCST90094840 | Inverse variance weighted | 27 | -0.040891863 | 0.047058909116042 | 0.384874198514995 | -0.133127325 | 0.0513435986106076 | 0.959932928356016 | 0.8753536305032 | 1.05268453209299 | 0.998735809224318 |
| ebi-fl187-GCST90094840 | ebi-met1400-GCST90199869 | 2-hydroxyhippurate levels \|\| id:ebi-met1400-GCST90199869 | F-small fish liking (derived food-liking factor) \|\| id:ebi-fl187-GCST90094840 | Inverse variance weighted | 27 | 0.0565308117809156 | 0.0491525389267885 | 0.250098691861167 | -0.039808165 | 0.152869788077421 | 1.05815921806794 | 0.960973770339865 | 1.1651732496155 | 0.998735809224318 |
| ebi-fl187-GCST90094840 | ebi-met1400-GCST90199902 | Hydantoin-5-propionate levels \|\| id:ebi-met1400-GCST90199902 | F-small fish liking (derived food-liking factor) \|\| id:ebi-fl187-GCST90094840 | Inverse variance weighted | 27 | 0.0999132919218295 | 0.0449249828373175 | 0.026148249415505 | 0.0118603255606872 | 0.187966258282972 | 1.10507509498367 | 1.01193093810836 | 1.20679279540161 | 0.998735809224318 |
| ebi-fl187-GCST90094840 | ebi-met1400-GCST90199904 | Pantoate levels \|\| id:ebi-met1400-GCST90199904 | F-small fish liking (derived food-liking factor) \|\| id:ebi-fl187-GCST90094840 | Inverse variance weighted | 27 | -0.01211123 | 0.0455715678909742 | 0.790421848560076 | -0.101431503 | 0.0772090428662949 | 0.987961815559743 | 0.903543066971469 | 1.08026787508395 | 0.998735809224318 |
| ebi-fl187-GCST90094840 | ebi-met1400-GCST90199936 | 3-methyl catechol sulfate (2) levels \|\| id:ebi-met1400-GCST90199936 | F-small fish liking (derived food-liking factor) \|\| id:ebi-fl187-GCST90094840 | Inverse variance weighted | 27 | -0.072746557 | 0.0511439221325552 | 0.154913327565356 | -0.172988644 | 0.0274955303427263 | 0.929836460672689 | 0.841147166506948 | 1.02787702083898 | 0.998735809224318 |
| ebi-fl187-GCST90094840 | ebi-met1400-GCST90199977 | 4-hydroxychlorothalonil levels \|\| id:ebi-met1400-GCST90199977 | F-small fish liking (derived food-liking factor) \|\| id:ebi-fl187-GCST90094840 | Inverse variance weighted | 27 | 0.0333168857194729 | 0.0517759536273037 | 0.519911496963139 | -0.068163983 | 0.134797754828988 | 1.03387810854582 | 0.934107282938277 | 1.14430533071961 | 0.998735809224318 |
| ebi-fl187-GCST90094840 | ebi-met1400-GCST90200040 | Sphingomyelin (d18:2/24:1, d18:1/24:2) levels \|\| id:ebi-met1400-GCST90200040 | F-small fish liking (derived food-liking factor) \|\| id:ebi-fl187-GCST90094840 | Inverse variance weighted | 27 | -0.022243561 | 0.0476518149107798 | 0.640647568440005 | -0.115641119 | 0.0711539958777147 | 0.978002002554758 | 0.890794855668356 | 1.07374656568203 | 0.998735809224318 |
| ebi-fl187-GCST90094840 | ebi-met1400-GCST90200077 | 1-stearoyl-2-oleoyl-GPI (18:0/18:1) levels \|\| id:ebi-met1400-GCST90200077 | F-small fish liking (derived food-liking factor) \|\| id:ebi-fl187-GCST90094840 | Inverse variance weighted | 27 | -0.011022102 | 0.0475778825819382 | 0.816798680207674 | -0.104274752 | 0.082230548014142 | 0.989038418958523 | 0.900977718245255 | 1.08570608836044 | 0.998735809224318 |
| ebi-fl187-GCST90094840 | ebi-met1400-GCST90200080 | 1-palmitoyl-2-oleoyl-GPI (16:0/18:1) levels \|\| id:ebi-met1400-GCST90200080 | F-small fish liking (derived food-liking factor) \|\| id:ebi-fl187-GCST90094840 | Inverse variance weighted | 27 | -0.013085478 | 0.0455784473498254 | 0.774037329401827 | -0.102419235 | 0.0762482784598646 | 0.986999764307253 | 0.902651049284314 | 1.07923049058101 | 0.998735809224318 |
| ebi-fl187-GCST90094840 | ebi-met1400-GCST90200082 | 1-oleoyl-2-linoleoyl-GPE (18:1/18:2) levels \|\| id:ebi-met1400-GCST90200082 | F-small fish liking (derived food-liking factor) \|\| id:ebi-fl187-GCST90094840 | Inverse variance weighted | 27 | -0.004444799 | 0.0550554333727108 | 0.93565416540434 | -0.112353448 | 0.103463850326156 | 0.995565064415923 | 0.893728314741232 | 1.109005702 | 0.998735809224318 |
| ebi-fl187-GCST90094840 | ebi-met1400-GCST90200100 | Perfluorooctanesulfonate (PFOS) levels \|\| id:ebi-met1400-GCST90200100 | F-small fish liking (derived food-liking factor) \|\| id:ebi-fl187-GCST90094840 | Inverse variance weighted (multiplicative random effects) | 27 | -0.036797691 | 0.0514167563950892 | 0.474191999621465 | -0.137574534 | 0.0639791511154284 | 0.963871115022957 | 0.871469393517359 | 1.06607017215584 | 0.998735809224318 |
| ebi-fl187-GCST90094840 | ebi-met1400-GCST90200102 | 1-palmitoleoyl-2-linolenoyl-GPC (16:1/18:3) levels \|\| id:ebi-met1400-GCST90200102 | F-small fish liking (derived food-liking factor) \|\| id:ebi-fl187-GCST90094840 | Inverse variance weighted | 27 | -0.041081739 | 0.0440301063596605 | 0.350800621770857 | -0.127380748 | 0.0452172693763997 | 0.959750677595923 | 0.880398399237363 | 1.04625515442071 | 0.998735809224318 |
| ebi-fl187-GCST90094840 | ebi-met1400-GCST90200114 | Glycosyl-N-tricosanoyl-sphingadienine (d18:2/23:0) levels \|\| id:ebi-met1400-GCST90200114 | F-small fish liking (derived food-liking factor) \|\| id:ebi-fl187-GCST90094840 | Inverse variance weighted | 27 | -0.07355713 | 0.049954945295042 | 0.140894155011513 | -0.171468823 | 0.0243545627231502 | 0.929083065708534 | 0.842426532082929 | 1.02465355744683 | 0.998735809224318 |
| ebi-fl187-GCST90094840 | ebi-met1400-GCST90200135 | Arachidoylcarnitine (C20) levels \|\| id:ebi-met1400-GCST90200135 | F-small fish liking (derived food-liking factor) \|\| id:ebi-fl187-GCST90094840 | Inverse variance weighted (multiplicative random effects) | 27 | 0.00211931229381104 | 0.0607041751890871 | 0.972149805041997 | -0.116860871 | 0.121099495664422 | 1.00212155962343 | 0.889708968803617 | 1.12873721123942 | 0.998955228396052 |
| ebi-fl187-GCST90094840 | ebi-met1400-GCST90200146 | Docosahexaenoylcarnitine (C22:6) levels \|\| id:ebi-met1400-GCST90200146 | F-small fish liking (derived food-liking factor) \|\| id:ebi-fl187-GCST90094840 | Inverse variance weighted | 27 | -0.0293546 | 0.0504091217492681 | 0.560346338020859 | -0.128156479 | 0.0694472781863245 | 0.971072060825155 | 0.87971571127678 | 1.07191554638329 | 0.998735809224318 |
| ebi-fl187-GCST90094840 | ebi-met1400-GCST90200162 | 3-hydroxyphenylacetoylglutamine levels \|\| id:ebi-met1400-GCST90200162 | F-small fish liking (derived food-liking factor) \|\| id:ebi-fl187-GCST90094840 | Inverse variance weighted | 27 | 0.0700581141629244 | 0.0549201698821508 | 0.202084425913869 | -0.037585419 | 0.17770164713194 | 1.0725705109805 | 0.963112146317439 | 1.19446889484644 | 0.998735809224318 |
| ebi-fl187-GCST90094840 | ebi-met1400-GCST90200172 | 3-carboxy-4-methyl-5-pentyl-2-furanpropionate (3-CMPFP) levels \|\| id:ebi-met1400-GCST90200172 | F-small fish liking (derived food-liking factor) \|\| id:ebi-fl187-GCST90094840 | Inverse variance weighted | 27 | 0.0234313762404454 | 0.0446544150613985 | 0.599773000746757 | -0.064091277 | 0.110954029760786 | 1.02370804764123 | 0.937919384893239 | 1.11734354112395 | 0.998735809224318 |
| ebi-fl187-GCST90094840 | ebi-met1400-GCST90200222 | Dihydrocaffeate sulfate (2) levels \|\| id:ebi-met1400-GCST90200222 | F-small fish liking (derived food-liking factor) \|\| id:ebi-fl187-GCST90094840 | Inverse variance weighted | 27 | 0.0143178663355147 | 0.0588250696417381 | 0.807697592918549 | -0.10097927 | 0.129615002833321 | 1.01442085793667 | 0.903951771465158 | 1.13839002201305 | 0.998735809224318 |
| ebi-fl187-GCST90094840 | ebi-met1400-GCST90200223 | 4-methylhexanoylglutamine levels \|\| id:ebi-met1400-GCST90200223 | F-small fish liking (derived food-liking factor) \|\| id:ebi-fl187-GCST90094840 | Inverse variance weighted | 27 | 0.0363926459184217 | 0.0638913663364722 | 0.568947715508757 | -0.088834432 | 0.161619723937907 | 1.03706296509936 | 0.914997055173819 | 1.17541317482859 | 0.998735809224318 |
| ebi-fl187-GCST90094840 | ebi-met1400-GCST90200224 | 2-ketocaprylate levels \|\| id:ebi-met1400-GCST90200224 | F-small fish liking (derived food-liking factor) \|\| id:ebi-fl187-GCST90094840 | Inverse variance weighted | 27 | -0.031631675 | 0.045509326447444 | 0.487018215510167 | -0.120829955 | 0.057566604763509 | 0.968863372898318 | 0.886184638127569 | 1.05925581978862 | 0.998735809224318 |
| ebi-fl187-GCST90094840 | ebi-met1400-GCST90200243 | Hydroxypalmitoyl sphingomyelin (d18:1/16:0(OH)) levels \|\| id:ebi-met1400-GCST90200243 | F-small fish liking (derived food-liking factor) \|\| id:ebi-fl187-GCST90094840 | Inverse variance weighted | 27 | -0.005547242 | 0.043932965370477 | 0.899521326619876 | -0.091655854 | 0.0805613699749107 | 0.994468115386069 | 0.912419100650271 | 1.08389536323242 | 0.998735809224318 |
| ebi-fl187-GCST90094840 | ebi-met1400-GCST90200273 | 2-methoxyhydroquinone sulfate (2) levels \|\| id:ebi-met1400-GCST90200273 | F-small fish liking (derived food-liking factor) \|\| id:ebi-fl187-GCST90094840 | Inverse variance weighted | 27 | -0.079952111 | 0.0649369888126542 | 0.218238338930845 | -0.207228609 | 0.047324387213834 | 0.923160554694077 | 0.812833807677295 | 1.0484620616093 | 0.998735809224318 |
| ebi-fl187-GCST90094840 | ebi-met1400-GCST90200276 | 2,4-di-tert-butylphenol levels \|\| id:ebi-met1400-GCST90200276 | F-small fish liking (derived food-liking factor) \|\| id:ebi-fl187-GCST90094840 | Inverse variance weighted | 27 | 0.00330752675927489 | 0.0447351185735759 | 0.941061469877963 | -0.084373306 | 0.0909883591634835 | 1.00331300266147 | 0.919088091273763 | 1.09525625548524 | 0.998735809224318 |
| ebi-fl187-GCST90094840 | ebi-met1400-GCST90200289 | N-acetylmethionine levels \|\| id:ebi-met1400-GCST90200289 | F-small fish liking (derived food-liking factor) \|\| id:ebi-fl187-GCST90094840 | Inverse variance weighted | 27 | -0.003759048 | 0.0447644325244507 | 0.933077103452785 | -0.091497336 | 0.0839792392754186 | 0.99624800790568 | 0.912563747017441 | 1.0876063140793 | 0.998735809224318 |
| ebi-fl187-GCST90094840 | ebi-met1400-GCST90200308 | 3-hydroxyisobutyrate levels \|\| id:ebi-met1400-GCST90200308 | F-small fish liking (derived food-liking factor) \|\| id:ebi-fl187-GCST90094840 | Inverse variance weighted | 27 | 0.0621938849471599 | 0.0466283940522975 | 0.182262852009193 | -0.029197767 | 0.153585537289663 | 1.06416865101022 | 0.971224368958374 | 1.1660075199796 | 0.998735809224318 |
| ebi-fl187-GCST90094840 | ebi-met1400-GCST90200383 | Cystathionine levels \|\| id:ebi-met1400-GCST90200383 | F-small fish liking (derived food-liking factor) \|\| id:ebi-fl187-GCST90094840 | Inverse variance weighted | 27 | -0.037386987 | 0.0505501691499425 | 0.459541758593683 | -0.136465319 | 0.0616913445551692 | 0.963303277383214 | 0.872436577133095 | 1.0636339976329 | 0.998735809224318 |
| ebi-fl187-GCST90094840 | ebi-met1400-GCST90200384 | Guanidinoacetate levels \|\| id:ebi-met1400-GCST90200384 | F-small fish liking (derived food-liking factor) \|\| id:ebi-fl187-GCST90094840 | Inverse variance weighted | 27 | -0.049363903 | 0.0430112916298461 | 0.251093937433044 | -0.133666034 | 0.0349382290702157 | 0.951834691620502 | 0.874882196623313 | 1.03555573958305 | 0.998735809224318 |
| ebi-fl187-GCST90094840 | ebi-met1400-GCST90200403 | Citrulline levels \|\| id:ebi-met1400-GCST90200403 | F-small fish liking (derived food-liking factor) \|\| id:ebi-fl187-GCST90094840 | Inverse variance weighted | 26 | -0.060831229 | 0.0456583809039537 | 0.18275753973293 | -0.150321656 | 0.028659197527845 | 0.940982036813491 | 0.860431169391658 | 1.02907382380359 | 0.998735809224318 |
| ebi-fl187-GCST90094840 | ebi-met1400-GCST90200434 | 12,13-DiHOME levels \|\| id:ebi-met1400-GCST90200434 | F-small fish liking (derived food-liking factor) \|\| id:ebi-fl187-GCST90094840 | Inverse variance weighted | 27 | 0.0204573913666641 | 0.0453936645846403 | 0.652230129901815 | -0.068514191 | 0.109428973952559 | 1.02066807804478 | 0.933780208529842 | 1.11564082856263 | 0.998735809224318 |
| ebi-fl187-GCST90094840 | ebi-met1400-GCST90200437 | Fructose levels \|\| id:ebi-met1400-GCST90200437 | F-small fish liking (derived food-liking factor) \|\| id:ebi-fl187-GCST90094840 | Inverse variance weighted | 26 | -0.043824149 | 0.0464536321368079 | 0.345478625393561 | -0.134873268 | 0.047224969776798 | 0.957122253378225 | 0.873826646295308 | 1.04835783137955 | 0.998735809224318 |
| ebi-fl187-GCST90094840 | ebi-met1400-GCST90200448 | N-stearoyl-sphinganine (d18:0/18:0) levels \|\| id:ebi-met1400-GCST90200448 | F-small fish liking (derived food-liking factor) \|\| id:ebi-fl187-GCST90094840 | Inverse variance weighted (multiplicative random effects) | 27 | -0.02694817 | 0.0640608151610259 | 0.673999415685427 | -0.152507367 | 0.0986110280185144 | 0.973411692439752 | 0.85855256862199 | 1.103636932 | 0.998735809224318 |
| ebi-fl187-GCST90094840 | ebi-met1400-GCST90200468 | X-11847 levels \|\| id:ebi-met1400-GCST90200468 | F-small fish liking (derived food-liking factor) \|\| id:ebi-fl187-GCST90094840 | Inverse variance weighted (multiplicative random effects) | 27 | 0.074006480296298 | 0.0636352997818852 | 0.244838258750661 | -0.050718707 | 0.198731667868793 | 1.07681378354599 | 0.950546014607242 | 1.21985459579643 | 0.998735809224318 |
| ebi-fl187-GCST90094840 | ebi-met1400-GCST90200476 | X-11843 levels \|\| id:ebi-met1400-GCST90200476 | F-small fish liking (derived food-liking factor) \|\| id:ebi-fl187-GCST90094840 | Inverse variance weighted | 27 | -0.077838853 | 0.049219884489591 | 0.113774874505383 | -0.174309826 | 0.0186321208698576 | 0.925113494046552 | 0.840036591881949 | 1.0188067819161 | 0.998735809224318 |
| ebi-fl187-GCST90094840 | ebi-met1400-GCST90200511 | X-12906 levels \|\| id:ebi-met1400-GCST90200511 | F-small fish liking (derived food-liking factor) \|\| id:ebi-fl187-GCST90094840 | Inverse variance weighted | 27 | -0.011278743 | 0.0450776321802411 | 0.802427206817706 | -0.099630902 | 0.0770734161483747 | 0.988784623640607 | 0.905171453361031 | 1.08012137183268 | 0.998735809224318 |
| ebi-fl187-GCST90094840 | ebi-met1400-GCST90200515 | X-12849 levels \|\| id:ebi-met1400-GCST90200515 | F-small fish liking (derived food-liking factor) \|\| id:ebi-fl187-GCST90094840 | Inverse variance weighted | 27 | 0.045236990919653 | 0.0473334869343808 | 0.33921979254006 | -0.047536643 | 0.138010625311039 | 1.04627578839046 | 0.953575530174429 | 1.14798774793627 | 0.998735809224318 |
| ebi-fl187-GCST90094840 | ebi-met1400-GCST90200551 | X-18886 levels \|\| id:ebi-met1400-GCST90200551 | F-small fish liking (derived food-liking factor) \|\| id:ebi-fl187-GCST90094840 | Inverse variance weighted | 27 | 0.0589979403403638 | 0.0444979481824345 | 0.184886887775415 | -0.028218038 | 0.146213918777935 | 1.06077305590646 | 0.972176372204292 | 1.15744376052443 | 0.998735809224318 |
| ebi-fl187-GCST90094840 | ebi-met1400-GCST90200564 | X-21319 levels \|\| id:ebi-met1400-GCST90200564 | F-small fish liking (derived food-liking factor) \|\| id:ebi-fl187-GCST90094840 | Inverse variance weighted | 27 | 0.0180658221744921 | 0.0454305040625893 | 0.690881995853943 | -0.070977966 | 0.107109610137167 | 1.01822999629656 | 0.93148241638234 | 1.11305625004147 | 0.998735809224318 |
| ebi-fl187-GCST90094840 | ebi-met1400-GCST90200579 | X-21442 levels \|\| id:ebi-met1400-GCST90200579 | F-small fish liking (derived food-liking factor) \|\| id:ebi-fl187-GCST90094840 | Inverse variance weighted | 27 | -0.022715394 | 0.0554995433632118 | 0.682327042338367 | -0.131494499 | 0.0860637111886087 | 0.977540658315196 | 0.876784098493437 | 1.08987576337353 | 0.998735809224318 |
| ebi-fl187-GCST90094840 | ebi-met1400-GCST90200581 | X-21441 levels \|\| id:ebi-met1400-GCST90200581 | F-small fish liking (derived food-liking factor) \|\| id:ebi-fl187-GCST90094840 | Inverse variance weighted (multiplicative random effects) | 27 | -0.014444819 | 0.057166800431093 | 0.800516699604211 | -0.126491748 | 0.0976021101419791 | 0.985659007174208 | 0.881181421420977 | 1.10252401469948 | 0.998735809224318 |
| ebi-fl187-GCST90094840 | ebi-met1400-GCST90200648 | X-24585 levels \|\| id:ebi-met1400-GCST90200648 | F-small fish liking (derived food-liking factor) \|\| id:ebi-fl187-GCST90094840 | Inverse variance weighted | 27 | -0.052799423 | 0.0514263358747906 | 0.304561714937416 | -0.153595041 | 0.0479961955203566 | 0.948570254973723 | 0.857619251240763 | 1.04916666378366 | 0.998735809224318 |
| ebi-fl187-GCST90094840 | ebi-met1400-GCST90200720 | Adenosine 5'-diphosphate (ADP) to pantothenate ratio \|\| id:ebi-met1400-GCST90200720 | F-small fish liking (derived food-liking factor) \|\| id:ebi-fl187-GCST90094840 | Inverse variance weighted | 27 | -0.112192206 | 0.0598448235705609 | 0.0608314411641579 | -0.22948806 | 0.00510364794704406 | 0.893872433118556 | 0.794940459812902 | 1.0051166937425 | 0.998735809224318 |
| ebi-fl187-GCST90094840 | ebi-met1400-GCST90200724 | Cholate to taurocholate ratio \|\| id:ebi-met1400-GCST90200724 | F-small fish liking (derived food-liking factor) \|\| id:ebi-fl187-GCST90094840 | Inverse variance weighted | 27 | -0.021879996 | 0.0531292440124778 | 0.680467073320287 | -0.126013314 | 0.0822533222200382 | 0.978357634793001 | 0.881603108769179 | 1.085730815 | 0.998735809224318 |
| ebi-fl187-GCST90094840 | ebi-met1400-GCST90200726 | N-acetylputrescine to (N(1) + N(8))-acetylspermidine ratio \|\| id:ebi-met1400-GCST90200726 | F-small fish liking (derived food-liking factor) \|\| id:ebi-fl187-GCST90094840 | Inverse variance weighted | 27 | 0.01131328042382 | 0.0494968278700306 | 0.81920643424703 | -0.085700502 | 0.10832706304908 | 1.01137751759662 | 0.917869089830092 | 1.11441216883037 | 0.998735809224318 |
| ebi-fl187-GCST90094840 | ebi-met1400-GCST90200744 | Arginine to glutamate ratio \|\| id:ebi-met1400-GCST90200744 | F-small fish liking (derived food-liking factor) \|\| id:ebi-fl187-GCST90094840 | Inverse variance weighted | 27 | -0.008074652 | 0.0484816040708788 | 0.867723476051836 | -0.103098596 | 0.0869492920207374 | 0.991957860476222 | 0.902038031811892 | 1.09084136395455 | 0.998735809224318 |
| ebi-fl187-GCST90094840 | ebi-met1400-GCST90200759 | Histidine to trans-urocanate ratio \|\| id:ebi-met1400-GCST90200759 | F-small fish liking (derived food-liking factor) \|\| id:ebi-fl187-GCST90094840 | Inverse variance weighted | 27 | 8.00690115334069e-05 | 0.0473459977692096 | 0.998650661076767 | -0.092718087 | 0.0928782246391843 | 1.00008007221714 | 0.911450414151206 | 1.09732809960622 | 0.999882738470636 |
| ebi-fl187-GCST90094840 | ebi-met1400-GCST90200795 | Oleoyl-linoleoyl-glycerol (18:1 to 18:2) [2] to linoleoyl-arachidonoyl-glycerol (18:2 to 20:4) [2] ratio \|\| id:ebi-met1400-GCST90200795 | F-small fish liking (derived food-liking factor) \|\| id:ebi-fl187-GCST90094840 | Inverse variance weighted | 27 | 0.0578374516123673 | 0.046722394628952 | 0.215754735308096 | -0.033738442 | 0.149413345085113 | 1.05954275474551 | 0.966824352347817 | 1.16115284685117 | 0.998735809224318 |
| ebi-fl187-GCST90094840 | ebi-met1400-GCST90200811 | Choline to taurocholate ratio \|\| id:ebi-met1400-GCST90200811 | F-small fish liking (derived food-liking factor) \|\| id:ebi-fl187-GCST90094840 | Inverse variance weighted | 27 | -0.003671925 | 0.0470457362790855 | 0.937788195731489 | -0.095881568 | 0.0885377184055113 | 0.996334808570134 | 0.908571613824418 | 1.09257546203763 | 0.998735809224318 |
| ebi-fl187-GCST90094840 | ebi-met1400-GCST90200831 | Cholate to phosphate ratio \|\| id:ebi-met1400-GCST90200831 | F-small fish liking (derived food-liking factor) \|\| id:ebi-fl187-GCST90094840 | Inverse variance weighted | 27 | -0.043810155 | 0.0556128118641039 | 0.430830727295563 | -0.152811266 | 0.0651909565793161 | 0.957135647954755 | 0.858291695412608 | 1.06736282487898 | 0.998735809224318 |
| ebi-fl187-GCST90094840 | ebi-met1400-GCST90200841 | Adenosine 5'-diphosphate (ADP) to oxalate (ethanedioate) ratio \|\| id:ebi-met1400-GCST90200841 | F-small fish liking (derived food-liking factor) \|\| id:ebi-fl187-GCST90094840 | Inverse variance weighted | 27 | -0.06726061 | 0.0664159058775892 | 0.311194687462859 | -0.197435785 | 0.0629145657172836 | 0.934951512055072 | 0.820832848451743 | 1.06493585331394 | 0.998735809224318 |
| ebi-fl187-GCST90094840 | ebi-met1400-GCST90200844 | Aspartate to phosphate ratio \|\| id:ebi-met1400-GCST90200844 | F-small fish liking (derived food-liking factor) \|\| id:ebi-fl187-GCST90094840 | Inverse variance weighted | 27 | -0.008454745 | 0.0422728143007056 | 0.841477203688473 | -0.091309461 | 0.0743999706141457 | 0.991580895429296 | 0.912735210833181 | 1.0772375827189 | 0.998735809224318 |
| ebi-fl187-GCST90094840 | ebi-met1400-GCST90200870 | Adenosine 5'-monophosphate (AMP) to acetoacetate ratio \|\| id:ebi-met1400-GCST90200870 | F-small fish liking (derived food-liking factor) \|\| id:ebi-fl187-GCST90094840 | Inverse variance weighted | 27 | -0.055914019 | 0.0494903832333972 | 0.258562349676662 | -0.15291517 | 0.0410871323911103 | 0.945620438025857 | 0.858202520143106 | 1.04194288856563 | 0.998735809224318 |
| ebi-fl187-GCST90094840 | ebi-met1400-GCST90200887 | Cholesterol to cortisol ratio \|\| id:ebi-met1400-GCST90200887 | F-small fish liking (derived food-liking factor) \|\| id:ebi-fl187-GCST90094840 | Inverse variance weighted | 27 | 0.0803631504845753 | 0.0437045777729638 | 0.0659473929552146 | -0.005297822 | 0.166024122919584 | 1.08368053533818 | 0.994716186758809 | 1.18060158093677 | 0.998735809224318 |
| ebi-fl187-GCST90094840 | ebi-met1400-GCST90200890 | Cortisol to taurocholate ratio \|\| id:ebi-met1400-GCST90200890 | F-small fish liking (derived food-liking factor) \|\| id:ebi-fl187-GCST90094840 | Inverse variance weighted | 27 | -0.012655605 | 0.0469066334114034 | 0.787310914684881 | -0.104592607 | 0.07928139619867 | 0.987424140121622 | 0.900691383421176 | 1.08250889310329 | 0.998735809224318 |
| ebi-fl187-GCST90094840 | ebi-met1400-GCST90200948 | Adenosine 5'-diphosphate (ADP) to mannitol to sorbitol ratio \|\| id:ebi-met1400-GCST90200948 | F-small fish liking (derived food-liking factor) \|\| id:ebi-fl187-GCST90094840 | Inverse variance weighted | 27 | -0.021930888 | 0.0613412791923333 | 0.720700673930413 | -0.142159796 | 0.0982980188044654 | 0.978307845123096 | 0.867482625477692 | 1.1032915377439 | 0.998735809224318 |
| ebi-fl187-GCST90094840 | ebi-met1400-GCST90200972 | Leucine to N-palmitoyl-sphingosine (d18:1 to 16:0) ratio \|\| id:ebi-met1400-GCST90200972 | F-small fish liking (derived food-liking factor) \|\| id:ebi-fl187-GCST90094840 | Inverse variance weighted | 27 | 0.00447567253986629 | 0.0461290260786547 | 0.922706475827502 | -0.085937219 | 0.0948885636540295 | 1.00448570332146 | 0.917651840902594 | 1.09953631998903 | 0.998735809224318 |
| ebi-fl187-GCST90094840 | ebi-met1400-GCST90200973 | Proline to glutamate ratio \|\| id:ebi-met1400-GCST90200973 | F-small fish liking (derived food-liking factor) \|\| id:ebi-fl187-GCST90094840 | Inverse variance weighted | 27 | -0.028925335 | 0.0455584529463949 | 0.52548973106288 | -0.118219902 | 0.0603692331671841 | 0.971488998365815 | 0.888500647720954 | 1.06222868420713 | 0.998735809224318 |
| ebi-fl187-GCST90094840 | ebi-met1400-GCST90200983 | Cholesterol to linoleoyl-arachidonoyl-glycerol (18:2 to 20:4) [1] ratio \|\| id:ebi-met1400-GCST90200983 | F-small fish liking (derived food-liking factor) \|\| id:ebi-fl187-GCST90094840 | Inverse variance weighted | 27 | 0.00682353651570719 | 0.053862944308766 | 0.899191049558618 | -0.098747834 | 0.112394907360889 | 1.00684686988283 | 0.905971134040231 | 1.1189546568355 | 0.998735809224318 |
| ebi-fl187-GCST90094840 | ebi-met1400-GCST90201001 | Serine to alpha-ketobutyrate ratio \|\| id:ebi-met1400-GCST90201001 | F-small fish liking (derived food-liking factor) \|\| id:ebi-fl187-GCST90094840 | Inverse variance weighted | 27 | -0.080153229 | 0.0472898498938283 | 0.0900877209453676 | -0.172841335 | 0.0125348763079175 | 0.922974908581718 | 0.841271084300389 | 1.01261376715434 | 0.998735809224318 |
| ebi-fl187-GCST90094840 | ebi-met1400-GCST90201005 | Fructose to maltose ratio \|\| id:ebi-met1400-GCST90201005 | F-small fish liking (derived food-liking factor) \|\| id:ebi-fl187-GCST90094840 | Inverse variance weighted | 27 | 0.00840509192321955 | 0.0600109220956838 | 0.888613082384616 | -0.109216315 | 0.12602649923076 | 1.00844051388039 | 0.896536461891889 | 1.1343122262862 | 0.998735809224318 |
| ebi-fl187-GCST90094840 | ebi-met1400-GCST90201020 | Paraxanthine to 5-acetylamino-6-formylamino-3-methyluracil ratio \|\| id:ebi-met1400-GCST90201020 | F-small fish liking (derived food-liking factor) \|\| id:ebi-fl187-GCST90094840 | Inverse variance weighted | 27 | 0.0194744084063459 | 0.0496059154531111 | 0.694627954726268 | -0.077753186 | 0.116702002694444 | 1.01966527166746 | 0.925192748998331 | 1.12378449503666 | 0.998735809224318 |
| ebi-fl187-GCST90094840 | ebi-pw11-GCST90275197 | Placental weight (fetal genotype, adjusted for sex) \|\| id:ebi-pw11-GCST90275197 | F-small fish liking (derived food-liking factor) \|\| id:ebi-fl187-GCST90094840 | Inverse variance weighted | 23 | 0.0245774207696358 | 0.0175308195050395 | 0.160928672783181 | -0.009782985 | 0.0589378269995132 | 1.02488193518393 | 0.990264712273216 | 1.06070929121076 | 0.998735809224318 |
| ebi-fl187-GCST90094840 | prot-a-1017 | FAS-associated factor 2 \|\| id:prot-a-1017 | F-small fish liking (derived food-liking factor) \|\| id:ebi-fl187-GCST90094840 | Inverse variance weighted | 28 | -0.033819488 | 0.0686253902189562 | 0.622144687698851 | -0.168325253 | 0.100686276845971 | 0.966745998157821 | 0.845078925682394 | 1.10592963160155 | 0.998735809224318 |
| ebi-fl187-GCST90094840 | prot-a-1019 | Fas apoptotic inhibitory molecule 3 \|\| id:prot-a-1019 | F-small fish liking (derived food-liking factor) \|\| id:ebi-fl187-GCST90094840 | Inverse variance weighted | 28 | -0.047583044 | 0.0686567764099423 | 0.488273923232431 | -0.182150326 | 0.0869842378049324 | 0.953531284832119 | 0.833476038110658 | 1.09087948492753 | 0.998735809224318 |
| ebi-fl187-GCST90094840 | prot-a-1052 | MIP18 family protein FAM96A \|\| id:prot-a-1052 | F-small fish liking (derived food-liking factor) \|\| id:ebi-fl187-GCST90094840 | Inverse variance weighted | 28 | 0.00665384569384174 | 0.0686533542233212 | 0.922790358469591 | -0.127906729 | 0.141214419971551 | 1.00667603170524 | 0.879935448142592 | 1.15167156289581 | 0.998735809224318 |
| ebi-fl187-GCST90094840 | prot-a-1066 | F-box/LRR-repeat protein 4 \|\| id:prot-a-1066 | F-small fish liking (derived food-liking factor) \|\| id:ebi-fl187-GCST90094840 | Inverse variance weighted | 28 | 0.0302303802635188 | 0.0686151013575453 | 0.659517729179348 | -0.104255218 | 0.164715978924308 | 1.03069195768841 | 0.90099531749402 | 1.17905819377427 | 0.998735809224318 |
| ebi-fl187-GCST90094840 | prot-a-1076 | Low affinity immunoglobulin gamma Fc region receptor III-B \|\| id:prot-a-1076 | F-small fish liking (derived food-liking factor) \|\| id:ebi-fl187-GCST90094840 | Inverse variance weighted | 28 | -0.080579871 | 0.0832596574336946 | 0.333137217824549 | -0.243768799 | 0.0826090576736267 | 0.922581213252422 | 0.783668797963694 | 1.08611711638638 | 0.998735809224318 |
| ebi-fl187-GCST90094840 | prot-a-1081 | Fc receptor-like protein 3 \|\| id:prot-a-1081 | F-small fish liking (derived food-liking factor) \|\| id:ebi-fl187-GCST90094840 | Inverse variance weighted | 28 | -0.029917515 | 0.0686499701502168 | 0.66298334570603 | -0.164471456 | 0.104636426594294 | 0.970525584146706 | 0.848341971337266 | 1.11030685891743 | 0.998735809224318 |
| ebi-fl187-GCST90094840 | prot-a-1105 | Fibroblast growth factor receptor 4 \|\| id:prot-a-1105 | F-small fish liking (derived food-liking factor) \|\| id:ebi-fl187-GCST90094840 | Inverse variance weighted | 28 | 0.0883249258932561 | 0.0751896220485749 | 0.24011647931093 | -0.059046733 | 0.235696585108463 | 1.09234299489474 | 0.942662714367573 | 1.26579019230238 | 0.998735809224318 |
| ebi-fl187-GCST90094840 | prot-a-1143 | Follistatin-related protein 1 \|\| id:prot-a-1143 | F-small fish liking (derived food-liking factor) \|\| id:ebi-fl187-GCST90094840 | Inverse variance weighted | 28 | -0.153649389 | 0.0686240314741576 | 0.025155743494234 | -0.28815249 | -0.019146287 | 0.857572643073016 | 0.74964726961151 | 0.981035839066499 | 0.998735809224318 |
| ebi-fl187-GCST90094840 | prot-a-1151 | Alpha-(1,3)-fucosyltransferase 10 \|\| id:prot-a-1151 | F-small fish liking (derived food-liking factor) \|\| id:ebi-fl187-GCST90094840 | Inverse variance weighted | 28 | -0.034932168 | 0.0686633955927918 | 0.610930886368645 | -0.169512423 | 0.0996480874295928 | 0.965670917491071 | 0.84407626820746 | 1.10478206296264 | 0.998735809224318 |
| ebi-fl187-GCST90094840 | prot-a-1165 | Growth arrest and DNA damage-inducible proteins-interacting protein 1 \|\| id:prot-a-1165 | F-small fish liking (derived food-liking factor) \|\| id:ebi-fl187-GCST90094840 | Inverse variance weighted | 28 | 0.0647740422266186 | 0.0686216263398441 | 0.345205193438155 | -0.069724345 | 0.199272429852713 | 1.06691791874807 | 0.932650873979347 | 1.22051442517699 | 0.998735809224318 |
| ebi-fl187-GCST90094840 | prot-a-1194 | Growth/differentiation factor 11/8 \|\| id:prot-a-1194 | F-small fish liking (derived food-liking factor) \|\| id:ebi-fl187-GCST90094840 | Inverse variance weighted | 28 | -0.150967613 | 0.0688401631488269 | 0.0283061914531284 | -0.285894332 | -0.016040893 | 0.859875547377255 | 0.751342004311272 | 0.984087077169474 | 0.998735809224318 |
| ebi-fl187-GCST90094840 | prot-a-1216 | D-glucuronyl C5-epimerase \|\| id:prot-a-1216 | F-small fish liking (derived food-liking factor) \|\| id:ebi-fl187-GCST90094840 | Inverse variance weighted | 28 | 0.0541830052819384 | 0.083572890760983 | 0.516770041030815 | -0.109619861 | 0.217985871173465 | 1.05567777908794 | 0.896174741873517 | 1.24356949731723 | 0.998735809224318 |
| ebi-fl187-GCST90094840 | prot-a-1246 | Glypican-3 \|\| id:prot-a-1246 | F-small fish liking (derived food-liking factor) \|\| id:ebi-fl187-GCST90094840 | Inverse variance weighted | 28 | 0.0416414082468388 | 0.0686400755094723 | 0.544074445074377 | -0.09289314 | 0.176175956245405 | 1.04252057243513 | 0.911290875862503 | 1.19264789403473 | 0.998735809224318 |
| ebi-fl187-GCST90094840 | prot-a-1275 | Glutamate receptor 4 \|\| id:prot-a-1275 | F-small fish liking (derived food-liking factor) \|\| id:ebi-fl187-GCST90094840 | Inverse variance weighted | 27 | -0.041996437 | 0.069810045479394 | 0.547452693321025 | -0.178824127 | 0.09483125169691 | 0.958873196607104 | 0.836252961130855 | 1.09947330521638 | 0.998735809224318 |
| ebi-fl187-GCST90094840 | prot-a-1289 | Glutathione S-transferase P \|\| id:prot-a-1289 | F-small fish liking (derived food-liking factor) \|\| id:ebi-fl187-GCST90094840 | Inverse variance weighted | 28 | 0.0941211043722888 | 0.068650204447695 | 0.170367354403493 | -0.040433296 | 0.228675505089771 | 1.09869279436184 | 0.960373222778867 | 1.25693410410774 | 0.998735809224318 |
| ebi-fl187-GCST90094840 | prot-a-130 | Apolipoprotein D \|\| id:prot-a-130 | F-small fish liking (derived food-liking factor) \|\| id:ebi-fl187-GCST90094840 | Inverse variance weighted | 28 | 0.110613874331115 | 0.0749365265986701 | 0.139916807424866 | -0.036261718 | 0.257489466464509 | 1.11696353528582 | 0.964387862979743 | 1.29367818390348 | 0.998735809224318 |
| ebi-fl187-GCST90094840 | prot-a-1313 | Heparin-binding EGF-like growth factor \|\| id:prot-a-1313 | F-small fish liking (derived food-liking factor) \|\| id:ebi-fl187-GCST90094840 | Inverse variance weighted | 28 | 0.00668904845509198 | 0.0686335083415294 | 0.922360789041655 | -0.127832628 | 0.14121072480449 | 1.00671147 | 0.880000654381953 | 1.15166730728484 | 0.998735809224318 |
| ebi-fl187-GCST90094840 | prot-a-1347 | HLA class II histocompatibility antigen, DQ alpha 2 chain \|\| id:prot-a-1347 | F-small fish liking (derived food-liking factor) \|\| id:ebi-fl187-GCST90094840 | Inverse variance weighted | 27 | 0.107172275414894 | 0.0714641150370704 | 0.133701043966494 | -0.03289739 | 0.247241940887552 | 1.113126002 | 0.967637843758427 | 1.28048887791996 | 0.998735809224318 |
| ebi-fl187-GCST90094840 | prot-a-1357 | Non-histone chromosomal protein HMG-14 \|\| id:prot-a-1357 | F-small fish liking (derived food-liking factor) \|\| id:ebi-fl187-GCST90094840 | Inverse variance weighted | 28 | -0.022885344 | 0.0686335083415294 | 0.738800121917082 | -0.15740702 | 0.111636332775339 | 0.977374539620712 | 0.854356248024851 | 1.11810616813212 | 0.998735809224318 |
| ebi-fl187-GCST90094840 | prot-a-137 | Apolipoprotein M \|\| id:prot-a-137 | F-small fish liking (derived food-liking factor) \|\| id:ebi-fl187-GCST90094840 | Inverse variance weighted | 28 | -0.131091637 | 0.0686568217297395 | 0.0562136989040091 | -0.265659008 | 0.00347573320325309 | 0.877137392129403 | 0.766700521693497 | 1.00348178056822 | 0.998735809224318 |
| ebi-fl187-GCST90094840 | prot-a-1381 | 17-beta-hydroxysteroid dehydrogenase 14 \|\| id:prot-a-1381 | F-small fish liking (derived food-liking factor) \|\| id:ebi-fl187-GCST90094840 | Inverse variance weighted | 28 | 0.0986277681887035 | 0.0749614971591075 | 0.188270604781628 | -0.048296766 | 0.245552302620554 | 1.10365540743871 | 0.952850971111489 | 1.27832714170177 | 0.998735809224318 |
| ebi-fl187-GCST90094840 | prot-a-1392 | Serine protease HTRA2, mitochondrial \|\| id:prot-a-1392 | F-small fish liking (derived food-liking factor) \|\| id:ebi-fl187-GCST90094840 | Inverse variance weighted | 28 | -0.028832455 | 0.068623651040418 | 0.674373451058801 | -0.163334811 | 0.10566990102694 | 0.97157923406145 | 0.849306783509063 | 1.11145492581523 | 0.998735809224318 |
| ebi-fl187-GCST90094840 | prot-a-1416 | Interferon-induced protein with tetratricopeptide repeats 2 \|\| id:prot-a-1416 | F-small fish liking (derived food-liking factor) \|\| id:ebi-fl187-GCST90094840 | Inverse variance weighted | 28 | -0.122288706 | 0.0686382204275249 | 0.0748077019964116 | -0.256819618 | 0.0122422056984831 | 0.884892857442879 | 0.773507727761206 | 1.01231744823002 | 0.998735809224318 |
| ebi-fl187-GCST90094840 | prot-a-1428 | Interferon gamma \|\| id:prot-a-1428 | F-small fish liking (derived food-liking factor) \|\| id:ebi-fl187-GCST90094840 | Inverse variance weighted | 28 | 0.0181571232608562 | 0.0686413351615292 | 0.7913779392782 | -0.116379894 | 0.152694140177453 | 1.01832296604545 | 0.890137001657743 | 1.16496860735412 | 0.998735809224318 |
| ebi-fl187-GCST90094840 | prot-a-1483 | Interleukin-17C \|\| id:prot-a-1483 | F-small fish liking (derived food-liking factor) \|\| id:ebi-fl187-GCST90094840 | Inverse variance weighted | 28 | -0.108596403 | 0.0688214169133979 | 0.114578273095045 | -0.24348638 | 0.026293574639005 | 0.897092408687273 | 0.783890152808779 | 1.02664230037936 | 0.998735809224318 |
| ebi-fl187-GCST90094840 | prot-a-1503 | Interleukin-1 receptor-like 2 \|\| id:prot-a-1503 | F-small fish liking (derived food-liking factor) \|\| id:ebi-fl187-GCST90094840 | Inverse variance weighted | 28 | -0.088678342 | 0.0686457656567086 | 0.196417625667461 | -0.223224043 | 0.0458673587285945 | 0.915139888341691 | 0.799935609526277 | 1.04693553488636 | 0.998735809224318 |
| ebi-fl187-GCST90094840 | prot-a-1522 | Interleukin-31 receptor subunit alpha \|\| id:prot-a-1522 | F-small fish liking (derived food-liking factor) \|\| id:ebi-fl187-GCST90094840 | Inverse variance weighted | 28 | -0.098032411 | 0.0695100348457016 | 0.15844089058407 | -0.234272079 | 0.0382072573533004 | 0.906619518883023 | 0.791146532061145 | 1.03894653987582 | 0.998735809224318 |
| ebi-fl187-GCST90094840 | prot-a-1540 | Interleukin-6 receptor subunit alpha \|\| id:prot-a-1540 | F-small fish liking (derived food-liking factor) \|\| id:ebi-fl187-GCST90094840 | Inverse variance weighted | 28 | -0.04691025 | 0.0686568217297395 | 0.494444493998076 | -0.181477621 | 0.087657120197306 | 0.954173030402062 | 0.834036910020591 | 1.09161376553968 | 0.998735809224318 |
| ebi-fl187-GCST90094840 | prot-a-1541 | Interleukin-6 receptor subunit alpha \|\| id:prot-a-1541 | F-small fish liking (derived food-liking factor) \|\| id:ebi-fl187-GCST90094840 | Inverse variance weighted | 28 | -0.085937746 | 0.0686490198694757 | 0.210627337442743 | -0.220489825 | 0.0486143328164687 | 0.917651356792367 | 0.802125800392474 | 1.04981539330969 | 0.998735809224318 |
| ebi-fl187-GCST90094840 | prot-a-1545 | Interleukin-9 \|\| id:prot-a-1545 | F-small fish liking (derived food-liking factor) \|\| id:ebi-fl187-GCST90094840 | Inverse variance weighted | 28 | 0.127664456910093 | 0.068636541041219 | 0.0628845147471888 | -0.006863164 | 0.262192077350882 | 1.13617170416531 | 0.993160334189166 | 1.29977617601876 | 0.998735809224318 |
| ebi-fl187-GCST90094840 | prot-a-1548 | Inositol monophosphatase 3 \|\| id:prot-a-1548 | F-small fish liking (derived food-liking factor) \|\| id:ebi-fl187-GCST90094840 | Inverse variance weighted | 28 | -0.072541632 | 0.0686271140478594 | 0.290493152248509 | -0.207050775 | 0.061967511814521 | 0.930027027230182 | 0.812978369757093 | 1.06392777908353 | 0.998735809224318 |
| ebi-fl187-GCST90094840 | prot-a-1555 | Inhibin beta A chain:Inhibin beta B chain heterodimer \|\| id:prot-a-1555 | F-small fish liking (derived food-liking factor) \|\| id:ebi-fl187-GCST90094840 | Inverse variance weighted | 28 | -0.149380493 | 0.0686248795624112 | 0.0294978263360254 | -0.283885257 | -0.014875729 | 0.861241356422342 | 0.752853024567222 | 0.985234368206973 | 0.998735809224318 |
| ebi-fl187-GCST90094840 | prot-a-1603 | Junctophilin-3 \|\| id:prot-a-1603 | F-small fish liking (derived food-liking factor) \|\| id:ebi-fl187-GCST90094840 | Inverse variance weighted | 28 | -0.065364458 | 0.0686234800766176 | 0.340839086147229 | -0.199866479 | 0.0691375632573515 | 0.936726004159184 | 0.81884007841763 | 1.07158360954153 | 0.998735809224318 |
| ebi-fl187-GCST90094840 | prot-a-1627 | UPF0258 protein KIAA1024 \|\| id:prot-a-1627 | F-small fish liking (derived food-liking factor) \|\| id:ebi-fl187-GCST90094840 | Inverse variance weighted | 28 | -0.025707442 | 0.0686271140478594 | 0.707960948630922 | -0.160216586 | 0.10880170114833 | 0.97462018045903 | 0.85195924660568 | 1.11494123685195 | 0.998735809224318 |
| ebi-fl187-GCST90094840 | prot-a-1666 | Kallikrein-7 \|\| id:prot-a-1666 | F-small fish liking (derived food-liking factor) \|\| id:ebi-fl187-GCST90094840 | Inverse variance weighted | 28 | -0.069672723 | 0.0686555914914932 | 0.310194029937685 | -0.204237682 | 0.0648922363703023 | 0.932699020941868 | 0.815268573266708 | 1.0670440296505 | 0.998735809224318 |
| ebi-fl187-GCST90094840 | prot-a-1678 | Importin subunit alpha-3 \|\| id:prot-a-1678 | F-small fish liking (derived food-liking factor) \|\| id:ebi-fl187-GCST90094840 | Inverse variance weighted (multiplicative random effects) | 28 | -0.004076579 | 0.0869866820245558 | 0.962621299121315 | -0.174570476 | 0.166417317837917 | 0.995931719038071 | 0.839817665407099 | 1.18106587875276 | 0.998735809224318 |
| ebi-fl187-GCST90094840 | prot-a-1718 | Protein LDOC1 \|\| id:prot-a-1718 | F-small fish liking (derived food-liking factor) \|\| id:ebi-fl187-GCST90094840 | Inverse variance weighted | 28 | -0.021779613 | 0.0686276015573368 | 0.750970931097693 | -0.156289712 | 0.112730486488624 | 0.978455850667257 | 0.855311360836557 | 1.11933021767489 | 0.998735809224318 |
| ebi-fl187-GCST90094840 | prot-a-1723 | Leptin \|\| id:prot-a-1723 | F-small fish liking (derived food-liking factor) \|\| id:ebi-fl187-GCST90094840 | Inverse variance weighted | 28 | -0.128626441 | 0.0795041201342568 | 0.105693028990127 | -0.284454517 | 0.0272016343301445 | 0.879302375400664 | 0.752424577516651 | 1.02757497626816 | 0.998735809224318 |
| ebi-fl187-GCST90094840 | prot-a-1730 | Galectin-7 \|\| id:prot-a-1730 | F-small fish liking (derived food-liking factor) \|\| id:ebi-fl187-GCST90094840 | Inverse variance weighted | 28 | -0.031800362 | 0.0686322793560692 | 0.643117739265626 | -0.166319629 | 0.102718905741564 | 0.968699952294884 | 0.846775536629251 | 1.10817986229445 | 0.998735809224318 |
| ebi-fl187-GCST90094840 | prot-a-1753 | Lipase member N \|\| id:prot-a-1753 | F-small fish liking (derived food-liking factor) \|\| id:ebi-fl187-GCST90094840 | Inverse variance weighted | 28 | 0.078870602828205 | 0.0757366406948289 | 0.297699343863476 | -0.069573213 | 0.22731441859007 | 1.08206429695136 | 0.932791838457563 | 1.25522447181028 | 0.998735809224318 |
| ebi-fl187-GCST90094840 | prot-a-1756 | VIP36-like protein \|\| id:prot-a-1756 | F-small fish liking (derived food-liking factor) \|\| id:ebi-fl187-GCST90094840 | Inverse variance weighted | 28 | 0.00524472572309806 | 0.0686569929428568 | 0.939108616256549 | -0.12932298 | 0.139812431891097 | 1.00525850337319 | 0.878690119983885 | 1.15005806440914 | 0.998735809224318 |
| ebi-fl187-GCST90094840 | prot-a-1760 | Ig Kappa chain V-I region HK102- like \|\| id:prot-a-1760 | F-small fish liking (derived food-liking factor) \|\| id:ebi-fl187-GCST90094840 | Inverse variance weighted | 28 | -0.058840942 | 0.0717396122094325 | 0.412101210033918 | -0.199450582 | 0.0817686977330256 | 0.942856725949822 | 0.819180701979786 | 1.08520477047414 | 0.998735809224318 |
| ebi-fl187-GCST90094840 | prot-a-179 | Neutral ceramidase \|\| id:prot-a-179 | F-small fish liking (derived food-liking factor) \|\| id:ebi-fl187-GCST90094840 | Inverse variance weighted | 28 | 0.127721747285506 | 0.0814271654156704 | 0.116755216231059 | -0.031875497 | 0.28731899150022 | 1.13623679773338 | 0.968627171629478 | 1.33284931327247 | 0.998735809224318 |
| ebi-fl187-GCST90094840 | prot-a-1809 | Lactotransferrin \|\| id:prot-a-1809 | F-small fish liking (derived food-liking factor) \|\| id:ebi-fl187-GCST90094840 | Inverse variance weighted | 28 | -0.01616298 | 0.0698763107482654 | 0.817075191349689 | -0.153120549 | 0.120794588729359 | 0.98396693972255 | 0.858026281019999 | 1.12839310389887 | 0.998735809224318 |
| ebi-fl187-GCST90094840 | prot-a-1818 | Lysophospholipase-like protein 1 \|\| id:prot-a-1818 | F-small fish liking (derived food-liking factor) \|\| id:ebi-fl187-GCST90094840 | Inverse variance weighted | 28 | -0.118752082 | 0.0815172211712285 | 0.145179067373162 | -0.278525836 | 0.0410216714140954 | 0.888027931509983 | 0.756898712582237 | 1.04187468419854 | 0.998735809224318 |
| ebi-fl187-GCST90094840 | prot-a-1852 | MAP kinase-activated protein kinase 3 \|\| id:prot-a-1852 | F-small fish liking (derived food-liking factor) \|\| id:ebi-fl187-GCST90094840 | Inverse variance weighted (multiplicative random effects) | 28 | 0.0744618573254297 | 0.084768723683767 | 0.379720217938534 | -0.091684841 | 0.240608555745613 | 1.0773042514731 | 0.912392652907733 | 1.27202301174096 | 0.998735809224318 |
| ebi-fl187-GCST90094840 | prot-a-1856 | Megakaryocyte-associated tyrosine-protein kinase \|\| id:prot-a-1856 | F-small fish liking (derived food-liking factor) \|\| id:ebi-fl187-GCST90094840 | Inverse variance weighted | 28 | 0.0316389310944027 | 0.0686435012849297 | 0.644858422046398 | -0.102902331 | 0.166180193612865 | 1.03214476263641 | 0.902215087241237 | 1.18078585262333 | 0.998735809224318 |
| ebi-fl187-GCST90094840 | prot-a-1890 | Beta-1,3-N-acetylglucosaminyltransferase manic fringe \|\| id:prot-a-1890 | F-small fish liking (derived food-liking factor) \|\| id:ebi-fl187-GCST90094840 | Inverse variance weighted | 28 | -0.034474374 | 0.0835517654552944 | 0.67989167108338 | -0.198235834 | 0.129287086259966 | 0.966113096952186 | 0.820176404579242 | 1.13801678625633 | 0.998735809224318 |
| ebi-fl187-GCST90094840 | prot-a-1919 | Matrilysin \|\| id:prot-a-1919 | F-small fish liking (derived food-liking factor) \|\| id:ebi-fl187-GCST90094840 | Inverse variance weighted | 28 | -0.076992273 | 0.0777401783634431 | 0.321988696419031 | -0.229363023 | 0.0753784761563668 | 0.925897007581361 | 0.795039863332474 | 1.07829218154513 | 0.998735809224318 |
| ebi-fl187-GCST90094840 | prot-a-1923 | Multimerin-2 \|\| id:prot-a-1923 | F-small fish liking (derived food-liking factor) \|\| id:ebi-fl187-GCST90094840 | Inverse variance weighted | 28 | -0.03461296 | 0.0704057758621005 | 0.622987278020541 | -0.17260828 | 0.103382361057898 | 0.965979216866689 | 0.841467169542971 | 1.10891533406489 | 0.998735809224318 |
| ebi-fl187-GCST90094840 | prot-a-1948 | Mesothelin \|\| id:prot-a-1948 | F-small fish liking (derived food-liking factor) \|\| id:ebi-fl187-GCST90094840 | Inverse variance weighted | 28 | 0.079353987237195 | 0.0726502894693341 | 0.274712896799303 | -0.06304058 | 0.22174855459709 | 1.08258747640024 | 0.938905372020853 | 1.24825746979814 | 0.998735809224318 |
| ebi-fl187-GCST90094840 | prot-a-195 | ATPase family AAA domain-containing protein 1 \|\| id:prot-a-195 | F-small fish liking (derived food-liking factor) \|\| id:ebi-fl187-GCST90094840 | Inverse variance weighted | 28 | -0.065355045 | 0.0714478277146488 | 0.360336523442574 | -0.205392788 | 0.0746826970868158 | 0.936734821095715 | 0.814327395918641 | 1.07754218935904 | 0.998735809224318 |
| ebi-fl187-GCST90094840 | prot-a-1966 | Mucin-1 \|\| id:prot-a-1966 | F-small fish liking (derived food-liking factor) \|\| id:ebi-fl187-GCST90094840 | Inverse variance weighted | 28 | 0.0476265814024909 | 0.0686424048428343 | 0.487784973770406 | -0.086912532 | 0.182165694894446 | 1.04877894862965 | 0.916757278970027 | 1.19981298029559 | 0.998735809224318 |
| ebi-fl187-GCST90094840 | prot-a-1986 | Myocardial zonula adherens protein \|\| id:prot-a-1986 | F-small fish liking (derived food-liking factor) \|\| id:ebi-fl187-GCST90094840 | Inverse variance weighted | 28 | -0.135674746 | 0.0699849559210651 | 0.0525467371804207 | -0.27284526 | 0.00149576765671491 | 0.873126574265298 | 0.761210568578949 | 1.00149688687512 | 0.998735809224318 |
| ebi-fl187-GCST90094840 | prot-a-2071 | Epididymal secretory protein E1 \|\| id:prot-a-2071 | F-small fish liking (derived food-liking factor) \|\| id:ebi-fl187-GCST90094840 | Inverse variance weighted | 28 | 0.0254787008506758 | 0.0754039708746365 | 0.735441554926922 | -0.122313082 | 0.173270483764963 | 1.02580605724137 | 0.884871287801576 | 1.18918771755769 | 0.998735809224318 |
| ebi-fl187-GCST90094840 | prot-a-2098 | Neuritin \|\| id:prot-a-2098 | F-small fish liking (derived food-liking factor) \|\| id:ebi-fl187-GCST90094840 | Inverse variance weighted | 28 | -0.038151218 | 0.0686400755094723 | 0.578336987629513 | -0.172685766 | 0.096383330038014 | 0.962567372410243 | 0.841401970448864 | 1.10118109889198 | 0.998735809224318 |
| ebi-fl187-GCST90094840 | prot-a-2115 | Neurotrophin-4 \|\| id:prot-a-2115 | F-small fish liking (derived food-liking factor) \|\| id:ebi-fl187-GCST90094840 | Inverse variance weighted | 28 | -0.006009541 | 0.0686549652897362 | 0.930248252265216 | -0.140573273 | 0.128554191129332 | 0.994008480334265 | 0.868859998789797 | 1.13718304485493 | 0.998735809224318 |
| ebi-fl187-GCST90094840 | prot-a-2118 | Netrin-1 \|\| id:prot-a-2118 | F-small fish liking (derived food-liking factor) \|\| id:ebi-fl187-GCST90094840 | Inverse variance weighted | 28 | -0.057166073 | 0.0757145550663619 | 0.450236431027439 | -0.205566601 | 0.0912344550303587 | 0.944437210915704 | 0.814185867307026 | 1.09552582669171 | 0.998735809224318 |
| ebi-fl187-GCST90094840 | prot-a-2120 | Netrin-G1 \|\| id:prot-a-2120 | F-small fish liking (derived food-liking factor) \|\| id:ebi-fl187-GCST90094840 | Inverse variance weighted | 28 | -0.030086308 | 0.0725485329072181 | 0.678357182539651 | -0.172281432 | 0.112108816873951 | 0.970361780314419 | 0.841742246523774 | 1.11863458034047 | 0.998735809224318 |
| ebi-fl187-GCST90094840 | prot-a-2145 | Olfactomedin-like protein 3 \|\| id:prot-a-2145 | F-small fish liking (derived food-liking factor) \|\| id:ebi-fl187-GCST90094840 | Inverse variance weighted | 28 | -0.012617075 | 0.0686382204275249 | 0.854154691197826 | -0.147147987 | 0.121913836584849 | 0.987462186143351 | 0.863166230159295 | 1.12965676250223 | 0.998735809224318 |
| ebi-fl187-GCST90094840 | prot-a-2162 | Prolyl 4-hydroxylase subunit alpha-2 \|\| id:prot-a-2162 | F-small fish liking (derived food-liking factor) \|\| id:ebi-fl187-GCST90094840 | Inverse variance weighted | 28 | -0.050968227 | 0.0686424881582921 | 0.457774045650777 | -0.185507504 | 0.0835710499347457 | 0.950308864353608 | 0.83068260240955 | 1.08716245537041 | 0.998735809224318 |
| ebi-fl187-GCST90094840 | prot-a-2168 | Polyadenylate-binding protein-interacting protein 1 \|\| id:prot-a-2168 | F-small fish liking (derived food-liking factor) \|\| id:ebi-fl187-GCST90094840 | Inverse variance weighted (multiplicative random effects) | 28 | -0.069327829 | 0.0837194447998772 | 0.40761551411351 | -0.233417941 | 0.0947622827064948 | 0.933020758579211 | 0.791822569314627 | 1.09939747826741 | 0.998735809224318 |
| ebi-fl187-GCST90094840 | prot-a-219 | Beta-1,4-galactosyltransferase 5 \|\| id:prot-a-219 | F-small fish liking (derived food-liking factor) \|\| id:ebi-fl187-GCST90094840 | Inverse variance weighted | 28 | 0.0512745254748254 | 0.0735790494104786 | 0.485888463795653 | -0.092940411 | 0.195489462319363 | 1.05261182238254 | 0.911247798686517 | 1.21590598102575 | 0.998735809224318 |
| ebi-fl187-GCST90094840 | prot-a-2190 | Pyruvate carboxylase, mitochondrial \|\| id:prot-a-2190 | F-small fish liking (derived food-liking factor) \|\| id:ebi-fl187-GCST90094840 | Inverse variance weighted (multiplicative random effects) | 28 | -0.021707407 | 0.0899732228828639 | 0.809349587428034 | -0.198054924 | 0.154640110020251 | 0.978526503340477 | 0.820324796643409 | 1.16723780831407 | 0.998735809224318 |
| ebi-fl187-GCST90094840 | prot-a-2197 | Protocadherin-9 \|\| id:prot-a-2197 | F-small fish liking (derived food-liking factor) \|\| id:ebi-fl187-GCST90094840 | Inverse variance weighted | 28 | -0.089117987 | 0.0686485649975912 | 0.194226527830559 | -0.223669174 | 0.0454332008640882 | 0.914737640486101 | 0.799579612402485 | 1.04648109824602 | 0.998735809224318 |
| ebi-fl187-GCST90094840 | prot-a-2198 | Protocadherin alpha-4 \|\| id:prot-a-2198 | F-small fish liking (derived food-liking factor) \|\| id:ebi-fl187-GCST90094840 | Inverse variance weighted (multiplicative random effects) | 28 | -0.029401933 | 0.0875944953596779 | 0.737127574696508 | -0.201087144 | 0.142283277657514 | 0.971026098348227 | 0.817841158373821 | 1.15290319399944 | 0.998735809224318 |
| ebi-fl187-GCST90094840 | prot-a-2254 | Placenta growth factor \|\| id:prot-a-2254 | F-small fish liking (derived food-liking factor) \|\| id:ebi-fl187-GCST90094840 | Inverse variance weighted | 28 | -0.001253884 | 0.0774627887988684 | 0.987085269858902 | -0.15308095 | 0.150573182025458 | 0.998746901763782 | 0.858060258965234 | 1.16250037612238 | 0.998955228396052 |
| ebi-fl187-GCST90094840 | prot-a-2261 | Membrane-associated progesterone receptor component 2 \|\| id:prot-a-2261 | F-small fish liking (derived food-liking factor) \|\| id:ebi-fl187-GCST90094840 | Inverse variance weighted | 28 | -0.070267874 | 0.0686304249061346 | 0.305901907405128 | -0.204783507 | 0.0642477588573368 | 0.932144089332007 | 0.814823701128777 | 1.06635656531949 | 0.998735809224318 |
| ebi-fl187-GCST90094840 | prot-a-2268 | Polymeric immunoglobulin receptor \|\| id:prot-a-2268 | F-small fish liking (derived food-liking factor) \|\| id:ebi-fl187-GCST90094840 | Inverse variance weighted | 28 | 0.0616544492281769 | 0.0804595106722807 | 0.443510193667572 | -0.096046192 | 0.219355090145847 | 1.0635947552327 | 0.908422053550127 | 1.24527338249619 | 0.998735809224318 |
| ebi-fl187-GCST90094840 | prot-a-2283 | Group 10 secretory phospholipase A2 \|\| id:prot-a-2283 | F-small fish liking (derived food-liking factor) \|\| id:ebi-fl187-GCST90094840 | Inverse variance weighted | 28 | -0.046939805 | 0.0720334889718738 | 0.514634316943431 | -0.188125443 | 0.0942458338441969 | 0.954144831048104 | 0.828510769898891 | 1.09882984228065 | 0.998735809224318 |
| ebi-fl187-GCST90094840 | prot-a-2298 | Inactive phospholipase D5 \|\| id:prot-a-2298 | F-small fish liking (derived food-liking factor) \|\| id:ebi-fl187-GCST90094840 | Inverse variance weighted | 28 | 0.221896930846594 | 0.0700627150190086 | 0.00153957493156027 | 0.0845740094093368 | 0.359219852283851 | 1.24844269530112 | 1.08825338224171 | 1.43221164196164 | 0.998735809224318 |
| ebi-fl187-GCST90094840 | prot-a-2304 | Procollagen-lysine,2-oxoglutarate 5-dioxygenase 2 \|\| id:prot-a-2304 | F-small fish liking (derived food-liking factor) \|\| id:ebi-fl187-GCST90094840 | Inverse variance weighted | 28 | 0.00357401731952201 | 0.0686471640622565 | 0.958478030198841 | -0.130974424 | 0.138122458881545 | 1.00358041173507 | 0.87724021018702 | 1.14811613868408 | 0.998735809224318 |
| ebi-fl187-GCST90094840 | prot-a-2331 | Periostin \|\| id:prot-a-2331 | F-small fish liking (derived food-liking factor) \|\| id:ebi-fl187-GCST90094840 | Inverse variance weighted | 28 | -0.094212117 | 0.0763854289473867 | 0.217434724658291 | -0.243927557 | 0.0555033240596828 | 0.910089696562881 | 0.783544394189024 | 1.05707253083876 | 0.998735809224318 |
| ebi-fl187-GCST90094840 | prot-a-2333 | Periostin \|\| id:prot-a-2333 | F-small fish liking (derived food-liking factor) \|\| id:ebi-fl187-GCST90094840 | Inverse variance weighted | 28 | -0.0999723 | 0.0788346159683607 | 0.204752622771072 | -0.254488148 | 0.0545435470872629 | 0.904862482188902 | 0.775313242429764 | 1.05605846368248 | 0.998735809224318 |
| ebi-fl187-GCST90094840 | prot-a-2367 | cAMP-dependent protein kinase catalytic subunit alpha \|\| id:prot-a-2367 | F-small fish liking (derived food-liking factor) \|\| id:ebi-fl187-GCST90094840 | Inverse variance weighted | 28 | -0.015815882 | 0.068623651040418 | 0.817724431099222 | -0.150318238 | 0.118686474042833 | 0.984308532294462 | 0.86043410998908 | 1.12601682743606 | 0.998735809224318 |
| ebi-fl187-GCST90094840 | prot-a-2385 | Phosphoribosyl pyrophosphate synthase-associated protein 1 \|\| id:prot-a-2385 | F-small fish liking (derived food-liking factor) \|\| id:ebi-fl187-GCST90094840 | Inverse variance weighted | 28 | 0.154585942072546 | 0.0722634201086595 | 0.0324194363596116 | 0.0129496386595732 | 0.296222245485518 | 1.16717458314992 | 1.01303384833263 | 1.34476899246103 | 0.998735809224318 |
| ebi-fl187-GCST90094840 | prot-a-2393 | Trypsin-3 \|\| id:prot-a-2393 | F-small fish liking (derived food-liking factor) \|\| id:ebi-fl187-GCST90094840 | Inverse variance weighted | 27 | -0.009562862 | 0.0698003823946103 | 0.891028421783398 | -0.146371611 | 0.127245887524693 | 0.990482716792267 | 0.863836631937582 | 1.13569623698834 | 0.998735809224318 |
| ebi-fl187-GCST90094840 | prot-a-2405 | Pregnancy-specific beta-1-glycoprotein 6 \|\| id:prot-a-2405 | F-small fish liking (derived food-liking factor) \|\| id:ebi-fl187-GCST90094840 | Inverse variance weighted | 28 | 0.111378066715035 | 0.0739302774601587 | 0.131931567988302 | -0.033525277 | 0.256281410536946 | 1.11781743654346 | 0.967030467189996 | 1.29211629192144 | 0.998735809224318 |
| ebi-fl187-GCST90094840 | prot-a-244 | Beclin-1 \|\| id:prot-a-244 | F-small fish liking (derived food-liking factor) \|\| id:ebi-fl187-GCST90094840 | Inverse variance weighted | 28 | -0.142342186 | 0.0686568217297394 | 0.0381497911612854 | -0.276909557 | -0.007774815 | 0.867324419473992 | 0.75812306128447 | 0.992255330343567 | 0.998735809224318 |
| ebi-fl187-GCST90094840 | prot-a-2454 | tRNA pseudouridine synthase A, mitochondrial \|\| id:prot-a-2454 | F-small fish liking (derived food-liking factor) \|\| id:ebi-fl187-GCST90094840 | Inverse variance weighted | 28 | 0.0799306386747725 | 0.0819675676964633 | 0.329486071345101 | -0.080725794 | 0.240587071359841 | 1.08321193205413 | 0.922446597151015 | 1.27199568340143 | 0.998735809224318 |
| ebi-fl187-GCST90094840 | prot-a-2466 | Glutaminyl-peptide cyclotransferase-like protein \|\| id:prot-a-2466 | F-small fish liking (derived food-liking factor) \|\| id:ebi-fl187-GCST90094840 | Inverse variance weighted | 28 | -0.026026406 | 0.0686505280438652 | 0.704602889610191 | -0.160581441 | 0.108528629013477 | 0.974309361702341 | 0.851648461714603 | 1.11463681903407 | 0.998735809224318 |
| ebi-fl187-GCST90094840 | prot-a-2474 | Ras-related protein Rab-27A \|\| id:prot-a-2474 | F-small fish liking (derived food-liking factor) \|\| id:ebi-fl187-GCST90094840 | Inverse variance weighted | 28 | -0.060439038 | 0.0831912435038374 | 0.467527893941917 | -0.223493875 | 0.102615799708428 | 0.941351154333332 | 0.79971979027497 | 1.10806560815509 | 0.998735809224318 |
| ebi-fl187-GCST90094840 | prot-a-2490 | Rap1 GTPase-GDP dissociation stimulator 1 \|\| id:prot-a-2490 | F-small fish liking (derived food-liking factor) \|\| id:ebi-fl187-GCST90094840 | Inverse variance weighted | 28 | -0.035132688 | 0.0704344670095085 | 0.617920528529575 | -0.173184243 | 0.102918867283026 | 0.965477300452303 | 0.840982655071031 | 1.10840147780448 | 0.998735809224318 |
| ebi-fl187-GCST90094840 | prot-a-2545 | Receptor-interacting serine/threonine-protein kinase 2 \|\| id:prot-a-2545 | F-small fish liking (derived food-liking factor) \|\| id:ebi-fl187-GCST90094840 | Inverse variance weighted | 28 | 0.0438418094582496 | 0.0686400755094723 | 0.523004905849822 | -0.090692739 | 0.178376357456815 | 1.04481706163786 | 0.913298289157069 | 1.19527508728513 | 0.998735809224318 |
| ebi-fl187-GCST90094840 | prot-a-2610 | RUN and FYVE domain-containing protein 1 \|\| id:prot-a-2610 | F-small fish liking (derived food-liking factor) \|\| id:ebi-fl187-GCST90094840 | Inverse variance weighted | 28 | -0.050481295 | 0.0686234800766176 | 0.461957635716803 | -0.184983316 | 0.0840207259423976 | 0.950771712683497 | 0.831118150146624 | 1.08765143617623 | 0.998735809224318 |
| ebi-fl187-GCST90094840 | prot-a-2627 | Serine--tRNA ligase, mitochondrial \|\| id:prot-a-2627 | F-small fish liking (derived food-liking factor) \|\| id:ebi-fl187-GCST90094840 | Inverse variance weighted | 28 | -0.03079212 | 0.0705029625317829 | 0.662293165442857 | -0.168977927 | 0.107393686445197 | 0.969677128493566 | 0.844527544707552 | 1.11337248786732 | 0.998735809224318 |
| ebi-fl187-GCST90094840 | prot-a-264 | BolA-like protein 2 \|\| id:prot-a-264 | F-small fish liking (derived food-liking factor) \|\| id:ebi-fl187-GCST90094840 | Inverse variance weighted | 28 | -0.012158424 | 0.0686507426976883 | 0.85942552368468 | -0.14671388 | 0.122397031737421 | 0.987915191036978 | 0.863541018742158 | 1.13020273906993 | 0.998735809224318 |
| ebi-fl187-GCST90094840 | prot-a-2646 | Secretoglobin family 1C member 1 \|\| id:prot-a-2646 | F-small fish liking (derived food-liking factor) \|\| id:ebi-fl187-GCST90094840 | Inverse variance weighted | 28 | 0.00489652900787333 | 0.0826077169698723 | 0.952733491806338 | -0.157014596 | 0.166807654268823 | 1.00490853659654 | 0.854691583431984 | 1.18152698177934 | 0.998735809224318 |
| ebi-fl187-GCST90094840 | prot-a-2672 | Semaphorin-3E \|\| id:prot-a-2672 | F-small fish liking (derived food-liking factor) \|\| id:ebi-fl187-GCST90094840 | Inverse variance weighted | 28 | -0.065431953 | 0.0692450002382277 | 0.344692590235902 | -0.201152153 | 0.0702882478681402 | 0.936662782059225 | 0.817787993136998 | 1.07281737401096 | 0.998735809224318 |
| ebi-fl187-GCST90094840 | prot-a-2701 | Plasma protease C1 inhibitor \|\| id:prot-a-2701 | F-small fish liking (derived food-liking factor) \|\| id:ebi-fl187-GCST90094840 | Inverse variance weighted | 28 | -0.075131953 | 0.068651972675292 | 0.273784476320351 | -0.209689819 | 0.0594259136903264 | 0.927621076097587 | 0.8108357126407 | 1.06122713566484 | 0.998735809224318 |
| ebi-fl187-GCST90094840 | prot-a-2738 | Signaling threshold-regulating transmembrane adapter 1 \|\| id:prot-a-2738 | F-small fish liking (derived food-liking factor) \|\| id:ebi-fl187-GCST90094840 | Inverse variance weighted | 28 | -0.105844092 | 0.0816094696304357 | 0.194645480741389 | -0.265798652 | 0.0541104685850214 | 0.899564886604202 | 0.766593463742824 | 1.05560120648603 | 0.998735809224318 |
| ebi-fl187-GCST90094840 | prot-a-274 | Serine/threonine-protein kinase BRSK2 \|\| id:prot-a-274 | F-small fish liking (derived food-liking factor) \|\| id:ebi-fl187-GCST90094840 | Inverse variance weighted | 28 | 0.061933905517089 | 0.0686481841556856 | 0.366954396696818 | -0.072616535 | 0.196484346462233 | 1.06389202501092 | 0.929957367365509 | 1.2171162685536 | 0.998735809224318 |
| ebi-fl187-GCST90094840 | prot-a-2749 | Mitochondrial glutamate carrier 2 \|\| id:prot-a-2749 | F-small fish liking (derived food-liking factor) \|\| id:ebi-fl187-GCST90094840 | Inverse variance weighted | 28 | -0.072797923 | 0.0686490198694757 | 0.288946088994195 | -0.207350002 | 0.0617541563165898 | 0.929788700300448 | 0.8127351416243 | 1.06370080845603 | 0.998735809224318 |
| ebi-fl187-GCST90094840 | prot-a-2760 | Sodium-coupled monocarboxylate transporter 1 \|\| id:prot-a-2760 | F-small fish liking (derived food-liking factor) \|\| id:ebi-fl187-GCST90094840 | Inverse variance weighted | 28 | -0.041128729 | 0.0686563929621241 | 0.549138410892822 | -0.175695259 | 0.0934378014790436 | 0.959705580318396 | 0.838873583620529 | 1.09794231082965 | 0.998735809224318 |
| ebi-fl187-GCST90094840 | prot-a-2777 | Small integral membrane protein 10 \|\| id:prot-a-2777 | F-small fish liking (derived food-liking factor) \|\| id:ebi-fl187-GCST90094840 | Inverse variance weighted | 28 | 0.0828566184817873 | 0.0686400755094723 | 0.227386996515608 | -0.05167793 | 0.217391166480353 | 1.0863860297023 | 0.949634666891711 | 1.24283016056627 | 0.998735809224318 |
| ebi-fl187-GCST90094840 | prot-a-2799 | Superoxide dismutase [Mn], mitochondrial \|\| id:prot-a-2799 | F-small fish liking (derived food-liking factor) \|\| id:ebi-fl187-GCST90094840 | Inverse variance weighted | 28 | 0.00435969078341756 | 0.0691579740825578 | 0.949734973532246 | -0.131189938 | 0.139909319985231 | 1.00436920806105 | 0.877051172856904 | 1.15016949674129 | 0.998735809224318 |
| ebi-fl187-GCST90094840 | prot-a-2805 | Protein SPACA7 \|\| id:prot-a-2805 | F-small fish liking (derived food-liking factor) \|\| id:ebi-fl187-GCST90094840 | Inverse variance weighted | 28 | -0.08153498 | 0.0686545843413005 | 0.234986470559744 | -0.216097965 | 0.0530280054201408 | 0.921700468310092 | 0.805656371721559 | 1.05445917527808 | 0.998735809224318 |
| ebi-fl187-GCST90094840 | prot-a-2811 | SPARC-like protein 1 \|\| id:prot-a-2811 | F-small fish liking (derived food-liking factor) \|\| id:ebi-fl187-GCST90094840 | Inverse variance weighted | 28 | -0.07028104 | 0.0686222712847759 | 0.305753823632017 | -0.204780692 | 0.0642186112388338 | 0.932131816318412 | 0.814825994516234 | 1.06632548401811 | 0.998735809224318 |
| ebi-fl187-GCST90094840 | prot-a-2831 | Sepiapterin reductase \|\| id:prot-a-2831 | F-small fish liking (derived food-liking factor) \|\| id:ebi-fl187-GCST90094840 | Inverse variance weighted | 28 | 0.0572415161647205 | 0.0686533542233212 | 0.404407245842728 | -0.077319058 | 0.19180209044243 | 1.0589115237648 | 0.925594488058861 | 1.21143073951687 | 0.998735809224318 |
| ebi-fl187-GCST90094840 | prot-a-2834 | SPRY domain-containing SOCS box protein 1 \|\| id:prot-a-2834 | F-small fish liking (derived food-liking factor) \|\| id:ebi-fl187-GCST90094840 | Inverse variance weighted | 28 | 0.0284855461449347 | 0.0686469543816939 | 0.67817325477988 | -0.106062484 | 0.163033576733055 | 1.02889513922583 | 0.89936844978338 | 1.17707621139869 | 0.998735809224318 |
| ebi-fl187-GCST90094840 | prot-a-2835 | Spectrin alpha chain, non-erythrocytic 1 \|\| id:prot-a-2835 | F-small fish liking (derived food-liking factor) \|\| id:ebi-fl187-GCST90094840 | Inverse variance weighted | 28 | 0.101885750266569 | 0.0700799079624006 | 0.145987596628332 | -0.035470869 | 0.239242369872874 | 1.10725696068855 | 0.965150849301987 | 1.27028637842459 | 0.998735809224318 |
| ebi-fl187-GCST90094840 | prot-a-2859 | Sia-alpha-2,3-Gal-beta-1,4-GlcNAc-R:alpha 2,8-sialyltransferase \|\| id:prot-a-2859 | F-small fish liking (derived food-liking factor) \|\| id:ebi-fl187-GCST90094840 | Inverse variance weighted | 28 | -0.034876963 | 0.0686382204275249 | 0.611363976419164 | -0.169407875 | 0.0996539491625039 | 0.96572422888047 | 0.844164519627728 | 1.104788539 | 0.998735809224318 |
| ebi-fl187-GCST90094840 | prot-a-2861 | Alpha-2,8-sialyltransferase 8F \|\| id:prot-a-2861 | F-small fish liking (derived food-liking factor) \|\| id:ebi-fl187-GCST90094840 | Inverse variance weighted | 28 | -0.059361507 | 0.0686527650357419 | 0.387223777220082 | -0.193920926 | 0.0751979129669332 | 0.942366036121828 | 0.823723036790905 | 1.07809749924645 | 0.998735809224318 |
| ebi-fl187-GCST90094840 | prot-a-289 | Uncharacterized protein C17orf89 \|\| id:prot-a-289 | F-small fish liking (derived food-liking factor) \|\| id:ebi-fl187-GCST90094840 | Inverse variance weighted | 28 | 0.0426457114395343 | 0.070282523543518 | 0.543999927588048 | -0.095108035 | 0.18039945758483 | 1.04356810510664 | 0.90927469593751 | 1.19769569620873 | 0.998735809224318 |
| ebi-fl187-GCST90094840 | prot-a-2921 | Tachykinin-3 \|\| id:prot-a-2921 | F-small fish liking (derived food-liking factor) \|\| id:ebi-fl187-GCST90094840 | Inverse variance weighted | 28 | 0.104043690269507 | 0.0686400755094723 | 0.129573381930971 | -0.030490858 | 0.238578238268072 | 1.10964893471754 | 0.969969299748818 | 1.26944302117463 | 0.998735809224318 |
| ebi-fl187-GCST90094840 | prot-a-2928 | Tax1-binding protein 1 \|\| id:prot-a-2928 | F-small fish liking (derived food-liking factor) \|\| id:ebi-fl187-GCST90094840 | Inverse variance weighted | 28 | -0.013803002 | 0.0686491910242318 | 0.840647146895929 | -0.148355417 | 0.120749411948377 | 0.986291822189661 | 0.862124646842696 | 1.12834212788223 | 0.998735809224318 |
| ebi-fl187-GCST90094840 | prot-a-296 | Uncharacterized protein C1orf185 \|\| id:prot-a-296 | F-small fish liking (derived food-liking factor) \|\| id:ebi-fl187-GCST90094840 | Inverse variance weighted | 28 | 0.0348414716417707 | 0.0721863556610301 | 0.629337117848503 | -0.106643785 | 0.17632672873739 | 1.03545554671995 | 0.898845797918308 | 1.1928277260863 | 0.998735809224318 |
| ebi-fl187-GCST90094840 | prot-a-2964 | Transforming growth factor beta-2 \|\| id:prot-a-2964 | F-small fish liking (derived food-liking factor) \|\| id:ebi-fl187-GCST90094840 | Inverse variance weighted | 28 | 0.0423218857558845 | 0.0686543046840723 | 0.537598142289903 | -0.092240551 | 0.176884322936666 | 1.04323022566154 | 0.911885767739072 | 1.19349302537337 | 0.998735809224318 |
| ebi-fl187-GCST90094840 | prot-a-2977 | Thymocyte nuclear protein 1 \|\| id:prot-a-2977 | F-small fish liking (derived food-liking factor) \|\| id:ebi-fl187-GCST90094840 | Inverse variance weighted | 28 | 0.13200799961697 | 0.0686294557784071 | 0.0544186733116929 | -0.002505734 | 0.266521732942648 | 1.14111744773322 | 0.997497403021518 | 1.30541595955723 | 0.998735809224318 |
| ebi-fl187-GCST90094840 | prot-a-3015 | Transmembrane protein 70, mitochondrial \|\| id:prot-a-3015 | F-small fish liking (derived food-liking factor) \|\| id:ebi-fl187-GCST90094840 | Inverse variance weighted | 28 | -0.123946211 | 0.068646194225247 | 0.0709839335491732 | -0.258492752 | 0.0106003297344213 | 0.883427358325723 | 0.772214628327367 | 1.01065671227807 | 0.998735809224318 |
| ebi-fl187-GCST90094840 | prot-a-3030 | Tumor necrosis factor alpha-induced protein 3 \|\| id:prot-a-3030 | F-small fish liking (derived food-liking factor) \|\| id:ebi-fl187-GCST90094840 | Inverse variance weighted | 28 | -0.071056038 | 0.0686389591895543 | 0.300568799857117 | -0.205588398 | 0.0634763217293683 | 0.931409696065764 | 0.814168120313392 | 1.06553425549429 | 0.998735809224318 |
| ebi-fl187-GCST90094840 | prot-a-3032 | Tumor necrosis factor receptor superfamily member 10A \|\| id:prot-a-3032 | F-small fish liking (derived food-liking factor) \|\| id:ebi-fl187-GCST90094840 | Inverse variance weighted | 28 | 0.0260325487329638 | 0.0686193223096173 | 0.704408402687775 | -0.108461323 | 0.160526420459814 | 1.02637435511457 | 0.89721359568163 | 1.17412879375344 | 0.998735809224318 |
| ebi-fl187-GCST90094840 | prot-a-3081 | Tumor protein p53-inducible protein 11 \|\| id:prot-a-3081 | F-small fish liking (derived food-liking factor) \|\| id:ebi-fl187-GCST90094840 | Inverse variance weighted | 28 | -0.054280667 | 0.0686383996821694 | 0.42904863390812 | -0.18881193 | 0.0802505967036819 | 0.947166231197136 | 0.827942203101758 | 1.08355856986061 | 0.998735809224318 |
| ebi-fl187-GCST90094840 | prot-a-315 | Complement C4 \|\| id:prot-a-315 | F-small fish liking (derived food-liking factor) \|\| id:ebi-fl187-GCST90094840 | Inverse variance weighted | 27 | -0.002722125 | 0.0697777950432204 | 0.968881352110796 | -0.139486603 | 0.134042353402725 | 0.997281576740429 | 0.869804675754591 | 1.14344124724674 | 0.998735809224318 |
| ebi-fl187-GCST90094840 | prot-a-3157 | UDP-glucuronosyltransferase 1-8 \|\| id:prot-a-3157 | F-small fish liking (derived food-liking factor) \|\| id:ebi-fl187-GCST90094840 | Inverse variance weighted | 28 | 0.0164178931016508 | 0.0686490198694757 | 0.810983850391064 | -0.118134186 | 0.150969972045823 | 1.01655340731211 | 0.888576810186554 | 1.16296173619578 | 0.998735809224318 |
| ebi-fl187-GCST90094840 | prot-a-317 | Glutaredoxin-like protein C5orf63 \|\| id:prot-a-317 | F-small fish liking (derived food-liking factor) \|\| id:ebi-fl187-GCST90094840 | Inverse variance weighted | 28 | -0.01094123 | 0.0727009709173606 | 0.880373036489855 | -0.153435133 | 0.131552672811193 | 0.989118407371249 | 0.857756402320258 | 1.14059798464244 | 0.998735809224318 |
| ebi-fl187-GCST90094840 | prot-a-3192 | Guanine nucleotide exchange factor VAV3 \|\| id:prot-a-3192 | F-small fish liking (derived food-liking factor) \|\| id:ebi-fl187-GCST90094840 | Inverse variance weighted | 28 | 0.0633471686558414 | 0.0686568217297395 | 0.356182401201451 | -0.071220202 | 0.197914539246131 | 1.0653966473567 | 0.931256804998201 | 1.21885822482777 | 0.998735809224318 |
| ebi-fl187-GCST90094840 | prot-a-3233 | Serine/threonine-protein kinase WNK3 \|\| id:prot-a-3233 | F-small fish liking (derived food-liking factor) \|\| id:ebi-fl187-GCST90094840 | Inverse variance weighted | 28 | 0.134389643683842 | 0.0686633955927918 | 0.0503211418824708 | -0.000190612 | 0.268969899045714 | 1.14383842224245 | 0.999809406487222 | 1.30861574987075 | 0.998735809224318 |
| ebi-fl187-GCST90094840 | prot-a-3250 | 14-3-3 protein theta \|\| id:prot-a-3250 | F-small fish liking (derived food-liking factor) \|\| id:ebi-fl187-GCST90094840 | Inverse variance weighted | 28 | 0.155902390885182 | 0.0686479677061045 | 0.0231444593428418 | 0.0213523741812167 | 0.290452407589146 | 1.16871212056665 | 1.02158196733059 | 1.33703223474909 | 0.998735809224318 |
| ebi-fl187-GCST90094840 | prot-a-3278 | E3 ubiquitin-protein ligase ZNRF3 \|\| id:prot-a-3278 | F-small fish liking (derived food-liking factor) \|\| id:ebi-fl187-GCST90094840 | Inverse variance weighted | 28 | 0.076599750486087 | 0.0715025181873727 | 0.28404020033481 | -0.063545185 | 0.216744686133337 | 1.07960987657617 | 0.938431715154362 | 1.24202695494908 | 0.998735809224318 |
| ebi-fl187-GCST90094840 | prot-a-355 | Calpain-3 \|\| id:prot-a-355 | F-small fish liking (derived food-liking factor) \|\| id:ebi-fl187-GCST90094840 | Inverse variance weighted | 28 | 0.00468152542310462 | 0.0686442855725981 | 0.945626599214815 | -0.129861274 | 0.139224325145397 | 1.00469250088386 | 0.878217253774381 | 1.14938190634956 | 0.998735809224318 |
| ebi-fl187-GCST90094840 | prot-a-385 | Coiled-coil domain-containing protein 90B, mitochondrial \|\| id:prot-a-385 | F-small fish liking (derived food-liking factor) \|\| id:ebi-fl187-GCST90094840 | Inverse variance weighted | 28 | 0.0877577085207608 | 0.0756971795363923 | 0.246323326767213 | -0.060608763 | 0.23612418041209 | 1.09172357466088 | 0.941191396302694 | 1.26633155397759 | 0.998735809224318 |
| ebi-fl187-GCST90094840 | prot-a-390 | C-C motif chemokine 14 \|\| id:prot-a-390 | F-small fish liking (derived food-liking factor) \|\| id:ebi-fl187-GCST90094840 | Inverse variance weighted | 28 | -0.059710898 | 0.0686300443660551 | 0.38427842961103 | -0.194225785 | 0.0748039886615766 | 0.942036838675813 | 0.82347195545302 | 1.07767289407459 | 0.998735809224318 |
| ebi-fl187-GCST90094840 | prot-a-40 | Pituitary adenylate cyclase-activating polypeptide 27 \|\| id:prot-a-40 | F-small fish liking (derived food-liking factor) \|\| id:ebi-fl187-GCST90094840 | Inverse variance weighted | 28 | 0.0183693874042492 | 0.0686322793560692 | 0.788969213076165 | -0.11614988 | 0.152888654942145 | 1.01853914243997 | 0.890341768753338 | 1.16519523298896 | 0.998735809224318 |
| ebi-fl187-GCST90094840 | prot-a-401 | C-C motif chemokine 24 \|\| id:prot-a-401 | F-small fish liking (derived food-liking factor) \|\| id:ebi-fl187-GCST90094840 | Inverse variance weighted | 28 | 0.0687957093102287 | 0.0686188090103609 | 0.316064507856823 | -0.065697156 | 0.203288574970536 | 1.07121734706264 | 0.936414408511981 | 1.2254260445131 | 0.998735809224318 |
| ebi-fl187-GCST90094840 | prot-a-412 | Cerebral cavernous malformations 2 protein \|\| id:prot-a-412 | F-small fish liking (derived food-liking factor) \|\| id:ebi-fl187-GCST90094840 | Inverse variance weighted | 28 | -0.015839655 | 0.0717880740597213 | 0.825369123298654 | -0.15654428 | 0.124864969873149 | 0.984285132322864 | 0.855093652940569 | 1.13299545421743 | 0.998735809224318 |
| ebi-fl187-GCST90094840 | prot-a-44 | Adhesion G protein-coupled receptor F5 \|\| id:prot-a-44 | F-small fish liking (derived food-liking factor) \|\| id:ebi-fl187-GCST90094840 | Inverse variance weighted | 28 | 0.0686469646570018 | 0.0740413917816402 | 0.353852246872367 | -0.076474163 | 0.213768092549016 | 1.07105802105956 | 0.926376848560554 | 1.2383354422755 | 0.998735809224318 |
| ebi-fl187-GCST90094840 | prot-a-48 | Adrenomedullin \|\| id:prot-a-48 | F-small fish liking (derived food-liking factor) \|\| id:ebi-fl187-GCST90094840 | Inverse variance weighted | 28 | 0.0830875833759734 | 0.0686477334315269 | 0.226145741059824 | -0.051461974 | 0.217637140901766 | 1.08663697571544 | 0.949839767740194 | 1.24313590259675 | 0.998735809224318 |
| ebi-fl187-GCST90094840 | prot-a-502 | Carcinoembryonic antigen-related cell adhesion molecule 1 \|\| id:prot-a-502 | F-small fish liking (derived food-liking factor) \|\| id:ebi-fl187-GCST90094840 | Inverse variance weighted | 28 | 0.0421447853106145 | 0.0686228916578427 | 0.539115859838095 | -0.092356082 | 0.176645652959986 | 1.04304548548332 | 0.911780422828403 | 1.19320820841082 | 0.998735809224318 |
| ebi-fl187-GCST90094840 | prot-a-518 | Complement factor B \|\| id:prot-a-518 | F-small fish liking (derived food-liking factor) \|\| id:ebi-fl187-GCST90094840 | Inverse variance weighted | 28 | -0.133075095 | 0.0686403852393251 | 0.0525343642051255 | -0.26761025 | 0.0014600601783295 | 0.875399351621744 | 0.765205962045522 | 1.00146112658513 | 0.998735809224318 |
| ebi-fl187-GCST90094840 | prot-a-525 | Properdin \|\| id:prot-a-525 | F-small fish liking (derived food-liking factor) \|\| id:ebi-fl187-GCST90094840 | Inverse variance weighted | 28 | 0.117735713338771 | 0.071249431111278 | 0.0984440195546869 | -0.021913172 | 0.257384598316876 | 1.12494676365233 | 0.978325177734821 | 1.29354252538196 | 0.998735809224318 |
| ebi-fl187-GCST90094840 | prot-a-533 | Chondroadherin \|\| id:prot-a-533 | F-small fish liking (derived food-liking factor) \|\| id:ebi-fl187-GCST90094840 | Inverse variance weighted | 28 | 0.00269783881059217 | 0.0686438054868081 | 0.968649612459747 | -0.13184402 | 0.137239697564736 | 1.00270148125255 | 0.876477697458399 | 1.14710307338286 | 0.998735809224318 |
| ebi-fl187-GCST90094840 | prot-a-535 | Coiled-coil-helix-coiled-coil-helix domain-containing protein 2 \|\| id:prot-a-535 | F-small fish liking (derived food-liking factor) \|\| id:ebi-fl187-GCST90094840 | Inverse variance weighted | 28 | -0.118932673 | 0.0750744003729804 | 0.113148795637699 | -0.266078498 | 0.0282131518338053 | 0.887867576301221 | 0.766378966208581 | 1.02861491221101 | 0.998735809224318 |
| ebi-fl187-GCST90094840 | prot-a-591 | Caseinolytic peptidase B protein homolog \|\| id:prot-a-591 | F-small fish liking (derived food-liking factor) \|\| id:ebi-fl187-GCST90094840 | Inverse variance weighted | 28 | -0.047464967 | 0.0686228916578427 | 0.489139358850363 | -0.181965835 | 0.0870359005786617 | 0.953643881486095 | 0.833629821125394 | 1.09093584424335 | 0.998735809224318 |
| ebi-fl187-GCST90094840 | prot-a-605 | Ciliary neurotrophic factor receptor subunit alpha \|\| id:prot-a-605 | F-small fish liking (derived food-liking factor) \|\| id:ebi-fl187-GCST90094840 | Inverse variance weighted | 28 | -0.132375081 | 0.0703397205560731 | 0.0598442635893428 | -0.270240934 | 0.00549077088872424 | 0.876012357507802 | 0.763195592652532 | 1.00550587279892 | 0.998735809224318 |
| ebi-fl187-GCST90094840 | prot-a-606 | Ciliary neurotrophic factor receptor subunit alpha \|\| id:prot-a-606 | F-small fish liking (derived food-liking factor) \|\| id:ebi-fl187-GCST90094840 | Inverse variance weighted | 28 | -0.159580739 | 0.0686504209184344 | 0.0200965008658178 | -0.294135564 | -0.025025914 | 0.85250113494944 | 0.745175465996639 | 0.975284638656314 | 0.998735809224318 |
| ebi-fl187-GCST90094840 | prot-a-707 | Cancer/testis antigen 55 \|\| id:prot-a-707 | F-small fish liking (derived food-liking factor) \|\| id:ebi-fl187-GCST90094840 | Inverse variance weighted | 28 | -0.070733904 | 0.0686555914914932 | 0.302882537905041 | -0.205298864 | 0.0638310548393721 | 0.931709782940309 | 0.814403884191192 | 1.06591230282355 | 0.998735809224318 |
| ebi-fl187-GCST90094840 | prot-a-719 | Dipeptidyl peptidase 1 \|\| id:prot-a-719 | F-small fish liking (derived food-liking factor) \|\| id:ebi-fl187-GCST90094840 | Inverse variance weighted | 28 | -0.104400687 | 0.0763643258287673 | 0.17158158166128 | -0.254074765 | 0.0452733917506183 | 0.900864260610408 | 0.775633809230444 | 1.04631387439175 | 0.998735809224318 |
| ebi-fl187-GCST90094840 | prot-a-752 | Cytochrome b5 \|\| id:prot-a-752 | F-small fish liking (derived food-liking factor) \|\| id:ebi-fl187-GCST90094840 | Inverse variance weighted | 28 | -0.11165015 | 0.0686568217297395 | 0.103905793599328 | -0.246217521 | 0.0229172200996345 | 0.894357093164688 | 0.781752158855046 | 1.0231818371509 | 0.998735809224318 |
| ebi-fl187-GCST90094840 | prot-a-755 | Cytoglobin \|\| id:prot-a-755 | F-small fish liking (derived food-liking factor) \|\| id:ebi-fl187-GCST90094840 | Inverse variance weighted | 28 | -0.124895064 | 0.0686257144253588 | 0.0687672960706272 | -0.259401464 | 0.00961133613343178 | 0.882589513014768 | 0.771513225756519 | 1.00965767335981 | 0.998735809224318 |
| ebi-fl187-GCST90094840 | prot-a-807 | Desmin \|\| id:prot-a-807 | F-small fish liking (derived food-liking factor) \|\| id:ebi-fl187-GCST90094840 | Inverse variance weighted | 28 | 0.0184894650830085 | 0.0686400755094723 | 0.787646225909335 | -0.116045083 | 0.153024013081574 | 1.01866145359919 | 0.890435078983093 | 1.1653529623225 | 0.998735809224318 |
| ebi-fl187-GCST90094840 | prot-a-82 | Ameloblastin \|\| id:prot-a-82 | F-small fish liking (derived food-liking factor) \|\| id:ebi-fl187-GCST90094840 | Inverse variance weighted (multiplicative random effects) | 28 | -0.025652206 | 0.0843644289028646 | 0.761078948612555 | -0.191006487 | 0.13970207452914 | 0.974674016324425 | 0.826127229236413 | 1.14993115403793 | 0.998735809224318 |
| ebi-fl187-GCST90094840 | prot-a-84 | Muellerian-inhibiting factor \|\| id:prot-a-84 | F-small fish liking (derived food-liking factor) \|\| id:ebi-fl187-GCST90094840 | Inverse variance weighted | 28 | -0.055927392 | 0.076601691035908 | 0.465325095481447 | -0.206066706 | 0.0942119227697898 | 0.945607792409388 | 0.813778790470318 | 1.0987925804119 | 0.998735809224318 |
| ebi-fl187-GCST90094840 | prot-a-858 | Inactive dipeptidyl peptidase 10 \|\| id:prot-a-858 | F-small fish liking (derived food-liking factor) \|\| id:ebi-fl187-GCST90094840 | Inverse variance weighted | 28 | 0.0617676811858444 | 0.0686533542233212 | 0.368277919924593 | -0.072792893 | 0.196328255463554 | 1.06371519496768 | 0.929793376717686 | 1.21692630248611 | 0.998735809224318 |
| ebi-fl187-GCST90094840 | prot-a-863 | Dihydropyrimidinase-related protein 3 \|\| id:prot-a-863 | F-small fish liking (derived food-liking factor) \|\| id:ebi-fl187-GCST90094840 | Inverse variance weighted | 28 | 0.00114625445391327 | 0.0686463653588694 | 0.986677572399459 | -0.133400622 | 0.135693130557297 | 1.00114691165463 | 0.875114432085205 | 1.14533037276892 | 0.998955228396052 |
| ebi-fl187-GCST90094840 | prot-a-864 | Dihydropyrimidinase-related protein 5 \|\| id:prot-a-864 | F-small fish liking (derived food-liking factor) \|\| id:ebi-fl187-GCST90094840 | Inverse variance weighted | 28 | -0.035057437 | 0.0686335083415294 | 0.609496816485885 | -0.169579113 | 0.0994642396397331 | 0.965549956652346 | 0.844019978836549 | 1.10457896989177 | 0.998735809224318 |
| ebi-fl187-GCST90094840 | prot-a-884 | Endothelin-converting enzyme 1 \|\| id:prot-a-884 | F-small fish liking (derived food-liking factor) \|\| id:ebi-fl187-GCST90094840 | Inverse variance weighted | 28 | -0.067625451 | 0.0686072345180129 | 0.32428535582396 | -0.202095631 | 0.0668447283185386 | 0.934610465129046 | 0.817016792079825 | 1.06912945975704 | 0.998735809224318 |
| ebi-fl187-GCST90094840 | prot-a-893 | Early endosome antigen 1 \|\| id:prot-a-893 | F-small fish liking (derived food-liking factor) \|\| id:ebi-fl187-GCST90094840 | Inverse variance weighted | 28 | -0.029872368 | 0.0686258854058593 | 0.663349759266876 | -0.164379103 | 0.104634367295378 | 0.970569401260266 | 0.848420321795524 | 1.11030457246608 | 0.998735809224318 |
| ebi-fl187-GCST90094840 | prot-a-897 | Ephrin-A2 \|\| id:prot-a-897 | F-small fish liking (derived food-liking factor) \|\| id:ebi-fl187-GCST90094840 | Inverse variance weighted | 28 | -0.04454815 | 0.0686336793802921 | 0.516292303914988 | -0.179070162 | 0.0899738614060781 | 0.956429546730733 | 0.836047238789576 | 1.09414568390171 | 0.998735809224318 |
| ebi-fl187-GCST90094840 | prot-a-90 | Anaphase-promoting complex subunit 10 \|\| id:prot-a-90 | F-small fish liking (derived food-liking factor) \|\| id:ebi-fl187-GCST90094840 | Inverse variance weighted | 28 | 0.0172712272864227 | 0.0739608380015221 | 0.815358770645783 | -0.127692015 | 0.162234469769406 | 1.01742123730706 | 0.880124402348047 | 1.17613597732525 | 0.998735809224318 |
| ebi-fl187-GCST90094840 | prot-a-914 | Histone-lysine N-methyltransferase EHMT2 \|\| id:prot-a-914 | F-small fish liking (derived food-liking factor) \|\| id:ebi-fl187-GCST90094840 | Inverse variance weighted | 28 | 0.0646089531010582 | 0.0686547555376799 | 0.346668821638289 | -0.069954368 | 0.199172273954911 | 1.06674179674013 | 0.932436368102009 | 1.22039218958036 | 0.998735809224318 |
| ebi-fl187-GCST90094840 | prot-a-932 | RNA polymerase II elongation factor ELL \|\| id:prot-a-932 | F-small fish liking (derived food-liking factor) \|\| id:ebi-fl187-GCST90094840 | Inverse variance weighted | 28 | -0.05971621 | 0.0707802024074874 | 0.398845335017251 | -0.198445407 | 0.0790129868674843 | 0.942031835008339 | 0.820004536378805 | 1.0822183766057 | 0.998735809224318 |
| ebi-fl187-GCST90094840 | prot-a-991 | Estrogen receptor \|\| id:prot-a-991 | F-small fish liking (derived food-liking factor) \|\| id:ebi-fl187-GCST90094840 | Inverse variance weighted | 28 | 0.011522613040838 | 0.0738630753734774 | 0.876033306338071 | -0.133249015 | 0.156294240772854 | 1.01158925406008 | 0.875247115580028 | 1.16917017001726 | 0.998735809224318 |
| ebi-fl187-GCST90094840 | prot-a-992 | Persulfide dioxygenase ETHE1, mitochondrial \|\| id:prot-a-992 | F-small fish liking (derived food-liking factor) \|\| id:ebi-fl187-GCST90094840 | Inverse variance weighted | 28 | -0.072692792 | 0.0773666562772425 | 0.347428866337836 | -0.224331438 | 0.0789458542719109 | 0.929886454679154 | 0.799050254590267 | 1.08214572691571 | 0.998735809224318 |
| ebi-fl187-GCST90094840 | prot-a-993 | Ethanolamine kinase 1 \|\| id:prot-a-993 | F-small fish liking (derived food-liking factor) \|\| id:ebi-fl187-GCST90094840 | Inverse variance weighted | 28 | 0.0571966352248004 | 0.0751028624729381 | 0.446312327249222 | -0.090004975 | 0.204398245671759 | 1.05886399988679 | 0.913926638271855 | 1.22678661864623 | 0.998735809224318 |
| ebi-fl187-GCST90094840 | prot-b-10 | coagulation factor III, tissue factor \|\| id:prot-b-10 | F-small fish liking (derived food-liking factor) \|\| id:ebi-fl187-GCST90094840 | Inverse variance weighted (multiplicative random effects) | 19 | 0.023913373895597 | 0.121568490400853 | 0.844056826415611 | -0.214360867 | 0.262187615081268 | 1.02420159145367 | 0.807057091961062 | 1.29977037607997 | 0.998735809224318 |
| ebi-fl187-GCST90094840 | prot-b-28 | prolactin \|\| id:prot-b-28 | F-small fish liking (derived food-liking factor) \|\| id:ebi-fl187-GCST90094840 | Inverse variance weighted | 19 | -0.082534248 | 0.109930011343044 | 0.45277962472846 | -0.29799707 | 0.132928574238378 | 0.920779902452232 | 0.742303514525863 | 1.1421684151684 | 0.998735809224318 |
| ebi-fl187-GCST90094840 | prot-b-54 | matrix metallopeptidase 7 \|\| id:prot-b-54 | F-small fish liking (derived food-liking factor) \|\| id:ebi-fl187-GCST90094840 | Inverse variance weighted | 19 | 0.0396635224706305 | 0.106556862393566 | 0.709722578646674 | -0.169187928 | 0.24851497276202 | 1.04046062366674 | 0.844350211579979 | 1.28212001910352 | 0.998735809224318 |
| ebi-fl187-GCST90094840 | ukb-rsfMRI191-edge-pheno716 | Net100_Pair10_33 (OccipitalPrecuneus)&(Temporal\|Frontal\|Supp_Motor_Area) (Default_mode\|Central_executive)&(Default_mode\|Salience) \|\| id:ukb-rsfMRI191-edge-pheno716 | F-small fish liking (derived food-liking factor) \|\| id:ebi-fl187-GCST90094840 | Inverse variance weighted | 28 | -0.009212091 | 0.0237313195884968 | 0.697880741731594 | -0.055725477 | 0.0373012956978174 | 0.990830210617689 | 0.945798743678873 | 1.03800572038614 | 0.998735809224318 |
| ebi-fl187-GCST90094840 | ukb-rsfMRI191-edge-pheno903 | Net100_Pair27_38 (Precentral\|Frontal\|Supp_Motor_Area)&(Subcortical) (Attention\|Salience\|Motor)&(Subcortical-cerebellum) \|\| id:ukb-rsfMRI191-edge-pheno903 | F-small fish liking (derived food-liking factor) \|\| id:ebi-fl187-GCST90094840 | Inverse variance weighted | 28 | 0.027940408097415 | 0.0211963799756582 | 0.187447126216549 | -0.013604497 | 0.0694853128497052 | 1.02833440219196 | 0.986487626274523 | 1.07195631710561 | 0.998735809224318 |
| ebi-fl187-GCST90094840 | ukb-rsfMRI191-node-pheno23 | Net100_Node2 RolandicOper\|SupraMarginal\|Insula Salience\|Motor \|\| id:ukb-rsfMRI191-node-pheno23 | F-small fish liking (derived food-liking factor) \|\| id:ebi-fl187-GCST90094840 | Inverse variance weighted | 28 | -0.012851815 | 0.0234985768938989 | 0.584434461858126 | -0.058909026 | 0.0332053952755471 | 0.987230416490039 | 0.942792534723844 | 1.03376284744191 | 0.998735809224318 |
| ebi-fl187-GCST90094840 | ukb-rsfMRI191-node-pheno55 | Net100_Node34 Parietal\|Postcentral\|Precuneus Attention \|\| id:ukb-rsfMRI191-node-pheno55 | F-small fish liking (derived food-liking factor) \|\| id:ebi-fl187-GCST90094840 | Inverse variance weighted | 28 | 0.00489999066770864 | 0.0203798367797494 | 0.809994393770641 | -0.035044489 | 0.0448444707560175 | 1.00491201525408 | 0.96556245798767 | 1.04586518463719 | 0.998735809224318 |
| ebi-fl187-GCST90094840 | ebi-a-GCST90001414 | IgD- CD24- B cell Absolute Count \|\| id:ebi-a-GCST90001414 | F-small fish liking (derived food-liking factor) \|\| id:ebi-fl187-GCST90094840 | MR Egger | 25 | -0.311878346 | 0.371833155890778 | 0.410237674801488 | -1.040671332 | 0.416914639258977 | 0.732070581915853 | 0.353217476210097 | 1.51727299186024 | 0.999950574162623 |
| ebi-fl187-GCST90094840 | ebi-a-GCST90001422 | CD20- CD38- B cell %B cell \|\| id:ebi-a-GCST90001422 | F-small fish liking (derived food-liking factor) \|\| id:ebi-fl187-GCST90094840 | MR Egger | 25 | -0.272888096 | 0.35514413314021 | 0.450075499444126 | -0.968970597 | 0.4231944051081 | 0.761177961838494 | 0.379473468315889 | 1.52683109087957 | 0.999950574162623 |
| ebi-fl187-GCST90094840 | ebi-a-GCST90001444 | CD20- CD38- B cell %lymphocyte \|\| id:ebi-a-GCST90001444 | F-small fish liking (derived food-liking factor) \|\| id:ebi-fl187-GCST90094840 | MR Egger | 25 | -0.324822606 | 0.355804066436057 | 0.370752566078012 | -1.022198577 | 0.372553363769908 | 0.722655536707179 | 0.359803015426732 | 1.45143593116964 | 0.999950574162623 |
| ebi-fl187-GCST90094840 | ebi-a-GCST90001459 | Myeloid Dendritic Cell %Dendritic Cell \|\| id:ebi-a-GCST90001459 | F-small fish liking (derived food-liking factor) \|\| id:ebi-fl187-GCST90094840 | MR Egger | 25 | -0.429214276 | 0.399083682381473 | 0.293304330120588 | -1.211418293 | 0.352989741861625 | 0.651020416440186 | 0.29777464807227 | 1.42331654278067 | 0.999950574162623 |
| ebi-fl187-GCST90094840 | ebi-a-GCST90001476 | HLA DR++ monocyte %leukocyte \|\| id:ebi-a-GCST90001476 | F-small fish liking (derived food-liking factor) \|\| id:ebi-fl187-GCST90094840 | MR Egger | 25 | -0.363831948 | 0.369507540938293 | 0.335046277190142 | -1.088066729 | 0.360402831821279 | 0.695007982120538 | 0.336867120135188 | 1.43390692157019 | 0.999950574162623 |
| ebi-fl187-GCST90094840 | ebi-a-GCST90001478 | CD4 regulatory T cell %CD4+ T cell \|\| id:ebi-a-GCST90001478 | F-small fish liking (derived food-liking factor) \|\| id:ebi-fl187-GCST90094840 | MR Egger | 25 | 0.116807487309631 | 0.462346489600974 | 0.802790174830835 | -0.789391632 | 1.02300660692754 | 1.12390304326407 | 0.454120983796428 | 2.78154521752836 | 0.999950574162623 |
| ebi-fl187-GCST90094840 | ebi-a-GCST90001485 | CD39+ resting CD4 regulatory T cell %CD4 regulatory T cell \|\| id:ebi-a-GCST90001485 | F-small fish liking (derived food-liking factor) \|\| id:ebi-fl187-GCST90094840 | MR Egger | 25 | 0.37801860929658 | 0.364351182919983 | 0.310282719406288 | -0.336109709 | 1.09214692781975 | 1.45939010084631 | 0.714544709368303 | 2.98066648388047 | 0.999950574162623 |
| ebi-fl187-GCST90094840 | ebi-a-GCST90001515 | Immature Myeloid-Derived Suppressor Cells Absolute Count \|\| id:ebi-a-GCST90001515 | F-small fish liking (derived food-liking factor) \|\| id:ebi-fl187-GCST90094840 | MR Egger | 25 | 0.506442989429208 | 0.491143893239942 | 0.313192456220725 | -0.456199041 | 1.4690850201795 | 1.65937825898424 | 0.633687694509305 | 4.3452574986828 | 0.999950574162623 |
| ebi-fl187-GCST90094840 | ebi-a-GCST90001517 | CD33+ HLA DR+ Absolute Count \|\| id:ebi-a-GCST90001517 | F-small fish liking (derived food-liking factor) \|\| id:ebi-fl187-GCST90094840 | MR Egger | 25 | 0.633974352311469 | 0.480881189728025 | 0.200362149902788 | -0.30855278 | 1.5765014841784 | 1.88508771375137 | 0.734509184107879 | 4.83800034829035 | 0.999950574162623 |
| ebi-fl187-GCST90094840 | ebi-a-GCST90001518 | CD33+ HLA DR+ CD14- Absolute Count \|\| id:ebi-a-GCST90001518 | F-small fish liking (derived food-liking factor) \|\| id:ebi-fl187-GCST90094840 | MR Egger | 25 | 0.452122643003276 | 0.486819231172757 | 0.36267513215146 | -0.50204305 | 1.40628833610188 | 1.57164468798296 | 0.605292752174636 | 4.0807807732553 | 0.999950574162623 |
| ebi-fl187-GCST90094840 | ebi-a-GCST90001520 | CD33+ HLA DR+ CD14dim Absolute Count \|\| id:ebi-a-GCST90001520 | F-small fish liking (derived food-liking factor) \|\| id:ebi-fl187-GCST90094840 | MR Egger | 25 | 0.556953775524008 | 0.482853795967455 | 0.260566914663654 | -0.389439665 | 1.50334721562022 | 1.74534767330305 | 0.677436359761168 | 4.49671538412601 | 0.999950574162623 |
| ebi-fl187-GCST90094840 | ebi-a-GCST90001531 | CD33dim HLA DR- Absolute Count \|\| id:ebi-a-GCST90001531 | F-small fish liking (derived food-liking factor) \|\| id:ebi-fl187-GCST90094840 | MR Egger | 25 | 0.574375931707005 | 0.490129934442144 | 0.253239843033853 | -0.38627874 | 1.53503060321361 | 1.77602182208942 | 0.679581072985451 | 4.64146757160403 | 0.999950574162623 |
| ebi-fl187-GCST90094840 | ebi-a-GCST90001532 | Basophil Absolute Count \|\| id:ebi-a-GCST90001532 | F-small fish liking (derived food-liking factor) \|\| id:ebi-fl187-GCST90094840 | MR Egger | 25 | 0.574838818469796 | 0.488385293634873 | 0.251225403092568 | -0.382396357 | 1.53207399399415 | 1.77684410937957 | 0.682224595078888 | 4.62776483259416 | 0.999950574162623 |
| ebi-fl187-GCST90094840 | ebi-a-GCST90001588 | T/B cell \|\| id:ebi-a-GCST90001588 | F-small fish liking (derived food-liking factor) \|\| id:ebi-fl187-GCST90094840 | MR Egger | 25 | 0.24160770804865 | 0.457579840149808 | 0.602548566345646 | -0.655248779 | 1.13846419474227 | 1.27329459160785 | 0.519312852567588 | 3.12196994355497 | 0.999950574162623 |
| ebi-fl187-GCST90094840 | ebi-a-GCST90001624 | HLA DR+ CD4+ T cell Absolute Count \|\| id:ebi-a-GCST90001624 | F-small fish liking (derived food-liking factor) \|\| id:ebi-fl187-GCST90094840 | MR Egger | 25 | -0.182026474 | 0.354117820190448 | 0.612136039251479 | -0.876097401 | 0.512044454011075 | 0.833579272310909 | 0.416404805744204 | 1.66869928886755 | 0.999950574162623 |
| ebi-fl187-GCST90094840 | ebi-a-GCST90001631 | CD8+ Natural Killer T %T cell \|\| id:ebi-a-GCST90001631 | F-small fish liking (derived food-liking factor) \|\| id:ebi-fl187-GCST90094840 | MR Egger | 25 | -0.254891946 | 0.359305333105183 | 0.485204988588062 | -0.959130399 | 0.449346506823695 | 0.77500023526394 | 0.383225994846243 | 1.56728763898217 | 0.999950574162623 |
| ebi-fl187-GCST90094840 | ebi-a-GCST90001632 | CD8+ Natural Killer T %lymphocyte \|\| id:ebi-a-GCST90001632 | F-small fish liking (derived food-liking factor) \|\| id:ebi-fl187-GCST90094840 | MR Egger | 25 | -0.29430626 | 0.361492903874602 | 0.423913515720652 | -1.002832351 | 0.414219831730004 | 0.745048278114291 | 0.3668389515115 | 1.51318973744168 | 0.999950574162623 |
| ebi-fl187-GCST90094840 | ebi-a-GCST90001646 | Natural Killer %CD3- lymphocyte \|\| id:ebi-a-GCST90001646 | F-small fish liking (derived food-liking factor) \|\| id:ebi-fl187-GCST90094840 | MR Egger | 25 | 0.583222357126898 | 0.370315975905974 | 0.128928570779198 | -0.142596956 | 1.30904166990261 | 1.79180296712571 | 0.86710347963611 | 3.70262367572072 | 0.999950574162623 |
| ebi-fl187-GCST90094840 | ebi-a-GCST90001684 | CD127- CD8+ T cell Absolute Count \|\| id:ebi-a-GCST90001684 | F-small fish liking (derived food-liking factor) \|\| id:ebi-fl187-GCST90094840 | MR Egger | 25 | -0.522317102 | 0.381403910579181 | 0.184079959881773 | -1.269868767 | 0.225234562484933 | 0.593144577818061 | 0.280868478577046 | 1.25261649857393 | 0.999950574162623 |
| ebi-fl187-GCST90094840 | ebi-a-GCST90001687 | CD28- CD8+ T cell Absolute Count \|\| id:ebi-a-GCST90001687 | F-small fish liking (derived food-liking factor) \|\| id:ebi-fl187-GCST90094840 | MR Egger | 25 | -0.331791335 | 0.404517489457042 | 0.420513113697707 | -1.124645614 | 0.46106294436126 | 0.71763705298611 | 0.324767539935725 | 1.58575866270537 | 0.999950574162623 |
| ebi-fl187-GCST90094840 | ebi-a-GCST90001701 | BAFF-R on CD20- CD38- B cell \|\| id:ebi-a-GCST90001701 | F-small fish liking (derived food-liking factor) \|\| id:ebi-fl187-GCST90094840 | MR Egger | 25 | -0.555730538 | 0.360232592331861 | 0.136551777265689 | -1.261786419 | 0.150325343416555 | 0.573653033000599 | 0.283147754034222 | 1.16221229934606 | 0.999950574162623 |
| ebi-fl187-GCST90094840 | ebi-a-GCST90001732 | CD19 on IgD- CD27- B cell \|\| id:ebi-a-GCST90001732 | F-small fish liking (derived food-liking factor) \|\| id:ebi-fl187-GCST90094840 | MR Egger | 25 | 0.0653484304947558 | 0.384936170843088 | 0.866679844420715 | -0.689126464 | 0.819823325347208 | 1.06753091991715 | 0.502014405062897 | 2.27009873319547 | 0.999950574162623 |
| ebi-fl187-GCST90094840 | ebi-a-GCST90001798 | CD27 on CD24+ CD27+ B cell \|\| id:ebi-a-GCST90001798 | F-small fish liking (derived food-liking factor) \|\| id:ebi-fl187-GCST90094840 | MR Egger | 25 | 0.74572282684934 | 0.408437111839998 | 0.0808932777920895 | -0.054813912 | 1.54625956605574 | 2.10796457782445 | 0.946661293532497 | 4.69388015726458 | 0.999950574162623 |
| ebi-fl187-GCST90094840 | ebi-a-GCST90001836 | CD66b on CD66b++ myeloid cell \|\| id:ebi-a-GCST90001836 | F-small fish liking (derived food-liking factor) \|\| id:ebi-fl187-GCST90094840 | MR Egger | 25 | -0.529480215 | 0.575931807432557 | 0.367456810089426 | -1.658306558 | 0.599346127476637 | 0.588910997186204 | 0.190461242290306 | 1.82092775641053 | 0.999950574162623 |
| ebi-fl187-GCST90094840 | ebi-a-GCST90001943 | CD25 on activated & secreting CD4 regulatory T cell \|\| id:ebi-a-GCST90001943 | F-small fish liking (derived food-liking factor) \|\| id:ebi-fl187-GCST90094840 | MR Egger | 25 | 0.306639786148055 | 0.429350689367134 | 0.482292955874693 | -0.534887565 | 1.14816713730764 | 1.35885140280515 | 0.585735143566726 | 3.15240967728478 | 0.999950574162623 |
| ebi-fl187-GCST90094840 | ebi-a-GCST90001960 | CD25 on CD4+ T cell \|\| id:ebi-a-GCST90001960 | F-small fish liking (derived food-liking factor) \|\| id:ebi-fl187-GCST90094840 | MR Egger | 25 | 0.227664999547047 | 0.393148916190092 | 0.568163305319281 | -0.542906876 | 0.998236875279628 | 1.25566460675379 | 0.581056735063795 | 2.71349338112574 | 0.999950574162623 |
| ebi-fl187-GCST90094840 | ebi-a-GCST90001988 | HLA DR on CD14+ CD16- monocyte \|\| id:ebi-a-GCST90001988 | F-small fish liking (derived food-liking factor) \|\| id:ebi-fl187-GCST90094840 | MR Egger | 25 | -0.134232718 | 0.445890411326363 | 0.766085520886135 | -1.008177924 | 0.73971248828819 | 0.874386555511121 | 0.364883219156156 | 2.09533299510659 | 0.999950574162623 |
| ebi-fl187-GCST90094840 | ebi-a-GCST90001991 | HLA DR on CD14+ monocyte \|\| id:ebi-a-GCST90001991 | F-small fish liking (derived food-liking factor) \|\| id:ebi-fl187-GCST90094840 | MR Egger | 25 | -0.072397386 | 0.447936081420155 | 0.873013068919823 | -0.950352105 | 0.805557333861106 | 0.930161189581813 | 0.386604873859036 | 2.23794343296283 | 0.999950574162623 |
| ebi-fl187-GCST90094840 | ebi-a-GCST90002019 | CD14 on Monocytic Myeloid-Derived Suppressor Cells \|\| id:ebi-a-GCST90002019 | F-small fish liking (derived food-liking factor) \|\| id:ebi-fl187-GCST90094840 | MR Egger | 25 | -0.22012039 | 0.528779582371575 | 0.681064061734872 | -1.256528371 | 0.816287591634628 | 0.802422188689438 | 0.284640479277995 | 2.26208644158549 | 0.999950574162623 |
| ebi-fl187-GCST90094840 | ebi-a-GCST90002037 | CD80 on plasmacytoid Dendritic Cell \|\| id:ebi-a-GCST90002037 | F-small fish liking (derived food-liking factor) \|\| id:ebi-fl187-GCST90094840 | MR Egger | 25 | 0.426035597280526 | 0.409988463318394 | 0.309540822984656 | -0.377541791 | 1.22961298538458 | 1.5311752798391 | 0.68554455151427 | 3.41990572663979 | 0.999950574162623 |
| ebi-fl187-GCST90094840 | ebi-a-GCST90002042 | CD45 on CD33+ HLA DR+ CD14- \|\| id:ebi-a-GCST90002042 | F-small fish liking (derived food-liking factor) \|\| id:ebi-fl187-GCST90094840 | MR Egger | 25 | -0.608614494 | 0.538921536901371 | 0.270404418746497 | -1.664900707 | 0.44767171803499 | 0.544104206561398 | 0.189209444294846 | 1.5646649600454 | 0.999950574162623 |
| ebi-fl187-GCST90094840 | ebi-a-GCST90002056 | CD8 on Effector Memory CD8+ T cell \|\| id:ebi-a-GCST90002056 | F-small fish liking (derived food-liking factor) \|\| id:ebi-fl187-GCST90094840 | MR Egger | 25 | 0.00651806204406527 | 0.43774209652783 | 0.988248200214706 | -0.851456447 | 0.864492571238613 | 1.00653935083923 | 0.426792877793478 | 2.37380124529188 | 0.999950574162623 |
| ebi-fl187-GCST90094840 | ebi-a-GCST90002058 | CD8 on CD8+ T cell \|\| id:ebi-a-GCST90002058 | F-small fish liking (derived food-liking factor) \|\| id:ebi-fl187-GCST90094840 | MR Egger | 25 | -0.012311925 | 0.454525784981518 | 0.97862367912279 | -0.903182463 | 0.878558613967826 | 0.987763557055314 | 0.405277823477588 | 2.40742717248757 | 0.999950574162623 |
| ebi-fl187-GCST90094840 | ebi-a-GCST90002063 | CD4 on CD4 regulatory T cell \|\| id:ebi-a-GCST90002063 | F-small fish liking (derived food-liking factor) \|\| id:ebi-fl187-GCST90094840 | MR Egger | 25 | -0.033296656 | 0.426276184701661 | 0.938415713490654 | -0.868797978 | 0.802204665697889 | 0.967251575732925 | 0.419455440871069 | 2.23045291489114 | 0.999950574162623 |
| ebi-fl187-GCST90094840 | ebi-a-GCST90002066 | CD4 on activated CD4 regulatory T cell \|\| id:ebi-a-GCST90002066 | F-small fish liking (derived food-liking factor) \|\| id:ebi-fl187-GCST90094840 | MR Egger | 25 | -0.032310068 | 0.451079391570713 | 0.943516904294072 | -0.916425676 | 0.851805539414112 | 0.968206325670218 | 0.399946026174224 | 2.34387499242077 | 0.999950574162623 |
| ebi-fl187-GCST90094840 | ebi-a-GCST90002070 | CD4 on activated & secreting CD4 regulatory T cell \|\| id:ebi-a-GCST90002070 | F-small fish liking (derived food-liking factor) \|\| id:ebi-fl187-GCST90094840 | MR Egger | 25 | -0.077556036 | 0.41498472832286 | 0.853386492045489 | -0.890926104 | 0.73581403141175 | 0.925375168527291 | 0.410275619024447 | 2.08718033151243 | 0.999950574162623 |
| ebi-fl187-GCST90094840 | ebi-a-GCST90002081 | SSC-A on CD4+ T cell \|\| id:ebi-a-GCST90002081 | F-small fish liking (derived food-liking factor) \|\| id:ebi-fl187-GCST90094840 | MR Egger | 25 | 0.162172936098268 | 0.379072611151045 | 0.672765017303217 | -0.580809382 | 0.905155253954318 | 1.17606360758742 | 0.559445378386207 | 2.47231573005634 | 0.999950574162623 |
| ebi-fl187-GCST90094840 | ebi-a-GCST90002094 | CD11b on Monocytic Myeloid-Derived Suppressor Cells \|\| id:ebi-a-GCST90002094 | F-small fish liking (derived food-liking factor) \|\| id:ebi-fl187-GCST90094840 | MR Egger | 25 | -0.535104506 | 0.53617361943757 | 0.328661109430676 | -1.5860048 | 0.515795787961813 | 0.585608087308501 | 0.204741964951837 | 1.67497089324991 | 0.999950574162623 |
| ebi-fl187-GCST90094840 | ebi-a-GCST90002104 | HLA DR on myeloid Dendritic Cell \|\| id:ebi-a-GCST90002104 | F-small fish liking (derived food-liking factor) \|\| id:ebi-fl187-GCST90094840 | MR Egger | 24 | -0.349691047 | 0.417212657158396 | 0.410954938042454 | -1.167427855 | 0.468045760766931 | 0.704905838667728 | 0.311166277513938 | 1.59687047503274 | 0.999950574162623 |
| ebi-fl187-GCST90094840 | ebi-a-GCST90002105 | HLA DR on plasmacytoid Dendritic Cell \|\| id:ebi-a-GCST90002105 | F-small fish liking (derived food-liking factor) \|\| id:ebi-fl187-GCST90094840 | MR Egger | 25 | -0.672870535 | 0.467024540590974 | 0.163128275352934 | -1.588238634 | 0.242497564624651 | 0.510241806654221 | 0.204285115789004 | 1.27442814544875 | 0.999950574162623 |
| ebi-fl187-GCST90094840 | ebi-a-GCST90002106 | HLA DR on Dendritic Cell \|\| id:ebi-a-GCST90002106 | F-small fish liking (derived food-liking factor) \|\| id:ebi-fl187-GCST90094840 | MR Egger | 24 | -0.876520058 | 0.451808695031339 | 0.0653024830770122 | -1.7620651 | 0.00902498419187669 | 0.416228846553483 | 0.171689940520078 | 1.0090658321533 | 0.999950574162623 |
| ebi-fl187-GCST90094840 | ebi-a-GCST90002111 | HLA DR on CD33dim HLA DR+ CD11b- \|\| id:ebi-a-GCST90002111 | F-small fish liking (derived food-liking factor) \|\| id:ebi-fl187-GCST90094840 | MR Egger | 25 | 0.0754820791749723 | 0.601927707065137 | 0.901296105094183 | -1.104296227 | 1.25526038502264 | 1.07840390165725 | 0.331444061669722 | 3.50875188184378 | 0.999950574162623 |
| ebi-fl187-GCST90094840 | ebi-a-GCST90002112 | HLA DR on CD33- HLA DR+ \|\| id:ebi-a-GCST90002112 | F-small fish liking (derived food-liking factor) \|\| id:ebi-fl187-GCST90094840 | MR Egger | 25 | -0.311839327 | 0.52900196088519 | 0.561281709230123 | -1.34868317 | 0.725004516194682 | 0.732099147242546 | 0.259581860740293 | 2.06474042471516 | 0.999950574162623 |
| ebi-fl187-GCST90094840 | ebi-a-GCST90002115 | HLA DR on HLA DR+ CD8+ T cell \|\| id:ebi-a-GCST90002115 | F-small fish liking (derived food-liking factor) \|\| id:ebi-fl187-GCST90094840 | MR Egger | 25 | -0.213627699 | 0.378830594298731 | 0.578270741704329 | -0.956135664 | 0.528880265610747 | 0.80764901741975 | 0.38437537529666 | 1.69703102035516 | 0.999950574162623 |
| ebi-fl187-GCST90094840 | ebi-a-GCST90002116 | HLA DR on B cell \|\| id:ebi-a-GCST90002116 | F-small fish liking (derived food-liking factor) \|\| id:ebi-fl187-GCST90094840 | MR Egger | 25 | -0.743105518 | 0.454997219973522 | 0.116041368985731 | -1.634900069 | 0.14868903362952 | 0.47563452821858 | 0.194971854123035 | 1.16031211505509 | 0.999950574162623 |
| ebi-fl187-GCST90094840 | ebi-cfmet338-GCST90026023 | Sphingomyelin (d18:1/24:1, d18:2/24:0) levels \|\| id:ebi-cfmet338-GCST90026023 | F-small fish liking (derived food-liking factor) \|\| id:ebi-fl187-GCST90094840 | MR Egger | 23 | -0.189660026 | 0.206930466131694 | 0.369789993825672 | -0.595243739 | 0.215923687862818 | 0.827240326546734 | 0.551428144736149 | 1.24100767143938 | 0.999950574162623 |
| ebi-fl187-GCST90094840 | ebi-cfmet338-GCST90026046 | 1-arachidonoyl-gpc (20:4n6) levels \|\| id:ebi-cfmet338-GCST90026046 | F-small fish liking (derived food-liking factor) \|\| id:ebi-fl187-GCST90094840 | MR Egger | 23 | 0.00160718958226031 | 0.256632034230917 | 0.995062295384376 | -0.501391598 | 0.504605976674858 | 1.00160848180363 | 0.60568720017085 | 1.6563327581266 | 0.999950574162623 |
| ebi-fl187-GCST90094840 | ebi-cfmet338-GCST90026047 | 1-linoleoyl-gpc (18:2) levels \|\| id:ebi-cfmet338-GCST90026047 | F-small fish liking (derived food-liking factor) \|\| id:ebi-fl187-GCST90094840 | MR Egger | 23 | -0.127021016 | 0.276966006015722 | 0.651221052113797 | -0.669874387 | 0.415832356157715 | 0.880715163615925 | 0.511772858856374 | 1.51563176123943 | 0.999950574162623 |
| ebi-fl187-GCST90094840 | ebi-cfmet338-GCST90026050 | 1-myristoyl-2-palmitoyl-gpc (14:0/16:0) levels \|\| id:ebi-cfmet338-GCST90026050 | F-small fish liking (derived food-liking factor) \|\| id:ebi-fl187-GCST90094840 | MR Egger | 23 | -0.112166981 | 0.146286420532979 | 0.451757033465162 | -0.398888366 | 0.174554402773447 | 0.893894981138469 | 0.671065611101246 | 1.1907155188496 | 0.999950574162623 |
| ebi-fl187-GCST90094840 | ebi-cfmet338-GCST90026068 | 2,3-dihydroxyisovalerate levels \|\| id:ebi-cfmet338-GCST90026068 | F-small fish liking (derived food-liking factor) \|\| id:ebi-fl187-GCST90094840 | MR Egger | 23 | -0.493597757 | 0.490362742567573 | 0.325586842 | -1.454708733 | 0.467513217962812 | 0.610426273122808 | 0.233468355649198 | 1.59602029954966 | 0.999950574162623 |
| ebi-fl187-GCST90094840 | ebi-cfmet338-GCST90026082 | 3-hydroxyhexanoate levels \|\| id:ebi-cfmet338-GCST90026082 | F-small fish liking (derived food-liking factor) \|\| id:ebi-fl187-GCST90094840 | MR Egger | 23 | 0.0199910068775543 | 0.12278559066222 | 0.87222275847558 | -0.220668751 | 0.260650764575506 | 1.02019216527244 | 0.801982292272262 | 1.29777435750406 | 0.999950574162623 |
| ebi-fl187-GCST90094840 | ebi-cfmet338-GCST90026094 | 3-ureidopropionate levels \|\| id:ebi-cfmet338-GCST90026094 | F-small fish liking (derived food-liking factor) \|\| id:ebi-fl187-GCST90094840 | MR Egger | 23 | -0.134587288 | 0.230355462057523 | 0.565270234959127 | -0.586083994 | 0.316909417323912 | 0.87407657888021 | 0.556502289626549 | 1.37287820731094 | 0.999950574162623 |
| ebi-fl187-GCST90094840 | ebi-cfmet338-GCST90026173 | Glycerol levels \|\| id:ebi-cfmet338-GCST90026173 | F-small fish liking (derived food-liking factor) \|\| id:ebi-fl187-GCST90094840 | MR Egger | 22 | -0.077538483 | 0.150074098550199 | 0.611054180983077 | -0.371683716 | 0.216606750116613 | 0.92539141183504 | 0.689572308587043 | 1.24185564651332 | 0.999950574162623 |
| ebi-fl187-GCST90094840 | ebi-cfmet338-GCST90026179 | Histidine levels \|\| id:ebi-cfmet338-GCST90026179 | F-small fish liking (derived food-liking factor) \|\| id:ebi-fl187-GCST90094840 | MR Egger | 23 | 0.0749769176864987 | 0.0900986742683134 | 0.414679578679525 | -0.101616484 | 0.251570319252393 | 1.07785927111189 | 0.903375944478692 | 1.28604333048991 | 0.999950574162623 |
| ebi-fl187-GCST90094840 | ebi-cfmet338-GCST90026188 | Isocitrate levels \|\| id:ebi-cfmet338-GCST90026188 | F-small fish liking (derived food-liking factor) \|\| id:ebi-fl187-GCST90094840 | MR Egger | 22 | 0.168253678519829 | 0.180181770218592 | 0.361545051741428 | -0.184902591 | 0.521409948148269 | 1.18323673432211 | 0.831185244742135 | 1.68440089415157 | 0.999950574162623 |
| ebi-fl187-GCST90094840 | ebi-cfmet338-GCST90026207 | N-acetyl-isoputreanine levels \|\| id:ebi-cfmet338-GCST90026207 | F-small fish liking (derived food-liking factor) \|\| id:ebi-fl187-GCST90094840 | MR Egger | 23 | -6.13E-06 | 0.112674044924531 | 0.999957086204066 | -0.220847261 | 0.220834995390782 | 0.999993867357506 | 0.801839143276231 | 1.24711763342804 | 0.999974376584515 |
| ebi-fl187-GCST90094840 | ebi-cfmet338-GCST90026213 | N-acetylglutamate levels \|\| id:ebi-cfmet338-GCST90026213 | F-small fish liking (derived food-liking factor) \|\| id:ebi-fl187-GCST90094840 | MR Egger | 23 | 0.0291807274410395 | 0.191789541807101 | 0.880521150299305 | -0.346726775 | 0.405088229382958 | 1.02961065656059 | 0.706998471881024 | 1.49943478842699 | 0.999950574162623 |
| ebi-fl187-GCST90094840 | ebi-cfmet338-GCST90026224 | N-acetyltaurine levels \|\| id:ebi-cfmet338-GCST90026224 | F-small fish liking (derived food-liking factor) \|\| id:ebi-fl187-GCST90094840 | MR Egger | 23 | 0.159291187142914 | 0.170407834084586 | 0.360534879404709 | -0.174708168 | 0.493290541948703 | 1.17267936612401 | 0.839702037223617 | 1.63769627173928 | 0.999950574162623 |
| ebi-fl187-GCST90094840 | ebi-cfmet338-GCST90026250 | Phenyllactate (pla) levels \|\| id:ebi-cfmet338-GCST90026250 | F-small fish liking (derived food-liking factor) \|\| id:ebi-fl187-GCST90094840 | MR Egger | 23 | 0.070285664160095 | 0.246288791482007 | 0.778149501501587 | -0.412440367 | 0.553011695464829 | 1.07281460216766 | 0.662032674412455 | 1.73848091658859 | 0.999950574162623 |
| ebi-fl187-GCST90094840 | ebi-cfmet338-GCST90026251 | Pipecolate levels \|\| id:ebi-cfmet338-GCST90026251 | F-small fish liking (derived food-liking factor) \|\| id:ebi-fl187-GCST90094840 | MR Egger | 23 | 0.106417499019433 | 0.430795404862886 | 0.807287333591108 | -0.737941495 | 0.95077649255069 | 1.11228615796154 | 0.478097068700556 | 2.58771822332113 | 0.999950574162623 |
| ebi-fl187-GCST90094840 | ebi-cfmet338-GCST90026278 | Trigonelline (n'-methylnicotinate) levels \|\| id:ebi-cfmet338-GCST90026278 | F-small fish liking (derived food-liking factor) \|\| id:ebi-fl187-GCST90094840 | MR Egger | 23 | 0.181566055182048 | 0.474049633568712 | 0.705561411764586 | -0.747571227 | 1.11070333697672 | 1.1990937404491 | 0.473515218413045 | 3.03649332159371 | 0.999950574162623 |
| ebi-fl187-GCST90094840 | ebi-cfmet338-GCST90026299 | 1-carboxyethylvaline levels \|\| id:ebi-cfmet338-GCST90026299 | F-small fish liking (derived food-liking factor) \|\| id:ebi-fl187-GCST90094840 | MR Egger | 23 | -0.272259594 | 0.246787663389761 | 0.282416573357761 | -0.755963414 | 0.211444226191544 | 0.761656513922977 | 0.46955801783905 | 1.23546105733871 | 0.999950574162623 |
| ebi-fl187-GCST90094840 | ebi-gm430-GCST90011306 | C_Erysipelotrichia abundance \|\| id:ebi-gm430-GCST90011306 | F-small fish liking (derived food-liking factor) \|\| id:ebi-fl187-GCST90094840 | MR Egger | 21 | -0.003192919 | 0.261854578227326 | 0.99039837527217 | -0.516427893 | 0.51004205405835 | 0.996812172678681 | 0.59664803677038 | 1.66536122867156 | 0.999950574162623 |
| ebi-fl187-GCST90094840 | ebi-gm430-GCST90011312 | F_Erysipelotrichaceae abundance \|\| id:ebi-gm430-GCST90011312 | F-small fish liking (derived food-liking factor) \|\| id:ebi-fl187-GCST90094840 | MR Egger | 21 | -0.003192919 | 0.261854578227326 | 0.99039837527217 | -0.516427893 | 0.51004205405835 | 0.996812172678681 | 0.59664803677038 | 1.66536122867156 | 0.999950574162623 |
| ebi-fl187-GCST90094840 | ebi-gm430-GCST90011352 | O_Erysipelotrichales abundance \|\| id:ebi-gm430-GCST90011352 | F-small fish liking (derived food-liking factor) \|\| id:ebi-fl187-GCST90094840 | MR Egger | 21 | -0.003192919 | 0.261854578227326 | 0.99039837527217 | -0.516427893 | 0.51004205405835 | 0.996812172678681 | 0.59664803677038 | 1.66536122867156 | 0.999950574162623 |
| ebi-fl187-GCST90094840 | ebi-gm430-GCST90011362 | OTU97_11 (Parabacteroides) abundance \|\| id:ebi-gm430-GCST90011362 | F-small fish liking (derived food-liking factor) \|\| id:ebi-fl187-GCST90094840 | MR Egger | 21 | -0.043535085 | 0.314685280343947 | 0.891423610420855 | -0.660318235 | 0.573248064091664 | 0.9573989627929 | 0.516686880550255 | 1.77401983379326 | 0.999950574162623 |
| ebi-fl187-GCST90094840 | ebi-gm430-GCST90011384 | OTU97_235 (Phascolarctobacterium) abundance \|\| id:ebi-gm430-GCST90011384 | F-small fish liking (derived food-liking factor) \|\| id:ebi-fl187-GCST90094840 | MR Egger | 21 | 0.0657220697199316 | 1.06005715187654 | 0.95121149965005 | -2.011989948 | 2.14343408739795 | 1.06792986586921 | 0.133722309293756 | 8.52867561470298 | 0.999950574162623 |
| ebi-fl187-GCST90094840 | ebi-gm430-GCST90011390 | OTU97_3 (Bacteroides) abundance \|\| id:ebi-gm430-GCST90011390 | F-small fish liking (derived food-liking factor) \|\| id:ebi-fl187-GCST90094840 | MR Egger | 21 | -0.205511895 | 0.243589506771898 | 0.409344387033177 | -0.682947328 | 0.271923538540925 | 0.81423040945705 | 0.50512602418167 | 1.31248664283065 | 0.999950574162623 |
| ebi-fl187-GCST90094840 | ebi-gm430-GCST90011398 | OTU97_4 (Alistipes) abundance \|\| id:ebi-gm430-GCST90011398 | F-small fish liking (derived food-liking factor) \|\| id:ebi-fl187-GCST90094840 | MR Egger | 21 | -0.503831612 | 0.284393365770098 | 0.0924995002738955 | -1.061242609 | 0.0535793851110447 | 0.604211116313562 | 0.34602556868966 | 1.05504074296979 | 0.999950574162623 |
| ebi-fl187-GCST90094840 | ebi-gm430-GCST90011421 | OTU99_108 (Cytophagales) abundance \|\| id:ebi-gm430-GCST90011421 | F-small fish liking (derived food-liking factor) \|\| id:ebi-fl187-GCST90094840 | MR Egger | 21 | 0.911124510626429 | 0.897557666395212 | 0.322804057525772 | -0.848088516 | 2.67033753676104 | 2.48711775180494 | 0.428232710299036 | 14.4448440359069 | 0.999950574162623 |
| ebi-fl187-GCST90094840 | ebi-gm430-GCST90011428 | OTU99_123 (Phascolarctobacterium) abundance \|\| id:ebi-gm430-GCST90011428 | F-small fish liking (derived food-liking factor) \|\| id:ebi-fl187-GCST90094840 | MR Egger | 21 | 0.414649600974562 | 0.541216581032815 | 0.453004891996775 | -0.646134898 | 1.47543409979888 | 1.51384019961632 | 0.5240674414688 | 4.37293365058403 | 0.999950574162623 |
| ebi-fl187-GCST90094840 | ebi-gm430-GCST90011450 | OTU99_298 (Phascolarctobacterium) abundance \|\| id:ebi-gm430-GCST90011450 | F-small fish liking (derived food-liking factor) \|\| id:ebi-fl187-GCST90094840 | MR Egger | 21 | 0.084235701308133 | 1.05731155981319 | 0.93733310766096 | -1.988094956 | 2.15656635854199 | 1.0878852795759 | 0.136956084460374 | 8.6414151381523 | 0.999950574162623 |
| ebi-fl187-GCST90094840 | ebi-gm430-GCST90011451 | OTU99_3 (Bacteroides) abundance \|\| id:ebi-gm430-GCST90011451 | F-small fish liking (derived food-liking factor) \|\| id:ebi-fl187-GCST90094840 | MR Egger | 21 | -0.208021682 | 0.243588946699768 | 0.403751428120985 | -0.685456018 | 0.269412653437342 | 0.81218942655337 | 0.50386040795051 | 1.30919527352481 | 0.999950574162623 |
| ebi-fl187-GCST90094840 | ebi-gm430-GCST90011472 | OTU99_72 (Parabacteroides) abundance \|\| id:ebi-gm430-GCST90011472 | F-small fish liking (derived food-liking factor) \|\| id:ebi-fl187-GCST90094840 | MR Egger | 21 | 0.00526749779939391 | 0.758183780477498 | 0.994529154053489 | -1.480772712 | 1.49130770753529 | 1.00528139545717 | 0.227461857966681 | 4.44290173783929 | 0.999950574162623 |
| ebi-fl187-GCST90094840 | ebi-gm430-GCST90011507 | TestASV_3 (Bacteroides) abundance \|\| id:ebi-gm430-GCST90011507 | F-small fish liking (derived food-liking factor) \|\| id:ebi-fl187-GCST90094840 | MR Egger | 21 | -0.097129996 | 0.313148561846913 | 0.759806487718787 | -0.710901177 | 0.516641184985142 | 0.907438034937669 | 0.491201338371735 | 1.67638750737354 | 0.999950574162623 |
| ebi-fl187-GCST90094840 | ebi-gm430-GCST90011512 | TestASV_34 (Phascolarctobacterium) abundance \|\| id:ebi-gm430-GCST90011512 | F-small fish liking (derived food-liking factor) \|\| id:ebi-fl187-GCST90094840 | MR Egger | 21 | 0.809825573002649 | 0.849658081584093 | 0.352498864437162 | -0.855504267 | 2.47515541290747 | 2.2475159250301 | 0.425068788907464 | 11.8835538272455 | 0.999950574162623 |
| ebi-fl187-GCST90094840 | ebi-gm430-GCST90011519 | TestASV_41 (Barnesiella) abundance \|\| id:ebi-gm430-GCST90011519 | F-small fish liking (derived food-liking factor) \|\| id:ebi-fl187-GCST90094840 | MR Egger | 21 | 1.38924559863889 | 0.985554637514748 | 0.174814390981055 | -0.542441491 | 3.32093268816779 | 4.01182238683089 | 0.58132721325756 | 27.6861610749103 | 0.999950574162623 |
| ebi-fl187-GCST90094840 | ebi-gm430-GCST90011524 | TestASV_46 (Sutterella) abundance \|\| id:ebi-gm430-GCST90011524 | F-small fish liking (derived food-liking factor) \|\| id:ebi-fl187-GCST90094840 | MR Egger | 21 | -1.479902589 | 1.23466701278234 | 0.245410899952708 | -3.899849934 | 0.940044756229804 | 0.227659863918889 | 0.0202449492989113 | 2.56009599600994 | 0.999950574162623 |
| ebi-fl187-GCST90094840 | ebi-gm430-GCST90011581 | OTU97_19 (Bacteria) prevalence \|\| id:ebi-gm430-GCST90011581 | F-small fish liking (derived food-liking factor) \|\| id:ebi-fl187-GCST90094840 | MR Egger | 21 | -0.67287199 | 1.1648420579806 | 0.570282842220529 | -2.955962424 | 1.61021844358476 | 0.510241064189886 | 0.0520285629502429 | 5.00390417922184 | 0.999950574162623 |
| ebi-fl187-GCST90094840 | ebi-gm430-GCST90011614 | OTU97_80 (Ruminococcus) prevalence \|\| id:ebi-gm430-GCST90011614 | F-small fish liking (derived food-liking factor) \|\| id:ebi-fl187-GCST90094840 | MR Egger | 21 | 0.00601195493684969 | 0.539956905746818 | 0.991232506324073 | -1.05230358 | 1.06432749020061 | 1.00603006300805 | 0.349132567153374 | 2.89888879724978 | 0.999950574162623 |
| ebi-fl187-GCST90094840 | ebi-gm430-GCST90011623 | OTU99_11 (Parabacteroides) prevalence \|\| id:ebi-gm430-GCST90011623 | F-small fish liking (derived food-liking factor) \|\| id:ebi-fl187-GCST90094840 | MR Egger | 21 | 0.343736872458335 | 0.471331599989339 | 0.474720105841569 | -0.580073064 | 1.26754680843744 | 1.41020752224619 | 0.559857459913878 | 3.55212781500789 | 0.999950574162623 |
| ebi-fl187-GCST90094840 | ebi-gm430-GCST90011642 | OTU99_19 (Bacteria) prevalence \|\| id:ebi-gm430-GCST90011642 | F-small fish liking (derived food-liking factor) \|\| id:ebi-fl187-GCST90094840 | MR Egger | 21 | -0.67287199 | 1.1648420579806 | 0.570282842220529 | -2.955962424 | 1.61021844358476 | 0.510241064189886 | 0.0520285629502429 | 5.00390417922184 | 0.999950574162623 |
| ebi-fl187-GCST90094840 | ebi-gm430-GCST90011653 | OTU99_30 (Parasutterella) prevalence \|\| id:ebi-gm430-GCST90011653 | F-small fish liking (derived food-liking factor) \|\| id:ebi-fl187-GCST90094840 | MR Egger | 21 | 0.112309891230073 | 0.557392318945866 | 0.84245567193286 | -0.980179054 | 1.20479883636397 | 1.11885953168367 | 0.375243903949936 | 3.33608791098818 | 0.999950574162623 |
| ebi-fl187-GCST90094840 | ebi-gm430-GCST90011675 | OTU99_8 (Ruminococcaceae) prevalence \|\| id:ebi-gm430-GCST90011675 | F-small fish liking (derived food-liking factor) \|\| id:ebi-fl187-GCST90094840 | MR Egger | 21 | 0.1903938209594 | 0.676223198886468 | 0.781328573749405 | -1.135003649 | 1.51579129077688 | 1.20972591928052 | 0.321420948512827 | 4.55302246648896 | 0.999950574162623 |
| ebi-fl187-GCST90094840 | ebi-gm430-GCST90011679 | OTU99_92 (Ruminococcus) prevalence \|\| id:ebi-gm430-GCST90011679 | F-small fish liking (derived food-liking factor) \|\| id:ebi-fl187-GCST90094840 | MR Egger | 21 | -0.208998152 | 0.564592001835572 | 0.715346017792614 | -1.315598476 | 0.897602171669962 | 0.81139673516181 | 0.268313695998089 | 2.45371247033149 | 0.999950574162623 |
| ebi-fl187-GCST90094840 | ebi-gm430-GCST90011684 | TestASV_11 (Lachnospiraceae) prevalence \|\| id:ebi-gm430-GCST90011684 | F-small fish liking (derived food-liking factor) \|\| id:ebi-fl187-GCST90094840 | MR Egger | 21 | -0.338301483 | 0.692534354373687 | 0.630787764222118 | -1.695668818 | 1.01906585119913 | 0.712980303785762 | 0.183476475622939 | 2.77060539701627 | 0.999950574162623 |
| ebi-fl187-GCST90094840 | ebi-gm430-GCST90011688 | TestASV_15 (Bacteroides) prevalence \|\| id:ebi-gm430-GCST90011688 | F-small fish liking (derived food-liking factor) \|\| id:ebi-fl187-GCST90094840 | MR Egger | 21 | 0.685201589621119 | 0.538661074966809 | 0.218706037433768 | -0.370574117 | 1.74097729655606 | 1.98417178366121 | 0.69033788191381 | 5.70291413845497 | 0.999950574162623 |
| ebi-fl187-GCST90094840 | ebi-gm430-GCST90011692 | TestASV_19 (Prevotella) prevalence \|\| id:ebi-gm430-GCST90011692 | F-small fish liking (derived food-liking factor) \|\| id:ebi-fl187-GCST90094840 | MR Egger | 21 | 0.829042812866736 | 1.05720526059645 | 0.442599954328834 | -1.243079498 | 2.90116512363578 | 2.29112465531599 | 0.288494430599246 | 18.1953328363855 | 0.999950574162623 |
| ebi-fl187-GCST90094840 | ebi-gm430-GCST90011693 | TestASV_2 (Bacteroides) prevalence \|\| id:ebi-gm430-GCST90011693 | F-small fish liking (derived food-liking factor) \|\| id:ebi-fl187-GCST90094840 | MR Egger | 21 | 0.558143525470595 | 0.530329471465366 | 0.305796168347501 | -0.481302239 | 1.59758928954271 | 1.74742543636829 | 0.617978112633963 | 4.94110647811171 | 0.999950574162623 |
| ebi-fl187-GCST90094840 | ebi-gm430-GCST90011716 | TestASV_40 (Barnesiella) prevalence \|\| id:ebi-gm430-GCST90011716 | F-small fish liking (derived food-liking factor) \|\| id:ebi-fl187-GCST90094840 | MR Egger | 21 | 0.820224056245627 | 1.05953876666265 | 0.448378394909149 | -1.256471926 | 2.89692003890442 | 2.27100861419687 | 0.284656546220249 | 18.1182558217574 | 0.999950574162623 |
| ebi-fl187-GCST90094840 | ebi-gm430-GCST90011718 | TestASV_42 (Alphaproteobacteria) prevalence \|\| id:ebi-gm430-GCST90011718 | F-small fish liking (derived food-liking factor) \|\| id:ebi-fl187-GCST90094840 | MR Egger | 21 | 0.54266554922273 | 0.843803071560819 | 0.52783319427954 | -1.111188471 | 2.19651956948194 | 1.72058706460814 | 0.32916752244795 | 8.99365716544825 | 0.999950574162623 |
| ebi-fl187-GCST90094840 | ebi-hbsm44-GCST90104929 | Sulcal depth of the right inferior frontal sulcus \|\| id:ebi-hbsm44-GCST90104929 | F-small fish liking (derived food-liking factor) \|\| id:ebi-fl187-GCST90094840 | MR Egger | 24 | 0.365497635942122 | 0.381398959233069 | 0.348321406789455 | -0.382044324 | 1.11303959603894 | 1.44123103808994 | 0.682464802859491 | 3.04359564984256 | 0.999950574162623 |
| ebi-fl187-GCST90094840 | ebi-hbsm44-GCST90104935 | Sulcal opening distance of the left calloso-marginal posterior fissure \|\| id:ebi-hbsm44-GCST90104935 | F-small fish liking (derived food-liking factor) \|\| id:ebi-fl187-GCST90094840 | MR Egger | 27 | -0.154675663 | 0.0958924000846414 | 0.119295335651978 | -0.342624767 | 0.0332734413412042 | 0.856692989813167 | 0.709904541206637 | 1.03383319332985 | 0.999950574162623 |
| ebi-fl187-GCST90094840 | ebi-hbsm44-GCST90104936 | Sulcal opening distance of the left anterior inferior temporal sulcus \|\| id:ebi-hbsm44-GCST90104936 | F-small fish liking (derived food-liking factor) \|\| id:ebi-fl187-GCST90094840 | MR Egger | 26 | -0.010522694 | 0.0461235329553364 | 0.821468791465428 | -0.100924818 | 0.0798794307470879 | 0.989532476016087 | 0.904000994538073 | 1.08315646443605 | 0.999950574162623 |
| ebi-fl187-GCST90094840 | ebi-hbsm44-GCST90104941 | Sulcal opening distance of the right posterior inferior temporal sulcus \|\| id:ebi-hbsm44-GCST90104941 | F-small fish liking (derived food-liking factor) \|\| id:ebi-fl187-GCST90094840 | MR Egger | 26 | -0.036064745 | 0.0498779630031932 | 0.47663137765567 | -0.133825552 | 0.0616960629083593 | 0.964577840276272 | 0.874742648343384 | 1.06363901624561 | 0.999950574162623 |
| ebi-fl187-GCST90094840 | ebi-IPs91-GCST90274819 | Leukemia inhibitory factor levels \|\| id:ebi-IPs91-GCST90274819 | F-small fish liking (derived food-liking factor) \|\| id:ebi-fl187-GCST90094840 | MR Egger | 28 | 0.0463526535405754 | 0.144338176662248 | 0.750672140363684 | -0.236550173 | 0.329255479798582 | 1.04744373057231 | 0.789346277660696 | 1.38993291001096 | 0.999950574162623 |
| ebi-fl187-GCST90094840 | ebi-IPs91-GCST90274837 | STAM binding protein levels \|\| id:ebi-IPs91-GCST90274837 | F-small fish liking (derived food-liking factor) \|\| id:ebi-fl187-GCST90094840 | MR Egger | 27 | -0.054523515 | 0.133402722683952 | 0.686229824033586 | -0.315992852 | 0.206945821148417 | 0.946936241094735 | 0.729064661668935 | 1.22991593454273 | 0.999950574162623 |
| ebi-fl187-GCST90094840 | ebi-IPs91-GCST90274840 | TNF-beta levels \|\| id:ebi-IPs91-GCST90274840 | F-small fish liking (derived food-liking factor) \|\| id:ebi-fl187-GCST90094840 | MR Egger | 28 | -0.164818467 | 0.171067809713701 | 0.344188499415006 | -0.500111374 | 0.170474440285014 | 0.848047638956144 | 0.606463111854278 | 1.18586734111521 | 0.999950574162623 |
| ebi-fl187-GCST90094840 | ebi-IPs91-GCST90274842 | Tumor necrosis factor ligand superfamily member 14 levels \|\| id:ebi-IPs91-GCST90274842 | F-small fish liking (derived food-liking factor) \|\| id:ebi-fl187-GCST90094840 | MR Egger | 28 | -0.057741587 | 0.146136292023323 | 0.69597633977602 | -0.344168719 | 0.228685545462873 | 0.943893830452276 | 0.708809328 | 1.25694672425847 | 0.999950574162623 |
| ebi-fl187-GCST90094840 | ebi-met1400-GCST90199628 | 3-methylhistidine levels \|\| id:ebi-met1400-GCST90199628 | F-small fish liking (derived food-liking factor) \|\| id:ebi-fl187-GCST90094840 | MR Egger | 27 | 0.0897071180530448 | 0.201144506269813 | 0.659447942439554 | -0.304536114 | 0.483950350341879 | 1.09385386673515 | 0.73746539475453 | 1.62247108851761 | 0.999950574162623 |
| ebi-fl187-GCST90094840 | ebi-met1400-GCST90199651 | Docosahexaenoate DHA; 22:6n3 levels \|\| id:ebi-met1400-GCST90199651 | F-small fish liking (derived food-liking factor) \|\| id:ebi-fl187-GCST90094840 | MR Egger | 27 | -0.209905078 | 0.218544638819166 | 0.346017874903963 | -0.63825257 | 0.218442414313399 | 0.810661192084552 | 0.528214636234636 | 1.2441373700596 | 0.999950574162623 |
| ebi-fl187-GCST90094840 | ebi-met1400-GCST90199652 | 1-stearoyl-2-oleoyl-GPS (18:0/18:1) levels \|\| id:ebi-met1400-GCST90199652 | F-small fish liking (derived food-liking factor) \|\| id:ebi-fl187-GCST90094840 | MR Egger | 27 | -0.277972069 | 0.191278739083186 | 0.158598098836789 | -0.652878398 | 0.0969342592447628 | 0.757317973595927 | 0.520545281808264 | 1.10178793886887 | 0.999950574162623 |
| ebi-fl187-GCST90094840 | ebi-met1400-GCST90199653 | 1-myristoyl-2-palmitoyl-gpc (14:0/16:0) levels \|\| id:ebi-met1400-GCST90199653 | F-small fish liking (derived food-liking factor) \|\| id:ebi-fl187-GCST90094840 | MR Egger | 26 | 0.0736859937605967 | 0.24325109258133 | 0.76455976529308 | -0.403086148 | 0.550458135220004 | 1.07646873452164 | 0.66825452825396 | 1.73404726404224 | 0.999950574162623 |
| ebi-fl187-GCST90094840 | ebi-met1400-GCST90199667 | Erythritol levels in elite athletes \|\| id:ebi-met1400-GCST90199667 | F-small fish liking (derived food-liking factor) \|\| id:ebi-fl187-GCST90094840 | MR Egger | 27 | -0.334625765 | 0.182185305316748 | 0.0781676991132235 | -0.691708964 | 0.0224574329365426 | 0.715605840653206 | 0.500719625692203 | 1.02271149941259 | 0.999950574162623 |
| ebi-fl187-GCST90094840 | ebi-met1400-GCST90199674 | 3-hydroxymyristate levels \|\| id:ebi-met1400-GCST90199674 | F-small fish liking (derived food-liking factor) \|\| id:ebi-fl187-GCST90094840 | MR Egger | 27 | -0.060273699 | 0.233770083614727 | 0.798646202140676 | -0.518463063 | 0.397915664772895 | 0.941506808738862 | 0.595434991140279 | 1.48871847320239 | 0.999950574162623 |
| ebi-fl187-GCST90094840 | ebi-met1400-GCST90199685 | 2-linoleoylglycerol (18:2) levels \|\| id:ebi-met1400-GCST90199685 | F-small fish liking (derived food-liking factor) \|\| id:ebi-fl187-GCST90094840 | MR Egger | 27 | 0.129474597941217 | 0.224874206801798 | 0.569921419865897 | -0.311278847 | 0.57022804327274 | 1.13823019770516 | 0.732509589000959 | 1.76867033882096 | 0.999950574162623 |
| ebi-fl187-GCST90094840 | ebi-met1400-GCST90199703 | Gamma-glutamylvaline levels \|\| id:ebi-met1400-GCST90199703 | F-small fish liking (derived food-liking factor) \|\| id:ebi-fl187-GCST90094840 | MR Egger | 27 | -0.076285936 | 0.211570141376926 | 0.721449381174787 | -0.490963414 | 0.338391540622921 | 0.926551233883997 | 0.612036465818211 | 1.40268960586239 | 0.999950574162623 |
| ebi-fl187-GCST90094840 | ebi-met1400-GCST90199779 | Gamma-glutamyl-2-aminobutyrate levels \|\| id:ebi-met1400-GCST90199779 | F-small fish liking (derived food-liking factor) \|\| id:ebi-fl187-GCST90094840 | MR Egger | 27 | 0.117270128459906 | 0.227858978866911 | 0.611310303019837 | -0.32933347 | 0.563873727039051 | 1.12442312735767 | 0.71940307731301 | 1.75746727976075 | 0.999950574162623 |
| ebi-fl187-GCST90094840 | ebi-met1400-GCST90199824 | 4-vinylphenol sulfate levels \|\| id:ebi-met1400-GCST90199824 | F-small fish liking (derived food-liking factor) \|\| id:ebi-fl187-GCST90094840 | MR Egger | 27 | 0.129821163337052 | 0.189018325952843 | 0.498519389973861 | -0.240654756 | 0.500297082204624 | 1.13862473726711 | 0.786112980702837 | 1.64921114921353 | 0.999950574162623 |
| ebi-fl187-GCST90094840 | ebi-met1400-GCST90199830 | 4-ethylphenylsulfate levels \|\| id:ebi-met1400-GCST90199830 | F-small fish liking (derived food-liking factor) \|\| id:ebi-fl187-GCST90094840 | MR Egger | 27 | -0.192172756 | 0.190845311486673 | 0.32360349361524 | -0.566229567 | 0.181884054035538 | 0.825164303698382 | 0.567661739338739 | 1.19947511151835 | 0.999950574162623 |
| ebi-fl187-GCST90094840 | ebi-met1400-GCST90199833 | Hexanoylglutamine levels \|\| id:ebi-met1400-GCST90199833 | F-small fish liking (derived food-liking factor) \|\| id:ebi-fl187-GCST90094840 | MR Egger | 27 | 0.0252801790515825 | 0.215540259411689 | 0.907569430810812 | -0.397178729 | 0.447739087498494 | 1.025602433 | 0.672213870516743 | 1.56477037423513 | 0.999950574162623 |
| ebi-fl187-GCST90094840 | ebi-met1400-GCST90199837 | Carotenoid levels (cryptoxanthin) \|\| id:ebi-met1400-GCST90199837 | F-small fish liking (derived food-liking factor) \|\| id:ebi-fl187-GCST90094840 | MR Egger | 27 | -0.372051972 | 0.189677412943416 | 0.0610482066022491 | -0.743819701 | -0.000284242 | 0.68931841664303 | 0.475294959202987 | 0.999715798206739 | 0.999950574162623 |
| ebi-fl187-GCST90094840 | ebi-met1400-GCST90199869 | 2-hydroxyhippurate levels \|\| id:ebi-met1400-GCST90199869 | F-small fish liking (derived food-liking factor) \|\| id:ebi-fl187-GCST90094840 | MR Egger | 27 | 0.346548836004886 | 0.20188208421242 | 0.0984213353753614 | -0.049140049 | 0.742237721061228 | 1.41417855497483 | 0.952047786971955 | 2.10063088504361 | 0.999950574162623 |
| ebi-fl187-GCST90094840 | ebi-met1400-GCST90199902 | Hydantoin-5-propionate levels \|\| id:ebi-met1400-GCST90199902 | F-small fish liking (derived food-liking factor) \|\| id:ebi-fl187-GCST90094840 | MR Egger | 27 | -0.064302121 | 0.188648299174673 | 0.736059089262188 | -0.434052787 | 0.305448545598095 | 0.937721651529442 | 0.647878054935845 | 1.35723364767183 | 0.999950574162623 |
| ebi-fl187-GCST90094840 | ebi-met1400-GCST90199904 | Pantoate levels \|\| id:ebi-met1400-GCST90199904 | F-small fish liking (derived food-liking factor) \|\| id:ebi-fl187-GCST90094840 | MR Egger | 27 | -0.291278095 | 0.191312176085341 | 0.14042683427547 | -0.66624996 | 0.0836937697917574 | 0.74730782629745 | 0.513631107713384 | 1.08729587997823 | 0.999950574162623 |
| ebi-fl187-GCST90094840 | ebi-met1400-GCST90199936 | 3-methyl catechol sulfate (2) levels \|\| id:ebi-met1400-GCST90199936 | F-small fish liking (derived food-liking factor) \|\| id:ebi-fl187-GCST90094840 | MR Egger | 27 | -0.183966852 | 0.214673486880492 | 0.399606768935559 | -0.604726886 | 0.236793182290564 | 0.831963381295757 | 0.546223587425241 | 1.26717901561108 | 0.999950574162623 |
| ebi-fl187-GCST90094840 | ebi-met1400-GCST90199977 | 4-hydroxychlorothalonil levels \|\| id:ebi-met1400-GCST90199977 | F-small fish liking (derived food-liking factor) \|\| id:ebi-fl187-GCST90094840 | MR Egger | 27 | -0.033666654 | 0.221385965964721 | 0.880350593986342 | -0.467583148 | 0.400249838917699 | 0.966893760729957 | 0.626514633320122 | 1.49219746007247 | 0.999950574162623 |
| ebi-fl187-GCST90094840 | ebi-met1400-GCST90200040 | Sphingomyelin (d18:2/24:1, d18:1/24:2) levels \|\| id:ebi-met1400-GCST90200040 | F-small fish liking (derived food-liking factor) \|\| id:ebi-fl187-GCST90094840 | MR Egger | 27 | 0.104640222915394 | 0.202496350333991 | 0.609871390750459 | -0.292252624 | 0.501533069570016 | 1.11031107400679 | 0.746579908340374 | 1.65125081359686 | 0.999950574162623 |
| ebi-fl187-GCST90094840 | ebi-met1400-GCST90200077 | 1-stearoyl-2-oleoyl-GPI (18:0/18:1) levels \|\| id:ebi-met1400-GCST90200077 | F-small fish liking (derived food-liking factor) \|\| id:ebi-fl187-GCST90094840 | MR Egger | 27 | -0.072437876 | 0.203448762211012 | 0.724790441668885 | -0.47119745 | 0.326321698181928 | 0.930123528090495 | 0.624254307425392 | 1.38586112617398 | 0.999950574162623 |
| ebi-fl187-GCST90094840 | ebi-met1400-GCST90200080 | 1-palmitoyl-2-oleoyl-GPI (16:0/18:1) levels \|\| id:ebi-met1400-GCST90200080 | F-small fish liking (derived food-liking factor) \|\| id:ebi-fl187-GCST90094840 | MR Egger | 27 | 0.0403241827530311 | 0.194977009785926 | 0.837829520816092 | -0.341830756 | 0.422479121933446 | 1.04114824179218 | 0.710468436751107 | 1.52573936478293 | 0.999950574162623 |
| ebi-fl187-GCST90094840 | ebi-met1400-GCST90200082 | 1-oleoyl-2-linoleoyl-GPE (18:1/18:2) levels \|\| id:ebi-met1400-GCST90200082 | F-small fish liking (derived food-liking factor) \|\| id:ebi-fl187-GCST90094840 | MR Egger | 27 | -0.019699946 | 0.235681355994294 | 0.934050360440811 | -0.481635404 | 0.442235511777373 | 0.98049282999671 | 0.617772258176357 | 1.55618219651507 | 0.999950574162623 |
| ebi-fl187-GCST90094840 | ebi-met1400-GCST90200100 | Perfluorooctanesulfonate (PFOS) levels \|\| id:ebi-met1400-GCST90200100 | F-small fish liking (derived food-liking factor) \|\| id:ebi-fl187-GCST90094840 | MR Egger | 27 | -0.139721745 | 0.219250511779191 | 0.529742447648254 | -0.569452748 | 0.290009258281631 | 0.869600172502182 | 0.565835008386148 | 1.33643986110481 | 0.999950574162623 |
| ebi-fl187-GCST90094840 | ebi-met1400-GCST90200102 | 1-palmitoleoyl-2-linolenoyl-GPC (16:1/18:3) levels \|\| id:ebi-met1400-GCST90200102 | F-small fish liking (derived food-liking factor) \|\| id:ebi-fl187-GCST90094840 | MR Egger | 27 | -0.029467472 | 0.185054036007485 | 0.874761533967568 | -0.392173382 | 0.333238438936708 | 0.970962460945968 | 0.675586969041977 | 1.3954799955706 | 0.999950574162623 |
| ebi-fl187-GCST90094840 | ebi-met1400-GCST90200114 | Glycosyl-N-tricosanoyl-sphingadienine (d18:2/23:0) levels \|\| id:ebi-met1400-GCST90200114 | F-small fish liking (derived food-liking factor) \|\| id:ebi-fl187-GCST90094840 | MR Egger | 27 | -0.056181726 | 0.213975604203669 | 0.79504027041664 | -0.475573911 | 0.363210457741728 | 0.94536732198706 | 0.621528252350801 | 1.43793845267802 | 0.999950574162623 |
| ebi-fl187-GCST90094840 | ebi-met1400-GCST90200135 | Arachidoylcarnitine (C20) levels \|\| id:ebi-met1400-GCST90200135 | F-small fish liking (derived food-liking factor) \|\| id:ebi-fl187-GCST90094840 | MR Egger | 27 | 0.102595346913354 | 0.259225845076881 | 0.695627749265356 | -0.405487309 | 0.61067800326404 | 1.10804294534804 | 0.666651865944853 | 1.84167964038538 | 0.999950574162623 |
| ebi-fl187-GCST90094840 | ebi-met1400-GCST90200146 | Docosahexaenoylcarnitine (C22:6) levels \|\| id:ebi-met1400-GCST90200146 | F-small fish liking (derived food-liking factor) \|\| id:ebi-fl187-GCST90094840 | MR Egger | 27 | -0.110066979 | 0.210632708021947 | 0.6058849304132 | -0.522907087 | 0.302773128551456 | 0.895774135077695 | 0.592794734836431 | 1.35360733474733 | 0.999950574162623 |
| ebi-fl187-GCST90094840 | ebi-met1400-GCST90200162 | 3-hydroxyphenylacetoylglutamine levels \|\| id:ebi-met1400-GCST90200162 | F-small fish liking (derived food-liking factor) \|\| id:ebi-fl187-GCST90094840 | MR Egger | 27 | 0.00330134107090956 | 0.231018313248382 | 0.988711743057523 | -0.449494553 | 0.456097235037738 | 1.0033067964991 | 0.637950520387808 | 1.57790376483957 | 0.999950574162623 |
| ebi-fl187-GCST90094840 | ebi-met1400-GCST90200172 | 3-carboxy-4-methyl-5-pentyl-2-furanpropionate (3-CMPFP) levels \|\| id:ebi-met1400-GCST90200172 | F-small fish liking (derived food-liking factor) \|\| id:ebi-fl187-GCST90094840 | MR Egger | 27 | -0.087260257 | 0.187520805627993 | 0.645718743486805 | -0.454801036 | 0.280280522394064 | 0.916438555377398 | 0.634574213023288 | 1.32350103194532 | 0.999950574162623 |
| ebi-fl187-GCST90094840 | ebi-met1400-GCST90200222 | Dihydrocaffeate sulfate (2) levels \|\| id:ebi-met1400-GCST90200222 | F-small fish liking (derived food-liking factor) \|\| id:ebi-fl187-GCST90094840 | MR Egger | 27 | -0.12474953 | 0.250394702472062 | 0.622689723352722 | -0.615523147 | 0.366024087135908 | 0.882717969524493 | 0.540358134856701 | 1.44198997564473 | 0.999950574162623 |
| ebi-fl187-GCST90094840 | ebi-met1400-GCST90200223 | 4-methylhexanoylglutamine levels \|\| id:ebi-met1400-GCST90200223 | F-small fish liking (derived food-liking factor) \|\| id:ebi-fl187-GCST90094840 | MR Egger | 27 | -0.105047442 | 0.271846056982285 | 0.702453145298301 | -0.637865714 | 0.42777082968221 | 0.900281810400686 | 0.528419018856213 | 1.53383453133976 | 0.999950574162623 |
| ebi-fl187-GCST90094840 | ebi-met1400-GCST90200224 | 2-ketocaprylate levels \|\| id:ebi-met1400-GCST90200224 | F-small fish liking (derived food-liking factor) \|\| id:ebi-fl187-GCST90094840 | MR Egger | 27 | -0.125592763 | 0.191079022894236 | 0.517005925215994 | -0.500107648 | 0.248922121585393 | 0.881973945829674 | 0.606465371317275 | 1.28264213904377 | 0.999950574162623 |
| ebi-fl187-GCST90094840 | ebi-met1400-GCST90200243 | Hydroxypalmitoyl sphingomyelin (d18:1/16:0(OH)) levels \|\| id:ebi-met1400-GCST90200243 | F-small fish liking (derived food-liking factor) \|\| id:ebi-fl187-GCST90094840 | MR Egger | 27 | -0.007481918 | 0.184643650094897 | 0.967999758412572 | -0.369383472 | 0.354419636149251 | 0.99254600183721 | 0.691160318842182 | 1.42535319072329 | 0.999950574162623 |
| ebi-fl187-GCST90094840 | ebi-met1400-GCST90200273 | 2-methoxyhydroquinone sulfate (2) levels \|\| id:ebi-met1400-GCST90200273 | F-small fish liking (derived food-liking factor) \|\| id:ebi-fl187-GCST90094840 | MR Egger | 27 | -0.351440698 | 0.271432866899516 | 0.207233798283344 | -0.883449117 | 0.180567721017423 | 0.703673577900901 | 0.413354741174331 | 1.19789724155365 | 0.999950574162623 |
| ebi-fl187-GCST90094840 | ebi-met1400-GCST90200276 | 2,4-di-tert-butylphenol levels \|\| id:ebi-met1400-GCST90200276 | F-small fish liking (derived food-liking factor) \|\| id:ebi-fl187-GCST90094840 | MR Egger | 27 | -0.30114075 | 0.187879173548485 | 0.121530499411799 | -0.66938393 | 0.067102430089819 | 0.739973614082273 | 0.512023923104745 | 1.06940501181615 | 0.999950574162623 |
| ebi-fl187-GCST90094840 | ebi-met1400-GCST90200289 | N-acetylmethionine levels \|\| id:ebi-met1400-GCST90200289 | F-small fish liking (derived food-liking factor) \|\| id:ebi-fl187-GCST90094840 | MR Egger | 27 | 0.122352443118918 | 0.188266780452996 | 0.521691600280866 | -0.246650447 | 0.491355332806791 | 1.13015234601466 | 0.781413791669345 | 1.63453005157977 | 0.999950574162623 |
| ebi-fl187-GCST90094840 | ebi-met1400-GCST90200308 | 3-hydroxyisobutyrate levels \|\| id:ebi-met1400-GCST90200308 | F-small fish liking (derived food-liking factor) \|\| id:ebi-fl187-GCST90094840 | MR Egger | 27 | -0.148009687 | 0.194903142424782 | 0.454714093906155 | -0.530019846 | 0.234000472471876 | 0.862422760887673 | 0.588593288438177 | 1.2636450892444 | 0.999950574162623 |
| ebi-fl187-GCST90094840 | ebi-met1400-GCST90200383 | Cystathionine levels \|\| id:ebi-met1400-GCST90200383 | F-small fish liking (derived food-liking factor) \|\| id:ebi-fl187-GCST90094840 | MR Egger | 27 | 0.167818559657723 | 0.212897427 | 0.437955587110471 | -0.249460396 | 0.585097515783923 | 1.1827219976946 | 0.77922114012718 | 1.79516603412786 | 0.999950574162623 |
| ebi-fl187-GCST90094840 | ebi-met1400-GCST90200384 | Guanidinoacetate levels \|\| id:ebi-met1400-GCST90200384 | F-small fish liking (derived food-liking factor) \|\| id:ebi-fl187-GCST90094840 | MR Egger | 27 | -0.026395668 | 0.1808644784088 | 0.885137812545865 | -0.380890046 | 0.328098709830499 | 0.97394965279544 | 0.68325301221126 | 1.38832600695079 | 0.999950574162623 |
| ebi-fl187-GCST90094840 | ebi-met1400-GCST90200403 | Citrulline levels \|\| id:ebi-met1400-GCST90200403 | F-small fish liking (derived food-liking factor) \|\| id:ebi-fl187-GCST90094840 | MR Egger | 26 | 0.126728874631374 | 0.189301680177254 | 0.509595258401094 | -0.244302419 | 0.497760167778792 | 1.13510921915189 | 0.783250728919257 | 1.64503254428052 | 0.999950574162623 |
| ebi-fl187-GCST90094840 | ebi-met1400-GCST90200434 | 12,13-DiHOME levels \|\| id:ebi-met1400-GCST90200434 | F-small fish liking (derived food-liking factor) \|\| id:ebi-fl187-GCST90094840 | MR Egger | 27 | 0.116624083548228 | 0.190776854890092 | 0.54651239786575 | -0.257298552 | 0.490546719132808 | 1.12369693411965 | 0.773137357573387 | 1.63320888245922 | 0.999950574162623 |
| ebi-fl187-GCST90094840 | ebi-met1400-GCST90200437 | Fructose levels \|\| id:ebi-met1400-GCST90200437 | F-small fish liking (derived food-liking factor) \|\| id:ebi-fl187-GCST90094840 | MR Egger | 26 | -0.126963757 | 0.190416946143427 | 0.51128072267607 | -0.500180971 | 0.246253457656321 | 0.880765593795651 | 0.60642090504711 | 1.27922376151254 | 0.999950574162623 |
| ebi-fl187-GCST90094840 | ebi-met1400-GCST90200448 | N-stearoyl-sphinganine (d18:0/18:0) levels \|\| id:ebi-met1400-GCST90200448 | F-small fish liking (derived food-liking factor) \|\| id:ebi-fl187-GCST90094840 | MR Egger | 27 | -0.370176924 | 0.264689212313175 | 0.174235983398403 | -0.88896778 | 0.148613932293001 | 0.690612134076652 | 0.411079858691529 | 1.16022497733659 | 0.999950574162623 |
| ebi-fl187-GCST90094840 | ebi-met1400-GCST90200468 | X-11847 levels \|\| id:ebi-met1400-GCST90200468 | F-small fish liking (derived food-liking factor) \|\| id:ebi-fl187-GCST90094840 | MR Egger | 27 | -0.349104717 | 0.258318165926718 | 0.188653852410523 | -0.855408322 | 0.157198888648916 | 0.705319267790029 | 0.425109574139265 | 1.17022833588991 | 0.999950574162623 |
| ebi-fl187-GCST90094840 | ebi-met1400-GCST90200476 | X-11843 levels \|\| id:ebi-met1400-GCST90200476 | F-small fish liking (derived food-liking factor) \|\| id:ebi-fl187-GCST90094840 | MR Egger | 27 | -0.066061205 | 0.210996411750665 | 0.756808169296791 | -0.479614172 | 0.34749176195718 | 0.936073570086046 | 0.619022181762349 | 1.41551264951283 | 0.999950574162623 |
| ebi-fl187-GCST90094840 | ebi-met1400-GCST90200511 | X-12906 levels \|\| id:ebi-met1400-GCST90200511 | F-small fish liking (derived food-liking factor) \|\| id:ebi-fl187-GCST90094840 | MR Egger | 27 | -0.168560015 | 0.189163999359306 | 0.381382973628328 | -0.539321454 | 0.20220142384122 | 0.844880556472706 | 0.583143808261529 | 1.2240945451066 | 0.999950574162623 |
| ebi-fl187-GCST90094840 | ebi-met1400-GCST90200515 | X-12849 levels \|\| id:ebi-met1400-GCST90200515 | F-small fish liking (derived food-liking factor) \|\| id:ebi-fl187-GCST90094840 | MR Egger | 27 | -0.021434342 | 0.19883208459717 | 0.915013886036112 | -0.411145227 | 0.368276544184874 | 0.978793741365343 | 0.66289065469972 | 1.44524165689128 | 0.999950574162623 |
| ebi-fl187-GCST90094840 | ebi-met1400-GCST90200551 | X-18886 levels \|\| id:ebi-met1400-GCST90200551 | F-small fish liking (derived food-liking factor) \|\| id:ebi-fl187-GCST90094840 | MR Egger | 27 | -0.024294433 | 0.189724433178498 | 0.899133004966297 | -0.396154322 | 0.347565456138282 | 0.97599830144669 | 0.672902844264436 | 1.41561696840218 | 0.999950574162623 |
| ebi-fl187-GCST90094840 | ebi-met1400-GCST90200564 | X-21319 levels \|\| id:ebi-met1400-GCST90200564 | F-small fish liking (derived food-liking factor) \|\| id:ebi-fl187-GCST90094840 | MR Egger | 27 | 0.0989162420345366 | 0.190861660195664 | 0.608832660912812 | -0.275172612 | 0.473005096018037 | 1.10397382908452 | 0.759441023315431 | 1.60480956109389 | 0.999950574162623 |
| ebi-fl187-GCST90094840 | ebi-met1400-GCST90200579 | X-21442 levels \|\| id:ebi-met1400-GCST90200579 | F-small fish liking (derived food-liking factor) \|\| id:ebi-fl187-GCST90094840 | MR Egger | 27 | 0.135124969417279 | 0.234827152538785 | 0.570148718811013 | -0.32513625 | 0.595386188393299 | 1.144679825 | 0.722428916315134 | 1.81373125168425 | 0.999950574162623 |
| ebi-fl187-GCST90094840 | ebi-met1400-GCST90200581 | X-21441 levels \|\| id:ebi-met1400-GCST90200581 | F-small fish liking (derived food-liking factor) \|\| id:ebi-fl187-GCST90094840 | MR Egger | 27 | -0.119240482 | 0.244141194372812 | 0.629517723904889 | -0.597757223 | 0.359276258864998 | 0.887594324542189 | 0.550043879473836 | 1.43229243040232 | 0.999950574162623 |
| ebi-fl187-GCST90094840 | ebi-met1400-GCST90200648 | X-24585 levels \|\| id:ebi-met1400-GCST90200648 | F-small fish liking (derived food-liking factor) \|\| id:ebi-fl187-GCST90094840 | MR Egger | 27 | -0.059669327 | 0.220255855847435 | 0.78868515056761 | -0.491370804 | 0.372032150536747 | 0.942076001253372 | 0.611787178568418 | 1.45067962067187 | 0.999950574162623 |
| ebi-fl187-GCST90094840 | ebi-met1400-GCST90200720 | Adenosine 5'-diphosphate (ADP) to pantothenate ratio \|\| id:ebi-met1400-GCST90200720 | F-small fish liking (derived food-liking factor) \|\| id:ebi-fl187-GCST90094840 | MR Egger | 27 | -0.02467169 | 0.251198020366727 | 0.922543946735949 | -0.51701981 | 0.467676430256357 | 0.975630168923735 | 0.596294975163183 | 1.59628081094205 | 0.999950574162623 |
| ebi-fl187-GCST90094840 | ebi-met1400-GCST90200724 | Cholate to taurocholate ratio \|\| id:ebi-met1400-GCST90200724 | F-small fish liking (derived food-liking factor) \|\| id:ebi-fl187-GCST90094840 | MR Egger | 27 | -0.152089782 | 0.225501564903619 | 0.50621405597001 | -0.594072849 | 0.289893284927598 | 0.85891116227377 | 0.552074184516635 | 1.33628487867875 | 0.999950574162623 |
| ebi-fl187-GCST90094840 | ebi-met1400-GCST90200726 | N-acetylputrescine to (N(1) + N(8))-acetylspermidine ratio \|\| id:ebi-met1400-GCST90200726 | F-small fish liking (derived food-liking factor) \|\| id:ebi-fl187-GCST90094840 | MR Egger | 27 | -0.084507639 | 0.211232626495235 | 0.692503631711413 | -0.498523587 | 0.329508308898299 | 0.91896463534943 | 0.607426810867799 | 1.39028436992503 | 0.999950574162623 |
| ebi-fl187-GCST90094840 | ebi-met1400-GCST90200744 | Arginine to glutamate ratio \|\| id:ebi-met1400-GCST90200744 | F-small fish liking (derived food-liking factor) \|\| id:ebi-fl187-GCST90094840 | MR Egger | 27 | 0.124064636837724 | 0.206032861247894 | 0.55249080372398 | -0.279759771 | 0.527889044883596 | 1.13208904329012 | 0.755965324280674 | 1.69534972143999 | 0.999950574162623 |
| ebi-fl187-GCST90094840 | ebi-met1400-GCST90200759 | Histidine to trans-urocanate ratio \|\| id:ebi-met1400-GCST90200759 | F-small fish liking (derived food-liking factor) \|\| id:ebi-fl187-GCST90094840 | MR Egger | 27 | 0.0913975361763517 | 0.201903074954582 | 0.654683039793714 | -0.304332491 | 0.487127563087333 | 1.09570450086779 | 0.737615575329823 | 1.62763422218287 | 0.999950574162623 |
| ebi-fl187-GCST90094840 | ebi-met1400-GCST90200795 | Oleoyl-linoleoyl-glycerol (18:1 to 18:2) [2] to linoleoyl-arachidonoyl-glycerol (18:2 to 20:4) [2] ratio \|\| id:ebi-met1400-GCST90200795 | F-small fish liking (derived food-liking factor) \|\| id:ebi-fl187-GCST90094840 | MR Egger | 27 | 0.258967258378362 | 0.196020602756924 | 0.198425556849995 | -0.125233123 | 0.643167639781933 | 1.29559138437069 | 0.882291196215304 | 1.90249777222751 | 0.999950574162623 |
| ebi-fl187-GCST90094840 | ebi-met1400-GCST90200811 | Choline to taurocholate ratio \|\| id:ebi-met1400-GCST90200811 | F-small fish liking (derived food-liking factor) \|\| id:ebi-fl187-GCST90094840 | MR Egger | 27 | 0.112400969787351 | 0.1972375636201 | 0.573847704058293 | -0.274184655 | 0.498986594482748 | 1.11896144043639 | 0.76019168917333 | 1.64705129379282 | 0.999950574162623 |
| ebi-fl187-GCST90094840 | ebi-met1400-GCST90200831 | Cholate to phosphate ratio \|\| id:ebi-met1400-GCST90200831 | F-small fish liking (derived food-liking factor) \|\| id:ebi-fl187-GCST90094840 | MR Egger | 27 | -0.194013183 | 0.236422029563608 | 0.419617818280088 | -0.657400361 | 0.269373994971338 | 0.82364704607996 | 0.518196709407346 | 1.30914466302211 | 0.999950574162623 |
| ebi-fl187-GCST90094840 | ebi-met1400-GCST90200841 | Adenosine 5'-diphosphate (ADP) to oxalate (ethanedioate) ratio \|\| id:ebi-met1400-GCST90200841 | F-small fish liking (derived food-liking factor) \|\| id:ebi-fl187-GCST90094840 | MR Egger | 27 | 0.0206877548576889 | 0.28334257107893 | 0.942376626878409 | -0.534663684 | 0.576039194172392 | 1.02090322979056 | 0.585866292955816 | 1.77897827051709 | 0.999950574162623 |
| ebi-fl187-GCST90094840 | ebi-met1400-GCST90200844 | Aspartate to phosphate ratio \|\| id:ebi-met1400-GCST90200844 | F-small fish liking (derived food-liking factor) \|\| id:ebi-fl187-GCST90094840 | MR Egger | 27 | -0.211248333 | 0.17757238567702 | 0.245361424654218 | -0.559290209 | 0.136793542997357 | 0.809572998281943 | 0.571614646905991 | 1.14659140225811 | 0.999950574162623 |
| ebi-fl187-GCST90094840 | ebi-met1400-GCST90200870 | Adenosine 5'-monophosphate (AMP) to acetoacetate ratio \|\| id:ebi-met1400-GCST90200870 | F-small fish liking (derived food-liking factor) \|\| id:ebi-fl187-GCST90094840 | MR Egger | 27 | -0.029024033 | 0.207709009664295 | 0.889989740946482 | -0.436133692 | 0.37808562572605 | 0.971393118485459 | 0.6465312839947 | 1.45948790723736 | 0.999950574162623 |
| ebi-fl187-GCST90094840 | ebi-met1400-GCST90200887 | Cholesterol to cortisol ratio \|\| id:ebi-met1400-GCST90200887 | F-small fish liking (derived food-liking factor) \|\| id:ebi-fl187-GCST90094840 | MR Egger | 27 | 0.0559907727715278 | 0.183555825469846 | 0.762865359216822 | -0.303778645 | 0.415760190692426 | 1.05758792508617 | 0.738024213610646 | 1.51552238891467 | 0.999950574162623 |
| ebi-fl187-GCST90094840 | ebi-met1400-GCST90200890 | Cortisol to taurocholate ratio \|\| id:ebi-met1400-GCST90200890 | F-small fish liking (derived food-liking factor) \|\| id:ebi-fl187-GCST90094840 | MR Egger | 27 | 0.0670980853570598 | 0.196735188169337 | 0.735909803614705 | -0.318502883 | 0.452699054168961 | 1.06940036554725 | 0.727236980997563 | 1.57255086266911 | 0.999950574162623 |
| ebi-fl187-GCST90094840 | ebi-met1400-GCST90200948 | Adenosine 5'-diphosphate (ADP) to mannitol to sorbitol ratio \|\| id:ebi-met1400-GCST90200948 | F-small fish liking (derived food-liking factor) \|\| id:ebi-fl187-GCST90094840 | MR Egger | 27 | 0.130374724401258 | 0.26145789094376 | 0.622388470560101 | -0.382082742 | 0.642832190651028 | 1.1392552100749 | 0.682438584639276 | 1.90185968803164 | 0.999950574162623 |
| ebi-fl187-GCST90094840 | ebi-met1400-GCST90200972 | Leucine to N-palmitoyl-sphingosine (d18:1 to 16:0) ratio \|\| id:ebi-met1400-GCST90200972 | F-small fish liking (derived food-liking factor) \|\| id:ebi-fl187-GCST90094840 | MR Egger | 27 | -0.09724044 | 0.196552018770623 | 0.625109707502082 | -0.482482397 | 0.288001516515964 | 0.90733781934957 | 0.617249230716849 | 1.33375932678916 | 0.999950574162623 |
| ebi-fl187-GCST90094840 | ebi-met1400-GCST90200973 | Proline to glutamate ratio \|\| id:ebi-met1400-GCST90200973 | F-small fish liking (derived food-liking factor) \|\| id:ebi-fl187-GCST90094840 | MR Egger | 27 | 0.127629539829398 | 0.192528292131155 | 0.513452412810465 | -0.249725913 | 0.504984992406461 | 1.13613203305884 | 0.779014271694072 | 1.65696065328226 | 0.999950574162623 |
| ebi-fl187-GCST90094840 | ebi-met1400-GCST90200983 | Cholesterol to linoleoyl-arachidonoyl-glycerol (18:2 to 20:4) [1] ratio \|\| id:ebi-met1400-GCST90200983 | F-small fish liking (derived food-liking factor) \|\| id:ebi-fl187-GCST90094840 | MR Egger | 27 | -0.133652661 | 0.228632929404129 | 0.564073594816858 | -0.581773203 | 0.314467880297907 | 0.874893896312703 | 0.558906432830821 | 1.36953036294166 | 0.999950574162623 |
| ebi-fl187-GCST90094840 | ebi-met1400-GCST90201001 | Serine to alpha-ketobutyrate ratio \|\| id:ebi-met1400-GCST90201001 | F-small fish liking (derived food-liking factor) \|\| id:ebi-fl187-GCST90094840 | MR Egger | 27 | 0.0741662169287407 | 0.199835739849924 | 0.713662948425767 | -0.317511833 | 0.465844267034592 | 1.07698580389215 | 0.727958066666614 | 1.59335884152846 | 0.999950574162623 |
| ebi-fl187-GCST90094840 | ebi-met1400-GCST90201005 | Fructose to maltose ratio \|\| id:ebi-met1400-GCST90201005 | F-small fish liking (derived food-liking factor) \|\| id:ebi-fl187-GCST90094840 | MR Egger | 27 | -0.082619313 | 0.254973627697683 | 0.748610112387737 | -0.582367623 | 0.417128997262876 | 0.920701579612974 | 0.558574306196964 | 1.51759826633149 | 0.999950574162623 |
| ebi-fl187-GCST90094840 | ebi-met1400-GCST90201020 | Paraxanthine to 5-acetylamino-6-formylamino-3-methyluracil ratio \|\| id:ebi-met1400-GCST90201020 | F-small fish liking (derived food-liking factor) \|\| id:ebi-fl187-GCST90094840 | MR Egger | 27 | 0.0686445081431486 | 0.212409845362537 | 0.749254083473243 | -0.347678789 | 0.484967805053721 | 1.07105538999392 | 0.706325719535095 | 1.6241227194588 | 0.999950574162623 |
| ebi-fl187-GCST90094840 | ebi-pw11-GCST90275197 | Placental weight (fetal genotype, adjusted for sex) \|\| id:ebi-pw11-GCST90275197 | F-small fish liking (derived food-liking factor) \|\| id:ebi-fl187-GCST90094840 | MR Egger | 23 | 0.120000440853475 | 0.0716877140658251 | 0.108974717813083 | -0.020507479 | 0.260508360422492 | 1.12749734864039 | 0.979701369539261 | 1.297589562 | 0.999950574162623 |
| ebi-fl187-GCST90094840 | prot-a-1017 | FAS-associated factor 2 \|\| id:prot-a-1017 | F-small fish liking (derived food-liking factor) \|\| id:ebi-fl187-GCST90094840 | MR Egger | 28 | 0.119704648283448 | 0.269661965415459 | 0.660782877397921 | -0.408832804 | 0.648242100497748 | 1.12716389262128 | 0.664425312336353 | 1.91217645872284 | 0.999950574162623 |
| ebi-fl187-GCST90094840 | prot-a-1019 | Fas apoptotic inhibitory molecule 3 \|\| id:prot-a-1019 | F-small fish liking (derived food-liking factor) \|\| id:ebi-fl187-GCST90094840 | MR Egger | 28 | -0.102549443 | 0.266791129396941 | 0.703822037414935 | -0.625460056 | 0.42036117102235 | 0.90253352504961 | 0.535015234808683 | 1.52251134330737 | 0.999950574162623 |
| ebi-fl187-GCST90094840 | prot-a-1052 | MIP18 family protein FAM96A \|\| id:prot-a-1052 | F-small fish liking (derived food-liking factor) \|\| id:ebi-fl187-GCST90094840 | MR Egger | 28 | -0.062763188 | 0.266751375468785 | 0.815829173214982 | -0.585595884 | 0.460069508124469 | 0.939165853294181 | 0.556773990390817 | 1.58418409483292 | 0.999950574162623 |
| ebi-fl187-GCST90094840 | prot-a-1066 | F-box/LRR-repeat protein 4 \|\| id:prot-a-1066 | F-small fish liking (derived food-liking factor) \|\| id:ebi-fl187-GCST90094840 | MR Egger | 28 | 0.26909950490609 | 0.266526609891022 | 0.321965862367969 | -0.25329265 | 0.791491660292493 | 1.30878536513224 | 0.776240681371958 | 2.20668559776699 | 0.999950574162623 |
| ebi-fl187-GCST90094840 | prot-a-1076 | Low affinity immunoglobulin gamma Fc region receptor III-B \|\| id:prot-a-1076 | F-small fish liking (derived food-liking factor) \|\| id:ebi-fl187-GCST90094840 | MR Egger | 28 | 0.347245110083087 | 0.318079242002908 | 0.2849811568747 | -0.276190204 | 0.970680424408787 | 1.41516355371936 | 0.758668615047626 | 2.63973999194615 | 0.999950574162623 |
| ebi-fl187-GCST90094840 | prot-a-1081 | Fc receptor-like protein 3 \|\| id:prot-a-1081 | F-small fish liking (derived food-liking factor) \|\| id:ebi-fl187-GCST90094840 | MR Egger | 28 | -0.397944382 | 0.266751317996029 | 0.14778186438145 | -0.920776965 | 0.124888201363882 | 0.671699385261102 | 0.398209525922627 | 1.13302177569656 | 0.999950574162623 |
| ebi-fl187-GCST90094840 | prot-a-1105 | Fibroblast growth factor receptor 4 \|\| id:prot-a-1105 | F-small fish liking (derived food-liking factor) \|\| id:ebi-fl187-GCST90094840 | MR Egger | 28 | -0.020629705 | 0.296775901743264 | 0.945113379163585 | -0.602310472 | 0.561051062789838 | 0.979581631964118 | 0.547545085879407 | 1.75251353437007 | 0.999950574162623 |
| ebi-fl187-GCST90094840 | prot-a-1143 | Follistatin-related protein 1 \|\| id:prot-a-1143 | F-small fish liking (derived food-liking factor) \|\| id:ebi-fl187-GCST90094840 | MR Egger | 28 | -0.156808953 | 0.266482779402449 | 0.561314934943749 | -0.6791152 | 0.365497295011029 | 0.854867363389662 | 0.507065445321794 | 1.44123054672955 | 0.999950574162623 |
| ebi-fl187-GCST90094840 | prot-a-1151 | Alpha-(1,3)-fucosyltransferase 10 \|\| id:prot-a-1151 | F-small fish liking (derived food-liking factor) \|\| id:ebi-fl187-GCST90094840 | MR Egger | 28 | 0.283876850070417 | 0.266792501674945 | 0.297095079626511 | -0.239036453 | 0.806790153353309 | 1.32826934440395 | 0.787386179093841 | 2.24070411461086 | 0.999950574162623 |
| ebi-fl187-GCST90094840 | prot-a-1165 | Growth arrest and DNA damage-inducible proteins-interacting protein 1 \|\| id:prot-a-1165 | F-small fish liking (derived food-liking factor) \|\| id:ebi-fl187-GCST90094840 | MR Egger | 28 | 0.0407421946303811 | 0.268526292040444 | 0.880574895739097 | -0.485569338 | 0.567053727029652 | 1.04158354509797 | 0.615346756864769 | 1.76306492122665 | 0.999950574162623 |
| ebi-fl187-GCST90094840 | prot-a-1194 | Growth/differentiation factor 11/8 \|\| id:prot-a-1194 | F-small fish liking (derived food-liking factor) \|\| id:ebi-fl187-GCST90094840 | MR Egger | 28 | -0.14694343 | 0.272551900688327 | 0.594377867490769 | -0.681145156 | 0.387258295057249 | 0.863342815086005 | 0.506037169116257 | 1.47293689446237 | 0.999950574162623 |
| ebi-fl187-GCST90094840 | prot-a-1216 | D-glucuronyl C5-epimerase \|\| id:prot-a-1216 | F-small fish liking (derived food-liking factor) \|\| id:ebi-fl187-GCST90094840 | MR Egger | 28 | -0.215667146 | 0.326323961167861 | 0.514491127003488 | -0.85526211 | 0.423927817951197 | 0.806003538781154 | 0.425171734786245 | 1.52795129914821 | 0.999950574162623 |
| ebi-fl187-GCST90094840 | prot-a-1246 | Glypican-3 \|\| id:prot-a-1246 | F-small fish liking (derived food-liking factor) \|\| id:ebi-fl187-GCST90094840 | MR Egger | 28 | 0.083244211538666 | 0.266696164268246 | 0.757430273485958 | -0.43948027 | 0.605968693504429 | 1.08680718699805 | 0.644371232855458 | 1.83302699047641 | 0.999950574162623 |
| ebi-fl187-GCST90094840 | prot-a-1275 | Glutamate receptor 4 \|\| id:prot-a-1275 | F-small fish liking (derived food-liking factor) \|\| id:ebi-fl187-GCST90094840 | MR Egger | 27 | 0.0143145030006562 | 0.267375267051317 | 0.957729478808058 | -0.50974102 | 0.538370026421238 | 1.01441744610537 | 0.600651115044588 | 1.7132120946559 | 0.999950574162623 |
| ebi-fl187-GCST90094840 | prot-a-1289 | Glutathione S-transferase P \|\| id:prot-a-1289 | F-small fish liking (derived food-liking factor) \|\| id:ebi-fl187-GCST90094840 | MR Egger | 28 | 0.0604479554069745 | 0.266752632335638 | 0.822501697781485 | -0.462387204 | 0.583283114784825 | 1.06231230851983 | 0.629778440012704 | 1.79191183618474 | 0.999950574162623 |
| ebi-fl187-GCST90094840 | prot-a-130 | Apolipoprotein D \|\| id:prot-a-130 | F-small fish liking (derived food-liking factor) \|\| id:ebi-fl187-GCST90094840 | MR Egger | 28 | 0.425225657917938 | 0.289714385561824 | 0.154168740595301 | -0.142614538 | 0.993065853619113 | 1.52993562280022 | 0.867088234240193 | 2.69949806430508 | 0.999950574162623 |
| ebi-fl187-GCST90094840 | prot-a-1313 | Heparin-binding EGF-like growth factor \|\| id:prot-a-1313 | F-small fish liking (derived food-liking factor) \|\| id:ebi-fl187-GCST90094840 | MR Egger | 28 | -0.128806505 | 0.266537280299229 | 0.632956695310183 | -0.651219575 | 0.393606564232924 | 0.879144058933633 | 0.521409491100697 | 1.48231723731519 | 0.999950574162623 |
| ebi-fl187-GCST90094840 | prot-a-1347 | HLA class II histocompatibility antigen, DQ alpha 2 chain \|\| id:prot-a-1347 | F-small fish liking (derived food-liking factor) \|\| id:ebi-fl187-GCST90094840 | MR Egger | 27 | -0.391838005 | 0.267388236702031 | 0.155269220605066 | -0.915918949 | 0.132242938854267 | 0.675813583459865 | 0.400148740790509 | 1.14138557249134 | 0.999950574162623 |
| ebi-fl187-GCST90094840 | prot-a-1357 | Non-histone chromosomal protein HMG-14 \|\| id:prot-a-1357 | F-small fish liking (derived food-liking factor) \|\| id:ebi-fl187-GCST90094840 | MR Egger | 28 | 0.282997920293357 | 0.266537280299229 | 0.298109175798292 | -0.239415149 | 0.805410989679846 | 1.32710240183061 | 0.787088055643957 | 2.23761594692685 | 0.999950574162623 |
| ebi-fl187-GCST90094840 | prot-a-137 | Apolipoprotein M \|\| id:prot-a-137 | F-small fish liking (derived food-liking factor) \|\| id:ebi-fl187-GCST90094840 | MR Egger | 28 | 0.0287090484916058 | 0.268230952962304 | 0.915585937095415 | -0.497023619 | 0.554441716297721 | 1.02912512540419 | 0.608338615100409 | 1.74096875892612 | 0.999950574162623 |
| ebi-fl187-GCST90094840 | prot-a-1381 | 17-beta-hydroxysteroid dehydrogenase 14 \|\| id:prot-a-1381 | F-small fish liking (derived food-liking factor) \|\| id:ebi-fl187-GCST90094840 | MR Egger | 28 | 0.0777073655349403 | 0.296634480080115 | 0.795413989012845 | -0.503696215 | 0.659110946491965 | 1.08080633120562 | 0.604292929847712 | 1.9330729649088 | 0.999950574162623 |
| ebi-fl187-GCST90094840 | prot-a-1392 | Serine protease HTRA2, mitochondrial \|\| id:prot-a-1392 | F-small fish liking (derived food-liking factor) \|\| id:ebi-fl187-GCST90094840 | MR Egger | 28 | 0.0544215488851465 | 0.266490988219755 | 0.839775341767417 | -0.467900788 | 0.576743885795866 | 1.05592963430722 | 0.626315658588344 | 1.78023234341811 | 0.999950574162623 |
| ebi-fl187-GCST90094840 | prot-a-1416 | Interferon-induced protein with tetratricopeptide repeats 2 \|\| id:prot-a-1416 | F-small fish liking (derived food-liking factor) \|\| id:ebi-fl187-GCST90094840 | MR Egger | 28 | -0.153069725 | 0.26653537990398 | 0.570704498412069 | -0.675479069 | 0.369339619992916 | 0.858069891129447 | 0.508912557851066 | 1.44677887527856 | 0.999950574162623 |
| ebi-fl187-GCST90094840 | prot-a-1428 | Interferon gamma \|\| id:prot-a-1428 | F-small fish liking (derived food-liking factor) \|\| id:ebi-fl187-GCST90094840 | MR Egger | 28 | -0.019081982 | 0.266675331332995 | 0.943503554515793 | -0.541765632 | 0.503601667055049 | 0.981098926142657 | 0.581720241357038 | 1.65467012224437 | 0.999950574162623 |
| ebi-fl187-GCST90094840 | prot-a-1483 | Interleukin-17C \|\| id:prot-a-1483 | F-small fish liking (derived food-liking factor) \|\| id:ebi-fl187-GCST90094840 | MR Egger | 28 | -0.572604598 | 0.266527049849778 | 0.0411716188592846 | -1.094997616 | -0.05021158 | 0.564054388922579 | 0.334540404632744 | 0.951028184509118 | 0.999950574162623 |
| ebi-fl187-GCST90094840 | prot-a-1503 | Interleukin-1 receptor-like 2 \|\| id:prot-a-1503 | F-small fish liking (derived food-liking factor) \|\| id:ebi-fl187-GCST90094840 | MR Egger | 28 | -0.089130718 | 0.266771548331272 | 0.740977489916608 | -0.612002952 | 0.433741517165237 | 0.914725995005266 | 0.542263652390245 | 1.5430199723883 | 0.999950574162623 |
| ebi-fl187-GCST90094840 | prot-a-1522 | Interleukin-31 receptor subunit alpha \|\| id:prot-a-1522 | F-small fish liking (derived food-liking factor) \|\| id:ebi-fl187-GCST90094840 | MR Egger | 28 | -0.693398655 | 0.266760262041943 | 0.0151925614560706 | -1.216248768 | -0.170548541 | 0.499874278837799 | 0.296339723543972 | 0.843202158844333 | 0.999950574162623 |
| ebi-fl187-GCST90094840 | prot-a-1540 | Interleukin-6 receptor subunit alpha \|\| id:prot-a-1540 | F-small fish liking (derived food-liking factor) \|\| id:ebi-fl187-GCST90094840 | MR Egger | 28 | -0.467581032 | 0.266753999854067 | 0.0914126654998002 | -0.990418871 | 0.0552568079652318 | 0.626515958973403 | 0.371421080742596 | 1.05681197756352 | 0.999950574162623 |
| ebi-fl187-GCST90094840 | prot-a-1541 | Interleukin-6 receptor subunit alpha \|\| id:prot-a-1541 | F-small fish liking (derived food-liking factor) \|\| id:ebi-fl187-GCST90094840 | MR Egger | 28 | -0.411165444 | 0.26674510262836 | 0.135299585687162 | -0.933985845 | 0.111654956891037 | 0.66287725329115 | 0.392984210245597 | 1.11812699206467 | 0.999950574162623 |
| ebi-fl187-GCST90094840 | prot-a-1545 | Interleukin-9 \|\| id:prot-a-1545 | F-small fish liking (derived food-liking factor) \|\| id:ebi-fl187-GCST90094840 | MR Egger | 28 | -0.177120372 | 0.266702843300774 | 0.512466025434404 | -0.699857945 | 0.345617200840446 | 0.837678945339647 | 0.496655851277796 | 1.41286167002763 | 0.999950574162623 |
| ebi-fl187-GCST90094840 | prot-a-1548 | Inositol monophosphatase 3 \|\| id:prot-a-1548 | F-small fish liking (derived food-liking factor) \|\| id:ebi-fl187-GCST90094840 | MR Egger | 28 | -0.154175875 | 0.266493563966348 | 0.567881101703574 | -0.67650326 | 0.368151510264749 | 0.857121261458883 | 0.508391600886274 | 1.445060964 | 0.999950574162623 |
| ebi-fl187-GCST90094840 | prot-a-1555 | Inhibin beta A chain:Inhibin beta B chain heterodimer \|\| id:prot-a-1555 | F-small fish liking (derived food-liking factor) \|\| id:ebi-fl187-GCST90094840 | MR Egger | 28 | -0.266498081 | 0.266461432411524 | 0.326461726073541 | -0.788762489 | 0.25576632650699 | 0.766057473774064 | 0.45440678107498 | 1.2914509148319 | 0.999950574162623 |
| ebi-fl187-GCST90094840 | prot-a-1603 | Junctophilin-3 \|\| id:prot-a-1603 | F-small fish liking (derived food-liking factor) \|\| id:ebi-fl187-GCST90094840 | MR Egger | 28 | -0.142911546 | 0.268818098344493 | 0.599496175354353 | -0.669795019 | 0.383971926823787 | 0.866830740210236 | 0.511813479233933 | 1.46810422675482 | 0.999950574162623 |
| ebi-fl187-GCST90094840 | prot-a-1627 | UPF0258 protein KIAA1024 \|\| id:prot-a-1627 | F-small fish liking (derived food-liking factor) \|\| id:ebi-fl187-GCST90094840 | MR Egger | 28 | -0.301733502 | 0.266493563966348 | 0.267869833401507 | -0.824060887 | 0.220593883607724 | 0.73953512343463 | 0.438646737889368 | 1.24681697491937 | 0.999950574162623 |
| ebi-fl187-GCST90094840 | prot-a-1666 | Kallikrein-7 \|\| id:prot-a-1666 | F-small fish liking (derived food-liking factor) \|\| id:ebi-fl187-GCST90094840 | MR Egger | 28 | -0.046704018 | 0.266783609963219 | 0.862386090388569 | -0.569599893 | 0.476191858014903 | 0.954369832546959 | 0.565751754635373 | 1.60993186466162 | 0.999950574162623 |
| ebi-fl187-GCST90094840 | prot-a-1678 | Importin subunit alpha-3 \|\| id:prot-a-1678 | F-small fish liking (derived food-liking factor) \|\| id:ebi-fl187-GCST90094840 | MR Egger | 28 | 0.0455548886661022 | 0.344219298928968 | 0.89573217641008 | -0.629114937 | 0.720224714566879 | 1.04660844997914 | 0.533063386839833 | 2.05489492358787 | 0.999950574162623 |
| ebi-fl187-GCST90094840 | prot-a-1718 | Protein LDOC1 \|\| id:prot-a-1718 | F-small fish liking (derived food-liking factor) \|\| id:ebi-fl187-GCST90094840 | MR Egger | 28 | 0.262524971849785 | 0.26653356087177 | 0.333721327994171 | -0.259880807 | 0.784930751158454 | 1.30020893638534 | 0.771143494878739 | 2.19225512434899 | 0.999950574162623 |
| ebi-fl187-GCST90094840 | prot-a-1723 | Leptin \|\| id:prot-a-1723 | F-small fish liking (derived food-liking factor) \|\| id:ebi-fl187-GCST90094840 | MR Egger | 28 | -0.508812708 | 0.305023810538492 | 0.107295223 | -1.106659377 | 0.0890339601556378 | 0.60120896552408 | 0.330661734218269 | 1.09311777814587 | 0.999950574162623 |
| ebi-fl187-GCST90094840 | prot-a-1730 | Galectin-7 \|\| id:prot-a-1730 | F-small fish liking (derived food-liking factor) \|\| id:ebi-fl187-GCST90094840 | MR Egger | 28 | -0.341683182 | 0.266566893187639 | 0.211228757226997 | -0.864154293 | 0.180787928183186 | 0.710573291130458 | 0.421407789440161 | 1.19816105615596 | 0.999950574162623 |
| ebi-fl187-GCST90094840 | prot-a-1753 | Lipase member N \|\| id:prot-a-1753 | F-small fish liking (derived food-liking factor) \|\| id:ebi-fl187-GCST90094840 | MR Egger | 28 | -0.046634535 | 0.298736439128634 | 0.87715527966306 | -0.632157956 | 0.538888885884135 | 0.95443614704834 | 0.531443730788347 | 1.71410124161437 | 0.999950574162623 |
| ebi-fl187-GCST90094840 | prot-a-1756 | VIP36-like protein \|\| id:prot-a-1756 | F-small fish liking (derived food-liking factor) \|\| id:ebi-fl187-GCST90094840 | MR Egger | 28 | -0.155912125 | 0.266748690315478 | 0.563928087683011 | -0.678739558 | 0.366915307748907 | 0.855634375707756 | 0.507255956158608 | 1.44327567967262 | 0.999950574162623 |
| ebi-fl187-GCST90094840 | prot-a-1760 | Ig Kappa chain V-I region HK102- like \|\| id:prot-a-1760 | F-small fish liking (derived food-liking factor) \|\| id:ebi-fl187-GCST90094840 | MR Egger | 28 | -0.126211859 | 0.283748668935225 | 0.66014407571519 | -0.68235925 | 0.429935532505204 | 0.881428088873161 | 0.505423165189717 | 1.53715842360128 | 0.999950574162623 |
| ebi-fl187-GCST90094840 | prot-a-179 | Neutral ceramidase \|\| id:prot-a-179 | F-small fish liking (derived food-liking factor) \|\| id:ebi-fl187-GCST90094840 | MR Egger | 28 | 0.0132208059436194 | 0.321644837942841 | 0.967527214182863 | -0.617203076 | 0.643644688311587 | 1.01330858721834 | 0.539451133148519 | 1.9034055725076 | 0.999950574162623 |
| ebi-fl187-GCST90094840 | prot-a-1809 | Lactotransferrin \|\| id:prot-a-1809 | F-small fish liking (derived food-liking factor) \|\| id:ebi-fl187-GCST90094840 | MR Egger | 28 | -0.014690752 | 0.276652715067575 | 0.958056722481209 | -0.556930073 | 0.527548569587044 | 0.985416630664075 | 0.572965328125439 | 1.69477259499505 | 0.999950574162623 |
| ebi-fl187-GCST90094840 | prot-a-1818 | Lysophospholipase-like protein 1 \|\| id:prot-a-1818 | F-small fish liking (derived food-liking factor) \|\| id:ebi-fl187-GCST90094840 | MR Egger | 28 | -0.303736828 | 0.320486643424973 | 0.351989872954111 | -0.931890649 | 0.324416993413918 | 0.738055076546849 | 0.393808452600183 | 1.38322398216681 | 0.999950574162623 |
| ebi-fl187-GCST90094840 | prot-a-1852 | MAP kinase-activated protein kinase 3 \|\| id:prot-a-1852 | F-small fish liking (derived food-liking factor) \|\| id:ebi-fl187-GCST90094840 | MR Egger | 28 | -0.004685342 | 0.335317811872226 | 0.988958319390689 | -0.661908253 | 0.652537569253048 | 0.995325617076012 | 0.515865991675081 | 1.92040781907508 | 0.999950574162623 |
| ebi-fl187-GCST90094840 | prot-a-1856 | Megakaryocyte-associated tyrosine-protein kinase \|\| id:prot-a-1856 | F-small fish liking (derived food-liking factor) \|\| id:ebi-fl187-GCST90094840 | MR Egger | 28 | 0.0908393858535191 | 0.266711704568246 | 0.736148838947481 | -0.431915555 | 0.613594326807281 | 1.09509310368854 | 0.649264201420685 | 1.84705841338873 | 0.999950574162623 |
| ebi-fl187-GCST90094840 | prot-a-1890 | Beta-1,3-N-acetylglucosaminyltransferase manic fringe \|\| id:prot-a-1890 | F-small fish liking (derived food-liking factor) \|\| id:ebi-fl187-GCST90094840 | MR Egger | 28 | -0.098442624 | 0.330603756580368 | 0.768248020090301 | -0.746425987 | 0.54954073894133 | 0.906247688029662 | 0.47405781751135 | 1.73245718501297 | 0.999950574162623 |
| ebi-fl187-GCST90094840 | prot-a-1919 | Matrilysin \|\| id:prot-a-1919 | F-small fish liking (derived food-liking factor) \|\| id:ebi-fl187-GCST90094840 | MR Egger | 28 | -0.331351323 | 0.303447323578141 | 0.284867714441242 | -0.926108077 | 0.263405431536493 | 0.717952891596179 | 0.396092275120995 | 1.30135422205307 | 0.999950574162623 |
| ebi-fl187-GCST90094840 | prot-a-1923 | Multimerin-2 \|\| id:prot-a-1923 | F-small fish liking (derived food-liking factor) \|\| id:ebi-fl187-GCST90094840 | MR Egger | 28 | 0.0730644797828317 | 0.277791470940052 | 0.794608925486353 | -0.471406803 | 0.617535762825334 | 1.07579990202241 | 0.62412363123402 | 1.85435284176488 | 0.999950574162623 |
| ebi-fl187-GCST90094840 | prot-a-1948 | Mesothelin \|\| id:prot-a-1948 | F-small fish liking (derived food-liking factor) \|\| id:ebi-fl187-GCST90094840 | MR Egger | 28 | 0.117646633214495 | 0.287553666333521 | 0.685795539523579 | -0.445958553 | 0.681251819228196 | 1.12484655771806 | 0.640210306436877 | 1.97635021755922 | 0.999950574162623 |
| ebi-fl187-GCST90094840 | prot-a-195 | ATPase family AAA domain-containing protein 1 \|\| id:prot-a-195 | F-small fish liking (derived food-liking factor) \|\| id:ebi-fl187-GCST90094840 | MR Egger | 28 | 0.0923967820053434 | 0.280922390174325 | 0.744862155219023 | -0.458211103 | 0.64300466674702 | 1.09679992622855 | 0.632413957797822 | 1.90218774165565 | 0.999950574162623 |
| ebi-fl187-GCST90094840 | prot-a-1966 | Mucin-1 \|\| id:prot-a-1966 | F-small fish liking (derived food-liking factor) \|\| id:ebi-fl187-GCST90094840 | MR Egger | 28 | 0.39655970852573 | 0.266743733820921 | 0.14912934862862 | -0.12625801 | 0.919377426814734 | 1.48670120406181 | 0.8813874108871 | 2.50772865922175 | 0.999950574162623 |
| ebi-fl187-GCST90094840 | prot-a-1986 | Myocardial zonula adherens protein \|\| id:prot-a-1986 | F-small fish liking (derived food-liking factor) \|\| id:ebi-fl187-GCST90094840 | MR Egger | 28 | -0.255104966 | 0.275922536135785 | 0.363700511779856 | -0.795913137 | 0.285703204837398 | 0.774835162353492 | 0.451169067683255 | 1.33069745207101 | 0.999950574162623 |
| ebi-fl187-GCST90094840 | prot-a-2071 | Epididymal secretory protein E1 \|\| id:prot-a-2071 | F-small fish liking (derived food-liking factor) \|\| id:ebi-fl187-GCST90094840 | MR Egger | 28 | -0.285751402 | 0.291806058529613 | 0.336480482273886 | -0.857691277 | 0.286188472520553 | 0.751449401398584 | 0.424140175023821 | 1.33134335324537 | 0.999950574162623 |
| ebi-fl187-GCST90094840 | prot-a-2098 | Neuritin \|\| id:prot-a-2098 | F-small fish liking (derived food-liking factor) \|\| id:ebi-fl187-GCST90094840 | MR Egger | 28 | -0.288310337 | 0.266575744154902 | 0.289390014002051 | -0.810798795 | 0.234178121820714 | 0.749528949784169 | 0.44450285759375 | 1.26386959491272 | 0.999950574162623 |
| ebi-fl187-GCST90094840 | prot-a-2115 | Neurotrophin-4 \|\| id:prot-a-2115 | F-small fish liking (derived food-liking factor) \|\| id:ebi-fl187-GCST90094840 | MR Egger | 28 | 0.0387735835963175 | 0.267905999444979 | 0.886041456995755 | -0.486322175 | 0.563869342508477 | 1.03953508920618 | 0.614883675056364 | 1.75745957410863 | 0.999950574162623 |
| ebi-fl187-GCST90094840 | prot-a-2118 | Netrin-1 \|\| id:prot-a-2118 | F-small fish liking (derived food-liking factor) \|\| id:ebi-fl187-GCST90094840 | MR Egger | 28 | 0.13272098788067 | 0.297358593348668 | 0.659051901273839 | -0.450101855 | 0.715543830844059 | 1.14193134119472 | 0.637563209261057 | 2.04529867636833 | 0.999950574162623 |
| ebi-fl187-GCST90094840 | prot-a-2120 | Netrin-G1 \|\| id:prot-a-2120 | F-small fish liking (derived food-liking factor) \|\| id:ebi-fl187-GCST90094840 | MR Egger | 28 | -0.171553679 | 0.285668912677189 | 0.553350185702906 | -0.731464748 | 0.388357389669529 | 0.842355049879938 | 0.481203631561081 | 1.47455668145382 | 0.999950574162623 |
| ebi-fl187-GCST90094840 | prot-a-2145 | Olfactomedin-like protein 3 \|\| id:prot-a-2145 | F-small fish liking (derived food-liking factor) \|\| id:ebi-fl187-GCST90094840 | MR Egger | 28 | 0.0176777689770554 | 0.26653537990398 | 0.947627025922369 | -0.504731576 | 0.540087113588855 | 1.01783494554608 | 0.603667592771781 | 1.71615635621251 | 0.999950574162623 |
| ebi-fl187-GCST90094840 | prot-a-2162 | Prolyl 4-hydroxylase subunit alpha-2 \|\| id:prot-a-2162 | F-small fish liking (derived food-liking factor) \|\| id:ebi-fl187-GCST90094840 | MR Egger | 28 | 0.040547747498813 | 0.269416833848131 | 0.881530183192312 | -0.487509247 | 0.568604741841151 | 1.04138103185504 | 0.61415419720972 | 1.76580158278579 | 0.999950574162623 |
| ebi-fl187-GCST90094840 | prot-a-2168 | Polyadenylate-binding protein-interacting protein 1 \|\| id:prot-a-2168 | F-small fish liking (derived food-liking factor) \|\| id:ebi-fl187-GCST90094840 | MR Egger | 28 | -0.445589097 | 0.322594455256827 | 0.178953601014565 | -1.07787423 | 0.186696034816692 | 0.640446879234565 | 0.340318195539708 | 1.20526087202245 | 0.999950574162623 |
| ebi-fl187-GCST90094840 | prot-a-219 | Beta-1,4-galactosyltransferase 5 \|\| id:prot-a-219 | F-small fish liking (derived food-liking factor) \|\| id:ebi-fl187-GCST90094840 | MR Egger | 28 | -0.531973141 | 0.266566893187639 | 0.0565486410358348 | -1.054444252 | -0.009502031 | 0.587444713993597 | 0.348385988373575 | 0.990542971059377 | 0.999950574162623 |
| ebi-fl187-GCST90094840 | prot-a-2190 | Pyruvate carboxylase, mitochondrial \|\| id:prot-a-2190 | F-small fish liking (derived food-liking factor) \|\| id:ebi-fl187-GCST90094840 | MR Egger | 28 | -0.445234983 | 0.345503510715072 | 0.208867118902125 | -1.122421864 | 0.231951897563894 | 0.64067371063189 | 0.325490545304419 | 1.26105906735615 | 0.999950574162623 |
| ebi-fl187-GCST90094840 | prot-a-2197 | Protocadherin-9 \|\| id:prot-a-2197 | F-small fish liking (derived food-liking factor) \|\| id:ebi-fl187-GCST90094840 | MR Egger | 28 | -0.117297041 | 0.266669793224451 | 0.663674171576831 | -0.639969836 | 0.405375753826572 | 0.889320989224552 | 0.527308329735536 | 1.49986597456557 | 0.999950574162623 |
| ebi-fl187-GCST90094840 | prot-a-2198 | Protocadherin alpha-4 \|\| id:prot-a-2198 | F-small fish liking (derived food-liking factor) \|\| id:ebi-fl187-GCST90094840 | MR Egger | 28 | -0.222150694 | 0.344587348920244 | 0.524778494396512 | -0.897541898 | 0.453240510097988 | 0.800794680459773 | 0.407570278877053 | 1.57340256021493 | 0.999950574162623 |
| ebi-fl187-GCST90094840 | prot-a-2254 | Placenta growth factor \|\| id:prot-a-2254 | F-small fish liking (derived food-liking factor) \|\| id:ebi-fl187-GCST90094840 | MR Egger | 28 | -0.522524274 | 0.287737496939621 | 0.0809235359000616 | -1.086489768 | 0.0414412196206837 | 0.593021707520145 | 0.337398765282789 | 1.04231189256236 | 0.999950574162623 |
| ebi-fl187-GCST90094840 | prot-a-2261 | Membrane-associated progesterone receptor component 2 \|\| id:prot-a-2261 | F-small fish liking (derived food-liking factor) \|\| id:ebi-fl187-GCST90094840 | MR Egger | 28 | -0.251816767 | 0.266526534463754 | 0.353454269228782 | -0.774208774 | 0.27057524060264 | 0.777387168044302 | 0.461068445827648 | 1.31071821224965 | 0.999950574162623 |
| ebi-fl187-GCST90094840 | prot-a-2268 | Polymeric immunoglobulin receptor \|\| id:prot-a-2268 | F-small fish liking (derived food-liking factor) \|\| id:ebi-fl187-GCST90094840 | MR Egger | 28 | -0.227333117 | 0.312956189857048 | 0.474079595541929 | -0.84072725 | 0.386061014644686 | 0.796655358262412 | 0.431396676262229 | 1.47117443126156 | 0.999950574162623 |
| ebi-fl187-GCST90094840 | prot-a-2283 | Group 10 secretory phospholipase A2 \|\| id:prot-a-2283 | F-small fish liking (derived food-liking factor) \|\| id:ebi-fl187-GCST90094840 | MR Egger | 28 | 0.303795839268149 | 0.276089666346961 | 0.281262479329626 | -0.237339907 | 0.844931585308192 | 1.3549923916103 | 0.788723150109374 | 2.32781855213352 | 0.999950574162623 |
| ebi-fl187-GCST90094840 | prot-a-2298 | Inactive phospholipase D5 \|\| id:prot-a-2298 | F-small fish liking (derived food-liking factor) \|\| id:ebi-fl187-GCST90094840 | MR Egger | 28 | 0.514054678744552 | 0.270857285287343 | 0.0688678830982276 | -0.0168256 | 1.04493495790774 | 1.67205712332095 | 0.983315159434058 | 2.84321358907701 | 0.999950574162623 |
| ebi-fl187-GCST90094840 | prot-a-2304 | Procollagen-lysine,2-oxoglutarate 5-dioxygenase 2 \|\| id:prot-a-2304 | F-small fish liking (derived food-liking factor) \|\| id:ebi-fl187-GCST90094840 | MR Egger | 28 | -0.031397289 | 0.266704649472339 | 0.907191830932216 | -0.554138402 | 0.49134382361307 | 0.969090487253439 | 0.57456709366834 | 1.63451123956502 | 0.999950574162623 |
| ebi-fl187-GCST90094840 | prot-a-2331 | Periostin \|\| id:prot-a-2331 | F-small fish liking (derived food-liking factor) \|\| id:ebi-fl187-GCST90094840 | MR Egger | 28 | -0.303981672 | 0.29931888862409 | 0.319188456265704 | -0.890646694 | 0.282683349307451 | 0.737874389796458 | 0.410390269952661 | 1.32668499957443 | 0.999950574162623 |
| ebi-fl187-GCST90094840 | prot-a-2333 | Periostin \|\| id:prot-a-2333 | F-small fish liking (derived food-liking factor) \|\| id:ebi-fl187-GCST90094840 | MR Egger | 28 | -0.594548563 | 0.295438690320428 | 0.0546413245747491 | -1.173608396 | -0.01548873 | 0.55181161779183 | 0.309249032548028 | 0.984630603436883 | 0.999950574162623 |
| ebi-fl187-GCST90094840 | prot-a-2367 | cAMP-dependent protein kinase catalytic subunit alpha \|\| id:prot-a-2367 | F-small fish liking (derived food-liking factor) \|\| id:ebi-fl187-GCST90094840 | MR Egger | 28 | -0.22763355 | 0.266490988219755 | 0.400802621237473 | -0.749955887 | 0.294688787226733 | 0.796416053282692 | 0.472387390897821 | 1.34270842564377 | 0.999950574162623 |
| ebi-fl187-GCST90094840 | prot-a-2385 | Phosphoribosyl pyrophosphate synthase-associated protein 1 \|\| id:prot-a-2385 | F-small fish liking (derived food-liking factor) \|\| id:ebi-fl187-GCST90094840 | MR Egger | 28 | 0.200183613900651 | 0.285844014176802 | 0.489944467187152 | -0.360070654 | 0.760437881687182 | 1.22162704527535 | 0.697627034268857 | 2.13921273752278 | 0.999950574162623 |
| ebi-fl187-GCST90094840 | prot-a-2393 | Trypsin-3 \|\| id:prot-a-2393 | F-small fish liking (derived food-liking factor) \|\| id:ebi-fl187-GCST90094840 | MR Egger | 27 | 0.137670142563708 | 0.267415632655044 | 0.611202775539285 | -0.386464497 | 0.661804782567594 | 1.14759694444865 | 0.679454847332802 | 1.93828736681718 | 0.999950574162623 |
| ebi-fl187-GCST90094840 | prot-a-2405 | Pregnancy-specific beta-1-glycoprotein 6 \|\| id:prot-a-2405 | F-small fish liking (derived food-liking factor) \|\| id:ebi-fl187-GCST90094840 | MR Egger | 28 | -0.119186369 | 0.289009158200821 | 0.683429531044373 | -0.685644319 | 0.447271581259919 | 0.887642356492643 | 0.503765539332269 | 1.56403900529633 | 0.999950574162623 |
| ebi-fl187-GCST90094840 | prot-a-244 | Beclin-1 \|\| id:prot-a-244 | F-small fish liking (derived food-liking factor) \|\| id:ebi-fl187-GCST90094840 | MR Egger | 28 | 0.203884211103324 | 0.266753999854067 | 0.451560625924548 | -0.318953629 | 0.726722050817295 | 1.22615616996624 | 0.726909256316961 | 2.06828973504048 | 0.999950574162623 |
| ebi-fl187-GCST90094840 | prot-a-2454 | tRNA pseudouridine synthase A, mitochondrial \|\| id:prot-a-2454 | F-small fish liking (derived food-liking factor) \|\| id:ebi-fl187-GCST90094840 | MR Egger | 28 | -0.028154534 | 0.323843533874437 | 0.931386352161644 | -0.66288786 | 0.606578792340613 | 0.972238111295655 | 0.515360893094953 | 1.83414565932457 | 0.999950574162623 |
| ebi-fl187-GCST90094840 | prot-a-2466 | Glutaminyl-peptide cyclotransferase-like protein \|\| id:prot-a-2466 | F-small fish liking (derived food-liking factor) \|\| id:ebi-fl187-GCST90094840 | MR Egger | 28 | 0.093853262175627 | 0.266758450511827 | 0.727802353295898 | -0.428993301 | 0.616699825178808 | 1.09839855747662 | 0.6511642914283 | 1.85280336613725 | 0.999950574162623 |
| ebi-fl187-GCST90094840 | prot-a-2474 | Ras-related protein Rab-27A \|\| id:prot-a-2474 | F-small fish liking (derived food-liking factor) \|\| id:ebi-fl187-GCST90094840 | MR Egger | 28 | -0.179552284 | 0.328356290692442 | 0.589164193550858 | -0.823130614 | 0.464026045376309 | 0.835644258658258 | 0.439054988970209 | 1.59046439414424 | 0.999950574162623 |
| ebi-fl187-GCST90094840 | prot-a-2490 | Rap1 GTPase-GDP dissociation stimulator 1 \|\| id:prot-a-2490 | F-small fish liking (derived food-liking factor) \|\| id:ebi-fl187-GCST90094840 | MR Egger | 28 | -0.011528012 | 0.278873924778129 | 0.967342476246475 | -0.558120904 | 0.535064880918969 | 0.988538181278327 | 0.572283429497014 | 1.70755902665912 | 0.999950574162623 |
| ebi-fl187-GCST90094840 | prot-a-2545 | Receptor-interacting serine/threonine-protein kinase 2 \|\| id:prot-a-2545 | F-small fish liking (derived food-liking factor) \|\| id:ebi-fl187-GCST90094840 | MR Egger | 28 | -0.06419424 | 0.266575744154902 | 0.811590809091756 | -0.586682699 | 0.458294218453347 | 0.937822819048904 | 0.556169208812952 | 1.58137420409519 | 0.999950574162623 |
| ebi-fl187-GCST90094840 | prot-a-2610 | RUN and FYVE domain-containing protein 1 \|\| id:prot-a-2610 | F-small fish liking (derived food-liking factor) \|\| id:ebi-fl187-GCST90094840 | MR Egger | 28 | -0.412325056 | 0.266496287557093 | 0.1339001897603 | -0.934657779 | 0.110007667899049 | 0.662109018749633 | 0.392720239522979 | 1.11628662999924 | 0.999950574162623 |
| ebi-fl187-GCST90094840 | prot-a-2627 | Serine--tRNA ligase, mitochondrial \|\| id:prot-a-2627 | F-small fish liking (derived food-liking factor) \|\| id:ebi-fl187-GCST90094840 | MR Egger | 28 | -0.171861391 | 0.277740056998101 | 0.541441988339059 | -0.716231903 | 0.372509120265418 | 0.842095886768663 | 0.488589845527498 | 1.45137171597806 | 0.999950574162623 |
| ebi-fl187-GCST90094840 | prot-a-264 | BolA-like protein 2 \|\| id:prot-a-264 | F-small fish liking (derived food-liking factor) \|\| id:ebi-fl187-GCST90094840 | MR Egger | 28 | 0.0943560693288631 | 0.266783143102943 | 0.72643006431792 | -0.428538891 | 0.617251029810631 | 1.09895097899754 | 0.651460254021151 | 1.85382492145166 | 0.999950574162623 |
| ebi-fl187-GCST90094840 | prot-a-2646 | Secretoglobin family 1C member 1 \|\| id:prot-a-2646 | F-small fish liking (derived food-liking factor) \|\| id:ebi-fl187-GCST90094840 | MR Egger | 28 | 0.0541268977230585 | 0.326961321408376 | 0.86979533696269 | -0.586717292 | 0.694971087683476 | 1.05561854924643 | 0.556149969248654 | 2.00365114290767 | 0.999950574162623 |
| ebi-fl187-GCST90094840 | prot-a-2672 | Semaphorin-3E \|\| id:prot-a-2672 | F-small fish liking (derived food-liking factor) \|\| id:ebi-fl187-GCST90094840 | MR Egger | 28 | -0.303306418 | 0.269728426632481 | 0.271080740938966 | -0.831974134 | 0.225361298531775 | 0.738372811228523 | 0.435189315785258 | 1.25277526029737 | 0.999950574162623 |
| ebi-fl187-GCST90094840 | prot-a-2701 | Plasma protease C1 inhibitor \|\| id:prot-a-2701 | F-small fish liking (derived food-liking factor) \|\| id:ebi-fl187-GCST90094840 | MR Egger | 28 | 0.244510780284196 | 0.26675353061505 | 0.367767847281754 | -0.27832614 | 0.767347700289693 | 1.27699642853455 | 0.757049877211386 | 2.15404549631101 | 0.999950574162623 |
| ebi-fl187-GCST90094840 | prot-a-2738 | Signaling threshold-regulating transmembrane adapter 1 \|\| id:prot-a-2738 | F-small fish liking (derived food-liking factor) \|\| id:ebi-fl187-GCST90094840 | MR Egger | 28 | 0.364414791631852 | 0.308499173767195 | 0.248196023859893 | -0.240243589 | 0.969073172215554 | 1.43967125391468 | 0.786436270545995 | 2.63550067179544 | 0.999950574162623 |
| ebi-fl187-GCST90094840 | prot-a-274 | Serine/threonine-protein kinase BRSK2 \|\| id:prot-a-274 | F-small fish liking (derived food-liking factor) \|\| id:ebi-fl187-GCST90094840 | MR Egger | 28 | -0.256866215 | 0.266678015360785 | 0.344317116419218 | -0.779555125 | 0.265822695446201 | 0.773471686015529 | 0.458609990155023 | 1.30450374372673 | 0.999950574162623 |
| ebi-fl187-GCST90094840 | prot-a-2749 | Mitochondrial glutamate carrier 2 \|\| id:prot-a-2749 | F-small fish liking (derived food-liking factor) \|\| id:ebi-fl187-GCST90094840 | MR Egger | 28 | -0.143533852 | 0.269237967738297 | 0.598484279247517 | -0.671240268 | 0.384172565152105 | 0.866291474325455 | 0.511074315225912 | 1.46839881428446 | 0.999950574162623 |
| ebi-fl187-GCST90094840 | prot-a-2760 | Sodium-coupled monocarboxylate transporter 1 \|\| id:prot-a-2760 | F-small fish liking (derived food-liking factor) \|\| id:ebi-fl187-GCST90094840 | MR Egger | 28 | -0.302577804 | 0.266773354834176 | 0.267054763094942 | -0.825453579 | 0.220297971645687 | 0.738910995917417 | 0.438036263215098 | 1.24644808144471 | 0.999950574162623 |
| ebi-fl187-GCST90094840 | prot-a-2777 | Small integral membrane protein 10 \|\| id:prot-a-2777 | F-small fish liking (derived food-liking factor) \|\| id:ebi-fl187-GCST90094840 | MR Egger | 28 | 0.346915505039195 | 0.266575744154902 | 0.20454375461238 | -0.175572954 | 0.869403963582803 | 1.414697186 | 0.83897618868772 | 2.38548859163302 | 0.999950574162623 |
| ebi-fl187-GCST90094840 | prot-a-2799 | Superoxide dismutase [Mn], mitochondrial \|\| id:prot-a-2799 | F-small fish liking (derived food-liking factor) \|\| id:ebi-fl187-GCST90094840 | MR Egger | 28 | -0.132448248 | 0.2723270842179 | 0.630788317902742 | -0.666209333 | 0.401312836797681 | 0.875948264771838 | 0.513651975493161 | 1.49378450617292 | 0.999950574162623 |
| ebi-fl187-GCST90094840 | prot-a-2805 | Protein SPACA7 \|\| id:prot-a-2805 | F-small fish liking (derived food-liking factor) \|\| id:ebi-fl187-GCST90094840 | MR Egger | 28 | -0.022634543 | 0.266721768581918 | 0.933021187339567 | -0.545409209 | 0.500140123638736 | 0.977619696671067 | 0.57960455528744 | 1.64895231171062 | 0.999950574162623 |
| ebi-fl187-GCST90094840 | prot-a-2811 | SPARC-like protein 1 \|\| id:prot-a-2811 | F-small fish liking (derived food-liking factor) \|\| id:ebi-fl187-GCST90094840 | MR Egger | 28 | -0.186244093 | 0.266493097929518 | 0.490838014422178 | -0.708570565 | 0.336082378518887 | 0.830070955390635 | 0.492347473228621 | 1.39945430503546 | 0.999950574162623 |
| ebi-fl187-GCST90094840 | prot-a-2831 | Sepiapterin reductase \|\| id:prot-a-2831 | F-small fish liking (derived food-liking factor) \|\| id:ebi-fl187-GCST90094840 | MR Egger | 28 | 0.14739289758014 | 0.266751375468785 | 0.585292520210423 | -0.375439798 | 0.670225593498959 | 1.15880916691727 | 0.686987076566857 | 1.95467823360258 | 0.999950574162623 |
| ebi-fl187-GCST90094840 | prot-a-2834 | SPRY domain-containing SOCS box protein 1 \|\| id:prot-a-2834 | F-small fish liking (derived food-liking factor) \|\| id:ebi-fl187-GCST90094840 | MR Egger | 28 | -0.24073188 | 0.266707568317956 | 0.375021499599964 | -0.763478714 | 0.28201495411114 | 0.786052354657617 | 0.466042375847753 | 1.32579854597736 | 0.999950574162623 |
| ebi-fl187-GCST90094840 | prot-a-2835 | Spectrin alpha chain, non-erythrocytic 1 \|\| id:prot-a-2835 | F-small fish liking (derived food-liking factor) \|\| id:ebi-fl187-GCST90094840 | MR Egger | 28 | -0.338465271 | 0.266522659311427 | 0.215357778491726 | -0.860849683 | 0.183919141345863 | 0.712863536064795 | 0.422802681334701 | 1.20191863364395 | 0.999950574162623 |
| ebi-fl187-GCST90094840 | prot-a-2859 | Sia-alpha-2,3-Gal-beta-1,4-GlcNAc-R:alpha 2,8-sialyltransferase \|\| id:prot-a-2859 | F-small fish liking (derived food-liking factor) \|\| id:ebi-fl187-GCST90094840 | MR Egger | 28 | 0.144470859130323 | 0.26653537990398 | 0.592411655140415 | -0.377938485 | 0.666880203742123 | 1.15542802429568 | 0.685272653586691 | 1.9481499988952 | 0.999950574162623 |
| ebi-fl187-GCST90094840 | prot-a-2861 | Alpha-2,8-sialyltransferase 8F \|\| id:prot-a-2861 | F-small fish liking (derived food-liking factor) \|\| id:ebi-fl187-GCST90094840 | MR Egger | 28 | -0.220644546 | 0.266790690724259 | 0.415744784825356 | -0.7435543 | 0.302265207611964 | 0.802001704178058 | 0.475421119652452 | 1.35291998381291 | 0.999950574162623 |
| ebi-fl187-GCST90094840 | prot-a-289 | Uncharacterized protein C17orf89 \|\| id:prot-a-289 | F-small fish liking (derived food-liking factor) \|\| id:ebi-fl187-GCST90094840 | MR Egger | 28 | 0.036118708980311 | 0.278332566781751 | 0.897748588971078 | -0.509413122 | 0.581650539872542 | 1.03677891415387 | 0.600848099942805 | 1.78898879256903 | 0.999950574162623 |
| ebi-fl187-GCST90094840 | prot-a-2921 | Tachykinin-3 \|\| id:prot-a-2921 | F-small fish liking (derived food-liking factor) \|\| id:ebi-fl187-GCST90094840 | MR Egger | 28 | -0.11724568 | 0.266575744154902 | 0.663700974267413 | -0.639734138 | 0.405242778986637 | 0.889366667112058 | 0.527432629645293 | 1.49966654338764 | 0.999950574162623 |
| ebi-fl187-GCST90094840 | prot-a-2928 | Tax1-binding protein 1 \|\| id:prot-a-2928 | F-small fish liking (derived food-liking factor) \|\| id:ebi-fl187-GCST90094840 | MR Egger | 28 | -0.310655986 | 0.266739792935437 | 0.2547397024262 | -0.83346598 | 0.212154007910624 | 0.732965982884353 | 0.434540564154415 | 1.23633827629201 | 0.999950574162623 |
| ebi-fl187-GCST90094840 | prot-a-296 | Uncharacterized protein C1orf185 \|\| id:prot-a-296 | F-small fish liking (derived food-liking factor) \|\| id:ebi-fl187-GCST90094840 | MR Egger | 28 | -0.086377387 | 0.284649542852462 | 0.763960649392439 | -0.644290491 | 0.471535717372503 | 0.917248008770207 | 0.525034927196983 | 1.60245321979742 | 0.999950574162623 |
| ebi-fl187-GCST90094840 | prot-a-2964 | Transforming growth factor beta-2 \|\| id:prot-a-2964 | F-small fish liking (derived food-liking factor) \|\| id:ebi-fl187-GCST90094840 | MR Egger | 28 | -0.025514891 | 0.266757586186116 | 0.924533369261009 | -0.54835976 | 0.497329977941488 | 0.974807863010054 | 0.577896923124299 | 1.64432502019368 | 0.999950574162623 |
| ebi-fl187-GCST90094840 | prot-a-2977 | Thymocyte nuclear protein 1 \|\| id:prot-a-2977 | F-small fish liking (derived food-liking factor) \|\| id:ebi-fl187-GCST90094840 | MR Egger | 28 | -0.259416449 | 0.266573925273283 | 0.339446100772595 | -0.781901342 | 0.26306844481148 | 0.771501665249649 | 0.457535252651224 | 1.30091575683833 | 0.999950574162623 |
| ebi-fl187-GCST90094840 | prot-a-3015 | Transmembrane protein 70, mitochondrial \|\| id:prot-a-3015 | F-small fish liking (derived food-liking factor) \|\| id:ebi-fl187-GCST90094840 | MR Egger | 28 | 0.104335997240376 | 0.266752195719083 | 0.698884753965109 | -0.418498306 | 0.627170300849779 | 1.10997334024707 | 0.658034244058412 | 1.87230501631746 | 0.999950574162623 |
| ebi-fl187-GCST90094840 | prot-a-3030 | Tumor necrosis factor alpha-induced protein 3 \|\| id:prot-a-3030 | F-small fish liking (derived food-liking factor) \|\| id:ebi-fl187-GCST90094840 | MR Egger | 28 | -0.181722676 | 0.266708403065924 | 0.501672169709422 | -0.704471146 | 0.341025794307263 | 0.83383255038091 | 0.49436995480481 | 1.40638951723764 | 0.999950574162623 |
| ebi-fl187-GCST90094840 | prot-a-3032 | Tumor necrosis factor receptor superfamily member 10A \|\| id:prot-a-3032 | F-small fish liking (derived food-liking factor) \|\| id:ebi-fl187-GCST90094840 | MR Egger | 28 | -0.185064652 | 0.266484701841373 | 0.49354924744611 | -0.707374668 | 0.337245363632165 | 0.831050553054062 | 0.492936622689305 | 1.40108279633094 | 0.999950574162623 |
| ebi-fl187-GCST90094840 | prot-a-3081 | Tumor protein p53-inducible protein 11 \|\| id:prot-a-3081 | F-small fish liking (derived food-liking factor) \|\| id:ebi-fl187-GCST90094840 | MR Egger | 28 | 0.0307819202244895 | 0.266691824040047 | 0.908998009492381 | -0.491934055 | 0.553497895342982 | 1.03126058228571 | 0.61144268615568 | 1.73932637131174 | 0.999950574162623 |
| ebi-fl187-GCST90094840 | prot-a-315 | Complement C4 \|\| id:prot-a-315 | F-small fish liking (derived food-liking factor) \|\| id:ebi-fl187-GCST90094840 | MR Egger | 27 | -0.359410504 | 0.267126590269575 | 0.190551282780734 | -0.882978621 | 0.164157613272935 | 0.698087724962099 | 0.413549268951943 | 1.17840003194248 | 0.999950574162623 |
| ebi-fl187-GCST90094840 | prot-a-3157 | UDP-glucuronosyltransferase 1-8 \|\| id:prot-a-3157 | F-small fish liking (derived food-liking factor) \|\| id:ebi-fl187-GCST90094840 | MR Egger | 28 | 0.121091156486585 | 0.26674510262836 | 0.653625657803761 | -0.401729245 | 0.64391155763817 | 1.12872779853833 | 0.669161900319439 | 1.90391360085653 | 0.999950574162623 |
| ebi-fl187-GCST90094840 | prot-a-317 | Glutaredoxin-like protein C5orf63 \|\| id:prot-a-317 | F-small fish liking (derived food-liking factor) \|\| id:ebi-fl187-GCST90094840 | MR Egger | 28 | -0.255564733 | 0.283393025652558 | 0.375440006933548 | -0.811015063 | 0.299885597382373 | 0.774479000668716 | 0.444406736284478 | 1.3497043890281 | 0.999950574162623 |
| ebi-fl187-GCST90094840 | prot-a-3192 | Guanine nucleotide exchange factor VAV3 \|\| id:prot-a-3192 | F-small fish liking (derived food-liking factor) \|\| id:ebi-fl187-GCST90094840 | MR Egger | 28 | -0.190027733 | 0.266753999854067 | 0.482579577440286 | -0.712865573 | 0.332810106639782 | 0.826936200142364 | 0.490237371870509 | 1.39488239441386 | 0.999950574162623 |
| ebi-fl187-GCST90094840 | prot-a-3233 | Serine/threonine-protein kinase WNK3 \|\| id:prot-a-3233 | F-small fish liking (derived food-liking factor) \|\| id:ebi-fl187-GCST90094840 | MR Egger | 28 | 0.199397031467632 | 0.266792501674945 | 0.461536867203853 | -0.323516272 | 0.722310334750524 | 1.22066651272037 | 0.723600183538078 | 2.05918512622725 | 0.999950574162623 |
| ebi-fl187-GCST90094840 | prot-a-3250 | 14-3-3 protein theta \|\| id:prot-a-3250 | F-small fish liking (derived food-liking factor) \|\| id:ebi-fl187-GCST90094840 | MR Egger | 28 | 0.0399487612709039 | 0.266720400707628 | 0.88209585286787 | -0.482823224 | 0.562720746657854 | 1.04075744573734 | 0.61703889132842 | 1.75544212217443 | 0.999950574162623 |
| ebi-fl187-GCST90094840 | prot-a-3278 | E3 ubiquitin-protein ligase ZNRF3 \|\| id:prot-a-3278 | F-small fish liking (derived food-liking factor) \|\| id:ebi-fl187-GCST90094840 | MR Egger | 28 | -0.195705892 | 0.277497053922179 | 0.486923011564092 | -0.739600118 | 0.348188333435605 | 0.822254030400721 | 0.477304742968438 | 1.41649899874332 | 0.999950574162623 |
| ebi-fl187-GCST90094840 | prot-a-355 | Calpain-3 \|\| id:prot-a-355 | F-small fish liking (derived food-liking factor) \|\| id:ebi-fl187-GCST90094840 | MR Egger | 28 | -0.00228769 | 0.266733492620851 | 0.993222347599004 | -0.525085336 | 0.520509955441333 | 0.997714924673145 | 0.591504885769538 | 1.68288562759798 | 0.999950574162623 |
| ebi-fl187-GCST90094840 | prot-a-385 | Coiled-coil domain-containing protein 90B, mitochondrial \|\| id:prot-a-385 | F-small fish liking (derived food-liking factor) \|\| id:ebi-fl187-GCST90094840 | MR Egger | 28 | 0.050216511025786 | 0.299450714100774 | 0.868120483365885 | -0.536706889 | 0.637139910663303 | 1.05149873280149 | 0.584670470582575 | 1.89106452388719 | 0.999950574162623 |
| ebi-fl187-GCST90094840 | prot-a-390 | C-C motif chemokine 14 \|\| id:prot-a-390 | F-small fish liking (derived food-liking factor) \|\| id:ebi-fl187-GCST90094840 | MR Egger | 28 | -0.384883232 | 0.266534748583673 | 0.160674636920345 | -0.90729134 | 0.137524874880222 | 0.680530095469598 | 0.403616003434601 | 1.14743024780706 | 0.999950574162623 |
| ebi-fl187-GCST90094840 | prot-a-40 | Pituitary adenylate cyclase-activating polypeptide 27 \|\| id:prot-a-40 | F-small fish liking (derived food-liking factor) \|\| id:ebi-fl187-GCST90094840 | MR Egger | 28 | -0.222423252 | 0.266566893187639 | 0.411657174318079 | -0.744894363 | 0.300047858558206 | 0.800576446961941 | 0.474784452220777 | 1.34992341141823 | 0.999950574162623 |
| ebi-fl187-GCST90094840 | prot-a-401 | C-C motif chemokine 24 \|\| id:prot-a-401 | F-small fish liking (derived food-liking factor) \|\| id:ebi-fl187-GCST90094840 | MR Egger | 28 | 0.185900950191729 | 0.266490525100424 | 0.491625710619982 | -0.336420479 | 0.708222379388559 | 1.20430296849168 | 0.714322684968257 | 2.03037880559865 | 0.999950574162623 |
| ebi-fl187-GCST90094840 | prot-a-412 | Cerebral cavernous malformations 2 protein \|\| id:prot-a-412 | F-small fish liking (derived food-liking factor) \|\| id:ebi-fl187-GCST90094840 | MR Egger | 28 | 0.0679356582617275 | 0.283676030658281 | 0.812608765878643 | -0.488069362 | 0.623940678351957 | 1.07029644152977 | 0.61381029656172 | 1.86626793191324 | 0.999950574162623 |
| ebi-fl187-GCST90094840 | prot-a-44 | Adhesion G protein-coupled receptor F5 \|\| id:prot-a-44 | F-small fish liking (derived food-liking factor) \|\| id:ebi-fl187-GCST90094840 | MR Egger | 28 | -0.264025473 | 0.285215051574513 | 0.363111650544739 | -0.823046974 | 0.294996028190068 | 0.767953977395773 | 0.439091713133784 | 1.34312102405426 | 0.999950574162623 |
| ebi-fl187-GCST90094840 | prot-a-48 | Adrenomedullin \|\| id:prot-a-48 | F-small fish liking (derived food-liking factor) \|\| id:ebi-fl187-GCST90094840 | MR Egger | 28 | 0.278535629264546 | 0.266719088747421 | 0.305957877276101 | -0.244233785 | 0.801305043209491 | 1.32119367771647 | 0.783304488265887 | 2.22844725159477 | 0.999950574162623 |
| ebi-fl187-GCST90094840 | prot-a-502 | Carcinoembryonic antigen-related cell adhesion molecule 1 \|\| id:prot-a-502 | F-small fish liking (derived food-liking factor) \|\| id:ebi-fl187-GCST90094840 | MR Egger | 28 | -0.318015138 | 0.266535468245245 | 0.243587537322147 | -0.840424656 | 0.204394379907167 | 0.727591774153536 | 0.431527234051786 | 1.22678187618712 | 0.999950574162623 |
| ebi-fl187-GCST90094840 | prot-a-518 | Complement factor B \|\| id:prot-a-518 | F-small fish liking (derived food-liking factor) \|\| id:ebi-fl187-GCST90094840 | MR Egger | 28 | -0.056458122 | 0.266669117612359 | 0.83397946241995 | -0.579129592 | 0.466213348886089 | 0.945106063163891 | 0.560385918650221 | 1.59394702989794 | 0.999950574162623 |
| ebi-fl187-GCST90094840 | prot-a-525 | Properdin \|\| id:prot-a-525 | F-small fish liking (derived food-liking factor) \|\| id:ebi-fl187-GCST90094840 | MR Egger | 28 | 0.685204145557506 | 0.266496929106844 | 0.0162075966956361 | 0.162870164508092 | 1.20753812660692 | 1.98417685508456 | 1.17688387847124 | 3.34523895200884 | 0.999950574162623 |
| ebi-fl187-GCST90094840 | prot-a-533 | Chondroadherin \|\| id:prot-a-533 | F-small fish liking (derived food-liking factor) \|\| id:ebi-fl187-GCST90094840 | MR Egger | 28 | 0.0701590832486422 | 0.266708874383026 | 0.794581658532736 | -0.452590311 | 0.592908477039374 | 1.07267881291185 | 0.635978634002449 | 1.80924291187042 | 0.999950574162623 |
| ebi-fl187-GCST90094840 | prot-a-535 | Coiled-coil-helix-coiled-coil-helix domain-containing protein 2 \|\| id:prot-a-535 | F-small fish liking (derived food-liking factor) \|\| id:ebi-fl187-GCST90094840 | MR Egger | 28 | -0.621068706 | 0.279052928926818 | 0.0349217712222703 | -1.168012447 | -0.074124966 | 0.537369839970572 | 0.31098442538768 | 0.928555648888255 | 0.999950574162623 |
| ebi-fl187-GCST90094840 | prot-a-591 | Caseinolytic peptidase B protein homolog \|\| id:prot-a-591 | F-small fish liking (derived food-liking factor) \|\| id:ebi-fl187-GCST90094840 | MR Egger | 28 | -0.232601025 | 0.266535468245245 | 0.39082422031153 | -0.755010543 | 0.289808492367093 | 0.79246968574071 | 0.470005659362243 | 1.33617157646598 | 0.999950574162623 |
| ebi-fl187-GCST90094840 | prot-a-605 | Ciliary neurotrophic factor receptor subunit alpha \|\| id:prot-a-605 | F-small fish liking (derived food-liking factor) \|\| id:ebi-fl187-GCST90094840 | MR Egger | 28 | -0.429531393 | 0.27177743147505 | 0.126093499626042 | -0.962215159 | 0.103152372282574 | 0.650813999007418 | 0.38204565603556 | 1.10866032531097 | 0.999950574162623 |
| ebi-fl187-GCST90094840 | prot-a-606 | Ciliary neurotrophic factor receptor subunit alpha \|\| id:prot-a-606 | F-small fish liking (derived food-liking factor) \|\| id:ebi-fl187-GCST90094840 | MR Egger | 28 | -0.216792308 | 0.266710236770311 | 0.423691652245799 | -0.739544372 | 0.305959756461503 | 0.805097164497455 | 0.477331351664981 | 1.3579276576343 | 0.999950574162623 |
| ebi-fl187-GCST90094840 | prot-a-707 | Cancer/testis antigen 55 \|\| id:prot-a-707 | F-small fish liking (derived food-liking factor) \|\| id:ebi-fl187-GCST90094840 | MR Egger | 28 | -0.234123868 | 0.266783609963219 | 0.388209399938369 | -0.757019744 | 0.288772007312897 | 0.791263797390835 | 0.469062271759607 | 1.33478737207463 | 0.999950574162623 |
| ebi-fl187-GCST90094840 | prot-a-719 | Dipeptidyl peptidase 1 \|\| id:prot-a-719 | F-small fish liking (derived food-liking factor) \|\| id:ebi-fl187-GCST90094840 | MR Egger | 28 | -0.121039559 | 0.302217755920667 | 0.692057027528401 | -0.713386361 | 0.471307242517854 | 0.885998909591968 | 0.4899821286472 | 1.6020871413524 | 0.999950574162623 |
| ebi-fl187-GCST90094840 | prot-a-752 | Cytochrome b5 \|\| id:prot-a-752 | F-small fish liking (derived food-liking factor) \|\| id:ebi-fl187-GCST90094840 | MR Egger | 28 | -0.086408436 | 0.266753999854067 | 0.748585816752431 | -0.609246275 | 0.436429404133216 | 0.917219529613369 | 0.543760560426005 | 1.54717301461707 | 0.999950574162623 |
| ebi-fl187-GCST90094840 | prot-a-755 | Cytoglobin \|\| id:prot-a-755 | F-small fish liking (derived food-liking factor) \|\| id:ebi-fl187-GCST90094840 | MR Egger | 28 | -0.108881688 | 0.26652842382822 | 0.686238514492352 | -0.631277399 | 0.413514022343788 | 0.896836517421299 | 0.531911903082355 | 1.51212209074372 | 0.999950574162623 |
| ebi-fl187-GCST90094840 | prot-a-807 | Desmin \|\| id:prot-a-807 | F-small fish liking (derived food-liking factor) \|\| id:ebi-fl187-GCST90094840 | MR Egger | 28 | -0.036349881 | 0.266575744154902 | 0.892588192163518 | -0.55883834 | 0.48613857731428 | 0.964302842988619 | 0.571873000259224 | 1.62602531081976 | 0.999950574162623 |
| ebi-fl187-GCST90094840 | prot-a-82 | Ameloblastin \|\| id:prot-a-82 | F-small fish liking (derived food-liking factor) \|\| id:ebi-fl187-GCST90094840 | MR Egger | 28 | -0.007852705 | 0.334050724767351 | 0.981424928890611 | -0.662592125 | 0.646886715992601 | 0.992178047385039 | 0.515513326068755 | 1.90958647998461 | 0.999950574162623 |
| ebi-fl187-GCST90094840 | prot-a-84 | Muellerian-inhibiting factor \|\| id:prot-a-84 | F-small fish liking (derived food-liking factor) \|\| id:ebi-fl187-GCST90094840 | MR Egger | 28 | 0.114773968855664 | 0.301162173129581 | 0.706222874929886 | -0.47550389 | 0.705051828189644 | 1.12161988789119 | 0.621571773443256 | 2.02395157995038 | 0.999950574162623 |
| ebi-fl187-GCST90094840 | prot-a-858 | Inactive dipeptidyl peptidase 10 \|\| id:prot-a-858 | F-small fish liking (derived food-liking factor) \|\| id:ebi-fl187-GCST90094840 | MR Egger | 28 | -0.032370825 | 0.266751375468785 | 0.904345221167834 | -0.555203521 | 0.490461871000699 | 0.968147502287182 | 0.573955437417814 | 1.63307031361494 | 0.999950574162623 |
| ebi-fl187-GCST90094840 | prot-a-863 | Dihydropyrimidinase-related protein 3 \|\| id:prot-a-863 | F-small fish liking (derived food-liking factor) \|\| id:ebi-fl187-GCST90094840 | MR Egger | 28 | -0.118945232 | 0.266746886729407 | 0.659353219183976 | -0.64176913 | 0.403878665967599 | 0.887856425531544 | 0.526360399869802 | 1.49762222339036 | 0.999950574162623 |
| ebi-fl187-GCST90094840 | prot-a-864 | Dihydropyrimidinase-related protein 5 \|\| id:prot-a-864 | F-small fish liking (derived food-liking factor) \|\| id:ebi-fl187-GCST90094840 | MR Egger | 28 | -0.166564127 | 0.266537280299229 | 0.537470754455669 | -0.688977197 | 0.355848942097899 | 0.846568527050482 | 0.502089345182711 | 1.42739191314968 | 0.999950574162623 |
| ebi-fl187-GCST90094840 | prot-a-884 | Endothelin-converting enzyme 1 \|\| id:prot-a-884 | F-small fish liking (derived food-liking factor) \|\| id:ebi-fl187-GCST90094840 | MR Egger | 28 | -0.181492631 | 0.266444990369355 | 0.501790281306902 | -0.703724812 | 0.340739549753535 | 0.834024390897651 | 0.494739057243053 | 1.40598700350934 | 0.999950574162623 |
| ebi-fl187-GCST90094840 | prot-a-893 | Early endosome antigen 1 \|\| id:prot-a-893 | F-small fish liking (derived food-liking factor) \|\| id:ebi-fl187-GCST90094840 | MR Egger | 28 | -0.116515972 | 0.269554595074342 | 0.669121009454216 | -0.644842979 | 0.411811034150765 | 0.890015881355989 | 0.524744931852928 | 1.50954915613724 | 0.999950574162623 |
| ebi-fl187-GCST90094840 | prot-a-897 | Ephrin-A2 \|\| id:prot-a-897 | F-small fish liking (derived food-liking factor) \|\| id:ebi-fl187-GCST90094840 | MR Egger | 28 | -0.215028929 | 0.266531979607679 | 0.427119966169926 | -0.737431609 | 0.307373750851483 | 0.806518107932669 | 0.478340905527561 | 1.35984911787105 | 0.999950574162623 |
| ebi-fl187-GCST90094840 | prot-a-90 | Anaphase-promoting complex subunit 10 \|\| id:prot-a-90 | F-small fish liking (derived food-liking factor) \|\| id:ebi-fl187-GCST90094840 | MR Egger | 28 | -0.117975166 | 0.291424981421403 | 0.688919943570198 | -0.68916813 | 0.45321779739987 | 0.888718122601059 | 0.501993488860452 | 1.5733668244034 | 0.999950574162623 |
| ebi-fl187-GCST90094840 | prot-a-914 | Histone-lysine N-methyltransferase EHMT2 \|\| id:prot-a-914 | F-small fish liking (derived food-liking factor) \|\| id:ebi-fl187-GCST90094840 | MR Egger | 28 | 0.00741805965646947 | 0.266716460558203 | 0.978024055843578 | -0.515346203 | 0.530182322350547 | 1.00744564162037 | 0.597293773900454 | 1.69924209018967 | 0.999950574162623 |
| ebi-fl187-GCST90094840 | prot-a-932 | RNA polymerase II elongation factor ELL \|\| id:prot-a-932 | F-small fish liking (derived food-liking factor) \|\| id:ebi-fl187-GCST90094840 | MR Egger | 28 | -0.042912854 | 0.280099584509834 | 0.879418926279834 | -0.591908039 | 0.506082332128489 | 0.957994872319194 | 0.553270614977498 | 1.65877990000824 | 0.999950574162623 |
| ebi-fl187-GCST90094840 | prot-a-991 | Estrogen receptor \|\| id:prot-a-991 | F-small fish liking (derived food-liking factor) \|\| id:ebi-fl187-GCST90094840 | MR Egger | 28 | -0.275938594 | 0.286446166522756 | 0.3442628973529 | -0.83737308 | 0.285495892627037 | 0.758859528042699 | 0.4328460832352 | 1.33042161083452 | 0.999950574162623 |
| ebi-fl187-GCST90094840 | prot-a-992 | Persulfide dioxygenase ETHE1, mitochondrial \|\| id:prot-a-992 | F-small fish liking (derived food-liking factor) \|\| id:ebi-fl187-GCST90094840 | MR Egger | 28 | 0.200480204091217 | 0.301075180780901 | 0.511352367320738 | -0.38962715 | 0.790587558421783 | 1.22198942160948 | 0.677309362058807 | 2.20469143079087 | 0.999950574162623 |
| ebi-fl187-GCST90094840 | prot-a-993 | Ethanolamine kinase 1 \|\| id:prot-a-993 | F-small fish liking (derived food-liking factor) \|\| id:ebi-fl187-GCST90094840 | MR Egger | 28 | 0.0619718997087079 | 0.297259254748001 | 0.836480671218541 | -0.52065624 | 0.64460003901479 | 1.06393244749629 | 0.594130528032424 | 1.90522486125079 | 0.999950574162623 |
| ebi-fl187-GCST90094840 | prot-b-10 | coagulation factor III, tissue factor \|\| id:prot-b-10 | F-small fish liking (derived food-liking factor) \|\| id:ebi-fl187-GCST90094840 | MR Egger | 19 | -0.709346758 | 0.609172001162313 | 0.260325667628277 | -1.90332388 | 0.48463036435812 | 0.491965465060461 | 0.149072296366749 | 1.62357476681457 | 0.999950574162623 |
| ebi-fl187-GCST90094840 | prot-b-28 | prolactin \|\| id:prot-b-28 | F-small fish liking (derived food-liking factor) \|\| id:ebi-fl187-GCST90094840 | MR Egger | 19 | -0.804279257 | 0.546319763769025 | 0.159244732566119 | -1.875065994 | 0.266507479912209 | 0.447410278171609 | 0.153344846661622 | 1.30539735355642 | 0.999950574162623 |
| ebi-fl187-GCST90094840 | prot-b-54 | matrix metallopeptidase 7 \|\| id:prot-b-54 | F-small fish liking (derived food-liking factor) \|\| id:ebi-fl187-GCST90094840 | MR Egger | 19 | 0.0962636273264352 | 0.556834975728545 | 0.864790159398147 | -0.995132925 | 1.18766017975438 | 1.10104929241746 | 0.369674302283853 | 3.27939901919966 | 0.999950574162623 |
| ebi-fl187-GCST90094840 | ukb-rsfMRI191-edge-pheno716 | Net100_Pair10_33 (OccipitalPrecuneus)&(Temporal\|Frontal\|Supp_Motor_Area) (Default_mode\|Central_executive)&(Default_mode\|Salience) \|\| id:ukb-rsfMRI191-edge-pheno716 | F-small fish liking (derived food-liking factor) \|\| id:ebi-fl187-GCST90094840 | MR Egger | 28 | -0.031334022 | 0.0933086299049872 | 0.739708621162909 | -0.214218936 | 0.151550893097604 | 0.969151801451572 | 0.807171646639662 | 1.16363752142068 | 0.999950574162623 |
| ebi-fl187-GCST90094840 | ukb-rsfMRI191-edge-pheno903 | Net100_Pair27_38 (Precentral\|Frontal\|Supp_Motor_Area)&(Subcortical) (Attention\|Salience\|Motor)&(Subcortical-cerebellum) \|\| id:ukb-rsfMRI191-edge-pheno903 | F-small fish liking (derived food-liking factor) \|\| id:ebi-fl187-GCST90094840 | MR Egger | 28 | -0.038547625 | 0.0818816368506409 | 0.641728940482468 | -0.199035633 | 0.121940383563839 | 0.962185879869956 | 0.819520690921462 | 1.12968675187463 | 0.999950574162623 |
| ebi-fl187-GCST90094840 | ukb-rsfMRI191-node-pheno23 | Net100_Node2 RolandicOper\|SupraMarginal\|Insula Salience\|Motor \|\| id:ukb-rsfMRI191-node-pheno23 | F-small fish liking (derived food-liking factor) \|\| id:ebi-fl187-GCST90094840 | MR Egger | 28 | -0.042236693 | 0.0923093035176343 | 0.651072027543528 | -0.223162928 | 0.13868954173351 | 0.958642849510657 | 0.799984498757057 | 1.14876740030059 | 0.999950574162623 |
| ebi-fl187-GCST90094840 | ukb-rsfMRI191-node-pheno55 | Net100_Node34 Parietal\|Postcentral\|Precuneus Attention \|\| id:ukb-rsfMRI191-node-pheno55 | F-small fish liking (derived food-liking factor) \|\| id:ebi-fl187-GCST90094840 | MR Egger | 28 | -0.011189203 | 0.0801585845220388 | 0.890060948067657 | -0.168300029 | 0.145921622664811 | 0.988873163307003 | 0.845100242349463 | 1.15710549365153 | 0.999950574162623 |
| ebi-fl187-GCST90094840 | ebi-a-GCST90001414 | IgD- CD24- B cell Absolute Count \|\| id:ebi-a-GCST90001414 | F-small fish liking (derived food-liking factor) \|\| id:ebi-fl187-GCST90094840 | Weighted median | 25 | -0.024396215 | 0.105894828811198 | 0.817795295043296 | -0.231950079 | 0.183157649463552 | 0.97589896733081 | 0.792985708580441 | 1.20100373075101 | 0.999332001504091 |
| ebi-fl187-GCST90094840 | ebi-a-GCST90001422 | CD20- CD38- B cell %B cell \|\| id:ebi-a-GCST90001422 | F-small fish liking (derived food-liking factor) \|\| id:ebi-fl187-GCST90094840 | Weighted median | 25 | -0.084270848 | 0.102373553850152 | 0.410411274678393 | -0.284923013 | 0.116381317915902 | 0.919182264039075 | 0.752072151736543 | 1.12342417223284 | 0.999332001504091 |
| ebi-fl187-GCST90094840 | ebi-a-GCST90001444 | CD20- CD38- B cell %lymphocyte \|\| id:ebi-a-GCST90001444 | F-small fish liking (derived food-liking factor) \|\| id:ebi-fl187-GCST90094840 | Weighted median | 25 | -0.090414743 | 0.104186321385391 | 0.385494087857834 | -0.294619933 | 0.113790446433757 | 0.913552216862268 | 0.74481461283804 | 1.12051729188541 | 0.999332001504091 |
| ebi-fl187-GCST90094840 | ebi-a-GCST90001459 | Myeloid Dendritic Cell %Dendritic Cell \|\| id:ebi-a-GCST90001459 | F-small fish liking (derived food-liking factor) \|\| id:ebi-fl187-GCST90094840 | Weighted median | 25 | -0.032733688 | 0.116402761401046 | 0.778548893529554 | -0.2608831 | 0.195415724789546 | 0.967796261460097 | 0.770370970793036 | 1.21581632642771 | 0.999332001504091 |
| ebi-fl187-GCST90094840 | ebi-a-GCST90001476 | HLA DR++ monocyte %leukocyte \|\| id:ebi-a-GCST90001476 | F-small fish liking (derived food-liking factor) \|\| id:ebi-fl187-GCST90094840 | Weighted median | 25 | -0.024172092 | 0.109581576333674 | 0.825415294526154 | -0.238951982 | 0.190607797193881 | 0.9761177128433 | 0.787452693341437 | 1.20998479957361 | 0.999332001504091 |
| ebi-fl187-GCST90094840 | ebi-a-GCST90001478 | CD4 regulatory T cell %CD4+ T cell \|\| id:ebi-a-GCST90001478 | F-small fish liking (derived food-liking factor) \|\| id:ebi-fl187-GCST90094840 | Weighted median | 25 | -0.027640531 | 0.113607973256655 | 0.807774976509923 | -0.250312158 | 0.195031096927734 | 0.972737973442916 | 0.778557711931235 | 1.21534877951527 | 0.999332001504091 |
| ebi-fl187-GCST90094840 | ebi-a-GCST90001485 | CD39+ resting CD4 regulatory T cell %CD4 regulatory T cell \|\| id:ebi-a-GCST90001485 | F-small fish liking (derived food-liking factor) \|\| id:ebi-fl187-GCST90094840 | Weighted median | 25 | 0.00728820631112458 | 0.110477438624744 | 0.947401640144737 | -0.209247573 | 0.223823986015623 | 1.00731482992691 | 0.81119438063576 | 1.25085083281202 | 0.999332001504091 |
| ebi-fl187-GCST90094840 | ebi-a-GCST90001515 | Immature Myeloid-Derived Suppressor Cells Absolute Count \|\| id:ebi-a-GCST90001515 | F-small fish liking (derived food-liking factor) \|\| id:ebi-fl187-GCST90094840 | Weighted median | 25 | 0.0793406833307394 | 0.14791823485889 | 0.591694547127781 | -0.210579057 | 0.369260423654163 | 1.08257307385352 | 0.810115007365458 | 1.44666430022568 | 0.999332001504091 |
| ebi-fl187-GCST90094840 | ebi-a-GCST90001517 | CD33+ HLA DR+ Absolute Count \|\| id:ebi-a-GCST90001517 | F-small fish liking (derived food-liking factor) \|\| id:ebi-fl187-GCST90094840 | Weighted median | 25 | 0.0540806385447684 | 0.140033874094154 | 0.699350899814298 | -0.220385755 | 0.32854703176931 | 1.0555697183292 | 0.802209282282957 | 1.38894856350038 | 0.999332001504091 |
| ebi-fl187-GCST90094840 | ebi-a-GCST90001518 | CD33+ HLA DR+ CD14- Absolute Count \|\| id:ebi-a-GCST90001518 | F-small fish liking (derived food-liking factor) \|\| id:ebi-fl187-GCST90094840 | Weighted median | 25 | -0.124006674 | 0.137167504536133 | 0.365967374410702 | -0.392854983 | 0.144841634522603 | 0.883373944900093 | 0.675126645153386 | 1.15585650800534 | 0.999332001504091 |
| ebi-fl187-GCST90094840 | ebi-a-GCST90001520 | CD33+ HLA DR+ CD14dim Absolute Count \|\| id:ebi-a-GCST90001520 | F-small fish liking (derived food-liking factor) \|\| id:ebi-fl187-GCST90094840 | Weighted median | 25 | 0.0444242561318794 | 0.133061752398518 | 0.738483733371431 | -0.216376779 | 0.305225290832975 | 1.04542578911841 | 0.805431775264025 | 1.35693067261417 | 0.999332001504091 |
| ebi-fl187-GCST90094840 | ebi-a-GCST90001531 | CD33dim HLA DR- Absolute Count \|\| id:ebi-a-GCST90001531 | F-small fish liking (derived food-liking factor) \|\| id:ebi-fl187-GCST90094840 | Weighted median | 25 | -0.102148373 | 0.155428780311824 | 0.51104993103324 | -0.406788783 | 0.202492036086776 | 0.902895576111711 | 0.665784800695627 | 1.22445033366688 | 0.999332001504091 |
| ebi-fl187-GCST90094840 | ebi-a-GCST90001532 | Basophil Absolute Count \|\| id:ebi-a-GCST90001532 | F-small fish liking (derived food-liking factor) \|\| id:ebi-fl187-GCST90094840 | Weighted median | 25 | -0.11338827 | 0.147377395489809 | 0.441671585810562 | -0.402247965 | 0.175471425187894 | 0.892803943848216 | 0.668814882356342 | 1.19180793247695 | 0.999332001504091 |
| ebi-fl187-GCST90094840 | ebi-a-GCST90001588 | T/B cell \|\| id:ebi-a-GCST90001588 | F-small fish liking (derived food-liking factor) \|\| id:ebi-fl187-GCST90094840 | Weighted median | 25 | -0.002800617 | 0.120492300029854 | 0.981456346180162 | -0.238965525 | 0.233364291534948 | 0.997203301544369 | 0.787442029297784 | 1.26284143798849 | 0.999332001504091 |
| ebi-fl187-GCST90094840 | ebi-a-GCST90001624 | HLA DR+ CD4+ T cell Absolute Count \|\| id:ebi-a-GCST90001624 | F-small fish liking (derived food-liking factor) \|\| id:ebi-fl187-GCST90094840 | Weighted median | 25 | -0.095100653 | 0.106709638997329 | 0.372816742383539 | -0.304251546 | 0.114050239179986 | 0.909281407728879 | 0.737675284072354 | 1.12080843196627 | 0.999332001504091 |
| ebi-fl187-GCST90094840 | ebi-a-GCST90001631 | CD8+ Natural Killer T %T cell \|\| id:ebi-a-GCST90001631 | F-small fish liking (derived food-liking factor) \|\| id:ebi-fl187-GCST90094840 | Weighted median | 25 | -0.095215217 | 0.107462041502336 | 0.375598231006558 | -0.305840818 | 0.115410384411168 | 0.909177243072755 | 0.73650384807633 | 1.12233393142531 | 0.999332001504091 |
| ebi-fl187-GCST90094840 | ebi-a-GCST90001632 | CD8+ Natural Killer T %lymphocyte \|\| id:ebi-a-GCST90001632 | F-small fish liking (derived food-liking factor) \|\| id:ebi-fl187-GCST90094840 | Weighted median | 25 | -0.077946581 | 0.105663244466005 | 0.460703644795052 | -0.28504654 | 0.129153378467284 | 0.925013838828373 | 0.751979256511119 | 1.13786463471596 | 0.999332001504091 |
| ebi-fl187-GCST90094840 | ebi-a-GCST90001646 | Natural Killer %CD3- lymphocyte \|\| id:ebi-a-GCST90001646 | F-small fish liking (derived food-liking factor) \|\| id:ebi-fl187-GCST90094840 | Weighted median | 25 | 0.0410897580519957 | 0.109929596024911 | 0.708566148264365 | -0.17437225 | 0.256551766260822 | 1.04194562435791 | 0.839984155219316 | 1.29246567018296 | 0.999332001504091 |
| ebi-fl187-GCST90094840 | ebi-a-GCST90001684 | CD127- CD8+ T cell Absolute Count \|\| id:ebi-a-GCST90001684 | F-small fish liking (derived food-liking factor) \|\| id:ebi-fl187-GCST90094840 | Weighted median | 25 | -0.14531001 | 0.116516258854247 | 0.212352704992656 | -0.373681877 | 0.0830618577579717 | 0.864754169461417 | 0.68819580795814 | 1.08660902166726 | 0.999332001504091 |
| ebi-fl187-GCST90094840 | ebi-a-GCST90001687 | CD28- CD8+ T cell Absolute Count \|\| id:ebi-a-GCST90001687 | F-small fish liking (derived food-liking factor) \|\| id:ebi-fl187-GCST90094840 | Weighted median | 25 | -0.166601826 | 0.109767272035437 | 0.129071578632483 | -0.381745679 | 0.0485420273727321 | 0.846536613264602 | 0.682668648099075 | 1.04973948868602 | 0.999332001504091 |
| ebi-fl187-GCST90094840 | ebi-a-GCST90001701 | BAFF-R on CD20- CD38- B cell \|\| id:ebi-a-GCST90001701 | F-small fish liking (derived food-liking factor) \|\| id:ebi-fl187-GCST90094840 | Weighted median | 25 | -0.205746543 | 0.110685278646077 | 0.0630494112593049 | -0.422689689 | 0.0111966030678075 | 0.814039374051742 | 0.655281942613188 | 1.01125951962592 | 0.999332001504091 |
| ebi-fl187-GCST90094840 | ebi-a-GCST90001732 | CD19 on IgD- CD27- B cell \|\| id:ebi-a-GCST90001732 | F-small fish liking (derived food-liking factor) \|\| id:ebi-fl187-GCST90094840 | Weighted median | 25 | 0.0536601952503612 | 0.112749576720838 | 0.634128739995495 | -0.167328975 | 0.274649365623204 | 1.05512600440416 | 0.845921278501205 | 1.31606913487553 | 0.999332001504091 |
| ebi-fl187-GCST90094840 | ebi-a-GCST90001798 | CD27 on CD24+ CD27+ B cell \|\| id:ebi-a-GCST90001798 | F-small fish liking (derived food-liking factor) \|\| id:ebi-fl187-GCST90094840 | Weighted median | 25 | 0.0805151932790698 | 0.12098506023665 | 0.505732668880581 | -0.156615525 | 0.317645911342905 | 1.08384531368148 | 0.855032734524444 | 1.37388969633152 | 0.999332001504091 |
| ebi-fl187-GCST90094840 | ebi-a-GCST90001836 | CD66b on CD66b++ myeloid cell \|\| id:ebi-a-GCST90001836 | F-small fish liking (derived food-liking factor) \|\| id:ebi-fl187-GCST90094840 | Weighted median | 25 | -0.291479308 | 0.174767558736649 | 0.0953531932482168 | -0.634023723 | 0.0510651069040303 | 0.747157473461238 | 0.530453104591423 | 1.05239140899922 | 0.999332001504091 |
| ebi-fl187-GCST90094840 | ebi-a-GCST90001943 | CD25 on activated & secreting CD4 regulatory T cell \|\| id:ebi-a-GCST90001943 | F-small fish liking (derived food-liking factor) \|\| id:ebi-fl187-GCST90094840 | Weighted median | 25 | 0.109023448614645 | 0.119213500605553 | 0.360442272503188 | -0.124635013 | 0.342681909801529 | 1.11518849966025 | 0.882819061647504 | 1.40872059043855 | 0.999332001504091 |
| ebi-fl187-GCST90094840 | ebi-a-GCST90001960 | CD25 on CD4+ T cell \|\| id:ebi-a-GCST90001960 | F-small fish liking (derived food-liking factor) \|\| id:ebi-fl187-GCST90094840 | Weighted median | 25 | 0.0622548427559707 | 0.112061228166296 | 0.578523234362024 | -0.157385164 | 0.28189484996191 | 1.06423352237658 | 0.854374920589226 | 1.32563932163289 | 0.999332001504091 |
| ebi-fl187-GCST90094840 | ebi-a-GCST90001988 | HLA DR on CD14+ CD16- monocyte \|\| id:ebi-a-GCST90001988 | F-small fish liking (derived food-liking factor) \|\| id:ebi-fl187-GCST90094840 | Weighted median | 25 | 0.0928287709660023 | 0.126184246284418 | 0.461937308600722 | -0.154492352 | 0.340149893683462 | 1.09727383404283 | 0.856850045519148 | 1.40515819911707 | 0.999332001504091 |
| ebi-fl187-GCST90094840 | ebi-a-GCST90001991 | HLA DR on CD14+ monocyte \|\| id:ebi-a-GCST90001991 | F-small fish liking (derived food-liking factor) \|\| id:ebi-fl187-GCST90094840 | Weighted median | 25 | 0.0849976409318186 | 0.123366690423875 | 0.490833514418235 | -0.156801072 | 0.326796354162614 | 1.08871449834364 | 0.854874100043555 | 1.3865190895867 | 0.999332001504091 |
| ebi-fl187-GCST90094840 | ebi-a-GCST90002019 | CD14 on Monocytic Myeloid-Derived Suppressor Cells \|\| id:ebi-a-GCST90002019 | F-small fish liking (derived food-liking factor) \|\| id:ebi-fl187-GCST90094840 | Weighted median | 25 | -0.030572203 | 0.153586197649016 | 0.842219198018772 | -0.331601151 | 0.270456744027186 | 0.969890400188509 | 0.717773549206845 | 1.31056290583194 | 0.999332001504091 |
| ebi-fl187-GCST90094840 | ebi-a-GCST90002037 | CD80 on plasmacytoid Dendritic Cell \|\| id:ebi-a-GCST90002037 | F-small fish liking (derived food-liking factor) \|\| id:ebi-fl187-GCST90094840 | Weighted median | 25 | -0.022650363 | 0.121664486831569 | 0.852310896972263 | -0.261112757 | 0.215812030999902 | 0.977604230450791 | 0.770194069653417 | 1.24086911215163 | 0.999332001504091 |
| ebi-fl187-GCST90094840 | ebi-a-GCST90002042 | CD45 on CD33+ HLA DR+ CD14- \|\| id:ebi-a-GCST90002042 | F-small fish liking (derived food-liking factor) \|\| id:ebi-fl187-GCST90094840 | Weighted median | 25 | 0.168909174194186 | 0.166312396291495 | 0.309813303748562 | -0.157063123 | 0.494881470925517 | 1.18401259514212 | 0.854650109431714 | 1.64030380384242 | 0.999332001504091 |
| ebi-fl187-GCST90094840 | ebi-a-GCST90002056 | CD8 on Effector Memory CD8+ T cell \|\| id:ebi-a-GCST90002056 | F-small fish liking (derived food-liking factor) \|\| id:ebi-fl187-GCST90094840 | Weighted median | 25 | -0.043923277 | 0.12253539802302 | 0.720003747016363 | -0.284092657 | 0.196246102851999 | 0.957027380406729 | 0.752696898538714 | 1.21682633291873 | 0.999332001504091 |
| ebi-fl187-GCST90094840 | ebi-a-GCST90002058 | CD8 on CD8+ T cell \|\| id:ebi-a-GCST90002058 | F-small fish liking (derived food-liking factor) \|\| id:ebi-fl187-GCST90094840 | Weighted median | 25 | -0.169221215 | 0.121088781400889 | 0.162264153547092 | -0.406555226 | 0.0681127969109758 | 0.844322106319298 | 0.66594031726034 | 1.07048604918865 | 0.999332001504091 |
| ebi-fl187-GCST90094840 | ebi-a-GCST90002063 | CD4 on CD4 regulatory T cell \|\| id:ebi-a-GCST90002063 | F-small fish liking (derived food-liking factor) \|\| id:ebi-fl187-GCST90094840 | Weighted median | 25 | 0.0252585950312205 | 0.124325042722395 | 0.839005453895558 | -0.218418489 | 0.268935678767116 | 1.02558029620511 | 0.803788994656628 | 1.30857096944141 | 0.999332001504091 |
| ebi-fl187-GCST90094840 | ebi-a-GCST90002066 | CD4 on activated CD4 regulatory T cell \|\| id:ebi-a-GCST90002066 | F-small fish liking (derived food-liking factor) \|\| id:ebi-fl187-GCST90094840 | Weighted median | 25 | 0.0525613595636775 | 0.124246575698827 | 0.672265559414883 | -0.190961929 | 0.296084647933379 | 1.05396723106392 | 0.82616404060392 | 1.3445839682692 | 0.999332001504091 |
| ebi-fl187-GCST90094840 | ebi-a-GCST90002070 | CD4 on activated & secreting CD4 regulatory T cell \|\| id:ebi-a-GCST90002070 | F-small fish liking (derived food-liking factor) \|\| id:ebi-fl187-GCST90094840 | Weighted median | 25 | 0.00303841634248097 | 0.128982076312518 | 0.981206060887154 | -0.249766453 | 0.255843285915016 | 1.00304303700807 | 0.778982690719867 | 1.29155030795437 | 0.999332001504091 |
| ebi-fl187-GCST90094840 | ebi-a-GCST90002081 | SSC-A on CD4+ T cell \|\| id:ebi-a-GCST90002081 | F-small fish liking (derived food-liking factor) \|\| id:ebi-fl187-GCST90094840 | Weighted median | 25 | 0.0546027930586874 | 0.103050213203639 | 0.596204916470238 | -0.147375625 | 0.25658121093782 | 1.05612103274551 | 0.862969763666239 | 1.29250372697743 | 0.999332001504091 |
| ebi-fl187-GCST90094840 | ebi-a-GCST90002094 | CD11b on Monocytic Myeloid-Derived Suppressor Cells \|\| id:ebi-a-GCST90002094 | F-small fish liking (derived food-liking factor) \|\| id:ebi-fl187-GCST90094840 | Weighted median | 25 | -0.258197139 | 0.161997516879431 | 0.110973235523624 | -0.575712272 | 0.0593179939169039 | 0.772442938338638 | 0.562304212448845 | 1.06111261445247 | 0.999332001504091 |
| ebi-fl187-GCST90094840 | ebi-a-GCST90002104 | HLA DR on myeloid Dendritic Cell \|\| id:ebi-a-GCST90002104 | F-small fish liking (derived food-liking factor) \|\| id:ebi-fl187-GCST90094840 | Weighted median | 24 | 0.152681493817835 | 0.127253183066411 | 0.230207485338714 | -0.096734745 | 0.402097732628001 | 1.16495387483533 | 0.907796771839383 | 1.49495743165515 | 0.999332001504091 |
| ebi-fl187-GCST90094840 | ebi-a-GCST90002105 | HLA DR on plasmacytoid Dendritic Cell \|\| id:ebi-a-GCST90002105 | F-small fish liking (derived food-liking factor) \|\| id:ebi-fl187-GCST90094840 | Weighted median | 25 | 0.0449790950537344 | 0.119142896484483 | 0.705785528085072 | -0.188540982 | 0.278499172163322 | 1.04600599298108 | 0.828166562774836 | 1.32114551170282 | 0.999332001504091 |
| ebi-fl187-GCST90094840 | ebi-a-GCST90002106 | HLA DR on Dendritic Cell \|\| id:ebi-a-GCST90002106 | F-small fish liking (derived food-liking factor) \|\| id:ebi-fl187-GCST90094840 | Weighted median | 24 | -0.00469453 | 0.132485326881789 | 0.971733403460691 | -0.264365771 | 0.254976710724108 | 0.995316472118326 | 0.767692688840625 | 1.29043156730614 | 0.999332001504091 |
| ebi-fl187-GCST90094840 | ebi-a-GCST90002111 | HLA DR on CD33dim HLA DR+ CD11b- \|\| id:ebi-a-GCST90002111 | F-small fish liking (derived food-liking factor) \|\| id:ebi-fl187-GCST90094840 | Weighted median | 25 | -0.082970386 | 0.164232164909516 | 0.613417102787652 | -0.404865429 | 0.238924657095266 | 0.920378402785329 | 0.667066572400922 | 1.2698828563164 | 0.999332001504091 |
| ebi-fl187-GCST90094840 | ebi-a-GCST90002112 | HLA DR on CD33- HLA DR+ \|\| id:ebi-a-GCST90002112 | F-small fish liking (derived food-liking factor) \|\| id:ebi-fl187-GCST90094840 | Weighted median | 25 | 0.106445467295751 | 0.155796008474236 | 0.494457623889555 | -0.198914709 | 0.411805643905253 | 1.11231726712318 | 0.819619796286735 | 1.5095410193186 | 0.999332001504091 |
| ebi-fl187-GCST90094840 | ebi-a-GCST90002115 | HLA DR on HLA DR+ CD8+ T cell \|\| id:ebi-a-GCST90002115 | F-small fish liking (derived food-liking factor) \|\| id:ebi-fl187-GCST90094840 | Weighted median | 25 | 0.13331727781619 | 0.113740690938758 | 0.241150507743484 | -0.089614476 | 0.356249032056155 | 1.14261246641441 | 0.914283595217003 | 1.42796311257859 | 0.999332001504091 |
| ebi-fl187-GCST90094840 | ebi-a-GCST90002116 | HLA DR on B cell \|\| id:ebi-a-GCST90002116 | F-small fish liking (derived food-liking factor) \|\| id:ebi-fl187-GCST90094840 | Weighted median | 25 | -0.152586606 | 0.126239110229524 | 0.226773880700622 | -0.400015262 | 0.0948420504662307 | 0.858484541182027 | 0.670309815934832 | 1.09948517823908 | 0.999332001504091 |
| ebi-fl187-GCST90094840 | ebi-cfmet338-GCST90026023 | Sphingomyelin (d18:1/24:1, d18:2/24:0) levels \|\| id:ebi-cfmet338-GCST90026023 | F-small fish liking (derived food-liking factor) \|\| id:ebi-fl187-GCST90094840 | Weighted median | 23 | -0.012883636 | 0.0664721419886082 | 0.846316757314577 | -0.143169035 | 0.117401761887375 | 0.987199002356636 | 0.866607569756641 | 1.12457114876992 | 0.999332001504091 |
| ebi-fl187-GCST90094840 | ebi-cfmet338-GCST90026046 | 1-arachidonoyl-gpc (20:4n6) levels \|\| id:ebi-cfmet338-GCST90026046 | F-small fish liking (derived food-liking factor) \|\| id:ebi-fl187-GCST90094840 | Weighted median | 23 | 0.0689053992475607 | 0.081686806 | 0.398931219286747 | -0.09120074 | 0.229011538648881 | 1.07133485527092 | 0.912834449978123 | 1.25735654712185 | 0.999332001504091 |
| ebi-fl187-GCST90094840 | ebi-cfmet338-GCST90026047 | 1-linoleoyl-gpc (18:2) levels \|\| id:ebi-cfmet338-GCST90026047 | F-small fish liking (derived food-liking factor) \|\| id:ebi-fl187-GCST90094840 | Weighted median | 23 | -0.041466143 | 0.0957703189853844 | 0.665033011897299 | -0.229175969 | 0.146243681870904 | 0.959381816255153 | 0.79518859300777 | 1.15747821014334 | 0.999332001504091 |
| ebi-fl187-GCST90094840 | ebi-cfmet338-GCST90026050 | 1-myristoyl-2-palmitoyl-gpc (14:0/16:0) levels \|\| id:ebi-cfmet338-GCST90026050 | F-small fish liking (derived food-liking factor) \|\| id:ebi-fl187-GCST90094840 | Weighted median | 23 | -0.000328758 | 0.0530199070830053 | 0.995052620353948 | -0.104247776 | 0.103590259434522 | 0.999671295586968 | 0.90100202278596 | 1.10914589972894 | 0.999364451047625 |
| ebi-fl187-GCST90094840 | ebi-cfmet338-GCST90026068 | 2,3-dihydroxyisovalerate levels \|\| id:ebi-cfmet338-GCST90026068 | F-small fish liking (derived food-liking factor) \|\| id:ebi-fl187-GCST90094840 | Weighted median | 23 | 0.114296783267179 | 0.174772663994007 | 0.513128537759894 | -0.228257638 | 0.456851204695433 | 1.12108479472445 | 0.795919174265233 | 1.57909390500943 | 0.999332001504091 |
| ebi-fl187-GCST90094840 | ebi-cfmet338-GCST90026082 | 3-hydroxyhexanoate levels \|\| id:ebi-cfmet338-GCST90026082 | F-small fish liking (derived food-liking factor) \|\| id:ebi-fl187-GCST90094840 | Weighted median | 23 | 0.0356885550177765 | 0.0378134226604629 | 0.345268525927488 | -0.038425753 | 0.109802863432284 | 1.03633303550078 | 0.962303149827697 | 1.11605803292088 | 0.999332001504091 |
| ebi-fl187-GCST90094840 | ebi-cfmet338-GCST90026094 | 3-ureidopropionate levels \|\| id:ebi-cfmet338-GCST90026094 | F-small fish liking (derived food-liking factor) \|\| id:ebi-fl187-GCST90094840 | Weighted median | 23 | -0.072587605 | 0.0814064718378368 | 0.372570520195379 | -0.23214429 | 0.0869690796336301 | 0.929984271662666 | 0.792831717387805 | 1.09086294931475 | 0.999332001504091 |
| ebi-fl187-GCST90094840 | ebi-cfmet338-GCST90026173 | Glycerol levels \|\| id:ebi-cfmet338-GCST90026173 | F-small fish liking (derived food-liking factor) \|\| id:ebi-fl187-GCST90094840 | Weighted median | 22 | -0.015474911 | 0.0480974629043118 | 0.747649155262335 | -0.109745938 | 0.0787961167646697 | 0.984644210645396 | 0.896061761784068 | 1.08198370124305 | 0.999332001504091 |
| ebi-fl187-GCST90094840 | ebi-cfmet338-GCST90026179 | Histidine levels \|\| id:ebi-cfmet338-GCST90026179 | F-small fish liking (derived food-liking factor) \|\| id:ebi-fl187-GCST90094840 | Weighted median | 23 | 0.0284694092001517 | 0.0315231129315595 | 0.366457910058194 | -0.033315892 | 0.0902547105460084 | 1.02887853613574 | 0.967232970026604 | 1.09445301693108 | 0.999332001504091 |
| ebi-fl187-GCST90094840 | ebi-cfmet338-GCST90026188 | Isocitrate levels \|\| id:ebi-cfmet338-GCST90026188 | F-small fish liking (derived food-liking factor) \|\| id:ebi-fl187-GCST90094840 | Weighted median | 22 | 0.0127947271716668 | 0.0623175675008201 | 0.837326078038413 | -0.109347705 | 0.134937159473274 | 1.01287692990643 | 0.896418673932457 | 1.14446486331673 | 0.999332001504091 |
| ebi-fl187-GCST90094840 | ebi-cfmet338-GCST90026207 | N-acetyl-isoputreanine levels \|\| id:ebi-cfmet338-GCST90026207 | F-small fish liking (derived food-liking factor) \|\| id:ebi-fl187-GCST90094840 | Weighted median | 23 | 0.0313270520696528 | 0.0389205707753412 | 0.42087909876804 | -0.044957267 | 0.107611370789322 | 1.03182290852682 | 0.95603833568098 | 1.11361487800842 | 0.999332001504091 |
| ebi-fl187-GCST90094840 | ebi-cfmet338-GCST90026213 | N-acetylglutamate levels \|\| id:ebi-cfmet338-GCST90026213 | F-small fish liking (derived food-liking factor) \|\| id:ebi-fl187-GCST90094840 | Weighted median | 23 | 0.0609769193000645 | 0.0645697027334939 | 0.344986704994138 | -0.065579698 | 0.187533536657713 | 1.06287438201941 | 0.936524404609323 | 1.20627070303031 | 0.999332001504091 |
| ebi-fl187-GCST90094840 | ebi-cfmet338-GCST90026224 | N-acetyltaurine levels \|\| id:ebi-cfmet338-GCST90026224 | F-small fish liking (derived food-liking factor) \|\| id:ebi-fl187-GCST90094840 | Weighted median | 23 | 0.10239022960675 | 0.0545213446349059 | 0.0603833119606963 | -0.004471606 | 0.209252065091166 | 1.1078156898713 | 0.995538376866722 | 1.23275569404727 | 0.999332001504091 |
| ebi-fl187-GCST90094840 | ebi-cfmet338-GCST90026250 | Phenyllactate (pla) levels \|\| id:ebi-cfmet338-GCST90026250 | F-small fish liking (derived food-liking factor) \|\| id:ebi-fl187-GCST90094840 | Weighted median | 23 | 0.00836497271318383 | 0.0843068950359079 | 0.920963194435247 | -0.156876542 | 0.173606486983563 | 1.00840005685516 | 0.854809585763807 | 1.18958735559438 | 0.999332001504091 |
| ebi-fl187-GCST90094840 | ebi-cfmet338-GCST90026251 | Pipecolate levels \|\| id:ebi-cfmet338-GCST90026251 | F-small fish liking (derived food-liking factor) \|\| id:ebi-fl187-GCST90094840 | Weighted median | 23 | -0.033208879 | 0.136412285692248 | 0.807660743452152 | -0.300576959 | 0.234159200980075 | 0.967336482230348 | 0.74039092226984 | 1.26384568166376 | 0.999332001504091 |
| ebi-fl187-GCST90094840 | ebi-cfmet338-GCST90026278 | Trigonelline (n'-methylnicotinate) levels \|\| id:ebi-cfmet338-GCST90026278 | F-small fish liking (derived food-liking factor) \|\| id:ebi-fl187-GCST90094840 | Weighted median | 23 | 0.26801419953672 | 0.148234025224311 | 0.0705990652500745 | -0.02252449 | 0.558552888976369 | 1.30736570387045 | 0.977727292453652 | 1.74814091500643 | 0.999332001504091 |
| ebi-fl187-GCST90094840 | ebi-cfmet338-GCST90026299 | 1-carboxyethylvaline levels \|\| id:ebi-cfmet338-GCST90026299 | F-small fish liking (derived food-liking factor) \|\| id:ebi-fl187-GCST90094840 | Weighted median | 23 | -0.021607153 | 0.0866716202286714 | 0.803129461858085 | -0.191483529 | 0.148269222585548 | 0.97862460922671 | 0.8257332258852 | 1.15982510544789 | 0.999332001504091 |
| ebi-fl187-GCST90094840 | ebi-gm430-GCST90011306 | C_Erysipelotrichia abundance \|\| id:ebi-gm430-GCST90011306 | F-small fish liking (derived food-liking factor) \|\| id:ebi-fl187-GCST90094840 | Weighted median | 21 | 0.0293900073013552 | 0.0725583495703334 | 0.685438247953693 | -0.112824358 | 0.171604372459209 | 1.02982615588402 | 0.893307548790156 | 1.18720804808959 | 0.999332001504091 |
| ebi-fl187-GCST90094840 | ebi-gm430-GCST90011312 | F_Erysipelotrichaceae abundance \|\| id:ebi-gm430-GCST90011312 | F-small fish liking (derived food-liking factor) \|\| id:ebi-fl187-GCST90094840 | Weighted median | 21 | 0.0293900073013552 | 0.0752610402970241 | 0.69616124557249 | -0.118121632 | 0.176901646283522 | 1.02982615588402 | 0.888587965593543 | 1.1935137008461 | 0.999332001504091 |
| ebi-fl187-GCST90094840 | ebi-gm430-GCST90011352 | O_Erysipelotrichales abundance \|\| id:ebi-gm430-GCST90011352 | F-small fish liking (derived food-liking factor) \|\| id:ebi-fl187-GCST90094840 | Weighted median | 21 | 0.0293900073013552 | 0.0774146436559206 | 0.704209672284486 | -0.122342694 | 0.18112270886696 | 1.02982615588402 | 0.884845085203423 | 1.19856224448492 | 0.999332001504091 |
| ebi-fl187-GCST90094840 | ebi-gm430-GCST90011362 | OTU97_11 (Parabacteroides) abundance \|\| id:ebi-gm430-GCST90011362 | F-small fish liking (derived food-liking factor) \|\| id:ebi-fl187-GCST90094840 | Weighted median | 21 | 0.0669642225060022 | 0.0889607037551476 | 0.451606557559127 | -0.107398757 | 0.241327201866092 | 1.0692572221464 | 0.898167451146082 | 1.27293747469178 | 0.999332001504091 |
| ebi-fl187-GCST90094840 | ebi-gm430-GCST90011384 | OTU97_235 (Phascolarctobacterium) abundance \|\| id:ebi-gm430-GCST90011384 | F-small fish liking (derived food-liking factor) \|\| id:ebi-fl187-GCST90094840 | Weighted median | 21 | 0.279544335342483 | 0.281764169666234 | 0.321138182364503 | -0.272713437 | 0.831802107888303 | 1.32252704618469 | 0.761310919759462 | 2.29745527417712 | 0.999332001504091 |
| ebi-fl187-GCST90094840 | ebi-gm430-GCST90011390 | OTU97_3 (Bacteroides) abundance \|\| id:ebi-gm430-GCST90011390 | F-small fish liking (derived food-liking factor) \|\| id:ebi-fl187-GCST90094840 | Weighted median | 21 | -0.081493522 | 0.0746577943528769 | 0.275026189676923 | -0.227822799 | 0.0648357544574655 | 0.921738680420658 | 0.796265346027254 | 1.06698376266463 | 0.999332001504091 |
| ebi-fl187-GCST90094840 | ebi-gm430-GCST90011398 | OTU97_4 (Alistipes) abundance \|\| id:ebi-gm430-GCST90011398 | F-small fish liking (derived food-liking factor) \|\| id:ebi-fl187-GCST90094840 | Weighted median | 21 | -0.138969735 | 0.0831369840134641 | 0.0946080842425565 | -0.301918224 | 0.0239787535582427 | 0.870254366213973 | 0.739398527637994 | 1.02426855559723 | 0.999332001504091 |
| ebi-fl187-GCST90094840 | ebi-gm430-GCST90011421 | OTU99_108 (Cytophagales) abundance \|\| id:ebi-gm430-GCST90011421 | F-small fish liking (derived food-liking factor) \|\| id:ebi-fl187-GCST90094840 | Weighted median | 21 | 0.11482354394187 | 0.258124021805462 | 0.656436353830472 | -0.391099539 | 0.620746626680575 | 1.12167549367214 | 0.67631283332302 | 1.86031648537965 | 0.999332001504091 |
| ebi-fl187-GCST90094840 | ebi-gm430-GCST90011428 | OTU99_123 (Phascolarctobacterium) abundance \|\| id:ebi-gm430-GCST90011428 | F-small fish liking (derived food-liking factor) \|\| id:ebi-fl187-GCST90094840 | Weighted median | 21 | -0.015211088 | 0.160594764407964 | 0.924539445786756 | -0.329976827 | 0.299554649800725 | 0.984904015807533 | 0.718940393475774 | 1.34925778153052 | 0.999332001504091 |
| ebi-fl187-GCST90094840 | ebi-gm430-GCST90011450 | OTU99_298 (Phascolarctobacterium) abundance \|\| id:ebi-gm430-GCST90011450 | F-small fish liking (derived food-liking factor) \|\| id:ebi-fl187-GCST90094840 | Weighted median | 21 | 0.282853836628163 | 0.285034959300521 | 0.32102785598852 | -0.275814684 | 0.841522356857185 | 1.32691120182719 | 0.758953564271637 | 2.31989599946633 | 0.999332001504091 |
| ebi-fl187-GCST90094840 | ebi-gm430-GCST90011451 | OTU99_3 (Bacteroides) abundance \|\| id:ebi-gm430-GCST90011451 | F-small fish liking (derived food-liking factor) \|\| id:ebi-fl187-GCST90094840 | Weighted median | 21 | -0.071862765 | 0.0740927340190117 | 0.332094844159056 | -0.217084524 | 0.073358993613497 | 0.9306586059223 | 0.804861936487964 | 1.07611678663374 | 0.999332001504091 |
| ebi-fl187-GCST90094840 | ebi-gm430-GCST90011472 | OTU99_72 (Parabacteroides) abundance \|\| id:ebi-gm430-GCST90011472 | F-small fish liking (derived food-liking factor) \|\| id:ebi-fl187-GCST90094840 | Weighted median | 21 | -0.010370875 | 0.219158626338281 | 0.962257138732488 | -0.439921782 | 0.419180032828266 | 0.989682717301597 | 0.644086798025007 | 1.52071410860908 | 0.999332001504091 |
| ebi-fl187-GCST90094840 | ebi-gm430-GCST90011507 | TestASV_3 (Bacteroides) abundance \|\| id:ebi-gm430-GCST90011507 | F-small fish liking (derived food-liking factor) \|\| id:ebi-fl187-GCST90094840 | Weighted median | 21 | -0.069363666 | 0.0919329572626912 | 0.450547412278535 | -0.249552263 | 0.11082492985457 | 0.932987322253072 | 0.779149559371581 | 1.11719930148844 | 0.999332001504091 |
| ebi-fl187-GCST90094840 | ebi-gm430-GCST90011512 | TestASV_34 (Phascolarctobacterium) abundance \|\| id:ebi-gm430-GCST90011512 | F-small fish liking (derived food-liking factor) \|\| id:ebi-fl187-GCST90094840 | Weighted median | 21 | -0.135276617 | 0.245171112186819 | 0.581109989679482 | -0.615811997 | 0.345258762963218 | 0.873474260504957 | 0.540202074811982 | 1.41235533763959 | 0.999332001504091 |
| ebi-fl187-GCST90094840 | ebi-gm430-GCST90011519 | TestASV_41 (Barnesiella) abundance \|\| id:ebi-gm430-GCST90011519 | F-small fish liking (derived food-liking factor) \|\| id:ebi-fl187-GCST90094840 | Weighted median | 21 | 0.220000346468535 | 0.279809960492496 | 0.431721530583214 | -0.328427176 | 0.768427869033828 | 1.24607716231384 | 0.72005536355865 | 2.15637348601408 | 0.999332001504091 |
| ebi-fl187-GCST90094840 | ebi-gm430-GCST90011524 | TestASV_46 (Sutterella) abundance \|\| id:ebi-gm430-GCST90011524 | F-small fish liking (derived food-liking factor) \|\| id:ebi-fl187-GCST90094840 | Weighted median | 21 | -0.05570144 | 0.304882106761223 | 0.85503475566779 | -0.65327037 | 0.541867488959567 | 0.945821477924185 | 0.52034128283359 | 1.71921448021794 | 0.999332001504091 |
| ebi-fl187-GCST90094840 | ebi-gm430-GCST90011581 | OTU97_19 (Bacteria) prevalence \|\| id:ebi-gm430-GCST90011581 | F-small fish liking (derived food-liking factor) \|\| id:ebi-fl187-GCST90094840 | Weighted median | 21 | -0.085996962 | 0.351108505832891 | 0.806510729384653 | -0.774169634 | 0.602175709080115 | 0.917597018552344 | 0.461086492727369 | 1.82608751662998 | 0.999332001504091 |
| ebi-fl187-GCST90094840 | ebi-gm430-GCST90011614 | OTU97_80 (Ruminococcus) prevalence \|\| id:ebi-gm430-GCST90011614 | F-small fish liking (derived food-liking factor) \|\| id:ebi-fl187-GCST90094840 | Weighted median | 21 | 0.0151238640920288 | 0.162304680303415 | 0.925759026104914 | -0.302993309 | 0.333241037486723 | 1.01523880846119 | 0.73860403813002 | 1.39548362179987 | 0.999332001504091 |
| ebi-fl187-GCST90094840 | ebi-gm430-GCST90011623 | OTU99_11 (Parabacteroides) prevalence \|\| id:ebi-gm430-GCST90011623 | F-small fish liking (derived food-liking factor) \|\| id:ebi-fl187-GCST90094840 | Weighted median | 21 | 0.0552152897944797 | 0.135970576785657 | 0.684681886087624 | -0.211287041 | 0.321717620294368 | 1.05676810157421 | 0.809541662118306 | 1.37949517951006 | 0.999332001504091 |
| ebi-fl187-GCST90094840 | ebi-gm430-GCST90011642 | OTU99_19 (Bacteria) prevalence \|\| id:ebi-gm430-GCST90011642 | F-small fish liking (derived food-liking factor) \|\| id:ebi-fl187-GCST90094840 | Weighted median | 21 | -0.085996962 | 0.344368392197324 | 0.802800998966203 | -0.760959011 | 0.588965086354403 | 0.917597018552344 | 0.467218144722628 | 1.8021224089146 | 0.999332001504091 |
| ebi-fl187-GCST90094840 | ebi-gm430-GCST90011653 | OTU99_30 (Parasutterella) prevalence \|\| id:ebi-gm430-GCST90011653 | F-small fish liking (derived food-liking factor) \|\| id:ebi-fl187-GCST90094840 | Weighted median | 21 | 0.156409281477523 | 0.160783876542923 | 0.330656650428134 | -0.158727117 | 0.471545679501653 | 1.16930467991441 | 0.853229159323536 | 1.60246918372287 | 0.999332001504091 |
| ebi-fl187-GCST90094840 | ebi-gm430-GCST90011675 | OTU99_8 (Ruminococcaceae) prevalence \|\| id:ebi-gm430-GCST90011675 | F-small fish liking (derived food-liking factor) \|\| id:ebi-fl187-GCST90094840 | Weighted median | 21 | 0.24901816907642 | 0.175083879464254 | 0.154945199684565 | -0.094146235 | 0.592182572826358 | 1.28276533951956 | 0.910149657070754 | 1.80793005138146 | 0.999332001504091 |
| ebi-fl187-GCST90094840 | ebi-gm430-GCST90011679 | OTU99_92 (Ruminococcus) prevalence \|\| id:ebi-gm430-GCST90011679 | F-small fish liking (derived food-liking factor) \|\| id:ebi-fl187-GCST90094840 | Weighted median | 21 | -0.002173931 | 0.168461169110425 | 0.989703873012014 | -0.332357822 | 0.328009960770277 | 0.997828430589764 | 0.717230635930169 | 1.38820279978973 | 0.999332001504091 |
| ebi-fl187-GCST90094840 | ebi-gm430-GCST90011684 | TestASV_11 (Lachnospiraceae) prevalence \|\| id:ebi-gm430-GCST90011684 | F-small fish liking (derived food-liking factor) \|\| id:ebi-fl187-GCST90094840 | Weighted median | 21 | -0.139185478 | 0.179984242324757 | 0.439332814282216 | -0.491954592 | 0.213583637418135 | 0.870066635673527 | 0.611430128718762 | 1.2381070460145 | 0.999332001504091 |
| ebi-fl187-GCST90094840 | ebi-gm430-GCST90011688 | TestASV_15 (Bacteroides) prevalence \|\| id:ebi-gm430-GCST90011688 | F-small fish liking (derived food-liking factor) \|\| id:ebi-fl187-GCST90094840 | Weighted median | 21 | 0.10986104126348 | 0.159411252637646 | 0.490717918447891 | -0.202585014 | 0.422307096433265 | 1.1161229646455 | 0.81661705584116 | 1.52547692127969 | 0.999332001504091 |
| ebi-fl187-GCST90094840 | ebi-gm430-GCST90011692 | TestASV_19 (Prevotella) prevalence \|\| id:ebi-gm430-GCST90011692 | F-small fish liking (derived food-liking factor) \|\| id:ebi-fl187-GCST90094840 | Weighted median | 21 | 0.278106225431255 | 0.30912492669078 | 0.368303121152365 | -0.327778631 | 0.883991081745185 | 1.3206264738752 | 0.720522503482868 | 2.42054103108457 | 0.999332001504091 |
| ebi-fl187-GCST90094840 | ebi-gm430-GCST90011693 | TestASV_2 (Bacteroides) prevalence \|\| id:ebi-gm430-GCST90011693 | F-small fish liking (derived food-liking factor) \|\| id:ebi-fl187-GCST90094840 | Weighted median | 21 | 0.113719902406178 | 0.161662821765583 | 0.481782329623446 | -0.203139228 | 0.43057903306672 | 1.12043824887071 | 0.816164600342189 | 1.53814790424154 | 0.999332001504091 |
| ebi-fl187-GCST90094840 | ebi-gm430-GCST90011716 | TestASV_40 (Barnesiella) prevalence \|\| id:ebi-gm430-GCST90011716 | F-small fish liking (derived food-liking factor) \|\| id:ebi-fl187-GCST90094840 | Weighted median | 21 | -0.256395428 | 0.310068852621801 | 0.408294669071189 | -0.864130379 | 0.351339523244268 | 0.773835911979202 | 0.421417867139898 | 1.42096969626087 | 0.999332001504091 |
| ebi-fl187-GCST90094840 | ebi-gm430-GCST90011718 | TestASV_42 (Alphaproteobacteria) prevalence \|\| id:ebi-gm430-GCST90011718 | F-small fish liking (derived food-liking factor) \|\| id:ebi-fl187-GCST90094840 | Weighted median | 21 | 0.080093393559781 | 0.237492188742743 | 0.735931136769375 | -0.385391296 | 0.545578083495557 | 1.08338824443503 | 0.680184430422673 | 1.72560563823939 | 0.999332001504091 |
| ebi-fl187-GCST90094840 | ebi-hbsm44-GCST90104929 | Sulcal depth of the right inferior frontal sulcus \|\| id:ebi-hbsm44-GCST90104929 | F-small fish liking (derived food-liking factor) \|\| id:ebi-fl187-GCST90094840 | Weighted median | 24 | 0.0846015059163147 | 0.134451784382834 | 0.529196496887191 | -0.178923991 | 0.348127003306669 | 1.08828330581982 | 0.836169452989273 | 1.41641212734103 | 0.999332001504091 |
| ebi-fl187-GCST90094840 | ebi-hbsm44-GCST90104935 | Sulcal opening distance of the left calloso-marginal posterior fissure \|\| id:ebi-hbsm44-GCST90104935 | F-small fish liking (derived food-liking factor) \|\| id:ebi-fl187-GCST90094840 | Weighted median | 27 | -0.026175172 | 0.0281195274200513 | 0.351929146075945 | -0.081289445 | 0.0289391020992709 | 0.974164428677181 | 0.92192680536073 | 1.02936190658717 | 0.999332001504091 |
| ebi-fl187-GCST90094840 | ebi-hbsm44-GCST90104936 | Sulcal opening distance of the left anterior inferior temporal sulcus \|\| id:ebi-hbsm44-GCST90104936 | F-small fish liking (derived food-liking factor) \|\| id:ebi-fl187-GCST90094840 | Weighted median | 26 | -0.00914699 | 0.015749159656974 | 0.561380458424373 | -0.040015343 | 0.0217213625750444 | 0.990894716103908 | 0.960774697603747 | 1.02195898877387 | 0.999332001504091 |
| ebi-fl187-GCST90094840 | ebi-hbsm44-GCST90104941 | Sulcal opening distance of the right posterior inferior temporal sulcus \|\| id:ebi-hbsm44-GCST90104941 | F-small fish liking (derived food-liking factor) \|\| id:ebi-fl187-GCST90094840 | Weighted median | 26 | -0.007589186 | 0.0135197430592414 | 0.574565079426215 | -0.034087883 | 0.0189095099682891 | 0.992439538734689 | 0.966486563336637 | 1.01908942701034 | 0.999332001504091 |
| ebi-fl187-GCST90094840 | ebi-IPs91-GCST90274819 | Leukemia inhibitory factor levels \|\| id:ebi-IPs91-GCST90274819 | F-small fish liking (derived food-liking factor) \|\| id:ebi-fl187-GCST90094840 | Weighted median | 28 | -0.02768893 | 0.0512008697692497 | 0.588652179989922 | -0.128042635 | 0.0726647747799049 | 0.972690894733042 | 0.879815867645747 | 1.07536998534507 | 0.999332001504091 |
| ebi-fl187-GCST90094840 | ebi-IPs91-GCST90274837 | STAM binding protein levels \|\| id:ebi-IPs91-GCST90274837 | F-small fish liking (derived food-liking factor) \|\| id:ebi-fl187-GCST90094840 | Weighted median | 27 | -0.021396172 | 0.0493537081377028 | 0.664631851515302 | -0.11812944 | 0.0753370958686303 | 0.978831102189446 | 0.888581027214477 | 1.07824756242758 | 0.999332001504091 |
| ebi-fl187-GCST90094840 | ebi-IPs91-GCST90274840 | TNF-beta levels \|\| id:ebi-IPs91-GCST90274840 | F-small fish liking (derived food-liking factor) \|\| id:ebi-fl187-GCST90094840 | Weighted median | 28 | 0.0211127452320554 | 0.0545629076828614 | 0.698798270238683 | -0.085830554 | 0.128056044290464 | 1.02133719604559 | 0.917749727225321 | 1.13661670178864 | 0.999332001504091 |
| ebi-fl187-GCST90094840 | ebi-IPs91-GCST90274842 | Tumor necrosis factor ligand superfamily member 14 levels \|\| id:ebi-IPs91-GCST90274842 | F-small fish liking (derived food-liking factor) \|\| id:ebi-fl187-GCST90094840 | Weighted median | 28 | 0.015842175441753 | 0.0559805833114285 | 0.777181350838409 | -0.093879768 | 0.125564118732153 | 1.0159683279997 | 0.910392214075294 | 1.1337878636703 | 0.999332001504091 |
| ebi-fl187-GCST90094840 | ebi-met1400-GCST90199628 | 3-methylhistidine levels \|\| id:ebi-met1400-GCST90199628 | F-small fish liking (derived food-liking factor) \|\| id:ebi-fl187-GCST90094840 | Weighted median | 27 | 0.0520001205785258 | 0.065920909462752 | 0.430213770936435 | -0.077204862 | 0.18120510312552 | 1.05337586952787 | 0.925700193416244 | 1.19866100320091 | 0.999332001504091 |
| ebi-fl187-GCST90094840 | ebi-met1400-GCST90199651 | Docosahexaenoate DHA; 22:6n3 levels \|\| id:ebi-met1400-GCST90199651 | F-small fish liking (derived food-liking factor) \|\| id:ebi-fl187-GCST90094840 | Weighted median | 27 | -0.033847737 | 0.0628155009574458 | 0.589994703100371 | -0.156966119 | 0.0892706445344188 | 0.966718688588806 | 0.854733017349485 | 1.0933765326685 | 0.999332001504091 |
| ebi-fl187-GCST90094840 | ebi-met1400-GCST90199652 | 1-stearoyl-2-oleoyl-GPS (18:0/18:1) levels \|\| id:ebi-met1400-GCST90199652 | F-small fish liking (derived food-liking factor) \|\| id:ebi-fl187-GCST90094840 | Weighted median | 27 | -0.076333097 | 0.0637071565594207 | 0.230844164482589 | -0.201199124 | 0.0485329301236585 | 0.926507538520077 | 0.817749582108901 | 1.04972993898787 | 0.999332001504091 |
| ebi-fl187-GCST90094840 | ebi-met1400-GCST90199653 | 1-myristoyl-2-palmitoyl-gpc (14:0/16:0) levels \|\| id:ebi-met1400-GCST90199653 | F-small fish liking (derived food-liking factor) \|\| id:ebi-fl187-GCST90094840 | Weighted median | 26 | 0.0437911001606511 | 0.0701631618041168 | 0.532540659886555 | -0.093728697 | 0.18131089729672 | 1.04476408104186 | 0.91052975821125 | 1.19878782125648 | 0.999332001504091 |
| ebi-fl187-GCST90094840 | ebi-met1400-GCST90199667 | Erythritol levels in elite athletes \|\| id:ebi-met1400-GCST90199667 | F-small fish liking (derived food-liking factor) \|\| id:ebi-fl187-GCST90094840 | Weighted median | 27 | -0.075941854 | 0.0612581283437575 | 0.215085459326643 | -0.196007785 | 0.0441240778923666 | 0.926870099095162 | 0.822005835160984 | 1.04511202214085 | 0.999332001504091 |
| ebi-fl187-GCST90094840 | ebi-met1400-GCST90199674 | 3-hydroxymyristate levels \|\| id:ebi-met1400-GCST90199674 | F-small fish liking (derived food-liking factor) \|\| id:ebi-fl187-GCST90094840 | Weighted median | 27 | -0.00896263 | 0.0644524740507681 | 0.889404338028179 | -0.13528948 | 0.117364218731338 | 0.991077414239324 | 0.873463025405594 | 1.12452892961234 | 0.999332001504091 |
| ebi-fl187-GCST90094840 | ebi-met1400-GCST90199685 | 2-linoleoylglycerol (18:2) levels \|\| id:ebi-met1400-GCST90199685 | F-small fish liking (derived food-liking factor) \|\| id:ebi-fl187-GCST90094840 | Weighted median | 27 | 0.0443462586304815 | 0.0700663419025182 | 0.526787102731099 | -0.092983771 | 0.181676288759417 | 1.04534425169886 | 0.911208287721257 | 1.19922592812733 | 0.999332001504091 |
| ebi-fl187-GCST90094840 | ebi-met1400-GCST90199703 | Gamma-glutamylvaline levels \|\| id:ebi-met1400-GCST90199703 | F-small fish liking (derived food-liking factor) \|\| id:ebi-fl187-GCST90094840 | Weighted median | 27 | 0.042463676768896 | 0.0650460922231889 | 0.513869485068326 | -0.085026664 | 0.169954017526346 | 1.04337815681945 | 0.918487793526933 | 1.18525034932435 | 0.999332001504091 |
| ebi-fl187-GCST90094840 | ebi-met1400-GCST90199779 | Gamma-glutamyl-2-aminobutyrate levels \|\| id:ebi-met1400-GCST90199779 | F-small fish liking (derived food-liking factor) \|\| id:ebi-fl187-GCST90094840 | Weighted median | 27 | 0.00296565765697561 | 0.0702478116023461 | 0.966325644844633 | -0.134720053 | 0.140651368397574 | 1.00297005957009 | 0.873960540003175 | 1.15102329493089 | 0.999332001504091 |
| ebi-fl187-GCST90094840 | ebi-met1400-GCST90199824 | 4-vinylphenol sulfate levels \|\| id:ebi-met1400-GCST90199824 | F-small fish liking (derived food-liking factor) \|\| id:ebi-fl187-GCST90094840 | Weighted median | 27 | -0.045803424 | 0.0639432457595501 | 0.473797654603989 | -0.171132186 | 0.0795253372089521 | 0.955229718514821 | 0.84271017148017 | 1.08277299362747 | 0.999332001504091 |
| ebi-fl187-GCST90094840 | ebi-met1400-GCST90199830 | 4-ethylphenylsulfate levels \|\| id:ebi-met1400-GCST90199830 | F-small fish liking (derived food-liking factor) \|\| id:ebi-fl187-GCST90094840 | Weighted median | 27 | -0.041305517 | 0.0632657324128545 | 0.513827676465815 | -0.165306352 | 0.0826953186171574 | 0.959535930706878 | 0.847633989565883 | 1.08621080991461 | 0.999332001504091 |
| ebi-fl187-GCST90094840 | ebi-met1400-GCST90199833 | Hexanoylglutamine levels \|\| id:ebi-met1400-GCST90199833 | F-small fish liking (derived food-liking factor) \|\| id:ebi-fl187-GCST90094840 | Weighted median | 27 | 0.0256780872782258 | 0.0632254979262712 | 0.684643414075127 | -0.098243889 | 0.149600063213717 | 1.02601060943827 | 0.90642780933248 | 1.16136967538003 | 0.999332001504091 |
| ebi-fl187-GCST90094840 | ebi-met1400-GCST90199837 | Carotenoid levels (cryptoxanthin) \|\| id:ebi-met1400-GCST90199837 | F-small fish liking (derived food-liking factor) \|\| id:ebi-fl187-GCST90094840 | Weighted median | 27 | -0.022519621 | 0.0666989765450474 | 0.73564151427147 | -0.153249615 | 0.108210372787259 | 0.977732052688853 | 0.857915546261623 | 1.11428213536958 | 0.999332001504091 |
| ebi-fl187-GCST90094840 | ebi-met1400-GCST90199869 | 2-hydroxyhippurate levels \|\| id:ebi-met1400-GCST90199869 | F-small fish liking (derived food-liking factor) \|\| id:ebi-fl187-GCST90094840 | Weighted median | 27 | 0.10140508192743 | 0.0648407750580337 | 0.117838903506854 | -0.025682837 | 0.228493001041176 | 1.10672486521522 | 0.974644161477701 | 1.25670472947646 | 0.999332001504091 |
| ebi-fl187-GCST90094840 | ebi-met1400-GCST90199902 | Hydantoin-5-propionate levels \|\| id:ebi-met1400-GCST90199902 | F-small fish liking (derived food-liking factor) \|\| id:ebi-fl187-GCST90094840 | Weighted median | 27 | 0.0951707112470035 | 0.0617044188032052 | 0.122985018799557 | -0.02576995 | 0.216111372101286 | 1.09984659528476 | 0.97455926156319 | 1.24124061087798 | 0.999332001504091 |
| ebi-fl187-GCST90094840 | ebi-met1400-GCST90199904 | Pantoate levels \|\| id:ebi-met1400-GCST90199904 | F-small fish liking (derived food-liking factor) \|\| id:ebi-fl187-GCST90094840 | Weighted median | 27 | 0.0171656291290383 | 0.0634289799386037 | 0.786677508197081 | -0.107155172 | 0.141486429808702 | 1.01731380517153 | 0.898386258185237 | 1.15198487149966 | 0.999332001504091 |
| ebi-fl187-GCST90094840 | ebi-met1400-GCST90199936 | 3-methyl catechol sulfate (2) levels \|\| id:ebi-met1400-GCST90199936 | F-small fish liking (derived food-liking factor) \|\| id:ebi-fl187-GCST90094840 | Weighted median | 27 | -0.056342345 | 0.0722926039019907 | 0.43576457160523 | -0.198035849 | 0.0853511583358812 | 0.945215490402311 | 0.820340444258986 | 1.08909944590568 | 0.999332001504091 |
| ebi-fl187-GCST90094840 | ebi-met1400-GCST90199977 | 4-hydroxychlorothalonil levels \|\| id:ebi-met1400-GCST90199977 | F-small fish liking (derived food-liking factor) \|\| id:ebi-fl187-GCST90094840 | Weighted median | 27 | 0.0467537229557144 | 0.0663063902403388 | 0.480737803440459 | -0.083206802 | 0.176714247826778 | 1.04786391247215 | 0.920160836519072 | 1.19329005917617 | 0.999332001504091 |
| ebi-fl187-GCST90094840 | ebi-met1400-GCST90200040 | Sphingomyelin (d18:2/24:1, d18:1/24:2) levels \|\| id:ebi-met1400-GCST90200040 | F-small fish liking (derived food-liking factor) \|\| id:ebi-fl187-GCST90094840 | Weighted median | 27 | -0.078060835 | 0.0573156871979595 | 0.173215123477995 | -0.190399582 | 0.0342779115686818 | 0.924908157730376 | 0.826628761764005 | 1.0348721697158 | 0.999332001504091 |
| ebi-fl187-GCST90094840 | ebi-met1400-GCST90200077 | 1-stearoyl-2-oleoyl-GPI (18:0/18:1) levels \|\| id:ebi-met1400-GCST90200077 | F-small fish liking (derived food-liking factor) \|\| id:ebi-fl187-GCST90094840 | Weighted median | 27 | -0.044454758 | 0.064994770801967 | 0.493991348897332 | -0.171844508 | 0.0829349932171044 | 0.9565188743675 | 0.84211010409817 | 1.08647117825653 | 0.999332001504091 |
| ebi-fl187-GCST90094840 | ebi-met1400-GCST90200080 | 1-palmitoyl-2-oleoyl-GPI (16:0/18:1) levels \|\| id:ebi-met1400-GCST90200080 | F-small fish liking (derived food-liking factor) \|\| id:ebi-fl187-GCST90094840 | Weighted median | 27 | -0.033243321 | 0.0632236089184683 | 0.599023360507812 | -0.157161594 | 0.0906749528876166 | 0.967303166172562 | 0.85456595486649 | 1.09491304908542 | 0.999332001504091 |
| ebi-fl187-GCST90094840 | ebi-met1400-GCST90200082 | 1-oleoyl-2-linoleoyl-GPE (18:1/18:2) levels \|\| id:ebi-met1400-GCST90200082 | F-small fish liking (derived food-liking factor) \|\| id:ebi-fl187-GCST90094840 | Weighted median | 27 | 0.000974454899394371 | 0.0755624022159293 | 0.989710743089078 | -0.147127853 | 0.149076763242616 | 1.00097492983483 | 0.86318360936389 | 1.16076208965113 | 0.999332001504091 |
| ebi-fl187-GCST90094840 | ebi-met1400-GCST90200100 | Perfluorooctanesulfonate (PFOS) levels \|\| id:ebi-met1400-GCST90200100 | F-small fish liking (derived food-liking factor) \|\| id:ebi-fl187-GCST90094840 | Weighted median | 27 | -0.060117451 | 0.0653325704072474 | 0.357480889521982 | -0.188169289 | 0.0679343866482289 | 0.941653928563901 | 0.828474443461916 | 1.07029508052724 | 0.999332001504091 |
| ebi-fl187-GCST90094840 | ebi-met1400-GCST90200102 | 1-palmitoleoyl-2-linolenoyl-GPC (16:1/18:3) levels \|\| id:ebi-met1400-GCST90200102 | F-small fish liking (derived food-liking factor) \|\| id:ebi-fl187-GCST90094840 | Weighted median | 27 | -0.072944248 | 0.0648903590785215 | 0.260964099187474 | -0.200129352 | 0.0542408558529532 | 0.929652658630925 | 0.818624855683849 | 1.05573885241681 | 0.999332001504091 |
| ebi-fl187-GCST90094840 | ebi-met1400-GCST90200114 | Glycosyl-N-tricosanoyl-sphingadienine (d18:2/23:0) levels \|\| id:ebi-met1400-GCST90200114 | F-small fish liking (derived food-liking factor) \|\| id:ebi-fl187-GCST90094840 | Weighted median | 27 | -0.056711651 | 0.0696267360073758 | 0.415353025775442 | -0.193180053 | 0.0797567517078643 | 0.944866481520864 | 0.82433353668632 | 1.08302359199216 | 0.999332001504091 |
| ebi-fl187-GCST90094840 | ebi-met1400-GCST90200135 | Arachidoylcarnitine (C20) levels \|\| id:ebi-met1400-GCST90200135 | F-small fish liking (derived food-liking factor) \|\| id:ebi-fl187-GCST90094840 | Weighted median | 27 | 0.015811674615878 | 0.0711710560384173 | 0.82418591007807 | -0.123683595 | 0.155306944451176 | 1.01593734059921 | 0.883659390710593 | 1.16801642224817 | 0.999332001504091 |
| ebi-fl187-GCST90094840 | ebi-met1400-GCST90200146 | Docosahexaenoylcarnitine (C22:6) levels \|\| id:ebi-met1400-GCST90200146 | F-small fish liking (derived food-liking factor) \|\| id:ebi-fl187-GCST90094840 | Weighted median | 27 | -0.097940117 | 0.0688776417563185 | 0.15504264543906 | -0.232940295 | 0.0370600608996332 | 0.906703198287774 | 0.792200870635555 | 1.03775534748621 | 0.999332001504091 |
| ebi-fl187-GCST90094840 | ebi-met1400-GCST90200162 | 3-hydroxyphenylacetoylglutamine levels \|\| id:ebi-met1400-GCST90200162 | F-small fish liking (derived food-liking factor) \|\| id:ebi-fl187-GCST90094840 | Weighted median | 27 | 0.111978509803142 | 0.077521731417675 | 0.148604294577004 | -0.039964084 | 0.263921103381786 | 1.11848882384176 | 0.960823947701221 | 1.30202546684228 | 0.999332001504091 |
| ebi-fl187-GCST90094840 | ebi-met1400-GCST90200172 | 3-carboxy-4-methyl-5-pentyl-2-furanpropionate (3-CMPFP) levels \|\| id:ebi-met1400-GCST90200172 | F-small fish liking (derived food-liking factor) \|\| id:ebi-fl187-GCST90094840 | Weighted median | 27 | 0.00187897193611155 | 0.0613712285021521 | 0.975575386877019 | -0.118408636 | 0.12216657980033 | 1.00188073831003 | 0.888332973666956 | 1.12994231166856 | 0.999332001504091 |
| ebi-fl187-GCST90094840 | ebi-met1400-GCST90200222 | Dihydrocaffeate sulfate (2) levels \|\| id:ebi-met1400-GCST90200222 | F-small fish liking (derived food-liking factor) \|\| id:ebi-fl187-GCST90094840 | Weighted median | 27 | -0.000782407 | 0.0754661309392882 | 0.991727955948249 | -0.148696023 | 0.14713120979463 | 0.999217899154051 | 0.861831051483154 | 1.15850596038701 | 0.999332001504091 |
| ebi-fl187-GCST90094840 | ebi-met1400-GCST90200223 | 4-methylhexanoylglutamine levels \|\| id:ebi-met1400-GCST90200223 | F-small fish liking (derived food-liking factor) \|\| id:ebi-fl187-GCST90094840 | Weighted median | 27 | 0.0448908631142325 | 0.080084196693814 | 0.575107183383449 | -0.112074162 | 0.201855888634108 | 1.04591370591498 | 0.893977955486059 | 1.22367165041114 | 0.999332001504091 |
| ebi-fl187-GCST90094840 | ebi-met1400-GCST90200224 | 2-ketocaprylate levels \|\| id:ebi-met1400-GCST90200224 | F-small fish liking (derived food-liking factor) \|\| id:ebi-fl187-GCST90094840 | Weighted median | 27 | 0.0176884983599352 | 0.0627554481941956 | 0.778047834788016 | -0.10531218 | 0.140689176820559 | 1.01784586634551 | 0.900043503052788 | 1.15106681412918 | 0.999332001504091 |
| ebi-fl187-GCST90094840 | ebi-met1400-GCST90200243 | Hydroxypalmitoyl sphingomyelin (d18:1/16:0(OH)) levels \|\| id:ebi-met1400-GCST90200243 | F-small fish liking (derived food-liking factor) \|\| id:ebi-fl187-GCST90094840 | Weighted median | 27 | -0.068267477 | 0.0629250180616379 | 0.277965067304516 | -0.191600513 | 0.0550655581954348 | 0.93401061361406 | 0.825636634045878 | 1.05660988184209 | 0.999332001504091 |
| ebi-fl187-GCST90094840 | ebi-met1400-GCST90200273 | 2-methoxyhydroquinone sulfate (2) levels \|\| id:ebi-met1400-GCST90200273 | F-small fish liking (derived food-liking factor) \|\| id:ebi-fl187-GCST90094840 | Weighted median | 27 | -0.069965654 | 0.0816130306883868 | 0.391287213487711 | -0.229927194 | 0.0899958861296701 | 0.932425844435795 | 0.794591451288515 | 1.09416978242335 | 0.999332001504091 |
| ebi-fl187-GCST90094840 | ebi-met1400-GCST90200276 | 2,4-di-tert-butylphenol levels \|\| id:ebi-met1400-GCST90200276 | F-small fish liking (derived food-liking factor) \|\| id:ebi-fl187-GCST90094840 | Weighted median | 27 | -0.038101655 | 0.0632614256280046 | 0.546981842080804 | -0.162094049 | 0.0858907391919732 | 0.962615081243776 | 0.850361224927005 | 1.08968726168988 | 0.999332001504091 |
| ebi-fl187-GCST90094840 | ebi-met1400-GCST90200289 | N-acetylmethionine levels \|\| id:ebi-met1400-GCST90200289 | F-small fish liking (derived food-liking factor) \|\| id:ebi-fl187-GCST90094840 | Weighted median | 27 | 0.0211303335401718 | 0.0659778262484853 | 0.748768084578643 | -0.108186206 | 0.150446872987203 | 1.02135515979686 | 0.897460468430413 | 1.16235355109076 | 0.999332001504091 |
| ebi-fl187-GCST90094840 | ebi-met1400-GCST90200308 | 3-hydroxyisobutyrate levels \|\| id:ebi-met1400-GCST90200308 | F-small fish liking (derived food-liking factor) \|\| id:ebi-fl187-GCST90094840 | Weighted median | 27 | 0.0669071364907875 | 0.064056694559951 | 0.296254703769999 | -0.058643985 | 0.192458257828291 | 1.06919618425457 | 0.943042446801641 | 1.21222590170959 | 0.999332001504091 |
| ebi-fl187-GCST90094840 | ebi-met1400-GCST90200383 | Cystathionine levels \|\| id:ebi-met1400-GCST90200383 | F-small fish liking (derived food-liking factor) \|\| id:ebi-fl187-GCST90094840 | Weighted median | 27 | -0.045925711 | 0.070047570724445 | 0.512058340325627 | -0.183218949 | 0.0913675277643385 | 0.955112914076422 | 0.832585841545718 | 1.09567162100902 | 0.999332001504091 |
| ebi-fl187-GCST90094840 | ebi-met1400-GCST90200384 | Guanidinoacetate levels \|\| id:ebi-met1400-GCST90200384 | F-small fish liking (derived food-liking factor) \|\| id:ebi-fl187-GCST90094840 | Weighted median | 27 | -0.055448313 | 0.0632124100260211 | 0.380391875672469 | -0.179344636 | 0.068448010812477 | 0.946060921610347 | 0.835817796443242 | 1.0708449511448 | 0.999332001504091 |
| ebi-fl187-GCST90094840 | ebi-met1400-GCST90200403 | Citrulline levels \|\| id:ebi-met1400-GCST90200403 | F-small fish liking (derived food-liking factor) \|\| id:ebi-fl187-GCST90094840 | Weighted median | 26 | -0.071753938 | 0.0638708196203911 | 0.261257951533093 | -0.196940744 | 0.0534328685143249 | 0.930759892331317 | 0.821239294900035 | 1.05488617331451 | 0.999332001504091 |
| ebi-fl187-GCST90094840 | ebi-met1400-GCST90200434 | 12,13-DiHOME levels \|\| id:ebi-met1400-GCST90200434 | F-small fish liking (derived food-liking factor) \|\| id:ebi-fl187-GCST90094840 | Weighted median | 27 | -0.001593054 | 0.0622437513036364 | 0.979581331715288 | -0.123590807 | 0.120404698462687 | 0.998408214144684 | 0.883741388007608 | 1.12795324016555 | 0.999332001504091 |
| ebi-fl187-GCST90094840 | ebi-met1400-GCST90200437 | Fructose levels \|\| id:ebi-met1400-GCST90200437 | F-small fish liking (derived food-liking factor) \|\| id:ebi-fl187-GCST90094840 | Weighted median | 26 | -0.088360027 | 0.062582242790119 | 0.157978664428427 | -0.211021222 | 0.0343011693068988 | 0.915431237826448 | 0.809756881689885 | 1.0348962387817 | 0.999332001504091 |
| ebi-fl187-GCST90094840 | ebi-met1400-GCST90200448 | N-stearoyl-sphinganine (d18:0/18:0) levels \|\| id:ebi-met1400-GCST90200448 | F-small fish liking (derived food-liking factor) \|\| id:ebi-fl187-GCST90094840 | Weighted median | 27 | 0.0192844064981518 | 0.0776076983886089 | 0.803758600656085 | -0.132826682 | 0.171395495339825 | 1.01947155172428 | 0.875616838816676 | 1.18696009338933 | 0.999332001504091 |
| ebi-fl187-GCST90094840 | ebi-met1400-GCST90200468 | X-11847 levels \|\| id:ebi-met1400-GCST90200468 | F-small fish liking (derived food-liking factor) \|\| id:ebi-fl187-GCST90094840 | Weighted median | 27 | 0.0147591276081031 | 0.0712151469014163 | 0.835816940753109 | -0.12482256 | 0.154340815534879 | 1.01486858134962 | 0.882653506447221 | 1.16688851274865 | 0.999332001504091 |
| ebi-fl187-GCST90094840 | ebi-met1400-GCST90200476 | X-11843 levels \|\| id:ebi-met1400-GCST90200476 | F-small fish liking (derived food-liking factor) \|\| id:ebi-fl187-GCST90094840 | Weighted median | 27 | -0.105719156 | 0.0678538558190943 | 0.119223297123479 | -0.238712713 | 0.0272744015750504 | 0.899677281718036 | 0.787641128743566 | 1.02764975278872 | 0.999332001504091 |
| ebi-fl187-GCST90094840 | ebi-met1400-GCST90200511 | X-12906 levels \|\| id:ebi-met1400-GCST90200511 | F-small fish liking (derived food-liking factor) \|\| id:ebi-fl187-GCST90094840 | Weighted median | 27 | -0.044557214 | 0.0630638827321945 | 0.479851812131288 | -0.168162424 | 0.0790479961627817 | 0.956420877871437 | 0.845216539959083 | 1.08225626497164 | 0.999332001504091 |
| ebi-fl187-GCST90094840 | ebi-met1400-GCST90200515 | X-12849 levels \|\| id:ebi-met1400-GCST90200515 | F-small fish liking (derived food-liking factor) \|\| id:ebi-fl187-GCST90094840 | Weighted median | 27 | 0.0620860509201169 | 0.0687835624228645 | 0.366722731488697 | -0.072729731 | 0.196901833268931 | 1.06405390360607 | 0.929852105868457 | 1.21762450462148 | 0.999332001504091 |
| ebi-fl187-GCST90094840 | ebi-met1400-GCST90200551 | X-18886 levels \|\| id:ebi-met1400-GCST90200551 | F-small fish liking (derived food-liking factor) \|\| id:ebi-fl187-GCST90094840 | Weighted median | 27 | -0.00875733 | 0.0627110562574235 | 0.888939917792466 | -0.131671001 | 0.114156339868406 | 0.991280903331825 | 0.876629358120785 | 1.12092735682138 | 0.999332001504091 |
| ebi-fl187-GCST90094840 | ebi-met1400-GCST90200564 | X-21319 levels \|\| id:ebi-met1400-GCST90200564 | F-small fish liking (derived food-liking factor) \|\| id:ebi-fl187-GCST90094840 | Weighted median | 27 | 0.00121860526686242 | 0.0617720585167624 | 0.984260792186912 | -0.119854629 | 0.122291839959717 | 1.00121934806796 | 0.887049378222066 | 1.13008385728745 | 0.999332001504091 |
| ebi-fl187-GCST90094840 | ebi-met1400-GCST90200579 | X-21442 levels \|\| id:ebi-met1400-GCST90200579 | F-small fish liking (derived food-liking factor) \|\| id:ebi-fl187-GCST90094840 | Weighted median | 27 | 0.032959644559291 | 0.0720456834209026 | 0.647324226111388 | -0.108249895 | 0.17416918406426 | 1.03350883069539 | 0.897403311855944 | 1.19025692129027 | 0.999332001504091 |
| ebi-fl187-GCST90094840 | ebi-met1400-GCST90200581 | X-21441 levels \|\| id:ebi-met1400-GCST90200581 | F-small fish liking (derived food-liking factor) \|\| id:ebi-fl187-GCST90094840 | Weighted median | 27 | -0.063110514 | 0.066761999937814 | 0.344502715473677 | -0.193964034 | 0.0677430059130301 | 0.93883971305647 | 0.823687528610645 | 1.07009026626722 | 0.999332001504091 |
| ebi-fl187-GCST90094840 | ebi-met1400-GCST90200648 | X-24585 levels \|\| id:ebi-met1400-GCST90200648 | F-small fish liking (derived food-liking factor) \|\| id:ebi-fl187-GCST90094840 | Weighted median | 27 | -0.085500803 | 0.0673987743868824 | 0.204590550367141 | -0.217602401 | 0.046600794314841 | 0.91805240541395 | 0.804445224479894 | 1.04770367632085 | 0.999332001504091 |
| ebi-fl187-GCST90094840 | ebi-met1400-GCST90200720 | Adenosine 5'-diphosphate (ADP) to pantothenate ratio \|\| id:ebi-met1400-GCST90200720 | F-small fish liking (derived food-liking factor) \|\| id:ebi-fl187-GCST90094840 | Weighted median | 27 | -0.161257018 | 0.0842605455659656 | 0.0556469502447102 | -0.326407687 | 0.00389365112562456 | 0.851073301680329 | 0.721510976461587 | 1.00390124123305 | 0.999332001504091 |
| ebi-fl187-GCST90094840 | ebi-met1400-GCST90200724 | Cholate to taurocholate ratio \|\| id:ebi-met1400-GCST90200724 | F-small fish liking (derived food-liking factor) \|\| id:ebi-fl187-GCST90094840 | Weighted median | 27 | 0.0177982876250674 | 0.0718579709994696 | 0.804376565576346 | -0.123043336 | 0.158639910784028 | 1.01795762102981 | 0.884225343353199 | 1.17191587642468 | 0.999332001504091 |
| ebi-fl187-GCST90094840 | ebi-met1400-GCST90200726 | N-acetylputrescine to (N(1) + N(8))-acetylspermidine ratio \|\| id:ebi-met1400-GCST90200726 | F-small fish liking (derived food-liking factor) \|\| id:ebi-fl187-GCST90094840 | Weighted median | 27 | 0.0204132557460493 | 0.0645143730072019 | 0.751688234025445 | -0.106034915 | 0.146861426840165 | 1.0206230312198 | 0.899393244899507 | 1.15819345738214 | 0.999332001504091 |
| ebi-fl187-GCST90094840 | ebi-met1400-GCST90200744 | Arginine to glutamate ratio \|\| id:ebi-met1400-GCST90200744 | F-small fish liking (derived food-liking factor) \|\| id:ebi-fl187-GCST90094840 | Weighted median | 27 | -0.080962748 | 0.0614372092581327 | 0.187566279775262 | -0.201379679 | 0.0394541816478185 | 0.922228045184497 | 0.817601946616866 | 1.04024283558044 | 0.999332001504091 |
| ebi-fl187-GCST90094840 | ebi-met1400-GCST90200759 | Histidine to trans-urocanate ratio \|\| id:ebi-met1400-GCST90200759 | F-small fish liking (derived food-liking factor) \|\| id:ebi-fl187-GCST90094840 | Weighted median | 27 | -0.025969407 | 0.0626647055671406 | 0.678567721861289 | -0.148792229 | 0.0968534164061983 | 0.974364898380029 | 0.861748142213923 | 1.1016988708047 | 0.999332001504091 |
| ebi-fl187-GCST90094840 | ebi-met1400-GCST90200795 | Oleoyl-linoleoyl-glycerol (18:1 to 18:2) [2] to linoleoyl-arachidonoyl-glycerol (18:2 to 20:4) [2] ratio \|\| id:ebi-met1400-GCST90200795 | F-small fish liking (derived food-liking factor) \|\| id:ebi-fl187-GCST90094840 | Weighted median | 27 | 0.0783576182196817 | 0.0642053418994263 | 0.22230490359675 | -0.047484852 | 0.204200088342557 | 1.08150935697159 | 0.953624918625799 | 1.22654354597049 | 0.999332001504091 |
| ebi-fl187-GCST90094840 | ebi-met1400-GCST90200811 | Choline to taurocholate ratio \|\| id:ebi-met1400-GCST90200811 | F-small fish liking (derived food-liking factor) \|\| id:ebi-fl187-GCST90094840 | Weighted median | 27 | -0.025736184 | 0.0669132703830069 | 0.700518953966347 | -0.156886194 | 0.10541382607689 | 0.974592168827038 | 0.854801334953013 | 1.11117034648899 | 0.999332001504091 |
| ebi-fl187-GCST90094840 | ebi-met1400-GCST90200831 | Cholate to phosphate ratio \|\| id:ebi-met1400-GCST90200831 | F-small fish liking (derived food-liking factor) \|\| id:ebi-fl187-GCST90094840 | Weighted median | 27 | -0.082429167 | 0.0659387731417538 | 0.211267969033592 | -0.211669162 | 0.0468108285274004 | 0.920876664159583 | 0.809232377953761 | 1.04792375304852 | 0.999332001504091 |
| ebi-fl187-GCST90094840 | ebi-met1400-GCST90200841 | Adenosine 5'-diphosphate (ADP) to oxalate (ethanedioate) ratio \|\| id:ebi-met1400-GCST90200841 | F-small fish liking (derived food-liking factor) \|\| id:ebi-fl187-GCST90094840 | Weighted median | 27 | -0.069693306 | 0.0907091807712224 | 0.442299529121164 | -0.2474833 | 0.108096688212494 | 0.932679823259248 | 0.780763259127828 | 1.11415546587917 | 0.999332001504091 |
| ebi-fl187-GCST90094840 | ebi-met1400-GCST90200844 | Aspartate to phosphate ratio \|\| id:ebi-met1400-GCST90200844 | F-small fish liking (derived food-liking factor) \|\| id:ebi-fl187-GCST90094840 | Weighted median | 27 | -0.019995396 | 0.0596765234333741 | 0.737577556002077 | -0.136961381 | 0.0969705904119236 | 0.980203186624794 | 0.87200390101082 | 1.10182796883781 | 0.999332001504091 |
| ebi-fl187-GCST90094840 | ebi-met1400-GCST90200870 | Adenosine 5'-monophosphate (AMP) to acetoacetate ratio \|\| id:ebi-met1400-GCST90200870 | F-small fish liking (derived food-liking factor) \|\| id:ebi-fl187-GCST90094840 | Weighted median | 27 | -0.052889288 | 0.0684588250139077 | 0.43977644280318 | -0.187068585 | 0.0812900091693245 | 0.948485015477418 | 0.829386851032729 | 1.0846854196748 | 0.999332001504091 |
| ebi-fl187-GCST90094840 | ebi-met1400-GCST90200887 | Cholesterol to cortisol ratio \|\| id:ebi-met1400-GCST90200887 | F-small fish liking (derived food-liking factor) \|\| id:ebi-fl187-GCST90094840 | Weighted median | 27 | 0.0834962249557668 | 0.059276915302707 | 0.158959657702611 | -0.032686529 | 0.199678978949072 | 1.08708111150585 | 0.967841902374618 | 1.22101072509194 | 0.999332001504091 |
| ebi-fl187-GCST90094840 | ebi-met1400-GCST90200890 | Cortisol to taurocholate ratio \|\| id:ebi-met1400-GCST90200890 | F-small fish liking (derived food-liking factor) \|\| id:ebi-fl187-GCST90094840 | Weighted median | 27 | -0.032761889 | 0.067676384603266 | 0.628317507570824 | -0.165407603 | 0.099883824581652 | 0.967768968360375 | 0.847548170441974 | 1.1050425318397 | 0.999332001504091 |
| ebi-fl187-GCST90094840 | ebi-met1400-GCST90200948 | Adenosine 5'-diphosphate (ADP) to mannitol to sorbitol ratio \|\| id:ebi-met1400-GCST90200948 | F-small fish liking (derived food-liking factor) \|\| id:ebi-fl187-GCST90094840 | Weighted median | 27 | -0.057291579 | 0.0884046440875445 | 0.516945664828957 | -0.230564682 | 0.115981523265129 | 0.944318685584063 | 0.794085070681495 | 1.12297512302795 | 0.999332001504091 |
| ebi-fl187-GCST90094840 | ebi-met1400-GCST90200972 | Leucine to N-palmitoyl-sphingosine (d18:1 to 16:0) ratio \|\| id:ebi-met1400-GCST90200972 | F-small fish liking (derived food-liking factor) \|\| id:ebi-fl187-GCST90094840 | Weighted median | 27 | -0.016241967 | 0.06356632224976 | 0.798327574427633 | -0.140831958 | 0.10834802507872 | 0.983889222988698 | 0.868635266519617 | 1.11443552941612 | 0.999332001504091 |
| ebi-fl187-GCST90094840 | ebi-met1400-GCST90200973 | Proline to glutamate ratio \|\| id:ebi-met1400-GCST90200973 | F-small fish liking (derived food-liking factor) \|\| id:ebi-fl187-GCST90094840 | Weighted median | 27 | -0.055150353 | 0.0645594447116893 | 0.392962644305679 | -0.181686865 | 0.0713861584330455 | 0.946342851578597 | 0.833862411180615 | 1.07399587836791 | 0.999332001504091 |
| ebi-fl187-GCST90094840 | ebi-met1400-GCST90200983 | Cholesterol to linoleoyl-arachidonoyl-glycerol (18:2 to 20:4) [1] ratio \|\| id:ebi-met1400-GCST90200983 | F-small fish liking (derived food-liking factor) \|\| id:ebi-fl187-GCST90094840 | Weighted median | 27 | -0.040888206 | 0.0703017742209603 | 0.560829493528675 | -0.178679683 | 0.0969032717423491 | 0.959936439342178 | 0.836373761058006 | 1.10175379774143 | 0.999332001504091 |
| ebi-fl187-GCST90094840 | ebi-met1400-GCST90201001 | Serine to alpha-ketobutyrate ratio \|\| id:ebi-met1400-GCST90201001 | F-small fish liking (derived food-liking factor) \|\| id:ebi-fl187-GCST90094840 | Weighted median | 27 | -0.110029303 | 0.0669515075825539 | 0.100296509722771 | -0.241254257 | 0.0211956523091732 | 0.895807885454224 | 0.785641845727196 | 1.02142187563749 | 0.999332001504091 |
| ebi-fl187-GCST90094840 | ebi-met1400-GCST90201005 | Fructose to maltose ratio \|\| id:ebi-met1400-GCST90201005 | F-small fish liking (derived food-liking factor) \|\| id:ebi-fl187-GCST90094840 | Weighted median | 27 | 0.0127331006523089 | 0.0748644083481657 | 0.864945519431023 | -0.13400114 | 0.159467341014714 | 1.01281451175003 | 0.874589067824776 | 1.17288595633003 | 0.999332001504091 |
| ebi-fl187-GCST90094840 | ebi-met1400-GCST90201020 | Paraxanthine to 5-acetylamino-6-formylamino-3-methyluracil ratio \|\| id:ebi-met1400-GCST90201020 | F-small fish liking (derived food-liking factor) \|\| id:ebi-fl187-GCST90094840 | Weighted median | 27 | 0.064326422113222 | 0.0695782520841693 | 0.355216118376652 | -0.072046952 | 0.200699796198194 | 1.0664404516982 | 0.930487206576529 | 1.22225779030602 | 0.999332001504091 |
| ebi-fl187-GCST90094840 | ebi-pw11-GCST90275197 | Placental weight (fetal genotype, adjusted for sex) \|\| id:ebi-pw11-GCST90275197 | F-small fish liking (derived food-liking factor) \|\| id:ebi-fl187-GCST90094840 | Weighted median | 23 | 0.0410891697041295 | 0.0252350195623234 | 0.103469794486539 | -0.008371469 | 0.0905498080462835 | 1.04194501133161 | 0.991663474529026 | 1.0947760349391 | 0.999332001504091 |
| ebi-fl187-GCST90094840 | prot-a-1017 | FAS-associated factor 2 \|\| id:prot-a-1017 | F-small fish liking (derived food-liking factor) \|\| id:ebi-fl187-GCST90094840 | Weighted median | 28 | 0.00519858237255446 | 0.0933716911262171 | 0.955599747592854 | -0.177809932 | 0.18820709697994 | 1.00521211844787 | 0.837101514383322 | 1.20708347280775 | 0.999332001504091 |
| ebi-fl187-GCST90094840 | prot-a-1019 | Fas apoptotic inhibitory molecule 3 \|\| id:prot-a-1019 | F-small fish liking (derived food-liking factor) \|\| id:ebi-fl187-GCST90094840 | Weighted median | 28 | -0.136498405 | 0.0962000337689695 | 0.155927636152131 | -0.325050471 | 0.0520536613802641 | 0.872407711917261 | 0.722490887888511 | 1.05343226962629 | 0.999332001504091 |
| ebi-fl187-GCST90094840 | prot-a-1052 | MIP18 family protein FAM96A \|\| id:prot-a-1052 | F-small fish liking (derived food-liking factor) \|\| id:ebi-fl187-GCST90094840 | Weighted median | 28 | 0.0256329525959705 | 0.0969740392967194 | 0.791527196458747 | -0.164436164 | 0.215702069617541 | 1.02596430182047 | 0.848371911524104 | 1.24073267197044 | 0.999332001504091 |
| ebi-fl187-GCST90094840 | prot-a-1066 | F-box/LRR-repeat protein 4 \|\| id:prot-a-1066 | F-small fish liking (derived food-liking factor) \|\| id:ebi-fl187-GCST90094840 | Weighted median | 28 | 0.0399505233175187 | 0.0965206435033956 | 0.678942571344942 | -0.149229938 | 0.229130984584174 | 1.04075927960209 | 0.861371030238102 | 1.25750674222054 | 0.999332001504091 |
| ebi-fl187-GCST90094840 | prot-a-1076 | Low affinity immunoglobulin gamma Fc region receptor III-B \|\| id:prot-a-1076 | F-small fish liking (derived food-liking factor) \|\| id:ebi-fl187-GCST90094840 | Weighted median | 28 | -0.119597367 | 0.10468706183731 | 0.25327641124446 | -0.324784008 | 0.0855892742987617 | 0.887277612140446 | 0.72268343055053 | 1.08935880874691 | 0.999332001504091 |
| ebi-fl187-GCST90094840 | prot-a-1081 | Fc receptor-like protein 3 \|\| id:prot-a-1081 | F-small fish liking (derived food-liking factor) \|\| id:ebi-fl187-GCST90094840 | Weighted median | 28 | -0.093500074 | 0.0937141702388952 | 0.318417366100939 | -0.277179848 | 0.0901796994731141 | 0.910737949853885 | 0.757918174887894 | 1.09437092391503 | 0.999332001504091 |
| ebi-fl187-GCST90094840 | prot-a-1105 | Fibroblast growth factor receptor 4 \|\| id:prot-a-1105 | F-small fish liking (derived food-liking factor) \|\| id:ebi-fl187-GCST90094840 | Weighted median | 28 | 0.171254685895753 | 0.0997346380834549 | 0.0859602815560815 | -0.024225205 | 0.366734576539324 | 1.18679297 | 0.976065870336214 | 1.44301485828327 | 0.999332001504091 |
| ebi-fl187-GCST90094840 | prot-a-1143 | Follistatin-related protein 1 \|\| id:prot-a-1143 | F-small fish liking (derived food-liking factor) \|\| id:ebi-fl187-GCST90094840 | Weighted median | 28 | -0.127747066 | 0.0955831777946813 | 0.181385391877344 | -0.315090094 | 0.059595962516767 | 0.880075952160687 | 0.729723127312053 | 1.06140761143825 | 0.999332001504091 |
| ebi-fl187-GCST90094840 | prot-a-1151 | Alpha-(1,3)-fucosyltransferase 10 \|\| id:prot-a-1151 | F-small fish liking (derived food-liking factor) \|\| id:ebi-fl187-GCST90094840 | Weighted median | 28 | -0.062641075 | 0.0974131735821563 | 0.520194763923014 | -0.253570895 | 0.128288745442867 | 0.939280544671743 | 0.776024726702534 | 1.13688122458117 | 0.999332001504091 |
| ebi-fl187-GCST90094840 | prot-a-1165 | Growth arrest and DNA damage-inducible proteins-interacting protein 1 \|\| id:prot-a-1165 | F-small fish liking (derived food-liking factor) \|\| id:ebi-fl187-GCST90094840 | Weighted median | 28 | 0.0631680553062658 | 0.0979303057689383 | 0.518907280345948 | -0.128775344 | 0.255111454613385 | 1.06520583768335 | 0.879171454502746 | 1.29060545678937 | 0.999332001504091 |
| ebi-fl187-GCST90094840 | prot-a-1194 | Growth/differentiation factor 11/8 \|\| id:prot-a-1194 | F-small fish liking (derived food-liking factor) \|\| id:ebi-fl187-GCST90094840 | Weighted median | 28 | -0.184471711 | 0.0998275451136244 | 0.0646162445278123 | -0.3801337 | 0.0111902772895595 | 0.831543462988991 | 0.683769983357617 | 1.01125312264268 | 0.999332001504091 |
| ebi-fl187-GCST90094840 | prot-a-1216 | D-glucuronyl C5-epimerase \|\| id:prot-a-1216 | F-small fish liking (derived food-liking factor) \|\| id:ebi-fl187-GCST90094840 | Weighted median | 28 | 0.0575188035329602 | 0.10970668663375 | 0.600072510237795 | -0.157506302 | 0.272543909335111 | 1.05920518726713 | 0.854271429743002 | 1.31330112382561 | 0.999332001504091 |
| ebi-fl187-GCST90094840 | prot-a-1246 | Glypican-3 \|\| id:prot-a-1246 | F-small fish liking (derived food-liking factor) \|\| id:ebi-fl187-GCST90094840 | Weighted median | 28 | 0.0152223182759823 | 0.100171456260395 | 0.87921640760588 | -0.181113736 | 0.211558372546357 | 1.01533876789022 | 0.834340458757384 | 1.23560208876388 | 0.999332001504091 |
| ebi-fl187-GCST90094840 | prot-a-1275 | Glutamate receptor 4 \|\| id:prot-a-1275 | F-small fish liking (derived food-liking factor) \|\| id:ebi-fl187-GCST90094840 | Weighted median | 27 | 0.0333363199179109 | 0.0947836019780729 | 0.72505592431323 | -0.15243954 | 0.219112179794934 | 1.03389820133339 | 0.858610804031455 | 1.24497092943784 | 0.999332001504091 |
| ebi-fl187-GCST90094840 | prot-a-1289 | Glutathione S-transferase P \|\| id:prot-a-1289 | F-small fish liking (derived food-liking factor) \|\| id:ebi-fl187-GCST90094840 | Weighted median | 28 | 0.108288524321038 | 0.0940603761434106 | 0.249622770676708 | -0.076069813 | 0.292646861562123 | 1.11436922163044 | 0.926751505072182 | 1.3399695121299 | 0.999332001504091 |
| ebi-fl187-GCST90094840 | prot-a-130 | Apolipoprotein D \|\| id:prot-a-130 | F-small fish liking (derived food-liking factor) \|\| id:ebi-fl187-GCST90094840 | Weighted median | 28 | 0.121698071128613 | 0.0966829636000202 | 0.208126659460283 | -0.067800538 | 0.311196679784652 | 1.12941304788902 | 0.934446842067443 | 1.36505767403504 | 0.999332001504091 |
| ebi-fl187-GCST90094840 | prot-a-1313 | Heparin-binding EGF-like growth factor \|\| id:prot-a-1313 | F-small fish liking (derived food-liking factor) \|\| id:ebi-fl187-GCST90094840 | Weighted median | 28 | -0.068713662 | 0.098548270178081 | 0.485640762590893 | -0.261868271 | 0.124440947688125 | 0.933593965368095 | 0.769612396986488 | 1.13251514084827 | 0.999332001504091 |
| ebi-fl187-GCST90094840 | prot-a-1347 | HLA class II histocompatibility antigen, DQ alpha 2 chain \|\| id:prot-a-1347 | F-small fish liking (derived food-liking factor) \|\| id:ebi-fl187-GCST90094840 | Weighted median | 27 | 0.0243619270155829 | 0.0994011710815392 | 0.806389129281207 | -0.170464368 | 0.2191882223354 | 1.02466110332305 | 0.843273136345145 | 1.24506560378971 | 0.999332001504091 |
| ebi-fl187-GCST90094840 | prot-a-1357 | Non-histone chromosomal protein HMG-14 \|\| id:prot-a-1357 | F-small fish liking (derived food-liking factor) \|\| id:ebi-fl187-GCST90094840 | Weighted median | 28 | -0.030321284 | 0.100212152370383 | 0.762216875747748 | -0.226737102 | 0.166094535118408 | 0.970133795465039 | 0.797130318573812 | 1.18068471261673 | 0.999332001504091 |
| ebi-fl187-GCST90094840 | prot-a-137 | Apolipoprotein M \|\| id:prot-a-137 | F-small fish liking (derived food-liking factor) \|\| id:ebi-fl187-GCST90094840 | Weighted median | 28 | -0.075893806 | 0.10079849949774 | 0.451494413459232 | -0.273458865 | 0.121671253052586 | 0.926914634140048 | 0.76074362891774 | 1.12938275961018 | 0.999332001504091 |
| ebi-fl187-GCST90094840 | prot-a-1381 | 17-beta-hydroxysteroid dehydrogenase 14 \|\| id:prot-a-1381 | F-small fish liking (derived food-liking factor) \|\| id:ebi-fl187-GCST90094840 | Weighted median | 28 | 0.106744023977293 | 0.0978223548247502 | 0.275183671901796 | -0.084987791 | 0.298475839433803 | 1.11264940645402 | 0.918523498146222 | 1.34780297312045 | 0.999332001504091 |
| ebi-fl187-GCST90094840 | prot-a-1392 | Serine protease HTRA2, mitochondrial \|\| id:prot-a-1392 | F-small fish liking (derived food-liking factor) \|\| id:ebi-fl187-GCST90094840 | Weighted median | 28 | -0.035801648 | 0.10245543856343 | 0.726761795410011 | -0.236614308 | 0.165011011593875 | 0.964831650801258 | 0.789295654673155 | 1.17940610578094 | 0.999332001504091 |
| ebi-fl187-GCST90094840 | prot-a-1416 | Interferon-induced protein with tetratricopeptide repeats 2 \|\| id:prot-a-1416 | F-small fish liking (derived food-liking factor) \|\| id:ebi-fl187-GCST90094840 | Weighted median | 28 | -0.158555601 | 0.0973795248214954 | 0.103477569008102 | -0.349419469 | 0.0323082679694059 | 0.853375514212327 | 0.705097301535488 | 1.03283584644452 | 0.999332001504091 |
| ebi-fl187-GCST90094840 | prot-a-1428 | Interferon gamma \|\| id:prot-a-1428 | F-small fish liking (derived food-liking factor) \|\| id:ebi-fl187-GCST90094840 | Weighted median | 28 | 0.0304608030254787 | 0.0948434946851756 | 0.748082221796532 | -0.155432447 | 0.216354052608423 | 1.03092947994025 | 0.856044903755919 | 1.24154187233257 | 0.999332001504091 |
| ebi-fl187-GCST90094840 | prot-a-1483 | Interleukin-17C \|\| id:prot-a-1483 | F-small fish liking (derived food-liking factor) \|\| id:ebi-fl187-GCST90094840 | Weighted median | 28 | -0.100139507 | 0.100440367170676 | 0.318762283069576 | -0.297002627 | 0.0967236127867914 | 0.9047111958066 | 0.743042060742865 | 1.10155587558462 | 0.999332001504091 |
| ebi-fl187-GCST90094840 | prot-a-1503 | Interleukin-1 receptor-like 2 \|\| id:prot-a-1503 | F-small fish liking (derived food-liking factor) \|\| id:ebi-fl187-GCST90094840 | Weighted median | 28 | -0.022902955 | 0.091617908570649 | 0.802600195822698 | -0.202474056 | 0.156668145671784 | 0.977357326689143 | 0.816707671048193 | 1.16960741020955 | 0.999332001504091 |
| ebi-fl187-GCST90094840 | prot-a-1522 | Interleukin-31 receptor subunit alpha \|\| id:prot-a-1522 | F-small fish liking (derived food-liking factor) \|\| id:ebi-fl187-GCST90094840 | Weighted median | 28 | -0.093454974 | 0.0971925847283847 | 0.336278570039914 | -0.28395244 | 0.0970424917017309 | 0.910779024906117 | 0.752802446812826 | 1.1019071945381 | 0.999332001504091 |
| ebi-fl187-GCST90094840 | prot-a-1540 | Interleukin-6 receptor subunit alpha \|\| id:prot-a-1540 | F-small fish liking (derived food-liking factor) \|\| id:ebi-fl187-GCST90094840 | Weighted median | 28 | -0.168782706 | 0.0982078577332682 | 0.0856822555287323 | -0.361270108 | 0.0237046947664169 | 0.844692429712489 | 0.696790764601432 | 1.02398788425636 | 0.999332001504091 |
| ebi-fl187-GCST90094840 | prot-a-1541 | Interleukin-6 receptor subunit alpha \|\| id:prot-a-1541 | F-small fish liking (derived food-liking factor) \|\| id:ebi-fl187-GCST90094840 | Weighted median | 28 | -0.193991926 | 0.098029850020954 | 0.0478265123847128 | -0.386130433 | -0.00185342 | 0.823664554112918 | 0.679681867278123 | 0.998148296082796 | 0.999332001504091 |
| ebi-fl187-GCST90094840 | prot-a-1545 | Interleukin-9 \|\| id:prot-a-1545 | F-small fish liking (derived food-liking factor) \|\| id:ebi-fl187-GCST90094840 | Weighted median | 28 | 0.136913827921015 | 0.0931252385984594 | 0.141504390356141 | -0.04561164 | 0.319439295573996 | 1.14672932821244 | 0.95541293457394 | 1.37635581914009 | 0.999332001504091 |
| ebi-fl187-GCST90094840 | prot-a-1548 | Inositol monophosphatase 3 \|\| id:prot-a-1548 | F-small fish liking (derived food-liking factor) \|\| id:ebi-fl187-GCST90094840 | Weighted median | 28 | -0.068885344 | 0.0984067510829387 | 0.483923381073148 | -0.261762576 | 0.123991888138121 | 0.933433697731548 | 0.769693745700957 | 1.13200668828003 | 0.999332001504091 |
| ebi-fl187-GCST90094840 | prot-a-1555 | Inhibin beta A chain:Inhibin beta B chain heterodimer \|\| id:prot-a-1555 | F-small fish liking (derived food-liking factor) \|\| id:ebi-fl187-GCST90094840 | Weighted median | 28 | -0.200923149 | 0.09481717952786 | 0.0340855248637874 | -0.38676482 | -0.015081477 | 0.817975291740837 | 0.679250822076181 | 0.985031679245383 | 0.999332001504091 |
| ebi-fl187-GCST90094840 | prot-a-1603 | Junctophilin-3 \|\| id:prot-a-1603 | F-small fish liking (derived food-liking factor) \|\| id:ebi-fl187-GCST90094840 | Weighted median | 28 | -0.070985935 | 0.0959870678712739 | 0.459581316538887 | -0.259120588 | 0.11714871788768 | 0.931474993100817 | 0.771729955931065 | 1.12428661878932 | 0.999332001504091 |
| ebi-fl187-GCST90094840 | prot-a-1627 | UPF0258 protein KIAA1024 \|\| id:prot-a-1627 | F-small fish liking (derived food-liking factor) \|\| id:ebi-fl187-GCST90094840 | Weighted median | 28 | -0.053388575 | 0.0958330448381322 | 0.577459212580918 | -0.241221342 | 0.134444193335515 | 0.948011567737113 | 0.785667705541936 | 1.14390081993183 | 0.999332001504091 |
| ebi-fl187-GCST90094840 | prot-a-1666 | Kallikrein-7 \|\| id:prot-a-1666 | F-small fish liking (derived food-liking factor) \|\| id:ebi-fl187-GCST90094840 | Weighted median | 28 | -0.037032747 | 0.0947200570353321 | 0.695819051778487 | -0.222684059 | 0.148618565059983 | 0.963644578624239 | 0.800367678702941 | 1.16023035240101 | 0.999332001504091 |
| ebi-fl187-GCST90094840 | prot-a-1678 | Importin subunit alpha-3 \|\| id:prot-a-1678 | F-small fish liking (derived food-liking factor) \|\| id:ebi-fl187-GCST90094840 | Weighted median | 28 | -0.05213242 | 0.101638691502809 | 0.608007954482992 | -0.251344255 | 0.147079415319876 | 0.949203165012915 | 0.777754579275796 | 1.15844595773321 | 0.999332001504091 |
| ebi-fl187-GCST90094840 | prot-a-1718 | Protein LDOC1 \|\| id:prot-a-1718 | F-small fish liking (derived food-liking factor) \|\| id:ebi-fl187-GCST90094840 | Weighted median | 28 | -0.04160012 | 0.0975189568442587 | 0.669681653050812 | -0.232737276 | 0.149537034954161 | 0.959253289652304 | 0.792361718719704 | 1.16129647857745 | 0.999332001504091 |
| ebi-fl187-GCST90094840 | prot-a-1723 | Leptin \|\| id:prot-a-1723 | F-small fish liking (derived food-liking factor) \|\| id:ebi-fl187-GCST90094840 | Weighted median | 28 | -0.142188296 | 0.103662110979674 | 0.170171935040158 | -0.345366034 | 0.060989441386718 | 0.867457902138003 | 0.707961168422381 | 1.06288769150785 | 0.999332001504091 |
| ebi-fl187-GCST90094840 | prot-a-1730 | Galectin-7 \|\| id:prot-a-1730 | F-small fish liking (derived food-liking factor) \|\| id:ebi-fl187-GCST90094840 | Weighted median | 28 | -0.016961679 | 0.0981083492211316 | 0.862740079680432 | -0.209254044 | 0.17533068500908 | 0.983181359949402 | 0.811189131783499 | 1.19164020901842 | 0.999332001504091 |
| ebi-fl187-GCST90094840 | prot-a-1753 | Lipase member N \|\| id:prot-a-1753 | F-small fish liking (derived food-liking factor) \|\| id:ebi-fl187-GCST90094840 | Weighted median | 28 | 0.0460710586061878 | 0.100766077746062 | 0.647521536393536 | -0.151430454 | 0.243572570988469 | 1.04714881724873 | 0.859477653619808 | 1.27579890046851 | 0.999332001504091 |
| ebi-fl187-GCST90094840 | prot-a-1756 | VIP36-like protein \|\| id:prot-a-1756 | F-small fish liking (derived food-liking factor) \|\| id:ebi-fl187-GCST90094840 | Weighted median | 28 | 0.0761359420009711 | 0.0941340144927491 | 0.418628050622463 | -0.108366726 | 0.260638610406759 | 1.07910926045878 | 0.897298473041938 | 1.29775858423138 | 0.999332001504091 |
| ebi-fl187-GCST90094840 | prot-a-1760 | Ig Kappa chain V-I region HK102- like \|\| id:prot-a-1760 | F-small fish liking (derived food-liking factor) \|\| id:ebi-fl187-GCST90094840 | Weighted median | 28 | -0.122053876 | 0.0994934243015805 | 0.219915368917559 | -0.317060987 | 0.0729532358663782 | 0.885100681741976 | 0.728286337484125 | 1.07568023248436 | 0.999332001504091 |
| ebi-fl187-GCST90094840 | prot-a-179 | Neutral ceramidase \|\| id:prot-a-179 | F-small fish liking (derived food-liking factor) \|\| id:ebi-fl187-GCST90094840 | Weighted median | 28 | 0.0687909514675307 | 0.105154028280178 | 0.5129879471739 | -0.137310944 | 0.274892846896679 | 1.07121225039113 | 0.871699134404935 | 1.31638961207798 | 0.999332001504091 |
| ebi-fl187-GCST90094840 | prot-a-1809 | Lactotransferrin \|\| id:prot-a-1809 | F-small fish liking (derived food-liking factor) \|\| id:ebi-fl187-GCST90094840 | Weighted median | 28 | 0.0297036182156754 | 0.100152672549592 | 0.766784600524963 | -0.16659562 | 0.226002856412876 | 1.03014917125428 | 0.846541866747615 | 1.25357924601297 | 0.999332001504091 |
| ebi-fl187-GCST90094840 | prot-a-1818 | Lysophospholipase-like protein 1 \|\| id:prot-a-1818 | F-small fish liking (derived food-liking factor) \|\| id:ebi-fl187-GCST90094840 | Weighted median | 28 | -0.007292444 | 0.106591128557688 | 0.945455193399381 | -0.216211056 | 0.201626168111114 | 0.992734081489409 | 0.805565264680838 | 1.22339058020467 | 0.999332001504091 |
| ebi-fl187-GCST90094840 | prot-a-1852 | MAP kinase-activated protein kinase 3 \|\| id:prot-a-1852 | F-small fish liking (derived food-liking factor) \|\| id:ebi-fl187-GCST90094840 | Weighted median | 28 | -0.08959392 | 0.102996345264517 | 0.384368857384981 | -0.291466756 | 0.1122789169544 | 0.914302390026939 | 0.74716685164442 | 1.11882487635681 | 0.999332001504091 |
| ebi-fl187-GCST90094840 | prot-a-1856 | Megakaryocyte-associated tyrosine-protein kinase \|\| id:prot-a-1856 | F-small fish liking (derived food-liking factor) \|\| id:ebi-fl187-GCST90094840 | Weighted median | 28 | 0.0162873342763031 | 0.099482009436499 | 0.869950466807102 | -0.178697404 | 0.211272072771841 | 1.01642069595686 | 0.836358939797014 | 1.23524838679929 | 0.999332001504091 |
| ebi-fl187-GCST90094840 | prot-a-1890 | Beta-1,3-N-acetylglucosaminyltransferase manic fringe \|\| id:prot-a-1890 | F-small fish liking (derived food-liking factor) \|\| id:ebi-fl187-GCST90094840 | Weighted median | 28 | -0.124246226 | 0.108383349488297 | 0.251646648757033 | -0.336677591 | 0.0881851392005031 | 0.883162356753965 | 0.714139047793797 | 1.092190311 | 0.999332001504091 |
| ebi-fl187-GCST90094840 | prot-a-1919 | Matrilysin \|\| id:prot-a-1919 | F-small fish liking (derived food-liking factor) \|\| id:ebi-fl187-GCST90094840 | Weighted median | 28 | -0.0875313 | 0.0989186233446846 | 0.376220327607318 | -0.281411802 | 0.106349201748322 | 0.916190194442344 | 0.754717477490989 | 1.11221019444634 | 0.999332001504091 |
| ebi-fl187-GCST90094840 | prot-a-1923 | Multimerin-2 \|\| id:prot-a-1923 | F-small fish liking (derived food-liking factor) \|\| id:ebi-fl187-GCST90094840 | Weighted median | 28 | 0.0143124947961698 | 0.0995745346653188 | 0.885708550054264 | -0.180853593 | 0.209478582740195 | 1.01441540894975 | 0.834557534693458 | 1.2330349665978 | 0.999332001504091 |
| ebi-fl187-GCST90094840 | prot-a-1948 | Mesothelin \|\| id:prot-a-1948 | F-small fish liking (derived food-liking factor) \|\| id:ebi-fl187-GCST90094840 | Weighted median | 28 | 0.16533609878691 | 0.0949430410547278 | 0.0816092687408635 | -0.020752262 | 0.351424459254177 | 1.17978957792888 | 0.979461584682214 | 1.42109039288274 | 0.999332001504091 |
| ebi-fl187-GCST90094840 | prot-a-195 | ATPase family AAA domain-containing protein 1 \|\| id:prot-a-195 | F-small fish liking (derived food-liking factor) \|\| id:ebi-fl187-GCST90094840 | Weighted median | 28 | -0.076640625 | 0.0988785484331124 | 0.438281398074878 | -0.27044258 | 0.117161330346474 | 0.926222655456082 | 0.763041712966363 | 1.12430079889739 | 0.999332001504091 |
| ebi-fl187-GCST90094840 | prot-a-1966 | Mucin-1 \|\| id:prot-a-1966 | F-small fish liking (derived food-liking factor) \|\| id:ebi-fl187-GCST90094840 | Weighted median | 28 | 0.0313271447827633 | 0.09622724086546 | 0.744761964898613 | -0.157278247 | 0.219932536879065 | 1.03182300419034 | 0.854466272792644 | 1.24599266919776 | 0.999332001504091 |
| ebi-fl187-GCST90094840 | prot-a-1986 | Myocardial zonula adherens protein \|\| id:prot-a-1986 | F-small fish liking (derived food-liking factor) \|\| id:ebi-fl187-GCST90094840 | Weighted median | 28 | -0.122858185 | 0.102182123209825 | 0.229229811464078 | -0.323135147 | 0.0774187761351643 | 0.884389072989555 | 0.723876018195209 | 1.08049446695774 | 0.999332001504091 |
| ebi-fl187-GCST90094840 | prot-a-2071 | Epididymal secretory protein E1 \|\| id:prot-a-2071 | F-small fish liking (derived food-liking factor) \|\| id:ebi-fl187-GCST90094840 | Weighted median | 28 | 0.0573745518101505 | 0.103532324372051 | 0.579462219208612 | -0.145548804 | 0.260297907579371 | 1.0590524061138 | 0.864547695693981 | 1.2973165095248 | 0.999332001504091 |
| ebi-fl187-GCST90094840 | prot-a-2098 | Neuritin \|\| id:prot-a-2098 | F-small fish liking (derived food-liking factor) \|\| id:ebi-fl187-GCST90094840 | Weighted median | 28 | -0.035278762 | 0.0999431405249094 | 0.724097188179371 | -0.231167318 | 0.160609792992933 | 0.965336279254009 | 0.793606670352503 | 1.17422668792597 | 0.999332001504091 |
| ebi-fl187-GCST90094840 | prot-a-2115 | Neurotrophin-4 \|\| id:prot-a-2115 | F-small fish liking (derived food-liking factor) \|\| id:ebi-fl187-GCST90094840 | Weighted median | 28 | 0.00650139375531881 | 0.0970182045611932 | 0.946572069385415 | -0.183654287 | 0.196657074695258 | 1.00652257369052 | 0.832223464416739 | 1.21732651705346 | 0.999332001504091 |
| ebi-fl187-GCST90094840 | prot-a-2118 | Netrin-1 \|\| id:prot-a-2118 | F-small fish liking (derived food-liking factor) \|\| id:ebi-fl187-GCST90094840 | Weighted median | 28 | -0.08723334 | 0.104794282783251 | 0.405169317472602 | -0.292630134 | 0.118163454608089 | 0.916463223476566 | 0.746298120029786 | 1.12542805273519 | 0.999332001504091 |
| ebi-fl187-GCST90094840 | prot-a-2120 | Netrin-G1 \|\| id:prot-a-2120 | F-small fish liking (derived food-liking factor) \|\| id:ebi-fl187-GCST90094840 | Weighted median | 28 | -0.055893779 | 0.0973956984596508 | 0.566046675400605 | -0.246789348 | 0.135001789860525 | 0.945639577223505 | 0.781305259634089 | 1.14453883291436 | 0.999332001504091 |
| ebi-fl187-GCST90094840 | prot-a-2145 | Olfactomedin-like protein 3 \|\| id:prot-a-2145 | F-small fish liking (derived food-liking factor) \|\| id:ebi-fl187-GCST90094840 | Weighted median | 28 | -0.048804607 | 0.0973858094939123 | 0.616267663015558 | -0.239680794 | 0.142071579189058 | 0.9523671970045 | 0.786878997458258 | 1.15265915199156 | 0.999332001504091 |
| ebi-fl187-GCST90094840 | prot-a-2162 | Prolyl 4-hydroxylase subunit alpha-2 \|\| id:prot-a-2162 | F-small fish liking (derived food-liking factor) \|\| id:ebi-fl187-GCST90094840 | Weighted median | 28 | 0.0431935179640249 | 0.0961144407871012 | 0.653145470525425 | -0.145190786 | 0.231577821906743 | 1.04413993513517 | 0.864857274728056 | 1.26058742407745 | 0.999332001504091 |
| ebi-fl187-GCST90094840 | prot-a-2168 | Polyadenylate-binding protein-interacting protein 1 \|\| id:prot-a-2168 | F-small fish liking (derived food-liking factor) \|\| id:ebi-fl187-GCST90094840 | Weighted median | 28 | -0.040608529 | 0.106629711314241 | 0.7033242778274 | -0.249602763 | 0.168385705642582 | 0.960204949221514 | 0.779110213239045 | 1.18339296397672 | 0.999332001504091 |
| ebi-fl187-GCST90094840 | prot-a-219 | Beta-1,4-galactosyltransferase 5 \|\| id:prot-a-219 | F-small fish liking (derived food-liking factor) \|\| id:ebi-fl187-GCST90094840 | Weighted median | 28 | 0.159734918606875 | 0.107736948072168 | 0.138170552682169 | -0.0514295 | 0.370899336828324 | 1.17319983632185 | 0.949870613846064 | 1.44903720136423 | 0.999332001504091 |
| ebi-fl187-GCST90094840 | prot-a-2190 | Pyruvate carboxylase, mitochondrial \|\| id:prot-a-2190 | F-small fish liking (derived food-liking factor) \|\| id:ebi-fl187-GCST90094840 | Weighted median | 28 | -0.043309579 | 0.108292564704512 | 0.689207167575185 | -0.255563006 | 0.168943847957612 | 0.957614886845751 | 0.774480338359735 | 1.1840536500265 | 0.999332001504091 |
| ebi-fl187-GCST90094840 | prot-a-2197 | Protocadherin-9 \|\| id:prot-a-2197 | F-small fish liking (derived food-liking factor) \|\| id:ebi-fl187-GCST90094840 | Weighted median | 28 | -0.086944142 | 0.0975499653119196 | 0.372780026383585 | -0.278142074 | 0.104253790071234 | 0.916728300867342 | 0.757189237005111 | 1.10988209623145 | 0.999332001504091 |
| ebi-fl187-GCST90094840 | prot-a-2198 | Protocadherin alpha-4 \|\| id:prot-a-2198 | F-small fish liking (derived food-liking factor) \|\| id:ebi-fl187-GCST90094840 | Weighted median | 28 | -0.065542534 | 0.103866438936832 | 0.528022830446957 | -0.269120754 | 0.138035686584067 | 0.936559210553864 | 0.764050987827557 | 1.14801651833116 | 0.999332001504091 |
| ebi-fl187-GCST90094840 | prot-a-2254 | Placenta growth factor \|\| id:prot-a-2254 | F-small fish liking (derived food-liking factor) \|\| id:ebi-fl187-GCST90094840 | Weighted median | 28 | -0.142879794 | 0.0988013282052103 | 0.148140092599819 | -0.336530397 | 0.0507708094739907 | 0.866858264363664 | 0.714244172301476 | 1.05208173848201 | 0.999332001504091 |
| ebi-fl187-GCST90094840 | prot-a-2261 | Membrane-associated progesterone receptor component 2 \|\| id:prot-a-2261 | F-small fish liking (derived food-liking factor) \|\| id:ebi-fl187-GCST90094840 | Weighted median | 28 | -0.198115691 | 0.0966148450470826 | 0.0403086509184134 | -0.387480787 | -0.008750594 | 0.820274949525889 | 0.678764675287378 | 0.991287580684462 | 0.999332001504091 |
| ebi-fl187-GCST90094840 | prot-a-2268 | Polymeric immunoglobulin receptor \|\| id:prot-a-2268 | F-small fish liking (derived food-liking factor) \|\| id:ebi-fl187-GCST90094840 | Weighted median | 28 | 0.0832072035539525 | 0.100169654816243 | 0.406164160422605 | -0.11312532 | 0.279539726993789 | 1.08676696719852 | 0.893038737590418 | 1.32252095153295 | 0.999332001504091 |
| ebi-fl187-GCST90094840 | prot-a-2283 | Group 10 secretory phospholipase A2 \|\| id:prot-a-2283 | F-small fish liking (derived food-liking factor) \|\| id:ebi-fl187-GCST90094840 | Weighted median | 28 | 0.0335744825781048 | 0.0987611504853253 | 0.733889382043326 | -0.159997372 | 0.227146337529342 | 1.03414446660382 | 0.852146028085068 | 1.25501351007944 | 0.999332001504091 |
| ebi-fl187-GCST90094840 | prot-a-2298 | Inactive phospholipase D5 \|\| id:prot-a-2298 | F-small fish liking (derived food-liking factor) \|\| id:ebi-fl187-GCST90094840 | Weighted median | 28 | 0.24447960662227 | 0.0985292371827819 | 0.0130907836996986 | 0.0513623017440179 | 0.437596911500523 | 1.27695662050009 | 1.05270422077635 | 1.54898040537585 | 0.999332001504091 |
| ebi-fl187-GCST90094840 | prot-a-2304 | Procollagen-lysine,2-oxoglutarate 5-dioxygenase 2 \|\| id:prot-a-2304 | F-small fish liking (derived food-liking factor) \|\| id:ebi-fl187-GCST90094840 | Weighted median | 28 | 0.0304802386289447 | 0.098789837035889 | 0.757674376860195 | -0.163147842 | 0.224108319219287 | 1.03094951687154 | 0.849465592471313 | 1.25120654180417 | 0.999332001504091 |
| ebi-fl187-GCST90094840 | prot-a-2331 | Periostin \|\| id:prot-a-2331 | F-small fish liking (derived food-liking factor) \|\| id:ebi-fl187-GCST90094840 | Weighted median | 28 | -0.008577165 | 0.100185807643978 | 0.931774397806487 | -0.204941348 | 0.187787017513098 | 0.991459513472613 | 0.814695098139391 | 1.2065765083162 | 0.999332001504091 |
| ebi-fl187-GCST90094840 | prot-a-2333 | Periostin \|\| id:prot-a-2333 | F-small fish liking (derived food-liking factor) \|\| id:ebi-fl187-GCST90094840 | Weighted median | 28 | -0.105468533 | 0.105936088005679 | 0.319451126187848 | -0.313103265 | 0.102166199819825 | 0.899902789938133 | 0.731174403826656 | 1.10756753395654 | 0.999332001504091 |
| ebi-fl187-GCST90094840 | prot-a-2367 | cAMP-dependent protein kinase catalytic subunit alpha \|\| id:prot-a-2367 | F-small fish liking (derived food-liking factor) \|\| id:ebi-fl187-GCST90094840 | Weighted median | 28 | -0.088014141 | 0.0955662262734586 | 0.357063284049844 | -0.275323945 | 0.0992956624842816 | 0.915747927029617 | 0.759326103857929 | 1.10439277880528 | 0.999332001504091 |
| ebi-fl187-GCST90094840 | prot-a-2385 | Phosphoribosyl pyrophosphate synthase-associated protein 1 \|\| id:prot-a-2385 | F-small fish liking (derived food-liking factor) \|\| id:ebi-fl187-GCST90094840 | Weighted median | 28 | 0.205670635299956 | 0.0983572186042287 | 0.0365228773828312 | 0.0128904868356676 | 0.398450783764244 | 1.22834856270579 | 1.01297392730506 | 1.48951532791721 | 0.999332001504091 |
| ebi-fl187-GCST90094840 | prot-a-2393 | Trypsin-3 \|\| id:prot-a-2393 | F-small fish liking (derived food-liking factor) \|\| id:ebi-fl187-GCST90094840 | Weighted median | 27 | 0.00907864568294813 | 0.0968466898632257 | 0.925313750959568 | -0.180740866 | 0.198898157814871 | 1.0091199815833 | 0.834651616912059 | 1.22005770622976 | 0.999332001504091 |
| ebi-fl187-GCST90094840 | prot-a-2405 | Pregnancy-specific beta-1-glycoprotein 6 \|\| id:prot-a-2405 | F-small fish liking (derived food-liking factor) \|\| id:ebi-fl187-GCST90094840 | Weighted median | 28 | 0.0225266219955445 | 0.100477433791726 | 0.822604925514896 | -0.174409148 | 0.219462392227327 | 1.02278226230684 | 0.839953161989055 | 1.24540701009127 | 0.999332001504091 |
| ebi-fl187-GCST90094840 | prot-a-244 | Beclin-1 \|\| id:prot-a-244 | F-small fish liking (derived food-liking factor) \|\| id:ebi-fl187-GCST90094840 | Weighted median | 28 | -0.086467191 | 0.09466114001059 | 0.3610116240123 | -0.272003026 | 0.0990686433204565 | 0.917165639486556 | 0.761851956087216 | 1.10414208893688 | 0.999332001504091 |
| ebi-fl187-GCST90094840 | prot-a-2454 | tRNA pseudouridine synthase A, mitochondrial \|\| id:prot-a-2454 | F-small fish liking (derived food-liking factor) \|\| id:ebi-fl187-GCST90094840 | Weighted median | 28 | 0.093074027867889 | 0.101943989352854 | 0.361246846340724 | -0.106736191 | 0.292884246999483 | 1.09754298102762 | 0.898762743181845 | 1.34028763913645 | 0.999332001504091 |
| ebi-fl187-GCST90094840 | prot-a-2466 | Glutaminyl-peptide cyclotransferase-like protein \|\| id:prot-a-2466 | F-small fish liking (derived food-liking factor) \|\| id:ebi-fl187-GCST90094840 | Weighted median | 28 | -0.097535598 | 0.0991455632433307 | 0.325232744088817 | -0.291860902 | 0.096789706029557 | 0.907070051167268 | 0.746872417294256 | 1.10162868339055 | 0.999332001504091 |
[truncated: 135,727 more chars]
