# Supplementary material for: Mendelian Randomization Mediation Analysis Reveals the Impact of Dietary Preferences on Preeclampsia and Fetal Growth Restriction via Immune Modulation
Source: Food Sci Nutr. 2025 Nov 26;13(12):e71280. doi: 10.1002/fsn3.71280 (PMC12648301; doi:10.1002/fsn3.71280)
Supplement: Supplementary file 1 — Data S1: fsn371280‐sup‐0001‐Supinfo1.zip. [file FSN3-13-e71280-s001.zip › fsn371280-sup-0012-Supinfo12@Supplementary Material 3_PE - MR (Dietary Preferences to Immune Cells) MR Analysis - 5.docx]

| id.exposure | id.outcome | outcome | exposure | method | nsnp | b | se | pval | lo_ci | up_ci | or | or_lci95 | or_uci95 | p_fdr |
| --- | --- | --- | --- | --- | --- | --- | --- | --- | --- | --- | --- | --- | --- | --- |
| ebi-fl187-GCST90094840 | prot-a-289 | Uncharacterized protein C17orf89 \|\| id:prot-a-289 | F-small fish liking (derived food-liking factor) \|\| id:ebi-fl187-GCST90094840 | Weighted mode | 28 | -0.043526817 | 0.150932088324946 | 0.775253080382509 | -0.33935371 | 0.252300075880166 | 0.957406878739786 | 0.712230481234622 | 1.28698217165505 | 0.999714303421225 |
| ebi-fl187-GCST90094840 | prot-a-2921 | Tachykinin-3 \|\| id:prot-a-2921 | F-small fish liking (derived food-liking factor) \|\| id:ebi-fl187-GCST90094840 | Weighted mode | 28 | 0.0366009777934409 | 0.166698719516302 | 0.82786251458571 | -0.290128512 | 0.363330468045393 | 1.03727904087834 | 0.748167412566572 | 1.43811103046373 | 0.999714303421225 |
| ebi-fl187-GCST90094840 | prot-a-2928 | Tax1-binding protein 1 \|\| id:prot-a-2928 | F-small fish liking (derived food-liking factor) \|\| id:ebi-fl187-GCST90094840 | Weighted mode | 28 | -0.030157601 | 0.157438558044535 | 0.84952675131346 | -0.338737175 | 0.278421972489374 | 0.970292602143684 | 0.712669731866883 | 1.32104352363686 | 0.999714303421225 |
| ebi-fl187-GCST90094840 | prot-a-296 | Uncharacterized protein C1orf185 \|\| id:prot-a-296 | F-small fish liking (derived food-liking factor) \|\| id:ebi-fl187-GCST90094840 | Weighted mode | 28 | -0.068923223 | 0.183424475688018 | 0.710032955197898 | -0.428435196 | 0.290588748885333 | 0.933398340419299 | 0.651527810917082 | 1.33721453988459 | 0.999714303421225 |
| ebi-fl187-GCST90094840 | prot-a-2964 | Transforming growth factor beta-2 \|\| id:prot-a-2964 | F-small fish liking (derived food-liking factor) \|\| id:ebi-fl187-GCST90094840 | Weighted mode | 28 | -0.010846443 | 0.134805514854254 | 0.936464898726278 | -0.275065252 | 0.253372365972322 | 0.989212167425607 | 0.759522561047103 | 1.28836293004097 | 0.999714303421225 |
| ebi-fl187-GCST90094840 | prot-a-2977 | Thymocyte nuclear protein 1 \|\| id:prot-a-2977 | F-small fish liking (derived food-liking factor) \|\| id:ebi-fl187-GCST90094840 | Weighted mode | 28 | 0.124191571242358 | 0.175402898095785 | 0.484990828712318 | -0.219598109 | 0.467981251510097 | 1.13223275345951 | 0.802841387842981 | 1.59676746542771 | 0.999714303421225 |
| ebi-fl187-GCST90094840 | prot-a-3015 | Transmembrane protein 70, mitochondrial \|\| id:prot-a-3015 | F-small fish liking (derived food-liking factor) \|\| id:ebi-fl187-GCST90094840 | Weighted mode | 28 | -0.163456472 | 0.179711719178243 | 0.371109103808359 | -0.515691442 | 0.18877849740073 | 0.849203462165234 | 0.597087600541882 | 1.20777339790501 | 0.999714303421225 |
| ebi-fl187-GCST90094840 | prot-a-3030 | Tumor necrosis factor alpha-induced protein 3 \|\| id:prot-a-3030 | F-small fish liking (derived food-liking factor) \|\| id:ebi-fl187-GCST90094840 | Weighted mode | 28 | -0.138758404 | 0.142863992727199 | 0.340038968634223 | -0.41877183 | 0.141255021541219 | 0.87043829729042 | 0.65785428078919 | 1.15171832351827 | 0.999714303421225 |
| ebi-fl187-GCST90094840 | prot-a-3032 | Tumor necrosis factor receptor superfamily member 10A \|\| id:prot-a-3032 | F-small fish liking (derived food-liking factor) \|\| id:ebi-fl187-GCST90094840 | Weighted mode | 28 | -0.12490073 | 0.157577870852876 | 0.434904024834283 | -0.433753357 | 0.18395189714173 | 0.882584512638936 | 0.648072078474776 | 1.20195800409017 | 0.999714303421225 |
| ebi-fl187-GCST90094840 | prot-a-3081 | Tumor protein p53-inducible protein 11 \|\| id:prot-a-3081 | F-small fish liking (derived food-liking factor) \|\| id:ebi-fl187-GCST90094840 | Weighted mode | 28 | 0.00248849223072156 | 0.174778783711182 | 0.988744829093432 | -0.340077924 | 0.345054908304639 | 1.00249159109748 | 0.71171486104451 | 1.41206745176887 | 0.999714303421225 |
| ebi-fl187-GCST90094840 | prot-a-315 | Complement C4 \|\| id:prot-a-315 | F-small fish liking (derived food-liking factor) \|\| id:ebi-fl187-GCST90094840 | Weighted mode | 27 | -0.184834764 | 0.141114997727953 | 0.20171374733113 | -0.461420159 | 0.0917506318989556 | 0.831241623838727 | 0.630387758534979 | 1.09609145775267 | 0.999714303421225 |
| ebi-fl187-GCST90094840 | prot-a-3157 | UDP-glucuronosyltransferase 1-8 \|\| id:prot-a-3157 | F-small fish liking (derived food-liking factor) \|\| id:ebi-fl187-GCST90094840 | Weighted mode | 28 | 0.108604151904811 | 0.193639879570916 | 0.579520648097041 | -0.270930012 | 0.488138315863805 | 1.11472100280829 | 0.762669872234905 | 1.62928019 | 0.999714303421225 |
| ebi-fl187-GCST90094840 | prot-a-317 | Glutaredoxin-like protein C5orf63 \|\| id:prot-a-317 | F-small fish liking (derived food-liking factor) \|\| id:ebi-fl187-GCST90094840 | Weighted mode | 28 | 0.0508467873786906 | 0.204359755227458 | 0.805390526606483 | -0.349698333 | 0.451391907624508 | 1.0521616764848 | 0.70490070302192 | 1.57049665111326 | 0.999714303421225 |
| ebi-fl187-GCST90094840 | prot-a-3192 | Guanine nucleotide exchange factor VAV3 \|\| id:prot-a-3192 | F-small fish liking (derived food-liking factor) \|\| id:ebi-fl187-GCST90094840 | Weighted mode | 28 | 0.0358120269763829 | 0.140802653986183 | 0.801158477806648 | -0.240161175 | 0.311785228789302 | 1.03646100147039 | 0.786501086666327 | 1.36586131383789 | 0.999714303421225 |
| ebi-fl187-GCST90094840 | prot-a-3233 | Serine/threonine-protein kinase WNK3 \|\| id:prot-a-3233 | F-small fish liking (derived food-liking factor) \|\| id:ebi-fl187-GCST90094840 | Weighted mode | 28 | 0.160450762756052 | 0.153265733081166 | 0.304436965095844 | -0.139950074 | 0.460851599595138 | 1.1740399652253 | 0.869401639989378 | 1.58542355632441 | 0.999714303421225 |
| ebi-fl187-GCST90094840 | prot-a-3250 | 14-3-3 protein theta \|\| id:prot-a-3250 | F-small fish liking (derived food-liking factor) \|\| id:ebi-fl187-GCST90094840 | Weighted mode | 28 | 0.04852834711942 | 0.150879811792088 | 0.75020547496158 | -0.247196084 | 0.344252778231912 | 1.04972512808213 | 0.780987539361069 | 1.41093524415069 | 0.999714303421225 |
| ebi-fl187-GCST90094840 | prot-a-3278 | E3 ubiquitin-protein ligase ZNRF3 \|\| id:prot-a-3278 | F-small fish liking (derived food-liking factor) \|\| id:ebi-fl187-GCST90094840 | Weighted mode | 28 | 0.205234698478792 | 0.209413992658719 | 0.335765496117052 | -0.205216727 | 0.615686124089881 | 1.22781319703936 | 0.814470779365569 | 1.85092612898685 | 0.999714303421225 |
| ebi-fl187-GCST90094840 | prot-a-355 | Calpain-3 \|\| id:prot-a-355 | F-small fish liking (derived food-liking factor) \|\| id:ebi-fl187-GCST90094840 | Weighted mode | 28 | 0.0869523905500804 | 0.176090994233108 | 0.625445991613047 | -0.258185958 | 0.432090739246972 | 1.09084474396376 | 0.772451575086841 | 1.54047489035101 | 0.999714303421225 |
| ebi-fl187-GCST90094840 | prot-a-385 | Coiled-coil domain-containing protein 90B, mitochondrial \|\| id:prot-a-385 | F-small fish liking (derived food-liking factor) \|\| id:ebi-fl187-GCST90094840 | Weighted mode | 28 | 0.139334371710961 | 0.139357077630412 | 0.326266319301427 | -0.1338055 | 0.412474243866568 | 1.14950839884078 | 0.874760188525993 | 1.5105506358629 | 0.999714303421225 |
| ebi-fl187-GCST90094840 | prot-a-390 | C-C motif chemokine 14 \|\| id:prot-a-390 | F-small fish liking (derived food-liking factor) \|\| id:ebi-fl187-GCST90094840 | Weighted mode | 28 | -0.143872885 | 0.140740916123409 | 0.31573349897475 | -0.419725081 | 0.131979310349142 | 0.865997822157327 | 0.657227479397485 | 1.14108471037875 | 0.999714303421225 |
| ebi-fl187-GCST90094840 | prot-a-40 | Pituitary adenylate cyclase-activating polypeptide 27 \|\| id:prot-a-40 | F-small fish liking (derived food-liking factor) \|\| id:ebi-fl187-GCST90094840 | Weighted mode | 28 | 0.000407546605218245 | 0.154066508938616 | 0.997908835262433 | -0.301562811 | 0.302377904124906 | 1.00040762966362 | 0.739661366088747 | 1.35307246176908 | 0.999714303421225 |
| ebi-fl187-GCST90094840 | prot-a-401 | C-C motif chemokine 24 \|\| id:prot-a-401 | F-small fish liking (derived food-liking factor) \|\| id:ebi-fl187-GCST90094840 | Weighted mode | 28 | 0.173870927931916 | 0.170972811794528 | 0.318203551618158 | -0.161235783 | 0.508977639049191 | 1.18990197279984 | 0.851091374412337 | 1.66358953625936 | 0.999714303421225 |
| ebi-fl187-GCST90094840 | prot-a-412 | Cerebral cavernous malformations 2 protein \|\| id:prot-a-412 | F-small fish liking (derived food-liking factor) \|\| id:ebi-fl187-GCST90094840 | Weighted mode | 28 | 0.0546787377575686 | 0.180357308433772 | 0.764084805083607 | -0.298821587 | 0.408179062287762 | 1.05620124258504 | 0.741691725245633 | 1.50407646043069 | 0.999714303421225 |
| ebi-fl187-GCST90094840 | prot-a-44 | Adhesion G protein-coupled receptor F5 \|\| id:prot-a-44 | F-small fish liking (derived food-liking factor) \|\| id:ebi-fl187-GCST90094840 | Weighted mode | 28 | 0.000111348348175611 | 0.163917691392087 | 0.999462995840804 | -0.321167327 | 0.321390023476666 | 1.00011135454763 | 0.725301878407912 | 1.37904333529462 | 0.999714303421225 |
| ebi-fl187-GCST90094840 | prot-a-48 | Adrenomedullin \|\| id:prot-a-48 | F-small fish liking (derived food-liking factor) \|\| id:ebi-fl187-GCST90094840 | Weighted mode | 28 | 0.0394026096732405 | 0.150373733549065 | 0.795286184484877 | -0.255329908 | 0.334135127429408 | 1.0401891895867 | 0.774660888910874 | 1.39673186761016 | 0.999714303421225 |
| ebi-fl187-GCST90094840 | prot-a-502 | Carcinoembryonic antigen-related cell adhesion molecule 1 \|\| id:prot-a-502 | F-small fish liking (derived food-liking factor) \|\| id:ebi-fl187-GCST90094840 | Weighted mode | 28 | 0.0835072146396692 | 0.156939039518097 | 0.599008610936889 | -0.224093303 | 0.39110773209514 | 1.08709305824929 | 0.799240559495693 | 1.47861779942633 | 0.999714303421225 |
| ebi-fl187-GCST90094840 | prot-a-518 | Complement factor B \|\| id:prot-a-518 | F-small fish liking (derived food-liking factor) \|\| id:ebi-fl187-GCST90094840 | Weighted mode | 28 | -0.110710547 | 0.154766612866814 | 0.480540426480459 | -0.414053109 | 0.192632013853633 | 0.895197828801651 | 0.660965847373353 | 1.2124365515644 | 0.999714303421225 |
| ebi-fl187-GCST90094840 | prot-a-525 | Properdin \|\| id:prot-a-525 | F-small fish liking (derived food-liking factor) \|\| id:ebi-fl187-GCST90094840 | Weighted mode | 28 | -0.05672551 | 0.215728356364167 | 0.794586226286245 | -0.479553088 | 0.366102068568706 | 0.944853386670689 | 0.619059995098935 | 1.44210242847367 | 0.999714303421225 |
| ebi-fl187-GCST90094840 | prot-a-533 | Chondroadherin \|\| id:prot-a-533 | F-small fish liking (derived food-liking factor) \|\| id:ebi-fl187-GCST90094840 | Weighted mode | 28 | -0.049435468 | 0.146074163211332 | 0.737658949573404 | -0.335740828 | 0.23686989184503 | 0.951766575508591 | 0.714808340158837 | 1.26727622407716 | 0.999714303421225 |
| ebi-fl187-GCST90094840 | prot-a-535 | Coiled-coil-helix-coiled-coil-helix domain-containing protein 2 \|\| id:prot-a-535 | F-small fish liking (derived food-liking factor) \|\| id:ebi-fl187-GCST90094840 | Weighted mode | 28 | 0.015412695403574 | 0.196554369213314 | 0.93807666973507 | -0.369833868 | 0.40065925906167 | 1.01553208356924 | 0.690849093069943 | 1.49280852085316 | 0.999714303421225 |
| ebi-fl187-GCST90094840 | prot-a-591 | Caseinolytic peptidase B protein homolog \|\| id:prot-a-591 | F-small fish liking (derived food-liking factor) \|\| id:ebi-fl187-GCST90094840 | Weighted mode | 28 | -0.067939077 | 0.137280382675477 | 0.62467914774364 | -0.337008627 | 0.201130473409053 | 0.934317393603023 | 0.713902681267019 | 1.22278430225231 | 0.999714303421225 |
| ebi-fl187-GCST90094840 | prot-a-605 | Ciliary neurotrophic factor receptor subunit alpha \|\| id:prot-a-605 | F-small fish liking (derived food-liking factor) \|\| id:ebi-fl187-GCST90094840 | Weighted mode | 28 | -0.00357642 | 0.17726641951468 | 0.984051816328482 | -0.351018602 | 0.343865762201884 | 0.996429967725898 | 0.703970658263495 | 1.41038929524617 | 0.999714303421225 |
| ebi-fl187-GCST90094840 | prot-a-606 | Ciliary neurotrophic factor receptor subunit alpha \|\| id:prot-a-606 | F-small fish liking (derived food-liking factor) \|\| id:ebi-fl187-GCST90094840 | Weighted mode | 28 | -0.143762415 | 0.159510509456052 | 0.375413132762308 | -0.456403014 | 0.168878183362622 | 0.866093494291677 | 0.633558452900838 | 1.18397590217579 | 0.999714303421225 |
| ebi-fl187-GCST90094840 | prot-a-707 | Cancer/testis antigen 55 \|\| id:prot-a-707 | F-small fish liking (derived food-liking factor) \|\| id:ebi-fl187-GCST90094840 | Weighted mode | 28 | 0.0264664085839479 | 0.180211745490747 | 0.884331411387999 | -0.326748613 | 0.379681429745812 | 1.02681975435277 | 0.721265037196455 | 1.46181882325444 | 0.999714303421225 |
| ebi-fl187-GCST90094840 | prot-a-719 | Dipeptidyl peptidase 1 \|\| id:prot-a-719 | F-small fish liking (derived food-liking factor) \|\| id:ebi-fl187-GCST90094840 | Weighted mode | 28 | 0.00507829146826166 | 0.206282783064417 | 0.980540625077307 | -0.399235963 | 0.409392546274519 | 1.0050912078455 | 0.670832390826265 | 1.50590274098729 | 0.999714303421225 |
| ebi-fl187-GCST90094840 | prot-a-752 | Cytochrome b5 \|\| id:prot-a-752 | F-small fish liking (derived food-liking factor) \|\| id:ebi-fl187-GCST90094840 | Weighted mode | 28 | 0.0343425792500216 | 0.225141187294905 | 0.87989685248834 | -0.406934148 | 0.475619306348035 | 1.03493909466341 | 0.665688025847241 | 1.60901035931885 | 0.999714303421225 |
| ebi-fl187-GCST90094840 | prot-a-755 | Cytoglobin \|\| id:prot-a-755 | F-small fish liking (derived food-liking factor) \|\| id:ebi-fl187-GCST90094840 | Weighted mode | 28 | -0.060701649 | 0.165904280659385 | 0.717303149401641 | -0.385874039 | 0.264470741002287 | 0.941103977122684 | 0.679856155524336 | 1.30274130572968 | 0.999714303421225 |
| ebi-fl187-GCST90094840 | prot-a-807 | Desmin \|\| id:prot-a-807 | F-small fish liking (derived food-liking factor) \|\| id:ebi-fl187-GCST90094840 | Weighted mode | 28 | -0.041621974 | 0.156360821264988 | 0.792113759667737 | -0.348089184 | 0.264845235756825 | 0.959232326876079 | 0.706035906581257 | 1.30322926687894 | 0.999714303421225 |
| ebi-fl187-GCST90094840 | prot-a-82 | Ameloblastin \|\| id:prot-a-82 | F-small fish liking (derived food-liking factor) \|\| id:ebi-fl187-GCST90094840 | Weighted mode | 28 | -0.07775546 | 0.146200060188367 | 0.599184058350309 | -0.364307578 | 0.2087966575038 | 0.925190644572428 | 0.694677494063562 | 1.23219441556577 | 0.999714303421225 |
| ebi-fl187-GCST90094840 | prot-a-84 | Muellerian-inhibiting factor \|\| id:prot-a-84 | F-small fish liking (derived food-liking factor) \|\| id:ebi-fl187-GCST90094840 | Weighted mode | 28 | -0.145310885 | 0.174444489137188 | 0.412157390261291 | -0.487222084 | 0.196600313692349 | 0.864753412438492 | 0.6143305849688 | 1.21725742234044 | 0.999714303421225 |
| ebi-fl187-GCST90094840 | prot-a-858 | Inactive dipeptidyl peptidase 10 \|\| id:prot-a-858 | F-small fish liking (derived food-liking factor) \|\| id:ebi-fl187-GCST90094840 | Weighted mode | 28 | -0.011194201 | 0.142843258778722 | 0.938114015964471 | -0.291166988 | 0.268778586692666 | 0.988868221410643 | 0.74739086250103 | 1.30836541945882 | 0.999714303421225 |
| ebi-fl187-GCST90094840 | prot-a-863 | Dihydropyrimidinase-related protein 3 \|\| id:prot-a-863 | F-small fish liking (derived food-liking factor) \|\| id:ebi-fl187-GCST90094840 | Weighted mode | 28 | -0.008263038 | 0.137204901001187 | 0.952420742140306 | -0.277184643 | 0.260658568440807 | 0.991771007536624 | 0.75791454020868 | 1.29778448519986 | 0.999714303421225 |
| ebi-fl187-GCST90094840 | prot-a-864 | Dihydropyrimidinase-related protein 5 \|\| id:prot-a-864 | F-small fish liking (derived food-liking factor) \|\| id:ebi-fl187-GCST90094840 | Weighted mode | 28 | 0.0333690207097241 | 0.174639932599805 | 0.849897761435269 | -0.308925247 | 0.375663288605343 | 1.03393201117603 | 0.734235654156231 | 1.45595681397819 | 0.999714303421225 |
| ebi-fl187-GCST90094840 | prot-a-884 | Endothelin-converting enzyme 1 \|\| id:prot-a-884 | F-small fish liking (derived food-liking factor) \|\| id:ebi-fl187-GCST90094840 | Weighted mode | 28 | -0.08323164 | 0.158771660320591 | 0.604401330064694 | -0.394424094 | 0.227960814136491 | 0.920137981685627 | 0.674068127150108 | 1.25603610560859 | 0.999714303421225 |
| ebi-fl187-GCST90094840 | prot-a-893 | Early endosome antigen 1 \|\| id:prot-a-893 | F-small fish liking (derived food-liking factor) \|\| id:ebi-fl187-GCST90094840 | Weighted mode | 28 | -0.008247474 | 0.166066940225238 | 0.960756076017021 | -0.333738677 | 0.317243728367926 | 0.991786442636505 | 0.716240927775803 | 1.37333725238538 | 0.999714303421225 |
| ebi-fl187-GCST90094840 | prot-a-897 | Ephrin-A2 \|\| id:prot-a-897 | F-small fish liking (derived food-liking factor) \|\| id:ebi-fl187-GCST90094840 | Weighted mode | 28 | -0.268516041 | 0.195376396324962 | 0.180633580331886 | -0.651453778 | 0.114421695589594 | 0.764513158997397 | 0.521287389490481 | 1.12122484077634 | 0.999714303421225 |
| ebi-fl187-GCST90094840 | prot-a-90 | Anaphase-promoting complex subunit 10 \|\| id:prot-a-90 | F-small fish liking (derived food-liking factor) \|\| id:ebi-fl187-GCST90094840 | Weighted mode | 28 | 0.16530406885357 | 0.147143904510869 | 0.271150134115269 | -0.123097984 | 0.453706121694874 | 1.17975178995252 | 0.884177023125663 | 1.57413532527226 | 0.999714303421225 |
| ebi-fl187-GCST90094840 | prot-a-914 | Histone-lysine N-methyltransferase EHMT2 \|\| id:prot-a-914 | F-small fish liking (derived food-liking factor) \|\| id:ebi-fl187-GCST90094840 | Weighted mode | 28 | 0.0497332759532218 | 0.167440341169247 | 0.768723685185942 | -0.278449793 | 0.377916344644946 | 1.05099073448623 | 0.756956271497332 | 1.45924086445699 | 0.999714303421225 |
| ebi-fl187-GCST90094840 | prot-a-932 | RNA polymerase II elongation factor ELL \|\| id:prot-a-932 | F-small fish liking (derived food-liking factor) \|\| id:ebi-fl187-GCST90094840 | Weighted mode | 28 | 0.046100850643672 | 0.186288427557146 | 0.806416508474394 | -0.319024467 | 0.411226168655679 | 1.04718001441025 | 0.726857764792111 | 1.50866653105631 | 0.999714303421225 |
| ebi-fl187-GCST90094840 | prot-a-991 | Estrogen receptor \|\| id:prot-a-991 | F-small fish liking (derived food-liking factor) \|\| id:ebi-fl187-GCST90094840 | Weighted mode | 28 | -0.044197306 | 0.15285263139338 | 0.774675375466202 | -0.343788463 | 0.255393851630292 | 0.95676516343621 | 0.709078908304674 | 1.29096997138687 | 0.999714303421225 |
| ebi-fl187-GCST90094840 | prot-a-992 | Persulfide dioxygenase ETHE1, mitochondrial \|\| id:prot-a-992 | F-small fish liking (derived food-liking factor) \|\| id:ebi-fl187-GCST90094840 | Weighted mode | 28 | -0.20937976 | 0.227004793527578 | 0.364510976567742 | -0.654309155 | 0.235549635397596 | 0.811087158757838 | 0.519801040403735 | 1.26560419846581 | 0.999714303421225 |
| ebi-fl187-GCST90094840 | prot-a-993 | Ethanolamine kinase 1 \|\| id:prot-a-993 | F-small fish liking (derived food-liking factor) \|\| id:ebi-fl187-GCST90094840 | Weighted mode | 28 | 0.159689843977382 | 0.19303072501328 | 0.415332236393911 | -0.218650377 | 0.538030065003411 | 1.1731469559657 | 0.803602626966836 | 1.71262976763313 | 0.999714303421225 |
| ebi-fl187-GCST90094840 | prot-b-10 | coagulation factor III, tissue factor \|\| id:prot-b-10 | F-small fish liking (derived food-liking factor) \|\| id:ebi-fl187-GCST90094840 | Weighted mode | 19 | -0.119391882 | 0.196147917278626 | 0.550339716250173 | -0.5038418 | 0.265058035586113 | 0.887459952778973 | 0.604204960431952 | 1.30350662335411 | 0.999714303421225 |
| ebi-fl187-GCST90094840 | prot-b-28 | prolactin \|\| id:prot-b-28 | F-small fish liking (derived food-liking factor) \|\| id:ebi-fl187-GCST90094840 | Weighted mode | 19 | 0.234318933817241 | 0.273120929841811 | 0.402210161129172 | -0.300998089 | 0.76963595630719 | 1.26404757544433 | 0.740079187278904 | 2.15898014759946 | 0.999714303421225 |
| ebi-fl187-GCST90094840 | prot-b-54 | matrix metallopeptidase 7 \|\| id:prot-b-54 | F-small fish liking (derived food-liking factor) \|\| id:ebi-fl187-GCST90094840 | Weighted mode | 19 | 0.159120042989666 | 0.191946149518625 | 0.417971995990608 | -0.21709441 | 0.535334496046172 | 1.17247868607996 | 0.804853979399961 | 1.70801947247209 | 0.999714303421225 |
| ebi-fl187-GCST90094840 | ukb-rsfMRI191-edge-pheno716 | Net100_Pair10_33 (OccipitalPrecuneus)&(Temporal\|Frontal\|Supp_Motor_Area) (Default_mode\|Central_executive)&(Default_mode\|Salience) \|\| id:ukb-rsfMRI191-edge-pheno716 | F-small fish liking (derived food-liking factor) \|\| id:ebi-fl187-GCST90094840 | Weighted mode | 28 | -0.05078876 | 0.057615740857575 | 0.385822985914277 | -0.163715612 | 0.0621380922498582 | 0.950479428762753 | 0.848983428326086 | 1.06410927982705 | 0.999714303421225 |
| ebi-fl187-GCST90094840 | ukb-rsfMRI191-edge-pheno903 | Net100_Pair27_38 (Precentral\|Frontal\|Supp_Motor_Area)&(Subcortical) (Attention\|Salience\|Motor)&(Subcortical-cerebellum) \|\| id:ukb-rsfMRI191-edge-pheno903 | F-small fish liking (derived food-liking factor) \|\| id:ebi-fl187-GCST90094840 | Weighted mode | 28 | 0.0123411697727139 | 0.0442592836991594 | 0.782493762928967 | -0.074407026 | 0.0990893658230663 | 1.01241763624653 | 0.92829377697486 | 1.10416496976127 | 0.999714303421225 |
| ebi-fl187-GCST90094840 | ukb-rsfMRI191-node-pheno23 | Net100_Node2 RolandicOper\|SupraMarginal\|Insula Salience\|Motor \|\| id:ukb-rsfMRI191-node-pheno23 | F-small fish liking (derived food-liking factor) \|\| id:ebi-fl187-GCST90094840 | Weighted mode | 28 | -0.069479437 | 0.0449830061771762 | 0.134091471723711 | -0.157646129 | 0.0186872554136879 | 0.932879316270559 | 0.854151988282629 | 1.0188629549118 | 0.999714303421225 |
| ebi-fl187-GCST90094840 | ukb-rsfMRI191-node-pheno55 | Net100_Node34 Parietal\|Postcentral\|Precuneus Attention \|\| id:ukb-rsfMRI191-node-pheno55 | F-small fish liking (derived food-liking factor) \|\| id:ebi-fl187-GCST90094840 | Weighted mode | 28 | 0.0258274350373739 | 0.0555268733056568 | 0.645565240885976 | -0.083005237 | 0.134660106716461 | 1.02616385326668 | 0.920346327683535 | 1.14414783009077 | 0.999714303421225 |
| ebi-fl187-GCST90094846 | ebi-a-GCST90001414 | IgD- CD24- B cell Absolute Count \|\| id:ebi-a-GCST90001414 | Spicy food liking \|\| id:ebi-fl187-GCST90094846 | Inverse variance weighted | 15 | 0.181707961533322 | 0.121655302848545 | 0.135272457941968 | -0.056736432 | 0.42015235511647 | 1.19926391154053 | 0.944843066901576 | 1.52219345191355 | 0.998735809224318 |
| ebi-fl187-GCST90094846 | ebi-a-GCST90001422 | CD20- CD38- B cell %B cell \|\| id:ebi-a-GCST90001422 | Spicy food liking \|\| id:ebi-fl187-GCST90094846 | Inverse variance weighted | 15 | 0.159605577890639 | 0.116786151139162 | 0.171735615846121 | -0.069295278 | 0.388506434123397 | 1.17304810362756 | 0.933051129607499 | 1.474776472 | 0.998735809224318 |
| ebi-fl187-GCST90094846 | ebi-a-GCST90001444 | CD20- CD38- B cell %lymphocyte \|\| id:ebi-a-GCST90001444 | Spicy food liking \|\| id:ebi-fl187-GCST90094846 | Inverse variance weighted | 15 | 0.206825590614506 | 0.115307977983516 | 0.0728642916573989 | -0.019178046 | 0.432829227462197 | 1.22976806998235 | 0.981004682504276 | 1.54161293306723 | 0.998735809224318 |
| ebi-fl187-GCST90094846 | ebi-a-GCST90001459 | Myeloid Dendritic Cell %Dendritic Cell \|\| id:ebi-a-GCST90001459 | Spicy food liking \|\| id:ebi-fl187-GCST90094846 | Inverse variance weighted | 15 | 0.0925271883989135 | 0.124122244169708 | 0.45599845384562 | -0.15075241 | 0.335806786971541 | 1.0969429652778 | 0.860060614558367 | 1.39906868039798 | 0.998735809224318 |
| ebi-fl187-GCST90094846 | ebi-a-GCST90001476 | HLA DR++ monocyte %leukocyte \|\| id:ebi-a-GCST90001476 | Spicy food liking \|\| id:ebi-fl187-GCST90094846 | Inverse variance weighted | 15 | -0.142767131 | 0.12130734627824 | 0.239233725769284 | -0.38052953 | 0.09499526737425 | 0.86695593226469 | 0.68349937991656 | 1.0996536508646 | 0.998735809224318 |
| ebi-fl187-GCST90094846 | ebi-a-GCST90001478 | CD4 regulatory T cell %CD4+ T cell \|\| id:ebi-a-GCST90001478 | Spicy food liking \|\| id:ebi-fl187-GCST90094846 | Inverse variance weighted | 15 | -0.086341478 | 0.122276936308693 | 0.480117136583502 | -0.326004273 | 0.153321317030624 | 0.917280946346937 | 0.721802102948595 | 1.1656994778679 | 0.998735809224318 |
| ebi-fl187-GCST90094846 | ebi-a-GCST90001485 | CD39+ resting CD4 regulatory T cell %CD4 regulatory T cell \|\| id:ebi-a-GCST90001485 | Spicy food liking \|\| id:ebi-fl187-GCST90094846 | Inverse variance weighted | 15 | 0.170548114471878 | 0.134783831478455 | 0.205747503179969 | -0.093628195 | 0.434724424169651 | 1.18595471214575 | 0.910621272643547 | 1.54453736313194 | 0.998735809224318 |
| ebi-fl187-GCST90094846 | ebi-a-GCST90001515 | Immature Myeloid-Derived Suppressor Cells Absolute Count \|\| id:ebi-a-GCST90001515 | Spicy food liking \|\| id:ebi-fl187-GCST90094846 | Inverse variance weighted | 15 | 0.141199099482717 | 0.177300549823116 | 0.425809601302072 | -0.206309978 | 0.488708177136024 | 1.15165391885965 | 0.813580844889902 | 1.6302089179647 | 0.998735809224318 |
| ebi-fl187-GCST90094846 | ebi-a-GCST90001517 | CD33+ HLA DR+ Absolute Count \|\| id:ebi-a-GCST90001517 | Spicy food liking \|\| id:ebi-fl187-GCST90094846 | Inverse variance weighted | 15 | 0.409237940202467 | 0.156467278246485 | 0.00891006330871377 | 0.102562074839357 | 0.715913805565577 | 1.50566993727651 | 1.10800607907448 | 2.04605552517537 | 0.998735809224318 |
| ebi-fl187-GCST90094846 | ebi-a-GCST90001518 | CD33+ HLA DR+ CD14- Absolute Count \|\| id:ebi-a-GCST90001518 | Spicy food liking \|\| id:ebi-fl187-GCST90094846 | Inverse variance weighted | 15 | 0.278450799874534 | 0.158627228560322 | 0.0791946158464171 | -0.032458568 | 0.589360167852765 | 1.32108160641624 | 0.968062557667934 | 1.80283453480076 | 0.998735809224318 |
| ebi-fl187-GCST90094846 | ebi-a-GCST90001520 | CD33+ HLA DR+ CD14dim Absolute Count \|\| id:ebi-a-GCST90001520 | Spicy food liking \|\| id:ebi-fl187-GCST90094846 | Inverse variance weighted | 15 | 0.434761280465544 | 0.15689534296049 | 0.00558797394965165 | 0.127246408262983 | 0.742276152668105 | 1.54459429010707 | 1.13569682838901 | 2.1007116172153 | 0.998735809224318 |
| ebi-fl187-GCST90094846 | ebi-a-GCST90001531 | CD33dim HLA DR- Absolute Count \|\| id:ebi-a-GCST90001531 | Spicy food liking \|\| id:ebi-fl187-GCST90094846 | Inverse variance weighted | 15 | 0.120640205856549 | 0.159921046296161 | 0.450624955128755 | -0.192805045 | 0.434085456597024 | 1.12821891277624 | 0.824642726787344 | 1.54355076907656 | 0.998735809224318 |
| ebi-fl187-GCST90094846 | ebi-a-GCST90001532 | Basophil Absolute Count \|\| id:ebi-a-GCST90001532 | Spicy food liking \|\| id:ebi-fl187-GCST90094846 | Inverse variance weighted | 15 | 0.069019005413774 | 0.159413289987934 | 0.665046439140143 | -0.243431043 | 0.381469053790124 | 1.07145657243034 | 0.783933531902382 | 1.46443434281763 | 0.998735809224318 |
| ebi-fl187-GCST90094846 | ebi-a-GCST90001588 | T/B cell \|\| id:ebi-a-GCST90001588 | Spicy food liking \|\| id:ebi-fl187-GCST90094846 | Inverse variance weighted | 15 | -0.06727643 | 0.119546337482836 | 0.57359528663187 | -0.301587251 | 0.167034391866268 | 0.934936721428659 | 0.739643288873453 | 1.18179490874191 | 0.998735809224318 |
| ebi-fl187-GCST90094846 | ebi-a-GCST90001624 | HLA DR+ CD4+ T cell Absolute Count \|\| id:ebi-a-GCST90001624 | Spicy food liking \|\| id:ebi-fl187-GCST90094846 | Inverse variance weighted | 15 | 0.131278839507421 | 0.116011681469371 | 0.257802564850809 | -0.096104056 | 0.358661735187388 | 1.14028569368781 | 0.90836948969841 | 1.43141252318018 | 0.998735809224318 |
| ebi-fl187-GCST90094846 | ebi-a-GCST90001631 | CD8+ Natural Killer T %T cell \|\| id:ebi-a-GCST90001631 | Spicy food liking \|\| id:ebi-fl187-GCST90094846 | Inverse variance weighted | 15 | -0.002026039 | 0.13349510879054 | 0.98789106646813 | -0.263676453 | 0.259624373956356 | 0.997976011759075 | 0.768222055774778 | 1.29644301743211 | 0.998955228396052 |
| ebi-fl187-GCST90094846 | ebi-a-GCST90001632 | CD8+ Natural Killer T %lymphocyte \|\| id:ebi-a-GCST90001632 | Spicy food liking \|\| id:ebi-fl187-GCST90094846 | Inverse variance weighted | 15 | 0.00305831595273133 | 0.129923616514579 | 0.981220059802535 | -0.251591972 | 0.257708604321305 | 1.00306299737217 | 0.777561940070902 | 1.29396170883247 | 0.998955228396052 |
| ebi-fl187-GCST90094846 | ebi-a-GCST90001646 | Natural Killer %CD3- lymphocyte \|\| id:ebi-a-GCST90001646 | Spicy food liking \|\| id:ebi-fl187-GCST90094846 | Inverse variance weighted | 15 | -0.119492159 | 0.115350560838251 | 0.300246724313658 | -0.345579258 | 0.10659494068615 | 0.887370966060778 | 0.707810230099129 | 1.11248354138278 | 0.998735809224318 |
| ebi-fl187-GCST90094846 | ebi-a-GCST90001684 | CD127- CD8+ T cell Absolute Count \|\| id:ebi-a-GCST90001684 | Spicy food liking \|\| id:ebi-fl187-GCST90094846 | Inverse variance weighted | 15 | -0.001038955 | 0.124589103597418 | 0.993346473430438 | -0.245233599 | 0.243155687593735 | 0.998961584070154 | 0.78252172098317 | 1.27526715193815 | 0.999107451094609 |
| ebi-fl187-GCST90094846 | ebi-a-GCST90001687 | CD28- CD8+ T cell Absolute Count \|\| id:ebi-a-GCST90001687 | Spicy food liking \|\| id:ebi-fl187-GCST90094846 | Inverse variance weighted | 15 | -0.02811642 | 0.121164513594641 | 0.816497934722018 | -0.265598866 | 0.209366027 | 0.97227516841684 | 0.766746633848978 | 1.23289618941606 | 0.998735809224318 |
| ebi-fl187-GCST90094846 | ebi-a-GCST90001701 | BAFF-R on CD20- CD38- B cell \|\| id:ebi-a-GCST90001701 | Spicy food liking \|\| id:ebi-fl187-GCST90094846 | Inverse variance weighted (multiplicative random effects) | 15 | 0.018680807494209 | 0.158332082609864 | 0.906079559505377 | -0.291650074 | 0.329011689409542 | 1.01885638538668 | 0.747029895111051 | 1.38959409902719 | 0.998735809224318 |
| ebi-fl187-GCST90094846 | ebi-a-GCST90001732 | CD19 on IgD- CD27- B cell \|\| id:ebi-a-GCST90001732 | Spicy food liking \|\| id:ebi-fl187-GCST90094846 | Inverse variance weighted | 15 | -0.023489542 | 0.11686050304593 | 0.840694687074831 | -0.252536128 | 0.205557043940112 | 0.976784189795269 | 0.776828147085089 | 1.22820904084658 | 0.998735809224318 |
| ebi-fl187-GCST90094846 | ebi-a-GCST90001798 | CD27 on CD24+ CD27+ B cell \|\| id:ebi-a-GCST90001798 | Spicy food liking \|\| id:ebi-fl187-GCST90094846 | Inverse variance weighted | 15 | -0.005498344 | 0.117559268654965 | 0.962695879451626 | -0.235914511 | 0.224917822227931 | 0.994516743893324 | 0.789848194458278 | 1.25221980732961 | 0.998735809224318 |
| ebi-fl187-GCST90094846 | ebi-a-GCST90001836 | CD66b on CD66b++ myeloid cell \|\| id:ebi-a-GCST90001836 | Spicy food liking \|\| id:ebi-fl187-GCST90094846 | Inverse variance weighted | 15 | -0.225619632 | 0.182856660031605 | 0.217254845020112 | -0.584018686 | 0.132779421323938 | 0.798021585549003 | 0.557652825921755 | 1.14199807012455 | 0.998735809224318 |
| ebi-fl187-GCST90094846 | ebi-a-GCST90001943 | CD25 on activated & secreting CD4 regulatory T cell \|\| id:ebi-a-GCST90001943 | Spicy food liking \|\| id:ebi-fl187-GCST90094846 | Inverse variance weighted | 15 | 0.0451421309137717 | 0.11929428361326 | 0.705126158598596 | -0.188674665 | 0.278958926795761 | 1.04617654337028 | 0.828055858456605 | 1.32175305412147 | 0.998735809224318 |
| ebi-fl187-GCST90094846 | ebi-a-GCST90001960 | CD25 on CD4+ T cell \|\| id:ebi-a-GCST90001960 | Spicy food liking \|\| id:ebi-fl187-GCST90094846 | Inverse variance weighted | 15 | 0.260368073227286 | 0.127387529099579 | 0.0409628822512503 | 0.0106885161921111 | 0.510047630262462 | 1.2974075397718 | 1.01074584244344 | 1.66537051509158 | 0.998735809224318 |
| ebi-fl187-GCST90094846 | ebi-a-GCST90001988 | HLA DR on CD14+ CD16- monocyte \|\| id:ebi-a-GCST90001988 | Spicy food liking \|\| id:ebi-fl187-GCST90094846 | Inverse variance weighted | 15 | 0.0764795726010985 | 0.119708159312149 | 0.522898696575528 | -0.15814842 | 0.311107564852911 | 1.07948013914053 | 0.853723063286083 | 1.36493603243372 | 0.998735809224318 |
| ebi-fl187-GCST90094846 | ebi-a-GCST90001991 | HLA DR on CD14+ monocyte \|\| id:ebi-a-GCST90001991 | Spicy food liking \|\| id:ebi-fl187-GCST90094846 | Inverse variance weighted | 15 | 0.0595965360114566 | 0.120550655009895 | 0.621045431781535 | -0.176682748 | 0.295875819830851 | 1.06140822015006 | 0.838045614161388 | 1.34430321066648 | 0.998735809224318 |
| ebi-fl187-GCST90094846 | ebi-a-GCST90002019 | CD14 on Monocytic Myeloid-Derived Suppressor Cells \|\| id:ebi-a-GCST90002019 | Spicy food liking \|\| id:ebi-fl187-GCST90094846 | Inverse variance weighted | 15 | 0.034920422698101 | 0.171491216734042 | 0.838644168714036 | -0.301202362 | 0.371043207496823 | 1.03553730025637 | 0.739928024206218 | 1.44924569031243 | 0.998735809224318 |
| ebi-fl187-GCST90094846 | ebi-a-GCST90002037 | CD80 on plasmacytoid Dendritic Cell \|\| id:ebi-a-GCST90002037 | Spicy food liking \|\| id:ebi-fl187-GCST90094846 | Inverse variance weighted | 15 | 0.0068371000421931 | 0.132750300098638 | 0.958924343204147 | -0.253353488 | 0.267027688235524 | 1.00686052636964 | 0.776193458133393 | 1.30607660878677 | 0.998735809224318 |
| ebi-fl187-GCST90094846 | ebi-a-GCST90002042 | CD45 on CD33+ HLA DR+ CD14- \|\| id:ebi-a-GCST90002042 | Spicy food liking \|\| id:ebi-fl187-GCST90094846 | Inverse variance weighted | 15 | -0.086146249 | 0.178374398509638 | 0.629129912348188 | -0.43576007 | 0.263467572152619 | 0.9174600438617 | 0.646772887536303 | 1.30143509151884 | 0.998735809224318 |
| ebi-fl187-GCST90094846 | ebi-a-GCST90002056 | CD8 on Effector Memory CD8+ T cell \|\| id:ebi-a-GCST90002056 | Spicy food liking \|\| id:ebi-fl187-GCST90094846 | Inverse variance weighted | 15 | -0.076795156 | 0.130043494356541 | 0.554832434621812 | -0.331680405 | 0.178090092896265 | 0.926079535975246 | 0.717716664875085 | 1.19493297135771 | 0.998735809224318 |
| ebi-fl187-GCST90094846 | ebi-a-GCST90002058 | CD8 on CD8+ T cell \|\| id:ebi-a-GCST90002058 | Spicy food liking \|\| id:ebi-fl187-GCST90094846 | Inverse variance weighted (multiplicative random effects) | 15 | -0.355764144 | 0.197233760753785 | 0.071267527312894 | -0.742342315 | 0.0308140274348494 | 0.700637850640496 | 0.475997672380318 | 1.03129369371772 | 0.998735809224318 |
| ebi-fl187-GCST90094846 | ebi-a-GCST90002063 | CD4 on CD4 regulatory T cell \|\| id:ebi-a-GCST90002063 | Spicy food liking \|\| id:ebi-fl187-GCST90094846 | Inverse variance weighted | 15 | -0.259944528 | 0.129484589540515 | 0.0446929881454702 | -0.513734324 | -0.006154733 | 0.771094358387051 | 0.598257315569014 | 0.993864168582403 | 0.998735809224318 |
| ebi-fl187-GCST90094846 | ebi-a-GCST90002066 | CD4 on activated CD4 regulatory T cell \|\| id:ebi-a-GCST90002066 | Spicy food liking \|\| id:ebi-fl187-GCST90094846 | Inverse variance weighted | 15 | -0.272339699 | 0.129510243758814 | 0.0354795028253082 | -0.526179777 | -0.018499621 | 0.76159550381629 | 0.590857872540724 | 0.981670446293683 | 0.998735809224318 |
| ebi-fl187-GCST90094846 | ebi-a-GCST90002070 | CD4 on activated & secreting CD4 regulatory T cell \|\| id:ebi-a-GCST90002070 | Spicy food liking \|\| id:ebi-fl187-GCST90094846 | Inverse variance weighted | 15 | -0.251860541 | 0.130101702889503 | 0.0528830998451801 | -0.506859879 | 0.00313879652035337 | 0.77735313929025 | 0.60238417135879 | 1.00314372770013 | 0.998735809224318 |
| ebi-fl187-GCST90094846 | ebi-a-GCST90002081 | SSC-A on CD4+ T cell \|\| id:ebi-a-GCST90002081 | Spicy food liking \|\| id:ebi-fl187-GCST90094846 | Inverse variance weighted | 15 | 0.142855985803186 | 0.125552823419539 | 0.255197407392082 | -0.103227548 | 0.388939519705483 | 1.15356366015894 | 0.90192172 | 1.47541531508177 | 0.998735809224318 |
| ebi-fl187-GCST90094846 | ebi-a-GCST90002094 | CD11b on Monocytic Myeloid-Derived Suppressor Cells \|\| id:ebi-a-GCST90002094 | Spicy food liking \|\| id:ebi-fl187-GCST90094846 | Inverse variance weighted | 15 | 0.106301644863012 | 0.196323738367424 | 0.588189881894735 | -0.278492882 | 0.491096172063162 | 1.11215730245138 | 0.756923655258123 | 1.6341065004424 | 0.998735809224318 |
| ebi-fl187-GCST90094846 | ebi-a-GCST90002104 | HLA DR on myeloid Dendritic Cell \|\| id:ebi-a-GCST90002104 | Spicy food liking \|\| id:ebi-fl187-GCST90094846 | Inverse variance weighted | 15 | 0.0153469995527429 | 0.132054863880985 | 0.907480687401038 | -0.243480534 | 0.274174532759473 | 1.01546536951641 | 0.783894735449908 | 1.31544437034045 | 0.998735809224318 |
| ebi-fl187-GCST90094846 | ebi-a-GCST90002105 | HLA DR on plasmacytoid Dendritic Cell \|\| id:ebi-a-GCST90002105 | Spicy food liking \|\| id:ebi-fl187-GCST90094846 | Inverse variance weighted | 15 | 0.0339105597640032 | 0.129497524931969 | 0.793427367479707 | -0.219904589 | 0.287725708630663 | 1.03449207737472 | 0.802595370654004 | 1.3333915161746 | 0.998735809224318 |
| ebi-fl187-GCST90094846 | ebi-a-GCST90002106 | HLA DR on Dendritic Cell \|\| id:ebi-a-GCST90002106 | Spicy food liking \|\| id:ebi-fl187-GCST90094846 | Inverse variance weighted | 15 | 0.0195993783816007 | 0.13187246984948 | 0.881850546971421 | -0.238870663 | 0.278069419286581 | 1.01979270717386 | 0.787516731212921 | 1.32057786760066 | 0.998735809224318 |
| ebi-fl187-GCST90094846 | ebi-a-GCST90002111 | HLA DR on CD33dim HLA DR+ CD11b- \|\| id:ebi-a-GCST90002111 | Spicy food liking \|\| id:ebi-fl187-GCST90094846 | Inverse variance weighted | 15 | 0.0145778084655718 | 0.193814511579661 | 0.940043444725367 | -0.365298634 | 0.394454251161707 | 1.01468458293039 | 0.693989370947266 | 1.48357431098876 | 0.998735809224318 |
| ebi-fl187-GCST90094846 | ebi-a-GCST90002112 | HLA DR on CD33- HLA DR+ \|\| id:ebi-a-GCST90002112 | Spicy food liking \|\| id:ebi-fl187-GCST90094846 | Inverse variance weighted | 15 | -0.028836218 | 0.171645912485419 | 0.86658463407182 | -0.365262206 | 0.307589770811121 | 0.971575578357644 | 0.694014652121135 | 1.36014290415331 | 0.998735809224318 |
| ebi-fl187-GCST90094846 | ebi-a-GCST90002115 | HLA DR on HLA DR+ CD8+ T cell \|\| id:ebi-a-GCST90002115 | Spicy food liking \|\| id:ebi-fl187-GCST90094846 | Inverse variance weighted | 15 | -0.305391324 | 0.140574317985013 | 0.0298213905046621 | -0.580916987 | -0.02986566 | 0.736834976948712 | 0.559385182375316 | 0.970575911484793 | 0.998735809224318 |
| ebi-fl187-GCST90094846 | ebi-a-GCST90002116 | HLA DR on B cell \|\| id:ebi-a-GCST90002116 | Spicy food liking \|\| id:ebi-fl187-GCST90094846 | Inverse variance weighted | 15 | -0.043322458 | 0.128256827481689 | 0.735529767689449 | -0.294705839 | 0.20806092427625 | 0.957602554066737 | 0.74475063150118 | 1.23128818260517 | 0.998735809224318 |
| ebi-fl187-GCST90094846 | ebi-cfmet338-GCST90026023 | Sphingomyelin (d18:1/24:1, d18:2/24:0) levels \|\| id:ebi-cfmet338-GCST90026023 | Spicy food liking \|\| id:ebi-fl187-GCST90094846 | Inverse variance weighted | 15 | -0.089033967 | 0.0770258600715561 | 0.247723254639469 | -0.240004653 | 0.0619367185223976 | 0.914814499343309 | 0.786624200928589 | 1.06389501774904 | 0.998735809224318 |
| ebi-fl187-GCST90094846 | ebi-cfmet338-GCST90026046 | 1-arachidonoyl-gpc (20:4n6) levels \|\| id:ebi-cfmet338-GCST90026046 | Spicy food liking \|\| id:ebi-fl187-GCST90094846 | Inverse variance weighted | 15 | -0.076966352 | 0.0883936544070311 | 0.383905636044926 | -0.250217915 | 0.0962852101385934 | 0.925921008010228 | 0.778631089082207 | 1.10107305641396 | 0.998735809224318 |
| ebi-fl187-GCST90094846 | ebi-cfmet338-GCST90026047 | 1-linoleoyl-gpc (18:2) levels \|\| id:ebi-cfmet338-GCST90026047 | Spicy food liking \|\| id:ebi-fl187-GCST90094846 | Inverse variance weighted | 15 | -0.015620428 | 0.0994762726651233 | 0.87522383124062 | -0.210593922 | 0.179353066838686 | 0.984500938542019 | 0.810102965082486 | 1.1964430939855 | 0.998735809224318 |
| ebi-fl187-GCST90094846 | ebi-cfmet338-GCST90026050 | 1-myristoyl-2-palmitoyl-gpc (14:0/16:0) levels \|\| id:ebi-cfmet338-GCST90026050 | Spicy food liking \|\| id:ebi-fl187-GCST90094846 | Inverse variance weighted | 15 | -0.116097354 | 0.0515907723725832 | 0.0244266633394089 | -0.217215268 | -0.01497944 | 0.89038853623507 | 0.804756712448523 | 0.98513219361254 | 0.998735809224318 |
| ebi-fl187-GCST90094846 | ebi-cfmet338-GCST90026068 | 2,3-dihydroxyisovalerate levels \|\| id:ebi-cfmet338-GCST90026068 | Spicy food liking \|\| id:ebi-fl187-GCST90094846 | Inverse variance weighted | 15 | -0.120985112 | 0.167294757444712 | 0.469566037773092 | -0.448882837 | 0.206912612228896 | 0.886047150643257 | 0.638340884274777 | 1.22987509104163 | 0.998735809224318 |
| ebi-fl187-GCST90094846 | ebi-cfmet338-GCST90026082 | 3-hydroxyhexanoate levels \|\| id:ebi-cfmet338-GCST90026082 | Spicy food liking \|\| id:ebi-fl187-GCST90094846 | Inverse variance weighted (multiplicative random effects) | 15 | 0.0374071946468273 | 0.0529748412233364 | 0.480106475034288 | -0.066423494 | 0.141237883444567 | 1.0381156499216 | 0.935734502280524 | 1.15169858542747 | 0.998735809224318 |
| ebi-fl187-GCST90094846 | ebi-cfmet338-GCST90026094 | 3-ureidopropionate levels \|\| id:ebi-cfmet338-GCST90026094 | Spicy food liking \|\| id:ebi-fl187-GCST90094846 | Inverse variance weighted (multiplicative random effects) | 15 | -0.045936965 | 0.109802478239786 | 0.675683928030537 | -0.261149822 | 0.169275892643407 | 0.95510216543848 | 0.770165523188259 | 1.18444687402904 | 0.998735809224318 |
| ebi-fl187-GCST90094846 | ebi-cfmet338-GCST90026173 | Glycerol levels \|\| id:ebi-cfmet338-GCST90026173 | Spicy food liking \|\| id:ebi-fl187-GCST90094846 | Inverse variance weighted | 15 | 0.0164758547364973 | 0.0460626265214319 | 0.720579966873793 | -0.073806893 | 0.106758602718504 | 1.01661233011712 | 0.928851043934372 | 1.11266562760001 | 0.998735809224318 |
| ebi-fl187-GCST90094846 | ebi-cfmet338-GCST90026179 | Histidine levels \|\| id:ebi-cfmet338-GCST90026179 | Spicy food liking \|\| id:ebi-fl187-GCST90094846 | Inverse variance weighted (multiplicative random effects) | 15 | 0.0333980694119461 | 0.0455168831885015 | 0.46310036402313 | -0.055815022 | 0.122611160461409 | 1.03396204599537 | 0.945714056349168 | 1.13044477385267 | 0.998735809224318 |
| ebi-fl187-GCST90094846 | ebi-cfmet338-GCST90026188 | Isocitrate levels \|\| id:ebi-cfmet338-GCST90026188 | Spicy food liking \|\| id:ebi-fl187-GCST90094846 | Inverse variance weighted | 15 | -0.059557366 | 0.0708498209202167 | 0.400564099815148 | -0.198423015 | 0.0793082833175214 | 0.94218148315371 | 0.820022898027672 | 1.08253799903984 | 0.998735809224318 |
| ebi-fl187-GCST90094846 | ebi-cfmet338-GCST90026207 | N-acetyl-isoputreanine levels \|\| id:ebi-cfmet338-GCST90026207 | Spicy food liking \|\| id:ebi-fl187-GCST90094846 | Inverse variance weighted | 14 | -0.01214435 | 0.0444042798819108 | 0.784472715353927 | -0.099176738 | 0.0748880388892076 | 0.987929095320662 | 0.905582642789541 | 1.07776347653334 | 0.998735809224318 |
| ebi-fl187-GCST90094846 | ebi-cfmet338-GCST90026213 | N-acetylglutamate levels \|\| id:ebi-cfmet338-GCST90026213 | Spicy food liking \|\| id:ebi-fl187-GCST90094846 | Inverse variance weighted | 15 | 0.000394854645797902 | 0.0734685184673265 | 0.995711810779252 | -0.143603442 | 0.144393150841758 | 1.00039493261116 | 0.866231191255459 | 1.15533824144983 | 0.999107451094609 |
| ebi-fl187-GCST90094846 | ebi-cfmet338-GCST90026224 | N-acetyltaurine levels \|\| id:ebi-cfmet338-GCST90026224 | Spicy food liking \|\| id:ebi-fl187-GCST90094846 | Inverse variance weighted | 15 | 0.0477347578297501 | 0.0576497264708297 | 0.407662795181248 | -0.065258706 | 0.160728221712576 | 1.04889240792599 | 0.936825069708194 | 1.17436575832399 | 0.998735809224318 |
| ebi-fl187-GCST90094846 | ebi-cfmet338-GCST90026250 | Phenyllactate (pla) levels \|\| id:ebi-cfmet338-GCST90026250 | Spicy food liking \|\| id:ebi-fl187-GCST90094846 | Inverse variance weighted | 15 | 0.166653767735398 | 0.086813865094788 | 0.0548998939289252 | -0.003501408 | 0.336808943321183 | 1.18134517467716 | 0.99650471492988 | 1.40047146874885 | 0.998735809224318 |
| ebi-fl187-GCST90094846 | ebi-cfmet338-GCST90026251 | Pipecolate levels \|\| id:ebi-cfmet338-GCST90026251 | Spicy food liking \|\| id:ebi-fl187-GCST90094846 | Inverse variance weighted | 14 | 0.023863632990281 | 0.148592178263776 | 0.872409890567112 | -0.267377036 | 0.315104302387281 | 1.02415064800629 | 0.765384439257631 | 1.3704022397281 | 0.998735809224318 |
| ebi-fl187-GCST90094846 | ebi-cfmet338-GCST90026278 | Trigonelline (n'-methylnicotinate) levels \|\| id:ebi-cfmet338-GCST90026278 | Spicy food liking \|\| id:ebi-fl187-GCST90094846 | Inverse variance weighted | 15 | 0.177669774116704 | 0.158491265209377 | 0.262284999950068 | -0.132973106 | 0.488312653927083 | 1.19443082412787 | 0.875488637451752 | 1.62956425999922 | 0.998735809224318 |
| ebi-fl187-GCST90094846 | ebi-cfmet338-GCST90026299 | 1-carboxyethylvaline levels \|\| id:ebi-cfmet338-GCST90026299 | Spicy food liking \|\| id:ebi-fl187-GCST90094846 | Inverse variance weighted | 15 | -0.050284869 | 0.101240747901793 | 0.619410445936292 | -0.248716735 | 0.148146996513454 | 0.950958486962704 | 0.77980083218122 | 1.15968335324402 | 0.998735809224318 |
| ebi-fl187-GCST90094846 | ebi-gm430-GCST90011306 | C_Erysipelotrichia abundance \|\| id:ebi-gm430-GCST90011306 | Spicy food liking \|\| id:ebi-fl187-GCST90094846 | Inverse variance weighted | 14 | -0.12360851 | 0.0756993985810221 | 0.102492913531106 | -0.271979332 | 0.0247623109030053 | 0.883725742681764 | 0.761870007610999 | 1.02507144326016 | 0.998735809224318 |
| ebi-fl187-GCST90094846 | ebi-gm430-GCST90011312 | F_Erysipelotrichaceae abundance \|\| id:ebi-gm430-GCST90011312 | Spicy food liking \|\| id:ebi-fl187-GCST90094846 | Inverse variance weighted | 14 | -0.12360851 | 0.0756993985810221 | 0.102492913531106 | -0.271979332 | 0.0247623109030053 | 0.883725742681764 | 0.761870007610999 | 1.02507144326016 | 0.998735809224318 |
| ebi-fl187-GCST90094846 | ebi-gm430-GCST90011352 | O_Erysipelotrichales abundance \|\| id:ebi-gm430-GCST90011352 | Spicy food liking \|\| id:ebi-fl187-GCST90094846 | Inverse variance weighted | 14 | -0.12360851 | 0.0756993985810221 | 0.102492913531106 | -0.271979332 | 0.0247623109030053 | 0.883725742681764 | 0.761870007610999 | 1.02507144326016 | 0.998735809224318 |
| ebi-fl187-GCST90094846 | ebi-gm430-GCST90011362 | OTU97_11 (Parabacteroides) abundance \|\| id:ebi-gm430-GCST90011362 | Spicy food liking \|\| id:ebi-fl187-GCST90094846 | Inverse variance weighted | 14 | -0.085004332 | 0.0986917115931691 | 0.389066360394664 | -0.278440087 | 0.108431422905203 | 0.918508305582571 | 0.756963618700801 | 1.1145284747927 | 0.998735809224318 |
| ebi-fl187-GCST90094846 | ebi-gm430-GCST90011384 | OTU97_235 (Phascolarctobacterium) abundance \|\| id:ebi-gm430-GCST90011384 | Spicy food liking \|\| id:ebi-fl187-GCST90094846 | Inverse variance weighted | 14 | 0.2639931336884 | 0.300891293741268 | 0.380285451011812 | -0.325753802 | 0.853740069421285 | 1.30211925551365 | 0.721982916270482 | 2.34841367762256 | 0.998735809224318 |
| ebi-fl187-GCST90094846 | ebi-gm430-GCST90011390 | OTU97_3 (Bacteroides) abundance \|\| id:ebi-gm430-GCST90011390 | Spicy food liking \|\| id:ebi-fl187-GCST90094846 | Inverse variance weighted | 14 | -0.12969249 | 0.0915189267632477 | 0.156450437647202 | -0.309069587 | 0.0496846063181722 | 0.878365495447153 | 0.734129682664793 | 1.05093958439548 | 0.998735809224318 |
| ebi-fl187-GCST90094846 | ebi-gm430-GCST90011398 | OTU97_4 (Alistipes) abundance \|\| id:ebi-gm430-GCST90011398 | Spicy food liking \|\| id:ebi-fl187-GCST90094846 | Inverse variance weighted | 14 | 0.0122385612394264 | 0.0915914761650968 | 0.893702134159262 | -0.167280732 | 0.191757854523016 | 1.01231375888725 | 0.845962089332106 | 1.21137715194956 | 0.998735809224318 |
| ebi-fl187-GCST90094846 | ebi-gm430-GCST90011421 | OTU99_108 (Cytophagales) abundance \|\| id:ebi-gm430-GCST90011421 | Spicy food liking \|\| id:ebi-fl187-GCST90094846 | Inverse variance weighted | 14 | 0.158186848368634 | 0.288791126321722 | 0.583859986730913 | -0.407843759 | 0.724217455959208 | 1.17138504564588 | 0.665082783756705 | 2.06311598897853 | 0.998735809224318 |
| ebi-fl187-GCST90094846 | ebi-gm430-GCST90011428 | OTU99_123 (Phascolarctobacterium) abundance \|\| id:ebi-gm430-GCST90011428 | Spicy food liking \|\| id:ebi-fl187-GCST90094846 | Inverse variance weighted | 14 | -0.174287608 | 0.172771997549805 | 0.313083841143399 | -0.512920723 | 0.164345507209803 | 0.840055256309185 | 0.598744256261657 | 1.17862146696618 | 0.998735809224318 |
| ebi-fl187-GCST90094846 | ebi-gm430-GCST90011450 | OTU99_298 (Phascolarctobacterium) abundance \|\| id:ebi-gm430-GCST90011450 | Spicy food liking \|\| id:ebi-fl187-GCST90094846 | Inverse variance weighted | 14 | 0.255508907887537 | 0.300892245341831 | 0.39578626031556 | -0.334239893 | 0.845257708757526 | 1.29111851410521 | 0.715882026552108 | 2.32857783215195 | 0.998735809224318 |
| ebi-fl187-GCST90094846 | ebi-gm430-GCST90011451 | OTU99_3 (Bacteroides) abundance \|\| id:ebi-gm430-GCST90011451 | Spicy food liking \|\| id:ebi-fl187-GCST90094846 | Inverse variance weighted | 14 | -0.135582248 | 0.0913033204228547 | 0.137553059809077 | -0.314536756 | 0.0433722603993506 | 0.873207340741128 | 0.730127023152473 | 1.04432658393053 | 0.998735809224318 |
| ebi-fl187-GCST90094846 | ebi-gm430-GCST90011472 | OTU99_72 (Parabacteroides) abundance \|\| id:ebi-gm430-GCST90011472 | Spicy food liking \|\| id:ebi-fl187-GCST90094846 | Inverse variance weighted | 14 | -0.219899649 | 0.263136124741005 | 0.40333055998762 | -0.735646454 | 0.295847155117936 | 0.8025993352668 | 0.47919558098849 | 1.34426467715315 | 0.998735809224318 |
| ebi-fl187-GCST90094846 | ebi-gm430-GCST90011507 | TestASV_3 (Bacteroides) abundance \|\| id:ebi-gm430-GCST90011507 | Spicy food liking \|\| id:ebi-fl187-GCST90094846 | Inverse variance weighted | 14 | -0.001356081 | 0.124758001048411 | 0.991327409528881 | -0.245881763 | 0.243169600724048 | 0.998644837731962 | 0.782014682227792 | 1.27528489501965 | 0.999107451094609 |
| ebi-fl187-GCST90094846 | ebi-gm430-GCST90011512 | TestASV_34 (Phascolarctobacterium) abundance \|\| id:ebi-gm430-GCST90011512 | Spicy food liking \|\| id:ebi-fl187-GCST90094846 | Inverse variance weighted | 14 | 0.172725116032918 | 0.253931394245991 | 0.496375384875352 | -0.324980417 | 0.67043064875506 | 1.18853934976469 | 0.722541503258331 | 1.95507909174599 | 0.998735809224318 |
| ebi-fl187-GCST90094846 | ebi-gm430-GCST90011519 | TestASV_41 (Barnesiella) abundance \|\| id:ebi-gm430-GCST90011519 | Spicy food liking \|\| id:ebi-fl187-GCST90094846 | Inverse variance weighted | 14 | -0.103426766 | 0.317622051840401 | 0.744705509486463 | -0.725965988 | 0.519112455707339 | 0.901742058592019 | 0.483856943369003 | 1.68053543795826 | 0.998735809224318 |
| ebi-fl187-GCST90094846 | ebi-gm430-GCST90011524 | TestASV_46 (Sutterella) abundance \|\| id:ebi-gm430-GCST90011524 | Spicy food liking \|\| id:ebi-fl187-GCST90094846 | Inverse variance weighted | 14 | -0.455186902 | 0.402764399452313 | 0.258410183325249 | -1.244605125 | 0.334231321013659 | 0.634329399490081 | 0.288054631294859 | 1.39686623071708 | 0.998735809224318 |
| ebi-fl187-GCST90094846 | ebi-gm430-GCST90011581 | OTU97_19 (Bacteria) prevalence \|\| id:ebi-gm430-GCST90011581 | Spicy food liking \|\| id:ebi-fl187-GCST90094846 | Inverse variance weighted | 14 | -0.679253186 | 0.376312087848444 | 0.0710705790524474 | -1.416824878 | 0.0583185065287287 | 0.506995482516649 | 0.242482708127644 | 1.06005257561286 | 0.998735809224318 |
| ebi-fl187-GCST90094846 | ebi-gm430-GCST90011614 | OTU97_80 (Ruminococcus) prevalence \|\| id:ebi-gm430-GCST90011614 | Spicy food liking \|\| id:ebi-fl187-GCST90094846 | Inverse variance weighted | 14 | 0.29588627346945 | 0.176088245745658 | 0.0928932249621666 | -0.049246688 | 0.641019235130939 | 1.34431726359986 | 0.951946266827265 | 1.89841482464739 | 0.998735809224318 |
| ebi-fl187-GCST90094846 | ebi-gm430-GCST90011623 | OTU99_11 (Parabacteroides) prevalence \|\| id:ebi-gm430-GCST90011623 | Spicy food liking \|\| id:ebi-fl187-GCST90094846 | Inverse variance weighted | 14 | 0.00717376953551276 | 0.185252031225209 | 0.969110137782598 | -0.355920212 | 0.370267750736923 | 1.00719956266128 | 0.700528512008431 | 1.44812229857228 | 0.998735809224318 |
| ebi-fl187-GCST90094846 | ebi-gm430-GCST90011642 | OTU99_19 (Bacteria) prevalence \|\| id:ebi-gm430-GCST90011642 | Spicy food liking \|\| id:ebi-fl187-GCST90094846 | Inverse variance weighted | 14 | -0.679253186 | 0.376312087848444 | 0.0710705790524474 | -1.416824878 | 0.0583185065287287 | 0.506995482516649 | 0.242482708127644 | 1.06005257561286 | 0.998735809224318 |
| ebi-fl187-GCST90094846 | ebi-gm430-GCST90011653 | OTU99_30 (Parasutterella) prevalence \|\| id:ebi-gm430-GCST90011653 | Spicy food liking \|\| id:ebi-fl187-GCST90094846 | Inverse variance weighted | 14 | -0.055140822 | 0.209497341226484 | 0.79239230256178 | -0.465755611 | 0.355473966454574 | 0.946351871075741 | 0.627660658569568 | 1.42685677628669 | 0.998735809224318 |
| ebi-fl187-GCST90094846 | ebi-gm430-GCST90011675 | OTU99_8 (Ruminococcaceae) prevalence \|\| id:ebi-gm430-GCST90011675 | Spicy food liking \|\| id:ebi-fl187-GCST90094846 | Inverse variance weighted | 14 | -0.228974319 | 0.209525136782774 | 0.274470618642024 | -0.639643587 | 0.181694948937616 | 0.795348958474862 | 0.527480391280474 | 1.19924830610564 | 0.998735809224318 |
| ebi-fl187-GCST90094846 | ebi-gm430-GCST90011679 | OTU99_92 (Ruminococcus) prevalence \|\| id:ebi-gm430-GCST90011679 | Spicy food liking \|\| id:ebi-fl187-GCST90094846 | Inverse variance weighted | 14 | 0.238873879311127 | 0.184119920668435 | 0.194499767057345 | -0.122001165 | 0.59974892382126 | 1.26981837611594 | 0.885147337129229 | 1.82166136719259 | 0.998735809224318 |
| ebi-fl187-GCST90094846 | ebi-gm430-GCST90011684 | TestASV_11 (Lachnospiraceae) prevalence \|\| id:ebi-gm430-GCST90011684 | Spicy food liking \|\| id:ebi-fl187-GCST90094846 | Inverse variance weighted | 14 | 0.135815603394803 | 0.191911956289757 | 0.479133157089267 | -0.240331831 | 0.511963037722727 | 1.14547065321965 | 0.786366876913099 | 1.66856343509551 | 0.998735809224318 |
| ebi-fl187-GCST90094846 | ebi-gm430-GCST90011688 | TestASV_15 (Bacteroides) prevalence \|\| id:ebi-gm430-GCST90011688 | Spicy food liking \|\| id:ebi-fl187-GCST90094846 | Inverse variance weighted | 14 | -0.151383541 | 0.184202521008156 | 0.411172474169166 | -0.512420482 | 0.20965340055441 | 0.859517975373579 | 0.599043847847197 | 1.23325054191816 | 0.998735809224318 |
| ebi-fl187-GCST90094846 | ebi-gm430-GCST90011692 | TestASV_19 (Prevotella) prevalence \|\| id:ebi-gm430-GCST90011692 | Spicy food liking \|\| id:ebi-fl187-GCST90094846 | Inverse variance weighted | 14 | 0.0387356387988417 | 0.343561006600448 | 0.910230755126468 | -0.634643934 | 0.712115211735721 | 1.03949564500611 | 0.530124213851367 | 2.03829813419846 | 0.998735809224318 |
| ebi-fl187-GCST90094846 | ebi-gm430-GCST90011693 | TestASV_2 (Bacteroides) prevalence \|\| id:ebi-gm430-GCST90011693 | Spicy food liking \|\| id:ebi-fl187-GCST90094846 | Inverse variance weighted | 14 | -0.216558356 | 0.1728156242146 | 0.210162793832084 | -0.55527698 | 0.122160267188855 | 0.805285540089641 | 0.573913276833905 | 1.12993517880427 | 0.998735809224318 |
| ebi-fl187-GCST90094846 | ebi-gm430-GCST90011716 | TestASV_40 (Barnesiella) prevalence \|\| id:ebi-gm430-GCST90011716 | Spicy food liking \|\| id:ebi-fl187-GCST90094846 | Inverse variance weighted | 14 | -0.277764194 | 0.361655875250354 | 0.442466680829601 | -0.98660971 | 0.431081321423132 | 0.757475417653652 | 0.372838581805435 | 1.5389206920892 | 0.998735809224318 |
| ebi-fl187-GCST90094846 | ebi-gm430-GCST90011718 | TestASV_42 (Alphaproteobacteria) prevalence \|\| id:ebi-gm430-GCST90011718 | Spicy food liking \|\| id:ebi-fl187-GCST90094846 | Inverse variance weighted | 14 | 0.326897487403907 | 0.296475163192346 | 0.270195088988131 | -0.254193832 | 0.907988807260905 | 1.38665931984719 | 0.775541462372467 | 2.47933110299087 | 0.998735809224318 |
| ebi-fl187-GCST90094846 | ebi-hbsm44-GCST90104929 | Sulcal depth of the right inferior frontal sulcus \|\| id:ebi-hbsm44-GCST90104929 | Spicy food liking \|\| id:ebi-fl187-GCST90094846 | Inverse variance weighted | 12 | 0.212352269112043 | 0.154752617182251 | 0.169999721745113 | -0.090962861 | 0.515667398789255 | 1.23658341850432 | 0.913051620490728 | 1.6747558589272 | 0.998735809224318 |
| ebi-fl187-GCST90094846 | ebi-hbsm44-GCST90104935 | Sulcal opening distance of the left calloso-marginal posterior fissure \|\| id:ebi-hbsm44-GCST90104935 | Spicy food liking \|\| id:ebi-fl187-GCST90094846 | Inverse variance weighted | 13 | -0.068390427 | 0.0351153895562815 | 0.0514638849100274 | -0.137216591 | 0.0004357361355775 | 0.933895783891566 | 0.871781385745514 | 1.00043583108236 | 0.998735809224318 |
| ebi-fl187-GCST90094846 | ebi-hbsm44-GCST90104936 | Sulcal opening distance of the left anterior inferior temporal sulcus \|\| id:ebi-hbsm44-GCST90104936 | Spicy food liking \|\| id:ebi-fl187-GCST90094846 | Inverse variance weighted | 14 | -0.007903665 | 0.0208987069914629 | 0.705290043246298 | -0.048865131 | 0.0330578002948505 | 0.992127486429765 | 0.952309557969237 | 1.03361028049373 | 0.998735809224318 |
| ebi-fl187-GCST90094846 | ebi-hbsm44-GCST90104941 | Sulcal opening distance of the right posterior inferior temporal sulcus \|\| id:ebi-hbsm44-GCST90104941 | Spicy food liking \|\| id:ebi-fl187-GCST90094846 | Inverse variance weighted | 14 | -0.006864652 | 0.0152563289720825 | 0.65274334344387 | -0.036767057 | 0.0230377529119009 | 0.993158856027348 | 0.963900643435806 | 1.02330517156797 | 0.998735809224318 |
| ebi-fl187-GCST90094846 | ebi-IPs91-GCST90274819 | Leukemia inhibitory factor levels \|\| id:ebi-IPs91-GCST90274819 | Spicy food liking \|\| id:ebi-fl187-GCST90094846 | Inverse variance weighted | 16 | -0.045581743 | 0.0757499199297083 | 0.547347341758441 | -0.194051586 | 0.102888100145445 | 0.955441498804855 | 0.823615416165119 | 1.10836737598833 | 0.998735809224318 |
| ebi-fl187-GCST90094846 | ebi-IPs91-GCST90274837 | STAM binding protein levels \|\| id:ebi-IPs91-GCST90274837 | Spicy food liking \|\| id:ebi-fl187-GCST90094846 | Inverse variance weighted | 16 | -0.005042027 | 0.0525101657294363 | 0.923504679975475 | -0.107961951 | 0.0978778981691719 | 0.994970663019706 | 0.897661750472569 | 1.10282811955473 | 0.998735809224318 |
| ebi-fl187-GCST90094846 | ebi-IPs91-GCST90274840 | TNF-beta levels \|\| id:ebi-IPs91-GCST90274840 | Spicy food liking \|\| id:ebi-fl187-GCST90094846 | Inverse variance weighted | 16 | -0.060329301 | 0.0598386364933915 | 0.313358558470299 | -0.177613028 | 0.0569544267513045 | 0.94145446084917 | 0.837266359191785 | 1.05860756511033 | 0.998735809224318 |
| ebi-fl187-GCST90094846 | ebi-IPs91-GCST90274842 | Tumor necrosis factor ligand superfamily member 14 levels \|\| id:ebi-IPs91-GCST90274842 | Spicy food liking \|\| id:ebi-fl187-GCST90094846 | Inverse variance weighted | 16 | -0.035205808 | 0.0599480833366974 | 0.557021293971986 | -0.152704052 | 0.0822924349402737 | 0.965406707000869 | 0.858383721393292 | 1.0857732814521 | 0.998735809224318 |
| ebi-fl187-GCST90094846 | ebi-met1400-GCST90199628 | 3-methylhistidine levels \|\| id:ebi-met1400-GCST90199628 | Spicy food liking \|\| id:ebi-fl187-GCST90094846 | Inverse variance weighted (multiplicative random effects) | 16 | -0.036243071 | 0.0923306136060938 | 0.694662370781828 | -0.217211073 | 0.144724932124921 | 0.964405846337916 | 0.804760088081095 | 1.15572162465024 | 0.998735809224318 |
| ebi-fl187-GCST90094846 | ebi-met1400-GCST90199651 | Docosahexaenoate DHA; 22:6n3 levels \|\| id:ebi-met1400-GCST90199651 | Spicy food liking \|\| id:ebi-fl187-GCST90094846 | Inverse variance weighted | 16 | 0.143805321166714 | 0.0663162249059044 | 0.030122283521398 | 0.0138255203511409 | 0.273785121982286 | 1.15465929891763 | 1.01392153483075 | 1.3149322218504 | 0.998735809224318 |
| ebi-fl187-GCST90094846 | ebi-met1400-GCST90199652 | 1-stearoyl-2-oleoyl-GPS (18:0/18:1) levels \|\| id:ebi-met1400-GCST90199652 | Spicy food liking \|\| id:ebi-fl187-GCST90094846 | Inverse variance weighted | 16 | 0.0282593099076154 | 0.072776582990165 | 0.697792382703096 | -0.114382793 | 0.170901412568339 | 1.02866239218983 | 0.891916471366063 | 1.18637378171192 | 0.998735809224318 |
| ebi-fl187-GCST90094846 | ebi-met1400-GCST90199653 | 1-myristoyl-2-palmitoyl-gpc (14:0/16:0) levels \|\| id:ebi-met1400-GCST90199653 | Spicy food liking \|\| id:ebi-fl187-GCST90094846 | Inverse variance weighted | 16 | -0.052818637 | 0.0688886724665095 | 0.443245298367946 | -0.187840435 | 0.0822031609831333 | 0.948552029076168 | 0.828746935616749 | 1.0856763545013 | 0.998735809224318 |
| ebi-fl187-GCST90094846 | ebi-met1400-GCST90199667 | Erythritol levels in elite athletes \|\| id:ebi-met1400-GCST90199667 | Spicy food liking \|\| id:ebi-fl187-GCST90094846 | Inverse variance weighted (multiplicative random effects) | 16 | 0.0172458038619225 | 0.089061083506234 | 0.846457637852651 | -0.15731392 | 0.191805527534141 | 1.01739537130385 | 0.854435792390958 | 1.21143490332258 | 0.998735809224318 |
| ebi-fl187-GCST90094846 | ebi-met1400-GCST90199674 | 3-hydroxymyristate levels \|\| id:ebi-met1400-GCST90199674 | Spicy food liking \|\| id:ebi-fl187-GCST90094846 | Inverse variance weighted | 16 | 0.0910335251607374 | 0.0676163769862061 | 0.17819817287961 | -0.041494574 | 0.223561624053701 | 1.09530572494343 | 0.959354541041978 | 1.2505227001801 | 0.998735809224318 |
| ebi-fl187-GCST90094846 | ebi-met1400-GCST90199685 | 2-linoleoylglycerol (18:2) levels \|\| id:ebi-met1400-GCST90199685 | Spicy food liking \|\| id:ebi-fl187-GCST90094846 | Inverse variance weighted | 16 | -0.12723199 | 0.0838340787435254 | 0.129099043296507 | -0.291546784 | 0.0370828042828098 | 0.880529374842891 | 0.74710705983592 | 1.03777894982212 | 0.998735809224318 |
| ebi-fl187-GCST90094846 | ebi-met1400-GCST90199703 | Gamma-glutamylvaline levels \|\| id:ebi-met1400-GCST90199703 | Spicy food liking \|\| id:ebi-fl187-GCST90094846 | Inverse variance weighted | 16 | -0.079980837 | 0.0676875555991936 | 0.237357293210497 | -0.212648446 | 0.0526867723697591 | 0.923134036599628 | 0.808440298025388 | 1.05409942034082 | 0.998735809224318 |
| ebi-fl187-GCST90094846 | ebi-met1400-GCST90199779 | Gamma-glutamyl-2-aminobutyrate levels \|\| id:ebi-met1400-GCST90199779 | Spicy food liking \|\| id:ebi-fl187-GCST90094846 | Inverse variance weighted | 16 | 0.0490969368782667 | 0.0718176602602547 | 0.494206814877212 | -0.091665677 | 0.189859550988366 | 1.05032216075681 | 0.912410138043005 | 1.20907977167267 | 0.998735809224318 |
| ebi-fl187-GCST90094846 | ebi-met1400-GCST90199824 | 4-vinylphenol sulfate levels \|\| id:ebi-met1400-GCST90199824 | Spicy food liking \|\| id:ebi-fl187-GCST90094846 | Inverse variance weighted | 16 | -0.060128728 | 0.0694193899245071 | 0.386399037452789 | -0.196190732 | 0.0759332765246472 | 0.941643310178681 | 0.821855465608471 | 1.07889058442629 | 0.998735809224318 |
| ebi-fl187-GCST90094846 | ebi-met1400-GCST90199830 | 4-ethylphenylsulfate levels \|\| id:ebi-met1400-GCST90199830 | Spicy food liking \|\| id:ebi-fl187-GCST90094846 | Inverse variance weighted | 16 | 0.00838423503341573 | 0.0884644006564956 | 0.924493351882253 | -0.16500599 | 0.181774460320147 | 1.00841948116706 | 0.847888625005056 | 1.19934366378742 | 0.998735809224318 |
| ebi-fl187-GCST90094846 | ebi-met1400-GCST90199833 | Hexanoylglutamine levels \|\| id:ebi-met1400-GCST90199833 | Spicy food liking \|\| id:ebi-fl187-GCST90094846 | Inverse variance weighted | 15 | 0.0145444038056513 | 0.0704202170810796 | 0.836371206024781 | -0.123479222 | 0.152568029284567 | 1.01465068830309 | 0.883840005769707 | 1.16482170138628 | 0.998735809224318 |
| ebi-fl187-GCST90094846 | ebi-met1400-GCST90199837 | Carotenoid levels (cryptoxanthin) \|\| id:ebi-met1400-GCST90199837 | Spicy food liking \|\| id:ebi-fl187-GCST90094846 | Inverse variance weighted | 16 | 0.207007458090701 | 0.0730134090402077 | 0.00457973178702299 | 0.0639011763718938 | 0.350113739809508 | 1.22999174513654 | 1.06598704884838 | 1.41922896224532 | 0.998735809224318 |
| ebi-fl187-GCST90094846 | ebi-met1400-GCST90199869 | 2-hydroxyhippurate levels \|\| id:ebi-met1400-GCST90199869 | Spicy food liking \|\| id:ebi-fl187-GCST90094846 | Inverse variance weighted | 16 | 0.150497143574527 | 0.0698663140513204 | 0.0312344443356735 | 0.0135591680339391 | 0.287435119115115 | 1.16241198475519 | 1.01365151044289 | 1.33300410287173 | 0.998735809224318 |
| ebi-fl187-GCST90094846 | ebi-met1400-GCST90199902 | Hydantoin-5-propionate levels \|\| id:ebi-met1400-GCST90199902 | Spicy food liking \|\| id:ebi-fl187-GCST90094846 | Inverse variance weighted | 16 | -0.064081453 | 0.069305863099663 | 0.355164614510118 | -0.199920944 | 0.0717580388676164 | 0.9379285995014 | 0.818795480819408 | 1.07439535069526 | 0.998735809224318 |
| ebi-fl187-GCST90094846 | ebi-met1400-GCST90199904 | Pantoate levels \|\| id:ebi-met1400-GCST90199904 | Spicy food liking \|\| id:ebi-fl187-GCST90094846 | Inverse variance weighted | 16 | 0.017839331116249 | 0.0878357128414376 | 0.839057780805875 | -0.154318666 | 0.189997328285467 | 1.01799940242188 | 0.85699888104273 | 1.20924636689184 | 0.998735809224318 |
| ebi-fl187-GCST90094846 | ebi-met1400-GCST90199936 | 3-methyl catechol sulfate (2) levels \|\| id:ebi-met1400-GCST90199936 | Spicy food liking \|\| id:ebi-fl187-GCST90094846 | Inverse variance weighted | 16 | -0.090406726 | 0.0810831302079651 | 0.2648555178514 | -0.249329661 | 0.0685162092777096 | 0.913559541343767 | 0.779323018520235 | 1.07091798361729 | 0.998735809224318 |
| ebi-fl187-GCST90094846 | ebi-met1400-GCST90199977 | 4-hydroxychlorothalonil levels \|\| id:ebi-met1400-GCST90199977 | Spicy food liking \|\| id:ebi-fl187-GCST90094846 | Inverse variance weighted | 16 | 0.110822761074646 | 0.0688332329611431 | 0.107393872837879 | -0.024090376 | 0.245735897678486 | 1.11719687853165 | 0.976197481407147 | 1.2785618577931 | 0.998735809224318 |
| ebi-fl187-GCST90094846 | ebi-met1400-GCST90200040 | Sphingomyelin (d18:2/24:1, d18:1/24:2) levels \|\| id:ebi-met1400-GCST90200040 | Spicy food liking \|\| id:ebi-fl187-GCST90094846 | Inverse variance weighted | 16 | 0.0517680701765043 | 0.0632400175059905 | 0.413016545545044 | -0.072182364 | 0.175718504488246 | 1.05313146159244 | 0.930361215821322 | 1.19210243992891 | 0.998735809224318 |
| ebi-fl187-GCST90094846 | ebi-met1400-GCST90200077 | 1-stearoyl-2-oleoyl-GPI (18:0/18:1) levels \|\| id:ebi-met1400-GCST90200077 | Spicy food liking \|\| id:ebi-fl187-GCST90094846 | Inverse variance weighted | 16 | 0.047903345222169 | 0.0686596685400559 | 0.485369433620292 | -0.086669605 | 0.182476295560679 | 1.04906925286847 | 0.916980011093621 | 1.20018570088726 | 0.998735809224318 |
| ebi-fl187-GCST90094846 | ebi-met1400-GCST90200080 | 1-palmitoyl-2-oleoyl-GPI (16:0/18:1) levels \|\| id:ebi-met1400-GCST90200080 | Spicy food liking \|\| id:ebi-fl187-GCST90094846 | Inverse variance weighted | 16 | -0.035042498 | 0.069229330708906 | 0.61273033750536 | -0.170731987 | 0.100646989877863 | 0.965564380529691 | 0.84304749130368 | 1.10588618383285 | 0.998735809224318 |
| ebi-fl187-GCST90094846 | ebi-met1400-GCST90200082 | 1-oleoyl-2-linoleoyl-GPE (18:1/18:2) levels \|\| id:ebi-met1400-GCST90200082 | Spicy food liking \|\| id:ebi-fl187-GCST90094846 | Inverse variance weighted | 16 | -0.167566021 | 0.069617911505337 | 0.0160868853472832 | -0.304017127 | -0.031114914 | 0.845720780212037 | 0.737848228895103 | 0.96936417283742 | 0.998735809224318 |
| ebi-fl187-GCST90094846 | ebi-met1400-GCST90200100 | Perfluorooctanesulfonate (PFOS) levels \|\| id:ebi-met1400-GCST90200100 | Spicy food liking \|\| id:ebi-fl187-GCST90094846 | Inverse variance weighted (multiplicative random effects) | 16 | 0.119459557084952 | 0.092398004069097 | 0.196052469961541 | -0.061640531 | 0.300559645060382 | 1.12688766852336 | 0.940220806387648 | 1.35061446081896 | 0.998735809224318 |
| ebi-fl187-GCST90094846 | ebi-met1400-GCST90200102 | 1-palmitoleoyl-2-linolenoyl-GPC (16:1/18:3) levels \|\| id:ebi-met1400-GCST90200102 | Spicy food liking \|\| id:ebi-fl187-GCST90094846 | Inverse variance weighted | 16 | -0.114684459 | 0.0679685711692341 | 0.0915426017058097 | -0.247902859 | 0.0185339403263081 | 0.891647450703379 | 0.780435752172907 | 1.01870675982267 | 0.998735809224318 |
| ebi-fl187-GCST90094846 | ebi-met1400-GCST90200114 | Glycosyl-N-tricosanoyl-sphingadienine (d18:2/23:0) levels \|\| id:ebi-met1400-GCST90200114 | Spicy food liking \|\| id:ebi-fl187-GCST90094846 | Inverse variance weighted | 16 | -0.031096509 | 0.081393621490891 | 0.702423588624596 | -0.190628007 | 0.128434989396917 | 0.969382014738998 | 0.826439960983896 | 1.13704749874474 | 0.998735809224318 |
| ebi-fl187-GCST90094846 | ebi-met1400-GCST90200135 | Arachidoylcarnitine (C20) levels \|\| id:ebi-met1400-GCST90200135 | Spicy food liking \|\| id:ebi-fl187-GCST90094846 | Inverse variance weighted | 16 | 0.00490950689052659 | 0.0780714584602829 | 0.94985825056607 | -0.148110552 | 0.157929565472681 | 1.00492157826623 | 0.862335776993359 | 1.17108370707536 | 0.998735809224318 |
| ebi-fl187-GCST90094846 | ebi-met1400-GCST90200146 | Docosahexaenoylcarnitine (C22:6) levels \|\| id:ebi-met1400-GCST90200146 | Spicy food liking \|\| id:ebi-fl187-GCST90094846 | Inverse variance weighted | 16 | 0.133745151223593 | 0.0800947068357746 | 0.0949515034327797 | -0.023240474 | 0.290730776621712 | 1.14310146451094 | 0.977027505638416 | 1.33740447492645 | 0.998735809224318 |
| ebi-fl187-GCST90094846 | ebi-met1400-GCST90200162 | 3-hydroxyphenylacetoylglutamine levels \|\| id:ebi-met1400-GCST90200162 | Spicy food liking \|\| id:ebi-fl187-GCST90094846 | Inverse variance weighted | 16 | 0.0445037344717994 | 0.0888645799851717 | 0.616509091177091 | -0.129670842 | 0.218678311242736 | 1.0455088811266 | 0.878384510367494 | 1.24443089286407 | 0.998735809224318 |
| ebi-fl187-GCST90094846 | ebi-met1400-GCST90200172 | 3-carboxy-4-methyl-5-pentyl-2-furanpropionate (3-CMPFP) levels \|\| id:ebi-met1400-GCST90200172 | Spicy food liking \|\| id:ebi-fl187-GCST90094846 | Inverse variance weighted | 16 | 0.0767440007787913 | 0.0689056857377568 | 0.265384626048366 | -0.058311143 | 0.211799144824795 | 1.07976562184975 | 0.94335638278175 | 1.23589962331163 | 0.998735809224318 |
| ebi-fl187-GCST90094846 | ebi-met1400-GCST90200222 | Dihydrocaffeate sulfate (2) levels \|\| id:ebi-met1400-GCST90200222 | Spicy food liking \|\| id:ebi-fl187-GCST90094846 | Inverse variance weighted | 16 | 0.0448291980189422 | 0.0787930455367202 | 0.56939097861463 | -0.109605171 | 0.199263567270914 | 1.04584921153519 | 0.89618790621845 | 1.22050360831599 | 0.998735809224318 |
| ebi-fl187-GCST90094846 | ebi-met1400-GCST90200223 | 4-methylhexanoylglutamine levels \|\| id:ebi-met1400-GCST90200223 | Spicy food liking \|\| id:ebi-fl187-GCST90094846 | Inverse variance weighted | 16 | -0.044552245 | 0.10404336490133 | 0.668499386268078 | -0.24847724 | 0.159372750257176 | 0.956425630379607 | 0.779987613029479 | 1.17277501740589 | 0.998735809224318 |
| ebi-fl187-GCST90094846 | ebi-met1400-GCST90200224 | 2-ketocaprylate levels \|\| id:ebi-met1400-GCST90200224 | Spicy food liking \|\| id:ebi-fl187-GCST90094846 | Inverse variance weighted | 16 | -0.043005248 | 0.0702416582763338 | 0.540374318789246 | -0.180678898 | 0.0946684023863062 | 0.957906363119006 | 0.834703340533281 | 1.09929427132477 | 0.998735809224318 |
| ebi-fl187-GCST90094846 | ebi-met1400-GCST90200243 | Hydroxypalmitoyl sphingomyelin (d18:1/16:0(OH)) levels \|\| id:ebi-met1400-GCST90200243 | Spicy food liking \|\| id:ebi-fl187-GCST90094846 | Inverse variance weighted | 16 | -0.0253078 | 0.0677695812683062 | 0.708821786537535 | -0.158136179 | 0.107520579079525 | 0.975009757632313 | 0.853733513055629 | 1.11351377559929 | 0.998735809224318 |
| ebi-fl187-GCST90094846 | ebi-met1400-GCST90200273 | 2-methoxyhydroquinone sulfate (2) levels \|\| id:ebi-met1400-GCST90200273 | Spicy food liking \|\| id:ebi-fl187-GCST90094846 | Inverse variance weighted | 16 | 0.0082471527726211 | 0.0993050144738774 | 0.933812812988335 | -0.186390676 | 0.202884981141421 | 1.0082812542192 | 0.82994929070323 | 1.22493156991366 | 0.998735809224318 |
| ebi-fl187-GCST90094846 | ebi-met1400-GCST90200276 | 2,4-di-tert-butylphenol levels \|\| id:ebi-met1400-GCST90200276 | Spicy food liking \|\| id:ebi-fl187-GCST90094846 | Inverse variance weighted | 16 | 0.0810957387195786 | 0.0887051822699884 | 0.360603110877941 | -0.092766419 | 0.254957895968756 | 1.08447471781772 | 0.911406363073192 | 1.2904072883803 | 0.998735809224318 |
| ebi-fl187-GCST90094846 | ebi-met1400-GCST90200289 | N-acetylmethionine levels \|\| id:ebi-met1400-GCST90200289 | Spicy food liking \|\| id:ebi-fl187-GCST90094846 | Inverse variance weighted | 16 | -0.012494883 | 0.0781294659114539 | 0.872939870717224 | -0.165628636 | 0.140638870172382 | 0.987582853928523 | 0.847360854913024 | 1.15100890927245 | 0.998735809224318 |
| ebi-fl187-GCST90094846 | ebi-met1400-GCST90200308 | 3-hydroxyisobutyrate levels \|\| id:ebi-met1400-GCST90200308 | Spicy food liking \|\| id:ebi-fl187-GCST90094846 | Inverse variance weighted | 16 | 0.0391511617392657 | 0.0765890139658151 | 0.609221460392562 | -0.110963306 | 0.189265629112263 | 1.03992766904481 | 0.894971588741786 | 1.20836188595144 | 0.998735809224318 |
| ebi-fl187-GCST90094846 | ebi-met1400-GCST90200383 | Cystathionine levels \|\| id:ebi-met1400-GCST90200383 | Spicy food liking \|\| id:ebi-fl187-GCST90094846 | Inverse variance weighted | 16 | -0.023878893 | 0.0713793076382564 | 0.737975709748396 | -0.163782336 | 0.11602454975572 | 0.976403951741012 | 0.848926782412695 | 1.123023442 | 0.998735809224318 |
| ebi-fl187-GCST90094846 | ebi-met1400-GCST90200384 | Guanidinoacetate levels \|\| id:ebi-met1400-GCST90200384 | Spicy food liking \|\| id:ebi-fl187-GCST90094846 | Inverse variance weighted | 16 | 0.0484481879936315 | 0.0803318851921927 | 0.546441934304892 | -0.109002307 | 0.205898682970329 | 1.04964098640596 | 0.896728348758734 | 1.22862871667693 | 0.998735809224318 |
| ebi-fl187-GCST90094846 | ebi-met1400-GCST90200403 | Citrulline levels \|\| id:ebi-met1400-GCST90200403 | Spicy food liking \|\| id:ebi-fl187-GCST90094846 | Inverse variance weighted | 16 | -0.055781893 | 0.0699666385391277 | 0.425297633341689 | -0.192916505 | 0.081352718569048 | 0.945745387116907 | 0.824550817544225 | 1.08475344177915 | 0.998735809224318 |
| ebi-fl187-GCST90094846 | ebi-met1400-GCST90200434 | 12,13-DiHOME levels \|\| id:ebi-met1400-GCST90200434 | Spicy food liking \|\| id:ebi-fl187-GCST90094846 | Inverse variance weighted | 16 | -0.016609511 | 0.0700384710209875 | 0.812541664520053 | -0.153884915 | 0.120665891787181 | 0.98352766598749 | 0.857370686169318 | 1.12824789250115 | 0.998735809224318 |
| ebi-fl187-GCST90094846 | ebi-met1400-GCST90200437 | Fructose levels \|\| id:ebi-met1400-GCST90200437 | Spicy food liking \|\| id:ebi-fl187-GCST90094846 | Inverse variance weighted | 16 | 0.0440254630863012 | 0.0697849105367134 | 0.528123124689573 | -0.092752962 | 0.18080388773826 | 1.0450089637031 | 0.91141862791828 | 1.19818017842591 | 0.998735809224318 |
| ebi-fl187-GCST90094846 | ebi-met1400-GCST90200448 | N-stearoyl-sphinganine (d18:0/18:0) levels \|\| id:ebi-met1400-GCST90200448 | Spicy food liking \|\| id:ebi-fl187-GCST90094846 | Inverse variance weighted | 16 | -0.043511906 | 0.0772889055740266 | 0.573450281517581 | -0.194998161 | 0.107974348741757 | 0.957421154791308 | 0.822836171161128 | 1.11401916902657 | 0.998735809224318 |
| ebi-fl187-GCST90094846 | ebi-met1400-GCST90200468 | X-11847 levels \|\| id:ebi-met1400-GCST90200468 | Spicy food liking \|\| id:ebi-fl187-GCST90094846 | Inverse variance weighted | 16 | 0.0519314886208363 | 0.0815168850304521 | 0.524083030648151 | -0.107841606 | 0.211704583280522 | 1.05330357676058 | 0.897769786481797 | 1.23578276026015 | 0.998735809224318 |
| ebi-fl187-GCST90094846 | ebi-met1400-GCST90200476 | X-11843 levels \|\| id:ebi-met1400-GCST90200476 | Spicy food liking \|\| id:ebi-fl187-GCST90094846 | Inverse variance weighted | 16 | 0.0952954614490899 | 0.0731895312331382 | 0.192903813384351 | -0.04815602 | 0.238746942666041 | 1.09998380992839 | 0.952985090965372 | 1.26965719986121 | 0.998735809224318 |
| ebi-fl187-GCST90094846 | ebi-met1400-GCST90200511 | X-12906 levels \|\| id:ebi-met1400-GCST90200511 | Spicy food liking \|\| id:ebi-fl187-GCST90094846 | Inverse variance weighted | 16 | 0.0542291418895717 | 0.0791262116308584 | 0.493123145205369 | -0.100858233 | 0.209316516686054 | 1.05572648560299 | 0.904061189928295 | 1.23283514967835 | 0.998735809224318 |
| ebi-fl187-GCST90094846 | ebi-met1400-GCST90200515 | X-12849 levels \|\| id:ebi-met1400-GCST90200515 | Spicy food liking \|\| id:ebi-fl187-GCST90094846 | Inverse variance weighted | 16 | -0.078124652 | 0.0909906574831132 | 0.390560791059512 | -0.256466341 | 0.100217036447234 | 0.924849134860488 | 0.773781038904914 | 1.10541080647661 | 0.998735809224318 |
| ebi-fl187-GCST90094846 | ebi-met1400-GCST90200551 | X-18886 levels \|\| id:ebi-met1400-GCST90200551 | Spicy food liking \|\| id:ebi-fl187-GCST90094846 | Inverse variance weighted | 16 | -0.092590581 | 0.0684002326979256 | 0.175845237190095 | -0.226655037 | 0.0414738746802779 | 0.911566636235568 | 0.797195737500907 | 1.04234592987507 | 0.998735809224318 |
| ebi-fl187-GCST90094846 | ebi-met1400-GCST90200564 | X-21319 levels \|\| id:ebi-met1400-GCST90200564 | Spicy food liking \|\| id:ebi-fl187-GCST90094846 | Inverse variance weighted | 16 | -0.001048407 | 0.0701061439368417 | 0.988068431879982 | -0.138456449 | 0.136359635552998 | 0.998952142822939 | 0.870701170629201 | 1.14609399563506 | 0.998955228396052 |
| ebi-fl187-GCST90094846 | ebi-met1400-GCST90200579 | X-21442 levels \|\| id:ebi-met1400-GCST90200579 | Spicy food liking \|\| id:ebi-fl187-GCST90094846 | Inverse variance weighted | 16 | -0.018817756 | 0.0904631903276518 | 0.835216777452169 | -0.196125609 | 0.158490097152639 | 0.981358192697765 | 0.821908989083918 | 1.1717403206022 | 0.998735809224318 |
| ebi-fl187-GCST90094846 | ebi-met1400-GCST90200581 | X-21441 levels \|\| id:ebi-met1400-GCST90200581 | Spicy food liking \|\| id:ebi-fl187-GCST90094846 | Inverse variance weighted | 16 | -0.005446748 | 0.0695520346978567 | 0.937579994630315 | -0.141768736 | 0.130875239775161 | 0.994568058405642 | 0.867821929042816 | 1.13982556754678 | 0.998735809224318 |
| ebi-fl187-GCST90094846 | ebi-met1400-GCST90200648 | X-24585 levels \|\| id:ebi-met1400-GCST90200648 | Spicy food liking \|\| id:ebi-fl187-GCST90094846 | Inverse variance weighted | 16 | 0.0368108665451251 | 0.0678132305658822 | 0.587248778868885 | -0.096103065 | 0.169724798454254 | 1.03749677693076 | 0.908370389719105 | 1.18497869847404 | 0.998735809224318 |
| ebi-fl187-GCST90094846 | ebi-met1400-GCST90200720 | Adenosine 5'-diphosphate (ADP) to pantothenate ratio \|\| id:ebi-met1400-GCST90200720 | Spicy food liking \|\| id:ebi-fl187-GCST90094846 | Inverse variance weighted | 16 | -0.104441564 | 0.0922930828167922 | 0.257790284601457 | -0.285336006 | 0.0764528786285121 | 0.900827436898036 | 0.7517616154543 | 1.07945132391189 | 0.998735809224318 |
| ebi-fl187-GCST90094846 | ebi-met1400-GCST90200724 | Cholate to taurocholate ratio \|\| id:ebi-met1400-GCST90200724 | Spicy food liking \|\| id:ebi-fl187-GCST90094846 | Inverse variance weighted | 16 | 0.00749322050365357 | 0.0799114498952887 | 0.925292620208451 | -0.149133221 | 0.164119662298419 | 1.00752136493397 | 0.861454343194276 | 1.1783553113615 | 0.998735809224318 |
| ebi-fl187-GCST90094846 | ebi-met1400-GCST90200726 | N-acetylputrescine to (N(1) + N(8))-acetylspermidine ratio \|\| id:ebi-met1400-GCST90200726 | Spicy food liking \|\| id:ebi-fl187-GCST90094846 | Inverse variance weighted | 16 | 0.0488061343404854 | 0.0690357973442862 | 0.479586051154126 | -0.086504028 | 0.184116297135286 | 1.05001676881351 | 0.917131854153488 | 1.20215562222206 | 0.998735809224318 |
| ebi-fl187-GCST90094846 | ebi-met1400-GCST90200744 | Arginine to glutamate ratio \|\| id:ebi-met1400-GCST90200744 | Spicy food liking \|\| id:ebi-fl187-GCST90094846 | Inverse variance weighted | 16 | 0.123959838474979 | 0.0724092572863481 | 0.0869089109367523 | -0.017962306 | 0.265881982756221 | 1.13197040842838 | 0.982198054824297 | 1.3045810865373 | 0.998735809224318 |
| ebi-fl187-GCST90094846 | ebi-met1400-GCST90200759 | Histidine to trans-urocanate ratio \|\| id:ebi-met1400-GCST90200759 | Spicy food liking \|\| id:ebi-fl187-GCST90094846 | Inverse variance weighted | 16 | -0.008408537 | 0.0886265640474293 | 0.924413291327525 | -0.182116603 | 0.165299528472573 | 0.991626715809807 | 0.833504146004294 | 1.17974643344207 | 0.998735809224318 |
| ebi-fl187-GCST90094846 | ebi-met1400-GCST90200795 | Oleoyl-linoleoyl-glycerol (18:1 to 18:2) [2] to linoleoyl-arachidonoyl-glycerol (18:2 to 20:4) [2] ratio \|\| id:ebi-met1400-GCST90200795 | Spicy food liking \|\| id:ebi-fl187-GCST90094846 | Inverse variance weighted | 16 | 0.121418830309889 | 0.0816973460019224 | 0.137225467966204 | -0.038707968 | 0.281545628473657 | 1.12909771369399 | 0.962031612284369 | 1.32517646072129 | 0.998735809224318 |
| ebi-fl187-GCST90094846 | ebi-met1400-GCST90200811 | Choline to taurocholate ratio \|\| id:ebi-met1400-GCST90200811 | Spicy food liking \|\| id:ebi-fl187-GCST90094846 | Inverse variance weighted | 16 | 0.104018112928375 | 0.0726183174409185 | 0.152030816101922 | -0.038313789 | 0.246350015112575 | 1.10962055321117 | 0.962410899305085 | 1.27934728606845 | 0.998735809224318 |
| ebi-fl187-GCST90094846 | ebi-met1400-GCST90200831 | Cholate to phosphate ratio \|\| id:ebi-met1400-GCST90200831 | Spicy food liking \|\| id:ebi-fl187-GCST90094846 | Inverse variance weighted | 16 | -0.029827583 | 0.0723524844787815 | 0.680153443548078 | -0.171638453 | 0.111983286465306 | 0.970612869171632 | 0.842283643509257 | 1.11849416649777 | 0.998735809224318 |
| ebi-fl187-GCST90094846 | ebi-met1400-GCST90200841 | Adenosine 5'-diphosphate (ADP) to oxalate (ethanedioate) ratio \|\| id:ebi-met1400-GCST90200841 | Spicy food liking \|\| id:ebi-fl187-GCST90094846 | Inverse variance weighted | 16 | -0.110545858 | 0.0935900035357803 | 0.237534546460671 | -0.293982265 | 0.0728905493048752 | 0.895345270840148 | 0.745289709370439 | 1.07561280390277 | 0.998735809224318 |
| ebi-fl187-GCST90094846 | ebi-met1400-GCST90200844 | Aspartate to phosphate ratio \|\| id:ebi-met1400-GCST90200844 | Spicy food liking \|\| id:ebi-fl187-GCST90094846 | Inverse variance weighted | 16 | -0.138305329 | 0.0709306202007839 | 0.0511918828810391 | -0.277329345 | 0.000718686184765366 | 0.870832760297678 | 0.757804876758272 | 1.00071894450156 | 0.998735809224318 |
| ebi-fl187-GCST90094846 | ebi-met1400-GCST90200870 | Adenosine 5'-monophosphate (AMP) to acetoacetate ratio \|\| id:ebi-met1400-GCST90200870 | Spicy food liking \|\| id:ebi-fl187-GCST90094846 | Inverse variance weighted | 16 | 0.0514718259094877 | 0.0845781003399662 | 0.542808464572305 | -0.114301251 | 0.217244902575821 | 1.05281952364172 | 0.891989202980937 | 1.24264839266768 | 0.998735809224318 |
| ebi-fl187-GCST90094846 | ebi-met1400-GCST90200887 | Cholesterol to cortisol ratio \|\| id:ebi-met1400-GCST90200887 | Spicy food liking \|\| id:ebi-fl187-GCST90094846 | Inverse variance weighted | 16 | -0.05964747 | 0.0674280688619142 | 0.376367605591674 | -0.191806485 | 0.0725115452372213 | 0.942096592614534 | 0.82546659345082 | 1.07520521951782 | 0.998735809224318 |
| ebi-fl187-GCST90094846 | ebi-met1400-GCST90200890 | Cortisol to taurocholate ratio \|\| id:ebi-met1400-GCST90200890 | Spicy food liking \|\| id:ebi-fl187-GCST90094846 | Inverse variance weighted | 16 | 0.0973424097914308 | 0.0723727834415886 | 0.178620700831726 | -0.044508246 | 0.239193065336944 | 1.10223772600262 | 0.956467713263553 | 1.27022374908817 | 0.998735809224318 |
| ebi-fl187-GCST90094846 | ebi-met1400-GCST90200948 | Adenosine 5'-diphosphate (ADP) to mannitol to sorbitol ratio \|\| id:ebi-met1400-GCST90200948 | Spicy food liking \|\| id:ebi-fl187-GCST90094846 | Inverse variance weighted | 16 | 0.00940809993604674 | 0.103360813181128 | 0.927475171654736 | -0.193179094 | 0.211995293771057 | 1.00945249522413 | 0.82433432766942 | 1.23614206749724 | 0.998735809224318 |
| ebi-fl187-GCST90094846 | ebi-met1400-GCST90200972 | Leucine to N-palmitoyl-sphingosine (d18:1 to 16:0) ratio \|\| id:ebi-met1400-GCST90200972 | Spicy food liking \|\| id:ebi-fl187-GCST90094846 | Inverse variance weighted | 16 | 0.0122588589169335 | 0.0726297979115768 | 0.865965340598831 | -0.130095545 | 0.154613262823624 | 1.012334307 | 0.878011537309479 | 1.16720647167178 | 0.998735809224318 |
| ebi-fl187-GCST90094846 | ebi-met1400-GCST90200973 | Proline to glutamate ratio \|\| id:ebi-met1400-GCST90200973 | Spicy food liking \|\| id:ebi-fl187-GCST90094846 | Inverse variance weighted | 16 | 0.0597102236976051 | 0.0679898021717943 | 0.379822587662156 | -0.073549789 | 0.192970235954322 | 1.06152889605422 | 0.929089886593201 | 1.21284669375749 | 0.998735809224318 |
| ebi-fl187-GCST90094846 | ebi-met1400-GCST90200983 | Cholesterol to linoleoyl-arachidonoyl-glycerol (18:2 to 20:4) [1] ratio \|\| id:ebi-met1400-GCST90200983 | Spicy food liking \|\| id:ebi-fl187-GCST90094846 | Inverse variance weighted | 16 | 0.0256539369811377 | 0.0715141557328113 | 0.719800599633693 | -0.114513808 | 0.165821682217448 | 1.02598583127644 | 0.891799624136333 | 1.180362603 | 0.998735809224318 |
| ebi-fl187-GCST90094846 | ebi-met1400-GCST90201001 | Serine to alpha-ketobutyrate ratio \|\| id:ebi-met1400-GCST90201001 | Spicy food liking \|\| id:ebi-fl187-GCST90094846 | Inverse variance weighted | 16 | 0.00919169406879485 | 0.0706235255122109 | 0.896447279217522 | -0.129230416 | 0.147613804072728 | 1.00923406741683 | 0.878771459268582 | 1.15906518366275 | 0.998735809224318 |
| ebi-fl187-GCST90094846 | ebi-met1400-GCST90201005 | Fructose to maltose ratio \|\| id:ebi-met1400-GCST90201005 | Spicy food liking \|\| id:ebi-fl187-GCST90094846 | Inverse variance weighted | 16 | 0.0484245093984437 | 0.0921854646289159 | 0.599378541680867 | -0.132259001 | 0.229108020071119 | 1.0496161326762 | 0.876114051035653 | 1.25747786452212 | 0.998735809224318 |
| ebi-fl187-GCST90094846 | ebi-met1400-GCST90201020 | Paraxanthine to 5-acetylamino-6-formylamino-3-methyluracil ratio \|\| id:ebi-met1400-GCST90201020 | Spicy food liking \|\| id:ebi-fl187-GCST90094846 | Inverse variance weighted | 16 | -0.046306188 | 0.0847257032782937 | 0.584693161346598 | -0.212368566 | 0.119756190543697 | 0.95474958467883 | 0.808666595373838 | 1.12722199069321 | 0.998735809224318 |
| ebi-fl187-GCST90094846 | ebi-pw11-GCST90275197 | Placental weight (fetal genotype, adjusted for sex) \|\| id:ebi-pw11-GCST90275197 | Spicy food liking \|\| id:ebi-fl187-GCST90094846 | Inverse variance weighted | 15 | -0.012650092 | 0.0288548442808134 | 0.661093131293782 | -0.069205587 | 0.0439054030558121 | 0.987429584352058 | 0.933134820411934 | 1.04488350742635 | 0.998735809224318 |
| ebi-fl187-GCST90094846 | prot-a-1017 | FAS-associated factor 2 \|\| id:prot-a-1017 | Spicy food liking \|\| id:ebi-fl187-GCST90094846 | Inverse variance weighted | 16 | -0.06568353 | 0.140274286325752 | 0.639605333750182 | -0.340621131 | 0.209254071257291 | 0.936427168564544 | 0.711328357324315 | 1.23275816716246 | 0.998735809224318 |
| ebi-fl187-GCST90094846 | prot-a-1019 | Fas apoptotic inhibitory molecule 3 \|\| id:prot-a-1019 | Spicy food liking \|\| id:ebi-fl187-GCST90094846 | Inverse variance weighted | 16 | 0.158152301607894 | 0.111404853105634 | 0.155719008885295 | -0.06020121 | 0.376505813694937 | 1.17134457878598 | 0.9415750597539 | 1.45718401102289 | 0.998735809224318 |
| ebi-fl187-GCST90094846 | prot-a-1052 | MIP18 family protein FAM96A \|\| id:prot-a-1052 | Spicy food liking \|\| id:ebi-fl187-GCST90094846 | Inverse variance weighted | 16 | 0.0131553348948489 | 0.11786050057662 | 0.91112642345667 | -0.217851246 | 0.244161916025023 | 1.01324224701411 | 0.804245067250435 | 1.27655100782174 | 0.998735809224318 |
| ebi-fl187-GCST90094846 | prot-a-1066 | F-box/LRR-repeat protein 4 \|\| id:prot-a-1066 | Spicy food liking \|\| id:ebi-fl187-GCST90094846 | Inverse variance weighted | 16 | -0.089716249 | 0.111404853105634 | 0.420636634921358 | -0.308069761 | 0.128637263110778 | 0.914190550976574 | 0.734864051323273 | 1.13727751682767 | 0.998735809224318 |
| ebi-fl187-GCST90094846 | prot-a-1076 | Low affinity immunoglobulin gamma Fc region receptor III-B \|\| id:prot-a-1076 | Spicy food liking \|\| id:ebi-fl187-GCST90094846 | Inverse variance weighted | 16 | -0.025757859 | 0.111389291759574 | 0.817126968676311 | -0.244080871 | 0.192565153029778 | 0.974571044824135 | 0.783424275656667 | 1.21235548976765 | 0.998735809224318 |
| ebi-fl187-GCST90094846 | prot-a-1081 | Fc receptor-like protein 3 \|\| id:prot-a-1081 | Spicy food liking \|\| id:ebi-fl187-GCST90094846 | Inverse variance weighted | 16 | -0.01361782 | 0.11131746101108 | 0.9026351506841 | -0.231800043 | 0.204564403910816 | 0.986474483371621 | 0.79310469408747 | 1.22699047628633 | 0.998735809224318 |
| ebi-fl187-GCST90094846 | prot-a-1105 | Fibroblast growth factor receptor 4 \|\| id:prot-a-1105 | Spicy food liking \|\| id:ebi-fl187-GCST90094846 | Inverse variance weighted | 16 | 0.0527772344756766 | 0.13161745443863 | 0.688427758125605 | -0.205192976 | 0.310747445175391 | 1.05419478070742 | 0.814490124016056 | 1.36444458060593 | 0.998735809224318 |
| ebi-fl187-GCST90094846 | prot-a-1143 | Follistatin-related protein 1 \|\| id:prot-a-1143 | Spicy food liking \|\| id:ebi-fl187-GCST90094846 | Inverse variance weighted | 16 | -0.060079306 | 0.111414361711451 | 0.589719868186227 | -0.278451455 | 0.158292843248126 | 0.941689849244214 | 0.7569550134959 | 1.17150921304307 | 0.998735809224318 |
| ebi-fl187-GCST90094846 | prot-a-1151 | Alpha-(1,3)-fucosyltransferase 10 \|\| id:prot-a-1151 | Spicy food liking \|\| id:ebi-fl187-GCST90094846 | Inverse variance weighted | 16 | 0.00908142222471269 | 0.111434900508632 | 0.935048051248432 | -0.209330983 | 0.227493827221631 | 1.00912278345097 | 0.811126722238071 | 1.25544969011752 | 0.998735809224318 |
| ebi-fl187-GCST90094846 | prot-a-1165 | Growth arrest and DNA damage-inducible proteins-interacting protein 1 \|\| id:prot-a-1165 | Spicy food liking \|\| id:ebi-fl187-GCST90094846 | Inverse variance weighted | 16 | -0.002752739 | 0.111277939894882 | 0.980264338483421 | -0.220857501 | 0.21535202344897 | 0.99725104656618 | 0.80183093230456 | 1.24029843425845 | 0.998955228396052 |
| ebi-fl187-GCST90094846 | prot-a-1194 | Growth/differentiation factor 11/8 \|\| id:prot-a-1194 | Spicy food liking \|\| id:ebi-fl187-GCST90094846 | Inverse variance weighted | 16 | -0.026182209 | 0.113323895564565 | 0.817284712855513 | -0.248297044 | 0.19593262627695 | 0.974157573130613 | 0.780128176200982 | 1.21644494614849 | 0.998735809224318 |
| ebi-fl187-GCST90094846 | prot-a-1216 | D-glucuronyl C5-epimerase \|\| id:prot-a-1216 | Spicy food liking \|\| id:ebi-fl187-GCST90094846 | Inverse variance weighted | 16 | -0.043946061 | 0.123948780808517 | 0.722926611696892 | -0.286885672 | 0.19899354896402 | 0.957005575602085 | 0.750597538393208 | 1.2201740944875 | 0.998735809224318 |
| ebi-fl187-GCST90094846 | prot-a-1246 | Glypican-3 \|\| id:prot-a-1246 | Spicy food liking \|\| id:ebi-fl187-GCST90094846 | Inverse variance weighted (multiplicative random effects) | 16 | -0.016129814 | 0.175584986383581 | 0.926806661149088 | -0.360276387 | 0.328016759389388 | 0.983999574919426 | 0.697483523886126 | 1.3882122376839 | 0.998735809224318 |
| ebi-fl187-GCST90094846 | prot-a-1275 | Glutamate receptor 4 \|\| id:prot-a-1275 | Spicy food liking \|\| id:ebi-fl187-GCST90094846 | Inverse variance weighted (multiplicative random effects) | 16 | 0.071929253406499 | 0.178872318134393 | 0.687591119550883 | -0.27866049 | 0.42251899694991 | 1.07457931854834 | 0.756796799580966 | 1.52580020487821 | 0.998735809224318 |
| ebi-fl187-GCST90094846 | prot-a-1289 | Glutathione S-transferase P \|\| id:prot-a-1289 | Spicy food liking \|\| id:ebi-fl187-GCST90094846 | Inverse variance weighted | 16 | 0.0730592830086574 | 0.111410863773654 | 0.51197569784927 | -0.14530601 | 0.291424576005019 | 1.07579431134779 | 0.86475762814659 | 1.33833268728573 | 0.998735809224318 |
| ebi-fl187-GCST90094846 | prot-a-130 | Apolipoprotein D \|\| id:prot-a-130 | Spicy food liking \|\| id:ebi-fl187-GCST90094846 | Inverse variance weighted | 16 | 0.0149821987436608 | 0.111373217922163 | 0.892989455607394 | -0.203309308 | 0.2332737058711 | 1.01509499448868 | 0.81602579876537 | 1.26272704783963 | 0.998735809224318 |
| ebi-fl187-GCST90094846 | prot-a-1313 | Heparin-binding EGF-like growth factor \|\| id:prot-a-1313 | Spicy food liking \|\| id:ebi-fl187-GCST90094846 | Inverse variance weighted | 15 | 0.17482419047889 | 0.114846951697078 | 0.12794990080508 | -0.050275835 | 0.399924215805163 | 1.19103680259452 | 0.950967078461334 | 1.49171164519157 | 0.998735809224318 |
| ebi-fl187-GCST90094846 | prot-a-1347 | HLA class II histocompatibility antigen, DQ alpha 2 chain \|\| id:prot-a-1347 | Spicy food liking \|\| id:ebi-fl187-GCST90094846 | Inverse variance weighted | 15 | 0.0497150450145616 | 0.11482550521288 | 0.665042600333263 | -0.175342945 | 0.274773035231806 | 1.05097157411327 | 0.839169182370297 | 1.31623190269484 | 0.998735809224318 |
| ebi-fl187-GCST90094846 | prot-a-1357 | Non-histone chromosomal protein HMG-14 \|\| id:prot-a-1357 | Spicy food liking \|\| id:ebi-fl187-GCST90094846 | Inverse variance weighted | 16 | -0.064210214 | 0.12178051429013 | 0.5980117937533 | -0.302900022 | 0.174479594132983 | 0.937807838588088 | 0.738672943807878 | 1.19062644637219 | 0.998735809224318 |
| ebi-fl187-GCST90094846 | prot-a-137 | Apolipoprotein M \|\| id:prot-a-137 | Spicy food liking \|\| id:ebi-fl187-GCST90094846 | Inverse variance weighted (multiplicative random effects) | 16 | -0.110445907 | 0.143943335320844 | 0.442910658467266 | -0.392574845 | 0.171683029789198 | 0.89543476523856 | 0.67531580067383 | 1.18730143437751 | 0.998735809224318 |
| ebi-fl187-GCST90094846 | prot-a-1381 | 17-beta-hydroxysteroid dehydrogenase 14 \|\| id:prot-a-1381 | Spicy food liking \|\| id:ebi-fl187-GCST90094846 | Inverse variance weighted | 16 | -0.06392052 | 0.136584389613715 | 0.639789688687522 | -0.331625924 | 0.203784883180652 | 0.938079554697285 | 0.717755767773069 | 1.22603438446946 | 0.998735809224318 |
| ebi-fl187-GCST90094846 | prot-a-1392 | Serine protease HTRA2, mitochondrial \|\| id:prot-a-1392 | Spicy food liking \|\| id:ebi-fl187-GCST90094846 | Inverse variance weighted | 16 | -0.157935177 | 0.117749771634518 | 0.179830213812488 | -0.388724729 | 0.0728543756298787 | 0.853905133060059 | 0.677920856163645 | 1.07557389573851 | 0.998735809224318 |
| ebi-fl187-GCST90094846 | prot-a-1416 | Interferon-induced protein with tetratricopeptide repeats 2 \|\| id:prot-a-1416 | Spicy food liking \|\| id:ebi-fl187-GCST90094846 | Inverse variance weighted | 16 | -0.09792293 | 0.124414384049362 | 0.431240417857256 | -0.341775122 | 0.145929263144237 | 0.906718782247129 | 0.710507964121355 | 1.15711433452602 | 0.998735809224318 |
| ebi-fl187-GCST90094846 | prot-a-1428 | Interferon gamma \|\| id:prot-a-1428 | Spicy food liking \|\| id:ebi-fl187-GCST90094846 | Inverse variance weighted | 16 | -0.021719849 | 0.122691436514384 | 0.859486206135407 | -0.262195065 | 0.21875536627331 | 0.978514328134727 | 0.769360933784213 | 1.24452678621905 | 0.998735809224318 |
| ebi-fl187-GCST90094846 | prot-a-1483 | Interleukin-17C \|\| id:prot-a-1483 | Spicy food liking \|\| id:ebi-fl187-GCST90094846 | Inverse variance weighted | 16 | 0.126540258468431 | 0.130167297029449 | 0.330983112437125 | -0.128587644 | 0.38166816064615 | 1.13489513939654 | 0.879336490729281 | 1.4647259507651 | 0.998735809224318 |
| ebi-fl187-GCST90094846 | prot-a-1503 | Interleukin-1 receptor-like 2 \|\| id:prot-a-1503 | Spicy food liking \|\| id:ebi-fl187-GCST90094846 | Inverse variance weighted | 16 | -0.01945145 | 0.111370049161256 | 0.861349923515415 | -0.237736746 | 0.198833846265296 | 0.98073650870132 | 0.788410215567399 | 1.21997924495111 | 0.998735809224318 |
| ebi-fl187-GCST90094846 | prot-a-1522 | Interleukin-31 receptor subunit alpha \|\| id:prot-a-1522 | Spicy food liking \|\| id:ebi-fl187-GCST90094846 | Inverse variance weighted | 16 | -0.068767485 | 0.113134857585076 | 0.543295981659168 | -0.290511806 | 0.152976835779611 | 0.933543717681155 | 0.747880699814961 | 1.16529798541074 | 0.998735809224318 |
| ebi-fl187-GCST90094846 | prot-a-1540 | Interleukin-6 receptor subunit alpha \|\| id:prot-a-1540 | Spicy food liking \|\| id:ebi-fl187-GCST90094846 | Inverse variance weighted | 16 | 0.110396237353327 | 0.116507257044729 | 0.343359526352269 | -0.117957986 | 0.338750461160997 | 1.11672046916875 | 0.888733390671146 | 1.4031931503313 | 0.998735809224318 |
| ebi-fl187-GCST90094846 | prot-a-1541 | Interleukin-6 receptor subunit alpha \|\| id:prot-a-1541 | Spicy food liking \|\| id:ebi-fl187-GCST90094846 | Inverse variance weighted | 16 | 0.13483824174154 | 0.11133656322948 | 0.225862140999531 | -0.083381422 | 0.353057905671321 | 1.14435166104735 | 0.920000171810761 | 1.42341356476528 | 0.998735809224318 |
| ebi-fl187-GCST90094846 | prot-a-1545 | Interleukin-9 \|\| id:prot-a-1545 | Spicy food liking \|\| id:ebi-fl187-GCST90094846 | Inverse variance weighted | 16 | 0.0326831196065673 | 0.111296986664368 | 0.769020149893134 | -0.185458974 | 0.250825213468729 | 1.03322307922537 | 0.830722915907853 | 1.28508544907213 | 0.998735809224318 |
| ebi-fl187-GCST90094846 | prot-a-1548 | Inositol monophosphatase 3 \|\| id:prot-a-1548 | Spicy food liking \|\| id:ebi-fl187-GCST90094846 | Inverse variance weighted | 16 | -0.226754743 | 0.136116011454104 | 0.095734681419342 | -0.493542125 | 0.0400326398821604 | 0.797116257004637 | 0.610460233577609 | 1.04084474668784 | 0.998735809224318 |
| ebi-fl187-GCST90094846 | prot-a-1555 | Inhibin beta A chain:Inhibin beta B chain heterodimer \|\| id:prot-a-1555 | Spicy food liking \|\| id:ebi-fl187-GCST90094846 | Inverse variance weighted | 16 | -0.258137484 | 0.111429996711869 | 0.0205261413717437 | -0.476540277 | -0.03973469 | 0.772489020104882 | 0.620927918333359 | 0.961044379811937 | 0.998735809224318 |
| ebi-fl187-GCST90094846 | prot-a-1603 | Junctophilin-3 \|\| id:prot-a-1603 | Spicy food liking \|\| id:ebi-fl187-GCST90094846 | Inverse variance weighted (multiplicative random effects) | 16 | 0.148882716464062 | 0.169549904683625 | 0.379886428066277 | -0.183435097 | 0.481200529643967 | 1.16053686935931 | 0.832405899861356 | 1.6180157124867 | 0.998735809224318 |
| ebi-fl187-GCST90094846 | prot-a-1627 | UPF0258 protein KIAA1024 \|\| id:prot-a-1627 | Spicy food liking \|\| id:ebi-fl187-GCST90094846 | Inverse variance weighted (multiplicative random effects) | 16 | 0.0958293494491413 | 0.156004818214469 | 0.539035780885749 | -0.209940094 | 0.4015987931495 | 1.10057123488037 | 0.810632806080893 | 1.49421172442068 | 0.998735809224318 |
| ebi-fl187-GCST90094846 | prot-a-1666 | Kallikrein-7 \|\| id:prot-a-1666 | Spicy food liking \|\| id:ebi-fl187-GCST90094846 | Inverse variance weighted | 16 | 0.0946354380020614 | 0.139929642288071 | 0.498845561468495 | -0.179626661 | 0.368897536886681 | 1.09925803436328 | 0.835582108672928 | 1.44613942013592 | 0.998735809224318 |
| ebi-fl187-GCST90094846 | prot-a-1678 | Importin subunit alpha-3 \|\| id:prot-a-1678 | Spicy food liking \|\| id:ebi-fl187-GCST90094846 | Inverse variance weighted | 16 | 0.221979265028006 | 0.133201684212115 | 0.0956158727941372 | -0.039096036 | 0.483054566083751 | 1.24854548904013 | 0.961658350863518 | 1.62101835522219 | 0.998735809224318 |
| ebi-fl187-GCST90094846 | prot-a-1718 | Protein LDOC1 \|\| id:prot-a-1718 | Spicy food liking \|\| id:ebi-fl187-GCST90094846 | Inverse variance weighted | 16 | -0.087262498 | 0.11141191088415 | 0.433484961485995 | -0.305629843 | 0.131104847188848 | 0.916436501176002 | 0.736659248221271 | 1.14008730999525 | 0.998735809224318 |
| ebi-fl187-GCST90094846 | prot-a-1723 | Leptin \|\| id:prot-a-1723 | Spicy food liking \|\| id:ebi-fl187-GCST90094846 | Inverse variance weighted | 16 | -0.083568275 | 0.122900546699861 | 0.496525568511133 | -0.324453346 | 0.157316796687986 | 0.919828283 | 0.722922433814928 | 1.17036632335302 | 0.998735809224318 |
| ebi-fl187-GCST90094846 | prot-a-1730 | Galectin-7 \|\| id:prot-a-1730 | Spicy food liking \|\| id:ebi-fl187-GCST90094846 | Inverse variance weighted | 16 | 0.0394314039898264 | 0.111348483061148 | 0.723244383270182 | -0.178811623 | 0.257674430789677 | 1.04021914155476 | 0.836263417512831 | 1.29391749034665 | 0.998735809224318 |
| ebi-fl187-GCST90094846 | prot-a-1753 | Lipase member N \|\| id:prot-a-1753 | Spicy food liking \|\| id:ebi-fl187-GCST90094846 | Inverse variance weighted | 16 | 0.153145788166933 | 0.112463928904068 | 0.173282317564871 | -0.067283512 | 0.373575088818906 | 1.16549488191992 | 0.934930099402878 | 1.45291965746862 | 0.998735809224318 |
| ebi-fl187-GCST90094846 | prot-a-1756 | VIP36-like protein \|\| id:prot-a-1756 | Spicy food liking \|\| id:ebi-fl187-GCST90094846 | Inverse variance weighted | 16 | 0.0961779572535961 | 0.111413314531851 | 0.387997988301735 | -0.122192139 | 0.314548053736023 | 1.10095496948476 | 0.884978313115381 | 1.3696401673011 | 0.998735809224318 |
| ebi-fl187-GCST90094846 | prot-a-1760 | Ig Kappa chain V-I region HK102- like \|\| id:prot-a-1760 | Spicy food liking \|\| id:ebi-fl187-GCST90094846 | Inverse variance weighted | 16 | 0.151502620719386 | 0.111369575858615 | 0.173716301143202 | -0.066781748 | 0.369786989402272 | 1.16358135122588 | 0.935399331869136 | 1.44742626468977 | 0.998735809224318 |
| ebi-fl187-GCST90094846 | prot-a-179 | Neutral ceramidase \|\| id:prot-a-179 | Spicy food liking \|\| id:ebi-fl187-GCST90094846 | Inverse variance weighted (multiplicative random effects) | 16 | 0.0986190237151891 | 0.150440911574681 | 0.512124468754162 | -0.196245163 | 0.393483210401565 | 1.10364575659543 | 0.821810732417837 | 1.48213439908181 | 0.998735809224318 |
| ebi-fl187-GCST90094846 | prot-a-1809 | Lactotransferrin \|\| id:prot-a-1809 | Spicy food liking \|\| id:ebi-fl187-GCST90094846 | Inverse variance weighted | 16 | 0.0817319821999009 | 0.111416356207914 | 0.463209286800184 | -0.136644076 | 0.300108040367412 | 1.08516492733388 | 0.872280636529101 | 1.35000465469607 | 0.998735809224318 |
| ebi-fl187-GCST90094846 | prot-a-1818 | Lysophospholipase-like protein 1 \|\| id:prot-a-1818 | Spicy food liking \|\| id:ebi-fl187-GCST90094846 | Inverse variance weighted | 16 | -0.113911437 | 0.128078612206205 | 0.373795195112373 | -0.364945517 | 0.137122642974544 | 0.892336980467988 | 0.694234473912081 | 1.14696880756107 | 0.998735809224318 |
| ebi-fl187-GCST90094846 | prot-a-1852 | MAP kinase-activated protein kinase 3 \|\| id:prot-a-1852 | Spicy food liking \|\| id:ebi-fl187-GCST90094846 | Inverse variance weighted | 16 | -0.12650261 | 0.125075594141053 | 0.311820608618395 | -0.371650774 | 0.11864555501161 | 0.881171850118295 | 0.689595024975523 | 1.12597075286101 | 0.998735809224318 |
| ebi-fl187-GCST90094846 | prot-a-1856 | Megakaryocyte-associated tyrosine-protein kinase \|\| id:prot-a-1856 | Spicy food liking \|\| id:ebi-fl187-GCST90094846 | Inverse variance weighted | 16 | 0.0666187685102812 | 0.119493493489991 | 0.577179308999043 | -0.167588479 | 0.300826015750663 | 1.06888790676097 | 0.845701787358275 | 1.35097427284475 | 0.998735809224318 |
| ebi-fl187-GCST90094846 | prot-a-1890 | Beta-1,3-N-acetylglucosaminyltransferase manic fringe \|\| id:prot-a-1890 | Spicy food liking \|\| id:ebi-fl187-GCST90094846 | Inverse variance weighted | 16 | 0.0129388076561309 | 0.126124191789378 | 0.91829009891839 | -0.234264608 | 0.260142223563311 | 1.01302287621895 | 0.791152442731688 | 1.29711455380154 | 0.998735809224318 |
| ebi-fl187-GCST90094846 | prot-a-1919 | Matrilysin \|\| id:prot-a-1919 | Spicy food liking \|\| id:ebi-fl187-GCST90094846 | Inverse variance weighted | 16 | -0.071803456 | 0.115431664158958 | 0.533913607170044 | -0.298049518 | 0.154442605446556 | 0.930713803765892 | 0.742264583338109 | 1.16700729627268 | 0.998735809224318 |
| ebi-fl187-GCST90094846 | prot-a-1923 | Multimerin-2 \|\| id:prot-a-1923 | Spicy food liking \|\| id:ebi-fl187-GCST90094846 | Inverse variance weighted | 16 | -0.140429242 | 0.12254892456948 | 0.251835081852357 | -0.380625134 | 0.0997666502606953 | 0.86898515049955 | 0.68343403775498 | 1.10491305681713 | 0.998735809224318 |
| ebi-fl187-GCST90094846 | prot-a-1948 | Mesothelin \|\| id:prot-a-1948 | Spicy food liking \|\| id:ebi-fl187-GCST90094846 | Inverse variance weighted | 16 | 0.00947393392992036 | 0.115350994987318 | 0.934542256826667 | -0.216614016 | 0.235561884105063 | 1.00951895370111 | 0.805240719165228 | 1.26561970057634 | 0.998735809224318 |
| ebi-fl187-GCST90094846 | prot-a-195 | ATPase family AAA domain-containing protein 1 \|\| id:prot-a-195 | Spicy food liking \|\| id:ebi-fl187-GCST90094846 | Inverse variance weighted | 16 | -0.140702073 | 0.11138677951609 | 0.206522818173687 | -0.359020161 | 0.0776160147086632 | 0.868748096536313 | 0.698360271572113 | 1.08070760316373 | 0.998735809224318 |
| ebi-fl187-GCST90094846 | prot-a-1966 | Mucin-1 \|\| id:prot-a-1966 | Spicy food liking \|\| id:ebi-fl187-GCST90094846 | Inverse variance weighted | 16 | -0.064347059 | 0.111343220374562 | 0.563320650798615 | -0.28257977 | 0.153885653427976 | 0.937679513401448 | 0.753836505690826 | 1.16635751016996 | 0.998735809224318 |
| ebi-fl187-GCST90094846 | prot-a-1986 | Myocardial zonula adherens protein \|\| id:prot-a-1986 | Spicy food liking \|\| id:ebi-fl187-GCST90094846 | Inverse variance weighted | 16 | -0.087244727 | 0.112170128614993 | 0.436693231167768 | -0.307098179 | 0.132608725176954 | 0.916452787529741 | 0.735578379016849 | 1.14180315209047 | 0.998735809224318 |
| ebi-fl187-GCST90094846 | prot-a-2071 | Epididymal secretory protein E1 \|\| id:prot-a-2071 | Spicy food liking \|\| id:ebi-fl187-GCST90094846 | Inverse variance weighted | 16 | -0.122177197 | 0.121953740921569 | 0.316424592169496 | -0.36120653 | 0.116852134902459 | 0.884991536493766 | 0.696835066599376 | 1.12395322394974 | 0.998735809224318 |
| ebi-fl187-GCST90094846 | prot-a-2098 | Neuritin \|\| id:prot-a-2098 | Spicy food liking \|\| id:ebi-fl187-GCST90094846 | Inverse variance weighted | 16 | 0.0307323881124338 | 0.118098168580527 | 0.794688312787139 | -0.200740022 | 0.262204798530267 | 1.03120950303603 | 0.818125098185293 | 1.29979271080983 | 0.998735809224318 |
| ebi-fl187-GCST90094846 | prot-a-2115 | Neurotrophin-4 \|\| id:prot-a-2115 | Spicy food liking \|\| id:ebi-fl187-GCST90094846 | Inverse variance weighted | 16 | 0.212993736812737 | 0.111371503345454 | 0.0558169980975902 | -0.00529441 | 0.431281883369827 | 1.23737690129608 | 0.994719580941221 | 1.53922937197269 | 0.998735809224318 |
| ebi-fl187-GCST90094846 | prot-a-2118 | Netrin-1 \|\| id:prot-a-2118 | Spicy food liking \|\| id:ebi-fl187-GCST90094846 | Inverse variance weighted | 16 | 0.196644439866444 | 0.11138677951609 | 0.0774932172337386 | -0.021673648 | 0.41496252771798 | 1.21731113643847 | 0.978559537822701 | 1.51431399482778 | 0.998735809224318 |
| ebi-fl187-GCST90094846 | prot-a-2120 | Netrin-G1 \|\| id:prot-a-2120 | Spicy food liking \|\| id:ebi-fl187-GCST90094846 | Inverse variance weighted | 16 | 0.0880579800753235 | 0.111414361711451 | 0.429314803564982 | -0.130314169 | 0.306430129029767 | 1.09205143741726 | 0.877819603993656 | 1.35856653979868 | 0.998735809224318 |
| ebi-fl187-GCST90094846 | prot-a-2145 | Olfactomedin-like protein 3 \|\| id:prot-a-2145 | Spicy food liking \|\| id:ebi-fl187-GCST90094846 | Inverse variance weighted (multiplicative random effects) | 16 | -0.07090159 | 0.146565104198263 | 0.628559814229662 | -0.358169194 | 0.216366014516359 | 0.931553562070996 | 0.698954806083511 | 1.24155672363097 | 0.998735809224318 |
| ebi-fl187-GCST90094846 | prot-a-2162 | Prolyl 4-hydroxylase subunit alpha-2 \|\| id:prot-a-2162 | Spicy food liking \|\| id:ebi-fl187-GCST90094846 | Inverse variance weighted | 16 | 0.258124583184741 | 0.13605943129086 | 0.0578086934731202 | -0.008551902 | 0.524801068514827 | 1.29450008152151 | 0.991484561351686 | 1.69012259633643 | 0.998735809224318 |
| ebi-fl187-GCST90094846 | prot-a-2168 | Polyadenylate-binding protein-interacting protein 1 \|\| id:prot-a-2168 | Spicy food liking \|\| id:ebi-fl187-GCST90094846 | Inverse variance weighted (multiplicative random effects) | 16 | -0.017045908 | 0.146986802094737 | 0.907677132671915 | -0.30514004 | 0.271048223782878 | 0.983098551193278 | 0.73702015454607 | 1.31133830655361 | 0.998735809224318 |
| ebi-fl187-GCST90094846 | prot-a-219 | Beta-1,4-galactosyltransferase 5 \|\| id:prot-a-219 | Spicy food liking \|\| id:ebi-fl187-GCST90094846 | Inverse variance weighted | 16 | -0.05023602 | 0.111414361711451 | 0.652066256601698 | -0.268608168 | 0.168136129432381 | 0.951004942278706 | 0.764442729732088 | 1.18309765409829 | 0.998735809224318 |
| ebi-fl187-GCST90094846 | prot-a-2190 | Pyruvate carboxylase, mitochondrial \|\| id:prot-a-2190 | Spicy food liking \|\| id:ebi-fl187-GCST90094846 | Inverse variance weighted | 16 | -0.078091053 | 0.11143244873294 | 0.483432360768778 | -0.296498652 | 0.140316546890907 | 0.924880209937994 | 0.743416629283318 | 1.15063797208786 | 0.998735809224318 |
| ebi-fl187-GCST90094846 | prot-a-2197 | Protocadherin-9 \|\| id:prot-a-2197 | Spicy food liking \|\| id:ebi-fl187-GCST90094846 | Inverse variance weighted | 16 | -0.159016501 | 0.111404853105634 | 0.153471834960393 | -0.377370013 | 0.0593370106811243 | 0.852982283446099 | 0.685662322642229 | 1.06113279357274 | 0.998735809224318 |
| ebi-fl187-GCST90094846 | prot-a-2198 | Protocadherin alpha-4 \|\| id:prot-a-2198 | Spicy food liking \|\| id:ebi-fl187-GCST90094846 | Inverse variance weighted | 16 | 0.09006791732875 | 0.138987269844365 | 0.51696556160942 | -0.182347132 | 0.362482966223705 | 1.09424859962339 | 0.833312021295651 | 1.43689274506809 | 0.998735809224318 |
| ebi-fl187-GCST90094846 | prot-a-2254 | Placenta growth factor \|\| id:prot-a-2254 | Spicy food liking \|\| id:ebi-fl187-GCST90094846 | Inverse variance weighted | 16 | -0.13464634 | 0.111363720252227 | 0.226636620538201 | -0.352919232 | 0.0836265517945457 | 0.874024964791547 | 0.702633941708819 | 1.08722279658309 | 0.998735809224318 |
| ebi-fl187-GCST90094846 | prot-a-2261 | Membrane-associated progesterone receptor component 2 \|\| id:prot-a-2261 | Spicy food liking \|\| id:ebi-fl187-GCST90094846 | Inverse variance weighted | 16 | 0.0995835857923237 | 0.118474744640611 | 0.400601786358671 | -0.132626914 | 0.331794085287921 | 1.10471080500891 | 0.875791777074952 | 1.39346588384214 | 0.998735809224318 |
| ebi-fl187-GCST90094846 | prot-a-2268 | Polymeric immunoglobulin receptor \|\| id:prot-a-2268 | Spicy food liking \|\| id:ebi-fl187-GCST90094846 | Inverse variance weighted | 16 | 0.0996018017492858 | 0.143344399232986 | 0.487153918649338 | -0.181353221 | 0.380556824245939 | 1.10473092855667 | 0.834140670862754 | 1.4630990516832 | 0.998735809224318 |
| ebi-fl187-GCST90094846 | prot-a-2283 | Group 10 secretory phospholipase A2 \|\| id:prot-a-2283 | Spicy food liking \|\| id:ebi-fl187-GCST90094846 | Inverse variance weighted | 16 | 0.0476106851603908 | 0.129589414289106 | 0.713323351493093 | -0.206384567 | 0.301605937167038 | 1.04876227711808 | 0.813520163235244 | 1.35202833760352 | 0.998735809224318 |
| ebi-fl187-GCST90094846 | prot-a-2298 | Inactive phospholipase D5 \|\| id:prot-a-2298 | Spicy food liking \|\| id:ebi-fl187-GCST90094846 | Inverse variance weighted (multiplicative random effects) | 16 | 0.0443435910665002 | 0.147342110893113 | 0.763447535765936 | -0.244446946 | 0.333134128417002 | 1.0453414631799 | 0.78313753561964 | 1.39533443991864 | 0.998735809224318 |
| ebi-fl187-GCST90094846 | prot-a-2304 | Procollagen-lysine,2-oxoglutarate 5-dioxygenase 2 \|\| id:prot-a-2304 | Spicy food liking \|\| id:ebi-fl187-GCST90094846 | Inverse variance weighted | 16 | -0.223078417 | 0.128634066828827 | 0.0828809224616074 | -0.475201188 | 0.0290443535225899 | 0.800052108778844 | 0.621759952961776 | 1.02947025409471 | 0.998735809224318 |
| ebi-fl187-GCST90094846 | prot-a-2331 | Periostin \|\| id:prot-a-2331 | Spicy food liking \|\| id:ebi-fl187-GCST90094846 | Inverse variance weighted | 16 | -0.027477928 | 0.127719893612117 | 0.829656520277244 | -0.277808919 | 0.222853063552703 | 0.972896156151404 | 0.757441540066235 | 1.24963694303247 | 0.998735809224318 |
| ebi-fl187-GCST90094846 | prot-a-2333 | Periostin \|\| id:prot-a-2333 | Spicy food liking \|\| id:ebi-fl187-GCST90094846 | Inverse variance weighted | 16 | -0.015615174 | 0.141974523967964 | 0.91242067768948 | -0.293885241 | 0.262654892744478 | 0.984506110485799 | 0.745362023379382 | 1.30037787166752 | 0.998735809224318 |
| ebi-fl187-GCST90094846 | prot-a-2367 | cAMP-dependent protein kinase catalytic subunit alpha \|\| id:prot-a-2367 | Spicy food liking \|\| id:ebi-fl187-GCST90094846 | Inverse variance weighted | 16 | -0.177002548 | 0.111408413584144 | 0.112111917489238 | -0.395363039 | 0.041357942576805 | 0.837777649822511 | 0.673435511730524 | 1.0422250955233 | 0.998735809224318 |
| ebi-fl187-GCST90094846 | prot-a-2385 | Phosphoribosyl pyrophosphate synthase-associated protein 1 \|\| id:prot-a-2385 | Spicy food liking \|\| id:ebi-fl187-GCST90094846 | Inverse variance weighted | 16 | 0.18444660401416 | 0.113179340404967 | 0.103168218793747 | -0.037384903 | 0.406278111207896 | 1.20255276807995 | 0.96330528471585 | 1.5012200005145 | 0.998735809224318 |
| ebi-fl187-GCST90094846 | prot-a-2393 | Trypsin-3 \|\| id:prot-a-2393 | Spicy food liking \|\| id:ebi-fl187-GCST90094846 | Inverse variance weighted (multiplicative random effects) | 16 | 0.117917123615373 | 0.159131736772739 | 0.458691509798326 | -0.19398108 | 0.42981532768994 | 1.12515085906783 | 0.823673487645321 | 1.53697366086185 | 0.998735809224318 |
| ebi-fl187-GCST90094846 | prot-a-2405 | Pregnancy-specific beta-1-glycoprotein 6 \|\| id:prot-a-2405 | Spicy food liking \|\| id:ebi-fl187-GCST90094846 | Inverse variance weighted | 16 | 0.183474322364204 | 0.119774714044784 | 0.125564757434873 | -0.051284117 | 0.418232761891981 | 1.20138411631184 | 0.950008718402783 | 1.51927426240149 | 0.998735809224318 |
| ebi-fl187-GCST90094846 | prot-a-244 | Beclin-1 \|\| id:prot-a-244 | Spicy food liking \|\| id:ebi-fl187-GCST90094846 | Inverse variance weighted | 16 | 0.0942626698115709 | 0.111362773900349 | 0.397303508990282 | -0.124008367 | 0.312533706656254 | 1.09884834229975 | 0.883372449645293 | 1.3668840134869 | 0.998735809224318 |
| ebi-fl187-GCST90094846 | prot-a-2454 | tRNA pseudouridine synthase A, mitochondrial \|\| id:prot-a-2454 | Spicy food liking \|\| id:ebi-fl187-GCST90094846 | Inverse variance weighted | 16 | -0.213062003 | 0.111385648118938 | 0.0557695961495637 | -0.431377874 | 0.00525386698278024 | 0.808106030397959 | 0.649613392610746 | 1.00526769274422 | 0.998735809224318 |
| ebi-fl187-GCST90094846 | prot-a-2466 | Glutaminyl-peptide cyclotransferase-like protein \|\| id:prot-a-2466 | Spicy food liking \|\| id:ebi-fl187-GCST90094846 | Inverse variance weighted | 16 | -0.058940972 | 0.117964047824855 | 0.617320138707343 | -0.290150506 | 0.17226856172723 | 0.942762416885698 | 0.748150958086372 | 1.18799684085889 | 0.998735809224318 |
| ebi-fl187-GCST90094846 | prot-a-2474 | Ras-related protein Rab-27A \|\| id:prot-a-2474 | Spicy food liking \|\| id:ebi-fl187-GCST90094846 | Inverse variance weighted (multiplicative random effects) | 16 | 0.00651098272133527 | 0.153398522646845 | 0.966144047157896 | -0.294150122 | 0.307172087109152 | 1.00653222524755 | 0.745164617691951 | 1.35957491325843 | 0.998735809224318 |
| ebi-fl187-GCST90094846 | prot-a-2490 | Rap1 GTPase-GDP dissociation stimulator 1 \|\| id:prot-a-2490 | Spicy food liking \|\| id:ebi-fl187-GCST90094846 | Inverse variance weighted | 16 | -0.16398592 | 0.132292319171961 | 0.215133560773992 | -0.423278865 | 0.0953070259631406 | 0.848753972579707 | 0.654895979952687 | 1.09999653078017 | 0.998735809224318 |
| ebi-fl187-GCST90094846 | prot-a-2545 | Receptor-interacting serine/threonine-protein kinase 2 \|\| id:prot-a-2545 | Spicy food liking \|\| id:ebi-fl187-GCST90094846 | Inverse variance weighted | 16 | 0.107828874573173 | 0.111435948297019 | 0.333228705174188 | -0.110585584 | 0.32624333323533 | 1.11385711980144 | 0.895309702645224 | 1.38575252749605 | 0.998735809224318 |
| ebi-fl187-GCST90094846 | prot-a-2610 | RUN and FYVE domain-containing protein 1 \|\| id:prot-a-2610 | Spicy food liking \|\| id:ebi-fl187-GCST90094846 | Inverse variance weighted | 16 | 0.129199107969277 | 0.111434900508632 | 0.246287749885908 | -0.089213297 | 0.347611512966195 | 1.1379166698888 | 0.914650460542113 | 1.41568216873073 | 0.998735809224318 |
| ebi-fl187-GCST90094846 | prot-a-2627 | Serine--tRNA ligase, mitochondrial \|\| id:prot-a-2627 | Spicy food liking \|\| id:ebi-fl187-GCST90094846 | Inverse variance weighted | 16 | -0.123420962 | 0.111361728174677 | 0.267737089548178 | -0.341689949 | 0.0948480256336033 | 0.883891499863139 | 0.710568483161591 | 1.09949174786667 | 0.998735809224318 |
| ebi-fl187-GCST90094846 | prot-a-264 | BolA-like protein 2 \|\| id:prot-a-264 | Spicy food liking \|\| id:ebi-fl187-GCST90094846 | Inverse variance weighted | 16 | -0.017974299 | 0.139274053514276 | 0.897312505459895 | -0.290951444 | 0.255002846154663 | 0.982186275465306 | 0.747551975553669 | 1.29046529373148 | 0.998735809224318 |
| ebi-fl187-GCST90094846 | prot-a-2646 | Secretoglobin family 1C member 1 \|\| id:prot-a-2646 | Spicy food liking \|\| id:ebi-fl187-GCST90094846 | Inverse variance weighted | 16 | -0.258748473 | 0.122800979156716 | 0.0351127729501157 | -0.499438392 | -0.018058554 | 0.772017181797747 | 0.606871387768802 | 0.982103524738912 | 0.998735809224318 |
| ebi-fl187-GCST90094846 | prot-a-2672 | Semaphorin-3E \|\| id:prot-a-2672 | Spicy food liking \|\| id:ebi-fl187-GCST90094846 | Inverse variance weighted (multiplicative random effects) | 16 | 0.112349212512886 | 0.168068278408912 | 0.503831307777668 | -0.217064613 | 0.441763038194354 | 1.11890352754072 | 0.804877961909398 | 1.55544711520378 | 0.998735809224318 |
| ebi-fl187-GCST90094846 | prot-a-2701 | Plasma protease C1 inhibitor \|\| id:prot-a-2701 | Spicy food liking \|\| id:ebi-fl187-GCST90094846 | Inverse variance weighted (multiplicative random effects) | 16 | -0.07480169 | 0.14385316022032 | 0.603073036885028 | -0.356753885 | 0.207150503562295 | 0.927927484947459 | 0.699944743787169 | 1.23016770247043 | 0.998735809224318 |
| ebi-fl187-GCST90094846 | prot-a-2738 | Signaling threshold-regulating transmembrane adapter 1 \|\| id:prot-a-2738 | Spicy food liking \|\| id:ebi-fl187-GCST90094846 | Inverse variance weighted | 16 | 0.0872354919139053 | 0.111371225741454 | 0.4334593408043 | -0.131052111 | 0.305523094367156 | 1.09115360731626 | 0.877172063290723 | 1.35733483154111 | 0.998735809224318 |
| ebi-fl187-GCST90094846 | prot-a-274 | Serine/threonine-protein kinase BRSK2 \|\| id:prot-a-274 | Spicy food liking \|\| id:ebi-fl187-GCST90094846 | Inverse variance weighted | 16 | -0.012858061 | 0.12209135519351 | 0.916125794180033 | -0.252157118 | 0.226440994842956 | 0.987224250367006 | 0.777122628899682 | 1.25412860759522 | 0.998735809224318 |
| ebi-fl187-GCST90094846 | prot-a-2749 | Mitochondrial glutamate carrier 2 \|\| id:prot-a-2749 | Spicy food liking \|\| id:ebi-fl187-GCST90094846 | Inverse variance weighted (multiplicative random effects) | 16 | 0.116492685729719 | 0.149171846066543 | 0.434843997677765 | -0.175884133 | 0.408869504020144 | 1.12354929249395 | 0.8387151574849 | 1.50511529617418 | 0.998735809224318 |
| ebi-fl187-GCST90094846 | prot-a-2760 | Sodium-coupled monocarboxylate transporter 1 \|\| id:prot-a-2760 | Spicy food liking \|\| id:ebi-fl187-GCST90094846 | Inverse variance weighted | 16 | -0.08945285 | 0.11139160999535 | 0.421946780428573 | -0.307780406 | 0.128874705287757 | 0.914431379270228 | 0.735076718801983 | 1.13754758653879 | 0.998735809224318 |
| ebi-fl187-GCST90094846 | prot-a-2777 | Small integral membrane protein 10 \|\| id:prot-a-2777 | Spicy food liking \|\| id:ebi-fl187-GCST90094846 | Inverse variance weighted | 16 | 0.222584537834668 | 0.111296986664368 | 0.0455094191241819 | 0.00444244397250654 | 0.440726631696829 | 1.24930142842423 | 1.00445232625514 | 1.55383587480126 | 0.998735809224318 |
| ebi-fl187-GCST90094846 | prot-a-2799 | Superoxide dismutase [Mn], mitochondrial \|\| id:prot-a-2799 | Spicy food liking \|\| id:ebi-fl187-GCST90094846 | Inverse variance weighted | 16 | 0.284179236161709 | 0.111380835805082 | 0.0107285105781414 | 0.0658727979837481 | 0.502485674339669 | 1.32867105531202 | 1.06809084521553 | 1.65282455245435 | 0.998735809224318 |
| ebi-fl187-GCST90094846 | prot-a-2805 | Protein SPACA7 \|\| id:prot-a-2805 | Spicy food liking \|\| id:ebi-fl187-GCST90094846 | Inverse variance weighted | 16 | -0.098170126 | 0.111392331468482 | 0.378155152827803 | -0.316499096 | 0.120158843464871 | 0.906494672128876 | 0.728695670379378 | 1.12767596131074 | 0.998735809224318 |
| ebi-fl187-GCST90094846 | prot-a-2811 | SPARC-like protein 1 \|\| id:prot-a-2811 | Spicy food liking \|\| id:ebi-fl187-GCST90094846 | Inverse variance weighted (multiplicative random effects) | 16 | -0.013613053 | 0.162427758168738 | 0.933207583277618 | -0.331971459 | 0.304745353194502 | 0.986479185763332 | 0.71750780107652 | 1.35627958676439 | 0.998735809224318 |
| ebi-fl187-GCST90094846 | prot-a-2831 | Sepiapterin reductase \|\| id:prot-a-2831 | Spicy food liking \|\| id:ebi-fl187-GCST90094846 | Inverse variance weighted | 16 | 0.0657905154122792 | 0.12281712057375 | 0.592180627387138 | -0.174931041 | 0.30651207173683 | 1.06800296356984 | 0.839514910955551 | 1.35867786897992 | 0.998735809224318 |
| ebi-fl187-GCST90094846 | prot-a-2834 | SPRY domain-containing SOCS box protein 1 \|\| id:prot-a-2834 | Spicy food liking \|\| id:ebi-fl187-GCST90094846 | Inverse variance weighted | 16 | 0.174705300144378 | 0.114649881414552 | 0.12755473287427 | -0.050008467 | 0.3994190677169 | 1.19089520824791 | 0.951221370068013 | 1.49095830019717 | 0.998735809224318 |
| ebi-fl187-GCST90094846 | prot-a-2835 | Spectrin alpha chain, non-erythrocytic 1 \|\| id:prot-a-2835 | Spicy food liking \|\| id:ebi-fl187-GCST90094846 | Inverse variance weighted | 16 | 0.121899430099036 | 0.111389291759574 | 0.273799305217584 | -0.096423582 | 0.3402224419478 | 1.12964048823534 | 0.908079288778696 | 1.40526014460347 | 0.998735809224318 |
| ebi-fl187-GCST90094846 | prot-a-2859 | Sia-alpha-2,3-Gal-beta-1,4-GlcNAc-R:alpha 2,8-sialyltransferase \|\| id:prot-a-2859 | Spicy food liking \|\| id:ebi-fl187-GCST90094846 | Inverse variance weighted | 16 | 0.0586262462190107 | 0.111322142545478 | 0.598446397522497 | -0.159565153 | 0.276817645608147 | 1.06037884606477 | 0.852514421569806 | 1.31892583718548 | 0.998735809224318 |
| ebi-fl187-GCST90094846 | prot-a-2861 | Alpha-2,8-sialyltransferase 8F \|\| id:prot-a-2861 | Spicy food liking \|\| id:ebi-fl187-GCST90094846 | Inverse variance weighted (multiplicative random effects) | 16 | -0.058753819 | 0.1485793989723 | 0.692520271800008 | -0.349969441 | 0.23246180289698 | 0.942938874137328 | 0.704709624558631 | 1.26170225206765 | 0.998735809224318 |
| ebi-fl187-GCST90094846 | prot-a-289 | Uncharacterized protein C17orf89 \|\| id:prot-a-289 | Spicy food liking \|\| id:ebi-fl187-GCST90094846 | Inverse variance weighted (multiplicative random effects) | 16 | -0.133502641 | 0.162692687533373 | 0.411884579727904 | -0.452380308 | 0.185375026811187 | 0.875025158248182 | 0.636112204953629 | 1.20366976392643 | 0.998735809224318 |
| ebi-fl187-GCST90094846 | prot-a-2921 | Tachykinin-3 \|\| id:prot-a-2921 | Spicy food liking \|\| id:ebi-fl187-GCST90094846 | Inverse variance weighted | 16 | 0.0569576077966436 | 0.130081751576889 | 0.661487706211921 | -0.198002625 | 0.311917840887345 | 1.05861093259434 | 0.820367699428557 | 1.36604245558288 | 0.998735809224318 |
| ebi-fl187-GCST90094846 | prot-a-2928 | Tax1-binding protein 1 \|\| id:prot-a-2928 | Spicy food liking \|\| id:ebi-fl187-GCST90094846 | Inverse variance weighted (multiplicative random effects) | 16 | -0.030924822 | 0.150028610808088 | 0.836692361944234 | -0.324980899 | 0.263131255199755 | 0.969548459065754 | 0.722541154647511 | 1.30099747042833 | 0.998735809224318 |
| ebi-fl187-GCST90094846 | prot-a-296 | Uncharacterized protein C1orf185 \|\| id:prot-a-296 | Spicy food liking \|\| id:ebi-fl187-GCST90094846 | Inverse variance weighted | 16 | 0.22258264303546 | 0.123538237949178 | 0.071587776463738 | -0.019552303 | 0.46471758941585 | 1.24929906125111 | 0.980637603220495 | 1.5915646507102 | 0.998735809224318 |
| ebi-fl187-GCST90094846 | prot-a-2964 | Transforming growth factor beta-2 \|\| id:prot-a-2964 | Spicy food liking \|\| id:ebi-fl187-GCST90094846 | Inverse variance weighted | 16 | 0.108827562381519 | 0.111414361711451 | 0.328677003280435 | -0.109544587 | 0.327199711335963 | 1.11497007098011 | 0.896242203102901 | 1.38707846481389 | 0.998735809224318 |
| ebi-fl187-GCST90094846 | prot-a-2977 | Thymocyte nuclear protein 1 \|\| id:prot-a-2977 | Spicy food liking \|\| id:ebi-fl187-GCST90094846 | Inverse variance weighted | 16 | -0.08919946 | 0.111320882267279 | 0.422968198919781 | -0.307388389 | 0.128989469655152 | 0.914663117049566 | 0.735364937906931 | 1.13767814395947 | 0.998735809224318 |
| ebi-fl187-GCST90094846 | prot-a-3015 | Transmembrane protein 70, mitochondrial \|\| id:prot-a-3015 | Spicy food liking \|\| id:ebi-fl187-GCST90094846 | Inverse variance weighted | 16 | 0.0057784604251221 | 0.125591816076055 | 0.963302400290141 | -0.240381499 | 0.25193841993419 | 1.00579518793179 | 0.786327820494391 | 1.2865168110556 | 0.998735809224318 |
| ebi-fl187-GCST90094846 | prot-a-3030 | Tumor necrosis factor alpha-induced protein 3 \|\| id:prot-a-3030 | Spicy food liking \|\| id:ebi-fl187-GCST90094846 | Inverse variance weighted | 16 | -0.109374041 | 0.127328418504063 | 0.390345153453333 | -0.358937741 | 0.140189659038562 | 0.896395066071973 | 0.698417832446488 | 1.1504919793693 | 0.998735809224318 |
| ebi-fl187-GCST90094846 | prot-a-3032 | Tumor necrosis factor receptor superfamily member 10A \|\| id:prot-a-3032 | Spicy food liking \|\| id:ebi-fl187-GCST90094846 | Inverse variance weighted | 16 | 0.179672066611183 | 0.111429996711869 | 0.106870358375466 | -0.038730727 | 0.398074860166447 | 1.1968248199423 | 0.962009717569281 | 1.48895548919105 | 0.998735809224318 |
| ebi-fl187-GCST90094846 | prot-a-3081 | Tumor protein p53-inducible protein 11 \|\| id:prot-a-3081 | Spicy food liking \|\| id:ebi-fl187-GCST90094846 | Inverse variance weighted | 16 | -0.027280744 | 0.137533990649734 | 0.842766245612391 | -0.296847366 | 0.242285877615098 | 0.973088014494349 | 0.743157434996834 | 1.27415839411818 | 0.998735809224318 |
| ebi-fl187-GCST90094846 | prot-a-315 | Complement C4 \|\| id:prot-a-315 | Spicy food liking \|\| id:ebi-fl187-GCST90094846 | Inverse variance weighted | 15 | -0.048188903 | 0.145799070597303 | 0.741010223800626 | -0.333955081 | 0.237577275674553 | 0.952953754540176 | 0.716085947321573 | 1.26817299192775 | 0.998735809224318 |
| ebi-fl187-GCST90094846 | prot-a-3157 | UDP-glucuronosyltransferase 1-8 \|\| id:prot-a-3157 | Spicy food liking \|\| id:ebi-fl187-GCST90094846 | Inverse variance weighted | 16 | 0.161122273257546 | 0.115236003542339 | 0.162054858381261 | -0.064740294 | 0.386984840200529 | 1.17482861015317 | 0.937310857318917 | 1.47253416778123 | 0.998735809224318 |
| ebi-fl187-GCST90094846 | prot-a-317 | Glutaredoxin-like protein C5orf63 \|\| id:prot-a-317 | Spicy food liking \|\| id:ebi-fl187-GCST90094846 | Inverse variance weighted | 16 | 0.0147812950708319 | 0.11137003136989 | 0.894412946695676 | -0.203503966 | 0.233066556555816 | 1.01489107866042 | 0.815866968249967 | 1.26246550188675 | 0.998735809224318 |
| ebi-fl187-GCST90094846 | prot-a-3192 | Guanine nucleotide exchange factor VAV3 \|\| id:prot-a-3192 | Spicy food liking \|\| id:ebi-fl187-GCST90094846 | Inverse variance weighted | 16 | 0.0557033498441035 | 0.139164698303452 | 0.688958211726575 | -0.217059459 | 0.328466158518869 | 1.05728399374925 | 0.804882110533089 | 1.38883623925743 | 0.998735809224318 |
| ebi-fl187-GCST90094846 | prot-a-3233 | Serine/threonine-protein kinase WNK3 \|\| id:prot-a-3233 | Spicy food liking \|\| id:ebi-fl187-GCST90094846 | Inverse variance weighted | 16 | 0.212127967418209 | 0.111429996711869 | 0.0569508070003381 | -0.006274826 | 0.430530760973473 | 1.23630608185368 | 0.993744819472066 | 1.53807365641454 | 0.998735809224318 |
| ebi-fl187-GCST90094846 | prot-a-3250 | 14-3-3 protein theta \|\| id:prot-a-3250 | Spicy food liking \|\| id:ebi-fl187-GCST90094846 | Inverse variance weighted | 16 | -0.099136093 | 0.116361842169766 | 0.394234109985204 | -0.327205303 | 0.128933117854659 | 0.905619451351434 | 0.720935717241454 | 1.13761403555401 | 0.998735809224318 |
| ebi-fl187-GCST90094846 | prot-a-3278 | E3 ubiquitin-protein ligase ZNRF3 \|\| id:prot-a-3278 | Spicy food liking \|\| id:ebi-fl187-GCST90094846 | Inverse variance weighted | 16 | 0.183210758451056 | 0.111302610410783 | 0.0997514370810144 | -0.034942358 | 0.40136387485619 | 1.20106751653692 | 0.965661077333507 | 1.49386074797954 | 0.998735809224318 |
| ebi-fl187-GCST90094846 | prot-a-355 | Calpain-3 \|\| id:prot-a-355 | Spicy food liking \|\| id:ebi-fl187-GCST90094846 | Inverse variance weighted | 16 | 0.12667040496008 | 0.111345581361743 | 0.255273771463469 | -0.091566935 | 0.344907744429096 | 1.13504285162923 | 0.912500236352636 | 1.41185966174011 | 0.998735809224318 |
| ebi-fl187-GCST90094846 | prot-a-385 | Coiled-coil domain-containing protein 90B, mitochondrial \|\| id:prot-a-385 | Spicy food liking \|\| id:ebi-fl187-GCST90094846 | Inverse variance weighted | 16 | -0.042709415 | 0.11145404583536 | 0.701569980564616 | -0.261159345 | 0.175740514362028 | 0.958189784739672 | 0.77015818873972 | 1.1921286782419 | 0.998735809224318 |
| ebi-fl187-GCST90094846 | prot-a-390 | C-C motif chemokine 14 \|\| id:prot-a-390 | Spicy food liking \|\| id:ebi-fl187-GCST90094846 | Inverse variance weighted (multiplicative random effects) | 16 | -0.045155808 | 0.14531214263194 | 0.755990373263152 | -0.329967607 | 0.239655992002176 | 0.955848541804797 | 0.718947021822912 | 1.27081190565861 | 0.998735809224318 |
| ebi-fl187-GCST90094846 | prot-a-40 | Pituitary adenylate cyclase-activating polypeptide 27 \|\| id:prot-a-40 | Spicy food liking \|\| id:ebi-fl187-GCST90094846 | Inverse variance weighted | 16 | 0.10931773383158 | 0.111429996711869 | 0.326571025759901 | -0.10908506 | 0.327720527386844 | 1.11551673144431 | 0.896654145100605 | 1.38780106569693 | 0.998735809224318 |
| ebi-fl187-GCST90094846 | prot-a-401 | C-C motif chemokine 24 \|\| id:prot-a-401 | Spicy food liking \|\| id:ebi-fl187-GCST90094846 | Inverse variance weighted | 16 | 0.114009187242278 | 0.111392787259033 | 0.306077144501359 | -0.104320676 | 0.332339050269981 | 1.12076242155276 | 0.900936342623988 | 1.39422548091064 | 0.998735809224318 |
| ebi-fl187-GCST90094846 | prot-a-412 | Cerebral cavernous malformations 2 protein \|\| id:prot-a-412 | Spicy food liking \|\| id:ebi-fl187-GCST90094846 | Inverse variance weighted | 16 | -0.062475041 | 0.130275004622873 | 0.631538332883953 | -0.31781405 | 0.192863968495131 | 0.939436510324715 | 0.727738099004976 | 1.21271781446892 | 0.998735809224318 |
| ebi-fl187-GCST90094846 | prot-a-44 | Adhesion G protein-coupled receptor F5 \|\| id:prot-a-44 | Spicy food liking \|\| id:ebi-fl187-GCST90094846 | Inverse variance weighted | 16 | 0.0224804220565959 | 0.111429996711869 | 0.840116239707613 | -0.195922371 | 0.24088321561186 | 1.02273501092028 | 0.822076048732904 | 1.27237243339509 | 0.998735809224318 |
| ebi-fl187-GCST90094846 | prot-a-48 | Adrenomedullin \|\| id:prot-a-48 | Spicy food liking \|\| id:ebi-fl187-GCST90094846 | Inverse variance weighted | 16 | 0.0867348244660592 | 0.111380835805082 | 0.436142873456569 | -0.131571614 | 0.30504126264402 | 1.09060743896024 | 0.876716487967624 | 1.35668098209571 | 0.998735809224318 |
| ebi-fl187-GCST90094846 | prot-a-502 | Carcinoembryonic antigen-related cell adhesion molecule 1 \|\| id:prot-a-502 | Spicy food liking \|\| id:ebi-fl187-GCST90094846 | Inverse variance weighted | 16 | -0.089354435 | 0.111435948297019 | 0.422642223614787 | -0.307768894 | 0.129060023491302 | 0.914521377583901 | 0.735085181098722 | 1.13775841434849 | 0.998735809224318 |
| ebi-fl187-GCST90094846 | prot-a-518 | Complement factor B \|\| id:prot-a-518 | Spicy food liking \|\| id:ebi-fl187-GCST90094846 | Inverse variance weighted | 16 | 0.180571534155265 | 0.111380835805082 | 0.10497297584678 | -0.037734904 | 0.398877972333226 | 1.19790180930967 | 0.962968186049866 | 1.49015176776885 | 0.998735809224318 |
| ebi-fl187-GCST90094846 | prot-a-525 | Properdin \|\| id:prot-a-525 | Spicy food liking \|\| id:ebi-fl187-GCST90094846 | Inverse variance weighted | 16 | 0.2185096674279 | 0.124579042083083 | 0.0794332200598947 | -0.025665255 | 0.462684589910743 | 1.24422104498627 | 0.974661297950038 | 1.5883322873728 | 0.998735809224318 |
| ebi-fl187-GCST90094846 | prot-a-533 | Chondroadherin \|\| id:prot-a-533 | Spicy food liking \|\| id:ebi-fl187-GCST90094846 | Inverse variance weighted (multiplicative random effects) | 16 | -0.041364726 | 0.160548717416495 | 0.796680165610315 | -0.356040213 | 0.273310759735188 | 0.959479118756563 | 0.700444453020105 | 1.31430861556619 | 0.998735809224318 |
| ebi-fl187-GCST90094846 | prot-a-535 | Coiled-coil-helix-coiled-coil-helix domain-containing protein 2 \|\| id:prot-a-535 | Spicy food liking \|\| id:ebi-fl187-GCST90094846 | Inverse variance weighted | 16 | -0.023893819 | 0.130298559169405 | 0.854501857169806 | -0.279278995 | 0.231491356631111 | 0.976389377921699 | 0.75632886156917 | 1.26047843175047 | 0.998735809224318 |
| ebi-fl187-GCST90094846 | prot-a-591 | Caseinolytic peptidase B protein homolog \|\| id:prot-a-591 | Spicy food liking \|\| id:ebi-fl187-GCST90094846 | Inverse variance weighted | 16 | 0.11239828939169 | 0.129494448450472 | 0.38540655815239 | -0.14141083 | 0.366207408354616 | 1.11895844118102 | 0.868132583888304 | 1.44225434723615 | 0.998735809224318 |
| ebi-fl187-GCST90094846 | prot-a-605 | Ciliary neurotrophic factor receptor subunit alpha \|\| id:prot-a-605 | Spicy food liking \|\| id:ebi-fl187-GCST90094846 | Inverse variance weighted | 16 | -0.020766939 | 0.1149614067547 | 0.856648040113968 | -0.246091296 | 0.204557418717586 | 0.979447209385498 | 0.781850841999565 | 1.22698190555069 | 0.998735809224318 |
| ebi-fl187-GCST90094846 | prot-a-606 | Ciliary neurotrophic factor receptor subunit alpha \|\| id:prot-a-606 | Spicy food liking \|\| id:ebi-fl187-GCST90094846 | Inverse variance weighted | 16 | 0.0948521560683644 | 0.127642818889803 | 0.457417452832188 | -0.155327769 | 0.345032081092378 | 1.09949628925499 | 0.856134517173669 | 1.41203521857332 | 0.998735809224318 |
| ebi-fl187-GCST90094846 | prot-a-707 | Cancer/testis antigen 55 \|\| id:prot-a-707 | Spicy food liking \|\| id:ebi-fl187-GCST90094846 | Inverse variance weighted | 16 | 0.181624469899582 | 0.136261021537335 | 0.182559433628784 | -0.085447132 | 0.448696072112758 | 1.1991637872171 | 0.918101679682828 | 1.56626855216041 | 0.998735809224318 |
| ebi-fl187-GCST90094846 | prot-a-719 | Dipeptidyl peptidase 1 \|\| id:prot-a-719 | Spicy food liking \|\| id:ebi-fl187-GCST90094846 | Inverse variance weighted | 16 | -0.092974691 | 0.111366555395346 | 0.403800539902486 | -0.31125314 | 0.12530375747044 | 0.911216561889107 | 0.732528420387825 | 1.13349270765687 | 0.998735809224318 |
| ebi-fl187-GCST90094846 | prot-a-752 | Cytochrome b5 \|\| id:prot-a-752 | Spicy food liking \|\| id:ebi-fl187-GCST90094846 | Inverse variance weighted | 16 | -0.088520668 | 0.113307849252252 | 0.43466112373905 | -0.310604053 | 0.133562716451925 | 0.915284193371295 | 0.733004049453986 | 1.14289294207771 | 0.998735809224318 |
| ebi-fl187-GCST90094846 | prot-a-755 | Cytoglobin \|\| id:prot-a-755 | Spicy food liking \|\| id:ebi-fl187-GCST90094846 | Inverse variance weighted | 16 | -0.009753947 | 0.11792310149776 | 0.934078577626069 | -0.240883226 | 0.221375331904793 | 0.99029346842269 | 0.785933397642956 | 1.24779167871189 | 0.998735809224318 |
| ebi-fl187-GCST90094846 | prot-a-807 | Desmin \|\| id:prot-a-807 | Spicy food liking \|\| id:ebi-fl187-GCST90094846 | Inverse variance weighted | 16 | -0.026202026 | 0.121276996711843 | 0.828947930259214 | -0.26390494 | 0.211500887229102 | 0.974138268152423 | 0.768046546782506 | 1.23553106182733 | 0.998735809224318 |
| ebi-fl187-GCST90094846 | prot-a-82 | Ameloblastin \|\| id:prot-a-82 | Spicy food liking \|\| id:ebi-fl187-GCST90094846 | Inverse variance weighted | 16 | -0.203122203 | 0.114493138194358 | 0.0760467172080583 | -0.427528754 | 0.0212843480570213 | 0.816178496030436 | 0.652118650925421 | 1.02151247543246 | 0.998735809224318 |
| ebi-fl187-GCST90094846 | prot-a-84 | Muellerian-inhibiting factor \|\| id:prot-a-84 | Spicy food liking \|\| id:ebi-fl187-GCST90094846 | Inverse variance weighted | 16 | 0.0237299641124706 | 0.111343675562523 | 0.831230563377904 | -0.19450364 | 0.241963568215016 | 1.02401376008749 | 0.823243181654059 | 1.27374778706538 | 0.998735809224318 |
| ebi-fl187-GCST90094846 | prot-a-858 | Inactive dipeptidyl peptidase 10 \|\| id:prot-a-858 | Spicy food liking \|\| id:ebi-fl187-GCST90094846 | Inverse variance weighted (multiplicative random effects) | 16 | 0.00350446496080007 | 0.148847261160845 | 0.981216314245258 | -0.288236167 | 0.295245096836057 | 1.00351061277764 | 0.749584544264484 | 1.34345559505296 | 0.998955228396052 |
| ebi-fl187-GCST90094846 | prot-a-863 | Dihydropyrimidinase-related protein 3 \|\| id:prot-a-863 | Spicy food liking \|\| id:ebi-fl187-GCST90094846 | Inverse variance weighted | 16 | -0.164875705 | 0.111414361711451 | 0.138915288892823 | -0.383247854 | 0.0534964435565362 | 0.847999099248371 | 0.681643929932173 | 1.05495323987937 | 0.998735809224318 |
| ebi-fl187-GCST90094846 | prot-a-864 | Dihydropyrimidinase-related protein 5 \|\| id:prot-a-864 | Spicy food liking \|\| id:ebi-fl187-GCST90094846 | Inverse variance weighted | 16 | -0.041704599 | 0.122524951084061 | 0.73357310289447 | -0.281853504 | 0.198444304642381 | 0.959153073042309 | 0.754384191001306 | 1.21950410480555 | 0.998735809224318 |
| ebi-fl187-GCST90094846 | prot-a-884 | Endothelin-converting enzyme 1 \|\| id:prot-a-884 | Spicy food liking \|\| id:ebi-fl187-GCST90094846 | Inverse variance weighted | 16 | -0.08501263 | 0.11139136361089 | 0.445350865129124 | -0.303339703 | 0.133314442360952 | 0.918500683373958 | 0.738348234657925 | 1.1426092265925 | 0.998735809224318 |
| ebi-fl187-GCST90094846 | prot-a-893 | Early endosome antigen 1 \|\| id:prot-a-893 | Spicy food liking \|\| id:ebi-fl187-GCST90094846 | Inverse variance weighted | 16 | -0.013188913 | 0.113616080056553 | 0.907586638977445 | -0.23587643 | 0.209498603888087 | 0.986897679585111 | 0.789878273213173 | 1.23305965362035 | 0.998735809224318 |
| ebi-fl187-GCST90094846 | prot-a-897 | Ephrin-A2 \|\| id:prot-a-897 | Spicy food liking \|\| id:ebi-fl187-GCST90094846 | Inverse variance weighted | 16 | 0.120537297204339 | 0.117078893954675 | 0.303226423729228 | -0.108937335 | 0.350011929355501 | 1.12810281526235 | 0.896786612918258 | 1.4190844772555 | 0.998735809224318 |
| ebi-fl187-GCST90094846 | prot-a-90 | Anaphase-promoting complex subunit 10 \|\| id:prot-a-90 | Spicy food liking \|\| id:ebi-fl187-GCST90094846 | Inverse variance weighted | 16 | 0.166201586370868 | 0.114359757145432 | 0.146134285889267 | -0.057943538 | 0.390346710375914 | 1.18081111315952 | 0.943703229649544 | 1.47749296723168 | 0.998735809224318 |
| ebi-fl187-GCST90094846 | prot-a-914 | Histone-lysine N-methyltransferase EHMT2 \|\| id:prot-a-914 | Spicy food liking \|\| id:ebi-fl187-GCST90094846 | Inverse variance weighted | 16 | -0.168571981 | 0.137689252674508 | 0.220841735144307 | -0.438442917 | 0.101298953767874 | 0.844870446209912 | 0.645040020569792 | 1.10660741677454 | 0.998735809224318 |
| ebi-fl187-GCST90094846 | prot-a-932 | RNA polymerase II elongation factor ELL \|\| id:prot-a-932 | Spicy food liking \|\| id:ebi-fl187-GCST90094846 | Inverse variance weighted | 16 | -0.092537766 | 0.111408413584144 | 0.406189806982812 | -0.310898256 | 0.125822724786574 | 0.911614782417846 | 0.732788428563038 | 1.13408110599176 | 0.998735809224318 |
| ebi-fl187-GCST90094846 | prot-a-991 | Estrogen receptor \|\| id:prot-a-991 | Spicy food liking \|\| id:ebi-fl187-GCST90094846 | Inverse variance weighted | 16 | 0.070426370905355 | 0.111435948297019 | 0.527393611368901 | -0.147988088 | 0.288840829567512 | 1.0729655650391 | 0.862441388492419 | 1.3348792383122 | 0.998735809224318 |
| ebi-fl187-GCST90094846 | prot-a-992 | Persulfide dioxygenase ETHE1, mitochondrial \|\| id:prot-a-992 | Spicy food liking \|\| id:ebi-fl187-GCST90094846 | Inverse variance weighted | 16 | 0.0639628263342952 | 0.117013784578857 | 0.584635413199826 | -0.165384191 | 0.293309844108855 | 1.06605276893566 | 0.847568013152392 | 1.34085818308371 | 0.998735809224318 |
| ebi-fl187-GCST90094846 | prot-a-993 | Ethanolamine kinase 1 \|\| id:prot-a-993 | Spicy food liking \|\| id:ebi-fl187-GCST90094846 | Inverse variance weighted (multiplicative random effects) | 16 | 0.114679949730131 | 0.145955350409361 | 0.432031897617892 | -0.171392537 | 0.400752436532478 | 1.12151443912732 | 0.842490799683503 | 1.49294762345605 | 0.998735809224318 |
| ebi-fl187-GCST90094846 | prot-b-10 | coagulation factor III, tissue factor \|\| id:prot-b-10 | Spicy food liking \|\| id:ebi-fl187-GCST90094846 | Inverse variance weighted | 12 | 0.0437391718227781 | 0.185547228987753 | 0.813641672521541 | -0.319933397 | 0.407411740638774 | 1.04470982958827 | 0.726197402393552 | 1.5029227926746 | 0.998735809224318 |
| ebi-fl187-GCST90094846 | prot-b-28 | prolactin \|\| id:prot-b-28 | Spicy food liking \|\| id:ebi-fl187-GCST90094846 | Inverse variance weighted | 12 | 0.017920891658566 | 0.148907592049841 | 0.904206626659617 | -0.273937989 | 0.309779772076253 | 1.01808243439124 | 0.760379225858267 | 1.36312488291097 | 0.998735809224318 |
| ebi-fl187-GCST90094846 | prot-b-54 | matrix metallopeptidase 7 \|\| id:prot-b-54 | Spicy food liking \|\| id:ebi-fl187-GCST90094846 | Inverse variance weighted | 12 | 0.0522259405103165 | 0.157180040540084 | 0.739686695264722 | -0.255846939 | 0.360298819968882 | 1.05361376965516 | 0.774260468844837 | 1.43375778601118 | 0.998735809224318 |
| ebi-fl187-GCST90094846 | prot-c-2421_7_3 | BDNF \|\| id:prot-c-2421_7_3 | Spicy food liking \|\| id:ebi-fl187-GCST90094846 | Inverse variance weighted | 2 | -0.0677194 | 0.626570370330157 | 0.913932644773922 | -1.295797326 | 1.16035852555866 | 0.934522663580115 | 0.273679565445011 | 3.19107715376853 | 0.998735809224318 |
| ebi-fl187-GCST90094846 | prot-c-2443_10_4 | FGF-8B \|\| id:prot-c-2443_10_4 | Spicy food liking \|\| id:ebi-fl187-GCST90094846 | Inverse variance weighted | 2 | 0.155312384598855 | 1.14292972183561 | 0.891908472910456 | -2.08482987 | 2.39545463939665 | 1.16802277644728 | 0.124328270310487 | 10.9731857677469 | 0.998735809224318 |
| ebi-fl187-GCST90094846 | prot-c-2605_49_2 | CD30 \|\| id:prot-c-2605_49_2 | Spicy food liking \|\| id:ebi-fl187-GCST90094846 | Inverse variance weighted | 2 | -0.083550214 | 0.62572859208666 | 0.89377844162181 | -1.309978255 | 1.14287782604407 | 0.919844896009863 | 0.26982592370297 | 3.13577962081514 | 0.998735809224318 |
| ebi-fl187-GCST90094846 | prot-c-2624_31_2 | HPV E7 Type18 \|\| id:prot-c-2624_31_2 | Spicy food liking \|\| id:ebi-fl187-GCST90094846 | Inverse variance weighted | 2 | 0.402826318051911 | 0.978954598873031 | 0.680716050593502 | -1.515924696 | 2.32157733184305 | 1.49604703273475 | 0.219605023101662 | 10.1917373862544 | 0.998735809224318 |
| ebi-fl187-GCST90094846 | prot-c-2774_10_3 | IL-16 \|\| id:prot-c-2774_10_3 | Spicy food liking \|\| id:ebi-fl187-GCST90094846 | Inverse variance weighted | 2 | -1.487356668 | 0.984718180129636 | 0.13093146286872 | -3.417404301 | 0.442690964687625 | 0.225969178265521 | 0.032797456857821 | 1.55689112565502 | 0.998735809224318 |
| ebi-fl187-GCST90094846 | prot-c-2851_63_3 | C5a \|\| id:prot-c-2851_63_3 | Spicy food liking \|\| id:ebi-fl187-GCST90094846 | Inverse variance weighted | 2 | -0.486851172 | 0.549918976664167 | 0.375987102192295 | -1.564692366 | 0.59099002234535 | 0.614558489701067 | 0.209152345612781 | 1.80577528861611 | 0.998735809224318 |
| ebi-fl187-GCST90094846 | prot-c-2891_1_3 | CD22 \|\| id:prot-c-2891_1_3 | Spicy food liking \|\| id:ebi-fl187-GCST90094846 | Inverse variance weighted | 2 | -0.621446996 | 1.17349665299422 | 0.596411060366948 | -2.921500436 | 1.67860644412843 | 0.53716659718659 | 0.0538528239557028 | 5.35808397662429 | 0.998735809224318 |
| ebi-fl187-GCST90094846 | prot-c-2961_1_2 | Protein C \|\| id:prot-c-2961_1_2 | Spicy food liking \|\| id:ebi-fl187-GCST90094846 | Inverse variance weighted | 2 | 0.779277358396711 | 1.08963018373843 | 0.474500295829098 | -1.356397802 | 2.91495251852403 | 2.17989641233983 | 0.257586987154983 | 18.4479364467001 | 0.998735809224318 |
| ebi-fl187-GCST90094846 | prot-c-2982_82_2 | Galectin-4 \|\| id:prot-c-2982_82_2 | Spicy food liking \|\| id:ebi-fl187-GCST90094846 | Inverse variance weighted | 2 | -0.891645941 | 0.583747497151883 | 0.126648799410763 | -2.035791036 | 0.252499153095012 | 0.40998039343287 | 0.130577150446859 | 1.28723840598571 | 0.998735809224318 |
| ebi-fl187-GCST90094846 | prot-c-2994_71_2 | IL-1Rrp2 \|\| id:prot-c-2994_71_2 | Spicy food liking \|\| id:ebi-fl187-GCST90094846 | Inverse variance weighted | 2 | -0.230097322 | 0.809411294879935 | 0.776197819371175 | -1.81654346 | 1.35634881557254 | 0.794456280355156 | 0.162586768396181 | 3.88199352027085 | 0.998735809224318 |
| ebi-fl187-GCST90094846 | prot-c-3336_50_1 | TFPI \|\| id:prot-c-3336_50_1 | Spicy food liking \|\| id:ebi-fl187-GCST90094846 | Inverse variance weighted | 2 | -0.424159971 | 0.872608721930979 | 0.626908558855716 | -2.134473066 | 1.28615312417989 | 0.654319201566895 | 0.118306913915266 | 3.61883852236883 | 0.998735809224318 |
| ebi-fl187-GCST90094846 | prot-c-3472_40_2 | Survivin \|\| id:prot-c-3472_40_2 | Spicy food liking \|\| id:ebi-fl187-GCST90094846 | Inverse variance weighted | 2 | 0.491668910202402 | 0.63328511190562 | 0.43752556805252 | -0.749569909 | 1.73290772953742 | 1.6350426836271 | 0.472569756976418 | 5.65707927309432 | 0.998735809224318 |
| ebi-fl187-GCST90094846 | prot-c-3473_78_2 | Thrombopoietin Receptor \|\| id:prot-c-3473_78_2 | Spicy food liking \|\| id:ebi-fl187-GCST90094846 | Inverse variance weighted | 2 | -0.506570557 | 0.631593309712259 | 0.422522988844783 | -1.744493445 | 0.731352329540449 | 0.602558479151187 | 0.1747334787516 | 2.07788869878302 | 0.998735809224318 |
| ebi-fl187-GCST90094846 | prot-c-3497_13_2 | IFN-aA \|\| id:prot-c-3497_13_2 | Spicy food liking \|\| id:ebi-fl187-GCST90094846 | Inverse variance weighted | 2 | -0.112523529 | 0.76468993595225 | 0.883014239239768 | -1.611315804 | 1.38626874539432 | 0.893576321839469 | 0.199624774205979 | 3.99989753841003 | 0.998735809224318 |
| ebi-fl187-GCST90094846 | prot-c-3665_64_3 | COLEC12 \|\| id:prot-c-3665_64_3 | Spicy food liking \|\| id:ebi-fl187-GCST90094846 | Inverse variance weighted | 2 | 0.132138676589193 | 0.626973414295747 | 0.833077341033772 | -1.096729215 | 1.36100656860886 | 1.1412665752498 | 0.333961615777819 | 3.90011706210253 | 0.998735809224318 |
| ebi-fl187-GCST90094846 | prot-c-3741_4_3 | IL-5 \|\| id:prot-c-3741_4_3 | Spicy food liking \|\| id:ebi-fl187-GCST90094846 | Inverse variance weighted | 2 | -0.37745535 | 0.617400168023472 | 0.540959580195293 | -1.587559679 | 0.832648979353835 | 0.685603813130221 | 0.204423863325733 | 2.29940174758222 | 0.998735809224318 |
| ebi-fl187-GCST90094846 | prot-c-3848_14_2 | GAPDH, liver \|\| id:prot-c-3848_14_2 | Spicy food liking \|\| id:ebi-fl187-GCST90094846 | Inverse variance weighted | 2 | -0.770700811 | 0.625237451364336 | 0.217705259226974 | -1.996166216 | 0.454764593182833 | 0.462688697107159 | 0.135855125302079 | 1.57580238474406 | 0.998735809224318 |
| ebi-fl187-GCST90094846 | prot-c-3859_50_2 | PSA1 \|\| id:prot-c-3859_50_2 | Spicy food liking \|\| id:ebi-fl187-GCST90094846 | Inverse variance weighted | 2 | -1.487595372 | 0.809261298056049 | 0.0660308913376911 | -3.073747517 | 0.0985567717753872 | 0.225915244945252 | 0.0462475163685843 | 1.10357705464466 | 0.998735809224318 |
| ebi-fl187-GCST90094846 | prot-c-3868_8_1 | SGTA \|\| id:prot-c-3868_8_1 | Spicy food liking \|\| id:ebi-fl187-GCST90094846 | Inverse variance weighted | 2 | -1.092986308 | 0.608542645127975 | 0.0724830924955934 | -2.285729892 | 0.0997572767246862 | 0.335213945497714 | 0.101699805117628 | 1.10490269992334 | 0.998735809224318 |
| ebi-fl187-GCST90094846 | prot-c-3882_10_1 | EF-1-beta \|\| id:prot-c-3882_10_1 | Spicy food liking \|\| id:ebi-fl187-GCST90094846 | Inverse variance weighted | 2 | -1.026611636 | 0.933792378731325 | 0.271593493051811 | -2.856844698 | 0.803621426159054 | 0.358218681763817 | 0.0574497458543801 | 2.23361517194289 | 0.998735809224318 |
| ebi-fl187-GCST90094846 | prot-c-4134_4_2 | HB-EGF \|\| id:prot-c-4134_4_2 | Spicy food liking \|\| id:ebi-fl187-GCST90094846 | Inverse variance weighted | 2 | -0.571359494 | 0.635640588013671 | 0.368720870735448 | -1.817215047 | 0.674496058137717 | 0.564757132422416 | 0.16247761397177 | 1.96304346688282 | 0.998735809224318 |
| ebi-fl187-GCST90094846 | prot-c-4220_39_2 | FER \|\| id:prot-c-4220_39_2 | Spicy food liking \|\| id:ebi-fl187-GCST90094846 | Inverse variance weighted | 2 | -0.572824743 | 0.622854022368237 | 0.357741386924371 | -1.793618627 | 0.647969140704249 | 0.563930228687463 | 0.166357094902011 | 1.91165458266037 | 0.998735809224318 |
| ebi-fl187-GCST90094846 | prot-c-4228_37_2 | HSP70 protein 8 \|\| id:prot-c-4228_37_2 | Spicy food liking \|\| id:ebi-fl187-GCST90094846 | Inverse variance weighted | 2 | -0.078946055 | 0.634145916838643 | 0.900925782527424 | -1.321872052 | 1.16397994224805 | 0.924089773349111 | 0.266635678660059 | 3.20265432405663 | 0.998735809224318 |
| ebi-fl187-GCST90094846 | prot-c-4246_40_2 | NCAM-L1 \|\| id:prot-c-4246_40_2 | Spicy food liking \|\| id:ebi-fl187-GCST90094846 | Inverse variance weighted | 2 | -0.111798097 | 0.628081886438925 | 0.858723574646968 | -1.342838594 | 1.11924240061206 | 0.894224786113562 | 0.261103448905348 | 3.06253315094938 | 0.998735809224318 |
| ebi-fl187-GCST90094846 | prot-c-4267_81_3 | PESC \|\| id:prot-c-4267_81_3 | Spicy food liking \|\| id:ebi-fl187-GCST90094846 | Inverse variance weighted | 2 | -0.205473837 | 0.636365914612156 | 0.746781350762201 | -1.452751029 | 1.04180335590477 | 0.814261398025161 | 0.233925865160412 | 2.83432370276467 | 0.998735809224318 |
| ebi-fl187-GCST90094846 | prot-c-4280_47_2 | PSA2 \|\| id:prot-c-4280_47_2 | Spicy food liking \|\| id:ebi-fl187-GCST90094846 | Inverse variance weighted | 2 | 0.115056872686678 | 0.662586131012412 | 0.862141968647975 | -1.183611944 | 1.413725689 | 1.12193724334283 | 0.306170866964571 | 4.11124412482187 | 0.998735809224318 |
| ebi-fl187-GCST90094846 | prot-c-4324_33_2 | CYTT \|\| id:prot-c-4324_33_2 | Spicy food liking \|\| id:ebi-fl187-GCST90094846 | Inverse variance weighted | 2 | -0.07718129 | 0.611517213411167 | 0.899563492413042 | -1.275755028 | 1.12139244854236 | 0.925722014486615 | 0.279220069635304 | 3.06912482768324 | 0.998735809224318 |
| ebi-fl187-GCST90094846 | prot-c-4462_8_2 | RASA1 \|\| id:prot-c-4462_8_2 | Spicy food liking \|\| id:ebi-fl187-GCST90094846 | Inverse variance weighted | 2 | 1.22303899368137 | 1.03751727835888 | 0.238472593562925 | -0.810494872 | 3.25657285926477 | 3.39749703275462 | 0.444637972929061 | 25.9604145177633 | 0.998735809224318 |
| ebi-fl187-GCST90094846 | prot-c-4499_21_1 | PDGF-AA \|\| id:prot-c-4499_21_1 | Spicy food liking \|\| id:ebi-fl187-GCST90094846 | Inverse variance weighted | 2 | -0.45953257 | 0.6325317992039 | 0.467534034424809 | -1.699294896 | 0.780229756805936 | 0.63157879562827 | 0.182812380346079 | 2.18197353118056 | 0.998735809224318 |
| ebi-fl187-GCST90094846 | prot-c-4535_50_2 | BST1 \|\| id:prot-c-4535_50_2 | Spicy food liking \|\| id:ebi-fl187-GCST90094846 | Inverse variance weighted | 2 | 0.138739704827136 | 0.628994472118715 | 0.825424298310384 | -1.094089461 | 1.37156887017982 | 1.14882502747261 | 0.334844357189009 | 3.94152959550235 | 0.998735809224318 |
| ebi-fl187-GCST90094846 | prot-c-4542_24_2 | Clusterin \|\| id:prot-c-4542_24_2 | Spicy food liking \|\| id:ebi-fl187-GCST90094846 | Inverse variance weighted | 2 | -0.204394626 | 0.630037591526959 | 0.745622686957666 | -1.439268305 | 1.03047905368291 | 0.815140632257543 | 0.237101180952519 | 2.8024080170663 | 0.998735809224318 |
| ebi-fl187-GCST90094846 | prot-c-4696_2_2 | FABP \|\| id:prot-c-4696_2_2 | Spicy food liking \|\| id:ebi-fl187-GCST90094846 | Inverse variance weighted | 2 | 0.0582332861187712 | 0.601992402128784 | 0.922937436380036 | -1.121671822 | 1.23813839429119 | 1.05996224134701 | 0.325734768586949 | 3.44918645914051 | 0.998735809224318 |
| ebi-fl187-GCST90094846 | prot-c-4906_35_1 | Coagulation Factor V \|\| id:prot-c-4906_35_1 | Spicy food liking \|\| id:ebi-fl187-GCST90094846 | Inverse variance weighted | 2 | 0.206030444412755 | 1.87821003509386 | 0.912651183730688 | -3.475261224 | 3.88732211319673 | 1.22879061323448 | 0.0309537468939022 | 48.780083921621 | 0.998735809224318 |
| ebi-fl187-GCST90094846 | prot-c-4916_2_1 | IgD \|\| id:prot-c-4916_2_1 | Spicy food liking \|\| id:ebi-fl187-GCST90094846 | Inverse variance weighted | 2 | -0.075871456 | 0.63074954993173 | 0.904255192995782 | -1.312140574 | 1.16039766195464 | 0.9269353509613 | 0.269243104371757 | 3.19120204347146 | 0.998735809224318 |
| ebi-fl187-GCST90094846 | prot-c-4922_13_1 | MIP-3b \|\| id:prot-c-4922_13_1 | Spicy food liking \|\| id:ebi-fl187-GCST90094846 | Inverse variance weighted | 2 | 0.301268713383358 | 0.635014777098117 | 0.635194816862302 | -0.94336025 | 1.54589767649567 | 1.35157247835948 | 0.389317431156331 | 4.69218179836715 | 0.998735809224318 |
| ebi-fl187-GCST90094846 | prot-c-4973_18_1 | cIAP-2 \|\| id:prot-c-4973_18_1 | Spicy food liking \|\| id:ebi-fl187-GCST90094846 | Inverse variance weighted | 2 | 1.07131800493787 | 0.633813026702374 | 0.0909754487875207 | -0.170955527 | 2.31359153727452 | 2.9192245178734 | 0.842859056772835 | 10.1106723802459 | 0.998735809224318 |
| ebi-fl187-GCST90094846 | prot-c-4997_19_1 | IF4A3 \|\| id:prot-c-4997_19_1 | Spicy food liking \|\| id:ebi-fl187-GCST90094846 | Inverse variance weighted | 2 | -0.563399851 | 0.63441925260645 | 0.37451060560738 | -1.806861587 | 0.680061883630121 | 0.569270335430363 | 0.164168557951591 | 1.97399988672958 | 0.998735809224318 |
| ebi-fl187-GCST90094846 | prot-c-5000_52_1 | LG3BP \|\| id:prot-c-5000_52_1 | Spicy food liking \|\| id:ebi-fl187-GCST90094846 | Inverse variance weighted | 2 | -0.621463268 | 0.585783015342225 | 0.288730641844903 | -1.769597978 | 0.526671441936145 | 0.537157856270991 | 0.170401480165844 | 1.69328671483854 | 0.998735809224318 |
| ebi-fl187-GCST90094846 | prot-c-5006_71_1 | MK13 \|\| id:prot-c-5006_71_1 | Spicy food liking \|\| id:ebi-fl187-GCST90094846 | Inverse variance weighted | 2 | 0.370847419237494 | 0.644231878475202 | 0.564856669621511 | -0.891847063 | 1.63354190104889 | 1.44896197279657 | 0.409897945954414 | 5.12198418980131 | 0.998735809224318 |
| ebi-fl187-GCST90094846 | prot-c-5109_24_3 | Nr-CAM \|\| id:prot-c-5109_24_3 | Spicy food liking \|\| id:ebi-fl187-GCST90094846 | Inverse variance weighted | 2 | 0.285652790464819 | 0.621021370156301 | 0.645535915346021 | -0.931549095 | 1.50285467597117 | 1.3306303674849 | 0.393942982335263 | 4.49450111886031 | 0.998735809224318 |
| ebi-fl187-GCST90094846 | prot-c-5255_22_3 | PDE4D \|\| id:prot-c-5255_22_3 | Spicy food liking \|\| id:ebi-fl187-GCST90094846 | Inverse variance weighted | 2 | 0.889674753073333 | 0.62306329164148 | 0.15331940589474 | -0.331529299 | 2.11087880469063 | 2.43433776164157 | 0.717825124677664 | 8.2554930637391 | 0.998735809224318 |
| ebi-fl187-GCST90094846 | prot-c-5440_26_3 | Troponin I, skeletal, fast twitch \|\| id:prot-c-5440_26_3 | Spicy food liking \|\| id:ebi-fl187-GCST90094846 | Inverse variance weighted | 2 | -0.150178461 | 0.661124314187498 | 0.820302314788579 | -1.445982117 | 1.14562519440073 | 0.860554386974068 | 0.235514659933607 | 3.14440660784802 | 0.998735809224318 |
| ebi-fl187-GCST90094846 | ukb-rsfMRI191-edge-pheno716 | Net100_Pair10_33 (OccipitalPrecuneus)&(Temporal\|Frontal\|Supp_Motor_Area) (Default_mode\|Central_executive)&(Default_mode\|Salience) \|\| id:ukb-rsfMRI191-edge-pheno716 | Spicy food liking \|\| id:ebi-fl187-GCST90094846 | Inverse variance weighted | 15 | 0.0119020303848223 | 0.0353378740422849 | 0.736262672286149 | -0.057360203 | 0.0811642635077007 | 1.0119731413902 | 0.944253885267686 | 1.0845490337642 | 0.998735809224318 |
| ebi-fl187-GCST90094846 | ukb-rsfMRI191-edge-pheno903 | Net100_Pair27_38 (Precentral\|Frontal\|Supp_Motor_Area)&(Subcortical) (Attention\|Salience\|Motor)&(Subcortical-cerebellum) \|\| id:ukb-rsfMRI191-edge-pheno903 | Spicy food liking \|\| id:ebi-fl187-GCST90094846 | Inverse variance weighted | 15 | -0.044975602 | 0.0426094795989736 | 0.29118280390947 | -0.128490182 | 0.0385389780679336 | 0.956020806595772 | 0.879422196578296 | 1.03929123713295 | 0.998735809224318 |
| ebi-fl187-GCST90094846 | ukb-rsfMRI191-node-pheno23 | Net100_Node2 RolandicOper\|SupraMarginal\|Insula Salience\|Motor \|\| id:ukb-rsfMRI191-node-pheno23 | Spicy food liking \|\| id:ebi-fl187-GCST90094846 | Inverse variance weighted (multiplicative random effects) | 15 | -0.038647533 | 0.0424788255589552 | 0.362924298845179 | -0.121906031 | 0.0446109649715351 | 0.962089754161853 | 0.885231548723426 | 1.04562099757744 | 0.998735809224318 |
| ebi-fl187-GCST90094846 | ukb-rsfMRI191-node-pheno55 | Net100_Node34 Parietal\|Postcentral\|Precuneus Attention \|\| id:ukb-rsfMRI191-node-pheno55 | Spicy food liking \|\| id:ebi-fl187-GCST90094846 | Inverse variance weighted (multiplicative random effects) | 15 | -0.000301665 | 0.0478483699462257 | 0.994969690387101 | -0.09408447 | 0.0934811402653378 | 0.999698380666995 | 0.910205873972562 | 1.09798989534794 | 0.999107451094609 |
| ebi-fl187-GCST90094846 | ebi-a-GCST90001414 | IgD- CD24- B cell Absolute Count \|\| id:ebi-a-GCST90001414 | Spicy food liking \|\| id:ebi-fl187-GCST90094846 | MR Egger | 15 | 0.907661347228474 | 0.746051580198073 | 0.245376535592105 | -0.55459975 | 2.3699224444167 | 2.4785193540624 | 0.574302079631438 | 10.6965626737835 | 0.999950574162623 |
| ebi-fl187-GCST90094846 | ebi-a-GCST90001422 | CD20- CD38- B cell %B cell \|\| id:ebi-a-GCST90001422 | Spicy food liking \|\| id:ebi-fl187-GCST90094846 | MR Egger | 15 | -0.207354785 | 0.737249305494081 | 0.782941955037065 | -1.652363424 | 1.23765385400701 | 0.812731254167291 | 0.1915965492989 | 3.44751559418679 | 0.999950574162623 |
| ebi-fl187-GCST90094846 | ebi-a-GCST90001444 | CD20- CD38- B cell %lymphocyte \|\| id:ebi-a-GCST90001444 | Spicy food liking \|\| id:ebi-fl187-GCST90094846 | MR Egger | 15 | -0.109597334 | 0.708755598846258 | 0.879485245076787 | -1.498758308 | 1.27956363963782 | 0.896194929789058 | 0.223407391201248 | 3.59507063692496 | 0.999950574162623 |
| ebi-fl187-GCST90094846 | ebi-a-GCST90001459 | Myeloid Dendritic Cell %Dendritic Cell \|\| id:ebi-a-GCST90001459 | Spicy food liking \|\| id:ebi-fl187-GCST90094846 | MR Egger | 15 | 1.38094812443414 | 0.760479328746955 | 0.0925112217645222 | -0.10959136 | 2.87148760877818 | 3.97867211601478 | 0.896200283844693 | 17.663274707796 | 0.999950574162623 |
| ebi-fl187-GCST90094846 | ebi-a-GCST90001476 | HLA DR++ monocyte %leukocyte \|\| id:ebi-a-GCST90001476 | Spicy food liking \|\| id:ebi-fl187-GCST90094846 | MR Egger | 15 | 0.65333753695884 | 0.743470021430539 | 0.395479801132539 | -0.803863705 | 2.1105387789627 | 1.92194469795706 | 0.44759623905651 | 8.25268646088625 | 0.999950574162623 |
| ebi-fl187-GCST90094846 | ebi-a-GCST90001478 | CD4 regulatory T cell %CD4+ T cell \|\| id:ebi-a-GCST90001478 | Spicy food liking \|\| id:ebi-fl187-GCST90094846 | MR Egger | 15 | 1.41899070003359 | 0.75002458555274 | 0.0809856597195462 | -0.051057488 | 2.88903888771696 | 4.13294695123547 | 0.950224042815247 | 17.9760243185592 | 0.999950574162623 |
| ebi-fl187-GCST90094846 | ebi-a-GCST90001485 | CD39+ resting CD4 regulatory T cell %CD4 regulatory T cell \|\| id:ebi-a-GCST90001485 | Spicy food liking \|\| id:ebi-fl187-GCST90094846 | MR Egger | 15 | 0.0346047356078426 | 0.856290200063412 | 0.968378410275643 | -1.643724057 | 1.71293352773213 | 1.03521044609373 | 0.19325899309764 | 5.54520465270227 | 0.999950574162623 |
| ebi-fl187-GCST90094846 | ebi-a-GCST90001515 | Immature Myeloid-Derived Suppressor Cells Absolute Count \|\| id:ebi-a-GCST90001515 | Spicy food liking \|\| id:ebi-fl187-GCST90094846 | MR Egger | 15 | 0.324976164967846 | 1.12987011236945 | 0.778169582668752 | -1.889569255 | 2.53952158521196 | 1.38399765795894 | 0.151136896237953 | 12.6736062795686 | 0.999950574162623 |
| ebi-fl187-GCST90094846 | ebi-a-GCST90001517 | CD33+ HLA DR+ Absolute Count \|\| id:ebi-a-GCST90001517 | Spicy food liking \|\| id:ebi-fl187-GCST90094846 | MR Egger | 15 | -0.503573062 | 0.961959953398666 | 0.609444033987747 | -2.389014571 | 1.38186844616512 | 0.604367354872808 | 0.0917200229148487 | 3.98233545989249 | 0.999950574162623 |
| ebi-fl187-GCST90094846 | ebi-a-GCST90001518 | CD33+ HLA DR+ CD14- Absolute Count \|\| id:ebi-a-GCST90001518 | Spicy food liking \|\| id:ebi-fl187-GCST90094846 | MR Egger | 15 | -0.956952136 | 0.97479171825408 | 0.344165975614096 | -2.867543904 | 0.953639631502218 | 0.384061671557344 | 0.0568383557391804 | 2.59513783678549 | 0.999950574162623 |
| ebi-fl187-GCST90094846 | ebi-a-GCST90001520 | CD33+ HLA DR+ CD14dim Absolute Count \|\| id:ebi-a-GCST90001520 | Spicy food liking \|\| id:ebi-fl187-GCST90094846 | MR Egger | 15 | 0.0101995585220538 | 0.965043347337707 | 0.991727776318925 | -1.881285402 | 1.90168451930396 | 1.01025175131597 | 0.152394092095304 | 6.6971664518249 | 0.999950574162623 |
| ebi-fl187-GCST90094846 | ebi-a-GCST90001531 | CD33dim HLA DR- Absolute Count \|\| id:ebi-a-GCST90001531 | Spicy food liking \|\| id:ebi-fl187-GCST90094846 | MR Egger | 15 | 0.508470978030135 | 0.981994301647619 | 0.613300110821904 | -1.416237853 | 2.43317980925947 | 1.66274687377977 | 0.242625093239293 | 11.3950586452229 | 0.999950574162623 |
| ebi-fl187-GCST90094846 | ebi-a-GCST90001532 | Basophil Absolute Count \|\| id:ebi-a-GCST90001532 | Spicy food liking \|\| id:ebi-fl187-GCST90094846 | MR Egger | 15 | 0.365209973855265 | 0.979315083712518 | 0.715214906331969 | -1.55424759 | 2.2846675379318 | 1.44081651018672 | 0.211348343391947 | 9.82242009901526 | 0.999950574162623 |
| ebi-fl187-GCST90094846 | ebi-a-GCST90001588 | T/B cell \|\| id:ebi-a-GCST90001588 | Spicy food liking \|\| id:ebi-fl187-GCST90094846 | MR Egger | 15 | 0.139486018589268 | 0.732773293930928 | 0.851972412940596 | -1.296749638 | 1.57572167469389 | 1.14968273141921 | 0.273419061340618 | 4.83422910035125 | 0.999950574162623 |
| ebi-fl187-GCST90094846 | ebi-a-GCST90001624 | HLA DR+ CD4+ T cell Absolute Count \|\| id:ebi-a-GCST90001624 | Spicy food liking \|\| id:ebi-fl187-GCST90094846 | MR Egger | 15 | 0.868450926757931 | 0.711329248114177 | 0.243811356043681 | -0.5257544 | 2.26265625306172 | 2.38321621616063 | 0.591109263558869 | 9.6085781142649 | 0.999950574162623 |
| ebi-fl187-GCST90094846 | ebi-a-GCST90001631 | CD8+ Natural Killer T %T cell \|\| id:ebi-a-GCST90001631 | Spicy food liking \|\| id:ebi-fl187-GCST90094846 | MR Egger | 15 | 0.218007314926245 | 0.847635132481232 | 0.801054383418435 | -1.443357545 | 1.87937217458946 | 1.24359616440001 | 0.2361335970955 | 6.54939169662061 | 0.999950574162623 |
| ebi-fl187-GCST90094846 | ebi-a-GCST90001632 | CD8+ Natural Killer T %lymphocyte \|\| id:ebi-a-GCST90001632 | Spicy food liking \|\| id:ebi-fl187-GCST90094846 | MR Egger | 15 | 0.188661096951789 | 0.825506795786059 | 0.822782405970775 | -1.429332223 | 1.80665441669246 | 1.20763161308884 | 0.239468780657407 | 6.09003858009351 | 0.999950574162623 |
| ebi-fl187-GCST90094846 | ebi-a-GCST90001646 | Natural Killer %CD3- lymphocyte \|\| id:ebi-a-GCST90001646 | Spicy food liking \|\| id:ebi-fl187-GCST90094846 | MR Egger | 15 | 0.0297290653495394 | 0.707039178484358 | 0.967099961304974 | -1.356067724 | 1.41552585517888 | 1.03017538593169 | 0.257672024793269 | 4.11865171095293 | 0.999950574162623 |
| ebi-fl187-GCST90094846 | ebi-a-GCST90001684 | CD127- CD8+ T cell Absolute Count \|\| id:ebi-a-GCST90001684 | Spicy food liking \|\| id:ebi-fl187-GCST90094846 | MR Egger | 15 | 0.347022839845215 | 0.764307444276307 | 0.657290665682525 | -1.151019751 | 1.84506543062678 | 1.41484903993447 | 0.316314043315067 | 6.32851385548378 | 0.999950574162623 |
| ebi-fl187-GCST90094846 | ebi-a-GCST90001687 | CD28- CD8+ T cell Absolute Count \|\| id:ebi-a-GCST90001687 | Spicy food liking \|\| id:ebi-fl187-GCST90094846 | MR Egger | 15 | 0.11324669877151 | 0.743964822518243 | 0.881350108973323 | -1.344924353 | 1.57141775090727 | 1.11990817884435 | 0.260559417557832 | 4.81346765662036 | 0.999950574162623 |
| ebi-fl187-GCST90094846 | ebi-a-GCST90001701 | BAFF-R on CD20- CD38- B cell \|\| id:ebi-a-GCST90001701 | Spicy food liking \|\| id:ebi-fl187-GCST90094846 | MR Egger | 15 | -0.86090694 | 0.983097053322426 | 0.397080527486676 | -2.787777165 | 1.06596328420223 | 0.422778473549168 | 0.0615578950034727 | 2.90363466272655 | 0.999950574162623 |
| ebi-fl187-GCST90094846 | ebi-a-GCST90001732 | CD19 on IgD- CD27- B cell \|\| id:ebi-a-GCST90001732 | Spicy food liking \|\| id:ebi-fl187-GCST90094846 | MR Egger | 15 | 0.888147855577597 | 0.719885480381897 | 0.239142383651765 | -0.522827686 | 2.29912339712612 | 2.43062361369275 | 0.592841805154604 | 9.96544288893411 | 0.999950574162623 |
| ebi-fl187-GCST90094846 | ebi-a-GCST90001798 | CD27 on CD24+ CD27+ B cell \|\| id:ebi-a-GCST90001798 | Spicy food liking \|\| id:ebi-fl187-GCST90094846 | MR Egger | 15 | 0.648561764622917 | 0.722894671812339 | 0.385940251803633 | -0.768311792 | 2.0654353213751 | 1.91278781064612 | 0.463795390793085 | 7.88873128363772 | 0.999950574162623 |
| ebi-fl187-GCST90094846 | ebi-a-GCST90001836 | CD66b on CD66b++ myeloid cell \|\| id:ebi-a-GCST90001836 | Spicy food liking \|\| id:ebi-fl187-GCST90094846 | MR Egger | 15 | 0.143886098700688 | 1.16743701468733 | 0.903794609671011 | -2.14429045 | 2.43206264748785 | 1.15475257321556 | 0.117151132136259 | 11.3823356294757 | 0.999950574162623 |
| ebi-fl187-GCST90094846 | ebi-a-GCST90001943 | CD25 on activated & secreting CD4 regulatory T cell \|\| id:ebi-a-GCST90001943 | Spicy food liking \|\| id:ebi-fl187-GCST90094846 | MR Egger | 15 | 0.0189185707141732 | 0.73579958721911 | 0.979877882633971 | -1.42324862 | 1.46108576166363 | 1.0190986607625 | 0.240930053939023 | 4.31063731314638 | 0.999950574162623 |
| ebi-fl187-GCST90094846 | ebi-a-GCST90001960 | CD25 on CD4+ T cell \|\| id:ebi-a-GCST90001960 | Spicy food liking \|\| id:ebi-fl187-GCST90094846 | MR Egger | 15 | 0.0338285087175549 | 0.785604518100288 | 0.966307689557763 | -1.505956347 | 1.57361336419412 | 1.03440719969943 | 0.221805069808603 | 4.82404778084342 | 0.999950574162623 |
| ebi-fl187-GCST90094846 | ebi-a-GCST90001988 | HLA DR on CD14+ CD16- monocyte \|\| id:ebi-a-GCST90001988 | Spicy food liking \|\| id:ebi-fl187-GCST90094846 | MR Egger | 15 | 0.12173730100987 | 0.764062509291096 | 0.87585845872717 | -1.375825217 | 1.61929981922042 | 1.12945735549787 | 0.252631034287387 | 5.04955347820434 | 0.999950574162623 |
| ebi-fl187-GCST90094846 | ebi-a-GCST90001991 | HLA DR on CD14+ monocyte \|\| id:ebi-a-GCST90001991 | Spicy food liking \|\| id:ebi-fl187-GCST90094846 | MR Egger | 15 | 0.10353452027993 | 0.769465742374722 | 0.895025585228881 | -1.404618335 | 1.61168737533438 | 1.10908407859711 | 0.245460722399232 | 5.0112599742005 | 0.999950574162623 |
| ebi-fl187-GCST90094846 | ebi-a-GCST90002019 | CD14 on Monocytic Myeloid-Derived Suppressor Cells \|\| id:ebi-a-GCST90002019 | Spicy food liking \|\| id:ebi-fl187-GCST90094846 | MR Egger | 15 | 0.524935961420637 | 1.05695845253481 | 0.627729702095487 | -1.546702606 | 2.59657452838886 | 1.69035059726213 | 0.212948994253436 | 13.4176972832467 | 0.999950574162623 |
| ebi-fl187-GCST90094846 | ebi-a-GCST90002037 | CD80 on plasmacytoid Dendritic Cell \|\| id:ebi-a-GCST90002037 | Spicy food liking \|\| id:ebi-fl187-GCST90094846 | MR Egger | 15 | -0.119946421 | 0.818480601785424 | 0.885737254932477 | -1.7241684 | 1.48427555858577 | 0.886967958376884 | 0.17832128203472 | 4.4117681872323 | 0.999950574162623 |
| ebi-fl187-GCST90094846 | ebi-a-GCST90002042 | CD45 on CD33+ HLA DR+ CD14- \|\| id:ebi-a-GCST90002042 | Spicy food liking \|\| id:ebi-fl187-GCST90094846 | MR Egger | 15 | 0.930775513799339 | 1.10548698809768 | 0.415031426661293 | -1.235978983 | 3.09753001047079 | 2.53647548681106 | 0.290550179440514 | 22.143190231657 | 0.999950574162623 |
| ebi-fl187-GCST90094846 | ebi-a-GCST90002056 | CD8 on Effector Memory CD8+ T cell \|\| id:ebi-a-GCST90002056 | Spicy food liking \|\| id:ebi-fl187-GCST90094846 | MR Egger | 15 | -1.17246019 | 0.802120187669738 | 0.167571902432079 | -2.744615758 | 0.39969537774739 | 0.309604318077433 | 0.0642729925889046 | 1.49137032385107 | 0.999950574162623 |
| ebi-fl187-GCST90094846 | ebi-a-GCST90002058 | CD8 on CD8+ T cell \|\| id:ebi-a-GCST90002058 | Spicy food liking \|\| id:ebi-fl187-GCST90094846 | MR Egger | 15 | -1.015497532 | 1.2474393256942 | 0.430267061705364 | -3.46047861 | 1.42948354659274 | 0.362222167957612 | 0.0314147230151255 | 4.17654164567169 | 0.999950574162623 |
| ebi-fl187-GCST90094846 | ebi-a-GCST90002063 | CD4 on CD4 regulatory T cell \|\| id:ebi-a-GCST90002063 | Spicy food liking \|\| id:ebi-fl187-GCST90094846 | MR Egger | 15 | -0.814162415 | 0.799208357413363 | 0.326925044170098 | -2.380610796 | 0.752285965080355 | 0.443010230532943 | 0.0924940652501382 | 2.1218449402791 | 0.999950574162623 |
| ebi-fl187-GCST90094846 | ebi-a-GCST90002066 | CD4 on activated CD4 regulatory T cell \|\| id:ebi-a-GCST90002066 | Spicy food liking \|\| id:ebi-fl187-GCST90094846 | MR Egger | 15 | -0.704820037 | 0.798624531960568 | 0.393510520107278 | -2.270124119 | 0.860484045995358 | 0.494197503693516 | 0.103299357844413 | 2.36430484906554 | 0.999950574162623 |
| ebi-fl187-GCST90094846 | ebi-a-GCST90002070 | CD4 on activated & secreting CD4 regulatory T cell \|\| id:ebi-a-GCST90002070 | Spicy food liking \|\| id:ebi-fl187-GCST90094846 | MR Egger | 15 | -0.735458868 | 0.802260170955744 | 0.375975738638954 | -2.307888803 | 0.836971067042305 | 0.479285479723686 | 0.0994710329728257 | 2.30936147146194 | 0.999950574162623 |
| ebi-fl187-GCST90094846 | ebi-a-GCST90002081 | SSC-A on CD4+ T cell \|\| id:ebi-a-GCST90002081 | Spicy food liking \|\| id:ebi-fl187-GCST90094846 | MR Egger | 15 | 0.462142505451835 | 0.797132531775799 | 0.571990318220628 | -1.100237257 | 2.0245222677324 | 1.5874715104522 | 0.332792117128434 | 7.57249245637881 | 0.999950574162623 |
| ebi-fl187-GCST90094846 | ebi-a-GCST90002094 | CD11b on Monocytic Myeloid-Derived Suppressor Cells \|\| id:ebi-a-GCST90002094 | Spicy food liking \|\| id:ebi-fl187-GCST90094846 | MR Egger | 15 | 0.928126167300235 | 1.23584259442446 | 0.466026512090365 | -1.494125318 | 3.35037765237218 | 2.52976437831138 | 0.224444836807656 | 28.5134998015461 | 0.999950574162623 |
| ebi-fl187-GCST90094846 | ebi-a-GCST90002104 | HLA DR on myeloid Dendritic Cell \|\| id:ebi-a-GCST90002104 | Spicy food liking \|\| id:ebi-fl187-GCST90094846 | MR Egger | 15 | -0.458722844 | 0.813412063773877 | 0.582387687476926 | -2.053010489 | 1.1355648007165 | 0.632090408096605 | 0.128347931321157 | 3.11293123227669 | 0.999950574162623 |
| ebi-fl187-GCST90094846 | ebi-a-GCST90002105 | HLA DR on plasmacytoid Dendritic Cell \|\| id:ebi-a-GCST90002105 | Spicy food liking \|\| id:ebi-fl187-GCST90094846 | MR Egger | 15 | 0.260228709757527 | 0.796876272878302 | 0.749197822026956 | -1.301648785 | 1.822106205 | 1.29722674115402 | 0.272082816913035 | 6.18487134563496 | 0.999950574162623 |
| ebi-fl187-GCST90094846 | ebi-a-GCST90002106 | HLA DR on Dendritic Cell \|\| id:ebi-a-GCST90002106 | Spicy food liking \|\| id:ebi-fl187-GCST90094846 | MR Egger | 15 | 0.102180744080007 | 0.812257944892741 | 0.901816211808391 | -1.489844828 | 1.69420631606978 | 1.10758364282407 | 0.225407629798925 | 5.44232476489707 | 0.999950574162623 |
| ebi-fl187-GCST90094846 | ebi-a-GCST90002111 | HLA DR on CD33dim HLA DR+ CD11b- \|\| id:ebi-a-GCST90002111 | Spicy food liking \|\| id:ebi-fl187-GCST90094846 | MR Egger | 15 | -0.686011699 | 1.22372852551682 | 0.58460877287032 | -3.084519609 | 1.71249621140548 | 0.503580500081301 | 0.0457520074918295 | 5.54278017434362 | 0.999950574162623 |
| ebi-fl187-GCST90094846 | ebi-a-GCST90002112 | HLA DR on CD33- HLA DR+ \|\| id:ebi-a-GCST90002112 | Spicy food liking \|\| id:ebi-fl187-GCST90094846 | MR Egger | 15 | 0.221024279742821 | 1.09088904102722 | 0.842576941259548 | -1.917118241 | 2.35916680015616 | 1.24735371562383 | 0.147030057447874 | 10.5821307485524 | 0.999950574162623 |
| ebi-fl187-GCST90094846 | ebi-a-GCST90002115 | HLA DR on HLA DR+ CD8+ T cell \|\| id:ebi-a-GCST90002115 | Spicy food liking \|\| id:ebi-fl187-GCST90094846 | MR Egger | 15 | -0.213727218 | 0.900125467709685 | 0.816015227188268 | -1.977973135 | 1.55051869833159 | 0.807568644863591 | 0.138349368801538 | 4.71391464822908 | 0.999950574162623 |
| ebi-fl187-GCST90094846 | ebi-a-GCST90002116 | HLA DR on B cell \|\| id:ebi-a-GCST90002116 | Spicy food liking \|\| id:ebi-fl187-GCST90094846 | MR Egger | 15 | -0.64761732 | 0.788909291170711 | 0.426499728611607 | -2.19387953 | 0.898644891184331 | 0.523291128096046 | 0.11148340533832 | 2.45627233858731 | 0.999950574162623 |
| ebi-fl187-GCST90094846 | ebi-cfmet338-GCST90026023 | Sphingomyelin (d18:1/24:1, d18:2/24:0) levels \|\| id:ebi-cfmet338-GCST90026023 | Spicy food liking \|\| id:ebi-fl187-GCST90094846 | MR Egger | 15 | 0.43583578824826 | 0.462157796308268 | 0.362854399249841 | -0.469993493 | 1.34166506901246 | 1.54625486068116 | 0.62500633548823 | 3.8254077733667 | 0.999950574162623 |
| ebi-fl187-GCST90094846 | ebi-cfmet338-GCST90026046 | 1-arachidonoyl-gpc (20:4n6) levels \|\| id:ebi-cfmet338-GCST90026046 | Spicy food liking \|\| id:ebi-fl187-GCST90094846 | MR Egger | 15 | 0.312827631387226 | 0.546453544081449 | 0.576771350863354 | -0.758221315 | 1.38387657778687 | 1.36728583355237 | 0.46849899848452 | 3.9903405486037 | 0.999950574162623 |
| ebi-fl187-GCST90094846 | ebi-cfmet338-GCST90026047 | 1-linoleoyl-gpc (18:2) levels \|\| id:ebi-cfmet338-GCST90026047 | Spicy food liking \|\| id:ebi-fl187-GCST90094846 | MR Egger | 15 | 0.239822526918697 | 0.622873853332193 | 0.706443928381766 | -0.981010226 | 1.46065527944979 | 1.27102355783648 | 0.374932141415205 | 4.30878205980821 | 0.999950574162623 |
| ebi-fl187-GCST90094846 | ebi-cfmet338-GCST90026050 | 1-myristoyl-2-palmitoyl-gpc (14:0/16:0) levels \|\| id:ebi-cfmet338-GCST90026050 | Spicy food liking \|\| id:ebi-fl187-GCST90094846 | MR Egger | 15 | -0.431267421 | 0.312809607429148 | 0.191252831110765 | -1.044374252 | 0.18183940936375 | 0.649685147961667 | 0.351911958784329 | 1.19942156254103 | 0.999950574162623 |
| ebi-fl187-GCST90094846 | ebi-cfmet338-GCST90026068 | 2,3-dihydroxyisovalerate levels \|\| id:ebi-cfmet338-GCST90026068 | Spicy food liking \|\| id:ebi-fl187-GCST90094846 | MR Egger | 15 | 0.037348631351981 | 1.01639061468135 | 0.971245539291369 | -1.954776973 | 2.02947423612743 | 1.03805485622887 | 0.141596052855761 | 7.61008419943748 | 0.999950574162623 |
| ebi-fl187-GCST90094846 | ebi-cfmet338-GCST90026082 | 3-hydroxyhexanoate levels \|\| id:ebi-cfmet338-GCST90026082 | Spicy food liking \|\| id:ebi-fl187-GCST90094846 | MR Egger | 15 | 0.212788479183357 | 0.330074328224007 | 0.530354201087702 | -0.434157204 | 0.859734162502411 | 1.23712294631066 | 0.647810409004774 | 2.36253256047496 | 0.999950574162623 |
| ebi-fl187-GCST90094846 | ebi-cfmet338-GCST90026094 | 3-ureidopropionate levels \|\| id:ebi-cfmet338-GCST90026094 | Spicy food liking \|\| id:ebi-fl187-GCST90094846 | MR Egger | 15 | 0.299483837302251 | 0.685815691487645 | 0.669506786637138 | -1.044714918 | 1.64368259261804 | 1.34916224059871 | 0.35179209467322 | 5.17418890025986 | 0.999950574162623 |
| ebi-fl187-GCST90094846 | ebi-cfmet338-GCST90026173 | Glycerol levels \|\| id:ebi-cfmet338-GCST90026173 | Spicy food liking \|\| id:ebi-fl187-GCST90094846 | MR Egger | 15 | 0.0862790985134954 | 0.280047935524026 | 0.762897085808502 | -0.462614855 | 0.635173052140587 | 1.09011053408107 | 0.629635086549084 | 1.88734872293665 | 0.999950574162623 |
| ebi-fl187-GCST90094846 | ebi-cfmet338-GCST90026179 | Histidine levels \|\| id:ebi-cfmet338-GCST90026179 | Spicy food liking \|\| id:ebi-fl187-GCST90094846 | MR Egger | 15 | -0.13226096 | 0.282785728861739 | 0.647737524917129 | -0.686520988 | 0.421999068888426 | 0.876112335250011 | 0.503324097045893 | 1.52500710473085 | 0.999950574162623 |
| ebi-fl187-GCST90094846 | ebi-cfmet338-GCST90026188 | Isocitrate levels \|\| id:ebi-cfmet338-GCST90026188 | Spicy food liking \|\| id:ebi-fl187-GCST90094846 | MR Egger | 15 | -0.023432738 | 0.44656716217594 | 0.958949532566961 | -0.898704376 | 0.851838899605063 | 0.976839676395764 | 0.407096762488481 | 2.34395318584235 | 0.999950574162623 |
| ebi-fl187-GCST90094846 | ebi-cfmet338-GCST90026207 | N-acetyl-isoputreanine levels \|\| id:ebi-cfmet338-GCST90026207 | Spicy food liking \|\| id:ebi-fl187-GCST90094846 | MR Egger | 14 | -0.016787504 | 0.281225548520802 | 0.95338188696783 | -0.56798958 | 0.534414570670517 | 0.983352620510504 | 0.566663526251151 | 1.70644894451226 | 0.999950574162623 |
| ebi-fl187-GCST90094846 | ebi-cfmet338-GCST90026213 | N-acetylglutamate levels \|\| id:ebi-cfmet338-GCST90026213 | Spicy food liking \|\| id:ebi-fl187-GCST90094846 | MR Egger | 15 | -0.557213153 | 0.436167487504903 | 0.223762008358625 | -1.412101428 | 0.297675122621416 | 0.572803156393029 | 0.243630772216208 | 1.34672419657499 | 0.999950574162623 |
| ebi-fl187-GCST90094846 | ebi-cfmet338-GCST90026224 | N-acetyltaurine levels \|\| id:ebi-cfmet338-GCST90026224 | Spicy food liking \|\| id:ebi-fl187-GCST90094846 | MR Egger | 15 | -0.126448404 | 0.360102477945302 | 0.731104574177733 | -0.832249261 | 0.579352452507236 | 0.88121961554385 | 0.435069599849353 | 1.78488226042026 | 0.999950574162623 |
| ebi-fl187-GCST90094846 | ebi-cfmet338-GCST90026250 | Phenyllactate (pla) levels \|\| id:ebi-cfmet338-GCST90026250 | Spicy food liking \|\| id:ebi-fl187-GCST90094846 | MR Egger | 15 | -0.277305245 | 0.52826351043762 | 0.608463140941591 | -1.312701726 | 0.758091235100126 | 0.757823139806618 | 0.269092060438398 | 2.13419864670378 | 0.999950574162623 |
| ebi-fl187-GCST90094846 | ebi-cfmet338-GCST90026251 | Pipecolate levels \|\| id:ebi-cfmet338-GCST90026251 | Spicy food liking \|\| id:ebi-fl187-GCST90094846 | MR Egger | 14 | -0.865565297 | 0.898017656671019 | 0.354137408744798 | -2.625679904 | 0.894549309991064 | 0.42081360067546 | 0.0723905216817 | 2.44623304818939 | 0.999950574162623 |
| ebi-fl187-GCST90094846 | ebi-cfmet338-GCST90026278 | Trigonelline (n'-methylnicotinate) levels \|\| id:ebi-cfmet338-GCST90026278 | Spicy food liking \|\| id:ebi-fl187-GCST90094846 | MR Egger | 15 | -0.368025048 | 0.988320746790163 | 0.715614371099528 | -2.305133711 | 1.5690836159948 | 0.692099845952676 | 0.0997454626542037 | 4.80224547585002 | 0.999950574162623 |
| ebi-fl187-GCST90094846 | ebi-cfmet338-GCST90026299 | 1-carboxyethylvaline levels \|\| id:ebi-cfmet338-GCST90026299 | Spicy food liking \|\| id:ebi-fl187-GCST90094846 | MR Egger | 15 | 0.335750012704928 | 0.629325885079582 | 0.602685132351449 | -0.897728722 | 1.56922874746091 | 1.39898925155449 | 0.407494141924051 | 4.80294248335418 | 0.999950574162623 |
| ebi-fl187-GCST90094846 | ebi-gm430-GCST90011306 | C_Erysipelotrichia abundance \|\| id:ebi-gm430-GCST90011306 | Spicy food liking \|\| id:ebi-fl187-GCST90094846 | MR Egger | 14 | -0.091143416 | 0.479587571165254 | 0.852450468803241 | -1.031135056 | 0.848848223159732 | 0.912886778644407 | 0.356601967633308 | 2.33695365214783 | 0.999950574162623 |
| ebi-fl187-GCST90094846 | ebi-gm430-GCST90011312 | F_Erysipelotrichaceae abundance \|\| id:ebi-gm430-GCST90011312 | Spicy food liking \|\| id:ebi-fl187-GCST90094846 | MR Egger | 14 | -0.091143416 | 0.479587571165254 | 0.852450468803241 | -1.031135056 | 0.848848223159732 | 0.912886778644407 | 0.356601967633308 | 2.33695365214783 | 0.999950574162623 |
| ebi-fl187-GCST90094846 | ebi-gm430-GCST90011352 | O_Erysipelotrichales abundance \|\| id:ebi-gm430-GCST90011352 | Spicy food liking \|\| id:ebi-fl187-GCST90094846 | MR Egger | 14 | -0.091143416 | 0.479587571165254 | 0.852450468803241 | -1.031135056 | 0.848848223159732 | 0.912886778644407 | 0.356601967633308 | 2.33695365214783 | 0.999950574162623 |
| ebi-fl187-GCST90094846 | ebi-gm430-GCST90011362 | OTU97_11 (Parabacteroides) abundance \|\| id:ebi-gm430-GCST90011362 | Spicy food liking \|\| id:ebi-fl187-GCST90094846 | MR Egger | 14 | 0.470160292523442 | 0.624854654222809 | 0.466295691904309 | -0.75455483 | 1.69487541480015 | 1.60025068088006 | 0.470219896051318 | 5.44596743600492 | 0.999950574162623 |
| ebi-fl187-GCST90094846 | ebi-gm430-GCST90011384 | OTU97_235 (Phascolarctobacterium) abundance \|\| id:ebi-gm430-GCST90011384 | Spicy food liking \|\| id:ebi-fl187-GCST90094846 | MR Egger | 14 | -2.630764222 | 1.90431420084907 | 0.192313251870154 | -6.363220055 | 1.10169161213372 | 0.0720233993688371 | 0.00172380701166792 | 3.00925220835702 | 0.999950574162623 |
| ebi-fl187-GCST90094846 | ebi-gm430-GCST90011390 | OTU97_3 (Bacteroides) abundance \|\| id:ebi-gm430-GCST90011390 | Spicy food liking \|\| id:ebi-fl187-GCST90094846 | MR Egger | 14 | -0.063092436 | 0.603016468666279 | 0.918399413281232 | -1.245004715 | 1.11881984214435 | 0.938856685106879 | 0.287939550484682 | 3.06123932501163 | 0.999950574162623 |
| ebi-fl187-GCST90094846 | ebi-gm430-GCST90011398 | OTU97_4 (Alistipes) abundance \|\| id:ebi-gm430-GCST90011398 | Spicy food liking \|\| id:ebi-fl187-GCST90094846 | MR Egger | 14 | -0.629522645 | 0.581153816909408 | 0.299992525532288 | -1.768584126 | 0.509538836213101 | 0.532846097093635 | 0.170574329677196 | 1.66452340000535 | 0.999950574162623 |
| ebi-fl187-GCST90094846 | ebi-gm430-GCST90011421 | OTU99_108 (Cytophagales) abundance \|\| id:ebi-gm430-GCST90011421 | Spicy food liking \|\| id:ebi-fl187-GCST90094846 | MR Egger | 14 | 0.160395232996298 | 1.83230708527198 | 0.931688248929393 | -3.430926654 | 3.75171712012937 | 1.17397477287817 | 0.0323569431811052 | 42.5941585285211 | 0.999950574162623 |
| ebi-fl187-GCST90094846 | ebi-gm430-GCST90011428 | OTU99_123 (Phascolarctobacterium) abundance \|\| id:ebi-gm430-GCST90011428 | Spicy food liking \|\| id:ebi-fl187-GCST90094846 | MR Egger | 14 | 0.326162241924491 | 1.08913962019565 | 0.769706683635942 | -1.808551414 | 2.46087589750796 | 1.38564015956319 | 0.163891375738984 | 11.715068246496 | 0.999950574162623 |
| ebi-fl187-GCST90094846 | ebi-gm430-GCST90011450 | OTU99_298 (Phascolarctobacterium) abundance \|\| id:ebi-gm430-GCST90011450 | Spicy food liking \|\| id:ebi-fl187-GCST90094846 | MR Egger | 14 | -2.660072354 | 1.90428503100237 | 0.187744569520073 | -6.392471014 | 1.07232630711954 | 0.0699431609189301 | 0.00167411432555727 | 2.92216946277125 | 0.999950574162623 |
| ebi-fl187-GCST90094846 | ebi-gm430-GCST90011451 | OTU99_3 (Bacteroides) abundance \|\| id:ebi-gm430-GCST90011451 | Spicy food liking \|\| id:ebi-fl187-GCST90094846 | MR Egger | 14 | -0.100425516 | 0.601825605203432 | 0.870252646091626 | -1.280003702 | 1.07915267021073 | 0.904452477153104 | 0.278036271109097 | 2.94218549315605 | 0.999950574162623 |
| ebi-fl187-GCST90094846 | ebi-gm430-GCST90011472 | OTU99_72 (Parabacteroides) abundance \|\| id:ebi-gm430-GCST90011472 | Spicy food liking \|\| id:ebi-fl187-GCST90094846 | MR Egger | 14 | 0.465291679210823 | 1.73716197116569 | 0.793366345790251 | -2.939545784 | 3.87012914269558 | 1.59247861405763 | 0.0528897466379545 | 47.9485778895949 | 0.999950574162623 |
| ebi-fl187-GCST90094846 | ebi-gm430-GCST90011507 | TestASV_3 (Bacteroides) abundance \|\| id:ebi-gm430-GCST90011507 | Spicy food liking \|\| id:ebi-fl187-GCST90094846 | MR Egger | 14 | 0.561985230047113 | 0.8070923002602 | 0.499501105629242 | -1.019915678 | 2.1438861385571 | 1.75415144005163 | 0.360625347374685 | 8.53253188395044 | 0.999950574162623 |
| ebi-fl187-GCST90094846 | ebi-gm430-GCST90011512 | TestASV_34 (Phascolarctobacterium) abundance \|\| id:ebi-gm430-GCST90011512 | Spicy food liking \|\| id:ebi-fl187-GCST90094846 | MR Egger | 14 | -0.694792416 | 1.61564548441578 | 0.674787938412778 | -3.861457566 | 2.47187273307184 | 0.499178058417696 | 0.021037313895255 | 11.8446078832271 | 0.999950574162623 |
| ebi-fl187-GCST90094846 | ebi-gm430-GCST90011519 | TestASV_41 (Barnesiella) abundance \|\| id:ebi-gm430-GCST90011519 | Spicy food liking \|\| id:ebi-fl187-GCST90094846 | MR Egger | 14 | -0.92410821 | 2.01636598157118 | 0.654923052522077 | -4.876185533 | 3.02796911430215 | 0.396885199722443 | 0.00762604792261989 | 20.6552415296925 | 0.999950574162623 |
| ebi-fl187-GCST90094846 | ebi-gm430-GCST90011524 | TestASV_46 (Sutterella) abundance \|\| id:ebi-gm430-GCST90011524 | Spicy food liking \|\| id:ebi-fl187-GCST90094846 | MR Egger | 14 | -0.784919604 | 2.63747340874746 | 0.771095614817276 | -5.954367485 | 4.38452827752072 | 0.456156373630671 | 0.00259448436659461 | 80.2003819652991 | 0.999950574162623 |
| ebi-fl187-GCST90094846 | ebi-gm430-GCST90011581 | OTU97_19 (Bacteria) prevalence \|\| id:ebi-gm430-GCST90011581 | Spicy food liking \|\| id:ebi-fl187-GCST90094846 | MR Egger | 14 | -1.614665707 | 2.35866609684595 | 0.506623770182704 | -6.237651257 | 3.00831984282679 | 0.198957169315882 | 0.00195444061522156 | 20.2533425236363 | 0.999950574162623 |
| ebi-fl187-GCST90094846 | ebi-gm430-GCST90011614 | OTU97_80 (Ruminococcus) prevalence \|\| id:ebi-gm430-GCST90011614 | Spicy food liking \|\| id:ebi-fl187-GCST90094846 | MR Egger | 14 | -0.099614087 | 1.1032483241234 | 0.929545074937189 | -2.261980802 | 2.06275262809275 | 0.905186673774141 | 0.104143991635271 | 7.86759659883053 | 0.999950574162623 |
| ebi-fl187-GCST90094846 | ebi-gm430-GCST90011623 | OTU99_11 (Parabacteroides) prevalence \|\| id:ebi-gm430-GCST90011623 | Spicy food liking \|\| id:ebi-fl187-GCST90094846 | MR Egger | 14 | 0.446698366260453 | 1.2005427933797 | 0.716320641244177 | -1.906365509 | 2.79976224128467 | 1.56314273158206 | 0.148619562684667 | 16.4407373777712 | 0.999950574162623 |
| ebi-fl187-GCST90094846 | ebi-gm430-GCST90011642 | OTU99_19 (Bacteria) prevalence \|\| id:ebi-gm430-GCST90011642 | Spicy food liking \|\| id:ebi-fl187-GCST90094846 | MR Egger | 14 | -1.614665707 | 2.35866609684595 | 0.506623770182704 | -6.237651257 | 3.00831984282679 | 0.198957169315882 | 0.00195444061522156 | 20.2533425236363 | 0.999950574162623 |
| ebi-fl187-GCST90094846 | ebi-gm430-GCST90011653 | OTU99_30 (Parasutterella) prevalence \|\| id:ebi-gm430-GCST90011653 | Spicy food liking \|\| id:ebi-fl187-GCST90094846 | MR Egger | 14 | 1.20118630887391 | 1.31689428543639 | 0.379657172176444 | -1.379926491 | 3.78229910832925 | 3.32405794407652 | 0.251597047132655 | 43.9168954544702 | 0.999950574162623 |
| ebi-fl187-GCST90094846 | ebi-gm430-GCST90011675 | OTU99_8 (Ruminococcaceae) prevalence \|\| id:ebi-gm430-GCST90011675 | Spicy food liking \|\| id:ebi-fl187-GCST90094846 | MR Egger | 14 | -2.209950374 | 1.2394997418476 | 0.0998897165146593 | -4.639369868 | 0.219469120299707 | 0.109706092685522 | 0.00966378517212005 | 1.24541538930797 | 0.999950574162623 |
| ebi-fl187-GCST90094846 | ebi-gm430-GCST90011679 | OTU99_92 (Ruminococcus) prevalence \|\| id:ebi-gm430-GCST90011679 | Spicy food liking \|\| id:ebi-fl187-GCST90094846 | MR Egger | 14 | 0.796972119094215 | 1.15370744868208 | 0.502840009455977 | -1.46429448 | 3.05823871851109 | 2.21881244727054 | 0.231241079071305 | 21.2900263912218 | 0.999950574162623 |
| ebi-fl187-GCST90094846 | ebi-gm430-GCST90011684 | TestASV_11 (Lachnospiraceae) prevalence \|\| id:ebi-gm430-GCST90011684 | Spicy food liking \|\| id:ebi-fl187-GCST90094846 | MR Egger | 14 | -0.677418334 | 1.23051161932139 | 0.592065269454855 | -3.089221108 | 1.73438443958007 | 0.507926597837349 | 0.0455374093115975 | 5.66543931002504 | 0.999950574162623 |
| ebi-fl187-GCST90094846 | ebi-gm430-GCST90011688 | TestASV_15 (Bacteroides) prevalence \|\| id:ebi-gm430-GCST90011688 | Spicy food liking \|\| id:ebi-fl187-GCST90094846 | MR Egger | 14 | -1.25373013 | 1.15672989183429 | 0.299724860201138 | -3.520920718 | 1.01346045760491 | 0.285438087330112 | 0.0295721949659341 | 2.7551185088739 | 0.999950574162623 |
| ebi-fl187-GCST90094846 | ebi-gm430-GCST90011692 | TestASV_19 (Prevotella) prevalence \|\| id:ebi-gm430-GCST90011692 | Spicy food liking \|\| id:ebi-fl187-GCST90094846 | MR Egger | 14 | -1.524751784 | 2.13193455595879 | 0.488169869009128 | -5.703343513 | 2.65383994608723 | 0.217675080686798 | 0.00333479685914213 | 14.2084938763535 | 0.999950574162623 |
| ebi-fl187-GCST90094846 | ebi-gm430-GCST90011693 | TestASV_2 (Bacteroides) prevalence \|\| id:ebi-gm430-GCST90011693 | Spicy food liking \|\| id:ebi-fl187-GCST90094846 | MR Egger | 14 | -1.766574567 | 1.08202272161722 | 0.128486294901153 | -3.887339101 | 0.354189967314172 | 0.170917453507653 | 0.0204998214747323 | 1.42502586910563 | 0.999950574162623 |
| ebi-fl187-GCST90094846 | ebi-gm430-GCST90011716 | TestASV_40 (Barnesiella) prevalence \|\| id:ebi-gm430-GCST90011716 | Spicy food liking \|\| id:ebi-fl187-GCST90094846 | MR Egger | 14 | 0.00942073386012964 | 2.35957300852542 | 0.99688001352268 | -4.615342363 | 4.63418383056996 | 1.00946524865088 | 0.00989879384750063 | 102.943864063912 | 0.999950574162623 |
| ebi-fl187-GCST90094846 | ebi-gm430-GCST90011718 | TestASV_42 (Alphaproteobacteria) prevalence \|\| id:ebi-gm430-GCST90011718 | Spicy food liking \|\| id:ebi-fl187-GCST90094846 | MR Egger | 14 | -2.215759009 | 1.78219793437728 | 0.237509798667255 | -5.708866961 | 1.2773489420744 | 0.109070697148919 | 0.00331642806030406 | 3.58711745294439 | 0.999950574162623 |
| ebi-fl187-GCST90094846 | ebi-hbsm44-GCST90104929 | Sulcal depth of the right inferior frontal sulcus \|\| id:ebi-hbsm44-GCST90104929 | Spicy food liking \|\| id:ebi-fl187-GCST90094846 | MR Egger | 12 | 1.40816256009406 | 0.939276901464797 | 0.16471172033924 | -0.432820167 | 3.24914528696506 | 4.08843624227825 | 0.648677135016411 | 25.7683059951719 | 0.999950574162623 |
| ebi-fl187-GCST90094846 | ebi-hbsm44-GCST90104935 | Sulcal opening distance of the left calloso-marginal posterior fissure \|\| id:ebi-hbsm44-GCST90104935 | Spicy food liking \|\| id:ebi-fl187-GCST90094846 | MR Egger | 13 | -0.321799272 | 0.251310141412376 | 0.226715610765941 | -0.814367149 | 0.170768605067088 | 0.724843672075568 | 0.442919540640289 | 1.18621623283649 | 0.999950574162623 |
| ebi-fl187-GCST90094846 | ebi-hbsm44-GCST90104936 | Sulcal opening distance of the left anterior inferior temporal sulcus \|\| id:ebi-hbsm44-GCST90104936 | Spicy food liking \|\| id:ebi-fl187-GCST90094846 | MR Egger | 14 | -0.015736351 | 0.137022828317708 | 0.910467470627832 | -0.284301095 | 0.25282839224023 | 0.984386818187453 | 0.752540024728826 | 1.28766228503314 | 0.999950574162623 |
| ebi-fl187-GCST90094846 | ebi-hbsm44-GCST90104941 | Sulcal opening distance of the right posterior inferior temporal sulcus \|\| id:ebi-hbsm44-GCST90104941 | Spicy food liking \|\| id:ebi-fl187-GCST90094846 | MR Egger | 14 | -0.015926939 | 0.0961508278625132 | 0.871193998405818 | -0.204382562 | 0.17252868362033 | 0.984199224017721 | 0.815150466262471 | 1.18830590504157 | 0.999950574162623 |
| ebi-fl187-GCST90094846 | ebi-IPs91-GCST90274819 | Leukemia inhibitory factor levels \|\| id:ebi-IPs91-GCST90274819 | Spicy food liking \|\| id:ebi-fl187-GCST90094846 | MR Egger | 16 | 0.37845395490557 | 0.472560982841372 | 0.4365951885836 | -0.547765571 | 1.30467348127466 | 1.46002557823463 | 0.578240404834497 | 3.68648519072181 | 0.999950574162623 |
| ebi-fl187-GCST90094846 | ebi-IPs91-GCST90274837 | STAM binding protein levels \|\| id:ebi-IPs91-GCST90274837 | Spicy food liking \|\| id:ebi-fl187-GCST90094846 | MR Egger | 16 | -0.199560058 | 0.325821797282317 | 0.550038384913251 | -0.83817078 | 0.439050664973755 | 0.819091026612884 | 0.432500939493643 | 1.55123387862053 | 0.999950574162623 |
| ebi-fl187-GCST90094846 | ebi-IPs91-GCST90274840 | TNF-beta levels \|\| id:ebi-IPs91-GCST90274840 | Spicy food liking \|\| id:ebi-fl187-GCST90094846 | MR Egger | 16 | -0.097892051 | 0.371534137443726 | 0.796018531297914 | -0.826098961 | 0.630314858269516 | 0.906746780770226 | 0.437753654048577 | 1.87820185356107 | 0.999950574162623 |
| ebi-fl187-GCST90094846 | ebi-IPs91-GCST90274842 | Tumor necrosis factor ligand superfamily member 14 levels \|\| id:ebi-IPs91-GCST90274842 | Spicy food liking \|\| id:ebi-fl187-GCST90094846 | MR Egger | 16 | 0.15114877398359 | 0.372101873470657 | 0.690734578002015 | -0.578170898 | 0.880468445986079 | 1.16316969459889 | 0.560923414950134 | 2.41202934727472 | 0.999950574162623 |
| ebi-fl187-GCST90094846 | ebi-met1400-GCST90199628 | 3-methylhistidine levels \|\| id:ebi-met1400-GCST90199628 | Spicy food liking \|\| id:ebi-fl187-GCST90094846 | MR Egger | 16 | 0.967576302897897 | 0.528415007875828 | 0.0884491936892221 | -0.068117113 | 2.00326971833452 | 2.63155862280531 | 0.934151066367925 | 7.41325577263051 | 0.999950574162623 |
| ebi-fl187-GCST90094846 | ebi-met1400-GCST90199651 | Docosahexaenoate DHA; 22:6n3 levels \|\| id:ebi-met1400-GCST90199651 | Spicy food liking \|\| id:ebi-fl187-GCST90094846 | MR Egger | 16 | -0.13725265 | 0.412354492835168 | 0.744178566302578 | -0.945467456 | 0.670962155731404 | 0.871749950485344 | 0.388497922692939 | 1.95611850612609 | 0.999950574162623 |
| ebi-fl187-GCST90094846 | ebi-met1400-GCST90199652 | 1-stearoyl-2-oleoyl-GPS (18:0/18:1) levels \|\| id:ebi-met1400-GCST90199652 | Spicy food liking \|\| id:ebi-fl187-GCST90094846 | MR Egger | 16 | -0.198321414 | 0.464339196802917 | 0.67579871292201 | -1.10842624 | 0.711783411827986 | 0.820106217229583 | 0.330078016231679 | 2.03762193925251 | 0.999950574162623 |
| ebi-fl187-GCST90094846 | ebi-met1400-GCST90199653 | 1-myristoyl-2-palmitoyl-gpc (14:0/16:0) levels \|\| id:ebi-met1400-GCST90199653 | Spicy food liking \|\| id:ebi-fl187-GCST90094846 | MR Egger | 16 | -0.185692115 | 0.433930667539442 | 0.675211495301357 | -1.036196223 | 0.66481199354224 | 0.830529263260879 | 0.35480170493672 | 1.94412497892501 | 0.999950574162623 |
| ebi-fl187-GCST90094846 | ebi-met1400-GCST90199667 | Erythritol levels in elite athletes \|\| id:ebi-met1400-GCST90199667 | Spicy food liking \|\| id:ebi-fl187-GCST90094846 | MR Egger | 16 | 0.093558515522684 | 0.572798133338408 | 0.872588331185394 | -1.029125826 | 1.21624285686596 | 1.09807485588553 | 0.35731918328458 | 3.37448546155364 | 0.999950574162623 |
| ebi-fl187-GCST90094846 | ebi-met1400-GCST90199674 | 3-hydroxymyristate levels \|\| id:ebi-met1400-GCST90199674 | Spicy food liking \|\| id:ebi-fl187-GCST90094846 | MR Egger | 16 | -0.283236926 | 0.420428761526189 | 0.511480856296702 | -1.107277299 | 0.54080344630197 | 0.753341280359815 | 0.330457474263755 | 1.71738613556429 | 0.999950574162623 |
| ebi-fl187-GCST90094846 | ebi-met1400-GCST90199685 | 2-linoleoylglycerol (18:2) levels \|\| id:ebi-met1400-GCST90199685 | Spicy food liking \|\| id:ebi-fl187-GCST90094846 | MR Egger | 16 | -0.487334089 | 0.53012378818741 | 0.373515169645791 | -1.526376714 | 0.551708535352297 | 0.614261780252177 | 0.217321660973646 | 1.73621687312766 | 0.999950574162623 |
| ebi-fl187-GCST90094846 | ebi-met1400-GCST90199703 | Gamma-glutamylvaline levels \|\| id:ebi-met1400-GCST90199703 | Spicy food liking \|\| id:ebi-fl187-GCST90094846 | MR Egger | 16 | 0.645021678233511 | 0.420887791272048 | 0.147674615053967 | -0.179918393 | 1.46996174912673 | 1.90602834815167 | 0.835338378373078 | 4.34906878220222 | 0.999950574162623 |
| ebi-fl187-GCST90094846 | ebi-met1400-GCST90199779 | Gamma-glutamyl-2-aminobutyrate levels \|\| id:ebi-met1400-GCST90199779 | Spicy food liking \|\| id:ebi-fl187-GCST90094846 | MR Egger | 16 | 0.666632213534307 | 0.446442636242296 | 0.157574032724144 | -0.208395354 | 1.54165978056921 | 1.94766693667194 | 0.811885991284168 | 4.67233889601298 | 0.999950574162623 |
| ebi-fl187-GCST90094846 | ebi-met1400-GCST90199824 | 4-vinylphenol sulfate levels \|\| id:ebi-met1400-GCST90199824 | Spicy food liking \|\| id:ebi-fl187-GCST90094846 | MR Egger | 16 | 0.181126630790035 | 0.431609255658933 | 0.681106733623796 | -0.66482751 | 1.02708077188154 | 1.19856694516306 | 0.514362242243338 | 2.7929008081388 | 0.999950574162623 |
| ebi-fl187-GCST90094846 | ebi-met1400-GCST90199830 | 4-ethylphenylsulfate levels \|\| id:ebi-met1400-GCST90199830 | Spicy food liking \|\| id:ebi-fl187-GCST90094846 | MR Egger | 16 | 0.126122626086156 | 0.568454239877012 | 0.827619305592334 | -0.988047684 | 1.2402929362451 | 1.13442126939446 | 0.372302834714413 | 3.45662588747598 | 0.999950574162623 |
| ebi-fl187-GCST90094846 | ebi-met1400-GCST90199833 | Hexanoylglutamine levels \|\| id:ebi-met1400-GCST90199833 | Spicy food liking \|\| id:ebi-fl187-GCST90094846 | MR Egger | 15 | -0.088516302 | 0.433105576666562 | 0.841224743122243 | -0.937403232 | 0.760370628299814 | 0.915288189616837 | 0.391643523312352 | 2.13906887305762 | 0.999950574162623 |
| ebi-fl187-GCST90094846 | ebi-met1400-GCST90199837 | Carotenoid levels (cryptoxanthin) \|\| id:ebi-met1400-GCST90199837 | Spicy food liking \|\| id:ebi-fl187-GCST90094846 | MR Egger | 16 | -0.375665296 | 0.44261203292612 | 0.410300656479002 | -1.243184881 | 0.491854288305927 | 0.686832179895322 | 0.288464029832323 | 1.63534581283487 | 0.999950574162623 |
| ebi-fl187-GCST90094846 | ebi-met1400-GCST90199869 | 2-hydroxyhippurate levels \|\| id:ebi-met1400-GCST90199869 | Spicy food liking \|\| id:ebi-fl187-GCST90094846 | MR Egger | 16 | 0.774807387628471 | 0.434330598695396 | 0.0961201447359638 | -0.076480586 | 1.62609536107145 | 2.17017408454759 | 0.926370898850711 | 5.08398478739447 | 0.999950574162623 |
| ebi-fl187-GCST90094846 | ebi-met1400-GCST90199902 | Hydantoin-5-propionate levels \|\| id:ebi-met1400-GCST90199902 | Spicy food liking \|\| id:ebi-fl187-GCST90094846 | MR Egger | 16 | -0.119585864 | 0.433645128209047 | 0.786754260386488 | -0.969530315 | 0.730358587706299 | 0.887287818836506 | 0.379261129588419 | 2.07582483949783 | 0.999950574162623 |
| ebi-fl187-GCST90094846 | ebi-met1400-GCST90199904 | Pantoate levels \|\| id:ebi-met1400-GCST90199904 | Spicy food liking \|\| id:ebi-fl187-GCST90094846 | MR Egger | 16 | -0.528039657 | 0.545700783591575 | 0.349646190092492 | -1.597613193 | 0.541533878442645 | 0.589759968803959 | 0.202378981513919 | 1.71864102784669 | 0.999950574162623 |
| ebi-fl187-GCST90094846 | ebi-met1400-GCST90199936 | 3-methyl catechol sulfate (2) levels \|\| id:ebi-met1400-GCST90199936 | Spicy food liking \|\| id:ebi-fl187-GCST90094846 | MR Egger | 16 | 0.449561942654729 | 0.500852300475281 | 0.384578544816515 | -0.532108566 | 1.43123245158628 | 1.5676253252707 | 0.587365164653807 | 4.18385241126531 | 0.999950574162623 |
| ebi-fl187-GCST90094846 | ebi-met1400-GCST90199977 | 4-hydroxychlorothalonil levels \|\| id:ebi-met1400-GCST90199977 | Spicy food liking \|\| id:ebi-fl187-GCST90094846 | MR Egger | 16 | -0.369485368 | 0.42800827739011 | 0.402538029233531 | -1.208381591 | 0.469410856155027 | 0.691089896437333 | 0.298680275263743 | 1.59905184410328 | 0.999950574162623 |
| ebi-fl187-GCST90094846 | ebi-met1400-GCST90200040 | Sphingomyelin (d18:2/24:1, d18:1/24:2) levels \|\| id:ebi-met1400-GCST90200040 | Spicy food liking \|\| id:ebi-fl187-GCST90094846 | MR Egger | 16 | -0.488754957 | 0.393179589358727 | 0.234253379935861 | -1.259386953 | 0.281877037650493 | 0.613389615108979 | 0.283827973166409 | 1.32561570914276 | 0.999950574162623 |
| ebi-fl187-GCST90094846 | ebi-met1400-GCST90200077 | 1-stearoyl-2-oleoyl-GPI (18:0/18:1) levels \|\| id:ebi-met1400-GCST90200077 | Spicy food liking \|\| id:ebi-fl187-GCST90094846 | MR Egger | 16 | 0.279391248050812 | 0.426874481496485 | 0.523394511616408 | -0.557282736 | 1.11606523178392 | 1.32232459959743 | 0.572763300535592 | 3.05281840695001 | 0.999950574162623 |
| ebi-fl187-GCST90094846 | ebi-met1400-GCST90200080 | 1-palmitoyl-2-oleoyl-GPI (16:0/18:1) levels \|\| id:ebi-met1400-GCST90200080 | Spicy food liking \|\| id:ebi-fl187-GCST90094846 | MR Egger | 16 | 0.401700584323118 | 0.43049583551017 | 0.366577102857478 | -0.442071253 | 1.24547242192305 | 1.4943638297271 | 0.642703839069425 | 3.47457587748348 | 0.999950574162623 |
| ebi-fl187-GCST90094846 | ebi-met1400-GCST90200082 | 1-oleoyl-2-linoleoyl-GPE (18:1/18:2) levels \|\| id:ebi-met1400-GCST90200082 | Spicy food liking \|\| id:ebi-fl187-GCST90094846 | MR Egger | 16 | -0.204366767 | 0.432793085491188 | 0.644056937695638 | -1.052641214 | 0.643907681023304 | 0.815163341715736 | 0.349014708104385 | 1.90390622013106 | 0.999950574162623 |
| ebi-fl187-GCST90094846 | ebi-met1400-GCST90200100 | Perfluorooctanesulfonate (PFOS) levels \|\| id:ebi-met1400-GCST90200100 | Spicy food liking \|\| id:ebi-fl187-GCST90094846 | MR Egger | 16 | -0.367951793 | 0.580092806014368 | 0.536113937629401 | -1.504933693 | 0.769030106758965 | 0.692150547365724 | 0.222032015661278 | 2.1576725266032 | 0.999950574162623 |
| ebi-fl187-GCST90094846 | ebi-met1400-GCST90200102 | 1-palmitoleoyl-2-linolenoyl-GPC (16:1/18:3) levels \|\| id:ebi-met1400-GCST90200102 | Spicy food liking \|\| id:ebi-fl187-GCST90094846 | MR Egger | 16 | -0.27213515 | 0.423064260811982 | 0.530459079831064 | -1.101341102 | 0.557070800744267 | 0.761751303103302 | 0.332424968951727 | 1.74555193495059 | 0.999950574162623 |
| ebi-fl187-GCST90094846 | ebi-met1400-GCST90200114 | Glycosyl-N-tricosanoyl-sphingadienine (d18:2/23:0) levels \|\| id:ebi-met1400-GCST90200114 | Spicy food liking \|\| id:ebi-fl187-GCST90094846 | MR Egger | 16 | -1.074156914 | 0.451620610834207 | 0.0321683358141811 | -1.959333311 | -0.188980516 | 0.341585620146403 | 0.140952361022912 | 0.827802635188468 | 0.999950574162623 |
| ebi-fl187-GCST90094846 | ebi-met1400-GCST90200135 | Arachidoylcarnitine (C20) levels \|\| id:ebi-met1400-GCST90200135 | Spicy food liking \|\| id:ebi-fl187-GCST90094846 | MR Egger | 16 | -0.954280677 | 0.441900843778059 | 0.0486488667022263 | -1.820406331 | -0.088155023 | 0.385089048434037 | 0.161959928285458 | 0.915618923728231 | 0.999950574162623 |
| ebi-fl187-GCST90094846 | ebi-met1400-GCST90200146 | Docosahexaenoylcarnitine (C22:6) levels \|\| id:ebi-met1400-GCST90200146 | Spicy food liking \|\| id:ebi-fl187-GCST90094846 | MR Egger | 16 | 0.481306748734752 | 0.505987007102767 | 0.357625145182447 | -0.510427785 | 1.47304128265618 | 1.6181875857725 | 0.600238750636549 | 4.36248252877927 | 0.999950574162623 |
| ebi-fl187-GCST90094846 | ebi-met1400-GCST90200162 | 3-hydroxyphenylacetoylglutamine levels \|\| id:ebi-met1400-GCST90200162 | Spicy food liking \|\| id:ebi-fl187-GCST90094846 | MR Egger | 16 | 0.337510595046387 | 0.566654561693271 | 0.560935404343716 | -0.773132346 | 1.4481535359652 | 1.40145445678829 | 0.461565020315378 | 4.25525009046344 | 0.999950574162623 |
| ebi-fl187-GCST90094846 | ebi-met1400-GCST90200172 | 3-carboxy-4-methyl-5-pentyl-2-furanpropionate (3-CMPFP) levels \|\| id:ebi-met1400-GCST90200172 | Spicy food liking \|\| id:ebi-fl187-GCST90094846 | MR Egger | 16 | 0.830846301050448 | 0.428449733008195 | 0.0729023014307183 | -0.008915176 | 1.67060777774651 | 2.29526039982145 | 0.991124446698636 | 5.31539739589373 | 0.999950574162623 |
| ebi-fl187-GCST90094846 | ebi-met1400-GCST90200222 | Dihydrocaffeate sulfate (2) levels \|\| id:ebi-met1400-GCST90200222 | Spicy food liking \|\| id:ebi-fl187-GCST90094846 | MR Egger | 16 | 0.290883459010493 | 0.489670737391576 | 0.561962861690588 | -0.668871186 | 1.25063810429798 | 1.33760868862597 | 0.512286527588791 | 3.49257087104945 | 0.999950574162623 |
| ebi-fl187-GCST90094846 | ebi-met1400-GCST90200223 | 4-methylhexanoylglutamine levels \|\| id:ebi-met1400-GCST90200223 | Spicy food liking \|\| id:ebi-fl187-GCST90094846 | MR Egger | 16 | -0.155955752 | 0.669445631599661 | 0.819162013261987 | -1.46806919 | 1.15615768544776 | 0.855597047574464 | 0.230369856375561 | 3.17770006603954 | 0.999950574162623 |
| ebi-fl187-GCST90094846 | ebi-met1400-GCST90200224 | 2-ketocaprylate levels \|\| id:ebi-met1400-GCST90200224 | Spicy food liking \|\| id:ebi-fl187-GCST90094846 | MR Egger | 16 | -0.320902087 | 0.436735157463307 | 0.474605319828426 | -1.176902995 | 0.535098821782787 | 0.725494282946566 | 0.308231857390301 | 1.70761698367105 | 0.999950574162623 |
| ebi-fl187-GCST90094846 | ebi-met1400-GCST90200243 | Hydroxypalmitoyl sphingomyelin (d18:1/16:0(OH)) levels \|\| id:ebi-met1400-GCST90200243 | Spicy food liking \|\| id:ebi-fl187-GCST90094846 | MR Egger | 16 | -0.724599739 | 0.421366374469411 | 0.10751680228251 | -1.550477833 | 0.101278354490511 | 0.484518463765881 | 0.212146578876365 | 1.10658462169622 | 0.999950574162623 |
| ebi-fl187-GCST90094846 | ebi-met1400-GCST90200273 | 2-methoxyhydroquinone sulfate (2) levels \|\| id:ebi-met1400-GCST90200273 | Spicy food liking \|\| id:ebi-fl187-GCST90094846 | MR Egger | 16 | 0.183610854474728 | 0.636402754315789 | 0.777181248849438 | -1.063738544 | 1.43096025293367 | 1.20154815501881 | 0.345162988186558 | 4.18271372725742 | 0.999950574162623 |
| ebi-fl187-GCST90094846 | ebi-met1400-GCST90200276 | 2,4-di-tert-butylphenol levels \|\| id:ebi-met1400-GCST90200276 | Spicy food liking \|\| id:ebi-fl187-GCST90094846 | MR Egger | 16 | -0.819947039 | 0.516202970043743 | 0.134513207168402 | -1.83170486 | 0.191810782367409 | 0.440454980860514 | 0.160140318059749 | 1.21144126922774 | 0.999950574162623 |
| ebi-fl187-GCST90094846 | ebi-met1400-GCST90200289 | N-acetylmethionine levels \|\| id:ebi-met1400-GCST90200289 | Spicy food liking \|\| id:ebi-fl187-GCST90094846 | MR Egger | 16 | 0.564833646056368 | 0.477932723847305 | 0.25696025885555 | -0.371914493 | 1.50158178479709 | 1.75915511598835 | 0.689413189875212 | 4.48878374762186 | 0.999950574162623 |
| ebi-fl187-GCST90094846 | ebi-met1400-GCST90200308 | 3-hydroxyisobutyrate levels \|\| id:ebi-met1400-GCST90200308 | Spicy food liking \|\| id:ebi-fl187-GCST90094846 | MR Egger | 16 | -0.119344135 | 0.491095249978002 | 0.811517216618396 | -1.081890825 | 0.843202554731162 | 0.887502327389125 | 0.338954016555048 | 2.32379716023573 | 0.999950574162623 |
| ebi-fl187-GCST90094846 | ebi-met1400-GCST90200383 | Cystathionine levels \|\| id:ebi-met1400-GCST90200383 | Spicy food liking \|\| id:ebi-fl187-GCST90094846 | MR Egger | 16 | 0.418258751254703 | 0.443538167973894 | 0.361668057841982 | -0.451076058 | 1.28759356048353 | 1.51931374788447 | 0.636942395787379 | 3.62405498484248 | 0.999950574162623 |
| ebi-fl187-GCST90094846 | ebi-met1400-GCST90200384 | Guanidinoacetate levels \|\| id:ebi-met1400-GCST90200384 | Spicy food liking \|\| id:ebi-fl187-GCST90094846 | MR Egger | 16 | 0.793978695942178 | 0.476093204138879 | 0.117579588729655 | -0.139163984 | 1.72712137605438 | 2.21218053373477 | 0.870085336537201 | 5.62443993518077 | 0.999950574162623 |
| ebi-fl187-GCST90094846 | ebi-met1400-GCST90200403 | Citrulline levels \|\| id:ebi-met1400-GCST90200403 | Spicy food liking \|\| id:ebi-fl187-GCST90094846 | MR Egger | 16 | 0.709841819692159 | 0.432050930417283 | 0.12265339509519 | -0.136978004 | 1.55666164331003 | 2.03366954672823 | 0.871989406264921 | 4.74296109055399 | 0.999950574162623 |
| ebi-fl187-GCST90094846 | ebi-met1400-GCST90200434 | 12,13-DiHOME levels \|\| id:ebi-met1400-GCST90200434 | Spicy food liking \|\| id:ebi-fl187-GCST90094846 | MR Egger | 16 | 0.853634702436415 | 0.435486790481702 | 0.0701867401699161 | 8.05930922784803e-05 | 1.70718881178055 | 2.34816624538994 | 1.00008059633999 | 5.51344035287551 | 0.999950574162623 |
| ebi-fl187-GCST90094846 | ebi-met1400-GCST90200437 | Fructose levels \|\| id:ebi-met1400-GCST90200437 | Spicy food liking \|\| id:ebi-fl187-GCST90094846 | MR Egger | 16 | -0.358553598 | 0.433804490835462 | 0.422365569593542 | -1.2088104 | 0.491703204530743 | 0.698686176997924 | 0.298552226129914 | 1.63509875727924 | 0.999950574162623 |
| ebi-fl187-GCST90094846 | ebi-met1400-GCST90200448 | N-stearoyl-sphinganine (d18:0/18:0) levels \|\| id:ebi-met1400-GCST90200448 | Spicy food liking \|\| id:ebi-fl187-GCST90094846 | MR Egger | 16 | 0.191107669433437 | 0.480212649885431 | 0.696658555411266 | -0.750109124 | 1.13232446320888 | 1.21058978858121 | 0.472315008864157 | 3.1028606093663 | 0.999950574162623 |
| ebi-fl187-GCST90094846 | ebi-met1400-GCST90200468 | X-11847 levels \|\| id:ebi-met1400-GCST90200468 | Spicy food liking \|\| id:ebi-fl187-GCST90094846 | MR Egger | 16 | -0.229168672 | 0.51911593836562 | 0.665622128931575 | -1.246635911 | 0.788298567484194 | 0.795194395392381 | 0.287470247542362 | 2.19965068339906 | 0.999950574162623 |
| ebi-fl187-GCST90094846 | ebi-met1400-GCST90200476 | X-11843 levels \|\| id:ebi-met1400-GCST90200476 | Spicy food liking \|\| id:ebi-fl187-GCST90094846 | MR Egger | 16 | -0.328195299 | 0.455004151201356 | 0.482596701639947 | -1.220003436 | 0.563612837066045 | 0.720222347057091 | 0.295229152620221 | 1.75700883397413 | 0.999950574162623 |
| ebi-fl187-GCST90094846 | ebi-met1400-GCST90200511 | X-12906 levels \|\| id:ebi-met1400-GCST90200511 | Spicy food liking \|\| id:ebi-fl187-GCST90094846 | MR Egger | 16 | 0.460633124556951 | 0.497141159348731 | 0.369851041172532 | -0.513763548 | 1.43502979688046 | 1.58507721868684 | 0.59823983248491 | 4.19977014697259 | 0.999950574162623 |
| ebi-fl187-GCST90094846 | ebi-met1400-GCST90200515 | X-12849 levels \|\| id:ebi-met1400-GCST90200515 | Spicy food liking \|\| id:ebi-fl187-GCST90094846 | MR Egger | 16 | 0.585342299494572 | 0.557286799267619 | 0.311354041114135 | -0.506939827 | 1.6776244260591 | 1.79560551531765 | 0.602336013715438 | 5.35282482405636 | 0.999950574162623 |
| ebi-fl187-GCST90094846 | ebi-met1400-GCST90200551 | X-18886 levels \|\| id:ebi-met1400-GCST90200551 | Spicy food liking \|\| id:ebi-fl187-GCST90094846 | MR Egger | 16 | -0.493213789 | 0.425279916638917 | 0.265552235755989 | -1.326762426 | 0.340334847672328 | 0.61066070260527 | 0.265334913730672 | 1.40541811276627 | 0.999950574162623 |
| ebi-fl187-GCST90094846 | ebi-met1400-GCST90200564 | X-21319 levels \|\| id:ebi-met1400-GCST90200564 | Spicy food liking \|\| id:ebi-fl187-GCST90094846 | MR Egger | 16 | -0.306545014 | 0.435925223745201 | 0.493456896245806 | -1.160958453 | 0.547868424221736 | 0.735985387552779 | 0.313185863099349 | 1.72956239253808 | 0.999950574162623 |
| ebi-fl187-GCST90094846 | ebi-met1400-GCST90200579 | X-21442 levels \|\| id:ebi-met1400-GCST90200579 | Spicy food liking \|\| id:ebi-fl187-GCST90094846 | MR Egger | 16 | -0.048146618 | 0.581912417054289 | 0.935230736588898 | -1.188694956 | 1.09240171928373 | 0.952994050616125 | 0.304618545511725 | 2.98142602901626 | 0.999950574162623 |
| ebi-fl187-GCST90094846 | ebi-met1400-GCST90200581 | X-21441 levels \|\| id:ebi-met1400-GCST90200581 | Spicy food liking \|\| id:ebi-fl187-GCST90094846 | MR Egger | 16 | 0.296856493408671 | 0.432413420607869 | 0.503603648838039 | -0.550673811 | 1.14438679780009 | 1.34562217993921 | 0.576561186206078 | 3.14051499557092 | 0.999950574162623 |
| ebi-fl187-GCST90094846 | ebi-met1400-GCST90200648 | X-24585 levels \|\| id:ebi-met1400-GCST90200648 | Spicy food liking \|\| id:ebi-fl187-GCST90094846 | MR Egger | 16 | 0.69755117790671 | 0.421675189860776 | 0.120316789364075 | -0.128932194 | 1.52403455003383 | 2.00882741838966 | 0.879033567081198 | 4.59070932896617 | 0.999950574162623 |
| ebi-fl187-GCST90094846 | ebi-met1400-GCST90200720 | Adenosine 5'-diphosphate (ADP) to pantothenate ratio \|\| id:ebi-met1400-GCST90200720 | Spicy food liking \|\| id:ebi-fl187-GCST90094846 | MR Egger | 16 | -0.600547857 | 0.573938976903116 | 0.313124314426906 | -1.725468252 | 0.524372537634115 | 0.548511048091983 | 0.178089641446087 | 1.68939848177552 | 0.999950574162623 |
| ebi-fl187-GCST90094846 | ebi-met1400-GCST90200724 | Cholate to taurocholate ratio \|\| id:ebi-met1400-GCST90200724 | Spicy food liking \|\| id:ebi-fl187-GCST90094846 | MR Egger | 16 | 0.29854892243518 | 0.507826668278704 | 0.56596557375842 | -0.696791347 | 1.29388919226144 | 1.34790147820647 | 0.498181232543214 | 3.64694267119666 | 0.999950574162623 |
| ebi-fl187-GCST90094846 | ebi-met1400-GCST90200726 | N-acetylputrescine to (N(1) + N(8))-acetylspermidine ratio \|\| id:ebi-met1400-GCST90200726 | Spicy food liking \|\| id:ebi-fl187-GCST90094846 | MR Egger | 16 | -0.418930367 | 0.429324144147851 | 0.345728789358611 | -1.26040569 | 0.422544955328601 | 0.657749994646275 | 0.283538974313393 | 1.52583981269181 | 0.999950574162623 |
| ebi-fl187-GCST90094846 | ebi-met1400-GCST90200744 | Arginine to glutamate ratio \|\| id:ebi-met1400-GCST90200744 | Spicy food liking \|\| id:ebi-fl187-GCST90094846 | MR Egger | 16 | -0.564347117 | 0.427105737145961 | 0.207580158765779 | -1.401474361 | 0.272780128229488 | 0.568731340835928 | 0.24623365878824 | 1.31361138700864 | 0.999950574162623 |
| ebi-fl187-GCST90094846 | ebi-met1400-GCST90200759 | Histidine to trans-urocanate ratio \|\| id:ebi-met1400-GCST90200759 | Spicy food liking \|\| id:ebi-fl187-GCST90094846 | MR Egger | 16 | -0.661274655 | 0.542357849998537 | 0.242890636870594 | -1.724296041 | 0.401746731358401 | 0.516192947237982 | 0.178298522519103 | 1.49443279177866 | 0.999950574162623 |
| ebi-fl187-GCST90094846 | ebi-met1400-GCST90200795 | Oleoyl-linoleoyl-glycerol (18:1 to 18:2) [2] to linoleoyl-arachidonoyl-glycerol (18:2 to 20:4) [2] ratio \|\| id:ebi-met1400-GCST90200795 | Spicy food liking \|\| id:ebi-fl187-GCST90094846 | MR Egger | 16 | -0.794733451 | 0.463368441811356 | 0.108361537800643 | -1.702935597 | 0.113468695168906 | 0.451701619591424 | 0.182148025285387 | 1.12015682202339 | 0.999950574162623 |
| ebi-fl187-GCST90094846 | ebi-met1400-GCST90200811 | Choline to taurocholate ratio \|\| id:ebi-met1400-GCST90200811 | Spicy food liking \|\| id:ebi-fl187-GCST90094846 | MR Egger | 16 | 0.288410911986877 | 0.451243299645801 | 0.533045843072057 | -0.596025955 | 1.17284777929265 | 1.33430547360423 | 0.550996977503482 | 3.23118123978992 | 0.999950574162623 |
| ebi-fl187-GCST90094846 | ebi-met1400-GCST90200831 | Cholate to phosphate ratio \|\| id:ebi-met1400-GCST90200831 | Spicy food liking \|\| id:ebi-fl187-GCST90094846 | MR Egger | 16 | 0.231058973225208 | 0.449872446975151 | 0.615534499825581 | -0.650691023 | 1.1128089692965 | 1.25993353960273 | 0.521685155815577 | 3.04289379622877 | 0.999950574162623 |
| ebi-fl187-GCST90094846 | ebi-met1400-GCST90200841 | Adenosine 5'-diphosphate (ADP) to oxalate (ethanedioate) ratio \|\| id:ebi-met1400-GCST90200841 | Spicy food liking \|\| id:ebi-fl187-GCST90094846 | MR Egger | 16 | -0.008437908 | 0.582019691734664 | 0.988637545449547 | -1.149196504 | 1.13232068791976 | 0.991597591347939 | 0.316891288096719 | 3.1028488951925 | 0.999950574162623 |
| ebi-fl187-GCST90094846 | ebi-met1400-GCST90200844 | Aspartate to phosphate ratio \|\| id:ebi-met1400-GCST90200844 | Spicy food liking \|\| id:ebi-fl187-GCST90094846 | MR Egger | 16 | 0.472733966906434 | 0.425503655555338 | 0.285285743136201 | -0.361253198 | 1.3067211317949 | 1.60437450948354 | 0.696802547130449 | 3.69404155779969 | 0.999950574162623 |
| ebi-fl187-GCST90094846 | ebi-met1400-GCST90200870 | Adenosine 5'-monophosphate (AMP) to acetoacetate ratio \|\| id:ebi-met1400-GCST90200870 | Spicy food liking \|\| id:ebi-fl187-GCST90094846 | MR Egger | 16 | -0.072982817 | 0.54367729402014 | 0.895124122251094 | -1.138590313 | 0.992624679297984 | 0.929616803511298 | 0.320270184374474 | 2.69830737774864 | 0.999950574162623 |
| ebi-fl187-GCST90094846 | ebi-met1400-GCST90200887 | Cholesterol to cortisol ratio \|\| id:ebi-met1400-GCST90200887 | Spicy food liking \|\| id:ebi-fl187-GCST90094846 | MR Egger | 16 | 0.577700317797396 | 0.419203610916105 | 0.189803322981808 | -0.24393876 | 1.39933939519296 | 1.78193582910577 | 0.783535616829635 | 4.05252196690029 | 0.999950574162623 |
| ebi-fl187-GCST90094846 | ebi-met1400-GCST90200890 | Cortisol to taurocholate ratio \|\| id:ebi-met1400-GCST90200890 | Spicy food liking \|\| id:ebi-fl187-GCST90094846 | MR Egger | 16 | -0.39054463 | 0.449568531190456 | 0.399653563611794 | -1.271698951 | 0.490609690887885 | 0.676688229243242 | 0.280354907579166 | 1.63331173172722 | 0.999950574162623 |
| ebi-fl187-GCST90094846 | ebi-met1400-GCST90200948 | Adenosine 5'-diphosphate (ADP) to mannitol to sorbitol ratio \|\| id:ebi-met1400-GCST90200948 | Spicy food liking \|\| id:ebi-fl187-GCST90094846 | MR Egger | 16 | 0.241290567560811 | 0.662631836607773 | 0.72119447774744 | -1.057467832 | 1.54004896731205 | 1.2728908423659 | 0.347334206233985 | 4.66481868902795 | 0.999950574162623 |
| ebi-fl187-GCST90094846 | ebi-met1400-GCST90200972 | Leucine to N-palmitoyl-sphingosine (d18:1 to 16:0) ratio \|\| id:ebi-met1400-GCST90200972 | Spicy food liking \|\| id:ebi-fl187-GCST90094846 | MR Egger | 16 | 0.00487098799133235 | 0.467434157969952 | 0.991832680415821 | -0.911299962 | 0.921041937612438 | 1.00488287053875 | 0.402001297952899 | 2.51190627653276 | 0.999950574162623 |
| ebi-fl187-GCST90094846 | ebi-met1400-GCST90200973 | Proline to glutamate ratio \|\| id:ebi-met1400-GCST90200973 | Spicy food liking \|\| id:ebi-fl187-GCST90094846 | MR Egger | 16 | -0.555270765 | 0.422654980341951 | 0.210046214921095 | -1.383674526 | 0.273132996703136 | 0.573916843696083 | 0.25065581741221 | 1.31407500084628 | 0.999950574162623 |
| ebi-fl187-GCST90094846 | ebi-met1400-GCST90200983 | Cholesterol to linoleoyl-arachidonoyl-glycerol (18:2 to 20:4) [1] ratio \|\| id:ebi-met1400-GCST90200983 | Spicy food liking \|\| id:ebi-fl187-GCST90094846 | MR Egger | 16 | -0.705135999 | 0.444548215558648 | 0.135019369349977 | -1.576450501 | 0.16617850349689 | 0.494041380554352 | 0.206707505578916 | 1.18078385695998 | 0.999950574162623 |
| ebi-fl187-GCST90094846 | ebi-met1400-GCST90201001 | Serine to alpha-ketobutyrate ratio \|\| id:ebi-met1400-GCST90201001 | Spicy food liking \|\| id:ebi-fl187-GCST90094846 | MR Egger | 16 | 0.0502487119336792 | 0.439034449499435 | 0.910504122365339 | -0.810258809 | 0.910756232952571 | 1.05153259256049 | 0.44474294781126 | 2.48620197050595 | 0.999950574162623 |
| ebi-fl187-GCST90094846 | ebi-met1400-GCST90201005 | Fructose to maltose ratio \|\| id:ebi-met1400-GCST90201005 | Spicy food liking \|\| id:ebi-fl187-GCST90094846 | MR Egger | 16 | 0.217186609772846 | 0.591455376427552 | 0.718955777038943 | -0.942065928 | 1.37643914757085 | 1.24257595732101 | 0.389821659403531 | 3.96077275971454 | 0.999950574162623 |
| ebi-fl187-GCST90094846 | ebi-met1400-GCST90201020 | Paraxanthine to 5-acetylamino-6-formylamino-3-methyluracil ratio \|\| id:ebi-met1400-GCST90201020 | Spicy food liking \|\| id:ebi-fl187-GCST90094846 | MR Egger | 16 | 0.58991275988222 | 0.517277397616183 | 0.273246457586474 | -0.423950939 | 1.60377645920994 | 1.80383104210076 | 0.654455989094976 | 4.97177271300684 | 0.999950574162623 |
| ebi-fl187-GCST90094846 | ebi-pw11-GCST90275197 | Placental weight (fetal genotype, adjusted for sex) \|\| id:ebi-pw11-GCST90275197 | Spicy food liking \|\| id:ebi-fl187-GCST90094846 | MR Egger | 15 | -0.168174866 | 0.176517043561803 | 0.358101503638765 | -0.514148271 | 0.177798539474473 | 0.845206024043805 | 0.598009719817731 | 1.19458463534284 | 0.999950574162623 |
| ebi-fl187-GCST90094846 | prot-a-1017 | FAS-associated factor 2 \|\| id:prot-a-1017 | Spicy food liking \|\| id:ebi-fl187-GCST90094846 | MR Egger | 16 | 0.194355751692474 | 0.899979273319281 | 0.832136977648797 | -1.569603624 | 1.95831512739826 | 1.2145282766004 | 0.208127662817009 | 7.08737567460633 | 0.999950574162623 |
| ebi-fl187-GCST90094846 | prot-a-1019 | Fas apoptotic inhibitory molecule 3 \|\| id:prot-a-1019 | Spicy food liking \|\| id:ebi-fl187-GCST90094846 | MR Egger | 16 | 1.18731486576964 | 0.692270757168543 | 0.108365330328503 | -0.169535818 | 2.54416554981999 | 3.27826679235492 | 0.844056521285508 | 12.7325989324615 | 0.999950574162623 |
| ebi-fl187-GCST90094846 | prot-a-1052 | MIP18 family protein FAM96A \|\| id:prot-a-1052 | Spicy food liking \|\| id:ebi-fl187-GCST90094846 | MR Egger | 16 | -0.412879567 | 0.749523205530371 | 0.590415078401594 | -1.88194505 | 1.0561859160875 | 0.661741973764256 | 0.152293598887768 | 2.87538309580643 | 0.999950574162623 |
| ebi-fl187-GCST90094846 | prot-a-1066 | F-box/LRR-repeat protein 4 \|\| id:prot-a-1066 | Spicy food liking \|\| id:ebi-fl187-GCST90094846 | MR Egger | 16 | -0.43230433 | 0.692270757168543 | 0.542360652615399 | -1.789155014 | 0.924546353603131 | 0.649011832566075 | 0.167101308211509 | 2.52072448336323 | 0.999950574162623 |
| ebi-fl187-GCST90094846 | prot-a-1076 | Low affinity immunoglobulin gamma Fc region receptor III-B \|\| id:prot-a-1076 | Spicy food liking \|\| id:ebi-fl187-GCST90094846 | MR Egger | 16 | -0.596385927 | 0.692361353673592 | 0.403541410272419 | -1.95341418 | 0.760642326081367 | 0.550798669824434 | 0.141789150526398 | 2.13965013228486 | 0.999950574162623 |
| ebi-fl187-GCST90094846 | prot-a-1081 | Fc receptor-like protein 3 \|\| id:prot-a-1081 | Spicy food liking \|\| id:ebi-fl187-GCST90094846 | MR Egger | 16 | -0.748032108 | 0.691390441186045 | 0.297570374198107 | -2.103157373 | 0.607093156301015 | 0.473297034144087 | 0.122070397357069 | 1.83508932042168 | 0.999950574162623 |
| ebi-fl187-GCST90094846 | prot-a-1105 | Fibroblast growth factor receptor 4 \|\| id:prot-a-1105 | Spicy food liking \|\| id:ebi-fl187-GCST90094846 | MR Egger | 16 | 0.279915082542712 | 0.84433548024644 | 0.745161336326933 | -1.374982459 | 1.93481262382573 | 1.3230174602885 | 0.252844030968656 | 6.92274677603614 | 0.999950574162623 |
| ebi-fl187-GCST90094846 | prot-a-1143 | Follistatin-related protein 1 \|\| id:prot-a-1143 | Spicy food liking \|\| id:ebi-fl187-GCST90094846 | MR Egger | 16 | 0.0126391350784542 | 0.692573430002273 | 0.985697331953335 | -1.344804788 | 1.37008305788291 | 1.01271934652442 | 0.260590573373439 | 3.93567757094235 | 0.999950574162623 |
| ebi-fl187-GCST90094846 | prot-a-1151 | Alpha-(1,3)-fucosyltransferase 10 \|\| id:prot-a-1151 | Spicy food liking \|\| id:ebi-fl187-GCST90094846 | MR Egger | 16 | -0.174840433 | 0.692511781109501 | 0.804344628912872 | -1.532163524 | 1.1824826580246 | 0.839590981137143 | 0.216067693637897 | 3.26246373874003 | 0.999950574162623 |
| ebi-fl187-GCST90094846 | prot-a-1165 | Growth arrest and DNA damage-inducible proteins-interacting protein 1 \|\| id:prot-a-1165 | Spicy food liking \|\| id:ebi-fl187-GCST90094846 | MR Egger | 16 | 0.676516503101848 | 0.691409015761852 | 0.344452773629741 | -0.678645168 | 2.03167817399508 | 1.96701369763561 | 0.507303838559891 | 7.62687484815739 | 0.999950574162623 |
| ebi-fl187-GCST90094846 | prot-a-1194 | Growth/differentiation factor 11/8 \|\| id:prot-a-1194 | Spicy food liking \|\| id:ebi-fl187-GCST90094846 | MR Egger | 16 | 0.056288787717055 | 0.7286894065629 | 0.939520485782334 | -1.371942449 | 1.48452002458034 | 1.05790314906245 | 0.25361384878251 | 4.41284684637237 | 0.999950574162623 |
| ebi-fl187-GCST90094846 | prot-a-1216 | D-glucuronyl C5-epimerase \|\| id:prot-a-1216 | Spicy food liking \|\| id:ebi-fl187-GCST90094846 | MR Egger | 16 | -0.290833638 | 0.794515933180146 | 0.719798966141861 | -1.848084867 | 1.26641759091525 | 0.747640046478031 | 0.157538584912133 | 3.54811895390221 | 0.999950574162623 |
| ebi-fl187-GCST90094846 | prot-a-1246 | Glypican-3 \|\| id:prot-a-1246 | Spicy food liking \|\| id:ebi-fl187-GCST90094846 | MR Egger | 16 | 1.44481525882166 | 1.0579144712176 | 0.193567764921139 | -0.628697105 | 3.51832762240815 | 4.24106857043849 | 0.533286164569983 | 33.7279753613424 | 0.999950574162623 |
| ebi-fl187-GCST90094846 | prot-a-1275 | Glutamate receptor 4 \|\| id:prot-a-1275 | Spicy food liking \|\| id:ebi-fl187-GCST90094846 | MR Egger | 16 | -0.012384558 | 1.15052503672143 | 0.991563412799491 | -2.26741363 | 2.24264451424946 | 0.987691815303296 | 0.103579729479208 | 9.41820496077802 | 0.999950574162623 |
| ebi-fl187-GCST90094846 | prot-a-1289 | Glutathione S-transferase P \|\| id:prot-a-1289 | Spicy food liking \|\| id:ebi-fl187-GCST90094846 | MR Egger | 16 | -0.502701496 | 0.692424876513628 | 0.479799969251676 | -1.859854254 | 0.8544512616571 | 0.604894330637406 | 0.155695320641633 | 2.35008444524462 | 0.999950574162623 |
| ebi-fl187-GCST90094846 | prot-a-130 | Apolipoprotein D \|\| id:prot-a-130 | Spicy food liking \|\| id:ebi-fl187-GCST90094846 | MR Egger | 16 | 0.596226287734573 | 0.692335442579752 | 0.403646955702282 | -0.76075118 | 1.95320375519089 | 1.81525560632905 | 0.467315257385664 | 7.05124188485388 | 0.999950574162623 |
| ebi-fl187-GCST90094846 | prot-a-1313 | Heparin-binding EGF-like growth factor \|\| id:prot-a-1313 | Spicy food liking \|\| id:ebi-fl187-GCST90094846 | MR Egger | 15 | -1.232232566 | 0.705459329385075 | 0.10425517111021 | -2.614932852 | 0.150467719229212 | 0.291640743005867 | 0.073172701934519 | 1.16237778204675 | 0.999950574162623 |
| ebi-fl187-GCST90094846 | prot-a-1347 | HLA class II histocompatibility antigen, DQ alpha 2 chain \|\| id:prot-a-1347 | Spicy food liking \|\| id:ebi-fl187-GCST90094846 | MR Egger | 15 | -0.231603759 | 0.705457229588114 | 0.747910495589494 | -1.614299929 | 1.15109241110425 | 0.793260383417253 | 0.199029956804494 | 3.16164483981378 | 0.999950574162623 |
| ebi-fl187-GCST90094846 | prot-a-1357 | Non-histone chromosomal protein HMG-14 \|\| id:prot-a-1357 | Spicy food liking \|\| id:ebi-fl187-GCST90094846 | MR Egger | 16 | -0.174450261 | 0.782898832212531 | 0.826889097783322 | -1.708931972 | 1.36003144993797 | 0.839918629736197 | 0.181059065479272 | 3.89631583875977 | 0.999950574162623 |
| ebi-fl187-GCST90094846 | prot-a-137 | Apolipoprotein M \|\| id:prot-a-137 | Spicy food liking \|\| id:ebi-fl187-GCST90094846 | MR Egger | 16 | 1.59324062686919 | 0.802954504267657 | 0.0671905730214286 | 0.0194497985045785 | 3.1670314552338 | 4.91966592545822 | 1.01964017811406 | 23.7369155684715 | 0.999950574162623 |
| ebi-fl187-GCST90094846 | prot-a-1381 | 17-beta-hydroxysteroid dehydrogenase 14 \|\| id:prot-a-1381 | Spicy food liking \|\| id:ebi-fl187-GCST90094846 | MR Egger | 16 | 0.102725170736968 | 0.877439925419184 | 0.908464568515561 | -1.617057083 | 1.82250742455857 | 1.10818680505794 | 0.198481956330532 | 6.18735333734522 | 0.999950574162623 |
| ebi-fl187-GCST90094846 | prot-a-1392 | Serine protease HTRA2, mitochondrial \|\| id:prot-a-1392 | Spicy food liking \|\| id:ebi-fl187-GCST90094846 | MR Egger | 16 | 0.50755197317115 | 0.73579393382833 | 0.501593815376202 | -0.934604137 | 1.94970808347468 | 1.66121950326147 | 0.392741306462653 | 7.02663608997975 | 0.999950574162623 |
| ebi-fl187-GCST90094846 | prot-a-1416 | Interferon-induced protein with tetratricopeptide repeats 2 \|\| id:prot-a-1416 | Spicy food liking \|\| id:ebi-fl187-GCST90094846 | MR Egger | 16 | -0.696376784 | 0.78357591057421 | 0.38917026894153 | -2.232185569 | 0.839432000366859 | 0.49838780288096 | 0.107293675952423 | 2.31505165477463 | 0.999950574162623 |
| ebi-fl187-GCST90094846 | prot-a-1428 | Interferon gamma \|\| id:prot-a-1428 | Spicy food liking \|\| id:ebi-fl187-GCST90094846 | MR Egger | 16 | 0.827238749868536 | 0.755022064693166 | 0.291723419458217 | -0.652604497 | 2.30708199666714 | 2.28699504825675 | 0.520687879225665 | 10.0450702991764 | 0.999950574162623 |
| ebi-fl187-GCST90094846 | prot-a-1483 | Interleukin-17C \|\| id:prot-a-1483 | Spicy food liking \|\| id:ebi-fl187-GCST90094846 | MR Egger | 16 | 0.20665628663457 | 0.836764317395106 | 0.808515686469137 | -1.433401775 | 1.84671434872898 | 1.22955988297764 | 0.238496230106541 | 6.3389576646668 | 0.999950574162623 |
| ebi-fl187-GCST90094846 | prot-a-1503 | Interleukin-1 receptor-like 2 \|\| id:prot-a-1503 | Spicy food liking \|\| id:ebi-fl187-GCST90094846 | MR Egger | 16 | 0.0373119924331037 | 0.702960161868207 | 0.958419409552914 | -1.340489925 | 1.41511390969479 | 1.03801682371794 | 0.261717415305829 | 4.11695540039779 | 0.999950574162623 |
| ebi-fl187-GCST90094846 | prot-a-1522 | Interleukin-31 receptor subunit alpha \|\| id:prot-a-1522 | Spicy food liking \|\| id:ebi-fl187-GCST90094846 | MR Egger | 16 | 0.709466225141012 | 0.696457313177442 | 0.325637397194977 | -0.655590109 | 2.0745225589688 | 2.03290585495585 | 0.519135625738055 | 7.9607447653748 | 0.999950574162623 |
| ebi-fl187-GCST90094846 | prot-a-1540 | Interleukin-6 receptor subunit alpha \|\| id:prot-a-1540 | Spicy food liking \|\| id:ebi-fl187-GCST90094846 | MR Egger | 16 | 0.84945112050879 | 0.722084004468244 | 0.259055701434487 | -0.565833528 | 2.26473576926655 | 2.33836302 | 0.567886599904387 | 9.62858009817572 | 0.999950574162623 |
| ebi-fl187-GCST90094846 | prot-a-1541 | Interleukin-6 receptor subunit alpha \|\| id:prot-a-1541 | Spicy food liking \|\| id:ebi-fl187-GCST90094846 | MR Egger | 16 | 0.82084187052069 | 0.691769732699303 | 0.255138553870363 | -0.535026806 | 2.17671054661132 | 2.2724121092425 | 0.585653591156085 | 8.81725455492977 | 0.999950574162623 |
| ebi-fl187-GCST90094846 | prot-a-1545 | Interleukin-9 \|\| id:prot-a-1545 | Spicy food liking \|\| id:ebi-fl187-GCST90094846 | MR Egger | 16 | -0.667216998 | 0.691281251323486 | 0.350827786376556 | -2.022128251 | 0.68769425454835 | 0.513134647215146 | 0.132373441216888 | 1.989123828 | 0.999950574162623 |
| ebi-fl187-GCST90094846 | prot-a-1548 | Inositol monophosphatase 3 \|\| id:prot-a-1548 | Spicy food liking \|\| id:ebi-fl187-GCST90094846 | MR Egger | 16 | 1.2278083127575 | 0.782243387225562 | 0.138828423918669 | -0.305388726 | 2.7610053517196 | 3.41373948208765 | 0.736836890905406 | 15.8157353349184 | 0.999950574162623 |
| ebi-fl187-GCST90094846 | prot-a-1555 | Inhibin beta A chain:Inhibin beta B chain heterodimer \|\| id:prot-a-1555 | Spicy food liking \|\| id:ebi-fl187-GCST90094846 | MR Egger | 16 | 0.752959145333005 | 0.692636237058576 | 0.295358724196025 | -0.604607879 | 2.11052616996781 | 2.12327380527998 | 0.546288595712469 | 8.25258240346094 | 0.999950574162623 |
| ebi-fl187-GCST90094846 | prot-a-1603 | Junctophilin-3 \|\| id:prot-a-1603 | Spicy food liking \|\| id:ebi-fl187-GCST90094846 | MR Egger | 16 | -0.362089452 | 1.08185543641858 | 0.742818246110101 | -2.482526107 | 1.75834720331931 | 0.696220086734486 | 0.0835319481739307 | 5.80283855182312 | 0.999950574162623 |
| ebi-fl187-GCST90094846 | prot-a-1627 | UPF0258 protein KIAA1024 \|\| id:prot-a-1627 | Spicy food liking \|\| id:ebi-fl187-GCST90094846 | MR Egger | 16 | -0.374865729 | 0.995973671756955 | 0.712276195395848 | -2.326974125 | 1.57724266810556 | 0.687381568322987 | 0.0975905977061369 | 4.84158752560295 | 0.999950574162623 |
| ebi-fl187-GCST90094846 | prot-a-1666 | Kallikrein-7 \|\| id:prot-a-1666 | Spicy food liking \|\| id:ebi-fl187-GCST90094846 | MR Egger | 16 | 0.306750915587147 | 0.898568388410779 | 0.737889082190054 | -1.454443126 | 2.06794495687227 | 1.35900241959041 | 0.233530374762384 | 7.90855398717095 | 0.999950574162623 |
| ebi-fl187-GCST90094846 | prot-a-1678 | Importin subunit alpha-3 \|\| id:prot-a-1678 | Spicy food liking \|\| id:ebi-fl187-GCST90094846 | MR Egger | 16 | -0.583695645 | 0.828826791076439 | 0.492830220282006 | -2.208196156 | 1.04080486525927 | 0.557832999608703 | 0.109898709980731 | 2.83149506947809 | 0.999950574162623 |
| ebi-fl187-GCST90094846 | prot-a-1718 | Protein LDOC1 \|\| id:prot-a-1718 | Spicy food liking \|\| id:ebi-fl187-GCST90094846 | MR Egger | 16 | 1.02279824649493 | 0.692550125249203 | 0.161852318241651 | -0.334599999 | 2.38019649198336 | 2.78096571393841 | 0.715624279542047 | 10.8070261493225 | 0.999950574162623 |
| ebi-fl187-GCST90094846 | prot-a-1723 | Leptin \|\| id:prot-a-1723 | Spicy food liking \|\| id:ebi-fl187-GCST90094846 | MR Egger | 16 | -0.539935996 | 0.780696166084877 | 0.500492527784022 | -2.070100482 | 0.990228489216882 | 0.582785551606419 | 0.126173102963039 | 2.69184946066272 | 0.999950574162623 |
| ebi-fl187-GCST90094846 | prot-a-1730 | Galectin-7 \|\| id:prot-a-1730 | Spicy food liking \|\| id:ebi-fl187-GCST90094846 | MR Egger | 16 | 0.465741835409336 | 0.69175535947599 | 0.511734189109625 | -0.890098669 | 1.82158233998228 | 1.59319563955137 | 0.410615235691618 | 6.18163215889974 | 0.999950574162623 |
| ebi-fl187-GCST90094846 | prot-a-1753 | Lipase member N \|\| id:prot-a-1753 | Spicy food liking \|\| id:ebi-fl187-GCST90094846 | MR Egger | 16 | 0.716834107957129 | 0.707261353507572 | 0.32800169281222 | -0.669398145 | 2.10306636083197 | 2.0479393816943 | 0.51201664489131 | 8.1912487668926 | 0.999950574162623 |
| ebi-fl187-GCST90094846 | prot-a-1756 | VIP36-like protein \|\| id:prot-a-1756 | Spicy food liking \|\| id:ebi-fl187-GCST90094846 | MR Egger | 16 | 1.02997069142597 | 0.692448156573136 | 0.159074219121682 | -0.327227695 | 2.38716907830932 | 2.80098374065675 | 0.72091957422489 | 10.8826423860924 | 0.999950574162623 |
| ebi-fl187-GCST90094846 | prot-a-1760 | Ig Kappa chain V-I region HK102- like \|\| id:prot-a-1760 | Spicy food liking \|\| id:ebi-fl187-GCST90094846 | MR Egger | 16 | -0.372666564 | 0.692026506988542 | 0.598680217273854 | -1.729038517 | 0.983705390104632 | 0.688894897191983 | 0.177454947836204 | 2.67434740571563 | 0.999950574162623 |
| ebi-fl187-GCST90094846 | prot-a-179 | Neutral ceramidase \|\| id:prot-a-179 | Spicy food liking \|\| id:ebi-fl187-GCST90094846 | MR Egger | 16 | 0.750901884836446 | 0.951191086121913 | 0.443024125884769 | -1.113432644 | 2.6152364136354 | 2.11891016806525 | 0.328429641881423 | 13.6704478761735 | 0.999950574162623 |
| ebi-fl187-GCST90094846 | prot-a-1809 | Lactotransferrin \|\| id:prot-a-1809 | Spicy food liking \|\| id:ebi-fl187-GCST90094846 | MR Egger | 16 | 0.429329646865685 | 0.692633564002857 | 0.54531302283133 | -0.928232139 | 1.78689143231128 | 1.53622736347322 | 0.395251843573871 | 5.97086275662822 | 0.999950574162623 |
| ebi-fl187-GCST90094846 | prot-a-1818 | Lysophospholipase-like protein 1 \|\| id:prot-a-1818 | Spicy food liking \|\| id:ebi-fl187-GCST90094846 | MR Egger | 16 | -0.562800994 | 0.814590048130267 | 0.50092362985314 | -2.159397489 | 1.03379550006319 | 0.569611349172195 | 0.115394626674201 | 2.81171748162784 | 0.999950574162623 |
| ebi-fl187-GCST90094846 | prot-a-1852 | MAP kinase-activated protein kinase 3 \|\| id:prot-a-1852 | Spicy food liking \|\| id:ebi-fl187-GCST90094846 | MR Egger | 16 | 0.0648005810958587 | 0.802941939179673 | 0.936819754210029 | -1.50896562 | 1.63856678188802 | 1.06694623391893 | 0.221138601110728 | 5.14778632204419 | 0.999950574162623 |
| ebi-fl187-GCST90094846 | prot-a-1856 | Megakaryocyte-associated tyrosine-protein kinase \|\| id:prot-a-1856 | Spicy food liking \|\| id:ebi-fl187-GCST90094846 | MR Egger | 16 | -0.852431784 | 0.727251884852672 | 0.260706601452073 | -2.277845478 | 0.572981910705749 | 0.426376814075068 | 0.102504817868137 | 1.77354773523592 | 0.999950574162623 |
| ebi-fl187-GCST90094846 | prot-a-1890 | Beta-1,3-N-acetylglucosaminyltransferase manic fringe \|\| id:prot-a-1890 | Spicy food liking \|\| id:ebi-fl187-GCST90094846 | MR Egger | 16 | 0.08325513121612 | 0.810991874429693 | 0.919690049832825 | -1.506288943 | 1.67279920509832 | 1.08681905464678 | 0.2217313106169 | 5.32705847567067 | 0.999950574162623 |
| ebi-fl187-GCST90094846 | prot-a-1919 | Matrilysin \|\| id:prot-a-1919 | Spicy food liking \|\| id:ebi-fl187-GCST90094846 | MR Egger | 16 | 0.3417914517855 | 0.734045241277727 | 0.64864326664806 | -1.096937221 | 1.78052012468985 | 1.40746674222889 | 0.333892157086191 | 5.93294148556189 | 0.999950574162623 |
| ebi-fl187-GCST90094846 | prot-a-1923 | Multimerin-2 \|\| id:prot-a-1923 | Spicy food liking \|\| id:ebi-fl187-GCST90094846 | MR Egger | 16 | 0.220649347601581 | 0.782329033755489 | 0.782038031854339 | -1.312715559 | 1.75401425376234 | 1.24688613028621 | 0.269088338182604 | 5.77774953905687 | 0.999950574162623 |
| ebi-fl187-GCST90094846 | prot-a-1948 | Mesothelin \|\| id:prot-a-1948 | Spicy food liking \|\| id:ebi-fl187-GCST90094846 | MR Egger | 16 | 0.0421024893350177 | 0.741683867360506 | 0.955533753638711 | -1.411597891 | 1.49580286936161 | 1.04300136978989 | 0.24375348038792 | 4.46291825516634 | 0.999950574162623 |
| ebi-fl187-GCST90094846 | prot-a-195 | ATPase family AAA domain-containing protein 1 \|\| id:prot-a-195 | Spicy food liking \|\| id:ebi-fl187-GCST90094846 | MR Egger | 16 | -1.145149 | 0.692184497454188 | 0.120283846817025 | -2.501830615 | 0.211532615090048 | 0.318176505984341 | 0.0819348700555833 | 1.23557026320694 | 0.999950574162623 |
| ebi-fl187-GCST90094846 | prot-a-1966 | Mucin-1 \|\| id:prot-a-1966 | Spicy food liking \|\| id:ebi-fl187-GCST90094846 | MR Egger | 16 | -0.736064347 | 0.692093841008879 | 0.305541510109339 | -2.092568276 | 0.620439581065373 | 0.478995370132528 | 0.123369880727093 | 1.85974537104349 | 0.999950574162623 |
| ebi-fl187-GCST90094846 | prot-a-1986 | Myocardial zonula adherens protein \|\| id:prot-a-1986 | Spicy food liking \|\| id:ebi-fl187-GCST90094846 | MR Egger | 16 | 1.02786901017342 | 0.691904931614698 | 0.159562445003613 | -0.328264656 | 2.38400267613823 | 2.79510314737122 | 0.720172396686092 | 10.8482380613233 | 0.999950574162623 |
| ebi-fl187-GCST90094846 | prot-a-2071 | Epididymal secretory protein E1 \|\| id:prot-a-2071 | Spicy food liking \|\| id:ebi-fl187-GCST90094846 | MR Egger | 16 | -1.101656436 | 0.73819014858484 | 0.157789988664754 | -2.548509127 | 0.345196255057717 | 0.332320160406173 | 0.078198162637202 | 1.41226705702475 | 0.999950574162623 |
| ebi-fl187-GCST90094846 | prot-a-2098 | Neuritin \|\| id:prot-a-2098 | Spicy food liking \|\| id:ebi-fl187-GCST90094846 | MR Egger | 16 | -0.484311114 | 0.746940921688149 | 0.527223465492373 | -1.94831532 | 0.979693092901765 | 0.616121488312897 | 0.142513959863702 | 2.66363862686818 | 0.999950574162623 |
| ebi-fl187-GCST90094846 | prot-a-2115 | Neurotrophin-4 \|\| id:prot-a-2115 | Spicy food liking \|\| id:ebi-fl187-GCST90094846 | MR Egger | 16 | -0.205781618 | 0.692261307201161 | 0.770633025770115 | -1.56261378 | 1.15105054391214 | 0.814010822020791 | 0.209587538857903 | 3.16151247339282 | 0.999950574162623 |
| ebi-fl187-GCST90094846 | prot-a-2118 | Netrin-1 \|\| id:prot-a-2118 | Spicy food liking \|\| id:ebi-fl187-GCST90094846 | MR Egger | 16 | 0.425756979725994 | 0.692184497454188 | 0.548362425938608 | -0.930924635 | 1.7824385947362 | 1.53074872695239 | 0.39418906069943 | 5.94433458125075 | 0.999950574162623 |
| ebi-fl187-GCST90094846 | prot-a-2120 | Netrin-G1 \|\| id:prot-a-2120 | Spicy food liking \|\| id:ebi-fl187-GCST90094846 | MR Egger | 16 | 0.852217271999725 | 0.692573430002273 | 0.238781814539538 | -0.505226651 | 2.20966119480418 | 2.34484024083023 | 0.603368805902759 | 9.11262846409544 | 0.999950574162623 |
| ebi-fl187-GCST90094846 | prot-a-2145 | Olfactomedin-like protein 3 \|\| id:prot-a-2145 | Spicy food liking \|\| id:ebi-fl187-GCST90094846 | MR Egger | 16 | -0.677834482 | 0.928785239328174 | 0.477542253056964 | -2.498253551 | 1.14258458701695 | 0.507715269288081 | 0.0822284811313501 | 3.13486022265818 | 0.999950574162623 |
| ebi-fl187-GCST90094846 | prot-a-2162 | Prolyl 4-hydroxylase subunit alpha-2 \|\| id:prot-a-2162 | Spicy food liking \|\| id:ebi-fl187-GCST90094846 | MR Egger | 16 | -0.246681726 | 0.864497334659198 | 0.779556372717978 | -1.941096502 | 1.44773305008604 | 0.781389349993135 | 0.143546464498486 | 4.2534611940173 | 0.999950574162623 |
| ebi-fl187-GCST90094846 | prot-a-2168 | Polyadenylate-binding protein-interacting protein 1 \|\| id:prot-a-2168 | Spicy food liking \|\| id:ebi-fl187-GCST90094846 | MR Egger | 16 | -1.009056998 | 0.906658559059425 | 0.284478998793332 | -2.786107774 | 0.767993777589894 | 0.364562600729253 | 0.0616607450176835 | 2.15543762587301 | 0.999950574162623 |
| ebi-fl187-GCST90094846 | prot-a-219 | Beta-1,4-galactosyltransferase 5 \|\| id:prot-a-219 | Spicy food liking \|\| id:ebi-fl187-GCST90094846 | MR Egger | 16 | -0.74436438 | 0.692573430002273 | 0.30064809062427 | -2.101808302 | 0.613079543184327 | 0.475036146655779 | 0.122235190089906 | 1.84610782266214 | 0.999950574162623 |
| ebi-fl187-GCST90094846 | prot-a-2190 | Pyruvate carboxylase, mitochondrial \|\| id:prot-a-2190 | Spicy food liking \|\| id:ebi-fl187-GCST90094846 | MR Egger | 16 | -0.112620342 | 0.692659567365193 | 0.873163802386419 | -1.470233094 | 1.24499240957759 | 0.893489815877554 | 0.229871897067896 | 3.47290843839476 | 0.999950574162623 |
| ebi-fl187-GCST90094846 | prot-a-2197 | Protocadherin-9 \|\| id:prot-a-2197 | Spicy food liking \|\| id:ebi-fl187-GCST90094846 | MR Egger | 16 | 0.123226825970424 | 0.692270757168543 | 0.861269943355858 | -1.233623858 | 1.48007751002077 | 1.13114096399792 | 0.291235267789107 | 4.39328619142604 | 0.999950574162623 |
| ebi-fl187-GCST90094846 | prot-a-2198 | Protocadherin alpha-4 \|\| id:prot-a-2198 | Spicy food liking \|\| id:ebi-fl187-GCST90094846 | MR Egger | 16 | -0.193168977 | 0.890765770090925 | 0.831447959670699 | -1.939069886 | 1.55273193234692 | 0.824342667392913 | 0.14383767295363 | 4.72435919832722 | 0.999950574162623 |
| ebi-fl187-GCST90094846 | prot-a-2254 | Placenta growth factor \|\| id:prot-a-2254 | Spicy food liking \|\| id:ebi-fl187-GCST90094846 | MR Egger | 16 | -0.272057467 | 0.692203293138859 | 0.700216149591902 | -1.628775922 | 1.08466098729561 | 0.761810480673539 | 0.196169554092781 | 2.95843670108747 | 0.999950574162623 |
| ebi-fl187-GCST90094846 | prot-a-2261 | Membrane-associated progesterone receptor component 2 \|\| id:prot-a-2261 | Spicy food liking \|\| id:ebi-fl187-GCST90094846 | MR Egger | 16 | 0.464668965347587 | 0.755646859114285 | 0.548467046201882 | -1.016398879 | 1.94573680921159 | 1.59148726424342 | 0.361895827279429 | 6.99878672625128 | 0.999950574162623 |
| ebi-fl187-GCST90094846 | prot-a-2268 | Polymeric immunoglobulin receptor \|\| id:prot-a-2268 | Spicy food liking \|\| id:ebi-fl187-GCST90094846 | MR Egger | 16 | -0.392110995 | 0.912367824385672 | 0.673902008332976 | -2.180351931 | 1.39612994035151 | 0.675629118044389 | 0.113001754793956 | 4.03953643004715 | 0.999950574162623 |
| ebi-fl187-GCST90094846 | prot-a-2283 | Group 10 secretory phospholipase A2 \|\| id:prot-a-2283 | Spicy food liking \|\| id:ebi-fl187-GCST90094846 | MR Egger | 16 | 0.210403423347434 | 0.832793361964631 | 0.804212103084258 | -1.421871566 | 1.84267841279811 | 1.23417585489365 | 0.241262056205414 | 6.31342559521913 | 0.999950574162623 |
| ebi-fl187-GCST90094846 | prot-a-2298 | Inactive phospholipase D5 \|\| id:prot-a-2298 | Spicy food liking \|\| id:ebi-fl187-GCST90094846 | MR Egger | 16 | 0.0662188793987277 | 0.947706733322921 | 0.945283190914616 | -1.791286318 | 1.92372407671165 | 1.06846055557782 | 0.166745543877933 | 6.84640759972208 | 0.999950574162623 |
| ebi-fl187-GCST90094846 | prot-a-2304 | Procollagen-lysine,2-oxoglutarate 5-dioxygenase 2 \|\| id:prot-a-2304 | Spicy food liking \|\| id:ebi-fl187-GCST90094846 | MR Egger | 16 | -0.186218499 | 0.827694057270434 | 0.825241965165651 | -1.808498851 | 1.43606185321625 | 0.830092200821587 | 0.16389999048536 | 4.20410678380347 | 0.999950574162623 |
| ebi-fl187-GCST90094846 | prot-a-2331 | Periostin \|\| id:prot-a-2331 | Spicy food liking \|\| id:ebi-fl187-GCST90094846 | MR Egger | 16 | 1.76250521999613 | 0.691943084007442 | 0.0232440484223846 | 0.406296775341541 | 3.11871366465071 | 5.8270170838287 | 1.5012480197467 | 22.6172675324898 | 0.999950574162623 |
| ebi-fl187-GCST90094846 | prot-a-2333 | Periostin \|\| id:prot-a-2333 | Spicy food liking \|\| id:ebi-fl187-GCST90094846 | MR Egger | 16 | 1.626986545 | 0.797422340914798 | 0.0606488777720451 | 0.0640387564219938 | 3.189934333 | 5.08851757044349 | 1.06613371748907 | 24.2868325426332 | 0.999950574162623 |
| ebi-fl187-GCST90094846 | prot-a-2367 | cAMP-dependent protein kinase catalytic subunit alpha \|\| id:prot-a-2367 | Spicy food liking \|\| id:ebi-fl187-GCST90094846 | MR Egger | 16 | 0.0660881896295039 | 0.692572538897733 | 0.925330307053077 | -1.291353987 | 1.42353036586906 | 1.06832092783855 | 0.274898322345643 | 4.15175180088146 | 0.999950574162623 |
| ebi-fl187-GCST90094846 | prot-a-2385 | Phosphoribosyl pyrophosphate synthase-associated protein 1 \|\| id:prot-a-2385 | Spicy food liking \|\| id:ebi-fl187-GCST90094846 | MR Egger | 16 | 0.959383143763972 | 0.696738043900167 | 0.190143537699301 | -0.406223422 | 2.3249897098083 | 2.61008592895514 | 0.666161315516892 | 10.2265748518339 | 0.999950574162623 |
| ebi-fl187-GCST90094846 | prot-a-2393 | Trypsin-3 \|\| id:prot-a-2393 | Spicy food liking \|\| id:ebi-fl187-GCST90094846 | MR Egger | 16 | -0.690353793 | 0.999640675228803 | 0.501105865702671 | -2.649649517 | 1.26894193032717 | 0.501398646290347 | 0.0706759794797912 | 3.55708692475464 | 0.999950574162623 |
| ebi-fl187-GCST90094846 | prot-a-2405 | Pregnancy-specific beta-1-glycoprotein 6 \|\| id:prot-a-2405 | Spicy food liking \|\| id:ebi-fl187-GCST90094846 | MR Egger | 16 | 0.683430382387029 | 0.758410595375259 | 0.382757195800943 | -0.803054385 | 2.16991514932254 | 1.98066051475472 | 0.447958634494199 | 8.7575409259339 | 0.999950574162623 |
| ebi-fl187-GCST90094846 | prot-a-244 | Beclin-1 \|\| id:prot-a-244 | Spicy food liking \|\| id:ebi-fl187-GCST90094846 | MR Egger | 16 | 0.935977409809215 | 0.692097428399818 | 0.197696106035629 | -0.42053355 | 2.29248836947286 | 2.54970434626991 | 0.656696346085909 | 9.89954077274123 | 0.999950574162623 |
| ebi-fl187-GCST90094846 | prot-a-2454 | tRNA pseudouridine synthase A, mitochondrial \|\| id:prot-a-2454 | Spicy food liking \|\| id:ebi-fl187-GCST90094846 | MR Egger | 16 | -0.575230891 | 0.692052501284118 | 0.419814090237482 | -1.931653794 | 0.781192011099298 | 0.562574960080104 | 0.144908351656656 | 2.18407415508404 | 0.999950574162623 |
| ebi-fl187-GCST90094846 | prot-a-2466 | Glutaminyl-peptide cyclotransferase-like protein \|\| id:prot-a-2466 | Spicy food liking \|\| id:ebi-fl187-GCST90094846 | MR Egger | 16 | 1.30773060982388 | 0.69181892094964 | 0.0796026195385271 | -0.048234475 | 2.66369569488517 | 3.69777249442288 | 0.952910327005443 | 14.3492215720654 | 0.999950574162623 |
| ebi-fl187-GCST90094846 | prot-a-2474 | Ras-related protein Rab-27A \|\| id:prot-a-2474 | Spicy food liking \|\| id:ebi-fl187-GCST90094846 | MR Egger | 16 | 1.01630651922525 | 0.948149552120595 | 0.301902542536192 | -0.842066603 | 2.87467964138162 | 2.7629709148367 | 0.430819270446182 | 17.7197465385597 | 0.999950574162623 |
| ebi-fl187-GCST90094846 | prot-a-2490 | Rap1 GTPase-GDP dissociation stimulator 1 \|\| id:prot-a-2490 | Spicy food liking \|\| id:ebi-fl187-GCST90094846 | MR Egger | 16 | -0.112516678 | 0.850967285897509 | 0.896690142219106 | -1.780412558 | 1.55537920273024 | 0.893582444147867 | 0.168568588603337 | 4.73688242338091 | 0.999950574162623 |
| ebi-fl187-GCST90094846 | prot-a-2545 | Receptor-interacting serine/threonine-protein kinase 2 \|\| id:prot-a-2545 | Spicy food liking \|\| id:ebi-fl187-GCST90094846 | MR Egger | 16 | -0.806922557 | 0.692637138011826 | 0.263485944062474 | -2.164491348 | 0.550646233398118 | 0.446229200229271 | 0.114808317272951 | 1.73437346585139 | 0.999950574162623 |
| ebi-fl187-GCST90094846 | prot-a-2610 | RUN and FYVE domain-containing protein 1 \|\| id:prot-a-2610 | Spicy food liking \|\| id:ebi-fl187-GCST90094846 | MR Egger | 16 | 0.764603532805045 | 0.692511781109501 | 0.288164869095227 | -0.592719558 | 2.12192662377967 | 2.14814253775738 | 0.552821807483042 | 8.34720392730574 | 0.999950574162623 |
| ebi-fl187-GCST90094846 | prot-a-2627 | Serine--tRNA ligase, mitochondrial \|\| id:prot-a-2627 | Spicy food liking \|\| id:ebi-fl187-GCST90094846 | MR Egger | 16 | 0.477557461346341 | 0.691972282909588 | 0.50138778988591 | -0.878708213 | 1.83382313584913 | 1.61213189482175 | 0.415319069015758 | 6.25776527059239 | 0.999950574162623 |
| ebi-fl187-GCST90094846 | prot-a-264 | BolA-like protein 2 \|\| id:prot-a-264 | Spicy food liking \|\| id:ebi-fl187-GCST90094846 | MR Egger | 16 | -1.722835487 | 0.768171434713013 | 0.0416168064612168 | -3.228451499 | -0.217219475 | 0.178559127326453 | 0.0396188010610151 | 0.804753326646166 | 0.999950574162623 |
| ebi-fl187-GCST90094846 | prot-a-2646 | Secretoglobin family 1C member 1 \|\| id:prot-a-2646 | Spicy food liking \|\| id:ebi-fl187-GCST90094846 | MR Egger | 16 | 0.570589695157086 | 0.757582388583399 | 0.46382488944736 | -0.914271786 | 2.05545117678055 | 1.76931009745977 | 0.400808393940135 | 7.8103609313149 | 0.999950574162623 |
| ebi-fl187-GCST90094846 | prot-a-2672 | Semaphorin-3E \|\| id:prot-a-2672 | Spicy food liking \|\| id:ebi-fl187-GCST90094846 | MR Egger | 16 | 0.231099387894683 | 1.08041714895418 | 0.833710250389814 | -1.886518224 | 2.34871699984488 | 1.25998446042926 | 0.151598723793873 | 10.4721253635472 | 0.999950574162623 |
| ebi-fl187-GCST90094846 | prot-a-2701 | Plasma protease C1 inhibitor \|\| id:prot-a-2701 | Spicy food liking \|\| id:ebi-fl187-GCST90094846 | MR Egger | 16 | 0.949010918265665 | 0.883265336315222 | 0.300797644428662 | -0.782189141 | 2.6802109774435 | 2.58315344548212 | 0.457403593568657 | 14.5881707462025 | 0.999950574162623 |
| ebi-fl187-GCST90094846 | prot-a-2738 | Signaling threshold-regulating transmembrane adapter 1 \|\| id:prot-a-2738 | Spicy food liking \|\| id:ebi-fl187-GCST90094846 | MR Egger | 16 | 0.416326299025688 | 0.707781087565738 | 0.565757983062226 | -0.970924633 | 1.80357723065453 | 1.5163805816604 | 0.37873268756479 | 6.07132720236449 | 0.999950574162623 |
| ebi-fl187-GCST90094846 | prot-a-274 | Serine/threonine-protein kinase BRSK2 \|\| id:prot-a-274 | Spicy food liking \|\| id:ebi-fl187-GCST90094846 | MR Egger | 16 | 0.330327245222079 | 0.779896434666152 | 0.678327043172628 | -1.198269767 | 1.85892425716774 | 1.39142339062501 | 0.301715799264091 | 6.41683019815531 | 0.999950574162623 |
| ebi-fl187-GCST90094846 | prot-a-2749 | Mitochondrial glutamate carrier 2 \|\| id:prot-a-2749 | Spicy food liking \|\| id:ebi-fl187-GCST90094846 | MR Egger | 16 | -0.697565243 | 0.934159785039737 | 0.46758238251052 | -2.528518421 | 1.13338793609955 | 0.497795841630288 | 0.0797771288690823 | 3.1061621727583 | 0.999950574162623 |
| ebi-fl187-GCST90094846 | prot-a-2760 | Sodium-coupled monocarboxylate transporter 1 \|\| id:prot-a-2760 | Spicy food liking \|\| id:ebi-fl187-GCST90094846 | MR Egger | 16 | -0.141839355 | 0.692222031665064 | 0.84059599414062 | -1.498594537 | 1.21491582672219 | 0.86776064640233 | 0.223443981722948 | 3.37001038756218 | 0.999950574162623 |
| ebi-fl187-GCST90094846 | prot-a-2777 | Small integral membrane protein 10 \|\| id:prot-a-2777 | Spicy food liking \|\| id:ebi-fl187-GCST90094846 | MR Egger | 16 | 0.561513038882248 | 0.691281251323486 | 0.430227787284272 | -0.793398214 | 1.91642429147628 | 1.75332334076577 | 0.452305151177492 | 6.79661226336045 | 0.999950574162623 |
| ebi-fl187-GCST90094846 | prot-a-2799 | Superoxide dismutase [Mn], mitochondrial \|\| id:prot-a-2799 | Spicy food liking \|\| id:ebi-fl187-GCST90094846 | MR Egger | 16 | 0.249716319968037 | 0.692183570296804 | 0.723659796813437 | -1.106963478 | 1.60639611774977 | 1.28366121597717 | 0.330561195054957 | 4.9848141344299 | 0.999950574162623 |
| ebi-fl187-GCST90094846 | prot-a-2805 | Protein SPACA7 \|\| id:prot-a-2805 | Spicy food liking \|\| id:ebi-fl187-GCST90094846 | MR Egger | 16 | -0.415959076 | 0.692546568830243 | 0.557690336987742 | -1.773350351 | 0.941432199167237 | 0.659707267963692 | 0.169763268500388 | 2.56365044834846 | 0.999950574162623 |
| ebi-fl187-GCST90094846 | prot-a-2811 | SPARC-like protein 1 \|\| id:prot-a-2811 | Spicy food liking \|\| id:ebi-fl187-GCST90094846 | MR Egger | 16 | 0.41527027913448 | 1.03842353601723 | 0.695261724985089 | -1.620039851 | 2.45058040972825 | 1.51478009882311 | 0.197890812688807 | 11.5950746606861 | 0.999950574162623 |
| ebi-fl187-GCST90094846 | prot-a-2831 | Sepiapterin reductase \|\| id:prot-a-2831 | Spicy food liking \|\| id:ebi-fl187-GCST90094846 | MR Egger | 16 | 0.977625657265785 | 0.750088559538464 | 0.213483993562009 | -0.492547919 | 2.44779923396117 | 2.65813741411075 | 0.611067458356398 | 11.5628715220741 | 0.999950574162623 |
| ebi-fl187-GCST90094846 | prot-a-2834 | SPRY domain-containing SOCS box protein 1 \|\| id:prot-a-2834 | Spicy food liking \|\| id:ebi-fl187-GCST90094846 | MR Egger | 16 | -0.36438133 | 0.723103842322957 | 0.622159124901287 | -1.781664861 | 1.05290220122689 | 0.694626262590408 | 0.168357621830892 | 2.86595664296668 | 0.999950574162623 |
| ebi-fl187-GCST90094846 | prot-a-2835 | Spectrin alpha chain, non-erythrocytic 1 \|\| id:prot-a-2835 | Spicy food liking \|\| id:ebi-fl187-GCST90094846 | MR Egger | 16 | 0.151660404404417 | 0.692361353673592 | 0.829773906684906 | -1.205367849 | 1.50868865760466 | 1.16376495986416 | 0.299581778445694 | 4.52079859073648 | 0.999950574162623 |
| ebi-fl187-GCST90094846 | prot-a-2859 | Sia-alpha-2,3-Gal-beta-1,4-GlcNAc-R:alpha 2,8-sialyltransferase \|\| id:prot-a-2859 | Spicy food liking \|\| id:ebi-fl187-GCST90094846 | MR Egger | 16 | 0.056058416474114 | 0.691822859525116 | 0.936565081533335 | -1.299914388 | 1.41203122114334 | 1.05765946666888 | 0.272555125971477 | 4.10428365068198 | 0.999950574162623 |
| ebi-fl187-GCST90094846 | prot-a-2861 | Alpha-2,8-sialyltransferase 8F \|\| id:prot-a-2861 | Spicy food liking \|\| id:ebi-fl187-GCST90094846 | MR Egger | 16 | -1.252410092 | 0.899525029564636 | 0.185552481743016 | -3.01547915 | 0.510658966005433 | 0.28581512537782 | 0.0490223408096248 | 1.66638892687679 | 0.999950574162623 |
| ebi-fl187-GCST90094846 | prot-a-289 | Uncharacterized protein C17orf89 \|\| id:prot-a-289 | Spicy food liking \|\| id:ebi-fl187-GCST90094846 | MR Egger | 16 | -1.48066212 | 0.981160258080873 | 0.15350938736643 | -3.403736226 | 0.442411986162194 | 0.227487014878713 | 0.0332488125428554 | 1.55645684704455 | 0.999950574162623 |
| ebi-fl187-GCST90094846 | prot-a-2921 | Tachykinin-3 \|\| id:prot-a-2921 | Spicy food liking \|\| id:ebi-fl187-GCST90094846 | MR Egger | 16 | 0.218663505440376 | 0.83559330281423 | 0.79737384257253 | -1.419099368 | 1.85642637895627 | 1.24441246820262 | 0.241931810318382 | 6.40082173972997 | 0.999950574162623 |
| ebi-fl187-GCST90094846 | prot-a-2928 | Tax1-binding protein 1 \|\| id:prot-a-2928 | Spicy food liking \|\| id:ebi-fl187-GCST90094846 | MR Egger | 16 | -1.078801526 | 0.922478622968443 | 0.261744285626051 | -2.886859628 | 0.729256574521852 | 0.340002765868965 | 0.0557510169521312 | 2.07353851316836 | 0.999950574162623 |
| ebi-fl187-GCST90094846 | prot-a-296 | Uncharacterized protein C1orf185 \|\| id:prot-a-296 | Spicy food liking \|\| id:ebi-fl187-GCST90094846 | MR Egger | 16 | -0.685344087 | 0.755811616102519 | 0.37987327805598 | -2.166734855 | 0.796046680108108 | 0.503916808289491 | 0.114551032680794 | 2.21676002157284 | 0.999950574162623 |
| ebi-fl187-GCST90094846 | prot-a-2964 | Transforming growth factor beta-2 \|\| id:prot-a-2964 | Spicy food liking \|\| id:ebi-fl187-GCST90094846 | MR Egger | 16 | 0.46658453350439 | 0.692573430002273 | 0.511474101127106 | -0.890859389 | 1.82402845630884 | 1.59453878833684 | 0.410302991193886 | 6.19677165918897 | 0.999950574162623 |
| ebi-fl187-GCST90094846 | prot-a-2977 | Thymocyte nuclear protein 1 \|\| id:prot-a-2977 | Spicy food liking \|\| id:ebi-fl187-GCST90094846 | MR Egger | 16 | -1.004177891 | 0.691529535038217 | 0.168512012414468 | -2.359575779 | 0.351219998052472 | 0.366345687262965 | 0.0944602867070166 | 1.42079986473516 | 0.999950574162623 |
| ebi-fl187-GCST90094846 | prot-a-3015 | Transmembrane protein 70, mitochondrial \|\| id:prot-a-3015 | Spicy food liking \|\| id:ebi-fl187-GCST90094846 | MR Egger | 16 | 0.12296888108651 | 0.807088586854872 | 0.881076153179055 | -1.458924749 | 1.70486251132206 | 1.13084922960059 | 0.232486121281573 | 5.50062934096361 | 0.999950574162623 |
| ebi-fl187-GCST90094846 | prot-a-3030 | Tumor necrosis factor alpha-induced protein 3 \|\| id:prot-a-3030 | Spicy food liking \|\| id:ebi-fl187-GCST90094846 | MR Egger | 16 | -0.591411712 | 0.808207568799205 | 0.476388239878964 | -2.175498547 | 0.992675122590707 | 0.553545286221274 | 0.11355152877123 | 2.69844349269057 | 0.999950574162623 |
| ebi-fl187-GCST90094846 | prot-a-3032 | Tumor necrosis factor receptor superfamily member 10A \|\| id:prot-a-3032 | Spicy food liking \|\| id:ebi-fl187-GCST90094846 | MR Egger | 16 | 0.163293788796317 | 0.692636237058576 | 0.817035225077684 | -1.194273236 | 1.52086081343113 | 1.17738254068184 | 0.302924028528977 | 4.57616272249534 | 0.999950574162623 |
| ebi-fl187-GCST90094846 | prot-a-3081 | Tumor protein p53-inducible protein 11 \|\| id:prot-a-3081 | Spicy food liking \|\| id:ebi-fl187-GCST90094846 | MR Egger | 16 | 0.00310358102539074 | 0.884919534481551 | 0.997251158431923 | -1.731338707 | 1.73754586860923 | 1.00310840211924 | 0.177047236955496 | 5.68337853617651 | 0.999950574162623 |
| ebi-fl187-GCST90094846 | prot-a-315 | Complement C4 \|\| id:prot-a-315 | Spicy food liking \|\| id:ebi-fl187-GCST90094846 | MR Egger | 15 | -0.439135454 | 0.922819735755948 | 0.642069567994529 | -2.247862136 | 1.3695912280521 | 0.644593460934388 | 0.105624794791271 | 3.93374236324395 | 0.999950574162623 |
| ebi-fl187-GCST90094846 | prot-a-3157 | UDP-glucuronosyltransferase 1-8 \|\| id:prot-a-3157 | Spicy food liking \|\| id:ebi-fl187-GCST90094846 | MR Egger | 16 | 1.04688837373729 | 0.700966026721056 | 0.157500408845697 | -0.327005039 | 2.42078178611056 | 2.84877299565728 | 0.721080109757267 | 11.254654609067 | 0.999950574162623 |
| ebi-fl187-GCST90094846 | prot-a-317 | Glutaredoxin-like protein C5orf63 \|\| id:prot-a-317 | Spicy food liking \|\| id:ebi-fl187-GCST90094846 | MR Egger | 16 | 0.313744222552873 | 0.69181892094964 | 0.657134197347051 | -1.042220863 | 1.66970930761417 | 1.36853965019917 | 0.352670578722214 | 5.31062381487308 | 0.999950574162623 |
| ebi-fl187-GCST90094846 | prot-a-3192 | Guanine nucleotide exchange factor VAV3 \|\| id:prot-a-3192 | Spicy food liking \|\| id:ebi-fl187-GCST90094846 | MR Egger | 16 | -0.314307998 | 0.889491785465672 | 0.729085223784321 | -2.057711897 | 1.42909590170714 | 0.730294064547746 | 0.127745931556884 | 4.17492294442413 | 0.999950574162623 |
| ebi-fl187-GCST90094846 | prot-a-3233 | Serine/threonine-protein kinase WNK3 \|\| id:prot-a-3233 | Spicy food liking \|\| id:ebi-fl187-GCST90094846 | MR Egger | 16 | 0.471390763730796 | 0.692636237058576 | 0.507240762426291 | -0.886176261 | 1.82895778836561 | 1.60222095520175 | 0.412228999134119 | 6.22739301378554 | 0.999950574162623 |
| ebi-fl187-GCST90094846 | prot-a-3250 | 14-3-3 protein theta \|\| id:prot-a-3250 | Spicy food liking \|\| id:ebi-fl187-GCST90094846 | MR Egger | 16 | 0.0308056766421359 | 0.747343651489207 | 0.967702417879876 | -1.43398788 | 1.49559923356098 | 1.03128508163381 | 0.238356487273244 | 4.46200953776115 | 0.999950574162623 |
| ebi-fl187-GCST90094846 | prot-a-3278 | E3 ubiquitin-protein ligase ZNRF3 \|\| id:prot-a-3278 | Spicy food liking \|\| id:ebi-fl187-GCST90094846 | MR Egger | 16 | 0.291905067292673 | 0.696641819897774 | 0.681561204150783 | -1.0735129 | 1.65732303429231 | 1.33897589899839 | 0.341805676864463 | 5.24525067735924 | 0.999950574162623 |
| ebi-fl187-GCST90094846 | prot-a-355 | Calpain-3 \|\| id:prot-a-355 | Spicy food liking \|\| id:ebi-fl187-GCST90094846 | MR Egger | 16 | -0.095664496 | 0.691939496030376 | 0.892007333472999 | -1.451865908 | 1.26053691617554 | 0.908768860475372 | 0.234133009542563 | 3.52731485143094 | 0.999950574162623 |
| ebi-fl187-GCST90094846 | prot-a-385 | Coiled-coil domain-containing protein 90B, mitochondrial \|\| id:prot-a-385 | Spicy food liking \|\| id:ebi-fl187-GCST90094846 | MR Egger | 16 | -1.069321049 | 0.692723367592415 | 0.144971851728093 | -2.42705885 | 0.288416751427029 | 0.343241482453025 | 0.0882961433024332 | 1.33431326522393 | 0.999950574162623 |
| ebi-fl187-GCST90094846 | prot-a-390 | C-C motif chemokine 14 \|\| id:prot-a-390 | Spicy food liking \|\| id:ebi-fl187-GCST90094846 | MR Egger | 16 | -0.199538998 | 0.933960103611587 | 0.833901452458663 | -2.030100801 | 1.63102280492165 | 0.819108276476827 | 0.131322283032881 | 5.10909765728674 | 0.999950574162623 |
| ebi-fl187-GCST90094846 | prot-a-40 | Pituitary adenylate cyclase-activating polypeptide 27 \|\| id:prot-a-40 | Spicy food liking \|\| id:ebi-fl187-GCST90094846 | MR Egger | 16 | -0.418316935 | 0.692636237058576 | 0.55553831042878 | -1.775883959 | 0.93925008981384 | 0.658153603571815 | 0.169333699198036 | 2.55806238182973 | 0.999950574162623 |
| ebi-fl187-GCST90094846 | prot-a-401 | C-C motif chemokine 24 \|\| id:prot-a-401 | Spicy food liking \|\| id:ebi-fl187-GCST90094846 | MR Egger | 16 | -0.128824675 | 0.692338971422612 | 0.855057694312979 | -1.485809059 | 1.22815970890213 | 0.879128085090439 | 0.226319161013924 | 3.41493926776812 | 0.999950574162623 |
| ebi-fl187-GCST90094846 | prot-a-412 | Cerebral cavernous malformations 2 protein \|\| id:prot-a-412 | Spicy food liking \|\| id:ebi-fl187-GCST90094846 | MR Egger | 16 | 0.0484105196941244 | 0.837204952561802 | 0.954706061243944 | -1.592511187 | 1.68933222671526 | 1.04960144895956 | 0.203414158768249 | 5.41586292876078 | 0.999950574162623 |
| ebi-fl187-GCST90094846 | prot-a-44 | Adhesion G protein-coupled receptor F5 \|\| id:prot-a-44 | Spicy food liking \|\| id:ebi-fl187-GCST90094846 | MR Egger | 16 | 0.272781827731804 | 0.692636237058576 | 0.699638668664971 | -1.084785197 | 1.63034885236661 | 1.31361361949613 | 0.337974376040818 | 5.10565552791291 | 0.999950574162623 |
| ebi-fl187-GCST90094846 | prot-a-48 | Adrenomedullin \|\| id:prot-a-48 | Spicy food liking \|\| id:ebi-fl187-GCST90094846 | MR Egger | 16 | 0.738637855616311 | 0.692183570296805 | 0.303977656452532 | -0.618041942 | 2.09531765339805 | 2.09308249126064 | 0.538998795825603 | 8.12802245413432 | 0.999950574162623 |
| ebi-fl187-GCST90094846 | prot-a-502 | Carcinoembryonic antigen-related cell adhesion molecule 1 \|\| id:prot-a-502 | Spicy food liking \|\| id:ebi-fl187-GCST90094846 | MR Egger | 16 | -0.464735742 | 0.692637138011826 | 0.513160787588705 | -1.822304533 | 0.89283304828306 | 0.628301116711956 | 0.161652787207456 | 2.4420382727758 | 0.999950574162623 |
| ebi-fl187-GCST90094846 | prot-a-518 | Complement factor B \|\| id:prot-a-518 | Spicy food liking \|\| id:ebi-fl187-GCST90094846 | MR Egger | 16 | -0.689743623 | 0.692183570296804 | 0.335931591136063 | -2.046423421 | 0.666936174708328 | 0.501704678082582 | 0.129196158524891 | 1.94825904178454 | 0.999950574162623 |
| ebi-fl187-GCST90094846 | prot-a-525 | Properdin \|\| id:prot-a-525 | Spicy food liking \|\| id:ebi-fl187-GCST90094846 | MR Egger | 16 | 0.276405373075512 | 0.801476525742793 | 0.735318144119303 | -1.294488617 | 1.84729936353139 | 1.31838219236284 | 0.27403796675874 | 6.34266713367381 | 0.999950574162623 |
| ebi-fl187-GCST90094846 | prot-a-533 | Chondroadherin \|\| id:prot-a-533 | Spicy food liking \|\| id:ebi-fl187-GCST90094846 | MR Egger | 16 | 2.28246165625154 | 0.819288279870987 | 0.0145790066756776 | 0.6766566277044 | 3.88826668479867 | 9.80077688242664 | 1.96728934396017 | 48.8261819716607 | 0.999950574162623 |
| ebi-fl187-GCST90094846 | prot-a-535 | Coiled-coil-helix-coiled-coil-helix domain-containing protein 2 \|\| id:prot-a-535 | Spicy food liking \|\| id:ebi-fl187-GCST90094846 | MR Egger | 16 | -1.003013826 | 0.795211206334927 | 0.227807280629708 | -2.56162779 | 0.555600138419337 | 0.366772385622194 | 0.077179006889582 | 1.74298670424008 | 0.999950574162623 |
| ebi-fl187-GCST90094846 | prot-a-591 | Caseinolytic peptidase B protein homolog \|\| id:prot-a-591 | Spicy food liking \|\| id:ebi-fl187-GCST90094846 | MR Egger | 16 | 0.774388599766212 | 0.813009548995146 | 0.357001856679996 | -0.819110116 | 2.3678873157967 | 2.16926543226154 | 0.440823761911169 | 10.6748159291674 | 0.999950574162623 |
| ebi-fl187-GCST90094846 | prot-a-605 | Ciliary neurotrophic factor receptor subunit alpha \|\| id:prot-a-605 | Spicy food liking \|\| id:ebi-fl187-GCST90094846 | MR Egger | 16 | 1.48902482120344 | 0.692097428399818 | 0.0493818724144541 | 0.132513861539793 | 2.84553578086708 | 4.4327706666301 | 1.14169484162798 | 17.2107774043347 | 0.999950574162623 |
| ebi-fl187-GCST90094846 | prot-a-606 | Ciliary neurotrophic factor receptor subunit alpha \|\| id:prot-a-606 | Spicy food liking \|\| id:ebi-fl187-GCST90094846 | MR Egger | 16 | 1.45385399222371 | 0.734034643870497 | 0.0676303584044048 | 0.0151460902375318 | 2.89256189420988 | 4.27957622663721 | 1.01526137355743 | 18.0394656554541 | 0.999950574162623 |
| ebi-fl187-GCST90094846 | prot-a-707 | Cancer/testis antigen 55 \|\| id:prot-a-707 | Spicy food liking \|\| id:ebi-fl187-GCST90094846 | MR Egger | 16 | 0.463264036281088 | 0.873126281667454 | 0.604024337443437 | -1.248063476 | 2.1745915483493 | 1.58925290745084 | 0.287060157897908 | 8.79859058929109 | 0.999950574162623 |
| ebi-fl187-GCST90094846 | prot-a-719 | Dipeptidyl peptidase 1 \|\| id:prot-a-719 | Spicy food liking \|\| id:ebi-fl187-GCST90094846 | MR Egger | 16 | -0.511489403 | 0.692009816747456 | 0.472036037781378 | -1.867828644 | 0.844849837954645 | 0.599601864688941 | 0.154458682793231 | 2.32762826690511 | 0.999950574162623 |
| ebi-fl187-GCST90094846 | prot-a-752 | Cytochrome b5 \|\| id:prot-a-752 | Spicy food liking \|\| id:ebi-fl187-GCST90094846 | MR Egger | 16 | 0.994694691711541 | 0.692270757168543 | 0.172729523457444 | -0.362155992 | 2.35154537576189 | 2.70389869256517 | 0.696173761597835 | 10.5017863972287 | 0.999950574162623 |
| ebi-fl187-GCST90094846 | prot-a-755 | Cytoglobin \|\| id:prot-a-755 | Spicy food liking \|\| id:ebi-fl187-GCST90094846 | MR Egger | 16 | 0.196254238969375 | 0.756670062597961 | 0.799128067333228 | -1.286819084 | 1.67932756166138 | 1.21683623320088 | 0.276147790508474 | 5.36194917838773 | 0.999950574162623 |
| ebi-fl187-GCST90094846 | prot-a-807 | Desmin \|\| id:prot-a-807 | Spicy food liking \|\| id:ebi-fl187-GCST90094846 | MR Egger | 16 | 1.46141464341732 | 0.691822859525116 | 0.0530987102679197 | 0.105441838748093 | 2.81738744808655 | 4.31205523625719 | 1.11120147377453 | 16.733077483576 | 0.999950574162623 |
| ebi-fl187-GCST90094846 | prot-a-82 | Ameloblastin \|\| id:prot-a-82 | Spicy food liking \|\| id:ebi-fl187-GCST90094846 | MR Egger | 16 | 0.0792838496574083 | 0.732730777542615 | 0.915369938199816 | -1.356868474 | 1.51543617364093 | 1.08251154899745 | 0.257465776546662 | 4.55140589724348 | 0.999950574162623 |
| ebi-fl187-GCST90094846 | prot-a-84 | Muellerian-inhibiting factor \|\| id:prot-a-84 | Spicy food liking \|\| id:ebi-fl187-GCST90094846 | MR Egger | 16 | 0.0474577593735326 | 0.691886347708018 | 0.946284562998199 | -1.308639482 | 1.40355500088125 | 1.04860190658433 | 0.2701874012249 | 4.06964186156492 | 0.999950574162623 |
| ebi-fl187-GCST90094846 | prot-a-858 | Inactive dipeptidyl peptidase 10 \|\| id:prot-a-858 | Spicy food liking \|\| id:ebi-fl187-GCST90094846 | MR Egger | 16 | -0.494970478 | 0.948307664031149 | 0.609863703141616 | -2.3536535 | 1.36371254303743 | 0.609588903031652 | 0.0950213667070691 | 3.9106849709381 | 0.999950574162623 |
| ebi-fl187-GCST90094846 | prot-a-863 | Dihydropyrimidinase-related protein 3 \|\| id:prot-a-863 | Spicy food liking \|\| id:ebi-fl187-GCST90094846 | MR Egger | 16 | 1.15480462640576 | 0.692573430002273 | 0.117636953087291 | -0.202639296 | 2.51224854921021 | 3.17340335773793 | 0.816572729035134 | 12.3326294313082 | 0.999950574162623 |
| ebi-fl187-GCST90094846 | prot-a-864 | Dihydropyrimidinase-related protein 5 \|\| id:prot-a-864 | Spicy food liking \|\| id:ebi-fl187-GCST90094846 | MR Egger | 16 | -0.82460172 | 0.759148342558884 | 0.295731194521112 | -2.312532471 | 0.663329031881289 | 0.43840956766455 | 0.0990101933566373 | 1.94124405279664 | 0.999950574162623 |
| ebi-fl187-GCST90094846 | prot-a-884 | Endothelin-converting enzyme 1 \|\| id:prot-a-884 | Spicy food liking \|\| id:ebi-fl187-GCST90094846 | MR Egger | 16 | 1.78685250048307 | 0.692275540267002 | 0.021761584452641 | 0.429992441559743 | 3.14371255940639 | 5.970630305 | 1.53724590432304 | 23.1898007555983 | 0.999950574162623 |
| ebi-fl187-GCST90094846 | prot-a-893 | Early endosome antigen 1 \|\| id:prot-a-893 | Spicy food liking \|\| id:ebi-fl187-GCST90094846 | MR Egger | 16 | 0.104825561635344 | 0.730247283760088 | 0.887903098959185 | -1.326459115 | 1.53611023780512 | 1.11051687671101 | 0.265415404939731 | 4.64648136659594 | 0.999950574162623 |
| ebi-fl187-GCST90094846 | prot-a-897 | Ephrin-A2 \|\| id:prot-a-897 | Spicy food liking \|\| id:ebi-fl187-GCST90094846 | MR Egger | 16 | 1.66628925812303 | 0.692550125249202 | 0.0305149756779409 | 0.308891012634594 | 3.02368750361147 | 5.29249224170955 | 1.3619139310033 | 20.5669928847272 | 0.999950574162623 |
| ebi-fl187-GCST90094846 | prot-a-90 | Anaphase-promoting complex subunit 10 \|\| id:prot-a-90 | Spicy food liking \|\| id:ebi-fl187-GCST90094846 | MR Egger | 16 | -0.123589348 | 0.731529870243929 | 0.868255464200382 | -1.557387893 | 1.3102091980826 | 0.883742677433248 | 0.210685686560289 | 3.70694911774842 | 0.999950574162623 |
| ebi-fl187-GCST90094846 | prot-a-914 | Histone-lysine N-methyltransferase EHMT2 \|\| id:prot-a-914 | Spicy food liking \|\| id:ebi-fl187-GCST90094846 | MR Egger | 16 | 0.0131482839374095 | 0.884528548386317 | 0.988349852022897 | -1.720527671 | 1.74682423877459 | 1.01323510271134 | 0.178971684841005 | 5.73635642017069 | 0.999950574162623 |
| ebi-fl187-GCST90094846 | prot-a-932 | RNA polymerase II elongation factor ELL \|\| id:prot-a-932 | Spicy food liking \|\| id:ebi-fl187-GCST90094846 | MR Egger | 16 | 1.05375564891994 | 0.692572538897733 | 0.15039397689988 | -0.303686527 | 2.4111978251595 | 2.86840363109166 | 0.73809220193093 | 11.1473056744607 | 0.999950574162623 |
| ebi-fl187-GCST90094846 | prot-a-991 | Estrogen receptor \|\| id:prot-a-991 | Spicy food liking \|\| id:ebi-fl187-GCST90094846 | MR Egger | 16 | -0.429695069 | 0.692637138011826 | 0.544977610194314 | -1.787263859 | 0.9278737218704 | 0.650707485597224 | 0.167417621751213 | 2.52912583145802 | 0.999950574162623 |
| ebi-fl187-GCST90094846 | prot-a-992 | Persulfide dioxygenase ETHE1, mitochondrial \|\| id:prot-a-992 | Spicy food liking \|\| id:ebi-fl187-GCST90094846 | MR Egger | 16 | 1.4059515513603 | 0.691822859525116 | 0.0615521782533341 | 0.0499787466910755 | 2.76192435602953 | 4.07940665996033 | 1.05124875362408 | 15.8302767446416 | 0.999950574162623 |
| ebi-fl187-GCST90094846 | prot-a-993 | Ethanolamine kinase 1 \|\| id:prot-a-993 | Spicy food liking \|\| id:ebi-fl187-GCST90094846 | MR Egger | 16 | -0.162735397 | 0.93620691440105 | 0.8644920234339 | -1.997700949 | 1.67223015511696 | 0.849816022443687 | 0.135646783850155 | 5.32402797547923 | 0.999950574162623 |
| ebi-fl187-GCST90094846 | prot-b-10 | coagulation factor III, tissue factor \|\| id:prot-b-10 | Spicy food liking \|\| id:ebi-fl187-GCST90094846 | MR Egger | 12 | 0.448481013796169 | 1.30231081984625 | 0.737697812123137 | -2.104048193 | 3.00101022069483 | 1.56593174929968 | 0.121961703032011 | 20.105838000812 | 0.999950574162623 |
| ebi-fl187-GCST90094846 | prot-b-28 | prolactin \|\| id:prot-b-28 | Spicy food liking \|\| id:ebi-fl187-GCST90094846 | MR Egger | 12 | -0.781911683 | 1.01867381590871 | 0.460477594339808 | -2.778512362 | 1.21468899606712 | 0.45753052137037 | 0.062130866881802 | 3.36924605258898 | 0.999950574162623 |
| ebi-fl187-GCST90094846 | prot-b-54 | matrix metallopeptidase 7 \|\| id:prot-b-54 | Spicy food liking \|\| id:ebi-fl187-GCST90094846 | MR Egger | 12 | 1.11760911200475 | 1.05499460318261 | 0.314356989487103 | -0.95018031 | 3.18539853424267 | 3.05753523307665 | 0.386671296376828 | 24.1769218173219 | 0.999950574162623 |
| ebi-fl187-GCST90094846 | ukb-rsfMRI191-edge-pheno716 | Net100_Pair10_33 (OccipitalPrecuneus)&(Temporal\|Frontal\|Supp_Motor_Area) (Default_mode\|Central_executive)&(Default_mode\|Salience) \|\| id:ukb-rsfMRI191-edge-pheno716 | Spicy food liking \|\| id:ebi-fl187-GCST90094846 | MR Egger | 15 | -0.099265034 | 0.216142065474616 | 0.653633817647222 | -0.522903482 | 0.324373414798602 | 0.905502687643008 | 0.592796871884677 | 1.38316370449442 | 0.999950574162623 |
| ebi-fl187-GCST90094846 | ukb-rsfMRI191-edge-pheno903 | Net100_Pair27_38 (Precentral\|Frontal\|Supp_Motor_Area)&(Subcortical) (Attention\|Salience\|Motor)&(Subcortical-cerebellum) \|\| id:ukb-rsfMRI191-edge-pheno903 | Spicy food liking \|\| id:ebi-fl187-GCST90094846 | MR Egger | 15 | 0.167101947756014 | 0.263802188677437 | 0.537433869076511 | -0.349950342 | 0.684154237563791 | 1.18187474864527 | 0.70472308395226 | 1.98209474514665 | 0.999950574162623 |
| ebi-fl187-GCST90094846 | ukb-rsfMRI191-node-pheno23 | Net100_Node2 RolandicOper\|SupraMarginal\|Insula Salience\|Motor \|\| id:ukb-rsfMRI191-node-pheno23 | Spicy food liking \|\| id:ebi-fl187-GCST90094846 | MR Egger | 15 | -0.179346064 | 0.266709499728585 | 0.513079044588713 | -0.702096684 | 0.343404555164644 | 0.835816603051863 | 0.495545212196441 | 1.40973896375821 | 0.999950574162623 |
| ebi-fl187-GCST90094846 | ukb-rsfMRI191-node-pheno55 | Net100_Node34 Parietal\|Postcentral\|Precuneus Attention \|\| id:ukb-rsfMRI191-node-pheno55 | Spicy food liking \|\| id:ebi-fl187-GCST90094846 | MR Egger | 15 | -0.26591623 | 0.294382771866543 | 0.382799412664059 | -0.842906463 | 0.311074002415017 | 0.766503334456142 | 0.430457594313978 | 1.36489022262165 | 0.999950574162623 |
| ebi-fl187-GCST90094846 | ebi-a-GCST90001414 | IgD- CD24- B cell Absolute Count \|\| id:ebi-a-GCST90001414 | Spicy food liking \|\| id:ebi-fl187-GCST90094846 | Weighted median | 15 | 0.192159240967891 | 0.176207938341655 | 0.275481749528902 | -0.153208318 | 0.537526800117534 | 1.21186347991418 | 0.857950976406643 | 1.71176807805582 | 0.999332001504091 |
| ebi-fl187-GCST90094846 | ebi-a-GCST90001422 | CD20- CD38- B cell %B cell \|\| id:ebi-a-GCST90001422 | Spicy food liking \|\| id:ebi-fl187-GCST90094846 | Weighted median | 15 | 0.258648987067087 | 0.159134840762403 | 0.104089109101636 | -0.053255301 | 0.570553274961397 | 1.29517910041447 | 0.948137921184958 | 1.76924566001321 | 0.999332001504091 |
| ebi-fl187-GCST90094846 | ebi-a-GCST90001444 | CD20- CD38- B cell %lymphocyte \|\| id:ebi-a-GCST90001444 | Spicy food liking \|\| id:ebi-fl187-GCST90094846 | Weighted median | 15 | 0.203716113260032 | 0.159214688265572 | 0.200719006932355 | -0.108344676 | 0.515776902260553 | 1.22595007308124 | 0.897318259287518 | 1.67493926054871 | 0.999332001504091 |
| ebi-fl187-GCST90094846 | ebi-a-GCST90001459 | Myeloid Dendritic Cell %Dendritic Cell \|\| id:ebi-a-GCST90001459 | Spicy food liking \|\| id:ebi-fl187-GCST90094846 | Weighted median | 15 | 0.238969341161307 | 0.167012274471049 | 0.152473504391347 | -0.088374717 | 0.566313399124563 | 1.2699396011136 | 0.915417790020441 | 1.76176015810285 | 0.999332001504091 |
| ebi-fl187-GCST90094846 | ebi-a-GCST90001476 | HLA DR++ monocyte %leukocyte \|\| id:ebi-a-GCST90001476 | Spicy food liking \|\| id:ebi-fl187-GCST90094846 | Weighted median | 15 | -0.027941726 | 0.166556366340161 | 0.866771005113696 | -0.354392204 | 0.298508751948214 | 0.972445033335674 | 0.701599743087419 | 1.34784733343523 | 0.999332001504091 |
| ebi-fl187-GCST90094846 | ebi-a-GCST90001478 | CD4 regulatory T cell %CD4+ T cell \|\| id:ebi-a-GCST90001478 | Spicy food liking \|\| id:ebi-fl187-GCST90094846 | Weighted median | 15 | -0.19332225 | 0.169418902838185 | 0.253832388754372 | -0.525383299 | 0.13873879972482 | 0.82421632776104 | 0.591328664999716 | 1.14882398766889 | 0.999332001504091 |
| ebi-fl187-GCST90094846 | ebi-a-GCST90001485 | CD39+ resting CD4 regulatory T cell %CD4 regulatory T cell \|\| id:ebi-a-GCST90001485 | Spicy food liking \|\| id:ebi-fl187-GCST90094846 | Weighted median | 15 | 0.140325806475952 | 0.168794905154865 | 0.405782085621286 | -0.190512208 | 0.471163820579487 | 1.15064862656735 | 0.826535667627854 | 1.60185738338571 | 0.999332001504091 |
| ebi-fl187-GCST90094846 | ebi-a-GCST90001515 | Immature Myeloid-Derived Suppressor Cells Absolute Count \|\| id:ebi-a-GCST90001515 | Spicy food liking \|\| id:ebi-fl187-GCST90094846 | Weighted median | 15 | 0.0651743169971064 | 0.222539503691381 | 0.769624362602081 | -0.37100311 | 0.501351744232213 | 1.06734506455527 | 0.690041795361238 | 1.65095142712929 | 0.999332001504091 |
| ebi-fl187-GCST90094846 | ebi-a-GCST90001517 | CD33+ HLA DR+ Absolute Count \|\| id:ebi-a-GCST90001517 | Spicy food liking \|\| id:ebi-fl187-GCST90094846 | Weighted median | 15 | 0.41459652213195 | 0.214220938834262 | 0.0529450546712063 | -0.005276518 | 0.834469562247103 | 1.51375984886311 | 0.994737378385587 | 2.30359181209116 | 0.999332001504091 |
| ebi-fl187-GCST90094846 | ebi-a-GCST90001518 | CD33+ HLA DR+ CD14- Absolute Count \|\| id:ebi-a-GCST90001518 | Spicy food liking \|\| id:ebi-fl187-GCST90094846 | Weighted median | 15 | 0.203140822757159 | 0.216684905759297 | 0.348504452380271 | -0.221561593 | 0.62784323804538 | 1.225244998 | 0.80126656859203 | 1.87356538403104 | 0.999332001504091 |
| ebi-fl187-GCST90094846 | ebi-a-GCST90001520 | CD33+ HLA DR+ CD14dim Absolute Count \|\| id:ebi-a-GCST90001520 | Spicy food liking \|\| id:ebi-fl187-GCST90094846 | Weighted median | 15 | 0.287517448995657 | 0.221971606009161 | 0.195220444563358 | -0.147546899 | 0.722581796773613 | 1.3331138534581 | 0.862821972072643 | 2.05974419266675 | 0.999332001504091 |
| ebi-fl187-GCST90094846 | ebi-a-GCST90001531 | CD33dim HLA DR- Absolute Count \|\| id:ebi-a-GCST90001531 | Spicy food liking \|\| id:ebi-fl187-GCST90094846 | Weighted median | 15 | 0.0324131531597458 | 0.202200353822095 | 0.872643058656446 | -0.36389954 | 0.428725846651051 | 1.03294418131013 | 0.694961006788525 | 1.53530006903989 | 0.999332001504091 |
| ebi-fl187-GCST90094846 | ebi-a-GCST90001532 | Basophil Absolute Count \|\| id:ebi-a-GCST90001532 | Spicy food liking \|\| id:ebi-fl187-GCST90094846 | Weighted median | 15 | -0.044750327 | 0.217433571250895 | 0.836938165713224 | -0.470920127 | 0.381419472402971 | 0.956236198153786 | 0.624427451375562 | 1.46436173593151 | 0.999332001504091 |
| ebi-fl187-GCST90094846 | ebi-a-GCST90001588 | T/B cell \|\| id:ebi-a-GCST90001588 | Spicy food liking \|\| id:ebi-fl187-GCST90094846 | Weighted median | 15 | -0.142534248 | 0.157974631431673 | 0.366918576217657 | -0.452164526 | 0.167096029393144 | 0.867157855176815 | 0.636249481646312 | 1.18186775390234 | 0.999332001504091 |
| ebi-fl187-GCST90094846 | ebi-a-GCST90001624 | HLA DR+ CD4+ T cell Absolute Count \|\| id:ebi-a-GCST90001624 | Spicy food liking \|\| id:ebi-fl187-GCST90094846 | Weighted median | 15 | 0.261250792508025 | 0.154355065799869 | 0.0905447022051828 | -0.041285136 | 0.563786721475768 | 1.29855329203654 | 0.959555486682442 | 1.75731437698192 | 0.999332001504091 |
| ebi-fl187-GCST90094846 | ebi-a-GCST90001631 | CD8+ Natural Killer T %T cell \|\| id:ebi-a-GCST90001631 | Spicy food liking \|\| id:ebi-fl187-GCST90094846 | Weighted median | 15 | 0.00236514698933008 | 0.16210912340532 | 0.988359400879702 | -0.315368735 | 0.320099028863757 | 1.00236794615585 | 0.729519825259285 | 1.37726414648648 | 0.999332001504091 |
| ebi-fl187-GCST90094846 | ebi-a-GCST90001632 | CD8+ Natural Killer T %lymphocyte \|\| id:ebi-a-GCST90001632 | Spicy food liking \|\| id:ebi-fl187-GCST90094846 | Weighted median | 15 | 0.0890959731361983 | 0.163948293569214 | 0.586827258792108 | -0.232242682 | 0.410434628531857 | 1.09318556773929 | 0.792753712698114 | 1.50747283345561 | 0.999332001504091 |
| ebi-fl187-GCST90094846 | ebi-a-GCST90001646 | Natural Killer %CD3- lymphocyte \|\| id:ebi-a-GCST90001646 | Spicy food liking \|\| id:ebi-fl187-GCST90094846 | Weighted median | 15 | -0.182053179 | 0.14495118042564 | 0.209129736190171 | -0.466157493 | 0.102051134532757 | 0.833557011424144 | 0.627408463990398 | 1.10744009871213 | 0.999332001504091 |
| ebi-fl187-GCST90094846 | ebi-a-GCST90001684 | CD127- CD8+ T cell Absolute Count \|\| id:ebi-a-GCST90001684 | Spicy food liking \|\| id:ebi-fl187-GCST90094846 | Weighted median | 15 | -0.06603724 | 0.168733870220993 | 0.69552435469782 | -0.396755626 | 0.264681145710961 | 0.936096003500188 | 0.672498346963952 | 1.30301543747288 | 0.999332001504091 |
| ebi-fl187-GCST90094846 | ebi-a-GCST90001687 | CD28- CD8+ T cell Absolute Count \|\| id:ebi-a-GCST90001687 | Spicy food liking \|\| id:ebi-fl187-GCST90094846 | Weighted median | 15 | 0.0184272397457777 | 0.170327614885709 | 0.913847328636405 | -0.315414885 | 0.352269364921768 | 1.0185980690188 | 0.729486158298556 | 1.4222915875865 | 0.999332001504091 |
| ebi-fl187-GCST90094846 | ebi-a-GCST90001701 | BAFF-R on CD20- CD38- B cell \|\| id:ebi-a-GCST90001701 | Spicy food liking \|\| id:ebi-fl187-GCST90094846 | Weighted median | 15 | 0.162609376445114 | 0.172508210107697 | 0.345876233270825 | -0.175506715 | 0.500725468256201 | 1.17657700122093 | 0.839031762749196 | 1.64991779961474 | 0.999332001504091 |
| ebi-fl187-GCST90094846 | ebi-a-GCST90001732 | CD19 on IgD- CD27- B cell \|\| id:ebi-a-GCST90001732 | Spicy food liking \|\| id:ebi-fl187-GCST90094846 | Weighted median | 15 | 0.0634601854416037 | 0.156794168726694 | 0.685671854734682 | -0.243856385 | 0.370776756145923 | 1.06551706186565 | 0.783600162713801 | 1.44885958828145 | 0.999332001504091 |
| ebi-fl187-GCST90094846 | ebi-a-GCST90001798 | CD27 on CD24+ CD27+ B cell \|\| id:ebi-a-GCST90001798 | Spicy food liking \|\| id:ebi-fl187-GCST90094846 | Weighted median | 15 | 0.0720835390228582 | 0.155354896942017 | 0.642652971463785 | -0.232412059 | 0.376579137029212 | 1.07474512347116 | 0.792619450042096 | 1.45729086053046 | 0.999332001504091 |
| ebi-fl187-GCST90094846 | ebi-a-GCST90001836 | CD66b on CD66b++ myeloid cell \|\| id:ebi-a-GCST90001836 | Spicy food liking \|\| id:ebi-fl187-GCST90094846 | Weighted median | 15 | -0.082948893 | 0.241877200387569 | 0.731645073037422 | -0.557028206 | 0.391130419635467 | 0.920398184693886 | 0.572909104417992 | 1.47865134600783 | 0.999332001504091 |
| ebi-fl187-GCST90094846 | ebi-a-GCST90001943 | CD25 on activated & secreting CD4 regulatory T cell \|\| id:ebi-a-GCST90001943 | Spicy food liking \|\| id:ebi-fl187-GCST90094846 | Weighted median | 15 | -0.019173615 | 0.159913274683235 | 0.904562297363198 | -0.332603634 | 0.294256403196663 | 0.981009029395399 | 0.71705435411671 | 1.34212798545905 | 0.999332001504091 |
| ebi-fl187-GCST90094846 | ebi-a-GCST90001960 | CD25 on CD4+ T cell \|\| id:ebi-a-GCST90001960 | Spicy food liking \|\| id:ebi-fl187-GCST90094846 | Weighted median | 15 | 0.330736181829071 | 0.167986642588658 | 0.0489732723087911 | 0.00148236235530164 | 0.659990001302841 | 1.39199251094439 | 1.00148346159747 | 1.93477298909613 | 0.999332001504091 |
| ebi-fl187-GCST90094846 | ebi-a-GCST90001988 | HLA DR on CD14+ CD16- monocyte \|\| id:ebi-a-GCST90001988 | Spicy food liking \|\| id:ebi-fl187-GCST90094846 | Weighted median | 15 | 0.00108099369549947 | 0.163048304224539 | 0.994710145159514 | -0.318493683 | 0.320655669975595 | 1.00108157817977 | 0.727243672241445 | 1.37803100174406 | 0.999332001504091 |
| ebi-fl187-GCST90094846 | ebi-a-GCST90001991 | HLA DR on CD14+ monocyte \|\| id:ebi-a-GCST90001991 | Spicy food liking \|\| id:ebi-fl187-GCST90094846 | Weighted median | 15 | -0.014819642 | 0.158434678051199 | 0.925476269090467 | -0.325351611 | 0.295712327347461 | 0.985289628807297 | 0.722273350013978 | 1.34408344516163 | 0.999332001504091 |
| ebi-fl187-GCST90094846 | ebi-a-GCST90002019 | CD14 on Monocytic Myeloid-Derived Suppressor Cells \|\| id:ebi-a-GCST90002019 | Spicy food liking \|\| id:ebi-fl187-GCST90094846 | Weighted median | 15 | 0.181497198918771 | 0.224096563978054 | 0.417994082157884 | -0.257732066 | 0.620726464315757 | 1.19901117817731 | 0.772802264002534 | 1.86027897737812 | 0.999332001504091 |
| ebi-fl187-GCST90094846 | ebi-a-GCST90002037 | CD80 on plasmacytoid Dendritic Cell \|\| id:ebi-a-GCST90002037 | Spicy food liking \|\| id:ebi-fl187-GCST90094846 | Weighted median | 15 | 0.0437023036841695 | 0.177591819481908 | 0.805618087284686 | -0.304377663 | 0.39178226986871 | 1.04467131379148 | 0.737582256684384 | 1.4796155194468 | 0.999332001504091 |
| ebi-fl187-GCST90094846 | ebi-a-GCST90002042 | CD45 on CD33+ HLA DR+ CD14- \|\| id:ebi-a-GCST90002042 | Spicy food liking \|\| id:ebi-fl187-GCST90094846 | Weighted median | 15 | -0.077387624 | 0.237185484187395 | 0.74421652335617 | -0.542271173 | 0.3874959247775 | 0.925531025814784 | 0.581426231976186 | 1.47328694963466 | 0.999332001504091 |
| ebi-fl187-GCST90094846 | ebi-a-GCST90002056 | CD8 on Effector Memory CD8+ T cell \|\| id:ebi-a-GCST90002056 | Spicy food liking \|\| id:ebi-fl187-GCST90094846 | Weighted median | 15 | -0.130110555 | 0.184459177734059 | 0.480584782094299 | -0.491650543 | 0.231429433453899 | 0.877998358529754 | 0.611616061844372 | 1.26040038133775 | 0.999332001504091 |
| ebi-fl187-GCST90094846 | ebi-a-GCST90002058 | CD8 on CD8+ T cell \|\| id:ebi-a-GCST90002058 | Spicy food liking \|\| id:ebi-fl187-GCST90094846 | Weighted median | 15 | -0.59981377 | 0.195896853061849 | 0.00219947434200111 | -0.983771602 | -0.215855938 | 0.548913850831943 | 0.373898240823926 | 0.805851386118293 | 0.999332001504091 |
| ebi-fl187-GCST90094846 | ebi-a-GCST90002063 | CD4 on CD4 regulatory T cell \|\| id:ebi-a-GCST90002063 | Spicy food liking \|\| id:ebi-fl187-GCST90094846 | Weighted median | 15 | -0.443404598 | 0.187760261401966 | 0.0181988868223588 | -0.81141471 | -0.075394486 | 0.641847464254909 | 0.444229165811601 | 0.92737757688151 | 0.999332001504091 |
| ebi-fl187-GCST90094846 | ebi-a-GCST90002066 | CD4 on activated CD4 regulatory T cell \|\| id:ebi-a-GCST90002066 | Spicy food liking \|\| id:ebi-fl187-GCST90094846 | Weighted median | 15 | -0.414271953 | 0.173495725302311 | 0.0169498659501524 | -0.754323574 | -0.074220331 | 0.66082121474224 | 0.470328649564887 | 0.928467101158727 | 0.999332001504091 |
| ebi-fl187-GCST90094846 | ebi-a-GCST90002070 | CD4 on activated & secreting CD4 regulatory T cell \|\| id:ebi-a-GCST90002070 | Spicy food liking \|\| id:ebi-fl187-GCST90094846 | Weighted median | 15 | -0.384634605 | 0.17753029319108 | 0.0302664543600075 | -0.732593979 | -0.03667523 | 0.68069931507042 | 0.480660548015308 | 0.963989159190536 | 0.999332001504091 |
| ebi-fl187-GCST90094846 | ebi-a-GCST90002081 | SSC-A on CD4+ T cell \|\| id:ebi-a-GCST90002081 | Spicy food liking \|\| id:ebi-fl187-GCST90094846 | Weighted median | 15 | 0.223318607821576 | 0.166986306859548 | 0.181109452060571 | -0.103974554 | 0.55061176926629 | 1.25021883978831 | 0.901248230631542 | 1.73431369320564 | 0.999332001504091 |
| ebi-fl187-GCST90094846 | ebi-a-GCST90002094 | CD11b on Monocytic Myeloid-Derived Suppressor Cells \|\| id:ebi-a-GCST90002094 | Spicy food liking \|\| id:ebi-fl187-GCST90094846 | Weighted median | 15 | 0.157363610912494 | 0.235436290400047 | 0.503883731721928 | -0.304091518 | 0.618818740096585 | 1.17042111442733 | 0.737793341789332 | 1.85673346112747 | 0.999332001504091 |
| ebi-fl187-GCST90094846 | ebi-a-GCST90002104 | HLA DR on myeloid Dendritic Cell \|\| id:ebi-a-GCST90002104 | Spicy food liking \|\| id:ebi-fl187-GCST90094846 | Weighted median | 15 | -0.06336195 | 0.180584227910799 | 0.725684376754885 | -0.417307036 | 0.290583137088651 | 0.938603684955812 | 0.658818607645151 | 1.33720703572953 | 0.999332001504091 |
| ebi-fl187-GCST90094846 | ebi-a-GCST90002105 | HLA DR on plasmacytoid Dendritic Cell \|\| id:ebi-a-GCST90002105 | Spicy food liking \|\| id:ebi-fl187-GCST90094846 | Weighted median | 15 | 0.0184968900015548 | 0.164188713663562 | 0.910303062558623 | -0.303312989 | 0.340306768782136 | 1.01866901710559 | 0.738367959314659 | 1.40537865073944 | 0.999332001504091 |
| ebi-fl187-GCST90094846 | ebi-a-GCST90002106 | HLA DR on Dendritic Cell \|\| id:ebi-a-GCST90002106 | Spicy food liking \|\| id:ebi-fl187-GCST90094846 | Weighted median | 15 | -0.06379837 | 0.170430920481531 | 0.708154610003808 | -0.397842974 | 0.270246234280485 | 0.938194148675439 | 0.671767504339859 | 1.31028704860293 | 0.999332001504091 |
| ebi-fl187-GCST90094846 | ebi-a-GCST90002111 | HLA DR on CD33dim HLA DR+ CD11b- \|\| id:ebi-a-GCST90002111 | Spicy food liking \|\| id:ebi-fl187-GCST90094846 | Weighted median | 15 | 0.134817835553526 | 0.262288323777584 | 0.607247626768382 | -0.379267279 | 0.64890295015759 | 1.14432830943046 | 0.684362672414013 | 1.91344053752217 | 0.999332001504091 |
| ebi-fl187-GCST90094846 | ebi-a-GCST90002112 | HLA DR on CD33- HLA DR+ \|\| id:ebi-a-GCST90002112 | Spicy food liking \|\| id:ebi-fl187-GCST90094846 | Weighted median | 15 | -0.03030084 | 0.230974847212247 | 0.895627577076228 | -0.48301154 | 0.422409860921923 | 0.970153628999137 | 0.616922703951874 | 1.52563369419067 | 0.999332001504091 |
| ebi-fl187-GCST90094846 | ebi-a-GCST90002115 | HLA DR on HLA DR+ CD8+ T cell \|\| id:ebi-a-GCST90002115 | Spicy food liking \|\| id:ebi-fl187-GCST90094846 | Weighted median | 15 | -0.417393388 | 0.171169174796506 | 0.0147489893141779 | -0.75288497 | -0.081901805 | 0.658761720333106 | 0.471005753232663 | 0.921362427524036 | 0.999332001504091 |
| ebi-fl187-GCST90094846 | ebi-a-GCST90002116 | HLA DR on B cell \|\| id:ebi-a-GCST90002116 | Spicy food liking \|\| id:ebi-fl187-GCST90094846 | Weighted median | 15 | -0.058289581 | 0.170187930600427 | 0.731973696352324 | -0.391857925 | 0.275278763405106 | 0.943376724307469 | 0.675800121747663 | 1.31689772659939 | 0.999332001504091 |
| ebi-fl187-GCST90094846 | ebi-cfmet338-GCST90026023 | Sphingomyelin (d18:1/24:1, d18:2/24:0) levels \|\| id:ebi-cfmet338-GCST90026023 | Spicy food liking \|\| id:ebi-fl187-GCST90094846 | Weighted median | 15 | -0.121018438 | 0.0980074197901344 | 0.216909217610863 | -0.313112981 | 0.0710761050689846 | 0.886017623297705 | 0.731167300248914 | 1.07366293395077 | 0.999332001504091 |
| ebi-fl187-GCST90094846 | ebi-cfmet338-GCST90026046 | 1-arachidonoyl-gpc (20:4n6) levels \|\| id:ebi-cfmet338-GCST90026046 | Spicy food liking \|\| id:ebi-fl187-GCST90094846 | Weighted median | 15 | -0.114379293 | 0.112671033758016 | 0.310028859486734 | -0.335214519 | 0.106455932882905 | 0.891919592606727 | 0.715184648879021 | 1.1123289082374 | 0.999332001504091 |
| ebi-fl187-GCST90094846 | ebi-cfmet338-GCST90026047 | 1-linoleoyl-gpc (18:2) levels \|\| id:ebi-cfmet338-GCST90026047 | Spicy food liking \|\| id:ebi-fl187-GCST90094846 | Weighted median | 15 | 0.0111525505692032 | 0.136494029788757 | 0.934879520234309 | -0.256375748 | 0.278680848955167 | 1.01121497209856 | 0.773851141279935 | 1.32138555498544 | 0.999332001504091 |
| ebi-fl187-GCST90094846 | ebi-cfmet338-GCST90026050 | 1-myristoyl-2-palmitoyl-gpc (14:0/16:0) levels \|\| id:ebi-cfmet338-GCST90026050 | Spicy food liking \|\| id:ebi-fl187-GCST90094846 | Weighted median | 15 | -0.055558647 | 0.0719191334396605 | 0.439809159508776 | -0.196520148 | 0.0854028549108722 | 0.945956544879329 | 0.821584777696588 | 1.08915575007221 | 0.999332001504091 |
| ebi-fl187-GCST90094846 | ebi-cfmet338-GCST90026068 | 2,3-dihydroxyisovalerate levels \|\| id:ebi-cfmet338-GCST90026068 | Spicy food liking \|\| id:ebi-fl187-GCST90094846 | Weighted median | 15 | 0.0476562364734251 | 0.234831423419849 | 0.839183435699897 | -0.412613353 | 0.507925826376328 | 1.04881005070493 | 0.661918161744513 | 1.66184067160292 | 0.999332001504091 |
| ebi-fl187-GCST90094846 | ebi-cfmet338-GCST90026082 | 3-hydroxyhexanoate levels \|\| id:ebi-cfmet338-GCST90026082 | Spicy food liking \|\| id:ebi-fl187-GCST90094846 | Weighted median | 15 | 0.041260861895536 | 0.0595052460390572 | 0.488059360483189 | -0.07536942 | 0.157891144132088 | 1.04212392051214 | 0.927400822310884 | 1.17103871333375 | 0.999332001504091 |
| ebi-fl187-GCST90094846 | ebi-cfmet338-GCST90026094 | 3-ureidopropionate levels \|\| id:ebi-cfmet338-GCST90026094 | Spicy food liking \|\| id:ebi-fl187-GCST90094846 | Weighted median | 15 | -0.074672676 | 0.119252411096476 | 0.531201275018593 | -0.308407402 | 0.159062049413302 | 0.928047208430948 | 0.734615972957337 | 1.17241069181937 | 0.999332001504091 |
| ebi-fl187-GCST90094846 | ebi-cfmet338-GCST90026173 | Glycerol levels \|\| id:ebi-cfmet338-GCST90026173 | Spicy food liking \|\| id:ebi-fl187-GCST90094846 | Weighted median | 15 | -0.017673799 | 0.0592801478644503 | 0.765596116468156 | -0.133862888 | 0.0985152912348156 | 0.982481466942476 | 0.874709989273177 | 1.10353127862129 | 0.999332001504091 |
| ebi-fl187-GCST90094846 | ebi-cfmet338-GCST90026179 | Histidine levels \|\| id:ebi-cfmet338-GCST90026179 | Spicy food liking \|\| id:ebi-fl187-GCST90094846 | Weighted median | 15 | 0.0031095007749622 | 0.0493265444565787 | 0.94973537205114 | -0.093570526 | 0.0997895279098565 | 1.00311434028735 | 0.910673788653916 | 1.10493833491954 | 0.999332001504091 |
| ebi-fl187-GCST90094846 | ebi-cfmet338-GCST90026188 | Isocitrate levels \|\| id:ebi-cfmet338-GCST90026188 | Spicy food liking \|\| id:ebi-fl187-GCST90094846 | Weighted median | 15 | -0.017908165 | 0.0935919266386954 | 0.848256834077931 | -0.201348341 | 0.165532011391863 | 0.982251233435269 | 0.8176275687111 | 1.18002073622106 | 0.999332001504091 |
| ebi-fl187-GCST90094846 | ebi-cfmet338-GCST90026207 | N-acetyl-isoputreanine levels \|\| id:ebi-cfmet338-GCST90026207 | Spicy food liking \|\| id:ebi-fl187-GCST90094846 | Weighted median | 14 | 0.0219962335811268 | 0.0575626237271914 | 0.702367154508601 | -0.090826509 | 0.134818976086422 | 1.02223993427941 | 0.913176125065508 | 1.14432961457528 | 0.999332001504091 |
| ebi-fl187-GCST90094846 | ebi-cfmet338-GCST90026213 | N-acetylglutamate levels \|\| id:ebi-cfmet338-GCST90026213 | Spicy food liking \|\| id:ebi-fl187-GCST90094846 | Weighted median | 15 | 0.109932553840605 | 0.100480432079896 | 0.273924595035061 | -0.087009093 | 0.306874200717202 | 1.11620278432911 | 0.916668760293226 | 1.35916997470877 | 0.999332001504091 |
| ebi-fl187-GCST90094846 | ebi-cfmet338-GCST90026224 | N-acetyltaurine levels \|\| id:ebi-cfmet338-GCST90026224 | Spicy food liking \|\| id:ebi-fl187-GCST90094846 | Weighted median | 15 | 0.0494463629544573 | 0.0776329706551484 | 0.524173839229765 | -0.10271426 | 0.201606985438548 | 1.05068923483708 | 0.902384784499016 | 1.22336711252884 | 0.999332001504091 |
| ebi-fl187-GCST90094846 | ebi-cfmet338-GCST90026250 | Phenyllactate (pla) levels \|\| id:ebi-cfmet338-GCST90026250 | Spicy food liking \|\| id:ebi-fl187-GCST90094846 | Weighted median | 15 | 0.200627617107163 | 0.122024053556743 | 0.100142227506433 | -0.038539528 | 0.43979476207838 | 1.22216957203351 | 0.962193670527516 | 1.55238857680872 | 0.999332001504091 |
| ebi-fl187-GCST90094846 | ebi-cfmet338-GCST90026251 | Pipecolate levels \|\| id:ebi-cfmet338-GCST90026251 | Spicy food liking \|\| id:ebi-fl187-GCST90094846 | Weighted median | 14 | 0.077206920485931 | 0.188730216819697 | 0.682476429404921 | -0.292704304 | 0.447118145452537 | 1.08026558234706 | 0.746242768719294 | 1.56379904411857 | 0.999332001504091 |
| ebi-fl187-GCST90094846 | ebi-cfmet338-GCST90026278 | Trigonelline (n'-methylnicotinate) levels \|\| id:ebi-cfmet338-GCST90026278 | Spicy food liking \|\| id:ebi-fl187-GCST90094846 | Weighted median | 15 | 0.192670493061094 | 0.209548681840348 | 0.357857857749499 | -0.218044923 | 0.603385909468176 | 1.21248320605961 | 0.804089318472519 | 1.82829878622102 | 0.999332001504091 |
| ebi-fl187-GCST90094846 | ebi-cfmet338-GCST90026299 | 1-carboxyethylvaline levels \|\| id:ebi-cfmet338-GCST90026299 | Spicy food liking \|\| id:ebi-fl187-GCST90094846 | Weighted median | 15 | -0.073463659 | 0.118472928952372 | 0.535199753679373 | -0.3056706 | 0.158743281459274 | 0.929169911874721 | 0.736629225138453 | 1.17203702442162 | 0.999332001504091 |
| ebi-fl187-GCST90094846 | ebi-gm430-GCST90011306 | C_Erysipelotrichia abundance \|\| id:ebi-gm430-GCST90011306 | Spicy food liking \|\| id:ebi-fl187-GCST90094846 | Weighted median | 14 | -0.108777248 | 0.101807107061451 | 0.28531117777156 | -0.308319177 | 0.0907646822021673 | 0.896930188565475 | 0.73468078702129 | 1.0950112992907 | 0.999332001504091 |
| ebi-fl187-GCST90094846 | ebi-gm430-GCST90011312 | F_Erysipelotrichaceae abundance \|\| id:ebi-gm430-GCST90011312 | Spicy food liking \|\| id:ebi-fl187-GCST90094846 | Weighted median | 14 | -0.108777248 | 0.0964132830174003 | 0.259218902719896 | -0.297747282 | 0.0801927870758284 | 0.896930188565475 | 0.742488956102163 | 1.0834959315535 | 0.999332001504091 |
| ebi-fl187-GCST90094846 | ebi-gm430-GCST90011352 | O_Erysipelotrichales abundance \|\| id:ebi-gm430-GCST90011352 | Spicy food liking \|\| id:ebi-fl187-GCST90094846 | Weighted median | 14 | -0.108777248 | 0.0968889621700986 | 0.261564884986739 | -0.298679613 | 0.0811251182151172 | 0.896930188565475 | 0.741797033128599 | 1.0845065796059 | 0.999332001504091 |
| ebi-fl187-GCST90094846 | ebi-gm430-GCST90011362 | OTU97_11 (Parabacteroides) abundance \|\| id:ebi-gm430-GCST90011362 | Spicy food liking \|\| id:ebi-fl187-GCST90094846 | Weighted median | 14 | -0.078190249 | 0.132703764402152 | 0.55572109553338 | -0.338289628 | 0.181909128883494 | 0.924788469405891 | 0.712988756788009 | 1.19950518855149 | 0.999332001504091 |
| ebi-fl187-GCST90094846 | ebi-gm430-GCST90011384 | OTU97_235 (Phascolarctobacterium) abundance \|\| id:ebi-gm430-GCST90011384 | Spicy food liking \|\| id:ebi-fl187-GCST90094846 | Weighted median | 14 | 0.16207465638879 | 0.398988429591525 | 0.684585422701099 | -0.619942666 | 0.94409197838818 | 1.17594803007729 | 0.537975281194727 | 2.57047826876292 | 0.999332001504091 |
| ebi-fl187-GCST90094846 | ebi-gm430-GCST90011390 | OTU97_3 (Bacteroides) abundance \|\| id:ebi-gm430-GCST90011390 | Spicy food liking \|\| id:ebi-fl187-GCST90094846 | Weighted median | 14 | -0.107330517 | 0.114355485319459 | 0.347952059408177 | -0.331467268 | 0.116806234215459 | 0.898228744043587 | 0.717869652971664 | 1.12390163490859 | 0.999332001504091 |
| ebi-fl187-GCST90094846 | ebi-gm430-GCST90011398 | OTU97_4 (Alistipes) abundance \|\| id:ebi-gm430-GCST90011398 | Spicy food liking \|\| id:ebi-fl187-GCST90094846 | Weighted median | 14 | -0.065315976 | 0.118280784049145 | 0.580803646789178 | -0.297146313 | 0.166514360453258 | 0.9367714190573 | 0.742935303301922 | 1.18118049803591 | 0.999332001504091 |
| ebi-fl187-GCST90094846 | ebi-gm430-GCST90011421 | OTU99_108 (Cytophagales) abundance \|\| id:ebi-gm430-GCST90011421 | Spicy food liking \|\| id:ebi-fl187-GCST90094846 | Weighted median | 14 | 0.0510908839709998 | 0.376959085100321 | 0.892189450651046 | -0.687748923 | 0.78993069076763 | 1.05241853691269 | 0.502706427290813 | 2.20324371583321 | 0.999332001504091 |
| ebi-fl187-GCST90094846 | ebi-gm430-GCST90011428 | OTU99_123 (Phascolarctobacterium) abundance \|\| id:ebi-gm430-GCST90011428 | Spicy food liking \|\| id:ebi-fl187-GCST90094846 | Weighted median | 14 | -0.177992789 | 0.241794105536499 | 0.461649336346391 | -0.651909236 | 0.295923657577796 | 0.836948458473236 | 0.521050018975818 | 1.3443675206415 | 0.999332001504091 |
| ebi-fl187-GCST90094846 | ebi-gm430-GCST90011450 | OTU99_298 (Phascolarctobacterium) abundance \|\| id:ebi-gm430-GCST90011450 | Spicy food liking \|\| id:ebi-fl187-GCST90094846 | Weighted median | 14 | 0.171130981231166 | 0.40645320769429 | 0.673729605382839 | -0.625517306 | 0.967779268311974 | 1.186646167 | 0.53498460625799 | 2.63209279239799 | 0.999332001504091 |
| ebi-fl187-GCST90094846 | ebi-gm430-GCST90011451 | OTU99_3 (Bacteroides) abundance \|\| id:ebi-gm430-GCST90011451 | Spicy food liking \|\| id:ebi-fl187-GCST90094846 | Weighted median | 14 | -0.109035382 | 0.114994111262629 | 0.343036651151155 | -0.33442384 | 0.116353076005927 | 0.896698689882183 | 0.715750354196372 | 1.12339244503653 | 0.999332001504091 |
| ebi-fl187-GCST90094846 | ebi-gm430-GCST90011472 | OTU99_72 (Parabacteroides) abundance \|\| id:ebi-gm430-GCST90011472 | Spicy food liking \|\| id:ebi-fl187-GCST90094846 | Weighted median | 14 | -0.101965177 | 0.332207236264415 | 0.758894797211278 | -0.75309136 | 0.549161005772249 | 0.903060998138147 | 0.470908552256609 | 1.73179943844781 | 0.999332001504091 |
| ebi-fl187-GCST90094846 | ebi-gm430-GCST90011507 | TestASV_3 (Bacteroides) abundance \|\| id:ebi-gm430-GCST90011507 | Spicy food liking \|\| id:ebi-fl187-GCST90094846 | Weighted median | 14 | 0.0597417879185976 | 0.145089805913625 | 0.680517315703927 | -0.224634232 | 0.344117807509303 | 1.06156240291569 | 0.798808344123354 | 1.41074482205222 | 0.999332001504091 |
| ebi-fl187-GCST90094846 | ebi-gm430-GCST90011512 | TestASV_34 (Phascolarctobacterium) abundance \|\| id:ebi-gm430-GCST90011512 | Spicy food liking \|\| id:ebi-fl187-GCST90094846 | Weighted median | 14 | 0.347898030691376 | 0.327937379448074 | 0.288750227963585 | -0.294859233 | 0.9906552944096 | 1.41608784487922 | 0.744636400300812 | 2.69299860120267 | 0.999332001504091 |
| ebi-fl187-GCST90094846 | ebi-gm430-GCST90011519 | TestASV_41 (Barnesiella) abundance \|\| id:ebi-gm430-GCST90011519 | Spicy food liking \|\| id:ebi-fl187-GCST90094846 | Weighted median | 14 | -0.336945388 | 0.423548824387293 | 0.426305827829442 | -1.167101084 | 0.493210307940856 | 0.713947829058121 | 0.311267974442643 | 1.63756487807495 | 0.999332001504091 |
| ebi-fl187-GCST90094846 | ebi-gm430-GCST90011524 | TestASV_46 (Sutterella) abundance \|\| id:ebi-gm430-GCST90011524 | Spicy food liking \|\| id:ebi-fl187-GCST90094846 | Weighted median | 14 | -0.303328814 | 0.445503969523067 | 0.495955845604721 | -1.176516594 | 0.569858966284553 | 0.738356274585281 | 0.308350981571736 | 1.76801768374661 | 0.999332001504091 |
| ebi-fl187-GCST90094846 | ebi-gm430-GCST90011581 | OTU97_19 (Bacteria) prevalence \|\| id:ebi-gm430-GCST90011581 | Spicy food liking \|\| id:ebi-fl187-GCST90094846 | Weighted median | 14 | -1.165722984 | 0.509153268507189 | 0.0220484285571441 | -2.163663391 | -0.167782578 | 0.311697228337372 | 0.11490341298523 | 0.845537653139063 | 0.999332001504091 |
| ebi-fl187-GCST90094846 | ebi-gm430-GCST90011614 | OTU97_80 (Ruminococcus) prevalence \|\| id:ebi-gm430-GCST90011614 | Spicy food liking \|\| id:ebi-fl187-GCST90094846 | Weighted median | 14 | 0.147599628183259 | 0.237346263881282 | 0.534024285779357 | -0.317599049 | 0.612798305390572 | 1.15904875299928 | 0.727894579955854 | 1.84558870037289 | 0.999332001504091 |
| ebi-fl187-GCST90094846 | ebi-gm430-GCST90011623 | OTU99_11 (Parabacteroides) prevalence \|\| id:ebi-gm430-GCST90011623 | Spicy food liking \|\| id:ebi-fl187-GCST90094846 | Weighted median | 14 | -0.013751117 | 0.228425308551261 | 0.951996647325071 | -0.461464722 | 0.433962487689403 | 0.986342997651203 | 0.630359667420089 | 1.54336097199444 | 0.999332001504091 |
| ebi-fl187-GCST90094846 | ebi-gm430-GCST90011642 | OTU99_19 (Bacteria) prevalence \|\| id:ebi-gm430-GCST90011642 | Spicy food liking \|\| id:ebi-fl187-GCST90094846 | Weighted median | 14 | -1.165722984 | 0.481566582615669 | 0.0154911192324681 | -2.109593486 | -0.221852482 | 0.311697228337372 | 0.121287261351491 | 0.801033522157317 | 0.999332001504091 |
| ebi-fl187-GCST90094846 | ebi-gm430-GCST90011653 | OTU99_30 (Parasutterella) prevalence \|\| id:ebi-gm430-GCST90011653 | Spicy food liking \|\| id:ebi-fl187-GCST90094846 | Weighted median | 14 | 0.0402624692049413 | 0.23556144846803 | 0.864285647455301 | -0.42143797 | 0.50196290820228 | 1.04108399082269 | 0.656102685317103 | 1.65196073755355 | 0.999332001504091 |
| ebi-fl187-GCST90094846 | ebi-gm430-GCST90011675 | OTU99_8 (Ruminococcaceae) prevalence \|\| id:ebi-gm430-GCST90011675 | Spicy food liking \|\| id:ebi-fl187-GCST90094846 | Weighted median | 14 | -0.208393826 | 0.263815220551546 | 0.429572482611191 | -0.725471658 | 0.308684006331719 | 0.811887231482601 | 0.484096187149571 | 1.3616320354137 | 0.999332001504091 |
| ebi-fl187-GCST90094846 | ebi-gm430-GCST90011679 | OTU99_92 (Ruminococcus) prevalence \|\| id:ebi-gm430-GCST90011679 | Spicy food liking \|\| id:ebi-fl187-GCST90094846 | Weighted median | 14 | 0.158159063880536 | 0.244837988812513 | 0.518295958562012 | -0.321723394 | 0.638041521953062 | 1.17135249976416 | 0.724898673784569 | 1.89277029786857 | 0.999332001504091 |
| ebi-fl187-GCST90094846 | ebi-gm430-GCST90011684 | TestASV_11 (Lachnospiraceae) prevalence \|\| id:ebi-gm430-GCST90011684 | Spicy food liking \|\| id:ebi-fl187-GCST90094846 | Weighted median | 14 | 0.22873047150666 | 0.265736762885334 | 0.389380821508072 | -0.292113584 | 0.749574526761914 | 1.25700319517054 | 0.746683720020657 | 2.11609948135099 | 0.999332001504091 |
| ebi-fl187-GCST90094846 | ebi-gm430-GCST90011688 | TestASV_15 (Bacteroides) prevalence \|\| id:ebi-gm430-GCST90011688 | Spicy food liking \|\| id:ebi-fl187-GCST90094846 | Weighted median | 14 | -0.202348109 | 0.250764888313705 | 0.419710495808024 | -0.69384729 | 0.289151072356226 | 0.816810539559437 | 0.499650067872881 | 1.33529343921779 | 0.999332001504091 |
| ebi-fl187-GCST90094846 | ebi-gm430-GCST90011692 | TestASV_19 (Prevotella) prevalence \|\| id:ebi-gm430-GCST90011692 | Spicy food liking \|\| id:ebi-fl187-GCST90094846 | Weighted median | 14 | 0.232146425948454 | 0.465966383927593 | 0.6183400976266 | -0.681147687 | 1.14544053844654 | 1.26130440300095 | 0.506035888384035 | 3.1438260280509 | 0.999332001504091 |
| ebi-fl187-GCST90094846 | ebi-gm430-GCST90011693 | TestASV_2 (Bacteroides) prevalence \|\| id:ebi-gm430-GCST90011693 | Spicy food liking \|\| id:ebi-fl187-GCST90094846 | Weighted median | 14 | -0.453728103 | 0.246860049486605 | 0.0660628040866303 | -0.9375738 | 0.0301175940980248 | 0.635255433879412 | 0.391576727291234 | 1.03057571645003 | 0.999332001504091 |
| ebi-fl187-GCST90094846 | ebi-gm430-GCST90011716 | TestASV_40 (Barnesiella) prevalence \|\| id:ebi-gm430-GCST90011716 | Spicy food liking \|\| id:ebi-fl187-GCST90094846 | Weighted median | 14 | -0.046655344 | 0.504742463768152 | 0.926353259897919 | -1.035950573 | 0.94263988501751 | 0.954416286240414 | 0.354888872773298 | 2.56674840302145 | 0.999332001504091 |
| ebi-fl187-GCST90094846 | ebi-gm430-GCST90011718 | TestASV_42 (Alphaproteobacteria) prevalence \|\| id:ebi-gm430-GCST90011718 | Spicy food liking \|\| id:ebi-fl187-GCST90094846 | Weighted median | 14 | 0.226911052174323 | 0.397097559232517 | 0.56771230436685 | -0.551400164 | 1.00522226827006 | 1.25471825851571 | 0.576142551350895 | 2.73251455661687 | 0.999332001504091 |
| ebi-fl187-GCST90094846 | ebi-hbsm44-GCST90104929 | Sulcal depth of the right inferior frontal sulcus \|\| id:ebi-hbsm44-GCST90104929 | Spicy food liking \|\| id:ebi-fl187-GCST90094846 | Weighted median | 12 | 0.261334360248142 | 0.206105811238633 | 0.20481148025576 | -0.14263303 | 0.665301750275862 | 1.29866181373497 | 0.86707220019599 | 1.94507736042303 | 0.999332001504091 |
| ebi-fl187-GCST90094846 | ebi-hbsm44-GCST90104935 | Sulcal opening distance of the left calloso-marginal posterior fissure \|\| id:ebi-hbsm44-GCST90104935 | Spicy food liking \|\| id:ebi-fl187-GCST90094846 | Weighted median | 13 | -0.060594692 | 0.0467267535735649 | 0.194704153722271 | -0.152179129 | 0.0309897454576247 | 0.941204640675565 | 0.858834425195655 | 1.03147492652895 | 0.999332001504091 |
| ebi-fl187-GCST90094846 | ebi-hbsm44-GCST90104936 | Sulcal opening distance of the left anterior inferior temporal sulcus \|\| id:ebi-hbsm44-GCST90104936 | Spicy food liking \|\| id:ebi-fl187-GCST90094846 | Weighted median | 14 | -0.023053461 | 0.0277977373157523 | 0.406918437447752 | -0.077537026 | 0.031430103908321 | 0.977210239514854 | 0.925392759828103 | 1.03192924525373 | 0.999332001504091 |
| ebi-fl187-GCST90094846 | ebi-hbsm44-GCST90104941 | Sulcal opening distance of the right posterior inferior temporal sulcus \|\| id:ebi-hbsm44-GCST90104941 | Spicy food liking \|\| id:ebi-fl187-GCST90094846 | Weighted median | 14 | -0.010518173 | 0.01913694085356 | 0.582575447511081 | -0.048026578 | 0.026990230584545 | 0.989536949066191 | 0.953108455442375 | 1.02735776602963 | 0.999332001504091 |
| ebi-fl187-GCST90094846 | ebi-IPs91-GCST90274819 | Leukemia inhibitory factor levels \|\| id:ebi-IPs91-GCST90274819 | Spicy food liking \|\| id:ebi-fl187-GCST90094846 | Weighted median | 16 | -0.071866653 | 0.0837978348325102 | 0.391102633462508 | -0.236110409 | 0.0923771035523998 | 0.930654987849235 | 0.789693479859044 | 1.09677834311518 | 0.999332001504091 |
| ebi-fl187-GCST90094846 | ebi-IPs91-GCST90274837 | STAM binding protein levels \|\| id:ebi-IPs91-GCST90274837 | Spicy food liking \|\| id:ebi-fl187-GCST90094846 | Weighted median | 16 | -0.025494799 | 0.0685754730560265 | 0.710058717916614 | -0.159902727 | 0.10891312773725 | 0.974827448588949 | 0.852226683885413 | 1.11506547787257 | 0.999332001504091 |
| ebi-fl187-GCST90094846 | ebi-IPs91-GCST90274840 | TNF-beta levels \|\| id:ebi-IPs91-GCST90274840 | Spicy food liking \|\| id:ebi-fl187-GCST90094846 | Weighted median | 16 | -0.099651968 | 0.0869324019404002 | 0.251664593325808 | -0.270039476 | 0.0707355399224008 | 0.905152385326292 | 0.763349360003978 | 1.07329734403342 | 0.999332001504091 |
| ebi-fl187-GCST90094846 | ebi-IPs91-GCST90274842 | Tumor necrosis factor ligand superfamily member 14 levels \|\| id:ebi-IPs91-GCST90274842 | Spicy food liking \|\| id:ebi-fl187-GCST90094846 | Weighted median | 16 | -0.029800168 | 0.0790513176721305 | 0.706194086244115 | -0.184740751 | 0.125140414527094 | 0.970639478890932 | 0.831319774948079 | 1.13330757474223 | 0.999332001504091 |
| ebi-fl187-GCST90094846 | ebi-met1400-GCST90199628 | 3-methylhistidine levels \|\| id:ebi-met1400-GCST90199628 | Spicy food liking \|\| id:ebi-fl187-GCST90094846 | Weighted median | 16 | -0.044268829 | 0.104926403006234 | 0.673095368414857 | -0.249924579 | 0.161386920739732 | 0.9566967349277 | 0.778859523185539 | 1.17513956673197 | 0.999332001504091 |
| ebi-fl187-GCST90094846 | ebi-met1400-GCST90199651 | Docosahexaenoate DHA; 22:6n3 levels \|\| id:ebi-met1400-GCST90199651 | Spicy food liking \|\| id:ebi-fl187-GCST90094846 | Weighted median | 16 | 0.14906728487085 | 0.0943129641660168 | 0.113978711234889 | -0.035786125 | 0.333920694636243 | 1.16075108756865 | 0.964846628091751 | 1.39643239460405 | 0.999332001504091 |
| ebi-fl187-GCST90094846 | ebi-met1400-GCST90199652 | 1-stearoyl-2-oleoyl-GPS (18:0/18:1) levels \|\| id:ebi-met1400-GCST90199652 | Spicy food liking \|\| id:ebi-fl187-GCST90094846 | Weighted median | 16 | 0.00777547437370805 | 0.0998180344741444 | 0.937910392702569 | -0.187867873 | 0.203418821943031 | 1.00780578187539 | 0.8287241966791 | 1.2255856629401 | 0.999332001504091 |
| ebi-fl187-GCST90094846 | ebi-met1400-GCST90199653 | 1-myristoyl-2-palmitoyl-gpc (14:0/16:0) levels \|\| id:ebi-met1400-GCST90199653 | Spicy food liking \|\| id:ebi-fl187-GCST90094846 | Weighted median | 16 | -0.09858544 | 0.0929174457910887 | 0.288689755144887 | -0.280703634 | 0.0835327539098934 | 0.906118270706209 | 0.755252133678052 | 1.08712082216719 | 0.999332001504091 |
| ebi-fl187-GCST90094846 | ebi-met1400-GCST90199667 | Erythritol levels in elite athletes \|\| id:ebi-met1400-GCST90199667 | Spicy food liking \|\| id:ebi-fl187-GCST90094846 | Weighted median | 16 | 0.152232059466489 | 0.0971069648283149 | 0.116957243303934 | -0.038097592 | 0.342561710529986 | 1.16443042218385 | 0.962618992782185 | 1.40855127342587 | 0.999332001504091 |
| ebi-fl187-GCST90094846 | ebi-met1400-GCST90199674 | 3-hydroxymyristate levels \|\| id:ebi-met1400-GCST90199674 | Spicy food liking \|\| id:ebi-fl187-GCST90094846 | Weighted median | 16 | 0.0376068913935228 | 0.0914980180224588 | 0.681062869991621 | -0.141729224 | 0.216943006717542 | 1.03832297894036 | 0.86785621936929 | 1.24227329888712 | 0.999332001504091 |
| ebi-fl187-GCST90094846 | ebi-met1400-GCST90199685 | 2-linoleoylglycerol (18:2) levels \|\| id:ebi-met1400-GCST90199685 | Spicy food liking \|\| id:ebi-fl187-GCST90094846 | Weighted median | 16 | -0.13080579 | 0.112840108457126 | 0.246369124747373 | -0.351972402 | 0.0903608229857051 | 0.87738815575882 | 0.703299531252069 | 1.09456915817275 | 0.999332001504091 |
| ebi-fl187-GCST90094846 | ebi-met1400-GCST90199703 | Gamma-glutamylvaline levels \|\| id:ebi-met1400-GCST90199703 | Spicy food liking \|\| id:ebi-fl187-GCST90094846 | Weighted median | 16 | 0.0212841566625807 | 0.089999210997167 | 0.813050369000729 | -0.155114297 | 0.197682610217028 | 1.02151227992067 | 0.856317297484476 | 1.21857556900239 | 0.999332001504091 |
| ebi-fl187-GCST90094846 | ebi-met1400-GCST90199779 | Gamma-glutamyl-2-aminobutyrate levels \|\| id:ebi-met1400-GCST90199779 | Spicy food liking \|\| id:ebi-fl187-GCST90094846 | Weighted median | 16 | 0.0103302477615978 | 0.097584401349739 | 0.915693731609357 | -0.180935179 | 0.201595674407086 | 1.01038378897686 | 0.834489449480155 | 1.2233532750632 | 0.999332001504091 |
| ebi-fl187-GCST90094846 | ebi-met1400-GCST90199824 | 4-vinylphenol sulfate levels \|\| id:ebi-met1400-GCST90199824 | Spicy food liking \|\| id:ebi-fl187-GCST90094846 | Weighted median | 16 | -0.039527292 | 0.0946787694256861 | 0.676321175447581 | -0.22509768 | 0.146043095989461 | 0.961243719287419 | 0.798438223377345 | 1.15724605964015 | 0.999332001504091 |
| ebi-fl187-GCST90094846 | ebi-met1400-GCST90199830 | 4-ethylphenylsulfate levels \|\| id:ebi-met1400-GCST90199830 | Spicy food liking \|\| id:ebi-fl187-GCST90094846 | Weighted median | 16 | -0.003991288 | 0.097466451310952 | 0.967335454704514 | -0.195025533 | 0.187042956477098 | 0.996016666511392 | 0.822813649165157 | 1.20567907566315 | 0.999332001504091 |
| ebi-fl187-GCST90094846 | ebi-met1400-GCST90199833 | Hexanoylglutamine levels \|\| id:ebi-met1400-GCST90199833 | Spicy food liking \|\| id:ebi-fl187-GCST90094846 | Weighted median | 15 | -0.033542412 | 0.0929665085842223 | 0.718248195310145 | -0.215756769 | 0.148671945088718 | 0.967013897623199 | 0.805931305866258 | 1.16029228718358 | 0.999332001504091 |
| ebi-fl187-GCST90094846 | ebi-met1400-GCST90199837 | Carotenoid levels (cryptoxanthin) \|\| id:ebi-met1400-GCST90199837 | Spicy food liking \|\| id:ebi-fl187-GCST90094846 | Weighted median | 16 | 0.113260662864997 | 0.0982031807111535 | 0.24877500928448 | -0.079217571 | 0.305738897058858 | 1.11992381745604 | 0.923838901719985 | 1.35762777965965 | 0.999332001504091 |
| ebi-fl187-GCST90094846 | ebi-met1400-GCST90199869 | 2-hydroxyhippurate levels \|\| id:ebi-met1400-GCST90199869 | Spicy food liking \|\| id:ebi-fl187-GCST90094846 | Weighted median | 16 | 0.140304743564378 | 0.097345558964012 | 0.149498237431042 | -0.050492552 | 0.331102039133841 | 1.15062439081231 | 0.950761009909123 | 1.39250187474426 | 0.999332001504091 |
| ebi-fl187-GCST90094846 | ebi-met1400-GCST90199902 | Hydantoin-5-propionate levels \|\| id:ebi-met1400-GCST90199902 | Spicy food liking \|\| id:ebi-fl187-GCST90094846 | Weighted median | 16 | -0.073569485 | 0.0939380603421181 | 0.433527197723624 | -0.257688084 | 0.110549112900189 | 0.929071586665296 | 0.772836254786279 | 1.11689120147123 | 0.999332001504091 |
| ebi-fl187-GCST90094846 | ebi-met1400-GCST90199904 | Pantoate levels \|\| id:ebi-met1400-GCST90199904 | Spicy food liking \|\| id:ebi-fl187-GCST90094846 | Weighted median | 16 | -0.031340553 | 0.0978750222599803 | 0.748809201610897 | -0.223175597 | 0.160494490308963 | 0.969145471162218 | 0.799974363902005 | 1.17409130424246 | 0.999332001504091 |
| ebi-fl187-GCST90094846 | ebi-met1400-GCST90199936 | 3-methyl catechol sulfate (2) levels \|\| id:ebi-met1400-GCST90199936 | Spicy food liking \|\| id:ebi-fl187-GCST90094846 | Weighted median | 16 | -0.153003796 | 0.112235376542937 | 0.172806769281084 | -0.372985134 | 0.0669775422033798 | 0.858126464510949 | 0.688675470724195 | 1.06927146442387 | 0.999332001504091 |
| ebi-fl187-GCST90094846 | ebi-met1400-GCST90199977 | 4-hydroxychlorothalonil levels \|\| id:ebi-met1400-GCST90199977 | Spicy food liking \|\| id:ebi-fl187-GCST90094846 | Weighted median | 16 | 0.0907166434101998 | 0.095584425949425 | 0.342583243306275 | -0.096628831 | 0.278062118271073 | 1.09495869753417 | 0.907892924902459 | 1.32056822607636 | 0.999332001504091 |
| ebi-fl187-GCST90094846 | ebi-met1400-GCST90200040 | Sphingomyelin (d18:2/24:1, d18:1/24:2) levels \|\| id:ebi-met1400-GCST90200040 | Spicy food liking \|\| id:ebi-fl187-GCST90094846 | Weighted median | 16 | 0.0589693357778293 | 0.085110594542848 | 0.488400295698866 | -0.10784743 | 0.225786101081811 | 1.06074271339122 | 0.897764558346067 | 1.25330755547474 | 0.999332001504091 |
| ebi-fl187-GCST90094846 | ebi-met1400-GCST90200077 | 1-stearoyl-2-oleoyl-GPI (18:0/18:1) levels \|\| id:ebi-met1400-GCST90200077 | Spicy food liking \|\| id:ebi-fl187-GCST90094846 | Weighted median | 16 | -0.028059982 | 0.0907300982070278 | 0.757117114409824 | -0.205890975 | 0.149771010099045 | 0.972330042375415 | 0.813921809374804 | 1.16156822487898 | 0.999332001504091 |
| ebi-fl187-GCST90094846 | ebi-met1400-GCST90200080 | 1-palmitoyl-2-oleoyl-GPI (16:0/18:1) levels \|\| id:ebi-met1400-GCST90200080 | Spicy food liking \|\| id:ebi-fl187-GCST90094846 | Weighted median | 16 | -0.049891887 | 0.0969128879506699 | 0.606684620804088 | -0.239841147 | 0.140057373464974 | 0.951332270404557 | 0.786752828950359 | 1.15033979594396 | 0.999332001504091 |
| ebi-fl187-GCST90094846 | ebi-met1400-GCST90200082 | 1-oleoyl-2-linoleoyl-GPE (18:1/18:2) levels \|\| id:ebi-met1400-GCST90200082 | Spicy food liking \|\| id:ebi-fl187-GCST90094846 | Weighted median | 16 | -0.154580678 | 0.0926828786877905 | 0.0953464954328663 | -0.33623912 | 0.027077764200834 | 0.856774366487999 | 0.714452245385457 | 1.0274476983061 | 0.999332001504091 |
| ebi-fl187-GCST90094846 | ebi-met1400-GCST90200100 | Perfluorooctanesulfonate (PFOS) levels \|\| id:ebi-met1400-GCST90200100 | Spicy food liking \|\| id:ebi-fl187-GCST90094846 | Weighted median | 16 | 0.152912729179188 | 0.0943225702975822 | 0.104981621472738 | -0.031959509 | 0.337784966962449 | 1.16522328451286 | 0.96854579905664 | 1.40183902928841 | 0.999332001504091 |
| ebi-fl187-GCST90094846 | ebi-met1400-GCST90200102 | 1-palmitoleoyl-2-linolenoyl-GPC (16:1/18:3) levels \|\| id:ebi-met1400-GCST90200102 | Spicy food liking \|\| id:ebi-fl187-GCST90094846 | Weighted median | 16 | -0.125683403 | 0.0894344974506148 | 0.159928418517182 | -0.300975018 | 0.0496082115194561 | 0.881894007160853 | 0.740096261240177 | 1.05085930114412 | 0.999332001504091 |
| ebi-fl187-GCST90094846 | ebi-met1400-GCST90200114 | Glycosyl-N-tricosanoyl-sphingadienine (d18:2/23:0) levels \|\| id:ebi-met1400-GCST90200114 | Spicy food liking \|\| id:ebi-fl187-GCST90094846 | Weighted median | 16 | -0.029830976 | 0.107570444394838 | 0.781537686398989 | -0.240669047 | 0.181007095281165 | 0.970609576256962 | 0.786101746272642 | 1.19842368241601 | 0.999332001504091 |
| ebi-fl187-GCST90094846 | ebi-met1400-GCST90200135 | Arachidoylcarnitine (C20) levels \|\| id:ebi-met1400-GCST90200135 | Spicy food liking \|\| id:ebi-fl187-GCST90094846 | Weighted median | 16 | -0.137863126 | 0.100782468737595 | 0.171334723286945 | -0.335396765 | 0.059670512912703 | 0.871217930831233 | 0.715054321864302 | 1.06148674274554 | 0.999332001504091 |
| ebi-fl187-GCST90094846 | ebi-met1400-GCST90200146 | Docosahexaenoylcarnitine (C22:6) levels \|\| id:ebi-met1400-GCST90200146 | Spicy food liking \|\| id:ebi-fl187-GCST90094846 | Weighted median | 16 | 0.0675303137052203 | 0.107674326212317 | 0.530546548763605 | -0.143511366 | 0.278571993081362 | 1.06986269060858 | 0.866310953926072 | 1.32124172223488 | 0.999332001504091 |
| ebi-fl187-GCST90094846 | ebi-met1400-GCST90200162 | 3-hydroxyphenylacetoylglutamine levels \|\| id:ebi-met1400-GCST90200162 | Spicy food liking \|\| id:ebi-fl187-GCST90094846 | Weighted median | 16 | 0.00859491983960996 | 0.120185309748032 | 0.94298876481981 | -0.226968287 | 0.244158126945752 | 1.00863196221249 | 0.796946055227238 | 1.27654617087794 | 0.999332001504091 |
| ebi-fl187-GCST90094846 | ebi-met1400-GCST90200172 | 3-carboxy-4-methyl-5-pentyl-2-furanpropionate (3-CMPFP) levels \|\| id:ebi-met1400-GCST90200172 | Spicy food liking \|\| id:ebi-fl187-GCST90094846 | Weighted median | 16 | 0.13407575387321 | 0.0978614090159765 | 0.170668964380294 | -0.057732608 | 0.325884115544524 | 1.14347943936018 | 0.94390230581189 | 1.38525483006931 | 0.999332001504091 |
| ebi-fl187-GCST90094846 | ebi-met1400-GCST90200222 | Dihydrocaffeate sulfate (2) levels \|\| id:ebi-met1400-GCST90200222 | Spicy food liking \|\| id:ebi-fl187-GCST90094846 | Weighted median | 16 | 0.0416771766501017 | 0.109638257732766 | 0.703846306010683 | -0.173213809 | 0.256568161806322 | 1.04255786239828 | 0.840957791692298 | 1.29248686103638 | 0.999332001504091 |
| ebi-fl187-GCST90094846 | ebi-met1400-GCST90200223 | 4-methylhexanoylglutamine levels \|\| id:ebi-met1400-GCST90200223 | Spicy food liking \|\| id:ebi-fl187-GCST90094846 | Weighted median | 16 | -0.052029467 | 0.118875000857645 | 0.661616702756692 | -0.285024469 | 0.180965534401997 | 0.949300893116477 | 0.751995853537821 | 1.19837387590917 | 0.999332001504091 |
| ebi-fl187-GCST90094846 | ebi-met1400-GCST90200224 | 2-ketocaprylate levels \|\| id:ebi-met1400-GCST90200224 | Spicy food liking \|\| id:ebi-fl187-GCST90094846 | Weighted median | 16 | -0.026733801 | 0.0942477437340352 | 0.776675132852008 | -0.211459379 | 0.157991776926077 | 0.973620384005545 | 0.809402159505579 | 1.17115656416107 | 0.999332001504091 |
| ebi-fl187-GCST90094846 | ebi-met1400-GCST90200243 | Hydroxypalmitoyl sphingomyelin (d18:1/16:0(OH)) levels \|\| id:ebi-met1400-GCST90200243 | Spicy food liking \|\| id:ebi-fl187-GCST90094846 | Weighted median | 16 | 0.0463374914033994 | 0.0903933683792314 | 0.608216828136325 | -0.130833511 | 0.223508493426693 | 1.04742784920719 | 0.877363833992869 | 1.25045626088995 | 0.999332001504091 |
| ebi-fl187-GCST90094846 | ebi-met1400-GCST90200273 | 2-methoxyhydroquinone sulfate (2) levels \|\| id:ebi-met1400-GCST90200273 | Spicy food liking \|\| id:ebi-fl187-GCST90094846 | Weighted median | 16 | 0.0139511363054756 | 0.12580230155249 | 0.911697782086204 | -0.232621375 | 0.260523647348355 | 1.01404890755182 | 0.792453559666688 | 1.29760939851106 | 0.999332001504091 |
| ebi-fl187-GCST90094846 | ebi-met1400-GCST90200276 | 2,4-di-tert-butylphenol levels \|\| id:ebi-met1400-GCST90200276 | Spicy food liking \|\| id:ebi-fl187-GCST90094846 | Weighted median | 16 | 0.171319767617784 | 0.108852087608061 | 0.115515950767883 | -0.042030324 | 0.384669859329582 | 1.18687021100864 | 0.95884070415597 | 1.46912922206373 | 0.999332001504091 |
| ebi-fl187-GCST90094846 | ebi-met1400-GCST90200289 | N-acetylmethionine levels \|\| id:ebi-met1400-GCST90200289 | Spicy food liking \|\| id:ebi-fl187-GCST90094846 | Weighted median | 16 | 0.0810283481450412 | 0.101734028985672 | 0.425757500456135 | -0.118370349 | 0.280427044956958 | 1.08440163690593 | 0.888366986154697 | 1.32369496891624 | 0.999332001504091 |
| ebi-fl187-GCST90094846 | ebi-met1400-GCST90200308 | 3-hydroxyisobutyrate levels \|\| id:ebi-met1400-GCST90200308 | Spicy food liking \|\| id:ebi-fl187-GCST90094846 | Weighted median | 16 | -0.023209936 | 0.0979348053360454 | 0.812661645100371 | -0.215162154 | 0.168742282464614 | 0.977057342736311 | 0.806410666494582 | 1.18381500972043 | 0.999332001504091 |
| ebi-fl187-GCST90094846 | ebi-met1400-GCST90200383 | Cystathionine levels \|\| id:ebi-met1400-GCST90200383 | Spicy food liking \|\| id:ebi-fl187-GCST90094846 | Weighted median | 16 | -0.056613331 | 0.101108816012845 | 0.575530748337348 | -0.254786611 | 0.141559948213002 | 0.944959385071643 | 0.77508187460487 | 1.15206956670248 | 0.999332001504091 |
| ebi-fl187-GCST90094846 | ebi-met1400-GCST90200384 | Guanidinoacetate levels \|\| id:ebi-met1400-GCST90200384 | Spicy food liking \|\| id:ebi-fl187-GCST90094846 | Weighted median | 16 | 0.12708793756978 | 0.0918765681767852 | 0.166589308825443 | -0.052990136 | 0.307166011196279 | 1.13551686798494 | 0.948389367295087 | 1.3595666526248 | 0.999332001504091 |
| ebi-fl187-GCST90094846 | ebi-met1400-GCST90200403 | Citrulline levels \|\| id:ebi-met1400-GCST90200403 | Spicy food liking \|\| id:ebi-fl187-GCST90094846 | Weighted median | 16 | -0.01441661 | 0.0958590041336183 | 0.880453847918383 | -0.202300258 | 0.173467037925941 | 0.985686811555095 | 0.816849625255263 | 1.18942148032453 | 0.999332001504091 |
| ebi-fl187-GCST90094846 | ebi-met1400-GCST90200434 | 12,13-DiHOME levels \|\| id:ebi-met1400-GCST90200434 | Spicy food liking \|\| id:ebi-fl187-GCST90094846 | Weighted median | 16 | 0.0719712269368922 | 0.0994437923737003 | 0.469226750269555 | -0.122938606 | 0.266881059989345 | 1.07462442338262 | 0.884317952608456 | 1.30588511510378 | 0.999332001504091 |
| ebi-fl187-GCST90094846 | ebi-met1400-GCST90200437 | Fructose levels \|\| id:ebi-met1400-GCST90200437 | Spicy food liking \|\| id:ebi-fl187-GCST90094846 | Weighted median | 16 | 0.0662152506984434 | 0.0980420230824128 | 0.499436771433549 | -0.125947115 | 0.258377615939972 | 1.06845667846174 | 0.881661472620314 | 1.29482767388784 | 0.999332001504091 |
| ebi-fl187-GCST90094846 | ebi-met1400-GCST90200448 | N-stearoyl-sphinganine (d18:0/18:0) levels \|\| id:ebi-met1400-GCST90200448 | Spicy food liking \|\| id:ebi-fl187-GCST90094846 | Weighted median | 16 | 0.0325338582948699 | 0.102374760566425 | 0.750642879622179 | -0.168120672 | 0.233188389005063 | 1.03306887050227 | 0.845251829950328 | 1.26261932052079 | 0.999332001504091 |
| ebi-fl187-GCST90094846 | ebi-met1400-GCST90200468 | X-11847 levels \|\| id:ebi-met1400-GCST90200468 | Spicy food liking \|\| id:ebi-fl187-GCST90094846 | Weighted median | 16 | -0.039807056 | 0.100167215313572 | 0.69106806833611 | -0.236134798 | 0.15652068647461 | 0.960974836036917 | 0.789674220604276 | 1.1694349535553 | 0.999332001504091 |
| ebi-fl187-GCST90094846 | ebi-met1400-GCST90200476 | X-11843 levels \|\| id:ebi-met1400-GCST90200476 | Spicy food liking \|\| id:ebi-fl187-GCST90094846 | Weighted median | 16 | 0.0737923478909594 | 0.0979249511496024 | 0.451113337459653 | -0.118140556 | 0.26572525214418 | 1.07658322750605 | 0.888571149508475 | 1.3043766347675 | 0.999332001504091 |
| ebi-fl187-GCST90094846 | ebi-met1400-GCST90200511 | X-12906 levels \|\| id:ebi-met1400-GCST90200511 | Spicy food liking \|\| id:ebi-fl187-GCST90094846 | Weighted median | 16 | 0.0434490775535265 | 0.0996592691712837 | 0.662853938643077 | -0.15188309 | 0.238781245129243 | 1.04440680920804 | 0.859088710912495 | 1.26970075297757 | 0.999332001504091 |
| ebi-fl187-GCST90094846 | ebi-met1400-GCST90200515 | X-12849 levels \|\| id:ebi-met1400-GCST90200515 | Spicy food liking \|\| id:ebi-fl187-GCST90094846 | Weighted median | 16 | -0.07382939 | 0.111827637436478 | 0.509120997309482 | -0.293011559 | 0.14535277973768 | 0.928830148371931 | 0.746013517467203 | 1.15644746954944 | 0.999332001504091 |
| ebi-fl187-GCST90094846 | ebi-met1400-GCST90200551 | X-18886 levels \|\| id:ebi-met1400-GCST90200551 | Spicy food liking \|\| id:ebi-fl187-GCST90094846 | Weighted median | 16 | -0.0971991 | 0.0920977083163996 | 0.291246498324195 | -0.277710609 | 0.0833124079629999 | 0.90737532941347 | 0.757516008387356 | 1.08688130588944 | 0.999332001504091 |
| ebi-fl187-GCST90094846 | ebi-met1400-GCST90200564 | X-21319 levels \|\| id:ebi-met1400-GCST90200564 | Spicy food liking \|\| id:ebi-fl187-GCST90094846 | Weighted median | 16 | -0.052992816 | 0.0866076017664943 | 0.54062207311898 | -0.222743715 | 0.116758083847796 | 0.948386826034353 | 0.800319932946009 | 1.1238475199345 | 0.999332001504091 |
| ebi-fl187-GCST90094846 | ebi-met1400-GCST90200579 | X-21442 levels \|\| id:ebi-met1400-GCST90200579 | Spicy food liking \|\| id:ebi-fl187-GCST90094846 | Weighted median | 16 | 0.106100619171187 | 0.110152024694433 | 0.335437194394496 | -0.109797349 | 0.321998587572275 | 1.11193375273061 | 0.89601569516993 | 1.37988282697111 | 0.999332001504091 |
| ebi-fl187-GCST90094846 | ebi-met1400-GCST90200581 | X-21441 levels \|\| id:ebi-met1400-GCST90200581 | Spicy food liking \|\| id:ebi-fl187-GCST90094846 | Weighted median | 16 | -0.055739107 | 0.0887134304113644 | 0.529804047859069 | -0.22961743 | 0.11813921695377 | 0.945785852942766 | 0.794837625169263 | 1.12540077532966 | 0.999332001504091 |
| ebi-fl187-GCST90094846 | ebi-met1400-GCST90200648 | X-24585 levels \|\| id:ebi-met1400-GCST90200648 | Spicy food liking \|\| id:ebi-fl187-GCST90094846 | Weighted median | 16 | -0.036642884 | 0.0968604289045414 | 0.705203272249243 | -0.226489324 | 0.153203557089335 | 0.964020341393545 | 0.797327854367128 | 1.16556221324812 | 0.999332001504091 |
| ebi-fl187-GCST90094846 | ebi-met1400-GCST90200720 | Adenosine 5'-diphosphate (ADP) to pantothenate ratio \|\| id:ebi-met1400-GCST90200720 | Spicy food liking \|\| id:ebi-fl187-GCST90094846 | Weighted median | 16 | -0.096102921 | 0.129139048144612 | 0.456766537278388 | -0.349215455 | 0.157009613637923 | 0.908370521104434 | 0.705241166101637 | 1.17000686186918 | 0.999332001504091 |
| ebi-fl187-GCST90094846 | ebi-met1400-GCST90200724 | Cholate to taurocholate ratio \|\| id:ebi-met1400-GCST90200724 | Spicy food liking \|\| id:ebi-fl187-GCST90094846 | Weighted median | 16 | 0.0272317158329646 | 0.102566957030615 | 0.790622892454958 | -0.17379952 | 0.22826295161297 | 1.02760588773263 | 0.840465377312817 | 1.2564156585236 | 0.999332001504091 |
| ebi-fl187-GCST90094846 | ebi-met1400-GCST90200726 | N-acetylputrescine to (N(1) + N(8))-acetylspermidine ratio \|\| id:ebi-met1400-GCST90200726 | Spicy food liking \|\| id:ebi-fl187-GCST90094846 | Weighted median | 16 | 0.0802568854393715 | 0.0945239586192744 | 0.395845710148468 | -0.105010073 | 0.265523844333149 | 1.08356538409549 | 0.900315453253916 | 1.30411394957904 | 0.999332001504091 |
| ebi-fl187-GCST90094846 | ebi-met1400-GCST90200744 | Arginine to glutamate ratio \|\| id:ebi-met1400-GCST90200744 | Spicy food liking \|\| id:ebi-fl187-GCST90094846 | Weighted median | 16 | 0.0429080874960453 | 0.0974392123592958 | 0.659678204671379 | -0.148072769 | 0.233888943720265 | 1.04384194831413 | 0.862368359210028 | 1.26350416434386 | 0.999332001504091 |
| ebi-fl187-GCST90094846 | ebi-met1400-GCST90200759 | Histidine to trans-urocanate ratio \|\| id:ebi-met1400-GCST90200759 | Spicy food liking \|\| id:ebi-fl187-GCST90094846 | Weighted median | 16 | -0.016620485 | 0.104186829626365 | 0.87325464887954 | -0.220826671 | 0.18758570080517 | 0.983516872963059 | 0.801855652819575 | 1.20633362875436 | 0.999332001504091 |
| ebi-fl187-GCST90094846 | ebi-met1400-GCST90200795 | Oleoyl-linoleoyl-glycerol (18:1 to 18:2) [2] to linoleoyl-arachidonoyl-glycerol (18:2 to 20:4) [2] ratio \|\| id:ebi-met1400-GCST90200795 | Spicy food liking \|\| id:ebi-fl187-GCST90094846 | Weighted median | 16 | 0.208334620640656 | 0.104139960797081 | 0.0454435766537519 | 0.00422029747837843 | 0.412448943802934 | 1.2316252278262 | 1.0042292154749 | 1.51051241931914 | 0.999332001504091 |
| ebi-fl187-GCST90094846 | ebi-met1400-GCST90200811 | Choline to taurocholate ratio \|\| id:ebi-met1400-GCST90200811 | Spicy food liking \|\| id:ebi-fl187-GCST90094846 | Weighted median | 16 | 0.167957812276084 | 0.0987679242287806 | 0.0890312896477454 | -0.025627319 | 0.361542943764494 | 1.18288670629735 | 0.974698273248982 | 1.43554266826692 | 0.999332001504091 |
| ebi-fl187-GCST90094846 | ebi-met1400-GCST90200831 | Cholate to phosphate ratio \|\| id:ebi-met1400-GCST90200831 | Spicy food liking \|\| id:ebi-fl187-GCST90094846 | Weighted median | 16 | -0.077717808 | 0.0979994920775491 | 0.427752790828689 | -0.269796812 | 0.114361196705628 | 0.925225481153155 | 0.763534619466816 | 1.12115700997667 | 0.999332001504091 |
| ebi-fl187-GCST90094846 | ebi-met1400-GCST90200841 | Adenosine 5'-diphosphate (ADP) to oxalate (ethanedioate) ratio \|\| id:ebi-met1400-GCST90200841 | Spicy food liking \|\| id:ebi-fl187-GCST90094846 | Weighted median | 16 | -0.074143214 | 0.122314341110078 | 0.544402252162579 | -0.313879323 | 0.165592894488117 | 0.928538704495142 | 0.730607190569583 | 1.18009258172419 | 0.999332001504091 |
| ebi-fl187-GCST90094846 | ebi-met1400-GCST90200844 | Aspartate to phosphate ratio \|\| id:ebi-met1400-GCST90200844 | Spicy food liking \|\| id:ebi-fl187-GCST90094846 | Weighted median | 16 | -0.124000767 | 0.0881768578553565 | 0.159642963531874 | -0.296827409 | 0.0488258740274661 | 0.883379163004677 | 0.743172266312734 | 1.05003749602042 | 0.999332001504091 |
| ebi-fl187-GCST90094846 | ebi-met1400-GCST90200870 | Adenosine 5'-monophosphate (AMP) to acetoacetate ratio \|\| id:ebi-met1400-GCST90200870 | Spicy food liking \|\| id:ebi-fl187-GCST90094846 | Weighted median | 16 | -0.009155866 | 0.108846369023661 | 0.932963147272212 | -0.222494749 | 0.204183017500442 | 0.990885921522968 | 0.800519210207682 | 1.22652260801798 | 0.999332001504091 |
| ebi-fl187-GCST90094846 | ebi-met1400-GCST90200887 | Cholesterol to cortisol ratio \|\| id:ebi-met1400-GCST90200887 | Spicy food liking \|\| id:ebi-fl187-GCST90094846 | Weighted median | 16 | 0.0464805676370549 | 0.094439644654243 | 0.622597615615802 | -0.138621136 | 0.231582271159371 | 1.04757772196023 | 0.870557789093165 | 1.26059303276184 | 0.999332001504091 |
| ebi-fl187-GCST90094846 | ebi-met1400-GCST90200890 | Cortisol to taurocholate ratio \|\| id:ebi-met1400-GCST90200890 | Spicy food liking \|\| id:ebi-fl187-GCST90094846 | Weighted median | 16 | 0.0889674341174055 | 0.0974158798501789 | 0.361098216364888 | -0.10196769 | 0.279902558623756 | 1.09304505976963 | 0.903058728673808 | 1.32300089102881 | 0.999332001504091 |
| ebi-fl187-GCST90094846 | ebi-met1400-GCST90200948 | Adenosine 5'-diphosphate (ADP) to mannitol to sorbitol ratio \|\| id:ebi-met1400-GCST90200948 | Spicy food liking \|\| id:ebi-fl187-GCST90094846 | Weighted median | 16 | 0.0367395175717315 | 0.137721477799632 | 0.789648719883012 | -0.233194579 | 0.306673614059011 | 1.03742275524154 | 0.791999452135903 | 1.3588973706869 | 0.999332001504091 |
| ebi-fl187-GCST90094846 | ebi-met1400-GCST90200972 | Leucine to N-palmitoyl-sphingosine (d18:1 to 16:0) ratio \|\| id:ebi-met1400-GCST90200972 | Spicy food liking \|\| id:ebi-fl187-GCST90094846 | Weighted median | 16 | 0.156967427928287 | 0.0984794233267471 | 0.11095598894373 | -0.036052242 | 0.349987097648711 | 1.16995750534051 | 0.964589900261751 | 1.41904923940336 | 0.999332001504091 |
| ebi-fl187-GCST90094846 | ebi-met1400-GCST90200973 | Proline to glutamate ratio \|\| id:ebi-met1400-GCST90200973 | Spicy food liking \|\| id:ebi-fl187-GCST90094846 | Weighted median | 16 | -0.020924702 | 0.0925812292313224 | 0.821190131225522 | -0.202383911 | 0.160534507689021 | 0.979292700962542 | 0.816781296502009 | 1.17413828924051 | 0.999332001504091 |
| ebi-fl187-GCST90094846 | ebi-met1400-GCST90200983 | Cholesterol to linoleoyl-arachidonoyl-glycerol (18:2 to 20:4) [1] ratio \|\| id:ebi-met1400-GCST90200983 | Spicy food liking \|\| id:ebi-fl187-GCST90094846 | Weighted median | 16 | -0.026249638 | 0.0995149330724322 | 0.791952629744251 | -0.221298907 | 0.16879963084355 | 0.974091888924005 | 0.801477077533705 | 1.18388290153892 | 0.999332001504091 |
| ebi-fl187-GCST90094846 | ebi-met1400-GCST90201001 | Serine to alpha-ketobutyrate ratio \|\| id:ebi-met1400-GCST90201001 | Spicy food liking \|\| id:ebi-fl187-GCST90094846 | Weighted median | 16 | -0.006650238 | 0.0933628263320925 | 0.94321467185924 | -0.189641378 | 0.176340901544546 | 0.993371825829667 | 0.827255753132743 | 1.19284463192343 | 0.999332001504091 |
| ebi-fl187-GCST90094846 | ebi-met1400-GCST90201005 | Fructose to maltose ratio \|\| id:ebi-met1400-GCST90201005 | Spicy food liking \|\| id:ebi-fl187-GCST90094846 | Weighted median | 16 | -0.063113046 | 0.117810098426946 | 0.592153137274636 | -0.294020838 | 0.167794747348771 | 0.938837336290083 | 0.74526096117222 | 1.1826938346883 | 0.999332001504091 |
| ebi-fl187-GCST90094846 | ebi-met1400-GCST90201020 | Paraxanthine to 5-acetylamino-6-formylamino-3-methyluracil ratio \|\| id:ebi-met1400-GCST90201020 | Spicy food liking \|\| id:ebi-fl187-GCST90094846 | Weighted median | 16 | 0.0462772087692849 | 0.105938281541829 | 0.662233344954598 | -0.161361823 | 0.25391624059127 | 1.04736470940053 | 0.850984109728367 | 1.28906382852178 | 0.999332001504091 |
| ebi-fl187-GCST90094846 | ebi-pw11-GCST90275197 | Placental weight (fetal genotype, adjusted for sex) \|\| id:ebi-pw11-GCST90275197 | Spicy food liking \|\| id:ebi-fl187-GCST90094846 | Weighted median | 15 | -0.025796531 | 0.0370958684175234 | 0.486803586243304 | -0.098504433 | 0.0469113715911882 | 0.974533357245314 | 0.906191675815159 | 1.04802911981013 | 0.999332001504091 |
| ebi-fl187-GCST90094846 | prot-a-1017 | FAS-associated factor 2 \|\| id:prot-a-1017 | Spicy food liking \|\| id:ebi-fl187-GCST90094846 | Weighted median | 16 | 0.0215655917020675 | 0.166851909443656 | 0.897159917126918 | -0.305464151 | 0.348595334211634 | 1.02179980972809 | 0.736781317371339 | 1.41707563227224 | 0.999332001504091 |
| ebi-fl187-GCST90094846 | prot-a-1019 | Fas apoptotic inhibitory molecule 3 \|\| id:prot-a-1019 | Spicy food liking \|\| id:ebi-fl187-GCST90094846 | Weighted median | 16 | 0.258219646328083 | 0.146176334416884 | 0.0773129744601913 | -0.028285969 | 0.544725261785174 | 1.2946231466177 | 0.972110333503299 | 1.72413463162988 | 0.999332001504091 |
| ebi-fl187-GCST90094846 | prot-a-1052 | MIP18 family protein FAM96A \|\| id:prot-a-1052 | Spicy food liking \|\| id:ebi-fl187-GCST90094846 | Weighted median | 16 | -0.070396225 | 0.159934612759162 | 0.659823917246163 | -0.383868066 | 0.24307561568899 | 0.932024455047938 | 0.681221297278897 | 1.27516504295632 | 0.999332001504091 |
| ebi-fl187-GCST90094846 | prot-a-1066 | F-box/LRR-repeat protein 4 \|\| id:prot-a-1066 | Spicy food liking \|\| id:ebi-fl187-GCST90094846 | Weighted median | 16 | -0.024850651 | 0.14741471507738 | 0.866129799292012 | -0.313783492 | 0.264082191023141 | 0.975455584926035 | 0.730677208437619 | 1.3022352239479 | 0.999332001504091 |
| ebi-fl187-GCST90094846 | prot-a-1076 | Low affinity immunoglobulin gamma Fc region receptor III-B \|\| id:prot-a-1076 | Spicy food liking \|\| id:ebi-fl187-GCST90094846 | Weighted median | 16 | 0.026356006174846 | 0.156557165063757 | 0.866309913344848 | -0.280496037 | 0.33320804969981 | 1.02670639723574 | 0.755408937457427 | 1.39543758864279 | 0.999332001504091 |
| ebi-fl187-GCST90094846 | prot-a-1081 | Fc receptor-like protein 3 \|\| id:prot-a-1081 | Spicy food liking \|\| id:ebi-fl187-GCST90094846 | Weighted median | 16 | 0.0932588455751363 | 0.156651922833837 | 0.55162607894898 | -0.213778923 | 0.400296614329456 | 1.09774584515084 | 0.807526890767948 | 1.49226725985553 | 0.999332001504091 |
| ebi-fl187-GCST90094846 | prot-a-1105 | Fibroblast growth factor receptor 4 \|\| id:prot-a-1105 | Spicy food liking \|\| id:ebi-fl187-GCST90094846 | Weighted median | 16 | 0.0374371557483897 | 0.163783350958302 | 0.819197471365694 | -0.283578212 | 0.358452523626661 | 1.03814675347597 | 0.753084219515857 | 1.43111308645607 | 0.999332001504091 |
| ebi-fl187-GCST90094846 | prot-a-1143 | Follistatin-related protein 1 \|\| id:prot-a-1143 | Spicy food liking \|\| id:ebi-fl187-GCST90094846 | Weighted median | 16 | -0.121480642 | 0.148009412328708 | 0.411781307189034 | -0.41157909 | 0.168617806515356 | 0.885608197097336 | 0.662603113765591 | 1.18366766239414 | 0.999332001504091 |
| ebi-fl187-GCST90094846 | prot-a-1151 | Alpha-(1,3)-fucosyltransferase 10 \|\| id:prot-a-1151 | Spicy food liking \|\| id:ebi-fl187-GCST90094846 | Weighted median | 16 | 0.077049169845746 | 0.146879770433987 | 0.599880022297094 | -0.21083518 | 0.364933519896361 | 1.08009518320051 | 0.809907544676671 | 1.44041824581184 | 0.999332001504091 |
| ebi-fl187-GCST90094846 | prot-a-1165 | Growth arrest and DNA damage-inducible proteins-interacting protein 1 \|\| id:prot-a-1165 | Spicy food liking \|\| id:ebi-fl187-GCST90094846 | Weighted median | 16 | 0.0845387153886915 | 0.155617353418412 | 0.586959506945513 | -0.220471297 | 0.389548728088778 | 1.08821497408216 | 0.802140662124856 | 1.47631442430519 | 0.999332001504091 |
| ebi-fl187-GCST90094846 | prot-a-1194 | Growth/differentiation factor 11/8 \|\| id:prot-a-1194 | Spicy food liking \|\| id:ebi-fl187-GCST90094846 | Weighted median | 16 | -0.000448134 | 0.150944969976878 | 0.997631198639391 | -0.296300275 | 0.295404007185076 | 0.999551966427425 | 0.743564120686286 | 1.34366910101417 | 0.999364451047625 |
| ebi-fl187-GCST90094846 | prot-a-1216 | D-glucuronyl C5-epimerase \|\| id:prot-a-1216 | Spicy food liking \|\| id:ebi-fl187-GCST90094846 | Weighted median | 16 | 0.0362404515542066 | 0.163190981798612 | 0.824256385024984 | -0.283613873 | 0.356094775879486 | 1.03690514197093 | 0.753057364528553 | 1.42774285743671 | 0.999332001504091 |
| ebi-fl187-GCST90094846 | prot-a-1246 | Glypican-3 \|\| id:prot-a-1246 | Spicy food liking \|\| id:ebi-fl187-GCST90094846 | Weighted median | 16 | 0.115303148909059 | 0.174307925073184 | 0.508297391613911 | -0.226340384 | 0.456946682052499 | 1.12221358383552 | 0.797446617207523 | 1.57924467991972 | 0.999332001504091 |
| ebi-fl187-GCST90094846 | prot-a-1275 | Glutamate receptor 4 \|\| id:prot-a-1275 | Spicy food liking \|\| id:ebi-fl187-GCST90094846 | Weighted median | 16 | 0.00230914388600442 | 0.172325604317849 | 0.989308755786961 | -0.335449041 | 0.340067328348989 | 1.00231181201205 | 0.715016942634205 | 1.40504218654974 | 0.999332001504091 |
| ebi-fl187-GCST90094846 | prot-a-1289 | Glutathione S-transferase P \|\| id:prot-a-1289 | Spicy food liking \|\| id:ebi-fl187-GCST90094846 | Weighted median | 16 | 0.0360838038983501 | 0.154587467225396 | 0.815435297872932 | -0.266907632 | 0.339075239660127 | 1.03674272593247 | 0.765743798526623 | 1.40364895130983 | 0.999332001504091 |
| ebi-fl187-GCST90094846 | prot-a-130 | Apolipoprotein D \|\| id:prot-a-130 | Spicy food liking \|\| id:ebi-fl187-GCST90094846 | Weighted median | 16 | 0.019342156836673 | 0.158875140589015 | 0.903101457754755 | -0.292053119 | 0.330737432391143 | 1.01953042825156 | 0.746728869639757 | 1.39199425171852 | 0.999332001504091 |
| ebi-fl187-GCST90094846 | prot-a-1313 | Heparin-binding EGF-like growth factor \|\| id:prot-a-1313 | Spicy food liking \|\| id:ebi-fl187-GCST90094846 | Weighted median | 15 | 0.209381153469514 | 0.161289170643887 | 0.194228092073469 | -0.106745621 | 0.525507927931533 | 1.23291483875236 | 0.898754268132879 | 1.69131769774362 | 0.999332001504091 |
| ebi-fl187-GCST90094846 | prot-a-1347 | HLA class II histocompatibility antigen, DQ alpha 2 chain \|\| id:prot-a-1347 | Spicy food liking \|\| id:ebi-fl187-GCST90094846 | Weighted median | 15 | -0.004779312 | 0.156293706639641 | 0.975605257974442 | -0.311114977 | 0.301556353503784 | 0.995232091226366 | 0.732629635817992 | 1.3519613007477 | 0.999332001504091 |
| ebi-fl187-GCST90094846 | prot-a-1357 | Non-histone chromosomal protein HMG-14 \|\| id:prot-a-1357 | Spicy food liking \|\| id:ebi-fl187-GCST90094846 | Weighted median | 16 | -0.030031619 | 0.156132511416178 | 0.847470413265148 | -0.336051341 | 0.275988103851512 | 0.970414849978011 | 0.714586417364529 | 1.31783218680668 | 0.999332001504091 |
| ebi-fl187-GCST90094846 | prot-a-137 | Apolipoprotein M \|\| id:prot-a-137 | Spicy food liking \|\| id:ebi-fl187-GCST90094846 | Weighted median | 16 | 0.0541755290621024 | 0.162955611266597 | 0.739545151792254 | -0.265217469 | 0.373568527144633 | 1.05566988663829 | 0.767039124589782 | 1.45291012391436 | 0.999332001504091 |
| ebi-fl187-GCST90094846 | prot-a-1381 | 17-beta-hydroxysteroid dehydrogenase 14 \|\| id:prot-a-1381 | Spicy food liking \|\| id:ebi-fl187-GCST90094846 | Weighted median | 16 | -0.19497995 | 0.153707823511653 | 0.204615791815923 | -0.496247284 | 0.106287384090258 | 0.822851156062409 | 0.608811073173806 | 1.11214144234191 | 0.999332001504091 |
| ebi-fl187-GCST90094846 | prot-a-1392 | Serine protease HTRA2, mitochondrial \|\| id:prot-a-1392 | Spicy food liking \|\| id:ebi-fl187-GCST90094846 | Weighted median | 16 | -0.067776585 | 0.157343308835339 | 0.666645748477755 | -0.37616947 | 0.240616300768504 | 0.934469225120785 | 0.686485984495973 | 1.27203286362646 | 0.999332001504091 |
| ebi-fl187-GCST90094846 | prot-a-1416 | Interferon-induced protein with tetratricopeptide repeats 2 \|\| id:prot-a-1416 | Spicy food liking \|\| id:ebi-fl187-GCST90094846 | Weighted median | 16 | -0.156520008 | 0.169659190955749 | 0.356238929726652 | -0.489052023 | 0.176012005830016 | 0.855114408023635 | 0.613207425448071 | 1.19245237494525 | 0.999332001504091 |
| ebi-fl187-GCST90094846 | prot-a-1428 | Interferon gamma \|\| id:prot-a-1428 | Spicy food liking \|\| id:ebi-fl187-GCST90094846 | Weighted median | 16 | -0.073293165 | 0.16166317728571 | 0.650282644183805 | -0.390152993 | 0.24356666221466 | 0.929328343295579 | 0.676953297631651 | 1.27579136208365 | 0.999332001504091 |
| ebi-fl187-GCST90094846 | prot-a-1483 | Interleukin-17C \|\| id:prot-a-1483 | Spicy food liking \|\| id:ebi-fl187-GCST90094846 | Weighted median | 16 | 0.143518301767778 | 0.160815347658489 | 0.372156296606904 | -0.17167978 | 0.458716383178417 | 1.15432793685563 | 0.84224883521337 | 1.5820419454398 | 0.999332001504091 |
| ebi-fl187-GCST90094846 | prot-a-1503 | Interleukin-1 receptor-like 2 \|\| id:prot-a-1503 | Spicy food liking \|\| id:ebi-fl187-GCST90094846 | Weighted median | 16 | -0.10431759 | 0.157934763351001 | 0.508926198105567 | -0.413869726 | 0.205234545858641 | 0.900939122445834 | 0.661087067797584 | 1.22781300965034 | 0.999332001504091 |
| ebi-fl187-GCST90094846 | prot-a-1522 | Interleukin-31 receptor subunit alpha \|\| id:prot-a-1522 | Spicy food liking \|\| id:ebi-fl187-GCST90094846 | Weighted median | 16 | 0.0492395513056996 | 0.152210667254001 | 0.746319590137426 | -0.249093357 | 0.347572459123542 | 1.05047196253208 | 0.779507197914534 | 1.41562688198165 | 0.999332001504091 |
| ebi-fl187-GCST90094846 | prot-a-1540 | Interleukin-6 receptor subunit alpha \|\| id:prot-a-1540 | Spicy food liking \|\| id:ebi-fl187-GCST90094846 | Weighted median | 16 | 0.242296185088706 | 0.158767918946135 | 0.126984234392794 | -0.068888936 | 0.55348130622313 | 1.27417152753989 | 0.933430344786528 | 1.73929751765744 | 0.999332001504091 |
| ebi-fl187-GCST90094846 | prot-a-1541 | Interleukin-6 receptor subunit alpha \|\| id:prot-a-1541 | Spicy food liking \|\| id:ebi-fl187-GCST90094846 | Weighted median | 16 | 0.142260507170759 | 0.161367817989091 | 0.377997718289721 | -0.174020416 | 0.458541430429376 | 1.15287694213142 | 0.840279742258265 | 1.58176518706287 | 0.999332001504091 |
| ebi-fl187-GCST90094846 | prot-a-1545 | Interleukin-9 \|\| id:prot-a-1545 | Spicy food liking \|\| id:ebi-fl187-GCST90094846 | Weighted median | 16 | 0.0399475154395884 | 0.16072125886196 | 0.803707702305789 | -0.275066152 | 0.354961182809029 | 1.04075614912993 | 0.759521877725092 | 1.42612529502912 | 0.999332001504091 |
| ebi-fl187-GCST90094846 | prot-a-1548 | Inositol monophosphatase 3 \|\| id:prot-a-1548 | Spicy food liking \|\| id:ebi-fl187-GCST90094846 | Weighted median | 16 | -0.048118444 | 0.166283965715424 | 0.772294326375737 | -0.374035017 | 0.2777981289821 | 0.953020900956131 | 0.687952821623045 | 1.3202196562207 | 0.999332001504091 |
| ebi-fl187-GCST90094846 | prot-a-1555 | Inhibin beta A chain:Inhibin beta B chain heterodimer \|\| id:prot-a-1555 | Spicy food liking \|\| id:ebi-fl187-GCST90094846 | Weighted median | 16 | -0.118919025 | 0.148497105548336 | 0.423237524445337 | -0.409973352 | 0.172135301979682 | 0.887879694002529 | 0.663667935476601 | 1.18783853924764 | 0.999332001504091 |
| ebi-fl187-GCST90094846 | prot-a-1603 | Junctophilin-3 \|\| id:prot-a-1603 | Spicy food liking \|\| id:ebi-fl187-GCST90094846 | Weighted median | 16 | 0.246073789441788 | 0.17248586407709 | 0.153686102103993 | -0.091998504 | 0.584146083032883 | 1.27899394630919 | 0.91210651391923 | 1.79345886662575 | 0.999332001504091 |
| ebi-fl187-GCST90094846 | prot-a-1627 | UPF0258 protein KIAA1024 \|\| id:prot-a-1627 | Spicy food liking \|\| id:ebi-fl187-GCST90094846 | Weighted median | 16 | 0.173559129211676 | 0.168339005816204 | 0.302536323082511 | -0.156385322 | 0.503503580611436 | 1.18953102072171 | 0.855229587937393 | 1.65450782949621 | 0.999332001504091 |
| ebi-fl187-GCST90094846 | prot-a-1666 | Kallikrein-7 \|\| id:prot-a-1666 | Spicy food liking \|\| id:ebi-fl187-GCST90094846 | Weighted median | 16 | -0.059061148 | 0.15747812908911 | 0.707628117661339 | -0.367718281 | 0.249595984759939 | 0.942649126045862 | 0.692312191530274 | 1.28350675563131 | 0.999332001504091 |
| ebi-fl187-GCST90094846 | prot-a-1678 | Importin subunit alpha-3 \|\| id:prot-a-1678 | Spicy food liking \|\| id:ebi-fl187-GCST90094846 | Weighted median | 16 | 0.0737765049178988 | 0.157319685971726 | 0.639098841191077 | -0.23457008 | 0.382123089422481 | 1.07656617136209 | 0.790910805246858 | 1.4653924483425 | 0.999332001504091 |
| ebi-fl187-GCST90094846 | prot-a-1718 | Protein LDOC1 \|\| id:prot-a-1718 | Spicy food liking \|\| id:ebi-fl187-GCST90094846 | Weighted median | 16 | -0.048083749 | 0.154030631580262 | 0.75491129704864 | -0.349983787 | 0.253816288920522 | 0.953053966440588 | 0.70469951500805 | 1.2889349908773 | 0.999332001504091 |
| ebi-fl187-GCST90094846 | prot-a-1723 | Leptin \|\| id:prot-a-1723 | Spicy food liking \|\| id:ebi-fl187-GCST90094846 | Weighted median | 16 | -0.177755207 | 0.162720957060855 | 0.274660471869955 | -0.496688283 | 0.141177868576735 | 0.837147325994009 | 0.608542647275408 | 1.15162946846312 | 0.999332001504091 |
| ebi-fl187-GCST90094846 | prot-a-1730 | Galectin-7 \|\| id:prot-a-1730 | Spicy food liking \|\| id:ebi-fl187-GCST90094846 | Weighted median | 16 | 0.00769950544325708 | 0.15754813550837 | 0.961022249040464 | -0.30109484 | 0.316493851039662 | 1.00772922285612 | 0.740007586985666 | 1.37230780394402 | 0.999332001504091 |
| ebi-fl187-GCST90094846 | prot-a-1753 | Lipase member N \|\| id:prot-a-1753 | Spicy food liking \|\| id:ebi-fl187-GCST90094846 | Weighted median | 16 | 0.125464310776676 | 0.154861124153274 | 0.417840718791097 | -0.178063493 | 0.428992114117093 | 1.13367470826869 | 0.836889285555547 | 1.53570892392885 | 0.999332001504091 |
| ebi-fl187-GCST90094846 | prot-a-1756 | VIP36-like protein \|\| id:prot-a-1756 | Spicy food liking \|\| id:ebi-fl187-GCST90094846 | Weighted median | 16 | 0.220458179832326 | 0.151869186171701 | 0.146603938936289 | -0.077205425 | 0.518121784728861 | 1.24664778862851 | 0.925699672158554 | 1.67887140466241 | 0.999332001504091 |
| ebi-fl187-GCST90094846 | prot-a-1760 | Ig Kappa chain V-I region HK102- like \|\| id:prot-a-1760 | Spicy food liking \|\| id:ebi-fl187-GCST90094846 | Weighted median | 16 | 0.132496582493525 | 0.149943275279886 | 0.376888033862243 | -0.161392237 | 0.426385402042102 | 1.14167511440042 | 0.850958228289233 | 1.53171098593361 | 0.999332001504091 |
| ebi-fl187-GCST90094846 | prot-a-179 | Neutral ceramidase \|\| id:prot-a-179 | Spicy food liking \|\| id:ebi-fl187-GCST90094846 | Weighted median | 16 | 0.0973591886375445 | 0.179621958477834 | 0.587802830877801 | -0.25469985 | 0.4494182272541 | 1.10225622043496 | 0.775149124073829 | 1.56740004955723 | 0.999332001504091 |
| ebi-fl187-GCST90094846 | prot-a-1809 | Lactotransferrin \|\| id:prot-a-1809 | Spicy food liking \|\| id:ebi-fl187-GCST90094846 | Weighted median | 16 | 0.0203567001576873 | 0.150506776514437 | 0.892410756113434 | -0.274636582 | 0.315349982125984 | 1.02056531091599 | 0.759848215716181 | 1.3707389611534 | 0.999332001504091 |
| ebi-fl187-GCST90094846 | prot-a-1818 | Lysophospholipase-like protein 1 \|\| id:prot-a-1818 | Spicy food liking \|\| id:ebi-fl187-GCST90094846 | Weighted median | 16 | -0.225960392 | 0.169968861916395 | 0.183708691290546 | -0.559099361 | 0.107178577416656 | 0.797749698358262 | 0.571723748577717 | 1.11313301715013 | 0.999332001504091 |
| ebi-fl187-GCST90094846 | prot-a-1852 | MAP kinase-activated protein kinase 3 \|\| id:prot-a-1852 | Spicy food liking \|\| id:ebi-fl187-GCST90094846 | Weighted median | 16 | -0.135897285 | 0.163183689549597 | 0.404964763678041 | -0.455737317 | 0.183942746140349 | 0.872932290795306 | 0.633980351155116 | 1.20194700502114 | 0.999332001504091 |
| ebi-fl187-GCST90094846 | prot-a-1856 | Megakaryocyte-associated tyrosine-protein kinase \|\| id:prot-a-1856 | Spicy food liking \|\| id:ebi-fl187-GCST90094846 | Weighted median | 16 | -0.070977338 | 0.146715373240947 | 0.628545035748036 | -0.358539469 | 0.216584793642547 | 0.931483001240281 | 0.698696048137262 | 1.24182838004137 | 0.999332001504091 |
| ebi-fl187-GCST90094846 | prot-a-1890 | Beta-1,3-N-acetylglucosaminyltransferase manic fringe \|\| id:prot-a-1890 | Spicy food liking \|\| id:ebi-fl187-GCST90094846 | Weighted median | 16 | 0.0186603430775774 | 0.166064985351044 | 0.910531852238654 | -0.306827028 | 0.344147714365623 | 1.01883553529847 | 0.73577785871385 | 1.41078701362583 | 0.999332001504091 |
| ebi-fl187-GCST90094846 | prot-a-1919 | Matrilysin \|\| id:prot-a-1919 | Spicy food liking \|\| id:ebi-fl187-GCST90094846 | Weighted median | 16 | 0.0408279997402579 | 0.154653991601788 | 0.791783177500258 | -0.262293824 | 0.343949823279762 | 1.04167292212295 | 0.769284956268651 | 1.41050785907379 | 0.999332001504091 |
| ebi-fl187-GCST90094846 | prot-a-1923 | Multimerin-2 \|\| id:prot-a-1923 | Spicy food liking \|\| id:ebi-fl187-GCST90094846 | Weighted median | 16 | -0.158746789 | 0.161093964320756 | 0.324413009909096 | -0.474490959 | 0.15699738097288 | 0.853212374296028 | 0.622201701939659 | 1.16999254965468 | 0.999332001504091 |
| ebi-fl187-GCST90094846 | prot-a-1948 | Mesothelin \|\| id:prot-a-1948 | Spicy food liking \|\| id:ebi-fl187-GCST90094846 | Weighted median | 16 | -0.037943468 | 0.150560989491417 | 0.801030287390372 | -0.333043008 | 0.257156070932504 | 0.962767366064405 | 0.716739368056496 | 1.29324694926696 | 0.999332001504091 |
| ebi-fl187-GCST90094846 | prot-a-195 | ATPase family AAA domain-containing protein 1 \|\| id:prot-a-195 | Spicy food liking \|\| id:ebi-fl187-GCST90094846 | Weighted median | 16 | -0.116730286 | 0.157309771327854 | 0.458062548501917 | -0.425057438 | 0.191596865696719 | 0.889825159009622 | 0.653732235039179 | 1.2111821494607 | 0.999332001504091 |
| ebi-fl187-GCST90094846 | prot-a-1966 | Mucin-1 \|\| id:prot-a-1966 | Spicy food liking \|\| id:ebi-fl187-GCST90094846 | Weighted median | 16 | 0.00625265681712599 | 0.158179855937097 | 0.968468807860236 | -0.303779861 | 0.316285174453837 | 1.00627224548155 | 0.738023316417137 | 1.37202146531391 | 0.999332001504091 |
| ebi-fl187-GCST90094846 | prot-a-1986 | Myocardial zonula adherens protein \|\| id:prot-a-1986 | Spicy food liking \|\| id:ebi-fl187-GCST90094846 | Weighted median | 16 | -0.065888359 | 0.158462491551536 | 0.677557951395114 | -0.376474843 | 0.244698124293085 | 0.936235380572995 | 0.686276382406655 | 1.27723568857607 | 0.999332001504091 |
| ebi-fl187-GCST90094846 | prot-a-2071 | Epididymal secretory protein E1 \|\| id:prot-a-2071 | Spicy food liking \|\| id:ebi-fl187-GCST90094846 | Weighted median | 16 | -0.095162927 | 0.160998402619064 | 0.554466857502604 | -0.410719796 | 0.22039394237948 | 0.909224785357002 | 0.663172729295085 | 1.24656770972204 | 0.999332001504091 |
| ebi-fl187-GCST90094846 | prot-a-2098 | Neuritin \|\| id:prot-a-2098 | Spicy food liking \|\| id:ebi-fl187-GCST90094846 | Weighted median | 16 | 0.0490582201986408 | 0.15243502844397 | 0.747581212381871 | -0.249714436 | 0.347830875948823 | 1.05028149655741 | 0.77902321264496 | 1.4159927510576 | 0.999332001504091 |
| ebi-fl187-GCST90094846 | prot-a-2115 | Neurotrophin-4 \|\| id:prot-a-2115 | Spicy food liking \|\| id:ebi-fl187-GCST90094846 | Weighted median | 16 | 0.190600173158523 | 0.15724722581984 | 0.225472130376564 | -0.117604389 | 0.498804735765409 | 1.20997557464188 | 0.889047699703293 | 1.64675179039161 | 0.999332001504091 |
| ebi-fl187-GCST90094846 | prot-a-2118 | Netrin-1 \|\| id:prot-a-2118 | Spicy food liking \|\| id:ebi-fl187-GCST90094846 | Weighted median | 16 | 0.250179389220351 | 0.153347051582533 | 0.102793556841767 | -0.050380832 | 0.550739610322116 | 1.28425577766767 | 0.95086723498036 | 1.73453542387215 | 0.999332001504091 |
| ebi-fl187-GCST90094846 | prot-a-2120 | Netrin-G1 \|\| id:prot-a-2120 | Spicy food liking \|\| id:ebi-fl187-GCST90094846 | Weighted median | 16 | 0.204191948267952 | 0.15000904448326 | 0.173451258875147 | -0.089825779 | 0.498209675455142 | 1.22653356185515 | 0.914090425221145 | 1.64577216525721 | 0.999332001504091 |
| ebi-fl187-GCST90094846 | prot-a-2145 | Olfactomedin-like protein 3 \|\| id:prot-a-2145 | Spicy food liking \|\| id:ebi-fl187-GCST90094846 | Weighted median | 16 | -0.255481964 | 0.15759657783065 | 0.104993266998484 | -0.564371256 | 0.053407328979462 | 0.774543106428132 | 0.568717612088627 | 1.05485923235634 | 0.999332001504091 |
| ebi-fl187-GCST90094846 | prot-a-2162 | Prolyl 4-hydroxylase subunit alpha-2 \|\| id:prot-a-2162 | Spicy food liking \|\| id:ebi-fl187-GCST90094846 | Weighted median | 16 | 0.299525975868496 | 0.16257424910321 | 0.0654173996608302 | -0.019119552 | 0.618171504110788 | 1.34921909355901 | 0.981062066932536 | 1.85553210523771 | 0.999332001504091 |
| ebi-fl187-GCST90094846 | prot-a-2168 | Polyadenylate-binding protein-interacting protein 1 \|\| id:prot-a-2168 | Spicy food liking \|\| id:ebi-fl187-GCST90094846 | Weighted median | 16 | -0.100572609 | 0.171437625094737 | 0.557443785767436 | -0.436590354 | 0.235445136185749 | 0.904319448298133 | 0.646236105111247 | 1.26547195073457 | 0.999332001504091 |
| ebi-fl187-GCST90094846 | prot-a-219 | Beta-1,4-galactosyltransferase 5 \|\| id:prot-a-219 | Spicy food liking \|\| id:ebi-fl187-GCST90094846 | Weighted median | 16 | 0.0325292255713458 | 0.149142515995523 | 0.827344704758397 | -0.259790106 | 0.324848556922571 | 1.0330640845909 | 0.771213442060593 | 1.38382105999104 | 0.999332001504091 |
| ebi-fl187-GCST90094846 | prot-a-2190 | Pyruvate carboxylase, mitochondrial \|\| id:prot-a-2190 | Spicy food liking \|\| id:ebi-fl187-GCST90094846 | Weighted median | 16 | -0.059284231 | 0.148284566219518 | 0.689303523217969 | -0.349921981 | 0.23135351831114 | 0.9424388602936 | 0.70474307073371 | 1.26030470149469 | 0.999332001504091 |
| ebi-fl187-GCST90094846 | prot-a-2197 | Protocadherin-9 \|\| id:prot-a-2197 | Spicy food liking \|\| id:ebi-fl187-GCST90094846 | Weighted median | 16 | -0.139932591 | 0.1490438097766 | 0.347798081000148 | -0.432058458 | 0.152193275966788 | 0.869416839773472 | 0.6491714260806 | 1.16438526237266 | 0.999332001504091 |
| ebi-fl187-GCST90094846 | prot-a-2198 | Protocadherin alpha-4 \|\| id:prot-a-2198 | Spicy food liking \|\| id:ebi-fl187-GCST90094846 | Weighted median | 16 | 0.0570827630919316 | 0.157690366590614 | 0.717357478372472 | -0.251990355 | 0.366155881609536 | 1.05874343164951 | 0.777252234299742 | 1.44218003447862 | 0.999332001504091 |
| ebi-fl187-GCST90094846 | prot-a-2254 | Placenta growth factor \|\| id:prot-a-2254 | Spicy food liking \|\| id:ebi-fl187-GCST90094846 | Weighted median | 16 | -0.095337921 | 0.143864450632511 | 0.507527389242672 | -0.377312244 | 0.186636402731395 | 0.90906569061888 | 0.685701934323872 | 1.20518900194622 | 0.999332001504091 |
| ebi-fl187-GCST90094846 | prot-a-2261 | Membrane-associated progesterone receptor component 2 \|\| id:prot-a-2261 | Spicy food liking \|\| id:ebi-fl187-GCST90094846 | Weighted median | 16 | 0.06190908583369 | 0.159614076061658 | 0.698114214538351 | -0.250934503 | 0.37475267491454 | 1.06386561987537 | 0.778073331166758 | 1.45463160323926 | 0.999332001504091 |
| ebi-fl187-GCST90094846 | prot-a-2268 | Polymeric immunoglobulin receptor \|\| id:prot-a-2268 | Spicy food liking \|\| id:ebi-fl187-GCST90094846 | Weighted median | 16 | 0.258719456945488 | 0.166575839751762 | 0.120384161071944 | -0.067769189 | 0.585208102858941 | 1.29527037474419 | 0.934476136088995 | 1.79536456726614 | 0.999332001504091 |
| ebi-fl187-GCST90094846 | prot-a-2283 | Group 10 secretory phospholipase A2 \|\| id:prot-a-2283 | Spicy food liking \|\| id:ebi-fl187-GCST90094846 | Weighted median | 16 | 0.0193420500217771 | 0.15820927656867 | 0.902696193817985 | -0.290748132 | 0.329432232096371 | 1.01953031935053 | 0.74770397696964 | 1.39017860555956 | 0.999332001504091 |
| ebi-fl187-GCST90094846 | prot-a-2298 | Inactive phospholipase D5 \|\| id:prot-a-2298 | Spicy food liking \|\| id:ebi-fl187-GCST90094846 | Weighted median | 16 | -0.018990361 | 0.162242346251149 | 0.906820949445028 | -0.336985359 | 0.299004638068107 | 0.981188820284293 | 0.713919292149794 | 1.34851587796689 | 0.999332001504091 |
| ebi-fl187-GCST90094846 | prot-a-2304 | Procollagen-lysine,2-oxoglutarate 5-dioxygenase 2 \|\| id:prot-a-2304 | Spicy food liking \|\| id:ebi-fl187-GCST90094846 | Weighted median | 16 | -0.214049839 | 0.15928880018109 | 0.179018046723011 | -0.526255887 | 0.0981562097131733 | 0.807308148878408 | 0.590812903997628 | 1.10313509206647 | 0.999332001504091 |
| ebi-fl187-GCST90094846 | prot-a-2331 | Periostin \|\| id:prot-a-2331 | Spicy food liking \|\| id:ebi-fl187-GCST90094846 | Weighted median | 16 | -0.049815766 | 0.165170810659422 | 0.762955858022199 | -0.373550555 | 0.273919023264105 | 0.951404689800471 | 0.688286189438281 | 1.31510830474901 | 0.999332001504091 |
| ebi-fl187-GCST90094846 | prot-a-2333 | Periostin \|\| id:prot-a-2333 | Spicy food liking \|\| id:ebi-fl187-GCST90094846 | Weighted median | 16 | -0.11441149 | 0.167066805357476 | 0.493454491338288 | -0.441862428 | 0.213039448919066 | 0.891890876559313 | 0.642838065838151 | 1.23743346569339 | 0.999332001504091 |
| ebi-fl187-GCST90094846 | prot-a-2367 | cAMP-dependent protein kinase catalytic subunit alpha \|\| id:prot-a-2367 | Spicy food liking \|\| id:ebi-fl187-GCST90094846 | Weighted median | 16 | -0.019114094 | 0.152545882889509 | 0.900285559874034 | -0.318104024 | 0.279875836471825 | 0.981067421958859 | 0.727527103867629 | 1.32296553807035 | 0.999332001504091 |
| ebi-fl187-GCST90094846 | prot-a-2385 | Phosphoribosyl pyrophosphate synthase-associated protein 1 \|\| id:prot-a-2385 | Spicy food liking \|\| id:ebi-fl187-GCST90094846 | Weighted median | 16 | 0.165008932378832 | 0.157415834687821 | 0.2945299246849 | -0.143526104 | 0.473543968366961 | 1.17940365354457 | 0.866298186382703 | 1.60567458163855 | 0.999332001504091 |
| ebi-fl187-GCST90094846 | prot-a-2393 | Trypsin-3 \|\| id:prot-a-2393 | Spicy food liking \|\| id:ebi-fl187-GCST90094846 | Weighted median | 16 | 0.178323088571939 | 0.157521220068186 | 0.25761023446082 | -0.130418503 | 0.487064679905584 | 1.19521141800992 | 0.877728022443774 | 1.62753187458226 | 0.999332001504091 |
| ebi-fl187-GCST90094846 | prot-a-2405 | Pregnancy-specific beta-1-glycoprotein 6 \|\| id:prot-a-2405 | Spicy food liking \|\| id:ebi-fl187-GCST90094846 | Weighted median | 16 | 0.198152125461202 | 0.158889588937459 | 0.212358712235228 | -0.113271469 | 0.50957571977862 | 1.21914784314341 | 0.892908230435427 | 1.66458479469546 | 0.999332001504091 |
| ebi-fl187-GCST90094846 | prot-a-244 | Beclin-1 \|\| id:prot-a-244 | Spicy food liking \|\| id:ebi-fl187-GCST90094846 | Weighted median | 16 | -0.076572315 | 0.1591243809633 | 0.630366933608514 | -0.388456102 | 0.235311471510484 | 0.926285927335435 | 0.678102988682624 | 1.26530281314133 | 0.999332001504091 |
| ebi-fl187-GCST90094846 | prot-a-2454 | tRNA pseudouridine synthase A, mitochondrial \|\| id:prot-a-2454 | Spicy food liking \|\| id:ebi-fl187-GCST90094846 | Weighted median | 16 | -0.271530313 | 0.15892879230552 | 0.0875430563653373 | -0.583030746 | 0.0399701197574185 | 0.762212178057067 | 0.558204025641107 | 1.0407796749786 | 0.999332001504091 |
| ebi-fl187-GCST90094846 | prot-a-2466 | Glutaminyl-peptide cyclotransferase-like protein \|\| id:prot-a-2466 | Spicy food liking \|\| id:ebi-fl187-GCST90094846 | Weighted median | 16 | -0.017749439 | 0.159903309088216 | 0.911615494138522 | -0.331159925 | 0.295661046580589 | 0.982407154213512 | 0.718090319230361 | 1.34401452129907 | 0.999332001504091 |
| ebi-fl187-GCST90094846 | prot-a-2474 | Ras-related protein Rab-27A \|\| id:prot-a-2474 | Spicy food liking \|\| id:ebi-fl187-GCST90094846 | Weighted median | 16 | 0.0498158270398379 | 0.171438291464533 | 0.771375733381948 | -0.286203224 | 0.385834878310322 | 1.05107749849457 | 0.751109956692302 | 1.47084178288182 | 0.999332001504091 |
| ebi-fl187-GCST90094846 | prot-a-2490 | Rap1 GTPase-GDP dissociation stimulator 1 \|\| id:prot-a-2490 | Spicy food liking \|\| id:ebi-fl187-GCST90094846 | Weighted median | 16 | -0.302818678 | 0.155786047832549 | 0.051918212590893 | -0.608159331 | 0.00252197616786071 | 0.738733033085638 | 0.544351919010899 | 1.00252515902489 | 0.999332001504091 |
| ebi-fl187-GCST90094846 | prot-a-2545 | Receptor-interacting serine/threonine-protein kinase 2 \|\| id:prot-a-2545 | Spicy food liking \|\| id:ebi-fl187-GCST90094846 | Weighted median | 16 | 0.103889564254833 | 0.152748711217922 | 0.496419732690093 | -0.19549791 | 0.40327703824196 | 1.10947792212863 | 0.822425062650935 | 1.4967214833205 | 0.999332001504091 |
| ebi-fl187-GCST90094846 | prot-a-2610 | RUN and FYVE domain-containing protein 1 \|\| id:prot-a-2610 | Spicy food liking \|\| id:ebi-fl187-GCST90094846 | Weighted median | 16 | 0.07080694192575 | 0.150329470786952 | 0.637632368716151 | -0.223838821 | 0.365452704668175 | 1.073373982 | 0.799443977713096 | 1.44116628319812 | 0.999332001504091 |
| ebi-fl187-GCST90094846 | prot-a-2627 | Serine--tRNA ligase, mitochondrial \|\| id:prot-a-2627 | Spicy food liking \|\| id:ebi-fl187-GCST90094846 | Weighted median | 16 | -0.038946926 | 0.149177234054986 | 0.79403219011922 | -0.331334305 | 0.253440452565279 | 0.96180175428256 | 0.717965109640375 | 1.28845065326972 | 0.999332001504091 |
| ebi-fl187-GCST90094846 | prot-a-264 | BolA-like protein 2 \|\| id:prot-a-264 | Spicy food liking \|\| id:ebi-fl187-GCST90094846 | Weighted median | 16 | -0.117091037 | 0.156837895310242 | 0.455320970860823 | -0.424493312 | 0.190311237942012 | 0.88950421180153 | 0.654101126584109 | 1.20962602058892 | 0.999332001504091 |
| ebi-fl187-GCST90094846 | prot-a-2646 | Secretoglobin family 1C member 1 \|\| id:prot-a-2646 | Spicy food liking \|\| id:ebi-fl187-GCST90094846 | Weighted median | 16 | -0.19843554 | 0.158164558831461 | 0.20961875756365 | -0.508438075 | 0.111566995316358 | 0.820012627056258 | 0.601434240555921 | 1.11802864417924 | 0.999332001504091 |
| ebi-fl187-GCST90094846 | prot-a-2672 | Semaphorin-3E \|\| id:prot-a-2672 | Spicy food liking \|\| id:ebi-fl187-GCST90094846 | Weighted median | 16 | 0.102625152620377 | 0.168469202181862 | 0.54241660696869 | -0.227574484 | 0.432824788896826 | 1.10807597184362 | 0.79646309580482 | 1.54160609053264 | 0.999332001504091 |
| ebi-fl187-GCST90094846 | prot-a-2701 | Plasma protease C1 inhibitor \|\| id:prot-a-2701 | Spicy food liking \|\| id:ebi-fl187-GCST90094846 | Weighted median | 16 | 0.173613656926121 | 0.170519112846146 | 0.308607729611355 | -0.160603804 | 0.507831118104568 | 1.18959588489797 | 0.851629416228691 | 1.66168328899781 | 0.999332001504091 |
| ebi-fl187-GCST90094846 | prot-a-2738 | Signaling threshold-regulating transmembrane adapter 1 \|\| id:prot-a-2738 | Spicy food liking \|\| id:ebi-fl187-GCST90094846 | Weighted median | 16 | 0.174834302358088 | 0.152247378677447 | 0.250821327505935 | -0.12357056 | 0.473239164565884 | 1.19104884627568 | 0.88375928112199 | 1.60518524050308 | 0.999332001504091 |
| ebi-fl187-GCST90094846 | prot-a-274 | Serine/threonine-protein kinase BRSK2 \|\| id:prot-a-274 | Spicy food liking \|\| id:ebi-fl187-GCST90094846 | Weighted median | 16 | -0.01987596 | 0.159970892132262 | 0.901119431883167 | -0.333418908 | 0.29366698897267 | 0.980320265076828 | 0.716469996136125 | 1.34133714922196 | 0.999332001504091 |
| ebi-fl187-GCST90094846 | prot-a-2749 | Mitochondrial glutamate carrier 2 \|\| id:prot-a-2749 | Spicy food liking \|\| id:ebi-fl187-GCST90094846 | Weighted median | 16 | 0.213550502457485 | 0.163549347303137 | 0.191646028719305 | -0.107006218 | 0.534107223171633 | 1.23806602206591 | 0.898520085744424 | 1.70592455228664 | 0.999332001504091 |
| ebi-fl187-GCST90094846 | prot-a-2760 | Sodium-coupled monocarboxylate transporter 1 \|\| id:prot-a-2760 | Spicy food liking \|\| id:ebi-fl187-GCST90094846 | Weighted median | 16 | -0.10042399 | 0.155650975670964 | 0.518805994981691 | -0.405499902 | 0.204651922749135 | 0.904453857730358 | 0.666643471221613 | 1.22709786576667 | 0.999332001504091 |
| ebi-fl187-GCST90094846 | prot-a-2777 | Small integral membrane protein 10 \|\| id:prot-a-2777 | Spicy food liking \|\| id:ebi-fl187-GCST90094846 | Weighted median | 16 | 0.31153130901986 | 0.150488226817602 | 0.0384394779158831 | 0.0165743844573596 | 0.60648823358236 | 1.36551453867639 | 1.01671250158109 | 1.8339795688918 | 0.999332001504091 |
| ebi-fl187-GCST90094846 | prot-a-2799 | Superoxide dismutase [Mn], mitochondrial \|\| id:prot-a-2799 | Spicy food liking \|\| id:ebi-fl187-GCST90094846 | Weighted median | 16 | 0.303261976006718 | 0.152337136859317 | 0.0465106743318031 | 0.00468118776245657 | 0.601842764250979 | 1.35426920401172 | 1.0046921616388 | 1.8254796314355 | 0.999332001504091 |
| ebi-fl187-GCST90094846 | prot-a-2805 | Protein SPACA7 \|\| id:prot-a-2805 | Spicy food liking \|\| id:ebi-fl187-GCST90094846 | Weighted median | 16 | -0.240146192 | 0.147191392995404 | 0.102779941315409 | -0.528641322 | 0.0483489387004173 | 0.786512871109573 | 0.589405237924988 | 1.04953681544946 | 0.999332001504091 |
| ebi-fl187-GCST90094846 | prot-a-2811 | SPARC-like protein 1 \|\| id:prot-a-2811 | Spicy food liking \|\| id:ebi-fl187-GCST90094846 | Weighted median | 16 | 0.194184618090331 | 0.16897698780571 | 0.250482636405224 | -0.137010278 | 0.525379514189522 | 1.21432044778528 | 0.871961264060456 | 1.69110052325351 | 0.999332001504091 |
| ebi-fl187-GCST90094846 | prot-a-2831 | Sepiapterin reductase \|\| id:prot-a-2831 | Spicy food liking \|\| id:ebi-fl187-GCST90094846 | Weighted median | 16 | 0.0142735762274335 | 0.163378163506336 | 0.930381179860724 | -0.305947624 | 0.334494776699853 | 1.01437593012216 | 0.736425189271329 | 1.39723429155014 | 0.999332001504091 |
| ebi-fl187-GCST90094846 | prot-a-2834 | SPRY domain-containing SOCS box protein 1 \|\| id:prot-a-2834 | Spicy food liking \|\| id:ebi-fl187-GCST90094846 | Weighted median | 16 | 0.0880594418723077 | 0.150540263352504 | 0.558577250950101 | -0.206999474 | 0.383118358043215 | 1.09205303377593 | 0.813020077393252 | 1.46685163348354 | 0.999332001504091 |
| ebi-fl187-GCST90094846 | prot-a-2835 | Spectrin alpha chain, non-erythrocytic 1 \|\| id:prot-a-2835 | Spicy food liking \|\| id:ebi-fl187-GCST90094846 | Weighted median | 16 | 0.0454634738097935 | 0.149198033165575 | 0.760580185962817 | -0.246964671 | 0.337891618814321 | 1.04651277879102 | 0.78116829078631 | 1.40198854598988 | 0.999332001504091 |
| ebi-fl187-GCST90094846 | prot-a-2859 | Sia-alpha-2,3-Gal-beta-1,4-GlcNAc-R:alpha 2,8-sialyltransferase \|\| id:prot-a-2859 | Spicy food liking \|\| id:ebi-fl187-GCST90094846 | Weighted median | 16 | 0.00322979096718025 | 0.144102853154519 | 0.98211843451266 | -0.279211801 | 0.285671383150038 | 1.00323501236185 | 0.756379684111737 | 1.33065510770646 | 0.999332001504091 |
| ebi-fl187-GCST90094846 | prot-a-2861 | Alpha-2,8-sialyltransferase 8F \|\| id:prot-a-2861 | Spicy food liking \|\| id:ebi-fl187-GCST90094846 | Weighted median | 16 | -0.15970413 | 0.169494960947132 | 0.34607220219503 | -0.491914253 | 0.172505993838041 | 0.85239595037599 | 0.611454793953132 | 1.18827894294517 | 0.999332001504091 |
| ebi-fl187-GCST90094846 | prot-a-289 | Uncharacterized protein C17orf89 \|\| id:prot-a-289 | Spicy food liking \|\| id:ebi-fl187-GCST90094846 | Weighted median | 16 | -0.070479341 | 0.16138148259375 | 0.662310735714256 | -0.386787047 | 0.245828364397304 | 0.931946991966502 | 0.679235724560262 | 1.27868008767898 | 0.999332001504091 |
| ebi-fl187-GCST90094846 | prot-a-2921 | Tachykinin-3 \|\| id:prot-a-2921 | Spicy food liking \|\| id:ebi-fl187-GCST90094846 | Weighted median | 16 | 0.112636569437456 | 0.153869951466039 | 0.464153633279543 | -0.188948535 | 0.414221674310893 | 1.11922509841788 | 0.827829109462658 | 1.51319252561874 | 0.999332001504091 |
| ebi-fl187-GCST90094846 | prot-a-2928 | Tax1-binding protein 1 \|\| id:prot-a-2928 | Spicy food liking \|\| id:ebi-fl187-GCST90094846 | Weighted median | 16 | -0.012316881 | 0.165024825027348 | 0.940503905552631 | -0.335765538 | 0.311131776152533 | 0.987758661409871 | 0.714790677454914 | 1.36496907970902 | 0.999332001504091 |
| ebi-fl187-GCST90094846 | prot-a-296 | Uncharacterized protein C1orf185 \|\| id:prot-a-296 | Spicy food liking \|\| id:ebi-fl187-GCST90094846 | Weighted median | 16 | 0.155448091565186 | 0.156917697382966 | 0.32186406366425 | -0.152110595 | 0.463006778435799 | 1.16818129603074 | 0.858893285922943 | 1.58884411225739 | 0.999332001504091 |
| ebi-fl187-GCST90094846 | prot-a-2964 | Transforming growth factor beta-2 \|\| id:prot-a-2964 | Spicy food liking \|\| id:ebi-fl187-GCST90094846 | Weighted median | 16 | 0.113641733175297 | 0.149415006163196 | 0.44690929775655 | -0.179211679 | 0.406495145255162 | 1.12035066849764 | 0.835928932146927 | 1.50154585172617 | 0.999332001504091 |
| ebi-fl187-GCST90094846 | prot-a-2977 | Thymocyte nuclear protein 1 \|\| id:prot-a-2977 | Spicy food liking \|\| id:ebi-fl187-GCST90094846 | Weighted median | 16 | -0.121717523 | 0.154818541830843 | 0.43175340828925 | -0.425161865 | 0.18172681903543 | 0.885398437917672 | 0.653663971285847 | 1.1992865268755 | 0.999332001504091 |
| ebi-fl187-GCST90094846 | prot-a-3015 | Transmembrane protein 70, mitochondrial \|\| id:prot-a-3015 | Spicy food liking \|\| id:ebi-fl187-GCST90094846 | Weighted median | 16 | 0.134396330384072 | 0.156691901023865 | 0.391052193970235 | -0.172719796 | 0.441512456390848 | 1.14384607077267 | 0.841373338310042 | 1.55505739729054 | 0.999332001504091 |
| ebi-fl187-GCST90094846 | prot-a-3030 | Tumor necrosis factor alpha-induced protein 3 \|\| id:prot-a-3030 | Spicy food liking \|\| id:ebi-fl187-GCST90094846 | Weighted median | 16 | -0.046458583 | 0.157377952973418 | 0.767838134780362 | -0.354919371 | 0.262002204780006 | 0.954604096543383 | 0.701229980488462 | 1.29952940760268 | 0.999332001504091 |
| ebi-fl187-GCST90094846 | prot-a-3032 | Tumor necrosis factor receptor superfamily member 10A \|\| id:prot-a-3032 | Spicy food liking \|\| id:ebi-fl187-GCST90094846 | Weighted median | 16 | 0.162629304490969 | 0.158624201331423 | 0.305245711068285 | -0.14827413 | 0.473532739100557 | 1.17660044833499 | 0.862194728999939 | 1.60565655119215 | 0.999332001504091 |
| ebi-fl187-GCST90094846 | prot-a-3081 | Tumor protein p53-inducible protein 11 \|\| id:prot-a-3081 | Spicy food liking \|\| id:ebi-fl187-GCST90094846 | Weighted median | 16 | -0.030706658 | 0.160985337014178 | 0.848727768354005 | -0.346237918 | 0.284824602966096 | 0.969760003100778 | 0.707344177081781 | 1.32952881225923 | 0.999332001504091 |
| ebi-fl187-GCST90094846 | prot-a-315 | Complement C4 \|\| id:prot-a-315 | Spicy food liking \|\| id:ebi-fl187-GCST90094846 | Weighted median | 15 | -0.00967695 | 0.167380005695132 | 0.953896585693996 | -0.337741761 | 0.318387861329275 | 0.990369721180179 | 0.713379486521952 | 1.37490943202265 | 0.999332001504091 |
| ebi-fl187-GCST90094846 | prot-a-3157 | UDP-glucuronosyltransferase 1-8 \|\| id:prot-a-3157 | Spicy food liking \|\| id:ebi-fl187-GCST90094846 | Weighted median | 16 | 0.250868834475481 | 0.153028954756101 | 0.101139276502025 | -0.049067917 | 0.550805585797439 | 1.28514150701582 | 0.952116462754914 | 1.7346498644463 | 0.999332001504091 |
| ebi-fl187-GCST90094846 | prot-a-317 | Glutaredoxin-like protein C5orf63 \|\| id:prot-a-317 | Spicy food liking \|\| id:ebi-fl187-GCST90094846 | Weighted median | 16 | 0.0611240149764607 | 0.154324526480222 | 0.692050043958824 | -0.241352057 | 0.363600086877695 | 1.0630307377449 | 0.785565014096494 | 1.43849882455643 | 0.999332001504091 |
| ebi-fl187-GCST90094846 | prot-a-3192 | Guanine nucleotide exchange factor VAV3 \|\| id:prot-a-3192 | Spicy food liking \|\| id:ebi-fl187-GCST90094846 | Weighted median | 16 | -0.02090695 | 0.174718561216218 | 0.904751913730097 | -0.36335533 | 0.321541430294816 | 0.979310085438025 | 0.695339314906189 | 1.37925214766552 | 0.999332001504091 |
| ebi-fl187-GCST90094846 | prot-a-3233 | Serine/threonine-protein kinase WNK3 \|\| id:prot-a-3233 | Spicy food liking \|\| id:ebi-fl187-GCST90094846 | Weighted median | 16 | 0.214101335385335 | 0.144806820179511 | 0.139265774740951 | -0.069720032 | 0.497922702937176 | 1.2387481774573 | 0.932654896728521 | 1.6452999416358 | 0.999332001504091 |
| ebi-fl187-GCST90094846 | prot-a-3250 | 14-3-3 protein theta \|\| id:prot-a-3250 | Spicy food liking \|\| id:ebi-fl187-GCST90094846 | Weighted median | 16 | 0.103360603008613 | 0.148486104338048 | 0.48636862820012 | -0.187672161 | 0.394393367511187 | 1.10889120649288 | 0.828886403574209 | 1.4834839883185 | 0.999332001504091 |
| ebi-fl187-GCST90094846 | prot-a-3278 | E3 ubiquitin-protein ligase ZNRF3 \|\| id:prot-a-3278 | Spicy food liking \|\| id:ebi-fl187-GCST90094846 | Weighted median | 16 | 0.264833673402222 | 0.156189584733717 | 0.0899633400406252 | -0.041297913 | 0.570965259480308 | 1.30321419856714 | 0.959543227272454 | 1.76997471200403 | 0.999332001504091 |
| ebi-fl187-GCST90094846 | prot-a-355 | Calpain-3 \|\| id:prot-a-355 | Spicy food liking \|\| id:ebi-fl187-GCST90094846 | Weighted median | 16 | 0.17340194921174 | 0.150477278480864 | 0.249178759668692 | -0.121533517 | 0.468337415034234 | 1.18934406492919 | 0.88556137183566 | 1.59733627704439 | 0.999332001504091 |
| ebi-fl187-GCST90094846 | prot-a-385 | Coiled-coil domain-containing protein 90B, mitochondrial \|\| id:prot-a-385 | Spicy food liking \|\| id:ebi-fl187-GCST90094846 | Weighted median | 16 | -0.158957901 | 0.149966235333026 | 0.289163735835087 | -0.452891723 | 0.13497591981971 | 0.853032269649371 | 0.635786971205249 | 1.14450922403103 | 0.999332001504091 |
| ebi-fl187-GCST90094846 | prot-a-390 | C-C motif chemokine 14 \|\| id:prot-a-390 | Spicy food liking \|\| id:ebi-fl187-GCST90094846 | Weighted median | 16 | 0.076532368005689 | 0.171836031940034 | 0.656045011298627 | -0.260266255 | 0.413330990608155 | 1.0795371322357 | 0.770846317102575 | 1.51184534973993 | 0.999332001504091 |
| ebi-fl187-GCST90094846 | prot-a-40 | Pituitary adenylate cyclase-activating polypeptide 27 \|\| id:prot-a-40 | Spicy food liking \|\| id:ebi-fl187-GCST90094846 | Weighted median | 16 | 0.176175674335465 | 0.152401800689479 | 0.247683323612261 | -0.122531855 | 0.474883203686844 | 1.19264755781548 | 0.884677723071671 | 1.60782639832335 | 0.999332001504091 |
| ebi-fl187-GCST90094846 | prot-a-401 | C-C motif chemokine 24 \|\| id:prot-a-401 | Spicy food liking \|\| id:ebi-fl187-GCST90094846 | Weighted median | 16 | 0.177316225159478 | 0.150511028469533 | 0.238758907107564 | -0.117685391 | 0.472317840959762 | 1.19400860899678 | 0.888975688696006 | 1.60370702651009 | 0.999332001504091 |
| ebi-fl187-GCST90094846 | prot-a-412 | Cerebral cavernous malformations 2 protein \|\| id:prot-a-412 | Spicy food liking \|\| id:ebi-fl187-GCST90094846 | Weighted median | 16 | -0.158349237 | 0.163204021868289 | 0.331920260130534 | -0.47822912 | 0.161530645745808 | 0.853551637997598 | 0.619880153783023 | 1.17530847581127 | 0.999332001504091 |
| ebi-fl187-GCST90094846 | prot-a-44 | Adhesion G protein-coupled receptor F5 \|\| id:prot-a-44 | Spicy food liking \|\| id:ebi-fl187-GCST90094846 | Weighted median | 16 | 0.0406162413127273 | 0.141660389554584 | 0.774330104094084 | -0.237038122 | 0.318270604839712 | 1.04145236245647 | 0.788961210495995 | 1.37474822442069 | 0.999332001504091 |
| ebi-fl187-GCST90094846 | prot-a-48 | Adrenomedullin \|\| id:prot-a-48 | Spicy food liking \|\| id:ebi-fl187-GCST90094846 | Weighted median | 16 | 0.115253688342765 | 0.153076395883456 | 0.451500311668923 | -0.184776048 | 0.415283424274339 | 1.1221580798888 | 0.831290432503888 | 1.51480001095024 | 0.999332001504091 |
| ebi-fl187-GCST90094846 | prot-a-502 | Carcinoembryonic antigen-related cell adhesion molecule 1 \|\| id:prot-a-502 | Spicy food liking \|\| id:ebi-fl187-GCST90094846 | Weighted median | 16 | -0.018086577 | 0.151488617498692 | 0.904964545898284 | -0.315004268 | 0.278831112871608 | 0.982076003065232 | 0.729785759738746 | 1.32158412647275 | 0.999332001504091 |
| ebi-fl187-GCST90094846 | prot-a-518 | Complement factor B \|\| id:prot-a-518 | Spicy food liking \|\| id:ebi-fl187-GCST90094846 | Weighted median | 16 | 0.287002713463476 | 0.162383672050533 | 0.0771552737735424 | -0.031269284 | 0.605274710682521 | 1.33242782896502 | 0.96921454419977 | 1.83175534253487 | 0.999332001504091 |
| ebi-fl187-GCST90094846 | prot-a-525 | Properdin \|\| id:prot-a-525 | Spicy food liking \|\| id:ebi-fl187-GCST90094846 | Weighted median | 16 | 0.225840848825843 | 0.155100555325717 | 0.145366884005105 | -0.07815624 | 0.529837937264248 | 1.25337617311429 | 0.924819921748882 | 1.69865699731021 | 0.999332001504091 |
| ebi-fl187-GCST90094846 | prot-a-533 | Chondroadherin \|\| id:prot-a-533 | Spicy food liking \|\| id:ebi-fl187-GCST90094846 | Weighted median | 16 | -0.01612993 | 0.16474889195848 | 0.922006816840982 | -0.339037758 | 0.306777898589544 | 0.983999461044461 | 0.712455547764764 | 1.35903909005069 | 0.999332001504091 |
| ebi-fl187-GCST90094846 | prot-a-535 | Coiled-coil-helix-coiled-coil-helix domain-containing protein 2 \|\| id:prot-a-535 | Spicy food liking \|\| id:ebi-fl187-GCST90094846 | Weighted median | 16 | -0.060510788 | 0.158612251466837 | 0.702831272147504 | -0.3713908 | 0.250369225344785 | 0.9412836148382 | 0.689774324793413 | 1.2844995989496 | 0.999332001504091 |
| ebi-fl187-GCST90094846 | prot-a-591 | Caseinolytic peptidase B protein homolog \|\| id:prot-a-591 | Spicy food liking \|\| id:ebi-fl187-GCST90094846 | Weighted median | 16 | 0.0854098436500839 | 0.15976942787945 | 0.592939005263923 | -0.227738235 | 0.398557922293806 | 1.08916336192431 | 0.796332684585357 | 1.48967492094833 | 0.999332001504091 |
| ebi-fl187-GCST90094846 | prot-a-605 | Ciliary neurotrophic factor receptor subunit alpha \|\| id:prot-a-605 | Spicy food liking \|\| id:ebi-fl187-GCST90094846 | Weighted median | 16 | 0.126891254809435 | 0.153974739250046 | 0.409880259826479 | -0.174899234 | 0.428681743739525 | 1.13529355335472 | 0.839541613656044 | 1.53523235932988 | 0.999332001504091 |
| ebi-fl187-GCST90094846 | prot-a-606 | Ciliary neurotrophic factor receptor subunit alpha \|\| id:prot-a-606 | Spicy food liking \|\| id:ebi-fl187-GCST90094846 | Weighted median | 16 | 0.199458151316931 | 0.157086154884417 | 0.204178218916228 | -0.108430712 | 0.507347014890388 | 1.22074112195371 | 0.897241060471719 | 1.6608790574579 | 0.999332001504091 |
| ebi-fl187-GCST90094846 | prot-a-707 | Cancer/testis antigen 55 \|\| id:prot-a-707 | Spicy food liking \|\| id:ebi-fl187-GCST90094846 | Weighted median | 16 | 0.124889981983645 | 0.16586970812017 | 0.45148581071214 | -0.200214646 | 0.449994609899178 | 1.13302379317933 | 0.818555034711813 | 1.56830373215215 | 0.999332001504091 |
| ebi-fl187-GCST90094846 | prot-a-719 | Dipeptidyl peptidase 1 \|\| id:prot-a-719 | Spicy food liking \|\| id:ebi-fl187-GCST90094846 | Weighted median | 16 | -0.110668691 | 0.154695802978248 | 0.474364654727116 | -0.413872465 | 0.192535083101881 | 0.895235299549995 | 0.66108525768049 | 1.21231903487359 | 0.999332001504091 |
| ebi-fl187-GCST90094846 | prot-a-752 | Cytochrome b5 \|\| id:prot-a-752 | Spicy food liking \|\| id:ebi-fl187-GCST90094846 | Weighted median | 16 | 0.05217822431044 | 0.159509971526813 | 0.743580338157329 | -0.26046132 | 0.364817768502993 | 1.05356349640937 | 0.770695966410355 | 1.44025152504212 | 0.999332001504091 |
| ebi-fl187-GCST90094846 | prot-a-755 | Cytoglobin \|\| id:prot-a-755 | Spicy food liking \|\| id:ebi-fl187-GCST90094846 | Weighted median | 16 | -0.026142521 | 0.158328648260084 | 0.868853037572091 | -0.336466672 | 0.284181629496031 | 0.97419623620112 | 0.714289689252285 | 1.32867423526986 | 0.999332001504091 |
| ebi-fl187-GCST90094846 | prot-a-807 | Desmin \|\| id:prot-a-807 | Spicy food liking \|\| id:ebi-fl187-GCST90094846 | Weighted median | 16 | 0.0201001641380819 | 0.162532070663852 | 0.901577428295097 | -0.298462694 | 0.338663022639233 | 1.02030353273259 | 0.741957960548111 | 1.40307046256038 | 0.999332001504091 |
| ebi-fl187-GCST90094846 | prot-a-82 | Ameloblastin \|\| id:prot-a-82 | Spicy food liking \|\| id:ebi-fl187-GCST90094846 | Weighted median | 16 | -0.200770282 | 0.158997324254002 | 0.206687154503066 | -0.512405037 | 0.110864473704692 | 0.818100342480729 | 0.599053099807361 | 1.11724348072368 | 0.999332001504091 |
| ebi-fl187-GCST90094846 | prot-a-84 | Muellerian-inhibiting factor \|\| id:prot-a-84 | Spicy food liking \|\| id:ebi-fl187-GCST90094846 | Weighted median | 16 | 0.0367903678389417 | 0.155418855550141 | 0.812875933270333 | -0.267830589 | 0.341411324717217 | 1.03747550980713 | 0.765037375841956 | 1.40693182769665 | 0.999332001504091 |
| ebi-fl187-GCST90094846 | prot-a-858 | Inactive dipeptidyl peptidase 10 \|\| id:prot-a-858 | Spicy food liking \|\| id:ebi-fl187-GCST90094846 | Weighted median | 16 | 0.05338997754535 | 0.155917085750691 | 0.732030741929741 | -0.252207511 | 0.358987465616703 | 1.05484092919467 | 0.777083468337678 | 1.43187885374079 | 0.999332001504091 |
| ebi-fl187-GCST90094846 | prot-a-863 | Dihydropyrimidinase-related protein 3 \|\| id:prot-a-863 | Spicy food liking \|\| id:ebi-fl187-GCST90094846 | Weighted median | 16 | -0.200772971 | 0.148428229515284 | 0.17616418468033 | -0.491692301 | 0.0901463590923893 | 0.818098142673701 | 0.611590522915359 | 1.09433443778001 | 0.999332001504091 |
| ebi-fl187-GCST90094846 | prot-a-864 | Dihydropyrimidinase-related protein 5 \|\| id:prot-a-864 | Spicy food liking \|\| id:ebi-fl187-GCST90094846 | Weighted median | 16 | 0.0205721303584444 | 0.15928394065943 | 0.897235931620194 | -0.291624393 | 0.332768654050927 | 1.02078519518982 | 0.747049079897185 | 1.39482457412587 | 0.999332001504091 |
| ebi-fl187-GCST90094846 | prot-a-884 | Endothelin-converting enzyme 1 \|\| id:prot-a-884 | Spicy food liking \|\| id:ebi-fl187-GCST90094846 | Weighted median | 16 | -0.07320673 | 0.15787197087538 | 0.642855900007946 | -0.382635793 | 0.236222332456406 | 0.92940867308223 | 0.682061265286389 | 1.26645585310841 | 0.999332001504091 |
| ebi-fl187-GCST90094846 | prot-a-893 | Early endosome antigen 1 \|\| id:prot-a-893 | Spicy food liking \|\| id:ebi-fl187-GCST90094846 | Weighted median | 16 | 0.0382808487810298 | 0.156368916470393 | 0.806602687288781 | -0.268202228 | 0.344763925062999 | 1.03902300024841 | 0.764753111353608 | 1.4116566235793 | 0.999332001504091 |
| ebi-fl187-GCST90094846 | prot-a-897 | Ephrin-A2 \|\| id:prot-a-897 | Spicy food liking \|\| id:ebi-fl187-GCST90094846 | Weighted median | 16 | 0.218194133927097 | 0.15669000890661 | 0.163764675332144 | -0.088918284 | 0.525306551384053 | 1.24382851349585 | 0.91492033458001 | 1.69097714031624 | 0.999332001504091 |
| ebi-fl187-GCST90094846 | prot-a-90 | Anaphase-promoting complex subunit 10 \|\| id:prot-a-90 | Spicy food liking \|\| id:ebi-fl187-GCST90094846 | Weighted median | 16 | 0.138740737221969 | 0.15261417007802 | 0.363300125963388 | -0.160383036 | 0.437864510574888 | 1.14882621351424 | 0.851817449610145 | 1.54939496656416 | 0.999332001504091 |
| ebi-fl187-GCST90094846 | prot-a-914 | Histone-lysine N-methyltransferase EHMT2 \|\| id:prot-a-914 | Spicy food liking \|\| id:ebi-fl187-GCST90094846 | Weighted median | 16 | -0.034813239 | 0.157795999539877 | 0.825386917681816 | -0.344093398 | 0.274466920094741 | 0.965785770528459 | 0.708862718525447 | 1.31582904584897 | 0.999332001504091 |
| ebi-fl187-GCST90094846 | prot-a-932 | RNA polymerase II elongation factor ELL \|\| id:prot-a-932 | Spicy food liking \|\| id:ebi-fl187-GCST90094846 | Weighted median | 16 | -0.040976908 | 0.145225318812223 | 0.777820132870105 | -0.325618532 | 0.243664717250548 | 0.95985129494137 | 0.722080585181147 | 1.27591646598488 | 0.999332001504091 |
| ebi-fl187-GCST90094846 | prot-a-991 | Estrogen receptor \|\| id:prot-a-991 | Spicy food liking \|\| id:ebi-fl187-GCST90094846 | Weighted median | 16 | 0.134997555458027 | 0.147054975014517 | 0.358614991052475 | -0.153230196 | 0.423225306486479 | 1.14453398648653 | 0.857932206884985 | 1.52687827279382 | 0.999332001504091 |
| ebi-fl187-GCST90094846 | prot-a-992 | Persulfide dioxygenase ETHE1, mitochondrial \|\| id:prot-a-992 | Spicy food liking \|\| id:ebi-fl187-GCST90094846 | Weighted median | 16 | -0.027186112 | 0.154015462519009 | 0.859888995406476 | -0.329056418 | 0.274684194628965 | 0.973180104262642 | 0.719602416752025 | 1.31611497305325 | 0.999332001504091 |
| ebi-fl187-GCST90094846 | prot-a-993 | Ethanolamine kinase 1 \|\| id:prot-a-993 | Spicy food liking \|\| id:ebi-fl187-GCST90094846 | Weighted median | 16 | -0.139162908 | 0.16444428091539 | 0.397406729082001 | -0.461473699 | 0.183147882167001 | 0.870086272525793 | 0.630354008587118 | 1.2009920002487 | 0.999332001504091 |
| ebi-fl187-GCST90094846 | prot-b-10 | coagulation factor III, tissue factor \|\| id:prot-b-10 | Spicy food liking \|\| id:ebi-fl187-GCST90094846 | Weighted median | 12 | 0.132171920145504 | 0.209991308364571 | 0.529076649826938 | -0.279411044 | 0.543754884540063 | 1.14130451564009 | 0.756228995741392 | 1.72246238210351 | 0.999332001504091 |
| ebi-fl187-GCST90094846 | prot-b-28 | prolactin \|\| id:prot-b-28 | Spicy food liking \|\| id:ebi-fl187-GCST90094846 | Weighted median | 12 | -0.20562614 | 0.205293593662789 | 0.31652722742111 | -0.608001583 | 0.196749303900699 | 0.814137393061015 | 0.544437796253347 | 1.21743879528847 | 0.999332001504091 |
| ebi-fl187-GCST90094846 | prot-b-54 | matrix metallopeptidase 7 \|\| id:prot-b-54 | Spicy food liking \|\| id:ebi-fl187-GCST90094846 | Weighted median | 12 | 0.0156949779408202 | 0.215699678522758 | 0.94199463257845 | -0.407076392 | 0.438466347845426 | 1.0158187910068 | 0.665593342376853 | 1.55032773085982 | 0.999332001504091 |
| ebi-fl187-GCST90094846 | ukb-rsfMRI191-edge-pheno716 | Net100_Pair10_33 (OccipitalPrecuneus)&(Temporal\|Frontal\|Supp_Motor_Area) (Default_mode\|Central_executive)&(Default_mode\|Salience) \|\| id:ukb-rsfMRI191-edge-pheno716 | Spicy food liking \|\| id:ebi-fl187-GCST90094846 | Weighted median | 15 | 0.00511899174051107 | 0.0497289249622444 | 0.918012247463633 | -0.092349701 | 0.10258768466651 | 1.00513211616378 | 0.911786241057593 | 1.10803445528201 | 0.999332001504091 |
| ebi-fl187-GCST90094846 | ukb-rsfMRI191-edge-pheno903 | Net100_Pair27_38 (Precentral\|Frontal\|Supp_Motor_Area)&(Subcortical) (Attention\|Salience\|Motor)&(Subcortical-cerebellum) \|\| id:ukb-rsfMRI191-edge-pheno903 | Spicy food liking \|\| id:ebi-fl187-GCST90094846 | Weighted median | 15 | -0.01341484 | 0.0512118201436421 | 0.793361445249517 | -0.113790007 | 0.0869603278163191 | 0.986674738291045 | 0.892445343350718 | 1.09085340232328 | 0.999332001504091 |
| ebi-fl187-GCST90094846 | ukb-rsfMRI191-node-pheno23 | Net100_Node2 RolandicOper\|SupraMarginal\|Insula Salience\|Motor \|\| id:ukb-rsfMRI191-node-pheno23 | Spicy food liking \|\| id:ebi-fl187-GCST90094846 | Weighted median | 15 | -0.064601868 | 0.0498167851286131 | 0.194703734709022 | -0.162242767 | 0.0330390309320597 | 0.93744061427242 | 0.850234770732791 | 1.03359088046946 | 0.999332001504091 |
| ebi-fl187-GCST90094846 | ukb-rsfMRI191-node-pheno55 | Net100_Node34 Parietal\|Postcentral\|Precuneus Attention \|\| id:ukb-rsfMRI191-node-pheno55 | Spicy food liking \|\| id:ebi-fl187-GCST90094846 | Weighted median | 15 | 0.0330211903806392 | 0.0517870495707119 | 0.523711872089176 | -0.068481427 | 0.134523807539235 | 1.03357244080269 | 0.933810803817864 | 1.1439918943101 | 0.999332001504091 |
| ebi-fl187-GCST90094846 | ebi-a-GCST90001414 | IgD- CD24- B cell Absolute Count \|\| id:ebi-a-GCST90001414 | Spicy food liking \|\| id:ebi-fl187-GCST90094846 | Weighted mode | 15 | 0.250130390915969 | 0.27719586630826 | 0.38212867744491 | -0.293173507 | 0.793434288880159 | 1.28419285285379 | 0.745892711826417 | 2.21097653479224 | 0.999714303421225 |
| ebi-fl187-GCST90094846 | ebi-a-GCST90001422 | CD20- CD38- B cell %B cell \|\| id:ebi-a-GCST90001422 | Spicy food liking \|\| id:ebi-fl187-GCST90094846 | Weighted mode | 15 | 0.309548607328305 | 0.253023206916959 | 0.241371703235281 | -0.186376878 | 0.805474092885545 | 1.36280981290893 | 0.829960741897386 | 2.23775715212143 | 0.999714303421225 |
| ebi-fl187-GCST90094846 | ebi-a-GCST90001444 | CD20- CD38- B cell %lymphocyte \|\| id:ebi-a-GCST90001444 | Spicy food liking \|\| id:ebi-fl187-GCST90094846 | Weighted mode | 15 | 0.299082080431755 | 0.253249377665199 | 0.257286187419056 | -0.1972867 | 0.795450860655546 | 1.34862031426774 | 0.82095523187525 | 2.215439626 | 0.999714303421225 |
| ebi-fl187-GCST90094846 | ebi-a-GCST90001459 | Myeloid Dendritic Cell %Dendritic Cell \|\| id:ebi-a-GCST90001459 | Spicy food liking \|\| id:ebi-fl187-GCST90094846 | Weighted mode | 15 | 0.317984554607628 | 0.258131499248249 | 0.23828797245492 | -0.187953184 | 0.823922293134196 | 1.37435503361087 | 0.828653500634052 | 2.27942289143324 | 0.999714303421225 |
| ebi-fl187-GCST90094846 | ebi-a-GCST90001476 | HLA DR++ monocyte %leukocyte \|\| id:ebi-a-GCST90001476 | Spicy food liking \|\| id:ebi-fl187-GCST90094846 | Weighted mode | 15 | 0.061737542336241 | 0.242878649687099 | 0.803043482737378 | -0.414304611 | 0.537779695722956 | 1.06368313629851 | 0.660799633735112 | 1.71220103142395 | 0.999714303421225 |
| ebi-fl187-GCST90094846 | ebi-a-GCST90001478 | CD4 regulatory T cell %CD4+ T cell \|\| id:ebi-a-GCST90001478 | Spicy food liking \|\| id:ebi-fl187-GCST90094846 | Weighted mode | 15 | -0.289430559 | 0.345879905405077 | 0.416763334717423 | -0.967355173 | 0.388494055688604 | 0.748689780943762 | 0.380086974001456 | 1.47475821701659 | 0.999714303421225 |
| ebi-fl187-GCST90094846 | ebi-a-GCST90001485 | CD39+ resting CD4 regulatory T cell %CD4 regulatory T cell \|\| id:ebi-a-GCST90001485 | Spicy food liking \|\| id:ebi-fl187-GCST90094846 | Weighted mode | 15 | 0.0905857489666901 | 0.33572700727203 | 0.791235394901272 | -0.567439185 | 0.748610683219868 | 1.09481538290454 | 0.566975500441115 | 2.1140608751381 | 0.999714303421225 |
| ebi-fl187-GCST90094846 | ebi-a-GCST90001515 | Immature Myeloid-Derived Suppressor Cells Absolute Count \|\| id:ebi-a-GCST90001515 | Spicy food liking \|\| id:ebi-fl187-GCST90094846 | Weighted mode | 15 | -0.006857424 | 0.346588292151636 | 0.984493703311662 | -0.686170476 | 0.67245562901824 | 0.993166034878043 | 0.5035005491211 | 1.95904209947174 | 0.999714303421225 |
| ebi-fl187-GCST90094846 | ebi-a-GCST90001517 | CD33+ HLA DR+ Absolute Count \|\| id:ebi-a-GCST90001517 | Spicy food liking \|\| id:ebi-fl187-GCST90094846 | Weighted mode | 15 | 0.317567992007802 | 0.317651800901437 | 0.334405215058213 | -0.305029538 | 0.940165521774619 | 1.3737826479305 | 0.73710160174059 | 2.56040518606705 | 0.999714303421225 |
| ebi-fl187-GCST90094846 | ebi-a-GCST90001518 | CD33+ HLA DR+ CD14- Absolute Count \|\| id:ebi-a-GCST90001518 | Spicy food liking \|\| id:ebi-fl187-GCST90094846 | Weighted mode | 15 | 0.0984906428292376 | 0.314764313102492 | 0.758967116341688 | -0.518447411 | 0.715428696510123 | 1.10350407866998 | 0.595444311048147 | 2.04506320582315 | 0.999714303421225 |
| ebi-fl187-GCST90094846 | ebi-a-GCST90001520 | CD33+ HLA DR+ CD14dim Absolute Count \|\| id:ebi-a-GCST90001520 | Spicy food liking \|\| id:ebi-fl187-GCST90094846 | Weighted mode | 15 | 0.286483841476566 | 0.342399168573409 | 0.416817395308239 | -0.384618529 | 0.957586211880446 | 1.3317366488225 | 0.680710257954635 | 2.60539999374475 | 0.999714303421225 |
| ebi-fl187-GCST90094846 | ebi-a-GCST90001531 | CD33dim HLA DR- Absolute Count \|\| id:ebi-a-GCST90001531 | Spicy food liking \|\| id:ebi-fl187-GCST90094846 | Weighted mode | 15 | -0.000231902 | 0.335037816272613 | 0.999457497583746 | -0.656906022 | 0.656442218059078 | 0.999768125051909 | 0.518452937674746 | 1.92792099578549 | 0.999714303421225 |
| ebi-fl187-GCST90094846 | ebi-a-GCST90001532 | Basophil Absolute Count \|\| id:ebi-a-GCST90001532 | Spicy food liking \|\| id:ebi-fl187-GCST90094846 | Weighted mode | 15 | -0.065883869 | 0.345335375994402 | 0.851435122933138 | -0.742741206 | 0.610973467640899 | 0.936239584129304 | 0.475807838801115 | 1.84222387150919 | 0.999714303421225 |
| ebi-fl187-GCST90094846 | ebi-a-GCST90001588 | T/B cell \|\| id:ebi-a-GCST90001588 | Spicy food liking \|\| id:ebi-fl187-GCST90094846 | Weighted mode | 15 | -0.142818972 | 0.225802615616414 | 0.53725748002292 | -0.585392098 | 0.299754154859399 | 0.86691099007198 | 0.556887464338557 | 1.34952699213694 | 0.999714303421225 |
| ebi-fl187-GCST90094846 | ebi-a-GCST90001624 | HLA DR+ CD4+ T cell Absolute Count \|\| id:ebi-a-GCST90001624 | Spicy food liking \|\| id:ebi-fl187-GCST90094846 | Weighted mode | 15 | 0.316308260577664 | 0.241982606984752 | 0.212223331404429 | -0.157977649 | 0.790594170267779 | 1.37205314033698 | 0.85386886648222 | 2.20470600791926 | 0.999714303421225 |
| ebi-fl187-GCST90094846 | ebi-a-GCST90001631 | CD8+ Natural Killer T %T cell \|\| id:ebi-a-GCST90001631 | Spicy food liking \|\| id:ebi-fl187-GCST90094846 | Weighted mode | 15 | -0.030088921 | 0.240437503503604 | 0.902190057923275 | -0.501346428 | 0.441168586063348 | 0.970359244588201 | 0.605714559582409 | 1.5545227511238 | 0.999714303421225 |
| ebi-fl187-GCST90094846 | ebi-a-GCST90001632 | CD8+ Natural Killer T %lymphocyte \|\| id:ebi-a-GCST90001632 | Spicy food liking \|\| id:ebi-fl187-GCST90094846 | Weighted mode | 15 | -0.022312665 | 0.254369142179635 | 0.931343433234132 | -0.520876184 | 0.476250853747565 | 0.977934421453039 | 0.59399986695761 | 1.61002684657325 | 0.999714303421225 |
| ebi-fl187-GCST90094846 | ebi-a-GCST90001646 | Natural Killer %CD3- lymphocyte \|\| id:ebi-a-GCST90001646 | Spicy food liking \|\| id:ebi-fl187-GCST90094846 | Weighted mode | 15 | -0.200403079 | 0.213063882667756 | 0.36286836598879 | -0.618008289 | 0.2172021311782 | 0.818400806528785 | 0.539016935211468 | 1.2425952439958 | 0.999714303421225 |
| ebi-fl187-GCST90094846 | ebi-a-GCST90001684 | CD127- CD8+ T cell Absolute Count \|\| id:ebi-a-GCST90001684 | Spicy food liking \|\| id:ebi-fl187-GCST90094846 | Weighted mode | 15 | -0.152619755 | 0.274177205518202 | 0.586557575556145 | -0.690007078 | 0.384767567814194 | 0.85845608339094 | 0.501572519014912 | 1.4692727754668 | 0.999714303421225 |
| ebi-fl187-GCST90094846 | ebi-a-GCST90001687 | CD28- CD8+ T cell Absolute Count \|\| id:ebi-a-GCST90001687 | Spicy food liking \|\| id:ebi-fl187-GCST90094846 | Weighted mode | 15 | 0.012038686529281 | 0.238025559619839 | 0.960376892582894 | -0.45449141 | 0.478568783384165 | 1.01211144318768 | 0.634770723701834 | 1.61376310403475 | 0.999714303421225 |
| ebi-fl187-GCST90094846 | ebi-a-GCST90001701 | BAFF-R on CD20- CD38- B cell \|\| id:ebi-a-GCST90001701 | Spicy food liking \|\| id:ebi-fl187-GCST90094846 | Weighted mode | 15 | 0.360622781771962 | 0.340288107642522 | 0.307197064801127 | -0.306341909 | 1.0275874727513 | 1.43422234401418 | 0.736134885128311 | 2.79431633200074 | 0.999714303421225 |
| ebi-fl187-GCST90094846 | ebi-a-GCST90001732 | CD19 on IgD- CD27- B cell \|\| id:ebi-a-GCST90001732 | Spicy food liking \|\| id:ebi-fl187-GCST90094846 | Weighted mode | 15 | 0.2178798842574 | 0.263546109126171 | 0.42226071104763 | -0.29867049 | 0.734430258144694 | 1.2434377022057 | 0.74180380121307 | 2.08429414454089 | 0.999714303421225 |
| ebi-fl187-GCST90094846 | ebi-a-GCST90001798 | CD27 on CD24+ CD27+ B cell \|\| id:ebi-a-GCST90001798 | Spicy food liking \|\| id:ebi-fl187-GCST90094846 | Weighted mode | 15 | 0.159596179212528 | 0.255117529590832 | 0.541655383107028 | -0.340434179 | 0.659626537210559 | 1.17303707857783 | 0.711461354266953 | 1.93406989637009 | 0.999714303421225 |
| ebi-fl187-GCST90094846 | ebi-a-GCST90001836 | CD66b on CD66b++ myeloid cell \|\| id:ebi-a-GCST90001836 | Spicy food liking \|\| id:ebi-fl187-GCST90094846 | Weighted mode | 15 | 0.0190053580795402 | 0.406578741730798 | 0.96337704997602 | -0.777888976 | 0.815899691871905 | 1.01918710948838 | 0.459374739673277 | 2.26120914895334 | 0.999714303421225 |
| ebi-fl187-GCST90094846 | ebi-a-GCST90001943 | CD25 on activated & secreting CD4 regulatory T cell \|\| id:ebi-a-GCST90001943 | Spicy food liking \|\| id:ebi-fl187-GCST90094846 | Weighted mode | 15 | -0.120303398 | 0.248012779583978 | 0.635129416917838 | -0.606408446 | 0.365801649701548 | 0.886651387396071 | 0.545305851527079 | 1.44166925876521 | 0.999714303421225 |
| ebi-fl187-GCST90094846 | ebi-a-GCST90001960 | CD25 on CD4+ T cell \|\| id:ebi-a-GCST90001960 | Spicy food liking \|\| id:ebi-fl187-GCST90094846 | Weighted mode | 15 | 0.345569767052268 | 0.253362310792745 | 0.194116015413599 | -0.151020362 | 0.842159896206049 | 1.41279465423587 | 0.859830190531332 | 2.32137549601978 | 0.999714303421225 |
| ebi-fl187-GCST90094846 | ebi-a-GCST90001988 | HLA DR on CD14+ CD16- monocyte \|\| id:ebi-a-GCST90001988 | Spicy food liking \|\| id:ebi-fl187-GCST90094846 | Weighted mode | 15 | -0.050408747 | 0.216931100932695 | 0.819611539623937 | -0.475593705 | 0.374776210828312 | 0.950840691779434 | 0.621515949885647 | 1.45466583972614 | 0.999714303421225 |
| ebi-fl187-GCST90094846 | ebi-a-GCST90001991 | HLA DR on CD14+ monocyte \|\| id:ebi-a-GCST90001991 | Spicy food liking \|\| id:ebi-fl187-GCST90094846 | Weighted mode | 15 | -0.072771913 | 0.231857540427307 | 0.758251678588934 | -0.527212693 | 0.38166886576601 | 0.92981288363436 | 0.590247881187794 | 1.46472698357282 | 0.999714303421225 |
| ebi-fl187-GCST90094846 | ebi-a-GCST90002019 | CD14 on Monocytic Myeloid-Derived Suppressor Cells \|\| id:ebi-a-GCST90002019 | Spicy food liking \|\| id:ebi-fl187-GCST90094846 | Weighted mode | 15 | 0.179924504772466 | 0.336487569198873 | 0.601237601073593 | -0.479591131 | 0.839440140402258 | 1.19712698233628 | 0.61903644497034 | 2.31507049945375 | 0.999714303421225 |
| ebi-fl187-GCST90094846 | ebi-a-GCST90002037 | CD80 on plasmacytoid Dendritic Cell \|\| id:ebi-a-GCST90002037 | Spicy food liking \|\| id:ebi-fl187-GCST90094846 | Weighted mode | 15 | 0.0993347259371007 | 0.287906832075036 | 0.735205120808321 | -0.464962665 | 0.663632116804172 | 1.10443592104312 | 0.628158557095567 | 1.94183250377148 | 0.999714303421225 |
| ebi-fl187-GCST90094846 | ebi-a-GCST90002042 | CD45 on CD33+ HLA DR+ CD14- \|\| id:ebi-a-GCST90002042 | Spicy food liking \|\| id:ebi-fl187-GCST90094846 | Weighted mode | 15 | -0.099529313 | 0.372033587102284 | 0.792963762542134 | -0.828715144 | 0.629656517426809 | 0.905263413227293 | 0.436609906938447 | 1.87696576349896 | 0.999714303421225 |
| ebi-fl187-GCST90094846 | ebi-a-GCST90002056 | CD8 on Effector Memory CD8+ T cell \|\| id:ebi-a-GCST90002056 | Spicy food liking \|\| id:ebi-fl187-GCST90094846 | Weighted mode | 15 | -0.339347591 | 0.312899550515458 | 0.296455469354663 | -0.95263071 | 0.273935527805449 | 0.712234839492231 | 0.385724956967711 | 1.31513001018751 | 0.999714303421225 |
| ebi-fl187-GCST90094846 | ebi-a-GCST90002058 | CD8 on CD8+ T cell \|\| id:ebi-a-GCST90002058 | Spicy food liking \|\| id:ebi-fl187-GCST90094846 | Weighted mode | 15 | -0.715386751 | 0.327594475994629 | 0.0464931147457308 | -1.357471924 | -0.073301578 | 0.489002952856169 | 0.257310455798669 | 0.929320525121429 | 0.999714303421225 |
| ebi-fl187-GCST90094846 | ebi-a-GCST90002063 | CD4 on CD4 regulatory T cell \|\| id:ebi-a-GCST90002063 | Spicy food liking \|\| id:ebi-fl187-GCST90094846 | Weighted mode | 15 | -0.530765316 | 0.33512967581555 | 0.135570522378251 | -1.18761948 | 0.126088848814122 | 0.588154673335738 | 0.30494633142991 | 1.13438295238576 | 0.999714303421225 |
| ebi-fl187-GCST90094846 | ebi-a-GCST90002066 | CD4 on activated CD4 regulatory T cell \|\| id:ebi-a-GCST90002066 | Spicy food liking \|\| id:ebi-fl187-GCST90094846 | Weighted mode | 15 | -0.574246142 | 0.322178831252531 | 0.0963782395736271 | -1.205716651 | 0.0572243672502125 | 0.563129228304577 | 0.299477301805152 | 1.05889336473731 | 0.999714303421225 |
| ebi-fl187-GCST90094846 | ebi-a-GCST90002070 | CD4 on activated & secreting CD4 regulatory T cell \|\| id:ebi-a-GCST90002070 | Spicy food liking \|\| id:ebi-fl187-GCST90094846 | Weighted mode | 15 | -0.496951447 | 0.327786338168564 | 0.151748777421331 | -1.139412669 | 0.145509776193091 | 0.608382522123506 | 0.320006916478165 | 1.15662904195577 | 0.999714303421225 |
| ebi-fl187-GCST90094846 | ebi-a-GCST90002081 | SSC-A on CD4+ T cell \|\| id:ebi-a-GCST90002081 | Spicy food liking \|\| id:ebi-fl187-GCST90094846 | Weighted mode | 15 | 0.304411863740512 | 0.294362589288715 | 0.318605233199256 | -0.272538811 | 0.881362538746393 | 1.35582735723662 | 0.761443876001376 | 2.41418688962956 | 0.999714303421225 |
| ebi-fl187-GCST90094846 | ebi-a-GCST90002094 | CD11b on Monocytic Myeloid-Derived Suppressor Cells \|\| id:ebi-a-GCST90002094 | Spicy food liking \|\| id:ebi-fl187-GCST90094846 | Weighted mode | 15 | 0.18345065513804 | 0.371867745356119 | 0.629433119503059 | -0.545410126 | 0.912311436036033 | 1.20135568321872 | 0.57960402404674 | 2.49007152767028 | 0.999714303421225 |
| ebi-fl187-GCST90094846 | ebi-a-GCST90002104 | HLA DR on myeloid Dendritic Cell \|\| id:ebi-a-GCST90002104 | Spicy food liking \|\| id:ebi-fl187-GCST90094846 | Weighted mode | 15 | -0.063692788 | 0.303257155986725 | 0.836670922583261 | -0.658076814 | 0.53069123784887 | 0.938293210299066 | 0.51784629237721 | 1.7001070809097 | 0.999714303421225 |
| ebi-fl187-GCST90094846 | ebi-a-GCST90002105 | HLA DR on plasmacytoid Dendritic Cell \|\| id:ebi-a-GCST90002105 | Spicy food liking \|\| id:ebi-fl187-GCST90094846 | Weighted mode | 15 | 0.319766632850765 | 0.288500398482376 | 0.286378435672698 | -0.245694148 | 0.885227413876222 | 1.37680642545189 | 0.782161413841538 | 2.42353547441762 | 0.999714303421225 |
| ebi-fl187-GCST90094846 | ebi-a-GCST90002106 | HLA DR on Dendritic Cell \|\| id:ebi-a-GCST90002106 | Spicy food liking \|\| id:ebi-fl187-GCST90094846 | Weighted mode | 15 | -0.094433687 | 0.283733583430986 | 0.744197706611446 | -0.650551511 | 0.461684136538293 | 0.909888070045423 | 0.52175794240695 | 1.58674402960072 | 0.999714303421225 |
| ebi-fl187-GCST90094846 | ebi-a-GCST90002111 | HLA DR on CD33dim HLA DR+ CD11b- \|\| id:ebi-a-GCST90002111 | Spicy food liking \|\| id:ebi-fl187-GCST90094846 | Weighted mode | 15 | 0.36640406468837 | 0.514782392102946 | 0.488302036816977 | -0.642569424 | 1.37537755321014 | 1.44253800357891 | 0.525939325407903 | 3.95657025676019 | 0.999714303421225 |
| ebi-fl187-GCST90094846 | ebi-a-GCST90002112 | HLA DR on CD33- HLA DR+ \|\| id:ebi-a-GCST90002112 | Spicy food liking \|\| id:ebi-fl187-GCST90094846 | Weighted mode | 15 | -0.060306344 | 0.352406322308859 | 0.866572381484554 | -0.751022736 | 0.630410047833844 | 0.941476073958313 | 0.471883693604009 | 1.87838064728675 | 0.999714303421225 |
| ebi-fl187-GCST90094846 | ebi-a-GCST90002115 | HLA DR on HLA DR+ CD8+ T cell \|\| id:ebi-a-GCST90002115 | Spicy food liking \|\| id:ebi-fl187-GCST90094846 | Weighted mode | 15 | -0.504979005 | 0.266020096943283 | 0.0784707300685956 | -1.026378395 | 0.0164203855010053 | 0.603518246456642 | 0.358302243020402 | 1.01655594097233 | 0.999714303421225 |
| ebi-fl187-GCST90094846 | ebi-a-GCST90002116 | HLA DR on B cell \|\| id:ebi-a-GCST90002116 | Spicy food liking \|\| id:ebi-fl187-GCST90094846 | Weighted mode | 15 | -0.105065549 | 0.264150021846014 | 0.696813537501031 | -0.622799592 | 0.412668494065967 | 0.900265509371358 | 0.536440519042959 | 1.51084408912588 | 0.999714303421225 |
| ebi-fl187-GCST90094846 | ebi-cfmet338-GCST90026023 | Sphingomyelin (d18:1/24:1, d18:2/24:0) levels \|\| id:ebi-cfmet338-GCST90026023 | Spicy food liking \|\| id:ebi-fl187-GCST90094846 | Weighted mode | 15 | -0.291844854 | 0.190929364415264 | 0.148651583537253 | -0.666066408 | 0.0823767004002142 | 0.746884403221835 | 0.513725394565909 | 1.08586477849202 | 0.999714303421225 |
| ebi-fl187-GCST90094846 | ebi-cfmet338-GCST90026046 | 1-arachidonoyl-gpc (20:4n6) levels \|\| id:ebi-cfmet338-GCST90026046 | Spicy food liking \|\| id:ebi-fl187-GCST90094846 | Weighted mode | 15 | -0.07014654 | 0.161526024405632 | 0.670707810201892 | -0.386737548 | 0.246444467515626 | 0.932257196628415 | 0.67926934702807 | 1.27946812920083 | 0.999714303421225 |
| ebi-fl187-GCST90094846 | ebi-cfmet338-GCST90026047 | 1-linoleoyl-gpc (18:2) levels \|\| id:ebi-cfmet338-GCST90026047 | Spicy food liking \|\| id:ebi-fl187-GCST90094846 | Weighted mode | 15 | 0.0578519525389509 | 0.227273914313707 | 0.802773668834801 | -0.38760492 | 0.503308824593817 | 1.05955811920861 | 0.678680423694276 | 1.65418563551585 | 0.999714303421225 |
| ebi-fl187-GCST90094846 | ebi-cfmet338-GCST90026050 | 1-myristoyl-2-palmitoyl-gpc (14:0/16:0) levels \|\| id:ebi-cfmet338-GCST90026050 | Spicy food liking \|\| id:ebi-fl187-GCST90094846 | Weighted mode | 15 | -0.064701551 | 0.122452795644413 | 0.605511239480148 | -0.30470903 | 0.175305928707955 | 0.93734717219164 | 0.737337886225793 | 1.19161070877973 | 0.999714303421225 |
| ebi-fl187-GCST90094846 | ebi-cfmet338-GCST90026068 | 2,3-dihydroxyisovalerate levels \|\| id:ebi-cfmet338-GCST90026068 | Spicy food liking \|\| id:ebi-fl187-GCST90094846 | Weighted mode | 15 | 0.184422055042154 | 0.388835264288455 | 0.642603218804702 | -0.577695063 | 0.946539173047527 | 1.20252324700807 | 0.561190385486451 | 2.57677643272763 | 0.999714303421225 |
| ebi-fl187-GCST90094846 | ebi-cfmet338-GCST90026082 | 3-hydroxyhexanoate levels \|\| id:ebi-cfmet338-GCST90026082 | Spicy food liking \|\| id:ebi-fl187-GCST90094846 | Weighted mode | 15 | -0.118636774 | 0.124162857361467 | 0.355537233284124 | -0.361995975 | 0.12472242612589 | 0.888130333942175 | 0.6962851705712 | 1.13283396430972 | 0.999714303421225 |
| ebi-fl187-GCST90094846 | ebi-cfmet338-GCST90026094 | 3-ureidopropionate levels \|\| id:ebi-cfmet338-GCST90026094 | Spicy food liking \|\| id:ebi-fl187-GCST90094846 | Weighted mode | 15 | -0.105134422 | 0.232486612400516 | 0.658040210241813 | -0.560808183 | 0.350539337982123 | 0.900203507006366 | 0.570747609101485 | 1.41983311205158 | 0.999714303421225 |
| ebi-fl187-GCST90094846 | ebi-cfmet338-GCST90026173 | Glycerol levels \|\| id:ebi-cfmet338-GCST90026173 | Spicy food liking \|\| id:ebi-fl187-GCST90094846 | Weighted mode | 15 | -0.030450628 | 0.101188192032069 | 0.767890293782618 | -0.228779485 | 0.167878228049067 | 0.97000832181209 | 0.79550393494066 | 1.18279257091908 | 0.999714303421225 |
| ebi-fl187-GCST90094846 | ebi-cfmet338-GCST90026179 | Histidine levels \|\| id:ebi-cfmet338-GCST90026179 | Spicy food liking \|\| id:ebi-fl187-GCST90094846 | Weighted mode | 15 | -0.013462061 | 0.0811772484020043 | 0.870657506524126 | -0.172569468 | 0.145645345489098 | 0.986628146919197 | 0.841499829263469 | 1.15678585597007 | 0.999714303421225 |
| ebi-fl187-GCST90094846 | ebi-cfmet338-GCST90026188 | Isocitrate levels \|\| id:ebi-cfmet338-GCST90026188 | Spicy food liking \|\| id:ebi-fl187-GCST90094846 | Weighted mode | 15 | -0.026688583 | 0.154321259561124 | 0.865172641448486 | -0.329158251 | 0.275781086167185 | 0.973664410381669 | 0.719529141305909 | 1.31755940047581 | 0.999714303421225 |
| ebi-fl187-GCST90094846 | ebi-cfmet338-GCST90026207 | N-acetyl-isoputreanine levels \|\| id:ebi-cfmet338-GCST90026207 | Spicy food liking \|\| id:ebi-fl187-GCST90094846 | Weighted mode | 14 | 0.027669517699929 | 0.088389712903176 | 0.759215761828839 | -0.14557432 | 0.200913354990154 | 1.02805587400401 | 0.864525636495229 | 1.22251884207714 | 0.999714303421225 |
| ebi-fl187-GCST90094846 | ebi-cfmet338-GCST90026213 | N-acetylglutamate levels \|\| id:ebi-cfmet338-GCST90026213 | Spicy food liking \|\| id:ebi-fl187-GCST90094846 | Weighted mode | 15 | -0.271648743 | 0.214264818187768 | 0.225543528163259 | -0.691607787 | 0.148310300442692 | 0.762121914580393 | 0.500770289590653 | 1.15987274955644 | 0.999714303421225 |
| ebi-fl187-GCST90094846 | ebi-cfmet338-GCST90026224 | N-acetyltaurine levels \|\| id:ebi-cfmet338-GCST90026224 | Spicy food liking \|\| id:ebi-fl187-GCST90094846 | Weighted mode | 15 | -0.05002912 | 0.136987510831662 | 0.720413507281688 | -0.318524642 | 0.218466400812517 | 0.951201724706013 | 0.727221157807294 | 1.24416721291744 | 0.999714303421225 |
| ebi-fl187-GCST90094846 | ebi-cfmet338-GCST90026250 | Phenyllactate (pla) levels \|\| id:ebi-cfmet338-GCST90026250 | Spicy food liking \|\| id:ebi-fl187-GCST90094846 | Weighted mode | 15 | 0.180223435145865 | 0.184855309330777 | 0.346134298072055 | -0.182092971 | 0.542539841434188 | 1.1974848934447 | 0.833523843149323 | 1.72037078700743 | 0.999714303421225 |
| ebi-fl187-GCST90094846 | ebi-cfmet338-GCST90026251 | Pipecolate levels \|\| id:ebi-cfmet338-GCST90026251 | Spicy food liking \|\| id:ebi-fl187-GCST90094846 | Weighted mode | 14 | 0.428864401614658 | 0.454925949977516 | 0.363018368169466 | -0.46279046 | 1.32051926357059 | 1.5355128072227 | 0.629524529043879 | 3.74536570437687 | 0.999714303421225 |
| ebi-fl187-GCST90094846 | ebi-cfmet338-GCST90026278 | Trigonelline (n'-methylnicotinate) levels \|\| id:ebi-cfmet338-GCST90026278 | Spicy food liking \|\| id:ebi-fl187-GCST90094846 | Weighted mode | 15 | -0.03989641 | 0.35007747133886 | 0.910884144401671 | -0.726048254 | 0.646255433727865 | 0.96088897239303 | 0.483817139830926 | 1.90838137232838 | 0.999714303421225 |
| ebi-fl187-GCST90094846 | ebi-cfmet338-GCST90026299 | 1-carboxyethylvaline levels \|\| id:ebi-cfmet338-GCST90026299 | Spicy food liking \|\| id:ebi-fl187-GCST90094846 | Weighted mode | 15 | -0.025151336 | 0.178509888528493 | 0.889959281669699 | -0.375030717 | 0.324728045776883 | 0.975162323950035 | 0.687268167475311 | 1.38365430417784 | 0.999714303421225 |
| ebi-fl187-GCST90094846 | ebi-gm430-GCST90011306 | C_Erysipelotrichia abundance \|\| id:ebi-gm430-GCST90011306 | Spicy food liking \|\| id:ebi-fl187-GCST90094846 | Weighted mode | 14 | -0.152431368 | 0.151303307294953 | 0.33210055825439 | -0.448985851 | 0.144123113969877 | 0.858617820310682 | 0.638275129823186 | 1.15502629964494 | 0.999714303421225 |
| ebi-fl187-GCST90094846 | ebi-gm430-GCST90011312 | F_Erysipelotrichaceae abundance \|\| id:ebi-gm430-GCST90011312 | Spicy food liking \|\| id:ebi-fl187-GCST90094846 | Weighted mode | 14 | -0.152431368 | 0.148257652738452 | 0.322629388903136 | -0.443016368 | 0.138153631039134 | 0.858617820310682 | 0.642096697358498 | 1.14815192849911 | 0.999714303421225 |
| ebi-fl187-GCST90094846 | ebi-gm430-GCST90011352 | O_Erysipelotrichales abundance \|\| id:ebi-gm430-GCST90011352 | Spicy food liking \|\| id:ebi-fl187-GCST90094846 | Weighted mode | 14 | -0.152431368 | 0.145123621373265 | 0.312689181826091 | -0.436873666 | 0.132010929563367 | 0.858617820310682 | 0.646053044578577 | 1.14112079115108 | 0.999714303421225 |
| ebi-fl187-GCST90094846 | ebi-gm430-GCST90011362 | OTU97_11 (Parabacteroides) abundance \|\| id:ebi-gm430-GCST90011362 | Spicy food liking \|\| id:ebi-fl187-GCST90094846 | Weighted mode | 14 | -0.0397206 | 0.243164491568238 | 0.872755977461086 | -0.516323004 | 0.436881803367386 | 0.961057921124604 | 0.596710621876052 | 1.54787311285402 | 0.999714303421225 |
| ebi-fl187-GCST90094846 | ebi-gm430-GCST90011384 | OTU97_235 (Phascolarctobacterium) abundance \|\| id:ebi-gm430-GCST90011384 | Spicy food liking \|\| id:ebi-fl187-GCST90094846 | Weighted mode | 14 | 0.0614212466307862 | 0.611449556478625 | 0.921518263270961 | -1.137019884 | 1.25986237732889 | 1.06334675109193 | 0.320773541161516 | 3.52493634282772 | 0.999714303421225 |
| ebi-fl187-GCST90094846 | ebi-gm430-GCST90011390 | OTU97_3 (Bacteroides) abundance \|\| id:ebi-gm430-GCST90011390 | Spicy food liking \|\| id:ebi-fl187-GCST90094846 | Weighted mode | 14 | -0.127417943 | 0.234591723855887 | 0.596218786546365 | -0.587217722 | 0.332381835612701 | 0.880365652907278 | 0.555871724932506 | 1.39428513460181 | 0.999714303421225 |
| ebi-fl187-GCST90094846 | ebi-gm430-GCST90011398 | OTU97_4 (Alistipes) abundance \|\| id:ebi-gm430-GCST90011398 | Spicy food liking \|\| id:ebi-fl187-GCST90094846 | Weighted mode | 14 | -0.137808567 | 0.195353061645084 | 0.492988010587792 | -0.520700567 | 0.245083434282056 | 0.871265465142844 | 0.594104192135153 | 1.27772791506896 | 0.999714303421225 |
| ebi-fl187-GCST90094846 | ebi-gm430-GCST90011421 | OTU99_108 (Cytophagales) abundance \|\| id:ebi-gm430-GCST90011421 | Spicy food liking \|\| id:ebi-fl187-GCST90094846 | Weighted mode | 14 | -0.153546997 | 0.657955062139048 | 0.819108543608244 | -1.443138918 | 1.13604492523203 | 0.85766045616015 | 0.236185227773668 | 3.11442618572967 | 0.999714303421225 |
| ebi-fl187-GCST90094846 | ebi-gm430-GCST90011428 | OTU99_123 (Phascolarctobacterium) abundance \|\| id:ebi-gm430-GCST90011428 | Spicy food liking \|\| id:ebi-fl187-GCST90094846 | Weighted mode | 14 | 0.0959784622249074 | 0.421564583294161 | 0.82344283044811 | -0.730288121 | 0.922245045481462 | 1.10073535634813 | 0.481770161975428 | 2.51493018942225 | 0.999714303421225 |
| ebi-fl187-GCST90094846 | ebi-gm430-GCST90011450 | OTU99_298 (Phascolarctobacterium) abundance \|\| id:ebi-gm430-GCST90011450 | Spicy food liking \|\| id:ebi-fl187-GCST90094846 | Weighted mode | 14 | 0.0672518988299604 | 0.55661112639681 | 0.905678062003071 | -1.023705909 | 1.15820970656771 | 1.0695648663823 | 0.359261081278542 | 3.18422746858138 | 0.999714303421225 |
| ebi-fl187-GCST90094846 | ebi-gm430-GCST90011451 | OTU99_3 (Bacteroides) abundance \|\| id:ebi-gm430-GCST90011451 | Spicy food liking \|\| id:ebi-fl187-GCST90094846 | Weighted mode | 14 | -0.133401975 | 0.228193975665474 | 0.56882473734744 | -0.580662167 | 0.313858217495347 | 0.87511324791659 | 0.559527743001177 | 1.36869566569019 | 0.999714303421225 |
| ebi-fl187-GCST90094846 | ebi-gm430-GCST90011472 | OTU99_72 (Parabacteroides) abundance \|\| id:ebi-gm430-GCST90011472 | Spicy food liking \|\| id:ebi-fl187-GCST90094846 | Weighted mode | 14 | 0.348144383743183 | 0.629340966864026 | 0.589521332898669 | -0.885363911 | 1.58165267879667 | 1.41643674541603 | 0.41256400924832 | 4.86298612770453 | 0.999714303421225 |
| ebi-fl187-GCST90094846 | ebi-gm430-GCST90011507 | TestASV_3 (Bacteroides) abundance \|\| id:ebi-gm430-GCST90011507 | Spicy food liking \|\| id:ebi-fl187-GCST90094846 | Weighted mode | 14 | 0.0940466292410076 | 0.24511472651251 | 0.707414224474303 | -0.386378235 | 0.574471493205528 | 1.09861097211864 | 0.679513461481856 | 1.77619154950573 | 0.999714303421225 |
| ebi-fl187-GCST90094846 | ebi-gm430-GCST90011512 | TestASV_34 (Phascolarctobacterium) abundance \|\| id:ebi-gm430-GCST90011512 | Spicy food liking \|\| id:ebi-fl187-GCST90094846 | Weighted mode | 14 | 0.378772763618387 | 0.534599783104129 | 0.491134915063122 | -0.669042811 | 1.42658833850248 | 1.46049112131556 | 0.512198613963581 | 4.16446717599521 | 0.999714303421225 |
| ebi-fl187-GCST90094846 | ebi-gm430-GCST90011519 | TestASV_41 (Barnesiella) abundance \|\| id:ebi-gm430-GCST90011519 | Spicy food liking \|\| id:ebi-fl187-GCST90094846 | Weighted mode | 14 | -0.74277063 | 0.804729380492834 | 0.372817993846048 | -2.320040216 | 0.834498955350414 | 0.475793838761876 | 0.0982696334954765 | 2.30365952279839 | 0.999714303421225 |
| ebi-fl187-GCST90094846 | ebi-gm430-GCST90011524 | TestASV_46 (Sutterella) abundance \|\| id:ebi-gm430-GCST90011524 | Spicy food liking \|\| id:ebi-fl187-GCST90094846 | Weighted mode | 14 | -0.1936105 | 0.59879429693355 | 0.751584612723888 | -1.367247322 | 0.980026321800122 | 0.823978781352819 | 0.254807397742476 | 2.66452637613708 | 0.999714303421225 |
| ebi-fl187-GCST90094846 | ebi-gm430-GCST90011581 | OTU97_19 (Bacteria) prevalence \|\| id:ebi-gm430-GCST90011581 | Spicy food liking \|\| id:ebi-fl187-GCST90094846 | Weighted mode | 14 | -1.25897104 | 0.728266793566008 | 0.107518068400162 | -2.686373955 | 0.168431875637672 | 0.283946045429478 | 0.0681275254989883 | 1.18344760248538 | 0.999714303421225 |
| ebi-fl187-GCST90094846 | ebi-gm430-GCST90011614 | OTU97_80 (Ruminococcus) prevalence \|\| id:ebi-gm430-GCST90011614 | Spicy food liking \|\| id:ebi-fl187-GCST90094846 | Weighted mode | 14 | 0.00417397032418032 | 0.342754927302978 | 0.990468735664269 | -0.667625687 | 0.675973627838017 | 1.00418269347081 | 0.512924977503218 | 1.9659461443559 | 0.999714303421225 |
| ebi-fl187-GCST90094846 | ebi-gm430-GCST90011623 | OTU99_11 (Parabacteroides) prevalence \|\| id:ebi-gm430-GCST90011623 | Spicy food liking \|\| id:ebi-fl187-GCST90094846 | Weighted mode | 14 | 0.0601515483044264 | 0.346394154497336 | 0.86481489807283 | -0.618780995 | 0.739084091119204 | 1.06199747826772 | 0.538600594665511 | 2.09401670740343 | 0.999714303421225 |
| ebi-fl187-GCST90094846 | ebi-gm430-GCST90011642 | OTU99_19 (Bacteria) prevalence \|\| id:ebi-gm430-GCST90011642 | Spicy food liking \|\| id:ebi-fl187-GCST90094846 | Weighted mode | 14 | -1.25897104 | 0.805491050511615 | 0.142063289980435 | -2.837733499 | 0.319791419251061 | 0.283946045429478 | 0.0585582379897584 | 1.37684055195002 | 0.999714303421225 |
| ebi-fl187-GCST90094846 | ebi-gm430-GCST90011653 | OTU99_30 (Parasutterella) prevalence \|\| id:ebi-gm430-GCST90011653 | Spicy food liking \|\| id:ebi-fl187-GCST90094846 | Weighted mode | 14 | 0.174700608020443 | 0.34825919582761 | 0.624308698102016 | -0.507887416 | 0.857288631842559 | 1.19088962043311 | 0.601765517236841 | 2.35676197361294 | 0.999714303421225 |
| ebi-fl187-GCST90094846 | ebi-gm430-GCST90011675 | OTU99_8 (Ruminococcaceae) prevalence \|\| id:ebi-gm430-GCST90011675 | Spicy food liking \|\| id:ebi-fl187-GCST90094846 | Weighted mode | 14 | -0.178604659 | 0.380892933742202 | 0.646899941843082 | -0.925154809 | 0.56794549089658 | 0.836436511488215 | 0.39647003704607 | 1.76463786005922 | 0.999714303421225 |
| ebi-fl187-GCST90094846 | ebi-gm430-GCST90011679 | OTU99_92 (Ruminococcus) prevalence \|\| id:ebi-gm430-GCST90011679 | Spicy food liking \|\| id:ebi-fl187-GCST90094846 | Weighted mode | 14 | 0.218631972515305 | 0.373491107042883 | 0.568319053208237 | -0.513410597 | 0.950674542319354 | 1.24437322885617 | 0.598451018786595 | 2.58745441829735 | 0.999714303421225 |
| ebi-fl187-GCST90094846 | ebi-gm430-GCST90011684 | TestASV_11 (Lachnospiraceae) prevalence \|\| id:ebi-gm430-GCST90011684 | Spicy food liking \|\| id:ebi-fl187-GCST90094846 | Weighted mode | 14 | 0.238273316954188 | 0.448479198665234 | 0.604176262471489 | -0.640745912 | 1.11729254633805 | 1.26905599994922 | 0.526899256722309 | 3.0565674755846 | 0.999714303421225 |
| ebi-fl187-GCST90094846 | ebi-gm430-GCST90011688 | TestASV_15 (Bacteroides) prevalence \|\| id:ebi-gm430-GCST90011688 | Spicy food liking \|\| id:ebi-fl187-GCST90094846 | Weighted mode | 14 | -0.223003777 | 0.423102482155518 | 0.607024059601684 | -1.052284642 | 0.606277088452096 | 0.800111827608348 | 0.349139179343194 | 1.83359237391543 | 0.999714303421225 |
| ebi-fl187-GCST90094846 | ebi-gm430-GCST90011692 | TestASV_19 (Prevotella) prevalence \|\| id:ebi-gm430-GCST90011692 | Spicy food liking \|\| id:ebi-fl187-GCST90094846 | Weighted mode | 14 | 0.371352733928107 | 0.724695599571862 | 0.616947791128149 | -1.049050641 | 1.79175610908896 | 1.44969433958983 | 0.350270123327493 | 5.99997983919928 | 0.999714303421225 |
| ebi-fl187-GCST90094846 | ebi-gm430-GCST90011693 | TestASV_2 (Bacteroides) prevalence \|\| id:ebi-gm430-GCST90011693 | Spicy food liking \|\| id:ebi-fl187-GCST90094846 | Weighted mode | 14 | -0.453539803 | 0.345191332167465 | 0.211600662800064 | -1.130114815 | 0.223035207568952 | 0.635375063369632 | 0.322996169640638 | 1.24986457765464 | 0.999714303421225 |
| ebi-fl187-GCST90094846 | ebi-gm430-GCST90011716 | TestASV_40 (Barnesiella) prevalence \|\| id:ebi-gm430-GCST90011716 | Spicy food liking \|\| id:ebi-fl187-GCST90094846 | Weighted mode | 14 | 0.686938579157987 | 0.939477856819933 | 0.4776353117178 | -1.15443802 | 2.52831517852506 | 1.98762126427736 | 0.315234642632388 | 12.5323735272794 | 0.999714303421225 |
| ebi-fl187-GCST90094846 | ebi-gm430-GCST90011718 | TestASV_42 (Alphaproteobacteria) prevalence \|\| id:ebi-gm430-GCST90011718 | Spicy food liking \|\| id:ebi-fl187-GCST90094846 | Weighted mode | 14 | 0.253765144982202 | 0.608197072757675 | 0.68330987358093 | -0.938301118 | 1.44583140758724 | 1.28886907135133 | 0.391292030138693 | 4.24538031734824 | 0.999714303421225 |
| ebi-fl187-GCST90094846 | ebi-hbsm44-GCST90104929 | Sulcal depth of the right inferior frontal sulcus \|\| id:ebi-hbsm44-GCST90104929 | Spicy food liking \|\| id:ebi-fl187-GCST90094846 | Weighted mode | 12 | 0.237957328426527 | 0.280686477563504 | 0.414632564607101 | -0.312188168 | 0.788102824450995 | 1.26865505616243 | 0.731843805980247 | 2.19922015923974 | 0.999714303421225 |
| ebi-fl187-GCST90094846 | ebi-hbsm44-GCST90104935 | Sulcal opening distance of the left calloso-marginal posterior fissure \|\| id:ebi-hbsm44-GCST90104935 | Spicy food liking \|\| id:ebi-fl187-GCST90094846 | Weighted mode | 13 | -0.047308577 | 0.0760867640433696 | 0.545723938895306 | -0.196438635 | 0.101821480128572 | 0.953793033204687 | 0.821651750472469 | 1.10718579941768 | 0.999714303421225 |
| ebi-fl187-GCST90094846 | ebi-hbsm44-GCST90104936 | Sulcal opening distance of the left anterior inferior temporal sulcus \|\| id:ebi-hbsm44-GCST90104936 | Spicy food liking \|\| id:ebi-fl187-GCST90094846 | Weighted mode | 14 | -0.069573386 | 0.0611847516605883 | 0.276020773339777 | -0.1894955 | 0.0503487269868096 | 0.932791676772772 | 0.827376440477969 | 1.05163776690802 | 0.999714303421225 |
| ebi-fl187-GCST90094846 | ebi-hbsm44-GCST90104941 | Sulcal opening distance of the right posterior inferior temporal sulcus \|\| id:ebi-hbsm44-GCST90104941 | Spicy food liking \|\| id:ebi-fl187-GCST90094846 | Weighted mode | 14 | -0.017347665 | 0.0273988601928997 | 0.537613418706963 | -0.071049431 | 0.0363541014015296 | 0.982801939811826 | 0.93141585058758 | 1.03702299277874 | 0.999714303421225 |
| ebi-fl187-GCST90094846 | ebi-IPs91-GCST90274819 | Leukemia inhibitory factor levels \|\| id:ebi-IPs91-GCST90274819 | Spicy food liking \|\| id:ebi-fl187-GCST90094846 | Weighted mode | 16 | -0.085957206 | 0.118582752298983 | 0.479686868691559 | -0.3183794 | 0.146464988634779 | 0.917633499706068 | 0.727326788002836 | 1.1577343962471 | 0.999714303421225 |
| ebi-fl187-GCST90094846 | ebi-IPs91-GCST90274837 | STAM binding protein levels \|\| id:ebi-IPs91-GCST90274837 | Spicy food liking \|\| id:ebi-fl187-GCST90094846 | Weighted mode | 16 | -0.010688622 | 0.109087032618601 | 0.923243504658192 | -0.224499206 | 0.203121962297172 | 0.98936829870046 | 0.798916211384479 | 1.22522189001165 | 0.999714303421225 |
| ebi-fl187-GCST90094846 | ebi-IPs91-GCST90274840 | TNF-beta levels \|\| id:ebi-IPs91-GCST90274840 | Spicy food liking \|\| id:ebi-fl187-GCST90094846 | Weighted mode | 16 | -0.084701143 | 0.135752254368893 | 0.54204081922236 | -0.350775561 | 0.181373275945821 | 0.918786829601721 | 0.704141772870603 | 1.19886260235367 | 0.999714303421225 |
| ebi-fl187-GCST90094846 | ebi-IPs91-GCST90274842 | Tumor necrosis factor ligand superfamily member 14 levels \|\| id:ebi-IPs91-GCST90274842 | Spicy food liking \|\| id:ebi-fl187-GCST90094846 | Weighted mode | 16 | -0.000576626 | 0.11728695321452 | 0.996142110247616 | -0.230459054 | 0.229305802288409 | 0.99942354020478 | 0.794168952130239 | 1.25772659587887 | 0.999714303421225 |
| ebi-fl187-GCST90094846 | ebi-met1400-GCST90199628 | 3-methylhistidine levels \|\| id:ebi-met1400-GCST90199628 | Spicy food liking \|\| id:ebi-fl187-GCST90094846 | Weighted mode | 16 | -0.072715394 | 0.186763292979564 | 0.702492565025975 | -0.438771448 | 0.293340660137727 | 0.929865437557246 | 0.64482813932977 | 1.34089950364486 | 0.999714303421225 |
| ebi-fl187-GCST90094846 | ebi-met1400-GCST90199651 | Docosahexaenoate DHA; 22:6n3 levels \|\| id:ebi-met1400-GCST90199651 | Spicy food liking \|\| id:ebi-fl187-GCST90094846 | Weighted mode | 16 | 0.177989710037075 | 0.158863904789104 | 0.280165678954765 | -0.133383543 | 0.48936296342372 | 1.1948130265898 | 0.875129377679546 | 1.63127670595858 | 0.999714303421225 |
| ebi-fl187-GCST90094846 | ebi-met1400-GCST90199652 | 1-stearoyl-2-oleoyl-GPS (18:0/18:1) levels \|\| id:ebi-met1400-GCST90199652 | Spicy food liking \|\| id:ebi-fl187-GCST90094846 | Weighted mode | 16 | 0.0254806409811702 | 0.163792982644245 | 0.878449198119976 | -0.295553605 | 0.34651488696389 | 1.02580804744092 | 0.744119525125687 | 1.41413054578403 | 0.999714303421225 |
| ebi-fl187-GCST90094846 | ebi-met1400-GCST90199653 | 1-myristoyl-2-palmitoyl-gpc (14:0/16:0) levels \|\| id:ebi-met1400-GCST90199653 | Spicy food liking \|\| id:ebi-fl187-GCST90094846 | Weighted mode | 16 | 0.000436935331847077 | 0.172928878681816 | 0.998017303227247 | -0.338503667 | 0.339377537548206 | 1.00043703080199 | 0.7128361654962 | 1.40407333556541 | 0.999714303421225 |
| ebi-fl187-GCST90094846 | ebi-met1400-GCST90199667 | Erythritol levels in elite athletes \|\| id:ebi-met1400-GCST90199667 | Spicy food liking \|\| id:ebi-fl187-GCST90094846 | Weighted mode | 16 | 0.183709602585809 | 0.128356473305766 | 0.172864528260859 | -0.067869085 | 0.435288290265111 | 1.20166681148794 | 0.934382790206125 | 1.54540852096929 | 0.999714303421225 |
| ebi-fl187-GCST90094846 | ebi-met1400-GCST90199674 | 3-hydroxymyristate levels \|\| id:ebi-met1400-GCST90199674 | Spicy food liking \|\| id:ebi-fl187-GCST90094846 | Weighted mode | 16 | -0.011017716 | 0.142799655109297 | 0.939519938614663 | -0.29090504 | 0.268869607587464 | 0.989042756316599 | 0.747586665147636 | 1.30848451346996 | 0.999714303421225 |
| ebi-fl187-GCST90094846 | ebi-met1400-GCST90199685 | 2-linoleoylglycerol (18:2) levels \|\| id:ebi-met1400-GCST90199685 | Spicy food liking \|\| id:ebi-fl187-GCST90094846 | Weighted mode | 16 | -0.216570525 | 0.185023916288594 | 0.260060599395288 | -0.579217401 | 0.146076350611269 | 0.805275740595212 | 0.560336713835434 | 1.15728454406009 | 0.999714303421225 |
| ebi-fl187-GCST90094846 | ebi-met1400-GCST90199703 | Gamma-glutamylvaline levels \|\| id:ebi-met1400-GCST90199703 | Spicy food liking \|\| id:ebi-fl187-GCST90094846 | Weighted mode | 16 | 0.0535855839122874 | 0.134705093234562 | 0.696382083709552 | -0.210436399 | 0.317607566652029 | 1.05504728297792 | 0.810230585129894 | 1.37383701596583 | 0.999714303421225 |
| ebi-fl187-GCST90094846 | ebi-met1400-GCST90199779 | Gamma-glutamyl-2-aminobutyrate levels \|\| id:ebi-met1400-GCST90199779 | Spicy food liking \|\| id:ebi-fl187-GCST90094846 | Weighted mode | 16 | -0.028881884 | 0.15807103708219 | 0.857468571936689 | -0.338701117 | 0.280937348476712 | 0.971531210871731 | 0.712695429889218 | 1.32437062749877 | 0.999714303421225 |
| ebi-fl187-GCST90094846 | ebi-met1400-GCST90199824 | 4-vinylphenol sulfate levels \|\| id:ebi-met1400-GCST90199824 | Spicy food liking \|\| id:ebi-fl187-GCST90094846 | Weighted mode | 16 | -0.046107754 | 0.136772199379197 | 0.740702092887172 | -0.314181265 | 0.221965756716191 | 0.954939058079367 | 0.73038662273776 | 1.24852862341227 | 0.999714303421225 |
| ebi-fl187-GCST90094846 | ebi-met1400-GCST90199830 | 4-ethylphenylsulfate levels \|\| id:ebi-met1400-GCST90199830 | Spicy food liking \|\| id:ebi-fl187-GCST90094846 | Weighted mode | 16 | 0.0349033996395176 | 0.12265220523018 | 0.779863038480968 | -0.205494923 | 0.275301721890671 | 1.03551967239428 | 0.814244228790831 | 1.31692796092391 | 0.999714303421225 |
| ebi-fl187-GCST90094846 | ebi-met1400-GCST90199833 | Hexanoylglutamine levels \|\| id:ebi-met1400-GCST90199833 | Spicy food liking \|\| id:ebi-fl187-GCST90094846 | Weighted mode | 15 | -0.046767742 | 0.149149864077735 | 0.758476773377656 | -0.339101476 | 0.245565991353265 | 0.954309017528521 | 0.712410153008487 | 1.2783446405001 | 0.999714303421225 |
| ebi-fl187-GCST90094846 | ebi-met1400-GCST90199837 | Carotenoid levels (cryptoxanthin) \|\| id:ebi-met1400-GCST90199837 | Spicy food liking \|\| id:ebi-fl187-GCST90094846 | Weighted mode | 16 | 0.08827638915234 | 0.132643699455256 | 0.515825003322512 | -0.171705262 | 0.348258040084642 | 1.09228997741247 | 0.842227373186528 | 1.41659774158336 | 0.999714303421225 |
| ebi-fl187-GCST90094846 | ebi-met1400-GCST90199869 | 2-hydroxyhippurate levels \|\| id:ebi-met1400-GCST90199869 | Spicy food liking \|\| id:ebi-fl187-GCST90094846 | Weighted mode | 16 | -0.040159694 | 0.17069540823108 | 0.817180079193211 | -0.374722694 | 0.294403306533868 | 0.960636019479254 | 0.687479894844227 | 1.34232516302174 | 0.999714303421225 |
| ebi-fl187-GCST90094846 | ebi-met1400-GCST90199902 | Hydantoin-5-propionate levels \|\| id:ebi-met1400-GCST90199902 | Spicy food liking \|\| id:ebi-fl187-GCST90094846 | Weighted mode | 16 | -0.101586186 | 0.131984687004866 | 0.453439860161276 | -0.360276172 | 0.157103800980425 | 0.903403315676077 | 0.697483673953629 | 1.17011706689609 | 0.999714303421225 |
| ebi-fl187-GCST90094846 | ebi-met1400-GCST90199904 | Pantoate levels \|\| id:ebi-met1400-GCST90199904 | Spicy food liking \|\| id:ebi-fl187-GCST90094846 | Weighted mode | 16 | -0.041379828 | 0.146076740580976 | 0.780838497668225 | -0.327690239 | 0.24493058363343 | 0.959464629288085 | 0.720586194318372 | 1.27753262845359 | 0.999714303421225 |
| ebi-fl187-GCST90094846 | ebi-met1400-GCST90199936 | 3-methyl catechol sulfate (2) levels \|\| id:ebi-met1400-GCST90199936 | Spicy food liking \|\| id:ebi-fl187-GCST90094846 | Weighted mode | 16 | -0.19204546 | 0.202403227781406 | 0.357746462189056 | -0.588755786 | 0.204664866430203 | 0.8252693508766 | 0.555017415486587 | 1.22711374903288 | 0.999714303421225 |
| ebi-fl187-GCST90094846 | ebi-met1400-GCST90199977 | 4-hydroxychlorothalonil levels \|\| id:ebi-met1400-GCST90199977 | Spicy food liking \|\| id:ebi-fl187-GCST90094846 | Weighted mode | 16 | 0.187533435737225 | 0.158974270375742 | 0.256512442260605 | -0.124056134 | 0.499123005673679 | 1.20627058129289 | 0.883330254454528 | 1.6472759853462 | 0.999714303421225 |
| ebi-fl187-GCST90094846 | ebi-met1400-GCST90200040 | Sphingomyelin (d18:2/24:1, d18:1/24:2) levels \|\| id:ebi-met1400-GCST90200040 | Spicy food liking \|\| id:ebi-fl187-GCST90094846 | Weighted mode | 16 | 0.106798539081917 | 0.152036396286658 | 0.493154026510641 | -0.191192798 | 0.404789875803768 | 1.11271006430619 | 0.825973327090998 | 1.49898749342045 | 0.999714303421225 |
| ebi-fl187-GCST90094846 | ebi-met1400-GCST90200077 | 1-stearoyl-2-oleoyl-GPI (18:0/18:1) levels \|\| id:ebi-met1400-GCST90200077 | Spicy food liking \|\| id:ebi-fl187-GCST90094846 | Weighted mode | 16 | -0.055981687 | 0.159858631677881 | 0.731061283312184 | -0.369304605 | 0.257341230994488 | 0.945556451618112 | 0.691214830760223 | 1.29348642912262 | 0.999714303421225 |
| ebi-fl187-GCST90094846 | ebi-met1400-GCST90200080 | 1-palmitoyl-2-oleoyl-GPI (16:0/18:1) levels \|\| id:ebi-met1400-GCST90200080 | Spicy food liking \|\| id:ebi-fl187-GCST90094846 | Weighted mode | 16 | -0.044030319 | 0.148018522676229 | 0.770191624741479 | -0.334146624 | 0.246085985036454 | 0.956924943634503 | 0.715948799358412 | 1.27900954449605 | 0.999714303421225 |
| ebi-fl187-GCST90094846 | ebi-met1400-GCST90200082 | 1-oleoyl-2-linoleoyl-GPE (18:1/18:2) levels \|\| id:ebi-met1400-GCST90200082 | Spicy food liking \|\| id:ebi-fl187-GCST90094846 | Weighted mode | 16 | -0.194991861 | 0.149776381553982 | 0.212593885182381 | -0.488553569 | 0.0985698464282676 | 0.822841354790987 | 0.613513156996942 | 1.1035914836259 | 0.999714303421225 |
| ebi-fl187-GCST90094846 | ebi-met1400-GCST90200100 | Perfluorooctanesulfonate (PFOS) levels \|\| id:ebi-met1400-GCST90200100 | Spicy food liking \|\| id:ebi-fl187-GCST90094846 | Weighted mode | 16 | 0.188423816808041 | 0.149455206313065 | 0.226661602663914 | -0.104508388 | 0.48135602118165 | 1.20734510007936 | 0.900767242130894 | 1.61826731979872 | 0.999714303421225 |
| ebi-fl187-GCST90094846 | ebi-met1400-GCST90200102 | 1-palmitoleoyl-2-linolenoyl-GPC (16:1/18:3) levels \|\| id:ebi-met1400-GCST90200102 | Spicy food liking \|\| id:ebi-fl187-GCST90094846 | Weighted mode | 16 | -0.168126833 | 0.127703393027855 | 0.207754661931719 | -0.418425483 | 0.082171817733707 | 0.845246623058243 | 0.658082166116357 | 1.0856423264098 | 0.999714303421225 |
| ebi-fl187-GCST90094846 | ebi-met1400-GCST90200114 | Glycosyl-N-tricosanoyl-sphingadienine (d18:2/23:0) levels \|\| id:ebi-met1400-GCST90200114 | Spicy food liking \|\| id:ebi-fl187-GCST90094846 | Weighted mode | 16 | 0.00155647093938072 | 0.209049971656588 | 0.994157566510961 | -0.408181474 | 0.411294415386293 | 1.00155768286897 | 0.664858213721918 | 1.50876949612812 | 0.999714303421225 |
| ebi-fl187-GCST90094846 | ebi-met1400-GCST90200135 | Arachidoylcarnitine (C20) levels \|\| id:ebi-met1400-GCST90200135 | Spicy food liking \|\| id:ebi-fl187-GCST90094846 | Weighted mode | 16 | -0.226973448 | 0.154138134103986 | 0.161548351770996 | -0.52908419 | 0.0751372952187712 | 0.796941942710491 | 0.589144266628617 | 1.07803214938445 | 0.999714303421225 |
| ebi-fl187-GCST90094846 | ebi-met1400-GCST90200146 | Docosahexaenoylcarnitine (C22:6) levels \|\| id:ebi-met1400-GCST90200146 | Spicy food liking \|\| id:ebi-fl187-GCST90094846 | Weighted mode | 16 | 0.0298379323488112 | 0.18435267841394 | 0.873582065771929 | -0.331493317 | 0.391169182040133 | 1.03028754413973 | 0.717850953352756 | 1.47870866320053 | 0.999714303421225 |
| ebi-fl187-GCST90094846 | ebi-met1400-GCST90200162 | 3-hydroxyphenylacetoylglutamine levels \|\| id:ebi-met1400-GCST90200162 | Spicy food liking \|\| id:ebi-fl187-GCST90094846 | Weighted mode | 16 | -0.022446547 | 0.182193531268122 | 0.903583038327912 | -0.379545868 | 0.334652774668023 | 0.977803502701084 | 0.684172043157253 | 1.39745506917001 | 0.999714303421225 |
| ebi-fl187-GCST90094846 | ebi-met1400-GCST90200172 | 3-carboxy-4-methyl-5-pentyl-2-furanpropionate (3-CMPFP) levels \|\| id:ebi-met1400-GCST90200172 | Spicy food liking \|\| id:ebi-fl187-GCST90094846 | Weighted mode | 16 | 0.266729845505048 | 0.167540026979048 | 0.132226836866238 | -0.061648607 | 0.595108298383983 | 1.30568766128887 | 0.94021321274057 | 1.81322730391409 | 0.999714303421225 |
| ebi-fl187-GCST90094846 | ebi-met1400-GCST90200222 | Dihydrocaffeate sulfate (2) levels \|\| id:ebi-met1400-GCST90200222 | Spicy food liking \|\| id:ebi-fl187-GCST90094846 | Weighted mode | 16 | 0.19549666642475 | 0.180077226184584 | 0.294783875989926 | -0.157454697 | 0.548448029746534 | 1.21591474057213 | 0.854315515875587 | 1.73056514702923 | 0.999714303421225 |
| ebi-fl187-GCST90094846 | ebi-met1400-GCST90200223 | 4-methylhexanoylglutamine levels \|\| id:ebi-met1400-GCST90200223 | Spicy food liking \|\| id:ebi-fl187-GCST90094846 | Weighted mode | 16 | -0.160759882 | 0.210666667998674 | 0.457237755059135 | -0.573666551 | 0.252146787283228 | 0.851496506204933 | 0.563455707389867 | 1.28678490708327 | 0.999714303421225 |
| ebi-fl187-GCST90094846 | ebi-met1400-GCST90200224 | 2-ketocaprylate levels \|\| id:ebi-met1400-GCST90200224 | Spicy food liking \|\| id:ebi-fl187-GCST90094846 | Weighted mode | 16 | 0.00820361725511365 | 0.145712938873626 | 0.955846028528759 | -0.277393743 | 0.29380097744742 | 1.00823735912851 | 0.757756077260473 | 1.3415168849817 | 0.999714303421225 |
| ebi-fl187-GCST90094846 | ebi-met1400-GCST90200243 | Hydroxypalmitoyl sphingomyelin (d18:1/16:0(OH)) levels \|\| id:ebi-met1400-GCST90200243 | Spicy food liking \|\| id:ebi-fl187-GCST90094846 | Weighted mode | 16 | 0.0456165982346014 | 0.176138504430478 | 0.799169909193492 | -0.29961487 | 0.390848066918339 | 1.04667303772779 | 0.741103586618221 | 1.47823390371835 | 0.999714303421225 |
| ebi-fl187-GCST90094846 | ebi-met1400-GCST90200273 | 2-methoxyhydroquinone sulfate (2) levels \|\| id:ebi-met1400-GCST90200273 | Spicy food liking \|\| id:ebi-fl187-GCST90094846 | Weighted mode | 16 | -0.283505005 | 0.253203536751126 | 0.280462324244178 | -0.779783937 | 0.212773927184222 | 0.75313935278266 | 0.458505066638882 | 1.23710494382961 | 0.999714303421225 |
| ebi-fl187-GCST90094846 | ebi-met1400-GCST90200276 | 2,4-di-tert-butylphenol levels \|\| id:ebi-met1400-GCST90200276 | Spicy food liking \|\| id:ebi-fl187-GCST90094846 | Weighted mode | 16 | 0.218016610254799 | 0.251698767954976 | 0.400034141204641 | -0.275312975 | 0.711346195446552 | 1.24360772408867 | 0.759334433385012 | 2.03673125228715 | 0.999714303421225 |
| ebi-fl187-GCST90094846 | ebi-met1400-GCST90200289 | N-acetylmethionine levels \|\| id:ebi-met1400-GCST90200289 | Spicy food liking \|\| id:ebi-fl187-GCST90094846 | Weighted mode | 16 | 0.127381594260644 | 0.152151157732027 | 0.41561783679592 | -0.170834675 | 0.425597863415418 | 1.13585036907583 | 0.842960924556284 | 1.53050517923926 | 0.999714303421225 |
| ebi-fl187-GCST90094846 | ebi-met1400-GCST90200308 | 3-hydroxyisobutyrate levels \|\| id:ebi-met1400-GCST90200308 | Spicy food liking \|\| id:ebi-fl187-GCST90094846 | Weighted mode | 16 | -0.092685348 | 0.155567849897657 | 0.560200471607754 | -0.397598334 | 0.212227637467679 | 0.911480253962521 | 0.671931865562643 | 1.23642931068303 | 0.999714303421225 |
| ebi-fl187-GCST90094846 | ebi-met1400-GCST90200383 | Cystathionine levels \|\| id:ebi-met1400-GCST90200383 | Spicy food liking \|\| id:ebi-fl187-GCST90094846 | Weighted mode | 16 | -0.14109804 | 0.204287635164281 | 0.50031375919376 | -0.541501804 | 0.259305725396123 | 0.868404169591042 | 0.581873735297777 | 1.29602997354259 | 0.999714303421225 |
| ebi-fl187-GCST90094846 | ebi-met1400-GCST90200384 | Guanidinoacetate levels \|\| id:ebi-met1400-GCST90200384 | Spicy food liking \|\| id:ebi-fl187-GCST90094846 | Weighted mode | 16 | 0.16193331110262 | 0.158012396928034 | 0.321695002643261 | -0.147770987 | 0.471637609081568 | 1.17578182711274 | 0.862628645603325 | 1.60261650481324 | 0.999714303421225 |
| ebi-fl187-GCST90094846 | ebi-met1400-GCST90200403 | Citrulline levels \|\| id:ebi-met1400-GCST90200403 | Spicy food liking \|\| id:ebi-fl187-GCST90094846 | Weighted mode | 16 | 0.00471598726146472 | 0.152924779699977 | 0.975804840273928 | -0.295016581 | 0.304448555473419 | 1.00472712503103 | 0.744519242526866 | 1.35587710580455 | 0.999714303421225 |
| ebi-fl187-GCST90094846 | ebi-met1400-GCST90200434 | 12,13-DiHOME levels \|\| id:ebi-met1400-GCST90200434 | Spicy food liking \|\| id:ebi-fl187-GCST90094846 | Weighted mode | 16 | 0.101126621970552 | 0.146890043888312 | 0.501678284839095 | -0.186777864 | 0.389031107991644 | 1.10641672956069 | 0.829628006123019 | 1.47555045203024 | 0.999714303421225 |
| ebi-fl187-GCST90094846 | ebi-met1400-GCST90200437 | Fructose levels \|\| id:ebi-met1400-GCST90200437 | Spicy food liking \|\| id:ebi-fl187-GCST90094846 | Weighted mode | 16 | 0.112591577472887 | 0.166620873138254 | 0.509493334890639 | -0.213985334 | 0.439168488823864 | 1.1191747434147 | 0.807360225779349 | 1.55141666173643 | 0.999714303421225 |
| ebi-fl187-GCST90094846 | ebi-met1400-GCST90200448 | N-stearoyl-sphinganine (d18:0/18:0) levels \|\| id:ebi-met1400-GCST90200448 | Spicy food liking \|\| id:ebi-fl187-GCST90094846 | Weighted mode | 16 | 0.0874709795998248 | 0.163493866467789 | 0.600484493995121 | -0.232976999 | 0.407918957876691 | 1.09141059081126 | 0.792171794314042 | 1.50368529438296 | 0.999714303421225 |
| ebi-fl187-GCST90094846 | ebi-met1400-GCST90200468 | X-11847 levels \|\| id:ebi-met1400-GCST90200468 | Spicy food liking \|\| id:ebi-fl187-GCST90094846 | Weighted mode | 16 | -0.10123839 | 0.160085673091141 | 0.536646055233314 | -0.415006309 | 0.212529529438454 | 0.903717570135781 | 0.660336114579247 | 1.23680263511335 | 0.999714303421225 |
| ebi-fl187-GCST90094846 | ebi-met1400-GCST90200476 | X-11843 levels \|\| id:ebi-met1400-GCST90200476 | Spicy food liking \|\| id:ebi-fl187-GCST90094846 | Weighted mode | 16 | 0.0829498662227625 | 0.15314041456015 | 0.596008188790266 | -0.217205346 | 0.383105078760657 | 1.08648733746868 | 0.804764696871489 | 1.46683215487556 | 0.999714303421225 |
| ebi-fl187-GCST90094846 | ebi-met1400-GCST90200511 | X-12906 levels \|\| id:ebi-met1400-GCST90200511 | Spicy food liking \|\| id:ebi-fl187-GCST90094846 | Weighted mode | 16 | -0.118915754 | 0.18839230288891 | 0.53740153782055 | -0.488164668 | 0.250333159162391 | 0.887882597724752 | 0.61375179934043 | 1.28445327278819 | 0.999714303421225 |
| ebi-fl187-GCST90094846 | ebi-met1400-GCST90200515 | X-12849 levels \|\| id:ebi-met1400-GCST90200515 | Spicy food liking \|\| id:ebi-fl187-GCST90094846 | Weighted mode | 16 | -0.141708434 | 0.202775220119813 | 0.495342742868472 | -0.539147865 | 0.255730997931096 | 0.86787426265802 | 0.583245044228641 | 1.29140529051616 | 0.999714303421225 |
| ebi-fl187-GCST90094846 | ebi-met1400-GCST90200551 | X-18886 levels \|\| id:ebi-met1400-GCST90200551 | Spicy food liking \|\| id:ebi-fl187-GCST90094846 | Weighted mode | 16 | -0.11996501 | 0.157611829191105 | 0.458371832637622 | -0.428884195 | 0.188954175280842 | 0.886951470664956 | 0.651235341026514 | 1.20798559561389 | 0.999714303421225 |
| ebi-fl187-GCST90094846 | ebi-met1400-GCST90200564 | X-21319 levels \|\| id:ebi-met1400-GCST90200564 | Spicy food liking \|\| id:ebi-fl187-GCST90094846 | Weighted mode | 16 | -0.108061634 | 0.138596410174432 | 0.447704162004016 | -0.379710598 | 0.163587329450628 | 0.897572273315114 | 0.684059348416271 | 1.17772820105341 | 0.999714303421225 |
| ebi-fl187-GCST90094846 | ebi-met1400-GCST90200579 | X-21442 levels \|\| id:ebi-met1400-GCST90200579 | Spicy food liking \|\| id:ebi-fl187-GCST90094846 | Weighted mode | 16 | 0.224446136451114 | 0.174946686634476 | 0.21898261528034 | -0.118449369 | 0.567341642254688 | 1.25162929233675 | 0.888296789559929 | 1.763572608 | 0.999714303421225 |
| ebi-fl187-GCST90094846 | ebi-met1400-GCST90200581 | X-21441 levels \|\| id:ebi-met1400-GCST90200581 | Spicy food liking \|\| id:ebi-fl187-GCST90094846 | Weighted mode | 16 | -0.089754805 | 0.139405497777299 | 0.52940145334559 | -0.362989581 | 0.183479970520141 | 0.914155303990713 | 0.695593681014065 | 1.20139090193583 | 0.999714303421225 |
| ebi-fl187-GCST90094846 | ebi-met1400-GCST90200648 | X-24585 levels \|\| id:ebi-met1400-GCST90200648 | Spicy food liking \|\| id:ebi-fl187-GCST90094846 | Weighted mode | 16 | -0.050623667 | 0.182948613279832 | 0.785779538089061 | -0.409202949 | 0.307955614936923 | 0.950636358969091 | 0.664179424013426 | 1.36064059547822 | 0.999714303421225 |
| ebi-fl187-GCST90094846 | ebi-met1400-GCST90200720 | Adenosine 5'-diphosphate (ADP) to pantothenate ratio \|\| id:ebi-met1400-GCST90200720 | Spicy food liking \|\| id:ebi-fl187-GCST90094846 | Weighted mode | 16 | -0.244706585 | 0.224192804308256 | 0.292275104809526 | -0.684124481 | 0.194711311917011 | 0.782934229559888 | 0.504531763420412 | 1.21496019132843 | 0.999714303421225 |
| ebi-fl187-GCST90094846 | ebi-met1400-GCST90200724 | Cholate to taurocholate ratio \|\| id:ebi-met1400-GCST90200724 | Spicy food liking \|\| id:ebi-fl187-GCST90094846 | Weighted mode | 16 | 0.0312802922614308 | 0.162781241141475 | 0.850192974443667 | -0.28777094 | 0.350331524898723 | 1.03177466181351 | 0.749933352018418 | 1.41953808281118 | 0.999714303421225 |
| ebi-fl187-GCST90094846 | ebi-met1400-GCST90200726 | N-acetylputrescine to (N(1) + N(8))-acetylspermidine ratio \|\| id:ebi-met1400-GCST90200726 | Spicy food liking \|\| id:ebi-fl187-GCST90094846 | Weighted mode | 16 | 0.0714170229887573 | 0.157397272156646 | 0.656513442691175 | -0.23708163 | 0.379915676415784 | 1.0740290272851 | 0.78892688494164 | 1.46216128955512 | 0.999714303421225 |
| ebi-fl187-GCST90094846 | ebi-met1400-GCST90200744 | Arginine to glutamate ratio \|\| id:ebi-met1400-GCST90200744 | Spicy food liking \|\| id:ebi-fl187-GCST90094846 | Weighted mode | 16 | 0.0122153762405149 | 0.155667199327382 | 0.938490565804924 | -0.292892334 | 0.317323086922184 | 1.01229028866593 | 0.746102465911963 | 1.37344624276884 | 0.999714303421225 |
| ebi-fl187-GCST90094846 | ebi-met1400-GCST90200759 | Histidine to trans-urocanate ratio \|\| id:ebi-met1400-GCST90200759 | Spicy food liking \|\| id:ebi-fl187-GCST90094846 | Weighted mode | 16 | -0.017205976 | 0.169858497737482 | 0.920657164787451 | -0.350128632 | 0.315716679253337 | 0.982941201178571 | 0.704597450196307 | 1.3712416993635 | 0.999714303421225 |
| ebi-fl187-GCST90094846 | ebi-met1400-GCST90200795 | Oleoyl-linoleoyl-glycerol (18:1 to 18:2) [2] to linoleoyl-arachidonoyl-glycerol (18:2 to 20:4) [2] ratio \|\| id:ebi-met1400-GCST90200795 | Spicy food liking \|\| id:ebi-fl187-GCST90094846 | Weighted mode | 16 | 0.141100853308836 | 0.182092438664176 | 0.450450386757854 | -0.215800326 | 0.49800203309062 | 1.15154077882636 | 0.805896201946283 | 1.64543046870992 | 0.999714303421225 |
| ebi-fl187-GCST90094846 | ebi-met1400-GCST90200811 | Choline to taurocholate ratio \|\| id:ebi-met1400-GCST90200811 | Spicy food liking \|\| id:ebi-fl187-GCST90094846 | Weighted mode | 16 | 0.213361755857313 | 0.162137522255303 | 0.207954596340376 | -0.104427788 | 0.531151299477708 | 1.2378323633653 | 0.900839846718614 | 1.70088941488966 | 0.999714303421225 |
| ebi-fl187-GCST90094846 | ebi-met1400-GCST90200831 | Cholate to phosphate ratio \|\| id:ebi-met1400-GCST90200831 | Spicy food liking \|\| id:ebi-fl187-GCST90094846 | Weighted mode | 16 | -0.100671603 | 0.161585173402752 | 0.5426253652032 | -0.417378543 | 0.216035336786459 | 0.904229930454552 | 0.658771499430606 | 1.2411462363453 | 0.999714303421225 |
| ebi-fl187-GCST90094846 | ebi-met1400-GCST90200841 | Adenosine 5'-diphosphate (ADP) to oxalate (ethanedioate) ratio \|\| id:ebi-met1400-GCST90200841 | Spicy food liking \|\| id:ebi-fl187-GCST90094846 | Weighted mode | 16 | -0.083490324 | 0.18935614061094 | 0.665561730365862 | -0.454628359 | 0.287647712075393 | 0.91989998802012 | 0.634683798569108 | 1.33328752028523 | 0.999714303421225 |
| ebi-fl187-GCST90094846 | ebi-met1400-GCST90200844 | Aspartate to phosphate ratio \|\| id:ebi-met1400-GCST90200844 | Spicy food liking \|\| id:ebi-fl187-GCST90094846 | Weighted mode | 16 | -0.07232595 | 0.132448480538962 | 0.593048349338208 | -0.331924972 | 0.187273071636428 | 0.930227638487139 | 0.717541156458044 | 1.20595655262035 | 0.999714303421225 |
| ebi-fl187-GCST90094846 | ebi-met1400-GCST90200870 | Adenosine 5'-monophosphate (AMP) to acetoacetate ratio \|\| id:ebi-met1400-GCST90200870 | Spicy food liking \|\| id:ebi-fl187-GCST90094846 | Weighted mode | 16 | -0.026064236 | 0.154923010562396 | 0.868642005988429 | -0.329713337 | 0.27758486446363 | 0.974272503997547 | 0.719129851850425 | 1.31993813023226 | 0.999714303421225 |
| ebi-fl187-GCST90094846 | ebi-met1400-GCST90200887 | Cholesterol to cortisol ratio \|\| id:ebi-met1400-GCST90200887 | Spicy food liking \|\| id:ebi-fl187-GCST90094846 | Weighted mode | 16 | 0.0803063762230385 | 0.130521809056355 | 0.547597201170708 | -0.17551637 | 0.336129121973495 | 1.08361901192254 | 0.839023662640201 | 1.39951972189313 | 0.999714303421225 |
| ebi-fl187-GCST90094846 | ebi-met1400-GCST90200890 | Cortisol to taurocholate ratio \|\| id:ebi-met1400-GCST90200890 | Spicy food liking \|\| id:ebi-fl187-GCST90094846 | Weighted mode | 16 | 0.066335407042233 | 0.149113571020175 | 0.662769481264622 | -0.225927192 | 0.358598006241776 | 1.06858506802297 | 0.797776183914128 | 1.43132130367604 | 0.999714303421225 |
| ebi-fl187-GCST90094846 | ebi-met1400-GCST90200948 | Adenosine 5'-diphosphate (ADP) to mannitol to sorbitol ratio \|\| id:ebi-met1400-GCST90200948 | Spicy food liking \|\| id:ebi-fl187-GCST90094846 | Weighted mode | 16 | 0.00580818198725885 | 0.256720783590233 | 0.982248019137301 | -0.497364554 | 0.508980917824115 | 1.00582508218021 | 0.608131246809009 | 1.66359499080396 | 0.999714303421225 |
| ebi-fl187-GCST90094846 | ebi-met1400-GCST90200972 | Leucine to N-palmitoyl-sphingosine (d18:1 to 16:0) ratio \|\| id:ebi-met1400-GCST90200972 | Spicy food liking \|\| id:ebi-fl187-GCST90094846 | Weighted mode | 16 | 0.181548382808013 | 0.184387439684827 | 0.340435423097875 | -0.179850999 | 0.542947764590275 | 1.19907254980325 | 0.835394676802057 | 1.72107270924394 | 0.999714303421225 |
| ebi-fl187-GCST90094846 | ebi-met1400-GCST90200973 | Proline to glutamate ratio \|\| id:ebi-met1400-GCST90200973 | Spicy food liking \|\| id:ebi-fl187-GCST90094846 | Weighted mode | 16 | -0.062672331 | 0.142648361913954 | 0.666672457250901 | -0.342263121 | 0.216918457878374 | 0.939251186325237 | 0.710161321891456 | 1.24224280289402 | 0.999714303421225 |
| ebi-fl187-GCST90094846 | ebi-met1400-GCST90200983 | Cholesterol to linoleoyl-arachidonoyl-glycerol (18:2 to 20:4) [1] ratio \|\| id:ebi-met1400-GCST90200983 | Spicy food liking \|\| id:ebi-fl187-GCST90094846 | Weighted mode | 16 | -0.101748592 | 0.142234061924127 | 0.485373328696333 | -0.380527354 | 0.177030169005759 | 0.903256608733063 | 0.683500867417629 | 1.19366710433367 | 0.999714303421225 |
| ebi-fl187-GCST90094846 | ebi-met1400-GCST90201001 | Serine to alpha-ketobutyrate ratio \|\| id:ebi-met1400-GCST90201001 | Spicy food liking \|\| id:ebi-fl187-GCST90094846 | Weighted mode | 16 | 0.0300033288057854 | 0.143647643314738 | 0.83736156471313 | -0.251546052 | 0.311552709702672 | 1.03045796414224 | 0.777597646787523 | 1.3655437619326 | 0.999714303421225 |
| ebi-fl187-GCST90094846 | ebi-met1400-GCST90201005 | Fructose to maltose ratio \|\| id:ebi-met1400-GCST90201005 | Spicy food liking \|\| id:ebi-fl187-GCST90094846 | Weighted mode | 16 | -0.077557703 | 0.182408078644776 | 0.676736466944205 | -0.435077538 | 0.279962130653765 | 0.925373625568258 | 0.647214481652991 | 1.32307970722519 | 0.999714303421225 |
| ebi-fl187-GCST90094846 | ebi-met1400-GCST90201020 | Paraxanthine to 5-acetylamino-6-formylamino-3-methyluracil ratio \|\| id:ebi-met1400-GCST90201020 | Spicy food liking \|\| id:ebi-fl187-GCST90094846 | Weighted mode | 16 | 0.0948158840472642 | 0.144701335033408 | 0.522228259597344 | -0.188798733 | 0.378430500712743 | 1.09945640902566 | 0.827953129884957 | 1.45999133491476 | 0.999714303421225 |
| ebi-fl187-GCST90094846 | ebi-pw11-GCST90275197 | Placental weight (fetal genotype, adjusted for sex) \|\| id:ebi-pw11-GCST90275197 | Spicy food liking \|\| id:ebi-fl187-GCST90094846 | Weighted mode | 15 | -0.077917373 | 0.0690721380977916 | 0.278256997655186 | -0.213298764 | 0.0574640171895437 | 0.925040856290779 | 0.80791472519644 | 1.05914715887759 | 0.999714303421225 |
| ebi-fl187-GCST90094846 | prot-a-1017 | FAS-associated factor 2 \|\| id:prot-a-1017 | Spicy food liking \|\| id:ebi-fl187-GCST90094846 | Weighted mode | 16 | 0.107108404580792 | 0.257903252842783 | 0.683798660939777 | -0.398381971 | 0.612598780152646 | 1.11305490819022 | 0.671405521243769 | 1.84522049558263 | 0.999714303421225 |
| ebi-fl187-GCST90094846 | prot-a-1019 | Fas apoptotic inhibitory molecule 3 \|\| id:prot-a-1019 | Spicy food liking \|\| id:ebi-fl187-GCST90094846 | Weighted mode | 16 | 0.275439184531442 | 0.241214296651231 | 0.271402760796247 | -0.197340837 | 0.748219205967855 | 1.31710900176201 | 0.820910788932164 | 2.11323343036958 | 0.999714303421225 |
| ebi-fl187-GCST90094846 | prot-a-1052 | MIP18 family protein FAM96A \|\| id:prot-a-1052 | Spicy food liking \|\| id:ebi-fl187-GCST90094846 | Weighted mode | 16 | -0.110104746 | 0.255504140922691 | 0.672646712428885 | -0.610892862 | 0.390683370638941 | 0.895740305554031 | 0.542865948367254 | 1.47799046414167 | 0.999714303421225 |
| ebi-fl187-GCST90094846 | prot-a-1066 | F-box/LRR-repeat protein 4 \|\| id:prot-a-1066 | Spicy food liking \|\| id:ebi-fl187-GCST90094846 | Weighted mode | 16 | 0.0322201812722136 | 0.245122932574721 | 0.89717012574336 | -0.448220767 | 0.512661129118667 | 1.03274487135297 | 0.638763650801841 | 1.6697286515396 | 0.999714303421225 |
| ebi-fl187-GCST90094846 | prot-a-1076 | Low affinity immunoglobulin gamma Fc region receptor III-B \|\| id:prot-a-1076 | Spicy food liking \|\| id:ebi-fl187-GCST90094846 | Weighted mode | 16 | 0.0513390230444892 | 0.256222172274535 | 0.84388320747895 | -0.450856435 | 0.553534480702577 | 1.05267971547633 | 0.637082298579203 | 1.73939000635654 | 0.999714303421225 |
| ebi-fl187-GCST90094846 | prot-a-1081 | Fc receptor-like protein 3 \|\| id:prot-a-1081 | Spicy food liking \|\| id:ebi-fl187-GCST90094846 | Weighted mode | 16 | 0.239336235568937 | 0.266743441274069 | 0.38375940638646 | -0.283480909 | 0.762153380466111 | 1.27040562033597 | 0.753157500285443 | 2.1428857039458 | 0.999714303421225 |
| ebi-fl187-GCST90094846 | prot-a-1105 | Fibroblast growth factor receptor 4 \|\| id:prot-a-1105 | Spicy food liking \|\| id:ebi-fl187-GCST90094846 | Weighted mode | 16 | -0.036873504 | 0.310180998977352 | 0.90694993395876 | -0.644828262 | 0.571081254100197 | 0.963798044336601 | 0.52475265439706 | 1.77018003145571 | 0.999714303421225 |
| ebi-fl187-GCST90094846 | prot-a-1143 | Follistatin-related protein 1 \|\| id:prot-a-1143 | Spicy food liking \|\| id:ebi-fl187-GCST90094846 | Weighted mode | 16 | -0.072165404 | 0.205503246196316 | 0.730348731089897 | -0.474951767 | 0.330620958297607 | 0.930376994777207 | 0.621915052699733 | 1.39183212989152 | 0.999714303421225 |
| ebi-fl187-GCST90094846 | prot-a-1151 | Alpha-(1,3)-fucosyltransferase 10 \|\| id:prot-a-1151 | Spicy food liking \|\| id:ebi-fl187-GCST90094846 | Weighted mode | 16 | 0.0868532471957513 | 0.229039825018161 | 0.709849197650916 | -0.36206481 | 0.535771304231347 | 1.09073659931779 | 0.696237243355296 | 1.70876571232807 | 0.999714303421225 |
| ebi-fl187-GCST90094846 | prot-a-1165 | Growth arrest and DNA damage-inducible proteins-interacting protein 1 \|\| id:prot-a-1165 | Spicy food liking \|\| id:ebi-fl187-GCST90094846 | Weighted mode | 16 | 0.164460794916738 | 0.307622149577064 | 0.600748476885407 | -0.438478618 | 0.767400208087784 | 1.17875735536544 | 0.645016992059995 | 2.15415860346649 | 0.999714303421225 |
| ebi-fl187-GCST90094846 | prot-a-1194 | Growth/differentiation factor 11/8 \|\| id:prot-a-1194 | Spicy food liking \|\| id:ebi-fl187-GCST90094846 | Weighted mode | 16 | 0.000723337088403442 | 0.227000732331759 | 0.997499540382776 | -0.444198098 | 0.445644772458652 | 1.00072359875976 | 0.641338360215251 | 1.56149668137515 | 0.999714303421225 |
| ebi-fl187-GCST90094846 | prot-a-1216 | D-glucuronyl C5-epimerase \|\| id:prot-a-1216 | Spicy food liking \|\| id:ebi-fl187-GCST90094846 | Weighted mode | 16 | 0.175888495206573 | 0.303430769769506 | 0.570744434336066 | -0.418835814 | 0.770612803954805 | 1.19230510350397 | 0.657812190255688 | 2.16109017269663 | 0.999714303421225 |
| ebi-fl187-GCST90094846 | prot-a-1246 | Glypican-3 \|\| id:prot-a-1246 | Spicy food liking \|\| id:ebi-fl187-GCST90094846 | Weighted mode | 16 | 0.264393446766174 | 0.259028698049005 | 0.323572543186294 | -0.243302801 | 0.772089694942224 | 1.30264061522705 | 0.784034071202249 | 2.1642842253489 | 0.999714303421225 |
| ebi-fl187-GCST90094846 | prot-a-1275 | Glutamate receptor 4 \|\| id:prot-a-1275 | Spicy food liking \|\| id:ebi-fl187-GCST90094846 | Weighted mode | 16 | -0.125947468 | 0.285317447816392 | 0.665199087503553 | -0.685169665 | 0.433274730193828 | 0.881661161408668 | 0.504004710236837 | 1.54229987884678 | 0.999714303421225 |
| ebi-fl187-GCST90094846 | prot-a-1289 | Glutathione S-transferase P \|\| id:prot-a-1289 | Spicy food liking \|\| id:ebi-fl187-GCST90094846 | Weighted mode | 16 | 0.0305286433647825 | 0.195253569138261 | 0.877839047832123 | -0.352168352 | 0.413225638875773 | 1.03099942091835 | 0.7031617332242 | 1.51168608260293 | 0.999714303421225 |
| ebi-fl187-GCST90094846 | prot-a-130 | Apolipoprotein D \|\| id:prot-a-130 | Spicy food liking \|\| id:ebi-fl187-GCST90094846 | Weighted mode | 16 | -0.214793255 | 0.26843880535319 | 0.436109611522167 | -0.740933314 | 0.311346803112866 | 0.80670820551971 | 0.476668826221644 | 1.3652626164192 | 0.999714303421225 |
| ebi-fl187-GCST90094846 | prot-a-1313 | Heparin-binding EGF-like growth factor \|\| id:prot-a-1313 | Spicy food liking \|\| id:ebi-fl187-GCST90094846 | Weighted mode | 15 | 0.236307207630499 | 0.271596051375665 | 0.398935666591349 | -0.296021053 | 0.768635468326802 | 1.26656334833119 | 0.743771769179347 | 2.1568211940955 | 0.999714303421225 |
| ebi-fl187-GCST90094846 | prot-a-1347 | HLA class II histocompatibility antigen, DQ alpha 2 chain \|\| id:prot-a-1347 | Spicy food liking \|\| id:ebi-fl187-GCST90094846 | Weighted mode | 15 | -0.061350987 | 0.217151599148714 | 0.781674157664861 | -0.486968122 | 0.364266147156755 | 0.940493080829333 | 0.614486621540355 | 1.43945727064094 | 0.999714303421225 |
| ebi-fl187-GCST90094846 | prot-a-1357 | Non-histone chromosomal protein HMG-14 \|\| id:prot-a-1357 | Spicy food liking \|\| id:ebi-fl187-GCST90094846 | Weighted mode | 16 | 0.0321128670390769 | 0.238554276456561 | 0.894706334327187 | -0.435453515 | 0.499679248893937 | 1.03263404907559 | 0.646971189515023 | 1.64819252633117 | 0.999714303421225 |
| ebi-fl187-GCST90094846 | prot-a-137 | Apolipoprotein M \|\| id:prot-a-137 | Spicy food liking \|\| id:ebi-fl187-GCST90094846 | Weighted mode | 16 | 0.180558222890344 | 0.201005862669481 | 0.383232382143895 | -0.213413268 | 0.574529713722527 | 1.19788586382746 | 0.807822221196122 | 1.7762949633064 | 0.999714303421225 |
| ebi-fl187-GCST90094846 | prot-a-1381 | 17-beta-hydroxysteroid dehydrogenase 14 \|\| id:prot-a-1381 | Spicy food liking \|\| id:ebi-fl187-GCST90094846 | Weighted mode | 16 | -0.182675205 | 0.197195971752302 | 0.368922496693043 | -0.56917931 | 0.20382889936846 | 0.83303867837846 | 0.565989750329974 | 1.22608835101687 | 0.999714303421225 |
| ebi-fl187-GCST90094846 | prot-a-1392 | Serine protease HTRA2, mitochondrial \|\| id:prot-a-1392 | Spicy food liking \|\| id:ebi-fl187-GCST90094846 | Weighted mode | 16 | -0.032975389 | 0.208687930318666 | 0.876554178101297 | -0.442003733 | 0.376052954192379 | 0.967562371748869 | 0.642747236296216 | 1.45652426079488 | 0.999714303421225 |
| ebi-fl187-GCST90094846 | prot-a-1416 | Interferon-induced protein with tetratricopeptide repeats 2 \|\| id:prot-a-1416 | Spicy food liking \|\| id:ebi-fl187-GCST90094846 | Weighted mode | 16 | -0.167667159 | 0.254270985654595 | 0.519632847302971 | -0.666038291 | 0.330703972472648 | 0.845635249584003 | 0.513739839090472 | 1.39194767648352 | 0.999714303421225 |
| ebi-fl187-GCST90094846 | prot-a-1428 | Interferon gamma \|\| id:prot-a-1428 | Spicy food liking \|\| id:ebi-fl187-GCST90094846 | Weighted mode | 16 | -0.141032407 | 0.30343856345129 | 0.648763567024034 | -0.735771991 | 0.453707177352485 | 0.868461167010129 | 0.479135427744383 | 1.57413698702107 | 0.999714303421225 |
| ebi-fl187-GCST90094846 | prot-a-1483 | Interleukin-17C \|\| id:prot-a-1483 | Spicy food liking \|\| id:ebi-fl187-GCST90094846 | Weighted mode | 16 | 0.175251240833136 | 0.295340139131381 | 0.561763211125335 | -0.403615432 | 0.754117913530643 | 1.19154554390433 | 0.667900925300068 | 2.12573561349748 | 0.999714303421225 |
| ebi-fl187-GCST90094846 | prot-a-1503 | Interleukin-1 receptor-like 2 \|\| id:prot-a-1503 | Spicy food liking \|\| id:ebi-fl187-GCST90094846 | Weighted mode | 16 | -0.117568403 | 0.239283443858332 | 0.630300656341114 | -0.586563953 | 0.35142714697952 | 0.889079693963389 | 0.556235255429999 | 1.42109421238854 | 0.999714303421225 |
| ebi-fl187-GCST90094846 | prot-a-1522 | Interleukin-31 receptor subunit alpha \|\| id:prot-a-1522 | Spicy food liking \|\| id:ebi-fl187-GCST90094846 | Weighted mode | 16 | 0.073611707551728 | 0.238843679712958 | 0.762168425549786 | -0.394521905 | 0.541745319789125 | 1.07638877071053 | 0.674002199524513 | 1.71900445804046 | 0.999714303421225 |
| ebi-fl187-GCST90094846 | prot-a-1540 | Interleukin-6 receptor subunit alpha \|\| id:prot-a-1540 | Spicy food liking \|\| id:ebi-fl187-GCST90094846 | Weighted mode | 16 | 0.330467830470274 | 0.299624538516331 | 0.287434736044859 | -0.256796265 | 0.917731925962282 | 1.39161901797856 | 0.773525791973223 | 2.50360558276852 | 0.999714303421225 |
| ebi-fl187-GCST90094846 | prot-a-1541 | Interleukin-6 receptor subunit alpha \|\| id:prot-a-1541 | Spicy food liking \|\| id:ebi-fl187-GCST90094846 | Weighted mode | 16 | 0.378370756971761 | 0.279813441884969 | 0.196339850956365 | -0.170063589 | 0.926805103066301 | 1.45990411217614 | 0.843611170396453 | 2.52642460358509 | 0.999714303421225 |
| ebi-fl187-GCST90094846 | prot-a-1545 | Interleukin-9 \|\| id:prot-a-1545 | Spicy food liking \|\| id:ebi-fl187-GCST90094846 | Weighted mode | 16 | 0.135283346872949 | 0.298985638883059 | 0.657402371666066 | -0.450728505 | 0.721295199083745 | 1.1448611312193 | 0.63716380526969 | 2.05709583459776 | 0.999714303421225 |
| ebi-fl187-GCST90094846 | prot-a-1548 | Inositol monophosphatase 3 \|\| id:prot-a-1548 | Spicy food liking \|\| id:ebi-fl187-GCST90094846 | Weighted mode | 16 | 0.0622336162291033 | 0.223422090950674 | 0.784395550703945 | -0.375673682 | 0.500140914492425 | 1.06421093263487 | 0.686826420278778 | 1.64895361579115 | 0.999714303421225 |
| ebi-fl187-GCST90094846 | prot-a-1555 | Inhibin beta A chain:Inhibin beta B chain heterodimer \|\| id:prot-a-1555 | Spicy food liking \|\| id:ebi-fl187-GCST90094846 | Weighted mode | 16 | -0.079336138 | 0.226974520688461 | 0.731544619064653 | -0.524206198 | 0.365533923015534 | 0.923729372140772 | 0.59202512857412 | 1.44128333709531 | 0.999714303421225 |
| ebi-fl187-GCST90094846 | prot-a-1603 | Junctophilin-3 \|\| id:prot-a-1603 | Spicy food liking \|\| id:ebi-fl187-GCST90094846 | Weighted mode | 16 | 0.193369148963299 | 0.270534070130822 | 0.485728633500851 | -0.336877628 | 0.723615926419709 | 1.21333061059547 | 0.713996207348774 | 2.06187533694958 | 0.999714303421225 |
| ebi-fl187-GCST90094846 | prot-a-1627 | UPF0258 protein KIAA1024 \|\| id:prot-a-1627 | Spicy food liking \|\| id:ebi-fl187-GCST90094846 | Weighted mode | 16 | 0.0921159059928951 | 0.26139905493534 | 0.729443908106879 | -0.420226242 | 0.604458053666162 | 1.09649190469877 | 0.656898185252759 | 1.83026003734098 | 0.999714303421225 |
| ebi-fl187-GCST90094846 | prot-a-1666 | Kallikrein-7 \|\| id:prot-a-1666 | Spicy food liking \|\| id:ebi-fl187-GCST90094846 | Weighted mode | 16 | -0.109031495 | 0.21732529708748 | 0.623163785483622 | -0.534989078 | 0.316926086919768 | 0.896702175085183 | 0.585675687056996 | 1.37290109282656 | 0.999714303421225 |
| ebi-fl187-GCST90094846 | prot-a-1678 | Importin subunit alpha-3 \|\| id:prot-a-1678 | Spicy food liking \|\| id:ebi-fl187-GCST90094846 | Weighted mode | 16 | 0.0117779163280547 | 0.26016298647706 | 0.964488264146904 | -0.498141537 | 0.521697369823093 | 1.01184754909233 | 0.607658922493276 | 1.68488509705951 | 0.999714303421225 |
| ebi-fl187-GCST90094846 | prot-a-1718 | Protein LDOC1 \|\| id:prot-a-1718 | Spicy food liking \|\| id:ebi-fl187-GCST90094846 | Weighted mode | 16 | 0.152110932065388 | 0.253819219935904 | 0.55792438730472 | -0.345374739 | 0.64959660313976 | 1.16428938629488 | 0.707955005395642 | 1.9147682616939 | 0.999714303421225 |
| ebi-fl187-GCST90094846 | prot-a-1723 | Leptin \|\| id:prot-a-1723 | Spicy food liking \|\| id:ebi-fl187-GCST90094846 | Weighted mode | 16 | -0.314073833 | 0.296535901437506 | 0.306293053952676 | -0.895284199 | 0.267136534281283 | 0.730465094077882 | 0.408491489125288 | 1.30621877779822 | 0.999714303421225 |
| ebi-fl187-GCST90094846 | prot-a-1730 | Galectin-7 \|\| id:prot-a-1730 | Spicy food liking \|\| id:ebi-fl187-GCST90094846 | Weighted mode | 16 | -0.164037829 | 0.315937997574284 | 0.611190933718029 | -0.783276304 | 0.455200646586115 | 0.848709915714544 | 0.456906591518976 | 1.57648966857217 | 0.999714303421225 |
| ebi-fl187-GCST90094846 | prot-a-1753 | Lipase member N \|\| id:prot-a-1753 | Spicy food liking \|\| id:ebi-fl187-GCST90094846 | Weighted mode | 16 | 0.100538769496077 | 0.225338085973812 | 0.661848671544064 | -0.341123879 | 0.542201418004748 | 1.10576651088347 | 0.710970828386698 | 1.71978867173206 | 0.999714303421225 |
| ebi-fl187-GCST90094846 | prot-a-1756 | VIP36-like protein \|\| id:prot-a-1756 | Spicy food liking \|\| id:ebi-fl187-GCST90094846 | Weighted mode | 16 | 0.244942864879387 | 0.220003629518064 | 0.283078376731463 | -0.186264249 | 0.676149978734793 | 1.27754831824237 | 0.830054225020064 | 1.96629287129338 | 0.999714303421225 |
| ebi-fl187-GCST90094846 | prot-a-1760 | Ig Kappa chain V-I region HK102- like \|\| id:prot-a-1760 | Spicy food liking \|\| id:ebi-fl187-GCST90094846 | Weighted mode | 16 | 0.164191575971498 | 0.269946674882206 | 0.552129599112805 | -0.364903907 | 0.693287058740622 | 1.17844005426718 | 0.694263361662414 | 2.00027977592817 | 0.999714303421225 |
| ebi-fl187-GCST90094846 | prot-a-179 | Neutral ceramidase \|\| id:prot-a-179 | Spicy food liking \|\| id:ebi-fl187-GCST90094846 | Weighted mode | 16 | 0.478243895765905 | 0.327888821605647 | 0.165308504416025 | -0.164418195 | 1.12090598611297 | 1.61323889754215 | 0.848387156772356 | 3.06763217685219 | 0.999714303421225 |
| ebi-fl187-GCST90094846 | prot-a-1809 | Lactotransferrin \|\| id:prot-a-1809 | Spicy food liking \|\| id:ebi-fl187-GCST90094846 | Weighted mode | 16 | -0.019444365 | 0.221100319956908 | 0.931084830222334 | -0.452800992 | 0.413912262346892 | 0.980743457560017 | 0.635844659283775 | 1.51272439817333 | 0.999714303421225 |
| ebi-fl187-GCST90094846 | prot-a-1818 | Lysophospholipase-like protein 1 \|\| id:prot-a-1818 | Spicy food liking \|\| id:ebi-fl187-GCST90094846 | Weighted mode | 16 | -0.317172329 | 0.335440615345564 | 0.359366906648975 | -0.974635935 | 0.34029127666734 | 0.728205253130564 | 0.377329700913693 | 1.4053568786207 | 0.999714303421225 |
| ebi-fl187-GCST90094846 | prot-a-1852 | MAP kinase-activated protein kinase 3 \|\| id:prot-a-1852 | Spicy food liking \|\| id:ebi-fl187-GCST90094846 | Weighted mode | 16 | -0.23149763 | 0.30449426960592 | 0.458877853015671 | -0.828306399 | 0.365311138015461 | 0.793344575400571 | 0.436788405608753 | 1.44096227655201 | 0.999714303421225 |
| ebi-fl187-GCST90094846 | prot-a-1856 | Megakaryocyte-associated tyrosine-protein kinase \|\| id:prot-a-1856 | Spicy food liking \|\| id:ebi-fl187-GCST90094846 | Weighted mode | 16 | -0.074736045 | 0.202061337140083 | 0.716650749352569 | -0.470776266 | 0.321304176037005 | 0.927988401407298 | 0.624517288812617 | 1.37892495303658 | 0.999714303421225 |
| ebi-fl187-GCST90094846 | prot-a-1890 | Beta-1,3-N-acetylglucosaminyltransferase manic fringe \|\| id:prot-a-1890 | Spicy food liking \|\| id:ebi-fl187-GCST90094846 | Weighted mode | 16 | -0.051112273 | 0.254671681121304 | 0.843630107659881 | -0.550268768 | 0.448044222209166 | 0.95017198608514 | 0.576794765693559 | 1.56524791284364 | 0.999714303421225 |
| ebi-fl187-GCST90094846 | prot-a-1919 | Matrilysin \|\| id:prot-a-1919 | Spicy food liking \|\| id:ebi-fl187-GCST90094846 | Weighted mode | 16 | 0.0970221664230375 | 0.235360243374898 | 0.686003090222582 | -0.364283911 | 0.558328243437838 | 1.10188479819488 | 0.694693935775881 | 1.74774824705636 | 0.999714303421225 |
| ebi-fl187-GCST90094846 | prot-a-1923 | Multimerin-2 \|\| id:prot-a-1923 | Spicy food liking \|\| id:ebi-fl187-GCST90094846 | Weighted mode | 16 | -0.16194436 | 0.224689391564213 | 0.482147275673902 | -0.602335567 | 0.278446847534378 | 0.850488524463555 | 0.54753134521429 | 1.32107638506267 | 0.999714303421225 |
| ebi-fl187-GCST90094846 | prot-a-1948 | Mesothelin \|\| id:prot-a-1948 | Spicy food liking \|\| id:ebi-fl187-GCST90094846 | Weighted mode | 16 | 0.120935546977303 | 0.247051299191592 | 0.631557728990299 | -0.363284999 | 0.605156093392822 | 1.12855217142446 | 0.695388220003026 | 1.8315380775667 | 0.999714303421225 |
| ebi-fl187-GCST90094846 | prot-a-195 | ATPase family AAA domain-containing protein 1 \|\| id:prot-a-195 | Spicy food liking \|\| id:ebi-fl187-GCST90094846 | Weighted mode | 16 | -0.11930768 | 0.251280716897233 | 0.641769605032041 | -0.611817886 | 0.373202524644569 | 0.887534681655846 | 0.54236401662209 | 1.45237845247911 | 0.999714303421225 |
| ebi-fl187-GCST90094846 | prot-a-1966 | Mucin-1 \|\| id:prot-a-1966 | Spicy food liking \|\| id:ebi-fl187-GCST90094846 | Weighted mode | 16 | -0.025486621 | 0.288971426757064 | 0.930886268188091 | -0.591870617 | 0.540897375815516 | 0.974835421563913 | 0.553291319900951 | 1.71754745638485 | 0.999714303421225 |
| ebi-fl187-GCST90094846 | prot-a-1986 | Myocardial zonula adherens protein \|\| id:prot-a-1986 | Spicy food liking \|\| id:ebi-fl187-GCST90094846 | Weighted mode | 16 | -0.037251186 | 0.30242105876309 | 0.903602579722807 | -0.629996461 | 0.555494089156946 | 0.963434103776035 | 0.532593685749097 | 1.7428018715866 | 0.999714303421225 |
| ebi-fl187-GCST90094846 | prot-a-2071 | Epididymal secretory protein E1 \|\| id:prot-a-2071 | Spicy food liking \|\| id:ebi-fl187-GCST90094846 | Weighted mode | 16 | -0.164275527 | 0.291641322241175 | 0.581567797553237 | -0.735892518 | 0.407341464902595 | 0.84850820301331 | 0.479077682513604 | 1.50281717738006 | 0.999714303421225 |
| ebi-fl187-GCST90094846 | prot-a-2098 | Neuritin \|\| id:prot-a-2098 | Spicy food liking \|\| id:ebi-fl187-GCST90094846 | Weighted mode | 16 | 0.286490690063979 | 0.306558895088667 | 0.364828394560759 | -0.314364744 | 0.887346124437766 | 1.33174576936859 | 0.730252624088335 | 2.42867568801312 | 0.999714303421225 |
| ebi-fl187-GCST90094846 | prot-a-2115 | Neurotrophin-4 \|\| id:prot-a-2115 | Spicy food liking \|\| id:ebi-fl187-GCST90094846 | Weighted mode | 16 | 0.228334852077958 | 0.245760220389734 | 0.367550391018467 | -0.25335518 | 0.710024884041836 | 1.25650599864138 | 0.776192145021032 | 2.03404187320007 | 0.999714303421225 |
| ebi-fl187-GCST90094846 | prot-a-2118 | Netrin-1 \|\| id:prot-a-2118 | Spicy food liking \|\| id:ebi-fl187-GCST90094846 | Weighted mode | 16 | 0.308477429912462 | 0.247061443285919 | 0.230953265661724 | -0.175762999 | 0.792717858752864 | 1.3613507833945 | 0.838816760252415 | 2.20939309187281 | 0.999714303421225 |
| ebi-fl187-GCST90094846 | prot-a-2120 | Netrin-G1 \|\| id:prot-a-2120 | Spicy food liking \|\| id:ebi-fl187-GCST90094846 | Weighted mode | 16 | 0.236556322115801 | 0.231677584509173 | 0.323413317979275 | -0.217531744 | 0.69064438775378 | 1.26687890691129 | 0.804502066785295 | 1.99500067313696 | 0.999714303421225 |
| ebi-fl187-GCST90094846 | prot-a-2145 | Olfactomedin-like protein 3 \|\| id:prot-a-2145 | Spicy food liking \|\| id:ebi-fl187-GCST90094846 | Weighted mode | 16 | -0.396623724 | 0.221818617688669 | 0.09398158392324 | -0.831388215 | 0.0381407666745839 | 0.672587056395425 | 0.435444376277245 | 1.03887746191178 | 0.999714303421225 |
| ebi-fl187-GCST90094846 | prot-a-2162 | Prolyl 4-hydroxylase subunit alpha-2 \|\| id:prot-a-2162 | Spicy food liking \|\| id:ebi-fl187-GCST90094846 | Weighted mode | 16 | 0.295635310554848 | 0.275778303641125 | 0.300663694469179 | -0.244890165 | 0.836160785691452 | 1.34397993215185 | 0.782790511643563 | 2.30749099683693 | 0.999714303421225 |
| ebi-fl187-GCST90094846 | prot-a-2168 | Polyadenylate-binding protein-interacting protein 1 \|\| id:prot-a-2168 | Spicy food liking \|\| id:ebi-fl187-GCST90094846 | Weighted mode | 16 | -0.185236632 | 0.300058518026357 | 0.54627166804582 | -0.773351327 | 0.402878063427506 | 0.830907641329752 | 0.461463957243804 | 1.4961244482533 | 0.999714303421225 |
| ebi-fl187-GCST90094846 | prot-a-219 | Beta-1,4-galactosyltransferase 5 \|\| id:prot-a-219 | Spicy food liking \|\| id:ebi-fl187-GCST90094846 | Weighted mode | 16 | 0.0761394328725891 | 0.265370650085897 | 0.778100974101864 | -0.443987041 | 0.596265907040947 | 1.07911302749724 | 0.641473733441843 | 1.81532752692178 | 0.999714303421225 |
| ebi-fl187-GCST90094846 | prot-a-2190 | Pyruvate carboxylase, mitochondrial \|\| id:prot-a-2190 | Spicy food liking \|\| id:ebi-fl187-GCST90094846 | Weighted mode | 16 | -0.059493593 | 0.203246440529265 | 0.773749107873812 | -0.457856616 | 0.338869430569011 | 0.942241570637681 | 0.632638179703863 | 1.40336009732032 | 0.999714303421225 |
| ebi-fl187-GCST90094846 | prot-a-2197 | Protocadherin-9 \|\| id:prot-a-2197 | Spicy food liking \|\| id:ebi-fl187-GCST90094846 | Weighted mode | 16 | -0.14551345 | 0.247415356087356 | 0.565192856873546 | -0.630447548 | 0.339420648047799 | 0.864578261518929 | 0.532353494041123 | 1.40413386717314 | 0.999714303421225 |
| ebi-fl187-GCST90094846 | prot-a-2198 | Protocadherin alpha-4 \|\| id:prot-a-2198 | Spicy food liking \|\| id:ebi-fl187-GCST90094846 | Weighted mode | 16 | 0.0386837340703425 | 0.232024855732094 | 0.869815177065014 | -0.416084983 | 0.493452451305246 | 1.03944169166711 | 0.659624211149489 | 1.63796145155582 | 0.999714303421225 |
| ebi-fl187-GCST90094846 | prot-a-2254 | Placenta growth factor \|\| id:prot-a-2254 | Spicy food liking \|\| id:ebi-fl187-GCST90094846 | Weighted mode | 16 | -0.113841831 | 0.220326791800325 | 0.612902556750171 | -0.545682343 | 0.317998681178043 | 0.892399094815193 | 0.579446267497888 | 1.37437444867117 | 0.999714303421225 |
| ebi-fl187-GCST90094846 | prot-a-2261 | Membrane-associated progesterone receptor component 2 \|\| id:prot-a-2261 | Spicy food liking \|\| id:ebi-fl187-GCST90094846 | Weighted mode | 16 | -0.217788728 | 0.301010117179357 | 0.480488471268012 | -0.807768558 | 0.37219110130475 | 0.804295348509543 | 0.445851849551444 | 1.45091022563863 | 0.999714303421225 |
| ebi-fl187-GCST90094846 | prot-a-2268 | Polymeric immunoglobulin receptor \|\| id:prot-a-2268 | Spicy food liking \|\| id:ebi-fl187-GCST90094846 | Weighted mode | 16 | 0.25834624874507 | 0.28841200830329 | 0.384533865165446 | -0.306941288 | 0.823633785019518 | 1.29478705941281 | 0.735693794039522 | 2.27876535428924 | 0.999714303421225 |
| ebi-fl187-GCST90094846 | prot-a-2283 | Group 10 secretory phospholipase A2 \|\| id:prot-a-2283 | Spicy food liking \|\| id:ebi-fl187-GCST90094846 | Weighted mode | 16 | -0.027285522 | 0.246340567287079 | 0.913272404027438 | -0.510113034 | 0.455541990186201 | 0.973083365443087 | 0.600427706483727 | 1.57702788508429 | 0.999714303421225 |
| ebi-fl187-GCST90094846 | prot-a-2298 | Inactive phospholipase D5 \|\| id:prot-a-2298 | Spicy food liking \|\| id:ebi-fl187-GCST90094846 | Weighted mode | 16 | -0.048553616 | 0.271559285266829 | 0.860490726280196 | -0.580809816 | 0.483702582654737 | 0.952606262553141 | 0.55944513568014 | 1.62206914240523 | 0.999714303421225 |
| ebi-fl187-GCST90094846 | prot-a-2304 | Procollagen-lysine,2-oxoglutarate 5-dioxygenase 2 \|\| id:prot-a-2304 | Spicy food liking \|\| id:ebi-fl187-GCST90094846 | Weighted mode | 16 | -0.05277822 | 0.276427760071326 | 0.85114102907357 | -0.594576629 | 0.489020190217594 | 0.948590367980107 | 0.551796130757173 | 1.63071764383324 | 0.999714303421225 |
| ebi-fl187-GCST90094846 | prot-a-2331 | Periostin \|\| id:prot-a-2331 | Spicy food liking \|\| id:ebi-fl187-GCST90094846 | Weighted mode | 16 | -0.050828612 | 0.283737375704637 | 0.860225353783371 | -0.606953868 | 0.505296644504871 | 0.950441550968328 | 0.545008510820018 | 1.65747712902303 | 0.999714303421225 |
| ebi-fl187-GCST90094846 | prot-a-2333 | Periostin \|\| id:prot-a-2333 | Spicy food liking \|\| id:ebi-fl187-GCST90094846 | Weighted mode | 16 | -0.222604562 | 0.31426354292892 | 0.489598065341057 | -0.838561106 | 0.39335198240065 | 0.800431307884126 | 0.432332156287153 | 1.48193991430873 | 0.999714303421225 |
| ebi-fl187-GCST90094846 | prot-a-2367 | cAMP-dependent protein kinase catalytic subunit alpha \|\| id:prot-a-2367 | Spicy food liking \|\| id:ebi-fl187-GCST90094846 | Weighted mode | 16 | 0.0396685086162101 | 0.259732688802512 | 0.880648112609632 | -0.469407561 | 0.548744578669134 | 1.04046581156782 | 0.625372653432963 | 1.73107842036064 | 0.999714303421225 |
| ebi-fl187-GCST90094846 | prot-a-2385 | Phosphoribosyl pyrophosphate synthase-associated protein 1 \|\| id:prot-a-2385 | Spicy food liking \|\| id:ebi-fl187-GCST90094846 | Weighted mode | 16 | 0.180784488477083 | 0.239250236385274 | 0.46157336062407 | -0.288145975 | 0.649714951792221 | 1.19815693484115 | 0.749652153899851 | 1.91499488534749 | 0.999714303421225 |
| ebi-fl187-GCST90094846 | prot-a-2393 | Trypsin-3 \|\| id:prot-a-2393 | Spicy food liking \|\| id:ebi-fl187-GCST90094846 | Weighted mode | 16 | 0.228425751937976 | 0.264701346246513 | 0.401748753729689 | -0.290388887 | 0.747240390581141 | 1.25662022005204 | 0.747972634398846 | 2.11116597696489 | 0.999714303421225 |
| ebi-fl187-GCST90094846 | prot-a-2405 | Pregnancy-specific beta-1-glycoprotein 6 \|\| id:prot-a-2405 | Spicy food liking \|\| id:ebi-fl187-GCST90094846 | Weighted mode | 16 | 0.250507944353276 | 0.227636922394482 | 0.288474101389941 | -0.195660424 | 0.696676312246461 | 1.28467779581973 | 0.822291418082364 | 2.00707073280794 | 0.999714303421225 |
| ebi-fl187-GCST90094846 | prot-a-244 | Beclin-1 \|\| id:prot-a-244 | Spicy food liking \|\| id:ebi-fl187-GCST90094846 | Weighted mode | 16 | -0.126627484 | 0.28000325935969 | 0.657570403599869 | -0.675433872 | 0.422178904821391 | 0.881061821518163 | 0.508935559876027 | 1.52528138046789 | 0.999714303421225 |
| ebi-fl187-GCST90094846 | prot-a-2454 | tRNA pseudouridine synthase A, mitochondrial \|\| id:prot-a-2454 | Spicy food liking \|\| id:ebi-fl187-GCST90094846 | Weighted mode | 16 | -0.292108494 | 0.291639016876906 | 0.332416960545904 | -0.863720967 | 0.279503979496697 | 0.74668752077483 | 0.421590436152197 | 1.32247367556409 | 0.999714303421225 |
| ebi-fl187-GCST90094846 | prot-a-2466 | Glutaminyl-peptide cyclotransferase-like protein \|\| id:prot-a-2466 | Spicy food liking \|\| id:ebi-fl187-GCST90094846 | Weighted mode | 16 | -0.028126654 | 0.239182875046257 | 0.907948935947755 | -0.496925089 | 0.44067178117591 | 0.972265217806747 | 0.608398557845228 | 1.55375064843148 | 0.999714303421225 |
| ebi-fl187-GCST90094846 | prot-a-2474 | Ras-related protein Rab-27A \|\| id:prot-a-2474 | Spicy food liking \|\| id:ebi-fl187-GCST90094846 | Weighted mode | 16 | 0.13352562178936 | 0.290257617823674 | 0.652096261167202 | -0.435379309 | 0.702430552723762 | 1.14285054763599 | 0.647019200227403 | 2.01865319262988 | 0.999714303421225 |
| ebi-fl187-GCST90094846 | prot-a-2490 | Rap1 GTPase-GDP dissociation stimulator 1 \|\| id:prot-a-2490 | Spicy food liking \|\| id:ebi-fl187-GCST90094846 | Weighted mode | 16 | -0.33304614 | 0.285655619599307 | 0.261858910864977 | -0.892931154 | 0.226838874854587 | 0.71673712345722 | 0.409453819896118 | 1.25462769958298 | 0.999714303421225 |
| ebi-fl187-GCST90094846 | prot-a-2545 | Receptor-interacting serine/threonine-protein kinase 2 \|\| id:prot-a-2545 | Spicy food liking \|\| id:ebi-fl187-GCST90094846 | Weighted mode | 16 | 0.302348984299101 | 0.273794759351202 | 0.286867111462378 | -0.234288744 | 0.838986712627458 | 1.353033332 | 0.791133347882238 | 2.31402102013819 | 0.999714303421225 |
| ebi-fl187-GCST90094846 | prot-a-2610 | RUN and FYVE domain-containing protein 1 \|\| id:prot-a-2610 | Spicy food liking \|\| id:ebi-fl187-GCST90094846 | Weighted mode | 16 | -0.110617323 | 0.25001598765052 | 0.664483218825314 | -0.600648659 | 0.379414012506938 | 0.895281286683271 | 0.548455759875012 | 1.46142795996511 | 0.999714303421225 |
| ebi-fl187-GCST90094846 | prot-a-2627 | Serine--tRNA ligase, mitochondrial \|\| id:prot-a-2627 | Spicy food liking \|\| id:ebi-fl187-GCST90094846 | Weighted mode | 16 | 0.00766531214197253 | 0.211220467722891 | 0.971529222512858 | -0.406326805 | 0.421657428878838 | 1.0076947658563 | 0.66609244977805 | 1.52448619027665 | 0.999714303421225 |
| ebi-fl187-GCST90094846 | prot-a-264 | BolA-like protein 2 \|\| id:prot-a-264 | Spicy food liking \|\| id:ebi-fl187-GCST90094846 | Weighted mode | 16 | -0.17431035 | 0.208136058227141 | 0.41546621321113 | -0.582257024 | 0.233636323985642 | 0.840036151862315 | 0.558636087399476 | 1.26318501857012 | 0.999714303421225 |
| ebi-fl187-GCST90094846 | prot-a-2646 | Secretoglobin family 1C member 1 \|\| id:prot-a-2646 | Spicy food liking \|\| id:ebi-fl187-GCST90094846 | Weighted mode | 16 | -0.137107398 | 0.245854615262346 | 0.585292452710173 | -0.618982444 | 0.344767648074853 | 0.87187658344245 | 0.538492104911289 | 1.41166187920343 | 0.999714303421225 |
| ebi-fl187-GCST90094846 | prot-a-2672 | Semaphorin-3E \|\| id:prot-a-2672 | Spicy food liking \|\| id:ebi-fl187-GCST90094846 | Weighted mode | 16 | 0.112439247444391 | 0.240155802468311 | 0.64637743188937 | -0.358266125 | 0.583144620282281 | 1.1190042724784 | 0.698887058662289 | 1.79166368343067 | 0.999714303421225 |
| ebi-fl187-GCST90094846 | prot-a-2701 | Plasma protease C1 inhibitor \|\| id:prot-a-2701 | Spicy food liking \|\| id:ebi-fl187-GCST90094846 | Weighted mode | 16 | 0.299934863840424 | 0.248214296216969 | 0.245610324306886 | -0.186565157 | 0.786434884425684 | 1.34977088582078 | 0.829804492830278 | 2.19555504935312 | 0.999714303421225 |
| ebi-fl187-GCST90094846 | prot-a-2738 | Signaling threshold-regulating transmembrane adapter 1 \|\| id:prot-a-2738 | Spicy food liking \|\| id:ebi-fl187-GCST90094846 | Weighted mode | 16 | 0.314611678606586 | 0.297088872428364 | 0.306364007692294 | -0.267682511 | 0.89690586856618 | 1.36972731325176 | 0.765150669194426 | 2.45200453741111 | 0.999714303421225 |
| ebi-fl187-GCST90094846 | prot-a-274 | Serine/threonine-protein kinase BRSK2 \|\| id:prot-a-274 | Spicy food liking \|\| id:ebi-fl187-GCST90094846 | Weighted mode | 16 | -0.054577991 | 0.258926117269542 | 0.835891047635147 | -0.562073181 | 0.452917199178792 | 0.946884657809604 | 0.570026071048574 | 1.57289394421191 | 0.999714303421225 |
| ebi-fl187-GCST90094846 | prot-a-2749 | Mitochondrial glutamate carrier 2 \|\| id:prot-a-2749 | Spicy food liking \|\| id:ebi-fl187-GCST90094846 | Weighted mode | 16 | 0.307620946317435 | 0.252849002213979 | 0.242546690255962 | -0.187963098 | 0.803204990656834 | 1.36018530795799 | 0.8286452853186 | 2.23268520893524 | 0.999714303421225 |
| ebi-fl187-GCST90094846 | prot-a-2760 | Sodium-coupled monocarboxylate transporter 1 \|\| id:prot-a-2760 | Spicy food liking \|\| id:ebi-fl187-GCST90094846 | Weighted mode | 16 | -0.067108048 | 0.245086541410706 | 0.787962328808374 | -0.547477669 | 0.413261573479074 | 0.935094161118076 | 0.578406905724644 | 1.51174040541865 | 0.999714303421225 |
| ebi-fl187-GCST90094846 | prot-a-2777 | Small integral membrane protein 10 \|\| id:prot-a-2777 | Spicy food liking \|\| id:ebi-fl187-GCST90094846 | Weighted mode | 16 | 0.370861243090006 | 0.24701904729366 | 0.154021513837058 | -0.11329609 | 0.85501857578558 | 1.44898200317163 | 0.892886246636322 | 2.3514180596069 | 0.999714303421225 |
| ebi-fl187-GCST90094846 | prot-a-2799 | Superoxide dismutase [Mn], mitochondrial \|\| id:prot-a-2799 | Spicy food liking \|\| id:ebi-fl187-GCST90094846 | Weighted mode | 16 | 0.42793448299904 | 0.238494311870491 | 0.0929391137808012 | -0.039514368 | 0.895383334265202 | 1.5340855689893 | 0.961256142306349 | 2.44827411696392 | 0.999714303421225 |
| ebi-fl187-GCST90094846 | prot-a-2805 | Protein SPACA7 \|\| id:prot-a-2805 | Spicy food liking \|\| id:ebi-fl187-GCST90094846 | Weighted mode | 16 | -0.356902077 | 0.253550355007842 | 0.179629221326896 | -0.853860773 | 0.140056618443831 | 0.699841024652026 | 0.425767961175826 | 1.15033892741342 | 0.999714303421225 |
| ebi-fl187-GCST90094846 | prot-a-2811 | SPARC-like protein 1 \|\| id:prot-a-2811 | Spicy food liking \|\| id:ebi-fl187-GCST90094846 | Weighted mode | 16 | 0.290277427940736 | 0.237690445327278 | 0.240842901589643 | -0.175595845 | 0.756150700782201 | 1.33679830178609 | 0.838956983571103 | 2.13006117673817 | 0.999714303421225 |
| ebi-fl187-GCST90094846 | prot-a-2831 | Sepiapterin reductase \|\| id:prot-a-2831 | Spicy food liking \|\| id:ebi-fl187-GCST90094846 | Weighted mode | 16 | 0.143476549883819 | 0.330447714001407 | 0.670332227122695 | -0.50420097 | 0.791154069326576 | 1.15427974249566 | 0.60398798745844 | 2.20594076637581 | 0.999714303421225 |
| ebi-fl187-GCST90094846 | prot-a-2834 | SPRY domain-containing SOCS box protein 1 \|\| id:prot-a-2834 | Spicy food liking \|\| id:ebi-fl187-GCST90094846 | Weighted mode | 16 | -0.021562666 | 0.223113647285865 | 0.924288307332782 | -0.458865415 | 0.415740082666729 | 0.978668146316143 | 0.632000297129445 | 1.51549191505792 | 0.999714303421225 |
| ebi-fl187-GCST90094846 | prot-a-2835 | Spectrin alpha chain, non-erythrocytic 1 \|\| id:prot-a-2835 | Spicy food liking \|\| id:ebi-fl187-GCST90094846 | Weighted mode | 16 | -0.11581564 | 0.273887386960669 | 0.678398073957725 | -0.652634919 | 0.421003638012918 | 0.890639406060607 | 0.520672039129712 | 1.52348982087432 | 0.999714303421225 |
| ebi-fl187-GCST90094846 | prot-a-2859 | Sia-alpha-2,3-Gal-beta-1,4-GlcNAc-R:alpha 2,8-sialyltransferase \|\| id:prot-a-2859 | Spicy food liking \|\| id:ebi-fl187-GCST90094846 | Weighted mode | 16 | -0.013543965 | 0.243484647109537 | 0.956374285520295 | -0.490773873 | 0.463685943716324 | 0.986547342186874 | 0.612152482584961 | 1.58992356653704 | 0.999714303421225 |
| ebi-fl187-GCST90094846 | prot-a-2861 | Alpha-2,8-sialyltransferase 8F \|\| id:prot-a-2861 | Spicy food liking \|\| id:ebi-fl187-GCST90094846 | Weighted mode | 16 | -0.114842958 | 0.301715400413168 | 0.708811626102143 | -0.706205143 | 0.476519226492269 | 0.891506136537809 | 0.493513461373839 | 1.61045899188253 | 0.999714303421225 |
| ebi-fl187-GCST90094846 | prot-a-289 | Uncharacterized protein C17orf89 \|\| id:prot-a-289 | Spicy food liking \|\| id:ebi-fl187-GCST90094846 | Weighted mode | 16 | -0.064308743 | 0.230760571982298 | 0.784294355937179 | -0.516599464 | 0.387981977738048 | 0.937715441429299 | 0.596545677550234 | 1.47400321917663 | 0.999714303421225 |
| ebi-fl187-GCST90094846 | prot-a-2921 | Tachykinin-3 \|\| id:prot-a-2921 | Spicy food liking \|\| id:ebi-fl187-GCST90094846 | Weighted mode | 16 | 0.107486707386307 | 0.226289534453299 | 0.641631733821531 | -0.33604078 | 0.551014194914773 | 1.11347605964105 | 0.714593963978421 | 1.73501176597007 | 0.999714303421225 |
| ebi-fl187-GCST90094846 | prot-a-2928 | Tax1-binding protein 1 \|\| id:prot-a-2928 | Spicy food liking \|\| id:ebi-fl187-GCST90094846 | Weighted mode | 16 | 0.0532740812712278 | 0.257565756966931 | 0.838918834533615 | -0.451554802 | 0.558102964926412 | 1.05471868414519 | 0.636637536156758 | 1.74735456127905 | 0.999714303421225 |
| ebi-fl187-GCST90094846 | prot-a-296 | Uncharacterized protein C1orf185 \|\| id:prot-a-296 | Spicy food liking \|\| id:ebi-fl187-GCST90094846 | Weighted mode | 16 | -0.049666529 | 0.282536141038284 | 0.862811467150026 | -0.603437365 | 0.504104307832109 | 0.951546685201583 | 0.546928408687979 | 1.65550203597976 | 0.999714303421225 |
| ebi-fl187-GCST90094846 | prot-a-2964 | Transforming growth factor beta-2 \|\| id:prot-a-2964 | Spicy food liking \|\| id:ebi-fl187-GCST90094846 | Weighted mode | 16 | 0.102168014937637 | 0.21887092837939 | 0.647354010866509 | -0.326819005 | 0.531155034561241 | 1.10756954432393 | 0.721214267616933 | 1.70089576786557 | 0.999714303421225 |
| ebi-fl187-GCST90094846 | prot-a-2977 | Thymocyte nuclear protein 1 \|\| id:prot-a-2977 | Spicy food liking \|\| id:ebi-fl187-GCST90094846 | Weighted mode | 16 | -0.123474718 | 0.258329952812211 | 0.639560520366282 | -0.629801426 | 0.38285198934994 | 0.883843986162098 | 0.532697570566066 | 1.46646096216432 | 0.999714303421225 |
| ebi-fl187-GCST90094846 | prot-a-3015 | Transmembrane protein 70, mitochondrial \|\| id:prot-a-3015 | Spicy food liking \|\| id:ebi-fl187-GCST90094846 | Weighted mode | 16 | 0.246569381872187 | 0.285620447897962 | 0.401578441938976 | -0.313246696 | 0.806385459752192 | 1.27962796312149 | 0.731069538384445 | 2.23979749945673 | 0.999714303421225 |
| ebi-fl187-GCST90094846 | prot-a-3030 | Tumor necrosis factor alpha-induced protein 3 \|\| id:prot-a-3030 | Spicy food liking \|\| id:ebi-fl187-GCST90094846 | Weighted mode | 16 | -0.042593263 | 0.236354396085362 | 0.859400289454769 | -0.50584788 | 0.420661353060376 | 0.958301087063063 | 0.602994092233349 | 1.52296844246837 | 0.999714303421225 |
| ebi-fl187-GCST90094846 | prot-a-3032 | Tumor necrosis factor receptor superfamily member 10A \|\| id:prot-a-3032 | Spicy food liking \|\| id:ebi-fl187-GCST90094846 | Weighted mode | 16 | 0.213604800511417 | 0.264440817227908 | 0.431852430073069 | -0.304699201 | 0.731908802278116 | 1.23813324846667 | 0.73734513352811 | 2.07904530897697 | 0.999714303421225 |
| ebi-fl187-GCST90094846 | prot-a-3081 | Tumor protein p53-inducible protein 11 \|\| id:prot-a-3081 | Spicy food liking \|\| id:ebi-fl187-GCST90094846 | Weighted mode | 16 | 0.0366546443217124 | 0.241814747212408 | 0.881536902307965 | -0.43730226 | 0.510611548858032 | 1.03733470953707 | 0.645776209452538 | 1.66630991334073 | 0.999714303421225 |
| ebi-fl187-GCST90094846 | prot-a-315 | Complement C4 \|\| id:prot-a-315 | Spicy food liking \|\| id:ebi-fl187-GCST90094846 | Weighted mode | 15 | -0.140386349 | 0.270199592176456 | 0.611483166730837 | -0.669977549 | 0.389204851936758 | 0.869022424823603 | 0.511720066082683 | 1.47580684225947 | 0.999714303421225 |
| ebi-fl187-GCST90094846 | prot-a-3157 | UDP-glucuronosyltransferase 1-8 \|\| id:prot-a-3157 | Spicy food liking \|\| id:ebi-fl187-GCST90094846 | Weighted mode | 16 | 0.332680160991814 | 0.21750877567996 | 0.146952631346551 | -0.093637039 | 0.758997361324536 | 1.39470114729367 | 0.910613219039891 | 2.13613337648798 | 0.999714303421225 |
| ebi-fl187-GCST90094846 | prot-a-317 | Glutaredoxin-like protein C5orf63 \|\| id:prot-a-317 | Spicy food liking \|\| id:ebi-fl187-GCST90094846 | Weighted mode | 16 | 0.12057868315274 | 0.2675988475742 | 0.658726214676713 | -0.403915058 | 0.645072424398172 | 1.12814950383337 | 0.66770083464263 | 1.90612507423429 | 0.999714303421225 |
| ebi-fl187-GCST90094846 | prot-a-3192 | Guanine nucleotide exchange factor VAV3 \|\| id:prot-a-3192 | Spicy food liking \|\| id:ebi-fl187-GCST90094846 | Weighted mode | 16 | -0.163072811 | 0.371350618797969 | 0.666824112023907 | -0.890920024 | 0.564774401980758 | 0.84952933119871 | 0.410278113469467 | 1.75905089955679 | 0.999714303421225 |
| ebi-fl187-GCST90094846 | prot-a-3233 | Serine/threonine-protein kinase WNK3 \|\| id:prot-a-3233 | Spicy food liking \|\| id:ebi-fl187-GCST90094846 | Weighted mode | 16 | 0.208267482619288 | 0.252724928281545 | 0.422800429678324 | -0.287073377 | 0.703608342051115 | 1.23154254172106 | 0.750456660699019 | 2.02103214149107 | 0.999714303421225 |
| ebi-fl187-GCST90094846 | prot-a-3250 | 14-3-3 protein theta \|\| id:prot-a-3250 | Spicy food liking \|\| id:ebi-fl187-GCST90094846 | Weighted mode | 16 | 0.122997735149079 | 0.225854349512238 | 0.594040797176778 | -0.31967679 | 0.565672260193066 | 1.13088185966577 | 0.726383773712859 | 1.76063098709396 | 0.999714303421225 |
| ebi-fl187-GCST90094846 | prot-a-3278 | E3 ubiquitin-protein ligase ZNRF3 \|\| id:prot-a-3278 | Spicy food liking \|\| id:ebi-fl187-GCST90094846 | Weighted mode | 16 | 0.313807960070388 | 0.237232300694742 | 0.205719100293825 | -0.151167349 | 0.778783269432082 | 1.36862688029898 | 0.859703815795887 | 2.17881961561706 | 0.999714303421225 |
| ebi-fl187-GCST90094846 | prot-a-355 | Calpain-3 \|\| id:prot-a-355 | Spicy food liking \|\| id:ebi-fl187-GCST90094846 | Weighted mode | 16 | 0.170232236178942 | 0.259111581106558 | 0.521144482064313 | -0.337626463 | 0.678090935147797 | 1.1855801539563 | 0.713461742638654 | 1.9701130662684 | 0.999714303421225 |
| ebi-fl187-GCST90094846 | prot-a-385 | Coiled-coil domain-containing protein 90B, mitochondrial \|\| id:prot-a-385 | Spicy food liking \|\| id:ebi-fl187-GCST90094846 | Weighted mode | 16 | -0.236854341 | 0.238128757650858 | 0.335682819624893 | -0.703586706 | 0.22987802411186 | 0.789106220161549 | 0.494807388697878 | 1.25844650043787 | 0.999714303421225 |
| ebi-fl187-GCST90094846 | prot-a-390 | C-C motif chemokine 14 \|\| id:prot-a-390 | Spicy food liking \|\| id:ebi-fl187-GCST90094846 | Weighted mode | 16 | 0.316852253372731 | 0.319351554753568 | 0.336849083582953 | -0.309076794 | 0.942781300689725 | 1.37279973041117 | 0.734124391553922 | 2.5671114071389 | 0.999714303421225 |
| ebi-fl187-GCST90094846 | prot-a-40 | Pituitary adenylate cyclase-activating polypeptide 27 \|\| id:prot-a-40 | Spicy food liking \|\| id:ebi-fl187-GCST90094846 | Weighted mode | 16 | 0.261763879007115 | 0.267043404598097 | 0.342519145807852 | -0.261641194 | 0.785168952019385 | 1.29921973315538 | 0.769787178416001 | 2.19277738360582 | 0.999714303421225 |
| ebi-fl187-GCST90094846 | prot-a-401 | C-C motif chemokine 24 \|\| id:prot-a-401 | Spicy food liking \|\| id:ebi-fl187-GCST90094846 | Weighted mode | 16 | 0.375549642917159 | 0.250817973979472 | 0.155060683870781 | -0.116053586 | 0.867152871916924 | 1.45579136016584 | 0.890427507497062 | 2.38012467774137 | 0.999714303421225 |
| ebi-fl187-GCST90094846 | prot-a-412 | Cerebral cavernous malformations 2 protein \|\| id:prot-a-412 | Spicy food liking \|\| id:ebi-fl187-GCST90094846 | Weighted mode | 16 | -0.158690304 | 0.284053751968769 | 0.584635926856977 | -0.715435658 | 0.398055050194365 | 0.853260569726205 | 0.488979037989506 | 1.48892599331653 | 0.999714303421225 |
| ebi-fl187-GCST90094846 | prot-a-44 | Adhesion G protein-coupled receptor F5 \|\| id:prot-a-44 | Spicy food liking \|\| id:ebi-fl187-GCST90094846 | Weighted mode | 16 | 0.0224757375839355 | 0.206167414743688 | 0.914634088490959 | -0.381612395 | 0.426563870481564 | 1.0227302199573 | 0.682759642761157 | 1.53198437239767 | 0.999714303421225 |
| ebi-fl187-GCST90094846 | prot-a-48 | Adrenomedullin \|\| id:prot-a-48 | Spicy food liking \|\| id:ebi-fl187-GCST90094846 | Weighted mode | 16 | 0.0583943057421268 | 0.267210841728536 | 0.829959497667851 | -0.465338944 | 0.582127555530058 | 1.06013292980961 | 0.627922238612744 | 1.78984237180335 | 0.999714303421225 |
| ebi-fl187-GCST90094846 | prot-a-502 | Carcinoembryonic antigen-related cell adhesion molecule 1 \|\| id:prot-a-502 | Spicy food liking \|\| id:ebi-fl187-GCST90094846 | Weighted mode | 16 | 0.237044640087145 | 0.290657753877435 | 0.427520862717687 | -0.332644558 | 0.806733837686918 | 1.26749769772009 | 0.717025010019894 | 2.24057793141854 | 0.999714303421225 |
| ebi-fl187-GCST90094846 | prot-a-518 | Complement factor B \|\| id:prot-a-518 | Spicy food liking \|\| id:ebi-fl187-GCST90094846 | Weighted mode | 16 | 0.378713830965691 | 0.28725316439769 | 0.207148189731365 | -0.184302371 | 0.941730033185164 | 1.46040505323568 | 0.831684288382039 | 2.56441410437778 | 0.999714303421225 |
| ebi-fl187-GCST90094846 | prot-a-525 | Properdin \|\| id:prot-a-525 | Spicy food liking \|\| id:ebi-fl187-GCST90094846 | Weighted mode | 16 | 0.0188908782987764 | 0.242391335492758 | 0.938909523046088 | -0.456196139 | 0.493977895864581 | 1.01907043984981 | 0.633689533507914 | 1.63882233564256 | 0.999714303421225 |
| ebi-fl187-GCST90094846 | prot-a-533 | Chondroadherin \|\| id:prot-a-533 | Spicy food liking \|\| id:ebi-fl187-GCST90094846 | Weighted mode | 16 | 0.0474812741713482 | 0.270252482644756 | 0.862885534125945 | -0.482213592 | 0.577176140155071 | 1.04862656453606 | 0.617415172854228 | 1.78100202294563 | 0.999714303421225 |
| ebi-fl187-GCST90094846 | prot-a-535 | Coiled-coil-helix-coiled-coil-helix domain-containing protein 2 \|\| id:prot-a-535 | Spicy food liking \|\| id:ebi-fl187-GCST90094846 | Weighted mode | 16 | 0.0316835230447499 | 0.269347247951444 | 0.907920893215451 | -0.496237083 | 0.559604129029581 | 1.03219078901061 | 0.608817283769638 | 1.74997959703371 | 0.999714303421225 |
| ebi-fl187-GCST90094846 | prot-a-591 | Caseinolytic peptidase B protein homolog \|\| id:prot-a-591 | Spicy food liking \|\| id:ebi-fl187-GCST90094846 | Weighted mode | 16 | -0.03601244 | 0.283634005832883 | 0.900652127477818 | -0.591935092 | 0.519910211297251 | 0.964628293302095 | 0.553255647872229 | 1.68187662939829 | 0.999714303421225 |
| ebi-fl187-GCST90094846 | prot-a-605 | Ciliary neurotrophic factor receptor subunit alpha \|\| id:prot-a-605 | Spicy food liking \|\| id:ebi-fl187-GCST90094846 | Weighted mode | 16 | 0.211845376125967 | 0.215401682511625 | 0.340964849079259 | -0.210341922 | 0.634032673848752 | 1.23595676188012 | 0.810307137087905 | 1.88519765817079 | 0.999714303421225 |
| ebi-fl187-GCST90094846 | prot-a-606 | Ciliary neurotrophic factor receptor subunit alpha \|\| id:prot-a-606 | Spicy food liking \|\| id:ebi-fl187-GCST90094846 | Weighted mode | 16 | 0.211871745634044 | 0.222798870597112 | 0.356699425971578 | -0.224814041 | 0.648557532004383 | 1.23598935388165 | 0.798664724055013 | 1.91277971456212 | 0.999714303421225 |
| ebi-fl187-GCST90094846 | prot-a-707 | Cancer/testis antigen 55 \|\| id:prot-a-707 | Spicy food liking \|\| id:ebi-fl187-GCST90094846 | Weighted mode | 16 | 0.0811207013122051 | 0.316750478934281 | 0.801350391654842 | -0.539710237 | 0.701951640023395 | 1.08450178945621 | 0.582917135490158 | 2.01768666543784 | 0.999714303421225 |
| ebi-fl187-GCST90094846 | prot-a-719 | Dipeptidyl peptidase 1 \|\| id:prot-a-719 | Spicy food liking \|\| id:ebi-fl187-GCST90094846 | Weighted mode | 16 | -0.160738782 | 0.269895548384185 | 0.56034891298319 | -0.689734057 | 0.368256492726924 | 0.851514472875473 | 0.501709477479881 | 1.4452126779795 | 0.999714303421225 |
| ebi-fl187-GCST90094846 | prot-a-752 | Cytochrome b5 \|\| id:prot-a-752 | Spicy food liking \|\| id:ebi-fl187-GCST90094846 | Weighted mode | 16 | 0.132006318264042 | 0.246956561079417 | 0.60080714073077 | -0.352028541 | 0.616041177979698 | 1.14111552911367 | 0.703260049627216 | 1.85158342418941 | 0.999714303421225 |
| ebi-fl187-GCST90094846 | prot-a-755 | Cytoglobin \|\| id:prot-a-755 | Spicy food liking \|\| id:ebi-fl187-GCST90094846 | Weighted mode | 16 | 0.147496207866448 | 0.247766212326285 | 0.560515070951975 | -0.338125568 | 0.633117984025968 | 1.15892889000829 | 0.713105738805735 | 1.88347407545088 | 0.999714303421225 |
| ebi-fl187-GCST90094846 | prot-a-807 | Desmin \|\| id:prot-a-807 | Spicy food liking \|\| id:ebi-fl187-GCST90094846 | Weighted mode | 16 | 0.232890381739387 | 0.280416791075121 | 0.419270796629033 | -0.316726529 | 0.782507292246624 | 1.26224310684948 | 0.72852995987186 | 2.1869487166585 | 0.999714303421225 |
| ebi-fl187-GCST90094846 | prot-a-82 | Ameloblastin \|\| id:prot-a-82 | Spicy food liking \|\| id:ebi-fl187-GCST90094846 | Weighted mode | 16 | -0.076448346 | 0.257736539404945 | 0.77082851611138 | -0.581611963 | 0.428715271324212 | 0.926400765441953 | 0.558996558070877 | 1.53528383282571 | 0.999714303421225 |
| ebi-fl187-GCST90094846 | prot-a-84 | Muellerian-inhibiting factor \|\| id:prot-a-84 | Spicy food liking \|\| id:ebi-fl187-GCST90094846 | Weighted mode | 16 | 0.0408766734324222 | 0.223928265766271 | 0.857600377498187 | -0.398022727 | 0.479776074334313 | 1.04172362542405 | 0.671646762657251 | 1.61571256217073 | 0.999714303421225 |
| ebi-fl187-GCST90094846 | prot-a-858 | Inactive dipeptidyl peptidase 10 \|\| id:prot-a-858 | Spicy food liking \|\| id:ebi-fl187-GCST90094846 | Weighted mode | 16 | 0.0209600661762805 | 0.229805280200069 | 0.928534242328817 | -0.429458283 | 0.471378415368415 | 1.02118127115041 | 0.650861582013825 | 1.60220117051894 | 0.999714303421225 |
| ebi-fl187-GCST90094846 | prot-a-863 | Dihydropyrimidinase-related protein 3 \|\| id:prot-a-863 | Spicy food liking \|\| id:ebi-fl187-GCST90094846 | Weighted mode | 16 | -0.167561276 | 0.234807264059494 | 0.486422966207311 | -0.627783513 | 0.292660961775352 | 0.845724793253647 | 0.533773592859304 | 1.33998840611895 | 0.999714303421225 |
| ebi-fl187-GCST90094846 | prot-a-864 | Dihydropyrimidinase-related protein 5 \|\| id:prot-a-864 | Spicy food liking \|\| id:ebi-fl187-GCST90094846 | Weighted mode | 16 | 0.110704250269397 | 0.29812489761277 | 0.715580516617287 | -0.473620549 | 0.695029049590426 | 1.11706448647506 | 0.622743508355946 | 2.00376728171456 | 0.999714303421225 |
| ebi-fl187-GCST90094846 | prot-a-884 | Endothelin-converting enzyme 1 \|\| id:prot-a-884 | Spicy food liking \|\| id:ebi-fl187-GCST90094846 | Weighted mode | 16 | 0.0284706601149578 | 0.237834569848387 | 0.906303198455745 | -0.437685097 | 0.494626417017796 | 1.02887982317594 | 0.645529030019092 | 1.63988549129579 | 0.999714303421225 |
| ebi-fl187-GCST90094846 | prot-a-893 | Early endosome antigen 1 \|\| id:prot-a-893 | Spicy food liking \|\| id:ebi-fl187-GCST90094846 | Weighted mode | 16 | 0.105812629019694 | 0.290448543383933 | 0.720712786133977 | -0.463466516 | 0.675091774052202 | 1.11161357286724 | 0.629099079245724 | 1.96421323150597 | 0.999714303421225 |
| ebi-fl187-GCST90094846 | prot-a-897 | Ephrin-A2 \|\| id:prot-a-897 | Spicy food liking \|\| id:ebi-fl187-GCST90094846 | Weighted mode | 16 | 0.282179871843685 | 0.244426056681346 | 0.266373710997282 | -0.196895199 | 0.761254942939124 | 1.3260172116978 | 0.821276699215289 | 2.14096131961232 | 0.999714303421225 |
| ebi-fl187-GCST90094846 | prot-a-90 | Anaphase-promoting complex subunit 10 \|\| id:prot-a-90 | Spicy food liking \|\| id:ebi-fl187-GCST90094846 | Weighted mode | 16 | 0.0754420152111477 | 0.277395747519759 | 0.789356429973516 | -0.46825365 | 0.619137680349876 | 1.07836069738782 | 0.626094694640969 | 1.85732574261404 | 0.999714303421225 |
| ebi-fl187-GCST90094846 | prot-a-914 | Histone-lysine N-methyltransferase EHMT2 \|\| id:prot-a-914 | Spicy food liking \|\| id:ebi-fl187-GCST90094846 | Weighted mode | 16 | -0.104033455 | 0.271509307536617 | 0.706971459654711 | -0.636191698 | 0.428124787724147 | 0.901195147390299 | 0.529304341494561 | 1.53437754050269 | 0.999714303421225 |
| ebi-fl187-GCST90094846 | prot-a-932 | RNA polymerase II elongation factor ELL \|\| id:prot-a-932 | Spicy food liking \|\| id:ebi-fl187-GCST90094846 | Weighted mode | 16 | -0.044107808 | 0.224084978802715 | 0.846598691334549 | -0.483314367 | 0.395098750117797 | 0.956850795420683 | 0.616735911607337 | 1.48453078127241 | 0.999714303421225 |
| ebi-fl187-GCST90094846 | prot-a-991 | Estrogen receptor \|\| id:prot-a-991 | Spicy food liking \|\| id:ebi-fl187-GCST90094846 | Weighted mode | 16 | 0.16779169739875 | 0.243795006913209 | 0.501801913567786 | -0.310046516 | 0.64562991094864 | 1.18269022753671 | 0.733412839888306 | 1.90718800958524 | 0.999714303421225 |
| ebi-fl187-GCST90094846 | prot-a-992 | Persulfide dioxygenase ETHE1, mitochondrial \|\| id:prot-a-992 | Spicy food liking \|\| id:ebi-fl187-GCST90094846 | Weighted mode | 16 | -0.151734026 | 0.276896455054845 | 0.591766926748501 | -0.694451077 | 0.390983026360092 | 0.859216780065025 | 0.499348476404627 | 1.4784334188037 | 0.999714303421225 |
| ebi-fl187-GCST90094846 | prot-a-993 | Ethanolamine kinase 1 \|\| id:prot-a-993 | Spicy food liking \|\| id:ebi-fl187-GCST90094846 | Weighted mode | 16 | -0.155599927 | 0.296987688952281 | 0.607984334028014 | -0.737695797 | 0.426495943298572 | 0.855901544940843 | 0.478214550204042 | 1.53188031254916 | 0.999714303421225 |
| ebi-fl187-GCST90094846 | prot-b-10 | coagulation factor III, tissue factor \|\| id:prot-b-10 | Spicy food liking \|\| id:ebi-fl187-GCST90094846 | Weighted mode | 12 | 0.18310982537828 | 0.255599610279045 | 0.488679375631038 | -0.317865411 | 0.684085061525208 | 1.20094629521959 | 0.727700722504908 | 1.98195763642646 | 0.999714303421225 |
| ebi-fl187-GCST90094846 | prot-b-28 | prolactin \|\| id:prot-b-28 | Spicy food liking \|\| id:ebi-fl187-GCST90094846 | Weighted mode | 12 | -0.295939954 | 0.247615192914719 | 0.257155592011141 | -0.781265732 | 0.18938582412116 | 0.743832090827162 | 0.457826159145962 | 1.20850713374792 | 0.999714303421225 |
| ebi-fl187-GCST90094846 | prot-b-54 | matrix metallopeptidase 7 \|\| id:prot-b-54 | Spicy food liking \|\| id:ebi-fl187-GCST90094846 | Weighted mode | 12 | 0.14220771266478 | 0.303439122017672 | 0.648467918578785 | -0.452532966 | 0.736948391819417 | 1.15281607816946 | 0.636015104640132 | 2.08954928961628 | 0.999714303421225 |
| ebi-fl187-GCST90094846 | ukb-rsfMRI191-edge-pheno716 | Net100_Pair10_33 (OccipitalPrecuneus)&(Temporal\|Frontal\|Supp_Motor_Area) (Default_mode\|Central_executive)&(Default_mode\|Salience) \|\| id:ukb-rsfMRI191-edge-pheno716 | Spicy food liking \|\| id:ebi-fl187-GCST90094846 | Weighted mode | 15 | -0.010200699 | 0.0914763912789993 | 0.912793467747709 | -0.189494426 | 0.169093028013777 | 0.989851151781751 | 0.827377328851391 | 1.18423030039254 | 0.999714303421225 |
| ebi-fl187-GCST90094846 | ukb-rsfMRI191-edge-pheno903 | Net100_Pair27_38 (Precentral\|Frontal\|Supp_Motor_Area)&(Subcortical) (Attention\|Salience\|Motor)&(Subcortical-cerebellum) \|\| id:ukb-rsfMRI191-edge-pheno903 | Spicy food liking \|\| id:ebi-fl187-GCST90094846 | Weighted mode | 15 | 0.0655493589640281 | 0.0869370375017917 | 0.463350961378344 | -0.104847235 | 0.23594595246754 | 1.06774543882155 | 0.900462071582673 | 1.26610587841905 | 0.999714303421225 |
| ebi-fl187-GCST90094846 | ukb-rsfMRI191-node-pheno23 | Net100_Node2 RolandicOper\|SupraMarginal\|Insula Salience\|Motor \|\| id:ukb-rsfMRI191-node-pheno23 | Spicy food liking \|\| id:ebi-fl187-GCST90094846 | Weighted mode | 15 | -0.150166566 | 0.112035462569586 | 0.201479320140608 | -0.369756072 | 0.0694229410272181 | 0.860564624015768 | 0.690902840462818 | 1.07188945932153 | 0.999714303421225 |
| ebi-fl187-GCST90094846 | ukb-rsfMRI191-node-pheno55 | Net100_Node34 Parietal\|Postcentral\|Precuneus Attention \|\| id:ukb-rsfMRI191-node-pheno55 | Spicy food liking \|\| id:ebi-fl187-GCST90094846 | Weighted mode | 15 | 0.0787409152857836 | 0.0984190624520059 | 0.437043341335769 | -0.114160447 | 0.271642277691715 | 1.08192397579109 | 0.892114807147166 | 1.31211754363192 | 0.999714303421225 |
| ebi-fl187-GCST90094852 | ebi-a-GCST90001414 | IgD- CD24- B cell Absolute Count \|\| id:ebi-a-GCST90001414 | F-strong vegetable liking (derived food-liking factor) \|\| id:ebi-fl187-GCST90094852 | Inverse variance weighted | 23 | -0.12365976 | 0.0875704788655622 | 0.157915519470196 | -0.295297899 | 0.0479783783073741 | 0.883680452939253 | 0.74430982539844 | 1.04914797072429 | 0.998735809224318 |
| ebi-fl187-GCST90094852 | ebi-a-GCST90001422 | CD20- CD38- B cell %B cell \|\| id:ebi-a-GCST90001422 | F-strong vegetable liking (derived food-liking factor) \|\| id:ebi-fl187-GCST90094852 | Inverse variance weighted | 23 | -0.043534628 | 0.0874476013213239 | 0.618599120266367 | -0.214931927 | 0.127862670106271 | 0.95739940022758 | 0.806596345682418 | 1.1363969307109 | 0.998735809224318 |
| ebi-fl187-GCST90094852 | ebi-a-GCST90001444 | CD20- CD38- B cell %lymphocyte \|\| id:ebi-a-GCST90001444 | F-strong vegetable liking (derived food-liking factor) \|\| id:ebi-fl187-GCST90094852 | Inverse variance weighted | 23 | -0.082750158 | 0.0939602910754791 | 0.37848408092866 | -0.266912328 | 0.10141201278527 | 0.920581118573778 | 0.765740202321023 | 1.10673253579451 | 0.998735809224318 |
| ebi-fl187-GCST90094852 | ebi-a-GCST90001459 | Myeloid Dendritic Cell %Dendritic Cell \|\| id:ebi-a-GCST90001459 | F-strong vegetable liking (derived food-liking factor) \|\| id:ebi-fl187-GCST90094852 | Inverse variance weighted | 23 | -0.137160383 | 0.0891613003554751 | 0.123965535992785 | -0.311916532 | 0.0375957654695706 | 0.871830387947404 | 0.73204262786812 | 1.03831142670213 | 0.998735809224318 |
| ebi-fl187-GCST90094852 | ebi-a-GCST90001476 | HLA DR++ monocyte %leukocyte \|\| id:ebi-a-GCST90001476 | F-strong vegetable liking (derived food-liking factor) \|\| id:ebi-fl187-GCST90094852 | Inverse variance weighted | 23 | -0.006004959 | 0.087072349978844 | 0.945017350288894 | -0.176666765 | 0.164656847252241 | 0.994013035023023 | 0.838059008871417 | 1.17898847615309 | 0.998735809224318 |
| ebi-fl187-GCST90094852 | ebi-a-GCST90001478 | CD4 regulatory T cell %CD4+ T cell \|\| id:ebi-a-GCST90001478 | F-strong vegetable liking (derived food-liking factor) \|\| id:ebi-fl187-GCST90094852 | Inverse variance weighted | 23 | 0.109770023614695 | 0.105455922749883 | 0.297917764423933 | -0.096923585 | 0.316463632204465 | 1.11602138238046 | 0.907625359697895 | 1.37226633502722 | 0.998735809224318 |
| ebi-fl187-GCST90094852 | ebi-a-GCST90001485 | CD39+ resting CD4 regulatory T cell %CD4 regulatory T cell \|\| id:ebi-a-GCST90001485 | F-strong vegetable liking (derived food-liking factor) \|\| id:ebi-fl187-GCST90094852 | Inverse variance weighted | 23 | 0.00747276355296493 | 0.0860889441978392 | 0.930828242120016 | -0.161261567 | 0.17620709418073 | 1.0075007543299 | 0.85106943024934 | 1.1926850312059 | 0.998735809224318 |
| ebi-fl187-GCST90094852 | ebi-a-GCST90001515 | Immature Myeloid-Derived Suppressor Cells Absolute Count \|\| id:ebi-a-GCST90001515 | F-strong vegetable liking (derived food-liking factor) \|\| id:ebi-fl187-GCST90094852 | Inverse variance weighted | 22 | 0.0151426745442971 | 0.127668854784379 | 0.905585138773258 | -0.235088281 | 0.265373629921679 | 1.01525790574195 | 0.790501060456086 | 1.30391806758213 | 0.998735809224318 |
| ebi-fl187-GCST90094852 | ebi-a-GCST90001517 | CD33+ HLA DR+ Absolute Count \|\| id:ebi-a-GCST90001517 | F-strong vegetable liking (derived food-liking factor) \|\| id:ebi-fl187-GCST90094852 | Inverse variance weighted | 23 | 0.0476594336134219 | 0.117982795446416 | 0.686247721040792 | -0.183586845 | 0.278905712688398 | 1.04881340390285 | 0.832279592893835 | 1.32168272008394 | 0.998735809224318 |
| ebi-fl187-GCST90094852 | ebi-a-GCST90001518 | CD33+ HLA DR+ CD14- Absolute Count \|\| id:ebi-a-GCST90001518 | F-strong vegetable liking (derived food-liking factor) \|\| id:ebi-fl187-GCST90094852 | Inverse variance weighted | 23 | 0.0371702491664547 | 0.113156063844686 | 0.742543784491846 | -0.184615636 | 0.258956134302039 | 1.0378697022495 | 0.831423791844497 | 1.29557697219341 | 0.998735809224318 |
| ebi-fl187-GCST90094852 | ebi-a-GCST90001520 | CD33+ HLA DR+ CD14dim Absolute Count \|\| id:ebi-a-GCST90001520 | F-strong vegetable liking (derived food-liking factor) \|\| id:ebi-fl187-GCST90094852 | Inverse variance weighted | 23 | 0.066049671902357 | 0.122455808804158 | 0.589627733134292 | -0.173963713 | 0.306063057158506 | 1.06827977933702 | 0.840327389767891 | 1.35806793975331 | 0.998735809224318 |
| ebi-fl187-GCST90094852 | ebi-a-GCST90001531 | CD33dim HLA DR- Absolute Count \|\| id:ebi-a-GCST90001531 | F-strong vegetable liking (derived food-liking factor) \|\| id:ebi-fl187-GCST90094852 | Inverse variance weighted | 23 | -0.046190675 | 0.114113592354897 | 0.685640681994614 | -0.269853316 | 0.177471966303789 | 0.954859877199862 | 0.763491478315637 | 1.19419457974514 | 0.998735809224318 |
| ebi-fl187-GCST90094852 | ebi-a-GCST90001532 | Basophil Absolute Count \|\| id:ebi-a-GCST90001532 | F-strong vegetable liking (derived food-liking factor) \|\| id:ebi-fl187-GCST90094852 | Inverse variance weighted | 23 | -0.039977678 | 0.113746016061263 | 0.72523995638401 | -0.26291987 | 0.182964513262538 | 0.96081088592454 | 0.768803499299909 | 1.20077179585128 | 0.998735809224318 |
| ebi-fl187-GCST90094852 | ebi-a-GCST90001588 | T/B cell \|\| id:ebi-a-GCST90001588 | F-strong vegetable liking (derived food-liking factor) \|\| id:ebi-fl187-GCST90094852 | Inverse variance weighted | 23 | 0.0247379718053227 | 0.106353569288606 | 0.816071066454854 | -0.183715024 | 0.233190967610991 | 1.02504649424979 | 0.832172919348527 | 1.26262257632265 | 0.998735809224318 |
| ebi-fl187-GCST90094852 | ebi-a-GCST90001624 | HLA DR+ CD4+ T cell Absolute Count \|\| id:ebi-a-GCST90001624 | F-strong vegetable liking (derived food-liking factor) \|\| id:ebi-fl187-GCST90094852 | Inverse variance weighted | 23 | -0.133123105 | 0.085986858006317 | 0.121579044914424 | -0.301657347 | 0.0354111363210784 | 0.875357324287079 | 0.739591444656578 | 1.03604557721572 | 0.998735809224318 |
| ebi-fl187-GCST90094852 | ebi-a-GCST90001631 | CD8+ Natural Killer T %T cell \|\| id:ebi-a-GCST90001631 | F-strong vegetable liking (derived food-liking factor) \|\| id:ebi-fl187-GCST90094852 | Inverse variance weighted | 23 | -0.017777049 | 0.084271156570342 | 0.83292583817238 | -0.182948516 | 0.147394417870639 | 0.982380030547549 | 0.832811031172197 | 1.15881092864518 | 0.998735809224318 |
| ebi-fl187-GCST90094852 | ebi-a-GCST90001632 | CD8+ Natural Killer T %lymphocyte \|\| id:ebi-a-GCST90001632 | F-strong vegetable liking (derived food-liking factor) \|\| id:ebi-fl187-GCST90094852 | Inverse variance weighted | 23 | -0.036219417 | 0.0847845711721375 | 0.669238397583237 | -0.202397177 | 0.129958342058885 | 0.964428657799978 | 0.816770461122056 | 1.13878094306703 | 0.998735809224318 |
| ebi-fl187-GCST90094852 | ebi-a-GCST90001646 | Natural Killer %CD3- lymphocyte \|\| id:ebi-a-GCST90001646 | F-strong vegetable liking (derived food-liking factor) \|\| id:ebi-fl187-GCST90094852 | Inverse variance weighted | 23 | 0.116731917200307 | 0.0827617321101607 | 0.158404596479592 | -0.045481078 | 0.278944912136222 | 1.12381811299735 | 0.95553768333757 | 1.32173453033223 | 0.998735809224318 |
| ebi-fl187-GCST90094852 | ebi-a-GCST90001684 | CD127- CD8+ T cell Absolute Count \|\| id:ebi-a-GCST90001684 | F-strong vegetable liking (derived food-liking factor) \|\| id:ebi-fl187-GCST90094852 | Inverse variance weighted | 23 | -0.049360263 | 0.0893786724277504 | 0.58077025178062 | -0.224542461 | 0.125821934948345 | 0.951838155842717 | 0.798881654691022 | 1.13408021025151 | 0.998735809224318 |
| ebi-fl187-GCST90094852 | ebi-a-GCST90001687 | CD28- CD8+ T cell Absolute Count \|\| id:ebi-a-GCST90001687 | F-strong vegetable liking (derived food-liking factor) \|\| id:ebi-fl187-GCST90094852 | Inverse variance weighted | 23 | -0.04499562 | 0.0868420473622866 | 0.604366361679531 | -0.215206033 | 0.12521479239467 | 0.956001668694986 | 0.8063752829282 | 1.13339187087779 | 0.998735809224318 |
| ebi-fl187-GCST90094852 | ebi-a-GCST90001701 | BAFF-R on CD20- CD38- B cell \|\| id:ebi-a-GCST90001701 | F-strong vegetable liking (derived food-liking factor) \|\| id:ebi-fl187-GCST90094852 | Inverse variance weighted | 23 | 0.0466278484260322 | 0.0919365634315351 | 0.612032630936053 | -0.133567816 | 0.226823512751841 | 1.04773202139603 | 0.874968130214557 | 1.25460842601139 | 0.998735809224318 |
| ebi-fl187-GCST90094852 | ebi-a-GCST90001732 | CD19 on IgD- CD27- B cell \|\| id:ebi-a-GCST90001732 | F-strong vegetable liking (derived food-liking factor) \|\| id:ebi-fl187-GCST90094852 | Inverse variance weighted | 23 | 0.0253435194095285 | 0.0834054999368504 | 0.761235245516572 | -0.13813126 | 0.188818299285755 | 1.0256673966726 | 0.870984358428866 | 1.20782147051962 | 0.998735809224318 |
| ebi-fl187-GCST90094852 | ebi-a-GCST90001798 | CD27 on CD24+ CD27+ B cell \|\| id:ebi-a-GCST90001798 | F-strong vegetable liking (derived food-liking factor) \|\| id:ebi-fl187-GCST90094852 | Inverse variance weighted | 23 | 0.0943319558449114 | 0.103418793737038 | 0.361697378794813 | -0.10836888 | 0.297032791569505 | 1.09892447978023 | 0.897296540734311 | 1.34585943156761 | 0.998735809224318 |
| ebi-fl187-GCST90094852 | ebi-a-GCST90001836 | CD66b on CD66b++ myeloid cell \|\| id:ebi-a-GCST90001836 | F-strong vegetable liking (derived food-liking factor) \|\| id:ebi-fl187-GCST90094852 | Inverse variance weighted | 23 | 0.0761200738131627 | 0.125316265184203 | 0.543569741147734 | -0.169499806 | 0.3217399535742 | 1.07909213708622 | 0.844086918277207 | 1.37952598850596 | 0.998735809224318 |
| ebi-fl187-GCST90094852 | ebi-a-GCST90001943 | CD25 on activated & secreting CD4 regulatory T cell \|\| id:ebi-a-GCST90001943 | F-strong vegetable liking (derived food-liking factor) \|\| id:ebi-fl187-GCST90094852 | Inverse variance weighted | 23 | -0.003786338 | 0.0851259487113131 | 0.9645223922439 | -0.170633198 | 0.163060521177375 | 0.996220820843558 | 0.843130779008616 | 1.17710792749042 | 0.998735809224318 |
| ebi-fl187-GCST90094852 | ebi-a-GCST90001960 | CD25 on CD4+ T cell \|\| id:ebi-a-GCST90001960 | F-strong vegetable liking (derived food-liking factor) \|\| id:ebi-fl187-GCST90094852 | Inverse variance weighted | 23 | 0.141052995137133 | 0.096911200331759 | 0.145534478322994 | -0.048892958 | 0.33099894778738 | 1.15148566950877 | 0.952283058989878 | 1.39235832725042 | 0.998735809224318 |
| ebi-fl187-GCST90094852 | ebi-a-GCST90001988 | HLA DR on CD14+ CD16- monocyte \|\| id:ebi-a-GCST90001988 | F-strong vegetable liking (derived food-liking factor) \|\| id:ebi-fl187-GCST90094852 | Inverse variance weighted | 23 | -0.08864901 | 0.0853423995109736 | 0.298923243208802 | -0.255920113 | 0.0786220932821705 | 0.915166731800894 | 0.774203815296222 | 1.08179542705389 | 0.998735809224318 |
| ebi-fl187-GCST90094852 | ebi-a-GCST90001991 | HLA DR on CD14+ monocyte \|\| id:ebi-a-GCST90001991 | F-strong vegetable liking (derived food-liking factor) \|\| id:ebi-fl187-GCST90094852 | Inverse variance weighted | 23 | -0.099440447 | 0.0854959697790657 | 0.244788530192051 | -0.267012548 | 0.0681316536102476 | 0.90534386406437 | 0.765663463918379 | 1.07050623521247 | 0.998735809224318 |
| ebi-fl187-GCST90094852 | ebi-a-GCST90002019 | CD14 on Monocytic Myeloid-Derived Suppressor Cells \|\| id:ebi-a-GCST90002019 | F-strong vegetable liking (derived food-liking factor) \|\| id:ebi-fl187-GCST90094852 | Inverse variance weighted | 23 | 0.0581701008871595 | 0.133903215610028 | 0.663984203684909 | -0.204280202 | 0.320620403482815 | 1.05989526950313 | 0.81523390924688 | 1.37798240428062 | 0.998735809224318 |
| ebi-fl187-GCST90094852 | ebi-a-GCST90002037 | CD80 on plasmacytoid Dendritic Cell \|\| id:ebi-a-GCST90002037 | F-strong vegetable liking (derived food-liking factor) \|\| id:ebi-fl187-GCST90094852 | Inverse variance weighted | 23 | -0.057741494 | 0.0947598412950303 | 0.542295375469432 | -0.243470783 | 0.127987794819271 | 0.943893918030386 | 0.783902378928624 | 1.13653913094691 | 0.998735809224318 |
| ebi-fl187-GCST90094852 | ebi-a-GCST90002042 | CD45 on CD33+ HLA DR+ CD14- \|\| id:ebi-a-GCST90002042 | F-strong vegetable liking (derived food-liking factor) \|\| id:ebi-fl187-GCST90094852 | Inverse variance weighted | 23 | 0.0547075634076131 | 0.124446946152221 | 0.660222848264557 | -0.189208451 | 0.298623577865967 | 1.05623168871125 | 0.827613971710798 | 1.3480021101283 | 0.998735809224318 |
| ebi-fl187-GCST90094852 | ebi-a-GCST90002056 | CD8 on Effector Memory CD8+ T cell \|\| id:ebi-a-GCST90002056 | F-strong vegetable liking (derived food-liking factor) \|\| id:ebi-fl187-GCST90094852 | Inverse variance weighted | 23 | 0.085912687059795 | 0.0926886534097075 | 0.353980842531836 | -0.095757074 | 0.267582447742822 | 1.08971117826433 | 0.90868473274842 | 1.30680136821776 | 0.998735809224318 |
| ebi-fl187-GCST90094852 | ebi-a-GCST90002058 | CD8 on CD8+ T cell \|\| id:ebi-a-GCST90002058 | F-strong vegetable liking (derived food-liking factor) \|\| id:ebi-fl187-GCST90094852 | Inverse variance weighted | 23 | 0.0221036018308138 | 0.0896102027252962 | 0.805168349202408 | -0.153532396 | 0.197739599172394 | 1.02234969628428 | 0.857672978994547 | 1.21864501632995 | 0.998735809224318 |
| ebi-fl187-GCST90094852 | ebi-a-GCST90002063 | CD4 on CD4 regulatory T cell \|\| id:ebi-a-GCST90002063 | F-strong vegetable liking (derived food-liking factor) \|\| id:ebi-fl187-GCST90094852 | Inverse variance weighted | 23 | 0.0652454795600162 | 0.0922810087648789 | 0.479547579339871 | -0.115625298 | 0.246116256739179 | 1.06742102226821 | 0.890808949003737 | 1.2790482628788 | 0.998735809224318 |
| ebi-fl187-GCST90094852 | ebi-a-GCST90002066 | CD4 on activated CD4 regulatory T cell \|\| id:ebi-a-GCST90002066 | F-strong vegetable liking (derived food-liking factor) \|\| id:ebi-fl187-GCST90094852 | Inverse variance weighted | 23 | 0.0873022164773966 | 0.0922933834848707 | 0.344189064627131 | -0.093592815 | 0.268197248107743 | 1.09122641649346 | 0.910653491060539 | 1.3076050371985 | 0.998735809224318 |
| ebi-fl187-GCST90094852 | ebi-a-GCST90002070 | CD4 on activated & secreting CD4 regulatory T cell \|\| id:ebi-a-GCST90002070 | F-strong vegetable liking (derived food-liking factor) \|\| id:ebi-fl187-GCST90094852 | Inverse variance weighted | 23 | 0.0528678099798066 | 0.0927206311218979 | 0.568552982634692 | -0.128864627 | 0.234600246978727 | 1.05429026925553 | 0.879092962925754 | 1.26440321868527 | 0.998735809224318 |
| ebi-fl187-GCST90094852 | ebi-a-GCST90002081 | SSC-A on CD4+ T cell \|\| id:ebi-a-GCST90002081 | F-strong vegetable liking (derived food-liking factor) \|\| id:ebi-fl187-GCST90094852 | Inverse variance weighted | 23 | -0.104067448 | 0.0893883842379978 | 0.244336214068401 | -0.279268681 | 0.0711337855812362 | 0.901164514055077 | 0.756336662900525 | 1.07372486516488 | 0.998735809224318 |
| ebi-fl187-GCST90094852 | ebi-a-GCST90002094 | CD11b on Monocytic Myeloid-Derived Suppressor Cells \|\| id:ebi-a-GCST90002094 | F-strong vegetable liking (derived food-liking factor) \|\| id:ebi-fl187-GCST90094852 | Inverse variance weighted | 23 | -0.058460821 | 0.124495818470465 | 0.63865469026572 | -0.302472625 | 0.185550983515417 | 0.943215194199358 | 0.738988717880373 | 1.20388157632543 | 0.998735809224318 |
| ebi-fl187-GCST90094852 | ebi-a-GCST90002104 | HLA DR on myeloid Dendritic Cell \|\| id:ebi-a-GCST90002104 | F-strong vegetable liking (derived food-liking factor) \|\| id:ebi-fl187-GCST90094852 | Inverse variance weighted | 23 | -0.102399566 | 0.0940409803916849 | 0.27620576194654 | -0.286719887 | 0.0819207560170499 | 0.90266880424473 | 0.750721986286676 | 1.08536979739589 | 0.998735809224318 |
| ebi-fl187-GCST90094852 | ebi-a-GCST90002105 | HLA DR on plasmacytoid Dendritic Cell \|\| id:ebi-a-GCST90002105 | F-strong vegetable liking (derived food-liking factor) \|\| id:ebi-fl187-GCST90094852 | Inverse variance weighted | 23 | -0.060961319 | 0.101907246931868 | 0.549703849725494 | -0.260699523 | 0.138776885359539 | 0.940859632814609 | 0.770512406389293 | 1.14886774219284 | 0.998735809224318 |
| ebi-fl187-GCST90094852 | ebi-a-GCST90002106 | HLA DR on Dendritic Cell \|\| id:ebi-a-GCST90002106 | F-strong vegetable liking (derived food-liking factor) \|\| id:ebi-fl187-GCST90094852 | Inverse variance weighted | 23 | -0.05121808 | 0.106561483085545 | 0.630769918330637 | -0.260078587 | 0.157642426395585 | 0.9500714559259 | 0.770990993322398 | 1.17074749145314 | 0.998735809224318 |
| ebi-fl187-GCST90094852 | ebi-a-GCST90002111 | HLA DR on CD33dim HLA DR+ CD11b- \|\| id:ebi-a-GCST90002111 | F-strong vegetable liking (derived food-liking factor) \|\| id:ebi-fl187-GCST90094852 | Inverse variance weighted | 23 | -0.229195206 | 0.120330469024697 | 0.056817174586326 | -0.465042926 | 0.00665251300183778 | 0.795173295527674 | 0.628108142707783 | 1.00667469011703 | 0.998735809224318 |
| ebi-fl187-GCST90094852 | ebi-a-GCST90002112 | HLA DR on CD33- HLA DR+ \|\| id:ebi-a-GCST90002112 | F-strong vegetable liking (derived food-liking factor) \|\| id:ebi-fl187-GCST90094852 | Inverse variance weighted (multiplicative random effects) | 23 | -0.175439858 | 0.162767974458327 | 0.281099615322683 | -0.494465087 | 0.143585372424969 | 0.8390878604864 | 0.609897061647969 | 1.15440536098538 | 0.998735809224318 |
| ebi-fl187-GCST90094852 | ebi-a-GCST90002115 | HLA DR on HLA DR+ CD8+ T cell \|\| id:ebi-a-GCST90002115 | F-strong vegetable liking (derived food-liking factor) \|\| id:ebi-fl187-GCST90094852 | Inverse variance weighted | 23 | -0.041031128 | 0.0890737659860399 | 0.645055387138854 | -0.21561571 | 0.133553452909002 | 0.959799252445046 | 0.806044997591726 | 1.14288235488893 | 0.998735809224318 |
| ebi-fl187-GCST90094852 | ebi-a-GCST90002116 | HLA DR on B cell \|\| id:ebi-a-GCST90002116 | F-strong vegetable liking (derived food-liking factor) \|\| id:ebi-fl187-GCST90094852 | Inverse variance weighted | 23 | 0.123680892750706 | 0.0913052842543759 | 0.175549381286615 | -0.055277464 | 0.302639249889283 | 1.13165469415856 | 0.946222568461192 | 1.3534261277384 | 0.998735809224318 |
| ebi-fl187-GCST90094852 | ebi-cfmet338-GCST90026023 | Sphingomyelin (d18:1/24:1, d18:2/24:0) levels \|\| id:ebi-cfmet338-GCST90026023 | F-strong vegetable liking (derived food-liking factor) \|\| id:ebi-fl187-GCST90094852 | Inverse variance weighted | 20 | 0.138861381616218 | 0.0620820557505799 | 0.0253033757271337 | 0.0171805523450813 | 0.260542210887355 | 1.14896482131783 | 1.01732898687851 | 1.29763348695732 | 0.998735809224318 |
| ebi-fl187-GCST90094852 | ebi-cfmet338-GCST90026046 | 1-arachidonoyl-gpc (20:4n6) levels \|\| id:ebi-cfmet338-GCST90026046 | F-strong vegetable liking (derived food-liking factor) \|\| id:ebi-fl187-GCST90094852 | Inverse variance weighted | 19 | 0.0960551997776114 | 0.0689012690527045 | 0.16328766953484 | -0.038991288 | 0.231101687120912 | 1.10081982732656 | 0.961759088372745 | 1.25998735742191 | 0.998735809224318 |
| ebi-fl187-GCST90094852 | ebi-cfmet338-GCST90026047 | 1-linoleoyl-gpc (18:2) levels \|\| id:ebi-cfmet338-GCST90026047 | F-strong vegetable liking (derived food-liking factor) \|\| id:ebi-fl187-GCST90094852 | Inverse variance weighted | 20 | 0.102525773240408 | 0.0905049425729231 | 0.257290016964474 | -0.074863914 | 0.279915460683337 | 1.10796585741223 | 0.927869747631736 | 1.32301796057525 | 0.998735809224318 |
| ebi-fl187-GCST90094852 | ebi-cfmet338-GCST90026050 | 1-myristoyl-2-palmitoyl-gpc (14:0/16:0) levels \|\| id:ebi-cfmet338-GCST90026050 | F-strong vegetable liking (derived food-liking factor) \|\| id:ebi-fl187-GCST90094852 | Inverse variance weighted | 20 | 0.0724608910654089 | 0.0433442351718911 | 0.0945728749469087 | -0.01249381 | 0.157415592002315 | 1.07515075726728 | 0.987583913746293 | 1.17048195577369 | 0.998735809224318 |
| ebi-fl187-GCST90094852 | ebi-cfmet338-GCST90026068 | 2,3-dihydroxyisovalerate levels \|\| id:ebi-cfmet338-GCST90026068 | F-strong vegetable liking (derived food-liking factor) \|\| id:ebi-fl187-GCST90094852 | Inverse variance weighted | 20 | 0.110463465029047 | 0.141738197021681 | 0.435774394765257 | -0.167343401 | 0.388270331191543 | 1.11679554621392 | 0.845909075319586 | 1.47442831438123 | 0.998735809224318 |
| ebi-fl187-GCST90094852 | ebi-cfmet338-GCST90026082 | 3-hydroxyhexanoate levels \|\| id:ebi-cfmet338-GCST90026082 | F-strong vegetable liking (derived food-liking factor) \|\| id:ebi-fl187-GCST90094852 | Inverse variance weighted | 20 | -0.029424207 | 0.0300448278746913 | 0.327410285352968 | -0.088312069 | 0.029463655843527 | 0.971004470397136 | 0.915475140339962 | 1.02990200387211 | 0.998735809224318 |
| ebi-fl187-GCST90094852 | ebi-cfmet338-GCST90026094 | 3-ureidopropionate levels \|\| id:ebi-cfmet338-GCST90026094 | F-strong vegetable liking (derived food-liking factor) \|\| id:ebi-fl187-GCST90094852 | Inverse variance weighted | 20 | 0.0894915150617471 | 0.0683743387962987 | 0.190586346913379 | -0.044522189 | 0.223505219102493 | 1.09361805399132 | 0.956454377112077 | 1.25045216649745 | 0.998735809224318 |
| ebi-fl187-GCST90094852 | ebi-cfmet338-GCST90026173 | Glycerol levels \|\| id:ebi-cfmet338-GCST90026173 | F-strong vegetable liking (derived food-liking factor) \|\| id:ebi-fl187-GCST90094852 | Inverse variance weighted (multiplicative random effects) | 20 | -0.007269893 | 0.0534943725532977 | 0.891900226320365 | -0.112118863 | 0.0975790775882068 | 0.992756469132027 | 0.893937995193506 | 1.10249862104826 | 0.998735809224318 |
| ebi-fl187-GCST90094852 | ebi-cfmet338-GCST90026179 | Histidine levels \|\| id:ebi-cfmet338-GCST90026179 | F-strong vegetable liking (derived food-liking factor) \|\| id:ebi-fl187-GCST90094852 | Inverse variance weighted | 20 | 0.0121101068805371 | 0.0271490886921442 | 0.655554321298153 | -0.041102107 | 0.0653223207171397 | 1.01218373112385 | 0.959731129720335 | 1.06750304728611 | 0.998735809224318 |
| ebi-fl187-GCST90094852 | ebi-cfmet338-GCST90026188 | Isocitrate levels \|\| id:ebi-cfmet338-GCST90026188 | F-strong vegetable liking (derived food-liking factor) \|\| id:ebi-fl187-GCST90094852 | Inverse variance weighted | 20 | -0.029417973 | 0.0650371625357367 | 0.651034442560133 | -0.156890812 | 0.0980548655763961 | 0.971010523460972 | 0.854797387712499 | 1.10302330145757 | 0.998735809224318 |
| ebi-fl187-GCST90094852 | ebi-cfmet338-GCST90026207 | N-acetyl-isoputreanine levels \|\| id:ebi-cfmet338-GCST90026207 | F-strong vegetable liking (derived food-liking factor) \|\| id:ebi-fl187-GCST90094852 | Inverse variance weighted | 20 | 0.00854350133573484 | 0.0347119441294769 | 0.80558493387954 | -0.059491909 | 0.0765789118295096 | 1.00858010119935 | 0.942243157100862 | 1.07958737919112 | 0.998735809224318 |
| ebi-fl187-GCST90094852 | ebi-cfmet338-GCST90026213 | N-acetylglutamate levels \|\| id:ebi-cfmet338-GCST90026213 | F-strong vegetable liking (derived food-liking factor) \|\| id:ebi-fl187-GCST90094852 | Inverse variance weighted | 19 | 0.0749970535134462 | 0.059989788186429 | 0.211239766609125 | -0.042582931 | 0.192577038358847 | 1.07788097491815 | 0.958310988218734 | 1.21236989909723 | 0.998735809224318 |
| ebi-fl187-GCST90094852 | ebi-cfmet338-GCST90026224 | N-acetyltaurine levels \|\| id:ebi-cfmet338-GCST90026224 | F-strong vegetable liking (derived food-liking factor) \|\| id:ebi-fl187-GCST90094852 | Inverse variance weighted | 20 | 0.0465616531313334 | 0.0524736477156199 | 0.374899091085366 | -0.056286696 | 0.149410002653948 | 1.04766266876155 | 0.945268092087817 | 1.16114896578419 | 0.998735809224318 |
| ebi-fl187-GCST90094852 | ebi-cfmet338-GCST90026250 | Phenyllactate (pla) levels \|\| id:ebi-cfmet338-GCST90026250 | F-strong vegetable liking (derived food-liking factor) \|\| id:ebi-fl187-GCST90094852 | Inverse variance weighted | 20 | 0.0878779422994282 | 0.0730307044617005 | 0.228859742585735 | -0.055262238 | 0.231018123044361 | 1.0918548446029 | 0.946236975701166 | 1.25988207214102 | 0.998735809224318 |
| ebi-fl187-GCST90094852 | ebi-cfmet338-GCST90026251 | Pipecolate levels \|\| id:ebi-cfmet338-GCST90026251 | F-strong vegetable liking (derived food-liking factor) \|\| id:ebi-fl187-GCST90094852 | Inverse variance weighted | 20 | 0.10423283131207 | 0.117082516679278 | 0.373331108251871 | -0.125248901 | 0.333714564003454 | 1.10985883472366 | 0.882277275222212 | 1.39614457677596 | 0.998735809224318 |
| ebi-fl187-GCST90094852 | ebi-cfmet338-GCST90026278 | Trigonelline (n'-methylnicotinate) levels \|\| id:ebi-cfmet338-GCST90026278 | F-strong vegetable liking (derived food-liking factor) \|\| id:ebi-fl187-GCST90094852 | Inverse variance weighted | 20 | 0.0668157822454557 | 0.121603358861766 | 0.5826920608817 | -0.171526801 | 0.305158365614516 | 1.06909851310545 | 0.842377691048861 | 1.35683986277124 | 0.998735809224318 |
| ebi-fl187-GCST90094852 | ebi-cfmet338-GCST90026299 | 1-carboxyethylvaline levels \|\| id:ebi-cfmet338-GCST90026299 | F-strong vegetable liking (derived food-liking factor) \|\| id:ebi-fl187-GCST90094852 | Inverse variance weighted | 20 | 0.042704803681401 | 0.073277444774729 | 0.560039807785265 | -0.100918988 | 0.18632859543987 | 1.04362977370756 | 0.904006265205366 | 1.20481809307091 | 0.998735809224318 |
| ebi-fl187-GCST90094852 | ebi-gm430-GCST90011306 | C_Erysipelotrichia abundance \|\| id:ebi-gm430-GCST90011306 | F-strong vegetable liking (derived food-liking factor) \|\| id:ebi-fl187-GCST90094852 | Inverse variance weighted | 19 | -0.053227092 | 0.0602535477093025 | 0.377028187119726 | -0.171324046 | 0.0648698615184329 | 0.948164667428774 | 0.842548505177386 | 1.06702015496549 | 0.998735809224318 |
| ebi-fl187-GCST90094852 | ebi-gm430-GCST90011312 | F_Erysipelotrichaceae abundance \|\| id:ebi-gm430-GCST90011312 | F-strong vegetable liking (derived food-liking factor) \|\| id:ebi-fl187-GCST90094852 | Inverse variance weighted | 19 | -0.053227092 | 0.0602535477093025 | 0.377028187119726 | -0.171324046 | 0.0648698615184329 | 0.948164667428774 | 0.842548505177386 | 1.06702015496549 | 0.998735809224318 |
| ebi-fl187-GCST90094852 | ebi-gm430-GCST90011352 | O_Erysipelotrichales abundance \|\| id:ebi-gm430-GCST90011352 | F-strong vegetable liking (derived food-liking factor) \|\| id:ebi-fl187-GCST90094852 | Inverse variance weighted | 19 | -0.053227092 | 0.0602535477093025 | 0.377028187119726 | -0.171324046 | 0.0648698615184329 | 0.948164667428774 | 0.842548505177386 | 1.06702015496549 | 0.998735809224318 |
| ebi-fl187-GCST90094852 | ebi-gm430-GCST90011362 | OTU97_11 (Parabacteroides) abundance \|\| id:ebi-gm430-GCST90011362 | F-strong vegetable liking (derived food-liking factor) \|\| id:ebi-fl187-GCST90094852 | Inverse variance weighted | 19 | -0.075852045 | 0.0801641741993883 | 0.344042111107325 | -0.232973827 | 0.0812697363236653 | 0.926953343696734 | 0.792174307197154 | 1.08466343023759 | 0.998735809224318 |
| ebi-fl187-GCST90094852 | ebi-gm430-GCST90011384 | OTU97_235 (Phascolarctobacterium) abundance \|\| id:ebi-gm430-GCST90011384 | F-strong vegetable liking (derived food-liking factor) \|\| id:ebi-fl187-GCST90094852 | Inverse variance weighted | 19 | -0.254669011 | 0.267836823229397 | 0.341687411821856 | -0.779629185 | 0.2702911623743 | 0.775173029129601 | 0.458576026794958 | 1.31034591862483 | 0.998735809224318 |
| ebi-fl187-GCST90094852 | ebi-gm430-GCST90011390 | OTU97_3 (Bacteroides) abundance \|\| id:ebi-gm430-GCST90011390 | F-strong vegetable liking (derived food-liking factor) \|\| id:ebi-fl187-GCST90094852 | Inverse variance weighted | 19 | 0.0322256832669605 | 0.0622563864584441 | 0.604717455795876 | -0.089796834 | 0.154248200725511 | 1.03275055352546 | 0.914116883702462 | 1.16678044659583 | 0.998735809224318 |
| ebi-fl187-GCST90094852 | ebi-gm430-GCST90011398 | OTU97_4 (Alistipes) abundance \|\| id:ebi-gm430-GCST90011398 | F-strong vegetable liking (derived food-liking factor) \|\| id:ebi-fl187-GCST90094852 | Inverse variance weighted | 19 | 0.049839052422754 | 0.0729578102051547 | 0.49453003891486 | -0.093158256 | 0.192836360424857 | 1.05110191045543 | 0.911049310250593 | 1.21268433413244 | 0.998735809224318 |
| ebi-fl187-GCST90094852 | ebi-gm430-GCST90011421 | OTU99_108 (Cytophagales) abundance \|\| id:ebi-gm430-GCST90011421 | F-strong vegetable liking (derived food-liking factor) \|\| id:ebi-fl187-GCST90094852 | Inverse variance weighted | 19 | -0.236544818 | 0.230225007430245 | 0.304208343622824 | -0.687785833 | 0.214696196533667 | 0.78935050437491 | 0.502687872856002 | 1.23948527983568 | 0.998735809224318 |
| ebi-fl187-GCST90094852 | ebi-gm430-GCST90011428 | OTU99_123 (Phascolarctobacterium) abundance \|\| id:ebi-gm430-GCST90011428 | F-strong vegetable liking (derived food-liking factor) \|\| id:ebi-fl187-GCST90094852 | Inverse variance weighted | 19 | 0.17170286381219 | 0.144332038241372 | 0.23418874030559 | -0.111187931 | 0.454593658765279 | 1.18732498357499 | 0.894770577871695 | 1.57553304890129 | 0.998735809224318 |
| ebi-fl187-GCST90094852 | ebi-gm430-GCST90011450 | OTU99_298 (Phascolarctobacterium) abundance \|\| id:ebi-gm430-GCST90011450 | F-strong vegetable liking (derived food-liking factor) \|\| id:ebi-fl187-GCST90094852 | Inverse variance weighted | 19 | -0.256350412 | 0.267240856733096 | 0.337433534011699 | -0.780142491 | 0.267441667651586 | 0.773870748030914 | 0.458340697345894 | 1.30661740955108 | 0.998735809224318 |
| ebi-fl187-GCST90094852 | ebi-gm430-GCST90011451 | OTU99_3 (Bacteroides) abundance \|\| id:ebi-gm430-GCST90011451 | F-strong vegetable liking (derived food-liking factor) \|\| id:ebi-fl187-GCST90094852 | Inverse variance weighted | 19 | 0.0286211178181813 | 0.0622567701121029 | 0.645712190766135 | -0.093402152 | 0.150644387237903 | 1.02903463771726 | 0.910827136042606 | 1.16258315515575 | 0.998735809224318 |
| ebi-fl187-GCST90094852 | ebi-gm430-GCST90011472 | OTU99_72 (Parabacteroides) abundance \|\| id:ebi-gm430-GCST90011472 | F-strong vegetable liking (derived food-liking factor) \|\| id:ebi-fl187-GCST90094852 | Inverse variance weighted | 19 | -0.269783359 | 0.1833757120137 | 0.141235546007592 | -0.629199754 | 0.0896330366007704 | 0.763544891590317 | 0.533018175782249 | 1.09377283545362 | 0.998735809224318 |
| ebi-fl187-GCST90094852 | ebi-gm430-GCST90011507 | TestASV_3 (Bacteroides) abundance \|\| id:ebi-gm430-GCST90011507 | F-strong vegetable liking (derived food-liking factor) \|\| id:ebi-fl187-GCST90094852 | Inverse variance weighted | 19 | -0.016991378 | 0.0800592811691024 | 0.831923734634215 | -0.173907569 | 0.139924812840769 | 0.983152161089853 | 0.840374570443121 | 1.15018731628918 | 0.998735809224318 |
| ebi-fl187-GCST90094852 | ebi-gm430-GCST90011512 | TestASV_34 (Phascolarctobacterium) abundance \|\| id:ebi-gm430-GCST90011512 | F-strong vegetable liking (derived food-liking factor) \|\| id:ebi-fl187-GCST90094852 | Inverse variance weighted | 19 | 0.173556393964704 | 0.236898150817217 | 0.463790047222079 | -0.290763982 | 0.637876769566449 | 1.18952776706504 | 0.747692126266398 | 1.89245848513134 | 0.998735809224318 |
| ebi-fl187-GCST90094852 | ebi-gm430-GCST90011519 | TestASV_41 (Barnesiella) abundance \|\| id:ebi-gm430-GCST90011519 | F-strong vegetable liking (derived food-liking factor) \|\| id:ebi-fl187-GCST90094852 | Inverse variance weighted | 19 | 0.14121858063667 | 0.257457981724377 | 0.583340956662194 | -0.363399064 | 0.645836224816449 | 1.15167635462547 | 0.695308905691702 | 1.90758152951305 | 0.998735809224318 |
| ebi-fl187-GCST90094852 | ebi-gm430-GCST90011524 | TestASV_46 (Sutterella) abundance \|\| id:ebi-gm430-GCST90011524 | F-strong vegetable liking (derived food-liking factor) \|\| id:ebi-fl187-GCST90094852 | Inverse variance weighted | 19 | -0.044643695 | 0.261886443665206 | 0.864640786239833 | -0.557941125 | 0.468653734347731 | 0.956338168980803 | 0.572386323512523 | 1.59784162528046 | 0.998735809224318 |
| ebi-fl187-GCST90094852 | ebi-gm430-GCST90011581 | OTU97_19 (Bacteria) prevalence \|\| id:ebi-gm430-GCST90011581 | F-strong vegetable liking (derived food-liking factor) \|\| id:ebi-fl187-GCST90094852 | Inverse variance weighted | 19 | 0.317945471543806 | 0.298778573118311 | 0.287260369870118 | -0.267660532 | 0.903551474855695 | 1.37430132065502 | 0.765167487073363 | 2.46835385959499 | 0.998735809224318 |
| ebi-fl187-GCST90094852 | ebi-gm430-GCST90011614 | OTU97_80 (Ruminococcus) prevalence \|\| id:ebi-gm430-GCST90011614 | F-strong vegetable liking (derived food-liking factor) \|\| id:ebi-fl187-GCST90094852 | Inverse variance weighted | 19 | 0.00894975386265703 | 0.138649570301689 | 0.948532731721525 | -0.262803404 | 0.280702911653967 | 1.00898992265393 | 0.768893043804968 | 1.324060183 | 0.998735809224318 |
| ebi-fl187-GCST90094852 | ebi-gm430-GCST90011623 | OTU99_11 (Parabacteroides) prevalence \|\| id:ebi-gm430-GCST90011623 | F-strong vegetable liking (derived food-liking factor) \|\| id:ebi-fl187-GCST90094852 | Inverse variance weighted | 19 | 0.268307332657879 | 0.133085836109927 | 0.0437950361741738 | 0.00745909388242255 | 0.529155571433335 | 1.30774899223424 | 1.00748698222065 | 1.69749828719386 | 0.998735809224318 |
| ebi-fl187-GCST90094852 | ebi-gm430-GCST90011642 | OTU99_19 (Bacteria) prevalence \|\| id:ebi-gm430-GCST90011642 | F-strong vegetable liking (derived food-liking factor) \|\| id:ebi-fl187-GCST90094852 | Inverse variance weighted | 19 | 0.317945471543806 | 0.298778573118311 | 0.287260369870118 | -0.267660532 | 0.903551474855695 | 1.37430132065502 | 0.765167487073363 | 2.46835385959499 | 0.998735809224318 |
| ebi-fl187-GCST90094852 | ebi-gm430-GCST90011653 | OTU99_30 (Parasutterella) prevalence \|\| id:ebi-gm430-GCST90011653 | F-strong vegetable liking (derived food-liking factor) \|\| id:ebi-fl187-GCST90094852 | Inverse variance weighted | 19 | -0.024137849 | 0.131409981889225 | 0.854261732 | -0.281701414 | 0.233425715444613 | 0.976151138967659 | 0.754498934053024 | 1.26291900902923 | 0.998735809224318 |
| ebi-fl187-GCST90094852 | ebi-gm430-GCST90011675 | OTU99_8 (Ruminococcaceae) prevalence \|\| id:ebi-gm430-GCST90011675 | F-strong vegetable liking (derived food-liking factor) \|\| id:ebi-fl187-GCST90094852 | Inverse variance weighted | 19 | 0.098400811882094 | 0.172198672042002 | 0.567702984590702 | -0.239108585 | 0.435910209084418 | 1.1034049543057 | 0.787329385317478 | 1.54636993854285 | 0.998735809224318 |
| ebi-fl187-GCST90094852 | ebi-gm430-GCST90011679 | OTU99_92 (Ruminococcus) prevalence \|\| id:ebi-gm430-GCST90011679 | F-strong vegetable liking (derived food-liking factor) \|\| id:ebi-fl187-GCST90094852 | Inverse variance weighted | 19 | -0.059706817 | 0.144945778043115 | 0.680394256418233 | -0.343800542 | 0.224386907717798 | 0.942040683182331 | 0.70907034354865 | 1.25155516211452 | 0.998735809224318 |
| ebi-fl187-GCST90094852 | ebi-gm430-GCST90011684 | TestASV_11 (Lachnospiraceae) prevalence \|\| id:ebi-gm430-GCST90011684 | F-strong vegetable liking (derived food-liking factor) \|\| id:ebi-fl187-GCST90094852 | Inverse variance weighted | 19 | 0.10869353197083 | 0.146568484365884 | 0.458336571272279 | -0.178580697 | 0.395967761327963 | 1.11482064109789 | 0.836456554296101 | 1.48582141586992 | 0.998735809224318 |
| ebi-fl187-GCST90094852 | ebi-gm430-GCST90011688 | TestASV_15 (Bacteroides) prevalence \|\| id:ebi-gm430-GCST90011688 | F-strong vegetable liking (derived food-liking factor) \|\| id:ebi-fl187-GCST90094852 | Inverse variance weighted | 19 | 0.00507498617697527 | 0.148875426925394 | 0.972806331440157 | -0.286720851 | 0.296870822950747 | 1.00508788573178 | 0.750721262982575 | 1.34564146222701 | 0.998955228396052 |
| ebi-fl187-GCST90094852 | ebi-gm430-GCST90011692 | TestASV_19 (Prevotella) prevalence \|\| id:ebi-gm430-GCST90011692 | F-strong vegetable liking (derived food-liking factor) \|\| id:ebi-fl187-GCST90094852 | Inverse variance weighted | 19 | -0.113684337 | 0.275799679061736 | 0.680193608967859 | -0.654251708 | 0.426883033695233 | 0.892539652926691 | 0.519830902273775 | 1.53247340348948 | 0.998735809224318 |
| ebi-fl187-GCST90094852 | ebi-gm430-GCST90011693 | TestASV_2 (Bacteroides) prevalence \|\| id:ebi-gm430-GCST90011693 | F-strong vegetable liking (derived food-liking factor) \|\| id:ebi-fl187-GCST90094852 | Inverse variance weighted | 19 | 0.132542913734144 | 0.165766399948707 | 0.423956222000245 | -0.19235923 | 0.457445057633608 | 1.14172801085023 | 0.825010446613901 | 1.58003193306237 | 0.998735809224318 |
| ebi-fl187-GCST90094852 | ebi-gm430-GCST90011716 | TestASV_40 (Barnesiella) prevalence \|\| id:ebi-gm430-GCST90011716 | F-strong vegetable liking (derived food-liking factor) \|\| id:ebi-fl187-GCST90094852 | Inverse variance weighted | 19 | 0.417220664643335 | 0.315316532542186 | 0.185775382941608 | -0.200799739 | 1.03524106842602 | 1.51773738696547 | 0.818076243803383 | 2.81578495065846 | 0.998735809224318 |
| ebi-fl187-GCST90094852 | ebi-gm430-GCST90011718 | TestASV_42 (Alphaproteobacteria) prevalence \|\| id:ebi-gm430-GCST90011718 | F-strong vegetable liking (derived food-liking factor) \|\| id:ebi-fl187-GCST90094852 | Inverse variance weighted | 19 | 0.0676361516232627 | 0.216875135060097 | 0.755141764381813 | -0.357439113 | 0.492711416341054 | 1.06997592864068 | 0.699465285922966 | 1.63674811446834 | 0.998735809224318 |
| ebi-fl187-GCST90094852 | ebi-hbsm44-GCST90104929 | Sulcal depth of the right inferior frontal sulcus \|\| id:ebi-hbsm44-GCST90104929 | F-strong vegetable liking (derived food-liking factor) \|\| id:ebi-fl187-GCST90094852 | Inverse variance weighted | 24 | 0.00966011286552271 | 0.100192582426949 | 0.923190622405963 | -0.186717349 | 0.206037574422342 | 1.00970692236274 | 0.829678212878972 | 1.22879937455456 | 0.998735809224318 |
| ebi-fl187-GCST90094852 | ebi-hbsm44-GCST90104935 | Sulcal opening distance of the left calloso-marginal posterior fissure \|\| id:ebi-hbsm44-GCST90104935 | F-strong vegetable liking (derived food-liking factor) \|\| id:ebi-fl187-GCST90094852 | Inverse variance weighted (multiplicative random effects) | 24 | -0.000609458 | 0.0313854243052442 | 0.984507253017807 | -0.062124889 | 0.0609059738036677 | 0.999390727847091 | 0.93976551264233 | 1.06279897854357 | 0.998955228396052 |
| ebi-fl187-GCST90094852 | ebi-hbsm44-GCST90104936 | Sulcal opening distance of the left anterior inferior temporal sulcus \|\| id:ebi-hbsm44-GCST90104936 | F-strong vegetable liking (derived food-liking factor) \|\| id:ebi-fl187-GCST90094852 | Inverse variance weighted (multiplicative random effects) | 22 | -0.006300754 | 0.0198090851840157 | 0.750428833569372 | -0.045126561 | 0.0325250527393912 | 0.993719053906716 | 0.955876497317991 | 1.03305977379707 | 0.998735809224318 |
| ebi-fl187-GCST90094852 | ebi-hbsm44-GCST90104941 | Sulcal opening distance of the right posterior inferior temporal sulcus \|\| id:ebi-hbsm44-GCST90104941 | F-strong vegetable liking (derived food-liking factor) \|\| id:ebi-fl187-GCST90094852 | Inverse variance weighted | 22 | -0.00996304 | 0.0116810154573866 | 0.393700410270963 | -0.032857831 | 0.0129317499381267 | 0.990086426312458 | 0.967676123690944 | 1.01301572661439 | 0.998735809224318 |
| ebi-fl187-GCST90094852 | ebi-IPs91-GCST90274819 | Leukemia inhibitory factor levels \|\| id:ebi-IPs91-GCST90274819 | F-strong vegetable liking (derived food-liking factor) \|\| id:ebi-fl187-GCST90094852 | Inverse variance weighted | 27 | -0.034800916 | 0.0449133829382349 | 0.438431181133579 | -0.122831146 | 0.053229314741511 | 0.965797672159468 | 0.884412986291061 | 1.05467146910671 | 0.998735809224318 |
| ebi-fl187-GCST90094852 | ebi-IPs91-GCST90274837 | STAM binding protein levels \|\| id:ebi-IPs91-GCST90274837 | F-strong vegetable liking (derived food-liking factor) \|\| id:ebi-fl187-GCST90094852 | Inverse variance weighted | 27 | -0.005273592 | 0.0377438629688503 | 0.888880811492191 | -0.079251564 | 0.0687043791943953 | 0.994740288751336 | 0.923807498831099 | 1.07111951712573 | 0.998735809224318 |
| ebi-fl187-GCST90094852 | ebi-IPs91-GCST90274840 | TNF-beta levels \|\| id:ebi-IPs91-GCST90274840 | F-strong vegetable liking (derived food-liking factor) \|\| id:ebi-fl187-GCST90094852 | Inverse variance weighted | 27 | -0.044015527 | 0.0486946084018454 | 0.36604330273221 | -0.13945696 | 0.0514259051989841 | 0.956939098707238 | 0.869830460131167 | 1.0527711785312 | 0.998735809224318 |
| ebi-fl187-GCST90094852 | ebi-IPs91-GCST90274842 | Tumor necrosis factor ligand superfamily member 14 levels \|\| id:ebi-IPs91-GCST90274842 | F-strong vegetable liking (derived food-liking factor) \|\| id:ebi-fl187-GCST90094852 | Inverse variance weighted | 27 | -0.013668852 | 0.0420906622002744 | 0.745371917040469 | -0.09616655 | 0.0688288458806205 | 0.986424142534168 | 0.90831272403648 | 1.07125284411979 | 0.998735809224318 |
| ebi-fl187-GCST90094852 | ebi-met1400-GCST90199628 | 3-methylhistidine levels \|\| id:ebi-met1400-GCST90199628 | F-strong vegetable liking (derived food-liking factor) \|\| id:ebi-fl187-GCST90094852 | Inverse variance weighted | 26 | -0.145838897 | 0.0482119360528604 | 0.00248670813076056 | -0.240334291 | -0.051343502 | 0.864296933188136 | 0.786364942233535 | 0.94995230407483 | 0.998735809224318 |
| ebi-fl187-GCST90094852 | ebi-met1400-GCST90199651 | Docosahexaenoate DHA; 22:6n3 levels \|\| id:ebi-met1400-GCST90199651 | F-strong vegetable liking (derived food-liking factor) \|\| id:ebi-fl187-GCST90094852 | Inverse variance weighted | 26 | 0.0651505712623501 | 0.0462469289141865 | 0.158907760373666 | -0.025493409 | 0.155794551934156 | 1.06731971996339 | 0.974828803642066 | 1.16858609467289 | 0.998735809224318 |
| ebi-fl187-GCST90094852 | ebi-met1400-GCST90199652 | 1-stearoyl-2-oleoyl-GPS (18:0/18:1) levels \|\| id:ebi-met1400-GCST90199652 | F-strong vegetable liking (derived food-liking factor) \|\| id:ebi-fl187-GCST90094852 | Inverse variance weighted | 26 | 0.045349839934331 | 0.0490023838639748 | 0.354725686281718 | -0.050694832 | 0.141394512307722 | 1.04639386624462 | 0.950568709009444 | 1.15187898879542 | 0.998735809224318 |
| ebi-fl187-GCST90094852 | ebi-met1400-GCST90199653 | 1-myristoyl-2-palmitoyl-gpc (14:0/16:0) levels \|\| id:ebi-met1400-GCST90199653 | F-strong vegetable liking (derived food-liking factor) \|\| id:ebi-fl187-GCST90094852 | Inverse variance weighted | 26 | -0.055083662 | 0.0480367178654376 | 0.25150599498287 | -0.149235629 | 0.0390683049908588 | 0.946405966401287 | 0.861366128109824 | 1.03984150758916 | 0.998735809224318 |
| ebi-fl187-GCST90094852 | ebi-met1400-GCST90199667 | Erythritol levels in elite athletes \|\| id:ebi-met1400-GCST90199667 | F-strong vegetable liking (derived food-liking factor) \|\| id:ebi-fl187-GCST90094852 | Inverse variance weighted | 26 | -0.080509346 | 0.0455760479137331 | 0.0773147320852354 | -0.1698384 | 0.00881970768753269 | 0.922646280285219 | 0.843801163733983 | 1.0088587159054 | 0.998735809224318 |
| ebi-fl187-GCST90094852 | ebi-met1400-GCST90199674 | 3-hydroxymyristate levels \|\| id:ebi-met1400-GCST90199674 | F-strong vegetable liking (derived food-liking factor) \|\| id:ebi-fl187-GCST90094852 | Inverse variance weighted | 26 | 0.00979205586977077 | 0.0471525558893916 | 0.835488554775674 | -0.082626954 | 0.102211065412978 | 1.00984015491684 | 0.920694544882379 | 1.10761722674565 | 0.998735809224318 |
| ebi-fl187-GCST90094852 | ebi-met1400-GCST90199685 | 2-linoleoylglycerol (18:2) levels \|\| id:ebi-met1400-GCST90199685 | F-strong vegetable liking (derived food-liking factor) \|\| id:ebi-fl187-GCST90094852 | Inverse variance weighted | 26 | -0.065860101 | 0.055312210918121 | 0.233772433176219 | -0.174272034 | 0.0425518324865223 | 0.93626183730614 | 0.840068339158858 | 1.04347014062401 | 0.998735809224318 |
| ebi-fl187-GCST90094852 | ebi-met1400-GCST90199703 | Gamma-glutamylvaline levels \|\| id:ebi-met1400-GCST90199703 | F-strong vegetable liking (derived food-liking factor) \|\| id:ebi-fl187-GCST90094852 | Inverse variance weighted | 26 | -0.069876165 | 0.0471959580864966 | 0.138725456453456 | -0.162380243 | 0.0226279125245275 | 0.93250928974104 | 0.850117891549534 | 1.02288586571016 | 0.998735809224318 |
| ebi-fl187-GCST90094852 | ebi-met1400-GCST90199779 | Gamma-glutamyl-2-aminobutyrate levels \|\| id:ebi-met1400-GCST90199779 | F-strong vegetable liking (derived food-liking factor) \|\| id:ebi-fl187-GCST90094852 | Inverse variance weighted | 26 | 0.0674161773140852 | 0.0577561467121085 | 0.243107221871294 | -0.04578587 | 0.180618224869818 | 1.06974058731041 | 0.955246486991937 | 1.19795774150685 | 0.998735809224318 |
| ebi-fl187-GCST90094852 | ebi-met1400-GCST90199824 | 4-vinylphenol sulfate levels \|\| id:ebi-met1400-GCST90199824 | F-strong vegetable liking (derived food-liking factor) \|\| id:ebi-fl187-GCST90094852 | Inverse variance weighted | 26 | 0.0914081707220094 | 0.0491655471973363 | 0.062999989764202 | -0.004956302 | 0.187772643228788 | 1.09571615324929 | 0.995055960412174 | 1.20655916476708 | 0.998735809224318 |
| ebi-fl187-GCST90094852 | ebi-met1400-GCST90199830 | 4-ethylphenylsulfate levels \|\| id:ebi-met1400-GCST90199830 | F-strong vegetable liking (derived food-liking factor) \|\| id:ebi-fl187-GCST90094852 | Inverse variance weighted | 26 | 0.0389877044081427 | 0.0484464921063557 | 0.420959315068785 | -0.05596742 | 0.1339428289366 | 1.03975769913526 | 0.945569941943507 | 1.14332745252984 | 0.998735809224318 |
| ebi-fl187-GCST90094852 | ebi-met1400-GCST90199833 | Hexanoylglutamine levels \|\| id:ebi-met1400-GCST90199833 | F-strong vegetable liking (derived food-liking factor) \|\| id:ebi-fl187-GCST90094852 | Inverse variance weighted | 26 | -0.026375735 | 0.048907018985313 | 0.589677340549413 | -0.122233492 | 0.0694820226405098 | 0.973969067000114 | 0.884941717759369 | 1.07195279015089 | 0.998735809224318 |
| ebi-fl187-GCST90094852 | ebi-met1400-GCST90199837 | Carotenoid levels (cryptoxanthin) \|\| id:ebi-met1400-GCST90199837 | F-strong vegetable liking (derived food-liking factor) \|\| id:ebi-fl187-GCST90094852 | Inverse variance weighted | 26 | 0.134930766566508 | 0.0479178496830357 | 0.00486442998510722 | 0.0410117811877579 | 0.228849751945258 | 1.14445754688295 | 1.04186437987305 | 1.25715314000554 | 0.998735809224318 |
| ebi-fl187-GCST90094852 | ebi-met1400-GCST90199869 | 2-hydroxyhippurate levels \|\| id:ebi-met1400-GCST90199869 | F-strong vegetable liking (derived food-liking factor) \|\| id:ebi-fl187-GCST90094852 | Inverse variance weighted | 26 | 0.0241563689514918 | 0.0487132891528356 | 0.619972948960717 | -0.071321678 | 0.11963441569105 | 1.02445049761689 | 0.931162309713552 | 1.12708473175891 | 0.998735809224318 |
| ebi-fl187-GCST90094852 | ebi-met1400-GCST90199902 | Hydantoin-5-propionate levels \|\| id:ebi-met1400-GCST90199902 | F-strong vegetable liking (derived food-liking factor) \|\| id:ebi-fl187-GCST90094852 | Inverse variance weighted | 25 | -0.08116904 | 0.0596197123754115 | 0.173372674251737 | -0.198023677 | 0.0356855959240359 | 0.922037816691981 | 0.820350429809098 | 1.03632996889872 | 0.998735809224318 |
| ebi-fl187-GCST90094852 | ebi-met1400-GCST90199904 | Pantoate levels \|\| id:ebi-met1400-GCST90199904 | F-strong vegetable liking (derived food-liking factor) \|\| id:ebi-fl187-GCST90094852 | Inverse variance weighted | 26 | -0.002290956 | 0.0517818384195312 | 0.964711133996991 | -0.103783359 | 0.0992014471931692 | 0.997711666127976 | 0.901420560550302 | 1.10428873301955 | 0.998735809224318 |
| ebi-fl187-GCST90094852 | ebi-met1400-GCST90199936 | 3-methyl catechol sulfate (2) levels \|\| id:ebi-met1400-GCST90199936 | F-strong vegetable liking (derived food-liking factor) \|\| id:ebi-fl187-GCST90094852 | Inverse variance weighted | 26 | 0.00778734201907103 | 0.0655718121250004 | 0.905465093608111 | -0.12073341 | 0.136308093784072 | 1.00781774222797 | 0.886270199099454 | 1.14603492544547 | 0.998735809224318 |
| ebi-fl187-GCST90094852 | ebi-met1400-GCST90199977 | 4-hydroxychlorothalonil levels \|\| id:ebi-met1400-GCST90199977 | F-strong vegetable liking (derived food-liking factor) \|\| id:ebi-fl187-GCST90094852 | Inverse variance weighted | 26 | 0.041351328358482 | 0.0563657418673266 | 0.463177201148616 | -0.069125526 | 0.151828182418442 | 1.04221820204178 | 0.933209530944744 | 1.16396023041851 | 0.998735809224318 |
| ebi-fl187-GCST90094852 | ebi-met1400-GCST90200040 | Sphingomyelin (d18:2/24:1, d18:1/24:2) levels \|\| id:ebi-met1400-GCST90200040 | F-strong vegetable liking (derived food-liking factor) \|\| id:ebi-fl187-GCST90094852 | Inverse variance weighted | 26 | -0.01293228 | 0.0440913539177793 | 0.769287899646245 | -0.099351334 | 0.0734867737827595 | 0.987150982723922 | 0.905424546093723 | 1.07625430180455 | 0.998735809224318 |
| ebi-fl187-GCST90094852 | ebi-met1400-GCST90200077 | 1-stearoyl-2-oleoyl-GPI (18:0/18:1) levels \|\| id:ebi-met1400-GCST90200077 | F-strong vegetable liking (derived food-liking factor) \|\| id:ebi-fl187-GCST90094852 | Inverse variance weighted | 26 | -0.030478889 | 0.0570356526622208 | 0.593076506010881 | -0.142268768 | 0.0813109900906199 | 0.969980908994526 | 0.867388098690595 | 1.08470817761296 | 0.998735809224318 |
| ebi-fl187-GCST90094852 | ebi-met1400-GCST90200080 | 1-palmitoyl-2-oleoyl-GPI (16:0/18:1) levels \|\| id:ebi-met1400-GCST90200080 | F-strong vegetable liking (derived food-liking factor) \|\| id:ebi-fl187-GCST90094852 | Inverse variance weighted | 26 | -0.077946789 | 0.0482725664163586 | 0.106370849115673 | -0.172561019 | 0.0166674413783377 | 0.925013646322247 | 0.841506939355269 | 1.0168071181183 | 0.998735809224318 |
| ebi-fl187-GCST90094852 | ebi-met1400-GCST90200082 | 1-oleoyl-2-linoleoyl-GPE (18:1/18:2) levels \|\| id:ebi-met1400-GCST90200082 | F-strong vegetable liking (derived food-liking factor) \|\| id:ebi-fl187-GCST90094852 | Inverse variance weighted (multiplicative random effects) | 26 | 0.0318960793252227 | 0.0605812711916025 | 0.598540392883308 | -0.086843212 | 0.150635370860764 | 1.03241021096439 | 0.916820830676484 | 1.16257267291482 | 0.998735809224318 |
| ebi-fl187-GCST90094852 | ebi-met1400-GCST90200100 | Perfluorooctanesulfonate (PFOS) levels \|\| id:ebi-met1400-GCST90200100 | F-strong vegetable liking (derived food-liking factor) \|\| id:ebi-fl187-GCST90094852 | Inverse variance weighted (multiplicative random effects) | 26 | -0.078472876 | 0.0589655859751708 | 0.183246600205995 | -0.194045424 | 0.0370996726154064 | 0.92452713656173 | 0.823620490946233 | 1.03779645557025 | 0.998735809224318 |
| ebi-fl187-GCST90094852 | ebi-met1400-GCST90200102 | 1-palmitoleoyl-2-linolenoyl-GPC (16:1/18:3) levels \|\| id:ebi-met1400-GCST90200102 | F-strong vegetable liking (derived food-liking factor) \|\| id:ebi-fl187-GCST90094852 | Inverse variance weighted | 26 | -0.04649726 | 0.0473446013851733 | 0.326049265901663 | -0.139292679 | 0.0462981588677855 | 0.954567176226351 | 0.869973368638742 | 1.0473866520242 | 0.998735809224318 |
| ebi-fl187-GCST90094852 | ebi-met1400-GCST90200114 | Glycosyl-N-tricosanoyl-sphingadienine (d18:2/23:0) levels \|\| id:ebi-met1400-GCST90200114 | F-strong vegetable liking (derived food-liking factor) \|\| id:ebi-fl187-GCST90094852 | Inverse variance weighted | 26 | -0.052058108 | 0.0526371004356517 | 0.322662993644426 | -0.155226825 | 0.0511106085980013 | 0.949273704600891 | 0.856220943046443 | 1.05243929568051 | 0.998735809224318 |
| ebi-fl187-GCST90094852 | ebi-met1400-GCST90200135 | Arachidoylcarnitine (C20) levels \|\| id:ebi-met1400-GCST90200135 | F-strong vegetable liking (derived food-liking factor) \|\| id:ebi-fl187-GCST90094852 | Inverse variance weighted | 26 | 0.00208299142423429 | 0.0495599485660413 | 0.966474995248797 | -0.095054508 | 0.0992204906136752 | 1.00208516235795 | 0.909323367932727 | 1.10430976265449 | 0.998735809224318 |
| ebi-fl187-GCST90094852 | ebi-met1400-GCST90200146 | Docosahexaenoylcarnitine (C22:6) levels \|\| id:ebi-met1400-GCST90200146 | F-strong vegetable liking (derived food-liking factor) \|\| id:ebi-fl187-GCST90094852 | Inverse variance weighted | 26 | 0.0951375813971541 | 0.0646855711584181 | 0.141353395485069 | -0.031646138 | 0.221921300867654 | 1.09981015813579 | 0.968849360328819 | 1.24847312024662 | 0.998735809224318 |
| ebi-fl187-GCST90094852 | ebi-met1400-GCST90200162 | 3-hydroxyphenylacetoylglutamine levels \|\| id:ebi-met1400-GCST90200162 | F-strong vegetable liking (derived food-liking factor) \|\| id:ebi-fl187-GCST90094852 | Inverse variance weighted | 26 | 0.0453385564640022 | 0.0590341325195244 | 0.442483736178443 | -0.070368343 | 0.16104545620227 | 1.04638205935709 | 0.932050442157743 | 1.17473836674504 | 0.998735809224318 |
| ebi-fl187-GCST90094852 | ebi-met1400-GCST90200172 | 3-carboxy-4-methyl-5-pentyl-2-furanpropionate (3-CMPFP) levels \|\| id:ebi-met1400-GCST90200172 | F-strong vegetable liking (derived food-liking factor) \|\| id:ebi-fl187-GCST90094852 | Inverse variance weighted | 26 | 0.0161886364348213 | 0.0547490585845574 | 0.767468406879711 | -0.091119518 | 0.123496791260554 | 1.01632038237858 | 0.912908595012393 | 1.13144637401964 | 0.998735809224318 |
| ebi-fl187-GCST90094852 | ebi-met1400-GCST90200222 | Dihydrocaffeate sulfate (2) levels \|\| id:ebi-met1400-GCST90200222 | F-strong vegetable liking (derived food-liking factor) \|\| id:ebi-fl187-GCST90094852 | Inverse variance weighted (multiplicative random effects) | 26 | 0.0123407539258264 | 0.0794391544323161 | 0.876546577807754 | -0.143359989 | 0.168041496613166 | 1.0124172152359 | 0.866442103327109 | 1.18298569952925 | 0.998735809224318 |
| ebi-fl187-GCST90094852 | ebi-met1400-GCST90200223 | 4-methylhexanoylglutamine levels \|\| id:ebi-met1400-GCST90200223 | F-strong vegetable liking (derived food-liking factor) \|\| id:ebi-fl187-GCST90094852 | Inverse variance weighted | 26 | -0.015681008 | 0.0625737390650823 | 0.802123022160734 | -0.138325537 | 0.106963520460079 | 0.984441298767258 | 0.870815163326006 | 1.11289365589027 | 0.998735809224318 |
| ebi-fl187-GCST90094852 | ebi-met1400-GCST90200224 | 2-ketocaprylate levels \|\| id:ebi-met1400-GCST90200224 | F-strong vegetable liking (derived food-liking factor) \|\| id:ebi-fl187-GCST90094852 | Inverse variance weighted | 26 | -0.027885781 | 0.0519197853308686 | 0.591203004753213 | -0.12964856 | 0.0738769980938922 | 0.972499438220877 | 0.878404082657877 | 1.07667436435206 | 0.998735809224318 |
| ebi-fl187-GCST90094852 | ebi-met1400-GCST90200243 | Hydroxypalmitoyl sphingomyelin (d18:1/16:0(OH)) levels \|\| id:ebi-met1400-GCST90200243 | F-strong vegetable liking (derived food-liking factor) \|\| id:ebi-fl187-GCST90094852 | Inverse variance weighted | 26 | 0.0328677950857804 | 0.0495376699116813 | 0.507016181255112 | -0.064226038 | 0.129961628112676 | 1.0334139078128 | 0.93779299877289 | 1.13878468516861 | 0.998735809224318 |
| ebi-fl187-GCST90094852 | ebi-met1400-GCST90200273 | 2-methoxyhydroquinone sulfate (2) levels \|\| id:ebi-met1400-GCST90200273 | F-strong vegetable liking (derived food-liking factor) \|\| id:ebi-fl187-GCST90094852 | Inverse variance weighted (multiplicative random effects) | 26 | -0.003196468 | 0.0715838256732099 | 0.964383499911308 | -0.143500767 | 0.137107830102471 | 0.996808635048588 | 0.866320136120975 | 1.14695181778469 | 0.998735809224318 |
| ebi-fl187-GCST90094852 | ebi-met1400-GCST90200276 | 2,4-di-tert-butylphenol levels \|\| id:ebi-met1400-GCST90200276 | F-strong vegetable liking (derived food-liking factor) \|\| id:ebi-fl187-GCST90094852 | Inverse variance weighted | 26 | 0.0196166188856536 | 0.0481325571739976 | 0.683601100788817 | -0.074723193 | 0.113956430946689 | 1.01981028906573 | 0.928000327603133 | 1.1207032958388 | 0.998735809224318 |
| ebi-fl187-GCST90094852 | ebi-met1400-GCST90200289 | N-acetylmethionine levels \|\| id:ebi-met1400-GCST90200289 | F-strong vegetable liking (derived food-liking factor) \|\| id:ebi-fl187-GCST90094852 | Inverse variance weighted | 26 | -0.022666247 | 0.0481749319199754 | 0.637998706132187 | -0.117089113 | 0.0717566199007023 | 0.97758870282422 | 0.889505922889469 | 1.07439382616488 | 0.998735809224318 |
| ebi-fl187-GCST90094852 | ebi-met1400-GCST90200308 | 3-hydroxyisobutyrate levels \|\| id:ebi-met1400-GCST90200308 | F-strong vegetable liking (derived food-liking factor) \|\| id:ebi-fl187-GCST90094852 | Inverse variance weighted | 26 | -0.016286593 | 0.0488409566162771 | 0.738785726689316 | -0.112014868 | 0.0794416820867351 | 0.983845316583218 | 0.894030965084089 | 1.08268241790897 | 0.998735809224318 |
| ebi-fl187-GCST90094852 | ebi-met1400-GCST90200383 | Cystathionine levels \|\| id:ebi-met1400-GCST90200383 | F-strong vegetable liking (derived food-liking factor) \|\| id:ebi-fl187-GCST90094852 | Inverse variance weighted (multiplicative random effects) | 26 | -0.00051768 | 0.0593687482508581 | 0.993042740148456 | -0.116880427 | 0.115845066366847 | 0.999482453768442 | 0.88969157009231 | 1.12282189578051 | 0.999107451094609 |
| ebi-fl187-GCST90094852 | ebi-met1400-GCST90200384 | Guanidinoacetate levels \|\| id:ebi-met1400-GCST90200384 | F-strong vegetable liking (derived food-liking factor) \|\| id:ebi-fl187-GCST90094852 | Inverse variance weighted (multiplicative random effects) | 26 | -0.013879418 | 0.06176185928867 | 0.822193413237519 | -0.134932662 | 0.10717382612945 | 0.98621645697088 | 0.873774747704508 | 1.11312772834803 | 0.998735809224318 |
| ebi-fl187-GCST90094852 | ebi-met1400-GCST90200403 | Citrulline levels \|\| id:ebi-met1400-GCST90200403 | F-strong vegetable liking (derived food-liking factor) \|\| id:ebi-fl187-GCST90094852 | Inverse variance weighted | 26 | -0.042989259 | 0.0484340704703013 | 0.374765087911337 | -0.137920037 | 0.0519415193320692 | 0.957921679249959 | 0.871168350272587 | 1.05331414219759 | 0.998735809224318 |
| ebi-fl187-GCST90094852 | ebi-met1400-GCST90200434 | 12,13-DiHOME levels \|\| id:ebi-met1400-GCST90200434 | F-strong vegetable liking (derived food-liking factor) \|\| id:ebi-fl187-GCST90094852 | Inverse variance weighted | 26 | 0.0411501224811462 | 0.0488286114771389 | 0.399370451883103 | -0.054553956 | 0.136854200976339 | 1.04200852270912 | 0.946907416129612 | 1.14666095428471 | 0.998735809224318 |
| ebi-fl187-GCST90094852 | ebi-met1400-GCST90200437 | Fructose levels \|\| id:ebi-met1400-GCST90200437 | F-strong vegetable liking (derived food-liking factor) \|\| id:ebi-fl187-GCST90094852 | Inverse variance weighted | 25 | -0.033785279 | 0.049551504902357 | 0.495351862341291 | -0.130906228 | 0.0633356709335897 | 0.966779070435265 | 0.877300036464229 | 1.06538439779238 | 0.998735809224318 |
| ebi-fl187-GCST90094852 | ebi-met1400-GCST90200448 | N-stearoyl-sphinganine (d18:0/18:0) levels \|\| id:ebi-met1400-GCST90200448 | F-strong vegetable liking (derived food-liking factor) \|\| id:ebi-fl187-GCST90094852 | Inverse variance weighted | 26 | -0.045126117 | 0.0539993163521582 | 0.403335281210679 | -0.150964777 | 0.060712543182876 | 0.955876922027965 | 0.859877985678958 | 1.06259342055862 | 0.998735809224318 |
| ebi-fl187-GCST90094852 | ebi-met1400-GCST90200468 | X-11847 levels \|\| id:ebi-met1400-GCST90200468 | F-strong vegetable liking (derived food-liking factor) \|\| id:ebi-fl187-GCST90094852 | Inverse variance weighted (multiplicative random effects) | 26 | 0.101339093830025 | 0.0638883389546463 | 0.112696039972702 | -0.023882051 | 0.226560238181132 | 1.10665183695654 | 0.976400869 | 1.25427816299346 | 0.998735809224318 |
| ebi-fl187-GCST90094852 | ebi-met1400-GCST90200476 | X-11843 levels \|\| id:ebi-met1400-GCST90200476 | F-strong vegetable liking (derived food-liking factor) \|\| id:ebi-fl187-GCST90094852 | Inverse variance weighted (multiplicative random effects) | 26 | 0.100383092742814 | 0.0696504565579345 | 0.149516612775303 | -0.036131802 | 0.236897987596365 | 1.10559438214176 | 0.964513160234738 | 1.26731183 | 0.998735809224318 |
| ebi-fl187-GCST90094852 | ebi-met1400-GCST90200511 | X-12906 levels \|\| id:ebi-met1400-GCST90200511 | F-strong vegetable liking (derived food-liking factor) \|\| id:ebi-fl187-GCST90094852 | Inverse variance weighted | 26 | -0.07533534 | 0.055454026589451 | 0.174299109586441 | -0.184025232 | 0.0333545524152468 | 0.927432429263905 | 0.831914812840866 | 1.03391705205143 | 0.998735809224318 |
| ebi-fl187-GCST90094852 | ebi-met1400-GCST90200515 | X-12849 levels \|\| id:ebi-met1400-GCST90200515 | F-strong vegetable liking (derived food-liking factor) \|\| id:ebi-fl187-GCST90094852 | Inverse variance weighted | 26 | -0.048411127 | 0.0512655133578346 | 0.345005372712826 | -0.148891534 | 0.052069278738849 | 0.952742008162307 | 0.861662571246987 | 1.05344872158426 | 0.998735809224318 |
| ebi-fl187-GCST90094852 | ebi-met1400-GCST90200551 | X-18886 levels \|\| id:ebi-met1400-GCST90200551 | F-strong vegetable liking (derived food-liking factor) \|\| id:ebi-fl187-GCST90094852 | Inverse variance weighted | 26 | 0.0038793154040065 | 0.0477052953625327 | 0.935188789481868 | -0.089623064 | 0.0973816943145706 | 1.00388684968748 | 0.914275744221794 | 1.10228102773661 | 0.998735809224318 |
| ebi-fl187-GCST90094852 | ebi-met1400-GCST90200564 | X-21319 levels \|\| id:ebi-met1400-GCST90200564 | F-strong vegetable liking (derived food-liking factor) \|\| id:ebi-fl187-GCST90094852 | Inverse variance weighted | 26 | -0.026080021 | 0.0488988282390516 | 0.593793720252105 | -0.121921724 | 0.0697616827408723 | 0.974257125842201 | 0.885217657126865 | 1.07225261449826 | 0.998735809224318 |
| ebi-fl187-GCST90094852 | ebi-met1400-GCST90200579 | X-21442 levels \|\| id:ebi-met1400-GCST90200579 | F-strong vegetable liking (derived food-liking factor) \|\| id:ebi-fl187-GCST90094852 | Inverse variance weighted | 26 | 0.0127763523297766 | 0.0527655666604558 | 0.808676119149546 | -0.090644158 | 0.11619686298427 | 1.01285831862398 | 0.913342658462589 | 1.12321697021427 | 0.998735809224318 |
| ebi-fl187-GCST90094852 | ebi-met1400-GCST90200581 | X-21441 levels \|\| id:ebi-met1400-GCST90200581 | F-strong vegetable liking (derived food-liking factor) \|\| id:ebi-fl187-GCST90094852 | Inverse variance weighted | 26 | -0.029707054 | 0.0575988861057105 | 0.606023826088314 | -0.142600871 | 0.0831867623039036 | 0.970729862880653 | 0.867100084428266 | 1.08674475255025 | 0.998735809224318 |
| ebi-fl187-GCST90094852 | ebi-met1400-GCST90200648 | X-24585 levels \|\| id:ebi-met1400-GCST90200648 | F-strong vegetable liking (derived food-liking factor) \|\| id:ebi-fl187-GCST90094852 | Inverse variance weighted | 26 | -0.079358342 | 0.0472850250954009 | 0.0932890234389602 | -0.172036992 | 0.013320306771578 | 0.923708861067196 | 0.841948027585768 | 1.013409417 | 0.998735809224318 |
| ebi-fl187-GCST90094852 | ebi-met1400-GCST90200720 | Adenosine 5'-diphosphate (ADP) to pantothenate ratio \|\| id:ebi-met1400-GCST90200720 | F-strong vegetable liking (derived food-liking factor) \|\| id:ebi-fl187-GCST90094852 | Inverse variance weighted | 26 | 0.0599424610788989 | 0.0643346336690611 | 0.351476457272511 | -0.066153421 | 0.186038343070259 | 1.06177545137378 | 0.935987253256986 | 1.20446844251036 | 0.998735809224318 |
| ebi-fl187-GCST90094852 | ebi-met1400-GCST90200724 | Cholate to taurocholate ratio \|\| id:ebi-met1400-GCST90200724 | F-strong vegetable liking (derived food-liking factor) \|\| id:ebi-fl187-GCST90094852 | Inverse variance weighted | 26 | -0.002619762 | 0.0569736399821875 | 0.963324594668866 | -0.114288096 | 0.109048572309658 | 0.997383666526508 | 0.892000936584088 | 1.11521651766795 | 0.998735809224318 |
| ebi-fl187-GCST90094852 | ebi-met1400-GCST90200726 | N-acetylputrescine to (N(1) + N(8))-acetylspermidine ratio \|\| id:ebi-met1400-GCST90200726 | F-strong vegetable liking (derived food-liking factor) \|\| id:ebi-fl187-GCST90094852 | Inverse variance weighted | 26 | -0.024705126 | 0.0506168954654997 | 0.625492727426878 | -0.123914242 | 0.0745039886592904 | 0.975597547527396 | 0.883455601403507 | 1.07734964069434 | 0.998735809224318 |
| ebi-fl187-GCST90094852 | ebi-met1400-GCST90200744 | Arginine to glutamate ratio \|\| id:ebi-met1400-GCST90200744 | F-strong vegetable liking (derived food-liking factor) \|\| id:ebi-fl187-GCST90094852 | Inverse variance weighted | 26 | 0.112343163453541 | 0.0501106178440074 | 0.0249676176365035 | 0.0141263524792868 | 0.210559974427796 | 1.11889675924735 | 1.01422660088838 | 1.23436908158161 | 0.998735809224318 |
| ebi-fl187-GCST90094852 | ebi-met1400-GCST90200759 | Histidine to trans-urocanate ratio \|\| id:ebi-met1400-GCST90200759 | F-strong vegetable liking (derived food-liking factor) \|\| id:ebi-fl187-GCST90094852 | Inverse variance weighted | 26 | 0.022005115749191 | 0.0504281574114387 | 0.662571456202876 | -0.076834073 | 0.120844304275611 | 1.02224901402663 | 0.926043496684929 | 1.12844920397293 | 0.998735809224318 |
| ebi-fl187-GCST90094852 | ebi-met1400-GCST90200795 | Oleoyl-linoleoyl-glycerol (18:1 to 18:2) [2] to linoleoyl-arachidonoyl-glycerol (18:2 to 20:4) [2] ratio \|\| id:ebi-met1400-GCST90200795 | F-strong vegetable liking (derived food-liking factor) \|\| id:ebi-fl187-GCST90094852 | Inverse variance weighted | 26 | 0.058296769411142 | 0.0502721078191027 | 0.246201709217728 | -0.040236562 | 0.156830100736583 | 1.06002953337581 | 0.960562179844867 | 1.16979684939336 | 0.998735809224318 |
| ebi-fl187-GCST90094852 | ebi-met1400-GCST90200811 | Choline to taurocholate ratio \|\| id:ebi-met1400-GCST90200811 | F-strong vegetable liking (derived food-liking factor) \|\| id:ebi-fl187-GCST90094852 | Inverse variance weighted | 26 | -0.018217925 | 0.0506296718170844 | 0.718976479099007 | -0.117452082 | 0.0810162317038137 | 0.981947018179054 | 0.889183118763102 | 1.08438849789683 | 0.998735809224318 |
| ebi-fl187-GCST90094852 | ebi-met1400-GCST90200831 | Cholate to phosphate ratio \|\| id:ebi-met1400-GCST90200831 | F-strong vegetable liking (derived food-liking factor) \|\| id:ebi-fl187-GCST90094852 | Inverse variance weighted | 26 | 0.0214803937387235 | 0.0524840907591242 | 0.682338314437328 | -0.081388424 | 0.124349211626607 | 1.02171275817372 | 0.921835558702854 | 1.13241125313483 | 0.998735809224318 |
| ebi-fl187-GCST90094852 | ebi-met1400-GCST90200841 | Adenosine 5'-diphosphate (ADP) to oxalate (ethanedioate) ratio \|\| id:ebi-met1400-GCST90200841 | F-strong vegetable liking (derived food-liking factor) \|\| id:ebi-fl187-GCST90094852 | Inverse variance weighted | 26 | -0.009202627 | 0.0699716480257788 | 0.895364465563621 | -0.146347057 | 0.12794180314777 | 0.990839587594685 | 0.863857843144044 | 1.13648686081455 | 0.998735809224318 |
| ebi-fl187-GCST90094852 | ebi-met1400-GCST90200844 | Aspartate to phosphate ratio \|\| id:ebi-met1400-GCST90200844 | F-strong vegetable liking (derived food-liking factor) \|\| id:ebi-fl187-GCST90094852 | Inverse variance weighted | 26 | -0.026651997 | 0.0454739109975154 | 0.557812186405552 | -0.115780863 | 0.0624768682204424 | 0.973700032777409 | 0.890670380846944 | 1.06446983555149 | 0.998735809224318 |
| ebi-fl187-GCST90094852 | ebi-met1400-GCST90200870 | Adenosine 5'-monophosphate (AMP) to acetoacetate ratio \|\| id:ebi-met1400-GCST90200870 | F-strong vegetable liking (derived food-liking factor) \|\| id:ebi-fl187-GCST90094852 | Inverse variance weighted | 26 | 0.034645137147552 | 0.0532938215030564 | 0.515641884620079 | -0.069810753 | 0.139101027293543 | 1.03525227103457 | 0.932570289338269 | 1.14924019876585 | 0.998735809224318 |
| ebi-fl187-GCST90094852 | ebi-met1400-GCST90200887 | Cholesterol to cortisol ratio \|\| id:ebi-met1400-GCST90200887 | F-strong vegetable liking (derived food-liking factor) \|\| id:ebi-fl187-GCST90094852 | Inverse variance weighted (multiplicative random effects) | 25 | -0.067792954 | 0.0609219932539874 | 0.265801666361388 | -0.187200061 | 0.05161415233821 | 0.934453928086777 | 0.829277813459645 | 1.0529693783485 | 0.998735809224318 |
| ebi-fl187-GCST90094852 | ebi-met1400-GCST90200890 | Cortisol to taurocholate ratio \|\| id:ebi-met1400-GCST90200890 | F-strong vegetable liking (derived food-liking factor) \|\| id:ebi-fl187-GCST90094852 | Inverse variance weighted | 26 | 0.0296930741510036 | 0.050449115212956 | 0.556146592539121 | -0.069187192 | 0.128573339968397 | 1.03013830935207 | 0.933151985452871 | 1.13720482079854 | 0.998735809224318 |
| ebi-fl187-GCST90094852 | ebi-met1400-GCST90200948 | Adenosine 5'-diphosphate (ADP) to mannitol to sorbitol ratio \|\| id:ebi-met1400-GCST90200948 | F-strong vegetable liking (derived food-liking factor) \|\| id:ebi-fl187-GCST90094852 | Inverse variance weighted | 26 | 0.0549608947228471 | 0.0691744151995433 | 0.426889082846194 | -0.080620959 | 0.190542748513952 | 1.05649929916977 | 0.92254330685575 | 1.20990609421953 | 0.998735809224318 |
| ebi-fl187-GCST90094852 | ebi-met1400-GCST90200972 | Leucine to N-palmitoyl-sphingosine (d18:1 to 16:0) ratio \|\| id:ebi-met1400-GCST90200972 | F-strong vegetable liking (derived food-liking factor) \|\| id:ebi-fl187-GCST90094852 | Inverse variance weighted | 26 | -0.010526512 | 0.0519163127070369 | 0.839323214766692 | -0.112282485 | 0.0912294605898103 | 0.989528697522623 | 0.89379173887807 | 1.09552035516676 | 0.998735809224318 |
| ebi-fl187-GCST90094852 | ebi-met1400-GCST90200973 | Proline to glutamate ratio \|\| id:ebi-met1400-GCST90200973 | F-strong vegetable liking (derived food-liking factor) \|\| id:ebi-fl187-GCST90094852 | Inverse variance weighted | 26 | 0.0227673992230577 | 0.0486476941941183 | 0.639780498111133 | -0.072582081 | 0.11811687984353 | 1.02302855463391 | 0.929989408697112 | 1.12537563740923 | 0.998735809224318 |
| ebi-fl187-GCST90094852 | ebi-met1400-GCST90200983 | Cholesterol to linoleoyl-arachidonoyl-glycerol (18:2 to 20:4) [1] ratio \|\| id:ebi-met1400-GCST90200983 | F-strong vegetable liking (derived food-liking factor) \|\| id:ebi-fl187-GCST90094852 | Inverse variance weighted | 26 | 0.0439012649163463 | 0.0498608582489397 | 0.378601762915234 | -0.053826017 | 0.141628547084268 | 1.04487918356162 | 0.947596957683848 | 1.15214860008515 | 0.998735809224318 |
| ebi-fl187-GCST90094852 | ebi-met1400-GCST90201001 | Serine to alpha-ketobutyrate ratio \|\| id:ebi-met1400-GCST90201001 | F-strong vegetable liking (derived food-liking factor) \|\| id:ebi-fl187-GCST90094852 | Inverse variance weighted | 26 | 0.0897277599181104 | 0.0595883090577629 | 0.13211988450504 | -0.027065326 | 0.206520845671326 | 1.09387644615211 | 0.973297657965223 | 1.2293933614799 | 0.998735809224318 |
| ebi-fl187-GCST90094852 | ebi-met1400-GCST90201005 | Fructose to maltose ratio \|\| id:ebi-met1400-GCST90201005 | F-strong vegetable liking (derived food-liking factor) \|\| id:ebi-fl187-GCST90094852 | Inverse variance weighted | 26 | 0.0232207647075483 | 0.0552274837109711 | 0.674151865925404 | -0.085025103 | 0.131466632781052 | 1.0234924656229 | 0.918489226940859 | 1.1404998517792 | 0.998735809224318 |
| ebi-fl187-GCST90094852 | ebi-met1400-GCST90201020 | Paraxanthine to 5-acetylamino-6-formylamino-3-methyluracil ratio \|\| id:ebi-met1400-GCST90201020 | F-strong vegetable liking (derived food-liking factor) \|\| id:ebi-fl187-GCST90094852 | Inverse variance weighted | 25 | -0.05306467 | 0.0518638026182637 | 0.306234918607552 | -0.154717723 | 0.0485883828866992 | 0.948318682497579 | 0.856656957590721 | 1.04978815102743 | 0.998735809224318 |
| ebi-fl187-GCST90094852 | ebi-pw11-GCST90275197 | Placental weight (fetal genotype, adjusted for sex) \|\| id:ebi-pw11-GCST90275197 | F-strong vegetable liking (derived food-liking factor) \|\| id:ebi-fl187-GCST90094852 | Inverse variance weighted (multiplicative random effects) | 20 | 0.0121419289610167 | 0.0311505095810593 | 0.69669725566391 | -0.04891307 | 0.073196927739893 | 1.0122159414285 | 0.952263906575393 | 1.07594239895814 | 0.998735809224318 |
| ebi-fl187-GCST90094852 | prot-a-1017 | FAS-associated factor 2 \|\| id:prot-a-1017 | F-strong vegetable liking (derived food-liking factor) \|\| id:ebi-fl187-GCST90094852 | Inverse variance weighted | 27 | -0.075888231 | 0.0866253309042544 | 0.381002166231081 | -0.245673879 | 0.0938974180464507 | 0.92691980210869 | 0.782177267691715 | 1.09844705929225 | 0.998735809224318 |
| ebi-fl187-GCST90094852 | prot-a-1019 | Fas apoptotic inhibitory molecule 3 \|\| id:prot-a-1019 | F-strong vegetable liking (derived food-liking factor) \|\| id:ebi-fl187-GCST90094852 | Inverse variance weighted | 27 | 0.066162940171056 | 0.0765753376785977 | 0.387574630448249 | -0.083924722 | 0.216250602021107 | 1.06840078839123 | 0.919500471941556 | 1.24141344073997 | 0.998735809224318 |
| ebi-fl187-GCST90094852 | prot-a-1052 | MIP18 family protein FAM96A \|\| id:prot-a-1052 | F-strong vegetable liking (derived food-liking factor) \|\| id:ebi-fl187-GCST90094852 | Inverse variance weighted | 27 | -0.06933204 | 0.0790609018981997 | 0.380516670245244 | -0.224291407 | 0.0856273280712744 | 0.933016830058855 | 0.799082241983449 | 1.08940026374795 | 0.998735809224318 |
| ebi-fl187-GCST90094852 | prot-a-1066 | F-box/LRR-repeat protein 4 \|\| id:prot-a-1066 | F-strong vegetable liking (derived food-liking factor) \|\| id:ebi-fl187-GCST90094852 | Inverse variance weighted | 27 | 0.0432372657239546 | 0.0761752829132372 | 0.570305045592392 | -0.106066289 | 0.1925408202339 | 1.04418561491758 | 0.899365028283997 | 1.21232599012789 | 0.998735809224318 |
| ebi-fl187-GCST90094852 | prot-a-1076 | Low affinity immunoglobulin gamma Fc region receptor III-B \|\| id:prot-a-1076 | F-strong vegetable liking (derived food-liking factor) \|\| id:ebi-fl187-GCST90094852 | Inverse variance weighted | 27 | -0.057839351 | 0.0832110929983167 | 0.486997751001893 | -0.220933094 | 0.105254390905488 | 0.943801555684412 | 0.801770322023147 | 1.11099320097633 | 0.998735809224318 |
| ebi-fl187-GCST90094852 | prot-a-1081 | Fc receptor-like protein 3 \|\| id:prot-a-1081 | F-strong vegetable liking (derived food-liking factor) \|\| id:ebi-fl187-GCST90094852 | Inverse variance weighted | 27 | 0.140871527285766 | 0.0801538339192022 | 0.0788301484047494 | -0.016229987 | 0.297973041767402 | 1.15127673083684 | 0.983901009397841 | 1.34712547126825 | 0.998735809224318 |
| ebi-fl187-GCST90094852 | prot-a-1105 | Fibroblast growth factor receptor 4 \|\| id:prot-a-1105 | F-strong vegetable liking (derived food-liking factor) \|\| id:ebi-fl187-GCST90094852 | Inverse variance weighted | 27 | 0.0291661832987937 | 0.0847596862960734 | 0.730767753600823 | -0.136962802 | 0.195295168439097 | 1.02959568186564 | 0.872002662422061 | 1.21566976088347 | 0.998735809224318 |
| ebi-fl187-GCST90094852 | prot-a-1143 | Follistatin-related protein 1 \|\| id:prot-a-1143 | F-strong vegetable liking (derived food-liking factor) \|\| id:ebi-fl187-GCST90094852 | Inverse variance weighted (multiplicative random effects) | 27 | 0.057158096480801 | 0.0981919501439207 | 0.560495468115629 | -0.135298126 | 0.249614318762886 | 1.05882319338449 | 0.873455473255405 | 1.28353028766367 | 0.998735809224318 |
| ebi-fl187-GCST90094852 | prot-a-1151 | Alpha-(1,3)-fucosyltransferase 10 \|\| id:prot-a-1151 | F-strong vegetable liking (derived food-liking factor) \|\| id:ebi-fl187-GCST90094852 | Inverse variance weighted | 27 | 0.0886921172821582 | 0.0766969421318219 | 0.247518789518428 | -0.061633889 | 0.239018123860529 | 1.09274416748525 | 0.940227050973501 | 1.27000155370629 | 0.998735809224318 |
| ebi-fl187-GCST90094852 | prot-a-1165 | Growth arrest and DNA damage-inducible proteins-interacting protein 1 \|\| id:prot-a-1165 | F-strong vegetable liking (derived food-liking factor) \|\| id:ebi-fl187-GCST90094852 | Inverse variance weighted | 27 | 0.202542132541804 | 0.0876898490900662 | 0.0209016807705637 | 0.0306700283252743 | 0.374414236758334 | 1.22451167582445 | 1.03114519903592 | 1.45413938369914 | 0.998735809224318 |
| ebi-fl187-GCST90094852 | prot-a-1194 | Growth/differentiation factor 11/8 \|\| id:prot-a-1194 | F-strong vegetable liking (derived food-liking factor) \|\| id:ebi-fl187-GCST90094852 | Inverse variance weighted | 27 | -0.046120923 | 0.0868287525600453 | 0.59530022500736 | -0.216305278 | 0.124063431915325 | 0.954926482535979 | 0.805489366056586 | 1.13208767921149 | 0.998735809224318 |
| ebi-fl187-GCST90094852 | prot-a-1216 | D-glucuronyl C5-epimerase \|\| id:prot-a-1216 | F-strong vegetable liking (derived food-liking factor) \|\| id:ebi-fl187-GCST90094852 | Inverse variance weighted | 27 | 0.0219011660634348 | 0.0761752829132372 | 0.7737217408759 | -0.127402388 | 0.17120472057338 | 1.02214275708564 | 0.880379346835929 | 1.18673367295308 | 0.998735809224318 |
| ebi-fl187-GCST90094852 | prot-a-1246 | Glypican-3 \|\| id:prot-a-1246 | F-strong vegetable liking (derived food-liking factor) \|\| id:ebi-fl187-GCST90094852 | Inverse variance weighted (multiplicative random effects) | 27 | 0.0450030075392564 | 0.0968075620761905 | 0.642023991954348 | -0.144739814 | 0.23474582920859 | 1.0460310058833 | 0.865247388970689 | 1.26458730672494 | 0.998735809224318 |
| ebi-fl187-GCST90094852 | prot-a-1275 | Glutamate receptor 4 \|\| id:prot-a-1275 | F-strong vegetable liking (derived food-liking factor) \|\| id:ebi-fl187-GCST90094852 | Inverse variance weighted | 27 | -0.025805736 | 0.088159679969853 | 0.76973935503418 | -0.198598708 | 0.146987237231719 | 0.974524386705064 | 0.819878837940712 | 1.15833917932098 | 0.998735809224318 |
| ebi-fl187-GCST90094852 | prot-a-1289 | Glutathione S-transferase P \|\| id:prot-a-1289 | F-strong vegetable liking (derived food-liking factor) \|\| id:ebi-fl187-GCST90094852 | Inverse variance weighted | 27 | 0.0883607106518554 | 0.0795369983077389 | 0.266594838074753 | -0.067531806 | 0.244253227335024 | 1.09238208482453 | 0.934697991109668 | 1.2766675766885 | 0.998735809224318 |
| ebi-fl187-GCST90094852 | prot-a-130 | Apolipoprotein D \|\| id:prot-a-130 | F-strong vegetable liking (derived food-liking factor) \|\| id:ebi-fl187-GCST90094852 | Inverse variance weighted | 27 | 0.146243224907948 | 0.0854902810837873 | 0.087147308189545 | -0.021317726 | 0.313804175832171 | 1.1574776812188 | 0.978907890649542 | 1.36862170109863 | 0.998735809224318 |
| ebi-fl187-GCST90094852 | prot-a-1313 | Heparin-binding EGF-like growth factor \|\| id:prot-a-1313 | F-strong vegetable liking (derived food-liking factor) \|\| id:ebi-fl187-GCST90094852 | Inverse variance weighted | 27 | 0.0128804842972051 | 0.0886992056657214 | 0.884540863745661 | -0.160969959 | 0.186730927402019 | 1.01296379504506 | 0.851317645320178 | 1.20530292742397 | 0.998735809224318 |
| ebi-fl187-GCST90094852 | prot-a-1347 | HLA class II histocompatibility antigen, DQ alpha 2 chain \|\| id:prot-a-1347 | F-strong vegetable liking (derived food-liking factor) \|\| id:ebi-fl187-GCST90094852 | Inverse variance weighted (multiplicative random effects) | 27 | -0.074392892 | 0.105883156588213 | 0.482308674466227 | -0.281923879 | 0.133138094605292 | 0.928306897544008 | 0.754331102619237 | 1.14240774778548 | 0.998735809224318 |
| ebi-fl187-GCST90094852 | prot-a-1357 | Non-histone chromosomal protein HMG-14 \|\| id:prot-a-1357 | F-strong vegetable liking (derived food-liking factor) \|\| id:ebi-fl187-GCST90094852 | Inverse variance weighted | 27 | -0.270882188 | 0.0812041899294577 | 0.000850496140563221 | -0.430042401 | -0.111721976 | 0.762706346800057 | 0.650481513319914 | 0.894292857732595 | 0.998735809224318 |
| ebi-fl187-GCST90094852 | prot-a-137 | Apolipoprotein M \|\| id:prot-a-137 | F-strong vegetable liking (derived food-liking factor) \|\| id:ebi-fl187-GCST90094852 | Inverse variance weighted | 27 | 0.0132477986913664 | 0.0780300295712633 | 0.865184564586597 | -0.139691059 | 0.166186656651042 | 1.01333593957058 | 0.869626857060101 | 1.18079348411203 | 0.998735809224318 |
| ebi-fl187-GCST90094852 | prot-a-1381 | 17-beta-hydroxysteroid dehydrogenase 14 \|\| id:prot-a-1381 | F-strong vegetable liking (derived food-liking factor) \|\| id:ebi-fl187-GCST90094852 | Inverse variance weighted | 27 | 0.0839701502386669 | 0.084908982815768 | 0.322690995696188 | -0.082451456 | 0.250391756557572 | 1.08759642883047 | 0.920856138738329 | 1.28452854060943 | 0.998735809224318 |
| ebi-fl187-GCST90094852 | prot-a-1392 | Serine protease HTRA2, mitochondrial \|\| id:prot-a-1392 | F-strong vegetable liking (derived food-liking factor) \|\| id:ebi-fl187-GCST90094852 | Inverse variance weighted | 27 | 0.0505668880318396 | 0.0761752829132372 | 0.506803744302335 | -0.098736666 | 0.199870442541785 | 1.05186721833001 | 0.905981251847697 | 1.22124452657363 | 0.998735809224318 |
| ebi-fl187-GCST90094852 | prot-a-1416 | Interferon-induced protein with tetratricopeptide repeats 2 \|\| id:prot-a-1416 | F-strong vegetable liking (derived food-liking factor) \|\| id:ebi-fl187-GCST90094852 | Inverse variance weighted (multiplicative random effects) | 27 | -0.109489171 | 0.0971729786014174 | 0.259850298467392 | -0.299948209 | 0.0809698675208163 | 0.89629187066834 | 0.740856589690511 | 1.08433822227557 | 0.998735809224318 |
| ebi-fl187-GCST90094852 | prot-a-1428 | Interferon gamma \|\| id:prot-a-1428 | F-strong vegetable liking (derived food-liking factor) \|\| id:ebi-fl187-GCST90094852 | Inverse variance weighted | 27 | 0.0156184387628022 | 0.0804277211541789 | 0.846025364311762 | -0.142019895 | 0.173256772224993 | 1.0157410440469 | 0.867603995593534 | 1.18917141207455 | 0.998735809224318 |
| ebi-fl187-GCST90094852 | prot-a-1483 | Interleukin-17C \|\| id:prot-a-1483 | F-strong vegetable liking (derived food-liking factor) \|\| id:ebi-fl187-GCST90094852 | Inverse variance weighted (multiplicative random effects) | 27 | 0.0406297351842421 | 0.0939013587905733 | 0.665243429274894 | -0.143416928 | 0.224676398413766 | 1.04146641577566 | 0.866392770138831 | 1.25191752813771 | 0.998735809224318 |
| ebi-fl187-GCST90094852 | prot-a-1503 | Interleukin-1 receptor-like 2 \|\| id:prot-a-1503 | F-strong vegetable liking (derived food-liking factor) \|\| id:ebi-fl187-GCST90094852 | Inverse variance weighted | 27 | -0.091950799 | 0.0761752829132372 | 0.227395582387914 | -0.241254353 | 0.0573527557634013 | 0.912150027365746 | 0.785641770429674 | 1.0590293232096 | 0.998735809224318 |
| ebi-fl187-GCST90094852 | prot-a-1522 | Interleukin-31 receptor subunit alpha \|\| id:prot-a-1522 | F-strong vegetable liking (derived food-liking factor) \|\| id:ebi-fl187-GCST90094852 | Inverse variance weighted | 27 | -0.052133774 | 0.0829495015804884 | 0.529676349322626 | -0.214714797 | 0.110447249026495 | 0.949201879749385 | 0.806771500884792 | 1.11677743640132 | 0.998735809224318 |
| ebi-fl187-GCST90094852 | prot-a-1540 | Interleukin-6 receptor subunit alpha \|\| id:prot-a-1540 | F-strong vegetable liking (derived food-liking factor) \|\| id:ebi-fl187-GCST90094852 | Inverse variance weighted | 27 | 0.0209802922094263 | 0.0761752829132372 | 0.782992614273798 | -0.128323262 | 0.170283846719371 | 1.02120192580552 | 0.879569001683915 | 1.18564134396778 | 0.998735809224318 |
| ebi-fl187-GCST90094852 | prot-a-1541 | Interleukin-6 receptor subunit alpha \|\| id:prot-a-1541 | F-strong vegetable liking (derived food-liking factor) \|\| id:ebi-fl187-GCST90094852 | Inverse variance weighted | 27 | 0.00610758183871267 | 0.0761752829132372 | 0.936095687222297 | -0.143195973 | 0.155411136348658 | 1.00612627114612 | 0.866584225428189 | 1.16813812643568 | 0.998735809224318 |
| ebi-fl187-GCST90094852 | prot-a-1545 | Interleukin-9 \|\| id:prot-a-1545 | F-strong vegetable liking (derived food-liking factor) \|\| id:ebi-fl187-GCST90094852 | Inverse variance weighted | 27 | 0.142007669641513 | 0.0761752829132372 | 0.0622904772927655 | -0.007295885 | 0.291311224151458 | 1.15258548842061 | 0.992730665490881 | 1.3381809933925 | 0.998735809224318 |
| ebi-fl187-GCST90094852 | prot-a-1548 | Inositol monophosphatase 3 \|\| id:prot-a-1548 | F-strong vegetable liking (derived food-liking factor) \|\| id:ebi-fl187-GCST90094852 | Inverse variance weighted | 27 | 0.0426175061127541 | 0.0879797380006823 | 0.628101042358729 | -0.12982278 | 0.215057792594091 | 1.04353867134231 | 0.878251060459075 | 1.239933554 | 0.998735809224318 |
| ebi-fl187-GCST90094852 | prot-a-1555 | Inhibin beta A chain:Inhibin beta B chain heterodimer \|\| id:prot-a-1555 | F-strong vegetable liking (derived food-liking factor) \|\| id:ebi-fl187-GCST90094852 | Inverse variance weighted | 27 | -0.100937913 | 0.08613366037745 | 0.241247107076576 | -0.269759887 | 0.0678840613835007 | 0.903989157157855 | 0.763562813459082 | 1.07024121899928 | 0.998735809224318 |
| ebi-fl187-GCST90094852 | prot-a-1603 | Junctophilin-3 \|\| id:prot-a-1603 | F-strong vegetable liking (derived food-liking factor) \|\| id:ebi-fl187-GCST90094852 | Inverse variance weighted (multiplicative random effects) | 27 | -0.111673049 | 0.100413311018578 | 0.266080574329036 | -0.308483139 | 0.0851370403771372 | 0.894336613758798 | 0.734560337651994 | 1.08886627511937 | 0.998735809224318 |
| ebi-fl187-GCST90094852 | prot-a-1627 | UPF0258 protein KIAA1024 \|\| id:prot-a-1627 | F-strong vegetable liking (derived food-liking factor) \|\| id:ebi-fl187-GCST90094852 | Inverse variance weighted | 27 | -0.042008834 | 0.0784974230169912 | 0.592537872800714 | -0.195863783 | 0.111846114889088 | 0.958861309739266 | 0.82212421406787 | 1.11834075141229 | 0.998735809224318 |
| ebi-fl187-GCST90094852 | prot-a-1666 | Kallikrein-7 \|\| id:prot-a-1666 | F-strong vegetable liking (derived food-liking factor) \|\| id:ebi-fl187-GCST90094852 | Inverse variance weighted | 27 | 0.0108153461217015 | 0.0859658055468565 | 0.899882435159758 | -0.157677633 | 0.17930832499354 | 1.01087404339716 | 0.854125079545568 | 1.19638956411138 | 0.998735809224318 |
| ebi-fl187-GCST90094852 | prot-a-1678 | Importin subunit alpha-3 \|\| id:prot-a-1678 | F-strong vegetable liking (derived food-liking factor) \|\| id:ebi-fl187-GCST90094852 | Inverse variance weighted | 27 | -0.066833952 | 0.0761752829132372 | 0.380285388708186 | -0.216137506 | 0.0824696025341281 | 0.935350501545474 | 0.805624515660751 | 1.08596566233317 | 0.998735809224318 |
| ebi-fl187-GCST90094852 | prot-a-1718 | Protein LDOC1 \|\| id:prot-a-1718 | F-strong vegetable liking (derived food-liking factor) \|\| id:ebi-fl187-GCST90094852 | Inverse variance weighted | 27 | 0.046433367320852 | 0.0761752829132372 | 0.542153647281007 | -0.102870187 | 0.195736921830797 | 1.04752827712743 | 0.902244088721104 | 1.21620690575757 | 0.998735809224318 |
| ebi-fl187-GCST90094852 | prot-a-1723 | Leptin \|\| id:prot-a-1723 | F-strong vegetable liking (derived food-liking factor) \|\| id:ebi-fl187-GCST90094852 | Inverse variance weighted | 26 | -0.227511118 | 0.0772993557639285 | 0.0032478889042287 | -0.379017855 | -0.07600438 | 0.796513566062054 | 0.68453339022621 | 0.926812146754793 | 0.998735809224318 |
| ebi-fl187-GCST90094852 | prot-a-1730 | Galectin-7 \|\| id:prot-a-1730 | F-strong vegetable liking (derived food-liking factor) \|\| id:ebi-fl187-GCST90094852 | Inverse variance weighted | 26 | 0.0449075845795434 | 0.0938043113945026 | 0.632125816426808 | -0.138948866 | 0.228764034912769 | 1.04593119527095 | 0.870272528050329 | 1.25704538518727 | 0.998735809224318 |
| ebi-fl187-GCST90094852 | prot-a-1753 | Lipase member N \|\| id:prot-a-1753 | F-strong vegetable liking (derived food-liking factor) \|\| id:ebi-fl187-GCST90094852 | Inverse variance weighted | 27 | 0.0404023199761031 | 0.078895649956927 | 0.608582287390472 | -0.114233154 | 0.19503779389168 | 1.04122959740301 | 0.892049946674993 | 1.21535691868948 | 0.998735809224318 |
| ebi-fl187-GCST90094852 | prot-a-1756 | VIP36-like protein \|\| id:prot-a-1756 | F-strong vegetable liking (derived food-liking factor) \|\| id:ebi-fl187-GCST90094852 | Inverse variance weighted | 27 | 0.0612288620865292 | 0.0761752829132372 | 0.421518785934135 | -0.088074692 | 0.210532416596474 | 1.06314219928877 | 0.915692478878606 | 1.23433506551538 | 0.998735809224318 |
| ebi-fl187-GCST90094852 | prot-a-1760 | Ig Kappa chain V-I region HK102- like \|\| id:prot-a-1760 | F-strong vegetable liking (derived food-liking factor) \|\| id:ebi-fl187-GCST90094852 | Inverse variance weighted | 26 | -0.036648251 | 0.0772993557639286 | 0.635422818982676 | -0.188154988 | 0.114858486195193 | 0.964015166991096 | 0.828486291517212 | 1.12171468822615 | 0.998735809224318 |
| ebi-fl187-GCST90094852 | prot-a-179 | Neutral ceramidase \|\| id:prot-a-179 | F-strong vegetable liking (derived food-liking factor) \|\| id:ebi-fl187-GCST90094852 | Inverse variance weighted | 27 | 0.116176549373022 | 0.0761683430909296 | 0.127194187166937 | -0.033113403 | 0.265466501831244 | 1.12319415385313 | 0.967428843952488 | 1.30403917056643 | 0.998735809224318 |
| ebi-fl187-GCST90094852 | prot-a-1809 | Lactotransferrin \|\| id:prot-a-1809 | F-strong vegetable liking (derived food-liking factor) \|\| id:ebi-fl187-GCST90094852 | Inverse variance weighted | 27 | -0.036709087 | 0.0761752829132372 | 0.629874953886889 | -0.186012641 | 0.112594467765362 | 0.963956522292918 | 0.83026309934843 | 1.11917797816172 | 0.998735809224318 |
| ebi-fl187-GCST90094852 | prot-a-1818 | Lysophospholipase-like protein 1 \|\| id:prot-a-1818 | F-strong vegetable liking (derived food-liking factor) \|\| id:ebi-fl187-GCST90094852 | Inverse variance weighted | 27 | -0.031465349 | 0.084836090158818 | 0.710715151358006 | -0.197744085 | 0.134813388075675 | 0.969024533894212 | 0.820579824670467 | 1.14432322006697 | 0.998735809224318 |
| ebi-fl187-GCST90094852 | prot-a-1852 | MAP kinase-activated protein kinase 3 \|\| id:prot-a-1852 | F-strong vegetable liking (derived food-liking factor) \|\| id:ebi-fl187-GCST90094852 | Inverse variance weighted | 27 | -0.096408216 | 0.0789933756468227 | 0.222290734324913 | -0.251235232 | 0.0584188006830583 | 0.908093242582101 | 0.777839377439086 | 1.06015889802115 | 0.998735809224318 |
| ebi-fl187-GCST90094852 | prot-a-1856 | Megakaryocyte-associated tyrosine-protein kinase \|\| id:prot-a-1856 | F-strong vegetable liking (derived food-liking factor) \|\| id:ebi-fl187-GCST90094852 | Inverse variance weighted | 27 | -0.05693165 | 0.080206798265176 | 0.477821482089316 | -0.214136975 | 0.10027367440839 | 0.944658634396891 | 0.807237806219726 | 1.10547341646397 | 0.998735809224318 |
| ebi-fl187-GCST90094852 | prot-a-1890 | Beta-1,3-N-acetylglucosaminyltransferase manic fringe \|\| id:prot-a-1890 | F-strong vegetable liking (derived food-liking factor) \|\| id:ebi-fl187-GCST90094852 | Inverse variance weighted | 27 | -0.065907973 | 0.0763731025193977 | 0.388151968554218 | -0.215599254 | 0.0837833078190219 | 0.936217017459412 | 0.806058261734929 | 1.08739323866497 | 0.998735809224318 |
| ebi-fl187-GCST90094852 | prot-a-1919 | Matrilysin \|\| id:prot-a-1919 | F-strong vegetable liking (derived food-liking factor) \|\| id:ebi-fl187-GCST90094852 | Inverse variance weighted | 27 | -0.045131485 | 0.0761752829132372 | 0.553536651477615 | -0.194435039 | 0.104172069695548 | 0.95587179094504 | 0.823299658621537 | 1.1097913999555 | 0.998735809224318 |
| ebi-fl187-GCST90094852 | prot-a-1923 | Multimerin-2 \|\| id:prot-a-1923 | F-strong vegetable liking (derived food-liking factor) \|\| id:ebi-fl187-GCST90094852 | Inverse variance weighted | 27 | 0.00485375858759461 | 0.0761752829132372 | 0.949194542906594 | -0.144449796 | 0.15415731309754 | 1.00486555715522 | 0.865498362859417 | 1.16667440550766 | 0.998735809224318 |
| ebi-fl187-GCST90094852 | prot-a-1948 | Mesothelin \|\| id:prot-a-1948 | F-strong vegetable liking (derived food-liking factor) \|\| id:ebi-fl187-GCST90094852 | Inverse variance weighted | 27 | -0.033499524 | 0.0796035074571512 | 0.673879407342495 | -0.189522399 | 0.12252335062339 | 0.967055371556781 | 0.827354185107313 | 1.13034551403825 | 0.998735809224318 |
| ebi-fl187-GCST90094852 | prot-a-195 | ATPase family AAA domain-containing protein 1 \|\| id:prot-a-195 | F-strong vegetable liking (derived food-liking factor) \|\| id:ebi-fl187-GCST90094852 | Inverse variance weighted | 27 | -0.080580035 | 0.0761752829132372 | 0.290135733597679 | -0.22988359 | 0.0687235194959859 | 0.922581061840671 | 0.794626099922006 | 1.07114001887253 | 0.998735809224318 |
| ebi-fl187-GCST90094852 | prot-a-1966 | Mucin-1 \|\| id:prot-a-1966 | F-strong vegetable liking (derived food-liking factor) \|\| id:ebi-fl187-GCST90094852 | Inverse variance weighted | 27 | 0.0550346726215256 | 0.0761652331449943 | 0.469944426793038 | -0.094249184 | 0.204318529585714 | 1.05657724834344 | 0.910055962287693 | 1.22668882791638 | 0.998735809224318 |
| ebi-fl187-GCST90094852 | prot-a-1986 | Myocardial zonula adherens protein \|\| id:prot-a-1986 | F-strong vegetable liking (derived food-liking factor) \|\| id:ebi-fl187-GCST90094852 | Inverse variance weighted | 27 | -0.065415032 | 0.0761752829132372 | 0.390482024448255 | -0.214718586 | 0.0838885225643284 | 0.936678631139385 | 0.806768443802193 | 1.08750765448663 | 0.998735809224318 |
| ebi-fl187-GCST90094852 | prot-a-2071 | Epididymal secretory protein E1 \|\| id:prot-a-2071 | F-strong vegetable liking (derived food-liking factor) \|\| id:ebi-fl187-GCST90094852 | Inverse variance weighted (multiplicative random effects) | 26 | -0.023620007 | 0.0961214047268302 | 0.805890491156586 | -0.21201796 | 0.164777946576591 | 0.976656762292498 | 0.808950168729359 | 1.17913125950628 | 0.998735809224318 |
| ebi-fl187-GCST90094852 | prot-a-2098 | Neuritin \|\| id:prot-a-2098 | F-strong vegetable liking (derived food-liking factor) \|\| id:ebi-fl187-GCST90094852 | Inverse variance weighted | 27 | -0.144336458 | 0.0776532054491481 | 0.0630652072855104 | -0.296536741 | 0.00786382424254964 | 0.865596461825803 | 0.743388313844503 | 1.00789482531746 | 0.998735809224318 |
| ebi-fl187-GCST90094852 | prot-a-2115 | Neurotrophin-4 \|\| id:prot-a-2115 | F-strong vegetable liking (derived food-liking factor) \|\| id:ebi-fl187-GCST90094852 | Inverse variance weighted | 27 | 0.0539162713809614 | 0.0906909850599783 | 0.552174197752393 | -0.123838059 | 0.231670602098519 | 1.05539623158655 | 0.883522907584067 | 1.26070438704625 | 0.998735809224318 |
| ebi-fl187-GCST90094852 | prot-a-2118 | Netrin-1 \|\| id:prot-a-2118 | F-strong vegetable liking (derived food-liking factor) \|\| id:ebi-fl187-GCST90094852 | Inverse variance weighted | 27 | -0.071679528 | 0.0761752829132372 | 0.34671436961666 | -0.220983082 | 0.077624026966185 | 0.930829153122068 | 0.80173024380467 | 1.08071626210604 | 0.998735809224318 |
| ebi-fl187-GCST90094852 | prot-a-2120 | Netrin-G1 \|\| id:prot-a-2120 | F-strong vegetable liking (derived food-liking factor) \|\| id:ebi-fl187-GCST90094852 | Inverse variance weighted | 27 | -0.026439298 | 0.0793719067059233 | 0.739053889475649 | -0.182008236 | 0.129129638729171 | 0.973907159750089 | 0.833594475271777 | 1.13783762242816 | 0.998735809224318 |
| ebi-fl187-GCST90094852 | prot-a-2145 | Olfactomedin-like protein 3 \|\| id:prot-a-2145 | F-strong vegetable liking (derived food-liking factor) \|\| id:ebi-fl187-GCST90094852 | Inverse variance weighted | 27 | 0.04758515496334 | 0.082090782934658 | 0.562140539840131 | -0.11331278 | 0.20848308951527 | 1.04873550235227 | 0.892871344504384 | 1.23180809941276 | 0.998735809224318 |
| ebi-fl187-GCST90094852 | prot-a-2162 | Prolyl 4-hydroxylase subunit alpha-2 \|\| id:prot-a-2162 | F-strong vegetable liking (derived food-liking factor) \|\| id:ebi-fl187-GCST90094852 | Inverse variance weighted | 27 | 0.0403345621728001 | 0.0859876548148182 | 0.639016834377797 | -0.128201241 | 0.208870365609844 | 1.04115904836291 | 0.879676334153252 | 1.23228524162969 | 0.998735809224318 |
| ebi-fl187-GCST90094852 | prot-a-2168 | Polyadenylate-binding protein-interacting protein 1 \|\| id:prot-a-2168 | F-strong vegetable liking (derived food-liking factor) \|\| id:ebi-fl187-GCST90094852 | Inverse variance weighted | 26 | 0.0959653174600058 | 0.0857550162560213 | 0.263113006000368 | -0.072114514 | 0.264045149321808 | 1.10072088753575 | 0.930424342723367 | 1.30218698783305 | 0.998735809224318 |
| ebi-fl187-GCST90094852 | prot-a-219 | Beta-1,4-galactosyltransferase 5 \|\| id:prot-a-219 | F-strong vegetable liking (derived food-liking factor) \|\| id:ebi-fl187-GCST90094852 | Inverse variance weighted | 27 | 0.0554703774061038 | 0.0761652331449943 | 0.466436084432939 | -0.09381348 | 0.204754234370293 | 1.05703770440999 | 0.910452564419144 | 1.22722341856128 | 0.998735809224318 |
| ebi-fl187-GCST90094852 | prot-a-2190 | Pyruvate carboxylase, mitochondrial \|\| id:prot-a-2190 | F-strong vegetable liking (derived food-liking factor) \|\| id:ebi-fl187-GCST90094852 | Inverse variance weighted | 27 | 0.0179207383324587 | 0.079884819089441 | 0.822498811735951 | -0.138653507 | 0.174494983747763 | 1.01808227829263 | 0.870529608551087 | 1.19064476979554 | 0.998735809224318 |
| ebi-fl187-GCST90094852 | prot-a-2197 | Protocadherin-9 \|\| id:prot-a-2197 | F-strong vegetable liking (derived food-liking factor) \|\| id:ebi-fl187-GCST90094852 | Inverse variance weighted | 27 | 0.116916222066438 | 0.076166166324255 | 0.124780097530711 | -0.032369464 | 0.266201908061977 | 1.12402525723248 | 0.968148819926196 | 1.30499852181079 | 0.998735809224318 |
| ebi-fl187-GCST90094852 | prot-a-2198 | Protocadherin alpha-4 \|\| id:prot-a-2198 | F-strong vegetable liking (derived food-liking factor) \|\| id:ebi-fl187-GCST90094852 | Inverse variance weighted | 27 | 0.0692838552554625 | 0.0788537044651578 | 0.379597625906928 | -0.085269405 | 0.223837116007172 | 1.07174038511614 | 0.918264865473176 | 1.25086725658071 | 0.998735809224318 |
| ebi-fl187-GCST90094852 | prot-a-2254 | Placenta growth factor \|\| id:prot-a-2254 | F-strong vegetable liking (derived food-liking factor) \|\| id:ebi-fl187-GCST90094852 | Inverse variance weighted | 27 | -0.059656507 | 0.0761752829132372 | 0.433540309117202 | -0.208960062 | 0.0896470473217283 | 0.942088078496422 | 0.811427642038367 | 1.09378816010697 | 0.998735809224318 |
| ebi-fl187-GCST90094852 | prot-a-2261 | Membrane-associated progesterone receptor component 2 \|\| id:prot-a-2261 | F-strong vegetable liking (derived food-liking factor) \|\| id:ebi-fl187-GCST90094852 | Inverse variance weighted | 27 | 0.0336734208497802 | 0.0852339700966553 | 0.692790914137362 | -0.133385161 | 0.200732002239225 | 1.03424678813168 | 0.87512796243013 | 1.22229715502446 | 0.998735809224318 |
| ebi-fl187-GCST90094852 | prot-a-2268 | Polymeric immunoglobulin receptor \|\| id:prot-a-2268 | F-strong vegetable liking (derived food-liking factor) \|\| id:ebi-fl187-GCST90094852 | Inverse variance weighted (multiplicative random effects) | 27 | 0.00523967117198225 | 0.0951097337500979 | 0.956066126792131 | -0.181175407 | 0.191654749322174 | 1.00525342225554 | 0.834289005747049 | 1.21125225910368 | 0.998735809224318 |
| ebi-fl187-GCST90094852 | prot-a-2283 | Group 10 secretory phospholipase A2 \|\| id:prot-a-2283 | F-strong vegetable liking (derived food-liking factor) \|\| id:ebi-fl187-GCST90094852 | Inverse variance weighted | 27 | 0.193097689527111 | 0.0908553244280656 | 0.0335589555881361 | 0.0150212536481024 | 0.37117412540612 | 1.21300128525327 | 1.01513463970085 | 1.44943543494848 | 0.998735809224318 |
| ebi-fl187-GCST90094852 | prot-a-2298 | Inactive phospholipase D5 \|\| id:prot-a-2298 | F-strong vegetable liking (derived food-liking factor) \|\| id:ebi-fl187-GCST90094852 | Inverse variance weighted | 27 | 0.112877911517943 | 0.0883232499562649 | 0.201246240292075 | -0.060235658 | 0.285991481432223 | 1.11949524712948 | 0.941542625012874 | 1.33108111629929 | 0.998735809224318 |
| ebi-fl187-GCST90094852 | prot-a-2304 | Procollagen-lysine,2-oxoglutarate 5-dioxygenase 2 \|\| id:prot-a-2304 | F-strong vegetable liking (derived food-liking factor) \|\| id:ebi-fl187-GCST90094852 | Inverse variance weighted | 27 | -0.017981703 | 0.0828844718444492 | 0.828248093122398 | -0.180435268 | 0.144471861823851 | 0.98217900313169 | 0.834906724291468 | 1.15542918283646 | 0.998735809224318 |
| ebi-fl187-GCST90094852 | prot-a-2331 | Periostin \|\| id:prot-a-2331 | F-strong vegetable liking (derived food-liking factor) \|\| id:ebi-fl187-GCST90094852 | Inverse variance weighted | 27 | -0.063833305 | 0.092969420627529 | 0.492331432763224 | -0.246053369 | 0.118386759385494 | 0.938161373265415 | 0.781880495260851 | 1.12567939425783 | 0.998735809224318 |
| ebi-fl187-GCST90094852 | prot-a-2333 | Periostin \|\| id:prot-a-2333 | F-strong vegetable liking (derived food-liking factor) \|\| id:ebi-fl187-GCST90094852 | Inverse variance weighted (multiplicative random effects) | 27 | -0.00829481 | 0.0982635371285276 | 0.932727341441242 | -0.200891343 | 0.184301722559696 | 0.99173949680375 | 0.818001308306368 | 1.2023785531064 | 0.998735809224318 |
| ebi-fl187-GCST90094852 | prot-a-2367 | cAMP-dependent protein kinase catalytic subunit alpha \|\| id:prot-a-2367 | F-strong vegetable liking (derived food-liking factor) \|\| id:ebi-fl187-GCST90094852 | Inverse variance weighted | 27 | -0.031803018 | 0.0837465317689404 | 0.704128582335251 | -0.195946221 | 0.132340183992668 | 0.96869737896807 | 0.822056443239729 | 1.1414965720863 | 0.998735809224318 |
| ebi-fl187-GCST90094852 | prot-a-2385 | Phosphoribosyl pyrophosphate synthase-associated protein 1 \|\| id:prot-a-2385 | F-strong vegetable liking (derived food-liking factor) \|\| id:ebi-fl187-GCST90094852 | Inverse variance weighted | 27 | -0.027783882 | 0.0862181249215068 | 0.747262504451622 | -0.196771407 | 0.141203642574889 | 0.972598539876774 | 0.821378373103849 | 1.15165915094143 | 0.998735809224318 |
| ebi-fl187-GCST90094852 | prot-a-2393 | Trypsin-3 \|\| id:prot-a-2393 | F-strong vegetable liking (derived food-liking factor) \|\| id:ebi-fl187-GCST90094852 | Inverse variance weighted | 26 | 0.0247491346967455 | 0.0851308165762965 | 0.771266339375035 | -0.142107266 | 0.191605535186287 | 1.02505793679637 | 0.867528195395216 | 1.21119264983722 | 0.998735809224318 |
| ebi-fl187-GCST90094852 | prot-a-2405 | Pregnancy-specific beta-1-glycoprotein 6 \|\| id:prot-a-2405 | F-strong vegetable liking (derived food-liking factor) \|\| id:ebi-fl187-GCST90094852 | Inverse variance weighted | 27 | -0.126168198 | 0.0761752829132372 | 0.0976635323462329 | -0.275471753 | 0.0231353561371924 | 0.881466573070841 | 0.759213877394616 | 1.02340505432753 | 0.998735809224318 |
| ebi-fl187-GCST90094852 | prot-a-244 | Beclin-1 \|\| id:prot-a-244 | F-strong vegetable liking (derived food-liking factor) \|\| id:ebi-fl187-GCST90094852 | Inverse variance weighted | 26 | -0.062144606 | 0.0773088853940751 | 0.421484669562064 | -0.213670021 | 0.0893808097535175 | 0.93974698427095 | 0.807614837001844 | 1.09349699136883 | 0.998735809224318 |
| ebi-fl187-GCST90094852 | prot-a-2454 | tRNA pseudouridine synthase A, mitochondrial \|\| id:prot-a-2454 | F-strong vegetable liking (derived food-liking factor) \|\| id:ebi-fl187-GCST90094852 | Inverse variance weighted | 27 | 0.0551559279898956 | 0.0761752829132372 | 0.469025252386486 | -0.094147627 | 0.204459482499841 | 1.05670537177457 | 0.910148390282993 | 1.22686174546773 | 0.998735809224318 |
| ebi-fl187-GCST90094852 | prot-a-2466 | Glutaminyl-peptide cyclotransferase-like protein \|\| id:prot-a-2466 | F-strong vegetable liking (derived food-liking factor) \|\| id:ebi-fl187-GCST90094852 | Inverse variance weighted | 27 | 0.0948153808545624 | 0.0764820050800312 | 0.215083284997822 | -0.055089349 | 0.244720110811424 | 1.09945585578736 | 0.946400584133082 | 1.27726377085068 | 0.998735809224318 |
| ebi-fl187-GCST90094852 | prot-a-2474 | Ras-related protein Rab-27A \|\| id:prot-a-2474 | F-strong vegetable liking (derived food-liking factor) \|\| id:ebi-fl187-GCST90094852 | Inverse variance weighted | 27 | -0.146414426 | 0.0808455213677525 | 0.0701347510510435 | -0.304871647 | 0.0120427963770094 | 0.863799648391794 | 0.737217992176708 | 1.01211560282015 | 0.998735809224318 |
| ebi-fl187-GCST90094852 | prot-a-2490 | Rap1 GTPase-GDP dissociation stimulator 1 \|\| id:prot-a-2490 | F-strong vegetable liking (derived food-liking factor) \|\| id:ebi-fl187-GCST90094852 | Inverse variance weighted | 27 | 0.123813137658262 | 0.0761752829132372 | 0.104083414146073 | -0.025490417 | 0.273116692168207 | 1.13180435962501 | 0.974831720877944 | 1.31405357563919 | 0.998735809224318 |
| ebi-fl187-GCST90094852 | prot-a-2545 | Receptor-interacting serine/threonine-protein kinase 2 \|\| id:prot-a-2545 | F-strong vegetable liking (derived food-liking factor) \|\| id:ebi-fl187-GCST90094852 | Inverse variance weighted | 27 | -0.004014573 | 0.0919733885754646 | 0.965183959592811 | -0.184282415 | 0.1762532682922 | 0.995993474310878 | 0.831700885913899 | 1.19274010364893 | 0.998735809224318 |
| ebi-fl187-GCST90094852 | prot-a-2610 | RUN and FYVE domain-containing protein 1 \|\| id:prot-a-2610 | F-strong vegetable liking (derived food-liking factor) \|\| id:ebi-fl187-GCST90094852 | Inverse variance weighted (multiplicative random effects) | 27 | -0.061904502 | 0.103549101438262 | 0.549955030006446 | -0.264860741 | 0.141051736934766 | 0.939972648121775 | 0.767312797976551 | 1.15148422070769 | 0.998735809224318 |
| ebi-fl187-GCST90094852 | prot-a-2627 | Serine--tRNA ligase, mitochondrial \|\| id:prot-a-2627 | F-strong vegetable liking (derived food-liking factor) \|\| id:ebi-fl187-GCST90094852 | Inverse variance weighted | 27 | -0.06101661 | 0.0761699167101254 | 0.423097390923742 | -0.210309647 | 0.0882764263521779 | 0.940807612455768 | 0.810333289723309 | 1.09229001804548 | 0.998735809224318 |
| ebi-fl187-GCST90094852 | prot-a-264 | BolA-like protein 2 \|\| id:prot-a-264 | F-strong vegetable liking (derived food-liking factor) \|\| id:ebi-fl187-GCST90094852 | Inverse variance weighted | 26 | -0.077928609 | 0.0773088853940751 | 0.313446685662721 | -0.229454024 | 0.0735968065719231 | 0.925030463220669 | 0.794967517086816 | 1.07637273158269 | 0.998735809224318 |
| ebi-fl187-GCST90094852 | prot-a-2646 | Secretoglobin family 1C member 1 \|\| id:prot-a-2646 | F-strong vegetable liking (derived food-liking factor) \|\| id:ebi-fl187-GCST90094852 | Inverse variance weighted | 26 | 0.0725583385181624 | 0.0839058396200811 | 0.387171022927838 | -0.091897107 | 0.237013784173521 | 1.07525553307488 | 0.912199003483472 | 1.26745858852399 | 0.998735809224318 |
| ebi-fl187-GCST90094852 | prot-a-2672 | Semaphorin-3E \|\| id:prot-a-2672 | F-strong vegetable liking (derived food-liking factor) \|\| id:ebi-fl187-GCST90094852 | Inverse variance weighted | 27 | -0.036188578 | 0.0919527588087719 | 0.693908725579374 | -0.216415985 | 0.144038829125943 | 0.964458400562586 | 0.805400197452162 | 1.15492895253603 | 0.998735809224318 |
| ebi-fl187-GCST90094852 | prot-a-2701 | Plasma protease C1 inhibitor \|\| id:prot-a-2701 | F-strong vegetable liking (derived food-liking factor) \|\| id:ebi-fl187-GCST90094852 | Inverse variance weighted | 27 | 0.112801943454411 | 0.0761752829132372 | 0.138654332042993 | -0.036501611 | 0.262105497964356 | 1.11941020447373 | 0.964156540585343 | 1.29966364706624 | 0.998735809224318 |
| ebi-fl187-GCST90094852 | prot-a-2738 | Signaling threshold-regulating transmembrane adapter 1 \|\| id:prot-a-2738 | F-strong vegetable liking (derived food-liking factor) \|\| id:ebi-fl187-GCST90094852 | Inverse variance weighted | 27 | -0.111144827 | 0.0761752829132372 | 0.144546720823926 | -0.260448381 | 0.0381587276498215 | 0.894809147145157 | 0.770705938133909 | 1.03889612133172 | 0.998735809224318 |
| ebi-fl187-GCST90094852 | prot-a-274 | Serine/threonine-protein kinase BRSK2 \|\| id:prot-a-274 | F-strong vegetable liking (derived food-liking factor) \|\| id:ebi-fl187-GCST90094852 | Inverse variance weighted | 27 | -0.037550131 | 0.0761752829132372 | 0.622052322092656 | -0.186853685 | 0.111753423702849 | 0.963146133217552 | 0.829565105061519 | 1.11823709588548 | 0.998735809224318 |
| ebi-fl187-GCST90094852 | prot-a-2749 | Mitochondrial glutamate carrier 2 \|\| id:prot-a-2749 | F-strong vegetable liking (derived food-liking factor) \|\| id:ebi-fl187-GCST90094852 | Inverse variance weighted | 27 | 0.0271483389709846 | 0.0778885145162716 | 0.727424305034173 | -0.125513149 | 0.179809827422877 | 1.02752021275006 | 0.882044165927857 | 1.19698970685816 | 0.998735809224318 |
| ebi-fl187-GCST90094852 | prot-a-2760 | Sodium-coupled monocarboxylate transporter 1 \|\| id:prot-a-2760 | F-strong vegetable liking (derived food-liking factor) \|\| id:ebi-fl187-GCST90094852 | Inverse variance weighted | 27 | 0.026924230689304 | 0.0843304743928588 | 0.749521418894086 | -0.138363499 | 0.192211960499307 | 1.02728996276222 | 0.870782105680171 | 1.21192737047311 | 0.998735809224318 |
| ebi-fl187-GCST90094852 | prot-a-2777 | Small integral membrane protein 10 \|\| id:prot-a-2777 | F-strong vegetable liking (derived food-liking factor) \|\| id:ebi-fl187-GCST90094852 | Inverse variance weighted | 27 | 0.146181008186776 | 0.0839397093242292 | 0.0815959640897025 | -0.018340822 | 0.310702838462265 | 1.15740566899285 | 0.981826347221083 | 1.36438371857538 | 0.998735809224318 |
| ebi-fl187-GCST90094852 | prot-a-2799 | Superoxide dismutase [Mn], mitochondrial \|\| id:prot-a-2799 | F-strong vegetable liking (derived food-liking factor) \|\| id:ebi-fl187-GCST90094852 | Inverse variance weighted | 26 | 0.198399771033491 | 0.0826718549181534 | 0.0164019431638798 | 0.0363629353939103 | 0.360436606673071 | 1.21944979709597 | 1.03703215387242 | 1.43395535238182 | 0.998735809224318 |
| ebi-fl187-GCST90094852 | prot-a-2805 | Protein SPACA7 \|\| id:prot-a-2805 | F-strong vegetable liking (derived food-liking factor) \|\| id:ebi-fl187-GCST90094852 | Inverse variance weighted | 27 | 0.095541049012446 | 0.0825317376453741 | 0.247015884510082 | -0.066221157 | 0.257303254797379 | 1.10025398544651 | 0.935923855502588 | 1.29343730835974 | 0.998735809224318 |
| ebi-fl187-GCST90094852 | prot-a-2811 | SPARC-like protein 1 \|\| id:prot-a-2811 | F-strong vegetable liking (derived food-liking factor) \|\| id:ebi-fl187-GCST90094852 | Inverse variance weighted | 27 | 0.0778663487282679 | 0.0761598690653345 | 0.306588523274819 | -0.071406995 | 0.227139692096324 | 1.08097817490729 | 0.931082869265698 | 1.25500516999893 | 0.998735809224318 |
| ebi-fl187-GCST90094852 | prot-a-2831 | Sepiapterin reductase \|\| id:prot-a-2831 | F-strong vegetable liking (derived food-liking factor) \|\| id:ebi-fl187-GCST90094852 | Inverse variance weighted | 27 | 0.162673869495017 | 0.0834845775377594 | 0.051349194013126 | -0.000955902 | 0.326303641469026 | 1.17665288470713 | 0.999044554250242 | 1.38583610230343 | 0.998735809224318 |
| ebi-fl187-GCST90094852 | prot-a-2834 | SPRY domain-containing SOCS box protein 1 \|\| id:prot-a-2834 | F-strong vegetable liking (derived food-liking factor) \|\| id:ebi-fl187-GCST90094852 | Inverse variance weighted | 27 | -0.11905836 | 0.0761752829132372 | 0.118063732104052 | -0.268361915 | 0.0302451941087704 | 0.887755989454411 | 0.764630999659938 | 1.03070722631267 | 0.998735809224318 |
| ebi-fl187-GCST90094852 | prot-a-2835 | Spectrin alpha chain, non-erythrocytic 1 \|\| id:prot-a-2835 | F-strong vegetable liking (derived food-liking factor) \|\| id:ebi-fl187-GCST90094852 | Inverse variance weighted | 27 | -0.004001306 | 0.0761752829132372 | 0.958108293191734 | -0.15330486 | 0.145302248679571 | 0.996006688727356 | 0.857868151965527 | 1.15638903451156 | 0.998735809224318 |
| ebi-fl187-GCST90094852 | prot-a-2859 | Sia-alpha-2,3-Gal-beta-1,4-GlcNAc-R:alpha 2,8-sialyltransferase \|\| id:prot-a-2859 | F-strong vegetable liking (derived food-liking factor) \|\| id:ebi-fl187-GCST90094852 | Inverse variance weighted | 27 | 0.083521932298658 | 0.0761752829132372 | 0.272884515205623 | -0.065781622 | 0.232825486808603 | 1.08710905783195 | 0.93633531680284 | 1.26216119632825 | 0.998735809224318 |
| ebi-fl187-GCST90094852 | prot-a-2861 | Alpha-2,8-sialyltransferase 8F \|\| id:prot-a-2861 | F-strong vegetable liking (derived food-liking factor) \|\| id:ebi-fl187-GCST90094852 | Inverse variance weighted | 27 | -0.013163309 | 0.0852058634511958 | 0.877224738281406 | -0.180166801 | 0.153840183646385 | 0.986922948737601 | 0.835130899055042 | 1.16630447735442 | 0.998735809224318 |
| ebi-fl187-GCST90094852 | prot-a-289 | Uncharacterized protein C17orf89 \|\| id:prot-a-289 | F-strong vegetable liking (derived food-liking factor) \|\| id:ebi-fl187-GCST90094852 | Inverse variance weighted | 27 | 0.057368505485981 | 0.081920739577622 | 0.483744456695812 | -0.103196144 | 0.21793315505812 | 1.05904600275899 | 0.901950043963111 | 1.24350394289207 | 0.998735809224318 |
| ebi-fl187-GCST90094852 | prot-a-2921 | Tachykinin-3 \|\| id:prot-a-2921 | F-strong vegetable liking (derived food-liking factor) \|\| id:ebi-fl187-GCST90094852 | Inverse variance weighted | 27 | 0.119987409531149 | 0.0812987811865957 | 0.139975566647369 | -0.039358202 | 0.279333020656876 | 1.12748265595475 | 0.961406270201736 | 1.32224760632364 | 0.998735809224318 |
| ebi-fl187-GCST90094852 | prot-a-2928 | Tax1-binding protein 1 \|\| id:prot-a-2928 | F-strong vegetable liking (derived food-liking factor) \|\| id:ebi-fl187-GCST90094852 | Inverse variance weighted | 27 | 0.00451960184753624 | 0.0761752829132372 | 0.952687987764626 | -0.144783953 | 0.153823156357481 | 1.0045298306522 | 0.865209199063665 | 1.16628461852021 | 0.998735809224318 |
| ebi-fl187-GCST90094852 | prot-a-296 | Uncharacterized protein C1orf185 \|\| id:prot-a-296 | F-strong vegetable liking (derived food-liking factor) \|\| id:ebi-fl187-GCST90094852 | Inverse variance weighted | 27 | -0.053109954 | 0.0761752829132372 | 0.48567314745994 | -0.202413509 | 0.0961936004752929 | 0.948275740006237 | 0.81675712205541 | 1.10097219210214 | 0.998735809224318 |
| ebi-fl187-GCST90094852 | prot-a-2964 | Transforming growth factor beta-2 \|\| id:prot-a-2964 | F-strong vegetable liking (derived food-liking factor) \|\| id:ebi-fl187-GCST90094852 | Inverse variance weighted | 27 | 0.069115541007216 | 0.0763928432855078 | 0.365603977331954 | -0.080614432 | 0.218845513846811 | 1.07156001111908 | 0.922549328533174 | 1.24463898234602 | 0.998735809224318 |
| ebi-fl187-GCST90094852 | prot-a-2977 | Thymocyte nuclear protein 1 \|\| id:prot-a-2977 | F-strong vegetable liking (derived food-liking factor) \|\| id:ebi-fl187-GCST90094852 | Inverse variance weighted | 26 | -0.07298493 | 0.0775967669187644 | 0.346926987622121 | -0.225074593 | 0.079104733328318 | 0.929614839371608 | 0.798456657265933 | 1.08231767086648 | 0.998735809224318 |
| ebi-fl187-GCST90094852 | prot-a-3015 | Transmembrane protein 70, mitochondrial \|\| id:prot-a-3015 | F-strong vegetable liking (derived food-liking factor) \|\| id:ebi-fl187-GCST90094852 | Inverse variance weighted | 27 | -0.060336396 | 0.0761752829132372 | 0.428318029212518 | -0.20963995 | 0.0889671587912607 | 0.941447781307184 | 0.810876149189316 | 1.09304475882579 | 0.998735809224318 |
| ebi-fl187-GCST90094852 | prot-a-3030 | Tumor necrosis factor alpha-induced protein 3 \|\| id:prot-a-3030 | F-strong vegetable liking (derived food-liking factor) \|\| id:ebi-fl187-GCST90094852 | Inverse variance weighted | 27 | -0.087826301 | 0.0841658924987084 | 0.296721225306298 | -0.25279145 | 0.0771388485763854 | 0.915919957542985 | 0.776629831072757 | 1.08019204910885 | 0.998735809224318 |
| ebi-fl187-GCST90094852 | prot-a-3032 | Tumor necrosis factor receptor superfamily member 10A \|\| id:prot-a-3032 | F-strong vegetable liking (derived food-liking factor) \|\| id:ebi-fl187-GCST90094852 | Inverse variance weighted | 27 | 0.0513241170999364 | 0.0761752829132372 | 0.500461751481469 | -0.097979437 | 0.200627671609881 | 1.0526640244078 | 0.906667546995271 | 1.22216963864507 | 0.998735809224318 |
| ebi-fl187-GCST90094852 | prot-a-3081 | Tumor protein p53-inducible protein 11 \|\| id:prot-a-3081 | F-strong vegetable liking (derived food-liking factor) \|\| id:ebi-fl187-GCST90094852 | Inverse variance weighted | 27 | -0.129771483 | 0.076166166324255 | 0.0884195163011575 | -0.279057169 | 0.0195142031284755 | 0.878296113699733 | 0.756496653925534 | 1.01970585 | 0.998735809224318 |
| ebi-fl187-GCST90094852 | prot-a-315 | Complement C4 \|\| id:prot-a-315 | F-strong vegetable liking (derived food-liking factor) \|\| id:ebi-fl187-GCST90094852 | Inverse variance weighted | 27 | -0.123553137 | 0.0761752829132372 | 0.104812263073397 | -0.272856692 | 0.0257504171409436 | 0.883774678535145 | 0.761201866222014 | 1.02608482332871 | 0.998735809224318 |
| ebi-fl187-GCST90094852 | prot-a-3157 | UDP-glucuronosyltransferase 1-8 \|\| id:prot-a-3157 | F-strong vegetable liking (derived food-liking factor) \|\| id:ebi-fl187-GCST90094852 | Inverse variance weighted | 26 | -0.010069752 | 0.0795763706712533 | 0.899302918481009 | -0.166039438 | 0.145899934769432 | 0.989980778452807 | 0.84701282881692 | 1.15708039874077 | 0.998735809224318 |
| ebi-fl187-GCST90094852 | prot-a-317 | Glutaredoxin-like protein C5orf63 \|\| id:prot-a-317 | F-strong vegetable liking (derived food-liking factor) \|\| id:ebi-fl187-GCST90094852 | Inverse variance weighted (multiplicative random effects) | 27 | -0.037795943 | 0.0981766572757319 | 0.700253036380447 | -0.230222191 | 0.15463030514913 | 0.962909409143293 | 0.794357083603515 | 1.1672263637539 | 0.998735809224318 |
| ebi-fl187-GCST90094852 | prot-a-3192 | Guanine nucleotide exchange factor VAV3 \|\| id:prot-a-3192 | F-strong vegetable liking (derived food-liking factor) \|\| id:ebi-fl187-GCST90094852 | Inverse variance weighted | 27 | -0.032557141 | 0.0808017797403984 | 0.687002639506909 | -0.190928629 | 0.125814347733227 | 0.967967138068699 | 0.826191552288922 | 1.13407160577363 | 0.998735809224318 |
| ebi-fl187-GCST90094852 | prot-a-3233 | Serine/threonine-protein kinase WNK3 \|\| id:prot-a-3233 | F-strong vegetable liking (derived food-liking factor) \|\| id:ebi-fl187-GCST90094852 | Inverse variance weighted | 26 | -0.11512842 | 0.0853788354511192 | 0.177516216987497 | -0.282470937 | 0.0522140976145262 | 0.891251682132545 | 0.753918552509039 | 1.05360129189097 | 0.998735809224318 |
| ebi-fl187-GCST90094852 | prot-a-3250 | 14-3-3 protein theta \|\| id:prot-a-3250 | F-strong vegetable liking (derived food-liking factor) \|\| id:ebi-fl187-GCST90094852 | Inverse variance weighted | 27 | -0.016415289 | 0.0761752829132372 | 0.829382473887003 | -0.165718843 | 0.132888265883304 | 0.983718708023937 | 0.847284420534065 | 1.14212237716624 | 0.998735809224318 |
| ebi-fl187-GCST90094852 | prot-a-3278 | E3 ubiquitin-protein ligase ZNRF3 \|\| id:prot-a-3278 | F-strong vegetable liking (derived food-liking factor) \|\| id:ebi-fl187-GCST90094852 | Inverse variance weighted | 27 | 0.0268939241272503 | 0.0761752829132372 | 0.724048589560089 | -0.12240963 | 0.176197478637195 | 1.02725882960699 | 0.884785859090199 | 1.1926735629462 | 0.998735809224318 |
| ebi-fl187-GCST90094852 | prot-a-355 | Calpain-3 \|\| id:prot-a-355 | F-strong vegetable liking (derived food-liking factor) \|\| id:ebi-fl187-GCST90094852 | Inverse variance weighted | 27 | 0.151600315165333 | 0.0860090382890463 | 0.0779664380921831 | -0.0169774 | 0.320178030211864 | 1.16369503221422 | 0.983165904050061 | 1.37737295650876 | 0.998735809224318 |
| ebi-fl187-GCST90094852 | prot-a-385 | Coiled-coil domain-containing protein 90B, mitochondrial \|\| id:prot-a-385 | F-strong vegetable liking (derived food-liking factor) \|\| id:ebi-fl187-GCST90094852 | Inverse variance weighted (multiplicative random effects) | 27 | -0.012493367 | 0.107122501875784 | 0.907155710910305 | -0.22245347 | 0.197466737124251 | 0.987584351561313 | 0.800552255396896 | 1.21831253971702 | 0.998735809224318 |
| ebi-fl187-GCST90094852 | prot-a-390 | C-C motif chemokine 14 \|\| id:prot-a-390 | F-strong vegetable liking (derived food-liking factor) \|\| id:ebi-fl187-GCST90094852 | Inverse variance weighted (multiplicative random effects) | 27 | 0.0329088488334197 | 0.0959838739600153 | 0.731705213189893 | -0.155219544 | 0.22103724179505 | 1.03345633419745 | 0.856227177198021 | 1.24736988399263 | 0.998735809224318 |
| ebi-fl187-GCST90094852 | prot-a-40 | Pituitary adenylate cyclase-activating polypeptide 27 \|\| id:prot-a-40 | F-strong vegetable liking (derived food-liking factor) \|\| id:ebi-fl187-GCST90094852 | Inverse variance weighted | 27 | 0.0877124725346963 | 0.0764956667391043 | 0.25153324154291 | -0.062219034 | 0.237643979343341 | 1.09167419058545 | 0.939677042769655 | 1.26825758654032 | 0.998735809224318 |
| ebi-fl187-GCST90094852 | prot-a-401 | C-C motif chemokine 24 \|\| id:prot-a-401 | F-strong vegetable liking (derived food-liking factor) \|\| id:ebi-fl187-GCST90094852 | Inverse variance weighted | 27 | 0.170940813541573 | 0.0818474899307338 | 0.0367501893180309 | 0.0105197332773347 | 0.331361893805811 | 1.18642052691444 | 1.01057526021014 | 1.39286376988017 | 0.998735809224318 |
| ebi-fl187-GCST90094852 | prot-a-412 | Cerebral cavernous malformations 2 protein \|\| id:prot-a-412 | F-strong vegetable liking (derived food-liking factor) \|\| id:ebi-fl187-GCST90094852 | Inverse variance weighted | 27 | 0.0133623601388869 | 0.0845336959173573 | 0.874400532286102 | -0.152323684 | 0.179048404136907 | 1.01345203545257 | 0.858710285093242 | 1.19607863792087 | 0.998735809224318 |
| ebi-fl187-GCST90094852 | prot-a-44 | Adhesion G protein-coupled receptor F5 \|\| id:prot-a-44 | F-strong vegetable liking (derived food-liking factor) \|\| id:ebi-fl187-GCST90094852 | Inverse variance weighted | 27 | 0.0265203892612888 | 0.0761752829132372 | 0.727727885552555 | -0.122783165 | 0.175823943771234 | 1.02687518427468 | 0.884455422441566 | 1.19222814098222 | 0.998735809224318 |
| ebi-fl187-GCST90094852 | prot-a-48 | Adrenomedullin \|\| id:prot-a-48 | F-strong vegetable liking (derived food-liking factor) \|\| id:ebi-fl187-GCST90094852 | Inverse variance weighted | 27 | 0.0652157527907531 | 0.0920790807383437 | 0.478785005259215 | -0.115259245 | 0.245690751037907 | 1.0673892917614 | 0.891135091235006 | 1.27850413632343 | 0.998735809224318 |
| ebi-fl187-GCST90094852 | prot-a-502 | Carcinoembryonic antigen-related cell adhesion molecule 1 \|\| id:prot-a-502 | F-strong vegetable liking (derived food-liking factor) \|\| id:ebi-fl187-GCST90094852 | Inverse variance weighted | 27 | -0.016642667 | 0.076166166324255 | 0.827035881465111 | -0.165928353 | 0.132643019181245 | 0.98349505727456 | 0.847106924846151 | 1.1418423097641 | 0.998735809224318 |
| ebi-fl187-GCST90094852 | prot-a-518 | Complement factor B \|\| id:prot-a-518 | F-strong vegetable liking (derived food-liking factor) \|\| id:ebi-fl187-GCST90094852 | Inverse variance weighted | 27 | -0.12321383 | 0.0838080954568972 | 0.141510282533262 | -0.287477697 | 0.0410500371155643 | 0.884074600693849 | 0.750153297196784 | 1.04190423812396 | 0.998735809224318 |
| ebi-fl187-GCST90094852 | prot-a-525 | Properdin \|\| id:prot-a-525 | F-strong vegetable liking (derived food-liking factor) \|\| id:ebi-fl187-GCST90094852 | Inverse variance weighted (multiplicative random effects) | 27 | -0.015242546 | 0.0953222971933348 | 0.872955626381524 | -0.202074249 | 0.171589156273996 | 0.984873033395039 | 0.817034261938766 | 1.18718998344949 | 0.998735809224318 |
| ebi-fl187-GCST90094852 | prot-a-533 | Chondroadherin \|\| id:prot-a-533 | F-strong vegetable liking (derived food-liking factor) \|\| id:ebi-fl187-GCST90094852 | Inverse variance weighted (multiplicative random effects) | 27 | 0.116126244872794 | 0.0935323051807677 | 0.214397858245154 | -0.067197073 | 0.299449563027099 | 1.12313765355368 | 0.935010917508902 | 1.34911599983329 | 0.998735809224318 |
| ebi-fl187-GCST90094852 | prot-a-535 | Coiled-coil-helix-coiled-coil-helix domain-containing protein 2 \|\| id:prot-a-535 | F-strong vegetable liking (derived food-liking factor) \|\| id:ebi-fl187-GCST90094852 | Inverse variance weighted | 27 | -0.010278792 | 0.0802600969819019 | 0.898094751322789 | -0.167588582 | 0.147030998559129 | 0.989773854717892 | 0.845701700352628 | 1.15838987089021 | 0.998735809224318 |
| ebi-fl187-GCST90094852 | prot-a-591 | Caseinolytic peptidase B protein homolog \|\| id:prot-a-591 | F-strong vegetable liking (derived food-liking factor) \|\| id:ebi-fl187-GCST90094852 | Inverse variance weighted | 27 | -0.032226342 | 0.0880041684354604 | 0.714222495840575 | -0.204714512 | 0.14026182842264 | 0.968287393449129 | 0.814879921772728 | 1.15057501266303 | 0.998735809224318 |
| ebi-fl187-GCST90094852 | prot-a-605 | Ciliary neurotrophic factor receptor subunit alpha \|\| id:prot-a-605 | F-strong vegetable liking (derived food-liking factor) \|\| id:ebi-fl187-GCST90094852 | Inverse variance weighted | 27 | -0.008750374 | 0.0766913143221424 | 0.909159611269436 | -0.15906535 | 0.141564602467951 | 0.99128779949156 | 0.852940617755782 | 1.15207492874044 | 0.998735809224318 |
| ebi-fl187-GCST90094852 | prot-a-606 | Ciliary neurotrophic factor receptor subunit alpha \|\| id:prot-a-606 | F-strong vegetable liking (derived food-liking factor) \|\| id:ebi-fl187-GCST90094852 | Inverse variance weighted | 27 | -0.065268934 | 0.0761683430909296 | 0.391498118604405 | -0.214558886 | 0.0840210189314898 | 0.936815488403469 | 0.806897295390895 | 1.08765175484629 | 0.998735809224318 |
| ebi-fl187-GCST90094852 | prot-a-707 | Cancer/testis antigen 55 \|\| id:prot-a-707 | F-strong vegetable liking (derived food-liking factor) \|\| id:ebi-fl187-GCST90094852 | Inverse variance weighted | 27 | 0.117257330795525 | 0.0918719751259227 | 0.201845148040072 | -0.06281174 | 0.297326402042333 | 1.12440873745995 | 0.939120255403661 | 1.34625464800867 | 0.998735809224318 |
| ebi-fl187-GCST90094852 | prot-a-719 | Dipeptidyl peptidase 1 \|\| id:prot-a-719 | F-strong vegetable liking (derived food-liking factor) \|\| id:ebi-fl187-GCST90094852 | Inverse variance weighted (multiplicative random effects) | 27 | 0.0919927696676272 | 0.108728120003832 | 0.397507696921458 | -0.121114346 | 0.305099884875138 | 1.09635689502741 | 0.885932651353594 | 1.356760516 | 0.998735809224318 |
| ebi-fl187-GCST90094852 | prot-a-752 | Cytochrome b5 \|\| id:prot-a-752 | F-strong vegetable liking (derived food-liking factor) \|\| id:ebi-fl187-GCST90094852 | Inverse variance weighted | 27 | 0.0293980005117252 | 0.0838064000820529 | 0.725749897849065 | -0.134862544 | 0.193658544672549 | 1.02983438753403 | 0.873836017743461 | 1.21368179408112 | 0.998735809224318 |
| ebi-fl187-GCST90094852 | prot-a-755 | Cytoglobin \|\| id:prot-a-755 | F-strong vegetable liking (derived food-liking factor) \|\| id:ebi-fl187-GCST90094852 | Inverse variance weighted | 27 | -0.025005824 | 0.0845093809286865 | 0.767310987919847 | -0.19064421 | 0.140632562836049 | 0.975304232050439 | 0.826426569825352 | 1.15100164949503 | 0.998735809224318 |
| ebi-fl187-GCST90094852 | prot-a-807 | Desmin \|\| id:prot-a-807 | F-strong vegetable liking (derived food-liking factor) \|\| id:ebi-fl187-GCST90094852 | Inverse variance weighted (multiplicative random effects) | 27 | 0.0941228548683728 | 0.0972622319261255 | 0.333182954190859 | -0.09651112 | 0.284756829443579 | 1.09869471762095 | 0.907999800852023 | 1.32943870846169 | 0.998735809224318 |
| ebi-fl187-GCST90094852 | prot-a-82 | Ameloblastin \|\| id:prot-a-82 | F-strong vegetable liking (derived food-liking factor) \|\| id:ebi-fl187-GCST90094852 | Inverse variance weighted | 27 | 0.0533791560850886 | 0.0789937471197844 | 0.499206366753545 | -0.101448588 | 0.208206900439866 | 1.05482951433723 | 0.90352763 | 1.23146793444979 | 0.998735809224318 |
| ebi-fl187-GCST90094852 | prot-a-84 | Muellerian-inhibiting factor \|\| id:prot-a-84 | F-strong vegetable liking (derived food-liking factor) \|\| id:ebi-fl187-GCST90094852 | Inverse variance weighted | 27 | 0.0171949296102048 | 0.0761752829132372 | 0.821412618285289 | -0.132108625 | 0.16649848412015 | 1.01734361339222 | 0.876245807796593 | 1.18116174536973 | 0.998735809224318 |
| ebi-fl187-GCST90094852 | prot-a-858 | Inactive dipeptidyl peptidase 10 \|\| id:prot-a-858 | F-strong vegetable liking (derived food-liking factor) \|\| id:ebi-fl187-GCST90094852 | Inverse variance weighted | 27 | 0.0649937188888053 | 0.0768090974013953 | 0.397456717312934 | -0.085552112 | 0.21553954979554 | 1.06715232146075 | 0.918005302698847 | 1.2405310447021 | 0.998735809224318 |
| ebi-fl187-GCST90094852 | prot-a-863 | Dihydropyrimidinase-related protein 3 \|\| id:prot-a-863 | F-strong vegetable liking (derived food-liking factor) \|\| id:ebi-fl187-GCST90094852 | Inverse variance weighted | 27 | 0.00153814635703249 | 0.0761752829132372 | 0.98389005211294 | -0.147765408 | 0.150841700866977 | 1.00153932991089 | 0.862633457983371 | 1.16281257128991 | 0.998955228396052 |
| ebi-fl187-GCST90094852 | prot-a-864 | Dihydropyrimidinase-related protein 5 \|\| id:prot-a-864 | F-strong vegetable liking (derived food-liking factor) \|\| id:ebi-fl187-GCST90094852 | Inverse variance weighted | 27 | 0.0185255120773016 | 0.0761752829132372 | 0.807853687694102 | -0.130778042 | 0.167829066587247 | 1.01869817394462 | 0.877412501124026 | 1.18273442453656 | 0.998735809224318 |
| ebi-fl187-GCST90094852 | prot-a-884 | Endothelin-converting enzyme 1 \|\| id:prot-a-884 | F-strong vegetable liking (derived food-liking factor) \|\| id:ebi-fl187-GCST90094852 | Inverse variance weighted | 27 | 0.0282860749985973 | 0.0807497452557119 | 0.726118776415957 | -0.129983426 | 0.186555575699793 | 1.0286899248008 | 0.878109984855991 | 1.20509159403333 | 0.998735809224318 |
| ebi-fl187-GCST90094852 | prot-a-893 | Early endosome antigen 1 \|\| id:prot-a-893 | F-strong vegetable liking (derived food-liking factor) \|\| id:ebi-fl187-GCST90094852 | Inverse variance weighted | 27 | 0.0653443987279775 | 0.0761752829132372 | 0.39099391212035 | -0.083959156 | 0.214647953237922 | 1.06752661589013 | 0.919468810312746 | 1.23942548442313 | 0.998735809224318 |
| ebi-fl187-GCST90094852 | prot-a-897 | Ephrin-A2 \|\| id:prot-a-897 | F-strong vegetable liking (derived food-liking factor) \|\| id:ebi-fl187-GCST90094852 | Inverse variance weighted | 27 | 2.38431314119584e-06 | 0.0808821622143976 | 0.999976479280584 | -0.158526654 | 0.15853142225336 | 1.00000238431598 | 0.853400217276657 | 1.17178874388951 | 0.999997242828181 |
| ebi-fl187-GCST90094852 | prot-a-90 | Anaphase-promoting complex subunit 10 \|\| id:prot-a-90 | F-strong vegetable liking (derived food-liking factor) \|\| id:ebi-fl187-GCST90094852 | Inverse variance weighted | 27 | 0.10772725620195 | 0.0819734247089508 | 0.188788014022483 | -0.052940656 | 0.268395168631494 | 1.11374393720596 | 0.948436294599894 | 1.30786386468511 | 0.998735809224318 |
| ebi-fl187-GCST90094852 | prot-a-914 | Histone-lysine N-methyltransferase EHMT2 \|\| id:prot-a-914 | F-strong vegetable liking (derived food-liking factor) \|\| id:ebi-fl187-GCST90094852 | Inverse variance weighted | 27 | 0.124997940212392 | 0.0761752829132372 | 0.100812946333885 | -0.024305614 | 0.274301494722336 | 1.13314611902409 | 0.975987388474457 | 1.31561139234225 | 0.998735809224318 |
| ebi-fl187-GCST90094852 | prot-a-932 | RNA polymerase II elongation factor ELL \|\| id:prot-a-932 | F-strong vegetable liking (derived food-liking factor) \|\| id:ebi-fl187-GCST90094852 | Inverse variance weighted | 27 | 0.0382465494453437 | 0.0811831911017896 | 0.637559226411629 | -0.120872505 | 0.197365604004851 | 1.0389873630609 | 0.886146931592924 | 1.21818933419966 | 0.998735809224318 |
| ebi-fl187-GCST90094852 | prot-a-991 | Estrogen receptor \|\| id:prot-a-991 | F-strong vegetable liking (derived food-liking factor) \|\| id:ebi-fl187-GCST90094852 | Inverse variance weighted | 27 | -0.081610082 | 0.0828681380079183 | 0.324713191306487 | -0.244031633 | 0.0808114683050616 | 0.921631249082784 | 0.783462850836545 | 1.08416647755403 | 0.998735809224318 |
| ebi-fl187-GCST90094852 | prot-a-992 | Persulfide dioxygenase ETHE1, mitochondrial \|\| id:prot-a-992 | F-strong vegetable liking (derived food-liking factor) \|\| id:ebi-fl187-GCST90094852 | Inverse variance weighted | 27 | -0.217823815 | 0.0877790536447793 | 0.0130830317954614 | -0.389870761 | -0.04577687 | 0.80426712862946 | 0.677144382583472 | 0.955255084190467 | 0.998735809224318 |
| ebi-fl187-GCST90094852 | prot-a-993 | Ethanolamine kinase 1 \|\| id:prot-a-993 | F-strong vegetable liking (derived food-liking factor) \|\| id:ebi-fl187-GCST90094852 | Inverse variance weighted | 26 | 0.0437755614851403 | 0.0773088853940751 | 0.571229074768847 | -0.107749854 | 0.195300976857528 | 1.04474784691795 | 0.897852162570384 | 1.21567682202262 | 0.998735809224318 |
| ebi-fl187-GCST90094852 | prot-b-10 | coagulation factor III, tissue factor \|\| id:prot-b-10 | F-strong vegetable liking (derived food-liking factor) \|\| id:ebi-fl187-GCST90094852 | Inverse variance weighted | 20 | -0.18482571 | 0.105703307278918 | 0.08037179443902 | -0.392004192 | 0.0223527722399284 | 0.831249149619484 | 0.675701281216646 | 1.02260446731575 | 0.998735809224318 |
| ebi-fl187-GCST90094852 | prot-b-28 | prolactin \|\| id:prot-b-28 | F-strong vegetable liking (derived food-liking factor) \|\| id:ebi-fl187-GCST90094852 | Inverse variance weighted | 20 | -0.016865308 | 0.111595172960138 | 0.879873665610757 | -0.235591847 | 0.20186123095001 | 0.983276115091553 | 0.790103091035046 | 1.22367818766912 | 0.998735809224318 |
| ebi-fl187-GCST90094852 | prot-b-54 | matrix metallopeptidase 7 \|\| id:prot-b-54 | F-strong vegetable liking (derived food-liking factor) \|\| id:ebi-fl187-GCST90094852 | Inverse variance weighted | 20 | -0.078082675 | 0.105684442569947 | 0.460011160951854 | -0.285224183 | 0.129058832302836 | 0.924887958146451 | 0.751845684726042 | 1.1377570590646 | 0.998735809224318 |
| ebi-fl187-GCST90094852 | prot-c-2421_7_3 | BDNF \|\| id:prot-c-2421_7_3 | F-strong vegetable liking (derived food-liking factor) \|\| id:ebi-fl187-GCST90094852 | Inverse variance weighted | 2 | -0.377779855 | 1.06164400607564 | 0.721957250799142 | -2.458602107 | 1.70304239679293 | 0.685381367261055 | 0.0855544633985506 | 5.49062667134432 | 0.998735809224318 |
| ebi-fl187-GCST90094852 | prot-c-2443_10_4 | FGF-8B \|\| id:prot-c-2443_10_4 | F-strong vegetable liking (derived food-liking factor) \|\| id:ebi-fl187-GCST90094852 | Inverse variance weighted | 2 | 0.535453100806072 | 0.561934512140906 | 0.340653631335399 | -0.565938543 | 1.63684474460225 | 1.70822206372543 | 0.567826966571348 | 5.13892927033383 | 0.998735809224318 |
| ebi-fl187-GCST90094852 | prot-c-2605_49_2 | CD30 \|\| id:prot-c-2605_49_2 | F-strong vegetable liking (derived food-liking factor) \|\| id:ebi-fl187-GCST90094852 | Inverse variance weighted | 2 | 0.10568780434908 | 0.511106623013199 | 0.836179877972393 | -0.896081177 | 1.10745678545495 | 1.11147482472893 | 0.408166060336038 | 3.02665117474282 | 0.998735809224318 |
| ebi-fl187-GCST90094852 | prot-c-2624_31_2 | HPV E7 Type18 \|\| id:prot-c-2624_31_2 | F-strong vegetable liking (derived food-liking factor) \|\| id:ebi-fl187-GCST90094852 | Inverse variance weighted | 2 | 0.301061724305645 | 0.602419237013776 | 0.617247952461272 | -0.87967998 | 1.48180342885265 | 1.35129274657044 | 0.414915671650641 | 4.40087519391496 | 0.998735809224318 |
| ebi-fl187-GCST90094852 | prot-c-2774_10_3 | IL-16 \|\| id:prot-c-2774_10_3 | F-strong vegetable liking (derived food-liking factor) \|\| id:ebi-fl187-GCST90094852 | Inverse variance weighted | 2 | -0.994078953 | 0.496937364390065 | 0.0454559051968809 | -1.968076187 | -0.020081719 | 0.370064133981551 | 0.139725403294783 | 0.980118575650793 | 0.998735809224318 |
| ebi-fl187-GCST90094852 | prot-c-2851_63_3 | C5a \|\| id:prot-c-2851_63_3 | F-strong vegetable liking (derived food-liking factor) \|\| id:ebi-fl187-GCST90094852 | Inverse variance weighted | 2 | -0.480217345 | 0.44953606268621 | 0.285407308505023 | -1.361308028 | 0.400873337784553 | 0.618648916894347 | 0.256325276954338 | 1.49312813360474 | 0.998735809224318 |
| ebi-fl187-GCST90094852 | prot-c-2891_1_3 | CD22 \|\| id:prot-c-2891_1_3 | F-strong vegetable liking (derived food-liking factor) \|\| id:ebi-fl187-GCST90094852 | Inverse variance weighted | 2 | -0.12206192 | 0.519702165067711 | 0.814310423298266 | -1.140678163 | 0.896554323954882 | 0.88509356218614 | 0.319602205880251 | 2.45114269992514 | 0.998735809224318 |
| ebi-fl187-GCST90094852 | prot-c-2961_1_2 | Protein C \|\| id:prot-c-2961_1_2 | F-strong vegetable liking (derived food-liking factor) \|\| id:ebi-fl187-GCST90094852 | Inverse variance weighted | 2 | -0.505877643 | 1.32505469300362 | 0.702625527619638 | -3.102984841 | 2.09122955529152 | 0.60297614534526 | 0.0449149381529094 | 8.09486212844484 | 0.998735809224318 |
| ebi-fl187-GCST90094852 | prot-c-2982_82_2 | Galectin-4 \|\| id:prot-c-2982_82_2 | F-strong vegetable liking (derived food-liking factor) \|\| id:ebi-fl187-GCST90094852 | Inverse variance weighted | 2 | -0.111024272 | 0.477205746190263 | 0.816029365178999 | -1.046347535 | 0.824298990405242 | 0.894917027125076 | 0.351218221627966 | 2.28028170556232 | 0.998735809224318 |
| ebi-fl187-GCST90094852 | prot-c-2994_71_2 | IL-1Rrp2 \|\| id:prot-c-2994_71_2 | F-strong vegetable liking (derived food-liking factor) \|\| id:ebi-fl187-GCST90094852 | Inverse variance weighted | 2 | 0.0800860460302262 | 0.51484652163672 | 0.876385182952786 | -0.929013136 | 1.0891852284382 | 1.08338028423713 | 0.394943273266501 | 2.9718517056036 | 0.998735809224318 |
| ebi-fl187-GCST90094852 | prot-c-3336_50_1 | TFPI \|\| id:prot-c-3336_50_1 | F-strong vegetable liking (derived food-liking factor) \|\| id:ebi-fl187-GCST90094852 | Inverse variance weighted | 2 | 0.145333976762153 | 0.500224652126024 | 0.771405132418185 | -0.835106341 | 1.12577429492916 | 1.1564257251004 | 0.433828345064252 | 3.08260276878848 | 0.998735809224318 |
| ebi-fl187-GCST90094852 | prot-c-3472_40_2 | Survivin \|\| id:prot-c-3472_40_2 | F-strong vegetable liking (derived food-liking factor) \|\| id:ebi-fl187-GCST90094852 | Inverse variance weighted | 2 | -0.050594025 | 0.5172249480542 | 0.922076637313257 | -1.064354923 | 0.963166873134393 | 0.950664538187436 | 0.344950302435717 | 2.61998049511944 | 0.998735809224318 |
| ebi-fl187-GCST90094852 | prot-c-3473_78_2 | Thrombopoietin Receptor \|\| id:prot-c-3473_78_2 | F-strong vegetable liking (derived food-liking factor) \|\| id:ebi-fl187-GCST90094852 | Inverse variance weighted | 2 | -0.182518717 | 0.52844637377031 | 0.729803152034867 | -1.218273609 | 0.853236176078633 | 0.833169049764567 | 0.29574028980636 | 2.34723062569564 | 0.998735809224318 |
| ebi-fl187-GCST90094852 | prot-c-3497_13_2 | IFN-aA \|\| id:prot-c-3497_13_2 | F-strong vegetable liking (derived food-liking factor) \|\| id:ebi-fl187-GCST90094852 | Inverse variance weighted | 2 | -0.428531914 | 0.589776887267316 | 0.467470999038499 | -1.584494613 | 0.727430784647703 | 0.651464799116677 | 0.205051397154743 | 2.06975612152426 | 0.998735809224318 |
| ebi-fl187-GCST90094852 | prot-c-3665_64_3 | COLEC12 \|\| id:prot-c-3665_64_3 | F-strong vegetable liking (derived food-liking factor) \|\| id:ebi-fl187-GCST90094852 | Inverse variance weighted | 2 | 0.128310375103796 | 0.512113431976587 | 0.802161507379873 | -0.875431952 | 1.13205270177791 | 1.13690581520252 | 0.416681994358413 | 3.10201748609637 | 0.998735809224318 |
| ebi-fl187-GCST90094852 | prot-c-3741_4_3 | IL-5 \|\| id:prot-c-3741_4_3 | F-strong vegetable liking (derived food-liking factor) \|\| id:ebi-fl187-GCST90094852 | Inverse variance weighted | 2 | 0.382942053932433 | 0.504160595245649 | 0.44751546353122 | -0.605212713 | 1.37109682061391 | 1.46659304430649 | 0.5459582820004 | 3.93966943724575 | 0.998735809224318 |
| ebi-fl187-GCST90094852 | prot-c-3848_14_2 | GAPDH, liver \|\| id:prot-c-3848_14_2 | F-strong vegetable liking (derived food-liking factor) \|\| id:ebi-fl187-GCST90094852 | Inverse variance weighted | 2 | -0.493174161 | 0.510659504044982 | 0.334164642620875 | -1.494066789 | 0.507718466778331 | 0.61068490221891 | 0.224457973655202 | 1.66149610871477 | 0.998735809224318 |
| ebi-fl187-GCST90094852 | prot-c-3859_50_2 | PSA1 \|\| id:prot-c-3859_50_2 | F-strong vegetable liking (derived food-liking factor) \|\| id:ebi-fl187-GCST90094852 | Inverse variance weighted | 2 | -0.057065381 | 0.509484617254749 | 0.910818575809723 | -1.055655231 | 0.94152446862271 | 0.944532312694854 | 0.347964355556325 | 2.5638870058927 | 0.998735809224318 |
| ebi-fl187-GCST90094852 | prot-c-3868_8_1 | SGTA \|\| id:prot-c-3868_8_1 | F-strong vegetable liking (derived food-liking factor) \|\| id:ebi-fl187-GCST90094852 | Inverse variance weighted | 2 | -0.833499916 | 0.901041533230464 | 0.3549448377118 | -2.599541321 | 0.932541488936176 | 0.434525817923281 | 0.07430765373487 | 2.54095879699796 | 0.998735809224318 |
| ebi-fl187-GCST90094852 | prot-c-3882_10_1 | EF-1-beta \|\| id:prot-c-3882_10_1 | F-strong vegetable liking (derived food-liking factor) \|\| id:ebi-fl187-GCST90094852 | Inverse variance weighted | 2 | -0.825270406 | 0.513858753067343 | 0.10826824525653 | -1.832433562 | 0.181892750182285 | 0.438116507188481 | 0.160023666055142 | 1.19948554237529 | 0.998735809224318 |
| ebi-fl187-GCST90094852 | prot-c-4134_4_2 | HB-EGF \|\| id:prot-c-4134_4_2 | F-strong vegetable liking (derived food-liking factor) \|\| id:ebi-fl187-GCST90094852 | Inverse variance weighted | 2 | -0.611549409 | 0.949958701360478 | 0.51972834778542 | -2.473468464 | 1.25036964537431 | 0.542509648055141 | 0.0842919876671043 | 3.49163338507644 | 0.998735809224318 |
| ebi-fl187-GCST90094852 | prot-c-4220_39_2 | FER \|\| id:prot-c-4220_39_2 | F-strong vegetable liking (derived food-liking factor) \|\| id:ebi-fl187-GCST90094852 | Inverse variance weighted | 2 | -0.694788948 | 0.512556536847603 | 0.175246778604893 | -1.69939976 | 0.309821864587751 | 0.499179789944361 | 0.182793210953675 | 1.36318226146837 | 0.998735809224318 |
| ebi-fl187-GCST90094852 | prot-c-4228_37_2 | HSP70 protein 8 \|\| id:prot-c-4228_37_2 | F-strong vegetable liking (derived food-liking factor) \|\| id:ebi-fl187-GCST90094852 | Inverse variance weighted | 2 | -0.179437649 | 0.518086490693432 | 0.729082027999398 | -1.19488717 | 0.836011873162928 | 0.835740058884524 | 0.302738110088598 | 2.30714740810101 | 0.998735809224318 |
| ebi-fl187-GCST90094852 | prot-c-4246_40_2 | NCAM-L1 \|\| id:prot-c-4246_40_2 | F-strong vegetable liking (derived food-liking factor) \|\| id:ebi-fl187-GCST90094852 | Inverse variance weighted | 2 | -0.055929077 | 0.513466733177183 | 0.913262420420542 | -1.062323874 | 0.950465719855042 | 0.945606198577785 | 0.345651625385391 | 2.58691415610083 | 0.998735809224318 |
| ebi-fl187-GCST90094852 | prot-c-4267_81_3 | PESC \|\| id:prot-c-4267_81_3 | F-strong vegetable liking (derived food-liking factor) \|\| id:ebi-fl187-GCST90094852 | Inverse variance weighted | 2 | 0.232815442026661 | 0.519355416988054 | 0.653952806100843 | -0.785121175 | 1.25075205932325 | 1.26214851825793 | 0.456064434706227 | 3.49296888972898 | 0.998735809224318 |
| ebi-fl187-GCST90094852 | prot-c-4280_47_2 | PSA2 \|\| id:prot-c-4280_47_2 | F-strong vegetable liking (derived food-liking factor) \|\| id:ebi-fl187-GCST90094852 | Inverse variance weighted | 2 | -0.386068013 | 0.514631025992407 | 0.453143833881778 | -1.394744824 | 0.622608798114362 | 0.679724294134792 | 0.247896285535162 | 1.86378394109301 | 0.998735809224318 |
| ebi-fl187-GCST90094852 | prot-c-4324_33_2 | CYTT \|\| id:prot-c-4324_33_2 | F-strong vegetable liking (derived food-liking factor) \|\| id:ebi-fl187-GCST90094852 | Inverse variance weighted | 2 | 0.494160429866563 | 0.499570925697427 | 0.322580113418142 | -0.484998585 | 1.47331944423352 | 1.63912150374537 | 0.615698068284042 | 4.36369617258732 | 0.998735809224318 |
| ebi-fl187-GCST90094852 | prot-c-4462_8_2 | RASA1 \|\| id:prot-c-4462_8_2 | F-strong vegetable liking (derived food-liking factor) \|\| id:ebi-fl187-GCST90094852 | Inverse variance weighted | 2 | 0.868986141027973 | 0.516293267806579 | 0.0923508827879038 | -0.142948664 | 1.88092094592887 | 2.3844920888903 | 0.866798565834655 | 6.55954306581655 | 0.998735809224318 |
| ebi-fl187-GCST90094852 | prot-c-4499_21_1 | PDGF-AA \|\| id:prot-c-4499_21_1 | F-strong vegetable liking (derived food-liking factor) \|\| id:ebi-fl187-GCST90094852 | Inverse variance weighted | 2 | -0.635573491 | 0.689225146196453 | 0.356446917292768 | -1.986454778 | 0.715307795525319 | 0.529631662203084 | 0.13718090118587 | 2.04481597061337 | 0.998735809224318 |
| ebi-fl187-GCST90094852 | prot-c-4535_50_2 | BST1 \|\| id:prot-c-4535_50_2 | F-strong vegetable liking (derived food-liking factor) \|\| id:ebi-fl187-GCST90094852 | Inverse variance weighted | 2 | 0.521602258020798 | 0.528999402965556 | 0.324124906456392 | -0.515236572 | 1.55844108783329 | 1.68472485222198 | 0.597359259550636 | 4.75140844025668 | 0.998735809224318 |
| ebi-fl187-GCST90094852 | prot-c-4542_24_2 | Clusterin \|\| id:prot-c-4542_24_2 | F-strong vegetable liking (derived food-liking factor) \|\| id:ebi-fl187-GCST90094852 | Inverse variance weighted | 2 | 0.673887304925358 | 0.514170296128379 | 0.189982608152612 | -0.333886475 | 1.68166108533698 | 1.96184882152535 | 0.716135076499003 | 5.37447602390378 | 0.998735809224318 |
| ebi-fl187-GCST90094852 | prot-c-4696_2_2 | FABP \|\| id:prot-c-4696_2_2 | F-strong vegetable liking (derived food-liking factor) \|\| id:ebi-fl187-GCST90094852 | Inverse variance weighted | 2 | -0.062928603 | 0.491637328383409 | 0.898150543163637 | -1.026537766 | 0.900680561028037 | 0.939010514201898 | 0.358245144326354 | 2.46127759090704 | 0.998735809224318 |
| ebi-fl187-GCST90094852 | prot-c-4906_35_1 | Coagulation Factor V \|\| id:prot-c-4906_35_1 | F-strong vegetable liking (derived food-liking factor) \|\| id:ebi-fl187-GCST90094852 | Inverse variance weighted | 2 | -0.328798526 | 0.518854117558419 | 0.52627578049771 | -1.345752596 | 0.68815554487355 | 0.719788021041424 | 0.260343700496032 | 1.99004160364781 | 0.998735809224318 |
| ebi-fl187-GCST90094852 | prot-c-4916_2_1 | IgD \|\| id:prot-c-4916_2_1 | F-strong vegetable liking (derived food-liking factor) \|\| id:ebi-fl187-GCST90094852 | Inverse variance weighted | 2 | -0.385131077 | 0.862967884537518 | 0.655390172857047 | -2.07654813 | 1.30628597700268 | 0.680361450832091 | 0.125362200156252 | 3.69243442761372 | 0.998735809224318 |
| ebi-fl187-GCST90094852 | prot-c-4922_13_1 | MIP-3b \|\| id:prot-c-4922_13_1 | F-strong vegetable liking (derived food-liking factor) \|\| id:ebi-fl187-GCST90094852 | Inverse variance weighted | 2 | 0.315858440661485 | 0.518642117205743 | 0.542517548770393 | -0.700680109 | 1.33239699038474 | 1.3714361022968 | 0.49624768644768 | 3.79011738300838 | 0.998735809224318 |
| ebi-fl187-GCST90094852 | prot-c-4973_18_1 | cIAP-2 \|\| id:prot-c-4973_18_1 | F-strong vegetable liking (derived food-liking factor) \|\| id:ebi-fl187-GCST90094852 | Inverse variance weighted | 2 | 0.146803885902581 | 0.728362206818375 | 0.840265871692522 | -1.280786039 | 1.5743938112666 | 1.15812681576202 | 0.277818838034577 | 4.82781416435178 | 0.998735809224318 |
| ebi-fl187-GCST90094852 | prot-c-4997_19_1 | IF4A3 \|\| id:prot-c-4997_19_1 | F-strong vegetable liking (derived food-liking factor) \|\| id:ebi-fl187-GCST90094852 | Inverse variance weighted | 2 | 0.508397257306648 | 0.517728842232045 | 0.326111710309053 | -0.506351273 | 1.52314578808146 | 1.66262429939545 | 0.602690625089468 | 4.58663109373879 | 0.998735809224318 |
| ebi-fl187-GCST90094852 | prot-c-5000_52_1 | LG3BP \|\| id:prot-c-5000_52_1 | F-strong vegetable liking (derived food-liking factor) \|\| id:ebi-fl187-GCST90094852 | Inverse variance weighted | 2 | 0.443635817266267 | 0.478299740585601 | 0.353653095160749 | -0.493831674 | 1.38110330881404 | 1.55836285342316 | 0.61028350085418 | 3.97928959168999 | 0.998735809224318 |
| ebi-fl187-GCST90094852 | prot-c-5006_71_1 | MK13 \|\| id:prot-c-5006_71_1 | F-strong vegetable liking (derived food-liking factor) \|\| id:ebi-fl187-GCST90094852 | Inverse variance weighted | 2 | -0.178619567 | 0.51679829541023 | 0.729622873577278 | -1.191544226 | 0.834305091841138 | 0.836424042048567 | 0.3037518401718 | 2.30321297056561 | 0.998735809224318 |
| ebi-fl187-GCST90094852 | prot-c-5109_24_3 | Nr-CAM \|\| id:prot-c-5109_24_3 | F-strong vegetable liking (derived food-liking factor) \|\| id:ebi-fl187-GCST90094852 | Inverse variance weighted | 2 | -0.051860788 | 0.853326021736931 | 0.951538494533695 | -1.724379791 | 1.62065821426092 | 0.949461033686413 | 0.178283590587502 | 5.05641742752779 | 0.998735809224318 |
| ebi-fl187-GCST90094852 | prot-c-5255_22_3 | PDE4D \|\| id:prot-c-5255_22_3 | F-strong vegetable liking (derived food-liking factor) \|\| id:ebi-fl187-GCST90094852 | Inverse variance weighted | 2 | -0.53408267 | 0.802688919649157 | 0.505815825388882 | -2.107352952 | 1.03918761287876 | 0.586206788863573 | 0.121559314256156 | 2.82691952823632 | 0.998735809224318 |
| ebi-fl187-GCST90094852 | prot-c-5440_26_3 | Troponin I, skeletal, fast twitch \|\| id:prot-c-5440_26_3 | F-strong vegetable liking (derived food-liking factor) \|\| id:ebi-fl187-GCST90094852 | Inverse variance weighted | 2 | 0.454402368483771 | 0.494114560008893 | 0.357766360178887 | -0.514062169 | 1.4228669061012 | 1.57523169356493 | 0.598061211959561 | 4.14899819415009 | 0.998735809224318 |
| ebi-fl187-GCST90094852 | ukb-rsfMRI191-edge-pheno716 | Net100_Pair10_33 (OccipitalPrecuneus)&(Temporal\|Frontal\|Supp_Motor_Area) (Default_mode\|Central_executive)&(Default_mode\|Salience) \|\| id:ukb-rsfMRI191-edge-pheno716 | F-strong vegetable liking (derived food-liking factor) \|\| id:ebi-fl187-GCST90094852 | Inverse variance weighted | 27 | -0.009153934 | 0.0286518917776666 | 0.749356526162126 | -0.065311642 | 0.0470037736659407 | 0.990887835488004 | 0.936775479202565 | 1.04812596434945 | 0.998735809224318 |
| ebi-fl187-GCST90094852 | ukb-rsfMRI191-edge-pheno903 | Net100_Pair27_38 (Precentral\|Frontal\|Supp_Motor_Area)&(Subcortical) (Attention\|Salience\|Motor)&(Subcortical-cerebellum) \|\| id:ukb-rsfMRI191-edge-pheno903 | F-strong vegetable liking (derived food-liking factor) \|\| id:ebi-fl187-GCST90094852 | Inverse variance weighted | 27 | 0.00104849717021393 | 0.0235831905203843 | 0.964538123832059 | -0.045174556 | 0.0472715505901672 | 1.00104904703553 | 0.95583062106163 | 1.0484066658774 | 0.998735809224318 |
| ebi-fl187-GCST90094852 | ukb-rsfMRI191-node-pheno23 | Net100_Node2 RolandicOper\|SupraMarginal\|Insula Salience\|Motor \|\| id:ukb-rsfMRI191-node-pheno23 | F-strong vegetable liking (derived food-liking factor) \|\| id:ebi-fl187-GCST90094852 | Inverse variance weighted | 27 | -0.036473183 | 0.0249354234796191 | 0.14354796427967 | -0.085346613 | 0.0124002474691415 | 0.964183950503622 | 0.918193971665882 | 1.0124774493151 | 0.998735809224318 |
| ebi-fl187-GCST90094852 | ukb-rsfMRI191-node-pheno55 | Net100_Node34 Parietal\|Postcentral\|Precuneus Attention \|\| id:ukb-rsfMRI191-node-pheno55 | F-strong vegetable liking (derived food-liking factor) \|\| id:ebi-fl187-GCST90094852 | Inverse variance weighted | 27 | 0.0211324206532786 | 0.0272879746465453 | 0.438681001606725 | -0.03235201 | 0.0746168509605074 | 1.02135729148283 | 0.968165718409562 | 1.07747123971585 | 0.998735809224318 |
| ebi-fl187-GCST90094852 | ebi-a-GCST90001414 | IgD- CD24- B cell Absolute Count \|\| id:ebi-a-GCST90001414 | F-strong vegetable liking (derived food-liking factor) \|\| id:ebi-fl187-GCST90094852 | MR Egger | 23 | -0.493078917 | 0.358189054541013 | 0.183140547912655 | -1.195129464 | 0.208971629801236 | 0.61074306909267 | 0.302664767454249 | 1.23241003431665 | 0.999950574162623 |
| ebi-fl187-GCST90094852 | ebi-a-GCST90001422 | CD20- CD38- B cell %B cell \|\| id:ebi-a-GCST90001422 | F-strong vegetable liking (derived food-liking factor) \|\| id:ebi-fl187-GCST90094852 | MR Egger | 23 | -0.254999481 | 0.3669226738558 | 0.494693936722999 | -0.974167922 | 0.464168959613566 | 0.774916900031413 | 0.377506337671137 | 1.59069171039299 | 0.999950574162623 |
| ebi-fl187-GCST90094852 | ebi-a-GCST90001444 | CD20- CD38- B cell %lymphocyte \|\| id:ebi-a-GCST90001444 | F-strong vegetable liking (derived food-liking factor) \|\| id:ebi-fl187-GCST90094852 | MR Egger | 23 | -0.18316057 | 0.397626284965308 | 0.649795106159596 | -0.962508088 | 0.596186948568532 | 0.832634448919681 | 0.381933760021966 | 1.81518419709195 | 0.999950574162623 |
| ebi-fl187-GCST90094852 | ebi-a-GCST90001459 | Myeloid Dendritic Cell %Dendritic Cell \|\| id:ebi-a-GCST90001459 | F-strong vegetable liking (derived food-liking factor) \|\| id:ebi-fl187-GCST90094852 | MR Egger | 23 | 0.10968169504642 | 0.364508562405688 | 0.766447524481449 | -0.604755087 | 0.824118477361569 | 1.11592281016303 | 0.546208183597737 | 2.27987012212042 | 0.999950574162623 |
| ebi-fl187-GCST90094852 | ebi-a-GCST90001476 | HLA DR++ monocyte %leukocyte \|\| id:ebi-a-GCST90001476 | F-strong vegetable liking (derived food-liking factor) \|\| id:ebi-fl187-GCST90094852 | MR Egger | 23 | 0.0836801322027354 | 0.356642991852183 | 0.816766373834414 | -0.615340132 | 0.782700396233014 | 1.08728105198499 | 0.540457037403286 | 2.18737106595107 | 0.999950574162623 |
| ebi-fl187-GCST90094852 | ebi-a-GCST90001478 | CD4 regulatory T cell %CD4+ T cell \|\| id:ebi-a-GCST90001478 | F-strong vegetable liking (derived food-liking factor) \|\| id:ebi-fl187-GCST90094852 | MR Egger | 23 | 0.218156527634295 | 0.442194951811011 | 0.626885700476453 | -0.648545578 | 1.08485863318388 | 1.24378173859609 | 0.52280560408885 | 2.95902148172499 | 0.999950574162623 |
| ebi-fl187-GCST90094852 | ebi-a-GCST90001485 | CD39+ resting CD4 regulatory T cell %CD4 regulatory T cell \|\| id:ebi-a-GCST90001485 | F-strong vegetable liking (derived food-liking factor) \|\| id:ebi-fl187-GCST90094852 | MR Egger | 23 | 0.287662943250449 | 0.351932624925583 | 0.422888029820216 | -0.402125002 | 0.97745088810459 | 1.33330782797551 | 0.668897127250624 | 2.6576728942576 | 0.999950574162623 |
| ebi-fl187-GCST90094852 | ebi-a-GCST90001515 | Immature Myeloid-Derived Suppressor Cells Absolute Count \|\| id:ebi-a-GCST90001515 | F-strong vegetable liking (derived food-liking factor) \|\| id:ebi-fl187-GCST90094852 | MR Egger | 22 | -0.818350359 | 0.496724210807085 | 0.115078764825764 | -1.791929812 | 0.155229094417864 | 0.441158808331468 | 0.166638278631607 | 1.16792549567016 | 0.999950574162623 |
| ebi-fl187-GCST90094852 | ebi-a-GCST90001517 | CD33+ HLA DR+ Absolute Count \|\| id:ebi-a-GCST90001517 | F-strong vegetable liking (derived food-liking factor) \|\| id:ebi-fl187-GCST90094852 | MR Egger | 23 | -0.896239777 | 0.461284660935306 | 0.0655529670254511 | -1.800357713 | 0.00787815796598701 | 0.408101330042168 | 0.165239769251285 | 1.00790927230668 | 0.999950574162623 |
| ebi-fl187-GCST90094852 | ebi-a-GCST90001518 | CD33+ HLA DR+ CD14- Absolute Count \|\| id:ebi-a-GCST90001518 | F-strong vegetable liking (derived food-liking factor) \|\| id:ebi-fl187-GCST90094852 | MR Egger | 23 | -0.916407137 | 0.467134698673011 | 0.06318110635059 | -1.831991146 | -0.000823127 | 0.399953440776618 | 0.160094478706171 | 0.999177211368064 | 0.999950574162623 |
| ebi-fl187-GCST90094852 | ebi-a-GCST90001520 | CD33+ HLA DR+ CD14dim Absolute Count \|\| id:ebi-a-GCST90001520 | F-strong vegetable liking (derived food-liking factor) \|\| id:ebi-fl187-GCST90094852 | MR Egger | 23 | -0.716433816 | 0.488041932166858 | 0.156930168623747 | -1.672996003 | 0.240128370833639 | 0.488491202822374 | 0.1876839208294 | 1.27141235210955 | 0.999950574162623 |
| ebi-fl187-GCST90094852 | ebi-a-GCST90001531 | CD33dim HLA DR- Absolute Count \|\| id:ebi-a-GCST90001531 | F-strong vegetable liking (derived food-liking factor) \|\| id:ebi-fl187-GCST90094852 | MR Egger | 23 | -0.284493868 | 0.470505675795002 | 0.551885803056727 | -1.206684993 | 0.637697256315389 | 0.752394968953105 | 0.299187445855561 | 1.8921187942466 | 0.999950574162623 |
| ebi-fl187-GCST90094852 | ebi-a-GCST90001532 | Basophil Absolute Count \|\| id:ebi-a-GCST90001532 | F-strong vegetable liking (derived food-liking factor) \|\| id:ebi-fl187-GCST90094852 | MR Egger | 23 | -0.27205679 | 0.469078258586074 | 0.568098296195892 | -1.191450177 | 0.647336597028341 | 0.761810996766941 | 0.303780409235435 | 1.91044576000046 | 0.999950574162623 |
| ebi-fl187-GCST90094852 | ebi-a-GCST90001588 | T/B cell \|\| id:ebi-a-GCST90001588 | F-strong vegetable liking (derived food-liking factor) \|\| id:ebi-fl187-GCST90094852 | MR Egger | 23 | -0.569856934 | 0.425340915511853 | 0.194640622659426 | -1.403525128 | 0.263811260645953 | 0.565606352087064 | 0.245729208413044 | 1.30188245665734 | 0.999950574162623 |
| ebi-fl187-GCST90094852 | ebi-a-GCST90001624 | HLA DR+ CD4+ T cell Absolute Count \|\| id:ebi-a-GCST90001624 | F-strong vegetable liking (derived food-liking factor) \|\| id:ebi-fl187-GCST90094852 | MR Egger | 23 | -0.082825018 | 0.360953893062022 | 0.82073039275635 | -0.790294648 | 0.624644612701173 | 0.920512206471161 | 0.453711090473745 | 1.86758212451375 | 0.999950574162623 |
| ebi-fl187-GCST90094852 | ebi-a-GCST90001631 | CD8+ Natural Killer T %T cell \|\| id:ebi-a-GCST90001631 | F-strong vegetable liking (derived food-liking factor) \|\| id:ebi-fl187-GCST90094852 | MR Egger | 23 | -0.225990107 | 0.34638053979828 | 0.521200293609796 | -0.904895965 | 0.452915751458358 | 0.797725993891842 | 0.404583973989045 | 1.57289166710285 | 0.999950574162623 |
| ebi-fl187-GCST90094852 | ebi-a-GCST90001632 | CD8+ Natural Killer T %lymphocyte \|\| id:ebi-a-GCST90001632 | F-strong vegetable liking (derived food-liking factor) \|\| id:ebi-fl187-GCST90094852 | MR Egger | 23 | -0.28270005 | 0.348514313605383 | 0.426373624101592 | -0.965788105 | 0.400388004256288 | 0.753745839711331 | 0.38068306323053 | 1.49240364428321 | 0.999950574162623 |
| ebi-fl187-GCST90094852 | ebi-a-GCST90001646 | Natural Killer %CD3- lymphocyte \|\| id:ebi-a-GCST90001646 | F-strong vegetable liking (derived food-liking factor) \|\| id:ebi-fl187-GCST90094852 | MR Egger | 23 | -0.067014508 | 0.338970225154345 | 0.845180726850659 | -0.731396149 | 0.597367133513151 | 0.935181633820197 | 0.481236642749096 | 1.81732771477792 | 0.999950574162623 |
| ebi-fl187-GCST90094852 | ebi-a-GCST90001684 | CD127- CD8+ T cell Absolute Count \|\| id:ebi-a-GCST90001684 | F-strong vegetable liking (derived food-liking factor) \|\| id:ebi-fl187-GCST90094852 | MR Egger | 23 | -0.417487396 | 0.367340809546166 | 0.268545617285504 | -1.137475382 | 0.302500591109864 | 0.65869979431099 | 0.320627462648643 | 1.35323847633355 | 0.999950574162623 |
| ebi-fl187-GCST90094852 | ebi-a-GCST90001687 | CD28- CD8+ T cell Absolute Count \|\| id:ebi-a-GCST90001687 | F-strong vegetable liking (derived food-liking factor) \|\| id:ebi-fl187-GCST90094852 | MR Egger | 23 | -0.317534548 | 0.357515778326353 | 0.384510022903786 | -1.018265473 | 0.383196377933329 | 0.72794153171699 | 0.361220944548422 | 1.46696608155135 | 0.999950574162623 |
| ebi-fl187-GCST90094852 | ebi-a-GCST90001701 | BAFF-R on CD20- CD38- B cell \|\| id:ebi-a-GCST90001701 | F-strong vegetable liking (derived food-liking factor) \|\| id:ebi-fl187-GCST90094852 | MR Egger | 23 | -0.067353123 | 0.392721695851416 | 0.86546979774784 | -0.837087647 | 0.702381400479976 | 0.93486502033884 | 0.432969649375403 | 2.01855397373447 | 0.999950574162623 |
| ebi-fl187-GCST90094852 | ebi-a-GCST90001732 | CD19 on IgD- CD27- B cell \|\| id:ebi-a-GCST90001732 | F-strong vegetable liking (derived food-liking factor) \|\| id:ebi-fl187-GCST90094852 | MR Egger | 23 | 0.00920252388453188 | 0.347365002701278 | 0.979114757945579 | -0.671632881 | 0.690037929179037 | 1.009244997 | 0.510873700176119 | 1.99379115467061 | 0.999950574162623 |
| ebi-fl187-GCST90094852 | ebi-a-GCST90001798 | CD27 on CD24+ CD27+ B cell \|\| id:ebi-a-GCST90001798 | F-strong vegetable liking (derived food-liking factor) \|\| id:ebi-fl187-GCST90094852 | MR Egger | 23 | -0.306428903 | 0.429927318123361 | 0.48384821310461 | -1.149086447 | 0.536228640175817 | 0.73607084849354 | 0.316926166060998 | 1.70954737103569 | 0.999950574162623 |
| ebi-fl187-GCST90094852 | ebi-a-GCST90001836 | CD66b on CD66b++ myeloid cell \|\| id:ebi-a-GCST90001836 | F-strong vegetable liking (derived food-liking factor) \|\| id:ebi-fl187-GCST90094852 | MR Egger | 23 | 0.135461643379234 | 0.522545869792864 | 0.797978984874581 | -0.888728261 | 1.15965154817325 | 1.14506527415762 | 0.411178331739863 | 3.18882193167515 | 0.999950574162623 |
| ebi-fl187-GCST90094852 | ebi-a-GCST90001943 | CD25 on activated & secreting CD4 regulatory T cell \|\| id:ebi-a-GCST90001943 | F-strong vegetable liking (derived food-liking factor) \|\| id:ebi-fl187-GCST90094852 | MR Egger | 23 | 0.497100231564336 | 0.354859575469163 | 0.175867209858141 | -0.198424536 | 1.1926249994839 | 1.64394728587064 | 0.820021650228071 | 3.29572112878944 | 0.999950574162623 |
| ebi-fl187-GCST90094852 | ebi-a-GCST90001960 | CD25 on CD4+ T cell \|\| id:ebi-a-GCST90001960 | F-strong vegetable liking (derived food-liking factor) \|\| id:ebi-fl187-GCST90094852 | MR Egger | 23 | 0.45750882707863 | 0.406946443441866 | 0.273594983554004 | -0.340106202 | 1.25512385622469 | 1.58013269403456 | 0.711694735296643 | 3.50827286886731 | 0.999950574162623 |
| ebi-fl187-GCST90094852 | ebi-a-GCST90001988 | HLA DR on CD14+ CD16- monocyte \|\| id:ebi-a-GCST90001988 | F-strong vegetable liking (derived food-liking factor) \|\| id:ebi-fl187-GCST90094852 | MR Egger | 23 | -0.391069645 | 0.354556568008922 | 0.282514835319479 | -1.086000518 | 0.303861228462533 | 0.676333051295276 | 0.337563878100567 | 1.35508099636805 | 0.999950574162623 |
| ebi-fl187-GCST90094852 | ebi-a-GCST90001991 | HLA DR on CD14+ monocyte \|\| id:ebi-a-GCST90001991 | F-strong vegetable liking (derived food-liking factor) \|\| id:ebi-fl187-GCST90094852 | MR Egger | 23 | -0.415154895 | 0.355203769602032 | 0.255589433568782 | -1.111354283 | 0.281044493520714 | 0.660238005284895 | 0.329112946954406 | 1.32451253485013 | 0.999950574162623 |
| ebi-fl187-GCST90094852 | ebi-a-GCST90002019 | CD14 on Monocytic Myeloid-Derived Suppressor Cells \|\| id:ebi-a-GCST90002019 | F-strong vegetable liking (derived food-liking factor) \|\| id:ebi-fl187-GCST90094852 | MR Egger | 23 | -0.660225934 | 0.544457205427413 | 0.238736640413857 | -1.727362056 | 0.406910189003034 | 0.516734573581706 | 0.177752693648596 | 1.5021691882911 | 0.999950574162623 |
| ebi-fl187-GCST90094852 | ebi-a-GCST90002037 | CD80 on plasmacytoid Dendritic Cell \|\| id:ebi-a-GCST90002037 | F-strong vegetable liking (derived food-liking factor) \|\| id:ebi-fl187-GCST90094852 | MR Egger | 23 | -0.398144703 | 0.396087604022417 | 0.32624635647235 | -1.174476406 | 0.378187001359669 | 0.671564843502717 | 0.308980717665598 | 1.45963587124856 | 0.999950574162623 |
| ebi-fl187-GCST90094852 | ebi-a-GCST90002042 | CD45 on CD33+ HLA DR+ CD14- \|\| id:ebi-a-GCST90002042 | F-strong vegetable liking (derived food-liking factor) \|\| id:ebi-fl187-GCST90094852 | MR Egger | 23 | -0.087334697 | 0.528606272651367 | 0.870353378160715 | -1.123402991 | 0.948733597735929 | 0.916370338208464 | 0.325171354475349 | 2.58243718332192 | 0.999950574162623 |
| ebi-fl187-GCST90094852 | ebi-a-GCST90002056 | CD8 on Effector Memory CD8+ T cell \|\| id:ebi-a-GCST90002056 | F-strong vegetable liking (derived food-liking factor) \|\| id:ebi-fl187-GCST90094852 | MR Egger | 23 | 0.293159142800858 | 0.386484922070194 | 0.456565165894723 | -0.464351304 | 1.05066959005844 | 1.34065612922696 | 0.628542705823017 | 2.85956521360046 | 0.999950574162623 |
| ebi-fl187-GCST90094852 | ebi-a-GCST90002058 | CD8 on CD8+ T cell \|\| id:ebi-a-GCST90002058 | F-strong vegetable liking (derived food-liking factor) \|\| id:ebi-fl187-GCST90094852 | MR Egger | 23 | 0.399193536539895 | 0.372690023375213 | 0.296274577802073 | -0.331278909 | 1.12966598235531 | 1.4906220805323 | 0.718004882889328 | 3.09462266890032 | 0.999950574162623 |
| ebi-fl187-GCST90094852 | ebi-a-GCST90002063 | CD4 on CD4 regulatory T cell \|\| id:ebi-a-GCST90002063 | F-strong vegetable liking (derived food-liking factor) \|\| id:ebi-fl187-GCST90094852 | MR Egger | 23 | 0.313384663344259 | 0.384459255888126 | 0.424146038753465 | -0.440155478 | 1.06692480488499 | 1.36804766761932 | 0.643936295245825 | 2.90642791017734 | 0.999950574162623 |
| ebi-fl187-GCST90094852 | ebi-a-GCST90002066 | CD4 on activated CD4 regulatory T cell \|\| id:ebi-a-GCST90002066 | F-strong vegetable liking (derived food-liking factor) \|\| id:ebi-fl187-GCST90094852 | MR Egger | 23 | 0.272704802444945 | 0.384471975030833 | 0.485941383843128 | -0.480860269 | 1.02626987350538 | 1.31351244192693 | 0.618251300777806 | 2.79063696740512 | 0.999950574162623 |
| ebi-fl187-GCST90094852 | ebi-a-GCST90002070 | CD4 on activated & secreting CD4 regulatory T cell \|\| id:ebi-a-GCST90002070 | F-strong vegetable liking (derived food-liking factor) \|\| id:ebi-fl187-GCST90094852 | MR Egger | 23 | 0.206052940181541 | 0.386314883173577 | 0.599370429102812 | -0.551124231 | 0.963230111201752 | 1.22881825613492 | 0.576301550076682 | 2.6201461828613 | 0.999950574162623 |
| ebi-fl187-GCST90094852 | ebi-a-GCST90002081 | SSC-A on CD4+ T cell \|\| id:ebi-a-GCST90002081 | F-strong vegetable liking (derived food-liking factor) \|\| id:ebi-fl187-GCST90094852 | MR Egger | 23 | 0.184922109991462 | 0.374098302322855 | 0.626215587744064 | -0.548310563 | 0.918154782544259 | 1.2031247250829 | 0.57792535481899 | 2.50466447273139 | 0.999950574162623 |
| ebi-fl187-GCST90094852 | ebi-a-GCST90002094 | CD11b on Monocytic Myeloid-Derived Suppressor Cells \|\| id:ebi-a-GCST90002094 | F-strong vegetable liking (derived food-liking factor) \|\| id:ebi-fl187-GCST90094852 | MR Egger | 23 | -0.904486747 | 0.511290706201849 | 0.0914125598750356 | -1.906616531 | 0.0976430376137785 | 0.404749570917689 | 0.148582260596672 | 1.1025691391434 | 0.999950574162623 |
| ebi-fl187-GCST90094852 | ebi-a-GCST90002104 | HLA DR on myeloid Dendritic Cell \|\| id:ebi-a-GCST90002104 | F-strong vegetable liking (derived food-liking factor) \|\| id:ebi-fl187-GCST90094852 | MR Egger | 23 | -0.519264089 | 0.39008837147432 | 0.197413304495927 | -1.283837297 | 0.245309119259309 | 0.59495822330736 | 0.276972433189117 | 1.27801631160656 | 0.999950574162623 |
| ebi-fl187-GCST90094852 | ebi-a-GCST90002105 | HLA DR on plasmacytoid Dendritic Cell \|\| id:ebi-a-GCST90002105 | F-strong vegetable liking (derived food-liking factor) \|\| id:ebi-fl187-GCST90094852 | MR Egger | 23 | -0.242456126 | 0.429886561025452 | 0.578728217920125 | -1.085033786 | 0.600121533378118 | 0.784698174489338 | 0.337890369791333 | 1.82234026210088 | 0.999950574162623 |
| ebi-fl187-GCST90094852 | ebi-a-GCST90002106 | HLA DR on Dendritic Cell \|\| id:ebi-a-GCST90002106 | F-strong vegetable liking (derived food-liking factor) \|\| id:ebi-fl187-GCST90094852 | MR Egger | 23 | -0.628056149 | 0.43309654588298 | 0.161788395914787 | -1.476925379 | 0.220813081321208 | 0.533628087186749 | 0.228338665172532 | 1.2470903043049 | 0.999950574162623 |
| ebi-fl187-GCST90094852 | ebi-a-GCST90002111 | HLA DR on CD33dim HLA DR+ CD11b- \|\| id:ebi-a-GCST90002111 | F-strong vegetable liking (derived food-liking factor) \|\| id:ebi-fl187-GCST90094852 | MR Egger | 23 | -0.292028178 | 0.499280529869276 | 0.564848496097115 | -1.270618016 | 0.686561660758305 | 0.74674749398621 | 0.280658116870305 | 1.98687223442169 | 0.999950574162623 |
| ebi-fl187-GCST90094852 | ebi-a-GCST90002112 | HLA DR on CD33- HLA DR+ \|\| id:ebi-a-GCST90002112 | F-strong vegetable liking (derived food-liking factor) \|\| id:ebi-fl187-GCST90094852 | MR Egger | 23 | 0.425920106139829 | 0.677708044440065 | 0.536471449929773 | -0.902387661 | 1.75422787324236 | 1.5309984528706 | 0.405600067225149 | 5.77898391074731 | 0.999950574162623 |
| ebi-fl187-GCST90094852 | ebi-a-GCST90002115 | HLA DR on HLA DR+ CD8+ T cell \|\| id:ebi-a-GCST90002115 | F-strong vegetable liking (derived food-liking factor) \|\| id:ebi-fl187-GCST90094852 | MR Egger | 23 | -0.392008438 | 0.37280975877703 | 0.30498025979652 | -1.122715565 | 0.338698688961553 | 0.675698412230237 | 0.325394962441154 | 1.40312050581616 | 0.999950574162623 |
| ebi-fl187-GCST90094852 | ebi-a-GCST90002116 | HLA DR on B cell \|\| id:ebi-a-GCST90002116 | F-strong vegetable liking (derived food-liking factor) \|\| id:ebi-fl187-GCST90094852 | MR Egger | 23 | -0.139977971 | 0.378519783258784 | 0.715231992942196 | -0.881876747 | 0.601920803725485 | 0.869377386300896 | 0.414005199254907 | 1.82562209646554 | 0.999950574162623 |
| ebi-fl187-GCST90094852 | ebi-cfmet338-GCST90026023 | Sphingomyelin (d18:1/24:1, d18:2/24:0) levels \|\| id:ebi-cfmet338-GCST90026023 | F-strong vegetable liking (derived food-liking factor) \|\| id:ebi-fl187-GCST90094852 | MR Egger | 20 | 0.145533153216033 | 0.374610338288786 | 0.702208682216719 | -0.58870311 | 0.879769416262054 | 1.15665608081546 | 0.55504665271085 | 2.41034385623856 | 0.999950574162623 |
| ebi-fl187-GCST90094852 | ebi-cfmet338-GCST90026046 | 1-arachidonoyl-gpc (20:4n6) levels \|\| id:ebi-cfmet338-GCST90026046 | F-strong vegetable liking (derived food-liking factor) \|\| id:ebi-fl187-GCST90094852 | MR Egger | 19 | 0.143843986558519 | 0.407117722912247 | 0.728189864210947 | -0.65410675 | 0.941794723466523 | 1.15470394513495 | 0.51990626131973 | 2.56458000241365 | 0.999950574162623 |
| ebi-fl187-GCST90094852 | ebi-cfmet338-GCST90026047 | 1-linoleoyl-gpc (18:2) levels \|\| id:ebi-cfmet338-GCST90026047 | F-strong vegetable liking (derived food-liking factor) \|\| id:ebi-fl187-GCST90094852 | MR Egger | 20 | 0.20558068096904 | 0.543164879186474 | 0.70949607392001 | -0.859022482 | 1.27018384417453 | 1.22823807240231 | 0.423575933010323 | 3.56150726453522 | 0.999950574162623 |
| ebi-fl187-GCST90094852 | ebi-cfmet338-GCST90026050 | 1-myristoyl-2-palmitoyl-gpc (14:0/16:0) levels \|\| id:ebi-cfmet338-GCST90026050 | F-strong vegetable liking (derived food-liking factor) \|\| id:ebi-fl187-GCST90094852 | MR Egger | 20 | -0.058081819 | 0.251407799344367 | 0.819900000157711 | -0.550841105 | 0.434677468100028 | 0.943572742463565 | 0.576464738846621 | 1.54446483943071 | 0.999950574162623 |
| ebi-fl187-GCST90094852 | ebi-cfmet338-GCST90026068 | 2,3-dihydroxyisovalerate levels \|\| id:ebi-cfmet338-GCST90026068 | F-strong vegetable liking (derived food-liking factor) \|\| id:ebi-fl187-GCST90094852 | MR Egger | 20 | 0.270996233330633 | 0.845330986273743 | 0.752220508678323 | -1.3858525 | 1.92784496642717 | 1.31127013125425 | 0.250110489747274 | 6.87467910225177 | 0.999950574162623 |
| ebi-fl187-GCST90094852 | ebi-cfmet338-GCST90026082 | 3-hydroxyhexanoate levels \|\| id:ebi-cfmet338-GCST90026082 | F-strong vegetable liking (derived food-liking factor) \|\| id:ebi-fl187-GCST90094852 | MR Egger | 20 | -0.283791851 | 0.173812970422608 | 0.119893162997804 | -0.624465273 | 0.0568815713107651 | 0.752923348851509 | 0.535547723931069 | 1.05853044259924 | 0.999950574162623 |
| ebi-fl187-GCST90094852 | ebi-cfmet338-GCST90026094 | 3-ureidopropionate levels \|\| id:ebi-cfmet338-GCST90026094 | F-strong vegetable liking (derived food-liking factor) \|\| id:ebi-fl187-GCST90094852 | MR Egger | 20 | 0.188186322838051 | 0.39870729207543 | 0.642604423452114 | -0.59327997 | 0.969652615305895 | 1.20705839694488 | 0.552512086600344 | 2.6370282369751 | 0.999950574162623 |
| ebi-fl187-GCST90094852 | ebi-cfmet338-GCST90026173 | Glycerol levels \|\| id:ebi-cfmet338-GCST90026173 | F-strong vegetable liking (derived food-liking factor) \|\| id:ebi-fl187-GCST90094852 | MR Egger | 20 | 0.0256502264075059 | 0.32070630022392 | 0.937135346787602 | -0.602934122 | 0.654234574846389 | 1.02598202428753 | 0.547203715852148 | 1.92366952867249 | 0.999950574162623 |
| ebi-fl187-GCST90094852 | ebi-cfmet338-GCST90026179 | Histidine levels \|\| id:ebi-cfmet338-GCST90026179 | F-strong vegetable liking (derived food-liking factor) \|\| id:ebi-fl187-GCST90094852 | MR Egger | 20 | 0.0638653044530572 | 0.162465973172081 | 0.698862752422048 | -0.254568003 | 0.382298611870336 | 1.06594881053333 | 0.775251331909605 | 1.46564968018643 | 0.999950574162623 |
| ebi-fl187-GCST90094852 | ebi-cfmet338-GCST90026188 | Isocitrate levels \|\| id:ebi-cfmet338-GCST90026188 | F-strong vegetable liking (derived food-liking factor) \|\| id:ebi-fl187-GCST90094852 | MR Egger | 20 | -0.455044969 | 0.375778871685871 | 0.241579189937161 | -1.191571557 | 0.281481619656315 | 0.634419438195478 | 0.303743538387409 | 1.32509164045792 | 0.999950574162623 |
| ebi-fl187-GCST90094852 | ebi-cfmet338-GCST90026207 | N-acetyl-isoputreanine levels \|\| id:ebi-cfmet338-GCST90026207 | F-strong vegetable liking (derived food-liking factor) \|\| id:ebi-fl187-GCST90094852 | MR Egger | 20 | -0.01127714 | 0.207175617791234 | 0.957189993675886 | -0.417341351 | 0.394787070938823 | 0.988786208656611 | 0.65879600101434 | 1.48406815603641 | 0.999950574162623 |
| ebi-fl187-GCST90094852 | ebi-cfmet338-GCST90026213 | N-acetylglutamate levels \|\| id:ebi-cfmet338-GCST90026213 | F-strong vegetable liking (derived food-liking factor) \|\| id:ebi-fl187-GCST90094852 | MR Egger | 19 | 0.474578202617475 | 0.343959092877395 | 0.18554197507785 | -0.199581619 | 1.14873802465717 | 1.60733608432945 | 0.819073365789757 | 3.1542098618925 | 0.999950574162623 |
| ebi-fl187-GCST90094852 | ebi-cfmet338-GCST90026224 | N-acetyltaurine levels \|\| id:ebi-cfmet338-GCST90026224 | F-strong vegetable liking (derived food-liking factor) \|\| id:ebi-fl187-GCST90094852 | MR Egger | 20 | -0.02933674 | 0.313669555495591 | 0.926517665373999 | -0.644129069 | 0.585455588619388 | 0.971089404608918 | 0.525119686060822 | 1.79580894941821 | 0.999950574162623 |
| ebi-fl187-GCST90094852 | ebi-cfmet338-GCST90026250 | Phenyllactate (pla) levels \|\| id:ebi-cfmet338-GCST90026250 | F-strong vegetable liking (derived food-liking factor) \|\| id:ebi-fl187-GCST90094852 | MR Egger | 20 | -0.079019966 | 0.430779635752442 | 0.856507362852951 | -0.923348052 | 0.76530811977194 | 0.924021474968297 | 0.397187009561412 | 2.14965662433271 | 0.999950574162623 |
| ebi-fl187-GCST90094852 | ebi-cfmet338-GCST90026251 | Pipecolate levels \|\| id:ebi-cfmet338-GCST90026251 | F-strong vegetable liking (derived food-liking factor) \|\| id:ebi-fl187-GCST90094852 | MR Egger | 20 | 1.44978708383348 | 0.628556346857017 | 0.0331861063995258 | 0.217816643993724 | 2.68175752367323 | 4.26220692579245 | 1.24335906936395 | 14.6107494816974 | 0.999950574162623 |
| ebi-fl187-GCST90094852 | ebi-cfmet338-GCST90026278 | Trigonelline (n'-methylnicotinate) levels \|\| id:ebi-cfmet338-GCST90026278 | F-strong vegetable liking (derived food-liking factor) \|\| id:ebi-fl187-GCST90094852 | MR Egger | 20 | -0.337989168 | 0.70599214182167 | 0.637884961560991 | -1.721733766 | 1.0457554298466 | 0.713203013183132 | 0.178755958071613 | 2.84554732329381 | 0.999950574162623 |
| ebi-fl187-GCST90094852 | ebi-cfmet338-GCST90026299 | 1-carboxyethylvaline levels \|\| id:ebi-cfmet338-GCST90026299 | F-strong vegetable liking (derived food-liking factor) \|\| id:ebi-fl187-GCST90094852 | MR Egger | 20 | 0.338687878121484 | 0.425576243292504 | 0.436498980689323 | -0.495441559 | 1.17281731497479 | 1.40310533698677 | 0.609301805356055 | 3.23108280555696 | 0.999950574162623 |
| ebi-fl187-GCST90094852 | ebi-gm430-GCST90011306 | C_Erysipelotrichia abundance \|\| id:ebi-gm430-GCST90011306 | F-strong vegetable liking (derived food-liking factor) \|\| id:ebi-fl187-GCST90094852 | MR Egger | 19 | 0.261168798940636 | 0.355969180007473 | 0.473139366781496 | -0.436530794 | 0.958868391755282 | 1.29844682338461 | 0.646274596281302 | 2.60874272771779 | 0.999950574162623 |
| ebi-fl187-GCST90094852 | ebi-gm430-GCST90011312 | F_Erysipelotrichaceae abundance \|\| id:ebi-gm430-GCST90011312 | F-strong vegetable liking (derived food-liking factor) \|\| id:ebi-fl187-GCST90094852 | MR Egger | 19 | 0.261168798940636 | 0.355969180007473 | 0.473139366781496 | -0.436530794 | 0.958868391755282 | 1.29844682338461 | 0.646274596281302 | 2.60874272771779 | 0.999950574162623 |
| ebi-fl187-GCST90094852 | ebi-gm430-GCST90011352 | O_Erysipelotrichales abundance \|\| id:ebi-gm430-GCST90011352 | F-strong vegetable liking (derived food-liking factor) \|\| id:ebi-fl187-GCST90094852 | MR Egger | 19 | 0.261168798940636 | 0.355969180007473 | 0.473139366781496 | -0.436530794 | 0.958868391755282 | 1.29844682338461 | 0.646274596281302 | 2.60874272771779 | 0.999950574162623 |
| ebi-fl187-GCST90094852 | ebi-gm430-GCST90011362 | OTU97_11 (Parabacteroides) abundance \|\| id:ebi-gm430-GCST90011362 | F-strong vegetable liking (derived food-liking factor) \|\| id:ebi-fl187-GCST90094852 | MR Egger | 19 | 0.590165669548182 | 0.46861512889783 | 0.224909742878627 | -0.328319983 | 1.50865132218793 | 1.80428730610142 | 0.720132552593963 | 4.52062980798796 | 0.999950574162623 |
| ebi-fl187-GCST90094852 | ebi-gm430-GCST90011384 | OTU97_235 (Phascolarctobacterium) abundance \|\| id:ebi-gm430-GCST90011384 | F-strong vegetable liking (derived food-liking factor) \|\| id:ebi-fl187-GCST90094852 | MR Egger | 19 | 0.496497061239256 | 1.59819141612459 | 0.759834358914528 | -2.635958114 | 3.62895223684346 | 1.64295600463757 | 0.0716502873416529 | 37.6733232108819 | 0.999950574162623 |
| ebi-fl187-GCST90094852 | ebi-gm430-GCST90011390 | OTU97_3 (Bacteroides) abundance \|\| id:ebi-gm430-GCST90011390 | F-strong vegetable liking (derived food-liking factor) \|\| id:ebi-fl187-GCST90094852 | MR Egger | 19 | -0.258313934 | 0.366963270337483 | 0.49101109629234 | -0.977561944 | 0.460934075728579 | 0.772352726160102 | 0.376227244683558 | 1.58555432132162 | 0.999950574162623 |
| ebi-fl187-GCST90094852 | ebi-gm430-GCST90011398 | OTU97_4 (Alistipes) abundance \|\| id:ebi-gm430-GCST90011398 | F-strong vegetable liking (derived food-liking factor) \|\| id:ebi-fl187-GCST90094852 | MR Egger | 19 | 0.30600517973255 | 0.433875261102011 | 0.490185463032987 | -0.544390332 | 1.15640069149249 | 1.35798934055126 | 0.580195402088389 | 3.17847236019619 | 0.999950574162623 |
| ebi-fl187-GCST90094852 | ebi-gm430-GCST90011421 | OTU99_108 (Cytophagales) abundance \|\| id:ebi-gm430-GCST90011421 | F-strong vegetable liking (derived food-liking factor) \|\| id:ebi-fl187-GCST90094852 | MR Egger | 19 | -0.550611404 | 1.47258822770258 | 0.713095767157274 | -3.436884331 | 2.335661522 | 0.576597168539298 | 0.032164744077528 | 10.3362953538876 | 0.999950574162623 |
| ebi-fl187-GCST90094852 | ebi-gm430-GCST90011428 | OTU99_123 (Phascolarctobacterium) abundance \|\| id:ebi-gm430-GCST90011428 | F-strong vegetable liking (derived food-liking factor) \|\| id:ebi-fl187-GCST90094852 | MR Egger | 19 | 1.17330703929253 | 0.83304718944366 | 0.177023714139882 | -0.459465452 | 2.8060795306021 | 3.23266553289786 | 0.63162118711436 | 16.5449270239469 | 0.999950574162623 |
| ebi-fl187-GCST90094852 | ebi-gm430-GCST90011450 | OTU99_298 (Phascolarctobacterium) abundance \|\| id:ebi-gm430-GCST90011450 | F-strong vegetable liking (derived food-liking factor) \|\| id:ebi-fl187-GCST90094852 | MR Egger | 19 | 0.542820438194362 | 1.59316051912951 | 0.737489342156789 | -2.579774179 | 3.6654150556882 | 1.7208535852092 | 0.0757911172937085 | 39.0723499991624 | 0.999950574162623 |
| ebi-fl187-GCST90094852 | ebi-gm430-GCST90011451 | OTU99_3 (Bacteroides) abundance \|\| id:ebi-gm430-GCST90011451 | F-strong vegetable liking (derived food-liking factor) \|\| id:ebi-fl187-GCST90094852 | MR Egger | 19 | -0.269624595 | 0.366965662937891 | 0.472511911363124 | -0.988877294 | 0.44962810462881 | 0.763666124820383 | 0.371994096637953 | 1.56772904588798 | 0.999950574162623 |
| ebi-fl187-GCST90094852 | ebi-gm430-GCST90011472 | OTU99_72 (Parabacteroides) abundance \|\| id:ebi-gm430-GCST90011472 | F-strong vegetable liking (derived food-liking factor) \|\| id:ebi-fl187-GCST90094852 | MR Egger | 19 | -1.336683459 | 1.01053079883773 | 0.203436156107396 | -3.317323825 | 0.643956906343479 | 0.262715532048174 | 0.0362497126322016 | 1.90399994283108 | 0.999950574162623 |
| ebi-fl187-GCST90094852 | ebi-gm430-GCST90011507 | TestASV_3 (Bacteroides) abundance \|\| id:ebi-gm430-GCST90011507 | F-strong vegetable liking (derived food-liking factor) \|\| id:ebi-fl187-GCST90094852 | MR Egger | 19 | 0.389669495700122 | 0.470583493659135 | 0.419112387513881 | -0.532674152 | 1.31201314327203 | 1.47649272603813 | 0.587033053305248 | 3.7136422860161 | 0.999950574162623 |
| ebi-fl187-GCST90094852 | ebi-gm430-GCST90011512 | TestASV_34 (Phascolarctobacterium) abundance \|\| id:ebi-gm430-GCST90011512 | F-strong vegetable liking (derived food-liking factor) \|\| id:ebi-fl187-GCST90094852 | MR Egger | 19 | -0.255114324 | 1.41190508419985 | 0.858749037297603 | -3.022448289 | 2.51221964144797 | 0.774827911793781 | 0.0486818850354982 | 12.3322729277417 | 0.999950574162623 |
| ebi-fl187-GCST90094852 | ebi-gm430-GCST90011519 | TestASV_41 (Barnesiella) abundance \|\| id:ebi-gm430-GCST90011519 | F-strong vegetable liking (derived food-liking factor) \|\| id:ebi-fl187-GCST90094852 | MR Egger | 19 | 0.820704628358538 | 1.5736178869501 | 0.608719011327827 | -2.26358643 | 3.90499568678073 | 2.27210025989126 | 0.103976909340557 | 49.6498657609576 | 0.999950574162623 |
| ebi-fl187-GCST90094852 | ebi-gm430-GCST90011524 | TestASV_46 (Sutterella) abundance \|\| id:ebi-gm430-GCST90011524 | F-strong vegetable liking (derived food-liking factor) \|\| id:ebi-fl187-GCST90094852 | MR Egger | 19 | 2.11186438494613 | 1.74441909046921 | 0.242596569867337 | -1.307197032 | 5.53092580226577 | 8.26363352558236 | 0.270577414196565 | 252.377454518507 | 0.999950574162623 |
| ebi-fl187-GCST90094852 | ebi-gm430-GCST90011581 | OTU97_19 (Bacteria) prevalence \|\| id:ebi-gm430-GCST90011581 | F-strong vegetable liking (derived food-liking factor) \|\| id:ebi-fl187-GCST90094852 | MR Egger | 19 | 0.0118582352154295 | 1.76415538283706 | 0.994715099566692 | -3.445886315 | 3.46960278557606 | 1.01192882282553 | 0.0318764968965695 | 32.1239798020363 | 0.999950574162623 |
| ebi-fl187-GCST90094852 | ebi-gm430-GCST90011614 | OTU97_80 (Ruminococcus) prevalence \|\| id:ebi-gm430-GCST90011614 | F-strong vegetable liking (derived food-liking factor) \|\| id:ebi-fl187-GCST90094852 | MR Egger | 19 | -0.356167902 | 0.807809606465238 | 0.664836986839257 | -1.93947473 | 1.22713892714882 | 0.700355019688394 | 0.143779452951426 | 3.41145513864516 | 0.999950574162623 |
| ebi-fl187-GCST90094852 | ebi-gm430-GCST90011623 | OTU99_11 (Parabacteroides) prevalence \|\| id:ebi-gm430-GCST90011623 | F-strong vegetable liking (derived food-liking factor) \|\| id:ebi-fl187-GCST90094852 | MR Egger | 19 | 1.7688937610891 | 0.713295558822902 | 0.0239109594505629 | 0.370834465796209 | 3.16695305638198 | 5.8643623859008 | 1.4489432038743 | 23.7350546944915 | 0.999950574162623 |
| ebi-fl187-GCST90094852 | ebi-gm430-GCST90011642 | OTU99_19 (Bacteria) prevalence \|\| id:ebi-gm430-GCST90011642 | F-strong vegetable liking (derived food-liking factor) \|\| id:ebi-fl187-GCST90094852 | MR Egger | 19 | 0.0118582352154295 | 1.76415538283706 | 0.994715099566692 | -3.445886315 | 3.46960278557606 | 1.01192882282553 | 0.0318764968965695 | 32.1239798020363 | 0.999950574162623 |
| ebi-fl187-GCST90094852 | ebi-gm430-GCST90011653 | OTU99_30 (Parasutterella) prevalence \|\| id:ebi-gm430-GCST90011653 | F-strong vegetable liking (derived food-liking factor) \|\| id:ebi-fl187-GCST90094852 | MR Egger | 19 | -0.936860046 | 0.758053091950518 | 0.233310130743822 | -2.422644107 | 0.548924013754174 | 0.391856316286707 | 0.0886868097806894 | 1.73138906443359 | 0.999950574162623 |
| ebi-fl187-GCST90094852 | ebi-gm430-GCST90011675 | OTU99_8 (Ruminococcaceae) prevalence \|\| id:ebi-gm430-GCST90011675 | F-strong vegetable liking (derived food-liking factor) \|\| id:ebi-fl187-GCST90094852 | MR Egger | 19 | 0.599330451109227 | 1.01721442363104 | 0.563483691825595 | -1.394409819 | 2.59307072142606 | 1.82089921110174 | 0.247979345835286 | 13.3707665282466 | 0.999950574162623 |
| ebi-fl187-GCST90094852 | ebi-gm430-GCST90011679 | OTU99_92 (Ruminococcus) prevalence \|\| id:ebi-gm430-GCST90011679 | F-strong vegetable liking (derived food-liking factor) \|\| id:ebi-fl187-GCST90094852 | MR Egger | 19 | -0.760193587 | 0.842967602242992 | 0.379759387876008 | -2.412410088 | 0.89202291308175 | 0.467575901484796 | 0.0895990924722228 | 2.44006069276983 | 0.999950574162623 |
| ebi-fl187-GCST90094852 | ebi-gm430-GCST90011684 | TestASV_11 (Lachnospiraceae) prevalence \|\| id:ebi-gm430-GCST90011684 | F-strong vegetable liking (derived food-liking factor) \|\| id:ebi-fl187-GCST90094852 | MR Egger | 19 | -0.069098104 | 0.844540838165486 | 0.935747535826658 | -1.724398147 | 1.58620193895288 | 0.933235121627399 | 0.17828031809601 | 4.88515951474734 | 0.999950574162623 |
| ebi-fl187-GCST90094852 | ebi-gm430-GCST90011688 | TestASV_15 (Bacteroides) prevalence \|\| id:ebi-gm430-GCST90011688 | F-strong vegetable liking (derived food-liking factor) \|\| id:ebi-fl187-GCST90094852 | MR Egger | 19 | -0.792644457 | 0.877197655276707 | 0.378829015929502 | -2.511951861 | 0.926662947423948 | 0.452646207778593 | 0.081109769618658 | 2.52606548359879 | 0.999950574162623 |
| ebi-fl187-GCST90094852 | ebi-gm430-GCST90011692 | TestASV_19 (Prevotella) prevalence \|\| id:ebi-gm430-GCST90011692 | F-strong vegetable liking (derived food-liking factor) \|\| id:ebi-fl187-GCST90094852 | MR Egger | 19 | -1.355854785 | 1.79769266081669 | 0.461037875955866 | -4.8793324 | 2.16762283037953 | 0.257726899228526 | 0.00760208748730831 | 8.73748884064338 | 0.999950574162623 |
| ebi-fl187-GCST90094852 | ebi-gm430-GCST90011693 | TestASV_2 (Bacteroides) prevalence \|\| id:ebi-gm430-GCST90011693 | F-strong vegetable liking (derived food-liking factor) \|\| id:ebi-fl187-GCST90094852 | MR Egger | 19 | -0.41063131 | 1.00218968996871 | 0.687117737181276 | -2.374923103 | 1.55366048184184 | 0.663231412989239 | 0.0930216420361542 | 4.72874803698626 | 0.999950574162623 |
| ebi-fl187-GCST90094852 | ebi-gm430-GCST90011716 | TestASV_40 (Barnesiella) prevalence \|\| id:ebi-gm430-GCST90011716 | F-strong vegetable liking (derived food-liking factor) \|\| id:ebi-fl187-GCST90094852 | MR Egger | 19 | 0.815461154446477 | 1.83140929971093 | 0.661746570981287 | -2.774101073 | 4.4050233818799 | 2.26021774151533 | 0.0624055495174335 | 81.861056886191 | 0.999950574162623 |
| ebi-fl187-GCST90094852 | ebi-gm430-GCST90011718 | TestASV_42 (Alphaproteobacteria) prevalence \|\| id:ebi-gm430-GCST90011718 | F-strong vegetable liking (derived food-liking factor) \|\| id:ebi-fl187-GCST90094852 | MR Egger | 19 | -1.117954992 | 1.27325365845958 | 0.392168147831279 | -3.613532162 | 1.3776221787581 | 0.326947722197038 | 0.0269564639031311 | 3.96546125018331 | 0.999950574162623 |
| ebi-fl187-GCST90094852 | ebi-hbsm44-GCST90104929 | Sulcal depth of the right inferior frontal sulcus \|\| id:ebi-hbsm44-GCST90104929 | F-strong vegetable liking (derived food-liking factor) \|\| id:ebi-fl187-GCST90094852 | MR Egger | 24 | -0.032856838 | 0.441785477768983 | 0.941385838924513 | -0.898756375 | 0.833042697931526 | 0.96767708378014 | 0.407075594496414 | 2.30030724301131 | 0.999950574162623 |
| ebi-fl187-GCST90094852 | ebi-hbsm44-GCST90104935 | Sulcal opening distance of the left calloso-marginal posterior fissure \|\| id:ebi-hbsm44-GCST90104935 | F-strong vegetable liking (derived food-liking factor) \|\| id:ebi-fl187-GCST90094852 | MR Egger | 24 | 0.0630159833856398 | 0.137766984233581 | 0.651861507754046 | -0.207007306 | 0.333039272483458 | 1.06504386210203 | 0.81301371032171 | 1.39520209044491 | 0.999950574162623 |
| ebi-fl187-GCST90094852 | ebi-hbsm44-GCST90104936 | Sulcal opening distance of the left anterior inferior temporal sulcus \|\| id:ebi-hbsm44-GCST90104936 | F-strong vegetable liking (derived food-liking factor) \|\| id:ebi-fl187-GCST90094852 | MR Egger | 22 | 0.0288420959231181 | 0.0850710108256696 | 0.738116850385448 | -0.137897085 | 0.195581277141431 | 1.02926205696783 | 0.871188345223768 | 1.21601762434223 | 0.999950574162623 |
| ebi-fl187-GCST90094852 | ebi-hbsm44-GCST90104941 | Sulcal opening distance of the right posterior inferior temporal sulcus \|\| id:ebi-hbsm44-GCST90104941 | F-strong vegetable liking (derived food-liking factor) \|\| id:ebi-fl187-GCST90094852 | MR Egger | 22 | 0.0945144604513288 | 0.0651544696034863 | 0.162389104039392 | -0.0331883 | 0.222217220874162 | 1.09912505686246 | 0.967356389257704 | 1.24884262308943 | 0.999950574162623 |
| ebi-fl187-GCST90094852 | ebi-IPs91-GCST90274819 | Leukemia inhibitory factor levels \|\| id:ebi-IPs91-GCST90274819 | F-strong vegetable liking (derived food-liking factor) \|\| id:ebi-fl187-GCST90094852 | MR Egger | 27 | -0.060668802 | 0.209947150700641 | 0.774985688444363 | -0.472165217 | 0.350827613730958 | 0.941134890494165 | 0.623650466736632 | 1.42024247450698 | 0.999950574162623 |
| ebi-fl187-GCST90094852 | ebi-IPs91-GCST90274837 | STAM binding protein levels \|\| id:ebi-IPs91-GCST90274837 | F-strong vegetable liking (derived food-liking factor) \|\| id:ebi-fl187-GCST90094852 | MR Egger | 27 | -0.116055272 | 0.174040093502525 | 0.510988366197548 | -0.457173855 | 0.225063311618133 | 0.890426006625587 | 0.633070268119157 | 1.25240200527939 | 0.999950574162623 |
| ebi-fl187-GCST90094852 | ebi-IPs91-GCST90274840 | TNF-beta levels \|\| id:ebi-IPs91-GCST90274840 | F-strong vegetable liking (derived food-liking factor) \|\| id:ebi-fl187-GCST90094852 | MR Egger | 27 | 0.0100516662258676 | 0.228291246325914 | 0.965230282747186 | -0.437399177 | 0.457502509024659 | 1.01010235391237 | 0.64571362620675 | 1.58012271070245 | 0.999950574162623 |
| ebi-fl187-GCST90094852 | ebi-IPs91-GCST90274842 | Tumor necrosis factor ligand superfamily member 14 levels \|\| id:ebi-IPs91-GCST90274842 | F-strong vegetable liking (derived food-liking factor) \|\| id:ebi-fl187-GCST90094852 | MR Egger | 27 | -0.198089197 | 0.193740156276323 | 0.316361634104227 | -0.577819903 | 0.181641509664347 | 0.820296682168984 | 0.561120330865308 | 1.19918422086004 | 0.999950574162623 |
| ebi-fl187-GCST90094852 | ebi-met1400-GCST90199628 | 3-methylhistidine levels \|\| id:ebi-met1400-GCST90199628 | F-strong vegetable liking (derived food-liking factor) \|\| id:ebi-fl187-GCST90094852 | MR Egger | 26 | -0.132097001 | 0.215777588457359 | 0.546168549862878 | -0.555021074 | 0.290827072636054 | 0.87625599347671 | 0.574060163258033 | 1.33753326784797 | 0.999950574162623 |
| ebi-fl187-GCST90094852 | ebi-met1400-GCST90199651 | Docosahexaenoate DHA; 22:6n3 levels \|\| id:ebi-met1400-GCST90199651 | F-strong vegetable liking (derived food-liking factor) \|\| id:ebi-fl187-GCST90094852 | MR Egger | 26 | 0.344097628913954 | 0.203469904206058 | 0.103755913690365 | -0.054703383 | 0.742898641157827 | 1.41071635549053 | 0.946765932867035 | 2.1020196931061 | 0.999950574162623 |
| ebi-fl187-GCST90094852 | ebi-met1400-GCST90199652 | 1-stearoyl-2-oleoyl-GPS (18:0/18:1) levels \|\| id:ebi-met1400-GCST90199652 | F-strong vegetable liking (derived food-liking factor) \|\| id:ebi-fl187-GCST90094852 | MR Egger | 26 | 0.00611362500357851 | 0.2155541194258 | 0.977607669875234 | -0.416372449 | 0.428599699078146 | 1.00613235135143 | 0.659434618929684 | 1.53510640687776 | 0.999950574162623 |
| ebi-fl187-GCST90094852 | ebi-met1400-GCST90199653 | 1-myristoyl-2-palmitoyl-gpc (14:0/16:0) levels \|\| id:ebi-met1400-GCST90199653 | F-strong vegetable liking (derived food-liking factor) \|\| id:ebi-fl187-GCST90094852 | MR Egger | 26 | -0.06669308 | 0.211390650138493 | 0.755111557489671 | -0.481018754 | 0.347632594440763 | 0.935482275658511 | 0.618153324683635 | 1.41571201371295 | 0.999950574162623 |
| ebi-fl187-GCST90094852 | ebi-met1400-GCST90199667 | Erythritol levels in elite athletes \|\| id:ebi-met1400-GCST90199667 | F-strong vegetable liking (derived food-liking factor) \|\| id:ebi-fl187-GCST90094852 | MR Egger | 26 | -0.045000939 | 0.204447483717176 | 0.827647019379301 | -0.445718007 | 0.355716129444124 | 0.95599658449457 | 0.640364325034885 | 1.42720235003019 | 0.999950574162623 |
| ebi-fl187-GCST90094852 | ebi-met1400-GCST90199674 | 3-hydroxymyristate levels \|\| id:ebi-met1400-GCST90199674 | F-strong vegetable liking (derived food-liking factor) \|\| id:ebi-fl187-GCST90094852 | MR Egger | 26 | 0.24203326835632 | 0.207499604770814 | 0.254901928462135 | -0.164665957 | 0.648732493707116 | 1.27383657056019 | 0.848176984360457 | 1.91311440703626 | 0.999950574162623 |
| ebi-fl187-GCST90094852 | ebi-met1400-GCST90199685 | 2-linoleoylglycerol (18:2) levels \|\| id:ebi-met1400-GCST90199685 | F-strong vegetable liking (derived food-liking factor) \|\| id:ebi-fl187-GCST90094852 | MR Egger | 26 | -0.192880797 | 0.243226056621429 | 0.43554172282556 | -0.669603868 | 0.283842274144703 | 0.824580260859242 | 0.511911322179577 | 1.32822341905573 | 0.999950574162623 |
| ebi-fl187-GCST90094852 | ebi-met1400-GCST90199703 | Gamma-glutamylvaline levels \|\| id:ebi-met1400-GCST90199703 | F-strong vegetable liking (derived food-liking factor) \|\| id:ebi-fl187-GCST90094852 | MR Egger | 26 | 0.0755227073620145 | 0.207612257686519 | 0.719216174035278 | -0.331397318 | 0.482442732427592 | 1.07844771614272 | 0.717919870093035 | 1.6200268649797 | 0.999950574162623 |
| ebi-fl187-GCST90094852 | ebi-met1400-GCST90199779 | Gamma-glutamyl-2-aminobutyrate levels \|\| id:ebi-met1400-GCST90199779 | F-strong vegetable liking (derived food-liking factor) \|\| id:ebi-fl187-GCST90094852 | MR Egger | 26 | 0.393023722058972 | 0.250280810849459 | 0.129429813846393 | -0.097526667 | 0.883574111323912 | 1.48145353204049 | 0.907078151993361 | 2.41953194746476 | 0.999950574162623 |
| ebi-fl187-GCST90094852 | ebi-met1400-GCST90199824 | 4-vinylphenol sulfate levels \|\| id:ebi-met1400-GCST90199824 | F-strong vegetable liking (derived food-liking factor) \|\| id:ebi-fl187-GCST90094852 | MR Egger | 26 | -0.034678485 | 0.219190797708632 | 0.875613901096242 | -0.464292449 | 0.394935478426248 | 0.965915922716702 | 0.628579700336403 | 1.48428841920657 | 0.999950574162623 |
| ebi-fl187-GCST90094852 | ebi-met1400-GCST90199830 | 4-ethylphenylsulfate levels \|\| id:ebi-met1400-GCST90199830 | F-strong vegetable liking (derived food-liking factor) \|\| id:ebi-fl187-GCST90094852 | MR Egger | 26 | -0.196828855 | 0.213006758809888 | 0.364658367727899 | -0.614322102 | 0.220664392258368 | 0.821331188003468 | 0.541007518794109 | 1.2469048894012 | 0.999950574162623 |
| ebi-fl187-GCST90094852 | ebi-met1400-GCST90199833 | Hexanoylglutamine levels \|\| id:ebi-met1400-GCST90199833 | F-strong vegetable liking (derived food-liking factor) \|\| id:ebi-fl187-GCST90094852 | MR Egger | 26 | 0.148608657981889 | 0.216551486005674 | 0.499131701653372 | -0.275832255 | 0.573049570553009 | 1.16021885796524 | 0.758940228824543 | 1.7736677372644 | 0.999950574162623 |
| ebi-fl187-GCST90094852 | ebi-met1400-GCST90199837 | Carotenoid levels (cryptoxanthin) \|\| id:ebi-met1400-GCST90199837 | F-strong vegetable liking (derived food-liking factor) \|\| id:ebi-fl187-GCST90094852 | MR Egger | 26 | 0.430230574005224 | 0.210753312488794 | 0.0523532398512121 | 0.0171540815271891 | 0.84330706648326 | 1.53761201603935 | 1.01730205770458 | 2.32404003703998 | 0.999950574162623 |
| ebi-fl187-GCST90094852 | ebi-met1400-GCST90199869 | 2-hydroxyhippurate levels \|\| id:ebi-met1400-GCST90199869 | F-strong vegetable liking (derived food-liking factor) \|\| id:ebi-fl187-GCST90094852 | MR Egger | 26 | 0.0186332298526489 | 0.217172668634403 | 0.93233785984778 | -0.407025201 | 0.44429166037608 | 1.01880791175592 | 0.665627415832788 | 1.5593852301859 | 0.999950574162623 |
| ebi-fl187-GCST90094852 | ebi-met1400-GCST90199902 | Hydantoin-5-propionate levels \|\| id:ebi-met1400-GCST90199902 | F-strong vegetable liking (derived food-liking factor) \|\| id:ebi-fl187-GCST90094852 | MR Egger | 25 | 0.0762344516807054 | 0.26223687561134 | 0.773879622306916 | -0.437749825 | 0.590218727878931 | 1.07921556840252 | 0.645487247742813 | 1.80438304111382 | 0.999950574162623 |
| ebi-fl187-GCST90094852 | ebi-met1400-GCST90199904 | Pantoate levels \|\| id:ebi-met1400-GCST90199904 | F-strong vegetable liking (derived food-liking factor) \|\| id:ebi-fl187-GCST90094852 | MR Egger | 26 | 0.105787640796218 | 0.231275048843075 | 0.651489829075383 | -0.347511455 | 0.559086736528645 | 1.11158579596591 | 0.706443921613158 | 1.74907440490342 | 0.999950574162623 |
| ebi-fl187-GCST90094852 | ebi-met1400-GCST90199936 | 3-methyl catechol sulfate (2) levels \|\| id:ebi-met1400-GCST90199936 | F-strong vegetable liking (derived food-liking factor) \|\| id:ebi-fl187-GCST90094852 | MR Egger | 26 | 0.00739092150160907 | 0.294395066471408 | 0.98017843189604 | -0.569623409 | 0.584405251785569 | 1.00741830177552 | 0.565738450719819 | 1.79392373536033 | 0.999950574162623 |
| ebi-fl187-GCST90094852 | ebi-met1400-GCST90199977 | 4-hydroxychlorothalonil levels \|\| id:ebi-met1400-GCST90199977 | F-strong vegetable liking (derived food-liking factor) \|\| id:ebi-fl187-GCST90094852 | MR Egger | 26 | 0.586511625458573 | 0.225650465311335 | 0.0157326987321584 | 0.144236713448355 | 1.02878653746879 | 1.79770639153298 | 1.15515751748321 | 2.79766890769987 | 0.999950574162623 |
| ebi-fl187-GCST90094852 | ebi-met1400-GCST90200040 | Sphingomyelin (d18:2/24:1, d18:1/24:2) levels \|\| id:ebi-met1400-GCST90200040 | F-strong vegetable liking (derived food-liking factor) \|\| id:ebi-fl187-GCST90094852 | MR Egger | 26 | 0.294922005822093 | 0.193912312684272 | 0.141350746573185 | -0.085146127 | 0.674990138683266 | 1.34302160673518 | 0.918378074727049 | 1.96401360811409 | 0.999950574162623 |
| ebi-fl187-GCST90094852 | ebi-met1400-GCST90200077 | 1-stearoyl-2-oleoyl-GPI (18:0/18:1) levels \|\| id:ebi-met1400-GCST90200077 | F-strong vegetable liking (derived food-liking factor) \|\| id:ebi-fl187-GCST90094852 | MR Egger | 26 | -0.175759724 | 0.254169289213433 | 0.49588273144122 | -0.673931531 | 0.322412082937692 | 0.838819507387929 | 0.509700729308425 | 1.38045352010623 | 0.999950574162623 |
| ebi-fl187-GCST90094852 | ebi-met1400-GCST90200080 | 1-palmitoyl-2-oleoyl-GPI (16:0/18:1) levels \|\| id:ebi-met1400-GCST90200080 | F-strong vegetable liking (derived food-liking factor) \|\| id:ebi-fl187-GCST90094852 | MR Egger | 26 | -0.122878037 | 0.212278084545086 | 0.568081056065594 | -0.538943082 | 0.293187009097386 | 0.8843715169309 | 0.583364494906739 | 1.34069348886873 | 0.999950574162623 |
| ebi-fl187-GCST90094852 | ebi-met1400-GCST90200082 | 1-oleoyl-2-linoleoyl-GPE (18:1/18:2) levels \|\| id:ebi-met1400-GCST90200082 | F-strong vegetable liking (derived food-liking factor) \|\| id:ebi-fl187-GCST90094852 | MR Egger | 26 | -0.398206898 | 0.256397599179967 | 0.133491835776601 | -0.900746192 | 0.10433239630005 | 0.671523076444424 | 0.406266393677201 | 1.1099693433065 | 0.999950574162623 |
| ebi-fl187-GCST90094852 | ebi-met1400-GCST90200100 | Perfluorooctanesulfonate (PFOS) levels \|\| id:ebi-met1400-GCST90200100 | F-strong vegetable liking (derived food-liking factor) \|\| id:ebi-fl187-GCST90094852 | MR Egger | 26 | -0.271681686 | 0.261620670543065 | 0.309407338739426 | -0.784458201 | 0.24109482781902 | 0.762096808228755 | 0.456366894074572 | 1.2726417114243 | 0.999950574162623 |
| ebi-fl187-GCST90094852 | ebi-met1400-GCST90200102 | 1-palmitoleoyl-2-linolenoyl-GPC (16:1/18:3) levels \|\| id:ebi-met1400-GCST90200102 | F-strong vegetable liking (derived food-liking factor) \|\| id:ebi-fl187-GCST90094852 | MR Egger | 26 | -0.169427065 | 0.207902182475122 | 0.423122268281601 | -0.576915342 | 0.23806121282027 | 0.844148320335608 | 0.561628127759892 | 1.26878685646969 | 0.999950574162623 |
| ebi-fl187-GCST90094852 | ebi-met1400-GCST90200114 | Glycosyl-N-tricosanoyl-sphingadienine (d18:2/23:0) levels \|\| id:ebi-met1400-GCST90200114 | F-strong vegetable liking (derived food-liking factor) \|\| id:ebi-fl187-GCST90094852 | MR Egger | 26 | -0.013144623 | 0.236210174381832 | 0.956082908206719 | -0.476116565 | 0.449827318827803 | 0.986941390312661 | 0.621191069046099 | 1.56804139088485 | 0.999950574162623 |
| ebi-fl187-GCST90094852 | ebi-met1400-GCST90200135 | Arachidoylcarnitine (C20) levels \|\| id:ebi-met1400-GCST90200135 | F-strong vegetable liking (derived food-liking factor) \|\| id:ebi-fl187-GCST90094852 | MR Egger | 26 | 0.164152806715062 | 0.218178589527237 | 0.459139940697798 | -0.263477229 | 0.591782842188446 | 1.17839436790814 | 0.768375119095553 | 1.80720751076915 | 0.999950574162623 |
| ebi-fl187-GCST90094852 | ebi-met1400-GCST90200146 | Docosahexaenoylcarnitine (C22:6) levels \|\| id:ebi-met1400-GCST90200146 | F-strong vegetable liking (derived food-liking factor) \|\| id:ebi-fl187-GCST90094852 | MR Egger | 26 | 0.235818767125519 | 0.289201641127556 | 0.422854823460687 | -0.331016449 | 0.802653983735528 | 1.26594485854977 | 0.718193355032948 | 2.23145532280102 | 0.999950574162623 |
| ebi-fl187-GCST90094852 | ebi-met1400-GCST90200162 | 3-hydroxyphenylacetoylglutamine levels \|\| id:ebi-met1400-GCST90200162 | F-strong vegetable liking (derived food-liking factor) \|\| id:ebi-fl187-GCST90094852 | MR Egger | 26 | 0.00346872679409034 | 0.260618371771481 | 0.989490811582854 | -0.507343282 | 0.514280735466193 | 1.0034747497889 | 0.602093047370907 | 1.6724351457983 | 0.999950574162623 |
| ebi-fl187-GCST90094852 | ebi-met1400-GCST90200172 | 3-carboxy-4-methyl-5-pentyl-2-furanpropionate (3-CMPFP) levels \|\| id:ebi-met1400-GCST90200172 | F-strong vegetable liking (derived food-liking factor) \|\| id:ebi-fl187-GCST90094852 | MR Egger | 26 | 0.395742835742144 | 0.232505981994599 | 0.101661527777984 | -0.059968889 | 0.851454560451558 | 1.4854872541997 | 0.941793833307247 | 2.34305248595725 | 0.999950574162623 |
| ebi-fl187-GCST90094852 | ebi-met1400-GCST90200222 | Dihydrocaffeate sulfate (2) levels \|\| id:ebi-met1400-GCST90200222 | F-strong vegetable liking (derived food-liking factor) \|\| id:ebi-fl187-GCST90094852 | MR Egger | 26 | -0.193455991 | 0.354120541809903 | 0.589902892641788 | -0.887532253 | 0.500620271059723 | 0.824106103575128 | 0.411670398751932 | 1.64974424201685 | 0.999950574162623 |
| ebi-fl187-GCST90094852 | ebi-met1400-GCST90200223 | 4-methylhexanoylglutamine levels \|\| id:ebi-met1400-GCST90200223 | F-strong vegetable liking (derived food-liking factor) \|\| id:ebi-fl187-GCST90094852 | MR Egger | 26 | -0.023922818 | 0.281533365143327 | 0.932987497625891 | -0.575728213 | 0.527882578081815 | 0.976361064740949 | 0.562295248812217 | 1.69533875798484 | 0.999950574162623 |
| ebi-fl187-GCST90094852 | ebi-met1400-GCST90200224 | 2-ketocaprylate levels \|\| id:ebi-met1400-GCST90200224 | F-strong vegetable liking (derived food-liking factor) \|\| id:ebi-fl187-GCST90094852 | MR Egger | 26 | -0.147927389 | 0.231777617942386 | 0.529367567497348 | -0.60221152 | 0.306356741946505 | 0.862493739011068 | 0.547599269053162 | 1.35846684222121 | 0.999950574162623 |
| ebi-fl187-GCST90094852 | ebi-met1400-GCST90200243 | Hydroxypalmitoyl sphingomyelin (d18:1/16:0(OH)) levels \|\| id:ebi-met1400-GCST90200243 | F-strong vegetable liking (derived food-liking factor) \|\| id:ebi-fl187-GCST90094852 | MR Egger | 26 | 0.04514985826252 | 0.222337575002684 | 0.840792958541757 | -0.390631789 | 0.48093150526778 | 1.04618462757252 | 0.67662925268421 | 1.61758048536496 | 0.999950574162623 |
| ebi-fl187-GCST90094852 | ebi-met1400-GCST90200273 | 2-methoxyhydroquinone sulfate (2) levels \|\| id:ebi-met1400-GCST90200273 | F-strong vegetable liking (derived food-liking factor) \|\| id:ebi-fl187-GCST90094852 | MR Egger | 26 | 0.339636881129863 | 0.312835113369104 | 0.288408405240331 | -0.273519941 | 0.952793703333306 | 1.4044375201956 | 0.760697167086438 | 2.59294346485851 | 0.999950574162623 |
| ebi-fl187-GCST90094852 | ebi-met1400-GCST90200276 | 2,4-di-tert-butylphenol levels \|\| id:ebi-met1400-GCST90200276 | F-strong vegetable liking (derived food-liking factor) \|\| id:ebi-fl187-GCST90094852 | MR Egger | 26 | 0.102229853587954 | 0.211742248112824 | 0.633610223251759 | -0.312784953 | 0.517244659889089 | 1.10763803704741 | 0.731407182788228 | 1.67739947047999 | 0.999950574162623 |
| ebi-fl187-GCST90094852 | ebi-met1400-GCST90200289 | N-acetylmethionine levels \|\| id:ebi-met1400-GCST90200289 | F-strong vegetable liking (derived food-liking factor) \|\| id:ebi-fl187-GCST90094852 | MR Egger | 26 | -0.119694682 | 0.212140853145808 | 0.577837939181804 | -0.535490754 | 0.296101389977104 | 0.887191270666822 | 0.585381940905093 | 1.34460647954123 | 0.999950574162623 |
| ebi-fl187-GCST90094852 | ebi-met1400-GCST90200308 | 3-hydroxyisobutyrate levels \|\| id:ebi-met1400-GCST90200308 | F-strong vegetable liking (derived food-liking factor) \|\| id:ebi-fl187-GCST90094852 | MR Egger | 26 | 0.113250892149471 | 0.214817716184583 | 0.602897724173246 | -0.307791832 | 0.534293615871254 | 1.11991287505247 | 0.735068320099852 | 1.70624255380495 | 0.999950574162623 |
| ebi-fl187-GCST90094852 | ebi-met1400-GCST90200383 | Cystathionine levels \|\| id:ebi-met1400-GCST90200383 | F-strong vegetable liking (derived food-liking factor) \|\| id:ebi-fl187-GCST90094852 | MR Egger | 26 | -0.219340881 | 0.262425430280495 | 0.411499564595114 | -0.733694724 | 0.295012962775238 | 0.803047928052322 | 0.480131754634109 | 1.34314376944423 | 0.999950574162623 |
| ebi-fl187-GCST90094852 | ebi-met1400-GCST90200384 | Guanidinoacetate levels \|\| id:ebi-met1400-GCST90200384 | F-strong vegetable liking (derived food-liking factor) \|\| id:ebi-fl187-GCST90094852 | MR Egger | 26 | -0.181614552 | 0.274918388881227 | 0.515152216109908 | -0.720454594 | 0.357225490641795 | 0.833922712679711 | 0.486531031703001 | 1.42935814040245 | 0.999950574162623 |
| ebi-fl187-GCST90094852 | ebi-met1400-GCST90200403 | Citrulline levels \|\| id:ebi-met1400-GCST90200403 | F-strong vegetable liking (derived food-liking factor) \|\| id:ebi-fl187-GCST90094852 | MR Egger | 26 | 0.00723125759683201 | 0.213030539701565 | 0.973201988889278 | -0.4103086 | 0.424771115411899 | 1.00725746627586 | 0.663445479122111 | 1.52924036005336 | 0.999950574162623 |
| ebi-fl187-GCST90094852 | ebi-met1400-GCST90200434 | 12,13-DiHOME levels \|\| id:ebi-met1400-GCST90200434 | F-strong vegetable liking (derived food-liking factor) \|\| id:ebi-fl187-GCST90094852 | MR Egger | 26 | 0.106890640750079 | 0.214961837911791 | 0.62353490020872 | -0.314434562 | 0.528215843057189 | 1.11281255147884 | 0.730201641640092 | 1.69590384917159 | 0.999950574162623 |
| ebi-fl187-GCST90094852 | ebi-met1400-GCST90200437 | Fructose levels \|\| id:ebi-met1400-GCST90200437 | F-strong vegetable liking (derived food-liking factor) \|\| id:ebi-fl187-GCST90094852 | MR Egger | 25 | -0.192665192 | 0.215087276672392 | 0.379664003096705 | -0.614236255 | 0.228905869986557 | 0.824758063275514 | 0.541053965042783 | 1.25722369095694 | 0.999950574162623 |
| ebi-fl187-GCST90094852 | ebi-met1400-GCST90200448 | N-stearoyl-sphinganine (d18:0/18:0) levels \|\| id:ebi-met1400-GCST90200448 | F-strong vegetable liking (derived food-liking factor) \|\| id:ebi-fl187-GCST90094852 | MR Egger | 26 | 0.174180473433413 | 0.237328821596519 | 0.470106940055342 | -0.290984017 | 0.63934496376259 | 1.19027035861589 | 0.747527625734571 | 1.8952390223805 | 0.999950574162623 |
| ebi-fl187-GCST90094852 | ebi-met1400-GCST90200468 | X-11847 levels \|\| id:ebi-met1400-GCST90200468 | F-strong vegetable liking (derived food-liking factor) \|\| id:ebi-fl187-GCST90094852 | MR Egger | 26 | 0.320102391319383 | 0.283277863419573 | 0.269645906088558 | -0.235122221 | 0.875327003621746 | 1.37726877748384 | 0.7904742311869 | 2.39965986314783 | 0.999950574162623 |
| ebi-fl187-GCST90094852 | ebi-met1400-GCST90200476 | X-11843 levels \|\| id:ebi-met1400-GCST90200476 | F-strong vegetable liking (derived food-liking factor) \|\| id:ebi-fl187-GCST90094852 | MR Egger | 26 | 0.455324848925951 | 0.304356303569265 | 0.147682328140126 | -0.141213506 | 1.05186320392171 | 1.57668548443787 | 0.868303903751622 | 2.86298046812444 | 0.999950574162623 |
| ebi-fl187-GCST90094852 | ebi-met1400-GCST90200511 | X-12906 levels \|\| id:ebi-met1400-GCST90200511 | F-strong vegetable liking (derived food-liking factor) \|\| id:ebi-fl187-GCST90094852 | MR Egger | 26 | -0.485149534 | 0.234073595925125 | 0.0491119742687415 | -0.943933782 | -0.026365286 | 0.615605135831585 | 0.389094208963716 | 0.973979243411365 | 0.999950574162623 |
| ebi-fl187-GCST90094852 | ebi-met1400-GCST90200515 | X-12849 levels \|\| id:ebi-met1400-GCST90200515 | F-strong vegetable liking (derived food-liking factor) \|\| id:ebi-fl187-GCST90094852 | MR Egger | 26 | -0.08618081 | 0.22988019033692 | 0.711031460561593 | -0.536745983 | 0.364384362847015 | 0.917428335809681 | 0.584647613534956 | 1.43962744713436 | 0.999950574162623 |
| ebi-fl187-GCST90094852 | ebi-met1400-GCST90200551 | X-18886 levels \|\| id:ebi-met1400-GCST90200551 | F-strong vegetable liking (derived food-liking factor) \|\| id:ebi-fl187-GCST90094852 | MR Egger | 26 | -0.066776317 | 0.209883659772465 | 0.753116524383977 | -0.47814829 | 0.344595655857988 | 0.935404411725638 | 0.619930260428171 | 1.41141910522558 | 0.999950574162623 |
| ebi-fl187-GCST90094852 | ebi-met1400-GCST90200564 | X-21319 levels \|\| id:ebi-met1400-GCST90200564 | F-strong vegetable liking (derived food-liking factor) \|\| id:ebi-fl187-GCST90094852 | MR Egger | 26 | -0.129349661 | 0.218333288816938 | 0.559096134816603 | -0.557282907 | 0.298583585234154 | 0.878666676490868 | 0.572763202452209 | 1.34794820105422 | 0.999950574162623 |
| ebi-fl187-GCST90094852 | ebi-met1400-GCST90200579 | X-21442 levels \|\| id:ebi-met1400-GCST90200579 | F-strong vegetable liking (derived food-liking factor) \|\| id:ebi-fl187-GCST90094852 | MR Egger | 26 | -0.02658994 | 0.234656776208946 | 0.91072402142801 | -0.486517221 | 0.433337341391548 | 0.973760459902618 | 0.614763756130835 | 1.54239644711254 | 0.999950574162623 |
| ebi-fl187-GCST90094852 | ebi-met1400-GCST90200581 | X-21441 levels \|\| id:ebi-met1400-GCST90200581 | F-strong vegetable liking (derived food-liking factor) \|\| id:ebi-fl187-GCST90094852 | MR Egger | 26 | 0.060669280407764 | 0.25785611984356 | 0.815984751415175 | -0.444728714 | 0.566067275301142 | 1.06254745081282 | 0.640998145958803 | 1.76132660031343 | 0.999950574162623 |
| ebi-fl187-GCST90094852 | ebi-met1400-GCST90200648 | X-24585 levels \|\| id:ebi-met1400-GCST90200648 | F-strong vegetable liking (derived food-liking factor) \|\| id:ebi-fl187-GCST90094852 | MR Egger | 26 | -0.250739383 | 0.208060313673228 | 0.239902595784142 | -0.658537598 | 0.157058831886295 | 0.778225163907301 | 0.517607732009349 | 1.17006444897467 | 0.999950574162623 |
| ebi-fl187-GCST90094852 | ebi-met1400-GCST90200720 | Adenosine 5'-diphosphate (ADP) to pantothenate ratio \|\| id:ebi-met1400-GCST90200720 | F-strong vegetable liking (derived food-liking factor) \|\| id:ebi-fl187-GCST90094852 | MR Egger | 26 | 0.233652351751777 | 0.281855432997159 | 0.41528497126352 | -0.318784297 | 0.786089000426209 | 1.26320526476643 | 0.727032355510353 | 2.19479577330986 | 0.999950574162623 |
| ebi-fl187-GCST90094852 | ebi-met1400-GCST90200724 | Cholate to taurocholate ratio \|\| id:ebi-met1400-GCST90200724 | F-strong vegetable liking (derived food-liking factor) \|\| id:ebi-fl187-GCST90094852 | MR Egger | 26 | -0.322106874 | 0.247137201630942 | 0.204819942677323 | -0.806495789 | 0.162282041334045 | 0.724620743173694 | 0.446419677234896 | 1.17619192928477 | 0.999950574162623 |
| ebi-fl187-GCST90094852 | ebi-met1400-GCST90200726 | N-acetylputrescine to (N(1) + N(8))-acetylspermidine ratio \|\| id:ebi-met1400-GCST90200726 | F-strong vegetable liking (derived food-liking factor) \|\| id:ebi-fl187-GCST90094852 | MR Egger | 26 | -0.069853902 | 0.227039873269915 | 0.760985821544918 | -0.514852053 | 0.375144249729054 | 0.932530050841454 | 0.597588999285943 | 1.45520131187398 | 0.999950574162623 |
| ebi-fl187-GCST90094852 | ebi-met1400-GCST90200744 | Arginine to glutamate ratio \|\| id:ebi-met1400-GCST90200744 | F-strong vegetable liking (derived food-liking factor) \|\| id:ebi-fl187-GCST90094852 | MR Egger | 26 | 0.317843358255514 | 0.220712980633139 | 0.162764222601217 | -0.114754084 | 0.750440800296466 | 1.37416099339283 | 0.891585372249526 | 2.11793339654958 | 0.999950574162623 |
| ebi-fl187-GCST90094852 | ebi-met1400-GCST90200759 | Histidine to trans-urocanate ratio \|\| id:ebi-met1400-GCST90200759 | F-strong vegetable liking (derived food-liking factor) \|\| id:ebi-fl187-GCST90094852 | MR Egger | 26 | 0.108159840323877 | 0.225361852171092 | 0.635616266836012 | -0.33354939 | 0.549869070579216 | 1.11422582937103 | 0.716376515979172 | 1.7330260989092 | 0.999950574162623 |
| ebi-fl187-GCST90094852 | ebi-met1400-GCST90200795 | Oleoyl-linoleoyl-glycerol (18:1 to 18:2) [2] to linoleoyl-arachidonoyl-glycerol (18:2 to 20:4) [2] ratio \|\| id:ebi-met1400-GCST90200795 | F-strong vegetable liking (derived food-liking factor) \|\| id:ebi-fl187-GCST90094852 | MR Egger | 26 | 0.543160574419622 | 0.220774830529548 | 0.0214666127509332 | 0.110441906581707 | 0.975879242257537 | 1.72143900940824 | 1.11677147009547 | 2.65349925429158 | 0.999950574162623 |
| ebi-fl187-GCST90094852 | ebi-met1400-GCST90200811 | Choline to taurocholate ratio \|\| id:ebi-met1400-GCST90200811 | F-strong vegetable liking (derived food-liking factor) \|\| id:ebi-fl187-GCST90094852 | MR Egger | 26 | -0.185438915 | 0.22280960148609 | 0.41345888409307 | -0.622145734 | 0.251267904120406 | 0.830739579930801 | 0.536791389592857 | 1.28565447032794 | 0.999950574162623 |
| ebi-fl187-GCST90094852 | ebi-met1400-GCST90200831 | Cholate to phosphate ratio \|\| id:ebi-met1400-GCST90200831 | F-strong vegetable liking (derived food-liking factor) \|\| id:ebi-fl187-GCST90094852 | MR Egger | 26 | 0.00840396929919142 | 0.235829105543875 | 0.971867470631923 | -0.453821078 | 0.470629016165187 | 1.00843938178147 | 0.635196373959975 | 1.6010009320234 | 0.999950574162623 |
| ebi-fl187-GCST90094852 | ebi-met1400-GCST90200841 | Adenosine 5'-diphosphate (ADP) to oxalate (ethanedioate) ratio \|\| id:ebi-met1400-GCST90200841 | F-strong vegetable liking (derived food-liking factor) \|\| id:ebi-fl187-GCST90094852 | MR Egger | 26 | 0.516100968879732 | 0.292974704892001 | 0.0908747229596166 | -0.058129453 | 1.09033139046805 | 1.67548214041207 | 0.943527797301647 | 2.97525988197499 | 0.999950574162623 |
| ebi-fl187-GCST90094852 | ebi-met1400-GCST90200844 | Aspartate to phosphate ratio \|\| id:ebi-met1400-GCST90200844 | F-strong vegetable liking (derived food-liking factor) \|\| id:ebi-fl187-GCST90094852 | MR Egger | 26 | -0.106219504 | 0.200020194164579 | 0.600268919939462 | -0.498259084 | 0.285820076745294 | 0.899227242598736 | 0.607587498078513 | 1.33085298230945 | 0.999950574162623 |
| ebi-fl187-GCST90094852 | ebi-met1400-GCST90200870 | Adenosine 5'-monophosphate (AMP) to acetoacetate ratio \|\| id:ebi-met1400-GCST90200870 | F-strong vegetable liking (derived food-liking factor) \|\| id:ebi-fl187-GCST90094852 | MR Egger | 26 | 0.227920766346675 | 0.234674631304869 | 0.341125822971259 | -0.232041511 | 0.687883043704217 | 1.25598580514585 | 0.79291320799472 | 1.98949938886423 | 0.999950574162623 |
| ebi-fl187-GCST90094852 | ebi-met1400-GCST90200887 | Cholesterol to cortisol ratio \|\| id:ebi-met1400-GCST90200887 | F-strong vegetable liking (derived food-liking factor) \|\| id:ebi-fl187-GCST90094852 | MR Egger | 25 | -0.51732213 | 0.253808204762203 | 0.0531849520049045 | -1.014786212 | -0.019858049 | 0.596114730145696 | 0.362479915575232 | 0.980337823497763 | 0.999950574162623 |
| ebi-fl187-GCST90094852 | ebi-met1400-GCST90200890 | Cortisol to taurocholate ratio \|\| id:ebi-met1400-GCST90200890 | F-strong vegetable liking (derived food-liking factor) \|\| id:ebi-fl187-GCST90094852 | MR Egger | 26 | 0.159060379459591 | 0.221881875993227 | 0.48037344879437 | -0.275828097 | 0.593948856406316 | 1.17240873394942 | 0.758943383823118 | 1.81112619035789 | 0.999950574162623 |
| ebi-fl187-GCST90094852 | ebi-met1400-GCST90200948 | Adenosine 5'-diphosphate (ADP) to mannitol to sorbitol ratio \|\| id:ebi-met1400-GCST90200948 | F-strong vegetable liking (derived food-liking factor) \|\| id:ebi-fl187-GCST90094852 | MR Egger | 26 | 0.132340144711811 | 0.309263370875196 | 0.672524507544752 | -0.473816062 | 0.738496351627194 | 1.14149652724734 | 0.622621765711309 | 2.09278633269288 | 0.999950574162623 |
| ebi-fl187-GCST90094852 | ebi-met1400-GCST90200972 | Leucine to N-palmitoyl-sphingosine (d18:1 to 16:0) ratio \|\| id:ebi-met1400-GCST90200972 | F-strong vegetable liking (derived food-liking factor) \|\| id:ebi-fl187-GCST90094852 | MR Egger | 26 | -0.072536017 | 0.232813050190173 | 0.758062786632403 | -0.528849595 | 0.383777561756038 | 0.930032249442006 | 0.589282493423348 | 1.46781890630638 | 0.999950574162623 |
| ebi-fl187-GCST90094852 | ebi-met1400-GCST90200973 | Proline to glutamate ratio \|\| id:ebi-met1400-GCST90200973 | F-strong vegetable liking (derived food-liking factor) \|\| id:ebi-fl187-GCST90094852 | MR Egger | 26 | 0.0265334833023337 | 0.218363963732166 | 0.904298471924932 | -0.401459886 | 0.454526852217378 | 1.02688863030852 | 0.669342169412203 | 1.575427796 | 0.999950574162623 |
| ebi-fl187-GCST90094852 | ebi-met1400-GCST90200983 | Cholesterol to linoleoyl-arachidonoyl-glycerol (18:2 to 20:4) [1] ratio \|\| id:ebi-met1400-GCST90200983 | F-strong vegetable liking (derived food-liking factor) \|\| id:ebi-fl187-GCST90094852 | MR Egger | 26 | 0.20396982680405 | 0.218913166024874 | 0.360750579288344 | -0.225099979 | 0.633039632212804 | 1.22626115267995 | 0.798436388212733 | 1.8833265076232 | 0.999950574162623 |
| ebi-fl187-GCST90094852 | ebi-met1400-GCST90201001 | Serine to alpha-ketobutyrate ratio \|\| id:ebi-met1400-GCST90201001 | F-strong vegetable liking (derived food-liking factor) \|\| id:ebi-fl187-GCST90094852 | MR Egger | 26 | 0.0333059659834748 | 0.267256224638933 | 0.901860875629959 | -0.490516234 | 0.557128166275783 | 1.03386681893146 | 0.612310217039008 | 1.74565207233732 | 0.999950574162623 |
| ebi-fl187-GCST90094852 | ebi-met1400-GCST90201005 | Fructose to maltose ratio \|\| id:ebi-met1400-GCST90201005 | F-strong vegetable liking (derived food-liking factor) \|\| id:ebi-fl187-GCST90094852 | MR Egger | 26 | -0.016768137 | 0.242608815285213 | 0.945469979934005 | -0.492281415 | 0.458745141267736 | 0.983371666011812 | 0.611230332456928 | 1.58208744259757 | 0.999950574162623 |
| ebi-fl187-GCST90094852 | ebi-met1400-GCST90201020 | Paraxanthine to 5-acetylamino-6-formylamino-3-methyluracil ratio \|\| id:ebi-met1400-GCST90201020 | F-strong vegetable liking (derived food-liking factor) \|\| id:ebi-fl187-GCST90094852 | MR Egger | 25 | -0.069544689 | 0.225137458851136 | 0.760179266875772 | -0.510814108 | 0.371724730275645 | 0.932818445661848 | 0.600006909246892 | 1.45023372090693 | 0.999950574162623 |
| ebi-fl187-GCST90094852 | ebi-pw11-GCST90275197 | Placental weight (fetal genotype, adjusted for sex) \|\| id:ebi-pw11-GCST90275197 | F-strong vegetable liking (derived food-liking factor) \|\| id:ebi-fl187-GCST90094852 | MR Egger | 20 | 0.184254639283386 | 0.183058123622979 | 0.327497682963705 | -0.174539283 | 0.543048561584424 | 1.20232194251747 | 0.839843861979942 | 1.72124619694311 | 0.999950574162623 |
| ebi-fl187-GCST90094852 | prot-a-1017 | FAS-associated factor 2 \|\| id:prot-a-1017 | F-strong vegetable liking (derived food-liking factor) \|\| id:ebi-fl187-GCST90094852 | MR Egger | 27 | 0.35133173886292 | 0.383675482229163 | 0.368572910041415 | -0.400672206 | 1.10333568403208 | 1.42095863493392 | 0.669869604085416 | 3.01420370454041 | 0.999950574162623 |
| ebi-fl187-GCST90094852 | prot-a-1019 | Fas apoptotic inhibitory molecule 3 \|\| id:prot-a-1019 | F-strong vegetable liking (derived food-liking factor) \|\| id:ebi-fl187-GCST90094852 | MR Egger | 27 | -0.033053763 | 0.347310091404593 | 0.924937944256302 | -0.713781542 | 0.647674016225122 | 0.96748654328152 | 0.48978853508347 | 1.91109048984028 | 0.999950574162623 |
| ebi-fl187-GCST90094852 | prot-a-1052 | MIP18 family protein FAM96A \|\| id:prot-a-1052 | F-strong vegetable liking (derived food-liking factor) \|\| id:ebi-fl187-GCST90094852 | MR Egger | 27 | -0.583486345 | 0.343349507054975 | 0.101656553111146 | -1.256451379 | 0.089478688732272 | 0.557949766361247 | 0.284662395257858 | 1.0936040269758 | 0.999950574162623 |
| ebi-fl187-GCST90094852 | prot-a-1066 | F-box/LRR-repeat protein 4 \|\| id:prot-a-1066 | F-strong vegetable liking (derived food-liking factor) \|\| id:ebi-fl187-GCST90094852 | MR Egger | 27 | 0.17210691874915 | 0.342023542755167 | 0.619227144452083 | -0.498259225 | 0.842473062549278 | 1.18780482503101 | 0.607587412608503 | 2.32210258653933 | 0.999950574162623 |
| ebi-fl187-GCST90094852 | prot-a-1076 | Low affinity immunoglobulin gamma Fc region receptor III-B \|\| id:prot-a-1076 | F-strong vegetable liking (derived food-liking factor) \|\| id:ebi-fl187-GCST90094852 | MR Egger | 27 | -0.506141649 | 0.366721346741115 | 0.179749268339338 | -1.224915489 | 0.212632190646621 | 0.602816977054502 | 0.293782527253414 | 1.23692961328388 | 0.999950574162623 |
| ebi-fl187-GCST90094852 | prot-a-1081 | Fc receptor-like protein 3 \|\| id:prot-a-1081 | F-strong vegetable liking (derived food-liking factor) \|\| id:ebi-fl187-GCST90094852 | MR Egger | 27 | -0.044834672 | 0.362164786070356 | 0.902466498661712 | -0.754677653 | 0.665008308623307 | 0.956155547979483 | 0.470162145770592 | 1.94450667744321 | 0.999950574162623 |
| ebi-fl187-GCST90094852 | prot-a-1105 | Fibroblast growth factor receptor 4 \|\| id:prot-a-1105 | F-strong vegetable liking (derived food-liking factor) \|\| id:ebi-fl187-GCST90094852 | MR Egger | 27 | -0.544333922 | 0.36666157625648 | 0.150158930893055 | -1.262990611 | 0.174322767683742 | 0.580228131978302 | 0.28280699478197 | 1.19043973929492 | 0.999950574162623 |
| ebi-fl187-GCST90094852 | prot-a-1143 | Follistatin-related protein 1 \|\| id:prot-a-1143 | F-strong vegetable liking (derived food-liking factor) \|\| id:ebi-fl187-GCST90094852 | MR Egger | 27 | -0.56717503 | 0.427358370483406 | 0.196448131837928 | -1.404797436 | 0.270447376307812 | 0.567125289890751 | 0.245416764022818 | 1.31055062890392 | 0.999950574162623 |
| ebi-fl187-GCST90094852 | prot-a-1151 | Alpha-(1,3)-fucosyltransferase 10 \|\| id:prot-a-1151 | F-strong vegetable liking (derived food-liking factor) \|\| id:ebi-fl187-GCST90094852 | MR Egger | 27 | -0.131508849 | 0.345515903350455 | 0.706703272023261 | -0.808720019 | 0.54570232181485 | 0.876771516769722 | 0.445427840525783 | 1.7258200379017 | 0.999950574162623 |
| ebi-fl187-GCST90094852 | prot-a-1165 | Growth arrest and DNA damage-inducible proteins-interacting protein 1 \|\| id:prot-a-1165 | F-strong vegetable liking (derived food-liking factor) \|\| id:ebi-fl187-GCST90094852 | MR Egger | 27 | 0.785803991932605 | 0.380049398751761 | 0.0491721759532497 | 0.0409071703791532 | 1.53070081348606 | 2.19417032700573 | 1.0417553952984 | 4.62141443724745 | 0.999950574162623 |
| ebi-fl187-GCST90094852 | prot-a-1194 | Growth/differentiation factor 11/8 \|\| id:prot-a-1194 | F-strong vegetable liking (derived food-liking factor) \|\| id:ebi-fl187-GCST90094852 | MR Egger | 27 | -0.573594859 | 0.379346290368455 | 0.143057421380241 | -1.317113588 | 0.169923870310774 | 0.563496104363792 | 0.267907478398392 | 1.18521461786516 | 0.999950574162623 |
| ebi-fl187-GCST90094852 | prot-a-1216 | D-glucuronyl C5-epimerase \|\| id:prot-a-1216 | F-strong vegetable liking (derived food-liking factor) \|\| id:ebi-fl187-GCST90094852 | MR Egger | 27 | 0.39275028524905 | 0.339368131924189 | 0.258088581971165 | -0.272411253 | 1.05791182382046 | 1.48104850349002 | 0.761541010410916 | 2.88035002672599 | 0.999950574162623 |
| ebi-fl187-GCST90094852 | prot-a-1246 | Glypican-3 \|\| id:prot-a-1246 | F-strong vegetable liking (derived food-liking factor) \|\| id:ebi-fl187-GCST90094852 | MR Egger | 27 | -0.318625561 | 0.4334502024542 | 0.469124019008004 | -1.168187958 | 0.530936835796134 | 0.727147770811889 | 0.31092984910534 | 1.70052467499692 | 0.999950574162623 |
| ebi-fl187-GCST90094852 | prot-a-1275 | Glutamate receptor 4 \|\| id:prot-a-1275 | F-strong vegetable liking (derived food-liking factor) \|\| id:ebi-fl187-GCST90094852 | MR Egger | 27 | -0.115131897 | 0.400151265939815 | 0.775932046988503 | -0.899428378 | 0.669164584551408 | 0.891248583415402 | 0.406802130366355 | 1.95260540234785 | 0.999950574162623 |
| ebi-fl187-GCST90094852 | prot-a-1289 | Glutathione S-transferase P \|\| id:prot-a-1289 | F-strong vegetable liking (derived food-liking factor) \|\| id:ebi-fl187-GCST90094852 | MR Egger | 27 | 0.329235513729106 | 0.357964739050364 | 0.366497072861645 | -0.372375375 | 1.03084640226782 | 1.38990515879095 | 0.689095524867767 | 2.80343766679431 | 0.999950574162623 |
| ebi-fl187-GCST90094852 | prot-a-130 | Apolipoprotein D \|\| id:prot-a-130 | F-strong vegetable liking (derived food-liking factor) \|\| id:ebi-fl187-GCST90094852 | MR Egger | 27 | 0.347193097064843 | 0.386214178076243 | 0.3772485880251 | -0.409786692 | 1.10417288609428 | 1.41508994870585 | 0.663791827166596 | 3.01672825873126 | 0.999950574162623 |
| ebi-fl187-GCST90094852 | prot-a-1313 | Heparin-binding EGF-like growth factor \|\| id:prot-a-1313 | F-strong vegetable liking (derived food-liking factor) \|\| id:ebi-fl187-GCST90094852 | MR Egger | 27 | 0.43526595047956 | 0.393616952678213 | 0.279336668696323 | -0.336223277 | 1.20675517772886 | 1.54537399725997 | 0.714463564888965 | 3.34262082598767 | 0.999950574162623 |
| ebi-fl187-GCST90094852 | prot-a-1347 | HLA class II histocompatibility antigen, DQ alpha 2 chain \|\| id:prot-a-1347 | F-strong vegetable liking (derived food-liking factor) \|\| id:ebi-fl187-GCST90094852 | MR Egger | 27 | -0.471011945 | 0.474198055329355 | 0.330090238907405 | -1.400440133 | 0.458416243881477 | 0.62437012053797 | 0.246488452359227 | 1.58156718373345 | 0.999950574162623 |
| ebi-fl187-GCST90094852 | prot-a-1357 | Non-histone chromosomal protein HMG-14 \|\| id:prot-a-1357 | F-strong vegetable liking (derived food-liking factor) \|\| id:ebi-fl187-GCST90094852 | MR Egger | 27 | -0.536375298 | 0.364890914203202 | 0.154046346854597 | -1.25156149 | 0.178810893997845 | 0.584864374061398 | 0.286057771680425 | 1.19579459085061 | 0.999950574162623 |
| ebi-fl187-GCST90094852 | prot-a-137 | Apolipoprotein M \|\| id:prot-a-137 | F-strong vegetable liking (derived food-liking factor) \|\| id:ebi-fl187-GCST90094852 | MR Egger | 27 | -0.206213787 | 0.35164271949159 | 0.5628448606478 | -0.895433517 | 0.483005943513045 | 0.813659108199639 | 0.4084304987345 | 1.62093953905874 | 0.999950574162623 |
| ebi-fl187-GCST90094852 | prot-a-1381 | 17-beta-hydroxysteroid dehydrogenase 14 \|\| id:prot-a-1381 | F-strong vegetable liking (derived food-liking factor) \|\| id:ebi-fl187-GCST90094852 | MR Egger | 27 | 0.26393167584216 | 0.383996770001826 | 0.498203494761819 | -0.488701993 | 1.01656534504574 | 1.3020392325277 | 0.613422103617382 | 2.76368613560552 | 0.999950574162623 |
| ebi-fl187-GCST90094852 | prot-a-1392 | Serine protease HTRA2, mitochondrial \|\| id:prot-a-1392 | F-strong vegetable liking (derived food-liking factor) \|\| id:ebi-fl187-GCST90094852 | MR Egger | 27 | 0.677569768487545 | 0.339368131924189 | 0.0568715244784856 | 0.0124082299161352 | 1.34273130705896 | 1.96908657653069 | 1.01248553139493 | 3.82948874393443 | 0.999950574162623 |
| ebi-fl187-GCST90094852 | prot-a-1416 | Interferon-induced protein with tetratricopeptide repeats 2 \|\| id:prot-a-1416 | F-strong vegetable liking (derived food-liking factor) \|\| id:ebi-fl187-GCST90094852 | MR Egger | 27 | -0.443278214 | 0.436250220647155 | 0.319310507057208 | -1.298328646 | 0.411772218626398 | 0.641928588822907 | 0.272987670912285 | 1.50949056333234 | 0.999950574162623 |
| ebi-fl187-GCST90094852 | prot-a-1428 | Interferon gamma \|\| id:prot-a-1428 | F-strong vegetable liking (derived food-liking factor) \|\| id:ebi-fl187-GCST90094852 | MR Egger | 27 | 0.11855793410546 | 0.36471840188765 | 0.747835085144242 | -0.596290134 | 0.833406001805254 | 1.1258720986055 | 0.550851435297582 | 2.30114310537028 | 0.999950574162623 |
| ebi-fl187-GCST90094852 | prot-a-1483 | Interleukin-17C \|\| id:prot-a-1483 | F-strong vegetable liking (derived food-liking factor) \|\| id:ebi-fl187-GCST90094852 | MR Egger | 27 | 0.0731342754890056 | 0.426544018096046 | 0.865244138071991 | -0.762892 | 0.909160550957256 | 1.07587499085668 | 0.46631588953117 | 2.48223794628704 | 0.999950574162623 |
| ebi-fl187-GCST90094852 | prot-a-1503 | Interleukin-1 receptor-like 2 \|\| id:prot-a-1503 | F-strong vegetable liking (derived food-liking factor) \|\| id:ebi-fl187-GCST90094852 | MR Egger | 27 | -0.185626431 | 0.339368131924189 | 0.589244454846555 | -0.85078797 | 0.479535107185459 | 0.830583817079103 | 0.427078274478429 | 1.6153232754258 | 0.999950574162623 |
| ebi-fl187-GCST90094852 | prot-a-1522 | Interleukin-31 receptor subunit alpha \|\| id:prot-a-1522 | F-strong vegetable liking (derived food-liking factor) \|\| id:ebi-fl187-GCST90094852 | MR Egger | 27 | -0.259526242 | 0.374372593724944 | 0.494555950832161 | -0.993296526 | 0.474244041406641 | 0.7714169639774 | 0.370353795561682 | 1.60679906468786 | 0.999950574162623 |
| ebi-fl187-GCST90094852 | prot-a-1540 | Interleukin-6 receptor subunit alpha \|\| id:prot-a-1540 | F-strong vegetable liking (derived food-liking factor) \|\| id:ebi-fl187-GCST90094852 | MR Egger | 27 | -0.34543644 | 0.339368131924189 | 0.318484485014383 | -1.010597979 | 0.319725098197485 | 0.707911324953077 | 0.364001249395766 | 1.37674924146194 | 0.999950574162623 |
| ebi-fl187-GCST90094852 | prot-a-1541 | Interleukin-6 receptor subunit alpha \|\| id:prot-a-1541 | F-strong vegetable liking (derived food-liking factor) \|\| id:ebi-fl187-GCST90094852 | MR Egger | 27 | -0.370371381 | 0.339368131924189 | 0.285523135022263 | -1.035532919 | 0.294790157700859 | 0.690477852748762 | 0.355037124314002 | 1.34284454353254 | 0.999950574162623 |
| ebi-fl187-GCST90094852 | prot-a-1545 | Interleukin-9 \|\| id:prot-a-1545 | F-strong vegetable liking (derived food-liking factor) \|\| id:ebi-fl187-GCST90094852 | MR Egger | 27 | 0.420854893164318 | 0.339368131924189 | 0.226447413372564 | -0.244306645 | 1.08601643173573 | 1.52326322646436 | 0.783247418210759 | 2.96244941655749 | 0.999950574162623 |
| ebi-fl187-GCST90094852 | prot-a-1548 | Inositol monophosphatase 3 \|\| id:prot-a-1548 | F-strong vegetable liking (derived food-liking factor) \|\| id:ebi-fl187-GCST90094852 | MR Egger | 27 | 0.12453583954131 | 0.399340352374934 | 0.75773759334612 | -0.658171251 | 0.907242930196181 | 1.13262261240775 | 0.517797390579955 | 2.47748251628021 | 0.999950574162623 |
| ebi-fl187-GCST90094852 | prot-a-1555 | Inhibin beta A chain:Inhibin beta B chain heterodimer \|\| id:prot-a-1555 | F-strong vegetable liking (derived food-liking factor) \|\| id:ebi-fl187-GCST90094852 | MR Egger | 27 | 0.165257538666997 | 0.387533363829238 | 0.673440936637336 | -0.594307854 | 0.924822931772303 | 1.17969689715872 | 0.551944459597569 | 2.52142175714673 | 0.999950574162623 |
| ebi-fl187-GCST90094852 | prot-a-1603 | Junctophilin-3 \|\| id:prot-a-1603 | F-strong vegetable liking (derived food-liking factor) \|\| id:ebi-fl187-GCST90094852 | MR Egger | 27 | 0.0472079261326113 | 0.455142805233897 | 0.918218254094469 | -0.844871972 | 0.939287824391048 | 1.04833996369395 | 0.429612357046764 | 2.55815891105341 | 0.999950574162623 |
| ebi-fl187-GCST90094852 | prot-a-1627 | UPF0258 protein KIAA1024 \|\| id:prot-a-1627 | F-strong vegetable liking (derived food-liking factor) \|\| id:ebi-fl187-GCST90094852 | MR Egger | 27 | -0.360162197 | 0.35061064359542 | 0.314143316157176 | -1.047359058 | 0.327034664421822 | 0.697563174223084 | 0.350863135652946 | 1.38684955068491 | 0.999950574162623 |
| ebi-fl187-GCST90094852 | prot-a-1666 | Kallikrein-7 \|\| id:prot-a-1666 | F-strong vegetable liking (derived food-liking factor) \|\| id:ebi-fl187-GCST90094852 | MR Egger | 27 | -0.139358608 | 0.389352450686592 | 0.723404523541239 | -0.902489412 | 0.623772195048452 | 0.869916013415666 | 0.405558799241941 | 1.86595352341388 | 0.999950574162623 |
| ebi-fl187-GCST90094852 | prot-a-1678 | Importin subunit alpha-3 \|\| id:prot-a-1678 | F-strong vegetable liking (derived food-liking factor) \|\| id:ebi-fl187-GCST90094852 | MR Egger | 27 | -0.463976911 | 0.339368131924189 | 0.18373742207051 | -1.12913845 | 0.201184627081265 | 0.628778071848214 | 0.323311685627549 | 1.22285052230562 | 0.999950574162623 |
| ebi-fl187-GCST90094852 | prot-a-1718 | Protein LDOC1 \|\| id:prot-a-1718 | F-strong vegetable liking (derived food-liking factor) \|\| id:ebi-fl187-GCST90094852 | MR Egger | 27 | 0.371943005065364 | 0.339368131924189 | 0.28353055316773 | -0.293218534 | 1.03710454363677 | 1.4505503049173 | 0.745859127675779 | 2.8210369881138 | 0.999950574162623 |
| ebi-fl187-GCST90094852 | prot-a-1723 | Leptin \|\| id:prot-a-1723 | F-strong vegetable liking (derived food-liking factor) \|\| id:ebi-fl187-GCST90094852 | MR Egger | 26 | 0.042423435676433 | 0.339662439731016 | 0.90164394780502 | -0.623314946 | 0.708161817549224 | 1.04333617098735 | 0.536164133164412 | 2.03025584584701 | 0.999950574162623 |
| ebi-fl187-GCST90094852 | prot-a-1730 | Galectin-7 \|\| id:prot-a-1730 | F-strong vegetable liking (derived food-liking factor) \|\| id:ebi-fl187-GCST90094852 | MR Egger | 26 | -0.688524091 | 0.391647948930435 | 0.0914932344675231 | -1.456154071 | 0.0791058885962918 | 0.502316896108466 | 0.233131158631437 | 1.08231892123414 | 0.999950574162623 |
| ebi-fl187-GCST90094852 | prot-a-1753 | Lipase member N \|\| id:prot-a-1753 | F-strong vegetable liking (derived food-liking factor) \|\| id:ebi-fl187-GCST90094852 | MR Egger | 27 | -0.069402327 | 0.35773932216184 | 0.84774167348753 | -0.770571398 | 0.631766744588848 | 0.932951253223734 | 0.462748579009365 | 1.88093076969583 | 0.999950574162623 |
| ebi-fl187-GCST90094852 | prot-a-1756 | VIP36-like protein \|\| id:prot-a-1756 | F-strong vegetable liking (derived food-liking factor) \|\| id:ebi-fl187-GCST90094852 | MR Egger | 27 | 0.128248134706304 | 0.339368131924189 | 0.708692525606275 | -0.536913404 | 0.793409673277714 | 1.13683505593474 | 0.584549739679018 | 2.21092211094269 | 0.999950574162623 |
| ebi-fl187-GCST90094852 | prot-a-1760 | Ig Kappa chain V-I region HK102- like \|\| id:prot-a-1760 | F-strong vegetable liking (derived food-liking factor) \|\| id:ebi-fl187-GCST90094852 | MR Egger | 26 | -0.33332122 | 0.339662439731016 | 0.336219320176441 | -0.999059602 | 0.332417161480243 | 0.716539989927691 | 0.368225556882256 | 1.39433438980377 | 0.999950574162623 |
| ebi-fl187-GCST90094852 | prot-a-179 | Neutral ceramidase \|\| id:prot-a-179 | F-strong vegetable liking (derived food-liking factor) \|\| id:ebi-fl187-GCST90094852 | MR Egger | 27 | 0.316965111852277 | 0.339366268259351 | 0.359242494008262 | -0.348192774 | 0.982122997640604 | 1.37295467124449 | 0.705962771873882 | 2.67011888500662 | 0.999950574162623 |
| ebi-fl187-GCST90094852 | prot-a-1809 | Lactotransferrin \|\| id:prot-a-1809 | F-strong vegetable liking (derived food-liking factor) \|\| id:ebi-fl187-GCST90094852 | MR Egger | 27 | 0.0749275688130738 | 0.339368131924189 | 0.827052672227016 | -0.59023397 | 0.740089107384484 | 1.07780608128359 | 0.554197604084925 | 2.09612228614735 | 0.999950574162623 |
| ebi-fl187-GCST90094852 | prot-a-1818 | Lysophospholipase-like protein 1 \|\| id:prot-a-1818 | F-strong vegetable liking (derived food-liking factor) \|\| id:ebi-fl187-GCST90094852 | MR Egger | 27 | -0.224801797 | 0.383389887333272 | 0.562895712499837 | -0.976245976 | 0.526642382013538 | 0.798674502627618 | 0.376722673483565 | 1.69323750877262 | 0.999950574162623 |
| ebi-fl187-GCST90094852 | prot-a-1852 | MAP kinase-activated protein kinase 3 \|\| id:prot-a-1852 | F-strong vegetable liking (derived food-liking factor) \|\| id:ebi-fl187-GCST90094852 | MR Egger | 27 | -0.115231809 | 0.35887165820385 | 0.750807538251336 | -0.818620259 | 0.58815664108873 | 0.891159541167677 | 0.441039755500877 | 1.80066608034524 | 0.999950574162623 |
| ebi-fl187-GCST90094852 | prot-a-1856 | Megakaryocyte-associated tyrosine-protein kinase \|\| id:prot-a-1856 | F-strong vegetable liking (derived food-liking factor) \|\| id:ebi-fl187-GCST90094852 | MR Egger | 27 | -0.559461304 | 0.349505256733974 | 0.122000569813164 | -1.244491608 | 0.125568998861096 | 0.57151685458922 | 0.288087332335845 | 1.13379339671477 | 0.999950574162623 |
| ebi-fl187-GCST90094852 | prot-a-1890 | Beta-1,3-N-acetylglucosaminyltransferase manic fringe \|\| id:prot-a-1890 | F-strong vegetable liking (derived food-liking factor) \|\| id:ebi-fl187-GCST90094852 | MR Egger | 27 | 0.29506703331348 | 0.339368131924189 | 0.392866911471225 | -0.370094505 | 0.96022857188489 | 1.34321639591424 | 0.690669055695738 | 2.61229350203819 | 0.999950574162623 |
| ebi-fl187-GCST90094852 | prot-a-1919 | Matrilysin \|\| id:prot-a-1919 | F-strong vegetable liking (derived food-liking factor) \|\| id:ebi-fl187-GCST90094852 | MR Egger | 27 | -0.404354869 | 0.339368131924189 | 0.244649047746169 | -1.069516407 | 0.260806669738519 | 0.667407237212698 | 0.343174433912753 | 1.29797670299977 | 0.999950574162623 |
| ebi-fl187-GCST90094852 | prot-a-1923 | Multimerin-2 \|\| id:prot-a-1923 | F-strong vegetable liking (derived food-liking factor) \|\| id:ebi-fl187-GCST90094852 | MR Egger | 27 | 0.348414135443667 | 0.339368131924189 | 0.314414395396814 | -0.316747403 | 1.01357567401508 | 1.41681888317557 | 0.728514752434009 | 2.75543596202563 | 0.999950574162623 |
| ebi-fl187-GCST90094852 | prot-a-1948 | Mesothelin \|\| id:prot-a-1948 | F-strong vegetable liking (derived food-liking factor) \|\| id:ebi-fl187-GCST90094852 | MR Egger | 27 | 0.0474041121246637 | 0.361283113141991 | 0.896659051054935 | -0.66071079 | 0.755519013882967 | 1.04854565348581 | 0.516484092452389 | 2.12871606988618 | 0.999950574162623 |
| ebi-fl187-GCST90094852 | prot-a-195 | ATPase family AAA domain-containing protein 1 \|\| id:prot-a-195 | F-strong vegetable liking (derived food-liking factor) \|\| id:ebi-fl187-GCST90094852 | MR Egger | 27 | 0.25798365467621 | 0.339368131924189 | 0.454252058638049 | -0.407177884 | 0.923145193247621 | 1.29431766241006 | 0.665525793450892 | 2.5171950174013 | 0.999950574162623 |
| ebi-fl187-GCST90094852 | prot-a-1966 | Mucin-1 \|\| id:prot-a-1966 | F-strong vegetable liking (derived food-liking factor) \|\| id:ebi-fl187-GCST90094852 | MR Egger | 27 | 0.30514244262153 | 0.339368117886296 | 0.377152666009077 | -0.360019068 | 0.97030395367867 | 1.35681825799163 | 0.697663022601769 | 2.63874639414604 | 0.999950574162623 |
| ebi-fl187-GCST90094852 | prot-a-1986 | Myocardial zonula adherens protein \|\| id:prot-a-1986 | F-strong vegetable liking (derived food-liking factor) \|\| id:ebi-fl187-GCST90094852 | MR Egger | 27 | -0.374493153 | 0.339368131924189 | 0.280318646291803 | -1.039654691 | 0.290668385974874 | 0.687637717872618 | 0.353576754057265 | 1.33732103599911 | 0.999950574162623 |
| ebi-fl187-GCST90094852 | prot-a-2071 | Epididymal secretory protein E1 \|\| id:prot-a-2071 | F-strong vegetable liking (derived food-liking factor) \|\| id:ebi-fl187-GCST90094852 | MR Egger | 26 | 0.0131340818911621 | 0.430971839957179 | 0.975939853276389 | -0.831570724 | 0.857838888207233 | 1.01322071280174 | 0.43536491068052 | 2.35805915374703 | 0.999950574162623 |
| ebi-fl187-GCST90094852 | prot-a-2098 | Neuritin \|\| id:prot-a-2098 | F-strong vegetable liking (derived food-liking factor) \|\| id:ebi-fl187-GCST90094852 | MR Egger | 27 | -0.180771628 | 0.352724368194648 | 0.612798892663792 | -0.872111389 | 0.510568133867113 | 0.834625942300259 | 0.418067912542901 | 1.66623757208134 | 0.999950574162623 |
| ebi-fl187-GCST90094852 | prot-a-2115 | Neurotrophin-4 \|\| id:prot-a-2115 | F-strong vegetable liking (derived food-liking factor) \|\| id:ebi-fl187-GCST90094852 | MR Egger | 27 | 0.0508444016826509 | 0.412126355 | 0.902799905484876 | -0.756923254 | 0.858612057623771 | 1.05215916634985 | 0.469107533519992 | 2.35988303796198 | 0.999950574162623 |
| ebi-fl187-GCST90094852 | prot-a-2118 | Netrin-1 \|\| id:prot-a-2118 | F-strong vegetable liking (derived food-liking factor) \|\| id:ebi-fl187-GCST90094852 | MR Egger | 27 | -0.118373909 | 0.339368131924189 | 0.730154972063693 | -0.783535447 | 0.546787630012824 | 0.888363823670057 | 0.456788202611867 | 1.72769410132131 | 0.999950574162623 |
| ebi-fl187-GCST90094852 | prot-a-2120 | Netrin-G1 \|\| id:prot-a-2120 | F-strong vegetable liking (derived food-liking factor) \|\| id:ebi-fl187-GCST90094852 | MR Egger | 27 | -0.242626939 | 0.357872206964574 | 0.504017302842604 | -0.944056465 | 0.458802586561726 | 0.784564149399144 | 0.389046476849101 | 1.5821783286863 | 0.999950574162623 |
| ebi-fl187-GCST90094852 | prot-a-2145 | Olfactomedin-like protein 3 \|\| id:prot-a-2145 | F-strong vegetable liking (derived food-liking factor) \|\| id:ebi-fl187-GCST90094852 | MR Egger | 27 | -0.467667999 | 0.357659071454831 | 0.202913512489528 | -1.168679779 | 0.233343780620504 | 0.626461474701793 | 0.310776964670419 | 1.26281553622144 | 0.999950574162623 |
| ebi-fl187-GCST90094852 | prot-a-2162 | Prolyl 4-hydroxylase subunit alpha-2 \|\| id:prot-a-2162 | F-strong vegetable liking (derived food-liking factor) \|\| id:ebi-fl187-GCST90094852 | MR Egger | 27 | -0.09698727 | 0.38970347628923 | 0.805491073438024 | -0.860806083 | 0.666831543719067 | 0.907567559569181 | 0.422821115857463 | 1.94805520417771 | 0.999950574162623 |
| ebi-fl187-GCST90094852 | prot-a-2168 | Polyadenylate-binding protein-interacting protein 1 \|\| id:prot-a-2168 | F-strong vegetable liking (derived food-liking factor) \|\| id:ebi-fl187-GCST90094852 | MR Egger | 26 | 0.194028162594415 | 0.384021569832392 | 0.617989547632423 | -0.558654114 | 0.946710439465902 | 1.2141304755389 | 0.571978363551272 | 2.57721778579162 | 0.999950574162623 |
| ebi-fl187-GCST90094852 | prot-a-219 | Beta-1,4-galactosyltransferase 5 \|\| id:prot-a-219 | F-strong vegetable liking (derived food-liking factor) \|\| id:ebi-fl187-GCST90094852 | MR Egger | 27 | -0.450673501 | 0.339368117886296 | 0.196183102501652 | -1.115835012 | 0.214488009750222 | 0.637198852811211 | 0.327641579874282 | 1.239227262 | 0.999950574162623 |
| ebi-fl187-GCST90094852 | prot-a-2190 | Pyruvate carboxylase, mitochondrial \|\| id:prot-a-2190 | F-strong vegetable liking (derived food-liking factor) \|\| id:ebi-fl187-GCST90094852 | MR Egger | 27 | -0.261760579 | 0.358405103889175 | 0.471966536189527 | -0.964234583 | 0.440713424374672 | 0.769695282672289 | 0.381274922436852 | 1.5538153529258 | 0.999950574162623 |
| ebi-fl187-GCST90094852 | prot-a-2197 | Protocadherin-9 \|\| id:prot-a-2197 | F-strong vegetable liking (derived food-liking factor) \|\| id:ebi-fl187-GCST90094852 | MR Egger | 27 | 0.520479812113613 | 0.339355534357722 | 0.137655124678629 | -0.144657035 | 1.18561665945475 | 1.68283490058954 | 0.865319016164556 | 3.27270434341599 | 0.999950574162623 |
| ebi-fl187-GCST90094852 | prot-a-2198 | Protocadherin alpha-4 \|\| id:prot-a-2198 | F-strong vegetable liking (derived food-liking factor) \|\| id:ebi-fl187-GCST90094852 | MR Egger | 27 | -0.064522923 | 0.357203781515753 | 0.858111703912678 | -0.764642335 | 0.635596488447882 | 0.93751462306521 | 0.465500394358335 | 1.88814806413357 | 0.999950574162623 |
| ebi-fl187-GCST90094852 | prot-a-2254 | Placenta growth factor \|\| id:prot-a-2254 | F-strong vegetable liking (derived food-liking factor) \|\| id:ebi-fl187-GCST90094852 | MR Egger | 27 | -0.159554122 | 0.339368131924189 | 0.642323068626351 | -0.824715661 | 0.505607416148671 | 0.8525238254929 | 0.438359617484073 | 1.65799230596202 | 0.999950574162623 |
| ebi-fl187-GCST90094852 | prot-a-2261 | Membrane-associated progesterone receptor component 2 \|\| id:prot-a-2261 | F-strong vegetable liking (derived food-liking factor) \|\| id:ebi-fl187-GCST90094852 | MR Egger | 27 | -0.279534143 | 0.381872910758742 | 0.470970602105977 | -1.028005048 | 0.468936761969245 | 0.756135910536897 | 0.357719883132586 | 1.59829392259851 | 0.999950574162623 |
| ebi-fl187-GCST90094852 | prot-a-2268 | Polymeric immunoglobulin receptor \|\| id:prot-a-2268 | F-strong vegetable liking (derived food-liking factor) \|\| id:ebi-fl187-GCST90094852 | MR Egger | 27 | 0.533009587552386 | 0.4183550049769 | 0.214359092344731 | -0.286966222 | 1.35298539730711 | 1.70405309601073 | 0.750537079898536 | 3.8689586854474 | 0.999950574162623 |
| ebi-fl187-GCST90094852 | prot-a-2283 | Group 10 secretory phospholipase A2 \|\| id:prot-a-2283 | F-strong vegetable liking (derived food-liking factor) \|\| id:ebi-fl187-GCST90094852 | MR Egger | 27 | 0.0964747137828181 | 0.412425259612715 | 0.816951707809353 | -0.711878795 | 0.904828222623739 | 1.10128173354262 | 0.490721365949529 | 2.47150733754557 | 0.999950574162623 |
| ebi-fl187-GCST90094852 | prot-a-2298 | Inactive phospholipase D5 \|\| id:prot-a-2298 | F-strong vegetable liking (derived food-liking factor) \|\| id:ebi-fl187-GCST90094852 | MR Egger | 27 | 0.37973624812193 | 0.397559382693524 | 0.348638052801219 | -0.399480142 | 1.15895263820124 | 1.46189895998497 | 0.670668607896195 | 3.18659401087686 | 0.999950574162623 |
| ebi-fl187-GCST90094852 | prot-a-2304 | Procollagen-lysine,2-oxoglutarate 5-dioxygenase 2 \|\| id:prot-a-2304 | F-strong vegetable liking (derived food-liking factor) \|\| id:ebi-fl187-GCST90094852 | MR Egger | 27 | 0.523055764324194 | 0.359827176215354 | 0.158487979980347 | -0.182205501 | 1.22831702970629 | 1.68717539091946 | 0.833430052058985 | 3.41547655102167 | 0.999950574162623 |
| ebi-fl187-GCST90094852 | prot-a-2331 | Periostin \|\| id:prot-a-2331 | F-strong vegetable liking (derived food-liking factor) \|\| id:ebi-fl187-GCST90094852 | MR Egger | 27 | -0.969599469 | 0.379283292147042 | 0.0170326322732073 | -1.712994721 | -0.226204216 | 0.379234903192209 | 0.180324960225785 | 0.79755521154214 | 0.999950574162623 |
| ebi-fl187-GCST90094852 | prot-a-2333 | Periostin \|\| id:prot-a-2333 | F-strong vegetable liking (derived food-liking factor) \|\| id:ebi-fl187-GCST90094852 | MR Egger | 27 | -0.709999427 | 0.42259522128048 | 0.105394469459221 | -1.538286061 | 0.118287206224667 | 0.491644478934688 | 0.214748852577271 | 1.12556733489408 | 0.999950574162623 |
| ebi-fl187-GCST90094852 | prot-a-2367 | cAMP-dependent protein kinase catalytic subunit alpha \|\| id:prot-a-2367 | F-strong vegetable liking (derived food-liking factor) \|\| id:ebi-fl187-GCST90094852 | MR Egger | 27 | 0.573056521534309 | 0.359666729369695 | 0.123659541215885 | -0.131890268 | 1.27800331109891 | 1.77368006603853 | 0.876437162979148 | 3.58946551966017 | 0.999950574162623 |
| ebi-fl187-GCST90094852 | prot-a-2385 | Phosphoribosyl pyrophosphate synthase-associated protein 1 \|\| id:prot-a-2385 | F-strong vegetable liking (derived food-liking factor) \|\| id:ebi-fl187-GCST90094852 | MR Egger | 27 | -0.397102372 | 0.384226502127997 | 0.311259595615891 | -1.150186317 | 0.355981571730716 | 0.672265200680801 | 0.316577780184759 | 1.42758124016992 | 0.999950574162623 |
| ebi-fl187-GCST90094852 | prot-a-2393 | Trypsin-3 \|\| id:prot-a-2393 | F-strong vegetable liking (derived food-liking factor) \|\| id:ebi-fl187-GCST90094852 | MR Egger | 26 | -0.270657493 | 0.374748874342413 | 0.477127754 | -1.005165287 | 0.463850300379543 | 0.762877742377216 | 0.365984137368685 | 1.59018490254478 | 0.999950574162623 |
| ebi-fl187-GCST90094852 | prot-a-2405 | Pregnancy-specific beta-1-glycoprotein 6 \|\| id:prot-a-2405 | F-strong vegetable liking (derived food-liking factor) \|\| id:ebi-fl187-GCST90094852 | MR Egger | 27 | -0.49748134 | 0.339368131924189 | 0.155140059760001 | -1.162642879 | 0.167680198204177 | 0.608060229425446 | 0.312658768714301 | 1.18255836588028 | 0.999950574162623 |
| ebi-fl187-GCST90094852 | prot-a-244 | Beclin-1 \|\| id:prot-a-244 | F-strong vegetable liking (derived food-liking factor) \|\| id:ebi-fl187-GCST90094852 | MR Egger | 26 | 0.154478881385772 | 0.339674701007878 | 0.653351141502761 | -0.511283533 | 0.820241295361213 | 1.16704963132629 | 0.59972531760034 | 2.27104776471432 | 0.999950574162623 |
| ebi-fl187-GCST90094852 | prot-a-2454 | tRNA pseudouridine synthase A, mitochondrial \|\| id:prot-a-2454 | F-strong vegetable liking (derived food-liking factor) \|\| id:ebi-fl187-GCST90094852 | MR Egger | 27 | 0.688843714909245 | 0.339368131924189 | 0.0531491624737632 | 0.0236821763378347 | 1.35400525348066 | 1.9914115619773 | 1.02396482591793 | 3.87290647959688 | 0.999950574162623 |
| ebi-fl187-GCST90094852 | prot-a-2466 | Glutaminyl-peptide cyclotransferase-like protein \|\| id:prot-a-2466 | F-strong vegetable liking (derived food-liking factor) \|\| id:ebi-fl187-GCST90094852 | MR Egger | 27 | 0.154358757900895 | 0.347267527190464 | 0.66050883252143 | -0.526285595 | 0.835003111194204 | 1.16690944967728 | 0.590795352154803 | 2.30482121902228 | 0.999950574162623 |
| ebi-fl187-GCST90094852 | prot-a-2474 | Ras-related protein Rab-27A \|\| id:prot-a-2474 | F-strong vegetable liking (derived food-liking factor) \|\| id:ebi-fl187-GCST90094852 | MR Egger | 27 | -0.476746829 | 0.360996301033905 | 0.19858356245486 | -1.184299579 | 0.230805921390687 | 0.620799678101229 | 0.305960405662307 | 1.25961475144582 | 0.999950574162623 |
| ebi-fl187-GCST90094852 | prot-a-2490 | Rap1 GTPase-GDP dissociation stimulator 1 \|\| id:prot-a-2490 | F-strong vegetable liking (derived food-liking factor) \|\| id:ebi-fl187-GCST90094852 | MR Egger | 27 | 0.3102990169549 | 0.34318711163057 | 0.374536851151667 | -0.362347722 | 0.982945755750817 | 1.36383286231681 | 0.696040297343996 | 2.6723166509654 | 0.999950574162623 |
| ebi-fl187-GCST90094852 | prot-a-2545 | Receptor-interacting serine/threonine-protein kinase 2 \|\| id:prot-a-2545 | F-strong vegetable liking (derived food-liking factor) \|\| id:ebi-fl187-GCST90094852 | MR Egger | 27 | 0.328453172644264 | 0.412256056924432 | 0.433114473641109 | -0.479568699 | 1.13647504421615 | 1.38881820412128 | 0.619050331308105 | 3.11576604768616 | 0.999950574162623 |
| ebi-fl187-GCST90094852 | prot-a-2610 | RUN and FYVE domain-containing protein 1 \|\| id:prot-a-2610 | F-strong vegetable liking (derived food-liking factor) \|\| id:ebi-fl187-GCST90094852 | MR Egger | 27 | -0.790226662 | 0.446185393375338 | 0.0887414819824055 | -1.664750033 | 0.0842967089748492 | 0.453741937552256 | 0.189237955303638 | 1.08795165094303 | 0.999950574162623 |
| ebi-fl187-GCST90094852 | prot-a-2627 | Serine--tRNA ligase, mitochondrial \|\| id:prot-a-2627 | F-strong vegetable liking (derived food-liking factor) \|\| id:ebi-fl187-GCST90094852 | MR Egger | 27 | 0.0242703002841744 | 0.342215083400985 | 0.944024781614268 | -0.646471263 | 0.695011863750106 | 1.02456722127647 | 0.523891192993459 | 2.00373284558592 | 0.999950574162623 |
| ebi-fl187-GCST90094852 | prot-a-264 | BolA-like protein 2 \|\| id:prot-a-264 | F-strong vegetable liking (derived food-liking factor) \|\| id:ebi-fl187-GCST90094852 | MR Egger | 26 | -0.012933667 | 0.339674701007878 | 0.969941545826429 | -0.678696081 | 0.652828746862885 | 0.987149613332772 | 0.507278010706579 | 1.92096708025196 | 0.999950574162623 |
| ebi-fl187-GCST90094852 | prot-a-2646 | Secretoglobin family 1C member 1 \|\| id:prot-a-2646 | F-strong vegetable liking (derived food-liking factor) \|\| id:ebi-fl187-GCST90094852 | MR Egger | 26 | 0.327177120259698 | 0.372457033728367 | 0.388420859143967 | -0.402838666 | 1.0571929063673 | 1.38704712957246 | 0.66841992958756 | 2.87828003698559 | 0.999950574162623 |
| ebi-fl187-GCST90094852 | prot-a-2672 | Semaphorin-3E \|\| id:prot-a-2672 | F-strong vegetable liking (derived food-liking factor) \|\| id:ebi-fl187-GCST90094852 | MR Egger | 27 | -0.757738619 | 0.390643586446315 | 0.0637761740883801 | -1.523400048 | 0.00792281048432919 | 0.468725195693982 | 0.217969518713287 | 1.00795427899877 | 0.999950574162623 |
| ebi-fl187-GCST90094852 | prot-a-2701 | Plasma protease C1 inhibitor \|\| id:prot-a-2701 | F-strong vegetable liking (derived food-liking factor) \|\| id:ebi-fl187-GCST90094852 | MR Egger | 27 | -0.063077749 | 0.339368131924189 | 0.854047992333455 | -0.728239287 | 0.602083789797882 | 0.938870474823464 | 0.482758240771456 | 1.82591967169037 | 0.999950574162623 |
| ebi-fl187-GCST90094852 | prot-a-2738 | Signaling threshold-regulating transmembrane adapter 1 \|\| id:prot-a-2738 | F-strong vegetable liking (derived food-liking factor) \|\| id:ebi-fl187-GCST90094852 | MR Egger | 27 | -0.548059772 | 0.339368131924189 | 0.118875254658711 | -1.21322131 | 0.117101766814009 | 0.578070311359463 | 0.297238238966429 | 1.12423383356462 | 0.999950574162623 |
| ebi-fl187-GCST90094852 | prot-a-274 | Serine/threonine-protein kinase BRSK2 \|\| id:prot-a-274 | F-strong vegetable liking (derived food-liking factor) \|\| id:ebi-fl187-GCST90094852 | MR Egger | 27 | -0.460411078 | 0.339368131924189 | 0.187010955572056 | -1.125572616 | 0.204750460746865 | 0.631024192130679 | 0.324466619247469 | 1.22721878749098 | 0.999950574162623 |
| ebi-fl187-GCST90094852 | prot-a-2749 | Mitochondrial glutamate carrier 2 \|\| id:prot-a-2749 | F-strong vegetable liking (derived food-liking factor) \|\| id:ebi-fl187-GCST90094852 | MR Egger | 27 | -0.723081915 | 0.339368131924189 | 0.0431347961292674 | -1.388243453 | -0.057920376 | 0.485254436265432 | 0.249513201511734 | 0.943725087444757 | 0.999950574162623 |
| ebi-fl187-GCST90094852 | prot-a-2760 | Sodium-coupled monocarboxylate transporter 1 \|\| id:prot-a-2760 | F-strong vegetable liking (derived food-liking factor) \|\| id:ebi-fl187-GCST90094852 | MR Egger | 27 | -0.553911723 | 0.364035531690024 | 0.14066165805053 | -1.267421365 | 0.159597918962253 | 0.574697350822003 | 0.28155671843157 | 1.17303911937054 | 0.999950574162623 |
| ebi-fl187-GCST90094852 | prot-a-2777 | Small integral membrane protein 10 \|\| id:prot-a-2777 | F-strong vegetable liking (derived food-liking factor) \|\| id:ebi-fl187-GCST90094852 | MR Egger | 27 | -0.301532547 | 0.370163660418145 | 0.422997809739831 | -1.027053322 | 0.423988227288334 | 0.739683751378732 | 0.358060496739925 | 1.5280436044614 | 0.999950574162623 |
| ebi-fl187-GCST90094852 | prot-a-2799 | Superoxide dismutase [Mn], mitochondrial \|\| id:prot-a-2799 | F-strong vegetable liking (derived food-liking factor) \|\| id:ebi-fl187-GCST90094852 | MR Egger | 26 | 0.745072541219427 | 0.352625417847169 | 0.0451967336510967 | 0.053926722238976 | 1.43621836019988 | 2.10659424435349 | 1.05540726144035 | 4.20476480736634 | 0.999950574162623 |
| ebi-fl187-GCST90094852 | prot-a-2805 | Protein SPACA7 \|\| id:prot-a-2805 | F-strong vegetable liking (derived food-liking factor) \|\| id:ebi-fl187-GCST90094852 | MR Egger | 27 | 0.268484565675135 | 0.373316912550304 | 0.478695170398969 | -0.463216583 | 1.00018571427373 | 1.30798078907437 | 0.62925633157257 | 2.718786699 | 0.999950574162623 |
| ebi-fl187-GCST90094852 | prot-a-2811 | SPARC-like protein 1 \|\| id:prot-a-2811 | F-strong vegetable liking (derived food-liking factor) \|\| id:ebi-fl187-GCST90094852 | MR Egger | 27 | 0.132366871132485 | 0.344638078654662 | 0.704170227472859 | -0.543123763 | 0.807857505295621 | 1.14152703577142 | 0.580930725167131 | 2.24309701130403 | 0.999950574162623 |
| ebi-fl187-GCST90094852 | prot-a-2831 | Sepiapterin reductase \|\| id:prot-a-2831 | F-strong vegetable liking (derived food-liking factor) \|\| id:ebi-fl187-GCST90094852 | MR Egger | 27 | -0.188131446 | 0.372452445021031 | 0.617901922801661 | -0.918138238 | 0.541875346069503 | 0.828505796159715 | 0.399261679620359 | 1.71922798832819 | 0.999950574162623 |
| ebi-fl187-GCST90094852 | prot-a-2834 | SPRY domain-containing SOCS box protein 1 \|\| id:prot-a-2834 | F-strong vegetable liking (derived food-liking factor) \|\| id:ebi-fl187-GCST90094852 | MR Egger | 27 | -0.728000271 | 0.339368131924189 | 0.0418442332084829 | -1.39316181 | -0.062838733 | 0.482873641633681 | 0.248289019626284 | 0.939094906957734 | 0.999950574162623 |
| ebi-fl187-GCST90094852 | prot-a-2835 | Spectrin alpha chain, non-erythrocytic 1 \|\| id:prot-a-2835 | F-strong vegetable liking (derived food-liking factor) \|\| id:ebi-fl187-GCST90094852 | MR Egger | 27 | -0.438675053 | 0.339368131924189 | 0.20796117977679 | -1.103836592 | 0.226486485234559 | 0.644890300537654 | 0.331596439899414 | 1.25418565969435 | 0.999950574162623 |
| ebi-fl187-GCST90094852 | prot-a-2859 | Sia-alpha-2,3-Gal-beta-1,4-GlcNAc-R:alpha 2,8-sialyltransferase \|\| id:prot-a-2859 | F-strong vegetable liking (derived food-liking factor) \|\| id:ebi-fl187-GCST90094852 | MR Egger | 27 | -0.41877779 | 0.339368131924189 | 0.228680768182101 | -1.083939329 | 0.246383748581513 | 0.65785035996272 | 0.338260378809107 | 1.27939044361831 | 0.999950574162623 |
| ebi-fl187-GCST90094852 | prot-a-2861 | Alpha-2,8-sialyltransferase 8F \|\| id:prot-a-2861 | F-strong vegetable liking (derived food-liking factor) \|\| id:ebi-fl187-GCST90094852 | MR Egger | 27 | -0.2555623 | 0.383991583305377 | 0.511798563827323 | -1.008185804 | 0.497061203019455 | 0.774480884697712 | 0.364880344096988 | 1.64388312625224 | 0.999950574162623 |
| ebi-fl187-GCST90094852 | prot-a-289 | Uncharacterized protein C17orf89 \|\| id:prot-a-289 | F-strong vegetable liking (derived food-liking factor) \|\| id:ebi-fl187-GCST90094852 | MR Egger | 27 | 0.344820275888536 | 0.367486996687044 | 0.357057732886118 | -0.375454238 | 1.06509478939514 | 1.41173617383676 | 0.686977157040136 | 2.90111396586481 | 0.999950574162623 |
| ebi-fl187-GCST90094852 | prot-a-2921 | Tachykinin-3 \|\| id:prot-a-2921 | F-strong vegetable liking (derived food-liking factor) \|\| id:ebi-fl187-GCST90094852 | MR Egger | 27 | 0.127410953612467 | 0.369394010959836 | 0.733040418496218 | -0.596601308 | 0.851423215093745 | 1.13588371739598 | 0.550680051168408 | 2.34297904328975 | 0.999950574162623 |
| ebi-fl187-GCST90094852 | prot-a-2928 | Tax1-binding protein 1 \|\| id:prot-a-2928 | F-strong vegetable liking (derived food-liking factor) \|\| id:ebi-fl187-GCST90094852 | MR Egger | 27 | 0.0104829888645608 | 0.339368131924189 | 0.97560277021274 | -0.65467855 | 0.675644527435971 | 1.01053812789785 | 0.519609064230267 | 1.96529925714065 | 0.999950574162623 |
| ebi-fl187-GCST90094852 | prot-a-296 | Uncharacterized protein C1orf185 \|\| id:prot-a-296 | F-strong vegetable liking (derived food-liking factor) \|\| id:ebi-fl187-GCST90094852 | MR Egger | 27 | -0.579092532 | 0.339368131924189 | 0.100330796567366 | -1.244254071 | 0.0860690063651458 | 0.560406686908112 | 0.288155771795028 | 1.08988153447338 | 0.999950574162623 |
| ebi-fl187-GCST90094852 | prot-a-2964 | Transforming growth factor beta-2 \|\| id:prot-a-2964 | F-strong vegetable liking (derived food-liking factor) \|\| id:ebi-fl187-GCST90094852 | MR Egger | 27 | -0.301143044 | 0.339368117886296 | 0.383340569538922 | -0.966304555 | 0.364018466888108 | 0.739971916507925 | 0.380486510164965 | 1.43910078962592 | 0.999950574162623 |
| ebi-fl187-GCST90094852 | prot-a-2977 | Thymocyte nuclear protein 1 \|\| id:prot-a-2977 | F-strong vegetable liking (derived food-liking factor) \|\| id:ebi-fl187-GCST90094852 | MR Egger | 26 | 0.203808488454846 | 0.343098309255948 | 0.558054602192525 | -0.468664198 | 0.876281174596505 | 1.22606332568886 | 0.62583770562376 | 2.40195064166192 | 0.999950574162623 |
| ebi-fl187-GCST90094852 | prot-a-3015 | Transmembrane protein 70, mitochondrial \|\| id:prot-a-3015 | F-strong vegetable liking (derived food-liking factor) \|\| id:ebi-fl187-GCST90094852 | MR Egger | 27 | -0.352712602 | 0.339368131924189 | 0.308603225812307 | -1.017874141 | 0.31244893616619 | 0.702779141391062 | 0.361362329572262 | 1.36676814697032 | 0.999950574162623 |
| ebi-fl187-GCST90094852 | prot-a-3030 | Tumor necrosis factor alpha-induced protein 3 \|\| id:prot-a-3030 | F-strong vegetable liking (derived food-liking factor) \|\| id:ebi-fl187-GCST90094852 | MR Egger | 27 | 0.200178955395156 | 0.377796757940136 | 0.600884393054483 | -0.54030269 | 0.940660600957823 | 1.2216213543323 | 0.582571886901256 | 2.56167310320903 | 0.999950574162623 |
| ebi-fl187-GCST90094852 | prot-a-3032 | Tumor necrosis factor receptor superfamily member 10A \|\| id:prot-a-3032 | F-strong vegetable liking (derived food-liking factor) \|\| id:ebi-fl187-GCST90094852 | MR Egger | 27 | -0.424279026 | 0.339368131924189 | 0.222801794730159 | -1.089440565 | 0.240882512617226 | 0.654241306133642 | 0.336404637762716 | 1.27237153892441 | 0.999950574162623 |
| ebi-fl187-GCST90094852 | prot-a-3081 | Tumor protein p53-inducible protein 11 \|\| id:prot-a-3081 | F-strong vegetable liking (derived food-liking factor) \|\| id:ebi-fl187-GCST90094852 | MR Egger | 27 | -0.233058826 | 0.339355534357722 | 0.498549727696706 | -0.898195674 | 0.432078020930256 | 0.792106975343334 | 0.407303906260238 | 1.54045529822805 | 0.999950574162623 |
| ebi-fl187-GCST90094852 | prot-a-315 | Complement C4 \|\| id:prot-a-315 | F-strong vegetable liking (derived food-liking factor) \|\| id:ebi-fl187-GCST90094852 | MR Egger | 27 | -0.547511072 | 0.339368131924189 | 0.119227263895699 | -1.212672611 | 0.117650466428349 | 0.578387585352466 | 0.297401378226649 | 1.12485086950374 | 0.999950574162623 |
| ebi-fl187-GCST90094852 | prot-a-3157 | UDP-glucuronosyltransferase 1-8 \|\| id:prot-a-3157 | F-strong vegetable liking (derived food-liking factor) \|\| id:ebi-fl187-GCST90094852 | MR Egger | 26 | 0.588989692392457 | 0.339674701007878 | 0.0957507014595753 | -0.076772722 | 1.2547521063679 | 1.80216675255273 | 0.926100312302207 | 3.50696891131876 | 0.999950574162623 |
| ebi-fl187-GCST90094852 | prot-a-317 | Glutaredoxin-like protein C5orf63 \|\| id:prot-a-317 | F-strong vegetable liking (derived food-liking factor) \|\| id:ebi-fl187-GCST90094852 | MR Egger | 27 | -0.396320494 | 0.439974550995659 | 0.376301092813654 | -1.258670614 | 0.466029626131302 | 0.672791036011076 | 0.284031363013618 | 1.59365421245806 | 0.999950574162623 |
| ebi-fl187-GCST90094852 | prot-a-3192 | Guanine nucleotide exchange factor VAV3 \|\| id:prot-a-3192 | F-strong vegetable liking (derived food-liking factor) \|\| id:ebi-fl187-GCST90094852 | MR Egger | 27 | -0.008674008 | 0.367075890989795 | 0.981335334341648 | -0.728142755 | 0.710794738001794 | 0.991363502337799 | 0.482804844961204 | 2.03560839130859 | 0.999950574162623 |
| ebi-fl187-GCST90094852 | prot-a-3233 | Serine/threonine-protein kinase WNK3 \|\| id:prot-a-3233 | F-strong vegetable liking (derived food-liking factor) \|\| id:ebi-fl187-GCST90094852 | MR Egger | 26 | -0.386058086 | 0.378631636520905 | 0.318080379974544 | -1.128176094 | 0.356059921331563 | 0.67973104150678 | 0.323622976405109 | 1.4276930949721 | 0.999950574162623 |
| ebi-fl187-GCST90094852 | prot-a-3250 | 14-3-3 protein theta \|\| id:prot-a-3250 | F-strong vegetable liking (derived food-liking factor) \|\| id:ebi-fl187-GCST90094852 | MR Egger | 27 | -0.068235224 | 0.339368131924189 | 0.842274271602896 | -0.733396763 | 0.596926314497379 | 0.934040738866872 | 0.48027483662113 | 1.81652677871025 | 0.999950574162623 |
| ebi-fl187-GCST90094852 | prot-a-3278 | E3 ubiquitin-protein ligase ZNRF3 \|\| id:prot-a-3278 | F-strong vegetable liking (derived food-liking factor) \|\| id:ebi-fl187-GCST90094852 | MR Egger | 27 | -0.335712075 | 0.339368131924189 | 0.332030461424182 | -1.000873613 | 0.329449464002404 | 0.714828893606145 | 0.367558197199455 | 1.39020256119306 | 0.999950574162623 |
| ebi-fl187-GCST90094852 | prot-a-355 | Calpain-3 \|\| id:prot-a-355 | F-strong vegetable liking (derived food-liking factor) \|\| id:ebi-fl187-GCST90094852 | MR Egger | 27 | 0.34181410705166 | 0.388811926543302 | 0.387705844229642 | -0.420257269 | 1.10388548307653 | 1.40749862912375 | 0.656877803796592 | 3.01586136650568 | 0.999950574162623 |
| ebi-fl187-GCST90094852 | prot-a-385 | Coiled-coil domain-containing protein 90B, mitochondrial \|\| id:prot-a-385 | F-strong vegetable liking (derived food-liking factor) \|\| id:ebi-fl187-GCST90094852 | MR Egger | 27 | -0.029920435 | 0.486683141260755 | 0.951467189414837 | -0.983819392 | 0.92397852189636 | 0.970522750143748 | 0.373880372691978 | 2.51929354238282 | 0.999950574162623 |
| ebi-fl187-GCST90094852 | prot-a-390 | C-C motif chemokine 14 \|\| id:prot-a-390 | F-strong vegetable liking (derived food-liking factor) \|\| id:ebi-fl187-GCST90094852 | MR Egger | 27 | -0.121378418 | 0.434934599998939 | 0.782484604997169 | -0.973850234 | 0.731093397811768 | 0.88569873166119 | 0.377626285849764 | 2.07735073712092 | 0.999950574162623 |
| ebi-fl187-GCST90094852 | prot-a-40 | Pituitary adenylate cyclase-activating polypeptide 27 \|\| id:prot-a-40 | F-strong vegetable liking (derived food-liking factor) \|\| id:ebi-fl187-GCST90094852 | MR Egger | 27 | -0.227855973 | 0.34145649337976 | 0.510689824026202 | -0.8971107 | 0.441398753930192 | 0.796238931406978 | 0.407746060078614 | 1.55488059348971 | 0.999950574162623 |
| ebi-fl187-GCST90094852 | prot-a-401 | C-C motif chemokine 24 \|\| id:prot-a-401 | F-strong vegetable liking (derived food-liking factor) \|\| id:ebi-fl187-GCST90094852 | MR Egger | 27 | 0.497305188533029 | 0.365808490859389 | 0.186135066732851 | -0.219679454 | 1.21428983061743 | 1.64428425885434 | 0.802776083746941 | 3.36790143435371 | 0.999950574162623 |
| ebi-fl187-GCST90094852 | prot-a-412 | Cerebral cavernous malformations 2 protein \|\| id:prot-a-412 | F-strong vegetable liking (derived food-liking factor) \|\| id:ebi-fl187-GCST90094852 | MR Egger | 27 | 0.291478778608705 | 0.379798618002684 | 0.449998374506721 | -0.452926513 | 1.03588406989397 | 1.33840523036798 | 0.635764852567124 | 2.81759608673433 | 0.999950574162623 |
| ebi-fl187-GCST90094852 | prot-a-44 | Adhesion G protein-coupled receptor F5 \|\| id:prot-a-44 | F-strong vegetable liking (derived food-liking factor) \|\| id:ebi-fl187-GCST90094852 | MR Egger | 27 | -0.064366502 | 0.339368131924189 | 0.85110261993285 | -0.72952804 | 0.600795036705088 | 0.937661281938199 | 0.482136485326271 | 1.82356802773598 | 0.999950574162623 |
| ebi-fl187-GCST90094852 | prot-a-48 | Adrenomedullin \|\| id:prot-a-48 | F-strong vegetable liking (derived food-liking factor) \|\| id:ebi-fl187-GCST90094852 | MR Egger | 27 | 0.0178166599673452 | 0.418231920547575 | 0.966358852795556 | -0.801917904 | 0.837551224240593 | 1.01797632346745 | 0.448468020030265 | 2.31070165286341 | 0.999950574162623 |
| ebi-fl187-GCST90094852 | prot-a-502 | Carcinoembryonic antigen-related cell adhesion molecule 1 \|\| id:prot-a-502 | F-strong vegetable liking (derived food-liking factor) \|\| id:ebi-fl187-GCST90094852 | MR Egger | 27 | -0.104229439 | 0.339355534357721 | 0.761281200826791 | -0.769366286 | 0.560907408468745 | 0.901018545024622 | 0.46330657906702 | 1.75226179631015 | 0.999950574162623 |
| ebi-fl187-GCST90094852 | prot-a-518 | Complement factor B \|\| id:prot-a-518 | F-strong vegetable liking (derived food-liking factor) \|\| id:ebi-fl187-GCST90094852 | MR Egger | 27 | 0.0780404710177391 | 0.3785203196364 | 0.838326010811038 | -0.663859355 | 0.819940297505082 | 1.08116641368978 | 0.514860465673292 | 2.27036428707379 | 0.999950574162623 |
| ebi-fl187-GCST90094852 | prot-a-525 | Properdin \|\| id:prot-a-525 | F-strong vegetable liking (derived food-liking factor) \|\| id:ebi-fl187-GCST90094852 | MR Egger | 27 | 0.409335718651256 | 0.424259480891737 | 0.34387391228405 | -0.422212864 | 1.24088430119906 | 1.50581716654517 | 0.655594472145658 | 3.4586706194166 | 0.999950574162623 |
| ebi-fl187-GCST90094852 | prot-a-533 | Chondroadherin \|\| id:prot-a-533 | F-strong vegetable liking (derived food-liking factor) \|\| id:ebi-fl187-GCST90094852 | MR Egger | 27 | -0.576554543 | 0.400499496102923 | 0.162388932565929 | -1.361533556 | 0.208424468909027 | 0.561830799202962 | 0.256267474979067 | 1.23173589219163 | 0.999950574162623 |
| ebi-fl187-GCST90094852 | prot-a-535 | Coiled-coil-helix-coiled-coil-helix domain-containing protein 2 \|\| id:prot-a-535 | F-strong vegetable liking (derived food-liking factor) \|\| id:ebi-fl187-GCST90094852 | MR Egger | 27 | -0.742939258 | 0.339368131924189 | 0.0381341974782999 | -1.408100797 | -0.077777719 | 0.475713613581388 | 0.244607401512722 | 0.925170051057889 | 0.999950574162623 |
| ebi-fl187-GCST90094852 | prot-a-591 | Caseinolytic peptidase B protein homolog \|\| id:prot-a-591 | F-strong vegetable liking (derived food-liking factor) \|\| id:ebi-fl187-GCST90094852 | MR Egger | 27 | -0.060249974 | 0.399790028690339 | 0.881418579301318 | -0.843838431 | 0.72333848189338 | 0.941529146038478 | 0.430056608806268 | 2.06130336027294 | 0.999950574162623 |
| ebi-fl187-GCST90094852 | prot-a-605 | Ciliary neurotrophic factor receptor subunit alpha \|\| id:prot-a-605 | F-strong vegetable liking (derived food-liking factor) \|\| id:ebi-fl187-GCST90094852 | MR Egger | 27 | -0.198976506 | 0.346239265574098 | 0.570644081320767 | -0.877605467 | 0.479652454450901 | 0.819569148003385 | 0.415777313306345 | 1.61551284031722 | 0.999950574162623 |
| ebi-fl187-GCST90094852 | prot-a-606 | Ciliary neurotrophic factor receptor subunit alpha \|\| id:prot-a-606 | F-strong vegetable liking (derived food-liking factor) \|\| id:ebi-fl187-GCST90094852 | MR Egger | 27 | -0.039919057 | 0.342104267280553 | 0.908040581514309 | -0.71044342 | 0.630605307355107 | 0.960867211945651 | 0.491426240728611 | 1.87874745480285 | 0.999950574162623 |
| ebi-fl187-GCST90094852 | prot-a-707 | Cancer/testis antigen 55 \|\| id:prot-a-707 | F-strong vegetable liking (derived food-liking factor) \|\| id:ebi-fl187-GCST90094852 | MR Egger | 27 | -0.415240711 | 0.402908064878408 | 0.312592326941837 | -1.204940519 | 0.374459095765367 | 0.660181348403149 | 0.299709826155708 | 1.4542046164111 | 0.999950574162623 |
| ebi-fl187-GCST90094852 | prot-a-719 | Dipeptidyl peptidase 1 \|\| id:prot-a-719 | F-strong vegetable liking (derived food-liking factor) \|\| id:ebi-fl187-GCST90094852 | MR Egger | 27 | 0.0224877611483099 | 0.49381367577608 | 0.964039535337727 | -0.945387043 | 0.990362565669427 | 1.02274251689387 | 0.388529164158534 | 2.69221039848528 | 0.999950574162623 |
| ebi-fl187-GCST90094852 | prot-a-752 | Cytochrome b5 \|\| id:prot-a-752 | F-strong vegetable liking (derived food-liking factor) \|\| id:ebi-fl187-GCST90094852 | MR Egger | 27 | 0.725990050522827 | 0.352906973281613 | 0.0502443401292006 | 0.034292382890866 | 1.41768771815479 | 2.06677630033029 | 1.03488714579275 | 4.12756530310834 | 0.999950574162623 |
| ebi-fl187-GCST90094852 | prot-a-755 | Cytoglobin \|\| id:prot-a-755 | F-strong vegetable liking (derived food-liking factor) \|\| id:ebi-fl187-GCST90094852 | MR Egger | 27 | -0.196387368 | 0.382370771696701 | 0.612038188255838 | -0.945834081 | 0.553059344396915 | 0.821693875002315 | 0.388355515953768 | 1.73856375532129 | 0.999950574162623 |
| ebi-fl187-GCST90094852 | prot-a-807 | Desmin \|\| id:prot-a-807 | F-strong vegetable liking (derived food-liking factor) \|\| id:ebi-fl187-GCST90094852 | MR Egger | 27 | 0.223601757128034 | 0.441064903274847 | 0.616626345517516 | -0.640885453 | 1.08808896754673 | 1.25057288850768 | 0.526825737875929 | 2.96859556591891 | 0.999950574162623 |
| ebi-fl187-GCST90094852 | prot-a-82 | Ameloblastin \|\| id:prot-a-82 | F-strong vegetable liking (derived food-liking factor) \|\| id:ebi-fl187-GCST90094852 | MR Egger | 27 | 0.196472591054104 | 0.357690536064075 | 0.587685997885548 | -0.50460086 | 0.897546041739691 | 1.21710196093922 | 0.603746506944368 | 2.45357474748685 | 0.999950574162623 |
| ebi-fl187-GCST90094852 | prot-a-84 | Muellerian-inhibiting factor \|\| id:prot-a-84 | F-strong vegetable liking (derived food-liking factor) \|\| id:ebi-fl187-GCST90094852 | MR Egger | 27 | 0.417359189870979 | 0.339368131924189 | 0.230215618758141 | -0.247802349 | 1.08252072844239 | 1.51794764644529 | 0.780514197678744 | 2.95211165178211 | 0.999950574162623 |
| ebi-fl187-GCST90094852 | prot-a-858 | Inactive dipeptidyl peptidase 10 \|\| id:prot-a-858 | F-strong vegetable liking (derived food-liking factor) \|\| id:ebi-fl187-GCST90094852 | MR Egger | 27 | 0.153287032369826 | 0.348526618209812 | 0.663850319318457 | -0.529825139 | 0.836399204061059 | 1.16565951294181 | 0.58870790254197 | 2.30804121066621 | 0.999950574162623 |
| ebi-fl187-GCST90094852 | prot-a-863 | Dihydropyrimidinase-related protein 3 \|\| id:prot-a-863 | F-strong vegetable liking (derived food-liking factor) \|\| id:ebi-fl187-GCST90094852 | MR Egger | 27 | -0.146421487 | 0.339368131924189 | 0.66983674614835 | -0.811583026 | 0.518740051459884 | 0.863793548599048 | 0.444154401585393 | 1.67990971594117 | 0.999950574162623 |
| ebi-fl187-GCST90094852 | prot-a-864 | Dihydropyrimidinase-related protein 5 \|\| id:prot-a-864 | F-strong vegetable liking (derived food-liking factor) \|\| id:ebi-fl187-GCST90094852 | MR Egger | 27 | 0.0320835759221232 | 0.339368131924189 | 0.925434546275986 | -0.633077963 | 0.697245114493533 | 1.03260380251387 | 0.530955023598162 | 2.00821268389217 | 0.999950574162623 |
| ebi-fl187-GCST90094852 | prot-a-884 | Endothelin-converting enzyme 1 \|\| id:prot-a-884 | F-strong vegetable liking (derived food-liking factor) \|\| id:ebi-fl187-GCST90094852 | MR Egger | 27 | 0.442840238629514 | 0.356845255325449 | 0.226129995346142 | -0.256576462 | 1.14225693906739 | 1.55712354627718 | 0.773695834115142 | 3.13383326038431 | 0.999950574162623 |
| ebi-fl187-GCST90094852 | prot-a-893 | Early endosome antigen 1 \|\| id:prot-a-893 | F-strong vegetable liking (derived food-liking factor) \|\| id:ebi-fl187-GCST90094852 | MR Egger | 27 | 0.25426355177264 | 0.339368131924189 | 0.460712973788748 | -0.410897987 | 0.91942509034405 | 1.28951161255863 | 0.663054568469862 | 2.50784818926881 | 0.999950574162623 |
| ebi-fl187-GCST90094852 | prot-a-897 | Ephrin-A2 \|\| id:prot-a-897 | F-strong vegetable liking (derived food-liking factor) \|\| id:ebi-fl187-GCST90094852 | MR Egger | 27 | -0.06576175 | 0.367240444222704 | 0.859326090983042 | -0.785553021 | 0.654029520323544 | 0.936353923710482 | 0.455867527733806 | 1.92327511197503 | 0.999950574162623 |
| ebi-fl187-GCST90094852 | prot-a-90 | Anaphase-promoting complex subunit 10 \|\| id:prot-a-90 | F-strong vegetable liking (derived food-liking factor) \|\| id:ebi-fl187-GCST90094852 | MR Egger | 27 | -0.009650581 | 0.3716518370941 | 0.97949004461103 | -0.738088182 | 0.718787019224447 | 0.990395835943082 | 0.478026942897693 | 2.05194273341058 | 0.999950574162623 |
| ebi-fl187-GCST90094852 | prot-a-914 | Histone-lysine N-methyltransferase EHMT2 \|\| id:prot-a-914 | F-strong vegetable liking (derived food-liking factor) \|\| id:ebi-fl187-GCST90094852 | MR Egger | 27 | -0.079751698 | 0.339368131924189 | 0.816122524494567 | -0.744913236 | 0.585409840645309 | 0.92334558654912 | 0.474775491337456 | 1.79572679667611 | 0.999950574162623 |
| ebi-fl187-GCST90094852 | prot-a-932 | RNA polymerase II elongation factor ELL \|\| id:prot-a-932 | F-strong vegetable liking (derived food-liking factor) \|\| id:ebi-fl187-GCST90094852 | MR Egger | 27 | 0.107964922702124 | 0.368612533250652 | 0.772019460682577 | -0.614515642 | 0.830445487873402 | 1.11400866828722 | 0.540902822226459 | 2.294340614 | 0.999950574162623 |
| ebi-fl187-GCST90094852 | prot-a-991 | Estrogen receptor \|\| id:prot-a-991 | F-strong vegetable liking (derived food-liking factor) \|\| id:ebi-fl187-GCST90094852 | MR Egger | 27 | -0.49569214 | 0.366862812024985 | 0.188744706903912 | -1.214743252 | 0.223358971096424 | 0.609149144576844 | 0.296786203764804 | 1.2502693037334 | 0.999950574162623 |
| ebi-fl187-GCST90094852 | prot-a-992 | Persulfide dioxygenase ETHE1, mitochondrial \|\| id:prot-a-992 | F-strong vegetable liking (derived food-liking factor) \|\| id:ebi-fl187-GCST90094852 | MR Egger | 27 | -0.493269808 | 0.394781567202736 | 0.223063203468089 | -1.267041679 | 0.280502063937016 | 0.610626495058977 | 0.281663641806937 | 1.32379427488759 | 0.999950574162623 |
| ebi-fl187-GCST90094852 | prot-a-993 | Ethanolamine kinase 1 \|\| id:prot-a-993 | F-strong vegetable liking (derived food-liking factor) \|\| id:ebi-fl187-GCST90094852 | MR Egger | 26 | 0.284517931972485 | 0.339674701007878 | 0.410508966533651 | -0.381244482 | 0.950280345947926 | 1.32912114685011 | 0.683010885336673 | 2.58643465416128 | 0.999950574162623 |
| ebi-fl187-GCST90094852 | prot-b-10 | coagulation factor III, tissue factor \|\| id:prot-b-10 | F-strong vegetable liking (derived food-liking factor) \|\| id:ebi-fl187-GCST90094852 | MR Egger | 20 | 0.728123069755367 | 0.480426637279972 | 0.146990833874176 | -0.213513139 | 1.66975927882411 | 2.07118947895245 | 0.807741546911524 | 5.31088919980142 | 0.999950574162623 |
| ebi-fl187-GCST90094852 | prot-b-28 | prolactin \|\| id:prot-b-28 | F-strong vegetable liking (derived food-liking factor) \|\| id:ebi-fl187-GCST90094852 | MR Egger | 20 | 0.28670358165739 | 0.515916011889756 | 0.58524576528894 | -0.724491802 | 1.29789896496131 | 1.33202931702883 | 0.484570764456625 | 3.66159543986091 | 0.999950574162623 |
| ebi-fl187-GCST90094852 | prot-b-54 | matrix metallopeptidase 7 \|\| id:prot-b-54 | F-strong vegetable liking (derived food-liking factor) \|\| id:ebi-fl187-GCST90094852 | MR Egger | 20 | 0.0425122291273847 | 0.480342845371392 | 0.930453419918542 | -0.898959748 | 0.983984206055313 | 1.04342881651957 | 0.406992814779198 | 2.67509316038932 | 0.999950574162623 |
| ebi-fl187-GCST90094852 | ukb-rsfMRI191-edge-pheno716 | Net100_Pair10_33 (OccipitalPrecuneus)&(Temporal\|Frontal\|Supp_Motor_Area) (Default_mode\|Central_executive)&(Default_mode\|Salience) \|\| id:ukb-rsfMRI191-edge-pheno716 | F-strong vegetable liking (derived food-liking factor) \|\| id:ebi-fl187-GCST90094852 | MR Egger | 27 | -0.131482311 | 0.128811341914221 | 0.317156458217054 | -0.383952541 | 0.120987918944508 | 0.876794784441751 | 0.681163753518337 | 1.12861127746951 | 0.999950574162623 |
| ebi-fl187-GCST90094852 | ukb-rsfMRI191-edge-pheno903 | Net100_Pair27_38 (Precentral\|Frontal\|Supp_Motor_Area)&(Subcortical) (Attention\|Salience\|Motor)&(Subcortical-cerebellum) \|\| id:ukb-rsfMRI191-edge-pheno903 | F-strong vegetable liking (derived food-liking factor) \|\| id:ebi-fl187-GCST90094852 | MR Egger | 27 | 0.0729623272471853 | 0.105916854731703 | 0.497252550859761 | -0.134634708 | 0.280559362521322 | 1.07569001194744 | 0.874035131397948 | 1.32387012859859 | 0.999950574162623 |
| ebi-fl187-GCST90094852 | ukb-rsfMRI191-node-pheno23 | Net100_Node2 RolandicOper\|SupraMarginal\|Insula Salience\|Motor \|\| id:ukb-rsfMRI191-node-pheno23 | F-strong vegetable liking (derived food-liking factor) \|\| id:ebi-fl187-GCST90094852 | MR Egger | 27 | -0.126742375 | 0.112696848723982 | 0.271427845074901 | -0.347628199 | 0.0941434480727431 | 0.88096060046398 | 0.706361453345635 | 1.09871734349878 | 0.999950574162623 |
| ebi-fl187-GCST90094852 | ukb-rsfMRI191-node-pheno55 | Net100_Node34 Parietal\|Postcentral\|Precuneus Attention \|\| id:ukb-rsfMRI191-node-pheno55 | F-strong vegetable liking (derived food-liking factor) \|\| id:ebi-fl187-GCST90094852 | MR Egger | 27 | -0.022486863 | 0.124664578501498 | 0.858309325528782 | -0.266829437 | 0.221855710719216 | 0.977764081855186 | 0.765803678094377 | 1.24839123539479 | 0.999950574162623 |
| ebi-fl187-GCST90094852 | ebi-a-GCST90001414 | IgD- CD24- B cell Absolute Count \|\| id:ebi-a-GCST90001414 | F-strong vegetable liking (derived food-liking factor) \|\| id:ebi-fl187-GCST90094852 | Weighted median | 23 | -0.163003971 | 0.133882719553289 | 0.223409037793553 | -0.425414101 | 0.099406159244574 | 0.84958781462682 | 0.653499114190199 | 1.10451481737173 | 0.999332001504091 |
| ebi-fl187-GCST90094852 | ebi-a-GCST90001422 | CD20- CD38- B cell %B cell \|\| id:ebi-a-GCST90001422 | F-strong vegetable liking (derived food-liking factor) \|\| id:ebi-fl187-GCST90094852 | Weighted median | 23 | -0.127018147 | 0.122469116107576 | 0.299668608840821 | -0.367057614 | 0.113021321021921 | 0.880717690465486 | 0.69276973057635 | 1.11965580490006 | 0.999332001504091 |
| ebi-fl187-GCST90094852 | ebi-a-GCST90001444 | CD20- CD38- B cell %lymphocyte \|\| id:ebi-a-GCST90001444 | F-strong vegetable liking (derived food-liking factor) \|\| id:ebi-fl187-GCST90094852 | Weighted median | 23 | -0.197395929 | 0.121295910156305 | 0.103653982583993 | -0.435135913 | 0.0403440546992134 | 0.820865564312698 | 0.647176701299858 | 1.04116893163958 | 0.999332001504091 |
| ebi-fl187-GCST90094852 | ebi-a-GCST90001459 | Myeloid Dendritic Cell %Dendritic Cell \|\| id:ebi-a-GCST90001459 | F-strong vegetable liking (derived food-liking factor) \|\| id:ebi-fl187-GCST90094852 | Weighted median | 23 | -0.084597845 | 0.124584082899088 | 0.497111146120182 | -0.328782648 | 0.159586957148412 | 0.918881742687298 | 0.719799449728365 | 1.17302626080456 | 0.999332001504091 |
| ebi-fl187-GCST90094852 | ebi-a-GCST90001476 | HLA DR++ monocyte %leukocyte \|\| id:ebi-a-GCST90001476 | F-strong vegetable liking (derived food-liking factor) \|\| id:ebi-fl187-GCST90094852 | Weighted median | 23 | 0.0157482170330542 | 0.126614399703602 | 0.901014920830496 | -0.232416006 | 0.263912440452115 | 1.01587287371675 | 0.792616321260264 | 1.30201418753609 | 0.999332001504091 |
| ebi-fl187-GCST90094852 | ebi-a-GCST90001478 | CD4 regulatory T cell %CD4+ T cell \|\| id:ebi-a-GCST90001478 | F-strong vegetable liking (derived food-liking factor) \|\| id:ebi-fl187-GCST90094852 | Weighted median | 23 | 0.0999157505149351 | 0.131152670787851 | 0.44616278663322 | -0.157143484 | 0.356974985259124 | 1.10507781191702 | 0.85458143106235 | 1.42900012333899 | 0.999332001504091 |
| ebi-fl187-GCST90094852 | ebi-a-GCST90001485 | CD39+ resting CD4 regulatory T cell %CD4 regulatory T cell \|\| id:ebi-a-GCST90001485 | F-strong vegetable liking (derived food-liking factor) \|\| id:ebi-fl187-GCST90094852 | Weighted median | 23 | -0.018712412 | 0.119692445661819 | 0.875767036167657 | -0.253309606 | 0.215884781416451 | 0.981461578153037 | 0.776227520247043 | 1.24095938918022 | 0.999332001504091 |
| ebi-fl187-GCST90094852 | ebi-a-GCST90001515 | Immature Myeloid-Derived Suppressor Cells Absolute Count \|\| id:ebi-a-GCST90001515 | F-strong vegetable liking (derived food-liking factor) \|\| id:ebi-fl187-GCST90094852 | Weighted median | 22 | 0.231997666942897 | 0.175098053013882 | 0.185184891012265 | -0.111194517 | 0.575189850850106 | 1.26111678656742 | 0.894764685090078 | 1.77746794868421 | 0.999332001504091 |
| ebi-fl187-GCST90094852 | ebi-a-GCST90001517 | CD33+ HLA DR+ Absolute Count \|\| id:ebi-a-GCST90001517 | F-strong vegetable liking (derived food-liking factor) \|\| id:ebi-fl187-GCST90094852 | Weighted median | 23 | 0.0388729777803588 | 0.159097945792423 | 0.806972443712668 | -0.272958996 | 0.350704951533509 | 1.03963841808322 | 0.76112399613814 | 1.4200682751282 | 0.999332001504091 |
| ebi-fl187-GCST90094852 | ebi-a-GCST90001518 | CD33+ HLA DR+ CD14- Absolute Count \|\| id:ebi-a-GCST90001518 | F-strong vegetable liking (derived food-liking factor) \|\| id:ebi-fl187-GCST90094852 | Weighted median | 23 | -0.01678509 | 0.158877212704041 | 0.915861488553828 | -0.328184427 | 0.294614246940008 | 0.983354994789109 | 0.720230177665833 | 1.34260834350285 | 0.999332001504091 |
| ebi-fl187-GCST90094852 | ebi-a-GCST90001520 | CD33+ HLA DR+ CD14dim Absolute Count \|\| id:ebi-a-GCST90001520 | F-strong vegetable liking (derived food-liking factor) \|\| id:ebi-fl187-GCST90094852 | Weighted median | 23 | 0.00122325614377295 | 0.160497725959832 | 0.993918868719851 | -0.313352287 | 0.315798799025044 | 1.00122400462673 | 0.73099234829379 | 1.37135431004252 | 0.999332001504091 |
| ebi-fl187-GCST90094852 | ebi-a-GCST90001531 | CD33dim HLA DR- Absolute Count \|\| id:ebi-a-GCST90001531 | F-strong vegetable liking (derived food-liking factor) \|\| id:ebi-fl187-GCST90094852 | Weighted median | 23 | -0.091869264 | 0.159820647722596 | 0.565408133536405 | -0.405117733 | 0.221379205687811 | 0.91222440245729 | 0.666898290043324 | 1.24779651239547 | 0.999332001504091 |
| ebi-fl187-GCST90094852 | ebi-a-GCST90001532 | Basophil Absolute Count \|\| id:ebi-a-GCST90001532 | F-strong vegetable liking (derived food-liking factor) \|\| id:ebi-fl187-GCST90094852 | Weighted median | 23 | -0.072699392 | 0.156081742159911 | 0.641374643448359 | -0.378619606 | 0.233220822958917 | 0.929880317780749 | 0.684806059032203 | 1.26266027292169 | 0.999332001504091 |
| ebi-fl187-GCST90094852 | ebi-a-GCST90001588 | T/B cell \|\| id:ebi-a-GCST90001588 | F-strong vegetable liking (derived food-liking factor) \|\| id:ebi-fl187-GCST90094852 | Weighted median | 23 | -0.044350548 | 0.130307202821034 | 0.733590124907425 | -0.299752666 | 0.211051569108338 | 0.95661855756478 | 0.741001472913792 | 1.23497604003249 | 0.999332001504091 |
| ebi-fl187-GCST90094852 | ebi-a-GCST90001624 | HLA DR+ CD4+ T cell Absolute Count \|\| id:ebi-a-GCST90001624 | F-strong vegetable liking (derived food-liking factor) \|\| id:ebi-fl187-GCST90094852 | Weighted median | 23 | -0.124736839 | 0.118166808088876 | 0.291151168490015 | -0.356343783 | 0.10687010455883 | 0.882729171652021 | 0.700231850940268 | 1.11278969878211 | 0.999332001504091 |
| ebi-fl187-GCST90094852 | ebi-a-GCST90001631 | CD8+ Natural Killer T %T cell \|\| id:ebi-a-GCST90001631 | F-strong vegetable liking (derived food-liking factor) \|\| id:ebi-fl187-GCST90094852 | Weighted median | 23 | -0.018657118 | 0.125135519075565 | 0.881478432720822 | -0.263922735 | 0.226608499349232 | 0.981515848631006 | 0.768032879096724 | 1.25433869738345 | 0.999332001504091 |
| ebi-fl187-GCST90094852 | ebi-a-GCST90001632 | CD8+ Natural Killer T %lymphocyte \|\| id:ebi-a-GCST90001632 | F-strong vegetable liking (derived food-liking factor) \|\| id:ebi-fl187-GCST90094852 | Weighted median | 23 | -0.036838725 | 0.128735046325877 | 0.774756147814026 | -0.289159416 | 0.21548196561975 | 0.963831564578388 | 0.748892810406633 | 1.24045961180107 | 0.999332001504091 |
| ebi-fl187-GCST90094852 | ebi-a-GCST90001646 | Natural Killer %CD3- lymphocyte \|\| id:ebi-a-GCST90001646 | F-strong vegetable liking (derived food-liking factor) \|\| id:ebi-fl187-GCST90094852 | Weighted median | 23 | 0.146283901888175 | 0.117629586472575 | 0.213647604574695 | -0.084270088 | 0.376837891374422 | 1.15752476487316 | 0.919182962647572 | 1.45766798966266 | 0.999332001504091 |
| ebi-fl187-GCST90094852 | ebi-a-GCST90001684 | CD127- CD8+ T cell Absolute Count \|\| id:ebi-a-GCST90001684 | F-strong vegetable liking (derived food-liking factor) \|\| id:ebi-fl187-GCST90094852 | Weighted median | 23 | -0.086377469 | 0.118853559053127 | 0.467375792400768 | -0.319330445 | 0.146575506650924 | 0.917247933120289 | 0.726635396714463 | 1.15786235382647 | 0.999332001504091 |
| ebi-fl187-GCST90094852 | ebi-a-GCST90001687 | CD28- CD8+ T cell Absolute Count \|\| id:ebi-a-GCST90001687 | F-strong vegetable liking (derived food-liking factor) \|\| id:ebi-fl187-GCST90094852 | Weighted median | 23 | -0.141850912 | 0.128712549310823 | 0.270428969587207 | -0.394127508 | 0.110425685021757 | 0.867750618369966 | 0.674268075998582 | 1.11675335446705 | 0.999332001504091 |
| ebi-fl187-GCST90094852 | ebi-a-GCST90001701 | BAFF-R on CD20- CD38- B cell \|\| id:ebi-a-GCST90001701 | F-strong vegetable liking (derived food-liking factor) \|\| id:ebi-fl187-GCST90094852 | Weighted median | 23 | -0.054610285 | 0.12471032508834 | 0.661460905663405 | -0.299042522 | 0.189821952247453 | 0.946854079367645 | 0.741527877442914 | 1.20903431265019 | 0.999332001504091 |
| ebi-fl187-GCST90094852 | ebi-a-GCST90001732 | CD19 on IgD- CD27- B cell \|\| id:ebi-a-GCST90001732 | F-strong vegetable liking (derived food-liking factor) \|\| id:ebi-fl187-GCST90094852 | Weighted median | 23 | 0.0240539485233843 | 0.116444564094007 | 0.836345725521436 | -0.204177397 | 0.252285294147638 | 1.02434557833138 | 0.815317723357208 | 1.28696314796943 | 0.999332001504091 |
| ebi-fl187-GCST90094852 | ebi-a-GCST90001798 | CD27 on CD24+ CD27+ B cell \|\| id:ebi-a-GCST90001798 | F-strong vegetable liking (derived food-liking factor) \|\| id:ebi-fl187-GCST90094852 | Weighted median | 23 | 0.127337012552248 | 0.125109216585643 | 0.308769775379652 | -0.117877052 | 0.372551077060107 | 1.13579973205465 | 0.88880532277348 | 1.45143261216066 | 0.999332001504091 |
| ebi-fl187-GCST90094852 | ebi-a-GCST90001836 | CD66b on CD66b++ myeloid cell \|\| id:ebi-a-GCST90001836 | F-strong vegetable liking (derived food-liking factor) \|\| id:ebi-fl187-GCST90094852 | Weighted median | 23 | 0.128629412252383 | 0.18036260033354 | 0.47573982485619 | -0.224881284 | 0.482140108906122 | 1.13726858825798 | 0.798611020717438 | 1.61953668091929 | 0.999332001504091 |
| ebi-fl187-GCST90094852 | ebi-a-GCST90001943 | CD25 on activated & secreting CD4 regulatory T cell \|\| id:ebi-a-GCST90001943 | F-strong vegetable liking (derived food-liking factor) \|\| id:ebi-fl187-GCST90094852 | Weighted median | 23 | 0.129618077720092 | 0.115721612226845 | 0.262677410644513 | -0.097196282 | 0.356432437684707 | 1.13839352243885 | 0.907377886484792 | 1.42822503306891 | 0.999332001504091 |
| ebi-fl187-GCST90094852 | ebi-a-GCST90001960 | CD25 on CD4+ T cell \|\| id:ebi-a-GCST90001960 | F-strong vegetable liking (derived food-liking factor) \|\| id:ebi-fl187-GCST90094852 | Weighted median | 23 | 0.133749988280821 | 0.1255962107661 | 0.286911875918342 | -0.112418585 | 0.379918561382376 | 1.14310699377151 | 0.893670102458377 | 1.46216550784767 | 0.999332001504091 |
| ebi-fl187-GCST90094852 | ebi-a-GCST90001988 | HLA DR on CD14+ CD16- monocyte \|\| id:ebi-a-GCST90001988 | F-strong vegetable liking (derived food-liking factor) \|\| id:ebi-fl187-GCST90094852 | Weighted median | 23 | -0.171139769 | 0.121405338561183 | 0.158641270298616 | -0.409094233 | 0.0668146944937633 | 0.842703781302547 | 0.664251635170388 | 1.06909735019237 | 0.999332001504091 |
| ebi-fl187-GCST90094852 | ebi-a-GCST90001991 | HLA DR on CD14+ monocyte \|\| id:ebi-a-GCST90001991 | F-strong vegetable liking (derived food-liking factor) \|\| id:ebi-fl187-GCST90094852 | Weighted median | 23 | -0.146078923 | 0.115546516795573 | 0.206142672542904 | -0.372550096 | 0.0803922499653395 | 0.864089503997276 | 0.688975135882223 | 1.0837120703379 | 0.999332001504091 |
| ebi-fl187-GCST90094852 | ebi-a-GCST90002019 | CD14 on Monocytic Myeloid-Derived Suppressor Cells \|\| id:ebi-a-GCST90002019 | F-strong vegetable liking (derived food-liking factor) \|\| id:ebi-fl187-GCST90094852 | Weighted median | 23 | 0.0240948837488826 | 0.170933771563064 | 0.887901268023784 | -0.310935309 | 0.359125076012488 | 1.02438751100688 | 0.732761277751478 | 1.43207590871466 | 0.999332001504091 |
| ebi-fl187-GCST90094852 | ebi-a-GCST90002037 | CD80 on plasmacytoid Dendritic Cell \|\| id:ebi-a-GCST90002037 | F-strong vegetable liking (derived food-liking factor) \|\| id:ebi-fl187-GCST90094852 | Weighted median | 23 | -0.015304339 | 0.14655318466681 | 0.916829252801245 | -0.302548581 | 0.271939902767463 | 0.98481217706072 | 0.738932589209402 | 1.31250812083514 | 0.999332001504091 |
| ebi-fl187-GCST90094852 | ebi-a-GCST90002042 | CD45 on CD33+ HLA DR+ CD14- \|\| id:ebi-a-GCST90002042 | F-strong vegetable liking (derived food-liking factor) \|\| id:ebi-fl187-GCST90094852 | Weighted median | 23 | 0.00138225476336555 | 0.190719213019972 | 0.994217310033253 | -0.372427403 | 0.37519191228251 | 1.0013832105178 | 0.689059673575534 | 1.45527067213723 | 0.999332001504091 |
| ebi-fl187-GCST90094852 | ebi-a-GCST90002056 | CD8 on Effector Memory CD8+ T cell \|\| id:ebi-a-GCST90002056 | F-strong vegetable liking (derived food-liking factor) \|\| id:ebi-fl187-GCST90094852 | Weighted median | 23 | 0.0503383500339866 | 0.131104630226712 | 0.701011395085183 | -0.206626725 | 0.307303425278342 | 1.05162685416915 | 0.813323186374102 | 1.35975348906506 | 0.999332001504091 |
| ebi-fl187-GCST90094852 | ebi-a-GCST90002058 | CD8 on CD8+ T cell \|\| id:ebi-a-GCST90002058 | F-strong vegetable liking (derived food-liking factor) \|\| id:ebi-fl187-GCST90094852 | Weighted median | 23 | 0.0212732843614319 | 0.131815534085347 | 0.871788779646237 | -0.237085162 | 0.279631731168713 | 1.02150117379191 | 0.788924098450823 | 1.3226426345795 | 0.999332001504091 |
| ebi-fl187-GCST90094852 | ebi-a-GCST90002063 | CD4 on CD4 regulatory T cell \|\| id:ebi-a-GCST90002063 | F-strong vegetable liking (derived food-liking factor) \|\| id:ebi-fl187-GCST90094852 | Weighted median | 23 | 0.0486304660867304 | 0.127283276793283 | 0.702413263057998 | -0.200844756 | 0.298105688601565 | 1.04983233040178 | 0.818039417057819 | 1.34730417504921 | 0.999332001504091 |
| ebi-fl187-GCST90094852 | ebi-a-GCST90002066 | CD4 on activated CD4 regulatory T cell \|\| id:ebi-a-GCST90002066 | F-strong vegetable liking (derived food-liking factor) \|\| id:ebi-fl187-GCST90094852 | Weighted median | 23 | 0.006562704911376 | 0.134730455413071 | 0.961150498597814 | -0.257508988 | 0.270634397520995 | 1.00658428664494 | 0.772974679019099 | 1.31079575259342 | 0.999332001504091 |
| ebi-fl187-GCST90094852 | ebi-a-GCST90002070 | CD4 on activated & secreting CD4 regulatory T cell \|\| id:ebi-a-GCST90002070 | F-strong vegetable liking (derived food-liking factor) \|\| id:ebi-fl187-GCST90094852 | Weighted median | 23 | 0.0663832521990122 | 0.132498375491962 | 0.61636280456681 | -0.193313564 | 0.326080068163257 | 1.06863619586619 | 0.82422348699515 | 1.38552630097772 | 0.999332001504091 |
| ebi-fl187-GCST90094852 | ebi-a-GCST90002081 | SSC-A on CD4+ T cell \|\| id:ebi-a-GCST90002081 | F-strong vegetable liking (derived food-liking factor) \|\| id:ebi-fl187-GCST90094852 | Weighted median | 23 | -0.097967773 | 0.124328034891793 | 0.430709493579971 | -0.341650722 | 0.145715174920699 | 0.906678122375346 | 0.710596357146918 | 1.15686663648927 | 0.999332001504091 |
| ebi-fl187-GCST90094852 | ebi-a-GCST90002094 | CD11b on Monocytic Myeloid-Derived Suppressor Cells \|\| id:ebi-a-GCST90002094 | F-strong vegetable liking (derived food-liking factor) \|\| id:ebi-fl187-GCST90094852 | Weighted median | 23 | 0.0187944992594192 | 0.176376487295382 | 0.91513885940242 | -0.326903416 | 0.364492414358368 | 1.01897222755266 | 0.721153391657979 | 1.43978300946003 | 0.999332001504091 |
| ebi-fl187-GCST90094852 | ebi-a-GCST90002104 | HLA DR on myeloid Dendritic Cell \|\| id:ebi-a-GCST90002104 | F-strong vegetable liking (derived food-liking factor) \|\| id:ebi-fl187-GCST90094852 | Weighted median | 23 | -0.108814935 | 0.136415434678718 | 0.42506016710577 | -0.376189187 | 0.158559317319124 | 0.896896386582852 | 0.686472449353148 | 1.17182143146949 | 0.999332001504091 |
| ebi-fl187-GCST90094852 | ebi-a-GCST90002105 | HLA DR on plasmacytoid Dendritic Cell \|\| id:ebi-a-GCST90002105 | F-strong vegetable liking (derived food-liking factor) \|\| id:ebi-fl187-GCST90094852 | Weighted median | 23 | 0.0106528197263473 | 0.134771729202469 | 0.936998079060395 | -0.25349977 | 0.274805408963187 | 1.01070976303304 | 0.776079923803396 | 1.31627451472265 | 0.999332001504091 |
| ebi-fl187-GCST90094852 | ebi-a-GCST90002106 | HLA DR on Dendritic Cell \|\| id:ebi-a-GCST90002106 | F-strong vegetable liking (derived food-liking factor) \|\| id:ebi-fl187-GCST90094852 | Weighted median | 23 | 0.119910322338396 | 0.135944061381964 | 0.377746336385289 | -0.146540038 | 0.386360682647046 | 1.12739574483184 | 0.863691151201867 | 1.4716153612275 | 0.999332001504091 |
| ebi-fl187-GCST90094852 | ebi-a-GCST90002111 | HLA DR on CD33dim HLA DR+ CD11b- \|\| id:ebi-a-GCST90002111 | F-strong vegetable liking (derived food-liking factor) \|\| id:ebi-fl187-GCST90094852 | Weighted median | 23 | -0.142559585 | 0.165593362443414 | 0.3892922576065 | -0.467122576 | 0.18200340489206 | 0.867135884030228 | 0.626803254738999 | 1.19961827844367 | 0.999332001504091 |
| ebi-fl187-GCST90094852 | ebi-a-GCST90002112 | HLA DR on CD33- HLA DR+ \|\| id:ebi-a-GCST90002112 | F-strong vegetable liking (derived food-liking factor) \|\| id:ebi-fl187-GCST90094852 | Weighted median | 23 | -0.3792568 | 0.185253808900976 | 0.0406356951597421 | -0.742354266 | -0.016159335 | 0.684369843688223 | 0.47599198373356 | 0.983970526722174 | 0.999332001504091 |
| ebi-fl187-GCST90094852 | ebi-a-GCST90002115 | HLA DR on HLA DR+ CD8+ T cell \|\| id:ebi-a-GCST90002115 | F-strong vegetable liking (derived food-liking factor) \|\| id:ebi-fl187-GCST90094852 | Weighted median | 23 | -0.076607035 | 0.122805585395125 | 0.53275406830183 | -0.317305982 | 0.164091912398506 | 0.926253767433117 | 0.728107932860972 | 1.17832261257324 | 0.999332001504091 |
| ebi-fl187-GCST90094852 | ebi-a-GCST90002116 | HLA DR on B cell \|\| id:ebi-a-GCST90002116 | F-strong vegetable liking (derived food-liking factor) \|\| id:ebi-fl187-GCST90094852 | Weighted median | 23 | 0.0790259539774667 | 0.128778545242445 | 0.53944150459302 | -0.173379995 | 0.331431902652659 | 1.0822324099414 | 0.840818047731674 | 1.39296128607998 | 0.999332001504091 |
| ebi-fl187-GCST90094852 | ebi-cfmet338-GCST90026023 | Sphingomyelin (d18:1/24:1, d18:2/24:0) levels \|\| id:ebi-cfmet338-GCST90026023 | F-strong vegetable liking (derived food-liking factor) \|\| id:ebi-fl187-GCST90094852 | Weighted median | 20 | 0.194544986088698 | 0.0809801220439621 | 0.0162887995766047 | 0.0358239468825319 | 0.353266025294863 | 1.21475812887281 | 1.03647335606188 | 1.42370983588937 | 0.999332001504091 |
| ebi-fl187-GCST90094852 | ebi-cfmet338-GCST90026046 | 1-arachidonoyl-gpc (20:4n6) levels \|\| id:ebi-cfmet338-GCST90026046 | F-strong vegetable liking (derived food-liking factor) \|\| id:ebi-fl187-GCST90094852 | Weighted median | 19 | 0.0273938537372278 | 0.0943541828195888 | 0.771563758944389 | -0.157540345 | 0.212328052063622 | 1.02777251510562 | 0.854242348856638 | 1.2365534724664 | 0.999332001504091 |
| ebi-fl187-GCST90094852 | ebi-cfmet338-GCST90026047 | 1-linoleoyl-gpc (18:2) levels \|\| id:ebi-cfmet338-GCST90026047 | F-strong vegetable liking (derived food-liking factor) \|\| id:ebi-fl187-GCST90094852 | Weighted median | 20 | 0.122079848367302 | 0.118412444416533 | 0.302554212906242 | -0.110008543 | 0.354168239423708 | 1.12984431440242 | 0.89582648249671 | 1.42499490663601 | 0.999332001504091 |
| ebi-fl187-GCST90094852 | ebi-cfmet338-GCST90026050 | 1-myristoyl-2-palmitoyl-gpc (14:0/16:0) levels \|\| id:ebi-cfmet338-GCST90026050 | F-strong vegetable liking (derived food-liking factor) \|\| id:ebi-fl187-GCST90094852 | Weighted median | 20 | 0.0359914065157971 | 0.0563439861257533 | 0.522966117903569 | -0.074442806 | 0.146425619322274 | 1.03664693804356 | 0.928260563205612 | 1.15768881793705 | 0.999332001504091 |
| ebi-fl187-GCST90094852 | ebi-cfmet338-GCST90026068 | 2,3-dihydroxyisovalerate levels \|\| id:ebi-cfmet338-GCST90026068 | F-strong vegetable liking (derived food-liking factor) \|\| id:ebi-fl187-GCST90094852 | Weighted median | 20 | 0.176175372717422 | 0.18950157227287 | 0.352538019811057 | -0.195247709 | 0.547598454372248 | 1.19264719809151 | 0.82263085979958 | 1.72909552586209 | 0.999332001504091 |
| ebi-fl187-GCST90094852 | ebi-cfmet338-GCST90026082 | 3-hydroxyhexanoate levels \|\| id:ebi-cfmet338-GCST90026082 | F-strong vegetable liking (derived food-liking factor) \|\| id:ebi-fl187-GCST90094852 | Weighted median | 20 | -0.028567291 | 0.0399728855497334 | 0.474815008408599 | -0.106914147 | 0.049779564787551 | 0.97183689617621 | 0.898602817815134 | 1.05103938474819 | 0.999332001504091 |
| ebi-fl187-GCST90094852 | ebi-cfmet338-GCST90026094 | 3-ureidopropionate levels \|\| id:ebi-cfmet338-GCST90026094 | F-strong vegetable liking (derived food-liking factor) \|\| id:ebi-fl187-GCST90094852 | Weighted median | 20 | 0.118754625286461 | 0.0920547182642943 | 0.197035425594344 | -0.061672623 | 0.299181873084478 | 1.1260935694982 | 0.940190633661949 | 1.34875490338181 | 0.999332001504091 |
| ebi-fl187-GCST90094852 | ebi-cfmet338-GCST90026173 | Glycerol levels \|\| id:ebi-cfmet338-GCST90026173 | F-strong vegetable liking (derived food-liking factor) \|\| id:ebi-fl187-GCST90094852 | Weighted median | 20 | -0.013415681 | 0.0581824191188086 | 0.817641387586994 | -0.127453223 | 0.100621860311097 | 0.986673908008007 | 0.880334594604074 | 1.10585839374134 | 0.999332001504091 |
| ebi-fl187-GCST90094852 | ebi-cfmet338-GCST90026179 | Histidine levels \|\| id:ebi-cfmet338-GCST90026179 | F-strong vegetable liking (derived food-liking factor) \|\| id:ebi-fl187-GCST90094852 | Weighted median | 20 | 0.0163688919626694 | 0.038016242381523 | 0.666776496405089 | -0.058142943 | 0.0908807270304544 | 1.01650359625773 | 0.943515068823383 | 1.09513837706212 | 0.999332001504091 |
| ebi-fl187-GCST90094852 | ebi-cfmet338-GCST90026188 | Isocitrate levels \|\| id:ebi-cfmet338-GCST90026188 | F-strong vegetable liking (derived food-liking factor) \|\| id:ebi-fl187-GCST90094852 | Weighted median | 20 | -0.011128606 | 0.0792699962380436 | 0.888352938809121 | -0.166497799 | 0.144240586251546 | 0.988933087497219 | 0.846624680352968 | 1.15516199118959 | 0.999332001504091 |
| ebi-fl187-GCST90094852 | ebi-cfmet338-GCST90026207 | N-acetyl-isoputreanine levels \|\| id:ebi-cfmet338-GCST90026207 | F-strong vegetable liking (derived food-liking factor) \|\| id:ebi-fl187-GCST90094852 | Weighted median | 20 | 0.0230153815605168 | 0.0471325087828556 | 0.625328683666062 | -0.069364336 | 0.115395098774914 | 1.02328227910447 | 0.93298669782952 | 1.12231677596819 | 0.999332001504091 |
| ebi-fl187-GCST90094852 | ebi-cfmet338-GCST90026213 | N-acetylglutamate levels \|\| id:ebi-cfmet338-GCST90026213 | F-strong vegetable liking (derived food-liking factor) \|\| id:ebi-fl187-GCST90094852 | Weighted median | 19 | 0.0924060981718662 | 0.0853989669461644 | 0.279229551725764 | -0.074975877 | 0.259788073386348 | 1.0968101442469 | 0.927765866515074 | 1.29665526178674 | 0.999332001504091 |
| ebi-fl187-GCST90094852 | ebi-cfmet338-GCST90026224 | N-acetyltaurine levels \|\| id:ebi-cfmet338-GCST90026224 | F-strong vegetable liking (derived food-liking factor) \|\| id:ebi-fl187-GCST90094852 | Weighted median | 20 | 0.0258304008519834 | 0.0658816201785662 | 0.695004317084489 | -0.103297575 | 0.154958376401973 | 1.02616689668294 | 0.901858563257846 | 1.16760936 | 0.999332001504091 |
| ebi-fl187-GCST90094852 | ebi-cfmet338-GCST90026250 | Phenyllactate (pla) levels \|\| id:ebi-cfmet338-GCST90026250 | F-strong vegetable liking (derived food-liking factor) \|\| id:ebi-fl187-GCST90094852 | Weighted median | 20 | 0.0887583727180075 | 0.10299853636082 | 0.388828396435198 | -0.113118759 | 0.290635503985214 | 1.09281657012484 | 0.893044597137472 | 1.33727706294559 | 0.999332001504091 |
| ebi-fl187-GCST90094852 | ebi-cfmet338-GCST90026251 | Pipecolate levels \|\| id:ebi-cfmet338-GCST90026251 | F-strong vegetable liking (derived food-liking factor) \|\| id:ebi-fl187-GCST90094852 | Weighted median | 20 | 0.116383313477849 | 0.155514559183033 | 0.454233589508332 | -0.188425223 | 0.421191849476593 | 1.12342641409761 | 0.828262436500053 | 1.52377658610881 | 0.999332001504091 |
| ebi-fl187-GCST90094852 | ebi-cfmet338-GCST90026278 | Trigonelline (n'-methylnicotinate) levels \|\| id:ebi-cfmet338-GCST90026278 | F-strong vegetable liking (derived food-liking factor) \|\| id:ebi-fl187-GCST90094852 | Weighted median | 20 | 0.145467957568038 | 0.168213676158822 | 0.387159110851089 | -0.184230848 | 0.475166762839329 | 1.15658067433088 | 0.831743775522604 | 1.60828237685959 | 0.999332001504091 |
| ebi-fl187-GCST90094852 | ebi-cfmet338-GCST90026299 | 1-carboxyethylvaline levels \|\| id:ebi-cfmet338-GCST90026299 | F-strong vegetable liking (derived food-liking factor) \|\| id:ebi-fl187-GCST90094852 | Weighted median | 20 | 0.00588578488760442 | 0.0996526045942606 | 0.952901904095501 | -0.18943332 | 0.201204889892355 | 1.00590314015255 | 0.827427887852671 | 1.22287530094578 | 0.999332001504091 |
| ebi-fl187-GCST90094852 | ebi-gm430-GCST90011306 | C_Erysipelotrichia abundance \|\| id:ebi-gm430-GCST90011306 | F-strong vegetable liking (derived food-liking factor) \|\| id:ebi-fl187-GCST90094852 | Weighted median | 19 | 0.00584932490114192 | 0.085348646936639 | 0.945360175714189 | -0.161434023 | 0.173132672896954 | 1.00586646560626 | 0.850922670857899 | 1.18902384585802 | 0.999332001504091 |
| ebi-fl187-GCST90094852 | ebi-gm430-GCST90011312 | F_Erysipelotrichaceae abundance \|\| id:ebi-gm430-GCST90011312 | F-strong vegetable liking (derived food-liking factor) \|\| id:ebi-fl187-GCST90094852 | Weighted median | 19 | 0.00584932490114192 | 0.0885251303754598 | 0.947317872427061 | -0.167659931 | 0.179358580437043 | 1.00586646560626 | 0.845641362513556 | 1.19644969071036 | 0.999332001504091 |
| ebi-fl187-GCST90094852 | ebi-gm430-GCST90011352 | O_Erysipelotrichales abundance \|\| id:ebi-gm430-GCST90011352 | F-strong vegetable liking (derived food-liking factor) \|\| id:ebi-fl187-GCST90094852 | Weighted median | 19 | 0.00584932490114192 | 0.0869366386265368 | 0.946356712027607 | -0.164546487 | 0.176245136609154 | 1.00586646560626 | 0.848278322277211 | 1.19273040470389 | 0.999332001504091 |
| ebi-fl187-GCST90094852 | ebi-gm430-GCST90011362 | OTU97_11 (Parabacteroides) abundance \|\| id:ebi-gm430-GCST90011362 | F-strong vegetable liking (derived food-liking factor) \|\| id:ebi-fl187-GCST90094852 | Weighted median | 19 | -0.063905088 | 0.108139534991174 | 0.554553737344934 | -0.275858577 | 0.148048400419515 | 0.938094031533204 | 0.758920252143864 | 1.15956901863175 | 0.999332001504091 |
| ebi-fl187-GCST90094852 | ebi-gm430-GCST90011384 | OTU97_235 (Phascolarctobacterium) abundance \|\| id:ebi-gm430-GCST90011384 | F-strong vegetable liking (derived food-liking factor) \|\| id:ebi-fl187-GCST90094852 | Weighted median | 19 | -0.537030377 | 0.359441064909784 | 0.135157131593818 | -1.241534864 | 0.167474110512752 | 0.584481367231675 | 0.28894039323238 | 1.18231468026785 | 0.999332001504091 |
| ebi-fl187-GCST90094852 | ebi-gm430-GCST90011390 | OTU97_3 (Bacteroides) abundance \|\| id:ebi-gm430-GCST90011390 | F-strong vegetable liking (derived food-liking factor) \|\| id:ebi-fl187-GCST90094852 | Weighted median | 19 | -0.035555055 | 0.0842544661677258 | 0.673027871744434 | -0.200693809 | 0.129583698440527 | 0.965069600620965 | 0.818162907375524 | 1.13835438596244 | 0.999332001504091 |
| ebi-fl187-GCST90094852 | ebi-gm430-GCST90011398 | OTU97_4 (Alistipes) abundance \|\| id:ebi-gm430-GCST90011398 | F-strong vegetable liking (derived food-liking factor) \|\| id:ebi-fl187-GCST90094852 | Weighted median | 19 | 0.0485207075747296 | 0.102880563401546 | 0.637196825410707 | -0.153125197 | 0.25016661184176 | 1.04971710869074 | 0.858022293533631 | 1.28423936835023 | 0.999332001504091 |
| ebi-fl187-GCST90094852 | ebi-gm430-GCST90011421 | OTU99_108 (Cytophagales) abundance \|\| id:ebi-gm430-GCST90011421 | F-strong vegetable liking (derived food-liking factor) \|\| id:ebi-fl187-GCST90094852 | Weighted median | 19 | -0.153155406 | 0.314353767843948 | 0.626111533742017 | -0.769288791 | 0.462977979358118 | 0.857996373995118 | 0.463342484690419 | 1.58879835567125 | 0.999332001504091 |
| ebi-fl187-GCST90094852 | ebi-gm430-GCST90011428 | OTU99_123 (Phascolarctobacterium) abundance \|\| id:ebi-gm430-GCST90011428 | F-strong vegetable liking (derived food-liking factor) \|\| id:ebi-fl187-GCST90094852 | Weighted median | 19 | 0.058995804063481 | 0.199976318521255 | 0.767983221526243 | -0.33295778 | 0.450949388365142 | 1.06077078980393 | 0.716800456661409 | 1.5698018298456 | 0.999332001504091 |
| ebi-fl187-GCST90094852 | ebi-gm430-GCST90011450 | OTU99_298 (Phascolarctobacterium) abundance \|\| id:ebi-gm430-GCST90011450 | F-strong vegetable liking (derived food-liking factor) \|\| id:ebi-fl187-GCST90094852 | Weighted median | 19 | -0.543460958 | 0.362002762728867 | 0.133287833491969 | -1.252986373 | 0.16606445705791 | 0.580734871334887 | 0.285650463031146 | 1.18064920044458 | 0.999332001504091 |
| ebi-fl187-GCST90094852 | ebi-gm430-GCST90011451 | OTU99_3 (Bacteroides) abundance \|\| id:ebi-gm430-GCST90011451 | F-strong vegetable liking (derived food-liking factor) \|\| id:ebi-fl187-GCST90094852 | Weighted median | 19 | -0.034424456 | 0.0854864336797557 | 0.687176941829072 | -0.201977866 | 0.133128953611291 | 0.966161324233317 | 0.817113013383772 | 1.14239730509084 | 0.999332001504091 |
| ebi-fl187-GCST90094852 | ebi-gm430-GCST90011472 | OTU99_72 (Parabacteroides) abundance \|\| id:ebi-gm430-GCST90011472 | F-strong vegetable liking (derived food-liking factor) \|\| id:ebi-fl187-GCST90094852 | Weighted median | 19 | -0.19206748 | 0.255968239681587 | 0.453039911256673 | -0.693765229 | 0.309630270058373 | 0.825251178896294 | 0.499691071009698 | 1.3629211082231 | 0.999332001504091 |
| ebi-fl187-GCST90094852 | ebi-gm430-GCST90011507 | TestASV_3 (Bacteroides) abundance \|\| id:ebi-gm430-GCST90011507 | F-strong vegetable liking (derived food-liking factor) \|\| id:ebi-fl187-GCST90094852 | Weighted median | 19 | -0.12647878 | 0.10924799652409 | 0.246977609718822 | -0.340604854 | 0.0876472927058661 | 0.881192847833198 | 0.711339936045323 | 1.09160303776746 | 0.999332001504091 |
| ebi-fl187-GCST90094852 | ebi-gm430-GCST90011512 | TestASV_34 (Phascolarctobacterium) abundance \|\| id:ebi-gm430-GCST90011512 | F-strong vegetable liking (derived food-liking factor) \|\| id:ebi-fl187-GCST90094852 | Weighted median | 19 | 0.0348672936142216 | 0.2964421481316 | 0.906369376967365 | -0.546159317 | 0.615893903952157 | 1.03548228456977 | 0.579169952570881 | 1.85131075412031 | 0.999332001504091 |
| ebi-fl187-GCST90094852 | ebi-gm430-GCST90011519 | TestASV_41 (Barnesiella) abundance \|\| id:ebi-gm430-GCST90011519 | F-strong vegetable liking (derived food-liking factor) \|\| id:ebi-fl187-GCST90094852 | Weighted median | 19 | -0.027126114 | 0.357214051752588 | 0.93946843931303 | -0.727265655 | 0.673013427424712 | 0.973238494774844 | 0.483228498486228 | 1.96013515485697 | 0.999332001504091 |
| ebi-fl187-GCST90094852 | ebi-gm430-GCST90011524 | TestASV_46 (Sutterella) abundance \|\| id:ebi-gm430-GCST90011524 | F-strong vegetable liking (derived food-liking factor) \|\| id:ebi-fl187-GCST90094852 | Weighted median | 19 | 0.00910907179229127 | 0.361187574128719 | 0.979879658414746 | -0.698818573 | 0.71703671708458 | 1.0091506856453 | 0.49717232952442 | 2.04835435494276 | 0.999332001504091 |
| ebi-fl187-GCST90094852 | ebi-gm430-GCST90011581 | OTU97_19 (Bacteria) prevalence \|\| id:ebi-gm430-GCST90011581 | F-strong vegetable liking (derived food-liking factor) \|\| id:ebi-fl187-GCST90094852 | Weighted median | 19 | 0.167944708389827 | 0.400900771289243 | 0.675275771580162 | -0.617820803 | 0.953710220116743 | 1.18287120598606 | 0.539118002567921 | 2.59532103043551 | 0.999332001504091 |
| ebi-fl187-GCST90094852 | ebi-gm430-GCST90011614 | OTU97_80 (Ruminococcus) prevalence \|\| id:ebi-gm430-GCST90011614 | F-strong vegetable liking (derived food-liking factor) \|\| id:ebi-fl187-GCST90094852 | Weighted median | 19 | -0.071653972 | 0.189546293972812 | 0.705409143781739 | -0.443164708 | 0.299856763935855 | 0.930852941037671 | 0.642001455322307 | 1.34966547295983 | 0.999332001504091 |
| ebi-fl187-GCST90094852 | ebi-gm430-GCST90011623 | OTU99_11 (Parabacteroides) prevalence \|\| id:ebi-gm430-GCST90011623 | F-strong vegetable liking (derived food-liking factor) \|\| id:ebi-fl187-GCST90094852 | Weighted median | 19 | 0.187706414848275 | 0.171920837395027 | 0.274912722078064 | -0.149258426 | 0.524671256142529 | 1.20647925895365 | 0.861346491421766 | 1.68990321175245 | 0.999332001504091 |
| ebi-fl187-GCST90094852 | ebi-gm430-GCST90011642 | OTU99_19 (Bacteria) prevalence \|\| id:ebi-gm430-GCST90011642 | F-strong vegetable liking (derived food-liking factor) \|\| id:ebi-fl187-GCST90094852 | Weighted median | 19 | 0.167944708389827 | 0.415796946661249 | 0.686278613040263 | -0.647017307 | 0.982906723845874 | 1.18287120598606 | 0.523605203499914 | 2.6722123473915 | 0.999332001504091 |
| ebi-fl187-GCST90094852 | ebi-gm430-GCST90011653 | OTU99_30 (Parasutterella) prevalence \|\| id:ebi-gm430-GCST90011653 | F-strong vegetable liking (derived food-liking factor) \|\| id:ebi-fl187-GCST90094852 | Weighted median | 19 | 0.0275058481912259 | 0.18346630561408 | 0.880825239622278 | -0.332088111 | 0.387099807194822 | 1.02788762637307 | 0.717424107248591 | 1.47270347034041 | 0.999332001504091 |
| ebi-fl187-GCST90094852 | ebi-gm430-GCST90011675 | OTU99_8 (Ruminococcaceae) prevalence \|\| id:ebi-gm430-GCST90011675 | F-strong vegetable liking (derived food-liking factor) \|\| id:ebi-fl187-GCST90094852 | Weighted median | 19 | 0.282141925635326 | 0.213469646943423 | 0.186269483653937 | -0.136258582 | 0.700542433644436 | 1.32596689532706 | 0.8726169599476 | 2.01484533100167 | 0.999332001504091 |
| ebi-fl187-GCST90094852 | ebi-gm430-GCST90011679 | OTU99_92 (Ruminococcus) prevalence \|\| id:ebi-gm430-GCST90011679 | F-strong vegetable liking (derived food-liking factor) \|\| id:ebi-fl187-GCST90094852 | Weighted median | 19 | -0.035681002 | 0.187325317592822 | 0.848936046432187 | -0.402838624 | 0.331476620975532 | 0.964948061369747 | 0.668419957567332 | 1.39302357836531 | 0.999332001504091 |
| ebi-fl187-GCST90094852 | ebi-gm430-GCST90011684 | TestASV_11 (Lachnospiraceae) prevalence \|\| id:ebi-gm430-GCST90011684 | F-strong vegetable liking (derived food-liking factor) \|\| id:ebi-fl187-GCST90094852 | Weighted median | 19 | 0.0424230898588175 | 0.194864192436942 | 0.827658244544978 | -0.339510727 | 0.424356907035224 | 1.04333581018338 | 0.712118657746135 | 1.52860706705297 | 0.999332001504091 |
| ebi-fl187-GCST90094852 | ebi-gm430-GCST90011688 | TestASV_15 (Bacteroides) prevalence \|\| id:ebi-gm430-GCST90011688 | F-strong vegetable liking (derived food-liking factor) \|\| id:ebi-fl187-GCST90094852 | Weighted median | 19 | -0.326475912 | 0.204798747105459 | 0.110906827648923 | -0.727881456 | 0.0749296323766435 | 0.721461753446055 | 0.482931017579035 | 1.07780830540724 | 0.999332001504091 |
| ebi-fl187-GCST90094852 | ebi-gm430-GCST90011692 | TestASV_19 (Prevotella) prevalence \|\| id:ebi-gm430-GCST90011692 | F-strong vegetable liking (derived food-liking factor) \|\| id:ebi-fl187-GCST90094852 | Weighted median | 19 | -0.25191298 | 0.355141595735001 | 0.478119031854066 | -0.947990507 | 0.444164548047834 | 0.77731237716552 | 0.387518958108944 | 1.55918702569604 | 0.999332001504091 |
| ebi-fl187-GCST90094852 | ebi-gm430-GCST90011693 | TestASV_2 (Bacteroides) prevalence \|\| id:ebi-gm430-GCST90011693 | F-strong vegetable liking (derived food-liking factor) \|\| id:ebi-fl187-GCST90094852 | Weighted median | 19 | 0.366520561019011 | 0.201318723585375 | 0.0686679149213704 | -0.028064137 | 0.761105259246346 | 1.44270606375214 | 0.972326002525951 | 2.14064087659905 | 0.999332001504091 |
| ebi-fl187-GCST90094852 | ebi-gm430-GCST90011716 | TestASV_40 (Barnesiella) prevalence \|\| id:ebi-gm430-GCST90011716 | F-strong vegetable liking (derived food-liking factor) \|\| id:ebi-fl187-GCST90094852 | Weighted median | 19 | 0.0706142660498123 | 0.39219296990302 | 0.85711347172255 | -0.698083955 | 0.839312487059732 | 1.07316718900043 | 0.497537695721168 | 2.31477499182798 | 0.999332001504091 |
| ebi-fl187-GCST90094852 | ebi-gm430-GCST90011718 | TestASV_42 (Alphaproteobacteria) prevalence \|\| id:ebi-gm430-GCST90011718 | F-strong vegetable liking (derived food-liking factor) \|\| id:ebi-fl187-GCST90094852 | Weighted median | 19 | 0.0692583901334048 | 0.289617702907865 | 0.810999133853101 | -0.498392308 | 0.63690908783282 | 1.07171309346391 | 0.607506558727769 | 1.89062807339446 | 0.999332001504091 |
| ebi-fl187-GCST90094852 | ebi-hbsm44-GCST90104929 | Sulcal depth of the right inferior frontal sulcus \|\| id:ebi-hbsm44-GCST90104929 | F-strong vegetable liking (derived food-liking factor) \|\| id:ebi-fl187-GCST90094852 | Weighted median | 24 | 0.163619750993024 | 0.139417857562451 | 0.240558289625052 | -0.10963925 | 0.436878751815428 | 1.1777663854372 | 0.896157365912929 | 1.547868389 | 0.999332001504091 |
| ebi-fl187-GCST90094852 | ebi-hbsm44-GCST90104935 | Sulcal opening distance of the left calloso-marginal posterior fissure \|\| id:ebi-hbsm44-GCST90104935 | F-strong vegetable liking (derived food-liking factor) \|\| id:ebi-fl187-GCST90094852 | Weighted median | 24 | -0.02248278 | 0.0321382947909841 | 0.484199895010951 | -0.085473838 | 0.0405082772937685 | 0.977768073729086 | 0.918077161211285 | 1.04133992914335 | 0.999332001504091 |
| ebi-fl187-GCST90094852 | ebi-hbsm44-GCST90104936 | Sulcal opening distance of the left anterior inferior temporal sulcus \|\| id:ebi-hbsm44-GCST90104936 | F-strong vegetable liking (derived food-liking factor) \|\| id:ebi-fl187-GCST90094852 | Weighted median | 22 | -0.021654496 | 0.0216190279841611 | 0.316517206697479 | -0.064027791 | 0.020718798772296 | 0.978578279284831 | 0.937978931865784 | 1.02093492311519 | 0.999332001504091 |
| ebi-fl187-GCST90094852 | ebi-hbsm44-GCST90104941 | Sulcal opening distance of the right posterior inferior temporal sulcus \|\| id:ebi-hbsm44-GCST90104941 | F-strong vegetable liking (derived food-liking factor) \|\| id:ebi-fl187-GCST90094852 | Weighted median | 22 | -0.004054678 | 0.0157006708873252 | 0.796214974554994 | -0.034827993 | 0.0267186370827345 | 0.995953531250994 | 0.96577152163104 | 1.02707878022342 | 0.999332001504091 |
| ebi-fl187-GCST90094852 | ebi-IPs91-GCST90274819 | Leukemia inhibitory factor levels \|\| id:ebi-IPs91-GCST90274819 | F-strong vegetable liking (derived food-liking factor) \|\| id:ebi-fl187-GCST90094852 | Weighted median | 27 | 0.0153166734189105 | 0.0614415896778774 | 0.803137730863168 | -0.105108842 | 0.13574218918755 | 1.01543457484466 | 0.900226534482433 | 1.14538656248648 | 0.999332001504091 |
| ebi-fl187-GCST90094852 | ebi-IPs91-GCST90274837 | STAM binding protein levels \|\| id:ebi-IPs91-GCST90274837 | F-strong vegetable liking (derived food-liking factor) \|\| id:ebi-fl187-GCST90094852 | Weighted median | 27 | -0.019054261 | 0.0525540398915717 | 0.716929776917596 | -0.122060179 | 0.083951656897994 | 0.981126123629804 | 0.885095102339554 | 1.08757631572517 | 0.999332001504091 |
| ebi-fl187-GCST90094852 | ebi-IPs91-GCST90274840 | TNF-beta levels \|\| id:ebi-IPs91-GCST90274840 | F-strong vegetable liking (derived food-liking factor) \|\| id:ebi-fl187-GCST90094852 | Weighted median | 27 | -0.084449621 | 0.0618993083030738 | 0.172471159808452 | -0.205772265 | 0.0368730232443539 | 0.919017953388805 | 0.814018435416943 | 1.03756126631007 | 0.999332001504091 |
| ebi-fl187-GCST90094852 | ebi-IPs91-GCST90274842 | Tumor necrosis factor ligand superfamily member 14 levels \|\| id:ebi-IPs91-GCST90274842 | F-strong vegetable liking (derived food-liking factor) \|\| id:ebi-fl187-GCST90094852 | Weighted median | 27 | -0.008362692 | 0.0606052654321786 | 0.890251300562373 | -0.127149012 | 0.110423628171462 | 0.991672177963599 | 0.880602442204658 | 1.11675105747494 | 0.999332001504091 |
| ebi-fl187-GCST90094852 | ebi-met1400-GCST90199628 | 3-methylhistidine levels \|\| id:ebi-met1400-GCST90199628 | F-strong vegetable liking (derived food-liking factor) \|\| id:ebi-fl187-GCST90094852 | Weighted median | 26 | -0.12116175 | 0.0688945659750969 | 0.0786361826315862 | -0.256195099 | 0.013871599728465 | 0.885890655559628 | 0.773990949282753 | 1.01396825678018 | 0.999332001504091 |
| ebi-fl187-GCST90094852 | ebi-met1400-GCST90199651 | Docosahexaenoate DHA; 22:6n3 levels \|\| id:ebi-met1400-GCST90199651 | F-strong vegetable liking (derived food-liking factor) \|\| id:ebi-fl187-GCST90094852 | Weighted median | 26 | 0.0489574158975044 | 0.0657048413324562 | 0.456204473840543 | -0.079824073 | 0.177738904909119 | 1.05017562900119 | 0.923278761656957 | 1.19451339893143 | 0.999332001504091 |
| ebi-fl187-GCST90094852 | ebi-met1400-GCST90199652 | 1-stearoyl-2-oleoyl-GPS (18:0/18:1) levels \|\| id:ebi-met1400-GCST90199652 | F-strong vegetable liking (derived food-liking factor) \|\| id:ebi-fl187-GCST90094852 | Weighted median | 26 | 0.0299408666330528 | 0.0738360565296044 | 0.685106577482295 | -0.114777804 | 0.174659537431077 | 1.03039360150903 | 0.891564223756936 | 1.19084071089891 | 0.999332001504091 |
| ebi-fl187-GCST90094852 | ebi-met1400-GCST90199653 | 1-myristoyl-2-palmitoyl-gpc (14:0/16:0) levels \|\| id:ebi-met1400-GCST90199653 | F-strong vegetable liking (derived food-liking factor) \|\| id:ebi-fl187-GCST90094852 | Weighted median | 26 | -0.073204319 | 0.0650073195577006 | 0.260125948462211 | -0.200618666 | 0.0542100268518767 | 0.929410913868909 | 0.818224389001151 | 1.05570630554429 | 0.999332001504091 |
| ebi-fl187-GCST90094852 | ebi-met1400-GCST90199667 | Erythritol levels in elite athletes \|\| id:ebi-met1400-GCST90199667 | F-strong vegetable liking (derived food-liking factor) \|\| id:ebi-fl187-GCST90094852 | Weighted median | 26 | -0.059774226 | 0.0628947807526088 | 0.341916904914611 | -0.183047997 | 0.0634995440346822 | 0.941977183308085 | 0.832728186726554 | 1.06555899994341 | 0.999332001504091 |
| ebi-fl187-GCST90094852 | ebi-met1400-GCST90199674 | 3-hydroxymyristate levels \|\| id:ebi-met1400-GCST90199674 | F-strong vegetable liking (derived food-liking factor) \|\| id:ebi-fl187-GCST90094852 | Weighted median | 26 | 0.0306200214604756 | 0.0685348839184913 | 0.655033849686461 | -0.103708351 | 0.164948393940719 | 1.03109363598663 | 0.901488177192603 | 1.17933225645063 | 0.999332001504091 |
| ebi-fl187-GCST90094852 | ebi-met1400-GCST90199685 | 2-linoleoylglycerol (18:2) levels \|\| id:ebi-met1400-GCST90199685 | F-strong vegetable liking (derived food-liking factor) \|\| id:ebi-fl187-GCST90094852 | Weighted median | 26 | -0.115340912 | 0.0757894682950032 | 0.128044321751613 | -0.26388827 | 0.0332064454486333 | 0.891062317918868 | 0.768059349928335 | 1.0337639330724 | 0.999332001504091 |
| ebi-fl187-GCST90094852 | ebi-met1400-GCST90199703 | Gamma-glutamylvaline levels \|\| id:ebi-met1400-GCST90199703 | F-strong vegetable liking (derived food-liking factor) \|\| id:ebi-fl187-GCST90094852 | Weighted median | 26 | -0.101011139 | 0.067511759603431 | 0.134601373012997 | -0.233334188 | 0.0313119096441666 | 0.903922963870486 | 0.791888889534439 | 1.03180728434361 | 0.999332001504091 |
| ebi-fl187-GCST90094852 | ebi-met1400-GCST90199779 | Gamma-glutamyl-2-aminobutyrate levels \|\| id:ebi-met1400-GCST90199779 | F-strong vegetable liking (derived food-liking factor) \|\| id:ebi-fl187-GCST90094852 | Weighted median | 26 | 0.0769450136254593 | 0.0717528676942416 | 0.283557688459001 | -0.063690607 | 0.217580634306173 | 1.07998269042719 | 0.938295256559143 | 1.24306565920366 | 0.999332001504091 |
| ebi-fl187-GCST90094852 | ebi-met1400-GCST90199824 | 4-vinylphenol sulfate levels \|\| id:ebi-met1400-GCST90199824 | F-strong vegetable liking (derived food-liking factor) \|\| id:ebi-fl187-GCST90094852 | Weighted median | 26 | 0.0669980248353168 | 0.0692612367002024 | 0.333382318287799 | -0.068753999 | 0.202750048767714 | 1.06929336614202 | 0.933556307527343 | 1.22476629813981 | 0.999332001504091 |
| ebi-fl187-GCST90094852 | ebi-met1400-GCST90199830 | 4-ethylphenylsulfate levels \|\| id:ebi-met1400-GCST90199830 | F-strong vegetable liking (derived food-liking factor) \|\| id:ebi-fl187-GCST90094852 | Weighted median | 26 | -0.00087399 | 0.069386764918488 | 0.989950177166348 | -0.136872049 | 0.135124069232162 | 0.99912639180995 | 0.872081802515983 | 1.14467879496072 | 0.999332001504091 |
| ebi-fl187-GCST90094852 | ebi-met1400-GCST90199833 | Hexanoylglutamine levels \|\| id:ebi-met1400-GCST90199833 | F-strong vegetable liking (derived food-liking factor) \|\| id:ebi-fl187-GCST90094852 | Weighted median | 26 | 0.0449028957133445 | 0.0685808228445602 | 0.512632464906973 | -0.089515517 | 0.179321308488682 | 1.04592629105102 | 0.914374076614991 | 1.19640509753032 | 0.999332001504091 |
| ebi-fl187-GCST90094852 | ebi-met1400-GCST90199837 | Carotenoid levels (cryptoxanthin) \|\| id:ebi-met1400-GCST90199837 | F-strong vegetable liking (derived food-liking factor) \|\| id:ebi-fl187-GCST90094852 | Weighted median | 26 | 0.132784691556499 | 0.070890847123658 | 0.0610564357266697 | -0.006161369 | 0.271730751918869 | 1.14200408873582 | 0.993857573503426 | 1.31223363735311 | 0.999332001504091 |
| ebi-fl187-GCST90094852 | ebi-met1400-GCST90199869 | 2-hydroxyhippurate levels \|\| id:ebi-met1400-GCST90199869 | F-strong vegetable liking (derived food-liking factor) \|\| id:ebi-fl187-GCST90094852 | Weighted median | 26 | -0.014891998 | 0.0706882704346295 | 0.833143678646668 | -0.153441008 | 0.123657011835637 | 0.985218339195315 | 0.85775136294479 | 1.13162766953161 | 0.999332001504091 |
| ebi-fl187-GCST90094852 | ebi-met1400-GCST90199902 | Hydantoin-5-propionate levels \|\| id:ebi-met1400-GCST90199902 | F-strong vegetable liking (derived food-liking factor) \|\| id:ebi-fl187-GCST90094852 | Weighted median | 25 | -0.056738602 | 0.0724858925411332 | 0.43377175039664 | -0.198810951 | 0.085333747468355 | 0.944841016724318 | 0.819704842826714 | 1.08908048390458 | 0.999332001504091 |
| ebi-fl187-GCST90094852 | ebi-met1400-GCST90199904 | Pantoate levels \|\| id:ebi-met1400-GCST90199904 | F-strong vegetable liking (derived food-liking factor) \|\| id:ebi-fl187-GCST90094852 | Weighted median | 26 | 0.0197946199393158 | 0.0685849149780009 | 0.772876186138994 | -0.114631813 | 0.154221053296198 | 1.0199918325287 | 0.891694393385873 | 1.16674877193608 | 0.999332001504091 |
| ebi-fl187-GCST90094852 | ebi-met1400-GCST90199936 | 3-methyl catechol sulfate (2) levels \|\| id:ebi-met1400-GCST90199936 | F-strong vegetable liking (derived food-liking factor) \|\| id:ebi-fl187-GCST90094852 | Weighted median | 26 | 0.0585742914203034 | 0.083905255687802 | 0.485114416436147 | -0.10588001 | 0.223028592568395 | 1.06032375572639 | 0.899532576759347 | 1.24985630982711 | 0.999332001504091 |
| ebi-fl187-GCST90094852 | ebi-met1400-GCST90199977 | 4-hydroxychlorothalonil levels \|\| id:ebi-met1400-GCST90199977 | F-strong vegetable liking (derived food-liking factor) \|\| id:ebi-fl187-GCST90094852 | Weighted median | 26 | 0.0139320821158831 | 0.0729768539873553 | 0.848595343997864 | -0.129102552 | 0.156966715931099 | 1.01402958585576 | 0.878883829893608 | 1.16995667233436 | 0.999332001504091 |
| ebi-fl187-GCST90094852 | ebi-met1400-GCST90200040 | Sphingomyelin (d18:2/24:1, d18:1/24:2) levels \|\| id:ebi-met1400-GCST90200040 | F-strong vegetable liking (derived food-liking factor) \|\| id:ebi-fl187-GCST90094852 | Weighted median | 26 | 0.028940485156391 | 0.0625682049920592 | 0.643691974615883 | -0.093693197 | 0.151574166940827 | 1.02936333025447 | 0.910562082907725 | 1.16366460405308 | 0.999332001504091 |
| ebi-fl187-GCST90094852 | ebi-met1400-GCST90200077 | 1-stearoyl-2-oleoyl-GPI (18:0/18:1) levels \|\| id:ebi-met1400-GCST90200077 | F-strong vegetable liking (derived food-liking factor) \|\| id:ebi-fl187-GCST90094852 | Weighted median | 26 | -0.085727518 | 0.0682831025464971 | 0.209307494933351 | -0.219562399 | 0.0481073630439323 | 0.917844293247069 | 0.802870057890725 | 1.04928330352665 | 0.999332001504091 |
| ebi-fl187-GCST90094852 | ebi-met1400-GCST90200080 | 1-palmitoyl-2-oleoyl-GPI (16:0/18:1) levels \|\| id:ebi-met1400-GCST90200080 | F-strong vegetable liking (derived food-liking factor) \|\| id:ebi-fl187-GCST90094852 | Weighted median | 26 | -0.132060633 | 0.0668309161781434 | 0.0481501292044409 | -0.263049229 | -0.001072037 | 0.876287861388861 | 0.768704053968037 | 0.998928537001566 | 0.999332001504091 |
| ebi-fl187-GCST90094852 | ebi-met1400-GCST90200082 | 1-oleoyl-2-linoleoyl-GPE (18:1/18:2) levels \|\| id:ebi-met1400-GCST90200082 | F-strong vegetable liking (derived food-liking factor) \|\| id:ebi-fl187-GCST90094852 | Weighted median | 26 | -0.068427168 | 0.07805726567853 | 0.380688844639624 | -0.221419408 | 0.0845650731284668 | 0.933861472997712 | 0.80138050413755 | 1.08824365734728 | 0.999332001504091 |
| ebi-fl187-GCST90094852 | ebi-met1400-GCST90200100 | Perfluorooctanesulfonate (PFOS) levels \|\| id:ebi-met1400-GCST90200100 | F-strong vegetable liking (derived food-liking factor) \|\| id:ebi-fl187-GCST90094852 | Weighted median | 26 | -0.048497573 | 0.0709609571019425 | 0.494328704970111 | -0.187581049 | 0.0905859029289258 | 0.95265965141669 | 0.828961928996284 | 1.09481555146477 | 0.999332001504091 |
| ebi-fl187-GCST90094852 | ebi-met1400-GCST90200102 | 1-palmitoleoyl-2-linolenoyl-GPC (16:1/18:3) levels \|\| id:ebi-met1400-GCST90200102 | F-strong vegetable liking (derived food-liking factor) \|\| id:ebi-fl187-GCST90094852 | Weighted median | 26 | 0.0181384754409272 | 0.0670918574988622 | 0.786888803744229 | -0.113361565 | 0.149638516138697 | 1.0183039767192 | 0.892827786241696 | 1.16141433429966 | 0.999332001504091 |
| ebi-fl187-GCST90094852 | ebi-met1400-GCST90200114 | Glycosyl-N-tricosanoyl-sphingadienine (d18:2/23:0) levels \|\| id:ebi-met1400-GCST90200114 | F-strong vegetable liking (derived food-liking factor) \|\| id:ebi-fl187-GCST90094852 | Weighted median | 26 | -0.050290792 | 0.072913682275551 | 0.490363667029905 | -0.19320161 | 0.0926200250187847 | 0.95095285457852 | 0.824315767494392 | 1.09704480648211 | 0.999332001504091 |
| ebi-fl187-GCST90094852 | ebi-met1400-GCST90200135 | Arachidoylcarnitine (C20) levels \|\| id:ebi-met1400-GCST90200135 | F-strong vegetable liking (derived food-liking factor) \|\| id:ebi-fl187-GCST90094852 | Weighted median | 26 | -0.01025211 | 0.0696728614811695 | 0.883016471967816 | -0.146810919 | 0.12630669823687 | 0.989800263482944 | 0.863457225537105 | 1.13463010397706 | 0.999332001504091 |
| ebi-fl187-GCST90094852 | ebi-met1400-GCST90200146 | Docosahexaenoylcarnitine (C22:6) levels \|\| id:ebi-met1400-GCST90200146 | F-strong vegetable liking (derived food-liking factor) \|\| id:ebi-fl187-GCST90094852 | Weighted median | 26 | 0.148310743685212 | 0.0838635823352166 | 0.0769813389697256 | -0.016061878 | 0.312683365062236 | 1.15987326366147 | 0.984066426412268 | 1.36708859447777 | 0.999332001504091 |
| ebi-fl187-GCST90094852 | ebi-met1400-GCST90200162 | 3-hydroxyphenylacetoylglutamine levels \|\| id:ebi-met1400-GCST90200162 | F-strong vegetable liking (derived food-liking factor) \|\| id:ebi-fl187-GCST90094852 | Weighted median | 26 | 0.00726529839499526 | 0.0812920523978189 | 0.928785638872761 | -0.152067124 | 0.16659772109472 | 1.00729175470757 | 0.858930623685124 | 1.18127896610404 | 0.999332001504091 |
| ebi-fl187-GCST90094852 | ebi-met1400-GCST90200172 | 3-carboxy-4-methyl-5-pentyl-2-furanpropionate (3-CMPFP) levels \|\| id:ebi-met1400-GCST90200172 | F-strong vegetable liking (derived food-liking factor) \|\| id:ebi-fl187-GCST90094852 | Weighted median | 26 | 0.0781386815291761 | 0.0682625961494192 | 0.252342631811354 | -0.055656007 | 0.211933369982038 | 1.08127260081048 | 0.94586445075635 | 1.23606552326663 | 0.999332001504091 |
| ebi-fl187-GCST90094852 | ebi-met1400-GCST90200222 | Dihydrocaffeate sulfate (2) levels \|\| id:ebi-met1400-GCST90200222 | F-strong vegetable liking (derived food-liking factor) \|\| id:ebi-fl187-GCST90094852 | Weighted median | 26 | -0.034285053 | 0.0909167859124499 | 0.706096569684158 | -0.212481953 | 0.143911847458784 | 0.966296019864161 | 0.808574908284011 | 1.15478230704303 | 0.999332001504091 |
| ebi-fl187-GCST90094852 | ebi-met1400-GCST90200223 | 4-methylhexanoylglutamine levels \|\| id:ebi-met1400-GCST90200223 | F-strong vegetable liking (derived food-liking factor) \|\| id:ebi-fl187-GCST90094852 | Weighted median | 26 | -0.015592448 | 0.0854858208470353 | 0.855270348718998 | -0.183144657 | 0.151959761040458 | 0.984528485032513 | 0.832647698973186 | 1.16411339277793 | 0.999332001504091 |
| ebi-fl187-GCST90094852 | ebi-met1400-GCST90200224 | 2-ketocaprylate levels \|\| id:ebi-met1400-GCST90200224 | F-strong vegetable liking (derived food-liking factor) \|\| id:ebi-fl187-GCST90094852 | Weighted median | 26 | -0.085526995 | 0.0686156659304103 | 0.212593897469942 | -0.2200137 | 0.0489597103383773 | 0.918028360649431 | 0.802507803442921 | 1.05017803856984 | 0.999332001504091 |
| ebi-fl187-GCST90094852 | ebi-met1400-GCST90200243 | Hydroxypalmitoyl sphingomyelin (d18:1/16:0(OH)) levels \|\| id:ebi-met1400-GCST90200243 | F-strong vegetable liking (derived food-liking factor) \|\| id:ebi-fl187-GCST90094852 | Weighted median | 26 | 0.0373824260827944 | 0.0687357539711545 | 0.586539829635633 | -0.097339652 | 0.172104503866257 | 1.03808993760608 | 0.907247805535729 | 1.18780195662492 | 0.999332001504091 |
| ebi-fl187-GCST90094852 | ebi-met1400-GCST90200273 | 2-methoxyhydroquinone sulfate (2) levels \|\| id:ebi-met1400-GCST90200273 | F-strong vegetable liking (derived food-liking factor) \|\| id:ebi-fl187-GCST90094852 | Weighted median | 26 | 0.00210684561203006 | 0.0885975463685055 | 0.981028129604828 | -0.171544345 | 0.175758036494301 | 1.00210906657071 | 0.842362912380767 | 1.1921495670613 | 0.999332001504091 |
| ebi-fl187-GCST90094852 | ebi-met1400-GCST90200276 | 2,4-di-tert-butylphenol levels \|\| id:ebi-met1400-GCST90200276 | F-strong vegetable liking (derived food-liking factor) \|\| id:ebi-fl187-GCST90094852 | Weighted median | 26 | 0.0179655585731721 | 0.072225926594676 | 0.80356103162025 | -0.123597258 | 0.159528374698737 | 1.01812791000802 | 0.883735687094412 | 1.17295754406549 | 0.999332001504091 |
| ebi-fl187-GCST90094852 | ebi-met1400-GCST90200289 | N-acetylmethionine levels \|\| id:ebi-met1400-GCST90200289 | F-strong vegetable liking (derived food-liking factor) \|\| id:ebi-fl187-GCST90094852 | Weighted median | 26 | -0.019036587 | 0.0692128701853217 | 0.783282412616508 | -0.154693812 | 0.11662063894368 | 0.981143464863454 | 0.856677441526419 | 1.12369306343475 | 0.999332001504091 |
| ebi-fl187-GCST90094852 | ebi-met1400-GCST90200308 | 3-hydroxyisobutyrate levels \|\| id:ebi-met1400-GCST90200308 | F-strong vegetable liking (derived food-liking factor) \|\| id:ebi-fl187-GCST90094852 | Weighted median | 26 | 0.005130554788705 | 0.0677067062024601 | 0.93959717625356 | -0.127574589 | 0.137835698945527 | 1.00514373862208 | 0.880227757753304 | 1.14778695217462 | 0.999332001504091 |
| ebi-fl187-GCST90094852 | ebi-met1400-GCST90200383 | Cystathionine levels \|\| id:ebi-met1400-GCST90200383 | F-strong vegetable liking (derived food-liking factor) \|\| id:ebi-fl187-GCST90094852 | Weighted median | 26 | -0.048492612 | 0.0779062561040996 | 0.533647167286383 | -0.201188874 | 0.104203649815817 | 0.952664377423055 | 0.817757963657402 | 1.10982644785479 | 0.999332001504091 |
| ebi-fl187-GCST90094852 | ebi-met1400-GCST90200384 | Guanidinoacetate levels \|\| id:ebi-met1400-GCST90200384 | F-strong vegetable liking (derived food-liking factor) \|\| id:ebi-fl187-GCST90094852 | Weighted median | 26 | 0.0237940321854885 | 0.0744494998610713 | 0.749271859207599 | -0.122126988 | 0.169715051913188 | 1.02407936877753 | 0.885035972823395 | 1.18496714908678 | 0.999332001504091 |
| ebi-fl187-GCST90094852 | ebi-met1400-GCST90200403 | Citrulline levels \|\| id:ebi-met1400-GCST90200403 | F-strong vegetable liking (derived food-liking factor) \|\| id:ebi-fl187-GCST90094852 | Weighted median | 26 | -0.070767312 | 0.0707522848981268 | 0.317207736503184 | -0.20944179 | 0.0679071667241055 | 0.931678657652359 | 0.811036848451925 | 1.07026594757286 | 0.999332001504091 |
| ebi-fl187-GCST90094852 | ebi-met1400-GCST90200434 | 12,13-DiHOME levels \|\| id:ebi-met1400-GCST90200434 | F-strong vegetable liking (derived food-liking factor) \|\| id:ebi-fl187-GCST90094852 | Weighted median | 26 | 0.00140294442891907 | 0.0707676433178964 | 0.98418324635025 | -0.137301636 | 0.140107525331996 | 1.00140392901584 | 0.871707247771455 | 1.15039748907913 | 0.999332001504091 |
| ebi-fl187-GCST90094852 | ebi-met1400-GCST90200437 | Fructose levels \|\| id:ebi-met1400-GCST90200437 | F-strong vegetable liking (derived food-liking factor) \|\| id:ebi-fl187-GCST90094852 | Weighted median | 25 | -0.043822906 | 0.0666917322719285 | 0.511118846815168 | -0.174538701 | 0.0868928890701812 | 0.957123443109248 | 0.839844350418051 | 1.09077983901815 | 0.999332001504091 |
| ebi-fl187-GCST90094852 | ebi-met1400-GCST90200448 | N-stearoyl-sphinganine (d18:0/18:0) levels \|\| id:ebi-met1400-GCST90200448 | F-strong vegetable liking (derived food-liking factor) \|\| id:ebi-fl187-GCST90094852 | Weighted median | 26 | -0.038194948 | 0.0761631891153468 | 0.616027516088965 | -0.187474798 | 0.111084903092169 | 0.962525280631551 | 0.82905001143655 | 1.11748978116473 | 0.999332001504091 |
| ebi-fl187-GCST90094852 | ebi-met1400-GCST90200468 | X-11847 levels \|\| id:ebi-met1400-GCST90200468 | F-strong vegetable liking (derived food-liking factor) \|\| id:ebi-fl187-GCST90094852 | Weighted median | 26 | 0.130860407430636 | 0.0776566496124066 | 0.0919663307282418 | -0.021346626 | 0.283067440670953 | 1.13980866138664 | 0.978879600822526 | 1.32719466569776 | 0.999332001504091 |
| ebi-fl187-GCST90094852 | ebi-met1400-GCST90200476 | X-11843 levels \|\| id:ebi-met1400-GCST90200476 | F-strong vegetable liking (derived food-liking factor) \|\| id:ebi-fl187-GCST90094852 | Weighted median | 26 | 0.145974433836139 | 0.0786905183044463 | 0.0635898347221548 | -0.008258982 | 0.300207849712854 | 1.15716660336162 | 0.9917750296532 | 1.35013940450154 | 0.999332001504091 |
| ebi-fl187-GCST90094852 | ebi-met1400-GCST90200511 | X-12906 levels \|\| id:ebi-met1400-GCST90200511 | F-strong vegetable liking (derived food-liking factor) \|\| id:ebi-fl187-GCST90094852 | Weighted median | 26 | -0.131781983 | 0.0708199375366609 | 0.0627719404767401 | -0.27058906 | 0.00702509465108978 | 0.876532073211925 | 0.76292995005325 | 1.00704982851384 | 0.999332001504091 |
| ebi-fl187-GCST90094852 | ebi-met1400-GCST90200515 | X-12849 levels \|\| id:ebi-met1400-GCST90200515 | F-strong vegetable liking (derived food-liking factor) \|\| id:ebi-fl187-GCST90094852 | Weighted median | 26 | 0.0277598329862427 | 0.0767027299012508 | 0.71741589627396 | -0.122577518 | 0.178097183592694 | 1.02814872735759 | 0.88463732730517 | 1.1949414442947 | 0.999332001504091 |
| ebi-fl187-GCST90094852 | ebi-met1400-GCST90200551 | X-18886 levels \|\| id:ebi-met1400-GCST90200551 | F-strong vegetable liking (derived food-liking factor) \|\| id:ebi-fl187-GCST90094852 | Weighted median | 26 | 0.0450864888843266 | 0.0660514669952695 | 0.494861954522676 | -0.084374386 | 0.174547364195055 | 1.04611833360372 | 0.919087097940924 | 1.19070713793457 | 0.999332001504091 |
| ebi-fl187-GCST90094852 | ebi-met1400-GCST90200564 | X-21319 levels \|\| id:ebi-met1400-GCST90200564 | F-strong vegetable liking (derived food-liking factor) \|\| id:ebi-fl187-GCST90094852 | Weighted median | 26 | 0.000254451702692532 | 0.0714763745118542 | 0.997159583742027 | -0.139839242 | 0.140348145745927 | 1.00025448407827 | 0.869498002627985 | 1.15067433150477 | 0.999364451047625 |
| ebi-fl187-GCST90094852 | ebi-met1400-GCST90200579 | X-21442 levels \|\| id:ebi-met1400-GCST90200579 | F-strong vegetable liking (derived food-liking factor) \|\| id:ebi-fl187-GCST90094852 | Weighted median | 26 | 0.0253973377288376 | 0.0772023362422716 | 0.742177529739541 | -0.125919241 | 0.17671391676369 | 1.02572259785346 | 0.881686047722049 | 1.19328966412194 | 0.999332001504091 |
| ebi-fl187-GCST90094852 | ebi-met1400-GCST90200581 | X-21441 levels \|\| id:ebi-met1400-GCST90200581 | F-strong vegetable liking (derived food-liking factor) \|\| id:ebi-fl187-GCST90094852 | Weighted median | 26 | -0.063036486 | 0.0732024263609146 | 0.389168847476134 | -0.206513242 | 0.0804402692119303 | 0.938909215594881 | 0.813415490037792 | 1.08376411062449 | 0.999332001504091 |
| ebi-fl187-GCST90094852 | ebi-met1400-GCST90200648 | X-24585 levels \|\| id:ebi-met1400-GCST90200648 | F-strong vegetable liking (derived food-liking factor) \|\| id:ebi-fl187-GCST90094852 | Weighted median | 26 | -0.032605889 | 0.0702686030784566 | 0.642634930554745 | -0.170332351 | 0.105120573321362 | 0.967919952607252 | 0.843384470554244 | 1.11084454049711 | 0.999332001504091 |
| ebi-fl187-GCST90094852 | ebi-met1400-GCST90200720 | Adenosine 5'-diphosphate (ADP) to pantothenate ratio \|\| id:ebi-met1400-GCST90200720 | F-strong vegetable liking (derived food-liking factor) \|\| id:ebi-fl187-GCST90094852 | Weighted median | 26 | 0.0597923030044296 | 0.0880717964922087 | 0.497198632963309 | -0.112828418 | 0.232413024129159 | 1.06161602918604 | 0.893303921733216 | 1.26164070928744 | 0.999332001504091 |
| ebi-fl187-GCST90094852 | ebi-met1400-GCST90200724 | Cholate to taurocholate ratio \|\| id:ebi-met1400-GCST90200724 | F-strong vegetable liking (derived food-liking factor) \|\| id:ebi-fl187-GCST90094852 | Weighted median | 26 | 0.00230281308913252 | 0.0743560603095141 | 0.97529340142855 | -0.143435065 | 0.14804069129578 | 1.00230546659965 | 0.866377056453077 | 1.15956007940516 | 0.999332001504091 |
| ebi-fl187-GCST90094852 | ebi-met1400-GCST90200726 | N-acetylputrescine to (N(1) + N(8))-acetylspermidine ratio \|\| id:ebi-met1400-GCST90200726 | F-strong vegetable liking (derived food-liking factor) \|\| id:ebi-fl187-GCST90094852 | Weighted median | 26 | -0.086330073 | 0.0701960685799397 | 0.218756280413822 | -0.223914367 | 0.0512542216833419 | 0.917291408363688 | 0.799383584933119 | 1.05259045058855 | 0.999332001504091 |
| ebi-fl187-GCST90094852 | ebi-met1400-GCST90200744 | Arginine to glutamate ratio \|\| id:ebi-met1400-GCST90200744 | F-strong vegetable liking (derived food-liking factor) \|\| id:ebi-fl187-GCST90094852 | Weighted median | 26 | 0.101401252684412 | 0.0698908465807244 | 0.146821163369897 | -0.035584807 | 0.238387311982632 | 1.10672062730487 | 0.965040888909563 | 1.26920067426995 | 0.999332001504091 |
| ebi-fl187-GCST90094852 | ebi-met1400-GCST90200759 | Histidine to trans-urocanate ratio \|\| id:ebi-met1400-GCST90200759 | F-strong vegetable liking (derived food-liking factor) \|\| id:ebi-fl187-GCST90094852 | Weighted median | 26 | 0.0562846242041124 | 0.0678715172149597 | 0.406944881679791 | -0.07674355 | 0.189312797945433 | 1.05789874447817 | 0.926127328936974 | 1.20841888431591 | 0.999332001504091 |
| ebi-fl187-GCST90094852 | ebi-met1400-GCST90200795 | Oleoyl-linoleoyl-glycerol (18:1 to 18:2) [2] to linoleoyl-arachidonoyl-glycerol (18:2 to 20:4) [2] ratio \|\| id:ebi-met1400-GCST90200795 | F-strong vegetable liking (derived food-liking factor) \|\| id:ebi-fl187-GCST90094852 | Weighted median | 26 | 0.0742844063994876 | 0.0729353241805098 | 0.308441841902183 | -0.068668829 | 0.217237641793287 | 1.07711309979668 | 0.933635822000074 | 1.24263937010069 | 0.999332001504091 |
| ebi-fl187-GCST90094852 | ebi-met1400-GCST90200811 | Choline to taurocholate ratio \|\| id:ebi-met1400-GCST90200811 | F-strong vegetable liking (derived food-liking factor) \|\| id:ebi-fl187-GCST90094852 | Weighted median | 26 | -0.027968876 | 0.0702013408643822 | 0.690328411881796 | -0.165563504 | 0.109625752273032 | 0.972418632061668 | 0.847416047259251 | 1.1158603840923 | 0.999332001504091 |
| ebi-fl187-GCST90094852 | ebi-met1400-GCST90200831 | Cholate to phosphate ratio \|\| id:ebi-met1400-GCST90200831 | F-strong vegetable liking (derived food-liking factor) \|\| id:ebi-fl187-GCST90094852 | Weighted median | 26 | 0.000505377769505905 | 0.0730308242252679 | 0.994478634115455 | -0.142635038 | 0.143645793251031 | 1.00050550549437 | 0.867070459175338 | 1.15447511321812 | 0.999332001504091 |
| ebi-fl187-GCST90094852 | ebi-met1400-GCST90200841 | Adenosine 5'-diphosphate (ADP) to oxalate (ethanedioate) ratio \|\| id:ebi-met1400-GCST90200841 | F-strong vegetable liking (derived food-liking factor) \|\| id:ebi-fl187-GCST90094852 | Weighted median | 26 | -0.026296109 | 0.0944565162799881 | 0.780710075318668 | -0.211430881 | 0.158838663032934 | 0.974046623051533 | 0.809425225955797 | 1.17214882048896 | 0.999332001504091 |
| ebi-fl187-GCST90094852 | ebi-met1400-GCST90200844 | Aspartate to phosphate ratio \|\| id:ebi-met1400-GCST90200844 | F-strong vegetable liking (derived food-liking factor) \|\| id:ebi-fl187-GCST90094852 | Weighted median | 26 | -0.071012362 | 0.0650935411021027 | 0.275304596459666 | -0.198595703 | 0.0565709785504136 | 0.931450377457812 | 0.819881302238067 | 1.05820172175896 | 0.999332001504091 |
| ebi-fl187-GCST90094852 | ebi-met1400-GCST90200870 | Adenosine 5'-monophosphate (AMP) to acetoacetate ratio \|\| id:ebi-met1400-GCST90200870 | F-strong vegetable liking (derived food-liking factor) \|\| id:ebi-fl187-GCST90094852 | Weighted median | 26 | 0.0574268596466463 | 0.0752719723826257 | 0.445508204012259 | -0.090106206 | 0.204959925516593 | 1.05910780430276 | 0.913834125245965 | 1.227475874 | 0.999332001504091 |
| ebi-fl187-GCST90094852 | ebi-met1400-GCST90200887 | Cholesterol to cortisol ratio \|\| id:ebi-met1400-GCST90200887 | F-strong vegetable liking (derived food-liking factor) \|\| id:ebi-fl187-GCST90094852 | Weighted median | 25 | 0.03767915161803 | 0.0699249530672677 | 0.589989465294677 | -0.099373756 | 0.174732059629875 | 1.03839801110282 | 0.905404244150732 | 1.19092707641735 | 0.999332001504091 |
| ebi-fl187-GCST90094852 | ebi-met1400-GCST90200890 | Cortisol to taurocholate ratio \|\| id:ebi-met1400-GCST90200890 | F-strong vegetable liking (derived food-liking factor) \|\| id:ebi-fl187-GCST90094852 | Weighted median | 26 | 0.0337320257827592 | 0.068388078648664 | 0.621840023100966 | -0.100308608 | 0.167772659934141 | 1.0343074018715 | 0.904558220720041 | 1.18266771232768 | 0.999332001504091 |
| ebi-fl187-GCST90094852 | ebi-met1400-GCST90200948 | Adenosine 5'-diphosphate (ADP) to mannitol to sorbitol ratio \|\| id:ebi-met1400-GCST90200948 | F-strong vegetable liking (derived food-liking factor) \|\| id:ebi-fl187-GCST90094852 | Weighted median | 26 | -0.045012999 | 0.0964868892792036 | 0.640843816373211 | -0.234127302 | 0.144101304277586 | 0.95598505518017 | 0.791261080605588 | 1.15500110915146 | 0.999332001504091 |
| ebi-fl187-GCST90094852 | ebi-met1400-GCST90200972 | Leucine to N-palmitoyl-sphingosine (d18:1 to 16:0) ratio \|\| id:ebi-met1400-GCST90200972 | F-strong vegetable liking (derived food-liking factor) \|\| id:ebi-fl187-GCST90094852 | Weighted median | 26 | -0.044941878 | 0.0689163840600096 | 0.514322584554993 | -0.180017991 | 0.0901342344902815 | 0.956053047677941 | 0.835255184179231 | 1.09432116949081 | 0.999332001504091 |
| ebi-fl187-GCST90094852 | ebi-met1400-GCST90200973 | Proline to glutamate ratio \|\| id:ebi-met1400-GCST90200973 | F-strong vegetable liking (derived food-liking factor) \|\| id:ebi-fl187-GCST90094852 | Weighted median | 26 | 0.0419894252039568 | 0.0729176264136659 | 0.564718335169405 | -0.100929123 | 0.184907972974742 | 1.04288345041267 | 0.903997103609551 | 1.20310771660877 | 0.999332001504091 |
| ebi-fl187-GCST90094852 | ebi-met1400-GCST90200983 | Cholesterol to linoleoyl-arachidonoyl-glycerol (18:2 to 20:4) [1] ratio \|\| id:ebi-met1400-GCST90200983 | F-strong vegetable liking (derived food-liking factor) \|\| id:ebi-fl187-GCST90094852 | Weighted median | 26 | 0.034011325680474 | 0.068940038915682 | 0.621767790140946 | -0.101111151 | 0.169133801955211 | 1.03459632416915 | 0.903832565775678 | 1.18427858711386 | 0.999332001504091 |
| ebi-fl187-GCST90094852 | ebi-met1400-GCST90201001 | Serine to alpha-ketobutyrate ratio \|\| id:ebi-met1400-GCST90201001 | F-strong vegetable liking (derived food-liking factor) \|\| id:ebi-fl187-GCST90094852 | Weighted median | 26 | 0.0758479817261039 | 0.0721688837976669 | 0.293268232303797 | -0.065603031 | 0.217298993969531 | 1.0787985645957 | 0.936502553446279 | 1.24271561106908 | 0.999332001504091 |
| ebi-fl187-GCST90094852 | ebi-met1400-GCST90201005 | Fructose to maltose ratio \|\| id:ebi-met1400-GCST90201005 | F-strong vegetable liking (derived food-liking factor) \|\| id:ebi-fl187-GCST90094852 | Weighted median | 26 | 0.0616001105074433 | 0.0786867528935118 | 0.433713813674809 | -0.092625925 | 0.215826146178726 | 1.06353696242453 | 0.911534418615981 | 1.24088662736466 | 0.999332001504091 |
| ebi-fl187-GCST90094852 | ebi-met1400-GCST90201020 | Paraxanthine to 5-acetylamino-6-formylamino-3-methyluracil ratio \|\| id:ebi-met1400-GCST90201020 | F-strong vegetable liking (derived food-liking factor) \|\| id:ebi-fl187-GCST90094852 | Weighted median | 25 | -0.06262452 | 0.0715411937884878 | 0.381376377520744 | -0.20284526 | 0.0775962195434514 | 0.939296094116633 | 0.816404562006168 | 1.08068621058991 | 0.999332001504091 |
| ebi-fl187-GCST90094852 | ebi-pw11-GCST90275197 | Placental weight (fetal genotype, adjusted for sex) \|\| id:ebi-pw11-GCST90275197 | F-strong vegetable liking (derived food-liking factor) \|\| id:ebi-fl187-GCST90094852 | Weighted median | 20 | 0.00558780375494461 | 0.0322697203477572 | 0.862526176009464 | -0.057660848 | 0.0688364556365487 | 1.00560344464953 | 0.943970042361578 | 1.07126099612348 | 0.999332001504091 |
| ebi-fl187-GCST90094852 | prot-a-1017 | FAS-associated factor 2 \|\| id:prot-a-1017 | F-strong vegetable liking (derived food-liking factor) \|\| id:ebi-fl187-GCST90094852 | Weighted median | 27 | -0.144307363 | 0.112275458440855 | 0.198688352581239 | -0.364367262 | 0.0757525354278924 | 0.865621646999618 | 0.694636034707148 | 1.07869560217997 | 0.999332001504091 |
| ebi-fl187-GCST90094852 | prot-a-1019 | Fas apoptotic inhibitory molecule 3 \|\| id:prot-a-1019 | F-strong vegetable liking (derived food-liking factor) \|\| id:ebi-fl187-GCST90094852 | Weighted median | 27 | -0.03587715 | 0.111591756132418 | 0.747828793497782 | -0.254596992 | 0.182842692009873 | 0.964758806813384 | 0.77522885842411 | 1.20062552523656 | 0.999332001504091 |
| ebi-fl187-GCST90094852 | prot-a-1052 | MIP18 family protein FAM96A \|\| id:prot-a-1052 | F-strong vegetable liking (derived food-liking factor) \|\| id:ebi-fl187-GCST90094852 | Weighted median | 27 | -0.046499032 | 0.114173834664758 | 0.683813205680825 | -0.270279748 | 0.177281684238054 | 0.954565484870631 | 0.763165970586711 | 1.1939673675515 | 0.999332001504091 |
| ebi-fl187-GCST90094852 | prot-a-1066 | F-box/LRR-repeat protein 4 \|\| id:prot-a-1066 | F-strong vegetable liking (derived food-liking factor) \|\| id:ebi-fl187-GCST90094852 | Weighted median | 27 | 0.0793124787239978 | 0.109740064547193 | 0.469845546163283 | -0.135778048 | 0.294403005236495 | 1.0825425407363 | 0.873036383342212 | 1.34232475858276 | 0.999332001504091 |
| ebi-fl187-GCST90094852 | prot-a-1076 | Low affinity immunoglobulin gamma Fc region receptor III-B \|\| id:prot-a-1076 | F-strong vegetable liking (derived food-liking factor) \|\| id:ebi-fl187-GCST90094852 | Weighted median | 27 | -0.095981066 | 0.109109483255864 | 0.379034494314559 | -0.309835653 | 0.117873520878959 | 0.908481216814381 | 0.733567505581858 | 1.12510180048105 | 0.999332001504091 |
| ebi-fl187-GCST90094852 | prot-a-1081 | Fc receptor-like protein 3 \|\| id:prot-a-1081 | F-strong vegetable liking (derived food-liking factor) \|\| id:ebi-fl187-GCST90094852 | Weighted median | 27 | 0.0340834593229338 | 0.113959742823389 | 0.764876465524963 | -0.189277637 | 0.257444555256776 | 1.03467095606219 | 0.827556714755263 | 1.29362008455849 | 0.999332001504091 |
| ebi-fl187-GCST90094852 | prot-a-1105 | Fibroblast growth factor receptor 4 \|\| id:prot-a-1105 | F-strong vegetable liking (derived food-liking factor) \|\| id:ebi-fl187-GCST90094852 | Weighted median | 27 | -0.005732254 | 0.105955408413089 | 0.95685499291681 | -0.213404854 | 0.201940346488349 | 0.99428414401916 | 0.807829017797289 | 1.22377500345746 | 0.999332001504091 |
| ebi-fl187-GCST90094852 | prot-a-1143 | Follistatin-related protein 1 \|\| id:prot-a-1143 | F-strong vegetable liking (derived food-liking factor) \|\| id:ebi-fl187-GCST90094852 | Weighted median | 27 | 0.166098889951728 | 0.121069372150905 | 0.170084751320183 | -0.071197079 | 0.403394859367502 | 1.18068985431305 | 0.93127833820506 | 1.4968978391193 | 0.999332001504091 |
| ebi-fl187-GCST90094852 | prot-a-1151 | Alpha-(1,3)-fucosyltransferase 10 \|\| id:prot-a-1151 | F-strong vegetable liking (derived food-liking factor) \|\| id:ebi-fl187-GCST90094852 | Weighted median | 27 | 0.0698910926929069 | 0.110676428188201 | 0.527719978684387 | -0.147034707 | 0.28681689194178 | 1.07239138363653 | 0.863264015975509 | 1.33218025820099 | 0.999332001504091 |
| ebi-fl187-GCST90094852 | prot-a-1165 | Growth arrest and DNA damage-inducible proteins-interacting protein 1 \|\| id:prot-a-1165 | F-strong vegetable liking (derived food-liking factor) \|\| id:ebi-fl187-GCST90094852 | Weighted median | 27 | 0.187853147210814 | 0.116958061234695 | 0.108239158511964 | -0.041384653 | 0.417090947230815 | 1.20665630149429 | 0.959459999974616 | 1.51754052276738 | 0.999332001504091 |
| ebi-fl187-GCST90094852 | prot-a-1194 | Growth/differentiation factor 11/8 \|\| id:prot-a-1194 | F-strong vegetable liking (derived food-liking factor) \|\| id:ebi-fl187-GCST90094852 | Weighted median | 27 | -0.123052658 | 0.119284180898355 | 0.302263088756762 | -0.356849652 | 0.110744336686823 | 0.884217100342281 | 0.699877714735251 | 1.11710926648588 | 0.999332001504091 |
| ebi-fl187-GCST90094852 | prot-a-1216 | D-glucuronyl C5-epimerase \|\| id:prot-a-1216 | F-strong vegetable liking (derived food-liking factor) \|\| id:ebi-fl187-GCST90094852 | Weighted median | 27 | 0.0275745197651503 | 0.107462319726515 | 0.797489812753431 | -0.183051627 | 0.238200666429119 | 1.0279582154579 | 0.83272516360956 | 1.2689638057135 | 0.999332001504091 |
| ebi-fl187-GCST90094852 | prot-a-1246 | Glypican-3 \|\| id:prot-a-1246 | F-strong vegetable liking (derived food-liking factor) \|\| id:ebi-fl187-GCST90094852 | Weighted median | 27 | 0.0885855725860029 | 0.113290883809567 | 0.434255548096664 | -0.13346456 | 0.310635704852755 | 1.09262774759202 | 0.875058480780002 | 1.36429212564611 | 0.999332001504091 |
| ebi-fl187-GCST90094852 | prot-a-1275 | Glutamate receptor 4 \|\| id:prot-a-1275 | F-strong vegetable liking (derived food-liking factor) \|\| id:ebi-fl187-GCST90094852 | Weighted median | 27 | -0.117097648 | 0.113001878530279 | 0.300087789848979 | -0.33858133 | 0.104386033813367 | 0.889498331095219 | 0.712780806550404 | 1.11002888089865 | 0.999332001504091 |
| ebi-fl187-GCST90094852 | prot-a-1289 | Glutathione S-transferase P \|\| id:prot-a-1289 | F-strong vegetable liking (derived food-liking factor) \|\| id:ebi-fl187-GCST90094852 | Weighted median | 27 | 0.173742979702136 | 0.10881192502061 | 0.11032649016066 | -0.039528393 | 0.387014352742533 | 1.18974973668819 | 0.961242660715111 | 1.47257762664899 | 0.999332001504091 |
| ebi-fl187-GCST90094852 | prot-a-130 | Apolipoprotein D \|\| id:prot-a-130 | F-strong vegetable liking (derived food-liking factor) \|\| id:ebi-fl187-GCST90094852 | Weighted median | 27 | 0.150940155997874 | 0.113434559669577 | 0.183308673851758 | -0.071391581 | 0.373271892950244 | 1.16292706178982 | 0.93109722079463 | 1.45247920500603 | 0.999332001504091 |
| ebi-fl187-GCST90094852 | prot-a-1313 | Heparin-binding EGF-like growth factor \|\| id:prot-a-1313 | F-strong vegetable liking (derived food-liking factor) \|\| id:ebi-fl187-GCST90094852 | Weighted median | 27 | 0.0203039286392406 | 0.110717233517291 | 0.854495598948503 | -0.196701849 | 0.237309706333131 | 1.02051145555589 | 0.821435508579375 | 1.26783371310782 | 0.999332001504091 |
| ebi-fl187-GCST90094852 | prot-a-1347 | HLA class II histocompatibility antigen, DQ alpha 2 chain \|\| id:prot-a-1347 | F-strong vegetable liking (derived food-liking factor) \|\| id:ebi-fl187-GCST90094852 | Weighted median | 27 | -0.116782004 | 0.115388520818163 | 0.311501504520152 | -0.342943505 | 0.109379497021421 | 0.889779140510078 | 0.709678303997429 | 1.11558563144368 | 0.999332001504091 |
| ebi-fl187-GCST90094852 | prot-a-1357 | Non-histone chromosomal protein HMG-14 \|\| id:prot-a-1357 | F-strong vegetable liking (derived food-liking factor) \|\| id:ebi-fl187-GCST90094852 | Weighted median | 27 | -0.306141528 | 0.112239815528317 | 0.0063803452338244 | -0.526131566 | -0.086151489 | 0.73628240759439 | 0.590886358826827 | 0.917455235909195 | 0.999332001504091 |
| ebi-fl187-GCST90094852 | prot-a-137 | Apolipoprotein M \|\| id:prot-a-137 | F-strong vegetable liking (derived food-liking factor) \|\| id:ebi-fl187-GCST90094852 | Weighted median | 27 | -0.080509805 | 0.11613330243891 | 0.488150551843524 | -0.308131078 | 0.147111467880756 | 0.922645857089496 | 0.7348189933275 | 1.15848308948787 | 0.999332001504091 |
| ebi-fl187-GCST90094852 | prot-a-1381 | 17-beta-hydroxysteroid dehydrogenase 14 \|\| id:prot-a-1381 | F-strong vegetable liking (derived food-liking factor) \|\| id:ebi-fl187-GCST90094852 | Weighted median | 27 | 0.127435865542388 | 0.111523464181812 | 0.253170606997354 | -0.091150124 | 0.34602185533874 | 1.13591201480401 | 0.912880655084519 | 1.41343350654818 | 0.999332001504091 |
| ebi-fl187-GCST90094852 | prot-a-1392 | Serine protease HTRA2, mitochondrial \|\| id:prot-a-1392 | F-strong vegetable liking (derived food-liking factor) \|\| id:ebi-fl187-GCST90094852 | Weighted median | 27 | 0.10518752606588 | 0.110720655490549 | 0.342098928759102 | -0.111824959 | 0.322200010827356 | 1.11091891707766 | 0.8942007658707 | 1.38016079545547 | 0.999332001504091 |
| ebi-fl187-GCST90094852 | prot-a-1416 | Interferon-induced protein with tetratricopeptide repeats 2 \|\| id:prot-a-1416 | F-strong vegetable liking (derived food-liking factor) \|\| id:ebi-fl187-GCST90094852 | Weighted median | 27 | -0.093385672 | 0.112888312832019 | 0.408101322144922 | -0.314646766 | 0.127875420663546 | 0.910842145790789 | 0.730046706311365 | 1.13641142049743 | 0.999332001504091 |
| ebi-fl187-GCST90094852 | prot-a-1428 | Interferon gamma \|\| id:prot-a-1428 | F-strong vegetable liking (derived food-liking factor) \|\| id:ebi-fl187-GCST90094852 | Weighted median | 27 | 0.123806031657339 | 0.111303756431804 | 0.265998126671978 | -0.094349331 | 0.341961394263674 | 1.13179631705076 | 0.909964827834976 | 1.4077059509403 | 0.999332001504091 |
| ebi-fl187-GCST90094852 | prot-a-1483 | Interleukin-17C \|\| id:prot-a-1483 | F-strong vegetable liking (derived food-liking factor) \|\| id:ebi-fl187-GCST90094852 | Weighted median | 27 | -0.066829175 | 0.109415045963999 | 0.541341280872133 | -0.281282665 | 0.147624314788174 | 0.935354969421083 | 0.754814945261204 | 1.15907736633111 | 0.999332001504091 |
| ebi-fl187-GCST90094852 | prot-a-1503 | Interleukin-1 receptor-like 2 \|\| id:prot-a-1503 | F-strong vegetable liking (derived food-liking factor) \|\| id:ebi-fl187-GCST90094852 | Weighted median | 27 | -0.065171913 | 0.104058560493585 | 0.531117958309426 | -0.269126691 | 0.138782865946196 | 0.936906383499733 | 0.764046451563079 | 1.14887461311648 | 0.999332001504091 |
| ebi-fl187-GCST90094852 | prot-a-1522 | Interleukin-31 receptor subunit alpha \|\| id:prot-a-1522 | F-strong vegetable liking (derived food-liking factor) \|\| id:ebi-fl187-GCST90094852 | Weighted median | 27 | -0.033401492 | 0.108046836641382 | 0.757215785068048 | -0.245173292 | 0.178370307594681 | 0.967150178353698 | 0.782568913527763 | 1.19526785605752 | 0.999332001504091 |
| ebi-fl187-GCST90094852 | prot-a-1540 | Interleukin-6 receptor subunit alpha \|\| id:prot-a-1540 | F-strong vegetable liking (derived food-liking factor) \|\| id:ebi-fl187-GCST90094852 | Weighted median | 27 | 0.0201569922137877 | 0.11358906346022 | 0.859150698136423 | -0.202477572 | 0.242791556595818 | 1.02036151626652 | 0.816704799310542 | 1.27480287217197 | 0.999332001504091 |
| ebi-fl187-GCST90094852 | prot-a-1541 | Interleukin-6 receptor subunit alpha \|\| id:prot-a-1541 | F-strong vegetable liking (derived food-liking factor) \|\| id:ebi-fl187-GCST90094852 | Weighted median | 27 | -0.021796494 | 0.100754808613986 | 0.828728929084784 | -0.219275919 | 0.175682930701033 | 0.97843933288817 | 0.803100096951787 | 1.19206003308467 | 0.999332001504091 |
| ebi-fl187-GCST90094852 | prot-a-1545 | Interleukin-9 \|\| id:prot-a-1545 | F-strong vegetable liking (derived food-liking factor) \|\| id:ebi-fl187-GCST90094852 | Weighted median | 27 | 0.190110081608214 | 0.111855360513145 | 0.0892049941277264 | -0.029126425 | 0.409346588213977 | 1.20938272112478 | 0.971293660905349 | 1.50583353420826 | 0.999332001504091 |
| ebi-fl187-GCST90094852 | prot-a-1548 | Inositol monophosphatase 3 \|\| id:prot-a-1548 | F-strong vegetable liking (derived food-liking factor) \|\| id:ebi-fl187-GCST90094852 | Weighted median | 27 | 0.00405359667156511 | 0.116299438971631 | 0.97219550259438 | -0.223893304 | 0.232000497055962 | 1.00406182360702 | 0.799400422876382 | 1.26112035567557 | 0.999332001504091 |
| ebi-fl187-GCST90094852 | prot-a-1555 | Inhibin beta A chain:Inhibin beta B chain heterodimer \|\| id:prot-a-1555 | F-strong vegetable liking (derived food-liking factor) \|\| id:ebi-fl187-GCST90094852 | Weighted median | 27 | -0.181201942 | 0.11349492506374 | 0.110362869042991 | -0.403651995 | 0.0412481110888434 | 0.834266868133707 | 0.667876505086849 | 1.0421106326762 | 0.999332001504091 |
| ebi-fl187-GCST90094852 | prot-a-1603 | Junctophilin-3 \|\| id:prot-a-1603 | F-strong vegetable liking (derived food-liking factor) \|\| id:ebi-fl187-GCST90094852 | Weighted median | 27 | -0.209118498 | 0.118540731929574 | 0.0777140191252023 | -0.441458332 | 0.0232213368928028 | 0.811299092879451 | 0.643097886500465 | 1.02349305125036 | 0.999332001504091 |
| ebi-fl187-GCST90094852 | prot-a-1627 | UPF0258 protein KIAA1024 \|\| id:prot-a-1627 | F-strong vegetable liking (derived food-liking factor) \|\| id:ebi-fl187-GCST90094852 | Weighted median | 27 | 0.00291675229321512 | 0.113742793649809 | 0.979541766871505 | -0.220019123 | 0.225852627846842 | 1.00292101015389 | 0.802503451333259 | 1.2533909367455 | 0.999332001504091 |
| ebi-fl187-GCST90094852 | prot-a-1666 | Kallikrein-7 \|\| id:prot-a-1666 | F-strong vegetable liking (derived food-liking factor) \|\| id:ebi-fl187-GCST90094852 | Weighted median | 27 | 0.00824344123540441 | 0.116939301817939 | 0.943801006222911 | -0.22095759 | 0.237444472798566 | 1.00827751195274 | 0.801750681552824 | 1.26800458608981 | 0.999332001504091 |
| ebi-fl187-GCST90094852 | prot-a-1678 | Importin subunit alpha-3 \|\| id:prot-a-1678 | F-strong vegetable liking (derived food-liking factor) \|\| id:ebi-fl187-GCST90094852 | Weighted median | 27 | -0.065679835 | 0.105197692336434 | 0.532399901736756 | -0.271867312 | 0.140507641877287 | 0.936430628518614 | 0.761955356652847 | 1.15085787424589 | 0.999332001504091 |
| ebi-fl187-GCST90094852 | prot-a-1718 | Protein LDOC1 \|\| id:prot-a-1718 | F-strong vegetable liking (derived food-liking factor) \|\| id:ebi-fl187-GCST90094852 | Weighted median | 27 | 0.0318284954011785 | 0.10966230295741 | 0.771631879498088 | -0.183109618 | 0.246766609197702 | 1.03234043898887 | 0.832676874031333 | 1.27988036561183 | 0.999332001504091 |
| ebi-fl187-GCST90094852 | prot-a-1723 | Leptin \|\| id:prot-a-1723 | F-strong vegetable liking (derived food-liking factor) \|\| id:ebi-fl187-GCST90094852 | Weighted median | 26 | -0.208800212 | 0.108969452635498 | 0.0553478798971635 | -0.422380339 | 0.00477991556930163 | 0.811557359196995 | 0.655484685743231 | 1.00479135758909 | 0.999332001504091 |
| ebi-fl187-GCST90094852 | prot-a-1730 | Galectin-7 \|\| id:prot-a-1730 | F-strong vegetable liking (derived food-liking factor) \|\| id:ebi-fl187-GCST90094852 | Weighted median | 26 | 0.0477003111133518 | 0.112233724741512 | 0.670830321780288 | -0.172277789 | 0.267678411606715 | 1.04885627764897 | 0.841745312779473 | 1.3069267799438 | 0.999332001504091 |
| ebi-fl187-GCST90094852 | prot-a-1753 | Lipase member N \|\| id:prot-a-1753 | F-strong vegetable liking (derived food-liking factor) \|\| id:ebi-fl187-GCST90094852 | Weighted median | 27 | 0.0397470875696038 | 0.108359908622628 | 0.713763573952763 | -0.172638333 | 0.252132508469954 | 1.04054757349467 | 0.841441881302369 | 1.28676653345303 | 0.999332001504091 |
| ebi-fl187-GCST90094852 | prot-a-1756 | VIP36-like protein \|\| id:prot-a-1756 | F-strong vegetable liking (derived food-liking factor) \|\| id:ebi-fl187-GCST90094852 | Weighted median | 27 | 0.104524054835283 | 0.108170737082832 | 0.333900229430424 | -0.10749059 | 0.316538699517634 | 1.11018209879254 | 0.898084973527986 | 1.37236935124047 | 0.999332001504091 |
| ebi-fl187-GCST90094852 | prot-a-1760 | Ig Kappa chain V-I region HK102- like \|\| id:prot-a-1760 | F-strong vegetable liking (derived food-liking factor) \|\| id:ebi-fl187-GCST90094852 | Weighted median | 26 | -0.132052004 | 0.109879578827413 | 0.229445518319739 | -0.347415978 | 0.0833119705400552 | 0.876295423060824 | 0.706511373607015 | 1.08688083046272 | 0.999332001504091 |
| ebi-fl187-GCST90094852 | prot-a-179 | Neutral ceramidase \|\| id:prot-a-179 | F-strong vegetable liking (derived food-liking factor) \|\| id:ebi-fl187-GCST90094852 | Weighted median | 27 | 0.146275507628825 | 0.111853797836117 | 0.190962050970492 | -0.072957936 | 0.365508951387614 | 1.15751504835086 | 0.929639933456708 | 1.44124734635346 | 0.999332001504091 |
| ebi-fl187-GCST90094852 | prot-a-1809 | Lactotransferrin \|\| id:prot-a-1809 | F-strong vegetable liking (derived food-liking factor) \|\| id:ebi-fl187-GCST90094852 | Weighted median | 27 | 0.0297052404276413 | 0.10775522684383 | 0.782798994423245 | -0.181495004 | 0.240905485041549 | 1.03015084237595 | 0.834022411913692 | 1.27240076871903 | 0.999332001504091 |
| ebi-fl187-GCST90094852 | prot-a-1818 | Lysophospholipase-like protein 1 \|\| id:prot-a-1818 | F-strong vegetable liking (derived food-liking factor) \|\| id:ebi-fl187-GCST90094852 | Weighted median | 27 | -0.005477564 | 0.116071109355992 | 0.962360642717265 | -0.232976938 | 0.222021810511425 | 0.994537410672683 | 0.792171842250718 | 1.24859861014156 | 0.999332001504091 |
| ebi-fl187-GCST90094852 | prot-a-1852 | MAP kinase-activated protein kinase 3 \|\| id:prot-a-1852 | F-strong vegetable liking (derived food-liking factor) \|\| id:ebi-fl187-GCST90094852 | Weighted median | 27 | -0.173095814 | 0.106044171153759 | 0.102616474164953 | -0.38094239 | 0.034750760965323 | 0.841057025528827 | 0.683217248660943 | 1.03536162410684 | 0.999332001504091 |
| ebi-fl187-GCST90094852 | prot-a-1856 | Megakaryocyte-associated tyrosine-protein kinase \|\| id:prot-a-1856 | F-strong vegetable liking (derived food-liking factor) \|\| id:ebi-fl187-GCST90094852 | Weighted median | 27 | -0.108982403 | 0.113585537254499 | 0.337319863459028 | -0.331610056 | 0.113645249826341 | 0.896746197229628 | 0.717767157135739 | 1.12035460838691 | 0.999332001504091 |
| ebi-fl187-GCST90094852 | prot-a-1890 | Beta-1,3-N-acetylglucosaminyltransferase manic fringe \|\| id:prot-a-1890 | F-strong vegetable liking (derived food-liking factor) \|\| id:ebi-fl187-GCST90094852 | Weighted median | 27 | -0.089721367 | 0.108756924012837 | 0.409387748958101 | -0.302884938 | 0.123442204051274 | 0.914185872126915 | 0.738684085890761 | 1.13138461320533 | 0.999332001504091 |
| ebi-fl187-GCST90094852 | prot-a-1919 | Matrilysin \|\| id:prot-a-1919 | F-strong vegetable liking (derived food-liking factor) \|\| id:ebi-fl187-GCST90094852 | Weighted median | 27 | -0.031630047 | 0.111239568342721 | 0.776148538455708 | -0.249659601 | 0.186399506866953 | 0.968864950198225 | 0.779065931176235 | 1.20490353147051 | 0.999332001504091 |
| ebi-fl187-GCST90094852 | prot-a-1923 | Multimerin-2 \|\| id:prot-a-1923 | F-strong vegetable liking (derived food-liking factor) \|\| id:ebi-fl187-GCST90094852 | Weighted median | 27 | 0.0092976114973617 | 0.107943560653608 | 0.93135988974174 | -0.202271767 | 0.220866990378432 | 1.00934096855532 | 0.816872898362993 | 1.24715753557965 | 0.999332001504091 |
| ebi-fl187-GCST90094852 | prot-a-1948 | Mesothelin \|\| id:prot-a-1948 | F-strong vegetable liking (derived food-liking factor) \|\| id:ebi-fl187-GCST90094852 | Weighted median | 27 | 0.0774843006192939 | 0.108013190398165 | 0.473152231970718 | -0.134221553 | 0.289190153799698 | 1.08056526811987 | 0.874396318397876 | 1.3353456254326 | 0.999332001504091 |
| ebi-fl187-GCST90094852 | prot-a-195 | ATPase family AAA domain-containing protein 1 \|\| id:prot-a-195 | F-strong vegetable liking (derived food-liking factor) \|\| id:ebi-fl187-GCST90094852 | Weighted median | 27 | -0.023304101 | 0.112022732181409 | 0.835205460707097 | -0.242868656 | 0.196260454275356 | 0.976965342652667 | 0.784374529991834 | 1.2168437962339 | 0.999332001504091 |
| ebi-fl187-GCST90094852 | prot-a-1966 | Mucin-1 \|\| id:prot-a-1966 | F-strong vegetable liking (derived food-liking factor) \|\| id:ebi-fl187-GCST90094852 | Weighted median | 27 | 0.0353673107347863 | 0.109037659122156 | 0.745666501772957 | -0.178346501 | 0.249081122614212 | 1.03600017290582 | 0.836652472218157 | 1.28284609667779 | 0.999332001504091 |
| ebi-fl187-GCST90094852 | prot-a-1986 | Myocardial zonula adherens protein \|\| id:prot-a-1986 | F-strong vegetable liking (derived food-liking factor) \|\| id:ebi-fl187-GCST90094852 | Weighted median | 27 | -0.036728127 | 0.104030752243655 | 0.724050449118157 | -0.240628402 | 0.167172146926516 | 0.963938168035191 | 0.786133697931536 | 1.18195771818442 | 0.999332001504091 |
| ebi-fl187-GCST90094852 | prot-a-2071 | Epididymal secretory protein E1 \|\| id:prot-a-2071 | F-strong vegetable liking (derived food-liking factor) \|\| id:ebi-fl187-GCST90094852 | Weighted median | 26 | -0.102460001 | 0.118892512675702 | 0.388804836069602 | -0.335489326 | 0.130569323785564 | 0.902614252645289 | 0.714988138523623 | 1.13947693000993 | 0.999332001504091 |
| ebi-fl187-GCST90094852 | prot-a-2098 | Neuritin \|\| id:prot-a-2098 | F-strong vegetable liking (derived food-liking factor) \|\| id:ebi-fl187-GCST90094852 | Weighted median | 27 | -0.165097852 | 0.109391617183191 | 0.131238283487817 | -0.379505422 | 0.0493097173228879 | 0.847810739750386 | 0.684199715648874 | 1.0505456725518 | 0.999332001504091 |
| ebi-fl187-GCST90094852 | prot-a-2115 | Neurotrophin-4 \|\| id:prot-a-2115 | F-strong vegetable liking (derived food-liking factor) \|\| id:ebi-fl187-GCST90094852 | Weighted median | 27 | -0.051921736 | 0.11879962934732 | 0.662072904947092 | -0.284769009 | 0.180925538016635 | 0.949403168495638 | 0.752187982889401 | 1.19832594624433 | 0.999332001504091 |
| ebi-fl187-GCST90094852 | prot-a-2118 | Netrin-1 \|\| id:prot-a-2118 | F-strong vegetable liking (derived food-liking factor) \|\| id:ebi-fl187-GCST90094852 | Weighted median | 27 | -0.059638682 | 0.104380835637474 | 0.567757923106264 | -0.26422512 | 0.144947755593705 | 0.942104871302469 | 0.767800672831302 | 1.15597917524468 | 0.999332001504091 |
| ebi-fl187-GCST90094852 | prot-a-2120 | Netrin-G1 \|\| id:prot-a-2120 | F-strong vegetable liking (derived food-liking factor) \|\| id:ebi-fl187-GCST90094852 | Weighted median | 27 | -0.042242851 | 0.11336919374058 | 0.70943639159362 | -0.264446471 | 0.179960768450349 | 0.958636946091002 | 0.767630738263848 | 1.1971703953507 | 0.999332001504091 |
| ebi-fl187-GCST90094852 | prot-a-2145 | Olfactomedin-like protein 3 \|\| id:prot-a-2145 | F-strong vegetable liking (derived food-liking factor) \|\| id:ebi-fl187-GCST90094852 | Weighted median | 27 | -0.119927663 | 0.115021986692606 | 0.297110467402666 | -0.345370757 | 0.105515430521977 | 0.886984595850484 | 0.707957824262829 | 1.1112832520712 | 0.999332001504091 |
| ebi-fl187-GCST90094852 | prot-a-2162 | Prolyl 4-hydroxylase subunit alpha-2 \|\| id:prot-a-2162 | F-strong vegetable liking (derived food-liking factor) \|\| id:ebi-fl187-GCST90094852 | Weighted median | 27 | 0.0608717724226157 | 0.106867790196378 | 0.568949795777398 | -0.148589096 | 0.270332641207517 | 1.06276262997231 | 0.861923209526815 | 1.31040027137188 | 0.999332001504091 |
| ebi-fl187-GCST90094852 | prot-a-2168 | Polyadenylate-binding protein-interacting protein 1 \|\| id:prot-a-2168 | F-strong vegetable liking (derived food-liking factor) \|\| id:ebi-fl187-GCST90094852 | Weighted median | 26 | 0.0767064041410771 | 0.107816490542171 | 0.476803344734118 | -0.134613917 | 0.288026725603733 | 1.07972502705597 | 0.874053303393754 | 1.3337929500689 | 0.999332001504091 |
| ebi-fl187-GCST90094852 | prot-a-219 | Beta-1,4-galactosyltransferase 5 \|\| id:prot-a-219 | F-strong vegetable liking (derived food-liking factor) \|\| id:ebi-fl187-GCST90094852 | Weighted median | 27 | 0.0788308779778645 | 0.111125568610981 | 0.47808522238883 | -0.138975236 | 0.296636992455387 | 1.08202131296287 | 0.87024957861716 | 1.34532684700207 | 0.999332001504091 |
| ebi-fl187-GCST90094852 | prot-a-2190 | Pyruvate carboxylase, mitochondrial \|\| id:prot-a-2190 | F-strong vegetable liking (derived food-liking factor) \|\| id:ebi-fl187-GCST90094852 | Weighted median | 27 | -0.01333898 | 0.107762829614823 | 0.901488752993673 | -0.224554126 | 0.197876165723514 | 0.986749589628168 | 0.798872335472797 | 1.21881145384151 | 0.999332001504091 |
| ebi-fl187-GCST90094852 | prot-a-2197 | Protocadherin-9 \|\| id:prot-a-2197 | F-strong vegetable liking (derived food-liking factor) \|\| id:ebi-fl187-GCST90094852 | Weighted median | 27 | 0.0925660344834418 | 0.10592988271775 | 0.382204027074437 | -0.115056536 | 0.300188604610231 | 1.09698557804461 | 0.891315751372928 | 1.35011342118017 | 0.999332001504091 |
| ebi-fl187-GCST90094852 | prot-a-2198 | Protocadherin alpha-4 \|\| id:prot-a-2198 | F-strong vegetable liking (derived food-liking factor) \|\| id:ebi-fl187-GCST90094852 | Weighted median | 27 | -0.046144664 | 0.115180478598036 | 0.688693135381456 | -0.271898402 | 0.179609073711031 | 0.954903811667481 | 0.761931667591532 | 1.19674943085043 | 0.999332001504091 |
| ebi-fl187-GCST90094852 | prot-a-2254 | Placenta growth factor \|\| id:prot-a-2254 | F-strong vegetable liking (derived food-liking factor) \|\| id:ebi-fl187-GCST90094852 | Weighted median | 27 | -0.097131393 | 0.107184671995503 | 0.364826826611806 | -0.30721335 | 0.112950563990856 | 0.907436767351503 | 0.735493666422836 | 1.11957658418223 | 0.999332001504091 |
| ebi-fl187-GCST90094852 | prot-a-2261 | Membrane-associated progesterone receptor component 2 \|\| id:prot-a-2261 | F-strong vegetable liking (derived food-liking factor) \|\| id:ebi-fl187-GCST90094852 | Weighted median | 27 | 0.0149373494759118 | 0.114104597157583 | 0.895847081683399 | -0.208707661 | 0.238582359904775 | 1.01504946924237 | 0.811632472828536 | 1.26944825336836 | 0.999332001504091 |
| ebi-fl187-GCST90094852 | prot-a-2268 | Polymeric immunoglobulin receptor \|\| id:prot-a-2268 | F-strong vegetable liking (derived food-liking factor) \|\| id:ebi-fl187-GCST90094852 | Weighted median | 27 | 0.0674365104044461 | 0.115783632977244 | 0.560273304202131 | -0.15949941 | 0.294372431039844 | 1.06976233866357 | 0.85257047021595 | 1.342283719 | 0.999332001504091 |
| ebi-fl187-GCST90094852 | prot-a-2283 | Group 10 secretory phospholipase A2 \|\| id:prot-a-2283 | F-strong vegetable liking (derived food-liking factor) \|\| id:ebi-fl187-GCST90094852 | Weighted median | 27 | 0.24598848549012 | 0.111772191738573 | 0.0277500297818514 | 0.026914989682517 | 0.465061981297724 | 1.27888484772474 | 1.02728046961257 | 1.59211286705059 | 0.999332001504091 |
| ebi-fl187-GCST90094852 | prot-a-2298 | Inactive phospholipase D5 \|\| id:prot-a-2298 | F-strong vegetable liking (derived food-liking factor) \|\| id:ebi-fl187-GCST90094852 | Weighted median | 27 | 0.0827634186897097 | 0.111202544682388 | 0.456720213757742 | -0.135193569 | 0.300720406267189 | 1.08628478346835 | 0.873546803838319 | 1.3508316046833 | 0.999332001504091 |
| ebi-fl187-GCST90094852 | prot-a-2304 | Procollagen-lysine,2-oxoglutarate 5-dioxygenase 2 \|\| id:prot-a-2304 | F-strong vegetable liking (derived food-liking factor) \|\| id:ebi-fl187-GCST90094852 | Weighted median | 27 | -0.07272857 | 0.115004021948635 | 0.527125381945873 | -0.298136453 | 0.152679312779969 | 0.929853185603439 | 0.742200057221365 | 1.16495133402959 | 0.999332001504091 |
| ebi-fl187-GCST90094852 | prot-a-2331 | Periostin \|\| id:prot-a-2331 | F-strong vegetable liking (derived food-liking factor) \|\| id:ebi-fl187-GCST90094852 | Weighted median | 27 | 0.013205104362458 | 0.121402863237681 | 0.913384168263863 | -0.224744508 | 0.251154716308313 | 1.01329267679623 | 0.798720259662146 | 1.28550895814659 | 0.999332001504091 |
| ebi-fl187-GCST90094852 | prot-a-2333 | Periostin \|\| id:prot-a-2333 | F-strong vegetable liking (derived food-liking factor) \|\| id:ebi-fl187-GCST90094852 | Weighted median | 27 | 0.126819850512549 | 0.113946902650314 | 0.265719933592992 | -0.096516079 | 0.350155779707164 | 1.1352124914109 | 0.907995298114664 | 1.4192886277398 | 0.999332001504091 |
| ebi-fl187-GCST90094852 | prot-a-2367 | cAMP-dependent protein kinase catalytic subunit alpha \|\| id:prot-a-2367 | F-strong vegetable liking (derived food-liking factor) \|\| id:ebi-fl187-GCST90094852 | Weighted median | 27 | 0.0370794732751831 | 0.113280367237077 | 0.743422238797132 | -0.184950047 | 0.259108993059853 | 1.03777549297828 | 0.83114580144905 | 1.2957750276169 | 0.999332001504091 |
| ebi-fl187-GCST90094852 | prot-a-2385 | Phosphoribosyl pyrophosphate synthase-associated protein 1 \|\| id:prot-a-2385 | F-strong vegetable liking (derived food-liking factor) \|\| id:ebi-fl187-GCST90094852 | Weighted median | 27 | 0.0384224990447393 | 0.113206721759044 | 0.734307502526083 | -0.183462676 | 0.260307673692465 | 1.03917018855478 | 0.832382943349547 | 1.29732917932641 | 0.999332001504091 |
| ebi-fl187-GCST90094852 | prot-a-2393 | Trypsin-3 \|\| id:prot-a-2393 | F-strong vegetable liking (derived food-liking factor) \|\| id:ebi-fl187-GCST90094852 | Weighted median | 26 | -0.033938554 | 0.116178331167696 | 0.770191322939838 | -0.261648083 | 0.1937709751589 | 0.966630898482789 | 0.769781875360146 | 1.21381825658664 | 0.999332001504091 |
| ebi-fl187-GCST90094852 | prot-a-2405 | Pregnancy-specific beta-1-glycoprotein 6 \|\| id:prot-a-2405 | F-strong vegetable liking (derived food-liking factor) \|\| id:ebi-fl187-GCST90094852 | Weighted median | 27 | -0.09155791 | 0.109150513788158 | 0.401568906666297 | -0.305492917 | 0.122377097062823 | 0.912508471291037 | 0.736760123292729 | 1.13018020907067 | 0.999332001504091 |
| ebi-fl187-GCST90094852 | prot-a-244 | Beclin-1 \|\| id:prot-a-244 | F-strong vegetable liking (derived food-liking factor) \|\| id:ebi-fl187-GCST90094852 | Weighted median | 26 | -0.011748286 | 0.110995149159294 | 0.915705298156911 | -0.229298779 | 0.205802205990398 | 0.988320455292177 | 0.795090941764063 | 1.22851018800663 | 0.999332001504091 |
| ebi-fl187-GCST90094852 | prot-a-2454 | tRNA pseudouridine synthase A, mitochondrial \|\| id:prot-a-2454 | F-strong vegetable liking (derived food-liking factor) \|\| id:ebi-fl187-GCST90094852 | Weighted median | 27 | 0.0565306345144928 | 0.113265533589862 | 0.617710139780449 | -0.165469811 | 0.278531080350623 | 1.05815903049186 | 0.847495447586222 | 1.32118766773382 | 0.999332001504091 |
| ebi-fl187-GCST90094852 | prot-a-2466 | Glutaminyl-peptide cyclotransferase-like protein \|\| id:prot-a-2466 | F-strong vegetable liking (derived food-liking factor) \|\| id:ebi-fl187-GCST90094852 | Weighted median | 27 | 0.102634517761486 | 0.106599016474795 | 0.335643264172682 | -0.106299555 | 0.311568590052085 | 1.10808634918005 | 0.899155261698967 | 1.36556544741686 | 0.999332001504091 |
| ebi-fl187-GCST90094852 | prot-a-2474 | Ras-related protein Rab-27A \|\| id:prot-a-2474 | F-strong vegetable liking (derived food-liking factor) \|\| id:ebi-fl187-GCST90094852 | Weighted median | 27 | -0.268840246 | 0.112065586975412 | 0.0164419567528876 | -0.488488797 | -0.049191696 | 0.764265340120911 | 0.613552897066816 | 0.951998618053177 | 0.999332001504091 |
| ebi-fl187-GCST90094852 | prot-a-2490 | Rap1 GTPase-GDP dissociation stimulator 1 \|\| id:prot-a-2490 | F-strong vegetable liking (derived food-liking factor) \|\| id:ebi-fl187-GCST90094852 | Weighted median | 27 | 0.0977261717388897 | 0.11236676083727 | 0.384460481290537 | -0.12251268 | 0.31796502297994 | 1.10266080407435 | 0.884694687384167 | 1.37432819048219 | 0.999332001504091 |
| ebi-fl187-GCST90094852 | prot-a-2545 | Receptor-interacting serine/threonine-protein kinase 2 \|\| id:prot-a-2545 | F-strong vegetable liking (derived food-liking factor) \|\| id:ebi-fl187-GCST90094852 | Weighted median | 27 | 0.0941912575492647 | 0.116624071189987 | 0.419292951486216 | -0.134391922 | 0.322774437081639 | 1.09876987385554 | 0.874247360691792 | 1.38095382379785 | 0.999332001504091 |
| ebi-fl187-GCST90094852 | prot-a-2610 | RUN and FYVE domain-containing protein 1 \|\| id:prot-a-2610 | F-strong vegetable liking (derived food-liking factor) \|\| id:ebi-fl187-GCST90094852 | Weighted median | 27 | -0.108916359 | 0.118246867585997 | 0.357001886858816 | -0.34068022 | 0.122847501247698 | 0.89680542386579 | 0.711286327205046 | 1.13071197563348 | 0.999332001504091 |
| ebi-fl187-GCST90094852 | prot-a-2627 | Serine--tRNA ligase, mitochondrial \|\| id:prot-a-2627 | F-strong vegetable liking (derived food-liking factor) \|\| id:ebi-fl187-GCST90094852 | Weighted median | 27 | 0.0394074323864331 | 0.108442080882191 | 0.716308993218541 | -0.173139046 | 0.251953910915527 | 1.04019420613292 | 0.841020666034661 | 1.28653674061787 | 0.999332001504091 |
| ebi-fl187-GCST90094852 | prot-a-264 | BolA-like protein 2 \|\| id:prot-a-264 | F-strong vegetable liking (derived food-liking factor) \|\| id:ebi-fl187-GCST90094852 | Weighted median | 26 | -0.043681886 | 0.115638229433011 | 0.705619246429596 | -0.270332815 | 0.182969043953067 | 0.957258426602679 | 0.763125472140114 | 1.20077723618901 | 0.999332001504091 |
| ebi-fl187-GCST90094852 | prot-a-2646 | Secretoglobin family 1C member 1 \|\| id:prot-a-2646 | F-strong vegetable liking (derived food-liking factor) \|\| id:ebi-fl187-GCST90094852 | Weighted median | 26 | 0.0440918019491709 | 0.115849244832277 | 0.703502775905919 | -0.182972718 | 0.271156321820433 | 1.04507829070895 | 0.832790875692718 | 1.31148006731301 | 0.999332001504091 |
| ebi-fl187-GCST90094852 | prot-a-2672 | Semaphorin-3E \|\| id:prot-a-2672 | F-strong vegetable liking (derived food-liking factor) \|\| id:ebi-fl187-GCST90094852 | Weighted median | 27 | -0.166422946 | 0.11342928732756 | 0.142322867237099 | -0.388744349 | 0.0558984575980994 | 0.8466880554926 | 0.677907555792998 | 1.05749029817975 | 0.999332001504091 |
| ebi-fl187-GCST90094852 | prot-a-2701 | Plasma protease C1 inhibitor \|\| id:prot-a-2701 | F-strong vegetable liking (derived food-liking factor) \|\| id:ebi-fl187-GCST90094852 | Weighted median | 27 | 0.0660008034565036 | 0.106213884197112 | 0.534339594534838 | -0.14217841 | 0.274180016482843 | 1.06822757544004 | 0.867466478358124 | 1.31545158389327 | 0.999332001504091 |
| ebi-fl187-GCST90094852 | prot-a-2738 | Signaling threshold-regulating transmembrane adapter 1 \|\| id:prot-a-2738 | F-strong vegetable liking (derived food-liking factor) \|\| id:ebi-fl187-GCST90094852 | Weighted median | 27 | -0.124643232 | 0.104799635334398 | 0.234302770993425 | -0.330050517 | 0.0807640530443235 | 0.882811805223464 | 0.718887416143632 | 1.08411507273651 | 0.999332001504091 |
| ebi-fl187-GCST90094852 | prot-a-274 | Serine/threonine-protein kinase BRSK2 \|\| id:prot-a-274 | F-strong vegetable liking (derived food-liking factor) \|\| id:ebi-fl187-GCST90094852 | Weighted median | 27 | -0.10169323 | 0.107092582325059 | 0.342324492761149 | -0.311594691 | 0.108208231681545 | 0.903306616832923 | 0.732278267036999 | 1.11427974957628 | 0.999332001504091 |
| ebi-fl187-GCST90094852 | prot-a-2749 | Mitochondrial glutamate carrier 2 \|\| id:prot-a-2749 | F-strong vegetable liking (derived food-liking factor) \|\| id:ebi-fl187-GCST90094852 | Weighted median | 27 | 0.145987308656281 | 0.105236296569098 | 0.165370594728103 | -0.060275833 | 0.352250449931713 | 1.15718150176942 | 0.941504800029466 | 1.42226468520969 | 0.999332001504091 |
| ebi-fl187-GCST90094852 | prot-a-2760 | Sodium-coupled monocarboxylate transporter 1 \|\| id:prot-a-2760 | F-strong vegetable liking (derived food-liking factor) \|\| id:ebi-fl187-GCST90094852 | Weighted median | 27 | -0.07610921 | 0.11274794444915 | 0.499651267059437 | -0.297095181 | 0.144876761107945 | 0.92671499447677 | 0.742973291956859 | 1.15589711001071 | 0.999332001504091 |
| ebi-fl187-GCST90094852 | prot-a-2777 | Small integral membrane protein 10 \|\| id:prot-a-2777 | F-strong vegetable liking (derived food-liking factor) \|\| id:ebi-fl187-GCST90094852 | Weighted median | 27 | 0.232750250915325 | 0.108242249228214 | 0.0315337254353183 | 0.0205954424280257 | 0.444905059402625 | 1.26206624007529 | 1.02080899208269 | 1.56034204899396 | 0.999332001504091 |
| ebi-fl187-GCST90094852 | prot-a-2799 | Superoxide dismutase [Mn], mitochondrial \|\| id:prot-a-2799 | F-strong vegetable liking (derived food-liking factor) \|\| id:ebi-fl187-GCST90094852 | Weighted median | 26 | 0.268370983790391 | 0.114929535895735 | 0.0195386406278773 | 0.0431090934347512 | 0.493632874146031 | 1.30783223458784 | 1.0440517878336 | 1.6382570038754 | 0.999332001504091 |
| ebi-fl187-GCST90094852 | prot-a-2805 | Protein SPACA7 \|\| id:prot-a-2805 | F-strong vegetable liking (derived food-liking factor) \|\| id:ebi-fl187-GCST90094852 | Weighted median | 27 | 0.0290829136299714 | 0.114376981640757 | 0.79928509378808 | -0.19509597 | 0.253261797645856 | 1.02950995134362 | 0.822755694085504 | 1.28822048578297 | 0.999332001504091 |
| ebi-fl187-GCST90094852 | prot-a-2811 | SPARC-like protein 1 \|\| id:prot-a-2811 | F-strong vegetable liking (derived food-liking factor) \|\| id:ebi-fl187-GCST90094852 | Weighted median | 27 | 0.0988969593111142 | 0.109005847196745 | 0.364267796642608 | -0.114754501 | 0.312548419816735 | 1.10395254166775 | 0.891585000093785 | 1.3669041248187 | 0.999332001504091 |
| ebi-fl187-GCST90094852 | prot-a-2831 | Sepiapterin reductase \|\| id:prot-a-2831 | F-strong vegetable liking (derived food-liking factor) \|\| id:ebi-fl187-GCST90094852 | Weighted median | 27 | 0.0686230045314653 | 0.112463483604401 | 0.541742364182044 | -0.151805423 | 0.289051432396091 | 1.07103235868235 | 0.859155436079382 | 1.33516039726099 | 0.999332001504091 |
| ebi-fl187-GCST90094852 | prot-a-2834 | SPRY domain-containing SOCS box protein 1 \|\| id:prot-a-2834 | F-strong vegetable liking (derived food-liking factor) \|\| id:ebi-fl187-GCST90094852 | Weighted median | 27 | -0.141761149 | 0.108514447029743 | 0.191423221163384 | -0.354449465 | 0.070927167345116 | 0.867828513586199 | 0.701559570000485 | 1.07350303694483 | 0.999332001504091 |
| ebi-fl187-GCST90094852 | prot-a-2835 | Spectrin alpha chain, non-erythrocytic 1 \|\| id:prot-a-2835 | F-strong vegetable liking (derived food-liking factor) \|\| id:ebi-fl187-GCST90094852 | Weighted median | 27 | -0.050462467 | 0.106292648366461 | 0.634964959760671 | -0.258796058 | 0.157871123884672 | 0.950789614071373 | 0.771980446449166 | 1.17101526908366 | 0.999332001504091 |
| ebi-fl187-GCST90094852 | prot-a-2859 | Sia-alpha-2,3-Gal-beta-1,4-GlcNAc-R:alpha 2,8-sialyltransferase \|\| id:prot-a-2859 | F-strong vegetable liking (derived food-liking factor) \|\| id:ebi-fl187-GCST90094852 | Weighted median | 27 | 0.0410968534484853 | 0.106116136734358 | 0.698547562888084 | -0.166890775 | 0.249084481447828 | 1.04195301740147 | 0.84629204291765 | 1.28285040555162 | 0.999332001504091 |
| ebi-fl187-GCST90094852 | prot-a-2861 | Alpha-2,8-sialyltransferase 8F \|\| id:prot-a-2861 | F-strong vegetable liking (derived food-liking factor) \|\| id:ebi-fl187-GCST90094852 | Weighted median | 27 | -0.01249621 | 0.113455104634686 | 0.912296491643986 | -0.234868216 | 0.209875794618251 | 0.987581542960888 | 0.790675041438841 | 1.23352484001792 | 0.999332001504091 |
| ebi-fl187-GCST90094852 | prot-a-289 | Uncharacterized protein C17orf89 \|\| id:prot-a-289 | F-strong vegetable liking (derived food-liking factor) \|\| id:ebi-fl187-GCST90094852 | Weighted median | 27 | 0.0907679652063277 | 0.119355795911912 | 0.446966370360463 | -0.143169395 | 0.324705325193676 | 1.09501489422326 | 0.866607257714665 | 1.38362286710223 | 0.999332001504091 |
| ebi-fl187-GCST90094852 | prot-a-2921 | Tachykinin-3 \|\| id:prot-a-2921 | F-strong vegetable liking (derived food-liking factor) \|\| id:ebi-fl187-GCST90094852 | Weighted median | 27 | 0.115423031475898 | 0.109650333475875 | 0.292503071650925 | -0.099491622 | 0.330337685088613 | 1.12234812574495 | 0.90529753429562 | 1.39143791697532 | 0.999332001504091 |
| ebi-fl187-GCST90094852 | prot-a-2928 | Tax1-binding protein 1 \|\| id:prot-a-2928 | F-strong vegetable liking (derived food-liking factor) \|\| id:ebi-fl187-GCST90094852 | Weighted median | 27 | 0.0577720357225199 | 0.108692751838318 | 0.595060625584629 | -0.155265758 | 0.270809829325623 | 1.05947344608035 | 0.856187608641592 | 1.31102572802971 | 0.999332001504091 |
| ebi-fl187-GCST90094852 | prot-a-296 | Uncharacterized protein C1orf185 \|\| id:prot-a-296 | F-strong vegetable liking (derived food-liking factor) \|\| id:ebi-fl187-GCST90094852 | Weighted median | 27 | -0.030563856 | 0.108218397788778 | 0.777615697840336 | -0.242671916 | 0.181544203449049 | 0.969898496040943 | 0.784528863012463 | 1.19906753845912 | 0.999332001504091 |
| ebi-fl187-GCST90094852 | prot-a-2964 | Transforming growth factor beta-2 \|\| id:prot-a-2964 | F-strong vegetable liking (derived food-liking factor) \|\| id:ebi-fl187-GCST90094852 | Weighted median | 27 | 0.0236183668546724 | 0.1096558110878 | 0.829466097543942 | -0.191307023 | 0.23854375658676 | 1.02389948933619 | 0.825878985479756 | 1.26939924939959 | 0.999332001504091 |
| ebi-fl187-GCST90094852 | prot-a-2977 | Thymocyte nuclear protein 1 \|\| id:prot-a-2977 | F-strong vegetable liking (derived food-liking factor) \|\| id:ebi-fl187-GCST90094852 | Weighted median | 26 | -0.007489964 | 0.110650627060962 | 0.946032247273751 | -0.224365193 | 0.209385265489328 | 0.992538016327167 | 0.799023283699599 | 1.23291990853303 | 0.999332001504091 |
| ebi-fl187-GCST90094852 | prot-a-3015 | Transmembrane protein 70, mitochondrial \|\| id:prot-a-3015 | F-strong vegetable liking (derived food-liking factor) \|\| id:ebi-fl187-GCST90094852 | Weighted median | 27 | -0.056432236 | 0.107452638880054 | 0.599456387532579 | -0.267039409 | 0.154174935715393 | 0.945130527687651 | 0.76564289788383 | 1.16669496554603 | 0.999332001504091 |
| ebi-fl187-GCST90094852 | prot-a-3030 | Tumor necrosis factor alpha-induced protein 3 \|\| id:prot-a-3030 | F-strong vegetable liking (derived food-liking factor) \|\| id:ebi-fl187-GCST90094852 | Weighted median | 27 | -0.125628286 | 0.118007626555294 | 0.287067080395153 | -0.356923234 | 0.105666661626018 | 0.881942615906559 | 0.69982621820268 | 1.11145132537295 | 0.999332001504091 |
| ebi-fl187-GCST90094852 | prot-a-3032 | Tumor necrosis factor receptor superfamily member 10A \|\| id:prot-a-3032 | F-strong vegetable liking (derived food-liking factor) \|\| id:ebi-fl187-GCST90094852 | Weighted median | 27 | -0.089274458 | 0.110418600205122 | 0.418797493433268 | -0.305694914 | 0.1271459987104 | 0.914594521623268 | 0.736611314909265 | 1.1355827993035 | 0.999332001504091 |
| ebi-fl187-GCST90094852 | prot-a-3081 | Tumor protein p53-inducible protein 11 \|\| id:prot-a-3081 | F-strong vegetable liking (derived food-liking factor) \|\| id:ebi-fl187-GCST90094852 | Weighted median | 27 | -0.086293991 | 0.109663696183651 | 0.43134239346407 | -0.301234835 | 0.128646853996722 | 0.917324506862144 | 0.739903996956102 | 1.13728842437893 | 0.999332001504091 |
| ebi-fl187-GCST90094852 | prot-a-315 | Complement C4 \|\| id:prot-a-315 | F-strong vegetable liking (derived food-liking factor) \|\| id:ebi-fl187-GCST90094852 | Weighted median | 27 | -0.115827484 | 0.110907147698886 | 0.296316796386497 | -0.333205494 | 0.10155052519206 | 0.890628857507726 | 0.716622917466422 | 1.10688584259892 | 0.999332001504091 |
| ebi-fl187-GCST90094852 | prot-a-3157 | UDP-glucuronosyltransferase 1-8 \|\| id:prot-a-3157 | F-strong vegetable liking (derived food-liking factor) \|\| id:ebi-fl187-GCST90094852 | Weighted median | 26 | 0.106599984779409 | 0.111311093607875 | 0.338226063278326 | -0.111569759 | 0.324769728250844 | 1.11248915286766 | 0.894428995030145 | 1.38371197951437 | 0.999332001504091 |
| ebi-fl187-GCST90094852 | prot-a-317 | Glutaredoxin-like protein C5orf63 \|\| id:prot-a-317 | F-strong vegetable liking (derived food-liking factor) \|\| id:ebi-fl187-GCST90094852 | Weighted median | 27 | -0.090390859 | 0.119197673916721 | 0.448255023963287 | -0.3240183 | 0.143236581888187 | 0.913574036854397 | 0.723237007118888 | 1.15400278553119 | 0.999332001504091 |
| ebi-fl187-GCST90094852 | prot-a-3192 | Guanine nucleotide exchange factor VAV3 \|\| id:prot-a-3192 | F-strong vegetable liking (derived food-liking factor) \|\| id:ebi-fl187-GCST90094852 | Weighted median | 27 | -0.02335358 | 0.116541968510551 | 0.841177359185005 | -0.251775838 | 0.205068678396856 | 0.976917004498658 | 0.777418986205222 | 1.22760937231175 | 0.999332001504091 |
| ebi-fl187-GCST90094852 | prot-a-3233 | Serine/threonine-protein kinase WNK3 \|\| id:prot-a-3233 | F-strong vegetable liking (derived food-liking factor) \|\| id:ebi-fl187-GCST90094852 | Weighted median | 26 | -0.164633216 | 0.112616571281531 | 0.143770925985163 | -0.385361696 | 0.0560952637414235 | 0.848204754998158 | 0.680204564651728 | 1.05769843924798 | 0.999332001504091 |
| ebi-fl187-GCST90094852 | prot-a-3250 | 14-3-3 protein theta \|\| id:prot-a-3250 | F-strong vegetable liking (derived food-liking factor) \|\| id:ebi-fl187-GCST90094852 | Weighted median | 27 | 0.00070274904996652 | 0.104749551470326 | 0.994647152153969 | -0.204606372 | 0.206011869931806 | 1.00070299603593 | 0.81496804766253 | 1.22876778929861 | 0.999332001504091 |
| ebi-fl187-GCST90094852 | prot-a-3278 | E3 ubiquitin-protein ligase ZNRF3 \|\| id:prot-a-3278 | F-strong vegetable liking (derived food-liking factor) \|\| id:ebi-fl187-GCST90094852 | Weighted median | 27 | 0.0700993173310189 | 0.109653032755729 | 0.522638859884184 | -0.144820627 | 0.285019261532248 | 1.07261470519402 | 0.865177468783552 | 1.32978764162234 | 0.999332001504091 |
| ebi-fl187-GCST90094852 | prot-a-355 | Calpain-3 \|\| id:prot-a-355 | F-strong vegetable liking (derived food-liking factor) \|\| id:ebi-fl187-GCST90094852 | Weighted median | 27 | 0.110879272560673 | 0.115805638372299 | 0.338335100310912 | -0.116099779 | 0.337858323770379 | 1.11726001477139 | 0.890386377315235 | 1.40194186749672 | 0.999332001504091 |
| ebi-fl187-GCST90094852 | prot-a-385 | Coiled-coil domain-containing protein 90B, mitochondrial \|\| id:prot-a-385 | F-strong vegetable liking (derived food-liking factor) \|\| id:ebi-fl187-GCST90094852 | Weighted median | 27 | 0.0913524166322756 | 0.125705101 | 0.467397472824641 | -0.155029582 | 0.337734415295916 | 1.09565506429555 | 0.856389843357692 | 1.40176816578042 | 0.999332001504091 |
| ebi-fl187-GCST90094852 | prot-a-390 | C-C motif chemokine 14 \|\| id:prot-a-390 | F-strong vegetable liking (derived food-liking factor) \|\| id:ebi-fl187-GCST90094852 | Weighted median | 27 | -0.027779487 | 0.118109245324877 | 0.81405224390011 | -0.259273607 | 0.203714634271114 | 0.972602815143038 | 0.771611875438344 | 1.22594825991601 | 0.999332001504091 |
| ebi-fl187-GCST90094852 | prot-a-40 | Pituitary adenylate cyclase-activating polypeptide 27 \|\| id:prot-a-40 | F-strong vegetable liking (derived food-liking factor) \|\| id:ebi-fl187-GCST90094852 | Weighted median | 27 | 0.0841857608162806 | 0.114138569248888 | 0.460772722112199 | -0.139525835 | 0.3078973565441 | 1.08783095140656 | 0.869770552468853 | 1.36056132905291 | 0.999332001504091 |
| ebi-fl187-GCST90094852 | prot-a-401 | C-C motif chemokine 24 \|\| id:prot-a-401 | F-strong vegetable liking (derived food-liking factor) \|\| id:ebi-fl187-GCST90094852 | Weighted median | 27 | 0.234972420825928 | 0.111947010894472 | 0.0358203302272856 | 0.0155562794727632 | 0.454388562179093 | 1.26487388407482 | 1.015677908 | 1.57520994558636 | 0.999332001504091 |
| ebi-fl187-GCST90094852 | prot-a-412 | Cerebral cavernous malformations 2 protein \|\| id:prot-a-412 | F-strong vegetable liking (derived food-liking factor) \|\| id:ebi-fl187-GCST90094852 | Weighted median | 27 | 0.139407571798332 | 0.112833908518295 | 0.216640264331184 | -0.081746889 | 0.360562032494191 | 1.14959254603576 | 0.921505172370732 | 1.43413521868904 | 0.999332001504091 |
| ebi-fl187-GCST90094852 | prot-a-44 | Adhesion G protein-coupled receptor F5 \|\| id:prot-a-44 | F-strong vegetable liking (derived food-liking factor) \|\| id:ebi-fl187-GCST90094852 | Weighted median | 27 | 0.00731971367523729 | 0.108034121329571 | 0.945981684642326 | -0.204427164 | 0.219066591481197 | 1.00734656826202 | 0.815114109300156 | 1.2449141746062 | 0.999332001504091 |
| ebi-fl187-GCST90094852 | prot-a-48 | Adrenomedullin \|\| id:prot-a-48 | F-strong vegetable liking (derived food-liking factor) \|\| id:ebi-fl187-GCST90094852 | Weighted median | 27 | 0.0985864656343471 | 0.114485146266972 | 0.389167065458265 | -0.125804421 | 0.322977352317611 | 1.1036098245926 | 0.881787288952889 | 1.3812340688009 | 0.999332001504091 |
| ebi-fl187-GCST90094852 | prot-a-502 | Carcinoembryonic antigen-related cell adhesion molecule 1 \|\| id:prot-a-502 | F-strong vegetable liking (derived food-liking factor) \|\| id:ebi-fl187-GCST90094852 | Weighted median | 27 | -0.04171098 | 0.110801828409129 | 0.706584849041762 | -0.258882564 | 0.175460603369897 | 0.959146952869497 | 0.77191366817935 | 1.19179503501821 | 0.999332001504091 |
| ebi-fl187-GCST90094852 | prot-a-518 | Complement factor B \|\| id:prot-a-518 | F-strong vegetable liking (derived food-liking factor) \|\| id:ebi-fl187-GCST90094852 | Weighted median | 27 | -0.050485518 | 0.110711606206863 | 0.648383316202977 | -0.267480266 | 0.166509230054436 | 0.950767697484873 | 0.765305432799643 | 1.18117443812442 | 0.999332001504091 |
| ebi-fl187-GCST90094852 | prot-a-525 | Properdin \|\| id:prot-a-525 | F-strong vegetable liking (derived food-liking factor) \|\| id:ebi-fl187-GCST90094852 | Weighted median | 27 | 0.123194272466765 | 0.1201143501388 | 0.305060558228111 | -0.112229854 | 0.358618398738813 | 1.13110414199576 | 0.893838781641288 | 1.43135049218909 | 0.999332001504091 |
| ebi-fl187-GCST90094852 | prot-a-533 | Chondroadherin \|\| id:prot-a-533 | F-strong vegetable liking (derived food-liking factor) \|\| id:ebi-fl187-GCST90094852 | Weighted median | 27 | -0.02616935 | 0.113962950213109 | 0.81837903528943 | -0.249536732 | 0.19719803235357 | 0.974170099869751 | 0.779161659762106 | 1.21798521730393 | 0.999332001504091 |
| ebi-fl187-GCST90094852 | prot-a-535 | Coiled-coil-helix-coiled-coil-helix domain-containing protein 2 \|\| id:prot-a-535 | F-strong vegetable liking (derived food-liking factor) \|\| id:ebi-fl187-GCST90094852 | Weighted median | 27 | 0.0326556833231121 | 0.114097212673152 | 0.774717821511079 | -0.190974854 | 0.25628622016249 | 1.03319473181297 | 0.826153362742087 | 1.29212250653191 | 0.999332001504091 |
| ebi-fl187-GCST90094852 | prot-a-591 | Caseinolytic peptidase B protein homolog \|\| id:prot-a-591 | F-strong vegetable liking (derived food-liking factor) \|\| id:ebi-fl187-GCST90094852 | Weighted median | 27 | 0.00320046752396074 | 0.116729980472922 | 0.978126581418061 | -0.225590294 | 0.231991229250888 | 1.00320559448825 | 0.798044998357488 | 1.26110866791209 | 0.999332001504091 |
| ebi-fl187-GCST90094852 | prot-a-605 | Ciliary neurotrophic factor receptor subunit alpha \|\| id:prot-a-605 | F-strong vegetable liking (derived food-liking factor) \|\| id:ebi-fl187-GCST90094852 | Weighted median | 27 | -0.07004518 | 0.111463146941459 | 0.529731724094685 | -0.288512948 | 0.148422588286439 | 0.932351695566925 | 0.749377102356736 | 1.16000299647894 | 0.999332001504091 |
| ebi-fl187-GCST90094852 | prot-a-606 | Ciliary neurotrophic factor receptor subunit alpha \|\| id:prot-a-606 | F-strong vegetable liking (derived food-liking factor) \|\| id:ebi-fl187-GCST90094852 | Weighted median | 27 | -0.082776582 | 0.112137097014517 | 0.460409304472243 | -0.302565292 | 0.137012128512964 | 0.92055679353994 | 0.738920241263153 | 1.14684205792482 | 0.999332001504091 |
| ebi-fl187-GCST90094852 | prot-a-707 | Cancer/testis antigen 55 \|\| id:prot-a-707 | F-strong vegetable liking (derived food-liking factor) \|\| id:ebi-fl187-GCST90094852 | Weighted median | 27 | -0.043914053 | 0.112812136170007 | 0.697078574566435 | -0.265025839 | 0.177197734318175 | 0.957036208736089 | 0.767186126038188 | 1.19386713829384 | 0.999332001504091 |
| ebi-fl187-GCST90094852 | prot-a-719 | Dipeptidyl peptidase 1 \|\| id:prot-a-719 | F-strong vegetable liking (derived food-liking factor) \|\| id:ebi-fl187-GCST90094852 | Weighted median | 27 | -0.008772281 | 0.129950712248562 | 0.946179935677662 | -0.263475677 | 0.245931114639077 | 0.991266082829626 | 0.768376311140604 | 1.27881147911727 | 0.999332001504091 |
| ebi-fl187-GCST90094852 | prot-a-752 | Cytochrome b5 \|\| id:prot-a-752 | F-strong vegetable liking (derived food-liking factor) \|\| id:ebi-fl187-GCST90094852 | Weighted median | 27 | 0.0492254819254675 | 0.112649988508256 | 0.662127848656288 | -0.171568496 | 0.27001945940165 | 1.05045718314659 | 0.842342569325819 | 1.30998994210566 | 0.999332001504091 |
| ebi-fl187-GCST90094852 | prot-a-755 | Cytoglobin \|\| id:prot-a-755 | F-strong vegetable liking (derived food-liking factor) \|\| id:ebi-fl187-GCST90094852 | Weighted median | 27 | -0.03283831 | 0.111028250056397 | 0.767409281183713 | -0.25045368 | 0.184777060114777 | 0.967695013551014 | 0.778447536785821 | 1.20295022464583 | 0.999332001504091 |
| ebi-fl187-GCST90094852 | prot-a-807 | Desmin \|\| id:prot-a-807 | F-strong vegetable liking (derived food-liking factor) \|\| id:ebi-fl187-GCST90094852 | Weighted median | 27 | 0.145516499124991 | 0.120413549986443 | 0.226865454610987 | -0.090494059 | 0.381527057098419 | 1.15663681792019 | 0.91347976100654 | 1.4645192873178 | 0.999332001504091 |
| ebi-fl187-GCST90094852 | prot-a-82 | Ameloblastin \|\| id:prot-a-82 | F-strong vegetable liking (derived food-liking factor) \|\| id:ebi-fl187-GCST90094852 | Weighted median | 27 | 0.0169259414444271 | 0.113919505421276 | 0.881886570250704 | -0.206356289 | 0.240208172070127 | 1.01706999680115 | 0.813543168011136 | 1.27151381643577 | 0.999332001504091 |
| ebi-fl187-GCST90094852 | prot-a-84 | Muellerian-inhibiting factor \|\| id:prot-a-84 | F-strong vegetable liking (derived food-liking factor) \|\| id:ebi-fl187-GCST90094852 | Weighted median | 27 | 0.00618679617605939 | 0.10748586333119 | 0.954099780041453 | -0.204485496 | 0.216859088305193 | 1.00620597392874 | 0.815066563595464 | 1.24216905365828 | 0.999332001504091 |
| ebi-fl187-GCST90094852 | prot-a-858 | Inactive dipeptidyl peptidase 10 \|\| id:prot-a-858 | F-strong vegetable liking (derived food-liking factor) \|\| id:ebi-fl187-GCST90094852 | Weighted median | 27 | 0.13862746699424 | 0.11071776114241 | 0.210540804687838 | -0.078379345 | 0.355634278833363 | 1.14869609307694 | 0.924613612600567 | 1.42708553742682 | 0.999332001504091 |
| ebi-fl187-GCST90094852 | prot-a-863 | Dihydropyrimidinase-related protein 3 \|\| id:prot-a-863 | F-strong vegetable liking (derived food-liking factor) \|\| id:ebi-fl187-GCST90094852 | Weighted median | 27 | 0.00851114339394773 | 0.10965875757895 | 0.938134462988017 | -0.206420021 | 0.22344230824869 | 1.00854746615115 | 0.813491320702741 | 1.25037350195847 | 0.999332001504091 |
| ebi-fl187-GCST90094852 | prot-a-864 | Dihydropyrimidinase-related protein 5 \|\| id:prot-a-864 | F-strong vegetable liking (derived food-liking factor) \|\| id:ebi-fl187-GCST90094852 | Weighted median | 27 | -0.004404836 | 0.109165475708014 | 0.967814024912871 | -0.218369169 | 0.209559496260023 | 0.995604850934444 | 0.803828638659603 | 1.23313473983346 | 0.999332001504091 |
| ebi-fl187-GCST90094852 | prot-a-884 | Endothelin-converting enzyme 1 \|\| id:prot-a-884 | F-strong vegetable liking (derived food-liking factor) \|\| id:ebi-fl187-GCST90094852 | Weighted median | 27 | 0.0512602873846593 | 0.111332753053307 | 0.64521180877644 | -0.166951909 | 0.269472483369141 | 1.05259683530719 | 0.846240307239881 | 1.309273605 | 0.999332001504091 |
| ebi-fl187-GCST90094852 | prot-a-893 | Early endosome antigen 1 \|\| id:prot-a-893 | F-strong vegetable liking (derived food-liking factor) \|\| id:ebi-fl187-GCST90094852 | Weighted median | 27 | 0.12906938607589 | 0.10909781774761 | 0.236784926648001 | -0.084762337 | 0.342901108861206 | 1.13776906675775 | 0.918730606996007 | 1.40902941451306 | 0.999332001504091 |
| ebi-fl187-GCST90094852 | prot-a-897 | Ephrin-A2 \|\| id:prot-a-897 | F-strong vegetable liking (derived food-liking factor) \|\| id:ebi-fl187-GCST90094852 | Weighted median | 27 | 0.0965081034685632 | 0.107219302139675 | 0.368066988331391 | -0.113641729 | 0.306657935662325 | 1.10131850560752 | 0.892577683548913 | 1.35887606552189 | 0.999332001504091 |
| ebi-fl187-GCST90094852 | prot-a-90 | Anaphase-promoting complex subunit 10 \|\| id:prot-a-90 | F-strong vegetable liking (derived food-liking factor) \|\| id:ebi-fl187-GCST90094852 | Weighted median | 27 | 0.163937699797119 | 0.111564266244998 | 0.14171177321482 | -0.054728262 | 0.382603661637314 | 1.17814091438832 | 0.946742378841962 | 1.46609684448005 | 0.999332001504091 |
| ebi-fl187-GCST90094852 | prot-a-914 | Histone-lysine N-methyltransferase EHMT2 \|\| id:prot-a-914 | F-strong vegetable liking (derived food-liking factor) \|\| id:ebi-fl187-GCST90094852 | Weighted median | 27 | 0.124853273575168 | 0.113114089849944 | 0.26968780210974 | -0.096850343 | 0.346556889681058 | 1.13298220244248 | 0.907691838832314 | 1.41418994435683 | 0.999332001504091 |
| ebi-fl187-GCST90094852 | prot-a-932 | RNA polymerase II elongation factor ELL \|\| id:prot-a-932 | F-strong vegetable liking (derived food-liking factor) \|\| id:ebi-fl187-GCST90094852 | Weighted median | 27 | 0.0380647543939583 | 0.113670234862822 | 0.737723134475958 | -0.184728906 | 0.260858414725089 | 1.03879849746778 | 0.831329621831583 | 1.29804386852456 | 0.999332001504091 |
| ebi-fl187-GCST90094852 | prot-a-991 | Estrogen receptor \|\| id:prot-a-991 | F-strong vegetable liking (derived food-liking factor) \|\| id:ebi-fl187-GCST90094852 | Weighted median | 27 | -0.060756549 | 0.108494180594056 | 0.575480647194287 | -0.273405143 | 0.151892045215284 | 0.941052312253501 | 0.760784498886481 | 1.16403456654787 | 0.999332001504091 |
| ebi-fl187-GCST90094852 | prot-a-992 | Persulfide dioxygenase ETHE1, mitochondrial \|\| id:prot-a-992 | F-strong vegetable liking (derived food-liking factor) \|\| id:ebi-fl187-GCST90094852 | Weighted median | 27 | -0.267857189 | 0.111024307465944 | 0.0158392134843739 | -0.485464831 | -0.050249546 | 0.765017026367067 | 0.615411067912612 | 0.950992078541258 | 0.999332001504091 |
| ebi-fl187-GCST90094852 | prot-a-993 | Ethanolamine kinase 1 \|\| id:prot-a-993 | F-strong vegetable liking (derived food-liking factor) \|\| id:ebi-fl187-GCST90094852 | Weighted median | 26 | 0.0770048115880092 | 0.106692891698604 | 0.470452708042816 | -0.132113256 | 0.286122879317274 | 1.08004727312261 | 0.876241749700028 | 1.33125602903413 | 0.999332001504091 |
| ebi-fl187-GCST90094852 | prot-b-10 | coagulation factor III, tissue factor \|\| id:prot-b-10 | F-strong vegetable liking (derived food-liking factor) \|\| id:ebi-fl187-GCST90094852 | Weighted median | 20 | -0.144275633 | 0.142250939418966 | 0.310471464063364 | -0.423087475 | 0.13453620798571 | 0.865649113472351 | 0.655021332918105 | 1.14400608040831 | 0.999332001504091 |
| ebi-fl187-GCST90094852 | prot-b-28 | prolactin \|\| id:prot-b-28 | F-strong vegetable liking (derived food-liking factor) \|\| id:ebi-fl187-GCST90094852 | Weighted median | 20 | 0.0206528355645659 | 0.144279082672256 | 0.886175631647956 | -0.262134166 | 0.303439837602188 | 1.02086758119385 | 0.769407788053084 | 1.35451009791529 | 0.999332001504091 |
| ebi-fl187-GCST90094852 | prot-b-54 | matrix metallopeptidase 7 \|\| id:prot-b-54 | F-strong vegetable liking (derived food-liking factor) \|\| id:ebi-fl187-GCST90094852 | Weighted median | 20 | -0.116025565 | 0.142119140214672 | 0.414273413804755 | -0.394579079 | 0.162527950151092 | 0.890452458883528 | 0.673963664681974 | 1.17648120081647 | 0.999332001504091 |
| ebi-fl187-GCST90094852 | ukb-rsfMRI191-edge-pheno716 | Net100_Pair10_33 (OccipitalPrecuneus)&(Temporal\|Frontal\|Supp_Motor_Area) (Default_mode\|Central_executive)&(Default_mode\|Salience) \|\| id:ukb-rsfMRI191-edge-pheno716 | F-strong vegetable liking (derived food-liking factor) \|\| id:ebi-fl187-GCST90094852 | Weighted median | 27 | -0.039467258 | 0.0353148367843226 | 0.263745175458827 | -0.108684338 | 0.0297498223942303 | 0.961301428692163 | 0.897013526075335 | 1.03019676955014 | 0.999332001504091 |
| ebi-fl187-GCST90094852 | ukb-rsfMRI191-edge-pheno903 | Net100_Pair27_38 (Precentral\|Frontal\|Supp_Motor_Area)&(Subcortical) (Attention\|Salience\|Motor)&(Subcortical-cerebellum) \|\| id:ukb-rsfMRI191-edge-pheno903 | F-strong vegetable liking (derived food-liking factor) \|\| id:ebi-fl187-GCST90094852 | Weighted median | 27 | -0.015611099 | 0.0352397519358645 | 0.657768022962382 | -0.084681012 | 0.0534588151705872 | 0.984510122955965 | 0.918805325149789 | 1.05491354443854 | 0.999332001504091 |
| ebi-fl187-GCST90094852 | ukb-rsfMRI191-node-pheno23 | Net100_Node2 RolandicOper\|SupraMarginal\|Insula Salience\|Motor \|\| id:ukb-rsfMRI191-node-pheno23 | F-strong vegetable liking (derived food-liking factor) \|\| id:ebi-fl187-GCST90094852 | Weighted median | 27 | -0.074361677 | 0.0321684992457876 | 0.0207980668935444 | -0.137411935 | -0.011311418 | 0.92833587567432 | 0.871611104869395 | 0.988752315395456 | 0.999332001504091 |
| ebi-fl187-GCST90094852 | ukb-rsfMRI191-node-pheno55 | Net100_Node34 Parietal\|Postcentral\|Precuneus Attention \|\| id:ukb-rsfMRI191-node-pheno55 | F-strong vegetable liking (derived food-liking factor) \|\| id:ebi-fl187-GCST90094852 | Weighted median | 27 | -0.017320484 | 0.0356305193850619 | 0.626886546169802 | -0.087156302 | 0.0525153339884536 | 0.982828653292071 | 0.916533828365616 | 1.05391872273216 | 0.999332001504091 |
| ebi-fl187-GCST90094852 | ebi-a-GCST90001414 | IgD- CD24- B cell Absolute Count \|\| id:ebi-a-GCST90001414 | F-strong vegetable liking (derived food-liking factor) \|\| id:ebi-fl187-GCST90094852 | Weighted mode | 23 | -0.182088277 | 0.210103645462971 | 0.395482866207696 | -0.593891422 | 0.229714867999875 | 0.833527755748524 | 0.552174354920627 | 1.25824119394868 | 0.999714303421225 |
| ebi-fl187-GCST90094852 | ebi-a-GCST90001422 | CD20- CD38- B cell %B cell \|\| id:ebi-a-GCST90001422 | F-strong vegetable liking (derived food-liking factor) \|\| id:ebi-fl187-GCST90094852 | Weighted mode | 23 | -0.133449126 | 0.186965991544292 | 0.482878898713512 | -0.499902469 | 0.233004217780472 | 0.875071986566927 | 0.606589818094875 | 1.26238680378644 | 0.999714303421225 |
| ebi-fl187-GCST90094852 | ebi-a-GCST90001444 | CD20- CD38- B cell %lymphocyte \|\| id:ebi-a-GCST90001444 | F-strong vegetable liking (derived food-liking factor) \|\| id:ebi-fl187-GCST90094852 | Weighted mode | 23 | -0.230493786 | 0.172949698652355 | 0.196262557938038 | -0.569475195 | 0.108487623594392 | 0.794141369968611 | 0.565822307100104 | 1.11459111382127 | 0.999714303421225 |
| ebi-fl187-GCST90094852 | ebi-a-GCST90001459 | Myeloid Dendritic Cell %Dendritic Cell \|\| id:ebi-a-GCST90001459 | F-strong vegetable liking (derived food-liking factor) \|\| id:ebi-fl187-GCST90094852 | Weighted mode | 23 | 0.0421978913017573 | 0.238093039773145 | 0.860947158883798 | -0.424464467 | 0.508860249257121 | 1.04310087891848 | 0.654119994416655 | 1.6633942592916 | 0.999714303421225 |
| ebi-fl187-GCST90094852 | ebi-a-GCST90001476 | HLA DR++ monocyte %leukocyte \|\| id:ebi-a-GCST90001476 | F-strong vegetable liking (derived food-liking factor) \|\| id:ebi-fl187-GCST90094852 | Weighted mode | 23 | 0.0514948198603371 | 0.206125152348025 | 0.805042130442151 | -0.352510479 | 0.455500118462466 | 1.05284373240043 | 0.702921204042329 | 1.5769618535908 | 0.999714303421225 |
| ebi-fl187-GCST90094852 | ebi-a-GCST90001478 | CD4 regulatory T cell %CD4+ T cell \|\| id:ebi-a-GCST90001478 | F-strong vegetable liking (derived food-liking factor) \|\| id:ebi-fl187-GCST90094852 | Weighted mode | 23 | 0.133751246448258 | 0.180639794487451 | 0.46686809240022 | -0.220302751 | 0.487805243643661 | 1.14310843199241 | 0.802275871571726 | 1.62873761207877 | 0.999714303421225 |
| ebi-fl187-GCST90094852 | ebi-a-GCST90001485 | CD39+ resting CD4 regulatory T cell %CD4 regulatory T cell \|\| id:ebi-a-GCST90001485 | F-strong vegetable liking (derived food-liking factor) \|\| id:ebi-fl187-GCST90094852 | Weighted mode | 23 | 0.237496835412836 | 0.198992955879728 | 0.245387950624783 | -0.152529358 | 0.627523028937103 | 1.26807098386321 | 0.858533688658707 | 1.87296554737183 | 0.999714303421225 |
| ebi-fl187-GCST90094852 | ebi-a-GCST90001515 | Immature Myeloid-Derived Suppressor Cells Absolute Count \|\| id:ebi-a-GCST90001515 | F-strong vegetable liking (derived food-liking factor) \|\| id:ebi-fl187-GCST90094852 | Weighted mode | 22 | 0.348915725314171 | 0.395758204625006 | 0.387952222415232 | -0.426770356 | 1.12460180637918 | 1.41752972343594 | 0.652613403936089 | 3.0789905703824 | 0.999714303421225 |
| ebi-fl187-GCST90094852 | ebi-a-GCST90001517 | CD33+ HLA DR+ Absolute Count \|\| id:ebi-a-GCST90001517 | F-strong vegetable liking (derived food-liking factor) \|\| id:ebi-fl187-GCST90094852 | Weighted mode | 23 | -0.196311351 | 0.254747663297878 | 0.449134674402369 | -0.695616771 | 0.30299406914216 | 0.821756340249837 | 0.49876672825206 | 1.35390643459188 | 0.999714303421225 |
| ebi-fl187-GCST90094852 | ebi-a-GCST90001518 | CD33+ HLA DR+ CD14- Absolute Count \|\| id:ebi-a-GCST90001518 | F-strong vegetable liking (derived food-liking factor) \|\| id:ebi-fl187-GCST90094852 | Weighted mode | 23 | -0.080550365 | 0.258874761585877 | 0.758611438431042 | -0.587944898 | 0.426844167482877 | 0.922608435031742 | 0.555467655270303 | 1.53241384321019 | 0.999714303421225 |
| ebi-fl187-GCST90094852 | ebi-a-GCST90001520 | CD33+ HLA DR+ CD14dim Absolute Count \|\| id:ebi-a-GCST90001520 | F-strong vegetable liking (derived food-liking factor) \|\| id:ebi-fl187-GCST90094852 | Weighted mode | 23 | -0.174374194 | 0.254938848353413 | 0.50112683332639 | -0.674054337 | 0.325305948824333 | 0.83998252246682 | 0.509638138873499 | 1.38445415331221 | 0.999714303421225 |
| ebi-fl187-GCST90094852 | ebi-a-GCST90001531 | CD33dim HLA DR- Absolute Count \|\| id:ebi-a-GCST90001531 | F-strong vegetable liking (derived food-liking factor) \|\| id:ebi-fl187-GCST90094852 | Weighted mode | 23 | -0.145508969 | 0.232850499734785 | 0.538462783520731 | -0.601895948 | 0.310878010807062 | 0.864582135884622 | 0.547772103447462 | 1.36462274180509 | 0.999714303421225 |
| ebi-fl187-GCST90094852 | ebi-a-GCST90001532 | Basophil Absolute Count \|\| id:ebi-a-GCST90001532 | F-strong vegetable liking (derived food-liking factor) \|\| id:ebi-fl187-GCST90094852 | Weighted mode | 23 | -0.144793852 | 0.232286195618668 | 0.539468817571344 | -0.600074795 | 0.310487091465079 | 0.865200634153525 | 0.548770589065162 | 1.36408938863663 | 0.999714303421225 |
| ebi-fl187-GCST90094852 | ebi-a-GCST90001588 | T/B cell \|\| id:ebi-a-GCST90001588 | F-strong vegetable liking (derived food-liking factor) \|\| id:ebi-fl187-GCST90094852 | Weighted mode | 23 | -0.044146886 | 0.190247650559478 | 0.818644452551745 | -0.417032281 | 0.328738509433538 | 0.95681340497933 | 0.658999646591583 | 1.38921454159067 | 0.999714303421225 |
| ebi-fl187-GCST90094852 | ebi-a-GCST90001624 | HLA DR+ CD4+ T cell Absolute Count \|\| id:ebi-a-GCST90001624 | F-strong vegetable liking (derived food-liking factor) \|\| id:ebi-fl187-GCST90094852 | Weighted mode | 23 | -0.11789439 | 0.171756522883353 | 0.49962950612169 | -0.454537175 | 0.218748394577188 | 0.88878991251724 | 0.634741674211374 | 1.24451810978673 | 0.999714303421225 |
| ebi-fl187-GCST90094852 | ebi-a-GCST90001631 | CD8+ Natural Killer T %T cell \|\| id:ebi-a-GCST90001631 | F-strong vegetable liking (derived food-liking factor) \|\| id:ebi-fl187-GCST90094852 | Weighted mode | 23 | 0.0492258685561229 | 0.180612314815724 | 0.787741037415804 | -0.304774268 | 0.403226005594943 | 1.05045758928562 | 0.737289785150739 | 1.49664510361031 | 0.999714303421225 |
| ebi-fl187-GCST90094852 | ebi-a-GCST90001632 | CD8+ Natural Killer T %lymphocyte \|\| id:ebi-a-GCST90001632 | F-strong vegetable liking (derived food-liking factor) \|\| id:ebi-fl187-GCST90094852 | Weighted mode | 23 | -0.011865939 | 0.186170798672049 | 0.949755348189101 | -0.376760705 | 0.353028826228447 | 0.988204183456433 | 0.686080230120755 | 1.42337217329366 | 0.999714303421225 |
| ebi-fl187-GCST90094852 | ebi-a-GCST90001646 | Natural Killer %CD3- lymphocyte \|\| id:ebi-a-GCST90001646 | F-strong vegetable liking (derived food-liking factor) \|\| id:ebi-fl187-GCST90094852 | Weighted mode | 23 | 0.12406805815699 | 0.176232956348468 | 0.488817881479579 | -0.221348536 | 0.469484652599988 | 1.1320929165348 | 0.801437301625646 | 1.59916985279894 | 0.999714303421225 |
| ebi-fl187-GCST90094852 | ebi-a-GCST90001684 | CD127- CD8+ T cell Absolute Count \|\| id:ebi-a-GCST90001684 | F-strong vegetable liking (derived food-liking factor) \|\| id:ebi-fl187-GCST90094852 | Weighted mode | 23 | -0.143439322 | 0.181284537043313 | 0.437252620594076 | -0.498757014 | 0.211878371081562 | 0.866373368808582 | 0.607285037496791 | 1.23599754289138 | 0.999714303421225 |
| ebi-fl187-GCST90094852 | ebi-a-GCST90001687 | CD28- CD8+ T cell Absolute Count \|\| id:ebi-a-GCST90001687 | F-strong vegetable liking (derived food-liking factor) \|\| id:ebi-fl187-GCST90094852 | Weighted mode | 23 | -0.157791142 | 0.189620854093689 | 0.414271742748216 | -0.529448016 | 0.213865732229758 | 0.854028134126754 | 0.588929959997863 | 1.23845635885576 | 0.999714303421225 |
| ebi-fl187-GCST90094852 | ebi-a-GCST90001701 | BAFF-R on CD20- CD38- B cell \|\| id:ebi-a-GCST90001701 | F-strong vegetable liking (derived food-liking factor) \|\| id:ebi-fl187-GCST90094852 | Weighted mode | 23 | -0.114430902 | 0.207606828273773 | 0.58705804416108 | -0.521340285 | 0.29247848181577 | 0.891873563324502 | 0.59372425473635 | 1.3397439073975 | 0.999714303421225 |
| ebi-fl187-GCST90094852 | ebi-a-GCST90001732 | CD19 on IgD- CD27- B cell \|\| id:ebi-a-GCST90001732 | F-strong vegetable liking (derived food-liking factor) \|\| id:ebi-fl187-GCST90094852 | Weighted mode | 23 | 0.00273551825897256 | 0.192453112640181 | 0.988787408961888 | -0.374472583 | 0.379943619033727 | 1.00273926320305 | 0.687651862781486 | 1.46220214674023 | 0.999714303421225 |
| ebi-fl187-GCST90094852 | ebi-a-GCST90001798 | CD27 on CD24+ CD27+ B cell \|\| id:ebi-a-GCST90001798 | F-strong vegetable liking (derived food-liking factor) \|\| id:ebi-fl187-GCST90094852 | Weighted mode | 23 | 0.264112405883451 | 0.22265927295933 | 0.248206915641358 | -0.172299769 | 0.700524580883739 | 1.30227457139772 | 0.84172681164235 | 2.01480936077122 | 0.999714303421225 |
| ebi-fl187-GCST90094852 | ebi-a-GCST90001836 | CD66b on CD66b++ myeloid cell \|\| id:ebi-a-GCST90001836 | F-strong vegetable liking (derived food-liking factor) \|\| id:ebi-fl187-GCST90094852 | Weighted mode | 23 | 0.0862323091988428 | 0.275406873471804 | 0.757147069263482 | -0.453565163 | 0.626029781203578 | 1.09005952974952 | 0.635358950890179 | 1.87017083293305 | 0.999714303421225 |
| ebi-fl187-GCST90094852 | ebi-a-GCST90001943 | CD25 on activated & secreting CD4 regulatory T cell \|\| id:ebi-a-GCST90001943 | F-strong vegetable liking (derived food-liking factor) \|\| id:ebi-fl187-GCST90094852 | Weighted mode | 23 | 0.160885342058407 | 0.168172326665177 | 0.349129590513408 | -0.168732418 | 0.490503102322153 | 1.1745502895745 | 0.844734908830132 | 1.63313764865014 | 0.999714303421225 |
| ebi-fl187-GCST90094852 | ebi-a-GCST90001960 | CD25 on CD4+ T cell \|\| id:ebi-a-GCST90001960 | F-strong vegetable liking (derived food-liking factor) \|\| id:ebi-fl187-GCST90094852 | Weighted mode | 23 | 0.229276987370141 | 0.211674350926553 | 0.290466941117776 | -0.18560474 | 0.644158715186184 | 1.25769035511195 | 0.83060183341828 | 1.90438422563058 | 0.999714303421225 |
| ebi-fl187-GCST90094852 | ebi-a-GCST90001988 | HLA DR on CD14+ CD16- monocyte \|\| id:ebi-a-GCST90001988 | F-strong vegetable liking (derived food-liking factor) \|\| id:ebi-fl187-GCST90094852 | Weighted mode | 23 | -0.227907062 | 0.179027060902416 | 0.216295434551029 | -0.578800101 | 0.122985977621494 | 0.796198253671517 | 0.560570591210757 | 1.13086856336927 | 0.999714303421225 |
| ebi-fl187-GCST90094852 | ebi-a-GCST90001991 | HLA DR on CD14+ monocyte \|\| id:ebi-a-GCST90001991 | F-strong vegetable liking (derived food-liking factor) \|\| id:ebi-fl187-GCST90094852 | Weighted mode | 23 | -0.227669801 | 0.188533343885361 | 0.240030056177409 | -0.597195155 | 0.141855553198292 | 0.796387182821693 | 0.550353128572757 | 1.15241017455001 | 0.999714303421225 |
| ebi-fl187-GCST90094852 | ebi-a-GCST90002019 | CD14 on Monocytic Myeloid-Derived Suppressor Cells \|\| id:ebi-a-GCST90002019 | F-strong vegetable liking (derived food-liking factor) \|\| id:ebi-fl187-GCST90094852 | Weighted mode | 23 | 0.0429792705715002 | 0.361021191770872 | 0.906316799639292 | -0.664622265 | 0.75058080644241 | 1.04391625483896 | 0.514467823357436 | 2.11822994100033 | 0.999714303421225 |
| ebi-fl187-GCST90094852 | ebi-a-GCST90002037 | CD80 on plasmacytoid Dendritic Cell \|\| id:ebi-a-GCST90002037 | F-strong vegetable liking (derived food-liking factor) \|\| id:ebi-fl187-GCST90094852 | Weighted mode | 23 | 0.0252555531826699 | 0.23964043433698 | 0.917021865198737 | -0.444439698 | 0.49495080448315 | 1.02557717654992 | 0.641183431688732 | 1.64041753588342 | 0.999714303421225 |
| ebi-fl187-GCST90094852 | ebi-a-GCST90002042 | CD45 on CD33+ HLA DR+ CD14- \|\| id:ebi-a-GCST90002042 | F-strong vegetable liking (derived food-liking factor) \|\| id:ebi-fl187-GCST90094852 | Weighted mode | 23 | -0.019454576 | 0.276605116566719 | 0.944563734234406 | -0.561600604 | 0.522691452617839 | 0.98073344315704 | 0.570295515461269 | 1.68656084512379 | 0.999714303421225 |
| ebi-fl187-GCST90094852 | ebi-a-GCST90002056 | CD8 on Effector Memory CD8+ T cell \|\| id:ebi-a-GCST90002056 | F-strong vegetable liking (derived food-liking factor) \|\| id:ebi-fl187-GCST90094852 | Weighted mode | 23 | 0.0471848292269348 | 0.20932094187841 | 0.823734490547109 | -0.363084217 | 0.457453875308618 | 1.04831575056432 | 0.695527855864107 | 1.58004586533189 | 0.999714303421225 |
| ebi-fl187-GCST90094852 | ebi-a-GCST90002058 | CD8 on CD8+ T cell \|\| id:ebi-a-GCST90002058 | F-strong vegetable liking (derived food-liking factor) \|\| id:ebi-fl187-GCST90094852 | Weighted mode | 23 | 0.0206236127594779 | 0.193597776766722 | 0.916128765950284 | -0.35882803 | 0.400075255222253 | 1.02083774901539 | 0.698494461323363 | 1.49193696946492 | 0.999714303421225 |
| ebi-fl187-GCST90094852 | ebi-a-GCST90002063 | CD4 on CD4 regulatory T cell \|\| id:ebi-a-GCST90002063 | F-strong vegetable liking (derived food-liking factor) \|\| id:ebi-fl187-GCST90094852 | Weighted mode | 23 | 0.0445366996882961 | 0.216941967362897 | 0.839230751935513 | -0.380669556 | 0.469742955719573 | 1.0455433471213 | 0.683403678723374 | 1.59958297671397 | 0.999714303421225 |
| ebi-fl187-GCST90094852 | ebi-a-GCST90002066 | CD4 on activated CD4 regulatory T cell \|\| id:ebi-a-GCST90002066 | F-strong vegetable liking (derived food-liking factor) \|\| id:ebi-fl187-GCST90094852 | Weighted mode | 23 | -0.114884454 | 0.208457503097871 | 0.587107597934325 | -0.52346116 | 0.293692251864121 | 0.891469143464621 | 0.592466374027509 | 1.3413710357048 | 0.999714303421225 |
| ebi-fl187-GCST90094852 | ebi-a-GCST90002070 | CD4 on activated & secreting CD4 regulatory T cell \|\| id:ebi-a-GCST90002070 | F-strong vegetable liking (derived food-liking factor) \|\| id:ebi-fl187-GCST90094852 | Weighted mode | 23 | -0.263657148 | 0.231666062832443 | 0.26732513260423 | -0.717722631 | 0.190408335236809 | 0.768236886128036 | 0.487862033633022 | 1.20974347770552 | 0.999714303421225 |
| ebi-fl187-GCST90094852 | ebi-a-GCST90002081 | SSC-A on CD4+ T cell \|\| id:ebi-a-GCST90002081 | F-strong vegetable liking (derived food-liking factor) \|\| id:ebi-fl187-GCST90094852 | Weighted mode | 23 | 0.0180457621422614 | 0.19926688747413 | 0.928661085281711 | -0.372517337 | 0.408608861591555 | 1.01820957077489 | 0.688997706089555 | 1.50472305038824 | 0.999714303421225 |
| ebi-fl187-GCST90094852 | ebi-a-GCST90002094 | CD11b on Monocytic Myeloid-Derived Suppressor Cells \|\| id:ebi-a-GCST90002094 | F-strong vegetable liking (derived food-liking factor) \|\| id:ebi-fl187-GCST90094852 | Weighted mode | 23 | -0.030217128 | 0.265667608891093 | 0.910475220982458 | -0.550925641 | 0.49049138548592 | 0.970234845582273 | 0.576416008861719 | 1.63311851355587 | 0.999714303421225 |
| ebi-fl187-GCST90094852 | ebi-a-GCST90002104 | HLA DR on myeloid Dendritic Cell \|\| id:ebi-a-GCST90002104 | F-strong vegetable liking (derived food-liking factor) \|\| id:ebi-fl187-GCST90094852 | Weighted mode | 23 | -0.065742854 | 0.189107425188529 | 0.731408514823451 | -0.436393407 | 0.304907699664264 | 0.936371617827882 | 0.646363391979053 | 1.35649979184158 | 0.999714303421225 |
| ebi-fl187-GCST90094852 | ebi-a-GCST90002105 | HLA DR on plasmacytoid Dendritic Cell \|\| id:ebi-a-GCST90002105 | F-strong vegetable liking (derived food-liking factor) \|\| id:ebi-fl187-GCST90094852 | Weighted mode | 23 | 0.135815403674664 | 0.217008855945361 | 0.537851513936094 | -0.289521954 | 0.561152761327572 | 1.14547042444612 | 0.748621357513485 | 1.75269177149695 | 0.999714303421225 |
| ebi-fl187-GCST90094852 | ebi-a-GCST90002106 | HLA DR on Dendritic Cell \|\| id:ebi-a-GCST90002106 | F-strong vegetable liking (derived food-liking factor) \|\| id:ebi-fl187-GCST90094852 | Weighted mode | 23 | 0.141764427776842 | 0.206113354024606 | 0.498767931777071 | -0.262217746 | 0.54574660166507 | 1.15230516547174 | 0.769343483915726 | 1.72589645864642 | 0.999714303421225 |
| ebi-fl187-GCST90094852 | ebi-a-GCST90002111 | HLA DR on CD33dim HLA DR+ CD11b- \|\| id:ebi-a-GCST90002111 | F-strong vegetable liking (derived food-liking factor) \|\| id:ebi-fl187-GCST90094852 | Weighted mode | 23 | -0.154946149 | 0.253606427145686 | 0.547480110076253 | -0.652014747 | 0.342122447907533 | 0.856461297283895 | 0.520995045691393 | 1.40793268537089 | 0.999714303421225 |
| ebi-fl187-GCST90094852 | ebi-a-GCST90002112 | HLA DR on CD33- HLA DR+ \|\| id:ebi-a-GCST90002112 | F-strong vegetable liking (derived food-liking factor) \|\| id:ebi-fl187-GCST90094852 | Weighted mode | 23 | -0.606067939 | 0.512918269330347 | 0.249977240129677 | -1.611387747 | 0.399251868471754 | 0.545491563522226 | 0.199610412964801 | 1.49070903393398 | 0.999714303421225 |
| ebi-fl187-GCST90094852 | ebi-a-GCST90002115 | HLA DR on HLA DR+ CD8+ T cell \|\| id:ebi-a-GCST90002115 | F-strong vegetable liking (derived food-liking factor) \|\| id:ebi-fl187-GCST90094852 | Weighted mode | 23 | -0.187424532 | 0.192432274282726 | 0.340656506048798 | -0.564591789 | 0.189742725808231 | 0.829091685888283 | 0.56859220476645 | 1.20893852896106 | 0.999714303421225 |
| ebi-fl187-GCST90094852 | ebi-a-GCST90002116 | HLA DR on B cell \|\| id:ebi-a-GCST90002116 | F-strong vegetable liking (derived food-liking factor) \|\| id:ebi-fl187-GCST90094852 | Weighted mode | 23 | 0.0147138378017004 | 0.213595905259082 | 0.945702393628113 | -0.403934137 | 0.433361812109501 | 1.01482261918886 | 0.667688096091589 | 1.54243419112278 | 0.999714303421225 |
| ebi-fl187-GCST90094852 | ebi-cfmet338-GCST90026023 | Sphingomyelin (d18:1/24:1, d18:2/24:0) levels \|\| id:ebi-cfmet338-GCST90026023 | F-strong vegetable liking (derived food-liking factor) \|\| id:ebi-fl187-GCST90094852 | Weighted mode | 20 | 0.222166520061589 | 0.152767359559504 | 0.16218961826411 | -0.077257505 | 0.521590544798218 | 1.24877930735876 | 0.925651463335242 | 1.68470511878037 | 0.999714303421225 |
| ebi-fl187-GCST90094852 | ebi-cfmet338-GCST90026046 | 1-arachidonoyl-gpc (20:4n6) levels \|\| id:ebi-cfmet338-GCST90026046 | F-strong vegetable liking (derived food-liking factor) \|\| id:ebi-fl187-GCST90094852 | Weighted mode | 19 | 0.0105527165317446 | 0.148393933107889 | 0.944092096696144 | -0.280299392 | 0.301404825423206 | 1.01060859282076 | 0.75555749944719 | 1.35175645616703 | 0.999714303421225 |
| ebi-fl187-GCST90094852 | ebi-cfmet338-GCST90026047 | 1-linoleoyl-gpc (18:2) levels \|\| id:ebi-cfmet338-GCST90026047 | F-strong vegetable liking (derived food-liking factor) \|\| id:ebi-fl187-GCST90094852 | Weighted mode | 20 | 0.170063538776448 | 0.19802125021569 | 0.401148202399952 | -0.218058112 | 0.5581851891992 | 1.18538016653303 | 0.804078713971033 | 1.7474982421441 | 0.999714303421225 |
| ebi-fl187-GCST90094852 | ebi-cfmet338-GCST90026050 | 1-myristoyl-2-palmitoyl-gpc (14:0/16:0) levels \|\| id:ebi-cfmet338-GCST90026050 | F-strong vegetable liking (derived food-liking factor) \|\| id:ebi-fl187-GCST90094852 | Weighted mode | 20 | 7.18649497583601e-05 | 0.0957688526978477 | 0.99940909034144 | -0.187635086 | 0.18777881623754 | 1.00007186753211 | 0.828917135236564 | 1.20656661289035 | 0.999714303421225 |
| ebi-fl187-GCST90094852 | ebi-cfmet338-GCST90026068 | 2,3-dihydroxyisovalerate levels \|\| id:ebi-cfmet338-GCST90026068 | F-strong vegetable liking (derived food-liking factor) \|\| id:ebi-fl187-GCST90094852 | Weighted mode | 20 | 0.279185757372105 | 0.303367737772392 | 0.368959661894045 | -0.315415009 | 0.873786523405993 | 1.32205290213459 | 0.729486068402837 | 2.39596608043418 | 0.999714303421225 |
| ebi-fl187-GCST90094852 | ebi-cfmet338-GCST90026082 | 3-hydroxyhexanoate levels \|\| id:ebi-cfmet338-GCST90026082 | F-strong vegetable liking (derived food-liking factor) \|\| id:ebi-fl187-GCST90094852 | Weighted mode | 20 | -0.037057636 | 0.0710646415190281 | 0.608069259019743 | -0.176344334 | 0.102229061074967 | 0.963620594220578 | 0.838329268631025 | 1.10763715923023 | 0.999714303421225 |
| ebi-fl187-GCST90094852 | ebi-cfmet338-GCST90026094 | 3-ureidopropionate levels \|\| id:ebi-cfmet338-GCST90026094 | F-strong vegetable liking (derived food-liking factor) \|\| id:ebi-fl187-GCST90094852 | Weighted mode | 20 | 0.194680095164444 | 0.180502354251132 | 0.294292976387948 | -0.159104519 | 0.548464709496663 | 1.21492226480874 | 0.85290720915859 | 1.7305940126642 | 0.999714303421225 |
| ebi-fl187-GCST90094852 | ebi-cfmet338-GCST90026173 | Glycerol levels \|\| id:ebi-cfmet338-GCST90026173 | F-strong vegetable liking (derived food-liking factor) \|\| id:ebi-fl187-GCST90094852 | Weighted mode | 20 | -0.020511429 | 0.111882396822189 | 0.856481129830555 | -0.239800926 | 0.198778069204249 | 0.979697499871784 | 0.786784473543105 | 1.21991119999198 | 0.999714303421225 |
| ebi-fl187-GCST90094852 | ebi-cfmet338-GCST90026179 | Histidine levels \|\| id:ebi-cfmet338-GCST90026179 | F-strong vegetable liking (derived food-liking factor) \|\| id:ebi-fl187-GCST90094852 | Weighted mode | 20 | 0.0344388716540384 | 0.0730620165038706 | 0.642747493739633 | -0.108762681 | 0.177640424001625 | 1.0350387562351 | 0.89694325419312 | 1.19439576796019 | 0.999714303421225 |
| ebi-fl187-GCST90094852 | ebi-cfmet338-GCST90026188 | Isocitrate levels \|\| id:ebi-cfmet338-GCST90026188 | F-strong vegetable liking (derived food-liking factor) \|\| id:ebi-fl187-GCST90094852 | Weighted mode | 20 | 0.115298412209719 | 0.167129587603656 | 0.498613674250418 | -0.212275579 | 0.442872403912884 | 1.12220826825977 | 0.808741794200138 | 1.5571736324028 | 0.999714303421225 |
| ebi-fl187-GCST90094852 | ebi-cfmet338-GCST90026207 | N-acetyl-isoputreanine levels \|\| id:ebi-cfmet338-GCST90026207 | F-strong vegetable liking (derived food-liking factor) \|\| id:ebi-fl187-GCST90094852 | Weighted mode | 20 | 0.0504761438088664 | 0.0912950042644533 | 0.586785243478779 | -0.128462065 | 0.229414352167195 | 1.05177177178729 | 0.879446924000905 | 1.25786312935861 | 0.999714303421225 |
| ebi-fl187-GCST90094852 | ebi-cfmet338-GCST90026213 | N-acetylglutamate levels \|\| id:ebi-cfmet338-GCST90026213 | F-strong vegetable liking (derived food-liking factor) \|\| id:ebi-fl187-GCST90094852 | Weighted mode | 19 | 0.113181301010007 | 0.15072760487559 | 0.462417630469116 | -0.182244805 | 0.408607406566164 | 1.11983494175116 | 0.833397295994445 | 1.50472086097959 | 0.999714303421225 |
| ebi-fl187-GCST90094852 | ebi-cfmet338-GCST90026224 | N-acetyltaurine levels \|\| id:ebi-cfmet338-GCST90026224 | F-strong vegetable liking (derived food-liking factor) \|\| id:ebi-fl187-GCST90094852 | Weighted mode | 20 | -0.038849682 | 0.131199105971904 | 0.770356463007381 | -0.29599993 | 0.218300565644842 | 0.961895288397828 | 0.743787480260075 | 1.24396090334623 | 0.999714303421225 |
| ebi-fl187-GCST90094852 | ebi-cfmet338-GCST90026250 | Phenyllactate (pla) levels \|\| id:ebi-cfmet338-GCST90026250 | F-strong vegetable liking (derived food-liking factor) \|\| id:ebi-fl187-GCST90094852 | Weighted mode | 20 | 0.0805862816887515 | 0.204445528590111 | 0.697844657923424 | -0.320126954 | 0.481299517725369 | 1.08392236525987 | 0.726056855147808 | 1.61817588468519 | 0.999714303421225 |
| ebi-fl187-GCST90094852 | ebi-cfmet338-GCST90026251 | Pipecolate levels \|\| id:ebi-cfmet338-GCST90026251 | F-strong vegetable liking (derived food-liking factor) \|\| id:ebi-fl187-GCST90094852 | Weighted mode | 20 | 0.148055653774015 | 0.266727985673433 | 0.585315683357937 | -0.374731198 | 0.670842505693943 | 1.15957742942741 | 0.68747404825548 | 1.95588447047502 | 0.999714303421225 |
| ebi-fl187-GCST90094852 | ebi-cfmet338-GCST90026278 | Trigonelline (n'-methylnicotinate) levels \|\| id:ebi-cfmet338-GCST90026278 | F-strong vegetable liking (derived food-liking factor) \|\| id:ebi-fl187-GCST90094852 | Weighted mode | 20 | 0.106078997165753 | 0.286171972383108 | 0.714974388052906 | -0.454818069 | 0.666976063036644 | 1.11190971075288 | 0.634563404388992 | 1.94833675613078 | 0.999714303421225 |
| ebi-fl187-GCST90094852 | ebi-cfmet338-GCST90026299 | 1-carboxyethylvaline levels \|\| id:ebi-cfmet338-GCST90026299 | F-strong vegetable liking (derived food-liking factor) \|\| id:ebi-fl187-GCST90094852 | Weighted mode | 20 | -0.003483557 | 0.193600131732498 | 0.985831595678892 | -0.382939816 | 0.375972700818183 | 0.996522503169025 | 0.681853935039357 | 1.45640737449867 | 0.999714303421225 |
| ebi-fl187-GCST90094852 | ebi-gm430-GCST90011306 | C_Erysipelotrichia abundance \|\| id:ebi-gm430-GCST90011306 | F-strong vegetable liking (derived food-liking factor) \|\| id:ebi-fl187-GCST90094852 | Weighted mode | 19 | 0.11692073049433 | 0.171822612544567 | 0.504864185984702 | -0.21985159 | 0.453693051081681 | 1.12403032483073 | 0.80263790854101 | 1.57411475049277 | 0.999714303421225 |
| ebi-fl187-GCST90094852 | ebi-gm430-GCST90011312 | F_Erysipelotrichaceae abundance \|\| id:ebi-gm430-GCST90011312 | F-strong vegetable liking (derived food-liking factor) \|\| id:ebi-fl187-GCST90094852 | Weighted mode | 19 | 0.11692073049433 | 0.165441862353858 | 0.488790216922101 | -0.20734532 | 0.441186780707892 | 1.12403032483073 | 0.81273894673928 | 1.554551035 | 0.999714303421225 |
| ebi-fl187-GCST90094852 | ebi-gm430-GCST90011352 | O_Erysipelotrichales abundance \|\| id:ebi-gm430-GCST90011352 | F-strong vegetable liking (derived food-liking factor) \|\| id:ebi-fl187-GCST90094852 | Weighted mode | 19 | 0.11692073049433 | 0.174042682130598 | 0.510246337187031 | -0.224202926 | 0.458044387470302 | 1.12403032483073 | 0.799152948617874 | 1.58097917717026 | 0.999714303421225 |
| ebi-fl187-GCST90094852 | ebi-gm430-GCST90011362 | OTU97_11 (Parabacteroides) abundance \|\| id:ebi-gm430-GCST90011362 | F-strong vegetable liking (derived food-liking factor) \|\| id:ebi-fl187-GCST90094852 | Weighted mode | 19 | 0.0205695195671569 | 0.21235564011398 | 0.923905133884797 | -0.395647535 | 0.436786574190558 | 1.0207825301362 | 0.673243949013857 | 1.54772571718995 | 0.999714303421225 |
| ebi-fl187-GCST90094852 | ebi-gm430-GCST90011384 | OTU97_235 (Phascolarctobacterium) abundance \|\| id:ebi-gm430-GCST90011384 | F-strong vegetable liking (derived food-liking factor) \|\| id:ebi-fl187-GCST90094852 | Weighted mode | 19 | -0.616534733 | 0.746649671582443 | 0.419764693086779 | -2.079968089 | 0.846898623587859 | 0.539811792429301 | 0.12493419890828 | 2.33240196673181 | 0.999714303421225 |
| ebi-fl187-GCST90094852 | ebi-gm430-GCST90011390 | OTU97_3 (Bacteroides) abundance \|\| id:ebi-gm430-GCST90011390 | F-strong vegetable liking (derived food-liking factor) \|\| id:ebi-fl187-GCST90094852 | Weighted mode | 19 | -0.053086603 | 0.146498947321884 | 0.721296331489577 | -0.34022454 | 0.234051333862356 | 0.948297883590138 | 0.711610520052674 | 1.26370936162519 | 0.999714303421225 |
| ebi-fl187-GCST90094852 | ebi-gm430-GCST90011398 | OTU97_4 (Alistipes) abundance \|\| id:ebi-gm430-GCST90011398 | F-strong vegetable liking (derived food-liking factor) \|\| id:ebi-fl187-GCST90094852 | Weighted mode | 19 | 0.0488297897790703 | 0.166539409214436 | 0.772720787230371 | -0.277587452 | 0.375247031839364 | 1.05004160771448 | 0.757609307043652 | 1.4553508882225 | 0.999714303421225 |
| ebi-fl187-GCST90094852 | ebi-gm430-GCST90011421 | OTU99_108 (Cytophagales) abundance \|\| id:ebi-gm430-GCST90011421 | F-strong vegetable liking (derived food-liking factor) \|\| id:ebi-fl187-GCST90094852 | Weighted mode | 19 | -0.159085209 | 0.58077235817492 | 0.787264654011464 | -1.297399031 | 0.979228613417219 | 0.852923679435286 | 0.273241562524681 | 2.66240170865556 | 0.999714303421225 |
| ebi-fl187-GCST90094852 | ebi-gm430-GCST90011428 | OTU99_123 (Phascolarctobacterium) abundance \|\| id:ebi-gm430-GCST90011428 | F-strong vegetable liking (derived food-liking factor) \|\| id:ebi-fl187-GCST90094852 | Weighted mode | 19 | -0.03332215 | 0.394603949745987 | 0.933635109312565 | -0.806745891 | 0.740101591609059 | 0.967226917345966 | 0.446308040591674 | 2.09614845477205 | 0.999714303421225 |
| ebi-fl187-GCST90094852 | ebi-gm430-GCST90011450 | OTU99_298 (Phascolarctobacterium) abundance \|\| id:ebi-gm430-GCST90011450 | F-strong vegetable liking (derived food-liking factor) \|\| id:ebi-fl187-GCST90094852 | Weighted mode | 19 | -0.625349753 | 0.677585907956751 | 0.368261412526865 | -1.953418133 | 0.702718626685499 | 0.53507425201157 | 0.141788590150431 | 2.01923479782108 | 0.999714303421225 |
| ebi-fl187-GCST90094852 | ebi-gm430-GCST90011451 | OTU99_3 (Bacteroides) abundance \|\| id:ebi-gm430-GCST90011451 | F-strong vegetable liking (derived food-liking factor) \|\| id:ebi-fl187-GCST90094852 | Weighted mode | 19 | -0.05491751 | 0.145018144330218 | 0.709344743063097 | -0.339153073 | 0.229318052519082 | 0.946563226385181 | 0.712373395428126 | 1.25774200341417 | 0.999714303421225 |
| ebi-fl187-GCST90094852 | ebi-gm430-GCST90011472 | OTU99_72 (Parabacteroides) abundance \|\| id:ebi-gm430-GCST90011472 | F-strong vegetable liking (derived food-liking factor) \|\| id:ebi-fl187-GCST90094852 | Weighted mode | 19 | -0.233131453 | 0.439491643986045 | 0.6022786554433 | -1.094535075 | 0.628272169258923 | 0.79204944944113 | 0.334695178881536 | 1.87436918708065 | 0.999714303421225 |
| ebi-fl187-GCST90094852 | ebi-gm430-GCST90011507 | TestASV_3 (Bacteroides) abundance \|\| id:ebi-gm430-GCST90011507 | F-strong vegetable liking (derived food-liking factor) \|\| id:ebi-fl187-GCST90094852 | Weighted mode | 19 | -0.217920074 | 0.202392981369041 | 0.295826380881598 | -0.614610318 | 0.178770169215535 | 0.804189714549771 | 0.540851614523346 | 1.19574589336781 | 0.999714303421225 |
| ebi-fl187-GCST90094852 | ebi-gm430-GCST90011512 | TestASV_34 (Phascolarctobacterium) abundance \|\| id:ebi-gm430-GCST90011512 | F-strong vegetable liking (derived food-liking factor) \|\| id:ebi-fl187-GCST90094852 | Weighted mode | 19 | -0.030755052 | 0.591802278014778 | 0.959126217842793 | -1.190687517 | 1.12917741261133 | 0.969713072976494 | 0.304012178596211 | 3.09311109917895 | 0.999714303421225 |
| ebi-fl187-GCST90094852 | ebi-gm430-GCST90011519 | TestASV_41 (Barnesiella) abundance \|\| id:ebi-gm430-GCST90011519 | F-strong vegetable liking (derived food-liking factor) \|\| id:ebi-fl187-GCST90094852 | Weighted mode | 19 | -0.244014491 | 0.703629726284028 | 0.732767464600791 | -1.623128754 | 1.1350997729653 | 0.783476281177283 | 0.197280490339656 | 3.11148396940088 | 0.999714303421225 |
| ebi-fl187-GCST90094852 | ebi-gm430-GCST90011524 | TestASV_46 (Sutterella) abundance \|\| id:ebi-gm430-GCST90011524 | F-strong vegetable liking (derived food-liking factor) \|\| id:ebi-fl187-GCST90094852 | Weighted mode | 19 | 0.231011977003377 | 0.743737408906454 | 0.759665379081848 | -1.226713344 | 1.68873729846003 | 1.25987432887796 | 0.293254823121028 | 5.41264183713223 | 0.999714303421225 |
| ebi-fl187-GCST90094852 | ebi-gm430-GCST90011581 | OTU97_19 (Bacteria) prevalence \|\| id:ebi-gm430-GCST90011581 | F-strong vegetable liking (derived food-liking factor) \|\| id:ebi-fl187-GCST90094852 | Weighted mode | 19 | -0.194283126 | 0.781959272504234 | 0.806593526491962 | -1.7269233 | 1.33835704780807 | 0.823424738062786 | 0.177830700797053 | 3.81277415100309 | 0.999714303421225 |
| ebi-fl187-GCST90094852 | ebi-gm430-GCST90011614 | OTU97_80 (Ruminococcus) prevalence \|\| id:ebi-gm430-GCST90011614 | F-strong vegetable liking (derived food-liking factor) \|\| id:ebi-fl187-GCST90094852 | Weighted mode | 19 | -0.165044966 | 0.31197363350802 | 0.603244732804508 | -0.776513288 | 0.446423355429885 | 0.847855578348369 | 0.460007130780893 | 1.56271290950672 | 0.999714303421225 |
| ebi-fl187-GCST90094852 | ebi-gm430-GCST90011623 | OTU99_11 (Parabacteroides) prevalence \|\| id:ebi-gm430-GCST90011623 | F-strong vegetable liking (derived food-liking factor) \|\| id:ebi-fl187-GCST90094852 | Weighted mode | 19 | 0.141607630228542 | 0.322245462310228 | 0.665572781093339 | -0.489993476 | 0.773208736356588 | 1.15212450101114 | 0.612630391033616 | 2.16670750465159 | 0.999714303421225 |
| ebi-fl187-GCST90094852 | ebi-gm430-GCST90011642 | OTU99_19 (Bacteria) prevalence \|\| id:ebi-gm430-GCST90011642 | F-strong vegetable liking (derived food-liking factor) \|\| id:ebi-fl187-GCST90094852 | Weighted mode | 19 | -0.194283126 | 0.763591922372416 | 0.802044898656724 | -1.690923294 | 1.30235704154971 | 0.823424738062786 | 0.184349236820567 | 3.67795555299051 | 0.999714303421225 |
| ebi-fl187-GCST90094852 | ebi-gm430-GCST90011653 | OTU99_30 (Parasutterella) prevalence \|\| id:ebi-gm430-GCST90011653 | F-strong vegetable liking (derived food-liking factor) \|\| id:ebi-fl187-GCST90094852 | Weighted mode | 19 | 0.285371379496606 | 0.390469479884684 | 0.474282498745806 | -0.479948801 | 1.05069156007059 | 1.33025596618764 | 0.618815073660171 | 2.85962803897308 | 0.999714303421225 |
| ebi-fl187-GCST90094852 | ebi-gm430-GCST90011675 | OTU99_8 (Ruminococcaceae) prevalence \|\| id:ebi-gm430-GCST90011675 | F-strong vegetable liking (derived food-liking factor) \|\| id:ebi-fl187-GCST90094852 | Weighted mode | 19 | 0.386651253848047 | 0.391692079540546 | 0.336662890779621 | -0.381065222 | 1.15436772974752 | 1.47204303240188 | 0.68313333280951 | 3.17201721123913 | 0.999714303421225 |
| ebi-fl187-GCST90094852 | ebi-gm430-GCST90011679 | OTU99_92 (Ruminococcus) prevalence \|\| id:ebi-gm430-GCST90011679 | F-strong vegetable liking (derived food-liking factor) \|\| id:ebi-fl187-GCST90094852 | Weighted mode | 19 | 0.0391334268030885 | 0.329711331152303 | 0.906835536286765 | -0.607100782 | 0.685367635861603 | 1.03990922615752 | 0.544928447322052 | 1.98450127528103 | 0.999714303421225 |
| ebi-fl187-GCST90094852 | ebi-gm430-GCST90011684 | TestASV_11 (Lachnospiraceae) prevalence \|\| id:ebi-gm430-GCST90011684 | F-strong vegetable liking (derived food-liking factor) \|\| id:ebi-fl187-GCST90094852 | Weighted mode | 19 | -0.032768589 | 0.374623052537324 | 0.931263037240539 | -0.767029772 | 0.701492594223374 | 0.967762484805151 | 0.464390367252935 | 2.01676066740232 | 0.999714303421225 |
| ebi-fl187-GCST90094852 | ebi-gm430-GCST90011688 | TestASV_15 (Bacteroides) prevalence \|\| id:ebi-gm430-GCST90011688 | F-strong vegetable liking (derived food-liking factor) \|\| id:ebi-fl187-GCST90094852 | Weighted mode | 19 | -0.452567635 | 0.35060497624427 | 0.213100156695904 | -1.139753389 | 0.234618118324874 | 0.6359930552538 | 0.319897902574208 | 1.26442581547478 | 0.999714303421225 |
| ebi-fl187-GCST90094852 | ebi-gm430-GCST90011692 | TestASV_19 (Prevotella) prevalence \|\| id:ebi-gm430-GCST90011692 | F-strong vegetable liking (derived food-liking factor) \|\| id:ebi-fl187-GCST90094852 | Weighted mode | 19 | -0.446571711 | 0.641570200481036 | 0.495281256988594 | -1.704049304 | 0.810905882227841 | 0.6398178767427 | 0.181945278689132 | 2.24994524919206 | 0.999714303421225 |
| ebi-fl187-GCST90094852 | ebi-gm430-GCST90011693 | TestASV_2 (Bacteroides) prevalence \|\| id:ebi-gm430-GCST90011693 | F-strong vegetable liking (derived food-liking factor) \|\| id:ebi-fl187-GCST90094852 | Weighted mode | 19 | 0.517948015873558 | 0.337571396819417 | 0.142336509849625 | -0.143691922 | 1.17958795363962 | 1.67857969444603 | 0.866154550213777 | 3.25303352607351 | 0.999714303421225 |
| ebi-fl187-GCST90094852 | ebi-gm430-GCST90011716 | TestASV_40 (Barnesiella) prevalence \|\| id:ebi-gm430-GCST90011716 | F-strong vegetable liking (derived food-liking factor) \|\| id:ebi-fl187-GCST90094852 | Weighted mode | 19 | -0.217807493 | 0.649948975679075 | 0.741412358046197 | -1.491707485 | 1.05609249976925 | 0.804280256696421 | 0.224988163485475 | 2.87511450064982 | 0.999714303421225 |
| ebi-fl187-GCST90094852 | ebi-gm430-GCST90011718 | TestASV_42 (Alphaproteobacteria) prevalence \|\| id:ebi-gm430-GCST90011718 | F-strong vegetable liking (derived food-liking factor) \|\| id:ebi-fl187-GCST90094852 | Weighted mode | 19 | -0.000915671 | 0.494533415136721 | 0.998543016172573 | -0.970201164 | 0.968369822900416 | 0.999084748330993 | 0.379006787747707 | 2.63364764594151 | 0.999714303421225 |
| ebi-fl187-GCST90094852 | ebi-hbsm44-GCST90104929 | Sulcal depth of the right inferior frontal sulcus \|\| id:ebi-hbsm44-GCST90104929 | F-strong vegetable liking (derived food-liking factor) \|\| id:ebi-fl187-GCST90094852 | Weighted mode | 24 | 0.230267283539739 | 0.213742104530171 | 0.292510426952719 | -0.188667241 | 0.649201808418874 | 1.25893645795675 | 0.828062005658758 | 1.91401247049347 | 0.999714303421225 |
| ebi-fl187-GCST90094852 | ebi-hbsm44-GCST90104935 | Sulcal opening distance of the left calloso-marginal posterior fissure \|\| id:ebi-hbsm44-GCST90104935 | F-strong vegetable liking (derived food-liking factor) \|\| id:ebi-fl187-GCST90094852 | Weighted mode | 24 | -0.015699259 | 0.0474532850201493 | 0.743763403081191 | -0.108707697 | 0.0773091801373796 | 0.98442333248901 | 0.896992572674967 | 1.08037605557736 | 0.999714303421225 |
| ebi-fl187-GCST90094852 | ebi-hbsm44-GCST90104936 | Sulcal opening distance of the left anterior inferior temporal sulcus \|\| id:ebi-hbsm44-GCST90104936 | F-strong vegetable liking (derived food-liking factor) \|\| id:ebi-fl187-GCST90094852 | Weighted mode | 22 | -0.023313217 | 0.0308348615921806 | 0.458007180512932 | -0.083749546 | 0.0371231118128867 | 0.976956436572094 | 0.919661560511713 | 1.0378207809714 | 0.999714303421225 |
| ebi-fl187-GCST90094852 | ebi-hbsm44-GCST90104941 | Sulcal opening distance of the right posterior inferior temporal sulcus \|\| id:ebi-hbsm44-GCST90104941 | F-strong vegetable liking (derived food-liking factor) \|\| id:ebi-fl187-GCST90094852 | Weighted mode | 22 | 0.0059080260584487 | 0.027212394914987 | 0.830219686254246 | -0.047428268 | 0.0592443200918233 | 1.00592551286494 | 0.953678879996448 | 1.06103444110702 | 0.999714303421225 |
| ebi-fl187-GCST90094852 | ebi-IPs91-GCST90274819 | Leukemia inhibitory factor levels \|\| id:ebi-IPs91-GCST90274819 | F-strong vegetable liking (derived food-liking factor) \|\| id:ebi-fl187-GCST90094852 | Weighted mode | 27 | 0.0535160741347118 | 0.120120653031461 | 0.65963213156203 | -0.181920406 | 0.288952554076375 | 1.05497394942468 | 0.833667692882351 | 1.33502838537102 | 0.999714303421225 |
| ebi-fl187-GCST90094852 | ebi-IPs91-GCST90274837 | STAM binding protein levels \|\| id:ebi-IPs91-GCST90274837 | F-strong vegetable liking (derived food-liking factor) \|\| id:ebi-fl187-GCST90094852 | Weighted mode | 27 | -0.082127024 | 0.0911040591588322 | 0.375616925322057 | -0.26069098 | 0.0964369322451288 | 0.92115494274981 | 0.770518988870912 | 1.1012401262113 | 0.999714303421225 |
| ebi-fl187-GCST90094852 | ebi-IPs91-GCST90274840 | TNF-beta levels \|\| id:ebi-IPs91-GCST90274840 | F-strong vegetable liking (derived food-liking factor) \|\| id:ebi-fl187-GCST90094852 | Weighted mode | 27 | -0.086756891 | 0.0947658890734034 | 0.368349605456483 | -0.272498034 | 0.0989842513454879 | 0.916899974957535 | 0.761474926368788 | 1.10404891213709 | 0.999714303421225 |
| ebi-fl187-GCST90094852 | ebi-IPs91-GCST90274842 | Tumor necrosis factor ligand superfamily member 14 levels \|\| id:ebi-IPs91-GCST90274842 | F-strong vegetable liking (derived food-liking factor) \|\| id:ebi-fl187-GCST90094852 | Weighted mode | 27 | 0.0059601157754301 | 0.12655635299087 | 0.962797746805467 | -0.242090336 | 0.254010567637535 | 1.00597791260495 | 0.784985261852101 | 1.28918542784014 | 0.999714303421225 |
| ebi-fl187-GCST90094852 | ebi-met1400-GCST90199628 | 3-methylhistidine levels \|\| id:ebi-met1400-GCST90199628 | F-strong vegetable liking (derived food-liking factor) \|\| id:ebi-fl187-GCST90094852 | Weighted mode | 26 | -0.013469832 | 0.119617921191464 | 0.911241357764791 | -0.247920958 | 0.220981293085793 | 0.986620479791958 | 0.780421626938345 | 1.24730009720991 | 0.999714303421225 |
| ebi-fl187-GCST90094852 | ebi-met1400-GCST90199651 | Docosahexaenoate DHA; 22:6n3 levels \|\| id:ebi-met1400-GCST90199651 | F-strong vegetable liking (derived food-liking factor) \|\| id:ebi-fl187-GCST90094852 | Weighted mode | 26 | 0.0890830412719527 | 0.111086498996528 | 0.430153484437987 | -0.128646497 | 0.306812579305147 | 1.09317143090334 | 0.879284740615949 | 1.35908622331617 | 0.999714303421225 |
| ebi-fl187-GCST90094852 | ebi-met1400-GCST90199652 | 1-stearoyl-2-oleoyl-GPS (18:0/18:1) levels \|\| id:ebi-met1400-GCST90199652 | F-strong vegetable liking (derived food-liking factor) \|\| id:ebi-fl187-GCST90094852 | Weighted mode | 26 | 0.0533954570406142 | 0.116626805844214 | 0.651027766569843 | -0.175193082 | 0.281983996495273 | 1.05484670920638 | 0.839294952027966 | 1.32575750305055 | 0.999714303421225 |
| ebi-fl187-GCST90094852 | ebi-met1400-GCST90199653 | 1-myristoyl-2-palmitoyl-gpc (14:0/16:0) levels \|\| id:ebi-met1400-GCST90199653 | F-strong vegetable liking (derived food-liking factor) \|\| id:ebi-fl187-GCST90094852 | Weighted mode | 26 | 0.048121223359032 | 0.118588890657906 | 0.688355017468306 | -0.184313002 | 0.280555449048528 | 1.04929784702465 | 0.831675446729592 | 1.32386494767899 | 0.999714303421225 |
| ebi-fl187-GCST90094852 | ebi-met1400-GCST90199667 | Erythritol levels in elite athletes \|\| id:ebi-met1400-GCST90199667 | F-strong vegetable liking (derived food-liking factor) \|\| id:ebi-fl187-GCST90094852 | Weighted mode | 26 | -0.007860629 | 0.112186902236927 | 0.944697600252355 | -0.227746957 | 0.212025699674747 | 0.992170185240359 | 0.796325738922047 | 1.23617965408531 | 0.999714303421225 |
| ebi-fl187-GCST90094852 | ebi-met1400-GCST90199674 | 3-hydroxymyristate levels \|\| id:ebi-met1400-GCST90199674 | F-strong vegetable liking (derived food-liking factor) \|\| id:ebi-fl187-GCST90094852 | Weighted mode | 26 | 0.029878003866588 | 0.111228331976785 | 0.790427434648114 | -0.188129527 | 0.247885534541087 | 1.03032883015256 | 0.828507386365279 | 1.28131325768955 | 0.999714303421225 |
| ebi-fl187-GCST90094852 | ebi-met1400-GCST90199685 | 2-linoleoylglycerol (18:2) levels \|\| id:ebi-met1400-GCST90199685 | F-strong vegetable liking (derived food-liking factor) \|\| id:ebi-fl187-GCST90094852 | Weighted mode | 26 | -0.116890186 | 0.121191256290021 | 0.344028105605811 | -0.354425049 | 0.120644676022162 | 0.889682887163324 | 0.701576699752385 | 1.1282239561129 | 0.999714303421225 |
| ebi-fl187-GCST90094852 | ebi-met1400-GCST90199703 | Gamma-glutamylvaline levels \|\| id:ebi-met1400-GCST90199703 | F-strong vegetable liking (derived food-liking factor) \|\| id:ebi-fl187-GCST90094852 | Weighted mode | 26 | -0.162323868 | 0.115138515776344 | 0.170914714071978 | -0.387995359 | 0.0633476224665927 | 0.85016581805812 | 0.678415491527553 | 1.06539713084526 | 0.999714303421225 |
| ebi-fl187-GCST90094852 | ebi-met1400-GCST90199779 | Gamma-glutamyl-2-aminobutyrate levels \|\| id:ebi-met1400-GCST90199779 | F-strong vegetable liking (derived food-liking factor) \|\| id:ebi-fl187-GCST90094852 | Weighted mode | 26 | 0.131375745251186 | 0.132855643085273 | 0.332206008139707 | -0.129021315 | 0.391772805698321 | 1.14039619927529 | 0.878955230242949 | 1.47960151617968 | 0.999714303421225 |
| ebi-fl187-GCST90094852 | ebi-met1400-GCST90199824 | 4-vinylphenol sulfate levels \|\| id:ebi-met1400-GCST90199824 | F-strong vegetable liking (derived food-liking factor) \|\| id:ebi-fl187-GCST90094852 | Weighted mode | 26 | 0.0680615632955769 | 0.103835972139335 | 0.518150862514734 | -0.135456942 | 0.271580068688674 | 1.07043120572304 | 0.873316765307048 | 1.31203592064653 | 0.999714303421225 |
| ebi-fl187-GCST90094852 | ebi-met1400-GCST90199830 | 4-ethylphenylsulfate levels \|\| id:ebi-met1400-GCST90199830 | F-strong vegetable liking (derived food-liking factor) \|\| id:ebi-fl187-GCST90094852 | Weighted mode | 26 | 0.000642014301830529 | 0.10975924144736 | 0.995379390115723 | -0.214486099 | 0.215770127538655 | 1.00064222043712 | 0.806956029202152 | 1.24081711653028 | 0.999714303421225 |
| ebi-fl187-GCST90094852 | ebi-met1400-GCST90199833 | Hexanoylglutamine levels \|\| id:ebi-met1400-GCST90199833 | F-strong vegetable liking (derived food-liking factor) \|\| id:ebi-fl187-GCST90094852 | Weighted mode | 26 | 0.0667444485264803 | 0.110175500139646 | 0.550107836132073 | -0.149199532 | 0.282688428800186 | 1.06902225305255 | 0.861397221657757 | 1.32669173847836 | 0.999714303421225 |
| ebi-fl187-GCST90094852 | ebi-met1400-GCST90199837 | Carotenoid levels (cryptoxanthin) \|\| id:ebi-met1400-GCST90199837 | F-strong vegetable liking (derived food-liking factor) \|\| id:ebi-fl187-GCST90094852 | Weighted mode | 26 | 0.114207099701178 | 0.110513283130565 | 0.311299472625765 | -0.102398935 | 0.330813134637085 | 1.12098425635066 | 0.90266937321143 | 1.39209963279845 | 0.999714303421225 |
| ebi-fl187-GCST90094852 | ebi-met1400-GCST90199869 | 2-hydroxyhippurate levels \|\| id:ebi-met1400-GCST90199869 | F-strong vegetable liking (derived food-liking factor) \|\| id:ebi-fl187-GCST90094852 | Weighted mode | 26 | -0.033216344 | 0.124237884963687 | 0.791384669465485 | -0.276722599 | 0.210289910154964 | 0.967329260706303 | 0.75826481142919 | 1.23403576760332 | 0.999714303421225 |
| ebi-fl187-GCST90094852 | ebi-met1400-GCST90199902 | Hydantoin-5-propionate levels \|\| id:ebi-met1400-GCST90199902 | F-strong vegetable liking (derived food-liking factor) \|\| id:ebi-fl187-GCST90094852 | Weighted mode | 25 | -0.087450141 | 0.118979176505589 | 0.469459046342578 | -0.320649327 | 0.14574904493675 | 0.916264554533427 | 0.725677681971545 | 1.15690582024439 | 0.999714303421225 |
| ebi-fl187-GCST90094852 | ebi-met1400-GCST90199904 | Pantoate levels \|\| id:ebi-met1400-GCST90199904 | F-strong vegetable liking (derived food-liking factor) \|\| id:ebi-fl187-GCST90094852 | Weighted mode | 26 | 0.03717973336393 | 0.116567848684762 | 0.752411406130396 | -0.19129325 | 0.265652716786064 | 1.03787954565738 | 0.825890360240045 | 1.3042820247725 | 0.999714303421225 |
| ebi-fl187-GCST90094852 | ebi-met1400-GCST90199936 | 3-methyl catechol sulfate (2) levels \|\| id:ebi-met1400-GCST90199936 | F-strong vegetable liking (derived food-liking factor) \|\| id:ebi-fl187-GCST90094852 | Weighted mode | 26 | 0.252087721110418 | 0.185501591065645 | 0.186296678608465 | -0.111495397 | 0.615670839599082 | 1.28670890386821 | 0.894495508418279 | 1.85089783873966 | 0.999714303421225 |
| ebi-fl187-GCST90094852 | ebi-met1400-GCST90199977 | 4-hydroxychlorothalonil levels \|\| id:ebi-met1400-GCST90199977 | F-strong vegetable liking (derived food-liking factor) \|\| id:ebi-fl187-GCST90094852 | Weighted mode | 26 | -0.076446722 | 0.167158530787385 | 0.65138294120556 | -0.404077442 | 0.251183998763706 | 0.926402270223648 | 0.667592419626366 | 1.28554660155646 | 0.999714303421225 |
| ebi-fl187-GCST90094852 | ebi-met1400-GCST90200040 | Sphingomyelin (d18:2/24:1, d18:1/24:2) levels \|\| id:ebi-met1400-GCST90200040 | F-strong vegetable liking (derived food-liking factor) \|\| id:ebi-fl187-GCST90094852 | Weighted mode | 26 | 0.117650512004111 | 0.117812465986581 | 0.327544537625437 | -0.113261921 | 0.34856294533781 | 1.12485092076967 | 0.892916755541231 | 1.41702973553166 | 0.999714303421225 |
| ebi-fl187-GCST90094852 | ebi-met1400-GCST90200077 | 1-stearoyl-2-oleoyl-GPI (18:0/18:1) levels \|\| id:ebi-met1400-GCST90200077 | F-strong vegetable liking (derived food-liking factor) \|\| id:ebi-fl187-GCST90094852 | Weighted mode | 26 | -0.221799065 | 0.126905915286342 | 0.0927820288013126 | -0.470534658 | 0.0269365294242992 | 0.801076312803857 | 0.624668194824159 | 1.02730259720693 | 0.999714303421225 |
| ebi-fl187-GCST90094852 | ebi-met1400-GCST90200080 | 1-palmitoyl-2-oleoyl-GPI (16:0/18:1) levels \|\| id:ebi-met1400-GCST90200080 | F-strong vegetable liking (derived food-liking factor) \|\| id:ebi-fl187-GCST90094852 | Weighted mode | 26 | -0.174510547 | 0.123010169947724 | 0.168343096546419 | -0.41561048 | 0.0665893859754737 | 0.839867995992223 | 0.659937279050189 | 1.06885650058624 | 0.999714303421225 |
| ebi-fl187-GCST90094852 | ebi-met1400-GCST90200082 | 1-oleoyl-2-linoleoyl-GPE (18:1/18:2) levels \|\| id:ebi-met1400-GCST90200082 | F-strong vegetable liking (derived food-liking factor) \|\| id:ebi-fl187-GCST90094852 | Weighted mode | 26 | -0.208910029 | 0.14825555365503 | 0.171117219319994 | -0.499490914 | 0.0816708559876623 | 0.811468240825311 | 0.606839514383881 | 1.08509859733949 | 0.999714303421225 |
| ebi-fl187-GCST90094852 | ebi-met1400-GCST90200100 | Perfluorooctanesulfonate (PFOS) levels \|\| id:ebi-met1400-GCST90200100 | F-strong vegetable liking (derived food-liking factor) \|\| id:ebi-fl187-GCST90094852 | Weighted mode | 26 | -0.075658574 | 0.118943448273079 | 0.530499506475596 | -0.308787733 | 0.157470584644903 | 0.927132699763454 | 0.734336629221373 | 1.17054632543945 | 0.999714303421225 |
| ebi-fl187-GCST90094852 | ebi-met1400-GCST90200102 | 1-palmitoleoyl-2-linolenoyl-GPC (16:1/18:3) levels \|\| id:ebi-met1400-GCST90200102 | F-strong vegetable liking (derived food-liking factor) \|\| id:ebi-fl187-GCST90094852 | Weighted mode | 26 | 0.0852678052126021 | 0.140354746343403 | 0.548987368842963 | -0.189827498 | 0.360363108045672 | 1.08900866984859 | 0.827101798666392 | 1.43384996250472 | 0.999714303421225 |
| ebi-fl187-GCST90094852 | ebi-met1400-GCST90200114 | Glycosyl-N-tricosanoyl-sphingadienine (d18:2/23:0) levels \|\| id:ebi-met1400-GCST90200114 | F-strong vegetable liking (derived food-liking factor) \|\| id:ebi-fl187-GCST90094852 | Weighted mode | 26 | -0.091160417 | 0.120445414108677 | 0.456207466388401 | -0.327233429 | 0.144912594249231 | 0.912871258715535 | 0.720915440772693 | 1.15593853017727 | 0.999714303421225 |
| ebi-fl187-GCST90094852 | ebi-met1400-GCST90200135 | Arachidoylcarnitine (C20) levels \|\| id:ebi-met1400-GCST90200135 | F-strong vegetable liking (derived food-liking factor) \|\| id:ebi-fl187-GCST90094852 | Weighted mode | 26 | -0.030715197 | 0.109415744676856 | 0.781235208044007 | -0.245170056 | 0.183739662847726 | 0.969751722222398 | 0.782571445732342 | 1.20170293444997 | 0.999714303421225 |
| ebi-fl187-GCST90094852 | ebi-met1400-GCST90200146 | Docosahexaenoylcarnitine (C22:6) levels \|\| id:ebi-met1400-GCST90200146 | F-strong vegetable liking (derived food-liking factor) \|\| id:ebi-fl187-GCST90094852 | Weighted mode | 26 | 0.156922917978323 | 0.138904651606047 | 0.269318827438216 | -0.115330199 | 0.429176035126175 | 1.1699054317494 | 0.891071864134509 | 1.53599139903955 | 0.999714303421225 |
| ebi-fl187-GCST90094852 | ebi-met1400-GCST90200162 | 3-hydroxyphenylacetoylglutamine levels \|\| id:ebi-met1400-GCST90200162 | F-strong vegetable liking (derived food-liking factor) \|\| id:ebi-fl187-GCST90094852 | Weighted mode | 26 | -0.044393235 | 0.136639968951476 | 0.747965977818784 | -0.312207574 | 0.22342110422118 | 0.956577723735554 | 0.731829603612706 | 1.25034698928543 | 0.999714303421225 |
| ebi-fl187-GCST90094852 | ebi-met1400-GCST90200172 | 3-carboxy-4-methyl-5-pentyl-2-furanpropionate (3-CMPFP) levels \|\| id:ebi-met1400-GCST90200172 | F-strong vegetable liking (derived food-liking factor) \|\| id:ebi-fl187-GCST90094852 | Weighted mode | 26 | 0.0824056267205797 | 0.103262813269873 | 0.432375216495477 | -0.119989487 | 0.284800740729531 | 1.08589618901877 | 0.886929760704957 | 1.32949708710671 | 0.999714303421225 |
| ebi-fl187-GCST90094852 | ebi-met1400-GCST90200222 | Dihydrocaffeate sulfate (2) levels \|\| id:ebi-met1400-GCST90200222 | F-strong vegetable liking (derived food-liking factor) \|\| id:ebi-fl187-GCST90094852 | Weighted mode | 26 | -0.221407372 | 0.15981728724337 | 0.178174164481871 | -0.534649255 | 0.0918345113031022 | 0.801390150122206 | 0.585874746930388 | 1.09618340110706 | 0.999714303421225 |
| ebi-fl187-GCST90094852 | ebi-met1400-GCST90200223 | 4-methylhexanoylglutamine levels \|\| id:ebi-met1400-GCST90200223 | F-strong vegetable liking (derived food-liking factor) \|\| id:ebi-fl187-GCST90094852 | Weighted mode | 26 | -0.077518008 | 0.145158077888988 | 0.598041469007309 | -0.362027841 | 0.206991824229543 | 0.925410359056247 | 0.69626298284787 | 1.22997251576669 | 0.999714303421225 |
| ebi-fl187-GCST90094852 | ebi-met1400-GCST90200224 | 2-ketocaprylate levels \|\| id:ebi-met1400-GCST90200224 | F-strong vegetable liking (derived food-liking factor) \|\| id:ebi-fl187-GCST90094852 | Weighted mode | 26 | -0.083717244 | 0.0998482427104415 | 0.409726234709882 | -0.2794198 | 0.111985311484217 | 0.919691267347624 | 0.756222374462469 | 1.1184964314719 | 0.999714303421225 |
| ebi-fl187-GCST90094852 | ebi-met1400-GCST90200243 | Hydroxypalmitoyl sphingomyelin (d18:1/16:0(OH)) levels \|\| id:ebi-met1400-GCST90200243 | F-strong vegetable liking (derived food-liking factor) \|\| id:ebi-fl187-GCST90094852 | Weighted mode | 26 | 0.147798446294073 | 0.129424449306273 | 0.264287823354084 | -0.105873474 | 0.401470366934367 | 1.15927921579203 | 0.899538455567179 | 1.49401984078602 | 0.999714303421225 |
| ebi-fl187-GCST90094852 | ebi-met1400-GCST90200273 | 2-methoxyhydroquinone sulfate (2) levels \|\| id:ebi-met1400-GCST90200273 | F-strong vegetable liking (derived food-liking factor) \|\| id:ebi-fl187-GCST90094852 | Weighted mode | 26 | 0.0212649008036121 | 0.162436404049469 | 0.896892571780264 | -0.297110451 | 0.339640252740571 | 1.02149261001366 | 0.742961946840847 | 1.40444225542016 | 0.999714303421225 |
| ebi-fl187-GCST90094852 | ebi-met1400-GCST90200276 | 2,4-di-tert-butylphenol levels \|\| id:ebi-met1400-GCST90200276 | F-strong vegetable liking (derived food-liking factor) \|\| id:ebi-fl187-GCST90094852 | Weighted mode | 26 | -0.053707761 | 0.106535133666565 | 0.618582887813673 | -0.262516623 | 0.155101100646422 | 0.94770902325192 | 0.769113579036155 | 1.16777601804751 | 0.999714303421225 |
| ebi-fl187-GCST90094852 | ebi-met1400-GCST90200289 | N-acetylmethionine levels \|\| id:ebi-met1400-GCST90200289 | F-strong vegetable liking (derived food-liking factor) \|\| id:ebi-fl187-GCST90094852 | Weighted mode | 26 | -0.048067138 | 0.112804179490742 | 0.673673185558465 | -0.26916333 | 0.173029053812327 | 0.953069797739372 | 0.764018458481494 | 1.18890064667851 | 0.999714303421225 |
| ebi-fl187-GCST90094852 | ebi-met1400-GCST90200308 | 3-hydroxyisobutyrate levels \|\| id:ebi-met1400-GCST90200308 | F-strong vegetable liking (derived food-liking factor) \|\| id:ebi-fl187-GCST90094852 | Weighted mode | 26 | -0.020502851 | 0.11162484857983 | 0.855748960769353 | -0.239287554 | 0.198281852382151 | 0.979705903491327 | 0.787188490585112 | 1.2193060096983 | 0.999714303421225 |
| ebi-fl187-GCST90094852 | ebi-met1400-GCST90200383 | Cystathionine levels \|\| id:ebi-met1400-GCST90200383 | F-strong vegetable liking (derived food-liking factor) \|\| id:ebi-fl187-GCST90094852 | Weighted mode | 26 | -0.210769012 | 0.146637094539023 | 0.163017553624265 | -0.498177717 | 0.0766396936889808 | 0.809961136895939 | 0.60763693795097 | 1.07965300051377 | 0.999714303421225 |
| ebi-fl187-GCST90094852 | ebi-met1400-GCST90200384 | Guanidinoacetate levels \|\| id:ebi-met1400-GCST90200384 | F-strong vegetable liking (derived food-liking factor) \|\| id:ebi-fl187-GCST90094852 | Weighted mode | 26 | 0.108668140232276 | 0.130210107349374 | 0.411869742923261 | -0.14654367 | 0.363879950637049 | 1.11479233422302 | 0.863688014106627 | 1.43890146458484 | 0.999714303421225 |
| ebi-fl187-GCST90094852 | ebi-met1400-GCST90200403 | Citrulline levels \|\| id:ebi-met1400-GCST90200403 | F-strong vegetable liking (derived food-liking factor) \|\| id:ebi-fl187-GCST90094852 | Weighted mode | 26 | 0.0261135228659114 | 0.132800053711898 | 0.845700721110694 | -0.234174582 | 0.286401628141232 | 1.02645746825302 | 0.791223670102314 | 1.33162716681132 | 0.999714303421225 |
| ebi-fl187-GCST90094852 | ebi-met1400-GCST90200434 | 12,13-DiHOME levels \|\| id:ebi-met1400-GCST90200434 | F-strong vegetable liking (derived food-liking factor) \|\| id:ebi-fl187-GCST90094852 | Weighted mode | 26 | -0.032060106 | 0.109185566687711 | 0.771464878653078 | -0.246063816 | 0.18194360494783 | 0.968448371004364 | 0.781872327003006 | 1.19954654348241 | 0.999714303421225 |
| ebi-fl187-GCST90094852 | ebi-met1400-GCST90200437 | Fructose levels \|\| id:ebi-met1400-GCST90200437 | F-strong vegetable liking (derived food-liking factor) \|\| id:ebi-fl187-GCST90094852 | Weighted mode | 25 | -0.045589673 | 0.113131849129486 | 0.690531585987086 | -0.267328097 | 0.176148751189341 | 0.955433922004506 | 0.765421897329765 | 1.19261544842325 | 0.999714303421225 |
| ebi-fl187-GCST90094852 | ebi-met1400-GCST90200448 | N-stearoyl-sphinganine (d18:0/18:0) levels \|\| id:ebi-met1400-GCST90200448 | F-strong vegetable liking (derived food-liking factor) \|\| id:ebi-fl187-GCST90094852 | Weighted mode | 26 | -0.022395513 | 0.172777092788254 | 0.897903682567675 | -0.361038615 | 0.316247588410906 | 0.977853404380343 | 0.696952084920118 | 1.37196989742534 | 0.999714303421225 |
| ebi-fl187-GCST90094852 | ebi-met1400-GCST90200468 | X-11847 levels \|\| id:ebi-met1400-GCST90200468 | F-strong vegetable liking (derived food-liking factor) \|\| id:ebi-fl187-GCST90094852 | Weighted mode | 26 | 0.143676255567808 | 0.136871324987282 | 0.303890095190662 | -0.124591541 | 0.41194405254288 | 1.15451028174039 | 0.882857439654738 | 1.5097499672943 | 0.999714303421225 |
| ebi-fl187-GCST90094852 | ebi-met1400-GCST90200476 | X-11843 levels \|\| id:ebi-met1400-GCST90200476 | F-strong vegetable liking (derived food-liking factor) \|\| id:ebi-fl187-GCST90094852 | Weighted mode | 26 | 0.193391373926332 | 0.122246436636192 | 0.126224490046684 | -0.046211642 | 0.432994389733269 | 1.2133575771231 | 0.954839856701527 | 1.54186757038803 | 0.999714303421225 |
| ebi-fl187-GCST90094852 | ebi-met1400-GCST90200511 | X-12906 levels \|\| id:ebi-met1400-GCST90200511 | F-strong vegetable liking (derived food-liking factor) \|\| id:ebi-fl187-GCST90094852 | Weighted mode | 26 | -0.277108761 | 0.13103559731533 | 0.0445927121198785 | -0.533938532 | -0.020278991 | 0.757972054460067 | 0.58629128928108 | 0.97992524508918 | 0.999714303421225 |
| ebi-fl187-GCST90094852 | ebi-met1400-GCST90200515 | X-12849 levels \|\| id:ebi-met1400-GCST90200515 | F-strong vegetable liking (derived food-liking factor) \|\| id:ebi-fl187-GCST90094852 | Weighted mode | 26 | 0.034411542987661 | 0.123881891753512 | 0.783467824304452 | -0.208396965 | 0.277220050824544 | 1.03501047039275 | 0.811884683053842 | 1.31945668662354 | 0.999714303421225 |
| ebi-fl187-GCST90094852 | ebi-met1400-GCST90200551 | X-18886 levels \|\| id:ebi-met1400-GCST90200551 | F-strong vegetable liking (derived food-liking factor) \|\| id:ebi-fl187-GCST90094852 | Weighted mode | 26 | 0.068536948782797 | 0.114527165867303 | 0.554934121922394 | -0.155936296 | 0.29301019388271 | 1.07094019415658 | 0.855613694378405 | 1.34045645481791 | 0.999714303421225 |
| ebi-fl187-GCST90094852 | ebi-met1400-GCST90200564 | X-21319 levels \|\| id:ebi-met1400-GCST90200564 | F-strong vegetable liking (derived food-liking factor) \|\| id:ebi-fl187-GCST90094852 | Weighted mode | 26 | -0.029264976 | 0.113237923005597 | 0.798184770630247 | -0.251211305 | 0.192681353484869 | 0.971159096899726 | 0.777857989145421 | 1.21249637421256 | 0.999714303421225 |
| ebi-fl187-GCST90094852 | ebi-met1400-GCST90200579 | X-21442 levels \|\| id:ebi-met1400-GCST90200579 | F-strong vegetable liking (derived food-liking factor) \|\| id:ebi-fl187-GCST90094852 | Weighted mode | 26 | 0.0169056524813281 | 0.123244829195171 | 0.891994158471014 | -0.224654213 | 0.258465517703863 | 1.01704936171484 | 0.798792383238092 | 1.29494149652684 | 0.999714303421225 |
| ebi-fl187-GCST90094852 | ebi-met1400-GCST90200581 | X-21441 levels \|\| id:ebi-met1400-GCST90200581 | F-strong vegetable liking (derived food-liking factor) \|\| id:ebi-fl187-GCST90094852 | Weighted mode | 26 | -0.070968234 | 0.138405924327454 | 0.612624295949368 | -0.342243846 | 0.200307377567196 | 0.931491481309259 | 0.710175010402671 | 1.22177824767411 | 0.999714303421225 |
| ebi-fl187-GCST90094852 | ebi-met1400-GCST90200648 | X-24585 levels \|\| id:ebi-met1400-GCST90200648 | F-strong vegetable liking (derived food-liking factor) \|\| id:ebi-fl187-GCST90094852 | Weighted mode | 26 | -0.007522373 | 0.109430997103837 | 0.945742817765018 | -0.222007128 | 0.206962380839209 | 0.99250584875669 | 0.800909655584198 | 1.22993630173894 | 0.999714303421225 |
| ebi-fl187-GCST90094852 | ebi-met1400-GCST90200720 | Adenosine 5'-diphosphate (ADP) to pantothenate ratio \|\| id:ebi-met1400-GCST90200720 | F-strong vegetable liking (derived food-liking factor) \|\| id:ebi-fl187-GCST90094852 | Weighted mode | 26 | 0.108594481198046 | 0.159193628157451 | 0.501413853061223 | -0.20342503 | 0.420613992386649 | 1.11471022272047 | 0.815931372412453 | 1.52289631536487 | 0.999714303421225 |
| ebi-fl187-GCST90094852 | ebi-met1400-GCST90200724 | Cholate to taurocholate ratio \|\| id:ebi-met1400-GCST90200724 | F-strong vegetable liking (derived food-liking factor) \|\| id:ebi-fl187-GCST90094852 | Weighted mode | 26 | -0.171205097 | 0.144881242032005 | 0.248446692162501 | -0.455172331 | 0.112762137868158 | 0.842648731429752 | 0.634338642380979 | 1.11936564658111 | 0.999714303421225 |
| ebi-fl187-GCST90094852 | ebi-met1400-GCST90200726 | N-acetylputrescine to (N(1) + N(8))-acetylspermidine ratio \|\| id:ebi-met1400-GCST90200726 | F-strong vegetable liking (derived food-liking factor) \|\| id:ebi-fl187-GCST90094852 | Weighted mode | 26 | -0.12345526 | 0.108813673725652 | 0.267322945972225 | -0.336730061 | 0.0898195403124755 | 0.883861184141121 | 0.714101577973503 | 1.09397684717108 | 0.999714303421225 |
| ebi-fl187-GCST90094852 | ebi-met1400-GCST90200744 | Arginine to glutamate ratio \|\| id:ebi-met1400-GCST90200744 | F-strong vegetable liking (derived food-liking factor) \|\| id:ebi-fl187-GCST90094852 | Weighted mode | 26 | 0.284381323707625 | 0.128604687434788 | 0.0363858186955765 | 0.0323161363354406 | 0.536446511079809 | 1.32893959031779 | 1.03284397320699 | 1.70991987224395 | 0.999714303421225 |
| ebi-fl187-GCST90094852 | ebi-met1400-GCST90200759 | Histidine to trans-urocanate ratio \|\| id:ebi-met1400-GCST90200759 | F-strong vegetable liking (derived food-liking factor) \|\| id:ebi-fl187-GCST90094852 | Weighted mode | 26 | 0.0615142844271893 | 0.102635836421163 | 0.55433606859979 | -0.139651955 | 0.262680523812669 | 1.06344568713279 | 0.869660863883223 | 1.30041120216857 | 0.999714303421225 |
| ebi-fl187-GCST90094852 | ebi-met1400-GCST90200795 | Oleoyl-linoleoyl-glycerol (18:1 to 18:2) [2] to linoleoyl-arachidonoyl-glycerol (18:2 to 20:4) [2] ratio \|\| id:ebi-met1400-GCST90200795 | F-strong vegetable liking (derived food-liking factor) \|\| id:ebi-fl187-GCST90094852 | Weighted mode | 26 | 0.0676786091617597 | 0.166281269757269 | 0.687462411552719 | -0.25823268 | 0.393589897886007 | 1.07002135814926 | 0.772415485898787 | 1.48229253270774 | 0.999714303421225 |
| ebi-fl187-GCST90094852 | ebi-met1400-GCST90200811 | Choline to taurocholate ratio \|\| id:ebi-met1400-GCST90200811 | F-strong vegetable liking (derived food-liking factor) \|\| id:ebi-fl187-GCST90094852 | Weighted mode | 26 | -0.039288383 | 0.100092084127775 | 0.697999007010653 | -0.235468868 | 0.156892101910004 | 0.961473396598459 | 0.79020026324251 | 1.16986938041917 | 0.999714303421225 |
| ebi-fl187-GCST90094852 | ebi-met1400-GCST90200831 | Cholate to phosphate ratio \|\| id:ebi-met1400-GCST90200831 | F-strong vegetable liking (derived food-liking factor) \|\| id:ebi-fl187-GCST90094852 | Weighted mode | 26 | -0.052404853 | 0.116077368882112 | 0.655546717284726 | -0.279916496 | 0.175106790057136 | 0.948944606038726 | 0.755846855086028 | 1.19137343665669 | 0.999714303421225 |
| ebi-fl187-GCST90094852 | ebi-met1400-GCST90200841 | Adenosine 5'-diphosphate (ADP) to oxalate (ethanedioate) ratio \|\| id:ebi-met1400-GCST90200841 | F-strong vegetable liking (derived food-liking factor) \|\| id:ebi-fl187-GCST90094852 | Weighted mode | 26 | 0.0119304194349454 | 0.193663785741794 | 0.951368241345145 | -0.367650601 | 0.391511439488863 | 1.01200187075424 | 0.69235904925534 | 1.47921484887298 | 0.999714303421225 |
| ebi-fl187-GCST90094852 | ebi-met1400-GCST90200844 | Aspartate to phosphate ratio \|\| id:ebi-met1400-GCST90200844 | F-strong vegetable liking (derived food-liking factor) \|\| id:ebi-fl187-GCST90094852 | Weighted mode | 26 | -0.078896681 | 0.0944412432712349 | 0.411402915889568 | -0.264001518 | 0.106208155864737 | 0.924135400307275 | 0.76797237405922 | 1.11205333283933 | 0.999714303421225 |
| ebi-fl187-GCST90094852 | ebi-met1400-GCST90200870 | Adenosine 5'-monophosphate (AMP) to acetoacetate ratio \|\| id:ebi-met1400-GCST90200870 | F-strong vegetable liking (derived food-liking factor) \|\| id:ebi-fl187-GCST90094852 | Weighted mode | 26 | 0.102219310726304 | 0.120167835663558 | 0.40304451388135 | -0.133309647 | 0.337748268626876 | 1.10762635943438 | 0.875194048782894 | 1.40178758507326 | 0.999714303421225 |
| ebi-fl187-GCST90094852 | ebi-met1400-GCST90200887 | Cholesterol to cortisol ratio \|\| id:ebi-met1400-GCST90200887 | F-strong vegetable liking (derived food-liking factor) \|\| id:ebi-fl187-GCST90094852 | Weighted mode | 25 | 0.0541176098419167 | 0.153072254606575 | 0.726768258153277 | -0.245904009 | 0.354139228870803 | 1.05560874483234 | 0.781997285878098 | 1.42495356734552 | 0.999714303421225 |
| ebi-fl187-GCST90094852 | ebi-met1400-GCST90200890 | Cortisol to taurocholate ratio \|\| id:ebi-met1400-GCST90200890 | F-strong vegetable liking (derived food-liking factor) \|\| id:ebi-fl187-GCST90094852 | Weighted mode | 26 | 0.0695933747017546 | 0.109686197793446 | 0.531533300522473 | -0.145391573 | 0.284578322376908 | 1.07207216094959 | 0.864683640067388 | 1.3292014154374 | 0.999714303421225 |
| ebi-fl187-GCST90094852 | ebi-met1400-GCST90200948 | Adenosine 5'-diphosphate (ADP) to mannitol to sorbitol ratio \|\| id:ebi-met1400-GCST90200948 | F-strong vegetable liking (derived food-liking factor) \|\| id:ebi-fl187-GCST90094852 | Weighted mode | 26 | -0.073608564 | 0.146628983115917 | 0.620056455690119 | -0.361001371 | 0.213784243200276 | 0.929035280802535 | 0.696978043178271 | 1.23835544236087 | 0.999714303421225 |
| ebi-fl187-GCST90094852 | ebi-met1400-GCST90200972 | Leucine to N-palmitoyl-sphingosine (d18:1 to 16:0) ratio \|\| id:ebi-met1400-GCST90200972 | F-strong vegetable liking (derived food-liking factor) \|\| id:ebi-fl187-GCST90094852 | Weighted mode | 26 | -0.064748658 | 0.106328776585539 | 0.548053279752441 | -0.27315306 | 0.143655744283352 | 0.937303017553532 | 0.76097630373338 | 1.15448660149445 | 0.999714303421225 |
| ebi-fl187-GCST90094852 | ebi-met1400-GCST90200973 | Proline to glutamate ratio \|\| id:ebi-met1400-GCST90200973 | F-strong vegetable liking (derived food-liking factor) \|\| id:ebi-fl187-GCST90094852 | Weighted mode | 26 | 0.180257296227598 | 0.11862951247397 | 0.141184252261126 | -0.052256548 | 0.412771140676579 | 1.19752544226506 | 0.949085349448907 | 1.51099918011043 | 0.999714303421225 |
| ebi-fl187-GCST90094852 | ebi-met1400-GCST90200983 | Cholesterol to linoleoyl-arachidonoyl-glycerol (18:2 to 20:4) [1] ratio \|\| id:ebi-met1400-GCST90200983 | F-strong vegetable liking (derived food-liking factor) \|\| id:ebi-fl187-GCST90094852 | Weighted mode | 26 | -0.027902741 | 0.118768453284137 | 0.816173652786218 | -0.26068891 | 0.204883427263186 | 0.972482944751683 | 0.770520583882977 | 1.22738197734707 | 0.999714303421225 |
| ebi-fl187-GCST90094852 | ebi-met1400-GCST90201001 | Serine to alpha-ketobutyrate ratio \|\| id:ebi-met1400-GCST90201001 | F-strong vegetable liking (derived food-liking factor) \|\| id:ebi-fl187-GCST90094852 | Weighted mode | 26 | 0.0813646305358232 | 0.111074038038836 | 0.470660196968668 | -0.136340484 | 0.299069745091943 | 1.08476636340307 | 0.872545494108429 | 1.34860367868049 | 0.999714303421225 |
| ebi-fl187-GCST90094852 | ebi-met1400-GCST90201005 | Fructose to maltose ratio \|\| id:ebi-met1400-GCST90201005 | F-strong vegetable liking (derived food-liking factor) \|\| id:ebi-fl187-GCST90094852 | Weighted mode | 26 | 0.0968334372492308 | 0.128543419272816 | 0.458297919413529 | -0.155111665 | 0.348778539023949 | 1.10167686000991 | 0.856319551628307 | 1.41733527113031 | 0.999714303421225 |
| ebi-fl187-GCST90094852 | ebi-met1400-GCST90201020 | Paraxanthine to 5-acetylamino-6-formylamino-3-methyluracil ratio \|\| id:ebi-met1400-GCST90201020 | F-strong vegetable liking (derived food-liking factor) \|\| id:ebi-fl187-GCST90094852 | Weighted mode | 25 | -0.086597649 | 0.123637781042283 | 0.490403644694206 | -0.3289277 | 0.155732402181224 | 0.917045996098236 | 0.719695049174714 | 1.16851346959266 | 0.999714303421225 |
| ebi-fl187-GCST90094852 | ebi-pw11-GCST90275197 | Placental weight (fetal genotype, adjusted for sex) \|\| id:ebi-pw11-GCST90275197 | F-strong vegetable liking (derived food-liking factor) \|\| id:ebi-fl187-GCST90094852 | Weighted mode | 20 | -0.022432223 | 0.0669531215486078 | 0.741262844102764 | -0.153660342 | 0.108795894908778 | 0.977817508165498 | 0.857563250143673 | 1.11493476325484 | 0.999714303421225 |
| ebi-fl187-GCST90094852 | prot-a-1017 | FAS-associated factor 2 \|\| id:prot-a-1017 | F-strong vegetable liking (derived food-liking factor) \|\| id:ebi-fl187-GCST90094852 | Weighted mode | 27 | -0.130442029 | 0.202961084580819 | 0.526049722708199 | -0.528245755 | 0.267361697040783 | 0.877707373278354 | 0.589638433497847 | 1.30651292273674 | 0.999714303421225 |
| ebi-fl187-GCST90094852 | prot-a-1019 | Fas apoptotic inhibitory molecule 3 \|\| id:prot-a-1019 | F-strong vegetable liking (derived food-liking factor) \|\| id:ebi-fl187-GCST90094852 | Weighted mode | 27 | -0.172252169 | 0.205819356906756 | 0.410270223301154 | -0.575658109 | 0.231153770257028 | 0.841766878654648 | 0.56233466960021 | 1.26005298322393 | 0.999714303421225 |
| ebi-fl187-GCST90094852 | prot-a-1052 | MIP18 family protein FAM96A \|\| id:prot-a-1052 | F-strong vegetable liking (derived food-liking factor) \|\| id:ebi-fl187-GCST90094852 | Weighted mode | 27 | -0.135827797 | 0.205555701075438 | 0.514564011063198 | -0.538716971 | 0.267061376734255 | 0.872992951224734 | 0.58349641485518 | 1.3061206092881 | 0.999714303421225 |
| ebi-fl187-GCST90094852 | prot-a-1066 | F-box/LRR-repeat protein 4 \|\| id:prot-a-1066 | F-strong vegetable liking (derived food-liking factor) \|\| id:ebi-fl187-GCST90094852 | Weighted mode | 27 | 0.123811235991221 | 0.182187514162145 | 0.502775601666976 | -0.233276292 | 0.480898763749025 | 1.13180220731201 | 0.791934738246665 | 1.61752752419018 | 0.999714303421225 |
| ebi-fl187-GCST90094852 | prot-a-1076 | Low affinity immunoglobulin gamma Fc region receptor III-B \|\| id:prot-a-1076 | F-strong vegetable liking (derived food-liking factor) \|\| id:ebi-fl187-GCST90094852 | Weighted mode | 27 | -0.138722992 | 0.172301386302621 | 0.428053163676998 | -0.476433709 | 0.19898772551238 | 0.870469122287544 | 0.6209940932343 | 1.22016698888336 | 0.999714303421225 |
| ebi-fl187-GCST90094852 | prot-a-1081 | Fc receptor-like protein 3 \|\| id:prot-a-1081 | F-strong vegetable liking (derived food-liking factor) \|\| id:ebi-fl187-GCST90094852 | Weighted mode | 27 | -0.115588864 | 0.223814722608666 | 0.609905950329005 | -0.554265721 | 0.323087992061989 | 0.89084140476536 | 0.574493945450712 | 1.38138689663946 | 0.999714303421225 |
| ebi-fl187-GCST90094852 | prot-a-1105 | Fibroblast growth factor receptor 4 \|\| id:prot-a-1105 | F-strong vegetable liking (derived food-liking factor) \|\| id:ebi-fl187-GCST90094852 | Weighted mode | 27 | 0.0908548664507014 | 0.293492610284644 | 0.759359654806477 | -0.48439065 | 0.666100382608603 | 1.09511005651497 | 0.61607248636121 | 1.94663138255631 | 0.999714303421225 |
| ebi-fl187-GCST90094852 | prot-a-1143 | Follistatin-related protein 1 \|\| id:prot-a-1143 | F-strong vegetable liking (derived food-liking factor) \|\| id:ebi-fl187-GCST90094852 | Weighted mode | 27 | 0.267796907753367 | 0.351489804005714 | 0.452981395768108 | -0.421123108 | 0.956716923604567 | 1.30708165490704 | 0.656309299446141 | 2.60313613419205 | 0.999714303421225 |
| ebi-fl187-GCST90094852 | prot-a-1151 | Alpha-(1,3)-fucosyltransferase 10 \|\| id:prot-a-1151 | F-strong vegetable liking (derived food-liking factor) \|\| id:ebi-fl187-GCST90094852 | Weighted mode | 27 | 0.115792272054015 | 0.183395336761635 | 0.533303751178953 | -0.243662588 | 0.47524713210682 | 1.12276261873485 | 0.783752036997282 | 1.6084116385304 | 0.999714303421225 |
| ebi-fl187-GCST90094852 | prot-a-1165 | Growth arrest and DNA damage-inducible proteins-interacting protein 1 \|\| id:prot-a-1165 | F-strong vegetable liking (derived food-liking factor) \|\| id:ebi-fl187-GCST90094852 | Weighted mode | 27 | 0.00235830956080085 | 0.226245525219775 | 0.991762833830795 | -0.44108292 | 0.44579953899156 | 1.00236109256009 | 0.643339358745223 | 1.56173836750465 | 0.999714303421225 |
| ebi-fl187-GCST90094852 | prot-a-1194 | Growth/differentiation factor 11/8 \|\| id:prot-a-1194 | F-strong vegetable liking (derived food-liking factor) \|\| id:ebi-fl187-GCST90094852 | Weighted mode | 27 | -0.182978125 | 0.196512251787628 | 0.360353107927296 | -0.568142138 | 0.202185888607612 | 0.832786372826239 | 0.566577083298548 | 1.2240755286596 | 0.999714303421225 |
| ebi-fl187-GCST90094852 | prot-a-1216 | D-glucuronyl C5-epimerase \|\| id:prot-a-1216 | F-strong vegetable liking (derived food-liking factor) \|\| id:ebi-fl187-GCST90094852 | Weighted mode | 27 | 0.200908546055122 | 0.175969324061743 | 0.263974867760667 | -0.143991329 | 0.545808421216138 | 1.22251296307759 | 0.865895256112728 | 1.72600315608865 | 0.999714303421225 |
| ebi-fl187-GCST90094852 | prot-a-1246 | Glypican-3 \|\| id:prot-a-1246 | F-strong vegetable liking (derived food-liking factor) \|\| id:ebi-fl187-GCST90094852 | Weighted mode | 27 | 0.155377581533276 | 0.226687116306312 | 0.49914043628405 | -0.288929166 | 0.599684329493648 | 1.16809893043412 | 0.749065262492416 | 1.82154370200173 | 0.999714303421225 |
| ebi-fl187-GCST90094852 | prot-a-1275 | Glutamate receptor 4 \|\| id:prot-a-1275 | F-strong vegetable liking (derived food-liking factor) \|\| id:ebi-fl187-GCST90094852 | Weighted mode | 27 | -0.240807417 | 0.212743814367033 | 0.268003121966744 | -0.657785293 | 0.17617045922808 | 0.78599298075394 | 0.517997277208513 | 1.19264133804661 | 0.999714303421225 |
| ebi-fl187-GCST90094852 | prot-a-1289 | Glutathione S-transferase P \|\| id:prot-a-1289 | F-strong vegetable liking (derived food-liking factor) \|\| id:ebi-fl187-GCST90094852 | Weighted mode | 27 | 0.201540614460013 | 0.193211381919617 | 0.306499995256095 | -0.177153694 | 0.580234923022462 | 1.22328591915095 | 0.837651032605431 | 1.786458062 | 0.999714303421225 |
| ebi-fl187-GCST90094852 | prot-a-130 | Apolipoprotein D \|\| id:prot-a-130 | F-strong vegetable liking (derived food-liking factor) \|\| id:ebi-fl187-GCST90094852 | Weighted mode | 27 | 0.402942280548605 | 0.206750834042309 | 0.0621709480332258 | -0.002289354 | 0.808173915271531 | 1.49622052814313 | 0.997713264398285 | 2.24380686187132 | 0.999714303421225 |
| ebi-fl187-GCST90094852 | prot-a-1313 | Heparin-binding EGF-like growth factor \|\| id:prot-a-1313 | F-strong vegetable liking (derived food-liking factor) \|\| id:ebi-fl187-GCST90094852 | Weighted mode | 27 | 0.0587849568585435 | 0.177560083249238 | 0.743244540278133 | -0.289232806 | 0.40680272002705 | 1.06054715282526 | 0.74883785093121 | 1.50200776038108 | 0.999714303421225 |
| ebi-fl187-GCST90094852 | prot-a-1347 | HLA class II histocompatibility antigen, DQ alpha 2 chain \|\| id:prot-a-1347 | F-strong vegetable liking (derived food-liking factor) \|\| id:ebi-fl187-GCST90094852 | Weighted mode | 27 | -0.127294026 | 0.186735114741394 | 0.501467503564477 | -0.49329485 | 0.238706799329054 | 0.880474752448706 | 0.610611203528448 | 1.26960623260738 | 0.999714303421225 |
| ebi-fl187-GCST90094852 | prot-a-1357 | Non-histone chromosomal protein HMG-14 \|\| id:prot-a-1357 | F-strong vegetable liking (derived food-liking factor) \|\| id:ebi-fl187-GCST90094852 | Weighted mode | 27 | -0.278519628 | 0.185239154993446 | 0.144743567887438 | -0.641588371 | 0.0845491161590595 | 0.756903411385447 | 0.526455552636479 | 1.08822629241508 | 0.999714303421225 |
| ebi-fl187-GCST90094852 | prot-a-137 | Apolipoprotein M \|\| id:prot-a-137 | F-strong vegetable liking (derived food-liking factor) \|\| id:ebi-fl187-GCST90094852 | Weighted mode | 27 | -0.16395861 | 0.172528300097923 | 0.350696210944584 | -0.502114079 | 0.1741968578591 | 0.848777151757009 | 0.60524976070793 | 1.19028986067189 | 0.999714303421225 |
| ebi-fl187-GCST90094852 | prot-a-1381 | 17-beta-hydroxysteroid dehydrogenase 14 \|\| id:prot-a-1381 | F-strong vegetable liking (derived food-liking factor) \|\| id:ebi-fl187-GCST90094852 | Weighted mode | 27 | 0.122203529533348 | 0.173969997038291 | 0.48864638599663 | -0.218777665 | 0.463184723728398 | 1.12998406350666 | 0.803500344816369 | 1.58912686474433 | 0.999714303421225 |
| ebi-fl187-GCST90094852 | prot-a-1392 | Serine protease HTRA2, mitochondrial \|\| id:prot-a-1392 | F-strong vegetable liking (derived food-liking factor) \|\| id:ebi-fl187-GCST90094852 | Weighted mode | 27 | 0.324735366375463 | 0.195473169036595 | 0.108670451669801 | -0.058392045 | 0.707862777687189 | 1.38366443339265 | 0.943280066762961 | 2.02964880918757 | 0.999714303421225 |
| ebi-fl187-GCST90094852 | prot-a-1416 | Interferon-induced protein with tetratricopeptide repeats 2 \|\| id:prot-a-1416 | F-strong vegetable liking (derived food-liking factor) \|\| id:ebi-fl187-GCST90094852 | Weighted mode | 27 | -0.307836737 | 0.214223426678918 | 0.162646989749614 | -0.727714653 | 0.11204117932915 | 0.735035312311956 | 0.483011578652145 | 1.11855892120266 | 0.999714303421225 |
| ebi-fl187-GCST90094852 | prot-a-1428 | Interferon gamma \|\| id:prot-a-1428 | F-strong vegetable liking (derived food-liking factor) \|\| id:ebi-fl187-GCST90094852 | Weighted mode | 27 | 0.201198804662716 | 0.188620947335624 | 0.295920088465135 | -0.168498252 | 0.570895861440539 | 1.2228678594914 | 0.844932740262819 | 1.76985188349066 | 0.999714303421225 |
| ebi-fl187-GCST90094852 | prot-a-1483 | Interleukin-17C \|\| id:prot-a-1483 | F-strong vegetable liking (derived food-liking factor) \|\| id:ebi-fl187-GCST90094852 | Weighted mode | 27 | -0.038607022 | 0.180243130588963 | 0.832066654370274 | -0.391883558 | 0.314669513896048 | 0.962128730232565 | 0.675782798871515 | 1.36980653411826 | 0.999714303421225 |
| ebi-fl187-GCST90094852 | prot-a-1503 | Interleukin-1 receptor-like 2 \|\| id:prot-a-1503 | F-strong vegetable liking (derived food-liking factor) \|\| id:ebi-fl187-GCST90094852 | Weighted mode | 27 | 0.0151668193460826 | 0.198554496263564 | 0.939696628905854 | -0.373999993 | 0.404333632022668 | 1.01528241923878 | 0.68797691641744 | 1.49830374568832 | 0.999714303421225 |
| ebi-fl187-GCST90094852 | prot-a-1522 | Interleukin-31 receptor subunit alpha \|\| id:prot-a-1522 | F-strong vegetable liking (derived food-liking factor) \|\| id:ebi-fl187-GCST90094852 | Weighted mode | 27 | -0.032343237 | 0.16681045123835 | 0.847767229223713 | -0.359291721 | 0.294605247410299 | 0.968174211813301 | 0.69817065029075 | 1.34259626071355 | 0.999714303421225 |
| ebi-fl187-GCST90094852 | prot-a-1540 | Interleukin-6 receptor subunit alpha \|\| id:prot-a-1540 | F-strong vegetable liking (derived food-liking factor) \|\| id:ebi-fl187-GCST90094852 | Weighted mode | 27 | -0.02349422 | 0.205500107663822 | 0.909856851950611 | -0.426274431 | 0.379285990965836 | 0.976779620384762 | 0.652937131291563 | 1.461240878 | 0.999714303421225 |
| ebi-fl187-GCST90094852 | prot-a-1541 | Interleukin-6 receptor subunit alpha \|\| id:prot-a-1541 | F-strong vegetable liking (derived food-liking factor) \|\| id:ebi-fl187-GCST90094852 | Weighted mode | 27 | -0.03018671 | 0.192150314742968 | 0.876380087624622 | -0.406801327 | 0.346427906949927 | 0.970264358629169 | 0.665776449072275 | 1.41400755003858 | 0.999714303421225 |
| ebi-fl187-GCST90094852 | prot-a-1545 | Interleukin-9 \|\| id:prot-a-1545 | F-strong vegetable liking (derived food-liking factor) \|\| id:ebi-fl187-GCST90094852 | Weighted mode | 27 | 0.276355345768894 | 0.182080873314069 | 0.141141117621638 | -0.080523166 | 0.633233857464469 | 1.31831623890242 | 0.922633529675482 | 1.88369233271319 | 0.999714303421225 |
| ebi-fl187-GCST90094852 | prot-a-1548 | Inositol monophosphatase 3 \|\| id:prot-a-1548 | F-strong vegetable liking (derived food-liking factor) \|\| id:ebi-fl187-GCST90094852 | Weighted mode | 27 | 0.0554588844203274 | 0.182665482637267 | 0.763842136910531 | -0.302565462 | 0.413483230389371 | 1.0570255559605 | 0.738920115820535 | 1.51207553026608 | 0.999714303421225 |
| ebi-fl187-GCST90094852 | prot-a-1555 | Inhibin beta A chain:Inhibin beta B chain heterodimer \|\| id:prot-a-1555 | F-strong vegetable liking (derived food-liking factor) \|\| id:ebi-fl187-GCST90094852 | Weighted mode | 27 | -0.179438025 | 0.176766246129152 | 0.319405013909386 | -0.525899867 | 0.167023817547093 | 0.8357397444208 | 0.591023282488038 | 1.18178241213142 | 0.999714303421225 |
| ebi-fl187-GCST90094852 | prot-a-1603 | Junctophilin-3 \|\| id:prot-a-1603 | F-strong vegetable liking (derived food-liking factor) \|\| id:ebi-fl187-GCST90094852 | Weighted mode | 27 | -0.261515746 | 0.194380044520713 | 0.190118565758968 | -0.642500633 | 0.119469141526207 | 0.769883752943817 | 0.525975506459502 | 1.12689846916378 | 0.999714303421225 |
| ebi-fl187-GCST90094852 | prot-a-1627 | UPF0258 protein KIAA1024 \|\| id:prot-a-1627 | F-strong vegetable liking (derived food-liking factor) \|\| id:ebi-fl187-GCST90094852 | Weighted mode | 27 | 0.275917284773378 | 0.301830093272268 | 0.369039423205407 | -0.315669698 | 0.867504267587022 | 1.31773886245068 | 0.729300299707124 | 2.38096119021225 | 0.999714303421225 |
| ebi-fl187-GCST90094852 | prot-a-1666 | Kallikrein-7 \|\| id:prot-a-1666 | F-strong vegetable liking (derived food-liking factor) \|\| id:ebi-fl187-GCST90094852 | Weighted mode | 27 | -0.052670589 | 0.181269075170845 | 0.773688554170372 | -0.407957977 | 0.302616798070418 | 0.94869247050049 | 0.665006824083461 | 1.35339574120125 | 0.999714303421225 |
| ebi-fl187-GCST90094852 | prot-a-1678 | Importin subunit alpha-3 \|\| id:prot-a-1678 | F-strong vegetable liking (derived food-liking factor) \|\| id:ebi-fl187-GCST90094852 | Weighted mode | 27 | 0.0615241529720989 | 0.179288318725722 | 0.734239118280402 | -0.289880952 | 0.412929257674514 | 1.0634561818461 | 0.748352652364142 | 1.5112381136539 | 0.999714303421225 |
| ebi-fl187-GCST90094852 | prot-a-1718 | Protein LDOC1 \|\| id:prot-a-1718 | F-strong vegetable liking (derived food-liking factor) \|\| id:ebi-fl187-GCST90094852 | Weighted mode | 27 | -0.027783286 | 0.177403116871175 | 0.876761166243261 | -0.375493395 | 0.319926823401266 | 0.972599120134127 | 0.686950257522793 | 1.37702699449718 | 0.999714303421225 |
| ebi-fl187-GCST90094852 | prot-a-1723 | Leptin \|\| id:prot-a-1723 | F-strong vegetable liking (derived food-liking factor) \|\| id:ebi-fl187-GCST90094852 | Weighted mode | 26 | -0.228932566 | 0.156388370226672 | 0.155690518544298 | -0.535453771 | 0.077588640074782 | 0.795382167840195 | 0.585403590568104 | 1.08067801959368 | 0.999714303421225 |
| ebi-fl187-GCST90094852 | prot-a-1730 | Galectin-7 \|\| id:prot-a-1730 | F-strong vegetable liking (derived food-liking factor) \|\| id:ebi-fl187-GCST90094852 | Weighted mode | 26 | -0.00801726 | 0.204602688546464 | 0.969054510510266 | -0.40903853 | 0.393004009157784 | 0.992014792123832 | 0.664288636824911 | 1.48142432858124 | 0.999714303421225 |
| ebi-fl187-GCST90094852 | prot-a-1753 | Lipase member N \|\| id:prot-a-1753 | F-strong vegetable liking (derived food-liking factor) \|\| id:ebi-fl187-GCST90094852 | Weighted mode | 27 | 0.0646654949408316 | 0.175443475446668 | 0.715422796838618 | -0.279203717 | 0.408534706816301 | 1.06680211398909 | 0.756385798922393 | 1.5046114721257 | 0.999714303421225 |
| ebi-fl187-GCST90094852 | prot-a-1756 | VIP36-like protein \|\| id:prot-a-1756 | F-strong vegetable liking (derived food-liking factor) \|\| id:ebi-fl187-GCST90094852 | Weighted mode | 27 | 0.128363029756175 | 0.179904710968806 | 0.481888962376764 | -0.224250204 | 0.480976263255034 | 1.13696568015908 | 0.799115167748404 | 1.61765288663196 | 0.999714303421225 |
| ebi-fl187-GCST90094852 | prot-a-1760 | Ig Kappa chain V-I region HK102- like \|\| id:prot-a-1760 | F-strong vegetable liking (derived food-liking factor) \|\| id:ebi-fl187-GCST90094852 | Weighted mode | 26 | -0.125823044 | 0.175441327485578 | 0.479910651255153 | -0.469688046 | 0.21804195802789 | 0.881770867761943 | 0.625197270832426 | 1.2436392471746 | 0.999714303421225 |
| ebi-fl187-GCST90094852 | prot-a-179 | Neutral ceramidase \|\| id:prot-a-179 | F-strong vegetable liking (derived food-liking factor) \|\| id:ebi-fl187-GCST90094852 | Weighted mode | 27 | 0.195788881684224 | 0.167146396297315 | 0.252076693168682 | -0.131818055 | 0.523395818426962 | 1.21627010133193 | 0.876500455396504 | 1.68774921939407 | 0.999714303421225 |
| ebi-fl187-GCST90094852 | prot-a-1809 | Lactotransferrin \|\| id:prot-a-1809 | F-strong vegetable liking (derived food-liking factor) \|\| id:ebi-fl187-GCST90094852 | Weighted mode | 27 | 0.0296755944927494 | 0.182818710265117 | 0.87230683605326 | -0.328649078 | 0.388000266612379 | 1.03012030304384 | 0.719895599898258 | 1.47403017728278 | 0.999714303421225 |
| ebi-fl187-GCST90094852 | prot-a-1818 | Lysophospholipase-like protein 1 \|\| id:prot-a-1818 | F-strong vegetable liking (derived food-liking factor) \|\| id:ebi-fl187-GCST90094852 | Weighted mode | 27 | 0.0673049360236433 | 0.175379610329334 | 0.704271455010363 | -0.2764391 | 0.411048972269138 | 1.06962159460561 | 0.758479808977278 | 1.50839922448209 | 0.999714303421225 |
| ebi-fl187-GCST90094852 | prot-a-1852 | MAP kinase-activated protein kinase 3 \|\| id:prot-a-1852 | F-strong vegetable liking (derived food-liking factor) \|\| id:ebi-fl187-GCST90094852 | Weighted mode | 27 | -0.192365865 | 0.187359411418088 | 0.314009675994509 | -0.559590311 | 0.174858581604377 | 0.825004973009703 | 0.571443129774532 | 1.19107776439501 | 0.999714303421225 |
| ebi-fl187-GCST90094852 | prot-a-1856 | Megakaryocyte-associated tyrosine-protein kinase \|\| id:prot-a-1856 | F-strong vegetable liking (derived food-liking factor) \|\| id:ebi-fl187-GCST90094852 | Weighted mode | 27 | -0.141489537 | 0.174099647190844 | 0.423773600610541 | -0.482724845 | 0.199745771570404 | 0.868064258159986 | 0.617099597797438 | 1.22109228232262 | 0.999714303421225 |
| ebi-fl187-GCST90094852 | prot-a-1890 | Beta-1,3-N-acetylglucosaminyltransferase manic fringe \|\| id:prot-a-1890 | F-strong vegetable liking (derived food-liking factor) \|\| id:ebi-fl187-GCST90094852 | Weighted mode | 27 | -0.062431852 | 0.172785700092466 | 0.720775919536535 | -0.401091824 | 0.276228120070501 | 0.939477084012287 | 0.669588573718273 | 1.31814852586715 | 0.999714303421225 |
| ebi-fl187-GCST90094852 | prot-a-1919 | Matrilysin \|\| id:prot-a-1919 | F-strong vegetable liking (derived food-liking factor) \|\| id:ebi-fl187-GCST90094852 | Weighted mode | 27 | -0.064758561 | 0.206060539536993 | 0.755824221438114 | -0.468637218 | 0.339120096512286 | 0.937293735341571 | 0.625854590460972 | 1.40371191599551 | 0.999714303421225 |
| ebi-fl187-GCST90094852 | prot-a-1923 | Multimerin-2 \|\| id:prot-a-1923 | F-strong vegetable liking (derived food-liking factor) \|\| id:ebi-fl187-GCST90094852 | Weighted mode | 27 | 0.0156265651607044 | 0.181761097648624 | 0.932146458450012 | -0.340625186 | 0.371878316552008 | 1.01574929839633 | 0.711325472828986 | 1.45045647400945 | 0.999714303421225 |
| ebi-fl187-GCST90094852 | prot-a-1948 | Mesothelin \|\| id:prot-a-1948 | F-strong vegetable liking (derived food-liking factor) \|\| id:ebi-fl187-GCST90094852 | Weighted mode | 27 | 0.096843974007244 | 0.167220755962879 | 0.567479959774786 | -0.230908708 | 0.424596655694486 | 1.10168846817355 | 0.793811931660301 | 1.52897359248307 | 0.999714303421225 |
| ebi-fl187-GCST90094852 | prot-a-195 | ATPase family AAA domain-containing protein 1 \|\| id:prot-a-195 | F-strong vegetable liking (derived food-liking factor) \|\| id:ebi-fl187-GCST90094852 | Weighted mode | 27 | 0.0620332119875402 | 0.200439487510329 | 0.759418628231163 | -0.330828184 | 0.454894607507785 | 1.06399768161897 | 0.718328579117146 | 1.57600727494642 | 0.999714303421225 |
| ebi-fl187-GCST90094852 | prot-a-1966 | Mucin-1 \|\| id:prot-a-1966 | F-strong vegetable liking (derived food-liking factor) \|\| id:ebi-fl187-GCST90094852 | Weighted mode | 27 | 0.0364815864484358 | 0.167177963079695 | 0.828961383779568 | -0.291187221 | 0.364150394084638 | 1.03715520613105 | 0.747375740345036 | 1.43929065868279 | 0.999714303421225 |
| ebi-fl187-GCST90094852 | prot-a-1986 | Myocardial zonula adherens protein \|\| id:prot-a-1986 | F-strong vegetable liking (derived food-liking factor) \|\| id:ebi-fl187-GCST90094852 | Weighted mode | 27 | 0.0580967652956149 | 0.194903713049903 | 0.768011705853604 | -0.323914512 | 0.440108042873424 | 1.05981754430661 | 0.723312074035352 | 1.55287498652372 | 0.999714303421225 |
| ebi-fl187-GCST90094852 | prot-a-2071 | Epididymal secretory protein E1 \|\| id:prot-a-2071 | F-strong vegetable liking (derived food-liking factor) \|\| id:ebi-fl187-GCST90094852 | Weighted mode | 26 | -0.083223878 | 0.222026873693399 | 0.710942794326482 | -0.51839655 | 0.351948794542235 | 0.920145124003826 | 0.595474596423072 | 1.42183571610581 | 0.999714303421225 |
| ebi-fl187-GCST90094852 | prot-a-2098 | Neuritin \|\| id:prot-a-2098 | F-strong vegetable liking (derived food-liking factor) \|\| id:ebi-fl187-GCST90094852 | Weighted mode | 27 | -0.252677947 | 0.213394679093361 | 0.247090936064378 | -0.670931518 | 0.165575624192232 | 0.776717986037161 | 0.511232134052795 | 1.18007220135212 | 0.999714303421225 |
| ebi-fl187-GCST90094852 | prot-a-2115 | Neurotrophin-4 \|\| id:prot-a-2115 | F-strong vegetable liking (derived food-liking factor) \|\| id:ebi-fl187-GCST90094852 | Weighted mode | 27 | -0.147065077 | 0.17654409416392 | 0.41241961864181 | -0.493091501 | 0.198961347724879 | 0.863237798803366 | 0.610735383367919 | 1.22013480400229 | 0.999714303421225 |
| ebi-fl187-GCST90094852 | prot-a-2118 | Netrin-1 \|\| id:prot-a-2118 | F-strong vegetable liking (derived food-liking factor) \|\| id:ebi-fl187-GCST90094852 | Weighted mode | 27 | -0.094011666 | 0.191649666039381 | 0.627866948509519 | -0.469645012 | 0.281621679043819 | 0.910272142585934 | 0.625224176078809 | 1.325277245 | 0.999714303421225 |
| ebi-fl187-GCST90094852 | prot-a-2120 | Netrin-G1 \|\| id:prot-a-2120 | F-strong vegetable liking (derived food-liking factor) \|\| id:ebi-fl187-GCST90094852 | Weighted mode | 27 | -0.055386468 | 0.191572663804244 | 0.774786422217192 | -0.430868889 | 0.320095953118154 | 0.946119432463059 | 0.649944120015799 | 1.37725991037885 | 0.999714303421225 |
| ebi-fl187-GCST90094852 | prot-a-2145 | Olfactomedin-like protein 3 \|\| id:prot-a-2145 | F-strong vegetable liking (derived food-liking factor) \|\| id:ebi-fl187-GCST90094852 | Weighted mode | 27 | -0.23215833 | 0.196480229121457 | 0.248065046268879 | -0.617259579 | 0.152942919575768 | 0.792820586479994 | 0.539420653857523 | 1.16525846359695 | 0.999714303421225 |
| ebi-fl187-GCST90094852 | prot-a-2162 | Prolyl 4-hydroxylase subunit alpha-2 \|\| id:prot-a-2162 | F-strong vegetable liking (derived food-liking factor) \|\| id:ebi-fl187-GCST90094852 | Weighted mode | 27 | 0.0588194848500852 | 0.170821429221857 | 0.733365297557686 | -0.275990516 | 0.393629486124925 | 1.06058377202057 | 0.758820127054891 | 1.48235121522023 | 0.999714303421225 |
| ebi-fl187-GCST90094852 | prot-a-2168 | Polyadenylate-binding protein-interacting protein 1 \|\| id:prot-a-2168 | F-strong vegetable liking (derived food-liking factor) \|\| id:ebi-fl187-GCST90094852 | Weighted mode | 26 | 0.0516064655435915 | 0.171814779366972 | 0.76638532456054 | -0.285150502 | 0.388363433102856 | 1.05296128442026 | 0.751901083174749 | 1.47456559286574 | 0.999714303421225 |
| ebi-fl187-GCST90094852 | prot-a-219 | Beta-1,4-galactosyltransferase 5 \|\| id:prot-a-219 | F-strong vegetable liking (derived food-liking factor) \|\| id:ebi-fl187-GCST90094852 | Weighted mode | 27 | 0.186892115492216 | 0.236085848002582 | 0.435740786061099 | -0.275836147 | 0.649620377577277 | 1.20549722355964 | 0.758937275032179 | 1.91481378477344 | 0.999714303421225 |
| ebi-fl187-GCST90094852 | prot-a-2190 | Pyruvate carboxylase, mitochondrial \|\| id:prot-a-2190 | F-strong vegetable liking (derived food-liking factor) \|\| id:ebi-fl187-GCST90094852 | Weighted mode | 27 | -0.08893885 | 0.182181379176251 | 0.629508311406448 | -0.446014353 | 0.268136653043215 | 0.914901517961628 | 0.640174583360129 | 1.30752580518745 | 0.999714303421225 |
| ebi-fl187-GCST90094852 | prot-a-2197 | Protocadherin-9 \|\| id:prot-a-2197 | F-strong vegetable liking (derived food-liking factor) \|\| id:ebi-fl187-GCST90094852 | Weighted mode | 27 | 0.14026220592509 | 0.192682327110427 | 0.473152372074096 | -0.237395155 | 0.517919567061528 | 1.150575447 | 0.788679575589893 | 1.67853194152709 | 0.999714303421225 |
| ebi-fl187-GCST90094852 | prot-a-2198 | Protocadherin alpha-4 \|\| id:prot-a-2198 | F-strong vegetable liking (derived food-liking factor) \|\| id:ebi-fl187-GCST90094852 | Weighted mode | 27 | -0.122986616 | 0.210326081891511 | 0.563761203187102 | -0.535225737 | 0.289252504383915 | 0.88427549751572 | 0.585537098053329 | 1.33542888760817 | 0.999714303421225 |
| ebi-fl187-GCST90094852 | prot-a-2254 | Placenta growth factor \|\| id:prot-a-2254 | F-strong vegetable liking (derived food-liking factor) \|\| id:ebi-fl187-GCST90094852 | Weighted mode | 27 | -0.128350307 | 0.189547090853028 | 0.504296733374203 | -0.499862605 | 0.243161990932828 | 0.879545214203903 | 0.606613999589722 | 1.27527519040478 | 0.999714303421225 |
| ebi-fl187-GCST90094852 | prot-a-2261 | Membrane-associated progesterone receptor component 2 \|\| id:prot-a-2261 | F-strong vegetable liking (derived food-liking factor) \|\| id:ebi-fl187-GCST90094852 | Weighted mode | 27 | -0.173488292 | 0.183847245688603 | 0.354033158805352 | -0.533828894 | 0.18685230935434 | 0.840726994171697 | 0.586355572890426 | 1.20544923832601 | 0.999714303421225 |
| ebi-fl187-GCST90094852 | prot-a-2268 | Polymeric immunoglobulin receptor \|\| id:prot-a-2268 | F-strong vegetable liking (derived food-liking factor) \|\| id:ebi-fl187-GCST90094852 | Weighted mode | 27 | 0.245477919940396 | 0.209307073753614 | 0.251504615886615 | -0.164763945 | 0.655719784497481 | 1.27823205983912 | 0.848093877586329 | 1.92652870393376 | 0.999714303421225 |
| ebi-fl187-GCST90094852 | prot-a-2283 | Group 10 secretory phospholipase A2 \|\| id:prot-a-2283 | F-strong vegetable liking (derived food-liking factor) \|\| id:ebi-fl187-GCST90094852 | Weighted mode | 27 | 0.25958401932441 | 0.174751568860348 | 0.149450951559686 | -0.082929056 | 0.602097094290692 | 1.29639070100691 | 0.920416443257682 | 1.82594396478712 | 0.999714303421225 |
| ebi-fl187-GCST90094852 | prot-a-2298 | Inactive phospholipase D5 \|\| id:prot-a-2298 | F-strong vegetable liking (derived food-liking factor) \|\| id:ebi-fl187-GCST90094852 | Weighted mode | 27 | -0.011331573 | 0.253578181989338 | 0.964698477117449 | -0.50834481 | 0.485681663210001 | 0.988732386970923 | 0.601490336005223 | 1.62528252662854 | 0.999714303421225 |
| ebi-fl187-GCST90094852 | prot-a-2304 | Procollagen-lysine,2-oxoglutarate 5-dioxygenase 2 \|\| id:prot-a-2304 | F-strong vegetable liking (derived food-liking factor) \|\| id:ebi-fl187-GCST90094852 | Weighted mode | 27 | -0.270121542 | 0.214586634524007 | 0.219288438288017 | -0.690711346 | 0.15046826173132 | 0.763286717353674 | 0.501219402006675 | 1.16237841263932 | 0.999714303421225 |
| ebi-fl187-GCST90094852 | prot-a-2331 | Periostin \|\| id:prot-a-2331 | F-strong vegetable liking (derived food-liking factor) \|\| id:ebi-fl187-GCST90094852 | Weighted mode | 27 | 0.256696031240802 | 0.284190521611316 | 0.374684376241898 | -0.300317391 | 0.813709453598982 | 1.29265214116728 | 0.740583128868883 | 2.25626198184728 | 0.999714303421225 |
| ebi-fl187-GCST90094852 | prot-a-2333 | Periostin \|\| id:prot-a-2333 | F-strong vegetable liking (derived food-liking factor) \|\| id:ebi-fl187-GCST90094852 | Weighted mode | 27 | 0.321628854846137 | 0.280977955562636 | 0.262771798864469 | -0.229087938 | 0.872345647748904 | 1.37937273343669 | 0.795258596934564 | 2.39251627719928 | 0.999714303421225 |
| ebi-fl187-GCST90094852 | prot-a-2367 | cAMP-dependent protein kinase catalytic subunit alpha \|\| id:prot-a-2367 | F-strong vegetable liking (derived food-liking factor) \|\| id:ebi-fl187-GCST90094852 | Weighted mode | 27 | 0.315332070650691 | 0.245454738944224 | 0.210230662257156 | -0.165759218 | 0.796423358981371 | 1.37071440941624 | 0.847250212503025 | 2.21759518552449 | 0.999714303421225 |
| ebi-fl187-GCST90094852 | prot-a-2385 | Phosphoribosyl pyrophosphate synthase-associated protein 1 \|\| id:prot-a-2385 | F-strong vegetable liking (derived food-liking factor) \|\| id:ebi-fl187-GCST90094852 | Weighted mode | 27 | 0.179225906507292 | 0.23565411109629 | 0.45377041710699 | -0.282656151 | 0.64110796425602 | 1.19629096355786 | 0.753778929253496 | 1.89858327680702 | 0.999714303421225 |
| ebi-fl187-GCST90094852 | prot-a-2393 | Trypsin-3 \|\| id:prot-a-2393 | F-strong vegetable liking (derived food-liking factor) \|\| id:ebi-fl187-GCST90094852 | Weighted mode | 26 | -0.073923376 | 0.18455882264477 | 0.692160538578007 | -0.435658668 | 0.28781191640056 | 0.928742855123064 | 0.646838474691922 | 1.33350646983849 | 0.999714303421225 |
| ebi-fl187-GCST90094852 | prot-a-2405 | Pregnancy-specific beta-1-glycoprotein 6 \|\| id:prot-a-2405 | F-strong vegetable liking (derived food-liking factor) \|\| id:ebi-fl187-GCST90094852 | Weighted mode | 27 | -0.045922536 | 0.192475972271526 | 0.813295267098984 | -0.423175442 | 0.331330369612291 | 0.955115946388685 | 0.654963715088961 | 1.39281986166523 | 0.999714303421225 |
| ebi-fl187-GCST90094852 | prot-a-244 | Beclin-1 \|\| id:prot-a-244 | F-strong vegetable liking (derived food-liking factor) \|\| id:ebi-fl187-GCST90094852 | Weighted mode | 26 | 0.074414154641254 | 0.194547512901915 | 0.705324161665302 | -0.306898971 | 0.455727279929008 | 1.07725286239434 | 0.735724926966359 | 1.57732011924868 | 0.999714303421225 |
| ebi-fl187-GCST90094852 | prot-a-2454 | tRNA pseudouridine synthase A, mitochondrial \|\| id:prot-a-2454 | F-strong vegetable liking (derived food-liking factor) \|\| id:ebi-fl187-GCST90094852 | Weighted mode | 27 | 0.0630289486950508 | 0.18048044401084 | 0.72973026914289 | -0.290712722 | 0.416770618956297 | 1.06505767081476 | 0.747730454000093 | 1.51705448947947 | 0.999714303421225 |
| ebi-fl187-GCST90094852 | prot-a-2466 | Glutaminyl-peptide cyclotransferase-like protein \|\| id:prot-a-2466 | F-strong vegetable liking (derived food-liking factor) \|\| id:ebi-fl187-GCST90094852 | Weighted mode | 27 | 0.0837623429448771 | 0.186626751595659 | 0.657278077178363 | -0.28202609 | 0.449550776072368 | 1.08737044184154 | 0.754254005651632 | 1.56760782035113 | 0.999714303421225 |
| ebi-fl187-GCST90094852 | prot-a-2474 | Ras-related protein Rab-27A \|\| id:prot-a-2474 | F-strong vegetable liking (derived food-liking factor) \|\| id:ebi-fl187-GCST90094852 | Weighted mode | 27 | -0.421803686 | 0.208321183 | 0.0532741077152551 | -0.830113204 | -0.013494168 | 0.65586278184881 | 0.435999926738438 | 0.9865964699405 | 0.999714303421225 |
| ebi-fl187-GCST90094852 | prot-a-2490 | Rap1 GTPase-GDP dissociation stimulator 1 \|\| id:prot-a-2490 | F-strong vegetable liking (derived food-liking factor) \|\| id:ebi-fl187-GCST90094852 | Weighted mode | 27 | 0.085732325466604 | 0.190405611031277 | 0.656253952014143 | -0.287462672 | 0.458927323087907 | 1.08951465394335 | 0.750164568275315 | 1.58237569642404 | 0.999714303421225 |
| ebi-fl187-GCST90094852 | prot-a-2545 | Receptor-interacting serine/threonine-protein kinase 2 \|\| id:prot-a-2545 | F-strong vegetable liking (derived food-liking factor) \|\| id:ebi-fl187-GCST90094852 | Weighted mode | 27 | 0.230934417405739 | 0.214283266658702 | 0.291062738849231 | -0.189060785 | 0.650929620056795 | 1.25977661732122 | 0.827736191018095 | 1.91732238214362 | 0.999714303421225 |
| ebi-fl187-GCST90094852 | prot-a-2610 | RUN and FYVE domain-containing protein 1 \|\| id:prot-a-2610 | F-strong vegetable liking (derived food-liking factor) \|\| id:ebi-fl187-GCST90094852 | Weighted mode | 27 | -0.302485775 | 0.197831147127627 | 0.138338320516671 | -0.690234823 | 0.0852632736781024 | 0.738979000388027 | 0.501458301265433 | 1.08900373497941 | 0.999714303421225 |
| ebi-fl187-GCST90094852 | prot-a-2627 | Serine--tRNA ligase, mitochondrial \|\| id:prot-a-2627 | F-strong vegetable liking (derived food-liking factor) \|\| id:ebi-fl187-GCST90094852 | Weighted mode | 27 | 0.137696550964093 | 0.17416300408311 | 0.43631910939724 | -0.203662937 | 0.479056038966989 | 1.14762725104841 | 0.815737279677002 | 1.61454961071587 | 0.999714303421225 |
| ebi-fl187-GCST90094852 | prot-a-264 | BolA-like protein 2 \|\| id:prot-a-264 | F-strong vegetable liking (derived food-liking factor) \|\| id:ebi-fl187-GCST90094852 | Weighted mode | 26 | 0.0387950349170241 | 0.188577392386697 | 0.838671989200972 | -0.330816654 | 0.408406723994949 | 1.03955738884594 | 0.718336861042155 | 1.50441892002641 | 0.999714303421225 |
| ebi-fl187-GCST90094852 | prot-a-2646 | Secretoglobin family 1C member 1 \|\| id:prot-a-2646 | F-strong vegetable liking (derived food-liking factor) \|\| id:ebi-fl187-GCST90094852 | Weighted mode | 26 | 0.0412602557072435 | 0.1820715375942 | 0.822565378166009 | -0.315599958 | 0.398120469391876 | 1.04212328878901 | 0.729351162929464 | 1.4890234008463 | 0.999714303421225 |
| ebi-fl187-GCST90094852 | prot-a-2672 | Semaphorin-3E \|\| id:prot-a-2672 | F-strong vegetable liking (derived food-liking factor) \|\| id:ebi-fl187-GCST90094852 | Weighted mode | 27 | -0.276230404 | 0.193033731171572 | 0.164342021582337 | -0.654576517 | 0.102115709301478 | 0.758638117522039 | 0.519662084111026 | 1.10751161370938 | 0.999714303421225 |
| ebi-fl187-GCST90094852 | prot-a-2701 | Plasma protease C1 inhibitor \|\| id:prot-a-2701 | F-strong vegetable liking (derived food-liking factor) \|\| id:ebi-fl187-GCST90094852 | Weighted mode | 27 | 0.0883742890141095 | 0.15996133440131 | 0.58534316684438 | -0.225149926 | 0.401898504440677 | 1.0923969176849 | 0.798396509061456 | 1.49465962366278 | 0.999714303421225 |
| ebi-fl187-GCST90094852 | prot-a-2738 | Signaling threshold-regulating transmembrane adapter 1 \|\| id:prot-a-2738 | F-strong vegetable liking (derived food-liking factor) \|\| id:ebi-fl187-GCST90094852 | Weighted mode | 27 | -0.08346836 | 0.206567136927175 | 0.68946304286523 | -0.488339948 | 0.321403228289978 | 0.919920192405374 | 0.613644230167089 | 1.37906154542464 | 0.999714303421225 |
| ebi-fl187-GCST90094852 | prot-a-274 | Serine/threonine-protein kinase BRSK2 \|\| id:prot-a-274 | F-strong vegetable liking (derived food-liking factor) \|\| id:ebi-fl187-GCST90094852 | Weighted mode | 27 | -0.126730511 | 0.165886156730466 | 0.451768338656649 | -0.451867378 | 0.19840635655443 | 0.880971052937609 | 0.636438569993152 | 1.21945782783459 | 0.999714303421225 |
| ebi-fl187-GCST90094852 | prot-a-2749 | Mitochondrial glutamate carrier 2 \|\| id:prot-a-2749 | F-strong vegetable liking (derived food-liking factor) \|\| id:ebi-fl187-GCST90094852 | Weighted mode | 27 | 0.214387960821516 | 0.189860275826315 | 0.269129552960726 | -0.15773818 | 0.586514101441094 | 1.23910328508299 | 0.854073366 | 1.79771084262809 | 0.999714303421225 |
| ebi-fl187-GCST90094852 | prot-a-2760 | Sodium-coupled monocarboxylate transporter 1 \|\| id:prot-a-2760 | F-strong vegetable liking (derived food-liking factor) \|\| id:ebi-fl187-GCST90094852 | Weighted mode | 27 | -0.111288665 | 0.170305142792382 | 0.519193497047488 | -0.445086745 | 0.222509414459129 | 0.894680448347637 | 0.640768689957523 | 1.24920758 | 0.999714303421225 |
| ebi-fl187-GCST90094852 | prot-a-2777 | Small integral membrane protein 10 \|\| id:prot-a-2777 | F-strong vegetable liking (derived food-liking factor) \|\| id:ebi-fl187-GCST90094852 | Weighted mode | 27 | 0.237448021661538 | 0.186572948477206 | 0.214394530445217 | -0.128234957 | 0.603131000676862 | 1.26800908607232 | 0.879646675407194 | 1.82783279618226 | 0.999714303421225 |
| ebi-fl187-GCST90094852 | prot-a-2799 | Superoxide dismutase [Mn], mitochondrial \|\| id:prot-a-2799 | F-strong vegetable liking (derived food-liking factor) \|\| id:ebi-fl187-GCST90094852 | Weighted mode | 26 | 0.372774664391219 | 0.202236308799253 | 0.0771781322166939 | -0.023608501 | 0.769157829637754 | 1.45175717038691 | 0.976667999606438 | 2.15794812834974 | 0.999714303421225 |
| ebi-fl187-GCST90094852 | prot-a-2805 | Protein SPACA7 \|\| id:prot-a-2805 | F-strong vegetable liking (derived food-liking factor) \|\| id:ebi-fl187-GCST90094852 | Weighted mode | 27 | -0.020846795 | 0.190284591853158 | 0.913602825171769 | -0.393804595 | 0.352111005160464 | 0.979368997429143 | 0.674485841334919 | 1.42206637166322 | 0.999714303421225 |
| ebi-fl187-GCST90094852 | prot-a-2811 | SPARC-like protein 1 \|\| id:prot-a-2811 | F-strong vegetable liking (derived food-liking factor) \|\| id:ebi-fl187-GCST90094852 | Weighted mode | 27 | -0.122090488 | 0.197580689274171 | 0.541998608286269 | -0.509348639 | 0.265167662534272 | 0.885068276428605 | 0.600886845374456 | 1.30364953064024 | 0.999714303421225 |
| ebi-fl187-GCST90094852 | prot-a-2831 | Sepiapterin reductase \|\| id:prot-a-2831 | F-strong vegetable liking (derived food-liking factor) \|\| id:ebi-fl187-GCST90094852 | Weighted mode | 27 | 0.0716483356747508 | 0.205624771137412 | 0.730313866542707 | -0.331376216 | 0.474672887104078 | 1.07427749255971 | 0.717935019761353 | 1.60748828132663 | 0.999714303421225 |
| ebi-fl187-GCST90094852 | prot-a-2834 | SPRY domain-containing SOCS box protein 1 \|\| id:prot-a-2834 | F-strong vegetable liking (derived food-liking factor) \|\| id:ebi-fl187-GCST90094852 | Weighted mode | 27 | -0.250868081 | 0.175219837120555 | 0.164132606099183 | -0.594298962 | 0.0925627997599207 | 0.77812501426513 | 0.55194936788797 | 1.0969820296053 | 0.999714303421225 |
| ebi-fl187-GCST90094852 | prot-a-2835 | Spectrin alpha chain, non-erythrocytic 1 \|\| id:prot-a-2835 | F-strong vegetable liking (derived food-liking factor) \|\| id:ebi-fl187-GCST90094852 | Weighted mode | 27 | -0.176917705 | 0.201194334087856 | 0.387273475578829 | -0.571258599 | 0.217423190270697 | 0.837848732831511 | 0.564814116476635 | 1.24286996133605 | 0.999714303421225 |
| ebi-fl187-GCST90094852 | prot-a-2859 | Sia-alpha-2,3-Gal-beta-1,4-GlcNAc-R:alpha 2,8-sialyltransferase \|\| id:prot-a-2859 | F-strong vegetable liking (derived food-liking factor) \|\| id:ebi-fl187-GCST90094852 | Weighted mode | 27 | -0.111303971 | 0.191551311966073 | 0.56619943908096 | -0.486744543 | 0.264136600026787 | 0.894666754462064 | 0.614624022974325 | 1.30230607919652 | 0.999714303421225 |
| ebi-fl187-GCST90094852 | prot-a-2861 | Alpha-2,8-sialyltransferase 8F \|\| id:prot-a-2861 | F-strong vegetable liking (derived food-liking factor) \|\| id:ebi-fl187-GCST90094852 | Weighted mode | 27 | -0.041271733 | 0.165834214776802 | 0.805414129369332 | -0.366306794 | 0.283763327763633 | 0.959568347941091 | 0.693290071233235 | 1.32811856476253 | 0.999714303421225 |
| ebi-fl187-GCST90094852 | prot-a-289 | Uncharacterized protein C17orf89 \|\| id:prot-a-289 | F-strong vegetable liking (derived food-liking factor) \|\| id:ebi-fl187-GCST90094852 | Weighted mode | 27 | 0.0799844144603603 | 0.186041757645873 | 0.670790980550082 | -0.284657431 | 0.444626259446272 | 1.08327018419299 | 0.752271915578184 | 1.55990708633541 | 0.999714303421225 |
| ebi-fl187-GCST90094852 | prot-a-2921 | Tachykinin-3 \|\| id:prot-a-2921 | F-strong vegetable liking (derived food-liking factor) \|\| id:ebi-fl187-GCST90094852 | Weighted mode | 27 | 0.119519903415217 | 0.18641266367318 | 0.527032114992096 | -0.245848917 | 0.48488872421465 | 1.1269556741107 | 0.78204036870507 | 1.62399428754971 | 0.999714303421225 |
| ebi-fl187-GCST90094852 | prot-a-2928 | Tax1-binding protein 1 \|\| id:prot-a-2928 | F-strong vegetable liking (derived food-liking factor) \|\| id:ebi-fl187-GCST90094852 | Weighted mode | 27 | 0.0598643780239363 | 0.17634354202813 | 0.736978216707023 | -0.285768964 | 0.405497720399071 | 1.06169254793957 | 0.751436204444576 | 1.50004891923404 | 0.999714303421225 |
| ebi-fl187-GCST90094852 | prot-a-296 | Uncharacterized protein C1orf185 \|\| id:prot-a-296 | F-strong vegetable liking (derived food-liking factor) \|\| id:ebi-fl187-GCST90094852 | Weighted mode | 27 | -0.013688597 | 0.193079498904822 | 0.944022913035953 | -0.392124415 | 0.364747221124274 | 0.986404666080383 | 0.675620051744698 | 1.44014992265627 | 0.999714303421225 |
| ebi-fl187-GCST90094852 | prot-a-2964 | Transforming growth factor beta-2 \|\| id:prot-a-2964 | F-strong vegetable liking (derived food-liking factor) \|\| id:ebi-fl187-GCST90094852 | Weighted mode | 27 | 0.0276975701961768 | 0.176170729508264 | 0.876286005114673 | -0.31759706 | 0.372992200032375 | 1.02808471394207 | 0.727896028019149 | 1.45207301366611 | 0.999714303421225 |
| ebi-fl187-GCST90094852 | prot-a-2977 | Thymocyte nuclear protein 1 \|\| id:prot-a-2977 | F-strong vegetable liking (derived food-liking factor) \|\| id:ebi-fl187-GCST90094852 | Weighted mode | 26 | 0.0702826561598178 | 0.170537732307076 | 0.683761194545871 | -0.263971299 | 0.404536611481686 | 1.07281137514589 | 0.767995581457125 | 1.4986079014397 | 0.999714303421225 |
| ebi-fl187-GCST90094852 | prot-a-3015 | Transmembrane protein 70, mitochondrial \|\| id:prot-a-3015 | F-strong vegetable liking (derived food-liking factor) \|\| id:ebi-fl187-GCST90094852 | Weighted mode | 27 | -0.034989496 | 0.173552974179988 | 0.841793015667039 | -0.375153326 | 0.305174332981698 | 0.965615558633185 | 0.687183907688083 | 1.35686152810451 | 0.999714303421225 |
| ebi-fl187-GCST90094852 | prot-a-3030 | Tumor necrosis factor alpha-induced protein 3 \|\| id:prot-a-3030 | F-strong vegetable liking (derived food-liking factor) \|\| id:ebi-fl187-GCST90094852 | Weighted mode | 27 | -0.180386021 | 0.209437483561512 | 0.396953164940186 | -0.590883489 | 0.230111447022631 | 0.83494784199583 | 0.553837759208805 | 1.25874028496974 | 0.999714303421225 |
| ebi-fl187-GCST90094852 | prot-a-3032 | Tumor necrosis factor receptor superfamily member 10A \|\| id:prot-a-3032 | F-strong vegetable liking (derived food-liking factor) \|\| id:ebi-fl187-GCST90094852 | Weighted mode | 27 | -0.12505627 | 0.175495501784119 | 0.482446330896735 | -0.469027454 | 0.218914913408843 | 0.88244724580331 | 0.625610407672105 | 1.24472536274362 | 0.999714303421225 |
| ebi-fl187-GCST90094852 | prot-a-3081 | Tumor protein p53-inducible protein 11 \|\| id:prot-a-3081 | F-strong vegetable liking (derived food-liking factor) \|\| id:ebi-fl187-GCST90094852 | Weighted mode | 27 | -0.06695661 | 0.170374496387158 | 0.69752576843275 | -0.400890623 | 0.266977403023159 | 0.93523578043449 | 0.669723309482662 | 1.30601093409838 | 0.999714303421225 |
| ebi-fl187-GCST90094852 | prot-a-315 | Complement C4 \|\| id:prot-a-315 | F-strong vegetable liking (derived food-liking factor) \|\| id:ebi-fl187-GCST90094852 | Weighted mode | 27 | -0.312702028 | 0.217650805342561 | 0.162724403519592 | -0.739297607 | 0.113893550002821 | 0.731467836691147 | 0.477449154745187 | 1.12063282717335 | 0.999714303421225 |
| ebi-fl187-GCST90094852 | prot-a-3157 | UDP-glucuronosyltransferase 1-8 \|\| id:prot-a-3157 | F-strong vegetable liking (derived food-liking factor) \|\| id:ebi-fl187-GCST90094852 | Weighted mode | 26 | 0.146248889124667 | 0.186183461628376 | 0.439536457011183 | -0.218670696 | 0.511168473916285 | 1.1574842374418 | 0.803586299037672 | 1.66723818155021 | 0.999714303421225 |
| ebi-fl187-GCST90094852 | prot-a-317 | Glutaredoxin-like protein C5orf63 \|\| id:prot-a-317 | F-strong vegetable liking (derived food-liking factor) \|\| id:ebi-fl187-GCST90094852 | Weighted mode | 27 | -0.23370774 | 0.235932709167015 | 0.331025584943241 | -0.69613585 | 0.228720369598792 | 0.791593132808887 | 0.498507895925835 | 1.25699049710421 | 0.999714303421225 |
| ebi-fl187-GCST90094852 | prot-a-3192 | Guanine nucleotide exchange factor VAV3 \|\| id:prot-a-3192 | F-strong vegetable liking (derived food-liking factor) \|\| id:ebi-fl187-GCST90094852 | Weighted mode | 27 | -0.077560788 | 0.177849269103117 | 0.66636100361709 | -0.426145356 | 0.271023779231535 | 0.925370771053595 | 0.653021414867763 | 1.31130625186883 | 0.999714303421225 |
| ebi-fl187-GCST90094852 | prot-a-3233 | Serine/threonine-protein kinase WNK3 \|\| id:prot-a-3233 | F-strong vegetable liking (derived food-liking factor) \|\| id:ebi-fl187-GCST90094852 | Weighted mode | 26 | -0.261379103 | 0.208394095550723 | 0.221350698148582 | -0.66983153 | 0.147073324312725 | 0.769988959178297 | 0.511794792466784 | 1.15843890165208 | 0.999714303421225 |
| ebi-fl187-GCST90094852 | prot-a-3250 | 14-3-3 protein theta \|\| id:prot-a-3250 | F-strong vegetable liking (derived food-liking factor) \|\| id:ebi-fl187-GCST90094852 | Weighted mode | 27 | 0.00668982889487446 | 0.158885521342456 | 0.966736906920649 | -0.304725793 | 0.318105450726089 | 1.00671225578298 | 0.737325526542182 | 1.37452119784393 | 0.999714303421225 |
| ebi-fl187-GCST90094852 | prot-a-3278 | E3 ubiquitin-protein ligase ZNRF3 \|\| id:prot-a-3278 | F-strong vegetable liking (derived food-liking factor) \|\| id:ebi-fl187-GCST90094852 | Weighted mode | 27 | 0.225337678996576 | 0.221292141593825 | 0.317926619235915 | -0.208394919 | 0.659070276520472 | 1.25274567067746 | 0.811886344432951 | 1.9329943484851 | 0.999714303421225 |
| ebi-fl187-GCST90094852 | prot-a-355 | Calpain-3 \|\| id:prot-a-355 | F-strong vegetable liking (derived food-liking factor) \|\| id:ebi-fl187-GCST90094852 | Weighted mode | 27 | 0.0911654000884539 | 0.174738168680239 | 0.606277749364105 | -0.251321411 | 0.433652210701722 | 1.09545017783138 | 0.777772347162586 | 1.54288217688454 | 0.999714303421225 |
| ebi-fl187-GCST90094852 | prot-a-385 | Coiled-coil domain-containing protein 90B, mitochondrial \|\| id:prot-a-385 | F-strong vegetable liking (derived food-liking factor) \|\| id:ebi-fl187-GCST90094852 | Weighted mode | 27 | 0.183605178096998 | 0.189559121358575 | 0.341674005161701 | -0.1879307 | 0.555141055959805 | 1.20154133459698 | 0.828672132415712 | 1.74218671326193 | 0.999714303421225 |
| ebi-fl187-GCST90094852 | prot-a-390 | C-C motif chemokine 14 \|\| id:prot-a-390 | F-strong vegetable liking (derived food-liking factor) \|\| id:ebi-fl187-GCST90094852 | Weighted mode | 27 | -0.241521734 | 0.250188106608748 | 0.343258240969761 | -0.731890423 | 0.248846955286357 | 0.785431733291907 | 0.480998838990749 | 1.28254573120455 | 0.999714303421225 |
| ebi-fl187-GCST90094852 | prot-a-40 | Pituitary adenylate cyclase-activating polypeptide 27 \|\| id:prot-a-40 | F-strong vegetable liking (derived food-liking factor) \|\| id:ebi-fl187-GCST90094852 | Weighted mode | 27 | 0.0776469131000181 | 0.179998178052548 | 0.669750752744881 | -0.275149516 | 0.430443342083012 | 1.08074099580608 | 0.759458563617975 | 1.5379392055989 | 0.999714303421225 |
| ebi-fl187-GCST90094852 | prot-a-401 | C-C motif chemokine 24 \|\| id:prot-a-401 | F-strong vegetable liking (derived food-liking factor) \|\| id:ebi-fl187-GCST90094852 | Weighted mode | 27 | 0.263236061011801 | 0.168692205117782 | 0.130743928220873 | -0.067400661 | 0.593872783042653 | 1.30113382967018 | 0.934820580127466 | 1.81098841713707 | 0.999714303421225 |
| ebi-fl187-GCST90094852 | prot-a-412 | Cerebral cavernous malformations 2 protein \|\| id:prot-a-412 | F-strong vegetable liking (derived food-liking factor) \|\| id:ebi-fl187-GCST90094852 | Weighted mode | 27 | 0.185747718747882 | 0.175477931518661 | 0.299552012276866 | -0.158189027 | 0.529684464524457 | 1.20411844554671 | 0.853688396534831 | 1.69839631977083 | 0.999714303421225 |
| ebi-fl187-GCST90094852 | prot-a-44 | Adhesion G protein-coupled receptor F5 \|\| id:prot-a-44 | F-strong vegetable liking (derived food-liking factor) \|\| id:ebi-fl187-GCST90094852 | Weighted mode | 27 | -0.044729774 | 0.199405888073549 | 0.824265487596018 | -0.435565314 | 0.34610576686193 | 0.956255852343611 | 0.646898862456989 | 1.41355211488288 | 0.999714303421225 |
| ebi-fl187-GCST90094852 | prot-a-48 | Adrenomedullin \|\| id:prot-a-48 | F-strong vegetable liking (derived food-liking factor) \|\| id:ebi-fl187-GCST90094852 | Weighted mode | 27 | 0.0112643544200901 | 0.209018186694499 | 0.957433381056208 | -0.398411292 | 0.420940000341309 | 1.0113280361469 | 0.671385835580023 | 1.52339287261421 | 0.999714303421225 |
| ebi-fl187-GCST90094852 | prot-a-502 | Carcinoembryonic antigen-related cell adhesion molecule 1 \|\| id:prot-a-502 | F-strong vegetable liking (derived food-liking factor) \|\| id:ebi-fl187-GCST90094852 | Weighted mode | 27 | -0.076151617 | 0.17300080955635 | 0.663443997806287 | -0.415233204 | 0.262929969812101 | 0.926675696194414 | 0.660186304896635 | 1.30073562500187 | 0.999714303421225 |
| ebi-fl187-GCST90094852 | prot-a-518 | Complement factor B \|\| id:prot-a-518 | F-strong vegetable liking (derived food-liking factor) \|\| id:ebi-fl187-GCST90094852 | Weighted mode | 27 | 0.000747606914172305 | 0.21313588315518 | 0.997228078083244 | -0.416998724 | 0.418493937898325 | 1.00074788644188 | 0.659021760809233 | 1.51967111220746 | 0.999714303421225 |
| ebi-fl187-GCST90094852 | prot-a-525 | Properdin \|\| id:prot-a-525 | F-strong vegetable liking (derived food-liking factor) \|\| id:ebi-fl187-GCST90094852 | Weighted mode | 27 | 0.421176743836757 | 0.374287844024588 | 0.270753373774123 | -0.31242743 | 1.15478091812495 | 1.52375356866235 | 0.731668723889071 | 3.17332812269187 | 0.999714303421225 |
| ebi-fl187-GCST90094852 | prot-a-533 | Chondroadherin \|\| id:prot-a-533 | F-strong vegetable liking (derived food-liking factor) \|\| id:ebi-fl187-GCST90094852 | Weighted mode | 27 | -0.090296997 | 0.1649068237262 | 0.588661655876548 | -0.413514372 | 0.232920377067611 | 0.913659790356551 | 0.661322029832205 | 1.26228096881361 | 0.999714303421225 |
| ebi-fl187-GCST90094852 | prot-a-535 | Coiled-coil-helix-coiled-coil-helix domain-containing protein 2 \|\| id:prot-a-535 | F-strong vegetable liking (derived food-liking factor) \|\| id:ebi-fl187-GCST90094852 | Weighted mode | 27 | 0.166829619405295 | 0.306934146378934 | 0.591391529497576 | -0.434761307 | 0.768420546308006 | 1.18155293446576 | 0.647419182741385 | 2.15635769554009 | 0.999714303421225 |
| ebi-fl187-GCST90094852 | prot-a-591 | Caseinolytic peptidase B protein homolog \|\| id:prot-a-591 | F-strong vegetable liking (derived food-liking factor) \|\| id:ebi-fl187-GCST90094852 | Weighted mode | 27 | 0.0365595781846522 | 0.169216182731707 | 0.830632924289058 | -0.29510414 | 0.368223296338798 | 1.03723609882074 | 0.744454056006235 | 1.44516470293482 | 0.999714303421225 |
| ebi-fl187-GCST90094852 | prot-a-605 | Ciliary neurotrophic factor receptor subunit alpha \|\| id:prot-a-605 | F-strong vegetable liking (derived food-liking factor) \|\| id:ebi-fl187-GCST90094852 | Weighted mode | 27 | -0.276493782 | 0.214632180707326 | 0.209015838106368 | -0.697172856 | 0.144185292562605 | 0.758438335371936 | 0.497991208458993 | 1.15509811978763 | 0.999714303421225 |
| ebi-fl187-GCST90094852 | prot-a-606 | Ciliary neurotrophic factor receptor subunit alpha \|\| id:prot-a-606 | F-strong vegetable liking (derived food-liking factor) \|\| id:ebi-fl187-GCST90094852 | Weighted mode | 27 | -0.124048519 | 0.179665982091396 | 0.496035647133057 | -0.476193844 | 0.228096805493003 | 0.88333698085726 | 0.62114306553078 | 1.25620692727729 | 0.999714303421225 |
| ebi-fl187-GCST90094852 | prot-a-707 | Cancer/testis antigen 55 \|\| id:prot-a-707 | F-strong vegetable liking (derived food-liking factor) \|\| id:ebi-fl187-GCST90094852 | Weighted mode | 27 | -0.116073314 | 0.181267877238913 | 0.527554223818899 | -0.471358353 | 0.239211725287003 | 0.890409941299852 | 0.624153870613029 | 1.27024745162104 | 0.999714303421225 |
| ebi-fl187-GCST90094852 | prot-a-719 | Dipeptidyl peptidase 1 \|\| id:prot-a-719 | F-strong vegetable liking (derived food-liking factor) \|\| id:ebi-fl187-GCST90094852 | Weighted mode | 27 | 0.111660799583283 | 0.215317311 | 0.60843448748163 | -0.310361131 | 0.533682729764603 | 1.11813352495566 | 0.733182133906959 | 1.7052005522388 | 0.999714303421225 |
| ebi-fl187-GCST90094852 | prot-a-752 | Cytochrome b5 \|\| id:prot-a-752 | F-strong vegetable liking (derived food-liking factor) \|\| id:ebi-fl187-GCST90094852 | Weighted mode | 27 | 0.0588008961925937 | 0.211340525006096 | 0.783037907400148 | -0.355426533 | 0.473028325204541 | 1.06056405737533 | 0.700874433496246 | 1.60484683994747 | 0.999714303421225 |
| ebi-fl187-GCST90094852 | prot-a-755 | Cytoglobin \|\| id:prot-a-755 | F-strong vegetable liking (derived food-liking factor) \|\| id:ebi-fl187-GCST90094852 | Weighted mode | 27 | -0.104681328 | 0.229594673739332 | 0.65221880921993 | -0.554686889 | 0.345324232441173 | 0.900611476443011 | 0.574252037899673 | 1.41244780683315 | 0.999714303421225 |
| ebi-fl187-GCST90094852 | prot-a-807 | Desmin \|\| id:prot-a-807 | F-strong vegetable liking (derived food-liking factor) \|\| id:ebi-fl187-GCST90094852 | Weighted mode | 27 | 0.172540003510883 | 0.197820366523089 | 0.391079950487506 | -0.215187915 | 0.560267921896137 | 1.18831935661049 | 0.806389893283325 | 1.75114160663102 | 0.999714303421225 |
| ebi-fl187-GCST90094852 | prot-a-82 | Ameloblastin \|\| id:prot-a-82 | F-strong vegetable liking (derived food-liking factor) \|\| id:ebi-fl187-GCST90094852 | Weighted mode | 27 | 0.0307412644985576 | 0.180509260376896 | 0.866090168904722 | -0.323056886 | 0.384539414837274 | 1.03121865649038 | 0.723932671678324 | 1.46893759474686 | 0.999714303421225 |
| ebi-fl187-GCST90094852 | prot-a-84 | Muellerian-inhibiting factor \|\| id:prot-a-84 | F-strong vegetable liking (derived food-liking factor) \|\| id:ebi-fl187-GCST90094852 | Weighted mode | 27 | 0.0278999074217017 | 0.178163039794695 | 0.876771635586407 | -0.321299651 | 0.377099465419304 | 1.02829275479719 | 0.725205910060004 | 1.4580493276467 | 0.999714303421225 |
| ebi-fl187-GCST90094852 | prot-a-858 | Inactive dipeptidyl peptidase 10 \|\| id:prot-a-858 | F-strong vegetable liking (derived food-liking factor) \|\| id:ebi-fl187-GCST90094852 | Weighted mode | 27 | 0.194263256219396 | 0.166775433273279 | 0.254668591685406 | -0.132616593 | 0.521143105435023 | 1.21441594342812 | 0.875800815911957 | 1.68395148401036 | 0.999714303421225 |
| ebi-fl187-GCST90094852 | prot-a-863 | Dihydropyrimidinase-related protein 3 \|\| id:prot-a-863 | F-strong vegetable liking (derived food-liking factor) \|\| id:ebi-fl187-GCST90094852 | Weighted mode | 27 | 0.0360712790109816 | 0.170697060626111 | 0.834287147276136 | -0.29849496 | 0.370637517838159 | 1.03672974092792 | 0.74193402132452 | 1.44865786556828 | 0.999714303421225 |
| ebi-fl187-GCST90094852 | prot-a-864 | Dihydropyrimidinase-related protein 5 \|\| id:prot-a-864 | F-strong vegetable liking (derived food-liking factor) \|\| id:ebi-fl187-GCST90094852 | Weighted mode | 27 | -0.047469972 | 0.167656663912631 | 0.779313229615481 | -0.376077033 | 0.281137089241799 | 0.953639108552136 | 0.686549443838883 | 1.32463518472172 | 0.999714303421225 |
| ebi-fl187-GCST90094852 | prot-a-884 | Endothelin-converting enzyme 1 \|\| id:prot-a-884 | F-strong vegetable liking (derived food-liking factor) \|\| id:ebi-fl187-GCST90094852 | Weighted mode | 27 | 0.0572605003288225 | 0.169942659082494 | 0.738866793808959 | -0.275827111 | 0.390348112130511 | 1.05893162650575 | 0.758944132152492 | 1.47749503831569 | 0.999714303421225 |
| ebi-fl187-GCST90094852 | prot-a-893 | Early endosome antigen 1 \|\| id:prot-a-893 | F-strong vegetable liking (derived food-liking factor) \|\| id:ebi-fl187-GCST90094852 | Weighted mode | 27 | 0.189129126074611 | 0.192235537214828 | 0.33426123170336 | -0.187652527 | 0.565910779015673 | 1.20819695214066 | 0.828902678609768 | 1.76105098081026 | 0.999714303421225 |
| ebi-fl187-GCST90094852 | prot-a-897 | Ephrin-A2 \|\| id:prot-a-897 | F-strong vegetable liking (derived food-liking factor) \|\| id:ebi-fl187-GCST90094852 | Weighted mode | 27 | 0.234985934755624 | 0.231600802351457 | 0.319638185412207 | -0.218951638 | 0.688923507364481 | 1.26489097760707 | 0.80336056945592 | 1.99157046793488 | 0.999714303421225 |
| ebi-fl187-GCST90094852 | prot-a-90 | Anaphase-promoting complex subunit 10 \|\| id:prot-a-90 | F-strong vegetable liking (derived food-liking factor) \|\| id:ebi-fl187-GCST90094852 | Weighted mode | 27 | 0.181044002476407 | 0.192251021771185 | 0.355011223102087 | -0.195768 | 0.557856005147929 | 1.19846791368907 | 0.822202963479912 | 1.7469230882641 | 0.999714303421225 |
| ebi-fl187-GCST90094852 | prot-a-914 | Histone-lysine N-methyltransferase EHMT2 \|\| id:prot-a-914 | F-strong vegetable liking (derived food-liking factor) \|\| id:ebi-fl187-GCST90094852 | Weighted mode | 27 | 0.151077558089588 | 0.204923024293258 | 0.467577560680087 | -0.25057157 | 0.552726685704373 | 1.16308686137877 | 0.778355771467252 | 1.73798550316118 | 0.999714303421225 |
| ebi-fl187-GCST90094852 | prot-a-932 | RNA polymerase II elongation factor ELL \|\| id:prot-a-932 | F-strong vegetable liking (derived food-liking factor) \|\| id:ebi-fl187-GCST90094852 | Weighted mode | 27 | -0.009521796 | 0.178629268626596 | 0.957896529413383 | -0.359635162 | 0.340591570693589 | 0.990523392943451 | 0.697930911119649 | 1.40577896226752 | 0.999714303421225 |
| ebi-fl187-GCST90094852 | prot-a-991 | Estrogen receptor \|\| id:prot-a-991 | F-strong vegetable liking (derived food-liking factor) \|\| id:ebi-fl187-GCST90094852 | Weighted mode | 27 | -0.045296565 | 0.202782221633033 | 0.82498910031541 | -0.442749719 | 0.352156589646839 | 0.955714008711312 | 0.642267934335027 | 1.42213119730588 | 0.999714303421225 |
| ebi-fl187-GCST90094852 | prot-a-992 | Persulfide dioxygenase ETHE1, mitochondrial \|\| id:prot-a-992 | F-strong vegetable liking (derived food-liking factor) \|\| id:ebi-fl187-GCST90094852 | Weighted mode | 27 | -0.297400804 | 0.175967854782852 | 0.10296844081005 | -0.6422978 | 0.047496191047109 | 0.742746256781274 | 0.526082202624082 | 1.04864220688491 | 0.999714303421225 |
| ebi-fl187-GCST90094852 | prot-a-993 | Ethanolamine kinase 1 \|\| id:prot-a-993 | F-strong vegetable liking (derived food-liking factor) \|\| id:ebi-fl187-GCST90094852 | Weighted mode | 26 | 0.0963539477749518 | 0.188992261720436 | 0.61464207071226 | -0.274070885 | 0.466778780747007 | 1.10114874417459 | 0.760278180882043 | 1.59484855318423 | 0.999714303421225 |
| ebi-fl187-GCST90094852 | prot-b-10 | coagulation factor III, tissue factor \|\| id:prot-b-10 | F-strong vegetable liking (derived food-liking factor) \|\| id:ebi-fl187-GCST90094852 | Weighted mode | 20 | -0.010702267 | 0.252926253514156 | 0.966690067625107 | -0.506437724 | 0.485033190182399 | 0.989354798792811 | 0.602638524661711 | 1.62422891640362 | 0.999714303421225 |
| ebi-fl187-GCST90094852 | prot-b-28 | prolactin \|\| id:prot-b-28 | F-strong vegetable liking (derived food-liking factor) \|\| id:ebi-fl187-GCST90094852 | Weighted mode | 20 | 0.0089343555249497 | 0.238271113372798 | 0.970480227001077 | -0.458077027 | 0.475945737735635 | 1.00897438600598 | 0.63249875504814 | 1.60953567653845 | 0.999714303421225 |
| ebi-fl187-GCST90094852 | prot-b-54 | matrix metallopeptidase 7 \|\| id:prot-b-54 | F-strong vegetable liking (derived food-liking factor) \|\| id:ebi-fl187-GCST90094852 | Weighted mode | 20 | -0.146166292 | 0.243969551318539 | 0.556171880350162 | -0.624346613 | 0.332014028505372 | 0.864014012551266 | 0.535611275838666 | 1.39377240091898 | 0.999714303421225 |
| ebi-fl187-GCST90094852 | ukb-rsfMRI191-edge-pheno716 | Net100_Pair10_33 (OccipitalPrecuneus)&(Temporal\|Frontal\|Supp_Motor_Area) (Default_mode\|Central_executive)&(Default_mode\|Salience) \|\| id:ukb-rsfMRI191-edge-pheno716 | F-strong vegetable liking (derived food-liking factor) \|\| id:ebi-fl187-GCST90094852 | Weighted mode | 27 | -0.052309365 | 0.0527537743038997 | 0.330543538854987 | -0.155706763 | 0.0510880322142903 | 0.949035222742004 | 0.855810108720028 | 1.05241553567535 | 0.999714303421225 |
| ebi-fl187-GCST90094852 | ukb-rsfMRI191-edge-pheno903 | Net100_Pair27_38 (Precentral\|Frontal\|Supp_Motor_Area)&(Subcortical) (Attention\|Salience\|Motor)&(Subcortical-cerebellum) \|\| id:ukb-rsfMRI191-edge-pheno903 | F-strong vegetable liking (derived food-liking factor) \|\| id:ebi-fl187-GCST90094852 | Weighted mode | 27 | -0.033344418 | 0.0545442804564856 | 0.546291193507962 | -0.140251207 | 0.0735623719451679 | 0.967205379515601 | 0.869139873566556 | 1.07633566772757 | 0.999714303421225 |
| ebi-fl187-GCST90094852 | ukb-rsfMRI191-node-pheno23 | Net100_Node2 RolandicOper\|SupraMarginal\|Insula Salience\|Motor \|\| id:ukb-rsfMRI191-node-pheno23 | F-strong vegetable liking (derived food-liking factor) \|\| id:ebi-fl187-GCST90094852 | Weighted mode | 27 | -0.081354311 | 0.0563504448695873 | 0.160759898939387 | -0.191801183 | 0.0290925607438213 | 0.921867005768249 | 0.825470969720223 | 1.02951988319123 | 0.999714303421225 |
| ebi-fl187-GCST90094852 | ukb-rsfMRI191-node-pheno55 | Net100_Node34 Parietal\|Postcentral\|Precuneus Attention \|\| id:ukb-rsfMRI191-node-pheno55 | F-strong vegetable liking (derived food-liking factor) \|\| id:ebi-fl187-GCST90094852 | Weighted mode | 27 | -0.050308635 | 0.0541054104909008 | 0.361014295050649 | -0.156355239 | 0.055737969666085 | 0.950935887206391 | 0.855255315965019 | 1.0573205973665 | 0.999714303421225 |
| ebi-fl187-GCST90094853 | ebi-a-GCST90001414 | IgD- CD24- B cell Absolute Count \|\| id:ebi-a-GCST90001414 | F-sweetened coffee drink liking (derived food-liking factor) \|\| id:ebi-fl187-GCST90094853 | Inverse variance weighted (multiplicative random effects) | 4 | -0.066011551 | 0.563954329228803 | 0.906819452078979 | -1.171362037 | 1.03933893403181 | 0.936120050866212 | 0.309944497906048 | 2.8273473333261 | 0.998735809224318 |
| ebi-fl187-GCST90094853 | ebi-a-GCST90001422 | CD20- CD38- B cell %B cell \|\| id:ebi-a-GCST90001422 | F-sweetened coffee drink liking (derived food-liking factor) \|\| id:ebi-fl187-GCST90094853 | Inverse variance weighted | 4 | 0.102338704160436 | 0.432399143927262 | 0.812907788548701 | -0.745163618 | 0.94984102625787 | 1.10775861064398 | 0.474656631247303 | 2.58529863204738 | 0.998735809224318 |
| ebi-fl187-GCST90094853 | ebi-a-GCST90001444 | CD20- CD38- B cell %lymphocyte \|\| id:ebi-a-GCST90001444 | F-sweetened coffee drink liking (derived food-liking factor) \|\| id:ebi-fl187-GCST90094853 | Inverse variance weighted | 4 | 0.10954825701283 | 0.409724972401207 | 0.789184124165658 | -0.693512689 | 0.912609202919196 | 1.11577391355206 | 0.499817279228222 | 2.49081309891012 | 0.998735809224318 |
| ebi-fl187-GCST90094853 | ebi-a-GCST90001459 | Myeloid Dendritic Cell %Dendritic Cell \|\| id:ebi-a-GCST90001459 | F-sweetened coffee drink liking (derived food-liking factor) \|\| id:ebi-fl187-GCST90094853 | Inverse variance weighted | 4 | -0.103541103 | 0.382684794472195 | 0.786725635870188 | -0.8536033 | 0.646521094653345 | 0.901638962353812 | 0.425877599260763 | 1.90888842203859 | 0.998735809224318 |
| ebi-fl187-GCST90094853 | ebi-a-GCST90001476 | HLA DR++ monocyte %leukocyte \|\| id:ebi-a-GCST90001476 | F-sweetened coffee drink liking (derived food-liking factor) \|\| id:ebi-fl187-GCST90094853 | Inverse variance weighted | 4 | -0.408594411 | 0.301563280453907 | 0.175442739103865 | -0.999658441 | 0.182469618536448 | 0.664583725413613 | 0.368005115224636 | 1.20017768724501 | 0.998735809224318 |
| ebi-fl187-GCST90094853 | ebi-a-GCST90001478 | CD4 regulatory T cell %CD4+ T cell \|\| id:ebi-a-GCST90001478 | F-sweetened coffee drink liking (derived food-liking factor) \|\| id:ebi-fl187-GCST90094853 | Inverse variance weighted | 4 | -0.023153444 | 0.303603571141687 | 0.939210581564349 | -0.618216444 | 0.571909554964385 | 0.977112539750493 | 0.538904747800865 | 1.77164688051784 | 0.998735809224318 |
| ebi-fl187-GCST90094853 | ebi-a-GCST90001485 | CD39+ resting CD4 regulatory T cell %CD4 regulatory T cell \|\| id:ebi-a-GCST90001485 | F-sweetened coffee drink liking (derived food-liking factor) \|\| id:ebi-fl187-GCST90094853 | Inverse variance weighted | 4 | 0.213788575912563 | 0.298143922528563 | 0.473333982078175 | -0.370573512 | 0.798150664068546 | 1.23836080781034 | 0.690338299616958 | 2.22142895906482 | 0.998735809224318 |
| ebi-fl187-GCST90094853 | ebi-a-GCST90001515 | Immature Myeloid-Derived Suppressor Cells Absolute Count \|\| id:ebi-a-GCST90001515 | F-sweetened coffee drink liking (derived food-liking factor) \|\| id:ebi-fl187-GCST90094853 | Inverse variance weighted | 4 | -0.752325252 | 0.394555276763063 | 0.0565506775960755 | -1.525653594 | 0.0210030904538253 | 0.471269457474477 | 0.217478867420834 | 1.021225208 | 0.998735809224318 |
| ebi-fl187-GCST90094853 | ebi-a-GCST90001517 | CD33+ HLA DR+ Absolute Count \|\| id:ebi-a-GCST90001517 | F-sweetened coffee drink liking (derived food-liking factor) \|\| id:ebi-fl187-GCST90094853 | Inverse variance weighted | 4 | -0.050678388 | 0.464266056011668 | 0.913077115304913 | -0.960639857 | 0.859283082262092 | 0.950584341162719 | 0.382647967530291 | 2.36146710903993 | 0.998735809224318 |
| ebi-fl187-GCST90094853 | ebi-a-GCST90001518 | CD33+ HLA DR+ CD14- Absolute Count \|\| id:ebi-a-GCST90001518 | F-sweetened coffee drink liking (derived food-liking factor) \|\| id:ebi-fl187-GCST90094853 | Inverse variance weighted | 4 | -0.168925711 | 0.391030990629698 | 0.665740813524971 | -0.935346452 | 0.597495031064622 | 0.844571643801893 | 0.392449876851377 | 1.81756016140722 | 0.998735809224318 |
| ebi-fl187-GCST90094853 | ebi-a-GCST90001520 | CD33+ HLA DR+ CD14dim Absolute Count \|\| id:ebi-a-GCST90001520 | F-sweetened coffee drink liking (derived food-liking factor) \|\| id:ebi-fl187-GCST90094853 | Inverse variance weighted | 4 | -0.076263801 | 0.612279157345634 | 0.900874083808506 | -1.276330949 | 1.12380334767984 | 0.926571744025123 | 0.279059307207351 | 3.07653310479925 | 0.998735809224318 |
| ebi-fl187-GCST90094853 | ebi-a-GCST90001531 | CD33dim HLA DR- Absolute Count \|\| id:ebi-a-GCST90001531 | F-sweetened coffee drink liking (derived food-liking factor) \|\| id:ebi-fl187-GCST90094853 | Inverse variance weighted | 4 | -0.619139662 | 0.403251583486882 | 0.124693407687991 | -1.409512766 | 0.171233441361246 | 0.538407450634578 | 0.244262267068642 | 1.18676775736861 | 0.998735809224318 |
| ebi-fl187-GCST90094853 | ebi-a-GCST90001532 | Basophil Absolute Count \|\| id:ebi-a-GCST90001532 | F-sweetened coffee drink liking (derived food-liking factor) \|\| id:ebi-fl187-GCST90094853 | Inverse variance weighted | 4 | -0.58395319 | 0.393023334669671 | 0.137332623103674 | -1.354278926 | 0.186372546068623 | 0.557689351212061 | 0.258133360657108 | 1.2048710467473 | 0.998735809224318 |
| ebi-fl187-GCST90094853 | ebi-a-GCST90001588 | T/B cell \|\| id:ebi-a-GCST90001588 | F-sweetened coffee drink liking (derived food-liking factor) \|\| id:ebi-fl187-GCST90094853 | Inverse variance weighted | 4 | 0.103660759935081 | 0.313825871366731 | 0.74116348342381 | -0.511437948 | 0.718759467813873 | 1.10922409782653 | 0.599632717952682 | 2.05188620027264 | 0.998735809224318 |
| ebi-fl187-GCST90094853 | ebi-a-GCST90001624 | HLA DR+ CD4+ T cell Absolute Count \|\| id:ebi-a-GCST90001624 | F-sweetened coffee drink liking (derived food-liking factor) \|\| id:ebi-fl187-GCST90094853 | Inverse variance weighted | 4 | 0.0823421977257189 | 0.287989135324254 | 0.774938479829719 | -0.482116508 | 0.646800902961258 | 1.08582731389934 | 0.617475117085446 | 1.90942261961092 | 0.998735809224318 |
| ebi-fl187-GCST90094853 | ebi-a-GCST90001631 | CD8+ Natural Killer T %T cell \|\| id:ebi-a-GCST90001631 | F-sweetened coffee drink liking (derived food-liking factor) \|\| id:ebi-fl187-GCST90094853 | Inverse variance weighted | 4 | -0.130828306 | 0.291450877881178 | 0.653513679393887 | -0.702072027 | 0.440415414429983 | 0.877368400159517 | 0.495557430959377 | 1.55335236948868 | 0.998735809224318 |
| ebi-fl187-GCST90094853 | ebi-a-GCST90001632 | CD8+ Natural Killer T %lymphocyte \|\| id:ebi-a-GCST90001632 | F-sweetened coffee drink liking (derived food-liking factor) \|\| id:ebi-fl187-GCST90094853 | Inverse variance weighted | 4 | -0.098656406 | 0.293235943050024 | 0.736538818956254 | -0.673398854 | 0.476086042279175 | 0.906053969164669 | 0.509972307133177 | 1.60976151754975 | 0.998735809224318 |
| ebi-fl187-GCST90094853 | ebi-a-GCST90001646 | Natural Killer %CD3- lymphocyte \|\| id:ebi-a-GCST90001646 | F-sweetened coffee drink liking (derived food-liking factor) \|\| id:ebi-fl187-GCST90094853 | Inverse variance weighted | 4 | -0.323992614 | 0.286552545134376 | 0.258199525635073 | -0.885635603 | 0.237650374124441 | 0.723255584080723 | 0.412451934342689 | 1.2682656967959 | 0.998735809224318 |
| ebi-fl187-GCST90094853 | ebi-a-GCST90001684 | CD127- CD8+ T cell Absolute Count \|\| id:ebi-a-GCST90001684 | F-sweetened coffee drink liking (derived food-liking factor) \|\| id:ebi-fl187-GCST90094853 | Inverse variance weighted | 4 | -0.046411815 | 0.308833369936942 | 0.880542852849355 | -0.65172522 | 0.558901590123579 | 0.954648742602518 | 0.521145909388575 | 1.74875060004174 | 0.998735809224318 |
| ebi-fl187-GCST90094853 | ebi-a-GCST90001687 | CD28- CD8+ T cell Absolute Count \|\| id:ebi-a-GCST90001687 | F-sweetened coffee drink liking (derived food-liking factor) \|\| id:ebi-fl187-GCST90094853 | Inverse variance weighted | 4 | 0.225751910969711 | 0.300140824351683 | 0.451958875003107 | -0.362524105 | 0.81402792669901 | 1.25326470548145 | 0.695917538551503 | 2.25698065502808 | 0.998735809224318 |
| ebi-fl187-GCST90094853 | ebi-a-GCST90001701 | BAFF-R on CD20- CD38- B cell \|\| id:ebi-a-GCST90001701 | F-sweetened coffee drink liking (derived food-liking factor) \|\| id:ebi-fl187-GCST90094853 | Inverse variance weighted | 4 | 0.117423856048634 | 0.348284798519303 | 0.736004614363729 | -0.565214349 | 0.800062061146468 | 1.12459599550071 | 0.568238332356347 | 2.22567905240002 | 0.998735809224318 |
| ebi-fl187-GCST90094853 | ebi-a-GCST90001732 | CD19 on IgD- CD27- B cell \|\| id:ebi-a-GCST90001732 | F-sweetened coffee drink liking (derived food-liking factor) \|\| id:ebi-fl187-GCST90094853 | Inverse variance weighted | 4 | 0.00653694499344978 | 0.28743441989764 | 0.981855764135551 | -0.556834518 | 0.569908407992823 | 1.0065583574503 | 0.573020080713679 | 1.76810509972212 | 0.998955228396052 |
| ebi-fl187-GCST90094853 | ebi-a-GCST90001798 | CD27 on CD24+ CD27+ B cell \|\| id:ebi-a-GCST90001798 | F-sweetened coffee drink liking (derived food-liking factor) \|\| id:ebi-fl187-GCST90094853 | Inverse variance weighted | 4 | 0.0753015698853198 | 0.368758365804971 | 0.838194855832913 | -0.647464827 | 0.798067966863062 | 1.07820925730309 | 0.523370932110003 | 2.2212452606935 | 0.998735809224318 |
| ebi-fl187-GCST90094853 | ebi-a-GCST90001836 | CD66b on CD66b++ myeloid cell \|\| id:ebi-a-GCST90001836 | F-sweetened coffee drink liking (derived food-liking factor) \|\| id:ebi-fl187-GCST90094853 | Inverse variance weighted | 4 | 0.32842161567904 | 0.508173446476901 | 0.518098954412238 | -0.667598339 | 1.32444157077377 | 1.38877437792503 | 0.512939005051377 | 3.76008502723995 | 0.998735809224318 |
| ebi-fl187-GCST90094853 | ebi-a-GCST90001943 | CD25 on activated & secreting CD4 regulatory T cell \|\| id:ebi-a-GCST90001943 | F-sweetened coffee drink liking (derived food-liking factor) \|\| id:ebi-fl187-GCST90094853 | Inverse variance weighted | 4 | 0.166367697426523 | 0.319717376974053 | 0.6028136361433 | -0.460278361 | 0.793013756295667 | 1.18100727523195 | 0.631107944935926 | 2.21004694259142 | 0.998735809224318 |
| ebi-fl187-GCST90094853 | ebi-a-GCST90001960 | CD25 on CD4+ T cell \|\| id:ebi-a-GCST90001960 | F-sweetened coffee drink liking (derived food-liking factor) \|\| id:ebi-fl187-GCST90094853 | Inverse variance weighted | 4 | -0.133081659 | 0.312263966840681 | 0.669974351537019 | -0.745119035 | 0.478955715514354 | 0.875393604991718 | 0.474677793542529 | 1.61438764164926 | 0.998735809224318 |
| ebi-fl187-GCST90094853 | ebi-a-GCST90001988 | HLA DR on CD14+ CD16- monocyte \|\| id:ebi-a-GCST90001988 | F-sweetened coffee drink liking (derived food-liking factor) \|\| id:ebi-fl187-GCST90094853 | Inverse variance weighted | 4 | -0.292717169 | 0.293857332915878 | 0.3191918394246 | -0.868677541 | 0.28324320364629 | 0.746233168824408 | 0.419505961846707 | 1.32742795788252 | 0.998735809224318 |
| ebi-fl187-GCST90094853 | ebi-a-GCST90001991 | HLA DR on CD14+ monocyte \|\| id:ebi-a-GCST90001991 | F-sweetened coffee drink liking (derived food-liking factor) \|\| id:ebi-fl187-GCST90094853 | Inverse variance weighted | 4 | -0.319826235 | 0.294361989751585 | 0.277254936923059 | -0.896775735 | 0.257123264846093 | 0.726275227275855 | 0.407882663671482 | 1.29320452359171 | 0.998735809224318 |
| ebi-fl187-GCST90094853 | ebi-a-GCST90002019 | CD14 on Monocytic Myeloid-Derived Suppressor Cells \|\| id:ebi-a-GCST90002019 | F-sweetened coffee drink liking (derived food-liking factor) \|\| id:ebi-fl187-GCST90094853 | Inverse variance weighted | 4 | -0.619491921 | 0.416459231952156 | 0.136877218291132 | -1.435752015 | 0.196768173992889 | 0.538217825509326 | 0.237936364940865 | 1.21746176868753 | 0.998735809224318 |
| ebi-fl187-GCST90094853 | ebi-a-GCST90002037 | CD80 on plasmacytoid Dendritic Cell \|\| id:ebi-a-GCST90002037 | F-sweetened coffee drink liking (derived food-liking factor) \|\| id:ebi-fl187-GCST90094853 | Inverse variance weighted | 4 | -0.671044238 | 0.458778201277445 | 0.14355583338471 | -1.570249512 | 0.228161036580328 | 0.511174511178688 | 0.20799327897413 | 1.25628761740552 | 0.998735809224318 |
| ebi-fl187-GCST90094853 | ebi-a-GCST90002042 | CD45 on CD33+ HLA DR+ CD14- \|\| id:ebi-a-GCST90002042 | F-sweetened coffee drink liking (derived food-liking factor) \|\| id:ebi-fl187-GCST90094853 | Inverse variance weighted | 4 | -1.07308565 | 0.42515297178308 | 0.0116028278438856 | -1.906385475 | -0.239785825 | 0.341951744379892 | 0.148616595359705 | 0.786796354750533 | 0.998735809224318 |
| ebi-fl187-GCST90094853 | ebi-a-GCST90002056 | CD8 on Effector Memory CD8+ T cell \|\| id:ebi-a-GCST90002056 | F-sweetened coffee drink liking (derived food-liking factor) \|\| id:ebi-fl187-GCST90094853 | Inverse variance weighted | 4 | -0.29643532 | 0.319305021008074 | 0.353212235940841 | -0.922273161 | 0.329402521334567 | 0.743463713059969 | 0.397614171981982 | 1.39013730290771 | 0.998735809224318 |
| ebi-fl187-GCST90094853 | ebi-a-GCST90002058 | CD8 on CD8+ T cell \|\| id:ebi-a-GCST90002058 | F-sweetened coffee drink liking (derived food-liking factor) \|\| id:ebi-fl187-GCST90094853 | Inverse variance weighted | 4 | -0.44864116 | 0.318486936914334 | 0.15893447285004 | -1.072875556 | 0.175593236533322 | 0.638495175314785 | 0.342023593939856 | 1.19195311704708 | 0.998735809224318 |
| ebi-fl187-GCST90094853 | ebi-a-GCST90002063 | CD4 on CD4 regulatory T cell \|\| id:ebi-a-GCST90002063 | F-sweetened coffee drink liking (derived food-liking factor) \|\| id:ebi-fl187-GCST90094853 | Inverse variance weighted | 4 | -0.026275358 | 0.317524745017917 | 0.934049861717445 | -0.648623859 | 0.596073141819021 | 0.97406683517648 | 0.522764680083217 | 1.81497762863338 | 0.998735809224318 |
| ebi-fl187-GCST90094853 | ebi-a-GCST90002066 | CD4 on activated CD4 regulatory T cell \|\| id:ebi-a-GCST90002066 | F-sweetened coffee drink liking (derived food-liking factor) \|\| id:ebi-fl187-GCST90094853 | Inverse variance weighted | 4 | -0.089136793 | 0.317533822819095 | 0.778928312030282 | -0.711503086 | 0.533229499681434 | 0.914720437622718 | 0.490905769163702 | 1.70442787916326 | 0.998735809224318 |
| ebi-fl187-GCST90094853 | ebi-a-GCST90002070 | CD4 on activated & secreting CD4 regulatory T cell \|\| id:ebi-a-GCST90002070 | F-sweetened coffee drink liking (derived food-liking factor) \|\| id:ebi-fl187-GCST90094853 | Inverse variance weighted | 4 | -0.048755512 | 0.319013369264194 | 0.878530612109353 | -0.674021716 | 0.57651069194551 | 0.95241395519764 | 0.509654764127307 | 1.77981725258361 | 0.998735809224318 |
| ebi-fl187-GCST90094853 | ebi-a-GCST90002081 | SSC-A on CD4+ T cell \|\| id:ebi-a-GCST90002081 | F-sweetened coffee drink liking (derived food-liking factor) \|\| id:ebi-fl187-GCST90094853 | Inverse variance weighted | 4 | 0.170576512480728 | 0.302167778513377 | 0.572407592224428 | -0.421672333 | 0.762825358366947 | 1.18598839137637 | 0.655948936738451 | 2.14432615970588 | 0.998735809224318 |
| ebi-fl187-GCST90094853 | ebi-a-GCST90002094 | CD11b on Monocytic Myeloid-Derived Suppressor Cells \|\| id:ebi-a-GCST90002094 | F-sweetened coffee drink liking (derived food-liking factor) \|\| id:ebi-fl187-GCST90094853 | Inverse variance weighted | 4 | -0.64775221 | 0.421917346907851 | 0.124720333668013 | -1.47471021 | 0.179205789585648 | 0.523220545674962 | 0.228845034372598 | 1.19626689810834 | 0.998735809224318 |
| ebi-fl187-GCST90094853 | ebi-a-GCST90002104 | HLA DR on myeloid Dendritic Cell \|\| id:ebi-a-GCST90002104 | F-sweetened coffee drink liking (derived food-liking factor) \|\| id:ebi-fl187-GCST90094853 | Inverse variance weighted | 4 | -0.37155159 | 0.496570262408004 | 0.454318010354589 | -1.344829305 | 0.601726124064051 | 0.689663424998924 | 0.260584184591933 | 1.82526671956734 | 0.998735809224318 |
| ebi-fl187-GCST90094853 | ebi-a-GCST90002105 | HLA DR on plasmacytoid Dendritic Cell \|\| id:ebi-a-GCST90002105 | F-sweetened coffee drink liking (derived food-liking factor) \|\| id:ebi-fl187-GCST90094853 | Inverse variance weighted | 4 | -0.532486026 | 0.31866244217478 | 0.0947210049497629 | -1.157064413 | 0.092092360250659 | 0.587143499556912 | 0.314407798899587 | 1.09646608728697 | 0.998735809224318 |
| ebi-fl187-GCST90094853 | ebi-a-GCST90002106 | HLA DR on Dendritic Cell \|\| id:ebi-a-GCST90002106 | F-sweetened coffee drink liking (derived food-liking factor) \|\| id:ebi-fl187-GCST90094853 | Inverse variance weighted (multiplicative random effects) | 4 | -0.531866819 | 0.544098254841338 | 0.328311878799077 | -1.598299398 | 0.534565760631258 | 0.587507175831132 | 0.202240155659507 | 1.70670696196553 | 0.998735809224318 |
| ebi-fl187-GCST90094853 | ebi-a-GCST90002111 | HLA DR on CD33dim HLA DR+ CD11b- \|\| id:ebi-a-GCST90002111 | F-sweetened coffee drink liking (derived food-liking factor) \|\| id:ebi-fl187-GCST90094853 | Inverse variance weighted | 4 | 0.400454974790198 | 0.493216729046111 | 0.416834689151299 | -0.566249814 | 1.36715976372058 | 1.4925035946989 | 0.567650245923866 | 3.92418922775894 | 0.998735809224318 |
| ebi-fl187-GCST90094853 | ebi-a-GCST90002112 | HLA DR on CD33- HLA DR+ \|\| id:ebi-a-GCST90002112 | F-sweetened coffee drink liking (derived food-liking factor) \|\| id:ebi-fl187-GCST90094853 | Inverse variance weighted | 4 | -0.003899417 | 0.417197166443829 | 0.992542520060334 | -0.821605863 | 0.813807029005126 | 0.996108175630125 | 0.439724948995889 | 2.25648214826776 | 0.999107451094609 |
| ebi-fl187-GCST90094853 | ebi-a-GCST90002115 | HLA DR on HLA DR+ CD8+ T cell \|\| id:ebi-a-GCST90002115 | F-sweetened coffee drink liking (derived food-liking factor) \|\| id:ebi-fl187-GCST90094853 | Inverse variance weighted | 4 | -0.50864972 | 0.297851931648618 | 0.0876870508071889 | -1.092439506 | 0.075140066116569 | 0.601306963708786 | 0.335397291202147 | 1.0780351365055 | 0.998735809224318 |
| ebi-fl187-GCST90094853 | ebi-a-GCST90002116 | HLA DR on B cell \|\| id:ebi-a-GCST90002116 | F-sweetened coffee drink liking (derived food-liking factor) \|\| id:ebi-fl187-GCST90094853 | Inverse variance weighted | 4 | -0.126139344 | 0.313888645377165 | 0.687786937092371 | -0.741361089 | 0.489082400573939 | 0.881492007280843 | 0.476464962615183 | 1.63081909451457 | 0.998735809224318 |
| ebi-fl187-GCST90094853 | ebi-cfmet338-GCST90026023 | Sphingomyelin (d18:1/24:1, d18:2/24:0) levels \|\| id:ebi-cfmet338-GCST90026023 | F-sweetened coffee drink liking (derived food-liking factor) \|\| id:ebi-fl187-GCST90094853 | Inverse variance weighted | 4 | -0.141661887 | 0.1495307764099 | 0.343447153796652 | -0.434742209 | 0.151418434606323 | 0.86791465997447 | 0.647431547644529 | 1.1634833979579 | 0.998735809224318 |
| ebi-fl187-GCST90094853 | ebi-cfmet338-GCST90026046 | 1-arachidonoyl-gpc (20:4n6) levels \|\| id:ebi-cfmet338-GCST90026046 | F-sweetened coffee drink liking (derived food-liking factor) \|\| id:ebi-fl187-GCST90094853 | Inverse variance weighted | 4 | 0.0944133565758841 | 0.165906422637212 | 0.569304592583424 | -0.230763232 | 0.41958994494482 | 1.09901393667705 | 0.793927420555349 | 1.5213375955267 | 0.998735809224318 |
| ebi-fl187-GCST90094853 | ebi-cfmet338-GCST90026047 | 1-linoleoyl-gpc (18:2) levels \|\| id:ebi-cfmet338-GCST90026047 | F-sweetened coffee drink liking (derived food-liking factor) \|\| id:ebi-fl187-GCST90094853 | Inverse variance weighted | 4 | -0.152787587 | 0.202256179935959 | 0.449999320713884 | -0.549209699 | 0.243634526126671 | 0.858312019468584 | 0.577405954486111 | 1.2758779452143 | 0.998735809224318 |
| ebi-fl187-GCST90094853 | ebi-cfmet338-GCST90026050 | 1-myristoyl-2-palmitoyl-gpc (14:0/16:0) levels \|\| id:ebi-cfmet338-GCST90026050 | F-sweetened coffee drink liking (derived food-liking factor) \|\| id:ebi-fl187-GCST90094853 | Inverse variance weighted | 4 | 0.0268797666847484 | 0.121869442601218 | 0.825433500356308 | -0.211984341 | 0.265743874183136 | 1.02724428635212 | 0.808977365394642 | 1.30440092514617 | 0.998735809224318 |
| ebi-fl187-GCST90094853 | ebi-cfmet338-GCST90026068 | 2,3-dihydroxyisovalerate levels \|\| id:ebi-cfmet338-GCST90026068 | F-sweetened coffee drink liking (derived food-liking factor) \|\| id:ebi-fl187-GCST90094853 | Inverse variance weighted | 4 | -0.181110208 | 0.346296375050413 | 0.600979871243234 | -0.859851104 | 0.497630686608085 | 0.834343401901604 | 0.423225094323866 | 1.64481955733003 | 0.998735809224318 |
| ebi-fl187-GCST90094853 | ebi-cfmet338-GCST90026082 | 3-hydroxyhexanoate levels \|\| id:ebi-cfmet338-GCST90026082 | F-sweetened coffee drink liking (derived food-liking factor) \|\| id:ebi-fl187-GCST90094853 | Inverse variance weighted | 4 | 0.00668503923750941 | 0.0741808664138589 | 0.928193360666899 | -0.138709459 | 0.152079537408673 | 1.00670743398776 | 0.870480902170924 | 1.16425283440305 | 0.998735809224318 |
| ebi-fl187-GCST90094853 | ebi-cfmet338-GCST90026094 | 3-ureidopropionate levels \|\| id:ebi-cfmet338-GCST90026094 | F-sweetened coffee drink liking (derived food-liking factor) \|\| id:ebi-fl187-GCST90094853 | Inverse variance weighted | 4 | -0.247689222 | 0.168143249884974 | 0.140728241446907 | -0.577249992 | 0.0818715477892915 | 0.780602499680637 | 0.561440210757556 | 1.08531638958576 | 0.998735809224318 |
| ebi-fl187-GCST90094853 | ebi-cfmet338-GCST90026173 | Glycerol levels \|\| id:ebi-cfmet338-GCST90026173 | F-sweetened coffee drink liking (derived food-liking factor) \|\| id:ebi-fl187-GCST90094853 | Inverse variance weighted | 4 | -0.085082117 | 0.0951634641814807 | 0.371288272432489 | -0.271602506 | 0.101438273170919 | 0.918436862369584 | 0.762157153462045 | 1.10676159939932 | 0.998735809224318 |
| ebi-fl187-GCST90094853 | ebi-cfmet338-GCST90026179 | Histidine levels \|\| id:ebi-cfmet338-GCST90026179 | F-sweetened coffee drink liking (derived food-liking factor) \|\| id:ebi-fl187-GCST90094853 | Inverse variance weighted | 4 | -0.035961726 | 0.0658142401579692 | 0.584782394789947 | -0.164957637 | 0.0930341848328081 | 0.964677214951112 | 0.847929624520415 | 1.09749925245527 | 0.998735809224318 |
| ebi-fl187-GCST90094853 | ebi-cfmet338-GCST90026188 | Isocitrate levels \|\| id:ebi-cfmet338-GCST90026188 | F-sweetened coffee drink liking (derived food-liking factor) \|\| id:ebi-fl187-GCST90094853 | Inverse variance weighted | 4 | 0.303627385693861 | 0.135000046671677 | 0.0245067090519138 | 0.0390272942173744 | 0.568227477170348 | 1.35476415752276 | 1.03979886375906 | 1.76513553387922 | 0.998735809224318 |
| ebi-fl187-GCST90094853 | ebi-cfmet338-GCST90026207 | N-acetyl-isoputreanine levels \|\| id:ebi-cfmet338-GCST90026207 | F-sweetened coffee drink liking (derived food-liking factor) \|\| id:ebi-fl187-GCST90094853 | Inverse variance weighted | 4 | 0.0115698087034515 | 0.0821883206184822 | 0.888050117030246 | -0.1495193 | 0.172658917115677 | 1.01163699781185 | 0.86112181845901 | 1.18846067235085 | 0.998735809224318 |
| ebi-fl187-GCST90094853 | ebi-cfmet338-GCST90026213 | N-acetylglutamate levels \|\| id:ebi-cfmet338-GCST90026213 | F-sweetened coffee drink liking (derived food-liking factor) \|\| id:ebi-fl187-GCST90094853 | Inverse variance weighted | 4 | -0.101017201 | 0.14015414396861 | 0.47105850811443 | -0.375719323 | 0.173684921652321 | 0.903917484895808 | 0.686795073775844 | 1.18968066414384 | 0.998735809224318 |
| ebi-fl187-GCST90094853 | ebi-cfmet338-GCST90026224 | N-acetyltaurine levels \|\| id:ebi-cfmet338-GCST90026224 | F-sweetened coffee drink liking (derived food-liking factor) \|\| id:ebi-fl187-GCST90094853 | Inverse variance weighted | 4 | 0.0525033096125343 | 0.139422534166996 | 0.706488036402506 | -0.220764857 | 0.325771476579847 | 1.05390605009344 | 0.80190522023714 | 1.38509880518684 | 0.998735809224318 |
| ebi-fl187-GCST90094853 | ebi-cfmet338-GCST90026250 | Phenyllactate (pla) levels \|\| id:ebi-cfmet338-GCST90026250 | F-sweetened coffee drink liking (derived food-liking factor) \|\| id:ebi-fl187-GCST90094853 | Inverse variance weighted | 4 | -0.285270573 | 0.195610971639508 | 0.14474224594442 | -0.668668078 | 0.0981269310583624 | 0.751810806824505 | 0.512390587908737 | 1.10310279422772 | 0.998735809224318 |
| ebi-fl187-GCST90094853 | ebi-cfmet338-GCST90026251 | Pipecolate levels \|\| id:ebi-cfmet338-GCST90026251 | F-sweetened coffee drink liking (derived food-liking factor) \|\| id:ebi-fl187-GCST90094853 | Inverse variance weighted (multiplicative random effects) | 4 | -0.506103558 | 0.470291303558301 | 0.281860584522173 | -1.427874513 | 0.41566739712892 | 0.602839939466012 | 0.239818111235775 | 1.51538176471625 | 0.998735809224318 |
| ebi-fl187-GCST90094853 | ebi-cfmet338-GCST90026278 | Trigonelline (n'-methylnicotinate) levels \|\| id:ebi-cfmet338-GCST90026278 | F-sweetened coffee drink liking (derived food-liking factor) \|\| id:ebi-fl187-GCST90094853 | Inverse variance weighted | 4 | 0.269119618533694 | 0.300435665665363 | 0.370378527502013 | -0.319734286 | 0.857973523237806 | 1.30881168981843 | 0.726342010551904 | 2.35837665248603 | 0.998735809224318 |
| ebi-fl187-GCST90094853 | ebi-cfmet338-GCST90026299 | 1-carboxyethylvaline levels \|\| id:ebi-cfmet338-GCST90026299 | F-sweetened coffee drink liking (derived food-liking factor) \|\| id:ebi-fl187-GCST90094853 | Inverse variance weighted | 4 | 0.107611467566839 | 0.180824711506278 | 0.551766715834497 | -0.246804967 | 0.462027902119144 | 1.11361498578131 | 0.781293056613043 | 1.58728959135102 | 0.998735809224318 |
| ebi-fl187-GCST90094853 | ebi-gm430-GCST90011306 | C_Erysipelotrichia abundance \|\| id:ebi-gm430-GCST90011306 | F-sweetened coffee drink liking (derived food-liking factor) \|\| id:ebi-fl187-GCST90094853 | Inverse variance weighted | 4 | 0.176838901378447 | 0.172748730911729 | 0.305987865806463 | -0.161748611 | 0.515426413965435 | 1.19343881629158 | 0.85065502280148 | 1.67435231680731 | 0.998735809224318 |
| ebi-fl187-GCST90094853 | ebi-gm430-GCST90011312 | F_Erysipelotrichaceae abundance \|\| id:ebi-gm430-GCST90011312 | F-sweetened coffee drink liking (derived food-liking factor) \|\| id:ebi-fl187-GCST90094853 | Inverse variance weighted | 4 | 0.176838901378447 | 0.172748730911729 | 0.305987865806463 | -0.161748611 | 0.515426413965435 | 1.19343881629158 | 0.85065502280148 | 1.67435231680731 | 0.998735809224318 |
| ebi-fl187-GCST90094853 | ebi-gm430-GCST90011352 | O_Erysipelotrichales abundance \|\| id:ebi-gm430-GCST90011352 | F-sweetened coffee drink liking (derived food-liking factor) \|\| id:ebi-fl187-GCST90094853 | Inverse variance weighted | 4 | 0.176838901378447 | 0.172748730911729 | 0.305987865806463 | -0.161748611 | 0.515426413965435 | 1.19343881629158 | 0.85065502280148 | 1.67435231680731 | 0.998735809224318 |
| ebi-fl187-GCST90094853 | ebi-gm430-GCST90011362 | OTU97_11 (Parabacteroides) abundance \|\| id:ebi-gm430-GCST90011362 | F-sweetened coffee drink liking (derived food-liking factor) \|\| id:ebi-fl187-GCST90094853 | Inverse variance weighted | 4 | -0.458495069 | 0.184808707067728 | 0.0131044870666506 | -0.820720135 | -0.096270004 | 0.632234398819295 | 0.440114598325779 | 0.908218760684043 | 0.998735809224318 |
| ebi-fl187-GCST90094853 | ebi-gm430-GCST90011384 | OTU97_235 (Phascolarctobacterium) abundance \|\| id:ebi-gm430-GCST90011384 | F-sweetened coffee drink liking (derived food-liking factor) \|\| id:ebi-fl187-GCST90094853 | Inverse variance weighted (multiplicative random effects) | 4 | -0.57274758 | 1.16796975359032 | 0.623865891304084 | -2.861968297 | 1.7164731369844 | 0.563973744962463 | 0.0571561491936875 | 5.56486728889205 | 0.998735809224318 |
| ebi-fl187-GCST90094853 | ebi-gm430-GCST90011390 | OTU97_3 (Bacteroides) abundance \|\| id:ebi-gm430-GCST90011390 | F-sweetened coffee drink liking (derived food-liking factor) \|\| id:ebi-fl187-GCST90094853 | Inverse variance weighted | 4 | 0.089021502351197 | 0.146478614305559 | 0.543357260400017 | -0.198076582 | 0.376119586390092 | 1.09310416038318 | 0.820307030235514 | 1.45662131544084 | 0.998735809224318 |
| ebi-fl187-GCST90094853 | ebi-gm430-GCST90011398 | OTU97_4 (Alistipes) abundance \|\| id:ebi-gm430-GCST90011398 | F-sweetened coffee drink liking (derived food-liking factor) \|\| id:ebi-fl187-GCST90094853 | Inverse variance weighted | 4 | -0.060687509 | 0.170729139107626 | 0.722244437563834 | -0.395316622 | 0.273941603258576 | 0.941117284142538 | 0.673466771062785 | 1.31513800022252 | 0.998735809224318 |
| ebi-fl187-GCST90094853 | ebi-gm430-GCST90011421 | OTU99_108 (Cytophagales) abundance \|\| id:ebi-gm430-GCST90011421 | F-sweetened coffee drink liking (derived food-liking factor) \|\| id:ebi-fl187-GCST90094853 | Inverse variance weighted | 4 | 0.801303977723959 | 0.622926878767559 | 0.198319827052213 | -0.419632705 | 2.02224066010837 | 2.22844487721773 | 0.657288194374998 | 7.55523469506395 | 0.998735809224318 |
| ebi-fl187-GCST90094853 | ebi-gm430-GCST90011428 | OTU99_123 (Phascolarctobacterium) abundance \|\| id:ebi-gm430-GCST90011428 | F-sweetened coffee drink liking (derived food-liking factor) \|\| id:ebi-fl187-GCST90094853 | Inverse variance weighted | 4 | -0.187502388 | 0.339184702458023 | 0.580398179355278 | -0.852304405 | 0.477299629045925 | 0.829027138650414 | 0.426431128993292 | 1.61171628872738 | 0.998735809224318 |
| ebi-fl187-GCST90094853 | ebi-gm430-GCST90011450 | OTU99_298 (Phascolarctobacterium) abundance \|\| id:ebi-gm430-GCST90011450 | F-sweetened coffee drink liking (derived food-liking factor) \|\| id:ebi-fl187-GCST90094853 | Inverse variance weighted (multiplicative random effects) | 4 | -0.563223763 | 1.16007458556446 | 0.627317096062948 | -2.836969951 | 1.71052242453424 | 0.569370586105854 | 0.0586029670822286 | 5.53185069738273 | 0.998735809224318 |
| ebi-fl187-GCST90094853 | ebi-gm430-GCST90011451 | OTU99_3 (Bacteroides) abundance \|\| id:ebi-gm430-GCST90011451 | F-sweetened coffee drink liking (derived food-liking factor) \|\| id:ebi-fl187-GCST90094853 | Inverse variance weighted | 4 | 0.0898127768270357 | 0.146480330735695 | 0.539784555141441 | -0.197288671 | 0.376914225068998 | 1.09396944809962 | 0.820953613262718 | 1.45777926309268 | 0.998735809224318 |
| ebi-fl187-GCST90094853 | ebi-gm430-GCST90011472 | OTU99_72 (Parabacteroides) abundance \|\| id:ebi-gm430-GCST90011472 | F-sweetened coffee drink liking (derived food-liking factor) \|\| id:ebi-fl187-GCST90094853 | Inverse variance weighted | 4 | 0.372957388116442 | 0.438595701623701 | 0.395133651745557 | -0.486690187 | 1.2326049632989 | 1.45202246510228 | 0.614657432271511 | 3.43015333170229 | 0.998735809224318 |
| ebi-fl187-GCST90094853 | ebi-gm430-GCST90011507 | TestASV_3 (Bacteroides) abundance \|\| id:ebi-gm430-GCST90011507 | F-sweetened coffee drink liking (derived food-liking factor) \|\| id:ebi-fl187-GCST90094853 | Inverse variance weighted | 4 | 0.0882997221674528 | 0.275033874798584 | 0.74817223396329 | -0.450766672 | 0.627366116772678 | 1.09231546412836 | 0.637139487039146 | 1.87267167935023 | 0.998735809224318 |
| ebi-fl187-GCST90094853 | ebi-gm430-GCST90011512 | TestASV_34 (Phascolarctobacterium) abundance \|\| id:ebi-gm430-GCST90011512 | F-sweetened coffee drink liking (derived food-liking factor) \|\| id:ebi-fl187-GCST90094853 | Inverse variance weighted | 4 | -0.465083629 | 0.470449881674223 | 0.322862136314305 | -1.387165397 | 0.456998138929119 | 0.628082576979703 | 0.249782335781973 | 1.57932594500918 | 0.998735809224318 |
| ebi-fl187-GCST90094853 | ebi-gm430-GCST90011519 | TestASV_41 (Barnesiella) abundance \|\| id:ebi-gm430-GCST90011519 | F-sweetened coffee drink liking (derived food-liking factor) \|\| id:ebi-fl187-GCST90094853 | Inverse variance weighted | 4 | -0.800720614 | 0.638018456531067 | 0.209474520597186 | -2.051236789 | 0.449795560934734 | 0.449005288071942 | 0.128575784136843 | 1.56799159398475 | 0.998735809224318 |
| ebi-fl187-GCST90094853 | ebi-gm430-GCST90011524 | TestASV_46 (Sutterella) abundance \|\| id:ebi-gm430-GCST90011524 | F-sweetened coffee drink liking (derived food-liking factor) \|\| id:ebi-fl187-GCST90094853 | Inverse variance weighted | 4 | 0.191280126815162 | 0.725752975797253 | 0.792118270175546 | -1.231195706 | 1.61375595937778 | 1.21079858172993 | 0.29194329062967 | 5.02163691570795 | 0.998735809224318 |
| ebi-fl187-GCST90094853 | ebi-gm430-GCST90011581 | OTU97_19 (Bacteria) prevalence \|\| id:ebi-gm430-GCST90011581 | F-sweetened coffee drink liking (derived food-liking factor) \|\| id:ebi-fl187-GCST90094853 | Inverse variance weighted | 4 | -1.616112822 | 0.79732925090478 | 0.0426718498845749 | -3.178878154 | -0.05334749 | 0.198669463681466 | 0.041632334020214 | 0.948050517184967 | 0.998735809224318 |
| ebi-fl187-GCST90094853 | ebi-gm430-GCST90011614 | OTU97_80 (Ruminococcus) prevalence \|\| id:ebi-gm430-GCST90011614 | F-sweetened coffee drink liking (derived food-liking factor) \|\| id:ebi-fl187-GCST90094853 | Inverse variance weighted | 4 | -0.28535915 | 0.324328800035952 | 0.378943154360075 | -0.921043598 | 0.350325297930916 | 0.751744216789912 | 0.398103364264101 | 1.41952924342076 | 0.998735809224318 |
| ebi-fl187-GCST90094853 | ebi-gm430-GCST90011623 | OTU99_11 (Parabacteroides) prevalence \|\| id:ebi-gm430-GCST90011623 | F-sweetened coffee drink liking (derived food-liking factor) \|\| id:ebi-fl187-GCST90094853 | Inverse variance weighted | 4 | 0.173639759669588 | 0.314490982075889 | 0.580859607889643 | -0.442762565 | 0.79004208453833 | 1.18962693701945 | 0.642259683785552 | 2.20348915712855 | 0.998735809224318 |
| ebi-fl187-GCST90094853 | ebi-gm430-GCST90011642 | OTU99_19 (Bacteria) prevalence \|\| id:ebi-gm430-GCST90011642 | F-sweetened coffee drink liking (derived food-liking factor) \|\| id:ebi-fl187-GCST90094853 | Inverse variance weighted | 4 | -1.616112822 | 0.79732925090478 | 0.0426718498845749 | -3.178878154 | -0.05334749 | 0.198669463681466 | 0.041632334020214 | 0.948050517184967 | 0.998735809224318 |
| ebi-fl187-GCST90094853 | ebi-gm430-GCST90011653 | OTU99_30 (Parasutterella) prevalence \|\| id:ebi-gm430-GCST90011653 | F-sweetened coffee drink liking (derived food-liking factor) \|\| id:ebi-fl187-GCST90094853 | Inverse variance weighted | 4 | 0.181833634065239 | 0.307623218454718 | 0.554458756679272 | -0.421107874 | 0.784775142236486 | 1.19941463554347 | 0.656319297732799 | 2.19191401643281 | 0.998735809224318 |
| ebi-fl187-GCST90094853 | ebi-gm430-GCST90011675 | OTU99_8 (Ruminococcaceae) prevalence \|\| id:ebi-gm430-GCST90011675 | F-sweetened coffee drink liking (derived food-liking factor) \|\| id:ebi-fl187-GCST90094853 | Inverse variance weighted | 4 | -0.066811868 | 0.324624666168584 | 0.83693724631527 | -0.703076214 | 0.569452477424815 | 0.935371157782975 | 0.495060048358353 | 1.76729915030216 | 0.998735809224318 |
| ebi-fl187-GCST90094853 | ebi-gm430-GCST90011679 | OTU99_92 (Ruminococcus) prevalence \|\| id:ebi-gm430-GCST90011679 | F-sweetened coffee drink liking (derived food-liking factor) \|\| id:ebi-fl187-GCST90094853 | Inverse variance weighted | 4 | -0.42680271 | 0.338376574653007 | 0.207191908091154 | -1.090020797 | 0.236415375924973 | 0.652592289203264 | 0.336209501580904 | 1.26670035773833 | 0.998735809224318 |
| ebi-fl187-GCST90094853 | ebi-gm430-GCST90011684 | TestASV_11 (Lachnospiraceae) prevalence \|\| id:ebi-gm430-GCST90011684 | F-sweetened coffee drink liking (derived food-liking factor) \|\| id:ebi-fl187-GCST90094853 | Inverse variance weighted | 4 | 0.0502182126103523 | 0.444883006204549 | 0.910126015450848 | -0.82175248 | 0.922188904771269 | 1.05150052201703 | 0.4396604829666 | 2.51478900341851 | 0.998735809224318 |
| ebi-fl187-GCST90094853 | ebi-gm430-GCST90011688 | TestASV_15 (Bacteroides) prevalence \|\| id:ebi-gm430-GCST90011688 | F-sweetened coffee drink liking (derived food-liking factor) \|\| id:ebi-fl187-GCST90094853 | Inverse variance weighted | 4 | 0.129402570274739 | 0.322940474780849 | 0.68864027654133 | -0.50356076 | 0.762365900845203 | 1.1381482165926 | 0.604374789966943 | 2.143341159 | 0.998735809224318 |
| ebi-fl187-GCST90094853 | ebi-gm430-GCST90011692 | TestASV_19 (Prevotella) prevalence \|\| id:ebi-gm430-GCST90011692 | F-sweetened coffee drink liking (derived food-liking factor) \|\| id:ebi-fl187-GCST90094853 | Inverse variance weighted | 4 | 0.302440475376926 | 0.646909126480839 | 0.640130627880927 | -0.965501413 | 1.57038236327937 | 1.35315712785589 | 0.380792217875327 | 4.80848643095613 | 0.998735809224318 |
| ebi-fl187-GCST90094853 | ebi-gm430-GCST90011693 | TestASV_2 (Bacteroides) prevalence \|\| id:ebi-gm430-GCST90011693 | F-sweetened coffee drink liking (derived food-liking factor) \|\| id:ebi-fl187-GCST90094853 | Inverse variance weighted | 4 | -0.021075147 | 0.445257912107052 | 0.962248267312925 | -0.893780655 | 0.851630360770601 | 0.979145382006648 | 0.409106136283272 | 2.34346443154083 | 0.998735809224318 |
| ebi-fl187-GCST90094853 | ebi-gm430-GCST90011716 | TestASV_40 (Barnesiella) prevalence \|\| id:ebi-gm430-GCST90011716 | F-sweetened coffee drink liking (derived food-liking factor) \|\| id:ebi-fl187-GCST90094853 | Inverse variance weighted | 4 | -0.544822853 | 0.762758471852168 | 0.475054274073747 | -2.039829458 | 0.95018375161098 | 0.579944509543638 | 0.130050888119419 | 2.58618483128674 | 0.998735809224318 |
| ebi-fl187-GCST90094853 | ebi-gm430-GCST90011718 | TestASV_42 (Alphaproteobacteria) prevalence \|\| id:ebi-gm430-GCST90011718 | F-sweetened coffee drink liking (derived food-liking factor) \|\| id:ebi-fl187-GCST90094853 | Inverse variance weighted | 4 | 0.108129964681808 | 0.52913399322051 | 0.838077883451413 | -0.928972662 | 1.14523259139401 | 1.11419254165621 | 0.394959258661225 | 3.14317234666262 | 0.998735809224318 |
| ebi-fl187-GCST90094853 | ebi-hbsm44-GCST90104929 | Sulcal depth of the right inferior frontal sulcus \|\| id:ebi-hbsm44-GCST90104929 | F-sweetened coffee drink liking (derived food-liking factor) \|\| id:ebi-fl187-GCST90094853 | Inverse variance weighted (multiplicative random effects) | 3 | -0.300162984 | 0.828712726791005 | 0.717199663151096 | -1.924439929 | 1.32411396 | 0.740697488939798 | 0.145957480579496 | 3.75885338622922 | 0.998735809224318 |
| ebi-fl187-GCST90094853 | ebi-hbsm44-GCST90104935 | Sulcal opening distance of the left calloso-marginal posterior fissure \|\| id:ebi-hbsm44-GCST90104935 | F-sweetened coffee drink liking (derived food-liking factor) \|\| id:ebi-fl187-GCST90094853 | Inverse variance weighted (multiplicative random effects) | 3 | 0.0215732039994678 | 0.195369371979843 | 0.912074189985627 | -0.361350765 | 0.40449717307996 | 1.02180758800173 | 0.696734565443794 | 1.4985487999047 | 0.998735809224318 |
| ebi-fl187-GCST90094853 | ebi-hbsm44-GCST90104936 | Sulcal opening distance of the left anterior inferior temporal sulcus \|\| id:ebi-hbsm44-GCST90104936 | F-sweetened coffee drink liking (derived food-liking factor) \|\| id:ebi-fl187-GCST90094853 | Inverse variance weighted | 3 | 0.0312745860048892 | 0.0614735303353534 | 0.610928297841988 | -0.089213533 | 0.151762705462182 | 1.0317687742594 | 0.91465024429612 | 1.16388402034044 | 0.998735809224318 |
| ebi-fl187-GCST90094853 | ebi-hbsm44-GCST90104941 | Sulcal opening distance of the right posterior inferior temporal sulcus \|\| id:ebi-hbsm44-GCST90104941 | F-sweetened coffee drink liking (derived food-liking factor) \|\| id:ebi-fl187-GCST90094853 | Inverse variance weighted | 3 | -0.014416514 | 0.035079637436302 | 0.681097976727025 | -0.083172603 | 0.0543395755395058 | 0.985686906516468 | 0.920192305365754 | 1.05584307976997 | 0.998735809224318 |
| ebi-fl187-GCST90094853 | ebi-IPs91-GCST90274819 | Leukemia inhibitory factor levels \|\| id:ebi-IPs91-GCST90274819 | F-sweetened coffee drink liking (derived food-liking factor) \|\| id:ebi-fl187-GCST90094853 | Inverse variance weighted | 4 | 0.28021851230651 | 0.126563586199844 | 0.0268251198868445 | 0.0321538833548165 | 0.528283141258203 | 1.323418964 | 1.03267640478842 | 1.69601798429023 | 0.998735809224318 |
| ebi-fl187-GCST90094853 | ebi-IPs91-GCST90274837 | STAM binding protein levels \|\| id:ebi-IPs91-GCST90274837 | F-sweetened coffee drink liking (derived food-liking factor) \|\| id:ebi-fl187-GCST90094853 | Inverse variance weighted | 4 | 0.19658339475935 | 0.115312455614474 | 0.0882338018117033 | -0.029429018 | 0.42259580776372 | 1.21723682781789 | 0.970999798464885 | 1.52591740733481 | 0.998735809224318 |
| ebi-fl187-GCST90094853 | ebi-IPs91-GCST90274840 | TNF-beta levels \|\| id:ebi-IPs91-GCST90274840 | F-sweetened coffee drink liking (derived food-liking factor) \|\| id:ebi-fl187-GCST90094853 | Inverse variance weighted | 4 | -0.011593974 | 0.127603938225676 | 0.927604577463049 | -0.261697693 | 0.238509744479216 | 0.988472976685565 | 0.769743687161496 | 1.26935607519003 | 0.998735809224318 |
| ebi-fl187-GCST90094853 | ebi-IPs91-GCST90274842 | Tumor necrosis factor ligand superfamily member 14 levels \|\| id:ebi-IPs91-GCST90274842 | F-sweetened coffee drink liking (derived food-liking factor) \|\| id:ebi-fl187-GCST90094853 | Inverse variance weighted | 4 | 0.269910430344712 | 0.127603938225676 | 0.0344111823346285 | 0.0198067114223875 | 0.520014149267037 | 1.30984712292354 | 1.02000416581724 | 1.68205144932564 | 0.998735809224318 |
| ebi-fl187-GCST90094853 | ebi-met1400-GCST90199628 | 3-methylhistidine levels \|\| id:ebi-met1400-GCST90199628 | F-sweetened coffee drink liking (derived food-liking factor) \|\| id:ebi-fl187-GCST90094853 | Inverse variance weighted (multiplicative random effects) | 4 | 0.153573558002987 | 0.315607358509956 | 0.626544030423289 | -0.465016865 | 0.772163980682501 | 1.16599355212491 | 0.628124511983603 | 2.16444500677656 | 0.998735809224318 |
| ebi-fl187-GCST90094853 | ebi-met1400-GCST90199651 | Docosahexaenoate DHA; 22:6n3 levels \|\| id:ebi-met1400-GCST90199651 | F-sweetened coffee drink liking (derived food-liking factor) \|\| id:ebi-fl187-GCST90094853 | Inverse variance weighted | 4 | -0.008238486 | 0.139034338254279 | 0.9527489685537 | -0.280745789 | 0.264268817270417 | 0.991795357612401 | 0.755220296623521 | 1.30247827790024 | 0.998735809224318 |
| ebi-fl187-GCST90094853 | ebi-met1400-GCST90199652 | 1-stearoyl-2-oleoyl-GPS (18:0/18:1) levels \|\| id:ebi-met1400-GCST90199652 | F-sweetened coffee drink liking (derived food-liking factor) \|\| id:ebi-fl187-GCST90094853 | Inverse variance weighted | 4 | 0.109890324480699 | 0.147289103727068 | 0.455615522302605 | -0.178796319 | 0.398576967785753 | 1.11615564879526 | 0.83627621577412 | 1.48970329281022 | 0.998735809224318 |
| ebi-fl187-GCST90094853 | ebi-met1400-GCST90199653 | 1-myristoyl-2-palmitoyl-gpc (14:0/16:0) levels \|\| id:ebi-met1400-GCST90199653 | F-sweetened coffee drink liking (derived food-liking factor) \|\| id:ebi-fl187-GCST90094853 | Inverse variance weighted | 4 | 0.077513293857671 | 0.144458873166955 | 0.591559906314609 | -0.205626098 | 0.360652685264903 | 1.08059659766044 | 0.814137427359653 | 1.43426523291318 | 0.998735809224318 |
| ebi-fl187-GCST90094853 | ebi-met1400-GCST90199667 | Erythritol levels in elite athletes \|\| id:ebi-met1400-GCST90199667 | F-sweetened coffee drink liking (derived food-liking factor) \|\| id:ebi-fl187-GCST90094853 | Inverse variance weighted | 4 | -0.015066002 | 0.136587383152007 | 0.912169057301437 | -0.282777273 | 0.252645269267096 | 0.98504692267532 | 0.753687635987512 | 1.28742650607605 | 0.998735809224318 |
| ebi-fl187-GCST90094853 | ebi-met1400-GCST90199674 | 3-hydroxymyristate levels \|\| id:ebi-met1400-GCST90199674 | F-sweetened coffee drink liking (derived food-liking factor) \|\| id:ebi-fl187-GCST90094853 | Inverse variance weighted | 4 | 0.0566204930816797 | 0.141767317050423 | 0.689605630797917 | -0.221243448 | 0.334484434500508 | 1.05825411941841 | 0.801521527453295 | 1.39721984114929 | 0.998735809224318 |
| ebi-fl187-GCST90094853 | ebi-met1400-GCST90199685 | 2-linoleoylglycerol (18:2) levels \|\| id:ebi-met1400-GCST90199685 | F-sweetened coffee drink liking (derived food-liking factor) \|\| id:ebi-fl187-GCST90094853 | Inverse variance weighted | 4 | -0.106864193 | 0.173774262076134 | 0.538581241592956 | -0.447461746 | 0.233733361009999 | 0.898647707658989 | 0.639248669391257 | 1.26330760023292 | 0.998735809224318 |
| ebi-fl187-GCST90094853 | ebi-met1400-GCST90199703 | Gamma-glutamylvaline levels \|\| id:ebi-met1400-GCST90199703 | F-sweetened coffee drink liking (derived food-liking factor) \|\| id:ebi-fl187-GCST90094853 | Inverse variance weighted | 4 | -0.024321261 | 0.159460914431337 | 0.878775422157244 | -0.336864653 | 0.288222131538027 | 0.975972117856204 | 0.71400547183832 | 1.33405360659255 | 0.998735809224318 |
| ebi-fl187-GCST90094853 | ebi-met1400-GCST90199779 | Gamma-glutamyl-2-aminobutyrate levels \|\| id:ebi-met1400-GCST90199779 | F-sweetened coffee drink liking (derived food-liking factor) \|\| id:ebi-fl187-GCST90094853 | Inverse variance weighted | 4 | 0.0684472981868494 | 0.161817231958218 | 0.672301532506997 | -0.248714476 | 0.385609072824957 | 1.07084418803352 | 0.779802593605375 | 1.47050969623405 | 0.998735809224318 |
| ebi-fl187-GCST90094853 | ebi-met1400-GCST90199824 | 4-vinylphenol sulfate levels \|\| id:ebi-met1400-GCST90199824 | F-sweetened coffee drink liking (derived food-liking factor) \|\| id:ebi-fl187-GCST90094853 | Inverse variance weighted | 4 | -0.036440331 | 0.174809880551548 | 0.8348722084565 | -0.379067697 | 0.306187034775975 | 0.964215625860752 | 0.684499272560017 | 1.35823632021833 | 0.998735809224318 |
| ebi-fl187-GCST90094853 | ebi-met1400-GCST90199830 | 4-ethylphenylsulfate levels \|\| id:ebi-met1400-GCST90199830 | F-sweetened coffee drink liking (derived food-liking factor) \|\| id:ebi-fl187-GCST90094853 | Inverse variance weighted | 4 | 0.0226565253006165 | 0.145706442390453 | 0.876431625397337 | -0.262928102 | 0.308241152385905 | 1.02291513373312 | 0.768797170468622 | 1.36102916479574 | 0.998735809224318 |
| ebi-fl187-GCST90094853 | ebi-met1400-GCST90199833 | Hexanoylglutamine levels \|\| id:ebi-met1400-GCST90199833 | F-sweetened coffee drink liking (derived food-liking factor) \|\| id:ebi-fl187-GCST90094853 | Inverse variance weighted | 4 | 0.0798911910245703 | 0.143459603336201 | 0.577602718254882 | -0.201289632 | 0.361072013563524 | 1.08316920273153 | 0.817675572640234 | 1.43486678702859 | 0.998735809224318 |
| ebi-fl187-GCST90094853 | ebi-met1400-GCST90199837 | Carotenoid levels (cryptoxanthin) \|\| id:ebi-met1400-GCST90199837 | F-sweetened coffee drink liking (derived food-liking factor) \|\| id:ebi-fl187-GCST90094853 | Inverse variance weighted | 4 | -0.337347517 | 0.181409949596449 | 0.0629440320464863 | -0.692911018 | 0.0182159839866468 | 0.713660787389392 | 0.500118095008492 | 1.01838290703838 | 0.998735809224318 |
| ebi-fl187-GCST90094853 | ebi-met1400-GCST90199869 | 2-hydroxyhippurate levels \|\| id:ebi-met1400-GCST90199869 | F-sweetened coffee drink liking (derived food-liking factor) \|\| id:ebi-fl187-GCST90094853 | Inverse variance weighted | 4 | 0.0431581265553714 | 0.146489513061484 | 0.768287383295779 | -0.243961319 | 0.33027757215588 | 1.04410298220595 | 0.78351794089882 | 1.39135427607539 | 0.998735809224318 |
| ebi-fl187-GCST90094853 | ebi-met1400-GCST90199902 | Hydantoin-5-propionate levels \|\| id:ebi-met1400-GCST90199902 | F-sweetened coffee drink liking (derived food-liking factor) \|\| id:ebi-fl187-GCST90094853 | Inverse variance weighted | 4 | 0.177931044814798 | 0.145186246728648 | 0.220373146093054 | -0.106633999 | 0.462496088402949 | 1.19474293467401 | 0.898854594677994 | 1.58803291255894 | 0.998735809224318 |
| ebi-fl187-GCST90094853 | ebi-met1400-GCST90199904 | Pantoate levels \|\| id:ebi-met1400-GCST90199904 | F-sweetened coffee drink liking (derived food-liking factor) \|\| id:ebi-fl187-GCST90094853 | Inverse variance weighted | 4 | 0.0555447159537124 | 0.22926392423673 | 0.808567764188131 | -0.393812576 | 0.504902007457703 | 1.05711628597849 | 0.674480458523422 | 1.65682315619252 | 0.998735809224318 |
| ebi-fl187-GCST90094853 | ebi-met1400-GCST90199936 | 3-methyl catechol sulfate (2) levels \|\| id:ebi-met1400-GCST90199936 | F-sweetened coffee drink liking (derived food-liking factor) \|\| id:ebi-fl187-GCST90094853 | Inverse variance weighted | 4 | 0.0546226233904679 | 0.165723233197972 | 0.741701102074716 | -0.270194914 | 0.379440160458493 | 1.05614197618365 | 0.763230715732204 | 1.46146617381221 | 0.998735809224318 |
| ebi-fl187-GCST90094853 | ebi-met1400-GCST90199977 | 4-hydroxychlorothalonil levels \|\| id:ebi-met1400-GCST90199977 | F-sweetened coffee drink liking (derived food-liking factor) \|\| id:ebi-fl187-GCST90094853 | Inverse variance weighted | 4 | 0.0825331437919567 | 0.191533593133131 | 0.666536077826041 | -0.292872699 | 0.457938986332894 | 1.08603466814965 | 0.746117116294148 | 1.58081254894832 | 0.998735809224318 |
| ebi-fl187-GCST90094853 | ebi-met1400-GCST90200040 | Sphingomyelin (d18:2/24:1, d18:1/24:2) levels \|\| id:ebi-met1400-GCST90200040 | F-sweetened coffee drink liking (derived food-liking factor) \|\| id:ebi-fl187-GCST90094853 | Inverse variance weighted (multiplicative random effects) | 4 | -0.073158082 | 0.24810411697466 | 0.76809435706145 | -0.559442151 | 0.413125987305525 | 0.929453888514803 | 0.57152780101486 | 1.51153544821667 | 0.998735809224318 |
| ebi-fl187-GCST90094853 | ebi-met1400-GCST90200077 | 1-stearoyl-2-oleoyl-GPI (18:0/18:1) levels \|\| id:ebi-met1400-GCST90200077 | F-sweetened coffee drink liking (derived food-liking factor) \|\| id:ebi-fl187-GCST90094853 | Inverse variance weighted | 4 | -0.152670393 | 0.152163133305805 | 0.315699901534894 | -0.450910134 | 0.145569348086767 | 0.858412613828331 | 0.637048088268539 | 1.15669794659045 | 0.998735809224318 |
| ebi-fl187-GCST90094853 | ebi-met1400-GCST90200080 | 1-palmitoyl-2-oleoyl-GPI (16:0/18:1) levels \|\| id:ebi-met1400-GCST90200080 | F-sweetened coffee drink liking (derived food-liking factor) \|\| id:ebi-fl187-GCST90094853 | Inverse variance weighted | 4 | -0.077928765 | 0.157148504283158 | 0.619970256629679 | -0.385939833 | 0.230082303441726 | 0.925030318774584 | 0.679811426427225 | 1.25870360130496 | 0.998735809224318 |
| ebi-fl187-GCST90094853 | ebi-met1400-GCST90200082 | 1-oleoyl-2-linoleoyl-GPE (18:1/18:2) levels \|\| id:ebi-met1400-GCST90200082 | F-sweetened coffee drink liking (derived food-liking factor) \|\| id:ebi-fl187-GCST90094853 | Inverse variance weighted | 4 | 0.0026139777742186 | 0.177160592901831 | 0.98822776103714 | -0.344620784 | 0.349848739861807 | 1.0026173971929 | 0.708488972664513 | 1.4188529164728 | 0.998955228396052 |
| ebi-fl187-GCST90094853 | ebi-met1400-GCST90200100 | Perfluorooctanesulfonate (PFOS) levels \|\| id:ebi-met1400-GCST90200100 | F-sweetened coffee drink liking (derived food-liking factor) \|\| id:ebi-fl187-GCST90094853 | Inverse variance weighted | 4 | 0.320390988339817 | 0.180834657180921 | 0.0764386514491235 | -0.03404494 | 0.674826916414423 | 1.37766631051003 | 0.966528068146454 | 1.96369306351766 | 0.998735809224318 |
| ebi-fl187-GCST90094853 | ebi-met1400-GCST90200102 | 1-palmitoleoyl-2-linolenoyl-GPC (16:1/18:3) levels \|\| id:ebi-met1400-GCST90200102 | F-sweetened coffee drink liking (derived food-liking factor) \|\| id:ebi-fl187-GCST90094853 | Inverse variance weighted | 4 | 0.0703373762944367 | 0.142587853600117 | 0.621806632909474 | -0.209134817 | 0.349809569350666 | 1.07287008113495 | 0.811285853338658 | 1.41879734036731 | 0.998735809224318 |
| ebi-fl187-GCST90094853 | ebi-met1400-GCST90200114 | Glycosyl-N-tricosanoyl-sphingadienine (d18:2/23:0) levels \|\| id:ebi-met1400-GCST90200114 | F-sweetened coffee drink liking (derived food-liking factor) \|\| id:ebi-fl187-GCST90094853 | Inverse variance weighted | 4 | -0.116960949 | 0.152276140437551 | 0.442436963590118 | -0.415422184 | 0.181500286615861 | 0.889619933351814 | 0.660061554511379 | 1.19901488036635 | 0.998735809224318 |
| ebi-fl187-GCST90094853 | ebi-met1400-GCST90200135 | Arachidoylcarnitine (C20) levels \|\| id:ebi-met1400-GCST90200135 | F-sweetened coffee drink liking (derived food-liking factor) \|\| id:ebi-fl187-GCST90094853 | Inverse variance weighted (multiplicative random effects) | 4 | -0.00529001 | 0.308397457437483 | 0.986314379896434 | -0.609749027 | 0.599169006436151 | 0.994723957322137 | 0.543487252738576 | 1.82060526035293 | 0.998955228396052 |
| ebi-fl187-GCST90094853 | ebi-met1400-GCST90200146 | Docosahexaenoylcarnitine (C22:6) levels \|\| id:ebi-met1400-GCST90200146 | F-sweetened coffee drink liking (derived food-liking factor) \|\| id:ebi-fl187-GCST90094853 | Inverse variance weighted | 4 | 0.106936228336417 | 0.232816336043743 | 0.646007382585515 | -0.34938379 | 0.563256246982153 | 1.11286328307347 | 0.705122459166092 | 1.75638241374089 | 0.998735809224318 |
| ebi-fl187-GCST90094853 | ebi-met1400-GCST90200162 | 3-hydroxyphenylacetoylglutamine levels \|\| id:ebi-met1400-GCST90200162 | F-sweetened coffee drink liking (derived food-liking factor) \|\| id:ebi-fl187-GCST90094853 | Inverse variance weighted | 4 | -0.037373304 | 0.176909878712964 | 0.832687378122071 | -0.384116667 | 0.309370057913976 | 0.963316457981532 | 0.68105196649926 | 1.36256650573682 | 0.998735809224318 |
| ebi-fl187-GCST90094853 | ebi-met1400-GCST90200172 | 3-carboxy-4-methyl-5-pentyl-2-furanpropionate (3-CMPFP) levels \|\| id:ebi-met1400-GCST90200172 | F-sweetened coffee drink liking (derived food-liking factor) \|\| id:ebi-fl187-GCST90094853 | Inverse variance weighted | 4 | 0.212317362998158 | 0.144478968014778 | 0.14168686780497 | -0.070861414 | 0.495496140307122 | 1.23654025493602 | 0.931590988361109 | 1.64131235829919 | 0.998735809224318 |
| ebi-fl187-GCST90094853 | ebi-met1400-GCST90200222 | Dihydrocaffeate sulfate (2) levels \|\| id:ebi-met1400-GCST90200222 | F-sweetened coffee drink liking (derived food-liking factor) \|\| id:ebi-fl187-GCST90094853 | Inverse variance weighted | 4 | 0.235369463042762 | 0.164409453166754 | 0.152256779729618 | -0.086873065 | 0.557611991249601 | 1.26537619211792 | 0.916793461275152 | 1.74649686675535 | 0.998735809224318 |
| ebi-fl187-GCST90094853 | ebi-met1400-GCST90200223 | 4-methylhexanoylglutamine levels \|\| id:ebi-met1400-GCST90200223 | F-sweetened coffee drink liking (derived food-liking factor) \|\| id:ebi-fl187-GCST90094853 | Inverse variance weighted | 4 | 0.0676964099233608 | 0.205340574211496 | 0.741642763095763 | -0.334771116 | 0.470163935377894 | 1.07004040551389 | 0.715501834869519 | 1.60025651 | 0.998735809224318 |
| ebi-fl187-GCST90094853 | ebi-met1400-GCST90200224 | 2-ketocaprylate levels \|\| id:ebi-met1400-GCST90200224 | F-sweetened coffee drink liking (derived food-liking factor) \|\| id:ebi-fl187-GCST90094853 | Inverse variance weighted | 4 | -0.020977863 | 0.22972806660801 | 0.927241452135648 | -0.471244874 | 0.429289147153445 | 0.979240641389625 | 0.62422470332585 | 1.53616514796692 | 0.998735809224318 |
| ebi-fl187-GCST90094853 | ebi-met1400-GCST90200243 | Hydroxypalmitoyl sphingomyelin (d18:1/16:0(OH)) levels \|\| id:ebi-met1400-GCST90200243 | F-sweetened coffee drink liking (derived food-liking factor) \|\| id:ebi-fl187-GCST90094853 | Inverse variance weighted | 4 | -0.025159355 | 0.142078928245843 | 0.859445447358993 | -0.303634055 | 0.253315343938778 | 0.975154503487599 | 0.738130932515738 | 1.28828946706128 | 0.998735809224318 |
| ebi-fl187-GCST90094853 | ebi-met1400-GCST90200273 | 2-methoxyhydroquinone sulfate (2) levels \|\| id:ebi-met1400-GCST90200273 | F-sweetened coffee drink liking (derived food-liking factor) \|\| id:ebi-fl187-GCST90094853 | Inverse variance weighted | 4 | 0.130757706343084 | 0.213859804550691 | 0.540922974706341 | -0.288407511 | 0.549922923262439 | 1.13969160780839 | 0.749456118706597 | 1.73311942952775 | 0.998735809224318 |
| ebi-fl187-GCST90094853 | ebi-met1400-GCST90200276 | 2,4-di-tert-butylphenol levels \|\| id:ebi-met1400-GCST90200276 | F-sweetened coffee drink liking (derived food-liking factor) \|\| id:ebi-fl187-GCST90094853 | Inverse variance weighted | 4 | 0.149505912045424 | 0.144664536469016 | 0.301385769693391 | -0.134036579 | 0.433048403524696 | 1.16126033621556 | 0.87455807317904 | 1.54195085475062 | 0.998735809224318 |
| ebi-fl187-GCST90094853 | ebi-met1400-GCST90200289 | N-acetylmethionine levels \|\| id:ebi-met1400-GCST90200289 | F-sweetened coffee drink liking (derived food-liking factor) \|\| id:ebi-fl187-GCST90094853 | Inverse variance weighted | 4 | 0.155539296732782 | 0.2050118823343 | 0.44804142997436 | -0.246283993 | 0.557362586108009 | 1.16828784506047 | 0.781700196295375 | 1.7460613357711 | 0.998735809224318 |
| ebi-fl187-GCST90094853 | ebi-met1400-GCST90200308 | 3-hydroxyisobutyrate levels \|\| id:ebi-met1400-GCST90200308 | F-sweetened coffee drink liking (derived food-liking factor) \|\| id:ebi-fl187-GCST90094853 | Inverse variance weighted | 4 | 0.0974714936507072 | 0.215712845738429 | 0.651371554927739 | -0.325325684 | 0.520268671298029 | 1.10238001628562 | 0.722292076360977 | 1.68247962296425 | 0.998735809224318 |
| ebi-fl187-GCST90094853 | ebi-met1400-GCST90200383 | Cystathionine levels \|\| id:ebi-met1400-GCST90200383 | F-sweetened coffee drink liking (derived food-liking factor) \|\| id:ebi-fl187-GCST90094853 | Inverse variance weighted (multiplicative random effects) | 4 | -0.088657055 | 0.364020420392243 | 0.807579608042882 | -0.802137079 | 0.624822968814682 | 0.915159368952869 | 0.448369737904918 | 1.86791524890964 | 0.998735809224318 |
| ebi-fl187-GCST90094853 | ebi-met1400-GCST90200384 | Guanidinoacetate levels \|\| id:ebi-met1400-GCST90200384 | F-sweetened coffee drink liking (derived food-liking factor) \|\| id:ebi-fl187-GCST90094853 | Inverse variance weighted | 4 | 0.11176665800657 | 0.202169713786184 | 0.58037584321709 | -0.284485981 | 0.50801929702749 | 1.11825189507276 | 0.752400903287524 | 1.6619960121924 | 0.998735809224318 |
| ebi-fl187-GCST90094853 | ebi-met1400-GCST90200403 | Citrulline levels \|\| id:ebi-met1400-GCST90200403 | F-sweetened coffee drink liking (derived food-liking factor) \|\| id:ebi-fl187-GCST90094853 | Inverse variance weighted | 4 | -0.042607609 | 0.145630466549139 | 0.769848206079602 | -0.328043323 | 0.242828105667506 | 0.958287339851628 | 0.720331811946453 | 1.274849466 | 0.998735809224318 |
| ebi-fl187-GCST90094853 | ebi-met1400-GCST90200434 | 12,13-DiHOME levels \|\| id:ebi-met1400-GCST90200434 | F-sweetened coffee drink liking (derived food-liking factor) \|\| id:ebi-fl187-GCST90094853 | Inverse variance weighted | 4 | -0.174222436 | 0.146954524080897 | 0.235798713300708 | -0.462253303 | 0.113808431098713 | 0.840110006080292 | 0.629862773415355 | 1.1205374441947 | 0.998735809224318 |
| ebi-fl187-GCST90094853 | ebi-met1400-GCST90200437 | Fructose levels \|\| id:ebi-met1400-GCST90200437 | F-sweetened coffee drink liking (derived food-liking factor) \|\| id:ebi-fl187-GCST90094853 | Inverse variance weighted | 4 | 0.0063269055032258 | 0.146373031017029 | 0.965522551068004 | -0.280564235 | 0.293218046296603 | 1.00634696264742 | 0.755357421880659 | 1.34073510088539 | 0.998735809224318 |
| ebi-fl187-GCST90094853 | ebi-met1400-GCST90200448 | N-stearoyl-sphinganine (d18:0/18:0) levels \|\| id:ebi-met1400-GCST90200448 | F-sweetened coffee drink liking (derived food-liking factor) \|\| id:ebi-fl187-GCST90094853 | Inverse variance weighted | 4 | -0.034396285 | 0.161879284737472 | 0.831731736327535 | -0.351679683 | 0.2828871127435 | 0.966188542404448 | 0.703505430337671 | 1.32695535701204 | 0.998735809224318 |
| ebi-fl187-GCST90094853 | ebi-met1400-GCST90200468 | X-11847 levels \|\| id:ebi-met1400-GCST90200468 | F-sweetened coffee drink liking (derived food-liking factor) \|\| id:ebi-fl187-GCST90094853 | Inverse variance weighted | 4 | -0.059446634 | 0.243941784537723 | 0.807469456219962 | -0.537572532 | 0.418679263587822 | 0.942285818174459 | 0.584164573567046 | 1.51995277240276 | 0.998735809224318 |
| ebi-fl187-GCST90094853 | ebi-met1400-GCST90200476 | X-11843 levels \|\| id:ebi-met1400-GCST90200476 | F-sweetened coffee drink liking (derived food-liking factor) \|\| id:ebi-fl187-GCST90094853 | Inverse variance weighted | 4 | 0.0177473425092752 | 0.175769149531343 | 0.91957458442517 | -0.326760191 | 0.362254875590708 | 1.01790576238192 | 0.721256686442351 | 1.43656504066689 | 0.998735809224318 |
| ebi-fl187-GCST90094853 | ebi-met1400-GCST90200511 | X-12906 levels \|\| id:ebi-met1400-GCST90200511 | F-sweetened coffee drink liking (derived food-liking factor) \|\| id:ebi-fl187-GCST90094853 | Inverse variance weighted | 4 | -0.021061325 | 0.148093438087446 | 0.886909028390539 | -0.311324464 | 0.269201813697184 | 0.979158915852556 | 0.732476175447944 | 1.30891927223057 | 0.998735809224318 |
| ebi-fl187-GCST90094853 | ebi-met1400-GCST90200515 | X-12849 levels \|\| id:ebi-met1400-GCST90200515 | F-sweetened coffee drink liking (derived food-liking factor) \|\| id:ebi-fl187-GCST90094853 | Inverse variance weighted | 4 | 0.229147792485646 | 0.153228729822083 | 0.134793825454185 | -0.071180518 | 0.529476102936928 | 1.25752787844759 | 0.931293761697495 | 1.69804247608239 | 0.998735809224318 |
| ebi-fl187-GCST90094853 | ebi-met1400-GCST90200551 | X-18886 levels \|\| id:ebi-met1400-GCST90200551 | F-sweetened coffee drink liking (derived food-liking factor) \|\| id:ebi-fl187-GCST90094853 | Inverse variance weighted | 4 | 0.0184804509040571 | 0.180811076312216 | 0.91859108444364 | -0.335909259 | 0.372870160476001 | 1.01865227124394 | 0.714687954610827 | 1.45189581413259 | 0.998735809224318 |
| ebi-fl187-GCST90094853 | ebi-met1400-GCST90200564 | X-21319 levels \|\| id:ebi-met1400-GCST90200564 | F-sweetened coffee drink liking (derived food-liking factor) \|\| id:ebi-fl187-GCST90094853 | Inverse variance weighted | 4 | -0.081578393 | 0.146978440485841 | 0.578869724880102 | -0.369656136 | 0.206499350620502 | 0.921660455540966 | 0.690971890090929 | 1.22936693589112 | 0.998735809224318 |
| ebi-fl187-GCST90094853 | ebi-met1400-GCST90200579 | X-21442 levels \|\| id:ebi-met1400-GCST90200579 | F-sweetened coffee drink liking (derived food-liking factor) \|\| id:ebi-fl187-GCST90094853 | Inverse variance weighted | 4 | 0.124462332906468 | 0.158308533742854 | 0.431749720470901 | -0.185822393 | 0.434747059042463 | 1.1325393601908 | 0.830421070289666 | 1.54457232393438 | 0.998735809224318 |
| ebi-fl187-GCST90094853 | ebi-met1400-GCST90200581 | X-21441 levels \|\| id:ebi-met1400-GCST90200581 | F-sweetened coffee drink liking (derived food-liking factor) \|\| id:ebi-fl187-GCST90094853 | Inverse variance weighted | 4 | -0.109827546 | 0.145906596086437 | 0.451615170140268 | -0.395804474 | 0.176149382490097 | 0.895988638942455 | 0.673138298996535 | 1.19261620132252 | 0.998735809224318 |
| ebi-fl187-GCST90094853 | ebi-met1400-GCST90200648 | X-24585 levels \|\| id:ebi-met1400-GCST90200648 | F-sweetened coffee drink liking (derived food-liking factor) \|\| id:ebi-fl187-GCST90094853 | Inverse variance weighted | 4 | 0.092146111955581 | 0.142146230014523 | 0.516824093857244 | -0.186460499 | 0.370752722784047 | 1.09652502579256 | 0.829891342947328 | 1.44882476773309 | 0.998735809224318 |
| ebi-fl187-GCST90094853 | ebi-met1400-GCST90200720 | Adenosine 5'-diphosphate (ADP) to pantothenate ratio \|\| id:ebi-met1400-GCST90200720 | F-sweetened coffee drink liking (derived food-liking factor) \|\| id:ebi-fl187-GCST90094853 | Inverse variance weighted | 4 | -0.150891416 | 0.193353250392047 | 0.435159912809976 | -0.529863787 | 0.228080954734684 | 0.859941069401907 | 0.588685150904299 | 1.2561870156027 | 0.998735809224318 |
| ebi-fl187-GCST90094853 | ebi-met1400-GCST90200724 | Cholate to taurocholate ratio \|\| id:ebi-met1400-GCST90200724 | F-sweetened coffee drink liking (derived food-liking factor) \|\| id:ebi-fl187-GCST90094853 | Inverse variance weighted | 4 | 0.0676736522800019 | 0.195621456960486 | 0.729386402744724 | -0.315744403 | 0.451091707922555 | 1.07001605419306 | 0.729245819128212 | 1.57002525924606 | 0.998735809224318 |
| ebi-fl187-GCST90094853 | ebi-met1400-GCST90200726 | N-acetylputrescine to (N(1) + N(8))-acetylspermidine ratio \|\| id:ebi-met1400-GCST90200726 | F-sweetened coffee drink liking (derived food-liking factor) \|\| id:ebi-fl187-GCST90094853 | Inverse variance weighted | 4 | -0.047484194 | 0.171300184024659 | 0.781627512662599 | -0.383232555 | 0.288264166762447 | 0.953625546089564 | 0.681654358985583 | 1.33410968501391 | 0.998735809224318 |
| ebi-fl187-GCST90094853 | ebi-met1400-GCST90200744 | Arginine to glutamate ratio \|\| id:ebi-met1400-GCST90200744 | F-sweetened coffee drink liking (derived food-liking factor) \|\| id:ebi-fl187-GCST90094853 | Inverse variance weighted (multiplicative random effects) | 4 | -0.150526078 | 0.257779741554982 | 0.55926544204891 | -0.655774371 | 0.354722215575016 | 0.860255296086757 | 0.51903997725288 | 1.42578453852845 | 0.998735809224318 |
| ebi-fl187-GCST90094853 | ebi-met1400-GCST90200759 | Histidine to trans-urocanate ratio \|\| id:ebi-met1400-GCST90200759 | F-sweetened coffee drink liking (derived food-liking factor) \|\| id:ebi-fl187-GCST90094853 | Inverse variance weighted (multiplicative random effects) | 4 | -0.227191065 | 0.285883226178467 | 0.426788713462661 | -0.787522188 | 0.333140058121166 | 0.796768533015781 | 0.454970731488817 | 1.39534271386361 | 0.998735809224318 |
| ebi-fl187-GCST90094853 | ebi-met1400-GCST90200795 | Oleoyl-linoleoyl-glycerol (18:1 to 18:2) [2] to linoleoyl-arachidonoyl-glycerol (18:2 to 20:4) [2] ratio \|\| id:ebi-met1400-GCST90200795 | F-sweetened coffee drink liking (derived food-liking factor) \|\| id:ebi-fl187-GCST90094853 | Inverse variance weighted | 4 | -0.026579918 | 0.151140167121812 | 0.860401810753264 | -0.322814645 | 0.269654809899019 | 0.973770219288756 | 0.724108058820849 | 1.30951234200844 | 0.998735809224318 |
| ebi-fl187-GCST90094853 | ebi-met1400-GCST90200811 | Choline to taurocholate ratio \|\| id:ebi-met1400-GCST90200811 | F-sweetened coffee drink liking (derived food-liking factor) \|\| id:ebi-fl187-GCST90094853 | Inverse variance weighted | 4 | 0.0361757350201295 | 0.152178298450673 | 0.81209872970025 | -0.26209373 | 0.334445199983449 | 1.03683803923533 | 0.769438900863128 | 1.39716502297899 | 0.998735809224318 |
| ebi-fl187-GCST90094853 | ebi-met1400-GCST90200831 | Cholate to phosphate ratio \|\| id:ebi-met1400-GCST90200831 | F-sweetened coffee drink liking (derived food-liking factor) \|\| id:ebi-fl187-GCST90094853 | Inverse variance weighted | 4 | 0.12362548460901 | 0.151508506376147 | 0.414520734529359 | -0.173331188 | 0.420582157106259 | 1.13159199301201 | 0.840859086379419 | 1.52284783430538 | 0.998735809224318 |
| ebi-fl187-GCST90094853 | ebi-met1400-GCST90200841 | Adenosine 5'-diphosphate (ADP) to oxalate (ethanedioate) ratio \|\| id:ebi-met1400-GCST90200841 | F-sweetened coffee drink liking (derived food-liking factor) \|\| id:ebi-fl187-GCST90094853 | Inverse variance weighted | 4 | 0.220134603553557 | 0.195956039495189 | 0.261272873477886 | -0.163939234 | 0.604208440964127 | 1.24624446823211 | 0.848793598226299 | 1.82980323820144 | 0.998735809224318 |
| ebi-fl187-GCST90094853 | ebi-met1400-GCST90200844 | Aspartate to phosphate ratio \|\| id:ebi-met1400-GCST90200844 | F-sweetened coffee drink liking (derived food-liking factor) \|\| id:ebi-fl187-GCST90094853 | Inverse variance weighted | 4 | 0.0543840156667647 | 0.176362122286287 | 0.757804137323944 | -0.291285744 | 0.400053775347887 | 1.05589000261342 | 0.747302110401746 | 1.49190492319043 | 0.998735809224318 |
| ebi-fl187-GCST90094853 | ebi-met1400-GCST90200870 | Adenosine 5'-monophosphate (AMP) to acetoacetate ratio \|\| id:ebi-met1400-GCST90200870 | F-sweetened coffee drink liking (derived food-liking factor) \|\| id:ebi-fl187-GCST90094853 | Inverse variance weighted | 4 | 0.121315938126845 | 0.160600887260342 | 0.450016378948652 | -0.193461801 | 0.436093677157115 | 1.12898154434192 | 0.824101315519606 | 1.54665367408256 | 0.998735809224318 |
| ebi-fl187-GCST90094853 | ebi-met1400-GCST90200887 | Cholesterol to cortisol ratio \|\| id:ebi-met1400-GCST90200887 | F-sweetened coffee drink liking (derived food-liking factor) \|\| id:ebi-fl187-GCST90094853 | Inverse variance weighted | 4 | 0.0856216568903724 | 0.1620606281221 | 0.597269605467755 | -0.232017174 | 0.403260488009687 | 1.0893940855795 | 0.792932505185364 | 1.49669671243729 | 0.998735809224318 |
| ebi-fl187-GCST90094853 | ebi-met1400-GCST90200890 | Cortisol to taurocholate ratio \|\| id:ebi-met1400-GCST90200890 | F-sweetened coffee drink liking (derived food-liking factor) \|\| id:ebi-fl187-GCST90094853 | Inverse variance weighted | 4 | 0.00634183447008447 | 0.151684376289344 | 0.96665063247362 | -0.290959543 | 0.303643211997199 | 1.00636198648002 | 0.747545920829007 | 1.35478559860094 | 0.998735809224318 |
| ebi-fl187-GCST90094853 | ebi-met1400-GCST90200948 | Adenosine 5'-diphosphate (ADP) to mannitol to sorbitol ratio \|\| id:ebi-met1400-GCST90200948 | F-sweetened coffee drink liking (derived food-liking factor) \|\| id:ebi-fl187-GCST90094853 | Inverse variance weighted | 4 | -0.014825432 | 0.1953052778969 | 0.9394914822418 | -0.397623777 | 0.367972912823921 | 0.985283923779002 | 0.671914770220291 | 1.44480290281321 | 0.998735809224318 |
| ebi-fl187-GCST90094853 | ebi-met1400-GCST90200972 | Leucine to N-palmitoyl-sphingosine (d18:1 to 16:0) ratio \|\| id:ebi-met1400-GCST90200972 | F-sweetened coffee drink liking (derived food-liking factor) \|\| id:ebi-fl187-GCST90094853 | Inverse variance weighted | 4 | -0.079346027 | 0.140234562367693 | 0.571523387650988 | -0.354205769 | 0.195513715230731 | 0.923720236986396 | 0.701730557927348 | 1.21593547064334 | 0.998735809224318 |
| ebi-fl187-GCST90094853 | ebi-met1400-GCST90200973 | Proline to glutamate ratio \|\| id:ebi-met1400-GCST90200973 | F-sweetened coffee drink liking (derived food-liking factor) \|\| id:ebi-fl187-GCST90094853 | Inverse variance weighted | 4 | -0.119568405 | 0.15094546083984 | 0.428284968584648 | -0.415421508 | 0.176284698501904 | 0.887303309987132 | 0.660062000653122 | 1.19277759230965 | 0.998735809224318 |
| ebi-fl187-GCST90094853 | ebi-met1400-GCST90200983 | Cholesterol to linoleoyl-arachidonoyl-glycerol (18:2 to 20:4) [1] ratio \|\| id:ebi-met1400-GCST90200983 | F-sweetened coffee drink liking (derived food-liking factor) \|\| id:ebi-fl187-GCST90094853 | Inverse variance weighted | 4 | 0.0164280146316349 | 0.149934667076821 | 0.912752132786457 | -0.277443933 | 0.310299962102203 | 1.01656369643998 | 0.7577180465118 | 1.36383415134037 | 0.998735809224318 |
| ebi-fl187-GCST90094853 | ebi-met1400-GCST90201001 | Serine to alpha-ketobutyrate ratio \|\| id:ebi-met1400-GCST90201001 | F-sweetened coffee drink liking (derived food-liking factor) \|\| id:ebi-fl187-GCST90094853 | Inverse variance weighted | 4 | -0.074613709 | 0.158493521376162 | 0.637806280954448 | -0.385261011 | 0.236033593293849 | 0.928101934883887 | 0.680273054619665 | 1.266216846 | 0.998735809224318 |
| ebi-fl187-GCST90094853 | ebi-met1400-GCST90201005 | Fructose to maltose ratio \|\| id:ebi-met1400-GCST90201005 | F-sweetened coffee drink liking (derived food-liking factor) \|\| id:ebi-fl187-GCST90094853 | Inverse variance weighted | 4 | 0.0922797011888002 | 0.214905442293879 | 0.667634509365474 | -0.328934966 | 0.513494368084802 | 1.09667151951473 | 0.71968981974362 | 1.67112051431155 | 0.998735809224318 |
| ebi-fl187-GCST90094853 | ebi-met1400-GCST90201020 | Paraxanthine to 5-acetylamino-6-formylamino-3-methyluracil ratio \|\| id:ebi-met1400-GCST90201020 | F-sweetened coffee drink liking (derived food-liking factor) \|\| id:ebi-fl187-GCST90094853 | Inverse variance weighted | 4 | 0.0846749230827748 | 0.172282207074632 | 0.623080164794441 | -0.252998203 | 0.422348048949054 | 1.08836320742948 | 0.776469277305877 | 1.52553939467661 | 0.998735809224318 |
| ebi-fl187-GCST90094853 | ebi-pw11-GCST90275197 | Placental weight (fetal genotype, adjusted for sex) \|\| id:ebi-pw11-GCST90275197 | F-sweetened coffee drink liking (derived food-liking factor) \|\| id:ebi-fl187-GCST90094853 | Inverse variance weighted | 4 | 0.0416156302552669 | 0.0557470661958556 | 0.455360607664126 | -0.067648619 | 0.150879879999144 | 1.04249369869498 | 0.934588812182706 | 1.16285696731225 | 0.998735809224318 |
| ebi-fl187-GCST90094853 | prot-a-1017 | FAS-associated factor 2 \|\| id:prot-a-1017 | F-sweetened coffee drink liking (derived food-liking factor) \|\| id:ebi-fl187-GCST90094853 | Inverse variance weighted | 4 | -0.248801269 | 0.296075627059058 | 0.400723315507374 | -0.829109498 | 0.331506960510071 | 0.779734915857623 | 0.436437762218034 | 1.39306584269341 | 0.998735809224318 |
| ebi-fl187-GCST90094853 | prot-a-1019 | Fas apoptotic inhibitory molecule 3 \|\| id:prot-a-1019 | F-sweetened coffee drink liking (derived food-liking factor) \|\| id:ebi-fl187-GCST90094853 | Inverse variance weighted | 4 | -0.02316196 | 0.241296910446874 | 0.923528944928254 | -0.496103904 | 0.449779984743396 | 0.977104219419418 | 0.608898370682753 | 1.56796717083788 | 0.998735809224318 |
| ebi-fl187-GCST90094853 | prot-a-1052 | MIP18 family protein FAM96A \|\| id:prot-a-1052 | F-sweetened coffee drink liking (derived food-liking factor) \|\| id:ebi-fl187-GCST90094853 | Inverse variance weighted | 4 | 0.0158498507256824 | 0.308978982868247 | 0.959088456707983 | -0.589748956 | 0.621448657147446 | 1.01597612587501 | 0.55446646291106 | 1.86162294276319 | 0.998735809224318 |
| ebi-fl187-GCST90094853 | prot-a-1066 | F-box/LRR-repeat protein 4 \|\| id:prot-a-1066 | F-sweetened coffee drink liking (derived food-liking factor) \|\| id:ebi-fl187-GCST90094853 | Inverse variance weighted (multiplicative random effects) | 4 | -0.0353225 | 0.495012327580554 | 0.943113782881135 | -1.005546662 | 0.934901661644087 | 0.965294058320487 | 0.365844586623605 | 2.54696298127134 | 0.998735809224318 |
| ebi-fl187-GCST90094853 | prot-a-1076 | Low affinity immunoglobulin gamma Fc region receptor III-B \|\| id:prot-a-1076 | F-sweetened coffee drink liking (derived food-liking factor) \|\| id:ebi-fl187-GCST90094853 | Inverse variance weighted | 4 | 0.14893783613151 | 0.266420395667012 | 0.576139088416284 | -0.373246139 | 0.671121811638854 | 1.1606008395286 | 0.688495746072925 | 1.95643083693333 | 0.998735809224318 |
| ebi-fl187-GCST90094853 | prot-a-1081 | Fc receptor-like protein 3 \|\| id:prot-a-1081 | F-sweetened coffee drink liking (derived food-liking factor) \|\| id:ebi-fl187-GCST90094853 | Inverse variance weighted | 4 | -0.037535118 | 0.241296910446874 | 0.876383220824101 | -0.510477062 | 0.435406826945635 | 0.963160593305651 | 0.600209173508753 | 1.54559171942307 | 0.998735809224318 |
| ebi-fl187-GCST90094853 | prot-a-1105 | Fibroblast growth factor receptor 4 \|\| id:prot-a-1105 | F-sweetened coffee drink liking (derived food-liking factor) \|\| id:ebi-fl187-GCST90094853 | Inverse variance weighted | 4 | 0.263470483258898 | 0.241296910446874 | 0.274879996728979 | -0.209471461 | 0.736412427734771 | 1.30143888014022 | 0.811012784420711 | 2.08842966630969 | 0.998735809224318 |
| ebi-fl187-GCST90094853 | prot-a-1143 | Follistatin-related protein 1 \|\| id:prot-a-1143 | F-sweetened coffee drink liking (derived food-liking factor) \|\| id:ebi-fl187-GCST90094853 | Inverse variance weighted | 4 | 0.0295225348507084 | 0.241465588217237 | 0.902689987241157 | -0.443750018 | 0.502795087756494 | 1.02996264526482 | 0.641625795645389 | 1.65333603767265 | 0.998735809224318 |
| ebi-fl187-GCST90094853 | prot-a-1151 | Alpha-(1,3)-fucosyltransferase 10 \|\| id:prot-a-1151 | F-sweetened coffee drink liking (derived food-liking factor) \|\| id:ebi-fl187-GCST90094853 | Inverse variance weighted | 4 | -0.480033798 | 0.241296910446874 | 0.0466581097455264 | -0.952975743 | -0.007091854 | 0.618762478458707 | 0.385591892333651 | 0.992933234231669 | 0.998735809224318 |
| ebi-fl187-GCST90094853 | prot-a-1165 | Growth arrest and DNA damage-inducible proteins-interacting protein 1 \|\| id:prot-a-1165 | F-sweetened coffee drink liking (derived food-liking factor) \|\| id:ebi-fl187-GCST90094853 | Inverse variance weighted | 4 | -0.143253462 | 0.241296910446874 | 0.552725296790849 | -0.616195406 | 0.329688482477914 | 0.86653440751653 | 0.539994995816179 | 1.39053488500407 | 0.998735809224318 |
| ebi-fl187-GCST90094853 | prot-a-1194 | Growth/differentiation factor 11/8 \|\| id:prot-a-1194 | F-sweetened coffee drink liking (derived food-liking factor) \|\| id:ebi-fl187-GCST90094853 | Inverse variance weighted | 4 | -0.207741229 | 0.241296910446874 | 0.389273220387308 | -0.680683173 | 0.265200715848239 | 0.812417239837577 | 0.506271003461239 | 1.30369262128962 | 0.998735809224318 |
| ebi-fl187-GCST90094853 | prot-a-1216 | D-glucuronyl C5-epimerase \|\| id:prot-a-1216 | F-sweetened coffee drink liking (derived food-liking factor) \|\| id:ebi-fl187-GCST90094853 | Inverse variance weighted | 4 | -0.349808132 | 0.241296910446874 | 0.147142194669649 | -0.822750076 | 0.123133812461231 | 0.704823309774579 | 0.439222097716424 | 1.13103575750036 | 0.998735809224318 |
| ebi-fl187-GCST90094853 | prot-a-1246 | Glypican-3 \|\| id:prot-a-1246 | F-sweetened coffee drink liking (derived food-liking factor) \|\| id:ebi-fl187-GCST90094853 | Inverse variance weighted | 4 | 0.512262321856121 | 0.241296910446874 | 0.0337576787107768 | 0.0393203773802476 | 0.985204266331993 | 1.6690628843921 | 1.04010365596135 | 2.67835892710175 | 0.998735809224318 |
| ebi-fl187-GCST90094853 | prot-a-1275 | Glutamate receptor 4 \|\| id:prot-a-1275 | F-sweetened coffee drink liking (derived food-liking factor) \|\| id:ebi-fl187-GCST90094853 | Inverse variance weighted | 4 | -0.117706047 | 0.241296910446874 | 0.625687360213455 | -0.590647991 | 0.355235897539492 | 0.888957325941076 | 0.553968201768325 | 1.42651712647362 | 0.998735809224318 |
| ebi-fl187-GCST90094853 | prot-a-1289 | Glutathione S-transferase P \|\| id:prot-a-1289 | F-sweetened coffee drink liking (derived food-liking factor) \|\| id:ebi-fl187-GCST90094853 | Inverse variance weighted | 4 | -0.18347858 | 0.301344880551026 | 0.542613421389502 | -0.774114546 | 0.407157386216315 | 0.832369705185951 | 0.461111893870898 | 1.50254056622822 | 0.998735809224318 |
| ebi-fl187-GCST90094853 | prot-a-130 | Apolipoprotein D \|\| id:prot-a-130 | F-sweetened coffee drink liking (derived food-liking factor) \|\| id:ebi-fl187-GCST90094853 | Inverse variance weighted | 4 | -0.270099704 | 0.278837809418366 | 0.332713654475006 | -0.81662181 | 0.276422402548752 | 0.763303386209705 | 0.441922032125166 | 1.31840464390828 | 0.998735809224318 |
| ebi-fl187-GCST90094853 | prot-a-1313 | Heparin-binding EGF-like growth factor \|\| id:prot-a-1313 | F-sweetened coffee drink liking (derived food-liking factor) \|\| id:ebi-fl187-GCST90094853 | Inverse variance weighted | 4 | -0.260397032 | 0.247705627308454 | 0.293150288768486 | -0.745900061 | 0.22510599765912 | 0.770745514518144 | 0.474307202161961 | 1.25245546650373 | 0.998735809224318 |
| ebi-fl187-GCST90094853 | prot-a-1347 | HLA class II histocompatibility antigen, DQ alpha 2 chain \|\| id:prot-a-1347 | F-sweetened coffee drink liking (derived food-liking factor) \|\| id:ebi-fl187-GCST90094853 | Inverse variance weighted | 4 | 0.301120577598928 | 0.241296910446874 | 0.21205832609415 | -0.171821367 | 0.774062522074801 | 1.35137227693905 | 0.842129591972241 | 2.16855819851018 | 0.998735809224318 |
| ebi-fl187-GCST90094853 | prot-a-1357 | Non-histone chromosomal protein HMG-14 \|\| id:prot-a-1357 | F-sweetened coffee drink liking (derived food-liking factor) \|\| id:ebi-fl187-GCST90094853 | Inverse variance weighted | 4 | 0.178748645233609 | 0.241465588217237 | 0.459138908338029 | -0.294523908 | 0.652021198139394 | 1.1957201564318 | 0.744886137635135 | 1.91941643193474 | 0.998735809224318 |
| ebi-fl187-GCST90094853 | prot-a-137 | Apolipoprotein M \|\| id:prot-a-137 | F-sweetened coffee drink liking (derived food-liking factor) \|\| id:ebi-fl187-GCST90094853 | Inverse variance weighted | 4 | 0.213196756280773 | 0.241296910446874 | 0.37694170683442 | -0.259745188 | 0.686138700756646 | 1.23762813839849 | 0.771248083883768 | 1.98603204463398 | 0.998735809224318 |
| ebi-fl187-GCST90094853 | prot-a-1381 | 17-beta-hydroxysteroid dehydrogenase 14 \|\| id:prot-a-1381 | F-sweetened coffee drink liking (derived food-liking factor) \|\| id:ebi-fl187-GCST90094853 | Inverse variance weighted | 4 | -0.349052962 | 0.241296910446874 | 0.1480172888593 | -0.821994906 | 0.123888982607339 | 0.705355772321006 | 0.43955391040388 | 1.13189020452444 | 0.998735809224318 |
| ebi-fl187-GCST90094853 | prot-a-1392 | Serine protease HTRA2, mitochondrial \|\| id:prot-a-1392 | F-sweetened coffee drink liking (derived food-liking factor) \|\| id:ebi-fl187-GCST90094853 | Inverse variance weighted | 4 | 0.214364090813801 | 0.272916105822951 | 0.432185188873516 | -0.320551477 | 0.749279658226785 | 1.23907370803102 | 0.725748693272491 | 2.11547560218243 | 0.998735809224318 |
| ebi-fl187-GCST90094853 | prot-a-1416 | Interferon-induced protein with tetratricopeptide repeats 2 \|\| id:prot-a-1416 | F-sweetened coffee drink liking (derived food-liking factor) \|\| id:ebi-fl187-GCST90094853 | Inverse variance weighted | 4 | 0.109309960367326 | 0.241296910446874 | 0.65054140060241 | -0.363631984 | 0.582251904843199 | 1.11550806004857 | 0.695146972807716 | 1.79006495131143 | 0.998735809224318 |
| ebi-fl187-GCST90094853 | prot-a-1428 | Interferon gamma \|\| id:prot-a-1428 | F-sweetened coffee drink liking (derived food-liking factor) \|\| id:ebi-fl187-GCST90094853 | Inverse variance weighted | 4 | -0.068044627 | 0.241296910446874 | 0.777947012507467 | -0.540986571 | 0.404897317831274 | 0.93421878159739 | 0.582173613285466 | 1.49914855632826 | 0.998735809224318 |
| ebi-fl187-GCST90094853 | prot-a-1483 | Interleukin-17C \|\| id:prot-a-1483 | F-sweetened coffee drink liking (derived food-liking factor) \|\| id:ebi-fl187-GCST90094853 | Inverse variance weighted | 4 | 0.251308691659434 | 0.241296910446874 | 0.297647476013032 | -0.221633253 | 0.724250636135307 | 1.28570691007926 | 0.801209151658343 | 2.06318444466604 | 0.998735809224318 |
| ebi-fl187-GCST90094853 | prot-a-1503 | Interleukin-1 receptor-like 2 \|\| id:prot-a-1503 | F-sweetened coffee drink liking (derived food-liking factor) \|\| id:ebi-fl187-GCST90094853 | Inverse variance weighted | 4 | 0.106827373868523 | 0.241296910446874 | 0.657967058171616 | -0.366114571 | 0.579769318344396 | 1.11274214952603 | 0.693423350724054 | 1.78562647196539 | 0.998735809224318 |
| ebi-fl187-GCST90094853 | prot-a-1522 | Interleukin-31 receptor subunit alpha \|\| id:prot-a-1522 | F-sweetened coffee drink liking (derived food-liking factor) \|\| id:ebi-fl187-GCST90094853 | Inverse variance weighted | 4 | -0.047715617 | 0.241465588217237 | 0.843351498958781 | -0.52098817 | 0.425556935720244 | 0.953404880491757 | 0.593933350719174 | 1.53044254047166 | 0.998735809224318 |
| ebi-fl187-GCST90094853 | prot-a-1540 | Interleukin-6 receptor subunit alpha \|\| id:prot-a-1540 | F-sweetened coffee drink liking (derived food-liking factor) \|\| id:ebi-fl187-GCST90094853 | Inverse variance weighted | 4 | 0.395954193094965 | 0.241296910446874 | 0.100809491388382 | -0.076987751 | 0.868896137570838 | 1.48580125603552 | 0.925901194548089 | 2.38427748601644 | 0.998735809224318 |
| ebi-fl187-GCST90094853 | prot-a-1541 | Interleukin-6 receptor subunit alpha \|\| id:prot-a-1541 | F-sweetened coffee drink liking (derived food-liking factor) \|\| id:ebi-fl187-GCST90094853 | Inverse variance weighted | 4 | 0.532067680485499 | 0.241296910446874 | 0.0274517701682142 | 0.0591257360096257 | 1.00500962496137 | 1.70244879202849 | 1.06090862677158 | 2.73193356745444 | 0.998735809224318 |
| ebi-fl187-GCST90094853 | prot-a-1545 | Interleukin-9 \|\| id:prot-a-1545 | F-sweetened coffee drink liking (derived food-liking factor) \|\| id:ebi-fl187-GCST90094853 | Inverse variance weighted | 4 | -0.359226796 | 0.27889443856998 | 0.197732534112264 | -0.905859896 | 0.18740630323041 | 0.698215980545696 | 0.404194170689322 | 1.20611723483786 | 0.998735809224318 |
| ebi-fl187-GCST90094853 | prot-a-1548 | Inositol monophosphatase 3 \|\| id:prot-a-1548 | F-sweetened coffee drink liking (derived food-liking factor) \|\| id:ebi-fl187-GCST90094853 | Inverse variance weighted | 4 | 0.0875378840675566 | 0.284464919528457 | 0.758289142645331 | -0.470013358 | 0.645089126343332 | 1.09148361349866 | 0.624993919428027 | 1.90615691049661 | 0.998735809224318 |
| ebi-fl187-GCST90094853 | prot-a-1555 | Inhibin beta A chain:Inhibin beta B chain heterodimer \|\| id:prot-a-1555 | F-sweetened coffee drink liking (derived food-liking factor) \|\| id:ebi-fl187-GCST90094853 | Inverse variance weighted | 4 | -0.176574315 | 0.241296910446874 | 0.464307760328896 | -0.64951626 | 0.29636762901639 | 0.838136490342313 | 0.522298372309181 | 1.34496451393781 | 0.998735809224318 |
| ebi-fl187-GCST90094853 | prot-a-1603 | Junctophilin-3 \|\| id:prot-a-1603 | F-sweetened coffee drink liking (derived food-liking factor) \|\| id:ebi-fl187-GCST90094853 | Inverse variance weighted | 4 | -0.130981315 | 0.377607874232978 | 0.728688047178611 | -0.871092748 | 0.609130118545581 | 0.877234165401226 | 0.418493990636303 | 1.83883113775931 | 0.998735809224318 |
| ebi-fl187-GCST90094853 | prot-a-1627 | UPF0258 protein KIAA1024 \|\| id:prot-a-1627 | F-sweetened coffee drink liking (derived food-liking factor) \|\| id:ebi-fl187-GCST90094853 | Inverse variance weighted | 4 | 0.0417704525040097 | 0.241296910446874 | 0.862566449071584 | -0.431171492 | 0.514712396979883 | 1.04265511240861 | 0.649747474744173 | 1.67315722752111 | 0.998735809224318 |
| ebi-fl187-GCST90094853 | prot-a-1666 | Kallikrein-7 \|\| id:prot-a-1666 | F-sweetened coffee drink liking (derived food-liking factor) \|\| id:ebi-fl187-GCST90094853 | Inverse variance weighted | 4 | 0.166612591124054 | 0.241296910446874 | 0.489887431823037 | -0.306329353 | 0.639554535599927 | 1.18129653188751 | 0.736144127989629 | 1.89563625272731 | 0.998735809224318 |
| ebi-fl187-GCST90094853 | prot-a-1678 | Importin subunit alpha-3 \|\| id:prot-a-1678 | F-sweetened coffee drink liking (derived food-liking factor) \|\| id:ebi-fl187-GCST90094853 | Inverse variance weighted | 4 | 0.163868759857686 | 0.241296910446874 | 0.497063926291533 | -0.309073185 | 0.636810704333559 | 1.17805969622466 | 0.734127041253012 | 1.89044207593849 | 0.998735809224318 |
| ebi-fl187-GCST90094853 | prot-a-1718 | Protein LDOC1 \|\| id:prot-a-1718 | F-sweetened coffee drink liking (derived food-liking factor) \|\| id:ebi-fl187-GCST90094853 | Inverse variance weighted (multiplicative random effects) | 4 | -0.179917205 | 0.446274126986735 | 0.686834927796504 | -1.054614494 | 0.694780083710069 | 0.835339370317753 | 0.348326683425545 | 2.00326847412485 | 0.998735809224318 |
| ebi-fl187-GCST90094853 | prot-a-1723 | Leptin \|\| id:prot-a-1723 | F-sweetened coffee drink liking (derived food-liking factor) \|\| id:ebi-fl187-GCST90094853 | Inverse variance weighted | 4 | 0.0767574397097459 | 0.241296910446874 | 0.7504062780548 | -0.396184505 | 0.549699384185619 | 1.0797801328429 | 0.672882534448896 | 1.73273205290898 | 0.998735809224318 |
| ebi-fl187-GCST90094853 | prot-a-1730 | Galectin-7 \|\| id:prot-a-1730 | F-sweetened coffee drink liking (derived food-liking factor) \|\| id:ebi-fl187-GCST90094853 | Inverse variance weighted | 4 | 0.179039837478094 | 0.241296910446874 | 0.458093537800728 | -0.293902107 | 0.651981781953967 | 1.19606839156715 | 0.745349452367645 | 1.91934077735176 | 0.998735809224318 |
| ebi-fl187-GCST90094853 | prot-a-1753 | Lipase member N \|\| id:prot-a-1753 | F-sweetened coffee drink liking (derived food-liking factor) \|\| id:ebi-fl187-GCST90094853 | Inverse variance weighted | 4 | 0.272165629238966 | 0.278500001478476 | 0.328442729196929 | -0.273694374 | 0.818025632136778 | 1.31280442210234 | 0.760564488285068 | 2.26602145805876 | 0.998735809224318 |
| ebi-fl187-GCST90094853 | prot-a-1756 | VIP36-like protein \|\| id:prot-a-1756 | F-sweetened coffee drink liking (derived food-liking factor) \|\| id:ebi-fl187-GCST90094853 | Inverse variance weighted | 4 | -0.066010641 | 0.241296910446874 | 0.784418133778117 | -0.538952585 | 0.406931303883001 | 0.936120903357212 | 0.583358951366479 | 1.50220090674806 | 0.998735809224318 |
| ebi-fl187-GCST90094853 | prot-a-1760 | Ig Kappa chain V-I region HK102- like \|\| id:prot-a-1760 | F-sweetened coffee drink liking (derived food-liking factor) \|\| id:ebi-fl187-GCST90094853 | Inverse variance weighted | 4 | -0.276147965 | 0.241296910446874 | 0.252444422108026 | -0.749089909 | 0.196793979577438 | 0.758700661389178 | 0.472796644794242 | 1.21749318640551 | 0.998735809224318 |
| ebi-fl187-GCST90094853 | prot-a-179 | Neutral ceramidase \|\| id:prot-a-179 | F-sweetened coffee drink liking (derived food-liking factor) \|\| id:ebi-fl187-GCST90094853 | Inverse variance weighted | 4 | 0.1938871485765 | 0.241296910446874 | 0.421673630874204 | -0.279054796 | 0.666829093052373 | 1.21395927819317 | 0.756498449066409 | 1.94805043014956 | 0.998735809224318 |
| ebi-fl187-GCST90094853 | prot-a-1809 | Lactotransferrin \|\| id:prot-a-1809 | F-sweetened coffee drink liking (derived food-liking factor) \|\| id:ebi-fl187-GCST90094853 | Inverse variance weighted | 4 | 0.241300961992529 | 0.241296910446874 | 0.317302382212194 | -0.231640982 | 0.714242906468402 | 1.27290407341161 | 0.793230855963663 | 2.04263962745053 | 0.998735809224318 |
| ebi-fl187-GCST90094853 | prot-a-1818 | Lysophospholipase-like protein 1 \|\| id:prot-a-1818 | F-sweetened coffee drink liking (derived food-liking factor) \|\| id:ebi-fl187-GCST90094853 | Inverse variance weighted (multiplicative random effects) | 4 | -0.368870985 | 0.44715589679133 | 0.409413020603408 | -1.245296543 | 0.507554572751012 | 0.691514620481893 | 0.287855534023941 | 1.66122382173985 | 0.998735809224318 |
| ebi-fl187-GCST90094853 | prot-a-1852 | MAP kinase-activated protein kinase 3 \|\| id:prot-a-1852 | F-sweetened coffee drink liking (derived food-liking factor) \|\| id:ebi-fl187-GCST90094853 | Inverse variance weighted | 4 | -0.026281569 | 0.320703048850454 | 0.934686586464693 | -0.654859545 | 0.602296406303314 | 0.974060785239388 | 0.519515025846913 | 1.82630793362415 | 0.998735809224318 |
| ebi-fl187-GCST90094853 | prot-a-1856 | Megakaryocyte-associated tyrosine-protein kinase \|\| id:prot-a-1856 | F-sweetened coffee drink liking (derived food-liking factor) \|\| id:ebi-fl187-GCST90094853 | Inverse variance weighted | 4 | 0.00763318224103215 | 0.241296910446874 | 0.974763942832133 | -0.465308762 | 0.480575126716905 | 1.00766238924342 | 0.627941190729052 | 1.61700411708441 | 0.998955228396052 |
| ebi-fl187-GCST90094853 | prot-a-1890 | Beta-1,3-N-acetylglucosaminyltransferase manic fringe \|\| id:prot-a-1890 | F-sweetened coffee drink liking (derived food-liking factor) \|\| id:ebi-fl187-GCST90094853 | Inverse variance weighted | 4 | -0.358647054 | 0.29656680710282 | 0.226536197191098 | -0.939917996 | 0.222623888084568 | 0.698620883402962 | 0.390659869811337 | 1.24935058971489 | 0.998735809224318 |
| ebi-fl187-GCST90094853 | prot-a-1919 | Matrilysin \|\| id:prot-a-1919 | F-sweetened coffee drink liking (derived food-liking factor) \|\| id:ebi-fl187-GCST90094853 | Inverse variance weighted (multiplicative random effects) | 4 | 0.097815659601327 | 0.399334974221137 | 0.806497942948668 | -0.68488089 | 0.880512209074755 | 1.10275948324793 | 0.504150275402505 | 2.41213490743874 | 0.998735809224318 |
| ebi-fl187-GCST90094853 | prot-a-1923 | Multimerin-2 \|\| id:prot-a-1923 | F-sweetened coffee drink liking (derived food-liking factor) \|\| id:ebi-fl187-GCST90094853 | Inverse variance weighted | 4 | -0.002751066 | 0.244473249922254 | 0.991021566774302 | -0.481918636 | 0.476416503862245 | 0.997252714728862 | 0.617597310011125 | 1.61029356979578 | 0.999107451094609 |
| ebi-fl187-GCST90094853 | prot-a-1948 | Mesothelin \|\| id:prot-a-1948 | F-sweetened coffee drink liking (derived food-liking factor) \|\| id:ebi-fl187-GCST90094853 | Inverse variance weighted | 4 | 0.138880197775492 | 0.241296910446874 | 0.56491445128089 | -0.334061747 | 0.611822142251365 | 1.1489864406263 | 0.716009569633866 | 1.84378798375303 | 0.998735809224318 |
| ebi-fl187-GCST90094853 | prot-a-195 | ATPase family AAA domain-containing protein 1 \|\| id:prot-a-195 | F-sweetened coffee drink liking (derived food-liking factor) \|\| id:ebi-fl187-GCST90094853 | Inverse variance weighted | 4 | -0.382842879 | 0.268366332174081 | 0.15370439205328 | -0.90884089 | 0.143155132189526 | 0.681920034914449 | 0.402991064421404 | 1.15390879617981 | 0.998735809224318 |
| ebi-fl187-GCST90094853 | prot-a-1966 | Mucin-1 \|\| id:prot-a-1966 | F-sweetened coffee drink liking (derived food-liking factor) \|\| id:ebi-fl187-GCST90094853 | Inverse variance weighted | 4 | 0.0851594180982786 | 0.276340561885056 | 0.757954225757681 | -0.456468083 | 0.626786919392988 | 1.08889064173787 | 0.633517228915807 | 1.87158734687211 | 0.998735809224318 |
| ebi-fl187-GCST90094853 | prot-a-1986 | Myocardial zonula adherens protein \|\| id:prot-a-1986 | F-sweetened coffee drink liking (derived food-liking factor) \|\| id:ebi-fl187-GCST90094853 | Inverse variance weighted | 4 | -0.177062444 | 0.241296910446874 | 0.463073746184141 | -0.650004388 | 0.295879500965201 | 0.837727472245376 | 0.522043486036198 | 1.34430815923631 | 0.998735809224318 |
| ebi-fl187-GCST90094853 | prot-a-2071 | Epididymal secretory protein E1 \|\| id:prot-a-2071 | F-sweetened coffee drink liking (derived food-liking factor) \|\| id:ebi-fl187-GCST90094853 | Inverse variance weighted | 4 | 0.302677514184937 | 0.241465588217237 | 0.210023197560792 | -0.170595039 | 0.775950067090723 | 1.35347791662672 | 0.843162952692128 | 2.17265531525922 | 0.998735809224318 |
| ebi-fl187-GCST90094853 | prot-a-2098 | Neuritin \|\| id:prot-a-2098 | F-sweetened coffee drink liking (derived food-liking factor) \|\| id:ebi-fl187-GCST90094853 | Inverse variance weighted | 4 | -0.58795131 | 0.241296910446874 | 0.0148248364192479 | -1.060893254 | -0.115009365 | 0.555464093695168 | 0.346146475372633 | 0.891357796009463 | 0.998735809224318 |
| ebi-fl187-GCST90094853 | prot-a-2115 | Neurotrophin-4 \|\| id:prot-a-2115 | F-sweetened coffee drink liking (derived food-liking factor) \|\| id:ebi-fl187-GCST90094853 | Inverse variance weighted | 4 | -0.121556505 | 0.241296910446874 | 0.614427786611833 | -0.59449845 | 0.351385439357857 | 0.885541014335628 | 0.551839271681893 | 1.42103494316478 | 0.998735809224318 |
| ebi-fl187-GCST90094853 | prot-a-2118 | Netrin-1 \|\| id:prot-a-2118 | F-sweetened coffee drink liking (derived food-liking factor) \|\| id:ebi-fl187-GCST90094853 | Inverse variance weighted | 4 | -0.173441752 | 0.241296910446874 | 0.472270459382113 | -0.646383696 | 0.299500192775862 | 0.840766122932955 | 0.523937070585285 | 1.34918430696659 | 0.998735809224318 |
| ebi-fl187-GCST90094853 | prot-a-2120 | Netrin-G1 \|\| id:prot-a-2120 | F-sweetened coffee drink liking (derived food-liking factor) \|\| id:ebi-fl187-GCST90094853 | Inverse variance weighted | 4 | -0.02154979 | 0.293525297136733 | 0.941474172599873 | -0.596859372 | 0.553759792321344 | 0.97868074767637 | 0.550537958484407 | 1.7397819552882 | 0.998735809224318 |
| ebi-fl187-GCST90094853 | prot-a-2145 | Olfactomedin-like protein 3 \|\| id:prot-a-2145 | F-sweetened coffee drink liking (derived food-liking factor) \|\| id:ebi-fl187-GCST90094853 | Inverse variance weighted | 4 | 0.175487095688067 | 0.241296910446874 | 0.467062877771621 | -0.297454849 | 0.64842904016394 | 1.1918266088497 | 0.742706116545207 | 1.9125339537657 | 0.998735809224318 |
| ebi-fl187-GCST90094853 | prot-a-2162 | Prolyl 4-hydroxylase subunit alpha-2 \|\| id:prot-a-2162 | F-sweetened coffee drink liking (derived food-liking factor) \|\| id:ebi-fl187-GCST90094853 | Inverse variance weighted | 4 | -0.105992265 | 0.241296910446874 | 0.660472601716798 | -0.578934209 | 0.366949679600809 | 0.899431605264855 | 0.560495419118911 | 1.44332528858311 | 0.998735809224318 |
| ebi-fl187-GCST90094853 | prot-a-2168 | Polyadenylate-binding protein-interacting protein 1 \|\| id:prot-a-2168 | F-sweetened coffee drink liking (derived food-liking factor) \|\| id:ebi-fl187-GCST90094853 | Inverse variance weighted | 4 | -0.133299251 | 0.241296910446874 | 0.580654921298101 | -0.606241195 | 0.339642693807613 | 0.87520314779038 | 0.545397062170734 | 1.40444568376205 | 0.998735809224318 |
| ebi-fl187-GCST90094853 | prot-a-219 | Beta-1,4-galactosyltransferase 5 \|\| id:prot-a-219 | F-sweetened coffee drink liking (derived food-liking factor) \|\| id:ebi-fl187-GCST90094853 | Inverse variance weighted | 4 | -0.039601342 | 0.241296910446874 | 0.869637679092257 | -0.512543286 | 0.433340602521633 | 0.961172541950956 | 0.59897028700448 | 1.54240147707619 | 0.998735809224318 |
| ebi-fl187-GCST90094853 | prot-a-2190 | Pyruvate carboxylase, mitochondrial \|\| id:prot-a-2190 | F-sweetened coffee drink liking (derived food-liking factor) \|\| id:ebi-fl187-GCST90094853 | Inverse variance weighted | 4 | 0.223906036385151 | 0.259675515504964 | 0.388548216665214 | -0.285057974 | 0.732870046774881 | 1.25095346979572 | 0.751970658305289 | 2.08104474065615 | 0.998735809224318 |
| ebi-fl187-GCST90094853 | prot-a-2197 | Protocadherin-9 \|\| id:prot-a-2197 | F-sweetened coffee drink liking (derived food-liking factor) \|\| id:ebi-fl187-GCST90094853 | Inverse variance weighted | 4 | 0.00570857904266631 | 0.241296910446874 | 0.98112548467152 | -0.467233365 | 0.478650523518539 | 1.00572490402939 | 0.626733815336946 | 1.61389501863901 | 0.998955228396052 |
| ebi-fl187-GCST90094853 | prot-a-2198 | Protocadherin alpha-4 \|\| id:prot-a-2198 | F-sweetened coffee drink liking (derived food-liking factor) \|\| id:ebi-fl187-GCST90094853 | Inverse variance weighted | 4 | 0.307787198545016 | 0.241465588217237 | 0.202428625295025 | -0.165485354 | 0.781059751450801 | 1.36041146059402 | 0.847482275033687 | 2.18378530930573 | 0.998735809224318 |
| ebi-fl187-GCST90094853 | prot-a-2254 | Placenta growth factor \|\| id:prot-a-2254 | F-sweetened coffee drink liking (derived food-liking factor) \|\| id:ebi-fl187-GCST90094853 | Inverse variance weighted | 4 | -0.044876008 | 0.302152878100963 | 0.881931717261575 | -0.637095649 | 0.547343632954112 | 0.956116025103587 | 0.528826092293005 | 1.72865497142183 | 0.998735809224318 |
| ebi-fl187-GCST90094853 | prot-a-2261 | Membrane-associated progesterone receptor component 2 \|\| id:prot-a-2261 | F-sweetened coffee drink liking (derived food-liking factor) \|\| id:ebi-fl187-GCST90094853 | Inverse variance weighted | 4 | 0.545925357665682 | 0.241296910446874 | 0.0236688057619345 | 0.0729834131898093 | 1.01886730214156 | 1.72620500057088 | 1.07571269412445 | 2.7700553505332 | 0.998735809224318 |
| ebi-fl187-GCST90094853 | prot-a-2268 | Polymeric immunoglobulin receptor \|\| id:prot-a-2268 | F-sweetened coffee drink liking (derived food-liking factor) \|\| id:ebi-fl187-GCST90094853 | Inverse variance weighted | 4 | 0.254210761451348 | 0.241296910446874 | 0.292103415608325 | -0.218731183 | 0.727152705927221 | 1.28944354062308 | 0.80353769369588 | 2.06918064640768 | 0.998735809224318 |
| ebi-fl187-GCST90094853 | prot-a-2283 | Group 10 secretory phospholipase A2 \|\| id:prot-a-2283 | F-sweetened coffee drink liking (derived food-liking factor) \|\| id:ebi-fl187-GCST90094853 | Inverse variance weighted | 4 | -0.258296337 | 0.241465588217237 | 0.28475325267189 | -0.73156889 | 0.214976215952937 | 0.772366317509663 | 0.481153520741925 | 1.23983240838303 | 0.998735809224318 |
| ebi-fl187-GCST90094853 | prot-a-2298 | Inactive phospholipase D5 \|\| id:prot-a-2298 | F-sweetened coffee drink liking (derived food-liking factor) \|\| id:ebi-fl187-GCST90094853 | Inverse variance weighted | 4 | 0.132238024324031 | 0.241296910446874 | 0.583671080412322 | -0.34070392 | 0.605179968799904 | 1.14137996313122 | 0.711269469589936 | 1.83158180680591 | 0.998735809224318 |
| ebi-fl187-GCST90094853 | prot-a-2304 | Procollagen-lysine,2-oxoglutarate 5-dioxygenase 2 \|\| id:prot-a-2304 | F-sweetened coffee drink liking (derived food-liking factor) \|\| id:ebi-fl187-GCST90094853 | Inverse variance weighted | 4 | -0.116236783 | 0.241296910446874 | 0.630007119262934 | -0.589178727 | 0.356705161884548 | 0.890264399227309 | 0.554782725724453 | 1.42861459771769 | 0.998735809224318 |
| ebi-fl187-GCST90094853 | prot-a-2331 | Periostin \|\| id:prot-a-2331 | F-sweetened coffee drink liking (derived food-liking factor) \|\| id:ebi-fl187-GCST90094853 | Inverse variance weighted | 4 | -0.072880689 | 0.241296910446874 | 0.762623560454335 | -0.545822633 | 0.400061255543253 | 0.929711748309818 | 0.579364982260368 | 1.49191608297246 | 0.998735809224318 |
| ebi-fl187-GCST90094853 | prot-a-2333 | Periostin \|\| id:prot-a-2333 | F-sweetened coffee drink liking (derived food-liking factor) \|\| id:ebi-fl187-GCST90094853 | Inverse variance weighted | 4 | -0.169007228 | 0.241296910446874 | 0.483670104268377 | -0.641949173 | 0.30393471600892 | 0.844502798903398 | 0.526265640931163 | 1.35518058160472 | 0.998735809224318 |
| ebi-fl187-GCST90094853 | prot-a-2367 | cAMP-dependent protein kinase catalytic subunit alpha \|\| id:prot-a-2367 | F-sweetened coffee drink liking (derived food-liking factor) \|\| id:ebi-fl187-GCST90094853 | Inverse variance weighted | 4 | -0.018999724 | 0.279588348615347 | 0.945820608298201 | -0.566992887 | 0.528993439243585 | 0.981179633006652 | 0.567228596923169 | 1.6972230903892 | 0.998735809224318 |
| ebi-fl187-GCST90094853 | prot-a-2385 | Phosphoribosyl pyrophosphate synthase-associated protein 1 \|\| id:prot-a-2385 | F-sweetened coffee drink liking (derived food-liking factor) \|\| id:ebi-fl187-GCST90094853 | Inverse variance weighted | 4 | 0.123026020751359 | 0.241296910446874 | 0.610154256723295 | -0.349915924 | 0.595967965227232 | 1.13091384779268 | 0.704747339759403 | 1.81478674551062 | 0.998735809224318 |
| ebi-fl187-GCST90094853 | prot-a-2393 | Trypsin-3 \|\| id:prot-a-2393 | F-sweetened coffee drink liking (derived food-liking factor) \|\| id:ebi-fl187-GCST90094853 | Inverse variance weighted | 4 | 0.449667400203986 | 0.241296910446874 | 0.0623857735928508 | -0.023274544 | 0.922609344679859 | 1.56779065191298 | 0.97699421878322 | 2.51584654337766 | 0.998735809224318 |
| ebi-fl187-GCST90094853 | prot-a-2405 | Pregnancy-specific beta-1-glycoprotein 6 \|\| id:prot-a-2405 | F-sweetened coffee drink liking (derived food-liking factor) \|\| id:ebi-fl187-GCST90094853 | Inverse variance weighted | 4 | -0.048941312 | 0.241465588217237 | 0.83938170266556 | -0.522213865 | 0.424331240579294 | 0.95223701263357 | 0.593205815456451 | 1.52856783363054 | 0.998735809224318 |
| ebi-fl187-GCST90094853 | prot-a-244 | Beclin-1 \|\| id:prot-a-244 | F-sweetened coffee drink liking (derived food-liking factor) \|\| id:ebi-fl187-GCST90094853 | Inverse variance weighted | 4 | -0.301392636 | 0.241296910446874 | 0.211645680549756 | -0.77433458 | 0.171549308698158 | 0.739787248773621 | 0.461010444410719 | 1.1871426777491 | 0.998735809224318 |
| ebi-fl187-GCST90094853 | prot-a-2454 | tRNA pseudouridine synthase A, mitochondrial \|\| id:prot-a-2454 | F-sweetened coffee drink liking (derived food-liking factor) \|\| id:ebi-fl187-GCST90094853 | Inverse variance weighted | 4 | -0.279984348 | 0.340093444835999 | 0.410361795897749 | -0.9465675 | 0.386598803928127 | 0.75579557111289 | 0.388070792995116 | 1.47196582588747 | 0.998735809224318 |
| ebi-fl187-GCST90094853 | prot-a-2466 | Glutaminyl-peptide cyclotransferase-like protein \|\| id:prot-a-2466 | F-sweetened coffee drink liking (derived food-liking factor) \|\| id:ebi-fl187-GCST90094853 | Inverse variance weighted | 4 | -0.3087966 | 0.2412259660981 | 0.200505244031722 | -0.781599494 | 0.16400629329617 | 0.734330117394952 | 0.457673379804386 | 1.17822172996769 | 0.998735809224318 |
| ebi-fl187-GCST90094853 | prot-a-2474 | Ras-related protein Rab-27A \|\| id:prot-a-2474 | F-sweetened coffee drink liking (derived food-liking factor) \|\| id:ebi-fl187-GCST90094853 | Inverse variance weighted | 4 | 0.243273698376966 | 0.241296910446874 | 0.313362131339144 | -0.229668246 | 0.716215642852839 | 1.27541765609397 | 0.794797235853793 | 2.04667319423776 | 0.998735809224318 |
| ebi-fl187-GCST90094853 | prot-a-2490 | Rap1 GTPase-GDP dissociation stimulator 1 \|\| id:prot-a-2490 | F-sweetened coffee drink liking (derived food-liking factor) \|\| id:ebi-fl187-GCST90094853 | Inverse variance weighted | 4 | -0.273929129 | 0.241296910446874 | 0.256276120675056 | -0.746871073 | 0.199012815599435 | 0.760385962758847 | 0.473846867726153 | 1.22019760336339 | 0.998735809224318 |
| ebi-fl187-GCST90094853 | prot-a-2545 | Receptor-interacting serine/threonine-protein kinase 2 \|\| id:prot-a-2545 | F-sweetened coffee drink liking (derived food-liking factor) \|\| id:ebi-fl187-GCST90094853 | Inverse variance weighted | 4 | -0.3935769 | 0.359133183021403 | 0.273119072573691 | -1.097477938 | 0.310324138963173 | 0.674639435972169 | 0.333711664601966 | 1.36386712496763 | 0.998735809224318 |
| ebi-fl187-GCST90094853 | prot-a-2610 | RUN and FYVE domain-containing protein 1 \|\| id:prot-a-2610 | F-sweetened coffee drink liking (derived food-liking factor) \|\| id:ebi-fl187-GCST90094853 | Inverse variance weighted | 4 | -0.033960925 | 0.250248695654325 | 0.89205157156116 | -0.524448368 | 0.456526518892134 | 0.966609274552946 | 0.59188177521354 | 1.57858127886212 | 0.998735809224318 |
| ebi-fl187-GCST90094853 | prot-a-2627 | Serine--tRNA ligase, mitochondrial \|\| id:prot-a-2627 | F-sweetened coffee drink liking (derived food-liking factor) \|\| id:ebi-fl187-GCST90094853 | Inverse variance weighted | 4 | 0.0798516860903253 | 0.354976916706271 | 0.82201898533329 | -0.615903071 | 0.775606442834617 | 1.0831264130486 | 0.540152878772302 | 2.17190886644904 | 0.998735809224318 |
| ebi-fl187-GCST90094853 | prot-a-264 | BolA-like protein 2 \|\| id:prot-a-264 | F-sweetened coffee drink liking (derived food-liking factor) \|\| id:ebi-fl187-GCST90094853 | Inverse variance weighted | 4 | -0.195440669 | 0.329348455243846 | 0.552902729931191 | -0.840963642 | 0.450082302902125 | 0.822472139902047 | 0.431294709566298 | 1.56844126744631 | 0.998735809224318 |
| ebi-fl187-GCST90094853 | prot-a-2646 | Secretoglobin family 1C member 1 \|\| id:prot-a-2646 | F-sweetened coffee drink liking (derived food-liking factor) \|\| id:ebi-fl187-GCST90094853 | Inverse variance weighted | 4 | -0.180772114 | 0.241465588217237 | 0.454070931239948 | -0.654044667 | 0.292500439268401 | 0.834625536803196 | 0.519938540081344 | 1.33977332508385 | 0.998735809224318 |
| ebi-fl187-GCST90094853 | prot-a-2672 | Semaphorin-3E \|\| id:prot-a-2672 | F-sweetened coffee drink liking (derived food-liking factor) \|\| id:ebi-fl187-GCST90094853 | Inverse variance weighted | 4 | 0.263666518571669 | 0.241465588217237 | 0.274858411084415 | -0.209606034 | 0.736939071477454 | 1.30169403312682 | 0.810903651245631 | 2.08952981439311 | 0.998735809224318 |
| ebi-fl187-GCST90094853 | prot-a-2701 | Plasma protease C1 inhibitor \|\| id:prot-a-2701 | F-sweetened coffee drink liking (derived food-liking factor) \|\| id:ebi-fl187-GCST90094853 | Inverse variance weighted | 4 | 0.118238792964336 | 0.241465588217237 | 0.624366442073666 | -0.35503376 | 0.591511345870121 | 1.12551284382873 | 0.701149772033755 | 1.80671692718236 | 0.998735809224318 |
| ebi-fl187-GCST90094853 | prot-a-2738 | Signaling threshold-regulating transmembrane adapter 1 \|\| id:prot-a-2738 | F-sweetened coffee drink liking (derived food-liking factor) \|\| id:ebi-fl187-GCST90094853 | Inverse variance weighted | 4 | 0.061544376954726 | 0.282491628352368 | 0.827536123037402 | -0.492139215 | 0.615227968525367 | 1.06347768938293 | 0.61131725561131 | 1.85007831111242 | 0.998735809224318 |
| ebi-fl187-GCST90094853 | prot-a-274 | Serine/threonine-protein kinase BRSK2 \|\| id:prot-a-274 | F-sweetened coffee drink liking (derived food-liking factor) \|\| id:ebi-fl187-GCST90094853 | Inverse variance weighted | 4 | -0.35890554 | 0.241296910446874 | 0.136908633032175 | -0.831847485 | 0.114036404404756 | 0.698440322858919 | 0.435244435706793 | 1.1207929259408 | 0.998735809224318 |
| ebi-fl187-GCST90094853 | prot-a-2749 | Mitochondrial glutamate carrier 2 \|\| id:prot-a-2749 | F-sweetened coffee drink liking (derived food-liking factor) \|\| id:ebi-fl187-GCST90094853 | Inverse variance weighted | 4 | -0.013111658 | 0.340351077496748 | 0.969269959267492 | -0.68019977 | 0.653976453799298 | 0.98697392523985 | 0.506515795603501 | 1.92317305315764 | 0.998735809224318 |
| ebi-fl187-GCST90094853 | prot-a-2760 | Sodium-coupled monocarboxylate transporter 1 \|\| id:prot-a-2760 | F-sweetened coffee drink liking (derived food-liking factor) \|\| id:ebi-fl187-GCST90094853 | Inverse variance weighted | 4 | -0.47536132 | 0.241296910446874 | 0.048835327902061 | -0.948303264 | -0.002419375 | 0.621660397659501 | 0.387397777770092 | 0.997583548988559 | 0.998735809224318 |
| ebi-fl187-GCST90094853 | prot-a-2777 | Small integral membrane protein 10 \|\| id:prot-a-2777 | F-sweetened coffee drink liking (derived food-liking factor) \|\| id:ebi-fl187-GCST90094853 | Inverse variance weighted | 4 | -0.129826833 | 0.368921118554651 | 0.724906162082734 | -0.852912225 | 0.593258559616058 | 0.87824750145708 | 0.42617201415467 | 1.80987640717222 | 0.998735809224318 |
| ebi-fl187-GCST90094853 | prot-a-2799 | Superoxide dismutase [Mn], mitochondrial \|\| id:prot-a-2799 | F-sweetened coffee drink liking (derived food-liking factor) \|\| id:ebi-fl187-GCST90094853 | Inverse variance weighted | 4 | 0.241594093107195 | 0.241296910446874 | 0.316714849829567 | -0.231347851 | 0.714536037583068 | 1.27327725589454 | 0.793463410691507 | 2.04323847644773 | 0.998735809224318 |
| ebi-fl187-GCST90094853 | prot-a-2805 | Protein SPACA7 \|\| id:prot-a-2805 | F-sweetened coffee drink liking (derived food-liking factor) \|\| id:ebi-fl187-GCST90094853 | Inverse variance weighted | 4 | 0.231497676071474 | 0.241296910446874 | 0.33736265911714 | -0.241444268 | 0.704439620547347 | 1.26048639729391 | 0.785492579323912 | 2.02271288053481 | 0.998735809224318 |
| ebi-fl187-GCST90094853 | prot-a-2811 | SPARC-like protein 1 \|\| id:prot-a-2811 | F-sweetened coffee drink liking (derived food-liking factor) \|\| id:ebi-fl187-GCST90094853 | Inverse variance weighted | 4 | 0.24939234326369 | 0.241296910446874 | 0.301346736567201 | -0.223549601 | 0.722334287739563 | 1.28324540700677 | 0.799675226023304 | 2.05923445045674 | 0.998735809224318 |
| ebi-fl187-GCST90094853 | prot-a-2831 | Sepiapterin reductase \|\| id:prot-a-2831 | F-sweetened coffee drink liking (derived food-liking factor) \|\| id:ebi-fl187-GCST90094853 | Inverse variance weighted | 4 | -0.080186579 | 0.273653704934916 | 0.769505272588318 | -0.61654784 | 0.456174683107838 | 0.922944128730331 | 0.539804716880024 | 1.57802597517338 | 0.998735809224318 |
| ebi-fl187-GCST90094853 | prot-a-2834 | SPRY domain-containing SOCS box protein 1 \|\| id:prot-a-2834 | F-sweetened coffee drink liking (derived food-liking factor) \|\| id:ebi-fl187-GCST90094853 | Inverse variance weighted | 4 | -0.150570737 | 0.241296910446874 | 0.532623599331977 | -0.623512681 | 0.32237120791217 | 0.860216879069181 | 0.53605812531467 | 1.38039709518656 | 0.998735809224318 |
| ebi-fl187-GCST90094853 | prot-a-2835 | Spectrin alpha chain, non-erythrocytic 1 \|\| id:prot-a-2835 | F-sweetened coffee drink liking (derived food-liking factor) \|\| id:ebi-fl187-GCST90094853 | Inverse variance weighted | 4 | -0.41685939 | 0.299989494831594 | 0.164656465075604 | -1.0048388 | 0.171120020098104 | 0.659113591541185 | 0.366103646086476 | 1.18663316030375 | 0.998735809224318 |
| ebi-fl187-GCST90094853 | prot-a-2859 | Sia-alpha-2,3-Gal-beta-1,4-GlcNAc-R:alpha 2,8-sialyltransferase \|\| id:prot-a-2859 | F-sweetened coffee drink liking (derived food-liking factor) \|\| id:ebi-fl187-GCST90094853 | Inverse variance weighted | 4 | -0.342105332 | 0.241296910446874 | 0.156255733860778 | -0.815047276 | 0.130836612956866 | 0.710273386594173 | 0.442618401641423 | 1.13978154056201 | 0.998735809224318 |
| ebi-fl187-GCST90094853 | prot-a-2861 | Alpha-2,8-sialyltransferase 8F \|\| id:prot-a-2861 | F-sweetened coffee drink liking (derived food-liking factor) \|\| id:ebi-fl187-GCST90094853 | Inverse variance weighted (multiplicative random effects) | 4 | -0.09886664 | 0.399996227237121 | 0.804777488679409 | -0.882859245 | 0.685125965375382 | 0.905863505917223 | 0.413598639421108 | 1.98402173784028 | 0.998735809224318 |
| ebi-fl187-GCST90094853 | prot-a-289 | Uncharacterized protein C17orf89 \|\| id:prot-a-289 | F-sweetened coffee drink liking (derived food-liking factor) \|\| id:ebi-fl187-GCST90094853 | Inverse variance weighted | 4 | -0.153068007 | 0.241296910446874 | 0.525848884758068 | -0.626009952 | 0.319873937287603 | 0.858071364806213 | 0.53472111324059 | 1.37695417081673 | 0.998735809224318 |
| ebi-fl187-GCST90094853 | prot-a-2921 | Tachykinin-3 \|\| id:prot-a-2921 | F-sweetened coffee drink liking (derived food-liking factor) \|\| id:ebi-fl187-GCST90094853 | Inverse variance weighted | 4 | -0.081337282 | 0.241465588217237 | 0.736231231390667 | -0.554609835 | 0.391935270691688 | 0.921882704362685 | 0.574296287732206 | 1.4798419191582 | 0.998735809224318 |
| ebi-fl187-GCST90094853 | prot-a-2928 | Tax1-binding protein 1 \|\| id:prot-a-2928 | F-sweetened coffee drink liking (derived food-liking factor) \|\| id:ebi-fl187-GCST90094853 | Inverse variance weighted | 4 | -0.073783214 | 0.294485820220366 | 0.802162589025114 | -0.650975422 | 0.503408993342597 | 0.928873038417949 | 0.52153681013543 | 1.65435134152035 | 0.998735809224318 |
| ebi-fl187-GCST90094853 | prot-a-296 | Uncharacterized protein C1orf185 \|\| id:prot-a-296 | F-sweetened coffee drink liking (derived food-liking factor) \|\| id:ebi-fl187-GCST90094853 | Inverse variance weighted | 4 | -0.272759197 | 0.270118851519296 | 0.312603218036008 | -0.802192146 | 0.256673752058075 | 0.761276083184736 | 0.448345048309393 | 1.29262334225484 | 0.998735809224318 |
| ebi-fl187-GCST90094853 | prot-a-2964 | Transforming growth factor beta-2 \|\| id:prot-a-2964 | F-sweetened coffee drink liking (derived food-liking factor) \|\| id:ebi-fl187-GCST90094853 | Inverse variance weighted | 4 | 0.134908905977099 | 0.241465588217237 | 0.576360594300646 | -0.338363647 | 0.608181458882884 | 1.14443252863988 | 0.712935983772717 | 1.8370875400036 | 0.998735809224318 |
| ebi-fl187-GCST90094853 | prot-a-2977 | Thymocyte nuclear protein 1 \|\| id:prot-a-2977 | F-sweetened coffee drink liking (derived food-liking factor) \|\| id:ebi-fl187-GCST90094853 | Inverse variance weighted | 4 | -0.053598486 | 0.241296910446874 | 0.824215256171672 | -0.52654043 | 0.419343458893877 | 0.94781259053244 | 0.590644816200589 | 1.52096265324155 | 0.998735809224318 |
| ebi-fl187-GCST90094853 | prot-a-3015 | Transmembrane protein 70, mitochondrial \|\| id:prot-a-3015 | F-sweetened coffee drink liking (derived food-liking factor) \|\| id:ebi-fl187-GCST90094853 | Inverse variance weighted | 4 | 0.0911121213800278 | 0.294930516329237 | 0.757376743622091 | -0.486951691 | 0.669175933385332 | 1.09539181521552 | 0.614496718179812 | 1.95262756226803 | 0.998735809224318 |
| ebi-fl187-GCST90094853 | prot-a-3030 | Tumor necrosis factor alpha-induced protein 3 \|\| id:prot-a-3030 | F-sweetened coffee drink liking (derived food-liking factor) \|\| id:ebi-fl187-GCST90094853 | Inverse variance weighted | 4 | -0.351324249 | 0.292777966101651 | 0.230151690566267 | -0.925169063 | 0.222520564315835 | 0.703755524659638 | 0.396464386028507 | 1.24922150877217 | 0.998735809224318 |
| ebi-fl187-GCST90094853 | prot-a-3032 | Tumor necrosis factor receptor superfamily member 10A \|\| id:prot-a-3032 | F-sweetened coffee drink liking (derived food-liking factor) \|\| id:ebi-fl187-GCST90094853 | Inverse variance weighted | 4 | 0.0355727129611403 | 0.241296910446874 | 0.882798157287673 | -0.437369232 | 0.508514657437013 | 1.0362129915038 | 0.645732962428372 | 1.6628195031632 | 0.998735809224318 |
| ebi-fl187-GCST90094853 | prot-a-3081 | Tumor protein p53-inducible protein 11 \|\| id:prot-a-3081 | F-sweetened coffee drink liking (derived food-liking factor) \|\| id:ebi-fl187-GCST90094853 | Inverse variance weighted (multiplicative random effects) | 4 | -0.014841563 | 0.39754960741503 | 0.970219807093556 | -0.794038794 | 0.764355667333363 | 0.985268029951221 | 0.452015506314164 | 2.14761015337659 | 0.998735809224318 |
| ebi-fl187-GCST90094853 | prot-a-315 | Complement C4 \|\| id:prot-a-315 | F-sweetened coffee drink liking (derived food-liking factor) \|\| id:ebi-fl187-GCST90094853 | Inverse variance weighted | 4 | 0.327479081474661 | 0.241296910446874 | 0.174729631531156 | -0.145462863 | 0.800421025950534 | 1.38746602725115 | 0.864621998943863 | 2.22647813625789 | 0.998735809224318 |
| ebi-fl187-GCST90094853 | prot-a-3157 | UDP-glucuronosyltransferase 1-8 \|\| id:prot-a-3157 | F-sweetened coffee drink liking (derived food-liking factor) \|\| id:ebi-fl187-GCST90094853 | Inverse variance weighted | 4 | 0.418366342631957 | 0.241296910446874 | 0.0829493393287026 | -0.054575602 | 0.89130828710783 | 1.51947722173712 | 0.946886919754611 | 2.43831758493008 | 0.998735809224318 |
| ebi-fl187-GCST90094853 | prot-a-317 | Glutaredoxin-like protein C5orf63 \|\| id:prot-a-317 | F-sweetened coffee drink liking (derived food-liking factor) \|\| id:ebi-fl187-GCST90094853 | Inverse variance weighted | 4 | -0.038658244 | 0.241296910446874 | 0.872715437129971 | -0.511600188 | 0.434283700545202 | 0.962079449459698 | 0.599535441154322 | 1.54385679900853 | 0.998735809224318 |
| ebi-fl187-GCST90094853 | prot-a-3192 | Guanine nucleotide exchange factor VAV3 \|\| id:prot-a-3192 | F-sweetened coffee drink liking (derived food-liking factor) \|\| id:ebi-fl187-GCST90094853 | Inverse variance weighted | 4 | 0.0450669076721683 | 0.241296910446874 | 0.851841347406093 | -0.427875037 | 0.518008852148041 | 1.04609784953924 | 0.651892872325997 | 1.67868181608738 | 0.998735809224318 |
| ebi-fl187-GCST90094853 | prot-a-3233 | Serine/threonine-protein kinase WNK3 \|\| id:prot-a-3233 | F-sweetened coffee drink liking (derived food-liking factor) \|\| id:ebi-fl187-GCST90094853 | Inverse variance weighted | 4 | 0.157466024066215 | 0.241296910446874 | 0.514025783211056 | -0.31547592 | 0.630407968542088 | 1.170540987 | 0.729441635484618 | 1.87837674158941 | 0.998735809224318 |
| ebi-fl187-GCST90094853 | prot-a-3250 | 14-3-3 protein theta \|\| id:prot-a-3250 | F-sweetened coffee drink liking (derived food-liking factor) \|\| id:ebi-fl187-GCST90094853 | Inverse variance weighted | 4 | -0.548208069 | 0.252204333620845 | 0.0297302027793269 | -1.042528563 | -0.053887575 | 0.577984591392245 | 0.352562078486772 | 0.947538627298498 | 0.998735809224318 |
| ebi-fl187-GCST90094853 | prot-a-3278 | E3 ubiquitin-protein ligase ZNRF3 \|\| id:prot-a-3278 | F-sweetened coffee drink liking (derived food-liking factor) \|\| id:ebi-fl187-GCST90094853 | Inverse variance weighted | 4 | -0.399147332 | 0.299988763702931 | 0.183340106560941 | -0.987125309 | 0.188830644989627 | 0.670891850322059 | 0.37264639609266 | 1.20783638201787 | 0.998735809224318 |
| ebi-fl187-GCST90094853 | prot-a-355 | Calpain-3 \|\| id:prot-a-355 | F-sweetened coffee drink liking (derived food-liking factor) \|\| id:ebi-fl187-GCST90094853 | Inverse variance weighted | 4 | 0.0128226120227186 | 0.299026173265546 | 0.965796206182205 | -0.573268688 | 0.598913911623188 | 1.01290517422255 | 0.563679930561087 | 1.82014089262598 | 0.998735809224318 |
| ebi-fl187-GCST90094853 | prot-a-385 | Coiled-coil domain-containing protein 90B, mitochondrial \|\| id:prot-a-385 | F-sweetened coffee drink liking (derived food-liking factor) \|\| id:ebi-fl187-GCST90094853 | Inverse variance weighted | 4 | -0.07001333 | 0.273026761437279 | 0.7976157785131 | -0.605145782 | 0.465119122514988 | 0.932381391270466 | 0.545994824445804 | 1.59220384491711 | 0.998735809224318 |
| ebi-fl187-GCST90094853 | prot-a-390 | C-C motif chemokine 14 \|\| id:prot-a-390 | F-sweetened coffee drink liking (derived food-liking factor) \|\| id:ebi-fl187-GCST90094853 | Inverse variance weighted | 4 | -0.244256023 | 0.241296910446874 | 0.311412145400975 | -0.717197967 | 0.228685921473124 | 0.783287069081868 | 0.488118064237007 | 1.25694719688341 | 0.998735809224318 |
| ebi-fl187-GCST90094853 | prot-a-40 | Pituitary adenylate cyclase-activating polypeptide 27 \|\| id:prot-a-40 | F-sweetened coffee drink liking (derived food-liking factor) \|\| id:ebi-fl187-GCST90094853 | Inverse variance weighted (multiplicative random effects) | 4 | -0.159835879 | 0.434287789285814 | 0.712842375848297 | -1.011039946 | 0.691368187550492 | 0.852283654750898 | 0.363840408217818 | 1.99644517692133 | 0.998735809224318 |
| ebi-fl187-GCST90094853 | prot-a-401 | C-C motif chemokine 24 \|\| id:prot-a-401 | F-sweetened coffee drink liking (derived food-liking factor) \|\| id:ebi-fl187-GCST90094853 | Inverse variance weighted | 4 | 0.0458449071284106 | 0.248515942891004 | 0.853641063170715 | -0.441246341 | 0.532936155194779 | 1.04691202977218 | 0.643234232130317 | 1.70392796796869 | 0.998735809224318 |
| ebi-fl187-GCST90094853 | prot-a-412 | Cerebral cavernous malformations 2 protein \|\| id:prot-a-412 | F-sweetened coffee drink liking (derived food-liking factor) \|\| id:ebi-fl187-GCST90094853 | Inverse variance weighted | 4 | 0.100051438206546 | 0.241296910446874 | 0.67840519672542 | -0.372890506 | 0.572993382682419 | 1.10522776754771 | 0.688740641497797 | 1.77356808145086 | 0.998735809224318 |
| ebi-fl187-GCST90094853 | prot-a-44 | Adhesion G protein-coupled receptor F5 \|\| id:prot-a-44 | F-sweetened coffee drink liking (derived food-liking factor) \|\| id:ebi-fl187-GCST90094853 | Inverse variance weighted | 4 | 0.209069300656141 | 0.3697167817343 | 0.57174391242523 | -0.515575592 | 0.933714192855369 | 1.23253041073684 | 0.597156777287652 | 2.54394033722797 | 0.998735809224318 |
| ebi-fl187-GCST90094853 | prot-a-48 | Adrenomedullin \|\| id:prot-a-48 | F-sweetened coffee drink liking (derived food-liking factor) \|\| id:ebi-fl187-GCST90094853 | Inverse variance weighted | 4 | -0.275118151 | 0.241296910446874 | 0.254217809234725 | -0.748060095 | 0.197823793468686 | 0.759482384315054 | 0.473283788137225 | 1.21874762360894 | 0.998735809224318 |
| ebi-fl187-GCST90094853 | prot-a-502 | Carcinoembryonic antigen-related cell adhesion molecule 1 \|\| id:prot-a-502 | F-sweetened coffee drink liking (derived food-liking factor) \|\| id:ebi-fl187-GCST90094853 | Inverse variance weighted | 4 | 0.0315409686966734 | 0.280620502213676 | 0.910508447125309 | -0.518475216 | 0.581557153035479 | 1.03204365621305 | 0.595427755074002 | 1.78882173236488 | 0.998735809224318 |
| ebi-fl187-GCST90094853 | prot-a-518 | Complement factor B \|\| id:prot-a-518 | F-sweetened coffee drink liking (derived food-liking factor) \|\| id:ebi-fl187-GCST90094853 | Inverse variance weighted | 4 | 0.233136355108814 | 0.241296910446874 | 0.333953898695938 | -0.239805589 | 0.706078299584687 | 1.26255362322732 | 0.78678080475337 | 2.02603017497881 | 0.998735809224318 |
| ebi-fl187-GCST90094853 | prot-a-525 | Properdin \|\| id:prot-a-525 | F-sweetened coffee drink liking (derived food-liking factor) \|\| id:ebi-fl187-GCST90094853 | Inverse variance weighted | 4 | 0.0696336760562659 | 0.241465588217237 | 0.773056415164135 | -0.403638877 | 0.542906228962051 | 1.07211536778046 | 0.667885266556354 | 1.72100122489232 | 0.998735809224318 |
| ebi-fl187-GCST90094853 | prot-a-533 | Chondroadherin \|\| id:prot-a-533 | F-sweetened coffee drink liking (derived food-liking factor) \|\| id:ebi-fl187-GCST90094853 | Inverse variance weighted | 4 | -0.610170647 | 0.241296910446874 | 0.0114481580360378 | -1.083112592 | -0.137228703 | 0.543258155595814 | 0.338540146719414 | 0.871770826831868 | 0.998735809224318 |
| ebi-fl187-GCST90094853 | prot-a-535 | Coiled-coil-helix-coiled-coil-helix domain-containing protein 2 \|\| id:prot-a-535 | F-sweetened coffee drink liking (derived food-liking factor) \|\| id:ebi-fl187-GCST90094853 | Inverse variance weighted | 4 | -0.292207712 | 0.241296910446874 | 0.225899925206711 | -0.765149657 | 0.18073423200235 | 0.746613438941952 | 0.465264295728582 | 1.1980967212105 | 0.998735809224318 |
| ebi-fl187-GCST90094853 | prot-a-591 | Caseinolytic peptidase B protein homolog \|\| id:prot-a-591 | F-sweetened coffee drink liking (derived food-liking factor) \|\| id:ebi-fl187-GCST90094853 | Inverse variance weighted | 4 | -0.343776197 | 0.241296910446874 | 0.154243352888689 | -0.816718142 | 0.129165747006861 | 0.709087605893488 | 0.441879463130787 | 1.13787870852676 | 0.998735809224318 |
| ebi-fl187-GCST90094853 | prot-a-605 | Ciliary neurotrophic factor receptor subunit alpha \|\| id:prot-a-605 | F-sweetened coffee drink liking (derived food-liking factor) \|\| id:ebi-fl187-GCST90094853 | Inverse variance weighted | 4 | -0.073347185 | 0.241296910446874 | 0.761150232873357 | -0.546289129 | 0.399594759803593 | 0.929278142885525 | 0.579094773994838 | 1.49122027278469 | 0.998735809224318 |
| ebi-fl187-GCST90094853 | prot-a-606 | Ciliary neurotrophic factor receptor subunit alpha \|\| id:prot-a-606 | F-sweetened coffee drink liking (derived food-liking factor) \|\| id:ebi-fl187-GCST90094853 | Inverse variance weighted | 4 | 0.137924718231756 | 0.241296910446874 | 0.567594668570698 | -0.335017226 | 0.610866662707628 | 1.14788913189776 | 0.715325763870193 | 1.84202712341855 | 0.998735809224318 |
| ebi-fl187-GCST90094853 | prot-a-707 | Cancer/testis antigen 55 \|\| id:prot-a-707 | F-sweetened coffee drink liking (derived food-liking factor) \|\| id:ebi-fl187-GCST90094853 | Inverse variance weighted (multiplicative random effects) | 4 | 0.242602040882982 | 0.390684133439904 | 0.534621190228529 | -0.523138861 | 1.00834294242519 | 1.27456129988851 | 0.592657356490035 | 2.74105516346664 | 0.998735809224318 |
| ebi-fl187-GCST90094853 | prot-a-719 | Dipeptidyl peptidase 1 \|\| id:prot-a-719 | F-sweetened coffee drink liking (derived food-liking factor) \|\| id:ebi-fl187-GCST90094853 | Inverse variance weighted | 4 | 0.0296242274877066 | 0.277769359506638 | 0.915066292622005 | -0.514803717 | 0.574052172120717 | 1.03006739020803 | 0.597617885251325 | 1.77544691107057 | 0.998735809224318 |
| ebi-fl187-GCST90094853 | prot-a-752 | Cytochrome b5 \|\| id:prot-a-752 | F-sweetened coffee drink liking (derived food-liking factor) \|\| id:ebi-fl187-GCST90094853 | Inverse variance weighted | 4 | -0.037515464 | 0.316438372683535 | 0.905627635959997 | -0.657734675 | 0.582703746293104 | 0.963179522837023 | 0.518023498098841 | 1.79087395961243 | 0.998735809224318 |
| ebi-fl187-GCST90094853 | prot-a-755 | Cytoglobin \|\| id:prot-a-755 | F-sweetened coffee drink liking (derived food-liking factor) \|\| id:ebi-fl187-GCST90094853 | Inverse variance weighted | 4 | 0.154348665437165 | 0.241296910446874 | 0.522391823283165 | -0.318593279 | 0.627290609913039 | 1.16689767274541 | 0.727171244957226 | 1.87253028513078 | 0.998735809224318 |
| ebi-fl187-GCST90094853 | prot-a-807 | Desmin \|\| id:prot-a-807 | F-sweetened coffee drink liking (derived food-liking factor) \|\| id:ebi-fl187-GCST90094853 | Inverse variance weighted | 4 | 0.117669074929862 | 0.241296910446874 | 0.625795904351481 | -0.35527287 | 0.590611019405735 | 1.1248718014876 | 0.700982140430987 | 1.80509102415075 | 0.998735809224318 |
| ebi-fl187-GCST90094853 | prot-a-82 | Ameloblastin \|\| id:prot-a-82 | F-sweetened coffee drink liking (derived food-liking factor) \|\| id:ebi-fl187-GCST90094853 | Inverse variance weighted | 4 | -0.125923651 | 0.31559785949407 | 0.689892959874454 | -0.744495455 | 0.492648153993974 | 0.881682160104934 | 0.47497388508715 | 1.63664457321621 | 0.998735809224318 |
| ebi-fl187-GCST90094853 | prot-a-84 | Muellerian-inhibiting factor \|\| id:prot-a-84 | F-sweetened coffee drink liking (derived food-liking factor) \|\| id:ebi-fl187-GCST90094853 | Inverse variance weighted | 4 | 0.193115701114468 | 0.241296910446874 | 0.423523119234863 | -0.279826243 | 0.666057645590341 | 1.21302313352864 | 0.75591507530788 | 1.94654819111305 | 0.998735809224318 |
| ebi-fl187-GCST90094853 | prot-a-858 | Inactive dipeptidyl peptidase 10 \|\| id:prot-a-858 | F-sweetened coffee drink liking (derived food-liking factor) \|\| id:ebi-fl187-GCST90094853 | Inverse variance weighted | 4 | -0.217971208 | 0.241465588217237 | 0.366684649310935 | -0.691243761 | 0.255301344846286 | 0.804148594331735 | 0.500952616123818 | 1.29085055343014 | 0.998735809224318 |
| ebi-fl187-GCST90094853 | prot-a-863 | Dihydropyrimidinase-related protein 3 \|\| id:prot-a-863 | F-sweetened coffee drink liking (derived food-liking factor) \|\| id:ebi-fl187-GCST90094853 | Inverse variance weighted | 4 | 0.0565383924672332 | 0.279035586865596 | 0.839431491041319 | -0.490371358 | 0.603448142723801 | 1.05816723967145 | 0.612398932838369 | 1.82841257074729 | 0.998735809224318 |
| ebi-fl187-GCST90094853 | prot-a-864 | Dihydropyrimidinase-related protein 5 \|\| id:prot-a-864 | F-sweetened coffee drink liking (derived food-liking factor) \|\| id:ebi-fl187-GCST90094853 | Inverse variance weighted | 4 | -0.029606856 | 0.241465588217237 | 0.902413442830344 | -0.502879409 | 0.443665697128441 | 0.970827133610439 | 0.604786722024049 | 1.5584094177861 | 0.998735809224318 |
| ebi-fl187-GCST90094853 | prot-a-884 | Endothelin-converting enzyme 1 \|\| id:prot-a-884 | F-sweetened coffee drink liking (derived food-liking factor) \|\| id:ebi-fl187-GCST90094853 | Inverse variance weighted | 4 | 0.0812994949654588 | 0.241296910446874 | 0.736171772291104 | -0.39164245 | 0.554241439441332 | 1.08469570882836 | 0.675945755494338 | 1.74062011808952 | 0.998735809224318 |
| ebi-fl187-GCST90094853 | prot-a-893 | Early endosome antigen 1 \|\| id:prot-a-893 | F-sweetened coffee drink liking (derived food-liking factor) \|\| id:ebi-fl187-GCST90094853 | Inverse variance weighted | 4 | -0.156736629 | 0.258892091912098 | 0.54490463748524 | -0.66416513 | 0.350691870718766 | 0.854929192359211 | 0.514703058740851 | 1.42004969959966 | 0.998735809224318 |
| ebi-fl187-GCST90094853 | prot-a-897 | Ephrin-A2 \|\| id:prot-a-897 | F-sweetened coffee drink liking (derived food-liking factor) \|\| id:ebi-fl187-GCST90094853 | Inverse variance weighted | 4 | -0.060693 | 0.241296910446874 | 0.801405691115457 | -0.533634944 | 0.412248944678328 | 0.941112117041516 | 0.586469307272943 | 1.51021034836553 | 0.998735809224318 |
| ebi-fl187-GCST90094853 | prot-a-90 | Anaphase-promoting complex subunit 10 \|\| id:prot-a-90 | F-sweetened coffee drink liking (derived food-liking factor) \|\| id:ebi-fl187-GCST90094853 | Inverse variance weighted | 4 | -0.523261338 | 0.241296910446874 | 0.0301178336834253 | -0.996203283 | -0.050319394 | 0.592584773559708 | 0.369278830180788 | 0.950925656049373 | 0.998735809224318 |
| ebi-fl187-GCST90094853 | prot-a-914 | Histone-lysine N-methyltransferase EHMT2 \|\| id:prot-a-914 | F-sweetened coffee drink liking (derived food-liking factor) \|\| id:ebi-fl187-GCST90094853 | Inverse variance weighted | 4 | 0.159084750654679 | 0.241296910446874 | 0.509709268488015 | -0.313857194 | 0.632026695130552 | 1.17243730729958 | 0.730623358239695 | 1.8814197822251 | 0.998735809224318 |
| ebi-fl187-GCST90094853 | prot-a-932 | RNA polymerase II elongation factor ELL \|\| id:prot-a-932 | F-sweetened coffee drink liking (derived food-liking factor) \|\| id:ebi-fl187-GCST90094853 | Inverse variance weighted | 4 | 0.27232360984034 | 0.241296910446874 | 0.25907375260817 | -0.200618335 | 0.745265554316213 | 1.31301183611769 | 0.818224659979748 | 2.10700088387438 | 0.998735809224318 |
| ebi-fl187-GCST90094853 | prot-a-991 | Estrogen receptor \|\| id:prot-a-991 | F-sweetened coffee drink liking (derived food-liking factor) \|\| id:ebi-fl187-GCST90094853 | Inverse variance weighted | 4 | 0.365288199560961 | 0.241296910446874 | 0.130062971477702 | -0.107653745 | 0.838230144036834 | 1.44092922348349 | 0.897938458365872 | 2.31227096661755 | 0.998735809224318 |
| ebi-fl187-GCST90094853 | prot-a-992 | Persulfide dioxygenase ETHE1, mitochondrial \|\| id:prot-a-992 | F-sweetened coffee drink liking (derived food-liking factor) \|\| id:ebi-fl187-GCST90094853 | Inverse variance weighted | 4 | 0.228646061436503 | 0.241465588217237 | 0.343684892350116 | -0.244626491 | 0.701918614342288 | 1.25689709592063 | 0.782996939667748 | 2.01762003106178 | 0.998735809224318 |
| ebi-fl187-GCST90094853 | prot-a-993 | Ethanolamine kinase 1 \|\| id:prot-a-993 | F-sweetened coffee drink liking (derived food-liking factor) \|\| id:ebi-fl187-GCST90094853 | Inverse variance weighted | 4 | -0.228409986 | 0.241296910446874 | 0.343846170535786 | -0.701351931 | 0.244531958369111 | 0.795797926850232 | 0.495914408536418 | 1.27702347315972 | 0.998735809224318 |
| ebi-fl187-GCST90094853 | prot-b-10 | coagulation factor III, tissue factor \|\| id:prot-b-10 | F-sweetened coffee drink liking (derived food-liking factor) \|\| id:ebi-fl187-GCST90094853 | Inverse variance weighted | 4 | -0.463091395 | 0.236286941856928 | 0.050011132041637 | -0.926213801 | 3.10106885160755e-05 | 0.629335111576171 | 0.396050400676544 | 1.00003101116935 | 0.998735809224318 |
| ebi-fl187-GCST90094853 | prot-b-28 | prolactin \|\| id:prot-b-28 | F-sweetened coffee drink liking (derived food-liking factor) \|\| id:ebi-fl187-GCST90094853 | Inverse variance weighted | 4 | -0.052726445 | 0.236286941856928 | 0.823422011146269 | -0.515848852 | 0.410395960539692 | 0.948639481590383 | 0.596993620521976 | 1.50741454363488 | 0.998735809224318 |
| ebi-fl187-GCST90094853 | prot-b-54 | matrix metallopeptidase 7 \|\| id:prot-b-54 | F-sweetened coffee drink liking (derived food-liking factor) \|\| id:ebi-fl187-GCST90094853 | Inverse variance weighted | 4 | 0.262733747271069 | 0.236286941856928 | 0.266169689959308 | -0.200388659 | 0.725856153310648 | 1.30048041639198 | 0.818412608020687 | 2.06649958327171 | 0.998735809224318 |
| ebi-fl187-GCST90094853 | ukb-rsfMRI191-edge-pheno716 | Net100_Pair10_33 (OccipitalPrecuneus)&(Temporal\|Frontal\|Supp_Motor_Area) (Default_mode\|Central_executive)&(Default_mode\|Salience) \|\| id:ukb-rsfMRI191-edge-pheno716 | F-sweetened coffee drink liking (derived food-liking factor) \|\| id:ebi-fl187-GCST90094853 | Inverse variance weighted | 3 | -0.055465897 | 0.0854721714873558 | 0.516380285978401 | -0.222991354 | 0.112059558654135 | 0.946044285632393 | 0.800121767456459 | 1.11857947974951 | 0.998735809224318 |
| ebi-fl187-GCST90094853 | ukb-rsfMRI191-edge-pheno903 | Net100_Pair27_38 (Precentral\|Frontal\|Supp_Motor_Area)&(Subcortical) (Attention\|Salience\|Motor)&(Subcortical-cerebellum) \|\| id:ukb-rsfMRI191-edge-pheno903 | F-sweetened coffee drink liking (derived food-liking factor) \|\| id:ebi-fl187-GCST90094853 | Inverse variance weighted | 3 | -0.020389996 | 0.100653028752427 | 0.839465607689392 | -0.217669933 | 0.176889939906659 | 0.979816473838796 | 0.804390900904526 | 1.19349972920671 | 0.998735809224318 |
| ebi-fl187-GCST90094853 | ukb-rsfMRI191-node-pheno23 | Net100_Node2 RolandicOper\|SupraMarginal\|Insula Salience\|Motor \|\| id:ukb-rsfMRI191-node-pheno23 | F-sweetened coffee drink liking (derived food-liking factor) \|\| id:ebi-fl187-GCST90094853 | Inverse variance weighted | 3 | 0.0387327958802923 | 0.0785247885889741 | 0.621831958814671 | -0.11517579 | 0.192641381514682 | 1.03949268980886 | 0.891209464643292 | 1.21244790931226 | 0.998735809224318 |
| ebi-fl187-GCST90094853 | ukb-rsfMRI191-node-pheno55 | Net100_Node34 Parietal\|Postcentral\|Precuneus Attention \|\| id:ukb-rsfMRI191-node-pheno55 | F-sweetened coffee drink liking (derived food-liking factor) \|\| id:ebi-fl187-GCST90094853 | Inverse variance weighted | 3 | -0.028592043 | 0.0814205570826573 | 0.725464633324379 | -0.188176335 | 0.13099224925423 | 0.971812841821816 | 0.828468606746136 | 1.13995894574577 | 0.998735809224318 |
| ebi-fl187-GCST90094853 | ebi-a-GCST90001414 | IgD- CD24- B cell Absolute Count \|\| id:ebi-a-GCST90001414 | F-sweetened coffee drink liking (derived food-liking factor) \|\| id:ebi-fl187-GCST90094853 | MR Egger | 4 | 6.76353075573185 | 4.81636076405861 | 0.295389126346211 | -2.676536342 | 16.2035978532867 | 865.693357950991 | 0.0688010452326622 | 10892639.6025839 | 0.999950574162623 |
| ebi-fl187-GCST90094853 | ebi-a-GCST90001422 | CD20- CD38- B cell %B cell \|\| id:ebi-a-GCST90001422 | F-sweetened coffee drink liking (derived food-liking factor) \|\| id:ebi-fl187-GCST90094853 | MR Egger | 4 | -5.246522366 | 3.26783414250534 | 0.249604044600178 | -11.65147729 | 1.15843255334845 | 0.00526579911617934 | 8.70618134405267e-06 | 3.18493714249325 | 0.999950574162623 |
| ebi-fl187-GCST90094853 | ebi-a-GCST90001444 | CD20- CD38- B cell %lymphocyte \|\| id:ebi-a-GCST90001444 | F-sweetened coffee drink liking (derived food-liking factor) \|\| id:ebi-fl187-GCST90094853 | MR Egger | 4 | -5.647660323 | 2.67795116408233 | 0.169451945983659 | -10.8964446 | -0.398876041 | 0.00352575626336107 | 1.85239771194092e-05 | 0.671073881623661 | 0.999950574162623 |
| ebi-fl187-GCST90094853 | ebi-a-GCST90001459 | Myeloid Dendritic Cell %Dendritic Cell \|\| id:ebi-a-GCST90001459 | F-sweetened coffee drink liking (derived food-liking factor) \|\| id:ebi-fl187-GCST90094853 | MR Egger | 4 | 0.96910641388455 | 4.60784054052191 | 0.852901307958853 | -8.062261046 | 10.0004738733075 | 2.63558828169255 | 0.000315213287360227 | 22036.9060224825 | 0.999950574162623 |
| ebi-fl187-GCST90094853 | ebi-a-GCST90001476 | HLA DR++ monocyte %leukocyte \|\| id:ebi-a-GCST90001476 | F-sweetened coffee drink liking (derived food-liking factor) \|\| id:ebi-fl187-GCST90094853 | MR Egger | 4 | 1.09356783416897 | 2.97679171205841 | 0.748578310411535 | -4.740943921 | 6.92807958980346 | 2.98490474218333 | 0.00873040148154793 | 1020.53225601817 | 0.999950574162623 |
| ebi-fl187-GCST90094853 | ebi-a-GCST90001478 | CD4 regulatory T cell %CD4+ T cell \|\| id:ebi-a-GCST90001478 | F-sweetened coffee drink liking (derived food-liking factor) \|\| id:ebi-fl187-GCST90094853 | MR Egger | 4 | 0.32615807871612 | 2.96550985962875 | 0.922463825179259 | -5.486241246 | 6.13855740358848 | 1.38563439086649 | 0.00414338892054767 | 463.384611478415 | 0.999950574162623 |
| ebi-fl187-GCST90094853 | ebi-a-GCST90001485 | CD39+ resting CD4 regulatory T cell %CD4 regulatory T cell \|\| id:ebi-a-GCST90001485 | F-sweetened coffee drink liking (derived food-liking factor) \|\| id:ebi-fl187-GCST90094853 | MR Egger | 4 | 1.38710084130884 | 2.97428870441165 | 0.686820330253879 | -4.442505019 | 7.21670670195567 | 4.00322722192614 | 0.0117664264452411 | 1361.99620717062 | 0.999950574162623 |
| ebi-fl187-GCST90094853 | ebi-a-GCST90001515 | Immature Myeloid-Derived Suppressor Cells Absolute Count \|\| id:ebi-a-GCST90001515 | F-sweetened coffee drink liking (derived food-liking factor) \|\| id:ebi-fl187-GCST90094853 | MR Egger | 4 | 0.9964728309005 | 3.62380376543841 | 0.809134468522978 | -6.106182549 | 8.09912821115978 | 2.70871087789313 | 0.00222904385649599 | 3291.59724634148 | 0.999950574162623 |
| ebi-fl187-GCST90094853 | ebi-a-GCST90001517 | CD33+ HLA DR+ Absolute Count \|\| id:ebi-a-GCST90001517 | F-sweetened coffee drink liking (derived food-liking factor) \|\| id:ebi-fl187-GCST90094853 | MR Egger | 4 | 6.57297654625682 | 3.46747897048055 | 0.198482108466973 | -0.223282236 | 13.3692353283987 | 715.496384773886 | 0.799889060036333 | 640007.598805325 | 0.999950574162623 |
| ebi-fl187-GCST90094853 | ebi-a-GCST90001518 | CD33+ HLA DR+ CD14- Absolute Count \|\| id:ebi-a-GCST90001518 | F-sweetened coffee drink liking (derived food-liking factor) \|\| id:ebi-fl187-GCST90094853 | MR Egger | 4 | 4.99340411960384 | 3.55180694381864 | 0.29498657 | -1.96813749 | 11.9549457294884 | 147.437464974154 | 0.139716837999319 | 155584.726860988 | 0.999950574162623 |
| ebi-fl187-GCST90094853 | ebi-a-GCST90001520 | CD33+ HLA DR+ CD14dim Absolute Count \|\| id:ebi-a-GCST90001520 | F-sweetened coffee drink liking (derived food-liking factor) \|\| id:ebi-fl187-GCST90094853 | MR Egger | 4 | 7.09743250725718 | 4.30987569513154 | 0.241354873887969 | -1.349923855 | 15.544788869715 | 1208.85934906271 | 0.259260001195084 | 5636584.58335311 | 0.999950574162623 |
| ebi-fl187-GCST90094853 | ebi-a-GCST90001531 | CD33dim HLA DR- Absolute Count \|\| id:ebi-a-GCST90001531 | F-sweetened coffee drink liking (derived food-liking factor) \|\| id:ebi-fl187-GCST90094853 | MR Egger | 4 | 3.17307049647223 | 3.64202448435848 | 0.475486566384598 | -3.965297493 | 10.3114384858148 | 23.8806974959802 | 0.0189623945960437 | 30074.6675218698 | 0.999950574162623 |
| ebi-fl187-GCST90094853 | ebi-a-GCST90001532 | Basophil Absolute Count \|\| id:ebi-a-GCST90001532 | F-sweetened coffee drink liking (derived food-liking factor) \|\| id:ebi-fl187-GCST90094853 | MR Egger | 4 | 3.48521689183316 | 3.60550044295165 | 0.435706188924396 | -3.581563976 | 10.5519977600184 | 32.6295034152461 | 0.0278321353968983 | 38253.783906362 | 0.999950574162623 |
| ebi-fl187-GCST90094853 | ebi-a-GCST90001588 | T/B cell \|\| id:ebi-a-GCST90001588 | F-sweetened coffee drink liking (derived food-liking factor) \|\| id:ebi-fl187-GCST90094853 | MR Egger | 4 | -1.739276592 | 3.54666893993785 | 0.672375364770695 | -8.690747715 | 5.21219452993026 | 0.175647419355523 | 0.000168134262762764 | 183.496304794145 | 0.999950574162623 |
| ebi-fl187-GCST90094853 | ebi-a-GCST90001624 | HLA DR+ CD4+ T cell Absolute Count \|\| id:ebi-a-GCST90001624 | F-sweetened coffee drink liking (derived food-liking factor) \|\| id:ebi-fl187-GCST90094853 | MR Egger | 4 | 1.80118740235465 | 2.80899751840911 | 0.587053138980494 | -3.704447734 | 7.3068225384365 | 6.05683509651811 | 0.0246138069901934 | 1490.4338610045 | 0.999950574162623 |
| ebi-fl187-GCST90094853 | ebi-a-GCST90001631 | CD8+ Natural Killer T %T cell \|\| id:ebi-a-GCST90001631 | F-sweetened coffee drink liking (derived food-liking factor) \|\| id:ebi-fl187-GCST90094853 | MR Egger | 4 | -3.322961068 | 2.80754756597691 | 0.358194493471099 | -8.825754297 | 2.17983216128021 | 0.0360459390959004 | 0.000146900613666506 | 8.84482163059611 | 0.999950574162623 |
| ebi-fl187-GCST90094853 | ebi-a-GCST90001632 | CD8+ Natural Killer T %lymphocyte \|\| id:ebi-a-GCST90001632 | F-sweetened coffee drink liking (derived food-liking factor) \|\| id:ebi-fl187-GCST90094853 | MR Egger | 4 | -3.554428097 | 2.82456548294613 | 0.335246884598661 | -9.090576444 | 1.98172024933926 | 0.028597725358605 | 0.000112723071274567 | 7.2552130317152 | 0.999950574162623 |
| ebi-fl187-GCST90094853 | ebi-a-GCST90001646 | Natural Killer %CD3- lymphocyte \|\| id:ebi-a-GCST90001646 | F-sweetened coffee drink liking (derived food-liking factor) \|\| id:ebi-fl187-GCST90094853 | MR Egger | 4 | 0.536943524426019 | 3.0128764371592 | 0.874970937383428 | -5.368294292 | 6.44218134125805 | 1.71076993647054 | 0.00466207667170793 | 627.774698192428 | 0.999950574162623 |
| ebi-fl187-GCST90094853 | ebi-a-GCST90001684 | CD127- CD8+ T cell Absolute Count \|\| id:ebi-a-GCST90001684 | F-sweetened coffee drink liking (derived food-liking factor) \|\| id:ebi-fl187-GCST90094853 | MR Egger | 4 | 1.44976686441259 | 2.97803967306664 | 0.674511697732485 | -4.387190895 | 7.28672462362319 | 4.26212074730794 | 0.0124356131467751 | 1460.77825437532 | 0.999950574162623 |
| ebi-fl187-GCST90094853 | ebi-a-GCST90001687 | CD28- CD8+ T cell Absolute Count \|\| id:ebi-a-GCST90001687 | F-sweetened coffee drink liking (derived food-liking factor) \|\| id:ebi-fl187-GCST90094853 | MR Egger | 4 | 1.63021028438641 | 2.85905681423703 | 0.62606312273648 | -3.973541072 | 7.23396164 | 5.10494809655362 | 0.0188067191478383 | 1385.7012945026 | 0.999950574162623 |
| ebi-fl187-GCST90094853 | ebi-a-GCST90001701 | BAFF-R on CD20- CD38- B cell \|\| id:ebi-a-GCST90001701 | F-sweetened coffee drink liking (derived food-liking factor) \|\| id:ebi-fl187-GCST90094853 | MR Egger | 4 | 0.0808900560300775 | 3.74332664573194 | 0.984721816575994 | -7.25603017 | 7.41781028166469 | 1.08425168307905 | 0.000705904802930514 | 1665.38279294788 | 0.999950574162623 |
| ebi-fl187-GCST90094853 | ebi-a-GCST90001732 | CD19 on IgD- CD27- B cell \|\| id:ebi-a-GCST90001732 | F-sweetened coffee drink liking (derived food-liking factor) \|\| id:ebi-fl187-GCST90094853 | MR Egger | 4 | 1.02005113500217 | 2.84061677658541 | 0.753891480257842 | -4.547557747 | 6.58766001710957 | 2.77333657491028 | 0.0105930437095893 | 726.07986605139 | 0.999950574162623 |
| ebi-fl187-GCST90094853 | ebi-a-GCST90001798 | CD27 on CD24+ CD27+ B cell \|\| id:ebi-a-GCST90001798 | F-sweetened coffee drink liking (derived food-liking factor) \|\| id:ebi-fl187-GCST90094853 | MR Egger | 4 | -1.86577791 | 3.78856641172008 | 0.671136509736662 | -9.291368077 | 5.55981225729659 | 0.154775761479718 | 9.22168142726689e-05 | 259.774061059995 | 0.999950574162623 |
| ebi-fl187-GCST90094853 | ebi-a-GCST90001836 | CD66b on CD66b++ myeloid cell \|\| id:ebi-a-GCST90001836 | F-sweetened coffee drink liking (derived food-liking factor) \|\| id:ebi-fl187-GCST90094853 | MR Egger | 4 | -3.610407808 | 4.27362568935026 | 0.48716417786424 | -11.98671416 | 4.76589854319055 | 0.0270408171576703 | 6.22638805926294e-06 | 117.436591743868 | 0.999950574162623 |
| ebi-fl187-GCST90094853 | ebi-a-GCST90001943 | CD25 on activated & secreting CD4 regulatory T cell \|\| id:ebi-a-GCST90001943 | F-sweetened coffee drink liking (derived food-liking factor) \|\| id:ebi-fl187-GCST90094853 | MR Egger | 4 | -3.971429762 | 2.55635353976636 | 0.260508584427209 | -8.9818827 | 1.03902317548276 | 0.0188464678905175 | 0.000125666033155329 | 2.82645471516776 | 0.999950574162623 |
| ebi-fl187-GCST90094853 | ebi-a-GCST90001960 | CD25 on CD4+ T cell \|\| id:ebi-a-GCST90001960 | F-sweetened coffee drink liking (derived food-liking factor) \|\| id:ebi-fl187-GCST90094853 | MR Egger | 4 | 0.406880391144603 | 2.72643002564339 | 0.895057187464642 | -4.936922459 | 5.75068324140566 | 1.50212442753318 | 0.00717665085761791 | 314.405401705833 | 0.999950574162623 |
| ebi-fl187-GCST90094853 | ebi-a-GCST90001988 | HLA DR on CD14+ CD16- monocyte \|\| id:ebi-a-GCST90001988 | F-sweetened coffee drink liking (derived food-liking factor) \|\| id:ebi-fl187-GCST90094853 | MR Egger | 4 | 2.55159330165058 | 2.61939060098122 | 0.43274170506026 | -2.582412276 | 7.68559887957378 | 12.827525627018 | 0.0755914364810655 | 2176.77320833848 | 0.999950574162623 |
| ebi-fl187-GCST90094853 | ebi-a-GCST90001991 | HLA DR on CD14+ monocyte \|\| id:ebi-a-GCST90001991 | F-sweetened coffee drink liking (derived food-liking factor) \|\| id:ebi-fl187-GCST90094853 | MR Egger | 4 | 2.078837131 | 2.62341249340509 | 0.511181762606552 | -3.063051356 | 7.22072561817797 | 7.99516617634753 | 0.0467448422297622 | 1367.48096984083 | 0.999950574162623 |
| ebi-fl187-GCST90094853 | ebi-a-GCST90002019 | CD14 on Monocytic Myeloid-Derived Suppressor Cells \|\| id:ebi-a-GCST90002019 | F-sweetened coffee drink liking (derived food-liking factor) \|\| id:ebi-fl187-GCST90094853 | MR Egger | 4 | 2.02123728991263 | 3.53442802911888 | 0.625116302132894 | -4.906241647 | 8.94871622698563 | 7.54765779960027 | 0.00740024887811121 | 7698.00302640722 | 0.999950574162623 |
| ebi-fl187-GCST90094853 | ebi-a-GCST90002037 | CD80 on plasmacytoid Dendritic Cell \|\| id:ebi-a-GCST90002037 | F-sweetened coffee drink liking (derived food-liking factor) \|\| id:ebi-fl187-GCST90094853 | MR Egger | 4 | 5.99138902119287 | 2.84655600739773 | 0.16996153434569 | 0.412139246693308 | 11.5706387956924 | 399.969790774758 | 1.5100446904197 | 105941.125151694 | 0.999950574162623 |
| ebi-fl187-GCST90094853 | ebi-a-GCST90002042 | CD45 on CD33+ HLA DR+ CD14- \|\| id:ebi-a-GCST90002042 | F-sweetened coffee drink liking (derived food-liking factor) \|\| id:ebi-fl187-GCST90094853 | MR Egger | 4 | -0.720415755 | 3.58401733892212 | 0.859280268321939 | -7.745089739 | 6.30425822974092 | 0.48654992855875 | 0.000432862800199485 | 546.895766675784 | 0.999950574162623 |
| ebi-fl187-GCST90094853 | ebi-a-GCST90002056 | CD8 on Effector Memory CD8+ T cell \|\| id:ebi-a-GCST90002056 | F-sweetened coffee drink liking (derived food-liking factor) \|\| id:ebi-fl187-GCST90094853 | MR Egger | 4 | 0.336922993850605 | 2.78373447716314 | 0.914728646830121 | -5.119196581 | 5.79304256909036 | 1.40063120236998 | 0.00598082607224127 | 328.009499249192 | 0.999950574162623 |
| ebi-fl187-GCST90094853 | ebi-a-GCST90002058 | CD8 on CD8+ T cell \|\| id:ebi-a-GCST90002058 | F-sweetened coffee drink liking (derived food-liking factor) \|\| id:ebi-fl187-GCST90094853 | MR Egger | 4 | 0.03545994753917 | 3.41244400251076 | 0.992652394442272 | -6.652930297 | 6.72385019246027 | 1.03609614909657 | 0.00129023578610821 | 832.014769494786 | 0.999950574162623 |
| ebi-fl187-GCST90094853 | ebi-a-GCST90002063 | CD4 on CD4 regulatory T cell \|\| id:ebi-a-GCST90002063 | F-sweetened coffee drink liking (derived food-liking factor) \|\| id:ebi-fl187-GCST90094853 | MR Egger | 4 | 1.66961978929428 | 2.7715185560427 | 0.608099197441392 | -3.762556581 | 7.10179615913797 | 5.31014843802939 | 0.0232242896448083 | 1214.14591641598 | 0.999950574162623 |
| ebi-fl187-GCST90094853 | ebi-a-GCST90002066 | CD4 on activated CD4 regulatory T cell \|\| id:ebi-a-GCST90002066 | F-sweetened coffee drink liking (derived food-liking factor) \|\| id:ebi-fl187-GCST90094853 | MR Egger | 4 | 1.45520223464458 | 2.77206023993729 | 0.652003386477987 | -3.978035836 | 6.88844030492166 | 4.28535002421413 | 0.0187223770723676 | 980.870364860658 | 0.999950574162623 |
| ebi-fl187-GCST90094853 | ebi-a-GCST90002070 | CD4 on activated & secreting CD4 regulatory T cell \|\| id:ebi-a-GCST90002070 | F-sweetened coffee drink liking (derived food-liking factor) \|\| id:ebi-fl187-GCST90094853 | MR Egger | 4 | 1.50622354064742 | 2.7846229071812 | 0.642759492530163 | -3.951637357 | 6.96408443872256 | 4.50966801818827 | 0.019223200742679 | 1057.94585961526 | 0.999950574162623 |
| ebi-fl187-GCST90094853 | ebi-a-GCST90002081 | SSC-A on CD4+ T cell \|\| id:ebi-a-GCST90002081 | F-sweetened coffee drink liking (derived food-liking factor) \|\| id:ebi-fl187-GCST90094853 | MR Egger | 4 | 1.79956325102602 | 2.66391788629369 | 0.568975786780517 | -3.421715806 | 7.02084230816166 | 6.04700586399006 | 0.0326563548683133 | 1119.72937783729 | 0.999950574162623 |
| ebi-fl187-GCST90094853 | ebi-a-GCST90002094 | CD11b on Monocytic Myeloid-Derived Suppressor Cells \|\| id:ebi-a-GCST90002094 | F-sweetened coffee drink liking (derived food-liking factor) \|\| id:ebi-fl187-GCST90094853 | MR Egger | 4 | 1.24071150721042 | 3.53787622236179 | 0.759311915238492 | -5.693525889 | 8.17494890303952 | 3.45807303355598 | 0.00336769788368704 | 3550.87348046637 | 0.999950574162623 |
| ebi-fl187-GCST90094853 | ebi-a-GCST90002104 | HLA DR on myeloid Dendritic Cell \|\| id:ebi-a-GCST90002104 | F-sweetened coffee drink liking (derived food-liking factor) \|\| id:ebi-fl187-GCST90094853 | MR Egger | 4 | 2.801962082 | 4.83548135840267 | 0.620853306307753 | -6.67558138 | 12.2795055447222 | 16.4769441954236 | 0.00126133905506256 | 215239.264121284 | 0.999950574162623 |
| ebi-fl187-GCST90094853 | ebi-a-GCST90002105 | HLA DR on plasmacytoid Dendritic Cell \|\| id:ebi-a-GCST90002105 | F-sweetened coffee drink liking (derived food-liking factor) \|\| id:ebi-fl187-GCST90094853 | MR Egger | 4 | 4.27243982035308 | 2.78287977822119 | 0.264493017798425 | -1.182004545 | 9.72688418566661 | 71.6963486089749 | 0.306663401495709 | 16762.2428329831 | 0.999950574162623 |
| ebi-fl187-GCST90094853 | ebi-a-GCST90002106 | HLA DR on Dendritic Cell \|\| id:ebi-a-GCST90002106 | F-sweetened coffee drink liking (derived food-liking factor) \|\| id:ebi-fl187-GCST90094853 | MR Egger | 4 | 2.98369152713987 | 5.28888022546224 | 0.62948206618812 | -7.382513715 | 13.3498967690459 | 19.7606290660592 | 0.000622035299787112 | 627749.681119421 | 0.999950574162623 |
| ebi-fl187-GCST90094853 | ebi-a-GCST90002111 | HLA DR on CD33dim HLA DR+ CD11b- \|\| id:ebi-a-GCST90002111 | F-sweetened coffee drink liking (derived food-liking factor) \|\| id:ebi-fl187-GCST90094853 | MR Egger | 4 | 6.73826147153368 | 3.51950331522492 | 0.195645596643253 | -0.159965026 | 13.6364879693745 | 844.091981547278 | 0.852173592102505 | 836087.013157183 | 0.999950574162623 |
| ebi-fl187-GCST90094853 | ebi-a-GCST90002112 | HLA DR on CD33- HLA DR+ \|\| id:ebi-a-GCST90002112 | F-sweetened coffee drink liking (derived food-liking factor) \|\| id:ebi-fl187-GCST90094853 | MR Egger | 4 | 2.52771738159461 | 3.5602812002148 | 0.551335998757699 | -4.450433771 | 9.50586853401562 | 12.5248839516992 | 0.0116735022472934 | 13438.3593441208 | 0.999950574162623 |
| ebi-fl187-GCST90094853 | ebi-a-GCST90002115 | HLA DR on HLA DR+ CD8+ T cell \|\| id:ebi-a-GCST90002115 | F-sweetened coffee drink liking (derived food-liking factor) \|\| id:ebi-fl187-GCST90094853 | MR Egger | 4 | -0.513861055 | 2.59842060568709 | 0.861510747275915 | -5.606765442 | 4.57904333259222 | 0.598181502884253 | 0.0036729304723968 | 97.4211499732976 | 0.999950574162623 |
| ebi-fl187-GCST90094853 | ebi-a-GCST90002116 | HLA DR on B cell \|\| id:ebi-a-GCST90002116 | F-sweetened coffee drink liking (derived food-liking factor) \|\| id:ebi-fl187-GCST90094853 | MR Egger | 4 | -1.491487235 | 3.11778190879072 | 0.679569714657982 | -7.602339776 | 4.61936530672608 | 0.225037722673517 | 0.000499281858126382 | 101.429634988388 | 0.999950574162623 |
| ebi-fl187-GCST90094853 | ebi-cfmet338-GCST90026023 | Sphingomyelin (d18:1/24:1, d18:2/24:0) levels \|\| id:ebi-cfmet338-GCST90026023 | F-sweetened coffee drink liking (derived food-liking factor) \|\| id:ebi-fl187-GCST90094853 | MR Egger | 4 | -1.017830827 | 1.00709013355623 | 0.418566578833671 | -2.991727489 | 0.956065834828373 | 0.361377981971763 | 0.0502006407354247 | 2.60144181310875 | 0.999950574162623 |
| ebi-fl187-GCST90094853 | ebi-cfmet338-GCST90026046 | 1-arachidonoyl-gpc (20:4n6) levels \|\| id:ebi-cfmet338-GCST90026046 | F-sweetened coffee drink liking (derived food-liking factor) \|\| id:ebi-fl187-GCST90094853 | MR Egger | 4 | -1.186546974 | 1.08508234752918 | 0.388304797280147 | -3.313308375 | 0.94021442739495 | 0.305273563837389 | 0.0363955641811919 | 2.56053040733296 | 0.999950574162623 |
| ebi-fl187-GCST90094853 | ebi-cfmet338-GCST90026047 | 1-linoleoyl-gpc (18:2) levels \|\| id:ebi-cfmet338-GCST90026047 | F-sweetened coffee drink liking (derived food-liking factor) \|\| id:ebi-fl187-GCST90094853 | MR Egger | 4 | -0.381171791 | 1.59546061864968 | 0.833425209300534 | -3.508274604 | 2.74593102144274 | 0.683060535811937 | 0.0299485429906036 | 15.5791116693082 | 0.999950574162623 |
| ebi-fl187-GCST90094853 | ebi-cfmet338-GCST90026050 | 1-myristoyl-2-palmitoyl-gpc (14:0/16:0) levels \|\| id:ebi-cfmet338-GCST90026050 | F-sweetened coffee drink liking (derived food-liking factor) \|\| id:ebi-fl187-GCST90094853 | MR Egger | 4 | -1.063846831 | 0.709856554371179 | 0.272694969251699 | -2.455165677 | 0.327472015804665 | 0.345125613622087 | 0.0858489710345335 | 1.38745622390871 | 0.999950574162623 |
| ebi-fl187-GCST90094853 | ebi-cfmet338-GCST90026068 | 2,3-dihydroxyisovalerate levels \|\| id:ebi-cfmet338-GCST90026068 | F-sweetened coffee drink liking (derived food-liking factor) \|\| id:ebi-fl187-GCST90094853 | MR Egger | 4 | 0.0590445357426572 | 2.27770822637605 | 0.981672903112149 | -4.405263588 | 4.52335265943972 | 1.0608224842053 | 0.0122128868206844 | 92.144008170907 | 0.999950574162623 |
| ebi-fl187-GCST90094853 | ebi-cfmet338-GCST90026082 | 3-hydroxyhexanoate levels \|\| id:ebi-cfmet338-GCST90026082 | F-sweetened coffee drink liking (derived food-liking factor) \|\| id:ebi-fl187-GCST90094853 | MR Egger | 4 | 0.142065812414917 | 0.488624344574294 | 0.798622921168085 | -0.815637903 | 1.09976952778053 | 1.15265250488574 | 0.442357056468621 | 3.00347372691593 | 0.999950574162623 |
| ebi-fl187-GCST90094853 | ebi-cfmet338-GCST90026094 | 3-ureidopropionate levels \|\| id:ebi-cfmet338-GCST90026094 | F-sweetened coffee drink liking (derived food-liking factor) \|\| id:ebi-fl187-GCST90094853 | MR Egger | 4 | -1.167269603 | 1.10090460240619 | 0.400136850407187 | -3.325042624 | 0.990503417251963 | 0.311215524043304 | 0.0359709854869816 | 2.69258962728729 | 0.999950574162623 |
| ebi-fl187-GCST90094853 | ebi-cfmet338-GCST90026173 | Glycerol levels \|\| id:ebi-cfmet338-GCST90026173 | F-sweetened coffee drink liking (derived food-liking factor) \|\| id:ebi-fl187-GCST90094853 | MR Egger | 4 | -0.753761543 | 0.618801690353609 | 0.347383437711073 | -1.966612856 | 0.459089770505451 | 0.470593063454772 | 0.139930017593935 | 1.58263277014943 | 0.999950574162623 |
| ebi-fl187-GCST90094853 | ebi-cfmet338-GCST90026179 | Histidine levels \|\| id:ebi-cfmet338-GCST90026179 | F-sweetened coffee drink liking (derived food-liking factor) \|\| id:ebi-fl187-GCST90094853 | MR Egger | 4 | 0.275234829859598 | 0.433330364508906 | 0.590297606633608 | -0.574092685 | 1.12456234429705 | 1.31683987188408 | 0.563215651297914 | 3.078869069 | 0.999950574162623 |
| ebi-fl187-GCST90094853 | ebi-cfmet338-GCST90026188 | Isocitrate levels \|\| id:ebi-cfmet338-GCST90026188 | F-sweetened coffee drink liking (derived food-liking factor) \|\| id:ebi-fl187-GCST90094853 | MR Egger | 4 | -0.520761665 | 0.917155367293148 | 0.627412928459318 | -2.318386185 | 1.27686285448476 | 0.594067894640457 | 0.098432309001628 | 3.58537422338338 | 0.999950574162623 |
| ebi-fl187-GCST90094853 | ebi-cfmet338-GCST90026207 | N-acetyl-isoputreanine levels \|\| id:ebi-cfmet338-GCST90026207 | F-sweetened coffee drink liking (derived food-liking factor) \|\| id:ebi-fl187-GCST90094853 | MR Egger | 4 | -0.309058093 | 0.615781371866376 | 0.665543931856444 | -1.515989582 | 0.897873396163938 | 0.734138120726151 | 0.21959077431347 | 2.45437806751366 | 0.999950574162623 |
| ebi-fl187-GCST90094853 | ebi-cfmet338-GCST90026213 | N-acetylglutamate levels \|\| id:ebi-cfmet338-GCST90026213 | F-sweetened coffee drink liking (derived food-liking factor) \|\| id:ebi-fl187-GCST90094853 | MR Egger | 4 | -0.605305953 | 0.917187278795405 | 0.577118417652776 | -2.402993019 | 1.19238111340068 | 0.54590737906539 | 0.0904468386164146 | 3.29491744627942 | 0.999950574162623 |
| ebi-fl187-GCST90094853 | ebi-cfmet338-GCST90026224 | N-acetyltaurine levels \|\| id:ebi-cfmet338-GCST90026224 | F-sweetened coffee drink liking (derived food-liking factor) \|\| id:ebi-fl187-GCST90094853 | MR Egger | 4 | -0.96185052 | 0.857423386145548 | 0.378546069684943 | -2.642400356 | 0.71869931724107 | 0.382184990372661 | 0.0711901824898813 | 2.05176278185425 | 0.999950574162623 |
| ebi-fl187-GCST90094853 | ebi-cfmet338-GCST90026250 | Phenyllactate (pla) levels \|\| id:ebi-cfmet338-GCST90026250 | F-sweetened coffee drink liking (derived food-liking factor) \|\| id:ebi-fl187-GCST90094853 | MR Egger | 4 | -0.469751191 | 1.60992810200183 | 0.797933639071368 | -3.625210271 | 2.68570788859227 | 0.625157793612224 | 0.0266434943863073 | 14.6685814273279 | 0.999950574162623 |
| ebi-fl187-GCST90094853 | ebi-cfmet338-GCST90026251 | Pipecolate levels \|\| id:ebi-cfmet338-GCST90026251 | F-sweetened coffee drink liking (derived food-liking factor) \|\| id:ebi-fl187-GCST90094853 | MR Egger | 4 | -1.342154355 | 3.71271658593238 | 0.752342675058102 | -8.619078863 | 5.9347701538029 | 0.261282167361972 | 0.000180626559892464 | 377.953115101197 | 0.999950574162623 |
| ebi-fl187-GCST90094853 | ebi-cfmet338-GCST90026278 | Trigonelline (n'-methylnicotinate) levels \|\| id:ebi-cfmet338-GCST90026278 | F-sweetened coffee drink liking (derived food-liking factor) \|\| id:ebi-fl187-GCST90094853 | MR Egger | 4 | 0.292029151360915 | 1.98900449850253 | 0.896736346829483 | -3.606419666 | 4.19047796842588 | 1.33914205488366 | 0.0271488751145885 | 66.0543553126584 | 0.999950574162623 |
| ebi-fl187-GCST90094853 | ebi-cfmet338-GCST90026299 | 1-carboxyethylvaline levels \|\| id:ebi-cfmet338-GCST90026299 | F-sweetened coffee drink liking (derived food-liking factor) \|\| id:ebi-fl187-GCST90094853 | MR Egger | 4 | 0.0270589150912222 | 1.19382129907664 | 0.983974916990228 | -2.312830831 | 2.36694866128144 | 1.02742833201433 | 0.0989806570669947 | 10.6648006661672 | 0.999950574162623 |
| ebi-fl187-GCST90094853 | ebi-gm430-GCST90011306 | C_Erysipelotrichia abundance \|\| id:ebi-gm430-GCST90011306 | F-sweetened coffee drink liking (derived food-liking factor) \|\| id:ebi-fl187-GCST90094853 | MR Egger | 4 | 1.6056461553012 | 0.889795728035857 | 0.212915946173227 | -0.138353472 | 3.34964578225148 | 4.98107711250418 | 0.870790837466803 | 28.4926392575403 | 0.999950574162623 |
| ebi-fl187-GCST90094853 | ebi-gm430-GCST90011312 | F_Erysipelotrichaceae abundance \|\| id:ebi-gm430-GCST90011312 | F-sweetened coffee drink liking (derived food-liking factor) \|\| id:ebi-fl187-GCST90094853 | MR Egger | 4 | 1.6056461553012 | 0.889795728035857 | 0.212915946173227 | -0.138353472 | 3.34964578225148 | 4.98107711250418 | 0.870790837466803 | 28.4926392575403 | 0.999950574162623 |
| ebi-fl187-GCST90094853 | ebi-gm430-GCST90011352 | O_Erysipelotrichales abundance \|\| id:ebi-gm430-GCST90011352 | F-sweetened coffee drink liking (derived food-liking factor) \|\| id:ebi-fl187-GCST90094853 | MR Egger | 4 | 1.6056461553012 | 0.889795728035857 | 0.212915946173227 | -0.138353472 | 3.34964578225148 | 4.98107711250418 | 0.870790837466803 | 28.4926392575403 | 0.999950574162623 |
| ebi-fl187-GCST90094853 | ebi-gm430-GCST90011362 | OTU97_11 (Parabacteroides) abundance \|\| id:ebi-gm430-GCST90011362 | F-sweetened coffee drink liking (derived food-liking factor) \|\| id:ebi-fl187-GCST90094853 | MR Egger | 4 | 0.473910177713902 | 1.17039445208942 | 0.724742247904247 | -1.820062948 | 2.76788330380916 | 1.60626270235957 | 0.162015551996129 | 15.9248901553174 | 0.999950574162623 |
| ebi-fl187-GCST90094853 | ebi-gm430-GCST90011384 | OTU97_235 (Phascolarctobacterium) abundance \|\| id:ebi-gm430-GCST90011384 | F-sweetened coffee drink liking (derived food-liking factor) \|\| id:ebi-fl187-GCST90094853 | MR Egger | 4 | -10.28246163 | 5.3458368980961 | 0.194331093864549 | -20.76030195 | 0.195378689951847 | 3.42281678918994e-05 | 9.63641334125922e-10 | 1.21577129970118 | 0.999950574162623 |
| ebi-fl187-GCST90094853 | ebi-gm430-GCST90011390 | OTU97_3 (Bacteroides) abundance \|\| id:ebi-gm430-GCST90011390 | F-sweetened coffee drink liking (derived food-liking factor) \|\| id:ebi-fl187-GCST90094853 | MR Egger | 4 | 0.868008923596351 | 0.923610039863519 | 0.446527155332724 | -0.942266755 | 2.67828460172885 | 2.38216305982472 | 0.389743380740243 | 14.5600954987753 | 0.999950574162623 |
| ebi-fl187-GCST90094853 | ebi-gm430-GCST90011398 | OTU97_4 (Alistipes) abundance \|\| id:ebi-gm430-GCST90011398 | F-sweetened coffee drink liking (derived food-liking factor) \|\| id:ebi-fl187-GCST90094853 | MR Egger | 4 | -1.550934567 | 1.06994123623295 | 0.284222322283797 | -3.64801939 | 0.546150256204161 | 0.212049706575828 | 0.0260426580856599 | 1.72659326521106 | 0.999950574162623 |
| ebi-fl187-GCST90094853 | ebi-gm430-GCST90011421 | OTU99_108 (Cytophagales) abundance \|\| id:ebi-gm430-GCST90011421 | F-sweetened coffee drink liking (derived food-liking factor) \|\| id:ebi-fl187-GCST90094853 | MR Egger | 4 | 7.04163259222729 | 3.42472417180885 | 0.176076571667221 | 0.329173215481934 | 13.7540919689726 | 1143.25254909493 | 1.38981857283293 | 940429.503937254 | 0.999950574162623 |
| ebi-fl187-GCST90094853 | ebi-gm430-GCST90011428 | OTU99_123 (Phascolarctobacterium) abundance \|\| id:ebi-gm430-GCST90011428 | F-sweetened coffee drink liking (derived food-liking factor) \|\| id:ebi-fl187-GCST90094853 | MR Egger | 4 | -1.067086795 | 2.53497958166528 | 0.714716501021684 | -6.035646775 | 3.90147318555474 | 0.344009228646028 | 0.00239194897516711 | 49.4752817147229 | 0.999950574162623 |
| ebi-fl187-GCST90094853 | ebi-gm430-GCST90011450 | OTU99_298 (Phascolarctobacterium) abundance \|\| id:ebi-gm430-GCST90011450 | F-sweetened coffee drink liking (derived food-liking factor) \|\| id:ebi-fl187-GCST90094853 | MR Egger | 4 | -10.25278915 | 5.26759378234888 | 0.190998585836448 | -20.57727296 | 0.0716946682143433 | 3.52590210480776e-05 | 1.15718794693756e-09 | 1.07432726771726 | 0.999950574162623 |
| ebi-fl187-GCST90094853 | ebi-gm430-GCST90011451 | OTU99_3 (Bacteroides) abundance \|\| id:ebi-gm430-GCST90011451 | F-sweetened coffee drink liking (derived food-liking factor) \|\| id:ebi-fl187-GCST90094853 | MR Egger | 4 | 0.859584125703953 | 0.923610932963558 | 0.450270519427979 | -0.950693303 | 2.66986155431253 | 2.36217812024354 | 0.386472987705323 | 14.4379701797215 | 0.999950574162623 |
| ebi-fl187-GCST90094853 | ebi-gm430-GCST90011472 | OTU99_72 (Parabacteroides) abundance \|\| id:ebi-gm430-GCST90011472 | F-sweetened coffee drink liking (derived food-liking factor) \|\| id:ebi-fl187-GCST90094853 | MR Egger | 4 | 1.49969173187263 | 2.81834697559442 | 0.647839689918168 | -4.02426834 | 7.0236518040377 | 4.48030772136478 | 0.0178764988835496 | 1122.87967620957 | 0.999950574162623 |
| ebi-fl187-GCST90094853 | ebi-gm430-GCST90011507 | TestASV_3 (Bacteroides) abundance \|\| id:ebi-gm430-GCST90011507 | F-sweetened coffee drink liking (derived food-liking factor) \|\| id:ebi-fl187-GCST90094853 | MR Egger | 4 | -1.333504111 | 1.86916719344022 | 0.549599928156948 | -4.99707181 | 2.33006358800922 | 0.263552125420014 | 0.00675770590103468 | 10.278595107664 | 0.999950574162623 |
| ebi-fl187-GCST90094853 | ebi-gm430-GCST90011512 | TestASV_34 (Phascolarctobacterium) abundance \|\| id:ebi-gm430-GCST90011512 | F-sweetened coffee drink liking (derived food-liking factor) \|\| id:ebi-fl187-GCST90094853 | MR Egger | 4 | -2.716928617 | 2.96198410672851 | 0.455834481624743 | -8.522417466 | 3.08856023213509 | 0.0660773920533796 | 0.000198957869593181 | 21.9454588526901 | 0.999950574162623 |
| ebi-fl187-GCST90094853 | ebi-gm430-GCST90011519 | TestASV_41 (Barnesiella) abundance \|\| id:ebi-gm430-GCST90011519 | F-sweetened coffee drink liking (derived food-liking factor) \|\| id:ebi-fl187-GCST90094853 | MR Egger | 4 | 2.67210919666151 | 4.24994033906375 | 0.593753065162585 | -5.657773868 | 11.0019922612265 | 14.4704580698324 | 0.00349027807635431 | 59993.5455485241 | 0.999950574162623 |
| ebi-fl187-GCST90094853 | ebi-gm430-GCST90011524 | TestASV_46 (Sutterella) abundance \|\| id:ebi-gm430-GCST90011524 | F-sweetened coffee drink liking (derived food-liking factor) \|\| id:ebi-fl187-GCST90094853 | MR Egger | 4 | -5.034064756 | 4.26420817241945 | 0.359166183846735 | -13.39191277 | 3.32378326148479 | 0.00651228584285889 | 1.52744710588392e-06 | 27.7651950995436 | 0.999950574162623 |
| ebi-fl187-GCST90094853 | ebi-gm430-GCST90011581 | OTU97_19 (Bacteria) prevalence \|\| id:ebi-gm430-GCST90011581 | F-sweetened coffee drink liking (derived food-liking factor) \|\| id:ebi-fl187-GCST90094853 | MR Egger | 4 | 4.38793703339817 | 4.68836550837334 | 0.448115137011875 | -4.801259363 | 13.5771334298099 | 80.4742319674297 | 0.00821938933340636 | 787905.493711879 | 0.999950574162623 |
| ebi-fl187-GCST90094853 | ebi-gm430-GCST90011614 | OTU97_80 (Ruminococcus) prevalence \|\| id:ebi-gm430-GCST90011614 | F-sweetened coffee drink liking (derived food-liking factor) \|\| id:ebi-fl187-GCST90094853 | MR Egger | 4 | -1.359309283 | 2.03620329312315 | 0.573125747927019 | -5.350267738 | 2.63164917106328 | 0.256838118037012 | 0.00474687990922374 | 13.896668999065 | 0.999950574162623 |
| ebi-fl187-GCST90094853 | ebi-gm430-GCST90011623 | OTU99_11 (Parabacteroides) prevalence \|\| id:ebi-gm430-GCST90011623 | F-sweetened coffee drink liking (derived food-liking factor) \|\| id:ebi-fl187-GCST90094853 | MR Egger | 4 | -1.571744198 | 2.06767935205911 | 0.526552481224846 | -5.624395728 | 2.48090733186997 | 0.207682626607984 | 0.00360874313899833 | 11.9521040244398 | 0.999950574162623 |
| ebi-fl187-GCST90094853 | ebi-gm430-GCST90011642 | OTU99_19 (Bacteria) prevalence \|\| id:ebi-gm430-GCST90011642 | F-sweetened coffee drink liking (derived food-liking factor) \|\| id:ebi-fl187-GCST90094853 | MR Egger | 4 | 4.38793703339817 | 4.68836550837334 | 0.448115137011875 | -4.801259363 | 13.5771334298099 | 80.4742319674297 | 0.00821938933340636 | 787905.493711879 | 0.999950574162623 |
| ebi-fl187-GCST90094853 | ebi-gm430-GCST90011653 | OTU99_30 (Parasutterella) prevalence \|\| id:ebi-gm430-GCST90011653 | F-sweetened coffee drink liking (derived food-liking factor) \|\| id:ebi-fl187-GCST90094853 | MR Egger | 4 | -0.95820045 | 1.92409825351377 | 0.66785278972029 | -4.729433027 | 2.81303212723688 | 0.38358254135071 | 0.00883147683351926 | 16.6603580355469 | 0.999950574162623 |
| ebi-fl187-GCST90094853 | ebi-gm430-GCST90011675 | OTU99_8 (Ruminococcaceae) prevalence \|\| id:ebi-gm430-GCST90011675 | F-sweetened coffee drink liking (derived food-liking factor) \|\| id:ebi-fl187-GCST90094853 | MR Egger | 4 | 0.811849090464424 | 2.1627875544277 | 0.743455408066362 | -3.427214516 | 5.05091269714272 | 2.25206841722034 | 0.0324772798318934 | 156.164930748316 | 0.999950574162623 |
| ebi-fl187-GCST90094853 | ebi-gm430-GCST90011679 | OTU99_92 (Ruminococcus) prevalence \|\| id:ebi-gm430-GCST90011679 | F-sweetened coffee drink liking (derived food-liking factor) \|\| id:ebi-fl187-GCST90094853 | MR Egger | 4 | -0.376428021 | 2.12416505603963 | 0.875664394499528 | -4.53979153 | 3.78693548929205 | 0.686308516021432 | 0.010675631866443 | 44.1209836622512 | 0.999950574162623 |
| ebi-fl187-GCST90094853 | ebi-gm430-GCST90011684 | TestASV_11 (Lachnospiraceae) prevalence \|\| id:ebi-gm430-GCST90011684 | F-sweetened coffee drink liking (derived food-liking factor) \|\| id:ebi-fl187-GCST90094853 | MR Egger | 4 | 3.73934637645927 | 2.16015715786558 | 0.225581929194339 | -0.494561653 | 7.97325440587581 | 42.0704829182788 | 0.609838169473365 | 2902.28723221053 | 0.999950574162623 |
| ebi-fl187-GCST90094853 | ebi-gm430-GCST90011688 | TestASV_15 (Bacteroides) prevalence \|\| id:ebi-gm430-GCST90011688 | F-sweetened coffee drink liking (derived food-liking factor) \|\| id:ebi-fl187-GCST90094853 | MR Egger | 4 | 1.92657511105822 | 2.01434059577645 | 0.439788770591055 | -2.021532457 | 5.87468267878006 | 6.8659547945608 | 0.132452332014758 | 355.911704413781 | 0.999950574162623 |
| ebi-fl187-GCST90094853 | ebi-gm430-GCST90011692 | TestASV_19 (Prevotella) prevalence \|\| id:ebi-gm430-GCST90011692 | F-sweetened coffee drink liking (derived food-liking factor) \|\| id:ebi-fl187-GCST90094853 | MR Egger | 4 | 5.83456094043637 | 3.87701777761841 | 0.271275343464661 | -1.764393904 | 13.4335157845685 | 341.914580223059 | 0.171290573612891 | 682498.620346216 | 0.999950574162623 |
| ebi-fl187-GCST90094853 | ebi-gm430-GCST90011693 | TestASV_2 (Bacteroides) prevalence \|\| id:ebi-gm430-GCST90011693 | F-sweetened coffee drink liking (derived food-liking factor) \|\| id:ebi-fl187-GCST90094853 | MR Egger | 4 | -0.334108319 | 3.4034732871904 | 0.930752170897967 | -7.004915962 | 6.3366993236904 | 0.715976224052963 | 0.000907410188923725 | 564.928584301172 | 0.999950574162623 |
| ebi-fl187-GCST90094853 | ebi-gm430-GCST90011716 | TestASV_40 (Barnesiella) prevalence \|\| id:ebi-gm430-GCST90011716 | F-sweetened coffee drink liking (derived food-liking factor) \|\| id:ebi-fl187-GCST90094853 | MR Egger | 4 | -7.847206045 | 4.04688862963661 | 0.192053434078078 | -15.77910776 | 0.0846956693627297 | 0.000390842440476331 | 1.40352635141707e-07 | 1.0883857871515 | 0.999950574162623 |
| ebi-fl187-GCST90094853 | ebi-gm430-GCST90011718 | TestASV_42 (Alphaproteobacteria) prevalence \|\| id:ebi-gm430-GCST90011718 | F-sweetened coffee drink liking (derived food-liking factor) \|\| id:ebi-fl187-GCST90094853 | MR Egger | 4 | 1.49844178004818 | 3.9200750140818 | 0.739072934109291 | -6.184905248 | 9.18178880764851 | 4.47471105106716 | 0.00206029676154825 | 9718.5217994984 | 0.999950574162623 |
| ebi-fl187-GCST90094853 | ebi-hbsm44-GCST90104929 | Sulcal depth of the right inferior frontal sulcus \|\| id:ebi-hbsm44-GCST90104929 | F-sweetened coffee drink liking (derived food-liking factor) \|\| id:ebi-fl187-GCST90094853 | MR Egger | 3 | -0.856377465 | 6.50738588297191 | 0.916698991222202 | -13.6108538 | 11.898098865414 | 0.424697781564935 | 1.22710380417297e-06 | 146986.917531187 | 0.999950574162623 |
| ebi-fl187-GCST90094853 | ebi-hbsm44-GCST90104935 | Sulcal opening distance of the left calloso-marginal posterior fissure \|\| id:ebi-hbsm44-GCST90104935 | F-sweetened coffee drink liking (derived food-liking factor) \|\| id:ebi-fl187-GCST90094853 | MR Egger | 3 | -0.127282441 | 1.53291281864603 | 0.947260494560218 | -3.131791566 | 2.87722668339936 | 0.880484952294669 | 0.0436395440658092 | 17.7649370041137 | 0.999950574162623 |
| ebi-fl187-GCST90094853 | ebi-hbsm44-GCST90104936 | Sulcal opening distance of the left anterior inferior temporal sulcus \|\| id:ebi-hbsm44-GCST90104936 | F-sweetened coffee drink liking (derived food-liking factor) \|\| id:ebi-fl187-GCST90094853 | MR Egger | 3 | 0.253953056598493 | 0.428342331080326 | 0.659304422296975 | -0.585597912 | 1.09350402551593 | 1.28911128757863 | 0.556772860917029 | 2.98471428550873 | 0.999950574162623 |
| ebi-fl187-GCST90094853 | ebi-hbsm44-GCST90104941 | Sulcal opening distance of the right posterior inferior temporal sulcus \|\| id:ebi-hbsm44-GCST90104941 | F-sweetened coffee drink liking (derived food-liking factor) \|\| id:ebi-fl187-GCST90094853 | MR Egger | 3 | 0.0782502469789471 | 0.233562367204933 | 0.794195986933978 | -0.379531993 | 0.536032486700615 | 1.08139324020398 | 0.684181536219879 | 1.70921207026412 | 0.999950574162623 |
| ebi-fl187-GCST90094853 | ebi-IPs91-GCST90274819 | Leukemia inhibitory factor levels \|\| id:ebi-IPs91-GCST90274819 | F-sweetened coffee drink liking (derived food-liking factor) \|\| id:ebi-fl187-GCST90094853 | MR Egger | 4 | 0.223178894673029 | 0.793106052882367 | 0.804846980102474 | -1.331308969 | 1.77766675832247 | 1.25004417997925 | 0.264131295248629 | 5.91603675902588 | 0.999950574162623 |
| ebi-fl187-GCST90094853 | ebi-IPs91-GCST90274837 | STAM binding protein levels \|\| id:ebi-IPs91-GCST90274837 | F-sweetened coffee drink liking (derived food-liking factor) \|\| id:ebi-fl187-GCST90094853 | MR Egger | 4 | -0.250546171 | 0.705731221479146 | 0.756520128344553 | -1.633779365 | 1.13268702345303 | 0.778375541082454 | 0.195190482330388 | 3.10398578722646 | 0.999950574162623 |
| ebi-fl187-GCST90094853 | ebi-IPs91-GCST90274840 | TNF-beta levels \|\| id:ebi-IPs91-GCST90274840 | F-sweetened coffee drink liking (derived food-liking factor) \|\| id:ebi-fl187-GCST90094853 | MR Egger | 4 | 0.230201823689995 | 0.950885236455656 | 0.831269433568591 | -1.63353324 | 2.09393688714308 | 1.25885405086259 | 0.195238529496925 | 8.11680729954536 | 0.999950574162623 |
| ebi-fl187-GCST90094853 | ebi-IPs91-GCST90274842 | Tumor necrosis factor ligand superfamily member 14 levels \|\| id:ebi-IPs91-GCST90274842 | F-sweetened coffee drink liking (derived food-liking factor) \|\| id:ebi-fl187-GCST90094853 | MR Egger | 4 | 0.174865266920449 | 0.803023464101126 | 0.847814955576587 | -1.399060723 | 1.74879125655866 | 1.19108572715295 | 0.246828695680849 | 5.74765103998219 | 0.999950574162623 |
| ebi-fl187-GCST90094853 | ebi-met1400-GCST90199628 | 3-methylhistidine levels \|\| id:ebi-met1400-GCST90199628 | F-sweetened coffee drink liking (derived food-liking factor) \|\| id:ebi-fl187-GCST90094853 | MR Egger | 4 | 1.81545608715694 | 2.27533204244816 | 0.508620544121537 | -2.644194716 | 6.27510689035533 | 6.14387768223585 | 0.0710625562410115 | 531.183157080004 | 0.999950574162623 |
| ebi-fl187-GCST90094853 | ebi-met1400-GCST90199651 | Docosahexaenoate DHA; 22:6n3 levels \|\| id:ebi-met1400-GCST90199651 | F-sweetened coffee drink liking (derived food-liking factor) \|\| id:ebi-fl187-GCST90094853 | MR Egger | 4 | 0.376761527135879 | 0.923590390303596 | 0.722848639794473 | -1.433475638 | 2.18699869213093 | 1.45755668020666 | 0.238478614853314 | 8.9084359925598 | 0.999950574162623 |
| ebi-fl187-GCST90094853 | ebi-met1400-GCST90199652 | 1-stearoyl-2-oleoyl-GPS (18:0/18:1) levels \|\| id:ebi-met1400-GCST90199652 | F-sweetened coffee drink liking (derived food-liking factor) \|\| id:ebi-fl187-GCST90094853 | MR Egger | 4 | -0.434168832 | 0.979051085748124 | 0.700792626339456 | -2.35310896 | 1.48477129617302 | 0.647802876466157 | 0.0950731237331825 | 4.41395580874725 | 0.999950574162623 |
| ebi-fl187-GCST90094853 | ebi-met1400-GCST90199653 | 1-myristoyl-2-palmitoyl-gpc (14:0/16:0) levels \|\| id:ebi-met1400-GCST90199653 | F-sweetened coffee drink liking (derived food-liking factor) \|\| id:ebi-fl187-GCST90094853 | MR Egger | 4 | 0.138610809497532 | 0.960060140226872 | 0.898437780699788 | -1.743107065 | 2.0203286843422 | 1.14867695883492 | 0.174975894 | 7.54080307027408 | 0.999950574162623 |
| ebi-fl187-GCST90094853 | ebi-met1400-GCST90199667 | Erythritol levels in elite athletes \|\| id:ebi-met1400-GCST90199667 | F-sweetened coffee drink liking (derived food-liking factor) \|\| id:ebi-fl187-GCST90094853 | MR Egger | 4 | -1.219803463 | 0.907243502685561 | 0.310977670835394 | -2.998000728 | 0.558393802068014 | 0.295288196219818 | 0.0498867058047001 | 1.74786283079326 | 0.999950574162623 |
| ebi-fl187-GCST90094853 | ebi-met1400-GCST90199674 | 3-hydroxymyristate levels \|\| id:ebi-met1400-GCST90199674 | F-sweetened coffee drink liking (derived food-liking factor) \|\| id:ebi-fl187-GCST90094853 | MR Egger | 4 | -0.28308494 | 0.940971213493605 | 0.791927520292634 | -2.127388518 | 1.56121863886303 | 0.753455786920215 | 0.11914804088097 | 4.76462406470196 | 0.999950574162623 |
| ebi-fl187-GCST90094853 | ebi-met1400-GCST90199685 | 2-linoleoylglycerol (18:2) levels \|\| id:ebi-met1400-GCST90199685 | F-sweetened coffee drink liking (derived food-liking factor) \|\| id:ebi-fl187-GCST90094853 | MR Egger | 4 | -1.581277175 | 1.10552235354532 | 0.288894929994255 | -3.748100988 | 0.585546637964201 | 0.205712199881316 | 0.023562448771421 | 1.7959724640903 | 0.999950574162623 |
| ebi-fl187-GCST90094853 | ebi-met1400-GCST90199703 | Gamma-glutamylvaline levels \|\| id:ebi-met1400-GCST90199703 | F-sweetened coffee drink liking (derived food-liking factor) \|\| id:ebi-fl187-GCST90094853 | MR Egger | 4 | 0.584931516600745 | 1.22221206637035 | 0.679447448761876 | -1.810604133 | 2.98046716668664 | 1.79486806276471 | 0.163555297717686 | 19.697016285547 | 0.999950574162623 |
| ebi-fl187-GCST90094853 | ebi-met1400-GCST90199779 | Gamma-glutamyl-2-aminobutyrate levels \|\| id:ebi-met1400-GCST90199779 | F-sweetened coffee drink liking (derived food-liking factor) \|\| id:ebi-fl187-GCST90094853 | MR Egger | 4 | 1.69518740044302 | 1.00485357332783 | 0.233654333876823 | -0.274325603 | 3.66470040416556 | 5.4476667647258 | 0.760084548943529 | 39.0444368600143 | 0.999950574162623 |
| ebi-fl187-GCST90094853 | ebi-met1400-GCST90199824 | 4-vinylphenol sulfate levels \|\| id:ebi-met1400-GCST90199824 | F-sweetened coffee drink liking (derived food-liking factor) \|\| id:ebi-fl187-GCST90094853 | MR Egger | 4 | -1.946528246 | 0.967110984533503 | 0.18178275844768 | -3.842065776 | -0.050990716 | 0.142768870566147 | 0.0214492462140748 | 0.950287492590675 | 0.999950574162623 |
| ebi-fl187-GCST90094853 | ebi-met1400-GCST90199830 | 4-ethylphenylsulfate levels \|\| id:ebi-met1400-GCST90199830 | F-sweetened coffee drink liking (derived food-liking factor) \|\| id:ebi-fl187-GCST90094853 | MR Egger | 4 | 0.160066752120239 | 0.968055550783612 | 0.883871842064055 | -1.737322127 | 2.05745563165612 | 1.17358920794512 | 0.17599105177647 | 7.82603214824019 | 0.999950574162623 |
| ebi-fl187-GCST90094853 | ebi-met1400-GCST90199833 | Hexanoylglutamine levels \|\| id:ebi-met1400-GCST90199833 | F-sweetened coffee drink liking (derived food-liking factor) \|\| id:ebi-fl187-GCST90094853 | MR Egger | 4 | -1.446307532 | 0.953065228118222 | 0.268427894214512 | -3.314315379 | 0.421700315254161 | 0.23543803248308 | 0.0363589321480636 | 1.52455157136555 | 0.999950574162623 |
| ebi-fl187-GCST90094853 | ebi-met1400-GCST90199837 | Carotenoid levels (cryptoxanthin) \|\| id:ebi-met1400-GCST90199837 | F-sweetened coffee drink liking (derived food-liking factor) \|\| id:ebi-fl187-GCST90094853 | MR Egger | 4 | 0.20155863518036 | 1.42476789697176 | 0.900463997717904 | -2.590986443 | 2.99410371324501 | 1.22330796384303 | 0.0749460735884655 | 19.9674553015155 | 0.999950574162623 |
| ebi-fl187-GCST90094853 | ebi-met1400-GCST90199869 | 2-hydroxyhippurate levels \|\| id:ebi-met1400-GCST90199869 | F-sweetened coffee drink liking (derived food-liking factor) \|\| id:ebi-fl187-GCST90094853 | MR Egger | 4 | 0.262400753781547 | 0.972544603715434 | 0.812596712168477 | -1.64378667 | 2.1685881770638 | 1.30004743697374 | 0.193246892954164 | 8.74592761904256 | 0.999950574162623 |
| ebi-fl187-GCST90094853 | ebi-met1400-GCST90199902 | Hydantoin-5-propionate levels \|\| id:ebi-met1400-GCST90199902 | F-sweetened coffee drink liking (derived food-liking factor) \|\| id:ebi-fl187-GCST90094853 | MR Egger | 4 | 0.910473867438614 | 0.963703804403476 | 0.444503766646946 | -0.978385589 | 2.79933332406943 | 2.48550005191221 | 0.375917494499812 | 16.4336871745635 | 0.999950574162623 |
| ebi-fl187-GCST90094853 | ebi-met1400-GCST90199904 | Pantoate levels \|\| id:ebi-met1400-GCST90199904 | F-sweetened coffee drink liking (derived food-liking factor) \|\| id:ebi-fl187-GCST90094853 | MR Egger | 4 | -2.450933926 | 0.980721733273944 | 0.12968665801248 | -4.373148523 | -0.528719329 | 0.0862130322835251 | 0.0126114704827532 | 0.589359261926251 | 0.999950574162623 |
| ebi-fl187-GCST90094853 | ebi-met1400-GCST90199936 | 3-methyl catechol sulfate (2) levels \|\| id:ebi-met1400-GCST90199936 | F-sweetened coffee drink liking (derived food-liking factor) \|\| id:ebi-fl187-GCST90094853 | MR Egger | 4 | -0.172300633 | 1.10496755176879 | 0.890403092589828 | -2.338037035 | 1.99343576828692 | 0.841726084337609 | 0.0965169117548098 | 7.3407114688273 | 0.999950574162623 |
| ebi-fl187-GCST90094853 | ebi-met1400-GCST90199977 | 4-hydroxychlorothalonil levels \|\| id:ebi-met1400-GCST90199977 | F-sweetened coffee drink liking (derived food-liking factor) \|\| id:ebi-fl187-GCST90094853 | MR Egger | 4 | 1.40406376759061 | 1.2379559062612 | 0.374360825227755 | -1.022329809 | 3.83045734386256 | 4.07171288665275 | 0.359755800847632 | 46.0836094714029 | 0.999950574162623 |
| ebi-fl187-GCST90094853 | ebi-met1400-GCST90200040 | Sphingomyelin (d18:2/24:1, d18:1/24:2) levels \|\| id:ebi-met1400-GCST90200040 | F-sweetened coffee drink liking (derived food-liking factor) \|\| id:ebi-fl187-GCST90094853 | MR Egger | 4 | 0.814499708395108 | 1.91578954791868 | 0.712101194405504 | -2.940447806 | 4.56944722231571 | 2.25804570840615 | 0.0528420604726976 | 96.4907570908573 | 0.999950574162623 |
| ebi-fl187-GCST90094853 | ebi-met1400-GCST90200077 | 1-stearoyl-2-oleoyl-GPI (18:0/18:1) levels \|\| id:ebi-met1400-GCST90200077 | F-sweetened coffee drink liking (derived food-liking factor) \|\| id:ebi-fl187-GCST90094853 | MR Egger | 4 | -1.883447292 | 0.957240256318491 | 0.187987573301553 | -3.759638195 | -0.00725639 | 0.152064988722372 | 0.0232921660840718 | 0.992769874286111 | 0.999950574162623 |
| ebi-fl187-GCST90094853 | ebi-met1400-GCST90200080 | 1-palmitoyl-2-oleoyl-GPI (16:0/18:1) levels \|\| id:ebi-met1400-GCST90200080 | F-sweetened coffee drink liking (derived food-liking factor) \|\| id:ebi-fl187-GCST90094853 | MR Egger | 4 | -1.65709828 | 0.965166421090754 | 0.228134288438129 | -3.548824465 | 0.234627905417919 | 0.190691511456051 | 0.0287584263209186 | 1.26443819058844 | 0.999950574162623 |
| ebi-fl187-GCST90094853 | ebi-met1400-GCST90200082 | 1-oleoyl-2-linoleoyl-GPE (18:1/18:2) levels \|\| id:ebi-met1400-GCST90200082 | F-sweetened coffee drink liking (derived food-liking factor) \|\| id:ebi-fl187-GCST90094853 | MR Egger | 4 | -0.611747826 | 1.37366072906313 | 0.699636869605824 | -3.304122855 | 2.08062720289806 | 0.542402015719591 | 0.0367314164874825 | 8.00949091513893 | 0.999950574162623 |
| ebi-fl187-GCST90094853 | ebi-met1400-GCST90200100 | Perfluorooctanesulfonate (PFOS) levels \|\| id:ebi-met1400-GCST90200100 | F-sweetened coffee drink liking (derived food-liking factor) \|\| id:ebi-fl187-GCST90094853 | MR Egger | 4 | 1.11865723958378 | 1.35554784577419 | 0.495998780614204 | -1.538216538 | 3.7755310173012 | 3.06074160012562 | 0.214763783093887 | 43.6206654948154 | 0.999950574162623 |
| ebi-fl187-GCST90094853 | ebi-met1400-GCST90200102 | 1-palmitoleoyl-2-linolenoyl-GPC (16:1/18:3) levels \|\| id:ebi-met1400-GCST90200102 | F-sweetened coffee drink liking (derived food-liking factor) \|\| id:ebi-fl187-GCST90094853 | MR Egger | 4 | -0.152341626 | 0.951904620131259 | 0.887553231603235 | -2.018074682 | 1.71339142929759 | 0.85869487799345 | 0.132911115110534 | 5.54774439202449 | 0.999950574162623 |
| ebi-fl187-GCST90094853 | ebi-met1400-GCST90200114 | Glycosyl-N-tricosanoyl-sphingadienine (d18:2/23:0) levels \|\| id:ebi-met1400-GCST90200114 | F-sweetened coffee drink liking (derived food-liking factor) \|\| id:ebi-fl187-GCST90094853 | MR Egger | 4 | 0.273473659069653 | 1.01133693651745 | 0.812194910503855 | -1.708746737 | 2.25569405464385 | 1.31452273300427 | 0.181092607212125 | 9.54191362191242 | 0.999950574162623 |
| ebi-fl187-GCST90094853 | ebi-met1400-GCST90200135 | Arachidoylcarnitine (C20) levels \|\| id:ebi-met1400-GCST90200135 | F-sweetened coffee drink liking (derived food-liking factor) \|\| id:ebi-fl187-GCST90094853 | MR Egger | 4 | -1.261005865 | 2.34022665393476 | 0.643952059971323 | -5.847850107 | 3.32583837636799 | 0.283368852192311 | 0.00288609729324308 | 27.822314438526 | 0.999950574162623 |
| ebi-fl187-GCST90094853 | ebi-met1400-GCST90200146 | Docosahexaenoylcarnitine (C22:6) levels \|\| id:ebi-met1400-GCST90200146 | F-sweetened coffee drink liking (derived food-liking factor) \|\| id:ebi-fl187-GCST90094853 | MR Egger | 4 | -0.711033383 | 1.79831445656216 | 0.730743304309851 | -4.235729718 | 2.81366295182239 | 0.491136403104094 | 0.0144692478752779 | 16.6708711146048 | 0.999950574162623 |
| ebi-fl187-GCST90094853 | ebi-met1400-GCST90200162 | 3-hydroxyphenylacetoylglutamine levels \|\| id:ebi-met1400-GCST90200162 | F-sweetened coffee drink liking (derived food-liking factor) \|\| id:ebi-fl187-GCST90094853 | MR Egger | 4 | 1.3786732542566 | 1.17467428981374 | 0.361373420304675 | -0.923688354 | 3.68103486229153 | 3.96963144055215 | 0.397051869261238 | 39.6874438675725 | 0.999950574162623 |
| ebi-fl187-GCST90094853 | ebi-met1400-GCST90200172 | 3-carboxy-4-methyl-5-pentyl-2-furanpropionate (3-CMPFP) levels \|\| id:ebi-met1400-GCST90200172 | F-sweetened coffee drink liking (derived food-liking factor) \|\| id:ebi-fl187-GCST90094853 | MR Egger | 4 | -0.677503548 | 0.960743780054888 | 0.55375878755009 | -2.560561357 | 1.2055542605515 | 0.507883317190689 | 0.0772613570436687 | 3.33860902462308 | 0.999950574162623 |
| ebi-fl187-GCST90094853 | ebi-met1400-GCST90200222 | Dihydrocaffeate sulfate (2) levels \|\| id:ebi-met1400-GCST90200222 | F-sweetened coffee drink liking (derived food-liking factor) \|\| id:ebi-fl187-GCST90094853 | MR Egger | 4 | -0.468950895 | 1.08824691313987 | 0.708522415792905 | -2.601914845 | 1.66401305430963 | 0.625658305074972 | 0.0741314918897767 | 5.28045915076572 | 0.999950574162623 |
| ebi-fl187-GCST90094853 | ebi-met1400-GCST90200223 | 4-methylhexanoylglutamine levels \|\| id:ebi-met1400-GCST90200223 | F-sweetened coffee drink liking (derived food-liking factor) \|\| id:ebi-fl187-GCST90094853 | MR Egger | 4 | -1.844074264 | 1.15516730542829 | 0.251478378565508 | -4.108202183 | 0.420053654507181 | 0.158171678330919 | 0.0164372992380792 | 1.5220432179066 | 0.999950574162623 |
| ebi-fl187-GCST90094853 | ebi-met1400-GCST90200224 | 2-ketocaprylate levels \|\| id:ebi-met1400-GCST90200224 | F-sweetened coffee drink liking (derived food-liking factor) \|\| id:ebi-fl187-GCST90094853 | MR Egger | 4 | -1.597626633 | 1.48877151192237 | 0.395518510467814 | -4.515618796 | 1.32036553033562 | 0.202376261599991 | 0.0109368353581456 | 3.74478996144752 | 0.999950574162623 |
| ebi-fl187-GCST90094853 | ebi-met1400-GCST90200243 | Hydroxypalmitoyl sphingomyelin (d18:1/16:0(OH)) levels \|\| id:ebi-met1400-GCST90200243 | F-sweetened coffee drink liking (derived food-liking factor) \|\| id:ebi-fl187-GCST90094853 | MR Egger | 4 | -0.400404708 | 1.09964693313774 | 0.750659507527522 | -2.555712697 | 1.75490328102914 | 0.670048817091473 | 0.0776368807833092 | 5.7828883998931 | 0.999950574162623 |
| ebi-fl187-GCST90094853 | ebi-met1400-GCST90200273 | 2-methoxyhydroquinone sulfate (2) levels \|\| id:ebi-met1400-GCST90200273 | F-sweetened coffee drink liking (derived food-liking factor) \|\| id:ebi-fl187-GCST90094853 | MR Egger | 4 | -2.206107722 | 1.14589605909976 | 0.19407076221212 | -4.452063998 | 0.0398485536622681 | 0.110128465969388 | 0.0116544872901773 | 1.04065315914778 | 0.999950574162623 |
| ebi-fl187-GCST90094853 | ebi-met1400-GCST90200276 | 2,4-di-tert-butylphenol levels \|\| id:ebi-met1400-GCST90200276 | F-sweetened coffee drink liking (derived food-liking factor) \|\| id:ebi-fl187-GCST90094853 | MR Egger | 4 | -0.741507115 | 0.961207180146525 | 0.521126834709413 | -2.625473188 | 1.14245895848851 | 0.476395391758548 | 0.0724054875418456 | 3.1344664195185 | 0.999950574162623 |
| ebi-fl187-GCST90094853 | ebi-met1400-GCST90200289 | N-acetylmethionine levels \|\| id:ebi-met1400-GCST90200289 | F-sweetened coffee drink liking (derived food-liking factor) \|\| id:ebi-fl187-GCST90094853 | MR Egger | 4 | 0.181863744227947 | 1.66891890342218 | 0.923173609004656 | -3.089217306 | 3.45294479493541 | 1.19945075065701 | 0.0455375824305969 | 31.5932912214726 | 0.999950574162623 |
| ebi-fl187-GCST90094853 | ebi-met1400-GCST90200308 | 3-hydroxyisobutyrate levels \|\| id:ebi-met1400-GCST90200308 | F-sweetened coffee drink liking (derived food-liking factor) \|\| id:ebi-fl187-GCST90094853 | MR Egger | 4 | 0.00639246812368941 | 1.75279089357957 | 0.997421174114372 | -3.429077683 | 3.44186261953966 | 1.00641294355431 | 0.0324168255689988 | 31.2451017388414 | 0.999950574162623 |
| ebi-fl187-GCST90094853 | ebi-met1400-GCST90200383 | Cystathionine levels \|\| id:ebi-met1400-GCST90200383 | F-sweetened coffee drink liking (derived food-liking factor) \|\| id:ebi-fl187-GCST90094853 | MR Egger | 4 | 0.776938901649071 | 2.89058437291019 | 0.813284333138335 | -4.888606469 | 6.44248427255304 | 2.17480477447296 | 0.00753191111488281 | 627.964899602238 | 0.999950574162623 |
| ebi-fl187-GCST90094853 | ebi-met1400-GCST90200384 | Guanidinoacetate levels \|\| id:ebi-met1400-GCST90200384 | F-sweetened coffee drink liking (derived food-liking factor) \|\| id:ebi-fl187-GCST90094853 | MR Egger | 4 | -0.154446674 | 1.63373303436407 | 0.933301770863237 | -3.356563421 | 3.04767007347945 | 0.856889185504325 | 0.0348548347464258 | 21.0662044900259 | 0.999950574162623 |
| ebi-fl187-GCST90094853 | ebi-met1400-GCST90200403 | Citrulline levels \|\| id:ebi-met1400-GCST90200403 | F-sweetened coffee drink liking (derived food-liking factor) \|\| id:ebi-fl187-GCST90094853 | MR Egger | 4 | -0.482534071 | 0.965906936772672 | 0.666924007940259 | -2.375711667 | 1.41064352502472 | 0.617217335813536 | 0.0929483174055645 | 4.09859210217346 | 0.999950574162623 |
| ebi-fl187-GCST90094853 | ebi-met1400-GCST90200434 | 12,13-DiHOME levels \|\| id:ebi-met1400-GCST90200434 | F-sweetened coffee drink liking (derived food-liking factor) \|\| id:ebi-fl187-GCST90094853 | MR Egger | 4 | -1.170262814 | 0.974016907410131 | 0.352539473267062 | -3.079335953 | 0.738810324531054 | 0.310285383208186 | 0.0459897859130769 | 2.09344351405809 | 0.999950574162623 |
| ebi-fl187-GCST90094853 | ebi-met1400-GCST90200437 | Fructose levels \|\| id:ebi-met1400-GCST90200437 | F-sweetened coffee drink liking (derived food-liking factor) \|\| id:ebi-fl187-GCST90094853 | MR Egger | 4 | 1.50075263701261 | 0.973091421064485 | 0.262960642935566 | -0.406506548 | 3.408011822 | 4.48506342508427 | 0.665972734629991 | 30.2051313530197 | 0.999950574162623 |
| ebi-fl187-GCST90094853 | ebi-met1400-GCST90200448 | N-stearoyl-sphinganine (d18:0/18:0) levels \|\| id:ebi-met1400-GCST90200448 | F-sweetened coffee drink liking (derived food-liking factor) \|\| id:ebi-fl187-GCST90094853 | MR Egger | 4 | 0.521736125113412 | 1.07227651163744 | 0.674661480859284 | -1.579925838 | 2.62339808792279 | 1.68495039653595 | 0.20599037435919 | 13.7824781746166 | 0.999950574162623 |
| ebi-fl187-GCST90094853 | ebi-met1400-GCST90200468 | X-11847 levels \|\| id:ebi-met1400-GCST90200468 | F-sweetened coffee drink liking (derived food-liking factor) \|\| id:ebi-fl187-GCST90094853 | MR Egger | 4 | 0.178924538240705 | 1.97381643414338 | 0.936032732647764 | -3.689755673 | 4.04760474916174 | 1.19593049374365 | 0.024978104130025 | 57.2601402580704 | 0.999950574162623 |
| ebi-fl187-GCST90094853 | ebi-met1400-GCST90200476 | X-11843 levels \|\| id:ebi-met1400-GCST90200476 | F-sweetened coffee drink liking (derived food-liking factor) \|\| id:ebi-fl187-GCST90094853 | MR Egger | 4 | 0.431041863950673 | 1.39386520967366 | 0.786380301706326 | -2.300933947 | 3.16301767491104 | 1.53885997136632 | 0.100165250987644 | 23.6418317542646 | 0.999950574162623 |
| ebi-fl187-GCST90094853 | ebi-met1400-GCST90200511 | X-12906 levels \|\| id:ebi-met1400-GCST90200511 | F-sweetened coffee drink liking (derived food-liking factor) \|\| id:ebi-fl187-GCST90094853 | MR Egger | 4 | 1.35755609804705 | 0.968933450661448 | 0.296198847475561 | -0.541553465 | 3.25666566134349 | 3.88668301321577 | 0.581843676010671 | 25.9628238099866 | 0.999950574162623 |
| ebi-fl187-GCST90094853 | ebi-met1400-GCST90200515 | X-12849 levels \|\| id:ebi-met1400-GCST90200515 | F-sweetened coffee drink liking (derived food-liking factor) \|\| id:ebi-fl187-GCST90094853 | MR Egger | 4 | -0.834106257 | 1.01607859039597 | 0.497978383373481 | -2.825620295 | 1.15740777969605 | 0.4342624268412 | 0.0592718793941469 | 3.1816749746024 | 0.999950574162623 |
| ebi-fl187-GCST90094853 | ebi-met1400-GCST90200551 | X-18886 levels \|\| id:ebi-met1400-GCST90200551 | F-sweetened coffee drink liking (derived food-liking factor) \|\| id:ebi-fl187-GCST90094853 | MR Egger | 4 | -1.682744869 | 0.952675909813815 | 0.219378202953496 | -3.549989652 | 0.184499914108101 | 0.185863105323889 | 0.0287249368879485 | 1.20261687798982 | 0.999950574162623 |
| ebi-fl187-GCST90094853 | ebi-met1400-GCST90200564 | X-21319 levels \|\| id:ebi-met1400-GCST90200564 | F-sweetened coffee drink liking (derived food-liking factor) \|\| id:ebi-fl187-GCST90094853 | MR Egger | 4 | 0.356227018472263 | 0.976834961760981 | 0.750304054629071 | -1.558369507 | 2.27082354352379 | 1.42793167833881 | 0.210478976158489 | 9.68737550522931 | 0.999950574162623 |
| ebi-fl187-GCST90094853 | ebi-met1400-GCST90200579 | X-21442 levels \|\| id:ebi-met1400-GCST90200579 | F-sweetened coffee drink liking (derived food-liking factor) \|\| id:ebi-fl187-GCST90094853 | MR Egger | 4 | -0.331111647 | 1.23002271918868 | 0.813010241658224 | -2.741956176 | 2.07973288285417 | 0.718124988239617 | 0.0644441593694334 | 8.0023310689464 | 0.999950574162623 |
| ebi-fl187-GCST90094853 | ebi-met1400-GCST90200581 | X-21441 levels \|\| id:ebi-met1400-GCST90200581 | F-sweetened coffee drink liking (derived food-liking factor) \|\| id:ebi-fl187-GCST90094853 | MR Egger | 4 | -1.529236273 | 0.970833278877581 | 0.255896583683146 | -3.4320695 | 0.373596953231952 | 0.216701104538256 | 0.0323199853060733 | 1.45295142505142 | 0.999950574162623 |
| ebi-fl187-GCST90094853 | ebi-met1400-GCST90200648 | X-24585 levels \|\| id:ebi-met1400-GCST90200648 | F-sweetened coffee drink liking (derived food-liking factor) \|\| id:ebi-fl187-GCST90094853 | MR Egger | 4 | 1.28986942553736 | 0.943417985389406 | 0.304936658703073 | -0.559229826 | 3.13896867690059 | 3.63231223756799 | 0.57164916377286 | 23.0800515898745 | 0.999950574162623 |
| ebi-fl187-GCST90094853 | ebi-met1400-GCST90200720 | Adenosine 5'-diphosphate (ADP) to pantothenate ratio \|\| id:ebi-met1400-GCST90200720 | F-sweetened coffee drink liking (derived food-liking factor) \|\| id:ebi-fl187-GCST90094853 | MR Egger | 4 | 1.24012665354737 | 1.28259936268119 | 0.43560809598627 | -1.273768097 | 3.7540214044025 | 3.45605115818345 | 0.279775412100563 | 42.692420746709 | 0.999950574162623 |
| ebi-fl187-GCST90094853 | ebi-met1400-GCST90200724 | Cholate to taurocholate ratio \|\| id:ebi-met1400-GCST90200724 | F-sweetened coffee drink liking (derived food-liking factor) \|\| id:ebi-fl187-GCST90094853 | MR Egger | 4 | 2.13068657314071 | 1.03579366549442 | 0.175955625812802 | 0.100530988771654 | 4.16084215750977 | 8.42064621674071 | 1.10575790725244 | 64.1255036409354 | 0.999950574162623 |
| ebi-fl187-GCST90094853 | ebi-met1400-GCST90200726 | N-acetylputrescine to (N(1) + N(8))-acetylspermidine ratio \|\| id:ebi-met1400-GCST90200726 | F-sweetened coffee drink liking (derived food-liking factor) \|\| id:ebi-fl187-GCST90094853 | MR Egger | 4 | 1.56289713080004 | 0.960686747806606 | 0.245292925396058 | -0.320048895 | 3.44584315650099 | 4.77262816330575 | 0.726113532956469 | 31.3697218841736 | 0.999950574162623 |
| ebi-fl187-GCST90094853 | ebi-met1400-GCST90200744 | Arginine to glutamate ratio \|\| id:ebi-met1400-GCST90200744 | F-sweetened coffee drink liking (derived food-liking factor) \|\| id:ebi-fl187-GCST90094853 | MR Egger | 4 | -1.776820279 | 1.745623931 | 0.415832331480708 | -5.198243184 | 1.64460262526477 | 0.169175222907761 | 0.00552626452887573 | 5.17895151351238 | 0.999950574162623 |
| ebi-fl187-GCST90094853 | ebi-met1400-GCST90200759 | Histidine to trans-urocanate ratio \|\| id:ebi-met1400-GCST90200759 | F-sweetened coffee drink liking (derived food-liking factor) \|\| id:ebi-fl187-GCST90094853 | MR Egger | 4 | 0.418221057832443 | 2.27680541873141 | 0.871195172262919 | -4.044317563 | 4.880759679 | 1.51925648082913 | 0.0175216580064333 | 131.730698869585 | 0.999950574162623 |
| ebi-fl187-GCST90094853 | ebi-met1400-GCST90200795 | Oleoyl-linoleoyl-glycerol (18:1 to 18:2) [2] to linoleoyl-arachidonoyl-glycerol (18:2 to 20:4) [2] ratio \|\| id:ebi-met1400-GCST90200795 | F-sweetened coffee drink liking (derived food-liking factor) \|\| id:ebi-fl187-GCST90094853 | MR Egger | 4 | 0.832048897465236 | 1.00383551283706 | 0.494349507921828 | -1.135468708 | 2.79956650262587 | 2.29802233216138 | 0.321271503613252 | 16.4375196048188 | 0.999950574162623 |
| ebi-fl187-GCST90094853 | ebi-met1400-GCST90200811 | Choline to taurocholate ratio \|\| id:ebi-met1400-GCST90200811 | F-sweetened coffee drink liking (derived food-liking factor) \|\| id:ebi-fl187-GCST90094853 | MR Egger | 4 | 1.09879289440828 | 1.0136027211351 | 0.391633313278905 | -0.887868439 | 3.08545422783307 | 3.0005418661511 | 0.411532024113695 | 21.8774019103751 | 0.999950574162623 |
| ebi-fl187-GCST90094853 | ebi-met1400-GCST90200831 | Cholate to phosphate ratio \|\| id:ebi-met1400-GCST90200831 | F-sweetened coffee drink liking (derived food-liking factor) \|\| id:ebi-fl187-GCST90094853 | MR Egger | 4 | 0.671432090608794 | 1.00740107953001 | 0.573686170399946 | -1.303074025 | 2.64593820648762 | 1.95703797046368 | 0.271695309759406 | 14.0966644629537 | 0.999950574162623 |
| ebi-fl187-GCST90094853 | ebi-met1400-GCST90200841 | Adenosine 5'-diphosphate (ADP) to oxalate (ethanedioate) ratio \|\| id:ebi-met1400-GCST90200841 | F-sweetened coffee drink liking (derived food-liking factor) \|\| id:ebi-fl187-GCST90094853 | MR Egger | 4 | 0.602213924702698 | 1.29718899417231 | 0.688104336956993 | -1.940276504 | 3.14470435328042 | 1.82615730303478 | 0.143664220571627 | 23.2128116670817 | 0.999950574162623 |
| ebi-fl187-GCST90094853 | ebi-met1400-GCST90200844 | Aspartate to phosphate ratio \|\| id:ebi-met1400-GCST90200844 | F-sweetened coffee drink liking (derived food-liking factor) \|\| id:ebi-fl187-GCST90094853 | MR Egger | 4 | 0.368378775158317 | 1.4171581652563 | 0.819221746039602 | -2.409251229 | 3.14600877906066 | 1.44538941290521 | 0.0898825708673051 | 23.2431108142503 | 0.999950574162623 |
| ebi-fl187-GCST90094853 | ebi-met1400-GCST90200870 | Adenosine 5'-monophosphate (AMP) to acetoacetate ratio \|\| id:ebi-met1400-GCST90200870 | F-sweetened coffee drink liking (derived food-liking factor) \|\| id:ebi-fl187-GCST90094853 | MR Egger | 4 | -0.903727478 | 1.07002650678897 | 0.487265848013797 | -3.000979431 | 1.19352447534779 | 0.405057001247301 | 0.0497383292287282 | 3.29868688401159 | 0.999950574162623 |
| ebi-fl187-GCST90094853 | ebi-met1400-GCST90200887 | Cholesterol to cortisol ratio \|\| id:ebi-met1400-GCST90200887 | F-sweetened coffee drink liking (derived food-liking factor) \|\| id:ebi-fl187-GCST90094853 | MR Egger | 4 | 1.86566477873679 | 0.938855294762807 | 0.185260660899246 | 0.0255084010016919 | 3.70582115647189 | 6.46022908174885 | 1.02583652428862 | 40.6834410751898 | 0.999950574162623 |
| ebi-fl187-GCST90094853 | ebi-met1400-GCST90200890 | Cortisol to taurocholate ratio \|\| id:ebi-met1400-GCST90200890 | F-sweetened coffee drink liking (derived food-liking factor) \|\| id:ebi-fl187-GCST90094853 | MR Egger | 4 | 0.535260512328795 | 1.01047705623602 | 0.649236161409606 | -1.445274518 | 2.51579554255139 | 1.70789311151655 | 0.235681368921563 | 12.3764508569892 | 0.999950574162623 |
| ebi-fl187-GCST90094853 | ebi-met1400-GCST90200948 | Adenosine 5'-diphosphate (ADP) to mannitol to sorbitol ratio \|\| id:ebi-met1400-GCST90200948 | F-sweetened coffee drink liking (derived food-liking factor) \|\| id:ebi-fl187-GCST90094853 | MR Egger | 4 | -0.381386642 | 1.29327437653075 | 0.795865168133271 | -2.91620442 | 2.15343113613149 | 0.682913795502166 | 0.054138785920158 | 8.61436480631395 | 0.999950574162623 |
| ebi-fl187-GCST90094853 | ebi-met1400-GCST90200972 | Leucine to N-palmitoyl-sphingosine (d18:1 to 16:0) ratio \|\| id:ebi-met1400-GCST90200972 | F-sweetened coffee drink liking (derived food-liking factor) \|\| id:ebi-fl187-GCST90094853 | MR Egger | 4 | 0.0977715870066351 | 0.93146769423025 | 0.925982071871838 | -1.727905094 | 1.92344826769793 | 1.10271088284716 | 0.177656193489789 | 6.84451955917569 | 0.999950574162623 |
| ebi-fl187-GCST90094853 | ebi-met1400-GCST90200973 | Proline to glutamate ratio \|\| id:ebi-met1400-GCST90200973 | F-sweetened coffee drink liking (derived food-liking factor) \|\| id:ebi-fl187-GCST90094853 | MR Egger | 4 | -0.590225412 | 1.17988687072499 | 0.666525056520165 | -2.902803679 | 1.72235285462401 | 0.554202346796067 | 0.0548691686870215 | 5.59768351779161 | 0.999950574162623 |
| ebi-fl187-GCST90094853 | ebi-met1400-GCST90200983 | Cholesterol to linoleoyl-arachidonoyl-glycerol (18:2 to 20:4) [1] ratio \|\| id:ebi-met1400-GCST90200983 | F-sweetened coffee drink liking (derived food-liking factor) \|\| id:ebi-fl187-GCST90094853 | MR Egger | 4 | 0.153870111148121 | 0.994584439662563 | 0.891253728556569 | -1.795515391 | 2.10325561288674 | 1.166339382 | 0.166041853881286 | 8.19279912425243 | 0.999950574162623 |
| ebi-fl187-GCST90094853 | ebi-met1400-GCST90201001 | Serine to alpha-ketobutyrate ratio \|\| id:ebi-met1400-GCST90201001 | F-sweetened coffee drink liking (derived food-liking factor) \|\| id:ebi-fl187-GCST90094853 | MR Egger | 4 | -1.46313184 | 0.984360797443535 | 0.275525231903666 | -3.392479003 | 0.466215322990385 | 0.231510085622964 | 0.0336252164808632 | 1.59395017651872 | 0.999950574162623 |
| ebi-fl187-GCST90094853 | ebi-met1400-GCST90201005 | Fructose to maltose ratio \|\| id:ebi-met1400-GCST90201005 | F-sweetened coffee drink liking (derived food-liking factor) \|\| id:ebi-fl187-GCST90094853 | MR Egger | 4 | 2.1502194621099 | 1.09757700481164 | 0.18919049548086 | -0.001031467 | 4.30147039154071 | 8.58674265506899 | 0.998969064458654 | 73.8082409632345 | 0.999950574162623 |
| ebi-fl187-GCST90094853 | ebi-met1400-GCST90201020 | Paraxanthine to 5-acetylamino-6-formylamino-3-methyluracil ratio \|\| id:ebi-met1400-GCST90201020 | F-sweetened coffee drink liking (derived food-liking factor) \|\| id:ebi-fl187-GCST90094853 | MR Egger | 4 | -0.255069892 | 1.3781276157012 | 0.870232201853308 | -2.956200019 | 2.44606023481762 | 0.774862339423381 | 0.0520162026905963 | 11.5427811720139 | 0.999950574162623 |
| ebi-fl187-GCST90094853 | ebi-pw11-GCST90275197 | Placental weight (fetal genotype, adjusted for sex) \|\| id:ebi-pw11-GCST90275197 | F-sweetened coffee drink liking (derived food-liking factor) \|\| id:ebi-fl187-GCST90094853 | MR Egger | 4 | -0.451387873 | 0.340826105229834 | 0.31645225623628 | -1.119407039 | 0.216631293465925 | 0.636743818676239 | 0.32647332317668 | 1.24188612618429 | 0.999950574162623 |
| ebi-fl187-GCST90094853 | prot-a-1017 | FAS-associated factor 2 \|\| id:prot-a-1017 | F-sweetened coffee drink liking (derived food-liking factor) \|\| id:ebi-fl187-GCST90094853 | MR Egger | 4 | -3.110378092 | 1.56145492358888 | 0.184599973710845 | -6.170829742 | -0.049926442 | 0.044584095271107 | 0.00208950153372489 | 0.951299397995492 | 0.999950574162623 |
| ebi-fl187-GCST90094853 | prot-a-1019 | Fas apoptotic inhibitory molecule 3 \|\| id:prot-a-1019 | F-sweetened coffee drink liking (derived food-liking factor) \|\| id:ebi-fl187-GCST90094853 | MR Egger | 4 | 1.22988843553336 | 1.56145492358888 | 0.513422096269816 | -1.830563215 | 4.29034008576756 | 3.42084786993179 | 0.160323245933269 | 72.9912875771467 | 0.999950574162623 |
| ebi-fl187-GCST90094853 | prot-a-1052 | MIP18 family protein FAM96A \|\| id:prot-a-1052 | F-sweetened coffee drink liking (derived food-liking factor) \|\| id:ebi-fl187-GCST90094853 | MR Egger | 4 | 1.035681447 | 2.3374861715151 | 0.701028495924215 | -3.545791449 | 5.61715434337461 | 2.81702523567447 | 0.0288457835074348 | 275.105412767915 | 0.999950574162623 |
| ebi-fl187-GCST90094853 | prot-a-1066 | F-box/LRR-repeat protein 4 \|\| id:prot-a-1066 | F-sweetened coffee drink liking (derived food-liking factor) \|\| id:ebi-fl187-GCST90094853 | MR Egger | 4 | 4.60174603083111 | 2.09217374351574 | 0.158864361491551 | 0.50108549354027 | 8.70240656812196 | 99.6581700576737 | 1.65051191868176 | 6017.37603153847 | 0.999950574162623 |
| ebi-fl187-GCST90094853 | prot-a-1076 | Low affinity immunoglobulin gamma Fc region receptor III-B \|\| id:prot-a-1076 | F-sweetened coffee drink liking (derived food-liking factor) \|\| id:ebi-fl187-GCST90094853 | MR Egger | 4 | -1.096617121 | 1.91409120527851 | 0.62452675640884 | -4.848235883 | 2.65500164155868 | 0.333999053184234 | 0.00784219991064678 | 14.225009410499 | 0.999950574162623 |
| ebi-fl187-GCST90094853 | prot-a-1081 | Fc receptor-like protein 3 \|\| id:prot-a-1081 | F-sweetened coffee drink liking (derived food-liking factor) \|\| id:ebi-fl187-GCST90094853 | MR Egger | 4 | 0.304190978868165 | 1.56145492358888 | 0.863535429704222 | -2.756260671 | 3.36464262910236 | 1.35552790855695 | 0.063528880124482 | 28.9231591565343 | 0.999950574162623 |
| ebi-fl187-GCST90094853 | prot-a-1105 | Fibroblast growth factor receptor 4 \|\| id:prot-a-1105 | F-sweetened coffee drink liking (derived food-liking factor) \|\| id:ebi-fl187-GCST90094853 | MR Egger | 4 | -1.397655363 | 1.56145492358888 | 0.465191211512123 | -4.458107013 | 1.66279628720409 | 0.247175822641499 | 0.0115842714171623 | 5.27403796910244 | 0.999950574162623 |
| ebi-fl187-GCST90094853 | prot-a-1143 | Follistatin-related protein 1 \|\| id:prot-a-1143 | F-sweetened coffee drink liking (derived food-liking factor) \|\| id:ebi-fl187-GCST90094853 | MR Egger | 4 | -0.136067524 | 1.56463924156069 | 0.938622994335423 | -3.202760437 | 2.9306253897019 | 0.872783696864656 | 0.0406498376349688 | 18.7393462269931 | 0.999950574162623 |
| ebi-fl187-GCST90094853 | prot-a-1151 | Alpha-(1,3)-fucosyltransferase 10 \|\| id:prot-a-1151 | F-sweetened coffee drink liking (derived food-liking factor) \|\| id:ebi-fl187-GCST90094853 | MR Egger | 4 | 1.45682846950451 | 1.56145492358888 | 0.449316788770522 | -1.603623181 | 4.51728011973871 | 4.29232467948713 | 0.201166334014315 | 91.5861555285054 | 0.999950574162623 |
| ebi-fl187-GCST90094853 | prot-a-1165 | Growth arrest and DNA damage-inducible proteins-interacting protein 1 \|\| id:prot-a-1165 | F-sweetened coffee drink liking (derived food-liking factor) \|\| id:ebi-fl187-GCST90094853 | MR Egger | 4 | -0.826496683 | 1.56145492358888 | 0.649467778321524 | -3.886948333 | 2.23395496751583 | 0.437579584316515 | 0.0205078337240274 | 9.33671957688427 | 0.999950574162623 |
| ebi-fl187-GCST90094853 | prot-a-1194 | Growth/differentiation factor 11/8 \|\| id:prot-a-1194 | F-sweetened coffee drink liking (derived food-liking factor) \|\| id:ebi-fl187-GCST90094853 | MR Egger | 4 | -1.416974434 | 1.56145492358888 | 0.459944435730622 | -4.477426085 | 1.64347721593807 | 0.242446445984144 | 0.0113626219765037 | 5.1731263516371 | 0.999950574162623 |
| ebi-fl187-GCST90094853 | prot-a-1216 | D-glucuronyl C5-epimerase \|\| id:prot-a-1216 | F-sweetened coffee drink liking (derived food-liking factor) \|\| id:ebi-fl187-GCST90094853 | MR Egger | 4 | 1.73115772578455 | 1.56145492358888 | 0.383033943252293 | -1.329293924 | 4.79160937601875 | 5.64718802052503 | 0.264664068169772 | 120.495134680333 | 0.999950574162623 |
| ebi-fl187-GCST90094853 | prot-a-1246 | Glypican-3 \|\| id:prot-a-1246 | F-sweetened coffee drink liking (derived food-liking factor) \|\| id:ebi-fl187-GCST90094853 | MR Egger | 4 | 1.51805700678001 | 1.56145492358888 | 0.433496928284228 | -1.542394643 | 4.57850865701421 | 4.56335001758477 | 0.213868349300029 | 97.3690751864242 | 0.999950574162623 |
| ebi-fl187-GCST90094853 | prot-a-1275 | Glutamate receptor 4 \|\| id:prot-a-1275 | F-sweetened coffee drink liking (derived food-liking factor) \|\| id:ebi-fl187-GCST90094853 | MR Egger | 4 | -0.534962049 | 1.56145492358888 | 0.764552374688638 | -3.595413699 | 2.52548960127193 | 0.585691517323892 | 0.0274493250630348 | 12.4970123191523 | 0.999950574162623 |
| ebi-fl187-GCST90094853 | prot-a-1289 | Glutathione S-transferase P \|\| id:prot-a-1289 | F-sweetened coffee drink liking (derived food-liking factor) \|\| id:ebi-fl187-GCST90094853 | MR Egger | 4 | -3.420972306 | 1.56145492358888 | 0.159832240749606 | -6.481423956 | -0.360520656 | 0.03268064390679 | 0.00153162815463944 | 0.697313171560126 | 0.999950574162623 |
| ebi-fl187-GCST90094853 | prot-a-130 | Apolipoprotein D \|\| id:prot-a-130 | F-sweetened coffee drink liking (derived food-liking factor) \|\| id:ebi-fl187-GCST90094853 | MR Egger | 4 | -2.038087238 | 1.81179064408479 | 0.377490137496195 | -5.5891969 | 1.51302242475144 | 0.130277662917536 | 0.00373802867015065 | 4.54043319431555 | 0.999950574162623 |
| ebi-fl187-GCST90094853 | prot-a-1313 | Heparin-binding EGF-like growth factor \|\| id:prot-a-1313 | F-sweetened coffee drink liking (derived food-liking factor) \|\| id:ebi-fl187-GCST90094853 | MR Egger | 4 | -2.189093181 | 1.56145492358888 | 0.295977278393494 | -5.249544831 | 0.871358469204509 | 0.112018282877709 | 0.00524990744916804 | 2.39015560185904 | 0.999950574162623 |
| ebi-fl187-GCST90094853 | prot-a-1347 | HLA class II histocompatibility antigen, DQ alpha 2 chain \|\| id:prot-a-1347 | F-sweetened coffee drink liking (derived food-liking factor) \|\| id:ebi-fl187-GCST90094853 | MR Egger | 4 | -0.256454704 | 1.56145492358888 | 0.884639530246309 | -3.316906355 | 2.80399694594237 | 0.773790043133674 | 0.0362648489798258 | 16.5105066668249 | 0.999950574162623 |
| ebi-fl187-GCST90094853 | prot-a-1357 | Non-histone chromosomal protein HMG-14 \|\| id:prot-a-1357 | F-sweetened coffee drink liking (derived food-liking factor) \|\| id:ebi-fl187-GCST90094853 | MR Egger | 4 | -0.913200931 | 1.56463924156069 | 0.618509094065397 | -3.979893845 | 2.15349198211621 | 0.40123783155408 | 0.0186876230207696 | 8.61488897176985 | 0.999950574162623 |
| ebi-fl187-GCST90094853 | prot-a-137 | Apolipoprotein M \|\| id:prot-a-137 | F-sweetened coffee drink liking (derived food-liking factor) \|\| id:ebi-fl187-GCST90094853 | MR Egger | 4 | 1.56242332139162 | 1.56145492358888 | 0.422411093809231 | -1.498028329 | 4.62287497162582 | 4.77036738281091 | 0.223570533442544 | 101.786244441889 | 0.999950574162623 |
| ebi-fl187-GCST90094853 | prot-a-1381 | 17-beta-hydroxysteroid dehydrogenase 14 \|\| id:prot-a-1381 | F-sweetened coffee drink liking (derived food-liking factor) \|\| id:ebi-fl187-GCST90094853 | MR Egger | 4 | -0.645611282 | 1.56145492358888 | 0.719381570787333 | -3.706062932 | 2.41484036834973 | 0.524341923403329 | 0.0245740828985159 | 11.1879842586071 | 0.999950574162623 |
| ebi-fl187-GCST90094853 | prot-a-1392 | Serine protease HTRA2, mitochondrial \|\| id:prot-a-1392 | F-sweetened coffee drink liking (derived food-liking factor) \|\| id:ebi-fl187-GCST90094853 | MR Egger | 4 | -1.475578055 | 1.79320810104992 | 0.497081152600564 | -4.990265933 | 2.03910982261861 | 0.228646518473297 | 0.00680385487870132 | 7.68376623869713 | 0.999950574162623 |
| ebi-fl187-GCST90094853 | prot-a-1416 | Interferon-induced protein with tetratricopeptide repeats 2 \|\| id:prot-a-1416 | F-sweetened coffee drink liking (derived food-liking factor) \|\| id:ebi-fl187-GCST90094853 | MR Egger | 4 | 1.05932984070834 | 1.56145492358888 | 0.567474988739652 | -2.00112181 | 4.11978149094254 | 2.8844373089396 | 0.135183547951649 | 61.5457924819264 | 0.999950574162623 |
| ebi-fl187-GCST90094853 | prot-a-1428 | Interferon gamma \|\| id:prot-a-1428 | F-sweetened coffee drink liking (derived food-liking factor) \|\| id:ebi-fl187-GCST90094853 | MR Egger | 4 | -2.443610078 | 1.56145492358888 | 0.258063367410313 | -5.504061729 | 0.616841571745765 | 0.086846761233393 | 0.00407020574697664 | 1.85306601326787 | 0.999950574162623 |
| ebi-fl187-GCST90094853 | prot-a-1483 | Interleukin-17C \|\| id:prot-a-1483 | F-sweetened coffee drink liking (derived food-liking factor) \|\| id:ebi-fl187-GCST90094853 | MR Egger | 4 | 2.08783415291777 | 1.56145492358888 | 0.312979518135997 | -0.972617497 | 5.14828580315198 | 8.06742342344202 | 0.378092086740502 | 172.136161997408 | 0.999950574162623 |
| ebi-fl187-GCST90094853 | prot-a-1503 | Interleukin-1 receptor-like 2 \|\| id:prot-a-1503 | F-sweetened coffee drink liking (derived food-liking factor) \|\| id:ebi-fl187-GCST90094853 | MR Egger | 4 | 0.839935513713259 | 1.56145492358888 | 0.644483472116752 | -2.220516137 | 3.90038716394746 | 2.31621760769221 | 0.108553066161464 | 49.4215796558307 | 0.999950574162623 |
| ebi-fl187-GCST90094853 | prot-a-1522 | Interleukin-31 receptor subunit alpha \|\| id:prot-a-1522 | F-sweetened coffee drink liking (derived food-liking factor) \|\| id:ebi-fl187-GCST90094853 | MR Egger | 4 | -0.197704037 | 1.56463924156069 | 0.911006212040824 | -3.264396951 | 2.86898887605712 | 0.820612687864252 | 0.0382199766594059 | 17.619194 | 0.999950574162623 |
| ebi-fl187-GCST90094853 | prot-a-1540 | Interleukin-6 receptor subunit alpha \|\| id:prot-a-1540 | F-sweetened coffee drink liking (derived food-liking factor) \|\| id:ebi-fl187-GCST90094853 | MR Egger | 4 | 1.12159578680434 | 1.56145492358888 | 0.547149056291365 | -1.938855863 | 4.18204743703854 | 3.06974896164452 | 0.143868460815519 | 65.4998227832652 | 0.999950574162623 |
| ebi-fl187-GCST90094853 | prot-a-1541 | Interleukin-6 receptor subunit alpha \|\| id:prot-a-1541 | F-sweetened coffee drink liking (derived food-liking factor) \|\| id:ebi-fl187-GCST90094853 | MR Egger | 4 | 1.25999560622005 | 1.56145492358888 | 0.504409856161363 | -1.800456044 | 4.32044725645425 | 3.52540599747316 | 0.165223521839567 | 75.2222644127381 | 0.999950574162623 |
| ebi-fl187-GCST90094853 | prot-a-1545 | Interleukin-9 \|\| id:prot-a-1545 | F-sweetened coffee drink liking (derived food-liking factor) \|\| id:ebi-fl187-GCST90094853 | MR Egger | 4 | 2.05005157689439 | 1.56145492358888 | 0.319628813919962 | -1.010400073 | 5.11050322712859 | 7.7683017608539 | 0.36407329441226 | 165.7537457 | 0.999950574162623 |
| ebi-fl187-GCST90094853 | prot-a-1548 | Inositol monophosphatase 3 \|\| id:prot-a-1548 | F-sweetened coffee drink liking (derived food-liking factor) \|\| id:ebi-fl187-GCST90094853 | MR Egger | 4 | -1.638477505 | 1.88594958588332 | 0.476560026491974 | -5.334938694 | 2.05798368292552 | 0.194275600792682 | 0.00482020561200511 | 7.83016578574064 | 0.999950574162623 |
| ebi-fl187-GCST90094853 | prot-a-1555 | Inhibin beta A chain:Inhibin beta B chain heterodimer \|\| id:prot-a-1555 | F-sweetened coffee drink liking (derived food-liking factor) \|\| id:ebi-fl187-GCST90094853 | MR Egger | 4 | 2.25837945088567 | 1.56145492358888 | 0.28499926402195 | -0.802072199 | 5.31883110111987 | 9.56757187676385 | 0.448398828976053 | 204.145117475164 | 0.999950574162623 |
| ebi-fl187-GCST90094853 | prot-a-1603 | Junctophilin-3 \|\| id:prot-a-1603 | F-sweetened coffee drink liking (derived food-liking factor) \|\| id:ebi-fl187-GCST90094853 | MR Egger | 4 | 0.512380517076092 | 2.95983329895965 | 0.87849873154409 | -5.288892749 | 6.313653783 | 1.66926017130582 | 0.00504734584611377 | 552.058369777326 | 0.999950574162623 |
| ebi-fl187-GCST90094853 | prot-a-1627 | UPF0258 protein KIAA1024 \|\| id:prot-a-1627 | F-sweetened coffee drink liking (derived food-liking factor) \|\| id:ebi-fl187-GCST90094853 | MR Egger | 4 | -0.392991694 | 1.56145492358888 | 0.824786298712826 | -3.453443344 | 2.66745995648424 | 0.675034354566982 | 0.0316365132141366 | 14.4033375853205 | 0.999950574162623 |
| ebi-fl187-GCST90094853 | prot-a-1666 | Kallikrein-7 \|\| id:prot-a-1666 | F-sweetened coffee drink liking (derived food-liking factor) \|\| id:ebi-fl187-GCST90094853 | MR Egger | 4 | -0.783268268 | 1.56145492358888 | 0.66570292078294 | -3.843719918 | 2.27718338242812 | 0.45691026328033 | 0.0214137954374416 | 9.74918198414633 | 0.999950574162623 |
| ebi-fl187-GCST90094853 | prot-a-1678 | Importin subunit alpha-3 \|\| id:prot-a-1678 | F-sweetened coffee drink liking (derived food-liking factor) \|\| id:ebi-fl187-GCST90094853 | MR Egger | 4 | -2.226650922 | 1.56145492358888 | 0.289962389466812 | -5.287102573 | 0.833800727909951 | 0.10788915491115 | 0.0050563895776803 | 2.30205160591726 | 0.999950574162623 |
| ebi-fl187-GCST90094853 | prot-a-1718 | Protein LDOC1 \|\| id:prot-a-1718 | F-sweetened coffee drink liking (derived food-liking factor) \|\| id:ebi-fl187-GCST90094853 | MR Egger | 4 | 3.9408049948978 | 1.95241003139613 | 0.181018222372317 | 0.114081333361384 | 7.76752865643422 | 51.4600096767555 | 1.1208432831288 | 2362.625209 | 0.999950574162623 |
| ebi-fl187-GCST90094853 | prot-a-1723 | Leptin \|\| id:prot-a-1723 | F-sweetened coffee drink liking (derived food-liking factor) \|\| id:ebi-fl187-GCST90094853 | MR Egger | 4 | 1.34354487106897 | 1.56145492358888 | 0.480221183127362 | -1.716906779 | 4.40399652130317 | 3.83260554584028 | 0.179620896588779 | 81.7770401382312 | 0.999950574162623 |
| ebi-fl187-GCST90094853 | prot-a-1730 | Galectin-7 \|\| id:prot-a-1730 | F-sweetened coffee drink liking (derived food-liking factor) \|\| id:ebi-fl187-GCST90094853 | MR Egger | 4 | 0.680674535728413 | 1.87039730393937 | 0.750788848928998 | -2.98530418 | 4.34665325144959 | 1.97520963244099 | 0.0505241327811695 | 77.2195954156341 | 0.999950574162623 |
| ebi-fl187-GCST90094853 | prot-a-1753 | Lipase member N \|\| id:prot-a-1753 | F-sweetened coffee drink liking (derived food-liking factor) \|\| id:ebi-fl187-GCST90094853 | MR Egger | 4 | 0.190123145272276 | 2.20645356737252 | 0.939183620366139 | -4.134525847 | 4.51477213732241 | 1.20939852019757 | 0.0160102546442075 | 91.356746857568 | 0.999950574162623 |
| ebi-fl187-GCST90094853 | prot-a-1756 | VIP36-like protein \|\| id:prot-a-1756 | F-sweetened coffee drink liking (derived food-liking factor) \|\| id:ebi-fl187-GCST90094853 | MR Egger | 4 | -1.238941532 | 1.56145492358888 | 0.510695569561838 | -4.299393182 | 1.8215101186059 | 0.289690684144789 | 0.0135767951585781 | 6.18118572903806 | 0.999950574162623 |
| ebi-fl187-GCST90094853 | prot-a-1760 | Ig Kappa chain V-I region HK102- like \|\| id:prot-a-1760 | F-sweetened coffee drink liking (derived food-liking factor) \|\| id:ebi-fl187-GCST90094853 | MR Egger | 4 | -0.694984467 | 1.56145492358888 | 0.69979244577397 | -3.755436117 | 2.36546718325333 | 0.499082200178291 | 0.0233902474948986 | 10.6490126959592 | 0.999950574162623 |
| ebi-fl187-GCST90094853 | prot-a-179 | Neutral ceramidase \|\| id:prot-a-179 | F-sweetened coffee drink liking (derived food-liking factor) \|\| id:ebi-fl187-GCST90094853 | MR Egger | 4 | -0.21653459 | 1.56145492358888 | 0.902410103354974 | -3.27698624 | 2.84391705997924 | 0.805304678746741 | 0.0377418303797592 | 17.1829404956255 | 0.999950574162623 |
| ebi-fl187-GCST90094853 | prot-a-1809 | Lactotransferrin \|\| id:prot-a-1809 | F-sweetened coffee drink liking (derived food-liking factor) \|\| id:ebi-fl187-GCST90094853 | MR Egger | 4 | 0.755989456807093 | 1.56145492358888 | 0.676104405533715 | -2.304462193 | 3.81644110704129 | 2.12971774489571 | 0.0998124660218145 | 45.4421962877101 | 0.999950574162623 |
| ebi-fl187-GCST90094853 | prot-a-1818 | Lysophospholipase-like protein 1 \|\| id:prot-a-1818 | F-sweetened coffee drink liking (derived food-liking factor) \|\| id:ebi-fl187-GCST90094853 | MR Egger | 4 | 3.2464230891353 | 2.42161463646602 | 0.312033233416391 | -1.499941598 | 7.99278777660871 | 25.6982549581643 | 0.22314319170113 | 2959.53599507231 | 0.999950574162623 |
| ebi-fl187-GCST90094853 | prot-a-1852 | MAP kinase-activated protein kinase 3 \|\| id:prot-a-1852 | F-sweetened coffee drink liking (derived food-liking factor) \|\| id:ebi-fl187-GCST90094853 | MR Egger | 4 | -3.350577826 | 1.56145492358888 | 0.16502981196351 | -6.411029476 | -0.290126176 | 0.0350640872986434 | 0.00164333185957145 | 0.748169160675505 | 0.999950574162623 |
| ebi-fl187-GCST90094853 | prot-a-1856 | Megakaryocyte-associated tyrosine-protein kinase \|\| id:prot-a-1856 | F-sweetened coffee drink liking (derived food-liking factor) \|\| id:ebi-fl187-GCST90094853 | MR Egger | 4 | -1.500457591 | 1.56145492358888 | 0.437981823829211 | -4.560909242 | 1.55999405896764 | 0.223028081092864 | 0.0104525507285389 | 4.75879297291084 | 0.999950574162623 |
| ebi-fl187-GCST90094853 | prot-a-1890 | Beta-1,3-N-acetylglucosaminyltransferase manic fringe \|\| id:prot-a-1890 | F-sweetened coffee drink liking (derived food-liking factor) \|\| id:ebi-fl187-GCST90094853 | MR Egger | 4 | -1.663972682 | 2.15678094674428 | 0.521091281 | -5.891263337 | 2.56331797403722 | 0.189385116898034 | 0.00276348327946789 | 12.9788093052577 | 0.999950574162623 |
| ebi-fl187-GCST90094853 | prot-a-1919 | Matrilysin \|\| id:prot-a-1919 | F-sweetened coffee drink liking (derived food-liking factor) \|\| id:ebi-fl187-GCST90094853 | MR Egger | 4 | 3.62726423219556 | 1.90676177119718 | 0.197471120013933 | -0.109988839 | 7.36451730374203 | 37.6097841084373 | 0.895844133442745 | 1578.9530877959 | 0.999950574162623 |
| ebi-fl187-GCST90094853 | prot-a-1923 | Multimerin-2 \|\| id:prot-a-1923 | F-sweetened coffee drink liking (derived food-liking factor) \|\| id:ebi-fl187-GCST90094853 | MR Egger | 4 | -0.61153138 | 1.89059918428448 | 0.777037491696991 | -4.317105781 | 3.09404302162091 | 0.542519429437955 | 0.0133384320781405 | 22.0661116384165 | 0.999950574162623 |
| ebi-fl187-GCST90094853 | prot-a-1948 | Mesothelin \|\| id:prot-a-1948 | F-sweetened coffee drink liking (derived food-liking factor) \|\| id:ebi-fl187-GCST90094853 | MR Egger | 4 | -0.225193567 | 1.56145492358888 | 0.898546994955081 | -3.285645217 | 2.83525808298344 | 0.798361667128775 | 0.0374164355649448 | 17.0347961241342 | 0.999950574162623 |
| ebi-fl187-GCST90094853 | prot-a-195 | ATPase family AAA domain-containing protein 1 \|\| id:prot-a-195 | F-sweetened coffee drink liking (derived food-liking factor) \|\| id:ebi-fl187-GCST90094853 | MR Egger | 4 | 0.858795138306726 | 1.93238246975192 | 0.700200514299417 | -2.928674502 | 4.64626477902049 | 2.36031512651259 | 0.0534678627192615 | 104.195066215676 | 0.999950574162623 |
| ebi-fl187-GCST90094853 | prot-a-1966 | Mucin-1 \|\| id:prot-a-1966 | F-sweetened coffee drink liking (derived food-liking factor) \|\| id:ebi-fl187-GCST90094853 | MR Egger | 4 | -1.561538608 | 1.84948586732247 | 0.487388757327159 | -5.186530908 | 2.06345369202033 | 0.209813002776432 | 0.00559137018707499 | 7.87311422087969 | 0.999950574162623 |
| ebi-fl187-GCST90094853 | prot-a-1986 | Myocardial zonula adherens protein \|\| id:prot-a-1986 | F-sweetened coffee drink liking (derived food-liking factor) \|\| id:ebi-fl187-GCST90094853 | MR Egger | 4 | 0.404694963992089 | 1.56145492358888 | 0.819735625904252 | -2.655756686 | 3.46514661422629 | 1.49884522855318 | 0.0702456646217449 | 31.9811483207351 | 0.999950574162623 |
| ebi-fl187-GCST90094853 | prot-a-2071 | Epididymal secretory protein E1 \|\| id:prot-a-2071 | F-sweetened coffee drink liking (derived food-liking factor) \|\| id:ebi-fl187-GCST90094853 | MR Egger | 4 | -0.280153481 | 1.56463924156069 | 0.874393087528327 | -3.346846394 | 2.78653943256419 | 0.755667751992155 | 0.0351951709625328 | 16.2247756093804 | 0.999950574162623 |
| ebi-fl187-GCST90094853 | prot-a-2098 | Neuritin \|\| id:prot-a-2098 | F-sweetened coffee drink liking (derived food-liking factor) \|\| id:ebi-fl187-GCST90094853 | MR Egger | 4 | -2.057155595 | 1.5950240779747 | 0.326159633684564 | -5.183402787 | 1.06909159827344 | 0.127817016741965 | 0.00560888805147065 | 2.91273236671788 | 0.999950574162623 |
| ebi-fl187-GCST90094853 | prot-a-2115 | Neurotrophin-4 \|\| id:prot-a-2115 | F-sweetened coffee drink liking (derived food-liking factor) \|\| id:ebi-fl187-GCST90094853 | MR Egger | 4 | -0.519597876 | 1.56145492358888 | 0.770954745757175 | -3.580049526 | 2.54085377455476 | 0.594759667216216 | 0.027874317720009 | 12.6905011738895 | 0.999950574162623 |
| ebi-fl187-GCST90094853 | prot-a-2118 | Netrin-1 \|\| id:prot-a-2118 | F-sweetened coffee drink liking (derived food-liking factor) \|\| id:ebi-fl187-GCST90094853 | MR Egger | 4 | -1.183430572 | 1.76890442678532 | 0.572368779740059 | -4.650483248 | 2.28362210491102 | 0.306226402979233 | 0.00955698242097238 | 9.81215678243953 | 0.999950574162623 |
| ebi-fl187-GCST90094853 | prot-a-2120 | Netrin-G1 \|\| id:prot-a-2120 | F-sweetened coffee drink liking (derived food-liking factor) \|\| id:ebi-fl187-GCST90094853 | MR Egger | 4 | 2.98881124932717 | 1.56145492358888 | 0.195709313086604 | -0.071640401 | 6.04926289956137 | 19.8620574180663 | 0.930865574048711 | 423.800531329909 | 0.999950574162623 |
| ebi-fl187-GCST90094853 | prot-a-2145 | Olfactomedin-like protein 3 \|\| id:prot-a-2145 | F-sweetened coffee drink liking (derived food-liking factor) \|\| id:ebi-fl187-GCST90094853 | MR Egger | 4 | -0.735506781 | 1.56145492358888 | 0.683992700574114 | -3.795958431 | 2.32494486910063 | 0.479262516219443 | 0.0224613678175579 | 10.2261162952616 | 0.999950574162623 |
| ebi-fl187-GCST90094853 | prot-a-2162 | Prolyl 4-hydroxylase subunit alpha-2 \|\| id:prot-a-2162 | F-sweetened coffee drink liking (derived food-liking factor) \|\| id:ebi-fl187-GCST90094853 | MR Egger | 4 | -0.915600563 | 1.56145492358888 | 0.616987583113186 | -3.976052214 | 2.14485108678739 | 0.400276162659352 | 0.0187595520488727 | 8.54076930919704 | 0.999950574162623 |
| ebi-fl187-GCST90094853 | prot-a-2168 | Polyadenylate-binding protein-interacting protein 1 \|\| id:prot-a-2168 | F-sweetened coffee drink liking (derived food-liking factor) \|\| id:ebi-fl187-GCST90094853 | MR Egger | 4 | -0.870154443 | 1.56145492358888 | 0.633385992847961 | -3.930606093 | 2.19029720741662 | 0.418886850186226 | 0.0196317702669303 | 8.93786912097837 | 0.999950574162623 |
| ebi-fl187-GCST90094853 | prot-a-219 | Beta-1,4-galactosyltransferase 5 \|\| id:prot-a-219 | F-sweetened coffee drink liking (derived food-liking factor) \|\| id:ebi-fl187-GCST90094853 | MR Egger | 4 | 0.29706129117189 | 1.66586576500513 | 0.874897582951062 | -2.968035608 | 3.56215819058195 | 1.34589778857285 | 0.0514041891824415 | 35.2391679762947 | 0.999950574162623 |
| ebi-fl187-GCST90094853 | prot-a-2190 | Pyruvate carboxylase, mitochondrial \|\| id:prot-a-2190 | F-sweetened coffee drink liking (derived food-liking factor) \|\| id:ebi-fl187-GCST90094853 | MR Egger | 4 | 2.75435626068901 | 1.56145492358888 | 0.219787375221868 | -0.30609539 | 5.81480791092321 | 15.710923907468 | 0.736316379221532 | 335.226998871344 | 0.999950574162623 |
| ebi-fl187-GCST90094853 | prot-a-2197 | Protocadherin-9 \|\| id:prot-a-2197 | F-sweetened coffee drink liking (derived food-liking factor) \|\| id:ebi-fl187-GCST90094853 | MR Egger | 4 | -0.059079278 | 1.56145492358888 | 0.973255446144452 | -3.119530929 | 3.00137237191938 | 0.942632035915477 | 0.0441778861454218 | 20.1131206732995 | 0.999950574162623 |
| ebi-fl187-GCST90094853 | prot-a-2198 | Protocadherin alpha-4 \|\| id:prot-a-2198 | F-sweetened coffee drink liking (derived food-liking factor) \|\| id:ebi-fl187-GCST90094853 | MR Egger | 4 | -0.368523615 | 1.56463924156069 | 0.835716124768385 | -3.435216528 | 2.69816929856272 | 0.691754873685672 | 0.0322184332722263 | 14.8525162978794 | 0.999950574162623 |
| ebi-fl187-GCST90094853 | prot-a-2254 | Placenta growth factor \|\| id:prot-a-2254 | F-sweetened coffee drink liking (derived food-liking factor) \|\| id:ebi-fl187-GCST90094853 | MR Egger | 4 | 1.46333490877554 | 2.14122221788043 | 0.564896117337612 | -2.733460638 | 5.66013045582119 | 4.32034348181868 | 0.0649939793819123 | 287.18610521158 | 0.999950574162623 |
| ebi-fl187-GCST90094853 | prot-a-2261 | Membrane-associated progesterone receptor component 2 \|\| id:prot-a-2261 | F-sweetened coffee drink liking (derived food-liking factor) \|\| id:ebi-fl187-GCST90094853 | MR Egger | 4 | -1.092645252 | 1.56145492358888 | 0.55651469385422 | -4.153096902 | 1.9678063983959 | 0.335328291685627 | 0.0157156711494948 | 7.15496412053744 | 0.999950574162623 |
| ebi-fl187-GCST90094853 | prot-a-2268 | Polymeric immunoglobulin receptor \|\| id:prot-a-2268 | F-sweetened coffee drink liking (derived food-liking factor) \|\| id:ebi-fl187-GCST90094853 | MR Egger | 4 | 0.00968143854927991 | 1.56145492358888 | 0.995615790790088 | -3.050770212 | 3.07013308878348 | 1.00972845528285 | 0.0473224620378575 | 21.5447698514135 | 0.999950574162623 |
| ebi-fl187-GCST90094853 | prot-a-2283 | Group 10 secretory phospholipase A2 \|\| id:prot-a-2283 | F-sweetened coffee drink liking (derived food-liking factor) \|\| id:ebi-fl187-GCST90094853 | MR Egger | 4 | 1.88005366987463 | 1.56463924156069 | 0.352505601431136 | -1.186639244 | 4.94674658333358 | 6.55385659741419 | 0.305245397599434 | 140.716409280102 | 0.999950574162623 |
| ebi-fl187-GCST90094853 | prot-a-2298 | Inactive phospholipase D5 \|\| id:prot-a-2298 | F-sweetened coffee drink liking (derived food-liking factor) \|\| id:ebi-fl187-GCST90094853 | MR Egger | 4 | -0.215868194 | 1.56145492358888 | 0.902707593828429 | -3.276319845 | 2.84458345595066 | 0.805841509391446 | 0.0377669897656039 | 17.1943949541275 | 0.999950574162623 |
| ebi-fl187-GCST90094853 | prot-a-2304 | Procollagen-lysine,2-oxoglutarate 5-dioxygenase 2 \|\| id:prot-a-2304 | F-sweetened coffee drink liking (derived food-liking factor) \|\| id:ebi-fl187-GCST90094853 | MR Egger | 4 | -1.152336896 | 1.56145492358888 | 0.537365668404487 | -4.212788546 | 1.90811475400374 | 0.315897686023169 | 0.0148050262191441 | 6.74036955812714 | 0.999950574162623 |
| ebi-fl187-GCST90094853 | prot-a-2331 | Periostin \|\| id:prot-a-2331 | F-sweetened coffee drink liking (derived food-liking factor) \|\| id:ebi-fl187-GCST90094853 | MR Egger | 4 | 0.0312019127272107 | 1.56145492358888 | 0.98587158578638 | -3.029249738 | 3.09165356296141 | 1.03169379496529 | 0.0483519011388678 | 22.0134485201094 | 0.999950574162623 |
| ebi-fl187-GCST90094853 | prot-a-2333 | Periostin \|\| id:prot-a-2333 | F-sweetened coffee drink liking (derived food-liking factor) \|\| id:ebi-fl187-GCST90094853 | MR Egger | 4 | 0.00878192798360818 | 1.56266887220358 | 0.996026213927804 | -3.054049062 | 3.07161291750262 | 1.00882060224178 | 0.0471675528909802 | 21.5766760225987 | 0.999950574162623 |
| ebi-fl187-GCST90094853 | prot-a-2367 | cAMP-dependent protein kinase catalytic subunit alpha \|\| id:prot-a-2367 | F-sweetened coffee drink liking (derived food-liking factor) \|\| id:ebi-fl187-GCST90094853 | MR Egger | 4 | -2.612828461 | 1.56145492358888 | 0.23623560280719 | -5.673280112 | 0.447623188926189 | 0.0733268480193757 | 0.0034365744211701 | 1.56458903009174 | 0.999950574162623 |
| ebi-fl187-GCST90094853 | prot-a-2385 | Phosphoribosyl pyrophosphate synthase-associated protein 1 \|\| id:prot-a-2385 | F-sweetened coffee drink liking (derived food-liking factor) \|\| id:ebi-fl187-GCST90094853 | MR Egger | 4 | -1.430999859 | 1.56145492358888 | 0.456174340219937 | -4.491451509 | 1.62945179151222 | 0.239069766711398 | 0.0112043687591535 | 5.10107749789555 | 0.999950574162623 |
| ebi-fl187-GCST90094853 | prot-a-2393 | Trypsin-3 \|\| id:prot-a-2393 | F-sweetened coffee drink liking (derived food-liking factor) \|\| id:ebi-fl187-GCST90094853 | MR Egger | 4 | 0.177223382939129 | 1.56145492358888 | 0.920001395116882 | -2.883228267 | 3.23767503317333 | 1.19389775973223 | 0.0559538370107486 | 25.4744256487688 | 0.999950574162623 |
| ebi-fl187-GCST90094853 | prot-a-2405 | Pregnancy-specific beta-1-glycoprotein 6 \|\| id:prot-a-2405 | F-sweetened coffee drink liking (derived food-liking factor) \|\| id:ebi-fl187-GCST90094853 | MR Egger | 4 | 0.781290262144762 | 1.56463924156069 | 0.667056612175419 | -2.285402651 | 3.84798317560371 | 2.18428875319522 | 0.101733090895536 | 46.8983819850151 | 0.999950574162623 |
| ebi-fl187-GCST90094853 | prot-a-244 | Beclin-1 \|\| id:prot-a-244 | F-sweetened coffee drink liking (derived food-liking factor) \|\| id:ebi-fl187-GCST90094853 | MR Egger | 4 | -1.091008089 | 1.70150763479462 | 0.58706376816134 | -4.425963053 | 2.24394687527592 | 0.335877728365673 | 0.0119626850503495 | 9.43047885464386 | 0.999950574162623 |
| ebi-fl187-GCST90094853 | prot-a-2454 | tRNA pseudouridine synthase A, mitochondrial \|\| id:prot-a-2454 | F-sweetened coffee drink liking (derived food-liking factor) \|\| id:ebi-fl187-GCST90094853 | MR Egger | 4 | -2.884122063 | 1.94715308592042 | 0.276727716428718 | -6.700542111 | 0.932297985595644 | 0.0559038480655511 | 0.00123024479236987 | 2.54034014036827 | 0.999950574162623 |
| ebi-fl187-GCST90094853 | prot-a-2466 | Glutaminyl-peptide cyclotransferase-like protein \|\| id:prot-a-2466 | F-sweetened coffee drink liking (derived food-liking factor) \|\| id:ebi-fl187-GCST90094853 | MR Egger | 4 | 0.688554422553546 | 1.66379059812079 | 0.719144603802355 | -2.57247515 | 3.9495839948703 | 1.99083554515802 | 0.0763463427358663 | 51.9137659491666 | 0.999950574162623 |
| ebi-fl187-GCST90094853 | prot-a-2474 | Ras-related protein Rab-27A \|\| id:prot-a-2474 | F-sweetened coffee drink liking (derived food-liking factor) \|\| id:ebi-fl187-GCST90094853 | MR Egger | 4 | 0.13952223535005 | 1.56145492358888 | 0.936942922959732 | -2.920929415 | 3.19997388558425 | 1.14972436995767 | 0.0538835838156909 | 24.5318895527814 | 0.999950574162623 |
| ebi-fl187-GCST90094853 | prot-a-2490 | Rap1 GTPase-GDP dissociation stimulator 1 \|\| id:prot-a-2490 | F-sweetened coffee drink liking (derived food-liking factor) \|\| id:ebi-fl187-GCST90094853 | MR Egger | 4 | -1.606284598 | 1.56145492358888 | 0.411756779798018 | -4.666736249 | 1.45416705177862 | 0.200631658180587 | 0.00940290825535227 | 4.28091619860049 | 0.999950574162623 |
| ebi-fl187-GCST90094853 | prot-a-2545 | Receptor-interacting serine/threonine-protein kinase 2 \|\| id:prot-a-2545 | F-sweetened coffee drink liking (derived food-liking factor) \|\| id:ebi-fl187-GCST90094853 | MR Egger | 4 | -3.650305967 | 1.63354627499899 | 0.155005478408384 | -6.852056666 | -0.448555268 | 0.0259831775645664 | 0.00105727898543459 | 0.638550019108045 | 0.999950574162623 |
| ebi-fl187-GCST90094853 | prot-a-2610 | RUN and FYVE domain-containing protein 1 \|\| id:prot-a-2610 | F-sweetened coffee drink liking (derived food-liking factor) \|\| id:ebi-fl187-GCST90094853 | MR Egger | 4 | -1.992969305 | 1.56145492358888 | 0.330002921813405 | -5.053420955 | 1.06748234549723 | 0.136290137081335 | 0.00638744486641129 | 2.90804881359158 | 0.999950574162623 |
| ebi-fl187-GCST90094853 | prot-a-2627 | Serine--tRNA ligase, mitochondrial \|\| id:prot-a-2627 | F-sweetened coffee drink liking (derived food-liking factor) \|\| id:ebi-fl187-GCST90094853 | MR Egger | 4 | -2.257805965 | 2.26180431689968 | 0.423330747877165 | -6.690942426 | 2.17533049657427 | 0.104579684762162 | 0.00124211162330529 | 8.80509469499198 | 0.999950574162623 |
| ebi-fl187-GCST90094853 | prot-a-264 | BolA-like protein 2 \|\| id:prot-a-264 | F-sweetened coffee drink liking (derived food-liking factor) \|\| id:ebi-fl187-GCST90094853 | MR Egger | 4 | -3.583832877 | 1.56145492358888 | 0.148638195616269 | -6.644284527 | -0.523381227 | 0.0277690586281732 | 0.00130143922941208 | 0.592513733770935 | 0.999950574162623 |
| ebi-fl187-GCST90094853 | prot-a-2646 | Secretoglobin family 1C member 1 \|\| id:prot-a-2646 | F-sweetened coffee drink liking (derived food-liking factor) \|\| id:ebi-fl187-GCST90094853 | MR Egger | 4 | -0.25884578 | 1.70879627498288 | 0.893497773893871 | -3.608086479 | 3.09039491925179 | 0.771942062989495 | 0.0271036607096812 | 21.9857588609656 | 0.999950574162623 |
| ebi-fl187-GCST90094853 | prot-a-2672 | Semaphorin-3E \|\| id:prot-a-2672 | F-sweetened coffee drink liking (derived food-liking factor) \|\| id:ebi-fl187-GCST90094853 | MR Egger | 4 | -0.262818652 | 1.56463924156069 | 0.882053530981325 | -3.329511566 | 2.80387426098002 | 0.768881319390126 | 0.0358105919095965 | 16.5084812001858 | 0.999950574162623 |
| ebi-fl187-GCST90094853 | prot-a-2701 | Plasma protease C1 inhibitor \|\| id:prot-a-2701 | F-sweetened coffee drink liking (derived food-liking factor) \|\| id:ebi-fl187-GCST90094853 | MR Egger | 4 | 0.271490172118852 | 1.56463924156069 | 0.878218774814063 | -2.795202741 | 3.3381830855778 | 1.31191797841916 | 0.0611024850770158 | 28.1679015170994 | 0.999950574162623 |
| ebi-fl187-GCST90094853 | prot-a-2738 | Signaling threshold-regulating transmembrane adapter 1 \|\| id:prot-a-2738 | F-sweetened coffee drink liking (derived food-liking factor) \|\| id:ebi-fl187-GCST90094853 | MR Egger | 4 | -1.263042597 | 2.02825343610218 | 0.597006424178888 | -5.238419332 | 2.71233413734626 | 0.282792293110941 | 0.00530864140618336 | 15.0643968812427 | 0.999950574162623 |
| ebi-fl187-GCST90094853 | prot-a-274 | Serine/threonine-protein kinase BRSK2 \|\| id:prot-a-274 | F-sweetened coffee drink liking (derived food-liking factor) \|\| id:ebi-fl187-GCST90094853 | MR Egger | 4 | 0.578615928943446 | 1.56630596638647 | 0.74726482442307 | -2.491343765 | 3.64857562306093 | 1.78356813657786 | 0.0827986298714539 | 38.4199026331059 | 0.999950574162623 |
| ebi-fl187-GCST90094853 | prot-a-2749 | Mitochondrial glutamate carrier 2 \|\| id:prot-a-2749 | F-sweetened coffee drink liking (derived food-liking factor) \|\| id:ebi-fl187-GCST90094853 | MR Egger | 4 | 1.00856608469782 | 2.59643146113376 | 0.735138649875181 | -4.080439579 | 6.097571749 | 2.74166687699225 | 0.0169000351170325 | 444.776428708175 | 0.999950574162623 |
| ebi-fl187-GCST90094853 | prot-a-2760 | Sodium-coupled monocarboxylate transporter 1 \|\| id:prot-a-2760 | F-sweetened coffee drink liking (derived food-liking factor) \|\| id:ebi-fl187-GCST90094853 | MR Egger | 4 | -0.325213257 | 1.56145492358888 | 0.854298391060034 | -3.385664907 | 2.73523839302925 | 0.72237328590675 | 0.0338551243362836 | 15.4134174492592 | 0.999950574162623 |
| ebi-fl187-GCST90094853 | prot-a-2777 | Small integral membrane protein 10 \|\| id:prot-a-2777 | F-sweetened coffee drink liking (derived food-liking factor) \|\| id:ebi-fl187-GCST90094853 | MR Egger | 4 | -3.601474052 | 1.56145492358888 | 0.147491111761446 | -6.661925702 | -0.541022402 | 0.02728347553518 | 0.00127868163813675 | 0.582152754115919 | 0.999950574162623 |
| ebi-fl187-GCST90094853 | prot-a-2799 | Superoxide dismutase [Mn], mitochondrial \|\| id:prot-a-2799 | F-sweetened coffee drink liking (derived food-liking factor) \|\| id:ebi-fl187-GCST90094853 | MR Egger | 4 | 1.36739692741384 | 1.56145492358888 | 0.473534900221835 | -1.693054723 | 4.42784857764804 | 3.92512001333917 | 0.183956728022729 | 83.7510390879107 | 0.999950574162623 |
| ebi-fl187-GCST90094853 | prot-a-2805 | Protein SPACA7 \|\| id:prot-a-2805 | F-sweetened coffee drink liking (derived food-liking factor) \|\| id:ebi-fl187-GCST90094853 | MR Egger | 4 | 0.628763928400645 | 1.56145492358888 | 0.726148401418705 | -2.431687722 | 3.68921557863485 | 1.8752911519371 | 0.0878883762096357 | 40.0134472406834 | 0.999950574162623 |
| ebi-fl187-GCST90094853 | prot-a-2811 | SPARC-like protein 1 \|\| id:prot-a-2811 | F-sweetened coffee drink liking (derived food-liking factor) \|\| id:ebi-fl187-GCST90094853 | MR Egger | 4 | -1.128820709 | 1.56145492358888 | 0.544834768207269 | -4.189272359 | 1.93163094143503 | 0.323414431413106 | 0.0151573099410719 | 6.90075579722992 | 0.999950574162623 |
| ebi-fl187-GCST90094853 | prot-a-2831 | Sepiapterin reductase \|\| id:prot-a-2831 | F-sweetened coffee drink liking (derived food-liking factor) \|\| id:ebi-fl187-GCST90094853 | MR Egger | 4 | -0.07565709 | 2.1688232830605 | 0.975340833857566 | -4.326550725 | 4.17523654474879 | 0.927134075555729 | 0.0132130444050259 | 65.0552263132948 | 0.999950574162623 |
| ebi-fl187-GCST90094853 | prot-a-2834 | SPRY domain-containing SOCS box protein 1 \|\| id:prot-a-2834 | F-sweetened coffee drink liking (derived food-liking factor) \|\| id:ebi-fl187-GCST90094853 | MR Egger | 4 | 1.56257101170214 | 1.56145492358888 | 0.422374712296752 | -1.497880639 | 4.62302266193634 | 4.77107197188018 | 0.223603555082479 | 101.801278394094 | 0.999950574162623 |
| ebi-fl187-GCST90094853 | prot-a-2835 | Spectrin alpha chain, non-erythrocytic 1 \|\| id:prot-a-2835 | F-sweetened coffee drink liking (derived food-liking factor) \|\| id:ebi-fl187-GCST90094853 | MR Egger | 4 | 2.27442877515798 | 1.56463924156069 | 0.283238836269036 | -0.792264138 | 5.34112168861693 | 9.72236377035593 | 0.452818390298551 | 208.74672784559 | 0.999950574162623 |
| ebi-fl187-GCST90094853 | prot-a-2859 | Sia-alpha-2,3-Gal-beta-1,4-GlcNAc-R:alpha 2,8-sialyltransferase \|\| id:prot-a-2859 | F-sweetened coffee drink liking (derived food-liking factor) \|\| id:ebi-fl187-GCST90094853 | MR Egger | 4 | 1.43505206717794 | 1.56145492358888 | 0.455091173447009 | -1.625399583 | 4.49550371741214 | 4.1998636781446 | 0.196833008353221 | 89.6132973964661 | 0.999950574162623 |
| ebi-fl187-GCST90094853 | prot-a-2861 | Alpha-2,8-sialyltransferase 8F \|\| id:prot-a-2861 | F-sweetened coffee drink liking (derived food-liking factor) \|\| id:ebi-fl187-GCST90094853 | MR Egger | 4 | 2.73624775867137 | 2.43568554524226 | 0.377999874967388 | -2.03769591 | 7.5101914273462 | 15.4289830776438 | 0.13032865414587 | 1826.56316349112 | 0.999950574162623 |
| ebi-fl187-GCST90094853 | prot-a-289 | Uncharacterized protein C17orf89 \|\| id:prot-a-289 | F-sweetened coffee drink liking (derived food-liking factor) \|\| id:ebi-fl187-GCST90094853 | MR Egger | 4 | 0.755846993558194 | 1.56145492358888 | 0.676159040732474 | -2.304604657 | 3.81629864379239 | 2.12941435999765 | 0.0997982474264621 | 45.4357229059103 | 0.999950574162623 |
| ebi-fl187-GCST90094853 | prot-a-2921 | Tachykinin-3 \|\| id:prot-a-2921 | F-sweetened coffee drink liking (derived food-liking factor) \|\| id:ebi-fl187-GCST90094853 | MR Egger | 4 | -0.256664317 | 1.56463924156069 | 0.884778342460139 | -3.323357231 | 2.81002859595955 | 0.773627863518829 | 0.036031661859519 | 16.6103932021274 | 0.999950574162623 |
| ebi-fl187-GCST90094853 | prot-a-2928 | Tax1-binding protein 1 \|\| id:prot-a-2928 | F-sweetened coffee drink liking (derived food-liking factor) \|\| id:ebi-fl187-GCST90094853 | MR Egger | 4 | 3.17905872923314 | 1.56145492358888 | 0.178696738521273 | 0.118607078998944 | 6.23951037946734 | 24.0241296951661 | 1.1259274308295 | 512.607466348832 | 0.999950574162623 |
| ebi-fl187-GCST90094853 | prot-a-296 | Uncharacterized protein C1orf185 \|\| id:prot-a-296 | F-sweetened coffee drink liking (derived food-liking factor) \|\| id:ebi-fl187-GCST90094853 | MR Egger | 4 | -0.044931709 | 2.1345917023776 | 0.985117529898923 | -4.228731445 | 4.13886802799567 | 0.956062770407235 | 0.0145708627655172 | 62.7317706348813 | 0.999950574162623 |
| ebi-fl187-GCST90094853 | prot-a-2964 | Transforming growth factor beta-2 \|\| id:prot-a-2964 | F-sweetened coffee drink liking (derived food-liking factor) \|\| id:ebi-fl187-GCST90094853 | MR Egger | 4 | -0.871258119 | 1.56463924156069 | 0.633630188087865 | -3.937951033 | 2.19543479407011 | 0.418424789613284 | 0.0194881043508579 | 8.98390635696763 | 0.999950574162623 |
| ebi-fl187-GCST90094853 | prot-a-2977 | Thymocyte nuclear protein 1 \|\| id:prot-a-2977 | F-sweetened coffee drink liking (derived food-liking factor) \|\| id:ebi-fl187-GCST90094853 | MR Egger | 4 | -0.760477097 | 1.56145492358888 | 0.674385038621627 | -3.820928747 | 2.29997455338842 | 0.467443358049685 | 0.0219074449674277 | 9.97392864889722 | 0.999950574162623 |
| ebi-fl187-GCST90094853 | prot-a-3015 | Transmembrane protein 70, mitochondrial \|\| id:prot-a-3015 | F-sweetened coffee drink liking (derived food-liking factor) \|\| id:ebi-fl187-GCST90094853 | MR Egger | 4 | -1.06045087 | 2.19068707998965 | 0.676155191296448 | -5.354197546 | 3.23329580706069 | 0.34629963910528 | 0.00472826218612003 | 25.3631112920274 | 0.999950574162623 |
| ebi-fl187-GCST90094853 | prot-a-3030 | Tumor necrosis factor alpha-induced protein 3 \|\| id:prot-a-3030 | F-sweetened coffee drink liking (derived food-liking factor) \|\| id:ebi-fl187-GCST90094853 | MR Egger | 4 | -1.784117082 | 2.081506238 | 0.481685556888365 | -5.863869308 | 2.29563514315421 | 0.167945277398288 | 0.00284023265900649 | 9.9307414520944 | 0.999950574162623 |
| ebi-fl187-GCST90094853 | prot-a-3032 | Tumor necrosis factor receptor superfamily member 10A \|\| id:prot-a-3032 | F-sweetened coffee drink liking (derived food-liking factor) \|\| id:ebi-fl187-GCST90094853 | MR Egger | 4 | -0.256695671 | 1.64002787054735 | 0.889995964733503 | -3.471150298 | 2.95775895484479 | 0.773603607626287 | 0.0310812573911348 | 19.2547725531562 | 0.999950574162623 |
| ebi-fl187-GCST90094853 | prot-a-3081 | Tumor protein p53-inducible protein 11 \|\| id:prot-a-3081 | F-sweetened coffee drink liking (derived food-liking factor) \|\| id:ebi-fl187-GCST90094853 | MR Egger | 4 | 3.2235807192972 | 2.1343107746826 | 0.270040853695609 | -0.959668399 | 7.40682983767511 | 25.1178994955796 | 0.383019874661714 | 1647.19618173146 | 0.999950574162623 |
| ebi-fl187-GCST90094853 | prot-a-315 | Complement C4 \|\| id:prot-a-315 | F-sweetened coffee drink liking (derived food-liking factor) \|\| id:ebi-fl187-GCST90094853 | MR Egger | 4 | 0.949749567026669 | 1.59902060347818 | 0.612774521722839 | -2.184330816 | 4.0838299498439 | 2.58506219343448 | 0.11255302716737 | 59.3724283753571 | 0.999950574162623 |
| ebi-fl187-GCST90094853 | prot-a-3157 | UDP-glucuronosyltransferase 1-8 \|\| id:prot-a-3157 | F-sweetened coffee drink liking (derived food-liking factor) \|\| id:ebi-fl187-GCST90094853 | MR Egger | 4 | -0.945144668 | 1.56145492358888 | 0.60651685310274 | -4.005596318 | 2.11530698251988 | 0.38862334558372 | 0.0182134250274833 | 8.29213091468454 | 0.999950574162623 |
| ebi-fl187-GCST90094853 | prot-a-317 | Glutaredoxin-like protein C5orf63 \|\| id:prot-a-317 | F-sweetened coffee drink liking (derived food-liking factor) \|\| id:ebi-fl187-GCST90094853 | MR Egger | 4 | -0.010000671 | 1.56145492358888 | 0.995471230480778 | -3.070452321 | 3.05045097965672 | 0.990049169844265 | 0.0464001623510186 | 21.1248691608899 | 0.999950574162623 |
| ebi-fl187-GCST90094853 | prot-a-3192 | Guanine nucleotide exchange factor VAV3 \|\| id:prot-a-3192 | F-sweetened coffee drink liking (derived food-liking factor) \|\| id:ebi-fl187-GCST90094853 | MR Egger | 4 | 0.763975104287749 | 1.56145492358888 | 0.673047069102296 | -2.296476546 | 3.82442675452195 | 2.1467930078134 | 0.100612724226861 | 45.8065344499052 | 0.999950574162623 |
| ebi-fl187-GCST90094853 | prot-a-3233 | Serine/threonine-protein kinase WNK3 \|\| id:prot-a-3233 | F-sweetened coffee drink liking (derived food-liking factor) \|\| id:ebi-fl187-GCST90094853 | MR Egger | 4 | -0.498804651 | 1.56145492358888 | 0.779666878707924 | -3.559256301 | 2.56164699943523 | 0.607256109148443 | 0.0284599825052809 | 12.9571401538873 | 0.999950574162623 |
| ebi-fl187-GCST90094853 | prot-a-3250 | 14-3-3 protein theta \|\| id:prot-a-3250 | F-sweetened coffee drink liking (derived food-liking factor) \|\| id:ebi-fl187-GCST90094853 | MR Egger | 4 | -2.650318123 | 1.56145492358888 | 0.231726301470743 | -5.710769773 | 0.410133527665717 | 0.0706287408897063 | 0.00331012352088119 | 1.50701900040797 | 0.999950574162623 |
| ebi-fl187-GCST90094853 | prot-a-3278 | E3 ubiquitin-protein ligase ZNRF3 \|\| id:prot-a-3278 | F-sweetened coffee drink liking (derived food-liking factor) \|\| id:ebi-fl187-GCST90094853 | MR Egger | 4 | 1.16283743899067 | 2.09832458301151 | 0.635151746274398 | -2.949878744 | 5.27555362169322 | 3.19899737049757 | 0.0523460528516897 | 195.498678867818 | 0.999950574162623 |
| ebi-fl187-GCST90094853 | prot-a-355 | Calpain-3 \|\| id:prot-a-355 | F-sweetened coffee drink liking (derived food-liking factor) \|\| id:ebi-fl187-GCST90094853 | MR Egger | 4 | 3.30688370732741 | 1.56145492358888 | 0.168372624602837 | 0.246432057093208 | 6.36733535756161 | 27.2999182403487 | 1.2794522505594 | 582.503595272015 | 0.999950574162623 |
| ebi-fl187-GCST90094853 | prot-a-385 | Coiled-coil domain-containing protein 90B, mitochondrial \|\| id:prot-a-385 | F-sweetened coffee drink liking (derived food-liking factor) \|\| id:ebi-fl187-GCST90094853 | MR Egger | 4 | 2.02571565535782 | 1.56145492358888 | 0.324002661244797 | -1.034735995 | 5.08616730559202 | 7.58153477 | 0.355320174890059 | 161.768662531678 | 0.999950574162623 |
| ebi-fl187-GCST90094853 | prot-a-390 | C-C motif chemokine 14 \|\| id:prot-a-390 | F-sweetened coffee drink liking (derived food-liking factor) \|\| id:ebi-fl187-GCST90094853 | MR Egger | 4 | -0.144424608 | 1.56145492358888 | 0.934736573154902 | -3.204876258 | 2.91602704237658 | 0.86552016336276 | 0.0405639207842874 | 18.4677698482657 | 0.999950574162623 |
| ebi-fl187-GCST90094853 | prot-a-40 | Pituitary adenylate cyclase-activating polypeptide 27 \|\| id:prot-a-40 | F-sweetened coffee drink liking (derived food-liking factor) \|\| id:ebi-fl187-GCST90094853 | MR Egger | 4 | 0.471243859117417 | 3.41215765384454 | 0.902805757992555 | -6.216585142 | 7.15907286065271 | 1.60198559883967 | 0.00199604981813698 | 1285.71834007882 | 0.999950574162623 |
| ebi-fl187-GCST90094853 | prot-a-401 | C-C motif chemokine 24 \|\| id:prot-a-401 | F-sweetened coffee drink liking (derived food-liking factor) \|\| id:ebi-fl187-GCST90094853 | MR Egger | 4 | 1.42376179699092 | 1.70492326579115 | 0.49153388939896 | -1.917887804 | 4.76541139794157 | 4.15271275665168 | 0.146916952039719 | 117.379396998349 | 0.999950574162623 |
| ebi-fl187-GCST90094853 | prot-a-412 | Cerebral cavernous malformations 2 protein \|\| id:prot-a-412 | F-sweetened coffee drink liking (derived food-liking factor) \|\| id:ebi-fl187-GCST90094853 | MR Egger | 4 | -1.168500833 | 1.56145492358888 | 0.532288147603982 | -4.228952483 | 1.89195081743612 | 0.310832582153954 | 0.0145676424113345 | 6.63229446470496 | 0.999950574162623 |
| ebi-fl187-GCST90094853 | prot-a-44 | Adhesion G protein-coupled receptor F5 \|\| id:prot-a-44 | F-sweetened coffee drink liking (derived food-liking factor) \|\| id:ebi-fl187-GCST90094853 | MR Egger | 4 | -1.44877718 | 2.68349431440845 | 0.64334910162165 | -6.708426037 | 3.8108716758115 | 0.234857300679378 | 0.00122058376757344 | 45.1898125698163 | 0.999950574162623 |
| ebi-fl187-GCST90094853 | prot-a-48 | Adrenomedullin \|\| id:prot-a-48 | F-sweetened coffee drink liking (derived food-liking factor) \|\| id:ebi-fl187-GCST90094853 | MR Egger | 4 | -1.242625529 | 1.56145492358888 | 0.509590144612772 | -4.303077179 | 1.81782612150139 | 0.288625427908432 | 0.0135268703025023 | 6.15845615226421 | 0.999950574162623 |
| ebi-fl187-GCST90094853 | prot-a-502 | Carcinoembryonic antigen-related cell adhesion molecule 1 \|\| id:prot-a-502 | F-sweetened coffee drink liking (derived food-liking factor) \|\| id:ebi-fl187-GCST90094853 | MR Egger | 4 | 3.1346120832354 | 1.56145492358888 | 0.182487365004589 | 0.0741604330012038 | 6.1950637334696 | 22.9797198942311 | 1.07697957470231 | 490.322693968716 | 0.999950574162623 |
| ebi-fl187-GCST90094853 | prot-a-518 | Complement factor B \|\| id:prot-a-518 | F-sweetened coffee drink liking (derived food-liking factor) \|\| id:ebi-fl187-GCST90094853 | MR Egger | 4 | 1.20468117149611 | 1.56145492358888 | 0.521089141667323 | -1.855770479 | 4.26513282173031 | 3.33569539373818 | 0.156332445435354 | 71.1743728489632 | 0.999950574162623 |
| ebi-fl187-GCST90094853 | prot-a-525 | Properdin \|\| id:prot-a-525 | F-sweetened coffee drink liking (derived food-liking factor) \|\| id:ebi-fl187-GCST90094853 | MR Egger | 4 | 0.0670771798210819 | 1.56463924156069 | 0.96969975617001 | -2.999615734 | 3.13377009328003 | 1.06937800939312 | 0.0498062035397729 | 22.9603793443199 | 0.999950574162623 |
| ebi-fl187-GCST90094853 | prot-a-533 | Chondroadherin \|\| id:prot-a-533 | F-sweetened coffee drink liking (derived food-liking factor) \|\| id:ebi-fl187-GCST90094853 | MR Egger | 4 | -1.043794102 | 1.64250100767187 | 0.590121012625656 | -4.263096077 | 2.17550787275942 | 0.352116179558156 | 0.014078646286286 | 8.80665664762142 | 0.999950574162623 |
| ebi-fl187-GCST90094853 | prot-a-535 | Coiled-coil-helix-coiled-coil-helix domain-containing protein 2 \|\| id:prot-a-535 | F-sweetened coffee drink liking (derived food-liking factor) \|\| id:ebi-fl187-GCST90094853 | MR Egger | 4 | 1.54684764241028 | 1.56145492358888 | 0.426267317077514 | -1.513604008 | 4.60729929264448 | 4.69664132900644 | 0.2201152496342 | 100.213137481339 | 0.999950574162623 |
| ebi-fl187-GCST90094853 | prot-a-591 | Caseinolytic peptidase B protein homolog \|\| id:prot-a-591 | F-sweetened coffee drink liking (derived food-liking factor) \|\| id:ebi-fl187-GCST90094853 | MR Egger | 4 | -0.968015691 | 1.71299994474883 | 0.628940921971595 | -4.325495583 | 2.38946420051353 | 0.379836002724149 | 0.0132269934002096 | 10.9076480648409 | 0.999950574162623 |
| ebi-fl187-GCST90094853 | prot-a-605 | Ciliary neurotrophic factor receptor subunit alpha \|\| id:prot-a-605 | F-sweetened coffee drink liking (derived food-liking factor) \|\| id:ebi-fl187-GCST90094853 | MR Egger | 4 | 1.15329387011485 | 1.56145492358888 | 0.537063778664818 | -1.90715778 | 4.21374552034905 | 3.16861273829204 | 0.148501862293752 | 67.6092981608961 | 0.999950574162623 |
| ebi-fl187-GCST90094853 | prot-a-606 | Ciliary neurotrophic factor receptor subunit alpha \|\| id:prot-a-606 | F-sweetened coffee drink liking (derived food-liking factor) \|\| id:ebi-fl187-GCST90094853 | MR Egger | 4 | -0.108621968 | 1.56145492358888 | 0.950869810406353 | -3.169073618 | 2.95182968207592 | 0.897069474232568 | 0.042042527292944 | 19.1409435496746 | 0.999950574162623 |
| ebi-fl187-GCST90094853 | prot-a-707 | Cancer/testis antigen 55 \|\| id:prot-a-707 | F-sweetened coffee drink liking (derived food-liking factor) \|\| id:ebi-fl187-GCST90094853 | MR Egger | 4 | 3.06521516372274 | 2.34655312314602 | 0.321485763559564 | -1.534028958 | 7.66445928508894 | 21.4390744020121 | 0.215665009383803 | 2131.24007704483 | 0.999950574162623 |
| ebi-fl187-GCST90094853 | prot-a-719 | Dipeptidyl peptidase 1 \|\| id:prot-a-719 | F-sweetened coffee drink liking (derived food-liking factor) \|\| id:ebi-fl187-GCST90094853 | MR Egger | 4 | 2.48979824059391 | 1.56145492358888 | 0.25185655982809 | -0.57065341 | 5.55024989082811 | 12.0588428900098 | 0.565156039623662 | 257.301845243964 | 0.999950574162623 |
| ebi-fl187-GCST90094853 | prot-a-752 | Cytochrome b5 \|\| id:prot-a-752 | F-sweetened coffee drink liking (derived food-liking factor) \|\| id:ebi-fl187-GCST90094853 | MR Egger | 4 | -3.532431389 | 1.56145492358888 | 0.152051447292012 | -6.59288304 | -0.471979739 | 0.0292337507552727 | 0.00137008425691346 | 0.623766150810817 | 0.999950574162623 |
| ebi-fl187-GCST90094853 | prot-a-755 | Cytoglobin \|\| id:prot-a-755 | F-sweetened coffee drink liking (derived food-liking factor) \|\| id:ebi-fl187-GCST90094853 | MR Egger | 4 | 1.63661156548169 | 1.56145492358888 | 0.404564540603808 | -1.423840085 | 4.69706321571589 | 5.13773111902332 | 0.24078759449499 | 109.62475499098 | 0.999950574162623 |
| ebi-fl187-GCST90094853 | prot-a-807 | Desmin \|\| id:prot-a-807 | F-sweetened coffee drink liking (derived food-liking factor) \|\| id:ebi-fl187-GCST90094853 | MR Egger | 4 | 1.6096001674237 | 1.56145492358888 | 0.410963575673688 | -1.450851483 | 4.6700518176579 | 5.00081134076828 | 0.234370640535927 | 106.703271402815 | 0.999950574162623 |
| ebi-fl187-GCST90094853 | prot-a-82 | Ameloblastin \|\| id:prot-a-82 | F-sweetened coffee drink liking (derived food-liking factor) \|\| id:ebi-fl187-GCST90094853 | MR Egger | 4 | -0.307019714 | 2.501245908 | 0.913530060962001 | -5.209461693 | 4.59542226480357 | 0.735636098361931 | 0.0054646145362861 | 99.0299435796895 | 0.999950574162623 |
| ebi-fl187-GCST90094853 | prot-a-84 | Muellerian-inhibiting factor \|\| id:prot-a-84 | F-sweetened coffee drink liking (derived food-liking factor) \|\| id:ebi-fl187-GCST90094853 | MR Egger | 4 | 2.00970476517051 | 1.56145492358888 | 0.326919808510602 | -1.050746885 | 5.07015641540471 | 7.46111424132754 | 0.349676483412992 | 159.199226607391 | 0.999950574162623 |
| ebi-fl187-GCST90094853 | prot-a-858 | Inactive dipeptidyl peptidase 10 \|\| id:prot-a-858 | F-sweetened coffee drink liking (derived food-liking factor) \|\| id:ebi-fl187-GCST90094853 | MR Egger | 4 | 0.659599195829595 | 1.56463924156069 | 0.714329718160428 | -2.407093718 | 3.72629210928855 | 1.93401701695115 | 0.0900767028586262 | 41.5248527438571 | 0.999950574162623 |
| ebi-fl187-GCST90094853 | prot-a-863 | Dihydropyrimidinase-related protein 3 \|\| id:prot-a-863 | F-sweetened coffee drink liking (derived food-liking factor) \|\| id:ebi-fl187-GCST90094853 | MR Egger | 4 | -1.486656216 | 1.91592950178936 | 0.518972621619981 | -5.24187804 | 2.2685656073937 | 0.226127514332502 | 0.0052903120847745 | 9.66552670594958 | 0.999950574162623 |
| ebi-fl187-GCST90094853 | prot-a-864 | Dihydropyrimidinase-related protein 5 \|\| id:prot-a-864 | F-sweetened coffee drink liking (derived food-liking factor) \|\| id:ebi-fl187-GCST90094853 | MR Egger | 4 | -0.018662136 | 1.56463924156069 | 0.991566328255341 | -3.08535505 | 3.04803077724316 | 0.981510923223196 | 0.0457138003485855 | 21.0738045198699 | 0.999950574162623 |
| ebi-fl187-GCST90094853 | prot-a-884 | Endothelin-converting enzyme 1 \|\| id:prot-a-884 | F-sweetened coffee drink liking (derived food-liking factor) \|\| id:ebi-fl187-GCST90094853 | MR Egger | 4 | 1.06291077346544 | 1.56874449821493 | 0.56792594523364 | -2.011828443 | 4.13764998996671 | 2.894784801 | 0.133743907849013 | 62.6554074686857 | 0.999950574162623 |
| ebi-fl187-GCST90094853 | prot-a-893 | Early endosome antigen 1 \|\| id:prot-a-893 | F-sweetened coffee drink liking (derived food-liking factor) \|\| id:ebi-fl187-GCST90094853 | MR Egger | 4 | -0.529569796 | 2.03440847398234 | 0.818976465495055 | -4.517010405 | 3.45787081276139 | 0.588858244223041 | 0.0109216261463692 | 31.7493042832928 | 0.999950574162623 |
| ebi-fl187-GCST90094853 | prot-a-897 | Ephrin-A2 \|\| id:prot-a-897 | F-sweetened coffee drink liking (derived food-liking factor) \|\| id:ebi-fl187-GCST90094853 | MR Egger | 4 | 1.7238678296617 | 1.56145492358888 | 0.384646921096988 | -1.336583821 | 4.7843194798959 | 5.60617029559502 | 0.262741710014246 | 119.619931610812 | 0.999950574162623 |
| ebi-fl187-GCST90094853 | prot-a-90 | Anaphase-promoting complex subunit 10 \|\| id:prot-a-90 | F-sweetened coffee drink liking (derived food-liking factor) \|\| id:ebi-fl187-GCST90094853 | MR Egger | 4 | -1.174082441 | 1.64218808720872 | 0.548831199055133 | -4.392771092 | 2.04460621026378 | 0.309102469465015 | 0.0123664132322684 | 7.72611547381103 | 0.999950574162623 |
| ebi-fl187-GCST90094853 | prot-a-914 | Histone-lysine N-methyltransferase EHMT2 \|\| id:prot-a-914 | F-sweetened coffee drink liking (derived food-liking factor) \|\| id:ebi-fl187-GCST90094853 | MR Egger | 4 | -1.010035271 | 1.56145492358888 | 0.584050051545733 | -4.070486921 | 2.05041637940319 | 0.364206133491992 | 0.0170690751914103 | 7.77113617379444 | 0.999950574162623 |
| ebi-fl187-GCST90094853 | prot-a-932 | RNA polymerase II elongation factor ELL \|\| id:prot-a-932 | F-sweetened coffee drink liking (derived food-liking factor) \|\| id:ebi-fl187-GCST90094853 | MR Egger | 4 | -0.607348238 | 1.65816897347272 | 0.74927614144809 | -3.857359426 | 2.64266294967754 | 0.544793618150936 | 0.0211237046421126 | 14.0505697938174 | 0.999950574162623 |
| ebi-fl187-GCST90094853 | prot-a-991 | Estrogen receptor \|\| id:prot-a-991 | F-sweetened coffee drink liking (derived food-liking factor) \|\| id:ebi-fl187-GCST90094853 | MR Egger | 4 | -0.201938864 | 1.56145492358888 | 0.908931736605174 | -3.262390514 | 2.85851278627514 | 0.817144883418225 | 0.03829673960625 | 17.4355772152367 | 0.999950574162623 |
| ebi-fl187-GCST90094853 | prot-a-992 | Persulfide dioxygenase ETHE1, mitochondrial \|\| id:prot-a-992 | F-sweetened coffee drink liking (derived food-liking factor) \|\| id:ebi-fl187-GCST90094853 | MR Egger | 4 | 0.1126551182825 | 1.56463924156069 | 0.949153673315577 | -2.954037795 | 3.17934803174145 | 1.11924585894334 | 0.0521287950303148 | 24.0310809416048 | 0.999950574162623 |
| ebi-fl187-GCST90094853 | prot-a-993 | Ethanolamine kinase 1 \|\| id:prot-a-993 | F-sweetened coffee drink liking (derived food-liking factor) \|\| id:ebi-fl187-GCST90094853 | MR Egger | 4 | -0.366000219 | 1.56145492358888 | 0.836487063481251 | -3.426451869 | 2.69445143166816 | 0.693502649633609 | 0.0325020579926578 | 14.7973991418476 | 0.999950574162623 |
| ebi-fl187-GCST90094853 | prot-b-10 | coagulation factor III, tissue factor \|\| id:prot-b-10 | F-sweetened coffee drink liking (derived food-liking factor) \|\| id:ebi-fl187-GCST90094853 | MR Egger | 4 | -0.452138662 | 1.52455609865265 | 0.794757283701552 | -3.440268616 | 2.53599129114869 | 0.636265937566628 | 0.0320560734108439 | 12.6289436051327 | 0.999950574162623 |
| ebi-fl187-GCST90094853 | prot-b-28 | prolactin \|\| id:prot-b-28 | F-sweetened coffee drink liking (derived food-liking factor) \|\| id:ebi-fl187-GCST90094853 | MR Egger | 4 | -1.245810099 | 1.52455609865265 | 0.499694242767299 | -4.233940053 | 1.74231985414427 | 0.287707741883799 | 0.0144951661721313 | 5.71057577104713 | 0.999950574162623 |
| ebi-fl187-GCST90094853 | prot-b-54 | matrix metallopeptidase 7 \|\| id:prot-b-54 | F-sweetened coffee drink liking (derived food-liking factor) \|\| id:ebi-fl187-GCST90094853 | MR Egger | 4 | -0.854768259 | 1.52455609865265 | 0.631454853208018 | -3.842898213 | 2.13336169412797 | 0.425381757956464 | 0.0214313985011311 | 8.44320262126531 | 0.999950574162623 |
| ebi-fl187-GCST90094853 | ukb-rsfMRI191-edge-pheno716 | Net100_Pair10_33 (OccipitalPrecuneus)&(Temporal\|Frontal\|Supp_Motor_Area) (Default_mode\|Central_executive)&(Default_mode\|Salience) \|\| id:ukb-rsfMRI191-edge-pheno716 | F-sweetened coffee drink liking (derived food-liking factor) \|\| id:ebi-fl187-GCST90094853 | MR Egger | 3 | 0.215862472675173 | 0.476466575066518 | 0.729190749026794 | -0.718012014 | 1.14973695980555 | 1.24093170524707 | 0.487720874889928 | 3.15736228726102 | 0.999950574162623 |
| ebi-fl187-GCST90094853 | ukb-rsfMRI191-edge-pheno903 | Net100_Pair27_38 (Precentral\|Frontal\|Supp_Motor_Area)&(Subcortical) (Attention\|Salience\|Motor)&(Subcortical-cerebellum) \|\| id:ukb-rsfMRI191-edge-pheno903 | F-sweetened coffee drink liking (derived food-liking factor) \|\| id:ebi-fl187-GCST90094853 | MR Egger | 3 | -0.594528498 | 0.537616306637077 | 0.46802444964937 | -1.648256459 | 0.459199463435654 | 0.551822690276095 | 0.192385047670324 | 1.58280638329731 | 0.999950574162623 |
| ebi-fl187-GCST90094853 | ukb-rsfMRI191-node-pheno23 | Net100_Node2 RolandicOper\|SupraMarginal\|Insula Salience\|Motor \|\| id:ukb-rsfMRI191-node-pheno23 | F-sweetened coffee drink liking (derived food-liking factor) \|\| id:ebi-fl187-GCST90094853 | MR Egger | 3 | 0.0115979957707662 | 0.437742243127719 | 0.983136682463342 | -0.846376801 | 0.869572792301095 | 1.01166551329389 | 0.428966350257137 | 2.38589136461331 | 0.999950574162623 |
| ebi-fl187-GCST90094853 | ukb-rsfMRI191-node-pheno55 | Net100_Node34 Parietal\|Postcentral\|Precuneus Attention \|\| id:ukb-rsfMRI191-node-pheno55 | F-sweetened coffee drink liking (derived food-liking factor) \|\| id:ebi-fl187-GCST90094853 | MR Egger | 3 | -0.383045651 | 0.45386648984932 | 0.55374374084693 | -1.272623971 | 0.506532669450205 | 0.681781774791349 | 0.280095693763642 | 1.65952707873433 | 0.999950574162623 |
| ebi-fl187-GCST90094853 | ebi-a-GCST90001414 | IgD- CD24- B cell Absolute Count \|\| id:ebi-a-GCST90001414 | F-sweetened coffee drink liking (derived food-liking factor) \|\| id:ebi-fl187-GCST90094853 | Weighted median | 4 | 0.64300565006801 | 0.435640593835486 | 0.139943809924138 | -0.210849914 | 1.49686121398556 | 1.90218961211771 | 0.809895611874597 | 4.46764406103334 | 0.999332001504091 |
| ebi-fl187-GCST90094853 | ebi-a-GCST90001422 | CD20- CD38- B cell %B cell \|\| id:ebi-a-GCST90001422 | F-sweetened coffee drink liking (derived food-liking factor) \|\| id:ebi-fl187-GCST90094853 | Weighted median | 4 | 0.0693136969696567 | 0.385652515856629 | 0.857363786511303 | -0.686565234 | 0.82519262804865 | 1.07177236816361 | 0.503301827531159 | 2.28232036190634 | 0.999332001504091 |
| ebi-fl187-GCST90094853 | ebi-a-GCST90001444 | CD20- CD38- B cell %lymphocyte \|\| id:ebi-a-GCST90001444 | F-sweetened coffee drink liking (derived food-liking factor) \|\| id:ebi-fl187-GCST90094853 | Weighted median | 4 | 0.157506344651931 | 0.386334442180522 | 0.68349813282435 | -0.599709162 | 0.914721851325754 | 1.17058818493272 | 0.548971274573971 | 2.49608087375334 | 0.999332001504091 |
| ebi-fl187-GCST90094853 | ebi-a-GCST90001459 | Myeloid Dendritic Cell %Dendritic Cell \|\| id:ebi-a-GCST90001459 | F-sweetened coffee drink liking (derived food-liking factor) \|\| id:ebi-fl187-GCST90094853 | Weighted median | 4 | -0.100445804 | 0.386887607261594 | 0.795152832101042 | -0.858745515 | 0.65785390580879 | 0.904434127413108 | 0.423693266059321 | 1.93064454018248 | 0.999332001504091 |
| ebi-fl187-GCST90094853 | ebi-a-GCST90001476 | HLA DR++ monocyte %leukocyte \|\| id:ebi-a-GCST90001476 | F-sweetened coffee drink liking (derived food-liking factor) \|\| id:ebi-fl187-GCST90094853 | Weighted median | 4 | -0.426169625 | 0.350368661269996 | 0.223853030280325 | -1.112892202 | 0.260552950610864 | 0.653005566344203 | 0.32860718715584 | 1.29764742325702 | 0.999332001504091 |
| ebi-fl187-GCST90094853 | ebi-a-GCST90001478 | CD4 regulatory T cell %CD4+ T cell \|\| id:ebi-a-GCST90001478 | F-sweetened coffee drink liking (derived food-liking factor) \|\| id:ebi-fl187-GCST90094853 | Weighted median | 4 | 0.0189545847403365 | 0.371300810495429 | 0.959286368480402 | -0.708795004 | 0.746704173311377 | 1.01913536326923 | 0.492236983916539 | 2.11003423676514 | 0.999332001504091 |
| ebi-fl187-GCST90094853 | ebi-a-GCST90001485 | CD39+ resting CD4 regulatory T cell %CD4 regulatory T cell \|\| id:ebi-a-GCST90001485 | F-sweetened coffee drink liking (derived food-liking factor) \|\| id:ebi-fl187-GCST90094853 | Weighted median | 4 | 0.0940626750249377 | 0.362412557924961 | 0.795214111264636 | -0.616265939 | 0.804391288557862 | 1.09862860033435 | 0.539956910213834 | 2.23533541036565 | 0.999332001504091 |
| ebi-fl187-GCST90094853 | ebi-a-GCST90001515 | Immature Myeloid-Derived Suppressor Cells Absolute Count \|\| id:ebi-a-GCST90001515 | F-sweetened coffee drink liking (derived food-liking factor) \|\| id:ebi-fl187-GCST90094853 | Weighted median | 4 | -0.390054648 | 0.491465215895537 | 0.427395330570251 | -1.353326471 | 0.573217175530248 | 0.67701987595893 | 0.258379338204331 | 1.77396503771896 | 0.999332001504091 |
| ebi-fl187-GCST90094853 | ebi-a-GCST90001517 | CD33+ HLA DR+ Absolute Count \|\| id:ebi-a-GCST90001517 | F-sweetened coffee drink liking (derived food-liking factor) \|\| id:ebi-fl187-GCST90094853 | Weighted median | 4 | -0.283375707 | 0.478964446307505 | 0.55409002897407 | -1.222146022 | 0.655394607315687 | 0.753236738039231 | 0.294597275770941 | 1.9259023426039 | 0.999332001504091 |
| ebi-fl187-GCST90094853 | ebi-a-GCST90001518 | CD33+ HLA DR+ CD14- Absolute Count \|\| id:ebi-a-GCST90001518 | F-sweetened coffee drink liking (derived food-liking factor) \|\| id:ebi-fl187-GCST90094853 | Weighted median | 4 | -0.433259182 | 0.472181036496706 | 0.358843998859534 | -1.358734013 | 0.492215649891223 | 0.64839241861325 | 0.256985911880293 | 1.63593687077668 | 0.999332001504091 |
| ebi-fl187-GCST90094853 | ebi-a-GCST90001520 | CD33+ HLA DR+ CD14dim Absolute Count \|\| id:ebi-a-GCST90001520 | F-sweetened coffee drink liking (derived food-liking factor) \|\| id:ebi-fl187-GCST90094853 | Weighted median | 4 | -0.263965484 | 0.480634572519382 | 0.582866809658286 | -1.206009246 | 0.678078277962606 | 0.768000047354176 | 0.299389689046113 | 1.97008813034029 | 0.999332001504091 |
| ebi-fl187-GCST90094853 | ebi-a-GCST90001531 | CD33dim HLA DR- Absolute Count \|\| id:ebi-a-GCST90001531 | F-sweetened coffee drink liking (derived food-liking factor) \|\| id:ebi-fl187-GCST90094853 | Weighted median | 4 | -0.183171042 | 0.539167514420566 | 0.734060338372524 | -1.239939371 | 0.87359728580746 | 0.832625729206588 | 0.289401763627379 | 2.39551271646506 | 0.999332001504091 |
| ebi-fl187-GCST90094853 | ebi-a-GCST90001532 | Basophil Absolute Count \|\| id:ebi-a-GCST90001532 | F-sweetened coffee drink liking (derived food-liking factor) \|\| id:ebi-fl187-GCST90094853 | Weighted median | 4 | -0.426788835 | 0.486803229273851 | 0.380640183033711 | -1.380923164 | 0.527345494432634 | 0.652601344278292 | 0.25134641188439 | 1.69442846373206 | 0.999332001504091 |
| ebi-fl187-GCST90094853 | ebi-a-GCST90001588 | T/B cell \|\| id:ebi-a-GCST90001588 | F-sweetened coffee drink liking (derived food-liking factor) \|\| id:ebi-fl187-GCST90094853 | Weighted median | 4 | 0.218037321647601 | 0.380107603222883 | 0.566224977820089 | -0.526973581 | 0.963048223964451 | 1.24363348120347 | 0.590389033437572 | 2.61966965504925 | 0.999332001504091 |
| ebi-fl187-GCST90094853 | ebi-a-GCST90001624 | HLA DR+ CD4+ T cell Absolute Count \|\| id:ebi-a-GCST90001624 | F-sweetened coffee drink liking (derived food-liking factor) \|\| id:ebi-fl187-GCST90094853 | Weighted median | 4 | 0.0141701749403843 | 0.33205807919295 | 0.965961577399577 | -0.63666366 | 0.665004010158566 | 1.01427104776802 | 0.529054588657922 | 1.94449831906779 | 0.999332001504091 |
| ebi-fl187-GCST90094853 | ebi-a-GCST90001631 | CD8+ Natural Killer T %T cell \|\| id:ebi-a-GCST90001631 | F-sweetened coffee drink liking (derived food-liking factor) \|\| id:ebi-fl187-GCST90094853 | Weighted median | 4 | -0.122638393 | 0.356034153800115 | 0.730502763267578 | -0.820465334 | 0.575188548681403 | 0.884583476517274 | 0.440226754265012 | 1.77746563412259 | 0.999332001504091 |
| ebi-fl187-GCST90094853 | ebi-a-GCST90001632 | CD8+ Natural Killer T %lymphocyte \|\| id:ebi-a-GCST90001632 | F-sweetened coffee drink liking (derived food-liking factor) \|\| id:ebi-fl187-GCST90094853 | Weighted median | 4 | -0.154930203 | 0.369319222825919 | 0.674849192812363 | -0.87879588 | 0.568935473307187 | 0.856474954410203 | 0.415282660829057 | 1.76638568551725 | 0.999332001504091 |
| ebi-fl187-GCST90094853 | ebi-a-GCST90001646 | Natural Killer %CD3- lymphocyte \|\| id:ebi-a-GCST90001646 | F-sweetened coffee drink liking (derived food-liking factor) \|\| id:ebi-fl187-GCST90094853 | Weighted median | 4 | -0.42904867 | 0.347769159634779 | 0.217308434860663 | -1.110676223 | 0.25257888260205 | 0.651128237814797 | 0.329336181004066 | 1.28734104096072 | 0.999332001504091 |
| ebi-fl187-GCST90094853 | ebi-a-GCST90001684 | CD127- CD8+ T cell Absolute Count \|\| id:ebi-a-GCST90001684 | F-sweetened coffee drink liking (derived food-liking factor) \|\| id:ebi-fl187-GCST90094853 | Weighted median | 4 | -0.028808355 | 0.36372107170303 | 0.936869944095709 | -0.741701656 | 0.684084945191519 | 0.971602649078492 | 0.476302722200865 | 1.98195740585803 | 0.999332001504091 |
| ebi-fl187-GCST90094853 | ebi-a-GCST90001687 | CD28- CD8+ T cell Absolute Count \|\| id:ebi-a-GCST90001687 | F-sweetened coffee drink liking (derived food-liking factor) \|\| id:ebi-fl187-GCST90094853 | Weighted median | 4 | 0.267214412315018 | 0.359430632383329 | 0.457216508346633 | -0.437269627 | 0.971698451786343 | 1.30632050750947 | 0.645797283449082 | 2.6424286878784 | 0.999332001504091 |
| ebi-fl187-GCST90094853 | ebi-a-GCST90001701 | BAFF-R on CD20- CD38- B cell \|\| id:ebi-a-GCST90001701 | F-sweetened coffee drink liking (derived food-liking factor) \|\| id:ebi-fl187-GCST90094853 | Weighted median | 4 | -0.118066164 | 0.355201147364119 | 0.739592972052488 | -0.814260413 | 0.578128084857995 | 0.888637254895774 | 0.442966818827264 | 1.78269824561427 | 0.999332001504091 |
| ebi-fl187-GCST90094853 | ebi-a-GCST90001732 | CD19 on IgD- CD27- B cell \|\| id:ebi-a-GCST90001732 | F-sweetened coffee drink liking (derived food-liking factor) \|\| id:ebi-fl187-GCST90094853 | Weighted median | 4 | 0.0415986057159297 | 0.368891324029919 | 0.910215630252336 | -0.681428389 | 0.76462560081457 | 1.04247595087107 | 0.505893862610975 | 2.14818994351044 | 0.999332001504091 |
| ebi-fl187-GCST90094853 | ebi-a-GCST90001798 | CD27 on CD24+ CD27+ B cell \|\| id:ebi-a-GCST90001798 | F-sweetened coffee drink liking (derived food-liking factor) \|\| id:ebi-fl187-GCST90094853 | Weighted median | 4 | 0.275135240628329 | 0.349116555904601 | 0.430644107479732 | -0.409133209 | 0.959403690201347 | 1.31670873534354 | 0.664225745618129 | 2.61013955747316 | 0.999332001504091 |
| ebi-fl187-GCST90094853 | ebi-a-GCST90001836 | CD66b on CD66b++ myeloid cell \|\| id:ebi-a-GCST90001836 | F-sweetened coffee drink liking (derived food-liking factor) \|\| id:ebi-fl187-GCST90094853 | Weighted median | 4 | 0.20854965655259 | 0.570083013212373 | 0.714496922687467 | -0.908813049 | 1.32591236244884 | 1.23189009995772 | 0.403002284086265 | 3.7656193979511 | 0.999332001504091 |
| ebi-fl187-GCST90094853 | ebi-a-GCST90001943 | CD25 on activated & secreting CD4 regulatory T cell \|\| id:ebi-a-GCST90001943 | F-sweetened coffee drink liking (derived food-liking factor) \|\| id:ebi-fl187-GCST90094853 | Weighted median | 4 | 0.473373831556992 | 0.364946333320314 | 0.194594950272537 | -0.241920982 | 1.18866864486481 | 1.60540142052542 | 0.785118213767449 | 3.28270784683187 | 0.999332001504091 |
| ebi-fl187-GCST90094853 | ebi-a-GCST90001960 | CD25 on CD4+ T cell \|\| id:ebi-a-GCST90001960 | F-sweetened coffee drink liking (derived food-liking factor) \|\| id:ebi-fl187-GCST90094853 | Weighted median | 4 | -0.159932658 | 0.380699675316555 | 0.674411011929436 | -0.906104022 | 0.586238705217048 | 0.852201175621726 | 0.40409550840249 | 1.79721582801581 | 0.999332001504091 |
| ebi-fl187-GCST90094853 | ebi-a-GCST90001988 | HLA DR on CD14+ CD16- monocyte \|\| id:ebi-a-GCST90001988 | F-sweetened coffee drink liking (derived food-liking factor) \|\| id:ebi-fl187-GCST90094853 | Weighted median | 4 | -0.390511365 | 0.342900785377675 | 0.254767176759896 | -1.062596904 | 0.281574174772888 | 0.676710740110436 | 0.34555726510518 | 1.32521429014502 | 0.999332001504091 |
| ebi-fl187-GCST90094853 | ebi-a-GCST90001991 | HLA DR on CD14+ monocyte \|\| id:ebi-a-GCST90001991 | F-sweetened coffee drink liking (derived food-liking factor) \|\| id:ebi-fl187-GCST90094853 | Weighted median | 4 | -0.431346131 | 0.338728483262669 | 0.202866419989668 | -1.095253959 | 0.232561695841902 | 0.649634013155055 | 0.334454658543003 | 1.26182829351644 | 0.999332001504091 |
| ebi-fl187-GCST90094853 | ebi-a-GCST90002019 | CD14 on Monocytic Myeloid-Derived Suppressor Cells \|\| id:ebi-a-GCST90002019 | F-sweetened coffee drink liking (derived food-liking factor) \|\| id:ebi-fl187-GCST90094853 | Weighted median | 4 | -0.635933541 | 0.501694122397362 | 0.204950751343732 | -1.619254021 | 0.347386938419339 | 0.529441002405237 | 0.198046382359824 | 1.41536427824559 | 0.999332001504091 |
| ebi-fl187-GCST90094853 | ebi-a-GCST90002037 | CD80 on plasmacytoid Dendritic Cell \|\| id:ebi-a-GCST90002037 | F-sweetened coffee drink liking (derived food-liking factor) \|\| id:ebi-fl187-GCST90094853 | Weighted median | 4 | -0.578023685 | 0.46823188147355 | 0.217023464673808 | -1.495758173 | 0.339710802361225 | 0.561005996074381 | 0.224078649925472 | 1.40454134178373 | 0.999332001504091 |
| ebi-fl187-GCST90094853 | ebi-a-GCST90002042 | CD45 on CD33+ HLA DR+ CD14- \|\| id:ebi-a-GCST90002042 | F-sweetened coffee drink liking (derived food-liking factor) \|\| id:ebi-fl187-GCST90094853 | Weighted median | 4 | -0.8783296 | 0.536064417862855 | 0.101322080207396 | -1.929015859 | 0.172356659421472 | 0.415476344221193 | 0.145291115148322 | 1.18810150525162 | 0.999332001504091 |
| ebi-fl187-GCST90094853 | ebi-a-GCST90002056 | CD8 on Effector Memory CD8+ T cell \|\| id:ebi-a-GCST90002056 | F-sweetened coffee drink liking (derived food-liking factor) \|\| id:ebi-fl187-GCST90094853 | Weighted median | 4 | -0.28770115 | 0.385913640182237 | 0.455965546764807 | -1.044091885 | 0.468689584310615 | 0.749985691640396 | 0.352011340982153 | 1.59789890887022 | 0.999332001504091 |
| ebi-fl187-GCST90094853 | ebi-a-GCST90002058 | CD8 on CD8+ T cell \|\| id:ebi-a-GCST90002058 | F-sweetened coffee drink liking (derived food-liking factor) \|\| id:ebi-fl187-GCST90094853 | Weighted median | 4 | -0.597990767 | 0.387076424925668 | 0.122372704654071 | -1.35666056 | 0.160679025900553 | 0.549915435092804 | 0.257519312984816 | 1.17430798586799 | 0.999332001504091 |
| ebi-fl187-GCST90094853 | ebi-a-GCST90002063 | CD4 on CD4 regulatory T cell \|\| id:ebi-a-GCST90002063 | F-sweetened coffee drink liking (derived food-liking factor) \|\| id:ebi-fl187-GCST90094853 | Weighted median | 4 | -0.025458648 | 0.359479459845384 | 0.943540322545749 | -0.730038389 | 0.679121093598915 | 0.974862690948608 | 0.481890490443491 | 1.97214364062037 | 0.999332001504091 |
| ebi-fl187-GCST90094853 | ebi-a-GCST90002066 | CD4 on activated CD4 regulatory T cell \|\| id:ebi-a-GCST90002066 | F-sweetened coffee drink liking (derived food-liking factor) \|\| id:ebi-fl187-GCST90094853 | Weighted median | 4 | -0.064697245 | 0.380086785491385 | 0.864839455861322 | -0.809667345 | 0.680272854202927 | 0.937351207850069 | 0.445006075133674 | 1.97441638654946 | 0.999332001504091 |
| ebi-fl187-GCST90094853 | ebi-a-GCST90002070 | CD4 on activated & secreting CD4 regulatory T cell \|\| id:ebi-a-GCST90002070 | F-sweetened coffee drink liking (derived food-liking factor) \|\| id:ebi-fl187-GCST90094853 | Weighted median | 4 | -0.032596898 | 0.372260762893785 | 0.930222668658302 | -0.762227993 | 0.697034197447988 | 0.967928655106825 | 0.466625629315043 | 2.0077891622716 | 0.999332001504091 |
| ebi-fl187-GCST90094853 | ebi-a-GCST90002081 | SSC-A on CD4+ T cell \|\| id:ebi-a-GCST90002081 | F-sweetened coffee drink liking (derived food-liking factor) \|\| id:ebi-fl187-GCST90094853 | Weighted median | 4 | 0.0765560674336069 | 0.362032173530302 | 0.832526738333244 | -0.633026993 | 0.786139128 | 1.07956271695132 | 0.530982087046041 | 2.19490579487338 | 0.999332001504091 |
| ebi-fl187-GCST90094853 | ebi-a-GCST90002094 | CD11b on Monocytic Myeloid-Derived Suppressor Cells \|\| id:ebi-a-GCST90002094 | F-sweetened coffee drink liking (derived food-liking factor) \|\| id:ebi-fl187-GCST90094853 | Weighted median | 4 | -0.743364346 | 0.495135316667855 | 0.133268745829805 | -1.713829567 | 0.22710087449846 | 0.475511436305363 | 0.180174479535013 | 1.25495645465844 | 0.999332001504091 |
| ebi-fl187-GCST90094853 | ebi-a-GCST90002104 | HLA DR on myeloid Dendritic Cell \|\| id:ebi-a-GCST90002104 | F-sweetened coffee drink liking (derived food-liking factor) \|\| id:ebi-fl187-GCST90094853 | Weighted median | 4 | -0.260139996 | 0.405198942522632 | 0.520869537525124 | -1.054329923 | 0.534049931234407 | 0.770943649136514 | 0.348425821072233 | 1.70582681936396 | 0.999332001504091 |
| ebi-fl187-GCST90094853 | ebi-a-GCST90002105 | HLA DR on plasmacytoid Dendritic Cell \|\| id:ebi-a-GCST90002105 | F-sweetened coffee drink liking (derived food-liking factor) \|\| id:ebi-fl187-GCST90094853 | Weighted median | 4 | -0.630126648 | 0.394576473289234 | 0.110272475268374 | -1.403496535 | 0.143243239838547 | 0.532524353693671 | 0.245736234576322 | 1.15401046885003 | 0.999332001504091 |
| ebi-fl187-GCST90094853 | ebi-a-GCST90002106 | HLA DR on Dendritic Cell \|\| id:ebi-a-GCST90002106 | F-sweetened coffee drink liking (derived food-liking factor) \|\| id:ebi-fl187-GCST90094853 | Weighted median | 4 | -0.417611788 | 0.400057179941173 | 0.296540798103356 | -1.201723861 | 0.366500284780233 | 0.658617862 | 0.30067544235227 | 1.44267681139607 | 0.999332001504091 |
| ebi-fl187-GCST90094853 | ebi-a-GCST90002111 | HLA DR on CD33dim HLA DR+ CD11b- \|\| id:ebi-a-GCST90002111 | F-sweetened coffee drink liking (derived food-liking factor) \|\| id:ebi-fl187-GCST90094853 | Weighted median | 4 | 0.0474812973318767 | 0.520303618769383 | 0.927288460761721 | -0.972313795 | 1.06727639011987 | 1.04862658882281 | 0.378206931449007 | 2.90744994697279 | 0.999332001504091 |
| ebi-fl187-GCST90094853 | ebi-a-GCST90002112 | HLA DR on CD33- HLA DR+ \|\| id:ebi-a-GCST90002112 | F-sweetened coffee drink liking (derived food-liking factor) \|\| id:ebi-fl187-GCST90094853 | Weighted median | 4 | 0.0553189467043248 | 0.519742292515545 | 0.915236956441893 | -0.963375947 | 1.07401384003479 | 1.0568776485676 | 0.381602439493356 | 2.92710488309449 | 0.999332001504091 |
| ebi-fl187-GCST90094853 | ebi-a-GCST90002115 | HLA DR on HLA DR+ CD8+ T cell \|\| id:ebi-a-GCST90002115 | F-sweetened coffee drink liking (derived food-liking factor) \|\| id:ebi-fl187-GCST90094853 | Weighted median | 4 | -0.314870054 | 0.356386101075083 | 0.376961737545621 | -1.013386812 | 0.383646704539378 | 0.729883713891441 | 0.362987525008734 | 1.46762684417638 | 0.999332001504091 |
| ebi-fl187-GCST90094853 | ebi-a-GCST90002116 | HLA DR on B cell \|\| id:ebi-a-GCST90002116 | F-sweetened coffee drink liking (derived food-liking factor) \|\| id:ebi-fl187-GCST90094853 | Weighted median | 4 | -0.109650335 | 0.393280268597564 | 0.78039090632395 | -0.880479662 | 0.661178991357125 | 0.896147431826357 | 0.414584003977192 | 1.93707478307136 | 0.999332001504091 |
| ebi-fl187-GCST90094853 | ebi-cfmet338-GCST90026023 | Sphingomyelin (d18:1/24:1, d18:2/24:0) levels \|\| id:ebi-cfmet338-GCST90026023 | F-sweetened coffee drink liking (derived food-liking factor) \|\| id:ebi-fl187-GCST90094853 | Weighted median | 4 | -0.212553693 | 0.166497755596447 | 0.201737863474235 | -0.538889294 | 0.113781908415043 | 0.808516903818486 | 0.583395874224658 | 1.12050772492865 | 0.999332001504091 |
| ebi-fl187-GCST90094853 | ebi-cfmet338-GCST90026046 | 1-arachidonoyl-gpc (20:4n6) levels \|\| id:ebi-cfmet338-GCST90026046 | F-sweetened coffee drink liking (derived food-liking factor) \|\| id:ebi-fl187-GCST90094853 | Weighted median | 4 | 0.177588293483711 | 0.197188805403728 | 0.367800885346508 | -0.208901765 | 0.564078352075018 | 1.19433350511312 | 0.81147494688223 | 1.75782693836241 | 0.999332001504091 |
| ebi-fl187-GCST90094853 | ebi-cfmet338-GCST90026047 | 1-linoleoyl-gpc (18:2) levels \|\| id:ebi-cfmet338-GCST90026047 | F-sweetened coffee drink liking (derived food-liking factor) \|\| id:ebi-fl187-GCST90094853 | Weighted median | 4 | -0.093031377 | 0.240805796101755 | 0.69924959902531 | -0.565010737 | 0.37894798367137 | 0.91116491051044 | 0.568354044280405 | 1.46074705106857 | 0.999332001504091 |
| ebi-fl187-GCST90094853 | ebi-cfmet338-GCST90026050 | 1-myristoyl-2-palmitoyl-gpc (14:0/16:0) levels \|\| id:ebi-cfmet338-GCST90026050 | F-sweetened coffee drink liking (derived food-liking factor) \|\| id:ebi-fl187-GCST90094853 | Weighted median | 4 | 0.0472701354765394 | 0.128883154280584 | 0.713792549291229 | -0.205340847 | 0.299881117866483 | 1.04840518226389 | 0.814369693704212 | 1.34969834301939 | 0.999332001504091 |
| ebi-fl187-GCST90094853 | ebi-cfmet338-GCST90026068 | 2,3-dihydroxyisovalerate levels \|\| id:ebi-cfmet338-GCST90026068 | F-sweetened coffee drink liking (derived food-liking factor) \|\| id:ebi-fl187-GCST90094853 | Weighted median | 4 | -0.231072707 | 0.415631776922495 | 0.578241383546335 | -1.04571099 | 0.583565575769791 | 0.793681757719161 | 0.351441858963745 | 1.79241805285682 | 0.999332001504091 |
| ebi-fl187-GCST90094853 | ebi-cfmet338-GCST90026082 | 3-hydroxyhexanoate levels \|\| id:ebi-cfmet338-GCST90026082 | F-sweetened coffee drink liking (derived food-liking factor) \|\| id:ebi-fl187-GCST90094853 | Weighted median | 4 | 0.0466144871169575 | 0.0855378343679074 | 0.585782726018916 | -0.121039668 | 0.214268642478056 | 1.04771802241819 | 0.885998812878558 | 1.23895544615176 | 0.999332001504091 |
| ebi-fl187-GCST90094853 | ebi-cfmet338-GCST90026094 | 3-ureidopropionate levels \|\| id:ebi-cfmet338-GCST90026094 | F-sweetened coffee drink liking (derived food-liking factor) \|\| id:ebi-fl187-GCST90094853 | Weighted median | 4 | -0.233629627 | 0.193921839020405 | 0.228295263666407 | -0.613716432 | 0.146457177069456 | 0.791654968905107 | 0.541335290277491 | 1.15772535256456 | 0.999332001504091 |
| ebi-fl187-GCST90094853 | ebi-cfmet338-GCST90026173 | Glycerol levels \|\| id:ebi-cfmet338-GCST90026173 | F-sweetened coffee drink liking (derived food-liking factor) \|\| id:ebi-fl187-GCST90094853 | Weighted median | 4 | -0.050214289 | 0.116238578290563 | 0.66574627092642 | -0.278041902 | 0.177613324537617 | 0.951025608420927 | 0.757265089654066 | 1.19436340091363 | 0.999332001504091 |
| ebi-fl187-GCST90094853 | ebi-cfmet338-GCST90026179 | Histidine levels \|\| id:ebi-cfmet338-GCST90026179 | F-sweetened coffee drink liking (derived food-liking factor) \|\| id:ebi-fl187-GCST90094853 | Weighted median | 4 | -0.018559516 | 0.0752581317609673 | 0.80520895421396 | -0.166065454 | 0.1289464224042 | 0.981611651404107 | 0.84699079335594 | 1.13762917109699 | 0.999332001504091 |
| ebi-fl187-GCST90094853 | ebi-cfmet338-GCST90026188 | Isocitrate levels \|\| id:ebi-cfmet338-GCST90026188 | F-sweetened coffee drink liking (derived food-liking factor) \|\| id:ebi-fl187-GCST90094853 | Weighted median | 4 | 0.315977107696352 | 0.162982135934652 | 0.0525351570806822 | -0.003467879 | 0.635422094128271 | 1.37159885620914 | 0.996538127411028 | 1.88781881054741 | 0.999332001504091 |
| ebi-fl187-GCST90094853 | ebi-cfmet338-GCST90026207 | N-acetyl-isoputreanine levels \|\| id:ebi-cfmet338-GCST90026207 | F-sweetened coffee drink liking (derived food-liking factor) \|\| id:ebi-fl187-GCST90094853 | Weighted median | 4 | 0.00502247938157065 | 0.0954258925435226 | 0.958024921217168 | -0.18201227 | 0.192057228766875 | 1.00503511317327 | 0.833591112186926 | 1.21173986135867 | 0.999332001504091 |
| ebi-fl187-GCST90094853 | ebi-cfmet338-GCST90026213 | N-acetylglutamate levels \|\| id:ebi-cfmet338-GCST90026213 | F-sweetened coffee drink liking (derived food-liking factor) \|\| id:ebi-fl187-GCST90094853 | Weighted median | 4 | -0.109037188 | 0.159353980103302 | 0.493820375725638 | -0.421370989 | 0.203296612876304 | 0.896697070394393 | 0.656146632982263 | 1.22543589441176 | 0.999332001504091 |
| ebi-fl187-GCST90094853 | ebi-cfmet338-GCST90026224 | N-acetyltaurine levels \|\| id:ebi-cfmet338-GCST90026224 | F-sweetened coffee drink liking (derived food-liking factor) \|\| id:ebi-fl187-GCST90094853 | Weighted median | 4 | -0.028654345 | 0.132206375154095 | 0.828411355065316 | -0.28777884 | 0.230470150297385 | 0.971752297457953 | 0.749927427620229 | 1.25919188022153 | 0.999332001504091 |
| ebi-fl187-GCST90094853 | ebi-cfmet338-GCST90026250 | Phenyllactate (pla) levels \|\| id:ebi-cfmet338-GCST90026250 | F-sweetened coffee drink liking (derived food-liking factor) \|\| id:ebi-fl187-GCST90094853 | Weighted median | 4 | -0.258622236 | 0.218949612013476 | 0.23752517865872 | -0.687763475 | 0.170519003823348 | 0.77211464528869 | 0.502699111736988 | 1.18592018873746 | 0.999332001504091 |
| ebi-fl187-GCST90094853 | ebi-cfmet338-GCST90026251 | Pipecolate levels \|\| id:ebi-cfmet338-GCST90026251 | F-sweetened coffee drink liking (derived food-liking factor) \|\| id:ebi-fl187-GCST90094853 | Weighted median | 4 | -0.263885723 | 0.32023466667611 | 0.409917632483088 | -0.891545669 | 0.363774224088602 | 0.768061306693517 | 0.4100215050648 | 1.43874934254125 | 0.999332001504091 |
| ebi-fl187-GCST90094853 | ebi-cfmet338-GCST90026278 | Trigonelline (n'-methylnicotinate) levels \|\| id:ebi-cfmet338-GCST90026278 | F-sweetened coffee drink liking (derived food-liking factor) \|\| id:ebi-fl187-GCST90094853 | Weighted median | 4 | 0.0837840282475533 | 0.368935664170494 | 0.820348641925753 | -0.639329874 | 0.806897930021721 | 1.08739402205436 | 0.527645895077537 | 2.24094562324949 | 0.999332001504091 |
| ebi-fl187-GCST90094853 | ebi-cfmet338-GCST90026299 | 1-carboxyethylvaline levels \|\| id:ebi-cfmet338-GCST90026299 | F-sweetened coffee drink liking (derived food-liking factor) \|\| id:ebi-fl187-GCST90094853 | Weighted median | 4 | 0.1048097871831 | 0.20510952612918 | 0.609355087480647 | -0.297204884 | 0.506824458396293 | 1.11049935905374 | 0.742891790104639 | 1.66001138104523 | 0.999332001504091 |
| ebi-fl187-GCST90094853 | ebi-gm430-GCST90011306 | C_Erysipelotrichia abundance \|\| id:ebi-gm430-GCST90011306 | F-sweetened coffee drink liking (derived food-liking factor) \|\| id:ebi-fl187-GCST90094853 | Weighted median | 4 | 0.263760399305345 | 0.168001737134328 | 0.116418335068449 | -0.065523005 | 0.593043804088627 | 1.30181624285415 | 0.936577500098768 | 1.80948776794252 | 0.999332001504091 |
| ebi-fl187-GCST90094853 | ebi-gm430-GCST90011312 | F_Erysipelotrichaceae abundance \|\| id:ebi-gm430-GCST90011312 | F-sweetened coffee drink liking (derived food-liking factor) \|\| id:ebi-fl187-GCST90094853 | Weighted median | 4 | 0.263760399305345 | 0.169946774428977 | 0.120657992259498 | -0.069335279 | 0.596856077186141 | 1.30181624285415 | 0.933013808091043 | 1.81639919523414 | 0.999332001504091 |
| ebi-fl187-GCST90094853 | ebi-gm430-GCST90011352 | O_Erysipelotrichales abundance \|\| id:ebi-gm430-GCST90011352 | F-sweetened coffee drink liking (derived food-liking factor) \|\| id:ebi-fl187-GCST90094853 | Weighted median | 4 | 0.263760399305345 | 0.167609165797674 | 0.115565301896035 | -0.064753566 | 0.592274364268786 | 1.30181624285415 | 0.937298417437397 | 1.80809601150543 | 0.999332001504091 |
| ebi-fl187-GCST90094853 | ebi-gm430-GCST90011362 | OTU97_11 (Parabacteroides) abundance \|\| id:ebi-gm430-GCST90011362 | F-sweetened coffee drink liking (derived food-liking factor) \|\| id:ebi-fl187-GCST90094853 | Weighted median | 4 | -0.489484169 | 0.220219124364129 | 0.0262350794949126 | -0.921113653 | -0.057854685 | 0.612942487259882 | 0.398075476208042 | 0.943787083462492 | 0.999332001504091 |
| ebi-fl187-GCST90094853 | ebi-gm430-GCST90011384 | OTU97_235 (Phascolarctobacterium) abundance \|\| id:ebi-gm430-GCST90011384 | F-sweetened coffee drink liking (derived food-liking factor) \|\| id:ebi-fl187-GCST90094853 | Weighted median | 4 | -0.394124817 | 0.770266551696767 | 0.608879686318762 | -1.903847258 | 1.11559762473058 | 0.674269890915984 | 0.148994295661469 | 3.05139122123739 | 0.999332001504091 |
| ebi-fl187-GCST90094853 | ebi-gm430-GCST90011390 | OTU97_3 (Bacteroides) abundance \|\| id:ebi-gm430-GCST90011390 | F-sweetened coffee drink liking (derived food-liking factor) \|\| id:ebi-fl187-GCST90094853 | Weighted median | 4 | 0.0446557813234936 | 0.166750148932992 | 0.788852848607013 | -0.282174511 | 0.371486073232157 | 1.04566785954617 | 0.754142067275659 | 1.44988765371206 | 0.999332001504091 |
| ebi-fl187-GCST90094853 | ebi-gm430-GCST90011398 | OTU97_4 (Alistipes) abundance \|\| id:ebi-gm430-GCST90011398 | F-sweetened coffee drink liking (derived food-liking factor) \|\| id:ebi-fl187-GCST90094853 | Weighted median | 4 | 0.037564521024519 | 0.208191588863161 | 0.856813071227157 | -0.370490993 | 0.445620035196315 | 1.03827898574461 | 0.690395268059936 | 1.56145805469986 | 0.999332001504091 |
| ebi-fl187-GCST90094853 | ebi-gm430-GCST90011421 | OTU99_108 (Cytophagales) abundance \|\| id:ebi-gm430-GCST90011421 | F-sweetened coffee drink liking (derived food-liking factor) \|\| id:ebi-fl187-GCST90094853 | Weighted median | 4 | 0.20463378161947 | 0.672693112955286 | 0.760974917086499 | -1.11384472 | 1.52311228301183 | 1.2270756050271 | 0.328294331851499 | 4.58647742091906 | 0.999332001504091 |
| ebi-fl187-GCST90094853 | ebi-gm430-GCST90011428 | OTU99_123 (Phascolarctobacterium) abundance \|\| id:ebi-gm430-GCST90011428 | F-sweetened coffee drink liking (derived food-liking factor) \|\| id:ebi-fl187-GCST90094853 | Weighted median | 4 | -0.466050528 | 0.40021384000384 | 0.244219914141057 | -1.250469654 | 0.318368598653466 | 0.627475578314638 | 0.286370270282986 | 1.37488294784307 | 0.999332001504091 |
| ebi-fl187-GCST90094853 | ebi-gm430-GCST90011450 | OTU99_298 (Phascolarctobacterium) abundance \|\| id:ebi-gm430-GCST90011450 | F-sweetened coffee drink liking (derived food-liking factor) \|\| id:ebi-fl187-GCST90094853 | Weighted median | 4 | -0.379393416 | 0.809685501157097 | 0.639377837188795 | -1.966376998 | 1.20759016676362 | 0.68427635477745 | 0.139963025087606 | 3.34541304329794 | 0.999332001504091 |
| ebi-fl187-GCST90094853 | ebi-gm430-GCST90011451 | OTU99_3 (Bacteroides) abundance \|\| id:ebi-gm430-GCST90011451 | F-sweetened coffee drink liking (derived food-liking factor) \|\| id:ebi-fl187-GCST90094853 | Weighted median | 4 | 0.0447276043994595 | 0.175499686603605 | 0.798832313102947 | -0.299251781 | 0.388706990142526 | 1.04574296532541 | 0.741372722113717 | 1.47507227728814 | 0.999332001504091 |
| ebi-fl187-GCST90094853 | ebi-gm430-GCST90011472 | OTU99_72 (Parabacteroides) abundance \|\| id:ebi-gm430-GCST90011472 | F-sweetened coffee drink liking (derived food-liking factor) \|\| id:ebi-fl187-GCST90094853 | Weighted median | 4 | 0.442624219352255 | 0.501839460838642 | 0.377774851573267 | -0.540981124 | 1.42622956259599 | 1.55678721390255 | 0.582176784527086 | 4.16297333350248 | 0.999332001504091 |
| ebi-fl187-GCST90094853 | ebi-gm430-GCST90011507 | TestASV_3 (Bacteroides) abundance \|\| id:ebi-gm430-GCST90011507 | F-sweetened coffee drink liking (derived food-liking factor) \|\| id:ebi-fl187-GCST90094853 | Weighted median | 4 | -0.145983867 | 0.228929696167158 | 0.523682069384736 | -0.594686071 | 0.302718337684875 | 0.864171644923782 | 0.551735744373835 | 1.35353317146021 | 0.999332001504091 |
| ebi-fl187-GCST90094853 | ebi-gm430-GCST90011512 | TestASV_34 (Phascolarctobacterium) abundance \|\| id:ebi-gm430-GCST90011512 | F-sweetened coffee drink liking (derived food-liking factor) \|\| id:ebi-fl187-GCST90094853 | Weighted median | 4 | -0.569625382 | 0.561957734216299 | 0.31075240244327 | -1.671062541 | 0.531811777014879 | 0.565737334368065 | 0.188047151619342 | 1.70201318521308 | 0.999332001504091 |
| ebi-fl187-GCST90094853 | ebi-gm430-GCST90011519 | TestASV_41 (Barnesiella) abundance \|\| id:ebi-gm430-GCST90011519 | F-sweetened coffee drink liking (derived food-liking factor) \|\| id:ebi-fl187-GCST90094853 | Weighted median | 4 | -0.614491749 | 0.759948147965769 | 0.418747023846376 | -2.103990119 | 0.875006620845332 | 0.540915746335115 | 0.121968786032107 | 2.39889117660197 | 0.999332001504091 |
| ebi-fl187-GCST90094853 | ebi-gm430-GCST90011524 | TestASV_46 (Sutterella) abundance \|\| id:ebi-gm430-GCST90011524 | F-sweetened coffee drink liking (derived food-liking factor) \|\| id:ebi-fl187-GCST90094853 | Weighted median | 4 | -0.106991665 | 0.712189427463209 | 0.880583770470877 | -1.502882943 | 1.28889961312497 | 0.898533162499942 | 0.222487815086678 | 3.6287912836827 | 0.999332001504091 |
| ebi-fl187-GCST90094853 | ebi-gm430-GCST90011581 | OTU97_19 (Bacteria) prevalence \|\| id:ebi-gm430-GCST90011581 | F-sweetened coffee drink liking (derived food-liking factor) \|\| id:ebi-fl187-GCST90094853 | Weighted median | 4 | -1.065170163 | 0.864091236083649 | 0.217685596823942 | -2.758788986 | 0.628448659322894 | 0.344669199693628 | 0.0633684619984543 | 1.87470002381223 | 0.999332001504091 |
| ebi-fl187-GCST90094853 | ebi-gm430-GCST90011614 | OTU97_80 (Ruminococcus) prevalence \|\| id:ebi-gm430-GCST90011614 | F-sweetened coffee drink liking (derived food-liking factor) \|\| id:ebi-fl187-GCST90094853 | Weighted median | 4 | -0.283169914 | 0.378850337764836 | 0.454794794953088 | -1.025716576 | 0.459376748430119 | 0.753391765484888 | 0.358539452706104 | 1.58308701599346 | 0.999332001504091 |
| ebi-fl187-GCST90094853 | ebi-gm430-GCST90011623 | OTU99_11 (Parabacteroides) prevalence \|\| id:ebi-gm430-GCST90011623 | F-sweetened coffee drink liking (derived food-liking factor) \|\| id:ebi-fl187-GCST90094853 | Weighted median | 4 | 0.083865535082694 | 0.356367695449332 | 0.813949290406222 | -0.614615148 | 0.782346218163384 | 1.08748265571173 | 0.540849002082827 | 2.18659648426738 | 0.999332001504091 |
| ebi-fl187-GCST90094853 | ebi-gm430-GCST90011642 | OTU99_19 (Bacteria) prevalence \|\| id:ebi-gm430-GCST90011642 | F-sweetened coffee drink liking (derived food-liking factor) \|\| id:ebi-fl187-GCST90094853 | Weighted median | 4 | -1.065170163 | 0.869932298514598 | 0.220790509060809 | -2.770237468 | 0.639897141687554 | 0.344669199693628 | 0.0626471262571829 | 1.896285821 | 0.999332001504091 |
| ebi-fl187-GCST90094853 | ebi-gm430-GCST90011653 | OTU99_30 (Parasutterella) prevalence \|\| id:ebi-gm430-GCST90011653 | F-sweetened coffee drink liking (derived food-liking factor) \|\| id:ebi-fl187-GCST90094853 | Weighted median | 4 | 0.0052304261847208 | 0.356814776085809 | 0.988304501025855 | -0.694126535 | 0.704587387312907 | 1.00524412874342 | 0.499510562513734 | 2.02301179235889 | 0.999332001504091 |
| ebi-fl187-GCST90094853 | ebi-gm430-GCST90011675 | OTU99_8 (Ruminococcaceae) prevalence \|\| id:ebi-gm430-GCST90011675 | F-sweetened coffee drink liking (derived food-liking factor) \|\| id:ebi-fl187-GCST90094853 | Weighted median | 4 | -0.020532305 | 0.379379040368216 | 0.956838905003423 | -0.764115224 | 0.723050614561017 | 0.979677047926675 | 0.465745829608427 | 2.06071006377286 | 0.999332001504091 |
| ebi-fl187-GCST90094853 | ebi-gm430-GCST90011679 | OTU99_92 (Ruminococcus) prevalence \|\| id:ebi-gm430-GCST90011679 | F-sweetened coffee drink liking (derived food-liking factor) \|\| id:ebi-fl187-GCST90094853 | Weighted median | 4 | -0.373265603 | 0.392217564571623 | 0.341259306932215 | -1.14201203 | 0.39548082360202 | 0.688482345609562 | 0.319176183425528 | 1.48509808949026 | 0.999332001504091 |
| ebi-fl187-GCST90094853 | ebi-gm430-GCST90011684 | TestASV_11 (Lachnospiraceae) prevalence \|\| id:ebi-gm430-GCST90011684 | F-sweetened coffee drink liking (derived food-liking factor) \|\| id:ebi-fl187-GCST90094853 | Weighted median | 4 | 0.116082216287507 | 0.429617444400941 | 0.787007139725011 | -0.725967975 | 0.958132407313352 | 1.12308820448031 | 0.483855981834293 | 2.60682344002676 | 0.999332001504091 |
| ebi-fl187-GCST90094853 | ebi-gm430-GCST90011688 | TestASV_15 (Bacteroides) prevalence \|\| id:ebi-gm430-GCST90011688 | F-sweetened coffee drink liking (derived food-liking factor) \|\| id:ebi-fl187-GCST90094853 | Weighted median | 4 | 0.182612690123576 | 0.381012481093426 | 0.631737528793914 | -0.564171773 | 0.929397153066692 | 1.20034941085565 | 0.568831073069442 | 2.53298171699142 | 0.999332001504091 |
| ebi-fl187-GCST90094853 | ebi-gm430-GCST90011692 | TestASV_19 (Prevotella) prevalence \|\| id:ebi-gm430-GCST90011692 | F-sweetened coffee drink liking (derived food-liking factor) \|\| id:ebi-fl187-GCST90094853 | Weighted median | 4 | 0.614499067367242 | 0.740457879031337 | 0.406600614157828 | -0.836798376 | 2.06579651026866 | 1.84873027823987 | 0.433094913368848 | 7.89158112039537 | 0.999332001504091 |
| ebi-fl187-GCST90094853 | ebi-gm430-GCST90011693 | TestASV_2 (Bacteroides) prevalence \|\| id:ebi-gm430-GCST90011693 | F-sweetened coffee drink liking (derived food-liking factor) \|\| id:ebi-fl187-GCST90094853 | Weighted median | 4 | -0.212598622 | 0.38964061491343 | 0.585322039204771 | -0.976294227 | 0.551096983160481 | 0.808480578361491 | 0.376704496688703 | 1.73515541049641 | 0.999332001504091 |
| ebi-fl187-GCST90094853 | ebi-gm430-GCST90011716 | TestASV_40 (Barnesiella) prevalence \|\| id:ebi-gm430-GCST90011716 | F-sweetened coffee drink liking (derived food-liking factor) \|\| id:ebi-fl187-GCST90094853 | Weighted median | 4 | -0.433118744 | 0.783516110517962 | 0.580408201860337 | -1.96881032 | 1.10257283285814 | 0.64848348386769 | 0.139622863924252 | 3.01190519252866 | 0.999332001504091 |
| ebi-fl187-GCST90094853 | ebi-gm430-GCST90011718 | TestASV_42 (Alphaproteobacteria) prevalence \|\| id:ebi-gm430-GCST90011718 | F-sweetened coffee drink liking (derived food-liking factor) \|\| id:ebi-fl187-GCST90094853 | Weighted median | 4 | 0.575354128767952 | 0.616316525921734 | 0.350542903177485 | -0.632626262 | 1.78333451957455 | 1.77775997140365 | 0.53119491048099 | 5.94966264466533 | 0.999332001504091 |
| ebi-fl187-GCST90094853 | ebi-hbsm44-GCST90104929 | Sulcal depth of the right inferior frontal sulcus \|\| id:ebi-hbsm44-GCST90104929 | F-sweetened coffee drink liking (derived food-liking factor) \|\| id:ebi-fl187-GCST90094853 | Weighted median | 3 | -0.737863718 | 0.492679035609536 | 0.13422264691845 | -1.703514628 | 0.227787191828157 | 0.478134254884961 | 0.182042586455887 | 1.25581804865091 | 0.999332001504091 |
| ebi-fl187-GCST90094853 | ebi-hbsm44-GCST90104935 | Sulcal opening distance of the left calloso-marginal posterior fissure \|\| id:ebi-hbsm44-GCST90104935 | F-sweetened coffee drink liking (derived food-liking factor) \|\| id:ebi-fl187-GCST90094853 | Weighted median | 3 | -0.109330795 | 0.109467623019156 | 0.317915782265733 | -0.323887336 | 0.105225745739465 | 0.896433832277957 | 0.723331730876991 | 1.11096137684744 | 0.999332001504091 |
| ebi-fl187-GCST90094853 | ebi-hbsm44-GCST90104936 | Sulcal opening distance of the left anterior inferior temporal sulcus \|\| id:ebi-hbsm44-GCST90104936 | F-sweetened coffee drink liking (derived food-liking factor) \|\| id:ebi-fl187-GCST90094853 | Weighted median | 3 | 0.0604578787662255 | 0.0592407066268144 | 0.307469484095842 | -0.055653906 | 0.176569663754782 | 1.06232285027881 | 0.94586643773717 | 1.19311754091234 | 0.999332001504091 |
| ebi-fl187-GCST90094853 | ebi-hbsm44-GCST90104941 | Sulcal opening distance of the right posterior inferior temporal sulcus \|\| id:ebi-hbsm44-GCST90104941 | F-sweetened coffee drink liking (derived food-liking factor) \|\| id:ebi-fl187-GCST90094853 | Weighted median | 3 | -0.004137483 | 0.0429160616012937 | 0.923195968047707 | -0.088252963 | 0.0799779982380436 | 0.995871065087667 | 0.915529252183257 | 1.08326323371296 | 0.999332001504091 |
| ebi-fl187-GCST90094853 | ebi-IPs91-GCST90274819 | Leukemia inhibitory factor levels \|\| id:ebi-IPs91-GCST90274819 | F-sweetened coffee drink liking (derived food-liking factor) \|\| id:ebi-fl187-GCST90094853 | Weighted median | 4 | 0.285959260294104 | 0.140501242460789 | 0.0418227850257593 | 0.0105768250709578 | 0.56134169551725 | 1.33103822804175 | 1.01063295741134 | 1.75302294618068 | 0.999332001504091 |
| ebi-fl187-GCST90094853 | ebi-IPs91-GCST90274837 | STAM binding protein levels \|\| id:ebi-IPs91-GCST90274837 | F-sweetened coffee drink liking (derived food-liking factor) \|\| id:ebi-fl187-GCST90094853 | Weighted median | 4 | 0.166031723950269 | 0.132785257185252 | 0.211161608220977 | -0.09422738 | 0.426290828033363 | 1.18061055475972 | 0.910075805555192 | 1.53156613273522 | 0.999332001504091 |
| ebi-fl187-GCST90094853 | ebi-IPs91-GCST90274840 | TNF-beta levels \|\| id:ebi-IPs91-GCST90274840 | F-sweetened coffee drink liking (derived food-liking factor) \|\| id:ebi-fl187-GCST90094853 | Weighted median | 4 | 0.041800036668525 | 0.15015206723693 | 0.780717070920398 | -0.252498015 | 0.336098088452907 | 1.04268595894527 | 0.776857754810422 | 1.39947629054295 | 0.999332001504091 |
| ebi-fl187-GCST90094853 | ebi-IPs91-GCST90274842 | Tumor necrosis factor ligand superfamily member 14 levels \|\| id:ebi-IPs91-GCST90274842 | F-sweetened coffee drink liking (derived food-liking factor) \|\| id:ebi-fl187-GCST90094853 | Weighted median | 4 | 0.294284017915649 | 0.147959913760253 | 0.0467073581698199 | 0.00428258694555184 | 0.584285448885745 | 1.34216504845795 | 1.00429177032589 | 1.79370883096811 | 0.999332001504091 |
| ebi-fl187-GCST90094853 | ebi-met1400-GCST90199628 | 3-methylhistidine levels \|\| id:ebi-met1400-GCST90199628 | F-sweetened coffee drink liking (derived food-liking factor) \|\| id:ebi-fl187-GCST90094853 | Weighted median | 4 | -0.064356024 | 0.192794238940456 | 0.738525368336673 | -0.442232732 | 0.313520684584506 | 0.937671106924169 | 0.642600064413134 | 1.36823376381602 | 0.999332001504091 |
| ebi-fl187-GCST90094853 | ebi-met1400-GCST90199651 | Docosahexaenoate DHA; 22:6n3 levels \|\| id:ebi-met1400-GCST90199651 | F-sweetened coffee drink liking (derived food-liking factor) \|\| id:ebi-fl187-GCST90094853 | Weighted median | 4 | 0.0280084781803412 | 0.167224498626328 | 0.866984294549819 | -0.299751539 | 0.355768495487945 | 1.02840440338246 | 0.741002307891656 | 1.42727708892787 | 0.999332001504091 |
| ebi-fl187-GCST90094853 | ebi-met1400-GCST90199652 | 1-stearoyl-2-oleoyl-GPS (18:0/18:1) levels \|\| id:ebi-met1400-GCST90199652 | F-sweetened coffee drink liking (derived food-liking factor) \|\| id:ebi-fl187-GCST90094853 | Weighted median | 4 | 0.0209660330945598 | 0.166752476723543 | 0.899944494493926 | -0.305868821 | 0.347800887472705 | 1.02118736447378 | 0.73648322404372 | 1.41595028822951 | 0.999332001504091 |
| ebi-fl187-GCST90094853 | ebi-met1400-GCST90199653 | 1-myristoyl-2-palmitoyl-gpc (14:0/16:0) levels \|\| id:ebi-met1400-GCST90199653 | F-sweetened coffee drink liking (derived food-liking factor) \|\| id:ebi-fl187-GCST90094853 | Weighted median | 4 | 0.0742841308050525 | 0.159930650693432 | 0.642305935892254 | -0.239179945 | 0.38774820616418 | 1.07711280295034 | 0.787273204100307 | 1.47365867939757 | 0.999332001504091 |
| ebi-fl187-GCST90094853 | ebi-met1400-GCST90199667 | Erythritol levels in elite athletes \|\| id:ebi-met1400-GCST90199667 | F-sweetened coffee drink liking (derived food-liking factor) \|\| id:ebi-fl187-GCST90094853 | Weighted median | 4 | -0.03864766 | 0.163774769164607 | 0.813447761432259 | -0.359646208 | 0.282350887158369 | 0.962089631706842 | 0.69792320206569 | 1.32624400034015 | 0.999332001504091 |
| ebi-fl187-GCST90094853 | ebi-met1400-GCST90199674 | 3-hydroxymyristate levels \|\| id:ebi-met1400-GCST90199674 | F-sweetened coffee drink liking (derived food-liking factor) \|\| id:ebi-fl187-GCST90094853 | Weighted median | 4 | 0.0593258716095841 | 0.170379917609486 | 0.727691819994472 | -0.274618767 | 0.393270510124176 | 1.06112097360447 | 0.759861752460992 | 1.48181918220854 | 0.999332001504091 |
| ebi-fl187-GCST90094853 | ebi-met1400-GCST90199685 | 2-linoleoylglycerol (18:2) levels \|\| id:ebi-met1400-GCST90199685 | F-sweetened coffee drink liking (derived food-liking factor) \|\| id:ebi-fl187-GCST90094853 | Weighted median | 4 | -0.032802059 | 0.199265434016371 | 0.869247104347077 | -0.42336231 | 0.357758191591642 | 0.967730094016846 | 0.65484133472471 | 1.43011976368222 | 0.999332001504091 |
| ebi-fl187-GCST90094853 | ebi-met1400-GCST90199703 | Gamma-glutamylvaline levels \|\| id:ebi-met1400-GCST90199703 | F-sweetened coffee drink liking (derived food-liking factor) \|\| id:ebi-fl187-GCST90094853 | Weighted median | 4 | 0.106619494643407 | 0.170659233123737 | 0.532134617713399 | -0.227872602 | 0.441111591565931 | 1.11251085759146 | 0.79622569071259 | 1.55443415440566 | 0.999332001504091 |
| ebi-fl187-GCST90094853 | ebi-met1400-GCST90199779 | Gamma-glutamyl-2-aminobutyrate levels \|\| id:ebi-met1400-GCST90199779 | F-sweetened coffee drink liking (derived food-liking factor) \|\| id:ebi-fl187-GCST90094853 | Weighted median | 4 | 0.00203243543976842 | 0.194424902733299 | 0.99165940524583 | -0.379040374 | 0.383105244797035 | 1.00203450223665 | 0.684517975436104 | 1.46683239842308 | 0.999332001504091 |
| ebi-fl187-GCST90094853 | ebi-met1400-GCST90199824 | 4-vinylphenol sulfate levels \|\| id:ebi-met1400-GCST90199824 | F-sweetened coffee drink liking (derived food-liking factor) \|\| id:ebi-fl187-GCST90094853 | Weighted median | 4 | 0.0367242595683205 | 0.176554439072601 | 0.835224826839282 | -0.309322441 | 0.382770960150618 | 1.03740692636236 | 0.733944078195961 | 1.46634214082078 | 0.999332001504091 |
| ebi-fl187-GCST90094853 | ebi-met1400-GCST90199830 | 4-ethylphenylsulfate levels \|\| id:ebi-met1400-GCST90199830 | F-sweetened coffee drink liking (derived food-liking factor) \|\| id:ebi-fl187-GCST90094853 | Weighted median | 4 | 0.00186586619895683 | 0.166340158888207 | 0.991050179205175 | -0.324160845 | 0.327892577619843 | 1.00186760801045 | 0.723133920389266 | 1.38803985773516 | 0.999332001504091 |
| ebi-fl187-GCST90094853 | ebi-met1400-GCST90199833 | Hexanoylglutamine levels \|\| id:ebi-met1400-GCST90199833 | F-sweetened coffee drink liking (derived food-liking factor) \|\| id:ebi-fl187-GCST90094853 | Weighted median | 4 | 0.188504116736698 | 0.171845106611814 | 0.272666742598504 | -0.148312292 | 0.525320525695853 | 1.2074420536974 | 0.862161826462982 | 1.69100077072316 | 0.999332001504091 |
| ebi-fl187-GCST90094853 | ebi-met1400-GCST90199837 | Carotenoid levels (cryptoxanthin) \|\| id:ebi-met1400-GCST90199837 | F-sweetened coffee drink liking (derived food-liking factor) \|\| id:ebi-fl187-GCST90094853 | Weighted median | 4 | -0.214528539 | 0.186931955563693 | 0.251121739021474 | -0.580915172 | 0.151858093477774 | 0.806921782317926 | 0.559386197468883 | 1.16399504622271 | 0.999332001504091 |
| ebi-fl187-GCST90094853 | ebi-met1400-GCST90199869 | 2-hydroxyhippurate levels \|\| id:ebi-met1400-GCST90199869 | F-sweetened coffee drink liking (derived food-liking factor) \|\| id:ebi-fl187-GCST90094853 | Weighted median | 4 | 0.153117808284683 | 0.174929130978188 | 0.381403417796892 | -0.189743288 | 0.495978905001932 | 1.16546227196657 | 0.827171451169819 | 1.64210491725328 | 0.999332001504091 |
| ebi-fl187-GCST90094853 | ebi-met1400-GCST90199902 | Hydantoin-5-propionate levels \|\| id:ebi-met1400-GCST90199902 | F-sweetened coffee drink liking (derived food-liking factor) \|\| id:ebi-fl187-GCST90094853 | Weighted median | 4 | 0.263063815088704 | 0.172791587191187 | 0.127900330669972 | -0.075607696 | 0.601735325983431 | 1.30090973397296 | 0.927179871773292 | 1.82528351560182 | 0.999332001504091 |
| ebi-fl187-GCST90094853 | ebi-met1400-GCST90199904 | Pantoate levels \|\| id:ebi-met1400-GCST90199904 | F-sweetened coffee drink liking (derived food-liking factor) \|\| id:ebi-fl187-GCST90094853 | Weighted median | 4 | 0.197944817608043 | 0.186764274223317 | 0.289206263884033 | -0.16811316 | 0.564002795085744 | 1.21889513041694 | 0.845258179967151 | 1.75769412726875 | 0.999332001504091 |
| ebi-fl187-GCST90094853 | ebi-met1400-GCST90199936 | 3-methyl catechol sulfate (2) levels \|\| id:ebi-met1400-GCST90199936 | F-sweetened coffee drink liking (derived food-liking factor) \|\| id:ebi-fl187-GCST90094853 | Weighted median | 4 | 0.0302479943640651 | 0.187688792197583 | 0.871967096696499 | -0.337622038 | 0.398118027071328 | 1.03071011256008 | 0.7134648993191 | 1.48901976417829 | 0.999332001504091 |
| ebi-fl187-GCST90094853 | ebi-met1400-GCST90199977 | 4-hydroxychlorothalonil levels \|\| id:ebi-met1400-GCST90199977 | F-sweetened coffee drink liking (derived food-liking factor) \|\| id:ebi-fl187-GCST90094853 | Weighted median | 4 | 0.1526307076521 | 0.18457767030181 | 0.408283203498263 | -0.209141526 | 0.514402941443648 | 1.16489471279709 | 0.811280410133742 | 1.67263953985885 | 0.999332001504091 |
| ebi-fl187-GCST90094853 | ebi-met1400-GCST90200040 | Sphingomyelin (d18:2/24:1, d18:1/24:2) levels \|\| id:ebi-met1400-GCST90200040 | F-sweetened coffee drink liking (derived food-liking factor) \|\| id:ebi-fl187-GCST90094853 | Weighted median | 4 | 0.026981909877056 | 0.175105465952665 | 0.877539184385811 | -0.316224803 | 0.370188623144279 | 1.02734921772173 | 0.728895573552293 | 1.44800771667429 | 0.999332001504091 |
| ebi-fl187-GCST90094853 | ebi-met1400-GCST90200077 | 1-stearoyl-2-oleoyl-GPI (18:0/18:1) levels \|\| id:ebi-met1400-GCST90200077 | F-sweetened coffee drink liking (derived food-liking factor) \|\| id:ebi-fl187-GCST90094853 | Weighted median | 4 | -0.08470089 | 0.178130099927278 | 0.63443052159253 | -0.433835886 | 0.264434105433639 | 0.918787061313708 | 0.648018595500683 | 1.30269357993538 | 0.999332001504091 |
| ebi-fl187-GCST90094853 | ebi-met1400-GCST90200080 | 1-palmitoyl-2-oleoyl-GPI (16:0/18:1) levels \|\| id:ebi-met1400-GCST90200080 | F-sweetened coffee drink liking (derived food-liking factor) \|\| id:ebi-fl187-GCST90094853 | Weighted median | 4 | 0.0225316214233527 | 0.177844363117212 | 0.899183450796302 | -0.32604333 | 0.371106573133087 | 1.02278737564571 | 0.721773912083822 | 1.44933752559727 | 0.999332001504091 |
| ebi-fl187-GCST90094853 | ebi-met1400-GCST90200082 | 1-oleoyl-2-linoleoyl-GPE (18:1/18:2) levels \|\| id:ebi-met1400-GCST90200082 | F-sweetened coffee drink liking (derived food-liking factor) \|\| id:ebi-fl187-GCST90094853 | Weighted median | 4 | -0.120634975 | 0.181369619620848 | 0.505965231531887 | -0.47611943 | 0.234849479159233 | 0.886357442911377 | 0.621189289332813 | 1.26471838793003 | 0.999332001504091 |
| ebi-fl187-GCST90094853 | ebi-met1400-GCST90200100 | Perfluorooctanesulfonate (PFOS) levels \|\| id:ebi-met1400-GCST90200100 | F-sweetened coffee drink liking (derived food-liking factor) \|\| id:ebi-fl187-GCST90094853 | Weighted median | 4 | 0.305918459064172 | 0.17798354744669 | 0.0856504499270111 | -0.042929294 | 0.654766212059684 | 1.35787157991422 | 0.95797912261007 | 1.92469249487938 | 0.999332001504091 |
| ebi-fl187-GCST90094853 | ebi-met1400-GCST90200102 | 1-palmitoleoyl-2-linolenoyl-GPC (16:1/18:3) levels \|\| id:ebi-met1400-GCST90200102 | F-sweetened coffee drink liking (derived food-liking factor) \|\| id:ebi-fl187-GCST90094853 | Weighted median | 4 | 0.0649971845439165 | 0.165299122688293 | 0.694164757446865 | -0.258989096 | 0.388983465012972 | 1.06715601984905 | 0.771831439105611 | 1.47548015408614 | 0.999332001504091 |
| ebi-fl187-GCST90094853 | ebi-met1400-GCST90200114 | Glycosyl-N-tricosanoyl-sphingadienine (d18:2/23:0) levels \|\| id:ebi-met1400-GCST90200114 | F-sweetened coffee drink liking (derived food-liking factor) \|\| id:ebi-fl187-GCST90094853 | Weighted median | 4 | -0.054979376 | 0.184002624371488 | 0.765095172832882 | -0.41562452 | 0.305665767737847 | 0.94650466843582 | 0.659928013873809 | 1.35752850089208 | 0.999332001504091 |
| ebi-fl187-GCST90094853 | ebi-met1400-GCST90200135 | Arachidoylcarnitine (C20) levels \|\| id:ebi-met1400-GCST90200135 | F-sweetened coffee drink liking (derived food-liking factor) \|\| id:ebi-fl187-GCST90094853 | Weighted median | 4 | -0.251910515 | 0.192268308337254 | 0.190127170558187 | -0.628756399 | 0.124935369623911 | 0.777314293146241 | 0.53325454468118 | 1.13307521962272 | 0.999332001504091 |
| ebi-fl187-GCST90094853 | ebi-met1400-GCST90200146 | Docosahexaenoylcarnitine (C22:6) levels \|\| id:ebi-met1400-GCST90200146 | F-sweetened coffee drink liking (derived food-liking factor) \|\| id:ebi-fl187-GCST90094853 | Weighted median | 4 | 0.0822939095883839 | 0.207352244805604 | 0.691456128719662 | -0.32411649 | 0.488704309407368 | 1.0857748825868 | 0.723165995699379 | 1.63020261277115 | 0.999332001504091 |
| ebi-fl187-GCST90094853 | ebi-met1400-GCST90200162 | 3-hydroxyphenylacetoylglutamine levels \|\| id:ebi-met1400-GCST90200162 | F-sweetened coffee drink liking (derived food-liking factor) \|\| id:ebi-fl187-GCST90094853 | Weighted median | 4 | -0.067257092 | 0.201865759112489 | 0.739000437840751 | -0.46291398 | 0.328399795449608 | 0.93495480065175 | 0.629446775020255 | 1.38874407488008 | 0.999332001504091 |
| ebi-fl187-GCST90094853 | ebi-met1400-GCST90200172 | 3-carboxy-4-methyl-5-pentyl-2-furanpropionate (3-CMPFP) levels \|\| id:ebi-met1400-GCST90200172 | F-sweetened coffee drink liking (derived food-liking factor) \|\| id:ebi-fl187-GCST90094853 | Weighted median | 4 | 0.123261108405551 | 0.175085475648041 | 0.481429453099201 | -0.219906424 | 0.466428640675711 | 1.13117974292935 | 0.802593898083906 | 1.59429023054938 | 0.999332001504091 |
| ebi-fl187-GCST90094853 | ebi-met1400-GCST90200222 | Dihydrocaffeate sulfate (2) levels \|\| id:ebi-met1400-GCST90200222 | F-sweetened coffee drink liking (derived food-liking factor) \|\| id:ebi-fl187-GCST90094853 | Weighted median | 4 | 0.221123597949802 | 0.193599828192409 | 0.253384045562457 | -0.158332065 | 0.600579261206924 | 1.24747760671055 | 0.853566295148901 | 1.82317458888512 | 0.999332001504091 |
| ebi-fl187-GCST90094853 | ebi-met1400-GCST90200223 | 4-methylhexanoylglutamine levels \|\| id:ebi-met1400-GCST90200223 | F-sweetened coffee drink liking (derived food-liking factor) \|\| id:ebi-fl187-GCST90094853 | Weighted median | 4 | 0.14709911588355 | 0.213751201621353 | 0.491339984947127 | -0.271853239 | 0.566051471061402 | 1.15846877999636 | 0.761966079563913 | 1.76129876410554 | 0.999332001504091 |
| ebi-fl187-GCST90094853 | ebi-met1400-GCST90200224 | 2-ketocaprylate levels \|\| id:ebi-met1400-GCST90200224 | F-sweetened coffee drink liking (derived food-liking factor) \|\| id:ebi-fl187-GCST90094853 | Weighted median | 4 | -0.038096824 | 0.175984351929327 | 0.828614802644836 | -0.383026154 | 0.306832505833507 | 0.962619731736008 | 0.681795067569066 | 1.35911330545617 | 0.999332001504091 |
| ebi-fl187-GCST90094853 | ebi-met1400-GCST90200243 | Hydroxypalmitoyl sphingomyelin (d18:1/16:0(OH)) levels \|\| id:ebi-met1400-GCST90200243 | F-sweetened coffee drink liking (derived food-liking factor) \|\| id:ebi-fl187-GCST90094853 | Weighted median | 4 | -0.062479404 | 0.165179009992952 | 0.705242892320167 | -0.386230263 | 0.261271455916768 | 0.939432411474727 | 0.679614017525746 | 1.29858012485122 | 0.999332001504091 |
| ebi-fl187-GCST90094853 | ebi-met1400-GCST90200273 | 2-methoxyhydroquinone sulfate (2) levels \|\| id:ebi-met1400-GCST90200273 | F-sweetened coffee drink liking (derived food-liking factor) \|\| id:ebi-fl187-GCST90094853 | Weighted median | 4 | 0.129665477319335 | 0.217849047850388 | 0.551704500939293 | -0.297318656 | 0.556649611106095 | 1.13844748311445 | 0.742807274302879 | 1.74481688137204 | 0.999332001504091 |
| ebi-fl187-GCST90094853 | ebi-met1400-GCST90200276 | 2,4-di-tert-butylphenol levels \|\| id:ebi-met1400-GCST90200276 | F-sweetened coffee drink liking (derived food-liking factor) \|\| id:ebi-fl187-GCST90094853 | Weighted median | 4 | 0.123393752061923 | 0.170240255601648 | 0.468561707597712 | -0.210277149 | 0.457064653041154 | 1.13132979669809 | 0.810359624552259 | 1.57943099596567 | 0.999332001504091 |
| ebi-fl187-GCST90094853 | ebi-met1400-GCST90200289 | N-acetylmethionine levels \|\| id:ebi-met1400-GCST90200289 | F-sweetened coffee drink liking (derived food-liking factor) \|\| id:ebi-fl187-GCST90094853 | Weighted median | 4 | 0.138542314449315 | 0.182003204828859 | 0.446532778637092 | -0.218183967 | 0.495268595913878 | 1.14859828284573 | 0.803977522715692 | 1.64093892936194 | 0.999332001504091 |
| ebi-fl187-GCST90094853 | ebi-met1400-GCST90200308 | 3-hydroxyisobutyrate levels \|\| id:ebi-met1400-GCST90200308 | F-sweetened coffee drink liking (derived food-liking factor) \|\| id:ebi-fl187-GCST90094853 | Weighted median | 4 | -0.128297549 | 0.185363183589023 | 0.488848483277279 | -0.491609388 | 0.235014291207745 | 0.879591618925076 | 0.611641233300496 | 1.26492684593606 | 0.999332001504091 |
| ebi-fl187-GCST90094853 | ebi-met1400-GCST90200383 | Cystathionine levels \|\| id:ebi-met1400-GCST90200383 | F-sweetened coffee drink liking (derived food-liking factor) \|\| id:ebi-fl187-GCST90094853 | Weighted median | 4 | -0.012454082 | 0.190482360388346 | 0.947870004154221 | -0.385799508 | 0.360891344279544 | 0.987623149051871 | 0.679906827594807 | 1.43460757409018 | 0.999332001504091 |
| ebi-fl187-GCST90094853 | ebi-met1400-GCST90200384 | Guanidinoacetate levels \|\| id:ebi-met1400-GCST90200384 | F-sweetened coffee drink liking (derived food-liking factor) \|\| id:ebi-fl187-GCST90094853 | Weighted median | 4 | 0.000778220960805036 | 0.178709876202567 | 0.996525494056284 | -0.349493136 | 0.351049578317837 | 1.0007785238533 | 0.70504536099966 | 1.42055775303014 | 0.999364451047625 |
| ebi-fl187-GCST90094853 | ebi-met1400-GCST90200403 | Citrulline levels \|\| id:ebi-met1400-GCST90200403 | F-sweetened coffee drink liking (derived food-liking factor) \|\| id:ebi-fl187-GCST90094853 | Weighted median | 4 | 0.0141755030544175 | 0.181274870326339 | 0.93766980367533 | -0.341123243 | 0.369474248894042 | 1.01427645193422 | 0.710971280725954 | 1.44697366664069 | 0.999332001504091 |
| ebi-fl187-GCST90094853 | ebi-met1400-GCST90200434 | 12,13-DiHOME levels \|\| id:ebi-met1400-GCST90200434 | F-sweetened coffee drink liking (derived food-liking factor) \|\| id:ebi-fl187-GCST90094853 | Weighted median | 4 | -0.098440224 | 0.176684271392787 | 0.577422698315374 | -0.444741396 | 0.247860948264429 | 0.906249863290221 | 0.640990017442548 | 1.28128175535457 | 0.999332001504091 |
| ebi-fl187-GCST90094853 | ebi-met1400-GCST90200437 | Fructose levels \|\| id:ebi-met1400-GCST90200437 | F-sweetened coffee drink liking (derived food-liking factor) \|\| id:ebi-fl187-GCST90094853 | Weighted median | 4 | 0.0145961438331476 | 0.178104583391046 | 0.934684360993553 | -0.33448884 | 0.363681127279598 | 1.01470318771575 | 0.715703832314859 | 1.43861540580314 | 0.999332001504091 |
| ebi-fl187-GCST90094853 | ebi-met1400-GCST90200448 | N-stearoyl-sphinganine (d18:0/18:0) levels \|\| id:ebi-met1400-GCST90200448 | F-sweetened coffee drink liking (derived food-liking factor) \|\| id:ebi-fl187-GCST90094853 | Weighted median | 4 | -0.021264174 | 0.186471407020655 | 0.909210443951194 | -0.386748132 | 0.344219783903582 | 0.978960314684612 | 0.679262158044156 | 1.41088869205797 | 0.999332001504091 |
| ebi-fl187-GCST90094853 | ebi-met1400-GCST90200468 | X-11847 levels \|\| id:ebi-met1400-GCST90200468 | F-sweetened coffee drink liking (derived food-liking factor) \|\| id:ebi-fl187-GCST90094853 | Weighted median | 4 | -0.068128172 | 0.190525071092853 | 0.720656794377146 | -0.441557311 | 0.305300967212109 | 0.934140735096182 | 0.643034236335728 | 1.35703336409982 | 0.999332001504091 |
| ebi-fl187-GCST90094853 | ebi-met1400-GCST90200476 | X-11843 levels \|\| id:ebi-met1400-GCST90200476 | F-sweetened coffee drink liking (derived food-liking factor) \|\| id:ebi-fl187-GCST90094853 | Weighted median | 4 | -0.062595279 | 0.18379469674129 | 0.733426011772054 | -0.422832884 | 0.297642327085419 | 0.939323561183987 | 0.655188116288592 | 1.34668003075734 | 0.999332001504091 |
| ebi-fl187-GCST90094853 | ebi-met1400-GCST90200511 | X-12906 levels \|\| id:ebi-met1400-GCST90200511 | F-sweetened coffee drink liking (derived food-liking factor) \|\| id:ebi-fl187-GCST90094853 | Weighted median | 4 | -0.115860944 | 0.175333985958467 | 0.508739684442947 | -0.459515556 | 0.227793668971089 | 0.890599058268506 | 0.631589541178746 | 1.25582618279025 | 0.999332001504091 |
| ebi-fl187-GCST90094853 | ebi-met1400-GCST90200515 | X-12849 levels \|\| id:ebi-met1400-GCST90200515 | F-sweetened coffee drink liking (derived food-liking factor) \|\| id:ebi-fl187-GCST90094853 | Weighted median | 4 | 0.214513235381698 | 0.179597974527985 | 0.232318457260296 | -0.137498795 | 0.566525265456549 | 1.23925852292552 | 0.87153540046411 | 1.76213345530854 | 0.999332001504091 |
| ebi-fl187-GCST90094853 | ebi-met1400-GCST90200551 | X-18886 levels \|\| id:ebi-met1400-GCST90200551 | F-sweetened coffee drink liking (derived food-liking factor) \|\| id:ebi-fl187-GCST90094853 | Weighted median | 4 | 0.168357699108376 | 0.174043856218097 | 0.333379505531324 | -0.172768259 | 0.509483657295847 | 1.1833598217053 | 0.841332563438007 | 1.66443155594035 | 0.999332001504091 |
| ebi-fl187-GCST90094853 | ebi-met1400-GCST90200564 | X-21319 levels \|\| id:ebi-met1400-GCST90200564 | F-sweetened coffee drink liking (derived food-liking factor) \|\| id:ebi-fl187-GCST90094853 | Weighted median | 4 | -0.087898445 | 0.166174978343373 | 0.596839341261974 | -0.413601403 | 0.237804512511255 | 0.915853881503391 | 0.661264477046801 | 1.26846120029137 | 0.999332001504091 |
| ebi-fl187-GCST90094853 | ebi-met1400-GCST90200579 | X-21442 levels \|\| id:ebi-met1400-GCST90200579 | F-sweetened coffee drink liking (derived food-liking factor) \|\| id:ebi-fl187-GCST90094853 | Weighted median | 4 | -0.027138721 | 0.199227411713243 | 0.891647511718727 | -0.417624448 | 0.363347005954758 | 0.973226225241452 | 0.658609524135308 | 1.43813481401027 | 0.999332001504091 |
| ebi-fl187-GCST90094853 | ebi-met1400-GCST90200581 | X-21441 levels \|\| id:ebi-met1400-GCST90200581 | F-sweetened coffee drink liking (derived food-liking factor) \|\| id:ebi-fl187-GCST90094853 | Weighted median | 4 | -0.035076508 | 0.18038392066772 | 0.845819831666613 | -0.388628993 | 0.318475976155771 | 0.965531542203586 | 0.677985760915597 | 1.37503058726641 | 0.999332001504091 |
| ebi-fl187-GCST90094853 | ebi-met1400-GCST90200648 | X-24585 levels \|\| id:ebi-met1400-GCST90200648 | F-sweetened coffee drink liking (derived food-liking factor) \|\| id:ebi-fl187-GCST90094853 | Weighted median | 4 | 0.0619178991152767 | 0.176347651459913 | 0.72550380448233 | -0.283723498 | 0.407559295976706 | 1.06387499606397 | 0.752974815158552 | 1.50314457331722 | 0.999332001504091 |
| ebi-fl187-GCST90094853 | ebi-met1400-GCST90200720 | Adenosine 5'-diphosphate (ADP) to pantothenate ratio \|\| id:ebi-met1400-GCST90200720 | F-sweetened coffee drink liking (derived food-liking factor) \|\| id:ebi-fl187-GCST90094853 | Weighted median | 4 | -0.138285953 | 0.229830124087818 | 0.54738205691701 | -0.588752996 | 0.31218109064501 | 0.870849634449668 | 0.555018964372338 | 1.36640211326609 | 0.999332001504091 |
| ebi-fl187-GCST90094853 | ebi-met1400-GCST90200724 | Cholate to taurocholate ratio \|\| id:ebi-met1400-GCST90200724 | F-sweetened coffee drink liking (derived food-liking factor) \|\| id:ebi-fl187-GCST90094853 | Weighted median | 4 | -0.025665164 | 0.182505987766154 | 0.888165074784074 | -0.3833769 | 0.332046572168249 | 0.974661386840643 | 0.681555972510301 | 1.3938177601162 | 0.999332001504091 |
| ebi-fl187-GCST90094853 | ebi-met1400-GCST90200726 | N-acetylputrescine to (N(1) + N(8))-acetylspermidine ratio \|\| id:ebi-met1400-GCST90200726 | F-sweetened coffee drink liking (derived food-liking factor) \|\| id:ebi-fl187-GCST90094853 | Weighted median | 4 | -0.065362128 | 0.179374340250016 | 0.71556716743679 | -0.416935835 | 0.286211578890774 | 0.936728186446268 | 0.659063207451088 | 1.33137411611319 | 0.999332001504091 |
| ebi-fl187-GCST90094853 | ebi-met1400-GCST90200744 | Arginine to glutamate ratio \|\| id:ebi-met1400-GCST90200744 | F-sweetened coffee drink liking (derived food-liking factor) \|\| id:ebi-fl187-GCST90094853 | Weighted median | 4 | -0.14605171 | 0.178929288563715 | 0.414354998399863 | -0.496753115 | 0.204649695832806 | 0.864113018959372 | 0.608503195374299 | 1.22709513312544 | 0.999332001504091 |
| ebi-fl187-GCST90094853 | ebi-met1400-GCST90200759 | Histidine to trans-urocanate ratio \|\| id:ebi-met1400-GCST90200759 | F-sweetened coffee drink liking (derived food-liking factor) \|\| id:ebi-fl187-GCST90094853 | Weighted median | 4 | -0.140905051 | 0.197795082705992 | 0.476230153156304 | -0.528583413 | 0.246773310764215 | 0.868571777509395 | 0.589439370426532 | 1.27988894284397 | 0.999332001504091 |
| ebi-fl187-GCST90094853 | ebi-met1400-GCST90200795 | Oleoyl-linoleoyl-glycerol (18:1 to 18:2) [2] to linoleoyl-arachidonoyl-glycerol (18:2 to 20:4) [2] ratio \|\| id:ebi-met1400-GCST90200795 | F-sweetened coffee drink liking (derived food-liking factor) \|\| id:ebi-fl187-GCST90094853 | Weighted median | 4 | -0.047096703 | 0.174681899090952 | 0.787457153828541 | -0.389473225 | 0.295279819690544 | 0.953995139388229 | 0.677413625260696 | 1.343502244 | 0.999332001504091 |
| ebi-fl187-GCST90094853 | ebi-met1400-GCST90200811 | Choline to taurocholate ratio \|\| id:ebi-met1400-GCST90200811 | F-sweetened coffee drink liking (derived food-liking factor) \|\| id:ebi-fl187-GCST90094853 | Weighted median | 4 | -0.006326664 | 0.175961328237086 | 0.97131835471976 | -0.351210868 | 0.338557538986803 | 0.993693306843836 | 0.703835322069079 | 1.40292246936894 | 0.999332001504091 |
| ebi-fl187-GCST90094853 | ebi-met1400-GCST90200831 | Cholate to phosphate ratio \|\| id:ebi-met1400-GCST90200831 | F-sweetened coffee drink liking (derived food-liking factor) \|\| id:ebi-fl187-GCST90094853 | Weighted median | 4 | 0.183507922244219 | 0.175251675323555 | 0.295048478007053 | -0.159985361 | 0.527001205878387 | 1.20142448335216 | 0.852156263258149 | 1.69384519181869 | 0.999332001504091 |
| ebi-fl187-GCST90094853 | ebi-met1400-GCST90200841 | Adenosine 5'-diphosphate (ADP) to oxalate (ethanedioate) ratio \|\| id:ebi-met1400-GCST90200841 | F-sweetened coffee drink liking (derived food-liking factor) \|\| id:ebi-fl187-GCST90094853 | Weighted median | 4 | 0.307726621858758 | 0.240208820838057 | 0.200165705886096 | -0.163082667 | 0.778535910701351 | 1.36032905387178 | 0.849520958176453 | 2.17828073221395 | 0.999332001504091 |
| ebi-fl187-GCST90094853 | ebi-met1400-GCST90200844 | Aspartate to phosphate ratio \|\| id:ebi-met1400-GCST90200844 | F-sweetened coffee drink liking (derived food-liking factor) \|\| id:ebi-fl187-GCST90094853 | Weighted median | 4 | 0.00891390327912525 | 0.171044730619801 | 0.958437493505843 | -0.326333769 | 0.344161575293935 | 1.00895375042483 | 0.721564311627539 | 1.41080656857901 | 0.999332001504091 |
| ebi-fl187-GCST90094853 | ebi-met1400-GCST90200870 | Adenosine 5'-monophosphate (AMP) to acetoacetate ratio \|\| id:ebi-met1400-GCST90200870 | F-sweetened coffee drink liking (derived food-liking factor) \|\| id:ebi-fl187-GCST90094853 | Weighted median | 4 | 0.0802434892523381 | 0.193943358578202 | 0.679059378016625 | -0.299885494 | 0.460372472065615 | 1.08355086854817 | 0.740903053995048 | 1.584664118 | 0.999332001504091 |
| ebi-fl187-GCST90094853 | ebi-met1400-GCST90200887 | Cholesterol to cortisol ratio \|\| id:ebi-met1400-GCST90200887 | F-sweetened coffee drink liking (derived food-liking factor) \|\| id:ebi-fl187-GCST90094853 | Weighted median | 4 | 0.081227955547643 | 0.178967655579671 | 0.649922776470428 | -0.269548649 | 0.432004560483798 | 1.08461811310446 | 0.763724123906952 | 1.54034213985048 | 0.999332001504091 |
| ebi-fl187-GCST90094853 | ebi-met1400-GCST90200890 | Cortisol to taurocholate ratio \|\| id:ebi-met1400-GCST90200890 | F-sweetened coffee drink liking (derived food-liking factor) \|\| id:ebi-fl187-GCST90094853 | Weighted median | 4 | 0.0614360911856022 | 0.180000307962309 | 0.732869442371312 | -0.291364512 | 0.414236694791727 | 1.06336253611823 | 0.747243248923829 | 1.51321525466877 | 0.999332001504091 |
| ebi-fl187-GCST90094853 | ebi-met1400-GCST90200948 | Adenosine 5'-diphosphate (ADP) to mannitol to sorbitol ratio \|\| id:ebi-met1400-GCST90200948 | F-sweetened coffee drink liking (derived food-liking factor) \|\| id:ebi-fl187-GCST90094853 | Weighted median | 4 | 0.0069238765842382 | 0.222615264302994 | 0.975187848491182 | -0.429402041 | 0.443249794618106 | 1.00694790203546 | 0.650898188517987 | 1.55776140616129 | 0.999332001504091 |
| ebi-fl187-GCST90094853 | ebi-met1400-GCST90200972 | Leucine to N-palmitoyl-sphingosine (d18:1 to 16:0) ratio \|\| id:ebi-met1400-GCST90200972 | F-sweetened coffee drink liking (derived food-liking factor) \|\| id:ebi-fl187-GCST90094853 | Weighted median | 4 | -0.111485337 | 0.160762655719386 | 0.488009736468797 | -0.426580142 | 0.203609468177862 | 0.894504507397956 | 0.652737551628392 | 1.22581933850618 | 0.999332001504091 |
| ebi-fl187-GCST90094853 | ebi-met1400-GCST90200973 | Proline to glutamate ratio \|\| id:ebi-met1400-GCST90200973 | F-sweetened coffee drink liking (derived food-liking factor) \|\| id:ebi-fl187-GCST90094853 | Weighted median | 4 | -0.098819021 | 0.17519147538909 | 0.572711290766002 | -0.442194312 | 0.244556271111316 | 0.905906643582964 | 0.642624753355786 | 1.27705452147965 | 0.999332001504091 |
| ebi-fl187-GCST90094853 | ebi-met1400-GCST90200983 | Cholesterol to linoleoyl-arachidonoyl-glycerol (18:2 to 20:4) [1] ratio \|\| id:ebi-met1400-GCST90200983 | F-sweetened coffee drink liking (derived food-liking factor) \|\| id:ebi-fl187-GCST90094853 | Weighted median | 4 | 0.058757585291875 | 0.174525297931457 | 0.736365018991121 | -0.283311999 | 0.400827169237531 | 1.06051812438544 | 0.753284727371492 | 1.4930591996396 | 0.999332001504091 |
| ebi-fl187-GCST90094853 | ebi-met1400-GCST90201001 | Serine to alpha-ketobutyrate ratio \|\| id:ebi-met1400-GCST90201001 | F-sweetened coffee drink liking (derived food-liking factor) \|\| id:ebi-fl187-GCST90094853 | Weighted median | 4 | -0.089387182 | 0.177801400729396 | 0.615149829913297 | -0.437877927 | 0.259103563845503 | 0.914491430779264 | 0.645404564511182 | 1.29576799259562 | 0.999332001504091 |
| ebi-fl187-GCST90094853 | ebi-met1400-GCST90201005 | Fructose to maltose ratio \|\| id:ebi-met1400-GCST90201005 | F-sweetened coffee drink liking (derived food-liking factor) \|\| id:ebi-fl187-GCST90094853 | Weighted median | 4 | -0.019764282 | 0.208363985954887 | 0.924430320081059 | -0.428157694 | 0.388629130525854 | 0.980429751064361 | 0.651708635881529 | 1.47495743319698 | 0.999332001504091 |
| ebi-fl187-GCST90094853 | ebi-met1400-GCST90201020 | Paraxanthine to 5-acetylamino-6-formylamino-3-methyluracil ratio \|\| id:ebi-met1400-GCST90201020 | F-sweetened coffee drink liking (derived food-liking factor) \|\| id:ebi-fl187-GCST90094853 | Weighted median | 4 | -0.010220071 | 0.18911004329825 | 0.956900918004029 | -0.380875756 | 0.360435613825939 | 0.989831976426881 | 0.683262775712937 | 1.43395392868407 | 0.999332001504091 |
| ebi-fl187-GCST90094853 | ebi-pw11-GCST90275197 | Placental weight (fetal genotype, adjusted for sex) \|\| id:ebi-pw11-GCST90275197 | F-sweetened coffee drink liking (derived food-liking factor) \|\| id:ebi-fl187-GCST90094853 | Weighted median | 4 | 0.0617621626671027 | 0.062652753450134 | 0.324238486168716 | -0.061037234 | 0.184561559429365 | 1.06370932485164 | 0.940788209726131 | 1.20269101597873 | 0.999332001504091 |
| ebi-fl187-GCST90094853 | prot-a-1017 | FAS-associated factor 2 \|\| id:prot-a-1017 | F-sweetened coffee drink liking (derived food-liking factor) \|\| id:ebi-fl187-GCST90094853 | Weighted median | 4 | -0.333775506 | 0.294847846364283 | 0.257623392445484 | -0.911677285 | 0.244126272731381 | 0.716214549947884 | 0.401849642075097 | 1.27650550815021 | 0.999332001504091 |
| ebi-fl187-GCST90094853 | prot-a-1019 | Fas apoptotic inhibitory molecule 3 \|\| id:prot-a-1019 | F-sweetened coffee drink liking (derived food-liking factor) \|\| id:ebi-fl187-GCST90094853 | Weighted median | 4 | -0.097207419 | 0.286049011905003 | 0.733986177723162 | -0.657863482 | 0.463448644240371 | 0.907367781210635 | 0.517956776928439 | 1.58954632326948 | 0.999332001504091 |
| ebi-fl187-GCST90094853 | prot-a-1052 | MIP18 family protein FAM96A \|\| id:prot-a-1052 | F-sweetened coffee drink liking (derived food-liking factor) \|\| id:ebi-fl187-GCST90094853 | Weighted median | 4 | 0.238020815662334 | 0.288813550222012 | 0.409863916413041 | -0.328053743 | 0.804095374097477 | 1.26873560212193 | 0.720324306439482 | 2.23467404015324 | 0.999332001504091 |
| ebi-fl187-GCST90094853 | prot-a-1066 | F-box/LRR-repeat protein 4 \|\| id:prot-a-1066 | F-sweetened coffee drink liking (derived food-liking factor) \|\| id:ebi-fl187-GCST90094853 | Weighted median | 4 | -0.38928478 | 0.320929648491707 | 0.225133256313599 | -1.018306891 | 0.239737331448733 | 0.677541292602349 | 0.361205983977926 | 1.27091527699972 | 0.999332001504091 |
| ebi-fl187-GCST90094853 | prot-a-1076 | Low affinity immunoglobulin gamma Fc region receptor III-B \|\| id:prot-a-1076 | F-sweetened coffee drink liking (derived food-liking factor) \|\| id:ebi-fl187-GCST90094853 | Weighted median | 4 | -0.019344478 | 0.299490869107219 | 0.948499482520603 | -0.606346581 | 0.567657625462156 | 0.980841425759421 | 0.545339587824145 | 1.7641299549227 | 0.999332001504091 |
| ebi-fl187-GCST90094853 | prot-a-1081 | Fc receptor-like protein 3 \|\| id:prot-a-1081 | F-sweetened coffee drink liking (derived food-liking factor) \|\| id:ebi-fl187-GCST90094853 | Weighted median | 4 | -0.011776847 | 0.267777191638312 | 0.964920327784203 | -0.536620143 | 0.513066448560688 | 0.98829222858252 | 0.584721190577966 | 1.67040556219823 | 0.999332001504091 |
| ebi-fl187-GCST90094853 | prot-a-1105 | Fibroblast growth factor receptor 4 \|\| id:prot-a-1105 | F-sweetened coffee drink liking (derived food-liking factor) \|\| id:ebi-fl187-GCST90094853 | Weighted median | 4 | 0.175274212503013 | 0.293770712226573 | 0.550750362639151 | -0.400516383 | 0.751064808467096 | 1.1915729160096 | 0.669973993205911 | 2.11925541672667 | 0.999332001504091 |
| ebi-fl187-GCST90094853 | prot-a-1143 | Follistatin-related protein 1 \|\| id:prot-a-1143 | F-sweetened coffee drink liking (derived food-liking factor) \|\| id:ebi-fl187-GCST90094853 | Weighted median | 4 | -0.070000651 | 0.278137108049815 | 0.801290909599734 | -0.615149382 | 0.475148081226096 | 0.932393213335908 | 0.540560139145048 | 1.6082523318309 | 0.999332001504091 |
| ebi-fl187-GCST90094853 | prot-a-1151 | Alpha-(1,3)-fucosyltransferase 10 \|\| id:prot-a-1151 | F-sweetened coffee drink liking (derived food-liking factor) \|\| id:ebi-fl187-GCST90094853 | Weighted median | 4 | -0.417858482 | 0.285839528913593 | 0.143778515049757 | -0.978103959 | 0.142386994230546 | 0.65845540483382 | 0.376023379083853 | 1.15302277536894 | 0.999332001504091 |
| ebi-fl187-GCST90094853 | prot-a-1165 | Growth arrest and DNA damage-inducible proteins-interacting protein 1 \|\| id:prot-a-1165 | F-sweetened coffee drink liking (derived food-liking factor) \|\| id:ebi-fl187-GCST90094853 | Weighted median | 4 | -0.164975224 | 0.276645491973677 | 0.550947024056309 | -0.707200389 | 0.377249939991047 | 0.847914711527392 | 0.493022538697274 | 1.45826874350269 | 0.999332001504091 |
| ebi-fl187-GCST90094853 | prot-a-1194 | Growth/differentiation factor 11/8 \|\| id:prot-a-1194 | F-sweetened coffee drink liking (derived food-liking factor) \|\| id:ebi-fl187-GCST90094853 | Weighted median | 4 | -0.315550361 | 0.282965453022992 | 0.264783974474817 | -0.870162648 | 0.239061927381856 | 0.729387337772844 | 0.418883412961155 | 1.27005718546486 | 0.999332001504091 |
| ebi-fl187-GCST90094853 | prot-a-1216 | D-glucuronyl C5-epimerase \|\| id:prot-a-1216 | F-sweetened coffee drink liking (derived food-liking factor) \|\| id:ebi-fl187-GCST90094853 | Weighted median | 4 | -0.302831812 | 0.290250323891381 | 0.29678759926744 | -0.871722447 | 0.266058823011312 | 0.738723330458417 | 0.418230548678861 | 1.30481180938938 | 0.999332001504091 |
| ebi-fl187-GCST90094853 | prot-a-1246 | Glypican-3 \|\| id:prot-a-1246 | F-sweetened coffee drink liking (derived food-liking factor) \|\| id:ebi-fl187-GCST90094853 | Weighted median | 4 | 0.450799452698259 | 0.278779027418062 | 0.105867622858423 | -0.095607441 | 0.99720634643766 | 1.56956647820562 | 0.908820711764479 | 2.71069848829018 | 0.999332001504091 |
| ebi-fl187-GCST90094853 | prot-a-1275 | Glutamate receptor 4 \|\| id:prot-a-1275 | F-sweetened coffee drink liking (derived food-liking factor) \|\| id:ebi-fl187-GCST90094853 | Weighted median | 4 | -0.125685723 | 0.263995260183727 | 0.634009668308649 | -0.643116433 | 0.391744986923791 | 0.881891961563719 | 0.525651710448896 | 1.47956035605123 | 0.999332001504091 |
| ebi-fl187-GCST90094853 | prot-a-1289 | Glutathione S-transferase P \|\| id:prot-a-1289 | F-sweetened coffee drink liking (derived food-liking factor) \|\| id:ebi-fl187-GCST90094853 | Weighted median | 4 | -0.061553122 | 0.299338173414192 | 0.837079383592602 | -0.648255942 | 0.525149697449229 | 0.940302993220869 | 0.522957049124557 | 1.69071192469869 | 0.999332001504091 |
| ebi-fl187-GCST90094853 | prot-a-130 | Apolipoprotein D \|\| id:prot-a-130 | F-sweetened coffee drink liking (derived food-liking factor) \|\| id:ebi-fl187-GCST90094853 | Weighted median | 4 | -0.28593397 | 0.30117935674082 | 0.342426851009432 | -0.876245509 | 0.304377568958762 | 0.751312223264925 | 0.416343137290836 | 1.35578086023062 | 0.999332001504091 |
| ebi-fl187-GCST90094853 | prot-a-1313 | Heparin-binding EGF-like growth factor \|\| id:prot-a-1313 | F-sweetened coffee drink liking (derived food-liking factor) \|\| id:ebi-fl187-GCST90094853 | Weighted median | 4 | -0.418869165 | 0.291014422166344 | 0.150053761187159 | -0.989257432 | 0.151519102722034 | 0.657790251807223 | 0.371852714389516 | 1.16360052953484 | 0.999332001504091 |
| ebi-fl187-GCST90094853 | prot-a-1347 | HLA class II histocompatibility antigen, DQ alpha 2 chain \|\| id:prot-a-1347 | F-sweetened coffee drink liking (derived food-liking factor) \|\| id:ebi-fl187-GCST90094853 | Weighted median | 4 | 0.249884812602842 | 0.279190464889045 | 0.370769192516375 | -0.297328499 | 0.797098123785369 | 1.28387752166009 | 0.742799963546275 | 2.21909204566268 | 0.999332001504091 |
| ebi-fl187-GCST90094853 | prot-a-1357 | Non-histone chromosomal protein HMG-14 \|\| id:prot-a-1357 | F-sweetened coffee drink liking (derived food-liking factor) \|\| id:ebi-fl187-GCST90094853 | Weighted median | 4 | 0.0772981423004075 | 0.2736530267886 | 0.777584906807269 | -0.45906179 | 0.613658074806063 | 1.0803641306284 | 0.631876199933125 | 1.84717616341934 | 0.999332001504091 |
| ebi-fl187-GCST90094853 | prot-a-137 | Apolipoprotein M \|\| id:prot-a-137 | F-sweetened coffee drink liking (derived food-liking factor) \|\| id:ebi-fl187-GCST90094853 | Weighted median | 4 | 0.182765047039364 | 0.282803685781103 | 0.518110216292955 | -0.371530177 | 0.737060271170326 | 1.2005323063221 | 0.68967819303309 | 2.08978308011241 | 0.999332001504091 |
| ebi-fl187-GCST90094853 | prot-a-1381 | 17-beta-hydroxysteroid dehydrogenase 14 \|\| id:prot-a-1381 | F-sweetened coffee drink liking (derived food-liking factor) \|\| id:ebi-fl187-GCST90094853 | Weighted median | 4 | -0.291071023 | 0.269032054684539 | 0.279288304551997 | -0.81837385 | 0.236231804273372 | 0.747462589165905 | 0.441148445047809 | 1.26646784880325 | 0.999332001504091 |
| ebi-fl187-GCST90094853 | prot-a-1392 | Serine protease HTRA2, mitochondrial \|\| id:prot-a-1392 | F-sweetened coffee drink liking (derived food-liking factor) \|\| id:ebi-fl187-GCST90094853 | Weighted median | 4 | 0.0424370841884991 | 0.296188454206624 | 0.886071177019985 | -0.538092286 | 0.622966454433481 | 1.04335041107084 | 0.58386103043423 | 1.86445065441702 | 0.999332001504091 |
| ebi-fl187-GCST90094853 | prot-a-1416 | Interferon-induced protein with tetratricopeptide repeats 2 \|\| id:prot-a-1416 | F-sweetened coffee drink liking (derived food-liking factor) \|\| id:ebi-fl187-GCST90094853 | Weighted median | 4 | 0.06734030450068 | 0.268002369692251 | 0.801607245483338 | -0.45794434 | 0.592624949097491 | 1.06965942616144 | 0.632582684718923 | 1.80873001366518 | 0.999332001504091 |
| ebi-fl187-GCST90094853 | prot-a-1428 | Interferon gamma \|\| id:prot-a-1428 | F-sweetened coffee drink liking (derived food-liking factor) \|\| id:ebi-fl187-GCST90094853 | Weighted median | 4 | -0.020573586 | 0.291550467994275 | 0.943743074754836 | -0.592012503 | 0.550865331688925 | 0.979636606694416 | 0.553212821301468 | 1.7347535057449 | 0.999332001504091 |
| ebi-fl187-GCST90094853 | prot-a-1483 | Interleukin-17C \|\| id:prot-a-1483 | F-sweetened coffee drink liking (derived food-liking factor) \|\| id:ebi-fl187-GCST90094853 | Weighted median | 4 | 0.41384060924597 | 0.295665018382428 | 0.161604913544072 | -0.165662827 | 0.993344045275528 | 1.51261601066256 | 0.847331883646597 | 2.70024914661065 | 0.999332001504091 |
| ebi-fl187-GCST90094853 | prot-a-1503 | Interleukin-1 receptor-like 2 \|\| id:prot-a-1503 | F-sweetened coffee drink liking (derived food-liking factor) \|\| id:ebi-fl187-GCST90094853 | Weighted median | 4 | 0.060095526550842 | 0.266327323654122 | 0.821477253326559 | -0.461906028 | 0.582097080912922 | 1.06193798497316 | 0.630081547302045 | 1.78978782787344 | 0.999332001504091 |
| ebi-fl187-GCST90094853 | prot-a-1522 | Interleukin-31 receptor subunit alpha \|\| id:prot-a-1522 | F-sweetened coffee drink liking (derived food-liking factor) \|\| id:ebi-fl187-GCST90094853 | Weighted median | 4 | 0.0395192497769495 | 0.272936078997822 | 0.884874262625763 | -0.495435465 | 0.57447396461268 | 1.04031052443777 | 0.609305518253354 | 1.77619593920365 | 0.999332001504091 |
| ebi-fl187-GCST90094853 | prot-a-1540 | Interleukin-6 receptor subunit alpha \|\| id:prot-a-1540 | F-sweetened coffee drink liking (derived food-liking factor) \|\| id:ebi-fl187-GCST90094853 | Weighted median | 4 | 0.429339905764963 | 0.277768209365071 | 0.122182617940836 | -0.115085785 | 0.973765596120502 | 1.53624312355585 | 0.89128968170681 | 2.64789661892323 | 0.999332001504091 |
| ebi-fl187-GCST90094853 | prot-a-1541 | Interleukin-6 receptor subunit alpha \|\| id:prot-a-1541 | F-sweetened coffee drink liking (derived food-liking factor) \|\| id:ebi-fl187-GCST90094853 | Weighted median | 4 | 0.567230863823649 | 0.274060387059932 | 0.0384776839801992 | 0.0300725051861828 | 1.10438922246112 | 1.76337725255627 | 1.03052924995997 | 3.01738095736117 | 0.999332001504091 |
| ebi-fl187-GCST90094853 | prot-a-1545 | Interleukin-9 \|\| id:prot-a-1545 | F-sweetened coffee drink liking (derived food-liking factor) \|\| id:ebi-fl187-GCST90094853 | Weighted median | 4 | -0.374338418 | 0.303833370819226 | 0.217929776037687 | -0.969851825 | 0.221174988893372 | 0.687744127510173 | 0.379139213001236 | 1.24754171740919 | 0.999332001504091 |
| ebi-fl187-GCST90094853 | prot-a-1548 | Inositol monophosphatase 3 \|\| id:prot-a-1548 | F-sweetened coffee drink liking (derived food-liking factor) \|\| id:ebi-fl187-GCST90094853 | Weighted median | 4 | 0.154524244076549 | 0.299651298944658 | 0.606077780398235 | -0.432792302 | 0.741840790008078 | 1.16710257303862 | 0.648695210605995 | 2.09979724487391 | 0.999332001504091 |
| ebi-fl187-GCST90094853 | prot-a-1555 | Inhibin beta A chain:Inhibin beta B chain heterodimer \|\| id:prot-a-1555 | F-sweetened coffee drink liking (derived food-liking factor) \|\| id:ebi-fl187-GCST90094853 | Weighted median | 4 | -0.375248792 | 0.292199161500077 | 0.199064050198441 | -0.947959148 | 0.197461564993159 | 0.68711830829696 | 0.387531110563162 | 1.21830623846115 | 0.999332001504091 |
| ebi-fl187-GCST90094853 | prot-a-1603 | Junctophilin-3 \|\| id:prot-a-1603 | F-sweetened coffee drink liking (derived food-liking factor) \|\| id:ebi-fl187-GCST90094853 | Weighted median | 4 | 0.128140106934356 | 0.311418263016713 | 0.680725970799316 | -0.482239689 | 0.738519902447113 | 1.13671225280976 | 0.617399060525162 | 2.09283562010731 | 0.999332001504091 |
| ebi-fl187-GCST90094853 | prot-a-1627 | UPF0258 protein KIAA1024 \|\| id:prot-a-1627 | F-sweetened coffee drink liking (derived food-liking factor) \|\| id:ebi-fl187-GCST90094853 | Weighted median | 4 | -0.047544487 | 0.285602301689522 | 0.867786341908592 | -0.607324998 | 0.512236024536677 | 0.953568051021893 | 0.544806279433996 | 1.66901899308937 | 0.999332001504091 |
| ebi-fl187-GCST90094853 | prot-a-1666 | Kallikrein-7 \|\| id:prot-a-1666 | F-sweetened coffee drink liking (derived food-liking factor) \|\| id:ebi-fl187-GCST90094853 | Weighted median | 4 | 0.00610238614277835 | 0.287246824257677 | 0.98305069699219 | -0.556901389 | 0.569106161687825 | 1.00612104363353 | 0.572981763341931 | 1.76668721276236 | 0.999332001504091 |
| ebi-fl187-GCST90094853 | prot-a-1678 | Importin subunit alpha-3 \|\| id:prot-a-1678 | F-sweetened coffee drink liking (derived food-liking factor) \|\| id:ebi-fl187-GCST90094853 | Weighted median | 4 | 0.244089841120439 | 0.277410698911659 | 0.37892097023334 | -0.299635129 | 0.787814810987291 | 1.27645900384529 | 0.741088573273523 | 2.19858684543006 | 0.999332001504091 |
| ebi-fl187-GCST90094853 | prot-a-1718 | Protein LDOC1 \|\| id:prot-a-1718 | F-sweetened coffee drink liking (derived food-liking factor) \|\| id:ebi-fl187-GCST90094853 | Weighted median | 4 | -0.367913268 | 0.332062293450446 | 0.267877102526014 | -1.018755363 | 0.282928827500195 | 0.692177213232899 | 0.361044029459171 | 1.32701071178645 | 0.999332001504091 |
| ebi-fl187-GCST90094853 | prot-a-1723 | Leptin \|\| id:prot-a-1723 | F-sweetened coffee drink liking (derived food-liking factor) \|\| id:ebi-fl187-GCST90094853 | Weighted median | 4 | 0.0468489814345969 | 0.27130613175661 | 0.862903432933167 | -0.484911037 | 0.578608999677553 | 1.04796373514893 | 0.615751973588518 | 1.78355577780283 | 0.999332001504091 |
| ebi-fl187-GCST90094853 | prot-a-1730 | Galectin-7 \|\| id:prot-a-1730 | F-sweetened coffee drink liking (derived food-liking factor) \|\| id:ebi-fl187-GCST90094853 | Weighted median | 4 | 0.182239865903558 | 0.290750597648913 | 0.530796225630797 | -0.387631305 | 0.752111037295428 | 1.19990197493546 | 0.678662516287603 | 2.12147380310579 | 0.999332001504091 |
| ebi-fl187-GCST90094853 | prot-a-1753 | Lipase member N \|\| id:prot-a-1753 | F-sweetened coffee drink liking (derived food-liking factor) \|\| id:ebi-fl187-GCST90094853 | Weighted median | 4 | 0.27736140007627 | 0.315216530725102 | 0.378909403265375 | -0.340463 | 0.895185800297469 | 1.31964320402061 | 0.711440849279036 | 2.44779054742577 | 0.999332001504091 |
| ebi-fl187-GCST90094853 | prot-a-1756 | VIP36-like protein \|\| id:prot-a-1756 | F-sweetened coffee drink liking (derived food-liking factor) \|\| id:ebi-fl187-GCST90094853 | Weighted median | 4 | -0.008710739 | 0.283683316709543 | 0.975504122293418 | -0.564730039 | 0.547308562154867 | 0.991327089969672 | 0.568513602346712 | 1.72859434717344 | 0.999332001504091 |
| ebi-fl187-GCST90094853 | prot-a-1760 | Ig Kappa chain V-I region HK102- like \|\| id:prot-a-1760 | F-sweetened coffee drink liking (derived food-liking factor) \|\| id:ebi-fl187-GCST90094853 | Weighted median | 4 | -0.318322255 | 0.278299787845001 | 0.252702181366396 | -0.863789839 | 0.227145329140201 | 0.727368352527923 | 0.421561401143071 | 1.25501224453808 | 0.999332001504091 |
| ebi-fl187-GCST90094853 | prot-a-179 | Neutral ceramidase \|\| id:prot-a-179 | F-sweetened coffee drink liking (derived food-liking factor) \|\| id:ebi-fl187-GCST90094853 | Weighted median | 4 | 0.146979869060334 | 0.281825683990061 | 0.601999292671375 | -0.405398472 | 0.699358209680853 | 1.15833064451083 | 0.666711092512162 | 2.01246071511611 | 0.999332001504091 |
| ebi-fl187-GCST90094853 | prot-a-1809 | Lactotransferrin \|\| id:prot-a-1809 | F-sweetened coffee drink liking (derived food-liking factor) \|\| id:ebi-fl187-GCST90094853 | Weighted median | 4 | 0.289506978471045 | 0.271972222691312 | 0.287114661382275 | -0.243558578 | 0.822572534946017 | 1.33576876289817 | 0.783833559282116 | 2.27634829716739 | 0.999332001504091 |
| ebi-fl187-GCST90094853 | prot-a-1818 | Lysophospholipase-like protein 1 \|\| id:prot-a-1818 | F-sweetened coffee drink liking (derived food-liking factor) \|\| id:ebi-fl187-GCST90094853 | Weighted median | 4 | -0.47568311 | 0.331898910763463 | 0.151795877138898 | -1.126204975 | 0.174838755324293 | 0.621460385770694 | 0.324261504891471 | 1.19105414998775 | 0.999332001504091 |
| ebi-fl187-GCST90094853 | prot-a-1852 | MAP kinase-activated protein kinase 3 \|\| id:prot-a-1852 | F-sweetened coffee drink liking (derived food-liking factor) \|\| id:ebi-fl187-GCST90094853 | Weighted median | 4 | 0.0639901618804741 | 0.288283396163924 | 0.824337516975382 | -0.501045295 | 0.629025618361766 | 1.06608191046865 | 0.605896987733453 | 1.87578196102285 | 0.999332001504091 |
| ebi-fl187-GCST90094853 | prot-a-1856 | Megakaryocyte-associated tyrosine-protein kinase \|\| id:prot-a-1856 | F-sweetened coffee drink liking (derived food-liking factor) \|\| id:ebi-fl187-GCST90094853 | Weighted median | 4 | -0.013686341 | 0.289278980288155 | 0.962264637068851 | -0.580673142 | 0.553300460706523 | 0.986406891481772 | 0.5595216022684 | 1.73898300193956 | 0.999332001504091 |
| ebi-fl187-GCST90094853 | prot-a-1890 | Beta-1,3-N-acetylglucosaminyltransferase manic fringe \|\| id:prot-a-1890 | F-sweetened coffee drink liking (derived food-liking factor) \|\| id:ebi-fl187-GCST90094853 | Weighted median | 4 | -0.219922129 | 0.29449974950536 | 0.45520537837626 | -0.797141638 | 0.357297380335453 | 0.802581293581796 | 0.450615146388809 | 1.42946090021493 | 0.999332001504091 |
| ebi-fl187-GCST90094853 | prot-a-1919 | Matrilysin \|\| id:prot-a-1919 | F-sweetened coffee drink liking (derived food-liking factor) \|\| id:ebi-fl187-GCST90094853 | Weighted median | 4 | 0.0826511085976486 | 0.296129210893844 | 0.780164336310292 | -0.497762145 | 0.663064361949582 | 1.08616278957502 | 0.607889507416297 | 1.940730332 | 0.999332001504091 |
| ebi-fl187-GCST90094853 | prot-a-1923 | Multimerin-2 \|\| id:prot-a-1923 | F-sweetened coffee drink liking (derived food-liking factor) \|\| id:ebi-fl187-GCST90094853 | Weighted median | 4 | 0.0681272812743288 | 0.288192335006057 | 0.81312602965328 | -0.496729695 | 0.6329842578862 | 1.07050155460984 | 0.60851744668566 | 1.88322222257345 | 0.999332001504091 |
| ebi-fl187-GCST90094853 | prot-a-1948 | Mesothelin \|\| id:prot-a-1948 | F-sweetened coffee drink liking (derived food-liking factor) \|\| id:ebi-fl187-GCST90094853 | Weighted median | 4 | 0.214674885907816 | 0.271648522550602 | 0.429371805453131 | -0.317756218 | 0.747105990106995 | 1.23945886591008 | 0.727780186287864 | 2.11088225432324 | 0.999332001504091 |
| ebi-fl187-GCST90094853 | prot-a-195 | ATPase family AAA domain-containing protein 1 \|\| id:prot-a-195 | F-sweetened coffee drink liking (derived food-liking factor) \|\| id:ebi-fl187-GCST90094853 | Weighted median | 4 | -0.202909707 | 0.28804583913177 | 0.481161620779428 | -0.767479552 | 0.361660137452869 | 0.816351948764116 | 0.464181540617609 | 1.43571091466555 | 0.999332001504091 |
| ebi-fl187-GCST90094853 | prot-a-1966 | Mucin-1 \|\| id:prot-a-1966 | F-sweetened coffee drink liking (derived food-liking factor) \|\| id:ebi-fl187-GCST90094853 | Weighted median | 4 | -0.148954842 | 0.295164401312693 | 0.613804382308262 | -0.727477069 | 0.429567384264183 | 0.861608022249563 | 0.483126348287264 | 1.53659262558662 | 0.999332001504091 |
| ebi-fl187-GCST90094853 | prot-a-1986 | Myocardial zonula adherens protein \|\| id:prot-a-1986 | F-sweetened coffee drink liking (derived food-liking factor) \|\| id:ebi-fl187-GCST90094853 | Weighted median | 4 | -0.267350191 | 0.27937492571398 | 0.338588236519432 | -0.814925045 | 0.280224663629781 | 0.765404986766333 | 0.442672506560773 | 1.32342710487791 | 0.999332001504091 |
| ebi-fl187-GCST90094853 | prot-a-2071 | Epididymal secretory protein E1 \|\| id:prot-a-2071 | F-sweetened coffee drink liking (derived food-liking factor) \|\| id:ebi-fl187-GCST90094853 | Weighted median | 4 | 0.314172372322721 | 0.286065102988634 | 0.272093243305728 | -0.24651523 | 0.874859974180444 | 1.36912571558836 | 0.781519459268412 | 2.3985394130046 | 0.999332001504091 |
| ebi-fl187-GCST90094853 | prot-a-2098 | Neuritin \|\| id:prot-a-2098 | F-sweetened coffee drink liking (derived food-liking factor) \|\| id:ebi-fl187-GCST90094853 | Weighted median | 4 | -0.708170969 | 0.288897526813886 | 0.0142346108681788 | -1.274410121 | -0.141931816 | 0.492544252979723 | 0.279595847251317 | 0.867680416316381 | 0.999332001504091 |
| ebi-fl187-GCST90094853 | prot-a-2115 | Neurotrophin-4 \|\| id:prot-a-2115 | F-sweetened coffee drink liking (derived food-liking factor) \|\| id:ebi-fl187-GCST90094853 | Weighted median | 4 | -0.161495838 | 0.279167803690107 | 0.562933212691338 | -0.708664733 | 0.385673056975134 | 0.850870072560168 | 0.492301111972999 | 1.47060378855747 | 0.999332001504091 |
| ebi-fl187-GCST90094853 | prot-a-2118 | Netrin-1 \|\| id:prot-a-2118 | F-sweetened coffee drink liking (derived food-liking factor) \|\| id:ebi-fl187-GCST90094853 | Weighted median | 4 | -0.30842699 | 0.286819754199662 | 0.282225287018623 | -0.870593708 | 0.25373972814463 | 0.734601583439252 | 0.418702888051408 | 1.28883631279181 | 0.999332001504091 |
| ebi-fl187-GCST90094853 | prot-a-2120 | Netrin-G1 \|\| id:prot-a-2120 | F-sweetened coffee drink liking (derived food-liking factor) \|\| id:ebi-fl187-GCST90094853 | Weighted median | 4 | -0.162524492 | 0.310336076710587 | 0.600483879178981 | -0.770783202 | 0.445734218533354 | 0.849995272039771 | 0.462650577440742 | 1.5616363573706 | 0.999332001504091 |
| ebi-fl187-GCST90094853 | prot-a-2145 | Olfactomedin-like protein 3 \|\| id:prot-a-2145 | F-sweetened coffee drink liking (derived food-liking factor) \|\| id:ebi-fl187-GCST90094853 | Weighted median | 4 | 0.0800681571246066 | 0.277693970622317 | 0.77309234091174 | -0.464212025 | 0.624348339544349 | 1.08336090392282 | 0.62863025482084 | 1.8670288920201 | 0.999332001504091 |
| ebi-fl187-GCST90094853 | prot-a-2162 | Prolyl 4-hydroxylase subunit alpha-2 \|\| id:prot-a-2162 | F-sweetened coffee drink liking (derived food-liking factor) \|\| id:ebi-fl187-GCST90094853 | Weighted median | 4 | -0.11297906 | 0.275626586318979 | 0.681880043606436 | -0.65320717 | 0.427249048819995 | 0.893169362560467 | 0.520374169438762 | 1.53303441459646 | 0.999332001504091 |
| ebi-fl187-GCST90094853 | prot-a-2168 | Polyadenylate-binding protein-interacting protein 1 \|\| id:prot-a-2168 | F-sweetened coffee drink liking (derived food-liking factor) \|\| id:ebi-fl187-GCST90094853 | Weighted median | 4 | -0.100916053 | 0.27678449630736 | 0.715408867492576 | -0.643413665 | 0.441581560042347 | 0.904008918790369 | 0.525495492901799 | 1.55516486114801 | 0.999332001504091 |
| ebi-fl187-GCST90094853 | prot-a-219 | Beta-1,4-galactosyltransferase 5 \|\| id:prot-a-219 | F-sweetened coffee drink liking (derived food-liking factor) \|\| id:ebi-fl187-GCST90094853 | Weighted median | 4 | 0.0800466238997987 | 0.276807249156163 | 0.772445008310698 | -0.462495584 | 0.622588832245878 | 1.08333757592009 | 0.629710188024635 | 1.86374672939944 | 0.999332001504091 |
| ebi-fl187-GCST90094853 | prot-a-2190 | Pyruvate carboxylase, mitochondrial \|\| id:prot-a-2190 | F-sweetened coffee drink liking (derived food-liking factor) \|\| id:ebi-fl187-GCST90094853 | Weighted median | 4 | 0.139125574812857 | 0.276618328550127 | 0.614998443905508 | -0.403046349 | 0.681297498771105 | 1.14926841010808 | 0.668281124346739 | 1.97644049839577 | 0.999332001504091 |
| ebi-fl187-GCST90094853 | prot-a-2197 | Protocadherin-9 \|\| id:prot-a-2197 | F-sweetened coffee drink liking (derived food-liking factor) \|\| id:ebi-fl187-GCST90094853 | Weighted median | 4 | -0.021888789 | 0.273525628994795 | 0.936217661184543 | -0.557999022 | 0.514221443615178 | 0.978349031965623 | 0.572353184892352 | 1.67233598696248 | 0.999332001504091 |
| ebi-fl187-GCST90094853 | prot-a-2198 | Protocadherin alpha-4 \|\| id:prot-a-2198 | F-sweetened coffee drink liking (derived food-liking factor) \|\| id:ebi-fl187-GCST90094853 | Weighted median | 4 | 0.221881496235954 | 0.282932897948046 | 0.432911324005268 | -0.332666984 | 0.776429976214125 | 1.24842342622291 | 0.717008930032705 | 2.17369824260217 | 0.999332001504091 |
| ebi-fl187-GCST90094853 | prot-a-2254 | Placenta growth factor \|\| id:prot-a-2254 | F-sweetened coffee drink liking (derived food-liking factor) \|\| id:ebi-fl187-GCST90094853 | Weighted median | 4 | -0.028892532 | 0.293786441042004 | 0.921658064228657 | -0.604713956 | 0.546928892456135 | 0.971520866274447 | 0.546230650061286 | 1.7279381768503 | 0.999332001504091 |
| ebi-fl187-GCST90094853 | prot-a-2261 | Membrane-associated progesterone receptor component 2 \|\| id:prot-a-2261 | F-sweetened coffee drink liking (derived food-liking factor) \|\| id:ebi-fl187-GCST90094853 | Weighted median | 4 | 0.618335568004538 | 0.298628576845587 | 0.0383976840133128 | 0.0330235573871881 | 1.20364757862189 | 1.85583655603388 | 1.03357488727832 | 3.33224942392033 | 0.999332001504091 |
| ebi-fl187-GCST90094853 | prot-a-2268 | Polymeric immunoglobulin receptor \|\| id:prot-a-2268 | F-sweetened coffee drink liking (derived food-liking factor) \|\| id:ebi-fl187-GCST90094853 | Weighted median | 4 | 0.300762662485143 | 0.284368718376782 | 0.290214897970543 | -0.256600026 | 0.858125350503636 | 1.35088868692403 | 0.773677603174081 | 2.35873474554843 | 0.999332001504091 |
| ebi-fl187-GCST90094853 | prot-a-2283 | Group 10 secretory phospholipase A2 \|\| id:prot-a-2283 | F-sweetened coffee drink liking (derived food-liking factor) \|\| id:ebi-fl187-GCST90094853 | Weighted median | 4 | -0.201384765 | 0.29953058219903 | 0.501370727038938 | -0.788464706 | 0.385695176166592 | 0.817597788059114 | 0.45454211560802 | 1.47063631748398 | 0.999332001504091 |
| ebi-fl187-GCST90094853 | prot-a-2298 | Inactive phospholipase D5 \|\| id:prot-a-2298 | F-sweetened coffee drink liking (derived food-liking factor) \|\| id:ebi-fl187-GCST90094853 | Weighted median | 4 | 0.139942418270358 | 0.271974468896703 | 0.606872767025612 | -0.393127541 | 0.673012377307896 | 1.15020756600925 | 0.674942659391791 | 1.96013309648717 | 0.999332001504091 |
| ebi-fl187-GCST90094853 | prot-a-2304 | Procollagen-lysine,2-oxoglutarate 5-dioxygenase 2 \|\| id:prot-a-2304 | F-sweetened coffee drink liking (derived food-liking factor) \|\| id:ebi-fl187-GCST90094853 | Weighted median | 4 | -0.156399635 | 0.270443088345352 | 0.563055612074964 | -0.686468088 | 0.373668818381286 | 0.855217347476662 | 0.503350723754504 | 1.4530558453746 | 0.999332001504091 |
| ebi-fl187-GCST90094853 | prot-a-2331 | Periostin \|\| id:prot-a-2331 | F-sweetened coffee drink liking (derived food-liking factor) \|\| id:ebi-fl187-GCST90094853 | Weighted median | 4 | -0.014361704 | 0.273347388239326 | 0.958098329113006 | -0.550122585 | 0.521399176639156 | 0.985740933028895 | 0.576879089173326 | 1.6843827507097 | 0.999332001504091 |
| ebi-fl187-GCST90094853 | prot-a-2333 | Periostin \|\| id:prot-a-2333 | F-sweetened coffee drink liking (derived food-liking factor) \|\| id:ebi-fl187-GCST90094853 | Weighted median | 4 | -0.104124466 | 0.297246486979884 | 0.726116035312672 | -0.68672758 | 0.47847864851789 | 0.901113132527468 | 0.503220124956842 | 1.61361765426831 | 0.999332001504091 |
| ebi-fl187-GCST90094853 | prot-a-2367 | cAMP-dependent protein kinase catalytic subunit alpha \|\| id:prot-a-2367 | F-sweetened coffee drink liking (derived food-liking factor) \|\| id:ebi-fl187-GCST90094853 | Weighted median | 4 | -0.121956727 | 0.286624837146221 | 0.670477666602243 | -0.683741408 | 0.439827953937119 | 0.885186672472272 | 0.504725073089122 | 1.55244010432622 | 0.999332001504091 |
| ebi-fl187-GCST90094853 | prot-a-2385 | Phosphoribosyl pyrophosphate synthase-associated protein 1 \|\| id:prot-a-2385 | F-sweetened coffee drink liking (derived food-liking factor) \|\| id:ebi-fl187-GCST90094853 | Weighted median | 4 | 0.156506895559108 | 0.292780103114933 | 0.592958015217867 | -0.417342107 | 0.730355897664377 | 1.16941882608788 | 0.658795503133833 | 2.0758192554495 | 0.999332001504091 |
| ebi-fl187-GCST90094853 | prot-a-2393 | Trypsin-3 \|\| id:prot-a-2393 | F-sweetened coffee drink liking (derived food-liking factor) \|\| id:ebi-fl187-GCST90094853 | Weighted median | 4 | 0.391421826629119 | 0.281953627344578 | 0.165061251874146 | -0.161207283 | 0.944050936224492 | 1.47908229813941 | 0.851115631048644 | 2.57037277293797 | 0.999332001504091 |
| ebi-fl187-GCST90094853 | prot-a-2405 | Pregnancy-specific beta-1-glycoprotein 6 \|\| id:prot-a-2405 | F-sweetened coffee drink liking (derived food-liking factor) \|\| id:ebi-fl187-GCST90094853 | Weighted median | 4 | 0.0117729041281687 | 0.272405444220135 | 0.965527505960944 | -0.522141767 | 0.545687574799633 | 1.01184247752287 | 0.593248586359886 | 1.725794587 | 0.999332001504091 |
| ebi-fl187-GCST90094853 | prot-a-244 | Beclin-1 \|\| id:prot-a-244 | F-sweetened coffee drink liking (derived food-liking factor) \|\| id:ebi-fl187-GCST90094853 | Weighted median | 4 | -0.180453427 | 0.301010410486626 | 0.548844636142583 | -0.770433831 | 0.409526978021031 | 0.834891563586371 | 0.462812242414128 | 1.5061051957307 | 0.999332001504091 |
| ebi-fl187-GCST90094853 | prot-a-2454 | tRNA pseudouridine synthase A, mitochondrial \|\| id:prot-a-2454 | F-sweetened coffee drink liking (derived food-liking factor) \|\| id:ebi-fl187-GCST90094853 | Weighted median | 4 | -0.318150606 | 0.298591477379315 | 0.286647624833999 | -0.903389902 | 0.267088689634282 | 0.727493215299208 | 0.405193761959566 | 1.30615628371691 | 0.999332001504091 |
| ebi-fl187-GCST90094853 | prot-a-2466 | Glutaminyl-peptide cyclotransferase-like protein \|\| id:prot-a-2466 | F-sweetened coffee drink liking (derived food-liking factor) \|\| id:ebi-fl187-GCST90094853 | Weighted median | 4 | -0.273974997 | 0.292920710887012 | 0.349622712361414 | -0.84809959 | 0.300149596672458 | 0.760351086335315 | 0.428227967863669 | 1.35006075706701 | 0.999332001504091 |
| ebi-fl187-GCST90094853 | prot-a-2474 | Ras-related protein Rab-27A \|\| id:prot-a-2474 | F-sweetened coffee drink liking (derived food-liking factor) \|\| id:ebi-fl187-GCST90094853 | Weighted median | 4 | 0.298937382917024 | 0.277459061521029 | 0.281296791839651 | -0.244882378 | 0.84275714349824 | 1.34842518637755 | 0.78279660719248 | 2.32276234535372 | 0.999332001504091 |
| ebi-fl187-GCST90094853 | prot-a-2490 | Rap1 GTPase-GDP dissociation stimulator 1 \|\| id:prot-a-2490 | F-sweetened coffee drink liking (derived food-liking factor) \|\| id:ebi-fl187-GCST90094853 | Weighted median | 4 | -0.33947254 | 0.288359998947467 | 0.239094725569003 | -0.904658138 | 0.225713058086532 | 0.712145852273181 | 0.404680206328946 | 1.25321601348017 | 0.999332001504091 |
| ebi-fl187-GCST90094853 | prot-a-2545 | Receptor-interacting serine/threonine-protein kinase 2 \|\| id:prot-a-2545 | F-sweetened coffee drink liking (derived food-liking factor) \|\| id:ebi-fl187-GCST90094853 | Weighted median | 4 | -0.591746729 | 0.305677840332068 | 0.0528859621730563 | -1.190875296 | 0.00738183820331384 | 0.553359870426493 | 0.303955096946551 | 1.00740915113613 | 0.999332001504091 |
| ebi-fl187-GCST90094853 | prot-a-2610 | RUN and FYVE domain-containing protein 1 \|\| id:prot-a-2610 | F-sweetened coffee drink liking (derived food-liking factor) \|\| id:ebi-fl187-GCST90094853 | Weighted median | 4 | -0.00905195 | 0.277014332774159 | 0.973932301896195 | -0.552000042 | 0.533896142287863 | 0.99098889561255 | 0.575797039599406 | 1.70556450222568 | 0.999332001504091 |
| ebi-fl187-GCST90094853 | prot-a-2627 | Serine--tRNA ligase, mitochondrial \|\| id:prot-a-2627 | F-sweetened coffee drink liking (derived food-liking factor) \|\| id:ebi-fl187-GCST90094853 | Weighted median | 4 | -0.005973871 | 0.293742694184831 | 0.983774469120625 | -0.581709552 | 0.569761809274779 | 0.994043936763102 | 0.558942008936804 | 1.76784591677954 | 0.999332001504091 |
| ebi-fl187-GCST90094853 | prot-a-264 | BolA-like protein 2 \|\| id:prot-a-264 | F-sweetened coffee drink liking (derived food-liking factor) \|\| id:ebi-fl187-GCST90094853 | Weighted median | 4 | -0.217472395 | 0.30346936449138 | 0.473608236030709 | -0.81227235 | 0.37732755925721 | 0.804549814093525 | 0.443848340855765 | 1.4583819376454 | 0.999332001504091 |
| ebi-fl187-GCST90094853 | prot-a-2646 | Secretoglobin family 1C member 1 \|\| id:prot-a-2646 | F-sweetened coffee drink liking (derived food-liking factor) \|\| id:ebi-fl187-GCST90094853 | Weighted median | 4 | -0.190254055 | 0.27382545611751 | 0.487180355805243 | -0.726951949 | 0.346443839339544 | 0.826749067814652 | 0.483380114334472 | 1.41403007873725 | 0.999332001504091 |
| ebi-fl187-GCST90094853 | prot-a-2672 | Semaphorin-3E \|\| id:prot-a-2672 | F-sweetened coffee drink liking (derived food-liking factor) \|\| id:ebi-fl187-GCST90094853 | Weighted median | 4 | 0.261748412118616 | 0.285095224628254 | 0.358561949430727 | -0.297038228 | 0.820535052389995 | 1.29919963842404 | 0.743015607704858 | 2.27171499895548 | 0.999332001504091 |
| ebi-fl187-GCST90094853 | prot-a-2701 | Plasma protease C1 inhibitor \|\| id:prot-a-2701 | F-sweetened coffee drink liking (derived food-liking factor) \|\| id:ebi-fl187-GCST90094853 | Weighted median | 4 | 0.121919257221928 | 0.264511003960824 | 0.644853709390524 | -0.396522311 | 0.640360824985143 | 1.12966288597816 | 0.672655269230668 | 1.89716530046075 | 0.999332001504091 |
| ebi-fl187-GCST90094853 | prot-a-2738 | Signaling threshold-regulating transmembrane adapter 1 \|\| id:prot-a-2738 | F-sweetened coffee drink liking (derived food-liking factor) \|\| id:ebi-fl187-GCST90094853 | Weighted median | 4 | 0.189995167184159 | 0.289353039484717 | 0.51142489802475 | -0.37713679 | 0.757127124574205 | 1.20924375359076 | 0.685822253712038 | 2.13214203488394 | 0.999332001504091 |
| ebi-fl187-GCST90094853 | prot-a-274 | Serine/threonine-protein kinase BRSK2 \|\| id:prot-a-274 | F-sweetened coffee drink liking (derived food-liking factor) \|\| id:ebi-fl187-GCST90094853 | Weighted median | 4 | -0.389246772 | 0.290486193597685 | 0.180250635478383 | -0.958599712 | 0.180106167090028 | 0.677567044561893 | 0.383429421925074 | 1.19734447495281 | 0.999332001504091 |
| ebi-fl187-GCST90094853 | prot-a-2749 | Mitochondrial glutamate carrier 2 \|\| id:prot-a-2749 | F-sweetened coffee drink liking (derived food-liking factor) \|\| id:ebi-fl187-GCST90094853 | Weighted median | 4 | -0.131426005 | 0.302233458069312 | 0.663671823005579 | -0.723803583 | 0.460951572430923 | 0.876844154483095 | 0.484904369778498 | 1.58558206353633 | 0.999332001504091 |
| ebi-fl187-GCST90094853 | prot-a-2760 | Sodium-coupled monocarboxylate transporter 1 \|\| id:prot-a-2760 | F-sweetened coffee drink liking (derived food-liking factor) \|\| id:ebi-fl187-GCST90094853 | Weighted median | 4 | -0.461692113 | 0.265227603140152 | 0.0817292854790639 | -0.981538215 | 0.0581539888945063 | 0.630216345330474 | 0.374734233319238 | 1.0598781926159 | 0.999332001504091 |
| ebi-fl187-GCST90094853 | prot-a-2777 | Small integral membrane protein 10 \|\| id:prot-a-2777 | F-sweetened coffee drink liking (derived food-liking factor) \|\| id:ebi-fl187-GCST90094853 | Weighted median | 4 | 0.115080657670621 | 0.303869102856081 | 0.704897386030562 | -0.480502784 | 0.710664099268539 | 1.12196392891951 | 0.618472355660802 | 2.03534247937652 | 0.999332001504091 |
| ebi-fl187-GCST90094853 | prot-a-2799 | Superoxide dismutase [Mn], mitochondrial \|\| id:prot-a-2799 | F-sweetened coffee drink liking (derived food-liking factor) \|\| id:ebi-fl187-GCST90094853 | Weighted median | 4 | 0.182594266769585 | 0.285428674069214 | 0.5223550255549 | -0.376845934 | 0.742034467945245 | 1.20032729659725 | 0.686021758104269 | 2.10020396865821 | 0.999332001504091 |
| ebi-fl187-GCST90094853 | prot-a-2805 | Protein SPACA7 \|\| id:prot-a-2805 | F-sweetened coffee drink liking (derived food-liking factor) \|\| id:ebi-fl187-GCST90094853 | Weighted median | 4 | 0.26788414482615 | 0.26372246688996 | 0.309733913875613 | -0.24901189 | 0.784780179930472 | 1.30719568585829 | 0.779570704017067 | 2.19192505865269 | 0.999332001504091 |
| ebi-fl187-GCST90094853 | prot-a-2811 | SPARC-like protein 1 \|\| id:prot-a-2811 | F-sweetened coffee drink liking (derived food-liking factor) \|\| id:ebi-fl187-GCST90094853 | Weighted median | 4 | 0.0713435605846163 | 0.290859434629991 | 0.806235488057023 | -0.498740931 | 0.641428052459398 | 1.07395012942868 | 0.607294804442246 | 1.89919108818849 | 0.999332001504091 |
| ebi-fl187-GCST90094853 | prot-a-2831 | Sepiapterin reductase \|\| id:prot-a-2831 | F-sweetened coffee drink liking (derived food-liking factor) \|\| id:ebi-fl187-GCST90094853 | Weighted median | 4 | -0.185471812 | 0.29378167007355 | 0.527827822475314 | -0.761283886 | 0.39034026101329 | 0.830712251092985 | 0.467066382060563 | 1.47748343837449 | 0.999332001504091 |
| ebi-fl187-GCST90094853 | prot-a-2834 | SPRY domain-containing SOCS box protein 1 \|\| id:prot-a-2834 | F-sweetened coffee drink liking (derived food-liking factor) \|\| id:ebi-fl187-GCST90094853 | Weighted median | 4 | -0.0673414 | 0.276382996256826 | 0.807499985487653 | -0.609052073 | 0.474369272255539 | 0.93487597980791 | 0.543866170190388 | 1.60700029809879 | 0.999332001504091 |
| ebi-fl187-GCST90094853 | prot-a-2835 | Spectrin alpha chain, non-erythrocytic 1 \|\| id:prot-a-2835 | F-sweetened coffee drink liking (derived food-liking factor) \|\| id:ebi-fl187-GCST90094853 | Weighted median | 4 | -0.249403719 | 0.300445362693399 | 0.406474675571796 | -0.83827663 | 0.339469191468822 | 0.779265305340683 | 0.432455161728194 | 1.40420203028905 | 0.999332001504091 |
| ebi-fl187-GCST90094853 | prot-a-2859 | Sia-alpha-2,3-Gal-beta-1,4-GlcNAc-R:alpha 2,8-sialyltransferase \|\| id:prot-a-2859 | F-sweetened coffee drink liking (derived food-liking factor) \|\| id:ebi-fl187-GCST90094853 | Weighted median | 4 | -0.393277577 | 0.313786192392801 | 0.210085717819873 | -1.008298514 | 0.221743360120984 | 0.674841401155184 | 0.36483922056081 | 1.24825098577138 | 0.999332001504091 |
| ebi-fl187-GCST90094853 | prot-a-2861 | Alpha-2,8-sialyltransferase 8F \|\| id:prot-a-2861 | F-sweetened coffee drink liking (derived food-liking factor) \|\| id:ebi-fl187-GCST90094853 | Weighted median | 4 | -0.244527858 | 0.30185543730522 | 0.417892664673382 | -0.836164515 | 0.347108798991426 | 0.783074173081887 | 0.433369522058439 | 1.41497066437725 | 0.999332001504091 |
| ebi-fl187-GCST90094853 | prot-a-289 | Uncharacterized protein C17orf89 \|\| id:prot-a-289 | F-sweetened coffee drink liking (derived food-liking factor) \|\| id:ebi-fl187-GCST90094853 | Weighted median | 4 | -0.121218265 | 0.26876966961015 | 0.651981117660316 | -0.648006818 | 0.405570287278645 | 0.885840590355009 | 0.523087346893636 | 1.50015777705301 | 0.999332001504091 |
| ebi-fl187-GCST90094853 | prot-a-2921 | Tachykinin-3 \|\| id:prot-a-2921 | F-sweetened coffee drink liking (derived food-liking factor) \|\| id:ebi-fl187-GCST90094853 | Weighted median | 4 | -0.145163383 | 0.269573773730085 | 0.590237267002548 | -0.673527979 | 0.383201213581885 | 0.864880974779587 | 0.509906461229087 | 1.46697317530092 | 0.999332001504091 |
| ebi-fl187-GCST90094853 | prot-a-2928 | Tax1-binding protein 1 \|\| id:prot-a-2928 | F-sweetened coffee drink liking (derived food-liking factor) \|\| id:ebi-fl187-GCST90094853 | Weighted median | 4 | -0.24560535 | 0.292823153584202 | 0.401609748561661 | -0.819538731 | 0.328328031186863 | 0.782230871557796 | 0.440634858897442 | 1.38864441626141 | 0.999332001504091 |
| ebi-fl187-GCST90094853 | prot-a-296 | Uncharacterized protein C1orf185 \|\| id:prot-a-296 | F-sweetened coffee drink liking (derived food-liking factor) \|\| id:ebi-fl187-GCST90094853 | Weighted median | 4 | -0.27246623 | 0.300924146536318 | 0.365236783022188 | -0.862277558 | 0.317345096781949 | 0.761499144240249 | 0.422199402979012 | 1.37347647246071 | 0.999332001504091 |
| ebi-fl187-GCST90094853 | prot-a-2964 | Transforming growth factor beta-2 \|\| id:prot-a-2964 | F-sweetened coffee drink liking (derived food-liking factor) \|\| id:ebi-fl187-GCST90094853 | Weighted median | 4 | -0.009597219 | 0.286359797457441 | 0.973264265145193 | -0.570862422 | 0.55166798399107 | 0.990448687305916 | 0.565037927346321 | 1.73614646859758 | 0.999332001504091 |
| ebi-fl187-GCST90094853 | prot-a-2977 | Thymocyte nuclear protein 1 \|\| id:prot-a-2977 | F-sweetened coffee drink liking (derived food-liking factor) \|\| id:ebi-fl187-GCST90094853 | Weighted median | 4 | 0.00906733852536021 | 0.269709424097197 | 0.97318103785272 | -0.519563133 | 0.537697809755866 | 1.00910857136915 | 0.594780331295009 | 1.71206083192691 | 0.999332001504091 |
| ebi-fl187-GCST90094853 | prot-a-3015 | Transmembrane protein 70, mitochondrial \|\| id:prot-a-3015 | F-sweetened coffee drink liking (derived food-liking factor) \|\| id:ebi-fl187-GCST90094853 | Weighted median | 4 | 0.0588340066889979 | 0.295008550634609 | 0.841925198259853 | -0.519382753 | 0.637050765932832 | 1.06059917375909 | 0.594887627537334 | 1.89089595296362 | 0.999332001504091 |
| ebi-fl187-GCST90094853 | prot-a-3030 | Tumor necrosis factor alpha-induced protein 3 \|\| id:prot-a-3030 | F-sweetened coffee drink liking (derived food-liking factor) \|\| id:ebi-fl187-GCST90094853 | Weighted median | 4 | -0.44570938 | 0.309770478102734 | 0.150196569291238 | -1.052859517 | 0.16144075727441 | 0.640369849430683 | 0.348938525536933 | 1.17520283387703 | 0.999332001504091 |
| ebi-fl187-GCST90094853 | prot-a-3032 | Tumor necrosis factor receptor superfamily member 10A \|\| id:prot-a-3032 | F-sweetened coffee drink liking (derived food-liking factor) \|\| id:ebi-fl187-GCST90094853 | Weighted median | 4 | -0.119618946 | 0.288471801025475 | 0.678387699851806 | -0.685023676 | 0.445785784304367 | 0.887258465958048 | 0.504078295019247 | 1.56171688642966 | 0.999332001504091 |
| ebi-fl187-GCST90094853 | prot-a-3081 | Tumor protein p53-inducible protein 11 \|\| id:prot-a-3081 | F-sweetened coffee drink liking (derived food-liking factor) \|\| id:ebi-fl187-GCST90094853 | Weighted median | 4 | 0.0726383351948952 | 0.293509116015581 | 0.804534924576117 | -0.502639532 | 0.647916202585434 | 1.07534155338479 | 0.604931813539932 | 1.91155338594154 | 0.999332001504091 |
| ebi-fl187-GCST90094853 | prot-a-315 | Complement C4 \|\| id:prot-a-315 | F-sweetened coffee drink liking (derived food-liking factor) \|\| id:ebi-fl187-GCST90094853 | Weighted median | 4 | 0.381101780491157 | 0.290327828362305 | 0.189297479984054 | -0.187940763 | 0.950144324081274 | 1.46389659394187 | 0.828663793253931 | 2.58608286641761 | 0.999332001504091 |
| ebi-fl187-GCST90094853 | prot-a-3157 | UDP-glucuronosyltransferase 1-8 \|\| id:prot-a-3157 | F-sweetened coffee drink liking (derived food-liking factor) \|\| id:ebi-fl187-GCST90094853 | Weighted median | 4 | 0.293284526707825 | 0.28101910790661 | 0.296649105236482 | -0.257512925 | 0.84408197820478 | 1.34082423646909 | 0.772971635753525 | 2.32584166086552 | 0.999332001504091 |
| ebi-fl187-GCST90094853 | prot-a-317 | Glutaredoxin-like protein C5orf63 \|\| id:prot-a-317 | F-sweetened coffee drink liking (derived food-liking factor) \|\| id:ebi-fl187-GCST90094853 | Weighted median | 4 | -0.007234842 | 0.276930319252168 | 0.979157528642829 | -0.550018268 | 0.535548583609387 | 0.99279126634393 | 0.576939270838903 | 1.70838517734391 | 0.999332001504091 |
| ebi-fl187-GCST90094853 | prot-a-3192 | Guanine nucleotide exchange factor VAV3 \|\| id:prot-a-3192 | F-sweetened coffee drink liking (derived food-liking factor) \|\| id:ebi-fl187-GCST90094853 | Weighted median | 4 | 0.118198655409176 | 0.283262861588251 | 0.67647689843511 | -0.436996553 | 0.673393864122148 | 1.12546766940148 | 0.645973657881741 | 1.96088100406703 | 0.999332001504091 |
| ebi-fl187-GCST90094853 | prot-a-3233 | Serine/threonine-protein kinase WNK3 \|\| id:prot-a-3233 | F-sweetened coffee drink liking (derived food-liking factor) \|\| id:ebi-fl187-GCST90094853 | Weighted median | 4 | 0.0395261592467085 | 0.279652123913663 | 0.887600989622722 | -0.508592004 | 0.587644322117489 | 1.04031771245671 | 0.601341669917822 | 1.79974380122212 | 0.999332001504091 |
| ebi-fl187-GCST90094853 | prot-a-3250 | 14-3-3 protein theta \|\| id:prot-a-3250 | F-sweetened coffee drink liking (derived food-liking factor) \|\| id:ebi-fl187-GCST90094853 | Weighted median | 4 | -0.725456909 | 0.305609634690978 | 0.0176059658867253 | -1.324451793 | -0.126462025 | 0.484103327418616 | 0.265948714175046 | 0.88120761307206 | 0.999332001504091 |
| ebi-fl187-GCST90094853 | prot-a-3278 | E3 ubiquitin-protein ligase ZNRF3 \|\| id:prot-a-3278 | F-sweetened coffee drink liking (derived food-liking factor) \|\| id:ebi-fl187-GCST90094853 | Weighted median | 4 | -0.321629281 | 0.294463403614354 | 0.274720801240711 | -0.898777552 | 0.255518990376316 | 0.724966899734871 | 0.407066974001128 | 1.29113153185877 | 0.999332001504091 |
| ebi-fl187-GCST90094853 | prot-a-355 | Calpain-3 \|\| id:prot-a-355 | F-sweetened coffee drink liking (derived food-liking factor) \|\| id:ebi-fl187-GCST90094853 | Weighted median | 4 | -0.156996881 | 0.303734931164358 | 0.605234482533798 | -0.752317346 | 0.438323583851097 | 0.854706724446326 | 0.471273183198777 | 1.55010641567874 | 0.999332001504091 |
| ebi-fl187-GCST90094853 | prot-a-385 | Coiled-coil domain-containing protein 90B, mitochondrial \|\| id:prot-a-385 | F-sweetened coffee drink liking (derived food-liking factor) \|\| id:ebi-fl187-GCST90094853 | Weighted median | 4 | -0.172547255 | 0.296591964269766 | 0.560724004499666 | -0.753867505 | 0.40877299541502 | 0.841518522289987 | 0.470543201145046 | 1.50497004660542 | 0.999332001504091 |
| ebi-fl187-GCST90094853 | prot-a-390 | C-C motif chemokine 14 \|\| id:prot-a-390 | F-sweetened coffee drink liking (derived food-liking factor) \|\| id:ebi-fl187-GCST90094853 | Weighted median | 4 | -0.386214258 | 0.276937410575732 | 0.163139387058727 | -0.929011583 | 0.156583066315791 | 0.679624894728413 | 0.394943886707352 | 1.1695079049973 | 0.999332001504091 |
| ebi-fl187-GCST90094853 | prot-a-40 | Pituitary adenylate cyclase-activating polypeptide 27 \|\| id:prot-a-40 | F-sweetened coffee drink liking (derived food-liking factor) \|\| id:ebi-fl187-GCST90094853 | Weighted median | 4 | -0.179180247 | 0.318297216515877 | 0.57347999369629 | -0.803042792 | 0.44468229698227 | 0.835955207073224 | 0.447963827614011 | 1.55999450213417 | 0.999332001504091 |
| ebi-fl187-GCST90094853 | prot-a-401 | C-C motif chemokine 24 \|\| id:prot-a-401 | F-sweetened coffee drink liking (derived food-liking factor) \|\| id:ebi-fl187-GCST90094853 | Weighted median | 4 | 0.00147710531236964 | 0.295554515082091 | 0.996012395151941 | -0.577809744 | 0.580763954873268 | 1.00147819676975 | 0.56112603114193 | 1.787403405 | 0.999364451047625 |
| ebi-fl187-GCST90094853 | prot-a-412 | Cerebral cavernous malformations 2 protein \|\| id:prot-a-412 | F-sweetened coffee drink liking (derived food-liking factor) \|\| id:ebi-fl187-GCST90094853 | Weighted median | 4 | 0.0886048616940655 | 0.282706305960051 | 0.753964140572325 | -0.465499498 | 0.642709221375764 | 1.09264882360998 | 0.627821431314893 | 1.901625832 | 0.999332001504091 |
| ebi-fl187-GCST90094853 | prot-a-44 | Adhesion G protein-coupled receptor F5 \|\| id:prot-a-44 | F-sweetened coffee drink liking (derived food-liking factor) \|\| id:ebi-fl187-GCST90094853 | Weighted median | 4 | 0.106405341733755 | 0.315036691049104 | 0.735547526770574 | -0.511066573 | 0.723877256189998 | 1.11227263566316 | 0.599855448041623 | 2.06241423676996 | 0.999332001504091 |
| ebi-fl187-GCST90094853 | prot-a-48 | Adrenomedullin \|\| id:prot-a-48 | F-sweetened coffee drink liking (derived food-liking factor) \|\| id:ebi-fl187-GCST90094853 | Weighted median | 4 | -0.388106034 | 0.283537878702947 | 0.171062478545418 | -0.943840276 | 0.167628208385052 | 0.678340412389666 | 0.389130593357919 | 1.1824968864829 | 0.999332001504091 |
| ebi-fl187-GCST90094853 | prot-a-502 | Carcinoembryonic antigen-related cell adhesion molecule 1 \|\| id:prot-a-502 | F-sweetened coffee drink liking (derived food-liking factor) \|\| id:ebi-fl187-GCST90094853 | Weighted median | 4 | -0.100766547 | 0.309708594212789 | 0.744909215744182 | -0.707795391 | 0.506262298065614 | 0.904144083767806 | 0.492729276208871 | 1.6590784507511 | 0.999332001504091 |
| ebi-fl187-GCST90094853 | prot-a-518 | Complement factor B \|\| id:prot-a-518 | F-sweetened coffee drink liking (derived food-liking factor) \|\| id:ebi-fl187-GCST90094853 | Weighted median | 4 | 0.267410996729277 | 0.290730954245649 | 0.357683278949328 | -0.302421674 | 0.837243667050748 | 1.30657733500459 | 0.739026371272965 | 2.30999108923156 | 0.999332001504091 |
| ebi-fl187-GCST90094853 | prot-a-525 | Properdin \|\| id:prot-a-525 | F-sweetened coffee drink liking (derived food-liking factor) \|\| id:ebi-fl187-GCST90094853 | Weighted median | 4 | 0.178021976045327 | 0.283458658475393 | 0.529980971733138 | -0.377556995 | 0.733600946657097 | 1.19485157905874 | 0.685534128750384 | 2.08256633200971 | 0.999332001504091 |
| ebi-fl187-GCST90094853 | prot-a-533 | Chondroadherin \|\| id:prot-a-533 | F-sweetened coffee drink liking (derived food-liking factor) \|\| id:ebi-fl187-GCST90094853 | Weighted median | 4 | -0.482214224 | 0.302709160665017 | 0.111161279887953 | -1.075524179 | 0.111095731201909 | 0.617414782716402 | 0.341118901199586 | 1.11750188153222 | 0.999332001504091 |
| ebi-fl187-GCST90094853 | prot-a-535 | Coiled-coil-helix-coiled-coil-helix domain-containing protein 2 \|\| id:prot-a-535 | F-sweetened coffee drink liking (derived food-liking factor) \|\| id:ebi-fl187-GCST90094853 | Weighted median | 4 | -0.21307448 | 0.289473669967718 | 0.461684744282027 | -0.780442873 | 0.3542939131796 | 0.808095948023509 | 0.458203040565213 | 1.42517400235164 | 0.999332001504091 |
| ebi-fl187-GCST90094853 | prot-a-591 | Caseinolytic peptidase B protein homolog \|\| id:prot-a-591 | F-sweetened coffee drink liking (derived food-liking factor) \|\| id:ebi-fl187-GCST90094853 | Weighted median | 4 | -0.230838571 | 0.295565827222312 | 0.434799190746559 | -0.810147593 | 0.348470449938195 | 0.79386760861475 | 0.44479241323231 | 1.41689867286145 | 0.999332001504091 |
| ebi-fl187-GCST90094853 | prot-a-605 | Ciliary neurotrophic factor receptor subunit alpha \|\| id:prot-a-605 | F-sweetened coffee drink liking (derived food-liking factor) \|\| id:ebi-fl187-GCST90094853 | Weighted median | 4 | -0.122271597 | 0.276961758328579 | 0.658869405103093 | -0.665116643 | 0.420573449234853 | 0.884907997425786 | 0.514213544585441 | 1.522834574 | 0.999332001504091 |
| ebi-fl187-GCST90094853 | prot-a-606 | Ciliary neurotrophic factor receptor subunit alpha \|\| id:prot-a-606 | F-sweetened coffee drink liking (derived food-liking factor) \|\| id:ebi-fl187-GCST90094853 | Weighted median | 4 | 0.098456587710466 | 0.264025136552376 | 0.709218321292068 | -0.41903268 | 0.615945855353123 | 1.1034664993474 | 0.657682701890517 | 1.85140693480595 | 0.999332001504091 |
| ebi-fl187-GCST90094853 | prot-a-707 | Cancer/testis antigen 55 \|\| id:prot-a-707 | F-sweetened coffee drink liking (derived food-liking factor) \|\| id:ebi-fl187-GCST90094853 | Weighted median | 4 | 0.49423120994308 | 0.298405769590091 | 0.0976734712474104 | -0.090644098 | 1.07910651833966 | 1.63923752499677 | 0.913342713145529 | 2.94204970892387 | 0.999332001504091 |
| ebi-fl187-GCST90094853 | prot-a-719 | Dipeptidyl peptidase 1 \|\| id:prot-a-719 | F-sweetened coffee drink liking (derived food-liking factor) \|\| id:ebi-fl187-GCST90094853 | Weighted median | 4 | 0.202135968844643 | 0.288337071802472 | 0.483277732358008 | -0.363004692 | 0.767276629577488 | 1.22401442462451 | 0.695583169892971 | 2.15389241220343 | 0.999332001504091 |
| ebi-fl187-GCST90094853 | prot-a-752 | Cytochrome b5 \|\| id:prot-a-752 | F-sweetened coffee drink liking (derived food-liking factor) \|\| id:ebi-fl187-GCST90094853 | Weighted median | 4 | 0.0839399162521844 | 0.313321230229447 | 0.788773447097912 | -0.530169695 | 0.6980495275019 | 1.08756354695182 | 0.588505094833849 | 2.00982876621034 | 0.999332001504091 |
| ebi-fl187-GCST90094853 | prot-a-755 | Cytoglobin \|\| id:prot-a-755 | F-sweetened coffee drink liking (derived food-liking factor) \|\| id:ebi-fl187-GCST90094853 | Weighted median | 4 | 0.335376270491743 | 0.291455207818048 | 0.249857423414425 | -0.235875937 | 0.906628477815117 | 1.39846648791089 | 0.789878662703801 | 2.47596068884216 | 0.999332001504091 |
| ebi-fl187-GCST90094853 | prot-a-807 | Desmin \|\| id:prot-a-807 | F-sweetened coffee drink liking (derived food-liking factor) \|\| id:ebi-fl187-GCST90094853 | Weighted median | 4 | 0.0440453548449177 | 0.276372051205646 | 0.873377541389958 | -0.497643866 | 0.585734575207984 | 1.0450297509759 | 0.607961412375238 | 1.79631002592434 | 0.999332001504091 |
| ebi-fl187-GCST90094853 | prot-a-82 | Ameloblastin \|\| id:prot-a-82 | F-sweetened coffee drink liking (derived food-liking factor) \|\| id:ebi-fl187-GCST90094853 | Weighted median | 4 | -0.203426246 | 0.286952681128176 | 0.4783746607235 | -0.765853501 | 0.359001008891077 | 0.81593038013477 | 0.464936937379101 | 1.43189824620029 | 0.999332001504091 |
| ebi-fl187-GCST90094853 | prot-a-84 | Muellerian-inhibiting factor \|\| id:prot-a-84 | F-sweetened coffee drink liking (derived food-liking factor) \|\| id:ebi-fl187-GCST90094853 | Weighted median | 4 | 0.1572279646506 | 0.2856022553769 | 0.58196705808082 | -0.402552456 | 0.717008385189323 | 1.17026236194565 | 0.668611265408525 | 2.04829632200382 | 0.999332001504091 |
| ebi-fl187-GCST90094853 | prot-a-858 | Inactive dipeptidyl peptidase 10 \|\| id:prot-a-858 | F-sweetened coffee drink liking (derived food-liking factor) \|\| id:ebi-fl187-GCST90094853 | Weighted median | 4 | -0.209916883 | 0.280255192919997 | 0.453844852961131 | -0.759217061 | 0.339383294943579 | 0.810651621955366 | 0.468032724528531 | 1.40408141939401 | 0.999332001504091 |
| ebi-fl187-GCST90094853 | prot-a-863 | Dihydropyrimidinase-related protein 3 \|\| id:prot-a-863 | F-sweetened coffee drink liking (derived food-liking factor) \|\| id:ebi-fl187-GCST90094853 | Weighted median | 4 | -0.064021922 | 0.284763966058169 | 0.822115789033865 | -0.622159296 | 0.494115451085114 | 0.937984436445739 | 0.53678410959267 | 1.6390477797155 | 0.999332001504091 |
| ebi-fl187-GCST90094853 | prot-a-864 | Dihydropyrimidinase-related protein 5 \|\| id:prot-a-864 | F-sweetened coffee drink liking (derived food-liking factor) \|\| id:ebi-fl187-GCST90094853 | Weighted median | 4 | -0.034156801 | 0.274835765596855 | 0.901092971433347 | -0.572834902 | 0.504521299604726 | 0.966419957174436 | 0.563924500089162 | 1.65619251065944 | 0.999332001504091 |
| ebi-fl187-GCST90094853 | prot-a-884 | Endothelin-converting enzyme 1 \|\| id:prot-a-884 | F-sweetened coffee drink liking (derived food-liking factor) \|\| id:ebi-fl187-GCST90094853 | Weighted median | 4 | 0.131428938574734 | 0.287457111498479 | 0.647518429649164 | -0.431987 | 0.694844877111752 | 1.14045686235271 | 0.649217816486389 | 2.00339827690891 | 0.999332001504091 |
| ebi-fl187-GCST90094853 | prot-a-893 | Early endosome antigen 1 \|\| id:prot-a-893 | F-sweetened coffee drink liking (derived food-liking factor) \|\| id:ebi-fl187-GCST90094853 | Weighted median | 4 | -0.311271273 | 0.285980544704123 | 0.276403056729219 | -0.871793141 | 0.249250594376388 | 0.732515137156993 | 0.418200983241142 | 1.28306352128954 | 0.999332001504091 |
| ebi-fl187-GCST90094853 | prot-a-897 | Ephrin-A2 \|\| id:prot-a-897 | F-sweetened coffee drink liking (derived food-liking factor) \|\| id:ebi-fl187-GCST90094853 | Weighted median | 4 | -0.015050708 | 0.276774989008868 | 0.956633322853758 | -0.557529686 | 0.527428270650993 | 0.985061988004038 | 0.57262187376891 | 1.69456872791776 | 0.999332001504091 |
| ebi-fl187-GCST90094853 | prot-a-90 | Anaphase-promoting complex subunit 10 \|\| id:prot-a-90 | F-sweetened coffee drink liking (derived food-liking factor) \|\| id:ebi-fl187-GCST90094853 | Weighted median | 4 | -0.620238488 | 0.298283411263753 | 0.0375843067989345 | -1.204873974 | -0.035603002 | 0.537816159622257 | 0.299729770867315 | 0.965023329894303 | 0.999332001504091 |
| ebi-fl187-GCST90094853 | prot-a-914 | Histone-lysine N-methyltransferase EHMT2 \|\| id:prot-a-914 | F-sweetened coffee drink liking (derived food-liking factor) \|\| id:ebi-fl187-GCST90094853 | Weighted median | 4 | 0.200276359509275 | 0.278101120439547 | 0.471428577203921 | -0.344801837 | 0.745354555570788 | 1.2217403510734 | 0.708360710761203 | 2.1071884179417 | 0.999332001504091 |
| ebi-fl187-GCST90094853 | prot-a-932 | RNA polymerase II elongation factor ELL \|\| id:prot-a-932 | F-sweetened coffee drink liking (derived food-liking factor) \|\| id:ebi-fl187-GCST90094853 | Weighted median | 4 | 0.0669929600679655 | 0.28508523166623 | 0.814214367166467 | -0.491774094 | 0.625760014133777 | 1.06928795043361 | 0.611540500898764 | 1.86966639047146 | 0.999332001504091 |
| ebi-fl187-GCST90094853 | prot-a-991 | Estrogen receptor \|\| id:prot-a-991 | F-sweetened coffee drink liking (derived food-liking factor) \|\| id:ebi-fl187-GCST90094853 | Weighted median | 4 | 0.310089739299873 | 0.282887804573506 | 0.273009621905367 | -0.244370358 | 0.864549836263945 | 1.36354747243744 | 0.783197517339538 | 2.37393718497258 | 0.999332001504091 |
| ebi-fl187-GCST90094853 | prot-a-992 | Persulfide dioxygenase ETHE1, mitochondrial \|\| id:prot-a-992 | F-sweetened coffee drink liking (derived food-liking factor) \|\| id:ebi-fl187-GCST90094853 | Weighted median | 4 | 0.146912897851896 | 0.278178711520467 | 0.597413164362662 | -0.398317377 | 0.692143172432012 | 1.15825307230537 | 0.671448891589205 | 1.99799299143918 | 0.999332001504091 |
| ebi-fl187-GCST90094853 | prot-a-993 | Ethanolamine kinase 1 \|\| id:prot-a-993 | F-sweetened coffee drink liking (derived food-liking factor) \|\| id:ebi-fl187-GCST90094853 | Weighted median | 4 | -0.209915121 | 0.275260669126364 | 0.445699090856341 | -0.749426033 | 0.329595790008989 | 0.810653050082342 | 0.472637753392756 | 1.39040599886593 | 0.999332001504091 |
| ebi-fl187-GCST90094853 | prot-b-10 | coagulation factor III, tissue factor \|\| id:prot-b-10 | F-sweetened coffee drink liking (derived food-liking factor) \|\| id:ebi-fl187-GCST90094853 | Weighted median | 4 | -0.444579099 | 0.27264462181832 | 0.102971048819958 | -0.978962557 | 0.0898043601595728 | 0.64109405663601 | 0.375700664626651 | 1.09396024056131 | 0.999332001504091 |
| ebi-fl187-GCST90094853 | prot-b-28 | prolactin \|\| id:prot-b-28 | F-sweetened coffee drink liking (derived food-liking factor) \|\| id:ebi-fl187-GCST90094853 | Weighted median | 4 | -0.092368795 | 0.275495614314555 | 0.737412165498085 | -0.6323402 | 0.447602608571783 | 0.911768831304461 | 0.531346887160052 | 1.56455683062634 | 0.999332001504091 |
| ebi-fl187-GCST90094853 | prot-b-54 | matrix metallopeptidase 7 \|\| id:prot-b-54 | F-sweetened coffee drink liking (derived food-liking factor) \|\| id:ebi-fl187-GCST90094853 | Weighted median | 4 | 0.316891945755337 | 0.283767394162544 | 0.264109208381978 | -0.239292147 | 0.873076038313922 | 1.37285422118474 | 0.787184875231534 | 2.39426438683848 | 0.999332001504091 |
| ebi-fl187-GCST90094853 | ukb-rsfMRI191-edge-pheno716 | Net100_Pair10_33 (OccipitalPrecuneus)&(Temporal\|Frontal\|Supp_Motor_Area) (Default_mode\|Central_executive)&(Default_mode\|Salience) \|\| id:ukb-rsfMRI191-edge-pheno716 | F-sweetened coffee drink liking (derived food-liking factor) \|\| id:ebi-fl187-GCST90094853 | Weighted median | 3 | -0.02068812 | 0.10829399955604 | 0.848496898498221 | -0.232944359 | 0.191568118886276 | 0.979524410770395 | 0.792197650671735 | 1.21114733233748 | 0.999332001504091 |
| ebi-fl187-GCST90094853 | ukb-rsfMRI191-edge-pheno903 | Net100_Pair27_38 (Precentral\|Frontal\|Supp_Motor_Area)&(Subcortical) (Attention\|Salience\|Motor)&(Subcortical-cerebellum) \|\| id:ukb-rsfMRI191-edge-pheno903 | F-sweetened coffee drink liking (derived food-liking factor) \|\| id:ebi-fl187-GCST90094853 | Weighted median | 3 | -0.02003332 | 0.114762310344217 | 0.861422563774145 | -0.244967449 | 0.204900807906492 | 0.980166013270203 | 0.782730016751522 | 1.2274033102208 | 0.999332001504091 |
| ebi-fl187-GCST90094853 | ukb-rsfMRI191-node-pheno23 | Net100_Node2 RolandicOper\|SupraMarginal\|Insula Salience\|Motor \|\| id:ukb-rsfMRI191-node-pheno23 | F-sweetened coffee drink liking (derived food-liking factor) \|\| id:ebi-fl187-GCST90094853 | Weighted median | 3 | 0.019562792579405 | 0.0965437188575263 | 0.839423115764983 | -0.169662896 | 0.208788481540156 | 1.0197553979221 | 0.843949267000926 | 1.23218434123021 | 0.999332001504091 |
| ebi-fl187-GCST90094853 | ukb-rsfMRI191-node-pheno55 | Net100_Node34 Parietal\|Postcentral\|Precuneus Attention \|\| id:ukb-rsfMRI191-node-pheno55 | F-sweetened coffee drink liking (derived food-liking factor) \|\| id:ebi-fl187-GCST90094853 | Weighted median | 3 | 0.0159385368157823 | 0.10320201333314 | 0.877262679966119 | -0.186337409 | 0.218214482948736 | 1.0160662328208 | 0.829993500191138 | 1.24385382444671 | 0.999332001504091 |
| ebi-fl187-GCST90094853 | ebi-a-GCST90001414 | IgD- CD24- B cell Absolute Count \|\| id:ebi-a-GCST90001414 | F-sweetened coffee drink liking (derived food-liking factor) \|\| id:ebi-fl187-GCST90094853 | Weighted mode | 4 | 0.665493212365717 | 0.514520835935811 | 0.286452877599256 | -0.342967626 | 1.67395405079991 | 1.94544980465272 | 0.709661185710958 | 5.33321395988654 | 0.999714303421225 |
| ebi-fl187-GCST90094853 | ebi-a-GCST90001422 | CD20- CD38- B cell %B cell \|\| id:ebi-a-GCST90001422 | F-sweetened coffee drink liking (derived food-liking factor) \|\| id:ebi-fl187-GCST90094853 | Weighted mode | 4 | -0.048137275 | 0.528254843410152 | 0.933136768224042 | -1.083516768 | 0.987242217926596 | 0.953002954467192 | 0.338403344426727 | 2.68382285867109 | 0.999714303421225 |
| ebi-fl187-GCST90094853 | ebi-a-GCST90001444 | CD20- CD38- B cell %lymphocyte \|\| id:ebi-a-GCST90001444 | F-sweetened coffee drink liking (derived food-liking factor) \|\| id:ebi-fl187-GCST90094853 | Weighted mode | 4 | 0.291333751457896 | 0.462214094932689 | 0.57323775300177 | -0.614605875 | 1.19727337752597 | 1.33821113934536 | 0.540854017608632 | 3.31107654776425 | 0.999714303421225 |
| ebi-fl187-GCST90094853 | ebi-a-GCST90001459 | Myeloid Dendritic Cell %Dendritic Cell \|\| id:ebi-a-GCST90001459 | F-sweetened coffee drink liking (derived food-liking factor) \|\| id:ebi-fl187-GCST90094853 | Weighted mode | 4 | -0.119996141 | 0.430955611076029 | 0.798762266961344 | -0.964669139 | 0.724676856732271 | 0.886923859370352 | 0.381109273196771 | 2.06406400380148 | 0.999714303421225 |
| ebi-fl187-GCST90094853 | ebi-a-GCST90001476 | HLA DR++ monocyte %leukocyte \|\| id:ebi-a-GCST90001476 | F-sweetened coffee drink liking (derived food-liking factor) \|\| id:ebi-fl187-GCST90094853 | Weighted mode | 4 | -0.435254702 | 0.391644440466169 | 0.347470984342754 | -1.202877806 | 0.332368401127326 | 0.647099828345457 | 0.300328679566793 | 1.39426640322438 | 0.999714303421225 |
| ebi-fl187-GCST90094853 | ebi-a-GCST90001478 | CD4 regulatory T cell %CD4+ T cell \|\| id:ebi-a-GCST90001478 | F-sweetened coffee drink liking (derived food-liking factor) \|\| id:ebi-fl187-GCST90094853 | Weighted mode | 4 | 0.18788527290074 | 0.450934139945894 | 0.704945469856815 | -0.695945641 | 1.07171618719469 | 1.20669506678315 | 0.498602725604084 | 2.92038713273105 | 0.999714303421225 |
| ebi-fl187-GCST90094853 | ebi-a-GCST90001485 | CD39+ resting CD4 regulatory T cell %CD4 regulatory T cell \|\| id:ebi-a-GCST90001485 | F-sweetened coffee drink liking (derived food-liking factor) \|\| id:ebi-fl187-GCST90094853 | Weighted mode | 4 | -0.075297936 | 0.461555285321371 | 0.880778906633366 | -0.979946295 | 0.829350423406755 | 0.927467119481134 | 0.375331255455314 | 2.29182953781755 | 0.999714303421225 |
| ebi-fl187-GCST90094853 | ebi-a-GCST90001515 | Immature Myeloid-Derived Suppressor Cells Absolute Count \|\| id:ebi-a-GCST90001515 | F-sweetened coffee drink liking (derived food-liking factor) \|\| id:ebi-fl187-GCST90094853 | Weighted mode | 4 | -0.300642059 | 0.623160734489154 | 0.6625014614626 | -1.522037098 | 0.920752981054264 | 0.740342724677696 | 0.218266802867302 | 2.51118054959747 | 0.999714303421225 |
| ebi-fl187-GCST90094853 | ebi-a-GCST90001517 | CD33+ HLA DR+ Absolute Count \|\| id:ebi-a-GCST90001517 | F-sweetened coffee drink liking (derived food-liking factor) \|\| id:ebi-fl187-GCST90094853 | Weighted mode | 4 | -0.293420448 | 0.469412784245093 | 0.57623618447045 | -1.213469505 | 0.626628608934277 | 0.745708542971788 | 0.297164475082636 | 1.87129107847252 | 0.999714303421225 |
| ebi-fl187-GCST90094853 | ebi-a-GCST90001518 | CD33+ HLA DR+ CD14- Absolute Count \|\| id:ebi-a-GCST90001518 | F-sweetened coffee drink liking (derived food-liking factor) \|\| id:ebi-fl187-GCST90094853 | Weighted mode | 4 | -0.454602511 | 0.512715429822202 | 0.440570898947181 | -1.459524753 | 0.550319731889246 | 0.634700204441627 | 0.232346670549976 | 1.73380728273269 | 0.999714303421225 |
| ebi-fl187-GCST90094853 | ebi-a-GCST90001520 | CD33+ HLA DR+ CD14dim Absolute Count \|\| id:ebi-a-GCST90001520 | F-sweetened coffee drink liking (derived food-liking factor) \|\| id:ebi-fl187-GCST90094853 | Weighted mode | 4 | -0.382931849 | 0.573949051127403 | 0.552383724657402 | -1.507871989 | 0.742008291046172 | 0.681859366988776 | 0.221380577282622 | 2.10014899255044 | 0.999714303421225 |
| ebi-fl187-GCST90094853 | ebi-a-GCST90001531 | CD33dim HLA DR- Absolute Count \|\| id:ebi-a-GCST90001531 | F-sweetened coffee drink liking (derived food-liking factor) \|\| id:ebi-fl187-GCST90094853 | Weighted mode | 4 | -0.009682959 | 0.712462840270747 | 0.990009722741607 | -1.406110126 | 1.38674420791428 | 0.990363769884966 | 0.245094819253754 | 4.00179979196252 | 0.999714303421225 |
| ebi-fl187-GCST90094853 | ebi-a-GCST90001532 | Basophil Absolute Count \|\| id:ebi-a-GCST90001532 | F-sweetened coffee drink liking (derived food-liking factor) \|\| id:ebi-fl187-GCST90094853 | Weighted mode | 4 | -0.076952994 | 0.680163074159537 | 0.91706658065137 | -1.410072619 | 1.25616663122824 | 0.925933376892638 | 0.244125554239605 | 3.51193311619613 | 0.999714303421225 |
| ebi-fl187-GCST90094853 | ebi-a-GCST90001588 | T/B cell \|\| id:ebi-a-GCST90001588 | F-sweetened coffee drink liking (derived food-liking factor) \|\| id:ebi-fl187-GCST90094853 | Weighted mode | 4 | 0.0789582782892313 | 0.453086196019424 | 0.872751669752697 | -0.809090666 | 0.967007222487302 | 1.08215917159648 | 0.445262774808055 | 2.63006148038135 | 0.999714303421225 |
| ebi-fl187-GCST90094853 | ebi-a-GCST90001624 | HLA DR+ CD4+ T cell Absolute Count \|\| id:ebi-a-GCST90001624 | F-sweetened coffee drink liking (derived food-liking factor) \|\| id:ebi-fl187-GCST90094853 | Weighted mode | 4 | -0.033615266 | 0.397310697364243 | 0.937903679881775 | -0.812344233 | 0.745113701020619 | 0.966943449284556 | 0.443816436808581 | 2.10668095313823 | 0.999714303421225 |
| ebi-fl187-GCST90094853 | ebi-a-GCST90001631 | CD8+ Natural Killer T %T cell \|\| id:ebi-a-GCST90001631 | F-sweetened coffee drink liking (derived food-liking factor) \|\| id:ebi-fl187-GCST90094853 | Weighted mode | 4 | -0.13295974 | 0.453659404886047 | 0.788562709510527 | -1.022132174 | 0.75621269331535 | 0.875500338814126 | 0.359826908155397 | 2.13019322871936 | 0.999714303421225 |
| ebi-fl187-GCST90094853 | ebi-a-GCST90001632 | CD8+ Natural Killer T %lymphocyte \|\| id:ebi-a-GCST90001632 | F-sweetened coffee drink liking (derived food-liking factor) \|\| id:ebi-fl187-GCST90094853 | Weighted mode | 4 | -0.184678353 | 0.485934932503466 | 0.729220653743856 | -1.137110821 | 0.767754114766338 | 0.83137164909752 | 0.320744372439024 | 2.15492110950295 | 0.999714303421225 |
| ebi-fl187-GCST90094853 | ebi-a-GCST90001646 | Natural Killer %CD3- lymphocyte \|\| id:ebi-a-GCST90001646 | F-sweetened coffee drink liking (derived food-liking factor) \|\| id:ebi-fl187-GCST90094853 | Weighted mode | 4 | -0.486387903 | 0.440384084829271 | 0.35002117457269 | -1.34954071 | 0.376764902840619 | 0.614843261243379 | 0.259359354532776 | 1.45756160049596 | 0.999714303421225 |
| ebi-fl187-GCST90094853 | ebi-a-GCST90001684 | CD127- CD8+ T cell Absolute Count \|\| id:ebi-a-GCST90001684 | F-sweetened coffee drink liking (derived food-liking factor) \|\| id:ebi-fl187-GCST90094853 | Weighted mode | 4 | -0.044001852 | 0.418922353761528 | 0.92297629950621 | -0.865089666 | 0.77708596115707 | 0.956952184989711 | 0.421013800484785 | 2.17512462371094 | 0.999714303421225 |
| ebi-fl187-GCST90094853 | ebi-a-GCST90001687 | CD28- CD8+ T cell Absolute Count \|\| id:ebi-a-GCST90001687 | F-sweetened coffee drink liking (derived food-liking factor) \|\| id:ebi-fl187-GCST90094853 | Weighted mode | 4 | 0.250817577020147 | 0.358906786610406 | 0.534958651554073 | -0.452639725 | 0.954274878776543 | 1.28507563562064 | 0.635947208407189 | 2.59678691475349 | 0.999714303421225 |
| ebi-fl187-GCST90094853 | ebi-a-GCST90001701 | BAFF-R on CD20- CD38- B cell \|\| id:ebi-a-GCST90001701 | F-sweetened coffee drink liking (derived food-liking factor) \|\| id:ebi-fl187-GCST90094853 | Weighted mode | 4 | -0.216900293 | 0.408648434059942 | 0.632356283486081 | -1.017851224 | 0.584050637294239 | 0.805010230085766 | 0.36137061091945 | 1.79328769678834 | 0.999714303421225 |
| ebi-fl187-GCST90094853 | ebi-a-GCST90001732 | CD19 on IgD- CD27- B cell \|\| id:ebi-a-GCST90001732 | F-sweetened coffee drink liking (derived food-liking factor) \|\| id:ebi-fl187-GCST90094853 | Weighted mode | 4 | -0.362761935 | 0.543897228231012 | 0.55250641239288 | -1.428800502 | 0.703276632440473 | 0.695752048071037 | 0.239596144990586 | 2.02036185688239 | 0.999714303421225 |
| ebi-fl187-GCST90094853 | ebi-a-GCST90001798 | CD27 on CD24+ CD27+ B cell \|\| id:ebi-a-GCST90001798 | F-sweetened coffee drink liking (derived food-liking factor) \|\| id:ebi-fl187-GCST90094853 | Weighted mode | 4 | 0.368558575562103 | 0.403838490536853 | 0.428725156931521 | -0.422964866 | 1.16008201701433 | 1.44564931787009 | 0.655101649120799 | 3.19019491564872 | 0.999714303421225 |
| ebi-fl187-GCST90094853 | ebi-a-GCST90001836 | CD66b on CD66b++ myeloid cell \|\| id:ebi-a-GCST90001836 | F-sweetened coffee drink liking (derived food-liking factor) \|\| id:ebi-fl187-GCST90094853 | Weighted mode | 4 | 0.374319603100058 | 0.717994436139285 | 0.638169497813209 | -1.032949492 | 1.78158869793306 | 1.4540017796807 | 0.355955522856016 | 5.93928465655473 | 0.999714303421225 |
| ebi-fl187-GCST90094853 | ebi-a-GCST90001943 | CD25 on activated & secreting CD4 regulatory T cell \|\| id:ebi-a-GCST90001943 | F-sweetened coffee drink liking (derived food-liking factor) \|\| id:ebi-fl187-GCST90094853 | Weighted mode | 4 | 0.459804904368133 | 0.394372562656049 | 0.327913765266841 | -0.313165318 | 1.23277512717399 | 1.58376496922425 | 0.731129033467799 | 3.43073706954951 | 0.999714303421225 |
| ebi-fl187-GCST90094853 | ebi-a-GCST90001960 | CD25 on CD4+ T cell \|\| id:ebi-a-GCST90001960 | F-sweetened coffee drink liking (derived food-liking factor) \|\| id:ebi-fl187-GCST90094853 | Weighted mode | 4 | 0.196547373022798 | 0.484161949557906 | 0.71199645143204 | -0.752410048 | 1.14550479415629 | 1.21719298162327 | 0.471229497352486 | 3.14402804331393 | 0.999714303421225 |
| ebi-fl187-GCST90094853 | ebi-a-GCST90001988 | HLA DR on CD14+ CD16- monocyte \|\| id:ebi-a-GCST90001988 | F-sweetened coffee drink liking (derived food-liking factor) \|\| id:ebi-fl187-GCST90094853 | Weighted mode | 4 | -0.426151812 | 0.375008394952094 | 0.338359777022895 | -1.161168266 | 0.308864642502697 | 0.653017199007295 | 0.313120159573777 | 1.36187801762683 | 0.999714303421225 |
| ebi-fl187-GCST90094853 | ebi-a-GCST90001991 | HLA DR on CD14+ monocyte \|\| id:ebi-a-GCST90001991 | F-sweetened coffee drink liking (derived food-liking factor) \|\| id:ebi-fl187-GCST90094853 | Weighted mode | 4 | -0.462138901 | 0.387905831134386 | 0.319172320585381 | -1.22243433 | 0.298156527637661 | 0.629934835043266 | 0.29451235320334 | 1.34737267243596 | 0.999714303421225 |
| ebi-fl187-GCST90094853 | ebi-a-GCST90002019 | CD14 on Monocytic Myeloid-Derived Suppressor Cells \|\| id:ebi-a-GCST90002019 | F-sweetened coffee drink liking (derived food-liking factor) \|\| id:ebi-fl187-GCST90094853 | Weighted mode | 4 | -0.70282289 | 0.624369652851031 | 0.342235627788272 | -1.926587409 | 0.520941629962155 | 0.495185474999635 | 0.145644376032324 | 1.68361224326432 | 0.999714303421225 |
| ebi-fl187-GCST90094853 | ebi-a-GCST90002037 | CD80 on plasmacytoid Dendritic Cell \|\| id:ebi-a-GCST90002037 | F-sweetened coffee drink liking (derived food-liking factor) \|\| id:ebi-fl187-GCST90094853 | Weighted mode | 4 | -0.654046466 | 0.54567331606444 | 0.316730818557342 | -1.723566165 | 0.415473233660369 | 0.519937604565732 | 0.1784287057139 | 1.51508756149931 | 0.999714303421225 |
| ebi-fl187-GCST90094853 | ebi-a-GCST90002042 | CD45 on CD33+ HLA DR+ CD14- \|\| id:ebi-a-GCST90002042 | F-sweetened coffee drink liking (derived food-liking factor) \|\| id:ebi-fl187-GCST90094853 | Weighted mode | 4 | -0.581892523 | 0.746251604270064 | 0.492398527234827 | -2.044545667 | 0.880760621408468 | 0.558839748096776 | 0.129438984979739 | 2.41273418593133 | 0.999714303421225 |
| ebi-fl187-GCST90094853 | ebi-a-GCST90002056 | CD8 on Effector Memory CD8+ T cell \|\| id:ebi-a-GCST90002056 | F-sweetened coffee drink liking (derived food-liking factor) \|\| id:ebi-fl187-GCST90094853 | Weighted mode | 4 | -0.292971126 | 0.445818630285864 | 0.557998932226431 | -1.166775641 | 0.58083338950253 | 0.746043681757612 | 0.31136929073693 | 1.78752751683755 | 0.999714303421225 |
| ebi-fl187-GCST90094853 | ebi-a-GCST90002058 | CD8 on CD8+ T cell \|\| id:ebi-a-GCST90002058 | F-sweetened coffee drink liking (derived food-liking factor) \|\| id:ebi-fl187-GCST90094853 | Weighted mode | 4 | -0.828013749 | 0.562340059519278 | 0.237303628201555 | -1.930200266 | 0.274172767330464 | 0.436916250228607 | 0.145119133147164 | 1.31544204801885 | 0.999714303421225 |
| ebi-fl187-GCST90094853 | ebi-a-GCST90002063 | CD4 on CD4 regulatory T cell \|\| id:ebi-a-GCST90002063 | F-sweetened coffee drink liking (derived food-liking factor) \|\| id:ebi-fl187-GCST90094853 | Weighted mode | 4 | 0.140547153850775 | 0.489144537794568 | 0.792561986831827 | -0.81817614 | 1.09927044792813 | 1.15090334781008 | 0.441235673069273 | 3.00197512768305 | 0.999714303421225 |
| ebi-fl187-GCST90094853 | ebi-a-GCST90002066 | CD4 on activated CD4 regulatory T cell \|\| id:ebi-a-GCST90002066 | F-sweetened coffee drink liking (derived food-liking factor) \|\| id:ebi-fl187-GCST90094853 | Weighted mode | 4 | -0.027796616 | 0.478181968610281 | 0.957300546531192 | -0.965033274 | 0.909440042537835 | 0.972586155209644 | 0.380970522957351 | 2.48293180785373 | 0.999714303421225 |
| ebi-fl187-GCST90094853 | ebi-a-GCST90002070 | CD4 on activated & secreting CD4 regulatory T cell \|\| id:ebi-a-GCST90002070 | F-sweetened coffee drink liking (derived food-liking factor) \|\| id:ebi-fl187-GCST90094853 | Weighted mode | 4 | 0.00687954793354639 | 0.465354022632081 | 0.989133121074107 | -0.905214336 | 0.918973432292425 | 1.00690326638297 | 0.404455186333153 | 2.50671575519761 | 0.999714303421225 |
| ebi-fl187-GCST90094853 | ebi-a-GCST90002081 | SSC-A on CD4+ T cell \|\| id:ebi-a-GCST90002081 | F-sweetened coffee drink liking (derived food-liking factor) \|\| id:ebi-fl187-GCST90094853 | Weighted mode | 4 | 0.0144100764616349 | 0.433332236722173 | 0.975560738621969 | -0.834921108 | 0.863741260437094 | 1.01451440212471 | 0.433908712219778 | 2.3720184525752 | 0.999714303421225 |
| ebi-fl187-GCST90094853 | ebi-a-GCST90002094 | CD11b on Monocytic Myeloid-Derived Suppressor Cells \|\| id:ebi-a-GCST90002094 | F-sweetened coffee drink liking (derived food-liking factor) \|\| id:ebi-fl187-GCST90094853 | Weighted mode | 4 | -0.810676321 | 0.571755576389592 | 0.25124929111945 | -1.931317251 | 0.309964608815642 | 0.444557301130006 | 0.14495712779912 | 1.36337686175647 | 0.999714303421225 |
| ebi-fl187-GCST90094853 | ebi-a-GCST90002104 | HLA DR on myeloid Dendritic Cell \|\| id:ebi-a-GCST90002104 | F-sweetened coffee drink liking (derived food-liking factor) \|\| id:ebi-fl187-GCST90094853 | Weighted mode | 4 | -0.214565287 | 0.450312007370464 | 0.666284809836689 | -1.097176822 | 0.668046246964012 | 0.806892130056696 | 0.333812165838479 | 1.95042295091925 | 0.999714303421225 |
| ebi-fl187-GCST90094853 | ebi-a-GCST90002105 | HLA DR on plasmacytoid Dendritic Cell \|\| id:ebi-a-GCST90002105 | F-sweetened coffee drink liking (derived food-liking factor) \|\| id:ebi-fl187-GCST90094853 | Weighted mode | 4 | -0.679900284 | 0.411052538271478 | 0.196690635189657 | -1.485563259 | 0.125762691391916 | 0.506667512896844 | 0.226374797201206 | 1.13401302529673 | 0.999714303421225 |
| ebi-fl187-GCST90094853 | ebi-a-GCST90002106 | HLA DR on Dendritic Cell \|\| id:ebi-a-GCST90002106 | F-sweetened coffee drink liking (derived food-liking factor) \|\| id:ebi-fl187-GCST90094853 | Weighted mode | 4 | -0.408168216 | 0.448362114032869 | 0.429755116567552 | -1.28695796 | 0.470621527131725 | 0.664867027895327 | 0.276109442828107 | 1.60098894211874 | 0.999714303421225 |
| ebi-fl187-GCST90094853 | ebi-a-GCST90002111 | HLA DR on CD33dim HLA DR+ CD11b- \|\| id:ebi-a-GCST90002111 | F-sweetened coffee drink liking (derived food-liking factor) \|\| id:ebi-fl187-GCST90094853 | Weighted mode | 4 | 0.00872803119702059 | 0.525342072440328 | 0.987787714171111 | -1.020942431 | 1.03839849318006 | 1.00876623151829 | 0.360255264485729 | 2.82468963029388 | 0.999714303421225 |
| ebi-fl187-GCST90094853 | ebi-a-GCST90002112 | HLA DR on CD33- HLA DR+ \|\| id:ebi-a-GCST90002112 | F-sweetened coffee drink liking (derived food-liking factor) \|\| id:ebi-fl187-GCST90094853 | Weighted mode | 4 | 0.0286350405806344 | 0.63471046136937 | 0.966850629971916 | -1.215397464 | 1.2726675448646 | 1.02904896482181 | 0.296592106266582 | 3.57036397674402 | 0.999714303421225 |
| ebi-fl187-GCST90094853 | ebi-a-GCST90002115 | HLA DR on HLA DR+ CD8+ T cell \|\| id:ebi-a-GCST90002115 | F-sweetened coffee drink liking (derived food-liking factor) \|\| id:ebi-fl187-GCST90094853 | Weighted mode | 4 | -0.295568553 | 0.431668988470816 | 0.542682437164322 | -1.141639771 | 0.550502664243801 | 0.744108402 | 0.319295021736592 | 1.73412448119331 | 0.999714303421225 |
| ebi-fl187-GCST90094853 | ebi-a-GCST90002116 | HLA DR on B cell \|\| id:ebi-a-GCST90002116 | F-sweetened coffee drink liking (derived food-liking factor) \|\| id:ebi-fl187-GCST90094853 | Weighted mode | 4 | 0.0170145694777273 | 0.482708546255786 | 0.974096074093395 | -0.929094181 | 0.963123320139067 | 1.01716014170931 | 0.394911266462601 | 2.61986638960602 | 0.999714303421225 |
| ebi-fl187-GCST90094853 | ebi-cfmet338-GCST90026023 | Sphingomyelin (d18:1/24:1, d18:2/24:0) levels \|\| id:ebi-cfmet338-GCST90026023 | F-sweetened coffee drink liking (derived food-liking factor) \|\| id:ebi-fl187-GCST90094853 | Weighted mode | 4 | -0.235399588 | 0.267625491188272 | 0.443845037240631 | -0.75994555 | 0.289146375141808 | 0.79025501043733 | 0.467691891992249 | 1.33528716707292 | 0.999714303421225 |
| ebi-fl187-GCST90094853 | ebi-cfmet338-GCST90026046 | 1-arachidonoyl-gpc (20:4n6) levels \|\| id:ebi-cfmet338-GCST90026046 | F-sweetened coffee drink liking (derived food-liking factor) \|\| id:ebi-fl187-GCST90094853 | Weighted mode | 4 | 0.191820672375496 | 0.275226300606079 | 0.535982346231308 | -0.347622877 | 0.731264221563411 | 1.21145325045094 | 0.706365212691004 | 2.07770562827836 | 0.999714303421225 |
| ebi-fl187-GCST90094853 | ebi-cfmet338-GCST90026047 | 1-linoleoyl-gpc (18:2) levels \|\| id:ebi-cfmet338-GCST90026047 | F-sweetened coffee drink liking (derived food-liking factor) \|\| id:ebi-fl187-GCST90094853 | Weighted mode | 4 | 0.140483621260752 | 0.386122989961242 | 0.740113398605142 | -0.616317439 | 0.897284681584786 | 1.15083023026222 | 0.539929102849176 | 2.45293356460425 | 0.999714303421225 |
| ebi-fl187-GCST90094853 | ebi-cfmet338-GCST90026050 | 1-myristoyl-2-palmitoyl-gpc (14:0/16:0) levels \|\| id:ebi-cfmet338-GCST90026050 | F-sweetened coffee drink liking (derived food-liking factor) \|\| id:ebi-fl187-GCST90094853 | Weighted mode | 4 | 0.0680036115369199 | 0.202503610280721 | 0.759125439027959 | -0.328903465 | 0.464910687687133 | 1.07036917414958 | 0.719712491117302 | 1.59187200876709 | 0.999714303421225 |
| ebi-fl187-GCST90094853 | ebi-cfmet338-GCST90026068 | 2,3-dihydroxyisovalerate levels \|\| id:ebi-cfmet338-GCST90026068 | F-sweetened coffee drink liking (derived food-liking factor) \|\| id:ebi-fl187-GCST90094853 | Weighted mode | 4 | -0.31205478 | 0.511581823137393 | 0.584973488399252 | -1.314755153 | 0.690645593691051 | 0.731941431628607 | 0.268540066418018 | 1.99500307898408 | 0.999714303421225 |
| ebi-fl187-GCST90094853 | ebi-cfmet338-GCST90026082 | 3-hydroxyhexanoate levels \|\| id:ebi-cfmet338-GCST90026082 | F-sweetened coffee drink liking (derived food-liking factor) \|\| id:ebi-fl187-GCST90094853 | Weighted mode | 4 | 0.0509089018297408 | 0.11313039885436 | 0.683237514504035 | -0.17082668 | 0.272644483584287 | 1.05222703295952 | 0.842967664029958 | 1.31343321474244 | 0.999714303421225 |
| ebi-fl187-GCST90094853 | ebi-cfmet338-GCST90026094 | 3-ureidopropionate levels \|\| id:ebi-cfmet338-GCST90026094 | F-sweetened coffee drink liking (derived food-liking factor) \|\| id:ebi-fl187-GCST90094853 | Weighted mode | 4 | -0.199838838 | 0.265780410235537 | 0.506727758837512 | -0.720768442 | 0.321090766208125 | 0.818862712116647 | 0.486378358801707 | 1.37863070829679 | 0.999714303421225 |
| ebi-fl187-GCST90094853 | ebi-cfmet338-GCST90026173 | Glycerol levels \|\| id:ebi-cfmet338-GCST90026173 | F-sweetened coffee drink liking (derived food-liking factor) \|\| id:ebi-fl187-GCST90094853 | Weighted mode | 4 | -0.025943926 | 0.162795925871008 | 0.883506345592658 | -0.345023941 | 0.29313608883795 | 0.974389726133816 | 0.708203398467276 | 1.34062522214655 | 0.999714303421225 |
| ebi-fl187-GCST90094853 | ebi-cfmet338-GCST90026179 | Histidine levels \|\| id:ebi-cfmet338-GCST90026179 | F-sweetened coffee drink liking (derived food-liking factor) \|\| id:ebi-fl187-GCST90094853 | Weighted mode | 4 | -0.009249129 | 0.109963708849549 | 0.938266755613785 | -0.224777999 | 0.206279740072488 | 0.990793512356279 | 0.798693510142435 | 1.22909698358789 | 0.999714303421225 |
| ebi-fl187-GCST90094853 | ebi-cfmet338-GCST90026188 | Isocitrate levels \|\| id:ebi-cfmet338-GCST90026188 | F-sweetened coffee drink liking (derived food-liking factor) \|\| id:ebi-fl187-GCST90094853 | Weighted mode | 4 | 0.437533138409684 | 0.241843166080022 | 0.168131626368104 | -0.036479467 | 0.911545743926528 | 1.54888162525754 | 0.964177891054394 | 2.48816562930822 | 0.999714303421225 |
| ebi-fl187-GCST90094853 | ebi-cfmet338-GCST90026207 | N-acetyl-isoputreanine levels \|\| id:ebi-cfmet338-GCST90026207 | F-sweetened coffee drink liking (derived food-liking factor) \|\| id:ebi-fl187-GCST90094853 | Weighted mode | 4 | -0.105327499 | 0.157939787237524 | 0.552553194403105 | -0.414889482 | 0.204234484073281 | 0.900029715561557 | 0.660413264292533 | 1.22658573455757 | 0.999714303421225 |
| ebi-fl187-GCST90094853 | ebi-cfmet338-GCST90026213 | N-acetylglutamate levels \|\| id:ebi-cfmet338-GCST90026213 | F-sweetened coffee drink liking (derived food-liking factor) \|\| id:ebi-fl187-GCST90094853 | Weighted mode | 4 | -0.161550504 | 0.209160799620603 | 0.49616073747604 | -0.571505671 | 0.248404663276059 | 0.850823560403926 | 0.564674584027311 | 1.28197859690352 | 0.999714303421225 |
| ebi-fl187-GCST90094853 | ebi-cfmet338-GCST90026224 | N-acetyltaurine levels \|\| id:ebi-cfmet338-GCST90026224 | F-sweetened coffee drink liking (derived food-liking factor) \|\| id:ebi-fl187-GCST90094853 | Weighted mode | 4 | -0.122655782 | 0.191387341753616 | 0.567198669526992 | -0.497774972 | 0.25246340763986 | 0.884568094248217 | 0.60788170989737 | 1.28719239388536 | 0.999714303421225 |
| ebi-fl187-GCST90094853 | ebi-cfmet338-GCST90026250 | Phenyllactate (pla) levels \|\| id:ebi-cfmet338-GCST90026250 | F-sweetened coffee drink liking (derived food-liking factor) \|\| id:ebi-fl187-GCST90094853 | Weighted mode | 4 | -0.091730782 | 0.376581347112556 | 0.823256832239393 | -0.829830223 | 0.646368658083589 | 0.912350737491644 | 0.43612332392145 | 1.90859745981269 | 0.999714303421225 |
| ebi-fl187-GCST90094853 | ebi-cfmet338-GCST90026251 | Pipecolate levels \|\| id:ebi-cfmet338-GCST90026251 | F-sweetened coffee drink liking (derived food-liking factor) \|\| id:ebi-fl187-GCST90094853 | Weighted mode | 4 | 0.219295903013379 | 0.582949277421693 | 0.731808548344931 | -0.923284681 | 1.3618764867599 | 1.24519968051667 | 0.397212180752941 | 3.90351132087564 | 0.999714303421225 |
| ebi-fl187-GCST90094853 | ebi-cfmet338-GCST90026278 | Trigonelline (n'-methylnicotinate) levels \|\| id:ebi-cfmet338-GCST90026278 | F-sweetened coffee drink liking (derived food-liking factor) \|\| id:ebi-fl187-GCST90094853 | Weighted mode | 4 | 0.039347919589814 | 0.499699378324609 | 0.942195087423563 | -0.940062862 | 1.01875870110605 | 1.04013230310872 | 0.390603280512068 | 2.7697545359884 | 0.999714303421225 |
| ebi-fl187-GCST90094853 | ebi-cfmet338-GCST90026299 | 1-carboxyethylvaline levels \|\| id:ebi-cfmet338-GCST90026299 | F-sweetened coffee drink liking (derived food-liking factor) \|\| id:ebi-fl187-GCST90094853 | Weighted mode | 4 | 0.0329535518041084 | 0.270964852656308 | 0.910892282555216 | -0.498137559 | 0.564044663010473 | 1.03350253379829 | 0.607661339622311 | 1.75776771981474 | 0.999714303421225 |
| ebi-fl187-GCST90094853 | ebi-gm430-GCST90011306 | C_Erysipelotrichia abundance \|\| id:ebi-gm430-GCST90011306 | F-sweetened coffee drink liking (derived food-liking factor) \|\| id:ebi-fl187-GCST90094853 | Weighted mode | 4 | 0.387367192792036 | 0.261464443243718 | 0.235064603687285 | -0.125103116 | 0.899837501549723 | 1.47309730268755 | 0.882405907755892 | 2.45920346193516 | 0.999714303421225 |
| ebi-fl187-GCST90094853 | ebi-gm430-GCST90011312 | F_Erysipelotrichaceae abundance \|\| id:ebi-gm430-GCST90011312 | F-sweetened coffee drink liking (derived food-liking factor) \|\| id:ebi-fl187-GCST90094853 | Weighted mode | 4 | 0.387367192792036 | 0.245143067958569 | 0.212197924987077 | -0.09311322 | 0.867847605990832 | 1.47309730268755 | 0.911090340437411 | 2.38177880597824 | 0.999714303421225 |
| ebi-fl187-GCST90094853 | ebi-gm430-GCST90011352 | O_Erysipelotrichales abundance \|\| id:ebi-gm430-GCST90011352 | F-sweetened coffee drink liking (derived food-liking factor) \|\| id:ebi-fl187-GCST90094853 | Weighted mode | 4 | 0.387367192792036 | 0.256492690854298 | 0.22813869877362 | -0.115358481 | 0.890092866866459 | 1.47309730268755 | 0.891046663095854 | 2.43535580465093 | 0.999714303421225 |
| ebi-fl187-GCST90094853 | ebi-gm430-GCST90011362 | OTU97_11 (Parabacteroides) abundance \|\| id:ebi-gm430-GCST90011362 | F-sweetened coffee drink liking (derived food-liking factor) \|\| id:ebi-fl187-GCST90094853 | Weighted mode | 4 | -0.523093488 | 0.291070402655681 | 0.170167132886339 | -1.093591477 | 0.047404501511424 | 0.592684247721899 | 0.335011145721916 | 1.04854606177568 | 0.999714303421225 |
| ebi-fl187-GCST90094853 | ebi-gm430-GCST90011384 | OTU97_235 (Phascolarctobacterium) abundance \|\| id:ebi-gm430-GCST90011384 | F-sweetened coffee drink liking (derived food-liking factor) \|\| id:ebi-fl187-GCST90094853 | Weighted mode | 4 | -0.464209182 | 1.37988013731474 | 0.758716922231662 | -3.168774251 | 2.24035588729779 | 0.628632042305871 | 0.0420551153398539 | 9.39667484966227 | 0.999714303421225 |
| ebi-fl187-GCST90094853 | ebi-gm430-GCST90011390 | OTU97_3 (Bacteroides) abundance \|\| id:ebi-gm430-GCST90011390 | F-sweetened coffee drink liking (derived food-liking factor) \|\| id:ebi-fl187-GCST90094853 | Weighted mode | 4 | 0.00887749143954358 | 0.231493997858043 | 0.971818887858619 | -0.444850744 | 0.462605727241308 | 1.00891701323156 | 0.640919929806597 | 1.58820703218771 | 0.999714303421225 |
| ebi-fl187-GCST90094853 | ebi-gm430-GCST90011398 | OTU97_4 (Alistipes) abundance \|\| id:ebi-gm430-GCST90011398 | F-sweetened coffee drink liking (derived food-liking factor) \|\| id:ebi-fl187-GCST90094853 | Weighted mode | 4 | 0.0859985045115269 | 0.303002988897573 | 0.795008774481275 | -0.507887354 | 0.67988436275077 | 1.08980469851355 | 0.60176555459081 | 1.97364949163599 | 0.999714303421225 |
| ebi-fl187-GCST90094853 | ebi-gm430-GCST90011421 | OTU99_108 (Cytophagales) abundance \|\| id:ebi-gm430-GCST90011421 | F-sweetened coffee drink liking (derived food-liking factor) \|\| id:ebi-fl187-GCST90094853 | Weighted mode | 4 | 0.0852058442743794 | 0.925964404412299 | 0.932483689822502 | -1.729684388 | 1.90009607692249 | 1.08894119594007 | 0.177340371821462 | 6.68653683330033 | 0.999714303421225 |
| ebi-fl187-GCST90094853 | ebi-gm430-GCST90011428 | OTU99_123 (Phascolarctobacterium) abundance \|\| id:ebi-gm430-GCST90011428 | F-sweetened coffee drink liking (derived food-liking factor) \|\| id:ebi-fl187-GCST90094853 | Weighted mode | 4 | -0.501078673 | 0.538609339451221 | 0.420829809246687 | -1.556752978 | 0.554595632533131 | 0.60587676432592 | 0.210819496570756 | 1.74123674290647 | 0.999714303421225 |
| ebi-fl187-GCST90094853 | ebi-gm430-GCST90011450 | OTU99_298 (Phascolarctobacterium) abundance \|\| id:ebi-gm430-GCST90011450 | F-sweetened coffee drink liking (derived food-liking factor) \|\| id:ebi-fl187-GCST90094853 | Weighted mode | 4 | -0.406926118 | 1.38318991713168 | 0.787790850530474 | -3.117978356 | 2.3041261195327 | 0.66569337121214 | 0.0442465288079394 | 10.0154221453021 | 0.999714303421225 |
| ebi-fl187-GCST90094853 | ebi-gm430-GCST90011451 | OTU99_3 (Bacteroides) abundance \|\| id:ebi-gm430-GCST90011451 | F-sweetened coffee drink liking (derived food-liking factor) \|\| id:ebi-fl187-GCST90094853 | Weighted mode | 4 | 0.0167166864411147 | 0.22401960020232 | 0.945213086915408 | -0.42236173 | 0.455795102837663 | 1.0168571920816 | 0.655496883644351 | 1.57742710131492 | 0.999714303421225 |
| ebi-fl187-GCST90094853 | ebi-gm430-GCST90011472 | OTU99_72 (Parabacteroides) abundance \|\| id:ebi-gm430-GCST90011472 | F-sweetened coffee drink liking (derived food-liking factor) \|\| id:ebi-fl187-GCST90094853 | Weighted mode | 4 | 0.53071546562272 | 0.665485946833647 | 0.483455854843092 | -0.77363699 | 1.83506792141667 | 1.70014827121854 | 0.461332152921891 | 6.26555969667435 | 0.999714303421225 |
| ebi-fl187-GCST90094853 | ebi-gm430-GCST90011507 | TestASV_3 (Bacteroides) abundance \|\| id:ebi-gm430-GCST90011507 | F-sweetened coffee drink liking (derived food-liking factor) \|\| id:ebi-fl187-GCST90094853 | Weighted mode | 4 | -0.211934673 | 0.267449293211012 | 0.485991792643887 | -0.736135288 | 0.312265941228885 | 0.809017546153562 | 0.478961391000889 | 1.36651805820214 | 0.999714303421225 |
| ebi-fl187-GCST90094853 | ebi-gm430-GCST90011512 | TestASV_34 (Phascolarctobacterium) abundance \|\| id:ebi-gm430-GCST90011512 | F-sweetened coffee drink liking (derived food-liking factor) \|\| id:ebi-fl187-GCST90094853 | Weighted mode | 4 | -0.854120899 | 0.814521863360691 | 0.371384103194742 | -2.450583751 | 0.742341953355671 | 0.425657222414192 | 0.0862432272304514 | 2.10084985003195 | 0.999714303421225 |
| ebi-fl187-GCST90094853 | ebi-gm430-GCST90011519 | TestASV_41 (Barnesiella) abundance \|\| id:ebi-gm430-GCST90011519 | F-sweetened coffee drink liking (derived food-liking factor) \|\| id:ebi-fl187-GCST90094853 | Weighted mode | 4 | -0.341775331 | 1.11061485844917 | 0.77841159563526 | -2.518580453 | 1.83502979165186 | 0.710507815924154 | 0.0805739040111365 | 6.26532079691134 | 0.999714303421225 |
| ebi-fl187-GCST90094853 | ebi-gm430-GCST90011524 | TestASV_46 (Sutterella) abundance \|\| id:ebi-gm430-GCST90011524 | F-sweetened coffee drink liking (derived food-liking factor) \|\| id:ebi-fl187-GCST90094853 | Weighted mode | 4 | -0.753316389 | 1.166595747 | 0.564438706043048 | -3.039844054 | 1.53321127572142 | 0.470802596255754 | 0.0478423497441689 | 4.63303089890928 | 0.999714303421225 |
| ebi-fl187-GCST90094853 | ebi-gm430-GCST90011581 | OTU97_19 (Bacteria) prevalence \|\| id:ebi-gm430-GCST90011581 | F-sweetened coffee drink liking (derived food-liking factor) \|\| id:ebi-fl187-GCST90094853 | Weighted mode | 4 | -0.750173491 | 1.18010466577329 | 0.570157983693868 | -3.063178636 | 1.56283165422162 | 0.472284608648424 | 0.0467388929417059 | 4.77231567817391 | 0.999714303421225 |
| ebi-fl187-GCST90094853 | ebi-gm430-GCST90011614 | OTU97_80 (Ruminococcus) prevalence \|\| id:ebi-gm430-GCST90011614 | F-sweetened coffee drink liking (derived food-liking factor) \|\| id:ebi-fl187-GCST90094853 | Weighted mode | 4 | -0.30635722 | 0.486877396964225 | 0.573852370836886 | -1.260636918 | 0.647922478342909 | 0.736123614621732 | 0.283473419735424 | 1.91156538242464 | 0.999714303421225 |
| ebi-fl187-GCST90094853 | ebi-gm430-GCST90011623 | OTU99_11 (Parabacteroides) prevalence \|\| id:ebi-gm430-GCST90011623 | F-sweetened coffee drink liking (derived food-liking factor) \|\| id:ebi-fl187-GCST90094853 | Weighted mode | 4 | -0.005344276 | 0.516116226174535 | 0.992388319877433 | -1.01693208 | 1.00624352705919 | 0.994669978995436 | 0.361702915487041 | 2.73530658657416 | 0.999714303421225 |
| ebi-fl187-GCST90094853 | ebi-gm430-GCST90011642 | OTU99_19 (Bacteria) prevalence \|\| id:ebi-gm430-GCST90011642 | F-sweetened coffee drink liking (derived food-liking factor) \|\| id:ebi-fl187-GCST90094853 | Weighted mode | 4 | -0.750173491 | 1.13373664827536 | 0.555459145213318 | -2.972297321 | 1.47195033992568 | 0.472284608648424 | 0.0511855854213788 | 4.35772590525129 | 0.999714303421225 |
| ebi-fl187-GCST90094853 | ebi-gm430-GCST90011653 | OTU99_30 (Parasutterella) prevalence \|\| id:ebi-gm430-GCST90011653 | F-sweetened coffee drink liking (derived food-liking factor) \|\| id:ebi-fl187-GCST90094853 | Weighted mode | 4 | -0.16915409 | 0.497761388307351 | 0.756386110367857 | -1.144766411 | 0.806458230913462 | 0.844378782891837 | 0.318298259999579 | 2.2399604980525 | 0.999714303421225 |
| ebi-fl187-GCST90094853 | ebi-gm430-GCST90011675 | OTU99_8 (Ruminococcaceae) prevalence \|\| id:ebi-gm430-GCST90011675 | F-sweetened coffee drink liking (derived food-liking factor) \|\| id:ebi-fl187-GCST90094853 | Weighted mode | 4 | 0.328929174992279 | 0.558767006672657 | 0.59746784552455 | -0.766254158 | 1.42411250807069 | 1.38947944221037 | 0.464750694473867 | 4.15416941444461 | 0.999714303421225 |
| ebi-fl187-GCST90094853 | ebi-gm430-GCST90011679 | OTU99_92 (Ruminococcus) prevalence \|\| id:ebi-gm430-GCST90011679 | F-sweetened coffee drink liking (derived food-liking factor) \|\| id:ebi-fl187-GCST90094853 | Weighted mode | 4 | -0.350815316 | 0.502748980576949 | 0.53552620505676 | -1.336203318 | 0.634572686206513 | 0.704113780591878 | 0.26284170302403 | 1.8862159631269 | 0.999714303421225 |
| ebi-fl187-GCST90094853 | ebi-gm430-GCST90011684 | TestASV_11 (Lachnospiraceae) prevalence \|\| id:ebi-gm430-GCST90011684 | F-sweetened coffee drink liking (derived food-liking factor) \|\| id:ebi-fl187-GCST90094853 | Weighted mode | 4 | 0.647945284407686 | 0.772700294040354 | 0.463277088228494 | -0.866547292 | 2.16243786072678 | 1.9116089782057 | 0.420400566728141 | 8.69230247236967 | 0.999714303421225 |
| ebi-fl187-GCST90094853 | ebi-gm430-GCST90011688 | TestASV_15 (Bacteroides) prevalence \|\| id:ebi-gm430-GCST90011688 | F-sweetened coffee drink liking (derived food-liking factor) \|\| id:ebi-fl187-GCST90094853 | Weighted mode | 4 | 0.390074251079246 | 0.56582269293186 | 0.540112922504276 | -0.718938227 | 1.49908672922569 | 1.47709046537218 | 0.487269350800403 | 4.4775979431284 | 0.999714303421225 |
| ebi-fl187-GCST90094853 | ebi-gm430-GCST90011692 | TestASV_19 (Prevotella) prevalence \|\| id:ebi-gm430-GCST90011692 | F-sweetened coffee drink liking (derived food-liking factor) \|\| id:ebi-fl187-GCST90094853 | Weighted mode | 4 | 0.796778601235681 | 1.03527050765144 | 0.497564534524458 | -1.232351594 | 2.82590879623251 | 2.21838310898091 | 0.291606031833603 | 16.8762751142953 | 0.999714303421225 |
| ebi-fl187-GCST90094853 | ebi-gm430-GCST90011693 | TestASV_2 (Bacteroides) prevalence \|\| id:ebi-gm430-GCST90011693 | F-sweetened coffee drink liking (derived food-liking factor) \|\| id:ebi-fl187-GCST90094853 | Weighted mode | 4 | -0.296900112 | 0.522815279492656 | 0.609834370090092 | -1.32161806 | 0.727817835554156 | 0.743118237062415 | 0.266703410511294 | 2.07055737756067 | 0.999714303421225 |
| ebi-fl187-GCST90094853 | ebi-gm430-GCST90011716 | TestASV_40 (Barnesiella) prevalence \|\| id:ebi-gm430-GCST90011716 | F-sweetened coffee drink liking (derived food-liking factor) \|\| id:ebi-fl187-GCST90094853 | Weighted mode | 4 | 0.371859777838353 | 1.34892204416105 | 0.800698305368939 | -2.272027429 | 3.01574698439401 | 1.45042958466146 | 0.103102934189594 | 20.4043269631169 | 0.999714303421225 |
| ebi-fl187-GCST90094853 | ebi-gm430-GCST90011718 | TestASV_42 (Alphaproteobacteria) prevalence \|\| id:ebi-gm430-GCST90011718 | F-sweetened coffee drink liking (derived food-liking factor) \|\| id:ebi-fl187-GCST90094853 | Weighted mode | 4 | 0.609388204728006 | 0.761477961165386 | 0.482063992955796 | -0.883108599 | 2.10188500861216 | 1.83930577591392 | 0.413495519901557 | 8.1815777305537 | 0.999714303421225 |
| ebi-fl187-GCST90094853 | ebi-hbsm44-GCST90104929 | Sulcal depth of the right inferior frontal sulcus \|\| id:ebi-hbsm44-GCST90104929 | F-sweetened coffee drink liking (derived food-liking factor) \|\| id:ebi-fl187-GCST90094853 | Weighted mode | 3 | -1.083248135 | 0.609003213966843 | 0.217253206637204 | -2.276894434 | 0.11039816452337 | 0.338494263075525 | 0.102602350800061 | 1.11672262128105 | 0.999714303421225 |
| ebi-fl187-GCST90094853 | ebi-hbsm44-GCST90104935 | Sulcal opening distance of the left calloso-marginal posterior fissure \|\| id:ebi-hbsm44-GCST90104935 | F-sweetened coffee drink liking (derived food-liking factor) \|\| id:ebi-fl187-GCST90094853 | Weighted mode | 3 | -0.163708886 | 0.116093086071371 | 0.29391082445377 | -0.391251335 | 0.0638335626501326 | 0.848989138490737 | 0.676210179563501 | 1.06591497593324 | 0.999714303421225 |
| ebi-fl187-GCST90094853 | ebi-hbsm44-GCST90104936 | Sulcal opening distance of the left anterior inferior temporal sulcus \|\| id:ebi-hbsm44-GCST90104936 | F-sweetened coffee drink liking (derived food-liking factor) \|\| id:ebi-fl187-GCST90094853 | Weighted mode | 3 | 0.0844473475230072 | 0.0792689076892656 | 0.398314255978352 | -0.070919712 | 0.239814406593968 | 1.08811555074469 | 0.931536680763343 | 1.27101323675436 | 0.999714303421225 |
| ebi-fl187-GCST90094853 | ebi-hbsm44-GCST90104941 | Sulcal opening distance of the right posterior inferior temporal sulcus \|\| id:ebi-hbsm44-GCST90104941 | F-sweetened coffee drink liking (derived food-liking factor) \|\| id:ebi-fl187-GCST90094853 | Weighted mode | 3 | 0.00692226163049661 | 0.0544245215552733 | 0.910424544255964 | -0.099749801 | 0.113594323878832 | 1.00694627586249 | 0.90506383612256 | 1.1202975547197 | 0.999714303421225 |
| ebi-fl187-GCST90094853 | ebi-IPs91-GCST90274819 | Leukemia inhibitory factor levels \|\| id:ebi-IPs91-GCST90274819 | F-sweetened coffee drink liking (derived food-liking factor) \|\| id:ebi-fl187-GCST90094853 | Weighted mode | 4 | 0.296316408611951 | 0.18247200498377 | 0.202859432472063 | -0.061328721 | 0.65396153838014 | 1.3448956260757 | 0.940514022098796 | 1.92314436843937 | 0.999714303421225 |
| ebi-fl187-GCST90094853 | ebi-IPs91-GCST90274837 | STAM binding protein levels \|\| id:ebi-IPs91-GCST90274837 | F-sweetened coffee drink liking (derived food-liking factor) \|\| id:ebi-fl187-GCST90094853 | Weighted mode | 4 | 0.118542107105408 | 0.178086237348183 | 0.553244594183716 | -0.230506918 | 0.467591132307847 | 1.12585427956875 | 0.794130941108323 | 1.59614465727053 | 0.999714303421225 |
| ebi-fl187-GCST90094853 | ebi-IPs91-GCST90274840 | TNF-beta levels \|\| id:ebi-IPs91-GCST90274840 | F-sweetened coffee drink liking (derived food-liking factor) \|\| id:ebi-fl187-GCST90094853 | Weighted mode | 4 | 0.134794084271612 | 0.223980858371769 | 0.589740767045734 | -0.304208398 | 0.573796566680279 | 1.14430113048895 | 0.737707113642073 | 1.77499315517459 | 0.999714303421225 |
| ebi-fl187-GCST90094853 | ebi-IPs91-GCST90274842 | Tumor necrosis factor ligand superfamily member 14 levels \|\| id:ebi-IPs91-GCST90274842 | F-sweetened coffee drink liking (derived food-liking factor) \|\| id:ebi-fl187-GCST90094853 | Weighted mode | 4 | 0.308823070221043 | 0.228194166711133 | 0.26889237708736 | -0.138437497 | 0.756083636974863 | 1.36182140242713 | 0.870717672441843 | 2.12991833151573 | 0.999714303421225 |
| ebi-fl187-GCST90094853 | ebi-met1400-GCST90199628 | 3-methylhistidine levels \|\| id:ebi-met1400-GCST90199628 | F-sweetened coffee drink liking (derived food-liking factor) \|\| id:ebi-fl187-GCST90094853 | Weighted mode | 4 | -0.294391321 | 0.236196231021793 | 0.301080838967923 | -0.757335934 | 0.168553292049188 | 0.744984906340444 | 0.468913982492478 | 1.183591301 | 0.999714303421225 |
| ebi-fl187-GCST90094853 | ebi-met1400-GCST90199651 | Docosahexaenoate DHA; 22:6n3 levels \|\| id:ebi-met1400-GCST90199651 | F-sweetened coffee drink liking (derived food-liking factor) \|\| id:ebi-fl187-GCST90094853 | Weighted mode | 4 | 0.12323542000203 | 0.237671479768495 | 0.639919477419575 | -0.34260068 | 0.58907152034828 | 1.13115068510089 | 0.709921640632211 | 1.80231422620779 | 0.999714303421225 |
| ebi-fl187-GCST90094853 | ebi-met1400-GCST90199652 | 1-stearoyl-2-oleoyl-GPS (18:0/18:1) levels \|\| id:ebi-met1400-GCST90199652 | F-sweetened coffee drink liking (derived food-liking factor) \|\| id:ebi-fl187-GCST90094853 | Weighted mode | 4 | 0.00633502850445417 | 0.236688610449848 | 0.980327861363733 | -0.457574648 | 0.470244704986157 | 1.00635513723824 | 0.632816588785587 | 1.60038576768242 | 0.999714303421225 |
| ebi-fl187-GCST90094853 | ebi-met1400-GCST90199653 | 1-myristoyl-2-palmitoyl-gpc (14:0/16:0) levels \|\| id:ebi-met1400-GCST90199653 | F-sweetened coffee drink liking (derived food-liking factor) \|\| id:ebi-fl187-GCST90094853 | Weighted mode | 4 | 0.0706499796455283 | 0.21052282936678 | 0.759276589470746 | -0.341974766 | 0.483274725204418 | 1.07320551634395 | 0.710366129923481 | 1.62137527648616 | 0.999714303421225 |
| ebi-fl187-GCST90094853 | ebi-met1400-GCST90199667 | Erythritol levels in elite athletes \|\| id:ebi-met1400-GCST90199667 | F-sweetened coffee drink liking (derived food-liking factor) \|\| id:ebi-fl187-GCST90094853 | Weighted mode | 4 | -0.11040893 | 0.238146768255194 | 0.674488441539714 | -0.577176596 | 0.356358736001535 | 0.895467876933957 | 0.561481419848379 | 1.42811977435895 | 0.999714303421225 |
| ebi-fl187-GCST90094853 | ebi-met1400-GCST90199674 | 3-hydroxymyristate levels \|\| id:ebi-met1400-GCST90199674 | F-sweetened coffee drink liking (derived food-liking factor) \|\| id:ebi-fl187-GCST90094853 | Weighted mode | 4 | 0.212624249644823 | 0.233279730997736 | 0.429257925203636 | -0.244604023 | 0.669852522400385 | 1.2369197908626 | 0.783014532521366 | 1.95394913565775 | 0.999714303421225 |
| ebi-fl187-GCST90094853 | ebi-met1400-GCST90199685 | 2-linoleoylglycerol (18:2) levels \|\| id:ebi-met1400-GCST90199685 | F-sweetened coffee drink liking (derived food-liking factor) \|\| id:ebi-fl187-GCST90094853 | Weighted mode | 4 | 0.109037702512994 | 0.310047567953158 | 0.748329406710627 | -0.498655531 | 0.716730935701183 | 1.11520439555705 | 0.607346670006657 | 2.04772810206754 | 0.999714303421225 |
| ebi-fl187-GCST90094853 | ebi-met1400-GCST90199703 | Gamma-glutamylvaline levels \|\| id:ebi-met1400-GCST90199703 | F-sweetened coffee drink liking (derived food-liking factor) \|\| id:ebi-fl187-GCST90094853 | Weighted mode | 4 | 0.139182814745283 | 0.230295021888854 | 0.588245785659088 | -0.312195428 | 0.590561057647438 | 1.14933419603699 | 0.731838492404456 | 1.80500084088218 | 0.999714303421225 |
| ebi-fl187-GCST90094853 | ebi-met1400-GCST90199779 | Gamma-glutamyl-2-aminobutyrate levels \|\| id:ebi-met1400-GCST90199779 | F-sweetened coffee drink liking (derived food-liking factor) \|\| id:ebi-fl187-GCST90094853 | Weighted mode | 4 | -0.043666231 | 0.261759089380947 | 0.878123005252927 | -0.556714046 | 0.469381584408921 | 0.957273412560347 | 0.573089117771075 | 1.59900503774874 | 0.999714303421225 |
| ebi-fl187-GCST90094853 | ebi-met1400-GCST90199824 | 4-vinylphenol sulfate levels \|\| id:ebi-met1400-GCST90199824 | F-sweetened coffee drink liking (derived food-liking factor) \|\| id:ebi-fl187-GCST90094853 | Weighted mode | 4 | 0.183474987981064 | 0.303635813008945 | 0.588308536077376 | -0.411651206 | 0.778601181478597 | 1.20138491597363 | 0.662555331398991 | 2.17842291493054 | 0.999714303421225 |
| ebi-fl187-GCST90094853 | ebi-met1400-GCST90199830 | 4-ethylphenylsulfate levels \|\| id:ebi-met1400-GCST90199830 | F-sweetened coffee drink liking (derived food-liking factor) \|\| id:ebi-fl187-GCST90094853 | Weighted mode | 4 | -0.019844314 | 0.216457806732491 | 0.932732934797406 | -0.444101616 | 0.404412986728786 | 0.980351287939393 | 0.641400241205252 | 1.49842264785939 | 0.999714303421225 |
| ebi-fl187-GCST90094853 | ebi-met1400-GCST90199833 | Hexanoylglutamine levels \|\| id:ebi-met1400-GCST90199833 | F-sweetened coffee drink liking (derived food-liking factor) \|\| id:ebi-fl187-GCST90094853 | Weighted mode | 4 | 0.217878533967993 | 0.241574825506743 | 0.433581712955458 | -0.255608124 | 0.691365191961209 | 1.24343602320607 | 0.774445395879894 | 1.99643919640051 | 0.999714303421225 |
| ebi-fl187-GCST90094853 | ebi-met1400-GCST90199837 | Carotenoid levels (cryptoxanthin) \|\| id:ebi-met1400-GCST90199837 | F-sweetened coffee drink liking (derived food-liking factor) \|\| id:ebi-fl187-GCST90094853 | Weighted mode | 4 | -0.101545182 | 0.273754968703073 | 0.735331681221086 | -0.63810492 | 0.435014557084271 | 0.903440359562841 | 0.528292632686033 | 1.54498554927248 | 0.999714303421225 |
| ebi-fl187-GCST90094853 | ebi-met1400-GCST90199869 | 2-hydroxyhippurate levels \|\| id:ebi-met1400-GCST90199869 | F-sweetened coffee drink liking (derived food-liking factor) \|\| id:ebi-fl187-GCST90094853 | Weighted mode | 4 | 0.173445498992563 | 0.25613787788478 | 0.546848395579193 | -0.328584742 | 0.675475739646732 | 1.1893958617304 | 0.719941916566472 | 1.96496756661729 | 0.999714303421225 |
| ebi-fl187-GCST90094853 | ebi-met1400-GCST90199902 | Hydantoin-5-propionate levels \|\| id:ebi-met1400-GCST90199902 | F-sweetened coffee drink liking (derived food-liking factor) \|\| id:ebi-fl187-GCST90094853 | Weighted mode | 4 | 0.290749787043993 | 0.253125069879288 | 0.333983323912876 | -0.20537535 | 0.786874924007398 | 1.33742989979195 | 0.814341595986509 | 2.19652139307781 | 0.999714303421225 |
| ebi-fl187-GCST90094853 | ebi-met1400-GCST90199904 | Pantoate levels \|\| id:ebi-met1400-GCST90199904 | F-sweetened coffee drink liking (derived food-liking factor) \|\| id:ebi-fl187-GCST90094853 | Weighted mode | 4 | 0.25567242952707 | 0.251392575675232 | 0.384022361447761 | -0.237057019 | 0.748401877850525 | 1.29132965718423 | 0.788946301966545 | 2.11361949395924 | 0.999714303421225 |
| ebi-fl187-GCST90094853 | ebi-met1400-GCST90199936 | 3-methyl catechol sulfate (2) levels \|\| id:ebi-met1400-GCST90199936 | F-sweetened coffee drink liking (derived food-liking factor) \|\| id:ebi-fl187-GCST90094853 | Weighted mode | 4 | 0.0232451119599953 | 0.239889685440297 | 0.928917124570447 | -0.446938672 | 0.493428895422978 | 1.0235173851557 | 0.639583131744215 | 1.63792286838314 | 0.999714303421225 |
| ebi-fl187-GCST90094853 | ebi-met1400-GCST90199977 | 4-hydroxychlorothalonil levels \|\| id:ebi-met1400-GCST90199977 | F-sweetened coffee drink liking (derived food-liking factor) \|\| id:ebi-fl187-GCST90094853 | Weighted mode | 4 | 0.191031087520644 | 0.263419162161242 | 0.520778541824661 | -0.32527047 | 0.707332645356679 | 1.21049708284943 | 0.722331957866424 | 2.02857311189039 | 0.999714303421225 |
| ebi-fl187-GCST90094853 | ebi-met1400-GCST90200040 | Sphingomyelin (d18:2/24:1, d18:1/24:2) levels \|\| id:ebi-met1400-GCST90200040 | F-sweetened coffee drink liking (derived food-liking factor) \|\| id:ebi-fl187-GCST90094853 | Weighted mode | 4 | 0.231006539179243 | 0.347126704112673 | 0.553335288643716 | -0.449361801 | 0.911374879240082 | 1.25986747792156 | 0.638035215226008 | 2.48774052598675 | 0.999714303421225 |
| ebi-fl187-GCST90094853 | ebi-met1400-GCST90200077 | 1-stearoyl-2-oleoyl-GPI (18:0/18:1) levels \|\| id:ebi-met1400-GCST90200077 | F-sweetened coffee drink liking (derived food-liking factor) \|\| id:ebi-fl187-GCST90094853 | Weighted mode | 4 | -0.034609978 | 0.274460152265478 | 0.907627769955129 | -0.572551876 | 0.503331920870664 | 0.965982097481041 | 0.564084127705105 | 1.65422384148645 | 0.999714303421225 |
| ebi-fl187-GCST90094853 | ebi-met1400-GCST90200080 | 1-palmitoyl-2-oleoyl-GPI (16:0/18:1) levels \|\| id:ebi-met1400-GCST90200080 | F-sweetened coffee drink liking (derived food-liking factor) \|\| id:ebi-fl187-GCST90094853 | Weighted mode | 4 | 0.122042168853255 | 0.23194661708356 | 0.635192416946982 | -0.332573201 | 0.576657538337033 | 1.12980174321974 | 0.717076176514518 | 1.78007863151553 | 0.999714303421225 |
| ebi-fl187-GCST90094853 | ebi-met1400-GCST90200082 | 1-oleoyl-2-linoleoyl-GPE (18:1/18:2) levels \|\| id:ebi-met1400-GCST90200082 | F-sweetened coffee drink liking (derived food-liking factor) \|\| id:ebi-fl187-GCST90094853 | Weighted mode | 4 | -0.15460184 | 0.279573626689121 | 0.618807046228676 | -0.702566148 | 0.393362468779076 | 0.856756236045337 | 0.495312626123498 | 1.48195545457296 | 0.999714303421225 |
| ebi-fl187-GCST90094853 | ebi-met1400-GCST90200100 | Perfluorooctanesulfonate (PFOS) levels \|\| id:ebi-met1400-GCST90200100 | F-sweetened coffee drink liking (derived food-liking factor) \|\| id:ebi-fl187-GCST90094853 | Weighted mode | 4 | 0.321249506939087 | 0.27682800914878 | 0.329816283916097 | -0.221333391 | 0.863832404870695 | 1.37884957051387 | 0.801449439720707 | 2.3722346587064 | 0.999714303421225 |
| ebi-fl187-GCST90094853 | ebi-met1400-GCST90200102 | 1-palmitoleoyl-2-linolenoyl-GPC (16:1/18:3) levels \|\| id:ebi-met1400-GCST90200102 | F-sweetened coffee drink liking (derived food-liking factor) \|\| id:ebi-fl187-GCST90094853 | Weighted mode | 4 | 0.0547721077438087 | 0.22614891506013 | 0.824241966021345 | -0.388479766 | 0.498023981261664 | 1.05629986468463 | 0.678086942305478 | 1.64546658329562 | 0.999714303421225 |
| ebi-fl187-GCST90094853 | ebi-met1400-GCST90200114 | Glycosyl-N-tricosanoyl-sphingadienine (d18:2/23:0) levels \|\| id:ebi-met1400-GCST90200114 | F-sweetened coffee drink liking (derived food-liking factor) \|\| id:ebi-fl187-GCST90094853 | Weighted mode | 4 | -0.029835014 | 0.251262434327802 | 0.912985613332519 | -0.522309385 | 0.462639357373861 | 0.970605656772664 | 0.593149155167508 | 1.58826044469885 | 0.999714303421225 |
| ebi-fl187-GCST90094853 | ebi-met1400-GCST90200135 | Arachidoylcarnitine (C20) levels \|\| id:ebi-met1400-GCST90200135 | F-sweetened coffee drink liking (derived food-liking factor) \|\| id:ebi-fl187-GCST90094853 | Weighted mode | 4 | -0.301149728 | 0.218339308297097 | 0.261646694910259 | -0.729094772 | 0.126795316469287 | 0.739966970830399 | 0.482345425081591 | 1.13518464040018 | 0.999714303421225 |
| ebi-fl187-GCST90094853 | ebi-met1400-GCST90200146 | Docosahexaenoylcarnitine (C22:6) levels \|\| id:ebi-met1400-GCST90200146 | F-sweetened coffee drink liking (derived food-liking factor) \|\| id:ebi-fl187-GCST90094853 | Weighted mode | 4 | 0.0463555694898594 | 0.283117938234499 | 0.880350805647128 | -0.508555589 | 0.601266728429477 | 1.04744678486956 | 0.601363567676904 | 1.82442839258106 | 0.999714303421225 |
| ebi-fl187-GCST90094853 | ebi-met1400-GCST90200162 | 3-hydroxyphenylacetoylglutamine levels \|\| id:ebi-met1400-GCST90200162 | F-sweetened coffee drink liking (derived food-liking factor) \|\| id:ebi-fl187-GCST90094853 | Weighted mode | 4 | -0.141961327 | 0.336324154015434 | 0.701377921870403 | -0.801156669 | 0.517234014448266 | 0.867654810285444 | 0.448809539561442 | 1.67738161391823 | 0.999714303421225 |
| ebi-fl187-GCST90094853 | ebi-met1400-GCST90200172 | 3-carboxy-4-methyl-5-pentyl-2-furanpropionate (3-CMPFP) levels \|\| id:ebi-met1400-GCST90200172 | F-sweetened coffee drink liking (derived food-liking factor) \|\| id:ebi-fl187-GCST90094853 | Weighted mode | 4 | 0.0925229203565683 | 0.215711300348812 | 0.696916747694771 | -0.330271228 | 0.51531706904024 | 1.09693828348876 | 0.718728767391745 | 1.67416924488766 | 0.999714303421225 |
| ebi-fl187-GCST90094853 | ebi-met1400-GCST90200222 | Dihydrocaffeate sulfate (2) levels \|\| id:ebi-met1400-GCST90200222 | F-sweetened coffee drink liking (derived food-liking factor) \|\| id:ebi-fl187-GCST90094853 | Weighted mode | 4 | 0.198581351161545 | 0.267699966854436 | 0.512000969155362 | -0.326110584 | 0.72327328619624 | 1.21967124505095 | 0.72172537183167 | 2.06116897654402 | 0.999714303421225 |
| ebi-fl187-GCST90094853 | ebi-met1400-GCST90200223 | 4-methylhexanoylglutamine levels \|\| id:ebi-met1400-GCST90200223 | F-sweetened coffee drink liking (derived food-liking factor) \|\| id:ebi-fl187-GCST90094853 | Weighted mode | 4 | 0.187140265781768 | 0.341415067781487 | 0.621754200732161 | -0.482033267 | 0.856313798633483 | 1.20579640516419 | 0.617526518125096 | 2.35446564322659 | 0.999714303421225 |
| ebi-fl187-GCST90094853 | ebi-met1400-GCST90200224 | 2-ketocaprylate levels \|\| id:ebi-met1400-GCST90200224 | F-sweetened coffee drink liking (derived food-liking factor) \|\| id:ebi-fl187-GCST90094853 | Weighted mode | 4 | -0.333227584 | 0.361865660797366 | 0.425037274537008 | -1.04248428 | 0.376029110785364 | 0.716607087018293 | 0.352577691519705 | 1.45648953270812 | 0.999714303421225 |
| ebi-fl187-GCST90094853 | ebi-met1400-GCST90200243 | Hydroxypalmitoyl sphingomyelin (d18:1/16:0(OH)) levels \|\| id:ebi-met1400-GCST90200243 | F-sweetened coffee drink liking (derived food-liking factor) \|\| id:ebi-fl187-GCST90094853 | Weighted mode | 4 | -0.127821933 | 0.232605166514576 | 0.620909180342282 | -0.583728059 | 0.328084193679209 | 0.880010066219634 | 0.557814918410344 | 1.38830585394671 | 0.999714303421225 |
| ebi-fl187-GCST90094853 | ebi-met1400-GCST90200273 | 2-methoxyhydroquinone sulfate (2) levels \|\| id:ebi-met1400-GCST90200273 | F-sweetened coffee drink liking (derived food-liking factor) \|\| id:ebi-fl187-GCST90094853 | Weighted mode | 4 | 0.115247024304256 | 0.30023891039278 | 0.72667794175593 | -0.47322124 | 0.703715288674105 | 1.12215060180906 | 0.622992225088939 | 2.02124829561182 | 0.999714303421225 |
| ebi-fl187-GCST90094853 | ebi-met1400-GCST90200276 | 2,4-di-tert-butylphenol levels \|\| id:ebi-met1400-GCST90200276 | F-sweetened coffee drink liking (derived food-liking factor) \|\| id:ebi-fl187-GCST90094853 | Weighted mode | 4 | 0.0549855753364503 | 0.244948408627119 | 0.836805241797061 | -0.425113306 | 0.535084456245604 | 1.05652537454252 | 0.653695713566367 | 1.70759245301198 | 0.999714303421225 |
| ebi-fl187-GCST90094853 | ebi-met1400-GCST90200289 | N-acetylmethionine levels \|\| id:ebi-met1400-GCST90200289 | F-sweetened coffee drink liking (derived food-liking factor) \|\| id:ebi-fl187-GCST90094853 | Weighted mode | 4 | 0.0488485372639886 | 0.334916001557411 | 0.893286270468308 | -0.607586826 | 0.705283900316515 | 1.05006129353821 | 0.544663652730285 | 2.0244213372049 | 0.999714303421225 |
| ebi-fl187-GCST90094853 | ebi-met1400-GCST90200308 | 3-hydroxyisobutyrate levels \|\| id:ebi-met1400-GCST90200308 | F-sweetened coffee drink liking (derived food-liking factor) \|\| id:ebi-fl187-GCST90094853 | Weighted mode | 4 | -0.138043905 | 0.230878593951711 | 0.592028724502495 | -0.590565949 | 0.314478139206156 | 0.871060447050274 | 0.554013652473487 | 1.36954441290007 | 0.999714303421225 |
| ebi-fl187-GCST90094853 | ebi-met1400-GCST90200383 | Cystathionine levels \|\| id:ebi-met1400-GCST90200383 | F-sweetened coffee drink liking (derived food-liking factor) \|\| id:ebi-fl187-GCST90094853 | Weighted mode | 4 | 0.370961430290218 | 0.608471799978809 | 0.585159318523172 | -0.821643298 | 1.56356615824868 | 1.44912717989399 | 0.439708488546333 | 4.77582225089617 | 0.999714303421225 |
| ebi-fl187-GCST90094853 | ebi-met1400-GCST90200384 | Guanidinoacetate levels \|\| id:ebi-met1400-GCST90200384 | F-sweetened coffee drink liking (derived food-liking factor) \|\| id:ebi-fl187-GCST90094853 | Weighted mode | 4 | -0.047615885 | 0.235095893207666 | 0.85245391451073 | -0.508403836 | 0.413172065427907 | 0.953499970138807 | 0.601454833630034 | 1.51160509853671 | 0.999714303421225 |
| ebi-fl187-GCST90094853 | ebi-met1400-GCST90200403 | Citrulline levels \|\| id:ebi-met1400-GCST90200403 | F-sweetened coffee drink liking (derived food-liking factor) \|\| id:ebi-fl187-GCST90094853 | Weighted mode | 4 | 0.112765565797578 | 0.254711253685767 | 0.687945051585013 | -0.386468491 | 0.611999623021681 | 1.11936948369412 | 0.679452133604838 | 1.84411524970554 | 0.999714303421225 |
| ebi-fl187-GCST90094853 | ebi-met1400-GCST90200434 | 12,13-DiHOME levels \|\| id:ebi-met1400-GCST90200434 | F-sweetened coffee drink liking (derived food-liking factor) \|\| id:ebi-fl187-GCST90094853 | Weighted mode | 4 | -0.057135416 | 0.225546991384147 | 0.816389008671775 | -0.499207519 | 0.384936687567459 | 0.944466165305668 | 0.607011514452606 | 1.46952127952894 | 0.999714303421225 |
| ebi-fl187-GCST90094853 | ebi-met1400-GCST90200437 | Fructose levels \|\| id:ebi-met1400-GCST90200437 | F-sweetened coffee drink liking (derived food-liking factor) \|\| id:ebi-fl187-GCST90094853 | Weighted mode | 4 | 0.0312370151225012 | 0.244715547713493 | 0.906504698170126 | -0.448405458 | 0.510879488640947 | 1.03173001052433 | 0.638645687273321 | 1.66675644387616 | 0.999714303421225 |
| ebi-fl187-GCST90094853 | ebi-met1400-GCST90200448 | N-stearoyl-sphinganine (d18:0/18:0) levels \|\| id:ebi-met1400-GCST90200448 | F-sweetened coffee drink liking (derived food-liking factor) \|\| id:ebi-fl187-GCST90094853 | Weighted mode | 4 | 0.0723697196286792 | 0.263655239723354 | 0.80152750877285 | -0.44439455 | 0.589133989486453 | 1.07505273869635 | 0.641212380420434 | 1.80242681874096 | 0.999714303421225 |
| ebi-fl187-GCST90094853 | ebi-met1400-GCST90200468 | X-11847 levels \|\| id:ebi-met1400-GCST90200468 | F-sweetened coffee drink liking (derived food-liking factor) \|\| id:ebi-fl187-GCST90094853 | Weighted mode | 4 | -0.286023061 | 0.372496119885765 | 0.498476810435638 | -1.016115456 | 0.44406933401345 | 0.751245291307522 | 0.361998411264264 | 1.55903857627631 | 0.999714303421225 |
| ebi-fl187-GCST90094853 | ebi-met1400-GCST90200476 | X-11843 levels \|\| id:ebi-met1400-GCST90200476 | F-sweetened coffee drink liking (derived food-liking factor) \|\| id:ebi-fl187-GCST90094853 | Weighted mode | 4 | -0.086650329 | 0.295606055950352 | 0.788531458614176 | -0.666038199 | 0.492737540669991 | 0.916997687084053 | 0.513739886682294 | 1.636790874 | 0.999714303421225 |
| ebi-fl187-GCST90094853 | ebi-met1400-GCST90200511 | X-12906 levels \|\| id:ebi-met1400-GCST90200511 | F-sweetened coffee drink liking (derived food-liking factor) \|\| id:ebi-fl187-GCST90094853 | Weighted mode | 4 | -0.147443678 | 0.262674495639188 | 0.613780858645521 | -0.662285689 | 0.367398333836522 | 0.862911038159335 | 0.515671322130518 | 1.443972988 | 0.999714303421225 |
| ebi-fl187-GCST90094853 | ebi-met1400-GCST90200515 | X-12849 levels \|\| id:ebi-met1400-GCST90200515 | F-sweetened coffee drink liking (derived food-liking factor) \|\| id:ebi-fl187-GCST90094853 | Weighted mode | 4 | 0.00807931864017275 | 0.273564375831529 | 0.978293963891953 | -0.528106858 | 0.544265495269971 | 1.00811204440962 | 0.589720337916081 | 1.72334211445895 | 0.999714303421225 |
| ebi-fl187-GCST90094853 | ebi-met1400-GCST90200551 | X-18886 levels \|\| id:ebi-met1400-GCST90200551 | F-sweetened coffee drink liking (derived food-liking factor) \|\| id:ebi-fl187-GCST90094853 | Weighted mode | 4 | 0.231068161685473 | 0.236643338740211 | 0.400859153158976 | -0.232752782 | 0.694889105616287 | 1.25994511650518 | 0.792349432160975 | 2.0034868861782 | 0.999714303421225 |
| ebi-fl187-GCST90094853 | ebi-met1400-GCST90200564 | X-21319 levels \|\| id:ebi-met1400-GCST90200564 | F-sweetened coffee drink liking (derived food-liking factor) \|\| id:ebi-fl187-GCST90094853 | Weighted mode | 4 | -0.176348081 | 0.225577523508745 | 0.491378469980231 | -0.618480027 | 0.265783864724268 | 0.838326126852738 | 0.538762720109944 | 1.30445308988806 | 0.999714303421225 |
| ebi-fl187-GCST90094853 | ebi-met1400-GCST90200579 | X-21442 levels \|\| id:ebi-met1400-GCST90200579 | F-sweetened coffee drink liking (derived food-liking factor) \|\| id:ebi-fl187-GCST90094853 | Weighted mode | 4 | -0.028618923 | 0.257735029085722 | 0.918596676035047 | -0.53377958 | 0.476541733754017 | 0.971786719235157 | 0.586384488838952 | 1.61049523931251 | 0.999714303421225 |
| ebi-fl187-GCST90094853 | ebi-met1400-GCST90200581 | X-21441 levels \|\| id:ebi-met1400-GCST90200581 | F-sweetened coffee drink liking (derived food-liking factor) \|\| id:ebi-fl187-GCST90094853 | Weighted mode | 4 | 0.00270444487877664 | 0.222109358 | 0.991049517220499 | -0.432629896 | 0.438038785794376 | 1.00270810518879 | 0.64880057103775 | 1.54966501124239 | 0.999714303421225 |
| ebi-fl187-GCST90094853 | ebi-met1400-GCST90200648 | X-24585 levels \|\| id:ebi-met1400-GCST90200648 | F-sweetened coffee drink liking (derived food-liking factor) \|\| id:ebi-fl187-GCST90094853 | Weighted mode | 4 | 0.0358116780053207 | 0.240527433470016 | 0.891087166588405 | -0.435622092 | 0.507245447606552 | 1.03646063977555 | 0.646862134387399 | 1.6607103750497 | 0.999714303421225 |
| ebi-fl187-GCST90094853 | ebi-met1400-GCST90200720 | Adenosine 5'-diphosphate (ADP) to pantothenate ratio \|\| id:ebi-met1400-GCST90200720 | F-sweetened coffee drink liking (derived food-liking factor) \|\| id:ebi-fl187-GCST90094853 | Weighted mode | 4 | -0.093891061 | 0.312057792233646 | 0.783155607065836 | -0.705524334 | 0.517742211331343 | 0.910381932529704 | 0.493849564129888 | 1.67823427066656 | 0.999714303421225 |
| ebi-fl187-GCST90094853 | ebi-met1400-GCST90200724 | Cholate to taurocholate ratio \|\| id:ebi-met1400-GCST90200724 | F-sweetened coffee drink liking (derived food-liking factor) \|\| id:ebi-fl187-GCST90094853 | Weighted mode | 4 | -0.148297525 | 0.291085534435132 | 0.64553755902481 | -0.718825172 | 0.422230122679762 | 0.862174558453618 | 0.487324442034624 | 1.52535950411425 | 0.999714303421225 |
| ebi-fl187-GCST90094853 | ebi-met1400-GCST90200726 | N-acetylputrescine to (N(1) + N(8))-acetylspermidine ratio \|\| id:ebi-met1400-GCST90200726 | F-sweetened coffee drink liking (derived food-liking factor) \|\| id:ebi-fl187-GCST90094853 | Weighted mode | 4 | -0.101796351 | 0.286019114798517 | 0.745466076337557 | -0.662393816 | 0.458801113822057 | 0.903213471295629 | 0.515615567090458 | 1.58217599855123 | 0.999714303421225 |
| ebi-fl187-GCST90094853 | ebi-met1400-GCST90200744 | Arginine to glutamate ratio \|\| id:ebi-met1400-GCST90200744 | F-sweetened coffee drink liking (derived food-liking factor) \|\| id:ebi-fl187-GCST90094853 | Weighted mode | 4 | -0.460756681 | 0.418159201319911 | 0.350986208792751 | -1.280348716 | 0.358835353358665 | 0.630806145702953 | 0.277940361352341 | 1.43166106397978 | 0.999714303421225 |
| ebi-fl187-GCST90094853 | ebi-met1400-GCST90200759 | Histidine to trans-urocanate ratio \|\| id:ebi-met1400-GCST90200759 | F-sweetened coffee drink liking (derived food-liking factor) \|\| id:ebi-fl187-GCST90094853 | Weighted mode | 4 | 0.0150510268285018 | 0.374069546343493 | 0.970433012793258 | -0.718125284 | 0.748227337661748 | 1.01516486393754 | 0.487665634094736 | 2.11325061460683 | 0.999714303421225 |
| ebi-fl187-GCST90094853 | ebi-met1400-GCST90200795 | Oleoyl-linoleoyl-glycerol (18:1 to 18:2) [2] to linoleoyl-arachidonoyl-glycerol (18:2 to 20:4) [2] ratio \|\| id:ebi-met1400-GCST90200795 | F-sweetened coffee drink liking (derived food-liking factor) \|\| id:ebi-fl187-GCST90094853 | Weighted mode | 4 | -0.147221572 | 0.255585479634501 | 0.604975567138495 | -0.648169112 | 0.353725968118621 | 0.863102716863141 | 0.523002459606057 | 1.42436481162565 | 0.999714303421225 |
| ebi-fl187-GCST90094853 | ebi-met1400-GCST90200811 | Choline to taurocholate ratio \|\| id:ebi-met1400-GCST90200811 | F-sweetened coffee drink liking (derived food-liking factor) \|\| id:ebi-fl187-GCST90094853 | Weighted mode | 4 | -0.018897823 | 0.253529177905801 | 0.945273500438453 | -0.515815012 | 0.478019365519603 | 0.981279621156025 | 0.59701382292991 | 1.61287671727688 | 0.999714303421225 |
| ebi-fl187-GCST90094853 | ebi-met1400-GCST90200831 | Cholate to phosphate ratio \|\| id:ebi-met1400-GCST90200831 | F-sweetened coffee drink liking (derived food-liking factor) \|\| id:ebi-fl187-GCST90094853 | Weighted mode | 4 | 0.233588644835269 | 0.242092513530082 | 0.405783474216789 | -0.240912682 | 0.708089971354231 | 1.26312479241745 | 0.785910247751954 | 2.03010998492946 | 0.999714303421225 |
| ebi-fl187-GCST90094853 | ebi-met1400-GCST90200841 | Adenosine 5'-diphosphate (ADP) to oxalate (ethanedioate) ratio \|\| id:ebi-met1400-GCST90200841 | F-sweetened coffee drink liking (derived food-liking factor) \|\| id:ebi-fl187-GCST90094853 | Weighted mode | 4 | 0.402248431431258 | 0.32298303845741 | 0.30138985027831 | -0.230798324 | 1.03529518680778 | 1.49518273692722 | 0.793899560422299 | 2.81593734050689 | 0.999714303421225 |
| ebi-fl187-GCST90094853 | ebi-met1400-GCST90200844 | Aspartate to phosphate ratio \|\| id:ebi-met1400-GCST90200844 | F-sweetened coffee drink liking (derived food-liking factor) \|\| id:ebi-fl187-GCST90094853 | Weighted mode | 4 | -0.032051512 | 0.262437132868222 | 0.910517678125167 | -0.546428292 | 0.482325268683705 | 0.968456693906804 | 0.579014191029317 | 1.61983658173485 | 0.999714303421225 |
| ebi-fl187-GCST90094853 | ebi-met1400-GCST90200870 | Adenosine 5'-monophosphate (AMP) to acetoacetate ratio \|\| id:ebi-met1400-GCST90200870 | F-sweetened coffee drink liking (derived food-liking factor) \|\| id:ebi-fl187-GCST90094853 | Weighted mode | 4 | 0.0576244899175389 | 0.256461467633625 | 0.836654194168004 | -0.445039987 | 0.560288966479444 | 1.05931713674956 | 0.640798652132202 | 1.75117845906421 | 0.999714303421225 |
| ebi-fl187-GCST90094853 | ebi-met1400-GCST90200887 | Cholesterol to cortisol ratio \|\| id:ebi-met1400-GCST90200887 | F-sweetened coffee drink liking (derived food-liking factor) \|\| id:ebi-fl187-GCST90094853 | Weighted mode | 4 | 0.0875168159556052 | 0.268722906837455 | 0.766062422201651 | -0.439180081 | 0.614213713357016 | 1.09146061824193 | 0.644564695035577 | 1.84820281090219 | 0.999714303421225 |
| ebi-fl187-GCST90094853 | ebi-met1400-GCST90200890 | Cortisol to taurocholate ratio \|\| id:ebi-met1400-GCST90200890 | F-sweetened coffee drink liking (derived food-liking factor) \|\| id:ebi-fl187-GCST90094853 | Weighted mode | 4 | 0.146794468822945 | 0.279732729243381 | 0.636054185510608 | -0.40148168 | 0.695070618139971 | 1.15811590964092 | 0.669327581337998 | 2.00385057714531 | 0.999714303421225 |
| ebi-fl187-GCST90094853 | ebi-met1400-GCST90200948 | Adenosine 5'-diphosphate (ADP) to mannitol to sorbitol ratio \|\| id:ebi-met1400-GCST90200948 | F-sweetened coffee drink liking (derived food-liking factor) \|\| id:ebi-fl187-GCST90094853 | Weighted mode | 4 | 0.087310511314642 | 0.299254188491926 | 0.789481659099814 | -0.499227698 | 0.673848720758817 | 1.09123546807652 | 0.60699926540486 | 1.96177312668401 | 0.999714303421225 |
| ebi-fl187-GCST90094853 | ebi-met1400-GCST90200972 | Leucine to N-palmitoyl-sphingosine (d18:1 to 16:0) ratio \|\| id:ebi-met1400-GCST90200972 | F-sweetened coffee drink liking (derived food-liking factor) \|\| id:ebi-fl187-GCST90094853 | Weighted mode | 4 | -0.138151696 | 0.237893415550527 | 0.602171618133165 | -0.604422791 | 0.328119398378474 | 0.870966559493286 | 0.546389716928519 | 1.3883547296971 | 0.999714303421225 |
| ebi-fl187-GCST90094853 | ebi-met1400-GCST90200973 | Proline to glutamate ratio \|\| id:ebi-met1400-GCST90200973 | F-sweetened coffee drink liking (derived food-liking factor) \|\| id:ebi-fl187-GCST90094853 | Weighted mode | 4 | -0.068969145 | 0.276815639551628 | 0.819327554009096 | -0.611527799 | 0.473589508266988 | 0.933355478079937 | 0.542521372095702 | 1.60574770556349 | 0.999714303421225 |
| ebi-fl187-GCST90094853 | ebi-met1400-GCST90200983 | Cholesterol to linoleoyl-arachidonoyl-glycerol (18:2 to 20:4) [1] ratio \|\| id:ebi-met1400-GCST90200983 | F-sweetened coffee drink liking (derived food-liking factor) \|\| id:ebi-fl187-GCST90094853 | Weighted mode | 4 | 0.105257008283605 | 0.257535794973638 | 0.710178286824714 | -0.39951315 | 0.610027166431936 | 1.11099610886943 | 0.670646470894275 | 1.8404813974151 | 0.999714303421225 |
| ebi-fl187-GCST90094853 | ebi-met1400-GCST90201001 | Serine to alpha-ketobutyrate ratio \|\| id:ebi-met1400-GCST90201001 | F-sweetened coffee drink liking (derived food-liking factor) \|\| id:ebi-fl187-GCST90094853 | Weighted mode | 4 | -0.3048815 | 0.301334848006208 | 0.386164028571187 | -0.895497802 | 0.285734802410724 | 0.737210728921977 | 0.408404243672966 | 1.33073949954563 | 0.999714303421225 |
| ebi-fl187-GCST90094853 | ebi-met1400-GCST90201005 | Fructose to maltose ratio \|\| id:ebi-met1400-GCST90201005 | F-sweetened coffee drink liking (derived food-liking factor) \|\| id:ebi-fl187-GCST90094853 | Weighted mode | 4 | -0.058321638 | 0.283324706109706 | 0.850087538684801 | -0.613638062 | 0.496994786087319 | 0.943346482666472 | 0.541377716401687 | 1.64377394820389 | 0.999714303421225 |
| ebi-fl187-GCST90094853 | ebi-met1400-GCST90201020 | Paraxanthine to 5-acetylamino-6-formylamino-3-methyluracil ratio \|\| id:ebi-met1400-GCST90201020 | F-sweetened coffee drink liking (derived food-liking factor) \|\| id:ebi-fl187-GCST90094853 | Weighted mode | 4 | -0.114950468 | 0.253921019995492 | 0.681497318187094 | -0.612635667 | 0.382734731308357 | 0.891410296252604 | 0.541920662681156 | 1.46628901790496 | 0.999714303421225 |
| ebi-fl187-GCST90094853 | ebi-pw11-GCST90275197 | Placental weight (fetal genotype, adjusted for sex) \|\| id:ebi-pw11-GCST90275197 | F-sweetened coffee drink liking (derived food-liking factor) \|\| id:ebi-fl187-GCST90094853 | Weighted mode | 4 | 0.100071487047886 | 0.100382430277939 | 0.392285045316839 | -0.096678076 | 0.296821050392646 | 1.10524992630599 | 0.907848216955833 | 1.3455744878759 | 0.999714303421225 |
| ebi-fl187-GCST90094853 | prot-a-1017 | FAS-associated factor 2 \|\| id:prot-a-1017 | F-sweetened coffee drink liking (derived food-liking factor) \|\| id:ebi-fl187-GCST90094853 | Weighted mode | 4 | -0.413763471 | 0.460061733370119 | 0.434740481436613 | -1.315484468 | 0.487957526834014 | 0.661157315934901 | 0.268344287528633 | 1.62898566032489 | 0.999714303421225 |
| ebi-fl187-GCST90094853 | prot-a-1019 | Fas apoptotic inhibitory molecule 3 \|\| id:prot-a-1019 | F-sweetened coffee drink liking (derived food-liking factor) \|\| id:ebi-fl187-GCST90094853 | Weighted mode | 4 | -0.297476375 | 0.436133145942271 | 0.544132264648703 | -1.152297342 | 0.557344590569392 | 0.742690128713216 | 0.31591018151044 | 1.74602991473963 | 0.999714303421225 |
| ebi-fl187-GCST90094853 | prot-a-1052 | MIP18 family protein FAM96A \|\| id:prot-a-1052 | F-sweetened coffee drink liking (derived food-liking factor) \|\| id:ebi-fl187-GCST90094853 | Weighted mode | 4 | 0.357175671548942 | 0.4317880950636 | 0.468778752990841 | -0.489128995 | 1.2034803378736 | 1.4292869328503 | 0.613160227426309 | 3.3316921826312 | 0.999714303421225 |
| ebi-fl187-GCST90094853 | prot-a-1066 | F-box/LRR-repeat protein 4 \|\| id:prot-a-1066 | F-sweetened coffee drink liking (derived food-liking factor) \|\| id:ebi-fl187-GCST90094853 | Weighted mode | 4 | -0.526914526 | 0.376466164030422 | 0.256106001352491 | -1.264788208 | 0.210959155124451 | 0.590423899474655 | 0.282299078531364 | 1.23486191625003 | 0.999714303421225 |
| ebi-fl187-GCST90094853 | prot-a-1076 | Low affinity immunoglobulin gamma Fc region receptor III-B \|\| id:prot-a-1076 | F-sweetened coffee drink liking (derived food-liking factor) \|\| id:ebi-fl187-GCST90094853 | Weighted mode | 4 | -0.077761862 | 0.382349947600625 | 0.851852590337013 | -0.82716776 | 0.671644035074878 | 0.925184721745748 | 0.437286033317448 | 1.95745279779007 | 0.999714303421225 |
| ebi-fl187-GCST90094853 | prot-a-1081 | Fc receptor-like protein 3 \|\| id:prot-a-1081 | F-sweetened coffee drink liking (derived food-liking factor) \|\| id:ebi-fl187-GCST90094853 | Weighted mode | 4 | 0.177369918938592 | 0.397007988000428 | 0.685326460999161 | -0.600765738 | 0.955505575419431 | 1.1940727215525 | 0.548391551278531 | 2.59998473906397 | 0.999714303421225 |
| ebi-fl187-GCST90094853 | prot-a-1105 | Fibroblast growth factor receptor 4 \|\| id:prot-a-1105 | F-sweetened coffee drink liking (derived food-liking factor) \|\| id:ebi-fl187-GCST90094853 | Weighted mode | 4 | 0.139775763863943 | 0.383771676178131 | 0.739854046046254 | -0.612416721 | 0.891968249173079 | 1.15001589482195 | 0.542039326831418 | 2.43992731316052 | 0.999714303421225 |
| ebi-fl187-GCST90094853 | prot-a-1143 | Follistatin-related protein 1 \|\| id:prot-a-1143 | F-sweetened coffee drink liking (derived food-liking factor) \|\| id:ebi-fl187-GCST90094853 | Weighted mode | 4 | -0.110896019 | 0.369460458034525 | 0.783654740407339 | -0.835038517 | 0.61324647847504 | 0.895031810149301 | 0.433857770202619 | 1.84641602893274 | 0.999714303421225 |
| ebi-fl187-GCST90094853 | prot-a-1151 | Alpha-(1,3)-fucosyltransferase 10 \|\| id:prot-a-1151 | F-sweetened coffee drink liking (derived food-liking factor) \|\| id:ebi-fl187-GCST90094853 | Weighted mode | 4 | -0.16160411 | 0.427933603312203 | 0.730833652715461 | -1.000353973 | 0.677145752460308 | 0.850777952334955 | 0.36774924500159 | 1.96825182924884 | 0.999714303421225 |
| ebi-fl187-GCST90094853 | prot-a-1165 | Growth arrest and DNA damage-inducible proteins-interacting protein 1 \|\| id:prot-a-1165 | F-sweetened coffee drink liking (derived food-liking factor) \|\| id:ebi-fl187-GCST90094853 | Weighted mode | 4 | -0.42197233 | 0.441242384048729 | 0.409457652279806 | -1.286807402 | 0.442862743049101 | 0.655752184010978 | 0.276151016292697 | 1.55715858883312 | 0.999714303421225 |
| ebi-fl187-GCST90094853 | prot-a-1194 | Growth/differentiation factor 11/8 \|\| id:prot-a-1194 | F-sweetened coffee drink liking (derived food-liking factor) \|\| id:ebi-fl187-GCST90094853 | Weighted mode | 4 | -0.346086821 | 0.415807521222369 | 0.466287375585044 | -1.161069562 | 0.468895920710088 | 0.707451062912178 | 0.31315106706956 | 1.59822864759518 | 0.999714303421225 |
| ebi-fl187-GCST90094853 | prot-a-1216 | D-glucuronyl C5-epimerase \|\| id:prot-a-1216 | F-sweetened coffee drink liking (derived food-liking factor) \|\| id:ebi-fl187-GCST90094853 | Weighted mode | 4 | -0.056744069 | 0.453289163770975 | 0.908296331750343 | -0.94519083 | 0.831702691589081 | 0.944835850829854 | 0.388605406103106 | 2.29722688102933 | 0.999714303421225 |
| ebi-fl187-GCST90094853 | prot-a-1246 | Glypican-3 \|\| id:prot-a-1246 | F-sweetened coffee drink liking (derived food-liking factor) \|\| id:ebi-fl187-GCST90094853 | Weighted mode | 4 | 0.387947538169316 | 0.370924821801613 | 0.372457766935686 | -0.339065113 | 1.11496018890048 | 1.47395245601558 | 0.712436059041965 | 3.04944677493703 | 0.999714303421225 |
| ebi-fl187-GCST90094853 | prot-a-1275 | Glutamate receptor 4 \|\| id:prot-a-1275 | F-sweetened coffee drink liking (derived food-liking factor) \|\| id:ebi-fl187-GCST90094853 | Weighted mode | 4 | -0.165418776 | 0.359363136429591 | 0.676606775516836 | -0.869770524 | 0.538932971222666 | 0.847538700740699 | 0.419047699780475 | 1.7141768100136 | 0.999714303421225 |
| ebi-fl187-GCST90094853 | prot-a-1289 | Glutathione S-transferase P \|\| id:prot-a-1289 | F-sweetened coffee drink liking (derived food-liking factor) \|\| id:ebi-fl187-GCST90094853 | Weighted mode | 4 | 0.169626860199359 | 0.481799591308035 | 0.748065825013216 | -0.774700339 | 1.11395405916311 | 1.18486264941114 | 0.460841856750173 | 3.04638017880971 | 0.999714303421225 |
| ebi-fl187-GCST90094853 | prot-a-130 | Apolipoprotein D \|\| id:prot-a-130 | F-sweetened coffee drink liking (derived food-liking factor) \|\| id:ebi-fl187-GCST90094853 | Weighted mode | 4 | -0.3749208 | 0.485717212235393 | 0.496408112624048 | -1.326926536 | 0.577084936293456 | 0.687343714471979 | 0.265291373159742 | 1.78083959609068 | 0.999714303421225 |
| ebi-fl187-GCST90094853 | prot-a-1313 | Heparin-binding EGF-like growth factor \|\| id:prot-a-1313 | F-sweetened coffee drink liking (derived food-liking factor) \|\| id:ebi-fl187-GCST90094853 | Weighted mode | 4 | -0.532256841 | 0.409670953357203 | 0.284699171143129 | -1.33521191 | 0.270698227319017 | 0.587278079549683 | 0.263102415557097 | 1.31087942309187 | 0.999714303421225 |
| ebi-fl187-GCST90094853 | prot-a-1347 | HLA class II histocompatibility antigen, DQ alpha 2 chain \|\| id:prot-a-1347 | F-sweetened coffee drink liking (derived food-liking factor) \|\| id:ebi-fl187-GCST90094853 | Weighted mode | 4 | 0.244987812683219 | 0.353828385874088 | 0.538472293838916 | -0.448515824 | 0.938491448996432 | 1.2776057425241 | 0.638575206881934 | 2.55612246723563 | 0.999714303421225 |
| ebi-fl187-GCST90094853 | prot-a-1357 | Non-histone chromosomal protein HMG-14 \|\| id:prot-a-1357 | F-sweetened coffee drink liking (derived food-liking factor) \|\| id:ebi-fl187-GCST90094853 | Weighted mode | 4 | 0.0384967622227975 | 0.365274084954068 | 0.922716797925511 | -0.677440444 | 0.754433968732772 | 1.03924736350112 | 0.507915367705782 | 2.12640756947848 | 0.999714303421225 |
| ebi-fl187-GCST90094853 | prot-a-137 | Apolipoprotein M \|\| id:prot-a-137 | F-sweetened coffee drink liking (derived food-liking factor) \|\| id:ebi-fl187-GCST90094853 | Weighted mode | 4 | -0.054250521 | 0.408249217372503 | 0.902696273311207 | -0.854418987 | 0.74591794477671 | 0.947194784332389 | 0.425530357803941 | 2.10837591963262 | 0.999714303421225 |
| ebi-fl187-GCST90094853 | prot-a-1381 | 17-beta-hydroxysteroid dehydrogenase 14 \|\| id:prot-a-1381 | F-sweetened coffee drink liking (derived food-liking factor) \|\| id:ebi-fl187-GCST90094853 | Weighted mode | 4 | -0.254277407 | 0.353827542626813 | 0.524273783023509 | -0.947779391 | 0.439224576133592 | 0.775476649232736 | 0.387600778302361 | 1.55150367896351 | 0.999714303421225 |
| ebi-fl187-GCST90094853 | prot-a-1392 | Serine protease HTRA2, mitochondrial \|\| id:prot-a-1392 | F-sweetened coffee drink liking (derived food-liking factor) \|\| id:ebi-fl187-GCST90094853 | Weighted mode | 4 | -0.035956978 | 0.374447917598179 | 0.929554562608379 | -0.769874896 | 0.697960940797423 | 0.964681795424785 | 0.463070996634791 | 2.00965072998931 | 0.999714303421225 |
| ebi-fl187-GCST90094853 | prot-a-1416 | Interferon-induced protein with tetratricopeptide repeats 2 \|\| id:prot-a-1416 | F-sweetened coffee drink liking (derived food-liking factor) \|\| id:ebi-fl187-GCST90094853 | Weighted mode | 4 | -0.015140396 | 0.388504610223876 | 0.971361912489985 | -0.776609432 | 0.746328640251655 | 0.984973643745184 | 0.459962906025299 | 2.10924199791734 | 0.999714303421225 |
| ebi-fl187-GCST90094853 | prot-a-1428 | Interferon gamma \|\| id:prot-a-1428 | F-sweetened coffee drink liking (derived food-liking factor) \|\| id:ebi-fl187-GCST90094853 | Weighted mode | 4 | 0.259102477069426 | 0.476997582509725 | 0.624757474280049 | -0.675812785 | 1.19401773878849 | 1.29576658438673 | 0.508742754218235 | 3.30031440701957 | 0.999714303421225 |
| ebi-fl187-GCST90094853 | prot-a-1483 | Interleukin-17C \|\| id:prot-a-1483 | F-sweetened coffee drink liking (derived food-liking factor) \|\| id:ebi-fl187-GCST90094853 | Weighted mode | 4 | 0.513701552195917 | 0.393406038917765 | 0.28273471267249 | -0.257374284 | 1.28477738847474 | 1.67146677979899 | 0.773078808516083 | 3.61386338002757 | 0.999714303421225 |
| ebi-fl187-GCST90094853 | prot-a-1503 | Interleukin-1 receptor-like 2 \|\| id:prot-a-1503 | F-sweetened coffee drink liking (derived food-liking factor) \|\| id:ebi-fl187-GCST90094853 | Weighted mode | 4 | -0.059741548 | 0.379881712917081 | 0.885025430298378 | -0.804309705 | 0.684826609589097 | 0.942007966223789 | 0.447396655643923 | 1.98342789834203 | 0.999714303421225 |
| ebi-fl187-GCST90094853 | prot-a-1522 | Interleukin-31 receptor subunit alpha \|\| id:prot-a-1522 | F-sweetened coffee drink liking (derived food-liking factor) \|\| id:ebi-fl187-GCST90094853 | Weighted mode | 4 | 0.0754440418493712 | 0.372861500480043 | 0.852597116990638 | -0.655364499 | 0.806252582790256 | 1.07836288283704 | 0.519252760929378 | 2.23949990174203 | 0.999714303421225 |
| ebi-fl187-GCST90094853 | prot-a-1540 | Interleukin-6 receptor subunit alpha \|\| id:prot-a-1540 | F-sweetened coffee drink liking (derived food-liking factor) \|\| id:ebi-fl187-GCST90094853 | Weighted mode | 4 | 0.470261437771927 | 0.361522610465989 | 0.284232956185633 | -0.238322879 | 1.17884575428527 | 1.60041254681867 | 0.787948238281806 | 3.25062002245199 | 0.999714303421225 |
| ebi-fl187-GCST90094853 | prot-a-1541 | Interleukin-6 receptor subunit alpha \|\| id:prot-a-1541 | F-sweetened coffee drink liking (derived food-liking factor) \|\| id:ebi-fl187-GCST90094853 | Weighted mode | 4 | 0.590703289574972 | 0.372300183772783 | 0.210787879751682 | -0.139005071 | 1.32041164976963 | 1.80525758788935 | 0.870223615874069 | 3.74496267302369 | 0.999714303421225 |
| ebi-fl187-GCST90094853 | prot-a-1545 | Interleukin-9 \|\| id:prot-a-1545 | F-sweetened coffee drink liking (derived food-liking factor) \|\| id:ebi-fl187-GCST90094853 | Weighted mode | 4 | -0.403469541 | 0.508105834796718 | 0.48516974278491 | -1.399356977 | 0.592417895160978 | 0.667998373024456 | 0.246755582393608 | 1.80835554776442 | 0.999714303421225 |
| ebi-fl187-GCST90094853 | prot-a-1548 | Inositol monophosphatase 3 \|\| id:prot-a-1548 | F-sweetened coffee drink liking (derived food-liking factor) \|\| id:ebi-fl187-GCST90094853 | Weighted mode | 4 | 0.458870959217985 | 0.51503787940351 | 0.43859518760148 | -0.550603284 | 1.46834520284887 | 1.58228651011959 | 0.576601850522794 | 4.34204399073023 | 0.999714303421225 |
| ebi-fl187-GCST90094853 | prot-a-1555 | Inhibin beta A chain:Inhibin beta B chain heterodimer \|\| id:prot-a-1555 | F-sweetened coffee drink liking (derived food-liking factor) \|\| id:ebi-fl187-GCST90094853 | Weighted mode | 4 | -0.404811173 | 0.451184488908386 | 0.435720308243932 | -1.289132771 | 0.479510425179048 | 0.667102765925933 | 0.275509609345124 | 1.61528340649838 | 0.999714303421225 |
| ebi-fl187-GCST90094853 | prot-a-1603 | Junctophilin-3 \|\| id:prot-a-1603 | F-sweetened coffee drink liking (derived food-liking factor) \|\| id:ebi-fl187-GCST90094853 | Weighted mode | 4 | 0.192064590336865 | 0.368504529772038 | 0.638256308894097 | -0.530204288 | 0.91433346869006 | 1.21174878169931 | 0.588484737018162 | 2.49511162751607 | 0.999714303421225 |
| ebi-fl187-GCST90094853 | prot-a-1627 | UPF0258 protein KIAA1024 \|\| id:prot-a-1627 | F-sweetened coffee drink liking (derived food-liking factor) \|\| id:ebi-fl187-GCST90094853 | Weighted mode | 4 | -0.10906742 | 0.375899272798846 | 0.790600908616173 | -0.845829994 | 0.627695155169098 | 0.89666996240488 | 0.429200976011873 | 1.87328796162133 | 0.999714303421225 |
| ebi-fl187-GCST90094853 | prot-a-1666 | Kallikrein-7 \|\| id:prot-a-1666 | F-sweetened coffee drink liking (derived food-liking factor) \|\| id:ebi-fl187-GCST90094853 | Weighted mode | 4 | -0.052049802 | 0.416978995388454 | 0.908556037757673 | -0.869328633 | 0.76522902866595 | 0.949281589263489 | 0.419232913823662 | 2.1494866123361 | 0.999714303421225 |
| ebi-fl187-GCST90094853 | prot-a-1678 | Importin subunit alpha-3 \|\| id:prot-a-1678 | F-sweetened coffee drink liking (derived food-liking factor) \|\| id:ebi-fl187-GCST90094853 | Weighted mode | 4 | 0.285678861151914 | 0.424543969076535 | 0.549202538290084 | -0.546427318 | 1.11778504054192 | 1.33066505838506 | 0.579014754944038 | 3.05807318809699 | 0.999714303421225 |
| ebi-fl187-GCST90094853 | prot-a-1718 | Protein LDOC1 \|\| id:prot-a-1718 | F-sweetened coffee drink liking (derived food-liking factor) \|\| id:ebi-fl187-GCST90094853 | Weighted mode | 4 | -0.437171981 | 0.475239681603422 | 0.425467320081774 | -1.368641757 | 0.494297794565992 | 0.645860345907124 | 0.254452332968057 | 1.63934667664311 | 0.999714303421225 |
| ebi-fl187-GCST90094853 | prot-a-1723 | Leptin \|\| id:prot-a-1723 | F-sweetened coffee drink liking (derived food-liking factor) \|\| id:ebi-fl187-GCST90094853 | Weighted mode | 4 | -0.001863162 | 0.403538906513179 | 0.996605992985153 | -0.792799419 | 0.789073094351918 | 0.998138572195723 | 0.452576070133129 | 2.20135503189961 | 0.999714303421225 |
| ebi-fl187-GCST90094853 | prot-a-1730 | Galectin-7 \|\| id:prot-a-1730 | F-sweetened coffee drink liking (derived food-liking factor) \|\| id:ebi-fl187-GCST90094853 | Weighted mode | 4 | 0.173984672229587 | 0.443349725087668 | 0.720958807984684 | -0.694980789 | 1.04295013340142 | 1.19003732506167 | 0.499084035825278 | 2.83757590582543 | 0.999714303421225 |
| ebi-fl187-GCST90094853 | prot-a-1753 | Lipase member N \|\| id:prot-a-1753 | F-sweetened coffee drink liking (derived food-liking factor) \|\| id:ebi-fl187-GCST90094853 | Weighted mode | 4 | 0.303611625998502 | 0.442859796951349 | 0.542210065895667 | -0.564393576 | 1.17161682802315 | 1.3547428070206 | 0.56870491850468 | 3.22720626014585 | 0.999714303421225 |
| ebi-fl187-GCST90094853 | prot-a-1756 | VIP36-like protein \|\| id:prot-a-1756 | F-sweetened coffee drink liking (derived food-liking factor) \|\| id:ebi-fl187-GCST90094853 | Weighted mode | 4 | 0.0759836510185857 | 0.384345999959821 | 0.855920261939733 | -0.677334509 | 0.829301810939835 | 1.07894493436241 | 0.507969176765652 | 2.2917181290379 | 0.999714303421225 |
| ebi-fl187-GCST90094853 | prot-a-1760 | Ig Kappa chain V-I region HK102- like \|\| id:prot-a-1760 | F-sweetened coffee drink liking (derived food-liking factor) \|\| id:ebi-fl187-GCST90094853 | Weighted mode | 4 | -0.340005825 | 0.368808682329119 | 0.424571453065547 | -1.062870843 | 0.382859191945924 | 0.711766176414219 | 0.345462616500726 | 1.46647152452819 | 0.999714303421225 |
| ebi-fl187-GCST90094853 | prot-a-179 | Neutral ceramidase \|\| id:prot-a-179 | F-sweetened coffee drink liking (derived food-liking factor) \|\| id:ebi-fl187-GCST90094853 | Weighted mode | 4 | 0.0615395090222303 | 0.355210761246581 | 0.873486467338417 | -0.634673583 | 0.757752601065529 | 1.06347251245793 | 0.530108496493561 | 2.13347605675909 | 0.999714303421225 |
| ebi-fl187-GCST90094853 | prot-a-1809 | Lactotransferrin \|\| id:prot-a-1809 | F-sweetened coffee drink liking (derived food-liking factor) \|\| id:ebi-fl187-GCST90094853 | Weighted mode | 4 | 0.458793826362767 | 0.4004094409038 | 0.334988061730539 | -0.326008678 | 1.24359633053422 | 1.58216446855006 | 0.721798923771571 | 3.46806336654291 | 0.999714303421225 |
| ebi-fl187-GCST90094853 | prot-a-1818 | Lysophospholipase-like protein 1 \|\| id:prot-a-1818 | F-sweetened coffee drink liking (derived food-liking factor) \|\| id:ebi-fl187-GCST90094853 | Weighted mode | 4 | -0.570572576 | 0.562947437438002 | 0.385439240110175 | -1.673949554 | 0.532804401111865 | 0.565201724939421 | 0.18750504005397 | 1.70370348329063 | 0.999714303421225 |
| ebi-fl187-GCST90094853 | prot-a-1852 | MAP kinase-activated protein kinase 3 \|\| id:prot-a-1852 | F-sweetened coffee drink liking (derived food-liking factor) \|\| id:ebi-fl187-GCST90094853 | Weighted mode | 4 | 0.373750670817646 | 0.534058235304134 | 0.534418636659408 | -0.67300347 | 1.42050481201375 | 1.45317478640345 | 0.510173981940952 | 4.13920943558257 | 0.999714303421225 |
| ebi-fl187-GCST90094853 | prot-a-1856 | Megakaryocyte-associated tyrosine-protein kinase \|\| id:prot-a-1856 | F-sweetened coffee drink liking (derived food-liking factor) \|\| id:ebi-fl187-GCST90094853 | Weighted mode | 4 | -0.265043037 | 0.468554349184229 | 0.611169464260469 | -1.183409562 | 0.653323486988936 | 0.767172932127686 | 0.306232836794632 | 1.9219176948816 | 0.999714303421225 |
| ebi-fl187-GCST90094853 | prot-a-1890 | Beta-1,3-N-acetylglucosaminyltransferase manic fringe \|\| id:prot-a-1890 | F-sweetened coffee drink liking (derived food-liking factor) \|\| id:ebi-fl187-GCST90094853 | Weighted mode | 4 | -0.021312198 | 0.437002312518663 | 0.964168535431793 | -0.877836731 | 0.835212334555849 | 0.978913302102112 | 0.41568117013361 | 2.30530349191533 | 0.999714303421225 |
| ebi-fl187-GCST90094853 | prot-a-1919 | Matrilysin \|\| id:prot-a-1919 | F-sweetened coffee drink liking (derived food-liking factor) \|\| id:ebi-fl187-GCST90094853 | Weighted mode | 4 | -0.475582397 | 0.76234676661516 | 0.576949958533021 | -1.969782059 | 1.01861726567317 | 0.621522977987539 | 0.139487252830051 | 2.76936282225838 | 0.999714303421225 |
| ebi-fl187-GCST90094853 | prot-a-1923 | Multimerin-2 \|\| id:prot-a-1923 | F-sweetened coffee drink liking (derived food-liking factor) \|\| id:ebi-fl187-GCST90094853 | Weighted mode | 4 | 0.106661136901272 | 0.414825337015631 | 0.813710496769833 | -0.706396524 | 0.919718797451908 | 1.11255718602007 | 0.493419021547241 | 2.50858487028636 | 0.999714303421225 |
| ebi-fl187-GCST90094853 | prot-a-1948 | Mesothelin \|\| id:prot-a-1948 | F-sweetened coffee drink liking (derived food-liking factor) \|\| id:ebi-fl187-GCST90094853 | Weighted mode | 4 | 0.288773652556885 | 0.38024234739583 | 0.502809863133909 | -0.456501348 | 1.03404865345271 | 1.33478956812734 | 0.633496155225415 | 2.81242936754305 | 0.999714303421225 |
| ebi-fl187-GCST90094853 | prot-a-195 | ATPase family AAA domain-containing protein 1 \|\| id:prot-a-195 | F-sweetened coffee drink liking (derived food-liking factor) \|\| id:ebi-fl187-GCST90094853 | Weighted mode | 4 | -0.036718153 | 0.407654816802457 | 0.933906925277543 | -0.835721593 | 0.762285288424507 | 0.963947783330458 | 0.433561513384344 | 2.14316838626769 | 0.999714303421225 |
| ebi-fl187-GCST90094853 | prot-a-1966 | Mucin-1 \|\| id:prot-a-1966 | F-sweetened coffee drink liking (derived food-liking factor) \|\| id:ebi-fl187-GCST90094853 | Weighted mode | 4 | -0.187042052 | 0.375994762754994 | 0.653040671006472 | -0.923991787 | 0.549907682913329 | 0.829408857279063 | 0.396931408775921 | 1.73309301638387 | 0.999714303421225 |
| ebi-fl187-GCST90094853 | prot-a-1986 | Myocardial zonula adherens protein \|\| id:prot-a-1986 | F-sweetened coffee drink liking (derived food-liking factor) \|\| id:ebi-fl187-GCST90094853 | Weighted mode | 4 | -0.379025336 | 0.391173294131315 | 0.404043834504353 | -1.145724992 | 0.387674320794522 | 0.684528269441747 | 0.31799329154331 | 1.47354980160358 | 0.999714303421225 |
| ebi-fl187-GCST90094853 | prot-a-2071 | Epididymal secretory protein E1 \|\| id:prot-a-2071 | F-sweetened coffee drink liking (derived food-liking factor) \|\| id:ebi-fl187-GCST90094853 | Weighted mode | 4 | 0.477820290296321 | 0.392569877758405 | 0.31055639294054 | -0.29161667 | 1.24725725070279 | 1.61255566544205 | 0.747054849546763 | 3.48078293812948 | 0.999714303421225 |
| ebi-fl187-GCST90094853 | prot-a-2098 | Neuritin \|\| id:prot-a-2098 | F-sweetened coffee drink liking (derived food-liking factor) \|\| id:ebi-fl187-GCST90094853 | Weighted mode | 4 | -0.906893746 | 0.48140043033673 | 0.156097443134774 | -1.85043859 | 0.0366510973061384 | 0.403776510405464 | 0.157168218846499 | 1.03733103010123 | 0.999714303421225 |
| ebi-fl187-GCST90094853 | prot-a-2115 | Neurotrophin-4 \|\| id:prot-a-2115 | F-sweetened coffee drink liking (derived food-liking factor) \|\| id:ebi-fl187-GCST90094853 | Weighted mode | 4 | -0.246174025 | 0.384390245522348 | 0.567454549126511 | -0.999578906 | 0.507230856171631 | 0.781786162708952 | 0.368034385516074 | 1.66068614307913 | 0.999714303421225 |
| ebi-fl187-GCST90094853 | prot-a-2118 | Netrin-1 \|\| id:prot-a-2118 | F-sweetened coffee drink liking (derived food-liking factor) \|\| id:ebi-fl187-GCST90094853 | Weighted mode | 4 | -0.448028086 | 0.397174852947102 | 0.341369945059843 | -1.226490798 | 0.330434625342682 | 0.638886739729904 | 0.293320093142778 | 1.39157280985868 | 0.999714303421225 |
| ebi-fl187-GCST90094853 | prot-a-2120 | Netrin-G1 \|\| id:prot-a-2120 | F-sweetened coffee drink liking (derived food-liking factor) \|\| id:ebi-fl187-GCST90094853 | Weighted mode | 4 | -0.369209502 | 0.479154401151112 | 0.49709784971716 | -1.308352129 | 0.569933123810267 | 0.691280570308365 | 0.270265051657975 | 1.76814880042504 | 0.999714303421225 |
| ebi-fl187-GCST90094853 | prot-a-2145 | Olfactomedin-like protein 3 \|\| id:prot-a-2145 | F-sweetened coffee drink liking (derived food-liking factor) \|\| id:ebi-fl187-GCST90094853 | Weighted mode | 4 | 0.0599470134615145 | 0.397658222070788 | 0.88973867771686 | -0.719463102 | 0.839357128720259 | 1.06178028499289 | 0.487013662539473 | 2.31487832953393 | 0.999714303421225 |
| ebi-fl187-GCST90094853 | prot-a-2162 | Prolyl 4-hydroxylase subunit alpha-2 \|\| id:prot-a-2162 | F-sweetened coffee drink liking (derived food-liking factor) \|\| id:ebi-fl187-GCST90094853 | Weighted mode | 4 | -0.252466883 | 0.365932656520427 | 0.539820414576409 | -0.96969489 | 0.464761123976771 | 0.77688194056536 | 0.37919871793378 | 1.59163394028668 | 0.999714303421225 |
| ebi-fl187-GCST90094853 | prot-a-2168 | Polyadenylate-binding protein-interacting protein 1 \|\| id:prot-a-2168 | F-sweetened coffee drink liking (derived food-liking factor) \|\| id:ebi-fl187-GCST90094853 | Weighted mode | 4 | 0.0212658468578365 | 0.388265661345833 | 0.959764089438465 | -0.739734849 | 0.782266543095668 | 1.02149357640151 | 0.477240439344742 | 2.18642227398463 | 0.999714303421225 |
| ebi-fl187-GCST90094853 | prot-a-219 | Beta-1,4-galactosyltransferase 5 \|\| id:prot-a-219 | F-sweetened coffee drink liking (derived food-liking factor) \|\| id:ebi-fl187-GCST90094853 | Weighted mode | 4 | 0.244057832206726 | 0.41951492821187 | 0.601558438629923 | -0.578191427 | 1.06630709150199 | 1.27641814643308 | 0.560911899832483 | 2.90463312514894 | 0.999714303421225 |
| ebi-fl187-GCST90094853 | prot-a-2190 | Pyruvate carboxylase, mitochondrial \|\| id:prot-a-2190 | F-sweetened coffee drink liking (derived food-liking factor) \|\| id:ebi-fl187-GCST90094853 | Weighted mode | 4 | -0.082736193 | 0.452394035061572 | 0.866549434482701 | -0.969428501 | 0.803956115977591 | 0.920593974560065 | 0.379299745422843 | 2.23436286531517 | 0.999714303421225 |
| ebi-fl187-GCST90094853 | prot-a-2197 | Protocadherin-9 \|\| id:prot-a-2197 | F-sweetened coffee drink liking (derived food-liking factor) \|\| id:ebi-fl187-GCST90094853 | Weighted mode | 4 | -0.047179333 | 0.361424346782441 | 0.904402918696142 | -0.755571053 | 0.661212386329723 | 0.953916313228969 | 0.469742290352984 | 1.93713947271081 | 0.999714303421225 |
| ebi-fl187-GCST90094853 | prot-a-2198 | Protocadherin alpha-4 \|\| id:prot-a-2198 | F-sweetened coffee drink liking (derived food-liking factor) \|\| id:ebi-fl187-GCST90094853 | Weighted mode | 4 | 0.0448532152028016 | 0.415261075962565 | 0.920804887990882 | -0.769058494 | 0.858764924089429 | 1.04587433018963 | 0.46344920331923 | 2.36024381251582 | 0.999714303421225 |
| ebi-fl187-GCST90094853 | prot-a-2254 | Placenta growth factor \|\| id:prot-a-2254 | F-sweetened coffee drink liking (derived food-liking factor) \|\| id:ebi-fl187-GCST90094853 | Weighted mode | 4 | 0.269099840663685 | 0.520551498810784 | 0.640885948871831 | -0.751181097 | 1.28938077833282 | 1.30878580456694 | 0.471808971363762 | 3.63053775193114 | 0.999714303421225 |
| ebi-fl187-GCST90094853 | prot-a-2261 | Membrane-associated progesterone receptor component 2 \|\| id:prot-a-2261 | F-sweetened coffee drink liking (derived food-liking factor) \|\| id:ebi-fl187-GCST90094853 | Weighted mode | 4 | 0.791402346301939 | 0.441732448008993 | 0.171116264286705 | -0.074393252 | 1.65719794439957 | 2.20648851867145 | 0.928306563828801 | 5.24459459055036 | 0.999714303421225 |
| ebi-fl187-GCST90094853 | prot-a-2268 | Polymeric immunoglobulin receptor \|\| id:prot-a-2268 | F-sweetened coffee drink liking (derived food-liking factor) \|\| id:ebi-fl187-GCST90094853 | Weighted mode | 4 | 0.317529214362885 | 0.359012458734974 | 0.441589510589127 | -0.386135205 | 1.02119363348343 | 1.37372937690765 | 0.679678623684062 | 2.77650691844664 | 0.999714303421225 |
| ebi-fl187-GCST90094853 | prot-a-2283 | Group 10 secretory phospholipase A2 \|\| id:prot-a-2283 | F-sweetened coffee drink liking (derived food-liking factor) \|\| id:ebi-fl187-GCST90094853 | Weighted mode | 4 | -0.158534627 | 0.410369128629059 | 0.725028617332326 | -0.962858119 | 0.645788864754656 | 0.85339341251983 | 0.381800094770115 | 1.90749118847323 | 0.999714303421225 |
| ebi-fl187-GCST90094853 | prot-a-2298 | Inactive phospholipase D5 \|\| id:prot-a-2298 | F-sweetened coffee drink liking (derived food-liking factor) \|\| id:ebi-fl187-GCST90094853 | Weighted mode | 4 | 0.19894989408412 | 0.379652103632914 | 0.636507411285979 | -0.545168229 | 0.943068017204632 | 1.2201208290966 | 0.579744245319969 | 2.56784754590146 | 0.999714303421225 |
| ebi-fl187-GCST90094853 | prot-a-2304 | Procollagen-lysine,2-oxoglutarate 5-dioxygenase 2 \|\| id:prot-a-2304 | F-sweetened coffee drink liking (derived food-liking factor) \|\| id:ebi-fl187-GCST90094853 | Weighted mode | 4 | -0.257148551 | 0.384218590059587 | 0.551220325292681 | -1.010216988 | 0.49591988552216 | 0.773253337680769 | 0.364139957175281 | 1.64200800393525 | 0.999714303421225 |
| ebi-fl187-GCST90094853 | prot-a-2331 | Periostin \|\| id:prot-a-2331 | F-sweetened coffee drink liking (derived food-liking factor) \|\| id:ebi-fl187-GCST90094853 | Weighted mode | 4 | 0.104905872300392 | 0.384098123964886 | 0.802481613720556 | -0.647926451 | 0.857738195271569 | 1.11060606664133 | 0.523129387503144 | 2.35782172580223 | 0.999714303421225 |
| ebi-fl187-GCST90094853 | prot-a-2333 | Periostin \|\| id:prot-a-2333 | F-sweetened coffee drink liking (derived food-liking factor) \|\| id:ebi-fl187-GCST90094853 | Weighted mode | 4 | 0.0494776045164918 | 0.394909497799691 | 0.908219563793487 | -0.724545011 | 0.823500220203887 | 1.05072206052275 | 0.484544981362683 | 2.27846101174001 | 0.999714303421225 |
| ebi-fl187-GCST90094853 | prot-a-2367 | cAMP-dependent protein kinase catalytic subunit alpha \|\| id:prot-a-2367 | F-sweetened coffee drink liking (derived food-liking factor) \|\| id:ebi-fl187-GCST90094853 | Weighted mode | 4 | -0.361092705 | 0.490097729410168 | 0.514646038443327 | -1.321684255 | 0.599498844416072 | 0.696914387864926 | 0.266685756712749 | 1.82120586415977 | 0.999714303421225 |
| ebi-fl187-GCST90094853 | prot-a-2385 | Phosphoribosyl pyrophosphate synthase-associated protein 1 \|\| id:prot-a-2385 | F-sweetened coffee drink liking (derived food-liking factor) \|\| id:ebi-fl187-GCST90094853 | Weighted mode | 4 | 0.220389056373086 | 0.424532160875597 | 0.639534224790379 | -0.611693979 | 1.05247209168926 | 1.24656161899912 | 0.54243122329379 | 2.8647242327348 | 0.999714303421225 |
| ebi-fl187-GCST90094853 | prot-a-2393 | Trypsin-3 \|\| id:prot-a-2393 | F-sweetened coffee drink liking (derived food-liking factor) \|\| id:ebi-fl187-GCST90094853 | Weighted mode | 4 | 0.304547985916455 | 0.392145626232064 | 0.493993551204514 | -0.464057441 | 1.0731534133313 | 1.35601192796849 | 0.628727438383687 | 2.92458740709626 | 0.999714303421225 |
| ebi-fl187-GCST90094853 | prot-a-2405 | Pregnancy-specific beta-1-glycoprotein 6 \|\| id:prot-a-2405 | F-sweetened coffee drink liking (derived food-liking factor) \|\| id:ebi-fl187-GCST90094853 | Weighted mode | 4 | 0.0867435934463268 | 0.370666669162263 | 0.830030140101858 | -0.639763078 | 0.813250265004362 | 1.09061700251728 | 0.527417365959988 | 2.25522616991345 | 0.999714303421225 |
| ebi-fl187-GCST90094853 | prot-a-244 | Beclin-1 \|\| id:prot-a-244 | F-sweetened coffee drink liking (derived food-liking factor) \|\| id:ebi-fl187-GCST90094853 | Weighted mode | 4 | -0.079120045 | 0.389586023275466 | 0.852060939740979 | -0.842708651 | 0.684468560239377 | 0.923929004378581 | 0.430542752543752 | 1.98271786039471 | 0.999714303421225 |
| ebi-fl187-GCST90094853 | prot-a-2454 | tRNA pseudouridine synthase A, mitochondrial \|\| id:prot-a-2454 | F-sweetened coffee drink liking (derived food-liking factor) \|\| id:ebi-fl187-GCST90094853 | Weighted mode | 4 | -0.596646327 | 0.521635657193755 | 0.335704714125421 | -1.619052216 | 0.425759560677196 | 0.550655260356283 | 0.198086353312593 | 1.53075267774526 | 0.999714303421225 |
| ebi-fl187-GCST90094853 | prot-a-2466 | Glutaminyl-peptide cyclotransferase-like protein \|\| id:prot-a-2466 | F-sweetened coffee drink liking (derived food-liking factor) \|\| id:ebi-fl187-GCST90094853 | Weighted mode | 4 | -0.197785233 | 0.430959756955122 | 0.677485476058292 | -1.042466357 | 0.646895890520907 | 0.82054606033997 | 0.352584010754775 | 1.90960399962019 | 0.999714303421225 |
| ebi-fl187-GCST90094853 | prot-a-2474 | Ras-related protein Rab-27A \|\| id:prot-a-2474 | F-sweetened coffee drink liking (derived food-liking factor) \|\| id:ebi-fl187-GCST90094853 | Weighted mode | 4 | 0.324522215781276 | 0.358693247127685 | 0.432297955743124 | -0.378516549 | 1.02756098015154 | 1.38336953592642 | 0.684876637219321 | 2.79424230427713 | 0.999714303421225 |
| ebi-fl187-GCST90094853 | prot-a-2490 | Rap1 GTPase-GDP dissociation stimulator 1 \|\| id:prot-a-2490 | F-sweetened coffee drink liking (derived food-liking factor) \|\| id:ebi-fl187-GCST90094853 | Weighted mode | 4 | -0.509951267 | 0.409043121019386 | 0.30098236150182 | -1.311675784 | 0.291773250146022 | 0.600524843445168 | 0.269368274833801 | 1.3387994106483 | 0.999714303421225 |
| ebi-fl187-GCST90094853 | prot-a-2545 | Receptor-interacting serine/threonine-protein kinase 2 \|\| id:prot-a-2545 | F-sweetened coffee drink liking (derived food-liking factor) \|\| id:ebi-fl187-GCST90094853 | Weighted mode | 4 | -0.695341057 | 0.400116611248825 | 0.180628648249091 | -1.479569615 | 0.0888875012390724 | 0.498904264269447 | 0.22773568134888 | 1.09295769302364 | 0.999714303421225 |
| ebi-fl187-GCST90094853 | prot-a-2610 | RUN and FYVE domain-containing protein 1 \|\| id:prot-a-2610 | F-sweetened coffee drink liking (derived food-liking factor) \|\| id:ebi-fl187-GCST90094853 | Weighted mode | 4 | 0.185885590790224 | 0.477683594791345 | 0.723150373702893 | -0.750374255 | 1.12214543658126 | 1.20428447126091 | 0.472189800273746 | 3.07143671226987 | 0.999714303421225 |
| ebi-fl187-GCST90094853 | prot-a-2627 | Serine--tRNA ligase, mitochondrial \|\| id:prot-a-2627 | F-sweetened coffee drink liking (derived food-liking factor) \|\| id:ebi-fl187-GCST90094853 | Weighted mode | 4 | -0.349542545 | 0.505194070741253 | 0.538742350696003 | -1.339722924 | 0.640637833765889 | 0.705010526633033 | 0.261918229903342 | 1.89769090470264 | 0.999714303421225 |
| ebi-fl187-GCST90094853 | prot-a-264 | BolA-like protein 2 \|\| id:prot-a-264 | F-sweetened coffee drink liking (derived food-liking factor) \|\| id:ebi-fl187-GCST90094853 | Weighted mode | 4 | -0.354777848 | 0.527783562547657 | 0.549593493159262 | -1.389233631 | 0.679677934364666 | 0.701329227434172 | 0.249266261430459 | 1.97324211640505 | 0.999714303421225 |
| ebi-fl187-GCST90094853 | prot-a-2646 | Secretoglobin family 1C member 1 \|\| id:prot-a-2646 | F-sweetened coffee drink liking (derived food-liking factor) \|\| id:ebi-fl187-GCST90094853 | Weighted mode | 4 | -0.366432642 | 0.420019007822264 | 0.447185668203248 | -1.189669897 | 0.456804613701858 | 0.693202827922728 | 0.304321705007391 | 1.57902033516931 | 0.999714303421225 |
| ebi-fl187-GCST90094853 | prot-a-2672 | Semaphorin-3E \|\| id:prot-a-2672 | F-sweetened coffee drink liking (derived food-liking factor) \|\| id:ebi-fl187-GCST90094853 | Weighted mode | 4 | 0.255360479980319 | 0.362818975462052 | 0.532253409923758 | -0.455764712 | 0.966485671885941 | 1.2909268903077 | 0.63396298348149 | 2.62869012788059 | 0.999714303421225 |
| ebi-fl187-GCST90094853 | prot-a-2701 | Plasma protease C1 inhibitor \|\| id:prot-a-2701 | F-sweetened coffee drink liking (derived food-liking factor) \|\| id:ebi-fl187-GCST90094853 | Weighted mode | 4 | 0.16433312157391 | 0.368535644458331 | 0.685881253351684 | -0.557996742 | 0.886662984712238 | 1.17860686908024 | 0.572354490133831 | 2.42701712974824 | 0.999714303421225 |
| ebi-fl187-GCST90094853 | prot-a-2738 | Signaling threshold-regulating transmembrane adapter 1 \|\| id:prot-a-2738 | F-sweetened coffee drink liking (derived food-liking factor) \|\| id:ebi-fl187-GCST90094853 | Weighted mode | 4 | 0.419587673513632 | 0.460468703815666 | 0.429365017390747 | -0.482930986 | 1.32210633299234 | 1.52133413991696 | 0.616972401659158 | 3.75131457914951 | 0.999714303421225 |
| ebi-fl187-GCST90094853 | prot-a-274 | Serine/threonine-protein kinase BRSK2 \|\| id:prot-a-274 | F-sweetened coffee drink liking (derived food-liking factor) \|\| id:ebi-fl187-GCST90094853 | Weighted mode | 4 | -0.438743903 | 0.416543005256459 | 0.369545572354398 | -1.255168193 | 0.377680387204558 | 0.644845901522878 | 0.285027904380098 | 1.45889658633679 | 0.999714303421225 |
| ebi-fl187-GCST90094853 | prot-a-2749 | Mitochondrial glutamate carrier 2 \|\| id:prot-a-2749 | F-sweetened coffee drink liking (derived food-liking factor) \|\| id:ebi-fl187-GCST90094853 | Weighted mode | 4 | -0.189648945 | 0.439111045893592 | 0.694978247887482 | -1.050306594 | 0.671008705427317 | 0.827249493438824 | 0.349830476575911 | 1.95620956496708 | 0.999714303421225 |
| ebi-fl187-GCST90094853 | prot-a-2760 | Sodium-coupled monocarboxylate transporter 1 \|\| id:prot-a-2760 | F-sweetened coffee drink liking (derived food-liking factor) \|\| id:ebi-fl187-GCST90094853 | Weighted mode | 4 | -0.440865581 | 0.373780158791213 | 0.323226075223382 | -1.173474692 | 0.291743529962404 | 0.643479196417309 | 0.309290383020266 | 1.33875962187526 | 0.999714303421225 |
| ebi-fl187-GCST90094853 | prot-a-2777 | Small integral membrane protein 10 \|\| id:prot-a-2777 | F-sweetened coffee drink liking (derived food-liking factor) \|\| id:ebi-fl187-GCST90094853 | Weighted mode | 4 | 0.205188945105249 | 0.406855705040674 | 0.648740347074279 | -0.592248237 | 1.00262612698497 | 1.22775702172863 | 0.553082425641222 | 2.72542976330581 | 0.999714303421225 |
| ebi-fl187-GCST90094853 | prot-a-2799 | Superoxide dismutase [Mn], mitochondrial \|\| id:prot-a-2799 | F-sweetened coffee drink liking (derived food-liking factor) \|\| id:ebi-fl187-GCST90094853 | Weighted mode | 4 | 0.172003195145841 | 0.395309608741993 | 0.692883890268738 | -0.602803638 | 0.946810028280147 | 1.18768162802386 | 0.54727512186386 | 2.57747446063572 | 0.999714303421225 |
| ebi-fl187-GCST90094853 | prot-a-2805 | Protein SPACA7 \|\| id:prot-a-2805 | F-sweetened coffee drink liking (derived food-liking factor) \|\| id:ebi-fl187-GCST90094853 | Weighted mode | 4 | 0.297177283409797 | 0.34033036530944 | 0.44681951169424 | -0.369870233 | 0.9642247994163 | 1.34605391132368 | 0.690823971253897 | 2.62275370801206 | 0.999714303421225 |
| ebi-fl187-GCST90094853 | prot-a-2811 | SPARC-like protein 1 \|\| id:prot-a-2811 | F-sweetened coffee drink liking (derived food-liking factor) \|\| id:ebi-fl187-GCST90094853 | Weighted mode | 4 | 0.0619126331705314 | 0.369192167314426 | 0.877488597384838 | -0.661704015 | 0.785529281106806 | 1.06386939377178 | 0.515971362141899 | 2.19356764744835 | 0.999714303421225 |
| ebi-fl187-GCST90094853 | prot-a-2831 | Sepiapterin reductase \|\| id:prot-a-2831 | F-sweetened coffee drink liking (derived food-liking factor) \|\| id:ebi-fl187-GCST90094853 | Weighted mode | 4 | -0.330094776 | 0.4276312837919 | 0.496396161142517 | -1.168252092 | 0.508062540057571 | 0.718855599819449 | 0.31090990838878 | 1.66206788348991 | 0.999714303421225 |
| ebi-fl187-GCST90094853 | prot-a-2834 | SPRY domain-containing SOCS box protein 1 \|\| id:prot-a-2834 | F-sweetened coffee drink liking (derived food-liking factor) \|\| id:ebi-fl187-GCST90094853 | Weighted mode | 4 | 0.105415758951701 | 0.412246174817869 | 0.814704633483164 | -0.702586744 | 0.913418261594725 | 1.11117249424424 | 0.495302424844473 | 2.49282912828998 | 0.999714303421225 |
| ebi-fl187-GCST90094853 | prot-a-2835 | Spectrin alpha chain, non-erythrocytic 1 \|\| id:prot-a-2835 | F-sweetened coffee drink liking (derived food-liking factor) \|\| id:ebi-fl187-GCST90094853 | Weighted mode | 4 | -0.217752242 | 0.39537735085255 | 0.620167869095494 | -0.99269185 | 0.557187365305668 | 0.804324694566159 | 0.37057780731855 | 1.74575541630542 | 0.999714303421225 |
| ebi-fl187-GCST90094853 | prot-a-2859 | Sia-alpha-2,3-Gal-beta-1,4-GlcNAc-R:alpha 2,8-sialyltransferase \|\| id:prot-a-2859 | F-sweetened coffee drink liking (derived food-liking factor) \|\| id:ebi-fl187-GCST90094853 | Weighted mode | 4 | -0.471905565 | 0.437861060227201 | 0.360084872779023 | -1.330113243 | 0.386302112883542 | 0.623812419761224 | 0.26444731274232 | 1.4715291715879 | 0.999714303421225 |
| ebi-fl187-GCST90094853 | prot-a-2861 | Alpha-2,8-sialyltransferase 8F \|\| id:prot-a-2861 | F-sweetened coffee drink liking (derived food-liking factor) \|\| id:ebi-fl187-GCST90094853 | Weighted mode | 4 | -0.584590524 | 0.565685106084973 | 0.37741260273504 | -1.693333331 | 0.524152284383985 | 0.557334030258502 | 0.18390548322626 | 1.68902642724375 | 0.999714303421225 |
| ebi-fl187-GCST90094853 | prot-a-289 | Uncharacterized protein C17orf89 \|\| id:prot-a-289 | F-sweetened coffee drink liking (derived food-liking factor) \|\| id:ebi-fl187-GCST90094853 | Weighted mode | 4 | 0.0236947752705499 | 0.393875420772135 | 0.955813034744501 | -0.748301049 | 0.795690599983934 | 1.02397772686315 | 0.47316976227242 | 2.2159708179073 | 0.999714303421225 |
| ebi-fl187-GCST90094853 | prot-a-2921 | Tachykinin-3 \|\| id:prot-a-2921 | F-sweetened coffee drink liking (derived food-liking factor) \|\| id:ebi-fl187-GCST90094853 | Weighted mode | 4 | -0.175896405 | 0.393839835079111 | 0.685421782467332 | -0.947822482 | 0.596029671546203 | 0.838704864292037 | 0.387584076556918 | 1.81489873277553 | 0.999714303421225 |
| ebi-fl187-GCST90094853 | prot-a-2928 | Tax1-binding protein 1 \|\| id:prot-a-2928 | F-sweetened coffee drink liking (derived food-liking factor) \|\| id:ebi-fl187-GCST90094853 | Weighted mode | 4 | -0.319035862 | 0.456977293363045 | 0.535335996654936 | -1.214711357 | 0.576639633480699 | 0.726849482918319 | 0.29679567007173 | 1.7800467597487 | 0.999714303421225 |
| ebi-fl187-GCST90094853 | prot-a-296 | Uncharacterized protein C1orf185 \|\| id:prot-a-296 | F-sweetened coffee drink liking (derived food-liking factor) \|\| id:ebi-fl187-GCST90094853 | Weighted mode | 4 | -0.283747998 | 0.469312977809683 | 0.588108235030413 | -1.203601435 | 0.636105438221442 | 0.752956367095432 | 0.300111431549886 | 1.88910928124812 | 0.999714303421225 |
| ebi-fl187-GCST90094853 | prot-a-2964 | Transforming growth factor beta-2 \|\| id:prot-a-2964 | F-sweetened coffee drink liking (derived food-liking factor) \|\| id:ebi-fl187-GCST90094853 | Weighted mode | 4 | -0.054929509 | 0.379179138361549 | 0.894003032653342 | -0.79812062 | 0.688261601934287 | 0.946551868748882 | 0.450174217814227 | 1.99025267280353 | 0.999714303421225 |
| ebi-fl187-GCST90094853 | prot-a-2977 | Thymocyte nuclear protein 1 \|\| id:prot-a-2977 | F-sweetened coffee drink liking (derived food-liking factor) \|\| id:ebi-fl187-GCST90094853 | Weighted mode | 4 | 0.12713386646076 | 0.402375158601558 | 0.772740270356292 | -0.661521444 | 0.915789177319814 | 1.13556902221306 | 0.516065571822765 | 2.4987464280078 | 0.999714303421225 |
| ebi-fl187-GCST90094853 | prot-a-3015 | Transmembrane protein 70, mitochondrial \|\| id:prot-a-3015 | F-sweetened coffee drink liking (derived food-liking factor) \|\| id:ebi-fl187-GCST90094853 | Weighted mode | 4 | 0.0216430682608535 | 0.450757481319967 | 0.964722079510811 | -0.861841595 | 0.905127731647989 | 1.02187897832793 | 0.422383506220342 | 2.47224768716183 | 0.999714303421225 |
| ebi-fl187-GCST90094853 | prot-a-3030 | Tumor necrosis factor alpha-induced protein 3 \|\| id:prot-a-3030 | F-sweetened coffee drink liking (derived food-liking factor) \|\| id:ebi-fl187-GCST90094853 | Weighted mode | 4 | -0.492181608 | 0.446336558172077 | 0.350671534421281 | -1.367001262 | 0.382638045875269 | 0.611291340266613 | 0.254870103370249 | 1.46614725596949 | 0.999714303421225 |
| ebi-fl187-GCST90094853 | prot-a-3032 | Tumor necrosis factor receptor superfamily member 10A \|\| id:prot-a-3032 | F-sweetened coffee drink liking (derived food-liking factor) \|\| id:ebi-fl187-GCST90094853 | Weighted mode | 4 | -0.215563755 | 0.395218433067998 | 0.623396661425588 | -0.990191884 | 0.559064374037069 | 0.806086876731414 | 0.371505398392965 | 1.74903529167911 | 0.999714303421225 |
| ebi-fl187-GCST90094853 | prot-a-3081 | Tumor protein p53-inducible protein 11 \|\| id:prot-a-3081 | F-sweetened coffee drink liking (derived food-liking factor) \|\| id:ebi-fl187-GCST90094853 | Weighted mode | 4 | 0.567984289093538 | 0.697600678981423 | 0.475154224569094 | -0.799313042 | 1.93528161989713 | 1.76470632615464 | 0.449637740420113 | 6.92599427855079 | 0.999714303421225 |
| ebi-fl187-GCST90094853 | prot-a-315 | Complement C4 \|\| id:prot-a-315 | F-sweetened coffee drink liking (derived food-liking factor) \|\| id:ebi-fl187-GCST90094853 | Weighted mode | 4 | 0.602702494942017 | 0.456075848286373 | 0.278078480186967 | -0.291206168 | 1.49661115758331 | 1.82704972713353 | 0.747361580316113 | 4.46652703769812 | 0.999714303421225 |
| ebi-fl187-GCST90094853 | prot-a-3157 | UDP-glucuronosyltransferase 1-8 \|\| id:prot-a-3157 | F-sweetened coffee drink liking (derived food-liking factor) \|\| id:ebi-fl187-GCST90094853 | Weighted mode | 4 | 0.22875064413573 | 0.383976896560619 | 0.593301074680517 | -0.523844073 | 0.981345361394542 | 1.25702855248549 | 0.592239554472354 | 2.66804331090577 | 0.999714303421225 |
| ebi-fl187-GCST90094853 | prot-a-317 | Glutaredoxin-like protein C5orf63 \|\| id:prot-a-317 | F-sweetened coffee drink liking (derived food-liking factor) \|\| id:ebi-fl187-GCST90094853 | Weighted mode | 4 | 0.0144619404436912 | 0.349256066051137 | 0.96957247142811 | -0.670079949 | 0.69900382990392 | 1.01456702024594 | 0.511667668824332 | 2.01174766608934 | 0.999714303421225 |
| ebi-fl187-GCST90094853 | prot-a-3192 | Guanine nucleotide exchange factor VAV3 \|\| id:prot-a-3192 | F-sweetened coffee drink liking (derived food-liking factor) \|\| id:ebi-fl187-GCST90094853 | Weighted mode | 4 | 0.192142330195802 | 0.370381806597114 | 0.639760571342867 | -0.533806011 | 0.918090671126145 | 1.21184298654035 | 0.586368990624642 | 2.50450390028746 | 0.999714303421225 |
| ebi-fl187-GCST90094853 | prot-a-3233 | Serine/threonine-protein kinase WNK3 \|\| id:prot-a-3233 | F-sweetened coffee drink liking (derived food-liking factor) \|\| id:ebi-fl187-GCST90094853 | Weighted mode | 4 | 0.0266415120315814 | 0.377041275864406 | 0.948115448329313 | -0.712359389 | 0.765642412725817 | 1.02699956977455 | 0.490485585061368 | 2.15037535952284 | 0.999714303421225 |
| ebi-fl187-GCST90094853 | prot-a-3250 | 14-3-3 protein theta \|\| id:prot-a-3250 | F-sweetened coffee drink liking (derived food-liking factor) \|\| id:ebi-fl187-GCST90094853 | Weighted mode | 4 | -0.836464893 | 0.437985664257465 | 0.152155978951284 | -1.694916795 | 0.0219870088067243 | 0.433239366983401 | 0.183614506021967 | 1.02223050439012 | 0.999714303421225 |
| ebi-fl187-GCST90094853 | prot-a-3278 | E3 ubiquitin-protein ligase ZNRF3 \|\| id:prot-a-3278 | F-sweetened coffee drink liking (derived food-liking factor) \|\| id:ebi-fl187-GCST90094853 | Weighted mode | 4 | -0.222354054 | 0.474476065524128 | 0.671283937103383 | -1.152327143 | 0.707619034014548 | 0.800631846908943 | 0.315900767111663 | 2.02915415541946 | 0.999714303421225 |
| ebi-fl187-GCST90094853 | prot-a-355 | Calpain-3 \|\| id:prot-a-355 | F-sweetened coffee drink liking (derived food-liking factor) \|\| id:ebi-fl187-GCST90094853 | Weighted mode | 4 | -0.292630898 | 0.435208244462498 | 0.54948826764294 | -1.145639057 | 0.560377261600966 | 0.746297550124464 | 0.318020619632636 | 1.75133308640538 | 0.999714303421225 |
| ebi-fl187-GCST90094853 | prot-a-385 | Coiled-coil domain-containing protein 90B, mitochondrial \|\| id:prot-a-385 | F-sweetened coffee drink liking (derived food-liking factor) \|\| id:ebi-fl187-GCST90094853 | Weighted mode | 4 | -0.315142206 | 0.443288969570858 | 0.528422469881686 | -1.183988586 | 0.553704174768872 | 0.729685101590312 | 0.306055571916274 | 1.73968519556481 | 0.999714303421225 |
| ebi-fl187-GCST90094853 | prot-a-390 | C-C motif chemokine 14 \|\| id:prot-a-390 | F-sweetened coffee drink liking (derived food-liking factor) \|\| id:ebi-fl187-GCST90094853 | Weighted mode | 4 | -0.420643034 | 0.41001651457177 | 0.380423765803688 | -1.224275403 | 0.382989334237178 | 0.656624451970839 | 0.293970633441988 | 1.46666238691182 | 0.999714303421225 |
| ebi-fl187-GCST90094853 | prot-a-40 | Pituitary adenylate cyclase-activating polypeptide 27 \|\| id:prot-a-40 | F-sweetened coffee drink liking (derived food-liking factor) \|\| id:ebi-fl187-GCST90094853 | Weighted mode | 4 | -0.228872817 | 0.528285513090601 | 0.69410282273865 | -1.264312422 | 0.806566789001663 | 0.795429692480364 | 0.282433424314257 | 2.2402036770811 | 0.999714303421225 |
| ebi-fl187-GCST90094853 | prot-a-401 | C-C motif chemokine 24 \|\| id:prot-a-401 | F-sweetened coffee drink liking (derived food-liking factor) \|\| id:ebi-fl187-GCST90094853 | Weighted mode | 4 | -0.108012966 | 0.463578602654679 | 0.83075505790542 | -1.016627027 | 0.800601095306899 | 0.89761595795958 | 0.361813270677174 | 2.22687909284176 | 0.999714303421225 |
| ebi-fl187-GCST90094853 | prot-a-412 | Cerebral cavernous malformations 2 protein \|\| id:prot-a-412 | F-sweetened coffee drink liking (derived food-liking factor) \|\| id:ebi-fl187-GCST90094853 | Weighted mode | 4 | -0.002158047 | 0.415657624289102 | 0.996183440648366 | -0.816846991 | 0.812530896691887 | 0.997844279994329 | 0.441822531258848 | 2.25360441505881 | 0.999714303421225 |
| ebi-fl187-GCST90094853 | prot-a-44 | Adhesion G protein-coupled receptor F5 \|\| id:prot-a-44 | F-sweetened coffee drink liking (derived food-liking factor) \|\| id:ebi-fl187-GCST90094853 | Weighted mode | 4 | 0.00996799327217524 | 0.460662118495969 | 0.984095151265085 | -0.892929759 | 0.912865745524274 | 1.01001783920074 | 0.409454391082455 | 2.49145218056361 | 0.999714303421225 |
| ebi-fl187-GCST90094853 | prot-a-48 | Adrenomedullin \|\| id:prot-a-48 | F-sweetened coffee drink liking (derived food-liking factor) \|\| id:ebi-fl187-GCST90094853 | Weighted mode | 4 | -0.489273737 | 0.41155458230409 | 0.320029125842456 | -1.295920718 | 0.317373244450547 | 0.613071483756263 | 0.273645797646882 | 1.37351513316539 | 0.999714303421225 |
| ebi-fl187-GCST90094853 | prot-a-502 | Carcinoembryonic antigen-related cell adhesion molecule 1 \|\| id:prot-a-502 | F-sweetened coffee drink liking (derived food-liking factor) \|\| id:ebi-fl187-GCST90094853 | Weighted mode | 4 | -0.158920742 | 0.452131004496221 | 0.74845679799241 | -1.045097511 | 0.72725602646544 | 0.853063968137667 | 0.35165752717285 | 2.06939444631051 | 0.999714303421225 |
| ebi-fl187-GCST90094853 | prot-a-518 | Complement factor B \|\| id:prot-a-518 | F-sweetened coffee drink liking (derived food-liking factor) \|\| id:ebi-fl187-GCST90094853 | Weighted mode | 4 | 0.320965954311596 | 0.395063353071384 | 0.476020364795055 | -0.453358218 | 1.09529012633151 | 1.37845864952119 | 0.635490448916234 | 2.99005004981634 | 0.999714303421225 |
| ebi-fl187-GCST90094853 | prot-a-525 | Properdin \|\| id:prot-a-525 | F-sweetened coffee drink liking (derived food-liking factor) \|\| id:ebi-fl187-GCST90094853 | Weighted mode | 4 | 0.189236180402016 | 0.389869356436041 | 0.660644092894888 | -0.574907758 | 0.953380119016657 | 1.20832630177634 | 0.562756776103794 | 2.59446445349456 | 0.999714303421225 |
| ebi-fl187-GCST90094853 | prot-a-533 | Chondroadherin \|\| id:prot-a-533 | F-sweetened coffee drink liking (derived food-liking factor) \|\| id:ebi-fl187-GCST90094853 | Weighted mode | 4 | -0.445011901 | 0.379677671227529 | 0.325775240496013 | -1.189180136 | 0.299156334808005 | 0.640816649757466 | 0.304470786279381 | 1.34872045894602 | 0.999714303421225 |
| ebi-fl187-GCST90094853 | prot-a-535 | Coiled-coil-helix-coiled-coil-helix domain-containing protein 2 \|\| id:prot-a-535 | F-sweetened coffee drink liking (derived food-liking factor) \|\| id:ebi-fl187-GCST90094853 | Weighted mode | 4 | 0.0537298938979725 | 0.459951521664434 | 0.914386980114678 | -0.847775089 | 0.955234876360263 | 1.05519954782263 | 0.428366951004917 | 2.59928102089348 | 0.999714303421225 |
| ebi-fl187-GCST90094853 | prot-a-591 | Caseinolytic peptidase B protein homolog \|\| id:prot-a-591 | F-sweetened coffee drink liking (derived food-liking factor) \|\| id:ebi-fl187-GCST90094853 | Weighted mode | 4 | -0.176316556 | 0.398182561370088 | 0.68789058330598 | -0.956754376 | 0.604121264483444 | 0.838352555962351 | 0.384137631176544 | 1.8296437293476 | 0.999714303421225 |
| ebi-fl187-GCST90094853 | prot-a-605 | Ciliary neurotrophic factor receptor subunit alpha \|\| id:prot-a-605 | F-sweetened coffee drink liking (derived food-liking factor) \|\| id:ebi-fl187-GCST90094853 | Weighted mode | 4 | -0.32183426 | 0.432978414149877 | 0.511215396333168 | -1.170471951 | 0.526803432056213 | 0.724818311995876 | 0.310220497709372 | 1.6935102267057 | 0.999714303421225 |
| ebi-fl187-GCST90094853 | prot-a-606 | Ciliary neurotrophic factor receptor subunit alpha \|\| id:prot-a-606 | F-sweetened coffee drink liking (derived food-liking factor) \|\| id:ebi-fl187-GCST90094853 | Weighted mode | 4 | 0.0542984692795156 | 0.348559774032781 | 0.886098758897672 | -0.628878688 | 0.737475626383765 | 1.05579967890185 | 0.533189337627748 | 2.09065126269929 | 0.999714303421225 |
| ebi-fl187-GCST90094853 | prot-a-707 | Cancer/testis antigen 55 \|\| id:prot-a-707 | F-sweetened coffee drink liking (derived food-liking factor) \|\| id:ebi-fl187-GCST90094853 | Weighted mode | 4 | 0.603105908008962 | 0.399984144231365 | 0.228714081675556 | -0.180863015 | 1.38707483070244 | 1.82778693155637 | 0.834549671916091 | 4.00312309691272 | 0.999714303421225 |
| ebi-fl187-GCST90094853 | prot-a-719 | Dipeptidyl peptidase 1 \|\| id:prot-a-719 | F-sweetened coffee drink liking (derived food-liking factor) \|\| id:ebi-fl187-GCST90094853 | Weighted mode | 4 | 0.25211807373827 | 0.375569439624768 | 0.550097721809251 | -0.483998028 | 0.988234175402815 | 1.28674795945744 | 0.61631441732856 | 2.68648641767082 | 0.999714303421225 |
| ebi-fl187-GCST90094853 | prot-a-752 | Cytochrome b5 \|\| id:prot-a-752 | F-sweetened coffee drink liking (derived food-liking factor) \|\| id:ebi-fl187-GCST90094853 | Weighted mode | 4 | 0.201294672141079 | 0.490454006195218 | 0.709046305744203 | -0.75999518 | 1.16258452428371 | 1.22298509836907 | 0.467668681166796 | 3.19818839931971 | 0.999714303421225 |
| ebi-fl187-GCST90094853 | prot-a-755 | Cytoglobin \|\| id:prot-a-755 | F-sweetened coffee drink liking (derived food-liking factor) \|\| id:ebi-fl187-GCST90094853 | Weighted mode | 4 | 0.351785952107073 | 0.391153871361778 | 0.434745368491499 | -0.414875636 | 1.11844753997616 | 1.42160419976625 | 0.660422408527582 | 3.06009983110475 | 0.999714303421225 |
| ebi-fl187-GCST90094853 | prot-a-807 | Desmin \|\| id:prot-a-807 | F-sweetened coffee drink liking (derived food-liking factor) \|\| id:ebi-fl187-GCST90094853 | Weighted mode | 4 | -0.040053668 | 0.382016124895258 | 0.923113278385802 | -0.788805273 | 0.708697936676166 | 0.960737876774452 | 0.454387339984353 | 2.03134459666254 | 0.999714303421225 |
| ebi-fl187-GCST90094853 | prot-a-82 | Ameloblastin \|\| id:prot-a-82 | F-sweetened coffee drink liking (derived food-liking factor) \|\| id:ebi-fl187-GCST90094853 | Weighted mode | 4 | -0.479807685 | 0.494732136549391 | 0.403665133912727 | -1.449482672 | 0.489867302973418 | 0.61890240478607 | 0.234691669195576 | 1.63209963081723 | 0.999714303421225 |
| ebi-fl187-GCST90094853 | prot-a-84 | Muellerian-inhibiting factor \|\| id:prot-a-84 | F-sweetened coffee drink liking (derived food-liking factor) \|\| id:ebi-fl187-GCST90094853 | Weighted mode | 4 | 0.0895488982433211 | 0.393175929245171 | 0.834474482909531 | -0.681075923 | 0.860173719563856 | 1.09368081107527 | 0.506072204579625 | 2.3635712566112 | 0.999714303421225 |
| ebi-fl187-GCST90094853 | prot-a-858 | Inactive dipeptidyl peptidase 10 \|\| id:prot-a-858 | F-sweetened coffee drink liking (derived food-liking factor) \|\| id:ebi-fl187-GCST90094853 | Weighted mode | 4 | -0.184871643 | 0.386387203619372 | 0.665027161503238 | -0.942190563 | 0.572447275596319 | 0.831210968337766 | 0.389773077177588 | 1.77259978777399 | 0.999714303421225 |
| ebi-fl187-GCST90094853 | prot-a-863 | Dihydropyrimidinase-related protein 3 \|\| id:prot-a-863 | F-sweetened coffee drink liking (derived food-liking factor) \|\| id:ebi-fl187-GCST90094853 | Weighted mode | 4 | -0.160847528 | 0.414169220624639 | 0.723668554742208 | -0.9726192 | 0.650924144449232 | 0.85142187922885 | 0.378091442818872 | 1.91731188366742 | 0.999714303421225 |
| ebi-fl187-GCST90094853 | prot-a-864 | Dihydropyrimidinase-related protein 5 \|\| id:prot-a-864 | F-sweetened coffee drink liking (derived food-liking factor) \|\| id:ebi-fl187-GCST90094853 | Weighted mode | 4 | -0.182797943 | 0.382914227844142 | 0.665710165904888 | -0.93330983 | 0.567713943327776 | 0.832936439167685 | 0.393249963515245 | 1.76422930975414 | 0.999714303421225 |
| ebi-fl187-GCST90094853 | prot-a-884 | Endothelin-converting enzyme 1 \|\| id:prot-a-884 | F-sweetened coffee drink liking (derived food-liking factor) \|\| id:ebi-fl187-GCST90094853 | Weighted mode | 4 | 0.19591633870518 | 0.420493880246827 | 0.673015641987091 | -0.628251667 | 1.02008434398896 | 1.21642513337548 | 0.533523763505761 | 2.77342867613726 | 0.999714303421225 |
| ebi-fl187-GCST90094853 | prot-a-893 | Early endosome antigen 1 \|\| id:prot-a-893 | F-sweetened coffee drink liking (derived food-liking factor) \|\| id:ebi-fl187-GCST90094853 | Weighted mode | 4 | -0.385946818 | 0.432928811324541 | 0.438350396727326 | -1.234487288 | 0.462593652367628 | 0.679806678314347 | 0.290983915066308 | 1.5881878549042 | 0.999714303421225 |
| ebi-fl187-GCST90094853 | prot-a-897 | Ephrin-A2 \|\| id:prot-a-897 | F-sweetened coffee drink liking (derived food-liking factor) \|\| id:ebi-fl187-GCST90094853 | Weighted mode | 4 | 0.193802189487542 | 0.45201623435457 | 0.69702745471157 | -0.69214963 | 1.0797540088225 | 1.21385614569995 | 0.500499024215864 | 2.94395527496182 | 0.999714303421225 |
| ebi-fl187-GCST90094853 | prot-a-90 | Anaphase-promoting complex subunit 10 \|\| id:prot-a-90 | F-sweetened coffee drink liking (derived food-liking factor) \|\| id:ebi-fl187-GCST90094853 | Weighted mode | 4 | -0.80325246 | 0.424336264772017 | 0.154701562917321 | -1.634951539 | 0.0284466191208934 | 0.447869913747532 | 0.194961819156807 | 1.02885508817955 | 0.999714303421225 |
| ebi-fl187-GCST90094853 | prot-a-914 | Histone-lysine N-methyltransferase EHMT2 \|\| id:prot-a-914 | F-sweetened coffee drink liking (derived food-liking factor) \|\| id:ebi-fl187-GCST90094853 | Weighted mode | 4 | 0.32725448335921 | 0.408367892598109 | 0.481514417786019 | -0.473146586 | 1.1276555528515 | 1.38715443998845 | 0.62303873564454 | 3.08840739795907 | 0.999714303421225 |
| ebi-fl187-GCST90094853 | prot-a-932 | RNA polymerase II elongation factor ELL \|\| id:prot-a-932 | F-sweetened coffee drink liking (derived food-liking factor) \|\| id:ebi-fl187-GCST90094853 | Weighted mode | 4 | 0.0375118444561667 | 0.436533231769851 | 0.936934920791633 | -0.81809329 | 0.893116978725075 | 1.03822429421114 | 0.441272231141792 | 2.44273174022561 | 0.999714303421225 |
| ebi-fl187-GCST90094853 | prot-a-991 | Estrogen receptor \|\| id:prot-a-991 | F-sweetened coffee drink liking (derived food-liking factor) \|\| id:ebi-fl187-GCST90094853 | Weighted mode | 4 | 0.288965275579669 | 0.35177533575581 | 0.471590010456451 | -0.400514383 | 0.978444933661055 | 1.33504536904705 | 0.66997533379798 | 2.66031605568246 | 0.999714303421225 |
| ebi-fl187-GCST90094853 | prot-a-992 | Persulfide dioxygenase ETHE1, mitochondrial \|\| id:prot-a-992 | F-sweetened coffee drink liking (derived food-liking factor) \|\| id:ebi-fl187-GCST90094853 | Weighted mode | 4 | 0.0819280420425588 | 0.381213762144194 | 0.84361525857318 | -0.665250932 | 0.829107015845179 | 1.08537770545673 | 0.514144496334883 | 2.29127175706498 | 0.999714303421225 |
| ebi-fl187-GCST90094853 | prot-a-993 | Ethanolamine kinase 1 \|\| id:prot-a-993 | F-sweetened coffee drink liking (derived food-liking factor) \|\| id:ebi-fl187-GCST90094853 | Weighted mode | 4 | -0.198918949 | 0.366260647117943 | 0.62481043235186 | -0.916789817 | 0.518951919314089 | 0.819616321332931 | 0.399800415603346 | 1.68026567251449 | 0.999714303421225 |
| ebi-fl187-GCST90094853 | prot-b-10 | coagulation factor III, tissue factor \|\| id:prot-b-10 | F-sweetened coffee drink liking (derived food-liking factor) \|\| id:ebi-fl187-GCST90094853 | Weighted mode | 4 | -0.38604194 | 0.335073477299291 | 0.332754927260671 | -1.042785956 | 0.270702075065379 | 0.679742016402388 | 0.352471343190549 | 1.3108844670331 | 0.999714303421225 |
| ebi-fl187-GCST90094853 | prot-b-28 | prolactin \|\| id:prot-b-28 | F-sweetened coffee drink liking (derived food-liking factor) \|\| id:ebi-fl187-GCST90094853 | Weighted mode | 4 | -0.23666682 | 0.36692912457488 | 0.564861387336497 | -0.955847905 | 0.482514263683599 | 0.789254207551007 | 0.384485998836037 | 1.62014275168083 | 0.999714303421225 |
| ebi-fl187-GCST90094853 | prot-b-54 | matrix metallopeptidase 7 \|\| id:prot-b-54 | F-sweetened coffee drink liking (derived food-liking factor) \|\| id:ebi-fl187-GCST90094853 | Weighted mode | 4 | 0.52696043895846 | 0.422726141739193 | 0.301019606984198 | -0.301582799 | 1.35550367676728 | 1.69377614037491 | 0.739646581932536 | 3.87871408289019 | 0.999714303421225 |
| ebi-fl187-GCST90094853 | ukb-rsfMRI191-edge-pheno716 | Net100_Pair10_33 (OccipitalPrecuneus)&(Temporal\|Frontal\|Supp_Motor_Area) (Default_mode\|Central_executive)&(Default_mode\|Salience) \|\| id:ukb-rsfMRI191-edge-pheno716 | F-sweetened coffee drink liking (derived food-liking factor) \|\| id:ebi-fl187-GCST90094853 | Weighted mode | 3 | 0.00685943248838078 | 0.121330033136971 | 0.960055395875947 | -0.230947432 | 0.244666297436843 | 1.00688301227924 | 0.793781192063016 | 1.27719503882631 | 0.999714303421225 |
| ebi-fl187-GCST90094853 | ukb-rsfMRI191-edge-pheno903 | Net100_Pair27_38 (Precentral\|Frontal\|Supp_Motor_Area)&(Subcortical) (Attention\|Salience\|Motor)&(Subcortical-cerebellum) \|\| id:ukb-rsfMRI191-edge-pheno903 | F-sweetened coffee drink liking (derived food-liking factor) \|\| id:ebi-fl187-GCST90094853 | Weighted mode | 3 | -0.024743849 | 0.148490009193617 | 0.882979776623396 | -0.315784267 | 0.266296568925162 | 0.975559770544986 | 0.729216749233688 | 1.30512205994434 | 0.999714303421225 |
| ebi-fl187-GCST90094853 | ukb-rsfMRI191-node-pheno23 | Net100_Node2 RolandicOper\|SupraMarginal\|Insula Salience\|Motor \|\| id:ukb-rsfMRI191-node-pheno23 | F-sweetened coffee drink liking (derived food-liking factor) \|\| id:ebi-fl187-GCST90094853 | Weighted mode | 3 | 0.00752391128269062 | 0.101004714302719 | 0.947400041974511 | -0.190445329 | 0.205493151316019 | 1.00755228702406 | 0.826590947253559 | 1.22813056985491 | 0.999714303421225 |
| ebi-fl187-GCST90094853 | ukb-rsfMRI191-node-pheno55 | Net100_Node34 Parietal\|Postcentral\|Precuneus Attention \|\| id:ukb-rsfMRI191-node-pheno55 | F-sweetened coffee drink liking (derived food-liking factor) \|\| id:ebi-fl187-GCST90094853 | Weighted mode | 3 | 0.0209469283726375 | 0.113463704619016 | 0.870556828301891 | -0.201441933 | 0.243335789425909 | 1.02116785515951 | 0.817551049179681 | 1.27549685057268 | 0.999714303421225 |
