# Supplementary material for: Mendelian Randomization Mediation Analysis Reveals the Impact of Dietary Preferences on Preeclampsia and Fetal Growth Restriction via Immune Modulation
Source: Food Sci Nutr. 2025 Nov 26;13(12):e71280. doi: 10.1002/fsn3.71280 (PMC12648301; doi:10.1002/fsn3.71280)
Supplement: Supplementary file 1 — Data S1: fsn371280‐sup‐0001‐Supinfo1.zip. [file FSN3-13-e71280-s001.zip › fsn371280-sup-0013-Supinfo13@Supplementary Material 4_FGR - MR (Dietary Preferences to Immune Cells) MR Analysis -2.docx]

| id.exposure | id.outcome | outcome | exposure | method | nsnp | b | se | pval | lo_ci | up_ci | or | or_lci95 | or_uci95 | p_fdr |
| --- | --- | --- | --- | --- | --- | --- | --- | --- | --- | --- | --- | --- | --- | --- |
| ebi-fl187-GCST90094778 | prot-a-2186 | Prostate and testis expressed protein 1 \|\| id:prot-a-2186 | Hard cheese liking \|\| id:ebi-fl187-GCST90094778 | Weighted mode | 4 | 1.31136293959922 | 0.607633268264512 | 0.119784328523754 | 0.120401733800775 | 2.50232414539766 | 3.71122844699078 | 1.12794989617049 | 12.2108407762748 | 0.999228396881742 |
| ebi-fl187-GCST90094778 | prot-a-2227 | Platelet-derived growth factor C \|\| id:prot-a-2227 | Hard cheese liking \|\| id:ebi-fl187-GCST90094778 | Weighted mode | 4 | 0.272501548022562 | 0.50093760640232 | 0.624277138601088 | -0.709336161 | 1.25433925657111 | 1.31324549184457 | 0.491970678639967 | 3.50552135874784 | 0.999228396881742 |
| ebi-fl187-GCST90094778 | prot-a-2229 | Platelet-derived growth factor receptor alpha \|\| id:prot-a-2229 | Hard cheese liking \|\| id:ebi-fl187-GCST90094778 | Weighted mode | 4 | -0.059045504 | 0.567548857100771 | 0.923705956688353 | -1.171441264 | 1.05335025549943 | 0.942663872810144 | 0.309919942661043 | 2.86724103480262 | 0.999228396881742 |
| ebi-fl187-GCST90094778 | prot-a-2243 | Platelet endothelial aggregation receptor 1 \|\| id:prot-a-2243 | Hard cheese liking \|\| id:ebi-fl187-GCST90094778 | Weighted mode | 4 | 0.313487499407264 | 0.692365937689105 | 0.6814486760276 | -1.043549738 | 1.67052473727791 | 1.36818835948944 | 0.35220223452472 | 5.3149560211294 | 0.999228396881742 |
| ebi-fl187-GCST90094778 | prot-a-2246 | E3 ubiquitin-protein ligase pellino homolog 2 \|\| id:prot-a-2246 | Hard cheese liking \|\| id:ebi-fl187-GCST90094778 | Weighted mode | 4 | 1.05875894644805 | 0.626548367580417 | 0.189641933210187 | -0.169275854 | 2.28679374690567 | 2.88279107019461 | 0.844275974347601 | 9.84332683494344 | 0.999228396881742 |
| ebi-fl187-GCST90094778 | prot-a-2251 | Platelet factor 4 variant \|\| id:prot-a-2251 | Hard cheese liking \|\| id:ebi-fl187-GCST90094778 | Weighted mode | 4 | -0.188159139 | 0.518593040775181 | 0.740791272722408 | -1.204601499 | 0.828283220664446 | 0.828482852597468 | 0.299811450821661 | 2.28938499569292 | 0.999228396881742 |
| ebi-fl187-GCST90094778 | prot-a-2346 | Protein phosphatase 1L \|\| id:prot-a-2346 | Hard cheese liking \|\| id:ebi-fl187-GCST90094778 | Weighted mode | 4 | 0.123746674405306 | 0.59328125176362 | 0.848134953799098 | -1.039084579 | 1.286577928 | 1.1317291387253 | 0.353778389920949 | 3.62037614486881 | 0.999228396881742 |
| ebi-fl187-GCST90094778 | prot-a-2358 | PR domain zinc finger protein 4 \|\| id:prot-a-2358 | Hard cheese liking \|\| id:ebi-fl187-GCST90094778 | Weighted mode | 4 | 0.143827985714971 | 0.492908745212876 | 0.789458038128367 | -0.822273155 | 1.10992912633221 | 1.1546854690456 | 0.439431622176259 | 3.03414334594759 | 0.999228396881742 |
| ebi-fl187-GCST90094778 | prot-a-2390 | Trypsin-1 \|\| id:prot-a-2390 | Hard cheese liking \|\| id:ebi-fl187-GCST90094778 | Weighted mode | 4 | -0.692994141 | 0.586660260120637 | 0.322617766108334 | -1.84284825 | 0.456859969246259 | 0.500076525840484 | 0.158365717899676 | 1.57910774511888 | 0.999228396881742 |
| ebi-fl187-GCST90094778 | prot-a-242 | Brain-derived neurotrophic factor \|\| id:prot-a-242 | Hard cheese liking \|\| id:ebi-fl187-GCST90094778 | Weighted mode | 4 | 0.66552172939646 | 0.506370021462009 | 0.280200560617295 | -0.326963513 | 1.658006971 | 1.94550528389566 | 0.721110053927766 | 5.24883932632713 | 0.999228396881742 |
| ebi-fl187-GCST90094778 | prot-a-2464 | Dihydropteridine reductase \|\| id:prot-a-2464 | Hard cheese liking \|\| id:ebi-fl187-GCST90094778 | Weighted mode | 4 | 0.433358006624835 | 0.50177743741346 | 0.451305583821174 | -0.550125771 | 1.41684178395522 | 1.54242832142428 | 0.576877251558753 | 4.12407513089363 | 0.999228396881742 |
| ebi-fl187-GCST90094778 | prot-a-2474 | Ras-related protein Rab-27A \|\| id:prot-a-2474 | Hard cheese liking \|\| id:ebi-fl187-GCST90094778 | Weighted mode | 4 | -0.126898359 | 1.03353491677748 | 0.91004411693875 | -2.152626796 | 1.89883007775459 | 0.880823195684038 | 0.116178579174295 | 6.67807703940919 | 0.999228396881742 |
| ebi-fl187-GCST90094778 | prot-a-2478 | Ras-related protein Rab-7b \|\| id:prot-a-2478 | Hard cheese liking \|\| id:ebi-fl187-GCST90094778 | Weighted mode | 4 | 0.179195611317643 | 0.55879782534849 | 0.769492192772289 | -0.916048126 | 1.27443934900068 | 1.19625472224521 | 0.400097053975941 | 3.57669556992044 | 0.999228396881742 |
| ebi-fl187-GCST90094778 | prot-a-2528 | E3 ubiquitin-protein ligase rififylin \|\| id:prot-a-2528 | Hard cheese liking \|\| id:ebi-fl187-GCST90094778 | Weighted mode | 4 | 0.293604682799435 | 0.659689170108424 | 0.68642653833499 | -0.999386091 | 1.58659545621195 | 1.3412535782407 | 0.368105355151895 | 4.88708228762701 | 0.999228396881742 |
| ebi-fl187-GCST90094778 | prot-a-2543 | Protein RIC-3 \|\| id:prot-a-2543 | Hard cheese liking \|\| id:ebi-fl187-GCST90094778 | Weighted mode | 4 | -0.225123031 | 0.492448654817931 | 0.67863558885496 | -1.190322395 | 0.740076332079433 | 0.798417982263246 | 0.304123200519472 | 2.09609550771677 | 0.999228396881742 |
| ebi-fl187-GCST90094778 | prot-a-255 | Bone morphogenetic protein 6 \|\| id:prot-a-255 | Hard cheese liking \|\| id:ebi-fl187-GCST90094778 | Weighted mode | 4 | -0.498384949 | 0.519614908608647 | 0.408243896337515 | -1.51683017 | 0.520060272035232 | 0.607511029219921 | 0.219406266467307 | 1.68212903198388 | 0.999228396881742 |
| ebi-fl187-GCST90094778 | prot-a-2568 | RING finger protein 165 \|\| id:prot-a-2568 | Hard cheese liking \|\| id:ebi-fl187-GCST90094778 | Weighted mode | 4 | 0.794226066176074 | 0.465837334311418 | 0.186750687535232 | -0.118815109 | 1.70726724142645 | 2.21272782904031 | 0.887971963543708 | 5.5138727870077 | 0.999228396881742 |
| ebi-fl187-GCST90094778 | prot-a-2573 | E3 ubiquitin-protein ligase RNF8 \|\| id:prot-a-2573 | Hard cheese liking \|\| id:ebi-fl187-GCST90094778 | Weighted mode | 4 | -0.663850633 | 0.643782263564796 | 0.378312287065333 | -1.92566387 | 0.597962603252131 | 0.514864956375227 | 0.145778946467229 | 1.8184102006997 | 0.999228396881742 |
| ebi-fl187-GCST90094778 | prot-a-260 | Cytoplasmic tyrosine-protein kinase BMX \|\| id:prot-a-260 | Hard cheese liking \|\| id:ebi-fl187-GCST90094778 | Weighted mode | 4 | -0.048938943 | 0.608746891249019 | 0.940987456200404 | -1.24208285 | 1.14420496372977 | 0.95223926868397 | 0.288782101307427 | 3.13994399486164 | 0.999228396881742 |
| ebi-fl187-GCST90094778 | prot-a-2623 | Serum amyloid A-1 protein \|\| id:prot-a-2623 | Hard cheese liking \|\| id:ebi-fl187-GCST90094778 | Weighted mode | 4 | 1.11837003969189 | 0.684984545752237 | 0.201037909605646 | -0.22419967 | 2.46093974936627 | 3.05986268168764 | 0.799155551063005 | 11.7158162992558 | 0.999228396881742 |
| ebi-fl187-GCST90094778 | prot-a-2640 | Lysosome membrane protein 2 \|\| id:prot-a-2640 | Hard cheese liking \|\| id:ebi-fl187-GCST90094778 | Weighted mode | 4 | 0.808977938702756 | 0.516117697944447 | 0.215007620199179 | -0.202612749 | 1.82056862667387 | 2.24561166061642 | 0.816594406985544 | 6.17536893120759 | 0.999228396881742 |
| ebi-fl187-GCST90094778 | prot-a-2641 | Scavenger receptor class F member 2 \|\| id:prot-a-2641 | Hard cheese liking \|\| id:ebi-fl187-GCST90094778 | Weighted mode | 4 | 0.597080132745428 | 0.504409840324806 | 0.321773940389721 | -0.391563154 | 1.58572341978205 | 1.81680621516755 | 0.675999356886342 | 4.88282243148111 | 0.999228396881742 |
| ebi-fl187-GCST90094778 | prot-a-2669 | Semaphorin-3A \|\| id:prot-a-2669 | Hard cheese liking \|\| id:ebi-fl187-GCST90094778 | Weighted mode | 4 | 0.341505595777825 | 0.522764626537639 | 0.560184424120004 | -0.683113072 | 1.3661242637916 | 1.40706446690417 | 0.505042309395032 | 3.92012783324208 | 0.999228396881742 |
| ebi-fl187-GCST90094778 | prot-a-2683 | Sentrin-specific protease 7 \|\| id:prot-a-2683 | Hard cheese liking \|\| id:ebi-fl187-GCST90094778 | Weighted mode | 4 | -0.482091066 | 0.532800772481844 | 0.432257305956338 | -1.52638058 | 0.56219844824604 | 0.617490826896692 | 0.217320820909602 | 1.75452549693877 | 0.999228396881742 |
| ebi-fl187-GCST90094778 | prot-a-270 | Bromodomain testis-specific protein \|\| id:prot-a-270 | Hard cheese liking \|\| id:ebi-fl187-GCST90094778 | Weighted mode | 4 | -0.080199211 | 0.501619968310284 | 0.883133604294446 | -1.063374349 | 0.902975926563972 | 0.922932469472685 | 0.345288717635914 | 2.46693361149762 | 0.999228396881742 |
| ebi-fl187-GCST90094778 | prot-a-2708 | 14-3-3 protein sigma \|\| id:prot-a-2708 | Hard cheese liking \|\| id:ebi-fl187-GCST90094778 | Weighted mode | 4 | 0.803019612169921 | 0.535802633182765 | 0.230890665662417 | -0.247153549 | 1.85319277320814 | 2.2322713554904 | 0.781020759470009 | 6.3801574338746 | 0.999228396881742 |
| ebi-fl187-GCST90094778 | prot-a-2713 | Beta-sarcoglycan \|\| id:prot-a-2713 | Hard cheese liking \|\| id:ebi-fl187-GCST90094778 | Weighted mode | 4 | 0.290948373798803 | 0.530863173472284 | 0.621793464546184 | -0.749543446 | 1.33144019380448 | 1.33769552202919 | 0.472582262720272 | 3.78649274595422 | 0.999228396881742 |
| ebi-fl187-GCST90094778 | prot-a-2720 | Protein shisa-3 homolog \|\| id:prot-a-2720 | Hard cheese liking \|\| id:ebi-fl187-GCST90094778 | Weighted mode | 4 | 0.832103120356972 | 0.536031783674055 | 0.218389590245556 | -0.218519176 | 1.88272541635812 | 2.2981469409558 | 0.803708067676965 | 6.57139025304308 | 0.999228396881742 |
| ebi-fl187-GCST90094778 | prot-a-2730 | Sialic acid-binding Ig-like lectin 8 \|\| id:prot-a-2730 | Hard cheese liking \|\| id:ebi-fl187-GCST90094778 | Weighted mode | 4 | 0.685369437977423 | 0.696754400895153 | 0.397814101520598 | -0.680269188 | 2.05100806373192 | 1.9845048515854 | 0.506480635617194 | 7.77573559385313 | 0.999228396881742 |
| ebi-fl187-GCST90094778 | prot-a-2761 | Sodium- and chloride-dependent neutral and basic amino acid transporter B(0+) \|\| id:prot-a-2761 | Hard cheese liking \|\| id:ebi-fl187-GCST90094778 | Weighted mode | 4 | -0.368296162 | 0.618621282937019 | 0.593530746859287 | -1.580793876 | 0.844201552953986 | 0.691912233505424 | 0.205811644374678 | 2.32611978942706 | 0.999228396881742 |
| ebi-fl187-GCST90094778 | prot-a-2770 | Secreted Ly-6/uPAR-related protein 1 \|\| id:prot-a-2770 | Hard cheese liking \|\| id:ebi-fl187-GCST90094778 | Weighted mode | 4 | 0.181460322290659 | 0.499177194874294 | 0.740324077431517 | -0.79692698 | 1.15984762424428 | 1.19896696350324 | 0.450711884945596 | 3.18944724465317 | 0.999228396881742 |
| ebi-fl187-GCST90094778 | prot-a-2784 | N-lysine methyltransferase SMYD2 \|\| id:prot-a-2784 | Hard cheese liking \|\| id:ebi-fl187-GCST90094778 | Weighted mode | 4 | 1.10378321657629 | 0.712094711938757 | 0.218907512563311 | -0.291922419 | 2.49948885197625 | 3.01555296068856 | 0.746826473402216 | 12.1762684941941 | 0.999228396881742 |
| ebi-fl187-GCST90094778 | prot-a-2837 | Serine palmitoyltransferase 2 \|\| id:prot-a-2837 | Hard cheese liking \|\| id:ebi-fl187-GCST90094778 | Weighted mode | 4 | -0.650887054 | 0.622058422945053 | 0.372279659951757 | -1.870121563 | 0.568347455067552 | 0.521582899345242 | 0.154104927238116 | 1.76534732383366 | 0.999228396881742 |
| ebi-fl187-GCST90094778 | prot-a-2858 | Alpha-2,8-sialyltransferase 8B \|\| id:prot-a-2858 | Hard cheese liking \|\| id:ebi-fl187-GCST90094778 | Weighted mode | 4 | 0.0133804638751236 | 0.516678108627316 | 0.980965745012842 | -0.999308629 | 1.02606955678466 | 1.01347038288699 | 0.368133870278217 | 2.79007801214502 | 0.999228396881742 |
| ebi-fl187-GCST90094778 | prot-a-2911 | Synaptotagmin-11 \|\| id:prot-a-2911 | Hard cheese liking \|\| id:ebi-fl187-GCST90094778 | Weighted mode | 4 | 0.256861735354833 | 0.492690500242062 | 0.638166729774883 | -0.708811645 | 1.22253511582927 | 1.29286635669279 | 0.492228792526838 | 3.39578554047498 | 0.999228396881742 |
| ebi-fl187-GCST90094778 | prot-a-2962 | Transforming growth factor beta-1 \|\| id:prot-a-2962 | Hard cheese liking \|\| id:ebi-fl187-GCST90094778 | Weighted mode | 4 | 0.0978338006100088 | 0.6162081754758 | 0.883938082942685 | -1.109934223 | 1.30560182454258 | 1.10277948859875 | 0.329580639081615 | 3.68990910346818 | 0.999228396881742 |
| ebi-fl187-GCST90094778 | prot-a-2978 | Tyrosine-protein kinase receptor Tie-1, soluble \|\| id:prot-a-2978 | Hard cheese liking \|\| id:ebi-fl187-GCST90094778 | Weighted mode | 4 | 0.258243491479925 | 0.503984945298131 | 0.643708862123269 | -0.729567001 | 1.24605398426426 | 1.29465401747127 | 0.48211770123687 | 3.47659714765585 | 0.999228396881742 |
| ebi-fl187-GCST90094778 | prot-a-299 | Uncharacterized protein C1orf226 \|\| id:prot-a-299 | Hard cheese liking \|\| id:ebi-fl187-GCST90094778 | Weighted mode | 4 | -0.08515514 | 0.526549078727615 | 0.881802104909199 | -1.117191335 | 0.946881053852391 | 0.918369797041957 | 0.327197493491934 | 2.57765753373559 | 0.999228396881742 |
| ebi-fl187-GCST90094778 | prot-a-3015 | Transmembrane protein 70, mitochondrial \|\| id:prot-a-3015 | Hard cheese liking \|\| id:ebi-fl187-GCST90094778 | Weighted mode | 4 | 0.0696369273892776 | 0.532874670301818 | 0.904297971281607 | -0.974797426 | 1.11407128118084 | 1.07211885359021 | 0.377268770515016 | 3.04673730257203 | 0.999228396881742 |
| ebi-fl187-GCST90094778 | prot-a-3016 | Transmembrane protein 87B \|\| id:prot-a-3016 | Hard cheese liking \|\| id:ebi-fl187-GCST90094778 | Weighted mode | 4 | 0.793871479557834 | 0.67407703756722 | 0.32383121847649 | -0.527319514 | 2.11506247318959 | 2.21194336445087 | 0.590184833470987 | 8.29010365915942 | 0.999228396881742 |
| ebi-fl187-GCST90094778 | prot-a-3123 | Thioredoxin domain-containing protein 12 \|\| id:prot-a-3123 | Hard cheese liking \|\| id:ebi-fl187-GCST90094778 | Weighted mode | 4 | 0.417837211839354 | 0.569827006773987 | 0.516497073728025 | -0.699023721 | 1.53469814511637 | 1.51867343222408 | 0.497070346107556 | 4.63992473460532 | 0.999228396881742 |
| ebi-fl187-GCST90094778 | prot-a-3133 | PolyUbiquitin K48-linked \|\| id:prot-a-3133 | Hard cheese liking \|\| id:ebi-fl187-GCST90094778 | Weighted mode | 4 | 0.0631101630862649 | 0.656685150087686 | 0.929497943709372 | -1.223992731 | 1.35021305725813 | 1.06514417233764 | 0.294053742395392 | 3.85824747076102 | 0.999228396881742 |
| ebi-fl187-GCST90094778 | prot-a-3135 | Ubiquitin-conjugating enzyme E2 B \|\| id:prot-a-3135 | Hard cheese liking \|\| id:ebi-fl187-GCST90094778 | Weighted mode | 4 | -0.574960092 | 0.565310328724599 | 0.384003960720386 | -1.682968337 | 0.533048151936344 | 0.562727325476202 | 0.18582157559395 | 1.70411881303577 | 0.999228396881742 |
| ebi-fl187-GCST90094778 | prot-a-3162 | NKG2D ligand 3 \|\| id:prot-a-3162 | Hard cheese liking \|\| id:ebi-fl187-GCST90094778 | Weighted mode | 4 | 0.208672037012823 | 0.503945691578501 | 0.706642332047982 | -0.779061518 | 1.19640559250668 | 1.23204086846048 | 0.458836418808168 | 3.30820449148234 | 0.999228396881742 |
| ebi-fl187-GCST90094778 | prot-a-3188 | Vesicle-associated membrane protein 8 \|\| id:prot-a-3188 | Hard cheese liking \|\| id:ebi-fl187-GCST90094778 | Weighted mode | 4 | 0.22868828742378 | 0.538096691221105 | 0.699481173290914 | -0.825981227 | 1.28335780221714 | 1.25695017076197 | 0.43780519519494 | 3.60873682888817 | 0.999228396881742 |
| ebi-fl187-GCST90094778 | prot-a-3201 | Vitrin \|\| id:prot-a-3201 | Hard cheese liking \|\| id:ebi-fl187-GCST90094778 | Weighted mode | 4 | -0.456792106 | 0.566158643239144 | 0.478800218535462 | -1.566463047 | 0.652878834796074 | 0.633311988170313 | 0.208782331313258 | 1.92106329993244 | 0.999228396881742 |
| ebi-fl187-GCST90094778 | prot-a-3232 | WNT1-inducible-signaling pathway protein 3 \|\| id:prot-a-3232 | Hard cheese liking \|\| id:ebi-fl187-GCST90094778 | Weighted mode | 4 | -0.62432517 | 0.523855966518621 | 0.319030402375557 | -1.651082865 | 0.40243252407965 | 0.535622760735051 | 0.191842057117026 | 1.49545801441459 | 0.999228396881742 |
| ebi-fl187-GCST90094778 | prot-a-327 | Carbonic anhydrase-related protein 10 \|\| id:prot-a-327 | Hard cheese liking \|\| id:ebi-fl187-GCST90094778 | Weighted mode | 4 | -1.481671636 | 0.704820378664422 | 0.126300752816099 | -2.863119578 | -0.100223694 | 0.227257478899448 | 0.057090384263021 | 0.904635034120546 | 0.999228396881742 |
| ebi-fl187-GCST90094778 | prot-a-332 | Carbonic anhydrase 5A, mitochondrial \|\| id:prot-a-332 | Hard cheese liking \|\| id:ebi-fl187-GCST90094778 | Weighted mode | 4 | -0.292712893 | 0.585759129599346 | 0.651626205849512 | -1.440800787 | 0.855375001497755 | 0.746236359986836 | 0.236738106069751 | 2.3522563148424 | 0.999228396881742 |
| ebi-fl187-GCST90094778 | prot-a-338 | Cell adhesion molecule 3 \|\| id:prot-a-338 | Hard cheese liking \|\| id:ebi-fl187-GCST90094778 | Weighted mode | 4 | 0.716297690902478 | 0.587555651544057 | 0.309913130675482 | -0.435311386 | 1.86790676792883 | 2.04684112667077 | 0.647063149218793 | 6.47472909388975 | 0.999228396881742 |
| ebi-fl187-GCST90094778 | prot-a-34 | A disintegrin and metalloproteinase with thrombospondin motifs 3 \|\| id:prot-a-34 | Hard cheese liking \|\| id:ebi-fl187-GCST90094778 | Weighted mode | 4 | -0.124733042 | 0.534844078656566 | 0.830602108108219 | -1.173027436 | 0.923561352590514 | 0.882732524015744 | 0.309428746173236 | 2.51824278963066 | 0.999228396881742 |
| ebi-fl187-GCST90094778 | prot-a-358 | Cysteine--tRNA ligase, cytoplasmic \|\| id:prot-a-358 | Hard cheese liking \|\| id:ebi-fl187-GCST90094778 | Weighted mode | 4 | -0.591310845 | 0.629820984179476 | 0.417065758300225 | -1.825759974 | 0.643138283523161 | 0.553601123371832 | 0.16109516938241 | 1.90244192283036 | 0.999228396881742 |
| ebi-fl187-GCST90094778 | prot-a-382 | Coiled-coil domain-containing protein 167 \|\| id:prot-a-382 | Hard cheese liking \|\| id:ebi-fl187-GCST90094778 | Weighted mode | 4 | -0.45832529 | 0.511232575488714 | 0.436044124103768 | -1.460341138 | 0.54369055763954 | 0.632341748099957 | 0.232157063559071 | 1.72235158500085 | 0.999228396881742 |
| ebi-fl187-GCST90094778 | prot-a-40 | Pituitary adenylate cyclase-activating polypeptide 27 \|\| id:prot-a-40 | Hard cheese liking \|\| id:ebi-fl187-GCST90094778 | Weighted mode | 4 | 0.0864643661640954 | 0.567811049910698 | 0.888633473795717 | -1.026445292 | 1.19937402398906 | 1.0903125150083 | 0.358278274425304 | 3.31803925954091 | 0.999228396881742 |
| ebi-fl187-GCST90094778 | prot-a-403 | C-C motif chemokine 25 \|\| id:prot-a-403 | Hard cheese liking \|\| id:ebi-fl187-GCST90094778 | Weighted mode | 4 | 0.173480339307303 | 0.536767411599843 | 0.767765480260703 | -0.878583787 | 1.22554446604299 | 1.18943730137846 | 0.415370748608367 | 3.40602004029026 | 0.999228396881742 |
| ebi-fl187-GCST90094778 | prot-a-415 | Cyclin-C \|\| id:prot-a-415 | Hard cheese liking \|\| id:ebi-fl187-GCST90094778 | Weighted mode | 4 | -0.192436175 | 0.585859969580018 | 0.764149547727714 | -1.340721715 | 0.955849365852624 | 0.824946969156156 | 0.261656758837225 | 2.60087874260984 | 0.999228396881742 |
| ebi-fl187-GCST90094778 | prot-a-44 | Adhesion G protein-coupled receptor F5 \|\| id:prot-a-44 | Hard cheese liking \|\| id:ebi-fl187-GCST90094778 | Weighted mode | 4 | -0.28604493 | 0.519001683698924 | 0.619925895029069 | -1.30328823 | 0.731198370212817 | 0.751228862598223 | 0.271637117602393 | 2.07756881306139 | 0.999228396881742 |
| ebi-fl187-GCST90094778 | prot-a-455 | CD59 glycoprotein \|\| id:prot-a-455 | Hard cheese liking \|\| id:ebi-fl187-GCST90094778 | Weighted mode | 4 | 0.0649304999441296 | 0.507364469011646 | 0.906264859960589 | -0.929503859 | 1.05936485920696 | 1.06708485934964 | 0.394749513087105 | 2.88453831937213 | 0.999228396881742 |
| ebi-fl187-GCST90094778 | prot-a-478 | Cadherin-12 \|\| id:prot-a-478 | Hard cheese liking \|\| id:ebi-fl187-GCST90094778 | Weighted mode | 4 | -0.480620489 | 0.663110479744722 | 0.520992050723674 | -1.78031703 | 0.819076050810148 | 0.618399562308643 | 0.168584692426159 | 2.26840297989109 | 0.999228396881742 |
| ebi-fl187-GCST90094778 | prot-a-516 | Centrin-2 \|\| id:prot-a-516 | Hard cheese liking \|\| id:ebi-fl187-GCST90094778 | Weighted mode | 4 | -0.168650716 | 0.495679946706354 | 0.756105665326698 | -1.140183411 | 0.802881979954137 | 0.844803928700697 | 0.319760368825561 | 2.23196414417909 | 0.999228396881742 |
| ebi-fl187-GCST90094778 | prot-a-519 | Cryptic protein \|\| id:prot-a-519 | Hard cheese liking \|\| id:ebi-fl187-GCST90094778 | Weighted mode | 4 | -0.24243689 | 0.586963511367925 | 0.707327078067838 | -1.392885372 | 0.908011592374523 | 0.784713269343756 | 0.248357665500909 | 2.47938759547534 | 0.999228396881742 |
| ebi-fl187-GCST90094778 | prot-a-583 | Chloride intracellular channel protein 4 \|\| id:prot-a-583 | Hard cheese liking \|\| id:ebi-fl187-GCST90094778 | Weighted mode | 4 | 0.718188001256932 | 0.57850675685903 | 0.302659184801656 | -0.415685242 | 1.85206124470063 | 2.0507139509131 | 0.65988794268527 | 6.37294218675451 | 0.999228396881742 |
| ebi-fl187-GCST90094778 | prot-a-602 | CCR4-NOT transcription complex subunit 1 \|\| id:prot-a-602 | Hard cheese liking \|\| id:ebi-fl187-GCST90094778 | Weighted mode | 4 | 0.327127352441568 | 0.574586997358137 | 0.608972455166751 | -0.799063162 | 1.45331786726352 | 1.38697810098089 | 0.449750109636072 | 4.27728245393242 | 0.999228396881742 |
| ebi-fl187-GCST90094778 | prot-a-606 | Ciliary neurotrophic factor receptor subunit alpha \|\| id:prot-a-606 | Hard cheese liking \|\| id:ebi-fl187-GCST90094778 | Weighted mode | 4 | 0.418567589211567 | 0.553611423386644 | 0.504559727739588 | -0.666510801 | 1.50364597904939 | 1.51978304210241 | 0.513497149562874 | 4.49805903894162 | 0.999228396881742 |
| ebi-fl187-GCST90094778 | prot-a-626 | Collagen alpha-2(IX) chain \|\| id:prot-a-626 | Hard cheese liking \|\| id:ebi-fl187-GCST90094778 | Weighted mode | 4 | 0.445742133195599 | 0.564189930101621 | 0.487185574412873 | -0.66007013 | 1.55155439619478 | 1.56164871724383 | 0.516815089080089 | 4.71879936866814 | 0.999228396881742 |
| ebi-fl187-GCST90094778 | prot-a-647 | Probable serine carboxypeptidase CPVL \|\| id:prot-a-647 | Hard cheese liking \|\| id:ebi-fl187-GCST90094778 | Weighted mode | 4 | 0.239220145966539 | 0.563001557463986 | 0.699542251714074 | -0.864262907 | 1.34270319859595 | 1.27025814801279 | 0.421362021329499 | 3.82938110440454 | 0.999228396881742 |
| ebi-fl187-GCST90094778 | prot-a-651 | Cellular retinoic acid-binding protein 2 \|\| id:prot-a-651 | Hard cheese liking \|\| id:ebi-fl187-GCST90094778 | Weighted mode | 4 | -0.32611114 | 0.512668693373902 | 0.569917554029816 | -1.330941779 | 0.678719498775228 | 0.721724970289428 | 0.26422829935491 | 1.97135179695353 | 0.999228396881742 |
| ebi-fl187-GCST90094778 | prot-a-671 | Cytotoxic and regulatory T-cell molecule \|\| id:prot-a-671 | Hard cheese liking \|\| id:ebi-fl187-GCST90094778 | Weighted mode | 4 | 0.678890877645292 | 0.617398924325975 | 0.351833128242189 | -0.531211014 | 1.8889927693242 | 1.97168967394865 | 0.587892592236419 | 6.61270480644595 | 0.999228396881742 |
| ebi-fl187-GCST90094778 | prot-a-688 | Chorionic somatomammotropin hormone \|\| id:prot-a-688 | Hard cheese liking \|\| id:ebi-fl187-GCST90094778 | Weighted mode | 4 | -0.637144818 | 0.552428255968942 | 0.332316502799007 | -1.7199042 | 0.445614563233159 | 0.528800090942341 | 0.179083303240626 | 1.56144951048229 | 0.999228396881742 |
| ebi-fl187-GCST90094778 | prot-a-69 | Aldo-keto reductase family 1 member C1 \|\| id:prot-a-69 | Hard cheese liking \|\| id:ebi-fl187-GCST90094778 | Weighted mode | 4 | -0.232497703 | 0.532039366524349 | 0.691659552104908 | -1.275294861 | 0.810299455337368 | 0.792551569795733 | 0.2793485869504 | 2.24858123551994 | 0.999228396881742 |
| ebi-fl187-GCST90094778 | prot-a-741 | Stromal cell-derived factor 1 \|\| id:prot-a-741 | Hard cheese liking \|\| id:ebi-fl187-GCST90094778 | Weighted mode | 4 | 0.126083927064747 | 0.607084657754079 | 0.848772537784858 | -1.063802002 | 1.31596985626274 | 1.13437736925092 | 0.345141085477174 | 3.7283652106777 | 0.999228396881742 |
| ebi-fl187-GCST90094778 | prot-a-742 | Growth-regulated alpha protein \|\| id:prot-a-742 | Hard cheese liking \|\| id:ebi-fl187-GCST90094778 | Weighted mode | 4 | 0.383109839225461 | 0.578124754326226 | 0.554902678514499 | -0.750014679 | 1.51623435770486 | 1.46683913769503 | 0.472359618803326 | 4.55504020713031 | 0.999228396881742 |
| ebi-fl187-GCST90094778 | prot-a-788 | Beta-defensin 112 \|\| id:prot-a-788 | Hard cheese liking \|\| id:ebi-fl187-GCST90094778 | Weighted mode | 4 | 0.395199909711255 | 0.665344977287371 | 0.594338136028574 | -0.908876246 | 1.6992760651945 | 1.48468096339877 | 0.402976816586038 | 5.46998604473835 | 0.999228396881742 |
| ebi-fl187-GCST90094778 | prot-a-838 | DnaJ homolog subfamily B member 1 \|\| id:prot-a-838 | Hard cheese liking \|\| id:ebi-fl187-GCST90094778 | Weighted mode | 4 | 0.703708604104606 | 0.63917191559212 | 0.351321594179085 | -0.54906835 | 1.95648555866516 | 2.02123478448228 | 0.577487575873859 | 7.07442068830496 | 0.999228396881742 |
| ebi-fl187-GCST90094778 | prot-a-85 | Junctional adhesion molecule-like \|\| id:prot-a-85 | Hard cheese liking \|\| id:ebi-fl187-GCST90094778 | Weighted mode | 4 | -0.536890578 | 0.551534825974581 | 0.402127693261699 | -1.617898837 | 0.544117680915771 | 0.584563082688075 | 0.198314953683018 | 1.72308739858303 | 0.999228396881742 |
| ebi-fl187-GCST90094778 | prot-a-856 | Dipeptidase 1 \|\| id:prot-a-856 | Hard cheese liking \|\| id:ebi-fl187-GCST90094778 | Weighted mode | 4 | 0.714814719780378 | 0.654852750886451 | 0.354845448954879 | -0.568696672 | 1.99832611151782 | 2.04380796998615 | 0.566262984390374 | 7.37669798896697 | 0.999228396881742 |
| ebi-fl187-GCST90094778 | prot-a-860 | Dipeptidyl peptidase 2 \|\| id:prot-a-860 | Hard cheese liking \|\| id:ebi-fl187-GCST90094778 | Weighted mode | 4 | -0.054253519 | 0.604734202360463 | 0.93416796696066 | -1.239532555 | 1.13102551793636 | 0.947191945199131 | 0.289519520673979 | 3.09883277977791 | 0.999228396881742 |
| ebi-fl187-GCST90094778 | prot-a-861 | Dermatopontin \|\| id:prot-a-861 | Hard cheese liking \|\| id:ebi-fl187-GCST90094778 | Weighted mode | 4 | -0.100801048 | 0.689169916918658 | 0.892988152280496 | -1.451574085 | 1.24997198951271 | 0.904112890379874 | 0.234201345016825 | 3.49024519262401 | 0.999228396881742 |
| ebi-fl187-GCST90094778 | prot-a-877 | Dual specificity protein phosphatase 6 \|\| id:prot-a-877 | Hard cheese liking \|\| id:ebi-fl187-GCST90094778 | Weighted mode | 4 | 0.693928173271967 | 0.766766473049477 | 0.432174910917552 | -0.808934114 | 2.19679046044894 | 2.00156259553248 | 0.445332487044335 | 8.99609379595042 | 0.999228396881742 |
| ebi-fl187-GCST90094778 | prot-a-879 | Dynactin-associated protein \|\| id:prot-a-879 | Hard cheese liking \|\| id:ebi-fl187-GCST90094778 | Weighted mode | 4 | -0.393957243 | 0.544962727130278 | 0.521999089806206 | -1.462084188 | 0.674169702539152 | 0.674382890458594 | 0.231752754764806 | 1.9624029211857 | 0.999228396881742 |
| ebi-fl187-GCST90094778 | prot-a-937 | EMI domain-containing protein 1 \|\| id:prot-a-937 | Hard cheese liking \|\| id:ebi-fl187-GCST90094778 | Weighted mode | 4 | -0.031119118 | 0.574202694428082 | 0.960186694835568 | -1.156556399 | 1.0943181635344 | 0.969360098403834 | 0.314567563182716 | 2.98714524431654 | 0.999228396881742 |
| ebi-fl187-GCST90094778 | prot-b-1 | SRC proto-oncogene, non-receptor tyrosine kinase \|\| id:prot-b-1 | Hard cheese liking \|\| id:ebi-fl187-GCST90094778 | Weighted mode | 3 | -0.16025295 | 0.580041202226748 | 0.80826605140394 | -1.297133707 | 0.976627806141533 | 0.851928266264264 | 0.273314069698783 | 2.65548631162644 | 0.999228396881742 |
| ebi-fl187-GCST90094778 | prot-b-10 | coagulation factor III, tissue factor \|\| id:prot-b-10 | Hard cheese liking \|\| id:ebi-fl187-GCST90094778 | Weighted mode | 3 | -0.476275588 | 0.664315243759789 | 0.547830830836149 | -1.778333466 | 0.825782289664444 | 0.621092293011786 | 0.16891942280852 | 2.28366655547903 | 0.999228396881742 |
| ebi-fl187-GCST90094778 | prot-b-29 | myeloperoxidase \|\| id:prot-b-29 | Hard cheese liking \|\| id:ebi-fl187-GCST90094778 | Weighted mode | 3 | -0.273281619 | 0.599033927441293 | 0.692993571944703 | -1.447388117 | 0.900824878769639 | 0.760878479605558 | 0.235183759096984 | 2.46163282256292 | 0.999228396881742 |
| ebi-fl187-GCST90094778 | prot-b-33 | adrenomedullin \|\| id:prot-b-33 | Hard cheese liking \|\| id:ebi-fl187-GCST90094778 | Weighted mode | 3 | -0.133549413 | 0.598215131728363 | 0.844071781663156 | -1.306051072 | 1.03895224472073 | 0.874984231905034 | 0.270887663017232 | 2.82625423969102 | 0.999228396881742 |
| ebi-fl187-GCST90094778 | ukb-rsfMRI191-edge-pheno102 | Net25_Pair11_15 (Postcentral\|Precentral)&(Cerebellum) (Motor)&(Subcortical-cerebellum) \|\| id:ukb-rsfMRI191-edge-pheno102 | Hard cheese liking \|\| id:ebi-fl187-GCST90094778 | Weighted mode | 4 | -0.004690399 | 0.18622410661683 | 0.981487624475969 | -0.369689648 | 0.360308850105479 | 0.995320583879384 | 0.690948734802716 | 1.43377216686974 | 0.999228396881742 |
| ebi-fl187-GCST90094778 | ukb-rsfMRI191-edge-pheno1167 | Net100_Pair11_45 (Angular\|Temporal)&(Frontal) (Default_mode\|Central_executive)&(Salience\|Default_mode) \|\| id:ukb-rsfMRI191-edge-pheno1167 | Hard cheese liking \|\| id:ebi-fl187-GCST90094778 | Weighted mode | 4 | -0.260500494 | 0.227692409590125 | 0.335601175312502 | -0.706777617 | 0.185776628627548 | 0.770665775536746 | 0.493231018681548 | 1.20415325696927 | 0.999228396881742 |
| ebi-fl187-GCST90094778 | ukb-rsfMRI191-edge-pheno1317 | Net100_Pair26_48 (Parietal\|Frontal)&(Temporal) (Attention\|Central_executive)&(Default_mode\|Central_executive) \|\| id:ukb-rsfMRI191-edge-pheno1317 | Hard cheese liking \|\| id:ebi-fl187-GCST90094778 | Weighted mode | 4 | -0.136942922 | 0.198021335213333 | 0.538928888217894 | -0.525064739 | 0.251178894791167 | 0.872019997671181 | 0.59151706875872 | 1.28554004017866 | 0.999228396881742 |
| ebi-fl187-GCST90094778 | ukb-rsfMRI191-edge-pheno286 | Net100_Pair10_13 (OccipitalPrecuneus)&(Frontal_Sup) (Default_mode\|Central_executive)&(Default_mode) \|\| id:ukb-rsfMRI191-edge-pheno286 | Hard cheese liking \|\| id:ebi-fl187-GCST90094778 | Weighted mode | 4 | 0.0245704809094487 | 0.151365335213762 | 0.881366785121489 | -0.272105576 | 0.321246537928422 | 1.02487482267127 | 0.761773831726711 | 1.37884547670087 | 0.999228396881742 |
| ebi-fl187-GCST90094778 | ukb-rsfMRI191-edge-pheno599 | Net100_Pair11_29 (Angular\|Temporal)&(Parietal) (Default_mode\|Central_executive)&(Central_executive\|Salience) \|\| id:ukb-rsfMRI191-edge-pheno599 | Hard cheese liking \|\| id:ebi-fl187-GCST90094778 | Weighted mode | 4 | -0.156700875 | 0.210139973143178 | 0.509961451684435 | -0.568575222 | 0.255173472470289 | 0.854959760504498 | 0.566331761039771 | 1.29068549985593 | 0.999228396881742 |
| ebi-fl187-GCST90094778 | ukb-rsfMRI191-edge-pheno621 | Net100_Pair5_30 (Precuneus\|Angular\|Cingulate)&(Paracentral) (Default_mode\|Central_executive)&(Motor) \|\| id:ukb-rsfMRI191-edge-pheno621 | Hard cheese liking \|\| id:ebi-fl187-GCST90094778 | Weighted mode | 4 | -0.060421425 | 0.15072793181907 | 0.715362736717195 | -0.355848171 | 0.235005321850041 | 0.941367734538418 | 0.700578980450353 | 1.26491550040559 | 0.999228396881742 |
| ebi-fl187-GCST90094778 | ukb-rsfMRI191-edge-pheno624 | Net100_Pair8_30 (Calcarine\|Lingual)&(Paracentral) (Visual)&(Motor) \|\| id:ukb-rsfMRI191-edge-pheno624 | Hard cheese liking \|\| id:ebi-fl187-GCST90094778 | Weighted mode | 4 | -0.110546313 | 0.162718403612841 | 0.545624258722378 | -0.429474384 | 0.208381758096749 | 0.895344863136565 | 0.650851102543488 | 1.23168328487462 | 0.999228396881742 |
| ebi-fl187-GCST90094778 | ukb-rsfMRI191-edge-pheno882 | Net100_Pair6_38 (Postcentral\|Precentral)&(Subcortical) (Motor)&(Subcortical-cerebellum) \|\| id:ukb-rsfMRI191-edge-pheno882 | Hard cheese liking \|\| id:ebi-fl187-GCST90094778 | Weighted mode | 4 | -0.005600989 | 0.170143911095972 | 0.975806802538016 | -0.339083055 | 0.32788107630732 | 0.994414666856683 | 0.712423276182146 | 1.38802389354677 | 0.999228396881742 |
| ebi-fl187-GCST90094778 | ukb-rsfMRI191-edge-pheno899 | Net100_Pair23_38 (Cerebellum)&(Subcortical) (Subcortical-cerebellum)&(Subcortical-cerebellum) \|\| id:ukb-rsfMRI191-edge-pheno899 | Hard cheese liking \|\| id:ebi-fl187-GCST90094778 | Weighted mode | 4 | 0.0969135806378914 | 0.162403618778348 | 0.592710803711986 | -0.221397512 | 0.415224673443453 | 1.10176515566479 | 0.801398051488364 | 1.51471101780521 | 0.999228396881742 |
| ebi-fl187-GCST90094781 | ebi-a-GCST90001392 | IgD+ CD38+ B cell Absolute Count \|\| id:ebi-a-GCST90001392 | Herring liking \|\| id:ebi-fl187-GCST90094781 | Inverse variance weighted | 16 | -0.086578598 | 0.107122488934166 | 0.418963625443894 | -0.296538676 | 0.123381480581385 | 0.91706346684563 | 0.74338687544717 | 1.13131591369169 | 0.998074519377826 |
| ebi-fl187-GCST90094781 | ebi-a-GCST90001402 | Switched memory B cell %B cell \|\| id:ebi-a-GCST90001402 | Herring liking \|\| id:ebi-fl187-GCST90094781 | Inverse variance weighted | 16 | 0.0362780209063242 | 0.10603333975731 | 0.732247139894528 | -0.171547325 | 0.244103366830652 | 1.03694409855711 | 0.842360402355503 | 1.27647626897663 | 0.998074519377826 |
| ebi-fl187-GCST90094781 | ebi-a-GCST90001407 | Memory B cell Absolute Count \|\| id:ebi-a-GCST90001407 | Herring liking \|\| id:ebi-fl187-GCST90094781 | Inverse variance weighted | 16 | -0.042807379 | 0.105125116485388 | 0.683858089390312 | -0.248852607 | 0.163237849699193 | 0.958095922060241 | 0.779694886543829 | 1.17731668080763 | 0.998074519377826 |
| ebi-fl187-GCST90094781 | ebi-a-GCST90001411 | IgD+ CD24+ B cell %B cell \|\| id:ebi-a-GCST90001411 | Herring liking \|\| id:ebi-fl187-GCST90094781 | Inverse variance weighted | 16 | -0.059377431 | 0.106997399540899 | 0.578933822160383 | -0.269092334 | 0.150337472323325 | 0.942351029746611 | 0.76407270259609 | 1.16222639579618 | 0.998074519377826 |
| ebi-fl187-GCST90094781 | ebi-a-GCST90001418 | CD24+ CD27+ B cell Absolute Count \|\| id:ebi-a-GCST90001418 | Herring liking \|\| id:ebi-fl187-GCST90094781 | Inverse variance weighted | 16 | -0.040213373 | 0.10477551959735 | 0.701122949568804 | -0.245573391 | 0.165146645539835 | 0.960584454621139 | 0.782255870926688 | 1.17956608413396 | 0.998074519377826 |
| ebi-fl187-GCST90094781 | ebi-a-GCST90001436 | Memory B cell %lymphocyte \|\| id:ebi-a-GCST90001436 | Herring liking \|\| id:ebi-fl187-GCST90094781 | Inverse variance weighted | 16 | 0.0342312830099843 | 0.103671703046704 | 0.741256960758514 | -0.168965255 | 0.237427820981524 | 1.03482391624309 | 0.844538246390109 | 1.26798347168523 | 0.998074519377826 |
| ebi-fl187-GCST90094781 | ebi-a-GCST90001439 | IgD+ CD24+ B cell %lymphocyte \|\| id:ebi-a-GCST90001439 | Herring liking \|\| id:ebi-fl187-GCST90094781 | Inverse variance weighted | 16 | -0.030344561 | 0.10474283230109 | 0.772041645788146 | -0.235640513 | 0.174951390052923 | 0.970111213215637 | 0.790064641197906 | 1.1911883116042 | 0.998074519377826 |
| ebi-fl187-GCST90094781 | ebi-a-GCST90001457 | CD11c+ HLA DR++ monocyte %monocyte \|\| id:ebi-a-GCST90001457 | Herring liking \|\| id:ebi-fl187-GCST90094781 | Inverse variance weighted | 16 | 0.224137513392215 | 0.112094360845636 | 0.0455496160469921 | 0.00443256613476845 | 0.443842460649661 | 1.25124307027752 | 1.00444240448705 | 1.55868491207024 | 0.998074519377826 |
| ebi-fl187-GCST90094781 | ebi-a-GCST90001483 | CD39+ resting CD4 regulatory T cell Absolute Count \|\| id:ebi-a-GCST90001483 | Herring liking \|\| id:ebi-fl187-GCST90094781 | Inverse variance weighted | 15 | -0.008851664 | 0.108608534850778 | 0.935043827423653 | -0.221724392 | 0.204021064777841 | 0.991187397108433 | 0.801136133568279 | 1.22632398542645 | 0.998074519377826 |
| ebi-fl187-GCST90094781 | ebi-a-GCST90001525 | CD33dim HLA DR+ CD11b+ Absolute Count \|\| id:ebi-a-GCST90001525 | Herring liking \|\| id:ebi-fl187-GCST90094781 | Inverse variance weighted | 16 | 0.103988955553414 | 0.135825075873026 | 0.443908746542117 | -0.162228193 | 0.370206104264545 | 1.1095882000603 | 0.850247161816769 | 1.44803302969259 | 0.998074519377826 |
| ebi-fl187-GCST90094781 | ebi-a-GCST90001540 | Naive CD4+ T cell Absolute Count \|\| id:ebi-a-GCST90001540 | Herring liking \|\| id:ebi-fl187-GCST90094781 | Inverse variance weighted | 16 | -0.157127749 | 0.100171579751345 | 0.116744410760156 | -0.353464045 | 0.0392085475771914 | 0.854594878428968 | 0.702251241543776 | 1.03998734787783 | 0.998074519377826 |
| ebi-fl187-GCST90094781 | ebi-a-GCST90001624 | HLA DR+ CD4+ T cell Absolute Count \|\| id:ebi-a-GCST90001624 | Herring liking \|\| id:ebi-fl187-GCST90094781 | Inverse variance weighted | 16 | -0.16253586 | 0.101284741549165 | 0.108550634554154 | -0.361053954 | 0.0359822329951389 | 0.849985608819896 | 0.69694139476175 | 1.03663742838508 | 0.998074519377826 |
| ebi-fl187-GCST90094781 | ebi-a-GCST90001672 | CD39+ CD8+ T cell Absolute Count \|\| id:ebi-a-GCST90001672 | Herring liking \|\| id:ebi-fl187-GCST90094781 | Inverse variance weighted | 16 | 0.0833081447735424 | 0.110033949207886 | 0.448982104321556 | -0.132358396 | 0.298974685220999 | 1.08687667231839 | 0.876026974533027 | 1.3484754866819 | 0.998074519377826 |
| ebi-fl187-GCST90094781 | ebi-a-GCST90001722 | CD19 on CD20- CD38- B cell \|\| id:ebi-a-GCST90001722 | Herring liking \|\| id:ebi-fl187-GCST90094781 | Inverse variance weighted | 16 | -0.132697505 | 0.11605848208777 | 0.252886010225382 | -0.36017213 | 0.094777119964915 | 0.875729956043615 | 0.697556245506222 | 1.09941379043292 | 0.998074519377826 |
| ebi-fl187-GCST90094781 | ebi-a-GCST90001742 | CD19 on transitional B cell \|\| id:ebi-a-GCST90001742 | Herring liking \|\| id:ebi-fl187-GCST90094781 | Inverse variance weighted (multiplicative random effects) | 16 | -0.114806267 | 0.138828717678152 | 0.408257811349698 | -0.386910554 | 0.157298019441357 | 0.891538847487377 | 0.679151839722477 | 1.17034434730224 | 0.998074519377826 |
| ebi-fl187-GCST90094781 | ebi-a-GCST90001784 | CD25 on IgD+ CD38dim B cell \|\| id:ebi-a-GCST90001784 | Herring liking \|\| id:ebi-fl187-GCST90094781 | Inverse variance weighted | 16 | 0.0761805887162221 | 0.103973974959113 | 0.463748128810464 | -0.127608402 | 0.279969579636083 | 1.07915744021818 | 0.88019799526009 | 1.32308956285924 | 0.998074519377826 |
| ebi-fl187-GCST90094781 | ebi-a-GCST90001794 | CD25 on IgD+ B cell \|\| id:ebi-a-GCST90001794 | Herring liking \|\| id:ebi-fl187-GCST90094781 | Inverse variance weighted | 16 | 0.0752356107688654 | 0.113092527264707 | 0.505886041688796 | -0.146425743 | 0.296896964207691 | 1.07813814191851 | 0.863789872682948 | 1.34567663944601 | 0.998074519377826 |
| ebi-fl187-GCST90094781 | ebi-a-GCST90001802 | CD27 on IgD- CD38- B cell \|\| id:ebi-a-GCST90001802 | Herring liking \|\| id:ebi-fl187-GCST90094781 | Inverse variance weighted | 16 | -0.024445605 | 0.103871414190787 | 0.813941066173361 | -0.228033577 | 0.179142366602668 | 0.975850768671153 | 0.796097528799882 | 1.19619102969915 | 0.998074519377826 |
| ebi-fl187-GCST90094781 | ebi-a-GCST90001804 | CD27 on IgD- CD38dim B cell \|\| id:ebi-a-GCST90001804 | Herring liking \|\| id:ebi-fl187-GCST90094781 | Inverse variance weighted | 16 | -0.0291583 | 0.124727009469408 | 0.815158147089462 | -0.273623238 | 0.2153066390371 | 0.971262701874298 | 0.760618593402304 | 1.2402421453208 | 0.998074519377826 |
| ebi-fl187-GCST90094781 | ebi-a-GCST90001805 | CD27 on memory B cell \|\| id:ebi-a-GCST90001805 | Herring liking \|\| id:ebi-fl187-GCST90094781 | Inverse variance weighted | 16 | -0.090486884 | 0.118941593213089 | 0.446796020663452 | -0.323612407 | 0.142638638341337 | 0.913486314783397 | 0.723530623405345 | 1.15331296327047 | 0.998074519377826 |
| ebi-fl187-GCST90094781 | ebi-a-GCST90001806 | CD27 on unswitched memory B cell \|\| id:ebi-a-GCST90001806 | Herring liking \|\| id:ebi-fl187-GCST90094781 | Inverse variance weighted | 16 | -0.025511366 | 0.106837938751273 | 0.811271799676894 | -0.234913726 | 0.183890993528246 | 0.974811298783986 | 0.790639057982623 | 1.2018848027336 | 0.998074519377826 |
| ebi-fl187-GCST90094781 | ebi-a-GCST90001839 | CD3 on Effector Memory CD8+ T cell \|\| id:ebi-a-GCST90001839 | Herring liking \|\| id:ebi-fl187-GCST90094781 | Inverse variance weighted | 16 | 0.0320569276518702 | 0.114688226361612 | 0.779850644789385 | -0.192731996 | 0.256845851320629 | 1.03257628577531 | 0.824702968204503 | 1.29284582092246 | 0.998074519377826 |
| ebi-fl187-GCST90094781 | ebi-a-GCST90001859 | CD3 on CD8+ T cell \|\| id:ebi-a-GCST90001859 | Herring liking \|\| id:ebi-fl187-GCST90094781 | Inverse variance weighted | 16 | 0.0354659746570699 | 0.120040570183131 | 0.767650217643007 | -0.199813543 | 0.270745492216007 | 1.03610239378904 | 0.818883425471336 | 1.31094138313702 | 0.998074519377826 |
| ebi-fl187-GCST90094781 | ebi-a-GCST90001905 | CD86 on monocyte \|\| id:ebi-a-GCST90001905 | Herring liking \|\| id:ebi-fl187-GCST90094781 | Inverse variance weighted | 16 | 0.113302834796389 | 0.129082062554014 | 0.380074970116817 | -0.139698008 | 0.366303677402256 | 1.11997104780233 | 0.869620814443077 | 1.44239319837204 | 0.998074519377826 |
| ebi-fl187-GCST90094781 | ebi-a-GCST90001943 | CD25 on activated & secreting CD4 regulatory T cell \|\| id:ebi-a-GCST90001943 | Herring liking \|\| id:ebi-fl187-GCST90094781 | Inverse variance weighted | 16 | 0.0704812985206217 | 0.104584794709477 | 0.500365132622798 | -0.134504899 | 0.275467496151196 | 1.07302450209747 | 0.874148596315994 | 1.31714629177911 | 0.998074519377826 |
| ebi-fl187-GCST90094781 | ebi-a-GCST90001949 | CD33 on CD33dim HLA DR+ CD11b- \|\| id:ebi-a-GCST90001949 | Herring liking \|\| id:ebi-fl187-GCST90094781 | Inverse variance weighted | 16 | 0.230489320380213 | 0.149383120838821 | 0.122845126640877 | -0.062301596 | 0.523280237224302 | 1.25921601926554 | 0.939599464177752 | 1.6875541585824 | 0.998074519377826 |
| ebi-fl187-GCST90094781 | ebi-a-GCST90001955 | CD33 on Immature Myeloid-Derived Suppressor Cells \|\| id:ebi-a-GCST90001955 | Herring liking \|\| id:ebi-fl187-GCST90094781 | Inverse variance weighted | 16 | 0.0583442060352518 | 0.182653244697354 | 0.74940345314452 | -0.299656154 | 0.416344565642066 | 1.06007981879101 | 0.741072992179646 | 1.51640828105575 | 0.998074519377826 |
| ebi-fl187-GCST90094781 | ebi-a-GCST90001956 | CD33 on CD33+ HLA DR+ \|\| id:ebi-a-GCST90001956 | Herring liking \|\| id:ebi-fl187-GCST90094781 | Inverse variance weighted | 16 | 0.0922672630526054 | 0.183401653499344 | 0.614902050528892 | -0.267199978 | 0.45173450391132 | 1.09665787904984 | 0.765519969153388 | 1.57103478961124 | 0.998074519377826 |
| ebi-fl187-GCST90094781 | ebi-a-GCST90001957 | CD33 on CD33+ HLA DR+ CD14- \|\| id:ebi-a-GCST90001957 | Herring liking \|\| id:ebi-fl187-GCST90094781 | Inverse variance weighted | 16 | 0.0947481714274108 | 0.184314076497083 | 0.607211266473431 | -0.266507419 | 0.456003761361693 | 1.09938196447223 | 0.766050320755547 | 1.57775627926735 | 0.998074519377826 |
| ebi-fl187-GCST90094781 | ebi-a-GCST90002064 | CD4 on resting CD4 regulatory T cell \|\| id:ebi-a-GCST90002064 | Herring liking \|\| id:ebi-fl187-GCST90094781 | Inverse variance weighted | 16 | 0.0243355470003044 | 0.110782697800494 | 0.826128790783927 | -0.192798541 | 0.241469634689272 | 1.02463407310402 | 0.824648090442104 | 1.27311879568276 | 0.998074519377826 |
| ebi-fl187-GCST90094781 | ebi-a-GCST90002080 | SSC-A on T cell \|\| id:ebi-a-GCST90002080 | Herring liking \|\| id:ebi-fl187-GCST90094781 | Inverse variance weighted | 16 | -0.028810036 | 0.106230971300192 | 0.78623579229859 | -0.237022739 | 0.179402668095779 | 0.97160101648993 | 0.78897334703233 | 1.19650244053882 | 0.998074519377826 |
| ebi-fl187-GCST90094781 | ebi-a-GCST90002121 | CD8 on CD39+ CD8+ T cell \|\| id:ebi-a-GCST90002121 | Herring liking \|\| id:ebi-fl187-GCST90094781 | Inverse variance weighted | 16 | 0.0411687024877709 | 0.126938440837015 | 0.745695616361715 | -0.207630642 | 0.289968046528321 | 1.04202788341424 | 0.812507087651604 | 1.33638478520984 | 0.998074519377826 |
| ebi-fl187-GCST90094781 | ebi-cfmet338-GCST90026053 | 1-palmitoyl-2-docosahexaenoyl-gpc (16:0/22:6) levels \|\| id:ebi-cfmet338-GCST90026053 | Herring liking \|\| id:ebi-fl187-GCST90094781 | Inverse variance weighted | 15 | -0.050004776 | 0.0649722275375514 | 0.441517570328136 | -0.177350342 | 0.0773407897008719 | 0.951224881180405 | 0.837486326279766 | 1.08041020633263 | 0.998074519377826 |
| ebi-fl187-GCST90094781 | ebi-cfmet338-GCST90026063 | 1-stearoyl-2-oleoyl-gpc (18:0/18:1) levels \|\| id:ebi-cfmet338-GCST90026063 | Herring liking \|\| id:ebi-fl187-GCST90094781 | Inverse variance weighted | 14 | -0.034788658 | 0.0475118122774915 | 0.4640399648457 | -0.12791181 | 0.0583344939896844 | 0.965809510731902 | 0.879930976714268 | 1.0600695232975 | 0.998074519377826 |
| ebi-fl187-GCST90094781 | ebi-cfmet338-GCST90026113 | Adenosine 3',5'-cyclic monophosphate (camp) levels \|\| id:ebi-cfmet338-GCST90026113 | Herring liking \|\| id:ebi-fl187-GCST90094781 | Inverse variance weighted (multiplicative random effects) | 15 | 0.0810887286188744 | 0.11823153218612 | 0.492809664243601 | -0.150645074 | 0.312822531703669 | 1.08446711556739 | 0.860152934727712 | 1.36727886084507 | 0.998074519377826 |
| ebi-fl187-GCST90094781 | ebi-cfmet338-GCST90026117 | Alpha-ketoglutaramate levels \|\| id:ebi-cfmet338-GCST90026117 | Herring liking \|\| id:ebi-fl187-GCST90094781 | Inverse variance weighted | 15 | 0.0100939141661359 | 0.0359857112402313 | 0.779095584704883 | -0.06043808 | 0.0806259081969892 | 1.01014502955776 | 0.94135205586047 | 1.08396531816944 | 0.998074519377826 |
| ebi-fl187-GCST90094781 | ebi-cfmet338-GCST90026124 | Arginine levels \|\| id:ebi-cfmet338-GCST90026124 | Herring liking \|\| id:ebi-fl187-GCST90094781 | Inverse variance weighted | 15 | 0.0191369772112473 | 0.0293260646704278 | 0.514040849673215 | -0.03834211 | 0.0766160639652857 | 1.01932126283916 | 0.962383643938179 | 1.07962748891309 | 0.998074519377826 |
| ebi-fl187-GCST90094781 | ebi-cfmet338-GCST90026169 | Glutamate levels \|\| id:ebi-cfmet338-GCST90026169 | Herring liking \|\| id:ebi-fl187-GCST90094781 | Inverse variance weighted | 15 | 0.00787764625189853 | 0.0355266363845341 | 0.824516949236902 | -0.061754561 | 0.0775098535655854 | 1.00790875654544 | 0.940113598960583 | 1.08059288009892 | 0.998074519377826 |
| ebi-fl187-GCST90094781 | ebi-cfmet338-GCST90026170 | Glutamine levels \|\| id:ebi-cfmet338-GCST90026170 | Herring liking \|\| id:ebi-fl187-GCST90094781 | Inverse variance weighted | 15 | 0.00919297435391743 | 0.0196336325687564 | 0.639623086105613 | -0.029288945 | 0.0476748941886799 | 1.00923535952502 | 0.971135818616806 | 1.04882961928676 | 0.998074519377826 |
| ebi-fl187-GCST90094781 | ebi-cfmet338-GCST90026191 | Kynurenate levels \|\| id:ebi-cfmet338-GCST90026191 | Herring liking \|\| id:ebi-fl187-GCST90094781 | Inverse variance weighted | 15 | -0.111644905 | 0.140401195034293 | 0.426505974703257 | -0.386831248 | 0.163541437008753 | 0.894361784287609 | 0.679205702899115 | 1.17767415347059 | 0.998074519377826 |
| ebi-fl187-GCST90094781 | ebi-cfmet338-GCST90026202 | Methylsuccinoylcarnitine levels \|\| id:ebi-cfmet338-GCST90026202 | Herring liking \|\| id:ebi-fl187-GCST90094781 | Inverse variance weighted | 15 | -0.091529207 | 0.0944833453216063 | 0.332678040476796 | -0.276716564 | 0.0936581498348052 | 0.912534663366943 | 0.75826938762937 | 1.09818426726893 | 0.998074519377826 |
| ebi-fl187-GCST90094781 | ebi-cfmet338-GCST90026222 | N-acetylputrescine levels \|\| id:ebi-cfmet338-GCST90026222 | Herring liking \|\| id:ebi-fl187-GCST90094781 | Inverse variance weighted | 15 | 0.0778741700102699 | 0.0338816928769849 | 0.0215382160413556 | 0.0114660519713795 | 0.14428228804916 | 1.08098662957549 | 1.0115320391081 | 1.15521016452561 | 0.998074519377826 |
| ebi-fl187-GCST90094781 | ebi-cfmet338-GCST90026252 | Propionylcarnitine (c3) levels \|\| id:ebi-cfmet338-GCST90026252 | Herring liking \|\| id:ebi-fl187-GCST90094781 | Inverse variance weighted | 15 | 0.0328185225888539 | 0.0587741594377299 | 0.576582517338226 | -0.08237883 | 0.148015875086804 | 1.03336299018363 | 0.920923019422472 | 1.15953130387696 | 0.998074519377826 |
| ebi-fl187-GCST90094781 | ebi-cfmet338-GCST90026316 | Ethyl beta-glucopyranoside levels \|\| id:ebi-cfmet338-GCST90026316 | Herring liking \|\| id:ebi-fl187-GCST90094781 | Inverse variance weighted | 15 | -0.33939291 | 0.182488940617983 | 0.0629138444529772 | -0.697071233 | 0.0182854138601598 | 0.712202562776098 | 0.498041818116065 | 1.01845361568943 | 0.998074519377826 |
| ebi-fl187-GCST90094781 | ebi-gm430-GCST90011315 | F_Prevotellaceae abundance \|\| id:ebi-gm430-GCST90011315 | Herring liking \|\| id:ebi-fl187-GCST90094781 | Inverse variance weighted | 14 | 0.0217345255741005 | 0.0874249082393655 | 0.803664072865498 | -0.149618295 | 0.193087345723257 | 1.02197244090761 | 0.86103657603909 | 1.21298873827079 | 0.998074519377826 |
| ebi-fl187-GCST90094781 | ebi-gm430-GCST90011354 | OTU97_1 (Bacteroides) abundance \|\| id:ebi-gm430-GCST90011354 | Herring liking \|\| id:ebi-fl187-GCST90094781 | Inverse variance weighted | 14 | 0.0496511493738169 | 0.072273248701694 | 0.492087720032743 | -0.092004418 | 0.191306716829137 | 1.05090442375647 | 0.912101119799102 | 1.21083077730918 | 0.998074519377826 |
| ebi-fl187-GCST90094781 | ebi-gm430-GCST90011419 | OTU99_1 (Bacteroides) abundance \|\| id:ebi-gm430-GCST90011419 | Herring liking \|\| id:ebi-fl187-GCST90094781 | Inverse variance weighted | 14 | 0.0532455279947901 | 0.0722927388433222 | 0.461410261680367 | -0.08844824 | 0.194939296127702 | 1.05468856890099 | 0.915350487924694 | 1.21523721464595 | 0.998074519377826 |
| ebi-fl187-GCST90094781 | ebi-gm430-GCST90011435 | OTU99_155 (Butyrivibrio) abundance \|\| id:ebi-gm430-GCST90011435 | Herring liking \|\| id:ebi-fl187-GCST90094781 | Inverse variance weighted | 14 | 0.072166944188299 | 0.240231519363689 | 0.763867632409767 | -0.398686834 | 0.543020722141129 | 1.07483476650428 | 0.671200865891925 | 1.72119827907422 | 0.998074519377826 |
| ebi-fl187-GCST90094781 | ebi-gm430-GCST90011466 | OTU99_58 (Bacteroides) abundance \|\| id:ebi-gm430-GCST90011466 | Herring liking \|\| id:ebi-fl187-GCST90094781 | Inverse variance weighted | 14 | -0.187810769 | 0.133150562096606 | 0.158387882523747 | -0.448785871 | 0.0731643323131158 | 0.828771521330567 | 0.638402784541502 | 1.07590732872803 | 0.998074519377826 |
| ebi-fl187-GCST90094781 | ebi-gm430-GCST90011472 | OTU99_72 (Parabacteroides) abundance \|\| id:ebi-gm430-GCST90011472 | Herring liking \|\| id:ebi-fl187-GCST90094781 | Inverse variance weighted | 14 | 0.219877888201381 | 0.201402955885794 | 0.274951272732552 | -0.174871905 | 0.614627681737539 | 1.24592457920652 | 0.839564557622555 | 1.84896806681164 | 0.998074519377826 |
| ebi-fl187-GCST90094781 | ebi-gm430-GCST90011505 | TestASV_28 (Holdemanella) abundance \|\| id:ebi-gm430-GCST90011505 | Herring liking \|\| id:ebi-fl187-GCST90094781 | Inverse variance weighted | 14 | -0.00638629 | 0.169846016706393 | 0.970006235154493 | -0.339284482 | 0.326511903108813 | 0.993634059370679 | 0.712279789214139 | 1.38612474885853 | 0.998074519377826 |
| ebi-fl187-GCST90094781 | ebi-gm430-GCST90011513 | TestASV_35 (Alphaproteobacteria) abundance \|\| id:ebi-gm430-GCST90011513 | Herring liking \|\| id:ebi-fl187-GCST90094781 | Inverse variance weighted (multiplicative random effects) | 14 | 0.232979987789077 | 0.305258580688773 | 0.445331160317586 | -0.36532683 | 0.831286805939071 | 1.26235621653561 | 0.693969803408364 | 2.29627169597233 | 0.998074519377826 |
| ebi-fl187-GCST90094781 | ebi-gm430-GCST90011543 | G_Coprococcus prevalence \|\| id:ebi-gm430-GCST90011543 | Herring liking \|\| id:ebi-fl187-GCST90094781 | Inverse variance weighted | 14 | 0.335316915116341 | 0.217726520792278 | 0.123540121036113 | -0.091427066 | 0.762060895869206 | 1.39838348387091 | 0.912627875657946 | 2.14268752918945 | 0.998074519377826 |
| ebi-fl187-GCST90094781 | ebi-gm430-GCST90011547 | G_Parasutterella prevalence \|\| id:ebi-gm430-GCST90011547 | Herring liking \|\| id:ebi-fl187-GCST90094781 | Inverse variance weighted | 14 | 0.303431837699957 | 0.195254542003492 | 0.120176745563055 | -0.079267065 | 0.686130740026801 | 1.35449926201028 | 0.923793179017391 | 1.98601623443234 | 0.998074519377826 |
| ebi-fl187-GCST90094781 | ebi-gm430-GCST90011569 | OTU97_13 (Subdoligranulum) prevalence \|\| id:ebi-gm430-GCST90011569 | Herring liking \|\| id:ebi-fl187-GCST90094781 | Inverse variance weighted | 14 | 0.199858059540268 | 0.207691711511971 | 0.335907813508487 | -0.207217695 | 0.606933814103731 | 1.22122940399443 | 0.812842678919568 | 1.83479693655232 | 0.998074519377826 |
| ebi-fl187-GCST90094781 | ebi-gm430-GCST90011590 | OTU97_277 (Prevotella) prevalence \|\| id:ebi-gm430-GCST90011590 | Herring liking \|\| id:ebi-fl187-GCST90094781 | Inverse variance weighted | 14 | 0.100360582984254 | 0.173476540402763 | 0.562909461451115 | -0.239653436 | 0.440374602173669 | 1.10556949575924 | 0.786900525048213 | 1.55328897496728 | 0.998074519377826 |
| ebi-fl187-GCST90094781 | ebi-gm430-GCST90011616 | OTU97_86 (Phascolarctobacterium) prevalence \|\| id:ebi-gm430-GCST90011616 | Herring liking \|\| id:ebi-fl187-GCST90094781 | Inverse variance weighted | 14 | -0.092527396 | 0.213889161633599 | 0.665308974145184 | -0.511750153 | 0.326695360687947 | 0.91162423566095 | 0.599445538857377 | 1.38637906727694 | 0.998074519377826 |
| ebi-fl187-GCST90094781 | ebi-gm430-GCST90011665 | OTU99_55 (Barnesiella) prevalence \|\| id:ebi-gm430-GCST90011665 | Herring liking \|\| id:ebi-fl187-GCST90094781 | Inverse variance weighted | 14 | -0.150448888 | 0.1514340676048 | 0.320469110016831 | -0.447259661 | 0.146361884272599 | 0.860321701446189 | 0.639377865389799 | 1.15761503493404 | 0.998074519377826 |
| ebi-fl187-GCST90094781 | ebi-gm430-GCST90011687 | TestASV_14 (Lachnospiraceae) prevalence \|\| id:ebi-gm430-GCST90011687 | Herring liking \|\| id:ebi-fl187-GCST90094781 | Inverse variance weighted | 14 | 0.224391320033304 | 0.154413379560887 | 0.146172192525181 | -0.078258904 | 0.527041543972642 | 1.25156068438295 | 0.924724980638641 | 1.6939135196838 | 0.998074519377826 |
| ebi-fl187-GCST90094781 | ebi-gm430-GCST90011697 | TestASV_23 (Barnesiella) prevalence \|\| id:ebi-gm430-GCST90011697 | Herring liking \|\| id:ebi-fl187-GCST90094781 | Inverse variance weighted | 14 | -0.378680662 | 0.235918674215446 | 0.108464712922833 | -0.841081264 | 0.0837199393814587 | 0.684764248945384 | 0.431243982851041 | 1.08732433443762 | 0.998074519377826 |
| ebi-fl187-GCST90094781 | ebi-gm430-GCST90011727 | TestASV_6 (Subdoligranulum) prevalence \|\| id:ebi-gm430-GCST90011727 | Herring liking \|\| id:ebi-fl187-GCST90094781 | Inverse variance weighted | 14 | 0.0606029965665521 | 0.297754755573773 | 0.838718250699405 | -0.522996324 | 0.644202317491146 | 1.06247702342044 | 0.592741837698177 | 1.90446726298265 | 0.998074519377826 |
| ebi-fl187-GCST90094781 | ebi-hbsm44-GCST90104908 | Sulcal depth of the left posterior inferior temporal sulcus \|\| id:ebi-hbsm44-GCST90104908 | Herring liking \|\| id:ebi-fl187-GCST90094781 | Inverse variance weighted | 16 | 0.0558662754297189 | 0.0622512797634824 | 0.3694885726444 | -0.066146233 | 0.177878783766145 | 1.05745626639652 | 0.935993981162929 | 1.1946804977869 | 0.998074519377826 |
| ebi-fl187-GCST90094781 | ebi-hbsm44-GCST90104918 | Sulcal depth of the left inferior frontal sulcus \|\| id:ebi-hbsm44-GCST90104918 | Herring liking \|\| id:ebi-fl187-GCST90094781 | Inverse variance weighted | 15 | 0.111679631960255 | 0.103641477065542 | 0.281231358960549 | -0.091457663 | 0.314816927008717 | 1.11815458226599 | 0.912599951997814 | 1.37000847644732 | 0.998074519377826 |
| ebi-fl187-GCST90094781 | ebi-hbsm44-GCST90104925 | Sulcal depth of the right anterior inferior temporal sulcus \|\| id:ebi-hbsm44-GCST90104925 | Herring liking \|\| id:ebi-fl187-GCST90094781 | Inverse variance weighted | 15 | -0.05956213 | 0.0388563356629827 | 0.125305234969866 | -0.135720548 | 0.0165962881980436 | 0.942176994597401 | 0.873086584541356 | 1.01673477163197 | 0.998074519377826 |
| ebi-fl187-GCST90094781 | ebi-IPs91-GCST90274770 | C-C motif chemokine 4 levels \|\| id:ebi-IPs91-GCST90274770 | Herring liking \|\| id:ebi-fl187-GCST90094781 | Inverse variance weighted | 21 | 0.0212689301102575 | 0.0409200130491163 | 0.603224999735437 | -0.058934295 | 0.101472155686525 | 1.02149672592891 | 0.942768711300974 | 1.10679909990179 | 0.998074519377826 |
| ebi-fl187-GCST90094781 | ebi-IPs91-GCST90274779 | C-X-C motif chemokine 1 levels \|\| id:ebi-IPs91-GCST90274779 | Herring liking \|\| id:ebi-fl187-GCST90094781 | Inverse variance weighted (multiplicative random effects) | 21 | -0.019698715 | 0.0523533521247073 | 0.70672017119917 | -0.122311285 | 0.0829138554648671 | 0.980494037250707 | 0.884872878093353 | 1.08644821294067 | 0.998074519377826 |
| ebi-fl187-GCST90094781 | ebi-IPs91-GCST90274780 | C-X-C motif chemokine 10 levels \|\| id:ebi-IPs91-GCST90274780 | Herring liking \|\| id:ebi-fl187-GCST90094781 | Inverse variance weighted | 21 | -0.097300875 | 0.0419562020113932 | 0.0203892958267393 | -0.179535031 | -0.015066719 | 0.907282986347641 | 0.83565867656853 | 0.985046216113079 | 0.998074519377826 |
| ebi-fl187-GCST90094781 | ebi-IPs91-GCST90274796 | Interleukin-10 receptor subunit alpha levels \|\| id:ebi-IPs91-GCST90274796 | Herring liking \|\| id:ebi-fl187-GCST90094781 | Inverse variance weighted (multiplicative random effects) | 19 | -0.046585683 | 0.0618315551818606 | 0.451192202569771 | -0.167775531 | 0.0746041655844886 | 0.95448277452719 | 0.845543611942955 | 1.07745757167471 | 0.998074519377826 |
| ebi-fl187-GCST90094781 | ebi-IPs91-GCST90274807 | Interleukin-20 levels \|\| id:ebi-IPs91-GCST90274807 | Herring liking \|\| id:ebi-fl187-GCST90094781 | Inverse variance weighted | 19 | 0.0414407158640669 | 0.0556767442164493 | 0.456689405011411 | -0.067685703 | 0.150567134528308 | 1.04231136749098 | 0.934554155177202 | 1.16249334592593 | 0.998074519377826 |
| ebi-fl187-GCST90094781 | ebi-IPs91-GCST90274808 | Interleukin-20 receptor subunit alpha levels \|\| id:ebi-IPs91-GCST90274808 | Herring liking \|\| id:ebi-fl187-GCST90094781 | Inverse variance weighted | 19 | 0.01960025587169 | 0.0531435461439651 | 0.712263955530115 | -0.084561095 | 0.123761606313862 | 1.01979360203225 | 0.918915512913279 | 1.13174603772747 | 0.998074519377826 |
| ebi-fl187-GCST90094781 | ebi-IPs91-GCST90274810 | Interleukin-24 levels \|\| id:ebi-IPs91-GCST90274810 | Herring liking \|\| id:ebi-fl187-GCST90094781 | Inverse variance weighted | 19 | 0.0112608534300695 | 0.0472804379278446 | 0.811748070876501 | -0.081408805 | 0.103930511768645 | 1.01132449550374 | 0.921816771185627 | 1.10952335342131 | 0.998074519377826 |
| ebi-fl187-GCST90094781 | ebi-IPs91-GCST90274823 | Monocyte chemoattractant protein-3 levels \|\| id:ebi-IPs91-GCST90274823 | Herring liking \|\| id:ebi-fl187-GCST90094781 | Inverse variance weighted (multiplicative random effects) | 19 | -0.086859249 | 0.0609733219106799 | 0.154288731656319 | -0.20636696 | 0.0326484623587606 | 0.916806128310939 | 0.813534487267244 | 1.03318727117758 | 0.998074519377826 |
| ebi-fl187-GCST90094781 | ebi-IPs91-GCST90274824 | Monocyte chemoattractant protein-4 levels \|\| id:ebi-IPs91-GCST90274824 | Herring liking \|\| id:ebi-fl187-GCST90094781 | Inverse variance weighted | 21 | -0.095360908 | 0.0501339356315877 | 0.0571550863310893 | -0.193623422 | 0.00290160566306465 | 0.909044793560127 | 0.82396813411354 | 1.00290581939532 | 0.998074519377826 |
| ebi-fl187-GCST90094781 | ebi-IPs91-GCST90274834 | SIR2-like protein 2 levels \|\| id:ebi-IPs91-GCST90274834 | Herring liking \|\| id:ebi-fl187-GCST90094781 | Inverse variance weighted | 21 | -0.053992626 | 0.0456539239393285 | 0.236947774290615 | -0.143474317 | 0.0354890647061077 | 0.947439092688234 | 0.866343050072203 | 1.03612631772032 | 0.998074519377826 |
| ebi-fl187-GCST90094781 | ebi-met1400-GCST90199652 | 1-stearoyl-2-oleoyl-GPS (18:0/18:1) levels \|\| id:ebi-met1400-GCST90199652 | Herring liking \|\| id:ebi-fl187-GCST90094781 | Inverse variance weighted | 16 | -0.055112895 | 0.060643776259322 | 0.363457176266322 | -0.173974697 | 0.0637489064189639 | 0.946378300497425 | 0.84031816036522 | 1.06582474340806 | 0.998074519377826 |
| ebi-fl187-GCST90094781 | ebi-met1400-GCST90199665 | N-acetylaspartate (naa) levels \|\| id:ebi-met1400-GCST90199665 | Herring liking \|\| id:ebi-fl187-GCST90094781 | Inverse variance weighted | 16 | -0.007789693 | 0.0601906314251052 | 0.89702767352379 | -0.12576333 | 0.110183944879571 | 0.992240568316962 | 0.881823522991382 | 1.1164834229803 | 0.998074519377826 |
| ebi-fl187-GCST90094781 | ebi-met1400-GCST90199669 | Acetylcarnitine levels (Biocrates platform) \|\| id:ebi-met1400-GCST90199669 | Herring liking \|\| id:ebi-fl187-GCST90094781 | Inverse variance weighted | 16 | 0.0586858199935426 | 0.0582142377914471 | 0.313406071122872 | -0.055414086 | 0.172785726064779 | 1.06044201871676 | 0.94609330276539 | 1.18861138935569 | 0.998074519377826 |
| ebi-fl187-GCST90094781 | ebi-met1400-GCST90199670 | Iminodiacetate (IDA) levels \|\| id:ebi-met1400-GCST90199670 | Herring liking \|\| id:ebi-fl187-GCST90094781 | Inverse variance weighted | 16 | 0.0225839968928084 | 0.0652559435788934 | 0.729280060605947 | -0.105317653 | 0.150485646307439 | 1.02284094601753 | 0.900038577649177 | 1.16239862027096 | 0.998074519377826 |
| ebi-fl187-GCST90094781 | ebi-met1400-GCST90199686 | Indolepropionate levels \|\| id:ebi-met1400-GCST90199686 | Herring liking \|\| id:ebi-fl187-GCST90094781 | Inverse variance weighted | 16 | 0.118854517060631 | 0.0777640404304536 | 0.126413389913431 | -0.033563002 | 0.27127203630432 | 1.12620606260122 | 0.966993986580072 | 1.31163183333272 | 0.998074519377826 |
| ebi-fl187-GCST90094781 | ebi-met1400-GCST90199704 | 3-hydroxysebacate levels \|\| id:ebi-met1400-GCST90199704 | Herring liking \|\| id:ebi-fl187-GCST90094781 | Inverse variance weighted | 16 | 0.0165794214846884 | 0.061648734430347 | 0.787980712699969 | -0.104252098 | 0.137410940968169 | 1.01671762280264 | 0.900998128962824 | 1.1472995240372 | 0.998074519377826 |
| ebi-fl187-GCST90094781 | ebi-met1400-GCST90199713 | Docosapentaenoate n3 DPA; 22:5n3 levels \|\| id:ebi-met1400-GCST90199713 | Herring liking \|\| id:ebi-fl187-GCST90094781 | Inverse variance weighted | 16 | 0.00401304547573679 | 0.0598222379349453 | 0.946515756680066 | -0.113238541 | 0.121264631828229 | 1.00402110852492 | 0.892937632583374 | 1.12892362196358 | 0.998074519377826 |
| ebi-fl187-GCST90094781 | ebi-met1400-GCST90199715 | Myristoleate (14:1n5) levels \|\| id:ebi-met1400-GCST90199715 | Herring liking \|\| id:ebi-fl187-GCST90094781 | Inverse variance weighted | 16 | 0.0116645210158796 | 0.0557308107503037 | 0.834213178804898 | -0.097567868 | 0.120896910086475 | 1.0117328168288 | 0.907040780373495 | 1.12850856851977 | 0.998074519377826 |
| ebi-fl187-GCST90094781 | ebi-met1400-GCST90199725 | Campesterol levels \|\| id:ebi-met1400-GCST90199725 | Herring liking \|\| id:ebi-fl187-GCST90094781 | Inverse variance weighted | 16 | 0.14798528521723 | 0.0772959078440765 | 0.0555525509715843 | -0.003514694 | 0.29948526459162 | 1.15949583450812 | 0.99649147515049 | 1.34916416624501 | 0.998074519377826 |
| ebi-fl187-GCST90094781 | ebi-met1400-GCST90199737 | N-acetylproline levels \|\| id:ebi-met1400-GCST90199737 | Herring liking \|\| id:ebi-fl187-GCST90094781 | Inverse variance weighted | 16 | -0.064748919 | 0.0644994459863853 | 0.315442323287063 | -0.191167833 | 0.0616699950914758 | 0.93730277271358 | 0.825993947330416 | 1.06361128985989 | 0.998074519377826 |
| ebi-fl187-GCST90094781 | ebi-met1400-GCST90199775 | Gamma-glutamylisoleucine levels \|\| id:ebi-met1400-GCST90199775 | Herring liking \|\| id:ebi-fl187-GCST90094781 | Inverse variance weighted | 16 | 0.0351752877104692 | 0.0587319998433791 | 0.549231702571246 | -0.079939432 | 0.150290007403492 | 1.03580125611834 | 0.923172259406862 | 1.16217123212265 | 0.998074519377826 |
| ebi-fl187-GCST90094781 | ebi-met1400-GCST90199777 | 1-palmitoleoyl-GPC (16:1) levels \|\| id:ebi-met1400-GCST90199777 | Herring liking \|\| id:ebi-fl187-GCST90094781 | Inverse variance weighted | 16 | -0.100902155 | 0.067168714971623 | 0.133040422593476 | -0.232552837 | 0.0307485258980609 | 0.904021482137092 | 0.792507874667823 | 1.03122614460821 | 0.998074519377826 |
| ebi-fl187-GCST90094781 | ebi-met1400-GCST90199782 | Glutamine degradant levels \|\| id:ebi-met1400-GCST90199782 | Herring liking \|\| id:ebi-fl187-GCST90094781 | Inverse variance weighted | 16 | -0.101706779 | 0.050024756468917 | 0.0420394997218101 | -0.199755302 | -0.003658257 | 0.903294377559398 | 0.81893111948533 | 0.996348426767823 | 0.998074519377826 |
| ebi-fl187-GCST90094781 | ebi-met1400-GCST90199783 | 5-methyluridine (ribothymidine) levels \|\| id:ebi-met1400-GCST90199783 | Herring liking \|\| id:ebi-fl187-GCST90094781 | Inverse variance weighted | 16 | 0.0677454040937819 | 0.0623455907420124 | 0.277208765924305 | -0.054451954 | 0.189942761948126 | 1.07009283254018 | 0.947004007746095 | 1.20918038454688 | 0.998074519377826 |
| ebi-fl187-GCST90094781 | ebi-met1400-GCST90199787 | Homostachydrine levels \|\| id:ebi-met1400-GCST90199787 | Herring liking \|\| id:ebi-fl187-GCST90094781 | Inverse variance weighted | 16 | -0.018356222 | 0.0584111329922743 | 0.753324364917331 | -0.132842043 | 0.0961295987102792 | 0.981811227343455 | 0.875603389203732 | 1.10090173019348 | 0.998074519377826 |
| ebi-fl187-GCST90094781 | ebi-met1400-GCST90199802 | Beta-hydroxyisovaleroylcarnitine levels \|\| id:ebi-met1400-GCST90199802 | Herring liking \|\| id:ebi-fl187-GCST90094781 | Inverse variance weighted | 16 | 0.043315538779696 | 0.0589884089789025 | 0.462762301831131 | -0.072301743 | 0.158932820378345 | 1.04426734971519 | 0.930250157153148 | 1.17225919210639 | 0.998074519377826 |
| ebi-fl187-GCST90094781 | ebi-met1400-GCST90199873 | Pregnenolone sulfate levels \|\| id:ebi-met1400-GCST90199873 | Herring liking \|\| id:ebi-fl187-GCST90094781 | Inverse variance weighted | 16 | -0.039119134 | 0.0570821049448663 | 0.493146145138355 | -0.15100006 | 0.0727617913186031 | 0.961636138403121 | 0.859847647012221 | 1.07547431907983 | 0.998074519377826 |
| ebi-fl187-GCST90094781 | ebi-met1400-GCST90199911 | Beta-citrylglutamate levels \|\| id:ebi-met1400-GCST90199911 | Herring liking \|\| id:ebi-fl187-GCST90094781 | Inverse variance weighted | 16 | -0.011006698 | 0.0594173845531567 | 0.853037870807969 | -0.127464772 | 0.105451375767453 | 0.9890536541146 | 0.880324427637801 | 1.11121207137504 | 0.998074519377826 |
| ebi-fl187-GCST90094781 | ebi-met1400-GCST90199934 | 2-hydroxydecanoate levels \|\| id:ebi-met1400-GCST90199934 | Herring liking \|\| id:ebi-fl187-GCST90094781 | Inverse variance weighted | 16 | 0.0826662646849806 | 0.0652074688715051 | 0.204890003885771 | -0.045140374 | 0.210472903673131 | 1.08617925167786 | 0.955863293771254 | 1.23426160881308 | 0.998074519377826 |
| ebi-fl187-GCST90094781 | ebi-met1400-GCST90199945 | Sphingomyelin (d18:1/14:0, d16:1/16:0) levels \|\| id:ebi-met1400-GCST90199945 | Herring liking \|\| id:ebi-fl187-GCST90094781 | Inverse variance weighted | 16 | -0.04017368 | 0.0699461787609404 | 0.565730086630066 | -0.177268191 | 0.0969208301804595 | 0.960622583549212 | 0.837555130017848 | 1.10177314298713 | 0.998074519377826 |
| ebi-fl187-GCST90094781 | ebi-met1400-GCST90199979 | Carnitine C14:1 levels \|\| id:ebi-met1400-GCST90199979 | Herring liking \|\| id:ebi-fl187-GCST90094781 | Inverse variance weighted | 16 | 0.0254148862789887 | 0.0669151382506698 | 0.704088149231951 | -0.105738785 | 0.156568557250301 | 1.02574059795585 | 0.899659622250192 | 1.16949093665362 | 0.998074519377826 |
| ebi-fl187-GCST90094781 | ebi-met1400-GCST90199986 | Eugenol sulfate levels \|\| id:ebi-met1400-GCST90199986 | Herring liking \|\| id:ebi-fl187-GCST90094781 | Inverse variance weighted | 16 | -0.012059491 | 0.0718494007008744 | 0.866706006350381 | -0.152884316 | 0.128765334549413 | 0.9880129334097 | 0.858228999262495 | 1.13742317892275 | 0.998074519377826 |
| ebi-fl187-GCST90094781 | ebi-met1400-GCST90200002 | 6-hydroxyindole sulfate levels \|\| id:ebi-met1400-GCST90200002 | Herring liking \|\| id:ebi-fl187-GCST90094781 | Inverse variance weighted | 16 | 0.0183834186684749 | 0.0592848838165476 | 0.756495478957011 | -0.097814954 | 0.134581790948908 | 1.01855343393206 | 0.906816691382551 | 1.1440582287839 | 0.998074519377826 |
| ebi-fl187-GCST90094781 | ebi-met1400-GCST90200023 | Trans 3,4-methyleneheptanoate levels \|\| id:ebi-met1400-GCST90200023 | Herring liking \|\| id:ebi-fl187-GCST90094781 | Inverse variance weighted | 16 | 0.0275295401754193 | 0.0645132940290008 | 0.669578456124404 | -0.098916516 | 0.153975596472261 | 1.02791197935895 | 0.905818326094209 | 1.16646242063307 | 0.998074519377826 |
| ebi-fl187-GCST90094781 | ebi-met1400-GCST90200032 | 2-hydroxybutyrate/2-hydroxyisobutyrate levels \|\| id:ebi-met1400-GCST90200032 | Herring liking \|\| id:ebi-fl187-GCST90094781 | Inverse variance weighted | 16 | 0.0937967410278889 | 0.0603979184049764 | 0.120427985127396 | -0.024583179 | 0.212176661101643 | 1.09833647648394 | 0.975716526373244 | 1.23636628361637 | 0.998074519377826 |
| ebi-fl187-GCST90094781 | ebi-met1400-GCST90200051 | 1-palmitoyl-2-dihomo-linolenoyl-GPC (16:0/20:3n3 or 6) levels \|\| id:ebi-met1400-GCST90200051 | Herring liking \|\| id:ebi-fl187-GCST90094781 | Inverse variance weighted | 16 | -0.010158993 | 0.0587946942625925 | 0.862818394508242 | -0.125396594 | 0.105078607410851 | 0.989892434928555 | 0.882146978914493 | 1.11079792387269 | 0.998074519377826 |
| ebi-fl187-GCST90094781 | ebi-met1400-GCST90200069 | Phenylacetylglutamate levels \|\| id:ebi-met1400-GCST90200069 | Herring liking \|\| id:ebi-fl187-GCST90094781 | Inverse variance weighted | 16 | -0.055597035 | 0.0657487759012435 | 0.39777702854412 | -0.184464636 | 0.0732705658175924 | 0.945920231895688 | 0.831549346527276 | 1.07602163220537 | 0.998074519377826 |
| ebi-fl187-GCST90094781 | ebi-met1400-GCST90200072 | Thioproline levels \|\| id:ebi-met1400-GCST90200072 | Herring liking \|\| id:ebi-fl187-GCST90094781 | Inverse variance weighted | 16 | 0.0985958256378906 | 0.0691100902251795 | 0.153681157372603 | -0.036859951 | 0.234051602479243 | 1.10362015443282 | 0.963811106483114 | 1.26370970107891 | 0.998074519377826 |
| ebi-fl187-GCST90094781 | ebi-met1400-GCST90200081 | 1-oleoyl-2-docosahexaenoyl-GPC (18:1/22:6) levels \|\| id:ebi-met1400-GCST90200081 | Herring liking \|\| id:ebi-fl187-GCST90094781 | Inverse variance weighted | 16 | -0.084282703 | 0.0647435183253395 | 0.192986923919361 | -0.211179999 | 0.0426145927634507 | 0.919171366716447 | 0.809628321418223 | 1.04353563115408 | 0.998074519377826 |
| ebi-fl187-GCST90094781 | ebi-met1400-GCST90200097 | Furaneol sulfate levels \|\| id:ebi-met1400-GCST90200097 | Herring liking \|\| id:ebi-fl187-GCST90094781 | Inverse variance weighted | 16 | -0.063536764 | 0.0739993463981393 | 0.390554335180447 | -0.208575483 | 0.0815019545655212 | 0.938439617520594 | 0.811739759581828 | 1.08491533812004 | 0.998074519377826 |
| ebi-fl187-GCST90094781 | ebi-met1400-GCST90200124 | Sphingomyelin (d18:2/24:2) levels \|\| id:ebi-met1400-GCST90200124 | Herring liking \|\| id:ebi-fl187-GCST90094781 | Inverse variance weighted | 16 | -0.080222317 | 0.0585020587641484 | 0.170289745303335 | -0.194886352 | 0.0344417182971778 | 0.922911144850847 | 0.822928176835287 | 1.03504170262527 | 0.998074519377826 |
| ebi-fl187-GCST90094781 | ebi-met1400-GCST90200137 | Lignoceroylcarnitine (C24) levels \|\| id:ebi-met1400-GCST90200137 | Herring liking \|\| id:ebi-fl187-GCST90094781 | Inverse variance weighted | 16 | 0.0201324550927675 | 0.0718803616387416 | 0.779413957582444 | -0.120753054 | 0.161017963904701 | 1.02033647983967 | 0.886252789402381 | 1.17470607093224 | 0.998074519377826 |
| ebi-fl187-GCST90094781 | ebi-met1400-GCST90200165 | Octadecenedioate (C18:1-DC) levels \|\| id:ebi-met1400-GCST90200165 | Herring liking \|\| id:ebi-fl187-GCST90094781 | Inverse variance weighted | 16 | 0.0247802862808348 | 0.0586793541248091 | 0.672806119927081 | -0.090231248 | 0.139791820365461 | 1.02508986947226 | 0.913719865126419 | 1.15003436020215 | 0.998074519377826 |
| ebi-fl187-GCST90094781 | ebi-met1400-GCST90200176 | Glutamine conjugate of C6H10O2 (1) levels \|\| id:ebi-met1400-GCST90200176 | Herring liking \|\| id:ebi-fl187-GCST90094781 | Inverse variance weighted | 16 | 0.0439670347010827 | 0.0589094664359969 | 0.455456481997331 | -0.07149552 | 0.159429588915637 | 1.04494790730054 | 0.931000448920487 | 1.172841678 | 0.998074519377826 |
| ebi-fl187-GCST90094781 | ebi-met1400-GCST90200182 | Glycine conjugate of C10H12O2 levels \|\| id:ebi-met1400-GCST90200182 | Herring liking \|\| id:ebi-fl187-GCST90094781 | Inverse variance weighted | 16 | -0.011463212 | 0.085407700873203 | 0.893230552899753 | -0.178862306 | 0.155935881815484 | 0.988602240380991 | 0.836221034417483 | 1.16875126247827 | 0.998074519377826 |
| ebi-fl187-GCST90094781 | ebi-met1400-GCST90200208 | Glucuronide of piperine metabolite C17H21NO3 (4) levels \|\| id:ebi-met1400-GCST90200208 | Herring liking \|\| id:ebi-fl187-GCST90094781 | Inverse variance weighted | 16 | -0.018936075 | 0.0612978771328765 | 0.757383189425743 | -0.139079914 | 0.101207763988935 | 0.981242085950418 | 0.87015848751061 | 1.10650651008974 | 0.998074519377826 |
| ebi-fl187-GCST90094781 | ebi-met1400-GCST90200227 | 3-ethylcatechol sulfate (2) levels \|\| id:ebi-met1400-GCST90200227 | Herring liking \|\| id:ebi-fl187-GCST90094781 | Inverse variance weighted | 15 | -0.079562974 | 0.102839636947052 | 0.439131529600342 | -0.281128663 | 0.12200271392329 | 0.923519859942434 | 0.754931197587227 | 1.12975716785045 | 0.998074519377826 |
| ebi-fl187-GCST90094781 | ebi-met1400-GCST90200259 | Pentose acid levels \|\| id:ebi-met1400-GCST90200259 | Herring liking \|\| id:ebi-fl187-GCST90094781 | Inverse variance weighted | 16 | -0.032227721 | 0.0714469087900197 | 0.651937443742014 | -0.172263662 | 0.107808220013068 | 0.968286057693225 | 0.841757204145799 | 1.1138341138102 | 0.998074519377826 |
| ebi-fl187-GCST90094781 | ebi-met1400-GCST90200291 | Sarcosine levels \|\| id:ebi-met1400-GCST90200291 | Herring liking \|\| id:ebi-fl187-GCST90094781 | Inverse variance weighted | 16 | 0.0644246607125381 | 0.0600024339120505 | 0.282956807047711 | -0.05318011 | 0.182029431180157 | 1.06654522246061 | 0.948209215372101 | 1.19964950046088 | 0.998074519377826 |
| ebi-fl187-GCST90094781 | ebi-met1400-GCST90200306 | Butyrate/isobutyrate (4:0) levels \|\| id:ebi-met1400-GCST90200306 | Herring liking \|\| id:ebi-fl187-GCST90094781 | Inverse variance weighted | 16 | -0.004320521 | 0.0606030616475301 | 0.943165266795789 | -0.123102522 | 0.114461480100565 | 0.995688799293808 | 0.884173011119614 | 1.12126944904568 | 0.998074519377826 |
| ebi-fl187-GCST90094781 | ebi-met1400-GCST90200321 | Cholate levels \|\| id:ebi-met1400-GCST90200321 | Herring liking \|\| id:ebi-fl187-GCST90094781 | Inverse variance weighted | 16 | 0.00808555089568949 | 0.069528992027999 | 0.907422432423525 | -0.128191273 | 0.144362375270567 | 1.00811832724105 | 0.87968510262157 | 1.15530268580266 | 0.998074519377826 |
| ebi-fl187-GCST90094781 | ebi-met1400-GCST90200336 | Ceramide (d18:1/16:0) levels \|\| id:ebi-met1400-GCST90200336 | Herring liking \|\| id:ebi-fl187-GCST90094781 | Inverse variance weighted | 16 | 0.0427769764483203 | 0.0595562141063957 | 0.472595642036965 | -0.073953203 | 0.159507156096856 | 1.04370509807412 | 0.928715153721493 | 1.17293265581039 | 0.998074519377826 |
| ebi-fl187-GCST90094781 | ebi-met1400-GCST90200358 | Arachidonate (20:4n6) levels \|\| id:ebi-met1400-GCST90200358 | Herring liking \|\| id:ebi-fl187-GCST90094781 | Inverse variance weighted | 16 | -0.005095957 | 0.0595589654520625 | 0.931814968715108 | -0.121831529 | 0.111639615165283 | 0.994917005240836 | 0.885297502535334 | 1.11810983819858 | 0.998074519377826 |
| ebi-fl187-GCST90094781 | ebi-met1400-GCST90200413 | 2'-deoxyuridine levels \|\| id:ebi-met1400-GCST90200413 | Herring liking \|\| id:ebi-fl187-GCST90094781 | Inverse variance weighted | 16 | 0.00365121484498957 | 0.0596237431647106 | 0.951169986633901 | -0.113211322 | 0.120513751447822 | 1.00365788864994 | 0.89296193788976 | 1.12807625354084 | 0.998074519377826 |
| ebi-fl187-GCST90094781 | ebi-met1400-GCST90200432 | Threonine levels \|\| id:ebi-met1400-GCST90200432 | Herring liking \|\| id:ebi-fl187-GCST90094781 | Inverse variance weighted | 16 | -0.020968167 | 0.0726139958383221 | 0.772763470081525 | -0.163291599 | 0.121355265059788 | 0.979250136755086 | 0.849343484907774 | 1.12902594459647 | 0.998074519377826 |
| ebi-fl187-GCST90094781 | ebi-met1400-GCST90200436 | Caffeine levels \|\| id:ebi-met1400-GCST90200436 | Herring liking \|\| id:ebi-fl187-GCST90094781 | Inverse variance weighted | 16 | -0.034221765 | 0.0612525397707058 | 0.576366807243686 | -0.154276742 | 0.0858332134051376 | 0.966357177113141 | 0.857034810237252 | 1.08962457837572 | 0.998074519377826 |
| ebi-fl187-GCST90094781 | ebi-met1400-GCST90200442 | Valine levels \|\| id:ebi-met1400-GCST90200442 | Herring liking \|\| id:ebi-fl187-GCST90094781 | Inverse variance weighted | 16 | 0.0493902169419007 | 0.0587335576276359 | 0.400392813708851 | -0.065727556 | 0.164507989892067 | 1.05063024448216 | 0.936385942266725 | 1.17881298810252 | 0.998074519377826 |
| ebi-fl187-GCST90094781 | ebi-met1400-GCST90200459 | Margarate (17:0) levels \|\| id:ebi-met1400-GCST90200459 | Herring liking \|\| id:ebi-fl187-GCST90094781 | Inverse variance weighted | 16 | 0.0415530728450989 | 0.066825199430853 | 0.534061932959915 | -0.089424318 | 0.172530463729571 | 1.04242848502889 | 0.914457470439747 | 1.18830802035778 | 0.998074519377826 |
| ebi-fl187-GCST90094781 | ebi-met1400-GCST90200462 | X-11299 levels \|\| id:ebi-met1400-GCST90200462 | Herring liking \|\| id:ebi-fl187-GCST90094781 | Inverse variance weighted | 16 | 0.00288891569969476 | 0.059487881602104 | 0.961267480443426 | -0.113707332 | 0.119485163639819 | 1.00289309263796 | 0.892519129235962 | 1.12691652460372 | 0.998074519377826 |
| ebi-fl187-GCST90094781 | ebi-met1400-GCST90200497 | X-12740 levels \|\| id:ebi-met1400-GCST90200497 | Herring liking \|\| id:ebi-fl187-GCST90094781 | Inverse variance weighted | 16 | 0.0262248787964367 | 0.0755241853166223 | 0.728412408184776 | -0.121802524 | 0.174252282017016 | 1.02657177674389 | 0.885323180946442 | 1.19035583331332 | 0.998074519377826 |
| ebi-fl187-GCST90094781 | ebi-met1400-GCST90200527 | X-13866 levels \|\| id:ebi-met1400-GCST90200527 | Herring liking \|\| id:ebi-fl187-GCST90094781 | Inverse variance weighted | 16 | 0.0180746694159401 | 0.061677429964343 | 0.769482712467639 | -0.102813093 | 0.138962432146052 | 1.01823900486304 | 0.902295602802809 | 1.14908093068814 | 0.998074519377826 |
| ebi-fl187-GCST90094781 | ebi-met1400-GCST90200547 | X-17654 levels \|\| id:ebi-met1400-GCST90200547 | Herring liking \|\| id:ebi-fl187-GCST90094781 | Inverse variance weighted | 16 | -0.016019208 | 0.0605568965569228 | 0.79137037485866 | -0.134710726 | 0.102672308946331 | 0.984108416818879 | 0.873968691931567 | 1.10812822586738 | 0.998074519377826 |
| ebi-fl187-GCST90094781 | ebi-met1400-GCST90200553 | X-17653 levels \|\| id:ebi-met1400-GCST90200553 | Herring liking \|\| id:ebi-fl187-GCST90094781 | Inverse variance weighted | 16 | 0.0274623257333721 | 0.060301141478822 | 0.648807349456012 | -0.090727912 | 0.145652563031863 | 1.02784289115068 | 0.913266166258625 | 1.15679420515158 | 0.998074519377826 |
| ebi-fl187-GCST90094781 | ebi-met1400-GCST90200557 | X-17685 levels \|\| id:ebi-met1400-GCST90200557 | Herring liking \|\| id:ebi-fl187-GCST90094781 | Inverse variance weighted | 16 | -0.033267793 | 0.0635251089732022 | 0.600490573933567 | -0.157777006 | 0.0912414206940068 | 0.967279494328082 | 0.854040206166999 | 1.09553345778271 | 0.998074519377826 |
| ebi-fl187-GCST90094781 | ebi-met1400-GCST90200561 | X-18779 levels \|\| id:ebi-met1400-GCST90200561 | Herring liking \|\| id:ebi-fl187-GCST90094781 | Inverse variance weighted | 16 | -0.023567794 | 0.0649203417846943 | 0.716585204824066 | -0.150811664 | 0.103676075538223 | 0.976707757147196 | 0.860009653964237 | 1.10924108639271 | 0.998074519377826 |
| ebi-fl187-GCST90094781 | ebi-met1400-GCST90200570 | X-18921 levels \|\| id:ebi-met1400-GCST90200570 | Herring liking \|\| id:ebi-fl187-GCST90094781 | Inverse variance weighted | 16 | 0.00911197704805561 | 0.059957865132244 | 0.879208225749372 | -0.108405439 | 0.126629392707254 | 1.00915361749041 | 0.897263737310667 | 1.13499630191939 | 0.998074519377826 |
| ebi-fl187-GCST90094781 | ebi-met1400-GCST90200575 | X-21283 levels \|\| id:ebi-met1400-GCST90200575 | Herring liking \|\| id:ebi-fl187-GCST90094781 | Inverse variance weighted | 16 | -0.038306892 | 0.0635390511934016 | 0.546583613685898 | -0.162843432 | 0.0862296484032473 | 0.962417537383926 | 0.849724217387987 | 1.09005662932779 | 0.998074519377826 |
| ebi-fl187-GCST90094781 | ebi-met1400-GCST90200578 | X-18922 levels \|\| id:ebi-met1400-GCST90200578 | Herring liking \|\| id:ebi-fl187-GCST90094781 | Inverse variance weighted | 16 | -0.015782161 | 0.0601549227986204 | 0.793044992747198 | -0.13368581 | 0.102121487772167 | 0.98434172480407 | 0.874864895579312 | 1.10751801345127 | 0.998074519377826 |
| ebi-fl187-GCST90094781 | ebi-met1400-GCST90200623 | X-24241 levels \|\| id:ebi-met1400-GCST90200623 | Herring liking \|\| id:ebi-fl187-GCST90094781 | Inverse variance weighted | 16 | -0.006199329 | 0.0604616618296936 | 0.91833344550266 | -0.124704186 | 0.112305528271097 | 0.993819847277425 | 0.882757996049572 | 1.11885465015608 | 0.998074519377826 |
| ebi-fl187-GCST90094781 | ebi-met1400-GCST90200636 | X-24544 levels \|\| id:ebi-met1400-GCST90200636 | Herring liking \|\| id:ebi-fl187-GCST90094781 | Inverse variance weighted | 16 | -0.051339395 | 0.0561216341753699 | 0.360302954355832 | -0.161337798 | 0.0586590074927775 | 0.949956204961281 | 0.851004554508099 | 1.0604135859955 | 0.998074519377826 |
| ebi-fl187-GCST90094781 | ebi-met1400-GCST90200659 | X-25172 levels \|\| id:ebi-met1400-GCST90200659 | Herring liking \|\| id:ebi-fl187-GCST90094781 | Inverse variance weighted | 16 | 0.0154445131147731 | 0.0607367961767197 | 0.799274663608697 | -0.103599607 | 0.134488633621144 | 1.01556439598984 | 0.901586213618065 | 1.14395165634058 | 0.998074519377826 |
| ebi-fl187-GCST90094781 | ebi-met1400-GCST90200670 | X-26111 levels \|\| id:ebi-met1400-GCST90200670 | Herring liking \|\| id:ebi-fl187-GCST90094781 | Inverse variance weighted | 16 | -0.005482985 | 0.0603492966832611 | 0.927608468448572 | -0.123767607 | 0.112801636382573 | 0.994532019011281 | 0.883585156369555 | 1.11940986073443 | 0.998074519377826 |
| ebi-fl187-GCST90094781 | ebi-met1400-GCST90200673 | Carnitine C4 levels \|\| id:ebi-met1400-GCST90200673 | Herring liking \|\| id:ebi-fl187-GCST90094781 | Inverse variance weighted | 16 | -0.0254361 | 0.0649752965706943 | 0.695447349895056 | -0.152787681 | 0.101915481290185 | 0.974884672117334 | 0.858311938170021 | 1.10728988106077 | 0.998074519377826 |
| ebi-fl187-GCST90094781 | ebi-met1400-GCST90200710 | X-13431 levels \|\| id:ebi-met1400-GCST90200710 | Herring liking \|\| id:ebi-fl187-GCST90094781 | Inverse variance weighted | 16 | 0.0494133453363597 | 0.0591927601409533 | 0.403837659247798 | -0.066604465 | 0.165431155212628 | 1.05065454415389 | 0.935565177365508 | 1.17990172983955 | 0.998074519377826 |
| ebi-fl187-GCST90094781 | ebi-met1400-GCST90200730 | Adenosine 5'-diphosphate (ADP) to fructose ratio \|\| id:ebi-met1400-GCST90200730 | Herring liking \|\| id:ebi-fl187-GCST90094781 | Inverse variance weighted | 16 | 0.0118903640275718 | 0.0805772754240623 | 0.882686290357268 | -0.146041096 | 0.169821823858734 | 1.01196133541888 | 0.864122190659115 | 1.1850936770894 | 0.998074519377826 |
| ebi-fl187-GCST90094781 | ebi-met1400-GCST90200731 | Adenosine 5'-diphosphate (ADP) to mannose ratio \|\| id:ebi-met1400-GCST90200731 | Herring liking \|\| id:ebi-fl187-GCST90094781 | Inverse variance weighted | 16 | -0.112694128 | 0.0797841393444244 | 0.157806362992027 | -0.269071041 | 0.0436827849337215 | 0.893423891517516 | 0.764088971848884 | 1.0446509233118 | 0.998074519377826 |
| ebi-fl187-GCST90094781 | ebi-met1400-GCST90200748 | Aspartate to glutamate ratio \|\| id:ebi-met1400-GCST90200748 | Herring liking \|\| id:ebi-fl187-GCST90094781 | Inverse variance weighted | 16 | -0.025250495 | 0.0611792508120211 | 0.679804653534749 | -0.145161826 | 0.0946608370235203 | 0.975065632789802 | 0.864882321200941 | 1.09928595479625 | 0.998074519377826 |
| ebi-fl187-GCST90094781 | ebi-met1400-GCST90200774 | Uridine to pseudouridine ratio \|\| id:ebi-met1400-GCST90200774 | Herring liking \|\| id:ebi-fl187-GCST90094781 | Inverse variance weighted | 16 | 0.067082635307965 | 0.0571225625894689 | 0.240249579332968 | -0.044877587 | 0.179042857983324 | 1.06938384338674 | 0.956114515164649 | 1.19607200430344 | 0.998074519377826 |
| ebi-fl187-GCST90094781 | ebi-met1400-GCST90200799 | Spermidine to adenosine 5'-diphosphate (ADP) ratio \|\| id:ebi-met1400-GCST90200799 | Herring liking \|\| id:ebi-fl187-GCST90094781 | Inverse variance weighted | 16 | 0.0355858423894939 | 0.0835785474602234 | 0.670270180643706 | -0.128228111 | 0.199399795411532 | 1.03622659647734 | 0.879652698123389 | 1.2206698865788 | 0.998074519377826 |
| ebi-fl187-GCST90094781 | ebi-met1400-GCST90200811 | Choline to taurocholate ratio \|\| id:ebi-met1400-GCST90200811 | Herring liking \|\| id:ebi-fl187-GCST90094781 | Inverse variance weighted | 16 | -0.000359643 | 0.0626532796627902 | 0.995420004692374 | -0.123160071 | 0.12244078550804 | 0.99964042203263 | 0.884122129123371 | 1.13025219078317 | 0.999355215041207 |
| ebi-fl187-GCST90094781 | ebi-met1400-GCST90200812 | Glutamate to kynurenine ratio \|\| id:ebi-met1400-GCST90200812 | Herring liking \|\| id:ebi-fl187-GCST90094781 | Inverse variance weighted | 16 | 0.0521949772760021 | 0.0581757238536932 | 0.369614792874355 | -0.061829441 | 0.166219396029241 | 1.05358114687019 | 0.940043205499303 | 1.18083214318932 | 0.998074519377826 |
| ebi-fl187-GCST90094781 | ebi-met1400-GCST90200876 | Cortisone to 4-cholesten-3-one ratio \|\| id:ebi-met1400-GCST90200876 | Herring liking \|\| id:ebi-fl187-GCST90094781 | Inverse variance weighted (multiplicative random effects) | 16 | -0.069022547 | 0.0895420830755852 | 0.440802251818938 | -0.24452503 | 0.106479935583373 | 0.933305636370331 | 0.783076387660976 | 1.11235560745543 | 0.998074519377826 |
| ebi-fl187-GCST90094781 | ebi-met1400-GCST90200906 | 2'-deoxyuridine to cytidine ratio \|\| id:ebi-met1400-GCST90200906 | Herring liking \|\| id:ebi-fl187-GCST90094781 | Inverse variance weighted | 16 | 0.0183151784438668 | 0.0788237517822828 | 0.81626145389775 | -0.136179375 | 0.172809731937141 | 1.01848392998847 | 0.872686080339586 | 1.18863992335148 | 0.998074519377826 |
| ebi-fl187-GCST90094781 | ebi-met1400-GCST90200907 | Retinol (Vitamin A) to linoleoyl-arachidonoyl-glycerol (18:2 to 20:4) [1] ratio \|\| id:ebi-met1400-GCST90200907 | Herring liking \|\| id:ebi-fl187-GCST90094781 | Inverse variance weighted | 16 | -0.081425779 | 0.0725183652108505 | 0.261509803596366 | -0.223561774 | 0.0607102172286026 | 0.921801124699066 | 0.799665491487489 | 1.06259094901778 | 0.998074519377826 |
| ebi-fl187-GCST90094781 | ebi-met1400-GCST90200916 | Fructose to sucrose ratio \|\| id:ebi-met1400-GCST90200916 | Herring liking \|\| id:ebi-fl187-GCST90094781 | Inverse variance weighted | 16 | 0.0315475651674741 | 0.0616221842172681 | 0.608685004427278 | -0.089231916 | 0.15232704623332 | 1.03205046408135 | 0.914633430941961 | 1.16454103291805 | 0.998074519377826 |
| ebi-fl187-GCST90094781 | ebi-met1400-GCST90200917 | Alpha-ketobutyrate to 3-methyl-2-oxobutyrate ratio \|\| id:ebi-met1400-GCST90200917 | Herring liking \|\| id:ebi-fl187-GCST90094781 | Inverse variance weighted | 16 | 0.101408134758021 | 0.0601888269515926 | 0.0920207548236377 | -0.016561966 | 0.219378235583142 | 1.1067282438639 | 0.98357442926319 | 1.24530220522673 | 0.998074519377826 |
| ebi-fl187-GCST90094781 | ebi-met1400-GCST90200924 | Alpha-tocopherol to glycerol ratio \|\| id:ebi-met1400-GCST90200924 | Herring liking \|\| id:ebi-fl187-GCST90094781 | Inverse variance weighted | 16 | 0.00659463003708151 | 0.0558816290504508 | 0.90605918151707 | -0.102933363 | 0.116122622975965 | 1.00661642248779 | 0.902187090608229 | 1.12313358567234 | 0.998074519377826 |
| ebi-fl187-GCST90094781 | ebi-met1400-GCST90200938 | Alpha-ketoglutarate to alanine ratio \|\| id:ebi-met1400-GCST90200938 | Herring liking \|\| id:ebi-fl187-GCST90094781 | Inverse variance weighted | 16 | -0.000476002 | 0.0594810293471233 | 0.993614924267015 | -0.11705882 | 0.116106815164323 | 0.99952411091511 | 0.889532869411155 | 1.1231158315285 | 0.999355215041207 |
| ebi-fl187-GCST90094781 | ebi-met1400-GCST90200943 | Phosphate to EDTA ratio \|\| id:ebi-met1400-GCST90200943 | Herring liking \|\| id:ebi-fl187-GCST90094781 | Inverse variance weighted | 16 | -0.014902728 | 0.0569982282067495 | 0.793738178329828 | -0.126619255 | 0.096813799075102 | 0.985207767865271 | 0.881069070949541 | 1.10165522530033 | 0.998074519377826 |
| ebi-fl187-GCST90094781 | ebi-met1400-GCST90200947 | Adenosine 5'-diphosphate (ADP) to glucose ratio \|\| id:ebi-met1400-GCST90200947 | Herring liking \|\| id:ebi-fl187-GCST90094781 | Inverse variance weighted | 16 | -0.068512199 | 0.0800438361116928 | 0.392034578423987 | -0.225398117 | 0.0883737201067936 | 0.933782069132749 | 0.798198378790766 | 1.09239629621248 | 0.998074519377826 |
| ebi-fl187-GCST90094781 | ebi-met1400-GCST90200975 | Arachidonate (20:4n6) to caffeine ratio \|\| id:ebi-met1400-GCST90200975 | Herring liking \|\| id:ebi-fl187-GCST90094781 | Inverse variance weighted | 16 | 0.0389835131086864 | 0.0612654813949837 | 0.524577849074054 | -0.08109683 | 0.159063856642854 | 1.03975334120852 | 0.922104399360254 | 1.17241281063654 | 0.998074519377826 |
| ebi-fl187-GCST90094781 | ebi-met1400-GCST90200985 | Cholesterol to oleoyl-linoleoyl-glycerol (18:1 to 18:2) [2] ratio \|\| id:ebi-met1400-GCST90200985 | Herring liking \|\| id:ebi-fl187-GCST90094781 | Inverse variance weighted | 16 | -0.073953398 | 0.0612137356933955 | 0.227001991497826 | -0.19393232 | 0.0460255242496525 | 0.928714973077881 | 0.823713651595092 | 1.04710113708669 | 0.998074519377826 |
| ebi-fl187-GCST90094781 | ebi-met1400-GCST90201016 | Bilirubin (Z,Z) to etiocholanolone glucuronide ratio \|\| id:ebi-met1400-GCST90201016 | Herring liking \|\| id:ebi-fl187-GCST90094781 | Inverse variance weighted | 16 | 0.120566311799763 | 0.0618819336764884 | 0.0513757139495243 | -0.000722278 | 0.241854901805681 | 1.12813554718398 | 0.999277982573961 | 1.27360938098714 | 0.998074519377826 |
| ebi-fl187-GCST90094781 | ebi-pw11-GCST90275189 | Placental weight (fetal genotype, adjusted for sex and gestational age) \|\| id:ebi-pw11-GCST90275189 | Herring liking \|\| id:ebi-fl187-GCST90094781 | Inverse variance weighted | 15 | 0.0390932550107313 | 0.0260151127340138 | 0.132913000418673 | -0.011896366 | 0.0900828759693983 | 1.03986745197909 | 0.988174116043401 | 1.09426496821739 | 0.998074519377826 |
| ebi-fl187-GCST90094781 | ebi-pw11-GCST90275192 | Placental weight (fetal effect, conditional on maternal and paternal genotype) \|\| id:ebi-pw11-GCST90275192 | Herring liking \|\| id:ebi-fl187-GCST90094781 | Inverse variance weighted | 14 | 0.0427690282916435 | 0.0510032591757287 | 0.401719178591805 | -0.05719736 | 0.142735416276072 | 1.04369680257544 | 0.944407662966347 | 1.15342458391828 | 0.998074519377826 |
| ebi-fl187-GCST90094781 | ebi-pw11-GCST90275193 | Placental weight (maternal effect, conditional on fetal and paternal genotype) \|\| id:ebi-pw11-GCST90275193 | Herring liking \|\| id:ebi-fl187-GCST90094781 | Inverse variance weighted | 14 | -0.002750959 | 0.0384082479325108 | 0.942901000567026 | -0.078031125 | 0.0725292068741007 | 0.997252821346903 | 0.924935637454056 | 1.07522420956967 | 0.998074519377826 |
| ebi-fl187-GCST90094781 | ebi-pw11-GCST90275195 | Placental weight (fetal effect, conditional on maternal genotype) \|\| id:ebi-pw11-GCST90275195 | Herring liking \|\| id:ebi-fl187-GCST90094781 | Inverse variance weighted | 14 | 0.0418307505563892 | 0.0309766345943009 | 0.176888712686534 | -0.018883453 | 0.102544954361219 | 1.0427179843767 | 0.981293722174182 | 1.10798710964301 | 0.998074519377826 |
| ebi-fl187-GCST90094781 | ebi-pw11-GCST90275197 | Placental weight (fetal genotype, adjusted for sex) \|\| id:ebi-pw11-GCST90275197 | Herring liking \|\| id:ebi-fl187-GCST90094781 | Inverse variance weighted | 15 | 0.0385389500018567 | 0.0235185169993013 | 0.101283230097244 | -0.007557343 | 0.0846352433204873 | 1.03929120796412 | 0.992471141600296 | 1.08832002229292 | 0.998074519377826 |
| ebi-fl187-GCST90094781 | prot-a-1030 | Protein FAM171B \|\| id:prot-a-1030 | Herring liking \|\| id:ebi-fl187-GCST90094781 | Inverse variance weighted | 19 | -0.013419136 | 0.085777551488004 | 0.875685331817824 | -0.181543137 | 0.15470486520518 | 0.986670499499999 | 0.833982269344803 | 1.16731339546151 | 0.998074519377826 |
| ebi-fl187-GCST90094781 | prot-a-1036 | BRISC complex subunit Abro1 \|\| id:prot-a-1036 | Herring liking \|\| id:ebi-fl187-GCST90094781 | Inverse variance weighted | 19 | 0.0281492748153236 | 0.0857607434875144 | 0.742737537447162 | -0.139941782 | 0.196240332050852 | 1.02854920945572 | 0.869408848804527 | 1.2168193108762 | 0.998074519377826 |
| ebi-fl187-GCST90094781 | prot-a-1055 | Phenylalanine--tRNA ligase, mitochondrial \|\| id:prot-a-1055 | Herring liking \|\| id:ebi-fl187-GCST90094781 | Inverse variance weighted | 19 | -0.106717156 | 0.0857173747251311 | 0.213135935511064 | -0.27472321 | 0.0612888988917087 | 0.898779851917381 | 0.759782394268673 | 1.06320602886591 | 0.998074519377826 |
| ebi-fl187-GCST90094781 | prot-a-1083 | Fc receptor-like protein 5 \|\| id:prot-a-1083 | Herring liking \|\| id:ebi-fl187-GCST90094781 | Inverse variance weighted | 19 | -0.028227376 | 0.0857400578525198 | 0.741989102683429 | -0.19627789 | 0.139823137025118 | 0.972167293802577 | 0.821783837634308 | 1.15007037600075 | 0.998074519377826 |
| ebi-fl187-GCST90094781 | prot-a-1103 | Fibroblast growth factor receptor 3 \|\| id:prot-a-1103 | Herring liking \|\| id:ebi-fl187-GCST90094781 | Inverse variance weighted | 19 | -0.091423392 | 0.0925413411662748 | 0.323192092804728 | -0.272804421 | 0.0899576366469298 | 0.912631228291637 | 0.761241656161973 | 1.09412793179553 | 0.998074519377826 |
| ebi-fl187-GCST90094781 | prot-a-1105 | Fibroblast growth factor receptor 4 \|\| id:prot-a-1105 | Herring liking \|\| id:ebi-fl187-GCST90094781 | Inverse variance weighted | 19 | 0.0425726446116647 | 0.0985522282487456 | 0.665755557982617 | -0.150589723 | 0.235735011979206 | 1.04349185768114 | 0.860200546981246 | 1.26583883359305 | 0.998074519377826 |
| ebi-fl187-GCST90094781 | prot-a-1120 | Filamin-A \|\| id:prot-a-1120 | Herring liking \|\| id:ebi-fl187-GCST90094781 | Inverse variance weighted | 19 | -0.055731613 | 0.100710620691522 | 0.580000716713431 | -0.25312443 | 0.141661203401231 | 0.945792940214051 | 0.776371272161419 | 1.15218622562937 | 0.998074519377826 |
| ebi-fl187-GCST90094781 | prot-a-1166 | Galanin peptides \|\| id:prot-a-1166 | Herring liking \|\| id:ebi-fl187-GCST90094781 | Inverse variance weighted | 19 | -0.003622675 | 0.0857341572343688 | 0.966295625227711 | -0.171661623 | 0.164416273650229 | 0.996383879439551 | 0.842264128009024 | 1.17870487676328 | 0.998074519377826 |
| ebi-fl187-GCST90094781 | prot-a-1205 | ADP-ribosylation factor-binding protein GGA1 \|\| id:prot-a-1205 | Herring liking \|\| id:ebi-fl187-GCST90094781 | Inverse variance weighted | 19 | -0.026103735 | 0.0959895360938526 | 0.785665429659528 | -0.214243225 | 0.162035756172241 | 0.974234022617688 | 0.807152041335731 | 1.175902286 | 0.998074519377826 |
| ebi-fl187-GCST90094781 | prot-a-1227 | Guanine nucleotide-binding protein G(I)/G(S)/G(O) subunit gamma-T2 \|\| id:prot-a-1227 | Herring liking \|\| id:ebi-fl187-GCST90094781 | Inverse variance weighted | 19 | 0.0547968028690318 | 0.0857548385978801 | 0.52282702074057 | -0.113282681 | 0.222876286520877 | 1.05632595046416 | 0.892898219270013 | 1.2496659636484 | 0.998074519377826 |
| ebi-fl187-GCST90094781 | prot-a-1240 | Golgi SNAP receptor complex member 1 \|\| id:prot-a-1240 | Herring liking \|\| id:ebi-fl187-GCST90094781 | Inverse variance weighted | 19 | -0.110078769 | 0.0857400578525198 | 0.199188746240523 | -0.278129283 | 0.0579717439533041 | 0.895763573724561 | 0.757198922367656 | 1.05968505277692 | 0.998074519377826 |
| ebi-fl187-GCST90094781 | prot-a-1283 | Glutathione S-transferase A1 \|\| id:prot-a-1283 | Herring liking \|\| id:ebi-fl187-GCST90094781 | Inverse variance weighted | 19 | -0.012388227 | 0.0941912121833407 | 0.895362293611249 | -0.197003003 | 0.172226548477068 | 0.987688190799284 | 0.82118816704962 | 1.18794693029889 | 0.998074519377826 |
| ebi-fl187-GCST90094781 | prot-a-1324 | E3 ubiquitin-protein ligase HECW1 \|\| id:prot-a-1324 | Herring liking \|\| id:ebi-fl187-GCST90094781 | Inverse variance weighted | 19 | 0.0240278371469993 | 0.0857548385978801 | 0.77933004827581 | -0.144051647 | 0.192107320798844 | 1.02431883160764 | 0.865843029138227 | 1.21180056139083 | 0.998074519377826 |
| ebi-fl187-GCST90094781 | prot-a-1339 | Histidine triad nucleotide-binding protein 2, mitochondrial \|\| id:prot-a-1339 | Herring liking \|\| id:ebi-fl187-GCST90094781 | Inverse variance weighted (multiplicative random effects) | 19 | 0.113452986082523 | 0.116356910590105 | 0.329538952495471 | -0.114606559 | 0.341512530839129 | 1.12013922552132 | 0.891716913183407 | 1.40707422501634 | 0.998074519377826 |
| ebi-fl187-GCST90094781 | prot-a-1466 | Interleukin-11 \|\| id:prot-a-1466 | Herring liking \|\| id:ebi-fl187-GCST90094781 | Inverse variance weighted | 19 | 0.0684893108365147 | 0.0995812367043617 | 0.491595583707397 | -0.126689913 | 0.263668534777064 | 1.0708891779803 | 0.881006818915223 | 1.301696658 | 0.998074519377826 |
| ebi-fl187-GCST90094781 | prot-a-147 | Rho GTPase-activating protein 1 \|\| id:prot-a-147 | Herring liking \|\| id:ebi-fl187-GCST90094781 | Inverse variance weighted | 19 | -0.03196365 | 0.0857210102152413 | 0.709237759616357 | -0.19997683 | 0.136049529923401 | 0.968541787837945 | 0.818749723190768 | 1.14573864053662 | 0.998074519377826 |
| ebi-fl187-GCST90094781 | prot-a-1473 | Interleukin-12 receptor subunit beta-1 \|\| id:prot-a-1473 | Herring liking \|\| id:ebi-fl187-GCST90094781 | Inverse variance weighted | 19 | -0.036495582 | 0.0857607434875145 | 0.670434950121844 | -0.204586639 | 0.131595475125238 | 0.964162353449855 | 0.814984129166828 | 1.1406468059204 | 0.998074519377826 |
| ebi-fl187-GCST90094781 | prot-a-1501 | X-linked interleukin-1 receptor accessory protein-like 2 \|\| id:prot-a-1501 | Herring liking \|\| id:ebi-fl187-GCST90094781 | Inverse variance weighted | 19 | -0.161282341 | 0.0944689342379403 | 0.0877750982896875 | -0.346441452 | 0.0238767704698687 | 0.851051750689658 | 0.707200223415722 | 1.0241641028529 | 0.998074519377826 |
| ebi-fl187-GCST90094781 | prot-a-1503 | Interleukin-1 receptor-like 2 \|\| id:prot-a-1503 | Herring liking \|\| id:ebi-fl187-GCST90094781 | Inverse variance weighted | 19 | -0.095279921 | 0.103216910213822 | 0.355953120507764 | -0.297585065 | 0.107025223160057 | 0.909118417639179 | 0.742609410554928 | 1.11296232655219 | 0.998074519377826 |
| ebi-fl187-GCST90094781 | prot-a-1536 | Interleukin-5 receptor subunit alpha \|\| id:prot-a-1536 | Herring liking \|\| id:ebi-fl187-GCST90094781 | Inverse variance weighted | 19 | 0.112987210509606 | 0.0871232927517855 | 0.194677398233153 | -0.057774443 | 0.283748864303106 | 1.1196176135183 | 0.94386281802639 | 1.32809935571101 | 0.998074519377826 |
| ebi-fl187-GCST90094781 | prot-a-1559 | Insulin-induced gene 1 protein \|\| id:prot-a-1559 | Herring liking \|\| id:ebi-fl187-GCST90094781 | Inverse variance weighted | 19 | -0.003032549 | 0.0857247436047462 | 0.97178037989654 | -0.171053047 | 0.164987948167478 | 0.996972044235252 | 0.842776865701066 | 1.1793789049487 | 0.998074519377826 |
| ebi-fl187-GCST90094781 | prot-a-1567 | Interferon regulatory factor 1 \|\| id:prot-a-1567 | Herring liking \|\| id:ebi-fl187-GCST90094781 | Inverse variance weighted | 19 | 0.128300966645014 | 0.0857607434875144 | 0.134644860532035 | -0.039790091 | 0.296392023880542 | 1.13689511872134 | 0.96099113906475 | 1.34499732456463 | 0.998074519377826 |
| ebi-fl187-GCST90094781 | prot-a-1613 | Potassium voltage-gated channel subfamily E regulatory beta subunit 5 \|\| id:prot-a-1613 | Herring liking \|\| id:ebi-fl187-GCST90094781 | Inverse variance weighted (multiplicative random effects) | 19 | 0.00396189241873558 | 0.109446239727089 | 0.971123333748355 | -0.210552737 | 0.218476522283831 | 1.00396975108948 | 0.810136329505516 | 1.24417980578392 | 0.998074519377826 |
| ebi-fl187-GCST90094781 | prot-a-1649 | Kin of IRRE-like protein 2 \|\| id:prot-a-1649 | Herring liking \|\| id:ebi-fl187-GCST90094781 | Inverse variance weighted | 19 | 0.0652073713084108 | 0.0857188461475858 | 0.446828991308851 | -0.102801567 | 0.233216309757679 | 1.06738034549439 | 0.902306002878244 | 1.26265457429464 | 0.998074519377826 |
| ebi-fl187-GCST90094781 | prot-a-165 | Armadillo repeat-containing protein 5 \|\| id:prot-a-165 | Herring liking \|\| id:ebi-fl187-GCST90094781 | Inverse variance weighted | 19 | 0.025367524495883 | 0.0857551544698651 | 0.767372582929649 | -0.142712578 | 0.193447627256819 | 1.02569201820254 | 0.867003228658994 | 1.21342583444772 | 0.998074519377826 |
| ebi-fl187-GCST90094781 | prot-a-1656 | Kallikrein-11 \|\| id:prot-a-1656 | Herring liking \|\| id:ebi-fl187-GCST90094781 | Inverse variance weighted | 19 | -0.07105511 | 0.0861347482694567 | 0.409411519046379 | -0.239879216 | 0.0977689969187715 | 0.931410560966498 | 0.786722878630275 | 1.10270802673279 | 0.998074519377826 |
| ebi-fl187-GCST90094781 | prot-a-1697 | Laminin subunit gamma-2 \|\| id:prot-a-1697 | Herring liking \|\| id:ebi-fl187-GCST90094781 | Inverse variance weighted | 19 | -0.070828236 | 0.0870182178142215 | 0.415675188029438 | -0.241383943 | 0.0997274704550871 | 0.931621897059939 | 0.785539965614823 | 1.10486976738639 | 0.998074519377826 |
| ebi-fl187-GCST90094781 | prot-a-1698 | Lysosome-associated membrane glycoprotein 3 \|\| id:prot-a-1698 | Herring liking \|\| id:ebi-fl187-GCST90094781 | Inverse variance weighted | 19 | -0.081280822 | 0.0925586630509539 | 0.379859790411132 | -0.262695801 | 0.100134157796677 | 0.921934755726824 | 0.768975783120548 | 1.10531919531702 | 0.998074519377826 |
| ebi-fl187-GCST90094781 | prot-a-1724 | Leptin receptor \|\| id:prot-a-1724 | Herring liking \|\| id:ebi-fl187-GCST90094781 | Inverse variance weighted | 19 | 0.165329648265389 | 0.0857400578525198 | 0.0538220342568685 | -0.002720865 | 0.333380161656327 | 1.17978196769536 | 0.997282833073105 | 1.39567778080574 | 0.998074519377826 |
| ebi-fl187-GCST90094781 | prot-a-1743 | Leukocyte immunoglobulin-like receptor subfamily B member 2 \|\| id:prot-a-1743 | Herring liking \|\| id:ebi-fl187-GCST90094781 | Inverse variance weighted | 19 | -0.045290948 | 0.103319564150168 | 0.661126856763625 | -0.247797293 | 0.157215398113454 | 0.955719377099115 | 0.780518143457442 | 1.17024765589261 | 0.998074519377826 |
| ebi-fl187-GCST90094781 | prot-a-1746 | Leukocyte immunoglobulin-like receptor subfamily B member 4 \|\| id:prot-a-1746 | Herring liking \|\| id:ebi-fl187-GCST90094781 | Inverse variance weighted | 19 | 0.0719267118641324 | 0.0901041842819527 | 0.42471868694385 | -0.104677489 | 0.24853091305676 | 1.07457658746294 | 0.900614933680439 | 1.28214045663741 | 0.998074519377826 |
| ebi-fl187-GCST90094781 | prot-a-1750 | Leucine-rich repeat and immunoglobulin-like domain-containing nogo receptor-interacting protein 3 \|\| id:prot-a-1750 | Herring liking \|\| id:ebi-fl187-GCST90094781 | Inverse variance weighted | 19 | 0.0296506411415779 | 0.0857341572343688 | 0.729459979929214 | -0.138388307 | 0.197689589320941 | 1.03009459841088 | 0.870760503657845 | 1.21858407359759 | 0.998074519377826 |
| ebi-fl187-GCST90094781 | prot-a-1753 | Lipase member N \|\| id:prot-a-1753 | Herring liking \|\| id:ebi-fl187-GCST90094781 | Inverse variance weighted | 19 | -0.004233642 | 0.0857548385978801 | 0.960625126282554 | -0.172313126 | 0.163845841601436 | 0.995775307178365 | 0.841715569121374 | 1.17803269746004 | 0.998074519377826 |
| ebi-fl187-GCST90094781 | prot-a-1760 | Ig Kappa chain V-I region HK102- like \|\| id:prot-a-1760 | Herring liking \|\| id:ebi-fl187-GCST90094781 | Inverse variance weighted | 19 | -0.088701062 | 0.0929568447812112 | 0.33997348148502 | -0.270896478 | 0.0934943536531522 | 0.915119096453692 | 0.762695448161063 | 1.09800440361009 | 0.998074519377826 |
| ebi-fl187-GCST90094781 | prot-a-1792 | Leucine-rich repeat neuronal protein 1 \|\| id:prot-a-1792 | Herring liking \|\| id:ebi-fl187-GCST90094781 | Inverse variance weighted | 19 | 0.0319633765449865 | 0.0943006898504493 | 0.734646137000571 | -0.152865976 | 0.216792728651867 | 1.03247969163915 | 0.858244739872625 | 1.24208662648545 | 0.998074519377826 |
| ebi-fl187-GCST90094781 | prot-a-1795 | Leucine-rich repeat transmembrane neuronal protein 1 \|\| id:prot-a-1795 | Herring liking \|\| id:ebi-fl187-GCST90094781 | Inverse variance weighted | 19 | -0.008077791 | 0.10340273571534 | 0.937732833766418 | -0.210747153 | 0.194591570921361 | 0.991954746603851 | 0.809978841644983 | 1.21481471949509 | 0.998074519377826 |
| ebi-fl187-GCST90094781 | prot-a-1820 | LysM and putative peptidoglycan-binding domain-containing protein 4 \|\| id:prot-a-1820 | Herring liking \|\| id:ebi-fl187-GCST90094781 | Inverse variance weighted | 19 | 0.084963536962582 | 0.0866918361383568 | 0.327054589126525 | -0.084952462 | 0.254879535793761 | 1.088677369 | 0.918555949797006 | 1.29030617580102 | 0.998074519377826 |
| ebi-fl187-GCST90094781 | prot-a-1844 | Mitogen-activated protein kinase kinase kinase 7:TGF-beta-activated kinase 1 and MAP3K7-binding protein 1 fusion \|\| id:prot-a-1844 | Herring liking \|\| id:ebi-fl187-GCST90094781 | Inverse variance weighted | 19 | 0.000480562958514419 | 0.0857548385978801 | 0.995528744914148 | -0.167598921 | 0.168560046610359 | 1.00048067844739 | 0.845692956617413 | 1.18359929583685 | 0.999355215041207 |
| ebi-fl187-GCST90094781 | prot-a-1856 | Megakaryocyte-associated tyrosine-protein kinase \|\| id:prot-a-1856 | Herring liking \|\| id:ebi-fl187-GCST90094781 | Inverse variance weighted | 19 | 0.0335539422083136 | 0.0857716431260815 | 0.695648758421353 | -0.134558478 | 0.201666362735433 | 1.03412322511221 | 0.874101761380483 | 1.22343975491771 | 0.998074519377826 |
| ebi-fl187-GCST90094781 | prot-a-1910 | Stromelysin-2 \|\| id:prot-a-1910 | Herring liking \|\| id:ebi-fl187-GCST90094781 | Inverse variance weighted | 19 | -0.102751158 | 0.0967041768931817 | 0.287994823215936 | -0.292291345 | 0.0867890287954727 | 0.902351488571628 | 0.746551000664096 | 1.09066655620731 | 0.998074519377826 |
| ebi-fl187-GCST90094781 | prot-a-1940 | 39S ribosomal protein L14, mitochondrial \|\| id:prot-a-1940 | Herring liking \|\| id:ebi-fl187-GCST90094781 | Inverse variance weighted (multiplicative random effects) | 19 | -0.022343183 | 0.11166760095748 | 0.841412908188048 | -0.241211681 | 0.196525315134659 | 0.977904577484246 | 0.785675296551725 | 1.21716613321275 | 0.998074519377826 |
| ebi-fl187-GCST90094781 | prot-a-1942 | 39S ribosomal protein L33, mitochondrial \|\| id:prot-a-1942 | Herring liking \|\| id:ebi-fl187-GCST90094781 | Inverse variance weighted | 19 | 0.0917956933194621 | 0.0993207046553127 | 0.355363862062399 | -0.102872888 | 0.286464274443875 | 1.0961408503036 | 0.902241652109701 | 1.3317105909429 | 0.998074519377826 |
| ebi-fl187-GCST90094781 | prot-a-1948 | Mesothelin \|\| id:prot-a-1948 | Herring liking \|\| id:ebi-fl187-GCST90094781 | Inverse variance weighted | 19 | 0.0134119612850862 | 0.0968315954718877 | 0.889838860476792 | -0.176377966 | 0.203201888409986 | 1.01350230508185 | 0.838301074280891 | 1.22531982114823 | 0.998074519377826 |
| ebi-fl187-GCST90094781 | prot-a-1980 | Unconventional myosin-VI \|\| id:prot-a-1980 | Herring liking \|\| id:ebi-fl187-GCST90094781 | Inverse variance weighted | 19 | -0.045259124 | 0.0952537836181588 | 0.634685388157153 | -0.231956539 | 0.14143829239118 | 0.95574979251167 | 0.79298058597619 | 1.1519294192576 | 0.998074519377826 |
| ebi-fl187-GCST90094781 | prot-a-2039 | Neurofascin \|\| id:prot-a-2039 | Herring liking \|\| id:ebi-fl187-GCST90094781 | Inverse variance weighted | 19 | 0.0928896975881422 | 0.0857341572343688 | 0.278603428987292 | -0.075149251 | 0.260928645767505 | 1.09734068926772 | 0.927605030397289 | 1.29813503469986 | 0.998074519377826 |
| ebi-fl187-GCST90094781 | prot-a-2041 | NFU1 iron-sulfur cluster scaffold homolog, mitochondrial \|\| id:prot-a-2041 | Herring liking \|\| id:ebi-fl187-GCST90094781 | Inverse variance weighted | 19 | 0.085773464340129 | 0.0857173747251311 | 0.316993941837568 | -0.08223259 | 0.253779518801386 | 1.08955947627086 | 0.921057704857513 | 1.28888759745546 | 0.998074519377826 |
| ebi-fl187-GCST90094781 | prot-a-2088 | Glucocorticoid receptor \|\| id:prot-a-2088 | Herring liking \|\| id:ebi-fl187-GCST90094781 | Inverse variance weighted | 19 | 0.132320663715036 | 0.0899110111086774 | 0.141106028168522 | -0.043904918 | 0.308546245488043 | 1.14147428997378 | 0.957044950839599 | 1.36144446875569 | 0.998074519377826 |
| ebi-fl187-GCST90094781 | prot-a-2111 | Neuron-specific protein family member 2 \|\| id:prot-a-2111 | Herring liking \|\| id:ebi-fl187-GCST90094781 | Inverse variance weighted | 19 | 0.00388837664462104 | 0.0857400578525198 | 0.963827729715118 | -0.164162137 | 0.17193889003556 | 1.00389594618899 | 0.848604420765716 | 1.18760525648136 | 0.998074519377826 |
| ebi-fl187-GCST90094781 | prot-a-2125 | Nuclear migration protein nudC \|\| id:prot-a-2125 | Herring liking \|\| id:ebi-fl187-GCST90094781 | Inverse variance weighted | 19 | -0.008744482 | 0.0908515797564224 | 0.92332187157148 | -0.186813578 | 0.169324614693666 | 0.99129364015123 | 0.829598377399648 | 1.18450458411503 | 0.998074519377826 |
| ebi-fl187-GCST90094781 | prot-a-2142 | Ornithine decarboxylase \|\| id:prot-a-2142 | Herring liking \|\| id:ebi-fl187-GCST90094781 | Inverse variance weighted | 19 | -0.045782419 | 0.101662161606627 | 0.652466146336586 | -0.245040255 | 0.153475418094215 | 0.955249784113862 | 0.782673030788831 | 1.16587912723899 | 0.998074519377826 |
| ebi-fl187-GCST90094781 | prot-a-2186 | Prostate and testis expressed protein 1 \|\| id:prot-a-2186 | Herring liking \|\| id:ebi-fl187-GCST90094781 | Inverse variance weighted | 19 | 0.0863594796750846 | 0.0857275168142468 | 0.313756155888366 | -0.081666453 | 0.254385412631008 | 1.09019816195383 | 0.92157929718865 | 1.28966876312567 | 0.998074519377826 |
| ebi-fl187-GCST90094781 | prot-a-2227 | Platelet-derived growth factor C \|\| id:prot-a-2227 | Herring liking \|\| id:ebi-fl187-GCST90094781 | Inverse variance weighted | 19 | 0.0390000123881713 | 0.0857716431260815 | 0.649328048352118 | -0.129112408 | 0.207112432915291 | 1.03977049653101 | 0.878875167270901 | 1.23012087008155 | 0.998074519377826 |
| ebi-fl187-GCST90094781 | prot-a-2229 | Platelet-derived growth factor receptor alpha \|\| id:prot-a-2229 | Herring liking \|\| id:ebi-fl187-GCST90094781 | Inverse variance weighted | 19 | -0.114491634 | 0.0884480064166825 | 0.195509592751423 | -0.287849726 | 0.0588664587923624 | 0.891819399540345 | 0.749874270107909 | 1.06063359299159 | 0.998074519377826 |
| ebi-fl187-GCST90094781 | prot-a-2243 | Platelet endothelial aggregation receptor 1 \|\| id:prot-a-2243 | Herring liking \|\| id:ebi-fl187-GCST90094781 | Inverse variance weighted | 19 | -0.024149147 | 0.0857416820636833 | 0.778211874801785 | -0.192202844 | 0.143904549601059 | 0.976140110293326 | 0.825139476849921 | 1.1547738796368 | 0.998074519377826 |
| ebi-fl187-GCST90094781 | prot-a-2246 | E3 ubiquitin-protein ligase pellino homolog 2 \|\| id:prot-a-2246 | Herring liking \|\| id:ebi-fl187-GCST90094781 | Inverse variance weighted (multiplicative random effects) | 19 | 0.0723586404015481 | 0.109515634725166 | 0.508794998110204 | -0.142292004 | 0.287009284462873 | 1.07504082800886 | 0.867367944889476 | 1.33243658437625 | 0.998074519377826 |
| ebi-fl187-GCST90094781 | prot-a-2251 | Platelet factor 4 variant \|\| id:prot-a-2251 | Herring liking \|\| id:ebi-fl187-GCST90094781 | Inverse variance weighted | 19 | 0.104962145032759 | 0.0979552263226984 | 0.283930356928109 | -0.087030099 | 0.296954388625248 | 1.11066856523775 | 0.916649505388052 | 1.34575391636202 | 0.998074519377826 |
| ebi-fl187-GCST90094781 | prot-a-2346 | Protein phosphatase 1L \|\| id:prot-a-2346 | Herring liking \|\| id:ebi-fl187-GCST90094781 | Inverse variance weighted | 19 | 0.146680641149055 | 0.0857716431260815 | 0.087241877105267 | -0.021431779 | 0.314793061676175 | 1.15798409150325 | 0.978796249280389 | 1.36997578112959 | 0.998074519377826 |
| ebi-fl187-GCST90094781 | prot-a-2358 | PR domain zinc finger protein 4 \|\| id:prot-a-2358 | Herring liking \|\| id:ebi-fl187-GCST90094781 | Inverse variance weighted | 19 | 0.0982406148466216 | 0.0857341572343688 | 0.251846937484523 | -0.069798333 | 0.266279563025984 | 1.10322820626073 | 0.932581871621425 | 1.30509986535889 | 0.998074519377826 |
| ebi-fl187-GCST90094781 | prot-a-2390 | Trypsin-1 \|\| id:prot-a-2390 | Herring liking \|\| id:ebi-fl187-GCST90094781 | Inverse variance weighted | 19 | -0.09623846 | 0.0857548385978801 | 0.261755703058613 | -0.264317943 | 0.071841024087498 | 0.908247409962135 | 0.767729406491856 | 1.07448451332921 | 0.998074519377826 |
| ebi-fl187-GCST90094781 | prot-a-242 | Brain-derived neurotrophic factor \|\| id:prot-a-242 | Herring liking \|\| id:ebi-fl187-GCST90094781 | Inverse variance weighted | 19 | -0.022822783 | 0.0857400578525198 | 0.790096142652225 | -0.190873297 | 0.145227730177264 | 0.977435686436804 | 0.826237268586695 | 1.15630286534326 | 0.998074519377826 |
| ebi-fl187-GCST90094781 | prot-a-2464 | Dihydropteridine reductase \|\| id:prot-a-2464 | Herring liking \|\| id:ebi-fl187-GCST90094781 | Inverse variance weighted | 19 | 0.0165289830076701 | 0.0868520477089774 | 0.84906468600319 | -0.153701031 | 0.186758996517266 | 1.01666634240745 | 0.857528357513781 | 1.20533675968557 | 0.998074519377826 |
| ebi-fl187-GCST90094781 | prot-a-2474 | Ras-related protein Rab-27A \|\| id:prot-a-2474 | Herring liking \|\| id:ebi-fl187-GCST90094781 | Inverse variance weighted (multiplicative random effects) | 19 | -0.112518052 | 0.113042688265996 | 0.319561713411074 | -0.334081721 | 0.109045616855938 | 0.893581215904866 | 0.715995267881942 | 1.11521322170202 | 0.998074519377826 |
| ebi-fl187-GCST90094781 | prot-a-2478 | Ras-related protein Rab-7b \|\| id:prot-a-2478 | Herring liking \|\| id:ebi-fl187-GCST90094781 | Inverse variance weighted | 19 | -0.072245866 | 0.0857716431260815 | 0.399617372470265 | -0.240358286 | 0.0958665549682125 | 0.930302138435359 | 0.786346073732307 | 1.10061218296618 | 0.998074519377826 |
| ebi-fl187-GCST90094781 | prot-a-2528 | E3 ubiquitin-protein ligase rififylin \|\| id:prot-a-2528 | Herring liking \|\| id:ebi-fl187-GCST90094781 | Inverse variance weighted | 19 | -0.086883835 | 0.0864821386829809 | 0.315067893745464 | -0.256388827 | 0.0826211567374752 | 0.916783587538754 | 0.773841020082779 | 1.08613025746622 | 0.998074519377826 |
| ebi-fl187-GCST90094781 | prot-a-2543 | Protein RIC-3 \|\| id:prot-a-2543 | Herring liking \|\| id:ebi-fl187-GCST90094781 | Inverse variance weighted | 19 | 0.0684240334068017 | 0.0962581858877288 | 0.477184262346611 | -0.120242011 | 0.25709007774675 | 1.07081927536881 | 0.886705818245696 | 1.29316160659686 | 0.998074519377826 |
| ebi-fl187-GCST90094781 | prot-a-255 | Bone morphogenetic protein 6 \|\| id:prot-a-255 | Herring liking \|\| id:ebi-fl187-GCST90094781 | Inverse variance weighted (multiplicative random effects) | 19 | -0.019232711 | 0.110495427490801 | 0.861819187438946 | -0.235803748 | 0.197338327330293 | 0.980951058017556 | 0.789935684837207 | 1.21815610649881 | 0.998074519377826 |
| ebi-fl187-GCST90094781 | prot-a-2568 | RING finger protein 165 \|\| id:prot-a-2568 | Herring liking \|\| id:ebi-fl187-GCST90094781 | Inverse variance weighted (multiplicative random effects) | 19 | -0.041993769 | 0.112834201418697 | 0.709764538631369 | -0.263148804 | 0.179161265988064 | 0.958875755507589 | 0.768627514280462 | 1.19621363718806 | 0.998074519377826 |
| ebi-fl187-GCST90094781 | prot-a-2573 | E3 ubiquitin-protein ligase RNF8 \|\| id:prot-a-2573 | Herring liking \|\| id:ebi-fl187-GCST90094781 | Inverse variance weighted | 19 | 0.00252273233041655 | 0.0927894762995938 | 0.978310027042363 | -0.179344641 | 0.18439010587762 | 1.00252591709716 | 0.835817792492113 | 1.20248482800872 | 0.998074519377826 |
| ebi-fl187-GCST90094781 | prot-a-260 | Cytoplasmic tyrosine-protein kinase BMX \|\| id:prot-a-260 | Herring liking \|\| id:ebi-fl187-GCST90094781 | Inverse variance weighted | 19 | -0.054936076 | 0.0857188461475858 | 0.521596237358565 | -0.222945015 | 0.113072862069264 | 0.946545652644232 | 0.800158844955688 | 1.1197135146201 | 0.998074519377826 |
| ebi-fl187-GCST90094781 | prot-a-2623 | Serum amyloid A-1 protein \|\| id:prot-a-2623 | Herring liking \|\| id:ebi-fl187-GCST90094781 | Inverse variance weighted | 19 | -0.00010956 | 0.0857341572343688 | 0.998980378103348 | -0.168148508 | 0.167929387909502 | 0.999890445731646 | 0.845228301819201 | 1.18285308396984 | 0.999355215041207 |
| ebi-fl187-GCST90094781 | prot-a-2640 | Lysosome membrane protein 2 \|\| id:prot-a-2640 | Herring liking \|\| id:ebi-fl187-GCST90094781 | Inverse variance weighted | 19 | 0.0427939063394682 | 0.0857400578525198 | 0.617700364392766 | -0.125256607 | 0.210844419730407 | 1.04372276803739 | 0.882270476708972 | 1.23472024200916 | 0.998074519377826 |
| ebi-fl187-GCST90094781 | prot-a-2641 | Scavenger receptor class F member 2 \|\| id:prot-a-2641 | Herring liking \|\| id:ebi-fl187-GCST90094781 | Inverse variance weighted | 19 | 0.0295259623704037 | 0.0857341572343688 | 0.73055321913428 | -0.138512986 | 0.197564910549766 | 1.02996617548812 | 0.870651945075878 | 1.21843215150364 | 0.998074519377826 |
| ebi-fl187-GCST90094781 | prot-a-2669 | Semaphorin-3A \|\| id:prot-a-2669 | Herring liking \|\| id:ebi-fl187-GCST90094781 | Inverse variance weighted | 19 | 0.115705547538642 | 0.0908542731021105 | 0.202830404340537 | -0.062368828 | 0.293779922818778 | 1.12266525191297 | 0.939536295828794 | 1.34148864013922 | 0.998074519377826 |
| ebi-fl187-GCST90094781 | prot-a-2683 | Sentrin-specific protease 7 \|\| id:prot-a-2683 | Herring liking \|\| id:ebi-fl187-GCST90094781 | Inverse variance weighted | 19 | -0.012220721 | 0.0937687208356792 | 0.896306667488568 | -0.196007414 | 0.171565972089246 | 0.987853649000208 | 0.822006140641875 | 1.18716245973664 | 0.998074519377826 |
| ebi-fl187-GCST90094781 | prot-a-270 | Bromodomain testis-specific protein \|\| id:prot-a-270 | Herring liking \|\| id:ebi-fl187-GCST90094781 | Inverse variance weighted | 19 | 0.0370058125060378 | 0.0857607434875144 | 0.666104414451989 | -0.131085245 | 0.205096869741566 | 1.03769905245264 | 0.877142999386294 | 1.22764398075856 | 0.998074519377826 |
| ebi-fl187-GCST90094781 | prot-a-2708 | 14-3-3 protein sigma \|\| id:prot-a-2708 | Herring liking \|\| id:ebi-fl187-GCST90094781 | Inverse variance weighted | 19 | 0.118821430179539 | 0.0862372643442707 | 0.168251698028853 | -0.050203608 | 0.28784646829431 | 1.12616880057158 | 0.951035766357497 | 1.33355254580835 | 0.998074519377826 |
| ebi-fl187-GCST90094781 | prot-a-2713 | Beta-sarcoglycan \|\| id:prot-a-2713 | Herring liking \|\| id:ebi-fl187-GCST90094781 | Inverse variance weighted (multiplicative random effects) | 19 | 0.00875883009511352 | 0.118789337042032 | 0.941221929661311 | -0.224068271 | 0.241585930697497 | 1.00879730088511 | 0.79926056658238 | 1.27326686292634 | 0.998074519377826 |
| ebi-fl187-GCST90094781 | prot-a-2720 | Protein shisa-3 homolog \|\| id:prot-a-2720 | Herring liking \|\| id:ebi-fl187-GCST90094781 | Inverse variance weighted | 19 | 0.05596713610244 | 0.0983225448630812 | 0.56920700752145 | -0.136745052 | 0.248679324034079 | 1.05756292752579 | 0.872192561687072 | 1.28233075447643 | 0.998074519377826 |
| ebi-fl187-GCST90094781 | prot-a-2730 | Sialic acid-binding Ig-like lectin 8 \|\| id:prot-a-2730 | Herring liking \|\| id:ebi-fl187-GCST90094781 | Inverse variance weighted | 19 | -0.144894838 | 0.0857173747251311 | 0.0909554967956539 | -0.312900893 | 0.0231112162999449 | 0.865113265228905 | 0.73132238842135 | 1.02338034979426 | 0.998074519377826 |
| ebi-fl187-GCST90094781 | prot-a-2761 | Sodium- and chloride-dependent neutral and basic amino acid transporter B(0+) \|\| id:prot-a-2761 | Herring liking \|\| id:ebi-fl187-GCST90094781 | Inverse variance weighted | 19 | -0.120947669 | 0.0926327195887108 | 0.191665149096325 | -0.302507799 | 0.0606124613068871 | 0.886080327772176 | 0.738962724710841 | 1.06248707953717 | 0.998074519377826 |
| ebi-fl187-GCST90094781 | prot-a-2770 | Secreted Ly-6/uPAR-related protein 1 \|\| id:prot-a-2770 | Herring liking \|\| id:ebi-fl187-GCST90094781 | Inverse variance weighted | 19 | 0.134068501556594 | 0.0901111991883143 | 0.136800544324592 | -0.042549449 | 0.31068645196569 | 1.14347114651532 | 0.958343075383882 | 1.36436136128943 | 0.998074519377826 |
| ebi-fl187-GCST90094781 | prot-a-2784 | N-lysine methyltransferase SMYD2 \|\| id:prot-a-2784 | Herring liking \|\| id:ebi-fl187-GCST90094781 | Inverse variance weighted | 19 | -0.049126255 | 0.0857716431260815 | 0.566809665351361 | -0.217238675 | 0.118986165714743 | 0.952060919837323 | 0.80473787529627 | 1.12635433587338 | 0.998074519377826 |
| ebi-fl187-GCST90094781 | prot-a-2837 | Serine palmitoyltransferase 2 \|\| id:prot-a-2837 | Herring liking \|\| id:ebi-fl187-GCST90094781 | Inverse variance weighted | 19 | 0.0939323410607309 | 0.0857607434875144 | 0.273392383557216 | -0.074158716 | 0.262023398296259 | 1.09848542104444 | 0.928524310318782 | 1.29955694949215 | 0.998074519377826 |
| ebi-fl187-GCST90094781 | prot-a-2858 | Alpha-2,8-sialyltransferase 8B \|\| id:prot-a-2858 | Herring liking \|\| id:ebi-fl187-GCST90094781 | Inverse variance weighted | 19 | 0.00175078005251649 | 0.0882490935089123 | 0.984171749671553 | -0.171217443 | 0.174719003329985 | 1.00175231356273 | 0.842638327554139 | 1.1909115274178 | 0.999355215041207 |
| ebi-fl187-GCST90094781 | prot-a-2911 | Synaptotagmin-11 \|\| id:prot-a-2911 | Herring liking \|\| id:ebi-fl187-GCST90094781 | Inverse variance weighted | 19 | -0.015554154 | 0.0857607434875145 | 0.856079655004639 | -0.183645211 | 0.152536903279859 | 0.98456618715436 | 0.832231017705705 | 1.16478544570477 | 0.998074519377826 |
| ebi-fl187-GCST90094781 | prot-a-2962 | Transforming growth factor beta-1 \|\| id:prot-a-2962 | Herring liking \|\| id:ebi-fl187-GCST90094781 | Inverse variance weighted | 19 | -0.067890364 | 0.0857173747251311 | 0.428345689307892 | -0.235896418 | 0.100115690476499 | 0.934362907787872 | 0.789862484879296 | 1.10529878322202 | 0.998074519377826 |
| ebi-fl187-GCST90094781 | prot-a-2978 | Tyrosine-protein kinase receptor Tie-1, soluble \|\| id:prot-a-2978 | Herring liking \|\| id:ebi-fl187-GCST90094781 | Inverse variance weighted | 19 | 0.0554645517696936 | 0.093978695407574 | 0.55506851464802 | -0.128733691 | 0.239662794768539 | 1.05703154651059 | 0.879208075193298 | 1.2708205507245 | 0.998074519377826 |
| ebi-fl187-GCST90094781 | prot-a-299 | Uncharacterized protein C1orf226 \|\| id:prot-a-299 | Herring liking \|\| id:ebi-fl187-GCST90094781 | Inverse variance weighted | 19 | -0.036219064 | 0.0857551544698651 | 0.672766379728132 | -0.204299167 | 0.131861038675533 | 0.964428998583896 | 0.815218448370027 | 1.14094976036086 | 0.998074519377826 |
| ebi-fl187-GCST90094781 | prot-a-3015 | Transmembrane protein 70, mitochondrial \|\| id:prot-a-3015 | Herring liking \|\| id:ebi-fl187-GCST90094781 | Inverse variance weighted | 19 | -0.190753091 | 0.0857716431260815 | 0.0261508313054129 | -0.358865512 | -0.022640671 | 0.826336592629496 | 0.698468280733385 | 0.977613705810031 | 0.998074519377826 |
| ebi-fl187-GCST90094781 | prot-a-3016 | Transmembrane protein 87B \|\| id:prot-a-3016 | Herring liking \|\| id:ebi-fl187-GCST90094781 | Inverse variance weighted | 19 | -0.204997028 | 0.101972088518909 | 0.0443968137144891 | -0.404862322 | -0.005131735 | 0.814649737440434 | 0.667068645461847 | 0.99488141022171 | 0.998074519377826 |
| ebi-fl187-GCST90094781 | prot-a-3123 | Thioredoxin domain-containing protein 12 \|\| id:prot-a-3123 | Herring liking \|\| id:ebi-fl187-GCST90094781 | Inverse variance weighted | 19 | -0.032199003 | 0.0922231423277407 | 0.726982457713112 | -0.212956362 | 0.148558356016316 | 0.9683138655923 | 0.8081914043775 | 1.16016049814401 | 0.998074519377826 |
| ebi-fl187-GCST90094781 | prot-a-3133 | PolyUbiquitin K48-linked \|\| id:prot-a-3133 | Herring liking \|\| id:ebi-fl187-GCST90094781 | Inverse variance weighted | 19 | -0.044303657 | 0.102759479609472 | 0.666367040043739 | -0.245712237 | 0.157104923357163 | 0.956663416128535 | 0.782147265813607 | 1.17011838 | 0.998074519377826 |
| ebi-fl187-GCST90094781 | prot-a-3135 | Ubiquitin-conjugating enzyme E2 B \|\| id:prot-a-3135 | Herring liking \|\| id:ebi-fl187-GCST90094781 | Inverse variance weighted | 19 | 0.0225610179760404 | 0.0865294736662908 | 0.79429905101911 | -0.14703675 | 0.19215878636197 | 1.02281744251061 | 0.863262251591766 | 1.21186292899399 | 0.998074519377826 |
| ebi-fl187-GCST90094781 | prot-a-3162 | NKG2D ligand 3 \|\| id:prot-a-3162 | Herring liking \|\| id:ebi-fl187-GCST90094781 | Inverse variance weighted | 19 | 0.12914928940689 | 0.0857341572343688 | 0.13196645100033 | -0.038889659 | 0.297188237586253 | 1.13785998192826 | 0.961856835755152 | 1.3460686563165 | 0.998074519377826 |
| ebi-fl187-GCST90094781 | prot-a-3188 | Vesicle-associated membrane protein 8 \|\| id:prot-a-3188 | Herring liking \|\| id:ebi-fl187-GCST90094781 | Inverse variance weighted | 19 | 0.103876590352408 | 0.0986002942611139 | 0.292106410683478 | -0.089379986 | 0.297133167104191 | 1.1094635279637 | 0.914498010737723 | 1.34599452970782 | 0.998074519377826 |
| ebi-fl187-GCST90094781 | prot-a-3201 | Vitrin \|\| id:prot-a-3201 | Herring liking \|\| id:ebi-fl187-GCST90094781 | Inverse variance weighted | 19 | -0.051788083 | 0.0857176901833088 | 0.54573041776039 | -0.219794756 | 0.116218589275557 | 0.949530066627173 | 0.802683526839833 | 1.12324137381844 | 0.998074519377826 |
| ebi-fl187-GCST90094781 | prot-a-3232 | WNT1-inducible-signaling pathway protein 3 \|\| id:prot-a-3232 | Herring liking \|\| id:ebi-fl187-GCST90094781 | Inverse variance weighted | 19 | -0.00451463 | 0.102723249785986 | 0.964944700881213 | -0.2058522 | 0.196822939627359 | 0.995495545669817 | 0.813953370080601 | 1.21752844557952 | 0.998074519377826 |
| ebi-fl187-GCST90094781 | prot-a-327 | Carbonic anhydrase-related protein 10 \|\| id:prot-a-327 | Herring liking \|\| id:ebi-fl187-GCST90094781 | Inverse variance weighted | 19 | -0.21108138 | 0.0857607434875144 | 0.0138441478805885 | -0.379172437 | -0.042990322 | 0.809708170496463 | 0.684427581983625 | 0.957920660456982 | 0.998074519377826 |
| ebi-fl187-GCST90094781 | prot-a-332 | Carbonic anhydrase 5A, mitochondrial \|\| id:prot-a-332 | Herring liking \|\| id:ebi-fl187-GCST90094781 | Inverse variance weighted | 19 | -0.064635127 | 0.107603092174787 | 0.548052511728811 | -0.275537188 | 0.146266933368801 | 0.937409436103139 | 0.759164199803967 | 1.1575051235584 | 0.998074519377826 |
| ebi-fl187-GCST90094781 | prot-a-338 | Cell adhesion molecule 3 \|\| id:prot-a-338 | Herring liking \|\| id:ebi-fl187-GCST90094781 | Inverse variance weighted | 19 | 0.0108934031003057 | 0.085777551488004 | 0.898943590552577 | -0.157230598 | 0.179017404016794 | 1.01095295225039 | 0.854506988651088 | 1.19604155991414 | 0.998074519377826 |
| ebi-fl187-GCST90094781 | prot-a-34 | A disintegrin and metalloproteinase with thrombospondin motifs 3 \|\| id:prot-a-34 | Herring liking \|\| id:ebi-fl187-GCST90094781 | Inverse variance weighted | 19 | -0.024034312 | 0.102240771122846 | 0.814150047291542 | -0.224426223 | 0.176357599575285 | 0.97625221218769 | 0.798974520287961 | 1.19286455024606 | 0.998074519377826 |
| ebi-fl187-GCST90094781 | prot-a-358 | Cysteine--tRNA ligase, cytoplasmic \|\| id:prot-a-358 | Herring liking \|\| id:ebi-fl187-GCST90094781 | Inverse variance weighted | 19 | 0.00464138503656668 | 0.0857716431260815 | 0.95684491094883 | -0.163471035 | 0.172753805563686 | 1.00465217294792 | 0.849191095048871 | 1.18857344889008 | 0.998074519377826 |
| ebi-fl187-GCST90094781 | prot-a-382 | Coiled-coil domain-containing protein 167 \|\| id:prot-a-382 | Herring liking \|\| id:ebi-fl187-GCST90094781 | Inverse variance weighted | 19 | -0.079958697 | 0.0956955646470342 | 0.403406529632729 | -0.267522004 | 0.107604609406494 | 0.923154474369981 | 0.765273491352096 | 1.11360734845736 | 0.998074519377826 |
| ebi-fl187-GCST90094781 | prot-a-40 | Pituitary adenylate cyclase-activating polypeptide 27 \|\| id:prot-a-40 | Herring liking \|\| id:ebi-fl187-GCST90094781 | Inverse variance weighted | 19 | 0.0145561144261293 | 0.0857173747251311 | 0.865155460809988 | -0.15344994 | 0.182562168887386 | 1.01466257056179 | 0.857743701743672 | 1.20028876925141 | 0.998074519377826 |
| ebi-fl187-GCST90094781 | prot-a-403 | C-C motif chemokine 25 \|\| id:prot-a-403 | Herring liking \|\| id:ebi-fl187-GCST90094781 | Inverse variance weighted | 19 | 0.0223612138524328 | 0.0967135726455496 | 0.817151109597321 | -0.167197389 | 0.21191981623771 | 1.02261309978284 | 0.846032597721242 | 1.23604877006409 | 0.998074519377826 |
| ebi-fl187-GCST90094781 | prot-a-415 | Cyclin-C \|\| id:prot-a-415 | Herring liking \|\| id:ebi-fl187-GCST90094781 | Inverse variance weighted | 19 | 0.0725249935970529 | 0.0857716431260815 | 0.397798748601106 | -0.095587427 | 0.240637414124173 | 1.07521967936173 | 0.908838901185173 | 1.27205972079225 | 0.998074519377826 |
| ebi-fl187-GCST90094781 | prot-a-44 | Adhesion G protein-coupled receptor F5 \|\| id:prot-a-44 | Herring liking \|\| id:ebi-fl187-GCST90094781 | Inverse variance weighted | 19 | 0.0942864651289541 | 0.0944432274563837 | 0.318114448510188 | -0.090822261 | 0.279395190943466 | 1.09887449005591 | 0.913180004463821 | 1.32232981339166 | 0.998074519377826 |
| ebi-fl187-GCST90094781 | prot-a-455 | CD59 glycoprotein \|\| id:prot-a-455 | Herring liking \|\| id:ebi-fl187-GCST90094781 | Inverse variance weighted | 19 | 0.0430804481738975 | 0.0857341572343688 | 0.615323774853672 | -0.1249585 | 0.21111939635326 | 1.04402188112614 | 0.88253352696118 | 1.23505980789568 | 0.998074519377826 |
| ebi-fl187-GCST90094781 | prot-a-478 | Cadherin-12 \|\| id:prot-a-478 | Herring liking \|\| id:ebi-fl187-GCST90094781 | Inverse variance weighted | 19 | 0.0154719666321985 | 0.0857548385978801 | 0.856822134238246 | -0.152607517 | 0.183551450284043 | 1.0155922771874 | 0.858466589225179 | 1.2014767801431 | 0.998074519377826 |
| ebi-fl187-GCST90094781 | prot-a-516 | Centrin-2 \|\| id:prot-a-516 | Herring liking \|\| id:ebi-fl187-GCST90094781 | Inverse variance weighted | 19 | -0.018438466 | 0.0857341572343688 | 0.829716190215245 | -0.186477414 | 0.149600482045117 | 0.98173048240492 | 0.829877305088238 | 1.16137016179822 | 0.998074519377826 |
| ebi-fl187-GCST90094781 | prot-a-519 | Cryptic protein \|\| id:prot-a-519 | Herring liking \|\| id:ebi-fl187-GCST90094781 | Inverse variance weighted | 19 | 0.0236799004070007 | 0.101379995424307 | 0.815314417439594 | -0.175024891 | 0.222384691438642 | 1.02396249544747 | 0.83943612641964 | 1.24905178498221 | 0.998074519377826 |
| ebi-fl187-GCST90094781 | prot-a-583 | Chloride intracellular channel protein 4 \|\| id:prot-a-583 | Herring liking \|\| id:ebi-fl187-GCST90094781 | Inverse variance weighted | 19 | -0.004960009 | 0.0902207017602375 | 0.956157269294125 | -0.181792584 | 0.171872566500177 | 0.995052271582264 | 0.833774260270475 | 1.18752649291407 | 0.998074519377826 |
| ebi-fl187-GCST90094781 | prot-a-602 | CCR4-NOT transcription complex subunit 1 \|\| id:prot-a-602 | Herring liking \|\| id:ebi-fl187-GCST90094781 | Inverse variance weighted | 19 | 0.134733743537918 | 0.0857716431260815 | 0.116219234106524 | -0.033378677 | 0.302846164065038 | 1.14423208460233 | 0.967172244362301 | 1.3537061997645 | 0.998074519377826 |
| ebi-fl187-GCST90094781 | prot-a-606 | Ciliary neurotrophic factor receptor subunit alpha \|\| id:prot-a-606 | Herring liking \|\| id:ebi-fl187-GCST90094781 | Inverse variance weighted | 19 | -0.115612362 | 0.0862649289603764 | 0.180179938464382 | -0.284691623 | 0.0534668986215785 | 0.890820472118172 | 0.752246194052572 | 1.05492207181494 | 0.998074519377826 |
| ebi-fl187-GCST90094781 | prot-a-626 | Collagen alpha-2(IX) chain \|\| id:prot-a-626 | Herring liking \|\| id:ebi-fl187-GCST90094781 | Inverse variance weighted | 19 | 0.0420727800489122 | 0.085777551488004 | 0.623789275973006 | -0.126051221 | 0.2101967809654 | 1.04297038342408 | 0.881569690862569 | 1.23392084820364 | 0.998074519377826 |
| ebi-fl187-GCST90094781 | prot-a-647 | Probable serine carboxypeptidase CPVL \|\| id:prot-a-647 | Herring liking \|\| id:ebi-fl187-GCST90094781 | Inverse variance weighted | 19 | -0.149186733 | 0.105866324043549 | 0.158776290913188 | -0.356684728 | 0.0583112619418501 | 0.861408246375536 | 0.699993150974415 | 1.0600448959977 | 0.998074519377826 |
| ebi-fl187-GCST90094781 | prot-a-651 | Cellular retinoic acid-binding protein 2 \|\| id:prot-a-651 | Herring liking \|\| id:ebi-fl187-GCST90094781 | Inverse variance weighted | 19 | -0.045935379 | 0.0857173747251311 | 0.592032199356619 | -0.213941433 | 0.12207067576078 | 0.955103680237539 | 0.807395670249631 | 1.12983395083262 | 0.998074519377826 |
| ebi-fl187-GCST90094781 | prot-a-671 | Cytotoxic and regulatory T-cell molecule \|\| id:prot-a-671 | Herring liking \|\| id:ebi-fl187-GCST90094781 | Inverse variance weighted | 19 | -0.052932313 | 0.0857341572343688 | 0.536970578047268 | -0.220971262 | 0.115106634781879 | 0.948444207275777 | 0.801739720694474 | 1.12199307467987 | 0.998074519377826 |
| ebi-fl187-GCST90094781 | prot-a-688 | Chorionic somatomammotropin hormone \|\| id:prot-a-688 | Herring liking \|\| id:ebi-fl187-GCST90094781 | Inverse variance weighted | 19 | 0.0719077101047858 | 0.0967770765561608 | 0.45746702778429 | -0.11777536 | 0.261590780154861 | 1.07455616881123 | 0.88889571176938 | 1.29899485917402 | 0.998074519377826 |
| ebi-fl187-GCST90094781 | prot-a-69 | Aldo-keto reductase family 1 member C1 \|\| id:prot-a-69 | Herring liking \|\| id:ebi-fl187-GCST90094781 | Inverse variance weighted | 19 | -0.100576893 | 0.0857716431260815 | 0.240951271569129 | -0.268689313 | 0.0675355275949115 | 0.904315574263162 | 0.764380701556588 | 1.06986826876917 | 0.998074519377826 |
| ebi-fl187-GCST90094781 | prot-a-741 | Stromal cell-derived factor 1 \|\| id:prot-a-741 | Herring liking \|\| id:ebi-fl187-GCST90094781 | Inverse variance weighted | 19 | -0.050159416 | 0.0867918485782899 | 0.563312913783781 | -0.220271439 | 0.119952607320175 | 0.951077795498657 | 0.802300992538625 | 1.12744341774828 | 0.998074519377826 |
| ebi-fl187-GCST90094781 | prot-a-742 | Growth-regulated alpha protein \|\| id:prot-a-742 | Herring liking \|\| id:ebi-fl187-GCST90094781 | Inverse variance weighted | 19 | -0.057617154 | 0.085777551488004 | 0.501771935452494 | -0.225741155 | 0.110506846978133 | 0.944011289267482 | 0.79792461384968 | 1.11684399603236 | 0.998074519377826 |
| ebi-fl187-GCST90094781 | prot-a-788 | Beta-defensin 112 \|\| id:prot-a-788 | Herring liking \|\| id:ebi-fl187-GCST90094781 | Inverse variance weighted | 19 | -0.184192252 | 0.0857341572343687 | 0.0316810773809296 | -0.3522312 | -0.016153304 | 0.831775877714047 | 0.703117542142316 | 0.983976460947036 | 0.998074519377826 |
| ebi-fl187-GCST90094781 | prot-a-838 | DnaJ homolog subfamily B member 1 \|\| id:prot-a-838 | Herring liking \|\| id:ebi-fl187-GCST90094781 | Inverse variance weighted | 19 | -0.015766965 | 0.0857400578525198 | 0.854097684240419 | -0.183817478 | 0.152283548395771 | 0.984356682893816 | 0.832087663950697 | 1.16449037900306 | 0.998074519377826 |
| ebi-fl187-GCST90094781 | prot-a-85 | Junctional adhesion molecule-like \|\| id:prot-a-85 | Herring liking \|\| id:ebi-fl187-GCST90094781 | Inverse variance weighted | 19 | 0.0569363208179407 | 0.0857716431260815 | 0.5068100857304 | -0.1111761 | 0.22504874134506 | 1.05858839820581 | 0.894781164351318 | 1.25238375757311 | 0.998074519377826 |
| ebi-fl187-GCST90094781 | prot-a-856 | Dipeptidase 1 \|\| id:prot-a-856 | Herring liking \|\| id:ebi-fl187-GCST90094781 | Inverse variance weighted | 19 | 0.0726011969233131 | 0.098863000442829 | 0.462728348457934 | -0.121170284 | 0.266372677791258 | 1.07530161779971 | 0.885883095080425 | 1.30522139508453 | 0.998074519377826 |
| ebi-fl187-GCST90094781 | prot-a-860 | Dipeptidyl peptidase 2 \|\| id:prot-a-860 | Herring liking \|\| id:ebi-fl187-GCST90094781 | Inverse variance weighted | 19 | 0.0320185174367824 | 0.085777551488004 | 0.708944632172919 | -0.136105483 | 0.20014251835327 | 1.03253662505977 | 0.872750566866396 | 1.22157684287478 | 0.998074519377826 |
| ebi-fl187-GCST90094781 | prot-a-861 | Dermatopontin \|\| id:prot-a-861 | Herring liking \|\| id:ebi-fl187-GCST90094781 | Inverse variance weighted | 19 | -0.11770699 | 0.0906282130095082 | 0.194016107901344 | -0.295338288 | 0.0599243072560606 | 0.888956487382519 | 0.744279764153696 | 1.0617561762653 | 0.998074519377826 |
| ebi-fl187-GCST90094781 | prot-a-877 | Dual specificity protein phosphatase 6 \|\| id:prot-a-877 | Herring liking \|\| id:ebi-fl187-GCST90094781 | Inverse variance weighted | 19 | -0.022483102 | 0.0873644906891949 | 0.796909922814507 | -0.193717503 | 0.148751300082232 | 0.97776775969738 | 0.823890617678931 | 1.16038436582391 | 0.998074519377826 |
| ebi-fl187-GCST90094781 | prot-a-879 | Dynactin-associated protein \|\| id:prot-a-879 | Herring liking \|\| id:ebi-fl187-GCST90094781 | Inverse variance weighted | 19 | 0.0693001327306002 | 0.0857173747251311 | 0.41881868111116 | -0.098705922 | 0.237306187191857 | 1.07175783048559 | 0.906009106440667 | 1.26782925142972 | 0.998074519377826 |
| ebi-fl187-GCST90094781 | prot-a-937 | EMI domain-containing protein 1 \|\| id:prot-a-937 | Herring liking \|\| id:ebi-fl187-GCST90094781 | Inverse variance weighted | 19 | -0.072844872 | 0.100984034321566 | 0.470693965174277 | -0.270773579 | 0.125083835356702 | 0.929745048409573 | 0.762789188204373 | 1.13324345495379 | 0.998074519377826 |
| ebi-fl187-GCST90094781 | prot-b-1 | SRC proto-oncogene, non-receptor tyrosine kinase \|\| id:prot-b-1 | Herring liking \|\| id:ebi-fl187-GCST90094781 | Inverse variance weighted | 13 | -0.034355176 | 0.145278298437581 | 0.813061484718593 | -0.319100641 | 0.250390288811766 | 0.966228262474411 | 0.726802399458989 | 1.28452665524944 | 0.998074519377826 |
| ebi-fl187-GCST90094781 | prot-b-10 | coagulation factor III, tissue factor \|\| id:prot-b-10 | Herring liking \|\| id:ebi-fl187-GCST90094781 | Inverse variance weighted (multiplicative random effects) | 13 | 0.0112057930381162 | 0.17285270153317 | 0.948310495668771 | -0.327585502 | 0.349997088043129 | 1.01126881311358 | 0.720661670650929 | 1.41906341633578 | 0.998074519377826 |
| ebi-fl187-GCST90094781 | prot-b-29 | myeloperoxidase \|\| id:prot-b-29 | Herring liking \|\| id:ebi-fl187-GCST90094781 | Inverse variance weighted | 13 | -0.131200721 | 0.140587964594659 | 0.350701909430159 | -0.406753131 | 0.144351689895307 | 0.877041716286192 | 0.665808537292184 | 1.15529034102588 | 0.998074519377826 |
| ebi-fl187-GCST90094781 | prot-b-33 | adrenomedullin \|\| id:prot-b-33 | Herring liking \|\| id:ebi-fl187-GCST90094781 | Inverse variance weighted | 13 | 0.0184204702782138 | 0.12122078080818 | 0.879220043353638 | -0.21917226 | 0.256013200662247 | 1.01859117367555 | 0.803183349787467 | 1.29176978004372 | 0.998074519377826 |
| ebi-fl187-GCST90094781 | ukb-rsfMRI191-edge-pheno102 | Net25_Pair11_15 (Postcentral\|Precentral)&(Cerebellum) (Motor)&(Subcortical-cerebellum) \|\| id:ukb-rsfMRI191-edge-pheno102 | Herring liking \|\| id:ebi-fl187-GCST90094781 | Inverse variance weighted | 18 | 0.00107180563422658 | 0.0272352195248896 | 0.968608423324526 | -0.052309225 | 0.0544528359030101 | 1.00107238022315 | 0.949035356353641 | 1.05596267171337 | 0.998074519377826 |
| ebi-fl187-GCST90094781 | ukb-rsfMRI191-edge-pheno1167 | Net100_Pair11_45 (Angular\|Temporal)&(Frontal) (Default_mode\|Central_executive)&(Salience\|Default_mode) \|\| id:ukb-rsfMRI191-edge-pheno1167 | Herring liking \|\| id:ebi-fl187-GCST90094781 | Inverse variance weighted (multiplicative random effects) | 18 | -0.014144445 | 0.0372094628025031 | 0.703848674206427 | -0.087074992 | 0.0587861018003306 | 0.985955117401265 | 0.916608354408601 | 1.06054836709071 | 0.998074519377826 |
| ebi-fl187-GCST90094781 | ukb-rsfMRI191-edge-pheno1317 | Net100_Pair26_48 (Parietal\|Frontal)&(Temporal) (Attention\|Central_executive)&(Default_mode\|Central_executive) \|\| id:ukb-rsfMRI191-edge-pheno1317 | Herring liking \|\| id:ebi-fl187-GCST90094781 | Inverse variance weighted | 18 | -0.017702644 | 0.034299421851998 | 0.605770066544697 | -0.084929511 | 0.0495242230179105 | 0.982453127444868 | 0.918577032024754 | 1.05077104475239 | 0.998074519377826 |
| ebi-fl187-GCST90094781 | ukb-rsfMRI191-edge-pheno286 | Net100_Pair10_13 (OccipitalPrecuneus)&(Frontal_Sup) (Default_mode\|Central_executive)&(Default_mode) \|\| id:ukb-rsfMRI191-edge-pheno286 | Herring liking \|\| id:ebi-fl187-GCST90094781 | Inverse variance weighted | 18 | 0.0316052609159358 | 0.0268846675754949 | 0.239760024057638 | -0.021088688 | 0.0842992093639058 | 1.0321100107231 | 0.97913212390707 | 1.08795437124883 | 0.998074519377826 |
| ebi-fl187-GCST90094781 | ukb-rsfMRI191-edge-pheno599 | Net100_Pair11_29 (Angular\|Temporal)&(Parietal) (Default_mode\|Central_executive)&(Central_executive\|Salience) \|\| id:ukb-rsfMRI191-edge-pheno599 | Herring liking \|\| id:ebi-fl187-GCST90094781 | Inverse variance weighted | 18 | -0.051929401 | 0.0319493724327811 | 0.104084743919266 | -0.114550171 | 0.0106913684863795 | 0.94939589041996 | 0.891767196042343 | 1.01074872539213 | 0.998074519377826 |
| ebi-fl187-GCST90094781 | ukb-rsfMRI191-edge-pheno621 | Net100_Pair5_30 (Precuneus\|Angular\|Cingulate)&(Paracentral) (Default_mode\|Central_executive)&(Motor) \|\| id:ukb-rsfMRI191-edge-pheno621 | Herring liking \|\| id:ebi-fl187-GCST90094781 | Inverse variance weighted | 18 | -0.01816285 | 0.027493468342625 | 0.508853353103581 | -0.072050048 | 0.0357243477485475 | 0.982001100257646 | 0.930484325622511 | 1.03637012935395 | 0.998074519377826 |
| ebi-fl187-GCST90094781 | ukb-rsfMRI191-edge-pheno624 | Net100_Pair8_30 (Calcarine\|Lingual)&(Paracentral) (Visual)&(Motor) \|\| id:ukb-rsfMRI191-edge-pheno624 | Herring liking \|\| id:ebi-fl187-GCST90094781 | Inverse variance weighted | 18 | -0.01874186 | 0.0273989654262907 | 0.493952871076686 | -0.072443832 | 0.0349601125086625 | 0.981432676845311 | 0.930117988095246 | 1.03557840135129 | 0.998074519377826 |
| ebi-fl187-GCST90094781 | ukb-rsfMRI191-edge-pheno882 | Net100_Pair6_38 (Postcentral\|Precentral)&(Subcortical) (Motor)&(Subcortical-cerebellum) \|\| id:ukb-rsfMRI191-edge-pheno882 | Herring liking \|\| id:ebi-fl187-GCST90094781 | Inverse variance weighted | 17 | -0.007556033 | 0.0281344913008587 | 0.788261906146977 | -0.062699636 | 0.0475875703378316 | 0.992472442437959 | 0.939225541277774 | 1.04873803544431 | 0.998074519377826 |
| ebi-fl187-GCST90094781 | ukb-rsfMRI191-edge-pheno899 | Net100_Pair23_38 (Cerebellum)&(Subcortical) (Subcortical-cerebellum)&(Subcortical-cerebellum) \|\| id:ukb-rsfMRI191-edge-pheno899 | Herring liking \|\| id:ebi-fl187-GCST90094781 | Inverse variance weighted | 18 | 0.020965885587399 | 0.0295691602140462 | 0.478296124286768 | -0.036989668 | 0.0789214396069296 | 1.02118721384134 | 0.963686091685881 | 1.08211930701284 | 0.998074519377826 |
| ebi-fl187-GCST90094781 | ebi-a-GCST90001392 | IgD+ CD38+ B cell Absolute Count \|\| id:ebi-a-GCST90001392 | Herring liking \|\| id:ebi-fl187-GCST90094781 | MR Egger | 16 | -0.189546336 | 0.667172088673024 | 0.780489234977163 | -1.497203629 | 1.1181109582189 | 0.827334380990717 | 0.223754987990796 | 3.05907003064195 | 0.999279047927446 |
| ebi-fl187-GCST90094781 | ebi-a-GCST90001402 | Switched memory B cell %B cell \|\| id:ebi-a-GCST90001402 | Herring liking \|\| id:ebi-fl187-GCST90094781 | MR Egger | 16 | 0.1703397956936 | 0.647615895214688 | 0.796361935459904 | -1.098987359 | 1.43966695031439 | 1.18570768124051 | 0.333208333356992 | 4.21929034963986 | 0.999279047927446 |
| ebi-fl187-GCST90094781 | ebi-a-GCST90001407 | Memory B cell Absolute Count \|\| id:ebi-a-GCST90001407 | Herring liking \|\| id:ebi-fl187-GCST90094781 | MR Egger | 16 | -0.083360757 | 0.633662907729304 | 0.897209088295433 | -1.325340056 | 1.1586185424184 | 0.920019184231394 | 0.265712586596362 | 3.18552956108022 | 0.999279047927446 |
| ebi-fl187-GCST90094781 | ebi-a-GCST90001411 | IgD+ CD24+ B cell %B cell \|\| id:ebi-a-GCST90001411 | Herring liking \|\| id:ebi-fl187-GCST90094781 | MR Egger | 16 | -0.620444566 | 0.650273035515169 | 0.356203280479324 | -1.894979715 | 0.654090583732999 | 0.537705339005617 | 0.150321384211628 | 1.92339255729645 | 0.999279047927446 |
| ebi-fl187-GCST90094781 | ebi-a-GCST90001418 | CD24+ CD27+ B cell Absolute Count \|\| id:ebi-a-GCST90001418 | Herring liking \|\| id:ebi-fl187-GCST90094781 | MR Egger | 16 | -0.233997758 | 0.631540320699549 | 0.716541533389237 | -1.471816786 | 1.00382127093809 | 0.791363590421177 | 0.229508138966479 | 2.72868899144254 | 0.999279047927446 |
| ebi-fl187-GCST90094781 | ebi-a-GCST90001436 | Memory B cell %lymphocyte \|\| id:ebi-a-GCST90001436 | Herring liking \|\| id:ebi-fl187-GCST90094781 | MR Egger | 16 | -0.380450913 | 0.624619310427988 | 0.552219611404591 | -1.604704761 | 0.843802935471731 | 0.683553116746984 | 0.20094887401619 | 2.32519274219407 | 0.999279047927446 |
| ebi-fl187-GCST90094781 | ebi-a-GCST90001439 | IgD+ CD24+ B cell %lymphocyte \|\| id:ebi-a-GCST90001439 | Herring liking \|\| id:ebi-fl187-GCST90094781 | MR Egger | 16 | -0.408420005 | 0.631545073255806 | 0.528286996958355 | -1.646248349 | 0.829408338638235 | 0.664699643050517 | 0.192771766176106 | 2.29196227349941 | 0.999279047927446 |
| ebi-fl187-GCST90094781 | ebi-a-GCST90001457 | CD11c+ HLA DR++ monocyte %monocyte \|\| id:ebi-a-GCST90001457 | Herring liking \|\| id:ebi-fl187-GCST90094781 | MR Egger | 16 | 0.252666885076194 | 0.675931119722382 | 0.714148951214245 | -1.07215811 | 1.57749187973206 | 1.2874543351424 | 0.342269065647327 | 4.84279425586445 | 0.999279047927446 |
| ebi-fl187-GCST90094781 | ebi-a-GCST90001483 | CD39+ resting CD4 regulatory T cell Absolute Count \|\| id:ebi-a-GCST90001483 | Herring liking \|\| id:ebi-fl187-GCST90094781 | MR Egger | 15 | 1.42073172110786 | 0.660603517843801 | 0.0508952068691824 | 0.125948826134006 | 2.71551461608171 | 4.14014876641323 | 1.13422412416453 | 15.1123851475642 | 0.999279047927446 |
| ebi-fl187-GCST90094781 | ebi-a-GCST90001525 | CD33dim HLA DR+ CD11b+ Absolute Count \|\| id:ebi-a-GCST90001525 | Herring liking \|\| id:ebi-fl187-GCST90094781 | MR Egger | 16 | 0.568037644045891 | 0.817094340290153 | 0.498312010012649 | -1.033467263 | 2.16954255101459 | 1.76480048448847 | 0.355771267046698 | 8.75427848883015 | 0.999279047927446 |
| ebi-fl187-GCST90094781 | ebi-a-GCST90001540 | Naive CD4+ T cell Absolute Count \|\| id:ebi-a-GCST90001540 | Herring liking \|\| id:ebi-fl187-GCST90094781 | MR Egger | 16 | 0.369040136991958 | 0.60514789635665 | 0.551741647926427 | -0.81704974 | 1.55513001385099 | 1.44634565447364 | 0.441732961110179 | 4.73570219201508 | 0.999279047927446 |
| ebi-fl187-GCST90094781 | ebi-a-GCST90001624 | HLA DR+ CD4+ T cell Absolute Count \|\| id:ebi-a-GCST90001624 | Herring liking \|\| id:ebi-fl187-GCST90094781 | MR Egger | 16 | 0.219063134719462 | 0.610901274491526 | 0.725251569124525 | -0.978303363 | 1.41642963272285 | 1.24490987124196 | 0.375948405928074 | 4.12237573847348 | 0.999279047927446 |
| ebi-fl187-GCST90094781 | ebi-a-GCST90001672 | CD39+ CD8+ T cell Absolute Count \|\| id:ebi-a-GCST90001672 | Herring liking \|\| id:ebi-fl187-GCST90094781 | MR Egger | 16 | -0.161906079 | 0.665463427156517 | 0.81130309259271 | -1.466214396 | 1.14240223827222 | 0.850521082618316 | 0.230797541570007 | 3.13428863694724 | 0.999279047927446 |
| ebi-fl187-GCST90094781 | ebi-a-GCST90001722 | CD19 on CD20- CD38- B cell \|\| id:ebi-a-GCST90001722 | Herring liking \|\| id:ebi-fl187-GCST90094781 | MR Egger | 16 | -2.115756331 | 0.605557441 | 0.00357833613148217 | -3.302648916 | -0.928863746 | 0.120542085340322 | 0.0367855962783673 | 0.39500227828952 | 0.999279047927446 |
| ebi-fl187-GCST90094781 | ebi-a-GCST90001742 | CD19 on transitional B cell \|\| id:ebi-a-GCST90001742 | Herring liking \|\| id:ebi-fl187-GCST90094781 | MR Egger | 16 | -1.673439925 | 0.756240871000979 | 0.0440269855913945 | -3.155672032 | -0.191207818 | 0.18760062232819 | 0.0426097562377214 | 0.825960920817652 | 0.999279047927446 |
| ebi-fl187-GCST90094781 | ebi-a-GCST90001784 | CD25 on IgD+ CD38dim B cell \|\| id:ebi-a-GCST90001784 | Herring liking \|\| id:ebi-fl187-GCST90094781 | MR Egger | 16 | -1.20266925 | 0.627961859874423 | 0.0761229826590892 | -2.433474495 | 0.0281359954036122 | 0.300391321311595 | 0.0877314798015644 | 1.02853555101799 | 0.999279047927446 |
| ebi-fl187-GCST90094781 | ebi-a-GCST90001794 | CD25 on IgD+ B cell \|\| id:ebi-a-GCST90001794 | Herring liking \|\| id:ebi-fl187-GCST90094781 | MR Egger | 16 | -1.142436323 | 0.626390378274948 | 0.0895913961791536 | -2.370161464 | 0.0852888185746428 | 0.319040787826993 | 0.0934656336999596 | 1.08903155382247 | 0.999279047927446 |
| ebi-fl187-GCST90094781 | ebi-a-GCST90001802 | CD27 on IgD- CD38- B cell \|\| id:ebi-a-GCST90001802 | Herring liking \|\| id:ebi-fl187-GCST90094781 | MR Egger | 16 | 1.30927690417972 | 0.627317296667463 | 0.0556437548163905 | 0.079735002711492 | 2.53881880564795 | 3.70349476217518 | 1.08300003757213 | 12.6647026570814 | 0.999279047927446 |
| ebi-fl187-GCST90094781 | ebi-a-GCST90001804 | CD27 on IgD- CD38dim B cell \|\| id:ebi-a-GCST90001804 | Herring liking \|\| id:ebi-fl187-GCST90094781 | MR Egger | 16 | 1.07622788550925 | 0.721427126383114 | 0.157937910556443 | -0.337769282 | 2.49022505322015 | 2.93359280604208 | 0.7133598537283 | 12.0639908549432 | 0.999279047927446 |
| ebi-fl187-GCST90094781 | ebi-a-GCST90001805 | CD27 on memory B cell \|\| id:ebi-a-GCST90001805 | Herring liking \|\| id:ebi-fl187-GCST90094781 | MR Egger | 16 | 0.802084872585404 | 0.703688418333942 | 0.273483657501286 | -0.577144427 | 2.18131417251993 | 2.23018573799751 | 0.56149948199095 | 8.85793947366042 | 0.999279047927446 |
| ebi-fl187-GCST90094781 | ebi-a-GCST90001806 | CD27 on unswitched memory B cell \|\| id:ebi-a-GCST90001806 | Herring liking \|\| id:ebi-fl187-GCST90094781 | MR Egger | 16 | 1.05917845661357 | 0.62651393748404 | 0.113047526026268 | -0.168788861 | 2.28714577408229 | 2.88400068405854 | 0.844687231099033 | 9.84679256347725 | 0.999279047927446 |
| ebi-fl187-GCST90094781 | ebi-a-GCST90001839 | CD3 on Effector Memory CD8+ T cell \|\| id:ebi-a-GCST90001839 | Herring liking \|\| id:ebi-fl187-GCST90094781 | MR Egger | 16 | 0.270219335006056 | 0.693002100871535 | 0.702459279866396 | -1.088064783 | 1.62850345271426 | 1.31025180330607 | 0.336867775663973 | 5.09624224128603 | 0.999279047927446 |
| ebi-fl187-GCST90094781 | ebi-a-GCST90001859 | CD3 on CD8+ T cell \|\| id:ebi-a-GCST90001859 | Herring liking \|\| id:ebi-fl187-GCST90094781 | MR Egger | 16 | 0.287728600483821 | 0.748301971463787 | 0.706378988026685 | -1.178943264 | 1.75440046455284 | 1.33339537215265 | 0.307603622861986 | 5.77998139923019 | 0.999279047927446 |
| ebi-fl187-GCST90094781 | ebi-a-GCST90001905 | CD86 on monocyte \|\| id:ebi-a-GCST90001905 | Herring liking \|\| id:ebi-fl187-GCST90094781 | MR Egger | 16 | -0.344911566 | 0.79570232848501 | 0.671279201327396 | -1.90448813 | 1.21466499763873 | 0.708282986860051 | 0.148898839964772 | 3.36916519694905 | 0.999279047927446 |
| ebi-fl187-GCST90094781 | ebi-a-GCST90001943 | CD25 on activated & secreting CD4 regulatory T cell \|\| id:ebi-a-GCST90001943 | Herring liking \|\| id:ebi-fl187-GCST90094781 | MR Egger | 16 | 0.407617792207568 | 0.630462774874017 | 0.528388976857187 | -0.828089247 | 1.64332483096064 | 1.50323250418098 | 0.436883265512278 | 5.17233810495478 | 0.999279047927446 |
| ebi-fl187-GCST90094781 | ebi-a-GCST90001949 | CD33 on CD33dim HLA DR+ CD11b- \|\| id:ebi-a-GCST90001949 | Herring liking \|\| id:ebi-fl187-GCST90094781 | MR Egger | 16 | 0.263830390533837 | 0.924161406113097 | 0.779455930212598 | -1.547525965 | 2.07518674651551 | 1.30190736176099 | 0.212773732752318 | 7.96603394922107 | 0.999279047927446 |
| ebi-fl187-GCST90094781 | ebi-a-GCST90001955 | CD33 on Immature Myeloid-Derived Suppressor Cells \|\| id:ebi-a-GCST90001955 | Herring liking \|\| id:ebi-fl187-GCST90094781 | MR Egger | 16 | 1.07724185220652 | 1.10819719685208 | 0.347513655865588 | -1.094824654 | 3.2493083580366 | 2.93656888001571 | 0.334598272399696 | 25.7725084030785 | 0.999279047927446 |
| ebi-fl187-GCST90094781 | ebi-a-GCST90001956 | CD33 on CD33+ HLA DR+ \|\| id:ebi-a-GCST90001956 | Herring liking \|\| id:ebi-fl187-GCST90094781 | MR Egger | 16 | -0.126738378 | 1.14545912531163 | 0.913469029519292 | -2.371838263 | 2.11836150779238 | 0.880964122206024 | 0.0933090419264835 | 8.31749816085032 | 0.999279047927446 |
| ebi-fl187-GCST90094781 | ebi-a-GCST90001957 | CD33 on CD33+ HLA DR+ CD14- \|\| id:ebi-a-GCST90001957 | Herring liking \|\| id:ebi-fl187-GCST90094781 | MR Egger | 16 | -0.166299988 | 1.15094190910394 | 0.887172697895039 | -2.42214613 | 2.08954615400831 | 0.846792168733295 | 0.0887309847714447 | 8.08124669048865 | 0.999279047927446 |
| ebi-fl187-GCST90094781 | ebi-a-GCST90002064 | CD4 on resting CD4 regulatory T cell \|\| id:ebi-a-GCST90002064 | Herring liking \|\| id:ebi-fl187-GCST90094781 | MR Egger | 16 | 0.319665255267886 | 0.684701063108432 | 0.647776669771378 | -1.022348828 | 1.66167933896041 | 1.37666685521915 | 0.359748958449717 | 5.26815043030593 | 0.999279047927446 |
| ebi-fl187-GCST90094781 | ebi-a-GCST90002080 | SSC-A on T cell \|\| id:ebi-a-GCST90002080 | Herring liking \|\| id:ebi-fl187-GCST90094781 | MR Egger | 16 | 0.134555357288522 | 0.642137714287957 | 0.837043077771884 | -1.124034563 | 1.39314527729292 | 1.14402798753691 | 0.324966050302697 | 4.02749774952999 | 0.999279047927446 |
| ebi-fl187-GCST90094781 | ebi-a-GCST90002121 | CD8 on CD39+ CD8+ T cell \|\| id:ebi-a-GCST90002121 | Herring liking \|\| id:ebi-fl187-GCST90094781 | MR Egger | 16 | -0.420102623 | 0.780869970775279 | 0.599030959305404 | -1.950607766 | 1.11040251965559 | 0.656979395116965 | 0.142187628545145 | 3.03558002918103 | 0.999279047927446 |
| ebi-fl187-GCST90094781 | ebi-cfmet338-GCST90026053 | 1-palmitoyl-2-docosahexaenoyl-gpc (16:0/22:6) levels \|\| id:ebi-cfmet338-GCST90026053 | Herring liking \|\| id:ebi-fl187-GCST90094781 | MR Egger | 15 | 0.117697979196453 | 0.370063074845213 | 0.75549955303655 | -0.607625648 | 0.84302160589307 | 1.12490431555193 | 0.544642508365458 | 2.32337670988077 | 0.999279047927446 |
| ebi-fl187-GCST90094781 | ebi-cfmet338-GCST90026063 | 1-stearoyl-2-oleoyl-gpc (18:0/18:1) levels \|\| id:ebi-cfmet338-GCST90026063 | Herring liking \|\| id:ebi-fl187-GCST90094781 | MR Egger | 14 | 0.251066350554259 | 0.265742852282151 | 0.363410375964199 | -0.26978964 | 0.771922341027275 | 1.28539536819697 | 0.763540095800778 | 2.16392205421695 | 0.999279047927446 |
| ebi-fl187-GCST90094781 | ebi-cfmet338-GCST90026113 | Adenosine 3',5'-cyclic monophosphate (camp) levels \|\| id:ebi-cfmet338-GCST90026113 | Herring liking \|\| id:ebi-fl187-GCST90094781 | MR Egger | 15 | 0.115292281455233 | 0.680735953453028 | 0.868117219587391 | -1.218950187 | 1.44953475022317 | 1.12220138829748 | 0.295540266043439 | 4.26113156341173 | 0.999279047927446 |
| ebi-fl187-GCST90094781 | ebi-cfmet338-GCST90026117 | Alpha-ketoglutaramate levels \|\| id:ebi-cfmet338-GCST90026117 | Herring liking \|\| id:ebi-fl187-GCST90094781 | MR Egger | 15 | 0.138770456078186 | 0.199215589801312 | 0.498329880093986 | -0.2516921 | 0.529233012088757 | 1.14886035582263 | 0.777484088622486 | 1.69762974766393 | 0.999279047927446 |
| ebi-fl187-GCST90094781 | ebi-cfmet338-GCST90026124 | Arginine levels \|\| id:ebi-cfmet338-GCST90026124 | Herring liking \|\| id:ebi-fl187-GCST90094781 | MR Egger | 15 | 0.187636143387579 | 0.162610108671709 | 0.269313609189004 | -0.13107967 | 0.506351956384129 | 1.2063944808726 | 0.87714788957786 | 1.65922720760383 | 0.999279047927446 |
| ebi-fl187-GCST90094781 | ebi-cfmet338-GCST90026169 | Glutamate levels \|\| id:ebi-cfmet338-GCST90026169 | Herring liking \|\| id:ebi-fl187-GCST90094781 | MR Egger | 15 | 0.00078715998962107 | 0.204441321904057 | 0.996986372301261 | -0.399917831 | 0.401492150921572 | 1.00078746988135 | 0.67037512786514 | 1.4940523868496 | 0.999279047927446 |
| ebi-fl187-GCST90094781 | ebi-cfmet338-GCST90026170 | Glutamine levels \|\| id:ebi-cfmet338-GCST90026170 | Herring liking \|\| id:ebi-fl187-GCST90094781 | MR Egger | 15 | -0.050300208 | 0.111905703904642 | 0.660482814987697 | -0.269635387 | 0.169034971880993 | 0.95094390089477 | 0.763657882848721 | 1.18416155055667 | 0.999279047927446 |
| ebi-fl187-GCST90094781 | ebi-cfmet338-GCST90026191 | Kynurenate levels \|\| id:ebi-cfmet338-GCST90026191 | Herring liking \|\| id:ebi-fl187-GCST90094781 | MR Egger | 15 | 0.20513828178281 | 0.778721436096064 | 0.796348768984224 | -1.321155733 | 1.7314322965311 | 1.22769482105442 | 0.266826743231222 | 5.6487387860434 | 0.999279047927446 |
| ebi-fl187-GCST90094781 | ebi-cfmet338-GCST90026202 | Methylsuccinoylcarnitine levels \|\| id:ebi-cfmet338-GCST90026202 | Herring liking \|\| id:ebi-fl187-GCST90094781 | MR Egger | 15 | 0.130570602823158 | 0.538971844332885 | 0.812359640843906 | -0.925814212 | 1.18695541771561 | 1.1394783874447 | 0.396208689810587 | 3.27708863749124 | 0.999279047927446 |
| ebi-fl187-GCST90094781 | ebi-cfmet338-GCST90026222 | N-acetylputrescine levels \|\| id:ebi-cfmet338-GCST90026222 | Herring liking \|\| id:ebi-fl187-GCST90094781 | MR Egger | 15 | -0.068465781 | 0.188004938526199 | 0.721585663022173 | -0.436955461 | 0.300023898457384 | 0.933825414078254 | 0.646000203253738 | 1.34989106750467 | 0.999279047927446 |
| ebi-fl187-GCST90094781 | ebi-cfmet338-GCST90026252 | Propionylcarnitine (c3) levels \|\| id:ebi-cfmet338-GCST90026252 | Herring liking \|\| id:ebi-fl187-GCST90094781 | MR Egger | 15 | -0.00691755 | 0.337279533506959 | 0.983948119170157 | -0.667985435 | 0.654150336093171 | 0.993106321590578 | 0.512740486922407 | 1.92350748797494 | 0.999279047927446 |
| ebi-fl187-GCST90094781 | ebi-cfmet338-GCST90026316 | Ethyl beta-glucopyranoside levels \|\| id:ebi-cfmet338-GCST90026316 | Herring liking \|\| id:ebi-fl187-GCST90094781 | MR Egger | 15 | -0.432193798 | 1.01354353838725 | 0.67677911403694 | -2.418739133 | 1.55435153710853 | 0.649083573312278 | 0.0890338064724002 | 4.7320169926065 | 0.999279047927446 |
| ebi-fl187-GCST90094781 | ebi-gm430-GCST90011315 | F_Prevotellaceae abundance \|\| id:ebi-gm430-GCST90011315 | Herring liking \|\| id:ebi-fl187-GCST90094781 | MR Egger | 14 | -0.318899563 | 0.482025289208761 | 0.520740263024853 | -1.26366913 | 0.625870003932636 | 0.726948558232892 | 0.282615170082946 | 1.86987204601148 | 0.999279047927446 |
| ebi-fl187-GCST90094781 | ebi-gm430-GCST90011354 | OTU97_1 (Bacteroides) abundance \|\| id:ebi-gm430-GCST90011354 | Herring liking \|\| id:ebi-fl187-GCST90094781 | MR Egger | 14 | 0.18070663231676 | 0.390592740611716 | 0.651894151181365 | -0.584855139 | 0.946268403915724 | 1.19806365457401 | 0.557186570412816 | 2.57607881566094 | 0.999279047927446 |
| ebi-fl187-GCST90094781 | ebi-gm430-GCST90011419 | OTU99_1 (Bacteroides) abundance \|\| id:ebi-gm430-GCST90011419 | Herring liking \|\| id:ebi-fl187-GCST90094781 | MR Egger | 14 | 0.186016640133914 | 0.390690923011976 | 0.642538549795779 | -0.579737569 | 0.951770849237387 | 1.20444230229208 | 0.560045320552482 | 2.59029261796092 | 0.999279047927446 |
| ebi-fl187-GCST90094781 | ebi-gm430-GCST90011435 | OTU99_155 (Butyrivibrio) abundance \|\| id:ebi-gm430-GCST90011435 | Herring liking \|\| id:ebi-fl187-GCST90094781 | MR Egger | 14 | 0.237058863090893 | 1.35562253562481 | 0.864096926289533 | -2.419961307 | 2.89407903291551 | 1.2675157254728 | 0.0889250581937743 | 18.0668547983398 | 0.999279047927446 |
| ebi-fl187-GCST90094781 | ebi-gm430-GCST90011466 | OTU99_58 (Bacteroides) abundance \|\| id:ebi-gm430-GCST90011466 | Herring liking \|\| id:ebi-fl187-GCST90094781 | MR Egger | 14 | -0.007792208 | 0.721706214121782 | 0.991562891964085 | -1.422336388 | 1.40675197144884 | 0.992238072322861 | 0.241149938400317 | 4.08267320613046 | 0.999279047927446 |
| ebi-fl187-GCST90094781 | ebi-gm430-GCST90011472 | OTU99_72 (Parabacteroides) abundance \|\| id:ebi-gm430-GCST90011472 | Herring liking \|\| id:ebi-fl187-GCST90094781 | MR Egger | 14 | 0.409331372277138 | 1.08866762687325 | 0.713485005274866 | -1.724457176 | 2.5431199209487 | 1.50581062171463 | 0.178269794566038 | 12.719292317514 | 0.999279047927446 |
| ebi-fl187-GCST90094781 | ebi-gm430-GCST90011505 | TestASV_28 (Holdemanella) abundance \|\| id:ebi-gm430-GCST90011505 | Herring liking \|\| id:ebi-fl187-GCST90094781 | MR Egger | 14 | 0.532879382969753 | 0.91912371029511 | 0.572795030901586 | -1.268603089 | 2.33436185514817 | 1.70383123493256 | 0.281224192630784 | 10.3228703404731 | 0.999279047927446 |
| ebi-fl187-GCST90094781 | ebi-gm430-GCST90011513 | TestASV_35 (Alphaproteobacteria) abundance \|\| id:ebi-gm430-GCST90011513 | Herring liking \|\| id:ebi-fl187-GCST90094781 | MR Egger | 14 | 0.210777606298832 | 1.72819278139433 | 0.904946047667226 | -3.176480245 | 3.59803545783172 | 1.23463774886861 | 0.0417322843270256 | 36.5264062466909 | 0.999279047927446 |
| ebi-fl187-GCST90094781 | ebi-gm430-GCST90011543 | G_Coprococcus prevalence \|\| id:ebi-gm430-GCST90011543 | Herring liking \|\| id:ebi-fl187-GCST90094781 | MR Egger | 14 | -0.379691515 | 1.20737227445072 | 0.758558799781508 | -2.746141173 | 1.98675814274243 | 0.684072402617873 | 0.0641750243300661 | 7.29185624638948 | 0.999279047927446 |
| ebi-fl187-GCST90094781 | ebi-gm430-GCST90011547 | G_Parasutterella prevalence \|\| id:ebi-gm430-GCST90011547 | Herring liking \|\| id:ebi-fl187-GCST90094781 | MR Egger | 14 | 0.311575000176639 | 1.11486381832659 | 0.784640006253648 | -1.873558084 | 2.49670808409676 | 1.36557420088953 | 0.153576251363172 | 12.1424561517996 | 0.999279047927446 |
| ebi-fl187-GCST90094781 | ebi-gm430-GCST90011569 | OTU97_13 (Subdoligranulum) prevalence \|\| id:ebi-gm430-GCST90011569 | Herring liking \|\| id:ebi-fl187-GCST90094781 | MR Egger | 14 | 0.0401642792571076 | 1.18499643105353 | 0.973518931216495 | -2.282428726 | 2.36275728412202 | 1.04098177185846 | 0.102036087871673 | 10.6201940112055 | 0.999279047927446 |
| ebi-fl187-GCST90094781 | ebi-gm430-GCST90011590 | OTU97_277 (Prevotella) prevalence \|\| id:ebi-gm430-GCST90011590 | Herring liking \|\| id:ebi-fl187-GCST90094781 | MR Egger | 14 | 0.157914897010944 | 0.950129282013913 | 0.870764222303457 | -1.704338496 | 2.02016828975821 | 1.1710665292048 | 0.181892669163309 | 7.53959366329645 | 0.999279047927446 |
| ebi-fl187-GCST90094781 | ebi-gm430-GCST90011616 | OTU97_86 (Phascolarctobacterium) prevalence \|\| id:ebi-gm430-GCST90011616 | Herring liking \|\| id:ebi-fl187-GCST90094781 | MR Egger | 14 | -2.897993639 | 1.17459359900455 | 0.0296434322931256 | -5.200197093 | -0.595790185 | 0.0551337273086862 | 0.0055154772501794 | 0.551126901457112 | 0.999279047927446 |
| ebi-fl187-GCST90094781 | ebi-gm430-GCST90011665 | OTU99_55 (Barnesiella) prevalence \|\| id:ebi-gm430-GCST90011665 | Herring liking \|\| id:ebi-fl187-GCST90094781 | MR Egger | 14 | -0.637745154 | 0.853215063480999 | 0.469179606827386 | -2.310046678 | 1.03455637067586 | 0.528482728862548 | 0.0992566183342412 | 2.81385764892269 | 0.999279047927446 |
| ebi-fl187-GCST90094781 | ebi-gm430-GCST90011687 | TestASV_14 (Lachnospiraceae) prevalence \|\| id:ebi-gm430-GCST90011687 | Herring liking \|\| id:ebi-fl187-GCST90094781 | MR Egger | 14 | -0.070835826 | 0.846367916460039 | 0.934679796927903 | -1.729716943 | 1.58804528998215 | 0.931614826245443 | 0.177334598747268 | 4.89417288341596 | 0.999279047927446 |
| ebi-fl187-GCST90094781 | ebi-gm430-GCST90011697 | TestASV_23 (Barnesiella) prevalence \|\| id:ebi-gm430-GCST90011697 | Herring liking \|\| id:ebi-fl187-GCST90094781 | MR Egger | 14 | -0.088336621 | 1.34486666659834 | 0.948710787995314 | -2.724275287 | 2.54760204597103 | 0.915452664660769 | 0.065593722113922 | 12.7764297287321 | 0.999279047927446 |
| ebi-fl187-GCST90094781 | ebi-gm430-GCST90011727 | TestASV_6 (Subdoligranulum) prevalence \|\| id:ebi-gm430-GCST90011727 | Herring liking \|\| id:ebi-fl187-GCST90094781 | MR Egger | 14 | -0.644484605 | 1.68756004429984 | 0.709210095652148 | -3.952102292 | 2.66313308142161 | 0.52493302003988 | 0.0192142652849343 | 14.3411507773992 | 0.999279047927446 |
| ebi-fl187-GCST90094781 | ebi-hbsm44-GCST90104908 | Sulcal depth of the left posterior inferior temporal sulcus \|\| id:ebi-hbsm44-GCST90104908 | Herring liking \|\| id:ebi-fl187-GCST90094781 | MR Egger | 16 | 0.0184445692293183 | 0.293513846686462 | 0.950781866198473 | -0.55684257 | 0.593731708734784 | 1.01861572095022 | 0.573015466619723 | 1.81073295121983 | 0.999279047927446 |
| ebi-fl187-GCST90094781 | ebi-hbsm44-GCST90104918 | Sulcal depth of the left inferior frontal sulcus \|\| id:ebi-hbsm44-GCST90104918 | Herring liking \|\| id:ebi-fl187-GCST90094781 | MR Egger | 15 | -0.229116243 | 0.465227200511274 | 0.630591014592917 | -1.140961556 | 0.682729070444712 | 0.795236087855562 | 0.319511645861061 | 1.97927194085069 | 0.999279047927446 |
| ebi-fl187-GCST90094781 | ebi-hbsm44-GCST90104925 | Sulcal depth of the right anterior inferior temporal sulcus \|\| id:ebi-hbsm44-GCST90104925 | Herring liking \|\| id:ebi-fl187-GCST90094781 | MR Egger | 15 | 0.0355397859716407 | 0.180073705714955 | 0.846596312678869 | -0.317404677 | 0.388484249172953 | 1.03617887269122 | 0.728036075882445 | 1.47474375484797 | 0.999279047927446 |
| ebi-fl187-GCST90094781 | ebi-IPs91-GCST90274770 | C-C motif chemokine 4 levels \|\| id:ebi-IPs91-GCST90274770 | Herring liking \|\| id:ebi-fl187-GCST90094781 | MR Egger | 21 | 0.0485658911295268 | 0.170449053366453 | 0.778781027232376 | -0.285514253 | 0.382646035727775 | 1.04976453971278 | 0.751627627801105 | 1.46615897031662 | 0.999279047927446 |
| ebi-fl187-GCST90094781 | ebi-IPs91-GCST90274779 | C-X-C motif chemokine 1 levels \|\| id:ebi-IPs91-GCST90274779 | Herring liking \|\| id:ebi-fl187-GCST90094781 | MR Egger | 21 | -0.186448959 | 0.220088419493385 | 0.407453653729215 | -0.617822261 | 0.244924343536172 | 0.829900920116344 | 0.539117216782004 | 1.27752465655062 | 0.999279047927446 |
| ebi-fl187-GCST90094781 | ebi-IPs91-GCST90274780 | C-X-C motif chemokine 10 levels \|\| id:ebi-IPs91-GCST90274780 | Herring liking \|\| id:ebi-fl187-GCST90094781 | MR Egger | 21 | 0.206614021756481 | 0.169344638679575 | 0.237362531192998 | -0.12530147 | 0.538529513568447 | 1.22950791687726 | 0.882230896292888 | 1.71348535175536 | 0.999279047927446 |
| ebi-fl187-GCST90094781 | ebi-IPs91-GCST90274796 | Interleukin-10 receptor subunit alpha levels \|\| id:ebi-IPs91-GCST90274796 | Herring liking \|\| id:ebi-fl187-GCST90094781 | MR Egger | 19 | -0.25443846 | 0.28446234027115 | 0.383568234386045 | -0.811984647 | 0.303107727231762 | 0.775351767003177 | 0.443976055689598 | 1.35406032575606 | 0.999279047927446 |
| ebi-fl187-GCST90094781 | ebi-IPs91-GCST90274807 | Interleukin-20 levels \|\| id:ebi-IPs91-GCST90274807 | Herring liking \|\| id:ebi-fl187-GCST90094781 | MR Egger | 19 | -0.18687047 | 0.254347466876329 | 0.472532807505316 | -0.685391506 | 0.311650564625032 | 0.829551180815659 | 0.503892914089811 | 1.36567739364956 | 0.999279047927446 |
| ebi-fl187-GCST90094781 | ebi-IPs91-GCST90274808 | Interleukin-20 receptor subunit alpha levels \|\| id:ebi-IPs91-GCST90274808 | Herring liking \|\| id:ebi-fl187-GCST90094781 | MR Egger | 19 | -0.306182503 | 0.235076727451495 | 0.210122502092602 | -0.766932889 | 0.154567882450214 | 0.736252238690525 | 0.46443536076159 | 1.16715350460809 | 0.999279047927446 |
| ebi-fl187-GCST90094781 | ebi-IPs91-GCST90274810 | Interleukin-24 levels \|\| id:ebi-IPs91-GCST90274810 | Herring liking \|\| id:ebi-fl187-GCST90094781 | MR Egger | 19 | -0.260929914 | 0.21495677023162 | 0.241391989115965 | -0.682245183 | 0.160385356066987 | 0.770334907733642 | 0.505480820319235 | 1.17396317766959 | 0.999279047927446 |
| ebi-fl187-GCST90094781 | ebi-IPs91-GCST90274823 | Monocyte chemoattractant protein-3 levels \|\| id:ebi-IPs91-GCST90274823 | Herring liking \|\| id:ebi-fl187-GCST90094781 | MR Egger | 19 | -0.024418104 | 0.284182872484399 | 0.932530790632249 | -0.581416534 | 0.532580326321343 | 0.975877606364189 | 0.559105813066742 | 1.70332176905736 | 0.999279047927446 |
| ebi-fl187-GCST90094781 | ebi-IPs91-GCST90274824 | Monocyte chemoattractant protein-4 levels \|\| id:ebi-IPs91-GCST90274824 | Herring liking \|\| id:ebi-fl187-GCST90094781 | MR Egger | 21 | -0.373235714 | 0.203829733378607 | 0.0828120841694089 | -0.772741991 | 0.0262705633572974 | 0.688502923892657 | 0.461745229415706 | 1.02661867629598 | 0.999279047927446 |
| ebi-fl187-GCST90094781 | ebi-IPs91-GCST90274834 | SIR2-like protein 2 levels \|\| id:ebi-IPs91-GCST90274834 | Herring liking \|\| id:ebi-fl187-GCST90094781 | MR Egger | 21 | -0.307111773 | 0.185697064072743 | 0.114588706643008 | -0.671078019 | 0.0568544723437618 | 0.735568379452119 | 0.511157243536364 | 1.05850175790637 | 0.999279047927446 |
| ebi-fl187-GCST90094781 | ebi-met1400-GCST90199652 | 1-stearoyl-2-oleoyl-GPS (18:0/18:1) levels \|\| id:ebi-met1400-GCST90199652 | Herring liking \|\| id:ebi-fl187-GCST90094781 | MR Egger | 16 | -0.152459897 | 0.29907167120037 | 0.618148814788079 | -0.738640373 | 0.433720578557663 | 0.858593325438357 | 0.477763053891585 | 1.54298766403687 | 0.999279047927446 |
| ebi-fl187-GCST90094781 | ebi-met1400-GCST90199665 | N-acetylaspartate (naa) levels \|\| id:ebi-met1400-GCST90199665 | Herring liking \|\| id:ebi-fl187-GCST90094781 | MR Egger | 16 | -0.078711533 | 0.296812918230585 | 0.794728601730253 | -0.660464853 | 0.503041786475842 | 0.924306517683115 | 0.516611130438595 | 1.65374396387117 | 0.999279047927446 |
| ebi-fl187-GCST90094781 | ebi-met1400-GCST90199669 | Acetylcarnitine levels (Biocrates platform) \|\| id:ebi-met1400-GCST90199669 | Herring liking \|\| id:ebi-fl187-GCST90094781 | MR Egger | 16 | 0.0193657915713576 | 0.29626948964594 | 0.948807250149935 | -0.561322408 | 0.600053991277399 | 1.01955452486749 | 0.570454191570804 | 1.82221718156795 | 0.999279047927446 |
| ebi-fl187-GCST90094781 | ebi-met1400-GCST90199670 | Iminodiacetate (IDA) levels \|\| id:ebi-met1400-GCST90199670 | Herring liking \|\| id:ebi-fl187-GCST90094781 | MR Egger | 16 | -0.214154158 | 0.32686427690941 | 0.522973743141087 | -0.854808141 | 0.426499824902533 | 0.80722393553227 | 0.425364793495495 | 1.53188625871339 | 0.999279047927446 |
| ebi-fl187-GCST90094781 | ebi-met1400-GCST90199686 | Indolepropionate levels \|\| id:ebi-met1400-GCST90199686 | Herring liking \|\| id:ebi-fl187-GCST90094781 | MR Egger | 16 | 0.371228938961685 | 0.390926378197233 | 0.358414710435836 | -0.394986762 | 1.13744464022826 | 1.44951488583571 | 0.673688957278855 | 3.11878854708555 | 0.999279047927446 |
| ebi-fl187-GCST90094781 | ebi-met1400-GCST90199704 | 3-hydroxysebacate levels \|\| id:ebi-met1400-GCST90199704 | Herring liking \|\| id:ebi-fl187-GCST90094781 | MR Egger | 16 | -0.116690098 | 0.304544702887291 | 0.707354443389266 | -0.713597716 | 0.480217519692069 | 0.88986092014525 | 0.489878579449476 | 1.61642596843412 | 0.999279047927446 |
| ebi-fl187-GCST90094781 | ebi-met1400-GCST90199713 | Docosapentaenoate n3 DPA; 22:5n3 levels \|\| id:ebi-met1400-GCST90199713 | Herring liking \|\| id:ebi-fl187-GCST90094781 | MR Egger | 16 | -0.222636278 | 0.299103326357921 | 0.468977961552505 | -0.808878798 | 0.363606241245272 | 0.800405921266089 | 0.445357121660584 | 1.4385076776342 | 0.999279047927446 |
| ebi-fl187-GCST90094781 | ebi-met1400-GCST90199715 | Myristoleate (14:1n5) levels \|\| id:ebi-met1400-GCST90199715 | Herring liking \|\| id:ebi-fl187-GCST90094781 | MR Egger | 16 | -0.186154371 | 0.279368327483868 | 0.516024603213122 | -0.733716293 | 0.361407550878082 | 0.830145434717224 | 0.480121398815484 | 1.43534831915853 | 0.999279047927446 |
| ebi-fl187-GCST90094781 | ebi-met1400-GCST90199725 | Campesterol levels \|\| id:ebi-met1400-GCST90199725 | Herring liking \|\| id:ebi-fl187-GCST90094781 | MR Egger | 16 | 0.430639245139614 | 0.387435675018144 | 0.285072454454363 | -0.328734678 | 1.19001316817518 | 1.5382405221036 | 0.719833979278615 | 3.28712449252927 | 0.999279047927446 |
| ebi-fl187-GCST90094781 | ebi-met1400-GCST90199737 | N-acetylproline levels \|\| id:ebi-met1400-GCST90199737 | Herring liking \|\| id:ebi-fl187-GCST90094781 | MR Egger | 16 | -0.53998508 | 0.318024643891656 | 0.111626380590324 | -1.163313382 | 0.0833432223791665 | 0.582756947247628 | 0.312449200419889 | 1.08691479801834 | 0.999279047927446 |
| ebi-fl187-GCST90094781 | ebi-met1400-GCST90199775 | Gamma-glutamylisoleucine levels \|\| id:ebi-met1400-GCST90199775 | Herring liking \|\| id:ebi-fl187-GCST90094781 | MR Egger | 16 | -0.327085361 | 0.284522022331269 | 0.269570808919881 | -0.884748525 | 0.230577802820014 | 0.721022193260807 | 0.412817973743007 | 1.2593274427006 | 0.999279047927446 |
| ebi-fl187-GCST90094781 | ebi-met1400-GCST90199777 | 1-palmitoleoyl-GPC (16:1) levels \|\| id:ebi-met1400-GCST90199777 | Herring liking \|\| id:ebi-fl187-GCST90094781 | MR Egger | 16 | -1.053875149 | 0.290805513638404 | 0.00276379643479361 | -1.623853955 | -0.483896342 | 0.348584312395713 | 0.197137474134555 | 0.616377091072268 | 0.999279047927446 |
| ebi-fl187-GCST90094781 | ebi-met1400-GCST90199782 | Glutamine degradant levels \|\| id:ebi-met1400-GCST90199782 | Herring liking \|\| id:ebi-fl187-GCST90094781 | MR Egger | 16 | -0.109837724 | 0.246759011799933 | 0.663036904357981 | -0.593485387 | 0.37380993888699 | 0.895979519256707 | 0.552398602472994 | 1.4532609158198 | 0.999279047927446 |
| ebi-fl187-GCST90094781 | ebi-met1400-GCST90199783 | 5-methyluridine (ribothymidine) levels \|\| id:ebi-met1400-GCST90199783 | Herring liking \|\| id:ebi-fl187-GCST90094781 | MR Egger | 16 | -0.008896946 | 0.31749821730136 | 0.978040112534113 | -0.631193452 | 0.613399560288809 | 0.991142515085539 | 0.531956557647256 | 1.84669870328302 | 0.999279047927446 |
| ebi-fl187-GCST90094781 | ebi-met1400-GCST90199787 | Homostachydrine levels \|\| id:ebi-met1400-GCST90199787 | Herring liking \|\| id:ebi-fl187-GCST90094781 | MR Egger | 16 | 0.0624840657240346 | 0.288180467983775 | 0.831474337586386 | -0.502349652 | 0.627317782972234 | 1.06447749710452 | 0.605107196999149 | 1.87258116819838 | 0.999279047927446 |
| ebi-fl187-GCST90094781 | ebi-met1400-GCST90199802 | Beta-hydroxyisovaleroylcarnitine levels \|\| id:ebi-met1400-GCST90199802 | Herring liking \|\| id:ebi-fl187-GCST90094781 | MR Egger | 16 | 0.154815067539481 | 0.290942298510029 | 0.602988384911433 | -0.415431838 | 0.725061972619138 | 1.16744204321106 | 0.660055182544959 | 2.06485906072556 | 0.999279047927446 |
| ebi-fl187-GCST90094781 | ebi-met1400-GCST90199873 | Pregnenolone sulfate levels \|\| id:ebi-met1400-GCST90199873 | Herring liking \|\| id:ebi-fl187-GCST90094781 | MR Egger | 16 | -0.407807784 | 0.281594281077278 | 0.169582352781055 | -0.959732575 | 0.144117007155592 | 0.665106710850215 | 0.382995294925281 | 1.15501924613537 | 0.999279047927446 |
| ebi-fl187-GCST90094781 | ebi-met1400-GCST90199911 | Beta-citrylglutamate levels \|\| id:ebi-met1400-GCST90199911 | Herring liking \|\| id:ebi-fl187-GCST90094781 | MR Egger | 16 | -0.136762328 | 0.293149483619695 | 0.648014534440067 | -0.711335316 | 0.43781066001645 | 0.872177493775838 | 0.490988135332051 | 1.54931153302646 | 0.999279047927446 |
| ebi-fl187-GCST90094781 | ebi-met1400-GCST90199934 | 2-hydroxydecanoate levels \|\| id:ebi-met1400-GCST90199934 | Herring liking \|\| id:ebi-fl187-GCST90094781 | MR Egger | 16 | -0.055202829 | 0.330874509656838 | 0.869882338344853 | -0.703716868 | 0.59331120975093 | 0.94629319261811 | 0.494742987658912 | 1.80997169991773 | 0.999279047927446 |
| ebi-fl187-GCST90094781 | ebi-met1400-GCST90199945 | Sphingomyelin (d18:1/14:0, d16:1/16:0) levels \|\| id:ebi-met1400-GCST90199945 | Herring liking \|\| id:ebi-fl187-GCST90094781 | MR Egger | 16 | -0.017999857 | 0.357180818013931 | 0.960520159339619 | -0.718074261 | 0.682074545862763 | 0.982161172370726 | 0.487690517016512 | 1.97797688258101 | 0.999279047927446 |
| ebi-fl187-GCST90094781 | ebi-met1400-GCST90199979 | Carnitine C14:1 levels \|\| id:ebi-met1400-GCST90199979 | Herring liking \|\| id:ebi-fl187-GCST90094781 | MR Egger | 16 | 0.0370246570393476 | 0.341715465143125 | 0.91525612396725 | -0.632737655 | 0.706786968719872 | 1.03771860759125 | 0.531135742592955 | 2.02746646889925 | 0.999279047927446 |
| ebi-fl187-GCST90094781 | ebi-met1400-GCST90199986 | Eugenol sulfate levels \|\| id:ebi-met1400-GCST90199986 | Herring liking \|\| id:ebi-fl187-GCST90094781 | MR Egger | 16 | -0.559559407 | 0.335290906043363 | 0.117341899296277 | -1.216729582 | 0.0976107692349706 | 0.571460790237062 | 0.296197273400742 | 1.10253356159878 | 0.999279047927446 |
| ebi-fl187-GCST90094781 | ebi-met1400-GCST90200002 | 6-hydroxyindole sulfate levels \|\| id:ebi-met1400-GCST90200002 | Herring liking \|\| id:ebi-fl187-GCST90094781 | MR Egger | 16 | 0.148372011412081 | 0.292442717853836 | 0.619804296446934 | -0.424815716 | 0.721559738405599 | 1.15994432863677 | 0.653890275816511 | 2.05764008932005 | 0.999279047927446 |
| ebi-fl187-GCST90094781 | ebi-met1400-GCST90200023 | Trans 3,4-methyleneheptanoate levels \|\| id:ebi-met1400-GCST90200023 | Herring liking \|\| id:ebi-fl187-GCST90094781 | MR Egger | 16 | -0.298740249 | 0.318020193946727 | 0.3634638601858 | -0.922059829 | 0.324579331240563 | 0.741752055329927 | 0.397699004851602 | 1.38344854996927 | 0.999279047927446 |
| ebi-fl187-GCST90094781 | ebi-met1400-GCST90200032 | 2-hydroxybutyrate/2-hydroxyisobutyrate levels \|\| id:ebi-met1400-GCST90200032 | Herring liking \|\| id:ebi-fl187-GCST90094781 | MR Egger | 16 | 0.0754891985707981 | 0.298052627013748 | 0.803737264422093 | -0.50869395 | 0.659672347517744 | 1.07841157926882 | 0.601280368212493 | 1.9341584987356 | 0.999279047927446 |
| ebi-fl187-GCST90094781 | ebi-met1400-GCST90200051 | 1-palmitoyl-2-dihomo-linolenoyl-GPC (16:0/20:3n3 or 6) levels \|\| id:ebi-met1400-GCST90200051 | Herring liking \|\| id:ebi-fl187-GCST90094781 | MR Egger | 16 | -0.138686407 | 0.290144255156706 | 0.640034026196373 | -0.707369147 | 0.429996333171811 | 0.870500968726506 | 0.492939343974905 | 1.5372518866994 | 0.999279047927446 |
| ebi-fl187-GCST90094781 | ebi-met1400-GCST90200069 | Phenylacetylglutamate levels \|\| id:ebi-met1400-GCST90200069 | Herring liking \|\| id:ebi-fl187-GCST90094781 | MR Egger | 16 | 0.00843861701933272 | 0.323081734608668 | 0.979530967044233 | -0.624801583 | 0.641678816852322 | 1.00847432251226 | 0.535367644122747 | 1.89966739740697 | 0.999279047927446 |
| ebi-fl187-GCST90094781 | ebi-met1400-GCST90200072 | Thioproline levels \|\| id:ebi-met1400-GCST90200072 | Herring liking \|\| id:ebi-fl187-GCST90094781 | MR Egger | 16 | 0.352220016648946 | 0.346206145503711 | 0.326237404434556 | -0.326344029 | 1.03078406183622 | 1.42222140168499 | 0.721556908558084 | 2.80326290472763 | 0.999279047927446 |
| ebi-fl187-GCST90094781 | ebi-met1400-GCST90200081 | 1-oleoyl-2-docosahexaenoyl-GPC (18:1/22:6) levels \|\| id:ebi-met1400-GCST90200081 | Herring liking \|\| id:ebi-fl187-GCST90094781 | MR Egger | 16 | -0.542062937 | 0.306047523485059 | 0.098290263079473 | -1.141916083 | 0.0577902091360234 | 0.58154731865499 | 0.319206808762176 | 1.05949270050434 | 0.999279047927446 |
| ebi-fl187-GCST90094781 | ebi-met1400-GCST90200097 | Furaneol sulfate levels \|\| id:ebi-met1400-GCST90200097 | Herring liking \|\| id:ebi-fl187-GCST90094781 | MR Egger | 16 | -0.152798315 | 0.377728509124458 | 0.691943701024307 | -0.893146193 | 0.587549562550982 | 0.858302810872737 | 0.409365780723251 | 1.79957326635973 | 0.999279047927446 |
| ebi-fl187-GCST90094781 | ebi-met1400-GCST90200124 | Sphingomyelin (d18:2/24:2) levels \|\| id:ebi-met1400-GCST90200124 | Herring liking \|\| id:ebi-fl187-GCST90094781 | MR Egger | 16 | 0.121174253898344 | 0.293428760066418 | 0.68589119364886 | -0.453946116 | 0.696294623628524 | 1.12882159679411 | 0.635116955072724 | 2.00630480293648 | 0.999279047927446 |
| ebi-fl187-GCST90094781 | ebi-met1400-GCST90200137 | Lignoceroylcarnitine (C24) levels \|\| id:ebi-met1400-GCST90200137 | Herring liking \|\| id:ebi-fl187-GCST90094781 | MR Egger | 16 | -0.002384913 | 0.366884836828814 | 0.994905156290054 | -0.721479193 | 0.716709367143657 | 0.997617928604813 | 0.486032787568129 | 2.04768393600247 | 0.999279047927446 |
| ebi-fl187-GCST90094781 | ebi-met1400-GCST90200165 | Octadecenedioate (C18:1-DC) levels \|\| id:ebi-met1400-GCST90200165 | Herring liking \|\| id:ebi-fl187-GCST90094781 | MR Egger | 16 | -0.072934878 | 0.289420382140139 | 0.804700390255685 | -0.640198827 | 0.494329070566022 | 0.929661369063749 | 0.5271875942709 | 1.63939794965162 | 0.999279047927446 |
| ebi-fl187-GCST90094781 | ebi-met1400-GCST90200176 | Glutamine conjugate of C6H10O2 (1) levels \|\| id:ebi-met1400-GCST90200176 | Herring liking \|\| id:ebi-fl187-GCST90094781 | MR Egger | 16 | 0.0766485094826845 | 0.290513654054342 | 0.795748736258282 | -0.492758252 | 0.646055271429196 | 1.07966251855384 | 0.610938944199628 | 1.9079994245532 | 0.999279047927446 |
| ebi-fl187-GCST90094781 | ebi-met1400-GCST90200182 | Glycine conjugate of C10H12O2 levels \|\| id:ebi-met1400-GCST90200182 | Herring liking \|\| id:ebi-fl187-GCST90094781 | MR Egger | 16 | 0.511080591127546 | 0.411990641774668 | 0.235172693059304 | -0.296421067 | 1.3185822490059 | 1.66709166644749 | 0.743474309791039 | 3.73811789827654 | 0.999279047927446 |
| ebi-fl187-GCST90094781 | ebi-met1400-GCST90200208 | Glucuronide of piperine metabolite C17H21NO3 (4) levels \|\| id:ebi-met1400-GCST90200208 | Herring liking \|\| id:ebi-fl187-GCST90094781 | MR Egger | 16 | -0.21592729 | 0.302393810444867 | 0.486925429122102 | -0.808619158 | 0.376764578904815 | 0.805793889365997 | 0.445472769213612 | 1.45756112833965 | 0.999279047927446 |
| ebi-fl187-GCST90094781 | ebi-met1400-GCST90200227 | 3-ethylcatechol sulfate (2) levels \|\| id:ebi-met1400-GCST90200227 | Herring liking \|\| id:ebi-fl187-GCST90094781 | MR Egger | 15 | 0.926423070915141 | 0.471438613175294 | 0.0711397910742396 | 0.00240338909156579 | 1.85044275273872 | 2.52545961249959 | 1.00240627954629 | 6.36263597356289 | 0.999279047927446 |
| ebi-fl187-GCST90094781 | ebi-met1400-GCST90200259 | Pentose acid levels \|\| id:ebi-met1400-GCST90200259 | Herring liking \|\| id:ebi-fl187-GCST90094781 | MR Egger | 16 | -0.420637357 | 0.349147368950782 | 0.248269043392049 | -1.1049662 | 0.263691486084508 | 0.65662817981209 | 0.331222077291905 | 1.30172653359503 | 0.999279047927446 |
| ebi-fl187-GCST90094781 | ebi-met1400-GCST90200291 | Sarcosine levels \|\| id:ebi-met1400-GCST90200291 | Herring liking \|\| id:ebi-fl187-GCST90094781 | MR Egger | 16 | -0.021138659 | 0.296025877802458 | 0.944083001770475 | -0.601349379 | 0.559072061529031 | 0.979083196495459 | 0.548071580367025 | 1.74904873742554 | 0.999279047927446 |
| ebi-fl187-GCST90094781 | ebi-met1400-GCST90200306 | Butyrate/isobutyrate (4:0) levels \|\| id:ebi-met1400-GCST90200306 | Herring liking \|\| id:ebi-fl187-GCST90094781 | MR Egger | 16 | -0.147268839 | 0.298912004281256 | 0.629873567804629 | -0.733136367 | 0.438598689457793 | 0.86306192157838 | 0.480399914225345 | 1.55053291730847 | 0.999279047927446 |
| ebi-fl187-GCST90094781 | ebi-met1400-GCST90200321 | Cholate levels \|\| id:ebi-met1400-GCST90200321 | Herring liking \|\| id:ebi-fl187-GCST90094781 | MR Egger | 16 | 0.27964223721287 | 0.347459586832724 | 0.434379525521117 | -0.401378553 | 0.960663027405008 | 1.32265653039442 | 0.669396610987424 | 2.61342867394331 | 0.999279047927446 |
| ebi-fl187-GCST90094781 | ebi-met1400-GCST90200336 | Ceramide (d18:1/16:0) levels \|\| id:ebi-met1400-GCST90200336 | Herring liking \|\| id:ebi-fl187-GCST90094781 | MR Egger | 16 | -0.347530352 | 0.292790098597166 | 0.254994105183055 | -0.921398946 | 0.226338240860197 | 0.706430571747758 | 0.397961924388102 | 1.25399974750646 | 0.999279047927446 |
| ebi-fl187-GCST90094781 | ebi-met1400-GCST90200358 | Arachidonate (20:4n6) levels \|\| id:ebi-met1400-GCST90200358 | Herring liking \|\| id:ebi-fl187-GCST90094781 | MR Egger | 16 | -0.210435567 | 0.29889561895612 | 0.492950692867852 | -0.79627098 | 0.375399846421882 | 0.810231259319269 | 0.451007648841461 | 1.45557330405466 | 0.999279047927446 |
| ebi-fl187-GCST90094781 | ebi-met1400-GCST90200413 | 2'-deoxyuridine levels \|\| id:ebi-met1400-GCST90200413 | Herring liking \|\| id:ebi-fl187-GCST90094781 | MR Egger | 16 | 0.0809541090725088 | 0.29370961140152 | 0.786861579827561 | -0.494716729 | 0.656624947419488 | 1.08432113492239 | 0.609743605348477 | 1.92827331574459 | 0.999279047927446 |
| ebi-fl187-GCST90094781 | ebi-met1400-GCST90200432 | Threonine levels \|\| id:ebi-met1400-GCST90200432 | Herring liking \|\| id:ebi-fl187-GCST90094781 | MR Egger | 16 | -0.636052098 | 0.330526893924958 | 0.0748782647870389 | -1.28388481 | 0.011780614150484 | 0.529378237473323 | 0.276959273678584 | 1.01185027888103 | 0.999279047927446 |
| ebi-fl187-GCST90094781 | ebi-met1400-GCST90200436 | Caffeine levels \|\| id:ebi-met1400-GCST90200436 | Herring liking \|\| id:ebi-fl187-GCST90094781 | MR Egger | 16 | -0.087831044 | 0.301993752836709 | 0.775439831145528 | -0.679738799 | 0.504076711894055 | 0.915915613395477 | 0.506749338399887 | 1.65545635147848 | 0.999279047927446 |
| ebi-fl187-GCST90094781 | ebi-met1400-GCST90200442 | Valine levels \|\| id:ebi-met1400-GCST90200442 | Herring liking \|\| id:ebi-fl187-GCST90094781 | MR Egger | 16 | 0.157325603752897 | 0.289699408955053 | 0.595626193415306 | -0.410485238 | 0.725136445304801 | 1.17037663089059 | 0.663328300067122 | 2.06501284205151 | 0.999279047927446 |
| ebi-fl187-GCST90094781 | ebi-met1400-GCST90200459 | Margarate (17:0) levels \|\| id:ebi-met1400-GCST90200459 | Herring liking \|\| id:ebi-fl187-GCST90094781 | MR Egger | 16 | -0.063953721 | 0.339978542631768 | 0.853488943496438 | -0.730311664 | 0.602404222742696 | 0.938048410641608 | 0.481758819629169 | 1.82650485 | 0.999279047927446 |
| ebi-fl187-GCST90094781 | ebi-met1400-GCST90200462 | X-11299 levels \|\| id:ebi-met1400-GCST90200462 | Herring liking \|\| id:ebi-fl187-GCST90094781 | MR Egger | 16 | 0.224375363870914 | 0.29352665463742 | 0.457313131884828 | -0.350936879 | 0.799687606960257 | 1.25154071443676 | 0.704028191262825 | 2.22484579358005 | 0.999279047927446 |
| ebi-fl187-GCST90094781 | ebi-met1400-GCST90200497 | X-12740 levels \|\| id:ebi-met1400-GCST90200497 | Herring liking \|\| id:ebi-fl187-GCST90094781 | MR Egger | 16 | 0.665976281539398 | 0.344800190101443 | 0.0739229837972671 | -0.009832091 | 1.34178465413823 | 1.94638981850987 | 0.990216085925087 | 3.82586526259028 | 0.999279047927446 |
| ebi-fl187-GCST90094781 | ebi-met1400-GCST90200527 | X-13866 levels \|\| id:ebi-met1400-GCST90200527 | Herring liking \|\| id:ebi-fl187-GCST90094781 | MR Egger | 16 | -0.223156961 | 0.307948307760505 | 0.480598930913379 | -0.826735644 | 0.380421722570051 | 0.799989272610862 | 0.437475032295805 | 1.46290139790131 | 0.999279047927446 |
| ebi-fl187-GCST90094781 | ebi-met1400-GCST90200547 | X-17654 levels \|\| id:ebi-met1400-GCST90200547 | Herring liking \|\| id:ebi-fl187-GCST90094781 | MR Egger | 16 | 0.0531891393104175 | 0.298938455737121 | 0.861329341081025 | -0.532730234 | 0.639108512555175 | 1.05462909807692 | 0.58700013220398 | 1.89479094380176 | 0.999279047927446 |
| ebi-fl187-GCST90094781 | ebi-met1400-GCST90200553 | X-17653 levels \|\| id:ebi-met1400-GCST90200553 | Herring liking \|\| id:ebi-fl187-GCST90094781 | MR Egger | 16 | -0.093405861 | 0.29740140211066 | 0.758096561857875 | -0.67631261 | 0.489500886665133 | 0.910823756998406 | 0.508488535429696 | 1.63150171244597 | 0.999279047927446 |
| ebi-fl187-GCST90094781 | ebi-met1400-GCST90200557 | X-17685 levels \|\| id:ebi-met1400-GCST90200557 | Herring liking \|\| id:ebi-fl187-GCST90094781 | MR Egger | 16 | 0.263984497405369 | 0.313339896308749 | 0.413677574899696 | -0.350161699 | 0.878130694170518 | 1.30210801009178 | 0.704574151317893 | 2.40639720712688 | 0.999279047927446 |
| ebi-fl187-GCST90094781 | ebi-met1400-GCST90200561 | X-18779 levels \|\| id:ebi-met1400-GCST90200561 | Herring liking \|\| id:ebi-fl187-GCST90094781 | MR Egger | 16 | -0.41894454 | 0.313322213624551 | 0.20250951082351 | -1.033056079 | 0.195166998608084 | 0.657740672490825 | 0.355917584622674 | 1.21551395868043 | 0.999279047927446 |
| ebi-fl187-GCST90094781 | ebi-met1400-GCST90200570 | X-18921 levels \|\| id:ebi-met1400-GCST90200570 | Herring liking \|\| id:ebi-fl187-GCST90094781 | MR Egger | 16 | -0.100453326 | 0.295719554603404 | 0.739131386876883 | -0.680063653 | 0.479157000646637 | 0.904427324328512 | 0.506584745498506 | 1.61471262658548 | 0.999279047927446 |
| ebi-fl187-GCST90094781 | ebi-met1400-GCST90200575 | X-21283 levels \|\| id:ebi-met1400-GCST90200575 | Herring liking \|\| id:ebi-fl187-GCST90094781 | MR Egger | 16 | -0.210425969 | 0.320551003242841 | 0.522178443428981 | -0.838705935 | 0.417853997725758 | 0.810239036038782 | 0.432269546541662 | 1.51869892471776 | 0.999279047927446 |
| ebi-fl187-GCST90094781 | ebi-met1400-GCST90200578 | X-18922 levels \|\| id:ebi-met1400-GCST90200578 | Herring liking \|\| id:ebi-fl187-GCST90094781 | MR Egger | 16 | -0.00139865 | 0.296723107295428 | 0.996305571158839 | -0.58297594 | 0.580178640331307 | 0.998602327687282 | 0.55823461930515 | 1.78635751774712 | 0.999279047927446 |
| ebi-fl187-GCST90094781 | ebi-met1400-GCST90200623 | X-24241 levels \|\| id:ebi-met1400-GCST90200623 | Herring liking \|\| id:ebi-fl187-GCST90094781 | MR Egger | 16 | -0.075378258 | 0.298231176001025 | 0.804133866697121 | -0.659911363 | 0.509154846908638 | 0.927392626245397 | 0.516897148665871 | 1.66388436352216 | 0.999279047927446 |
| ebi-fl187-GCST90094781 | ebi-met1400-GCST90200636 | X-24544 levels \|\| id:ebi-met1400-GCST90200636 | Herring liking \|\| id:ebi-fl187-GCST90094781 | MR Egger | 16 | -0.75305661 | 0.276846536643985 | 0.0165894859139837 | -1.295675822 | -0.210437399 | 0.470924916581095 | 0.27371282058918 | 0.810229775059643 | 0.999279047927446 |
| ebi-fl187-GCST90094781 | ebi-met1400-GCST90200659 | X-25172 levels \|\| id:ebi-met1400-GCST90200659 | Herring liking \|\| id:ebi-fl187-GCST90094781 | MR Egger | 16 | -0.006644529 | 0.29982262881249 | 0.982631870502598 | -0.594296881 | 0.581007823713576 | 0.993377497311024 | 0.551950516231566 | 1.78783934998606 | 0.999279047927446 |
| ebi-fl187-GCST90094781 | ebi-met1400-GCST90200670 | X-26111 levels \|\| id:ebi-met1400-GCST90200670 | Herring liking \|\| id:ebi-fl187-GCST90094781 | MR Egger | 16 | 0.301389110579198 | 0.29771555768869 | 0.328553115362875 | -0.282133382 | 0.884911603649031 | 1.35173521369209 | 0.754173084339727 | 2.42277021797311 | 0.999279047927446 |
| ebi-fl187-GCST90094781 | ebi-met1400-GCST90200673 | Carnitine C4 levels \|\| id:ebi-met1400-GCST90200673 | Herring liking \|\| id:ebi-fl187-GCST90094781 | MR Egger | 16 | -0.421716803 | 0.313582773510202 | 0.200061843762459 | -1.036339039 | 0.192905433496477 | 0.655919767876968 | 0.354751037389303 | 1.21276810085728 | 0.999279047927446 |
| ebi-fl187-GCST90094781 | ebi-met1400-GCST90200710 | X-13431 levels \|\| id:ebi-met1400-GCST90200710 | Herring liking \|\| id:ebi-fl187-GCST90094781 | MR Egger | 16 | 0.555722857499734 | 0.29192377570858 | 0.077716776426192 | -0.016447743 | 1.12789345788855 | 1.74320061509078 | 0.983686782676877 | 3.08914223304252 | 0.999279047927446 |
| ebi-fl187-GCST90094781 | ebi-met1400-GCST90200730 | Adenosine 5'-diphosphate (ADP) to fructose ratio \|\| id:ebi-met1400-GCST90200730 | Herring liking \|\| id:ebi-fl187-GCST90094781 | MR Egger | 16 | 0.186783022453251 | 0.398031038411956 | 0.646103008820058 | -0.593357813 | 0.966923857740685 | 1.20536571937729 | 0.552469078963006 | 2.62984223511124 | 0.999279047927446 |
| ebi-fl187-GCST90094781 | ebi-met1400-GCST90200731 | Adenosine 5'-diphosphate (ADP) to mannose ratio \|\| id:ebi-met1400-GCST90200731 | Herring liking \|\| id:ebi-fl187-GCST90094781 | MR Egger | 16 | -0.189443741 | 0.3942203234679 | 0.638256905433515 | -0.962115575 | 0.583228092778595 | 0.827419265187727 | 0.382083703520299 | 1.79181324431291 | 0.999279047927446 |
| ebi-fl187-GCST90094781 | ebi-met1400-GCST90200748 | Aspartate to glutamate ratio \|\| id:ebi-met1400-GCST90200748 | Herring liking \|\| id:ebi-fl187-GCST90094781 | MR Egger | 16 | 0.0825813531547939 | 0.311099314517638 | 0.794531522544324 | -0.527173303 | 0.692336009609364 | 1.08608702645109 | 0.590271131161048 | 1.99837831591927 | 0.999279047927446 |
| ebi-fl187-GCST90094781 | ebi-met1400-GCST90200774 | Uridine to pseudouridine ratio \|\| id:ebi-met1400-GCST90200774 | Herring liking \|\| id:ebi-fl187-GCST90094781 | MR Egger | 16 | 0.277712737493124 | 0.281882868213831 | 0.341244211938785 | -0.274777684 | 0.830203159192234 | 1.32010692551167 | 0.759741006876711 | 2.29378469637702 | 0.999279047927446 |
| ebi-fl187-GCST90094781 | ebi-met1400-GCST90200799 | Spermidine to adenosine 5'-diphosphate (ADP) ratio \|\| id:ebi-met1400-GCST90200799 | Herring liking \|\| id:ebi-fl187-GCST90094781 | MR Egger | 16 | 0.135416904880138 | 0.420026394553841 | 0.751913112495634 | -0.687834828 | 0.958668638205667 | 1.14501404680182 | 0.502663243838496 | 2.60822167414077 | 0.999279047927446 |
| ebi-fl187-GCST90094781 | ebi-met1400-GCST90200811 | Choline to taurocholate ratio \|\| id:ebi-met1400-GCST90200811 | Herring liking \|\| id:ebi-fl187-GCST90094781 | MR Egger | 16 | 0.431963619867773 | 0.308873694477677 | 0.183719483812322 | -0.173428821 | 1.03735606104402 | 1.54027907858528 | 0.840776994438403 | 2.8217466172611 | 0.999279047927446 |
| ebi-fl187-GCST90094781 | ebi-met1400-GCST90200812 | Glutamate to kynurenine ratio \|\| id:ebi-met1400-GCST90200812 | Herring liking \|\| id:ebi-fl187-GCST90094781 | MR Egger | 16 | 0.118259795137908 | 0.286952669780153 | 0.686490164653511 | -0.444167438 | 0.680687027907008 | 1.12553648229306 | 0.64135802436812 | 1.97523430726661 | 0.999279047927446 |
| ebi-fl187-GCST90094781 | ebi-met1400-GCST90200876 | Cortisone to 4-cholesten-3-one ratio \|\| id:ebi-met1400-GCST90200876 | Herring liking \|\| id:ebi-fl187-GCST90094781 | MR Egger | 16 | -0.155229389 | 0.455968947551613 | 0.738580700022058 | -1.048928526 | 0.738469748320293 | 0.856218747894736 | 0.350312899228134 | 2.09273065839636 | 0.999279047927446 |
| ebi-fl187-GCST90094781 | ebi-met1400-GCST90200906 | 2'-deoxyuridine to cytidine ratio \|\| id:ebi-met1400-GCST90200906 | Herring liking \|\| id:ebi-fl187-GCST90094781 | MR Egger | 16 | -0.234889743 | 0.395765440146535 | 0.562308497255778 | -1.010590006 | 0.540810519778599 | 0.790658020476667 | 0.364004151716523 | 1.71739828349794 | 0.999279047927446 |
| ebi-fl187-GCST90094781 | ebi-met1400-GCST90200907 | Retinol (Vitamin A) to linoleoyl-arachidonoyl-glycerol (18:2 to 20:4) [1] ratio \|\| id:ebi-met1400-GCST90200907 | Herring liking \|\| id:ebi-fl187-GCST90094781 | MR Egger | 16 | -0.232822763 | 0.368132281848917 | 0.537290188691917 | -0.954362035 | 0.488716509669486 | 0.792293985084496 | 0.385057719489935 | 1.63022250179166 | 0.999279047927446 |
| ebi-fl187-GCST90094781 | ebi-met1400-GCST90200916 | Fructose to sucrose ratio \|\| id:ebi-met1400-GCST90200916 | Herring liking \|\| id:ebi-fl187-GCST90094781 | MR Egger | 16 | 0.0767189960232685 | 0.304020143231258 | 0.804438916573955 | -0.519160485 | 0.672598476756534 | 1.07973862291191 | 0.595019866623933 | 1.95932196419342 | 0.999279047927446 |
| ebi-fl187-GCST90094781 | ebi-met1400-GCST90200917 | Alpha-ketobutyrate to 3-methyl-2-oxobutyrate ratio \|\| id:ebi-met1400-GCST90200917 | Herring liking \|\| id:ebi-fl187-GCST90094781 | MR Egger | 16 | -0.394025323 | 0.296966814842727 | 0.205799547741592 | -0.97608028 | 0.188029633895951 | 0.674336979656862 | 0.376785100112535 | 1.20686927905834 | 0.999279047927446 |
| ebi-fl187-GCST90094781 | ebi-met1400-GCST90200924 | Alpha-tocopherol to glycerol ratio \|\| id:ebi-met1400-GCST90200924 | Herring liking \|\| id:ebi-fl187-GCST90094781 | MR Egger | 16 | 0.0138971649669652 | 0.27561133532768 | 0.96049761061377 | -0.526301052 | 0.554095382209218 | 1.01399417945185 | 0.590786220370817 | 1.74036590649808 | 0.999279047927446 |
| ebi-fl187-GCST90094781 | ebi-met1400-GCST90200938 | Alpha-ketoglutarate to alanine ratio \|\| id:ebi-met1400-GCST90200938 | Herring liking \|\| id:ebi-fl187-GCST90094781 | MR Egger | 16 | -0.080383398 | 0.293408437931852 | 0.788112846799206 | -0.655463936 | 0.494697140460417 | 0.922762493368472 | 0.519201130486446 | 1.64000147374453 | 0.999279047927446 |
| ebi-fl187-GCST90094781 | ebi-met1400-GCST90200943 | Phosphate to EDTA ratio \|\| id:ebi-met1400-GCST90200943 | Herring liking \|\| id:ebi-fl187-GCST90094781 | MR Egger | 16 | 0.705023857798836 | 0.281100429221202 | 0.0250718328021007 | 0.15406701652528 | 1.25598069907239 | 2.02389497002542 | 1.16656906356398 | 3.51128019560201 | 0.999279047927446 |
| ebi-fl187-GCST90094781 | ebi-met1400-GCST90200947 | Adenosine 5'-diphosphate (ADP) to glucose ratio \|\| id:ebi-met1400-GCST90200947 | Herring liking \|\| id:ebi-fl187-GCST90094781 | MR Egger | 16 | 0.138631323662158 | 0.395548659080367 | 0.73119694232927 | -0.636644048 | 0.913906695459677 | 1.14870052322486 | 0.529064964653619 | 2.49404700785838 | 0.999279047927446 |
| ebi-fl187-GCST90094781 | ebi-met1400-GCST90200975 | Arachidonate (20:4n6) to caffeine ratio \|\| id:ebi-met1400-GCST90200975 | Herring liking \|\| id:ebi-fl187-GCST90094781 | MR Egger | 16 | 0.0946475728289802 | 0.302033683203827 | 0.758621409499816 | -0.497338446 | 0.686633591908482 | 1.0992713737502 | 0.608147123863041 | 1.98701515756701 | 0.999279047927446 |
| ebi-fl187-GCST90094781 | ebi-met1400-GCST90200985 | Cholesterol to oleoyl-linoleoyl-glycerol (18:1 to 18:2) [2] ratio \|\| id:ebi-met1400-GCST90200985 | Herring liking \|\| id:ebi-fl187-GCST90094781 | MR Egger | 16 | -0.356509982 | 0.303123467902175 | 0.259158866209077 | -0.950631979 | 0.237612015007637 | 0.700115482825543 | 0.386496688399388 | 1.26821704817696 | 0.999279047927446 |
| ebi-fl187-GCST90094781 | ebi-met1400-GCST90201016 | Bilirubin (Z,Z) to etiocholanolone glucuronide ratio \|\| id:ebi-met1400-GCST90201016 | Herring liking \|\| id:ebi-fl187-GCST90094781 | MR Egger | 16 | 0.252451703176136 | 0.313687362011826 | 0.434397306653954 | -0.362375526 | 0.867278932719316 | 1.28717732807685 | 0.696020944542482 | 2.3804247370805 | 0.999279047927446 |
| ebi-fl187-GCST90094781 | ebi-pw11-GCST90275189 | Placental weight (fetal genotype, adjusted for sex and gestational age) \|\| id:ebi-pw11-GCST90275189 | Herring liking \|\| id:ebi-fl187-GCST90094781 | MR Egger | 15 | -0.019789528 | 0.151033182028789 | 0.897759397471666 | -0.315814565 | 0.276235508460912 | 0.980404999084763 | 0.729194655775269 | 1.31815826489906 | 0.999279047927446 |
| ebi-fl187-GCST90094781 | ebi-pw11-GCST90275192 | Placental weight (fetal effect, conditional on maternal and paternal genotype) \|\| id:ebi-pw11-GCST90275192 | Herring liking \|\| id:ebi-fl187-GCST90094781 | MR Egger | 14 | -0.026261665 | 0.290514870922643 | 0.929463115113077 | -0.595670812 | 0.543147481815281 | 0.974080173382191 | 0.551192695039569 | 1.72141647143594 | 0.999279047927446 |
| ebi-fl187-GCST90094781 | ebi-pw11-GCST90275193 | Placental weight (maternal effect, conditional on fetal and paternal genotype) \|\| id:ebi-pw11-GCST90275193 | Herring liking \|\| id:ebi-fl187-GCST90094781 | MR Egger | 14 | -0.089156682 | 0.211558763102265 | 0.680893404609465 | -0.503811858 | 0.325498493435133 | 0.91470224474471 | 0.604223051940864 | 1.38472074816321 | 0.999279047927446 |
| ebi-fl187-GCST90094781 | ebi-pw11-GCST90275195 | Placental weight (fetal effect, conditional on maternal genotype) \|\| id:ebi-pw11-GCST90275195 | Herring liking \|\| id:ebi-fl187-GCST90094781 | MR Egger | 14 | 0.050666690661528 | 0.1700037815067 | 0.770775387172942 | -0.282540721 | 0.383874102414661 | 1.05197220268326 | 0.753865943090153 | 1.46796061735067 | 0.999279047927446 |
| ebi-fl187-GCST90094781 | ebi-pw11-GCST90275197 | Placental weight (fetal genotype, adjusted for sex) \|\| id:ebi-pw11-GCST90275197 | Herring liking \|\| id:ebi-fl187-GCST90094781 | MR Egger | 15 | -0.001929547 | 0.136967508544934 | 0.98897398143583 | -0.270385864 | 0.266526769925944 | 0.998072313556588 | 0.763084990822495 | 1.30542253493218 | 0.999279047927446 |
| ebi-fl187-GCST90094781 | prot-a-1030 | Protein FAM171B \|\| id:prot-a-1030 | Herring liking \|\| id:ebi-fl187-GCST90094781 | MR Egger | 19 | -0.10293725 | 0.383197664332054 | 0.791449426721732 | -0.854004672 | 0.64813017227543 | 0.902183583891777 | 0.425706698119433 | 1.91196244418842 | 0.999279047927446 |
| ebi-fl187-GCST90094781 | prot-a-1036 | BRISC complex subunit Abro1 \|\| id:prot-a-1036 | Herring liking \|\| id:ebi-fl187-GCST90094781 | MR Egger | 19 | 0.301560524264076 | 0.38313548200677 | 0.442067198762872 | -0.44938502 | 1.05250606899734 | 1.35196693946597 | 0.638020400483435 | 2.86482157000626 | 0.999279047927446 |
| ebi-fl187-GCST90094781 | prot-a-1055 | Phenylalanine--tRNA ligase, mitochondrial \|\| id:prot-a-1055 | Herring liking \|\| id:ebi-fl187-GCST90094781 | MR Egger | 19 | -0.85248268 | 0.382630136376615 | 0.0396766598880553 | -1.602437747 | -0.102527612 | 0.426355113702916 | 0.201404944768371 | 0.902553227725782 | 0.999279047927446 |
| ebi-fl187-GCST90094781 | prot-a-1083 | Fc receptor-like protein 5 \|\| id:prot-a-1083 | Herring liking \|\| id:ebi-fl187-GCST90094781 | MR Egger | 19 | 0.176112152332308 | 0.382775484866168 | 0.651279833610825 | -0.574127798 | 0.926352102669998 | 1.19257180085969 | 0.563195875213167 | 2.52528039142229 | 0.999279047927446 |
| ebi-fl187-GCST90094781 | prot-a-1103 | Fibroblast growth factor receptor 3 \|\| id:prot-a-1103 | Herring liking \|\| id:ebi-fl187-GCST90094781 | MR Egger | 19 | -0.547989116 | 0.409600523968407 | 0.198564201847082 | -1.350806143 | 0.254827910641484 | 0.57811115660352 | 0.259031360056099 | 1.29023956526762 | 0.999279047927446 |
| ebi-fl187-GCST90094781 | prot-a-1105 | Fibroblast growth factor receptor 4 \|\| id:prot-a-1105 | Herring liking \|\| id:ebi-fl187-GCST90094781 | MR Egger | 19 | -0.194222794 | 0.448893322769904 | 0.67069330122955 | -1.074053706 | 0.685608118762347 | 0.823474418779756 | 0.341620876009277 | 1.9849785712927 | 0.999279047927446 |
| ebi-fl187-GCST90094781 | prot-a-1120 | Filamin-A \|\| id:prot-a-1120 | Herring liking \|\| id:ebi-fl187-GCST90094781 | MR Egger | 19 | 0.520636112703494 | 0.44010030841879 | 0.253092252737251 | -0.341960492 | 1.38323271720432 | 1.68309794923396 | 0.71037626984441 | 3.98777215254675 | 0.999279047927446 |
| ebi-fl187-GCST90094781 | prot-a-1166 | Galanin peptides \|\| id:prot-a-1166 | Herring liking \|\| id:ebi-fl187-GCST90094781 | MR Egger | 19 | 0.329749992298077 | 0.382691954006701 | 0.400866298428053 | -0.420326238 | 1.07982622215121 | 1.39062041918534 | 0.656832501428257 | 2.944167875 | 0.999279047927446 |
| ebi-fl187-GCST90094781 | prot-a-1205 | ADP-ribosylation factor-binding protein GGA1 \|\| id:prot-a-1205 | Herring liking \|\| id:ebi-fl187-GCST90094781 | MR Egger | 19 | 0.630168592517856 | 0.409843343329975 | 0.142554278899745 | -0.17312436 | 1.43346154544461 | 1.87792715704503 | 0.841033017130946 | 4.1931890131944 | 0.999279047927446 |
| ebi-fl187-GCST90094781 | prot-a-1227 | Guanine nucleotide-binding protein G(I)/G(S)/G(O) subunit gamma-T2 \|\| id:prot-a-1227 | Herring liking \|\| id:ebi-fl187-GCST90094781 | MR Egger | 19 | -0.093117665 | 0.383051678957426 | 0.810841339624064 | -0.843898956 | 0.657663625417721 | 0.911086290711735 | 0.430030580192961 | 1.9302772113332 | 0.999279047927446 |
| ebi-fl187-GCST90094781 | prot-a-1240 | Golgi SNAP receptor complex member 1 \|\| id:prot-a-1240 | Herring liking \|\| id:ebi-fl187-GCST90094781 | MR Egger | 19 | 0.208521915138182 | 0.382775484866168 | 0.59299171418326 | -0.541718035 | 0.958761865475872 | 1.23185592605797 | 0.581747929904616 | 2.60846484286235 | 0.999279047927446 |
| ebi-fl187-GCST90094781 | prot-a-1283 | Glutathione S-transferase A1 \|\| id:prot-a-1283 | Herring liking \|\| id:ebi-fl187-GCST90094781 | MR Egger | 19 | -0.474536328 | 0.417363140177056 | 0.271316987816365 | -1.292568083 | 0.343495426463524 | 0.622173473836935 | 0.274564771798721 | 1.40986707438963 | 0.999279047927446 |
| ebi-fl187-GCST90094781 | prot-a-1324 | E3 ubiquitin-protein ligase HECW1 \|\| id:prot-a-1324 | Herring liking \|\| id:ebi-fl187-GCST90094781 | MR Egger | 19 | 0.291071786943766 | 0.383051678957426 | 0.45773790709895 | -0.459709504 | 1.04185307770032 | 1.33786062142807 | 0.631467057637968 | 2.83446463393199 | 0.999279047927446 |
| ebi-fl187-GCST90094781 | prot-a-1339 | Histidine triad nucleotide-binding protein 2, mitochondrial \|\| id:prot-a-1339 | Herring liking \|\| id:ebi-fl187-GCST90094781 | MR Egger | 19 | 0.569331427797249 | 0.522709854503175 | 0.291269082312954 | -0.455179887 | 1.59384274262347 | 1.76708523234582 | 0.63433384925278 | 4.92262902579293 | 0.999279047927446 |
| ebi-fl187-GCST90094781 | prot-a-1466 | Interleukin-11 \|\| id:prot-a-1466 | Herring liking \|\| id:ebi-fl187-GCST90094781 | MR Egger | 19 | 0.0108847339314359 | 0.457218845675685 | 0.98128424689547 | -0.885264204 | 0.907033671455779 | 1.01094418816652 | 0.412605147114812 | 2.47696413564922 | 0.999279047927446 |
| ebi-fl187-GCST90094781 | prot-a-147 | Rho GTPase-activating protein 1 \|\| id:prot-a-147 | Herring liking \|\| id:ebi-fl187-GCST90094781 | MR Egger | 19 | -0.032291062 | 0.382643145771205 | 0.93373259244136 | -0.782271628 | 0.717689503577084 | 0.968224727506749 | 0.457365865304749 | 2.04969192952534 | 0.999279047927446 |
| ebi-fl187-GCST90094781 | prot-a-1473 | Interleukin-12 receptor subunit beta-1 \|\| id:prot-a-1473 | Herring liking \|\| id:ebi-fl187-GCST90094781 | MR Egger | 19 | -0.345991389 | 0.38313548200677 | 0.379116476761313 | -1.096936934 | 0.404954155378495 | 0.70751857927175 | 0.333892252923538 | 1.49923376667667 | 0.999279047927446 |
| ebi-fl187-GCST90094781 | prot-a-1501 | X-linked interleukin-1 receptor accessory protein-like 2 \|\| id:prot-a-1501 | Herring liking \|\| id:ebi-fl187-GCST90094781 | MR Egger | 19 | -0.264598086 | 0.433509753253088 | 0.54969509967998 | -1.114277202 | 0.585081030544277 | 0.767514362890706 | 0.328152381017318 | 1.79513644062951 | 0.999279047927446 |
| ebi-fl187-GCST90094781 | prot-a-1503 | Interleukin-1 receptor-like 2 \|\| id:prot-a-1503 | Herring liking \|\| id:ebi-fl187-GCST90094781 | MR Egger | 19 | 0.162759786033555 | 0.470048684853781 | 0.733394719819935 | -0.758535636 | 1.08405520834697 | 1.17675398299299 | 0.468351762426445 | 2.95664508512947 | 0.999279047927446 |
| ebi-fl187-GCST90094781 | prot-a-1536 | Interleukin-5 receptor subunit alpha \|\| id:prot-a-1536 | Herring liking \|\| id:ebi-fl187-GCST90094781 | MR Egger | 19 | 0.829754141180598 | 0.383113803584305 | 0.0448329010524292 | 0.078851086155361 | 1.58065719620584 | 2.29275497693212 | 1.08204317886256 | 4.85814751845014 | 0.999279047927446 |
| ebi-fl187-GCST90094781 | prot-a-1559 | Insulin-induced gene 1 protein \|\| id:prot-a-1559 | Herring liking \|\| id:ebi-fl187-GCST90094781 | MR Egger | 19 | -0.14026822 | 0.382750360112641 | 0.718532364279541 | -0.890458925 | 0.609922486183529 | 0.86912508771706 | 0.410467335610942 | 1.84028874544884 | 0.999279047927446 |
| ebi-fl187-GCST90094781 | prot-a-1567 | Interferon regulatory factor 1 \|\| id:prot-a-1567 | Herring liking \|\| id:ebi-fl187-GCST90094781 | MR Egger | 19 | -0.112157008 | 0.38313548200677 | 0.773267974093633 | -0.863102553 | 0.638788536383496 | 0.893903896106107 | 0.42185123403432 | 1.89418475283794 | 0.999279047927446 |
| ebi-fl187-GCST90094781 | prot-a-1613 | Potassium voltage-gated channel subfamily E regulatory beta subunit 5 \|\| id:prot-a-1613 | Herring liking \|\| id:ebi-fl187-GCST90094781 | MR Egger | 19 | -0.302736315 | 0.496946014181567 | 0.550451527260864 | -1.276750503 | 0.671277872477794 | 0.738793879317789 | 0.27894225131643 | 1.95673618299673 | 0.999279047927446 |
| ebi-fl187-GCST90094781 | prot-a-1649 | Kin of IRRE-like protein 2 \|\| id:prot-a-1649 | Herring liking \|\| id:ebi-fl187-GCST90094781 | MR Egger | 19 | 0.0333658814209363 | 0.382666831101993 | 0.931536901268704 | -0.716661108 | 0.783392870380843 | 1.03392876536995 | 0.488380185626457 | 2.18888628843159 | 0.999279047927446 |
| ebi-fl187-GCST90094781 | prot-a-165 | Armadillo repeat-containing protein 5 \|\| id:prot-a-165 | Herring liking \|\| id:ebi-fl187-GCST90094781 | MR Egger | 19 | -0.232319931 | 0.383095383892724 | 0.552243136902469 | -0.983186884 | 0.518547021270236 | 0.792692475711079 | 0.374116929918984 | 1.67958547394536 | 0.999279047927446 |
| ebi-fl187-GCST90094781 | prot-a-1656 | Kallikrein-11 \|\| id:prot-a-1656 | Herring liking \|\| id:ebi-fl187-GCST90094781 | MR Egger | 19 | 0.286814406248841 | 0.385695312417785 | 0.467254766995185 | -0.469148406 | 1.0427772185877 | 1.33217694681404 | 0.625534743102073 | 2.83708528933492 | 0.999279047927446 |
| ebi-fl187-GCST90094781 | prot-a-1697 | Laminin subunit gamma-2 \|\| id:prot-a-1697 | Herring liking \|\| id:ebi-fl187-GCST90094781 | MR Egger | 19 | -0.366105837 | 0.393156067598606 | 0.364788082451986 | -1.13669173 | 0.404480055328407 | 0.693429406723453 | 0.32087882168659 | 1.4985231483382 | 0.999279047927446 |
| ebi-fl187-GCST90094781 | prot-a-1698 | Lysosome-associated membrane glycoprotein 3 \|\| id:prot-a-1698 | Herring liking \|\| id:ebi-fl187-GCST90094781 | MR Egger | 19 | 0.233315832968848 | 0.418149503174504 | 0.584137398003316 | -0.586257193 | 1.05288885919088 | 1.26278024398589 | 0.556405912159578 | 2.86591840552502 | 0.999279047927446 |
| ebi-fl187-GCST90094781 | prot-a-1724 | Leptin receptor \|\| id:prot-a-1724 | Herring liking \|\| id:ebi-fl187-GCST90094781 | MR Egger | 19 | -0.09711413 | 0.382775484866168 | 0.802762271082494 | -0.847354081 | 0.653125819840703 | 0.907452432225832 | 0.428547334771229 | 1.92153783243611 | 0.999279047927446 |
| ebi-fl187-GCST90094781 | prot-a-1743 | Leukocyte immunoglobulin-like receptor subfamily B member 2 \|\| id:prot-a-1743 | Herring liking \|\| id:ebi-fl187-GCST90094781 | MR Egger | 19 | 0.406989801631773 | 0.46134325396715 | 0.389980898559137 | -0.497242976 | 1.31122257940739 | 1.50228878468962 | 0.608205186505412 | 3.71070757480974 | 0.999279047927446 |
| ebi-fl187-GCST90094781 | prot-a-1746 | Leukocyte immunoglobulin-like receptor subfamily B member 4 \|\| id:prot-a-1746 | Herring liking \|\| id:ebi-fl187-GCST90094781 | MR Egger | 19 | -0.143718682 | 0.410642311559253 | 0.730648428760886 | -0.948577613 | 0.661140248254226 | 0.86613137178692 | 0.387291510245982 | 1.9369997362375 | 0.999279047927446 |
| ebi-fl187-GCST90094781 | prot-a-1750 | Leucine-rich repeat and immunoglobulin-like domain-containing nogo receptor-interacting protein 3 \|\| id:prot-a-1750 | Herring liking \|\| id:ebi-fl187-GCST90094781 | MR Egger | 19 | -0.441620646 | 0.382691954006701 | 0.264472423254694 | -1.191696876 | 0.308455583594827 | 0.642993511189375 | 0.303705476009097 | 1.36132104321773 | 0.999279047927446 |
| ebi-fl187-GCST90094781 | prot-a-1753 | Lipase member N \|\| id:prot-a-1753 | Herring liking \|\| id:ebi-fl187-GCST90094781 | MR Egger | 19 | 0.0452624796208198 | 0.383051678957426 | 0.90732357995844 | -0.705518811 | 0.796043770377374 | 1.04630245694124 | 0.493852291712423 | 2.21675357140746 | 0.999279047927446 |
| ebi-fl187-GCST90094781 | prot-a-1760 | Ig Kappa chain V-I region HK102- like \|\| id:prot-a-1760 | Herring liking \|\| id:ebi-fl187-GCST90094781 | MR Egger | 19 | -0.171207343 | 0.426766060462643 | 0.693289830673053 | -1.007668821 | 0.665254135795478 | 0.842646838677051 | 0.365069029552953 | 1.94498474878005 | 0.999279047927446 |
| ebi-fl187-GCST90094781 | prot-a-1792 | Leucine-rich repeat neuronal protein 1 \|\| id:prot-a-1792 | Herring liking \|\| id:ebi-fl187-GCST90094781 | MR Egger | 19 | -0.475907302 | 0.414585722294128 | 0.266903265915373 | -1.288495318 | 0.336680713750634 | 0.621321074832695 | 0.275685289953021 | 1.4002918984072 | 0.999279047927446 |
| ebi-fl187-GCST90094781 | prot-a-1795 | Leucine-rich repeat transmembrane neuronal protein 1 \|\| id:prot-a-1795 | Herring liking \|\| id:ebi-fl187-GCST90094781 | MR Egger | 19 | -0.198168104 | 0.472967917716273 | 0.68047077192864 | -1.125185223 | 0.728849014534234 | 0.820231957119222 | 0.324592339851121 | 2.07269359402692 | 0.999279047927446 |
| ebi-fl187-GCST90094781 | prot-a-1820 | LysM and putative peptidoglycan-binding domain-containing protein 4 \|\| id:prot-a-1820 | Herring liking \|\| id:ebi-fl187-GCST90094781 | MR Egger | 19 | -0.17503219 | 0.393162068255622 | 0.661798527269795 | -0.945629843 | 0.595565464158539 | 0.839429999399526 | 0.388434840716882 | 1.81405643889056 | 0.999279047927446 |
| ebi-fl187-GCST90094781 | prot-a-1844 | Mitogen-activated protein kinase kinase kinase 7:TGF-beta-activated kinase 1 and MAP3K7-binding protein 1 fusion \|\| id:prot-a-1844 | Herring liking \|\| id:ebi-fl187-GCST90094781 | MR Egger | 19 | -0.853736044 | 0.383051678957426 | 0.0396124151427934 | -1.604517335 | -0.102954753 | 0.42582107027233 | 0.200986540763936 | 0.902167792921226 | 0.999279047927446 |
| ebi-fl187-GCST90094781 | prot-a-1856 | Megakaryocyte-associated tyrosine-protein kinase \|\| id:prot-a-1856 | Herring liking \|\| id:ebi-fl187-GCST90094781 | MR Egger | 19 | -0.219032613 | 0.383113803584305 | 0.574996068363518 | -0.969935668 | 0.531870442044907 | 0.803295519865438 | 0.37910742605575 | 1.70211303679657 | 0.999279047927446 |
| ebi-fl187-GCST90094781 | prot-a-1910 | Stromelysin-2 \|\| id:prot-a-1910 | Herring liking \|\| id:ebi-fl187-GCST90094781 | MR Egger | 19 | -0.537271408 | 0.431114776000019 | 0.229577938567142 | -1.382256369 | 0.307713553279555 | 0.584340506097381 | 0.251011539038898 | 1.36031127641996 | 0.999279047927446 |
| ebi-fl187-GCST90094781 | prot-a-1940 | 39S ribosomal protein L14, mitochondrial \|\| id:prot-a-1940 | Herring liking \|\| id:ebi-fl187-GCST90094781 | MR Egger | 19 | -0.23518949 | 0.510177211605507 | 0.650644740464146 | -1.235136824 | 0.764757845141286 | 0.790421058862658 | 0.290794971812084 | 2.14847404822838 | 0.999279047927446 |
| ebi-fl187-GCST90094781 | prot-a-1942 | 39S ribosomal protein L33, mitochondrial \|\| id:prot-a-1942 | Herring liking \|\| id:ebi-fl187-GCST90094781 | MR Egger | 19 | 0.276511473383807 | 0.45392121531673 | 0.550472080115904 | -0.613174109 | 1.1661970554046 | 1.31852208054086 | 0.54162894861505 | 3.20976284838387 | 0.999279047927446 |
| ebi-fl187-GCST90094781 | prot-a-1948 | Mesothelin \|\| id:prot-a-1948 | Herring liking \|\| id:ebi-fl187-GCST90094781 | MR Egger | 19 | 0.276011220834905 | 0.440296617336963 | 0.539069049367983 | -0.586970149 | 1.13899259081535 | 1.31786265146358 | 0.556009360664619 | 3.12362001612095 | 0.999279047927446 |
| ebi-fl187-GCST90094781 | prot-a-1980 | Unconventional myosin-VI \|\| id:prot-a-1980 | Herring liking \|\| id:ebi-fl187-GCST90094781 | MR Egger | 19 | -0.248505723 | 0.434592997273719 | 0.574932682508643 | -1.100307998 | 0.603296551672782 | 0.7799653970931 | 0.332768575976689 | 1.82813542077126 | 0.999279047927446 |
| ebi-fl187-GCST90094781 | prot-a-2039 | Neurofascin \|\| id:prot-a-2039 | Herring liking \|\| id:ebi-fl187-GCST90094781 | MR Egger | 19 | -0.760530299 | 0.382691954006701 | 0.0632436972288903 | -1.510606529 | -0.010454069 | 0.467418489685539 | 0.220776030294325 | 0.989600384646134 | 0.999279047927446 |
| ebi-fl187-GCST90094781 | prot-a-2041 | NFU1 iron-sulfur cluster scaffold homolog, mitochondrial \|\| id:prot-a-2041 | Herring liking \|\| id:ebi-fl187-GCST90094781 | MR Egger | 19 | 0.684954682061821 | 0.382630136376615 | 0.0912606949406468 | -0.065000385 | 1.43490974935999 | 1.98368193712478 | 0.937067102385029 | 4.19926600524097 | 0.999279047927446 |
| ebi-fl187-GCST90094781 | prot-a-2088 | Glucocorticoid receptor \|\| id:prot-a-2088 | Herring liking \|\| id:ebi-fl187-GCST90094781 | MR Egger | 19 | 0.00019499120646088 | 0.411949974723739 | 0.999627841815304 | -0.807226959 | 0.807616941664989 | 1.00019501021848 | 0.446093387774513 | 2.2425574686429 | 0.999947616389678 |
| ebi-fl187-GCST90094781 | prot-a-2111 | Neuron-specific protein family member 2 \|\| id:prot-a-2111 | Herring liking \|\| id:ebi-fl187-GCST90094781 | MR Egger | 19 | 0.0696347040768747 | 0.382775484866168 | 0.857796710935108 | -0.680605246 | 0.819874654414564 | 1.07211646993772 | 0.506310457099315 | 2.27021525823679 | 0.999279047927446 |
| ebi-fl187-GCST90094781 | prot-a-2125 | Nuclear migration protein nudC \|\| id:prot-a-2125 | Herring liking \|\| id:ebi-fl187-GCST90094781 | MR Egger | 19 | 0.702529825038258 | 0.383051678957426 | 0.0842147640182129 | -0.048251466 | 1.45331111579481 | 2.01885359895171 | 0.95289413673829 | 4.27725357609127 | 0.999279047927446 |
| ebi-fl187-GCST90094781 | prot-a-2142 | Ornithine decarboxylase \|\| id:prot-a-2142 | Herring liking \|\| id:ebi-fl187-GCST90094781 | MR Egger | 19 | -0.519388975 | 0.451842844155957 | 0.266269424525628 | -1.40500095 | 0.36622299958885 | 0.594883925918894 | 0.245366823476403 | 1.4422768339368 | 0.999279047927446 |
| ebi-fl187-GCST90094781 | prot-a-2186 | Prostate and testis expressed protein 1 \|\| id:prot-a-2186 | Herring liking \|\| id:ebi-fl187-GCST90094781 | MR Egger | 19 | 0.417936633276606 | 0.382761008854117 | 0.290110715359625 | -0.332274944 | 1.16814821063067 | 1.51882442842544 | 0.717290081080841 | 3.21603170770999 | 0.999279047927446 |
| ebi-fl187-GCST90094781 | prot-a-2227 | Platelet-derived growth factor C \|\| id:prot-a-2227 | Herring liking \|\| id:ebi-fl187-GCST90094781 | MR Egger | 19 | 0.342823051223591 | 0.383113803584305 | 0.383371003618667 | -0.408080004 | 1.09372610624883 | 1.40891943329812 | 0.66492568011216 | 2.98537720665303 | 0.999279047927446 |
| ebi-fl187-GCST90094781 | prot-a-2229 | Platelet-derived growth factor receptor alpha \|\| id:prot-a-2229 | Herring liking \|\| id:ebi-fl187-GCST90094781 | MR Egger | 19 | -0.001857066 | 0.405554412682765 | 0.996399741497061 | -0.796743715 | 0.793029582695344 | 0.998144657117577 | 0.450794492067024 | 2.21008191995442 | 0.999279047927446 |
| ebi-fl187-GCST90094781 | prot-a-2243 | Platelet endothelial aggregation receptor 1 \|\| id:prot-a-2243 | Herring liking \|\| id:ebi-fl187-GCST90094781 | MR Egger | 19 | -0.098528248 | 0.383002815989002 | 0.800072114141179 | -0.849213768 | 0.652157270992308 | 0.906170094446233 | 0.427751111520621 | 1.91967763017516 | 0.999279047927446 |
| ebi-fl187-GCST90094781 | prot-a-2246 | E3 ubiquitin-protein ligase pellino homolog 2 \|\| id:prot-a-2246 | Herring liking \|\| id:ebi-fl187-GCST90094781 | MR Egger | 19 | 1.00349178159863 | 0.446485144474922 | 0.0381688913955997 | 0.128380898427784 | 1.87860266476948 | 2.72779006561052 | 1.13698599640696 | 6.54435381399382 | 0.999279047927446 |
| ebi-fl187-GCST90094781 | prot-a-2251 | Platelet factor 4 variant \|\| id:prot-a-2251 | Herring liking \|\| id:ebi-fl187-GCST90094781 | MR Egger | 19 | -0.772719355 | 0.393344587708505 | 0.0660367375743997 | -1.543674747 | -0.001763963 | 0.46175568194584 | 0.213594750898257 | 0.998237592040973 | 0.999279047927446 |
| ebi-fl187-GCST90094781 | prot-a-2346 | Protein phosphatase 1L \|\| id:prot-a-2346 | Herring liking \|\| id:ebi-fl187-GCST90094781 | MR Egger | 19 | 0.404912109748422 | 0.383113803584305 | 0.305346796437742 | -0.345990945 | 1.15581516477366 | 1.4991707317735 | 0.707518893465226 | 3.17661182445408 | 0.999279047927446 |
| ebi-fl187-GCST90094781 | prot-a-2358 | PR domain zinc finger protein 4 \|\| id:prot-a-2358 | Herring liking \|\| id:ebi-fl187-GCST90094781 | MR Egger | 19 | 0.358640222138738 | 0.382691954006701 | 0.361804782497517 | -0.391436008 | 1.10871645199187 | 1.43138172946417 | 0.676085313354906 | 3.03046614823935 | 0.999279047927446 |
| ebi-fl187-GCST90094781 | prot-a-2390 | Trypsin-1 \|\| id:prot-a-2390 | Herring liking \|\| id:ebi-fl187-GCST90094781 | MR Egger | 19 | -0.179170989 | 0.383051678957426 | 0.645906369817478 | -0.92995228 | 0.571610301924593 | 0.835962946847895 | 0.394572539086266 | 1.7711167891231 | 0.999279047927446 |
| ebi-fl187-GCST90094781 | prot-a-242 | Brain-derived neurotrophic factor \|\| id:prot-a-242 | Herring liking \|\| id:ebi-fl187-GCST90094781 | MR Egger | 19 | -0.13611378 | 0.382775484866168 | 0.726516534809328 | -0.886353731 | 0.614126169864783 | 0.872743325690917 | 0.412155847383461 | 1.84804101985575 | 0.999279047927446 |
| ebi-fl187-GCST90094781 | prot-a-2464 | Dihydropteridine reductase \|\| id:prot-a-2464 | Herring liking \|\| id:ebi-fl187-GCST90094781 | MR Egger | 19 | 0.149483184876853 | 0.397813069717084 | 0.71174132322756 | -0.630230432 | 0.929196801522338 | 1.16123394435605 | 0.532469089075139 | 2.53247428102709 | 0.999279047927446 |
| ebi-fl187-GCST90094781 | prot-a-2474 | Ras-related protein Rab-27A \|\| id:prot-a-2474 | Herring liking \|\| id:ebi-fl187-GCST90094781 | MR Egger | 19 | 0.154269015081772 | 0.51510951501049 | 0.768198552490568 | -0.855345634 | 1.16388366450233 | 1.16680473263247 | 0.425136224007623 | 3.20234599456085 | 0.999279047927446 |
| ebi-fl187-GCST90094781 | prot-a-2478 | Ras-related protein Rab-7b \|\| id:prot-a-2478 | Herring liking \|\| id:ebi-fl187-GCST90094781 | MR Egger | 19 | -0.105329588 | 0.383113803584305 | 0.786683190603885 | -0.856232643 | 0.645573466764609 | 0.900027835087909 | 0.424759291569138 | 1.90708036295229 | 0.999279047927446 |
| ebi-fl187-GCST90094781 | prot-a-2528 | E3 ubiquitin-protein ligase rififylin \|\| id:prot-a-2528 | Herring liking \|\| id:ebi-fl187-GCST90094781 | MR Egger | 19 | -0.051008531 | 0.397386136868384 | 0.89937028592619 | -0.82988536 | 0.727868296847086 | 0.950270563345357 | 0.43609927801805 | 2.07066186319925 | 0.999279047927446 |
| ebi-fl187-GCST90094781 | prot-a-2543 | Protein RIC-3 \|\| id:prot-a-2543 | Herring liking \|\| id:ebi-fl187-GCST90094781 | MR Egger | 19 | -0.912369237 | 0.382611867105241 | 0.0290166066125894 | -1.662288497 | -0.162449978 | 0.401571677459592 | 0.189704345160383 | 0.850058610947341 | 0.999279047927446 |
| ebi-fl187-GCST90094781 | prot-a-255 | Bone morphogenetic protein 6 \|\| id:prot-a-255 | Herring liking \|\| id:ebi-fl187-GCST90094781 | MR Egger | 19 | -1.310574663 | 0.392809549892309 | 0.00391015925064006 | -2.080481381 | -0.540667945 | 0.269665045287159 | 0.124870087649301 | 0.582359138354724 | 0.999279047927446 |
| ebi-fl187-GCST90094781 | prot-a-2568 | RING finger protein 165 \|\| id:prot-a-2568 | Herring liking \|\| id:ebi-fl187-GCST90094781 | MR Egger | 19 | 0.571927582612126 | 0.495228975957164 | 0.264117503669043 | -0.39872121 | 1.54257637548817 | 1.77167881943162 | 0.671177792752361 | 4.67662350142853 | 0.999279047927446 |
| ebi-fl187-GCST90094781 | prot-a-2573 | E3 ubiquitin-protein ligase RNF8 \|\| id:prot-a-2573 | Herring liking \|\| id:ebi-fl187-GCST90094781 | MR Egger | 19 | 0.100272573275911 | 0.425574065902688 | 0.816544772099343 | -0.733852596 | 0.934397742445179 | 1.10547219919195 | 0.480055961271581 | 2.54567984105282 | 0.999279047927446 |
| ebi-fl187-GCST90094781 | prot-a-260 | Cytoplasmic tyrosine-protein kinase BMX \|\| id:prot-a-260 | Herring liking \|\| id:ebi-fl187-GCST90094781 | MR Egger | 19 | -0.192470878 | 0.384094328311202 | 0.622724872140114 | -0.945295761 | 0.560354005963827 | 0.824918341516639 | 0.388564631635046 | 1.75129235851219 | 0.999279047927446 |
| ebi-fl187-GCST90094781 | prot-a-2623 | Serum amyloid A-1 protein \|\| id:prot-a-2623 | Herring liking \|\| id:ebi-fl187-GCST90094781 | MR Egger | 19 | -0.198336107 | 0.382691954006701 | 0.610952663912047 | -0.948412336 | 0.551740123343148 | 0.820094167822006 | 0.387355525796762 | 1.73627171759658 | 0.999279047927446 |
| ebi-fl187-GCST90094781 | prot-a-2640 | Lysosome membrane protein 2 \|\| id:prot-a-2640 | Herring liking \|\| id:ebi-fl187-GCST90094781 | MR Egger | 19 | -0.147072923 | 0.382775484866168 | 0.705573425404032 | -0.897312874 | 0.603167026994675 | 0.863231025428845 | 0.407663632937734 | 1.82789864745367 | 0.999279047927446 |
| ebi-fl187-GCST90094781 | prot-a-2641 | Scavenger receptor class F member 2 \|\| id:prot-a-2641 | Herring liking \|\| id:ebi-fl187-GCST90094781 | MR Egger | 19 | -0.201980282 | 0.382691954006701 | 0.604467662690746 | -0.952056512 | 0.548095948074589 | 0.817111039759824 | 0.385946503291317 | 1.729955954 | 0.999279047927446 |
| ebi-fl187-GCST90094781 | prot-a-2669 | Semaphorin-3A \|\| id:prot-a-2669 | Herring liking \|\| id:ebi-fl187-GCST90094781 | MR Egger | 19 | 0.055178854453169 | 0.417323847290165 | 0.896362067749281 | -0.762775886 | 0.873133595141892 | 1.05672959856918 | 0.466370038358497 | 2.39440219706782 | 0.999279047927446 |
| ebi-fl187-GCST90094781 | prot-a-2683 | Sentrin-specific protease 7 \|\| id:prot-a-2683 | Herring liking \|\| id:ebi-fl187-GCST90094781 | MR Egger | 19 | -0.623460095 | 0.403018911544187 | 0.140282365022403 | -1.413377162 | 0.166456971528668 | 0.536086315177151 | 0.243320162490389 | 1.18111271330245 | 0.999279047927446 |
| ebi-fl187-GCST90094781 | prot-a-270 | Bromodomain testis-specific protein \|\| id:prot-a-270 | Herring liking \|\| id:ebi-fl187-GCST90094781 | MR Egger | 19 | 0.0532805069747474 | 0.38313548200677 | 0.891033397880693 | -0.697665038 | 0.804226051708016 | 1.05472546147653 | 0.497746166483289 | 2.23496608109834 | 0.999279047927446 |
| ebi-fl187-GCST90094781 | prot-a-2708 | 14-3-3 protein sigma \|\| id:prot-a-2708 | Herring liking \|\| id:ebi-fl187-GCST90094781 | MR Egger | 19 | -0.571059381 | 0.383113803584305 | 0.154394460838967 | -1.321962436 | 0.179843674243047 | 0.564926649147012 | 0.266611580137157 | 1.19703022183916 | 0.999279047927446 |
| ebi-fl187-GCST90094781 | prot-a-2713 | Beta-sarcoglycan \|\| id:prot-a-2713 | Herring liking \|\| id:ebi-fl187-GCST90094781 | MR Egger | 19 | 0.743363457013899 | 0.514181243767974 | 0.16643872896012 | -0.264431781 | 1.75115869479913 | 2.10299697229907 | 0.767642015027523 | 5.76127436867883 | 0.999279047927446 |
| ebi-fl187-GCST90094781 | prot-a-2720 | Protein shisa-3 homolog \|\| id:prot-a-2720 | Herring liking \|\| id:ebi-fl187-GCST90094781 | MR Egger | 19 | 0.895306956922127 | 0.400757308220181 | 0.0392024930890918 | 0.109822632810571 | 1.68079128103368 | 2.44808713143257 | 1.11608009691242 | 5.3698033139964 | 0.999279047927446 |
| ebi-fl187-GCST90094781 | prot-a-2730 | Sialic acid-binding Ig-like lectin 8 \|\| id:prot-a-2730 | Herring liking \|\| id:ebi-fl187-GCST90094781 | MR Egger | 19 | -0.944391793 | 0.382630136376615 | 0.0244902520896513 | -1.694346861 | -0.194436726 | 0.388916040333892 | 0.183719184092069 | 0.823298270000989 | 0.999279047927446 |
| ebi-fl187-GCST90094781 | prot-a-2761 | Sodium- and chloride-dependent neutral and basic amino acid transporter B(0+) \|\| id:prot-a-2761 | Herring liking \|\| id:ebi-fl187-GCST90094781 | MR Egger | 19 | -0.88494188 | 0.382630136376615 | 0.0335196471864665 | -1.634896948 | -0.134986813 | 0.412738160774596 | 0.194972462632475 | 0.873727433400237 | 0.999279047927446 |
| ebi-fl187-GCST90094781 | prot-a-2770 | Secreted Ly-6/uPAR-related protein 1 \|\| id:prot-a-2770 | Herring liking \|\| id:ebi-fl187-GCST90094781 | MR Egger | 19 | 0.656673084561359 | 0.393232642570525 | 0.113240375581685 | -0.114062895 | 1.42740906399959 | 1.92836613954488 | 0.892201839192881 | 4.16788646334465 | 0.999279047927446 |
| ebi-fl187-GCST90094781 | prot-a-2784 | N-lysine methyltransferase SMYD2 \|\| id:prot-a-2784 | Herring liking \|\| id:ebi-fl187-GCST90094781 | MR Egger | 19 | -0.403458692 | 0.383113803584305 | 0.307032342644006 | -1.154361747 | 0.347444362644731 | 0.668005619951051 | 0.31525868737926 | 1.41544555677657 | 0.999279047927446 |
| ebi-fl187-GCST90094781 | prot-a-2837 | Serine palmitoyltransferase 2 \|\| id:prot-a-2837 | Herring liking \|\| id:ebi-fl187-GCST90094781 | MR Egger | 19 | -0.324584808 | 0.38313548200677 | 0.408663599418658 | -1.075530353 | 0.426360736712697 | 0.722827403489057 | 0.341116795087206 | 1.53167320624351 | 0.999279047927446 |
| ebi-fl187-GCST90094781 | prot-a-2858 | Alpha-2,8-sialyltransferase 8B \|\| id:prot-a-2858 | Herring liking \|\| id:ebi-fl187-GCST90094781 | MR Egger | 19 | 0.0329425652834646 | 0.405594202037497 | 0.93621516455299 | -0.762022071 | 0.827907201276958 | 1.03349117926374 | 0.466721727871905 | 2.28852430437757 | 0.999279047927446 |
| ebi-fl187-GCST90094781 | prot-a-2911 | Synaptotagmin-11 \|\| id:prot-a-2911 | Herring liking \|\| id:ebi-fl187-GCST90094781 | MR Egger | 19 | -0.576831355 | 0.38313548200677 | 0.150535784924504 | -1.3277769 | 0.174114189519419 | 0.561675299353054 | 0.265065874744276 | 1.19019146545252 | 0.999279047927446 |
| ebi-fl187-GCST90094781 | prot-a-2962 | Transforming growth factor beta-1 \|\| id:prot-a-2962 | Herring liking \|\| id:ebi-fl187-GCST90094781 | MR Egger | 19 | -0.09073423 | 0.382630136376615 | 0.815387675274003 | -0.840689297 | 0.659220837216322 | 0.91326039578931 | 0.431413049032297 | 1.93328540336945 | 0.999279047927446 |
| ebi-fl187-GCST90094781 | prot-a-2978 | Tyrosine-protein kinase receptor Tie-1, soluble \|\| id:prot-a-2978 | Herring liking \|\| id:ebi-fl187-GCST90094781 | MR Egger | 19 | 0.0173152646909622 | 0.431852090262278 | 0.968484227082423 | -0.829114832 | 0.863745361605026 | 1.01746604288422 | 0.436435433976444 | 2.37202818064117 | 0.999279047927446 |
| ebi-fl187-GCST90094781 | prot-a-299 | Uncharacterized protein C1orf226 \|\| id:prot-a-299 | Herring liking \|\| id:ebi-fl187-GCST90094781 | MR Egger | 19 | 0.305917964556804 | 0.383095383892724 | 0.435570314899591 | -0.444948988 | 1.05678491698654 | 1.35787090843688 | 0.640856966675517 | 2.87710596881562 | 0.999279047927446 |
| ebi-fl187-GCST90094781 | prot-a-3015 | Transmembrane protein 70, mitochondrial \|\| id:prot-a-3015 | Herring liking \|\| id:ebi-fl187-GCST90094781 | MR Egger | 19 | -0.379096794 | 0.383113803584305 | 0.336287167566139 | -1.129999849 | 0.371806260808133 | 0.68447935581632 | 0.323033305121994 | 1.45035196405457 | 0.999279047927446 |
| ebi-fl187-GCST90094781 | prot-a-3016 | Transmembrane protein 87B \|\| id:prot-a-3016 | Herring liking \|\| id:ebi-fl187-GCST90094781 | MR Egger | 19 | -0.753293018 | 0.448069800745738 | 0.111003291093049 | -1.631509827 | 0.124923791490798 | 0.470813599548744 | 0.195633977487167 | 1.13306210080294 | 0.999279047927446 |
| ebi-fl187-GCST90094781 | prot-a-3123 | Thioredoxin domain-containing protein 12 \|\| id:prot-a-3123 | Herring liking \|\| id:ebi-fl187-GCST90094781 | MR Egger | 19 | -0.229581256 | 0.421031833235823 | 0.592642268552675 | -1.054803649 | 0.595641137171324 | 0.794866378374402 | 0.348260801910598 | 1.81419371920079 | 0.999279047927446 |
| ebi-fl187-GCST90094781 | prot-a-3133 | PolyUbiquitin K48-linked \|\| id:prot-a-3133 | Herring liking \|\| id:ebi-fl187-GCST90094781 | MR Egger | 19 | 0.0373888819323634 | 0.471547973857356 | 0.93772810488152 | -0.886845147 | 0.961622910692781 | 1.03809663938019 | 0.411953357155913 | 2.61593846481167 | 0.999279047927446 |
| ebi-fl187-GCST90094781 | prot-a-3135 | Ubiquitin-conjugating enzyme E2 B \|\| id:prot-a-3135 | Herring liking \|\| id:ebi-fl187-GCST90094781 | MR Egger | 19 | 0.455830323098629 | 0.38313548200677 | 0.250501238900672 | -0.295115222 | 1.2067758678319 | 1.57748265968747 | 0.744445806261381 | 3.34268998587245 | 0.999279047927446 |
| ebi-fl187-GCST90094781 | prot-a-3162 | NKG2D ligand 3 \|\| id:prot-a-3162 | Herring liking \|\| id:ebi-fl187-GCST90094781 | MR Egger | 19 | 0.797610856411585 | 0.382691954006701 | 0.052539110878527 | 0.0475346265584515 | 1.54768708626472 | 2.22023013829874 | 1.04868251275893 | 4.70058555095141 | 0.999279047927446 |
| ebi-fl187-GCST90094781 | prot-a-3188 | Vesicle-associated membrane protein 8 \|\| id:prot-a-3188 | Herring liking \|\| id:ebi-fl187-GCST90094781 | MR Egger | 19 | -0.427660264 | 0.433133682884375 | 0.337310241753877 | -1.276602283 | 0.421281754140117 | 0.652032896017771 | 0.278983599298032 | 1.52391358688848 | 0.999279047927446 |
| ebi-fl187-GCST90094781 | prot-a-3201 | Vitrin \|\| id:prot-a-3201 | Herring liking \|\| id:ebi-fl187-GCST90094781 | MR Egger | 19 | -0.391257286 | 0.382673656457142 | 0.320907346106298 | -1.141297652 | 0.358783081050416 | 0.676206155546109 | 0.319404277094759 | 1.43158622970723 | 0.999279047927446 |
| ebi-fl187-GCST90094781 | prot-a-3232 | WNT1-inducible-signaling pathway protein 3 \|\| id:prot-a-3232 | Herring liking \|\| id:ebi-fl187-GCST90094781 | MR Egger | 19 | -0.15619439 | 0.470391759299822 | 0.743909125515011 | -1.078162238 | 0.765773458462543 | 0.85539289458459 | 0.340220195220994 | 2.15065717551106 | 0.999279047927446 |
| ebi-fl187-GCST90094781 | prot-a-327 | Carbonic anhydrase-related protein 10 \|\| id:prot-a-327 | Herring liking \|\| id:ebi-fl187-GCST90094781 | MR Egger | 19 | -0.096737474 | 0.38313548200677 | 0.803690684844241 | -0.847683019 | 0.654208070564058 | 0.907794294304802 | 0.42840639242089 | 1.92361854386786 | 0.999279047927446 |
| ebi-fl187-GCST90094781 | prot-a-332 | Carbonic anhydrase 5A, mitochondrial \|\| id:prot-a-332 | Herring liking \|\| id:ebi-fl187-GCST90094781 | MR Egger | 19 | -0.341204015 | 0.489749497261704 | 0.495413249457742 | -1.30111303 | 0.618704999250646 | 0.710913856048358 | 0.272228625716721 | 1.85652228670272 | 0.999279047927446 |
| ebi-fl187-GCST90094781 | prot-a-338 | Cell adhesion molecule 3 \|\| id:prot-a-338 | Herring liking \|\| id:ebi-fl187-GCST90094781 | MR Egger | 19 | -0.220502142 | 0.383197664332054 | 0.57254142211598 | -0.971569564 | 0.530565280218659 | 0.802115920830025 | 0.378488509725022 | 1.69989295293649 | 0.999279047927446 |
| ebi-fl187-GCST90094781 | prot-a-34 | A disintegrin and metalloproteinase with thrombospondin motifs 3 \|\| id:prot-a-34 | Herring liking \|\| id:ebi-fl187-GCST90094781 | MR Egger | 19 | -0.87875812 | 0.418765878556981 | 0.0511132153887318 | -1.699539242 | -0.057976998 | 0.415298342385701 | 0.182767716399018 | 0.943671653760608 | 0.999279047927446 |
| ebi-fl187-GCST90094781 | prot-a-358 | Cysteine--tRNA ligase, cytoplasmic \|\| id:prot-a-358 | Herring liking \|\| id:ebi-fl187-GCST90094781 | MR Egger | 19 | 0.423285835338473 | 0.383113803584305 | 0.284614333802634 | -0.32761722 | 1.17418889036371 | 1.52697069577991 | 0.720638813268433 | 3.23551751978984 | 0.999279047927446 |
| ebi-fl187-GCST90094781 | prot-a-382 | Coiled-coil domain-containing protein 167 \|\| id:prot-a-382 | Herring liking \|\| id:ebi-fl187-GCST90094781 | MR Egger | 19 | -0.620330267 | 0.418532954416207 | 0.156598920732569 | -1.440654858 | 0.199994323453136 | 0.537766801525398 | 0.236772655468299 | 1.22139582482985 | 0.999279047927446 |
| ebi-fl187-GCST90094781 | prot-a-40 | Pituitary adenylate cyclase-activating polypeptide 27 \|\| id:prot-a-40 | Herring liking \|\| id:ebi-fl187-GCST90094781 | MR Egger | 19 | -0.550149486 | 0.382630136376615 | 0.168641528303804 | -1.300104554 | 0.199805580901471 | 0.576863570678251 | 0.272503300317619 | 1.22116531721926 | 0.999279047927446 |
| ebi-fl187-GCST90094781 | prot-a-403 | C-C motif chemokine 25 \|\| id:prot-a-403 | Herring liking \|\| id:ebi-fl187-GCST90094781 | MR Egger | 19 | 0.158520944281483 | 0.44328279253268 | 0.725038783282353 | -0.710313329 | 1.02735521764554 | 1.17177646598433 | 0.491490175166716 | 2.79366741312578 | 0.999279047927446 |
| ebi-fl187-GCST90094781 | prot-a-415 | Cyclin-C \|\| id:prot-a-415 | Herring liking \|\| id:ebi-fl187-GCST90094781 | MR Egger | 19 | -0.110904822 | 0.383113803584305 | 0.775712842992858 | -0.861807877 | 0.639998233518987 | 0.895023931904881 | 0.422397748638751 | 1.89647752921044 | 0.999279047927446 |
| ebi-fl187-GCST90094781 | prot-a-44 | Adhesion G protein-coupled receptor F5 \|\| id:prot-a-44 | Herring liking \|\| id:ebi-fl187-GCST90094781 | MR Egger | 19 | -0.449081308 | 0.41230388652579 | 0.291265772799731 | -1.257196926 | 0.359034309575952 | 0.638214204654229 | 0.284450245247302 | 1.43194593018651 | 0.999279047927446 |
| ebi-fl187-GCST90094781 | prot-a-455 | CD59 glycoprotein \|\| id:prot-a-455 | Herring liking \|\| id:ebi-fl187-GCST90094781 | MR Egger | 19 | -0.193779435 | 0.382691954006701 | 0.619108506514884 | -0.943855665 | 0.556296794884009 | 0.823839594436175 | 0.389124605193239 | 1.74420139025574 | 0.999279047927446 |
| ebi-fl187-GCST90094781 | prot-a-478 | Cadherin-12 \|\| id:prot-a-478 | Herring liking \|\| id:ebi-fl187-GCST90094781 | MR Egger | 19 | -0.665499498 | 0.383051678957426 | 0.100404664993537 | -1.416280789 | 0.0852817923110472 | 0.514016713023584 | 0.242614676111231 | 1.08902390202659 | 0.999279047927446 |
| ebi-fl187-GCST90094781 | prot-a-516 | Centrin-2 \|\| id:prot-a-516 | Herring liking \|\| id:ebi-fl187-GCST90094781 | MR Egger | 19 | 0.296270600247156 | 0.382691954006701 | 0.449460687938484 | -0.45380563 | 1.04634683010029 | 1.3448340200173 | 0.635206186524469 | 2.84723067842196 | 0.999279047927446 |
| ebi-fl187-GCST90094781 | prot-a-519 | Cryptic protein \|\| id:prot-a-519 | Herring liking \|\| id:ebi-fl187-GCST90094781 | MR Egger | 19 | 0.291364783423924 | 0.460876277370107 | 0.535668593924855 | -0.61195272 | 1.19468228706933 | 1.33825266731232 | 0.542290892101149 | 3.30250835419617 | 0.999279047927446 |
| ebi-fl187-GCST90094781 | prot-a-583 | Chloride intracellular channel protein 4 \|\| id:prot-a-583 | Herring liking \|\| id:ebi-fl187-GCST90094781 | MR Egger | 19 | 0.739686220783736 | 0.383051678957426 | 0.070328485066652 | -0.01109507 | 1.49046751154029 | 2.09527795666067 | 0.98896625331112 | 4.43917039734116 | 0.999279047927446 |
| ebi-fl187-GCST90094781 | prot-a-602 | CCR4-NOT transcription complex subunit 1 \|\| id:prot-a-602 | Herring liking \|\| id:ebi-fl187-GCST90094781 | MR Egger | 19 | 0.265415302082934 | 0.383113803584305 | 0.497800546287194 | -0.485487753 | 1.01631835710817 | 1.3039724057983 | 0.615396961871174 | 2.7630036227565 | 0.999279047927446 |
| ebi-fl187-GCST90094781 | prot-a-606 | Ciliary neurotrophic factor receptor subunit alpha \|\| id:prot-a-606 | Herring liking \|\| id:ebi-fl187-GCST90094781 | MR Egger | 19 | -0.24000301 | 0.395217904126626 | 0.551699114335964 | -1.014630102 | 0.534624082259998 | 0.786625493455406 | 0.362536506592626 | 1.70680650279799 | 0.999279047927446 |
| ebi-fl187-GCST90094781 | prot-a-626 | Collagen alpha-2(IX) chain \|\| id:prot-a-626 | Herring liking \|\| id:ebi-fl187-GCST90094781 | MR Egger | 19 | 0.398907253345835 | 0.383197664332054 | 0.312456831889222 | -0.352160169 | 1.14997467543666 | 1.49019540156037 | 0.703167487502329 | 3.15811293084611 | 0.999279047927446 |
| ebi-fl187-GCST90094781 | prot-a-647 | Probable serine carboxypeptidase CPVL \|\| id:prot-a-647 | Herring liking \|\| id:ebi-fl187-GCST90094781 | MR Egger | 19 | -0.510414114 | 0.477874270506009 | 0.300411808006708 | -1.447047684 | 0.426219456280697 | 0.600246956722023 | 0.23526383696826 | 1.53145682607675 | 0.999279047927446 |
| ebi-fl187-GCST90094781 | prot-a-651 | Cellular retinoic acid-binding protein 2 \|\| id:prot-a-651 | Herring liking \|\| id:ebi-fl187-GCST90094781 | MR Egger | 19 | -0.780649508 | 0.382630136376615 | 0.0571770287516407 | -1.530604575 | -0.030694441 | 0.458108369683301 | 0.216404794802776 | 0.96977185078156 | 0.999279047927446 |
| ebi-fl187-GCST90094781 | prot-a-671 | Cytotoxic and regulatory T-cell molecule \|\| id:prot-a-671 | Herring liking \|\| id:ebi-fl187-GCST90094781 | MR Egger | 19 | -0.155131438 | 0.382691954006701 | 0.690262248573466 | -0.905207668 | 0.594944791705216 | 0.856302619256221 | 0.404457883420931 | 1.81293085337626 | 0.999279047927446 |
| ebi-fl187-GCST90094781 | prot-a-688 | Chorionic somatomammotropin hormone \|\| id:prot-a-688 | Herring liking \|\| id:ebi-fl187-GCST90094781 | MR Egger | 19 | 0.301530878058154 | 0.441117972543625 | 0.503463880668879 | -0.563060348 | 1.16612210424366 | 1.3519268593698 | 0.569463637428444 | 3.20952228194753 | 0.999279047927446 |
| ebi-fl187-GCST90094781 | prot-a-69 | Aldo-keto reductase family 1 member C1 \|\| id:prot-a-69 | Herring liking \|\| id:ebi-fl187-GCST90094781 | MR Egger | 19 | -0.170887738 | 0.383113803584305 | 0.661190448796892 | -0.921790793 | 0.580015317118128 | 0.842916195696537 | 0.397806014634251 | 1.78606578792121 | 0.999279047927446 |
| ebi-fl187-GCST90094781 | prot-a-741 | Stromal cell-derived factor 1 \|\| id:prot-a-741 | Herring liking \|\| id:ebi-fl187-GCST90094781 | MR Egger | 19 | -0.248640933 | 0.395633000378624 | 0.538053160608517 | -1.024081614 | 0.526799747252381 | 0.779859944706381 | 0.359126130330068 | 1.69350398646422 | 0.999279047927446 |
| ebi-fl187-GCST90094781 | prot-a-742 | Growth-regulated alpha protein \|\| id:prot-a-742 | Herring liking \|\| id:ebi-fl187-GCST90094781 | MR Egger | 19 | -0.154662856 | 0.386412972398634 | 0.693955214810923 | -0.912032282 | 0.60270657029698 | 0.85670396173932 | 0.401707012181086 | 1.82705717302488 | 0.999279047927446 |
| ebi-fl187-GCST90094781 | prot-a-788 | Beta-defensin 112 \|\| id:prot-a-788 | Herring liking \|\| id:ebi-fl187-GCST90094781 | MR Egger | 19 | -0.141713365 | 0.382691954006701 | 0.715727413740558 | -0.891789595 | 0.60836286505154 | 0.867869982922082 | 0.409921502613311 | 1.83742082924516 | 0.999279047927446 |
| ebi-fl187-GCST90094781 | prot-a-838 | DnaJ homolog subfamily B member 1 \|\| id:prot-a-838 | Herring liking \|\| id:ebi-fl187-GCST90094781 | MR Egger | 19 | 0.407291382782462 | 0.382775484866168 | 0.302188254353928 | -0.342948568 | 1.15753133312015 | 1.50274191499432 | 0.709674710926951 | 3.18206810572574 | 0.999279047927446 |
| ebi-fl187-GCST90094781 | prot-a-85 | Junctional adhesion molecule-like \|\| id:prot-a-85 | Herring liking \|\| id:ebi-fl187-GCST90094781 | MR Egger | 19 | 0.037168881035195 | 0.383113803584305 | 0.923846834869773 | -0.713734174 | 0.788071936060432 | 1.03786828230848 | 0.489811735980778 | 2.19915222991765 | 0.999279047927446 |
| ebi-fl187-GCST90094781 | prot-a-856 | Dipeptidase 1 \|\| id:prot-a-856 | Herring liking \|\| id:ebi-fl187-GCST90094781 | MR Egger | 19 | -0.414446922 | 0.437613585509351 | 0.356876247811901 | -1.27216955 | 0.443275705342844 | 0.660705601234063 | 0.280223004026409 | 1.55780176941121 | 0.999279047927446 |
| ebi-fl187-GCST90094781 | prot-a-860 | Dipeptidyl peptidase 2 \|\| id:prot-a-860 | Herring liking \|\| id:ebi-fl187-GCST90094781 | MR Egger | 19 | 0.648445458437166 | 0.383197664332054 | 0.108854731335738 | -0.102621964 | 1.39951288052799 | 1.91256535452848 | 0.90246807473678 | 4.05322508101966 | 0.999279047927446 |
| ebi-fl187-GCST90094781 | prot-a-861 | Dermatopontin \|\| id:prot-a-861 | Herring liking \|\| id:ebi-fl187-GCST90094781 | MR Egger | 19 | 0.0637424993624485 | 0.414087824563373 | 0.879473816446716 | -0.747869637 | 0.87535463550666 | 1.06581791463057 | 0.473373937737474 | 2.39972617118911 | 0.999279047927446 |
| ebi-fl187-GCST90094781 | prot-a-877 | Dual specificity protein phosphatase 6 \|\| id:prot-a-877 | Herring liking \|\| id:ebi-fl187-GCST90094781 | MR Egger | 19 | 0.285381175423223 | 0.394283942780333 | 0.479033442905617 | -0.487415352 | 1.05817770327267 | 1.33026899734129 | 0.614211865567522 | 2.88111595443099 | 0.999279047927446 |
| ebi-fl187-GCST90094781 | prot-a-879 | Dynactin-associated protein \|\| id:prot-a-879 | Herring liking \|\| id:ebi-fl187-GCST90094781 | MR Egger | 19 | -0.373878852 | 0.382630136376615 | 0.342206514124501 | -1.123833919 | 0.376076215292401 | 0.688060263900794 | 0.325031259141439 | 1.45655814154544 | 0.999279047927446 |
| ebi-fl187-GCST90094781 | prot-a-937 | EMI domain-containing protein 1 \|\| id:prot-a-937 | Herring liking \|\| id:ebi-fl187-GCST90094781 | MR Egger | 19 | -0.576782023 | 0.446893143398527 | 0.21410339671741 | -1.452692584 | 0.299128538134811 | 0.561703008763908 | 0.233939537447776 | 1.34868296952524 | 0.999279047927446 |
| ebi-fl187-GCST90094781 | prot-b-1 | SRC proto-oncogene, non-receptor tyrosine kinase \|\| id:prot-b-1 | Herring liking \|\| id:ebi-fl187-GCST90094781 | MR Egger | 13 | -0.98214942 | 0.840803792715908 | 0.26745750663557 | -2.630124854 | 0.66582601386174 | 0.374505264070199 | 0.0720694635461531 | 1.94609736100604 | 0.999279047927446 |
| ebi-fl187-GCST90094781 | prot-b-10 | coagulation factor III, tissue factor \|\| id:prot-b-10 | Herring liking \|\| id:ebi-fl187-GCST90094781 | MR Egger | 13 | -0.8519569 | 1.02505686918552 | 0.423573379645549 | -2.861068363 | 1.15715456391745 | 0.426579341652386 | 0.0572076090959979 | 3.18086942628954 | 0.999279047927446 |
| ebi-fl187-GCST90094781 | prot-b-29 | myeloperoxidase \|\| id:prot-b-29 | Herring liking \|\| id:ebi-fl187-GCST90094781 | MR Egger | 13 | 1.0687079911847 | 0.778195573030638 | 0.196997682238637 | -0.456555332 | 2.59397131432475 | 2.9116152362177 | 0.633461957735035 | 13.3828135695577 | 0.999279047927446 |
| ebi-fl187-GCST90094781 | prot-b-33 | adrenomedullin \|\| id:prot-b-33 | Herring liking \|\| id:ebi-fl187-GCST90094781 | MR Egger | 13 | -0.897217361 | 0.710743786823398 | 0.232924686858228 | -2.290275183 | 0.495840461333444 | 0.407702571908754 | 0.10123859887755 | 1.64187759396059 | 0.999279047927446 |
| ebi-fl187-GCST90094781 | ukb-rsfMRI191-edge-pheno102 | Net25_Pair11_15 (Postcentral\|Precentral)&(Cerebellum) (Motor)&(Subcortical-cerebellum) \|\| id:ukb-rsfMRI191-edge-pheno102 | Herring liking \|\| id:ebi-fl187-GCST90094781 | MR Egger | 18 | -0.020090132 | 0.121220539540729 | 0.870443866061199 | -0.25768239 | 0.217502125371273 | 0.980110329895272 | 0.772840655338124 | 1.24296807127356 | 0.999279047927446 |
| ebi-fl187-GCST90094781 | ukb-rsfMRI191-edge-pheno1167 | Net100_Pair11_45 (Angular\|Temporal)&(Frontal) (Default_mode\|Central_executive)&(Salience\|Default_mode) \|\| id:ukb-rsfMRI191-edge-pheno1167 | Herring liking \|\| id:ebi-fl187-GCST90094781 | MR Egger | 18 | 0.0547404511613989 | 0.169798658153003 | 0.751335631638846 | -0.278064919 | 0.387545821141284 | 1.05626642637019 | 0.757247660295201 | 1.47336046313027 | 0.999279047927446 |
| ebi-fl187-GCST90094781 | ukb-rsfMRI191-edge-pheno1317 | Net100_Pair26_48 (Parietal\|Frontal)&(Temporal) (Attention\|Central_executive)&(Default_mode\|Central_executive) \|\| id:ukb-rsfMRI191-edge-pheno1317 | Herring liking \|\| id:ebi-fl187-GCST90094781 | MR Egger | 18 | -0.032872294 | 0.157316548101604 | 0.837119399554722 | -0.341212728 | 0.27546814022436 | 0.967662127905355 | 0.7109076619173 | 1.31714714011796 | 0.999279047927446 |
| ebi-fl187-GCST90094781 | ukb-rsfMRI191-edge-pheno286 | Net100_Pair10_13 (OccipitalPrecuneus)&(Frontal_Sup) (Default_mode\|Central_executive)&(Default_mode) \|\| id:ukb-rsfMRI191-edge-pheno286 | Herring liking \|\| id:ebi-fl187-GCST90094781 | MR Egger | 18 | 0.293661495452355 | 0.119663223745753 | 0.0259605743851695 | 0.0591215769106792 | 0.52820141399403 | 1.34132978057932 | 1.0609042143568 | 1.69587937904438 | 0.999279047927446 |
| ebi-fl187-GCST90094781 | ukb-rsfMRI191-edge-pheno599 | Net100_Pair11_29 (Angular\|Temporal)&(Parietal) (Default_mode\|Central_executive)&(Central_executive\|Salience) \|\| id:ukb-rsfMRI191-edge-pheno599 | Herring liking \|\| id:ebi-fl187-GCST90094781 | MR Egger | 18 | -0.169951071 | 0.143421228535577 | 0.253328880395489 | -0.451056679 | 0.111154537240243 | 0.84370609754408 | 0.636954739439731 | 1.11756759932299 | 0.999279047927446 |
| ebi-fl187-GCST90094781 | ukb-rsfMRI191-edge-pheno621 | Net100_Pair5_30 (Precuneus\|Angular\|Cingulate)&(Paracentral) (Default_mode\|Central_executive)&(Motor) \|\| id:ukb-rsfMRI191-edge-pheno621 | Herring liking \|\| id:ebi-fl187-GCST90094781 | MR Egger | 18 | -0.089985736 | 0.122374184982761 | 0.472778943656571 | -0.329839139 | 0.149867666067364 | 0.913944221222712 | 0.719039389477859 | 1.1616805028066 | 0.999279047927446 |
| ebi-fl187-GCST90094781 | ukb-rsfMRI191-edge-pheno624 | Net100_Pair8_30 (Calcarine\|Lingual)&(Paracentral) (Visual)&(Motor) \|\| id:ukb-rsfMRI191-edge-pheno624 | Herring liking \|\| id:ebi-fl187-GCST90094781 | MR Egger | 18 | -0.080107136 | 0.121949067027362 | 0.520589024932109 | -0.319127308 | 0.158913035002841 | 0.923017452349138 | 0.726783018309765 | 1.17223599874753 | 0.999279047927446 |
| ebi-fl187-GCST90094781 | ukb-rsfMRI191-edge-pheno882 | Net100_Pair6_38 (Postcentral\|Precentral)&(Subcortical) (Motor)&(Subcortical-cerebellum) \|\| id:ukb-rsfMRI191-edge-pheno882 | Herring liking \|\| id:ebi-fl187-GCST90094781 | MR Egger | 17 | -0.070713899 | 0.123440815137386 | 0.575229093908875 | -0.312657897 | 0.171230098537236 | 0.931728422308842 | 0.731500118298659 | 1.18676379021949 | 0.999279047927446 |
| ebi-fl187-GCST90094781 | ukb-rsfMRI191-edge-pheno899 | Net100_Pair23_38 (Cerebellum)&(Subcortical) (Subcortical-cerebellum)&(Subcortical-cerebellum) \|\| id:ukb-rsfMRI191-edge-pheno899 | Herring liking \|\| id:ebi-fl187-GCST90094781 | MR Egger | 18 | 0.190095206349506 | 0.128535963406279 | 0.158577665887601 | -0.061835282 | 0.442025694625813 | 1.20936473137773 | 0.940037715240405 | 1.55585571705098 | 0.999279047927446 |
| ebi-fl187-GCST90094781 | ebi-a-GCST90001392 | IgD+ CD38+ B cell Absolute Count \|\| id:ebi-a-GCST90001392 | Herring liking \|\| id:ebi-fl187-GCST90094781 | Weighted median | 16 | 0.00421312491778033 | 0.139818920005343 | 0.975961202645676 | -0.269831958 | 0.278258208128253 | 1.00422201260583 | 0.76350778470917 | 1.32082720150161 | 0.999489402457283 |
| ebi-fl187-GCST90094781 | ebi-a-GCST90001402 | Switched memory B cell %B cell \|\| id:ebi-a-GCST90001402 | Herring liking \|\| id:ebi-fl187-GCST90094781 | Weighted median | 16 | 0.272730524740275 | 0.140972680332394 | 0.0530352383660643 | -0.003575929 | 0.549036978191768 | 1.31354622891642 | 0.996430457307606 | 1.73158466087303 | 0.999489402457283 |
| ebi-fl187-GCST90094781 | ebi-a-GCST90001407 | Memory B cell Absolute Count \|\| id:ebi-a-GCST90001407 | Herring liking \|\| id:ebi-fl187-GCST90094781 | Weighted median | 16 | -0.029870233 | 0.146642463751912 | 0.838592403590346 | -0.317289462 | 0.257548996231704 | 0.970571473795087 | 0.728119961794561 | 1.2937551985569 | 0.999489402457283 |
| ebi-fl187-GCST90094781 | ebi-a-GCST90001411 | IgD+ CD24+ B cell %B cell \|\| id:ebi-a-GCST90001411 | Herring liking \|\| id:ebi-fl187-GCST90094781 | Weighted median | 16 | -0.095085834 | 0.148927193590141 | 0.523166515330772 | -0.386983133 | 0.196811465482914 | 0.909294882743613 | 0.679102548987586 | 1.21751447556242 | 0.999489402457283 |
| ebi-fl187-GCST90094781 | ebi-a-GCST90001418 | CD24+ CD27+ B cell Absolute Count \|\| id:ebi-a-GCST90001418 | Herring liking \|\| id:ebi-fl187-GCST90094781 | Weighted median | 16 | -0.040047491 | 0.136914215575698 | 0.769903897188972 | -0.308399353 | 0.228304371606141 | 0.960743811459251 | 0.734621885636441 | 1.25646770032937 | 0.999489402457283 |
| ebi-fl187-GCST90094781 | ebi-a-GCST90001436 | Memory B cell %lymphocyte \|\| id:ebi-a-GCST90001436 | Herring liking \|\| id:ebi-fl187-GCST90094781 | Weighted median | 16 | 0.0379209839681529 | 0.137498034909752 | 0.782707125131661 | -0.231575164 | 0.307417132391267 | 1.03864915970091 | 0.793283066572836 | 1.35990811149926 | 0.999489402457283 |
| ebi-fl187-GCST90094781 | ebi-a-GCST90001439 | IgD+ CD24+ B cell %lymphocyte \|\| id:ebi-a-GCST90001439 | Herring liking \|\| id:ebi-fl187-GCST90094781 | Weighted median | 16 | -0.091565693 | 0.145473512208548 | 0.52906623251926 | -0.376693777 | 0.193562391023199 | 0.912501369316718 | 0.686126149450925 | 1.21356509975786 | 0.999489402457283 |
| ebi-fl187-GCST90094781 | ebi-a-GCST90001457 | CD11c+ HLA DR++ monocyte %monocyte \|\| id:ebi-a-GCST90001457 | Herring liking \|\| id:ebi-fl187-GCST90094781 | Weighted median | 16 | 0.218160996289034 | 0.151775581090227 | 0.150606658931294 | -0.079319143 | 0.515641135225878 | 1.24378729663967 | 0.923745070949581 | 1.67471187444816 | 0.999489402457283 |
| ebi-fl187-GCST90094781 | ebi-a-GCST90001483 | CD39+ resting CD4 regulatory T cell Absolute Count \|\| id:ebi-a-GCST90001483 | Herring liking \|\| id:ebi-fl187-GCST90094781 | Weighted median | 15 | -0.029688178 | 0.142377761850007 | 0.834825339903641 | -0.308748592 | 0.249372234862169 | 0.970748186647018 | 0.73436537245074 | 1.28321960325231 | 0.999489402457283 |
| ebi-fl187-GCST90094781 | ebi-a-GCST90001525 | CD33dim HLA DR+ CD11b+ Absolute Count \|\| id:ebi-a-GCST90001525 | Herring liking \|\| id:ebi-fl187-GCST90094781 | Weighted median | 16 | 0.0322436866199433 | 0.178423514719402 | 0.856591807184148 | -0.317466402 | 0.381953775469972 | 1.03276914666559 | 0.72799113924223 | 1.46514435795827 | 0.999489402457283 |
| ebi-fl187-GCST90094781 | ebi-a-GCST90001540 | Naive CD4+ T cell Absolute Count \|\| id:ebi-a-GCST90001540 | Herring liking \|\| id:ebi-fl187-GCST90094781 | Weighted median | 16 | -0.139950433 | 0.139109031064293 | 0.314392235531908 | -0.412604134 | 0.13270326746935 | 0.869401327584187 | 0.661924264080106 | 1.14191110588095 | 0.999489402457283 |
| ebi-fl187-GCST90094781 | ebi-a-GCST90001624 | HLA DR+ CD4+ T cell Absolute Count \|\| id:ebi-a-GCST90001624 | Herring liking \|\| id:ebi-fl187-GCST90094781 | Weighted median | 16 | -0.095936395 | 0.139439220193218 | 0.49144279600305 | -0.369237266 | 0.177364477046063 | 0.908521801184729 | 0.69126137809231 | 1.19406622355476 | 0.999489402457283 |
| ebi-fl187-GCST90094781 | ebi-a-GCST90001672 | CD39+ CD8+ T cell Absolute Count \|\| id:ebi-a-GCST90001672 | Herring liking \|\| id:ebi-fl187-GCST90094781 | Weighted median | 16 | 0.0791797076206899 | 0.152628642421581 | 0.603918642139282 | -0.219972432 | 0.378331846766989 | 1.08239881990998 | 0.802540922486377 | 1.45984730811333 | 0.999489402457283 |
| ebi-fl187-GCST90094781 | ebi-a-GCST90001722 | CD19 on CD20- CD38- B cell \|\| id:ebi-a-GCST90001722 | Herring liking \|\| id:ebi-fl187-GCST90094781 | Weighted median | 16 | -0.225059569 | 0.14066207248603 | 0.109598184678303 | -0.500757231 | 0.050638093279633 | 0.798468653528762 | 0.606071549824191 | 1.05194211946259 | 0.999489402457283 |
| ebi-fl187-GCST90094781 | ebi-a-GCST90001742 | CD19 on transitional B cell \|\| id:ebi-a-GCST90001742 | Herring liking \|\| id:ebi-fl187-GCST90094781 | Weighted median | 16 | -0.220846353 | 0.156164687283511 | 0.157306473660469 | -0.52692914 | 0.0852364343526268 | 0.801839871338746 | 0.590415271423174 | 1.08897450724595 | 0.999489402457283 |
| ebi-fl187-GCST90094781 | ebi-a-GCST90001784 | CD25 on IgD+ CD38dim B cell \|\| id:ebi-a-GCST90001784 | Herring liking \|\| id:ebi-fl187-GCST90094781 | Weighted median | 16 | 0.0468184225302655 | 0.143052952527872 | 0.743455841821071 | -0.233565364 | 0.327202209484894 | 1.04793171101472 | 0.791705844652126 | 1.3870819299468 | 0.999489402457283 |
| ebi-fl187-GCST90094781 | ebi-a-GCST90001794 | CD25 on IgD+ B cell \|\| id:ebi-a-GCST90001794 | Herring liking \|\| id:ebi-fl187-GCST90094781 | Weighted median | 16 | 0.0114126511363321 | 0.14888858876457 | 0.938900160663818 | -0.280408983 | 0.303234285114888 | 1.01147802389477 | 0.755474702073248 | 1.3542317036089 | 0.999489402457283 |
| ebi-fl187-GCST90094781 | ebi-a-GCST90001802 | CD27 on IgD- CD38- B cell \|\| id:ebi-a-GCST90001802 | Herring liking \|\| id:ebi-fl187-GCST90094781 | Weighted median | 16 | 0.0451714141077513 | 0.144122944533374 | 0.753959478622069 | -0.237309557 | 0.327652385393165 | 1.0462071792095 | 0.788747087900191 | 1.38770650138742 | 0.999489402457283 |
| ebi-fl187-GCST90094781 | ebi-a-GCST90001804 | CD27 on IgD- CD38dim B cell \|\| id:ebi-a-GCST90001804 | Herring liking \|\| id:ebi-fl187-GCST90094781 | Weighted median | 16 | 0.00186469555314114 | 0.148842362591098 | 0.990004372084266 | -0.289866335 | 0.293595726231694 | 1.00186643517902 | 0.748363590819133 | 1.34124156526596 | 0.999911036302558 |
| ebi-fl187-GCST90094781 | ebi-a-GCST90001805 | CD27 on memory B cell \|\| id:ebi-a-GCST90001805 | Herring liking \|\| id:ebi-fl187-GCST90094781 | Weighted median | 16 | 0.0852490218038947 | 0.147772323539198 | 0.564010812635228 | -0.204384732 | 0.374882775940723 | 1.08898821474577 | 0.815148696791017 | 1.45482086461484 | 0.999489402457283 |
| ebi-fl187-GCST90094781 | ebi-a-GCST90001806 | CD27 on unswitched memory B cell \|\| id:ebi-a-GCST90001806 | Herring liking \|\| id:ebi-fl187-GCST90094781 | Weighted median | 16 | 0.0592614139713666 | 0.143304189149208 | 0.679214091365264 | -0.221614797 | 0.340137624703814 | 1.06105257845697 | 0.801223938955257 | 1.40514095936548 | 0.999489402457283 |
| ebi-fl187-GCST90094781 | ebi-a-GCST90001839 | CD3 on Effector Memory CD8+ T cell \|\| id:ebi-a-GCST90001839 | Herring liking \|\| id:ebi-fl187-GCST90094781 | Weighted median | 16 | 0.140501127562265 | 0.155114212883399 | 0.365046248162594 | -0.16352273 | 0.444524984813728 | 1.15085037721957 | 0.84914719793034 | 1.5597491153178 | 0.999489402457283 |
| ebi-fl187-GCST90094781 | ebi-a-GCST90001859 | CD3 on CD8+ T cell \|\| id:ebi-a-GCST90001859 | Herring liking \|\| id:ebi-fl187-GCST90094781 | Weighted median | 16 | 0.200344505413349 | 0.155791471772861 | 0.198450515437495 | -0.105006779 | 0.505695790088156 | 1.22182361051103 | 0.900318419071619 | 1.65813883574835 | 0.999489402457283 |
| ebi-fl187-GCST90094781 | ebi-a-GCST90001905 | CD86 on monocyte \|\| id:ebi-a-GCST90001905 | Herring liking \|\| id:ebi-fl187-GCST90094781 | Weighted median | 16 | -0.031411276 | 0.16262524393891 | 0.846840481790211 | -0.350156754 | 0.287334201746905 | 0.969076932659583 | 0.704577635351383 | 1.3328695863935 | 0.999489402457283 |
| ebi-fl187-GCST90094781 | ebi-a-GCST90001943 | CD25 on activated & secreting CD4 regulatory T cell \|\| id:ebi-a-GCST90001943 | Herring liking \|\| id:ebi-fl187-GCST90094781 | Weighted median | 16 | 0.186002581369228 | 0.137198998859824 | 0.17519023467185 | -0.082907456 | 0.454912619134483 | 1.2044253694402 | 0.92043632377342 | 1.57603566165677 | 0.999489402457283 |
| ebi-fl187-GCST90094781 | ebi-a-GCST90001949 | CD33 on CD33dim HLA DR+ CD11b- \|\| id:ebi-a-GCST90001949 | Herring liking \|\| id:ebi-fl187-GCST90094781 | Weighted median | 16 | 0.249600438196832 | 0.203718491020385 | 0.220491474415189 | -0.149687804 | 0.648888680596786 | 1.28351247166038 | 0.860976727786977 | 1.91341323376088 | 0.999489402457283 |
| ebi-fl187-GCST90094781 | ebi-a-GCST90001955 | CD33 on Immature Myeloid-Derived Suppressor Cells \|\| id:ebi-a-GCST90001955 | Herring liking \|\| id:ebi-fl187-GCST90094781 | Weighted median | 16 | 0.285245183630427 | 0.221505004432291 | 0.197829678853677 | -0.148904625 | 0.719394992317718 | 1.33008810397573 | 0.861651290923004 | 2.0531906386894 | 0.999489402457283 |
| ebi-fl187-GCST90094781 | ebi-a-GCST90001956 | CD33 on CD33+ HLA DR+ \|\| id:ebi-a-GCST90001956 | Herring liking \|\| id:ebi-fl187-GCST90094781 | Weighted median | 16 | 0.0250000590567175 | 0.235644853794113 | 0.915509273457157 | -0.436863854 | 0.48686397249318 | 1.02531518107618 | 0.646059383578836 | 1.62720524965023 | 0.999489402457283 |
| ebi-fl187-GCST90094781 | ebi-a-GCST90001957 | CD33 on CD33+ HLA DR+ CD14- \|\| id:ebi-a-GCST90001957 | Herring liking \|\| id:ebi-fl187-GCST90094781 | Weighted median | 16 | 0.108171356742313 | 0.236149054623255 | 0.646906534243565 | -0.35468079 | 0.571023503803892 | 1.1142386613358 | 0.701397300286291 | 1.77007780598619 | 0.999489402457283 |
| ebi-fl187-GCST90094781 | ebi-a-GCST90002064 | CD4 on resting CD4 regulatory T cell \|\| id:ebi-a-GCST90002064 | Herring liking \|\| id:ebi-fl187-GCST90094781 | Weighted median | 16 | -0.105204164 | 0.154830961112246 | 0.496835319310235 | -0.408672848 | 0.198264519635225 | 0.900140727362927 | 0.664531599656069 | 1.21928487595896 | 0.999489402457283 |
| ebi-fl187-GCST90094781 | ebi-a-GCST90002080 | SSC-A on T cell \|\| id:ebi-a-GCST90002080 | Herring liking \|\| id:ebi-fl187-GCST90094781 | Weighted median | 16 | -0.054177963 | 0.144679112636964 | 0.70805479600571 | -0.337749024 | 0.229393097537637 | 0.94726351342528 | 0.713374305262977 | 1.25783639422789 | 0.999489402457283 |
| ebi-fl187-GCST90094781 | ebi-a-GCST90002121 | CD8 on CD39+ CD8+ T cell \|\| id:ebi-a-GCST90002121 | Herring liking \|\| id:ebi-fl187-GCST90094781 | Weighted median | 16 | 0.0510601748436702 | 0.161017577498513 | 0.751160651656827 | -0.264534277 | 0.366654626740755 | 1.05238621855407 | 0.7675633386071 | 1.44289949414775 | 0.999489402457283 |
| ebi-fl187-GCST90094781 | ebi-cfmet338-GCST90026053 | 1-palmitoyl-2-docosahexaenoyl-gpc (16:0/22:6) levels \|\| id:ebi-cfmet338-GCST90026053 | Herring liking \|\| id:ebi-fl187-GCST90094781 | Weighted median | 15 | -0.092830366 | 0.0829385421967381 | 0.263026286861752 | -0.255389909 | 0.0697291766275635 | 0.911348082734099 | 0.774614410109111 | 1.07221776029977 | 0.999489402457283 |
| ebi-fl187-GCST90094781 | ebi-cfmet338-GCST90026063 | 1-stearoyl-2-oleoyl-gpc (18:0/18:1) levels \|\| id:ebi-cfmet338-GCST90026063 | Herring liking \|\| id:ebi-fl187-GCST90094781 | Weighted median | 14 | -0.081656005 | 0.0662946919625584 | 0.218056475554669 | -0.211593601 | 0.0482815913536074 | 0.921588926256869 | 0.809293526711046 | 1.04946613430968 | 0.999489402457283 |
| ebi-fl187-GCST90094781 | ebi-cfmet338-GCST90026113 | Adenosine 3',5'-cyclic monophosphate (camp) levels \|\| id:ebi-cfmet338-GCST90026113 | Herring liking \|\| id:ebi-fl187-GCST90094781 | Weighted median | 15 | 0.0892088053822601 | 0.135872953807164 | 0.511463597364961 | -0.177102184 | 0.355519794844303 | 1.09330892128126 | 0.837694181140378 | 1.42692216833351 | 0.999489402457283 |
| ebi-fl187-GCST90094781 | ebi-cfmet338-GCST90026117 | Alpha-ketoglutaramate levels \|\| id:ebi-cfmet338-GCST90026117 | Herring liking \|\| id:ebi-fl187-GCST90094781 | Weighted median | 15 | 0.00933374797868751 | 0.0468562153798243 | 0.84210646844626 | -0.082504434 | 0.101171930123143 | 1.00937744324541 | 0.9208073548353 | 1.10646686039436 | 0.999489402457283 |
| ebi-fl187-GCST90094781 | ebi-cfmet338-GCST90026124 | Arginine levels \|\| id:ebi-cfmet338-GCST90026124 | Herring liking \|\| id:ebi-fl187-GCST90094781 | Weighted median | 15 | 0.038744158747374 | 0.0409448135532292 | 0.344019483563029 | -0.041507676 | 0.118995993311703 | 1.03950450149323 | 0.95934197157984 | 1.12636540528422 | 0.999489402457283 |
| ebi-fl187-GCST90094781 | ebi-cfmet338-GCST90026169 | Glutamate levels \|\| id:ebi-cfmet338-GCST90026169 | Herring liking \|\| id:ebi-fl187-GCST90094781 | Weighted median | 15 | 0.0117486161031988 | 0.0430008394917425 | 0.784685416475435 | -0.072533029 | 0.0960302615070141 | 1.01181790216596 | 0.930035027746454 | 1.10079237512613 | 0.999489402457283 |
| ebi-fl187-GCST90094781 | ebi-cfmet338-GCST90026170 | Glutamine levels \|\| id:ebi-cfmet338-GCST90026170 | Herring liking \|\| id:ebi-fl187-GCST90094781 | Weighted median | 15 | 0.0260722516078429 | 0.0256609568367611 | 0.309616035445828 | -0.024223224 | 0.0763677270078947 | 1.02641510593613 | 0.976067803881291 | 1.07935941079561 | 0.999489402457283 |
| ebi-fl187-GCST90094781 | ebi-cfmet338-GCST90026191 | Kynurenate levels \|\| id:ebi-cfmet338-GCST90026191 | Herring liking \|\| id:ebi-fl187-GCST90094781 | Weighted median | 15 | -0.082293697 | 0.189411998056156 | 0.663947687774851 | -0.453541213 | 0.288953819077114 | 0.921001423511481 | 0.635374167603415 | 1.33503007418399 | 0.999489402457283 |
| ebi-fl187-GCST90094781 | ebi-cfmet338-GCST90026202 | Methylsuccinoylcarnitine levels \|\| id:ebi-cfmet338-GCST90026202 | Herring liking \|\| id:ebi-fl187-GCST90094781 | Weighted median | 15 | 0.0149237788552783 | 0.124191145047945 | 0.904350210903108 | -0.228490865 | 0.25833842314925 | 1.01503569448457 | 0.795733565848328 | 1.29477692697226 | 0.999489402457283 |
| ebi-fl187-GCST90094781 | ebi-cfmet338-GCST90026222 | N-acetylputrescine levels \|\| id:ebi-cfmet338-GCST90026222 | Herring liking \|\| id:ebi-fl187-GCST90094781 | Weighted median | 15 | 0.0525830371085845 | 0.0448858008939014 | 0.241403931589567 | -0.035393133 | 0.140559206860631 | 1.05399007873354 | 0.965225879856694 | 1.15091721974307 | 0.999489402457283 |
| ebi-fl187-GCST90094781 | ebi-cfmet338-GCST90026252 | Propionylcarnitine (c3) levels \|\| id:ebi-cfmet338-GCST90026252 | Herring liking \|\| id:ebi-fl187-GCST90094781 | Weighted median | 15 | 0.0802185529358145 | 0.0777343943295205 | 0.302092258338972 | -0.07214086 | 0.232577965821675 | 1.08352384911763 | 0.930399830506849 | 1.26184882360427 | 0.999489402457283 |
| ebi-fl187-GCST90094781 | ebi-cfmet338-GCST90026316 | Ethyl beta-glucopyranoside levels \|\| id:ebi-cfmet338-GCST90026316 | Herring liking \|\| id:ebi-fl187-GCST90094781 | Weighted median | 15 | -0.521296695 | 0.238720237319886 | 0.0289832793763045 | -0.98918836 | -0.05340503 | 0.593750135682835 | 0.371878399853132 | 0.947995967936332 | 0.999489402457283 |
| ebi-fl187-GCST90094781 | ebi-gm430-GCST90011315 | F_Prevotellaceae abundance \|\| id:ebi-gm430-GCST90011315 | Herring liking \|\| id:ebi-fl187-GCST90094781 | Weighted median | 14 | 0.135668294042268 | 0.115954639496659 | 0.241996228680931 | -0.091602799 | 0.36293938745572 | 1.14530192710715 | 0.91246751024422 | 1.43754872311483 | 0.999489402457283 |
| ebi-fl187-GCST90094781 | ebi-gm430-GCST90011354 | OTU97_1 (Bacteroides) abundance \|\| id:ebi-gm430-GCST90011354 | Herring liking \|\| id:ebi-fl187-GCST90094781 | Weighted median | 14 | 0.0179694638218419 | 0.0978483714602102 | 0.854291159598968 | -0.173813344 | 0.209752271883854 | 1.01813188605845 | 0.840453758553386 | 1.23337248106682 | 0.999489402457283 |
| ebi-fl187-GCST90094781 | ebi-gm430-GCST90011419 | OTU99_1 (Bacteroides) abundance \|\| id:ebi-gm430-GCST90011419 | Herring liking \|\| id:ebi-fl187-GCST90094781 | Weighted median | 14 | 0.0304657076477548 | 0.0989035785705856 | 0.758056132383298 | -0.163385306 | 0.224316721646103 | 1.03093453627234 | 0.849263898591759 | 1.25146732345675 | 0.999489402457283 |
| ebi-fl187-GCST90094781 | ebi-gm430-GCST90011435 | OTU99_155 (Butyrivibrio) abundance \|\| id:ebi-gm430-GCST90011435 | Herring liking \|\| id:ebi-fl187-GCST90094781 | Weighted median | 14 | 0.431571126693252 | 0.333076224425549 | 0.195074450518212 | -0.221258273 | 1.08440052656733 | 1.53967464818524 | 0.801509645110026 | 2.95766624485089 | 0.999489402457283 |
| ebi-fl187-GCST90094781 | ebi-gm430-GCST90011466 | OTU99_58 (Bacteroides) abundance \|\| id:ebi-gm430-GCST90011466 | Herring liking \|\| id:ebi-fl187-GCST90094781 | Weighted median | 14 | -0.185229396 | 0.175370836419741 | 0.290869770457756 | -0.528956235 | 0.158497443549321 | 0.830913653858012 | 0.589219655555285 | 1.17174892870303 | 0.999489402457283 |
| ebi-fl187-GCST90094781 | ebi-gm430-GCST90011472 | OTU99_72 (Parabacteroides) abundance \|\| id:ebi-gm430-GCST90011472 | Herring liking \|\| id:ebi-fl187-GCST90094781 | Weighted median | 14 | 0.214420900074655 | 0.275885090241062 | 0.437034350708485 | -0.326313877 | 0.755155676947137 | 1.23914410089198 | 0.721578665082745 | 2.12794276920495 | 0.999489402457283 |
| ebi-fl187-GCST90094781 | ebi-gm430-GCST90011505 | TestASV_28 (Holdemanella) abundance \|\| id:ebi-gm430-GCST90011505 | Herring liking \|\| id:ebi-fl187-GCST90094781 | Weighted median | 14 | -0.017589832 | 0.230154153396355 | 0.939079942127773 | -0.468691972 | 0.433512309042108 | 0.982563966392754 | 0.625820323482732 | 1.54266634020571 | 0.999489402457283 |
| ebi-fl187-GCST90094781 | ebi-gm430-GCST90011513 | TestASV_35 (Alphaproteobacteria) abundance \|\| id:ebi-gm430-GCST90011513 | Herring liking \|\| id:ebi-fl187-GCST90094781 | Weighted median | 14 | 0.324759855663399 | 0.318591028227867 | 0.308030740437566 | -0.29967856 | 0.949198270990017 | 1.38369831876428 | 0.741056387816278 | 2.58363745165607 | 0.999489402457283 |
| ebi-fl187-GCST90094781 | ebi-gm430-GCST90011543 | G_Coprococcus prevalence \|\| id:ebi-gm430-GCST90011543 | Herring liking \|\| id:ebi-fl187-GCST90094781 | Weighted median | 14 | 0.426296345479827 | 0.30906034088569 | 0.167792885844305 | -0.179461923 | 1.03205461361578 | 1.53157458309267 | 0.835719772326471 | 2.8068268590146 | 0.999489402457283 |
| ebi-fl187-GCST90094781 | ebi-gm430-GCST90011547 | G_Parasutterella prevalence \|\| id:ebi-gm430-GCST90011547 | Herring liking \|\| id:ebi-fl187-GCST90094781 | Weighted median | 14 | 0.391904356295572 | 0.277308371862978 | 0.157584031888426 | -0.151620053 | 0.935428765147009 | 1.47979617144606 | 0.859314713152587 | 2.54830584826443 | 0.999489402457283 |
| ebi-fl187-GCST90094781 | ebi-gm430-GCST90011569 | OTU97_13 (Subdoligranulum) prevalence \|\| id:ebi-gm430-GCST90011569 | Herring liking \|\| id:ebi-fl187-GCST90094781 | Weighted median | 14 | 0.347503188537764 | 0.290186564893832 | 0.231105315015069 | -0.221262479 | 0.916268855729674 | 1.4155288240746 | 0.801506274389683 | 2.49994531 | 0.999489402457283 |
| ebi-fl187-GCST90094781 | ebi-gm430-GCST90011590 | OTU97_277 (Prevotella) prevalence \|\| id:ebi-gm430-GCST90011590 | Herring liking \|\| id:ebi-fl187-GCST90094781 | Weighted median | 14 | 0.14502736652563 | 0.231629896853069 | 0.531238342937982 | -0.308967231 | 0.599021964357644 | 1.15607120748745 | 0.734204828565156 | 1.82033757445233 | 0.999489402457283 |
| ebi-fl187-GCST90094781 | ebi-gm430-GCST90011616 | OTU97_86 (Phascolarctobacterium) prevalence \|\| id:ebi-gm430-GCST90011616 | Herring liking \|\| id:ebi-fl187-GCST90094781 | Weighted median | 14 | 0.0182067488272007 | 0.30270369465401 | 0.952038478952128 | -0.575092493 | 0.61150599034906 | 1.01837350215329 | 0.56265282512421 | 1.84320515880998 | 0.999489402457283 |
| ebi-fl187-GCST90094781 | ebi-gm430-GCST90011665 | OTU99_55 (Barnesiella) prevalence \|\| id:ebi-gm430-GCST90011665 | Herring liking \|\| id:ebi-fl187-GCST90094781 | Weighted median | 14 | -0.024957301 | 0.19758562200039 | 0.899485494513474 | -0.41222512 | 0.362310518030629 | 0.975351557587459 | 0.662175190253155 | 1.43664497687476 | 0.999489402457283 |
| ebi-fl187-GCST90094781 | ebi-gm430-GCST90011687 | TestASV_14 (Lachnospiraceae) prevalence \|\| id:ebi-gm430-GCST90011687 | Herring liking \|\| id:ebi-fl187-GCST90094781 | Weighted median | 14 | 0.305514298317593 | 0.21730207909592 | 0.159741104148539 | -0.120397777 | 0.731426373345597 | 1.35732289240917 | 0.886567710581216 | 2.07804255926509 | 0.999489402457283 |
| ebi-fl187-GCST90094781 | ebi-gm430-GCST90011697 | TestASV_23 (Barnesiella) prevalence \|\| id:ebi-gm430-GCST90011697 | Herring liking \|\| id:ebi-fl187-GCST90094781 | Weighted median | 14 | -0.29610255 | 0.306495983973806 | 0.333999401118149 | -0.896834679 | 0.304629578497664 | 0.743711156462649 | 0.40785862226686 | 1.35612257299569 | 0.999489402457283 |
| ebi-fl187-GCST90094781 | ebi-gm430-GCST90011727 | TestASV_6 (Subdoligranulum) prevalence \|\| id:ebi-gm430-GCST90011727 | Herring liking \|\| id:ebi-fl187-GCST90094781 | Weighted median | 14 | 0.196436564735261 | 0.342249632072423 | 0.565996914184642 | -0.474372714 | 0.867245843597209 | 1.21705811402574 | 0.622275278553371 | 2.38034597221885 | 0.999489402457283 |
| ebi-fl187-GCST90094781 | ebi-hbsm44-GCST90104908 | Sulcal depth of the left posterior inferior temporal sulcus \|\| id:ebi-hbsm44-GCST90104908 | Herring liking \|\| id:ebi-fl187-GCST90094781 | Weighted median | 16 | 0.140443402374454 | 0.0830122993220545 | 0.0906767743177337 | -0.022260704 | 0.303147509045681 | 1.1507839460828 | 0.977985236859663 | 1.35411419380345 | 0.999489402457283 |
| ebi-fl187-GCST90094781 | ebi-hbsm44-GCST90104918 | Sulcal depth of the left inferior frontal sulcus \|\| id:ebi-hbsm44-GCST90104918 | Herring liking \|\| id:ebi-fl187-GCST90094781 | Weighted median | 15 | 0.150473477593004 | 0.142818364448438 | 0.292065932665322 | -0.129450517 | 0.430397471911942 | 1.16238447546016 | 0.878578062259667 | 1.53786866168239 | 0.999489402457283 |
| ebi-fl187-GCST90094781 | ebi-hbsm44-GCST90104925 | Sulcal depth of the right anterior inferior temporal sulcus \|\| id:ebi-hbsm44-GCST90104925 | Herring liking \|\| id:ebi-fl187-GCST90094781 | Weighted median | 15 | -0.029302029 | 0.0501930906615083 | 0.559364221016752 | -0.127680487 | 0.0690764282932125 | 0.971123112434143 | 0.880134548565714 | 1.07151810031846 | 0.999489402457283 |
| ebi-fl187-GCST90094781 | ebi-IPs91-GCST90274770 | C-C motif chemokine 4 levels \|\| id:ebi-IPs91-GCST90274770 | Herring liking \|\| id:ebi-fl187-GCST90094781 | Weighted median | 21 | 0.0263151680972166 | 0.0584492328780305 | 0.652549911138908 | -0.088245328 | 0.140875664538156 | 1.02666446937632 | 0.915536242179929 | 1.1512814939691 | 0.999489402457283 |
| ebi-fl187-GCST90094781 | ebi-IPs91-GCST90274779 | C-X-C motif chemokine 1 levels \|\| id:ebi-IPs91-GCST90274779 | Herring liking \|\| id:ebi-fl187-GCST90094781 | Weighted median | 21 | -0.004582007 | 0.0639042198244388 | 0.942839726579003 | -0.129834278 | 0.120670263650465 | 0.995428474174883 | 0.878240962656274 | 1.12825282505747 | 0.999489402457283 |
| ebi-fl187-GCST90094781 | ebi-IPs91-GCST90274780 | C-X-C motif chemokine 10 levels \|\| id:ebi-IPs91-GCST90274780 | Herring liking \|\| id:ebi-fl187-GCST90094781 | Weighted median | 21 | -0.10757498 | 0.0606361806693641 | 0.0760457050885378 | -0.226421894 | 0.0112719339089108 | 0.898009187015245 | 0.797381619918471 | 1.01133570152549 | 0.999489402457283 |
| ebi-fl187-GCST90094781 | ebi-IPs91-GCST90274796 | Interleukin-10 receptor subunit alpha levels \|\| id:ebi-IPs91-GCST90274796 | Herring liking \|\| id:ebi-fl187-GCST90094781 | Weighted median | 19 | -0.047094 | 0.0689338467952968 | 0.494495176428167 | -0.18220434 | 0.0880163398455275 | 0.953997717710939 | 0.833431020060103 | 1.09200596509121 | 0.999489402457283 |
| ebi-fl187-GCST90094781 | ebi-IPs91-GCST90274807 | Interleukin-20 levels \|\| id:ebi-IPs91-GCST90274807 | Herring liking \|\| id:ebi-fl187-GCST90094781 | Weighted median | 19 | -0.008164724 | 0.0629696032352211 | 0.896834363884386 | -0.131585146 | 0.115255698679677 | 0.991868517165967 | 0.876704624065706 | 1.12216033580688 | 0.999489402457283 |
| ebi-fl187-GCST90094781 | ebi-IPs91-GCST90274808 | Interleukin-20 receptor subunit alpha levels \|\| id:ebi-IPs91-GCST90274808 | Herring liking \|\| id:ebi-fl187-GCST90094781 | Weighted median | 19 | 0.00525045947658836 | 0.0701676298936666 | 0.940352061658175 | -0.132278095 | 0.142779014068175 | 1.00526426729416 | 0.876097322813173 | 1.15347487177971 | 0.999489402457283 |
| ebi-fl187-GCST90094781 | ebi-IPs91-GCST90274810 | Interleukin-24 levels \|\| id:ebi-IPs91-GCST90274810 | Herring liking \|\| id:ebi-fl187-GCST90094781 | Weighted median | 19 | -0.024901158 | 0.0657777619213122 | 0.705010919686768 | -0.153825572 | 0.104023255170622 | 0.975406318184726 | 0.857421566674077 | 1.10962625916352 | 0.999489402457283 |
| ebi-fl187-GCST90094781 | ebi-IPs91-GCST90274823 | Monocyte chemoattractant protein-3 levels \|\| id:ebi-IPs91-GCST90274823 | Herring liking \|\| id:ebi-fl187-GCST90094781 | Weighted median | 19 | -0.131259012 | 0.0708547671765436 | 0.0639529330876627 | -0.270134355 | 0.00761633210767629 | 0.876990594270696 | 0.763276937203306 | 1.00764541014084 | 0.999489402457283 |
| ebi-fl187-GCST90094781 | ebi-IPs91-GCST90274824 | Monocyte chemoattractant protein-4 levels \|\| id:ebi-IPs91-GCST90274824 | Herring liking \|\| id:ebi-fl187-GCST90094781 | Weighted median | 21 | 0.00758145755998328 | 0.062216568922911 | 0.903013122124911 | -0.114363018 | 0.129525932648889 | 1.00761026957568 | 0.891934109388636 | 1.13828862991941 | 0.999489402457283 |
| ebi-fl187-GCST90094781 | ebi-IPs91-GCST90274834 | SIR2-like protein 2 levels \|\| id:ebi-IPs91-GCST90274834 | Herring liking \|\| id:ebi-fl187-GCST90094781 | Weighted median | 21 | -0.081891529 | 0.0574352560534297 | 0.153924368829396 | -0.194464631 | 0.0306815732119217 | 0.9213718957269 | 0.823275296562746 | 1.03115710355908 | 0.999489402457283 |
| ebi-fl187-GCST90094781 | ebi-met1400-GCST90199652 | 1-stearoyl-2-oleoyl-GPS (18:0/18:1) levels \|\| id:ebi-met1400-GCST90199652 | Herring liking \|\| id:ebi-fl187-GCST90094781 | Weighted median | 16 | -0.035189104 | 0.0869108721669446 | 0.685560391283625 | -0.205534413 | 0.135156205478293 | 0.96542283370503 | 0.81421207426609 | 1.1447155812313 | 0.999489402457283 |
| ebi-fl187-GCST90094781 | ebi-met1400-GCST90199665 | N-acetylaspartate (naa) levels \|\| id:ebi-met1400-GCST90199665 | Herring liking \|\| id:ebi-fl187-GCST90094781 | Weighted median | 16 | -0.021528531 | 0.0799254388342506 | 0.787654451680842 | -0.178182391 | 0.135125329568105 | 0.9787015541801 | 0.836789786926126 | 1.14468023764146 | 0.999489402457283 |
| ebi-fl187-GCST90094781 | ebi-met1400-GCST90199669 | Acetylcarnitine levels (Biocrates platform) \|\| id:ebi-met1400-GCST90199669 | Herring liking \|\| id:ebi-fl187-GCST90094781 | Weighted median | 16 | 0.0441559235806416 | 0.0814207589724853 | 0.587599623341924 | -0.115428764 | 0.203740611166713 | 1.04514530498248 | 0.890984040110696 | 1.2259801066596 | 0.999489402457283 |
| ebi-fl187-GCST90094781 | ebi-met1400-GCST90199670 | Iminodiacetate (IDA) levels \|\| id:ebi-met1400-GCST90199670 | Herring liking \|\| id:ebi-fl187-GCST90094781 | Weighted median | 16 | -0.007999743 | 0.0812841105070916 | 0.921601126494416 | -0.1673166 | 0.151317113205548 | 0.992032169404038 | 0.845931746960424 | 1.16336551816222 | 0.999489402457283 |
| ebi-fl187-GCST90094781 | ebi-met1400-GCST90199686 | Indolepropionate levels \|\| id:ebi-met1400-GCST90199686 | Herring liking \|\| id:ebi-fl187-GCST90094781 | Weighted median | 16 | 0.162151545975819 | 0.0895468058271145 | 0.0701714877959548 | -0.013360193 | 0.337663285396964 | 1.17603845171189 | 0.986728657808993 | 1.40166846169844 | 0.999489402457283 |
| ebi-fl187-GCST90094781 | ebi-met1400-GCST90199704 | 3-hydroxysebacate levels \|\| id:ebi-met1400-GCST90199704 | Herring liking \|\| id:ebi-fl187-GCST90094781 | Weighted median | 16 | 0.0296056660190644 | 0.0867048011829162 | 0.7327621341231 | -0.140335744 | 0.19954707633758 | 1.03004827082191 | 0.869066402320477 | 1.22084968120993 | 0.999489402457283 |
| ebi-fl187-GCST90094781 | ebi-met1400-GCST90199713 | Docosapentaenoate n3 DPA; 22:5n3 levels \|\| id:ebi-met1400-GCST90199713 | Herring liking \|\| id:ebi-fl187-GCST90094781 | Weighted median | 16 | -0.068638546 | 0.0794473635869935 | 0.387615386435375 | -0.224355378 | 0.0870782869570729 | 0.933664096021368 | 0.799031125580868 | 1.09098208604287 | 0.999489402457283 |
| ebi-fl187-GCST90094781 | ebi-met1400-GCST90199715 | Myristoleate (14:1n5) levels \|\| id:ebi-met1400-GCST90199715 | Herring liking \|\| id:ebi-fl187-GCST90094781 | Weighted median | 16 | 0.00853555965682144 | 0.0746807791006616 | 0.909004794998419 | -0.137838767 | 0.154909886694118 | 1.00857209141183 | 0.871239152592864 | 1.1675527443269 | 0.999489402457283 |
| ebi-fl187-GCST90094781 | ebi-met1400-GCST90199725 | Campesterol levels \|\| id:ebi-met1400-GCST90199725 | Herring liking \|\| id:ebi-fl187-GCST90094781 | Weighted median | 16 | 0.120956257007938 | 0.104227685357479 | 0.245845183846823 | -0.083330006 | 0.325242520308597 | 1.12857554401653 | 0.920047475659549 | 1.38436634222506 | 0.999489402457283 |
| ebi-fl187-GCST90094781 | ebi-met1400-GCST90199737 | N-acetylproline levels \|\| id:ebi-met1400-GCST90199737 | Herring liking \|\| id:ebi-fl187-GCST90094781 | Weighted median | 16 | -0.026972242 | 0.0906177317191422 | 0.765971367938037 | -0.204582996 | 0.150638512070419 | 0.973388260364211 | 0.814987098222336 | 1.16257632480506 | 0.999489402457283 |
| ebi-fl187-GCST90094781 | ebi-met1400-GCST90199775 | Gamma-glutamylisoleucine levels \|\| id:ebi-met1400-GCST90199775 | Herring liking \|\| id:ebi-fl187-GCST90094781 | Weighted median | 16 | 0.0373529390305177 | 0.0829156196017383 | 0.652354715180036 | -0.125161675 | 0.199867553449925 | 1.03805932784512 | 0.882354236087818 | 1.2212409982911 | 0.999489402457283 |
| ebi-fl187-GCST90094781 | ebi-met1400-GCST90199777 | 1-palmitoleoyl-GPC (16:1) levels \|\| id:ebi-met1400-GCST90199777 | Herring liking \|\| id:ebi-fl187-GCST90094781 | Weighted median | 16 | -0.069886226 | 0.0848460017296625 | 0.410119587574849 | -0.23618439 | 0.0964119371659527 | 0.932499907906287 | 0.789635060004196 | 1.10121260097115 | 0.999489402457283 |
| ebi-fl187-GCST90094781 | ebi-met1400-GCST90199782 | Glutamine degradant levels \|\| id:ebi-met1400-GCST90199782 | Herring liking \|\| id:ebi-fl187-GCST90094781 | Weighted median | 16 | -0.131526048 | 0.0668163151670722 | 0.049013749784947 | -0.262486026 | -0.000566071 | 0.876756436729324 | 0.769137112109853 | 0.999434089505371 | 0.999489402457283 |
| ebi-fl187-GCST90094781 | ebi-met1400-GCST90199783 | 5-methyluridine (ribothymidine) levels \|\| id:ebi-met1400-GCST90199783 | Herring liking \|\| id:ebi-fl187-GCST90094781 | Weighted median | 16 | 0.0761468098086885 | 0.0850517298770574 | 0.370626765133692 | -0.090554581 | 0.242848200367721 | 1.07912098807445 | 0.91342447714701 | 1.27487508386023 | 0.999489402457283 |
| ebi-fl187-GCST90094781 | ebi-met1400-GCST90199787 | Homostachydrine levels \|\| id:ebi-met1400-GCST90199787 | Herring liking \|\| id:ebi-fl187-GCST90094781 | Weighted median | 16 | 0.000762407938682713 | 0.0771668708272339 | 0.992117036491717 | -0.150484659 | 0.152009474760061 | 1.00076269864549 | 0.860290927730217 | 1.16417126662328 | 0.999911036302558 |
| ebi-fl187-GCST90094781 | ebi-met1400-GCST90199802 | Beta-hydroxyisovaleroylcarnitine levels \|\| id:ebi-met1400-GCST90199802 | Herring liking \|\| id:ebi-fl187-GCST90094781 | Weighted median | 16 | 0.132417225466847 | 0.0792602325819182 | 0.0947880428045115 | -0.02293283 | 0.287767281327407 | 1.14158451805269 | 0.977328128314186 | 1.33344695000801 | 0.999489402457283 |
| ebi-fl187-GCST90094781 | ebi-met1400-GCST90199873 | Pregnenolone sulfate levels \|\| id:ebi-met1400-GCST90199873 | Herring liking \|\| id:ebi-fl187-GCST90094781 | Weighted median | 16 | -0.024057746 | 0.0777041463073997 | 0.756859844322811 | -0.176357873 | 0.128242380347824 | 0.976229334386221 | 0.838317918150777 | 1.13682851419711 | 0.999489402457283 |
| ebi-fl187-GCST90094781 | ebi-met1400-GCST90199911 | Beta-citrylglutamate levels \|\| id:ebi-met1400-GCST90199911 | Herring liking \|\| id:ebi-fl187-GCST90094781 | Weighted median | 16 | 0.0237190502987008 | 0.0801832999615622 | 0.767374915797551 | -0.133440218 | 0.180878318223363 | 1.024002584 | 0.875079781760737 | 1.19826936287681 | 0.999489402457283 |
| ebi-fl187-GCST90094781 | ebi-met1400-GCST90199934 | 2-hydroxydecanoate levels \|\| id:ebi-met1400-GCST90199934 | Herring liking \|\| id:ebi-fl187-GCST90094781 | Weighted median | 16 | 0.0978203127601187 | 0.0839771472054518 | 0.244081967808328 | -0.066774896 | 0.262415521282804 | 1.10276461457485 | 0.93540574143527 | 1.30006663556766 | 0.999489402457283 |
| ebi-fl187-GCST90094781 | ebi-met1400-GCST90199945 | Sphingomyelin (d18:1/14:0, d16:1/16:0) levels \|\| id:ebi-met1400-GCST90199945 | Herring liking \|\| id:ebi-fl187-GCST90094781 | Weighted median | 16 | 0.00823277184147524 | 0.0807777129561113 | 0.918821085463788 | -0.150091546 | 0.166557089235453 | 1.00826675430017 | 0.860629186044322 | 1.18123096951843 | 0.999489402457283 |
| ebi-fl187-GCST90094781 | ebi-met1400-GCST90199979 | Carnitine C14:1 levels \|\| id:ebi-met1400-GCST90199979 | Herring liking \|\| id:ebi-fl187-GCST90094781 | Weighted median | 16 | -0.025322225 | 0.0838122250033313 | 0.762552622637646 | -0.189594186 | 0.138949735962475 | 0.974995693376241 | 0.827294793598593 | 1.14906634183831 | 0.999489402457283 |
| ebi-fl187-GCST90094781 | ebi-met1400-GCST90199986 | Eugenol sulfate levels \|\| id:ebi-met1400-GCST90199986 | Herring liking \|\| id:ebi-fl187-GCST90094781 | Weighted median | 16 | -0.050969198 | 0.0932916632401932 | 0.584830546364124 | -0.233820858 | 0.131882462030672 | 0.950307941542759 | 0.791503594835095 | 1.1409742035946 | 0.999489402457283 |
| ebi-fl187-GCST90094781 | ebi-met1400-GCST90200002 | 6-hydroxyindole sulfate levels \|\| id:ebi-met1400-GCST90200002 | Herring liking \|\| id:ebi-fl187-GCST90094781 | Weighted median | 16 | 0.0151676749267751 | 0.0847243899825035 | 0.857919081725446 | -0.150892129 | 0.181227479292482 | 1.01528328789519 | 0.859940455915692 | 1.19868782493973 | 0.999489402457283 |
| ebi-fl187-GCST90094781 | ebi-met1400-GCST90200023 | Trans 3,4-methyleneheptanoate levels \|\| id:ebi-met1400-GCST90200023 | Herring liking \|\| id:ebi-fl187-GCST90094781 | Weighted median | 16 | -0.016363018 | 0.0858658178822931 | 0.848866430284835 | -0.184660021 | 0.151933985296336 | 0.983770129204167 | 0.831386890057188 | 1.1640833872757 | 0.999489402457283 |
| ebi-fl187-GCST90094781 | ebi-met1400-GCST90200032 | 2-hydroxybutyrate/2-hydroxyisobutyrate levels \|\| id:ebi-met1400-GCST90200032 | Herring liking \|\| id:ebi-fl187-GCST90094781 | Weighted median | 16 | 0.139260471537014 | 0.0832635200898385 | 0.0944204812395032 | -0.023936028 | 0.302456970913097 | 1.14942345310894 | 0.976348166862185 | 1.35317944909234 | 0.999489402457283 |
| ebi-fl187-GCST90094781 | ebi-met1400-GCST90200051 | 1-palmitoyl-2-dihomo-linolenoyl-GPC (16:0/20:3n3 or 6) levels \|\| id:ebi-met1400-GCST90200051 | Herring liking \|\| id:ebi-fl187-GCST90094781 | Weighted median | 16 | -0.029459847 | 0.0748944610941861 | 0.694059903096163 | -0.176252991 | 0.117333296880013 | 0.97096986434291 | 0.83840584769788 | 1.12449415763357 | 0.999489402457283 |
| ebi-fl187-GCST90094781 | ebi-met1400-GCST90200069 | Phenylacetylglutamate levels \|\| id:ebi-met1400-GCST90200069 | Herring liking \|\| id:ebi-fl187-GCST90094781 | Weighted median | 16 | -0.065266093 | 0.0886901715940386 | 0.461798450151037 | -0.239098829 | 0.108566643502563 | 0.936818149623672 | 0.787337066677555 | 1.11467919218865 | 0.999489402457283 |
| ebi-fl187-GCST90094781 | ebi-met1400-GCST90200072 | Thioproline levels \|\| id:ebi-met1400-GCST90200072 | Herring liking \|\| id:ebi-fl187-GCST90094781 | Weighted median | 16 | 0.140820873084073 | 0.0846987634448661 | 0.0963908544531474 | -0.025188703 | 0.30683044943601 | 1.15121841531009 | 0.975125885224528 | 1.35911051058184 | 0.999489402457283 |
| ebi-fl187-GCST90094781 | ebi-met1400-GCST90200081 | 1-oleoyl-2-docosahexaenoyl-GPC (18:1/22:6) levels \|\| id:ebi-met1400-GCST90200081 | Herring liking \|\| id:ebi-fl187-GCST90094781 | Weighted median | 16 | -0.05681731 | 0.0826359373085952 | 0.491728797461111 | -0.218783747 | 0.105149126814107 | 0.944766652727653 | 0.803495457320319 | 1.11087625944148 | 0.999489402457283 |
| ebi-fl187-GCST90094781 | ebi-met1400-GCST90200097 | Furaneol sulfate levels \|\| id:ebi-met1400-GCST90200097 | Herring liking \|\| id:ebi-fl187-GCST90094781 | Weighted median | 16 | -0.083850926 | 0.095420242513404 | 0.379534639900646 | -0.270874601 | 0.103172749785067 | 0.919568330028884 | 0.762712133848464 | 1.1086829172697 | 0.999489402457283 |
| ebi-fl187-GCST90094781 | ebi-met1400-GCST90200124 | Sphingomyelin (d18:2/24:2) levels \|\| id:ebi-met1400-GCST90200124 | Herring liking \|\| id:ebi-fl187-GCST90094781 | Weighted median | 16 | -0.095352578 | 0.0792124716941483 | 0.228683459744818 | -0.250609023 | 0.0599038661062517 | 0.90905236571714 | 0.778326619935635 | 1.06173447297006 | 0.999489402457283 |
| ebi-fl187-GCST90094781 | ebi-met1400-GCST90200137 | Lignoceroylcarnitine (C24) levels \|\| id:ebi-met1400-GCST90200137 | Herring liking \|\| id:ebi-fl187-GCST90094781 | Weighted median | 16 | -0.018941951 | 0.0894278452131229 | 0.832253181430769 | -0.194220528 | 0.156336625706867 | 0.981236320464244 | 0.823476285053312 | 1.16921972626798 | 0.999489402457283 |
| ebi-fl187-GCST90094781 | ebi-met1400-GCST90200165 | Octadecenedioate (C18:1-DC) levels \|\| id:ebi-met1400-GCST90200165 | Herring liking \|\| id:ebi-fl187-GCST90094781 | Weighted median | 16 | 0.0815635675766954 | 0.0778770043299681 | 0.294943587895868 | -0.071075361 | 0.234202496063433 | 1.08498218508019 | 0.931391698956676 | 1.26390040115243 | 0.999489402457283 |
| ebi-fl187-GCST90094781 | ebi-met1400-GCST90200176 | Glutamine conjugate of C6H10O2 (1) levels \|\| id:ebi-met1400-GCST90200176 | Herring liking \|\| id:ebi-fl187-GCST90094781 | Weighted median | 16 | 0.102166645604322 | 0.078817265127238 | 0.1948903924193 | -0.052315194 | 0.256648485253709 | 1.10756802769309 | 0.949029691188922 | 1.29259068220634 | 0.999489402457283 |
| ebi-fl187-GCST90094781 | ebi-met1400-GCST90200182 | Glycine conjugate of C10H12O2 levels \|\| id:ebi-met1400-GCST90200182 | Herring liking \|\| id:ebi-fl187-GCST90094781 | Weighted median | 16 | -0.033949352 | 0.0998870327478585 | 0.733948804110276 | -0.229727936 | 0.161829232218086 | 0.966620460822034 | 0.794749795779242 | 1.17565945941978 | 0.999489402457283 |
| ebi-fl187-GCST90094781 | ebi-met1400-GCST90200208 | Glucuronide of piperine metabolite C17H21NO3 (4) levels \|\| id:ebi-met1400-GCST90200208 | Herring liking \|\| id:ebi-fl187-GCST90094781 | Weighted median | 16 | 0.0114128259117499 | 0.0840449722182715 | 0.891983918512984 | -0.15331532 | 0.176140971459562 | 1.01147820067628 | 0.85785917931574 | 1.19260617023342 | 0.999489402457283 |
| ebi-fl187-GCST90094781 | ebi-met1400-GCST90200227 | 3-ethylcatechol sulfate (2) levels \|\| id:ebi-met1400-GCST90200227 | Herring liking \|\| id:ebi-fl187-GCST90094781 | Weighted median | 15 | -0.06705799 | 0.107034136856754 | 0.530980280573941 | -0.276844898 | 0.142728918627846 | 0.935140971302881 | 0.758172082110959 | 1.15341708939543 | 0.999489402457283 |
| ebi-fl187-GCST90094781 | ebi-met1400-GCST90200259 | Pentose acid levels \|\| id:ebi-met1400-GCST90200259 | Herring liking \|\| id:ebi-fl187-GCST90094781 | Weighted median | 16 | -0.071833247 | 0.0875922372845644 | 0.412166566267464 | -0.243514032 | 0.0998475382241985 | 0.930686077704128 | 0.783868476766493 | 1.10500243459888 | 0.999489402457283 |
| ebi-fl187-GCST90094781 | ebi-met1400-GCST90200291 | Sarcosine levels \|\| id:ebi-met1400-GCST90200291 | Herring liking \|\| id:ebi-fl187-GCST90094781 | Weighted median | 16 | 0.0460254964054815 | 0.0810446302714983 | 0.570100769925138 | -0.112821979 | 0.204872971737618 | 1.04710110793103 | 0.893309673908671 | 1.22736914449051 | 0.999489402457283 |
| ebi-fl187-GCST90094781 | ebi-met1400-GCST90200306 | Butyrate/isobutyrate (4:0) levels \|\| id:ebi-met1400-GCST90200306 | Herring liking \|\| id:ebi-fl187-GCST90094781 | Weighted median | 16 | -0.054449013 | 0.083182949179038 | 0.512745031837922 | -0.217487593 | 0.108589567367178 | 0.94700679263975 | 0.804537586422142 | 1.11470474523643 | 0.999489402457283 |
| ebi-fl187-GCST90094781 | ebi-met1400-GCST90200321 | Cholate levels \|\| id:ebi-met1400-GCST90200321 | Herring liking \|\| id:ebi-fl187-GCST90094781 | Weighted median | 16 | -0.006777849 | 0.0884909260287583 | 0.938946752786032 | -0.180220064 | 0.166664365560163 | 0.993245068358357 | 0.835086418336649 | 1.18135769443264 | 0.999489402457283 |
| ebi-fl187-GCST90094781 | ebi-met1400-GCST90200336 | Ceramide (d18:1/16:0) levels \|\| id:ebi-met1400-GCST90200336 | Herring liking \|\| id:ebi-fl187-GCST90094781 | Weighted median | 16 | 0.0214657218115283 | 0.079808247735046 | 0.787955686340365 | -0.134958444 | 0.177889887372219 | 1.02169776778849 | 0.873752220800052 | 1.19469376312218 | 0.999489402457283 |
| ebi-fl187-GCST90094781 | ebi-met1400-GCST90200358 | Arachidonate (20:4n6) levels \|\| id:ebi-met1400-GCST90200358 | Herring liking \|\| id:ebi-fl187-GCST90094781 | Weighted median | 16 | -0.044861447 | 0.0794856079363864 | 0.572483906092708 | -0.200653239 | 0.110930344248204 | 0.956129947035094 | 0.818196100978981 | 1.11731707658286 | 0.999489402457283 |
| ebi-fl187-GCST90094781 | ebi-met1400-GCST90200413 | 2'-deoxyuridine levels \|\| id:ebi-met1400-GCST90200413 | Herring liking \|\| id:ebi-fl187-GCST90094781 | Weighted median | 16 | -0.026283475 | 0.0793738461670552 | 0.740542361957562 | -0.181856213 | 0.129289263816826 | 0.974058929434223 | 0.833721209937559 | 1.13801926435529 | 0.999489402457283 |
| ebi-fl187-GCST90094781 | ebi-met1400-GCST90200432 | Threonine levels \|\| id:ebi-met1400-GCST90200432 | Herring liking \|\| id:ebi-fl187-GCST90094781 | Weighted median | 16 | -0.103662985 | 0.0855891133506782 | 0.225830175472549 | -0.271417647 | 0.0640916773456223 | 0.901529075211485 | 0.762298058423513 | 1.06619013976306 | 0.999489402457283 |
| ebi-fl187-GCST90094781 | ebi-met1400-GCST90200436 | Caffeine levels \|\| id:ebi-met1400-GCST90200436 | Herring liking \|\| id:ebi-fl187-GCST90094781 | Weighted median | 16 | -0.010947067 | 0.0817449968128245 | 0.893467987333482 | -0.171167261 | 0.14927312627419 | 0.989112633615023 | 0.842680613885682 | 1.16099004279404 | 0.999489402457283 |
| ebi-fl187-GCST90094781 | ebi-met1400-GCST90200442 | Valine levels \|\| id:ebi-met1400-GCST90200442 | Herring liking \|\| id:ebi-fl187-GCST90094781 | Weighted median | 16 | 0.0706326549622054 | 0.0788512962957426 | 0.370375417191747 | -0.083915886 | 0.225181195701861 | 1.0731869235593 | 0.919508596593092 | 1.25254965224468 | 0.999489402457283 |
| ebi-fl187-GCST90094781 | ebi-met1400-GCST90200459 | Margarate (17:0) levels \|\| id:ebi-met1400-GCST90200459 | Herring liking \|\| id:ebi-fl187-GCST90094781 | Weighted median | 16 | -0.033698705 | 0.0861005239319039 | 0.695510520670376 | -0.202455732 | 0.135058322003124 | 0.966862771768836 | 0.816722636630773 | 1.14460353797581 | 0.999489402457283 |
| ebi-fl187-GCST90094781 | ebi-met1400-GCST90200462 | X-11299 levels \|\| id:ebi-met1400-GCST90200462 | Herring liking \|\| id:ebi-fl187-GCST90094781 | Weighted median | 16 | 0.0276502576533448 | 0.0829463675840222 | 0.738869318234387 | -0.134924623 | 0.190225138118028 | 1.02803607379066 | 0.873781772419313 | 1.20952187648492 | 0.999489402457283 |
| ebi-fl187-GCST90094781 | ebi-met1400-GCST90200497 | X-12740 levels \|\| id:ebi-met1400-GCST90200497 | Herring liking \|\| id:ebi-fl187-GCST90094781 | Weighted median | 16 | -0.02624755 | 0.100435599080128 | 0.793832543936948 | -0.223101324 | 0.170606224074171 | 0.974093922689272 | 0.80003378230868 | 1.18602362950477 | 0.999489402457283 |
| ebi-fl187-GCST90094781 | ebi-met1400-GCST90200527 | X-13866 levels \|\| id:ebi-met1400-GCST90200527 | Herring liking \|\| id:ebi-fl187-GCST90094781 | Weighted median | 16 | -0.008485972 | 0.0802093142349715 | 0.915742756753007 | -0.165696227 | 0.148724284328329 | 0.991549932652026 | 0.847303582650367 | 1.1603530175889 | 0.999489402457283 |
| ebi-fl187-GCST90094781 | ebi-met1400-GCST90200547 | X-17654 levels \|\| id:ebi-met1400-GCST90200547 | Herring liking \|\| id:ebi-fl187-GCST90094781 | Weighted median | 16 | 0.00372548919967235 | 0.0809583841284304 | 0.963296432069703 | -0.154952944 | 0.162403922091396 | 1.00373243746044 | 0.856455478168131 | 1.17633529318438 | 0.999489402457283 |
| ebi-fl187-GCST90094781 | ebi-met1400-GCST90200553 | X-17653 levels \|\| id:ebi-met1400-GCST90200553 | Herring liking \|\| id:ebi-fl187-GCST90094781 | Weighted median | 16 | 0.0303534841064023 | 0.0818462949053677 | 0.710742108011711 | -0.130065254 | 0.190772222120923 | 1.03081884763939 | 0.878038133631449 | 1.2101837675932 | 0.999489402457283 |
| ebi-fl187-GCST90094781 | ebi-met1400-GCST90200557 | X-17685 levels \|\| id:ebi-met1400-GCST90200557 | Herring liking \|\| id:ebi-fl187-GCST90094781 | Weighted median | 16 | -0.049347408 | 0.087872768117066 | 0.574403611241129 | -0.221578034 | 0.122883217058228 | 0.951850391380924 | 0.801253394732444 | 1.13075236064935 | 0.999489402457283 |
| ebi-fl187-GCST90094781 | ebi-met1400-GCST90200561 | X-18779 levels \|\| id:ebi-met1400-GCST90200561 | Herring liking \|\| id:ebi-fl187-GCST90094781 | Weighted median | 16 | -0.044054721 | 0.0857639597067059 | 0.607479890741825 | -0.212152082 | 0.124042639628422 | 0.956901593048636 | 0.808841677610914 | 1.13206414076438 | 0.999489402457283 |
| ebi-fl187-GCST90094781 | ebi-met1400-GCST90200570 | X-18921 levels \|\| id:ebi-met1400-GCST90200570 | Herring liking \|\| id:ebi-fl187-GCST90094781 | Weighted median | 16 | -0.025210968 | 0.085282959389523 | 0.76752370239112 | -0.192365569 | 0.141943632065728 | 0.975104174220258 | 0.825005217239156 | 1.15251168200327 | 0.999489402457283 |
| ebi-fl187-GCST90094781 | ebi-met1400-GCST90200575 | X-21283 levels \|\| id:ebi-met1400-GCST90200575 | Herring liking \|\| id:ebi-fl187-GCST90094781 | Weighted median | 16 | -0.000477613 | 0.0905603697135142 | 0.995792000551176 | -0.177975937 | 0.177020711997311 | 0.999522501397585 | 0.836962562842544 | 1.19365581586716 | 0.999911036302558 |
| ebi-fl187-GCST90094781 | ebi-met1400-GCST90200578 | X-18922 levels \|\| id:ebi-met1400-GCST90200578 | Herring liking \|\| id:ebi-fl187-GCST90094781 | Weighted median | 16 | 0.0045769018814133 | 0.0820694888345716 | 0.955526136111502 | -0.156279296 | 0.165433099997174 | 1.00458739189464 | 0.855320269277321 | 1.17990402449643 | 0.999489402457283 |
| ebi-fl187-GCST90094781 | ebi-met1400-GCST90200623 | X-24241 levels \|\| id:ebi-met1400-GCST90200623 | Herring liking \|\| id:ebi-fl187-GCST90094781 | Weighted median | 16 | 0.00423825068190351 | 0.0779311069476426 | 0.956628762397624 | -0.148506719 | 0.156983220299283 | 1.00424724476823 | 0.861994215467527 | 1.16997598188938 | 0.999489402457283 |
| ebi-fl187-GCST90094781 | ebi-met1400-GCST90200636 | X-24544 levels \|\| id:ebi-met1400-GCST90200636 | Herring liking \|\| id:ebi-fl187-GCST90094781 | Weighted median | 16 | -0.059332252 | 0.0814724382643708 | 0.466460946563398 | -0.219018231 | 0.100353726817847 | 0.942393604805295 | 0.803307072785503 | 1.10556191581679 | 0.999489402457283 |
| ebi-fl187-GCST90094781 | ebi-met1400-GCST90200659 | X-25172 levels \|\| id:ebi-met1400-GCST90200659 | Herring liking \|\| id:ebi-fl187-GCST90094781 | Weighted median | 16 | -0.000228262 | 0.0808131787151464 | 0.997746326679884 | -0.158622092 | 0.158165568254943 | 0.99977176402305 | 0.853318773771746 | 1.17136011870416 | 0.999911036302558 |
| ebi-fl187-GCST90094781 | ebi-met1400-GCST90200670 | X-26111 levels \|\| id:ebi-met1400-GCST90200670 | Herring liking \|\| id:ebi-fl187-GCST90094781 | Weighted median | 16 | 0.0278302866845422 | 0.0815328897241562 | 0.732849022334498 | -0.131974177 | 0.187634750543888 | 1.02822116678962 | 0.876363624971884 | 1.20639280055483 | 0.999489402457283 |
| ebi-fl187-GCST90094781 | ebi-met1400-GCST90200673 | Carnitine C4 levels \|\| id:ebi-met1400-GCST90200673 | Herring liking \|\| id:ebi-fl187-GCST90094781 | Weighted median | 16 | 0.0546593645149338 | 0.0885225121048756 | 0.53692946362084 | -0.118844759 | 0.22816348824049 | 1.05618078074031 | 0.887945635444262 | 1.2562906973996 | 0.999489402457283 |
| ebi-fl187-GCST90094781 | ebi-met1400-GCST90200710 | X-13431 levels \|\| id:ebi-met1400-GCST90200710 | Herring liking \|\| id:ebi-fl187-GCST90094781 | Weighted median | 16 | 0.112360374032123 | 0.0825518384534422 | 0.173485661957036 | -0.049441229 | 0.27416197740087 | 1.11891601627366 | 0.951761092123568 | 1.31542785456834 | 0.999489402457283 |
| ebi-fl187-GCST90094781 | ebi-met1400-GCST90200730 | Adenosine 5'-diphosphate (ADP) to fructose ratio \|\| id:ebi-met1400-GCST90200730 | Herring liking \|\| id:ebi-fl187-GCST90094781 | Weighted median | 16 | -0.005119051 | 0.112305864586567 | 0.963643946542299 | -0.225238546 | 0.215000443398895 | 0.994894028823166 | 0.798325758802387 | 1.23986244671958 | 0.999489402457283 |
| ebi-fl187-GCST90094781 | ebi-met1400-GCST90200731 | Adenosine 5'-diphosphate (ADP) to mannose ratio \|\| id:ebi-met1400-GCST90200731 | Herring liking \|\| id:ebi-fl187-GCST90094781 | Weighted median | 16 | -0.117195879 | 0.111976512047104 | 0.295278903077754 | -0.336669843 | 0.102278084412523 | 0.889410958992602 | 0.714144580951362 | 1.10769146063158 | 0.999489402457283 |
| ebi-fl187-GCST90094781 | ebi-met1400-GCST90200748 | Aspartate to glutamate ratio \|\| id:ebi-met1400-GCST90200748 | Herring liking \|\| id:ebi-fl187-GCST90094781 | Weighted median | 16 | -0.071159105 | 0.0807279720121746 | 0.378064694831982 | -0.22938593 | 0.0870677202337697 | 0.931313703756052 | 0.795021651542542 | 1.09097055799795 | 0.999489402457283 |
| ebi-fl187-GCST90094781 | ebi-met1400-GCST90200774 | Uridine to pseudouridine ratio \|\| id:ebi-met1400-GCST90200774 | Herring liking \|\| id:ebi-fl187-GCST90094781 | Weighted median | 16 | 0.0813321088048859 | 0.0766378498052177 | 0.288575171444062 | -0.068878077 | 0.231542294423113 | 1.08473108549692 | 0.933440481178587 | 1.26054263937393 | 0.999489402457283 |
| ebi-fl187-GCST90094781 | ebi-met1400-GCST90200799 | Spermidine to adenosine 5'-diphosphate (ADP) ratio \|\| id:ebi-met1400-GCST90200799 | Herring liking \|\| id:ebi-fl187-GCST90094781 | Weighted median | 16 | 0.0551614860045809 | 0.116782013086572 | 0.636680029494868 | -0.17373126 | 0.284054231654263 | 1.05671124497487 | 0.840522749691347 | 1.32850497582175 | 0.999489402457283 |
| ebi-fl187-GCST90094781 | ebi-met1400-GCST90200811 | Choline to taurocholate ratio \|\| id:ebi-met1400-GCST90200811 | Herring liking \|\| id:ebi-fl187-GCST90094781 | Weighted median | 16 | -0.029419534 | 0.082917797674777 | 0.72273672539538 | -0.191938417 | 0.133099349679349 | 0.971009007938481 | 0.825357694759731 | 1.14236348613936 | 0.999489402457283 |
| ebi-fl187-GCST90094781 | ebi-met1400-GCST90200812 | Glutamate to kynurenine ratio \|\| id:ebi-met1400-GCST90200812 | Herring liking \|\| id:ebi-fl187-GCST90094781 | Weighted median | 16 | 0.0242905162124555 | 0.0792069205043714 | 0.759093318252494 | -0.130955048 | 0.179536080401023 | 1.0245879340633 | 0.877257207991696 | 1.1966620793363 | 0.999489402457283 |
| ebi-fl187-GCST90094781 | ebi-met1400-GCST90200876 | Cortisone to 4-cholesten-3-one ratio \|\| id:ebi-met1400-GCST90200876 | Herring liking \|\| id:ebi-fl187-GCST90094781 | Weighted median | 16 | -0.100830296 | 0.0917332171095729 | 0.271694417757878 | -0.280627401 | 0.0789668096498555 | 0.904086447058453 | 0.755309710382806 | 1.08216840392601 | 0.999489402457283 |
| ebi-fl187-GCST90094781 | ebi-met1400-GCST90200906 | 2'-deoxyuridine to cytidine ratio \|\| id:ebi-met1400-GCST90200906 | Herring liking \|\| id:ebi-fl187-GCST90094781 | Weighted median | 16 | -0.133719295 | 0.0874408624157158 | 0.126201470228753 | -0.305103385 | 0.0376647953056366 | 0.874835600841919 | 0.737047170562524 | 1.03838310364359 | 0.999489402457283 |
| ebi-fl187-GCST90094781 | ebi-met1400-GCST90200907 | Retinol (Vitamin A) to linoleoyl-arachidonoyl-glycerol (18:2 to 20:4) [1] ratio \|\| id:ebi-met1400-GCST90200907 | Herring liking \|\| id:ebi-fl187-GCST90094781 | Weighted median | 16 | -0.105862474 | 0.0926049770450319 | 0.252971409412526 | -0.287368229 | 0.07564328055143 | 0.899548350445112 | 0.750235419180412 | 1.07857775586297 | 0.999489402457283 |
| ebi-fl187-GCST90094781 | ebi-met1400-GCST90200916 | Fructose to sucrose ratio \|\| id:ebi-met1400-GCST90200916 | Herring liking \|\| id:ebi-fl187-GCST90094781 | Weighted median | 16 | 0.015737053764373 | 0.0850133606720351 | 0.853140631054304 | -0.150889133 | 0.182363240681562 | 1.01586153331821 | 0.859943032547201 | 1.20005002170767 | 0.999489402457283 |
| ebi-fl187-GCST90094781 | ebi-met1400-GCST90200917 | Alpha-ketobutyrate to 3-methyl-2-oxobutyrate ratio \|\| id:ebi-met1400-GCST90200917 | Herring liking \|\| id:ebi-fl187-GCST90094781 | Weighted median | 16 | 0.0428411211832404 | 0.0786041474700295 | 0.585737236046353 | -0.111223008 | 0.196905250224498 | 1.0437720484082 | 0.894739192807694 | 1.21762866519742 | 0.999489402457283 |
| ebi-fl187-GCST90094781 | ebi-met1400-GCST90200924 | Alpha-tocopherol to glycerol ratio \|\| id:ebi-met1400-GCST90200924 | Herring liking \|\| id:ebi-fl187-GCST90094781 | Weighted median | 16 | 0.0112103584220253 | 0.0697118680468904 | 0.872243128863082 | -0.125424903 | 0.14784561979393 | 1.01127342995449 | 0.882122006700251 | 1.15933390433986 | 0.999489402457283 |
| ebi-fl187-GCST90094781 | ebi-met1400-GCST90200938 | Alpha-ketoglutarate to alanine ratio \|\| id:ebi-met1400-GCST90200938 | Herring liking \|\| id:ebi-fl187-GCST90094781 | Weighted median | 16 | -0.006597259 | 0.080441716026738 | 0.934636450744171 | -0.164263022 | 0.151068504539444 | 0.993424455261859 | 0.848518813169642 | 1.16307633136122 | 0.999489402457283 |
| ebi-fl187-GCST90094781 | ebi-met1400-GCST90200943 | Phosphate to EDTA ratio \|\| id:ebi-met1400-GCST90200943 | Herring liking \|\| id:ebi-fl187-GCST90094781 | Weighted median | 16 | -0.022876168 | 0.0803144654065012 | 0.775772493187435 | -0.18029252 | 0.134540184480693 | 0.977383507911855 | 0.835025913974547 | 1.14401062955179 | 0.999489402457283 |
| ebi-fl187-GCST90094781 | ebi-met1400-GCST90200947 | Adenosine 5'-diphosphate (ADP) to glucose ratio \|\| id:ebi-met1400-GCST90200947 | Herring liking \|\| id:ebi-fl187-GCST90094781 | Weighted median | 16 | -0.038555385 | 0.108269916455079 | 0.721762440392318 | -0.250764421 | 0.173653651025207 | 0.962178412794474 | 0.778205678509528 | 1.18964346266507 | 0.999489402457283 |
| ebi-fl187-GCST90094781 | ebi-met1400-GCST90200975 | Arachidonate (20:4n6) to caffeine ratio \|\| id:ebi-met1400-GCST90200975 | Herring liking \|\| id:ebi-fl187-GCST90094781 | Weighted median | 16 | -0.009629328 | 0.081624696948232 | 0.906090817574988 | -0.169613734 | 0.150355077595501 | 0.990416885105869 | 0.843990758203852 | 1.16224685728832 | 0.999489402457283 |
| ebi-fl187-GCST90094781 | ebi-met1400-GCST90200985 | Cholesterol to oleoyl-linoleoyl-glycerol (18:1 to 18:2) [2] ratio \|\| id:ebi-met1400-GCST90200985 | Herring liking \|\| id:ebi-fl187-GCST90094781 | Weighted median | 16 | -0.096559378 | 0.0831802834325279 | 0.245705127210758 | -0.259592733 | 0.066473977885081 | 0.907955983713162 | 0.77136567349276 | 1.0687331530164 | 0.999489402457283 |
| ebi-fl187-GCST90094781 | ebi-met1400-GCST90201016 | Bilirubin (Z,Z) to etiocholanolone glucuronide ratio \|\| id:ebi-met1400-GCST90201016 | Herring liking \|\| id:ebi-fl187-GCST90094781 | Weighted median | 16 | 0.154064404154462 | 0.083113684320326 | 0.0637880036484076 | -0.008838417 | 0.316967225422301 | 1.16656601605698 | 0.991200526876294 | 1.3729575730834 | 0.999489402457283 |
| ebi-fl187-GCST90094781 | ebi-pw11-GCST90275189 | Placental weight (fetal genotype, adjusted for sex and gestational age) \|\| id:ebi-pw11-GCST90275189 | Herring liking \|\| id:ebi-fl187-GCST90094781 | Weighted median | 15 | 0.0362000324129453 | 0.0323256041188577 | 0.262774990044512 | -0.027158152 | 0.0995582164859065 | 1.03686323200251 | 0.973207315000383 | 1.10468277961749 | 0.999489402457283 |
| ebi-fl187-GCST90094781 | ebi-pw11-GCST90275192 | Placental weight (fetal effect, conditional on maternal and paternal genotype) \|\| id:ebi-pw11-GCST90275192 | Herring liking \|\| id:ebi-fl187-GCST90094781 | Weighted median | 14 | 0.0418282690073779 | 0.0705856819738931 | 0.553456524042992 | -0.096519668 | 0.180176205676208 | 1.04271539682413 | 0.907992039344104 | 1.19742833820381 | 0.999489402457283 |
| ebi-fl187-GCST90094781 | ebi-pw11-GCST90275193 | Placental weight (maternal effect, conditional on fetal and paternal genotype) \|\| id:ebi-pw11-GCST90275193 | Herring liking \|\| id:ebi-fl187-GCST90094781 | Weighted median | 14 | -0.036655291 | 0.0523570860565199 | 0.483863722248915 | -0.13927518 | 0.0659645976781758 | 0.964008380453772 | 0.8699885923478 | 1.06818890012938 | 0.999489402457283 |
| ebi-fl187-GCST90094781 | ebi-pw11-GCST90275195 | Placental weight (fetal effect, conditional on maternal genotype) \|\| id:ebi-pw11-GCST90275195 | Herring liking \|\| id:ebi-fl187-GCST90094781 | Weighted median | 14 | 0.0367686702896586 | 0.0419784987204496 | 0.381088242755311 | -0.045509187 | 0.11904652778174 | 1.03745299937534 | 0.955510824060272 | 1.12642232700129 | 0.999489402457283 |
| ebi-fl187-GCST90094781 | ebi-pw11-GCST90275197 | Placental weight (fetal genotype, adjusted for sex) \|\| id:ebi-pw11-GCST90275197 | Herring liking \|\| id:ebi-fl187-GCST90094781 | Weighted median | 15 | 0.0540706623803554 | 0.0318683120831653 | 0.0897551895717618 | -0.008391229 | 0.116532554063359 | 1.05555918784467 | 0.991643878793299 | 1.12359408742495 | 0.999489402457283 |
| ebi-fl187-GCST90094781 | prot-a-1030 | Protein FAM171B \|\| id:prot-a-1030 | Herring liking \|\| id:ebi-fl187-GCST90094781 | Weighted median | 19 | 0.0294260979206135 | 0.119225711621402 | 0.805055270758411 | -0.204256297 | 0.263108492698561 | 1.02986332361841 | 0.815253397525074 | 1.3009678568089 | 0.999489402457283 |
| ebi-fl187-GCST90094781 | prot-a-1036 | BRISC complex subunit Abro1 \|\| id:prot-a-1036 | Herring liking \|\| id:ebi-fl187-GCST90094781 | Weighted median | 19 | 0.0691065550579258 | 0.117767051151574 | 0.557333191993026 | -0.161716865 | 0.29992997531501 | 1.07155038217842 | 0.850682028132469 | 1.34976428744763 | 0.999489402457283 |
| ebi-fl187-GCST90094781 | prot-a-1055 | Phenylalanine--tRNA ligase, mitochondrial \|\| id:prot-a-1055 | Herring liking \|\| id:ebi-fl187-GCST90094781 | Weighted median | 19 | -0.279386537 | 0.120264932475526 | 0.0201741952479815 | -0.515105805 | -0.043667269 | 0.756247529046665 | 0.597437379608823 | 0.957272418347257 | 0.999489402457283 |
| ebi-fl187-GCST90094781 | prot-a-1083 | Fc receptor-like protein 5 \|\| id:prot-a-1083 | Herring liking \|\| id:ebi-fl187-GCST90094781 | Weighted median | 19 | -0.030706706 | 0.121362987549362 | 0.800256302583956 | -0.268578162 | 0.207164749148423 | 0.969759955711872 | 0.764465668274592 | 1.23018522705521 | 0.999489402457283 |
| ebi-fl187-GCST90094781 | prot-a-1103 | Fibroblast growth factor receptor 3 \|\| id:prot-a-1103 | Herring liking \|\| id:ebi-fl187-GCST90094781 | Weighted median | 19 | -0.129515688 | 0.120090258181275 | 0.280816831965971 | -0.364892594 | 0.105861218476436 | 0.87852080646128 | 0.69427121604951 | 1.1116675868791 | 0.999489402457283 |
| ebi-fl187-GCST90094781 | prot-a-1105 | Fibroblast growth factor receptor 4 \|\| id:prot-a-1105 | Herring liking \|\| id:ebi-fl187-GCST90094781 | Weighted median | 19 | 0.142239857287007 | 0.12360315968648 | 0.249823376726924 | -0.100022336 | 0.384502050272508 | 1.15285313560238 | 0.904817208085906 | 1.46888270955835 | 0.999489402457283 |
| ebi-fl187-GCST90094781 | prot-a-1120 | Filamin-A \|\| id:prot-a-1120 | Herring liking \|\| id:ebi-fl187-GCST90094781 | Weighted median | 19 | -0.084883995 | 0.123565779658438 | 0.492111776204733 | -0.327072923 | 0.157304933256037 | 0.918618842714818 | 0.721031161350411 | 1.17035243887414 | 0.999489402457283 |
| ebi-fl187-GCST90094781 | prot-a-1166 | Galanin peptides \|\| id:prot-a-1166 | Herring liking \|\| id:ebi-fl187-GCST90094781 | Weighted median | 19 | -0.035715018 | 0.114196296513965 | 0.754469763952121 | -0.259539759 | 0.188109722998347 | 0.964915237615373 | 0.771406536772258 | 1.2069659400063 | 0.999489402457283 |
| ebi-fl187-GCST90094781 | prot-a-1205 | ADP-ribosylation factor-binding protein GGA1 \|\| id:prot-a-1205 | Herring liking \|\| id:ebi-fl187-GCST90094781 | Weighted median | 19 | 0.0146933504789273 | 0.129834075301938 | 0.90989560731753 | -0.239781437 | 0.269168138070726 | 1.01480182840328 | 0.786799807512835 | 1.30887519429627 | 0.999489402457283 |
| ebi-fl187-GCST90094781 | prot-a-1227 | Guanine nucleotide-binding protein G(I)/G(S)/G(O) subunit gamma-T2 \|\| id:prot-a-1227 | Herring liking \|\| id:ebi-fl187-GCST90094781 | Weighted median | 19 | 0.0498323977599373 | 0.118934770536657 | 0.675223943009209 | -0.183279752 | 0.282944548011785 | 1.0510949157499 | 0.832535219354126 | 1.3270315732377 | 0.999489402457283 |
| ebi-fl187-GCST90094781 | prot-a-1240 | Golgi SNAP receptor complex member 1 \|\| id:prot-a-1240 | Herring liking \|\| id:ebi-fl187-GCST90094781 | Weighted median | 19 | -0.091719962 | 0.123716450427364 | 0.458467671293398 | -0.334204205 | 0.150764280431076 | 0.912360609043598 | 0.715907575218383 | 1.16272254931859 | 0.999489402457283 |
| ebi-fl187-GCST90094781 | prot-a-1283 | Glutathione S-transferase A1 \|\| id:prot-a-1283 | Herring liking \|\| id:ebi-fl187-GCST90094781 | Weighted median | 19 | -0.004027378 | 0.127040544474692 | 0.974710082031941 | -0.253026846 | 0.244972088700484 | 0.995980720542513 | 0.776447037326063 | 1.2775856536314 | 0.999489402457283 |
| ebi-fl187-GCST90094781 | prot-a-1324 | E3 ubiquitin-protein ligase HECW1 \|\| id:prot-a-1324 | Herring liking \|\| id:ebi-fl187-GCST90094781 | Weighted median | 19 | 0.0811036144236764 | 0.118348247559875 | 0.493156975408591 | -0.150858951 | 0.313066179641031 | 1.08448325885334 | 0.859968988048344 | 1.36761203610639 | 0.999489402457283 |
| ebi-fl187-GCST90094781 | prot-a-1339 | Histidine triad nucleotide-binding protein 2, mitochondrial \|\| id:prot-a-1339 | Herring liking \|\| id:ebi-fl187-GCST90094781 | Weighted median | 19 | 0.127299403535782 | 0.13197217188488 | 0.33474881950092 | -0.131366053 | 0.385964860430146 | 1.13575701654707 | 0.876896724642774 | 1.47103297844042 | 0.999489402457283 |
| ebi-fl187-GCST90094781 | prot-a-1466 | Interleukin-11 \|\| id:prot-a-1466 | Herring liking \|\| id:ebi-fl187-GCST90094781 | Weighted median | 19 | 0.0792711947083269 | 0.120462200736874 | 0.510500436835746 | -0.156834719 | 0.3153771081526 | 1.08249784995559 | 0.85484533705992 | 1.37077614435925 | 0.999489402457283 |
| ebi-fl187-GCST90094781 | prot-a-147 | Rho GTPase-activating protein 1 \|\| id:prot-a-147 | Herring liking \|\| id:ebi-fl187-GCST90094781 | Weighted median | 19 | -0.099215908 | 0.110708300652837 | 0.370150158 | -0.316204177 | 0.117772361440761 | 0.905547172182571 | 0.728910608105564 | 1.12498799157152 | 0.999489402457283 |
| ebi-fl187-GCST90094781 | prot-a-1473 | Interleukin-12 receptor subunit beta-1 \|\| id:prot-a-1473 | Herring liking \|\| id:ebi-fl187-GCST90094781 | Weighted median | 19 | -0.064464117 | 0.117695800785116 | 0.583885501350027 | -0.295147887 | 0.166219652480284 | 0.937569756419138 | 0.744421489364022 | 1.18083244601499 | 0.999489402457283 |
| ebi-fl187-GCST90094781 | prot-a-1501 | X-linked interleukin-1 receptor accessory protein-like 2 \|\| id:prot-a-1501 | Herring liking \|\| id:ebi-fl187-GCST90094781 | Weighted median | 19 | -0.136446635 | 0.120035794222532 | 0.255656932273838 | -0.371716792 | 0.0988235211993286 | 0.872452877049139 | 0.689549500703089 | 1.10387147245441 | 0.999489402457283 |
| ebi-fl187-GCST90094781 | prot-a-1503 | Interleukin-1 receptor-like 2 \|\| id:prot-a-1503 | Herring liking \|\| id:ebi-fl187-GCST90094781 | Weighted median | 19 | -0.036279103 | 0.125581455579953 | 0.772666312446758 | -0.282418756 | 0.209860550290596 | 0.964371097393084 | 0.753957894340784 | 1.23350603590442 | 0.999489402457283 |
| ebi-fl187-GCST90094781 | prot-a-1536 | Interleukin-5 receptor subunit alpha \|\| id:prot-a-1536 | Herring liking \|\| id:ebi-fl187-GCST90094781 | Weighted median | 19 | 0.0577987701932911 | 0.125252913292798 | 0.644471128115745 | -0.18769694 | 0.303294480247176 | 1.05950177092085 | 0.828865865377458 | 1.35431322421899 | 0.999489402457283 |
| ebi-fl187-GCST90094781 | prot-a-1559 | Insulin-induced gene 1 protein \|\| id:prot-a-1559 | Herring liking \|\| id:ebi-fl187-GCST90094781 | Weighted median | 19 | -0.005513207 | 0.114757725940014 | 0.96168265757246 | -0.23043835 | 0.219411935973423 | 0.994501962965037 | 0.794185395251803 | 1.24534417310423 | 0.999489402457283 |
| ebi-fl187-GCST90094781 | prot-a-1567 | Interferon regulatory factor 1 \|\| id:prot-a-1567 | Herring liking \|\| id:ebi-fl187-GCST90094781 | Weighted median | 19 | 0.154244239312739 | 0.114063549318686 | 0.176290293716813 | -0.069320317 | 0.377808795977364 | 1.16677582450602 | 0.933027767223638 | 1.45908393348534 | 0.999489402457283 |
| ebi-fl187-GCST90094781 | prot-a-1613 | Potassium voltage-gated channel subfamily E regulatory beta subunit 5 \|\| id:prot-a-1613 | Herring liking \|\| id:ebi-fl187-GCST90094781 | Weighted median | 19 | 0.022701593357936 | 0.116455134598932 | 0.845441066106723 | -0.20555047 | 0.250953657171844 | 1.02296123556985 | 0.814199000535336 | 1.28525052080701 | 0.999489402457283 |
| ebi-fl187-GCST90094781 | prot-a-1649 | Kin of IRRE-like protein 2 \|\| id:prot-a-1649 | Herring liking \|\| id:ebi-fl187-GCST90094781 | Weighted median | 19 | 0.0635124048152859 | 0.113156141308972 | 0.57460591566173 | -0.158273632 | 0.285298441780872 | 1.06557270395205 | 0.853616173179504 | 1.33015894389446 | 0.999489402457283 |
| ebi-fl187-GCST90094781 | prot-a-165 | Armadillo repeat-containing protein 5 \|\| id:prot-a-165 | Herring liking \|\| id:ebi-fl187-GCST90094781 | Weighted median | 19 | 0.0203605243490932 | 0.12043513527698 | 0.86575101232481 | -0.215692341 | 0.256413389491974 | 1.02056921376055 | 0.805983231893893 | 1.29228683533325 | 0.999489402457283 |
| ebi-fl187-GCST90094781 | prot-a-1656 | Kallikrein-11 \|\| id:prot-a-1656 | Herring liking \|\| id:ebi-fl187-GCST90094781 | Weighted median | 19 | -0.064466372 | 0.122179962903414 | 0.597752995763192 | -0.303939099 | 0.175006355379551 | 0.937567642339921 | 0.737905804134652 | 1.19125378745826 | 0.999489402457283 |
| ebi-fl187-GCST90094781 | prot-a-1697 | Laminin subunit gamma-2 \|\| id:prot-a-1697 | Herring liking \|\| id:ebi-fl187-GCST90094781 | Weighted median | 19 | -0.197173798 | 0.124752487761785 | 0.113987836987547 | -0.441688674 | 0.0473410776498943 | 0.821047924126381 | 0.642949771038722 | 1.04847956104434 | 0.999489402457283 |
| ebi-fl187-GCST90094781 | prot-a-1698 | Lysosome-associated membrane glycoprotein 3 \|\| id:prot-a-1698 | Herring liking \|\| id:ebi-fl187-GCST90094781 | Weighted median | 19 | -0.026873686 | 0.124158560953608 | 0.828639723884166 | -0.270224465 | 0.216477093575886 | 0.973484198545607 | 0.763208161312002 | 1.24169464224397 | 0.999489402457283 |
| ebi-fl187-GCST90094781 | prot-a-1724 | Leptin receptor \|\| id:prot-a-1724 | Herring liking \|\| id:ebi-fl187-GCST90094781 | Weighted median | 19 | 0.222520870609093 | 0.129263289394846 | 0.0851683827438209 | -0.030835177 | 0.47587691782299 | 1.24922189140035 | 0.969635378501014 | 1.60942491224524 | 0.999489402457283 |
| ebi-fl187-GCST90094781 | prot-a-1743 | Leukocyte immunoglobulin-like receptor subfamily B member 2 \|\| id:prot-a-1743 | Herring liking \|\| id:ebi-fl187-GCST90094781 | Weighted median | 19 | -0.097887888 | 0.123148464204448 | 0.426685023826745 | -0.339258878 | 0.143483101565231 | 0.906750555424114 | 0.712298026847414 | 1.15428730499357 | 0.999489402457283 |
| ebi-fl187-GCST90094781 | prot-a-1746 | Leukocyte immunoglobulin-like receptor subfamily B member 4 \|\| id:prot-a-1746 | Herring liking \|\| id:ebi-fl187-GCST90094781 | Weighted median | 19 | 0.0140835720774266 | 0.122085933040538 | 0.908161478076236 | -0.225204857 | 0.253372000836882 | 1.0141832127949 | 0.798352654130482 | 1.28836245961409 | 0.999489402457283 |
| ebi-fl187-GCST90094781 | prot-a-1750 | Leucine-rich repeat and immunoglobulin-like domain-containing nogo receptor-interacting protein 3 \|\| id:prot-a-1750 | Herring liking \|\| id:ebi-fl187-GCST90094781 | Weighted median | 19 | -0.065321599 | 0.120084255115541 | 0.586465744498959 | -0.300686739 | 0.170043540725695 | 0.936766151589838 | 0.740309646324244 | 1.18535646147733 | 0.999489402457283 |
| ebi-fl187-GCST90094781 | prot-a-1753 | Lipase member N \|\| id:prot-a-1753 | Herring liking \|\| id:ebi-fl187-GCST90094781 | Weighted median | 19 | 0.0453410334837873 | 0.121238844208097 | 0.708418395021335 | -0.192287101 | 0.282969168131658 | 1.04638465126936 | 0.825069955939575 | 1.3270642453163 | 0.999489402457283 |
| ebi-fl187-GCST90094781 | prot-a-1760 | Ig Kappa chain V-I region HK102- like \|\| id:prot-a-1760 | Herring liking \|\| id:ebi-fl187-GCST90094781 | Weighted median | 19 | -0.242471449 | 0.119974938763662 | 0.0432779553653578 | -0.477622329 | -0.007320569 | 0.784686151056921 | 0.620256405800518 | 0.99270616135894 | 0.999489402457283 |
| ebi-fl187-GCST90094781 | prot-a-1792 | Leucine-rich repeat neuronal protein 1 \|\| id:prot-a-1792 | Herring liking \|\| id:ebi-fl187-GCST90094781 | Weighted median | 19 | -0.057435827 | 0.118801228773135 | 0.628768052481406 | -0.290286235 | 0.175414581867474 | 0.94418247991034 | 0.748049419063608 | 1.19174018808219 | 0.999489402457283 |
| ebi-fl187-GCST90094781 | prot-a-1795 | Leucine-rich repeat transmembrane neuronal protein 1 \|\| id:prot-a-1795 | Herring liking \|\| id:ebi-fl187-GCST90094781 | Weighted median | 19 | -0.034105181 | 0.125902056322518 | 0.78647824526139 | -0.280873211 | 0.212662849401524 | 0.966469845035574 | 0.75512407054778 | 1.23696753658707 | 0.999489402457283 |
| ebi-fl187-GCST90094781 | prot-a-1820 | LysM and putative peptidoglycan-binding domain-containing protein 4 \|\| id:prot-a-1820 | Herring liking \|\| id:ebi-fl187-GCST90094781 | Weighted median | 19 | -0.019706567 | 0.116231531863681 | 0.865367357213623 | -0.24752037 | 0.208107235377908 | 0.980486338073729 | 0.780734317459631 | 1.23134520623777 | 0.999489402457283 |
| ebi-fl187-GCST90094781 | prot-a-1844 | Mitogen-activated protein kinase kinase kinase 7:TGF-beta-activated kinase 1 and MAP3K7-binding protein 1 fusion \|\| id:prot-a-1844 | Herring liking \|\| id:ebi-fl187-GCST90094781 | Weighted median | 19 | 0.0178609180673617 | 0.121572634460595 | 0.883198628569642 | -0.220421445 | 0.256143281610128 | 1.0180213781624 | 0.802180651306344 | 1.29193782561068 | 0.999489402457283 |
| ebi-fl187-GCST90094781 | prot-a-1856 | Megakaryocyte-associated tyrosine-protein kinase \|\| id:prot-a-1856 | Herring liking \|\| id:ebi-fl187-GCST90094781 | Weighted median | 19 | 0.0142856845704557 | 0.114993708007848 | 0.901133057945465 | -0.211101983 | 0.239673352265838 | 1.01438821260824 | 0.809691487802359 | 1.27083396747986 | 0.999489402457283 |
| ebi-fl187-GCST90094781 | prot-a-1910 | Stromelysin-2 \|\| id:prot-a-1910 | Herring liking \|\| id:ebi-fl187-GCST90094781 | Weighted median | 19 | -0.113304831 | 0.115643264480271 | 0.327195266921102 | -0.339965629 | 0.113355967280519 | 0.892878441509558 | 0.711794787097623 | 1.12003055622715 | 0.999489402457283 |
| ebi-fl187-GCST90094781 | prot-a-1940 | 39S ribosomal protein L14, mitochondrial \|\| id:prot-a-1940 | Herring liking \|\| id:ebi-fl187-GCST90094781 | Weighted median | 19 | -0.029001424 | 0.129941277823923 | 0.823388653958461 | -0.283686328 | 0.225683480949955 | 0.971415081573745 | 0.753002803471221 | 1.25317894748714 | 0.999489402457283 |
| ebi-fl187-GCST90094781 | prot-a-1942 | 39S ribosomal protein L33, mitochondrial \|\| id:prot-a-1942 | Herring liking \|\| id:ebi-fl187-GCST90094781 | Weighted median | 19 | 0.0381863832039381 | 0.124325154140934 | 0.758729739392247 | -0.205490919 | 0.281863685320169 | 1.03892485297692 | 0.814247488786441 | 1.3255980092021 | 0.999489402457283 |
| ebi-fl187-GCST90094781 | prot-a-1948 | Mesothelin \|\| id:prot-a-1948 | Herring liking \|\| id:ebi-fl187-GCST90094781 | Weighted median | 19 | 0.0690365397581804 | 0.121583184727423 | 0.570161801407561 | -0.169266502 | 0.30733958182393 | 1.07147535988361 | 0.844283869801836 | 1.35980265394289 | 0.999489402457283 |
| ebi-fl187-GCST90094781 | prot-a-1980 | Unconventional myosin-VI \|\| id:prot-a-1980 | Herring liking \|\| id:ebi-fl187-GCST90094781 | Weighted median | 19 | -0.212653157 | 0.131422674034455 | 0.105644117548761 | -0.470241598 | 0.0449352841010415 | 0.808436489126581 | 0.624851287152536 | 1.04596016746584 | 0.999489402457283 |
| ebi-fl187-GCST90094781 | prot-a-2039 | Neurofascin \|\| id:prot-a-2039 | Herring liking \|\| id:ebi-fl187-GCST90094781 | Weighted median | 19 | 0.130059081695104 | 0.12223617793228 | 0.287329483832151 | -0.109523827 | 0.369641990442372 | 1.1388956692236 | 0.896260808854644 | 1.44721640460198 | 0.999489402457283 |
| ebi-fl187-GCST90094781 | prot-a-2041 | NFU1 iron-sulfur cluster scaffold homolog, mitochondrial \|\| id:prot-a-2041 | Herring liking \|\| id:ebi-fl187-GCST90094781 | Weighted median | 19 | 0.0726540235687789 | 0.118661992362864 | 0.540354421356468 | -0.159923481 | 0.305231528599991 | 1.07535842387747 | 0.852208996257491 | 1.35693913685797 | 0.999489402457283 |
| ebi-fl187-GCST90094781 | prot-a-2088 | Glucocorticoid receptor \|\| id:prot-a-2088 | Herring liking \|\| id:ebi-fl187-GCST90094781 | Weighted median | 19 | 0.264953015578924 | 0.11796914518463 | 0.0247070945399639 | 0.0337334910170495 | 0.496172540140798 | 1.30336973626722 | 1.03430891737528 | 1.64242291725396 | 0.999489402457283 |
| ebi-fl187-GCST90094781 | prot-a-2111 | Neuron-specific protein family member 2 \|\| id:prot-a-2111 | Herring liking \|\| id:ebi-fl187-GCST90094781 | Weighted median | 19 | 0.0193046540776997 | 0.118000195722032 | 0.870047231908185 | -0.21197573 | 0.250585037692882 | 1.0194921937646 | 0.808984331752135 | 1.28477683973909 | 0.999489402457283 |
| ebi-fl187-GCST90094781 | prot-a-2125 | Nuclear migration protein nudC \|\| id:prot-a-2125 | Herring liking \|\| id:ebi-fl187-GCST90094781 | Weighted median | 19 | 0.0224002415284239 | 0.12568926234635 | 0.858550820780484 | -0.223950713 | 0.26875119572727 | 1.02265301077437 | 0.79935453144871 | 1.3083295825577 | 0.999489402457283 |
| ebi-fl187-GCST90094781 | prot-a-2142 | Ornithine decarboxylase \|\| id:prot-a-2142 | Herring liking \|\| id:ebi-fl187-GCST90094781 | Weighted median | 19 | -0.017851083 | 0.120742104987846 | 0.882465313781406 | -0.254505609 | 0.218803442783994 | 0.98230730373131 | 0.77529970460212 | 1.24458662016268 | 0.999489402457283 |
| ebi-fl187-GCST90094781 | prot-a-2186 | Prostate and testis expressed protein 1 \|\| id:prot-a-2186 | Herring liking \|\| id:ebi-fl187-GCST90094781 | Weighted median | 19 | 0.0617708641555774 | 0.113909352139895 | 0.587625022486552 | -0.161491466 | 0.285033194349772 | 1.06371858074634 | 0.850873792758479 | 1.32980616943997 | 0.999489402457283 |
| ebi-fl187-GCST90094781 | prot-a-2227 | Platelet-derived growth factor C \|\| id:prot-a-2227 | Herring liking \|\| id:ebi-fl187-GCST90094781 | Weighted median | 19 | -0.078994385 | 0.113514800589662 | 0.48649457581587 | -0.301483394 | 0.143494624643644 | 0.924045113394677 | 0.739720110290507 | 1.15430060601333 | 0.999489402457283 |
| ebi-fl187-GCST90094781 | prot-a-2229 | Platelet-derived growth factor receptor alpha \|\| id:prot-a-2229 | Herring liking \|\| id:ebi-fl187-GCST90094781 | Weighted median | 19 | -0.151528632 | 0.121731296792899 | 0.213212786760552 | -0.390121973 | 0.0870647100728348 | 0.859393276080701 | 0.676974296635686 | 1.09096727400593 | 0.999489402457283 |
| ebi-fl187-GCST90094781 | prot-a-2243 | Platelet endothelial aggregation receptor 1 \|\| id:prot-a-2243 | Herring liking \|\| id:ebi-fl187-GCST90094781 | Weighted median | 19 | -0.083568619 | 0.112349815701994 | 0.456982259866073 | -0.303774258 | 0.136637019715253 | 0.919827966774603 | 0.738027451560834 | 1.14641194805334 | 0.999489402457283 |
| ebi-fl187-GCST90094781 | prot-a-2246 | E3 ubiquitin-protein ligase pellino homolog 2 \|\| id:prot-a-2246 | Herring liking \|\| id:ebi-fl187-GCST90094781 | Weighted median | 19 | 0.273509271175764 | 0.123768998746053 | 0.0271165002313467 | 0.0309220336334994 | 0.516096508718028 | 1.31456954676088 | 1.03140508584468 | 1.67547466750746 | 0.999489402457283 |
| ebi-fl187-GCST90094781 | prot-a-2251 | Platelet factor 4 variant \|\| id:prot-a-2251 | Herring liking \|\| id:ebi-fl187-GCST90094781 | Weighted median | 19 | 0.0891838241930481 | 0.128537465462652 | 0.487785535092994 | -0.162749608 | 0.341117256499847 | 1.09328160946537 | 0.849803945790047 | 1.40651815458916 | 0.999489402457283 |
| ebi-fl187-GCST90094781 | prot-a-2346 | Protein phosphatase 1L \|\| id:prot-a-2346 | Herring liking \|\| id:ebi-fl187-GCST90094781 | Weighted median | 19 | 0.182170384298195 | 0.117656771181399 | 0.121545164461841 | -0.048436887 | 0.412777655813737 | 1.19981860671627 | 0.952717466058797 | 1.51100902450941 | 0.999489402457283 |
| ebi-fl187-GCST90094781 | prot-a-2358 | PR domain zinc finger protein 4 \|\| id:prot-a-2358 | Herring liking \|\| id:ebi-fl187-GCST90094781 | Weighted median | 19 | 0.0488458246918554 | 0.121446408602458 | 0.687536362618057 | -0.189189136 | 0.286880785552674 | 1.05005844517507 | 0.827629957131197 | 1.33226537872735 | 0.999489402457283 |
| ebi-fl187-GCST90094781 | prot-a-2390 | Trypsin-1 \|\| id:prot-a-2390 | Herring liking \|\| id:ebi-fl187-GCST90094781 | Weighted median | 19 | -0.224138306 | 0.121891668680308 | 0.0659398938642799 | -0.463045977 | 0.014769364636305 | 0.799204591953211 | 0.629363695846043 | 1.01487897064108 | 0.999489402457283 |
| ebi-fl187-GCST90094781 | prot-a-242 | Brain-derived neurotrophic factor \|\| id:prot-a-242 | Herring liking \|\| id:ebi-fl187-GCST90094781 | Weighted median | 19 | -0.000747789 | 0.116217855829701 | 0.994866149583085 | -0.228534786 | 0.227039208543106 | 0.99925249064132 | 0.795698617304818 | 1.25487906895579 | 0.999911036302558 |
| ebi-fl187-GCST90094781 | prot-a-2464 | Dihydropteridine reductase \|\| id:prot-a-2464 | Herring liking \|\| id:ebi-fl187-GCST90094781 | Weighted median | 19 | -0.034541678 | 0.123480086723655 | 0.779680975726665 | -0.276562648 | 0.207479292374764 | 0.966048076278674 | 0.75838610658764 | 1.2305722343476 | 0.999489402457283 |
| ebi-fl187-GCST90094781 | prot-a-2474 | Ras-related protein Rab-27A \|\| id:prot-a-2474 | Herring liking \|\| id:ebi-fl187-GCST90094781 | Weighted median | 19 | -0.047756164 | 0.134441494007317 | 0.722425583379419 | -0.311261492 | 0.215749164458626 | 0.953366223939431 | 0.732522302064432 | 1.2407911054544 | 0.999489402457283 |
| ebi-fl187-GCST90094781 | prot-a-2478 | Ras-related protein Rab-7b \|\| id:prot-a-2478 | Herring liking \|\| id:ebi-fl187-GCST90094781 | Weighted median | 19 | -0.040700322 | 0.117408710014186 | 0.72885048812202 | -0.270821393 | 0.189420750126722 | 0.960116813204769 | 0.762752717115962 | 1.20854934281189 | 0.999489402457283 |
| ebi-fl187-GCST90094781 | prot-a-2528 | E3 ubiquitin-protein ligase rififylin \|\| id:prot-a-2528 | Herring liking \|\| id:ebi-fl187-GCST90094781 | Weighted median | 19 | -0.166687601 | 0.121974857879055 | 0.171759047132266 | -0.405758323 | 0.0723831203709064 | 0.846464004484527 | 0.666471219050878 | 1.07506714529751 | 0.999489402457283 |
| ebi-fl187-GCST90094781 | prot-a-2543 | Protein RIC-3 \|\| id:prot-a-2543 | Herring liking \|\| id:ebi-fl187-GCST90094781 | Weighted median | 19 | 0.123249696350291 | 0.132887427365224 | 0.353680240776105 | -0.137209661 | 0.383709053986129 | 1.13116683391728 | 0.87178742689717 | 1.46771835275087 | 0.999489402457283 |
| ebi-fl187-GCST90094781 | prot-a-255 | Bone morphogenetic protein 6 \|\| id:prot-a-255 | Herring liking \|\| id:ebi-fl187-GCST90094781 | Weighted median | 19 | -0.060441535 | 0.124903870438082 | 0.628453702167733 | -0.305253121 | 0.184370050865063 | 0.941348803185162 | 0.736936816411952 | 1.20246071240222 | 0.999489402457283 |
| ebi-fl187-GCST90094781 | prot-a-2568 | RING finger protein 165 \|\| id:prot-a-2568 | Herring liking \|\| id:ebi-fl187-GCST90094781 | Weighted median | 19 | -0.078424546 | 0.123877575171871 | 0.526680710461395 | -0.321224594 | 0.164375500838695 | 0.924571819481191 | 0.725260343694847 | 1.17865681863123 | 0.999489402457283 |
| ebi-fl187-GCST90094781 | prot-a-2573 | E3 ubiquitin-protein ligase RNF8 \|\| id:prot-a-2573 | Herring liking \|\| id:ebi-fl187-GCST90094781 | Weighted median | 19 | -0.137676355 | 0.124609010303807 | 0.269217323091193 | -0.381910015 | 0.106557305449781 | 0.87138066433051 | 0.682556470326195 | 1.1124416735896 | 0.999489402457283 |
| ebi-fl187-GCST90094781 | prot-a-260 | Cytoplasmic tyrosine-protein kinase BMX \|\| id:prot-a-260 | Herring liking \|\| id:ebi-fl187-GCST90094781 | Weighted median | 19 | -0.053535308 | 0.119473951616266 | 0.654086887694187 | -0.287704253 | 0.1806336375236 | 0.947872473268863 | 0.7499833649142 | 1.19797620535706 | 0.999489402457283 |
| ebi-fl187-GCST90094781 | prot-a-2623 | Serum amyloid A-1 protein \|\| id:prot-a-2623 | Herring liking \|\| id:ebi-fl187-GCST90094781 | Weighted median | 19 | -0.037651165 | 0.120368890617693 | 0.75443402745295 | -0.273574191 | 0.198271860330782 | 0.963048827171468 | 0.760655900523698 | 1.21929382639088 | 0.999489402457283 |
| ebi-fl187-GCST90094781 | prot-a-2640 | Lysosome membrane protein 2 \|\| id:prot-a-2640 | Herring liking \|\| id:ebi-fl187-GCST90094781 | Weighted median | 19 | -0.021676562 | 0.118020127222137 | 0.85427364092561 | -0.252996011 | 0.20964288753449 | 0.978556686465035 | 0.776470979023415 | 1.2332375767988 | 0.999489402457283 |
| ebi-fl187-GCST90094781 | prot-a-2641 | Scavenger receptor class F member 2 \|\| id:prot-a-2641 | Herring liking \|\| id:ebi-fl187-GCST90094781 | Weighted median | 19 | 0.0188811887862425 | 0.116410826031628 | 0.871152737740436 | -0.20928403 | 0.247046407808233 | 1.01906056560185 | 0.811164807589164 | 1.28023852446361 | 0.999489402457283 |
| ebi-fl187-GCST90094781 | prot-a-2669 | Semaphorin-3A \|\| id:prot-a-2669 | Herring liking \|\| id:ebi-fl187-GCST90094781 | Weighted median | 19 | 0.0101581725761291 | 0.127003945905733 | 0.936250676669613 | -0.238769561 | 0.259085906551365 | 1.01020994195678 | 0.787596354064743 | 1.29574511304104 | 0.999489402457283 |
| ebi-fl187-GCST90094781 | prot-a-2683 | Sentrin-specific protease 7 \|\| id:prot-a-2683 | Herring liking \|\| id:ebi-fl187-GCST90094781 | Weighted median | 19 | 0.14508074557119 | 0.123997821313437 | 0.241990276480472 | -0.097954984 | 0.388116475345527 | 1.15613291911214 | 0.90668971819543 | 1.47420148241569 | 0.999489402457283 |
| ebi-fl187-GCST90094781 | prot-a-270 | Bromodomain testis-specific protein \|\| id:prot-a-270 | Herring liking \|\| id:ebi-fl187-GCST90094781 | Weighted median | 19 | 0.013132522292893 | 0.116234379235691 | 0.91004394138115 | -0.214686861 | 0.240951905594847 | 1.0132191325857 | 0.806794039297308 | 1.27245983563768 | 0.999489402457283 |
| ebi-fl187-GCST90094781 | prot-a-2708 | 14-3-3 protein sigma \|\| id:prot-a-2708 | Herring liking \|\| id:ebi-fl187-GCST90094781 | Weighted median | 19 | 0.117425788483438 | 0.118597450640544 | 0.322115136453288 | -0.115025215 | 0.349876791738905 | 1.12459816871126 | 0.891343668596075 | 1.41889271851869 | 0.999489402457283 |
| ebi-fl187-GCST90094781 | prot-a-2713 | Beta-sarcoglycan \|\| id:prot-a-2713 | Herring liking \|\| id:ebi-fl187-GCST90094781 | Weighted median | 19 | -0.04222591 | 0.131534216377277 | 0.74819065057883 | -0.300032974 | 0.21558115430697 | 0.958653186965558 | 0.740793793424484 | 1.24058265746378 | 0.999489402457283 |
| ebi-fl187-GCST90094781 | prot-a-2720 | Protein shisa-3 homolog \|\| id:prot-a-2720 | Herring liking \|\| id:ebi-fl187-GCST90094781 | Weighted median | 19 | -0.077816361 | 0.119946590122046 | 0.516494533939922 | -0.312911678 | 0.15727895575129 | 0.925134301786928 | 0.73131450122172 | 1.170322036 | 0.999489402457283 |
| ebi-fl187-GCST90094781 | prot-a-2730 | Sialic acid-binding Ig-like lectin 8 \|\| id:prot-a-2730 | Herring liking \|\| id:ebi-fl187-GCST90094781 | Weighted median | 19 | -0.129920367 | 0.124346988027681 | 0.296105624466236 | -0.373640463 | 0.113799729892561 | 0.878165359392921 | 0.68822430933447 | 1.12052769420982 | 0.999489402457283 |
| ebi-fl187-GCST90094781 | prot-a-2761 | Sodium- and chloride-dependent neutral and basic amino acid transporter B(0+) \|\| id:prot-a-2761 | Herring liking \|\| id:ebi-fl187-GCST90094781 | Weighted median | 19 | -0.24889721 | 0.120145972166206 | 0.0383005737278694 | -0.484383316 | -0.013411105 | 0.779660110373812 | 0.616077004692035 | 0.986678423441511 | 0.999489402457283 |
| ebi-fl187-GCST90094781 | prot-a-2770 | Secreted Ly-6/uPAR-related protein 1 \|\| id:prot-a-2770 | Herring liking \|\| id:ebi-fl187-GCST90094781 | Weighted median | 19 | 0.124040848618169 | 0.120747506850675 | 0.304291172081277 | -0.112624265 | 0.360705962045491 | 1.13206211322771 | 0.893486311303782 | 1.43434164798286 | 0.999489402457283 |
| ebi-fl187-GCST90094781 | prot-a-2784 | N-lysine methyltransferase SMYD2 \|\| id:prot-a-2784 | Herring liking \|\| id:ebi-fl187-GCST90094781 | Weighted median | 19 | -0.185791017 | 0.117905016698676 | 0.115079154122143 | -0.416884849 | 0.0453028161869733 | 0.830447126560524 | 0.659096811052303 | 1.04634466204071 | 0.999489402457283 |
| ebi-fl187-GCST90094781 | prot-a-2837 | Serine palmitoyltransferase 2 \|\| id:prot-a-2837 | Herring liking \|\| id:ebi-fl187-GCST90094781 | Weighted median | 19 | 0.0850635991254612 | 0.120132072761479 | 0.478893092318538 | -0.150395263 | 0.320522461737961 | 1.08878631035361 | 0.860367837215708 | 1.37784744888856 | 0.999489402457283 |
| ebi-fl187-GCST90094781 | prot-a-2858 | Alpha-2,8-sialyltransferase 8B \|\| id:prot-a-2858 | Herring liking \|\| id:ebi-fl187-GCST90094781 | Weighted median | 19 | -0.012488374 | 0.116617128554952 | 0.914718628016683 | -0.241057946 | 0.216081197670345 | 0.987589281846462 | 0.785796091120473 | 1.24120315771395 | 0.999489402457283 |
| ebi-fl187-GCST90094781 | prot-a-2911 | Synaptotagmin-11 \|\| id:prot-a-2911 | Herring liking \|\| id:ebi-fl187-GCST90094781 | Weighted median | 19 | -0.023780794 | 0.113465441066759 | 0.833990631285972 | -0.246173058 | 0.198611470966197 | 0.976499741364979 | 0.781786918725206 | 1.21970798186384 | 0.999489402457283 |
| ebi-fl187-GCST90094781 | prot-a-2962 | Transforming growth factor beta-1 \|\| id:prot-a-2962 | Herring liking \|\| id:ebi-fl187-GCST90094781 | Weighted median | 19 | -0.044741466 | 0.118607963879586 | 0.706009056261672 | -0.277213075 | 0.187730143529044 | 0.956244671949009 | 0.757892991947271 | 1.2065078874545 | 0.999489402457283 |
| ebi-fl187-GCST90094781 | prot-a-2978 | Tyrosine-protein kinase receptor Tie-1, soluble \|\| id:prot-a-2978 | Herring liking \|\| id:ebi-fl187-GCST90094781 | Weighted median | 19 | 0.173337728242585 | 0.125597420687187 | 0.167554073007593 | -0.072833216 | 0.419508672789472 | 1.18926768655326 | 0.929755885217729 | 1.52121395816551 | 0.999489402457283 |
| ebi-fl187-GCST90094781 | prot-a-299 | Uncharacterized protein C1orf226 \|\| id:prot-a-299 | Herring liking \|\| id:ebi-fl187-GCST90094781 | Weighted median | 19 | 0.0100013274046868 | 0.118957722548088 | 0.932997089552392 | -0.223155809 | 0.243158463598939 | 1.01005150783038 | 0.799990194079813 | 1.27527069209132 | 0.999489402457283 |
| ebi-fl187-GCST90094781 | prot-a-3015 | Transmembrane protein 70, mitochondrial \|\| id:prot-a-3015 | Herring liking \|\| id:ebi-fl187-GCST90094781 | Weighted median | 19 | -0.198224709 | 0.119878391538764 | 0.0982193264617093 | -0.433186357 | 0.0367369379464824 | 0.820185528973811 | 0.648439639352875 | 1.03742007908306 | 0.999489402457283 |
| ebi-fl187-GCST90094781 | prot-a-3016 | Transmembrane protein 87B \|\| id:prot-a-3016 | Herring liking \|\| id:ebi-fl187-GCST90094781 | Weighted median | 19 | -0.134776856 | 0.12606206127605 | 0.285010651582973 | -0.381858496 | 0.11230478438163 | 0.873910898150822 | 0.682591635941187 | 1.1188538178522 | 0.999489402457283 |
| ebi-fl187-GCST90094781 | prot-a-3123 | Thioredoxin domain-containing protein 12 \|\| id:prot-a-3123 | Herring liking \|\| id:ebi-fl187-GCST90094781 | Weighted median | 19 | 0.0229793911394185 | 0.122325277222229 | 0.850990472026931 | -0.216778152 | 0.262736934494987 | 1.02324545140707 | 0.805108561044204 | 1.30048456132088 | 0.999489402457283 |
| ebi-fl187-GCST90094781 | prot-a-3133 | PolyUbiquitin K48-linked \|\| id:prot-a-3133 | Herring liking \|\| id:ebi-fl187-GCST90094781 | Weighted median | 19 | 0.0837707237626313 | 0.130711492169274 | 0.521598947605773 | -0.172423801 | 0.339965248414408 | 1.08737955493323 | 0.841622417248347 | 1.4048987672555 | 0.999489402457283 |
| ebi-fl187-GCST90094781 | prot-a-3135 | Ubiquitin-conjugating enzyme E2 B \|\| id:prot-a-3135 | Herring liking \|\| id:ebi-fl187-GCST90094781 | Weighted median | 19 | 0.0994042530782095 | 0.120861386763295 | 0.410812333351494 | -0.137484065 | 0.336292571134269 | 1.10451271198475 | 0.871548238026983 | 1.39974849091271 | 0.999489402457283 |
| ebi-fl187-GCST90094781 | prot-a-3162 | NKG2D ligand 3 \|\| id:prot-a-3162 | Herring liking \|\| id:ebi-fl187-GCST90094781 | Weighted median | 19 | 0.131210915079651 | 0.116476969976929 | 0.259955362856887 | -0.097083946 | 0.359505776234431 | 1.140208243 | 0.907479823566256 | 1.43262120412141 | 0.999489402457283 |
| ebi-fl187-GCST90094781 | prot-a-3188 | Vesicle-associated membrane protein 8 \|\| id:prot-a-3188 | Herring liking \|\| id:ebi-fl187-GCST90094781 | Weighted median | 19 | 0.185707351611381 | 0.120435181593728 | 0.123081056135363 | -0.050345604 | 0.421760307535088 | 1.2040698397141 | 0.950900732311586 | 1.52464303543525 | 0.999489402457283 |
| ebi-fl187-GCST90094781 | prot-a-3201 | Vitrin \|\| id:prot-a-3201 | Herring liking \|\| id:ebi-fl187-GCST90094781 | Weighted median | 19 | -0.025360901 | 0.117103791522868 | 0.828545243084924 | -0.254884333 | 0.204162530053982 | 0.974957984892407 | 0.775006135631963 | 1.22649747995912 | 0.999489402457283 |
| ebi-fl187-GCST90094781 | prot-a-3232 | WNT1-inducible-signaling pathway protein 3 \|\| id:prot-a-3232 | Herring liking \|\| id:ebi-fl187-GCST90094781 | Weighted median | 19 | 0.0685508618124229 | 0.129032325630545 | 0.595232452855508 | -0.184352496 | 0.321454220048291 | 1.07095509428288 | 0.831642601110777 | 1.37913186799059 | 0.999489402457283 |
| ebi-fl187-GCST90094781 | prot-a-327 | Carbonic anhydrase-related protein 10 \|\| id:prot-a-327 | Herring liking \|\| id:ebi-fl187-GCST90094781 | Weighted median | 19 | -0.13701374 | 0.12063997234204 | 0.256071996610599 | -0.373468085 | 0.0994406060889307 | 0.871958245603818 | 0.688342954072602 | 1.10455286507704 | 0.999489402457283 |
| ebi-fl187-GCST90094781 | prot-a-332 | Carbonic anhydrase 5A, mitochondrial \|\| id:prot-a-332 | Herring liking \|\| id:ebi-fl187-GCST90094781 | Weighted median | 19 | -0.078869641 | 0.130904229255948 | 0.546842161509106 | -0.33544193 | 0.177702648257533 | 0.924160389139516 | 0.71502202653085 | 1.19447009066042 | 0.999489402457283 |
| ebi-fl187-GCST90094781 | prot-a-338 | Cell adhesion molecule 3 \|\| id:prot-a-338 | Herring liking \|\| id:ebi-fl187-GCST90094781 | Weighted median | 19 | 0.0279877060078632 | 0.118799500302084 | 0.81375258635373 | -0.204859315 | 0.260834726599948 | 1.02838304141068 | 0.814761933470122 | 1.29801312066314 | 0.999489402457283 |
| ebi-fl187-GCST90094781 | prot-a-34 | A disintegrin and metalloproteinase with thrombospondin motifs 3 \|\| id:prot-a-34 | Herring liking \|\| id:ebi-fl187-GCST90094781 | Weighted median | 19 | 0.0266615301084305 | 0.126034827480871 | 0.832465163255548 | -0.220366732 | 0.273689791970937 | 1.02702012853663 | 0.802224542795677 | 1.3148068753215 | 0.999489402457283 |
| ebi-fl187-GCST90094781 | prot-a-358 | Cysteine--tRNA ligase, cytoplasmic \|\| id:prot-a-358 | Herring liking \|\| id:ebi-fl187-GCST90094781 | Weighted median | 19 | 0.0632035877587513 | 0.115904962851163 | 0.585543576038005 | -0.163970139 | 0.29037731494703 | 1.06524368773162 | 0.84876736617956 | 1.33693183723559 | 0.999489402457283 |
| ebi-fl187-GCST90094781 | prot-a-382 | Coiled-coil domain-containing protein 167 \|\| id:prot-a-382 | Herring liking \|\| id:ebi-fl187-GCST90094781 | Weighted median | 19 | -0.065661497 | 0.122035166773339 | 0.590539837832954 | -0.304850424 | 0.173527429487017 | 0.936447800672544 | 0.737233638408733 | 1.18949331351354 | 0.999489402457283 |
| ebi-fl187-GCST90094781 | prot-a-40 | Pituitary adenylate cyclase-activating polypeptide 27 \|\| id:prot-a-40 | Herring liking \|\| id:ebi-fl187-GCST90094781 | Weighted median | 19 | 0.0251625741489632 | 0.118094445915766 | 0.831271105306875 | -0.20630254 | 0.256627688143865 | 1.02548182380802 | 0.813586896590864 | 1.29256380033547 | 0.999489402457283 |
| ebi-fl187-GCST90094781 | prot-a-403 | C-C motif chemokine 25 \|\| id:prot-a-403 | Herring liking \|\| id:ebi-fl187-GCST90094781 | Weighted median | 19 | 0.00452064790813866 | 0.116526216491011 | 0.969053739987943 | -0.223870736 | 0.232912032230519 | 1.00453088145183 | 0.79941846338798 | 1.26227043532851 | 0.999489402457283 |
| ebi-fl187-GCST90094781 | prot-a-415 | Cyclin-C \|\| id:prot-a-415 | Herring liking \|\| id:ebi-fl187-GCST90094781 | Weighted median | 19 | 0.0745048243668768 | 0.112598806559203 | 0.508173785667052 | -0.146188836 | 0.295198485222915 | 1.07735054104398 | 0.863994534084517 | 1.3433929758799 | 0.999489402457283 |
| ebi-fl187-GCST90094781 | prot-a-44 | Adhesion G protein-coupled receptor F5 \|\| id:prot-a-44 | Herring liking \|\| id:ebi-fl187-GCST90094781 | Weighted median | 19 | 0.0168633817491912 | 0.123485868896851 | 0.891377625830379 | -0.225168921 | 0.25889568478702 | 1.01700637120233 | 0.798381343762656 | 1.29549865756083 | 0.999489402457283 |
| ebi-fl187-GCST90094781 | prot-a-455 | CD59 glycoprotein \|\| id:prot-a-455 | Herring liking \|\| id:ebi-fl187-GCST90094781 | Weighted median | 19 | -0.01757782 | 0.117362208495106 | 0.880942839471431 | -0.247607748 | 0.212452108819562 | 0.982575768809672 | 0.780666100692385 | 1.23670688479446 | 0.999489402457283 |
| ebi-fl187-GCST90094781 | prot-a-478 | Cadherin-12 \|\| id:prot-a-478 | Herring liking \|\| id:ebi-fl187-GCST90094781 | Weighted median | 19 | -0.071325723 | 0.112307766364015 | 0.525368654155774 | -0.291448945 | 0.1487974994159 | 0.931158543291141 | 0.74718016011321 | 1.16043797604678 | 0.999489402457283 |
| ebi-fl187-GCST90094781 | prot-a-516 | Centrin-2 \|\| id:prot-a-516 | Herring liking \|\| id:ebi-fl187-GCST90094781 | Weighted median | 19 | -0.005356157 | 0.123145053317894 | 0.965307190643692 | -0.246720461 | 0.236008147557457 | 0.994658161687309 | 0.781359082991932 | 1.26618462643685 | 0.999489402457283 |
| ebi-fl187-GCST90094781 | prot-a-519 | Cryptic protein \|\| id:prot-a-519 | Herring liking \|\| id:ebi-fl187-GCST90094781 | Weighted median | 19 | 0.0841013489056777 | 0.127032589356613 | 0.507942088489012 | -0.164882526 | 0.333085224044638 | 1.08773912939303 | 0.847993315205843 | 1.39526620363216 | 0.999489402457283 |
| ebi-fl187-GCST90094781 | prot-a-583 | Chloride intracellular channel protein 4 \|\| id:prot-a-583 | Herring liking \|\| id:ebi-fl187-GCST90094781 | Weighted median | 19 | 0.0667244141291576 | 0.126136572355309 | 0.596815528774845 | -0.180503268 | 0.313952095945563 | 1.06900083605053 | 0.834849952663942 | 1.36882416274956 | 0.999489402457283 |
| ebi-fl187-GCST90094781 | prot-a-602 | CCR4-NOT transcription complex subunit 1 \|\| id:prot-a-602 | Herring liking \|\| id:ebi-fl187-GCST90094781 | Weighted median | 19 | 0.205375157764839 | 0.122815985809103 | 0.0944812530575095 | -0.035344174 | 0.446094489950681 | 1.22798566691663 | 0.965273136756842 | 1.56219907167327 | 0.999489402457283 |
| ebi-fl187-GCST90094781 | prot-a-606 | Ciliary neurotrophic factor receptor subunit alpha \|\| id:prot-a-606 | Herring liking \|\| id:ebi-fl187-GCST90094781 | Weighted median | 19 | -0.199132072 | 0.118026809690359 | 0.0915693990869961 | -0.430464619 | 0.0322004752828934 | 0.819441661124274 | 0.650206926233234 | 1.03272452029409 | 0.999489402457283 |
| ebi-fl187-GCST90094781 | prot-a-626 | Collagen alpha-2(IX) chain \|\| id:prot-a-626 | Herring liking \|\| id:ebi-fl187-GCST90094781 | Weighted median | 19 | 0.0453511027193294 | 0.11861934674728 | 0.702220557346451 | -0.187142817 | 0.277845022343999 | 1.04639518761593 | 0.829325286256361 | 1.32028156721042 | 0.999489402457283 |
| ebi-fl187-GCST90094781 | prot-a-647 | Probable serine carboxypeptidase CPVL \|\| id:prot-a-647 | Herring liking \|\| id:ebi-fl187-GCST90094781 | Weighted median | 19 | -0.086918887 | 0.12643752453701 | 0.491802135792168 | -0.334736435 | 0.160898661345034 | 0.916751453309522 | 0.715526649397945 | 1.17456593385065 | 0.999489402457283 |
| ebi-fl187-GCST90094781 | prot-a-651 | Cellular retinoic acid-binding protein 2 \|\| id:prot-a-651 | Herring liking \|\| id:ebi-fl187-GCST90094781 | Weighted median | 19 | -0.028842471 | 0.122747224399273 | 0.814228502290245 | -0.269427031 | 0.211742088517268 | 0.971569502487879 | 0.763817012354855 | 1.2358291094543 | 0.999489402457283 |
| ebi-fl187-GCST90094781 | prot-a-671 | Cytotoxic and regulatory T-cell molecule \|\| id:prot-a-671 | Herring liking \|\| id:ebi-fl187-GCST90094781 | Weighted median | 19 | -0.126101586 | 0.12099786355584 | 0.297328107535135 | -0.363257399 | 0.111054226174657 | 0.881525291258427 | 0.695407413312369 | 1.11745550054871 | 0.999489402457283 |
| ebi-fl187-GCST90094781 | prot-a-688 | Chorionic somatomammotropin hormone \|\| id:prot-a-688 | Herring liking \|\| id:ebi-fl187-GCST90094781 | Weighted median | 19 | 0.0406899555260011 | 0.127714292471962 | 0.750028773011091 | -0.209630058 | 0.291009968771046 | 1.04152913512762 | 0.810884170829071 | 1.33777791988536 | 0.999489402457283 |
| ebi-fl187-GCST90094781 | prot-a-69 | Aldo-keto reductase family 1 member C1 \|\| id:prot-a-69 | Herring liking \|\| id:ebi-fl187-GCST90094781 | Weighted median | 19 | -0.033754087 | 0.115424275546197 | 0.769954125882887 | -0.259985667 | 0.192477493349278 | 0.96680922663364 | 0.77106263752567 | 1.21224921973063 | 0.999489402457283 |
| ebi-fl187-GCST90094781 | prot-a-741 | Stromal cell-derived factor 1 \|\| id:prot-a-741 | Herring liking \|\| id:ebi-fl187-GCST90094781 | Weighted median | 19 | -0.074821546 | 0.120115978749514 | 0.533343110744332 | -0.310248864 | 0.16060577249591 | 0.927909060774197 | 0.733264450243228 | 1.17422196696055 | 0.999489402457283 |
| ebi-fl187-GCST90094781 | prot-a-742 | Growth-regulated alpha protein \|\| id:prot-a-742 | Herring liking \|\| id:ebi-fl187-GCST90094781 | Weighted median | 19 | -0.104270858 | 0.121814041146941 | 0.392006685961019 | -0.343026378 | 0.134484662860373 | 0.900981226586722 | 0.709619492661208 | 1.14394711399124 | 0.999489402457283 |
| ebi-fl187-GCST90094781 | prot-a-788 | Beta-defensin 112 \|\| id:prot-a-788 | Herring liking \|\| id:ebi-fl187-GCST90094781 | Weighted median | 19 | -0.175869827 | 0.117339204719018 | 0.133921483263682 | -0.405854669 | 0.0541150138105677 | 0.838727155493366 | 0.666407010192701 | 1.05560600444251 | 0.999489402457283 |
| ebi-fl187-GCST90094781 | prot-a-838 | DnaJ homolog subfamily B member 1 \|\| id:prot-a-838 | Herring liking \|\| id:ebi-fl187-GCST90094781 | Weighted median | 19 | 0.104429965662488 | 0.115131346626558 | 0.364380000165271 | -0.121227474 | 0.330087405050542 | 1.11007764759116 | 0.885832433068974 | 1.39108971141676 | 0.999489402457283 |
| ebi-fl187-GCST90094781 | prot-a-85 | Junctional adhesion molecule-like \|\| id:prot-a-85 | Herring liking \|\| id:ebi-fl187-GCST90094781 | Weighted median | 19 | 0.0407422668051135 | 0.111321416114756 | 0.714374268792714 | -0.177447709 | 0.258932242390036 | 1.04158362027399 | 0.837404787108997 | 1.29554601875217 | 0.999489402457283 |
| ebi-fl187-GCST90094781 | prot-a-856 | Dipeptidase 1 \|\| id:prot-a-856 | Herring liking \|\| id:ebi-fl187-GCST90094781 | Weighted median | 19 | 0.12123608616279 | 0.130573251000057 | 0.353152943028789 | -0.134687486 | 0.377159658122902 | 1.12889139654751 | 0.873989002989775 | 1.45813709421913 | 0.999489402457283 |
| ebi-fl187-GCST90094781 | prot-a-860 | Dipeptidyl peptidase 2 \|\| id:prot-a-860 | Herring liking \|\| id:ebi-fl187-GCST90094781 | Weighted median | 19 | 0.160890994946683 | 0.11685841299769 | 0.168573212426244 | -0.068151495 | 0.389933484422155 | 1.17455692919482 | 0.934118948947376 | 1.47688255491892 | 0.999489402457283 |
| ebi-fl187-GCST90094781 | prot-a-861 | Dermatopontin \|\| id:prot-a-861 | Herring liking \|\| id:ebi-fl187-GCST90094781 | Weighted median | 19 | -0.173767206 | 0.121214020783555 | 0.151698727055365 | -0.411346687 | 0.0638122749589351 | 0.840492536692887 | 0.662757122811617 | 1.06589228530591 | 0.999489402457283 |
| ebi-fl187-GCST90094781 | prot-a-877 | Dual specificity protein phosphatase 6 \|\| id:prot-a-877 | Herring liking \|\| id:ebi-fl187-GCST90094781 | Weighted median | 19 | -0.017785006 | 0.116117481206257 | 0.878269029747183 | -0.245375269 | 0.209805257532739 | 0.982372214149829 | 0.782410868758289 | 1.23343783383941 | 0.999489402457283 |
| ebi-fl187-GCST90094781 | prot-a-879 | Dynactin-associated protein \|\| id:prot-a-879 | Herring liking \|\| id:ebi-fl187-GCST90094781 | Weighted median | 19 | -0.030003434 | 0.122193492039649 | 0.806038059553817 | -0.269502679 | 0.209495810040249 | 0.97044220069737 | 0.763759233595624 | 1.23305620864411 | 0.999489402457283 |
| ebi-fl187-GCST90094781 | prot-a-937 | EMI domain-containing protein 1 \|\| id:prot-a-937 | Herring liking \|\| id:ebi-fl187-GCST90094781 | Weighted median | 19 | -0.051492464 | 0.123929803143177 | 0.677778093194765 | -0.294394879 | 0.191409949753237 | 0.949810807322533 | 0.744982255827064 | 1.21095578136307 | 0.999489402457283 |
| ebi-fl187-GCST90094781 | prot-b-1 | SRC proto-oncogene, non-receptor tyrosine kinase \|\| id:prot-b-1 | Herring liking \|\| id:ebi-fl187-GCST90094781 | Weighted median | 13 | -0.030830113 | 0.180211859261412 | 0.864163182669725 | -0.384045357 | 0.322385131214624 | 0.969640288424172 | 0.681100533740651 | 1.38041631500662 | 0.999489402457283 |
| ebi-fl187-GCST90094781 | prot-b-10 | coagulation factor III, tissue factor \|\| id:prot-b-10 | Herring liking \|\| id:ebi-fl187-GCST90094781 | Weighted median | 13 | -0.091943628 | 0.164076239101972 | 0.575226149258646 | -0.413533057 | 0.229645800201768 | 0.91215656778624 | 0.661309673053684 | 1.25815429300099 | 0.999489402457283 |
| ebi-fl187-GCST90094781 | prot-b-29 | myeloperoxidase \|\| id:prot-b-29 | Herring liking \|\| id:ebi-fl187-GCST90094781 | Weighted median | 13 | -0.168625325 | 0.16533112616207 | 0.307764099484148 | -0.492674333 | 0.155423681985639 | 0.844825378796762 | 0.610990216282502 | 1.16815278156449 | 0.999489402457283 |
| ebi-fl187-GCST90094781 | prot-b-33 | adrenomedullin \|\| id:prot-b-33 | Herring liking \|\| id:ebi-fl187-GCST90094781 | Weighted median | 13 | -0.062945698 | 0.169258161351965 | 0.709973515996331 | -0.394691695 | 0.26880029791209 | 0.938994461264845 | 0.673887770471869 | 1.3083938259759 | 0.999489402457283 |
| ebi-fl187-GCST90094781 | ukb-rsfMRI191-edge-pheno102 | Net25_Pair11_15 (Postcentral\|Precentral)&(Cerebellum) (Motor)&(Subcortical-cerebellum) \|\| id:ukb-rsfMRI191-edge-pheno102 | Herring liking \|\| id:ebi-fl187-GCST90094781 | Weighted median | 18 | 0.00929582491678416 | 0.037061000183314 | 0.801949421578813 | -0.063343735 | 0.0819353852760795 | 1.00933916528796 | 0.938620781002346 | 1.08538567566794 | 0.999489402457283 |
| ebi-fl187-GCST90094781 | ukb-rsfMRI191-edge-pheno1167 | Net100_Pair11_45 (Angular\|Temporal)&(Frontal) (Default_mode\|Central_executive)&(Salience\|Default_mode) \|\| id:ukb-rsfMRI191-edge-pheno1167 | Herring liking \|\| id:ebi-fl187-GCST90094781 | Weighted median | 18 | -0.037553483 | 0.0384191241130216 | 0.328337274623042 | -0.112854966 | 0.0377480003832192 | 0.963142904688542 | 0.893280206598056 | 1.0384695059848 | 0.999489402457283 |
| ebi-fl187-GCST90094781 | ukb-rsfMRI191-edge-pheno1317 | Net100_Pair26_48 (Parietal\|Frontal)&(Temporal) (Attention\|Central_executive)&(Default_mode\|Central_executive) \|\| id:ukb-rsfMRI191-edge-pheno1317 | Herring liking \|\| id:ebi-fl187-GCST90094781 | Weighted median | 18 | 0.00285447093562315 | 0.0416050384182235 | 0.945301029757356 | -0.078691404 | 0.0844003462353412 | 1.00285854881693 | 0.924325123136445 | 1.08806440911455 | 0.999489402457283 |
| ebi-fl187-GCST90094781 | ukb-rsfMRI191-edge-pheno286 | Net100_Pair10_13 (OccipitalPrecuneus)&(Frontal_Sup) (Default_mode\|Central_executive)&(Default_mode) \|\| id:ukb-rsfMRI191-edge-pheno286 | Herring liking \|\| id:ebi-fl187-GCST90094781 | Weighted median | 18 | 0.034641785837777 | 0.0391834421504258 | 0.376646302066513 | -0.042157761 | 0.111441332452612 | 1.03524880158933 | 0.958718520462574 | 1.11788815832517 | 0.999489402457283 |
| ebi-fl187-GCST90094781 | ukb-rsfMRI191-edge-pheno599 | Net100_Pair11_29 (Angular\|Temporal)&(Parietal) (Default_mode\|Central_executive)&(Central_executive\|Salience) \|\| id:ukb-rsfMRI191-edge-pheno599 | Herring liking \|\| id:ebi-fl187-GCST90094781 | Weighted median | 18 | -0.01795131 | 0.0391354450369801 | 0.646451798701355 | -0.094656782 | 0.0587541626761768 | 0.982208855339711 | 0.909685101314973 | 1.06051449464567 | 0.999489402457283 |
| ebi-fl187-GCST90094781 | ukb-rsfMRI191-edge-pheno621 | Net100_Pair5_30 (Precuneus\|Angular\|Cingulate)&(Paracentral) (Default_mode\|Central_executive)&(Motor) \|\| id:ukb-rsfMRI191-edge-pheno621 | Herring liking \|\| id:ebi-fl187-GCST90094781 | Weighted median | 18 | -0.028213571 | 0.039280621783181 | 0.472598999872678 | -0.10520359 | 0.0487764474374907 | 0.97218071476997 | 0.900141244216857 | 1.04998559753024 | 0.999489402457283 |
| ebi-fl187-GCST90094781 | ukb-rsfMRI191-edge-pheno624 | Net100_Pair8_30 (Calcarine\|Lingual)&(Paracentral) (Visual)&(Motor) \|\| id:ukb-rsfMRI191-edge-pheno624 | Herring liking \|\| id:ebi-fl187-GCST90094781 | Weighted median | 18 | 0.00451234969639543 | 0.0384157143424761 | 0.906494717140282 | -0.07078245 | 0.0798071498076485 | 1.00452254567646 | 0.93166455331943 | 1.08307817569866 | 0.999489402457283 |
| ebi-fl187-GCST90094781 | ukb-rsfMRI191-edge-pheno882 | Net100_Pair6_38 (Postcentral\|Precentral)&(Subcortical) (Motor)&(Subcortical-cerebellum) \|\| id:ukb-rsfMRI191-edge-pheno882 | Herring liking \|\| id:ebi-fl187-GCST90094781 | Weighted median | 17 | -0.016708662 | 0.0392540560733907 | 0.670359686855254 | -0.093646612 | 0.0602292878255887 | 0.983430153400347 | 0.910604502 | 1.0620800406523 | 0.999489402457283 |
| ebi-fl187-GCST90094781 | ukb-rsfMRI191-edge-pheno899 | Net100_Pair23_38 (Cerebellum)&(Subcortical) (Subcortical-cerebellum)&(Subcortical-cerebellum) \|\| id:ukb-rsfMRI191-edge-pheno899 | Herring liking \|\| id:ebi-fl187-GCST90094781 | Weighted median | 18 | 0.0393216177614345 | 0.0397414586380331 | 0.322450017293421 | -0.038571641 | 0.117214876691979 | 1.04010494608716 | 0.962162771804783 | 1.12436100270825 | 0.999489402457283 |
| ebi-fl187-GCST90094781 | ebi-a-GCST90001392 | IgD+ CD38+ B cell Absolute Count \|\| id:ebi-a-GCST90001392 | Herring liking \|\| id:ebi-fl187-GCST90094781 | Weighted mode | 16 | 0.0188910021751982 | 0.223202249145591 | 0.933669864765315 | -0.418585406 | 0.456367410500556 | 1.01907056608862 | 0.657976931915751 | 1.57833013331408 | 0.999228396881742 |
| ebi-fl187-GCST90094781 | ebi-a-GCST90001402 | Switched memory B cell %B cell \|\| id:ebi-a-GCST90001402 | Herring liking \|\| id:ebi-fl187-GCST90094781 | Weighted mode | 16 | 0.345939384840849 | 0.231947665396091 | 0.156571837938442 | -0.108678039 | 0.800556809017187 | 1.41331694478968 | 0.897019175901269 | 2.22678047481283 | 0.999228396881742 |
| ebi-fl187-GCST90094781 | ebi-a-GCST90001407 | Memory B cell Absolute Count \|\| id:ebi-a-GCST90001407 | Herring liking \|\| id:ebi-fl187-GCST90094781 | Weighted mode | 16 | -0.249680044 | 0.280188422260795 | 0.386938384480776 | -0.798849352 | 0.299489263221985 | 0.779050004603874 | 0.449846281140815 | 1.3491695610646 | 0.999228396881742 |
| ebi-fl187-GCST90094781 | ebi-a-GCST90001411 | IgD+ CD24+ B cell %B cell \|\| id:ebi-a-GCST90001411 | Herring liking \|\| id:ebi-fl187-GCST90094781 | Weighted mode | 16 | -0.238457423 | 0.266517768533937 | 0.38507160142775 | -0.760832249 | 0.283917403325844 | 0.787842231501211 | 0.467277373857749 | 1.32832321114218 | 0.999228396881742 |
| ebi-fl187-GCST90094781 | ebi-a-GCST90001418 | CD24+ CD27+ B cell Absolute Count \|\| id:ebi-a-GCST90001418 | Herring liking \|\| id:ebi-fl187-GCST90094781 | Weighted mode | 16 | -0.121300482 | 0.221745728917865 | 0.592407273568055 | -0.555922111 | 0.313321146469847 | 0.8857677621471 | 0.5735431469023 | 1.36796077626699 | 0.999228396881742 |
| ebi-fl187-GCST90094781 | ebi-a-GCST90001436 | Memory B cell %lymphocyte \|\| id:ebi-a-GCST90001436 | Herring liking \|\| id:ebi-fl187-GCST90094781 | Weighted mode | 16 | 0.301996911536931 | 0.281307170835827 | 0.29999254489562 | -0.249365143 | 0.853358966375151 | 1.35255704938054 | 0.779295366943829 | 2.34751886053602 | 0.999228396881742 |
| ebi-fl187-GCST90094781 | ebi-a-GCST90001439 | IgD+ CD24+ B cell %lymphocyte \|\| id:ebi-a-GCST90001439 | Herring liking \|\| id:ebi-fl187-GCST90094781 | Weighted mode | 16 | -0.343972853 | 0.302579895667848 | 0.273457204062762 | -0.937029448 | 0.249083742520208 | 0.708948173612308 | 0.391789940653927 | 1.28284945761838 | 0.999228396881742 |
| ebi-fl187-GCST90094781 | ebi-a-GCST90001457 | CD11c+ HLA DR++ monocyte %monocyte \|\| id:ebi-a-GCST90001457 | Herring liking \|\| id:ebi-fl187-GCST90094781 | Weighted mode | 16 | 0.286920735364563 | 0.242437082232071 | 0.25503429133383 | -0.188255946 | 0.762097416539421 | 1.33231860354177 | 0.828402653908253 | 2.14276578300301 | 0.999228396881742 |
| ebi-fl187-GCST90094781 | ebi-a-GCST90001483 | CD39+ resting CD4 regulatory T cell Absolute Count \|\| id:ebi-a-GCST90001483 | Herring liking \|\| id:ebi-fl187-GCST90094781 | Weighted mode | 15 | -0.078347859 | 0.24173493689597 | 0.750648866681194 | -0.552148335 | 0.395452617394986 | 0.924642725372608 | 0.575711659230822 | 1.48505620109684 | 0.999228396881742 |
| ebi-fl187-GCST90094781 | ebi-a-GCST90001525 | CD33dim HLA DR+ CD11b+ Absolute Count \|\| id:ebi-a-GCST90001525 | Herring liking \|\| id:ebi-fl187-GCST90094781 | Weighted mode | 16 | -0.034253946 | 0.307199498140772 | 0.912695142062547 | -0.636364962 | 0.567857070468346 | 0.966326078942607 | 0.529212639827214 | 1.76448183692187 | 0.999228396881742 |
| ebi-fl187-GCST90094781 | ebi-a-GCST90001540 | Naive CD4+ T cell Absolute Count \|\| id:ebi-a-GCST90001540 | Herring liking \|\| id:ebi-fl187-GCST90094781 | Weighted mode | 16 | -0.196814713 | 0.21947347916791 | 0.384014632560502 | -0.626982732 | 0.233353306319583 | 0.821342803482257 | 0.534201199968211 | 1.26282756547952 | 0.999228396881742 |
| ebi-fl187-GCST90094781 | ebi-a-GCST90001624 | HLA DR+ CD4+ T cell Absolute Count \|\| id:ebi-a-GCST90001624 | Herring liking \|\| id:ebi-fl187-GCST90094781 | Weighted mode | 16 | 0.0542585403152081 | 0.225922809372925 | 0.813454309930248 | -0.388550166 | 0.497067246686142 | 1.05575752275579 | 0.678039206474065 | 1.64389306136395 | 0.999228396881742 |
| ebi-fl187-GCST90094781 | ebi-a-GCST90001672 | CD39+ CD8+ T cell Absolute Count \|\| id:ebi-a-GCST90001672 | Herring liking \|\| id:ebi-fl187-GCST90094781 | Weighted mode | 16 | 0.0988989901309758 | 0.233518248605702 | 0.677928101835991 | -0.358796777 | 0.556594757398152 | 1.10395478359877 | 0.698516291409503 | 1.74472117432139 | 0.999228396881742 |
| ebi-fl187-GCST90094781 | ebi-a-GCST90001722 | CD19 on CD20- CD38- B cell \|\| id:ebi-a-GCST90001722 | Herring liking \|\| id:ebi-fl187-GCST90094781 | Weighted mode | 16 | -0.357753172 | 0.215859821673448 | 0.118212217598719 | -0.780838423 | 0.0653320782016978 | 0.699245646917849 | 0.458021834346698 | 1.06751346348143 | 0.999228396881742 |
| ebi-fl187-GCST90094781 | ebi-a-GCST90001742 | CD19 on transitional B cell \|\| id:ebi-a-GCST90001742 | Herring liking \|\| id:ebi-fl187-GCST90094781 | Weighted mode | 16 | -0.366148788 | 0.291594721210938 | 0.22844121938455 | -0.937674442 | 0.205376865306417 | 0.693399623805774 | 0.391537320228472 | 1.227987764 | 0.999228396881742 |
| ebi-fl187-GCST90094781 | ebi-a-GCST90001784 | CD25 on IgD+ CD38dim B cell \|\| id:ebi-a-GCST90001784 | Herring liking \|\| id:ebi-fl187-GCST90094781 | Weighted mode | 16 | 0.0321346584575752 | 0.228695367165873 | 0.890124255991577 | -0.416108261 | 0.480377578102686 | 1.03265655188149 | 0.65960885658066 | 1.61668471171198 | 0.999228396881742 |
| ebi-fl187-GCST90094781 | ebi-a-GCST90001794 | CD25 on IgD+ B cell \|\| id:ebi-a-GCST90001794 | Herring liking \|\| id:ebi-fl187-GCST90094781 | Weighted mode | 16 | -0.074529885 | 0.235118888217332 | 0.755621726468462 | -0.535362906 | 0.386303136147989 | 0.928179735217751 | 0.585456786004008 | 1.47153067735216 | 0.999228396881742 |
| ebi-fl187-GCST90094781 | ebi-a-GCST90001802 | CD27 on IgD- CD38- B cell \|\| id:ebi-a-GCST90001802 | Herring liking \|\| id:ebi-fl187-GCST90094781 | Weighted mode | 16 | 0.066908327706104 | 0.236178864325203 | 0.780823030807187 | -0.396002246 | 0.529818901783502 | 1.0691974578982 | 0.673005184116162 | 1.6986246628654 | 0.999228396881742 |
| ebi-fl187-GCST90094781 | ebi-a-GCST90001804 | CD27 on IgD- CD38dim B cell \|\| id:ebi-a-GCST90001804 | Herring liking \|\| id:ebi-fl187-GCST90094781 | Weighted mode | 16 | 0.057563265129736 | 0.25222804676975 | 0.822557802182107 | -0.436803707 | 0.551930236798446 | 1.05925228226802 | 0.646098243824436 | 1.73660183759128 | 0.999228396881742 |
| ebi-fl187-GCST90094781 | ebi-a-GCST90001805 | CD27 on memory B cell \|\| id:ebi-a-GCST90001805 | Herring liking \|\| id:ebi-fl187-GCST90094781 | Weighted mode | 16 | 0.17960337400672 | 0.235748447415324 | 0.457965650419223 | -0.282463583 | 0.641670330940755 | 1.19674260975198 | 0.753924097168044 | 1.89965127706582 | 0.999228396881742 |
| ebi-fl187-GCST90094781 | ebi-a-GCST90001806 | CD27 on unswitched memory B cell \|\| id:ebi-a-GCST90001806 | Herring liking \|\| id:ebi-fl187-GCST90094781 | Weighted mode | 16 | 0.079772225150109 | 0.210611853557921 | 0.710170359261653 | -0.333027008 | 0.492571458123634 | 1.08304035022481 | 0.716750836014277 | 1.63651905414969 | 0.999228396881742 |
| ebi-fl187-GCST90094781 | ebi-a-GCST90001839 | CD3 on Effector Memory CD8+ T cell \|\| id:ebi-a-GCST90001839 | Herring liking \|\| id:ebi-fl187-GCST90094781 | Weighted mode | 16 | 0.274889463588801 | 0.235839104577965 | 0.261984529096975 | -0.187355181 | 0.737134108561612 | 1.31638515833417 | 0.829149185723709 | 2.08993738994025 | 0.999228396881742 |
| ebi-fl187-GCST90094781 | ebi-a-GCST90001859 | CD3 on CD8+ T cell \|\| id:ebi-a-GCST90001859 | Herring liking \|\| id:ebi-fl187-GCST90094781 | Weighted mode | 16 | 0.234638238563369 | 0.22247790287752 | 0.308273793988326 | -0.201418451 | 0.670694928203308 | 1.26445125627968 | 0.817570246815116 | 1.95559584725056 | 0.999228396881742 |
| ebi-fl187-GCST90094781 | ebi-a-GCST90001905 | CD86 on monocyte \|\| id:ebi-a-GCST90001905 | Herring liking \|\| id:ebi-fl187-GCST90094781 | Weighted mode | 16 | -0.113708025 | 0.29132148335527 | 0.701788339807559 | -0.684698132 | 0.457282082660715 | 0.892518511188826 | 0.504242421207628 | 1.57977444838326 | 0.999228396881742 |
| ebi-fl187-GCST90094781 | ebi-a-GCST90001943 | CD25 on activated & secreting CD4 regulatory T cell \|\| id:ebi-a-GCST90001943 | Herring liking \|\| id:ebi-fl187-GCST90094781 | Weighted mode | 16 | 0.215726281198683 | 0.215779266159944 | 0.333285129494828 | -0.20720108 | 0.638653642872173 | 1.24076271243386 | 0.812856184045779 | 1.8939292568383 | 0.999228396881742 |
| ebi-fl187-GCST90094781 | ebi-a-GCST90001949 | CD33 on CD33dim HLA DR+ CD11b- \|\| id:ebi-a-GCST90001949 | Herring liking \|\| id:ebi-fl187-GCST90094781 | Weighted mode | 16 | 0.528873755588247 | 0.363048323461449 | 0.165797002723608 | -0.182700958 | 1.24044846957269 | 1.69701997268099 | 0.833017225301207 | 3.4571635498136 | 0.999228396881742 |
| ebi-fl187-GCST90094781 | ebi-a-GCST90001955 | CD33 on Immature Myeloid-Derived Suppressor Cells \|\| id:ebi-a-GCST90001955 | Herring liking \|\| id:ebi-fl187-GCST90094781 | Weighted mode | 16 | 0.555142183551164 | 0.358850289080991 | 0.142698225074842 | -0.148204383 | 1.25848875014991 | 1.74218867773772 | 0.862254866654142 | 3.5200977184568 | 0.999228396881742 |
| ebi-fl187-GCST90094781 | ebi-a-GCST90001956 | CD33 on CD33+ HLA DR+ \|\| id:ebi-a-GCST90001956 | Herring liking \|\| id:ebi-fl187-GCST90094781 | Weighted mode | 16 | -0.312672149 | 0.481174025770424 | 0.525641474431582 | -1.25577324 | 0.630428941083539 | 0.731489692575954 | 0.284855501395131 | 1.87841613633659 | 0.999228396881742 |
| ebi-fl187-GCST90094781 | ebi-a-GCST90001957 | CD33 on CD33+ HLA DR+ CD14- \|\| id:ebi-a-GCST90001957 | Herring liking \|\| id:ebi-fl187-GCST90094781 | Weighted mode | 16 | 0.591368921770688 | 0.443190853451842 | 0.201995168330469 | -0.277285151 | 1.4600229945363 | 1.80645962547449 | 0.757838367932642 | 4.30605854302627 | 0.999228396881742 |
| ebi-fl187-GCST90094781 | ebi-a-GCST90002064 | CD4 on resting CD4 regulatory T cell \|\| id:ebi-a-GCST90002064 | Herring liking \|\| id:ebi-fl187-GCST90094781 | Weighted mode | 16 | -0.250980304 | 0.30202529888541 | 0.419010226242604 | -0.84294989 | 0.340989281651937 | 0.778037695511316 | 0.43043890137694 | 1.40633816715942 | 0.999228396881742 |
| ebi-fl187-GCST90094781 | ebi-a-GCST90002080 | SSC-A on T cell \|\| id:ebi-a-GCST90002080 | Herring liking \|\| id:ebi-fl187-GCST90094781 | Weighted mode | 16 | -0.133388134 | 0.211801028763879 | 0.538313744577196 | -0.54851815 | 0.281741882508941 | 0.875125360391001 | 0.577805397084289 | 1.32543655747085 | 0.999228396881742 |
| ebi-fl187-GCST90094781 | ebi-a-GCST90002121 | CD8 on CD39+ CD8+ T cell \|\| id:ebi-a-GCST90002121 | Herring liking \|\| id:ebi-fl187-GCST90094781 | Weighted mode | 16 | -0.014829479 | 0.283667556266233 | 0.958997304135993 | -0.570817889 | 0.541158931778166 | 0.985279936688224 | 0.565063090885647 | 1.71799674991834 | 0.999228396881742 |
| ebi-fl187-GCST90094781 | ebi-cfmet338-GCST90026053 | 1-palmitoyl-2-docosahexaenoyl-gpc (16:0/22:6) levels \|\| id:ebi-cfmet338-GCST90026053 | Herring liking \|\| id:ebi-fl187-GCST90094781 | Weighted mode | 15 | -0.167916078 | 0.159111929347797 | 0.309145519382998 | -0.479775459 | 0.143943303928642 | 0.845424781790183 | 0.618922349576999 | 1.15481863298921 | 0.999228396881742 |
| ebi-fl187-GCST90094781 | ebi-cfmet338-GCST90026063 | 1-stearoyl-2-oleoyl-gpc (18:0/18:1) levels \|\| id:ebi-cfmet338-GCST90026063 | Herring liking \|\| id:ebi-fl187-GCST90094781 | Weighted mode | 14 | -0.128163241 | 0.128633259729616 | 0.337266805766155 | -0.38028443 | 0.123957948549108 | 0.879709763142977 | 0.68366692645108 | 1.13196826909024 | 0.999228396881742 |
| ebi-fl187-GCST90094781 | ebi-cfmet338-GCST90026113 | Adenosine 3',5'-cyclic monophosphate (camp) levels \|\| id:ebi-cfmet338-GCST90026113 | Herring liking \|\| id:ebi-fl187-GCST90094781 | Weighted mode | 15 | 0.119416670535324 | 0.252328512333778 | 0.643323074332599 | -0.375147214 | 0.613980554709529 | 1.12683934123574 | 0.687188107882329 | 1.8477719366675 | 0.999228396881742 |
| ebi-fl187-GCST90094781 | ebi-cfmet338-GCST90026117 | Alpha-ketoglutaramate levels \|\| id:ebi-cfmet338-GCST90026117 | Herring liking \|\| id:ebi-fl187-GCST90094781 | Weighted mode | 15 | 0.0134551384740761 | 0.0804169462380125 | 0.869513225761895 | -0.144162076 | 0.171072353100581 | 1.01354606620716 | 0.865747419676814 | 1.18657659841191 | 0.999228396881742 |
| ebi-fl187-GCST90094781 | ebi-cfmet338-GCST90026124 | Arginine levels \|\| id:ebi-cfmet338-GCST90026124 | Herring liking \|\| id:ebi-fl187-GCST90094781 | Weighted mode | 15 | 0.0666699903398959 | 0.0640689716502973 | 0.315700678759943 | -0.058905194 | 0.192245174774479 | 1.06894265855744 | 0.942796147562528 | 1.21196762443078 | 0.999228396881742 |
| ebi-fl187-GCST90094781 | ebi-cfmet338-GCST90026169 | Glutamate levels \|\| id:ebi-cfmet338-GCST90026169 | Herring liking \|\| id:ebi-fl187-GCST90094781 | Weighted mode | 15 | 0.0248735392963263 | 0.0601575565276126 | 0.685523683314595 | -0.093035271 | 0.142782350090447 | 1.02518546665103 | 0.911161361703396 | 1.15347871980399 | 0.999228396881742 |
| ebi-fl187-GCST90094781 | ebi-cfmet338-GCST90026170 | Glutamine levels \|\| id:ebi-cfmet338-GCST90026170 | Herring liking \|\| id:ebi-fl187-GCST90094781 | Weighted mode | 15 | 0.0188383330298695 | 0.0393775326126121 | 0.639748154829015 | -0.058341631 | 0.0960182969505892 | 1.01901689392632 | 0.943327622525813 | 1.10077920471244 | 0.999228396881742 |
| ebi-fl187-GCST90094781 | ebi-cfmet338-GCST90026191 | Kynurenate levels \|\| id:ebi-cfmet338-GCST90026191 | Herring liking \|\| id:ebi-fl187-GCST90094781 | Weighted mode | 15 | -0.028433822 | 0.313275545929547 | 0.928966743695524 | -0.642453892 | 0.585586247695937 | 0.971966614507606 | 0.526000091469589 | 1.79604360348671 | 0.999228396881742 |
| ebi-fl187-GCST90094781 | ebi-cfmet338-GCST90026202 | Methylsuccinoylcarnitine levels \|\| id:ebi-cfmet338-GCST90026202 | Herring liking \|\| id:ebi-fl187-GCST90094781 | Weighted mode | 15 | 0.0259674120718978 | 0.178940912446608 | 0.886686830593725 | -0.324756776 | 0.376691600467249 | 1.02630750269337 | 0.722703110774712 | 1.45745476168713 | 0.999228396881742 |
| ebi-fl187-GCST90094781 | ebi-cfmet338-GCST90026222 | N-acetylputrescine levels \|\| id:ebi-cfmet338-GCST90026222 | Herring liking \|\| id:ebi-fl187-GCST90094781 | Weighted mode | 15 | 0.0503336856078017 | 0.0665695336362322 | 0.462119013639149 | -0.0801426 | 0.180809971534817 | 1.05162194894475 | 0.922984719086241 | 1.19818746793253 | 0.999228396881742 |
| ebi-fl187-GCST90094781 | ebi-cfmet338-GCST90026252 | Propionylcarnitine (c3) levels \|\| id:ebi-cfmet338-GCST90026252 | Herring liking \|\| id:ebi-fl187-GCST90094781 | Weighted mode | 15 | 0.112387769063042 | 0.120939373524475 | 0.368485440870135 | -0.124653403 | 0.349428941171013 | 1.1189466694324 | 0.882802826336949 | 1.41825740888144 | 0.999228396881742 |
| ebi-fl187-GCST90094781 | ebi-cfmet338-GCST90026316 | Ethyl beta-glucopyranoside levels \|\| id:ebi-cfmet338-GCST90026316 | Herring liking \|\| id:ebi-fl187-GCST90094781 | Weighted mode | 15 | -0.69872976 | 0.410137383625404 | 0.110531660879569 | -1.502599032 | 0.105139511866657 | 0.497216487080513 | 0.222550990700298 | 1.11086557847597 | 0.999228396881742 |
| ebi-fl187-GCST90094781 | ebi-gm430-GCST90011315 | F_Prevotellaceae abundance \|\| id:ebi-gm430-GCST90011315 | Herring liking \|\| id:ebi-fl187-GCST90094781 | Weighted mode | 14 | 0.168965863656956 | 0.220989440578615 | 0.458171921121401 | -0.26417344 | 0.602105167191041 | 1.18407971808262 | 0.767840353970539 | 1.82595870551029 | 0.999228396881742 |
| ebi-fl187-GCST90094781 | ebi-gm430-GCST90011354 | OTU97_1 (Bacteroides) abundance \|\| id:ebi-gm430-GCST90011354 | Herring liking \|\| id:ebi-fl187-GCST90094781 | Weighted mode | 14 | -0.01817184 | 0.166354041935903 | 0.914683531120503 | -0.344225762 | 0.307882082564774 | 0.981992272670513 | 0.70876889702678 | 1.360540548 | 0.999228396881742 |
| ebi-fl187-GCST90094781 | ebi-gm430-GCST90011419 | OTU99_1 (Bacteroides) abundance \|\| id:ebi-gm430-GCST90011419 | Herring liking \|\| id:ebi-fl187-GCST90094781 | Weighted mode | 14 | -0.012159693 | 0.168961217509411 | 0.943723321165226 | -0.343323679 | 0.319004293715002 | 0.987913937715811 | 0.709408553798652 | 1.37575723200282 | 0.999228396881742 |
| ebi-fl187-GCST90094781 | ebi-gm430-GCST90011435 | OTU99_155 (Butyrivibrio) abundance \|\| id:ebi-gm430-GCST90011435 | Herring liking \|\| id:ebi-fl187-GCST90094781 | Weighted mode | 14 | 0.6194500608932 | 0.560050611398669 | 0.288748905628488 | -0.478249137 | 1.71714925923459 | 1.85790602566849 | 0.619867745474642 | 5.56863109173746 | 0.999228396881742 |
| ebi-fl187-GCST90094781 | ebi-gm430-GCST90011466 | OTU99_58 (Bacteroides) abundance \|\| id:ebi-gm430-GCST90011466 | Herring liking \|\| id:ebi-fl187-GCST90094781 | Weighted mode | 14 | -0.117505408 | 0.255320699191087 | 0.652956739050309 | -0.617933979 | 0.382923162168704 | 0.88913570306899 | 0.539056991166259 | 1.46656533803892 | 0.999228396881742 |
| ebi-fl187-GCST90094781 | ebi-gm430-GCST90011472 | OTU99_72 (Parabacteroides) abundance \|\| id:ebi-gm430-GCST90011472 | Herring liking \|\| id:ebi-fl187-GCST90094781 | Weighted mode | 14 | 0.372917283064882 | 0.450189571877812 | 0.422416701992815 | -0.509454278 | 1.25528884394539 | 1.45196423283416 | 0.600823372005113 | 3.50885173856345 | 0.999228396881742 |
| ebi-fl187-GCST90094781 | ebi-gm430-GCST90011505 | TestASV_28 (Holdemanella) abundance \|\| id:ebi-gm430-GCST90011505 | Herring liking \|\| id:ebi-fl187-GCST90094781 | Weighted mode | 14 | 0.109029709537693 | 0.378390865728533 | 0.777781895499085 | -0.632616387 | 0.850675806365619 | 1.11519548179149 | 0.531200155922989 | 2.34122853455722 | 0.999228396881742 |
| ebi-fl187-GCST90094781 | ebi-gm430-GCST90011513 | TestASV_35 (Alphaproteobacteria) abundance \|\| id:ebi-gm430-GCST90011513 | Herring liking \|\| id:ebi-fl187-GCST90094781 | Weighted mode | 14 | 0.477813695615292 | 0.561701388491631 | 0.410356193647736 | -0.623121026 | 1.57874841705889 | 1.61254503118686 | 0.536268116392359 | 4.84888323232503 | 0.999228396881742 |
| ebi-fl187-GCST90094781 | ebi-gm430-GCST90011543 | G_Coprococcus prevalence \|\| id:ebi-gm430-GCST90011543 | Herring liking \|\| id:ebi-fl187-GCST90094781 | Weighted mode | 14 | 0.499792651397031 | 0.441684551309822 | 0.278261728092653 | -0.365909069 | 1.36549437196428 | 1.64837944608762 | 0.693565864862096 | 3.91765935427687 | 0.999228396881742 |
| ebi-fl187-GCST90094781 | ebi-gm430-GCST90011547 | G_Parasutterella prevalence \|\| id:ebi-gm430-GCST90011547 | Herring liking \|\| id:ebi-fl187-GCST90094781 | Weighted mode | 14 | 0.396123442271157 | 0.419035788838702 | 0.361734691678386 | -0.425186704 | 1.21743358839501 | 1.48605274795589 | 0.653647735186138 | 3.37850596097971 | 0.999228396881742 |
| ebi-fl187-GCST90094781 | ebi-gm430-GCST90011569 | OTU97_13 (Subdoligranulum) prevalence \|\| id:ebi-gm430-GCST90011569 | Herring liking \|\| id:ebi-fl187-GCST90094781 | Weighted mode | 14 | 0.807590580363052 | 0.577989406008565 | 0.185733508877864 | -0.325268655 | 1.94044981613984 | 2.24249835268755 | 0.72233326882908 | 6.96188183324046 | 0.999228396881742 |
| ebi-fl187-GCST90094781 | ebi-gm430-GCST90011590 | OTU97_277 (Prevotella) prevalence \|\| id:ebi-gm430-GCST90011590 | Herring liking \|\| id:ebi-fl187-GCST90094781 | Weighted mode | 14 | 0.147512147008853 | 0.33576594781665 | 0.667636487270693 | -0.510589111 | 0.805613404729487 | 1.15894736248812 | 0.600141924615376 | 2.23806891991255 | 0.999228396881742 |
| ebi-fl187-GCST90094781 | ebi-gm430-GCST90011616 | OTU97_86 (Phascolarctobacterium) prevalence \|\| id:ebi-gm430-GCST90011616 | Herring liking \|\| id:ebi-fl187-GCST90094781 | Weighted mode | 14 | 0.191053235370372 | 0.458500693886942 | 0.683702397468187 | -0.707608125 | 1.08971459538878 | 1.21052389305381 | 0.492821556585632 | 2.97342532215212 | 0.999228396881742 |
| ebi-fl187-GCST90094781 | ebi-gm430-GCST90011665 | OTU99_55 (Barnesiella) prevalence \|\| id:ebi-gm430-GCST90011665 | Herring liking \|\| id:ebi-fl187-GCST90094781 | Weighted mode | 14 | 0.211383100972025 | 0.395264773822899 | 0.601823699818414 | -0.563335856 | 0.986102057664908 | 1.23538554181835 | 0.569306767482529 | 2.68076461427389 | 0.999228396881742 |
| ebi-fl187-GCST90094781 | ebi-gm430-GCST90011687 | TestASV_14 (Lachnospiraceae) prevalence \|\| id:ebi-gm430-GCST90011687 | Herring liking \|\| id:ebi-fl187-GCST90094781 | Weighted mode | 14 | 0.466465711382053 | 0.380717829878701 | 0.242225708587444 | -0.279741235 | 1.21267265794431 | 1.59434933310982 | 0.755979337004923 | 3.36245935776309 | 0.999228396881742 |
| ebi-fl187-GCST90094781 | ebi-gm430-GCST90011697 | TestASV_23 (Barnesiella) prevalence \|\| id:ebi-gm430-GCST90011697 | Herring liking \|\| id:ebi-fl187-GCST90094781 | Weighted mode | 14 | -0.096844898 | 0.469410228563629 | 0.839743354144424 | -1.016888946 | 0.823199150112114 | 0.907696780917511 | 0.361718517404109 | 2.27777513852724 | 0.999228396881742 |
| ebi-fl187-GCST90094781 | ebi-gm430-GCST90011727 | TestASV_6 (Subdoligranulum) prevalence \|\| id:ebi-gm430-GCST90011727 | Herring liking \|\| id:ebi-fl187-GCST90094781 | Weighted mode | 14 | 0.382893280925918 | 0.688631925718258 | 0.587639816009189 | -0.966825293 | 1.7326118553337 | 1.46652151589873 | 0.380288427862339 | 5.65540573685935 | 0.999228396881742 |
| ebi-fl187-GCST90094781 | ebi-hbsm44-GCST90104908 | Sulcal depth of the left posterior inferior temporal sulcus \|\| id:ebi-hbsm44-GCST90104908 | Herring liking \|\| id:ebi-fl187-GCST90094781 | Weighted mode | 16 | 0.217971015721499 | 0.151094519904255 | 0.169685216148969 | -0.078174243 | 0.51411627473384 | 1.24355102366751 | 0.924803271738427 | 1.67216011850562 | 0.999228396881742 |
| ebi-fl187-GCST90094781 | ebi-hbsm44-GCST90104918 | Sulcal depth of the left inferior frontal sulcus \|\| id:ebi-hbsm44-GCST90104918 | Herring liking \|\| id:ebi-fl187-GCST90094781 | Weighted mode | 15 | 0.157634712154717 | 0.209162544550641 | 0.46354847874309 | -0.252323875 | 0.567593299473973 | 1.17073846005983 | 0.776993048561708 | 1.76401647916983 | 0.999228396881742 |
| ebi-fl187-GCST90094781 | ebi-hbsm44-GCST90104925 | Sulcal depth of the right anterior inferior temporal sulcus \|\| id:ebi-hbsm44-GCST90104925 | Herring liking \|\| id:ebi-fl187-GCST90094781 | Weighted mode | 15 | 0.000504766519546451 | 0.0730398771506898 | 0.994583507316619 | -0.142653393 | 0.143662925734898 | 1.0005048939356 | 0.867054544257178 | 1.15449489241381 | 0.999228396881742 |
| ebi-fl187-GCST90094781 | ebi-IPs91-GCST90274770 | C-C motif chemokine 4 levels \|\| id:ebi-IPs91-GCST90274770 | Herring liking \|\| id:ebi-fl187-GCST90094781 | Weighted mode | 21 | 0.0225123576704853 | 0.0898782372745067 | 0.804774212995253 | -0.153648987 | 0.198673702728518 | 1.02276767311224 | 0.857572987121495 | 1.21978388880296 | 0.999228396881742 |
| ebi-fl187-GCST90094781 | ebi-IPs91-GCST90274779 | C-X-C motif chemokine 1 levels \|\| id:ebi-IPs91-GCST90274779 | Herring liking \|\| id:ebi-fl187-GCST90094781 | Weighted mode | 21 | -0.136428099 | 0.131379426601262 | 0.311463032937523 | -0.393931775 | 0.121075577265072 | 0.872469049512025 | 0.674400065607399 | 1.1287102139749 | 0.999228396881742 |
| ebi-fl187-GCST90094781 | ebi-IPs91-GCST90274780 | C-X-C motif chemokine 10 levels \|\| id:ebi-IPs91-GCST90274780 | Herring liking \|\| id:ebi-fl187-GCST90094781 | Weighted mode | 21 | -0.145929439 | 0.0978163453891873 | 0.151338871723975 | -0.337649476 | 0.0457905981772357 | 0.864218681352959 | 0.713445323962125 | 1.04685517462176 | 0.999228396881742 |
| ebi-fl187-GCST90094781 | ebi-IPs91-GCST90274796 | Interleukin-10 receptor subunit alpha levels \|\| id:ebi-IPs91-GCST90274796 | Herring liking \|\| id:ebi-fl187-GCST90094781 | Weighted mode | 19 | -0.017429661 | 0.117211048351981 | 0.883441103061189 | -0.247163316 | 0.212303993992457 | 0.982721357090336 | 0.7810131315282 | 1.23652372373281 | 0.999228396881742 |
| ebi-fl187-GCST90094781 | ebi-IPs91-GCST90274807 | Interleukin-20 levels \|\| id:ebi-IPs91-GCST90274807 | Herring liking \|\| id:ebi-fl187-GCST90094781 | Weighted mode | 19 | -0.009376063 | 0.0988366156075117 | 0.925470727759326 | -0.203095829 | 0.184343704021459 | 0.990667755650994 | 0.816200021915334 | 1.20242903177523 | 0.999228396881742 |
| ebi-fl187-GCST90094781 | ebi-IPs91-GCST90274808 | Interleukin-20 receptor subunit alpha levels \|\| id:ebi-IPs91-GCST90274808 | Herring liking \|\| id:ebi-fl187-GCST90094781 | Weighted mode | 19 | 0.0158903861203248 | 0.109723122563001 | 0.886460222331776 | -0.199166934 | 0.230947706343807 | 1.01601730970291 | 0.81941309392505 | 1.2597933585259 | 0.999228396881742 |
| ebi-fl187-GCST90094781 | ebi-IPs91-GCST90274810 | Interleukin-24 levels \|\| id:ebi-IPs91-GCST90274810 | Herring liking \|\| id:ebi-fl187-GCST90094781 | Weighted mode | 19 | -0.085204631 | 0.120401097035536 | 0.488210827470324 | -0.321190781 | 0.150781519565069 | 0.918324347888454 | 0.725284867352547 | 1.16274259382118 | 0.999228396881742 |
| ebi-fl187-GCST90094781 | ebi-IPs91-GCST90274823 | Monocyte chemoattractant protein-3 levels \|\| id:ebi-IPs91-GCST90274823 | Herring liking \|\| id:ebi-fl187-GCST90094781 | Weighted mode | 19 | -0.124117891 | 0.137761949141468 | 0.37950709675696 | -0.394131311 | 0.145895529405022 | 0.883275704565925 | 0.674265511793747 | 1.15707530139119 | 0.999228396881742 |
| ebi-fl187-GCST90094781 | ebi-IPs91-GCST90274824 | Monocyte chemoattractant protein-4 levels \|\| id:ebi-IPs91-GCST90274824 | Herring liking \|\| id:ebi-fl187-GCST90094781 | Weighted mode | 21 | 0.0568961392082414 | 0.126681734129023 | 0.658169242076971 | -0.19140006 | 0.305192338101127 | 1.05854586327453 | 0.82580215191002 | 1.35688595877831 | 0.999228396881742 |
| ebi-fl187-GCST90094781 | ebi-IPs91-GCST90274834 | SIR2-like protein 2 levels \|\| id:ebi-IPs91-GCST90274834 | Herring liking \|\| id:ebi-fl187-GCST90094781 | Weighted mode | 21 | -0.100757563 | 0.124045212097115 | 0.426202723071996 | -0.343886179 | 0.142371052336051 | 0.904152205926934 | 0.709009623581359 | 1.15300439414802 | 0.999228396881742 |
| ebi-fl187-GCST90094781 | ebi-met1400-GCST90199652 | 1-stearoyl-2-oleoyl-GPS (18:0/18:1) levels \|\| id:ebi-met1400-GCST90199652 | Herring liking \|\| id:ebi-fl187-GCST90094781 | Weighted mode | 16 | -0.001107978 | 0.142835943353665 | 0.993913102428194 | -0.281066427 | 0.278850471340491 | 0.998892635947893 | 0.754978183176284 | 1.32160971056557 | 0.999228396881742 |
| ebi-fl187-GCST90094781 | ebi-met1400-GCST90199665 | N-acetylaspartate (naa) levels \|\| id:ebi-met1400-GCST90199665 | Herring liking \|\| id:ebi-fl187-GCST90094781 | Weighted mode | 16 | -0.080687666 | 0.129274824415482 | 0.541902144867451 | -0.334066321 | 0.17269099027411 | 0.922481769262104 | 0.716006294087894 | 1.18849879064957 | 0.999228396881742 |
| ebi-fl187-GCST90094781 | ebi-met1400-GCST90199669 | Acetylcarnitine levels (Biocrates platform) \|\| id:ebi-met1400-GCST90199669 | Herring liking \|\| id:ebi-fl187-GCST90094781 | Weighted mode | 16 | 0.073399251474098 | 0.121882818925673 | 0.556027870300445 | -0.165491074 | 0.312289576568416 | 1.07616010966537 | 0.847477428076537 | 1.36655035670211 | 0.999228396881742 |
| ebi-fl187-GCST90094781 | ebi-met1400-GCST90199670 | Iminodiacetate (IDA) levels \|\| id:ebi-met1400-GCST90199670 | Herring liking \|\| id:ebi-fl187-GCST90094781 | Weighted mode | 16 | 0.00515338067241189 | 0.119755410093533 | 0.966243198801514 | -0.229567223 | 0.239873984455737 | 1.00516668217801 | 0.794877532701253 | 1.27108896326107 | 0.999228396881742 |
| ebi-fl187-GCST90094781 | ebi-met1400-GCST90199686 | Indolepropionate levels \|\| id:ebi-met1400-GCST90199686 | Herring liking \|\| id:ebi-fl187-GCST90094781 | Weighted mode | 16 | 0.0674344598745627 | 0.155815850532118 | 0.671330438517795 | -0.237964607 | 0.372833526917514 | 1.06976014508617 | 0.78823058831257 | 1.45184262699659 | 0.999228396881742 |
| ebi-fl187-GCST90094781 | ebi-met1400-GCST90199704 | 3-hydroxysebacate levels \|\| id:ebi-met1400-GCST90199704 | Herring liking \|\| id:ebi-fl187-GCST90094781 | Weighted mode | 16 | 0.0156666658150151 | 0.14050524049206 | 0.91269635259698 | -0.259723606 | 0.291056937179453 | 1.01579003142452 | 0.771264729637521 | 1.33784075466067 | 0.999228396881742 |
| ebi-fl187-GCST90094781 | ebi-met1400-GCST90199713 | Docosapentaenoate n3 DPA; 22:5n3 levels \|\| id:ebi-met1400-GCST90199713 | Herring liking \|\| id:ebi-fl187-GCST90094781 | Weighted mode | 16 | -0.093416062 | 0.127491090478474 | 0.475020681976128 | -0.3432986 | 0.15646647512139 | 0.91081446596522 | 0.709426345539697 | 1.16937155862234 | 0.999228396881742 |
| ebi-fl187-GCST90094781 | ebi-met1400-GCST90199715 | Myristoleate (14:1n5) levels \|\| id:ebi-met1400-GCST90199715 | Herring liking \|\| id:ebi-fl187-GCST90094781 | Weighted mode | 16 | 0.0759809698738341 | 0.112426045370278 | 0.509434355301065 | -0.144374079 | 0.296336018799579 | 1.07894204155874 | 0.865563898167856 | 1.34492199998987 | 0.999228396881742 |
| ebi-fl187-GCST90094781 | ebi-met1400-GCST90199725 | Campesterol levels \|\| id:ebi-met1400-GCST90199725 | Herring liking \|\| id:ebi-fl187-GCST90094781 | Weighted mode | 16 | 0.0961646626794335 | 0.18547514768184 | 0.61168886396862 | -0.267366627 | 0.459695952135841 | 1.10094033285457 | 0.765392406667726 | 1.5835924238952 | 0.999228396881742 |
| ebi-fl187-GCST90094781 | ebi-met1400-GCST90199737 | N-acetylproline levels \|\| id:ebi-met1400-GCST90199737 | Herring liking \|\| id:ebi-fl187-GCST90094781 | Weighted mode | 16 | -0.026262301 | 0.155618500803909 | 0.86823942080823 | -0.331274562 | 0.278749960701018 | 0.974079554177598 | 0.718008003937843 | 1.32147688140391 | 0.999228396881742 |
| ebi-fl187-GCST90094781 | ebi-met1400-GCST90199775 | Gamma-glutamylisoleucine levels \|\| id:ebi-met1400-GCST90199775 | Herring liking \|\| id:ebi-fl187-GCST90094781 | Weighted mode | 16 | 0.0387747364937059 | 0.155856473177637 | 0.80690133011741 | -0.266703951 | 0.344253423921875 | 1.03953628768416 | 0.765899781819726 | 1.41093615517771 | 0.999228396881742 |
| ebi-fl187-GCST90094781 | ebi-met1400-GCST90199777 | 1-palmitoleoyl-GPC (16:1) levels \|\| id:ebi-met1400-GCST90199777 | Herring liking \|\| id:ebi-fl187-GCST90094781 | Weighted mode | 16 | -0.04488877 | 0.145639755787026 | 0.762155389313492 | -0.330342691 | 0.240565151668678 | 0.95610382365887 | 0.718677406936306 | 1.27196780195447 | 0.999228396881742 |
| ebi-fl187-GCST90094781 | ebi-met1400-GCST90199782 | Glutamine degradant levels \|\| id:ebi-met1400-GCST90199782 | Herring liking \|\| id:ebi-fl187-GCST90094781 | Weighted mode | 16 | -0.199257847 | 0.113587487409022 | 0.099792919497924 | -0.421889322 | 0.0233736283683995 | 0.819338602131432 | 0.655806618561522 | 1.02364893238678 | 0.999228396881742 |
| ebi-fl187-GCST90094781 | ebi-met1400-GCST90199783 | 5-methyluridine (ribothymidine) levels \|\| id:ebi-met1400-GCST90199783 | Herring liking \|\| id:ebi-fl187-GCST90094781 | Weighted mode | 16 | 0.0728664760799651 | 0.152994525727004 | 0.640746135463333 | -0.227002794 | 0.372735746504893 | 1.07558691074549 | 0.796918555421678 | 1.45170067216578 | 0.999228396881742 |
| ebi-fl187-GCST90094781 | ebi-met1400-GCST90199787 | Homostachydrine levels \|\| id:ebi-met1400-GCST90199787 | Herring liking \|\| id:ebi-fl187-GCST90094781 | Weighted mode | 16 | -0.001894164 | 0.130162856289632 | 0.988581180686389 | -0.257013362 | 0.253225034811176 | 0.998107629279082 | 0.773357880208919 | 1.28817312801673 | 0.999228396881742 |
| ebi-fl187-GCST90094781 | ebi-met1400-GCST90199802 | Beta-hydroxyisovaleroylcarnitine levels \|\| id:ebi-met1400-GCST90199802 | Herring liking \|\| id:ebi-fl187-GCST90094781 | Weighted mode | 16 | 0.160636143062688 | 0.137363617623678 | 0.260481010871844 | -0.108596547 | 0.429868833605096 | 1.17425762928875 | 0.897092278637173 | 1.53705590024427 | 0.999228396881742 |
| ebi-fl187-GCST90094781 | ebi-met1400-GCST90199873 | Pregnenolone sulfate levels \|\| id:ebi-met1400-GCST90199873 | Herring liking \|\| id:ebi-fl187-GCST90094781 | Weighted mode | 16 | 0.00785989115575303 | 0.139079806314834 | 0.955678638288325 | -0.264736529 | 0.280456311532828 | 1.00789086118743 | 0.767408112955752 | 1.32373370950238 | 0.999228396881742 |
| ebi-fl187-GCST90094781 | ebi-met1400-GCST90199911 | Beta-citrylglutamate levels \|\| id:ebi-met1400-GCST90199911 | Herring liking \|\| id:ebi-fl187-GCST90094781 | Weighted mode | 16 | 0.0203538640212039 | 0.148247974797765 | 0.892622728608988 | -0.270212167 | 0.310919894624824 | 1.02056241645759 | 0.763217547898872 | 1.36467989861228 | 0.999228396881742 |
| ebi-fl187-GCST90094781 | ebi-met1400-GCST90199934 | 2-hydroxydecanoate levels \|\| id:ebi-met1400-GCST90199934 | Herring liking \|\| id:ebi-fl187-GCST90094781 | Weighted mode | 16 | 0.103154910196871 | 0.130963010312018 | 0.443162355323951 | -0.15353259 | 0.359842410408426 | 1.10866313899943 | 0.857672812173809 | 1.43310355456037 | 0.999228396881742 |
| ebi-fl187-GCST90094781 | ebi-met1400-GCST90199945 | Sphingomyelin (d18:1/14:0, d16:1/16:0) levels \|\| id:ebi-met1400-GCST90199945 | Herring liking \|\| id:ebi-fl187-GCST90094781 | Weighted mode | 16 | -0.003117513 | 0.133143039229407 | 0.981628073356888 | -0.26407787 | 0.257842843391061 | 0.996887340900768 | 0.767913739587392 | 1.29413542070777 | 0.999228396881742 |
| ebi-fl187-GCST90094781 | ebi-met1400-GCST90199979 | Carnitine C14:1 levels \|\| id:ebi-met1400-GCST90199979 | Herring liking \|\| id:ebi-fl187-GCST90094781 | Weighted mode | 16 | -0.092756243 | 0.14842539640238 | 0.541404310724778 | -0.38367002 | 0.198157533864463 | 0.911415637086058 | 0.681356223992685 | 1.21915443680442 | 0.999228396881742 |
| ebi-fl187-GCST90094781 | ebi-met1400-GCST90199986 | Eugenol sulfate levels \|\| id:ebi-met1400-GCST90199986 | Herring liking \|\| id:ebi-fl187-GCST90094781 | Weighted mode | 16 | -0.095587458 | 0.148811830130616 | 0.530349627351438 | -0.387258645 | 0.196083729059485 | 0.908838872950772 | 0.678915474087428 | 1.21662876825232 | 0.999228396881742 |
| ebi-fl187-GCST90094781 | ebi-met1400-GCST90200002 | 6-hydroxyindole sulfate levels \|\| id:ebi-met1400-GCST90200002 | Herring liking \|\| id:ebi-fl187-GCST90094781 | Weighted mode | 16 | 0.0128733092854656 | 0.127774450237705 | 0.921083039054049 | -0.237564613 | 0.263311231751367 | 1.01295652704401 | 0.788545938873976 | 1.30123164053865 | 0.999228396881742 |
| ebi-fl187-GCST90094781 | ebi-met1400-GCST90200023 | Trans 3,4-methyleneheptanoate levels \|\| id:ebi-met1400-GCST90200023 | Herring liking \|\| id:ebi-fl187-GCST90094781 | Weighted mode | 16 | -0.009229735 | 0.147575849853711 | 0.950956752794903 | -0.298478401 | 0.280018930303549 | 0.990812727856152 | 0.741946306884067 | 1.3231548598235 | 0.999228396881742 |
| ebi-fl187-GCST90094781 | ebi-met1400-GCST90200032 | 2-hydroxybutyrate/2-hydroxyisobutyrate levels \|\| id:ebi-met1400-GCST90200032 | Herring liking \|\| id:ebi-fl187-GCST90094781 | Weighted mode | 16 | 0.233377665998387 | 0.14272764156168 | 0.122828792799594 | -0.046368511 | 0.51312384345928 | 1.26285832792808 | 0.954690083120191 | 1.67050143770743 | 0.999228396881742 |
| ebi-fl187-GCST90094781 | ebi-met1400-GCST90200051 | 1-palmitoyl-2-dihomo-linolenoyl-GPC (16:0/20:3n3 or 6) levels \|\| id:ebi-met1400-GCST90200051 | Herring liking \|\| id:ebi-fl187-GCST90094781 | Weighted mode | 16 | -0.035646024 | 0.129513005914392 | 0.786893682351524 | -0.289491516 | 0.218199467490715 | 0.964981813339078 | 0.748644144610178 | 1.24383514755204 | 0.999228396881742 |
| ebi-fl187-GCST90094781 | ebi-met1400-GCST90200069 | Phenylacetylglutamate levels \|\| id:ebi-met1400-GCST90200069 | Herring liking \|\| id:ebi-fl187-GCST90094781 | Weighted mode | 16 | -0.055644847 | 0.143210777542812 | 0.703066924053947 | -0.336337971 | 0.225048277184079 | 0.945875006779671 | 0.714381624893949 | 1.25238317626557 | 0.999228396881742 |
| ebi-fl187-GCST90094781 | ebi-met1400-GCST90200072 | Thioproline levels \|\| id:ebi-met1400-GCST90200072 | Herring liking \|\| id:ebi-fl187-GCST90094781 | Weighted mode | 16 | 0.124813680155806 | 0.133226347978247 | 0.363673447140429 | -0.136309962 | 0.38593732219317 | 1.13293734469105 | 0.872572126469491 | 1.47099246934344 | 0.999228396881742 |
| ebi-fl187-GCST90094781 | ebi-met1400-GCST90200081 | 1-oleoyl-2-docosahexaenoyl-GPC (18:1/22:6) levels \|\| id:ebi-met1400-GCST90200081 | Herring liking \|\| id:ebi-fl187-GCST90094781 | Weighted mode | 16 | -0.039518197 | 0.13151834923351 | 0.767937984085611 | -0.297294162 | 0.21825776702781 | 0.961252461468734 | 0.742825469218403 | 1.24390766467921 | 0.999228396881742 |
| ebi-fl187-GCST90094781 | ebi-met1400-GCST90200097 | Furaneol sulfate levels \|\| id:ebi-met1400-GCST90200097 | Herring liking \|\| id:ebi-fl187-GCST90094781 | Weighted mode | 16 | -0.037115764 | 0.169701082642392 | 0.829821879010707 | -0.369729886 | 0.295498358146182 | 0.963564582962935 | 0.690920932981037 | 1.34379588346619 | 0.999228396881742 |
| ebi-fl187-GCST90094781 | ebi-met1400-GCST90200124 | Sphingomyelin (d18:2/24:2) levels \|\| id:ebi-met1400-GCST90200124 | Herring liking \|\| id:ebi-fl187-GCST90094781 | Weighted mode | 16 | -0.113084247 | 0.121640078163398 | 0.367266256883105 | -0.3514988 | 0.125330305940022 | 0.893075417789445 | 0.703632693996702 | 1.133522801 | 0.999228396881742 |
| ebi-fl187-GCST90094781 | ebi-met1400-GCST90200137 | Lignoceroylcarnitine (C24) levels \|\| id:ebi-met1400-GCST90200137 | Herring liking \|\| id:ebi-fl187-GCST90094781 | Weighted mode | 16 | -0.011573369 | 0.150316160773289 | 0.939646306258004 | -0.306193045 | 0.283046305651724 | 0.988493344360512 | 0.736244477731032 | 1.3271666157094 | 0.999228396881742 |
| ebi-fl187-GCST90094781 | ebi-met1400-GCST90200165 | Octadecenedioate (C18:1-DC) levels \|\| id:ebi-met1400-GCST90200165 | Herring liking \|\| id:ebi-fl187-GCST90094781 | Weighted mode | 16 | 0.131579445807559 | 0.152244203753072 | 0.401051935290888 | -0.166819194 | 0.42997808516358 | 1.14062852227693 | 0.846352623518584 | 1.53722383517026 | 0.999228396881742 |
| ebi-fl187-GCST90094781 | ebi-met1400-GCST90200176 | Glutamine conjugate of C6H10O2 (1) levels \|\| id:ebi-met1400-GCST90200176 | Herring liking \|\| id:ebi-fl187-GCST90094781 | Weighted mode | 16 | 0.159139804093302 | 0.13164662354375 | 0.245434909376207 | -0.098887578 | 0.417167186239052 | 1.17250185578171 | 0.905844539106682 | 1.51765622296217 | 0.999228396881742 |
| ebi-fl187-GCST90094781 | ebi-met1400-GCST90200182 | Glycine conjugate of C10H12O2 levels \|\| id:ebi-met1400-GCST90200182 | Herring liking \|\| id:ebi-fl187-GCST90094781 | Weighted mode | 16 | -0.084693847 | 0.151718760069184 | 0.584924599268651 | -0.382062617 | 0.212674922298635 | 0.918793532341697 | 0.68245231863284 | 1.23698247045901 | 0.999228396881742 |
| ebi-fl187-GCST90094781 | ebi-met1400-GCST90200208 | Glucuronide of piperine metabolite C17H21NO3 (4) levels \|\| id:ebi-met1400-GCST90200208 | Herring liking \|\| id:ebi-fl187-GCST90094781 | Weighted mode | 16 | 0.0168261497227057 | 0.134787311869724 | 0.902311956983469 | -0.247356982 | 0.281009280987364 | 1.01696850669907 | 0.780861890488848 | 1.32446589622946 | 0.999228396881742 |
| ebi-fl187-GCST90094781 | ebi-met1400-GCST90200227 | 3-ethylcatechol sulfate (2) levels \|\| id:ebi-met1400-GCST90200227 | Herring liking \|\| id:ebi-fl187-GCST90094781 | Weighted mode | 15 | -0.059782834 | 0.146012780158718 | 0.688415450103343 | -0.345967883 | 0.226402214744285 | 0.941969074684353 | 0.707535210311831 | 1.2540799733071 | 0.999228396881742 |
| ebi-fl187-GCST90094781 | ebi-met1400-GCST90200259 | Pentose acid levels \|\| id:ebi-met1400-GCST90200259 | Herring liking \|\| id:ebi-fl187-GCST90094781 | Weighted mode | 16 | -0.137518125 | 0.153090820498369 | 0.383229987961252 | -0.437576133 | 0.162539883592467 | 0.871518553942478 | 0.645599373293955 | 1.17649524036968 | 0.999228396881742 |
| ebi-fl187-GCST90094781 | ebi-met1400-GCST90200291 | Sarcosine levels \|\| id:ebi-met1400-GCST90200291 | Herring liking \|\| id:ebi-fl187-GCST90094781 | Weighted mode | 16 | 0.0562855089305577 | 0.119431187220074 | 0.644222202287397 | -0.177799618 | 0.290370635881903 | 1.05789968042958 | 0.837110148472045 | 1.3369229078106 | 0.999228396881742 |
| ebi-fl187-GCST90094781 | ebi-met1400-GCST90200306 | Butyrate/isobutyrate (4:0) levels \|\| id:ebi-met1400-GCST90200306 | Herring liking \|\| id:ebi-fl187-GCST90094781 | Weighted mode | 16 | -0.036902706 | 0.131541857314092 | 0.782895790563989 | -0.294724746 | 0.220919334710461 | 0.963769900177513 | 0.744736550999914 | 1.24722281891638 | 0.999228396881742 |
| ebi-fl187-GCST90094781 | ebi-met1400-GCST90200321 | Cholate levels \|\| id:ebi-met1400-GCST90200321 | Herring liking \|\| id:ebi-fl187-GCST90094781 | Weighted mode | 16 | -0.013744911 | 0.13484542305728 | 0.920161671295864 | -0.27804194 | 0.250552118655811 | 0.986349119442152 | 0.757265061357086 | 1.2847345468188 | 0.999228396881742 |
| ebi-fl187-GCST90094781 | ebi-met1400-GCST90200336 | Ceramide (d18:1/16:0) levels \|\| id:ebi-met1400-GCST90200336 | Herring liking \|\| id:ebi-fl187-GCST90094781 | Weighted mode | 16 | 0.0206895635244208 | 0.170433719676162 | 0.904990568708685 | -0.313360527 | 0.354739654089698 | 1.02090507626594 | 0.730986324719906 | 1.42580940230985 | 0.999228396881742 |
| ebi-fl187-GCST90094781 | ebi-met1400-GCST90200358 | Arachidonate (20:4n6) levels \|\| id:ebi-met1400-GCST90200358 | Herring liking \|\| id:ebi-fl187-GCST90094781 | Weighted mode | 16 | -0.048505732 | 0.126344417156184 | 0.706427478301095 | -0.296140789 | 0.199129326123142 | 0.952651879163104 | 0.743682718207127 | 1.22033977750741 | 0.999228396881742 |
| ebi-fl187-GCST90094781 | ebi-met1400-GCST90200413 | 2'-deoxyuridine levels \|\| id:ebi-met1400-GCST90200413 | Herring liking \|\| id:ebi-fl187-GCST90094781 | Weighted mode | 16 | -0.017449632 | 0.109888369961652 | 0.875949268931771 | -0.232830838 | 0.197931572633551 | 0.982701730656574 | 0.792287587445944 | 1.21887898628894 | 0.999228396881742 |
| ebi-fl187-GCST90094781 | ebi-met1400-GCST90200432 | Threonine levels \|\| id:ebi-met1400-GCST90200432 | Herring liking \|\| id:ebi-fl187-GCST90094781 | Weighted mode | 16 | -0.167443963 | 0.135362507684206 | 0.235102622314086 | -0.432754478 | 0.097866552500926 | 0.845824013773166 | 0.648719747469359 | 1.10281560730374 | 0.999228396881742 |
| ebi-fl187-GCST90094781 | ebi-met1400-GCST90200436 | Caffeine levels \|\| id:ebi-met1400-GCST90200436 | Herring liking \|\| id:ebi-fl187-GCST90094781 | Weighted mode | 16 | 0.0311125985344214 | 0.127710366977316 | 0.810826776018803 | -0.219199721 | 0.281424917809961 | 1.03160165418159 | 0.803161294165252 | 1.32501650744543 | 0.999228396881742 |
| ebi-fl187-GCST90094781 | ebi-met1400-GCST90200442 | Valine levels \|\| id:ebi-met1400-GCST90200442 | Herring liking \|\| id:ebi-fl187-GCST90094781 | Weighted mode | 16 | 0.0640862152549689 | 0.126112803129879 | 0.618728112221916 | -0.183094879 | 0.311267309389532 | 1.06618431615177 | 0.832689147375684 | 1.3651540909241 | 0.999228396881742 |
| ebi-fl187-GCST90094781 | ebi-met1400-GCST90200459 | Margarate (17:0) levels \|\| id:ebi-met1400-GCST90200459 | Herring liking \|\| id:ebi-fl187-GCST90094781 | Weighted mode | 16 | -0.088091968 | 0.128367924984912 | 0.503028545701899 | -0.339693101 | 0.163509165412711 | 0.915676660304689 | 0.711988798222035 | 1.17763614865928 | 0.999228396881742 |
| ebi-fl187-GCST90094781 | ebi-met1400-GCST90200462 | X-11299 levels \|\| id:ebi-met1400-GCST90200462 | Herring liking \|\| id:ebi-fl187-GCST90094781 | Weighted mode | 16 | 0.0462056674524681 | 0.132908634916739 | 0.732933375999282 | -0.214295257 | 0.306706591889277 | 1.04728978223025 | 0.807110044960723 | 1.35894218491268 | 0.999228396881742 |
| ebi-fl187-GCST90094781 | ebi-met1400-GCST90200497 | X-12740 levels \|\| id:ebi-met1400-GCST90200497 | Herring liking \|\| id:ebi-fl187-GCST90094781 | Weighted mode | 16 | 0.217178443908026 | 0.169423975820758 | 0.219350129099306 | -0.114892549 | 0.549249436516712 | 1.24256581065514 | 0.891461927503125 | 1.73195258953294 | 0.999228396881742 |
| ebi-fl187-GCST90094781 | ebi-met1400-GCST90200527 | X-13866 levels \|\| id:ebi-met1400-GCST90200527 | Herring liking \|\| id:ebi-fl187-GCST90094781 | Weighted mode | 16 | -0.06769818 | 0.136530377175282 | 0.627188432125084 | -0.335297719 | 0.199901359152363 | 0.934542494527094 | 0.715125148044266 | 1.22128228389874 | 0.999228396881742 |
| ebi-fl187-GCST90094781 | ebi-met1400-GCST90200547 | X-17654 levels \|\| id:ebi-met1400-GCST90200547 | Herring liking \|\| id:ebi-fl187-GCST90094781 | Weighted mode | 16 | 0.0252751418155015 | 0.138978056000088 | 0.858124117482932 | -0.247121848 | 0.297672131575675 | 1.02559726640144 | 0.781045518941902 | 1.34672016846734 | 0.999228396881742 |
| ebi-fl187-GCST90094781 | ebi-met1400-GCST90200553 | X-17653 levels \|\| id:ebi-met1400-GCST90200553 | Herring liking \|\| id:ebi-fl187-GCST90094781 | Weighted mode | 16 | 0.05613625708603 | 0.121824165406429 | 0.651554191048069 | -0.182639107 | 0.29491162128263 | 1.05774179873331 | 0.833068750080958 | 1.34300766014672 | 0.999228396881742 |
| ebi-fl187-GCST90094781 | ebi-met1400-GCST90200557 | X-17685 levels \|\| id:ebi-met1400-GCST90200557 | Herring liking \|\| id:ebi-fl187-GCST90094781 | Weighted mode | 16 | -0.085444101 | 0.162141016130912 | 0.605918083493519 | -0.403240492 | 0.232352290777833 | 0.918104462889189 | 0.668151394630767 | 1.26156408794575 | 0.999228396881742 |
| ebi-fl187-GCST90094781 | ebi-met1400-GCST90200561 | X-18779 levels \|\| id:ebi-met1400-GCST90200561 | Herring liking \|\| id:ebi-fl187-GCST90094781 | Weighted mode | 16 | -0.043648213 | 0.146906451076046 | 0.770453088429485 | -0.331584857 | 0.244288431597641 | 0.957290661123036 | 0.717785244802313 | 1.27671252162026 | 0.999228396881742 |
| ebi-fl187-GCST90094781 | ebi-met1400-GCST90200570 | X-18921 levels \|\| id:ebi-met1400-GCST90200570 | Herring liking \|\| id:ebi-fl187-GCST90094781 | Weighted mode | 16 | -0.13716006 | 0.151641630449426 | 0.380027756250301 | -0.434377656 | 0.16005753557592 | 0.871830669655207 | 0.647667613871425 | 1.17357839155802 | 0.999228396881742 |
| ebi-fl187-GCST90094781 | ebi-met1400-GCST90200575 | X-21283 levels \|\| id:ebi-met1400-GCST90200575 | Herring liking \|\| id:ebi-fl187-GCST90094781 | Weighted mode | 16 | -0.036798148 | 0.134712762371954 | 0.788455211216643 | -0.300835162 | 0.227238866470466 | 0.963870675151203 | 0.740199775721275 | 1.25512964052325 | 0.999228396881742 |
| ebi-fl187-GCST90094781 | ebi-met1400-GCST90200578 | X-18922 levels \|\| id:ebi-met1400-GCST90200578 | Herring liking \|\| id:ebi-fl187-GCST90094781 | Weighted mode | 16 | -0.019866658 | 0.162010298873431 | 0.904031223600668 | -0.337406844 | 0.297673527481633 | 0.98032938336846 | 0.713618449377527 | 1.34672204836336 | 0.999228396881742 |
| ebi-fl187-GCST90094781 | ebi-met1400-GCST90200623 | X-24241 levels \|\| id:ebi-met1400-GCST90200623 | Herring liking \|\| id:ebi-fl187-GCST90094781 | Weighted mode | 16 | -0.010362545 | 0.123261011431271 | 0.934112576019173 | -0.251954128 | 0.23122903722821 | 0.989690961014594 | 0.777280392981954 | 1.26014782716477 | 0.999228396881742 |
| ebi-fl187-GCST90094781 | ebi-met1400-GCST90200636 | X-24544 levels \|\| id:ebi-met1400-GCST90200636 | Herring liking \|\| id:ebi-fl187-GCST90094781 | Weighted mode | 16 | -0.100142827 | 0.14083999983671 | 0.487969807000982 | -0.376189227 | 0.175903572227916 | 0.904708191641793 | 0.686472421543782 | 1.19232308004898 | 0.999228396881742 |
| ebi-fl187-GCST90094781 | ebi-met1400-GCST90200659 | X-25172 levels \|\| id:ebi-met1400-GCST90200659 | Herring liking \|\| id:ebi-fl187-GCST90094781 | Weighted mode | 16 | 0.00201665417543784 | 0.131546713739281 | 0.987970697799186 | -0.255814905 | 0.259848213104429 | 1.00201868899008 | 0.774285272052714 | 1.29673324461354 | 0.999228396881742 |
| ebi-fl187-GCST90094781 | ebi-met1400-GCST90200670 | X-26111 levels \|\| id:ebi-met1400-GCST90200670 | Herring liking \|\| id:ebi-fl187-GCST90094781 | Weighted mode | 16 | 0.0467660338169066 | 0.128357363519459 | 0.720687357763612 | -0.204814399 | 0.298346466315046 | 1.04787681265873 | 0.814798530059963 | 1.34762861492522 | 0.999228396881742 |
| ebi-fl187-GCST90094781 | ebi-met1400-GCST90200673 | Carnitine C4 levels \|\| id:ebi-met1400-GCST90200673 | Herring liking \|\| id:ebi-fl187-GCST90094781 | Weighted mode | 16 | 0.121864947082461 | 0.17807514339441 | 0.504193801843034 | -0.227162334 | 0.470892228135506 | 1.12960153549526 | 0.796791425475097 | 1.60142239009717 | 0.999228396881742 |
| ebi-fl187-GCST90094781 | ebi-met1400-GCST90200710 | X-13431 levels \|\| id:ebi-met1400-GCST90200710 | Herring liking \|\| id:ebi-fl187-GCST90094781 | Weighted mode | 16 | 0.123432160655518 | 0.117629204247236 | 0.310638556694082 | -0.10712108 | 0.353985400980101 | 1.13137325031896 | 0.898416886385229 | 1.4247343866024 | 0.999228396881742 |
| ebi-fl187-GCST90094781 | ebi-met1400-GCST90200730 | Adenosine 5'-diphosphate (ADP) to fructose ratio \|\| id:ebi-met1400-GCST90200730 | Herring liking \|\| id:ebi-fl187-GCST90094781 | Weighted mode | 16 | 0.10678212062665 | 0.209042905311944 | 0.616916329445688 | -0.302941974 | 0.516506215038059 | 1.11269179547575 | 0.738641955724096 | 1.67616126070897 | 0.999228396881742 |
| ebi-fl187-GCST90094781 | ebi-met1400-GCST90200731 | Adenosine 5'-diphosphate (ADP) to mannose ratio \|\| id:ebi-met1400-GCST90200731 | Herring liking \|\| id:ebi-fl187-GCST90094781 | Weighted mode | 16 | -0.138951462 | 0.192305716207279 | 0.481067911488408 | -0.515870666 | 0.237967741677384 | 0.870270268534064 | 0.596980597656721 | 1.26866826705457 | 0.999228396881742 |
| ebi-fl187-GCST90094781 | ebi-met1400-GCST90200748 | Aspartate to glutamate ratio \|\| id:ebi-met1400-GCST90200748 | Herring liking \|\| id:ebi-fl187-GCST90094781 | Weighted mode | 16 | -0.129302025 | 0.144501545799394 | 0.385020243550318 | -0.412525054 | 0.153921005169188 | 0.878708533872782 | 0.661976611079802 | 1.16639874366766 | 0.999228396881742 |
| ebi-fl187-GCST90094781 | ebi-met1400-GCST90200774 | Uridine to pseudouridine ratio \|\| id:ebi-met1400-GCST90200774 | Herring liking \|\| id:ebi-fl187-GCST90094781 | Weighted mode | 16 | 0.156452327628145 | 0.120986038625936 | 0.215523352031338 | -0.080680308 | 0.393584963334981 | 1.16935501506314 | 0.922488556448114 | 1.48228521827765 | 0.999228396881742 |
| ebi-fl187-GCST90094781 | ebi-met1400-GCST90200799 | Spermidine to adenosine 5'-diphosphate (ADP) ratio \|\| id:ebi-met1400-GCST90200799 | Herring liking \|\| id:ebi-fl187-GCST90094781 | Weighted mode | 16 | 0.0847193335103661 | 0.23986283196987 | 0.728853821335201 | -0.385411817 | 0.55485048417131 | 1.0884115431782 | 0.680170472654514 | 1.74168055649377 | 0.999228396881742 |
| ebi-fl187-GCST90094781 | ebi-met1400-GCST90200811 | Choline to taurocholate ratio \|\| id:ebi-met1400-GCST90200811 | Herring liking \|\| id:ebi-fl187-GCST90094781 | Weighted mode | 16 | -0.049721213 | 0.155595076345307 | 0.753712898947846 | -0.354687563 | 0.255245136453708 | 0.951494651693324 | 0.70139255008872 | 1.29077799883458 | 0.999228396881742 |
| ebi-fl187-GCST90094781 | ebi-met1400-GCST90200812 | Glutamate to kynurenine ratio \|\| id:ebi-met1400-GCST90200812 | Herring liking \|\| id:ebi-fl187-GCST90094781 | Weighted mode | 16 | 0.0524788246727506 | 0.114820267926356 | 0.654183058895507 | -0.1725689 | 0.277526549808409 | 1.05388024558318 | 0.841500307053618 | 1.31986116073953 | 0.999228396881742 |
| ebi-fl187-GCST90094781 | ebi-met1400-GCST90200876 | Cortisone to 4-cholesten-3-one ratio \|\| id:ebi-met1400-GCST90200876 | Herring liking \|\| id:ebi-fl187-GCST90094781 | Weighted mode | 16 | 0.0202200968406097 | 0.158141769549856 | 0.899957750521215 | -0.289737771 | 0.330177965158326 | 1.02042590783091 | 0.748459809357597 | 1.39121569435539 | 0.999228396881742 |
| ebi-fl187-GCST90094781 | ebi-met1400-GCST90200906 | 2'-deoxyuridine to cytidine ratio \|\| id:ebi-met1400-GCST90200906 | Herring liking \|\| id:ebi-fl187-GCST90094781 | Weighted mode | 16 | -0.172673651 | 0.124331956002953 | 0.185167456576076 | -0.416364284 | 0.0710169831095692 | 0.841412164350347 | 0.659440003003854 | 1.07359945877077 | 0.999228396881742 |
| ebi-fl187-GCST90094781 | ebi-met1400-GCST90200907 | Retinol (Vitamin A) to linoleoyl-arachidonoyl-glycerol (18:2 to 20:4) [1] ratio \|\| id:ebi-met1400-GCST90200907 | Herring liking \|\| id:ebi-fl187-GCST90094781 | Weighted mode | 16 | -0.095916101 | 0.151479519324287 | 0.536141489216447 | -0.392815958 | 0.200983757339795 | 0.908540238910376 | 0.675152992381938 | 1.22260491330588 | 0.999228396881742 |
| ebi-fl187-GCST90094781 | ebi-met1400-GCST90200916 | Fructose to sucrose ratio \|\| id:ebi-met1400-GCST90200916 | Herring liking \|\| id:ebi-fl187-GCST90094781 | Weighted mode | 16 | -0.003901391 | 0.149184743952316 | 0.979481318071736 | -0.29630349 | 0.288500706786335 | 0.99610620917963 | 0.743561730590662 | 1.33442529267613 | 0.999228396881742 |
| ebi-fl187-GCST90094781 | ebi-met1400-GCST90200917 | Alpha-ketobutyrate to 3-methyl-2-oxobutyrate ratio \|\| id:ebi-met1400-GCST90200917 | Herring liking \|\| id:ebi-fl187-GCST90094781 | Weighted mode | 16 | 0.0292499516740291 | 0.110702400872924 | 0.795205046318576 | -0.187726754 | 0.246226657384961 | 1.02968193303557 | 0.828841153792791 | 1.27918947842801 | 0.999228396881742 |
| ebi-fl187-GCST90094781 | ebi-met1400-GCST90200924 | Alpha-tocopherol to glycerol ratio \|\| id:ebi-met1400-GCST90200924 | Herring liking \|\| id:ebi-fl187-GCST90094781 | Weighted mode | 16 | 0.020664246017395 | 0.121917831673407 | 0.867673369299876 | -0.218294704 | 0.259623196097273 | 1.02087922982168 | 0.803888497548067 | 1.29644149040582 | 0.999228396881742 |
| ebi-fl187-GCST90094781 | ebi-met1400-GCST90200938 | Alpha-ketoglutarate to alanine ratio \|\| id:ebi-met1400-GCST90200938 | Herring liking \|\| id:ebi-fl187-GCST90094781 | Weighted mode | 16 | -0.007337087 | 0.123947703283489 | 0.953578209767429 | -0.250274585 | 0.235600411429967 | 0.992689763708387 | 0.778586965071693 | 1.26566846245708 | 0.999228396881742 |
| ebi-fl187-GCST90094781 | ebi-met1400-GCST90200943 | Phosphate to EDTA ratio \|\| id:ebi-met1400-GCST90200943 | Herring liking \|\| id:ebi-fl187-GCST90094781 | Weighted mode | 16 | -0.082494936 | 0.140046734295539 | 0.564592345899179 | -0.356986536 | 0.19199666279393 | 0.920816100466084 | 0.699781919783661 | 1.21166647337744 | 0.999228396881742 |
| ebi-fl187-GCST90094781 | ebi-met1400-GCST90200947 | Adenosine 5'-diphosphate (ADP) to glucose ratio \|\| id:ebi-met1400-GCST90200947 | Herring liking \|\| id:ebi-fl187-GCST90094781 | Weighted mode | 16 | 0.052227289858689 | 0.215466381688095 | 0.811759512160958 | -0.370086818 | 0.474541397967356 | 1.05361519134814 | 0.690674364894667 | 1.60727692797586 | 0.999228396881742 |
| ebi-fl187-GCST90094781 | ebi-met1400-GCST90200975 | Arachidonate (20:4n6) to caffeine ratio \|\| id:ebi-met1400-GCST90200975 | Herring liking \|\| id:ebi-fl187-GCST90094781 | Weighted mode | 16 | -0.034855286 | 0.146370350302498 | 0.81500199496976 | -0.321741173 | 0.252030600171124 | 0.96574516258386 | 0.724885786054152 | 1.28663540794612 | 0.999228396881742 |
| ebi-fl187-GCST90094781 | ebi-met1400-GCST90200985 | Cholesterol to oleoyl-linoleoyl-glycerol (18:1 to 18:2) [2] ratio \|\| id:ebi-met1400-GCST90200985 | Herring liking \|\| id:ebi-fl187-GCST90094781 | Weighted mode | 16 | -0.103973733 | 0.135967127997507 | 0.456314378896955 | -0.370469304 | 0.162521837746499 | 0.901248970101083 | 0.690410242304398 | 1.17647400970936 | 0.999228396881742 |
| ebi-fl187-GCST90094781 | ebi-met1400-GCST90201016 | Bilirubin (Z,Z) to etiocholanolone glucuronide ratio \|\| id:ebi-met1400-GCST90201016 | Herring liking \|\| id:ebi-fl187-GCST90094781 | Weighted mode | 16 | 0.137176466972945 | 0.128490848296705 | 0.302583795301556 | -0.114665596 | 0.389018529634487 | 1.14703054366976 | 0.891664270433012 | 1.47553189214638 | 0.999228396881742 |
| ebi-fl187-GCST90094781 | ebi-pw11-GCST90275189 | Placental weight (fetal genotype, adjusted for sex and gestational age) \|\| id:ebi-pw11-GCST90275189 | Herring liking \|\| id:ebi-fl187-GCST90094781 | Weighted mode | 15 | 0.0421709603889385 | 0.051996260467366 | 0.430916388095228 | -0.05974171 | 0.144083630904976 | 1.04307278763792 | 0.942007813242915 | 1.15498069656687 | 0.999228396881742 |
| ebi-fl187-GCST90094781 | ebi-pw11-GCST90275192 | Placental weight (fetal effect, conditional on maternal and paternal genotype) \|\| id:ebi-pw11-GCST90275192 | Herring liking \|\| id:ebi-fl187-GCST90094781 | Weighted mode | 14 | 0.0505736320804209 | 0.10825855889498 | 0.648121555538053 | -0.161613143 | 0.262760407514582 | 1.05187431219756 | 0.850770267018362 | 1.30051508797874 | 0.999228396881742 |
| ebi-fl187-GCST90094781 | ebi-pw11-GCST90275193 | Placental weight (maternal effect, conditional on fetal and paternal genotype) \|\| id:ebi-pw11-GCST90275193 | Herring liking \|\| id:ebi-fl187-GCST90094781 | Weighted mode | 14 | -0.048338054 | 0.0765426830986152 | 0.53864869800104 | -0.198361713 | 0.101685604583935 | 0.952811630568721 | 0.820073168224375 | 1.10703537016423 | 0.999228396881742 |
| ebi-fl187-GCST90094781 | ebi-pw11-GCST90275195 | Placental weight (fetal effect, conditional on maternal genotype) \|\| id:ebi-pw11-GCST90275195 | Herring liking \|\| id:ebi-fl187-GCST90094781 | Weighted mode | 14 | 0.0460365672240262 | 0.0658272757677031 | 0.4966542336549 | -0.082984893 | 0.175058027728724 | 1.04711270026156 | 0.920365050811608 | 1.19131534393029 | 0.999228396881742 |
| ebi-fl187-GCST90094781 | ebi-pw11-GCST90275197 | Placental weight (fetal genotype, adjusted for sex) \|\| id:ebi-pw11-GCST90275197 | Herring liking \|\| id:ebi-fl187-GCST90094781 | Weighted mode | 15 | 0.0694186408240863 | 0.0503483575651633 | 0.189599120555089 | -0.02926414 | 0.168101421651806 | 1.07188484998903 | 0.971159908403002 | 1.1830565921171 | 0.999228396881742 |
| ebi-fl187-GCST90094781 | prot-a-1030 | Protein FAM171B \|\| id:prot-a-1030 | Herring liking \|\| id:ebi-fl187-GCST90094781 | Weighted mode | 19 | 0.0700683674235036 | 0.186168550835363 | 0.711041063541722 | -0.294821992 | 0.434958727060814 | 1.07258150838182 | 0.744664131682146 | 1.54489929510083 | 0.999228396881742 |
| ebi-fl187-GCST90094781 | prot-a-1036 | BRISC complex subunit Abro1 \|\| id:prot-a-1036 | Herring liking \|\| id:ebi-fl187-GCST90094781 | Weighted mode | 19 | 0.0938906411766503 | 0.191292990108265 | 0.629483191148971 | -0.281043619 | 0.468824901788849 | 1.09843961528477 | 0.75499540228866 | 1.59811514715112 | 0.999228396881742 |
| ebi-fl187-GCST90094781 | prot-a-1055 | Phenylalanine--tRNA ligase, mitochondrial \|\| id:prot-a-1055 | Herring liking \|\| id:ebi-fl187-GCST90094781 | Weighted mode | 19 | -0.31478148 | 0.170212207548893 | 0.0808988365962085 | -0.648397407 | 0.0188344469674417 | 0.72994836528447 | 0.522883074609498 | 1.01901293397075 | 0.999228396881742 |
| ebi-fl187-GCST90094781 | prot-a-1083 | Fc receptor-like protein 5 \|\| id:prot-a-1083 | Herring liking \|\| id:ebi-fl187-GCST90094781 | Weighted mode | 19 | -0.009013085 | 0.191057206089472 | 0.962893387244613 | -0.383485209 | 0.365459039286061 | 0.991027411441919 | 0.68148215805985 | 1.44117541246475 | 0.999228396881742 |
| ebi-fl187-GCST90094781 | prot-a-1103 | Fibroblast growth factor receptor 3 \|\| id:prot-a-1103 | Herring liking \|\| id:ebi-fl187-GCST90094781 | Weighted mode | 19 | -0.220679632 | 0.261303859680243 | 0.409456792647041 | -0.732835197 | 0.291475932911092 | 0.801973565756508 | 0.480544618195653 | 1.33840142167683 | 0.999228396881742 |
| ebi-fl187-GCST90094781 | prot-a-1105 | Fibroblast growth factor receptor 4 \|\| id:prot-a-1105 | Herring liking \|\| id:ebi-fl187-GCST90094781 | Weighted mode | 19 | 0.261652992933307 | 0.234804447346723 | 0.27979014378466 | -0.198563724 | 0.721869709732885 | 1.29907567576729 | 0.81990752139841 | 2.05827799761114 | 0.999228396881742 |
| ebi-fl187-GCST90094781 | prot-a-1120 | Filamin-A \|\| id:prot-a-1120 | Herring liking \|\| id:ebi-fl187-GCST90094781 | Weighted mode | 19 | -0.139840213 | 0.201012932659173 | 0.495515373283562 | -0.533825561 | 0.254145135026546 | 0.869497158654601 | 0.586357527068795 | 1.28935892183017 | 0.999228396881742 |
| ebi-fl187-GCST90094781 | prot-a-1166 | Galanin peptides \|\| id:prot-a-1166 | Herring liking \|\| id:ebi-fl187-GCST90094781 | Weighted mode | 19 | 0.239672056728892 | 0.211603280292994 | 0.272224791830188 | -0.175070373 | 0.654414486103159 | 1.27083232106856 | 0.839397948036553 | 1.9240156496096 | 0.999228396881742 |
| ebi-fl187-GCST90094781 | prot-a-1205 | ADP-ribosylation factor-binding protein GGA1 \|\| id:prot-a-1205 | Herring liking \|\| id:ebi-fl187-GCST90094781 | Weighted mode | 19 | -0.096166245 | 0.223953152842753 | 0.672725574585934 | -0.535114425 | 0.342781934268988 | 0.908313000746406 | 0.585602278843662 | 1.40886150401269 | 0.999228396881742 |
| ebi-fl187-GCST90094781 | prot-a-1227 | Guanine nucleotide-binding protein G(I)/G(S)/G(O) subunit gamma-T2 \|\| id:prot-a-1227 | Herring liking \|\| id:ebi-fl187-GCST90094781 | Weighted mode | 19 | 0.0478196392804393 | 0.22455749305258 | 0.833758705062094 | -0.392313047 | 0.487952325663496 | 1.04898144321386 | 0.67549261985106 | 1.62897718771474 | 0.999228396881742 |
| ebi-fl187-GCST90094781 | prot-a-1240 | Golgi SNAP receptor complex member 1 \|\| id:prot-a-1240 | Herring liking \|\| id:ebi-fl187-GCST90094781 | Weighted mode | 19 | -0.225474805 | 0.227876734010664 | 0.335553601146995 | -0.672113203 | 0.221163594123725 | 0.798137169629945 | 0.510628375329105 | 1.24752750203968 | 0.999228396881742 |
| ebi-fl187-GCST90094781 | prot-a-1283 | Glutathione S-transferase A1 \|\| id:prot-a-1283 | Herring liking \|\| id:ebi-fl187-GCST90094781 | Weighted mode | 19 | -0.075441762 | 0.239931112858288 | 0.756809108164166 | -0.545706744 | 0.394823218742203 | 0.927333734596875 | 0.579432128611835 | 1.48412180280992 | 0.999228396881742 |
| ebi-fl187-GCST90094781 | prot-a-1324 | E3 ubiquitin-protein ligase HECW1 \|\| id:prot-a-1324 | Herring liking \|\| id:ebi-fl187-GCST90094781 | Weighted mode | 19 | 0.11388350987297 | 0.186271419181122 | 0.54858927228268 | -0.251208472 | 0.478975491467969 | 1.12062157593074 | 0.777860192800811 | 1.61441956802006 | 0.999228396881742 |
| ebi-fl187-GCST90094781 | prot-a-1339 | Histidine triad nucleotide-binding protein 2, mitochondrial \|\| id:prot-a-1339 | Herring liking \|\| id:ebi-fl187-GCST90094781 | Weighted mode | 19 | 0.271328257307361 | 0.204615302406868 | 0.201408157594327 | -0.129717735 | 0.672374250024823 | 1.31170557666295 | 0.878343321150934 | 1.95888268108437 | 0.999228396881742 |
| ebi-fl187-GCST90094781 | prot-a-1466 | Interleukin-11 \|\| id:prot-a-1466 | Herring liking \|\| id:ebi-fl187-GCST90094781 | Weighted mode | 19 | 0.0575023712922915 | 0.176387512728343 | 0.748183685416747 | -0.288217154 | 0.403221896239843 | 1.05918778229558 | 0.749598796445204 | 1.49663895337675 | 0.999228396881742 |
| ebi-fl187-GCST90094781 | prot-a-147 | Rho GTPase-activating protein 1 \|\| id:prot-a-147 | Herring liking \|\| id:ebi-fl187-GCST90094781 | Weighted mode | 19 | -0.123996237 | 0.176034514168349 | 0.490206287682167 | -0.469023885 | 0.221031410826675 | 0.883383165097443 | 0.625612640399335 | 1.24736261063949 | 0.999228396881742 |
| ebi-fl187-GCST90094781 | prot-a-1473 | Interleukin-12 receptor subunit beta-1 \|\| id:prot-a-1473 | Herring liking \|\| id:ebi-fl187-GCST90094781 | Weighted mode | 19 | -0.05121027 | 0.190077124068933 | 0.790671914213256 | -0.423761433 | 0.321340892971343 | 0.950078876248868 | 0.654580024227581 | 1.37897558386304 | 0.999228396881742 |
| ebi-fl187-GCST90094781 | prot-a-1501 | X-linked interleukin-1 receptor accessory protein-like 2 \|\| id:prot-a-1501 | Herring liking \|\| id:ebi-fl187-GCST90094781 | Weighted mode | 19 | -0.190904142 | 0.201016517228105 | 0.354845862489975 | -0.584896516 | 0.203088231917431 | 0.826211783488732 | 0.557163516551824 | 1.22518056350902 | 0.999228396881742 |
| ebi-fl187-GCST90094781 | prot-a-1503 | Interleukin-1 receptor-like 2 \|\| id:prot-a-1503 | Herring liking \|\| id:ebi-fl187-GCST90094781 | Weighted mode | 19 | -0.018508883 | 0.168969363007936 | 0.91398628014122 | -0.349688835 | 0.312671068006894 | 0.98166135397555 | 0.704907398118056 | 1.36707178341703 | 0.999228396881742 |
| ebi-fl187-GCST90094781 | prot-a-1536 | Interleukin-5 receptor subunit alpha \|\| id:prot-a-1536 | Herring liking \|\| id:ebi-fl187-GCST90094781 | Weighted mode | 19 | -0.113444522 | 0.209009215033153 | 0.593943954809119 | -0.523102584 | 0.296213539045089 | 0.892753722853546 | 0.592678856577203 | 1.34475728436087 | 0.999228396881742 |
| ebi-fl187-GCST90094781 | prot-a-1559 | Insulin-induced gene 1 protein \|\| id:prot-a-1559 | Herring liking \|\| id:ebi-fl187-GCST90094781 | Weighted mode | 19 | -0.015127804 | 0.176281684982521 | 0.932559983429029 | -0.360639906 | 0.330384298778758 | 0.98498604661155 | 0.697230021369668 | 1.39150277854295 | 0.999228396881742 |
| ebi-fl187-GCST90094781 | prot-a-1567 | Interferon regulatory factor 1 \|\| id:prot-a-1567 | Herring liking \|\| id:ebi-fl187-GCST90094781 | Weighted mode | 19 | 0.16690312565654 | 0.177649113520408 | 0.359906539733172 | -0.181289137 | 0.51509538815654 | 1.18163978918477 | 0.834194127566189 | 1.67379815470331 | 0.999228396881742 |
| ebi-fl187-GCST90094781 | prot-a-1613 | Potassium voltage-gated channel subfamily E regulatory beta subunit 5 \|\| id:prot-a-1613 | Herring liking \|\| id:ebi-fl187-GCST90094781 | Weighted mode | 19 | -0.015533266 | 0.179387564516033 | 0.93195296200893 | -0.367132892 | 0.336066360819746 | 0.984586753306663 | 0.692717582244812 | 1.39943188917696 | 0.999228396881742 |
| ebi-fl187-GCST90094781 | prot-a-1649 | Kin of IRRE-like protein 2 \|\| id:prot-a-1649 | Herring liking \|\| id:ebi-fl187-GCST90094781 | Weighted mode | 19 | 0.0753646280805871 | 0.175825119367084 | 0.673275213489054 | -0.269252606 | 0.419981862040073 | 1.07827724937668 | 0.763950252947336 | 1.52193395059128 | 0.999228396881742 |
| ebi-fl187-GCST90094781 | prot-a-165 | Armadillo repeat-containing protein 5 \|\| id:prot-a-165 | Herring liking \|\| id:ebi-fl187-GCST90094781 | Weighted mode | 19 | 0.000891622756737487 | 0.209571289042043 | 0.996652201874711 | -0.409868104 | 0.411651349279142 | 1.00089202037047 | 0.663737788878115 | 1.5093081232192 | 0.999228396881742 |
| ebi-fl187-GCST90094781 | prot-a-1656 | Kallikrein-11 \|\| id:prot-a-1656 | Herring liking \|\| id:ebi-fl187-GCST90094781 | Weighted mode | 19 | 0.00142057166301712 | 0.22787552541567 | 0.995094607303337 | -0.445215458 | 0.44805660147773 | 1.0014215811529 | 0.640686220091416 | 1.56526728958786 | 0.999228396881742 |
| ebi-fl187-GCST90094781 | prot-a-1697 | Laminin subunit gamma-2 \|\| id:prot-a-1697 | Herring liking \|\| id:ebi-fl187-GCST90094781 | Weighted mode | 19 | -0.264367836 | 0.186939746583156 | 0.174371052238092 | -0.630769739 | 0.102034067351405 | 0.76769110332726 | 0.532182001930454 | 1.10742119799242 | 0.999228396881742 |
| ebi-fl187-GCST90094781 | prot-a-1698 | Lysosome-associated membrane glycoprotein 3 \|\| id:prot-a-1698 | Herring liking \|\| id:ebi-fl187-GCST90094781 | Weighted mode | 19 | 0.00103760766334737 | 0.19499978131101 | 0.995812949797047 | -0.381161964 | 0.383237179032928 | 1.00103814616441 | 0.683067248557041 | 1.46702593660162 | 0.999228396881742 |
| ebi-fl187-GCST90094781 | prot-a-1724 | Leptin receptor \|\| id:prot-a-1724 | Herring liking \|\| id:ebi-fl187-GCST90094781 | Weighted mode | 19 | 0.258119216895913 | 0.258603623092598 | 0.331447650426061 | -0.248743884 | 0.764982318157405 | 1.29449313487882 | 0.779779661574709 | 2.14895637681089 | 0.999228396881742 |
| ebi-fl187-GCST90094781 | prot-a-1743 | Leukocyte immunoglobulin-like receptor subfamily B member 2 \|\| id:prot-a-1743 | Herring liking \|\| id:ebi-fl187-GCST90094781 | Weighted mode | 19 | -0.115620889 | 0.18177235893703 | 0.532732623587623 | -0.471894713 | 0.240652934483018 | 0.890812876219885 | 0.623819189791882 | 1.2720794637688 | 0.999228396881742 |
| ebi-fl187-GCST90094781 | prot-a-1746 | Leukocyte immunoglobulin-like receptor subfamily B member 4 \|\| id:prot-a-1746 | Herring liking \|\| id:ebi-fl187-GCST90094781 | Weighted mode | 19 | -0.069181885 | 0.187125278111669 | 0.715913201666645 | -0.43594743 | 0.297583660299299 | 0.933156937579318 | 0.646651719588477 | 1.34660102768546 | 0.999228396881742 |
| ebi-fl187-GCST90094781 | prot-a-1750 | Leucine-rich repeat and immunoglobulin-like domain-containing nogo receptor-interacting protein 3 \|\| id:prot-a-1750 | Herring liking \|\| id:ebi-fl187-GCST90094781 | Weighted mode | 19 | -0.161210468 | 0.169318458558459 | 0.353650404133408 | -0.493074647 | 0.170653710982181 | 0.851112920397647 | 0.610745677296164 | 1.18607995143703 | 0.999228396881742 |
| ebi-fl187-GCST90094781 | prot-a-1753 | Lipase member N \|\| id:prot-a-1753 | Herring liking \|\| id:ebi-fl187-GCST90094781 | Weighted mode | 19 | 0.0978801648372844 | 0.201757847994515 | 0.63343107405374 | -0.297565217 | 0.493325546906535 | 1.1028306193029 | 0.742624149750013 | 1.6377536002316 | 0.999228396881742 |
| ebi-fl187-GCST90094781 | prot-a-1760 | Ig Kappa chain V-I region HK102- like \|\| id:prot-a-1760 | Herring liking \|\| id:ebi-fl187-GCST90094781 | Weighted mode | 19 | -0.246427351 | 0.194714382050031 | 0.221799348069476 | -0.628067539 | 0.135212838165604 | 0.781588141342988 | 0.533622008737957 | 1.14478041138661 | 0.999228396881742 |
| ebi-fl187-GCST90094781 | prot-a-1792 | Leucine-rich repeat neuronal protein 1 \|\| id:prot-a-1792 | Herring liking \|\| id:ebi-fl187-GCST90094781 | Weighted mode | 19 | 0.00620538468089871 | 0.199243764170025 | 0.975496893269328 | -0.384312393 | 0.396723162454148 | 1.00622467796719 | 0.680918679658872 | 1.48694423107532 | 0.999228396881742 |
| ebi-fl187-GCST90094781 | prot-a-1795 | Leucine-rich repeat transmembrane neuronal protein 1 \|\| id:prot-a-1795 | Herring liking \|\| id:ebi-fl187-GCST90094781 | Weighted mode | 19 | -0.007520979 | 0.171231436654252 | 0.965449463254106 | -0.343134595 | 0.328092636623724 | 0.992507232574518 | 0.70954270418943 | 1.38831757538548 | 0.999228396881742 |
| ebi-fl187-GCST90094781 | prot-a-1820 | LysM and putative peptidoglycan-binding domain-containing protein 4 \|\| id:prot-a-1820 | Herring liking \|\| id:ebi-fl187-GCST90094781 | Weighted mode | 19 | -0.064783972 | 0.1887466211833 | 0.735399782034182 | -0.43472735 | 0.305159405399257 | 0.937269917942051 | 0.647441168083434 | 1.35684127359336 | 0.999228396881742 |
| ebi-fl187-GCST90094781 | prot-a-1844 | Mitogen-activated protein kinase kinase kinase 7:TGF-beta-activated kinase 1 and MAP3K7-binding protein 1 fusion \|\| id:prot-a-1844 | Herring liking \|\| id:ebi-fl187-GCST90094781 | Weighted mode | 19 | 0.0586166816595102 | 0.205428934699978 | 0.778642565350489 | -0.34402403 | 0.461257393671466 | 1.06036870405671 | 0.708911892442197 | 1.58606704236462 | 0.999228396881742 |
| ebi-fl187-GCST90094781 | prot-a-1856 | Megakaryocyte-associated tyrosine-protein kinase \|\| id:prot-a-1856 | Herring liking \|\| id:ebi-fl187-GCST90094781 | Weighted mode | 19 | -0.050966705 | 0.17729206564078 | 0.777033490543002 | -0.398459154 | 0.29652574359245 | 0.950310310527163 | 0.671353702333601 | 1.34517718924484 | 0.999228396881742 |
| ebi-fl187-GCST90094781 | prot-a-1910 | Stromelysin-2 \|\| id:prot-a-1910 | Herring liking \|\| id:ebi-fl187-GCST90094781 | Weighted mode | 19 | -0.142826329 | 0.156182434951704 | 0.372550834876494 | -0.448943902 | 0.163291243107614 | 0.866904611668707 | 0.638301905211574 | 1.17737954343622 | 0.999228396881742 |
| ebi-fl187-GCST90094781 | prot-a-1940 | 39S ribosomal protein L14, mitochondrial \|\| id:prot-a-1940 | Herring liking \|\| id:ebi-fl187-GCST90094781 | Weighted mode | 19 | -0.227405949 | 0.222459095807936 | 0.320211884404946 | -0.663425777 | 0.208613878508622 | 0.796597338531713 | 0.515083746457312 | 1.23196921689005 | 0.999228396881742 |
| ebi-fl187-GCST90094781 | prot-a-1942 | 39S ribosomal protein L33, mitochondrial \|\| id:prot-a-1942 | Herring liking \|\| id:ebi-fl187-GCST90094781 | Weighted mode | 19 | 0.0322431997799568 | 0.23840860921565 | 0.893920314407708 | -0.435037674 | 0.499524073842631 | 1.03276864387239 | 0.647240282305317 | 1.64793678781396 | 0.999228396881742 |
| ebi-fl187-GCST90094781 | prot-a-1948 | Mesothelin \|\| id:prot-a-1948 | Herring liking \|\| id:ebi-fl187-GCST90094781 | Weighted mode | 19 | -0.071433566 | 0.213778972325582 | 0.742130426127797 | -0.490440352 | 0.34757321941138 | 0.931058129133223 | 0.612356682250685 | 1.41562795826596 | 0.999228396881742 |
| ebi-fl187-GCST90094781 | prot-a-1980 | Unconventional myosin-VI \|\| id:prot-a-1980 | Herring liking \|\| id:ebi-fl187-GCST90094781 | Weighted mode | 19 | -0.382204837 | 0.259951402661049 | 0.158746565126851 | -0.891709586 | 0.127299912688034 | 0.682355267605659 | 0.40995430129859 | 1.13575759482046 | 0.999228396881742 |
| ebi-fl187-GCST90094781 | prot-a-2039 | Neurofascin \|\| id:prot-a-2039 | Herring liking \|\| id:ebi-fl187-GCST90094781 | Weighted mode | 19 | 0.169309475916585 | 0.257465690614315 | 0.519116769789991 | -0.335323278 | 0.673942229520642 | 1.18448665229992 | 0.715106870885657 | 1.9619565782371 | 0.999228396881742 |
| ebi-fl187-GCST90094781 | prot-a-2041 | NFU1 iron-sulfur cluster scaffold homolog, mitochondrial \|\| id:prot-a-2041 | Herring liking \|\| id:ebi-fl187-GCST90094781 | Weighted mode | 19 | 0.0575931473122688 | 0.181810184784759 | 0.755057872249781 | -0.298754815 | 0.413941109490397 | 1.059283936 | 0.741741251069808 | 1.51276803658055 | 0.999228396881742 |
| ebi-fl187-GCST90094781 | prot-a-2088 | Glucocorticoid receptor \|\| id:prot-a-2088 | Herring liking \|\| id:ebi-fl187-GCST90094781 | Weighted mode | 19 | 0.349931055006672 | 0.202020013240635 | 0.100345695683836 | -0.046028171 | 0.745890280958317 | 1.41896971436322 | 0.955015058135109 | 2.10831759471082 | 0.999228396881742 |
| ebi-fl187-GCST90094781 | prot-a-2111 | Neuron-specific protein family member 2 \|\| id:prot-a-2111 | Herring liking \|\| id:ebi-fl187-GCST90094781 | Weighted mode | 19 | -0.008660028 | 0.215682236102933 | 0.968414114478372 | -0.431397211 | 0.414077154536727 | 0.991377361808645 | 0.649600830935007 | 1.51297385517815 | 0.999228396881742 |
| ebi-fl187-GCST90094781 | prot-a-2125 | Nuclear migration protein nudC \|\| id:prot-a-2125 | Herring liking \|\| id:ebi-fl187-GCST90094781 | Weighted mode | 19 | 0.18485826804081 | 0.197273882063646 | 0.361129317441597 | -0.201798541 | 0.571515076885556 | 1.20304791770537 | 0.817259555811871 | 1.77094814248753 | 0.999228396881742 |
| ebi-fl187-GCST90094781 | prot-a-2142 | Ornithine decarboxylase \|\| id:prot-a-2142 | Herring liking \|\| id:ebi-fl187-GCST90094781 | Weighted mode | 19 | -0.012991523 | 0.200323105548548 | 0.949005998847958 | -0.40562481 | 0.379641763575393 | 0.987092502272074 | 0.666560207123296 | 1.46176083964987 | 0.999228396881742 |
| ebi-fl187-GCST90094781 | prot-a-2186 | Prostate and testis expressed protein 1 \|\| id:prot-a-2186 | Herring liking \|\| id:ebi-fl187-GCST90094781 | Weighted mode | 19 | 0.184090621976858 | 0.175532875817995 | 0.308170150292105 | -0.159953815 | 0.528135058580129 | 1.20212475708224 | 0.852183146454319 | 1.69576685199969 | 0.999228396881742 |
| ebi-fl187-GCST90094781 | prot-a-2227 | Platelet-derived growth factor C \|\| id:prot-a-2227 | Herring liking \|\| id:ebi-fl187-GCST90094781 | Weighted mode | 19 | -0.165776828 | 0.187852008252722 | 0.38914746890931 | -0.533966764 | 0.202413108048309 | 0.847235292179408 | 0.586274737293323 | 1.22435369401768 | 0.999228396881742 |
| ebi-fl187-GCST90094781 | prot-a-2229 | Platelet-derived growth factor receptor alpha \|\| id:prot-a-2229 | Herring liking \|\| id:ebi-fl187-GCST90094781 | Weighted mode | 19 | -0.073992609 | 0.192714268186992 | 0.705513396161477 | -0.451712575 | 0.303727356485632 | 0.928678557529742 | 0.636537100233055 | 1.35489960113834 | 0.999228396881742 |
| ebi-fl187-GCST90094781 | prot-a-2243 | Platelet endothelial aggregation receptor 1 \|\| id:prot-a-2243 | Herring liking \|\| id:ebi-fl187-GCST90094781 | Weighted mode | 19 | -0.191234603 | 0.161920678971639 | 0.252957987635032 | -0.508599134 | 0.126129927531404 | 0.825938797491446 | 0.601337382118439 | 1.13442955233948 | 0.999228396881742 |
| ebi-fl187-GCST90094781 | prot-a-2246 | E3 ubiquitin-protein ligase pellino homolog 2 \|\| id:prot-a-2246 | Herring liking \|\| id:ebi-fl187-GCST90094781 | Weighted mode | 19 | 0.312772409179172 | 0.190240904106288 | 0.117510090576483 | -0.060099763 | 0.685644581227497 | 1.36721033109433 | 0.941670585138673 | 1.98505094982423 | 0.999228396881742 |
| ebi-fl187-GCST90094781 | prot-a-2251 | Platelet factor 4 variant \|\| id:prot-a-2251 | Herring liking \|\| id:ebi-fl187-GCST90094781 | Weighted mode | 19 | 0.458427129033731 | 0.284457004274027 | 0.124447431454168 | -0.099108599 | 1.01596285741082 | 1.58158439942674 | 0.90564435030109 | 2.7620215503784 | 0.999228396881742 |
| ebi-fl187-GCST90094781 | prot-a-2346 | Protein phosphatase 1L \|\| id:prot-a-2346 | Herring liking \|\| id:ebi-fl187-GCST90094781 | Weighted mode | 19 | 0.148527169733965 | 0.192038282342845 | 0.449309734738898 | -0.227867864 | 0.524922203125942 | 1.16012431761531 | 0.796229463733392 | 1.69032734108046 | 0.999228396881742 |
| ebi-fl187-GCST90094781 | prot-a-2358 | PR domain zinc finger protein 4 \|\| id:prot-a-2358 | Herring liking \|\| id:ebi-fl187-GCST90094781 | Weighted mode | 19 | 0.00848181186224895 | 0.210201305922381 | 0.968257573121626 | -0.403512748 | 0.420476371470116 | 1.00851788434302 | 0.6679695116393 | 1.52268674739897 | 0.999228396881742 |
| ebi-fl187-GCST90094781 | prot-a-2390 | Trypsin-1 \|\| id:prot-a-2390 | Herring liking \|\| id:ebi-fl187-GCST90094781 | Weighted mode | 19 | -0.321516084 | 0.224293872902911 | 0.168869073402115 | -0.761132074 | 0.118099907361327 | 0.725048968588001 | 0.46713729337753 | 1.12535653715335 | 0.999228396881742 |
| ebi-fl187-GCST90094781 | prot-a-242 | Brain-derived neurotrophic factor \|\| id:prot-a-242 | Herring liking \|\| id:ebi-fl187-GCST90094781 | Weighted mode | 19 | 0.0661585736825618 | 0.215422009484617 | 0.762283180802228 | -0.356068565 | 0.488385712272412 | 1.06839612324166 | 0.700424594041463 | 1.62968331761669 | 0.999228396881742 |
| ebi-fl187-GCST90094781 | prot-a-2464 | Dihydropteridine reductase \|\| id:prot-a-2464 | Herring liking \|\| id:ebi-fl187-GCST90094781 | Weighted mode | 19 | -0.067456469 | 0.198419076257492 | 0.737814367352687 | -0.456357858 | 0.32144492067471 | 0.934768411330401 | 0.633587062164087 | 1.37911904298776 | 0.999228396881742 |
| ebi-fl187-GCST90094781 | prot-a-2474 | Ras-related protein Rab-27A \|\| id:prot-a-2474 | Herring liking \|\| id:ebi-fl187-GCST90094781 | Weighted mode | 19 | 0.0677992981452547 | 0.353400185657223 | 0.850008518978765 | -0.624865066 | 0.760463662033412 | 1.07015050573248 | 0.53533365849524 | 2.1392678878787 | 0.999228396881742 |
| ebi-fl187-GCST90094781 | prot-a-2478 | Ras-related protein Rab-7b \|\| id:prot-a-2478 | Herring liking \|\| id:ebi-fl187-GCST90094781 | Weighted mode | 19 | 0.0877503180246736 | 0.232709033473372 | 0.710521731311947 | -0.368359388 | 0.543860023632483 | 1.09171550631189 | 0.691868488058935 | 1.72264348975567 | 0.999228396881742 |
| ebi-fl187-GCST90094781 | prot-a-2528 | E3 ubiquitin-protein ligase rififylin \|\| id:prot-a-2528 | Herring liking \|\| id:ebi-fl187-GCST90094781 | Weighted mode | 19 | -0.242688416 | 0.221580348149686 | 0.287841352380453 | -0.676985899 | 0.191609065931945 | 0.784515917954881 | 0.508146290815063 | 1.21119692625796 | 0.999228396881742 |
| ebi-fl187-GCST90094781 | prot-a-2543 | Protein RIC-3 \|\| id:prot-a-2543 | Herring liking \|\| id:ebi-fl187-GCST90094781 | Weighted mode | 19 | 0.273801160823352 | 0.250101584455599 | 0.288055274038039 | -0.216397945 | 0.764000266356326 | 1.31495331200845 | 0.8054147275624 | 2.14684702624589 | 0.999228396881742 |
| ebi-fl187-GCST90094781 | prot-a-255 | Bone morphogenetic protein 6 \|\| id:prot-a-255 | Herring liking \|\| id:ebi-fl187-GCST90094781 | Weighted mode | 19 | -0.09239183 | 0.22409224749276 | 0.684992914107204 | -0.531612635 | 0.346828974770239 | 0.911747829105572 | 0.587656529416536 | 1.41457477670516 | 0.999228396881742 |
| ebi-fl187-GCST90094781 | prot-a-2568 | RING finger protein 165 \|\| id:prot-a-2568 | Herring liking \|\| id:ebi-fl187-GCST90094781 | Weighted mode | 19 | 0.010218509005318 | 0.19685556276372 | 0.959173203565208 | -0.375618394 | 0.396055412022209 | 1.01027089625628 | 0.686864394603208 | 1.48595165485623 | 0.999228396881742 |
| ebi-fl187-GCST90094781 | prot-a-2573 | E3 ubiquitin-protein ligase RNF8 \|\| id:prot-a-2573 | Herring liking \|\| id:ebi-fl187-GCST90094781 | Weighted mode | 19 | -0.257248227 | 0.307820549432194 | 0.414275154189941 | -0.860576503 | 0.346080050293577 | 0.773176267032334 | 0.422918198211197 | 1.41351576364071 | 0.999228396881742 |
| ebi-fl187-GCST90094781 | prot-a-260 | Cytoplasmic tyrosine-protein kinase BMX \|\| id:prot-a-260 | Herring liking \|\| id:ebi-fl187-GCST90094781 | Weighted mode | 19 | -0.068105869 | 0.169448010874831 | 0.692469478826982 | -0.40022397 | 0.264012232135556 | 0.934161569423342 | 0.670169930935242 | 1.30214412420692 | 0.999228396881742 |
| ebi-fl187-GCST90094781 | prot-a-2623 | Serum amyloid A-1 protein \|\| id:prot-a-2623 | Herring liking \|\| id:ebi-fl187-GCST90094781 | Weighted mode | 19 | -0.202556547 | 0.205949409203979 | 0.338385010502851 | -0.606217389 | 0.201104295276498 | 0.816640302926556 | 0.545410046233745 | 1.22275229246174 | 0.999228396881742 |
| ebi-fl187-GCST90094781 | prot-a-2640 | Lysosome membrane protein 2 \|\| id:prot-a-2640 | Herring liking \|\| id:ebi-fl187-GCST90094781 | Weighted mode | 19 | -0.088837799 | 0.21742746314175 | 0.687663437977439 | -0.514995627 | 0.337320028659571 | 0.914993974386488 | 0.597503207580039 | 1.40118741212185 | 0.999228396881742 |
| ebi-fl187-GCST90094781 | prot-a-2641 | Scavenger receptor class F member 2 \|\| id:prot-a-2641 | Herring liking \|\| id:ebi-fl187-GCST90094781 | Weighted mode | 19 | -1.26E-05 | 0.174882063860898 | 0.99994334165495 | -0.342781437 | 0.342756253075878 | 0.999987407987799 | 0.709793328983786 | 1.40882532323293 | 0.99994334165495 |
| ebi-fl187-GCST90094781 | prot-a-2669 | Semaphorin-3A \|\| id:prot-a-2669 | Herring liking \|\| id:ebi-fl187-GCST90094781 | Weighted mode | 19 | -0.110823969 | 0.209020222925771 | 0.602448539680671 | -0.520503606 | 0.29885566786953 | 0.895096299700288 | 0.594221219233616 | 1.34831500425124 | 0.999228396881742 |
| ebi-fl187-GCST90094781 | prot-a-2683 | Sentrin-specific protease 7 \|\| id:prot-a-2683 | Herring liking \|\| id:ebi-fl187-GCST90094781 | Weighted mode | 19 | 0.209624351107691 | 0.222968886052678 | 0.359586736768702 | -0.227394666 | 0.64664336777094 | 1.2332147171926 | 0.796606327163248 | 1.90912184204729 | 0.999228396881742 |
| ebi-fl187-GCST90094781 | prot-a-270 | Bromodomain testis-specific protein \|\| id:prot-a-270 | Herring liking \|\| id:ebi-fl187-GCST90094781 | Weighted mode | 19 | 0.0520917382426052 | 0.185658904147492 | 0.782233761951753 | -0.311799714 | 0.41598319037169 | 1.05347238178548 | 0.732128148646297 | 1.51586038760672 | 0.999228396881742 |
| ebi-fl187-GCST90094781 | prot-a-2708 | 14-3-3 protein sigma \|\| id:prot-a-2708 | Herring liking \|\| id:ebi-fl187-GCST90094781 | Weighted mode | 19 | 0.131563534652477 | 0.177707710932295 | 0.468643428211883 | -0.216743579 | 0.479870648079774 | 1.140610374 | 0.805136396898991 | 1.61586537338517 | 0.999228396881742 |
| ebi-fl187-GCST90094781 | prot-a-2713 | Beta-sarcoglycan \|\| id:prot-a-2713 | Herring liking \|\| id:ebi-fl187-GCST90094781 | Weighted mode | 19 | -0.128263353 | 0.208271260258657 | 0.545704535914045 | -0.536475023 | 0.279948317215449 | 0.879621697721459 | 0.584806051277426 | 1.3230614310715 | 0.999228396881742 |
| ebi-fl187-GCST90094781 | prot-a-2720 | Protein shisa-3 homolog \|\| id:prot-a-2720 | Herring liking \|\| id:ebi-fl187-GCST90094781 | Weighted mode | 19 | -0.129501812 | 0.182797204273395 | 0.487742863295508 | -0.487784333 | 0.228780708063874 | 0.87853299623893 | 0.61398527531892 | 1.25706634426965 | 0.999228396881742 |
| ebi-fl187-GCST90094781 | prot-a-2730 | Sialic acid-binding Ig-like lectin 8 \|\| id:prot-a-2730 | Herring liking \|\| id:ebi-fl187-GCST90094781 | Weighted mode | 19 | -0.439332743 | 0.22781096409245 | 0.0697174106879157 | -0.885842232 | 0.00717674703641863 | 0.644466302565633 | 0.41236671844981 | 1.00720256160335 | 0.999228396881742 |
| ebi-fl187-GCST90094781 | prot-a-2761 | Sodium- and chloride-dependent neutral and basic amino acid transporter B(0+) \|\| id:prot-a-2761 | Herring liking \|\| id:ebi-fl187-GCST90094781 | Weighted mode | 19 | -0.386127982 | 0.199299041481406 | 0.0685459118745211 | -0.776754103 | 0.00449813931519355 | 0.67968353286369 | 0.459896367330629 | 1.00450827112958 | 0.999228396881742 |
| ebi-fl187-GCST90094781 | prot-a-2770 | Secreted Ly-6/uPAR-related protein 1 \|\| id:prot-a-2770 | Herring liking \|\| id:ebi-fl187-GCST90094781 | Weighted mode | 19 | 0.127143988627381 | 0.196277996803902 | 0.525307483729391 | -0.257560885 | 0.511848862363029 | 1.13558051669009 | 0.772934564676168 | 1.66837293714043 | 0.999228396881742 |
| ebi-fl187-GCST90094781 | prot-a-2784 | N-lysine methyltransferase SMYD2 \|\| id:prot-a-2784 | Herring liking \|\| id:ebi-fl187-GCST90094781 | Weighted mode | 19 | -0.240731055 | 0.181635070590318 | 0.201630711913117 | -0.596735793 | 0.115273683733203 | 0.786053003283317 | 0.550605997879676 | 1.12218051810207 | 0.999228396881742 |
| ebi-fl187-GCST90094781 | prot-a-2837 | Serine palmitoyltransferase 2 \|\| id:prot-a-2837 | Herring liking \|\| id:ebi-fl187-GCST90094781 | Weighted mode | 19 | 0.0165268090016433 | 0.197461362131234 | 0.934221604431179 | -0.370497461 | 0.403551078778862 | 1.0166641321711 | 0.690390802854402 | 1.49713170188507 | 0.999228396881742 |
| ebi-fl187-GCST90094781 | prot-a-2858 | Alpha-2,8-sialyltransferase 8B \|\| id:prot-a-2858 | Herring liking \|\| id:ebi-fl187-GCST90094781 | Weighted mode | 19 | -0.1148485 | 0.195605583 | 0.564400341575692 | -0.498235444 | 0.268538442978343 | 0.891501195728784 | 0.60760186195197 | 1.30805126145034 | 0.999228396881742 |
| ebi-fl187-GCST90094781 | prot-a-2911 | Synaptotagmin-11 \|\| id:prot-a-2911 | Herring liking \|\| id:ebi-fl187-GCST90094781 | Weighted mode | 19 | -0.048955767 | 0.186978343026984 | 0.796428371523815 | -0.415433319 | 0.317521785727901 | 0.952223248834067 | 0.660054204741453 | 1.37371917201145 | 0.999228396881742 |
| ebi-fl187-GCST90094781 | prot-a-2962 | Transforming growth factor beta-1 \|\| id:prot-a-2962 | Herring liking \|\| id:ebi-fl187-GCST90094781 | Weighted mode | 19 | 0.00883123378030915 | 0.20089535595936 | 0.965420783429371 | -0.384923664 | 0.402586131460655 | 1.00887034417157 | 0.680502581134984 | 1.49568774544733 | 0.999228396881742 |
| ebi-fl187-GCST90094781 | prot-a-2978 | Tyrosine-protein kinase receptor Tie-1, soluble \|\| id:prot-a-2978 | Herring liking \|\| id:ebi-fl187-GCST90094781 | Weighted mode | 19 | 0.322991666498159 | 0.268141415751684 | 0.243974243981035 | -0.202565508 | 0.848548841371459 | 1.38125384017625 | 0.816632984545941 | 2.33625411550383 | 0.999228396881742 |
| ebi-fl187-GCST90094781 | prot-a-299 | Uncharacterized protein C1orf226 \|\| id:prot-a-299 | Herring liking \|\| id:ebi-fl187-GCST90094781 | Weighted mode | 19 | 0.0449886319282771 | 0.170633245525794 | 0.795039014439607 | -0.289452529 | 0.379429793158833 | 1.04601596865657 | 0.74867333211279 | 1.46145102243299 | 0.999228396881742 |
| ebi-fl187-GCST90094781 | prot-a-3015 | Transmembrane protein 70, mitochondrial \|\| id:prot-a-3015 | Herring liking \|\| id:ebi-fl187-GCST90094781 | Weighted mode | 19 | -0.190639234 | 0.175615965497774 | 0.292004909796784 | -0.534846527 | 0.153568057906053 | 0.826430682080326 | 0.585759181556192 | 1.16598713906499 | 0.999228396881742 |
| ebi-fl187-GCST90094781 | prot-a-3016 | Transmembrane protein 87B \|\| id:prot-a-3016 | Herring liking \|\| id:ebi-fl187-GCST90094781 | Weighted mode | 19 | -0.070898905 | 0.25322168850981 | 0.782679326076172 | -0.567213415 | 0.425415604336571 | 0.931556062894708 | 0.5671035213278 | 1.53022625619403 | 0.999228396881742 |
| ebi-fl187-GCST90094781 | prot-a-3123 | Thioredoxin domain-containing protein 12 \|\| id:prot-a-3123 | Herring liking \|\| id:ebi-fl187-GCST90094781 | Weighted mode | 19 | -0.072587282 | 0.189937536777636 | 0.706814351629127 | -0.444864854 | 0.299690290431721 | 0.929984572527584 | 0.640910886891148 | 1.34944080812003 | 0.999228396881742 |
| ebi-fl187-GCST90094781 | prot-a-3133 | PolyUbiquitin K48-linked \|\| id:prot-a-3133 | Herring liking \|\| id:ebi-fl187-GCST90094781 | Weighted mode | 19 | 0.199611604631769 | 0.213923337216688 | 0.363117635574969 | -0.219678136 | 0.618901345576477 | 1.22092846309912 | 0.802777141195149 | 1.85688684382109 | 0.999228396881742 |
| ebi-fl187-GCST90094781 | prot-a-3135 | Ubiquitin-conjugating enzyme E2 B \|\| id:prot-a-3135 | Herring liking \|\| id:ebi-fl187-GCST90094781 | Weighted mode | 19 | 0.155425607940079 | 0.192608685858718 | 0.430229383702385 | -0.222087416 | 0.532938632223166 | 1.16815503137569 | 0.800845354302506 | 1.70393218865186 | 0.999228396881742 |
| ebi-fl187-GCST90094781 | prot-a-3162 | NKG2D ligand 3 \|\| id:prot-a-3162 | Herring liking \|\| id:ebi-fl187-GCST90094781 | Weighted mode | 19 | 0.0905036556040174 | 0.201442655168751 | 0.658594131743711 | -0.304323949 | 0.485331259734769 | 1.0947255095173 | 0.737621876222326 | 1.62471312174953 | 0.999228396881742 |
| ebi-fl187-GCST90094781 | prot-a-3188 | Vesicle-associated membrane protein 8 \|\| id:prot-a-3188 | Herring liking \|\| id:ebi-fl187-GCST90094781 | Weighted mode | 19 | 0.312260334723459 | 0.265074530307858 | 0.254133073952522 | -0.207285745 | 0.831806414126861 | 1.36651039683271 | 0.812787367136273 | 2.29746516758891 | 0.999228396881742 |
| ebi-fl187-GCST90094781 | prot-a-3201 | Vitrin \|\| id:prot-a-3201 | Herring liking \|\| id:ebi-fl187-GCST90094781 | Weighted mode | 19 | 0.158295743834221 | 0.236311282286383 | 0.511448894977641 | -0.304874369 | 0.621465857115532 | 1.17151261111134 | 0.737215985425982 | 1.86165496289377 | 0.999228396881742 |
| ebi-fl187-GCST90094781 | prot-a-3232 | WNT1-inducible-signaling pathway protein 3 \|\| id:prot-a-3232 | Herring liking \|\| id:ebi-fl187-GCST90094781 | Weighted mode | 19 | 0.00246616530081378 | 0.244960039913307 | 0.992078079497205 | -0.477655513 | 0.482587843530896 | 1.00246920878786 | 0.620235823406079 | 1.62026196592292 | 0.999228396881742 |
| ebi-fl187-GCST90094781 | prot-a-327 | Carbonic anhydrase-related protein 10 \|\| id:prot-a-327 | Herring liking \|\| id:ebi-fl187-GCST90094781 | Weighted mode | 19 | -0.0634308 | 0.194607671067699 | 0.748227073407077 | -0.444861835 | 0.318000235573329 | 0.938539064220171 | 0.640912821627505 | 1.37437658499399 | 0.999228396881742 |
| ebi-fl187-GCST90094781 | prot-a-332 | Carbonic anhydrase 5A, mitochondrial \|\| id:prot-a-332 | Herring liking \|\| id:ebi-fl187-GCST90094781 | Weighted mode | 19 | -0.411739082 | 0.280109206890832 | 0.158845716271124 | -0.960753127 | 0.137274963765709 | 0.662497111096502 | 0.382604627471542 | 1.14714352806372 | 0.999228396881742 |
| ebi-fl187-GCST90094781 | prot-a-338 | Cell adhesion molecule 3 \|\| id:prot-a-338 | Herring liking \|\| id:ebi-fl187-GCST90094781 | Weighted mode | 19 | 0.0525030532610709 | 0.184031125705326 | 0.778675419946982 | -0.308197953 | 0.41320405964351 | 1.05390577992312 | 0.734769853625967 | 1.51165346192981 | 0.999228396881742 |
| ebi-fl187-GCST90094781 | prot-a-34 | A disintegrin and metalloproteinase with thrombospondin motifs 3 \|\| id:prot-a-34 | Herring liking \|\| id:ebi-fl187-GCST90094781 | Weighted mode | 19 | 0.0424643977659711 | 0.255368730141563 | 0.869785017578114 | -0.458058313 | 0.542987108843436 | 1.04337890909232 | 0.632510591344799 | 1.72114042489642 | 0.999228396881742 |
| ebi-fl187-GCST90094781 | prot-a-358 | Cysteine--tRNA ligase, cytoplasmic \|\| id:prot-a-358 | Herring liking \|\| id:ebi-fl187-GCST90094781 | Weighted mode | 19 | 0.0752658664292929 | 0.187814840533135 | 0.693324659755685 | -0.292851221 | 0.443382953874237 | 1.07817076219349 | 0.746133141370695 | 1.55796885032261 | 0.999228396881742 |
| ebi-fl187-GCST90094781 | prot-a-382 | Coiled-coil domain-containing protein 167 \|\| id:prot-a-382 | Herring liking \|\| id:ebi-fl187-GCST90094781 | Weighted mode | 19 | -0.018756407 | 0.201022077767987 | 0.926691806012433 | -0.412759679 | 0.375246865389393 | 0.981418399744752 | 0.661821312972558 | 1.45535064597941 | 0.999228396881742 |
| ebi-fl187-GCST90094781 | prot-a-40 | Pituitary adenylate cyclase-activating polypeptide 27 \|\| id:prot-a-40 | Herring liking \|\| id:ebi-fl187-GCST90094781 | Weighted mode | 19 | 0.0116579985187302 | 0.19620122522408 | 0.953273430225818 | -0.372896403 | 0.396212399957927 | 1.01172621782591 | 0.688736580246482 | 1.48618494965086 | 0.999228396881742 |
| ebi-fl187-GCST90094781 | prot-a-403 | C-C motif chemokine 25 \|\| id:prot-a-403 | Herring liking \|\| id:ebi-fl187-GCST90094781 | Weighted mode | 19 | -0.026690138 | 0.182549705773396 | 0.885382546833565 | -0.384487562 | 0.331107284943137 | 0.973662895555659 | 0.68079941453567 | 1.3925091795627 | 0.999228396881742 |
| ebi-fl187-GCST90094781 | prot-a-415 | Cyclin-C \|\| id:prot-a-415 | Herring liking \|\| id:ebi-fl187-GCST90094781 | Weighted mode | 19 | 0.0874534064329682 | 0.204639078206026 | 0.674189800919406 | -0.313639187 | 0.488545999716779 | 1.09139141143936 | 0.730782656587937 | 1.62994455632688 | 0.999228396881742 |
| ebi-fl187-GCST90094781 | prot-a-44 | Adhesion G protein-coupled receptor F5 \|\| id:prot-a-44 | Herring liking \|\| id:ebi-fl187-GCST90094781 | Weighted mode | 19 | 0.00357875443654176 | 0.195167087635235 | 0.985571841194615 | -0.378948737 | 0.386106246201603 | 1.00358516582418 | 0.684580705202595 | 1.47124097627654 | 0.999228396881742 |
| ebi-fl187-GCST90094781 | prot-a-455 | CD59 glycoprotein \|\| id:prot-a-455 | Herring liking \|\| id:ebi-fl187-GCST90094781 | Weighted mode | 19 | -0.062681504 | 0.186190827227682 | 0.740272476329544 | -0.427615525 | 0.302252517448131 | 0.939242571134759 | 0.652062067988893 | 1.3529028151456 | 0.999228396881742 |
| ebi-fl187-GCST90094781 | prot-a-478 | Cadherin-12 \|\| id:prot-a-478 | Herring liking \|\| id:ebi-fl187-GCST90094781 | Weighted mode | 19 | -0.140792662 | 0.182439893387146 | 0.450293268219949 | -0.498374853 | 0.21678952937326 | 0.86866940149416 | 0.607517162763253 | 1.24208265271064 | 0.999228396881742 |
| ebi-fl187-GCST90094781 | prot-a-516 | Centrin-2 \|\| id:prot-a-516 | Herring liking \|\| id:ebi-fl187-GCST90094781 | Weighted mode | 19 | 0.0259793162587862 | 0.204888840910102 | 0.900506654907395 | -0.375602812 | 0.427561444442587 | 1.02631972012241 | 0.686875097467046 | 1.53351340264985 | 0.999228396881742 |
| ebi-fl187-GCST90094781 | prot-a-519 | Cryptic protein \|\| id:prot-a-519 | Herring liking \|\| id:ebi-fl187-GCST90094781 | Weighted mode | 19 | 0.145635561226368 | 0.206239289885825 | 0.489135786899681 | -0.258593447 | 0.549864569402585 | 1.1567745377287 | 0.772136873842075 | 1.73301829827018 | 0.999228396881742 |
| ebi-fl187-GCST90094781 | prot-a-583 | Chloride intracellular channel protein 4 \|\| id:prot-a-583 | Herring liking \|\| id:ebi-fl187-GCST90094781 | Weighted mode | 19 | 0.120468344318234 | 0.163409524782243 | 0.470491514679742 | -0.199814324 | 0.440751012891429 | 1.12802503199913 | 0.818882785634488 | 1.55387375963794 | 0.999228396881742 |
| ebi-fl187-GCST90094781 | prot-a-602 | CCR4-NOT transcription complex subunit 1 \|\| id:prot-a-602 | Herring liking \|\| id:ebi-fl187-GCST90094781 | Weighted mode | 19 | 0.309632036624621 | 0.276217829874511 | 0.277033668233011 | -0.23175491 | 0.851018983178663 | 1.36292351591565 | 0.793140490345753 | 2.34203212778371 | 0.999228396881742 |
| ebi-fl187-GCST90094781 | prot-a-606 | Ciliary neurotrophic factor receptor subunit alpha \|\| id:prot-a-606 | Herring liking \|\| id:ebi-fl187-GCST90094781 | Weighted mode | 19 | -0.176664373 | 0.184146777647782 | 0.350084165716955 | -0.537592057 | 0.184263311478585 | 0.83806101347221 | 0.58415316780629 | 1.20233236933326 | 0.999228396881742 |
| ebi-fl187-GCST90094781 | prot-a-626 | Collagen alpha-2(IX) chain \|\| id:prot-a-626 | Herring liking \|\| id:ebi-fl187-GCST90094781 | Weighted mode | 19 | 0.133221211749324 | 0.185466556992318 | 0.48179080812678 | -0.23029324 | 0.496735663454267 | 1.14250270540105 | 0.794300647662501 | 1.64334806435074 | 0.999228396881742 |
| ebi-fl187-GCST90094781 | prot-a-647 | Probable serine carboxypeptidase CPVL \|\| id:prot-a-647 | Herring liking \|\| id:ebi-fl187-GCST90094781 | Weighted mode | 19 | -0.057237802 | 0.231933563142214 | 0.807866991688532 | -0.511827586 | 0.397351981510234 | 0.944369469479141 | 0.599399123733183 | 1.48787954398363 | 0.999228396881742 |
| ebi-fl187-GCST90094781 | prot-a-651 | Cellular retinoic acid-binding protein 2 \|\| id:prot-a-651 | Herring liking \|\| id:ebi-fl187-GCST90094781 | Weighted mode | 19 | -0.080119231 | 0.209776794595158 | 0.706987704905712 | -0.491281749 | 0.331043286149756 | 0.92300628862582 | 0.611841664143367 | 1.39242006350712 | 0.999228396881742 |
| ebi-fl187-GCST90094781 | prot-a-671 | Cytotoxic and regulatory T-cell molecule \|\| id:prot-a-671 | Herring liking \|\| id:ebi-fl187-GCST90094781 | Weighted mode | 19 | 0.275319946510957 | 0.241017779669091 | 0.268288169674647 | -0.197074902 | 0.747714794662376 | 1.31695196165464 | 0.821129127090578 | 2.11216776032679 | 0.999228396881742 |
| ebi-fl187-GCST90094781 | prot-a-688 | Chorionic somatomammotropin hormone \|\| id:prot-a-688 | Herring liking \|\| id:ebi-fl187-GCST90094781 | Weighted mode | 19 | -0.066319535 | 0.225089118012575 | 0.771642437813609 | -0.507494206 | 0.374855136786891 | 0.935831785952811 | 0.60200218397021 | 1.45478065515285 | 0.999228396881742 |
| ebi-fl187-GCST90094781 | prot-a-69 | Aldo-keto reductase family 1 member C1 \|\| id:prot-a-69 | Herring liking \|\| id:ebi-fl187-GCST90094781 | Weighted mode | 19 | -0.029683883 | 0.194820769834026 | 0.880593878503085 | -0.411532591 | 0.352164826368448 | 0.970752356851953 | 0.662633924487903 | 1.42214291105288 | 0.999228396881742 |
| ebi-fl187-GCST90094781 | prot-a-741 | Stromal cell-derived factor 1 \|\| id:prot-a-741 | Herring liking \|\| id:ebi-fl187-GCST90094781 | Weighted mode | 19 | -0.133898472 | 0.18938571778561 | 0.488610223856235 | -0.505094479 | 0.237297534488551 | 0.874678864166454 | 0.603448559377804 | 1.26781828132683 | 0.999228396881742 |
| ebi-fl187-GCST90094781 | prot-a-742 | Growth-regulated alpha protein \|\| id:prot-a-742 | Herring liking \|\| id:ebi-fl187-GCST90094781 | Weighted mode | 19 | -0.244415075 | 0.212236881616238 | 0.264546949585494 | -0.660399363 | 0.171569213351456 | 0.783162495916645 | 0.516644964618056 | 1.18716630764769 | 0.999228396881742 |
| ebi-fl187-GCST90094781 | prot-a-788 | Beta-defensin 112 \|\| id:prot-a-788 | Herring liking \|\| id:ebi-fl187-GCST90094781 | Weighted mode | 19 | -0.225747478 | 0.193386874159866 | 0.25830634368379 | -0.604785752 | 0.1532907949094 | 0.797919568118246 | 0.5461914346381 | 1.16566389879011 | 0.999228396881742 |
| ebi-fl187-GCST90094781 | prot-a-838 | DnaJ homolog subfamily B member 1 \|\| id:prot-a-838 | Herring liking \|\| id:ebi-fl187-GCST90094781 | Weighted mode | 19 | 0.162395424212525 | 0.187103637813762 | 0.396849595519577 | -0.204327706 | 0.529118554327498 | 1.17632529687202 | 0.815195183136973 | 1.69743545188311 | 0.999228396881742 |
| ebi-fl187-GCST90094781 | prot-a-85 | Junctional adhesion molecule-like \|\| id:prot-a-85 | Herring liking \|\| id:ebi-fl187-GCST90094781 | Weighted mode | 19 | 0.0437714370358735 | 0.162581855568336 | 0.790816805757301 | -0.274889 | 0.362431873949812 | 1.04474353791735 | 0.759656440502793 | 1.43681933282582 | 0.999228396881742 |
| ebi-fl187-GCST90094781 | prot-a-856 | Dipeptidase 1 \|\| id:prot-a-856 | Herring liking \|\| id:ebi-fl187-GCST90094781 | Weighted mode | 19 | 0.283654653677178 | 0.271809433023669 | 0.310493905216087 | -0.249091835 | 0.816401142403569 | 1.32797424053309 | 0.779508383906741 | 2.26234331782431 | 0.999228396881742 |
| ebi-fl187-GCST90094781 | prot-a-860 | Dipeptidyl peptidase 2 \|\| id:prot-a-860 | Herring liking \|\| id:ebi-fl187-GCST90094781 | Weighted mode | 19 | 0.16844593595561 | 0.184030812215002 | 0.372126836448241 | -0.192254456 | 0.529146327897014 | 1.18346424225191 | 0.825096890935033 | 1.69748259637931 | 0.999228396881742 |
| ebi-fl187-GCST90094781 | prot-a-861 | Dermatopontin \|\| id:prot-a-861 | Herring liking \|\| id:ebi-fl187-GCST90094781 | Weighted mode | 19 | -0.17105579 | 0.18378027621577 | 0.364292588615844 | -0.531265131 | 0.189153551795521 | 0.842774554115403 | 0.58786077815108 | 1.20822646358264 | 0.999228396881742 |
| ebi-fl187-GCST90094781 | prot-a-877 | Dual specificity protein phosphatase 6 \|\| id:prot-a-877 | Herring liking \|\| id:ebi-fl187-GCST90094781 | Weighted mode | 19 | -0.010907148 | 0.189433516903757 | 0.954719369908726 | -0.382196841 | 0.360382544823776 | 0.989152118959844 | 0.682360723118339 | 1.43387783219912 | 0.999228396881742 |
| ebi-fl187-GCST90094781 | prot-a-879 | Dynactin-associated protein \|\| id:prot-a-879 | Herring liking \|\| id:ebi-fl187-GCST90094781 | Weighted mode | 19 | -0.076701856 | 0.200028306146165 | 0.705873763294708 | -0.468757336 | 0.315353624299281 | 0.926165943500335 | 0.625779418999043 | 1.37074395363133 | 0.999228396881742 |
| ebi-fl187-GCST90094781 | prot-a-937 | EMI domain-containing protein 1 \|\| id:prot-a-937 | Herring liking \|\| id:ebi-fl187-GCST90094781 | Weighted mode | 19 | -0.09049937 | 0.17854662481113 | 0.618401134647529 | -0.440450754 | 0.259452014826854 | 0.913474909569955 | 0.643746184229157 | 1.29621958289822 | 0.999228396881742 |
| ebi-fl187-GCST90094781 | prot-b-1 | SRC proto-oncogene, non-receptor tyrosine kinase \|\| id:prot-b-1 | Herring liking \|\| id:ebi-fl187-GCST90094781 | Weighted mode | 13 | 0.220491859890599 | 0.386456787402487 | 0.578833758703541 | -0.536963443 | 0.977947163199474 | 1.24668977650576 | 0.58452048980307 | 2.65899215845728 | 0.999228396881742 |
| ebi-fl187-GCST90094781 | prot-b-10 | coagulation factor III, tissue factor \|\| id:prot-b-10 | Herring liking \|\| id:ebi-fl187-GCST90094781 | Weighted mode | 13 | -0.108497757 | 0.223504447862433 | 0.63610718822167 | -0.546566475 | 0.329570960430139 | 0.897180906850353 | 0.578934186621235 | 1.39037147609914 | 0.999228396881742 |
| ebi-fl187-GCST90094781 | prot-b-29 | myeloperoxidase \|\| id:prot-b-29 | Herring liking \|\| id:ebi-fl187-GCST90094781 | Weighted mode | 13 | -0.249540174 | 0.225784028694806 | 0.290732818594609 | -0.69207687 | 0.192996522057994 | 0.779158978124474 | 0.5005354415603 | 1.21287857519044 | 0.999228396881742 |
| ebi-fl187-GCST90094781 | prot-b-33 | adrenomedullin \|\| id:prot-b-33 | Herring liking \|\| id:ebi-fl187-GCST90094781 | Weighted mode | 13 | -0.151503835 | 0.253396386616919 | 0.561026069198822 | -0.648160753 | 0.345153082751788 | 0.859414586396738 | 0.523006831538963 | 1.41220608751542 | 0.999228396881742 |
| ebi-fl187-GCST90094781 | ukb-rsfMRI191-edge-pheno102 | Net25_Pair11_15 (Postcentral\|Precentral)&(Cerebellum) (Motor)&(Subcortical-cerebellum) \|\| id:ukb-rsfMRI191-edge-pheno102 | Herring liking \|\| id:ebi-fl187-GCST90094781 | Weighted mode | 18 | 0.0174224947439892 | 0.0530094658865548 | 0.746420937147821 | -0.086476058 | 0.121321047881637 | 1.01757515167172 | 0.91715750674583 | 1.12898731317552 | 0.999228396881742 |
| ebi-fl187-GCST90094781 | ukb-rsfMRI191-edge-pheno1167 | Net100_Pair11_45 (Angular\|Temporal)&(Frontal) (Default_mode\|Central_executive)&(Salience\|Default_mode) \|\| id:ukb-rsfMRI191-edge-pheno1167 | Herring liking \|\| id:ebi-fl187-GCST90094781 | Weighted mode | 18 | -0.065361535 | 0.0612041559532602 | 0.300484767983917 | -0.185321681 | 0.0545986106989184 | 0.936728741954148 | 0.830836976692041 | 1.05611661567663 | 0.999228396881742 |
| ebi-fl187-GCST90094781 | ukb-rsfMRI191-edge-pheno1317 | Net100_Pair26_48 (Parietal\|Frontal)&(Temporal) (Attention\|Central_executive)&(Default_mode\|Central_executive) \|\| id:ukb-rsfMRI191-edge-pheno1317 | Herring liking \|\| id:ebi-fl187-GCST90094781 | Weighted mode | 18 | 0.0807518585384701 | 0.0871812023926149 | 0.367278085820888 | -0.090123298 | 0.251627015227995 | 1.08410185256951 | 0.913818506192596 | 1.28611624603819 | 0.999228396881742 |
| ebi-fl187-GCST90094781 | ukb-rsfMRI191-edge-pheno286 | Net100_Pair10_13 (OccipitalPrecuneus)&(Frontal_Sup) (Default_mode\|Central_executive)&(Default_mode) \|\| id:ukb-rsfMRI191-edge-pheno286 | Herring liking \|\| id:ebi-fl187-GCST90094781 | Weighted mode | 18 | 0.0146512274407724 | 0.064343603517151 | 0.822593267109021 | -0.111462235 | 0.140764690334388 | 1.01475908276743 | 0.894525172103452 | 1.15115373851091 | 0.999228396881742 |
| ebi-fl187-GCST90094781 | ukb-rsfMRI191-edge-pheno599 | Net100_Pair11_29 (Angular\|Temporal)&(Parietal) (Default_mode\|Central_executive)&(Central_executive\|Salience) \|\| id:ukb-rsfMRI191-edge-pheno599 | Herring liking \|\| id:ebi-fl187-GCST90094781 | Weighted mode | 18 | -0.022828872 | 0.0597098395299 | 0.706954153805472 | -0.139860157 | 0.0942024139276585 | 0.9774297354968 | 0.869479817537835 | 1.0987821322164 | 0.999228396881742 |
| ebi-fl187-GCST90094781 | ukb-rsfMRI191-edge-pheno621 | Net100_Pair5_30 (Precuneus\|Angular\|Cingulate)&(Paracentral) (Default_mode\|Central_executive)&(Motor) \|\| id:ukb-rsfMRI191-edge-pheno621 | Herring liking \|\| id:ebi-fl187-GCST90094781 | Weighted mode | 18 | -0.026775407 | 0.0726512369832307 | 0.717014728129419 | -0.169171831 | 0.115621017755526 | 0.973579876457947 | 0.844363802858688 | 1.12257035727348 | 0.999228396881742 |
| ebi-fl187-GCST90094781 | ukb-rsfMRI191-edge-pheno624 | Net100_Pair8_30 (Calcarine\|Lingual)&(Paracentral) (Visual)&(Motor) \|\| id:ukb-rsfMRI191-edge-pheno624 | Herring liking \|\| id:ebi-fl187-GCST90094781 | Weighted mode | 18 | 0.0161046801414352 | 0.0696276473122722 | 0.81984489210378 | -0.120365509 | 0.152574868873489 | 1.01623505946813 | 0.886596318915871 | 1.16482966831513 | 0.999228396881742 |
| ebi-fl187-GCST90094781 | ukb-rsfMRI191-edge-pheno882 | Net100_Pair6_38 (Postcentral\|Precentral)&(Subcortical) (Motor)&(Subcortical-cerebellum) \|\| id:ukb-rsfMRI191-edge-pheno882 | Herring liking \|\| id:ebi-fl187-GCST90094781 | Weighted mode | 17 | -0.023375461 | 0.0591956397622756 | 0.698142244711669 | -0.139398915 | 0.0926479926322174 | 0.976895628403185 | 0.869880950470941 | 1.09707548863623 | 0.999228396881742 |
| ebi-fl187-GCST90094781 | ukb-rsfMRI191-edge-pheno899 | Net100_Pair23_38 (Cerebellum)&(Subcortical) (Subcortical-cerebellum)&(Subcortical-cerebellum) \|\| id:ukb-rsfMRI191-edge-pheno899 | Herring liking \|\| id:ebi-fl187-GCST90094781 | Weighted mode | 18 | 0.0450152407580775 | 0.0686513252496125 | 0.520787941213808 | -0.089541357 | 0.179571838247318 | 1.04604380228776 | 0.91435044979661 | 1.19670487016005 | 0.999228396881742 |
| ebi-fl187-GCST90094785 | ebi-a-GCST90001392 | IgD+ CD38+ B cell Absolute Count \|\| id:ebi-a-GCST90001392 | F-highly palatable foods liking (derived food-liking factor) \|\| id:ebi-fl187-GCST90094785 | Inverse variance weighted | 14 | 0.32722083005724 | 0.302865354835701 | 0.279956599543173 | -0.266395265 | 0.920836925535215 | 1.3871077584467 | 0.766136240481147 | 2.51139135819326 | 0.998074519377826 |
| ebi-fl187-GCST90094785 | ebi-a-GCST90001402 | Switched memory B cell %B cell \|\| id:ebi-a-GCST90001402 | F-highly palatable foods liking (derived food-liking factor) \|\| id:ebi-fl187-GCST90094785 | Inverse variance weighted | 14 | -0.147916112 | 0.352101782291591 | 0.674416235093556 | -0.838035605 | 0.542203381781928 | 0.86250346602103 | 0.432559407007051 | 1.71979204901713 | 0.998074519377826 |
| ebi-fl187-GCST90094785 | ebi-a-GCST90001407 | Memory B cell Absolute Count \|\| id:ebi-a-GCST90001407 | F-highly palatable foods liking (derived food-liking factor) \|\| id:ebi-fl187-GCST90094785 | Inverse variance weighted | 14 | 0.0760609074859697 | 0.322740045877754 | 0.813686921241447 | -0.556509582 | 0.708631397406367 | 1.0790282930565 | 0.573206305574987 | 2.03120943697313 | 0.998074519377826 |
| ebi-fl187-GCST90094785 | ebi-a-GCST90001411 | IgD+ CD24+ B cell %B cell \|\| id:ebi-a-GCST90001411 | F-highly palatable foods liking (derived food-liking factor) \|\| id:ebi-fl187-GCST90094785 | Inverse variance weighted | 14 | -0.101358671 | 0.3774729126985 | 0.788299807922992 | -0.841205579 | 0.638488238314239 | 0.903608876841452 | 0.431190375690408 | 1.89361601821308 | 0.998074519377826 |
| ebi-fl187-GCST90094785 | ebi-a-GCST90001418 | CD24+ CD27+ B cell Absolute Count \|\| id:ebi-a-GCST90001418 | F-highly palatable foods liking (derived food-liking factor) \|\| id:ebi-fl187-GCST90094785 | Inverse variance weighted | 14 | 0.146589709273412 | 0.315396850295776 | 0.642090008064112 | -0.471588117 | 0.764767535853133 | 1.15787879862516 | 0.624010479111183 | 2.14849486857217 | 0.998074519377826 |
| ebi-fl187-GCST90094785 | ebi-a-GCST90001436 | Memory B cell %lymphocyte \|\| id:ebi-a-GCST90001436 | F-highly palatable foods liking (derived food-liking factor) \|\| id:ebi-fl187-GCST90094785 | Inverse variance weighted | 14 | -0.058820703 | 0.312045086339071 | 0.850484203768266 | -0.670429072 | 0.552787666722466 | 0.942875809275834 | 0.511489065200199 | 1.73809149051817 | 0.998074519377826 |
| ebi-fl187-GCST90094785 | ebi-a-GCST90001439 | IgD+ CD24+ B cell %lymphocyte \|\| id:ebi-a-GCST90001439 | F-highly palatable foods liking (derived food-liking factor) \|\| id:ebi-fl187-GCST90094785 | Inverse variance weighted | 14 | -0.015548089 | 0.331677860704275 | 0.962611197257513 | -0.665636696 | 0.634540518093818 | 0.984572158634435 | 0.513946196093465 | 1.88615528809514 | 0.998074519377826 |
| ebi-fl187-GCST90094785 | ebi-a-GCST90001457 | CD11c+ HLA DR++ monocyte %monocyte \|\| id:ebi-a-GCST90001457 | F-highly palatable foods liking (derived food-liking factor) \|\| id:ebi-fl187-GCST90094785 | Inverse variance weighted | 14 | -0.386189856 | 0.337626630128501 | 0.252691212775926 | -1.047938051 | 0.275558339421282 | 0.679641479668962 | 0.350660047429472 | 1.31726595109048 | 0.998074519377826 |
| ebi-fl187-GCST90094785 | ebi-a-GCST90001483 | CD39+ resting CD4 regulatory T cell Absolute Count \|\| id:ebi-a-GCST90001483 | F-highly palatable foods liking (derived food-liking factor) \|\| id:ebi-fl187-GCST90094785 | Inverse variance weighted | 14 | 0.194374002974758 | 0.339603880391721 | 0.567081447730234 | -0.471249603 | 0.859997608542532 | 1.21455044350111 | 0.624221751597017 | 2.36315504231426 | 0.998074519377826 |
| ebi-fl187-GCST90094785 | ebi-a-GCST90001525 | CD33dim HLA DR+ CD11b+ Absolute Count \|\| id:ebi-a-GCST90001525 | F-highly palatable foods liking (derived food-liking factor) \|\| id:ebi-fl187-GCST90094785 | Inverse variance weighted | 14 | 0.208867804623215 | 0.465618339064031 | 0.653733532681615 | -0.70374414 | 1.12147974918872 | 1.2322820857677 | 0.494729495292079 | 3.06939277596031 | 0.998074519377826 |
| ebi-fl187-GCST90094785 | ebi-a-GCST90001540 | Naive CD4+ T cell Absolute Count \|\| id:ebi-a-GCST90001540 | F-highly palatable foods liking (derived food-liking factor) \|\| id:ebi-fl187-GCST90094785 | Inverse variance weighted | 14 | 0.226166841604009 | 0.30344557681987 | 0.456072747748005 | -0.368586489 | 0.820920172170954 | 1.25378483130124 | 0.691711381610877 | 2.27259004982717 | 0.998074519377826 |
| ebi-fl187-GCST90094785 | ebi-a-GCST90001624 | HLA DR+ CD4+ T cell Absolute Count \|\| id:ebi-a-GCST90001624 | F-highly palatable foods liking (derived food-liking factor) \|\| id:ebi-fl187-GCST90094785 | Inverse variance weighted (multiplicative random effects) | 14 | 0.103264510511862 | 0.412037696497907 | 0.802108624793458 | -0.704329375 | 0.91085839564776 | 1.10878465548769 | 0.49444004713916 | 2.486455981 | 0.998074519377826 |
| ebi-fl187-GCST90094785 | ebi-a-GCST90001672 | CD39+ CD8+ T cell Absolute Count \|\| id:ebi-a-GCST90001672 | F-highly palatable foods liking (derived food-liking factor) \|\| id:ebi-fl187-GCST90094785 | Inverse variance weighted | 14 | 0.473652822806981 | 0.356903662149643 | 0.184470070293095 | -0.225878355 | 1.17318400062028 | 1.60584937595938 | 0.797815145981469 | 3.23226781449075 | 0.998074519377826 |
| ebi-fl187-GCST90094785 | ebi-a-GCST90001722 | CD19 on CD20- CD38- B cell \|\| id:ebi-a-GCST90001722 | F-highly palatable foods liking (derived food-liking factor) \|\| id:ebi-fl187-GCST90094785 | Inverse variance weighted | 14 | 0.0447709828242387 | 0.391603347168789 | 0.908978282452208 | -0.722771578 | 0.812313543275065 | 1.04578832899187 | 0.485405052100095 | 2.25311463966814 | 0.998074519377826 |
| ebi-fl187-GCST90094785 | ebi-a-GCST90001742 | CD19 on transitional B cell \|\| id:ebi-a-GCST90001742 | F-highly palatable foods liking (derived food-liking factor) \|\| id:ebi-fl187-GCST90094785 | Inverse variance weighted | 14 | -0.565768728 | 0.335527711164764 | 0.0917561925882405 | -1.223403042 | 0.0918655855730082 | 0.567923400113755 | 0.294227193757294 | 1.09621746473518 | 0.998074519377826 |
| ebi-fl187-GCST90094785 | ebi-a-GCST90001784 | CD25 on IgD+ CD38dim B cell \|\| id:ebi-a-GCST90001784 | F-highly palatable foods liking (derived food-liking factor) \|\| id:ebi-fl187-GCST90094785 | Inverse variance weighted | 14 | -0.07944821 | 0.390604917559072 | 0.838824061803592 | -0.845033848 | 0.686137428820443 | 0.92362585368665 | 0.429542818794568 | 1.98602951852953 | 0.998074519377826 |
| ebi-fl187-GCST90094785 | ebi-a-GCST90001794 | CD25 on IgD+ B cell \|\| id:ebi-a-GCST90001794 | F-highly palatable foods liking (derived food-liking factor) \|\| id:ebi-fl187-GCST90094785 | Inverse variance weighted | 14 | -0.160107872 | 0.381112954413085 | 0.674407955699569 | -0.907089263 | 0.586873518415054 | 0.852051871269273 | 0.40369757305938 | 1.79835708654777 | 0.998074519377826 |
| ebi-fl187-GCST90094785 | ebi-a-GCST90001802 | CD27 on IgD- CD38- B cell \|\| id:ebi-a-GCST90001802 | F-highly palatable foods liking (derived food-liking factor) \|\| id:ebi-fl187-GCST90094785 | Inverse variance weighted | 14 | 0.208605928189547 | 0.311952016143847 | 0.503679473881346 | -0.402820023 | 0.820031879831487 | 1.23195942238062 | 0.668432390652405 | 2.27057222183841 | 0.998074519377826 |
| ebi-fl187-GCST90094785 | ebi-a-GCST90001804 | CD27 on IgD- CD38dim B cell \|\| id:ebi-a-GCST90001804 | F-highly palatable foods liking (derived food-liking factor) \|\| id:ebi-fl187-GCST90094785 | Inverse variance weighted | 14 | 0.106895460963365 | 0.307804282552339 | 0.728378071095703 | -0.496400933 | 0.710191854765949 | 1.11281791548561 | 0.608717537291047 | 2.03438152699984 | 0.998074519377826 |
| ebi-fl187-GCST90094785 | ebi-a-GCST90001805 | CD27 on memory B cell \|\| id:ebi-a-GCST90001805 | F-highly palatable foods liking (derived food-liking factor) \|\| id:ebi-fl187-GCST90094785 | Inverse variance weighted | 14 | 0.428247370408725 | 0.311983542335834 | 0.169858028714478 | -0.183240373 | 1.03973511338696 | 1.53456564014941 | 0.832568005172007 | 2.8284676918862 | 0.998074519377826 |
| ebi-fl187-GCST90094785 | ebi-a-GCST90001806 | CD27 on unswitched memory B cell \|\| id:ebi-a-GCST90001806 | F-highly palatable foods liking (derived food-liking factor) \|\| id:ebi-fl187-GCST90094785 | Inverse variance weighted | 14 | 0.109569969802763 | 0.31170864251462 | 0.725202727897007 | -0.50137897 | 0.720518909131418 | 1.11579814037967 | 0.605694848827686 | 2.05549955143984 | 0.998074519377826 |
| ebi-fl187-GCST90094785 | ebi-a-GCST90001839 | CD3 on Effector Memory CD8+ T cell \|\| id:ebi-a-GCST90001839 | F-highly palatable foods liking (derived food-liking factor) \|\| id:ebi-fl187-GCST90094785 | Inverse variance weighted | 14 | -0.097993454 | 0.368658610766122 | 0.790384896984414 | -0.820564331 | 0.624577423360647 | 0.906654838931938 | 0.440183175458116 | 1.86745664711784 | 0.998074519377826 |
| ebi-fl187-GCST90094785 | ebi-a-GCST90001859 | CD3 on CD8+ T cell \|\| id:ebi-a-GCST90001859 | F-highly palatable foods liking (derived food-liking factor) \|\| id:ebi-fl187-GCST90094785 | Inverse variance weighted | 14 | -0.086346921 | 0.337677078666694 | 0.798175881914013 | -0.748193995 | 0.575500153328377 | 0.917275953853567 | 0.473220419887876 | 1.77801958697668 | 0.998074519377826 |
| ebi-fl187-GCST90094785 | ebi-a-GCST90001905 | CD86 on monocyte \|\| id:ebi-a-GCST90001905 | F-highly palatable foods liking (derived food-liking factor) \|\| id:ebi-fl187-GCST90094785 | Inverse variance weighted | 14 | -0.361359253 | 0.344239470869265 | 0.293841260789222 | -1.036068616 | 0.313350110201659 | 0.696728651849909 | 0.35484698322194 | 1.36800039808984 | 0.998074519377826 |
| ebi-fl187-GCST90094785 | ebi-a-GCST90001943 | CD25 on activated & secreting CD4 regulatory T cell \|\| id:ebi-a-GCST90001943 | F-highly palatable foods liking (derived food-liking factor) \|\| id:ebi-fl187-GCST90094785 | Inverse variance weighted | 14 | -0.853731467 | 0.314432666591693 | 0.00662460048946413 | -1.470019493 | -0.23744344 | 0.425823019334268 | 0.229921003263752 | 0.788641495213668 | 0.877504772527479 |
| ebi-fl187-GCST90094785 | ebi-a-GCST90001949 | CD33 on CD33dim HLA DR+ CD11b- \|\| id:ebi-a-GCST90001949 | F-highly palatable foods liking (derived food-liking factor) \|\| id:ebi-fl187-GCST90094785 | Inverse variance weighted | 14 | -0.902967095 | 0.483528038963686 | 0.0618380059300439 | -1.850682051 | 0.0447478618078201 | 0.405365116994327 | 0.157129959122678 | 1.04576414958227 | 0.998074519377826 |
| ebi-fl187-GCST90094785 | ebi-a-GCST90001955 | CD33 on Immature Myeloid-Derived Suppressor Cells \|\| id:ebi-a-GCST90001955 | F-highly palatable foods liking (derived food-liking factor) \|\| id:ebi-fl187-GCST90094785 | Inverse variance weighted | 14 | -0.349781956 | 0.483842805574839 | 0.469726096124399 | -1.298113855 | 0.598549943307644 | 0.70484175974984 | 0.273046312713531 | 1.81947853955631 | 0.998074519377826 |
| ebi-fl187-GCST90094785 | ebi-a-GCST90001956 | CD33 on CD33+ HLA DR+ \|\| id:ebi-a-GCST90001956 | F-highly palatable foods liking (derived food-liking factor) \|\| id:ebi-fl187-GCST90094785 | Inverse variance weighted | 14 | -0.680170869 | 0.490909222692542 | 0.16588958611695 | -1.642352945 | 0.282011207856153 | 0.506530434813808 | 0.193524154451014 | 1.32579357920725 | 0.998074519377826 |
| ebi-fl187-GCST90094785 | ebi-a-GCST90001957 | CD33 on CD33+ HLA DR+ CD14- \|\| id:ebi-a-GCST90001957 | F-highly palatable foods liking (derived food-liking factor) \|\| id:ebi-fl187-GCST90094785 | Inverse variance weighted | 14 | -0.711402233 | 0.510534955534181 | 0.163485353374099 | -1.712050746 | 0.289246279516334 | 0.49095528070436 | 0.180495262845357 | 1.33542057476606 | 0.998074519377826 |
| ebi-fl187-GCST90094785 | ebi-a-GCST90002064 | CD4 on resting CD4 regulatory T cell \|\| id:ebi-a-GCST90002064 | F-highly palatable foods liking (derived food-liking factor) \|\| id:ebi-fl187-GCST90094785 | Inverse variance weighted | 14 | 0.186198992289542 | 0.336480772855936 | 0.580008801427494 | -0.473303323 | 0.845701307087176 | 1.2046619549687 | 0.622941090464102 | 2.3296110145308 | 0.998074519377826 |
| ebi-fl187-GCST90094785 | ebi-a-GCST90002080 | SSC-A on T cell \|\| id:ebi-a-GCST90002080 | F-highly palatable foods liking (derived food-liking factor) \|\| id:ebi-fl187-GCST90094785 | Inverse variance weighted | 14 | -0.174908795 | 0.320040799826373 | 0.584708334442386 | -0.802188763 | 0.452371172286146 | 0.839533586624738 | 0.448346565002429 | 1.57203533625194 | 0.998074519377826 |
| ebi-fl187-GCST90094785 | ebi-a-GCST90002121 | CD8 on CD39+ CD8+ T cell \|\| id:ebi-a-GCST90002121 | F-highly palatable foods liking (derived food-liking factor) \|\| id:ebi-fl187-GCST90094785 | Inverse variance weighted | 14 | 0.00522780957145699 | 0.337741602062196 | 0.987650256997558 | -0.65674573 | 0.667201349613362 | 1.00524149841174 | 0.518536047809655 | 1.94877573969556 | 0.999355215041207 |
| ebi-fl187-GCST90094785 | ebi-cfmet338-GCST90026053 | 1-palmitoyl-2-docosahexaenoyl-gpc (16:0/22:6) levels \|\| id:ebi-cfmet338-GCST90026053 | F-highly palatable foods liking (derived food-liking factor) \|\| id:ebi-fl187-GCST90094785 | Inverse variance weighted | 13 | -0.178723341 | 0.179863823604861 | 0.320388818858921 | -0.531256435 | 0.173809753596954 | 0.836337247897049 | 0.587865890231911 | 1.18982918356443 | 0.998074519377826 |
| ebi-fl187-GCST90094785 | ebi-cfmet338-GCST90026063 | 1-stearoyl-2-oleoyl-gpc (18:0/18:1) levels \|\| id:ebi-cfmet338-GCST90026063 | F-highly palatable foods liking (derived food-liking factor) \|\| id:ebi-fl187-GCST90094785 | Inverse variance weighted | 13 | -0.274480426 | 0.175583468155368 | 0.117994380682426 | -0.618624023 | 0.0696631718215557 | 0.759966879875178 | 0.538685146063068 | 1.07214699111008 | 0.998074519377826 |
| ebi-fl187-GCST90094785 | ebi-cfmet338-GCST90026113 | Adenosine 3',5'-cyclic monophosphate (camp) levels \|\| id:ebi-cfmet338-GCST90026113 | F-highly palatable foods liking (derived food-liking factor) \|\| id:ebi-fl187-GCST90094785 | Inverse variance weighted (multiplicative random effects) | 13 | -0.03843321 | 0.380455202666466 | 0.919535364770809 | -0.784125407 | 0.707258987002234 | 0.962295974126045 | 0.456518795175201 | 2.02842369603603 | 0.998074519377826 |
| ebi-fl187-GCST90094785 | ebi-cfmet338-GCST90026117 | Alpha-ketoglutaramate levels \|\| id:ebi-cfmet338-GCST90026117 | F-highly palatable foods liking (derived food-liking factor) \|\| id:ebi-fl187-GCST90094785 | Inverse variance weighted | 13 | -0.028477949 | 0.122250005820829 | 0.815801436078576 | -0.268087961 | 0.211132062132142 | 0.971923725531004 | 0.764840502249078 | 1.23507545098917 | 0.998074519377826 |
| ebi-fl187-GCST90094785 | ebi-cfmet338-GCST90026124 | Arginine levels \|\| id:ebi-cfmet338-GCST90026124 | F-highly palatable foods liking (derived food-liking factor) \|\| id:ebi-fl187-GCST90094785 | Inverse variance weighted | 13 | -0.012833941 | 0.0979603093898931 | 0.895766096408574 | -0.204836148 | 0.179168265247523 | 0.987248062680463 | 0.814780809297518 | 1.19622200982698 | 0.998074519377826 |
| ebi-fl187-GCST90094785 | ebi-cfmet338-GCST90026169 | Glutamate levels \|\| id:ebi-cfmet338-GCST90026169 | F-highly palatable foods liking (derived food-liking factor) \|\| id:ebi-fl187-GCST90094785 | Inverse variance weighted | 13 | 0.152168120039965 | 0.0977732459474295 | 0.119627978084046 | -0.039467442 | 0.343803682096926 | 1.16435597155062 | 0.961301251510911 | 1.41030174084841 | 0.998074519377826 |
| ebi-fl187-GCST90094785 | ebi-cfmet338-GCST90026170 | Glutamine levels \|\| id:ebi-cfmet338-GCST90026170 | F-highly palatable foods liking (derived food-liking factor) \|\| id:ebi-fl187-GCST90094785 | Inverse variance weighted | 13 | -0.017355434 | 0.0584717298315685 | 0.766605831324444 | -0.131960024 | 0.0972491568161087 | 0.982794304377332 | 0.876376028278879 | 1.1021349438476 | 0.998074519377826 |
| ebi-fl187-GCST90094785 | ebi-cfmet338-GCST90026191 | Kynurenate levels \|\| id:ebi-cfmet338-GCST90026191 | F-highly palatable foods liking (derived food-liking factor) \|\| id:ebi-fl187-GCST90094785 | Inverse variance weighted (multiplicative random effects) | 13 | -0.072140934 | 0.627845386947792 | 0.908522492153684 | -1.302717893 | 1.15843602406001 | 0.930399761278014 | 0.271792086519519 | 3.18494819651057 | 0.998074519377826 |
| ebi-fl187-GCST90094785 | ebi-cfmet338-GCST90026202 | Methylsuccinoylcarnitine levels \|\| id:ebi-cfmet338-GCST90026202 | F-highly palatable foods liking (derived food-liking factor) \|\| id:ebi-fl187-GCST90094785 | Inverse variance weighted | 13 | 0.0992032871874789 | 0.299435160256125 | 0.740416835468798 | -0.487689627 | 0.686096201289483 | 1.1042907649064 | 0.614043426022779 | 1.98594764112388 | 0.998074519377826 |
| ebi-fl187-GCST90094785 | ebi-cfmet338-GCST90026222 | N-acetylputrescine levels \|\| id:ebi-cfmet338-GCST90026222 | F-highly palatable foods liking (derived food-liking factor) \|\| id:ebi-fl187-GCST90094785 | Inverse variance weighted | 13 | -0.075361091 | 0.107419955339236 | 0.482956233693608 | -0.285904204 | 0.135182021365169 | 0.927408546888267 | 0.751334587674777 | 1.14474513346071 | 0.998074519377826 |
| ebi-fl187-GCST90094785 | ebi-cfmet338-GCST90026252 | Propionylcarnitine (c3) levels \|\| id:ebi-cfmet338-GCST90026252 | F-highly palatable foods liking (derived food-liking factor) \|\| id:ebi-fl187-GCST90094785 | Inverse variance weighted | 13 | 0.158245797973371 | 0.178090592160726 | 0.37423470153109 | -0.190811763 | 0.507303358608394 | 1.17145410036667 | 0.826288111787929 | 1.66080655123609 | 0.998074519377826 |
| ebi-fl187-GCST90094785 | ebi-cfmet338-GCST90026316 | Ethyl beta-glucopyranoside levels \|\| id:ebi-cfmet338-GCST90026316 | F-highly palatable foods liking (derived food-liking factor) \|\| id:ebi-fl187-GCST90094785 | Inverse variance weighted (multiplicative random effects) | 13 | 0.370353745328746 | 0.813485166228429 | 0.648916461950646 | -1.22407718 | 1.96478467113647 | 1.44824683461289 | 0.29402891078375 | 7.13337640293764 | 0.998074519377826 |
| ebi-fl187-GCST90094785 | ebi-gm430-GCST90011315 | F_Prevotellaceae abundance \|\| id:ebi-gm430-GCST90011315 | F-highly palatable foods liking (derived food-liking factor) \|\| id:ebi-fl187-GCST90094785 | Inverse variance weighted | 9 | 0.0867052501036538 | 0.31135044699987 | 0.78064298484913 | -0.523541626 | 0.6969521262234 | 1.09057518541754 | 0.592418702702316 | 2.00762438731807 | 0.998074519377826 |
| ebi-fl187-GCST90094785 | ebi-gm430-GCST90011354 | OTU97_1 (Bacteroides) abundance \|\| id:ebi-gm430-GCST90011354 | F-highly palatable foods liking (derived food-liking factor) \|\| id:ebi-fl187-GCST90094785 | Inverse variance weighted | 9 | -0.026047874 | 0.26378514071166 | 0.921339503810008 | -0.54306675 | 0.490971001850893 | 0.9742884454618 | 0.580963846884284 | 1.63390197178559 | 0.998074519377826 |
| ebi-fl187-GCST90094785 | ebi-gm430-GCST90011419 | OTU99_1 (Bacteroides) abundance \|\| id:ebi-gm430-GCST90011419 | F-highly palatable foods liking (derived food-liking factor) \|\| id:ebi-fl187-GCST90094785 | Inverse variance weighted | 9 | -0.015008487 | 0.26384733900855 | 0.954638217539851 | -0.532149272 | 0.502132297023778 | 0.98510357856698 | 0.587341256081384 | 1.65224058493688 | 0.998074519377826 |
| ebi-fl187-GCST90094785 | ebi-gm430-GCST90011435 | OTU99_155 (Butyrivibrio) abundance \|\| id:ebi-gm430-GCST90011435 | F-highly palatable foods liking (derived food-liking factor) \|\| id:ebi-fl187-GCST90094785 | Inverse variance weighted | 9 | -1.725175978 | 0.833469844870694 | 0.0384643233000364 | -3.358776874 | -0.091575082 | 0.178141700024822 | 0.0347777705441071 | 0.912492801903052 | 0.998074519377826 |
| ebi-fl187-GCST90094785 | ebi-gm430-GCST90011466 | OTU99_58 (Bacteroides) abundance \|\| id:ebi-gm430-GCST90011466 | F-highly palatable foods liking (derived food-liking factor) \|\| id:ebi-fl187-GCST90094785 | Inverse variance weighted | 9 | 0.485988215933864 | 0.484791079705596 | 0.316116945204247 | -0.4642023 | 1.43617873215683 | 1.62578083778954 | 0.628636368283586 | 4.20459818406705 | 0.998074519377826 |
| ebi-fl187-GCST90094785 | ebi-gm430-GCST90011472 | OTU99_72 (Parabacteroides) abundance \|\| id:ebi-gm430-GCST90011472 | F-highly palatable foods liking (derived food-liking factor) \|\| id:ebi-fl187-GCST90094785 | Inverse variance weighted | 8 | -0.18139208 | 0.773567658163549 | 0.814606402494114 | -1.69758469 | 1.33480053044204 | 0.83410825777772 | 0.18312529476771 | 3.79923803849986 | 0.998074519377826 |
| ebi-fl187-GCST90094785 | ebi-gm430-GCST90011505 | TestASV_28 (Holdemanella) abundance \|\| id:ebi-gm430-GCST90011505 | F-highly palatable foods liking (derived food-liking factor) \|\| id:ebi-fl187-GCST90094785 | Inverse variance weighted | 9 | -1.736766428 | 0.619364930370778 | 0.00504559320704001 | -2.950721692 | -0.522811165 | 0.176088877034305 | 0.0523019464397621 | 0.592851599718466 | 0.877504772527479 |
| ebi-fl187-GCST90094785 | ebi-gm430-GCST90011513 | TestASV_35 (Alphaproteobacteria) abundance \|\| id:ebi-gm430-GCST90011513 | F-highly palatable foods liking (derived food-liking factor) \|\| id:ebi-fl187-GCST90094785 | Inverse variance weighted | 9 | 0.192585741436242 | 0.821732806784049 | 0.814701314188973 | -1.41801056 | 1.80318204273298 | 1.2123804504922 | 0.242195371118476 | 6.06892836121405 | 0.998074519377826 |
| ebi-fl187-GCST90094785 | ebi-gm430-GCST90011543 | G_Coprococcus prevalence \|\| id:ebi-gm430-GCST90011543 | F-highly palatable foods liking (derived food-liking factor) \|\| id:ebi-fl187-GCST90094785 | Inverse variance weighted | 9 | 0.230316407641805 | 0.829418285583535 | 0.781254739871838 | -1.395343432 | 1.85597624738553 | 1.25899830359885 | 0.247747937160359 | 6.39794117615123 | 0.998074519377826 |
| ebi-fl187-GCST90094785 | ebi-gm430-GCST90011547 | G_Parasutterella prevalence \|\| id:ebi-gm430-GCST90011547 | F-highly palatable foods liking (derived food-liking factor) \|\| id:ebi-fl187-GCST90094785 | Inverse variance weighted | 9 | 0.432925560148683 | 0.754418872352895 | 0.566067255946102 | -1.04573543 | 1.91158654996036 | 1.5417614479359 | 0.351433269866008 | 6.76381141503108 | 0.998074519377826 |
| ebi-fl187-GCST90094785 | ebi-gm430-GCST90011569 | OTU97_13 (Subdoligranulum) prevalence \|\| id:ebi-gm430-GCST90011569 | F-highly palatable foods liking (derived food-liking factor) \|\| id:ebi-fl187-GCST90094785 | Inverse variance weighted | 9 | 0.026874895952689 | 0.724534137968784 | 0.97041112720934 | -1.393212014 | 1.44696180637151 | 1.02723928293263 | 0.248276554635036 | 4.25018200349649 | 0.998074519377826 |
| ebi-fl187-GCST90094785 | ebi-gm430-GCST90011590 | OTU97_277 (Prevotella) prevalence \|\| id:ebi-gm430-GCST90011590 | F-highly palatable foods liking (derived food-liking factor) \|\| id:ebi-fl187-GCST90094785 | Inverse variance weighted | 9 | 0.402183439114284 | 0.791348051882932 | 0.61129505043072 | -1.148858743 | 1.95322562080483 | 1.4950855646946 | 0.316998339725912 | 7.05139606627238 | 0.998074519377826 |
| ebi-fl187-GCST90094785 | ebi-gm430-GCST90011616 | OTU97_86 (Phascolarctobacterium) prevalence \|\| id:ebi-gm430-GCST90011616 | F-highly palatable foods liking (derived food-liking factor) \|\| id:ebi-fl187-GCST90094785 | Inverse variance weighted | 9 | 0.179429077222856 | 0.763080398438014 | 0.814101749238071 | -1.316208504 | 1.67506665816136 | 1.19653403954107 | 0.268150066993948 | 5.33915103520293 | 0.998074519377826 |
| ebi-fl187-GCST90094785 | ebi-gm430-GCST90011665 | OTU99_55 (Barnesiella) prevalence \|\| id:ebi-gm430-GCST90011665 | F-highly palatable foods liking (derived food-liking factor) \|\| id:ebi-fl187-GCST90094785 | Inverse variance weighted | 9 | 0.712256726688768 | 0.508226749946204 | 0.161078168741788 | -0.283867703 | 1.70838115658333 | 2.03858660427415 | 0.752866239907995 | 5.52001819557466 | 0.998074519377826 |
| ebi-fl187-GCST90094785 | ebi-gm430-GCST90011687 | TestASV_14 (Lachnospiraceae) prevalence \|\| id:ebi-gm430-GCST90011687 | F-highly palatable foods liking (derived food-liking factor) \|\| id:ebi-fl187-GCST90094785 | Inverse variance weighted | 9 | -0.165689728 | 0.551010214280491 | 0.763641944190604 | -1.245669748 | 0.914290291500633 | 0.847309089280372 | 0.287748124710946 | 2.49500389793444 | 0.998074519377826 |
| ebi-fl187-GCST90094785 | ebi-gm430-GCST90011697 | TestASV_23 (Barnesiella) prevalence \|\| id:ebi-gm430-GCST90011697 | F-highly palatable foods liking (derived food-liking factor) \|\| id:ebi-fl187-GCST90094785 | Inverse variance weighted | 9 | 1.52269177358556 | 0.73030986153624 | 0.0370698334678472 | 0.0912844449745274 | 2.95409910219659 | 4.58454916938257 | 1.0955805933355 | 19.1844317198945 | 0.998074519377826 |
| ebi-fl187-GCST90094785 | ebi-gm430-GCST90011727 | TestASV_6 (Subdoligranulum) prevalence \|\| id:ebi-gm430-GCST90011727 | F-highly palatable foods liking (derived food-liking factor) \|\| id:ebi-fl187-GCST90094785 | Inverse variance weighted | 9 | 0.0801277431975771 | 1.03994276157378 | 0.938583651549337 | -1.958160069 | 2.11841555588219 | 1.08342545906797 | 0.141117829201128 | 8.31794771788664 | 0.998074519377826 |
| ebi-fl187-GCST90094785 | ebi-hbsm44-GCST90104908 | Sulcal depth of the left posterior inferior temporal sulcus \|\| id:ebi-hbsm44-GCST90104908 | F-highly palatable foods liking (derived food-liking factor) \|\| id:ebi-fl187-GCST90094785 | Inverse variance weighted | 12 | -0.010853373 | 0.216340246082645 | 0.959988455268878 | -0.434880255 | 0.413173509527954 | 0.989205312553271 | 0.6473421783512 | 1.51160728144728 | 0.998074519377826 |
| ebi-fl187-GCST90094785 | ebi-hbsm44-GCST90104918 | Sulcal depth of the left inferior frontal sulcus \|\| id:ebi-hbsm44-GCST90104918 | F-highly palatable foods liking (derived food-liking factor) \|\| id:ebi-fl187-GCST90094785 | Inverse variance weighted | 12 | 0.112905422286627 | 0.357794253307857 | 0.752336783419497 | -0.588371314 | 0.814182158770027 | 1.11952604572791 | 0.555230845321913 | 2.25732878067413 | 0.998074519377826 |
| ebi-fl187-GCST90094785 | ebi-hbsm44-GCST90104925 | Sulcal depth of the right anterior inferior temporal sulcus \|\| id:ebi-hbsm44-GCST90104925 | F-highly palatable foods liking (derived food-liking factor) \|\| id:ebi-fl187-GCST90094785 | Inverse variance weighted | 12 | -0.094550462 | 0.121865809083365 | 0.43783332286281 | -0.333407448 | 0.144306523864822 | 0.909781824113149 | 0.716478207247322 | 1.15523816232548 | 0.998074519377826 |
| ebi-fl187-GCST90094785 | ebi-IPs91-GCST90274770 | C-C motif chemokine 4 levels \|\| id:ebi-IPs91-GCST90274770 | F-highly palatable foods liking (derived food-liking factor) \|\| id:ebi-fl187-GCST90094785 | Inverse variance weighted | 17 | -0.025478852 | 0.134487565188332 | 0.849739076042633 | -0.28907448 | 0.238116776030512 | 0.974842994982209 | 0.748956421419722 | 1.26885735629913 | 0.998074519377826 |
| ebi-fl187-GCST90094785 | ebi-IPs91-GCST90274779 | C-X-C motif chemokine 1 levels \|\| id:ebi-IPs91-GCST90274779 | F-highly palatable foods liking (derived food-liking factor) \|\| id:ebi-fl187-GCST90094785 | Inverse variance weighted | 17 | 0.0304897790047221 | 0.132509658597567 | 0.818018131232836 | -0.229229152 | 0.290208709855953 | 1.03095935256425 | 0.795146303383042 | 1.33670644272328 | 0.998074519377826 |
| ebi-fl187-GCST90094785 | ebi-IPs91-GCST90274780 | C-X-C motif chemokine 10 levels \|\| id:ebi-IPs91-GCST90274780 | F-highly palatable foods liking (derived food-liking factor) \|\| id:ebi-fl187-GCST90094785 | Inverse variance weighted | 17 | 0.0503892655052293 | 0.156045897368771 | 0.746761049921663 | -0.255460693 | 0.35623922434802 | 1.05168039960913 | 0.774559581314177 | 1.42794910760183 | 0.998074519377826 |
| ebi-fl187-GCST90094785 | ebi-IPs91-GCST90274796 | Interleukin-10 receptor subunit alpha levels \|\| id:ebi-IPs91-GCST90274796 | F-highly palatable foods liking (derived food-liking factor) \|\| id:ebi-fl187-GCST90094785 | Inverse variance weighted (multiplicative random effects) | 17 | -0.237801017 | 0.192918458122548 | 0.217706219530665 | -0.615921195 | 0.140319160771167 | 0.788359545517541 | 0.540143088905943 | 1.15064097972167 | 0.998074519377826 |
| ebi-fl187-GCST90094785 | ebi-IPs91-GCST90274807 | Interleukin-20 levels \|\| id:ebi-IPs91-GCST90274807 | F-highly palatable foods liking (derived food-liking factor) \|\| id:ebi-fl187-GCST90094785 | Inverse variance weighted | 17 | -0.20342999 | 0.147062447419027 | 0.166576011574079 | -0.491672387 | 0.0848124073510134 | 0.815927325729481 | 0.611602702296755 | 1.08851285053518 | 0.998074519377826 |
| ebi-fl187-GCST90094785 | ebi-IPs91-GCST90274808 | Interleukin-20 receptor subunit alpha levels \|\| id:ebi-IPs91-GCST90274808 | F-highly palatable foods liking (derived food-liking factor) \|\| id:ebi-fl187-GCST90094785 | Inverse variance weighted | 17 | -0.295634509 | 0.149708860570184 | 0.0482988488077077 | -0.589063876 | -0.002205143 | 0.744059325079086 | 0.554846446751743 | 0.997797286939922 | 0.998074519377826 |
| ebi-fl187-GCST90094785 | ebi-IPs91-GCST90274810 | Interleukin-24 levels \|\| id:ebi-IPs91-GCST90274810 | F-highly palatable foods liking (derived food-liking factor) \|\| id:ebi-fl187-GCST90094785 | Inverse variance weighted | 17 | 0.191283951384593 | 0.150339991915886 | 0.203251477481658 | -0.103382433 | 0.485950335539728 | 1.21080321252203 | 0.901782036525468 | 1.62571925373705 | 0.998074519377826 |
| ebi-fl187-GCST90094785 | ebi-IPs91-GCST90274823 | Monocyte chemoattractant protein-3 levels \|\| id:ebi-IPs91-GCST90274823 | F-highly palatable foods liking (derived food-liking factor) \|\| id:ebi-fl187-GCST90094785 | Inverse variance weighted | 17 | 0.0689565952668273 | 0.149749000020616 | 0.645170881706153 | -0.224551445 | 0.362464635307235 | 1.07138970475484 | 0.798874477726141 | 1.43686640574861 | 0.998074519377826 |
| ebi-fl187-GCST90094785 | ebi-IPs91-GCST90274824 | Monocyte chemoattractant protein-4 levels \|\| id:ebi-IPs91-GCST90274824 | F-highly palatable foods liking (derived food-liking factor) \|\| id:ebi-fl187-GCST90094785 | Inverse variance weighted | 17 | -0.127981603 | 0.132077810389968 | 0.332551971379503 | -0.386854111 | 0.130890905451827 | 0.879869566033133 | 0.679190173886455 | 1.13984342382544 | 0.998074519377826 |
| ebi-fl187-GCST90094785 | ebi-IPs91-GCST90274834 | SIR2-like protein 2 levels \|\| id:ebi-IPs91-GCST90274834 | F-highly palatable foods liking (derived food-liking factor) \|\| id:ebi-fl187-GCST90094785 | Inverse variance weighted | 17 | -0.091589499 | 0.132473305863373 | 0.48932627126894 | -0.351237179 | 0.168058180272792 | 0.912479646281293 | 0.703816803695168 | 1.18300543622464 | 0.998074519377826 |
| ebi-fl187-GCST90094785 | ebi-met1400-GCST90199652 | 1-stearoyl-2-oleoyl-GPS (18:0/18:1) levels \|\| id:ebi-met1400-GCST90199652 | F-highly palatable foods liking (derived food-liking factor) \|\| id:ebi-fl187-GCST90094785 | Inverse variance weighted | 14 | 0.0363534978712872 | 0.197356554602567 | 0.853854888002496 | -0.350465349 | 0.423172344892318 | 1.03702236690419 | 0.704360240003538 | 1.52679740902776 | 0.998074519377826 |
| ebi-fl187-GCST90094785 | ebi-met1400-GCST90199665 | N-acetylaspartate (naa) levels \|\| id:ebi-met1400-GCST90199665 | F-highly palatable foods liking (derived food-liking factor) \|\| id:ebi-fl187-GCST90094785 | Inverse variance weighted | 14 | -0.273919191 | 0.206272341506971 | 0.18419489015468 | -0.67821298 | 0.130374598823953 | 0.760393519775745 | 0.507523136530155 | 1.13925506701031 | 0.998074519377826 |
| ebi-fl187-GCST90094785 | ebi-met1400-GCST90199669 | Acetylcarnitine levels (Biocrates platform) \|\| id:ebi-met1400-GCST90199669 | F-highly palatable foods liking (derived food-liking factor) \|\| id:ebi-fl187-GCST90094785 | Inverse variance weighted | 14 | 0.342439530198141 | 0.207804842486709 | 0.0993752376247931 | -0.064857961 | 0.74973702147209 | 1.40837918667688 | 0.937200572884939 | 2.11644336426174 | 0.998074519377826 |
| ebi-fl187-GCST90094785 | ebi-met1400-GCST90199670 | Iminodiacetate (IDA) levels \|\| id:ebi-met1400-GCST90199670 | F-highly palatable foods liking (derived food-liking factor) \|\| id:ebi-fl187-GCST90094785 | Inverse variance weighted | 14 | 0.154279525745206 | 0.19504904595095 | 0.428956765357415 | -0.228016604 | 0.536575655809068 | 1.16681699658876 | 0.796111040844301 | 1.71014071364285 | 0.998074519377826 |
| ebi-fl187-GCST90094785 | ebi-met1400-GCST90199686 | Indolepropionate levels \|\| id:ebi-met1400-GCST90199686 | F-highly palatable foods liking (derived food-liking factor) \|\| id:ebi-fl187-GCST90094785 | Inverse variance weighted | 14 | -0.24976746 | 0.196865389668098 | 0.204540217016907 | -0.635623624 | 0.136088703979222 | 0.778981906642872 | 0.529605111109323 | 1.14578352464522 | 0.998074519377826 |
| ebi-fl187-GCST90094785 | ebi-met1400-GCST90199704 | 3-hydroxysebacate levels \|\| id:ebi-met1400-GCST90199704 | F-highly palatable foods liking (derived food-liking factor) \|\| id:ebi-fl187-GCST90094785 | Inverse variance weighted (multiplicative random effects) | 14 | 0.587659746316525 | 0.290793554756829 | 0.0432918798866226 | 0.0177043789931397 | 1.15761511363991 | 1.79977156104281 | 1.01786203051072 | 3.1823347122136 | 0.998074519377826 |
| ebi-fl187-GCST90094785 | ebi-met1400-GCST90199713 | Docosapentaenoate n3 DPA; 22:5n3 levels \|\| id:ebi-met1400-GCST90199713 | F-highly palatable foods liking (derived food-liking factor) \|\| id:ebi-fl187-GCST90094785 | Inverse variance weighted | 14 | 0.381858714393311 | 0.18709269072834 | 0.041249481606039 | 0.0151570405657635 | 0.748560388220858 | 1.4650050863779 | 1.01527249106358 | 2.11395455112228 | 0.998074519377826 |
| ebi-fl187-GCST90094785 | ebi-met1400-GCST90199715 | Myristoleate (14:1n5) levels \|\| id:ebi-met1400-GCST90199715 | F-highly palatable foods liking (derived food-liking factor) \|\| id:ebi-fl187-GCST90094785 | Inverse variance weighted | 14 | 0.220574840772856 | 0.18407485839074 | 0.230804606122633 | -0.140211882 | 0.581361563218707 | 1.24679323221567 | 0.869174053834495 | 1.7884718912638 | 0.998074519377826 |
| ebi-fl187-GCST90094785 | ebi-met1400-GCST90199725 | Campesterol levels \|\| id:ebi-met1400-GCST90199725 | F-highly palatable foods liking (derived food-liking factor) \|\| id:ebi-fl187-GCST90094785 | Inverse variance weighted | 14 | 0.10364324853901 | 0.244326429736598 | 0.67142002933626 | -0.375236554 | 0.582523050822742 | 1.1092046739341 | 0.687126717166405 | 1.79055038603496 | 0.998074519377826 |
| ebi-fl187-GCST90094785 | ebi-met1400-GCST90199737 | N-acetylproline levels \|\| id:ebi-met1400-GCST90199737 | F-highly palatable foods liking (derived food-liking factor) \|\| id:ebi-fl187-GCST90094785 | Inverse variance weighted | 14 | 0.0528007914833207 | 0.210051650277029 | 0.801527661693885 | -0.358900443 | 0.464502026026297 | 1.05421961467443 | 0.698443882826322 | 1.59122160461482 | 0.998074519377826 |
| ebi-fl187-GCST90094785 | ebi-met1400-GCST90199775 | Gamma-glutamylisoleucine levels \|\| id:ebi-met1400-GCST90199775 | F-highly palatable foods liking (derived food-liking factor) \|\| id:ebi-fl187-GCST90094785 | Inverse variance weighted | 14 | 0.229697769119406 | 0.187867580267752 | 0.221458958626947 | -0.138522688 | 0.5979182264442 | 1.25821967961684 | 0.870643497706535 | 1.81832950724996 | 0.998074519377826 |
| ebi-fl187-GCST90094785 | ebi-met1400-GCST90199777 | 1-palmitoleoyl-GPC (16:1) levels \|\| id:ebi-met1400-GCST90199777 | F-highly palatable foods liking (derived food-liking factor) \|\| id:ebi-fl187-GCST90094785 | Inverse variance weighted | 14 | 0.331525911912144 | 0.194116160918411 | 0.087659755350335 | -0.048941763 | 0.71199358731223 | 1.39309224349448 | 0.952236583021035 | 2.03805024243814 | 0.998074519377826 |
| ebi-fl187-GCST90094785 | ebi-met1400-GCST90199782 | Glutamine degradant levels \|\| id:ebi-met1400-GCST90199782 | F-highly palatable foods liking (derived food-liking factor) \|\| id:ebi-fl187-GCST90094785 | Inverse variance weighted | 14 | -0.33174797 | 0.162924436907235 | 0.0417295172579623 | -0.651079867 | -0.012416074 | 0.71766817365432 | 0.521482341140042 | 0.987660687321364 | 0.998074519377826 |
| ebi-fl187-GCST90094785 | ebi-met1400-GCST90199783 | 5-methyluridine (ribothymidine) levels \|\| id:ebi-met1400-GCST90199783 | F-highly palatable foods liking (derived food-liking factor) \|\| id:ebi-fl187-GCST90094785 | Inverse variance weighted (multiplicative random effects) | 14 | 0.378120281603223 | 0.269011215438857 | 0.159845015559201 | -0.149141701 | 0.905382263863382 | 1.45953848794746 | 0.861447038638728 | 2.47287703393376 | 0.998074519377826 |
| ebi-fl187-GCST90094785 | ebi-met1400-GCST90199787 | Homostachydrine levels \|\| id:ebi-met1400-GCST90199787 | F-highly palatable foods liking (derived food-liking factor) \|\| id:ebi-fl187-GCST90094785 | Inverse variance weighted | 14 | 0.062313237098989 | 0.190200912324658 | 0.743200531865305 | -0.310480551 | 0.435107025255318 | 1.06429566940843 | 0.733094582187612 | 1.54512841786581 | 0.998074519377826 |
| ebi-fl187-GCST90094785 | ebi-met1400-GCST90199802 | Beta-hydroxyisovaleroylcarnitine levels \|\| id:ebi-met1400-GCST90199802 | F-highly palatable foods liking (derived food-liking factor) \|\| id:ebi-fl187-GCST90094785 | Inverse variance weighted | 14 | 0.250537872621615 | 0.192170849118301 | 0.192327482380448 | -0.126116992 | 0.627192736893485 | 1.28471624457688 | 0.881511711240717 | 1.87234702390587 | 0.998074519377826 |
| ebi-fl187-GCST90094785 | ebi-met1400-GCST90199873 | Pregnenolone sulfate levels \|\| id:ebi-met1400-GCST90199873 | F-highly palatable foods liking (derived food-liking factor) \|\| id:ebi-fl187-GCST90094785 | Inverse variance weighted | 14 | 0.114158440807202 | 0.199005984847392 | 0.566209235175272 | -0.275893289 | 0.504210171108091 | 1.12092971182363 | 0.758893908393785 | 1.65567730212564 | 0.998074519377826 |
| ebi-fl187-GCST90094785 | ebi-met1400-GCST90199911 | Beta-citrylglutamate levels \|\| id:ebi-met1400-GCST90199911 | F-highly palatable foods liking (derived food-liking factor) \|\| id:ebi-fl187-GCST90094785 | Inverse variance weighted | 14 | -0.103911815 | 0.193502735753685 | 0.591264593227428 | -0.483177177 | 0.275353546740826 | 0.901304775175196 | 0.616820527025765 | 1.31699621228669 | 0.998074519377826 |
| ebi-fl187-GCST90094785 | ebi-met1400-GCST90199934 | 2-hydroxydecanoate levels \|\| id:ebi-met1400-GCST90199934 | F-highly palatable foods liking (derived food-liking factor) \|\| id:ebi-fl187-GCST90094785 | Inverse variance weighted | 14 | 0.189246916406695 | 0.194152221940261 | 0.329691853739728 | -0.191291439 | 0.569785271409607 | 1.20833927444281 | 0.825891856310393 | 1.76788739470538 | 0.998074519377826 |
| ebi-fl187-GCST90094785 | ebi-met1400-GCST90199945 | Sphingomyelin (d18:1/14:0, d16:1/16:0) levels \|\| id:ebi-met1400-GCST90199945 | F-highly palatable foods liking (derived food-liking factor) \|\| id:ebi-fl187-GCST90094785 | Inverse variance weighted | 14 | -0.112266684 | 0.201061506531867 | 0.576591854794246 | -0.506347237 | 0.281813869066694 | 0.893805862227312 | 0.602693058114206 | 1.32553197452048 | 0.998074519377826 |
| ebi-fl187-GCST90094785 | ebi-met1400-GCST90199979 | Carnitine C14:1 levels \|\| id:ebi-met1400-GCST90199979 | F-highly palatable foods liking (derived food-liking factor) \|\| id:ebi-fl187-GCST90094785 | Inverse variance weighted | 14 | 0.488549228081972 | 0.198059073866403 | 0.0136370491771842 | 0.100353443303822 | 0.876745012860122 | 1.62994981839165 | 1.10556160237453 | 2.40306501670176 | 0.998074519377826 |
| ebi-fl187-GCST90094785 | ebi-met1400-GCST90199986 | Eugenol sulfate levels \|\| id:ebi-met1400-GCST90199986 | F-highly palatable foods liking (derived food-liking factor) \|\| id:ebi-fl187-GCST90094785 | Inverse variance weighted | 14 | 0.114247042593469 | 0.210570177239177 | 0.587432500627609 | -0.298470505 | 0.526964589982257 | 1.12102903259831 | 0.741952165558359 | 1.69378317129457 | 0.998074519377826 |
| ebi-fl187-GCST90094785 | ebi-met1400-GCST90200002 | 6-hydroxyindole sulfate levels \|\| id:ebi-met1400-GCST90200002 | F-highly palatable foods liking (derived food-liking factor) \|\| id:ebi-fl187-GCST90094785 | Inverse variance weighted | 14 | 0.352380028403285 | 0.193127784863967 | 0.0680621389698723 | -0.02615043 | 0.730910486736661 | 1.42244899203461 | 0.974188531472976 | 2.0769708014125 | 0.998074519377826 |
| ebi-fl187-GCST90094785 | ebi-met1400-GCST90200023 | Trans 3,4-methyleneheptanoate levels \|\| id:ebi-met1400-GCST90200023 | F-highly palatable foods liking (derived food-liking factor) \|\| id:ebi-fl187-GCST90094785 | Inverse variance weighted | 14 | 0.289539270672528 | 0.209795067992973 | 0.167554247068727 | -0.121659063 | 0.700737603938755 | 1.33581189850866 | 0.8854502001415 | 2.01523860733461 | 0.998074519377826 |
| ebi-fl187-GCST90094785 | ebi-met1400-GCST90200032 | 2-hydroxybutyrate/2-hydroxyisobutyrate levels \|\| id:ebi-met1400-GCST90200032 | F-highly palatable foods liking (derived food-liking factor) \|\| id:ebi-fl187-GCST90094785 | Inverse variance weighted | 14 | 0.127925473341923 | 0.196650708457274 | 0.515355551528747 | -0.257509915 | 0.51336086191818 | 1.1364683023563 | 0.772973962057519 | 1.670897424 | 0.998074519377826 |
| ebi-fl187-GCST90094785 | ebi-met1400-GCST90200051 | 1-palmitoyl-2-dihomo-linolenoyl-GPC (16:0/20:3n3 or 6) levels \|\| id:ebi-met1400-GCST90200051 | F-highly palatable foods liking (derived food-liking factor) \|\| id:ebi-fl187-GCST90094785 | Inverse variance weighted | 14 | -0.021135065 | 0.19145969794272 | 0.912100793271508 | -0.396396073 | 0.354125943267916 | 0.979086715585242 | 0.672740189161558 | 1.42493463610405 | 0.998074519377826 |
| ebi-fl187-GCST90094785 | ebi-met1400-GCST90200069 | Phenylacetylglutamate levels \|\| id:ebi-met1400-GCST90200069 | F-highly palatable foods liking (derived food-liking factor) \|\| id:ebi-fl187-GCST90094785 | Inverse variance weighted | 14 | 0.131915891161845 | 0.238506704836397 | 0.580201158628234 | -0.33555725 | 0.599389032641184 | 1.14101234600845 | 0.71493957502242 | 1.8210058852916 | 0.998074519377826 |
| ebi-fl187-GCST90094785 | ebi-met1400-GCST90200072 | Thioproline levels \|\| id:ebi-met1400-GCST90200072 | F-highly palatable foods liking (derived food-liking factor) \|\| id:ebi-fl187-GCST90094785 | Inverse variance weighted | 14 | 0.0119381674096508 | 0.194512993664408 | 0.951060838600725 | -0.3693073 | 0.393183634991891 | 1.01200971174951 | 0.691212967945825 | 1.48169045456276 | 0.998074519377826 |
| ebi-fl187-GCST90094785 | ebi-met1400-GCST90200081 | 1-oleoyl-2-docosahexaenoyl-GPC (18:1/22:6) levels \|\| id:ebi-met1400-GCST90200081 | F-highly palatable foods liking (derived food-liking factor) \|\| id:ebi-fl187-GCST90094785 | Inverse variance weighted | 14 | -0.36584704 | 0.187127763657754 | 0.0505753365072772 | -0.732617457 | 0.000923376882526417 | 0.693608887590108 | 0.480649263555575 | 1.00092380332621 | 0.998074519377826 |
| ebi-fl187-GCST90094785 | ebi-met1400-GCST90200097 | Furaneol sulfate levels \|\| id:ebi-met1400-GCST90200097 | F-highly palatable foods liking (derived food-liking factor) \|\| id:ebi-fl187-GCST90094785 | Inverse variance weighted | 13 | -0.475167379 | 0.241308879919035 | 0.0489387242138433 | -0.948132783 | -0.002201974 | 0.621780974968544 | 0.387463827407288 | 0.997800448676313 | 0.998074519377826 |
| ebi-fl187-GCST90094785 | ebi-met1400-GCST90200124 | Sphingomyelin (d18:2/24:2) levels \|\| id:ebi-met1400-GCST90200124 | F-highly palatable foods liking (derived food-liking factor) \|\| id:ebi-fl187-GCST90094785 | Inverse variance weighted | 14 | -0.249003854 | 0.177636824370759 | 0.160987500306876 | -0.59717203 | 0.099164321856161 | 0.779576968959025 | 0.55036585572138 | 1.10424773668919 | 0.998074519377826 |
| ebi-fl187-GCST90094785 | ebi-met1400-GCST90200137 | Lignoceroylcarnitine (C24) levels \|\| id:ebi-met1400-GCST90200137 | F-highly palatable foods liking (derived food-liking factor) \|\| id:ebi-fl187-GCST90094785 | Inverse variance weighted | 14 | 0.350671059557462 | 0.195852269179663 | 0.0733755587856238 | -0.033199388 | 0.734541507149602 | 1.42002014702382 | 0.967345663208415 | 2.08452603308886 | 0.998074519377826 |
| ebi-fl187-GCST90094785 | ebi-met1400-GCST90200165 | Octadecenedioate (C18:1-DC) levels \|\| id:ebi-met1400-GCST90200165 | F-highly palatable foods liking (derived food-liking factor) \|\| id:ebi-fl187-GCST90094785 | Inverse variance weighted | 14 | -0.045654335 | 0.191043900728286 | 0.81112651697372 | -0.42010038 | 0.328791710810094 | 0.955372144198982 | 0.656980868735997 | 1.38928845168266 | 0.998074519377826 |
| ebi-fl187-GCST90094785 | ebi-met1400-GCST90200176 | Glutamine conjugate of C6H10O2 (1) levels \|\| id:ebi-met1400-GCST90200176 | F-highly palatable foods liking (derived food-liking factor) \|\| id:ebi-fl187-GCST90094785 | Inverse variance weighted | 14 | 0.324818604218817 | 0.191790949275942 | 0.0903398779597256 | -0.051091656 | 0.700728864799664 | 1.38377961142954 | 0.950191575438042 | 2.01522099596108 | 0.998074519377826 |
| ebi-fl187-GCST90094785 | ebi-met1400-GCST90200182 | Glycine conjugate of C10H12O2 levels \|\| id:ebi-met1400-GCST90200182 | F-highly palatable foods liking (derived food-liking factor) \|\| id:ebi-fl187-GCST90094785 | Inverse variance weighted | 14 | 0.259771141406177 | 0.270643243970022 | 0.337141456980913 | -0.270689617 | 0.790231899587421 | 1.29663330703143 | 0.762853236510785 | 2.20390745222918 | 0.998074519377826 |
| ebi-fl187-GCST90094785 | ebi-met1400-GCST90200208 | Glucuronide of piperine metabolite C17H21NO3 (4) levels \|\| id:ebi-met1400-GCST90200208 | F-highly palatable foods liking (derived food-liking factor) \|\| id:ebi-fl187-GCST90094785 | Inverse variance weighted | 14 | 0.323987931856425 | 0.199435535227842 | 0.104263895869997 | -0.066905717 | 0.714881580902995 | 1.38263062123395 | 0.935283378324844 | 2.04394462584987 | 0.998074519377826 |
| ebi-fl187-GCST90094785 | ebi-met1400-GCST90200227 | 3-ethylcatechol sulfate (2) levels \|\| id:ebi-met1400-GCST90200227 | F-highly palatable foods liking (derived food-liking factor) \|\| id:ebi-fl187-GCST90094785 | Inverse variance weighted | 14 | 0.0563131586719603 | 0.254364066756121 | 0.824790314855875 | -0.442240412 | 0.554866729513958 | 1.05792893148656 | 0.642595129194218 | 1.74170885092102 | 0.998074519377826 |
| ebi-fl187-GCST90094785 | ebi-met1400-GCST90200259 | Pentose acid levels \|\| id:ebi-met1400-GCST90200259 | F-highly palatable foods liking (derived food-liking factor) \|\| id:ebi-fl187-GCST90094785 | Inverse variance weighted | 14 | -0.15648672 | 0.192171470615646 | 0.41546866352134 | -0.533142803 | 0.220169362028623 | 0.855142873601588 | 0.586758004184942 | 1.24628778654222 | 0.998074519377826 |
| ebi-fl187-GCST90094785 | ebi-met1400-GCST90200291 | Sarcosine levels \|\| id:ebi-met1400-GCST90200291 | F-highly palatable foods liking (derived food-liking factor) \|\| id:ebi-fl187-GCST90094785 | Inverse variance weighted | 14 | 0.334161410037773 | 0.195383938341623 | 0.0872139494301608 | -0.048791109 | 0.717113929187354 | 1.39676857784925 | 0.952380052436257 | 2.04851251879574 | 0.998074519377826 |
| ebi-fl187-GCST90094785 | ebi-met1400-GCST90200306 | Butyrate/isobutyrate (4:0) levels \|\| id:ebi-met1400-GCST90200306 | F-highly palatable foods liking (derived food-liking factor) \|\| id:ebi-fl187-GCST90094785 | Inverse variance weighted | 14 | 0.0977782184288372 | 0.241925532456127 | 0.686090217333792 | -0.376395825 | 0.571952262042847 | 1.10271819541284 | 0.686330612327184 | 1.77172254399585 | 0.998074519377826 |
| ebi-fl187-GCST90094785 | ebi-met1400-GCST90200321 | Cholate levels \|\| id:ebi-met1400-GCST90200321 | F-highly palatable foods liking (derived food-liking factor) \|\| id:ebi-fl187-GCST90094785 | Inverse variance weighted | 14 | -0.272423581 | 0.2051134113125 | 0.184125009769494 | -0.674445867 | 0.129598705192154 | 0.761531622451664 | 0.509438639090592 | 1.13837146909215 | 0.998074519377826 |
| ebi-fl187-GCST90094785 | ebi-met1400-GCST90200336 | Ceramide (d18:1/16:0) levels \|\| id:ebi-met1400-GCST90200336 | F-highly palatable foods liking (derived food-liking factor) \|\| id:ebi-fl187-GCST90094785 | Inverse variance weighted | 14 | 0.184056384450357 | 0.193228652075707 | 0.340827481041347 | -0.194671774 | 0.562784542518742 | 1.20208360000857 | 0.823104778426684 | 1.75555411568817 | 0.998074519377826 |
| ebi-fl187-GCST90094785 | ebi-met1400-GCST90200358 | Arachidonate (20:4n6) levels \|\| id:ebi-met1400-GCST90200358 | F-highly palatable foods liking (derived food-liking factor) \|\| id:ebi-fl187-GCST90094785 | Inverse variance weighted | 14 | 0.280408998697353 | 0.226743626092402 | 0.21620641242026 | -0.164008508 | 0.72482650583846 | 1.32367108138873 | 0.848734800437173 | 2.06437291224813 | 0.998074519377826 |
| ebi-fl187-GCST90094785 | ebi-met1400-GCST90200413 | 2'-deoxyuridine levels \|\| id:ebi-met1400-GCST90200413 | F-highly palatable foods liking (derived food-liking factor) \|\| id:ebi-fl187-GCST90094785 | Inverse variance weighted | 14 | 0.14903946936239 | 0.209257658225437 | 0.476322695647004 | -0.261105541 | 0.559184479484247 | 1.16071880113599 | 0.770199627871692 | 1.74924537296065 | 0.998074519377826 |
| ebi-fl187-GCST90094785 | ebi-met1400-GCST90200432 | Threonine levels \|\| id:ebi-met1400-GCST90200432 | F-highly palatable foods liking (derived food-liking factor) \|\| id:ebi-fl187-GCST90094785 | Inverse variance weighted | 14 | -0.112595626 | 0.222632758857501 | 0.613034974147353 | -0.548955834 | 0.323764580984732 | 0.893511899718212 | 0.577552556536957 | 1.38232184396359 | 0.998074519377826 |
| ebi-fl187-GCST90094785 | ebi-met1400-GCST90200436 | Caffeine levels \|\| id:ebi-met1400-GCST90200436 | F-highly palatable foods liking (derived food-liking factor) \|\| id:ebi-fl187-GCST90094785 | Inverse variance weighted | 14 | 0.338185141611562 | 0.199474765485971 | 0.090003724585346 | -0.052785399 | 0.729155681964064 | 1.40240012199012 | 0.94858355786681 | 2.07332931911734 | 0.998074519377826 |
| ebi-fl187-GCST90094785 | ebi-met1400-GCST90200442 | Valine levels \|\| id:ebi-met1400-GCST90200442 | F-highly palatable foods liking (derived food-liking factor) \|\| id:ebi-fl187-GCST90094785 | Inverse variance weighted | 14 | -0.043362384 | 0.191230561396012 | 0.82061467286992 | -0.418174284 | 0.3314495166498 | 0.957564321496062 | 0.658247496405622 | 1.39298582191217 | 0.998074519377826 |
| ebi-fl187-GCST90094785 | ebi-met1400-GCST90200459 | Margarate (17:0) levels \|\| id:ebi-met1400-GCST90200459 | F-highly palatable foods liking (derived food-liking factor) \|\| id:ebi-fl187-GCST90094785 | Inverse variance weighted | 14 | 0.293647728926757 | 0.210582050434961 | 0.163179481523953 | -0.11909309 | 0.706388547779281 | 1.34131131525567 | 0.887725158646315 | 2.02665884469957 | 0.998074519377826 |
| ebi-fl187-GCST90094785 | ebi-met1400-GCST90200462 | X-11299 levels \|\| id:ebi-met1400-GCST90200462 | F-highly palatable foods liking (derived food-liking factor) \|\| id:ebi-fl187-GCST90094785 | Inverse variance weighted | 14 | -0.126465702 | 0.19371165704064 | 0.513849503574927 | -0.506140549 | 0.253209146286345 | 0.881204373001662 | 0.602817639944364 | 1.28815266100859 | 0.998074519377826 |
| ebi-fl187-GCST90094785 | ebi-met1400-GCST90200497 | X-12740 levels \|\| id:ebi-met1400-GCST90200497 | F-highly palatable foods liking (derived food-liking factor) \|\| id:ebi-fl187-GCST90094785 | Inverse variance weighted (multiplicative random effects) | 14 | -0.168809169 | 0.293062069382318 | 0.564602688514209 | -0.743210825 | 0.405592487315097 | 0.844670077517725 | 0.475584443141551 | 1.50019108098001 | 0.998074519377826 |
| ebi-fl187-GCST90094785 | ebi-met1400-GCST90200527 | X-13866 levels \|\| id:ebi-met1400-GCST90200527 | F-highly palatable foods liking (derived food-liking factor) \|\| id:ebi-fl187-GCST90094785 | Inverse variance weighted | 14 | 0.0261220621378146 | 0.191009590518815 | 0.891222196350504 | -0.348256735 | 0.400500859554692 | 1.02646623348986 | 0.705917618990911 | 1.49257207944604 | 0.998074519377826 |
| ebi-fl187-GCST90094785 | ebi-met1400-GCST90200547 | X-17654 levels \|\| id:ebi-met1400-GCST90200547 | F-highly palatable foods liking (derived food-liking factor) \|\| id:ebi-fl187-GCST90094785 | Inverse variance weighted | 14 | -0.206775699 | 0.222945463085558 | 0.35368146577464 | -0.643748807 | 0.23019740853298 | 0.813202031468219 | 0.525319407178171 | 1.25884849283656 | 0.998074519377826 |
| ebi-fl187-GCST90094785 | ebi-met1400-GCST90200553 | X-17653 levels \|\| id:ebi-met1400-GCST90200553 | F-highly palatable foods liking (derived food-liking factor) \|\| id:ebi-fl187-GCST90094785 | Inverse variance weighted | 14 | -0.046982073 | 0.214987762073782 | 0.827013296714078 | -0.468358087 | 0.374393940562056 | 0.954104501570609 | 0.626029310704082 | 1.45410987050029 | 0.998074519377826 |
| ebi-fl187-GCST90094785 | ebi-met1400-GCST90200557 | X-17685 levels \|\| id:ebi-met1400-GCST90200557 | F-highly palatable foods liking (derived food-liking factor) \|\| id:ebi-fl187-GCST90094785 | Inverse variance weighted | 14 | 0.252452133807361 | 0.234566522512739 | 0.281815627627724 | -0.20729825 | 0.712202517932329 | 1.28717788237572 | 0.812777202775518 | 2.03847609802467 | 0.998074519377826 |
| ebi-fl187-GCST90094785 | ebi-met1400-GCST90200561 | X-18779 levels \|\| id:ebi-met1400-GCST90200561 | F-highly palatable foods liking (derived food-liking factor) \|\| id:ebi-fl187-GCST90094785 | Inverse variance weighted | 14 | 0.230758702030441 | 0.194477311878274 | 0.235401824590052 | -0.150416829 | 0.611934233311858 | 1.25955527464734 | 0.8603492829261 | 1.84399466748694 | 0.998074519377826 |
| ebi-fl187-GCST90094785 | ebi-met1400-GCST90200570 | X-18921 levels \|\| id:ebi-met1400-GCST90200570 | F-highly palatable foods liking (derived food-liking factor) \|\| id:ebi-fl187-GCST90094785 | Inverse variance weighted | 14 | 0.36616510368986 | 0.195287071245586 | 0.0607914237339293 | -0.016597556 | 0.748927763331209 | 1.44219333444007 | 0.983539424585874 | 2.11473130808063 | 0.998074519377826 |
| ebi-fl187-GCST90094785 | ebi-met1400-GCST90200575 | X-21283 levels \|\| id:ebi-met1400-GCST90200575 | F-highly palatable foods liking (derived food-liking factor) \|\| id:ebi-fl187-GCST90094785 | Inverse variance weighted | 13 | -0.176145692 | 0.212941737813283 | 0.408122949694151 | -0.593511498 | 0.24122011428195 | 0.83849581244653 | 0.552384179214846 | 1.27280116619146 | 0.998074519377826 |
| ebi-fl187-GCST90094785 | ebi-met1400-GCST90200578 | X-18922 levels \|\| id:ebi-met1400-GCST90200578 | F-highly palatable foods liking (derived food-liking factor) \|\| id:ebi-fl187-GCST90094785 | Inverse variance weighted | 14 | 0.272164999175123 | 0.201060176997092 | 0.175848240699663 | -0.121912948 | 0.666242946089424 | 1.312803595 | 0.885225426023236 | 1.94690892088503 | 0.998074519377826 |
| ebi-fl187-GCST90094785 | ebi-met1400-GCST90200623 | X-24241 levels \|\| id:ebi-met1400-GCST90200623 | F-highly palatable foods liking (derived food-liking factor) \|\| id:ebi-fl187-GCST90094785 | Inverse variance weighted | 14 | -0.030931356 | 0.233128670441278 | 0.894446989850852 | -0.48786355 | 0.426000838048911 | 0.969542124025893 | 0.613936638932478 | 1.53112205828788 | 0.998074519377826 |
| ebi-fl187-GCST90094785 | ebi-met1400-GCST90200636 | X-24544 levels \|\| id:ebi-met1400-GCST90200636 | F-highly palatable foods liking (derived food-liking factor) \|\| id:ebi-fl187-GCST90094785 | Inverse variance weighted | 14 | 0.244866362854394 | 0.192471287087066 | 0.203293963221895 | -0.13237736 | 0.622110085545044 | 1.27745058694736 | 0.876010361572793 | 1.86285468035136 | 0.998074519377826 |
| ebi-fl187-GCST90094785 | ebi-met1400-GCST90200659 | X-25172 levels \|\| id:ebi-met1400-GCST90200659 | F-highly palatable foods liking (derived food-liking factor) \|\| id:ebi-fl187-GCST90094785 | Inverse variance weighted | 14 | 0.409240280767391 | 0.227897504077421 | 0.0725389374279585 | -0.037438827 | 0.855919388759137 | 1.50567346139887 | 0.963253340799066 | 2.35353720183335 | 0.998074519377826 |
| ebi-fl187-GCST90094785 | ebi-met1400-GCST90200670 | X-26111 levels \|\| id:ebi-met1400-GCST90200670 | F-highly palatable foods liking (derived food-liking factor) \|\| id:ebi-fl187-GCST90094785 | Inverse variance weighted | 14 | -0.02588733 | 0.196446255673929 | 0.895159748025465 | -0.410921991 | 0.359147331618729 | 0.974444874612996 | 0.663038652815516 | 1.43210778078686 | 0.998074519377826 |
| ebi-fl187-GCST90094785 | ebi-met1400-GCST90200673 | Carnitine C4 levels \|\| id:ebi-met1400-GCST90200673 | F-highly palatable foods liking (derived food-liking factor) \|\| id:ebi-fl187-GCST90094785 | Inverse variance weighted | 14 | 0.389511989599714 | 0.195102531966612 | 0.0458852196159291 | 0.00711102694515436 | 0.771912952254274 | 1.47626018774015 | 1.00713637033415 | 2.16390173773936 | 0.998074519377826 |
| ebi-fl187-GCST90094785 | ebi-met1400-GCST90200710 | X-13431 levels \|\| id:ebi-met1400-GCST90200710 | F-highly palatable foods liking (derived food-liking factor) \|\| id:ebi-fl187-GCST90094785 | Inverse variance weighted | 14 | 0.0119017003993091 | 0.192766418474373 | 0.950768638828996 | -0.36592048 | 0.38972388060908 | 1.01197280745378 | 0.69355795087668 | 1.47657302714418 | 0.998074519377826 |
| ebi-fl187-GCST90094785 | ebi-met1400-GCST90200730 | Adenosine 5'-diphosphate (ADP) to fructose ratio \|\| id:ebi-met1400-GCST90200730 | F-highly palatable foods liking (derived food-liking factor) \|\| id:ebi-fl187-GCST90094785 | Inverse variance weighted | 14 | -0.324323677 | 0.271178871018568 | 0.231705557731973 | -0.855834264 | 0.207186910604616 | 0.723016181088511 | 0.424928540672804 | 1.23021249005332 | 0.998074519377826 |
| ebi-fl187-GCST90094785 | ebi-met1400-GCST90200731 | Adenosine 5'-diphosphate (ADP) to mannose ratio \|\| id:ebi-met1400-GCST90200731 | F-highly palatable foods liking (derived food-liking factor) \|\| id:ebi-fl187-GCST90094785 | Inverse variance weighted | 14 | -0.087627333 | 0.259424864084129 | 0.735532461764166 | -0.596100067 | 0.420845400621664 | 0.916102214195988 | 0.550956143931271 | 1.52324876689184 | 0.998074519377826 |
| ebi-fl187-GCST90094785 | ebi-met1400-GCST90200748 | Aspartate to glutamate ratio \|\| id:ebi-met1400-GCST90200748 | F-highly palatable foods liking (derived food-liking factor) \|\| id:ebi-fl187-GCST90094785 | Inverse variance weighted | 14 | -0.255586264 | 0.188995618882041 | 0.176266693922684 | -0.626017677 | 0.114845148709946 | 0.774462325229371 | 0.534716982191849 | 1.12169972747282 | 0.998074519377826 |
| ebi-fl187-GCST90094785 | ebi-met1400-GCST90200774 | Uridine to pseudouridine ratio \|\| id:ebi-met1400-GCST90200774 | F-highly palatable foods liking (derived food-liking factor) \|\| id:ebi-fl187-GCST90094785 | Inverse variance weighted | 14 | 0.160593476211475 | 0.186029129068221 | 0.387988709638933 | -0.204023617 | 0.525210569185188 | 1.17420752848203 | 0.815443112833769 | 1.69081484440097 | 0.998074519377826 |
| ebi-fl187-GCST90094785 | ebi-met1400-GCST90200799 | Spermidine to adenosine 5'-diphosphate (ADP) ratio \|\| id:ebi-met1400-GCST90200799 | F-highly palatable foods liking (derived food-liking factor) \|\| id:ebi-fl187-GCST90094785 | Inverse variance weighted | 14 | 0.000653730364826585 | 0.275364725266464 | 0.998105781797032 | -0.539061131 | 0.540368591887097 | 1.00065394409309 | 0.583295633470518 | 1.71663948497511 | 0.999355215041207 |
| ebi-fl187-GCST90094785 | ebi-met1400-GCST90200811 | Choline to taurocholate ratio \|\| id:ebi-met1400-GCST90200811 | F-highly palatable foods liking (derived food-liking factor) \|\| id:ebi-fl187-GCST90094785 | Inverse variance weighted | 14 | -0.007831178 | 0.20394314696669 | 0.969369694895207 | -0.407559746 | 0.391897390099616 | 0.992199405831221 | 0.665271702880946 | 1.47978586293186 | 0.998074519377826 |
| ebi-fl187-GCST90094785 | ebi-met1400-GCST90200812 | Glutamate to kynurenine ratio \|\| id:ebi-met1400-GCST90200812 | F-highly palatable foods liking (derived food-liking factor) \|\| id:ebi-fl187-GCST90094785 | Inverse variance weighted | 14 | 0.174634015486763 | 0.189454660266921 | 0.35664747584266 | -0.196697119 | 0.545965149609928 | 1.19081031871643 | 0.821439394322085 | 1.72627369099067 | 0.998074519377826 |
| ebi-fl187-GCST90094785 | ebi-met1400-GCST90200876 | Cortisone to 4-cholesten-3-one ratio \|\| id:ebi-met1400-GCST90200876 | F-highly palatable foods liking (derived food-liking factor) \|\| id:ebi-fl187-GCST90094785 | Inverse variance weighted | 14 | 0.166194393843805 | 0.206481991062109 | 0.420885663675672 | -0.238510309 | 0.570899096325538 | 1.18080262017418 | 0.787800567064476 | 1.76985760876723 | 0.998074519377826 |
| ebi-fl187-GCST90094785 | ebi-met1400-GCST90200906 | 2'-deoxyuridine to cytidine ratio \|\| id:ebi-met1400-GCST90200906 | F-highly palatable foods liking (derived food-liking factor) \|\| id:ebi-fl187-GCST90094785 | Inverse variance weighted | 14 | -0.066667018 | 0.221566992745639 | 0.763499321558039 | -0.500938324 | 0.367604288062081 | 0.935506656619417 | 0.60596180466742 | 1.44427041083815 | 0.998074519377826 |
| ebi-fl187-GCST90094785 | ebi-met1400-GCST90200907 | Retinol (Vitamin A) to linoleoyl-arachidonoyl-glycerol (18:2 to 20:4) [1] ratio \|\| id:ebi-met1400-GCST90200907 | F-highly palatable foods liking (derived food-liking factor) \|\| id:ebi-fl187-GCST90094785 | Inverse variance weighted | 14 | -0.132375992 | 0.203199874118189 | 0.514751489107695 | -0.530647745 | 0.265895761261174 | 0.876011559803167 | 0.588223827041208 | 1.3045990618381 | 0.998074519377826 |
| ebi-fl187-GCST90094785 | ebi-met1400-GCST90200916 | Fructose to sucrose ratio \|\| id:ebi-met1400-GCST90200916 | F-highly palatable foods liking (derived food-liking factor) \|\| id:ebi-fl187-GCST90094785 | Inverse variance weighted | 14 | -0.14728655 | 0.200615336420106 | 0.462842739738026 | -0.540492609 | 0.245919509294557 | 0.863046635889947 | 0.582461255759357 | 1.2787966381556 | 0.998074519377826 |
| ebi-fl187-GCST90094785 | ebi-met1400-GCST90200917 | Alpha-ketobutyrate to 3-methyl-2-oxobutyrate ratio \|\| id:ebi-met1400-GCST90200917 | F-highly palatable foods liking (derived food-liking factor) \|\| id:ebi-fl187-GCST90094785 | Inverse variance weighted | 14 | 0.126897112435231 | 0.196120052036466 | 0.517607395598869 | -0.25749819 | 0.511292414426705 | 1.135300203 | 0.77298302575451 | 1.66744483270738 | 0.998074519377826 |
| ebi-fl187-GCST90094785 | ebi-met1400-GCST90200924 | Alpha-tocopherol to glycerol ratio \|\| id:ebi-met1400-GCST90200924 | F-highly palatable foods liking (derived food-liking factor) \|\| id:ebi-fl187-GCST90094785 | Inverse variance weighted | 14 | -0.145405762 | 0.181845008526651 | 0.423934640691872 | -0.501821979 | 0.211010454499629 | 0.864671370951424 | 0.605426579744008 | 1.23492526551964 | 0.998074519377826 |
| ebi-fl187-GCST90094785 | ebi-met1400-GCST90200938 | Alpha-ketoglutarate to alanine ratio \|\| id:ebi-met1400-GCST90200938 | F-highly palatable foods liking (derived food-liking factor) \|\| id:ebi-fl187-GCST90094785 | Inverse variance weighted | 14 | 0.329450031081058 | 0.19369242605028 | 0.0889631400247106 | -0.050187124 | 0.709087186139607 | 1.39020334954748 | 0.951051443320088 | 2.03213545036656 | 0.998074519377826 |
| ebi-fl187-GCST90094785 | ebi-met1400-GCST90200943 | Phosphate to EDTA ratio \|\| id:ebi-met1400-GCST90200943 | F-highly palatable foods liking (derived food-liking factor) \|\| id:ebi-fl187-GCST90094785 | Inverse variance weighted | 14 | -0.059078927 | 0.232670614340546 | 0.799560002795694 | -0.515113332 | 0.396955476634461 | 0.942632366630263 | 0.597432882768531 | 1.48728970943374 | 0.998074519377826 |
| ebi-fl187-GCST90094785 | ebi-met1400-GCST90200947 | Adenosine 5'-diphosphate (ADP) to glucose ratio \|\| id:ebi-met1400-GCST90200947 | F-highly palatable foods liking (derived food-liking factor) \|\| id:ebi-fl187-GCST90094785 | Inverse variance weighted | 14 | -0.142859082 | 0.260274857305264 | 0.583089336923548 | -0.652997803 | 0.367279637868071 | 0.866876218361417 | 0.520483129909922 | 1.44380160427226 | 0.998074519377826 |
| ebi-fl187-GCST90094785 | ebi-met1400-GCST90200975 | Arachidonate (20:4n6) to caffeine ratio \|\| id:ebi-met1400-GCST90200975 | F-highly palatable foods liking (derived food-liking factor) \|\| id:ebi-fl187-GCST90094785 | Inverse variance weighted | 14 | -0.157728356 | 0.199553502224535 | 0.429290493293665 | -0.54885322 | 0.233396508471425 | 0.854081756739584 | 0.577611824260023 | 1.26288212352626 | 0.998074519377826 |
| ebi-fl187-GCST90094785 | ebi-met1400-GCST90200985 | Cholesterol to oleoyl-linoleoyl-glycerol (18:1 to 18:2) [2] ratio \|\| id:ebi-met1400-GCST90200985 | F-highly palatable foods liking (derived food-liking factor) \|\| id:ebi-fl187-GCST90094785 | Inverse variance weighted | 14 | -0.019891437 | 0.195078845358008 | 0.918783551140936 | -0.402245974 | 0.362463099998851 | 0.980305092487051 | 0.668816214187216 | 1.43686419971727 | 0.998074519377826 |
| ebi-fl187-GCST90094785 | ebi-met1400-GCST90201016 | Bilirubin (Z,Z) to etiocholanolone glucuronide ratio \|\| id:ebi-met1400-GCST90201016 | F-highly palatable foods liking (derived food-liking factor) \|\| id:ebi-fl187-GCST90094785 | Inverse variance weighted | 14 | 0.0920879840759053 | 0.203611547462567 | 0.6510723997895 | -0.306990649 | 0.491166617102536 | 1.09646128897026 | 0.735657480044435 | 1.63422161919389 | 0.998074519377826 |
| ebi-fl187-GCST90094785 | ebi-pw11-GCST90275189 | Placental weight (fetal genotype, adjusted for sex and gestational age) \|\| id:ebi-pw11-GCST90275189 | F-highly palatable foods liking (derived food-liking factor) \|\| id:ebi-fl187-GCST90094785 | Inverse variance weighted (multiplicative random effects) | 13 | 0.0473607037310303 | 0.105999593767071 | 0.655018890656391 | -0.1603985 | 0.25511990751449 | 1.0485001387912 | 0.851804277273837 | 1.29061636619577 | 0.998074519377826 |
| ebi-fl187-GCST90094785 | ebi-pw11-GCST90275192 | Placental weight (fetal effect, conditional on maternal and paternal genotype) \|\| id:ebi-pw11-GCST90275192 | F-highly palatable foods liking (derived food-liking factor) \|\| id:ebi-fl187-GCST90094785 | Inverse variance weighted | 13 | 0.195146685478952 | 0.159624327314278 | 0.221504527874829 | -0.117716996 | 0.508010367014937 | 1.2154892680392 | 0.888947592693345 | 1.66198117061342 | 0.998074519377826 |
| ebi-fl187-GCST90094785 | ebi-pw11-GCST90275193 | Placental weight (maternal effect, conditional on fetal and paternal genotype) \|\| id:ebi-pw11-GCST90275193 | F-highly palatable foods liking (derived food-liking factor) \|\| id:ebi-fl187-GCST90094785 | Inverse variance weighted | 13 | -0.117185592 | 0.121023450245692 | 0.332900402501166 | -0.354391554 | 0.120020370648288 | 0.889420108736199 | 0.701600198980341 | 1.12751981965512 | 0.998074519377826 |
| ebi-fl187-GCST90094785 | ebi-pw11-GCST90275195 | Placental weight (fetal effect, conditional on maternal genotype) \|\| id:ebi-pw11-GCST90275195 | F-highly palatable foods liking (derived food-liking factor) \|\| id:ebi-fl187-GCST90094785 | Inverse variance weighted (multiplicative random effects) | 13 | 0.0894592106163668 | 0.128869333076168 | 0.487565942130968 | -0.163124682 | 0.342043103445656 | 1.09358272583726 | 0.84948526610859 | 1.40782097814135 | 0.998074519377826 |
| ebi-fl187-GCST90094785 | ebi-pw11-GCST90275197 | Placental weight (fetal genotype, adjusted for sex) \|\| id:ebi-pw11-GCST90275197 | F-highly palatable foods liking (derived food-liking factor) \|\| id:ebi-fl187-GCST90094785 | Inverse variance weighted (multiplicative random effects) | 13 | 0.0654892174030791 | 0.0992491684115355 | 0.509352333061164 | -0.129039153 | 0.260017587489689 | 1.06768122487514 | 0.878939552029774 | 1.29695289661088 | 0.998074519377826 |
| ebi-fl187-GCST90094785 | prot-a-1030 | Protein FAM171B \|\| id:prot-a-1030 | F-highly palatable foods liking (derived food-liking factor) \|\| id:ebi-fl187-GCST90094785 | Inverse variance weighted | 17 | -0.002131199 | 0.267701604565209 | 0.993648030331807 | -0.526826344 | 0.522563946378677 | 0.997871070822077 | 0.590475967037379 | 1.68634581180264 | 0.999355215041207 |
| ebi-fl187-GCST90094785 | prot-a-1036 | BRISC complex subunit Abro1 \|\| id:prot-a-1036 | F-highly palatable foods liking (derived food-liking factor) \|\| id:ebi-fl187-GCST90094785 | Inverse variance weighted | 17 | 0.0162271863340349 | 0.313389005217548 | 0.958704236850553 | -0.598015264 | 0.63046963656043 | 1.01635956218207 | 0.549901964013154 | 1.87849258093252 | 0.998074519377826 |
| ebi-fl187-GCST90094785 | prot-a-1055 | Phenylalanine--tRNA ligase, mitochondrial \|\| id:prot-a-1055 | F-highly palatable foods liking (derived food-liking factor) \|\| id:ebi-fl187-GCST90094785 | Inverse variance weighted | 17 | -0.515918101 | 0.335812459239319 | 0.124457943347864 | -1.174110521 | 0.142274319493768 | 0.596952280696867 | 0.309093789971283 | 1.15289286615011 | 0.998074519377826 |
| ebi-fl187-GCST90094785 | prot-a-1083 | Fc receptor-like protein 5 \|\| id:prot-a-1083 | F-highly palatable foods liking (derived food-liking factor) \|\| id:ebi-fl187-GCST90094785 | Inverse variance weighted | 17 | -0.466495725 | 0.267701604565209 | 0.0814042222865115 | -0.99119087 | 0.0581994196218293 | 0.627196289884611 | 0.371134454761547 | 1.05992634474685 | 0.998074519377826 |
| ebi-fl187-GCST90094785 | prot-a-1103 | Fibroblast growth factor receptor 3 \|\| id:prot-a-1103 | F-highly palatable foods liking (derived food-liking factor) \|\| id:ebi-fl187-GCST90094785 | Inverse variance weighted (multiplicative random effects) | 17 | -0.075896303 | 0.369037124032658 | 0.837056234730229 | -0.799209066 | 0.647416460048472 | 0.926912319551307 | 0.449684494182284 | 1.91059834006132 | 0.998074519377826 |
| ebi-fl187-GCST90094785 | prot-a-1105 | Fibroblast growth factor receptor 4 \|\| id:prot-a-1105 | F-highly palatable foods liking (derived food-liking factor) \|\| id:ebi-fl187-GCST90094785 | Inverse variance weighted | 17 | 0.044251523950014 | 0.267701604565209 | 0.868706539571088 | -0.480443621 | 0.568946668897824 | 1.04524522603585 | 0.618508947379605 | 1.76640546135899 | 0.998074519377826 |
| ebi-fl187-GCST90094785 | prot-a-1120 | Filamin-A \|\| id:prot-a-1120 | F-highly palatable foods liking (derived food-liking factor) \|\| id:ebi-fl187-GCST90094785 | Inverse variance weighted | 17 | 0.104579915086727 | 0.306385036818513 | 0.732851454847773 | -0.495934757 | 0.705094587251012 | 1.11024411557585 | 0.609001372806109 | 2.02403812407045 | 0.998074519377826 |
| ebi-fl187-GCST90094785 | prot-a-1166 | Galanin peptides \|\| id:prot-a-1166 | F-highly palatable foods liking (derived food-liking factor) \|\| id:ebi-fl187-GCST90094785 | Inverse variance weighted | 17 | 0.156823048106644 | 0.30041511869734 | 0.60165559929952 | -0.431990585 | 0.745636680753429 | 1.16978859927817 | 0.64921548931875 | 2.10778299272729 | 0.998074519377826 |
| ebi-fl187-GCST90094785 | prot-a-1205 | ADP-ribosylation factor-binding protein GGA1 \|\| id:prot-a-1205 | F-highly palatable foods liking (derived food-liking factor) \|\| id:ebi-fl187-GCST90094785 | Inverse variance weighted | 17 | -0.059226525 | 0.288497485204477 | 0.837343183049556 | -0.624681596 | 0.506228545790936 | 0.942493246493447 | 0.535431884921686 | 1.65902245402453 | 0.998074519377826 |
| ebi-fl187-GCST90094785 | prot-a-1227 | Guanine nucleotide-binding protein G(I)/G(S)/G(O) subunit gamma-T2 \|\| id:prot-a-1227 | F-highly palatable foods liking (derived food-liking factor) \|\| id:ebi-fl187-GCST90094785 | Inverse variance weighted | 17 | -0.340485888 | 0.267701604565209 | 0.203413673480172 | -0.865181033 | 0.184209257098807 | 0.711424566217753 | 0.420975335386265 | 1.20226737975925 | 0.998074519377826 |
| ebi-fl187-GCST90094785 | prot-a-1240 | Golgi SNAP receptor complex member 1 \|\| id:prot-a-1240 | F-highly palatable foods liking (derived food-liking factor) \|\| id:ebi-fl187-GCST90094785 | Inverse variance weighted | 17 | 0.0396254638568887 | 0.267701604565209 | 0.882326181496978 | -0.485069681 | 0.564320608804698 | 1.04042102593128 | 0.615654295806648 | 1.75825283535396 | 0.998074519377826 |
| ebi-fl187-GCST90094785 | prot-a-1283 | Glutathione S-transferase A1 \|\| id:prot-a-1283 | F-highly palatable foods liking (derived food-liking factor) \|\| id:ebi-fl187-GCST90094785 | Inverse variance weighted | 17 | -0.060023811 | 0.267701604565209 | 0.822586827554633 | -0.584718956 | 0.464671333963192 | 0.941742109510396 | 0.557262455113424 | 1.59149103386945 | 0.998074519377826 |
| ebi-fl187-GCST90094785 | prot-a-1324 | E3 ubiquitin-protein ligase HECW1 \|\| id:prot-a-1324 | F-highly palatable foods liking (derived food-liking factor) \|\| id:ebi-fl187-GCST90094785 | Inverse variance weighted | 17 | -0.322110336 | 0.331321811292468 | 0.330952154507658 | -0.971501086 | 0.327280413878665 | 0.724618234256994 | 0.378514428587823 | 1.38719041 | 0.998074519377826 |
| ebi-fl187-GCST90094785 | prot-a-1339 | Histidine triad nucleotide-binding protein 2, mitochondrial \|\| id:prot-a-1339 | F-highly palatable foods liking (derived food-liking factor) \|\| id:ebi-fl187-GCST90094785 | Inverse variance weighted | 17 | 0.13197395833959 | 0.267765803270912 | 0.622103847224947 | -0.392847016 | 0.656794932750579 | 1.14107860329882 | 0.675132024035475 | 1.92860112178292 | 0.998074519377826 |
| ebi-fl187-GCST90094785 | prot-a-1466 | Interleukin-11 \|\| id:prot-a-1466 | F-highly palatable foods liking (derived food-liking factor) \|\| id:ebi-fl187-GCST90094785 | Inverse variance weighted (multiplicative random effects) | 17 | 0.0633088497009013 | 0.34372100501207 | 0.853866965307631 | -0.61038432 | 0.737002019524559 | 1.06535582325275 | 0.543142088523651 | 2.08966135035421 | 0.998074519377826 |
| ebi-fl187-GCST90094785 | prot-a-147 | Rho GTPase-activating protein 1 \|\| id:prot-a-147 | F-highly palatable foods liking (derived food-liking factor) \|\| id:ebi-fl187-GCST90094785 | Inverse variance weighted | 17 | -0.200485902 | 0.285650531799091 | 0.482768180074045 | -0.760360944 | 0.35938914038024 | 0.818333026847629 | 0.467497655952086 | 1.43245411886779 | 0.998074519377826 |
| ebi-fl187-GCST90094785 | prot-a-1473 | Interleukin-12 receptor subunit beta-1 \|\| id:prot-a-1473 | F-highly palatable foods liking (derived food-liking factor) \|\| id:ebi-fl187-GCST90094785 | Inverse variance weighted | 17 | 0.232455051617669 | 0.301063315527888 | 0.440047204529008 | -0.357629047 | 0.822539150052329 | 1.26169373399209 | 0.69933244649322 | 2.27627230279003 | 0.998074519377826 |
| ebi-fl187-GCST90094785 | prot-a-1501 | X-linked interleukin-1 receptor accessory protein-like 2 \|\| id:prot-a-1501 | F-highly palatable foods liking (derived food-liking factor) \|\| id:ebi-fl187-GCST90094785 | Inverse variance weighted | 17 | 0.456333985281768 | 0.267701903806385 | 0.0882625539975605 | -0.068361746 | 0.981029716742283 | 1.57827737816573 | 0.933922569542374 | 2.66720129019934 | 0.998074519377826 |
| ebi-fl187-GCST90094785 | prot-a-1503 | Interleukin-1 receptor-like 2 \|\| id:prot-a-1503 | F-highly palatable foods liking (derived food-liking factor) \|\| id:ebi-fl187-GCST90094785 | Inverse variance weighted | 17 | -0.057426286 | 0.267637751039723 | 0.830104758683338 | -0.581996278 | 0.467143705674972 | 0.944191487609944 | 0.558781768440793 | 1.59543065938369 | 0.998074519377826 |
| ebi-fl187-GCST90094785 | prot-a-1536 | Interleukin-5 receptor subunit alpha \|\| id:prot-a-1536 | F-highly palatable foods liking (derived food-liking factor) \|\| id:ebi-fl187-GCST90094785 | Inverse variance weighted | 17 | -0.721324883 | 0.30634522151348 | 0.0185419332140631 | -1.321761517 | -0.120888249 | 0.486107793312805 | 0.266665152825865 | 0.886132980688902 | 0.998074519377826 |
| ebi-fl187-GCST90094785 | prot-a-1559 | Insulin-induced gene 1 protein \|\| id:prot-a-1559 | F-highly palatable foods liking (derived food-liking factor) \|\| id:ebi-fl187-GCST90094785 | Inverse variance weighted | 17 | -0.077651178 | 0.270662585929442 | 0.774194316451191 | -0.608149846 | 0.452847490472834 | 0.925287130811937 | 0.544357082194367 | 1.57278430363195 | 0.998074519377826 |
| ebi-fl187-GCST90094785 | prot-a-1567 | Interferon regulatory factor 1 \|\| id:prot-a-1567 | F-highly palatable foods liking (derived food-liking factor) \|\| id:ebi-fl187-GCST90094785 | Inverse variance weighted | 17 | -0.084109638 | 0.302449256265007 | 0.780939404385117 | -0.67691018 | 0.508690904753233 | 0.919330457452471 | 0.508184768605478 | 1.66311259646569 | 0.998074519377826 |
| ebi-fl187-GCST90094785 | prot-a-1613 | Potassium voltage-gated channel subfamily E regulatory beta subunit 5 \|\| id:prot-a-1613 | F-highly palatable foods liking (derived food-liking factor) \|\| id:ebi-fl187-GCST90094785 | Inverse variance weighted | 17 | -0.096537128 | 0.267765803270912 | 0.71845215872478 | -0.621358102 | 0.428283846592159 | 0.907976185798603 | 0.537214349933516 | 1.53462161626808 | 0.998074519377826 |
| ebi-fl187-GCST90094785 | prot-a-1649 | Kin of IRRE-like protein 2 \|\| id:prot-a-1649 | F-highly palatable foods liking (derived food-liking factor) \|\| id:ebi-fl187-GCST90094785 | Inverse variance weighted | 17 | 0.285920542420215 | 0.267701604565209 | 0.285494851796502 | -0.238774603 | 0.810615687368024 | 1.33098669406915 | 0.787592383700333 | 2.24929242137409 | 0.998074519377826 |
| ebi-fl187-GCST90094785 | prot-a-165 | Armadillo repeat-containing protein 5 \|\| id:prot-a-165 | F-highly palatable foods liking (derived food-liking factor) \|\| id:ebi-fl187-GCST90094785 | Inverse variance weighted | 17 | -0.490030019 | 0.267938243568274 | 0.0674158398365015 | -1.015188977 | 0.0351289382726223 | 0.612608003954473 | 0.362333950754941 | 1.03575324842497 | 0.998074519377826 |
| ebi-fl187-GCST90094785 | prot-a-1656 | Kallikrein-11 \|\| id:prot-a-1656 | F-highly palatable foods liking (derived food-liking factor) \|\| id:ebi-fl187-GCST90094785 | Inverse variance weighted | 17 | -0.255277979 | 0.267701604565209 | 0.340290450931832 | -0.779973124 | 0.269417165498127 | 0.77470111703655 | 0.458418331408525 | 1.30920118070679 | 0.998074519377826 |
| ebi-fl187-GCST90094785 | prot-a-1697 | Laminin subunit gamma-2 \|\| id:prot-a-1697 | F-highly palatable foods liking (derived food-liking factor) \|\| id:ebi-fl187-GCST90094785 | Inverse variance weighted | 17 | -0.039136191 | 0.267701604565209 | 0.883768764953647 | -0.563831336 | 0.485558953920383 | 0.961619736248029 | 0.569024757091617 | 1.62508310160017 | 0.998074519377826 |
| ebi-fl187-GCST90094785 | prot-a-1698 | Lysosome-associated membrane glycoprotein 3 \|\| id:prot-a-1698 | F-highly palatable foods liking (derived food-liking factor) \|\| id:ebi-fl187-GCST90094785 | Inverse variance weighted | 17 | -0.423879875 | 0.267701604565209 | 0.11332934120013 | -0.948575019 | 0.10081527041389 | 0.654502499604586 | 0.387292514717373 | 1.10607229861196 | 0.998074519377826 |
| ebi-fl187-GCST90094785 | prot-a-1724 | Leptin receptor \|\| id:prot-a-1724 | F-highly palatable foods liking (derived food-liking factor) \|\| id:ebi-fl187-GCST90094785 | Inverse variance weighted | 17 | -0.017013422 | 0.267765803270912 | 0.949337748459442 | -0.541834396 | 0.507807552341976 | 0.983130488901082 | 0.581680240908833 | 1.66164413062531 | 0.998074519377826 |
| ebi-fl187-GCST90094785 | prot-a-1743 | Leukocyte immunoglobulin-like receptor subfamily B member 2 \|\| id:prot-a-1743 | F-highly palatable foods liking (derived food-liking factor) \|\| id:ebi-fl187-GCST90094785 | Inverse variance weighted | 17 | -0.281224872 | 0.267701604565209 | 0.293480846570782 | -0.805920017 | 0.24347027248377 | 0.754858569486553 | 0.446676787039053 | 1.27566839482408 | 0.998074519377826 |
| ebi-fl187-GCST90094785 | prot-a-1746 | Leukocyte immunoglobulin-like receptor subfamily B member 4 \|\| id:prot-a-1746 | F-highly palatable foods liking (derived food-liking factor) \|\| id:ebi-fl187-GCST90094785 | Inverse variance weighted | 17 | 0.357300218170606 | 0.315967069229484 | 0.258133076034822 | -0.261995238 | 0.976595673860395 | 1.42946495679508 | 0.769514688497792 | 2.65540098616458 | 0.998074519377826 |
| ebi-fl187-GCST90094785 | prot-a-1750 | Leucine-rich repeat and immunoglobulin-like domain-containing nogo receptor-interacting protein 3 \|\| id:prot-a-1750 | F-highly palatable foods liking (derived food-liking factor) \|\| id:ebi-fl187-GCST90094785 | Inverse variance weighted | 17 | 0.173685536288574 | 0.267701604565209 | 0.516465881990729 | -0.351009609 | 0.698380681236384 | 1.18968139536493 | 0.703976989548121 | 2.01049443872583 | 0.998074519377826 |
| ebi-fl187-GCST90094785 | prot-a-1753 | Lipase member N \|\| id:prot-a-1753 | F-highly palatable foods liking (derived food-liking factor) \|\| id:ebi-fl187-GCST90094785 | Inverse variance weighted | 17 | 0.0882011212046112 | 0.282697663494289 | 0.755042001383785 | -0.465886299 | 0.642288541653417 | 1.09220776608152 | 0.627578636156128 | 1.90082602491907 | 0.998074519377826 |
| ebi-fl187-GCST90094785 | prot-a-1760 | Ig Kappa chain V-I region HK102- like \|\| id:prot-a-1760 | F-highly palatable foods liking (derived food-liking factor) \|\| id:ebi-fl187-GCST90094785 | Inverse variance weighted | 17 | -0.363142762 | 0.267765803270912 | 0.175036992346885 | -0.887963736 | 0.161678212882953 | 0.695487137604954 | 0.41149280824689 | 1.17548192551574 | 0.998074519377826 |
| ebi-fl187-GCST90094785 | prot-a-1792 | Leucine-rich repeat neuronal protein 1 \|\| id:prot-a-1792 | F-highly palatable foods liking (derived food-liking factor) \|\| id:ebi-fl187-GCST90094785 | Inverse variance weighted | 16 | -0.000600687 | 0.33666169962034 | 0.998576379594698 | -0.660457618 | 0.659256244460925 | 0.999399493581252 | 0.516614868101222 | 1.93335385689048 | 0.999355215041207 |
| ebi-fl187-GCST90094785 | prot-a-1795 | Leucine-rich repeat transmembrane neuronal protein 1 \|\| id:prot-a-1795 | F-highly palatable foods liking (derived food-liking factor) \|\| id:ebi-fl187-GCST90094785 | Inverse variance weighted | 17 | 0.150787151362274 | 0.267701604565209 | 0.573253920729005 | -0.373907994 | 0.675482296310084 | 1.16274914217011 | 0.688040213029885 | 1.96498045029036 | 0.998074519377826 |
| ebi-fl187-GCST90094785 | prot-a-1820 | LysM and putative peptidoglycan-binding domain-containing protein 4 \|\| id:prot-a-1820 | F-highly palatable foods liking (derived food-liking factor) \|\| id:ebi-fl187-GCST90094785 | Inverse variance weighted | 17 | 0.153883974691285 | 0.267661399569964 | 0.565345193680966 | -0.370732368 | 0.678500317848415 | 1.16635555216446 | 0.690228643792471 | 1.97091976158828 | 0.998074519377826 |
| ebi-fl187-GCST90094785 | prot-a-1844 | Mitogen-activated protein kinase kinase kinase 7:TGF-beta-activated kinase 1 and MAP3K7-binding protein 1 fusion \|\| id:prot-a-1844 | F-highly palatable foods liking (derived food-liking factor) \|\| id:ebi-fl187-GCST90094785 | Inverse variance weighted | 17 | 0.361871596608845 | 0.267701604565209 | 0.176448188429552 | -0.162823548 | 0.886566741556655 | 1.43601454098494 | 0.849741113417856 | 2.42678355720108 | 0.998074519377826 |
| ebi-fl187-GCST90094785 | prot-a-1856 | Megakaryocyte-associated tyrosine-protein kinase \|\| id:prot-a-1856 | F-highly palatable foods liking (derived food-liking factor) \|\| id:ebi-fl187-GCST90094785 | Inverse variance weighted | 17 | 0.291095630233312 | 0.337761055283029 | 0.388776152772305 | -0.370916038 | 0.95310729858805 | 1.33789252080653 | 0.690101881376755 | 2.59375672713584 | 0.998074519377826 |
| ebi-fl187-GCST90094785 | prot-a-1910 | Stromelysin-2 \|\| id:prot-a-1910 | F-highly palatable foods liking (derived food-liking factor) \|\| id:ebi-fl187-GCST90094785 | Inverse variance weighted | 17 | 0.258726441430502 | 0.267765803270912 | 0.333923332433874 | -0.266094533 | 0.78354741584149 | 1.2952794215723 | 0.766366677150463 | 2.1892245970127 | 0.998074519377826 |
| ebi-fl187-GCST90094785 | prot-a-1940 | 39S ribosomal protein L14, mitochondrial \|\| id:prot-a-1940 | F-highly palatable foods liking (derived food-liking factor) \|\| id:ebi-fl187-GCST90094785 | Inverse variance weighted | 17 | -0.030247328 | 0.267765803270912 | 0.910060779172016 | -0.555068302 | 0.494573646583497 | 0.970205545042138 | 0.574033052114996 | 1.63979895610948 | 0.998074519377826 |
| ebi-fl187-GCST90094785 | prot-a-1942 | 39S ribosomal protein L33, mitochondrial \|\| id:prot-a-1942 | F-highly palatable foods liking (derived food-liking factor) \|\| id:ebi-fl187-GCST90094785 | Inverse variance weighted | 17 | -0.824035642 | 0.267714223917337 | 0.00208365501099223 | -1.348755521 | -0.299315764 | 0.438657811546252 | 0.2595630804602 | 0.741325288979426 | 0.598008988154769 |
| ebi-fl187-GCST90094785 | prot-a-1948 | Mesothelin \|\| id:prot-a-1948 | F-highly palatable foods liking (derived food-liking factor) \|\| id:ebi-fl187-GCST90094785 | Inverse variance weighted | 17 | -0.21189121 | 0.312244602900543 | 0.497387238067454 | -0.823890631 | 0.400108211875165 | 0.809052709777079 | 0.438721426330712 | 1.49198613952403 | 0.998074519377826 |
| ebi-fl187-GCST90094785 | prot-a-1980 | Unconventional myosin-VI \|\| id:prot-a-1980 | F-highly palatable foods liking (derived food-liking factor) \|\| id:ebi-fl187-GCST90094785 | Inverse variance weighted | 17 | 0.127839956133208 | 0.270340368058701 | 0.63629509201523 | -0.402027165 | 0.657707077528263 | 1.13637111891478 | 0.668962572900056 | 1.93036108777426 | 0.998074519377826 |
| ebi-fl187-GCST90094785 | prot-a-2039 | Neurofascin \|\| id:prot-a-2039 | F-highly palatable foods liking (derived food-liking factor) \|\| id:ebi-fl187-GCST90094785 | Inverse variance weighted | 17 | -0.666138606 | 0.280189708296565 | 0.0174325390167718 | -1.215310434 | -0.116966778 | 0.513688306040381 | 0.296617919650569 | 0.889614747731676 | 0.998074519377826 |
| ebi-fl187-GCST90094785 | prot-a-2041 | NFU1 iron-sulfur cluster scaffold homolog, mitochondrial \|\| id:prot-a-2041 | F-highly palatable foods liking (derived food-liking factor) \|\| id:ebi-fl187-GCST90094785 | Inverse variance weighted | 17 | 0.11710670573679 | 0.267765803270912 | 0.66185934917293 | -0.407714269 | 0.641927680147778 | 1.12423938608243 | 0.665168911266896 | 1.90014021372663 | 0.998074519377826 |
| ebi-fl187-GCST90094785 | prot-a-2088 | Glucocorticoid receptor \|\| id:prot-a-2088 | F-highly palatable foods liking (derived food-liking factor) \|\| id:ebi-fl187-GCST90094785 | Inverse variance weighted | 17 | -0.064187836 | 0.267742714454086 | 0.810534147577281 | -0.588963556 | 0.460587884573605 | 0.93782882519857 | 0.554902111711409 | 1.5850055114419 | 0.998074519377826 |
| ebi-fl187-GCST90094785 | prot-a-2111 | Neuron-specific protein family member 2 \|\| id:prot-a-2111 | F-highly palatable foods liking (derived food-liking factor) \|\| id:ebi-fl187-GCST90094785 | Inverse variance weighted | 17 | 0.108405636308629 | 0.286836430383316 | 0.705478596288691 | -0.453793767 | 0.670605039859928 | 1.1144997352671 | 0.63521372161572 | 1.95542006987982 | 0.998074519377826 |
| ebi-fl187-GCST90094785 | prot-a-2125 | Nuclear migration protein nudC \|\| id:prot-a-2125 | F-highly palatable foods liking (derived food-liking factor) \|\| id:ebi-fl187-GCST90094785 | Inverse variance weighted | 17 | -0.037569446 | 0.322512812141358 | 0.90726438585127 | -0.669694558 | 0.59455566547399 | 0.963127529732698 | 0.511864898888777 | 1.81222553166431 | 0.998074519377826 |
| ebi-fl187-GCST90094785 | prot-a-2142 | Ornithine decarboxylase \|\| id:prot-a-2142 | F-highly palatable foods liking (derived food-liking factor) \|\| id:ebi-fl187-GCST90094785 | Inverse variance weighted | 17 | -0.403665482 | 0.267765803270912 | 0.131674381563141 | -0.928486456 | 0.121155492692399 | 0.667867497792652 | 0.395151336874364 | 1.12880041893832 | 0.998074519377826 |
| ebi-fl187-GCST90094785 | prot-a-2186 | Prostate and testis expressed protein 1 \|\| id:prot-a-2186 | F-highly palatable foods liking (derived food-liking factor) \|\| id:ebi-fl187-GCST90094785 | Inverse variance weighted | 17 | 0.428360549842631 | 0.287774389293929 | 0.136611416358642 | -0.135677253 | 0.992398352858731 | 1.5347393312488 | 0.873124385143357 | 2.6976967485512 | 0.998074519377826 |
| ebi-fl187-GCST90094785 | prot-a-2227 | Platelet-derived growth factor C \|\| id:prot-a-2227 | F-highly palatable foods liking (derived food-liking factor) \|\| id:ebi-fl187-GCST90094785 | Inverse variance weighted | 17 | 0.140012490860352 | 0.267765803270913 | 0.601049686390168 | -0.384808484 | 0.664833465271341 | 1.15028816685635 | 0.680580966174169 | 1.94416672309805 | 0.998074519377826 |
| ebi-fl187-GCST90094785 | prot-a-2229 | Platelet-derived growth factor receptor alpha \|\| id:prot-a-2229 | F-highly palatable foods liking (derived food-liking factor) \|\| id:ebi-fl187-GCST90094785 | Inverse variance weighted | 17 | 0.115374401042888 | 0.318674386367766 | 0.717318560556642 | -0.509227396 | 0.739976198323709 | 1.12229354679671 | 0.600959703224608 | 2.09588562830941 | 0.998074519377826 |
| ebi-fl187-GCST90094785 | prot-a-2243 | Platelet endothelial aggregation receptor 1 \|\| id:prot-a-2243 | F-highly palatable foods liking (derived food-liking factor) \|\| id:ebi-fl187-GCST90094785 | Inverse variance weighted | 17 | -0.507661668 | 0.267765803270912 | 0.0579710554807921 | -1.032482642 | 0.0171593065451719 | 0.601901379895008 | 0.356121739294278 | 1.01730737314001 | 0.998074519377826 |
| ebi-fl187-GCST90094785 | prot-a-2246 | E3 ubiquitin-protein ligase pellino homolog 2 \|\| id:prot-a-2246 | F-highly palatable foods liking (derived food-liking factor) \|\| id:ebi-fl187-GCST90094785 | Inverse variance weighted | 17 | 0.449276886958843 | 0.284857568654951 | 0.114749892793695 | -0.109043948 | 1.00759772152255 | 1.56717852842674 | 0.896691009210137 | 2.73901323280273 | 0.998074519377826 |
| ebi-fl187-GCST90094785 | prot-a-2251 | Platelet factor 4 variant \|\| id:prot-a-2251 | F-highly palatable foods liking (derived food-liking factor) \|\| id:ebi-fl187-GCST90094785 | Inverse variance weighted | 17 | 0.285757303794409 | 0.288905996761628 | 0.322613570100019 | -0.28049845 | 0.8520130574472 | 1.33076944336258 | 0.755407115029322 | 2.3443614392205 | 0.998074519377826 |
| ebi-fl187-GCST90094785 | prot-a-2346 | Protein phosphatase 1L \|\| id:prot-a-2346 | F-highly palatable foods liking (derived food-liking factor) \|\| id:ebi-fl187-GCST90094785 | Inverse variance weighted | 17 | 0.122446894815313 | 0.27000556224593 | 0.650190416883753 | -0.406764007 | 0.651657796817336 | 1.1302590958622 | 0.66580129608383 | 1.91871904019001 | 0.998074519377826 |
| ebi-fl187-GCST90094785 | prot-a-2358 | PR domain zinc finger protein 4 \|\| id:prot-a-2358 | F-highly palatable foods liking (derived food-liking factor) \|\| id:ebi-fl187-GCST90094785 | Inverse variance weighted | 17 | 0.269418294881475 | 0.267765803270912 | 0.314333124174528 | -0.25540268 | 0.794239269292463 | 1.30920265929764 | 0.774604517768504 | 2.21275704413624 | 0.998074519377826 |
| ebi-fl187-GCST90094785 | prot-a-2390 | Trypsin-1 \|\| id:prot-a-2390 | F-highly palatable foods liking (derived food-liking factor) \|\| id:ebi-fl187-GCST90094785 | Inverse variance weighted | 17 | 0.128209166459733 | 0.326427503196581 | 0.694493355754475 | -0.51158874 | 0.768007072725031 | 1.13679075632911 | 0.599542305035651 | 2.15546628289802 | 0.998074519377826 |
| ebi-fl187-GCST90094785 | prot-a-242 | Brain-derived neurotrophic factor \|\| id:prot-a-242 | F-highly palatable foods liking (derived food-liking factor) \|\| id:ebi-fl187-GCST90094785 | Inverse variance weighted | 17 | 0.285319093266156 | 0.267765803270912 | 0.286625014725415 | -0.239501881 | 0.810140067677144 | 1.33018641393596 | 0.787019792842351 | 2.24822286797843 | 0.998074519377826 |
| ebi-fl187-GCST90094785 | prot-a-2464 | Dihydropteridine reductase \|\| id:prot-a-2464 | F-highly palatable foods liking (derived food-liking factor) \|\| id:ebi-fl187-GCST90094785 | Inverse variance weighted | 17 | 0.00438117583070196 | 0.289461525929703 | 0.987923994119011 | -0.562963415 | 0.57172576665292 | 1.00439078721279 | 0.56951884 | 1.7713213024488 | 0.999355215041207 |
| ebi-fl187-GCST90094785 | prot-a-2474 | Ras-related protein Rab-27A \|\| id:prot-a-2474 | F-highly palatable foods liking (derived food-liking factor) \|\| id:ebi-fl187-GCST90094785 | Inverse variance weighted | 17 | 0.197884528119708 | 0.340806367154668 | 0.561485641203809 | -0.470095952 | 0.865865007742858 | 1.21882164606838 | 0.624942301252414 | 2.37706137342244 | 0.998074519377826 |
| ebi-fl187-GCST90094785 | prot-a-2478 | Ras-related protein Rab-7b \|\| id:prot-a-2478 | F-highly palatable foods liking (derived food-liking factor) \|\| id:ebi-fl187-GCST90094785 | Inverse variance weighted | 17 | -0.110054971 | 0.314410453944711 | 0.726311697321103 | -0.72629946 | 0.506189519115992 | 0.895784892096074 | 0.483695617120282 | 1.65895770915789 | 0.998074519377826 |
| ebi-fl187-GCST90094785 | prot-a-2528 | E3 ubiquitin-protein ligase rififylin \|\| id:prot-a-2528 | F-highly palatable foods liking (derived food-liking factor) \|\| id:ebi-fl187-GCST90094785 | Inverse variance weighted | 17 | -0.039965512 | 0.267701604565209 | 0.881323804412128 | -0.564660657 | 0.484729632694024 | 0.960822575186056 | 0.568553048408285 | 1.62373594437967 | 0.998074519377826 |
| ebi-fl187-GCST90094785 | prot-a-2543 | Protein RIC-3 \|\| id:prot-a-2543 | F-highly palatable foods liking (derived food-liking factor) \|\| id:ebi-fl187-GCST90094785 | Inverse variance weighted | 17 | -0.304425388 | 0.267658250600128 | 0.255385324399539 | -0.829035559 | 0.220184783662216 | 0.737547056401079 | 0.436470033126522 | 1.24630700648402 | 0.998074519377826 |
| ebi-fl187-GCST90094785 | prot-a-255 | Bone morphogenetic protein 6 \|\| id:prot-a-255 | F-highly palatable foods liking (derived food-liking factor) \|\| id:ebi-fl187-GCST90094785 | Inverse variance weighted | 17 | -0.046780221 | 0.294856350415625 | 0.873941263145922 | -0.624698668 | 0.531138225604156 | 0.954297108807915 | 0.535422744205904 | 1.70086717782189 | 0.998074519377826 |
| ebi-fl187-GCST90094785 | prot-a-2568 | RING finger protein 165 \|\| id:prot-a-2568 | F-highly palatable foods liking (derived food-liking factor) \|\| id:ebi-fl187-GCST90094785 | Inverse variance weighted | 17 | -0.012669833 | 0.267765803270913 | 0.962260701835919 | -0.537490808 | 0.512151141093955 | 0.987410091121918 | 0.584212315825554 | 1.6688773270239 | 0.998074519377826 |
| ebi-fl187-GCST90094785 | prot-a-2573 | E3 ubiquitin-protein ligase RNF8 \|\| id:prot-a-2573 | F-highly palatable foods liking (derived food-liking factor) \|\| id:ebi-fl187-GCST90094785 | Inverse variance weighted | 17 | -0.072146278 | 0.283136335593387 | 0.79886900953541 | -0.627093496 | 0.482800939532815 | 0.930394789353548 | 0.534142032996893 | 1.62060727406051 | 0.998074519377826 |
| ebi-fl187-GCST90094785 | prot-a-260 | Cytoplasmic tyrosine-protein kinase BMX \|\| id:prot-a-260 | F-highly palatable foods liking (derived food-liking factor) \|\| id:ebi-fl187-GCST90094785 | Inverse variance weighted | 16 | 0.214809085677705 | 0.275367803202074 | 0.435343424998517 | -0.324911809 | 0.75452997995377 | 1.23962521216624 | 0.722591077152004 | 2.12661173826665 | 0.998074519377826 |
| ebi-fl187-GCST90094785 | prot-a-2623 | Serum amyloid A-1 protein \|\| id:prot-a-2623 | F-highly palatable foods liking (derived food-liking factor) \|\| id:ebi-fl187-GCST90094785 | Inverse variance weighted | 17 | 0.273098496857088 | 0.267701604565209 | 0.307652535215176 | -0.251596648 | 0.797793641804898 | 1.31402966624308 | 0.777558304452572 | 2.22063600102958 | 0.998074519377826 |
| ebi-fl187-GCST90094785 | prot-a-2640 | Lysosome membrane protein 2 \|\| id:prot-a-2640 | F-highly palatable foods liking (derived food-liking factor) \|\| id:ebi-fl187-GCST90094785 | Inverse variance weighted | 17 | -0.069476404 | 0.269582834032357 | 0.796624394113111 | -0.597858759 | 0.458905950292957 | 0.932882145029047 | 0.549988033033148 | 1.58234187699416 | 0.998074519377826 |
| ebi-fl187-GCST90094785 | prot-a-2641 | Scavenger receptor class F member 2 \|\| id:prot-a-2641 | F-highly palatable foods liking (derived food-liking factor) \|\| id:ebi-fl187-GCST90094785 | Inverse variance weighted | 17 | 0.412837209732423 | 0.30379920440249 | 0.174173614905111 | -0.182609231 | 1.0082836503613 | 1.51109901369756 | 0.833093639393104 | 2.74089264546682 | 0.998074519377826 |
| ebi-fl187-GCST90094785 | prot-a-2669 | Semaphorin-3A \|\| id:prot-a-2669 | F-highly palatable foods liking (derived food-liking factor) \|\| id:ebi-fl187-GCST90094785 | Inverse variance weighted | 17 | 0.421988609561058 | 0.267765803270912 | 0.115034665343066 | -0.102832365 | 0.946809583972047 | 1.52499115426572 | 0.902278214348387 | 2.57747331544319 | 0.998074519377826 |
| ebi-fl187-GCST90094785 | prot-a-2683 | Sentrin-specific protease 7 \|\| id:prot-a-2683 | F-highly palatable foods liking (derived food-liking factor) \|\| id:ebi-fl187-GCST90094785 | Inverse variance weighted | 17 | -0.114012903 | 0.318282237583607 | 0.720183760239658 | -0.737846088 | 0.509820282837372 | 0.892246443307085 | 0.478142684215713 | 1.66499194042877 | 0.998074519377826 |
| ebi-fl187-GCST90094785 | prot-a-270 | Bromodomain testis-specific protein \|\| id:prot-a-270 | F-highly palatable foods liking (derived food-liking factor) \|\| id:ebi-fl187-GCST90094785 | Inverse variance weighted | 17 | 0.275255968471129 | 0.267765803270912 | 0.303962612169945 | -0.249565006 | 0.800076942882117 | 1.31686770834479 | 0.779139630479036 | 2.22571217461378 | 0.998074519377826 |
| ebi-fl187-GCST90094785 | prot-a-2708 | 14-3-3 protein sigma \|\| id:prot-a-2708 | F-highly palatable foods liking (derived food-liking factor) \|\| id:ebi-fl187-GCST90094785 | Inverse variance weighted | 17 | 0.638897045134381 | 0.267765803270912 | 0.0170313952912684 | 0.114076070723393 | 1.16371801954537 | 1.89439029961103 | 1.12083738455188 | 3.20181558602736 | 0.998074519377826 |
| ebi-fl187-GCST90094785 | prot-a-2713 | Beta-sarcoglycan \|\| id:prot-a-2713 | F-highly palatable foods liking (derived food-liking factor) \|\| id:ebi-fl187-GCST90094785 | Inverse variance weighted | 17 | -0.002271216 | 0.267765803270912 | 0.993232346266266 | -0.52709219 | 0.52254975867783 | 0.997731361525756 | 0.590319011857024 | 1.68632188660246 | 0.999355215041207 |
| ebi-fl187-GCST90094785 | prot-a-2720 | Protein shisa-3 homolog \|\| id:prot-a-2720 | F-highly palatable foods liking (derived food-liking factor) \|\| id:ebi-fl187-GCST90094785 | Inverse variance weighted | 17 | 0.0163487012341242 | 0.267765803270912 | 0.951314633404699 | -0.508472273 | 0.541169675645113 | 1.01648307251675 | 0.601413673135288 | 1.71801520794599 | 0.998074519377826 |
| ebi-fl187-GCST90094785 | prot-a-2730 | Sialic acid-binding Ig-like lectin 8 \|\| id:prot-a-2730 | F-highly palatable foods liking (derived food-liking factor) \|\| id:ebi-fl187-GCST90094785 | Inverse variance weighted | 17 | -0.046528589 | 0.272166636154485 | 0.864258163329332 | -0.579975195 | 0.4869180182657 | 0.954537271298434 | 0.559912254759142 | 1.62729319559152 | 0.998074519377826 |
| ebi-fl187-GCST90094785 | prot-a-2761 | Sodium- and chloride-dependent neutral and basic amino acid transporter B(0+) \|\| id:prot-a-2761 | F-highly palatable foods liking (derived food-liking factor) \|\| id:ebi-fl187-GCST90094785 | Inverse variance weighted | 17 | -0.104423152 | 0.267765803270912 | 0.696551766829694 | -0.629244126 | 0.420397822425144 | 0.900844022821149 | 0.532994525275689 | 1.5225671465065 | 0.998074519377826 |
| ebi-fl187-GCST90094785 | prot-a-2770 | Secreted Ly-6/uPAR-related protein 1 \|\| id:prot-a-2770 | F-highly palatable foods liking (derived food-liking factor) \|\| id:ebi-fl187-GCST90094785 | Inverse variance weighted | 17 | 0.518912871884667 | 0.267701604565209 | 0.0525744032774711 | -0.005782273 | 1.04360801683248 | 1.68020006374016 | 0.99423441210286 | 2.83944331419942 | 0.998074519377826 |
| ebi-fl187-GCST90094785 | prot-a-2784 | N-lysine methyltransferase SMYD2 \|\| id:prot-a-2784 | F-highly palatable foods liking (derived food-liking factor) \|\| id:ebi-fl187-GCST90094785 | Inverse variance weighted | 17 | 0.238328990590507 | 0.267701604565209 | 0.373316347393418 | -0.286366154 | 0.763024135538317 | 1.26912665487822 | 0.750987588220951 | 2.1447524451609 | 0.998074519377826 |
| ebi-fl187-GCST90094785 | prot-a-2837 | Serine palmitoyltransferase 2 \|\| id:prot-a-2837 | F-highly palatable foods liking (derived food-liking factor) \|\| id:ebi-fl187-GCST90094785 | Inverse variance weighted | 17 | -0.274971732 | 0.267742714454086 | 0.304420526358427 | -0.799747453 | 0.249803987996138 | 0.759593594859624 | 0.449442455280494 | 1.28377375695778 | 0.998074519377826 |
| ebi-fl187-GCST90094785 | prot-a-2858 | Alpha-2,8-sialyltransferase 8B \|\| id:prot-a-2858 | F-highly palatable foods liking (derived food-liking factor) \|\| id:ebi-fl187-GCST90094785 | Inverse variance weighted | 17 | 0.31282021047324 | 0.267701604565209 | 0.242588747037097 | -0.211874934 | 0.83751535542105 | 1.36727568707945 | 0.809065877488384 | 2.31061877220923 | 0.998074519377826 |
| ebi-fl187-GCST90094785 | prot-a-2911 | Synaptotagmin-11 \|\| id:prot-a-2911 | F-highly palatable foods liking (derived food-liking factor) \|\| id:ebi-fl187-GCST90094785 | Inverse variance weighted | 17 | 0.160959840362385 | 0.267701604565209 | 0.547662912513345 | -0.363735305 | 0.685654985310195 | 1.17463779483846 | 0.695075153601247 | 1.9850716025659 | 0.998074519377826 |
| ebi-fl187-GCST90094785 | prot-a-2962 | Transforming growth factor beta-1 \|\| id:prot-a-2962 | F-highly palatable foods liking (derived food-liking factor) \|\| id:ebi-fl187-GCST90094785 | Inverse variance weighted | 17 | -0.374967162 | 0.267701604565209 | 0.161306569119763 | -0.899662307 | 0.14972798290842 | 0.687311848339804 | 0.406706978658425 | 1.16151824693675 | 0.998074519377826 |
| ebi-fl187-GCST90094785 | prot-a-2978 | Tyrosine-protein kinase receptor Tie-1, soluble \|\| id:prot-a-2978 | F-highly palatable foods liking (derived food-liking factor) \|\| id:ebi-fl187-GCST90094785 | Inverse variance weighted | 17 | -0.320943193 | 0.308759140106983 | 0.298590223446795 | -0.926111108 | 0.284224721428139 | 0.725464461147559 | 0.396091074606177 | 1.32873149164344 | 0.998074519377826 |
| ebi-fl187-GCST90094785 | prot-a-299 | Uncharacterized protein C1orf226 \|\| id:prot-a-299 | F-highly palatable foods liking (derived food-liking factor) \|\| id:ebi-fl187-GCST90094785 | Inverse variance weighted | 17 | -0.423861316 | 0.267701604565209 | 0.113345133409419 | -0.94855646 | 0.100833829425502 | 0.654514646636794 | 0.38729970255035 | 1.10609282641108 | 0.998074519377826 |
| ebi-fl187-GCST90094785 | prot-a-3015 | Transmembrane protein 70, mitochondrial \|\| id:prot-a-3015 | F-highly palatable foods liking (derived food-liking factor) \|\| id:ebi-fl187-GCST90094785 | Inverse variance weighted | 17 | 0.238338285532228 | 0.277614772780197 | 0.390604495367378 | -0.305786669 | 0.782463240181415 | 1.26913845139134 | 0.736543730221582 | 2.18685237917297 | 0.998074519377826 |
| ebi-fl187-GCST90094785 | prot-a-3016 | Transmembrane protein 87B \|\| id:prot-a-3016 | F-highly palatable foods liking (derived food-liking factor) \|\| id:ebi-fl187-GCST90094785 | Inverse variance weighted | 17 | 0.864809781311711 | 0.285726694133288 | 0.00247236922776942 | 0.304785460810466 | 1.42483410181296 | 2.37455435839963 | 1.35633398499608 | 4.15716811889146 | 0.637747204560213 |
| ebi-fl187-GCST90094785 | prot-a-3123 | Thioredoxin domain-containing protein 12 \|\| id:prot-a-3123 | F-highly palatable foods liking (derived food-liking factor) \|\| id:ebi-fl187-GCST90094785 | Inverse variance weighted | 17 | 0.334110536747599 | 0.279457355908647 | 0.231864815527629 | -0.213625881 | 0.881846954328547 | 1.39669752144353 | 0.80765048603505 | 2.41535664267756 | 0.998074519377826 |
| ebi-fl187-GCST90094785 | prot-a-3133 | PolyUbiquitin K48-linked \|\| id:prot-a-3133 | F-highly palatable foods liking (derived food-liking factor) \|\| id:ebi-fl187-GCST90094785 | Inverse variance weighted | 17 | -0.153355444 | 0.267701604565209 | 0.566739736496399 | -0.678050589 | 0.371339701235475 | 0.857824759199141 | 0.507605560525398 | 1.44967544629221 | 0.998074519377826 |
| ebi-fl187-GCST90094785 | prot-a-3135 | Ubiquitin-conjugating enzyme E2 B \|\| id:prot-a-3135 | F-highly palatable foods liking (derived food-liking factor) \|\| id:ebi-fl187-GCST90094785 | Inverse variance weighted | 17 | 0.142062026621483 | 0.284798772477631 | 0.617909405319277 | -0.416143567 | 0.700267620677639 | 1.15264814118972 | 0.659585568678462 | 2.0142917014544 | 0.998074519377826 |
| ebi-fl187-GCST90094785 | prot-a-3162 | NKG2D ligand 3 \|\| id:prot-a-3162 | F-highly palatable foods liking (derived food-liking factor) \|\| id:ebi-fl187-GCST90094785 | Inverse variance weighted | 17 | 0.432620847104193 | 0.267701604565209 | 0.106082155410518 | -0.092074298 | 0.957315992052003 | 1.54129172468026 | 0.912037384616753 | 2.60469605811837 | 0.998074519377826 |
| ebi-fl187-GCST90094785 | prot-a-3188 | Vesicle-associated membrane protein 8 \|\| id:prot-a-3188 | F-highly palatable foods liking (derived food-liking factor) \|\| id:ebi-fl187-GCST90094785 | Inverse variance weighted | 17 | 0.00941409264085706 | 0.267765803270912 | 0.971953804680691 | -0.515406882 | 0.534235067051845 | 1.00945854459308 | 0.597257531970878 | 1.70614265824222 | 0.998074519377826 |
| ebi-fl187-GCST90094785 | prot-a-3201 | Vitrin \|\| id:prot-a-3201 | F-highly palatable foods liking (derived food-liking factor) \|\| id:ebi-fl187-GCST90094785 | Inverse variance weighted | 17 | -0.00161511 | 0.271489072239451 | 0.995253349122916 | -0.533733692 | 0.53050347144999 | 0.99838619344914 | 0.586411397780528 | 1.6997878878932 | 0.999355215041207 |
| ebi-fl187-GCST90094785 | prot-a-3232 | WNT1-inducible-signaling pathway protein 3 \|\| id:prot-a-3232 | F-highly palatable foods liking (derived food-liking factor) \|\| id:ebi-fl187-GCST90094785 | Inverse variance weighted | 17 | -0.19511276 | 0.267765803270912 | 0.466203885007845 | -0.719933735 | 0.329708214003232 | 0.822741880115398 | 0.486784511755146 | 1.39056232264905 | 0.998074519377826 |
| ebi-fl187-GCST90094785 | prot-a-327 | Carbonic anhydrase-related protein 10 \|\| id:prot-a-327 | F-highly palatable foods liking (derived food-liking factor) \|\| id:ebi-fl187-GCST90094785 | Inverse variance weighted | 17 | -0.511465764 | 0.306781428786828 | 0.0954747945663745 | -1.112757364 | 0.0898258366558846 | 0.599616038907354 | 0.328651498675047 | 1.09398373524668 | 0.998074519377826 |
| ebi-fl187-GCST90094785 | prot-a-332 | Carbonic anhydrase 5A, mitochondrial \|\| id:prot-a-332 | F-highly palatable foods liking (derived food-liking factor) \|\| id:ebi-fl187-GCST90094785 | Inverse variance weighted | 17 | -0.418478959 | 0.267765803270912 | 0.11808684926712 | -0.943299934 | 0.10634201509286 | 0.65804697520358 | 0.38934091393463 | 1.11220220140359 | 0.998074519377826 |
| ebi-fl187-GCST90094785 | prot-a-338 | Cell adhesion molecule 3 \|\| id:prot-a-338 | F-highly palatable foods liking (derived food-liking factor) \|\| id:ebi-fl187-GCST90094785 | Inverse variance weighted | 17 | -0.216733668 | 0.267765803270912 | 0.418276454165261 | -0.741554643 | 0.308087306120466 | 0.80514437623015 | 0.476372750127443 | 1.36081979164763 | 0.998074519377826 |
| ebi-fl187-GCST90094785 | prot-a-34 | A disintegrin and metalloproteinase with thrombospondin motifs 3 \|\| id:prot-a-34 | F-highly palatable foods liking (derived food-liking factor) \|\| id:ebi-fl187-GCST90094785 | Inverse variance weighted | 17 | -0.123898131 | 0.267765803270912 | 0.643571671616153 | -0.648719105 | 0.40092284375725 | 0.883469834793375 | 0.522714890993482 | 1.4932020541951 | 0.998074519377826 |
| ebi-fl187-GCST90094785 | prot-a-358 | Cysteine--tRNA ligase, cytoplasmic \|\| id:prot-a-358 | F-highly palatable foods liking (derived food-liking factor) \|\| id:ebi-fl187-GCST90094785 | Inverse variance weighted | 17 | -0.12621736 | 0.271248214993908 | 0.641701944125566 | -0.657863861 | 0.405429141714403 | 0.881423240092566 | 0.517956580812183 | 1.49994605137953 | 0.998074519377826 |
| ebi-fl187-GCST90094785 | prot-a-382 | Coiled-coil domain-containing protein 167 \|\| id:prot-a-382 | F-highly palatable foods liking (derived food-liking factor) \|\| id:ebi-fl187-GCST90094785 | Inverse variance weighted | 17 | -0.252150038 | 0.267765803270912 | 0.346355854421149 | -0.776971012 | 0.272670936499973 | 0.777128130640085 | 0.459796622473823 | 1.31346795934008 | 0.998074519377826 |
| ebi-fl187-GCST90094785 | prot-a-40 | Pituitary adenylate cyclase-activating polypeptide 27 \|\| id:prot-a-40 | F-highly palatable foods liking (derived food-liking factor) \|\| id:ebi-fl187-GCST90094785 | Inverse variance weighted | 17 | -0.193147351 | 0.267701604565209 | 0.470600461770425 | -0.717842496 | 0.3315477935496 | 0.824360494517736 | 0.487803559418513 | 1.39312272696741 | 0.998074519377826 |
| ebi-fl187-GCST90094785 | prot-a-403 | C-C motif chemokine 25 \|\| id:prot-a-403 | F-highly palatable foods liking (derived food-liking factor) \|\| id:ebi-fl187-GCST90094785 | Inverse variance weighted | 17 | -0.115664534 | 0.267658250600128 | 0.665643201426702 | -0.640274705 | 0.408945637497056 | 0.890773997855999 | 0.527147594147572 | 1.50522989019699 | 0.998074519377826 |
| ebi-fl187-GCST90094785 | prot-a-415 | Cyclin-C \|\| id:prot-a-415 | F-highly palatable foods liking (derived food-liking factor) \|\| id:ebi-fl187-GCST90094785 | Inverse variance weighted | 17 | 0.131281650091609 | 0.267701604565209 | 0.623848806797327 | -0.393413495 | 0.655976795039419 | 1.14028889856126 | 0.674749684370797 | 1.92702390575334 | 0.998074519377826 |
| ebi-fl187-GCST90094785 | prot-a-44 | Adhesion G protein-coupled receptor F5 \|\| id:prot-a-44 | F-highly palatable foods liking (derived food-liking factor) \|\| id:ebi-fl187-GCST90094785 | Inverse variance weighted | 17 | -0.310330241 | 0.278143514258612 | 0.264542069707828 | -0.855491529 | 0.234831047054758 | 0.733204782037253 | 0.425074203495036 | 1.26469507672341 | 0.998074519377826 |
| ebi-fl187-GCST90094785 | prot-a-455 | CD59 glycoprotein \|\| id:prot-a-455 | F-highly palatable foods liking (derived food-liking factor) \|\| id:ebi-fl187-GCST90094785 | Inverse variance weighted | 17 | 0.125787813024116 | 0.267765803270912 | 0.638520760063636 | -0.399033161 | 0.650608787435105 | 1.1340415139127 | 0.670968450740143 | 1.916707341 | 0.998074519377826 |
| ebi-fl187-GCST90094785 | prot-a-478 | Cadherin-12 \|\| id:prot-a-478 | F-highly palatable foods liking (derived food-liking factor) \|\| id:ebi-fl187-GCST90094785 | Inverse variance weighted | 17 | 0.413275210796228 | 0.267701604565209 | 0.122639078269609 | -0.111419934 | 0.937970355744037 | 1.51176102164259 | 0.894563012482586 | 2.55479083604781 | 0.998074519377826 |
| ebi-fl187-GCST90094785 | prot-a-516 | Centrin-2 \|\| id:prot-a-516 | F-highly palatable foods liking (derived food-liking factor) \|\| id:ebi-fl187-GCST90094785 | Inverse variance weighted | 17 | -0.080806378 | 0.29632187492344 | 0.785085383521469 | -0.661597253 | 0.499984496555538 | 0.922372265447378 | 0.516026451021751 | 1.64869571003961 | 0.998074519377826 |
| ebi-fl187-GCST90094785 | prot-a-519 | Cryptic protein \|\| id:prot-a-519 | F-highly palatable foods liking (derived food-liking factor) \|\| id:ebi-fl187-GCST90094785 | Inverse variance weighted | 17 | 0.134214908612605 | 0.267701604565209 | 0.616117701074261 | -0.390480236 | 0.658910053560415 | 1.14363857101528 | 0.676731805247311 | 1.93268466321882 | 0.998074519377826 |
| ebi-fl187-GCST90094785 | prot-a-583 | Chloride intracellular channel protein 4 \|\| id:prot-a-583 | F-highly palatable foods liking (derived food-liking factor) \|\| id:ebi-fl187-GCST90094785 | Inverse variance weighted | 17 | 0.114495656349656 | 0.267765803270912 | 0.668945088475438 | -0.410325318 | 0.639316630760644 | 1.1213077704845 | 0.663434387837398 | 1.89518532533029 | 0.998074519377826 |
| ebi-fl187-GCST90094785 | prot-a-602 | CCR4-NOT transcription complex subunit 1 \|\| id:prot-a-602 | F-highly palatable foods liking (derived food-liking factor) \|\| id:ebi-fl187-GCST90094785 | Inverse variance weighted | 17 | 0.74122244529231 | 0.267658250600128 | 0.00561791682192842 | 0.216612274116059 | 1.26583261646856 | 2.09849924768957 | 1.24186250654218 | 3.54604400193647 | 0.877504772527479 |
| ebi-fl187-GCST90094785 | prot-a-606 | Ciliary neurotrophic factor receptor subunit alpha \|\| id:prot-a-606 | F-highly palatable foods liking (derived food-liking factor) \|\| id:ebi-fl187-GCST90094785 | Inverse variance weighted | 17 | -0.25284558 | 0.267765803270912 | 0.345027180808986 | -0.777666554 | 0.271975394807644 | 0.77658779356021 | 0.45947692594692 | 1.31255470525278 | 0.998074519377826 |
| ebi-fl187-GCST90094785 | prot-a-626 | Collagen alpha-2(IX) chain \|\| id:prot-a-626 | F-highly palatable foods liking (derived food-liking factor) \|\| id:ebi-fl187-GCST90094785 | Inverse variance weighted | 17 | -0.342346784 | 0.272791552529795 | 0.209487562582243 | -0.877018227 | 0.192324659206173 | 0.710101910201297 | 0.416021546034215 | 1.21206396081721 | 0.998074519377826 |
| ebi-fl187-GCST90094785 | prot-a-647 | Probable serine carboxypeptidase CPVL \|\| id:prot-a-647 | F-highly palatable foods liking (derived food-liking factor) \|\| id:ebi-fl187-GCST90094785 | Inverse variance weighted | 17 | 0.223097531712567 | 0.267765803270912 | 0.40474233647663 | -0.301723443 | 0.747918506123555 | 1.24994247682155 | 0.739542562506063 | 2.11259807693623 | 0.998074519377826 |
| ebi-fl187-GCST90094785 | prot-a-651 | Cellular retinoic acid-binding protein 2 \|\| id:prot-a-651 | F-highly palatable foods liking (derived food-liking factor) \|\| id:ebi-fl187-GCST90094785 | Inverse variance weighted | 17 | 0.308458164427673 | 0.269406349628598 | 0.252227772027788 | -0.219578281 | 0.836494609699725 | 1.36132455656433 | 0.802857306885157 | 2.30826142131648 | 0.998074519377826 |
| ebi-fl187-GCST90094785 | prot-a-671 | Cytotoxic and regulatory T-cell molecule \|\| id:prot-a-671 | F-highly palatable foods liking (derived food-liking factor) \|\| id:ebi-fl187-GCST90094785 | Inverse variance weighted | 17 | 0.372445203231814 | 0.317410567996466 | 0.240640939628441 | -0.24967951 | 0.994569916504888 | 1.45127895156796 | 0.779050420903311 | 2.70356133409446 | 0.998074519377826 |
| ebi-fl187-GCST90094785 | prot-a-688 | Chorionic somatomammotropin hormone \|\| id:prot-a-688 | F-highly palatable foods liking (derived food-liking factor) \|\| id:ebi-fl187-GCST90094785 | Inverse variance weighted | 17 | -0.763909443 | 0.267701604565209 | 0.0043228880394014 | -1.288604588 | -0.239214298 | 0.465841680911633 | 0.275655167351434 | 0.787246158886295 | 0.877504772527479 |
| ebi-fl187-GCST90094785 | prot-a-69 | Aldo-keto reductase family 1 member C1 \|\| id:prot-a-69 | F-highly palatable foods liking (derived food-liking factor) \|\| id:ebi-fl187-GCST90094785 | Inverse variance weighted | 17 | -0.050549051 | 0.267765803270912 | 0.850264596234793 | -0.575370025 | 0.474271923519952 | 0.950707294488702 | 0.56249669228559 | 1.60684386626603 | 0.998074519377826 |
| ebi-fl187-GCST90094785 | prot-a-741 | Stromal cell-derived factor 1 \|\| id:prot-a-741 | F-highly palatable foods liking (derived food-liking factor) \|\| id:ebi-fl187-GCST90094785 | Inverse variance weighted | 17 | 0.311056392939797 | 0.267765803270913 | 0.245368182076584 | -0.213764581 | 0.835877367350786 | 1.36486618783272 | 0.80753847216586 | 2.30683710423446 | 0.998074519377826 |
| ebi-fl187-GCST90094785 | prot-a-742 | Growth-regulated alpha protein \|\| id:prot-a-742 | F-highly palatable foods liking (derived food-liking factor) \|\| id:ebi-fl187-GCST90094785 | Inverse variance weighted | 17 | 0.319094235116681 | 0.267765803270912 | 0.23338222724453 | -0.205726739 | 0.84391520952767 | 1.37588097510137 | 0.814055495271463 | 2.32545381566969 | 0.998074519377826 |
| ebi-fl187-GCST90094785 | prot-a-788 | Beta-defensin 112 \|\| id:prot-a-788 | F-highly palatable foods liking (derived food-liking factor) \|\| id:ebi-fl187-GCST90094785 | Inverse variance weighted | 17 | 0.0942579026041759 | 0.282116542626612 | 0.738296702667853 | -0.458690521 | 0.647206326152335 | 1.0988431038743 | 0.632110839697655 | 1.91019690076765 | 0.998074519377826 |
| ebi-fl187-GCST90094785 | prot-a-838 | DnaJ homolog subfamily B member 1 \|\| id:prot-a-838 | F-highly palatable foods liking (derived food-liking factor) \|\| id:ebi-fl187-GCST90094785 | Inverse variance weighted | 17 | 0.0233129268396221 | 0.267765803270912 | 0.930620150889817 | -0.501508048 | 0.548133901250611 | 1.02358679721753 | 0.605616671966012 | 1.7300216125768 | 0.998074519377826 |
| ebi-fl187-GCST90094785 | prot-a-85 | Junctional adhesion molecule-like \|\| id:prot-a-85 | F-highly palatable foods liking (derived food-liking factor) \|\| id:ebi-fl187-GCST90094785 | Inverse variance weighted (multiplicative random effects) | 17 | -0.159217201 | 0.352613457554647 | 0.651604243369497 | -0.850339578 | 0.531905175696573 | 0.85281110733184 | 0.427269815916661 | 1.70217215842464 | 0.998074519377826 |
| ebi-fl187-GCST90094785 | prot-a-856 | Dipeptidase 1 \|\| id:prot-a-856 | F-highly palatable foods liking (derived food-liking factor) \|\| id:ebi-fl187-GCST90094785 | Inverse variance weighted (multiplicative random effects) | 17 | 0.109829757356978 | 0.385425630670185 | 0.775677034182987 | -0.645604479 | 0.865263993470541 | 1.11608804850519 | 0.524345490580624 | 2.37563315484371 | 0.998074519377826 |
| ebi-fl187-GCST90094785 | prot-a-860 | Dipeptidyl peptidase 2 \|\| id:prot-a-860 | F-highly palatable foods liking (derived food-liking factor) \|\| id:ebi-fl187-GCST90094785 | Inverse variance weighted | 17 | 0.0242269825084 | 0.267701604565209 | 0.927889931666176 | -0.500468162 | 0.54892212745621 | 1.02452284026456 | 0.606246771297537 | 1.73138579852101 | 0.998074519377826 |
| ebi-fl187-GCST90094785 | prot-a-861 | Dermatopontin \|\| id:prot-a-861 | F-highly palatable foods liking (derived food-liking factor) \|\| id:ebi-fl187-GCST90094785 | Inverse variance weighted | 17 | 0.256553772974044 | 0.267701604565209 | 0.337882645442865 | -0.268141372 | 0.781248917921853 | 1.29246826379351 | 0.764799652223477 | 2.18419844734093 | 0.998074519377826 |
| ebi-fl187-GCST90094785 | prot-a-877 | Dual specificity protein phosphatase 6 \|\| id:prot-a-877 | F-highly palatable foods liking (derived food-liking factor) \|\| id:ebi-fl187-GCST90094785 | Inverse variance weighted | 17 | 0.377046100266656 | 0.267765803270912 | 0.159095852263107 | -0.147774874 | 0.901867074677644 | 1.45797152069787 | 0.862625292341108 | 2.46419966356086 | 0.998074519377826 |
| ebi-fl187-GCST90094785 | prot-a-879 | Dynactin-associated protein \|\| id:prot-a-879 | F-highly palatable foods liking (derived food-liking factor) \|\| id:ebi-fl187-GCST90094785 | Inverse variance weighted | 17 | 0.146341487385857 | 0.267701604565209 | 0.584613037288733 | -0.378353658 | 0.671036632333667 | 1.15759142343209 | 0.684988206564725 | 1.95626419661125 | 0.998074519377826 |
| ebi-fl187-GCST90094785 | prot-a-937 | EMI domain-containing protein 1 \|\| id:prot-a-937 | F-highly palatable foods liking (derived food-liking factor) \|\| id:ebi-fl187-GCST90094785 | Inverse variance weighted | 17 | -0.409016117 | 0.330339776895697 | 0.215653800976206 | -1.05648208 | 0.238449845690028 | 0.66430352563905 | 0.347676760587671 | 1.26928004457518 | 0.998074519377826 |
| ebi-fl187-GCST90094785 | prot-b-1 | SRC proto-oncogene, non-receptor tyrosine kinase \|\| id:prot-b-1 | F-highly palatable foods liking (derived food-liking factor) \|\| id:ebi-fl187-GCST90094785 | Inverse variance weighted | 12 | -0.483933355 | 0.446286111269655 | 0.278206654104622 | -1.358654133 | 0.390787422792004 | 0.616354277291595 | 0.257006440681085 | 1.47814426023294 | 0.998074519377826 |
| ebi-fl187-GCST90094785 | prot-b-10 | coagulation factor III, tissue factor \|\| id:prot-b-10 | F-highly palatable foods liking (derived food-liking factor) \|\| id:ebi-fl187-GCST90094785 | Inverse variance weighted | 12 | -0.563791393 | 0.351439541732221 | 0.108662400466043 | -1.252612895 | 0.125030108504392 | 0.56904748592187 | 0.285757167048866 | 1.13318257098562 | 0.998074519377826 |
| ebi-fl187-GCST90094785 | prot-b-29 | myeloperoxidase \|\| id:prot-b-29 | F-highly palatable foods liking (derived food-liking factor) \|\| id:ebi-fl187-GCST90094785 | Inverse variance weighted | 12 | -0.000286654 | 0.351347753451682 | 0.999349029398804 | -0.688928251 | 0.688354942410891 | 0.999713386727028 | 0.502113920825988 | 1.99043845260682 | 0.999355215041207 |
| ebi-fl187-GCST90094785 | prot-b-33 | adrenomedullin \|\| id:prot-b-33 | F-highly palatable foods liking (derived food-liking factor) \|\| id:ebi-fl187-GCST90094785 | Inverse variance weighted | 12 | 0.0694248244127595 | 0.351508214427523 | 0.843431936055212 | -0.619531276 | 0.758380924690704 | 1.07189147810454 | 0.538196644238818 | 2.13481699139564 | 0.998074519377826 |
| ebi-fl187-GCST90094785 | ukb-rsfMRI191-edge-pheno102 | Net25_Pair11_15 (Postcentral\|Precentral)&(Cerebellum) (Motor)&(Subcortical-cerebellum) \|\| id:ukb-rsfMRI191-edge-pheno102 | F-highly palatable foods liking (derived food-liking factor) \|\| id:ebi-fl187-GCST90094785 | Inverse variance weighted | 17 | -0.123336159 | 0.0830558216294556 | 0.137549362305055 | -0.286125569 | 0.039453251676102 | 0.883966459578452 | 0.751168286490358 | 1.04024186818448 | 0.998074519377826 |
| ebi-fl187-GCST90094785 | ukb-rsfMRI191-edge-pheno1167 | Net100_Pair11_45 (Angular\|Temporal)&(Frontal) (Default_mode\|Central_executive)&(Salience\|Default_mode) \|\| id:ukb-rsfMRI191-edge-pheno1167 | F-highly palatable foods liking (derived food-liking factor) \|\| id:ebi-fl187-GCST90094785 | Inverse variance weighted | 17 | -0.025779368 | 0.105450117111519 | 0.806866997127855 | -0.232461597 | 0.180902861660539 | 0.974550082943417 | 0.79258018588903 | 1.19829877288655 | 0.998074519377826 |
| ebi-fl187-GCST90094785 | ukb-rsfMRI191-edge-pheno1317 | Net100_Pair26_48 (Parietal\|Frontal)&(Temporal) (Attention\|Central_executive)&(Default_mode\|Central_executive) \|\| id:ukb-rsfMRI191-edge-pheno1317 | F-highly palatable foods liking (derived food-liking factor) \|\| id:ebi-fl187-GCST90094785 | Inverse variance weighted (multiplicative random effects) | 17 | -0.03692097 | 0.113358026171414 | 0.744649601973928 | -0.259102701 | 0.185260761160443 | 0.963752297552929 | 0.771743759784667 | 1.20353223367522 | 0.998074519377826 |
| ebi-fl187-GCST90094785 | ukb-rsfMRI191-edge-pheno286 | Net100_Pair10_13 (OccipitalPrecuneus)&(Frontal_Sup) (Default_mode\|Central_executive)&(Default_mode) \|\| id:ukb-rsfMRI191-edge-pheno286 | F-highly palatable foods liking (derived food-liking factor) \|\| id:ebi-fl187-GCST90094785 | Inverse variance weighted | 17 | 0.0130366135455039 | 0.0889248247955532 | 0.883445674496089 | -0.161256043 | 0.187329270144788 | 1.01312196066775 | 0.851074131587747 | 1.20602432748416 | 0.998074519377826 |
| ebi-fl187-GCST90094785 | ukb-rsfMRI191-edge-pheno599 | Net100_Pair11_29 (Angular\|Temporal)&(Parietal) (Default_mode\|Central_executive)&(Central_executive\|Salience) \|\| id:ukb-rsfMRI191-edge-pheno599 | F-highly palatable foods liking (derived food-liking factor) \|\| id:ebi-fl187-GCST90094785 | Inverse variance weighted | 17 | -0.008928939 | 0.0942995034248208 | 0.924563428949046 | -0.193755966 | 0.175898087835767 | 0.991110805717534 | 0.823858929667233 | 1.19231654089957 | 0.998074519377826 |
| ebi-fl187-GCST90094785 | ukb-rsfMRI191-edge-pheno621 | Net100_Pair5_30 (Precuneus\|Angular\|Cingulate)&(Paracentral) (Default_mode\|Central_executive)&(Motor) \|\| id:ukb-rsfMRI191-edge-pheno621 | F-highly palatable foods liking (derived food-liking factor) \|\| id:ebi-fl187-GCST90094785 | Inverse variance weighted | 17 | -0.061408222 | 0.085814769840513 | 0.474244324866344 | -0.229605171 | 0.106788726437407 | 0.940439252989399 | 0.794847369080941 | 1.11269914573146 | 0.998074519377826 |
| ebi-fl187-GCST90094785 | ukb-rsfMRI191-edge-pheno624 | Net100_Pair8_30 (Calcarine\|Lingual)&(Paracentral) (Visual)&(Motor) \|\| id:ukb-rsfMRI191-edge-pheno624 | F-highly palatable foods liking (derived food-liking factor) \|\| id:ebi-fl187-GCST90094785 | Inverse variance weighted | 17 | -0.110994556 | 0.0835605734420348 | 0.184074886062706 | -0.27477328 | 0.0527841682400776 | 0.894943621251677 | 0.759744353203829 | 1.05420209 | 0.998074519377826 |
| ebi-fl187-GCST90094785 | ukb-rsfMRI191-edge-pheno882 | Net100_Pair6_38 (Postcentral\|Precentral)&(Subcortical) (Motor)&(Subcortical-cerebellum) \|\| id:ukb-rsfMRI191-edge-pheno882 | F-highly palatable foods liking (derived food-liking factor) \|\| id:ebi-fl187-GCST90094785 | Inverse variance weighted | 17 | 0.0146787520888323 | 0.0926086709067182 | 0.874060457534151 | -0.166834243 | 0.196191747 | 1.01478701403845 | 0.846339886566143 | 1.21676019316454 | 0.998074519377826 |
| ebi-fl187-GCST90094785 | ukb-rsfMRI191-edge-pheno899 | Net100_Pair23_38 (Cerebellum)&(Subcortical) (Subcortical-cerebellum)&(Subcortical-cerebellum) \|\| id:ukb-rsfMRI191-edge-pheno899 | F-highly palatable foods liking (derived food-liking factor) \|\| id:ebi-fl187-GCST90094785 | Inverse variance weighted | 17 | -0.027710294 | 0.0835143838165375 | 0.740038282071447 | -0.191398486 | 0.135977898299747 | 0.972670114374253 | 0.825803451247608 | 1.14565657235689 | 0.998074519377826 |
| ebi-fl187-GCST90094785 | ebi-a-GCST90001392 | IgD+ CD38+ B cell Absolute Count \|\| id:ebi-a-GCST90001392 | F-highly palatable foods liking (derived food-liking factor) \|\| id:ebi-fl187-GCST90094785 | MR Egger | 14 | 1.66518235328661 | 2.57064041923252 | 0.529332933200447 | -3.373272868 | 6.70363757498235 | 5.28663719753667 | 0.0342772685702188 | 815.366393653112 | 0.999279047927446 |
| ebi-fl187-GCST90094785 | ebi-a-GCST90001402 | Switched memory B cell %B cell \|\| id:ebi-a-GCST90001402 | F-highly palatable foods liking (derived food-liking factor) \|\| id:ebi-fl187-GCST90094785 | MR Egger | 14 | -0.813718953 | 3.11642318681879 | 0.798437449447358 | -6.921908399 | 5.29447049297613 | 0.44320673241885 | 0.000985946564200022 | 199.23210323348 | 0.999279047927446 |
| ebi-fl187-GCST90094785 | ebi-a-GCST90001407 | Memory B cell Absolute Count \|\| id:ebi-a-GCST90001407 | F-highly palatable foods liking (derived food-liking factor) \|\| id:ebi-fl187-GCST90094785 | MR Egger | 14 | -3.66800598 | 2.70544060588894 | 0.200132578216518 | -8.970669568 | 1.63465760728067 | 0.0255273212129831 | 0.000127083072928003 | 5.12770200859085 | 0.999279047927446 |
| ebi-fl187-GCST90094785 | ebi-a-GCST90001411 | IgD+ CD24+ B cell %B cell \|\| id:ebi-a-GCST90001411 | F-highly palatable foods liking (derived food-liking factor) \|\| id:ebi-fl187-GCST90094785 | MR Egger | 14 | -1.723680854 | 3.33792175845562 | 0.61496275632503 | -8.2660075 | 4.81864579268701 | 0.178408243153555 | 0.000257109755390383 | 123.797329964434 | 0.999279047927446 |
| ebi-fl187-GCST90094785 | ebi-a-GCST90001418 | CD24+ CD27+ B cell Absolute Count \|\| id:ebi-a-GCST90001418 | F-highly palatable foods liking (derived food-liking factor) \|\| id:ebi-fl187-GCST90094785 | MR Egger | 14 | -2.893350708 | 2.69646717576071 | 0.304368686398338 | -8.178426372 | 2.39172495658874 | 0.0553903045923789 | 0.000280643220751786 | 10.9323354920804 | 0.999279047927446 |
| ebi-fl187-GCST90094785 | ebi-a-GCST90001436 | Memory B cell %lymphocyte \|\| id:ebi-a-GCST90001436 | F-highly palatable foods liking (derived food-liking factor) \|\| id:ebi-fl187-GCST90094785 | MR Egger | 14 | -1.470948962 | 2.65826217184473 | 0.590184819117442 | -6.681142819 | 3.73924489479697 | 0.229707398128685 | 0.00125434366557617 | 42.0662137523634 | 0.999279047927446 |
| ebi-fl187-GCST90094785 | ebi-a-GCST90001439 | IgD+ CD24+ B cell %lymphocyte \|\| id:ebi-a-GCST90001439 | F-highly palatable foods liking (derived food-liking factor) \|\| id:ebi-fl187-GCST90094785 | MR Egger | 14 | 0.189109732344191 | 2.961105099 | 0.950129582535853 | -5.614656262 | 5.99287572689576 | 1.20817352092189 | 0.00364406208473086 | 400.564870388205 | 0.999279047927446 |
| ebi-fl187-GCST90094785 | ebi-a-GCST90001457 | CD11c+ HLA DR++ monocyte %monocyte \|\| id:ebi-a-GCST90001457 | F-highly palatable foods liking (derived food-liking factor) \|\| id:ebi-fl187-GCST90094785 | MR Egger | 14 | -5.881031786 | 2.8271545688414 | 0.0596023516612582 | -11.42225474 | -0.339808831 | 0.00279190314261557 | 1.09490841917811e-05 | 0.71190640433633 | 0.999279047927446 |
| ebi-fl187-GCST90094785 | ebi-a-GCST90001483 | CD39+ resting CD4 regulatory T cell Absolute Count \|\| id:ebi-a-GCST90001483 | F-highly palatable foods liking (derived food-liking factor) \|\| id:ebi-fl187-GCST90094785 | MR Egger | 14 | -1.948696439 | 2.95526944055004 | 0.522094316179458 | -7.741024542 | 3.84363166460863 | 0.142459655469807 | 0.000434626054092385 | 46.6947465424201 | 0.999279047927446 |
| ebi-fl187-GCST90094785 | ebi-a-GCST90001525 | CD33dim HLA DR+ CD11b+ Absolute Count \|\| id:ebi-a-GCST90001525 | F-highly palatable foods liking (derived food-liking factor) \|\| id:ebi-fl187-GCST90094785 | MR Egger | 14 | -1.823690926 | 3.97092977636301 | 0.65425538729374 | -9.606713288 | 5.9593314353061 | 0.16142882809466 | 6.72755767721981e-05 | 387.351068401161 | 0.999279047927446 |
| ebi-fl187-GCST90094785 | ebi-a-GCST90001540 | Naive CD4+ T cell Absolute Count \|\| id:ebi-a-GCST90001540 | F-highly palatable foods liking (derived food-liking factor) \|\| id:ebi-fl187-GCST90094785 | MR Egger | 14 | -4.548036069 | 2.58179283917756 | 0.103572237494886 | -9.608350033 | 0.512277896121201 | 0.0105879780399862 | 6.71655538445009e-05 | 1.66908887902232 | 0.999279047927446 |
| ebi-fl187-GCST90094785 | ebi-a-GCST90001624 | HLA DR+ CD4+ T cell Absolute Count \|\| id:ebi-a-GCST90001624 | F-highly palatable foods liking (derived food-liking factor) \|\| id:ebi-fl187-GCST90094785 | MR Egger | 14 | -6.37914843 | 3.07970593866091 | 0.0605432083008335 | -12.41537207 | -0.342924791 | 0.00169656708654593 | 4.05576160127476e-06 | 0.709691584990166 | 0.999279047927446 |
| ebi-fl187-GCST90094785 | ebi-a-GCST90001672 | CD39+ CD8+ T cell Absolute Count \|\| id:ebi-a-GCST90001672 | F-highly palatable foods liking (derived food-liking factor) \|\| id:ebi-fl187-GCST90094785 | MR Egger | 14 | -2.260012417 | 3.10195575345982 | 0.480238212284819 | -8.339845694 | 3.81982085961088 | 0.104349189025063 | 0.00023880918979644 | 45.5960394969295 | 0.999279047927446 |
| ebi-fl187-GCST90094785 | ebi-a-GCST90001722 | CD19 on CD20- CD38- B cell \|\| id:ebi-a-GCST90001722 | F-highly palatable foods liking (derived food-liking factor) \|\| id:ebi-fl187-GCST90094785 | MR Egger | 14 | 1.34507623170182 | 3.3426268121544 | 0.694464729951891 | -5.20647232 | 7.89662478352446 | 3.83847914324451 | 0.00548097474798046 | 2688.19376308056 | 0.999279047927446 |
| ebi-fl187-GCST90094785 | ebi-a-GCST90001742 | CD19 on transitional B cell \|\| id:ebi-a-GCST90001742 | F-highly palatable foods liking (derived food-liking factor) \|\| id:ebi-fl187-GCST90094785 | MR Egger | 14 | -0.616270354 | 2.93120678983354 | 0.837004290702016 | -6.361435662 | 5.12889495423366 | 0.539954526130019 | 0.00172688570730191 | 168.830449551761 | 0.999279047927446 |
| ebi-fl187-GCST90094785 | ebi-a-GCST90001784 | CD25 on IgD+ CD38dim B cell \|\| id:ebi-a-GCST90001784 | F-highly palatable foods liking (derived food-liking factor) \|\| id:ebi-fl187-GCST90094785 | MR Egger | 14 | -4.397294839 | 3.19663282553133 | 0.194078601707181 | -10.66269518 | 1.86810549936767 | 0.0123105970508575 | 2.34018570716065e-05 | 6.47601595398421 | 0.999279047927446 |
| ebi-fl187-GCST90094785 | ebi-a-GCST90001794 | CD25 on IgD+ B cell \|\| id:ebi-a-GCST90001794 | F-highly palatable foods liking (derived food-liking factor) \|\| id:ebi-fl187-GCST90094785 | MR Egger | 14 | -3.549502486 | 3.20321613615364 | 0.289532184595728 | -9.827806113 | 2.72880114098578 | 0.0287389341237483 | 5.39309457182281e-05 | 15.3145160643825 | 0.999279047927446 |
| ebi-fl187-GCST90094785 | ebi-a-GCST90001802 | CD27 on IgD- CD38- B cell \|\| id:ebi-a-GCST90001802 | F-highly palatable foods liking (derived food-liking factor) \|\| id:ebi-fl187-GCST90094785 | MR Egger | 14 | 0.0401632470748363 | 2.63513657327747 | 0.988090045036536 | -5.124704437 | 5.20503093069867 | 1.04098069737609 | 0.00594797510074409 | 182.186507837607 | 0.999279047927446 |
| ebi-fl187-GCST90094785 | ebi-a-GCST90001804 | CD27 on IgD- CD38dim B cell \|\| id:ebi-a-GCST90001804 | F-highly palatable foods liking (derived food-liking factor) \|\| id:ebi-fl187-GCST90094785 | MR Egger | 14 | 0.100280093900414 | 2.61809998137854 | 0.970076242529764 | -5.03119587 | 5.23175605740235 | 1.10548051306453 | 0.00653099567948431 | 187.121110584153 | 0.999279047927446 |
| ebi-fl187-GCST90094785 | ebi-a-GCST90001805 | CD27 on memory B cell \|\| id:ebi-a-GCST90001805 | F-highly palatable foods liking (derived food-liking factor) \|\| id:ebi-fl187-GCST90094785 | MR Egger | 14 | 2.14972799300358 | 2.64240380118733 | 0.431749540020532 | -3.029383457 | 7.32883944333075 | 8.58252357318919 | 0.0483454359637858 | 1523.61250686672 | 0.999279047927446 |
| ebi-fl187-GCST90094785 | ebi-a-GCST90001806 | CD27 on unswitched memory B cell \|\| id:ebi-a-GCST90001806 | F-highly palatable foods liking (derived food-liking factor) \|\| id:ebi-fl187-GCST90094785 | MR Egger | 14 | -1.206737244 | 2.6286079664972 | 0.654382383018867 | -6.358808859 | 3.94533436990226 | 0.299171813231892 | 0.00173142785917014 | 51.6936200133434 | 0.999279047927446 |
| ebi-fl187-GCST90094785 | ebi-a-GCST90001839 | CD3 on Effector Memory CD8+ T cell \|\| id:ebi-a-GCST90001839 | F-highly palatable foods liking (derived food-liking factor) \|\| id:ebi-fl187-GCST90094785 | MR Egger | 14 | -2.127705361 | 3.10193873773388 | 0.505795493706587 | -8.207505287 | 3.95209456519711 | 0.119110295670459 | 0.000272599932390339 | 52.044262851794 | 0.999279047927446 |
| ebi-fl187-GCST90094785 | ebi-a-GCST90001859 | CD3 on CD8+ T cell \|\| id:ebi-a-GCST90001859 | F-highly palatable foods liking (derived food-liking factor) \|\| id:ebi-fl187-GCST90094785 | MR Egger | 14 | -2.040255134 | 2.77920605563268 | 0.476978815484279 | -7.487499003 | 3.40698873477198 | 0.129995540330099 | 0.000560041873284526 | 30.1742446624683 | 0.999279047927446 |
| ebi-fl187-GCST90094785 | ebi-a-GCST90001905 | CD86 on monocyte \|\| id:ebi-a-GCST90001905 | F-highly palatable foods liking (derived food-liking factor) \|\| id:ebi-fl187-GCST90094785 | MR Egger | 14 | 1.72521489694503 | 2.79633575307543 | 0.548791188506116 | -3.755603179 | 7.20603297297287 | 5.6137272729207 | 0.023386340202854 | 1347.53593855989 | 0.999279047927446 |
| ebi-fl187-GCST90094785 | ebi-a-GCST90001943 | CD25 on activated & secreting CD4 regulatory T cell \|\| id:ebi-a-GCST90001943 | F-highly palatable foods liking (derived food-liking factor) \|\| id:ebi-fl187-GCST90094785 | MR Egger | 14 | 0.625165089529806 | 2.59717443912373 | 0.813843462470644 | -4.465296811 | 5.71562699021233 | 1.8685544107394 | 0.011501281544694 | 303.574481880624 | 0.999279047927446 |
| ebi-fl187-GCST90094785 | ebi-a-GCST90001949 | CD33 on CD33dim HLA DR+ CD11b- \|\| id:ebi-a-GCST90001949 | F-highly palatable foods liking (derived food-liking factor) \|\| id:ebi-fl187-GCST90094785 | MR Egger | 14 | 4.11408827441742 | 3.86343683426305 | 0.307889433991307 | -3.458247921 | 11.686424469573 | 61.1963945020376 | 0.0314848777220461 | 118945.950278432 | 0.999279047927446 |
| ebi-fl187-GCST90094785 | ebi-a-GCST90001955 | CD33 on Immature Myeloid-Derived Suppressor Cells \|\| id:ebi-a-GCST90001955 | F-highly palatable foods liking (derived food-liking factor) \|\| id:ebi-fl187-GCST90094785 | MR Egger | 14 | -0.051596189 | 4.12644326102077 | 0.990229154691028 | -8.139424981 | 8.03623260214917 | 0.949712293263892 | 0.000291804943374128 | 3090.9464025774 | 0.999279047927446 |
| ebi-fl187-GCST90094785 | ebi-a-GCST90001956 | CD33 on CD33+ HLA DR+ \|\| id:ebi-a-GCST90001956 | F-highly palatable foods liking (derived food-liking factor) \|\| id:ebi-fl187-GCST90094785 | MR Egger | 14 | 0.493466366444779 | 4.18046672611802 | 0.907988033612847 | -7.700248417 | 8.68718114963609 | 1.63798424417655 | 0.000452714707002305 | 5926.4528911294 | 0.999279047927446 |
| ebi-fl187-GCST90094785 | ebi-a-GCST90001957 | CD33 on CD33+ HLA DR+ CD14- \|\| id:ebi-a-GCST90001957 | F-highly palatable foods liking (derived food-liking factor) \|\| id:ebi-fl187-GCST90094785 | MR Egger | 14 | 0.908454524848453 | 4.34211432268552 | 0.837786600270601 | -7.602089548 | 9.41899859731208 | 2.48048604000726 | 0.000499406808118618 | 12320.2385202756 | 0.999279047927446 |
| ebi-fl187-GCST90094785 | ebi-a-GCST90002064 | CD4 on resting CD4 regulatory T cell \|\| id:ebi-a-GCST90002064 | F-highly palatable foods liking (derived food-liking factor) \|\| id:ebi-fl187-GCST90094785 | MR Egger | 14 | -2.336392685 | 2.76552663135046 | 0.414733845684578 | -7.756824883 | 3.08403951208963 | 0.0966757498269215 | 0.000427812782218305 | 21.8464734880884 | 0.999279047927446 |
| ebi-fl187-GCST90094785 | ebi-a-GCST90002080 | SSC-A on T cell \|\| id:ebi-a-GCST90002080 | F-highly palatable foods liking (derived food-liking factor) \|\| id:ebi-fl187-GCST90094785 | MR Egger | 14 | -2.547523152 | 2.63937054582755 | 0.35349348389731 | -7.720689421 | 2.62564311821184 | 0.0782753021545689 | 0.000443554702349316 | 13.813455014538 | 0.999279047927446 |
| ebi-fl187-GCST90094785 | ebi-a-GCST90002121 | CD8 on CD39+ CD8+ T cell \|\| id:ebi-a-GCST90002121 | F-highly palatable foods liking (derived food-liking factor) \|\| id:ebi-fl187-GCST90094785 | MR Egger | 14 | 1.37435145410311 | 2.72912415822124 | 0.623668335948761 | -3.974731896 | 6.72343480421675 | 3.9525125057362 | 0.0187843369753207 | 831.669232112159 | 0.999279047927446 |
| ebi-fl187-GCST90094785 | ebi-cfmet338-GCST90026053 | 1-palmitoyl-2-docosahexaenoyl-gpc (16:0/22:6) levels \|\| id:ebi-cfmet338-GCST90026053 | F-highly palatable foods liking (derived food-liking factor) \|\| id:ebi-fl187-GCST90094785 | MR Egger | 13 | 0.16549520430711 | 1.54791339940246 | 0.916781406251806 | -2.868415059 | 3.19940546713593 | 1.17997730389712 | 0.0567888623129678 | 24.5179491365578 | 0.999279047927446 |
| ebi-fl187-GCST90094785 | ebi-cfmet338-GCST90026063 | 1-stearoyl-2-oleoyl-gpc (18:0/18:1) levels \|\| id:ebi-cfmet338-GCST90026063 | F-highly palatable foods liking (derived food-liking factor) \|\| id:ebi-fl187-GCST90094785 | MR Egger | 13 | -0.190523947 | 1.5698119875283 | 0.905588757371544 | -3.267355443 | 2.88630754841302 | 0.826525964557047 | 0.0381070702691129 | 17.9269926883009 | 0.999279047927446 |
| ebi-fl187-GCST90094785 | ebi-cfmet338-GCST90026113 | Adenosine 3',5'-cyclic monophosphate (camp) levels \|\| id:ebi-cfmet338-GCST90026113 | F-highly palatable foods liking (derived food-liking factor) \|\| id:ebi-fl187-GCST90094785 | MR Egger | 13 | 4.05707903451271 | 3.09984122317339 | 0.217284521340119 | -2.018609763 | 10.1327678319326 | 57.8052171960724 | 0.132840015883208 | 25153.8899093694 | 0.999279047927446 |
| ebi-fl187-GCST90094785 | ebi-cfmet338-GCST90026117 | Alpha-ketoglutaramate levels \|\| id:ebi-cfmet338-GCST90026117 | F-highly palatable foods liking (derived food-liking factor) \|\| id:ebi-fl187-GCST90094785 | MR Egger | 13 | 0.028525501223181 | 1.08387476316718 | 0.979475022623519 | -2.095869035 | 2.15292003703084 | 1.0289362496329 | 0.122963337815076 | 8.60996313714918 | 0.999279047927446 |
| ebi-fl187-GCST90094785 | ebi-cfmet338-GCST90026124 | Arginine levels \|\| id:ebi-cfmet338-GCST90026124 | F-highly palatable foods liking (derived food-liking factor) \|\| id:ebi-fl187-GCST90094785 | MR Egger | 13 | -0.069241922 | 0.879888185239813 | 0.938689320223665 | -1.793822765 | 1.65533892110429 | 0.933100915162919 | 0.166323138553975 | 5.23485382399351 | 0.999279047927446 |
| ebi-fl187-GCST90094785 | ebi-cfmet338-GCST90026169 | Glutamate levels \|\| id:ebi-cfmet338-GCST90026169 | F-highly palatable foods liking (derived food-liking factor) \|\| id:ebi-fl187-GCST90094785 | MR Egger | 13 | -0.385513293 | 0.843623963649949 | 0.656582796628323 | -2.039016262 | 1.26798967538667 | 0.680101455030156 | 0.130156687984301 | 3.55370128340946 | 0.999279047927446 |
| ebi-fl187-GCST90094785 | ebi-cfmet338-GCST90026170 | Glutamine levels \|\| id:ebi-cfmet338-GCST90026170 | F-highly palatable foods liking (derived food-liking factor) \|\| id:ebi-fl187-GCST90094785 | MR Egger | 13 | 0.0779621007443984 | 0.522244454615967 | 0.884032286121968 | -0.94563703 | 1.10156123179169 | 1.08108168570253 | 0.388432049084791 | 3.00885988660092 | 0.999279047927446 |
| ebi-fl187-GCST90094785 | ebi-cfmet338-GCST90026191 | Kynurenate levels \|\| id:ebi-cfmet338-GCST90026191 | F-highly palatable foods liking (derived food-liking factor) \|\| id:ebi-fl187-GCST90094785 | MR Egger | 13 | 5.97677784966148 | 5.28884396652478 | 0.282484984085496 | -4.389356325 | 16.3429120240501 | 394.168250417129 | 0.0124087138326816 | 12520927.7715548 | 0.999279047927446 |
| ebi-fl187-GCST90094785 | ebi-cfmet338-GCST90026202 | Methylsuccinoylcarnitine levels \|\| id:ebi-cfmet338-GCST90026202 | F-highly palatable foods liking (derived food-liking factor) \|\| id:ebi-fl187-GCST90094785 | MR Egger | 13 | -1.112129417 | 2.58419177750979 | 0.675245293552994 | -6.177145301 | 3.95288646713037 | 0.328857939339193 | 0.00207634674788164 | 52.0854931271744 | 0.999279047927446 |
| ebi-fl187-GCST90094785 | ebi-cfmet338-GCST90026222 | N-acetylputrescine levels \|\| id:ebi-cfmet338-GCST90026222 | F-highly palatable foods liking (derived food-liking factor) \|\| id:ebi-fl187-GCST90094785 | MR Egger | 13 | -0.463318912 | 0.916225204178127 | 0.62305906349388 | -2.259120312 | 1.33248248820097 | 0.629191943655135 | 0.104442320993185 | 3.7904414436209 | 0.999279047927446 |
| ebi-fl187-GCST90094785 | ebi-cfmet338-GCST90026252 | Propionylcarnitine (c3) levels \|\| id:ebi-cfmet338-GCST90026252 | F-highly palatable foods liking (derived food-liking factor) \|\| id:ebi-fl187-GCST90094785 | MR Egger | 13 | 3.99447125653694 | 1.52635453830384 | 0.0239564618057639 | 1.0028163614614 | 6.98612615161248 | 54.2971237832977 | 2.72594828332852 | 1081.52369183574 | 0.999279047927446 |
| ebi-fl187-GCST90094785 | ebi-cfmet338-GCST90026316 | Ethyl beta-glucopyranoside levels \|\| id:ebi-cfmet338-GCST90026316 | F-highly palatable foods liking (derived food-liking factor) \|\| id:ebi-fl187-GCST90094785 | MR Egger | 13 | -2.304685655 | 7.2154929464753 | 0.755401146903025 | -16.44705183 | 11.8376805198998 | 0.0997901642439097 | 7.19674552680747e-08 | 138369.167601289 | 0.999279047927446 |
| ebi-fl187-GCST90094785 | ebi-gm430-GCST90011315 | F_Prevotellaceae abundance \|\| id:ebi-gm430-GCST90011315 | F-highly palatable foods liking (derived food-liking factor) \|\| id:ebi-fl187-GCST90094785 | MR Egger | 9 | 2.45065540139891 | 4.79742330606765 | 0.625183742959862 | -6.952294278 | 11.8536050812915 | 11.5959442273109 | 0.000956438298739783 | 140590.274040761 | 0.999279047927446 |
| ebi-fl187-GCST90094785 | ebi-gm430-GCST90011354 | OTU97_1 (Bacteroides) abundance \|\| id:ebi-gm430-GCST90011354 | F-highly palatable foods liking (derived food-liking factor) \|\| id:ebi-fl187-GCST90094785 | MR Egger | 9 | 0.0454522299931455 | 3.87635160952335 | 0.99097178817314 | -7.552196925 | 7.64310138465891 | 1.04650101205937 | 0.000524955571497521 | 2086.20391458489 | 0.999279047927446 |
| ebi-fl187-GCST90094785 | ebi-gm430-GCST90011419 | OTU99_1 (Bacteroides) abundance \|\| id:ebi-gm430-GCST90011419 | F-highly palatable foods liking (derived food-liking factor) \|\| id:ebi-fl187-GCST90094785 | MR Egger | 9 | 0.177807425573503 | 3.87740316947012 | 0.964704763162694 | -7.421902787 | 7.77751763773495 | 1.19459525058737 | 0.000598010176507159 | 2386.34369244521 | 0.999279047927446 |
| ebi-fl187-GCST90094785 | ebi-gm430-GCST90011435 | OTU99_155 (Butyrivibrio) abundance \|\| id:ebi-gm430-GCST90011435 | F-highly palatable foods liking (derived food-liking factor) \|\| id:ebi-fl187-GCST90094785 | MR Egger | 9 | -4.316084302 | 12.4417388373509 | 0.738858812609692 | -28.70189242 | 20.0697238196679 | 0.0133520639706889 | 3.42709781922461e-13 | 520199952.500051 | 0.999279047927446 |
| ebi-fl187-GCST90094785 | ebi-gm430-GCST90011466 | OTU99_58 (Bacteroides) abundance \|\| id:ebi-gm430-GCST90011466 | F-highly palatable foods liking (derived food-liking factor) \|\| id:ebi-fl187-GCST90094785 | MR Egger | 9 | -6.003295363 | 7.1191949058539 | 0.426957860470575 | -19.95691738 | 7.95032665215644 | 0.00247059723180381 | 2.15189415832501e-09 | 2836.50134844353 | 0.999279047927446 |
| ebi-fl187-GCST90094785 | ebi-gm430-GCST90011472 | OTU99_72 (Parabacteroides) abundance \|\| id:ebi-gm430-GCST90011472 | F-highly palatable foods liking (derived food-liking factor) \|\| id:ebi-fl187-GCST90094785 | MR Egger | 8 | 5.42721312489231 | 10.7419456326361 | 0.631411025063488 | -15.62700032 | 26.4814265648591 | 227.514307128905 | 1.63410513403697e-07 | 316765175447.852 | 0.999279047927446 |
| ebi-fl187-GCST90094785 | ebi-gm430-GCST90011505 | TestASV_28 (Holdemanella) abundance \|\| id:ebi-gm430-GCST90011505 | F-highly palatable foods liking (derived food-liking factor) \|\| id:ebi-fl187-GCST90094785 | MR Egger | 9 | -4.48936633 | 9.07565726123955 | 0.635985503088947 | -22.27765456 | 13.2989219021074 | 0.0112277562490584 | 2.11318516894003e-10 | 596552.125394211 | 0.999279047927446 |
| ebi-fl187-GCST90094785 | ebi-gm430-GCST90011513 | TestASV_35 (Alphaproteobacteria) abundance \|\| id:ebi-gm430-GCST90011513 | F-highly palatable foods liking (derived food-liking factor) \|\| id:ebi-fl187-GCST90094785 | MR Egger | 9 | 10.7589839488118 | 12.2512479531569 | 0.408967381232559 | -13.25346204 | 34.7714299369994 | 47050.8376761666 | 1.75426246125078e-06 | 1261944193031730 | 0.999279047927446 |
| ebi-fl187-GCST90094785 | ebi-gm430-GCST90011543 | G_Coprococcus prevalence \|\| id:ebi-gm430-GCST90011543 | F-highly palatable foods liking (derived food-liking factor) \|\| id:ebi-fl187-GCST90094785 | MR Egger | 9 | 3.91758259554901 | 12.9727537143468 | 0.771437950777345 | -21.50901468 | 29.3441798756687 | 50.2787536661069 | 4.55778265488419e-10 | 5546453750944.13 | 0.999279047927446 |
| ebi-fl187-GCST90094785 | ebi-gm430-GCST90011547 | G_Parasutterella prevalence \|\| id:ebi-gm430-GCST90011547 | F-highly palatable foods liking (derived food-liking factor) \|\| id:ebi-fl187-GCST90094785 | MR Egger | 9 | -6.826295915 | 11.5105219193069 | 0.571803167398809 | -29.38691888 | 15.7343270465066 | 0.0010848691315634 | 1.72752082528714e-13 | 6812890.56196187 | 0.999279047927446 |
| ebi-fl187-GCST90094785 | ebi-gm430-GCST90011569 | OTU97_13 (Subdoligranulum) prevalence \|\| id:ebi-gm430-GCST90011569 | F-highly palatable foods liking (derived food-liking factor) \|\| id:ebi-fl187-GCST90094785 | MR Egger | 9 | 9.74191615058272 | 10.8176489657224 | 0.397746511318617 | -11.46067582 | 30.9445081233987 | 17016.115602037 | 1.05363874294353e-05 | 27480784293578.8 | 0.999279047927446 |
| ebi-fl187-GCST90094785 | ebi-gm430-GCST90011590 | OTU97_277 (Prevotella) prevalence \|\| id:ebi-gm430-GCST90011590 | F-highly palatable foods liking (derived food-liking factor) \|\| id:ebi-fl187-GCST90094785 | MR Egger | 9 | 6.30822438586394 | 12.2005477722446 | 0.621055983782804 | -17.60484925 | 30.2212980194633 | 549.069147804869 | 2.26105494957238e-08 | 13333463175151.2 | 0.999279047927446 |
| ebi-fl187-GCST90094785 | ebi-gm430-GCST90011616 | OTU97_86 (Phascolarctobacterium) prevalence \|\| id:ebi-gm430-GCST90011616 | F-highly palatable foods liking (derived food-liking factor) \|\| id:ebi-fl187-GCST90094785 | MR Egger | 9 | -13.31857391 | 11.4063878867377 | 0.281179587372787 | -35.67509417 | 9.03794634529445 | 1.64367838059316e-06 | 3.20998837996742e-16 | 8416.47476261813 | 0.999279047927446 |
| ebi-fl187-GCST90094785 | ebi-gm430-GCST90011665 | OTU99_55 (Barnesiella) prevalence \|\| id:ebi-gm430-GCST90011665 | F-highly palatable foods liking (derived food-liking factor) \|\| id:ebi-fl187-GCST90094785 | MR Egger | 9 | -6.226553933 | 7.49991606077305 | 0.433814375827459 | -20.92638941 | 8.47328154647295 | 0.00197625046797284 | 8.16177376228546e-10 | 4785.19256464812 | 0.999279047927446 |
| ebi-fl187-GCST90094785 | ebi-gm430-GCST90011687 | TestASV_14 (Lachnospiraceae) prevalence \|\| id:ebi-gm430-GCST90011687 | F-highly palatable foods liking (derived food-liking factor) \|\| id:ebi-fl187-GCST90094785 | MR Egger | 9 | 5.99850285291684 | 8.14045906288805 | 0.485156742355178 | -9.95679691 | 21.9538026161774 | 402.825253158353 | 4.7404333454861e-05 | 3423066474.22024 | 0.999279047927446 |
| ebi-fl187-GCST90094785 | ebi-gm430-GCST90011697 | TestASV_23 (Barnesiella) prevalence \|\| id:ebi-gm430-GCST90011697 | F-highly palatable foods liking (derived food-liking factor) \|\| id:ebi-fl187-GCST90094785 | MR Egger | 9 | -3.005722633 | 10.7841528218853 | 0.78851642012787 | -24.14266216 | 18.1312168975488 | 0.0495029689040543 | 3.27321885048427e-11 | 74866485.9348838 | 0.999279047927446 |
| ebi-fl187-GCST90094785 | ebi-gm430-GCST90011727 | TestASV_6 (Subdoligranulum) prevalence \|\| id:ebi-gm430-GCST90011727 | F-highly palatable foods liking (derived food-liking factor) \|\| id:ebi-fl187-GCST90094785 | MR Egger | 9 | -0.486015173 | 16.2233015868213 | 0.976936913158868 | -32.28368628 | 31.3116559367718 | 0.615072474503287 | 9.53615258694744e-15 | 39671570420277.6 | 0.999279047927446 |
| ebi-fl187-GCST90094785 | ebi-hbsm44-GCST90104908 | Sulcal depth of the left posterior inferior temporal sulcus \|\| id:ebi-hbsm44-GCST90104908 | F-highly palatable foods liking (derived food-liking factor) \|\| id:ebi-fl187-GCST90094785 | MR Egger | 12 | -0.234417284 | 2.93076438703135 | 0.93782722347388 | -5.978715482 | 5.50988091498246 | 0.791031662477543 | 0.00253207670182182 | 247.121696823708 | 0.999279047927446 |
| ebi-fl187-GCST90094785 | ebi-hbsm44-GCST90104918 | Sulcal depth of the left inferior frontal sulcus \|\| id:ebi-hbsm44-GCST90104918 | F-highly palatable foods liking (derived food-liking factor) \|\| id:ebi-fl187-GCST90094785 | MR Egger | 12 | -1.734496682 | 4.73322532739446 | 0.721662770633897 | -11.01161832 | 7.54262495974339 | 0.17648900803861 | 1.65087779154204e-05 | 1886.77624219281 | 0.999279047927446 |
| ebi-fl187-GCST90094785 | ebi-hbsm44-GCST90104925 | Sulcal depth of the right anterior inferior temporal sulcus \|\| id:ebi-hbsm44-GCST90104925 | F-highly palatable foods liking (derived food-liking factor) \|\| id:ebi-fl187-GCST90094785 | MR Egger | 12 | 1.70555237769762 | 1.61166835134453 | 0.314833245641272 | -1.453317591 | 4.8644223463329 | 5.50442534940909 | 0.233793369293748 | 129.596055348981 | 0.999279047927446 |
| ebi-fl187-GCST90094785 | ebi-IPs91-GCST90274770 | C-C motif chemokine 4 levels \|\| id:ebi-IPs91-GCST90274770 | F-highly palatable foods liking (derived food-liking factor) \|\| id:ebi-fl187-GCST90094785 | MR Egger | 17 | 0.666183412027558 | 0.72425590371494 | 0.372224089613802 | -0.753358159 | 2.08572498330884 | 1.94679301693903 | 0.470782931145773 | 8.05042579088121 | 0.999279047927446 |
| ebi-fl187-GCST90094785 | ebi-IPs91-GCST90274779 | C-X-C motif chemokine 1 levels \|\| id:ebi-IPs91-GCST90274779 | F-highly palatable foods liking (derived food-liking factor) \|\| id:ebi-fl187-GCST90094785 | MR Egger | 17 | 0.0299255144399999 | 0.714307709150882 | 0.967135430178629 | -1.370117595 | 1.42996862437573 | 1.03037778282896 | 0.254077079475839 | 4.17856808468425 | 0.999279047927446 |
| ebi-fl187-GCST90094785 | ebi-IPs91-GCST90274780 | C-X-C motif chemokine 10 levels \|\| id:ebi-IPs91-GCST90274780 | F-highly palatable foods liking (derived food-liking factor) \|\| id:ebi-fl187-GCST90094785 | MR Egger | 17 | 0.286928797645005 | 0.861874710333194 | 0.74380785200677 | -1.402345635 | 1.97620322989807 | 1.33232934511129 | 0.246019215427096 | 7.21529609288059 | 0.999279047927446 |
| ebi-fl187-GCST90094785 | ebi-IPs91-GCST90274796 | Interleukin-10 receptor subunit alpha levels \|\| id:ebi-IPs91-GCST90274796 | F-highly palatable foods liking (derived food-liking factor) \|\| id:ebi-fl187-GCST90094785 | MR Egger | 17 | 1.36268116642498 | 1.02468808483969 | 0.203436869146748 | -0.64570748 | 3.37106981271077 | 3.90665366117706 | 0.524291485197448 | 29.1096522817656 | 0.999279047927446 |
| ebi-fl187-GCST90094785 | ebi-IPs91-GCST90274807 | Interleukin-20 levels \|\| id:ebi-IPs91-GCST90274807 | F-highly palatable foods liking (derived food-liking factor) \|\| id:ebi-fl187-GCST90094785 | MR Egger | 17 | -0.962082097 | 0.824816125503433 | 0.261655430653486 | -2.578721703 | 0.654557509330089 | 0.38209649534621 | 0.0758709276673105 | 1.9242908482159 | 0.999279047927446 |
| ebi-fl187-GCST90094785 | ebi-IPs91-GCST90274808 | Interleukin-20 receptor subunit alpha levels \|\| id:ebi-IPs91-GCST90274808 | F-highly palatable foods liking (derived food-liking factor) \|\| id:ebi-fl187-GCST90094785 | MR Egger | 17 | -0.339117156 | 0.828986522077704 | 0.688266286892924 | -1.963930739 | 1.2856964273519 | 0.712398982441505 | 0.140305829962272 | 3.6171861876314 | 0.999279047927446 |
| ebi-fl187-GCST90094785 | ebi-IPs91-GCST90274810 | Interleukin-24 levels \|\| id:ebi-IPs91-GCST90274810 | F-highly palatable foods liking (derived food-liking factor) \|\| id:ebi-fl187-GCST90094785 | MR Egger | 17 | 0.894293190382547 | 0.82920638294936 | 0.297852210124222 | -0.73095132 | 2.51953770096329 | 2.44560660016498 | 0.481450758331358 | 12.42285226 | 0.999279047927446 |
| ebi-fl187-GCST90094785 | ebi-IPs91-GCST90274823 | Monocyte chemoattractant protein-3 levels \|\| id:ebi-IPs91-GCST90274823 | F-highly palatable foods liking (derived food-liking factor) \|\| id:ebi-fl187-GCST90094785 | MR Egger | 17 | -0.19915646 | 0.825531840566278 | 0.812630903951963 | -1.817198868 | 1.41888594747192 | 0.81942167655615 | 0.162480242771643 | 4.13251403712991 | 0.999279047927446 |
| ebi-fl187-GCST90094785 | ebi-IPs91-GCST90274824 | Monocyte chemoattractant protein-4 levels \|\| id:ebi-IPs91-GCST90274824 | F-highly palatable foods liking (derived food-liking factor) \|\| id:ebi-fl187-GCST90094785 | MR Egger | 17 | -0.096581853 | 0.71144223261591 | 0.893820045832214 | -1.491008629 | 1.29784492286209 | 0.907935577248208 | 0.225145452745918 | 3.66139756490366 | 0.999279047927446 |
| ebi-fl187-GCST90094785 | ebi-IPs91-GCST90274834 | SIR2-like protein 2 levels \|\| id:ebi-IPs91-GCST90274834 | F-highly palatable foods liking (derived food-liking factor) \|\| id:ebi-fl187-GCST90094785 | MR Egger | 17 | 0.0221776567895339 | 0.711853483407197 | 0.975556844862097 | -1.373055171 | 1.41741048426764 | 1.02242540915226 | 0.253331804137211 | 4.12642116073976 | 0.999279047927446 |
| ebi-fl187-GCST90094785 | ebi-met1400-GCST90199652 | 1-stearoyl-2-oleoyl-GPS (18:0/18:1) levels \|\| id:ebi-met1400-GCST90199652 | F-highly palatable foods liking (derived food-liking factor) \|\| id:ebi-fl187-GCST90094785 | MR Egger | 14 | -0.295222345 | 1.72064925057836 | 0.866630131525603 | -3.667694876 | 3.07725018630139 | 0.744366063117459 | 0.0255352641077453 | 21.6986530306895 | 0.999279047927446 |
| ebi-fl187-GCST90094785 | ebi-met1400-GCST90199665 | N-acetylaspartate (naa) levels \|\| id:ebi-met1400-GCST90199665 | F-highly palatable foods liking (derived food-liking factor) \|\| id:ebi-fl187-GCST90094785 | MR Egger | 14 | -3.906665076 | 1.71043907100205 | 0.0413770575536468 | -7.259125656 | -0.554204497 | 0.020107446167386 | 0.000703723063017806 | 0.574529118941349 | 0.999279047927446 |
| ebi-fl187-GCST90094785 | ebi-met1400-GCST90199669 | Acetylcarnitine levels (Biocrates platform) \|\| id:ebi-met1400-GCST90199669 | F-highly palatable foods liking (derived food-liking factor) \|\| id:ebi-fl187-GCST90094785 | MR Egger | 14 | 1.98398811439013 | 1.82322384289118 | 0.297890060421734 | -1.589530618 | 5.55750684645684 | 7.27168554744762 | 0.204021353279589 | 259.175864932607 | 0.999279047927446 |
| ebi-fl187-GCST90094785 | ebi-met1400-GCST90199670 | Iminodiacetate (IDA) levels \|\| id:ebi-met1400-GCST90199670 | F-highly palatable foods liking (derived food-liking factor) \|\| id:ebi-fl187-GCST90094785 | MR Egger | 14 | -0.088527146 | 1.69870569774748 | 0.959294971990814 | -3.417990314 | 3.24093602111513 | 0.915278263824881 | 0.0327782427641257 | 25.557633039046 | 0.999279047927446 |
| ebi-fl187-GCST90094785 | ebi-met1400-GCST90199686 | Indolepropionate levels \|\| id:ebi-met1400-GCST90199686 | F-highly palatable foods liking (derived food-liking factor) \|\| id:ebi-fl187-GCST90094785 | MR Egger | 14 | 0.129741486956247 | 1.71920246307235 | 0.941087404037984 | -3.239895341 | 3.49937831457806 | 1.13853401938304 | 0.0391679941806819 | 33.0948709630839 | 0.999279047927446 |
| ebi-fl187-GCST90094785 | ebi-met1400-GCST90199704 | 3-hydroxysebacate levels \|\| id:ebi-met1400-GCST90199704 | F-highly palatable foods liking (derived food-liking factor) \|\| id:ebi-fl187-GCST90094785 | MR Egger | 14 | 4.17580653422213 | 2.43152902037509 | 0.111586208936795 | -0.589990346 | 8.94160341415732 | 65.0923176773189 | 0.554332636395011 | 7643.44283995172 | 0.999279047927446 |
| ebi-fl187-GCST90094785 | ebi-met1400-GCST90199713 | Docosapentaenoate n3 DPA; 22:5n3 levels \|\| id:ebi-met1400-GCST90199713 | F-highly palatable foods liking (derived food-liking factor) \|\| id:ebi-fl187-GCST90094785 | MR Egger | 14 | 0.0493687669799011 | 1.6308702396518 | 0.976348228760673 | -3.147136903 | 3.24587443669742 | 1.05060770874503 | 0.0429749924783272 | 25.684159415063 | 0.999279047927446 |
| ebi-fl187-GCST90094785 | ebi-met1400-GCST90199715 | Myristoleate (14:1n5) levels \|\| id:ebi-met1400-GCST90199715 | F-highly palatable foods liking (derived food-liking factor) \|\| id:ebi-fl187-GCST90094785 | MR Egger | 14 | 1.08720200516377 | 1.65121650289162 | 0.522697424072794 | -2.149182341 | 4.32358635083134 | 2.96596370067699 | 0.116579441100608 | 75.4587652049371 | 0.999279047927446 |
| ebi-fl187-GCST90094785 | ebi-met1400-GCST90199725 | Campesterol levels \|\| id:ebi-met1400-GCST90199725 | F-highly palatable foods liking (derived food-liking factor) \|\| id:ebi-fl187-GCST90094785 | MR Egger | 14 | 0.397750107674232 | 2.1422400093715 | 0.85580485104065 | -3.801040311 | 4.59654052604238 | 1.48847202569237 | 0.0223475114041188 | 99.1407468690416 | 0.999279047927446 |
| ebi-fl187-GCST90094785 | ebi-met1400-GCST90199737 | N-acetylproline levels \|\| id:ebi-met1400-GCST90199737 | F-highly palatable foods liking (derived food-liking factor) \|\| id:ebi-fl187-GCST90094785 | MR Egger | 14 | 1.32439718617768 | 1.83304048754306 | 0.483822269309907 | -2.268362169 | 4.91715654176208 | 3.75991814108838 | 0.103481526574876 | 136.613605303324 | 0.999279047927446 |
| ebi-fl187-GCST90094785 | ebi-met1400-GCST90199775 | Gamma-glutamylisoleucine levels \|\| id:ebi-met1400-GCST90199775 | F-highly palatable foods liking (derived food-liking factor) \|\| id:ebi-fl187-GCST90094785 | MR Egger | 14 | 1.57322107888713 | 1.63776872268059 | 0.355715715110055 | -1.636805618 | 4.78324777534109 | 4.82215574891122 | 0.194600679478885 | 119.491803055501 | 0.999279047927446 |
| ebi-fl187-GCST90094785 | ebi-met1400-GCST90199777 | 1-palmitoleoyl-GPC (16:1) levels \|\| id:ebi-met1400-GCST90199777 | F-highly palatable foods liking (derived food-liking factor) \|\| id:ebi-fl187-GCST90094785 | MR Egger | 14 | 1.83376712430519 | 1.70472750588923 | 0.303215883724666 | -1.507498787 | 5.17503303584807 | 6.25741477331397 | 0.221463212405676 | 176.802454998998 | 0.999279047927446 |
| ebi-fl187-GCST90094785 | ebi-met1400-GCST90199782 | Glutamine degradant levels \|\| id:ebi-met1400-GCST90199782 | F-highly palatable foods liking (derived food-liking factor) \|\| id:ebi-fl187-GCST90094785 | MR Egger | 14 | -0.320691673 | 1.42022916177699 | 0.825154873071712 | -3.10434083 | 2.46295748421579 | 0.725646953146194 | 0.044854075279788 | 11.7394795751733 | 0.999279047927446 |
| ebi-fl187-GCST90094785 | ebi-met1400-GCST90199783 | 5-methyluridine (ribothymidine) levels \|\| id:ebi-met1400-GCST90199783 | F-highly palatable foods liking (derived food-liking factor) \|\| id:ebi-fl187-GCST90094785 | MR Egger | 14 | -0.373606986 | 2.4344388019486 | 0.880580249179529 | -5.145107037 | 4.39789306619769 | 0.688247349786889 | 0.0058278505324151 | 81.279437736777 | 0.999279047927446 |
| ebi-fl187-GCST90094785 | ebi-met1400-GCST90199787 | Homostachydrine levels \|\| id:ebi-met1400-GCST90199787 | F-highly palatable foods liking (derived food-liking factor) \|\| id:ebi-fl187-GCST90094785 | MR Egger | 14 | 0.0265835034969463 | 1.65739200709108 | 0.987466584114335 | -3.22190483 | 3.27507183739546 | 1.02693999676232 | 0.0398790230910243 | 26.4451251612421 | 0.999279047927446 |
| ebi-fl187-GCST90094785 | ebi-met1400-GCST90199802 | Beta-hydroxyisovaleroylcarnitine levels \|\| id:ebi-met1400-GCST90199802 | F-highly palatable foods liking (derived food-liking factor) \|\| id:ebi-fl187-GCST90094785 | MR Egger | 14 | -1.048127918 | 1.67431246407839 | 0.543035547030807 | -4.329780348 | 2.2335245116195 | 0.350593474876132 | 0.0131704400915839 | 9.3327013957769 | 0.999279047927446 |
| ebi-fl187-GCST90094785 | ebi-met1400-GCST90199873 | Pregnenolone sulfate levels \|\| id:ebi-met1400-GCST90199873 | F-highly palatable foods liking (derived food-liking factor) \|\| id:ebi-fl187-GCST90094785 | MR Egger | 14 | 0.335620389069297 | 1.80395057778947 | 0.855515503453287 | -3.200122743 | 3.87136352153666 | 1.39880792123409 | 0.0407572009939852 | 48.0078011440481 | 0.999279047927446 |
| ebi-fl187-GCST90094785 | ebi-met1400-GCST90199911 | Beta-citrylglutamate levels \|\| id:ebi-met1400-GCST90199911 | F-highly palatable foods liking (derived food-liking factor) \|\| id:ebi-fl187-GCST90094785 | MR Egger | 14 | -1.834221335 | 1.68565461423243 | 0.297906977012569 | -5.138104378 | 1.46966170936797 | 0.15973783569186 | 0.00586880420745592 | 4.34776408439442 | 0.999279047927446 |
| ebi-fl187-GCST90094785 | ebi-met1400-GCST90199934 | 2-hydroxydecanoate levels \|\| id:ebi-met1400-GCST90199934 | F-highly palatable foods liking (derived food-liking factor) \|\| id:ebi-fl187-GCST90094785 | MR Egger | 14 | -0.922669115 | 1.69212704065226 | 0.595556429696742 | -4.239238115 | 2.39389988482683 | 0.397456766290681 | 0.0144185729606409 | 10.9561384126896 | 0.999279047927446 |
| ebi-fl187-GCST90094785 | ebi-met1400-GCST90199945 | Sphingomyelin (d18:1/14:0, d16:1/16:0) levels \|\| id:ebi-met1400-GCST90199945 | F-highly palatable foods liking (derived food-liking factor) \|\| id:ebi-fl187-GCST90094785 | MR Egger | 14 | 0.92683639189963 | 1.79813976338112 | 0.615607578159754 | -2.597517544 | 4.45119032812663 | 2.52650365370012 | 0.074458188128056 | 85.7289288477174 | 0.999279047927446 |
| ebi-fl187-GCST90094785 | ebi-met1400-GCST90199979 | Carnitine C14:1 levels \|\| id:ebi-met1400-GCST90199979 | F-highly palatable foods liking (derived food-liking factor) \|\| id:ebi-fl187-GCST90094785 | MR Egger | 14 | -0.108878437 | 1.78829003306355 | 0.952453779214958 | -3.613926902 | 3.39617002733042 | 0.896839432938772 | 0.0269458252118295 | 29.8495578499052 | 0.999279047927446 |
| ebi-fl187-GCST90094785 | ebi-met1400-GCST90199986 | Eugenol sulfate levels \|\| id:ebi-met1400-GCST90199986 | F-highly palatable foods liking (derived food-liking factor) \|\| id:ebi-fl187-GCST90094785 | MR Egger | 14 | -0.38616063 | 1.84043269669989 | 0.837327831407215 | -3.993408716 | 3.22108745506624 | 0.679661342593624 | 0.0184367612036156 | 25.0553519414016 | 0.999279047927446 |
| ebi-fl187-GCST90094785 | ebi-met1400-GCST90200002 | 6-hydroxyindole sulfate levels \|\| id:ebi-met1400-GCST90200002 | F-highly palatable foods liking (derived food-liking factor) \|\| id:ebi-fl187-GCST90094785 | MR Egger | 14 | 2.87507867321517 | 1.68429212932061 | 0.113542302182121 | -0.4261339 | 6.17629124668357 | 17.7268186924244 | 0.653028895531763 | 481.203975971388 | 0.999279047927446 |
| ebi-fl187-GCST90094785 | ebi-met1400-GCST90200023 | Trans 3,4-methyleneheptanoate levels \|\| id:ebi-met1400-GCST90200023 | F-highly palatable foods liking (derived food-liking factor) \|\| id:ebi-fl187-GCST90094785 | MR Egger | 14 | 0.0857805393019509 | 1.83653839010509 | 0.963514493947698 | -3.513834705 | 3.68539578390794 | 1.08956718488983 | 0.0297824881163895 | 39.8608956293162 | 0.999279047927446 |
| ebi-fl187-GCST90094785 | ebi-met1400-GCST90200032 | 2-hydroxybutyrate/2-hydroxyisobutyrate levels \|\| id:ebi-met1400-GCST90200032 | F-highly palatable foods liking (derived food-liking factor) \|\| id:ebi-fl187-GCST90094785 | MR Egger | 14 | -1.015480545 | 1.71200690328346 | 0.56409161867356 | -4.371014076 | 2.34005298531414 | 0.362228320949775 | 0.0126384177569278 | 10.381786630385 | 0.999279047927446 |
| ebi-fl187-GCST90094785 | ebi-met1400-GCST90200051 | 1-palmitoyl-2-dihomo-linolenoyl-GPC (16:0/20:3n3 or 6) levels \|\| id:ebi-met1400-GCST90200051 | F-highly palatable foods liking (derived food-liking factor) \|\| id:ebi-fl187-GCST90094785 | MR Egger | 14 | -2.538453423 | 1.6672825670239 | 0.153789182633962 | -5.806327254 | 0.729420408736177 | 0.0789884671487672 | 0.00300845911267598 | 2.07387825755166 | 0.999279047927446 |
| ebi-fl187-GCST90094785 | ebi-met1400-GCST90200069 | Phenylacetylglutamate levels \|\| id:ebi-met1400-GCST90200069 | F-highly palatable foods liking (derived food-liking factor) \|\| id:ebi-fl187-GCST90094785 | MR Egger | 14 | 3.95875932121358 | 1.88318093736112 | 0.0573239898826102 | 0.267724683985779 | 7.64979395844138 | 52.3922836142674 | 1.30698725595434 | 2100.21281371524 | 0.999279047927446 |
| ebi-fl187-GCST90094785 | ebi-met1400-GCST90200072 | Thioproline levels \|\| id:ebi-met1400-GCST90200072 | F-highly palatable foods liking (derived food-liking factor) \|\| id:ebi-fl187-GCST90094785 | MR Egger | 14 | -3.786763252 | 1.69504190774544 | 0.0452817172023318 | -7.109045391 | -0.464481113 | 0.0226688566066991 | 0.000817675179482137 | 0.628461120931348 | 0.999279047927446 |
| ebi-fl187-GCST90094785 | ebi-met1400-GCST90200081 | 1-oleoyl-2-docosahexaenoyl-GPC (18:1/22:6) levels \|\| id:ebi-met1400-GCST90200081 | F-highly palatable foods liking (derived food-liking factor) \|\| id:ebi-fl187-GCST90094785 | MR Egger | 14 | -1.34855906 | 1.63052640655916 | 0.424339031473884 | -4.544390817 | 1.84727269720203 | 0.259614079657551 | 0.0106266443211305 | 6.34249800027811 | 0.999279047927446 |
| ebi-fl187-GCST90094785 | ebi-met1400-GCST90200097 | Furaneol sulfate levels \|\| id:ebi-met1400-GCST90200097 | F-highly palatable foods liking (derived food-liking factor) \|\| id:ebi-fl187-GCST90094785 | MR Egger | 13 | -0.52225546 | 2.02537505091211 | 0.801278337760477 | -4.491990559 | 3.44747964014242 | 0.593181141921886 | 0.0111983306663954 | 31.4210999490886 | 0.999279047927446 |
| ebi-fl187-GCST90094785 | ebi-met1400-GCST90200124 | Sphingomyelin (d18:2/24:2) levels \|\| id:ebi-met1400-GCST90200124 | F-highly palatable foods liking (derived food-liking factor) \|\| id:ebi-fl187-GCST90094785 | MR Egger | 14 | 0.326619971054369 | 1.54748939432903 | 0.836378806574279 | -2.706459242 | 3.35969918393926 | 1.38627455260677 | 0.0667728150345877 | 28.7805319306913 | 0.999279047927446 |
| ebi-fl187-GCST90094785 | ebi-met1400-GCST90200137 | Lignoceroylcarnitine (C24) levels \|\| id:ebi-met1400-GCST90200137 | F-highly palatable foods liking (derived food-liking factor) \|\| id:ebi-fl187-GCST90094785 | MR Egger | 14 | -2.010024459 | 1.70810132266311 | 0.262106675353469 | -5.357903052 | 1.33785413304799 | 0.133985397430088 | 0.00471077400684537 | 3.81085713269451 | 0.999279047927446 |
| ebi-fl187-GCST90094785 | ebi-met1400-GCST90200165 | Octadecenedioate (C18:1-DC) levels \|\| id:ebi-met1400-GCST90200165 | F-highly palatable foods liking (derived food-liking factor) \|\| id:ebi-fl187-GCST90094785 | MR Egger | 14 | 0.388835124993623 | 1.66493864577454 | 0.819276110044868 | -2.874444621 | 3.65211487071172 | 1.47526129756453 | 0.0564474805607441 | 38.5561210964893 | 0.999279047927446 |
| ebi-fl187-GCST90094785 | ebi-met1400-GCST90200176 | Glutamine conjugate of C6H10O2 (1) levels \|\| id:ebi-met1400-GCST90200176 | F-highly palatable foods liking (derived food-liking factor) \|\| id:ebi-fl187-GCST90094785 | MR Egger | 14 | 1.79664745325245 | 1.66801004255558 | 0.30260415350332 | -1.47265223 | 5.06594713666139 | 6.02939969823828 | 0.229316477851971 | 158.530521058251 | 0.999279047927446 |
| ebi-fl187-GCST90094785 | ebi-met1400-GCST90200182 | Glycine conjugate of C10H12O2 levels \|\| id:ebi-met1400-GCST90200182 | F-highly palatable foods liking (derived food-liking factor) \|\| id:ebi-fl187-GCST90094785 | MR Egger | 14 | 1.21578757449401 | 2.4596682178447 | 0.63002628644987 | -3.605162132 | 6.03673728146963 | 3.37294946749064 | 0.0271830372024753 | 418.525274622711 | 0.999279047927446 |
| ebi-fl187-GCST90094785 | ebi-met1400-GCST90200208 | Glucuronide of piperine metabolite C17H21NO3 (4) levels \|\| id:ebi-met1400-GCST90200208 | F-highly palatable foods liking (derived food-liking factor) \|\| id:ebi-fl187-GCST90094785 | MR Egger | 14 | 0.140085682322615 | 1.73571494737696 | 0.93700478749274 | -3.261915615 | 3.54208697918146 | 1.15037236121042 | 0.038314931033964 | 34.5389260459261 | 0.999279047927446 |
| ebi-fl187-GCST90094785 | ebi-met1400-GCST90200227 | 3-ethylcatechol sulfate (2) levels \|\| id:ebi-met1400-GCST90200227 | F-highly palatable foods liking (derived food-liking factor) \|\| id:ebi-fl187-GCST90094785 | MR Egger | 14 | -2.787035655 | 2.21573531363682 | 0.232376654636669 | -7.12987687 | 1.55580555962242 | 0.0616035577082878 | 0.000800817986273569 | 4.73890245644649 | 0.999279047927446 |
| ebi-fl187-GCST90094785 | ebi-met1400-GCST90200259 | Pentose acid levels \|\| id:ebi-met1400-GCST90200259 | F-highly palatable foods liking (derived food-liking factor) \|\| id:ebi-fl187-GCST90094785 | MR Egger | 14 | -0.180555769 | 1.70152559544104 | 0.917245134748214 | -3.515545936 | 3.15443439769674 | 0.834806122789087 | 0.0297315669769981 | 23.4397757503097 | 0.999279047927446 |
| ebi-fl187-GCST90094785 | ebi-met1400-GCST90200291 | Sarcosine levels \|\| id:ebi-met1400-GCST90200291 | F-highly palatable foods liking (derived food-liking factor) \|\| id:ebi-fl187-GCST90094785 | MR Egger | 14 | -1.235856917 | 1.70068896500357 | 0.481357567620186 | -4.569207289 | 2.09749345414097 | 0.290585647788951 | 0.0103661738448918 | 8.14572666486143 | 0.999279047927446 |
| ebi-fl187-GCST90094785 | ebi-met1400-GCST90200306 | Butyrate/isobutyrate (4:0) levels \|\| id:ebi-met1400-GCST90200306 | F-highly palatable foods liking (derived food-liking factor) \|\| id:ebi-fl187-GCST90094785 | MR Egger | 14 | 1.71644243545555 | 2.14474257423649 | 0.439092722832676 | -2.48725301 | 5.92013788095908 | 5.5646964415811 | 0.0831380325242982 | 372.463065900617 | 0.999279047927446 |
| ebi-fl187-GCST90094785 | ebi-met1400-GCST90200321 | Cholate levels \|\| id:ebi-met1400-GCST90200321 | F-highly palatable foods liking (derived food-liking factor) \|\| id:ebi-fl187-GCST90094785 | MR Egger | 14 | -2.686049178 | 1.75268992712288 | 0.151321980666068 | -6.121321436 | 0.749223078700866 | 0.0681496553240421 | 0.00219555276364014 | 2.11535591296177 | 0.999279047927446 |
| ebi-fl187-GCST90094785 | ebi-met1400-GCST90200336 | Ceramide (d18:1/16:0) levels \|\| id:ebi-met1400-GCST90200336 | F-highly palatable foods liking (derived food-liking factor) \|\| id:ebi-fl187-GCST90094785 | MR Egger | 14 | 1.39873432594916 | 1.68403795410037 | 0.422426788195107 | -1.901980064 | 4.69944871598589 | 4.05007065218065 | 0.149272756781048 | 109.88657703773 | 0.999279047927446 |
| ebi-fl187-GCST90094785 | ebi-met1400-GCST90200358 | Arachidonate (20:4n6) levels \|\| id:ebi-met1400-GCST90200358 | F-highly palatable foods liking (derived food-liking factor) \|\| id:ebi-fl187-GCST90094785 | MR Egger | 14 | 0.143501672490853 | 2.05759520234732 | 0.945547422325018 | -3.889384924 | 4.1763882690916 | 1.1543087413763 | 0.0204579253455683 | 65.1301951645055 | 0.999279047927446 |
| ebi-fl187-GCST90094785 | ebi-met1400-GCST90200413 | 2'-deoxyuridine levels \|\| id:ebi-met1400-GCST90200413 | F-highly palatable foods liking (derived food-liking factor) \|\| id:ebi-fl187-GCST90094785 | MR Egger | 14 | 1.47588952540545 | 1.85521442589124 | 0.441754466448716 | -2.160330749 | 5.11210980015227 | 4.37492565011371 | 0.115286983637543 | 166.020255193752 | 0.999279047927446 |
| ebi-fl187-GCST90094785 | ebi-met1400-GCST90200432 | Threonine levels \|\| id:ebi-met1400-GCST90200432 | F-highly palatable foods liking (derived food-liking factor) \|\| id:ebi-fl187-GCST90094785 | MR Egger | 14 | -3.652336723 | 1.73759591872545 | 0.0573461833675082 | -7.058024724 | -0.246648723 | 0.0259304656030955 | 0.000860476094069844 | 0.781415138697321 | 0.999279047927446 |
| ebi-fl187-GCST90094785 | ebi-met1400-GCST90200436 | Caffeine levels \|\| id:ebi-met1400-GCST90200436 | F-highly palatable foods liking (derived food-liking factor) \|\| id:ebi-fl187-GCST90094785 | MR Egger | 14 | -0.654053724 | 1.73844903968096 | 0.713314594013106 | -4.061413842 | 2.75330639333497 | 0.519933830553166 | 0.0172246489558037 | 15.6944381767851 | 0.999279047927446 |
| ebi-fl187-GCST90094785 | ebi-met1400-GCST90200442 | Valine levels \|\| id:ebi-met1400-GCST90200442 | F-highly palatable foods liking (derived food-liking factor) \|\| id:ebi-fl187-GCST90094785 | MR Egger | 14 | 0.681167870846071 | 1.66540531700551 | 0.689737564570885 | -2.58302655 | 3.94536229217687 | 1.97618431311975 | 0.0755450168696238 | 51.6950634369488 | 0.999279047927446 |
| ebi-fl187-GCST90094785 | ebi-met1400-GCST90200459 | Margarate (17:0) levels \|\| id:ebi-met1400-GCST90200459 | F-highly palatable foods liking (derived food-liking factor) \|\| id:ebi-fl187-GCST90094785 | MR Egger | 14 | 0.00186152148711256 | 1.90872314550853 | 0.999237872407475 | -3.739235844 | 3.74295888668382 | 1.00186325519385 | 0.0237722619158268 | 42.222737813576 | 0.999947616389678 |
| ebi-fl187-GCST90094785 | ebi-met1400-GCST90200462 | X-11299 levels \|\| id:ebi-met1400-GCST90200462 | F-highly palatable foods liking (derived food-liking factor) \|\| id:ebi-fl187-GCST90094785 | MR Egger | 14 | -1.07207034 | 1.68999290963972 | 0.537749413063915 | -4.384456443 | 2.24031576310251 | 0.34229910784915 | 0.0124696642697838 | 9.39629782320961 | 0.999279047927446 |
| ebi-fl187-GCST90094785 | ebi-met1400-GCST90200497 | X-12740 levels \|\| id:ebi-met1400-GCST90200497 | F-highly palatable foods liking (derived food-liking factor) \|\| id:ebi-fl187-GCST90094785 | MR Egger | 14 | 1.16683152507992 | 2.63421851560255 | 0.665680215893152 | -3.996236766 | 6.32989981566091 | 3.21179999176066 | 0.0183846947881446 | 561.100377566554 | 0.999279047927446 |
| ebi-fl187-GCST90094785 | ebi-met1400-GCST90200527 | X-13866 levels \|\| id:ebi-met1400-GCST90200527 | F-highly palatable foods liking (derived food-liking factor) \|\| id:ebi-fl187-GCST90094785 | MR Egger | 14 | -1.607819845 | 1.66485886256862 | 0.353234761920049 | -4.870943216 | 1.65530352559889 | 0.200323875435178 | 0.00766613106176766 | 5.2346685369759 | 0.999279047927446 |
| ebi-fl187-GCST90094785 | ebi-met1400-GCST90200547 | X-17654 levels \|\| id:ebi-met1400-GCST90200547 | F-highly palatable foods liking (derived food-liking factor) \|\| id:ebi-fl187-GCST90094785 | MR Egger | 14 | 0.535700857631277 | 2.00424003058674 | 0.793788940102722 | -3.392609602 | 4.46401131758129 | 1.70864533983329 | 0.0336208253368508 | 86.8351347143785 | 0.999279047927446 |
| ebi-fl187-GCST90094785 | ebi-met1400-GCST90200553 | X-17653 levels \|\| id:ebi-met1400-GCST90200553 | F-highly palatable foods liking (derived food-liking factor) \|\| id:ebi-fl187-GCST90094785 | MR Egger | 14 | 1.48626095927106 | 1.89891612419453 | 0.448980211763121 | -2.235614644 | 5.20813656269234 | 4.42053601576087 | 0.106926387972383 | 182.753191585282 | 0.999279047927446 |
| ebi-fl187-GCST90094785 | ebi-met1400-GCST90200557 | X-17685 levels \|\| id:ebi-met1400-GCST90200557 | F-highly palatable foods liking (derived food-liking factor) \|\| id:ebi-fl187-GCST90094785 | MR Egger | 14 | 3.50135838886708 | 1.89882657564947 | 0.0900086616095491 | -0.220341699 | 7.22305847714003 | 33.1604661864524 | 0.802244624611113 | 1370.67483404565 | 0.999279047927446 |
| ebi-fl187-GCST90094785 | ebi-met1400-GCST90200561 | X-18779 levels \|\| id:ebi-met1400-GCST90200561 | F-highly palatable foods liking (derived food-liking factor) \|\| id:ebi-fl187-GCST90094785 | MR Egger | 14 | -0.219837416 | 1.73310509228027 | 0.901162977910266 | -3.616723397 | 3.17704856456082 | 0.802649285038385 | 0.0268705766142697 | 23.9758857437582 | 0.999279047927446 |
| ebi-fl187-GCST90094785 | ebi-met1400-GCST90200570 | X-18921 levels \|\| id:ebi-met1400-GCST90200570 | F-highly palatable foods liking (derived food-liking factor) \|\| id:ebi-fl187-GCST90094785 | MR Egger | 14 | 0.539864077920964 | 1.70531999479378 | 0.757004427208084 | -2.802563112 | 3.88229126771676 | 1.71577363481524 | 0.0606543992081899 | 48.5352951205164 | 0.999279047927446 |
| ebi-fl187-GCST90094785 | ebi-met1400-GCST90200575 | X-21283 levels \|\| id:ebi-met1400-GCST90200575 | F-highly palatable foods liking (derived food-liking factor) \|\| id:ebi-fl187-GCST90094785 | MR Egger | 13 | 0.466421346900304 | 1.82129573505831 | 0.802604958564246 | -3.103318294 | 4.03616098761459 | 1.59427860219692 | 0.0448999636498704 | 56.6086039900456 | 0.999279047927446 |
| ebi-fl187-GCST90094785 | ebi-met1400-GCST90200578 | X-18922 levels \|\| id:ebi-met1400-GCST90200578 | F-highly palatable foods liking (derived food-liking factor) \|\| id:ebi-fl187-GCST90094785 | MR Egger | 14 | -1.246907574 | 1.77072109595507 | 0.494759323335357 | -4.717520923 | 2.22370577361322 | 0.287392162961334 | 0.00893730738493082 | 9.24151444884349 | 0.999279047927446 |
| ebi-fl187-GCST90094785 | ebi-met1400-GCST90200623 | X-24241 levels \|\| id:ebi-met1400-GCST90200623 | F-highly palatable foods liking (derived food-liking factor) \|\| id:ebi-fl187-GCST90094785 | MR Egger | 14 | -2.528315607 | 1.98897361330673 | 0.227757465260734 | -6.426703889 | 1.3700726752909 | 0.0797933104657428 | 0.00161777442037199 | 3.93563670861998 | 0.999279047927446 |
| ebi-fl187-GCST90094785 | ebi-met1400-GCST90200636 | X-24544 levels \|\| id:ebi-met1400-GCST90200636 | F-highly palatable foods liking (derived food-liking factor) \|\| id:ebi-fl187-GCST90094785 | MR Egger | 14 | 1.70369220462943 | 1.6995738321064 | 0.335924949774582 | -1.627472506 | 5.03485691555798 | 5.49419568304298 | 0.196425411239962 | 153.677602164678 | 0.999279047927446 |
| ebi-fl187-GCST90094785 | ebi-met1400-GCST90200659 | X-25172 levels \|\| id:ebi-met1400-GCST90200659 | F-highly palatable foods liking (derived food-liking factor) \|\| id:ebi-fl187-GCST90094785 | MR Egger | 14 | -2.071022306 | 1.9389336992018 | 0.306481178416695 | -5.871332356 | 1.72928774481548 | 0.126056847187701 | 0.00281911476606603 | 5.63663775387118 | 0.999279047927446 |
| ebi-fl187-GCST90094785 | ebi-met1400-GCST90200670 | X-26111 levels \|\| id:ebi-met1400-GCST90200670 | F-highly palatable foods liking (derived food-liking factor) \|\| id:ebi-fl187-GCST90094785 | MR Egger | 14 | 0.589000649004681 | 1.71016758053931 | 0.73649766073476 | -2.762927809 | 3.94092910686173 | 1.80218649830317 | 0.0631067331659718 | 51.4663968759763 | 0.999279047927446 |
| ebi-fl187-GCST90094785 | ebi-met1400-GCST90200673 | Carnitine C4 levels \|\| id:ebi-met1400-GCST90200673 | F-highly palatable foods liking (derived food-liking factor) \|\| id:ebi-fl187-GCST90094785 | MR Egger | 14 | -0.421872363 | 1.70459812798303 | 0.808712252345337 | -3.762884694 | 2.9191399681713 | 0.655817740873462 | 0.0232166707040981 | 18.5253482174967 | 0.999279047927446 |
| ebi-fl187-GCST90094785 | ebi-met1400-GCST90200710 | X-13431 levels \|\| id:ebi-met1400-GCST90200710 | F-highly palatable foods liking (derived food-liking factor) \|\| id:ebi-fl187-GCST90094785 | MR Egger | 14 | 0.250100085116954 | 1.67886131903937 | 0.884051473531041 | -3.0404681 | 3.54066827043412 | 1.28415393495302 | 0.0478125032127694 | 34.4899601118334 | 0.999279047927446 |
| ebi-fl187-GCST90094785 | ebi-met1400-GCST90200730 | Adenosine 5'-diphosphate (ADP) to fructose ratio \|\| id:ebi-met1400-GCST90200730 | F-highly palatable foods liking (derived food-liking factor) \|\| id:ebi-fl187-GCST90094785 | MR Egger | 14 | -1.335162913 | 2.43287361073869 | 0.593206683332366 | -6.10359519 | 3.43326936363887 | 0.263115306952591 | 0.00223481866048469 | 30.9777549189346 | 0.999279047927446 |
| ebi-fl187-GCST90094785 | ebi-met1400-GCST90200731 | Adenosine 5'-diphosphate (ADP) to mannose ratio \|\| id:ebi-met1400-GCST90200731 | F-highly palatable foods liking (derived food-liking factor) \|\| id:ebi-fl187-GCST90094785 | MR Egger | 14 | -1.717586703 | 2.25521352575309 | 0.461000909410347 | -6.137805214 | 2.70263180709599 | 0.179498809501385 | 0.00215965840882186 | 14.9189438852004 | 0.999279047927446 |
| ebi-fl187-GCST90094785 | ebi-met1400-GCST90200748 | Aspartate to glutamate ratio \|\| id:ebi-met1400-GCST90200748 | F-highly palatable foods liking (derived food-liking factor) \|\| id:ebi-fl187-GCST90094785 | MR Egger | 14 | 0.337266029295934 | 1.64688959240361 | 0.841168909608922 | -2.890637572 | 3.56516963040702 | 1.40111175093611 | 0.0555407900781336 | 35.3454485586102 | 0.999279047927446 |
| ebi-fl187-GCST90094785 | ebi-met1400-GCST90200774 | Uridine to pseudouridine ratio \|\| id:ebi-met1400-GCST90200774 | F-highly palatable foods liking (derived food-liking factor) \|\| id:ebi-fl187-GCST90094785 | MR Egger | 14 | -1.34426803 | 1.62146709752812 | 0.423263786160676 | -4.522343541 | 1.83380748081119 | 0.26073048483329 | 0.0108635346686335 | 6.25766730580645 | 0.999279047927446 |
| ebi-fl187-GCST90094785 | ebi-met1400-GCST90200799 | Spermidine to adenosine 5'-diphosphate (ADP) ratio \|\| id:ebi-met1400-GCST90200799 | F-highly palatable foods liking (derived food-liking factor) \|\| id:ebi-fl187-GCST90094785 | MR Egger | 14 | 2.14793484676674 | 2.40751092252211 | 0.389836181271119 | -2.570786561 | 6.86665625491008 | 8.56714764310685 | 0.076475369189197 | 959.734088464645 | 0.999279047927446 |
| ebi-fl187-GCST90094785 | ebi-met1400-GCST90200811 | Choline to taurocholate ratio \|\| id:ebi-met1400-GCST90200811 | F-highly palatable foods liking (derived food-liking factor) \|\| id:ebi-fl187-GCST90094785 | MR Egger | 14 | -1.338916936 | 1.77394232841638 | 0.464943126219188 | -4.8158439 | 2.13801002749872 | 0.262129417777342 | 0.00810038328009091 | 8.48254079941721 | 0.999279047927446 |
| ebi-fl187-GCST90094785 | ebi-met1400-GCST90200812 | Glutamate to kynurenine ratio \|\| id:ebi-met1400-GCST90200812 | F-highly palatable foods liking (derived food-liking factor) \|\| id:ebi-fl187-GCST90094785 | MR Egger | 14 | -2.089015993 | 1.65013087295822 | 0.22954944638612 | -5.323272504 | 1.14524051782564 | 0.123808904704458 | 0.00487676833865606 | 3.14319726090206 | 0.999279047927446 |
| ebi-fl187-GCST90094785 | ebi-met1400-GCST90200876 | Cortisone to 4-cholesten-3-one ratio \|\| id:ebi-met1400-GCST90200876 | F-highly palatable foods liking (derived food-liking factor) \|\| id:ebi-fl187-GCST90094785 | MR Egger | 14 | -0.951643814 | 1.81642933963628 | 0.609881997566117 | -4.51184532 | 2.60855769170633 | 0.386105815378199 | 0.0109781832154188 | 13.5794509659381 | 0.999279047927446 |
| ebi-fl187-GCST90094785 | ebi-met1400-GCST90200906 | 2'-deoxyuridine to cytidine ratio \|\| id:ebi-met1400-GCST90200906 | F-highly palatable foods liking (derived food-liking factor) \|\| id:ebi-fl187-GCST90094785 | MR Egger | 14 | -0.245242963 | 2.00696384609677 | 0.90476633961219 | -4.178892101 | 3.68840617581398 | 0.782514393462527 | 0.0153154661621859 | 39.9810733471421 | 0.999279047927446 |
| ebi-fl187-GCST90094785 | ebi-met1400-GCST90200907 | Retinol (Vitamin A) to linoleoyl-arachidonoyl-glycerol (18:2 to 20:4) [1] ratio \|\| id:ebi-met1400-GCST90200907 | F-highly palatable foods liking (derived food-liking factor) \|\| id:ebi-fl187-GCST90094785 | MR Egger | 14 | -0.378351183 | 1.76832096976411 | 0.834170108641462 | -3.844260284 | 3.08755791783524 | 0.684989901679427 | 0.021402227284824 | 21.9234736253596 | 0.999279047927446 |
| ebi-fl187-GCST90094785 | ebi-met1400-GCST90200916 | Fructose to sucrose ratio \|\| id:ebi-met1400-GCST90200916 | F-highly palatable foods liking (derived food-liking factor) \|\| id:ebi-fl187-GCST90094785 | MR Egger | 14 | -0.674713309 | 1.7495439511496 | 0.70650466720147 | -4.103819454 | 2.75439283495955 | 0.509302411947588 | 0.0165094975671233 | 15.7114985335575 | 0.999279047927446 |
| ebi-fl187-GCST90094785 | ebi-met1400-GCST90200917 | Alpha-ketobutyrate to 3-methyl-2-oxobutyrate ratio \|\| id:ebi-met1400-GCST90200917 | F-highly palatable foods liking (derived food-liking factor) \|\| id:ebi-fl187-GCST90094785 | MR Egger | 14 | -2.144580719 | 1.70917840721996 | 0.233459679305063 | -5.494570397 | 1.20540895880623 | 0.117117131698891 | 0.00410902133227753 | 3.33812395414668 | 0.999279047927446 |
| ebi-fl187-GCST90094785 | ebi-met1400-GCST90200924 | Alpha-tocopherol to glycerol ratio \|\| id:ebi-met1400-GCST90200924 | F-highly palatable foods liking (derived food-liking factor) \|\| id:ebi-fl187-GCST90094785 | MR Egger | 14 | -0.265434913 | 1.58640515981527 | 0.869905992427553 | -3.374789026 | 2.843919201 | 0.766872355006699 | 0.0342253382128738 | 17.182977278872 | 0.999279047927446 |
| ebi-fl187-GCST90094785 | ebi-met1400-GCST90200938 | Alpha-ketoglutarate to alanine ratio \|\| id:ebi-met1400-GCST90200938 | F-highly palatable foods liking (derived food-liking factor) \|\| id:ebi-fl187-GCST90094785 | MR Egger | 14 | 0.64475970667919 | 1.74233756609866 | 0.717790646872704 | -2.770221923 | 4.05974133623257 | 1.90552908834151 | 0.0626481001528886 | 57.9593171645161 | 0.999279047927446 |
| ebi-fl187-GCST90094785 | ebi-met1400-GCST90200943 | Phosphate to EDTA ratio \|\| id:ebi-met1400-GCST90200943 | F-highly palatable foods liking (derived food-liking factor) \|\| id:ebi-fl187-GCST90094785 | MR Egger | 14 | -4.335869232 | 1.70847978557169 | 0.0260408983814046 | -7.684489612 | -0.987248852 | 0.0130904904523558 | 0.000459905459803368 | 0.372600360857825 | 0.999279047927446 |
| ebi-fl187-GCST90094785 | ebi-met1400-GCST90200947 | Adenosine 5'-diphosphate (ADP) to glucose ratio \|\| id:ebi-met1400-GCST90200947 | F-highly palatable foods liking (derived food-liking factor) \|\| id:ebi-fl187-GCST90094785 | MR Egger | 14 | -1.793230375 | 2.26255035538059 | 0.443415707825899 | -6.227829071 | 2.64136832161585 | 0.166421695924924 | 0.00197373208024398 | 14.0323913016118 | 0.999279047927446 |
| ebi-fl187-GCST90094785 | ebi-met1400-GCST90200975 | Arachidonate (20:4n6) to caffeine ratio \|\| id:ebi-met1400-GCST90200975 | F-highly palatable foods liking (derived food-liking factor) \|\| id:ebi-fl187-GCST90094785 | MR Egger | 14 | 1.32709843707452 | 1.74001746967683 | 0.460377065868592 | -2.083335803 | 4.73753267764111 | 3.77008835330235 | 0.124514163873994 | 114.152203648815 | 0.999279047927446 |
| ebi-fl187-GCST90094785 | ebi-met1400-GCST90200985 | Cholesterol to oleoyl-linoleoyl-glycerol (18:1 to 18:2) [2] ratio \|\| id:ebi-met1400-GCST90200985 | F-highly palatable foods liking (derived food-liking factor) \|\| id:ebi-fl187-GCST90094785 | MR Egger | 14 | 1.8400317687343 | 1.70069223771699 | 0.300545987422419 | -1.493325017 | 5.17338855465961 | 6.29673829725511 | 0.224624532036396 | 176.51194562175 | 0.999279047927446 |
| ebi-fl187-GCST90094785 | ebi-met1400-GCST90201016 | Bilirubin (Z,Z) to etiocholanolone glucuronide ratio \|\| id:ebi-met1400-GCST90201016 | F-highly palatable foods liking (derived food-liking factor) \|\| id:ebi-fl187-GCST90094785 | MR Egger | 14 | 2.91851174272639 | 1.74503344535735 | 0.120280995959959 | -0.50175381 | 6.33877729562679 | 18.5137137772789 | 0.605467852324402 | 566.103710562262 | 0.999279047927446 |
| ebi-fl187-GCST90094785 | ebi-pw11-GCST90275189 | Placental weight (fetal genotype, adjusted for sex and gestational age) \|\| id:ebi-pw11-GCST90275189 | F-highly palatable foods liking (derived food-liking factor) \|\| id:ebi-fl187-GCST90094785 | MR Egger | 13 | -0.735063015 | 0.913739993569404 | 0.438175084454373 | -2.525993402 | 1.05586737246543 | 0.479475243923685 | 0.0799788221621549 | 2.87446730672738 | 0.999279047927446 |
| ebi-fl187-GCST90094785 | ebi-pw11-GCST90275192 | Placental weight (fetal effect, conditional on maternal and paternal genotype) \|\| id:ebi-pw11-GCST90275192 | F-highly palatable foods liking (derived food-liking factor) \|\| id:ebi-fl187-GCST90094785 | MR Egger | 13 | 0.293205272602485 | 1.41741592621655 | 0.839899310734723 | -2.484929943 | 3.07134048798692 | 1.3407179748547 | 0.0833313922731107 | 21.5707986998138 | 0.999279047927446 |
| ebi-fl187-GCST90094785 | ebi-pw11-GCST90275193 | Placental weight (maternal effect, conditional on fetal and paternal genotype) \|\| id:ebi-pw11-GCST90275193 | F-highly palatable foods liking (derived food-liking factor) \|\| id:ebi-fl187-GCST90094785 | MR Egger | 13 | -0.067352192 | 1.03062622059929 | 0.949067304487444 | -2.087379585 | 1.9526752002456 | 0.934865890941447 | 0.124011672529665 | 7.04751590086558 | 0.999279047927446 |
| ebi-fl187-GCST90094785 | ebi-pw11-GCST90275195 | Placental weight (fetal effect, conditional on maternal genotype) \|\| id:ebi-pw11-GCST90275195 | F-highly palatable foods liking (derived food-liking factor) \|\| id:ebi-fl187-GCST90094785 | MR Egger | 13 | 0.249027386720545 | 1.14595705107024 | 0.831945320001543 | -1.997048433 | 2.49510320681821 | 1.28277716364846 | 0.1357353244252 | 12.1229846286976 | 0.999279047927446 |
| ebi-fl187-GCST90094785 | ebi-pw11-GCST90275197 | Placental weight (fetal genotype, adjusted for sex) \|\| id:ebi-pw11-GCST90275197 | F-highly palatable foods liking (derived food-liking factor) \|\| id:ebi-fl187-GCST90094785 | MR Egger | 13 | -0.83365484 | 0.841455811188226 | 0.343110710178147 | -2.48290823 | 0.815598550042608 | 0.434458504794163 | 0.0835000348453999 | 2.26052830681397 | 0.999279047927446 |
| ebi-fl187-GCST90094785 | prot-a-1030 | Protein FAM171B \|\| id:prot-a-1030 | F-highly palatable foods liking (derived food-liking factor) \|\| id:ebi-fl187-GCST90094785 | MR Egger | 17 | 1.3755651732274 | 1.28181846836121 | 0.300171777551957 | -1.136799025 | 3.88792937121537 | 3.95731265818255 | 0.320844394807499 | 48.809715014681 | 0.999279047927446 |
| ebi-fl187-GCST90094785 | prot-a-1036 | BRISC complex subunit Abro1 \|\| id:prot-a-1036 | F-highly palatable foods liking (derived food-liking factor) \|\| id:ebi-fl187-GCST90094785 | MR Egger | 17 | 2.65295316569187 | 1.38464153990747 | 0.0746254302565856 | -0.060944253 | 5.3668505839105 | 14.1958996475193 | 0.940875689756353 | 214.187239607204 | 0.999279047927446 |
| ebi-fl187-GCST90094785 | prot-a-1055 | Phenylalanine--tRNA ligase, mitochondrial \|\| id:prot-a-1055 | F-highly palatable foods liking (derived food-liking factor) \|\| id:ebi-fl187-GCST90094785 | MR Egger | 17 | -2.423530357 | 1.58246798893583 | 0.14646461561297 | -5.525167615 | 0.678106901200092 | 0.088608245876859 | 0.00398520065502824 | 1.97014452144775 | 0.999279047927446 |
| ebi-fl187-GCST90094785 | prot-a-1083 | Fc receptor-like protein 5 \|\| id:prot-a-1083 | F-highly palatable foods liking (derived food-liking factor) \|\| id:ebi-fl187-GCST90094785 | MR Egger | 17 | -1.109600427 | 1.28181846836121 | 0.400318399134124 | -3.621964625 | 1.40276377139392 | 0.329690670383747 | 0.0267301102414113 | 4.06642311447293 | 0.999279047927446 |
| ebi-fl187-GCST90094785 | prot-a-1103 | Fibroblast growth factor receptor 3 \|\| id:prot-a-1103 | F-highly palatable foods liking (derived food-liking factor) \|\| id:ebi-fl187-GCST90094785 | MR Egger | 17 | 2.29565270173644 | 1.71392570574564 | 0.20037907088336 | -1.063641682 | 5.6549470849979 | 9.93091582336568 | 0.345196423141688 | 285.701364438224 | 0.999279047927446 |
| ebi-fl187-GCST90094785 | prot-a-1105 | Fibroblast growth factor receptor 4 \|\| id:prot-a-1105 | F-highly palatable foods liking (derived food-liking factor) \|\| id:ebi-fl187-GCST90094785 | MR Egger | 17 | 0.204769688728312 | 1.28181846836121 | 0.875209784044557 | -2.307594509 | 2.71713388671628 | 1.22724238465792 | 0.099500310993501 | 15.1368760123697 | 0.999279047927446 |
| ebi-fl187-GCST90094785 | prot-a-1120 | Filamin-A \|\| id:prot-a-1120 | F-highly palatable foods liking (derived food-liking factor) \|\| id:ebi-fl187-GCST90094785 | MR Egger | 17 | 2.68177115138153 | 1.35377648103722 | 0.0662366424162535 | 0.0283692485485716 | 5.33517305421448 | 14.6109485940861 | 1.02877548815193 | 207.508655948361 | 0.999279047927446 |
| ebi-fl187-GCST90094785 | prot-a-1166 | Galanin peptides \|\| id:prot-a-1166 | F-highly palatable foods liking (derived food-liking factor) \|\| id:ebi-fl187-GCST90094785 | MR Egger | 17 | 0.460906868440859 | 1.48346265025905 | 0.760306800639148 | -2.446679926 | 3.3684936629486 | 1.58551118327584 | 0.0865805637156801 | 29.0347579688662 | 0.999279047927446 |
| ebi-fl187-GCST90094785 | prot-a-1205 | ADP-ribosylation factor-binding protein GGA1 \|\| id:prot-a-1205 | F-highly palatable foods liking (derived food-liking factor) \|\| id:ebi-fl187-GCST90094785 | MR Egger | 17 | 1.81216739650144 | 1.33804902120077 | 0.195680881948724 | -0.810408685 | 4.43474347805495 | 6.12370555723424 | 0.444676296526796 | 84.3304894922407 | 0.999279047927446 |
| ebi-fl187-GCST90094785 | prot-a-1227 | Guanine nucleotide-binding protein G(I)/G(S)/G(O) subunit gamma-T2 \|\| id:prot-a-1227 | F-highly palatable foods liking (derived food-liking factor) \|\| id:ebi-fl187-GCST90094785 | MR Egger | 17 | 1.73509694259278 | 1.28181846836121 | 0.195903665744037 | -0.777267255 | 4.24746114058075 | 5.66947739100336 | 0.45966043108322 | 69.9276503121037 | 0.999279047927446 |
| ebi-fl187-GCST90094785 | prot-a-1240 | Golgi SNAP receptor complex member 1 \|\| id:prot-a-1240 | F-highly palatable foods liking (derived food-liking factor) \|\| id:ebi-fl187-GCST90094785 | MR Egger | 17 | 0.720465387794132 | 1.28181846836121 | 0.582374006137104 | -1.79189881 | 3.23282958578209 | 2.05538954129903 | 0.166643444790287 | 25.3512892259153 | 0.999279047927446 |
| ebi-fl187-GCST90094785 | prot-a-1283 | Glutathione S-transferase A1 \|\| id:prot-a-1283 | F-highly palatable foods liking (derived food-liking factor) \|\| id:ebi-fl187-GCST90094785 | MR Egger | 17 | 0.264383999810601 | 1.28181846836121 | 0.839363618563514 | -2.247980198 | 2.77674819779856 | 1.30262830929715 | 0.105612325245867 | 16.0666902109398 | 0.999279047927446 |
| ebi-fl187-GCST90094785 | prot-a-1324 | E3 ubiquitin-protein ligase HECW1 \|\| id:prot-a-1324 | F-highly palatable foods liking (derived food-liking factor) \|\| id:ebi-fl187-GCST90094785 | MR Egger | 17 | 1.89294708970132 | 1.53055078308003 | 0.235185210816882 | -1.106932445 | 4.89282662453817 | 6.63890532829796 | 0.330571453413308 | 133.329915523579 | 0.999279047927446 |
| ebi-fl187-GCST90094785 | prot-a-1339 | Histidine triad nucleotide-binding protein 2, mitochondrial \|\| id:prot-a-1339 | F-highly palatable foods liking (derived food-liking factor) \|\| id:ebi-fl187-GCST90094785 | MR Egger | 17 | 2.63175576881035 | 1.28182232981614 | 0.05792753616945 | 0.119384002370716 | 5.14412753524998 | 13.8981504316294 | 1.12680253006394 | 171.421859879247 | 0.999279047927446 |
| ebi-fl187-GCST90094785 | prot-a-1466 | Interleukin-11 \|\| id:prot-a-1466 | F-highly palatable foods liking (derived food-liking factor) \|\| id:ebi-fl187-GCST90094785 | MR Egger | 17 | 1.58262840805317 | 1.65178345949926 | 0.353185271350675 | -1.654867173 | 4.82012398867172 | 4.86773340119346 | 0.191117439658806 | 123.980461999678 | 0.999279047927446 |
| ebi-fl187-GCST90094785 | prot-a-147 | Rho GTPase-activating protein 1 \|\| id:prot-a-147 | F-highly palatable foods liking (derived food-liking factor) \|\| id:ebi-fl187-GCST90094785 | MR Egger | 17 | -0.002826457 | 1.41165440916448 | 0.998428838697103 | -2.769669099 | 2.76401618537744 | 0.997177534082773 | 0.062682743121541 | 15.863425640951 | 0.999739542784661 |
| ebi-fl187-GCST90094785 | prot-a-1473 | Interleukin-12 receptor subunit beta-1 \|\| id:prot-a-1473 | F-highly palatable foods liking (derived food-liking factor) \|\| id:ebi-fl187-GCST90094785 | MR Egger | 17 | 0.144358695075328 | 1.48830483680568 | 0.924014198255381 | -2.772718785 | 3.06143617521446 | 1.15529843407104 | 0.0624918716021111 | 21.3582092766431 | 0.999279047927446 |
| ebi-fl187-GCST90094785 | prot-a-1501 | X-linked interleukin-1 receptor accessory protein-like 2 \|\| id:prot-a-1501 | F-highly palatable foods liking (derived food-liking factor) \|\| id:ebi-fl187-GCST90094785 | MR Egger | 17 | -0.372388622 | 1.28182229907844 | 0.775400261637254 | -2.884760328 | 2.13998308396326 | 0.689086396189791 | 0.0558681779462537 | 8.49929385330302 | 0.999279047927446 |
| ebi-fl187-GCST90094785 | prot-a-1503 | Interleukin-1 receptor-like 2 \|\| id:prot-a-1503 | F-highly palatable foods liking (derived food-liking factor) \|\| id:ebi-fl187-GCST90094785 | MR Egger | 17 | 2.12618701106927 | 1.28181843797619 | 0.11792903522297 | -0.386177127 | 4.6385511495026 | 8.38284211114865 | 0.679650130381907 | 103.394435929793 | 0.999279047927446 |
| ebi-fl187-GCST90094785 | prot-a-1536 | Interleukin-5 receptor subunit alpha \|\| id:prot-a-1536 | F-highly palatable foods liking (derived food-liking factor) \|\| id:ebi-fl187-GCST90094785 | MR Egger | 17 | -2.438047697 | 1.44556774340848 | 0.112367226425424 | -5.271360474 | 0.395265079757354 | 0.0873311820399173 | 0.00513661758047449 | 1.48477772327849 | 0.999279047927446 |
| ebi-fl187-GCST90094785 | prot-a-1559 | Insulin-induced gene 1 protein \|\| id:prot-a-1559 | F-highly palatable foods liking (derived food-liking factor) \|\| id:ebi-fl187-GCST90094785 | MR Egger | 17 | 1.42524565004557 | 1.28181846836121 | 0.283687857301392 | -1.087118548 | 3.93760984803353 | 4.15887934618559 | 0.33718668251933 | 51.2958497853413 | 0.999279047927446 |
| ebi-fl187-GCST90094785 | prot-a-1567 | Interferon regulatory factor 1 \|\| id:prot-a-1567 | F-highly palatable foods liking (derived food-liking factor) \|\| id:ebi-fl187-GCST90094785 | MR Egger | 17 | -2.187468895 | 1.38877823410977 | 0.136084805309947 | -4.909474234 | 0.534536443846545 | 0.112200380457936 | 0.00737636555523982 | 1.70665692753838 | 0.999279047927446 |
| ebi-fl187-GCST90094785 | prot-a-1613 | Potassium voltage-gated channel subfamily E regulatory beta subunit 5 \|\| id:prot-a-1613 | F-highly palatable foods liking (derived food-liking factor) \|\| id:ebi-fl187-GCST90094785 | MR Egger | 17 | 1.7973710747223 | 1.28182232981614 | 0.181210736474672 | -0.715000692 | 4.30974284116193 | 6.03376428027243 | 0.489191773413786 | 74.4213483718929 | 0.999279047927446 |
| ebi-fl187-GCST90094785 | prot-a-1649 | Kin of IRRE-like protein 2 \|\| id:prot-a-1649 | F-highly palatable foods liking (derived food-liking factor) \|\| id:ebi-fl187-GCST90094785 | MR Egger | 17 | 1.75679941342952 | 1.28181846836121 | 0.190676854951975 | -0.755564785 | 4.26916361141748 | 5.79386392419114 | 0.469745234941291 | 71.4618407491434 | 0.999279047927446 |
| ebi-fl187-GCST90094785 | prot-a-165 | Armadillo repeat-containing protein 5 \|\| id:prot-a-165 | F-highly palatable foods liking (derived food-liking factor) \|\| id:ebi-fl187-GCST90094785 | MR Egger | 17 | -2.656933558 | 1.28152241482559 | 0.0557867730640125 | -5.168717491 | -0.145149625 | 0.070163043102153 | 0.00569186400600103 | 0.864892873786922 | 0.999279047927446 |
| ebi-fl187-GCST90094785 | prot-a-1656 | Kallikrein-11 \|\| id:prot-a-1656 | F-highly palatable foods liking (derived food-liking factor) \|\| id:ebi-fl187-GCST90094785 | MR Egger | 17 | 2.25560690299104 | 1.28181846836121 | 0.0988324974900939 | -0.256757295 | 4.767971101 | 9.54108206460561 | 0.773555936879895 | 117.680238265268 | 0.999279047927446 |
| ebi-fl187-GCST90094785 | prot-a-1697 | Laminin subunit gamma-2 \|\| id:prot-a-1697 | F-highly palatable foods liking (derived food-liking factor) \|\| id:ebi-fl187-GCST90094785 | MR Egger | 17 | 1.73291003946659 | 1.28181846836121 | 0.196436719519667 | -0.779454159 | 4.24527423745455 | 5.65709234056186 | 0.458656296622086 | 69.7748924092775 | 0.999279047927446 |
| ebi-fl187-GCST90094785 | prot-a-1698 | Lysosome-associated membrane glycoprotein 3 \|\| id:prot-a-1698 | F-highly palatable foods liking (derived food-liking factor) \|\| id:ebi-fl187-GCST90094785 | MR Egger | 17 | -1.079849842 | 1.28181846836121 | 0.41277531437514 | -3.59221404 | 1.43251435571495 | 0.339646522365844 | 0.0275372942018154 | 4.18921914803113 | 0.999279047927446 |
| ebi-fl187-GCST90094785 | prot-a-1724 | Leptin receptor \|\| id:prot-a-1724 | F-highly palatable foods liking (derived food-liking factor) \|\| id:ebi-fl187-GCST90094785 | MR Egger | 17 | 0.308082532002719 | 1.28182232981614 | 0.813314877636583 | -2.204289234 | 2.82045429844235 | 1.36081329494923 | 0.110328915436387 | 16.7844741007925 | 0.999279047927446 |
| ebi-fl187-GCST90094785 | prot-a-1743 | Leukocyte immunoglobulin-like receptor subfamily B member 2 \|\| id:prot-a-1743 | F-highly palatable foods liking (derived food-liking factor) \|\| id:ebi-fl187-GCST90094785 | MR Egger | 17 | -0.549031843 | 1.28181846836121 | 0.674502999722775 | -3.061396041 | 1.9633323554214 | 0.577508659111249 | 0.0468222837650984 | 7.12302400757901 | 0.999279047927446 |
| ebi-fl187-GCST90094785 | prot-a-1746 | Leukocyte immunoglobulin-like receptor subfamily B member 4 \|\| id:prot-a-1746 | F-highly palatable foods liking (derived food-liking factor) \|\| id:ebi-fl187-GCST90094785 | MR Egger | 17 | 1.71158189782353 | 1.52070083412797 | 0.278054989438344 | -1.268991737 | 4.69215553271435 | 5.53771465123967 | 0.28111491668683 | 109.088069462773 | 0.999279047927446 |
| ebi-fl187-GCST90094785 | prot-a-1750 | Leucine-rich repeat and immunoglobulin-like domain-containing nogo receptor-interacting protein 3 \|\| id:prot-a-1750 | F-highly palatable foods liking (derived food-liking factor) \|\| id:ebi-fl187-GCST90094785 | MR Egger | 17 | 1.96414612496431 | 1.28181846836121 | 0.146262371007474 | -0.548218073 | 4.47651032295227 | 7.12882286671727 | 0.577978809339824 | 87.9272987933218 | 0.999279047927446 |
| ebi-fl187-GCST90094785 | prot-a-1753 | Lipase member N \|\| id:prot-a-1753 | F-highly palatable foods liking (derived food-liking factor) \|\| id:ebi-fl187-GCST90094785 | MR Egger | 17 | -1.773868069 | 1.30814459820283 | 0.195156745164686 | -4.337831482 | 0.790095343315804 | 0.169675401660324 | 0.0130648288283291 | 2.20360651539238 | 0.999279047927446 |
| ebi-fl187-GCST90094785 | prot-a-1760 | Ig Kappa chain V-I region HK102- like \|\| id:prot-a-1760 | F-highly palatable foods liking (derived food-liking factor) \|\| id:ebi-fl187-GCST90094785 | MR Egger | 17 | -0.644513432 | 1.28182232981614 | 0.622399452990091 | -3.156885199 | 1.86785833417923 | 0.524917888090281 | 0.0425580948548275 | 6.4744155060763 | 0.999279047927446 |
| ebi-fl187-GCST90094785 | prot-a-1792 | Leucine-rich repeat neuronal protein 1 \|\| id:prot-a-1792 | F-highly palatable foods liking (derived food-liking factor) \|\| id:ebi-fl187-GCST90094785 | MR Egger | 16 | 1.45372307902541 | 3.18455715318944 | 0.655038414588611 | -4.788008941 | 7.69545509927671 | 4.27901601029667 | 0.0083290244606684 | 2198.33404294094 | 0.999279047927446 |
| ebi-fl187-GCST90094785 | prot-a-1795 | Leucine-rich repeat transmembrane neuronal protein 1 \|\| id:prot-a-1795 | F-highly palatable foods liking (derived food-liking factor) \|\| id:ebi-fl187-GCST90094785 | MR Egger | 17 | 0.44874840292339 | 1.28181846836121 | 0.731140417703712 | -2.063615795 | 2.96111260091135 | 1.56635051840807 | 0.126993954621178 | 19.3194546451904 | 0.999279047927446 |
| ebi-fl187-GCST90094785 | prot-a-1820 | LysM and putative peptidoglycan-binding domain-containing protein 4 \|\| id:prot-a-1820 | F-highly palatable foods liking (derived food-liking factor) \|\| id:ebi-fl187-GCST90094785 | MR Egger | 17 | 1.32069928152323 | 1.2817607508099 | 0.319160975023989 | -1.19155179 | 3.83295035311063 | 3.74603999813353 | 0.303749542632794 | 46.1986396620871 | 0.999279047927446 |
| ebi-fl187-GCST90094785 | prot-a-1844 | Mitogen-activated protein kinase kinase kinase 7:TGF-beta-activated kinase 1 and MAP3K7-binding protein 1 fusion \|\| id:prot-a-1844 | F-highly palatable foods liking (derived food-liking factor) \|\| id:ebi-fl187-GCST90094785 | MR Egger | 17 | 0.256231992246064 | 1.28181846836121 | 0.844245703545457 | -2.256132206 | 2.76859619023403 | 1.29205243932043 | 0.104754872500699 | 15.9362468408592 | 0.999279047927446 |
| ebi-fl187-GCST90094785 | prot-a-1856 | Megakaryocyte-associated tyrosine-protein kinase \|\| id:prot-a-1856 | F-highly palatable foods liking (derived food-liking factor) \|\| id:ebi-fl187-GCST90094785 | MR Egger | 17 | -0.396209847 | 1.66043294300993 | 0.814630763508554 | -3.650658415 | 2.85823872144368 | 0.672865482415447 | 0.025974021458143 | 17.4307993914524 | 0.999279047927446 |
| ebi-fl187-GCST90094785 | prot-a-1910 | Stromelysin-2 \|\| id:prot-a-1910 | F-highly palatable foods liking (derived food-liking factor) \|\| id:ebi-fl187-GCST90094785 | MR Egger | 17 | 0.67622022220727 | 1.28182232981614 | 0.60553057288498 | -1.836151544 | 3.1885919886469 | 1.96643099538366 | 0.15942980554821 | 24.2542531260648 | 0.999279047927446 |
| ebi-fl187-GCST90094785 | prot-a-1940 | 39S ribosomal protein L14, mitochondrial \|\| id:prot-a-1940 | F-highly palatable foods liking (derived food-liking factor) \|\| id:ebi-fl187-GCST90094785 | MR Egger | 17 | 0.904395893309466 | 1.28182232981614 | 0.491277860978935 | -1.607975873 | 3.4167676597491 | 2.47043906341286 | 0.200292621720891 | 30.4707637934905 | 0.999279047927446 |
| ebi-fl187-GCST90094785 | prot-a-1942 | 39S ribosomal protein L33, mitochondrial \|\| id:prot-a-1942 | F-highly palatable foods liking (derived food-liking factor) \|\| id:ebi-fl187-GCST90094785 | MR Egger | 17 | -1.091401113 | 1.28161689747661 | 0.407836808967673 | -3.603370232 | 1.42056800610281 | 0.335745746285112 | 0.0272317901669658 | 4.13947101741739 | 0.999279047927446 |
| ebi-fl187-GCST90094785 | prot-a-1948 | Mesothelin \|\| id:prot-a-1948 | F-highly palatable foods liking (derived food-liking factor) \|\| id:ebi-fl187-GCST90094785 | MR Egger | 17 | 0.420054586339498 | 1.53509298151594 | 0.788097346458834 | -2.588727657 | 3.42883683011075 | 1.52204463619632 | 0.0751155520248674 | 30.8407488479492 | 0.999279047927446 |
| ebi-fl187-GCST90094785 | prot-a-1980 | Unconventional myosin-VI \|\| id:prot-a-1980 | F-highly palatable foods liking (derived food-liking factor) \|\| id:ebi-fl187-GCST90094785 | MR Egger | 17 | 0.884476888195985 | 1.32169627988674 | 0.513537973360785 | -1.70604782 | 3.47500159677399 | 2.42171723121156 | 0.181582021115857 | 32.2978801089843 | 0.999279047927446 |
| ebi-fl187-GCST90094785 | prot-a-2039 | Neurofascin \|\| id:prot-a-2039 | F-highly palatable foods liking (derived food-liking factor) \|\| id:ebi-fl187-GCST90094785 | MR Egger | 17 | 1.13409250097971 | 1.30154364668433 | 0.397298300679732 | -1.416933047 | 3.68511804848099 | 3.10835143668597 | 0.242456480510643 | 39.8498263836823 | 0.999279047927446 |
| ebi-fl187-GCST90094785 | prot-a-2041 | NFU1 iron-sulfur cluster scaffold homolog, mitochondrial \|\| id:prot-a-2041 | F-highly palatable foods liking (derived food-liking factor) \|\| id:ebi-fl187-GCST90094785 | MR Egger | 17 | 2.51202223996337 | 1.28182232981614 | 0.0688787002051202 | -0.000349526 | 5.024394006 | 12.3298387590195 | 0.999650534601003 | 152.078069846777 | 0.999279047927446 |
| ebi-fl187-GCST90094785 | prot-a-2088 | Glucocorticoid receptor \|\| id:prot-a-2088 | F-highly palatable foods liking (derived food-liking factor) \|\| id:ebi-fl187-GCST90094785 | MR Egger | 17 | 1.58237101236838 | 1.28152241482559 | 0.235913703310465 | -0.929412921 | 4.09415494542654 | 4.86648062885729 | 0.394785412698898 | 59.9886240708339 | 0.999279047927446 |
| ebi-fl187-GCST90094785 | prot-a-2111 | Neuron-specific protein family member 2 \|\| id:prot-a-2111 | F-highly palatable foods liking (derived food-liking factor) \|\| id:ebi-fl187-GCST90094785 | MR Egger | 17 | 1.24341219283044 | 1.38646869476594 | 0.383983112850785 | -1.474066449 | 3.96089083457168 | 3.46742482410973 | 0.22899240339842 | 52.5040775694805 | 0.999279047927446 |
| ebi-fl187-GCST90094785 | prot-a-2125 | Nuclear migration protein nudC \|\| id:prot-a-2125 | F-highly palatable foods liking (derived food-liking factor) \|\| id:ebi-fl187-GCST90094785 | MR Egger | 17 | 3.26199935645775 | 1.3355826847804 | 0.0274490905073876 | 0.644257294288171 | 5.87974141862733 | 26.1016715530939 | 1.90457196737094 | 357.716730865282 | 0.999279047927446 |
| ebi-fl187-GCST90094785 | prot-a-2142 | Ornithine decarboxylase \|\| id:prot-a-2142 | F-highly palatable foods liking (derived food-liking factor) \|\| id:ebi-fl187-GCST90094785 | MR Egger | 17 | -2.414778632 | 1.28182232981614 | 0.0791165980827433 | -4.927150399 | 0.0975931343442147 | 0.0893871241721374 | 0.00724712530403191 | 1.1025141187113 | 0.999279047927446 |
| ebi-fl187-GCST90094785 | prot-a-2186 | Prostate and testis expressed protein 1 \|\| id:prot-a-2186 | F-highly palatable foods liking (derived food-liking factor) \|\| id:ebi-fl187-GCST90094785 | MR Egger | 17 | 2.71945827358037 | 1.28816703464681 | 0.0519571300546098 | 0.194650885672624 | 5.24426566148812 | 15.1721008903989 | 1.21488677806506 | 189.476624146877 | 0.999279047927446 |
| ebi-fl187-GCST90094785 | prot-a-2227 | Platelet-derived growth factor C \|\| id:prot-a-2227 | F-highly palatable foods liking (derived food-liking factor) \|\| id:ebi-fl187-GCST90094785 | MR Egger | 17 | -0.885016688 | 1.28182232981614 | 0.500464586335876 | -3.397388455 | 1.62735507799385 | 0.412707285778416 | 0.033460539665235 | 5.09039320461273 | 0.999279047927446 |
| ebi-fl187-GCST90094785 | prot-a-2229 | Platelet-derived growth factor receptor alpha \|\| id:prot-a-2229 | F-highly palatable foods liking (derived food-liking factor) \|\| id:ebi-fl187-GCST90094785 | MR Egger | 17 | 0.0425459614721611 | 1.57544010226551 | 0.978811154460433 | -3.045316639 | 3.13040856191257 | 1.04346401441381 | 0.0475812435261673 | 22.8833268886255 | 0.999279047927446 |
| ebi-fl187-GCST90094785 | prot-a-2243 | Platelet endothelial aggregation receptor 1 \|\| id:prot-a-2243 | F-highly palatable foods liking (derived food-liking factor) \|\| id:ebi-fl187-GCST90094785 | MR Egger | 17 | -0.472859369 | 1.28182232981614 | 0.717360128287849 | -2.985231136 | 2.03951239698518 | 0.623217708461608 | 0.0505278234056974 | 7.68686014874615 | 0.999279047927446 |
| ebi-fl187-GCST90094785 | prot-a-2246 | E3 ubiquitin-protein ligase pellino homolog 2 \|\| id:prot-a-2246 | F-highly palatable foods liking (derived food-liking factor) \|\| id:ebi-fl187-GCST90094785 | MR Egger | 17 | 2.72859589675086 | 1.28152241482559 | 0.0502146315502198 | 0.216811963692704 | 5.24037982980902 | 15.3113731707912 | 1.24211051830222 | 188.741778546136 | 0.999279047927446 |
| ebi-fl187-GCST90094785 | prot-a-2251 | Platelet factor 4 variant \|\| id:prot-a-2251 | F-highly palatable foods liking (derived food-liking factor) \|\| id:ebi-fl187-GCST90094785 | MR Egger | 17 | 0.950686277399934 | 1.41789106354388 | 0.512735308764263 | -1.828380207 | 3.72975276194594 | 2.58748478246161 | 0.160673615080453 | 41.6688047761792 | 0.999279047927446 |
| ebi-fl187-GCST90094785 | prot-a-2346 | Protein phosphatase 1L \|\| id:prot-a-2346 | F-highly palatable foods liking (derived food-liking factor) \|\| id:ebi-fl187-GCST90094785 | MR Egger | 17 | -0.256956894 | 1.33117032252458 | 0.849524185250176 | -2.866050726 | 2.35213693796946 | 0.773401551155981 | 0.0569232889003759 | 10.508000695064 | 0.999279047927446 |
| ebi-fl187-GCST90094785 | prot-a-2358 | PR domain zinc finger protein 4 \|\| id:prot-a-2358 | F-highly palatable foods liking (derived food-liking factor) \|\| id:ebi-fl187-GCST90094785 | MR Egger | 17 | -0.365466997 | 1.28182232981614 | 0.779454908944095 | -2.877838764 | 2.14690476906058 | 0.693872538547165 | 0.0562562145102042 | 8.55832735888329 | 0.999279047927446 |
| ebi-fl187-GCST90094785 | prot-a-2390 | Trypsin-1 \|\| id:prot-a-2390 | F-highly palatable foods liking (derived food-liking factor) \|\| id:ebi-fl187-GCST90094785 | MR Egger | 17 | -0.386984879 | 1.60853082182733 | 0.813135663982663 | -3.53970529 | 2.76573553147761 | 0.679101363334296 | 0.0290218788574243 | 15.8907238207468 | 0.999279047927446 |
| ebi-fl187-GCST90094785 | prot-a-242 | Brain-derived neurotrophic factor \|\| id:prot-a-242 | F-highly palatable foods liking (derived food-liking factor) \|\| id:ebi-fl187-GCST90094785 | MR Egger | 17 | 1.45599230419317 | 1.28182232981614 | 0.273831414710131 | -1.056379462 | 3.9683640706328 | 4.28873708660454 | 0.347712440136429 | 52.8979227513413 | 0.999279047927446 |
| ebi-fl187-GCST90094785 | prot-a-2464 | Dihydropteridine reductase \|\| id:prot-a-2464 | F-highly palatable foods liking (derived food-liking factor) \|\| id:ebi-fl187-GCST90094785 | MR Egger | 17 | 1.24179419128137 | 1.39382211796711 | 0.387035759880249 | -1.49009716 | 3.97368554249691 | 3.46181906166214 | 0.225350759410792 | 53.1801678726157 | 0.999279047927446 |
| ebi-fl187-GCST90094785 | prot-a-2474 | Ras-related protein Rab-27A \|\| id:prot-a-2474 | F-highly palatable foods liking (derived food-liking factor) \|\| id:ebi-fl187-GCST90094785 | MR Egger | 17 | 3.05175230281131 | 1.50757077393962 | 0.0611274824139272 | 0.0969135858896508 | 6.00659101973297 | 21.152377336898 | 1.10176516145099 | 406.096582699414 | 0.999279047927446 |
| ebi-fl187-GCST90094785 | prot-a-2478 | Ras-related protein Rab-7b \|\| id:prot-a-2478 | F-highly palatable foods liking (derived food-liking factor) \|\| id:ebi-fl187-GCST90094785 | MR Egger | 17 | 0.504384721342011 | 1.54673134473153 | 0.748855980868561 | -2.527208714 | 3.53597815701581 | 1.6559663262099 | 0.0798816819791941 | 34.3285770354129 | 0.999279047927446 |
| ebi-fl187-GCST90094785 | prot-a-2528 | E3 ubiquitin-protein ligase rififylin \|\| id:prot-a-2528 | F-highly palatable foods liking (derived food-liking factor) \|\| id:ebi-fl187-GCST90094785 | MR Egger | 17 | 0.400939913052279 | 1.28181846836121 | 0.758746893986753 | -2.111424285 | 2.91330411104024 | 1.49322754231903 | 0.121065411936713 | 18.417551780237 | 0.999279047927446 |
| ebi-fl187-GCST90094785 | prot-a-2543 | Protein RIC-3 \|\| id:prot-a-2543 | F-highly palatable foods liking (derived food-liking factor) \|\| id:ebi-fl187-GCST90094785 | MR Egger | 17 | 0.937295027392056 | 1.2817302345621 | 0.475881705719308 | -1.574896232 | 3.44948628713377 | 2.55306609581138 | 0.207029034483035 | 31.4842143077072 | 0.999279047927446 |
| ebi-fl187-GCST90094785 | prot-a-255 | Bone morphogenetic protein 6 \|\| id:prot-a-255 | F-highly palatable foods liking (derived food-liking factor) \|\| id:ebi-fl187-GCST90094785 | MR Egger | 17 | -0.179995836 | 1.45771969406361 | 0.90336820316379 | -3.037126436 | 2.67713476445513 | 0.835273689559232 | 0.047972543781938 | 14.5433633797125 | 0.999279047927446 |
| ebi-fl187-GCST90094785 | prot-a-2568 | RING finger protein 165 \|\| id:prot-a-2568 | F-highly palatable foods liking (derived food-liking factor) \|\| id:ebi-fl187-GCST90094785 | MR Egger | 17 | 1.21384184547156 | 1.28182232981614 | 0.358663051767542 | -1.298529921 | 3.72621361191119 | 3.36639300244112 | 0.272932730941484 | 41.5215932797529 | 0.999279047927446 |
| ebi-fl187-GCST90094785 | prot-a-2573 | E3 ubiquitin-protein ligase RNF8 \|\| id:prot-a-2573 | F-highly palatable foods liking (derived food-liking factor) \|\| id:ebi-fl187-GCST90094785 | MR Egger | 17 | -1.28939986 | 1.36280357269706 | 0.359071551472225 | -3.960494863 | 1.38169514246042 | 0.275436033672187 | 0.0190536830047731 | 3.98164536620356 | 0.999279047927446 |
| ebi-fl187-GCST90094785 | prot-a-260 | Cytoplasmic tyrosine-protein kinase BMX \|\| id:prot-a-260 | F-highly palatable foods liking (derived food-liking factor) \|\| id:ebi-fl187-GCST90094785 | MR Egger | 16 | -0.506021757 | 1.29026480484436 | 0.700827481578448 | -3.034940775 | 2.02289726039548 | 0.60288925423967 | 0.048077510201318 | 7.56019709331166 | 0.999279047927446 |
| ebi-fl187-GCST90094785 | prot-a-2623 | Serum amyloid A-1 protein \|\| id:prot-a-2623 | F-highly palatable foods liking (derived food-liking factor) \|\| id:ebi-fl187-GCST90094785 | MR Egger | 17 | 1.75912420813899 | 1.28181846836121 | 0.190123720785932 | -0.75323999 | 4.27148840612696 | 5.80734913749219 | 0.470838566571654 | 71.6281681220326 | 0.999279047927446 |
| ebi-fl187-GCST90094785 | prot-a-2640 | Lysosome membrane protein 2 \|\| id:prot-a-2640 | F-highly palatable foods liking (derived food-liking factor) \|\| id:ebi-fl187-GCST90094785 | MR Egger | 17 | 1.06137356509323 | 1.29929660925741 | 0.426779441944077 | -1.485247789 | 3.60799491923775 | 2.89033833178276 | 0.226446222829338 | 36.8920071520416 | 0.999279047927446 |
| ebi-fl187-GCST90094785 | prot-a-2641 | Scavenger receptor class F member 2 \|\| id:prot-a-2641 | F-highly palatable foods liking (derived food-liking factor) \|\| id:ebi-fl187-GCST90094785 | MR Egger | 17 | 1.80319266781534 | 1.45683288197992 | 0.234834166310998 | -1.052199781 | 4.65858511649598 | 6.06899284442029 | 0.349168808806775 | 105.486725092926 | 0.999279047927446 |
| ebi-fl187-GCST90094785 | prot-a-2669 | Semaphorin-3A \|\| id:prot-a-2669 | F-highly palatable foods liking (derived food-liking factor) \|\| id:ebi-fl187-GCST90094785 | MR Egger | 17 | -0.185334101 | 1.28182232981614 | 0.886962205077417 | -2.697705868 | 2.32703766520813 | 0.830826657267655 | 0.0673598680672791 | 10.2475398814187 | 0.999279047927446 |
| ebi-fl187-GCST90094785 | prot-a-2683 | Sentrin-specific protease 7 \|\| id:prot-a-2683 | F-highly palatable foods liking (derived food-liking factor) \|\| id:ebi-fl187-GCST90094785 | MR Egger | 17 | -0.64038994 | 1.56784391590034 | 0.688712822465528 | -3.713364016 | 2.43258413470876 | 0.527086851477957 | 0.0243953188470211 | 11.3882729200266 | 0.999279047927446 |
| ebi-fl187-GCST90094785 | prot-a-270 | Bromodomain testis-specific protein \|\| id:prot-a-270 | F-highly palatable foods liking (derived food-liking factor) \|\| id:ebi-fl187-GCST90094785 | MR Egger | 17 | 1.07862721556971 | 1.28182232981614 | 0.413293986204311 | -1.433744551 | 3.59099898200934 | 2.94063991423531 | 0.238414493472918 | 36.2702912026451 | 0.999279047927446 |
| ebi-fl187-GCST90094785 | prot-a-2708 | 14-3-3 protein sigma \|\| id:prot-a-2708 | F-highly palatable foods liking (derived food-liking factor) \|\| id:ebi-fl187-GCST90094785 | MR Egger | 17 | 0.772543023783359 | 1.28182232981614 | 0.555717071346491 | -1.739828743 | 3.28491479022299 | 2.16526558023009 | 0.175550462348649 | 26.7067085452494 | 0.999279047927446 |
| ebi-fl187-GCST90094785 | prot-a-2713 | Beta-sarcoglycan \|\| id:prot-a-2713 | F-highly palatable foods liking (derived food-liking factor) \|\| id:ebi-fl187-GCST90094785 | MR Egger | 17 | 1.03929119988918 | 1.28182232981614 | 0.430162373976482 | -1.473080567 | 3.55166296632881 | 2.82721237554627 | 0.229218274292352 | 34.8712590264391 | 0.999279047927446 |
| ebi-fl187-GCST90094785 | prot-a-2720 | Protein shisa-3 homolog \|\| id:prot-a-2720 | F-highly palatable foods liking (derived food-liking factor) \|\| id:ebi-fl187-GCST90094785 | MR Egger | 17 | 1.38972061745938 | 1.28182232981614 | 0.295406112340129 | -1.122651149 | 3.90209238389901 | 4.01372853066037 | 0.325415923909213 | 49.5059262137755 | 0.999279047927446 |
| ebi-fl187-GCST90094785 | prot-a-2730 | Sialic acid-binding Ig-like lectin 8 \|\| id:prot-a-2730 | F-highly palatable foods liking (derived food-liking factor) \|\| id:ebi-fl187-GCST90094785 | MR Egger | 17 | 1.46942533826227 | 1.28476016431645 | 0.270658602462001 | -1.048704584 | 3.98755526032252 | 4.34673652003851 | 0.350391357883699 | 53.922900635317 | 0.999279047927446 |
| ebi-fl187-GCST90094785 | prot-a-2761 | Sodium- and chloride-dependent neutral and basic amino acid transporter B(0+) \|\| id:prot-a-2761 | F-highly palatable foods liking (derived food-liking factor) \|\| id:ebi-fl187-GCST90094785 | MR Egger | 17 | -1.259894337 | 1.28182232981614 | 0.341248990072547 | -3.772266104 | 1.25247742900991 | 0.283683999696776 | 0.0229998840615159 | 3.49900075446973 | 0.999279047927446 |
| ebi-fl187-GCST90094785 | prot-a-2770 | Secreted Ly-6/uPAR-related protein 1 \|\| id:prot-a-2770 | F-highly palatable foods liking (derived food-liking factor) \|\| id:ebi-fl187-GCST90094785 | MR Egger | 17 | -0.051613172 | 1.28181846836121 | 0.968412444497864 | -2.56397737 | 2.46075102614539 | 0.949696165015298 | 0.0769978815510817 | 11.7136054612933 | 0.999279047927446 |
| ebi-fl187-GCST90094785 | prot-a-2784 | N-lysine methyltransferase SMYD2 \|\| id:prot-a-2784 | F-highly palatable foods liking (derived food-liking factor) \|\| id:ebi-fl187-GCST90094785 | MR Egger | 17 | -0.799279846 | 1.28181846836121 | 0.542288639038172 | -3.311644044 | 1.71308435208461 | 0.449652666755374 | 0.0364561888837369 | 5.54604106767768 | 0.999279047927446 |
| ebi-fl187-GCST90094785 | prot-a-2837 | Serine palmitoyltransferase 2 \|\| id:prot-a-2837 | F-highly palatable foods liking (derived food-liking factor) \|\| id:ebi-fl187-GCST90094785 | MR Egger | 17 | -1.160957199 | 1.28152241482559 | 0.379300163791718 | -3.672741132 | 1.3508267344985 | 0.313186255928559 | 0.0254067312145885 | 3.8606159160777 | 0.999279047927446 |
| ebi-fl187-GCST90094785 | prot-a-2858 | Alpha-2,8-sialyltransferase 8B \|\| id:prot-a-2858 | F-highly palatable foods liking (derived food-liking factor) \|\| id:ebi-fl187-GCST90094785 | MR Egger | 17 | -0.294402405 | 1.29923720036363 | 0.823796625502803 | -2.840907317 | 2.25210250801872 | 0.744976649017852 | 0.0583726793822318 | 9.50770486219626 | 0.999279047927446 |
| ebi-fl187-GCST90094785 | prot-a-2911 | Synaptotagmin-11 \|\| id:prot-a-2911 | F-highly palatable foods liking (derived food-liking factor) \|\| id:ebi-fl187-GCST90094785 | MR Egger | 17 | 1.2343158687878 | 1.28181846836121 | 0.350844841009717 | -1.278048329 | 3.74668006677576 | 3.43602702309791 | 0.278580467603075 | 42.3801489208526 | 0.999279047927446 |
| ebi-fl187-GCST90094785 | prot-a-2962 | Transforming growth factor beta-1 \|\| id:prot-a-2962 | F-highly palatable foods liking (derived food-liking factor) \|\| id:ebi-fl187-GCST90094785 | MR Egger | 17 | -0.072584864 | 1.28181846836121 | 0.955590161123814 | -2.584949062 | 2.43977933392052 | 0.929986820847055 | 0.0753999202202687 | 11.4705093111852 | 0.999279047927446 |
| ebi-fl187-GCST90094785 | prot-a-2978 | Tyrosine-protein kinase receptor Tie-1, soluble \|\| id:prot-a-2978 | F-highly palatable foods liking (derived food-liking factor) \|\| id:ebi-fl187-GCST90094785 | MR Egger | 17 | 1.63432934517451 | 1.43742388181204 | 0.273382316312218 | -1.183021463 | 4.45168015352612 | 5.1260190545699 | 0.3063517084064 | 85.770931340642 | 0.999279047927446 |
| ebi-fl187-GCST90094785 | prot-a-299 | Uncharacterized protein C1orf226 \|\| id:prot-a-299 | F-highly palatable foods liking (derived food-liking factor) \|\| id:ebi-fl187-GCST90094785 | MR Egger | 17 | -1.813370657 | 1.28181846836121 | 0.177583228353641 | -4.325734855 | 0.698993541043327 | 0.163103443470508 | 0.013223828929238 | 2.01172696760453 | 0.999279047927446 |
| ebi-fl187-GCST90094785 | prot-a-3015 | Transmembrane protein 70, mitochondrial \|\| id:prot-a-3015 | F-highly palatable foods liking (derived food-liking factor) \|\| id:ebi-fl187-GCST90094785 | MR Egger | 17 | -0.556651998 | 1.35641066539945 | 0.687324710556655 | -3.215216903 | 2.10191290581488 | 0.573124677676521 | 0.0401466249738031 | 8.1818059768699 | 0.999279047927446 |
| ebi-fl187-GCST90094785 | prot-a-3016 | Transmembrane protein 87B \|\| id:prot-a-3016 | F-highly palatable foods liking (derived food-liking factor) \|\| id:ebi-fl187-GCST90094785 | MR Egger | 17 | 3.22777664574192 | 1.28152241482559 | 0.0236168319202991 | 0.715992712683764 | 5.73956057880008 | 25.2235137742292 | 2.04621697989039 | 310.927752712136 | 0.999279047927446 |
| ebi-fl187-GCST90094785 | prot-a-3123 | Thioredoxin domain-containing protein 12 \|\| id:prot-a-3123 | F-highly palatable foods liking (derived food-liking factor) \|\| id:ebi-fl187-GCST90094785 | MR Egger | 17 | 0.458252312331134 | 1.38174844598524 | 0.7447444770303 | -2.249974642 | 3.1664792664622 | 1.58130793622305 | 0.105401897330376 | 23.7238119283967 | 0.999279047927446 |
| ebi-fl187-GCST90094785 | prot-a-3133 | PolyUbiquitin K48-linked \|\| id:prot-a-3133 | F-highly palatable foods liking (derived food-liking factor) \|\| id:ebi-fl187-GCST90094785 | MR Egger | 17 | -2.385450494 | 1.28181846836121 | 0.0824609368132996 | -4.897814692 | 0.126913703749047 | 0.0920475033676117 | 0.0074628739405913 | 1.13531903977721 | 0.999279047927446 |
| ebi-fl187-GCST90094785 | prot-a-3135 | Ubiquitin-conjugating enzyme E2 B \|\| id:prot-a-3135 | F-highly palatable foods liking (derived food-liking factor) \|\| id:ebi-fl187-GCST90094785 | MR Egger | 17 | -1.453699345 | 1.34396123923069 | 0.296490258109208 | -4.087863374 | 1.1804646841586 | 0.233704134821452 | 0.0167750372824572 | 3.25588681044308 | 0.999279047927446 |
| ebi-fl187-GCST90094785 | prot-a-3162 | NKG2D ligand 3 \|\| id:prot-a-3162 | F-highly palatable foods liking (derived food-liking factor) \|\| id:ebi-fl187-GCST90094785 | MR Egger | 17 | 0.00693643102299902 | 1.28181846836121 | 0.995753663174772 | -2.505427767 | 2.51930062901096 | 1.00696054378059 | 0.0816406673342536 | 12.4199075024644 | 0.999279047927446 |
| ebi-fl187-GCST90094785 | prot-a-3188 | Vesicle-associated membrane protein 8 \|\| id:prot-a-3188 | F-highly palatable foods liking (derived food-liking factor) \|\| id:ebi-fl187-GCST90094785 | MR Egger | 17 | 0.775499186177426 | 1.28182232981614 | 0.554223722607657 | -1.73687258 | 3.28787095261706 | 2.17167592725666 | 0.17607018583874 | 26.7857747213692 | 0.999279047927446 |
| ebi-fl187-GCST90094785 | prot-a-3201 | Vitrin \|\| id:prot-a-3201 | F-highly palatable foods liking (derived food-liking factor) \|\| id:ebi-fl187-GCST90094785 | MR Egger | 17 | 0.152596929630767 | 1.34196896227121 | 0.910974722608735 | -2.477662236 | 2.78285609568234 | 1.16485536562316 | 0.0839392264675788 | 16.1651242204998 | 0.999279047927446 |
| ebi-fl187-GCST90094785 | prot-a-3232 | WNT1-inducible-signaling pathway protein 3 \|\| id:prot-a-3232 | F-highly palatable foods liking (derived food-liking factor) \|\| id:ebi-fl187-GCST90094785 | MR Egger | 17 | -0.647904051 | 1.28182232981614 | 0.62058483909779 | -3.160275818 | 1.86446771498459 | 0.52314110532239 | 0.0424140409155581 | 6.45250040246796 | 0.999279047927446 |
| ebi-fl187-GCST90094785 | prot-a-327 | Carbonic anhydrase-related protein 10 \|\| id:prot-a-327 | F-highly palatable foods liking (derived food-liking factor) \|\| id:ebi-fl187-GCST90094785 | MR Egger | 17 | -2.199984396 | 1.44981279479461 | 0.149950283383973 | -5.041617474 | 0.641648681813893 | 0.110804887350131 | 0.0064632856639356 | 1.8996101517195 | 0.999279047927446 |
| ebi-fl187-GCST90094785 | prot-a-332 | Carbonic anhydrase 5A, mitochondrial \|\| id:prot-a-332 | F-highly palatable foods liking (derived food-liking factor) \|\| id:ebi-fl187-GCST90094785 | MR Egger | 17 | 1.15614159368912 | 1.28182232981614 | 0.381337591202022 | -1.356230173 | 3.66851336012875 | 3.17764893166845 | 0.257630169818156 | 39.1935957658249 | 0.999279047927446 |
| ebi-fl187-GCST90094785 | prot-a-338 | Cell adhesion molecule 3 \|\| id:prot-a-338 | F-highly palatable foods liking (derived food-liking factor) \|\| id:ebi-fl187-GCST90094785 | MR Egger | 17 | 1.83963321794015 | 1.28182232981614 | 0.17176137308071 | -0.672738548 | 4.35200498437978 | 6.29422922723532 | 0.510309156095357 | 77.633961867579 | 0.999279047927446 |
| ebi-fl187-GCST90094785 | prot-a-34 | A disintegrin and metalloproteinase with thrombospondin motifs 3 \|\| id:prot-a-34 | F-highly palatable foods liking (derived food-liking factor) \|\| id:ebi-fl187-GCST90094785 | MR Egger | 17 | -0.148693969 | 1.28182232981614 | 0.909189722956781 | -2.661065735 | 2.36367779772671 | 0.861832822353402 | 0.0698737151750704 | 10.6299745451439 | 0.999279047927446 |
| ebi-fl187-GCST90094785 | prot-a-358 | Cysteine--tRNA ligase, cytoplasmic \|\| id:prot-a-358 | F-highly palatable foods liking (derived food-liking factor) \|\| id:ebi-fl187-GCST90094785 | MR Egger | 17 | 0.867127076804346 | 1.31563483736137 | 0.519826034460039 | -1.711517204 | 3.44577135803264 | 2.38006328294921 | 0.180591590299507 | 31.3674696670437 | 0.999279047927446 |
| ebi-fl187-GCST90094785 | prot-a-382 | Coiled-coil domain-containing protein 167 \|\| id:prot-a-382 | F-highly palatable foods liking (derived food-liking factor) \|\| id:ebi-fl187-GCST90094785 | MR Egger | 17 | -0.385775934 | 1.28182232981614 | 0.767577295240156 | -2.8981477 | 2.12659583252342 | 0.679922856265308 | 0.0551252339983648 | 8.38626989747904 | 0.999279047927446 |
| ebi-fl187-GCST90094785 | prot-a-40 | Pituitary adenylate cyclase-activating polypeptide 27 \|\| id:prot-a-40 | F-highly palatable foods liking (derived food-liking factor) \|\| id:ebi-fl187-GCST90094785 | MR Egger | 17 | -0.17641526 | 1.28181846836121 | 0.892364173850912 | -2.688779458 | 2.33594893785161 | 0.838269811015008 | 0.0679638414832827 | 10.3392665971064 | 0.999279047927446 |
| ebi-fl187-GCST90094785 | prot-a-403 | C-C motif chemokine 25 \|\| id:prot-a-403 | F-highly palatable foods liking (derived food-liking factor) \|\| id:ebi-fl187-GCST90094785 | MR Egger | 17 | 1.45273536246198 | 1.2817302345621 | 0.274830157074917 | -1.059455897 | 3.9649266222037 | 4.27479164189089 | 0.346644369171946 | 52.7164010343865 | 0.999279047927446 |
| ebi-fl187-GCST90094785 | prot-a-415 | Cyclin-C \|\| id:prot-a-415 | F-highly palatable foods liking (derived food-liking factor) \|\| id:ebi-fl187-GCST90094785 | MR Egger | 17 | -1.315949767 | 1.28181846836121 | 0.320867732733155 | -3.828313965 | 1.19641443145725 | 0.268219456363842 | 0.0217462496865678 | 3.30823373266754 | 0.999279047927446 |
| ebi-fl187-GCST90094785 | prot-a-44 | Adhesion G protein-coupled receptor F5 \|\| id:prot-a-44 | F-highly palatable foods liking (derived food-liking factor) \|\| id:ebi-fl187-GCST90094785 | MR Egger | 17 | -0.15692158 | 1.37489801243163 | 0.910645836332901 | -2.851721685 | 2.53787852394539 | 0.854771086978613 | 0.0577448168694429 | 12.65279987 | 0.999279047927446 |
| ebi-fl187-GCST90094785 | prot-a-455 | CD59 glycoprotein \|\| id:prot-a-455 | F-highly palatable foods liking (derived food-liking factor) \|\| id:ebi-fl187-GCST90094785 | MR Egger | 17 | -0.670416522 | 1.28182232981614 | 0.608601396170242 | -3.182788288 | 1.84195524479831 | 0.511495484471912 | 0.0414698638394042 | 6.30886157833391 | 0.999279047927446 |
| ebi-fl187-GCST90094785 | prot-a-478 | Cadherin-12 \|\| id:prot-a-478 | F-highly palatable foods liking (derived food-liking factor) \|\| id:ebi-fl187-GCST90094785 | MR Egger | 17 | 0.373714548884515 | 1.28181846836121 | 0.77462365495435 | -2.138649649 | 2.88607874687248 | 1.45312229586902 | 0.117813825661554 | 17.9228914339612 | 0.999279047927446 |
| ebi-fl187-GCST90094785 | prot-a-516 | Centrin-2 \|\| id:prot-a-516 | F-highly palatable foods liking (derived food-liking factor) \|\| id:ebi-fl187-GCST90094785 | MR Egger | 17 | 0.135882628719205 | 1.46427415114895 | 0.92729164206672 | -2.734094708 | 3.00585996497114 | 1.14554743133478 | 0.0649527817597839 | 20.2035830011246 | 0.999279047927446 |
| ebi-fl187-GCST90094785 | prot-a-519 | Cryptic protein \|\| id:prot-a-519 | F-highly palatable foods liking (derived food-liking factor) \|\| id:ebi-fl187-GCST90094785 | MR Egger | 17 | 2.00331235062963 | 1.28181846836121 | 0.138931711019124 | -0.509051847 | 4.51567654861759 | 7.41357182347527 | 0.601065210287746 | 91.4394083056565 | 0.999279047927446 |
| ebi-fl187-GCST90094785 | prot-a-583 | Chloride intracellular channel protein 4 \|\| id:prot-a-583 | F-highly palatable foods liking (derived food-liking factor) \|\| id:ebi-fl187-GCST90094785 | MR Egger | 17 | 2.89958965070385 | 1.28182232981614 | 0.0389720821365772 | 0.387217884264218 | 5.41196141714348 | 18.1666891516141 | 1.47287737311704 | 224.070652964771 | 0.999279047927446 |
| ebi-fl187-GCST90094785 | prot-a-602 | CCR4-NOT transcription complex subunit 1 \|\| id:prot-a-602 | F-highly palatable foods liking (derived food-liking factor) \|\| id:ebi-fl187-GCST90094785 | MR Egger | 17 | 1.20303634589961 | 1.2817302345621 | 0.362803314624284 | -1.309154914 | 3.71522760564132 | 3.33021326638007 | 0.270048173955382 | 41.0679333140249 | 0.999279047927446 |
| ebi-fl187-GCST90094785 | prot-a-606 | Ciliary neurotrophic factor receptor subunit alpha \|\| id:prot-a-606 | F-highly palatable foods liking (derived food-liking factor) \|\| id:ebi-fl187-GCST90094785 | MR Egger | 17 | 1.45612320321135 | 1.28182232981614 | 0.273790004612418 | -1.056248563 | 3.96849496965098 | 4.28929851482281 | 0.34775795833253 | 52.9048474907041 | 0.999279047927446 |
| ebi-fl187-GCST90094785 | prot-a-626 | Collagen alpha-2(IX) chain \|\| id:prot-a-626 | F-highly palatable foods liking (derived food-liking factor) \|\| id:ebi-fl187-GCST90094785 | MR Egger | 17 | -0.838903351 | 1.34264212172596 | 0.541480857314292 | -3.47048191 | 1.79267520736819 | 0.432184217940982 | 0.0311020386717969 | 6.00549694533794 | 0.999279047927446 |
| ebi-fl187-GCST90094785 | prot-a-647 | Probable serine carboxypeptidase CPVL \|\| id:prot-a-647 | F-highly palatable foods liking (derived food-liking factor) \|\| id:ebi-fl187-GCST90094785 | MR Egger | 17 | 0.175109469036705 | 1.2920366512075 | 0.893995051055105 | -2.357282367 | 2.7075013054034 | 1.19137662832606 | 0.0946771716672079 | 14.9917688237532 | 0.999279047927446 |
| ebi-fl187-GCST90094785 | prot-a-651 | Cellular retinoic acid-binding protein 2 \|\| id:prot-a-651 | F-highly palatable foods liking (derived food-liking factor) \|\| id:ebi-fl187-GCST90094785 | MR Egger | 17 | -1.229804501 | 1.28181846836121 | 0.352556944229055 | -3.742168699 | 1.28255969651231 | 0.2923497260345 | 0.0237026434410557 | 3.60585782446553 | 0.999279047927446 |
| ebi-fl187-GCST90094785 | prot-a-671 | Cytotoxic and regulatory T-cell molecule \|\| id:prot-a-671 | F-highly palatable foods liking (derived food-liking factor) \|\| id:ebi-fl187-GCST90094785 | MR Egger | 17 | -0.127605037 | 1.56411859094041 | 0.936057188123996 | -3.193277476 | 2.9380674008609 | 0.88020095697407 | 0.0410371520251813 | 18.8793248659815 | 0.999279047927446 |
| ebi-fl187-GCST90094785 | prot-a-688 | Chorionic somatomammotropin hormone \|\| id:prot-a-688 | F-highly palatable foods liking (derived food-liking factor) \|\| id:ebi-fl187-GCST90094785 | MR Egger | 17 | -0.251722184 | 1.28181846836121 | 0.846949394644249 | -2.764086382 | 2.2606420136016 | 0.777460698790037 | 0.0630336617133673 | 9.58924361576335 | 0.999279047927446 |
| ebi-fl187-GCST90094785 | prot-a-69 | Aldo-keto reductase family 1 member C1 \|\| id:prot-a-69 | F-highly palatable foods liking (derived food-liking factor) \|\| id:ebi-fl187-GCST90094785 | MR Egger | 17 | -0.314015488 | 1.28182232981614 | 0.809794918013667 | -2.826387254 | 2.19835627852622 | 0.730507714031587 | 0.0592264376797013 | 9.01019107624887 | 0.999279047927446 |
| ebi-fl187-GCST90094785 | prot-a-741 | Stromal cell-derived factor 1 \|\| id:prot-a-741 | F-highly palatable foods liking (derived food-liking factor) \|\| id:ebi-fl187-GCST90094785 | MR Egger | 17 | 2.83805209219932 | 1.28182232981614 | 0.0427310027999837 | 0.325680325759691 | 5.35042385863895 | 17.0824580544259 | 1.38497255804861 | 210.697584934371 | 0.999279047927446 |
| ebi-fl187-GCST90094785 | prot-a-742 | Growth-regulated alpha protein \|\| id:prot-a-742 | F-highly palatable foods liking (derived food-liking factor) \|\| id:ebi-fl187-GCST90094785 | MR Egger | 17 | 1.63396712358114 | 1.28182232981614 | 0.221800652879437 | -0.878404643 | 4.14633889002077 | 5.12416263601803 | 0.415445166688073 | 63.2021860542614 | 0.999279047927446 |
| ebi-fl187-GCST90094785 | prot-a-788 | Beta-defensin 112 \|\| id:prot-a-788 | F-highly palatable foods liking (derived food-liking factor) \|\| id:ebi-fl187-GCST90094785 | MR Egger | 17 | 0.517262654621483 | 1.39066486814502 | 0.715129597469085 | -2.208440487 | 3.24296579618573 | 1.67742965510616 | 0.109871861579063 | 25.6095619696477 | 0.999279047927446 |
| ebi-fl187-GCST90094785 | prot-a-838 | DnaJ homolog subfamily B member 1 \|\| id:prot-a-838 | F-highly palatable foods liking (derived food-liking factor) \|\| id:ebi-fl187-GCST90094785 | MR Egger | 17 | 1.82593454170124 | 1.28182232981614 | 0.17477879170133 | -0.686437225 | 4.33830630814087 | 6.20859449922037 | 0.503366259005325 | 76.5777303626178 | 0.999279047927446 |
| ebi-fl187-GCST90094785 | prot-a-85 | Junctional adhesion molecule-like \|\| id:prot-a-85 | F-highly palatable foods liking (derived food-liking factor) \|\| id:ebi-fl187-GCST90094785 | MR Egger | 17 | -0.373796673 | 1.74284781505791 | 0.833065371653952 | -3.78977839 | 3.04218504459733 | 0.688116810390303 | 0.0226006098076244 | 20.9509720654522 | 0.999279047927446 |
| ebi-fl187-GCST90094785 | prot-a-856 | Dipeptidase 1 \|\| id:prot-a-856 | F-highly palatable foods liking (derived food-liking factor) \|\| id:ebi-fl187-GCST90094785 | MR Egger | 17 | -0.825397491 | 1.89003410148169 | 0.668542404223941 | -4.52986433 | 2.87906934789515 | 0.438060832611412 | 0.010782138787224 | 17.7977020009785 | 0.999279047927446 |
| ebi-fl187-GCST90094785 | prot-a-860 | Dipeptidyl peptidase 2 \|\| id:prot-a-860 | F-highly palatable foods liking (derived food-liking factor) \|\| id:ebi-fl187-GCST90094785 | MR Egger | 17 | 1.84453455486578 | 1.28181846836121 | 0.170691030008691 | -0.667829643 | 4.35689875285375 | 6.3251550924966 | 0.512820374078724 | 78.0148156476991 | 0.999279047927446 |
| ebi-fl187-GCST90094785 | prot-a-861 | Dermatopontin \|\| id:prot-a-861 | F-highly palatable foods liking (derived food-liking factor) \|\| id:ebi-fl187-GCST90094785 | MR Egger | 17 | 3.50276320085467 | 1.28181846836121 | 0.0154146122152035 | 0.990399002866709 | 6.01512739884264 | 33.2070831432287 | 2.6923084968739 | 409.578015358078 | 0.999279047927446 |
| ebi-fl187-GCST90094785 | prot-a-877 | Dual specificity protein phosphatase 6 \|\| id:prot-a-877 | F-highly palatable foods liking (derived food-liking factor) \|\| id:ebi-fl187-GCST90094785 | MR Egger | 17 | 2.42456774625944 | 1.28182232981614 | 0.0780274039201072 | -0.08780402 | 4.93693951269907 | 11.2973450440572 | 0.915940364962397 | 139.343138403693 | 0.999279047927446 |
| ebi-fl187-GCST90094785 | prot-a-879 | Dynactin-associated protein \|\| id:prot-a-879 | F-highly palatable foods liking (derived food-liking factor) \|\| id:ebi-fl187-GCST90094785 | MR Egger | 17 | -1.895388861 | 1.28181846836121 | 0.159919495245352 | -4.407753059 | 0.616975336964298 | 0.150259893468321 | 0.0121825210055108 | 1.85331390562741 | 0.999279047927446 |
| ebi-fl187-GCST90094785 | prot-a-937 | EMI domain-containing protein 1 \|\| id:prot-a-937 | F-highly palatable foods liking (derived food-liking factor) \|\| id:ebi-fl187-GCST90094785 | MR Egger | 17 | -1.820487355 | 1.59029760679439 | 0.270252351456593 | -4.937470664 | 1.29649595478043 | 0.161946806187724 | 0.00717271766181789 | 3.65646178630672 | 0.999279047927446 |
| ebi-fl187-GCST90094785 | prot-b-1 | SRC proto-oncogene, non-receptor tyrosine kinase \|\| id:prot-b-1 | F-highly palatable foods liking (derived food-liking factor) \|\| id:ebi-fl187-GCST90094785 | MR Egger | 12 | 0.599233114737136 | 1.93517623563348 | 0.763181094199728 | -3.193712307 | 4.39217853657876 | 1.82072198000424 | 0.0410193116587302 | 80.8162885825764 | 0.999279047927446 |
| ebi-fl187-GCST90094785 | prot-b-10 | coagulation factor III, tissue factor \|\| id:prot-b-10 | F-highly palatable foods liking (derived food-liking factor) \|\| id:ebi-fl187-GCST90094785 | MR Egger | 12 | -2.910840355 | 1.47666734897807 | 0.0769878559066664 | -5.805108359 | -0.016572351 | 0.054429970135182 | 0.003012128343537 | 0.983564214743232 | 0.999279047927446 |
| ebi-fl187-GCST90094785 | prot-b-29 | myeloperoxidase \|\| id:prot-b-29 | F-highly palatable foods liking (derived food-liking factor) \|\| id:ebi-fl187-GCST90094785 | MR Egger | 12 | -1.481796652 | 1.47664746797189 | 0.339289352576572 | -4.376025689 | 1.41243238495084 | 0.22722906979599 | 0.0125752373364691 | 4.10593047103862 | 0.999279047927446 |
| ebi-fl187-GCST90094785 | prot-b-33 | adrenomedullin \|\| id:prot-b-33 | F-highly palatable foods liking (derived food-liking factor) \|\| id:ebi-fl187-GCST90094785 | MR Egger | 12 | -0.894293193 | 1.47720410986333 | 0.558404292560614 | -3.789613248 | 2.0010268624221 | 0.408896507477966 | 0.0226043424299657 | 7.39664753998916 | 0.999279047927446 |
| ebi-fl187-GCST90094785 | ukb-rsfMRI191-edge-pheno102 | Net25_Pair11_15 (Postcentral\|Precentral)&(Cerebellum) (Motor)&(Subcortical-cerebellum) \|\| id:ukb-rsfMRI191-edge-pheno102 | F-highly palatable foods liking (derived food-liking factor) \|\| id:ebi-fl187-GCST90094785 | MR Egger | 17 | 0.165937471546786 | 0.40850802084685 | 0.690328888758392 | -0.634738249 | 0.966613192406612 | 1.18049928462118 | 0.530074217451111 | 2.62902536118849 | 0.999279047927446 |
| ebi-fl187-GCST90094785 | ukb-rsfMRI191-edge-pheno1167 | Net100_Pair11_45 (Angular\|Temporal)&(Frontal) (Default_mode\|Central_executive)&(Salience\|Default_mode) \|\| id:ukb-rsfMRI191-edge-pheno1167 | F-highly palatable foods liking (derived food-liking factor) \|\| id:ebi-fl187-GCST90094785 | MR Egger | 17 | 0.388710375451366 | 0.524414838206392 | 0.470002092219035 | -0.639142707 | 1.41656345833589 | 1.47507727087181 | 0.527744661741041 | 4.12292745484992 | 0.999279047927446 |
| ebi-fl187-GCST90094785 | ukb-rsfMRI191-edge-pheno1317 | Net100_Pair26_48 (Parietal\|Frontal)&(Temporal) (Attention\|Central_executive)&(Default_mode\|Central_executive) \|\| id:ukb-rsfMRI191-edge-pheno1317 | F-highly palatable foods liking (derived food-liking factor) \|\| id:ebi-fl187-GCST90094785 | MR Egger | 17 | 0.846424439218325 | 0.52663963253936 | 0.128847878374659 | -0.185789241 | 1.87863811899547 | 2.33129624035552 | 0.83044860142232 | 6.54458584310609 | 0.999279047927446 |
| ebi-fl187-GCST90094785 | ukb-rsfMRI191-edge-pheno286 | Net100_Pair10_13 (OccipitalPrecuneus)&(Frontal_Sup) (Default_mode\|Central_executive)&(Default_mode) \|\| id:ukb-rsfMRI191-edge-pheno286 | F-highly palatable foods liking (derived food-liking factor) \|\| id:ebi-fl187-GCST90094785 | MR Egger | 17 | -0.055691016 | 0.451372358023638 | 0.903443029573204 | -0.940380838 | 0.828998805240035 | 0.945831337035292 | 0.390479097676195 | 2.29102383057598 | 0.999279047927446 |
| ebi-fl187-GCST90094785 | ukb-rsfMRI191-edge-pheno599 | Net100_Pair11_29 (Angular\|Temporal)&(Parietal) (Default_mode\|Central_executive)&(Central_executive\|Salience) \|\| id:ukb-rsfMRI191-edge-pheno599 | F-highly palatable foods liking (derived food-liking factor) \|\| id:ebi-fl187-GCST90094785 | MR Egger | 17 | -0.381751316 | 0.468841734600164 | 0.428243379599414 | -1.300681116 | 0.53717848400426 | 0.68266480003927 | 0.272346230572466 | 1.71117194547936 | 0.999279047927446 |
| ebi-fl187-GCST90094785 | ukb-rsfMRI191-edge-pheno621 | Net100_Pair5_30 (Precuneus\|Angular\|Cingulate)&(Paracentral) (Default_mode\|Central_executive)&(Motor) \|\| id:ukb-rsfMRI191-edge-pheno621 | F-highly palatable foods liking (derived food-liking factor) \|\| id:ebi-fl187-GCST90094785 | MR Egger | 17 | -0.160632523 | 0.435145217149457 | 0.717177088369448 | -1.013517149 | 0.692252102398163 | 0.851604958666668 | 0.362940217331278 | 1.99821064460237 | 0.999279047927446 |
| ebi-fl187-GCST90094785 | ukb-rsfMRI191-edge-pheno624 | Net100_Pair8_30 (Calcarine\|Lingual)&(Paracentral) (Visual)&(Motor) \|\| id:ukb-rsfMRI191-edge-pheno624 | F-highly palatable foods liking (derived food-liking factor) \|\| id:ebi-fl187-GCST90094785 | MR Egger | 17 | 0.160377868122748 | 0.411044481314082 | 0.701894343177706 | -0.645269315 | 0.966025051498347 | 1.17395438713168 | 0.524521261506768 | 2.6274795784383 | 0.999279047927446 |
| ebi-fl187-GCST90094785 | ukb-rsfMRI191-edge-pheno882 | Net100_Pair6_38 (Postcentral\|Precentral)&(Subcortical) (Motor)&(Subcortical-cerebellum) \|\| id:ukb-rsfMRI191-edge-pheno882 | F-highly palatable foods liking (derived food-liking factor) \|\| id:ebi-fl187-GCST90094785 | MR Egger | 17 | 0.0962058586633863 | 0.469946802613109 | 0.840545567781768 | -0.824889874 | 1.01730159178508 | 1.10098568810907 | 0.438283255988358 | 2.76572163973612 | 0.999279047927446 |
| ebi-fl187-GCST90094785 | ukb-rsfMRI191-edge-pheno899 | Net100_Pair23_38 (Cerebellum)&(Subcortical) (Subcortical-cerebellum)&(Subcortical-cerebellum) \|\| id:ukb-rsfMRI191-edge-pheno899 | F-highly palatable foods liking (derived food-liking factor) \|\| id:ebi-fl187-GCST90094785 | MR Egger | 17 | 0.102036842320867 | 0.410780877781477 | 0.807195672635365 | -0.703093678 | 0.907167362772562 | 1.10742427105669 | 0.49505140261863 | 2.47729530638297 | 0.999279047927446 |
| ebi-fl187-GCST90094785 | ebi-a-GCST90001392 | IgD+ CD38+ B cell Absolute Count \|\| id:ebi-a-GCST90001392 | F-highly palatable foods liking (derived food-liking factor) \|\| id:ebi-fl187-GCST90094785 | Weighted median | 14 | 0.497340834563765 | 0.405112575411888 | 0.219574321911622 | -0.296679813 | 1.29136148237106 | 1.64434287210623 | 0.743281963306507 | 3.63773589906362 | 0.999489402457283 |
| ebi-fl187-GCST90094785 | ebi-a-GCST90001402 | Switched memory B cell %B cell \|\| id:ebi-a-GCST90001402 | F-highly palatable foods liking (derived food-liking factor) \|\| id:ebi-fl187-GCST90094785 | Weighted median | 14 | 0.0102370946059337 | 0.443075644146817 | 0.981566820053938 | -0.858191168 | 0.878665357133694 | 1.01028967292216 | 0.423928204150498 | 2.40768416260128 | 0.999911036302558 |
| ebi-fl187-GCST90094785 | ebi-a-GCST90001407 | Memory B cell Absolute Count \|\| id:ebi-a-GCST90001407 | F-highly palatable foods liking (derived food-liking factor) \|\| id:ebi-fl187-GCST90094785 | Weighted median | 14 | 0.325616884543397 | 0.427576914171963 | 0.446334650715063 | -0.512433867 | 1.16366763632044 | 1.38488469649204 | 0.599035829437723 | 3.20165427229631 | 0.999489402457283 |
| ebi-fl187-GCST90094785 | ebi-a-GCST90001411 | IgD+ CD24+ B cell %B cell \|\| id:ebi-a-GCST90001411 | F-highly palatable foods liking (derived food-liking factor) \|\| id:ebi-fl187-GCST90094785 | Weighted median | 14 | -0.140742133 | 0.471121987644861 | 0.76513980984042 | -1.064141229 | 0.782656962444949 | 0.868713295014283 | 0.345024024162041 | 2.18727606220297 | 0.999489402457283 |
| ebi-fl187-GCST90094785 | ebi-a-GCST90001418 | CD24+ CD27+ B cell Absolute Count \|\| id:ebi-a-GCST90001418 | F-highly palatable foods liking (derived food-liking factor) \|\| id:ebi-fl187-GCST90094785 | Weighted median | 14 | 0.169247850001803 | 0.41116892359405 | 0.680613516941705 | -0.63664324 | 0.975138940246141 | 1.18441365947557 | 0.529065392081582 | 2.65153559039823 | 0.999489402457283 |
| ebi-fl187-GCST90094785 | ebi-a-GCST90001436 | Memory B cell %lymphocyte \|\| id:ebi-a-GCST90001436 | F-highly palatable foods liking (derived food-liking factor) \|\| id:ebi-fl187-GCST90094785 | Weighted median | 14 | 0.0632073592563362 | 0.415562693879804 | 0.879107545091649 | -0.751295521 | 0.877710239260752 | 1.06524770530319 | 0.471754988303985 | 2.40538563828075 | 0.999489402457283 |
| ebi-fl187-GCST90094785 | ebi-a-GCST90001439 | IgD+ CD24+ B cell %lymphocyte \|\| id:ebi-a-GCST90001439 | F-highly palatable foods liking (derived food-liking factor) \|\| id:ebi-fl187-GCST90094785 | Weighted median | 14 | 0.392520472998192 | 0.443914421819873 | 0.376574469510733 | -0.477551794 | 1.26259273976514 | 1.48070817950661 | 0.62030015708126 | 3.5345738475616 | 0.999489402457283 |
| ebi-fl187-GCST90094785 | ebi-a-GCST90001457 | CD11c+ HLA DR++ monocyte %monocyte \|\| id:ebi-a-GCST90001457 | F-highly palatable foods liking (derived food-liking factor) \|\| id:ebi-fl187-GCST90094785 | Weighted median | 14 | -0.272898904 | 0.460863002764549 | 0.553751779842432 | -1.176190389 | 0.630392581727451 | 0.761169735190013 | 0.308451583653271 | 1.87834783957702 | 0.999489402457283 |
| ebi-fl187-GCST90094785 | ebi-a-GCST90001483 | CD39+ resting CD4 regulatory T cell Absolute Count \|\| id:ebi-a-GCST90001483 | F-highly palatable foods liking (derived food-liking factor) \|\| id:ebi-fl187-GCST90094785 | Weighted median | 14 | -0.141387231 | 0.41482229657469 | 0.733225487953123 | -0.954438933 | 0.67166446980003 | 0.86815307039642 | 0.385028110616008 | 1.95749279820868 | 0.999489402457283 |
| ebi-fl187-GCST90094785 | ebi-a-GCST90001525 | CD33dim HLA DR+ CD11b+ Absolute Count \|\| id:ebi-a-GCST90001525 | F-highly palatable foods liking (derived food-liking factor) \|\| id:ebi-fl187-GCST90094785 | Weighted median | 14 | 0.339518939295987 | 0.60646993509067 | 0.575596663583478 | -0.849162133 | 1.5282000120737 | 1.40427188802658 | 0.427773198678506 | 4.60987163663745 | 0.999489402457283 |
| ebi-fl187-GCST90094785 | ebi-a-GCST90001540 | Naive CD4+ T cell Absolute Count \|\| id:ebi-a-GCST90001540 | F-highly palatable foods liking (derived food-liking factor) \|\| id:ebi-fl187-GCST90094785 | Weighted median | 14 | 0.265694009692578 | 0.428000686760663 | 0.534744778736976 | -0.573187336 | 1.10457535574348 | 1.3043358834802 | 0.563725788476079 | 3.01794264465563 | 0.999489402457283 |
| ebi-fl187-GCST90094785 | ebi-a-GCST90001624 | HLA DR+ CD4+ T cell Absolute Count \|\| id:ebi-a-GCST90001624 | F-highly palatable foods liking (derived food-liking factor) \|\| id:ebi-fl187-GCST90094785 | Weighted median | 14 | 0.128073968191414 | 0.463701463084943 | 0.782395079854142 | -0.780780899 | 1.0369288358379 | 1.1366370745764 | 0.458048182033318 | 2.8205413534588 | 0.999489402457283 |
| ebi-fl187-GCST90094785 | ebi-a-GCST90001672 | CD39+ CD8+ T cell Absolute Count \|\| id:ebi-a-GCST90001672 | F-highly palatable foods liking (derived food-liking factor) \|\| id:ebi-fl187-GCST90094785 | Weighted median | 14 | 0.317725600676906 | 0.4774454470413 | 0.505749953598676 | -0.618067476 | 1.25351867687785 | 1.37399918504889 | 0.538985033551755 | 3.50264597900704 | 0.999489402457283 |
| ebi-fl187-GCST90094785 | ebi-a-GCST90001722 | CD19 on CD20- CD38- B cell \|\| id:ebi-a-GCST90001722 | F-highly palatable foods liking (derived food-liking factor) \|\| id:ebi-fl187-GCST90094785 | Weighted median | 14 | 0.149229903535724 | 0.444756074866278 | 0.737223795866249 | -0.722492003 | 1.02095181027363 | 1.16093986270953 | 0.485540777909989 | 2.77583557580796 | 0.999489402457283 |
| ebi-fl187-GCST90094785 | ebi-a-GCST90001742 | CD19 on transitional B cell \|\| id:ebi-a-GCST90001742 | F-highly palatable foods liking (derived food-liking factor) \|\| id:ebi-fl187-GCST90094785 | Weighted median | 14 | -0.372590916 | 0.43030592731542 | 0.386559005326591 | -1.215990534 | 0.470808701142291 | 0.688947012131117 | 0.296416258467858 | 1.60128863368627 | 0.999489402457283 |
| ebi-fl187-GCST90094785 | ebi-a-GCST90001784 | CD25 on IgD+ CD38dim B cell \|\| id:ebi-a-GCST90001784 | F-highly palatable foods liking (derived food-liking factor) \|\| id:ebi-fl187-GCST90094785 | Weighted median | 14 | -0.061606647 | 0.430357882246343 | 0.886170035502148 | -0.905108096 | 0.781894801931863 | 0.940252665011446 | 0.404498157915155 | 2.18560964187769 | 0.999489402457283 |
| ebi-fl187-GCST90094785 | ebi-a-GCST90001794 | CD25 on IgD+ B cell \|\| id:ebi-a-GCST90001794 | F-highly palatable foods liking (derived food-liking factor) \|\| id:ebi-fl187-GCST90094785 | Weighted median | 14 | -0.260420267 | 0.447514560360927 | 0.560616663651382 | -1.137548805 | 0.616708271287821 | 0.770727606335357 | 0.320603922077573 | 1.85281901518254 | 0.999489402457283 |
| ebi-fl187-GCST90094785 | ebi-a-GCST90001802 | CD27 on IgD- CD38- B cell \|\| id:ebi-a-GCST90001802 | F-highly palatable foods liking (derived food-liking factor) \|\| id:ebi-fl187-GCST90094785 | Weighted median | 14 | 0.294222941826494 | 0.415965063858559 | 0.479363856317478 | -0.521068583 | 1.10951446698927 | 1.34208307676908 | 0.593885592531377 | 3.03288547087423 | 0.999489402457283 |
| ebi-fl187-GCST90094785 | ebi-a-GCST90001804 | CD27 on IgD- CD38dim B cell \|\| id:ebi-a-GCST90001804 | F-highly palatable foods liking (derived food-liking factor) \|\| id:ebi-fl187-GCST90094785 | Weighted median | 14 | 0.115839466136387 | 0.392652223571677 | 0.76798014404644 | -0.653758892 | 0.885437824336873 | 1.12281560773674 | 0.52008714647973 | 2.42404546528502 | 0.999489402457283 |
| ebi-fl187-GCST90094785 | ebi-a-GCST90001805 | CD27 on memory B cell \|\| id:ebi-a-GCST90001805 | F-highly palatable foods liking (derived food-liking factor) \|\| id:ebi-fl187-GCST90094785 | Weighted median | 14 | 0.438420972456851 | 0.388562844163292 | 0.259187250964436 | -0.323162202 | 1.2000041470169 | 1.5502573857326 | 0.723856433809262 | 3.3201306913461 | 0.999489402457283 |
| ebi-fl187-GCST90094785 | ebi-a-GCST90001806 | CD27 on unswitched memory B cell \|\| id:ebi-a-GCST90001806 | F-highly palatable foods liking (derived food-liking factor) \|\| id:ebi-fl187-GCST90094785 | Weighted median | 14 | 0.189189778844586 | 0.426636108800439 | 0.657443673359205 | -0.647016994 | 1.02539655209345 | 1.20827023485486 | 0.523605367211361 | 2.78820090827469 | 0.999489402457283 |
| ebi-fl187-GCST90094785 | ebi-a-GCST90001839 | CD3 on Effector Memory CD8+ T cell \|\| id:ebi-a-GCST90001839 | F-highly palatable foods liking (derived food-liking factor) \|\| id:ebi-fl187-GCST90094785 | Weighted median | 14 | -0.413718186 | 0.473599326296739 | 0.382357045545625 | -1.341972865 | 0.514536493924267 | 0.661187257091527 | 0.261329591626272 | 1.67286293993607 | 0.999489402457283 |
| ebi-fl187-GCST90094785 | ebi-a-GCST90001859 | CD3 on CD8+ T cell \|\| id:ebi-a-GCST90001859 | F-highly palatable foods liking (derived food-liking factor) \|\| id:ebi-fl187-GCST90094785 | Weighted median | 14 | -0.417237067 | 0.467577586053632 | 0.37221199968922 | -1.333689136 | 0.499215001816201 | 0.658864706448511 | 0.263503366362245 | 1.64742753535342 | 0.999489402457283 |
| ebi-fl187-GCST90094785 | ebi-a-GCST90001905 | CD86 on monocyte \|\| id:ebi-a-GCST90001905 | F-highly palatable foods liking (derived food-liking factor) \|\| id:ebi-fl187-GCST90094785 | Weighted median | 14 | -0.349138523 | 0.468368180812807 | 0.456008162132519 | -1.267140157 | 0.568863111437954 | 0.705295423896453 | 0.281635905542643 | 1.76625787117176 | 0.999489402457283 |
| ebi-fl187-GCST90094785 | ebi-a-GCST90001943 | CD25 on activated & secreting CD4 regulatory T cell \|\| id:ebi-a-GCST90001943 | F-highly palatable foods liking (derived food-liking factor) \|\| id:ebi-fl187-GCST90094785 | Weighted median | 14 | -0.865061136 | 0.403608819414102 | 0.0320877535944821 | -1.656134422 | -0.07398785 | 0.421025811825195 | 0.190875399494387 | 0.928682976919072 | 0.999489402457283 |
| ebi-fl187-GCST90094785 | ebi-a-GCST90001949 | CD33 on CD33dim HLA DR+ CD11b- \|\| id:ebi-a-GCST90001949 | F-highly palatable foods liking (derived food-liking factor) \|\| id:ebi-fl187-GCST90094785 | Weighted median | 14 | -0.605058464 | 0.636594139072472 | 0.341877613216234 | -1.852782976 | 0.642666048736847 | 0.546042501962193 | 0.156800187319442 | 1.90154373566976 | 0.999489402457283 |
| ebi-fl187-GCST90094785 | ebi-a-GCST90001955 | CD33 on Immature Myeloid-Derived Suppressor Cells \|\| id:ebi-a-GCST90001955 | F-highly palatable foods liking (derived food-liking factor) \|\| id:ebi-fl187-GCST90094785 | Weighted median | 14 | -0.345652841 | 0.634768117910073 | 0.586074038049545 | -1.589798352 | 0.898492670244887 | 0.707758149173323 | 0.203966737079879 | 2.45589847096036 | 0.999489402457283 |
| ebi-fl187-GCST90094785 | ebi-a-GCST90001956 | CD33 on CD33+ HLA DR+ \|\| id:ebi-a-GCST90001956 | F-highly palatable foods liking (derived food-liking factor) \|\| id:ebi-fl187-GCST90094785 | Weighted median | 14 | -0.122867979 | 0.645454784930814 | 0.84902800303186 | -1.387959358 | 1.14222339899554 | 0.884380411225666 | 0.249584097131217 | 3.13372815315424 | 0.999489402457283 |
| ebi-fl187-GCST90094785 | ebi-a-GCST90001957 | CD33 on CD33+ HLA DR+ CD14- \|\| id:ebi-a-GCST90001957 | F-highly palatable foods liking (derived food-liking factor) \|\| id:ebi-fl187-GCST90094785 | Weighted median | 14 | -0.098629664 | 0.630055090135328 | 0.875606341535548 | -1.333537641 | 1.13627831279075 | 0.906078199387194 | 0.263543288821909 | 3.11515313888156 | 0.999489402457283 |
| ebi-fl187-GCST90094785 | ebi-a-GCST90002064 | CD4 on resting CD4 regulatory T cell \|\| id:ebi-a-GCST90002064 | F-highly palatable foods liking (derived food-liking factor) \|\| id:ebi-fl187-GCST90094785 | Weighted median | 14 | -0.094505342 | 0.460136869152276 | 0.837271115553126 | -0.996373606 | 0.807362921212807 | 0.909822874042966 | 0.369215938888701 | 2.24198788552661 | 0.999489402457283 |
| ebi-fl187-GCST90094785 | ebi-a-GCST90002080 | SSC-A on T cell \|\| id:ebi-a-GCST90002080 | F-highly palatable foods liking (derived food-liking factor) \|\| id:ebi-fl187-GCST90094785 | Weighted median | 14 | -0.202033535 | 0.398473619166297 | 0.612141770357982 | -0.983041829 | 0.578974758446431 | 0.817067527025625 | 0.374171201350332 | 1.78420824828447 | 0.999489402457283 |
| ebi-fl187-GCST90094785 | ebi-a-GCST90002121 | CD8 on CD39+ CD8+ T cell \|\| id:ebi-a-GCST90002121 | F-highly palatable foods liking (derived food-liking factor) \|\| id:ebi-fl187-GCST90094785 | Weighted median | 14 | -0.31792002 | 0.435790377887263 | 0.465680518279332 | -1.172069161 | 0.536229120547852 | 0.727660984331936 | 0.309725406114819 | 1.70954819225463 | 0.999489402457283 |
| ebi-fl187-GCST90094785 | ebi-cfmet338-GCST90026053 | 1-palmitoyl-2-docosahexaenoyl-gpc (16:0/22:6) levels \|\| id:ebi-cfmet338-GCST90026053 | F-highly palatable foods liking (derived food-liking factor) \|\| id:ebi-fl187-GCST90094785 | Weighted median | 13 | -0.186203201 | 0.238819095987598 | 0.435577459355017 | -0.654288629 | 0.281882227450655 | 0.830104899958716 | 0.5198117101624 | 1.32562258884123 | 0.999489402457283 |
| ebi-fl187-GCST90094785 | ebi-cfmet338-GCST90026063 | 1-stearoyl-2-oleoyl-gpc (18:0/18:1) levels \|\| id:ebi-cfmet338-GCST90026063 | F-highly palatable foods liking (derived food-liking factor) \|\| id:ebi-fl187-GCST90094785 | Weighted median | 13 | -0.319645169 | 0.217968287864201 | 0.142518771878395 | -0.746863013 | 0.107572674981394 | 0.726406742812138 | 0.473850686902774 | 1.11357178661473 | 0.999489402457283 |
| ebi-fl187-GCST90094785 | ebi-cfmet338-GCST90026113 | Adenosine 3',5'-cyclic monophosphate (camp) levels \|\| id:ebi-cfmet338-GCST90026113 | F-highly palatable foods liking (derived food-liking factor) \|\| id:ebi-fl187-GCST90094785 | Weighted median | 13 | 0.532345873949467 | 0.400283769758156 | 0.183544233555747 | -0.252210315 | 1.31690206267545 | 1.7029224680389 | 0.777081289204018 | 3.73184243713061 | 0.999489402457283 |
| ebi-fl187-GCST90094785 | ebi-cfmet338-GCST90026117 | Alpha-ketoglutaramate levels \|\| id:ebi-cfmet338-GCST90026117 | F-highly palatable foods liking (derived food-liking factor) \|\| id:ebi-fl187-GCST90094785 | Weighted median | 13 | -0.115420326 | 0.16023999486421 | 0.471342387364432 | -0.429490716 | 0.198650063760963 | 0.890991558116541 | 0.650840472903299 | 1.21975505471199 | 0.999489402457283 |
| ebi-fl187-GCST90094785 | ebi-cfmet338-GCST90026124 | Arginine levels \|\| id:ebi-cfmet338-GCST90026124 | F-highly palatable foods liking (derived food-liking factor) \|\| id:ebi-fl187-GCST90094785 | Weighted median | 13 | 0.0543247986056396 | 0.126630136919286 | 0.66792052437835 | -0.19387027 | 0.302519866967439 | 1.05582747776188 | 0.823764764540693 | 1.35326456141709 | 0.999489402457283 |
| ebi-fl187-GCST90094785 | ebi-cfmet338-GCST90026169 | Glutamate levels \|\| id:ebi-cfmet338-GCST90026169 | F-highly palatable foods liking (derived food-liking factor) \|\| id:ebi-fl187-GCST90094785 | Weighted median | 13 | 0.163875773723998 | 0.122298447279282 | 0.180256365648673 | -0.075829183 | 0.403580730391392 | 1.17806795900685 | 0.926974536098109 | 1.49717609491236 | 0.999489402457283 |
| ebi-fl187-GCST90094785 | ebi-cfmet338-GCST90026170 | Glutamine levels \|\| id:ebi-cfmet338-GCST90026170 | F-highly palatable foods liking (derived food-liking factor) \|\| id:ebi-fl187-GCST90094785 | Weighted median | 13 | -0.035066641 | 0.0809855941444962 | 0.665015857867821 | -0.193798405 | 0.123665123548948 | 0.965541069515961 | 0.823823965912032 | 1.13163684900807 | 0.999489402457283 |
| ebi-fl187-GCST90094785 | ebi-cfmet338-GCST90026191 | Kynurenate levels \|\| id:ebi-cfmet338-GCST90026191 | F-highly palatable foods liking (derived food-liking factor) \|\| id:ebi-fl187-GCST90094785 | Weighted median | 13 | 0.481024986969398 | 0.73511586008849 | 0.512884653868819 | -0.959802099 | 1.92185207274284 | 1.61773170660934 | 0.382968668433642 | 6.83360308631259 | 0.999489402457283 |
| ebi-fl187-GCST90094785 | ebi-cfmet338-GCST90026202 | Methylsuccinoylcarnitine levels \|\| id:ebi-cfmet338-GCST90026202 | F-highly palatable foods liking (derived food-liking factor) \|\| id:ebi-fl187-GCST90094785 | Weighted median | 13 | 0.281869653145785 | 0.378924433239563 | 0.456956507532599 | -0.460822236 | 1.02456154229533 | 1.32560592016346 | 0.630764794703144 | 2.78587370495116 | 0.999489402457283 |
| ebi-fl187-GCST90094785 | ebi-cfmet338-GCST90026222 | N-acetylputrescine levels \|\| id:ebi-cfmet338-GCST90026222 | F-highly palatable foods liking (derived food-liking factor) \|\| id:ebi-fl187-GCST90094785 | Weighted median | 13 | -0.129358587 | 0.146795929285954 | 0.378202153536981 | -0.417078609 | 0.158361434162805 | 0.878658833203894 | 0.658969117243198 | 1.17158957068742 | 0.999489402457283 |
| ebi-fl187-GCST90094785 | ebi-cfmet338-GCST90026252 | Propionylcarnitine (c3) levels \|\| id:ebi-cfmet338-GCST90026252 | F-highly palatable foods liking (derived food-liking factor) \|\| id:ebi-fl187-GCST90094785 | Weighted median | 13 | 0.1830001566096 | 0.233426142005451 | 0.433055038838458 | -0.274515082 | 0.640515394940285 | 1.20081459613991 | 0.759940542951186 | 1.89745856788072 | 0.999489402457283 |
| ebi-fl187-GCST90094785 | ebi-cfmet338-GCST90026316 | Ethyl beta-glucopyranoside levels \|\| id:ebi-cfmet338-GCST90026316 | F-highly palatable foods liking (derived food-liking factor) \|\| id:ebi-fl187-GCST90094785 | Weighted median | 13 | 0.946312760838899 | 0.869296609577363 | 0.276332107455845 | -0.757508594 | 2.65013411561053 | 2.57619308512536 | 0.468833026616855 | 14.1559370502102 | 0.999489402457283 |
| ebi-fl187-GCST90094785 | ebi-gm430-GCST90011315 | F_Prevotellaceae abundance \|\| id:ebi-gm430-GCST90011315 | F-highly palatable foods liking (derived food-liking factor) \|\| id:ebi-fl187-GCST90094785 | Weighted median | 9 | -0.060928206 | 0.420065916369128 | 0.884675821118456 | -0.884257402 | 0.762400989849856 | 0.940890787444599 | 0.413020767691877 | 2.14341636825041 | 0.999489402457283 |
| ebi-fl187-GCST90094785 | ebi-gm430-GCST90011354 | OTU97_1 (Bacteroides) abundance \|\| id:ebi-gm430-GCST90011354 | F-highly palatable foods liking (derived food-liking factor) \|\| id:ebi-fl187-GCST90094785 | Weighted median | 9 | -0.276934513 | 0.357010234784959 | 0.437923371018875 | -0.976674573 | 0.422805547258353 | 0.758104141511394 | 0.376561245960236 | 1.52623748604607 | 0.999489402457283 |
| ebi-fl187-GCST90094785 | ebi-gm430-GCST90011419 | OTU99_1 (Bacteroides) abundance \|\| id:ebi-gm430-GCST90011419 | F-highly palatable foods liking (derived food-liking factor) \|\| id:ebi-fl187-GCST90094785 | Weighted median | 9 | -0.290929673 | 0.355405225824251 | 0.413022769032528 | -0.987523915 | 0.405664570054162 | 0.747568250729692 | 0.372497886435639 | 1.50029922275978 | 0.999489402457283 |
| ebi-fl187-GCST90094785 | ebi-gm430-GCST90011435 | OTU99_155 (Butyrivibrio) abundance \|\| id:ebi-gm430-GCST90011435 | F-highly palatable foods liking (derived food-liking factor) \|\| id:ebi-fl187-GCST90094785 | Weighted median | 9 | -1.713526779 | 1.08939879159558 | 0.115739547402087 | -3.848748411 | 0.421694852031224 | 0.180229042347073 | 0.0213063865959706 | 1.52454324242318 | 0.999489402457283 |
| ebi-fl187-GCST90094785 | ebi-gm430-GCST90011466 | OTU99_58 (Bacteroides) abundance \|\| id:ebi-gm430-GCST90011466 | F-highly palatable foods liking (derived food-liking factor) \|\| id:ebi-fl187-GCST90094785 | Weighted median | 9 | 0.560396440174806 | 0.599314147581404 | 0.349755871519499 | -0.614259289 | 1.73505216943436 | 1.75136667479839 | 0.541041502270364 | 5.66922355627647 | 0.999489402457283 |
| ebi-fl187-GCST90094785 | ebi-gm430-GCST90011472 | OTU99_72 (Parabacteroides) abundance \|\| id:ebi-gm430-GCST90011472 | F-highly palatable foods liking (derived food-liking factor) \|\| id:ebi-fl187-GCST90094785 | Weighted median | 8 | -0.26340755 | 0.9665683708056 | 0.785223537637287 | -2.157881557 | 1.6310664569447 | 0.768428660512446 | 0.11556968973906 | 5.10932068460319 | 0.999489402457283 |
| ebi-fl187-GCST90094785 | ebi-gm430-GCST90011505 | TestASV_28 (Holdemanella) abundance \|\| id:ebi-gm430-GCST90011505 | F-highly palatable foods liking (derived food-liking factor) \|\| id:ebi-fl187-GCST90094785 | Weighted median | 9 | -1.564686421 | 0.823689826859976 | 0.0574847990282792 | -3.179118482 | 0.0497456397714144 | 0.209153589090764 | 0.0416223298070099 | 1.05100372882492 | 0.999489402457283 |
| ebi-fl187-GCST90094785 | ebi-gm430-GCST90011513 | TestASV_35 (Alphaproteobacteria) abundance \|\| id:ebi-gm430-GCST90011513 | F-highly palatable foods liking (derived food-liking factor) \|\| id:ebi-fl187-GCST90094785 | Weighted median | 9 | -0.675528783 | 1.00620278598456 | 0.501988384349888 | -2.647686243 | 1.29662867770004 | 0.508887258605097 | 0.0708148720343193 | 3.65694711479683 | 0.999489402457283 |
| ebi-fl187-GCST90094785 | ebi-gm430-GCST90011543 | G_Coprococcus prevalence \|\| id:ebi-gm430-GCST90011543 | F-highly palatable foods liking (derived food-liking factor) \|\| id:ebi-fl187-GCST90094785 | Weighted median | 9 | 0.642744033915384 | 1.11646363980999 | 0.564820447999242 | -1.5455247 | 2.83101276794296 | 1.90169203367993 | 0.213199975818485 | 16.9626313374477 | 0.999489402457283 |
| ebi-fl187-GCST90094785 | ebi-gm430-GCST90011547 | G_Parasutterella prevalence \|\| id:ebi-gm430-GCST90011547 | F-highly palatable foods liking (derived food-liking factor) \|\| id:ebi-fl187-GCST90094785 | Weighted median | 9 | 0.60936851296829 | 0.975445271834037 | 0.532162689225192 | -1.30250422 | 2.521241246 | 1.83926955710314 | 0.271850167341066 | 12.4440331848027 | 0.999489402457283 |
| ebi-fl187-GCST90094785 | ebi-gm430-GCST90011569 | OTU97_13 (Subdoligranulum) prevalence \|\| id:ebi-gm430-GCST90011569 | F-highly palatable foods liking (derived food-liking factor) \|\| id:ebi-fl187-GCST90094785 | Weighted median | 9 | -0.4351134 | 0.952294212036965 | 0.647735244348834 | -2.301610055 | 1.4313832558043 | 0.647191271563505 | 0.100097551311715 | 4.18448340143328 | 0.999489402457283 |
| ebi-fl187-GCST90094785 | ebi-gm430-GCST90011590 | OTU97_277 (Prevotella) prevalence \|\| id:ebi-gm430-GCST90011590 | F-highly palatable foods liking (derived food-liking factor) \|\| id:ebi-fl187-GCST90094785 | Weighted median | 9 | 0.770637802563284 | 0.817087191632665 | 0.345602921030344 | -0.830853093 | 2.37212869816331 | 2.16114419761902 | 0.435677454337888 | 10.7201880574709 | 0.999489402457283 |
| ebi-fl187-GCST90094785 | ebi-gm430-GCST90011616 | OTU97_86 (Phascolarctobacterium) prevalence \|\| id:ebi-gm430-GCST90011616 | F-highly palatable foods liking (derived food-liking factor) \|\| id:ebi-fl187-GCST90094785 | Weighted median | 9 | 0.673814935309823 | 1.02804863468674 | 0.512190303733821 | -1.341160389 | 2.68879025929583 | 1.96170684841773 | 0.261542002051271 | 14.7138651877973 | 0.999489402457283 |
| ebi-fl187-GCST90094785 | ebi-gm430-GCST90011665 | OTU99_55 (Barnesiella) prevalence \|\| id:ebi-gm430-GCST90011665 | F-highly palatable foods liking (derived food-liking factor) \|\| id:ebi-fl187-GCST90094785 | Weighted median | 9 | 0.73598168650235 | 0.635210574497584 | 0.246602118398067 | -0.50903104 | 1.98099441251762 | 2.08753028725512 | 0.601077717289849 | 7.24994884165048 | 0.999489402457283 |
| ebi-fl187-GCST90094785 | ebi-gm430-GCST90011687 | TestASV_14 (Lachnospiraceae) prevalence \|\| id:ebi-gm430-GCST90011687 | F-highly palatable foods liking (derived food-liking factor) \|\| id:ebi-fl187-GCST90094785 | Weighted median | 9 | -0.202177918 | 0.767357415304059 | 0.79218607363081 | -1.706198452 | 1.30184261570923 | 0.816949564744331 | 0.181554671130831 | 3.67606400418643 | 0.999489402457283 |
| ebi-fl187-GCST90094785 | ebi-gm430-GCST90011697 | TestASV_23 (Barnesiella) prevalence \|\| id:ebi-gm430-GCST90011697 | F-highly palatable foods liking (derived food-liking factor) \|\| id:ebi-fl187-GCST90094785 | Weighted median | 9 | 1.55969122937321 | 0.990829674632286 | 0.115458211527592 | -0.382334933 | 3.50171739165249 | 4.75735208774722 | 0.682266501430735 | 33.1723730233449 | 0.999489402457283 |
| ebi-fl187-GCST90094785 | ebi-gm430-GCST90011727 | TestASV_6 (Subdoligranulum) prevalence \|\| id:ebi-gm430-GCST90011727 | F-highly palatable foods liking (derived food-liking factor) \|\| id:ebi-fl187-GCST90094785 | Weighted median | 9 | -1.156865539 | 1.19080801523479 | 0.331301212338674 | -3.490849249 | 1.17711817117816 | 0.314470332777562 | 0.0304749803458434 | 3.24500915422965 | 0.999489402457283 |
| ebi-fl187-GCST90094785 | ebi-hbsm44-GCST90104908 | Sulcal depth of the left posterior inferior temporal sulcus \|\| id:ebi-hbsm44-GCST90104908 | F-highly palatable foods liking (derived food-liking factor) \|\| id:ebi-fl187-GCST90094785 | Weighted median | 12 | -0.05678095 | 0.281119129972844 | 0.839930947399337 | -0.607774444 | 0.494212545047802 | 0.944801005645667 | 0.544561473252817 | 1.63920692908557 | 0.999489402457283 |
| ebi-fl187-GCST90094785 | ebi-hbsm44-GCST90104918 | Sulcal depth of the left inferior frontal sulcus \|\| id:ebi-hbsm44-GCST90104918 | F-highly palatable foods liking (derived food-liking factor) \|\| id:ebi-fl187-GCST90094785 | Weighted median | 12 | 0.0454204230439121 | 0.464481421766761 | 0.922101125660544 | -0.864963164 | 0.955804009706763 | 1.04646772658416 | 0.42106706292843 | 2.60076077944942 | 0.999489402457283 |
| ebi-fl187-GCST90094785 | ebi-hbsm44-GCST90104925 | Sulcal depth of the right anterior inferior temporal sulcus \|\| id:ebi-hbsm44-GCST90104925 | F-highly palatable foods liking (derived food-liking factor) \|\| id:ebi-fl187-GCST90094785 | Weighted median | 12 | -0.106646809 | 0.159667249213046 | 0.504177224572337 | -0.419594617 | 0.206300999585181 | 0.898843080335365 | 0.657313229204441 | 1.22912311386857 | 0.999489402457283 |
| ebi-fl187-GCST90094785 | ebi-IPs91-GCST90274770 | C-C motif chemokine 4 levels \|\| id:ebi-IPs91-GCST90274770 | F-highly palatable foods liking (derived food-liking factor) \|\| id:ebi-fl187-GCST90094785 | Weighted median | 17 | 0.0503478031770617 | 0.183014802324204 | 0.78323760209402 | -0.308361209 | 0.409056815732502 | 1.05163679539525 | 0.734649907641132 | 1.50539724830324 | 0.999489402457283 |
| ebi-fl187-GCST90094785 | ebi-IPs91-GCST90274779 | C-X-C motif chemokine 1 levels \|\| id:ebi-IPs91-GCST90274779 | F-highly palatable foods liking (derived food-liking factor) \|\| id:ebi-fl187-GCST90094785 | Weighted median | 17 | -0.046999073 | 0.167396366663479 | 0.778890502984272 | -0.375095952 | 0.281097805652476 | 0.95408828202222 | 0.687223335401764 | 1.3245831493192 | 0.999489402457283 |
| ebi-fl187-GCST90094785 | ebi-IPs91-GCST90274780 | C-X-C motif chemokine 10 levels \|\| id:ebi-IPs91-GCST90274780 | F-highly palatable foods liking (derived food-liking factor) \|\| id:ebi-fl187-GCST90094785 | Weighted median | 17 | -0.081919414 | 0.183776402266811 | 0.655773586406994 | -0.442121163 | 0.278282334360163 | 0.921346203233635 | 0.64267176300399 | 1.3208590684694 | 0.999489402457283 |
| ebi-fl187-GCST90094785 | ebi-IPs91-GCST90274796 | Interleukin-10 receptor subunit alpha levels \|\| id:ebi-IPs91-GCST90274796 | F-highly palatable foods liking (derived food-liking factor) \|\| id:ebi-fl187-GCST90094785 | Weighted median | 17 | -0.168870808 | 0.233810453746839 | 0.470137611476716 | -0.627139298 | 0.289397681057763 | 0.844618013986657 | 0.534117568977862 | 1.33562277480584 | 0.999489402457283 |
| ebi-fl187-GCST90094785 | ebi-IPs91-GCST90274807 | Interleukin-20 levels \|\| id:ebi-IPs91-GCST90274807 | F-highly palatable foods liking (derived food-liking factor) \|\| id:ebi-fl187-GCST90094785 | Weighted median | 17 | -0.193342953 | 0.205540903727938 | 0.346881999251666 | -0.596203125 | 0.209517217824058 | 0.824199263655696 | 0.55089936630781 | 1.23308260592741 | 0.999489402457283 |
| ebi-fl187-GCST90094785 | ebi-IPs91-GCST90274808 | Interleukin-20 receptor subunit alpha levels \|\| id:ebi-IPs91-GCST90274808 | F-highly palatable foods liking (derived food-liking factor) \|\| id:ebi-fl187-GCST90094785 | Weighted median | 17 | -0.301888755 | 0.190146461712272 | 0.112362263097543 | -0.67457582 | 0.07079831027119 | 0.73942031736057 | 0.509372440573347 | 1.07336471739656 | 0.999489402457283 |
| ebi-fl187-GCST90094785 | ebi-IPs91-GCST90274810 | Interleukin-24 levels \|\| id:ebi-IPs91-GCST90274810 | F-highly palatable foods liking (derived food-liking factor) \|\| id:ebi-fl187-GCST90094785 | Weighted median | 17 | 0.151401584113381 | 0.19673203647806 | 0.441547433323042 | -0.234193207 | 0.536996375610379 | 1.1634637928543 | 0.791208933719099 | 1.71086035507719 | 0.999489402457283 |
| ebi-fl187-GCST90094785 | ebi-IPs91-GCST90274823 | Monocyte chemoattractant protein-3 levels \|\| id:ebi-IPs91-GCST90274823 | F-highly palatable foods liking (derived food-liking factor) \|\| id:ebi-fl187-GCST90094785 | Weighted median | 17 | 0.111834070004162 | 0.191432220366845 | 0.559087986439726 | -0.263373082 | 0.487041221923179 | 1.11832728120776 | 0.768455147106067 | 1.62749369641597 | 0.999489402457283 |
| ebi-fl187-GCST90094785 | ebi-IPs91-GCST90274824 | Monocyte chemoattractant protein-4 levels \|\| id:ebi-IPs91-GCST90274824 | F-highly palatable foods liking (derived food-liking factor) \|\| id:ebi-fl187-GCST90094785 | Weighted median | 17 | -0.141602102 | 0.181244526131441 | 0.434639847282645 | -0.496841373 | 0.213637169473523 | 0.867966550161971 | 0.608449492697482 | 1.2381733262035 | 0.999489402457283 |
| ebi-fl187-GCST90094785 | ebi-IPs91-GCST90274834 | SIR2-like protein 2 levels \|\| id:ebi-IPs91-GCST90274834 | F-highly palatable foods liking (derived food-liking factor) \|\| id:ebi-fl187-GCST90094785 | Weighted median | 17 | -0.088938442 | 0.169078691010518 | 0.598875669952631 | -0.420332677 | 0.24245579203229 | 0.914901891052975 | 0.656828271983185 | 1.27437491039323 | 0.999489402457283 |
| ebi-fl187-GCST90094785 | ebi-met1400-GCST90199652 | 1-stearoyl-2-oleoyl-GPS (18:0/18:1) levels \|\| id:ebi-met1400-GCST90199652 | F-highly palatable foods liking (derived food-liking factor) \|\| id:ebi-fl187-GCST90094785 | Weighted median | 14 | 0.0609285652457483 | 0.264841026149033 | 0.818047380065589 | -0.458159846 | 0.580016976497852 | 1.06282298897635 | 0.632446374100061 | 1.78606875168503 | 0.999489402457283 |
| ebi-fl187-GCST90094785 | ebi-met1400-GCST90199665 | N-acetylaspartate (naa) levels \|\| id:ebi-met1400-GCST90199665 | F-highly palatable foods liking (derived food-liking factor) \|\| id:ebi-fl187-GCST90094785 | Weighted median | 14 | -0.563938336 | 0.275259910154296 | 0.040486711270948 | -1.10344776 | -0.024428912 | 0.568963874849554 | 0.33172540037409 | 0.975867059075842 | 0.999489402457283 |
| ebi-fl187-GCST90094785 | ebi-met1400-GCST90199669 | Acetylcarnitine levels (Biocrates platform) \|\| id:ebi-met1400-GCST90199669 | F-highly palatable foods liking (derived food-liking factor) \|\| id:ebi-fl187-GCST90094785 | Weighted median | 14 | 0.510350351294608 | 0.274666483510423 | 0.0631586617386162 | -0.027995956 | 1.04869665897504 | 1.66587473408781 | 0.972392298772584 | 2.85392905021471 | 0.999489402457283 |
| ebi-fl187-GCST90094785 | ebi-met1400-GCST90199670 | Iminodiacetate (IDA) levels \|\| id:ebi-met1400-GCST90199670 | F-highly palatable foods liking (derived food-liking factor) \|\| id:ebi-fl187-GCST90094785 | Weighted median | 14 | 0.072861849441916 | 0.254083260366578 | 0.774293278598195 | -0.425141341 | 0.570865039760408 | 1.07558193440568 | 0.65367738726529 | 1.76979733452268 | 0.999489402457283 |
| ebi-fl187-GCST90094785 | ebi-met1400-GCST90199686 | Indolepropionate levels \|\| id:ebi-met1400-GCST90199686 | F-highly palatable foods liking (derived food-liking factor) \|\| id:ebi-fl187-GCST90094785 | Weighted median | 14 | -0.111649532 | 0.269034039220941 | 0.678140798893619 | -0.638956249 | 0.415657184784201 | 0.894357646236905 | 0.527843073378449 | 1.5153662891943 | 0.999489402457283 |
| ebi-fl187-GCST90094785 | ebi-met1400-GCST90199704 | 3-hydroxysebacate levels \|\| id:ebi-met1400-GCST90199704 | F-highly palatable foods liking (derived food-liking factor) \|\| id:ebi-fl187-GCST90094785 | Weighted median | 14 | 0.70098870173961 | 0.294613058725835 | 0.0173430210077045 | 0.123547106636973 | 1.27843029684225 | 2.01574469285293 | 1.13150330460208 | 3.59099849751981 | 0.999489402457283 |
| ebi-fl187-GCST90094785 | ebi-met1400-GCST90199713 | Docosapentaenoate n3 DPA; 22:5n3 levels \|\| id:ebi-met1400-GCST90199713 | F-highly palatable foods liking (derived food-liking factor) \|\| id:ebi-fl187-GCST90094785 | Weighted median | 14 | 0.315044418277288 | 0.257143083975808 | 0.220510551035306 | -0.188956026 | 0.819044862869872 | 1.3703201768668 | 0.827822908317932 | 2.26833223417764 | 0.999489402457283 |
| ebi-fl187-GCST90094785 | ebi-met1400-GCST90199715 | Myristoleate (14:1n5) levels \|\| id:ebi-met1400-GCST90199715 | F-highly palatable foods liking (derived food-liking factor) \|\| id:ebi-fl187-GCST90094785 | Weighted median | 14 | 0.430388646802905 | 0.260757760383406 | 0.0988344724846796 | -0.080696564 | 0.941473857154381 | 1.53785508988365 | 0.922473561085049 | 2.56375724709037 | 0.999489402457283 |
| ebi-fl187-GCST90094785 | ebi-met1400-GCST90199725 | Campesterol levels \|\| id:ebi-met1400-GCST90199725 | F-highly palatable foods liking (derived food-liking factor) \|\| id:ebi-fl187-GCST90094785 | Weighted median | 14 | 0.0910125789413673 | 0.335693982135133 | 0.786300511807963 | -0.566947626 | 0.748972783926228 | 1.09528278266972 | 0.567254270999417 | 2.11482651668558 | 0.999489402457283 |
| ebi-fl187-GCST90094785 | ebi-met1400-GCST90199737 | N-acetylproline levels \|\| id:ebi-met1400-GCST90199737 | F-highly palatable foods liking (derived food-liking factor) \|\| id:ebi-fl187-GCST90094785 | Weighted median | 14 | -0.105562759 | 0.281815500349862 | 0.70797203333571 | -0.65792114 | 0.446795621333635 | 0.899817999080048 | 0.517926913639227 | 1.56329476253558 | 0.999489402457283 |
| ebi-fl187-GCST90094785 | ebi-met1400-GCST90199775 | Gamma-glutamylisoleucine levels \|\| id:ebi-met1400-GCST90199775 | F-highly palatable foods liking (derived food-liking factor) \|\| id:ebi-fl187-GCST90094785 | Weighted median | 14 | 0.149146431455447 | 0.247073513549461 | 0.546075003792222 | -0.335117655 | 0.633410518012392 | 1.16084296068848 | 0.715253928128325 | 1.88402513622857 | 0.999489402457283 |
| ebi-fl187-GCST90094785 | ebi-met1400-GCST90199777 | 1-palmitoleoyl-GPC (16:1) levels \|\| id:ebi-met1400-GCST90199777 | F-highly palatable foods liking (derived food-liking factor) \|\| id:ebi-fl187-GCST90094785 | Weighted median | 14 | 0.390758576771356 | 0.278178137942882 | 0.160108638158649 | -0.154470574 | 0.935987727139403 | 1.47810162226806 | 0.856868706335248 | 2.54973065254723 | 0.999489402457283 |
| ebi-fl187-GCST90094785 | ebi-met1400-GCST90199782 | Glutamine degradant levels \|\| id:ebi-met1400-GCST90199782 | F-highly palatable foods liking (derived food-liking factor) \|\| id:ebi-fl187-GCST90094785 | Weighted median | 14 | -0.340428463 | 0.213353476062492 | 0.110576392608936 | -0.758601276 | 0.0777443498087745 | 0.711465420644334 | 0.46832102078996 | 1.08084630478212 | 0.999489402457283 |
| ebi-fl187-GCST90094785 | ebi-met1400-GCST90199783 | 5-methyluridine (ribothymidine) levels \|\| id:ebi-met1400-GCST90199783 | F-highly palatable foods liking (derived food-liking factor) \|\| id:ebi-fl187-GCST90094785 | Weighted median | 14 | 0.430893824202953 | 0.282953379328993 | 0.127797766988775 | -0.123694799 | 0.985482447687778 | 1.5386321757862 | 0.883649490191056 | 2.6791041002612 | 0.999489402457283 |
| ebi-fl187-GCST90094785 | ebi-met1400-GCST90199787 | Homostachydrine levels \|\| id:ebi-met1400-GCST90199787 | F-highly palatable foods liking (derived food-liking factor) \|\| id:ebi-fl187-GCST90094785 | Weighted median | 14 | -0.056943602 | 0.257988660478377 | 0.825309417376876 | -0.562601376 | 0.448714172707691 | 0.944647344245784 | 0.569725065145638 | 1.5662969028096 | 0.999489402457283 |
| ebi-fl187-GCST90094785 | ebi-met1400-GCST90199802 | Beta-hydroxyisovaleroylcarnitine levels \|\| id:ebi-met1400-GCST90199802 | F-highly palatable foods liking (derived food-liking factor) \|\| id:ebi-fl187-GCST90094785 | Weighted median | 14 | 0.524052750773585 | 0.264675178838827 | 0.0477052707531195 | 0.00528940024948355 | 1.04281610129769 | 1.68885832071165 | 1.00530341382389 | 2.83719560504411 | 0.999489402457283 |
| ebi-fl187-GCST90094785 | ebi-met1400-GCST90199873 | Pregnenolone sulfate levels \|\| id:ebi-met1400-GCST90199873 | F-highly palatable foods liking (derived food-liking factor) \|\| id:ebi-fl187-GCST90094785 | Weighted median | 14 | -0.002349578 | 0.250174588958599 | 0.992506575776199 | -0.492691772 | 0.487992616517206 | 0.997653180255825 | 0.610979560931605 | 1.62904282191852 | 0.999911036302558 |
| ebi-fl187-GCST90094785 | ebi-met1400-GCST90199911 | Beta-citrylglutamate levels \|\| id:ebi-met1400-GCST90199911 | F-highly palatable foods liking (derived food-liking factor) \|\| id:ebi-fl187-GCST90094785 | Weighted median | 14 | -0.09319927 | 0.262185510059521 | 0.722236981776643 | -0.60708287 | 0.42068432952235 | 0.911011944680195 | 0.544938208355525 | 1.52300343529688 | 0.999489402457283 |
| ebi-fl187-GCST90094785 | ebi-met1400-GCST90199934 | 2-hydroxydecanoate levels \|\| id:ebi-met1400-GCST90199934 | F-highly palatable foods liking (derived food-liking factor) \|\| id:ebi-fl187-GCST90094785 | Weighted median | 14 | 0.116559241456798 | 0.268400178981454 | 0.664089422084798 | -0.409505109 | 0.642623592260447 | 1.12362407362255 | 0.663978765725014 | 1.90146300453682 | 0.999489402457283 |
| ebi-fl187-GCST90094785 | ebi-met1400-GCST90199945 | Sphingomyelin (d18:1/14:0, d16:1/16:0) levels \|\| id:ebi-met1400-GCST90199945 | F-highly palatable foods liking (derived food-liking factor) \|\| id:ebi-fl187-GCST90094785 | Weighted median | 14 | -0.299781529 | 0.255893696192305 | 0.241394617338147 | -0.801333174 | 0.201770115268656 | 0.740980085461191 | 0.448730329642918 | 1.22356669647667 | 0.999489402457283 |
| ebi-fl187-GCST90094785 | ebi-met1400-GCST90199979 | Carnitine C14:1 levels \|\| id:ebi-met1400-GCST90199979 | F-highly palatable foods liking (derived food-liking factor) \|\| id:ebi-fl187-GCST90094785 | Weighted median | 14 | 0.353741189266453 | 0.261805059768762 | 0.176643371699259 | -0.159396728 | 0.866879106413226 | 1.42438649225802 | 0.852658018650673 | 2.37947317089423 | 0.999489402457283 |
| ebi-fl187-GCST90094785 | ebi-met1400-GCST90199986 | Eugenol sulfate levels \|\| id:ebi-met1400-GCST90199986 | F-highly palatable foods liking (derived food-liking factor) \|\| id:ebi-fl187-GCST90094785 | Weighted median | 14 | 0.167626518609556 | 0.278275929792554 | 0.546924414853147 | -0.377794304 | 0.713047341002962 | 1.18249488833033 | 0.685371464484413 | 2.04019897732283 | 0.999489402457283 |
| ebi-fl187-GCST90094785 | ebi-met1400-GCST90200002 | 6-hydroxyindole sulfate levels \|\| id:ebi-met1400-GCST90200002 | F-highly palatable foods liking (derived food-liking factor) \|\| id:ebi-fl187-GCST90094785 | Weighted median | 14 | 0.401506624491676 | 0.255505812565254 | 0.11608541363927 | -0.099284768 | 0.902298017119573 | 1.49407401127805 | 0.90548481808185 | 2.46526182062912 | 0.999489402457283 |
| ebi-fl187-GCST90094785 | ebi-met1400-GCST90200023 | Trans 3,4-methyleneheptanoate levels \|\| id:ebi-met1400-GCST90200023 | F-highly palatable foods liking (derived food-liking factor) \|\| id:ebi-fl187-GCST90094785 | Weighted median | 14 | 0.375508537310481 | 0.272533160517861 | 0.168251052987224 | -0.158656457 | 0.90967353192549 | 1.45573152020867 | 0.853289449979276 | 2.48351161376777 | 0.999489402457283 |
| ebi-fl187-GCST90094785 | ebi-met1400-GCST90200032 | 2-hydroxybutyrate/2-hydroxyisobutyrate levels \|\| id:ebi-met1400-GCST90200032 | F-highly palatable foods liking (derived food-liking factor) \|\| id:ebi-fl187-GCST90094785 | Weighted median | 14 | 0.126214980148866 | 0.266339742403147 | 0.635580386563809 | -0.395810915 | 0.648240875259035 | 1.13452604264555 | 0.673133963466345 | 1.91217411585166 | 0.999489402457283 |
| ebi-fl187-GCST90094785 | ebi-met1400-GCST90200051 | 1-palmitoyl-2-dihomo-linolenoyl-GPC (16:0/20:3n3 or 6) levels \|\| id:ebi-met1400-GCST90200051 | F-highly palatable foods liking (derived food-liking factor) \|\| id:ebi-fl187-GCST90094785 | Weighted median | 14 | -0.051989191 | 0.264546861917554 | 0.844201874744927 | -0.57050104 | 0.466522658800824 | 0.949339128614074 | 0.565242158854424 | 1.59444012977426 | 0.999489402457283 |
| ebi-fl187-GCST90094785 | ebi-met1400-GCST90200069 | Phenylacetylglutamate levels \|\| id:ebi-met1400-GCST90200069 | F-highly palatable foods liking (derived food-liking factor) \|\| id:ebi-fl187-GCST90094785 | Weighted median | 14 | 0.0622980022213777 | 0.297855558733174 | 0.834327180403164 | -0.521498893 | 0.646094897338398 | 1.06427945511768 | 0.593630092859662 | 1.90807503226316 | 0.999489402457283 |
| ebi-fl187-GCST90094785 | ebi-met1400-GCST90200072 | Thioproline levels \|\| id:ebi-met1400-GCST90200072 | F-highly palatable foods liking (derived food-liking factor) \|\| id:ebi-fl187-GCST90094785 | Weighted median | 14 | 0.185264150038517 | 0.253406163089782 | 0.464720715434892 | -0.31141193 | 0.681940229694489 | 1.20353631230612 | 0.732412111479853 | 1.97771122614658 | 0.999489402457283 |
| ebi-fl187-GCST90094785 | ebi-met1400-GCST90200081 | 1-oleoyl-2-docosahexaenoyl-GPC (18:1/22:6) levels \|\| id:ebi-met1400-GCST90200081 | F-highly palatable foods liking (derived food-liking factor) \|\| id:ebi-fl187-GCST90094785 | Weighted median | 14 | -0.226212633 | 0.247092913318095 | 0.35993117662475 | -0.710514743 | 0.258089477571573 | 0.797548498878155 | 0.49139119235295 | 1.29445463810007 | 0.999489402457283 |
| ebi-fl187-GCST90094785 | ebi-met1400-GCST90200097 | Furaneol sulfate levels \|\| id:ebi-met1400-GCST90200097 | F-highly palatable foods liking (derived food-liking factor) \|\| id:ebi-fl187-GCST90094785 | Weighted median | 13 | -0.448128052 | 0.314437873988137 | 0.154107489194577 | -1.064426285 | 0.168170180542493 | 0.638822875944289 | 0.344925686883307 | 1.18313794057268 | 0.999489402457283 |
| ebi-fl187-GCST90094785 | ebi-met1400-GCST90200124 | Sphingomyelin (d18:2/24:2) levels \|\| id:ebi-met1400-GCST90200124 | F-highly palatable foods liking (derived food-liking factor) \|\| id:ebi-fl187-GCST90094785 | Weighted median | 14 | -0.062688434 | 0.230275053587626 | 0.785442983631549 | -0.514027539 | 0.388650670595355 | 0.93923606171952 | 0.598081922977898 | 1.47498920422478 | 0.999489402457283 |
| ebi-fl187-GCST90094785 | ebi-met1400-GCST90200137 | Lignoceroylcarnitine (C24) levels \|\| id:ebi-met1400-GCST90200137 | F-highly palatable foods liking (derived food-liking factor) \|\| id:ebi-fl187-GCST90094785 | Weighted median | 14 | 0.402888686965684 | 0.256478984601858 | 0.116218696067517 | -0.099810123 | 0.905587496785326 | 1.49614034247292 | 0.905009242294761 | 2.47338460179607 | 0.999489402457283 |
| ebi-fl187-GCST90094785 | ebi-met1400-GCST90200165 | Octadecenedioate (C18:1-DC) levels \|\| id:ebi-met1400-GCST90200165 | F-highly palatable foods liking (derived food-liking factor) \|\| id:ebi-fl187-GCST90094785 | Weighted median | 14 | -0.14098963 | 0.248018560303081 | 0.569719924530952 | -0.627106008 | 0.345126748304486 | 0.868498318074451 | 0.534135349805337 | 1.41216889833831 | 0.999489402457283 |
| ebi-fl187-GCST90094785 | ebi-met1400-GCST90200176 | Glutamine conjugate of C6H10O2 (1) levels \|\| id:ebi-met1400-GCST90200176 | F-highly palatable foods liking (derived food-liking factor) \|\| id:ebi-fl187-GCST90094785 | Weighted median | 14 | 0.243903443069595 | 0.252296401260484 | 0.333677156693057 | -0.250597503 | 0.738404389540143 | 1.2762210965484 | 0.778335585947127 | 2.09259388454308 | 0.999489402457283 |
| ebi-fl187-GCST90094785 | ebi-met1400-GCST90200182 | Glycine conjugate of C10H12O2 levels \|\| id:ebi-met1400-GCST90200182 | F-highly palatable foods liking (derived food-liking factor) \|\| id:ebi-fl187-GCST90094785 | Weighted median | 14 | 0.467899447717757 | 0.317127300132952 | 0.140096707715389 | -0.153670061 | 1.08946895597834 | 1.59663684913608 | 0.857554915543186 | 2.97269502140801 | 0.999489402457283 |
| ebi-fl187-GCST90094785 | ebi-met1400-GCST90200208 | Glucuronide of piperine metabolite C17H21NO3 (4) levels \|\| id:ebi-met1400-GCST90200208 | F-highly palatable foods liking (derived food-liking factor) \|\| id:ebi-fl187-GCST90094785 | Weighted median | 14 | 0.313908580939855 | 0.268856497701078 | 0.242980557479424 | -0.213050155 | 0.840867316433967 | 1.36876459965425 | 0.808115605522089 | 2.31837687140848 | 0.999489402457283 |
| ebi-fl187-GCST90094785 | ebi-met1400-GCST90200227 | 3-ethylcatechol sulfate (2) levels \|\| id:ebi-met1400-GCST90200227 | F-highly palatable foods liking (derived food-liking factor) \|\| id:ebi-fl187-GCST90094785 | Weighted median | 14 | 0.153262335942768 | 0.334111240595037 | 0.646437769991309 | -0.501595696 | 0.80812036750904 | 1.16563072567215 | 0.605563593170533 | 2.24368671425125 | 0.999489402457283 |
| ebi-fl187-GCST90094785 | ebi-met1400-GCST90200259 | Pentose acid levels \|\| id:ebi-met1400-GCST90200259 | F-highly palatable foods liking (derived food-liking factor) \|\| id:ebi-fl187-GCST90094785 | Weighted median | 14 | -0.268723322 | 0.25838540350768 | 0.29833540214845 | -0.775158712 | 0.237712069352185 | 0.764354706891138 | 0.460630667399324 | 1.26834394515066 | 0.999489402457283 |
| ebi-fl187-GCST90094785 | ebi-met1400-GCST90200291 | Sarcosine levels \|\| id:ebi-met1400-GCST90200291 | F-highly palatable foods liking (derived food-liking factor) \|\| id:ebi-fl187-GCST90094785 | Weighted median | 14 | 0.242475186761195 | 0.245383434867983 | 0.323080098158733 | -0.238476346 | 0.723426719102442 | 1.27439962678882 | 0.787827323635095 | 2.06148525195317 | 0.999489402457283 |
| ebi-fl187-GCST90094785 | ebi-met1400-GCST90200306 | Butyrate/isobutyrate (4:0) levels \|\| id:ebi-met1400-GCST90200306 | F-highly palatable foods liking (derived food-liking factor) \|\| id:ebi-fl187-GCST90094785 | Weighted median | 14 | 0.32111325497765 | 0.288738275153214 | 0.266084110233078 | -0.244813764 | 0.887040274277949 | 1.37866171235367 | 0.782850319326112 | 2.42793299074863 | 0.999489402457283 |
| ebi-fl187-GCST90094785 | ebi-met1400-GCST90200321 | Cholate levels \|\| id:ebi-met1400-GCST90200321 | F-highly palatable foods liking (derived food-liking factor) \|\| id:ebi-fl187-GCST90094785 | Weighted median | 14 | -0.315120408 | 0.272926393058666 | 0.248254875185893 | -0.850056138 | 0.219815322333408 | 0.72970100709541 | 0.427390938207628 | 1.24584662929232 | 0.999489402457283 |
| ebi-fl187-GCST90094785 | ebi-met1400-GCST90200336 | Ceramide (d18:1/16:0) levels \|\| id:ebi-met1400-GCST90200336 | F-highly palatable foods liking (derived food-liking factor) \|\| id:ebi-fl187-GCST90094785 | Weighted median | 14 | 0.261871156043696 | 0.256313108566475 | 0.306930201718291 | -0.240502537 | 0.764244848833988 | 1.29935911707444 | 0.786232650972587 | 2.14737217162879 | 0.999489402457283 |
| ebi-fl187-GCST90094785 | ebi-met1400-GCST90200358 | Arachidonate (20:4n6) levels \|\| id:ebi-met1400-GCST90200358 | F-highly palatable foods liking (derived food-liking factor) \|\| id:ebi-fl187-GCST90094785 | Weighted median | 14 | 0.682813189595649 | 0.281419686500882 | 0.0152529675383344 | 0.131230604053921 | 1.23439577513738 | 1.97943844252872 | 1.14023069281676 | 3.43630159444424 | 0.999489402457283 |
| ebi-fl187-GCST90094785 | ebi-met1400-GCST90200413 | 2'-deoxyuridine levels \|\| id:ebi-met1400-GCST90200413 | F-highly palatable foods liking (derived food-liking factor) \|\| id:ebi-fl187-GCST90094785 | Weighted median | 14 | 0.255465743359647 | 0.266585100561007 | 0.337916720162055 | -0.267041054 | 0.777972540459221 | 1.29106278478687 | 0.765641638367456 | 2.17705389928345 | 0.999489402457283 |
| ebi-fl187-GCST90094785 | ebi-met1400-GCST90200432 | Threonine levels \|\| id:ebi-met1400-GCST90200432 | F-highly palatable foods liking (derived food-liking factor) \|\| id:ebi-fl187-GCST90094785 | Weighted median | 14 | -0.044890833 | 0.269002271746304 | 0.867465248058229 | -0.572135286 | 0.482353619458026 | 0.956101850749426 | 0.564319168591855 | 1.61988250600721 | 0.999489402457283 |
| ebi-fl187-GCST90094785 | ebi-met1400-GCST90200436 | Caffeine levels \|\| id:ebi-met1400-GCST90200436 | F-highly palatable foods liking (derived food-liking factor) \|\| id:ebi-fl187-GCST90094785 | Weighted median | 14 | 0.312722244390262 | 0.257397622252392 | 0.224389602796004 | -0.191777095 | 0.817221584004951 | 1.36714174699694 | 0.825490853838827 | 2.26420020002641 | 0.999489402457283 |
| ebi-fl187-GCST90094785 | ebi-met1400-GCST90200442 | Valine levels \|\| id:ebi-met1400-GCST90200442 | F-highly palatable foods liking (derived food-liking factor) \|\| id:ebi-fl187-GCST90094785 | Weighted median | 14 | 0.0440699795270735 | 0.259024003429929 | 0.864901143036549 | -0.463617067 | 0.551757026249735 | 1.04505548481821 | 0.629004374764376 | 1.73630106588326 | 0.999489402457283 |
| ebi-fl187-GCST90094785 | ebi-met1400-GCST90200459 | Margarate (17:0) levels \|\| id:ebi-met1400-GCST90200459 | F-highly palatable foods liking (derived food-liking factor) \|\| id:ebi-fl187-GCST90094785 | Weighted median | 14 | 0.394183201401259 | 0.27818671684508 | 0.156490680063911 | -0.151062764 | 0.939429166417616 | 1.48317224301983 | 0.859793733202755 | 2.55852051197228 | 0.999489402457283 |
| ebi-fl187-GCST90094785 | ebi-met1400-GCST90200462 | X-11299 levels \|\| id:ebi-met1400-GCST90200462 | F-highly palatable foods liking (derived food-liking factor) \|\| id:ebi-fl187-GCST90094785 | Weighted median | 14 | -0.157738944 | 0.252189954800652 | 0.531658226868744 | -0.652031256 | 0.336553367180469 | 0.854072713479307 | 0.520986444585092 | 1.4001135873906 | 0.999489402457283 |
| ebi-fl187-GCST90094785 | ebi-met1400-GCST90200497 | X-12740 levels \|\| id:ebi-met1400-GCST90200497 | F-highly palatable foods liking (derived food-liking factor) \|\| id:ebi-fl187-GCST90094785 | Weighted median | 14 | -0.449714623 | 0.315278456051713 | 0.153751602428864 | -1.067660397 | 0.168231151096481 | 0.637810142147461 | 0.343811960806233 | 1.18321007934751 | 0.999489402457283 |
| ebi-fl187-GCST90094785 | ebi-met1400-GCST90200527 | X-13866 levels \|\| id:ebi-met1400-GCST90200527 | F-highly palatable foods liking (derived food-liking factor) \|\| id:ebi-fl187-GCST90094785 | Weighted median | 14 | 0.112446743664398 | 0.250936956848273 | 0.654075583118707 | -0.379389692 | 0.604283179087012 | 1.11901266081205 | 0.684278902853582 | 1.82993999937129 | 0.999489402457283 |
| ebi-fl187-GCST90094785 | ebi-met1400-GCST90200547 | X-17654 levels \|\| id:ebi-met1400-GCST90200547 | F-highly palatable foods liking (derived food-liking factor) \|\| id:ebi-fl187-GCST90094785 | Weighted median | 14 | -0.02226395 | 0.269702814991192 | 0.934209412361037 | -0.550881467 | 0.506353567858364 | 0.977982063080129 | 0.57644147229009 | 1.65922988140788 | 0.999489402457283 |
| ebi-fl187-GCST90094785 | ebi-met1400-GCST90200553 | X-17653 levels \|\| id:ebi-met1400-GCST90200553 | F-highly palatable foods liking (derived food-liking factor) \|\| id:ebi-fl187-GCST90094785 | Weighted median | 14 | 0.00229057884156489 | 0.283154273456332 | 0.99354557662996 | -0.552691797 | 0.557272954815975 | 1.00229320422144 | 0.575398866884219 | 1.74590484105112 | 0.999911036302558 |
| ebi-fl187-GCST90094785 | ebi-met1400-GCST90200557 | X-17685 levels \|\| id:ebi-met1400-GCST90200557 | F-highly palatable foods liking (derived food-liking factor) \|\| id:ebi-fl187-GCST90094785 | Weighted median | 14 | 0.243075290844352 | 0.298433694794867 | 0.415356564305084 | -0.341854751 | 0.828005332642291 | 1.27516462872587 | 0.710451389602105 | 2.2887488914115 | 0.999489402457283 |
| ebi-fl187-GCST90094785 | ebi-met1400-GCST90200561 | X-18779 levels \|\| id:ebi-met1400-GCST90200561 | F-highly palatable foods liking (derived food-liking factor) \|\| id:ebi-fl187-GCST90094785 | Weighted median | 14 | 0.128806975468995 | 0.263641742574172 | 0.625147420735789 | -0.38793084 | 0.645544790914372 | 1.13747054325602 | 0.678459263900284 | 1.9070256765855 | 0.999489402457283 |
| ebi-fl187-GCST90094785 | ebi-met1400-GCST90200570 | X-18921 levels \|\| id:ebi-met1400-GCST90200570 | F-highly palatable foods liking (derived food-liking factor) \|\| id:ebi-fl187-GCST90094785 | Weighted median | 14 | 0.614072674080174 | 0.258793255361468 | 0.0176523358832607 | 0.106837893571697 | 1.12130745458865 | 1.84794216009571 | 1.11275385530472 | 3.06886398171504 | 0.999489402457283 |
| ebi-fl187-GCST90094785 | ebi-met1400-GCST90200575 | X-21283 levels \|\| id:ebi-met1400-GCST90200575 | F-highly palatable foods liking (derived food-liking factor) \|\| id:ebi-fl187-GCST90094785 | Weighted median | 13 | -0.341544305 | 0.285026176290604 | 0.230803745298346 | -0.90019561 | 0.217107000926051 | 0.710671980882005 | 0.406490138373183 | 1.24247704121934 | 0.999489402457283 |
| ebi-fl187-GCST90094785 | ebi-met1400-GCST90200578 | X-18922 levels \|\| id:ebi-met1400-GCST90200578 | F-highly palatable foods liking (derived food-liking factor) \|\| id:ebi-fl187-GCST90094785 | Weighted median | 14 | 0.29849872168446 | 0.28071483222165 | 0.287622281625298 | -0.251702349 | 0.848699792838893 | 1.34783381423878 | 0.777476119810934 | 2.33660680310955 | 0.999489402457283 |
| ebi-fl187-GCST90094785 | ebi-met1400-GCST90200623 | X-24241 levels \|\| id:ebi-met1400-GCST90200623 | F-highly palatable foods liking (derived food-liking factor) \|\| id:ebi-fl187-GCST90094785 | Weighted median | 14 | -0.073017215 | 0.281457538364965 | 0.795307087685344 | -0.62467399 | 0.478639560304279 | 0.929584827186526 | 0.535435957498607 | 1.61387732525911 | 0.999489402457283 |
| ebi-fl187-GCST90094785 | ebi-met1400-GCST90200636 | X-24544 levels \|\| id:ebi-met1400-GCST90200636 | F-highly palatable foods liking (derived food-liking factor) \|\| id:ebi-fl187-GCST90094785 | Weighted median | 14 | 0.0275552818364054 | 0.251030082780668 | 0.912592701084383 | -0.46446368 | 0.519574244086514 | 1.02793843986122 | 0.628472076703529 | 1.68131166890772 | 0.999489402457283 |
| ebi-fl187-GCST90094785 | ebi-met1400-GCST90200659 | X-25172 levels \|\| id:ebi-met1400-GCST90200659 | F-highly palatable foods liking (derived food-liking factor) \|\| id:ebi-fl187-GCST90094785 | Weighted median | 14 | 0.482262018660814 | 0.278060977615072 | 0.0828515611328182 | -0.062737497 | 1.02726153478636 | 1.61973413027404 | 0.939189981084447 | 2.79340570663381 | 0.999489402457283 |
| ebi-fl187-GCST90094785 | ebi-met1400-GCST90200670 | X-26111 levels \|\| id:ebi-met1400-GCST90200670 | F-highly palatable foods liking (derived food-liking factor) \|\| id:ebi-fl187-GCST90094785 | Weighted median | 14 | -0.085097871 | 0.265519435649823 | 0.74859249361393 | -0.605515965 | 0.435320222888606 | 0.918422393098353 | 0.545792744100724 | 1.54545787070566 | 0.999489402457283 |
| ebi-fl187-GCST90094785 | ebi-met1400-GCST90200673 | Carnitine C4 levels \|\| id:ebi-met1400-GCST90200673 | F-highly palatable foods liking (derived food-liking factor) \|\| id:ebi-fl187-GCST90094785 | Weighted median | 14 | 0.384282663040403 | 0.251398157032786 | 0.126368135275144 | -0.108457725 | 0.877023050824663 | 1.46856049079305 | 0.897216824086285 | 2.40373325290088 | 0.999489402457283 |
| ebi-fl187-GCST90094785 | ebi-met1400-GCST90200710 | X-13431 levels \|\| id:ebi-met1400-GCST90200710 | F-highly palatable foods liking (derived food-liking factor) \|\| id:ebi-fl187-GCST90094785 | Weighted median | 14 | 0.0631636561310663 | 0.255418008437269 | 0.804679747086541 | -0.43745564 | 0.563782952668113 | 1.06520115166656 | 0.645677167769658 | 1.75730775401452 | 0.999489402457283 |
| ebi-fl187-GCST90094785 | ebi-met1400-GCST90200730 | Adenosine 5'-diphosphate (ADP) to fructose ratio \|\| id:ebi-met1400-GCST90200730 | F-highly palatable foods liking (derived food-liking factor) \|\| id:ebi-fl187-GCST90094785 | Weighted median | 14 | -0.333015451 | 0.364575341681512 | 0.361014419069831 | -1.047583121 | 0.38155221841813 | 0.716759119225992 | 0.350784529187912 | 1.46455613701941 | 0.999489402457283 |
| ebi-fl187-GCST90094785 | ebi-met1400-GCST90200731 | Adenosine 5'-diphosphate (ADP) to mannose ratio \|\| id:ebi-met1400-GCST90200731 | F-highly palatable foods liking (derived food-liking factor) \|\| id:ebi-fl187-GCST90094785 | Weighted median | 14 | -0.122863227 | 0.357690869201723 | 0.731229882412191 | -0.823937331 | 0.578210876403891 | 0.88438461402129 | 0.438700938794045 | 1.78284584406776 | 0.999489402457283 |
| ebi-fl187-GCST90094785 | ebi-met1400-GCST90200748 | Aspartate to glutamate ratio \|\| id:ebi-met1400-GCST90200748 | F-highly palatable foods liking (derived food-liking factor) \|\| id:ebi-fl187-GCST90094785 | Weighted median | 14 | -0.171881764 | 0.243785373170318 | 0.480776857650647 | -0.649701095 | 0.305937567465178 | 0.842078731346826 | 0.522201841987843 | 1.35789752691678 | 0.999489402457283 |
| ebi-fl187-GCST90094785 | ebi-met1400-GCST90200774 | Uridine to pseudouridine ratio \|\| id:ebi-met1400-GCST90200774 | F-highly palatable foods liking (derived food-liking factor) \|\| id:ebi-fl187-GCST90094785 | Weighted median | 14 | 0.303470221169585 | 0.241805493149002 | 0.209472263600506 | -0.170468545 | 0.777408987741628 | 1.35455125338936 | 0.843269613917781 | 2.17582736028432 | 0.999489402457283 |
| ebi-fl187-GCST90094785 | ebi-met1400-GCST90200799 | Spermidine to adenosine 5'-diphosphate (ADP) ratio \|\| id:ebi-met1400-GCST90200799 | F-highly palatable foods liking (derived food-liking factor) \|\| id:ebi-fl187-GCST90094785 | Weighted median | 14 | -0.120337736 | 0.375868548771288 | 0.748847725937126 | -0.857040092 | 0.616364619212795 | 0.88662094199844 | 0.424416458666424 | 1.85218239947675 | 0.999489402457283 |
| ebi-fl187-GCST90094785 | ebi-met1400-GCST90200811 | Choline to taurocholate ratio \|\| id:ebi-met1400-GCST90200811 | F-highly palatable foods liking (derived food-liking factor) \|\| id:ebi-fl187-GCST90094785 | Weighted median | 14 | 0.16049058611385 | 0.281846108359611 | 0.569066874233571 | -0.391927786 | 0.712908958498688 | 1.17408672036988 | 0.675752910836285 | 2.03991666901285 | 0.999489402457283 |
| ebi-fl187-GCST90094785 | ebi-met1400-GCST90200812 | Glutamate to kynurenine ratio \|\| id:ebi-met1400-GCST90200812 | F-highly palatable foods liking (derived food-liking factor) \|\| id:ebi-fl187-GCST90094785 | Weighted median | 14 | 0.0777861892314841 | 0.251568072340542 | 0.757165338460712 | -0.415287233 | 0.570859611018947 | 1.0808915277136 | 0.660150636715608 | 1.76978772677659 | 0.999489402457283 |
| ebi-fl187-GCST90094785 | ebi-met1400-GCST90200876 | Cortisone to 4-cholesten-3-one ratio \|\| id:ebi-met1400-GCST90200876 | F-highly palatable foods liking (derived food-liking factor) \|\| id:ebi-fl187-GCST90094785 | Weighted median | 14 | 0.111510328433031 | 0.272413508588538 | 0.682288594936605 | -0.422420148 | 0.645440805266566 | 1.11796529077554 | 0.65545859165415 | 1.90682738359513 | 0.999489402457283 |
| ebi-fl187-GCST90094785 | ebi-met1400-GCST90200906 | 2'-deoxyuridine to cytidine ratio \|\| id:ebi-met1400-GCST90200906 | F-highly palatable foods liking (derived food-liking factor) \|\| id:ebi-fl187-GCST90094785 | Weighted median | 14 | -0.022864275 | 0.275667649379962 | 0.933898094997195 | -0.563172867 | 0.517444318145531 | 0.977395132078155 | 0.569399565385278 | 1.67773441056928 | 0.999489402457283 |
| ebi-fl187-GCST90094785 | ebi-met1400-GCST90200907 | Retinol (Vitamin A) to linoleoyl-arachidonoyl-glycerol (18:2 to 20:4) [1] ratio \|\| id:ebi-met1400-GCST90200907 | F-highly palatable foods liking (derived food-liking factor) \|\| id:ebi-fl187-GCST90094785 | Weighted median | 14 | -0.063991044 | 0.257783854522844 | 0.803952381049438 | -0.569247399 | 0.441265310928872 | 0.938013400401248 | 0.565951214022362 | 1.55467311939991 | 0.999489402457283 |
| ebi-fl187-GCST90094785 | ebi-met1400-GCST90200916 | Fructose to sucrose ratio \|\| id:ebi-met1400-GCST90200916 | F-highly palatable foods liking (derived food-liking factor) \|\| id:ebi-fl187-GCST90094785 | Weighted median | 14 | -0.01007147 | 0.275824227573151 | 0.970872445438937 | -0.550686956 | 0.530544015869159 | 0.989979077243587 | 0.5765536072237 | 1.69985680620294 | 0.999489402457283 |
| ebi-fl187-GCST90094785 | ebi-met1400-GCST90200917 | Alpha-ketobutyrate to 3-methyl-2-oxobutyrate ratio \|\| id:ebi-met1400-GCST90200917 | F-highly palatable foods liking (derived food-liking factor) \|\| id:ebi-fl187-GCST90094785 | Weighted median | 14 | 0.365197995330017 | 0.274408690062758 | 0.183236944668484 | -0.172643037 | 0.903039027853024 | 1.44079925143314 | 0.841437923284983 | 2.467089283 | 0.999489402457283 |
| ebi-fl187-GCST90094785 | ebi-met1400-GCST90200924 | Alpha-tocopherol to glycerol ratio \|\| id:ebi-met1400-GCST90200924 | F-highly palatable foods liking (derived food-liking factor) \|\| id:ebi-fl187-GCST90094785 | Weighted median | 14 | -0.216249682 | 0.246619483929615 | 0.380565061775545 | -0.69962387 | 0.267124506950091 | 0.805534149745877 | 0.496772119526203 | 1.30620306756685 | 0.999489402457283 |
| ebi-fl187-GCST90094785 | ebi-met1400-GCST90200938 | Alpha-ketoglutarate to alanine ratio \|\| id:ebi-met1400-GCST90200938 | F-highly palatable foods liking (derived food-liking factor) \|\| id:ebi-fl187-GCST90094785 | Weighted median | 14 | 0.45579204323063 | 0.271978000534631 | 0.0937692264393755 | -0.077284838 | 0.988868924278507 | 1.57742227501525 | 0.925626162717935 | 2.68819220321946 | 0.999489402457283 |
| ebi-fl187-GCST90094785 | ebi-met1400-GCST90200943 | Phosphate to EDTA ratio \|\| id:ebi-met1400-GCST90200943 | F-highly palatable foods liking (derived food-liking factor) \|\| id:ebi-fl187-GCST90094785 | Weighted median | 14 | -0.078148811 | 0.27474253012397 | 0.776070292740721 | -0.61664417 | 0.460346548227052 | 0.924826792073523 | 0.53975272020021 | 1.58462303815671 | 0.999489402457283 |
| ebi-fl187-GCST90094785 | ebi-met1400-GCST90200947 | Adenosine 5'-diphosphate (ADP) to glucose ratio \|\| id:ebi-met1400-GCST90200947 | F-highly palatable foods liking (derived food-liking factor) \|\| id:ebi-fl187-GCST90094785 | Weighted median | 14 | -0.383965545 | 0.361474877183515 | 0.288136197498524 | -1.092456305 | 0.324525213992413 | 0.681154895771497 | 0.335391657037507 | 1.38337368356659 | 0.999489402457283 |
| ebi-fl187-GCST90094785 | ebi-met1400-GCST90200975 | Arachidonate (20:4n6) to caffeine ratio \|\| id:ebi-met1400-GCST90200975 | F-highly palatable foods liking (derived food-liking factor) \|\| id:ebi-fl187-GCST90094785 | Weighted median | 14 | -0.250245999 | 0.254772091990313 | 0.3259842169645 | -0.7495993 | 0.249107300985599 | 0.778609222174815 | 0.472555868126725 | 1.28287967993892 | 0.999489402457283 |
| ebi-fl187-GCST90094785 | ebi-met1400-GCST90200985 | Cholesterol to oleoyl-linoleoyl-glycerol (18:1 to 18:2) [2] ratio \|\| id:ebi-met1400-GCST90200985 | F-highly palatable foods liking (derived food-liking factor) \|\| id:ebi-fl187-GCST90094785 | Weighted median | 14 | -0.036961934 | 0.248599703779667 | 0.881805732263446 | -0.524217353 | 0.450293485896148 | 0.963712819813317 | 0.592018524667195 | 1.56877253054645 | 0.999489402457283 |
| ebi-fl187-GCST90094785 | ebi-met1400-GCST90201016 | Bilirubin (Z,Z) to etiocholanolone glucuronide ratio \|\| id:ebi-met1400-GCST90201016 | F-highly palatable foods liking (derived food-liking factor) \|\| id:ebi-fl187-GCST90094785 | Weighted median | 14 | 0.261942076625539 | 0.271070294093491 | 0.333881442292951 | -0.2693557 | 0.793239853048781 | 1.29945127164683 | 0.763871498381617 | 2.21054668352214 | 0.999489402457283 |
| ebi-fl187-GCST90094785 | ebi-pw11-GCST90275189 | Placental weight (fetal genotype, adjusted for sex and gestational age) \|\| id:ebi-pw11-GCST90275189 | F-highly palatable foods liking (derived food-liking factor) \|\| id:ebi-fl187-GCST90094785 | Weighted median | 13 | 0.00261726458967291 | 0.107621818131576 | 0.980598086693375 | -0.208321499 | 0.213556028127562 | 1.00262069261667 | 0.811945954974903 | 1.23807286322919 | 0.999911036302558 |
| ebi-fl187-GCST90094785 | ebi-pw11-GCST90275192 | Placental weight (fetal effect, conditional on maternal and paternal genotype) \|\| id:ebi-pw11-GCST90275192 | F-highly palatable foods liking (derived food-liking factor) \|\| id:ebi-fl187-GCST90094785 | Weighted median | 13 | 0.172995896696873 | 0.215383173609252 | 0.421858792273518 | -0.249155124 | 0.595146916971007 | 1.18886122681603 | 0.779459051529626 | 1.81329732954266 | 0.999489402457283 |
| ebi-fl187-GCST90094785 | ebi-pw11-GCST90275193 | Placental weight (maternal effect, conditional on fetal and paternal genotype) \|\| id:ebi-pw11-GCST90275193 | F-highly palatable foods liking (derived food-liking factor) \|\| id:ebi-fl187-GCST90094785 | Weighted median | 13 | -0.086020325 | 0.168309278931709 | 0.609291634365057 | -0.415906512 | 0.243865861845447 | 0.917575581434748 | 0.659741945842177 | 1.27617313549854 | 0.999489402457283 |
| ebi-fl187-GCST90094785 | ebi-pw11-GCST90275195 | Placental weight (fetal effect, conditional on maternal genotype) \|\| id:ebi-pw11-GCST90275195 | F-highly palatable foods liking (derived food-liking factor) \|\| id:ebi-fl187-GCST90094785 | Weighted median | 13 | 0.0813881189471501 | 0.147197331411388 | 0.580319259527878 | -0.207118651 | 0.36989488851347 | 1.08479184314084 | 0.812923190425309 | 1.44758244912319 | 0.999489402457283 |
| ebi-fl187-GCST90094785 | ebi-pw11-GCST90275197 | Placental weight (fetal genotype, adjusted for sex) \|\| id:ebi-pw11-GCST90275197 | F-highly palatable foods liking (derived food-liking factor) \|\| id:ebi-fl187-GCST90094785 | Weighted median | 13 | 0.133825902286321 | 0.110390144141991 | 0.225397804655662 | -0.08253878 | 0.350190584804624 | 1.14319377489603 | 0.920775729268031 | 1.41933802707848 | 0.999489402457283 |
| ebi-fl187-GCST90094785 | prot-a-1030 | Protein FAM171B \|\| id:prot-a-1030 | F-highly palatable foods liking (derived food-liking factor) \|\| id:ebi-fl187-GCST90094785 | Weighted median | 17 | -0.095794948 | 0.364895187324705 | 0.792914941669465 | -0.810989515 | 0.619399619223187 | 0.908650317592887 | 0.44441809017103 | 1.85781231214938 | 0.999489402457283 |
| ebi-fl187-GCST90094785 | prot-a-1036 | BRISC complex subunit Abro1 \|\| id:prot-a-1036 | F-highly palatable foods liking (derived food-liking factor) \|\| id:ebi-fl187-GCST90094785 | Weighted median | 17 | 0.446862038544637 | 0.395048702559209 | 0.257989087828717 | -0.327433418 | 1.22115749556069 | 1.5633985956618 | 0.720771279731517 | 3.39111065833219 | 0.999489402457283 |
| ebi-fl187-GCST90094785 | prot-a-1055 | Phenylalanine--tRNA ligase, mitochondrial \|\| id:prot-a-1055 | F-highly palatable foods liking (derived food-liking factor) \|\| id:ebi-fl187-GCST90094785 | Weighted median | 17 | -0.622607254 | 0.423918166216671 | 0.141915046203381 | -1.453486859 | 0.208272352211271 | 0.536543706861055 | 0.233753798808535 | 1.23154853884535 | 0.999489402457283 |
| ebi-fl187-GCST90094785 | prot-a-1083 | Fc receptor-like protein 5 \|\| id:prot-a-1083 | F-highly palatable foods liking (derived food-liking factor) \|\| id:ebi-fl187-GCST90094785 | Weighted median | 17 | -0.592350295 | 0.36575022265154 | 0.105329258341298 | -1.309220732 | 0.124520140930335 | 0.553025981652525 | 0.270030400503752 | 1.13260483194554 | 0.999489402457283 |
| ebi-fl187-GCST90094785 | prot-a-1103 | Fibroblast growth factor receptor 3 \|\| id:prot-a-1103 | F-highly palatable foods liking (derived food-liking factor) \|\| id:ebi-fl187-GCST90094785 | Weighted median | 17 | 0.164585869929699 | 0.405500135275936 | 0.68482810108423 | -0.630194395 | 0.959366135070533 | 1.17890479767742 | 0.532488277773828 | 2.61004153518127 | 0.999489402457283 |
| ebi-fl187-GCST90094785 | prot-a-1105 | Fibroblast growth factor receptor 4 \|\| id:prot-a-1105 | F-highly palatable foods liking (derived food-liking factor) \|\| id:ebi-fl187-GCST90094785 | Weighted median | 17 | 0.101752668705677 | 0.375500800078514 | 0.786407516811185 | -0.634228899 | 0.837734236859564 | 1.10710961500862 | 0.530344279454303 | 2.3111245791238 | 0.999489402457283 |
| ebi-fl187-GCST90094785 | prot-a-1120 | Filamin-A \|\| id:prot-a-1120 | F-highly palatable foods liking (derived food-liking factor) \|\| id:ebi-fl187-GCST90094785 | Weighted median | 17 | 0.230365348100172 | 0.406156092078892 | 0.570588991422313 | -0.565700592 | 1.0264312885748 | 1.2590599210607 | 0.567962097424223 | 2.79108745462168 | 0.999489402457283 |
| ebi-fl187-GCST90094785 | prot-a-1166 | Galanin peptides \|\| id:prot-a-1166 | F-highly palatable foods liking (derived food-liking factor) \|\| id:ebi-fl187-GCST90094785 | Weighted median | 17 | -0.11032061 | 0.36561571999873 | 0.762850851569535 | -0.826927421 | 0.606286201292449 | 0.895546968036254 | 0.437391142580862 | 1.83360908322614 | 0.999489402457283 |
| ebi-fl187-GCST90094785 | prot-a-1205 | ADP-ribosylation factor-binding protein GGA1 \|\| id:prot-a-1205 | F-highly palatable foods liking (derived food-liking factor) \|\| id:ebi-fl187-GCST90094785 | Weighted median | 17 | 0.308084530888889 | 0.385416763829657 | 0.42408502637579 | -0.447332326 | 1.06350138799502 | 1.36081601506282 | 0.639331406378938 | 2.89649500771414 | 0.999489402457283 |
| ebi-fl187-GCST90094785 | prot-a-1227 | Guanine nucleotide-binding protein G(I)/G(S)/G(O) subunit gamma-T2 \|\| id:prot-a-1227 | F-highly palatable foods liking (derived food-liking factor) \|\| id:ebi-fl187-GCST90094785 | Weighted median | 17 | -0.43027049 | 0.369669656120365 | 0.244451408479244 | -1.154823016 | 0.294282036311783 | 0.650333162518755 | 0.315113302081963 | 1.34216238882114 | 0.999489402457283 |
| ebi-fl187-GCST90094785 | prot-a-1240 | Golgi SNAP receptor complex member 1 \|\| id:prot-a-1240 | F-highly palatable foods liking (derived food-liking factor) \|\| id:ebi-fl187-GCST90094785 | Weighted median | 17 | -0.144569307 | 0.379325200071352 | 0.703112473947884 | -0.888046699 | 0.598908085636667 | 0.865394932827976 | 0.411458671106911 | 1.82013028854056 | 0.999489402457283 |
| ebi-fl187-GCST90094785 | prot-a-1283 | Glutathione S-transferase A1 \|\| id:prot-a-1283 | F-highly palatable foods liking (derived food-liking factor) \|\| id:ebi-fl187-GCST90094785 | Weighted median | 17 | 0.0503357484433817 | 0.359096189779824 | 0.888522955996363 | -0.653492784 | 0.754164280411838 | 1.05162411827016 | 0.520225564526741 | 2.12583417951321 | 0.999489402457283 |
| ebi-fl187-GCST90094785 | prot-a-1324 | E3 ubiquitin-protein ligase HECW1 \|\| id:prot-a-1324 | F-highly palatable foods liking (derived food-liking factor) \|\| id:ebi-fl187-GCST90094785 | Weighted median | 17 | -0.19849206 | 0.388560620121587 | 0.609463542785397 | -0.960070876 | 0.563086755302398 | 0.819966281135388 | 0.382865749183632 | 1.75608474676204 | 0.999489402457283 |
| ebi-fl187-GCST90094785 | prot-a-1339 | Histidine triad nucleotide-binding protein 2, mitochondrial \|\| id:prot-a-1339 | F-highly palatable foods liking (derived food-liking factor) \|\| id:ebi-fl187-GCST90094785 | Weighted median | 17 | 0.462872819951841 | 0.362182944488142 | 0.201246489247364 | -0.247005751 | 1.1727513911486 | 1.58863128736403 | 0.781136201012852 | 3.23086980723657 | 0.999489402457283 |
| ebi-fl187-GCST90094785 | prot-a-1466 | Interleukin-11 \|\| id:prot-a-1466 | F-highly palatable foods liking (derived food-liking factor) \|\| id:ebi-fl187-GCST90094785 | Weighted median | 17 | 0.238825281849216 | 0.388273131968984 | 0.538491270015716 | -0.522190057 | 0.999840620508425 | 1.26975666766522 | 0.593219938919134 | 2.71784862460609 | 0.999489402457283 |
| ebi-fl187-GCST90094785 | prot-a-147 | Rho GTPase-activating protein 1 \|\| id:prot-a-147 | F-highly palatable foods liking (derived food-liking factor) \|\| id:ebi-fl187-GCST90094785 | Weighted median | 17 | 0.0438110390830988 | 0.400674659788685 | 0.9129303038813 | -0.741511294 | 0.829133372268921 | 1.04478491271953 | 0.476393400666244 | 2.29133214759015 | 0.999489402457283 |
| ebi-fl187-GCST90094785 | prot-a-1473 | Interleukin-12 receptor subunit beta-1 \|\| id:prot-a-1473 | F-highly palatable foods liking (derived food-liking factor) \|\| id:ebi-fl187-GCST90094785 | Weighted median | 17 | 0.160810439206062 | 0.402078299709039 | 0.689194767089917 | -0.627263028 | 0.948883906635778 | 1.17446231570237 | 0.534051486382193 | 2.58282537578754 | 0.999489402457283 |
| ebi-fl187-GCST90094785 | prot-a-1501 | X-linked interleukin-1 receptor accessory protein-like 2 \|\| id:prot-a-1501 | F-highly palatable foods liking (derived food-liking factor) \|\| id:ebi-fl187-GCST90094785 | Weighted median | 17 | 0.301775079185754 | 0.374080147436119 | 0.419832220475184 | -0.43142201 | 1.03497216816055 | 1.3522570417471 | 0.649584721812191 | 2.81502788712957 | 0.999489402457283 |
| ebi-fl187-GCST90094785 | prot-a-1503 | Interleukin-1 receptor-like 2 \|\| id:prot-a-1503 | F-highly palatable foods liking (derived food-liking factor) \|\| id:ebi-fl187-GCST90094785 | Weighted median | 17 | 0.150029742172758 | 0.364937749950154 | 0.68099150840646 | -0.565248248 | 0.86530773207506 | 1.16186879871693 | 0.568219070153242 | 2.37573706399515 | 0.999489402457283 |
| ebi-fl187-GCST90094785 | prot-a-1536 | Interleukin-5 receptor subunit alpha \|\| id:prot-a-1536 | F-highly palatable foods liking (derived food-liking factor) \|\| id:ebi-fl187-GCST90094785 | Weighted median | 17 | -0.752895746 | 0.401870687893324 | 0.0610024583681727 | -1.540562294 | 0.0347708025922947 | 0.471000677904712 | 0.214260590115317 | 1.03538237464622 | 0.999489402457283 |
| ebi-fl187-GCST90094785 | prot-a-1559 | Insulin-induced gene 1 protein \|\| id:prot-a-1559 | F-highly palatable foods liking (derived food-liking factor) \|\| id:ebi-fl187-GCST90094785 | Weighted median | 17 | -0.039021573 | 0.373561328627004 | 0.916805676441135 | -0.771201777 | 0.693158631001458 | 0.961729961418756 | 0.462456963978526 | 2.00002290101414 | 0.999489402457283 |
| ebi-fl187-GCST90094785 | prot-a-1567 | Interferon regulatory factor 1 \|\| id:prot-a-1567 | F-highly palatable foods liking (derived food-liking factor) \|\| id:ebi-fl187-GCST90094785 | Weighted median | 17 | 0.108839479183107 | 0.3819920037292 | 0.775700784916146 | -0.639864848 | 0.857543806492339 | 1.11498335793639 | 0.527363693418225 | 2.35736343625992 | 0.999489402457283 |
| ebi-fl187-GCST90094785 | prot-a-1613 | Potassium voltage-gated channel subfamily E regulatory beta subunit 5 \|\| id:prot-a-1613 | F-highly palatable foods liking (derived food-liking factor) \|\| id:ebi-fl187-GCST90094785 | Weighted median | 17 | -0.097215288 | 0.36050057246859 | 0.78741606589478 | -0.80379641 | 0.609365833979366 | 0.907360641192838 | 0.447626361035439 | 1.83926462972697 | 0.999489402457283 |
| ebi-fl187-GCST90094785 | prot-a-1649 | Kin of IRRE-like protein 2 \|\| id:prot-a-1649 | F-highly palatable foods liking (derived food-liking factor) \|\| id:ebi-fl187-GCST90094785 | Weighted median | 17 | 0.505954495353705 | 0.384492802657794 | 0.188207245285371 | -0.247651398 | 1.25956038856298 | 1.65856786048926 | 0.780632025849216 | 3.52387201236765 | 0.999489402457283 |
| ebi-fl187-GCST90094785 | prot-a-165 | Armadillo repeat-containing protein 5 \|\| id:prot-a-165 | F-highly palatable foods liking (derived food-liking factor) \|\| id:ebi-fl187-GCST90094785 | Weighted median | 17 | -0.549271893 | 0.39442056479662 | 0.16373939141441 | -1.3223362 | 0.223792414370203 | 0.577370044758176 | 0.266511948993562 | 1.25081134201646 | 0.999489402457283 |
| ebi-fl187-GCST90094785 | prot-a-1656 | Kallikrein-11 \|\| id:prot-a-1656 | F-highly palatable foods liking (derived food-liking factor) \|\| id:ebi-fl187-GCST90094785 | Weighted median | 17 | -0.342037702 | 0.36258061923711 | 0.345505752095937 | -1.052695716 | 0.368620311718257 | 0.710321423675621 | 0.348995686766539 | 1.44573856945712 | 0.999489402457283 |
| ebi-fl187-GCST90094785 | prot-a-1697 | Laminin subunit gamma-2 \|\| id:prot-a-1697 | F-highly palatable foods liking (derived food-liking factor) \|\| id:ebi-fl187-GCST90094785 | Weighted median | 17 | 0.191694704065429 | 0.367909192689138 | 0.602340235369133 | -0.529407314 | 0.91279672173614 | 1.21130065534353 | 0.588953931237916 | 2.49128021703101 | 0.999489402457283 |
| ebi-fl187-GCST90094785 | prot-a-1698 | Lysosome-associated membrane glycoprotein 3 \|\| id:prot-a-1698 | F-highly palatable foods liking (derived food-liking factor) \|\| id:ebi-fl187-GCST90094785 | Weighted median | 17 | -0.633006096 | 0.361238617221141 | 0.0797185884587809 | -1.341033786 | 0.0750215936964014 | 0.53099318289752 | 0.26157511611436 | 1.07790742663905 | 0.999489402457283 |
| ebi-fl187-GCST90094785 | prot-a-1724 | Leptin receptor \|\| id:prot-a-1724 | F-highly palatable foods liking (derived food-liking factor) \|\| id:ebi-fl187-GCST90094785 | Weighted median | 17 | 0.099886333099261 | 0.372206622657379 | 0.788420232871827 | -0.629638647 | 0.829411313507724 | 1.10504530386183 | 0.532784289263268 | 2.29196909179819 | 0.999489402457283 |
| ebi-fl187-GCST90094785 | prot-a-1743 | Leukocyte immunoglobulin-like receptor subfamily B member 2 \|\| id:prot-a-1743 | F-highly palatable foods liking (derived food-liking factor) \|\| id:ebi-fl187-GCST90094785 | Weighted median | 17 | -0.436611544 | 0.348495619464612 | 0.210261546816083 | -1.119662958 | 0.246439869858428 | 0.646222411444382 | 0.326389783007373 | 1.2794622466585 | 0.999489402457283 |
| ebi-fl187-GCST90094785 | prot-a-1746 | Leukocyte immunoglobulin-like receptor subfamily B member 4 \|\| id:prot-a-1746 | F-highly palatable foods liking (derived food-liking factor) \|\| id:ebi-fl187-GCST90094785 | Weighted median | 17 | 0.529844404114627 | 0.405204612405786 | 0.191009977581534 | -0.264356636 | 1.32404544442997 | 1.69866798230638 | 0.767699701324508 | 3.75859585347569 | 0.999489402457283 |
| ebi-fl187-GCST90094785 | prot-a-1750 | Leucine-rich repeat and immunoglobulin-like domain-containing nogo receptor-interacting protein 3 \|\| id:prot-a-1750 | F-highly palatable foods liking (derived food-liking factor) \|\| id:ebi-fl187-GCST90094785 | Weighted median | 17 | 0.457145438343818 | 0.356361073991167 | 0.199556792234874 | -0.241322267 | 1.15561314336651 | 1.57955859593075 | 0.785588416620019 | 3.17597014568193 | 0.999489402457283 |
| ebi-fl187-GCST90094785 | prot-a-1753 | Lipase member N \|\| id:prot-a-1753 | F-highly palatable foods liking (derived food-liking factor) \|\| id:ebi-fl187-GCST90094785 | Weighted median | 17 | -0.028830872 | 0.374680153180639 | 0.938664946168973 | -0.763203973 | 0.7055422277899 | 0.971580771652994 | 0.46617043439474 | 2.02494436840776 | 0.999489402457283 |
| ebi-fl187-GCST90094785 | prot-a-1760 | Ig Kappa chain V-I region HK102- like \|\| id:prot-a-1760 | F-highly palatable foods liking (derived food-liking factor) \|\| id:ebi-fl187-GCST90094785 | Weighted median | 17 | -0.307042932 | 0.346676061793346 | 0.375791733206646 | -0.986528013 | 0.372442148771795 | 0.735619018381168 | 0.372869042507792 | 1.4512745187012 | 0.999489402457283 |
| ebi-fl187-GCST90094785 | prot-a-1792 | Leucine-rich repeat neuronal protein 1 \|\| id:prot-a-1792 | F-highly palatable foods liking (derived food-liking factor) \|\| id:ebi-fl187-GCST90094785 | Weighted median | 16 | 0.380974118940208 | 0.411145205167721 | 0.354125447503538 | -0.424870483 | 1.18681872106894 | 1.46370972256066 | 0.653854464791458 | 3.27664070108004 | 0.999489402457283 |
| ebi-fl187-GCST90094785 | prot-a-1795 | Leucine-rich repeat transmembrane neuronal protein 1 \|\| id:prot-a-1795 | F-highly palatable foods liking (derived food-liking factor) \|\| id:ebi-fl187-GCST90094785 | Weighted median | 17 | 0.194450829497901 | 0.367185036428295 | 0.596408871817107 | -0.525231842 | 0.914133500897359 | 1.21464375677328 | 0.591418232943284 | 2.49461273543421 | 0.999489402457283 |
| ebi-fl187-GCST90094785 | prot-a-1820 | LysM and putative peptidoglycan-binding domain-containing protein 4 \|\| id:prot-a-1820 | F-highly palatable foods liking (derived food-liking factor) \|\| id:ebi-fl187-GCST90094785 | Weighted median | 17 | 0.15887883372365 | 0.372430592625177 | 0.669670809402594 | -0.571085128 | 0.888842795268998 | 1.17219590746245 | 0.564912104145505 | 2.43231333757686 | 0.999489402457283 |
| ebi-fl187-GCST90094785 | prot-a-1844 | Mitogen-activated protein kinase kinase kinase 7:TGF-beta-activated kinase 1 and MAP3K7-binding protein 1 fusion \|\| id:prot-a-1844 | F-highly palatable foods liking (derived food-liking factor) \|\| id:ebi-fl187-GCST90094785 | Weighted median | 17 | 0.182855932102455 | 0.359895915500079 | 0.611397210647881 | -0.522540062 | 0.888251926482609 | 1.20064142173491 | 0.593012345028578 | 2.43087658405517 | 0.999489402457283 |
| ebi-fl187-GCST90094785 | prot-a-1856 | Megakaryocyte-associated tyrosine-protein kinase \|\| id:prot-a-1856 | F-highly palatable foods liking (derived food-liking factor) \|\| id:ebi-fl187-GCST90094785 | Weighted median | 17 | 0.122014024304732 | 0.397727466136088 | 0.759012378338593 | -0.657531809 | 0.901559857931465 | 1.12976994590722 | 0.518128597753621 | 2.46344273643458 | 0.999489402457283 |
| ebi-fl187-GCST90094785 | prot-a-1910 | Stromelysin-2 \|\| id:prot-a-1910 | F-highly palatable foods liking (derived food-liking factor) \|\| id:ebi-fl187-GCST90094785 | Weighted median | 17 | 0.310925603298349 | 0.378499958913415 | 0.411379745644333 | -0.430934316 | 1.05278552276864 | 1.36468768914653 | 0.6499015973976 | 2.86562226707179 | 0.999489402457283 |
| ebi-fl187-GCST90094785 | prot-a-1940 | 39S ribosomal protein L14, mitochondrial \|\| id:prot-a-1940 | F-highly palatable foods liking (derived food-liking factor) \|\| id:ebi-fl187-GCST90094785 | Weighted median | 17 | -0.045080627 | 0.363125297522705 | 0.901199516478579 | -0.75680621 | 0.666644956285667 | 0.955920405866324 | 0.46916244307519 | 1.94769175546558 | 0.999489402457283 |
| ebi-fl187-GCST90094785 | prot-a-1942 | 39S ribosomal protein L33, mitochondrial \|\| id:prot-a-1942 | F-highly palatable foods liking (derived food-liking factor) \|\| id:ebi-fl187-GCST90094785 | Weighted median | 17 | -1.019072407 | 0.360295987389783 | 0.00467767563366486 | -1.725252542 | -0.312892272 | 0.360929580746121 | 0.178128061249184 | 0.731328693210977 | 0.999489402457283 |
| ebi-fl187-GCST90094785 | prot-a-1948 | Mesothelin \|\| id:prot-a-1948 | F-highly palatable foods liking (derived food-liking factor) \|\| id:ebi-fl187-GCST90094785 | Weighted median | 17 | -0.03493505 | 0.404540589383111 | 0.93118235591659 | -0.827834605 | 0.757964504939374 | 0.965668134123213 | 0.436994528114341 | 2.13392819650367 | 0.999489402457283 |
| ebi-fl187-GCST90094785 | prot-a-1980 | Unconventional myosin-VI \|\| id:prot-a-1980 | F-highly palatable foods liking (derived food-liking factor) \|\| id:ebi-fl187-GCST90094785 | Weighted median | 17 | 0.228086376393886 | 0.367003276867067 | 0.534281735025343 | -0.491240046 | 0.947412799053336 | 1.25619382623906 | 0.611867179939804 | 2.57902855524358 | 0.999489402457283 |
| ebi-fl187-GCST90094785 | prot-a-2039 | Neurofascin \|\| id:prot-a-2039 | F-highly palatable foods liking (derived food-liking factor) \|\| id:ebi-fl187-GCST90094785 | Weighted median | 17 | -0.510907292 | 0.387971832702211 | 0.187883219299389 | -1.271332084 | 0.249517499774507 | 0.59995100086737 | 0.280457779400965 | 1.28340602357533 | 0.999489402457283 |
| ebi-fl187-GCST90094785 | prot-a-2041 | NFU1 iron-sulfur cluster scaffold homolog, mitochondrial \|\| id:prot-a-2041 | F-highly palatable foods liking (derived food-liking factor) \|\| id:ebi-fl187-GCST90094785 | Weighted median | 17 | 0.0876251959368779 | 0.360693893426039 | 0.80805569795485 | -0.619334835 | 0.794585227051915 | 1.0915789171338 | 0.538302378342267 | 2.21352269703958 | 0.999489402457283 |
| ebi-fl187-GCST90094785 | prot-a-2088 | Glucocorticoid receptor \|\| id:prot-a-2088 | F-highly palatable foods liking (derived food-liking factor) \|\| id:ebi-fl187-GCST90094785 | Weighted median | 17 | 0.0968563857482209 | 0.380031848647791 | 0.798828246397542 | -0.648006038 | 0.841718809097892 | 1.10170214213031 | 0.523087754897579 | 2.320351793 | 0.999489402457283 |
| ebi-fl187-GCST90094785 | prot-a-2111 | Neuron-specific protein family member 2 \|\| id:prot-a-2111 | F-highly palatable foods liking (derived food-liking factor) \|\| id:ebi-fl187-GCST90094785 | Weighted median | 17 | 0.298859550964336 | 0.383572373308375 | 0.435892685820836 | -0.452942301 | 1.05066140264875 | 1.34832023989639 | 0.635754815163196 | 2.85954180126437 | 0.999489402457283 |
| ebi-fl187-GCST90094785 | prot-a-2125 | Nuclear migration protein nudC \|\| id:prot-a-2125 | F-highly palatable foods liking (derived food-liking factor) \|\| id:ebi-fl187-GCST90094785 | Weighted median | 17 | -0.076841216 | 0.391971819621388 | 0.844580733761507 | -0.845105983 | 0.691423550352619 | 0.926036881676048 | 0.429511835033239 | 1.99655570878026 | 0.999489402457283 |
| ebi-fl187-GCST90094785 | prot-a-2142 | Ornithine decarboxylase \|\| id:prot-a-2142 | F-highly palatable foods liking (derived food-liking factor) \|\| id:ebi-fl187-GCST90094785 | Weighted median | 17 | -0.419181283 | 0.380847798698811 | 0.271047696775978 | -1.165642968 | 0.32728040246306 | 0.657584975493578 | 0.311722170068309 | 1.38719039425438 | 0.999489402457283 |
| ebi-fl187-GCST90094785 | prot-a-2186 | Prostate and testis expressed protein 1 \|\| id:prot-a-2186 | F-highly palatable foods liking (derived food-liking factor) \|\| id:ebi-fl187-GCST90094785 | Weighted median | 17 | 0.18314719918142 | 0.404692937108151 | 0.650866740020886 | -0.610050958 | 0.976345355913395 | 1.20099117998876 | 0.543323181950559 | 2.6547363748268 | 0.999489402457283 |
| ebi-fl187-GCST90094785 | prot-a-2227 | Platelet-derived growth factor C \|\| id:prot-a-2227 | F-highly palatable foods liking (derived food-liking factor) \|\| id:ebi-fl187-GCST90094785 | Weighted median | 17 | -0.138010283 | 0.372357004851144 | 0.710905919275437 | -0.867830013 | 0.59180944631619 | 0.871089734116712 | 0.419861655891499 | 1.80725559058818 | 0.999489402457283 |
| ebi-fl187-GCST90094785 | prot-a-2229 | Platelet-derived growth factor receptor alpha \|\| id:prot-a-2229 | F-highly palatable foods liking (derived food-liking factor) \|\| id:ebi-fl187-GCST90094785 | Weighted median | 17 | 0.173772330245485 | 0.388492451247582 | 0.654659104750825 | -0.587672874 | 0.935217534690746 | 1.18978465700188 | 0.555618776209306 | 2.54776762530395 | 0.999489402457283 |
| ebi-fl187-GCST90094785 | prot-a-2243 | Platelet endothelial aggregation receptor 1 \|\| id:prot-a-2243 | F-highly palatable foods liking (derived food-liking factor) \|\| id:ebi-fl187-GCST90094785 | Weighted median | 17 | -0.449698989 | 0.355342816721417 | 0.205679391299878 | -1.146170909 | 0.246772932204063 | 0.637820113873522 | 0.31785152449357 | 1.27988845832911 | 0.999489402457283 |
| ebi-fl187-GCST90094785 | prot-a-2246 | E3 ubiquitin-protein ligase pellino homolog 2 \|\| id:prot-a-2246 | F-highly palatable foods liking (derived food-liking factor) \|\| id:ebi-fl187-GCST90094785 | Weighted median | 17 | 0.110379304716656 | 0.38492294560412 | 0.774298478088538 | -0.644069669 | 0.864828278100731 | 1.11670156030687 | 0.525150879230989 | 2.37459828043683 | 0.999489402457283 |
| ebi-fl187-GCST90094785 | prot-a-2251 | Platelet factor 4 variant \|\| id:prot-a-2251 | F-highly palatable foods liking (derived food-liking factor) \|\| id:ebi-fl187-GCST90094785 | Weighted median | 17 | -0.135883898 | 0.399053989925785 | 0.7334686748548 | -0.918029718 | 0.646261922379148 | 0.872943977255859 | 0.399305009961894 | 1.90839375518979 | 0.999489402457283 |
| ebi-fl187-GCST90094785 | prot-a-2346 | Protein phosphatase 1L \|\| id:prot-a-2346 | F-highly palatable foods liking (derived food-liking factor) \|\| id:ebi-fl187-GCST90094785 | Weighted median | 17 | 0.128105493454908 | 0.388216602203383 | 0.741411611520748 | -0.632799047 | 0.889010033773538 | 1.1366729079245 | 0.53110313599015 | 2.43272014803824 | 0.999489402457283 |
| ebi-fl187-GCST90094785 | prot-a-2358 | PR domain zinc finger protein 4 \|\| id:prot-a-2358 | F-highly palatable foods liking (derived food-liking factor) \|\| id:ebi-fl187-GCST90094785 | Weighted median | 17 | 0.147365639905203 | 0.36746045281786 | 0.68839207701901 | -0.572856848 | 0.867588127428208 | 1.15877758090417 | 0.56391212429116 | 2.38116086561178 | 0.999489402457283 |
| ebi-fl187-GCST90094785 | prot-a-2390 | Trypsin-1 \|\| id:prot-a-2390 | F-highly palatable foods liking (derived food-liking factor) \|\| id:ebi-fl187-GCST90094785 | Weighted median | 17 | -0.303111372 | 0.386733813222429 | 0.433173545805506 | -1.061109646 | 0.454886902153027 | 0.738516841867519 | 0.346071580356188 | 1.57599513129805 | 0.999489402457283 |
| ebi-fl187-GCST90094785 | prot-a-242 | Brain-derived neurotrophic factor \|\| id:prot-a-242 | F-highly palatable foods liking (derived food-liking factor) \|\| id:ebi-fl187-GCST90094785 | Weighted median | 17 | 0.596764970728816 | 0.346440520100588 | 0.0849679208439493 | -0.082258449 | 1.27578839012597 | 1.81623371707655 | 0.921033887951307 | 3.58152393543644 | 0.999489402457283 |
| ebi-fl187-GCST90094785 | prot-a-2464 | Dihydropteridine reductase \|\| id:prot-a-2464 | F-highly palatable foods liking (derived food-liking factor) \|\| id:ebi-fl187-GCST90094785 | Weighted median | 17 | 0.0522530504956119 | 0.38975798862923 | 0.893350880000428 | -0.711672607 | 0.816178708208902 | 1.05364233349614 | 0.490822557160064 | 2.26184015127315 | 0.999489402457283 |
| ebi-fl187-GCST90094785 | prot-a-2474 | Ras-related protein Rab-27A \|\| id:prot-a-2474 | F-highly palatable foods liking (derived food-liking factor) \|\| id:ebi-fl187-GCST90094785 | Weighted median | 17 | 0.27094420091186 | 0.409947052554796 | 0.508660498277047 | -0.532552022 | 1.07444042391926 | 1.31120190447267 | 0.587104751898872 | 2.92835380523186 | 0.999489402457283 |
| ebi-fl187-GCST90094785 | prot-a-2478 | Ras-related protein Rab-7b \|\| id:prot-a-2478 | F-highly palatable foods liking (derived food-liking factor) \|\| id:ebi-fl187-GCST90094785 | Weighted median | 17 | -0.162653214 | 0.374595643426599 | 0.664135875107475 | -0.896860675 | 0.571554247098988 | 0.849885865821932 | 0.407848019526918 | 1.77101751226286 | 0.999489402457283 |
| ebi-fl187-GCST90094785 | prot-a-2528 | E3 ubiquitin-protein ligase rififylin \|\| id:prot-a-2528 | F-highly palatable foods liking (derived food-liking factor) \|\| id:ebi-fl187-GCST90094785 | Weighted median | 17 | 0.199997853379784 | 0.350044371153829 | 0.567762411206067 | -0.486089114 | 0.886084820841288 | 1.22140013627513 | 0.615026997305295 | 2.42561432169518 | 0.999489402457283 |
| ebi-fl187-GCST90094785 | prot-a-2543 | Protein RIC-3 \|\| id:prot-a-2543 | F-highly palatable foods liking (derived food-liking factor) \|\| id:ebi-fl187-GCST90094785 | Weighted median | 17 | -0.121896793 | 0.369935622034522 | 0.741771653402917 | -0.846970613 | 0.603177025862432 | 0.88523972643671 | 0.42871170061103 | 1.82791692446189 | 0.999489402457283 |
| ebi-fl187-GCST90094785 | prot-a-255 | Bone morphogenetic protein 6 \|\| id:prot-a-255 | F-highly palatable foods liking (derived food-liking factor) \|\| id:ebi-fl187-GCST90094785 | Weighted median | 17 | -0.40274007 | 0.411200649298625 | 0.327370180314554 | -1.208693343 | 0.403213202216785 | 0.668485835993192 | 0.298587175657186 | 1.49662594161975 | 0.999489402457283 |
| ebi-fl187-GCST90094785 | prot-a-2568 | RING finger protein 165 \|\| id:prot-a-2568 | F-highly palatable foods liking (derived food-liking factor) \|\| id:ebi-fl187-GCST90094785 | Weighted median | 17 | 0.225985775329242 | 0.384013152882555 | 0.556207157308779 | -0.526680004 | 0.978651554979049 | 1.2535578337039 | 0.590562383138752 | 2.66086579048368 | 0.999489402457283 |
| ebi-fl187-GCST90094785 | prot-a-2573 | E3 ubiquitin-protein ligase RNF8 \|\| id:prot-a-2573 | F-highly palatable foods liking (derived food-liking factor) \|\| id:ebi-fl187-GCST90094785 | Weighted median | 17 | -0.124943044 | 0.386432522586547 | 0.74644961538148 | -0.882350788 | 0.632464700348088 | 0.88254716757885 | 0.413808990101081 | 1.88224403440629 | 0.999489402457283 |
| ebi-fl187-GCST90094785 | prot-a-260 | Cytoplasmic tyrosine-protein kinase BMX \|\| id:prot-a-260 | F-highly palatable foods liking (derived food-liking factor) \|\| id:ebi-fl187-GCST90094785 | Weighted median | 16 | 0.0364086140037528 | 0.37631156214486 | 0.922923939138134 | -0.701162048 | 0.773979275807679 | 1.03707952514149 | 0.496008583084953 | 2.16837768164889 | 0.999489402457283 |
| ebi-fl187-GCST90094785 | prot-a-2623 | Serum amyloid A-1 protein \|\| id:prot-a-2623 | F-highly palatable foods liking (derived food-liking factor) \|\| id:ebi-fl187-GCST90094785 | Weighted median | 17 | 0.149764869978235 | 0.368165728987652 | 0.684164759606015 | -0.571839959 | 0.871369698794034 | 1.16156109273164 | 0.564485851862518 | 2.39018244247606 | 0.999489402457283 |
| ebi-fl187-GCST90094785 | prot-a-2640 | Lysosome membrane protein 2 \|\| id:prot-a-2640 | F-highly palatable foods liking (derived food-liking factor) \|\| id:ebi-fl187-GCST90094785 | Weighted median | 17 | 0.052000677315732 | 0.364469266253138 | 0.886546808163222 | -0.662359085 | 0.766360439171882 | 1.05337645598157 | 0.515633475579682 | 2.15191994035853 | 0.999489402457283 |
| ebi-fl187-GCST90094785 | prot-a-2641 | Scavenger receptor class F member 2 \|\| id:prot-a-2641 | F-highly palatable foods liking (derived food-liking factor) \|\| id:ebi-fl187-GCST90094785 | Weighted median | 17 | 0.717922146361247 | 0.397425939997412 | 0.0708511968708214 | -0.061032696 | 1.49687698875618 | 2.0501688310342 | 0.940792479090554 | 4.46771453764946 | 0.999489402457283 |
| ebi-fl187-GCST90094785 | prot-a-2669 | Semaphorin-3A \|\| id:prot-a-2669 | F-highly palatable foods liking (derived food-liking factor) \|\| id:ebi-fl187-GCST90094785 | Weighted median | 17 | 0.393438776941289 | 0.339413614393072 | 0.246386059755975 | -0.271811907 | 1.05868946115171 | 1.48206854418495 | 0.761997573815948 | 2.882590766 | 0.999489402457283 |
| ebi-fl187-GCST90094785 | prot-a-2683 | Sentrin-specific protease 7 \|\| id:prot-a-2683 | F-highly palatable foods liking (derived food-liking factor) \|\| id:ebi-fl187-GCST90094785 | Weighted median | 17 | 0.238795953291572 | 0.398889360162967 | 0.549404905187268 | -0.543027193 | 1.02061909921099 | 1.26971942807969 | 0.580986828590186 | 2.77491217822463 | 0.999489402457283 |
| ebi-fl187-GCST90094785 | prot-a-270 | Bromodomain testis-specific protein \|\| id:prot-a-270 | F-highly palatable foods liking (derived food-liking factor) \|\| id:ebi-fl187-GCST90094785 | Weighted median | 17 | 0.113982714528184 | 0.382025112303179 | 0.765425014850772 | -0.634786506 | 0.862751934642415 | 1.12073275232232 | 0.530048638662133 | 2.36967291397685 | 0.999489402457283 |
| ebi-fl187-GCST90094785 | prot-a-2708 | 14-3-3 protein sigma \|\| id:prot-a-2708 | F-highly palatable foods liking (derived food-liking factor) \|\| id:ebi-fl187-GCST90094785 | Weighted median | 17 | 0.894553681760351 | 0.36795945728939 | 0.015052218655429 | 0.173353145473146 | 1.61575421804756 | 2.44624374257928 | 1.18928602190872 | 5.03168147768542 | 0.999489402457283 |
| ebi-fl187-GCST90094785 | prot-a-2713 | Beta-sarcoglycan \|\| id:prot-a-2713 | F-highly palatable foods liking (derived food-liking factor) \|\| id:ebi-fl187-GCST90094785 | Weighted median | 17 | 0.100402059228048 | 0.36551292828357 | 0.783555858812018 | -0.61600328 | 0.816807398663845 | 1.10561535158014 | 0.54009875300535 | 2.26326259567862 | 0.999489402457283 |
| ebi-fl187-GCST90094785 | prot-a-2720 | Protein shisa-3 homolog \|\| id:prot-a-2720 | F-highly palatable foods liking (derived food-liking factor) \|\| id:ebi-fl187-GCST90094785 | Weighted median | 17 | -0.160036852 | 0.382405849295357 | 0.675581577862446 | -0.909552317 | 0.589478612144541 | 0.852112385937722 | 0.402704467591255 | 1.80304808290695 | 0.999489402457283 |
| ebi-fl187-GCST90094785 | prot-a-2730 | Sialic acid-binding Ig-like lectin 8 \|\| id:prot-a-2730 | F-highly palatable foods liking (derived food-liking factor) \|\| id:ebi-fl187-GCST90094785 | Weighted median | 17 | -0.035785948 | 0.377249261339768 | 0.92442585398377 | -0.7751945 | 0.703622604375961 | 0.964846798912613 | 0.460614182792292 | 2.02106096631355 | 0.999489402457283 |
| ebi-fl187-GCST90094785 | prot-a-2761 | Sodium- and chloride-dependent neutral and basic amino acid transporter B(0+) \|\| id:prot-a-2761 | F-highly palatable foods liking (derived food-liking factor) \|\| id:ebi-fl187-GCST90094785 | Weighted median | 17 | -0.33137715 | 0.357462865265774 | 0.353913355080201 | -1.032004366 | 0.369250066085679 | 0.717934349152448 | 0.356292104698314 | 1.44664931637871 | 0.999489402457283 |
| ebi-fl187-GCST90094785 | prot-a-2770 | Secreted Ly-6/uPAR-related protein 1 \|\| id:prot-a-2770 | F-highly palatable foods liking (derived food-liking factor) \|\| id:ebi-fl187-GCST90094785 | Weighted median | 17 | 0.30804253869134 | 0.37076944280699 | 0.406075520261384 | -0.418665569 | 1.03475064659304 | 1.36075887260766 | 0.657924188585409 | 2.81440436680359 | 0.999489402457283 |
| ebi-fl187-GCST90094785 | prot-a-2784 | N-lysine methyltransferase SMYD2 \|\| id:prot-a-2784 | F-highly palatable foods liking (derived food-liking factor) \|\| id:ebi-fl187-GCST90094785 | Weighted median | 17 | 0.245192010042871 | 0.369359012134507 | 0.506798399726678 | -0.478751654 | 0.969135673826504 | 1.2778666528811 | 0.619556330085508 | 2.63566539998092 | 0.999489402457283 |
| ebi-fl187-GCST90094785 | prot-a-2837 | Serine palmitoyltransferase 2 \|\| id:prot-a-2837 | F-highly palatable foods liking (derived food-liking factor) \|\| id:ebi-fl187-GCST90094785 | Weighted median | 17 | -0.279918701 | 0.367354691439565 | 0.446069060448651 | -0.999933896 | 0.4400964944928 | 0.755845188620902 | 0.367903760296095 | 1.55285705343586 | 0.999489402457283 |
| ebi-fl187-GCST90094785 | prot-a-2858 | Alpha-2,8-sialyltransferase 8B \|\| id:prot-a-2858 | F-highly palatable foods liking (derived food-liking factor) \|\| id:ebi-fl187-GCST90094785 | Weighted median | 17 | 0.255698903974369 | 0.386780547457422 | 0.50855103025369 | -0.502390969 | 1.01378877699092 | 1.29136384487573 | 0.605082195988131 | 2.75602321619946 | 0.999489402457283 |
| ebi-fl187-GCST90094785 | prot-a-2911 | Synaptotagmin-11 \|\| id:prot-a-2911 | F-highly palatable foods liking (derived food-liking factor) \|\| id:ebi-fl187-GCST90094785 | Weighted median | 17 | 0.33894047309092 | 0.401512433450989 | 0.398580368861819 | -0.448023896 | 1.12590484265486 | 1.40345979910227 | 0.638889416645791 | 3.08300522183828 | 0.999489402457283 |
| ebi-fl187-GCST90094785 | prot-a-2962 | Transforming growth factor beta-1 \|\| id:prot-a-2962 | F-highly palatable foods liking (derived food-liking factor) \|\| id:ebi-fl187-GCST90094785 | Weighted median | 17 | -0.477513226 | 0.375322566410478 | 0.203275704089832 | -1.213145457 | 0.258119003798913 | 0.62032408090882 | 0.297260786471105 | 1.29449285902625 | 0.999489402457283 |
| ebi-fl187-GCST90094785 | prot-a-2978 | Tyrosine-protein kinase receptor Tie-1, soluble \|\| id:prot-a-2978 | F-highly palatable foods liking (derived food-liking factor) \|\| id:ebi-fl187-GCST90094785 | Weighted median | 17 | -0.612082812 | 0.372547684612277 | 0.100390171897632 | -1.342276274 | 0.118110649610969 | 0.542220348978936 | 0.261250313927061 | 1.12536862607914 | 0.999489402457283 |
| ebi-fl187-GCST90094785 | prot-a-299 | Uncharacterized protein C1orf226 \|\| id:prot-a-299 | F-highly palatable foods liking (derived food-liking factor) \|\| id:ebi-fl187-GCST90094785 | Weighted median | 17 | -0.645636023 | 0.378502686269592 | 0.0880521429798528 | -1.387501288 | 0.0962292419407974 | 0.524328950682312 | 0.249698450231873 | 1.10101143306384 | 0.999489402457283 |
| ebi-fl187-GCST90094785 | prot-a-3015 | Transmembrane protein 70, mitochondrial \|\| id:prot-a-3015 | F-highly palatable foods liking (derived food-liking factor) \|\| id:ebi-fl187-GCST90094785 | Weighted median | 17 | 0.174002857810447 | 0.383778801678888 | 0.650265334156224 | -0.578203593 | 0.926209309101068 | 1.19005896677845 | 0.56090507560015 | 2.52491982336666 | 0.999489402457283 |
| ebi-fl187-GCST90094785 | prot-a-3016 | Transmembrane protein 87B \|\| id:prot-a-3016 | F-highly palatable foods liking (derived food-liking factor) \|\| id:ebi-fl187-GCST90094785 | Weighted median | 17 | 0.701621940652272 | 0.376977694581158 | 0.0627192878721503 | -0.037254341 | 1.44049822203134 | 2.01702154506403 | 0.96343106442747 | 4.2227991845687 | 0.999489402457283 |
| ebi-fl187-GCST90094785 | prot-a-3123 | Thioredoxin domain-containing protein 12 \|\| id:prot-a-3123 | F-highly palatable foods liking (derived food-liking factor) \|\| id:ebi-fl187-GCST90094785 | Weighted median | 17 | 0.1586997966921 | 0.411877284816746 | 0.700008904468816 | -0.648579682 | 0.965979274932922 | 1.1719860597726 | 0.522787774822194 | 2.62735930420037 | 0.999489402457283 |
| ebi-fl187-GCST90094785 | prot-a-3133 | PolyUbiquitin K48-linked \|\| id:prot-a-3133 | F-highly palatable foods liking (derived food-liking factor) \|\| id:ebi-fl187-GCST90094785 | Weighted median | 17 | -0.056835734 | 0.367845449400543 | 0.877207769652695 | -0.777812815 | 0.664141346974944 | 0.94474924694236 | 0.459409727462453 | 1.94282159528523 | 0.999489402457283 |
| ebi-fl187-GCST90094785 | prot-a-3135 | Ubiquitin-conjugating enzyme E2 B \|\| id:prot-a-3135 | F-highly palatable foods liking (derived food-liking factor) \|\| id:ebi-fl187-GCST90094785 | Weighted median | 17 | 0.0264051768185233 | 0.378853903778712 | 0.944434417243444 | -0.716148475 | 0.768958828224798 | 1.02675688229135 | 0.488630609584634 | 2.15751873634933 | 0.999489402457283 |
| ebi-fl187-GCST90094785 | prot-a-3162 | NKG2D ligand 3 \|\| id:prot-a-3162 | F-highly palatable foods liking (derived food-liking factor) \|\| id:ebi-fl187-GCST90094785 | Weighted median | 17 | 0.363899345126726 | 0.350697057981107 | 0.299434965872043 | -0.323466889 | 1.0512655787697 | 1.43892937161506 | 0.723635918184651 | 2.8612699901502 | 0.999489402457283 |
| ebi-fl187-GCST90094785 | prot-a-3188 | Vesicle-associated membrane protein 8 \|\| id:prot-a-3188 | F-highly palatable foods liking (derived food-liking factor) \|\| id:ebi-fl187-GCST90094785 | Weighted median | 17 | 0.268295718093412 | 0.378335103395602 | 0.478232415314182 | -0.473241085 | 1.00983252074879 | 1.30773380338747 | 0.622979862244655 | 2.74514122231875 | 0.999489402457283 |
| ebi-fl187-GCST90094785 | prot-a-3201 | Vitrin \|\| id:prot-a-3201 | F-highly palatable foods liking (derived food-liking factor) \|\| id:ebi-fl187-GCST90094785 | Weighted median | 17 | -0.195289973 | 0.376704480256282 | 0.604167418152122 | -0.933630754 | 0.54305080823892 | 0.822596092759982 | 0.393123780198596 | 1.72125006399296 | 0.999489402457283 |
| ebi-fl187-GCST90094785 | prot-a-3232 | WNT1-inducible-signaling pathway protein 3 \|\| id:prot-a-3232 | F-highly palatable foods liking (derived food-liking factor) \|\| id:ebi-fl187-GCST90094785 | Weighted median | 17 | -0.37839984 | 0.371017740159902 | 0.307777363204812 | -1.105594611 | 0.348794930244844 | 0.684956572548832 | 0.331013999088072 | 1.41735850317622 | 0.999489402457283 |
| ebi-fl187-GCST90094785 | prot-a-327 | Carbonic anhydrase-related protein 10 \|\| id:prot-a-327 | F-highly palatable foods liking (derived food-liking factor) \|\| id:ebi-fl187-GCST90094785 | Weighted median | 17 | -0.469281223 | 0.389735331597298 | 0.228550027247423 | -1.233162473 | 0.294600026814323 | 0.625451666954692 | 0.291369670385987 | 1.34258925157921 | 0.999489402457283 |
| ebi-fl187-GCST90094785 | prot-a-332 | Carbonic anhydrase 5A, mitochondrial \|\| id:prot-a-332 | F-highly palatable foods liking (derived food-liking factor) \|\| id:ebi-fl187-GCST90094785 | Weighted median | 17 | -0.21573324 | 0.386571282834258 | 0.576797637707242 | -0.973412954 | 0.541946474355457 | 0.805950268493832 | 0.377791450316471 | 1.71935027841725 | 0.999489402457283 |
| ebi-fl187-GCST90094785 | prot-a-338 | Cell adhesion molecule 3 \|\| id:prot-a-338 | F-highly palatable foods liking (derived food-liking factor) \|\| id:ebi-fl187-GCST90094785 | Weighted median | 17 | -0.346129093 | 0.357092471112995 | 0.332396422905716 | -1.046030336 | 0.353772150876548 | 0.707421158442327 | 0.351329645288132 | 1.42443059423995 | 0.999489402457283 |
| ebi-fl187-GCST90094785 | prot-a-34 | A disintegrin and metalloproteinase with thrombospondin motifs 3 \|\| id:prot-a-34 | F-highly palatable foods liking (derived food-liking factor) \|\| id:ebi-fl187-GCST90094785 | Weighted median | 17 | -0.103892636 | 0.361013848618005 | 0.773514998251648 | -0.811479779 | 0.603694507447195 | 0.90132206190897 | 0.444200261361328 | 1.82886308259737 | 0.999489402457283 |
| ebi-fl187-GCST90094785 | prot-a-358 | Cysteine--tRNA ligase, cytoplasmic \|\| id:prot-a-358 | F-highly palatable foods liking (derived food-liking factor) \|\| id:ebi-fl187-GCST90094785 | Weighted median | 17 | 0.186094572414664 | 0.396688296841862 | 0.638983578329836 | -0.591414489 | 0.963603634224714 | 1.20453617088538 | 0.553543748950846 | 2.62112505058756 | 0.999489402457283 |
| ebi-fl187-GCST90094785 | prot-a-382 | Coiled-coil domain-containing protein 167 \|\| id:prot-a-382 | F-highly palatable foods liking (derived food-liking factor) \|\| id:ebi-fl187-GCST90094785 | Weighted median | 17 | -0.492702413 | 0.373092156286781 | 0.186637534514837 | -1.223963039 | 0.238558213211507 | 0.610973059587624 | 0.294062473466672 | 1.26941760076085 | 0.999489402457283 |
| ebi-fl187-GCST90094785 | prot-a-40 | Pituitary adenylate cyclase-activating polypeptide 27 \|\| id:prot-a-40 | F-highly palatable foods liking (derived food-liking factor) \|\| id:ebi-fl187-GCST90094785 | Weighted median | 17 | -0.074805054 | 0.340150777194406 | 0.825935532539259 | -0.741500577 | 0.591890469627432 | 0.92792436414321 | 0.476398506262696 | 1.80740202635267 | 0.999489402457283 |
| ebi-fl187-GCST90094785 | prot-a-403 | C-C motif chemokine 25 \|\| id:prot-a-403 | F-highly palatable foods liking (derived food-liking factor) \|\| id:ebi-fl187-GCST90094785 | Weighted median | 17 | -0.02946733 | 0.362970418402824 | 0.935295755723912 | -0.74088935 | 0.681954689897391 | 0.970962598303978 | 0.476689782774193 | 1.97773982445896 | 0.999489402457283 |
| ebi-fl187-GCST90094785 | prot-a-415 | Cyclin-C \|\| id:prot-a-415 | F-highly palatable foods liking (derived food-liking factor) \|\| id:ebi-fl187-GCST90094785 | Weighted median | 17 | -0.202616591 | 0.397792733632779 | 0.610505397904406 | -0.982290349 | 0.57705716695885 | 0.816591269886523 | 0.374452489129119 | 1.78079014404669 | 0.999489402457283 |
| ebi-fl187-GCST90094785 | prot-a-44 | Adhesion G protein-coupled receptor F5 \|\| id:prot-a-44 | F-highly palatable foods liking (derived food-liking factor) \|\| id:ebi-fl187-GCST90094785 | Weighted median | 17 | -0.03336591 | 0.370410536822406 | 0.928225082295132 | -0.759370562 | 0.692638742243849 | 0.967184592388296 | 0.46796088664594 | 1.99898338183352 | 0.999489402457283 |
| ebi-fl187-GCST90094785 | prot-a-455 | CD59 glycoprotein \|\| id:prot-a-455 | F-highly palatable foods liking (derived food-liking factor) \|\| id:ebi-fl187-GCST90094785 | Weighted median | 17 | -0.094328441 | 0.36235389964219 | 0.794615937685415 | -0.804542084 | 0.615885202202031 | 0.909983837064408 | 0.447292701978929 | 1.85129464454681 | 0.999489402457283 |
| ebi-fl187-GCST90094785 | prot-a-478 | Cadherin-12 \|\| id:prot-a-478 | F-highly palatable foods liking (derived food-liking factor) \|\| id:ebi-fl187-GCST90094785 | Weighted median | 17 | 0.721708140643141 | 0.375801734905695 | 0.0548011841887838 | -0.01486326 | 1.4582795410583 | 2.05794547037241 | 0.985246653244447 | 4.29855767089378 | 0.999489402457283 |
| ebi-fl187-GCST90094785 | prot-a-516 | Centrin-2 \|\| id:prot-a-516 | F-highly palatable foods liking (derived food-liking factor) \|\| id:ebi-fl187-GCST90094785 | Weighted median | 17 | -0.120995857 | 0.376340839749513 | 0.747826927532392 | -0.858623903 | 0.616632188735599 | 0.886037630285495 | 0.423744795197578 | 1.8526780533455 | 0.999489402457283 |
| ebi-fl187-GCST90094785 | prot-a-519 | Cryptic protein \|\| id:prot-a-519 | F-highly palatable foods liking (derived food-liking factor) \|\| id:ebi-fl187-GCST90094785 | Weighted median | 17 | 0.371775103254727 | 0.367432820841557 | 0.311625132226141 | -0.348393226 | 1.09194343210418 | 1.45030677533978 | 0.705821274647448 | 2.98005999273273 | 0.999489402457283 |
| ebi-fl187-GCST90094785 | prot-a-583 | Chloride intracellular channel protein 4 \|\| id:prot-a-583 | F-highly palatable foods liking (derived food-liking factor) \|\| id:ebi-fl187-GCST90094785 | Weighted median | 17 | 0.0507382142890625 | 0.368507327733363 | 0.890488768063425 | -0.671536148 | 0.773012576646454 | 1.05204744624207 | 0.510923121086581 | 2.16628252561878 | 0.999489402457283 |
| ebi-fl187-GCST90094785 | prot-a-602 | CCR4-NOT transcription complex subunit 1 \|\| id:prot-a-602 | F-highly palatable foods liking (derived food-liking factor) \|\| id:ebi-fl187-GCST90094785 | Weighted median | 17 | 0.90861535212114 | 0.365417320763962 | 0.0129001426116972 | 0.192397403423774 | 1.62483330081851 | 2.48088500189312 | 1.21215213466875 | 5.0775725394405 | 0.999489402457283 |
| ebi-fl187-GCST90094785 | prot-a-606 | Ciliary neurotrophic factor receptor subunit alpha \|\| id:prot-a-606 | F-highly palatable foods liking (derived food-liking factor) \|\| id:ebi-fl187-GCST90094785 | Weighted median | 17 | -0.339848019 | 0.37347715945582 | 0.362845380079235 | -1.071863252 | 0.392167213393456 | 0.711878506449138 | 0.342370001267523 | 1.48018519750003 | 0.999489402457283 |
| ebi-fl187-GCST90094785 | prot-a-626 | Collagen alpha-2(IX) chain \|\| id:prot-a-626 | F-highly palatable foods liking (derived food-liking factor) \|\| id:ebi-fl187-GCST90094785 | Weighted median | 17 | -0.422057719 | 0.395088455268984 | 0.285402646501357 | -1.196431091 | 0.352315653496032 | 0.655696192283381 | 0.302271067054056 | 1.42235742496002 | 0.999489402457283 |
| ebi-fl187-GCST90094785 | prot-a-647 | Probable serine carboxypeptidase CPVL \|\| id:prot-a-647 | F-highly palatable foods liking (derived food-liking factor) \|\| id:ebi-fl187-GCST90094785 | Weighted median | 17 | 0.315769445043342 | 0.385674588483128 | 0.412931466548295 | -0.440152748 | 1.07169163847027 | 1.37131405592401 | 0.643938053073817 | 2.9203154418321 | 0.999489402457283 |
| ebi-fl187-GCST90094785 | prot-a-651 | Cellular retinoic acid-binding protein 2 \|\| id:prot-a-651 | F-highly palatable foods liking (derived food-liking factor) \|\| id:ebi-fl187-GCST90094785 | Weighted median | 17 | 0.646826105422535 | 0.365272928245388 | 0.0765935997607006 | -0.069108834 | 1.3627610447835 | 1.90947074236696 | 0.933225107987118 | 3.90696573072246 | 0.999489402457283 |
| ebi-fl187-GCST90094785 | prot-a-671 | Cytotoxic and regulatory T-cell molecule \|\| id:prot-a-671 | F-highly palatable foods liking (derived food-liking factor) \|\| id:ebi-fl187-GCST90094785 | Weighted median | 17 | 0.542879604505855 | 0.397562600937459 | 0.172089733325897 | -0.236343093 | 1.32210230234328 | 1.72095540478057 | 0.789509751928602 | 3.75129945894739 | 0.999489402457283 |
| ebi-fl187-GCST90094785 | prot-a-688 | Chorionic somatomammotropin hormone \|\| id:prot-a-688 | F-highly palatable foods liking (derived food-liking factor) \|\| id:ebi-fl187-GCST90094785 | Weighted median | 17 | -0.702569597 | 0.349097574746359 | 0.0441640206421605 | -1.386800844 | -0.018338351 | 0.495310917684279 | 0.249873411448859 | 0.981828773836768 | 0.999489402457283 |
| ebi-fl187-GCST90094785 | prot-a-69 | Aldo-keto reductase family 1 member C1 \|\| id:prot-a-69 | F-highly palatable foods liking (derived food-liking factor) \|\| id:ebi-fl187-GCST90094785 | Weighted median | 17 | -0.238999025 | 0.375898453457681 | 0.524901952590221 | -0.975759994 | 0.497761944038645 | 0.787415650308553 | 0.376905798724068 | 1.64503546628838 | 0.999489402457283 |
| ebi-fl187-GCST90094785 | prot-a-741 | Stromal cell-derived factor 1 \|\| id:prot-a-741 | F-highly palatable foods liking (derived food-liking factor) \|\| id:ebi-fl187-GCST90094785 | Weighted median | 17 | 0.0876438901975557 | 0.370421195935794 | 0.812962438410029 | -0.638381654 | 0.813669434231712 | 1.09159932358537 | 0.528146456588239 | 2.2561716894771 | 0.999489402457283 |
| ebi-fl187-GCST90094785 | prot-a-742 | Growth-regulated alpha protein \|\| id:prot-a-742 | F-highly palatable foods liking (derived food-liking factor) \|\| id:ebi-fl187-GCST90094785 | Weighted median | 17 | 0.272338657229648 | 0.351832974160372 | 0.438897284874659 | -0.417253972 | 0.961931286583978 | 1.3130315936666 | 0.658853568253104 | 2.61674528156209 | 0.999489402457283 |
| ebi-fl187-GCST90094785 | prot-a-788 | Beta-defensin 112 \|\| id:prot-a-788 | F-highly palatable foods liking (derived food-liking factor) \|\| id:ebi-fl187-GCST90094785 | Weighted median | 17 | 0.0357999069505594 | 0.383260963071695 | 0.925578881979145 | -0.715391581 | 0.786991394571082 | 1.03644843961241 | 0.489000591121798 | 2.19677723806152 | 0.999489402457283 |
| ebi-fl187-GCST90094785 | prot-a-838 | DnaJ homolog subfamily B member 1 \|\| id:prot-a-838 | F-highly palatable foods liking (derived food-liking factor) \|\| id:ebi-fl187-GCST90094785 | Weighted median | 17 | 0.217413228981589 | 0.362234343364049 | 0.548372629712157 | -0.492566084 | 0.927392541975125 | 1.2428575808107 | 0.611056358672045 | 2.52790915969777 | 0.999489402457283 |
| ebi-fl187-GCST90094785 | prot-a-85 | Junctional adhesion molecule-like \|\| id:prot-a-85 | F-highly palatable foods liking (derived food-liking factor) \|\| id:ebi-fl187-GCST90094785 | Weighted median | 17 | -0.284821265 | 0.411149513158035 | 0.488470364303961 | -1.09067431 | 0.521031781225507 | 0.752148677927598 | 0.335989855864554 | 1.68376402987682 | 0.999489402457283 |
| ebi-fl187-GCST90094785 | prot-a-856 | Dipeptidase 1 \|\| id:prot-a-856 | F-highly palatable foods liking (derived food-liking factor) \|\| id:ebi-fl187-GCST90094785 | Weighted median | 17 | 0.217295328392816 | 0.405497128876463 | 0.59204570298242 | -0.577479044 | 1.01206970099068 | 1.24271105580802 | 0.561311626231285 | 2.75128947283049 | 0.999489402457283 |
| ebi-fl187-GCST90094785 | prot-a-860 | Dipeptidyl peptidase 2 \|\| id:prot-a-860 | F-highly palatable foods liking (derived food-liking factor) \|\| id:ebi-fl187-GCST90094785 | Weighted median | 17 | -0.170059189 | 0.367112019194707 | 0.643195827560212 | -0.889598747 | 0.549480368320804 | 0.843614882143569 | 0.41082056269999 | 1.73235259865468 | 0.999489402457283 |
| ebi-fl187-GCST90094785 | prot-a-861 | Dermatopontin \|\| id:prot-a-861 | F-highly palatable foods liking (derived food-liking factor) \|\| id:ebi-fl187-GCST90094785 | Weighted median | 17 | -0.002490123 | 0.391300371150313 | 0.99492252715759 | -0.76943885 | 0.764458604803646 | 0.99751297513262 | 0.463272960958171 | 2.1478312343115 | 0.999911036302558 |
| ebi-fl187-GCST90094785 | prot-a-877 | Dual specificity protein phosphatase 6 \|\| id:prot-a-877 | F-highly palatable foods liking (derived food-liking factor) \|\| id:ebi-fl187-GCST90094785 | Weighted median | 17 | 0.721278865461563 | 0.376801824516689 | 0.0555929743239896 | -0.017252711 | 1.45981044151427 | 2.05706223504601 | 0.982895265203616 | 4.30514337453429 | 0.999489402457283 |
| ebi-fl187-GCST90094785 | prot-a-879 | Dynactin-associated protein \|\| id:prot-a-879 | F-highly palatable foods liking (derived food-liking factor) \|\| id:ebi-fl187-GCST90094785 | Weighted median | 17 | -0.011805668 | 0.350041068698116 | 0.973095228703851 | -0.697886163 | 0.674274826493601 | 0.988263745319581 | 0.497636114508153 | 1.96260922758468 | 0.999489402457283 |
| ebi-fl187-GCST90094785 | prot-a-937 | EMI domain-containing protein 1 \|\| id:prot-a-937 | F-highly palatable foods liking (derived food-liking factor) \|\| id:ebi-fl187-GCST90094785 | Weighted median | 17 | -0.64173848 | 0.372020673462571 | 0.0845261073885065 | -1.370899 | 0.0874220401936008 | 0.526376533178383 | 0.253878620106083 | 1.09135717913198 | 0.999489402457283 |
| ebi-fl187-GCST90094785 | prot-b-1 | SRC proto-oncogene, non-receptor tyrosine kinase \|\| id:prot-b-1 | F-highly palatable foods liking (derived food-liking factor) \|\| id:ebi-fl187-GCST90094785 | Weighted median | 12 | -0.358664964 | 0.516826438241492 | 0.487697479441313 | -1.371644782 | 0.654314855452954 | 0.698608371450129 | 0.253689352414959 | 1.92382396822825 | 0.999489402457283 |
| ebi-fl187-GCST90094785 | prot-b-10 | coagulation factor III, tissue factor \|\| id:prot-b-10 | F-highly palatable foods liking (derived food-liking factor) \|\| id:ebi-fl187-GCST90094785 | Weighted median | 12 | -0.411758244 | 0.508260875205253 | 0.417864490033844 | -1.407949559 | 0.584433071377245 | 0.662484416259858 | 0.244644398024317 | 1.79397364228034 | 0.999489402457283 |
| ebi-fl187-GCST90094785 | prot-b-29 | myeloperoxidase \|\| id:prot-b-29 | F-highly palatable foods liking (derived food-liking factor) \|\| id:ebi-fl187-GCST90094785 | Weighted median | 12 | -0.200088846 | 0.472535579323429 | 0.671976766245632 | -1.126258582 | 0.726080889391116 | 0.818658015288981 | 0.324244122771972 | 2.06696405247789 | 0.999489402457283 |
| ebi-fl187-GCST90094785 | prot-b-33 | adrenomedullin \|\| id:prot-b-33 | F-highly palatable foods liking (derived food-liking factor) \|\| id:ebi-fl187-GCST90094785 | Weighted median | 12 | 0.0949302522685465 | 0.471245887775567 | 0.840350425009901 | -0.828711688 | 1.01857219230866 | 1.0995821590903 | 0.436611415970805 | 2.76923800057152 | 0.999489402457283 |
| ebi-fl187-GCST90094785 | ukb-rsfMRI191-edge-pheno102 | Net25_Pair11_15 (Postcentral\|Precentral)&(Cerebellum) (Motor)&(Subcortical-cerebellum) \|\| id:ukb-rsfMRI191-edge-pheno102 | F-highly palatable foods liking (derived food-liking factor) \|\| id:ebi-fl187-GCST90094785 | Weighted median | 17 | -0.050409564 | 0.111357371863703 | 0.650777183191098 | -0.268670013 | 0.16785088451572 | 0.950839914622125 | 0.764395454452368 | 1.18276022963314 | 0.999489402457283 |
| ebi-fl187-GCST90094785 | ukb-rsfMRI191-edge-pheno1167 | Net100_Pair11_45 (Angular\|Temporal)&(Frontal) (Default_mode\|Central_executive)&(Salience\|Default_mode) \|\| id:ukb-rsfMRI191-edge-pheno1167 | F-highly palatable foods liking (derived food-liking factor) \|\| id:ebi-fl187-GCST90094785 | Weighted median | 17 | 0.073628485580297 | 0.117907622403382 | 0.532326134817684 | -0.157470454 | 0.304727425490925 | 1.07640683054358 | 0.854302054161893 | 1.35625527200394 | 0.999489402457283 |
| ebi-fl187-GCST90094785 | ukb-rsfMRI191-edge-pheno1317 | Net100_Pair26_48 (Parietal\|Frontal)&(Temporal) (Attention\|Central_executive)&(Default_mode\|Central_executive) \|\| id:ukb-rsfMRI191-edge-pheno1317 | F-highly palatable foods liking (derived food-liking factor) \|\| id:ebi-fl187-GCST90094785 | Weighted median | 17 | -0.065208014 | 0.128694439148676 | 0.612373283754804 | -0.317449115 | 0.187033086930036 | 0.936872560684135 | 0.728003724641477 | 1.20566717621549 | 0.999489402457283 |
| ebi-fl187-GCST90094785 | ukb-rsfMRI191-edge-pheno286 | Net100_Pair10_13 (OccipitalPrecuneus)&(Frontal_Sup) (Default_mode\|Central_executive)&(Default_mode) \|\| id:ukb-rsfMRI191-edge-pheno286 | F-highly palatable foods liking (derived food-liking factor) \|\| id:ebi-fl187-GCST90094785 | Weighted median | 17 | 0.0493386053401052 | 0.119957858428134 | 0.680852947496677 | -0.185778797 | 0.284456007859247 | 1.05057602117163 | 0.830457274157741 | 1.32903884474997 | 0.999489402457283 |
| ebi-fl187-GCST90094785 | ukb-rsfMRI191-edge-pheno599 | Net100_Pair11_29 (Angular\|Temporal)&(Parietal) (Default_mode\|Central_executive)&(Central_executive\|Salience) \|\| id:ukb-rsfMRI191-edge-pheno599 | F-highly palatable foods liking (derived food-liking factor) \|\| id:ebi-fl187-GCST90094785 | Weighted median | 17 | -0.121650118 | 0.124500475078947 | 0.328516850826969 | -0.365671049 | 0.122370813507148 | 0.885458120481248 | 0.693730967312505 | 1.13017310754271 | 0.999489402457283 |
| ebi-fl187-GCST90094785 | ukb-rsfMRI191-edge-pheno621 | Net100_Pair5_30 (Precuneus\|Angular\|Cingulate)&(Paracentral) (Default_mode\|Central_executive)&(Motor) \|\| id:ukb-rsfMRI191-edge-pheno621 | F-highly palatable foods liking (derived food-liking factor) \|\| id:ebi-fl187-GCST90094785 | Weighted median | 17 | -0.080340608 | 0.121609497585087 | 0.508840521878958 | -0.318695223 | 0.158014007076675 | 0.922801978939748 | 0.727097117686252 | 1.17118259943725 | 0.999489402457283 |
| ebi-fl187-GCST90094785 | ukb-rsfMRI191-edge-pheno624 | Net100_Pair8_30 (Calcarine\|Lingual)&(Paracentral) (Visual)&(Motor) \|\| id:ukb-rsfMRI191-edge-pheno624 | F-highly palatable foods liking (derived food-liking factor) \|\| id:ebi-fl187-GCST90094785 | Weighted median | 17 | -0.130953141 | 0.120900000446686 | 0.278740727764872 | -0.367917142 | 0.106010860002515 | 0.877258881013251 | 0.692174531684168 | 1.11183395096047 | 0.999489402457283 |
| ebi-fl187-GCST90094785 | ukb-rsfMRI191-edge-pheno882 | Net100_Pair6_38 (Postcentral\|Precentral)&(Subcortical) (Motor)&(Subcortical-cerebellum) \|\| id:ukb-rsfMRI191-edge-pheno882 | F-highly palatable foods liking (derived food-liking factor) \|\| id:ebi-fl187-GCST90094785 | Weighted median | 17 | 0.0378978238226952 | 0.115896880255153 | 0.743670861219878 | -0.189260061 | 0.265055709122795 | 1.03862510471385 | 0.827571259302818 | 1.30350359079729 | 0.999489402457283 |
| ebi-fl187-GCST90094785 | ukb-rsfMRI191-edge-pheno899 | Net100_Pair23_38 (Cerebellum)&(Subcortical) (Subcortical-cerebellum)&(Subcortical-cerebellum) \|\| id:ukb-rsfMRI191-edge-pheno899 | F-highly palatable foods liking (derived food-liking factor) \|\| id:ebi-fl187-GCST90094785 | Weighted median | 17 | 0.0678796645129934 | 0.115733796558086 | 0.557529154597681 | -0.158958577 | 0.294717905766843 | 1.07023651329757 | 0.853031693590191 | 1.34274752392215 | 0.999489402457283 |
| ebi-fl187-GCST90094785 | ebi-a-GCST90001392 | IgD+ CD38+ B cell Absolute Count \|\| id:ebi-a-GCST90001392 | F-highly palatable foods liking (derived food-liking factor) \|\| id:ebi-fl187-GCST90094785 | Weighted mode | 14 | 0.697232768169222 | 0.732182610806227 | 0.358332804027147 | -0.737845149 | 2.13231068534943 | 2.00818789000015 | 0.478143133421119 | 8.43433340282101 | 0.999228396881742 |
| ebi-fl187-GCST90094785 | ebi-a-GCST90001402 | Switched memory B cell %B cell \|\| id:ebi-a-GCST90001402 | F-highly palatable foods liking (derived food-liking factor) \|\| id:ebi-fl187-GCST90094785 | Weighted mode | 14 | -0.034456843 | 0.777949417462227 | 0.965344859830688 | -1.559237702 | 1.49032401489114 | 0.96613003373718 | 0.210296318670301 | 4.43853343696607 | 0.999228396881742 |
| ebi-fl187-GCST90094785 | ebi-a-GCST90001407 | Memory B cell Absolute Count \|\| id:ebi-a-GCST90001407 | F-highly palatable foods liking (derived food-liking factor) \|\| id:ebi-fl187-GCST90094785 | Weighted mode | 14 | 0.437022573990228 | 0.731994999906559 | 0.56074254368232 | -0.997687626 | 1.87173277380708 | 1.54809102325353 | 0.36873110038802 | 6.4995488955399 | 0.999228396881742 |
| ebi-fl187-GCST90094785 | ebi-a-GCST90001411 | IgD+ CD24+ B cell %B cell \|\| id:ebi-a-GCST90001411 | F-highly palatable foods liking (derived food-liking factor) \|\| id:ebi-fl187-GCST90094785 | Weighted mode | 14 | -0.224627838 | 0.897964617919879 | 0.806379314518826 | -1.984638489 | 1.53538281336887 | 0.798813451654746 | 0.137430287715273 | 4.64310263154353 | 0.999228396881742 |
| ebi-fl187-GCST90094785 | ebi-a-GCST90001418 | CD24+ CD27+ B cell Absolute Count \|\| id:ebi-a-GCST90001418 | F-highly palatable foods liking (derived food-liking factor) \|\| id:ebi-fl187-GCST90094785 | Weighted mode | 14 | 0.18275682878119 | 0.702628281427185 | 0.798857248252518 | -1.194394603 | 1.55990826037847 | 1.2005224400782 | 0.302887265786128 | 4.75838469270279 | 0.999228396881742 |
| ebi-fl187-GCST90094785 | ebi-a-GCST90001436 | Memory B cell %lymphocyte \|\| id:ebi-a-GCST90001436 | F-highly palatable foods liking (derived food-liking factor) \|\| id:ebi-fl187-GCST90094785 | Weighted mode | 14 | 0.104771412227114 | 0.643864231438215 | 0.873238878932471 | -1.157202481 | 1.36674530584602 | 1.11045674450738 | 0.314364392140462 | 3.92256315362513 | 0.999228396881742 |
| ebi-fl187-GCST90094785 | ebi-a-GCST90001439 | IgD+ CD24+ B cell %lymphocyte \|\| id:ebi-a-GCST90001439 | F-highly palatable foods liking (derived food-liking factor) \|\| id:ebi-fl187-GCST90094785 | Weighted mode | 14 | 0.48767976051144 | 0.790484609938507 | 0.547929090553412 | -1.061670075 | 2.03702959599091 | 1.62853324580417 | 0.345877686043518 | 7.66779887718968 | 0.999228396881742 |
| ebi-fl187-GCST90094785 | ebi-a-GCST90001457 | CD11c+ HLA DR++ monocyte %monocyte \|\| id:ebi-a-GCST90001457 | F-highly palatable foods liking (derived food-liking factor) \|\| id:ebi-fl187-GCST90094785 | Weighted mode | 14 | -0.009908236 | 0.773124606152385 | 0.989969317576407 | -1.525232464 | 1.50541599175521 | 0.990140688550195 | 0.217570473604692 | 4.50602771083691 | 0.999228396881742 |
| ebi-fl187-GCST90094785 | ebi-a-GCST90001483 | CD39+ resting CD4 regulatory T cell Absolute Count \|\| id:ebi-a-GCST90001483 | F-highly palatable foods liking (derived food-liking factor) \|\| id:ebi-fl187-GCST90094785 | Weighted mode | 14 | -0.382207324 | 0.737321053290438 | 0.612907488199864 | -1.827356589 | 1.06294194032222 | 0.682353570181207 | 0.160838167781756 | 2.89487502351326 | 0.999228396881742 |
| ebi-fl187-GCST90094785 | ebi-a-GCST90001525 | CD33dim HLA DR+ CD11b+ Absolute Count \|\| id:ebi-a-GCST90001525 | F-highly palatable foods liking (derived food-liking factor) \|\| id:ebi-fl187-GCST90094785 | Weighted mode | 14 | 0.856064465979378 | 1.09385064808769 | 0.447875017923799 | -1.287882804 | 3.00001173623124 | 2.35387867123745 | 0.275854202604338 | 20.0857726530769 | 0.999228396881742 |
| ebi-fl187-GCST90094785 | ebi-a-GCST90001540 | Naive CD4+ T cell Absolute Count \|\| id:ebi-a-GCST90001540 | F-highly palatable foods liking (derived food-liking factor) \|\| id:ebi-fl187-GCST90094785 | Weighted mode | 14 | 0.0690409969243562 | 0.754122527451898 | 0.928450149786574 | -1.409039157 | 1.54712115073008 | 1.07148013563798 | 0.24437797928204 | 4.69792607517141 | 0.999228396881742 |
| ebi-fl187-GCST90094785 | ebi-a-GCST90001624 | HLA DR+ CD4+ T cell Absolute Count \|\| id:ebi-a-GCST90001624 | F-highly palatable foods liking (derived food-liking factor) \|\| id:ebi-fl187-GCST90094785 | Weighted mode | 14 | 0.151832857506073 | 0.790706618675689 | 0.850692154767031 | -1.397952115 | 1.70161783011042 | 1.16396567204731 | 0.247102483587226 | 5.4828104761897 | 0.999228396881742 |
| ebi-fl187-GCST90094785 | ebi-a-GCST90001672 | CD39+ CD8+ T cell Absolute Count \|\| id:ebi-a-GCST90001672 | F-highly palatable foods liking (derived food-liking factor) \|\| id:ebi-fl187-GCST90094785 | Weighted mode | 14 | 0.435998385326038 | 0.844487388303276 | 0.614322729284219 | -1.219196896 | 2.09119366640046 | 1.54650629764391 | 0.29546736276002 | 8.09457161803287 | 0.999228396881742 |
| ebi-fl187-GCST90094785 | ebi-a-GCST90001722 | CD19 on CD20- CD38- B cell \|\| id:ebi-a-GCST90001722 | F-highly palatable foods liking (derived food-liking factor) \|\| id:ebi-fl187-GCST90094785 | Weighted mode | 14 | -0.894617807 | 0.987456994311321 | 0.3814309436967 | -2.830033516 | 1.04079790176379 | 0.408763795416241 | 0.0590108758315311 | 2.83147535244361 | 0.999228396881742 |
| ebi-fl187-GCST90094785 | ebi-a-GCST90001742 | CD19 on transitional B cell \|\| id:ebi-a-GCST90001742 | F-highly palatable foods liking (derived food-liking factor) \|\| id:ebi-fl187-GCST90094785 | Weighted mode | 14 | 0.0716078257386483 | 0.675163254490297 | 0.917153873779497 | -1.251712153 | 1.39492780453963 | 1.07423397452859 | 0.286014676495146 | 4.03468327630062 | 0.999228396881742 |
| ebi-fl187-GCST90094785 | ebi-a-GCST90001784 | CD25 on IgD+ CD38dim B cell \|\| id:ebi-a-GCST90001784 | F-highly palatable foods liking (derived food-liking factor) \|\| id:ebi-fl187-GCST90094785 | Weighted mode | 14 | 0.22008644365089 | 0.753440620333907 | 0.774813243139874 | -1.256657172 | 1.69683005950535 | 1.24618445066505 | 0.284603819677186 | 5.45662277772953 | 0.999228396881742 |
| ebi-fl187-GCST90094785 | ebi-a-GCST90001794 | CD25 on IgD+ B cell \|\| id:ebi-a-GCST90001794 | F-highly palatable foods liking (derived food-liking factor) \|\| id:ebi-fl187-GCST90094785 | Weighted mode | 14 | -0.003485975 | 0.777275094935891 | 0.996489699331897 | -1.526945161 | 1.51997321151318 | 0.996520094394029 | 0.217198160386093 | 4.57210271378832 | 0.999228396881742 |
| ebi-fl187-GCST90094785 | ebi-a-GCST90001802 | CD27 on IgD- CD38- B cell \|\| id:ebi-a-GCST90001802 | F-highly palatable foods liking (derived food-liking factor) \|\| id:ebi-fl187-GCST90094785 | Weighted mode | 14 | 0.467973835340293 | 0.698947045631887 | 0.514866651442415 | -0.901962374 | 1.83791004477879 | 1.59675562357296 | 0.4057726002914 | 6.2833925198027 | 0.999228396881742 |
| ebi-fl187-GCST90094785 | ebi-a-GCST90001804 | CD27 on IgD- CD38dim B cell \|\| id:ebi-a-GCST90001804 | F-highly palatable foods liking (derived food-liking factor) \|\| id:ebi-fl187-GCST90094785 | Weighted mode | 14 | 0.283495855513795 | 0.681775334274645 | 0.684323939183805 | -1.0527838 | 1.6197755106921 | 1.32776337740546 | 0.348964947193637 | 5.05195607913279 | 0.999228396881742 |
| ebi-fl187-GCST90094785 | ebi-a-GCST90001805 | CD27 on memory B cell \|\| id:ebi-a-GCST90001805 | F-highly palatable foods liking (derived food-liking factor) \|\| id:ebi-fl187-GCST90094785 | Weighted mode | 14 | 0.426957231071116 | 0.679659088219191 | 0.540756968285385 | -0.905174582 | 1.75908904398073 | 1.53258711321306 | 0.404471265601588 | 5.80714493053843 | 0.999228396881742 |
| ebi-fl187-GCST90094785 | ebi-a-GCST90001806 | CD27 on unswitched memory B cell \|\| id:ebi-a-GCST90001806 | F-highly palatable foods liking (derived food-liking factor) \|\| id:ebi-fl187-GCST90094785 | Weighted mode | 14 | 0.402925357994579 | 0.715445454095365 | 0.582895225724504 | -0.999347732 | 1.80519844802149 | 1.49619520848464 | 0.368119475421697 | 6.08117812655251 | 0.999228396881742 |
| ebi-fl187-GCST90094785 | ebi-a-GCST90001839 | CD3 on Effector Memory CD8+ T cell \|\| id:ebi-a-GCST90001839 | F-highly palatable foods liking (derived food-liking factor) \|\| id:ebi-fl187-GCST90094785 | Weighted mode | 14 | -0.478339856 | 0.809479153335767 | 0.564705138931151 | -2.064918997 | 1.10823928431959 | 0.619811514385658 | 0.126828563676924 | 3.02902045270849 | 0.999228396881742 |
| ebi-fl187-GCST90094785 | ebi-a-GCST90001859 | CD3 on CD8+ T cell \|\| id:ebi-a-GCST90001859 | F-highly palatable foods liking (derived food-liking factor) \|\| id:ebi-fl187-GCST90094785 | Weighted mode | 14 | -0.730175051 | 0.714895368859437 | 0.325709503572788 | -2.131369974 | 0.671019872373121 | 0.481824639019566 | 0.118674601370025 | 1.95623140997525 | 0.999228396881742 |
| ebi-fl187-GCST90094785 | ebi-a-GCST90001905 | CD86 on monocyte \|\| id:ebi-a-GCST90001905 | F-highly palatable foods liking (derived food-liking factor) \|\| id:ebi-fl187-GCST90094785 | Weighted mode | 14 | 0.00201069782552565 | 0.769256873406945 | 0.997954161657125 | -1.505732774 | 1.50975416970314 | 1.00201272063392 | 0.221854664912318 | 4.52561812350897 | 0.999264242604493 |
| ebi-fl187-GCST90094785 | ebi-a-GCST90001943 | CD25 on activated & secreting CD4 regulatory T cell \|\| id:ebi-a-GCST90001943 | F-highly palatable foods liking (derived food-liking factor) \|\| id:ebi-fl187-GCST90094785 | Weighted mode | 14 | -0.888274809 | 0.688929045665446 | 0.219743332332845 | -2.238575739 | 0.462026120416429 | 0.411364823790607 | 0.106610237146494 | 1.58728676327536 | 0.999228396881742 |
| ebi-fl187-GCST90094785 | ebi-a-GCST90001949 | CD33 on CD33dim HLA DR+ CD11b- \|\| id:ebi-a-GCST90001949 | F-highly palatable foods liking (derived food-liking factor) \|\| id:ebi-fl187-GCST90094785 | Weighted mode | 14 | -0.424975854 | 1.10919027603776 | 0.707807423144387 | -2.598988795 | 1.7490370865729 | 0.653785570944194 | 0.0743487219776701 | 5.7490641588056 | 0.999228396881742 |
| ebi-fl187-GCST90094785 | ebi-a-GCST90001955 | CD33 on Immature Myeloid-Derived Suppressor Cells \|\| id:ebi-a-GCST90001955 | F-highly palatable foods liking (derived food-liking factor) \|\| id:ebi-fl187-GCST90094785 | Weighted mode | 14 | -0.440437292 | 1.22704930551243 | 0.725402260337234 | -2.845453931 | 1.96457934686494 | 0.643754850716217 | 0.0581078837973477 | 7.13191189797855 | 0.999228396881742 |
| ebi-fl187-GCST90094785 | ebi-a-GCST90001956 | CD33 on CD33+ HLA DR+ \|\| id:ebi-a-GCST90001956 | F-highly palatable foods liking (derived food-liking factor) \|\| id:ebi-fl187-GCST90094785 | Weighted mode | 14 | 0.15671301155391 | 1.1004890792632 | 0.888945016074419 | -2.000245584 | 2.31367160690979 | 1.16965988685494 | 0.135302051164012 | 10.111481970507 | 0.999228396881742 |
| ebi-fl187-GCST90094785 | ebi-a-GCST90001957 | CD33 on CD33+ HLA DR+ CD14- \|\| id:ebi-a-GCST90001957 | F-highly palatable foods liking (derived food-liking factor) \|\| id:ebi-fl187-GCST90094785 | Weighted mode | 14 | 0.225500161125845 | 1.20699633185422 | 0.854679964682012 | -2.140212649 | 2.59121297156012 | 1.25294923599888 | 0.117629826460748 | 13.3459500470659 | 0.999228396881742 |
| ebi-fl187-GCST90094785 | ebi-a-GCST90002064 | CD4 on resting CD4 regulatory T cell \|\| id:ebi-a-GCST90002064 | F-highly palatable foods liking (derived food-liking factor) \|\| id:ebi-fl187-GCST90094785 | Weighted mode | 14 | -0.736991133 | 0.812864358448577 | 0.381085410371981 | -2.330205276 | 0.856223009291203 | 0.478551649599251 | 0.0972757766743561 | 2.35425189254271 | 0.999228396881742 |
| ebi-fl187-GCST90094785 | ebi-a-GCST90002080 | SSC-A on T cell \|\| id:ebi-a-GCST90002080 | F-highly palatable foods liking (derived food-liking factor) \|\| id:ebi-fl187-GCST90094785 | Weighted mode | 14 | -0.270327508 | 0.729089242682416 | 0.716777323722684 | -1.699342423 | 1.15868740802919 | 0.763129522665188 | 0.182803691989694 | 3.18574894207295 | 0.999228396881742 |
| ebi-fl187-GCST90094785 | ebi-a-GCST90002121 | CD8 on CD39+ CD8+ T cell \|\| id:ebi-a-GCST90002121 | F-highly palatable foods liking (derived food-liking factor) \|\| id:ebi-fl187-GCST90094785 | Weighted mode | 14 | -0.434742174 | 0.673322382745001 | 0.529727193066434 | -1.754454044 | 0.884969696117126 | 0.647431570212315 | 0.173001667769745 | 2.42291096676288 | 0.999228396881742 |
| ebi-fl187-GCST90094785 | ebi-cfmet338-GCST90026053 | 1-palmitoyl-2-docosahexaenoyl-gpc (16:0/22:6) levels \|\| id:ebi-cfmet338-GCST90026053 | F-highly palatable foods liking (derived food-liking factor) \|\| id:ebi-fl187-GCST90094785 | Weighted mode | 13 | -0.211183961 | 0.420863943248708 | 0.62489691396593 | -1.03607729 | 0.61370936798193 | 0.809625113908998 | 0.354843905311311 | 1.84727091338115 | 0.999228396881742 |
| ebi-fl187-GCST90094785 | ebi-cfmet338-GCST90026063 | 1-stearoyl-2-oleoyl-gpc (18:0/18:1) levels \|\| id:ebi-cfmet338-GCST90026063 | F-highly palatable foods liking (derived food-liking factor) \|\| id:ebi-fl187-GCST90094785 | Weighted mode | 13 | -0.575214068 | 0.388274594902053 | 0.164258715698429 | -1.336232274 | 0.185804137978818 | 0.562584424576756 | 0.262834092189438 | 1.20418638289983 | 0.999228396881742 |
| ebi-fl187-GCST90094785 | ebi-cfmet338-GCST90026113 | Adenosine 3',5'-cyclic monophosphate (camp) levels \|\| id:ebi-cfmet338-GCST90026113 | F-highly palatable foods liking (derived food-liking factor) \|\| id:ebi-fl187-GCST90094785 | Weighted mode | 13 | 0.66691689466936 | 0.514751024949689 | 0.219477792486797 | -0.341995114 | 1.67582890357075 | 1.94822147963639 | 0.710351675314161 | 5.34322233003532 | 0.999228396881742 |
| ebi-fl187-GCST90094785 | ebi-cfmet338-GCST90026117 | Alpha-ketoglutaramate levels \|\| id:ebi-cfmet338-GCST90026117 | F-highly palatable foods liking (derived food-liking factor) \|\| id:ebi-fl187-GCST90094785 | Weighted mode | 13 | -0.239209527 | 0.240406922430709 | 0.339368160147047 | -0.710407095 | 0.231988040498881 | 0.78724991461148 | 0.491444092088995 | 1.26110464655571 | 0.999228396881742 |
| ebi-fl187-GCST90094785 | ebi-cfmet338-GCST90026124 | Arginine levels \|\| id:ebi-cfmet338-GCST90026124 | F-highly palatable foods liking (derived food-liking factor) \|\| id:ebi-fl187-GCST90094785 | Weighted mode | 13 | 0.131056214068708 | 0.203173063724757 | 0.531035919978488 | -0.267162991 | 0.529275418969231 | 1.14003186534037 | 0.765548283944444 | 1.69770174037221 | 0.999228396881742 |
| ebi-fl187-GCST90094785 | ebi-cfmet338-GCST90026169 | Glutamate levels \|\| id:ebi-cfmet338-GCST90026169 | F-highly palatable foods liking (derived food-liking factor) \|\| id:ebi-fl187-GCST90094785 | Weighted mode | 13 | 0.157561384063379 | 0.195829213256385 | 0.436709648747066 | -0.226263874 | 0.541386642045893 | 1.17065261519055 | 0.797507632433715 | 1.71838799996235 | 0.999228396881742 |
| ebi-fl187-GCST90094785 | ebi-cfmet338-GCST90026170 | Glutamine levels \|\| id:ebi-cfmet338-GCST90026170 | F-highly palatable foods liking (derived food-liking factor) \|\| id:ebi-fl187-GCST90094785 | Weighted mode | 13 | -0.072559061 | 0.128409585981091 | 0.582443305605486 | -0.32424185 | 0.17912372731718 | 0.930010817477954 | 0.723075345655159 | 1.19616873376084 | 0.999228396881742 |
| ebi-fl187-GCST90094785 | ebi-cfmet338-GCST90026191 | Kynurenate levels \|\| id:ebi-cfmet338-GCST90026191 | F-highly palatable foods liking (derived food-liking factor) \|\| id:ebi-fl187-GCST90094785 | Weighted mode | 13 | 1.84245840359457 | 1.87665545825341 | 0.345589799434899 | -1.835786295 | 5.52070310177125 | 6.31203673626878 | 0.159488047864772 | 249.810617744774 | 0.999228396881742 |
| ebi-fl187-GCST90094785 | ebi-cfmet338-GCST90026202 | Methylsuccinoylcarnitine levels \|\| id:ebi-cfmet338-GCST90026202 | F-highly palatable foods liking (derived food-liking factor) \|\| id:ebi-fl187-GCST90094785 | Weighted mode | 13 | 0.408568278612948 | 0.619322516344436 | 0.521905377901873 | -0.805303853 | 1.62244041064804 | 1.50466198548399 | 0.446952097999902 | 5.06543699132855 | 0.999228396881742 |
| ebi-fl187-GCST90094785 | ebi-cfmet338-GCST90026222 | N-acetylputrescine levels \|\| id:ebi-cfmet338-GCST90026222 | F-highly palatable foods liking (derived food-liking factor) \|\| id:ebi-fl187-GCST90094785 | Weighted mode | 13 | -0.305179106 | 0.264354786705542 | 0.270796583516142 | -0.823314488 | 0.212956276342528 | 0.736991363289454 | 0.438974265855992 | 1.23733054944372 | 0.999228396881742 |
| ebi-fl187-GCST90094785 | ebi-cfmet338-GCST90026252 | Propionylcarnitine (c3) levels \|\| id:ebi-cfmet338-GCST90026252 | F-highly palatable foods liking (derived food-liking factor) \|\| id:ebi-fl187-GCST90094785 | Weighted mode | 13 | 0.195396193968395 | 0.358566866674451 | 0.595779879956099 | -0.507394865 | 0.898187252650318 | 1.21579258076838 | 0.602061990505287 | 2.45514851088818 | 0.999228396881742 |
| ebi-fl187-GCST90094785 | ebi-cfmet338-GCST90026316 | Ethyl beta-glucopyranoside levels \|\| id:ebi-cfmet338-GCST90026316 | F-highly palatable foods liking (derived food-liking factor) \|\| id:ebi-fl187-GCST90094785 | Weighted mode | 13 | -0.820654656 | 1.92746405071298 | 0.677812671309563 | -4.598484195 | 2.95717488331631 | 0.440143417603371 | 0.0100670839164083 | 19.2435296723635 | 0.999228396881742 |
| ebi-fl187-GCST90094785 | ebi-gm430-GCST90011315 | F_Prevotellaceae abundance \|\| id:ebi-gm430-GCST90011315 | F-highly palatable foods liking (derived food-liking factor) \|\| id:ebi-fl187-GCST90094785 | Weighted mode | 9 | -0.383151818 | 0.678317413870834 | 0.587656498196453 | -1.712653949 | 0.946350313129187 | 0.681709395633003 | 0.180386420379617 | 2.57628982889239 | 0.999228396881742 |
| ebi-fl187-GCST90094785 | ebi-gm430-GCST90011354 | OTU97_1 (Bacteroides) abundance \|\| id:ebi-gm430-GCST90011354 | F-highly palatable foods liking (derived food-liking factor) \|\| id:ebi-fl187-GCST90094785 | Weighted mode | 9 | -0.538035561 | 0.599019607749913 | 0.395310139614472 | -1.712113992 | 0.636042870031598 | 0.583894150831141 | 0.180483847572196 | 1.88899108679762 | 0.999228396881742 |
| ebi-fl187-GCST90094785 | ebi-gm430-GCST90011419 | OTU99_1 (Bacteroides) abundance \|\| id:ebi-gm430-GCST90011419 | F-highly palatable foods liking (derived food-liking factor) \|\| id:ebi-fl187-GCST90094785 | Weighted mode | 9 | -0.515533594 | 0.591266258628223 | 0.408659311721412 | -1.674415461 | 0.643348273091652 | 0.597181857039467 | 0.187417700474815 | 1.90284145773643 | 0.999228396881742 |
| ebi-fl187-GCST90094785 | ebi-gm430-GCST90011435 | OTU99_155 (Butyrivibrio) abundance \|\| id:ebi-gm430-GCST90011435 | F-highly palatable foods liking (derived food-liking factor) \|\| id:ebi-fl187-GCST90094785 | Weighted mode | 9 | -1.841096175 | 1.63311109131892 | 0.292267360792963 | -5.041993913 | 1.35980156448495 | 0.158643429876783 | 0.00646085308448026 | 3.89542023537508 | 0.999228396881742 |
| ebi-fl187-GCST90094785 | ebi-gm430-GCST90011466 | OTU99_58 (Bacteroides) abundance \|\| id:ebi-gm430-GCST90011466 | F-highly palatable foods liking (derived food-liking factor) \|\| id:ebi-fl187-GCST90094785 | Weighted mode | 9 | 0.886204931653355 | 0.961344404497512 | 0.383570641855048 | -0.998030101 | 2.77043996446848 | 2.42590568169852 | 0.368604840702486 | 15.9656567864966 | 0.999228396881742 |
| ebi-fl187-GCST90094785 | ebi-gm430-GCST90011472 | OTU99_72 (Parabacteroides) abundance \|\| id:ebi-gm430-GCST90011472 | F-highly palatable foods liking (derived food-liking factor) \|\| id:ebi-fl187-GCST90094785 | Weighted mode | 8 | -0.471327865 | 1.41698607191902 | 0.749155258215517 | -3.248620565 | 2.30596483642808 | 0.624172900703238 | 0.0388277312194458 | 10.033854612066 | 0.999228396881742 |
| ebi-fl187-GCST90094785 | ebi-gm430-GCST90011505 | TestASV_28 (Holdemanella) abundance \|\| id:ebi-gm430-GCST90011505 | F-highly palatable foods liking (derived food-liking factor) \|\| id:ebi-fl187-GCST90094785 | Weighted mode | 9 | -1.411831655 | 1.21868632694299 | 0.280076752521704 | -3.800456856 | 0.97679354583445 | 0.243696506190061 | 0.0223605539739209 | 2.65592646758692 | 0.999228396881742 |
| ebi-fl187-GCST90094785 | ebi-gm430-GCST90011513 | TestASV_35 (Alphaproteobacteria) abundance \|\| id:ebi-gm430-GCST90011513 | F-highly palatable foods liking (derived food-liking factor) \|\| id:ebi-fl187-GCST90094785 | Weighted mode | 9 | -1.291547767 | 1.70025039894201 | 0.46927080647165 | -4.624038549 | 2.04094301528928 | 0.274845057702294 | 0.00981308530668736 | 7.69786498155672 | 0.999228396881742 |
| ebi-fl187-GCST90094785 | ebi-gm430-GCST90011543 | G_Coprococcus prevalence \|\| id:ebi-gm430-GCST90011543 | F-highly palatable foods liking (derived food-liking factor) \|\| id:ebi-fl187-GCST90094785 | Weighted mode | 9 | 1.49113099350589 | 1.59485698899336 | 0.37716643771143 | -1.634788705 | 4.61705069193288 | 4.44211668413804 | 0.19499356812805 | 101.195135946943 | 0.999228396881742 |
| ebi-fl187-GCST90094785 | ebi-gm430-GCST90011547 | G_Parasutterella prevalence \|\| id:ebi-gm430-GCST90011547 | F-highly palatable foods liking (derived food-liking factor) \|\| id:ebi-fl187-GCST90094785 | Weighted mode | 9 | 0.647159378755688 | 1.36516432951067 | 0.648131095100901 | -2.028562707 | 3.3228814645966 | 1.91010722410113 | 0.131524424484691 | 27.7401678194608 | 0.999228396881742 |
| ebi-fl187-GCST90094785 | ebi-gm430-GCST90011569 | OTU97_13 (Subdoligranulum) prevalence \|\| id:ebi-gm430-GCST90011569 | F-highly palatable foods liking (derived food-liking factor) \|\| id:ebi-fl187-GCST90094785 | Weighted mode | 9 | -0.21686362 | 1.47367980372383 | 0.886648436028286 | -3.105276035 | 2.67154879554711 | 0.805039753340183 | 0.0448121471285848 | 14.4623510807992 | 0.999228396881742 |
| ebi-fl187-GCST90094785 | ebi-gm430-GCST90011590 | OTU97_277 (Prevotella) prevalence \|\| id:ebi-gm430-GCST90011590 | F-highly palatable foods liking (derived food-liking factor) \|\| id:ebi-fl187-GCST90094785 | Weighted mode | 9 | 0.767667128720939 | 1.12723336732002 | 0.515084221627313 | -1.441710271 | 2.97704452866818 | 2.15473366958994 | 0.236522894266402 | 19.6297157671135 | 0.999228396881742 |
| ebi-fl187-GCST90094785 | ebi-gm430-GCST90011616 | OTU97_86 (Phascolarctobacterium) prevalence \|\| id:ebi-gm430-GCST90011616 | F-highly palatable foods liking (derived food-liking factor) \|\| id:ebi-fl187-GCST90094785 | Weighted mode | 9 | 0.622120260254331 | 1.4350197604623 | 0.6760844539627 | -2.19051847 | 3.43475899076043 | 1.8628736344526 | 0.111858738152146 | 31.0239346095474 | 0.999228396881742 |
| ebi-fl187-GCST90094785 | ebi-gm430-GCST90011665 | OTU99_55 (Barnesiella) prevalence \|\| id:ebi-gm430-GCST90011665 | F-highly palatable foods liking (derived food-liking factor) \|\| id:ebi-fl187-GCST90094785 | Weighted mode | 9 | 0.978927848672777 | 0.945823949568645 | 0.330938991305334 | -0.874887092 | 2.83274278982732 | 2.66160107249366 | 0.416909089191797 | 16.9920024598945 | 0.999228396881742 |
| ebi-fl187-GCST90094785 | ebi-gm430-GCST90011687 | TestASV_14 (Lachnospiraceae) prevalence \|\| id:ebi-gm430-GCST90011687 | F-highly palatable foods liking (derived food-liking factor) \|\| id:ebi-fl187-GCST90094785 | Weighted mode | 9 | 0.621895495748846 | 1.29575676714241 | 0.644112219955325 | -1.917787768 | 3.16157875934796 | 1.86245497363318 | 0.146931649775154 | 23.607837617825 | 0.999228396881742 |
| ebi-fl187-GCST90094785 | ebi-gm430-GCST90011697 | TestASV_23 (Barnesiella) prevalence \|\| id:ebi-gm430-GCST90011697 | F-highly palatable foods liking (derived food-liking factor) \|\| id:ebi-fl187-GCST90094785 | Weighted mode | 9 | 1.29247243770559 | 1.60324167708146 | 0.44345133663501 | -1.849881249 | 4.43482612478525 | 3.64177950688421 | 0.157255839434246 | 84.3374594194787 | 0.999228396881742 |
| ebi-fl187-GCST90094785 | ebi-gm430-GCST90011727 | TestASV_6 (Subdoligranulum) prevalence \|\| id:ebi-gm430-GCST90011727 | F-highly palatable foods liking (derived food-liking factor) \|\| id:ebi-fl187-GCST90094785 | Weighted mode | 9 | -1.488591474 | 1.77771106298029 | 0.426693758029274 | -4.972905158 | 1.99572220930006 | 0.225690322421096 | 0.00692300636711065 | 7.35751477515925 | 0.999228396881742 |
| ebi-fl187-GCST90094785 | ebi-hbsm44-GCST90104908 | Sulcal depth of the left posterior inferior temporal sulcus \|\| id:ebi-hbsm44-GCST90104908 | F-highly palatable foods liking (derived food-liking factor) \|\| id:ebi-fl187-GCST90094785 | Weighted mode | 12 | 0.0213280945110168 | 0.453842662209515 | 0.963359989807291 | -0.868203523 | 0.910859712441666 | 1.02155716395845 | 0.419704862346242 | 2.48645925472724 | 0.999228396881742 |
| ebi-fl187-GCST90094785 | ebi-hbsm44-GCST90104918 | Sulcal depth of the left inferior frontal sulcus \|\| id:ebi-hbsm44-GCST90104918 | F-highly palatable foods liking (derived food-liking factor) \|\| id:ebi-fl187-GCST90094785 | Weighted mode | 12 | -0.004529506 | 0.733095959246643 | 0.99518085234475 | -1.441397587 | 1.43233857373162 | 0.995480736351584 | 0.236596862923092 | 4.18848282349889 | 0.999228396881742 |
| ebi-fl187-GCST90094785 | ebi-hbsm44-GCST90104925 | Sulcal depth of the right anterior inferior temporal sulcus \|\| id:ebi-hbsm44-GCST90104925 | F-highly palatable foods liking (derived food-liking factor) \|\| id:ebi-fl187-GCST90094785 | Weighted mode | 12 | 0.0763214063758444 | 0.272134468836419 | 0.784332996177843 | -0.457062153 | 0.609704965295226 | 1.07930941534341 | 0.63314098751705 | 1.83988848773998 | 0.999228396881742 |
| ebi-fl187-GCST90094785 | ebi-IPs91-GCST90274770 | C-C motif chemokine 4 levels \|\| id:ebi-IPs91-GCST90274770 | F-highly palatable foods liking (derived food-liking factor) \|\| id:ebi-fl187-GCST90094785 | Weighted mode | 17 | 0.233251144069105 | 0.354252080736883 | 0.519623118139969 | -0.461082934 | 0.927585222313395 | 1.26269855876339 | 0.630600376907205 | 2.52839628501801 | 0.999228396881742 |
| ebi-fl187-GCST90094785 | ebi-IPs91-GCST90274779 | C-X-C motif chemokine 1 levels \|\| id:ebi-IPs91-GCST90274779 | F-highly palatable foods liking (derived food-liking factor) \|\| id:ebi-fl187-GCST90094785 | Weighted mode | 17 | -0.090663891 | 0.288340414179614 | 0.757254781672341 | -0.655811103 | 0.474483320507076 | 0.913324635686036 | 0.519020912352969 | 1.60718358412453 | 0.999228396881742 |
| ebi-fl187-GCST90094785 | ebi-IPs91-GCST90274780 | C-X-C motif chemokine 10 levels \|\| id:ebi-IPs91-GCST90274780 | F-highly palatable foods liking (derived food-liking factor) \|\| id:ebi-fl187-GCST90094785 | Weighted mode | 17 | -0.193247886 | 0.325966810397354 | 0.561571272218328 | -0.832142834 | 0.445647062548243 | 0.824277622069196 | 0.435115905391275 | 1.56150025734654 | 0.999228396881742 |
| ebi-fl187-GCST90094785 | ebi-IPs91-GCST90274796 | Interleukin-10 receptor subunit alpha levels \|\| id:ebi-IPs91-GCST90274796 | F-highly palatable foods liking (derived food-liking factor) \|\| id:ebi-fl187-GCST90094785 | Weighted mode | 17 | -0.889842374 | 0.504062940493054 | 0.0965809055562979 | -1.877805737 | 0.0981209892370678 | 0.410720487824751 | 0.152925296117804 | 1.10309623980752 | 0.999228396881742 |
| ebi-fl187-GCST90094785 | ebi-IPs91-GCST90274807 | Interleukin-20 levels \|\| id:ebi-IPs91-GCST90274807 | F-highly palatable foods liking (derived food-liking factor) \|\| id:ebi-fl187-GCST90094785 | Weighted mode | 17 | -0.454267565 | 0.387644063591607 | 0.258405116088265 | -1.214049929 | 0.305514799931648 | 0.634912830250949 | 0.296992043723671 | 1.35732357326158 | 0.999228396881742 |
| ebi-fl187-GCST90094785 | ebi-IPs91-GCST90274808 | Interleukin-20 receptor subunit alpha levels \|\| id:ebi-IPs91-GCST90274808 | F-highly palatable foods liking (derived food-liking factor) \|\| id:ebi-fl187-GCST90094785 | Weighted mode | 17 | -0.337163112 | 0.331689348557411 | 0.32451569187133 | -0.987274235 | 0.312948011470787 | 0.713792402513366 | 0.37259090343243 | 1.36745043744258 | 0.999228396881742 |
| ebi-fl187-GCST90094785 | ebi-IPs91-GCST90274810 | Interleukin-24 levels \|\| id:ebi-IPs91-GCST90274810 | F-highly palatable foods liking (derived food-liking factor) \|\| id:ebi-fl187-GCST90094785 | Weighted mode | 17 | -0.136005597 | 0.354367304878244 | 0.706180109675835 | -0.830565514 | 0.558554320791158 | 0.872837747402754 | 0.435802763913402 | 1.74814341802224 | 0.999228396881742 |
| ebi-fl187-GCST90094785 | ebi-IPs91-GCST90274823 | Monocyte chemoattractant protein-3 levels \|\| id:ebi-IPs91-GCST90274823 | F-highly palatable foods liking (derived food-liking factor) \|\| id:ebi-fl187-GCST90094785 | Weighted mode | 17 | 0.151720897346897 | 0.340776667571623 | 0.662124760523065 | -0.516201371 | 0.819643165787277 | 1.16383536156032 | 0.59678320568697 | 2.26968979004532 | 0.999228396881742 |
| ebi-fl187-GCST90094785 | ebi-IPs91-GCST90274824 | Monocyte chemoattractant protein-4 levels \|\| id:ebi-IPs91-GCST90274824 | F-highly palatable foods liking (derived food-liking factor) \|\| id:ebi-fl187-GCST90094785 | Weighted mode | 17 | -0.056572013 | 0.298429093199214 | 0.852033193132968 | -0.641493035 | 0.528349009981291 | 0.944998430166572 | 0.52650574522487 | 1.69612970250852 | 0.999228396881742 |
| ebi-fl187-GCST90094785 | ebi-IPs91-GCST90274834 | SIR2-like protein 2 levels \|\| id:ebi-IPs91-GCST90274834 | F-highly palatable foods liking (derived food-liking factor) \|\| id:ebi-fl187-GCST90094785 | Weighted mode | 17 | -0.345886022 | 0.293444602887718 | 0.255746439667081 | -0.921037443 | 0.229265400084968 | 0.707593132861329 | 0.398105814587947 | 1.25767578197957 | 0.999228396881742 |
| ebi-fl187-GCST90094785 | ebi-met1400-GCST90199652 | 1-stearoyl-2-oleoyl-GPS (18:0/18:1) levels \|\| id:ebi-met1400-GCST90199652 | F-highly palatable foods liking (derived food-liking factor) \|\| id:ebi-fl187-GCST90094785 | Weighted mode | 14 | 0.101521080940973 | 0.428868798400842 | 0.816564524292085 | -0.739061764 | 0.942103925806625 | 1.10685325165406 | 0.477561771072879 | 2.56537309915914 | 0.999228396881742 |
| ebi-fl187-GCST90094785 | ebi-met1400-GCST90199665 | N-acetylaspartate (naa) levels \|\| id:ebi-met1400-GCST90199665 | F-highly palatable foods liking (derived food-liking factor) \|\| id:ebi-fl187-GCST90094785 | Weighted mode | 14 | -0.635704794 | 0.434080446852471 | 0.166822051196979 | -1.48650247 | 0.21509288185615 | 0.529562124566143 | 0.226162283275678 | 1.23997706298876 | 0.999228396881742 |
| ebi-fl187-GCST90094785 | ebi-met1400-GCST90199669 | Acetylcarnitine levels (Biocrates platform) \|\| id:ebi-met1400-GCST90199669 | F-highly palatable foods liking (derived food-liking factor) \|\| id:ebi-fl187-GCST90094785 | Weighted mode | 14 | 0.679863632456942 | 0.46053375292824 | 0.163685046206052 | -0.222782523 | 1.58250978819629 | 1.97360857772619 | 0.800288874568212 | 4.86715602559886 | 0.999228396881742 |
| ebi-fl187-GCST90094785 | ebi-met1400-GCST90199670 | Iminodiacetate (IDA) levels \|\| id:ebi-met1400-GCST90199670 | F-highly palatable foods liking (derived food-liking factor) \|\| id:ebi-fl187-GCST90094785 | Weighted mode | 14 | -0.100892323 | 0.431914495714106 | 0.818938855815344 | -0.947444734 | 0.745660088783497 | 0.904030371089691 | 0.387730513147816 | 2.10783233235243 | 0.999228396881742 |
| ebi-fl187-GCST90094785 | ebi-met1400-GCST90199686 | Indolepropionate levels \|\| id:ebi-met1400-GCST90199686 | F-highly palatable foods liking (derived food-liking factor) \|\| id:ebi-fl187-GCST90094785 | Weighted mode | 14 | -0.070298633 | 0.474646491168706 | 0.884530660906144 | -1.000605756 | 0.860008489638061 | 0.932115417865376 | 0.367656663568341 | 2.36318075616993 | 0.999228396881742 |
| ebi-fl187-GCST90094785 | ebi-met1400-GCST90199704 | 3-hydroxysebacate levels \|\| id:ebi-met1400-GCST90199704 | F-highly palatable foods liking (derived food-liking factor) \|\| id:ebi-fl187-GCST90094785 | Weighted mode | 14 | 0.783110035426291 | 0.586835937207717 | 0.204956400978477 | -0.367088402 | 1.93330847235342 | 2.18826728242008 | 0.692748402338991 | 6.91234174361466 | 0.999228396881742 |
| ebi-fl187-GCST90094785 | ebi-met1400-GCST90199713 | Docosapentaenoate n3 DPA; 22:5n3 levels \|\| id:ebi-met1400-GCST90199713 | F-highly palatable foods liking (derived food-liking factor) \|\| id:ebi-fl187-GCST90094785 | Weighted mode | 14 | 0.267053928591918 | 0.428410248620921 | 0.543830129780074 | -0.572630159 | 1.10673801588892 | 1.30611088115212 | 0.564039971407697 | 3.02447649163307 | 0.999228396881742 |
| ebi-fl187-GCST90094785 | ebi-met1400-GCST90199715 | Myristoleate (14:1n5) levels \|\| id:ebi-met1400-GCST90199715 | F-highly palatable foods liking (derived food-liking factor) \|\| id:ebi-fl187-GCST90094785 | Weighted mode | 14 | 0.681946495811892 | 0.475063866094965 | 0.174771125994227 | -0.249178682 | 1.61307167335802 | 1.97772361875614 | 0.779440689127225 | 5.01820185518624 | 0.999228396881742 |
| ebi-fl187-GCST90094785 | ebi-met1400-GCST90199725 | Campesterol levels \|\| id:ebi-met1400-GCST90199725 | F-highly palatable foods liking (derived food-liking factor) \|\| id:ebi-fl187-GCST90094785 | Weighted mode | 14 | -0.172915816 | 0.599983852353277 | 0.777736722348447 | -1.348884166 | 1.00305253493594 | 0.841208428426373 | 0.259529691116102 | 2.72659215603584 | 0.999228396881742 |
| ebi-fl187-GCST90094785 | ebi-met1400-GCST90199737 | N-acetylproline levels \|\| id:ebi-met1400-GCST90199737 | F-highly palatable foods liking (derived food-liking factor) \|\| id:ebi-fl187-GCST90094785 | Weighted mode | 14 | -0.172841515 | 0.495125809492017 | 0.732614093834144 | -1.143288101 | 0.797605072036642 | 0.841270933467377 | 0.318769151501626 | 2.22021729569231 | 0.999228396881742 |
| ebi-fl187-GCST90094785 | ebi-met1400-GCST90199775 | Gamma-glutamylisoleucine levels \|\| id:ebi-met1400-GCST90199775 | F-highly palatable foods liking (derived food-liking factor) \|\| id:ebi-fl187-GCST90094785 | Weighted mode | 14 | 0.0304377515237519 | 0.467034453900495 | 0.949028300424517 | -0.884949778 | 0.945825281168722 | 1.03090571574146 | 0.412734901080722 | 2.57493754941884 | 0.999228396881742 |
| ebi-fl187-GCST90094785 | ebi-met1400-GCST90199777 | 1-palmitoleoyl-GPC (16:1) levels \|\| id:ebi-met1400-GCST90199777 | F-highly palatable foods liking (derived food-liking factor) \|\| id:ebi-fl187-GCST90094785 | Weighted mode | 14 | 0.354700306501012 | 0.448042715161563 | 0.442761297251816 | -0.523463415 | 1.23286402821768 | 1.42575330125167 | 0.59246503805519 | 3.43104207921326 | 0.999228396881742 |
| ebi-fl187-GCST90094785 | ebi-met1400-GCST90199782 | Glutamine degradant levels \|\| id:ebi-met1400-GCST90199782 | F-highly palatable foods liking (derived food-liking factor) \|\| id:ebi-fl187-GCST90094785 | Weighted mode | 14 | -0.382867198 | 0.333726378951494 | 0.271954933068254 | -1.0369709 | 0.271236505219647 | 0.681903451738973 | 0.354526954630945 | 1.31158523045892 | 0.999228396881742 |
| ebi-fl187-GCST90094785 | ebi-met1400-GCST90199783 | 5-methyluridine (ribothymidine) levels \|\| id:ebi-met1400-GCST90199783 | F-highly palatable foods liking (derived food-liking factor) \|\| id:ebi-fl187-GCST90094785 | Weighted mode | 14 | 0.491451238015406 | 0.503971872523777 | 0.34728028983569 | -0.496333632 | 1.47923610816201 | 1.63468681904264 | 0.608758505790881 | 4.38959122695148 | 0.999228396881742 |
| ebi-fl187-GCST90094785 | ebi-met1400-GCST90199787 | Homostachydrine levels \|\| id:ebi-met1400-GCST90199787 | F-highly palatable foods liking (derived food-liking factor) \|\| id:ebi-fl187-GCST90094785 | Weighted mode | 14 | -0.194633127 | 0.427927909674773 | 0.656736050549339 | -1.03337183 | 0.644105575970779 | 0.82313658926411 | 0.355805220974948 | 1.90428303083573 | 0.999228396881742 |
| ebi-fl187-GCST90094785 | ebi-met1400-GCST90199802 | Beta-hydroxyisovaleroylcarnitine levels \|\| id:ebi-met1400-GCST90199802 | F-highly palatable foods liking (derived food-liking factor) \|\| id:ebi-fl187-GCST90094785 | Weighted mode | 14 | 0.648051123487753 | 0.426945349684196 | 0.152982716339656 | -0.188761762 | 1.48486400886878 | 1.91181131184861 | 0.827983740478233 | 4.41436505746021 | 0.999228396881742 |
| ebi-fl187-GCST90094785 | ebi-met1400-GCST90199873 | Pregnenolone sulfate levels \|\| id:ebi-met1400-GCST90199873 | F-highly palatable foods liking (derived food-liking factor) \|\| id:ebi-fl187-GCST90094785 | Weighted mode | 14 | -0.227352785 | 0.440510483968948 | 0.61444193057414 | -1.090753333 | 0.636047764026785 | 0.796639690534028 | 0.335963306061964 | 1.88900033153353 | 0.999228396881742 |
| ebi-fl187-GCST90094785 | ebi-met1400-GCST90199911 | Beta-citrylglutamate levels \|\| id:ebi-met1400-GCST90199911 | F-highly palatable foods liking (derived food-liking factor) \|\| id:ebi-fl187-GCST90094785 | Weighted mode | 14 | -0.422151349 | 0.4639820078245 | 0.379464934153568 | -1.331556084 | 0.487253386553362 | 0.655634802354725 | 0.264066032470069 | 1.62783902964665 | 0.999228396881742 |
| ebi-fl187-GCST90094785 | ebi-met1400-GCST90199934 | 2-hydroxydecanoate levels \|\| id:ebi-met1400-GCST90199934 | F-highly palatable foods liking (derived food-liking factor) \|\| id:ebi-fl187-GCST90094785 | Weighted mode | 14 | 0.00677251961803593 | 0.506523282919992 | 0.989535141820219 | -0.986013115 | 0.999558154141221 | 1.00679550498935 | 0.373061081674329 | 2.71708103219311 | 0.999228396881742 |
| ebi-fl187-GCST90094785 | ebi-met1400-GCST90199945 | Sphingomyelin (d18:1/14:0, d16:1/16:0) levels \|\| id:ebi-met1400-GCST90199945 | F-highly palatable foods liking (derived food-liking factor) \|\| id:ebi-fl187-GCST90094785 | Weighted mode | 14 | -0.346430769 | 0.422964246766889 | 0.427515767932096 | -1.175440693 | 0.482579154759301 | 0.707207778362243 | 0.308682915442606 | 1.62024788789794 | 0.999228396881742 |
| ebi-fl187-GCST90094785 | ebi-met1400-GCST90199979 | Carnitine C14:1 levels \|\| id:ebi-met1400-GCST90199979 | F-highly palatable foods liking (derived food-liking factor) \|\| id:ebi-fl187-GCST90094785 | Weighted mode | 14 | 0.157492694574966 | 0.477976800562415 | 0.747027459407329 | -0.779341835 | 1.0943272236773 | 1.17057220642296 | 0.458707817622665 | 2.98717230840193 | 0.999228396881742 |
| ebi-fl187-GCST90094785 | ebi-met1400-GCST90199986 | Eugenol sulfate levels \|\| id:ebi-met1400-GCST90199986 | F-highly palatable foods liking (derived food-liking factor) \|\| id:ebi-fl187-GCST90094785 | Weighted mode | 14 | 0.425762668453515 | 0.529412024667972 | 0.435731637578826 | -0.6118849 | 1.46341023680274 | 1.53075743498957 | 0.542327671693305 | 4.32066893702778 | 0.999228396881742 |
| ebi-fl187-GCST90094785 | ebi-met1400-GCST90200002 | 6-hydroxyindole sulfate levels \|\| id:ebi-met1400-GCST90200002 | F-highly palatable foods liking (derived food-liking factor) \|\| id:ebi-fl187-GCST90094785 | Weighted mode | 14 | 0.356367538928974 | 0.414712971582552 | 0.405736076667635 | -0.456469885 | 1.16920496323078 | 1.42813234604903 | 0.633516087207039 | 3.21943205392503 | 0.999228396881742 |
| ebi-fl187-GCST90094785 | ebi-met1400-GCST90200023 | Trans 3,4-methyleneheptanoate levels \|\| id:ebi-met1400-GCST90200023 | F-highly palatable foods liking (derived food-liking factor) \|\| id:ebi-fl187-GCST90094785 | Weighted mode | 14 | 0.437867198436315 | 0.492982242709847 | 0.390570933294801 | -0.528377997 | 1.40411239414761 | 1.54939913112872 | 0.589560463240228 | 4.07191088484548 | 0.999228396881742 |
| ebi-fl187-GCST90094785 | ebi-met1400-GCST90200032 | 2-hydroxybutyrate/2-hydroxyisobutyrate levels \|\| id:ebi-met1400-GCST90200032 | F-highly palatable foods liking (derived food-liking factor) \|\| id:ebi-fl187-GCST90094785 | Weighted mode | 14 | 0.150773579221498 | 0.45483949071161 | 0.74555935425384 | -0.740711823 | 1.04225898101625 | 1.16273336128216 | 0.476774415912029 | 2.83561538605704 | 0.999228396881742 |
| ebi-fl187-GCST90094785 | ebi-met1400-GCST90200051 | 1-palmitoyl-2-dihomo-linolenoyl-GPC (16:0/20:3n3 or 6) levels \|\| id:ebi-met1400-GCST90200051 | F-highly palatable foods liking (derived food-liking factor) \|\| id:ebi-fl187-GCST90094785 | Weighted mode | 14 | 0.364526948271251 | 0.50143409073636 | 0.480132161586706 | -0.61828387 | 1.34733776611452 | 1.43983273165959 | 0.538868413016998 | 3.84716981934683 | 0.999228396881742 |
| ebi-fl187-GCST90094785 | ebi-met1400-GCST90200069 | Phenylacetylglutamate levels \|\| id:ebi-met1400-GCST90200069 | F-highly palatable foods liking (derived food-liking factor) \|\| id:ebi-fl187-GCST90094785 | Weighted mode | 14 | -0.011574353 | 0.462794202320661 | 0.980427041544636 | -0.918650989 | 0.895502283794415 | 0.988492372385214 | 0.399057010315604 | 2.44856535533851 | 0.999228396881742 |
| ebi-fl187-GCST90094785 | ebi-met1400-GCST90200072 | Thioproline levels \|\| id:ebi-met1400-GCST90200072 | F-highly palatable foods liking (derived food-liking factor) \|\| id:ebi-fl187-GCST90094785 | Weighted mode | 14 | 0.265456193489572 | 0.405754866360739 | 0.524370519197093 | -0.529823345 | 1.06073573155662 | 1.30402572815439 | 0.588708959122852 | 2.88849536487814 | 0.999228396881742 |
| ebi-fl187-GCST90094785 | ebi-met1400-GCST90200081 | 1-oleoyl-2-docosahexaenoyl-GPC (18:1/22:6) levels \|\| id:ebi-met1400-GCST90200081 | F-highly palatable foods liking (derived food-liking factor) \|\| id:ebi-fl187-GCST90094785 | Weighted mode | 14 | -0.205138586 | 0.370525711739774 | 0.589219993286233 | -0.931368981 | 0.521091808618293 | 0.814534425202128 | 0.39401394323005 | 1.68386510487521 | 0.999228396881742 |
| ebi-fl187-GCST90094785 | ebi-met1400-GCST90200097 | Furaneol sulfate levels \|\| id:ebi-met1400-GCST90200097 | F-highly palatable foods liking (derived food-liking factor) \|\| id:ebi-fl187-GCST90094785 | Weighted mode | 13 | -0.474051533 | 0.508448478833043 | 0.369535904317522 | -1.470610551 | 0.522507485715439 | 0.622475173854167 | 0.229785146726795 | 1.68625060228748 | 0.999228396881742 |
| ebi-fl187-GCST90094785 | ebi-met1400-GCST90200124 | Sphingomyelin (d18:2/24:2) levels \|\| id:ebi-met1400-GCST90200124 | F-highly palatable foods liking (derived food-liking factor) \|\| id:ebi-fl187-GCST90094785 | Weighted mode | 14 | 0.0158217899158497 | 0.37897983218643 | 0.967333688518546 | -0.726978681 | 0.758622261001253 | 1.01594761716213 | 0.483367192534525 | 2.13533226242635 | 0.999228396881742 |
| ebi-fl187-GCST90094785 | ebi-met1400-GCST90200137 | Lignoceroylcarnitine (C24) levels \|\| id:ebi-met1400-GCST90200137 | F-highly palatable foods liking (derived food-liking factor) \|\| id:ebi-fl187-GCST90094785 | Weighted mode | 14 | 0.552978711151878 | 0.441577044960269 | 0.232528530509004 | -0.312512297 | 1.41846971927401 | 1.73842357493567 | 0.731606632346335 | 4.1307943261803 | 0.999228396881742 |
| ebi-fl187-GCST90094785 | ebi-met1400-GCST90200165 | Octadecenedioate (C18:1-DC) levels \|\| id:ebi-met1400-GCST90200165 | F-highly palatable foods liking (derived food-liking factor) \|\| id:ebi-fl187-GCST90094785 | Weighted mode | 14 | -0.284602619 | 0.399546656601107 | 0.488858300874846 | -1.067714066 | 0.498508827866796 | 0.752313149825859 | 0.343793509125472 | 1.64626457561869 | 0.999228396881742 |
| ebi-fl187-GCST90094785 | ebi-met1400-GCST90200176 | Glutamine conjugate of C6H10O2 (1) levels \|\| id:ebi-met1400-GCST90200176 | F-highly palatable foods liking (derived food-liking factor) \|\| id:ebi-fl187-GCST90094785 | Weighted mode | 14 | 0.189766091285643 | 0.446938289618145 | 0.678077380702602 | -0.686232956 | 1.06576513893721 | 1.20896677671696 | 0.503469091314146 | 2.90305937826361 | 0.999228396881742 |
| ebi-fl187-GCST90094785 | ebi-met1400-GCST90200182 | Glycine conjugate of C10H12O2 levels \|\| id:ebi-met1400-GCST90200182 | F-highly palatable foods liking (derived food-liking factor) \|\| id:ebi-fl187-GCST90094785 | Weighted mode | 14 | 0.626027966544718 | 0.566665121601927 | 0.289292538109193 | -0.484635672 | 1.7366916048845 | 1.87016743921406 | 0.615921553486028 | 5.67852550523839 | 0.999228396881742 |
| ebi-fl187-GCST90094785 | ebi-met1400-GCST90200208 | Glucuronide of piperine metabolite C17H21NO3 (4) levels \|\| id:ebi-met1400-GCST90200208 | F-highly palatable foods liking (derived food-liking factor) \|\| id:ebi-fl187-GCST90094785 | Weighted mode | 14 | 0.41235490782226 | 0.472705644590031 | 0.398854334993055 | -0.514148156 | 1.33885797121872 | 1.51037038348081 | 0.598009789015589 | 3.81468453727351 | 0.999228396881742 |
| ebi-fl187-GCST90094785 | ebi-met1400-GCST90200227 | 3-ethylcatechol sulfate (2) levels \|\| id:ebi-met1400-GCST90200227 | F-highly palatable foods liking (derived food-liking factor) \|\| id:ebi-fl187-GCST90094785 | Weighted mode | 14 | 0.142235923436211 | 0.513398018061723 | 0.786099551067117 | -0.864024192 | 1.14849603883719 | 1.15284860045908 | 0.421462618643765 | 3.15344668017598 | 0.999228396881742 |
| ebi-fl187-GCST90094785 | ebi-met1400-GCST90200259 | Pentose acid levels \|\| id:ebi-met1400-GCST90200259 | F-highly palatable foods liking (derived food-liking factor) \|\| id:ebi-fl187-GCST90094785 | Weighted mode | 14 | -0.314927609 | 0.443571888213766 | 0.490256157537787 | -1.18432851 | 0.554473292194027 | 0.729841706543051 | 0.305951554067688 | 1.74102373244304 | 0.999228396881742 |
| ebi-fl187-GCST90094785 | ebi-met1400-GCST90200291 | Sarcosine levels \|\| id:ebi-met1400-GCST90200291 | F-highly palatable foods liking (derived food-liking factor) \|\| id:ebi-fl187-GCST90094785 | Weighted mode | 14 | 0.0892578231500847 | 0.420977891549046 | 0.835375643162049 | -0.735858844 | 0.914374490586215 | 1.09336251415762 | 0.479093815245561 | 2.49521398382556 | 0.999228396881742 |
| ebi-fl187-GCST90094785 | ebi-met1400-GCST90200306 | Butyrate/isobutyrate (4:0) levels \|\| id:ebi-met1400-GCST90200306 | F-highly palatable foods liking (derived food-liking factor) \|\| id:ebi-fl187-GCST90094785 | Weighted mode | 14 | 0.449863622494168 | 0.517121493015996 | 0.400112477117051 | -0.563694504 | 1.46342174880552 | 1.56809831756966 | 0.569102623304294 | 4.3207186768669 | 0.999228396881742 |
| ebi-fl187-GCST90094785 | ebi-met1400-GCST90200321 | Cholate levels \|\| id:ebi-met1400-GCST90200321 | F-highly palatable foods liking (derived food-liking factor) \|\| id:ebi-fl187-GCST90094785 | Weighted mode | 14 | -0.62317162 | 0.507507737014721 | 0.241251555086934 | -1.617886785 | 0.371543544450204 | 0.536240984984653 | 0.198317343840226 | 1.44997098291604 | 0.999228396881742 |
| ebi-fl187-GCST90094785 | ebi-met1400-GCST90200336 | Ceramide (d18:1/16:0) levels \|\| id:ebi-met1400-GCST90200336 | F-highly palatable foods liking (derived food-liking factor) \|\| id:ebi-fl187-GCST90094785 | Weighted mode | 14 | 0.313350522964101 | 0.428848258040107 | 0.477937975778995 | -0.527192063 | 1.15389310872271 | 1.36800096274914 | 0.590260058076735 | 3.17051206239552 | 0.999228396881742 |
| ebi-fl187-GCST90094785 | ebi-met1400-GCST90200358 | Arachidonate (20:4n6) levels \|\| id:ebi-met1400-GCST90200358 | F-highly palatable foods liking (derived food-liking factor) \|\| id:ebi-fl187-GCST90094785 | Weighted mode | 14 | 0.887788508015302 | 0.507114565199693 | 0.103549274989957 | -0.10615604 | 1.8817330558067 | 2.42975033193755 | 0.899284313004434 | 6.56487229920301 | 0.999228396881742 |
| ebi-fl187-GCST90094785 | ebi-met1400-GCST90200413 | 2'-deoxyuridine levels \|\| id:ebi-met1400-GCST90200413 | F-highly palatable foods liking (derived food-liking factor) \|\| id:ebi-fl187-GCST90094785 | Weighted mode | 14 | 0.378844245849697 | 0.457362508041136 | 0.422434351204037 | -0.51758627 | 1.27527476161032 | 1.46059552421115 | 0.595957293365646 | 3.5796848349614 | 0.999228396881742 |
| ebi-fl187-GCST90094785 | ebi-met1400-GCST90200432 | Threonine levels \|\| id:ebi-met1400-GCST90200432 | F-highly palatable foods liking (derived food-liking factor) \|\| id:ebi-fl187-GCST90094785 | Weighted mode | 14 | 0.218080403355606 | 0.516360058167733 | 0.679677080273652 | -0.793985311 | 1.23014611736436 | 1.2436870602121 | 0.452039682142326 | 3.42172947385629 | 0.999228396881742 |
| ebi-fl187-GCST90094785 | ebi-met1400-GCST90200436 | Caffeine levels \|\| id:ebi-met1400-GCST90200436 | F-highly palatable foods liking (derived food-liking factor) \|\| id:ebi-fl187-GCST90094785 | Weighted mode | 14 | 0.431276616006019 | 0.432689357631412 | 0.337084975137186 | -0.416794525 | 1.27934775696359 | 1.53922126431296 | 0.65915634621246 | 3.59429460723048 | 0.999228396881742 |
| ebi-fl187-GCST90094785 | ebi-met1400-GCST90200442 | Valine levels \|\| id:ebi-met1400-GCST90200442 | F-highly palatable foods liking (derived food-liking factor) \|\| id:ebi-fl187-GCST90094785 | Weighted mode | 14 | 0.449709378102708 | 0.467640405068294 | 0.353769062560403 | -0.466865816 | 1.36628457203656 | 1.56785646585154 | 0.626964213440142 | 3.92075631242892 | 0.999228396881742 |
| ebi-fl187-GCST90094785 | ebi-met1400-GCST90200459 | Margarate (17:0) levels \|\| id:ebi-met1400-GCST90200459 | F-highly palatable foods liking (derived food-liking factor) \|\| id:ebi-fl187-GCST90094785 | Weighted mode | 14 | 0.860480609531604 | 0.528436477788957 | 0.12743125916682 | -0.175254887 | 1.89621610599796 | 2.36429672423158 | 0.83924308140866 | 6.66064353 | 0.999228396881742 |
| ebi-fl187-GCST90094785 | ebi-met1400-GCST90200462 | X-11299 levels \|\| id:ebi-met1400-GCST90200462 | F-highly palatable foods liking (derived food-liking factor) \|\| id:ebi-fl187-GCST90094785 | Weighted mode | 14 | -0.114607868 | 0.411879176184703 | 0.785192393866413 | -0.921891054 | 0.692675316966828 | 0.891715745319378 | 0.397766132306263 | 1.999056495 | 0.999228396881742 |
| ebi-fl187-GCST90094785 | ebi-met1400-GCST90200497 | X-12740 levels \|\| id:ebi-met1400-GCST90200497 | F-highly palatable foods liking (derived food-liking factor) \|\| id:ebi-fl187-GCST90094785 | Weighted mode | 14 | -0.479804817 | 0.557071103265941 | 0.404680157459258 | -1.571664179 | 0.61205454571863 | 0.618904179788813 | 0.207699245846037 | 1.84421653626998 | 0.999228396881742 |
| ebi-fl187-GCST90094785 | ebi-met1400-GCST90200527 | X-13866 levels \|\| id:ebi-met1400-GCST90200527 | F-highly palatable foods liking (derived food-liking factor) \|\| id:ebi-fl187-GCST90094785 | Weighted mode | 14 | 0.355510902621061 | 0.439035224849432 | 0.432653682099687 | -0.504998138 | 1.21601994332595 | 1.42690947987946 | 0.603506699104879 | 3.37373332688729 | 0.999228396881742 |
| ebi-fl187-GCST90094785 | ebi-met1400-GCST90200547 | X-17654 levels \|\| id:ebi-met1400-GCST90200547 | F-highly palatable foods liking (derived food-liking factor) \|\| id:ebi-fl187-GCST90094785 | Weighted mode | 14 | 0.167031818322841 | 0.550510453509159 | 0.766376670921591 | -0.911968671 | 1.24603230720079 | 1.18179186734531 | 0.401732565958717 | 3.47652178605563 | 0.999228396881742 |
| ebi-fl187-GCST90094785 | ebi-met1400-GCST90200553 | X-17653 levels \|\| id:ebi-met1400-GCST90200553 | F-highly palatable foods liking (derived food-liking factor) \|\| id:ebi-fl187-GCST90094785 | Weighted mode | 14 | -0.059827911 | 0.528293222379643 | 0.911564481428117 | -1.095282627 | 0.975626804685394 | 0.941926614678542 | 0.33444507036872 | 2.65282949592179 | 0.999228396881742 |
| ebi-fl187-GCST90094785 | ebi-met1400-GCST90200557 | X-17685 levels \|\| id:ebi-met1400-GCST90200557 | F-highly palatable foods liking (derived food-liking factor) \|\| id:ebi-fl187-GCST90094785 | Weighted mode | 14 | 0.46995453773803 | 0.549304654574801 | 0.407742110935789 | -0.606682585 | 1.54659166070464 | 1.59992145551562 | 0.545156382436137 | 4.69543922861269 | 0.999228396881742 |
| ebi-fl187-GCST90094785 | ebi-met1400-GCST90200561 | X-18779 levels \|\| id:ebi-met1400-GCST90200561 | F-highly palatable foods liking (derived food-liking factor) \|\| id:ebi-fl187-GCST90094785 | Weighted mode | 14 | -0.066022891 | 0.484202709506887 | 0.89363052394558 | -1.015060202 | 0.883014419246378 | 0.93610943520288 | 0.362380613130648 | 2.41817813349724 | 0.999228396881742 |
| ebi-fl187-GCST90094785 | ebi-met1400-GCST90200570 | X-18921 levels \|\| id:ebi-met1400-GCST90200570 | F-highly palatable foods liking (derived food-liking factor) \|\| id:ebi-fl187-GCST90094785 | Weighted mode | 14 | 0.685085183367953 | 0.428247554129165 | 0.133666887021092 | -0.154280023 | 1.52445038946112 | 1.98394082710089 | 0.857031998971269 | 4.5926187238777 | 0.999228396881742 |
| ebi-fl187-GCST90094785 | ebi-met1400-GCST90200575 | X-21283 levels \|\| id:ebi-met1400-GCST90200575 | F-highly palatable foods liking (derived food-liking factor) \|\| id:ebi-fl187-GCST90094785 | Weighted mode | 13 | -0.448065666 | 0.461389503793014 | 0.350655505040394 | -1.352389094 | 0.456257761102867 | 0.638862730882646 | 0.258621650608284 | 1.57815707985338 | 0.999228396881742 |
| ebi-fl187-GCST90094785 | ebi-met1400-GCST90200578 | X-18922 levels \|\| id:ebi-met1400-GCST90200578 | F-highly palatable foods liking (derived food-liking factor) \|\| id:ebi-fl187-GCST90094785 | Weighted mode | 14 | 0.41856218908485 | 0.487297533694249 | 0.405930407846284 | -0.536540977 | 1.37366535512558 | 1.51977483510356 | 0.584767482275941 | 3.94980162102127 | 0.999228396881742 |
| ebi-fl187-GCST90094785 | ebi-met1400-GCST90200623 | X-24241 levels \|\| id:ebi-met1400-GCST90200623 | F-highly palatable foods liking (derived food-liking factor) \|\| id:ebi-fl187-GCST90094785 | Weighted mode | 14 | -0.071748976 | 0.495785581408797 | 0.887153278982266 | -1.043488715 | 0.899990763781825 | 0.930764510924354 | 0.352223727660684 | 2.4595803938309 | 0.999228396881742 |
| ebi-fl187-GCST90094785 | ebi-met1400-GCST90200636 | X-24544 levels \|\| id:ebi-met1400-GCST90200636 | F-highly palatable foods liking (derived food-liking factor) \|\| id:ebi-fl187-GCST90094785 | Weighted mode | 14 | -0.030627749 | 0.387448628341795 | 0.938196856942144 | -0.790027061 | 0.728771562546935 | 0.969836528503528 | 0.453832514157432 | 2.07253306600569 | 0.999228396881742 |
| ebi-fl187-GCST90094785 | ebi-met1400-GCST90200659 | X-25172 levels \|\| id:ebi-met1400-GCST90200659 | F-highly palatable foods liking (derived food-liking factor) \|\| id:ebi-fl187-GCST90094785 | Weighted mode | 14 | 0.843127812174153 | 0.48404503529431 | 0.105130172887601 | -0.105600457 | 1.791856081 | 2.32362348018473 | 0.899784078694878 | 6.00057970074028 | 0.999228396881742 |
| ebi-fl187-GCST90094785 | ebi-met1400-GCST90200670 | X-26111 levels \|\| id:ebi-met1400-GCST90200670 | F-highly palatable foods liking (derived food-liking factor) \|\| id:ebi-fl187-GCST90094785 | Weighted mode | 14 | -0.124540044 | 0.464664082952883 | 0.792888288297652 | -1.035281647 | 0.78620155866142 | 0.882902905759677 | 0.355126346740926 | 2.1950428295526 | 0.999228396881742 |
| ebi-fl187-GCST90094785 | ebi-met1400-GCST90200673 | Carnitine C4 levels \|\| id:ebi-met1400-GCST90200673 | F-highly palatable foods liking (derived food-liking factor) \|\| id:ebi-fl187-GCST90094785 | Weighted mode | 14 | 0.334194093155539 | 0.423450082463907 | 0.444141329030927 | -0.495768068 | 1.1641562547848 | 1.39681422934718 | 0.609102894855727 | 3.20321904194677 | 0.999228396881742 |
| ebi-fl187-GCST90094785 | ebi-met1400-GCST90200710 | X-13431 levels \|\| id:ebi-met1400-GCST90200710 | F-highly palatable foods liking (derived food-liking factor) \|\| id:ebi-fl187-GCST90094785 | Weighted mode | 14 | 0.443775901392301 | 0.451277493938804 | 0.343370554931431 | -0.440727987 | 1.32827978951236 | 1.55858117061255 | 0.643567741733274 | 3.7745447881612 | 0.999228396881742 |
| ebi-fl187-GCST90094785 | ebi-met1400-GCST90200730 | Adenosine 5'-diphosphate (ADP) to fructose ratio \|\| id:ebi-met1400-GCST90200730 | F-highly palatable foods liking (derived food-liking factor) \|\| id:ebi-fl187-GCST90094785 | Weighted mode | 14 | -0.093401485 | 0.656489934438509 | 0.88904489346441 | -1.380121756 | 1.19331878664534 | 0.910827743334437 | 0.25154792363719 | 3.29800845116199 | 0.999228396881742 |
| ebi-fl187-GCST90094785 | ebi-met1400-GCST90200731 | Adenosine 5'-diphosphate (ADP) to mannose ratio \|\| id:ebi-met1400-GCST90200731 | F-highly palatable foods liking (derived food-liking factor) \|\| id:ebi-fl187-GCST90094785 | Weighted mode | 14 | -0.149982016 | 0.690090662400399 | 0.831319882865926 | -1.502559714 | 1.20259568216373 | 0.860723455415087 | 0.222559741020693 | 3.32874608545131 | 0.999228396881742 |
| ebi-fl187-GCST90094785 | ebi-met1400-GCST90200748 | Aspartate to glutamate ratio \|\| id:ebi-met1400-GCST90200748 | F-highly palatable foods liking (derived food-liking factor) \|\| id:ebi-fl187-GCST90094785 | Weighted mode | 14 | -0.100591293 | 0.391035366598838 | 0.801017591929609 | -0.867020612 | 0.665838025236278 | 0.904302551882368 | 0.420201629849739 | 1.94612073645071 | 0.999228396881742 |
| ebi-fl187-GCST90094785 | ebi-met1400-GCST90200774 | Uridine to pseudouridine ratio \|\| id:ebi-met1400-GCST90200774 | F-highly palatable foods liking (derived food-liking factor) \|\| id:ebi-fl187-GCST90094785 | Weighted mode | 14 | 0.34694979072585 | 0.409420267098669 | 0.412092715151261 | -0.455513933 | 1.14941351423924 | 1.41474569023292 | 0.63412198810855 | 3.15634121756711 | 0.999228396881742 |
| ebi-fl187-GCST90094785 | ebi-met1400-GCST90200799 | Spermidine to adenosine 5'-diphosphate (ADP) ratio \|\| id:ebi-met1400-GCST90200799 | F-highly palatable foods liking (derived food-liking factor) \|\| id:ebi-fl187-GCST90094785 | Weighted mode | 14 | -0.1168211 | 0.652394484416638 | 0.860647233639623 | -1.39551429 | 1.1618720892517 | 0.889744354008684 | 0.247705611168383 | 3.19591070931451 | 0.999228396881742 |
| ebi-fl187-GCST90094785 | ebi-met1400-GCST90200811 | Choline to taurocholate ratio \|\| id:ebi-met1400-GCST90200811 | F-highly palatable foods liking (derived food-liking factor) \|\| id:ebi-fl187-GCST90094785 | Weighted mode | 14 | 0.25213285733999 | 0.488050171796204 | 0.614102091769293 | -0.704445479 | 1.20871119406055 | 1.2867669823674 | 0.494382643630341 | 3.34916544551866 | 0.999228396881742 |
| ebi-fl187-GCST90094785 | ebi-met1400-GCST90200812 | Glutamate to kynurenine ratio \|\| id:ebi-met1400-GCST90200812 | F-highly palatable foods liking (derived food-liking factor) \|\| id:ebi-fl187-GCST90094785 | Weighted mode | 14 | 0.0548914562215086 | 0.379617042019716 | 0.887246794733364 | -0.689157946 | 0.798940858580152 | 1.05642593998877 | 0.501998601004892 | 2.22318501375721 | 0.999228396881742 |
| ebi-fl187-GCST90094785 | ebi-met1400-GCST90200876 | Cortisone to 4-cholesten-3-one ratio \|\| id:ebi-met1400-GCST90200876 | F-highly palatable foods liking (derived food-liking factor) \|\| id:ebi-fl187-GCST90094785 | Weighted mode | 14 | 0.0752612620885751 | 0.463971975303843 | 0.873634003864799 | -0.83412381 | 0.984646333684107 | 1.07816579793938 | 0.43425480472229 | 2.67686500000766 | 0.999228396881742 |
| ebi-fl187-GCST90094785 | ebi-met1400-GCST90200906 | 2'-deoxyuridine to cytidine ratio \|\| id:ebi-met1400-GCST90200906 | F-highly palatable foods liking (derived food-liking factor) \|\| id:ebi-fl187-GCST90094785 | Weighted mode | 14 | -0.02941689 | 0.479662887995452 | 0.952030633275305 | -0.969556151 | 0.91072237036605 | 0.97101157495778 | 0.379251331237106 | 2.48611778270203 | 0.999228396881742 |
| ebi-fl187-GCST90094785 | ebi-met1400-GCST90200907 | Retinol (Vitamin A) to linoleoyl-arachidonoyl-glycerol (18:2 to 20:4) [1] ratio \|\| id:ebi-met1400-GCST90200907 | F-highly palatable foods liking (derived food-liking factor) \|\| id:ebi-fl187-GCST90094785 | Weighted mode | 14 | 0.086195267286644 | 0.489918903753207 | 0.863053848604112 | -0.874045784 | 1.04643631864293 | 1.09001915260796 | 0.417259985901105 | 2.84748548434687 | 0.999228396881742 |
| ebi-fl187-GCST90094785 | ebi-met1400-GCST90200916 | Fructose to sucrose ratio \|\| id:ebi-met1400-GCST90200916 | F-highly palatable foods liking (derived food-liking factor) \|\| id:ebi-fl187-GCST90094785 | Weighted mode | 14 | 0.160147770725392 | 0.489698819648312 | 0.748848801646692 | -0.799661916 | 1.11995745723608 | 1.17368429435761 | 0.449480900829452 | 3.06472381869767 | 0.999228396881742 |
| ebi-fl187-GCST90094785 | ebi-met1400-GCST90200917 | Alpha-ketobutyrate to 3-methyl-2-oxobutyrate ratio \|\| id:ebi-met1400-GCST90200917 | F-highly palatable foods liking (derived food-liking factor) \|\| id:ebi-fl187-GCST90094785 | Weighted mode | 14 | 0.450813254853804 | 0.441834397739529 | 0.326189232092622 | -0.415182165 | 1.31680867442328 | 1.56958814175579 | 0.660220000961196 | 3.7314939431609 | 0.999228396881742 |
| ebi-fl187-GCST90094785 | ebi-met1400-GCST90200924 | Alpha-tocopherol to glycerol ratio \|\| id:ebi-met1400-GCST90200924 | F-highly palatable foods liking (derived food-liking factor) \|\| id:ebi-fl187-GCST90094785 | Weighted mode | 14 | -0.485361562 | 0.4595459211393 | 0.310120850500023 | -1.386071567 | 0.415348443501397 | 0.615474624335807 | 0.250055704643901 | 1.51489850527808 | 0.999228396881742 |
| ebi-fl187-GCST90094785 | ebi-met1400-GCST90200938 | Alpha-ketoglutarate to alanine ratio \|\| id:ebi-met1400-GCST90200938 | F-highly palatable foods liking (derived food-liking factor) \|\| id:ebi-fl187-GCST90094785 | Weighted mode | 14 | 0.589605078675958 | 0.44305379278267 | 0.206133768524513 | -0.278780355 | 1.45799051252999 | 1.80327612256336 | 0.756706091537903 | 4.29731544462435 | 0.999228396881742 |
| ebi-fl187-GCST90094785 | ebi-met1400-GCST90200943 | Phosphate to EDTA ratio \|\| id:ebi-met1400-GCST90200943 | F-highly palatable foods liking (derived food-liking factor) \|\| id:ebi-fl187-GCST90094785 | Weighted mode | 14 | 0.218539652597886 | 0.517891549009648 | 0.679934778628941 | -0.796527783 | 1.2336070886568 | 1.24425835372515 | 0.450891843335195 | 3.43359249828769 | 0.999228396881742 |
| ebi-fl187-GCST90094785 | ebi-met1400-GCST90200947 | Adenosine 5'-diphosphate (ADP) to glucose ratio \|\| id:ebi-met1400-GCST90200947 | F-highly palatable foods liking (derived food-liking factor) \|\| id:ebi-fl187-GCST90094785 | Weighted mode | 14 | -0.526045252 | 0.660865902332875 | 0.44032939968964 | -1.82134242 | 0.769251917003239 | 0.590937363233539 | 0.1618083902297 | 2.15815117355583 | 0.999228396881742 |
| ebi-fl187-GCST90094785 | ebi-met1400-GCST90200975 | Arachidonate (20:4n6) to caffeine ratio \|\| id:ebi-met1400-GCST90200975 | F-highly palatable foods liking (derived food-liking factor) \|\| id:ebi-fl187-GCST90094785 | Weighted mode | 14 | -0.475201531 | 0.427839702159446 | 0.286819087428709 | -1.313767347 | 0.36336428541125 | 0.621759740086839 | 0.268805462953006 | 1.43815966441292 | 0.999228396881742 |
| ebi-fl187-GCST90094785 | ebi-met1400-GCST90200985 | Cholesterol to oleoyl-linoleoyl-glycerol (18:1 to 18:2) [2] ratio \|\| id:ebi-met1400-GCST90200985 | F-highly palatable foods liking (derived food-liking factor) \|\| id:ebi-fl187-GCST90094785 | Weighted mode | 14 | 0.0511182102502428 | 0.406589189050949 | 0.901873581138168 | -0.7457966 | 0.848033020790104 | 1.05244729598845 | 0.474356277045652 | 2.33504933829893 | 0.999228396881742 |
| ebi-fl187-GCST90094785 | ebi-met1400-GCST90201016 | Bilirubin (Z,Z) to etiocholanolone glucuronide ratio \|\| id:ebi-met1400-GCST90201016 | F-highly palatable foods liking (derived food-liking factor) \|\| id:ebi-fl187-GCST90094785 | Weighted mode | 14 | 0.354308669811278 | 0.449851206841615 | 0.44504676584575 | -0.527399696 | 1.23601703522084 | 1.42519503327464 | 0.5901375134485 | 3.44187725162798 | 0.999228396881742 |
| ebi-fl187-GCST90094785 | ebi-pw11-GCST90275189 | Placental weight (fetal genotype, adjusted for sex and gestational age) \|\| id:ebi-pw11-GCST90275189 | F-highly palatable foods liking (derived food-liking factor) \|\| id:ebi-fl187-GCST90094785 | Weighted mode | 13 | -0.015013146 | 0.158581405891565 | 0.926138199515024 | -0.325832701 | 0.295806409852639 | 0.985098989707251 | 0.721925954644698 | 1.3442099058481 | 0.999228396881742 |
| ebi-fl187-GCST90094785 | ebi-pw11-GCST90275192 | Placental weight (fetal effect, conditional on maternal and paternal genotype) \|\| id:ebi-pw11-GCST90275192 | F-highly palatable foods liking (derived food-liking factor) \|\| id:ebi-fl187-GCST90094785 | Weighted mode | 13 | -0.037048271 | 0.401046502380216 | 0.927921252465539 | -0.823099415 | 0.749002873941879 | 0.963629619127624 | 0.439068687150506 | 2.11489015280597 | 0.999228396881742 |
| ebi-fl187-GCST90094785 | ebi-pw11-GCST90275193 | Placental weight (maternal effect, conditional on fetal and paternal genotype) \|\| id:ebi-pw11-GCST90275193 | F-highly palatable foods liking (derived food-liking factor) \|\| id:ebi-fl187-GCST90094785 | Weighted mode | 13 | -0.122237054 | 0.283935030702638 | 0.67445508902818 | -0.678749715 | 0.434275605762093 | 0.884938565042275 | 0.507250804338828 | 1.54384430187317 | 0.999228396881742 |
| ebi-fl187-GCST90094785 | ebi-pw11-GCST90275195 | Placental weight (fetal effect, conditional on maternal genotype) \|\| id:ebi-pw11-GCST90275195 | F-highly palatable foods liking (derived food-liking factor) \|\| id:ebi-fl187-GCST90094785 | Weighted mode | 13 | -0.007899645 | 0.25501618088659 | 0.97579710740181 | -0.50773136 | 0.491932069042596 | 0.992131474704256 | 0.601859433545194 | 1.63547301618382 | 0.999228396881742 |
| ebi-fl187-GCST90094785 | ebi-pw11-GCST90275197 | Placental weight (fetal genotype, adjusted for sex) \|\| id:ebi-pw11-GCST90275197 | F-highly palatable foods liking (derived food-liking factor) \|\| id:ebi-fl187-GCST90094785 | Weighted mode | 13 | 0.129201681174832 | 0.181953551116459 | 0.491223405951882 | -0.227427279 | 0.485830641363091 | 1.13791959798607 | 0.796580347500003 | 1.62552467625466 | 0.999228396881742 |
| ebi-fl187-GCST90094785 | prot-a-1030 | Protein FAM171B \|\| id:prot-a-1030 | F-highly palatable foods liking (derived food-liking factor) \|\| id:ebi-fl187-GCST90094785 | Weighted mode | 17 | -0.307844592 | 0.696760928669228 | 0.66453099117778 | -1.673496012 | 1.0578068282405 | 0.735029538639856 | 0.18759010065931 | 2.88004761858049 | 0.999228396881742 |
| ebi-fl187-GCST90094785 | prot-a-1036 | BRISC complex subunit Abro1 \|\| id:prot-a-1036 | F-highly palatable foods liking (derived food-liking factor) \|\| id:ebi-fl187-GCST90094785 | Weighted mode | 17 | 0.91397658268234 | 0.655833699763409 | 0.182492473502651 | -0.371457469 | 2.19941063421862 | 2.49422131596783 | 0.689728340142088 | 9.01969603242153 | 0.999228396881742 |
| ebi-fl187-GCST90094785 | prot-a-1055 | Phenylalanine--tRNA ligase, mitochondrial \|\| id:prot-a-1055 | F-highly palatable foods liking (derived food-liking factor) \|\| id:ebi-fl187-GCST90094785 | Weighted mode | 17 | -1.366445091 | 0.754211197628382 | 0.0888353190693246 | -2.844699039 | 0.11180885613254 | 0.255011894141563 | 0.0581517655449304 | 1.11829908420274 | 0.999228396881742 |
| ebi-fl187-GCST90094785 | prot-a-1083 | Fc receptor-like protein 5 \|\| id:prot-a-1083 | F-highly palatable foods liking (derived food-liking factor) \|\| id:ebi-fl187-GCST90094785 | Weighted mode | 17 | -1.131605121 | 0.655295950691824 | 0.103444622659549 | -2.415985185 | 0.152774942016146 | 0.322515164760122 | 0.0892793389423769 | 1.16506274276272 | 0.999228396881742 |
| ebi-fl187-GCST90094785 | prot-a-1103 | Fibroblast growth factor receptor 3 \|\| id:prot-a-1103 | F-highly palatable foods liking (derived food-liking factor) \|\| id:ebi-fl187-GCST90094785 | Weighted mode | 17 | 0.219767342485474 | 0.714719899389172 | 0.762438714313208 | -1.18108366 | 1.62061834528825 | 1.24578685519449 | 0.30694593318234 | 5.05621583737816 | 0.999228396881742 |
| ebi-fl187-GCST90094785 | prot-a-1105 | Fibroblast growth factor receptor 4 \|\| id:prot-a-1105 | F-highly palatable foods liking (derived food-liking factor) \|\| id:ebi-fl187-GCST90094785 | Weighted mode | 17 | 0.318929175195401 | 0.595182448732914 | 0.599432169613908 | -0.847628424 | 1.48548677471191 | 1.37565389103768 | 0.428429781726999 | 4.41711502944255 | 0.999228396881742 |
| ebi-fl187-GCST90094785 | prot-a-1120 | Filamin-A \|\| id:prot-a-1120 | F-highly palatable foods liking (derived food-liking factor) \|\| id:ebi-fl187-GCST90094785 | Weighted mode | 17 | 0.907317716193286 | 0.842489387299889 | 0.297473933869264 | -0.743961483 | 2.55859691530107 | 2.47766780420889 | 0.475227575714801 | 12.9176799952732 | 0.999228396881742 |
| ebi-fl187-GCST90094785 | prot-a-1166 | Galanin peptides \|\| id:prot-a-1166 | F-highly palatable foods liking (derived food-liking factor) \|\| id:ebi-fl187-GCST90094785 | Weighted mode | 17 | -0.201707821 | 0.547681775882823 | 0.717481476660684 | -1.275164102 | 0.871748459354865 | 0.817333700494814 | 0.279385116773359 | 2.39108792078737 | 0.999228396881742 |
| ebi-fl187-GCST90094785 | prot-a-1205 | ADP-ribosylation factor-binding protein GGA1 \|\| id:prot-a-1205 | F-highly palatable foods liking (derived food-liking factor) \|\| id:ebi-fl187-GCST90094785 | Weighted mode | 17 | 0.561154580680211 | 0.617660807117709 | 0.377087587323009 | -0.649460601 | 1.77176976263092 | 1.75269496026425 | 0.522327443548267 | 5.88125257763109 | 0.999228396881742 |
| ebi-fl187-GCST90094785 | prot-a-1227 | Guanine nucleotide-binding protein G(I)/G(S)/G(O) subunit gamma-T2 \|\| id:prot-a-1227 | F-highly palatable foods liking (derived food-liking factor) \|\| id:ebi-fl187-GCST90094785 | Weighted mode | 17 | -0.429944577 | 0.849805313997845 | 0.619802824757459 | -2.095562992 | 1.23567383890226 | 0.650545149191447 | 0.123000975595642 | 3.44069621470156 | 0.999228396881742 |
| ebi-fl187-GCST90094785 | prot-a-1240 | Golgi SNAP receptor complex member 1 \|\| id:prot-a-1240 | F-highly palatable foods liking (derived food-liking factor) \|\| id:ebi-fl187-GCST90094785 | Weighted mode | 17 | -0.400408336 | 0.584208855714355 | 0.502910685372442 | -1.545457693 | 0.744641020983077 | 0.670046385960286 | 0.213214262122873 | 2.10568540241323 | 0.999228396881742 |
| ebi-fl187-GCST90094785 | prot-a-1283 | Glutathione S-transferase A1 \|\| id:prot-a-1283 | F-highly palatable foods liking (derived food-liking factor) \|\| id:ebi-fl187-GCST90094785 | Weighted mode | 17 | 0.300721906168648 | 0.57095689394768 | 0.605628984685536 | -0.818353606 | 1.4197974183061 | 1.35083363079911 | 0.441157375800802 | 4.13628242027136 | 0.999228396881742 |
| ebi-fl187-GCST90094785 | prot-a-1324 | E3 ubiquitin-protein ligase HECW1 \|\| id:prot-a-1324 | F-highly palatable foods liking (derived food-liking factor) \|\| id:ebi-fl187-GCST90094785 | Weighted mode | 17 | -0.215463843 | 0.6928688761738 | 0.759835544141501 | -1.573486841 | 1.14255915388022 | 0.806167417987571 | 0.207321025187404 | 3.13478049434339 | 0.999228396881742 |
| ebi-fl187-GCST90094785 | prot-a-1339 | Histidine triad nucleotide-binding protein 2, mitochondrial \|\| id:prot-a-1339 | F-highly palatable foods liking (derived food-liking factor) \|\| id:ebi-fl187-GCST90094785 | Weighted mode | 17 | 0.708550627254512 | 0.664579046861032 | 0.302173884862672 | -0.594024305 | 2.01112555910213 | 2.0310453825039 | 0.552100985554053 | 7.47172248144183 | 0.999228396881742 |
| ebi-fl187-GCST90094785 | prot-a-1466 | Interleukin-11 \|\| id:prot-a-1466 | F-highly palatable foods liking (derived food-liking factor) \|\| id:ebi-fl187-GCST90094785 | Weighted mode | 17 | 0.643746418699196 | 0.639967770983113 | 0.329432771386401 | -0.610590412 | 1.8980832498261 | 1.90359921654384 | 0.543030162652639 | 6.67309152685926 | 0.999228396881742 |
| ebi-fl187-GCST90094785 | prot-a-147 | Rho GTPase-activating protein 1 \|\| id:prot-a-147 | F-highly palatable foods liking (derived food-liking factor) \|\| id:ebi-fl187-GCST90094785 | Weighted mode | 17 | 0.199417012939896 | 0.607958088101439 | 0.747156476397121 | -0.99218084 | 1.39101486561872 | 1.22069090367812 | 0.370767224787096 | 4.01892665452876 | 0.999228396881742 |
| ebi-fl187-GCST90094785 | prot-a-1473 | Interleukin-12 receptor subunit beta-1 \|\| id:prot-a-1473 | F-highly palatable foods liking (derived food-liking factor) \|\| id:ebi-fl187-GCST90094785 | Weighted mode | 17 | 0.0704903127793841 | 0.657788229554789 | 0.915992187496332 | -1.218774617 | 1.35975524270677 | 1.07303417466159 | 0.295592158651887 | 3.89523979676218 | 0.999228396881742 |
| ebi-fl187-GCST90094785 | prot-a-1501 | X-linked interleukin-1 receptor accessory protein-like 2 \|\| id:prot-a-1501 | F-highly palatable foods liking (derived food-liking factor) \|\| id:ebi-fl187-GCST90094785 | Weighted mode | 17 | -0.090888253 | 0.655625285371956 | 0.891473639416858 | -1.375913813 | 1.19413730603914 | 0.913119743325513 | 0.252608653326681 | 3.30070904013162 | 0.999228396881742 |
| ebi-fl187-GCST90094785 | prot-a-1503 | Interleukin-1 receptor-like 2 \|\| id:prot-a-1503 | F-highly palatable foods liking (derived food-liking factor) \|\| id:ebi-fl187-GCST90094785 | Weighted mode | 17 | 0.532610704112145 | 0.669036863274504 | 0.437634965578424 | -0.778701548 | 1.84392295613017 | 1.70337351299566 | 0.459001616149056 | 6.32128781837003 | 0.999228396881742 |
| ebi-fl187-GCST90094785 | prot-a-1536 | Interleukin-5 receptor subunit alpha \|\| id:prot-a-1536 | F-highly palatable foods liking (derived food-liking factor) \|\| id:ebi-fl187-GCST90094785 | Weighted mode | 17 | -1.107636885 | 0.658320206431244 | 0.111877118487892 | -2.397944489 | 0.182670720019634 | 0.330338667841754 | 0.0909046168016942 | 1.2004190690283 | 0.999228396881742 |
| ebi-fl187-GCST90094785 | prot-a-1559 | Insulin-induced gene 1 protein \|\| id:prot-a-1559 | F-highly palatable foods liking (derived food-liking factor) \|\| id:ebi-fl187-GCST90094785 | Weighted mode | 17 | -0.400324337 | 0.773657688038489 | 0.611926194795174 | -1.916693406 | 1.11604473114357 | 0.670102671420052 | 0.147092534188898 | 3.0527558228593 | 0.999228396881742 |
| ebi-fl187-GCST90094785 | prot-a-1567 | Interferon regulatory factor 1 \|\| id:prot-a-1567 | F-highly palatable foods liking (derived food-liking factor) \|\| id:ebi-fl187-GCST90094785 | Weighted mode | 17 | 0.664152585906668 | 0.699569468064131 | 0.356551514696652 | -0.707003571 | 2.03530874331236 | 1.9428434306472 | 0.493119583487037 | 7.65461507190009 | 0.999228396881742 |
| ebi-fl187-GCST90094785 | prot-a-1613 | Potassium voltage-gated channel subfamily E regulatory beta subunit 5 \|\| id:prot-a-1613 | F-highly palatable foods liking (derived food-liking factor) \|\| id:ebi-fl187-GCST90094785 | Weighted mode | 17 | 0.663360957524638 | 0.651960519780347 | 0.324061964323105 | -0.614481661 | 1.94120357629412 | 1.94130602925123 | 0.540921203078893 | 6.96713140057394 | 0.999228396881742 |
| ebi-fl187-GCST90094785 | prot-a-1649 | Kin of IRRE-like protein 2 \|\| id:prot-a-1649 | F-highly palatable foods liking (derived food-liking factor) \|\| id:ebi-fl187-GCST90094785 | Weighted mode | 17 | 0.665667835038933 | 0.613036043382575 | 0.293633096003436 | -0.53588281 | 1.86721848006878 | 1.94578955396132 | 0.585152483598638 | 6.47027414977517 | 0.999228396881742 |
| ebi-fl187-GCST90094785 | prot-a-165 | Armadillo repeat-containing protein 5 \|\| id:prot-a-165 | F-highly palatable foods liking (derived food-liking factor) \|\| id:ebi-fl187-GCST90094785 | Weighted mode | 17 | -0.664604587 | 0.728212829524269 | 0.374973086079143 | -2.091901733 | 0.762692558440868 | 0.514476918134519 | 0.123452139394283 | 2.14404141225802 | 0.999228396881742 |
| ebi-fl187-GCST90094785 | prot-a-1656 | Kallikrein-11 \|\| id:prot-a-1656 | F-highly palatable foods liking (derived food-liking factor) \|\| id:ebi-fl187-GCST90094785 | Weighted mode | 17 | -0.716372786 | 0.639061381122935 | 0.278841461235776 | -1.968933093 | 0.536187520929792 | 0.488521016419721 | 0.139605723100523 | 1.70947707718197 | 0.999228396881742 |
| ebi-fl187-GCST90094785 | prot-a-1697 | Laminin subunit gamma-2 \|\| id:prot-a-1697 | F-highly palatable foods liking (derived food-liking factor) \|\| id:ebi-fl187-GCST90094785 | Weighted mode | 17 | 0.338354793702251 | 0.578753316941516 | 0.566951804545477 | -0.796001708 | 1.47271129490762 | 1.40263806228576 | 0.451129109098023 | 4.36104320048491 | 0.999228396881742 |
| ebi-fl187-GCST90094785 | prot-a-1698 | Lysosome-associated membrane glycoprotein 3 \|\| id:prot-a-1698 | F-highly palatable foods liking (derived food-liking factor) \|\| id:ebi-fl187-GCST90094785 | Weighted mode | 17 | -0.696963529 | 0.497651795313574 | 0.18045661891705 | -1.672361047 | 0.278433990206299 | 0.498095462476405 | 0.187803129672713 | 1.32105939965937 | 0.999228396881742 |
| ebi-fl187-GCST90094785 | prot-a-1724 | Leptin receptor \|\| id:prot-a-1724 | F-highly palatable foods liking (derived food-liking factor) \|\| id:ebi-fl187-GCST90094785 | Weighted mode | 17 | 0.314512754842597 | 0.581711863648109 | 0.596183974682311 | -0.825642498 | 1.45466800759289 | 1.36959182137209 | 0.437953517832325 | 4.28306128571267 | 0.999228396881742 |
| ebi-fl187-GCST90094785 | prot-a-1743 | Leukocyte immunoglobulin-like receptor subfamily B member 2 \|\| id:prot-a-1743 | F-highly palatable foods liking (derived food-liking factor) \|\| id:ebi-fl187-GCST90094785 | Weighted mode | 17 | -0.437823299 | 0.542830801607881 | 0.431748530211213 | -1.50177167 | 0.626125071864294 | 0.645439822458287 | 0.222735197015298 | 1.87034905123851 | 0.999228396881742 |
| ebi-fl187-GCST90094785 | prot-a-1746 | Leukocyte immunoglobulin-like receptor subfamily B member 4 \|\| id:prot-a-1746 | F-highly palatable foods liking (derived food-liking factor) \|\| id:ebi-fl187-GCST90094785 | Weighted mode | 17 | 1.06988283593213 | 0.816823889683856 | 0.208756234865318 | -0.531091988 | 2.67085765971249 | 2.91503794226492 | 0.587962571013662 | 14.4523590850253 | 0.999228396881742 |
| ebi-fl187-GCST90094785 | prot-a-1750 | Leucine-rich repeat and immunoglobulin-like domain-containing nogo receptor-interacting protein 3 \|\| id:prot-a-1750 | F-highly palatable foods liking (derived food-liking factor) \|\| id:ebi-fl187-GCST90094785 | Weighted mode | 17 | 0.888679651275133 | 0.583497063845164 | 0.147271722768713 | -0.254974594 | 2.03233389641165 | 2.43191655263205 | 0.774936185847135 | 7.63187760099309 | 0.999228396881742 |
| ebi-fl187-GCST90094785 | prot-a-1753 | Lipase member N \|\| id:prot-a-1753 | F-highly palatable foods liking (derived food-liking factor) \|\| id:ebi-fl187-GCST90094785 | Weighted mode | 17 | 0.118312017788495 | 0.534980385406432 | 0.827771635823371 | -0.930249538 | 1.1668735731851 | 1.12559526232631 | 0.394455266665657 | 3.21193504470388 | 0.999228396881742 |
| ebi-fl187-GCST90094785 | prot-a-1760 | Ig Kappa chain V-I region HK102- like \|\| id:prot-a-1760 | F-highly palatable foods liking (derived food-liking factor) \|\| id:ebi-fl187-GCST90094785 | Weighted mode | 17 | -0.310743091 | 0.566963341423644 | 0.591200198823081 | -1.42199124 | 0.800505058222503 | 0.732902140864485 | 0.241233185124439 | 2.22666524013544 | 0.999228396881742 |
| ebi-fl187-GCST90094785 | prot-a-1792 | Leucine-rich repeat neuronal protein 1 \|\| id:prot-a-1792 | F-highly palatable foods liking (derived food-liking factor) \|\| id:ebi-fl187-GCST90094785 | Weighted mode | 16 | 0.981769788308013 | 0.771483154409112 | 0.222541735957027 | -0.530337194 | 2.49387677094987 | 2.66917594063549 | 0.588406528876142 | 12.1081256791544 | 0.999228396881742 |
| ebi-fl187-GCST90094785 | prot-a-1795 | Leucine-rich repeat transmembrane neuronal protein 1 \|\| id:prot-a-1795 | F-highly palatable foods liking (derived food-liking factor) \|\| id:ebi-fl187-GCST90094785 | Weighted mode | 17 | 0.513302160472409 | 0.578091607089915 | 0.387734440119564 | -0.619757389 | 1.64636171036864 | 1.67079934309424 | 0.538074964437586 | 5.18806974749698 | 0.999228396881742 |
| ebi-fl187-GCST90094785 | prot-a-1820 | LysM and putative peptidoglycan-binding domain-containing protein 4 \|\| id:prot-a-1820 | F-highly palatable foods liking (derived food-liking factor) \|\| id:ebi-fl187-GCST90094785 | Weighted mode | 17 | 0.272518207685548 | 0.542845611353028 | 0.62249290600786 | -0.791459191 | 1.33649560593748 | 1.31326737025412 | 0.453183032175138 | 3.8056834950247 | 0.999228396881742 |
| ebi-fl187-GCST90094785 | prot-a-1844 | Mitogen-activated protein kinase kinase kinase 7:TGF-beta-activated kinase 1 and MAP3K7-binding protein 1 fusion \|\| id:prot-a-1844 | F-highly palatable foods liking (derived food-liking factor) \|\| id:ebi-fl187-GCST90094785 | Weighted mode | 17 | 0.0732333053571614 | 0.572999153882036 | 0.899894287048043 | -1.049845036 | 1.19631164696595 | 1.07598153989086 | 0.349991980978332 | 3.3078937150208 | 0.999228396881742 |
| ebi-fl187-GCST90094785 | prot-a-1856 | Megakaryocyte-associated tyrosine-protein kinase \|\| id:prot-a-1856 | F-highly palatable foods liking (derived food-liking factor) \|\| id:ebi-fl187-GCST90094785 | Weighted mode | 17 | 0.165171105577654 | 0.704973491422505 | 0.817728445084766 | -1.216576938 | 1.54691914876576 | 1.17959493671785 | 0.296242489838439 | 4.69697718071853 | 0.999228396881742 |
| ebi-fl187-GCST90094785 | prot-a-1910 | Stromelysin-2 \|\| id:prot-a-1910 | F-highly palatable foods liking (derived food-liking factor) \|\| id:ebi-fl187-GCST90094785 | Weighted mode | 17 | 0.413117282855613 | 0.595440646640016 | 0.497757058356511 | -0.753946385 | 1.58018095027005 | 1.51152229118934 | 0.470506086144068 | 4.85583439629024 | 0.999228396881742 |
| ebi-fl187-GCST90094785 | prot-a-1940 | 39S ribosomal protein L14, mitochondrial \|\| id:prot-a-1940 | F-highly palatable foods liking (derived food-liking factor) \|\| id:ebi-fl187-GCST90094785 | Weighted mode | 17 | 0.683487606358205 | 0.644915297520345 | 0.304968661184424 | -0.580546377 | 1.94752158949808 | 1.9807738592579 | 0.55959253465515 | 7.01128917657431 | 0.999228396881742 |
| ebi-fl187-GCST90094785 | prot-a-1942 | 39S ribosomal protein L33, mitochondrial \|\| id:prot-a-1942 | F-highly palatable foods liking (derived food-liking factor) \|\| id:ebi-fl187-GCST90094785 | Weighted mode | 17 | -1.277722242 | 0.635827423773856 | 0.0616562177515114 | -2.523943992 | -0.031500491 | 0.278671324027204 | 0.0801428996369411 | 0.968990480589988 | 0.999228396881742 |
| ebi-fl187-GCST90094785 | prot-a-1948 | Mesothelin \|\| id:prot-a-1948 | F-highly palatable foods liking (derived food-liking factor) \|\| id:ebi-fl187-GCST90094785 | Weighted mode | 17 | 0.47272305942675 | 0.642620063586929 | 0.472609831572416 | -0.786812265 | 1.73225838405713 | 1.60435700989661 | 0.455293840487281 | 5.65340706663108 | 0.999228396881742 |
| ebi-fl187-GCST90094785 | prot-a-1980 | Unconventional myosin-VI \|\| id:prot-a-1980 | F-highly palatable foods liking (derived food-liking factor) \|\| id:ebi-fl187-GCST90094785 | Weighted mode | 17 | 0.359212335577804 | 0.550456763640725 | 0.523298671056309 | -0.719682921 | 1.43810759231363 | 1.4322008764882 | 0.486906619273001 | 4.21271609261791 | 0.999228396881742 |
| ebi-fl187-GCST90094785 | prot-a-2039 | Neurofascin \|\| id:prot-a-2039 | F-highly palatable foods liking (derived food-liking factor) \|\| id:ebi-fl187-GCST90094785 | Weighted mode | 17 | -0.440864164 | 0.566363791993584 | 0.447685837272447 | -1.550937196 | 0.669208868682015 | 0.643480108641712 | 0.212049149072349 | 1.95269187369515 | 0.999228396881742 |
| ebi-fl187-GCST90094785 | prot-a-2041 | NFU1 iron-sulfur cluster scaffold homolog, mitochondrial \|\| id:prot-a-2041 | F-highly palatable foods liking (derived food-liking factor) \|\| id:ebi-fl187-GCST90094785 | Weighted mode | 17 | 0.0719075727035317 | 0.717193078268723 | 0.921381503012077 | -1.333790861 | 1.47760600611023 | 1.07455602116587 | 0.263476562795788 | 4.38244157420095 | 0.999228396881742 |
| ebi-fl187-GCST90094785 | prot-a-2088 | Glucocorticoid receptor \|\| id:prot-a-2088 | F-highly palatable foods liking (derived food-liking factor) \|\| id:ebi-fl187-GCST90094785 | Weighted mode | 17 | 0.0963972869466216 | 0.768135242658444 | 0.901694858401193 | -1.409147789 | 1.60194236255717 | 1.10119646808319 | 0.244351433508415 | 4.96266235850481 | 0.999228396881742 |
| ebi-fl187-GCST90094785 | prot-a-2111 | Neuron-specific protein family member 2 \|\| id:prot-a-2111 | F-highly palatable foods liking (derived food-liking factor) \|\| id:ebi-fl187-GCST90094785 | Weighted mode | 17 | 0.38584060593197 | 0.647115448684808 | 0.559352150333233 | -0.882505673 | 1.65418688535419 | 1.47085020733119 | 0.413744902135159 | 5.22882655772148 | 0.999228396881742 |
| ebi-fl187-GCST90094785 | prot-a-2125 | Nuclear migration protein nudC \|\| id:prot-a-2125 | F-highly palatable foods liking (derived food-liking factor) \|\| id:ebi-fl187-GCST90094785 | Weighted mode | 17 | -0.293276133 | 0.87429578793799 | 0.741649205661997 | -2.006895878 | 1.42034361107505 | 0.745816167593334 | 0.134405238107035 | 4.13854224491339 | 0.999228396881742 |
| ebi-fl187-GCST90094785 | prot-a-2142 | Ornithine decarboxylase \|\| id:prot-a-2142 | F-highly palatable foods liking (derived food-liking factor) \|\| id:ebi-fl187-GCST90094785 | Weighted mode | 17 | 0.095967501389155 | 0.731910209676808 | 0.897315514614376 | -1.33857651 | 1.5305115123557 | 1.10072329143481 | 0.262218668799887 | 4.6205396810694 | 0.999228396881742 |
| ebi-fl187-GCST90094785 | prot-a-2186 | Prostate and testis expressed protein 1 \|\| id:prot-a-2186 | F-highly palatable foods liking (derived food-liking factor) \|\| id:ebi-fl187-GCST90094785 | Weighted mode | 17 | -0.415925273 | 0.998180442635305 | 0.682446433121644 | -2.372358941 | 1.54050839413191 | 0.659729567968023 | 0.0932604706474278 | 4.66696232422754 | 0.999228396881742 |
| ebi-fl187-GCST90094785 | prot-a-2227 | Platelet-derived growth factor C \|\| id:prot-a-2227 | F-highly palatable foods liking (derived food-liking factor) \|\| id:ebi-fl187-GCST90094785 | Weighted mode | 17 | -0.28586038 | 0.602436602884904 | 0.641549940485363 | -1.466636122 | 0.894915361310592 | 0.751367514297791 | 0.230700228809342 | 2.4471286589343 | 0.999228396881742 |
| ebi-fl187-GCST90094785 | prot-a-2229 | Platelet-derived growth factor receptor alpha \|\| id:prot-a-2229 | F-highly palatable foods liking (derived food-liking factor) \|\| id:ebi-fl187-GCST90094785 | Weighted mode | 17 | 0.302924839412971 | 0.571417248949792 | 0.603302509850282 | -0.817052969 | 1.42290264735456 | 1.35381270726047 | 0.441731534906257 | 4.14914648719582 | 0.999228396881742 |
| ebi-fl187-GCST90094785 | prot-a-2243 | Platelet endothelial aggregation receptor 1 \|\| id:prot-a-2243 | F-highly palatable foods liking (derived food-liking factor) \|\| id:ebi-fl187-GCST90094785 | Weighted mode | 17 | -0.459635604 | 0.563280337011144 | 0.426483267759182 | -1.563665065 | 0.644393856046177 | 0.63151372434661 | 0.209367318458023 | 1.90483207682715 | 0.999228396881742 |
| ebi-fl187-GCST90094785 | prot-a-2246 | E3 ubiquitin-protein ligase pellino homolog 2 \|\| id:prot-a-2246 | F-highly palatable foods liking (derived food-liking factor) \|\| id:ebi-fl187-GCST90094785 | Weighted mode | 17 | -0.016834587 | 0.684504709536154 | 0.980683069097303 | -1.358463818 | 1.32479464339676 | 0.983306322542887 | 0.257055357619342 | 3.7614128447174 | 0.999228396881742 |
| ebi-fl187-GCST90094785 | prot-a-2251 | Platelet factor 4 variant \|\| id:prot-a-2251 | F-highly palatable foods liking (derived food-liking factor) \|\| id:ebi-fl187-GCST90094785 | Weighted mode | 17 | -0.278850136 | 0.648353686928826 | 0.672867216779082 | -1.549623363 | 0.991923090257021 | 0.756653289713745 | 0.212327929428335 | 2.69641493879811 | 0.999228396881742 |
| ebi-fl187-GCST90094785 | prot-a-2346 | Protein phosphatase 1L \|\| id:prot-a-2346 | F-highly palatable foods liking (derived food-liking factor) \|\| id:ebi-fl187-GCST90094785 | Weighted mode | 17 | 0.145177502726682 | 0.56802059376181 | 0.801527974937178 | -0.968142861 | 1.25849786649983 | 1.15624478865677 | 0.37978770210704 | 3.52012980904564 | 0.999228396881742 |
| ebi-fl187-GCST90094785 | prot-a-2358 | PR domain zinc finger protein 4 \|\| id:prot-a-2358 | F-highly palatable foods liking (derived food-liking factor) \|\| id:ebi-fl187-GCST90094785 | Weighted mode | 17 | 0.064757511534228 | 0.557156779134389 | 0.908917768276782 | -1.027269776 | 1.15678479863763 | 1.06690028200192 | 0.357983001493963 | 3.1796934686436 | 0.999228396881742 |
| ebi-fl187-GCST90094785 | prot-a-2390 | Trypsin-1 \|\| id:prot-a-2390 | F-highly palatable foods liking (derived food-liking factor) \|\| id:ebi-fl187-GCST90094785 | Weighted mode | 17 | -0.750909952 | 0.692642947433195 | 0.294377025658821 | -2.108490129 | 0.606670224681945 | 0.471936917219548 | 0.121421158355199 | 1.8343133672234 | 0.999228396881742 |
| ebi-fl187-GCST90094785 | prot-a-242 | Brain-derived neurotrophic factor \|\| id:prot-a-242 | F-highly palatable foods liking (derived food-liking factor) \|\| id:ebi-fl187-GCST90094785 | Weighted mode | 17 | 0.751890555641169 | 0.591220507774195 | 0.221633847426359 | -0.40690164 | 1.91068275087859 | 2.12100610861314 | 0.665709666552914 | 6.75770105017195 | 0.999228396881742 |
| ebi-fl187-GCST90094785 | prot-a-2464 | Dihydropteridine reductase \|\| id:prot-a-2464 | F-highly palatable foods liking (derived food-liking factor) \|\| id:ebi-fl187-GCST90094785 | Weighted mode | 17 | 0.176312952092564 | 0.612813441390056 | 0.777261469023825 | -1.024801393 | 1.37742729721707 | 1.19281129303557 | 0.358867731960878 | 3.96468853028085 | 0.999228396881742 |
| ebi-fl187-GCST90094785 | prot-a-2474 | Ras-related protein Rab-27A \|\| id:prot-a-2474 | F-highly palatable foods liking (derived food-liking factor) \|\| id:ebi-fl187-GCST90094785 | Weighted mode | 17 | 0.463936343356884 | 0.998600611529573 | 0.648486929713325 | -1.493320855 | 2.42119354195485 | 1.59032173267487 | 0.224625466914397 | 11.2592897330819 | 0.999228396881742 |
| ebi-fl187-GCST90094785 | prot-a-2478 | Ras-related protein Rab-7b \|\| id:prot-a-2478 | F-highly palatable foods liking (derived food-liking factor) \|\| id:ebi-fl187-GCST90094785 | Weighted mode | 17 | -0.075413238 | 0.558770894744797 | 0.894324610761834 | -1.170604191 | 1.01977771614084 | 0.927360187077201 | 0.31017947691043 | 2.77257839603683 | 0.999228396881742 |
| ebi-fl187-GCST90094785 | prot-a-2528 | E3 ubiquitin-protein ligase rififylin \|\| id:prot-a-2528 | F-highly palatable foods liking (derived food-liking factor) \|\| id:ebi-fl187-GCST90094785 | Weighted mode | 17 | 0.316477108880676 | 0.602951769083133 | 0.606863854750611 | -0.865308359 | 1.49826257628362 | 1.37228482874091 | 0.420921737808537 | 4.47390923784759 | 0.999228396881742 |
| ebi-fl187-GCST90094785 | prot-a-2543 | Protein RIC-3 \|\| id:prot-a-2543 | F-highly palatable foods liking (derived food-liking factor) \|\| id:ebi-fl187-GCST90094785 | Weighted mode | 17 | -0.061161186 | 0.545983185491035 | 0.912200657749737 | -1.13128823 | 1.0089658571798 | 0.940671604102429 | 0.32261738323574 | 2.74276313907753 | 0.999228396881742 |
| ebi-fl187-GCST90094785 | prot-a-255 | Bone morphogenetic protein 6 \|\| id:prot-a-255 | F-highly palatable foods liking (derived food-liking factor) \|\| id:ebi-fl187-GCST90094785 | Weighted mode | 17 | -0.591301623 | 0.618536176952432 | 0.353309850637564 | -1.80363253 | 0.62102928372226 | 0.553606228906503 | 0.164699524325226 | 1.860842391 | 0.999228396881742 |
| ebi-fl187-GCST90094785 | prot-a-2568 | RING finger protein 165 \|\| id:prot-a-2568 | F-highly palatable foods liking (derived food-liking factor) \|\| id:ebi-fl187-GCST90094785 | Weighted mode | 17 | 0.801050907522309 | 0.776420122336714 | 0.317545914376296 | -0.720732532 | 2.32283434730227 | 2.22788099557711 | 0.486395824795598 | 10.2045566130003 | 0.999228396881742 |
| ebi-fl187-GCST90094785 | prot-a-2573 | E3 ubiquitin-protein ligase RNF8 \|\| id:prot-a-2573 | F-highly palatable foods liking (derived food-liking factor) \|\| id:ebi-fl187-GCST90094785 | Weighted mode | 17 | -0.117921451 | 0.59579290721508 | 0.845597642951165 | -1.285675549 | 1.04983264753237 | 0.888765861889869 | 0.276463755788571 | 2.85717292311159 | 0.999228396881742 |
| ebi-fl187-GCST90094785 | prot-a-260 | Cytoplasmic tyrosine-protein kinase BMX \|\| id:prot-a-260 | F-highly palatable foods liking (derived food-liking factor) \|\| id:ebi-fl187-GCST90094785 | Weighted mode | 16 | -0.529689918 | 0.65437635481744 | 0.430905708899061 | -1.812267573 | 0.752887737407107 | 0.588787513764269 | 0.163283459450545 | 2.12312219211468 | 0.999228396881742 |
| ebi-fl187-GCST90094785 | prot-a-2623 | Serum amyloid A-1 protein \|\| id:prot-a-2623 | F-highly palatable foods liking (derived food-liking factor) \|\| id:ebi-fl187-GCST90094785 | Weighted mode | 17 | -0.025713929 | 0.887639850050807 | 0.977247678279852 | -1.765488035 | 1.71406017721667 | 0.974613858608231 | 0.171103261727755 | 5.55145567536041 | 0.999228396881742 |
| ebi-fl187-GCST90094785 | prot-a-2640 | Lysosome membrane protein 2 \|\| id:prot-a-2640 | F-highly palatable foods liking (derived food-liking factor) \|\| id:ebi-fl187-GCST90094785 | Weighted mode | 17 | 0.233437055056438 | 0.662252065313882 | 0.72907135852473 | -1.064576993 | 1.53145110307165 | 1.26293333012176 | 0.344873707923376 | 4.62488313747251 | 0.999228396881742 |
| ebi-fl187-GCST90094785 | prot-a-2641 | Scavenger receptor class F member 2 \|\| id:prot-a-2641 | F-highly palatable foods liking (derived food-liking factor) \|\| id:ebi-fl187-GCST90094785 | Weighted mode | 17 | 0.761224875771562 | 0.595030235316049 | 0.219031877410879 | -0.405034385 | 1.92748413699102 | 2.14089694793732 | 0.666953876956396 | 6.87219896314809 | 0.999228396881742 |
| ebi-fl187-GCST90094785 | prot-a-2669 | Semaphorin-3A \|\| id:prot-a-2669 | F-highly palatable foods liking (derived food-liking factor) \|\| id:ebi-fl187-GCST90094785 | Weighted mode | 17 | 0.435672476851094 | 0.5243288831776 | 0.418249388547445 | -0.592012134 | 1.46335708787919 | 1.54600236025815 | 0.553213025255379 | 4.32043930422719 | 0.999228396881742 |
| ebi-fl187-GCST90094785 | prot-a-2683 | Sentrin-specific protease 7 \|\| id:prot-a-2683 | F-highly palatable foods liking (derived food-liking factor) \|\| id:ebi-fl187-GCST90094785 | Weighted mode | 17 | 0.455147237737188 | 0.689531466122797 | 0.518591542753194 | -0.896334436 | 1.80662891133787 | 1.57640547232206 | 0.408062701653026 | 6.08988325348087 | 0.999228396881742 |
| ebi-fl187-GCST90094785 | prot-a-270 | Bromodomain testis-specific protein \|\| id:prot-a-270 | F-highly palatable foods liking (derived food-liking factor) \|\| id:ebi-fl187-GCST90094785 | Weighted mode | 17 | 0.111928653739876 | 0.600813606492238 | 0.85455482990804 | -1.065666015 | 1.28952332246466 | 1.11843306178225 | 0.344498337289753 | 3.63105530066899 | 0.999228396881742 |
| ebi-fl187-GCST90094785 | prot-a-2708 | 14-3-3 protein sigma \|\| id:prot-a-2708 | F-highly palatable foods liking (derived food-liking factor) \|\| id:ebi-fl187-GCST90094785 | Weighted mode | 17 | 1.14880521640754 | 0.589288158535265 | 0.0689889030697196 | -0.006199574 | 2.30381000713666 | 3.15442180589452 | 0.993819603387626 | 10.0122566465635 | 0.999228396881742 |
| ebi-fl187-GCST90094785 | prot-a-2713 | Beta-sarcoglycan \|\| id:prot-a-2713 | F-highly palatable foods liking (derived food-liking factor) \|\| id:ebi-fl187-GCST90094785 | Weighted mode | 17 | 0.235556923248732 | 0.556067562905841 | 0.677488517891139 | -0.8543355 | 1.32544934654418 | 1.26561342203441 | 0.425565885657745 | 3.76387625986042 | 0.999228396881742 |
| ebi-fl187-GCST90094785 | prot-a-2720 | Protein shisa-3 homolog \|\| id:prot-a-2720 | F-highly palatable foods liking (derived food-liking factor) \|\| id:ebi-fl187-GCST90094785 | Weighted mode | 17 | -0.377276722 | 0.775351922896398 | 0.633148544044031 | -1.896966491 | 1.1424130464844 | 0.685726291818657 | 0.150023025809573 | 3.13432251318692 | 0.999228396881742 |
| ebi-fl187-GCST90094785 | prot-a-2730 | Sialic acid-binding Ig-like lectin 8 \|\| id:prot-a-2730 | F-highly palatable foods liking (derived food-liking factor) \|\| id:ebi-fl187-GCST90094785 | Weighted mode | 17 | -0.10900328 | 0.696114290255889 | 0.877528221209045 | -1.473387289 | 1.25538072923972 | 0.896727476530545 | 0.229147978783007 | 3.50917416525111 | 0.999228396881742 |
| ebi-fl187-GCST90094785 | prot-a-2761 | Sodium- and chloride-dependent neutral and basic amino acid transporter B(0+) \|\| id:prot-a-2761 | F-highly palatable foods liking (derived food-liking factor) \|\| id:ebi-fl187-GCST90094785 | Weighted mode | 17 | -0.675486928 | 0.493712784017951 | 0.190164489448329 | -1.643163985 | 0.292190128715431 | 0.50890855846087 | 0.193367262341972 | 1.33935764378097 | 0.999228396881742 |
| ebi-fl187-GCST90094785 | prot-a-2770 | Secreted Ly-6/uPAR-related protein 1 \|\| id:prot-a-2770 | F-highly palatable foods liking (derived food-liking factor) \|\| id:ebi-fl187-GCST90094785 | Weighted mode | 17 | 0.232410245997553 | 0.589433157674595 | 0.698569341259992 | -0.922878743 | 1.38769923503976 | 1.26163720428838 | 0.397373456879249 | 4.00562344487009 | 0.999228396881742 |
| ebi-fl187-GCST90094785 | prot-a-2784 | N-lysine methyltransferase SMYD2 \|\| id:prot-a-2784 | F-highly palatable foods liking (derived food-liking factor) \|\| id:ebi-fl187-GCST90094785 | Weighted mode | 17 | -0.355186691 | 0.630455059236855 | 0.580985596853486 | -1.590878607 | 0.880505225253257 | 0.701042552760181 | 0.203746519960557 | 2.41211806157794 | 0.999228396881742 |
| ebi-fl187-GCST90094785 | prot-a-2837 | Serine palmitoyltransferase 2 \|\| id:prot-a-2837 | F-highly palatable foods liking (derived food-liking factor) \|\| id:ebi-fl187-GCST90094785 | Weighted mode | 17 | -0.426443662 | 0.634534917618175 | 0.511136099302953 | -1.6701321 | 0.817244776972302 | 0.652826643774675 | 0.188222199826681 | 2.2642527141567 | 0.999228396881742 |
| ebi-fl187-GCST90094785 | prot-a-2858 | Alpha-2,8-sialyltransferase 8B \|\| id:prot-a-2858 | F-highly palatable foods liking (derived food-liking factor) \|\| id:ebi-fl187-GCST90094785 | Weighted mode | 17 | 0.167275108583038 | 0.559495634438194 | 0.768807596536343 | -0.929336335 | 1.2638865520819 | 1.18207942077427 | 0.394815648803089 | 3.53914988236682 | 0.999228396881742 |
| ebi-fl187-GCST90094785 | prot-a-2911 | Synaptotagmin-11 \|\| id:prot-a-2911 | F-highly palatable foods liking (derived food-liking factor) \|\| id:ebi-fl187-GCST90094785 | Weighted mode | 17 | 0.407192337643841 | 0.600811401770209 | 0.50762122300082 | -0.77039801 | 1.58478268511345 | 1.50259308308369 | 0.462828821229088 | 4.87823115106614 | 0.999228396881742 |
| ebi-fl187-GCST90094785 | prot-a-2962 | Transforming growth factor beta-1 \|\| id:prot-a-2962 | F-highly palatable foods liking (derived food-liking factor) \|\| id:ebi-fl187-GCST90094785 | Weighted mode | 17 | -0.395859646 | 0.579864055155133 | 0.504576228485806 | -1.532393194 | 0.740673902157617 | 0.673101161784409 | 0.216018075041677 | 2.09734844599513 | 0.999228396881742 |
| ebi-fl187-GCST90094785 | prot-a-2978 | Tyrosine-protein kinase receptor Tie-1, soluble \|\| id:prot-a-2978 | F-highly palatable foods liking (derived food-liking factor) \|\| id:ebi-fl187-GCST90094785 | Weighted mode | 17 | -0.919364003 | 0.732199461535772 | 0.227281710404167 | -2.354474948 | 0.51574694129146 | 0.398772578488025 | 0.094943343649171 | 1.67488907849704 | 0.999228396881742 |
| ebi-fl187-GCST90094785 | prot-a-299 | Uncharacterized protein C1orf226 \|\| id:prot-a-299 | F-highly palatable foods liking (derived food-liking factor) \|\| id:ebi-fl187-GCST90094785 | Weighted mode | 17 | -0.793550545 | 0.593261203549795 | 0.199728607864854 | -1.956342504 | 0.369241414052984 | 0.452236256241847 | 0.141374553352355 | 1.44663679997567 | 0.999228396881742 |
| ebi-fl187-GCST90094785 | prot-a-3015 | Transmembrane protein 70, mitochondrial \|\| id:prot-a-3015 | F-highly palatable foods liking (derived food-liking factor) \|\| id:ebi-fl187-GCST90094785 | Weighted mode | 17 | 0.0703464188667899 | 0.576767095296192 | 0.904444079981307 | -1.060117088 | 1.20080992564733 | 1.07287978268415 | 0.346415246916809 | 3.32280705984286 | 0.999228396881742 |
| ebi-fl187-GCST90094785 | prot-a-3016 | Transmembrane protein 87B \|\| id:prot-a-3016 | F-highly palatable foods liking (derived food-liking factor) \|\| id:ebi-fl187-GCST90094785 | Weighted mode | 17 | 0.142237232167218 | 0.703225035024901 | 0.842259659551857 | -1.236083836 | 1.52055830081602 | 1.15285010922878 | 0.290519715802604 | 4.57477858491312 | 0.999228396881742 |
| ebi-fl187-GCST90094785 | prot-a-3123 | Thioredoxin domain-containing protein 12 \|\| id:prot-a-3123 | F-highly palatable foods liking (derived food-liking factor) \|\| id:ebi-fl187-GCST90094785 | Weighted mode | 17 | 0.278910029661566 | 0.649815135305856 | 0.673490771338793 | -0.994727636 | 1.55254769486104 | 1.3216884257651 | 0.369824157785885 | 4.72348887444177 | 0.999228396881742 |
| ebi-fl187-GCST90094785 | prot-a-3133 | PolyUbiquitin K48-linked \|\| id:prot-a-3133 | F-highly palatable foods liking (derived food-liking factor) \|\| id:ebi-fl187-GCST90094785 | Weighted mode | 17 | 0.630760020405968 | 0.859615862954745 | 0.473705117028545 | -1.054087071 | 2.31560711179727 | 1.87903814403951 | 0.348510447418579 | 10.1310717452174 | 0.999228396881742 |
| ebi-fl187-GCST90094785 | prot-a-3135 | Ubiquitin-conjugating enzyme E2 B \|\| id:prot-a-3135 | F-highly palatable foods liking (derived food-liking factor) \|\| id:ebi-fl187-GCST90094785 | Weighted mode | 17 | -0.111719284 | 0.543963560397985 | 0.839865400711824 | -1.177887862 | 0.95444929482769 | 0.894295265657766 | 0.307928439609874 | 2.5972398755735 | 0.999228396881742 |
| ebi-fl187-GCST90094785 | prot-a-3162 | NKG2D ligand 3 \|\| id:prot-a-3162 | F-highly palatable foods liking (derived food-liking factor) \|\| id:ebi-fl187-GCST90094785 | Weighted mode | 17 | 0.439015606162701 | 0.63183184238961 | 0.497129085035835 | -0.799374805 | 1.67740601724634 | 1.5511794951584 | 0.449609970207128 | 5.35165584760361 | 0.999228396881742 |
| ebi-fl187-GCST90094785 | prot-a-3188 | Vesicle-associated membrane protein 8 \|\| id:prot-a-3188 | F-highly palatable foods liking (derived food-liking factor) \|\| id:ebi-fl187-GCST90094785 | Weighted mode | 17 | 0.50319498830111 | 0.58641903404783 | 0.403519251645301 | -0.646186318 | 1.65257629503486 | 1.65399733987329 | 0.524040494308315 | 5.22041183843782 | 0.999228396881742 |
| ebi-fl187-GCST90094785 | prot-a-3201 | Vitrin \|\| id:prot-a-3201 | F-highly palatable foods liking (derived food-liking factor) \|\| id:ebi-fl187-GCST90094785 | Weighted mode | 17 | -0.257365921 | 0.610230604020867 | 0.678818097160204 | -1.453417905 | 0.938686062698877 | 0.773085273724564 | 0.2337699176927 | 2.55661997210195 | 0.999228396881742 |
| ebi-fl187-GCST90094785 | prot-a-3232 | WNT1-inducible-signaling pathway protein 3 \|\| id:prot-a-3232 | F-highly palatable foods liking (derived food-liking factor) \|\| id:ebi-fl187-GCST90094785 | Weighted mode | 17 | -0.345120123 | 0.570953553063793 | 0.554011783809589 | -1.464189087 | 0.773948840663037 | 0.70813528478154 | 0.231265451541038 | 2.16831168776479 | 0.999228396881742 |
| ebi-fl187-GCST90094785 | prot-a-327 | Carbonic anhydrase-related protein 10 \|\| id:prot-a-327 | F-highly palatable foods liking (derived food-liking factor) \|\| id:ebi-fl187-GCST90094785 | Weighted mode | 17 | -0.363612978 | 0.901362750573296 | 0.691991071925438 | -2.13028397 | 1.40305801270804 | 0.695160184683167 | 0.118803552471813 | 4.06761980020242 | 0.999228396881742 |
| ebi-fl187-GCST90094785 | prot-a-332 | Carbonic anhydrase 5A, mitochondrial \|\| id:prot-a-332 | F-highly palatable foods liking (derived food-liking factor) \|\| id:ebi-fl187-GCST90094785 | Weighted mode | 17 | -0.007351945 | 0.615269186560563 | 0.990613909545289 | -1.213279551 | 1.19857566024317 | 0.992675014026589 | 0.297220928093926 | 3.31539131444097 | 0.999228396881742 |
| ebi-fl187-GCST90094785 | prot-a-338 | Cell adhesion molecule 3 \|\| id:prot-a-338 | F-highly palatable foods liking (derived food-liking factor) \|\| id:ebi-fl187-GCST90094785 | Weighted mode | 17 | -0.508497738 | 0.662675043946407 | 0.454054130480154 | -1.807340824 | 0.790345348113292 | 0.601398358424435 | 0.16408990106007 | 2.20415749646409 | 0.999228396881742 |
| ebi-fl187-GCST90094785 | prot-a-34 | A disintegrin and metalloproteinase with thrombospondin motifs 3 \|\| id:prot-a-34 | F-highly palatable foods liking (derived food-liking factor) \|\| id:ebi-fl187-GCST90094785 | Weighted mode | 17 | -0.113988931 | 0.543003761996152 | 0.836377619668212 | -1.178276305 | 0.950298442396237 | 0.892267832236682 | 0.307808850284838 | 2.58648145986582 | 0.999228396881742 |
| ebi-fl187-GCST90094785 | prot-a-358 | Cysteine--tRNA ligase, cytoplasmic \|\| id:prot-a-358 | F-highly palatable foods liking (derived food-liking factor) \|\| id:ebi-fl187-GCST90094785 | Weighted mode | 17 | 0.52734456014514 | 0.635845371052805 | 0.41910279287658 | -0.718912367 | 1.77360148740864 | 1.69442688064948 | 0.487281951723816 | 5.89203528616389 | 0.999228396881742 |
| ebi-fl187-GCST90094785 | prot-a-382 | Coiled-coil domain-containing protein 167 \|\| id:prot-a-382 | F-highly palatable foods liking (derived food-liking factor) \|\| id:ebi-fl187-GCST90094785 | Weighted mode | 17 | -0.67124476 | 0.601280726997267 | 0.280754111282891 | -1.849754985 | 0.507265464805228 | 0.511072019624564 | 0.157275696494192 | 1.66074361815194 | 0.999228396881742 |
| ebi-fl187-GCST90094785 | prot-a-40 | Pituitary adenylate cyclase-activating polypeptide 27 \|\| id:prot-a-40 | F-highly palatable foods liking (derived food-liking factor) \|\| id:ebi-fl187-GCST90094785 | Weighted mode | 17 | -0.097113563 | 0.555748465204915 | 0.863473087272896 | -1.186380555 | 0.99215342886782 | 0.907452947262562 | 0.305324371394343 | 2.69703609880507 | 0.999228396881742 |
| ebi-fl187-GCST90094785 | prot-a-403 | C-C motif chemokine 25 \|\| id:prot-a-403 | F-highly palatable foods liking (derived food-liking factor) \|\| id:ebi-fl187-GCST90094785 | Weighted mode | 17 | 0.0670376593313486 | 0.573077149754547 | 0.908332948133837 | -1.056193554 | 1.19026887285026 | 1.06933574788558 | 0.347777088690387 | 3.28796513310288 | 0.999228396881742 |
| ebi-fl187-GCST90094785 | prot-a-415 | Cyclin-C \|\| id:prot-a-415 | F-highly palatable foods liking (derived food-liking factor) \|\| id:ebi-fl187-GCST90094785 | Weighted mode | 17 | -0.46789676 | 0.657502464283736 | 0.486942400152359 | -1.75660159 | 0.820808070218741 | 0.626318181548344 | 0.172630537463656 | 2.27233530232511 | 0.999228396881742 |
| ebi-fl187-GCST90094785 | prot-a-44 | Adhesion G protein-coupled receptor F5 \|\| id:prot-a-44 | F-highly palatable foods liking (derived food-liking factor) \|\| id:ebi-fl187-GCST90094785 | Weighted mode | 17 | 0.00551535711322204 | 0.538856585503542 | 0.991960093580632 | -1.05064355 | 1.06167426470016 | 1.00553059469596 | 0.349712618956021 | 2.8912075860687 | 0.999228396881742 |
| ebi-fl187-GCST90094785 | prot-a-455 | CD59 glycoprotein \|\| id:prot-a-455 | F-highly palatable foods liking (derived food-liking factor) \|\| id:ebi-fl187-GCST90094785 | Weighted mode | 17 | -0.295645793 | 0.543489027653839 | 0.593956924237995 | -1.360884287 | 0.769592701426571 | 0.744050929565404 | 0.256433915491225 | 2.1588867631906 | 0.999228396881742 |
| ebi-fl187-GCST90094785 | prot-a-478 | Cadherin-12 \|\| id:prot-a-478 | F-highly palatable foods liking (derived food-liking factor) \|\| id:ebi-fl187-GCST90094785 | Weighted mode | 17 | 0.772040286709102 | 0.577372753571956 | 0.199871076038936 | -0.35961031 | 1.90369088371014 | 2.16417729453101 | 0.697948256335636 | 6.7106168969512 | 0.999228396881742 |
| ebi-fl187-GCST90094785 | prot-a-516 | Centrin-2 \|\| id:prot-a-516 | F-highly palatable foods liking (derived food-liking factor) \|\| id:ebi-fl187-GCST90094785 | Weighted mode | 17 | -0.107221125 | 0.610421877094541 | 0.862772038817438 | -1.303648004 | 1.08920575451589 | 0.898327008835367 | 0.271539407260134 | 2.97191270668866 | 0.999228396881742 |
| ebi-fl187-GCST90094785 | prot-a-519 | Cryptic protein \|\| id:prot-a-519 | F-highly palatable foods liking (derived food-liking factor) \|\| id:ebi-fl187-GCST90094785 | Weighted mode | 17 | 0.672080629878462 | 0.556778905753892 | 0.244941775653397 | -0.419206025 | 1.76336728515609 | 1.95830759809695 | 0.657568705456104 | 5.83204251805783 | 0.999228396881742 |
| ebi-fl187-GCST90094785 | prot-a-583 | Chloride intracellular channel protein 4 \|\| id:prot-a-583 | F-highly palatable foods liking (derived food-liking factor) \|\| id:ebi-fl187-GCST90094785 | Weighted mode | 17 | -0.445560659 | 0.850414748910178 | 0.607506940841442 | -2.112373567 | 1.22125224907594 | 0.640465092969369 | 0.120950541285618 | 3.39143199321124 | 0.999228396881742 |
| ebi-fl187-GCST90094785 | prot-a-602 | CCR4-NOT transcription complex subunit 1 \|\| id:prot-a-602 | F-highly palatable foods liking (derived food-liking factor) \|\| id:ebi-fl187-GCST90094785 | Weighted mode | 17 | 0.981097756897198 | 0.622300970669123 | 0.134459811094255 | -0.238612146 | 2.20080765940868 | 2.66738277316231 | 0.787720343921667 | 9.03230558086309 | 0.999228396881742 |
| ebi-fl187-GCST90094785 | prot-a-606 | Ciliary neurotrophic factor receptor subunit alpha \|\| id:prot-a-606 | F-highly palatable foods liking (derived food-liking factor) \|\| id:ebi-fl187-GCST90094785 | Weighted mode | 17 | -0.115146626 | 0.609295662812546 | 0.852482406482826 | -1.309366125 | 1.07907287359941 | 0.891235456469839 | 0.269991142891936 | 2.94195072609075 | 0.999228396881742 |
| ebi-fl187-GCST90094785 | prot-a-626 | Collagen alpha-2(IX) chain \|\| id:prot-a-626 | F-highly palatable foods liking (derived food-liking factor) \|\| id:ebi-fl187-GCST90094785 | Weighted mode | 17 | -0.7372662 | 0.610584093470985 | 0.244795827735318 | -1.934011023 | 0.45947862292277 | 0.478420033929134 | 0.144567171685963 | 1.58324830039529 | 0.999228396881742 |
| ebi-fl187-GCST90094785 | prot-a-647 | Probable serine carboxypeptidase CPVL \|\| id:prot-a-647 | F-highly palatable foods liking (derived food-liking factor) \|\| id:ebi-fl187-GCST90094785 | Weighted mode | 17 | 0.40704395898271 | 0.557825836765205 | 0.476123353502023 | -0.686294681 | 1.50038259904251 | 1.5023701468737 | 0.503438015788975 | 4.48340408834642 | 0.999228396881742 |
| ebi-fl187-GCST90094785 | prot-a-651 | Cellular retinoic acid-binding protein 2 \|\| id:prot-a-651 | F-highly palatable foods liking (derived food-liking factor) \|\| id:ebi-fl187-GCST90094785 | Weighted mode | 17 | 0.79948452955077 | 0.623135687602186 | 0.21776539571319 | -0.421861418 | 2.02083047725105 | 2.22439402353348 | 0.655824918527001 | 7.54458794131311 | 0.999228396881742 |
| ebi-fl187-GCST90094785 | prot-a-671 | Cytotoxic and regulatory T-cell molecule \|\| id:prot-a-671 | F-highly palatable foods liking (derived food-liking factor) \|\| id:ebi-fl187-GCST90094785 | Weighted mode | 17 | 0.773921911259496 | 0.658664363171476 | 0.257190526819699 | -0.517060241 | 2.06490406307559 | 2.16825329721056 | 0.596270866863086 | 7.88454144271309 | 0.999228396881742 |
| ebi-fl187-GCST90094785 | prot-a-688 | Chorionic somatomammotropin hormone \|\| id:prot-a-688 | F-highly palatable foods liking (derived food-liking factor) \|\| id:ebi-fl187-GCST90094785 | Weighted mode | 17 | -0.61440273 | 0.497148194954543 | 0.234350419330618 | -1.588813192 | 0.360007731761345 | 0.540963900158767 | 0.204167775860406 | 1.43334049676414 | 0.999228396881742 |
| ebi-fl187-GCST90094785 | prot-a-69 | Aldo-keto reductase family 1 member C1 \|\| id:prot-a-69 | F-highly palatable foods liking (derived food-liking factor) \|\| id:ebi-fl187-GCST90094785 | Weighted mode | 17 | -0.286626585 | 0.544226967206852 | 0.605649836682389 | -1.353311441 | 0.780058270353932 | 0.750792033227102 | 0.258383221593147 | 2.1815993843628 | 0.999228396881742 |
| ebi-fl187-GCST90094785 | prot-a-741 | Stromal cell-derived factor 1 \|\| id:prot-a-741 | F-highly palatable foods liking (derived food-liking factor) \|\| id:ebi-fl187-GCST90094785 | Weighted mode | 17 | -0.051147095 | 0.711174159715434 | 0.943557530265192 | -1.445048448 | 1.34275425787858 | 0.950138899415932 | 0.235734655358072 | 3.82957663484843 | 0.999228396881742 |
| ebi-fl187-GCST90094785 | prot-a-742 | Growth-regulated alpha protein \|\| id:prot-a-742 | F-highly palatable foods liking (derived food-liking factor) \|\| id:ebi-fl187-GCST90094785 | Weighted mode | 17 | 0.460329275171779 | 0.552893062989974 | 0.417334893049912 | -0.623341128 | 1.54399967863213 | 1.58459566711177 | 0.536150095449405 | 4.68328449354226 | 0.999228396881742 |
| ebi-fl187-GCST90094785 | prot-a-788 | Beta-defensin 112 \|\| id:prot-a-788 | F-highly palatable foods liking (derived food-liking factor) \|\| id:ebi-fl187-GCST90094785 | Weighted mode | 17 | 0.816613573324631 | 0.709423139553354 | 0.266605422295414 | -0.57385578 | 2.2070829268492 | 2.26282396054896 | 0.563349095357533 | 9.08916392806984 | 0.999228396881742 |
| ebi-fl187-GCST90094785 | prot-a-838 | DnaJ homolog subfamily B member 1 \|\| id:prot-a-838 | F-highly palatable foods liking (derived food-liking factor) \|\| id:ebi-fl187-GCST90094785 | Weighted mode | 17 | 0.529410740531194 | 0.5647794911249 | 0.362500189223125 | -0.577557062 | 1.636378543 | 1.69793149156826 | 0.561267835602857 | 5.1365340523435 | 0.999228396881742 |
| ebi-fl187-GCST90094785 | prot-a-85 | Junctional adhesion molecule-like \|\| id:prot-a-85 | F-highly palatable foods liking (derived food-liking factor) \|\| id:ebi-fl187-GCST90094785 | Weighted mode | 17 | -0.356782514 | 0.664581065968964 | 0.598755602098796 | -1.659361403 | 0.945796375162816 | 0.699924704911493 | 0.190260440979109 | 2.57486311933458 | 0.999228396881742 |
| ebi-fl187-GCST90094785 | prot-a-856 | Dipeptidase 1 \|\| id:prot-a-856 | F-highly palatable foods liking (derived food-liking factor) \|\| id:ebi-fl187-GCST90094785 | Weighted mode | 17 | 0.45201952670893 | 0.597054151370771 | 0.460004702017517 | -0.71820661 | 1.62224566339564 | 1.57148263416204 | 0.487625975825172 | 5.06445060744317 | 0.999228396881742 |
| ebi-fl187-GCST90094785 | prot-a-860 | Dipeptidyl peptidase 2 \|\| id:prot-a-860 | F-highly palatable foods liking (derived food-liking factor) \|\| id:ebi-fl187-GCST90094785 | Weighted mode | 17 | -0.536004781 | 0.641266504315136 | 0.415545888345239 | -1.79288713 | 0.72087756734329 | 0.585081116245284 | 0.166478829204336 | 2.05623690545459 | 0.999228396881742 |
| ebi-fl187-GCST90094785 | prot-a-861 | Dermatopontin \|\| id:prot-a-861 | F-highly palatable foods liking (derived food-liking factor) \|\| id:ebi-fl187-GCST90094785 | Weighted mode | 17 | -0.480694021 | 0.808847531346193 | 0.56062497540915 | -2.066035182 | 1.10464714064681 | 0.618354092255288 | 0.126687078453068 | 3.01815929515266 | 0.999228396881742 |
| ebi-fl187-GCST90094785 | prot-a-877 | Dual specificity protein phosphatase 6 \|\| id:prot-a-877 | F-highly palatable foods liking (derived food-liking factor) \|\| id:ebi-fl187-GCST90094785 | Weighted mode | 17 | 0.961626519869241 | 0.578018111765142 | 0.115639844062509 | -0.171288979 | 2.09454201892892 | 2.61594790621223 | 0.842578050763827 | 8.12172052406607 | 0.999228396881742 |
| ebi-fl187-GCST90094785 | prot-a-879 | Dynactin-associated protein \|\| id:prot-a-879 | F-highly palatable foods liking (derived food-liking factor) \|\| id:ebi-fl187-GCST90094785 | Weighted mode | 17 | -0.091366307 | 0.56845892888666 | 0.874320785869275 | -1.205545807 | 1.02281319403471 | 0.912683327748351 | 0.299528470093658 | 2.78100728284473 | 0.999228396881742 |
| ebi-fl187-GCST90094785 | prot-a-937 | EMI domain-containing protein 1 \|\| id:prot-a-937 | F-highly palatable foods liking (derived food-liking factor) \|\| id:ebi-fl187-GCST90094785 | Weighted mode | 17 | -0.704307196 | 0.520083533456352 | 0.194484702551317 | -1.723670922 | 0.31505652919777 | 0.49445101307443 | 0.178410015101407 | 1.37033677280599 | 0.999228396881742 |
| ebi-fl187-GCST90094785 | prot-b-1 | SRC proto-oncogene, non-receptor tyrosine kinase \|\| id:prot-b-1 | F-highly palatable foods liking (derived food-liking factor) \|\| id:ebi-fl187-GCST90094785 | Weighted mode | 12 | -0.101882068 | 0.748137624570516 | 0.894138563662787 | -1.568231812 | 1.36446767596107 | 0.903136053851813 | 0.2084133706951 | 3.91363917318095 | 0.999228396881742 |
| ebi-fl187-GCST90094785 | prot-b-10 | coagulation factor III, tissue factor \|\| id:prot-b-10 | F-highly palatable foods liking (derived food-liking factor) \|\| id:ebi-fl187-GCST90094785 | Weighted mode | 12 | -0.069899239 | 0.818809224351617 | 0.933503636385597 | -1.674765319 | 1.53496684050186 | 0.932487773361027 | 0.187352142319399 | 4.64117162848035 | 0.999228396881742 |
| ebi-fl187-GCST90094785 | prot-b-29 | myeloperoxidase \|\| id:prot-b-29 | F-highly palatable foods liking (derived food-liking factor) \|\| id:ebi-fl187-GCST90094785 | Weighted mode | 12 | -0.26232192 | 0.625777825884885 | 0.68314401183083 | -1.488846459 | 0.964202618481135 | 0.769263342392908 | 0.225632782145183 | 2.6226955335273 | 0.999228396881742 |
| ebi-fl187-GCST90094785 | prot-b-33 | adrenomedullin \|\| id:prot-b-33 | F-highly palatable foods liking (derived food-liking factor) \|\| id:ebi-fl187-GCST90094785 | Weighted mode | 12 | 0.138522013863982 | 0.674124494825111 | 0.840947022109351 | -1.182761996 | 1.4598060237212 | 1.14857496586495 | 0.306431206934634 | 4.30512435534373 | 0.999228396881742 |
| ebi-fl187-GCST90094785 | ukb-rsfMRI191-edge-pheno102 | Net25_Pair11_15 (Postcentral\|Precentral)&(Cerebellum) (Motor)&(Subcortical-cerebellum) \|\| id:ukb-rsfMRI191-edge-pheno102 | F-highly palatable foods liking (derived food-liking factor) \|\| id:ebi-fl187-GCST90094785 | Weighted mode | 17 | -0.022617794 | 0.179059788309055 | 0.901056906668717 | -0.37357498 | 0.328339390636878 | 0.977636070308362 | 0.688269378243921 | 1.38866019058784 | 0.999228396881742 |
| ebi-fl187-GCST90094785 | ukb-rsfMRI191-edge-pheno1167 | Net100_Pair11_45 (Angular\|Temporal)&(Frontal) (Default_mode\|Central_executive)&(Salience\|Default_mode) \|\| id:ukb-rsfMRI191-edge-pheno1167 | F-highly palatable foods liking (derived food-liking factor) \|\| id:ebi-fl187-GCST90094785 | Weighted mode | 17 | 0.0754100195403209 | 0.173793117488199 | 0.670150119803191 | -0.265224491 | 0.416044529817191 | 1.07832619506588 | 0.767033738677704 | 1.51595337249414 | 0.999228396881742 |
| ebi-fl187-GCST90094785 | ukb-rsfMRI191-edge-pheno1317 | Net100_Pair26_48 (Parietal\|Frontal)&(Temporal) (Attention\|Central_executive)&(Default_mode\|Central_executive) \|\| id:ukb-rsfMRI191-edge-pheno1317 | F-highly palatable foods liking (derived food-liking factor) \|\| id:ebi-fl187-GCST90094785 | Weighted mode | 17 | -0.220458848 | 0.244477547913191 | 0.380561464189496 | -0.699634842 | 0.258717146131123 | 0.802150648463393 | 0.496766669153935 | 1.29526738161826 | 0.999228396881742 |
| ebi-fl187-GCST90094785 | ukb-rsfMRI191-edge-pheno286 | Net100_Pair10_13 (OccipitalPrecuneus)&(Frontal_Sup) (Default_mode\|Central_executive)&(Default_mode) \|\| id:ukb-rsfMRI191-edge-pheno286 | F-highly palatable foods liking (derived food-liking factor) \|\| id:ebi-fl187-GCST90094785 | Weighted mode | 17 | 0.0759815723922059 | 0.196048810363531 | 0.703445473022515 | -0.308274096 | 0.460237240704727 | 1.07894269164134 | 0.734713908322673 | 1.58444983640489 | 0.999228396881742 |
| ebi-fl187-GCST90094785 | ukb-rsfMRI191-edge-pheno599 | Net100_Pair11_29 (Angular\|Temporal)&(Parietal) (Default_mode\|Central_executive)&(Central_executive\|Salience) \|\| id:ukb-rsfMRI191-edge-pheno599 | F-highly palatable foods liking (derived food-liking factor) \|\| id:ebi-fl187-GCST90094785 | Weighted mode | 17 | -0.229923888 | 0.196190019071638 | 0.258375017709794 | -0.614456325 | 0.15460854965091 | 0.794594078561265 | 0.540934908105097 | 1.16720097043905 | 0.999228396881742 |
| ebi-fl187-GCST90094785 | ukb-rsfMRI191-edge-pheno621 | Net100_Pair5_30 (Precuneus\|Angular\|Cingulate)&(Paracentral) (Default_mode\|Central_executive)&(Motor) \|\| id:ukb-rsfMRI191-edge-pheno621 | F-highly palatable foods liking (derived food-liking factor) \|\| id:ebi-fl187-GCST90094785 | Weighted mode | 17 | -0.333533214 | 0.209304989643683 | 0.130602804957818 | -0.743770993 | 0.0767045661373454 | 0.71638810444247 | 0.47531811027138 | 1.07972304251917 | 0.999228396881742 |
| ebi-fl187-GCST90094785 | ukb-rsfMRI191-edge-pheno624 | Net100_Pair8_30 (Calcarine\|Lingual)&(Paracentral) (Visual)&(Motor) \|\| id:ukb-rsfMRI191-edge-pheno624 | F-highly palatable foods liking (derived food-liking factor) \|\| id:ebi-fl187-GCST90094785 | Weighted mode | 17 | -0.169689155 | 0.177715270674101 | 0.353864784041737 | -0.518011086 | 0.178632775245852 | 0.843927106117511 | 0.595704175008386 | 1.19558161637838 | 0.999228396881742 |
| ebi-fl187-GCST90094785 | ukb-rsfMRI191-edge-pheno882 | Net100_Pair6_38 (Postcentral\|Precentral)&(Subcortical) (Motor)&(Subcortical-cerebellum) \|\| id:ukb-rsfMRI191-edge-pheno882 | F-highly palatable foods liking (derived food-liking factor) \|\| id:ebi-fl187-GCST90094785 | Weighted mode | 17 | 0.0685509036931322 | 0.179736138737066 | 0.707925608554693 | -0.283731928 | 0.420833735617781 | 1.07095513913524 | 0.752968467242151 | 1.5232309982927 | 0.999228396881742 |
| ebi-fl187-GCST90094785 | ukb-rsfMRI191-edge-pheno899 | Net100_Pair23_38 (Cerebellum)&(Subcortical) (Subcortical-cerebellum)&(Subcortical-cerebellum) \|\| id:ukb-rsfMRI191-edge-pheno899 | F-highly palatable foods liking (derived food-liking factor) \|\| id:ebi-fl187-GCST90094785 | Weighted mode | 17 | 0.100507242015313 | 0.181384576205941 | 0.587164464945059 | -0.255006527 | 0.456021011378958 | 1.10573164940062 | 0.774911439827679 | 1.57778349582515 | 0.999228396881742 |
| ebi-fl187-GCST90094803 | ebi-a-GCST90001392 | IgD+ CD38+ B cell Absolute Count \|\| id:ebi-a-GCST90001392 | Mushroom liking \|\| id:ebi-fl187-GCST90094803 | Inverse variance weighted | 6 | 0.168824585111729 | 0.258062845761187 | 0.512983282559066 | -0.336978593 | 0.674627762803656 | 1.18391244483895 | 0.713924123012542 | 1.96330202589319 | 0.998074519377826 |
| ebi-fl187-GCST90094803 | ebi-a-GCST90001402 | Switched memory B cell %B cell \|\| id:ebi-a-GCST90001402 | Mushroom liking \|\| id:ebi-fl187-GCST90094803 | Inverse variance weighted | 6 | 0.483743813294905 | 0.271698711128951 | 0.0750036433692418 | -0.048785661 | 1.01627328710765 | 1.62213602273312 | 0.952385241582603 | 2.76287909698799 | 0.998074519377826 |
| ebi-fl187-GCST90094803 | ebi-a-GCST90001407 | Memory B cell Absolute Count \|\| id:ebi-a-GCST90001407 | Mushroom liking \|\| id:ebi-fl187-GCST90094803 | Inverse variance weighted | 6 | 0.0510999971992682 | 0.269110816314744 | 0.84939956765544 | -0.476357203 | 0.578557197176166 | 1.05242812788675 | 0.621041604835936 | 1.78346338754522 | 0.998074519377826 |
| ebi-fl187-GCST90094803 | ebi-a-GCST90001411 | IgD+ CD24+ B cell %B cell \|\| id:ebi-a-GCST90001411 | Mushroom liking \|\| id:ebi-fl187-GCST90094803 | Inverse variance weighted | 6 | 0.0877962356301205 | 0.272904616074052 | 0.747672147101332 | -0.447096812 | 0.622689283135262 | 1.09176563642469 | 0.639481995826861 | 1.86393395381927 | 0.998074519377826 |
| ebi-fl187-GCST90094803 | ebi-a-GCST90001418 | CD24+ CD27+ B cell Absolute Count \|\| id:ebi-a-GCST90001418 | Mushroom liking \|\| id:ebi-fl187-GCST90094803 | Inverse variance weighted | 6 | -0.019928999 | 0.268197834837292 | 0.940766041694317 | -0.545596755 | 0.505738757675347 | 0.980268271249953 | 0.579495863147094 | 1.65821008350401 | 0.998074519377826 |
| ebi-fl187-GCST90094803 | ebi-a-GCST90001436 | Memory B cell %lymphocyte \|\| id:ebi-a-GCST90001436 | Mushroom liking \|\| id:ebi-fl187-GCST90094803 | Inverse variance weighted | 6 | 0.0380290428906394 | 0.285956757911348 | 0.894202246963588 | -0.522446203 | 0.598504288396882 | 1.03876140107418 | 0.593068007579094 | 1.81939547332216 | 0.998074519377826 |
| ebi-fl187-GCST90094803 | ebi-a-GCST90001439 | IgD+ CD24+ B cell %lymphocyte \|\| id:ebi-a-GCST90001439 | Mushroom liking \|\| id:ebi-fl187-GCST90094803 | Inverse variance weighted | 6 | -0.097172931 | 0.270207788417709 | 0.719129093687004 | -0.626780196 | 0.432434334284655 | 0.907399075122331 | 0.534309405742715 | 1.54100428082177 | 0.998074519377826 |
| ebi-fl187-GCST90094803 | ebi-a-GCST90001457 | CD11c+ HLA DR++ monocyte %monocyte \|\| id:ebi-a-GCST90001457 | Mushroom liking \|\| id:ebi-fl187-GCST90094803 | Inverse variance weighted | 6 | 0.142611506265716 | 0.290062205486606 | 0.622960884298102 | -0.425910416 | 0.711133429019463 | 1.15328167192044 | 0.653174853197101 | 2.03629795035335 | 0.998074519377826 |
| ebi-fl187-GCST90094803 | ebi-a-GCST90001483 | CD39+ resting CD4 regulatory T cell Absolute Count \|\| id:ebi-a-GCST90001483 | Mushroom liking \|\| id:ebi-fl187-GCST90094803 | Inverse variance weighted | 6 | 0.457016483422493 | 0.271948522455331 | 0.0928550766211897 | -0.076002621 | 0.990035587434942 | 1.57935491720925 | 0.926813777757322 | 2.69133024818516 | 0.998074519377826 |
| ebi-fl187-GCST90094803 | ebi-a-GCST90001525 | CD33dim HLA DR+ CD11b+ Absolute Count \|\| id:ebi-a-GCST90001525 | Mushroom liking \|\| id:ebi-fl187-GCST90094803 | Inverse variance weighted | 6 | 0.160949257775902 | 0.353352901850222 | 0.6487556518198 | -0.53162243 | 0.853520945402337 | 1.17462536419819 | 0.587650773672728 | 2.34789914015518 | 0.998074519377826 |
| ebi-fl187-GCST90094803 | ebi-a-GCST90001540 | Naive CD4+ T cell Absolute Count \|\| id:ebi-a-GCST90001540 | Mushroom liking \|\| id:ebi-fl187-GCST90094803 | Inverse variance weighted | 6 | -0.085265099 | 0.257511763446143 | 0.740560286458394 | -0.589988155 | 0.419457957273137 | 0.918268819911229 | 0.554333850538535 | 1.52113681097046 | 0.998074519377826 |
| ebi-fl187-GCST90094803 | ebi-a-GCST90001624 | HLA DR+ CD4+ T cell Absolute Count \|\| id:ebi-a-GCST90001624 | Mushroom liking \|\| id:ebi-fl187-GCST90094803 | Inverse variance weighted | 6 | 0.0318781624877081 | 0.260633462654843 | 0.902653258373055 | -0.478963424 | 0.542719749291201 | 1.0323917136041 | 0.619425140176799 | 1.72068032307214 | 0.998074519377826 |
| ebi-fl187-GCST90094803 | ebi-a-GCST90001672 | CD39+ CD8+ T cell Absolute Count \|\| id:ebi-a-GCST90001672 | Mushroom liking \|\| id:ebi-fl187-GCST90094803 | Inverse variance weighted | 6 | 0.321715756647079 | 0.282066615548291 | 0.254050328907933 | -0.23113481 | 0.874566323121729 | 1.379492609 | 0.793632469366953 | 2.39783518277064 | 0.998074519377826 |
| ebi-fl187-GCST90094803 | ebi-a-GCST90001722 | CD19 on CD20- CD38- B cell \|\| id:ebi-a-GCST90001722 | Mushroom liking \|\| id:ebi-fl187-GCST90094803 | Inverse variance weighted | 6 | 0.169295917251373 | 0.259859411963083 | 0.514729959692013 | -0.34002853 | 0.678620364699015 | 1.18447059235083 | 0.711750016105281 | 1.97115637850072 | 0.998074519377826 |
| ebi-fl187-GCST90094803 | ebi-a-GCST90001742 | CD19 on transitional B cell \|\| id:ebi-a-GCST90001742 | Mushroom liking \|\| id:ebi-fl187-GCST90094803 | Inverse variance weighted (multiplicative random effects) | 6 | -0.190900476 | 0.479859570926075 | 0.690758661814862 | -1.131425235 | 0.749624282900207 | 0.826214812167533 | 0.322573186009175 | 2.11620477290886 | 0.998074519377826 |
| ebi-fl187-GCST90094803 | ebi-a-GCST90001784 | CD25 on IgD+ CD38dim B cell \|\| id:ebi-a-GCST90001784 | Mushroom liking \|\| id:ebi-fl187-GCST90094803 | Inverse variance weighted | 6 | 0.110832553083204 | 0.267587038834005 | 0.678733110771117 | -0.413638043 | 0.635303149197853 | 1.11720781818661 | 0.661240248471477 | 1.88759427742415 | 0.998074519377826 |
| ebi-fl187-GCST90094803 | ebi-a-GCST90001794 | CD25 on IgD+ B cell \|\| id:ebi-a-GCST90001794 | Mushroom liking \|\| id:ebi-fl187-GCST90094803 | Inverse variance weighted | 6 | 0.0501190998815954 | 0.279489884679404 | 0.857683708102997 | -0.497681074 | 0.597919273853227 | 1.05139631009545 | 0.60793879142017 | 1.81833141178569 | 0.998074519377826 |
| ebi-fl187-GCST90094803 | ebi-a-GCST90001802 | CD27 on IgD- CD38- B cell \|\| id:ebi-a-GCST90001802 | Mushroom liking \|\| id:ebi-fl187-GCST90094803 | Inverse variance weighted | 6 | 0.770819788393465 | 0.305867828339927 | 0.0117319020036673 | 0.171318844847208 | 1.37032073193972 | 2.16153753102943 | 1.18686911580024 | 3.93661309056691 | 0.998074519377826 |
| ebi-fl187-GCST90094803 | ebi-a-GCST90001804 | CD27 on IgD- CD38dim B cell \|\| id:ebi-a-GCST90001804 | Mushroom liking \|\| id:ebi-fl187-GCST90094803 | Inverse variance weighted | 6 | 0.990742408225223 | 0.263466938624498 | 0.000169638451039403 | 0.474347208521207 | 1.50713760792924 | 2.69323320880493 | 1.60696484206229 | 4.51379204270638 | 0.443070605573995 |
| ebi-fl187-GCST90094803 | ebi-a-GCST90001805 | CD27 on memory B cell \|\| id:ebi-a-GCST90001805 | Mushroom liking \|\| id:ebi-fl187-GCST90094803 | Inverse variance weighted | 6 | 0.653198824229861 | 0.267221265785127 | 0.0145088538450828 | 0.129445143291013 | 1.17695250516871 | 1.92167811825249 | 1.13819667202658 | 3.24447161103998 | 0.998074519377826 |
| ebi-fl187-GCST90094803 | ebi-a-GCST90001806 | CD27 on unswitched memory B cell \|\| id:ebi-a-GCST90001806 | Mushroom liking \|\| id:ebi-fl187-GCST90094803 | Inverse variance weighted | 6 | 0.419837681286746 | 0.267088926379671 | 0.115973225311886 | -0.103656614 | 0.943331976990901 | 1.52171453282606 | 0.901534818334474 | 2.56852544385582 | 0.998074519377826 |
| ebi-fl187-GCST90094803 | ebi-a-GCST90001839 | CD3 on Effector Memory CD8+ T cell \|\| id:ebi-a-GCST90001839 | Mushroom liking \|\| id:ebi-fl187-GCST90094803 | Inverse variance weighted | 6 | 0.261008282295817 | 0.296541637451844 | 0.378764977396378 | -0.320213327 | 0.842229891701431 | 1.29823841778378 | 0.725994146320115 | 2.32153798753437 | 0.998074519377826 |
| ebi-fl187-GCST90094803 | ebi-a-GCST90001859 | CD3 on CD8+ T cell \|\| id:ebi-a-GCST90001859 | Mushroom liking \|\| id:ebi-fl187-GCST90094803 | Inverse variance weighted | 6 | 0.0762249218866091 | 0.290585396084232 | 0.793078562115304 | -0.493322454 | 0.645772298211704 | 1.07920528374937 | 0.610594348460853 | 1.90745958820028 | 0.998074519377826 |
| ebi-fl187-GCST90094803 | ebi-a-GCST90001905 | CD86 on monocyte \|\| id:ebi-a-GCST90001905 | Mushroom liking \|\| id:ebi-fl187-GCST90094803 | Inverse variance weighted | 6 | 0.0341419776525134 | 0.295830593041212 | 0.908119700531117 | -0.545685985 | 0.61396994001329 | 1.0347315050498 | 0.579444157141602 | 1.84775232323376 | 0.998074519377826 |
| ebi-fl187-GCST90094803 | ebi-a-GCST90001943 | CD25 on activated & secreting CD4 regulatory T cell \|\| id:ebi-a-GCST90001943 | Mushroom liking \|\| id:ebi-fl187-GCST90094803 | Inverse variance weighted | 6 | -0.220585588 | 0.271054151112403 | 0.415755899743503 | -0.751851724 | 0.310680548216649 | 0.802048990184096 | 0.471492669535519 | 1.36435330646613 | 0.998074519377826 |
| ebi-fl187-GCST90094803 | ebi-a-GCST90001949 | CD33 on CD33dim HLA DR+ CD11b- \|\| id:ebi-a-GCST90001949 | Mushroom liking \|\| id:ebi-fl187-GCST90094803 | Inverse variance weighted | 6 | -0.240958509 | 0.418791368727144 | 0.565043479451798 | -1.061789592 | 0.579872573465766 | 0.785874232231523 | 0.34583635025842 | 1.78581085656264 | 0.998074519377826 |
| ebi-fl187-GCST90094803 | ebi-a-GCST90001955 | CD33 on Immature Myeloid-Derived Suppressor Cells \|\| id:ebi-a-GCST90001955 | Mushroom liking \|\| id:ebi-fl187-GCST90094803 | Inverse variance weighted | 6 | -0.299669658 | 0.453320491983762 | 0.50857721235136 | -1.188177822 | 0.588838506532536 | 0.741062984661118 | 0.304776114712675 | 1.80189431101769 | 0.998074519377826 |
| ebi-fl187-GCST90094803 | ebi-a-GCST90001956 | CD33 on CD33+ HLA DR+ \|\| id:ebi-a-GCST90001956 | Mushroom liking \|\| id:ebi-fl187-GCST90094803 | Inverse variance weighted | 6 | -0.041801554 | 0.398309860711001 | 0.916417606380195 | -0.822488881 | 0.738885772600799 | 0.959060082950062 | 0.439336835361803 | 2.09360146628887 | 0.998074519377826 |
| ebi-fl187-GCST90094803 | ebi-a-GCST90001957 | CD33 on CD33+ HLA DR+ CD14- \|\| id:ebi-a-GCST90001957 | Mushroom liking \|\| id:ebi-fl187-GCST90094803 | Inverse variance weighted | 6 | -0.107571476 | 0.398937365335295 | 0.787433356752076 | -0.889488713 | 0.674345759590943 | 0.898012333408599 | 0.410865769580828 | 1.96274844647362 | 0.998074519377826 |
| ebi-fl187-GCST90094803 | ebi-a-GCST90002064 | CD4 on resting CD4 regulatory T cell \|\| id:ebi-a-GCST90002064 | Mushroom liking \|\| id:ebi-fl187-GCST90094803 | Inverse variance weighted | 6 | -0.281709604 | 0.286554273729846 | 0.325561491590947 | -0.843355981 | 0.279936772493411 | 0.754492754388057 | 0.430264139694226 | 1.3230461567832 | 0.998074519377826 |
| ebi-fl187-GCST90094803 | ebi-a-GCST90002080 | SSC-A on T cell \|\| id:ebi-a-GCST90002080 | Mushroom liking \|\| id:ebi-fl187-GCST90094803 | Inverse variance weighted | 6 | 0.260255982795062 | 0.273862969932764 | 0.341952415000158 | -0.276515438 | 0.797027403863279 | 1.29726212095008 | 0.75842191031667 | 2.21893511719515 | 0.998074519377826 |
| ebi-fl187-GCST90094803 | ebi-a-GCST90002121 | CD8 on CD39+ CD8+ T cell \|\| id:ebi-a-GCST90002121 | Mushroom liking \|\| id:ebi-fl187-GCST90094803 | Inverse variance weighted | 6 | 0.100160119149088 | 0.290826688914119 | 0.730547020826989 | -0.469860191 | 0.67018042942076 | 1.10534789127067 | 0.625089655256807 | 1.95458995435552 | 0.998074519377826 |
| ebi-fl187-GCST90094803 | ebi-cfmet338-GCST90026053 | 1-palmitoyl-2-docosahexaenoyl-gpc (16:0/22:6) levels \|\| id:ebi-cfmet338-GCST90026053 | Mushroom liking \|\| id:ebi-fl187-GCST90094803 | Inverse variance weighted | 6 | 0.324773303439753 | 0.14999424939996 | 0.0303694356513576 | 0.0307845746158308 | 0.618762032263675 | 1.38371692655493 | 1.0312633196585 | 1.85662817278196 | 0.998074519377826 |
| ebi-fl187-GCST90094803 | ebi-cfmet338-GCST90026063 | 1-stearoyl-2-oleoyl-gpc (18:0/18:1) levels \|\| id:ebi-cfmet338-GCST90026063 | Mushroom liking \|\| id:ebi-fl187-GCST90094803 | Inverse variance weighted | 6 | 0.255716811481442 | 0.122048735966707 | 0.0361529989546781 | 0.0165012889866972 | 0.494932333976187 | 1.29138697018998 | 1.01663818721831 | 1.64038723681972 | 0.998074519377826 |
| ebi-fl187-GCST90094803 | ebi-cfmet338-GCST90026113 | Adenosine 3',5'-cyclic monophosphate (camp) levels \|\| id:ebi-cfmet338-GCST90026113 | Mushroom liking \|\| id:ebi-fl187-GCST90094803 | Inverse variance weighted (multiplicative random effects) | 6 | 0.16875092738591 | 0.360464002736012 | 0.639677911492919 | -0.537758518 | 0.875260372748494 | 1.18382524375225 | 0.584055937134242 | 2.39949997704238 | 0.998074519377826 |
| ebi-fl187-GCST90094803 | ebi-cfmet338-GCST90026117 | Alpha-ketoglutaramate levels \|\| id:ebi-cfmet338-GCST90026117 | Mushroom liking \|\| id:ebi-fl187-GCST90094803 | Inverse variance weighted | 6 | 0.104518402855103 | 0.128195845538211 | 0.414899135711588 | -0.146745454 | 0.355782260109996 | 1.11017582408306 | 0.863513753070235 | 1.42729673499277 | 0.998074519377826 |
| ebi-fl187-GCST90094803 | ebi-cfmet338-GCST90026124 | Arginine levels \|\| id:ebi-cfmet338-GCST90026124 | Mushroom liking \|\| id:ebi-fl187-GCST90094803 | Inverse variance weighted | 6 | 0.0685549687835583 | 0.0772001994363655 | 0.374532448032344 | -0.082757422 | 0.219867359678835 | 1.07095949267357 | 0.920574431138671 | 1.24591146153055 | 0.998074519377826 |
| ebi-fl187-GCST90094803 | ebi-cfmet338-GCST90026169 | Glutamate levels \|\| id:ebi-cfmet338-GCST90026169 | Mushroom liking \|\| id:ebi-fl187-GCST90094803 | Inverse variance weighted | 6 | 0.0498098216021647 | 0.0815524434801067 | 0.541351566952382 | -0.110032968 | 0.209652610823174 | 1.05107118633311 | 0.895804602265028 | 1.23324956798207 | 0.998074519377826 |
| ebi-fl187-GCST90094803 | ebi-cfmet338-GCST90026170 | Glutamine levels \|\| id:ebi-cfmet338-GCST90026170 | Mushroom liking \|\| id:ebi-fl187-GCST90094803 | Inverse variance weighted | 6 | -0.02185823 | 0.0487626807383789 | 0.653967474074929 | -0.117433085 | 0.0737166239774069 | 0.97837892973651 | 0.889200011003901 | 1.07650170749735 | 0.998074519377826 |
| ebi-fl187-GCST90094803 | ebi-cfmet338-GCST90026191 | Kynurenate levels \|\| id:ebi-cfmet338-GCST90026191 | Mushroom liking \|\| id:ebi-fl187-GCST90094803 | Inverse variance weighted | 6 | -0.007099832 | 0.370531889579862 | 0.984712518309775 | -0.733342335 | 0.719142671872437 | 0.992925312559152 | 0.480300977426258 | 2.05267264206652 | 0.999355215041207 |
| ebi-fl187-GCST90094803 | ebi-cfmet338-GCST90026202 | Methylsuccinoylcarnitine levels \|\| id:ebi-cfmet338-GCST90026202 | Mushroom liking \|\| id:ebi-fl187-GCST90094803 | Inverse variance weighted | 6 | 0.606856288954619 | 0.249113897865902 | 0.0148482400323635 | 0.11859304913745 | 1.09511952877179 | 1.83465469915978 | 1.12591163433441 | 2.98953999808242 | 0.998074519377826 |
| ebi-fl187-GCST90094803 | ebi-cfmet338-GCST90026222 | N-acetylputrescine levels \|\| id:ebi-cfmet338-GCST90026222 | Mushroom liking \|\| id:ebi-fl187-GCST90094803 | Inverse variance weighted | 6 | 0.0583967418234852 | 0.0890498409786323 | 0.511968309007319 | -0.116140946 | 0.232934430141605 | 1.06013551238282 | 0.890349722780827 | 1.26229870786611 | 0.998074519377826 |
| ebi-fl187-GCST90094803 | ebi-cfmet338-GCST90026252 | Propionylcarnitine (c3) levels \|\| id:ebi-cfmet338-GCST90026252 | Mushroom liking \|\| id:ebi-fl187-GCST90094803 | Inverse variance weighted | 6 | 0.181313052009072 | 0.148854993142513 | 0.223204391706866 | -0.110442735 | 0.473068838568397 | 1.19879040430219 | 0.895437606358544 | 1.60491185900849 | 0.998074519377826 |
| ebi-fl187-GCST90094803 | ebi-cfmet338-GCST90026316 | Ethyl beta-glucopyranoside levels \|\| id:ebi-cfmet338-GCST90026316 | Mushroom liking \|\| id:ebi-fl187-GCST90094803 | Inverse variance weighted | 6 | -0.072502574 | 0.482267182105266 | 0.880498787397912 | -1.017746251 | 0.872741102762328 | 0.930063352521603 | 0.361408547114825 | 2.39346259685689 | 0.998074519377826 |
| ebi-fl187-GCST90094803 | ebi-gm430-GCST90011315 | F_Prevotellaceae abundance \|\| id:ebi-gm430-GCST90011315 | Mushroom liking \|\| id:ebi-fl187-GCST90094803 | Inverse variance weighted | 6 | 0.154808534866904 | 0.208525304166551 | 0.457846910360674 | -0.253901061 | 0.563518131033344 | 1.16743441671935 | 0.775768551782029 | 1.75684244251731 | 0.998074519377826 |
| ebi-fl187-GCST90094803 | ebi-gm430-GCST90011354 | OTU97_1 (Bacteroides) abundance \|\| id:ebi-gm430-GCST90011354 | Mushroom liking \|\| id:ebi-fl187-GCST90094803 | Inverse variance weighted (multiplicative random effects) | 6 | -0.074085688 | 0.313310412220205 | 0.81307497934266 | -0.688174096 | 0.54000271965804 | 0.928592120957844 | 0.502492735311395 | 1.71601152914306 | 0.998074519377826 |
| ebi-fl187-GCST90094803 | ebi-gm430-GCST90011419 | OTU99_1 (Bacteroides) abundance \|\| id:ebi-gm430-GCST90011419 | Mushroom liking \|\| id:ebi-fl187-GCST90094803 | Inverse variance weighted (multiplicative random effects) | 6 | -0.074042177 | 0.315092772515952 | 0.814220124421618 | -0.691624011 | 0.543539657029476 | 0.928632525986727 | 0.500762164969242 | 1.72209170070478 | 0.998074519377826 |
| ebi-fl187-GCST90094803 | ebi-gm430-GCST90011435 | OTU99_155 (Butyrivibrio) abundance \|\| id:ebi-gm430-GCST90011435 | Mushroom liking \|\| id:ebi-fl187-GCST90094803 | Inverse variance weighted | 6 | -0.971304307 | 0.592643256890878 | 0.101226611133512 | -2.13288509 | 0.190276476903233 | 0.378588919904622 | 0.118494931661698 | 1.20958397346271 | 0.998074519377826 |
| ebi-fl187-GCST90094803 | ebi-gm430-GCST90011466 | OTU99_58 (Bacteroides) abundance \|\| id:ebi-gm430-GCST90011466 | Mushroom liking \|\| id:ebi-fl187-GCST90094803 | Inverse variance weighted | 6 | -0.038051717 | 0.347348876865442 | 0.912767070653274 | -0.718855516 | 0.642752081553178 | 0.962663153454233 | 0.48730965515247 | 1.9017073378702 | 0.998074519377826 |
| ebi-fl187-GCST90094803 | ebi-gm430-GCST90011472 | OTU99_72 (Parabacteroides) abundance \|\| id:ebi-gm430-GCST90011472 | Mushroom liking \|\| id:ebi-fl187-GCST90094803 | Inverse variance weighted | 6 | -0.365031753 | 0.527580138273714 | 0.48900125536268 | -1.399088824 | 0.669025318156633 | 0.694174608498974 | 0.246821759605989 | 1.95233348896769 | 0.998074519377826 |
| ebi-fl187-GCST90094803 | ebi-gm430-GCST90011505 | TestASV_28 (Holdemanella) abundance \|\| id:ebi-gm430-GCST90011505 | Mushroom liking \|\| id:ebi-fl187-GCST90094803 | Inverse variance weighted | 6 | -0.074766546 | 0.443985640993633 | 0.866269734598039 | -0.944978402 | 0.795445310197013 | 0.92796009690008 | 0.388687965500203 | 2.21542732955635 | 0.998074519377826 |
| ebi-fl187-GCST90094803 | ebi-gm430-GCST90011513 | TestASV_35 (Alphaproteobacteria) abundance \|\| id:ebi-gm430-GCST90011513 | Mushroom liking \|\| id:ebi-fl187-GCST90094803 | Inverse variance weighted | 6 | -0.476960545 | 0.523951136069308 | 0.362656444236566 | -1.503904771 | 0.549983682162496 | 0.620667017517149 | 0.222260586766162 | 1.73322473515705 | 0.998074519377826 |
| ebi-fl187-GCST90094803 | ebi-gm430-GCST90011543 | G_Coprococcus prevalence \|\| id:ebi-gm430-GCST90011543 | Mushroom liking \|\| id:ebi-fl187-GCST90094803 | Inverse variance weighted | 6 | 0.624345032451146 | 0.552112260177785 | 0.258126707439703 | -0.457794997 | 1.7064850623996 | 1.86702271759176 | 0.632677163315692 | 5.50956163762209 | 0.998074519377826 |
| ebi-fl187-GCST90094803 | ebi-gm430-GCST90011547 | G_Parasutterella prevalence \|\| id:ebi-gm430-GCST90011547 | Mushroom liking \|\| id:ebi-fl187-GCST90094803 | Inverse variance weighted | 6 | -0.037491996 | 0.496595141509731 | 0.939818399529246 | -1.010818473 | 0.935834481666932 | 0.963202127456325 | 0.363920998113535 | 2.54933994780634 | 0.998074519377826 |
| ebi-fl187-GCST90094803 | ebi-gm430-GCST90011569 | OTU97_13 (Subdoligranulum) prevalence \|\| id:ebi-gm430-GCST90011569 | Mushroom liking \|\| id:ebi-fl187-GCST90094803 | Inverse variance weighted | 6 | 0.243502656372252 | 0.557417708938936 | 0.662226991738121 | -0.849036053 | 1.33604136589257 | 1.27570970659603 | 0.427827135866266 | 3.80395519374453 | 0.998074519377826 |
| ebi-fl187-GCST90094803 | ebi-gm430-GCST90011590 | OTU97_277 (Prevotella) prevalence \|\| id:ebi-gm430-GCST90011590 | Mushroom liking \|\| id:ebi-fl187-GCST90094803 | Inverse variance weighted | 6 | 0.463839159729178 | 0.482785256401868 | 0.336674525155439 | -0.482419943 | 1.41009826227684 | 1.59016718694945 | 0.617287781756215 | 4.09635790174986 | 0.998074519377826 |
| ebi-fl187-GCST90094803 | ebi-gm430-GCST90011616 | OTU97_86 (Phascolarctobacterium) prevalence \|\| id:ebi-gm430-GCST90011616 | Mushroom liking \|\| id:ebi-fl187-GCST90094803 | Inverse variance weighted | 6 | 0.892770316055606 | 0.574073238066399 | 0.119910217 | -0.232413231 | 2.01795386266575 | 2.44188508308044 | 0.792618521432646 | 7.52291625508961 | 0.998074519377826 |
| ebi-fl187-GCST90094803 | ebi-gm430-GCST90011665 | OTU99_55 (Barnesiella) prevalence \|\| id:ebi-gm430-GCST90011665 | Mushroom liking \|\| id:ebi-fl187-GCST90094803 | Inverse variance weighted | 6 | -0.141773748 | 0.419749707419047 | 0.735545695667372 | -0.964483174 | 0.680935678851182 | 0.867817580007756 | 0.381180152565098 | 1.97572551220883 | 0.998074519377826 |
| ebi-fl187-GCST90094803 | ebi-gm430-GCST90011687 | TestASV_14 (Lachnospiraceae) prevalence \|\| id:ebi-gm430-GCST90011687 | Mushroom liking \|\| id:ebi-fl187-GCST90094803 | Inverse variance weighted | 6 | 0.405365405144552 | 0.393643135134399 | 0.303113795352775 | -0.36617514 | 1.17690595000797 | 1.49985045300984 | 0.69338135195972 | 3.24432056765858 | 0.998074519377826 |
| ebi-fl187-GCST90094803 | ebi-gm430-GCST90011697 | TestASV_23 (Barnesiella) prevalence \|\| id:ebi-gm430-GCST90011697 | Mushroom liking \|\| id:ebi-fl187-GCST90094803 | Inverse variance weighted | 6 | 0.176702343950406 | 0.523857012148418 | 0.735883027571065 | -0.8500574 | 1.2034620877613 | 1.19327585448337 | 0.427390399095358 | 3.33163137942958 | 0.998074519377826 |
| ebi-fl187-GCST90094803 | ebi-gm430-GCST90011727 | TestASV_6 (Subdoligranulum) prevalence \|\| id:ebi-gm430-GCST90011727 | Mushroom liking \|\| id:ebi-fl187-GCST90094803 | Inverse variance weighted (multiplicative random effects) | 6 | 0.0055665498443744 | 0.936681682730787 | 0.995258327224361 | -1.830329548 | 1.84146264799672 | 1.00558207187097 | 0.160360712465005 | 6.3057546186008 | 0.999355215041207 |
| ebi-fl187-GCST90094803 | ebi-hbsm44-GCST90104908 | Sulcal depth of the left posterior inferior temporal sulcus \|\| id:ebi-hbsm44-GCST90104908 | Mushroom liking \|\| id:ebi-fl187-GCST90094803 | Inverse variance weighted | 5 | 0.0576695483453898 | 0.218661221364387 | 0.791980842454398 | -0.370906446 | 0.486245542219588 | 1.0593648689897 | 0.690108501274719 | 1.62619924776557 | 0.998074519377826 |
| ebi-fl187-GCST90094803 | ebi-hbsm44-GCST90104918 | Sulcal depth of the left inferior frontal sulcus \|\| id:ebi-hbsm44-GCST90104918 | Mushroom liking \|\| id:ebi-fl187-GCST90094803 | Inverse variance weighted | 5 | -0.10669093 | 0.344159353980385 | 0.756557574621024 | -0.781243264 | 0.567861403589147 | 0.898803423049067 | 0.457836445741133 | 1.76448948265138 | 0.998074519377826 |
| ebi-fl187-GCST90094803 | ebi-hbsm44-GCST90104925 | Sulcal depth of the right anterior inferior temporal sulcus \|\| id:ebi-hbsm44-GCST90104925 | Mushroom liking \|\| id:ebi-fl187-GCST90094803 | Inverse variance weighted | 4 | -0.181490601 | 0.129929657162103 | 0.162462480459976 | -0.436152729 | 0.0731715268711977 | 0.834026084138921 | 0.646518976065893 | 1.07591506943364 | 0.998074519377826 |
| ebi-fl187-GCST90094803 | ebi-IPs91-GCST90274770 | C-C motif chemokine 4 levels \|\| id:ebi-IPs91-GCST90274770 | Mushroom liking \|\| id:ebi-fl187-GCST90094803 | Inverse variance weighted | 7 | 0.00694968323141223 | 0.107699743766873 | 0.948549560202216 | -0.204141815 | 0.218041181014484 | 1.006973888 | 0.815346734956372 | 1.2436382808506 | 0.998074519377826 |
| ebi-fl187-GCST90094803 | ebi-IPs91-GCST90274779 | C-X-C motif chemokine 1 levels \|\| id:ebi-IPs91-GCST90274779 | Mushroom liking \|\| id:ebi-fl187-GCST90094803 | Inverse variance weighted | 7 | -0.138219524 | 0.140857804970006 | 0.32645966424967 | -0.414300821 | 0.137861774194314 | 0.870907486059131 | 0.660802138013439 | 1.14781688139515 | 0.998074519377826 |
| ebi-fl187-GCST90094803 | ebi-IPs91-GCST90274780 | C-X-C motif chemokine 10 levels \|\| id:ebi-IPs91-GCST90274780 | Mushroom liking \|\| id:ebi-fl187-GCST90094803 | Inverse variance weighted | 7 | 0.0259011455046578 | 0.107112239590823 | 0.808924967508509 | -0.184038844 | 0.235841135102671 | 1.02623949507158 | 0.831903488662273 | 1.26597317549214 | 0.998074519377826 |
| ebi-fl187-GCST90094803 | ebi-IPs91-GCST90274796 | Interleukin-10 receptor subunit alpha levels \|\| id:ebi-IPs91-GCST90274796 | Mushroom liking \|\| id:ebi-fl187-GCST90094803 | Inverse variance weighted | 7 | 0.0485462919946715 | 0.120831279012515 | 0.687853856705391 | -0.188283015 | 0.285375598859201 | 1.04974396543762 | 0.828380230130969 | 1.33026157903175 | 0.998074519377826 |
| ebi-fl187-GCST90094803 | ebi-IPs91-GCST90274807 | Interleukin-20 levels \|\| id:ebi-IPs91-GCST90274807 | Mushroom liking \|\| id:ebi-fl187-GCST90094803 | Inverse variance weighted | 7 | 0.169196879069646 | 0.119441505840174 | 0.156609602932569 | -0.064908472 | 0.403302230516388 | 1.18435329034583 | 0.937153234859998 | 1.4967591896138 | 0.998074519377826 |
| ebi-fl187-GCST90094803 | ebi-IPs91-GCST90274808 | Interleukin-20 receptor subunit alpha levels \|\| id:ebi-IPs91-GCST90274808 | Mushroom liking \|\| id:ebi-fl187-GCST90094803 | Inverse variance weighted | 7 | 0.225298468253569 | 0.121577542427552 | 0.0638643441864735 | -0.012993515 | 0.463590451411571 | 1.25269655055194 | 0.987090536375992 | 1.58977174832014 | 0.998074519377826 |
| ebi-fl187-GCST90094803 | ebi-IPs91-GCST90274810 | Interleukin-24 levels \|\| id:ebi-IPs91-GCST90274810 | Mushroom liking \|\| id:ebi-fl187-GCST90094803 | Inverse variance weighted | 7 | -0.019152479 | 0.122214839530882 | 0.875472171817938 | -0.258693564 | 0.220388606659656 | 0.981029764576115 | 0.772059573412853 | 1.24656105840374 | 0.998074519377826 |
| ebi-fl187-GCST90094803 | ebi-IPs91-GCST90274823 | Monocyte chemoattractant protein-3 levels \|\| id:ebi-IPs91-GCST90274823 | Mushroom liking \|\| id:ebi-fl187-GCST90094803 | Inverse variance weighted (multiplicative random effects) | 7 | 0.132728626871932 | 0.179411060089668 | 0.459420260985841 | -0.218917051 | 0.484374304647682 | 1.14194006443158 | 0.803388355727817 | 1.62315908795148 | 0.998074519377826 |
| ebi-fl187-GCST90094803 | ebi-IPs91-GCST90274824 | Monocyte chemoattractant protein-4 levels \|\| id:ebi-IPs91-GCST90274824 | Mushroom liking \|\| id:ebi-fl187-GCST90094803 | Inverse variance weighted | 7 | -0.131637087 | 0.106951845292974 | 0.218395031314271 | -0.341262704 | 0.0779885296237002 | 0.876659088203556 | 0.710872134774622 | 1.08111025785748 | 0.998074519377826 |
| ebi-fl187-GCST90094803 | ebi-IPs91-GCST90274834 | SIR2-like protein 2 levels \|\| id:ebi-IPs91-GCST90274834 | Mushroom liking \|\| id:ebi-fl187-GCST90094803 | Inverse variance weighted (multiplicative random effects) | 7 | -0.222557454 | 0.161351643370252 | 0.167792473467237 | -0.538806675 | 0.0936917668969154 | 0.800469015195166 | 0.583444075454471 | 1.09822118561819 | 0.998074519377826 |
| ebi-fl187-GCST90094803 | ebi-met1400-GCST90199652 | 1-stearoyl-2-oleoyl-GPS (18:0/18:1) levels \|\| id:ebi-met1400-GCST90199652 | Mushroom liking \|\| id:ebi-fl187-GCST90094803 | Inverse variance weighted | 5 | -0.057886718 | 0.19702566722145 | 0.76890849652645 | -0.444057026 | 0.328283589565576 | 0.943756851867348 | 0.641428841700101 | 1.38858270402347 | 0.998074519377826 |
| ebi-fl187-GCST90094803 | ebi-met1400-GCST90199665 | N-acetylaspartate (naa) levels \|\| id:ebi-met1400-GCST90199665 | Mushroom liking \|\| id:ebi-fl187-GCST90094803 | Inverse variance weighted | 5 | -0.222218235 | 0.212143913796032 | 0.294874497257857 | -0.638020306 | 0.193583836411322 | 0.800740595938295 | 0.528337335827342 | 1.2135911254115 | 0.998074519377826 |
| ebi-fl187-GCST90094803 | ebi-met1400-GCST90199669 | Acetylcarnitine levels (Biocrates platform) \|\| id:ebi-met1400-GCST90199669 | Mushroom liking \|\| id:ebi-fl187-GCST90094803 | Inverse variance weighted | 5 | 0.174829715478487 | 0.231241980915128 | 0.44962125144057 | -0.278404567 | 0.628063998072138 | 1.19104338309055 | 0.756990506090684 | 1.87397903803281 | 0.998074519377826 |
| ebi-fl187-GCST90094803 | ebi-met1400-GCST90199670 | Iminodiacetate (IDA) levels \|\| id:ebi-met1400-GCST90199670 | Mushroom liking \|\| id:ebi-fl187-GCST90094803 | Inverse variance weighted | 5 | -0.088295562 | 0.24007810438415 | 0.713038412705281 | -0.558848646 | 0.382257523032753 | 0.915490253004804 | 0.571867106349043 | 1.46558945958197 | 0.998074519377826 |
| ebi-fl187-GCST90094803 | ebi-met1400-GCST90199686 | Indolepropionate levels \|\| id:ebi-met1400-GCST90199686 | Mushroom liking \|\| id:ebi-fl187-GCST90094803 | Inverse variance weighted | 5 | 0.272778754371769 | 0.199149387277182 | 0.170774549419555 | -0.117554045 | 0.663111553435046 | 1.31360958229473 | 0.889092459720273 | 1.94082191996032 | 0.998074519377826 |
| ebi-fl187-GCST90094803 | ebi-met1400-GCST90199704 | 3-hydroxysebacate levels \|\| id:ebi-met1400-GCST90199704 | Mushroom liking \|\| id:ebi-fl187-GCST90094803 | Inverse variance weighted | 5 | 0.0501578443575587 | 0.200533691955267 | 0.802493171732511 | -0.342888192 | 0.443203880589882 | 1.05143704668367 | 0.709717559314018 | 1.55768988470205 | 0.998074519377826 |
| ebi-fl187-GCST90094803 | ebi-met1400-GCST90199713 | Docosapentaenoate n3 DPA; 22:5n3 levels \|\| id:ebi-met1400-GCST90199713 | Mushroom liking \|\| id:ebi-fl187-GCST90094803 | Inverse variance weighted | 5 | 0.15841064565134 | 0.186891154847026 | 0.396655698493093 | -0.207896018 | 0.524717309151511 | 1.17164722777283 | 0.812291496137871 | 1.6899810386723 | 0.998074519377826 |
| ebi-fl187-GCST90094803 | ebi-met1400-GCST90199715 | Myristoleate (14:1n5) levels \|\| id:ebi-met1400-GCST90199715 | Mushroom liking \|\| id:ebi-fl187-GCST90094803 | Inverse variance weighted | 5 | 0.158520176204777 | 0.179115833529297 | 0.376148611148549 | -0.192546858 | 0.509587209922199 | 1.17177556597047 | 0.824855666613224 | 1.66460392112363 | 0.998074519377826 |
| ebi-fl187-GCST90094803 | ebi-met1400-GCST90199725 | Campesterol levels \|\| id:ebi-met1400-GCST90199725 | Mushroom liking \|\| id:ebi-fl187-GCST90094803 | Inverse variance weighted | 5 | 0.0267998007558186 | 0.268639933352177 | 0.920534034487392 | -0.499734469 | 0.553334070126085 | 1.02716214509282 | 0.606691734023283 | 1.73904144913106 | 0.998074519377826 |
| ebi-fl187-GCST90094803 | ebi-met1400-GCST90199737 | N-acetylproline levels \|\| id:ebi-met1400-GCST90199737 | Mushroom liking \|\| id:ebi-fl187-GCST90094803 | Inverse variance weighted | 5 | -0.15887606 | 0.210250373722773 | 0.44985802871955 | -0.570966792 | 0.253214672735491 | 0.853102086093393 | 0.564978957293388 | 1.28815977993844 | 0.998074519377826 |
| ebi-fl187-GCST90094803 | ebi-met1400-GCST90199775 | Gamma-glutamylisoleucine levels \|\| id:ebi-met1400-GCST90199775 | Mushroom liking \|\| id:ebi-fl187-GCST90094803 | Inverse variance weighted | 5 | -0.059032561 | 0.187694020974183 | 0.753130021539469 | -0.426912842 | 0.308847719787392 | 0.942676073878173 | 0.652520421842981 | 1.36185497114787 | 0.998074519377826 |
| ebi-fl187-GCST90094803 | ebi-met1400-GCST90199777 | 1-palmitoleoyl-GPC (16:1) levels \|\| id:ebi-met1400-GCST90199777 | Mushroom liking \|\| id:ebi-fl187-GCST90094803 | Inverse variance weighted | 5 | -0.200318973 | 0.253088521240317 | 0.42865363905736 | -0.696372475 | 0.295734528406878 | 0.818469641535976 | 0.498389950689532 | 1.3441132855693 | 0.998074519377826 |
| ebi-fl187-GCST90094803 | ebi-met1400-GCST90199782 | Glutamine degradant levels \|\| id:ebi-met1400-GCST90199782 | Mushroom liking \|\| id:ebi-fl187-GCST90094803 | Inverse variance weighted (multiplicative random effects) | 5 | -0.40810554 | 0.296248390885727 | 0.168333331831667 | -0.988752386 | 0.172541306629155 | 0.664908700982785 | 0.372040564744119 | 1.18832090513217 | 0.998074519377826 |
| ebi-fl187-GCST90094803 | ebi-met1400-GCST90199783 | 5-methyluridine (ribothymidine) levels \|\| id:ebi-met1400-GCST90199783 | Mushroom liking \|\| id:ebi-fl187-GCST90094803 | Inverse variance weighted | 5 | -0.206771235 | 0.194076833438279 | 0.286690816815753 | -0.587161828 | 0.173619358912633 | 0.813205662007293 | 0.555902795548798 | 1.18960266797699 | 0.998074519377826 |
| ebi-fl187-GCST90094803 | ebi-met1400-GCST90199787 | Homostachydrine levels \|\| id:ebi-met1400-GCST90199787 | Mushroom liking \|\| id:ebi-fl187-GCST90094803 | Inverse variance weighted | 5 | -0.124734911 | 0.189933304713126 | 0.511354502458307 | -0.497004188 | 0.247534366668453 | 0.88273087419645 | 0.608350436151627 | 1.28086338063447 | 0.998074519377826 |
| ebi-fl187-GCST90094803 | ebi-met1400-GCST90199802 | Beta-hydroxyisovaleroylcarnitine levels \|\| id:ebi-met1400-GCST90199802 | Mushroom liking \|\| id:ebi-fl187-GCST90094803 | Inverse variance weighted | 5 | -0.182656324 | 0.19472730916099 | 0.348238846946087 | -0.56432185 | 0.199009201983415 | 0.833054407375091 | 0.568745710952594 | 1.22019319404575 | 0.998074519377826 |
| ebi-fl187-GCST90094803 | ebi-met1400-GCST90199873 | Pregnenolone sulfate levels \|\| id:ebi-met1400-GCST90199873 | Mushroom liking \|\| id:ebi-fl187-GCST90094803 | Inverse variance weighted | 5 | -0.480927485 | 0.185893640082847 | 0.00967844029875918 | -0.845279019 | -0.11657595 | 0.618209745736242 | 0.429437520148341 | 0.889962501625966 | 0.998074519377826 |
| ebi-fl187-GCST90094803 | ebi-met1400-GCST90199911 | Beta-citrylglutamate levels \|\| id:ebi-met1400-GCST90199911 | Mushroom liking \|\| id:ebi-fl187-GCST90094803 | Inverse variance weighted | 5 | -0.031025496 | 0.193300294849999 | 0.872483968262552 | -0.409894074 | 0.347843082185405 | 0.969450855912785 | 0.663720551923869 | 1.41601003510561 | 0.998074519377826 |
| ebi-fl187-GCST90094803 | ebi-met1400-GCST90199934 | 2-hydroxydecanoate levels \|\| id:ebi-met1400-GCST90199934 | Mushroom liking \|\| id:ebi-fl187-GCST90094803 | Inverse variance weighted | 5 | -0.088193964 | 0.193956139713735 | 0.649317263342593 | -0.468347998 | 0.291960069792994 | 0.915583269263862 | 0.626035626671988 | 1.33904954804613 | 0.998074519377826 |
| ebi-fl187-GCST90094803 | ebi-met1400-GCST90199945 | Sphingomyelin (d18:1/14:0, d16:1/16:0) levels \|\| id:ebi-met1400-GCST90199945 | Mushroom liking \|\| id:ebi-fl187-GCST90094803 | Inverse variance weighted | 5 | 0.0165633383364356 | 0.186405761561162 | 0.929196062224353 | -0.348791954 | 0.381918630996313 | 1.01670127091388 | 0.705539899527806 | 1.46509286713579 | 0.998074519377826 |
| ebi-fl187-GCST90094803 | ebi-met1400-GCST90199979 | Carnitine C14:1 levels \|\| id:ebi-met1400-GCST90199979 | Mushroom liking \|\| id:ebi-fl187-GCST90094803 | Inverse variance weighted (multiplicative random effects) | 5 | 0.100439698270459 | 0.385671902911272 | 0.794533775122492 | -0.655477231 | 0.856356627976552 | 1.10565696666642 | 0.519194227647807 | 2.35456648560286 | 0.998074519377826 |
| ebi-fl187-GCST90094803 | ebi-met1400-GCST90199986 | Eugenol sulfate levels \|\| id:ebi-met1400-GCST90199986 | Mushroom liking \|\| id:ebi-fl187-GCST90094803 | Inverse variance weighted | 5 | -0.110496514 | 0.210685147565471 | 0.599956713172796 | -0.523439404 | 0.302446374734184 | 0.895389451069226 | 0.59247926419621 | 1.35316511063676 | 0.998074519377826 |
| ebi-fl187-GCST90094803 | ebi-met1400-GCST90200002 | 6-hydroxyindole sulfate levels \|\| id:ebi-met1400-GCST90200002 | Mushroom liking \|\| id:ebi-fl187-GCST90094803 | Inverse variance weighted | 5 | -0.093305969 | 0.192839446916843 | 0.628490108562963 | -0.471271285 | 0.284659346730719 | 0.91091474577318 | 0.624208216999261 | 1.32930911748635 | 0.998074519377826 |
| ebi-fl187-GCST90094803 | ebi-met1400-GCST90200023 | Trans 3,4-methyleneheptanoate levels \|\| id:ebi-met1400-GCST90200023 | Mushroom liking \|\| id:ebi-fl187-GCST90094803 | Inverse variance weighted | 5 | -0.187797549 | 0.209901264708551 | 0.370950312013503 | -0.599204028 | 0.22360892951821 | 0.828782477833512 | 0.549248648614938 | 1.25058185813655 | 0.998074519377826 |
| ebi-fl187-GCST90094803 | ebi-met1400-GCST90200032 | 2-hydroxybutyrate/2-hydroxyisobutyrate levels \|\| id:ebi-met1400-GCST90200032 | Mushroom liking \|\| id:ebi-fl187-GCST90094803 | Inverse variance weighted | 5 | 0.409939284561498 | 0.198447793029496 | 0.0388541118287584 | 0.0209816102236864 | 0.79889695889931 | 1.50672630078757 | 1.02120327176511 | 2.22308741878686 | 0.998074519377826 |
| ebi-fl187-GCST90094803 | ebi-met1400-GCST90200051 | 1-palmitoyl-2-dihomo-linolenoyl-GPC (16:0/20:3n3 or 6) levels \|\| id:ebi-met1400-GCST90200051 | Mushroom liking \|\| id:ebi-fl187-GCST90094803 | Inverse variance weighted | 5 | -0.061122091 | 0.191278201696579 | 0.749312884617126 | -0.436027366 | 0.313783184270613 | 0.940708380686173 | 0.646600030591182 | 1.36859297189348 | 0.998074519377826 |
| ebi-fl187-GCST90094803 | ebi-met1400-GCST90200069 | Phenylacetylglutamate levels \|\| id:ebi-met1400-GCST90200069 | Mushroom liking \|\| id:ebi-fl187-GCST90094803 | Inverse variance weighted | 5 | -0.088113662 | 0.214230923598141 | 0.68085091550364 | -0.508006272 | 0.331778948056816 | 0.915656795246664 | 0.601693997656024 | 1.39344479078667 | 0.998074519377826 |
| ebi-fl187-GCST90094803 | ebi-met1400-GCST90200072 | Thioproline levels \|\| id:ebi-met1400-GCST90200072 | Mushroom liking \|\| id:ebi-fl187-GCST90094803 | Inverse variance weighted | 5 | -0.057354556 | 0.194258891724479 | 0.767804411765766 | -0.438101983 | 0.323392872178771 | 0.944259217613651 | 0.645259973707768 | 1.38180811824561 | 0.998074519377826 |
| ebi-fl187-GCST90094803 | ebi-met1400-GCST90200081 | 1-oleoyl-2-docosahexaenoyl-GPC (18:1/22:6) levels \|\| id:ebi-met1400-GCST90200081 | Mushroom liking \|\| id:ebi-fl187-GCST90094803 | Inverse variance weighted | 5 | 0.25617125717711 | 0.186886532382808 | 0.170458670925476 | -0.110126346 | 0.622468860647413 | 1.29197396880942 | 0.895720957124189 | 1.86352314613727 | 0.998074519377826 |
| ebi-fl187-GCST90094803 | ebi-met1400-GCST90200097 | Furaneol sulfate levels \|\| id:ebi-met1400-GCST90200097 | Mushroom liking \|\| id:ebi-fl187-GCST90094803 | Inverse variance weighted | 5 | -0.083937372 | 0.34800418325258 | 0.809402864900727 | -0.766025572 | 0.598150826809528 | 0.919488839702897 | 0.464856942372613 | 1.81875250054992 | 0.998074519377826 |
| ebi-fl187-GCST90094803 | ebi-met1400-GCST90200124 | Sphingomyelin (d18:2/24:2) levels \|\| id:ebi-met1400-GCST90200124 | Mushroom liking \|\| id:ebi-fl187-GCST90094803 | Inverse variance weighted | 5 | 0.18739059035051 | 0.177440349346085 | 0.290933247146032 | -0.160392494 | 0.535173675068836 | 1.2060982834115 | 0.851809392957041 | 1.70774480919762 | 0.998074519377826 |
| ebi-fl187-GCST90094803 | ebi-met1400-GCST90200137 | Lignoceroylcarnitine (C24) levels \|\| id:ebi-met1400-GCST90200137 | Mushroom liking \|\| id:ebi-fl187-GCST90094803 | Inverse variance weighted | 5 | 0.321828750640092 | 0.195613250240564 | 0.0999224281250456 | -0.06157322 | 0.705230721111596 | 1.37964849180495 | 0.940284095775895 | 2.02431368295027 | 0.998074519377826 |
| ebi-fl187-GCST90094803 | ebi-met1400-GCST90200165 | Octadecenedioate (C18:1-DC) levels \|\| id:ebi-met1400-GCST90200165 | Mushroom liking \|\| id:ebi-fl187-GCST90094803 | Inverse variance weighted | 5 | -0.134290375 | 0.205104342531722 | 0.51263404268891 | -0.536294887 | 0.267714136057806 | 0.874336142115321 | 0.584911405583186 | 1.30697347001961 | 0.998074519377826 |
| ebi-fl187-GCST90094803 | ebi-met1400-GCST90200176 | Glutamine conjugate of C6H10O2 (1) levels \|\| id:ebi-met1400-GCST90200176 | Mushroom liking \|\| id:ebi-fl187-GCST90094803 | Inverse variance weighted | 5 | 0.12457850283664 | 0.191701593838227 | 0.515784999808955 | -0.251156621 | 0.500313626759564 | 1.13267093485155 | 0.777900526391981 | 1.64923843490371 | 0.998074519377826 |
| ebi-fl187-GCST90094803 | ebi-met1400-GCST90200182 | Glycine conjugate of C10H12O2 levels \|\| id:ebi-met1400-GCST90200182 | Mushroom liking \|\| id:ebi-fl187-GCST90094803 | Inverse variance weighted | 5 | -0.153827601 | 0.224168015298494 | 0.492577128107665 | -0.593196911 | 0.285541708855116 | 0.857419826479602 | 0.552557979339537 | 1.33048256713086 | 0.998074519377826 |
| ebi-fl187-GCST90094803 | ebi-met1400-GCST90200208 | Glucuronide of piperine metabolite C17H21NO3 (4) levels \|\| id:ebi-met1400-GCST90200208 | Mushroom liking \|\| id:ebi-fl187-GCST90094803 | Inverse variance weighted | 5 | -0.023340098 | 0.208835642606005 | 0.911011324327202 | -0.432657958 | 0.385977761303383 | 0.976930175069331 | 0.648782364862218 | 1.47105195617282 | 0.998074519377826 |
| ebi-fl187-GCST90094803 | ebi-met1400-GCST90200227 | 3-ethylcatechol sulfate (2) levels \|\| id:ebi-met1400-GCST90200227 | Mushroom liking \|\| id:ebi-fl187-GCST90094803 | Inverse variance weighted | 4 | 0.0879624158803419 | 0.281238932951982 | 0.754457260275114 | -0.463265893 | 0.639190724466226 | 1.0919470813872 | 0.629225303844978 | 1.89494672458973 | 0.998074519377826 |
| ebi-fl187-GCST90094803 | ebi-met1400-GCST90200259 | Pentose acid levels \|\| id:ebi-met1400-GCST90200259 | Mushroom liking \|\| id:ebi-fl187-GCST90094803 | Inverse variance weighted | 5 | -0.273436091 | 0.220277483069507 | 0.214485470626853 | -0.705179958 | 0.158307775633791 | 0.760760954135318 | 0.494019663466333 | 1.17152670660106 | 0.998074519377826 |
| ebi-fl187-GCST90094803 | ebi-met1400-GCST90200291 | Sarcosine levels \|\| id:ebi-met1400-GCST90200291 | Mushroom liking \|\| id:ebi-fl187-GCST90094803 | Inverse variance weighted | 5 | 0.180109451007664 | 0.195152501664759 | 0.356050755426922 | -0.202389452 | 0.562608354270592 | 1.1973484069399 | 0.816776770437385 | 1.75524483493061 | 0.998074519377826 |
| ebi-fl187-GCST90094803 | ebi-met1400-GCST90200306 | Butyrate/isobutyrate (4:0) levels \|\| id:ebi-met1400-GCST90200306 | Mushroom liking \|\| id:ebi-fl187-GCST90094803 | Inverse variance weighted | 5 | 0.0284299296619198 | 0.197079249307363 | 0.885298059398001 | -0.357845399 | 0.414705258304351 | 1.02883791728805 | 0.699181160771488 | 1.51392445826435 | 0.998074519377826 |
| ebi-fl187-GCST90094803 | ebi-met1400-GCST90200321 | Cholate levels \|\| id:ebi-met1400-GCST90200321 | Mushroom liking \|\| id:ebi-fl187-GCST90094803 | Inverse variance weighted | 5 | -0.158111031 | 0.200678995126623 | 0.430766712947775 | -0.551441862 | 0.235219799330456 | 0.853754983335717 | 0.57611852806447 | 1.26518682539056 | 0.998074519377826 |
| ebi-fl187-GCST90094803 | ebi-met1400-GCST90200336 | Ceramide (d18:1/16:0) levels \|\| id:ebi-met1400-GCST90200336 | Mushroom liking \|\| id:ebi-fl187-GCST90094803 | Inverse variance weighted | 5 | 0.0289651125586503 | 0.192965024211127 | 0.880681376212836 | -0.349246335 | 0.407176560012459 | 1.02938868111143 | 0.705219388727345 | 1.50256937591093 | 0.998074519377826 |
| ebi-fl187-GCST90094803 | ebi-met1400-GCST90200358 | Arachidonate (20:4n6) levels \|\| id:ebi-met1400-GCST90200358 | Mushroom liking \|\| id:ebi-fl187-GCST90094803 | Inverse variance weighted | 5 | 0.194549644573562 | 0.191563102222791 | 0.309824485819011 | -0.180914036 | 0.570013324930233 | 1.21476378781835 | 0.834507093361209 | 1.76829061362581 | 0.998074519377826 |
| ebi-fl187-GCST90094803 | ebi-met1400-GCST90200413 | 2'-deoxyuridine levels \|\| id:ebi-met1400-GCST90200413 | Mushroom liking \|\| id:ebi-fl187-GCST90094803 | Inverse variance weighted | 5 | -0.218496581 | 0.194144645115178 | 0.260406050264276 | -0.599020086 | 0.162026923278949 | 0.803726227261944 | 0.549349688113603 | 1.17589189976045 | 0.998074519377826 |
| ebi-fl187-GCST90094803 | ebi-met1400-GCST90200432 | Threonine levels \|\| id:ebi-met1400-GCST90200432 | Mushroom liking \|\| id:ebi-fl187-GCST90094803 | Inverse variance weighted | 5 | 0.161485966981382 | 0.258976413961926 | 0.532920051007824 | -0.346107804 | 0.669079738346756 | 1.17525596565381 | 0.707436218269794 | 1.95243973821837 | 0.998074519377826 |
| ebi-fl187-GCST90094803 | ebi-met1400-GCST90200436 | Caffeine levels \|\| id:ebi-met1400-GCST90200436 | Mushroom liking \|\| id:ebi-fl187-GCST90094803 | Inverse variance weighted | 5 | -0.057971428 | 0.199168996828726 | 0.771000441295494 | -0.448342662 | 0.332399806009754 | 0.943676910001033 | 0.638685793461763 | 1.39431019068441 | 0.998074519377826 |
| ebi-fl187-GCST90094803 | ebi-met1400-GCST90200442 | Valine levels \|\| id:ebi-met1400-GCST90200442 | Mushroom liking \|\| id:ebi-fl187-GCST90094803 | Inverse variance weighted (multiplicative random effects) | 5 | 0.139925546204061 | 0.302858010685891 | 0.644069137330703 | -0.453676155 | 0.733527247148408 | 1.15018815979465 | 0.635288435084601 | 2.08241285354994 | 0.998074519377826 |
| ebi-fl187-GCST90094803 | ebi-met1400-GCST90200459 | Margarate (17:0) levels \|\| id:ebi-met1400-GCST90200459 | Mushroom liking \|\| id:ebi-fl187-GCST90094803 | Inverse variance weighted | 5 | 0.206726968942097 | 0.190994619030957 | 0.279087866728458 | -0.167622484 | 0.581076422242772 | 1.22964679417892 | 0.845673029226464 | 1.78796199734258 | 0.998074519377826 |
| ebi-fl187-GCST90094803 | ebi-met1400-GCST90200462 | X-11299 levels \|\| id:ebi-met1400-GCST90200462 | Mushroom liking \|\| id:ebi-fl187-GCST90094803 | Inverse variance weighted | 5 | 0.102399440342366 | 0.193525005329565 | 0.596716869268508 | -0.27690957 | 0.481708450788314 | 1.10782589371572 | 0.758123050999775 | 1.61883774562529 | 0.998074519377826 |
| ebi-fl187-GCST90094803 | ebi-met1400-GCST90200497 | X-12740 levels \|\| id:ebi-met1400-GCST90200497 | Mushroom liking \|\| id:ebi-fl187-GCST90094803 | Inverse variance weighted | 5 | -0.124376706 | 0.222549745522732 | 0.576249432662746 | -0.560574208 | 0.311820794872516 | 0.883047128756747 | 0.570881165426423 | 1.36590989303891 | 0.998074519377826 |
| ebi-fl187-GCST90094803 | ebi-met1400-GCST90200527 | X-13866 levels \|\| id:ebi-met1400-GCST90200527 | Mushroom liking \|\| id:ebi-fl187-GCST90094803 | Inverse variance weighted | 5 | 0.139334603807136 | 0.190803806468506 | 0.465236987243105 | -0.234640857 | 0.513310064485407 | 1.14950866563731 | 0.790854828708776 | 1.67081254916616 | 0.998074519377826 |
| ebi-fl187-GCST90094803 | ebi-met1400-GCST90200547 | X-17654 levels \|\| id:ebi-met1400-GCST90200547 | Mushroom liking \|\| id:ebi-fl187-GCST90094803 | Inverse variance weighted | 5 | 0.169289586219154 | 0.197174814170226 | 0.39057441955938 | -0.21717305 | 0.555752221992797 | 1.18446309345308 | 0.804790689 | 1.74325180404471 | 0.998074519377826 |
| ebi-fl187-GCST90094803 | ebi-met1400-GCST90200553 | X-17653 levels \|\| id:ebi-met1400-GCST90200553 | Mushroom liking \|\| id:ebi-fl187-GCST90094803 | Inverse variance weighted | 5 | 0.0130477022828341 | 0.259918995677324 | 0.959963720693598 | -0.496393529 | 0.522488933810389 | 1.01313319497335 | 0.60872204400554 | 1.6862193194166 | 0.998074519377826 |
| ebi-fl187-GCST90094803 | ebi-met1400-GCST90200557 | X-17685 levels \|\| id:ebi-met1400-GCST90200557 | Mushroom liking \|\| id:ebi-fl187-GCST90094803 | Inverse variance weighted | 5 | 0.25756631145498 | 0.2064719437306 | 0.21222742190151 | -0.147118698 | 0.662251321166955 | 1.29377760041098 | 0.863191512007279 | 1.93915308021596 | 0.998074519377826 |
| ebi-fl187-GCST90094803 | ebi-met1400-GCST90200561 | X-18779 levels \|\| id:ebi-met1400-GCST90200561 | Mushroom liking \|\| id:ebi-fl187-GCST90094803 | Inverse variance weighted | 5 | -0.17449329 | 0.194245926231051 | 0.369019666882338 | -0.555215305 | 0.206228725685193 | 0.839882490050538 | 0.573948673792546 | 1.22903428355766 | 0.998074519377826 |
| ebi-fl187-GCST90094803 | ebi-met1400-GCST90200570 | X-18921 levels \|\| id:ebi-met1400-GCST90200570 | Mushroom liking \|\| id:ebi-fl187-GCST90094803 | Inverse variance weighted | 5 | -0.052136785 | 0.240304178549782 | 0.828238251085523 | -0.523132975 | 0.41885940478831 | 0.949199021613807 | 0.592660844604371 | 1.52022660318323 | 0.998074519377826 |
| ebi-fl187-GCST90094803 | ebi-met1400-GCST90200575 | X-21283 levels \|\| id:ebi-met1400-GCST90200575 | Mushroom liking \|\| id:ebi-fl187-GCST90094803 | Inverse variance weighted | 5 | -0.079593763 | 0.206484096080788 | 0.699888015405526 | -0.484302591 | 0.325115065513736 | 0.923491426762853 | 0.616126739220832 | 1.38418990934074 | 0.998074519377826 |
| ebi-fl187-GCST90094803 | ebi-met1400-GCST90200578 | X-18922 levels \|\| id:ebi-met1400-GCST90200578 | Mushroom liking \|\| id:ebi-fl187-GCST90094803 | Inverse variance weighted | 5 | 0.0128924614907953 | 0.198910929157688 | 0.948321099679553 | -0.37697296 | 0.402757882639865 | 1.01297592758119 | 0.68593462155182 | 1.49594465364285 | 0.998074519377826 |
| ebi-fl187-GCST90094803 | ebi-met1400-GCST90200623 | X-24241 levels \|\| id:ebi-met1400-GCST90200623 | Mushroom liking \|\| id:ebi-fl187-GCST90094803 | Inverse variance weighted | 5 | -0.328754647 | 0.232837863531027 | 0.157965663097074 | -0.78511686 | 0.127607565332561 | 0.719819604846997 | 0.456066402884298 | 1.13610706740338 | 0.998074519377826 |
| ebi-fl187-GCST90094803 | ebi-met1400-GCST90200636 | X-24544 levels \|\| id:ebi-met1400-GCST90200636 | Mushroom liking \|\| id:ebi-fl187-GCST90094803 | Inverse variance weighted | 5 | -0.292835403 | 0.182625146135847 | 0.108829163646357 | -0.65078069 | 0.0651098833419557 | 0.746144943746846 | 0.521638380144889 | 1.06727629382705 | 0.998074519377826 |
| ebi-fl187-GCST90094803 | ebi-met1400-GCST90200659 | X-25172 levels \|\| id:ebi-met1400-GCST90200659 | Mushroom liking \|\| id:ebi-fl187-GCST90094803 | Inverse variance weighted | 5 | -0.026280111 | 0.197579654711264 | 0.894185307207122 | -0.413536234 | 0.360976012211469 | 0.974062205831098 | 0.661307572 | 1.43472904448885 | 0.998074519377826 |
| ebi-fl187-GCST90094803 | ebi-met1400-GCST90200670 | X-26111 levels \|\| id:ebi-met1400-GCST90200670 | Mushroom liking \|\| id:ebi-fl187-GCST90094803 | Inverse variance weighted | 5 | 0.0156961446791979 | 0.196295608807563 | 0.936267660250004 | -0.369043249 | 0.400435537942021 | 1.01581997620226 | 0.69139550792725 | 1.49247458541506 | 0.998074519377826 |
| ebi-fl187-GCST90094803 | ebi-met1400-GCST90200673 | Carnitine C4 levels \|\| id:ebi-met1400-GCST90200673 | Mushroom liking \|\| id:ebi-fl187-GCST90094803 | Inverse variance weighted (multiplicative random effects) | 5 | -0.021489899 | 0.305722325573261 | 0.943961079973506 | -0.620705657 | 0.577725859168741 | 0.978739363709718 | 0.537564967198208 | 1.78198134277173 | 0.998074519377826 |
| ebi-fl187-GCST90094803 | ebi-met1400-GCST90200710 | X-13431 levels \|\| id:ebi-met1400-GCST90200710 | Mushroom liking \|\| id:ebi-fl187-GCST90094803 | Inverse variance weighted | 5 | -0.07070927 | 0.192498535230338 | 0.713377593117039 | -0.448006399 | 0.306587858802577 | 0.931732735180822 | 0.638900595502031 | 1.35878084309091 | 0.998074519377826 |
| ebi-fl187-GCST90094803 | ebi-met1400-GCST90200730 | Adenosine 5'-diphosphate (ADP) to fructose ratio \|\| id:ebi-met1400-GCST90200730 | Mushroom liking \|\| id:ebi-fl187-GCST90094803 | Inverse variance weighted | 5 | 0.333435395654765 | 0.261750278572921 | 0.202710076591025 | -0.17959515 | 0.84646594165769 | 1.39575487179911 | 0.83560843872654 | 2.33139299684418 | 0.998074519377826 |
| ebi-fl187-GCST90094803 | ebi-met1400-GCST90200731 | Adenosine 5'-diphosphate (ADP) to mannose ratio \|\| id:ebi-met1400-GCST90200731 | Mushroom liking \|\| id:ebi-fl187-GCST90094803 | Inverse variance weighted | 5 | 0.176907052047489 | 0.277948784865884 | 0.524467810150253 | -0.367872566 | 0.721686670384621 | 1.1935201527169 | 0.692205386369201 | 2.05790128622547 | 0.998074519377826 |
| ebi-fl187-GCST90094803 | ebi-met1400-GCST90200748 | Aspartate to glutamate ratio \|\| id:ebi-met1400-GCST90200748 | Mushroom liking \|\| id:ebi-fl187-GCST90094803 | Inverse variance weighted | 5 | 0.193500502752636 | 0.189253111353539 | 0.306571317990922 | -0.177435596 | 0.564436601005571 | 1.21348999663664 | 0.837414930888684 | 1.75845679079842 | 0.998074519377826 |
| ebi-fl187-GCST90094803 | ebi-met1400-GCST90200774 | Uridine to pseudouridine ratio \|\| id:ebi-met1400-GCST90200774 | Mushroom liking \|\| id:ebi-fl187-GCST90094803 | Inverse variance weighted | 5 | 0.0830177225611173 | 0.18585775967695 | 0.655110870457818 | -0.281263486 | 0.447298931527939 | 1.08656106502248 | 0.75482942198454 | 1.5640817827673 | 0.998074519377826 |
| ebi-fl187-GCST90094803 | ebi-met1400-GCST90200799 | Spermidine to adenosine 5'-diphosphate (ADP) ratio \|\| id:ebi-met1400-GCST90200799 | Mushroom liking \|\| id:ebi-fl187-GCST90094803 | Inverse variance weighted | 5 | -0.210678868 | 0.414933229117242 | 0.611634559754353 | -1.023947997 | 0.602590261167373 | 0.810034153084718 | 0.359174118985442 | 1.82684468195291 | 0.998074519377826 |
| ebi-fl187-GCST90094803 | ebi-met1400-GCST90200811 | Choline to taurocholate ratio \|\| id:ebi-met1400-GCST90200811 | Mushroom liking \|\| id:ebi-fl187-GCST90094803 | Inverse variance weighted | 5 | 0.0374195050590286 | 0.203823325096929 | 0.85433672217695 | -0.362074212 | 0.43691322224901 | 1.03812842963183 | 0.696230697160828 | 1.54792174606012 | 0.998074519377826 |
| ebi-fl187-GCST90094803 | ebi-met1400-GCST90200812 | Glutamate to kynurenine ratio \|\| id:ebi-met1400-GCST90200812 | Mushroom liking \|\| id:ebi-fl187-GCST90094803 | Inverse variance weighted | 5 | 0.0641872335402233 | 0.224648330157943 | 0.775090155330297 | -0.376123494 | 0.504497960649792 | 1.06629202570338 | 0.686517547304827 | 1.65615385730814 | 0.998074519377826 |
| ebi-fl187-GCST90094803 | ebi-met1400-GCST90200876 | Cortisone to 4-cholesten-3-one ratio \|\| id:ebi-met1400-GCST90200876 | Mushroom liking \|\| id:ebi-fl187-GCST90094803 | Inverse variance weighted | 5 | -0.093624297 | 0.268658617036483 | 0.727473742958264 | -0.620195187 | 0.432946591923836 | 0.910624822032018 | 0.537839448156101 | 1.541793874 | 0.998074519377826 |
| ebi-fl187-GCST90094803 | ebi-met1400-GCST90200906 | 2'-deoxyuridine to cytidine ratio \|\| id:ebi-met1400-GCST90200906 | Mushroom liking \|\| id:ebi-fl187-GCST90094803 | Inverse variance weighted | 5 | 0.0839631947008958 | 0.200685184773119 | 0.675667386927084 | -0.309379767 | 0.477306156856209 | 1.08758886403873 | 0.733902005000441 | 1.61172680973988 | 0.998074519377826 |
| ebi-fl187-GCST90094803 | ebi-met1400-GCST90200907 | Retinol (Vitamin A) to linoleoyl-arachidonoyl-glycerol (18:2 to 20:4) [1] ratio \|\| id:ebi-met1400-GCST90200907 | Mushroom liking \|\| id:ebi-fl187-GCST90094803 | Inverse variance weighted | 5 | -0.215362557 | 0.202839478565889 | 0.288354251282264 | -0.612927935 | 0.182202820647103 | 0.806249075659185 | 0.541762299616902 | 1.19985752508241 | 0.998074519377826 |
| ebi-fl187-GCST90094803 | ebi-met1400-GCST90200916 | Fructose to sucrose ratio \|\| id:ebi-met1400-GCST90200916 | Mushroom liking \|\| id:ebi-fl187-GCST90094803 | Inverse variance weighted | 5 | -0.102296602 | 0.259121277294095 | 0.693003249668037 | -0.610174306 | 0.405581101172135 | 0.902761750722103 | 0.543256168109032 | 1.50017399968714 | 0.998074519377826 |
| ebi-fl187-GCST90094803 | ebi-met1400-GCST90200917 | Alpha-ketobutyrate to 3-methyl-2-oxobutyrate ratio \|\| id:ebi-met1400-GCST90200917 | Mushroom liking \|\| id:ebi-fl187-GCST90094803 | Inverse variance weighted | 5 | 0.401257212693361 | 0.252701993668593 | 0.112316366821873 | -0.094038695 | 0.896553120283803 | 1.49370141805859 | 0.91024753962444 | 2.45113974955734 | 0.998074519377826 |
| ebi-fl187-GCST90094803 | ebi-met1400-GCST90200924 | Alpha-tocopherol to glycerol ratio \|\| id:ebi-met1400-GCST90200924 | Mushroom liking \|\| id:ebi-fl187-GCST90094803 | Inverse variance weighted | 5 | 0.0423200153689002 | 0.181816374631459 | 0.815945912970497 | -0.314040079 | 0.398680109646559 | 1.04322827441913 | 0.730489750340664 | 1.48985695150407 | 0.998074519377826 |
| ebi-fl187-GCST90094803 | ebi-met1400-GCST90200938 | Alpha-ketoglutarate to alanine ratio \|\| id:ebi-met1400-GCST90200938 | Mushroom liking \|\| id:ebi-fl187-GCST90094803 | Inverse variance weighted | 5 | -0.109881425 | 0.19340391891951 | 0.569936671086733 | -0.488953106 | 0.269190256325407 | 0.895940365344962 | 0.613268085011902 | 1.30890414465133 | 0.998074519377826 |
| ebi-fl187-GCST90094803 | ebi-met1400-GCST90200943 | Phosphate to EDTA ratio \|\| id:ebi-met1400-GCST90200943 | Mushroom liking \|\| id:ebi-fl187-GCST90094803 | Inverse variance weighted | 5 | 0.0857711516706038 | 0.243594933857428 | 0.724759033926956 | -0.391674919 | 0.563217222031162 | 1.08955695648278 | 0.675923808446547 | 1.75631387234069 | 0.998074519377826 |
| ebi-fl187-GCST90094803 | ebi-met1400-GCST90200947 | Adenosine 5'-diphosphate (ADP) to glucose ratio \|\| id:ebi-met1400-GCST90200947 | Mushroom liking \|\| id:ebi-fl187-GCST90094803 | Inverse variance weighted | 5 | 0.322080088909754 | 0.260074510261474 | 0.215562343714104 | -0.187665951 | 0.831826129022243 | 1.37999529385011 | 0.828891551216153 | 2.29751046232082 | 0.998074519377826 |
| ebi-fl187-GCST90094803 | ebi-met1400-GCST90200975 | Arachidonate (20:4n6) to caffeine ratio \|\| id:ebi-met1400-GCST90200975 | Mushroom liking \|\| id:ebi-fl187-GCST90094803 | Inverse variance weighted | 5 | 0.0921024014277683 | 0.199237783199763 | 0.643885006230803 | -0.298403654 | 0.482608456499304 | 1.09647709715242 | 0.742001767572979 | 1.62029536467586 | 0.998074519377826 |
| ebi-fl187-GCST90094803 | ebi-met1400-GCST90200985 | Cholesterol to oleoyl-linoleoyl-glycerol (18:1 to 18:2) [2] ratio \|\| id:ebi-met1400-GCST90200985 | Mushroom liking \|\| id:ebi-fl187-GCST90094803 | Inverse variance weighted | 5 | -0.055174693 | 0.194698014302061 | 0.776880952090989 | -0.436782801 | 0.326433414770198 | 0.946319817817153 | 0.646111750807759 | 1.38601595849932 | 0.998074519377826 |
| ebi-fl187-GCST90094803 | ebi-met1400-GCST90201016 | Bilirubin (Z,Z) to etiocholanolone glucuronide ratio \|\| id:ebi-met1400-GCST90201016 | Mushroom liking \|\| id:ebi-fl187-GCST90094803 | Inverse variance weighted | 5 | 0.120403906502867 | 0.200442815102765 | 0.548046773565518 | -0.272464011 | 0.513271824104287 | 1.12795234687225 | 0.761500834260539 | 1.67074865787912 | 0.998074519377826 |
| ebi-fl187-GCST90094803 | ebi-pw11-GCST90275189 | Placental weight (fetal genotype, adjusted for sex and gestational age) \|\| id:ebi-pw11-GCST90275189 | Mushroom liking \|\| id:ebi-fl187-GCST90094803 | Inverse variance weighted | 6 | 0.0907194541705539 | 0.0602269869082953 | 0.131992121815472 | -0.02732544 | 0.208764348510813 | 1.09496177520499 | 0.973044522216099 | 1.23215460524816 | 0.998074519377826 |
| ebi-fl187-GCST90094803 | ebi-pw11-GCST90275192 | Placental weight (fetal effect, conditional on maternal and paternal genotype) \|\| id:ebi-pw11-GCST90275192 | Mushroom liking \|\| id:ebi-fl187-GCST90094803 | Inverse variance weighted | 6 | 0.154157967912062 | 0.128280107503391 | 0.229468412815385 | -0.097271043 | 0.405586978618709 | 1.16667516946326 | 0.907310052950553 | 1.50018281690559 | 0.998074519377826 |
| ebi-fl187-GCST90094803 | ebi-pw11-GCST90275193 | Placental weight (maternal effect, conditional on fetal and paternal genotype) \|\| id:ebi-pw11-GCST90275193 | Mushroom liking \|\| id:ebi-fl187-GCST90094803 | Inverse variance weighted | 6 | -0.013300659 | 0.102469491575343 | 0.896723741542915 | -0.214140863 | 0.187539544151586 | 0.986787403569366 | 0.807234667658872 | 1.20627794971594 | 0.998074519377826 |
| ebi-fl187-GCST90094803 | ebi-pw11-GCST90275195 | Placental weight (fetal effect, conditional on maternal genotype) \|\| id:ebi-pw11-GCST90275195 | Mushroom liking \|\| id:ebi-fl187-GCST90094803 | Inverse variance weighted | 6 | 0.082138437806329 | 0.0824856112066657 | 0.319351653786828 | -0.07953336 | 0.243810235771394 | 1.0856060883526 | 0.923547209773175 | 1.27610214897156 | 0.998074519377826 |
| ebi-fl187-GCST90094803 | ebi-pw11-GCST90275197 | Placental weight (fetal genotype, adjusted for sex) \|\| id:ebi-pw11-GCST90275197 | Mushroom liking \|\| id:ebi-fl187-GCST90094803 | Inverse variance weighted | 6 | 0.0840846552762245 | 0.0610105289390824 | 0.168141829100177 | -0.035495981 | 0.203665291996826 | 1.08772097123063 | 0.965126612637184 | 1.22588777033306 | 0.998074519377826 |
| ebi-fl187-GCST90094803 | prot-a-1030 | Protein FAM171B \|\| id:prot-a-1030 | Mushroom liking \|\| id:ebi-fl187-GCST90094803 | Inverse variance weighted (multiplicative random effects) | 7 | 0.071944058057076 | 0.34884195395797 | 0.836605921562718 | -0.611786172 | 0.755674287814698 | 1.07459522743743 | 0.542381217368709 | 2.12904662966288 | 0.998074519377826 |
| ebi-fl187-GCST90094803 | prot-a-1036 | BRISC complex subunit Abro1 \|\| id:prot-a-1036 | Mushroom liking \|\| id:ebi-fl187-GCST90094803 | Inverse variance weighted | 7 | 0.175279193054634 | 0.221360340499081 | 0.428461296673946 | -0.258587074 | 0.609145460432833 | 1.1915788507148 | 0.77214179439749 | 1.83885934911571 | 0.998074519377826 |
| ebi-fl187-GCST90094803 | prot-a-1055 | Phenylalanine--tRNA ligase, mitochondrial \|\| id:prot-a-1055 | Mushroom liking \|\| id:ebi-fl187-GCST90094803 | Inverse variance weighted | 7 | 0.00495591606245984 | 0.221444538580673 | 0.982144879831822 | -0.42907538 | 0.438987211680579 | 1.00496821692676 | 0.651110846884832 | 1.55113545084527 | 0.999355215041207 |
| ebi-fl187-GCST90094803 | prot-a-1083 | Fc receptor-like protein 5 \|\| id:prot-a-1083 | Mushroom liking \|\| id:ebi-fl187-GCST90094803 | Inverse variance weighted | 7 | -0.214933023 | 0.221444538580673 | 0.331749860318617 | -0.648964318 | 0.219098273022633 | 0.806595462038725 | 0.522586730143101 | 1.24495361603099 | 0.998074519377826 |
| ebi-fl187-GCST90094803 | prot-a-1103 | Fibroblast growth factor receptor 3 \|\| id:prot-a-1103 | Mushroom liking \|\| id:ebi-fl187-GCST90094803 | Inverse variance weighted | 7 | -0.210908228 | 0.221360340499081 | 0.340700352745382 | -0.644774495 | 0.222958039310705 | 0.809848384822385 | 0.524780869240464 | 1.24976813150348 | 0.998074519377826 |
| ebi-fl187-GCST90094803 | prot-a-1105 | Fibroblast growth factor receptor 4 \|\| id:prot-a-1105 | Mushroom liking \|\| id:ebi-fl187-GCST90094803 | Inverse variance weighted | 7 | -0.23838109 | 0.222783207988203 | 0.284613132047998 | -0.675036177 | 0.198273997996971 | 0.787902372426088 | 0.509138001027615 | 1.21929643283685 | 0.998074519377826 |
| ebi-fl187-GCST90094803 | prot-a-1120 | Filamin-A \|\| id:prot-a-1120 | Mushroom liking \|\| id:ebi-fl187-GCST90094803 | Inverse variance weighted | 7 | -0.046111413 | 0.221360340499081 | 0.834987487088545 | -0.47997768 | 0.387754854483707 | 0.954935564128514 | 0.61879720303684 | 1.47366847678391 | 0.998074519377826 |
| ebi-fl187-GCST90094803 | prot-a-1166 | Galanin peptides \|\| id:prot-a-1166 | Mushroom liking \|\| id:ebi-fl187-GCST90094803 | Inverse variance weighted | 7 | -0.035978983 | 0.221444538580673 | 0.870932591385209 | -0.470010278 | 0.398052312891187 | 0.964660567804638 | 0.624995844326744 | 1.48892191768026 | 0.998074519377826 |
| ebi-fl187-GCST90094803 | prot-a-1205 | ADP-ribosylation factor-binding protein GGA1 \|\| id:prot-a-1205 | Mushroom liking \|\| id:ebi-fl187-GCST90094803 | Inverse variance weighted | 7 | -0.008391447 | 0.221444538580673 | 0.969772098032657 | -0.442422742 | 0.425639848807903 | 0.991643663103274 | 0.642477975339002 | 1.5305694394489 | 0.998074519377826 |
| ebi-fl187-GCST90094803 | prot-a-1227 | Guanine nucleotide-binding protein G(I)/G(S)/G(O) subunit gamma-T2 \|\| id:prot-a-1227 | Mushroom liking \|\| id:ebi-fl187-GCST90094803 | Inverse variance weighted | 7 | -0.052953209 | 0.221276393275928 | 0.810866771065207 | -0.48665494 | 0.380748522141084 | 0.948424389473416 | 0.614679097831416 | 1.46337955157656 | 0.998074519377826 |
| ebi-fl187-GCST90094803 | prot-a-1240 | Golgi SNAP receptor complex member 1 \|\| id:prot-a-1240 | Mushroom liking \|\| id:ebi-fl187-GCST90094803 | Inverse variance weighted | 7 | 0.063533807529942 | 0.225905918198287 | 0.778526234671899 | -0.379241792 | 0.506309407198584 | 1.06559551034464 | 0.684380114927387 | 1.65915661033945 | 0.998074519377826 |
| ebi-fl187-GCST90094803 | prot-a-1283 | Glutathione S-transferase A1 \|\| id:prot-a-1283 | Mushroom liking \|\| id:ebi-fl187-GCST90094803 | Inverse variance weighted | 7 | 0.0146344375525279 | 0.26058053482603 | 0.955213628662762 | -0.496103411 | 0.525372285811547 | 1.01474204521887 | 0.608898671175304 | 1.69108829938392 | 0.998074519377826 |
| ebi-fl187-GCST90094803 | prot-a-1324 | E3 ubiquitin-protein ligase HECW1 \|\| id:prot-a-1324 | Mushroom liking \|\| id:ebi-fl187-GCST90094803 | Inverse variance weighted | 7 | 0.0305851541102241 | 0.221360340499081 | 0.890106792998627 | -0.403281113 | 0.464451421488423 | 1.03105768511044 | 0.668124254329389 | 1.59114108361824 | 0.998074519377826 |
| ebi-fl187-GCST90094803 | prot-a-1339 | Histidine triad nucleotide-binding protein 2, mitochondrial \|\| id:prot-a-1339 | Mushroom liking \|\| id:ebi-fl187-GCST90094803 | Inverse variance weighted | 7 | -0.107528343 | 0.221444538580673 | 0.62726720858268 | -0.541559639 | 0.326502952665588 | 0.898051068671277 | 0.581840084113823 | 1.38611234248318 | 0.998074519377826 |
| ebi-fl187-GCST90094803 | prot-a-1466 | Interleukin-11 \|\| id:prot-a-1466 | Mushroom liking \|\| id:ebi-fl187-GCST90094803 | Inverse variance weighted | 7 | -0.16902169 | 0.221360340499081 | 0.445130064008592 | -0.602887957 | 0.264844577464669 | 0.844490586259594 | 0.54722897795233 | 1.30322840897362 | 0.998074519377826 |
| ebi-fl187-GCST90094803 | prot-a-147 | Rho GTPase-activating protein 1 \|\| id:prot-a-147 | Mushroom liking \|\| id:ebi-fl187-GCST90094803 | Inverse variance weighted | 7 | -0.158160035 | 0.221444538580673 | 0.475091383725584 | -0.592191331 | 0.275871260412816 | 0.853713146876831 | 0.553113900218199 | 1.31767821575778 | 0.998074519377826 |
| ebi-fl187-GCST90094803 | prot-a-1473 | Interleukin-12 receptor subunit beta-1 \|\| id:prot-a-1473 | Mushroom liking \|\| id:ebi-fl187-GCST90094803 | Inverse variance weighted | 7 | -0.142577401 | 0.243975872414835 | 0.558957014142065 | -0.620770111 | 0.335615309271188 | 0.867120435999106 | 0.537530320362282 | 1.39880081559031 | 0.998074519377826 |
| ebi-fl187-GCST90094803 | prot-a-1501 | X-linked interleukin-1 receptor accessory protein-like 2 \|\| id:prot-a-1501 | Mushroom liking \|\| id:ebi-fl187-GCST90094803 | Inverse variance weighted | 7 | -0.309422503 | 0.221444538580673 | 0.162326024207948 | -0.743453798 | 0.124608792910604 | 0.733870642182662 | 0.475468902685002 | 1.13270524405755 | 0.998074519377826 |
| ebi-fl187-GCST90094803 | prot-a-1503 | Interleukin-1 receptor-like 2 \|\| id:prot-a-1503 | Mushroom liking \|\| id:ebi-fl187-GCST90094803 | Inverse variance weighted | 7 | -0.197280062 | 0.221360340499081 | 0.372812855531882 | -0.631146329 | 0.236586205744978 | 0.820960681524546 | 0.531981625371992 | 1.26691676641644 | 0.998074519377826 |
| ebi-fl187-GCST90094803 | prot-a-1536 | Interleukin-5 receptor subunit alpha \|\| id:prot-a-1536 | Mushroom liking \|\| id:ebi-fl187-GCST90094803 | Inverse variance weighted | 7 | 0.0244971415477655 | 0.221360340499081 | 0.911880928582983 | -0.409369126 | 0.458363408925965 | 1.02479966176213 | 0.664069062031669 | 1.58148362390912 | 0.998074519377826 |
| ebi-fl187-GCST90094803 | prot-a-1559 | Insulin-induced gene 1 protein \|\| id:prot-a-1559 | Mushroom liking \|\| id:ebi-fl187-GCST90094803 | Inverse variance weighted | 7 | 0.0323534324903198 | 0.221444538580673 | 0.883841072515005 | -0.401677863 | 0.466384728108439 | 1.03288249503413 | 0.669196283769844 | 1.59422022270951 | 0.998074519377826 |
| ebi-fl187-GCST90094803 | prot-a-1567 | Interferon regulatory factor 1 \|\| id:prot-a-1567 | Mushroom liking \|\| id:ebi-fl187-GCST90094803 | Inverse variance weighted | 7 | -0.013664391 | 0.305978571626979 | 0.964379911187624 | -0.613382392 | 0.586053609247874 | 0.986428542874477 | 0.541516148318348 | 1.79688320139519 | 0.998074519377826 |
| ebi-fl187-GCST90094803 | prot-a-1613 | Potassium voltage-gated channel subfamily E regulatory beta subunit 5 \|\| id:prot-a-1613 | Mushroom liking \|\| id:ebi-fl187-GCST90094803 | Inverse variance weighted | 7 | 0.230053257340517 | 0.221360340499081 | 0.298678980827869 | -0.20381301 | 0.663919524718716 | 1.25866704140372 | 0.815614868722662 | 1.94239068201036 | 0.998074519377826 |
| ebi-fl187-GCST90094803 | prot-a-1649 | Kin of IRRE-like protein 2 \|\| id:prot-a-1649 | Mushroom liking \|\| id:ebi-fl187-GCST90094803 | Inverse variance weighted | 7 | -0.288894219 | 0.293702874727831 | 0.325298706961338 | -0.864551854 | 0.286763414987316 | 0.749091440494508 | 0.42124028750649 | 1.33210901916283 | 0.998074519377826 |
| ebi-fl187-GCST90094803 | prot-a-165 | Armadillo repeat-containing protein 5 \|\| id:prot-a-165 | Mushroom liking \|\| id:ebi-fl187-GCST90094803 | Inverse variance weighted | 7 | 0.254205015681379 | 0.291015229353808 | 0.38238459134734 | -0.316184834 | 0.824594865214843 | 1.28943613179839 | 0.728924707753915 | 2.28095648329773 | 0.998074519377826 |
| ebi-fl187-GCST90094803 | prot-a-1656 | Kallikrein-11 \|\| id:prot-a-1656 | Mushroom liking \|\| id:ebi-fl187-GCST90094803 | Inverse variance weighted | 7 | -0.45037226 | 0.28438020083454 | 0.113262536677187 | -1.007757453 | 0.107012933899736 | 0.637390832509324 | 0.365036674132545 | 1.11294864915248 | 0.998074519377826 |
| ebi-fl187-GCST90094803 | prot-a-1697 | Laminin subunit gamma-2 \|\| id:prot-a-1697 | Mushroom liking \|\| id:ebi-fl187-GCST90094803 | Inverse variance weighted | 7 | -0.101624237 | 0.221444538580673 | 0.646295154156453 | -0.535655532 | 0.332407059005656 | 0.903368940873283 | 0.585285490858787 | 1.39432030364719 | 0.998074519377826 |
| ebi-fl187-GCST90094803 | prot-a-1698 | Lysosome-associated membrane glycoprotein 3 \|\| id:prot-a-1698 | Mushroom liking \|\| id:ebi-fl187-GCST90094803 | Inverse variance weighted | 7 | -0.21538804 | 0.221444538580673 | 0.330727261329206 | -0.649419336 | 0.218643255357916 | 0.80622853034175 | 0.522348998039794 | 1.24438726900267 | 0.998074519377826 |
| ebi-fl187-GCST90094803 | prot-a-1724 | Leptin receptor \|\| id:prot-a-1724 | Mushroom liking \|\| id:ebi-fl187-GCST90094803 | Inverse variance weighted | 7 | 0.0131502378603103 | 0.221360340499081 | 0.952628354691183 | -0.42071603 | 0.44701650523851 | 1.01323708249655 | 0.656576523290624 | 1.56364010732635 | 0.998074519377826 |
| ebi-fl187-GCST90094803 | prot-a-1743 | Leukocyte immunoglobulin-like receptor subfamily B member 2 \|\| id:prot-a-1743 | Mushroom liking \|\| id:ebi-fl187-GCST90094803 | Inverse variance weighted | 7 | -0.166857191 | 0.265890481204738 | 0.530304637560598 | -0.688002535 | 0.354288151769638 | 0.846320464555306 | 0.502578951210859 | 1.42516579136359 | 0.998074519377826 |
| ebi-fl187-GCST90094803 | prot-a-1746 | Leukocyte immunoglobulin-like receptor subfamily B member 4 \|\| id:prot-a-1746 | Mushroom liking \|\| id:ebi-fl187-GCST90094803 | Inverse variance weighted | 7 | 0.201759058448766 | 0.285898535738283 | 0.480373716318834 | -0.358602072 | 0.762120188495801 | 1.22355316779488 | 0.698652309641098 | 2.14281457852754 | 0.998074519377826 |
| ebi-fl187-GCST90094803 | prot-a-1750 | Leucine-rich repeat and immunoglobulin-like domain-containing nogo receptor-interacting protein 3 \|\| id:prot-a-1750 | Mushroom liking \|\| id:ebi-fl187-GCST90094803 | Inverse variance weighted | 7 | -0.034329028 | 0.221360340499081 | 0.876756589572766 | -0.468195295 | 0.399537239791065 | 0.966253528261912 | 0.626131231439279 | 1.49113450024276 | 0.998074519377826 |
| ebi-fl187-GCST90094803 | prot-a-1753 | Lipase member N \|\| id:prot-a-1753 | Mushroom liking \|\| id:ebi-fl187-GCST90094803 | Inverse variance weighted | 7 | 0.454861754771312 | 0.221360340499081 | 0.0398937311900794 | 0.0209954873931125 | 0.888728022149511 | 1.57595549964522 | 1.02121744327426 | 2.43203418940719 | 0.998074519377826 |
| ebi-fl187-GCST90094803 | prot-a-1760 | Ig Kappa chain V-I region HK102- like \|\| id:prot-a-1760 | Mushroom liking \|\| id:ebi-fl187-GCST90094803 | Inverse variance weighted | 7 | -0.078394564 | 0.221360340499081 | 0.723227149879697 | -0.512260832 | 0.355471702879203 | 0.924599540408282 | 0.599139492784453 | 1.42685354649249 | 0.998074519377826 |
| ebi-fl187-GCST90094803 | prot-a-1792 | Leucine-rich repeat neuronal protein 1 \|\| id:prot-a-1792 | Mushroom liking \|\| id:ebi-fl187-GCST90094803 | Inverse variance weighted | 7 | 0.175737623759443 | 0.238213775537274 | 0.460678091162991 | -0.291161376 | 0.642636623812499 | 1.19212523227664 | 0.747395056441548 | 1.90148778371239 | 0.998074519377826 |
| ebi-fl187-GCST90094803 | prot-a-1795 | Leucine-rich repeat transmembrane neuronal protein 1 \|\| id:prot-a-1795 | Mushroom liking \|\| id:ebi-fl187-GCST90094803 | Inverse variance weighted | 7 | -0.179390246 | 0.221444538580673 | 0.417887829973674 | -0.613421541 | 0.254641049974853 | 0.835779676370185 | 0.541494948521523 | 1.28999849276652 | 0.998074519377826 |
| ebi-fl187-GCST90094803 | prot-a-1820 | LysM and putative peptidoglycan-binding domain-containing protein 4 \|\| id:prot-a-1820 | Mushroom liking \|\| id:ebi-fl187-GCST90094803 | Inverse variance weighted (multiplicative random effects) | 7 | -0.011515411 | 0.337716997684588 | 0.972799151923898 | -0.673440727 | 0.650409904206836 | 0.988550637324609 | 0.509950953897483 | 1.91632617820605 | 0.998074519377826 |
| ebi-fl187-GCST90094803 | prot-a-1844 | Mitogen-activated protein kinase kinase kinase 7:TGF-beta-activated kinase 1 and MAP3K7-binding protein 1 fusion \|\| id:prot-a-1844 | Mushroom liking \|\| id:ebi-fl187-GCST90094803 | Inverse variance weighted | 7 | 0.160403198520313 | 0.234478228913152 | 0.493921064222798 | -0.29917413 | 0.619980527190092 | 1.17398412423966 | 0.741430292826082 | 1.85889184364639 | 0.998074519377826 |
| ebi-fl187-GCST90094803 | prot-a-1856 | Megakaryocyte-associated tyrosine-protein kinase \|\| id:prot-a-1856 | Mushroom liking \|\| id:ebi-fl187-GCST90094803 | Inverse variance weighted | 7 | 0.116810588601155 | 0.221360340499081 | 0.597711534235091 | -0.317055679 | 0.550676855979355 | 1.12390652882045 | 0.728290203688911 | 1.73442657765695 | 0.998074519377826 |
| ebi-fl187-GCST90094803 | prot-a-1910 | Stromelysin-2 \|\| id:prot-a-1910 | Mushroom liking \|\| id:ebi-fl187-GCST90094803 | Inverse variance weighted | 7 | -0.330268055 | 0.309420629406386 | 0.285802226209871 | -0.936732488 | 0.276196379071589 | 0.718731048469541 | 0.391906303987586 | 1.31810668718023 | 0.998074519377826 |
| ebi-fl187-GCST90094803 | prot-a-1940 | 39S ribosomal protein L14, mitochondrial \|\| id:prot-a-1940 | Mushroom liking \|\| id:ebi-fl187-GCST90094803 | Inverse variance weighted | 7 | -0.182237059 | 0.242988270396772 | 0.453264976578801 | -0.658494069 | 0.294019951329537 | 0.833403751429906 | 0.517630263491706 | 1.34181067430686 | 0.998074519377826 |
| ebi-fl187-GCST90094803 | prot-a-1942 | 39S ribosomal protein L33, mitochondrial \|\| id:prot-a-1942 | Mushroom liking \|\| id:ebi-fl187-GCST90094803 | Inverse variance weighted | 7 | -0.135322289 | 0.30672010717202 | 0.659074401113223 | -0.736493699 | 0.46584912065697 | 0.873434367682681 | 0.478789756586231 | 1.59336657510936 | 0.998074519377826 |
| ebi-fl187-GCST90094803 | prot-a-1948 | Mesothelin \|\| id:prot-a-1948 | Mushroom liking \|\| id:ebi-fl187-GCST90094803 | Inverse variance weighted | 7 | -0.110133646 | 0.221360340499081 | 0.618814601031685 | -0.543999914 | 0.323732621125471 | 0.895714418421314 | 0.580421965271247 | 1.38227766585797 | 0.998074519377826 |
| ebi-fl187-GCST90094803 | prot-a-1980 | Unconventional myosin-VI \|\| id:prot-a-1980 | Mushroom liking \|\| id:ebi-fl187-GCST90094803 | Inverse variance weighted | 7 | 0.0781019181159668 | 0.249582198083511 | 0.754333354993135 | -0.41107919 | 0.567283026359649 | 1.08123285026975 | 0.662934431659706 | 1.76346923718475 | 0.998074519377826 |
| ebi-fl187-GCST90094803 | prot-a-2039 | Neurofascin \|\| id:prot-a-2039 | Mushroom liking \|\| id:ebi-fl187-GCST90094803 | Inverse variance weighted | 7 | -0.465168825 | 0.221444538580673 | 0.0356751310231198 | -0.899200121 | -0.031137529 | 0.628029069162475 | 0.406894996481738 | 0.969342250761215 | 0.998074519377826 |
| ebi-fl187-GCST90094803 | prot-a-2041 | NFU1 iron-sulfur cluster scaffold homolog, mitochondrial \|\| id:prot-a-2041 | Mushroom liking \|\| id:ebi-fl187-GCST90094803 | Inverse variance weighted | 7 | 0.0612291694337949 | 0.307503681228603 | 0.842171327862902 | -0.541478046 | 0.663936384641858 | 1.06314252604267 | 0.581887560010152 | 1.94242343084404 | 0.998074519377826 |
| ebi-fl187-GCST90094803 | prot-a-2088 | Glucocorticoid receptor \|\| id:prot-a-2088 | Mushroom liking \|\| id:ebi-fl187-GCST90094803 | Inverse variance weighted | 7 | 0.119351314316948 | 0.221444538580673 | 0.589909576130975 | -0.314679981 | 0.553382609935067 | 1.12676569768424 | 0.730022457728564 | 1.73912586391954 | 0.998074519377826 |
| ebi-fl187-GCST90094803 | prot-a-2111 | Neuron-specific protein family member 2 \|\| id:prot-a-2111 | Mushroom liking \|\| id:ebi-fl187-GCST90094803 | Inverse variance weighted | 7 | 0.0140125847301368 | 0.221360340499081 | 0.949525906438143 | -0.419853683 | 0.447878852108336 | 1.01411122117423 | 0.657142964199455 | 1.56498908903992 | 0.998074519377826 |
| ebi-fl187-GCST90094803 | prot-a-2125 | Nuclear migration protein nudC \|\| id:prot-a-2125 | Mushroom liking \|\| id:ebi-fl187-GCST90094803 | Inverse variance weighted | 7 | 0.0758322296769637 | 0.221360340499081 | 0.731919194527819 | -0.358034038 | 0.509698497055163 | 1.07878157144154 | 0.699049280571013 | 1.66478918042983 | 0.998074519377826 |
| ebi-fl187-GCST90094803 | prot-a-2142 | Ornithine decarboxylase \|\| id:prot-a-2142 | Mushroom liking \|\| id:ebi-fl187-GCST90094803 | Inverse variance weighted | 7 | -0.006355063 | 0.221360340499081 | 0.977096573065494 | -0.440221331 | 0.427511204136943 | 0.993665087464212 | 0.643893891876732 | 1.53343636040313 | 0.998074519377826 |
| ebi-fl187-GCST90094803 | prot-a-2186 | Prostate and testis expressed protein 1 \|\| id:prot-a-2186 | Mushroom liking \|\| id:ebi-fl187-GCST90094803 | Inverse variance weighted | 7 | 0.235211130013945 | 0.24930483512475 | 0.345441584254158 | -0.253426347 | 0.723848606858454 | 1.26517585713307 | 0.776136907763172 | 2.0623551508271 | 0.998074519377826 |
| ebi-fl187-GCST90094803 | prot-a-2227 | Platelet-derived growth factor C \|\| id:prot-a-2227 | Mushroom liking \|\| id:ebi-fl187-GCST90094803 | Inverse variance weighted | 7 | -0.102238961 | 0.221444538580673 | 0.644302891722511 | -0.536270257 | 0.331792334553667 | 0.902813788546498 | 0.584925812119146 | 1.39346344425583 | 0.998074519377826 |
| ebi-fl187-GCST90094803 | prot-a-2229 | Platelet-derived growth factor receptor alpha \|\| id:prot-a-2229 | Mushroom liking \|\| id:ebi-fl187-GCST90094803 | Inverse variance weighted | 7 | 0.191732410664066 | 0.264377576934889 | 0.46831590500725 | -0.32644764 | 0.709912461456448 | 1.21134633023229 | 0.721482150772475 | 2.03381321380738 | 0.998074519377826 |
| ebi-fl187-GCST90094803 | prot-a-2243 | Platelet endothelial aggregation receptor 1 \|\| id:prot-a-2243 | Mushroom liking \|\| id:ebi-fl187-GCST90094803 | Inverse variance weighted | 7 | 0.0861567067562563 | 0.221360340499081 | 0.697117302577642 | -0.347709561 | 0.520022974134456 | 1.08997712170167 | 0.706303984917219 | 1.68206629327217 | 0.998074519377826 |
| ebi-fl187-GCST90094803 | prot-a-2246 | E3 ubiquitin-protein ligase pellino homolog 2 \|\| id:prot-a-2246 | Mushroom liking \|\| id:ebi-fl187-GCST90094803 | Inverse variance weighted | 7 | -0.090943785 | 0.221360340499081 | 0.681189519034982 | -0.524810053 | 0.342922482005224 | 0.913069037291984 | 0.591667739353126 | 1.40905953022349 | 0.998074519377826 |
| ebi-fl187-GCST90094803 | prot-a-2251 | Platelet factor 4 variant \|\| id:prot-a-2251 | Mushroom liking \|\| id:ebi-fl187-GCST90094803 | Inverse variance weighted | 7 | 0.375885518160331 | 0.221444538580673 | 0.0896163204133556 | -0.058145777 | 0.80991681377845 | 1.45628040656763 | 0.943512394572716 | 2.24772099948215 | 0.998074519377826 |
| ebi-fl187-GCST90094803 | prot-a-2346 | Protein phosphatase 1L \|\| id:prot-a-2346 | Mushroom liking \|\| id:ebi-fl187-GCST90094803 | Inverse variance weighted | 7 | 0.275591239432883 | 0.271662938071059 | 0.310363209750339 | -0.256868119 | 0.80805059805216 | 1.31730928986847 | 0.773470212920416 | 2.24353017890854 | 0.998074519377826 |
| ebi-fl187-GCST90094803 | prot-a-2358 | PR domain zinc finger protein 4 \|\| id:prot-a-2358 | Mushroom liking \|\| id:ebi-fl187-GCST90094803 | Inverse variance weighted | 7 | 0.170701484081391 | 0.221444538580673 | 0.440793272679672 | -0.263329812 | 0.60473277969951 | 1.18613661550572 | 0.768488399170264 | 1.83076292649625 | 0.998074519377826 |
| ebi-fl187-GCST90094803 | prot-a-2390 | Trypsin-1 \|\| id:prot-a-2390 | Mushroom liking \|\| id:ebi-fl187-GCST90094803 | Inverse variance weighted | 7 | -0.442386077 | 0.278319864133772 | 0.111950267228549 | -0.98789301 | 0.103120857023533 | 0.642501532707488 | 0.372360424622083 | 1.10862538614419 | 0.998074519377826 |
| ebi-fl187-GCST90094803 | prot-a-242 | Brain-derived neurotrophic factor \|\| id:prot-a-242 | Mushroom liking \|\| id:ebi-fl187-GCST90094803 | Inverse variance weighted | 7 | 0.107679766059848 | 0.221444538580673 | 0.626782372345387 | -0.32635153 | 0.541711061677967 | 1.11369104660402 | 0.721551496165622 | 1.71894556920337 | 0.998074519377826 |
| ebi-fl187-GCST90094803 | prot-a-2464 | Dihydropteridine reductase \|\| id:prot-a-2464 | Mushroom liking \|\| id:ebi-fl187-GCST90094803 | Inverse variance weighted | 7 | 0.0089763316712704 | 0.291659263964075 | 0.975447562160405 | -0.562675826 | 0.580628489040858 | 1.00901673975136 | 0.569682651074685 | 1.78716128914549 | 0.998074519377826 |
| ebi-fl187-GCST90094803 | prot-a-2474 | Ras-related protein Rab-27A \|\| id:prot-a-2474 | Mushroom liking \|\| id:ebi-fl187-GCST90094803 | Inverse variance weighted | 7 | 0.333233661339873 | 0.221444538580673 | 0.132370951721955 | -0.100797634 | 0.767264956957992 | 1.39547332854571 | 0.904115976456611 | 2.1538672707836 | 0.998074519377826 |
| ebi-fl187-GCST90094803 | prot-a-2478 | Ras-related protein Rab-7b \|\| id:prot-a-2478 | Mushroom liking \|\| id:ebi-fl187-GCST90094803 | Inverse variance weighted (multiplicative random effects) | 7 | -0.221410669 | 0.324273117171345 | 0.494739107121428 | -0.856985978 | 0.414164640997553 | 0.801387507971781 | 0.424439426014315 | 1.5131062256963 | 0.998074519377826 |
| ebi-fl187-GCST90094803 | prot-a-2528 | E3 ubiquitin-protein ligase rififylin \|\| id:prot-a-2528 | Mushroom liking \|\| id:ebi-fl187-GCST90094803 | Inverse variance weighted | 7 | 0.0151173789649757 | 0.283379581875184 | 0.957455634186149 | -0.540306602 | 0.570541359440336 | 1.01523222452987 | 0.582569608267314 | 1.76922457865488 | 0.998074519377826 |
| ebi-fl187-GCST90094803 | prot-a-2543 | Protein RIC-3 \|\| id:prot-a-2543 | Mushroom liking \|\| id:ebi-fl187-GCST90094803 | Inverse variance weighted | 7 | 0.464188810606997 | 0.221360340499081 | 0.0359951043681087 | 0.0303225432287975 | 0.898055077985196 | 1.5907232875171 | 1.03078695369301 | 2.45482402390088 | 0.998074519377826 |
| ebi-fl187-GCST90094803 | prot-a-255 | Bone morphogenetic protein 6 \|\| id:prot-a-255 | Mushroom liking \|\| id:ebi-fl187-GCST90094803 | Inverse variance weighted | 7 | 0.021848616749356 | 0.221360340499081 | 0.921375209338819 | -0.412017651 | 0.455714884127555 | 1.02208904559613 | 0.662312585715323 | 1.57730056722284 | 0.998074519377826 |
| ebi-fl187-GCST90094803 | prot-a-2568 | RING finger protein 165 \|\| id:prot-a-2568 | Mushroom liking \|\| id:ebi-fl187-GCST90094803 | Inverse variance weighted | 7 | -0.079882775 | 0.238918578159467 | 0.738114365640979 | -0.548163188 | 0.388397638593004 | 0.923224565412887 | 0.57801053271117 | 1.47461603196724 | 0.998074519377826 |
| ebi-fl187-GCST90094803 | prot-a-2573 | E3 ubiquitin-protein ligase RNF8 \|\| id:prot-a-2573 | Mushroom liking \|\| id:ebi-fl187-GCST90094803 | Inverse variance weighted (multiplicative random effects) | 7 | 0.161731479190884 | 0.357234844449109 | 0.650742069580198 | -0.538448816 | 0.861911774311138 | 1.17554454076557 | 0.583652903639181 | 2.36768284490203 | 0.998074519377826 |
| ebi-fl187-GCST90094803 | prot-a-260 | Cytoplasmic tyrosine-protein kinase BMX \|\| id:prot-a-260 | Mushroom liking \|\| id:ebi-fl187-GCST90094803 | Inverse variance weighted | 7 | 0.106726916983657 | 0.282207984031026 | 0.705292929388936 | -0.446400732 | 0.659854565684468 | 1.11263037253051 | 0.639927281514756 | 1.93451096966372 | 0.998074519377826 |
| ebi-fl187-GCST90094803 | prot-a-2623 | Serum amyloid A-1 protein \|\| id:prot-a-2623 | Mushroom liking \|\| id:ebi-fl187-GCST90094803 | Inverse variance weighted | 7 | 0.00294965336574269 | 0.221360340499081 | 0.989368406147908 | -0.430916614 | 0.436815920743942 | 1.00295400787361 | 0.649913102161158 | 1.5477711382718 | 0.999355215041207 |
| ebi-fl187-GCST90094803 | prot-a-2640 | Lysosome membrane protein 2 \|\| id:prot-a-2640 | Mushroom liking \|\| id:ebi-fl187-GCST90094803 | Inverse variance weighted | 7 | -0.209283888 | 0.275904251605352 | 0.448128911451776 | -0.750056221 | 0.331488445489271 | 0.811164923243856 | 0.472339996660283 | 1.39304005028914 | 0.998074519377826 |
| ebi-fl187-GCST90094803 | prot-a-2641 | Scavenger receptor class F member 2 \|\| id:prot-a-2641 | Mushroom liking \|\| id:ebi-fl187-GCST90094803 | Inverse variance weighted | 7 | -0.125955548 | 0.236177809754598 | 0.593820295900914 | -0.588864055 | 0.336952959397213 | 0.881654037442902 | 0.554957327909706 | 1.4006731736784 | 0.998074519377826 |
| ebi-fl187-GCST90094803 | prot-a-2669 | Semaphorin-3A \|\| id:prot-a-2669 | Mushroom liking \|\| id:ebi-fl187-GCST90094803 | Inverse variance weighted | 7 | 0.385615806339057 | 0.221444538580673 | 0.0816195895735491 | -0.048415489 | 0.819647101957176 | 1.47051959796516 | 0.952737852466451 | 2.26969872394753 | 0.998074519377826 |
| ebi-fl187-GCST90094803 | prot-a-2683 | Sentrin-specific protease 7 \|\| id:prot-a-2683 | Mushroom liking \|\| id:ebi-fl187-GCST90094803 | Inverse variance weighted | 7 | -0.22967315 | 0.221444538580673 | 0.299661858470355 | -0.663704446 | 0.204358145123893 | 0.794793337863508 | 0.514940228554956 | 1.22673742481706 | 0.998074519377826 |
| ebi-fl187-GCST90094803 | prot-a-270 | Bromodomain testis-specific protein \|\| id:prot-a-270 | Mushroom liking \|\| id:ebi-fl187-GCST90094803 | Inverse variance weighted | 7 | -0.067069603 | 0.221444538580673 | 0.761986539106122 | -0.501100899 | 0.36696169213239 | 0.935130110756219 | 0.60586329806922 | 1.44334262667786 | 0.998074519377826 |
| ebi-fl187-GCST90094803 | prot-a-2708 | 14-3-3 protein sigma \|\| id:prot-a-2708 | Mushroom liking \|\| id:ebi-fl187-GCST90094803 | Inverse variance weighted | 7 | 0.410953887739244 | 0.221360340499081 | 0.0633833422929421 | -0.02291238 | 0.844820155117443 | 1.50825580586949 | 0.977348115616432 | 2.32755917731959 | 0.998074519377826 |
| ebi-fl187-GCST90094803 | prot-a-2713 | Beta-sarcoglycan \|\| id:prot-a-2713 | Mushroom liking \|\| id:ebi-fl187-GCST90094803 | Inverse variance weighted | 7 | -0.033847458 | 0.221360340499081 | 0.878471927054834 | -0.467713726 | 0.400018809210133 | 0.966718958471672 | 0.626432829707087 | 1.49185275794939 | 0.998074519377826 |
| ebi-fl187-GCST90094803 | prot-a-2720 | Protein shisa-3 homolog \|\| id:prot-a-2720 | Mushroom liking \|\| id:ebi-fl187-GCST90094803 | Inverse variance weighted | 7 | -0.108430394 | 0.253345955802604 | 0.668655933436729 | -0.604988468 | 0.388127679122169 | 0.897241345799439 | 0.546080724211613 | 1.47421799913235 | 0.998074519377826 |
| ebi-fl187-GCST90094803 | prot-a-2730 | Sialic acid-binding Ig-like lectin 8 \|\| id:prot-a-2730 | Mushroom liking \|\| id:ebi-fl187-GCST90094803 | Inverse variance weighted | 7 | 0.0289574082926338 | 0.221360340499081 | 0.8959211015005 | -0.404908859 | 0.462823675670833 | 1.02938075045775 | 0.667037602505184 | 1.588553217 | 0.998074519377826 |
| ebi-fl187-GCST90094803 | prot-a-2761 | Sodium- and chloride-dependent neutral and basic amino acid transporter B(0+) \|\| id:prot-a-2761 | Mushroom liking \|\| id:ebi-fl187-GCST90094803 | Inverse variance weighted | 7 | 0.203987921299885 | 0.221444538580673 | 0.356962018534279 | -0.230043374 | 0.638019216918004 | 1.22628334145802 | 0.794499140897391 | 1.89272808003156 | 0.998074519377826 |
| ebi-fl187-GCST90094803 | prot-a-2770 | Secreted Ly-6/uPAR-related protein 1 \|\| id:prot-a-2770 | Mushroom liking \|\| id:ebi-fl187-GCST90094803 | Inverse variance weighted | 7 | 0.365399907513734 | 0.221360340499081 | 0.0987994847172946 | -0.06846636 | 0.799266174891934 | 1.44109019572791 | 0.933824873570456 | 2.22390836975967 | 0.998074519377826 |
| ebi-fl187-GCST90094803 | prot-a-2784 | N-lysine methyltransferase SMYD2 \|\| id:prot-a-2784 | Mushroom liking \|\| id:ebi-fl187-GCST90094803 | Inverse variance weighted | 7 | -0.067329561 | 0.221360340499081 | 0.761003933986165 | -0.501195828 | 0.366536706659665 | 0.934887048514576 | 0.605805786806294 | 1.44272935735386 | 0.998074519377826 |
| ebi-fl187-GCST90094803 | prot-a-2837 | Serine palmitoyltransferase 2 \|\| id:prot-a-2837 | Mushroom liking \|\| id:ebi-fl187-GCST90094803 | Inverse variance weighted | 7 | 0.253520506599988 | 0.28193281947725 | 0.368533833430414 | -0.29906782 | 0.806108832775397 | 1.28855380307176 | 0.741509118896074 | 2.23917799673535 | 0.998074519377826 |
| ebi-fl187-GCST90094803 | prot-a-2858 | Alpha-2,8-sialyltransferase 8B \|\| id:prot-a-2858 | Mushroom liking \|\| id:ebi-fl187-GCST90094803 | Inverse variance weighted | 7 | 0.00283488092112749 | 0.221360340499081 | 0.989782063553476 | -0.431031386 | 0.436701148299327 | 1.00283890299585 | 0.649838514326032 | 1.54759350698834 | 0.999355215041207 |
| ebi-fl187-GCST90094803 | prot-a-2911 | Synaptotagmin-11 \|\| id:prot-a-2911 | Mushroom liking \|\| id:ebi-fl187-GCST90094803 | Inverse variance weighted | 7 | 0.148622988865339 | 0.221444538580673 | 0.502123577020953 | -0.285408307 | 0.582654284483458 | 1.16023548504561 | 0.751707264498431 | 1.79078538193617 | 0.998074519377826 |
| ebi-fl187-GCST90094803 | prot-a-2962 | Transforming growth factor beta-1 \|\| id:prot-a-2962 | Mushroom liking \|\| id:ebi-fl187-GCST90094803 | Inverse variance weighted | 7 | 0.33169339548958 | 0.221360340499081 | 0.13402107367076 | -0.102172872 | 0.765559662867779 | 1.39332558310685 | 0.902873456737402 | 2.1501974236293 | 0.998074519377826 |
| ebi-fl187-GCST90094803 | prot-a-2978 | Tyrosine-protein kinase receptor Tie-1, soluble \|\| id:prot-a-2978 | Mushroom liking \|\| id:ebi-fl187-GCST90094803 | Inverse variance weighted | 7 | 0.15993060585886 | 0.221360340499081 | 0.46999334502307 | -0.273935662 | 0.593796873237059 | 1.17342943903828 | 0.76038099544511 | 1.81085095057599 | 0.998074519377826 |
| ebi-fl187-GCST90094803 | prot-a-299 | Uncharacterized protein C1orf226 \|\| id:prot-a-299 | Mushroom liking \|\| id:ebi-fl187-GCST90094803 | Inverse variance weighted | 7 | -0.090531732 | 0.229830748884224 | 0.693650373626569 | -0.541 | 0.35993653605109 | 0.913445348210692 | 0.582165795646022 | 1.43323845270203 | 0.998074519377826 |
| ebi-fl187-GCST90094803 | prot-a-3015 | Transmembrane protein 70, mitochondrial \|\| id:prot-a-3015 | Mushroom liking \|\| id:ebi-fl187-GCST90094803 | Inverse variance weighted | 7 | -0.023071973 | 0.221360340499081 | 0.916988316791815 | -0.45693824 | 0.410794294638929 | 0.977192150046419 | 0.633219446413772 | 1.50801511785595 | 0.998074519377826 |
| ebi-fl187-GCST90094803 | prot-a-3016 | Transmembrane protein 87B \|\| id:prot-a-3016 | Mushroom liking \|\| id:ebi-fl187-GCST90094803 | Inverse variance weighted | 7 | 0.279548099776695 | 0.221360340499081 | 0.206637905724663 | -0.154318168 | 0.713414367154894 | 1.32253202476012 | 0.856999308215184 | 2.04094792113523 | 0.998074519377826 |
| ebi-fl187-GCST90094803 | prot-a-3123 | Thioredoxin domain-containing protein 12 \|\| id:prot-a-3123 | Mushroom liking \|\| id:ebi-fl187-GCST90094803 | Inverse variance weighted | 7 | 0.0209453585348337 | 0.221360340499081 | 0.924615791014514 | -0.412920909 | 0.454811625913033 | 1.02116625209287 | 0.661714616532638 | 1.5758765007754 | 0.998074519377826 |
| ebi-fl187-GCST90094803 | prot-a-3133 | PolyUbiquitin K48-linked \|\| id:prot-a-3133 | Mushroom liking \|\| id:ebi-fl187-GCST90094803 | Inverse variance weighted | 7 | -0.101138369 | 0.236210083679417 | 0.668526440855851 | -0.564110133 | 0.361833395511477 | 0.903807965680721 | 0.568866137072483 | 1.43595968470143 | 0.998074519377826 |
| ebi-fl187-GCST90094803 | prot-a-3135 | Ubiquitin-conjugating enzyme E2 B \|\| id:prot-a-3135 | Mushroom liking \|\| id:ebi-fl187-GCST90094803 | Inverse variance weighted | 7 | -0.268754588 | 0.221444538580673 | 0.224884123921885 | -0.702785884 | 0.165276707324705 | 0.764330808361518 | 0.495203800030928 | 1.17971951058147 | 0.998074519377826 |
| ebi-fl187-GCST90094803 | prot-a-3162 | NKG2D ligand 3 \|\| id:prot-a-3162 | Mushroom liking \|\| id:ebi-fl187-GCST90094803 | Inverse variance weighted | 7 | 0.312136927892587 | 0.221360340499081 | 0.15851454297145 | -0.121729339 | 0.746003195270786 | 1.36634177052032 | 0.885387975639988 | 2.10855566738315 | 0.998074519377826 |
| ebi-fl187-GCST90094803 | prot-a-3188 | Vesicle-associated membrane protein 8 \|\| id:prot-a-3188 | Mushroom liking \|\| id:ebi-fl187-GCST90094803 | Inverse variance weighted | 7 | 0.514430679390559 | 0.283208227371405 | 0.0693029435482104 | -0.040657446 | 1.06951880503851 | 1.67268593608908 | 0.960157979329602 | 2.91397697152266 | 0.998074519377826 |
| ebi-fl187-GCST90094803 | prot-a-3201 | Vitrin \|\| id:prot-a-3201 | Mushroom liking \|\| id:ebi-fl187-GCST90094803 | Inverse variance weighted | 7 | -0.070641978 | 0.221360340499081 | 0.749630494888558 | -0.504508246 | 0.363224289099681 | 0.931795435422015 | 0.603802424897478 | 1.43795834145702 | 0.998074519377826 |
| ebi-fl187-GCST90094803 | prot-a-3232 | WNT1-inducible-signaling pathway protein 3 \|\| id:prot-a-3232 | Mushroom liking \|\| id:ebi-fl187-GCST90094803 | Inverse variance weighted | 7 | 0.162250319865455 | 0.284278120675206 | 0.568172500929478 | -0.394934797 | 0.719435436388858 | 1.1761546193412 | 0.673723966870998 | 2.05327367975691 | 0.998074519377826 |
| ebi-fl187-GCST90094803 | prot-a-327 | Carbonic anhydrase-related protein 10 \|\| id:prot-a-327 | Mushroom liking \|\| id:ebi-fl187-GCST90094803 | Inverse variance weighted | 7 | 0.0431293298465001 | 0.221360340499081 | 0.845520042042367 | -0.390736938 | 0.476995597224699 | 1.04407291590925 | 0.676558109678082 | 1.61122635017105 | 0.998074519377826 |
| ebi-fl187-GCST90094803 | prot-a-332 | Carbonic anhydrase 5A, mitochondrial \|\| id:prot-a-332 | Mushroom liking \|\| id:ebi-fl187-GCST90094803 | Inverse variance weighted | 7 | -0.163935447 | 0.29751591053107 | 0.58162404371318 | -0.747066631 | 0.419195738069414 | 0.848796812856099 | 0.473754212306742 | 1.52073799197842 | 0.998074519377826 |
| ebi-fl187-GCST90094803 | prot-a-338 | Cell adhesion molecule 3 \|\| id:prot-a-338 | Mushroom liking \|\| id:ebi-fl187-GCST90094803 | Inverse variance weighted | 7 | 0.165689224960046 | 0.221444538580673 | 0.454327490318253 | -0.268342071 | 0.599720520578165 | 1.18020626607505 | 0.764646173341538 | 1.82160962683674 | 0.998074519377826 |
| ebi-fl187-GCST90094803 | prot-a-34 | A disintegrin and metalloproteinase with thrombospondin motifs 3 \|\| id:prot-a-34 | Mushroom liking \|\| id:ebi-fl187-GCST90094803 | Inverse variance weighted | 7 | 0.0444338048353835 | 0.277992516578617 | 0.873008584455035 | -0.500431528 | 0.589299137329473 | 1.04543577162696 | 0.606268981421885 | 1.80272451022316 | 0.998074519377826 |
| ebi-fl187-GCST90094803 | prot-a-358 | Cysteine--tRNA ligase, cytoplasmic \|\| id:prot-a-358 | Mushroom liking \|\| id:ebi-fl187-GCST90094803 | Inverse variance weighted | 7 | 0.0265543739613764 | 0.224303153167852 | 0.905761744428485 | -0.413079806 | 0.466188554170366 | 1.02691008291285 | 0.661609480150889 | 1.59390750892441 | 0.998074519377826 |
| ebi-fl187-GCST90094803 | prot-a-382 | Coiled-coil domain-containing protein 167 \|\| id:prot-a-382 | Mushroom liking \|\| id:ebi-fl187-GCST90094803 | Inverse variance weighted (multiplicative random effects) | 7 | 0.163072566840755 | 0.331718196723209 | 0.623002353753458 | -0.487095099 | 0.813240232418244 | 1.17712210662167 | 0.614408600684494 | 2.25520354427618 | 0.998074519377826 |
| ebi-fl187-GCST90094803 | prot-a-40 | Pituitary adenylate cyclase-activating polypeptide 27 \|\| id:prot-a-40 | Mushroom liking \|\| id:ebi-fl187-GCST90094803 | Inverse variance weighted | 7 | -0.068271727 | 0.221360340499081 | 0.75776355291537 | -0.502137994 | 0.365594540277005 | 0.934006644174693 | 0.605235285755201 | 1.44137070639222 | 0.998074519377826 |
| ebi-fl187-GCST90094803 | prot-a-403 | C-C motif chemokine 25 \|\| id:prot-a-403 | Mushroom liking \|\| id:ebi-fl187-GCST90094803 | Inverse variance weighted | 7 | 0.35196155328476 | 0.319281835761221 | 0.27030787708298 | -0.273830845 | 0.977753951376753 | 1.42185385705735 | 0.760460700258126 | 2.65847845936371 | 0.998074519377826 |
| ebi-fl187-GCST90094803 | prot-a-415 | Cyclin-C \|\| id:prot-a-415 | Mushroom liking \|\| id:ebi-fl187-GCST90094803 | Inverse variance weighted | 7 | -0.104652137 | 0.233085970932824 | 0.653442375813247 | -0.56150064 | 0.35219636556149 | 0.900637766235059 | 0.570352527233891 | 1.42218776500001 | 0.998074519377826 |
| ebi-fl187-GCST90094803 | prot-a-44 | Adhesion G protein-coupled receptor F5 \|\| id:prot-a-44 | Mushroom liking \|\| id:ebi-fl187-GCST90094803 | Inverse variance weighted | 7 | -0.189166512 | 0.221444538580673 | 0.392972701568875 | -0.623197807 | 0.244864783742582 | 0.827648681886012 | 0.536226942414031 | 1.27744856971165 | 0.998074519377826 |
| ebi-fl187-GCST90094803 | prot-a-455 | CD59 glycoprotein \|\| id:prot-a-455 | Mushroom liking \|\| id:ebi-fl187-GCST90094803 | Inverse variance weighted | 7 | 0.137048295628214 | 0.221360340499081 | 0.535838256157746 | -0.296817972 | 0.570914563006413 | 1.14688353664378 | 0.743179279674057 | 1.76988498280175 | 0.998074519377826 |
| ebi-fl187-GCST90094803 | prot-a-478 | Cadherin-12 \|\| id:prot-a-478 | Mushroom liking \|\| id:ebi-fl187-GCST90094803 | Inverse variance weighted | 7 | 0.256408747496651 | 0.221444538580673 | 0.246908319370992 | -0.177622548 | 0.69044004311477 | 1.29228083655773 | 0.837258388605799 | 1.9945930470941 | 0.998074519377826 |
| ebi-fl187-GCST90094803 | prot-a-516 | Centrin-2 \|\| id:prot-a-516 | Mushroom liking \|\| id:ebi-fl187-GCST90094803 | Inverse variance weighted | 7 | 0.121047499221687 | 0.252520923964249 | 0.631685166613594 | -0.373893512 | 0.615988510191615 | 1.12867852244547 | 0.688050177188451 | 1.85148590795402 | 0.998074519377826 |
| ebi-fl187-GCST90094803 | prot-a-519 | Cryptic protein \|\| id:prot-a-519 | Mushroom liking \|\| id:ebi-fl187-GCST90094803 | Inverse variance weighted | 7 | -0.081229195 | 0.221444538580673 | 0.713756962126935 | -0.515260491 | 0.352802100601837 | 0.921982353466213 | 0.597344971579343 | 1.42304949492688 | 0.998074519377826 |
| ebi-fl187-GCST90094803 | prot-a-583 | Chloride intracellular channel protein 4 \|\| id:prot-a-583 | Mushroom liking \|\| id:ebi-fl187-GCST90094803 | Inverse variance weighted | 7 | 0.0381920870457608 | 0.264372838863968 | 0.885134890691134 | -0.479978677 | 0.556362851219139 | 1.03893077885685 | 0.618796586186107 | 1.74431660961275 | 0.998074519377826 |
| ebi-fl187-GCST90094803 | prot-a-602 | CCR4-NOT transcription complex subunit 1 \|\| id:prot-a-602 | Mushroom liking \|\| id:ebi-fl187-GCST90094803 | Inverse variance weighted | 7 | 0.155399982825848 | 0.221444538580673 | 0.482831485972066 | -0.278631313 | 0.589431278443967 | 1.1681250976531 | 0.75681888122415 | 1.80296273998869 | 0.998074519377826 |
| ebi-fl187-GCST90094803 | prot-a-606 | Ciliary neurotrophic factor receptor subunit alpha \|\| id:prot-a-606 | Mushroom liking \|\| id:ebi-fl187-GCST90094803 | Inverse variance weighted | 7 | -0.225616782 | 0.238818409417061 | 0.344801299879205 | -0.693700864 | 0.242467300423393 | 0.79802386015633 | 0.49972323466156 | 1.2743895764825 | 0.998074519377826 |
| ebi-fl187-GCST90094803 | prot-a-626 | Collagen alpha-2(IX) chain \|\| id:prot-a-626 | Mushroom liking \|\| id:ebi-fl187-GCST90094803 | Inverse variance weighted | 7 | -0.146072267 | 0.273373900403888 | 0.593112252187494 | -0.681885111 | 0.389740578261719 | 0.864095255762603 | 0.505662862540627 | 1.47659768265353 | 0.998074519377826 |
| ebi-fl187-GCST90094803 | prot-a-647 | Probable serine carboxypeptidase CPVL \|\| id:prot-a-647 | Mushroom liking \|\| id:ebi-fl187-GCST90094803 | Inverse variance weighted | 7 | 0.0642997575389276 | 0.226907565054123 | 0.776889977186434 | -0.38043907 | 0.509038585045008 | 1.06641201589666 | 0.683561212114463 | 1.66369092846997 | 0.998074519377826 |
| ebi-fl187-GCST90094803 | prot-a-651 | Cellular retinoic acid-binding protein 2 \|\| id:prot-a-651 | Mushroom liking \|\| id:ebi-fl187-GCST90094803 | Inverse variance weighted | 7 | 0.0582546539582333 | 0.221360340499081 | 0.792422152901694 | -0.375611613 | 0.492120921336433 | 1.059984891 | 0.686869051965947 | 1.63578190818099 | 0.998074519377826 |
| ebi-fl187-GCST90094803 | prot-a-671 | Cytotoxic and regulatory T-cell molecule \|\| id:prot-a-671 | Mushroom liking \|\| id:ebi-fl187-GCST90094803 | Inverse variance weighted | 7 | 0.0958945363946764 | 0.275668261033612 | 0.727943759859427 | -0.444415255 | 0.636204328020557 | 1.10064298009592 | 0.641199104254187 | 1.88929610412274 | 0.998074519377826 |
| ebi-fl187-GCST90094803 | prot-a-688 | Chorionic somatomammotropin hormone \|\| id:prot-a-688 | Mushroom liking \|\| id:ebi-fl187-GCST90094803 | Inverse variance weighted | 7 | 0.124716661913892 | 0.221444538580673 | 0.573301476903864 | -0.309314634 | 0.558747957532011 | 1.13282743443341 | 0.733949808347088 | 1.74848195559185 | 0.998074519377826 |
| ebi-fl187-GCST90094803 | prot-a-69 | Aldo-keto reductase family 1 member C1 \|\| id:prot-a-69 | Mushroom liking \|\| id:ebi-fl187-GCST90094803 | Inverse variance weighted | 7 | 0.00565589631614894 | 0.221360340499081 | 0.979615764176701 | -0.428210371 | 0.439522163694348 | 1.00567192109497 | 0.651674306961411 | 1.55196545586496 | 0.998460104120911 |
| ebi-fl187-GCST90094803 | prot-a-741 | Stromal cell-derived factor 1 \|\| id:prot-a-741 | Mushroom liking \|\| id:ebi-fl187-GCST90094803 | Inverse variance weighted | 7 | 0.148447192878913 | 0.266212079549496 | 0.577098731626602 | -0.373328483 | 0.670222868795925 | 1.16003153823107 | 0.68843905514584 | 1.95467290769212 | 0.998074519377826 |
| ebi-fl187-GCST90094803 | prot-a-742 | Growth-regulated alpha protein \|\| id:prot-a-742 | Mushroom liking \|\| id:ebi-fl187-GCST90094803 | Inverse variance weighted | 7 | 0.10679114756872 | 0.221444538580673 | 0.629629904990275 | -0.327240148 | 0.54082244318684 | 1.11270183972546 | 0.720910596963443 | 1.71741876086087 | 0.998074519377826 |
| ebi-fl187-GCST90094803 | prot-a-788 | Beta-defensin 112 \|\| id:prot-a-788 | Mushroom liking \|\| id:ebi-fl187-GCST90094803 | Inverse variance weighted | 7 | 0.0585074341497529 | 0.221360340499081 | 0.791542160260472 | -0.375358833 | 0.492373701527952 | 1.06025286774389 | 0.687042700803021 | 1.63619545371089 | 0.998074519377826 |
| ebi-fl187-GCST90094803 | prot-a-838 | DnaJ homolog subfamily B member 1 \|\| id:prot-a-838 | Mushroom liking \|\| id:ebi-fl187-GCST90094803 | Inverse variance weighted | 7 | -0.080784655 | 0.221360340499081 | 0.715151432920154 | -0.514650922 | 0.353081612651365 | 0.922392302881253 | 0.597709205276614 | 1.42344731000225 | 0.998074519377826 |
| ebi-fl187-GCST90094803 | prot-a-85 | Junctional adhesion molecule-like \|\| id:prot-a-85 | Mushroom liking \|\| id:ebi-fl187-GCST90094803 | Inverse variance weighted | 7 | 0.337505444316607 | 0.221360340499081 | 0.127336948097998 | -0.096360823 | 0.771371711694807 | 1.40144723829368 | 0.908136280431926 | 2.16273086324316 | 0.998074519377826 |
| ebi-fl187-GCST90094803 | prot-a-856 | Dipeptidase 1 \|\| id:prot-a-856 | Mushroom liking \|\| id:ebi-fl187-GCST90094803 | Inverse variance weighted | 7 | 0.229369051506795 | 0.283287626741775 | 0.418130759990465 | -0.325874697 | 0.784612799920674 | 1.2578061486188 | 0.721895637520904 | 2.19155820491774 | 0.998074519377826 |
| ebi-fl187-GCST90094803 | prot-a-860 | Dipeptidyl peptidase 2 \|\| id:prot-a-860 | Mushroom liking \|\| id:ebi-fl187-GCST90094803 | Inverse variance weighted | 7 | 0.0389151430208025 | 0.221444538580673 | 0.860503602320412 | -0.395116153 | 0.472946438638922 | 1.03968225561129 | 0.673601794106825 | 1.60471542993183 | 0.998074519377826 |
| ebi-fl187-GCST90094803 | prot-a-861 | Dermatopontin \|\| id:prot-a-861 | Mushroom liking \|\| id:ebi-fl187-GCST90094803 | Inverse variance weighted | 7 | -0.2814935 | 0.221444538580673 | 0.203668424670233 | -0.715524795 | 0.152537795869971 | 0.754655821112237 | 0.488935453395307 | 1.16478648538121 | 0.998074519377826 |
| ebi-fl187-GCST90094803 | prot-a-877 | Dual specificity protein phosphatase 6 \|\| id:prot-a-877 | Mushroom liking \|\| id:ebi-fl187-GCST90094803 | Inverse variance weighted | 7 | -0.318383721 | 0.221360340499081 | 0.150347487388759 | -0.752249988 | 0.115482546581837 | 0.727323645653056 | 0.471304928352317 | 1.12241492440029 | 0.998074519377826 |
| ebi-fl187-GCST90094803 | prot-a-879 | Dynactin-associated protein \|\| id:prot-a-879 | Mushroom liking \|\| id:ebi-fl187-GCST90094803 | Inverse variance weighted | 7 | -0.002239603 | 0.221444538580673 | 0.99193064800008 | -0.436270898 | 0.431791692948103 | 0.997762903368851 | 0.64644258202457 | 1.54001428591101 | 0.999355215041207 |
| ebi-fl187-GCST90094803 | prot-a-937 | EMI domain-containing protein 1 \|\| id:prot-a-937 | Mushroom liking \|\| id:ebi-fl187-GCST90094803 | Inverse variance weighted | 7 | 0.553164258290779 | 0.269446117522701 | 0.0400756806735121 | 0.0250498679462844 | 1.08127864863527 | 1.73874616438305 | 1.02536625215869 | 2.94844716977175 | 0.998074519377826 |
| ebi-fl187-GCST90094803 | prot-b-1 | SRC proto-oncogene, non-receptor tyrosine kinase \|\| id:prot-b-1 | Mushroom liking \|\| id:ebi-fl187-GCST90094803 | Inverse variance weighted | 7 | 0.00945379808494603 | 0.274597014814068 | 0.972535937069877 | -0.528756351 | 0.547663947120519 | 1.00949862638861 | 0.58933744306486 | 1.72920877278849 | 0.998074519377826 |
| ebi-fl187-GCST90094803 | prot-b-10 | coagulation factor III, tissue factor \|\| id:prot-b-10 | Mushroom liking \|\| id:ebi-fl187-GCST90094803 | Inverse variance weighted (multiplicative random effects) | 7 | 0.805934842001347 | 0.406475022420245 | 0.0473963262240409 | 0.00924379805766695 | 1.60262588594503 | 2.23878843431358 | 1.00928665390742 | 4.96605561384545 | 0.998074519377826 |
| ebi-fl187-GCST90094803 | prot-b-29 | myeloperoxidase \|\| id:prot-b-29 | Mushroom liking \|\| id:ebi-fl187-GCST90094803 | Inverse variance weighted | 7 | 0.275182316607693 | 0.365867961082825 | 0.451969520042822 | -0.441918887 | 0.992283520330029 | 1.31677072215583 | 0.642801772847503 | 2.69738698299782 | 0.998074519377826 |
| ebi-fl187-GCST90094803 | prot-b-33 | adrenomedullin \|\| id:prot-b-33 | Mushroom liking \|\| id:ebi-fl187-GCST90094803 | Inverse variance weighted | 7 | -0.228219576 | 0.274597014814068 | 0.40591303108755 | -0.766429725 | 0.309990572709046 | 0.795949468985689 | 0.46466910662272 | 1.36341226078752 | 0.998074519377826 |
| ebi-fl187-GCST90094803 | prot-c-2201_17_6 | Endostatin \|\| id:prot-c-2201_17_6 | Mushroom liking \|\| id:ebi-fl187-GCST90094803 | Inverse variance weighted | 2 | 1.47408201245557 | 0.846509464020134 | 0.0816196068275462 | -0.185076537 | 3.13324056193503 | 4.36702505770889 | 0.831040676037899 | 22.9482243222805 | 0.998074519377826 |
| ebi-fl187-GCST90094803 | prot-c-2515_14_3 | GFRa-2 \|\| id:prot-c-2515_14_3 | Mushroom liking \|\| id:ebi-fl187-GCST90094803 | Inverse variance weighted | 2 | 0.398577988090307 | 0.867591737045149 | 0.645941698195198 | -1.301901817 | 2.0990577926988 | 1.48970481276204 | 0.272013980116943 | 8.15847931129246 | 0.998074519377826 |
| ebi-fl187-GCST90094803 | prot-c-2570_72_5 | IGFBP-2 \|\| id:prot-c-2570_72_5 | Mushroom liking \|\| id:ebi-fl187-GCST90094803 | Inverse variance weighted | 2 | 1.31178703208256 | 0.747749226720321 | 0.0793766963916339 | -0.153801452 | 2.77737551645439 | 3.71280268486676 | 0.857442247307169 | 16.0767723074596 | 0.998074519377826 |
| ebi-fl187-GCST90094803 | prot-c-2585_2_5 | PRL \|\| id:prot-c-2585_2_5 | Mushroom liking \|\| id:ebi-fl187-GCST90094803 | Inverse variance weighted | 2 | 0.766703888800271 | 1.09203701681601 | 0.482625539595297 | -1.373688664 | 2.90709644175964 | 2.15265914339461 | 0.253171370915478 | 18.3035758383102 | 0.998074519377826 |
| ebi-fl187-GCST90094803 | prot-c-2614_28_2 | Ephrin-A4 \|\| id:prot-c-2614_28_2 | Mushroom liking \|\| id:ebi-fl187-GCST90094803 | Inverse variance weighted | 2 | 1.6403143654632 | 0.8386856826596 | 0.0504869052629092 | -0.003509573 | 3.28413830347602 | 5.15679037424446 | 0.996496578801819 | 26.6859791890856 | 0.998074519377826 |
| ebi-fl187-GCST90094803 | prot-c-2642_4_1 | PAFAH beta subunit \|\| id:prot-c-2642_4_1 | Mushroom liking \|\| id:ebi-fl187-GCST90094803 | Inverse variance weighted | 2 | -0.689483708 | 0.865777427761287 | 0.425813964381587 | -2.386407466 | 1.00744005039043 | 0.501835095627938 | 0.0919594586011947 | 2.73858140352982 | 0.998074519377826 |
| ebi-fl187-GCST90094803 | prot-c-2658_27_1 | TrkC \|\| id:prot-c-2658_27_1 | Mushroom liking \|\| id:ebi-fl187-GCST90094803 | Inverse variance weighted | 2 | 1.41573161002089 | 1.05457880677501 | 0.179446355389577 | -0.651242851 | 3.4827060712999 | 4.11949923067265 | 0.521397354540222 | 32.5476793538342 | 0.998074519377826 |
| ebi-fl187-GCST90094803 | prot-c-2685_21_2 | IGFBP-5 \|\| id:prot-c-2685_21_2 | Mushroom liking \|\| id:ebi-fl187-GCST90094803 | Inverse variance weighted | 2 | -0.105649919 | 0.8487315506531 | 0.90093540714801 | -1.769163758 | 1.55786392050985 | 0.899739574884574 | 0.170475487989667 | 4.74866687381203 | 0.998074519377826 |
| ebi-fl187-GCST90094803 | prot-c-2752_62_2 | BMP-14 \|\| id:prot-c-2752_62_2 | Mushroom liking \|\| id:ebi-fl187-GCST90094803 | Inverse variance weighted | 2 | 0.236217728687932 | 1.07024839685805 | 0.825315912707015 | -1.861469129 | 2.33390458652972 | 1.2664500226523 | 0.155444095083296 | 10.3181510948778 | 0.998074519377826 |
| ebi-fl187-GCST90094803 | prot-c-2768_56_2 | Hemopexin \|\| id:prot-c-2768_56_2 | Mushroom liking \|\| id:ebi-fl187-GCST90094803 | Inverse variance weighted | 2 | 1.42055814237075 | 1.31846136044376 | 0.28128539018156 | -1.163626124 | 4.00474240884051 | 4.13943018698579 | 0.312351499578315 | 54.8576917225044 | 0.998074519377826 |
| ebi-fl187-GCST90094803 | prot-c-2864_2_3 | MEK1 \|\| id:prot-c-2864_2_3 | Mushroom liking \|\| id:ebi-fl187-GCST90094803 | Inverse variance weighted | 2 | 0.696677066380979 | 0.864005392225589 | 0.420050196137568 | -0.996773502 | 2.39012763514313 | 2.00707224640983 | 0.369068320238756 | 10.9148869773016 | 0.998074519377826 |
| ebi-fl187-GCST90094803 | prot-c-2888_49_2 | C7 \|\| id:prot-c-2888_49_2 | Mushroom liking \|\| id:ebi-fl187-GCST90094803 | Inverse variance weighted | 2 | 0.240311280967071 | 0.845814396562297 | 0.776319784393927 | -1.417484936 | 1.89810749822917 | 1.27164492758207 | 0.242322708175729 | 6.67325334063422 | 0.998074519377826 |
| ebi-fl187-GCST90094803 | prot-c-2911_27_2 | Midkine \|\| id:prot-c-2911_27_2 | Mushroom liking \|\| id:ebi-fl187-GCST90094803 | Inverse variance weighted | 2 | -0.58737141 | 1.08569894647753 | 0.588502326034309 | -2.715341345 | 1.54059852493524 | 0.555786300539774 | 0.0661823581169454 | 4.66738298024768 | 0.998074519377826 |
| ebi-fl187-GCST90094803 | prot-c-2957_21_2 | NG36 \|\| id:prot-c-2957_21_2 | Mushroom liking \|\| id:ebi-fl187-GCST90094803 | Inverse variance weighted | 2 | -1.371998413 | 0.860260099510054 | 0.110742508561902 | -3.058108208 | 0.314111381927339 | 0.253599655942813 | 0.0469764809351405 | 1.36904221461604 | 0.998074519377826 |
| ebi-fl187-GCST90094803 | prot-c-2985_35_1 | Gro-a \|\| id:prot-c-2985_35_1 | Mushroom liking \|\| id:ebi-fl187-GCST90094803 | Inverse variance weighted | 2 | 0.21062120968315 | 0.865373002380214 | 0.807705013183707 | -1.485509875 | 1.90675229434837 | 1.23444467080187 | 0.226386882236764 | 6.73119232976327 | 0.998074519377826 |
| ebi-fl187-GCST90094803 | prot-c-2994_71_2 | IL-1Rrp2 \|\| id:prot-c-2994_71_2 | Mushroom liking \|\| id:ebi-fl187-GCST90094803 | Inverse variance weighted | 2 | 0.409661492390219 | 0.865273967367797 | 0.635894199058894 | -1.286275484 | 2.1055984684311 | 1.50630780214747 | 0.276297945275641 | 8.21201617169741 | 0.998074519377826 |
| ebi-fl187-GCST90094803 | prot-c-3060_43_2 | C9 \|\| id:prot-c-3060_43_2 | Mushroom liking \|\| id:ebi-fl187-GCST90094803 | Inverse variance weighted | 2 | 0.19641752612931 | 0.854733846251307 | 0.818247468145578 | -1.478860813 | 1.87169586478187 | 1.21703494315645 | 0.22789715815195 | 6.49930900795289 | 0.998074519377826 |
| ebi-fl187-GCST90094803 | prot-c-3173_49_2 | ASAHL \|\| id:prot-c-3173_49_2 | Mushroom liking \|\| id:ebi-fl187-GCST90094803 | Inverse variance weighted | 2 | 0.717035544599946 | 0.864541656387886 | 0.40688811578436 | -0.977466102 | 2.4115371911202 | 2.04835195328016 | 0.376263304811011 | 11.1510893325463 | 0.998074519377826 |
| ebi-fl187-GCST90094803 | prot-c-3197_70_2 | IDE \|\| id:prot-c-3197_70_2 | Mushroom liking \|\| id:ebi-fl187-GCST90094803 | Inverse variance weighted | 2 | -1.049266034 | 0.869406554834799 | 0.227479844854092 | -2.753302881 | 0.654770813928461 | 0.35019468595936 | 0.0637170633986993 | 1.92470135208205 | 0.998074519377826 |
| ebi-fl187-GCST90094803 | prot-c-3389_7_2 | PCI \|\| id:prot-c-3389_7_2 | Mushroom liking \|\| id:ebi-fl187-GCST90094803 | Inverse variance weighted | 2 | -0.05889124 | 0.841420855413876 | 0.944201435315171 | -1.708076116 | 1.59029363679722 | 0.942809303696418 | 0.181214092281357 | 4.90518906088393 | 0.998074519377826 |
| ebi-fl187-GCST90094803 | prot-c-3404_51_2 | TPSG1 \|\| id:prot-c-3404_51_2 | Mushroom liking \|\| id:ebi-fl187-GCST90094803 | Inverse variance weighted | 2 | -0.252702905 | 0.867741867215059 | 0.77088384412534 | -1.953476965 | 1.44807115452713 | 0.776698600653608 | 0.141780248625536 | 4.2548995512808 | 0.998074519377826 |
| ebi-fl187-GCST90094803 | prot-c-3452_17_2 | LCK \|\| id:prot-c-3452_17_2 | Mushroom liking \|\| id:ebi-fl187-GCST90094803 | Inverse variance weighted | 2 | -1.562509516 | 0.867795561370076 | 0.0717737964119895 | -3.263388816 | 0.138369784308784 | 0.209609392503541 | 0.0382585269689296 | 1.14840013211655 | 0.998074519377826 |
| ebi-fl187-GCST90094803 | prot-c-3580_25_8 | a1-Antitrypsin \|\| id:prot-c-3580_25_8 | Mushroom liking \|\| id:ebi-fl187-GCST90094803 | Inverse variance weighted | 2 | -0.024189109 | 0.861084355124858 | 0.97758921243977 | -1.711914445 | 1.66353622701299 | 0.976101102768616 | 0.180519866225141 | 5.27794188390229 | 0.998074519377826 |
| ebi-fl187-GCST90094803 | prot-c-3620_67_5 | IL22RA1 \|\| id:prot-c-3620_67_5 | Mushroom liking \|\| id:ebi-fl187-GCST90094803 | Inverse variance weighted | 2 | -0.236644966 | 0.868917716230593 | 0.785357138467949 | -1.93972369 | 1.46643375795311 | 0.789271456593685 | 0.143743662149588 | 4.33375233994832 | 0.998074519377826 |
| ebi-fl187-GCST90094803 | prot-c-3623_84_4 | LY86 \|\| id:prot-c-3623_84_4 | Mushroom liking \|\| id:ebi-fl187-GCST90094803 | Inverse variance weighted | 2 | -0.933074105 | 0.864569339751192 | 0.280482664585282 | -2.627630011 | 0.761481801169872 | 0.393342673320465 | 0.0722494900132387 | 2.14144706940548 | 0.998074519377826 |
| ebi-fl187-GCST90094803 | prot-c-3635_76_4 | OCAD1 \|\| id:prot-c-3635_76_4 | Mushroom liking \|\| id:ebi-fl187-GCST90094803 | Inverse variance weighted | 2 | -1.491889525 | 1.03587278411084 | 0.149803849396359 | -3.522200182 | 0.538421131629243 | 0.224947210292441 | 0.02953438261088 | 1.71329965095363 | 0.998074519377826 |
| ebi-fl187-GCST90094803 | prot-c-3707_12_2 | Albumin \|\| id:prot-c-3707_12_2 | Mushroom liking \|\| id:ebi-fl187-GCST90094803 | Inverse variance weighted | 2 | 0.454379995062713 | 0.794534541901295 | 0.567401926018753 | -1.102907707 | 2.01166769718925 | 1.57519645063724 | 0.3319045979064 | 7.47577428499466 | 0.998074519377826 |
| ebi-fl187-GCST90094803 | prot-c-4336_2_1 | SAA \|\| id:prot-c-4336_2_1 | Mushroom liking \|\| id:ebi-fl187-GCST90094803 | Inverse variance weighted | 2 | -1.571076879 | 0.860281066455477 | 0.0678146114248807 | -3.257227769 | 0.115074011401852 | 0.207821263488498 | 0.0384949671694958 | 1.12195647207047 | 0.998074519377826 |
| ebi-fl187-GCST90094803 | prot-c-4452_9_2 | LRRT1 \|\| id:prot-c-4452_9_2 | Mushroom liking \|\| id:ebi-fl187-GCST90094803 | Inverse variance weighted | 2 | -1.0580121 | 1.02580011898319 | 0.302352431994251 | -3.068580334 | 0.952556132842938 | 0.347145214715594 | 0.0464871042145369 | 2.5923275311748 | 0.998074519377826 |
| ebi-fl187-GCST90094803 | prot-c-4455_89_2 | MFGM \|\| id:prot-c-4455_89_2 | Mushroom liking \|\| id:ebi-fl187-GCST90094803 | Inverse variance weighted | 2 | 0.264989496780711 | 0.846296709010854 | 0.754192123886817 | -1.393752053 | 1.92373104644198 | 1.3034172856289 | 0.248142511955485 | 6.84645531750308 | 0.998074519377826 |
| ebi-fl187-GCST90094803 | prot-c-4462_8_2 | RASA1 \|\| id:prot-c-4462_8_2 | Mushroom liking \|\| id:ebi-fl187-GCST90094803 | Inverse variance weighted | 2 | 0.605713113437344 | 1.28663824355725 | 0.63780364267705 | -1.916097844 | 3.12752407080956 | 1.83255856517785 | 0.147180163009125 | 22.817415242287 | 0.998074519377826 |
| ebi-fl187-GCST90094803 | prot-c-4467_49_2 | SPARCL1 \|\| id:prot-c-4467_49_2 | Mushroom liking \|\| id:ebi-fl187-GCST90094803 | Inverse variance weighted | 2 | 1.01338133526796 | 0.856723413875944 | 0.236865803570255 | -0.665796556 | 2.69255922646481 | 2.75490052608268 | 0.513864043189379 | 14.7694259000986 | 0.998074519377826 |
| ebi-fl187-GCST90094803 | prot-c-4811_33_3 | ITI heavy chain H4 \|\| id:prot-c-4811_33_3 | Mushroom liking \|\| id:ebi-fl187-GCST90094803 | Inverse variance weighted | 2 | -0.775037789 | 0.868654082819661 | 0.372270555671589 | -2.477599791 | 0.927524213240633 | 0.460686371753152 | 0.0839444682169373 | 2.52824203461047 | 0.998074519377826 |
| ebi-fl187-GCST90094803 | prot-c-4962_52_1 | ARMEL \|\| id:prot-c-4962_52_1 | Mushroom liking \|\| id:ebi-fl187-GCST90094803 | Inverse variance weighted | 2 | 1.8425174468139 | 0.832735704199819 | 0.026924702417342 | 0.210355466582254 | 3.47467942704555 | 6.31240943024065 | 1.23411666923117 | 32.2874763856927 | 0.998074519377826 |
| ebi-fl187-GCST90094803 | prot-c-4984_83_1 | Esterase D \|\| id:prot-c-4984_83_1 | Mushroom liking \|\| id:ebi-fl187-GCST90094803 | Inverse variance weighted | 2 | -0.191730858 | 0.878776010946479 | 0.827289303909126 | -1.914131839 | 1.53067012371868 | 0.825529023344708 | 0.14746980453274 | 4.62127260928977 | 0.998074519377826 |
| ebi-fl187-GCST90094803 | prot-c-4988_49_2 | FGFR4 \|\| id:prot-c-4988_49_2 | Mushroom liking \|\| id:ebi-fl187-GCST90094803 | Inverse variance weighted | 2 | -0.68847404 | 0.872965610860524 | 0.430310095483279 | -2.399486637 | 1.02253855744122 | 0.502342038432679 | 0.0907645364741044 | 2.78024362134758 | 0.998074519377826 |
| ebi-fl187-GCST90094803 | prot-c-5066_134_3 | CLM6 \|\| id:prot-c-5066_134_3 | Mushroom liking \|\| id:ebi-fl187-GCST90094803 | Inverse variance weighted | 2 | -0.57033336 | 0.948168910529537 | 0.547500157480051 | -2.428744425 | 1.28807770439432 | 0.565336946421208 | 0.0881474388615932 | 3.62580997379507 | 0.998074519377826 |
| ebi-fl187-GCST90094803 | prot-c-5085_18_3 | IL-20 Ra \|\| id:prot-c-5085_18_3 | Mushroom liking \|\| id:ebi-fl187-GCST90094803 | Inverse variance weighted | 2 | 1.2008988321432 | 0.86874096510034 | 0.166866112001988 | -0.501833459 | 2.90363112373987 | 3.32310249210775 | 0.605419629166732 | 18.240257898892 | 0.998074519377826 |
| ebi-fl187-GCST90094803 | prot-c-5265_12_3 | GRB2-related adapter protein 2 \|\| id:prot-c-5265_12_3 | Mushroom liking \|\| id:ebi-fl187-GCST90094803 | Inverse variance weighted | 2 | -0.423457057 | 0.860422331623741 | 0.622613138429839 | -2.109884827 | 1.26297071316148 | 0.654779293367065 | 0.121251930601636 | 3.53591007495667 | 0.998074519377826 |
| ebi-fl187-GCST90094803 | prot-c-5340_24_3 | Caspase-10 \|\| id:prot-c-5340_24_3 | Mushroom liking \|\| id:ebi-fl187-GCST90094803 | Inverse variance weighted | 2 | -0.632255098 | 0.866883088100013 | 0.465791885338695 | -2.331345951 | 1.06683575476862 | 0.531392107572441 | 0.0971648799121058 | 2.90616910395727 | 0.998074519377826 |
| ebi-fl187-GCST90094803 | prot-c-5400_52_3 | sLeptin R \|\| id:prot-c-5400_52_3 | Mushroom liking \|\| id:ebi-fl187-GCST90094803 | Inverse variance weighted | 2 | -0.054396963 | 0.851629359717967 | 0.949070564554468 | -1.723590509 | 1.61479658155792 | 0.947056085185236 | 0.178424362236767 | 5.02686526235799 | 0.998074519377826 |
| ebi-fl187-GCST90094803 | prot-c-5468_67_3 | IL-17 RC \|\| id:prot-c-5468_67_3 | Mushroom liking \|\| id:ebi-fl187-GCST90094803 | Inverse variance weighted | 2 | 1.40582072593259 | 0.844563389435706 | 0.0960019209940192 | -0.249523517 | 3.06116496922658 | 4.07887300474783 | 0.779171956545309 | 21.3524175878026 | 0.998074519377826 |
| ebi-fl187-GCST90094803 | ukb-rsfMRI191-edge-pheno102 | Net25_Pair11_15 (Postcentral\|Precentral)&(Cerebellum) (Motor)&(Subcortical-cerebellum) \|\| id:ukb-rsfMRI191-edge-pheno102 | Mushroom liking \|\| id:ebi-fl187-GCST90094803 | Inverse variance weighted | 6 | -0.085841109 | 0.0939644998421586 | 0.360954245276733 | -0.270011528 | 0.0983293109986769 | 0.917740040551416 | 0.763370693856713 | 1.10332606269715 | 0.998074519377826 |
| ebi-fl187-GCST90094803 | ukb-rsfMRI191-edge-pheno1167 | Net100_Pair11_45 (Angular\|Temporal)&(Frontal) (Default_mode\|Central_executive)&(Salience\|Default_mode) \|\| id:ukb-rsfMRI191-edge-pheno1167 | Mushroom liking \|\| id:ebi-fl187-GCST90094803 | Inverse variance weighted | 6 | 0.00364983657835808 | 0.0886456958654108 | 0.967157732396652 | -0.170095727 | 0.177395400474563 | 1.00365650534271 | 0.843584058691759 | 1.19410314874717 | 0.998074519377826 |
| ebi-fl187-GCST90094803 | ukb-rsfMRI191-edge-pheno1317 | Net100_Pair26_48 (Parietal\|Frontal)&(Temporal) (Attention\|Central_executive)&(Default_mode\|Central_executive) \|\| id:ukb-rsfMRI191-edge-pheno1317 | Mushroom liking \|\| id:ebi-fl187-GCST90094803 | Inverse variance weighted | 6 | 0.0507317239313525 | 0.0756363052028137 | 0.502390989833026 | -0.097515434 | 0.198978882128867 | 1.05204061809998 | 0.907088341204869 | 1.22015619852644 | 0.998074519377826 |
| ebi-fl187-GCST90094803 | ukb-rsfMRI191-edge-pheno286 | Net100_Pair10_13 (OccipitalPrecuneus)&(Frontal_Sup) (Default_mode\|Central_executive)&(Default_mode) \|\| id:ukb-rsfMRI191-edge-pheno286 | Mushroom liking \|\| id:ebi-fl187-GCST90094803 | Inverse variance weighted | 6 | 0.0342078211781648 | 0.0748647876931216 | 0.647722764402819 | -0.112527163 | 0.180942805056683 | 1.03479963766322 | 0.89357307492119 | 1.19834663796508 | 0.998074519377826 |
| ebi-fl187-GCST90094803 | ukb-rsfMRI191-edge-pheno599 | Net100_Pair11_29 (Angular\|Temporal)&(Parietal) (Default_mode\|Central_executive)&(Central_executive\|Salience) \|\| id:ukb-rsfMRI191-edge-pheno599 | Mushroom liking \|\| id:ebi-fl187-GCST90094803 | Inverse variance weighted (multiplicative random effects) | 6 | 0.168263592862067 | 0.114788829845907 | 0.142688238221074 | -0.056722514 | 0.393249699360045 | 1.18324846539419 | 0.944856217709984 | 1.48178834473993 | 0.998074519377826 |
| ebi-fl187-GCST90094803 | ukb-rsfMRI191-edge-pheno621 | Net100_Pair5_30 (Precuneus\|Angular\|Cingulate)&(Paracentral) (Default_mode\|Central_executive)&(Motor) \|\| id:ukb-rsfMRI191-edge-pheno621 | Mushroom liking \|\| id:ebi-fl187-GCST90094803 | Inverse variance weighted | 6 | -0.042625289 | 0.0802843466271036 | 0.595467973185589 | -0.199982608 | 0.114732030636603 | 0.958270397496838 | 0.818744992451096 | 1.12157285013697 | 0.998074519377826 |
| ebi-fl187-GCST90094803 | ukb-rsfMRI191-edge-pheno624 | Net100_Pair8_30 (Calcarine\|Lingual)&(Paracentral) (Visual)&(Motor) \|\| id:ukb-rsfMRI191-edge-pheno624 | Mushroom liking \|\| id:ebi-fl187-GCST90094803 | Inverse variance weighted | 6 | 0.0521905702563655 | 0.086462820017939 | 0.546097213883036 | -0.117276557 | 0.221657697491526 | 1.05357650372762 | 0.889339206186285 | 1.24814406188947 | 0.998074519377826 |
| ebi-fl187-GCST90094803 | ukb-rsfMRI191-edge-pheno882 | Net100_Pair6_38 (Postcentral\|Precentral)&(Subcortical) (Motor)&(Subcortical-cerebellum) \|\| id:ukb-rsfMRI191-edge-pheno882 | Mushroom liking \|\| id:ebi-fl187-GCST90094803 | Inverse variance weighted | 6 | -0.129402933 | 0.0767316189043189 | 0.0917119301764753 | -0.279796906 | 0.0209910396069097 | 0.878619868880578 | 0.755937251810467 | 1.02121290112751 | 0.998074519377826 |
| ebi-fl187-GCST90094803 | ukb-rsfMRI191-edge-pheno899 | Net100_Pair23_38 (Cerebellum)&(Subcortical) (Subcortical-cerebellum)&(Subcortical-cerebellum) \|\| id:ukb-rsfMRI191-edge-pheno899 | Mushroom liking \|\| id:ebi-fl187-GCST90094803 | Inverse variance weighted | 6 | 0.0491863817822397 | 0.07625750108627 | 0.518924690844285 | -0.10027832 | 0.198651083911329 | 1.05041611092324 | 0.904585618414034 | 1.21975629904619 | 0.998074519377826 |
| ebi-fl187-GCST90094803 | ebi-a-GCST90001392 | IgD+ CD38+ B cell Absolute Count \|\| id:ebi-a-GCST90001392 | Mushroom liking \|\| id:ebi-fl187-GCST90094803 | MR Egger | 6 | -0.985490928 | 1.56762491463791 | 0.563688813367858 | -4.05803576 | 2.0870539050187 | 0.373255940280421 | 0.0172829336196134 | 8.06113128829673 | 0.999279047927446 |
| ebi-fl187-GCST90094803 | ebi-a-GCST90001402 | Switched memory B cell %B cell \|\| id:ebi-a-GCST90001402 | Mushroom liking \|\| id:ebi-fl187-GCST90094803 | MR Egger | 6 | 1.47556319594639 | 1.65084490716464 | 0.421935586851204 | -1.760092822 | 4.71121921398908 | 4.37349821591246 | 0.172028894999274 | 111.187638824688 | 0.999279047927446 |
| ebi-fl187-GCST90094803 | ebi-a-GCST90001407 | Memory B cell Absolute Count \|\| id:ebi-a-GCST90001407 | Mushroom liking \|\| id:ebi-fl187-GCST90094803 | MR Egger | 6 | 0.967984218393024 | 1.63535071556286 | 0.585744858727929 | -2.237303184 | 4.17327162089623 | 2.63263229531282 | 0.106745990840261 | 64.9275232518616 | 0.999279047927446 |
| ebi-fl187-GCST90094803 | ebi-a-GCST90001411 | IgD+ CD24+ B cell %B cell \|\| id:ebi-a-GCST90001411 | Mushroom liking \|\| id:ebi-fl187-GCST90094803 | MR Egger | 6 | -0.964946154 | 1.67623659213051 | 0.595679151505059 | -4.250369875 | 2.32047756647286 | 0.381003714673814 | 0.0142589589056702 | 10.1805350275271 | 0.999279047927446 |
| ebi-fl187-GCST90094803 | ebi-a-GCST90001418 | CD24+ CD27+ B cell Absolute Count \|\| id:ebi-a-GCST90001418 | Mushroom liking \|\| id:ebi-fl187-GCST90094803 | MR Egger | 6 | 0.565517989697994 | 1.62974315570448 | 0.746079759651091 | -2.628778595 | 3.75981457487877 | 1.76035939462983 | 0.0721665529844046 | 42.9404629999602 | 0.999279047927446 |
| ebi-fl187-GCST90094803 | ebi-a-GCST90001436 | Memory B cell %lymphocyte \|\| id:ebi-a-GCST90001436 | Mushroom liking \|\| id:ebi-fl187-GCST90094803 | MR Egger | 6 | -0.156075726 | 1.93998989928536 | 0.939742357115531 | -3.958455929 | 3.64630447658976 | 0.85549440474061 | 0.0190925718374397 | 38.3327444188177 | 0.999279047927446 |
| ebi-fl187-GCST90094803 | ebi-a-GCST90001439 | IgD+ CD24+ B cell %lymphocyte \|\| id:ebi-a-GCST90001439 | Mushroom liking \|\| id:ebi-fl187-GCST90094803 | MR Egger | 6 | -0.681205559 | 1.81230807180524 | 0.726093185566709 | -4.23332938 | 2.87091826196533 | 0.506006603809495 | 0.0145040206829856 | 17.65322104 | 0.999279047927446 |
| ebi-fl187-GCST90094803 | ebi-a-GCST90001457 | CD11c+ HLA DR++ monocyte %monocyte \|\| id:ebi-a-GCST90001457 | Mushroom liking \|\| id:ebi-fl187-GCST90094803 | MR Egger | 6 | 3.60632313283036 | 1.76299274897508 | 0.11024372542965 | 0.150857344839198 | 7.06178892082152 | 36.8303831214045 | 1.16283076243977 | 1166.5301303376 | 0.999279047927446 |
| ebi-fl187-GCST90094803 | ebi-a-GCST90001483 | CD39+ resting CD4 regulatory T cell Absolute Count \|\| id:ebi-a-GCST90001483 | Mushroom liking \|\| id:ebi-fl187-GCST90094803 | MR Egger | 6 | 2.15693444110747 | 1.66174314665491 | 0.264075364798589 | -1.100082126 | 5.41395100855108 | 8.64459647786766 | 0.332843747338098 | 224.516905793797 | 0.999279047927446 |
| ebi-fl187-GCST90094803 | ebi-a-GCST90001525 | CD33dim HLA DR+ CD11b+ Absolute Count \|\| id:ebi-a-GCST90001525 | Mushroom liking \|\| id:ebi-fl187-GCST90094803 | MR Egger | 6 | 0.322559388391064 | 2.16578172764135 | 0.888812401544687 | -3.922372798 | 4.56749157456811 | 1.38065688341576 | 0.0197940716489001 | 96.3022395561173 | 0.999279047927446 |
| ebi-fl187-GCST90094803 | ebi-a-GCST90001540 | Naive CD4+ T cell Absolute Count \|\| id:ebi-a-GCST90001540 | Mushroom liking \|\| id:ebi-fl187-GCST90094803 | MR Egger | 6 | 0.532440760888988 | 1.57475371116774 | 0.752280707113849 | -2.554076513 | 3.61895803477775 | 1.70308406080654 | 0.0777640129729 | 37.2986836364024 | 0.999279047927446 |
| ebi-fl187-GCST90094803 | ebi-a-GCST90001624 | HLA DR+ CD4+ T cell Absolute Count \|\| id:ebi-a-GCST90001624 | Mushroom liking \|\| id:ebi-fl187-GCST90094803 | MR Egger | 6 | -0.454000849 | 1.58704128771502 | 0.789030369446163 | -3.564601773 | 2.65660007463055 | 0.63508219387617 | 0.0283082563458469 | 14.2477653180409 | 0.999279047927446 |
| ebi-fl187-GCST90094803 | ebi-a-GCST90001672 | CD39+ CD8+ T cell Absolute Count \|\| id:ebi-a-GCST90001672 | Mushroom liking \|\| id:ebi-fl187-GCST90094803 | MR Egger | 6 | 1.76571031880475 | 1.72529706627291 | 0.363961887116091 | -1.615871931 | 5.14729256869965 | 5.8457232108211 | 0.198717327064443 | 171.965275310143 | 0.999279047927446 |
| ebi-fl187-GCST90094803 | ebi-a-GCST90001722 | CD19 on CD20- CD38- B cell \|\| id:ebi-a-GCST90001722 | Mushroom liking \|\| id:ebi-fl187-GCST90094803 | MR Egger | 6 | 2.04972412586328 | 1.57459593831197 | 0.262909511720267 | -1.036483913 | 5.13593216495473 | 7.7657584388618 | 0.354699646709891 | 170.022735263896 | 0.999279047927446 |
| ebi-fl187-GCST90094803 | ebi-a-GCST90001742 | CD19 on transitional B cell \|\| id:ebi-a-GCST90001742 | Mushroom liking \|\| id:ebi-fl187-GCST90094803 | MR Egger | 6 | 0.234075631101068 | 3.24835923678219 | 0.94601364766138 | -6.132708473 | 6.60085973519416 | 1.26374006664624 | 0.00217069372627479 | 735.727448195914 | 0.999279047927446 |
| ebi-fl187-GCST90094803 | ebi-a-GCST90001784 | CD25 on IgD+ CD38dim B cell \|\| id:ebi-a-GCST90001784 | Mushroom liking \|\| id:ebi-fl187-GCST90094803 | MR Egger | 6 | -1.285169009 | 1.6237275660305 | 0.472945706783332 | -4.467675038 | 1.89733702043666 | 0.276603831149059 | 0.0114739613831654 | 6.66811372736468 | 0.999279047927446 |
| ebi-fl187-GCST90094803 | ebi-a-GCST90001794 | CD25 on IgD+ B cell \|\| id:ebi-a-GCST90001794 | Mushroom liking \|\| id:ebi-fl187-GCST90094803 | MR Egger | 6 | -1.931910424 | 1.62028668276692 | 0.29903090818691 | -5.107672322 | 1.24385147466765 | 0.144871168652614 | 0.00605014934706563 | 3.46894833545777 | 0.999279047927446 |
| ebi-fl187-GCST90094803 | ebi-a-GCST90001802 | CD27 on IgD- CD38- B cell \|\| id:ebi-a-GCST90001802 | Mushroom liking \|\| id:ebi-fl187-GCST90094803 | MR Egger | 6 | -0.650822253 | 1.94608334676675 | 0.754857553018975 | -4.465145613 | 3.1635011061923 | 0.521616699238717 | 0.0115030206471549 | 23.6532637183426 | 0.999279047927446 |
| ebi-fl187-GCST90094803 | ebi-a-GCST90001804 | CD27 on IgD- CD38dim B cell \|\| id:ebi-a-GCST90001804 | Mushroom liking \|\| id:ebi-fl187-GCST90094803 | MR Egger | 6 | -0.296550314 | 1.59884628833218 | 0.861879812311129 | -3.430289039 | 2.83718841162792 | 0.743378224360548 | 0.032377581048506 | 17.0677106367383 | 0.999279047927446 |
| ebi-fl187-GCST90094803 | ebi-a-GCST90001805 | CD27 on memory B cell \|\| id:ebi-a-GCST90001805 | Mushroom liking \|\| id:ebi-fl187-GCST90094803 | MR Egger | 6 | -1.232713569 | 1.6216079495405 | 0.489487754989157 | -4.411065151 | 1.94563801160459 | 0.29150049662804 | 0.0121422381273503 | 6.99809529702721 | 0.999279047927446 |
| ebi-fl187-GCST90094803 | ebi-a-GCST90001806 | CD27 on unswitched memory B cell \|\| id:ebi-a-GCST90001806 | Mushroom liking \|\| id:ebi-fl187-GCST90094803 | MR Egger | 6 | -0.338459963 | 1.62048658980035 | 0.844760167365365 | -3.514613679 | 2.83769375303122 | 0.712867319902498 | 0.02975929737562 | 17.0763378372398 | 0.999279047927446 |
| ebi-fl187-GCST90094803 | ebi-a-GCST90001839 | CD3 on Effector Memory CD8+ T cell \|\| id:ebi-a-GCST90001839 | Mushroom liking \|\| id:ebi-fl187-GCST90094803 | MR Egger | 6 | 0.00806215220309978 | 1.81099278620709 | 0.996661174338712 | -3.541483709 | 3.557608013 | 1.00809473886619 | 0.0289703116736277 | 35.0791877553323 | 0.999279047927446 |
| ebi-fl187-GCST90094803 | ebi-a-GCST90001859 | CD3 on CD8+ T cell \|\| id:ebi-a-GCST90001859 | Mushroom liking \|\| id:ebi-fl187-GCST90094803 | MR Egger | 6 | -1.667170911 | 1.77377624702477 | 0.400484926289963 | -5.143772355 | 1.80943053305402 | 0.188780387371447 | 0.00583563405364661 | 6.10696872499121 | 0.999279047927446 |
| ebi-fl187-GCST90094803 | ebi-a-GCST90001905 | CD86 on monocyte \|\| id:ebi-a-GCST90001905 | Mushroom liking \|\| id:ebi-fl187-GCST90094803 | MR Egger | 6 | -0.712090902 | 1.80358693456847 | 0.71312427002062 | -4.247121294 | 2.82293948978936 | 0.49061729159636 | 0.0143053556089963 | 16.8262386054894 | 0.999279047927446 |
| ebi-fl187-GCST90094803 | ebi-a-GCST90001943 | CD25 on activated & secreting CD4 regulatory T cell \|\| id:ebi-a-GCST90001943 | Mushroom liking \|\| id:ebi-fl187-GCST90094803 | MR Egger | 6 | -0.682843759 | 1.65315329887528 | 0.700748358310277 | -3.923024224 | 2.55733670711248 | 0.505178342450682 | 0.0197811814612343 | 12.9014112823013 | 0.999279047927446 |
| ebi-fl187-GCST90094803 | ebi-a-GCST90001949 | CD33 on CD33dim HLA DR+ CD11b- \|\| id:ebi-a-GCST90001949 | Mushroom liking \|\| id:ebi-fl187-GCST90094803 | MR Egger | 6 | 1.05195379593381 | 2.79181578102943 | 0.725459438785422 | -4.420005135 | 6.52391272675148 | 2.86323984303412 | 0.0120341704904972 | 681.238678246388 | 0.999279047927446 |
| ebi-fl187-GCST90094803 | ebi-a-GCST90001955 | CD33 on Immature Myeloid-Derived Suppressor Cells \|\| id:ebi-a-GCST90001955 | Mushroom liking \|\| id:ebi-fl187-GCST90094803 | MR Egger | 6 | -3.448187075 | 2.66510252245504 | 0.265376074708796 | -8.671788019 | 1.77541386899811 | 0.0318032410361462 | 0.000171352448714894 | 5.90272358515352 | 0.999279047927446 |
| ebi-fl187-GCST90094803 | ebi-a-GCST90001956 | CD33 on CD33+ HLA DR+ \|\| id:ebi-a-GCST90001956 | Mushroom liking \|\| id:ebi-fl187-GCST90094803 | MR Egger | 6 | -0.499643033 | 2.51342463964058 | 0.852122539550255 | -5.425955327 | 4.42666926024955 | 0.60674720952036 | 0.00440085989204564 | 83.6523282475191 | 0.999279047927446 |
| ebi-fl187-GCST90094803 | ebi-a-GCST90001957 | CD33 on CD33+ HLA DR+ CD14- \|\| id:ebi-a-GCST90001957 | Mushroom liking \|\| id:ebi-fl187-GCST90094803 | MR Egger | 6 | -0.181887057 | 2.51045862498473 | 0.94572054922958 | -5.102385962 | 4.73861184756745 | 0.833695494833225 | 0.00608221729765044 | 114.275459769205 | 0.999279047927446 |
| ebi-fl187-GCST90094803 | ebi-a-GCST90002064 | CD4 on resting CD4 regulatory T cell \|\| id:ebi-a-GCST90002064 | Mushroom liking \|\| id:ebi-fl187-GCST90094803 | MR Egger | 6 | 0.635787912757338 | 1.79734901515899 | 0.741393912282764 | -2.887016157 | 4.15859198246895 | 1.88850953616906 | 0.0557422909596329 | 63.9813722544023 | 0.999279047927446 |
| ebi-fl187-GCST90094803 | ebi-a-GCST90002080 | SSC-A on T cell \|\| id:ebi-a-GCST90002080 | Mushroom liking \|\| id:ebi-fl187-GCST90094803 | MR Egger | 6 | -0.166209661 | 1.66589161732931 | 0.925325635070537 | -3.431357231 | 3.09893790924957 | 0.846868660485327 | 0.0323430140393143 | 22.1743875583284 | 0.999279047927446 |
| ebi-fl187-GCST90094803 | ebi-a-GCST90002121 | CD8 on CD39+ CD8+ T cell \|\| id:ebi-a-GCST90002121 | Mushroom liking \|\| id:ebi-fl187-GCST90094803 | MR Egger | 6 | 1.60019461905267 | 1.77147961969788 | 0.41744252745481 | -1.871905436 | 5.07229467366051 | 4.95399647268144 | 0.153830268719602 | 159.539999868783 | 0.999279047927446 |
| ebi-fl187-GCST90094803 | ebi-cfmet338-GCST90026053 | 1-palmitoyl-2-docosahexaenoyl-gpc (16:0/22:6) levels \|\| id:ebi-cfmet338-GCST90026053 | Mushroom liking \|\| id:ebi-fl187-GCST90094803 | MR Egger | 6 | -0.973148781 | 0.978926485283215 | 0.376442361514981 | -2.891844692 | 0.945547130328332 | 0.377891266000523 | 0.0554737861194057 | 2.57422142797503 | 0.999279047927446 |
| ebi-fl187-GCST90094803 | ebi-cfmet338-GCST90026063 | 1-stearoyl-2-oleoyl-gpc (18:0/18:1) levels \|\| id:ebi-cfmet338-GCST90026063 | Mushroom liking \|\| id:ebi-fl187-GCST90094803 | MR Egger | 6 | -0.541358052 | 0.797632665050038 | 0.53456256741556 | -2.104718075 | 1.02200197158907 | 0.581957387136956 | 0.12188003040389 | 2.77875218213323 | 0.999279047927446 |
| ebi-fl187-GCST90094803 | ebi-cfmet338-GCST90026113 | Adenosine 3',5'-cyclic monophosphate (camp) levels \|\| id:ebi-cfmet338-GCST90026113 | Mushroom liking \|\| id:ebi-fl187-GCST90094803 | MR Egger | 6 | -3.121798166 | 2.04038294640875 | 0.200757144312358 | -7.120948741 | 0.877352408758658 | 0.0440778378380036 | 0.000807999804689879 | 2.4045250719077 | 0.999279047927446 |
| ebi-fl187-GCST90094803 | ebi-cfmet338-GCST90026117 | Alpha-ketoglutaramate levels \|\| id:ebi-cfmet338-GCST90026117 | Mushroom liking \|\| id:ebi-fl187-GCST90094803 | MR Egger | 6 | -1.483507333 | 0.614299514919362 | 0.0731610594363578 | -2.687534382 | -0.279480283 | 0.226840685774044 | 0.0680485143461491 | 0.756176636872462 | 0.999279047927446 |
| ebi-fl187-GCST90094803 | ebi-cfmet338-GCST90026124 | Arginine levels \|\| id:ebi-cfmet338-GCST90026124 | Mushroom liking \|\| id:ebi-fl187-GCST90094803 | MR Egger | 6 | 0.285829810486618 | 0.504043448288391 | 0.60097330247395 | -0.702095348 | 1.27375496913186 | 1.33086593655117 | 0.495545874053402 | 3.57424858890327 | 0.999279047927446 |
| ebi-fl187-GCST90094803 | ebi-cfmet338-GCST90026169 | Glutamate levels \|\| id:ebi-cfmet338-GCST90026169 | Mushroom liking \|\| id:ebi-fl187-GCST90094803 | MR Egger | 6 | -0.568209704 | 0.530604197631508 | 0.344540748053215 | -1.608193932 | 0.471774522902388 | 0.566538803213138 | 0.200248950937162 | 1.60283594018374 | 0.999279047927446 |
| ebi-fl187-GCST90094803 | ebi-cfmet338-GCST90026170 | Glutamine levels \|\| id:ebi-cfmet338-GCST90026170 | Mushroom liking \|\| id:ebi-fl187-GCST90094803 | MR Egger | 6 | -0.18273759 | 0.317140419685857 | 0.595346199917524 | -0.804332812 | 0.438857632720986 | 0.832986711217015 | 0.447386317589119 | 1.55093446934913 | 0.999279047927446 |
| ebi-fl187-GCST90094803 | ebi-cfmet338-GCST90026191 | Kynurenate levels \|\| id:ebi-cfmet338-GCST90026191 | Mushroom liking \|\| id:ebi-fl187-GCST90094803 | MR Egger | 6 | -1.451968574 | 2.41441032314921 | 0.580009532847822 | -6.184212807 | 3.28027565978695 | 0.234108973435614 | 0.00206172388870537 | 26.5830995815314 | 0.999279047927446 |
| ebi-fl187-GCST90094803 | ebi-cfmet338-GCST90026202 | Methylsuccinoylcarnitine levels \|\| id:ebi-cfmet338-GCST90026202 | Mushroom liking \|\| id:ebi-fl187-GCST90094803 | MR Egger | 6 | -1.101629584 | 1.62494713161345 | 0.534995600936124 | -4.286525962 | 2.08326679440297 | 0.332329084189372 | 0.013752619533026 | 8.03066062672107 | 0.999279047927446 |
| ebi-fl187-GCST90094803 | ebi-cfmet338-GCST90026222 | N-acetylputrescine levels \|\| id:ebi-cfmet338-GCST90026222 | Mushroom liking \|\| id:ebi-fl187-GCST90094803 | MR Egger | 6 | 0.279592621482453 | 0.623012582512009 | 0.676832518952216 | -0.94151204 | 1.50069728320599 | 1.32259090745255 | 0.390037636666704 | 4.48481516662184 | 0.999279047927446 |
| ebi-fl187-GCST90094803 | ebi-cfmet338-GCST90026252 | Propionylcarnitine (c3) levels \|\| id:ebi-cfmet338-GCST90026252 | Mushroom liking \|\| id:ebi-fl187-GCST90094803 | MR Egger | 6 | 0.662836479368993 | 0.969363283349252 | 0.531667264694831 | -1.237115556 | 2.56278851473353 | 1.94028812360344 | 0.290220135510613 | 12.9719393727555 | 0.999279047927446 |
| ebi-fl187-GCST90094803 | ebi-cfmet338-GCST90026316 | Ethyl beta-glucopyranoside levels \|\| id:ebi-cfmet338-GCST90026316 | Mushroom liking \|\| id:ebi-fl187-GCST90094803 | MR Egger | 6 | 2.18208675229135 | 3.14366162480453 | 0.525809720931001 | -3.979490032 | 8.34366353690822 | 8.86478558275636 | 0.0186951708399443 | 4203.46110239018 | 0.999279047927446 |
| ebi-fl187-GCST90094803 | ebi-gm430-GCST90011315 | F_Prevotellaceae abundance \|\| id:ebi-gm430-GCST90011315 | Mushroom liking \|\| id:ebi-fl187-GCST90094803 | MR Egger | 6 | 2.13349094727807 | 1.38450792995364 | 0.198174334910076 | -0.580144595 | 4.84712648998721 | 8.44429400233172 | 0.559817413672579 | 127.373853432006 | 0.999279047927446 |
| ebi-fl187-GCST90094803 | ebi-gm430-GCST90011354 | OTU97_1 (Bacteroides) abundance \|\| id:ebi-gm430-GCST90011354 | Mushroom liking \|\| id:ebi-fl187-GCST90094803 | MR Egger | 6 | -0.033219356 | 2.33248775523128 | 0.989318930439678 | -4.604895356 | 4.53845664461594 | 0.967326347827384 | 0.0100027486786666 | 93.5463134444962 | 0.999279047927446 |
| ebi-fl187-GCST90094803 | ebi-gm430-GCST90011419 | OTU99_1 (Bacteroides) abundance \|\| id:ebi-gm430-GCST90011419 | Mushroom liking \|\| id:ebi-fl187-GCST90094803 | MR Egger | 6 | 0.0304754155719121 | 2.34539186469117 | 0.990255036656406 | -4.566492639 | 4.6274434703666 | 1.03094454455521 | 0.010394352603442 | 102.252318590404 | 0.999279047927446 |
| ebi-fl187-GCST90094803 | ebi-gm430-GCST90011435 | OTU99_155 (Butyrivibrio) abundance \|\| id:ebi-gm430-GCST90011435 | Mushroom liking \|\| id:ebi-fl187-GCST90094803 | MR Egger | 6 | 0.914760804242678 | 3.92219949370634 | 0.827034466879986 | -6.772750203 | 8.6022718119071 | 2.49617810527796 | 0.00114454259353162 | 5444.01332766732 | 0.999279047927446 |
| ebi-fl187-GCST90094803 | ebi-gm430-GCST90011466 | OTU99_58 (Bacteroides) abundance \|\| id:ebi-gm430-GCST90011466 | Mushroom liking \|\| id:ebi-fl187-GCST90094803 | MR Egger | 6 | 1.89455465201501 | 2.29433473337095 | 0.455347558267286 | -2.602341425 | 6.39145072942208 | 6.64958636522389 | 0.0740998756075478 | 596.721633687434 | 0.999279047927446 |
| ebi-fl187-GCST90094803 | ebi-gm430-GCST90011472 | OTU99_72 (Parabacteroides) abundance \|\| id:ebi-gm430-GCST90011472 | Mushroom liking \|\| id:ebi-fl187-GCST90094803 | MR Egger | 6 | -1.255213428 | 3.73450259380721 | 0.753677765522628 | -8.574838512 | 6.06441165548312 | 0.285015011440652 | 0.000188796939956615 | 430.269456513342 | 0.999279047927446 |
| ebi-fl187-GCST90094803 | ebi-gm430-GCST90011505 | TestASV_28 (Holdemanella) abundance \|\| id:ebi-gm430-GCST90011505 | Mushroom liking \|\| id:ebi-fl187-GCST90094803 | MR Egger | 6 | 1.33673187123788 | 2.92497521963909 | 0.671384752921778 | -4.396219559 | 7.0696833017305 | 3.80658275220633 | 0.0123238415019687 | 1175.77560917835 | 0.999279047927446 |
| ebi-fl187-GCST90094803 | ebi-gm430-GCST90011513 | TestASV_35 (Alphaproteobacteria) abundance \|\| id:ebi-gm430-GCST90011513 | Mushroom liking \|\| id:ebi-fl187-GCST90094803 | MR Egger | 6 | -3.424412715 | 3.46399733420099 | 0.378835126478724 | -10.21384749 | 3.36502206023143 | 0.0325684023143033 | 3.66591504406025e-05 | 28.9341355857369 | 0.999279047927446 |
| ebi-fl187-GCST90094803 | ebi-gm430-GCST90011543 | G_Coprococcus prevalence \|\| id:ebi-gm430-GCST90011543 | Mushroom liking \|\| id:ebi-fl187-GCST90094803 | MR Egger | 6 | -0.196358511 | 3.66150508394558 | 0.959803220410257 | -7.372908475 | 6.98019145373148 | 0.821717587233092 | 0.000628038884552804 | 1075.12418382974 | 0.999279047927446 |
| ebi-fl187-GCST90094803 | ebi-gm430-GCST90011547 | G_Parasutterella prevalence \|\| id:ebi-gm430-GCST90011547 | Mushroom liking \|\| id:ebi-fl187-GCST90094803 | MR Egger | 6 | 0.200883580492088 | 3.44855617667917 | 0.956342229821855 | -6.558286526 | 6.96005368678326 | 1.22248244273413 | 0.00141831387368647 | 1053.69012495719 | 0.999279047927446 |
| ebi-fl187-GCST90094803 | ebi-gm430-GCST90011569 | OTU97_13 (Subdoligranulum) prevalence \|\| id:ebi-gm430-GCST90011569 | Mushroom liking \|\| id:ebi-fl187-GCST90094803 | MR Egger | 6 | 2.53445439557955 | 3.95870511400774 | 0.556860337312149 | -5.224607628 | 10.2935164190347 | 12.6095491451104 | 0.00538247147740145 | 29540.4685952405 | 0.999279047927446 |
| ebi-fl187-GCST90094803 | ebi-gm430-GCST90011590 | OTU97_277 (Prevotella) prevalence \|\| id:ebi-gm430-GCST90011590 | Mushroom liking \|\| id:ebi-fl187-GCST90094803 | MR Egger | 6 | 4.32201642963954 | 3.05964846506702 | 0.230643046491433 | -1.674894562 | 10.3189274211709 | 75.3403938315108 | 0.187327929943255 | 30300.7402281473 | 0.999279047927446 |
| ebi-fl187-GCST90094803 | ebi-gm430-GCST90011616 | OTU97_86 (Phascolarctobacterium) prevalence \|\| id:ebi-gm430-GCST90011616 | Mushroom liking \|\| id:ebi-fl187-GCST90094803 | MR Egger | 6 | 2.50757810011861 | 4.19835865174002 | 0.582489842550711 | -5.721204857 | 10.736361057529 | 12.2751648106331 | 0.00327576169833915 | 45998.3616038377 | 0.999279047927446 |
| ebi-fl187-GCST90094803 | ebi-gm430-GCST90011665 | OTU99_55 (Barnesiella) prevalence \|\| id:ebi-gm430-GCST90011665 | Mushroom liking \|\| id:ebi-fl187-GCST90094803 | MR Egger | 6 | 2.82496111671535 | 2.7469510347617 | 0.3618811035939 | -2.559062911 | 8.20898514484829 | 16.8602893899117 | 0.0773772157855404 | 3673.81218651564 | 0.999279047927446 |
| ebi-fl187-GCST90094803 | ebi-gm430-GCST90011687 | TestASV_14 (Lachnospiraceae) prevalence \|\| id:ebi-gm430-GCST90011687 | Mushroom liking \|\| id:ebi-fl187-GCST90094803 | MR Egger | 6 | -2.79857788 | 2.6247519788133 | 0.346401244175158 | -7.943091758 | 2.34593599874649 | 0.0608966033691328 | 0.000355106874736355 | 10.4430428294319 | 0.999279047927446 |
| ebi-fl187-GCST90094803 | ebi-gm430-GCST90011697 | TestASV_23 (Barnesiella) prevalence \|\| id:ebi-gm430-GCST90011697 | Mushroom liking \|\| id:ebi-fl187-GCST90094803 | MR Egger | 6 | 2.06029322631229 | 3.51833925940447 | 0.58959894825091 | -4.835651722 | 8.95623817474505 | 7.848270792 | 0.00794151097967604 | 7756.12532542724 | 0.999279047927446 |
| ebi-fl187-GCST90094803 | ebi-gm430-GCST90011727 | TestASV_6 (Subdoligranulum) prevalence \|\| id:ebi-gm430-GCST90011727 | Mushroom liking \|\| id:ebi-fl187-GCST90094803 | MR Egger | 6 | 8.47750491395922 | 5.38650837697056 | 0.190637015013322 | -2.080051505 | 19.0350613328215 | 4805.44492782412 | 0.124923777845812 | 184851125.642808 | 0.999279047927446 |
| ebi-fl187-GCST90094803 | ebi-hbsm44-GCST90104908 | Sulcal depth of the left posterior inferior temporal sulcus \|\| id:ebi-hbsm44-GCST90104908 | Mushroom liking \|\| id:ebi-fl187-GCST90094803 | MR Egger | 5 | -1.139193281 | 1.52052193908675 | 0.508125495700774 | -4.119416281 | 1.84102971980131 | 0.320077130055377 | 0.0162539994348872 | 6.30302527048156 | 0.999279047927446 |
| ebi-fl187-GCST90094803 | ebi-hbsm44-GCST90104918 | Sulcal depth of the left inferior frontal sulcus \|\| id:ebi-hbsm44-GCST90104918 | Mushroom liking \|\| id:ebi-fl187-GCST90094803 | MR Egger | 5 | 2.42123794673719 | 2.22976361633374 | 0.356995853257958 | -1.949098741 | 6.79157463475131 | 11.2597897104924 | 0.142402355134247 | 890.314378613959 | 0.999279047927446 |
| ebi-fl187-GCST90094803 | ebi-hbsm44-GCST90104925 | Sulcal depth of the right anterior inferior temporal sulcus \|\| id:ebi-hbsm44-GCST90104925 | Mushroom liking \|\| id:ebi-fl187-GCST90094803 | MR Egger | 4 | -0.414737105 | 0.929234024286778 | 0.699035589892418 | -2.236035792 | 1.40656158291568 | 0.660513903891518 | 0.106881365604364 | 4.08189598595662 | 0.999279047927446 |
| ebi-fl187-GCST90094803 | ebi-IPs91-GCST90274770 | C-C motif chemokine 4 levels \|\| id:ebi-IPs91-GCST90274770 | Mushroom liking \|\| id:ebi-fl187-GCST90094803 | MR Egger | 7 | 0.0320589626930754 | 0.474822859663702 | 0.94878631880813 | -0.898593842 | 0.96271176763393 | 1.03257838711274 | 0.407141762958881 | 2.61878839887036 | 0.999279047927446 |
| ebi-fl187-GCST90094803 | ebi-IPs91-GCST90274779 | C-X-C motif chemokine 1 levels \|\| id:ebi-IPs91-GCST90274779 | Mushroom liking \|\| id:ebi-fl187-GCST90094803 | MR Egger | 7 | -0.227395327 | 0.673399472875299 | 0.749317851455003 | -1.547258293 | 1.09246764022979 | 0.796605800566616 | 0.212830693947402 | 2.98162257391882 | 0.999279047927446 |
| ebi-fl187-GCST90094803 | ebi-IPs91-GCST90274780 | C-X-C motif chemokine 10 levels \|\| id:ebi-IPs91-GCST90274780 | Mushroom liking \|\| id:ebi-fl187-GCST90094803 | MR Egger | 7 | 0.0861006792496165 | 0.467321826010845 | 0.861062384430488 | -0.8298501 | 1.00205145823087 | 1.08991605471198 | 0.436114655125852 | 2.72386399392181 | 0.999279047927446 |
| ebi-fl187-GCST90094803 | ebi-IPs91-GCST90274796 | Interleukin-10 receptor subunit alpha levels \|\| id:ebi-IPs91-GCST90274796 | Mushroom liking \|\| id:ebi-fl187-GCST90094803 | MR Egger | 7 | 0.0687737630945468 | 0.538478667567024 | 0.903349407913612 | -0.986644425 | 1.12419195152591 | 1.07119383815366 | 0.372825638648456 | 3.0777288897246 | 0.999279047927446 |
| ebi-fl187-GCST90094803 | ebi-IPs91-GCST90274807 | Interleukin-20 levels \|\| id:ebi-IPs91-GCST90274807 | Mushroom liking \|\| id:ebi-fl187-GCST90094803 | MR Egger | 7 | -0.076049001 | 0.536882735065005 | 0.892887531644221 | -1.128339162 | 0.976241159893407 | 0.926770792904956 | 0.323570208243146 | 2.65445977627291 | 0.999279047927446 |
| ebi-fl187-GCST90094803 | ebi-IPs91-GCST90274808 | Interleukin-20 receptor subunit alpha levels \|\| id:ebi-IPs91-GCST90274808 | Mushroom liking \|\| id:ebi-fl187-GCST90094803 | MR Egger | 7 | 0.555861324090619 | 0.541687942212735 | 0.351871473642701 | -0.505847043 | 1.61756969082758 | 1.74344200684914 | 0.602994596908198 | 5.04082465552988 | 0.999279047927446 |
| ebi-fl187-GCST90094803 | ebi-IPs91-GCST90274810 | Interleukin-24 levels \|\| id:ebi-IPs91-GCST90274810 | Mushroom liking \|\| id:ebi-fl187-GCST90094803 | MR Egger | 7 | 0.144437300485652 | 0.543113966035992 | 0.800900926068073 | -0.920066073 | 1.2089406739162 | 1.15538925034778 | 0.39849271062775 | 3.34993409971358 | 0.999279047927446 |
| ebi-fl187-GCST90094803 | ebi-IPs91-GCST90274823 | Monocyte chemoattractant protein-3 levels \|\| id:ebi-IPs91-GCST90274823 | Mushroom liking \|\| id:ebi-fl187-GCST90094803 | MR Egger | 7 | 0.329145770207647 | 0.864151450131965 | 0.718932125841992 | -1.364591072 | 2.0228826124663 | 1.38978042940442 | 0.255485129639676 | 7.56008635289108 | 0.999279047927446 |
| ebi-fl187-GCST90094803 | ebi-IPs91-GCST90274824 | Monocyte chemoattractant protein-4 levels \|\| id:ebi-IPs91-GCST90274824 | Mushroom liking \|\| id:ebi-fl187-GCST90094803 | MR Egger | 7 | -0.028343659 | 0.481037996816672 | 0.955296762817555 | -0.971178133 | 0.91449081491674 | 0.97205425435289 | 0.37863669099602 | 2.49550425480421 | 0.999279047927446 |
| ebi-fl187-GCST90094803 | ebi-IPs91-GCST90274834 | SIR2-like protein 2 levels \|\| id:ebi-IPs91-GCST90274834 | Mushroom liking \|\| id:ebi-fl187-GCST90094803 | MR Egger | 7 | -0.035025167 | 0.767983561443153 | 0.965389208054089 | -1.540272948 | 1.47022261309052 | 0.965581114845428 | 0.214322594569156 | 4.35020344551353 | 0.999279047927446 |
| ebi-fl187-GCST90094803 | ebi-met1400-GCST90199652 | 1-stearoyl-2-oleoyl-GPS (18:0/18:1) levels \|\| id:ebi-met1400-GCST90199652 | Mushroom liking \|\| id:ebi-fl187-GCST90094803 | MR Egger | 5 | -0.185253911 | 1.3245183513675 | 0.89762877726887 | -2.78130988 | 2.41080205745099 | 0.830893283930485 | 0.0619572975820736 | 11.1428948037356 | 0.999279047927446 |
| ebi-fl187-GCST90094803 | ebi-met1400-GCST90199665 | N-acetylaspartate (naa) levels \|\| id:ebi-met1400-GCST90199665 | Mushroom liking \|\| id:ebi-fl187-GCST90094803 | MR Egger | 5 | -0.793487578 | 1.6152324886646 | 0.656941539287587 | -3.959343256 | 2.37236809973449 | 0.452264733033828 | 0.0190756379927541 | 10.7227547945634 | 0.999279047927446 |
| ebi-fl187-GCST90094803 | ebi-met1400-GCST90199669 | Acetylcarnitine levels (Biocrates platform) \|\| id:ebi-met1400-GCST90199669 | Mushroom liking \|\| id:ebi-fl187-GCST90094803 | MR Egger | 5 | 2.16482294214065 | 1.37522213268696 | 0.213517391371407 | -0.530612438 | 4.86025832220709 | 8.7130590646818 | 0.588244596036116 | 129.057536229321 | 0.999279047927446 |
| ebi-fl187-GCST90094803 | ebi-met1400-GCST90199670 | Iminodiacetate (IDA) levels \|\| id:ebi-met1400-GCST90199670 | Mushroom liking \|\| id:ebi-fl187-GCST90094803 | MR Egger | 5 | -1.904927436 | 1.53824033981966 | 0.303645884843899 | -4.919878502 | 1.11002362981186 | 0.148833442145277 | 0.00730001772841162 | 3.0344300966008 | 0.999279047927446 |
| ebi-fl187-GCST90094803 | ebi-met1400-GCST90199686 | Indolepropionate levels \|\| id:ebi-met1400-GCST90199686 | Mushroom liking \|\| id:ebi-fl187-GCST90094803 | MR Egger | 5 | -0.916570608 | 1.38674896573061 | 0.55586965607658 | -3.634598581 | 1.80145736460489 | 0.399888065123512 | 0.0263945275244234 | 6.05847043407988 | 0.999279047927446 |
| ebi-fl187-GCST90094803 | ebi-met1400-GCST90199704 | 3-hydroxysebacate levels \|\| id:ebi-met1400-GCST90199704 | Mushroom liking \|\| id:ebi-fl187-GCST90094803 | MR Egger | 5 | 0.148351581084387 | 1.35306195107984 | 0.919616585879591 | -2.503649843 | 2.80035300520087 | 1.1599206308361 | 0.0817859473407331 | 16.45045284 | 0.999279047927446 |
| ebi-fl187-GCST90094803 | ebi-met1400-GCST90199713 | Docosapentaenoate n3 DPA; 22:5n3 levels \|\| id:ebi-met1400-GCST90199713 | Mushroom liking \|\| id:ebi-fl187-GCST90094803 | MR Egger | 5 | 0.474852504416716 | 1.25875051809057 | 0.731099710134167 | -1.992298511 | 2.94200351987424 | 1.60777703998407 | 0.136381590315835 | 18.9537825766195 | 0.999279047927446 |
| ebi-fl187-GCST90094803 | ebi-met1400-GCST90199715 | Myristoleate (14:1n5) levels \|\| id:ebi-met1400-GCST90199715 | Mushroom liking \|\| id:ebi-fl187-GCST90094803 | MR Egger | 5 | 0.852510972156414 | 1.20770551165645 | 0.531133580868905 | -1.514591831 | 3.21961377500306 | 2.34552902191897 | 0.219897922115606 | 25.0184555621763 | 0.999279047927446 |
| ebi-fl187-GCST90094803 | ebi-met1400-GCST90199725 | Campesterol levels \|\| id:ebi-met1400-GCST90199725 | Mushroom liking \|\| id:ebi-fl187-GCST90094803 | MR Egger | 5 | -0.89950013 | 2.01868015003956 | 0.686088244316996 | -4.856113224 | 3.05711296375408 | 0.406772942388086 | 0.00778066690210246 | 21.2660725283523 | 0.999279047927446 |
| ebi-fl187-GCST90094803 | ebi-met1400-GCST90199737 | N-acetylproline levels \|\| id:ebi-met1400-GCST90199737 | Mushroom liking \|\| id:ebi-fl187-GCST90094803 | MR Egger | 5 | -0.153276927 | 1.41919455308572 | 0.920811691936913 | -2.934898251 | 2.62834439746325 | 0.857892115779485 | 0.0531361255985076 | 13.8508194571354 | 0.999279047927446 |
| ebi-fl187-GCST90094803 | ebi-met1400-GCST90199775 | Gamma-glutamylisoleucine levels \|\| id:ebi-met1400-GCST90199775 | Mushroom liking \|\| id:ebi-fl187-GCST90094803 | MR Egger | 5 | -1.249696604 | 1.26558525965372 | 0.39622607270016 | -3.730243713 | 1.23085050492875 | 0.286591734459271 | 0.0239869891852579 | 3.4241405466115 | 0.999279047927446 |
| ebi-fl187-GCST90094803 | ebi-met1400-GCST90199777 | 1-palmitoleoyl-GPC (16:1) levels \|\| id:ebi-met1400-GCST90199777 | Mushroom liking \|\| id:ebi-fl187-GCST90094803 | MR Egger | 5 | -0.853726699 | 1.93060963952255 | 0.688277667269154 | -4.637721592 | 2.93026819469508 | 0.425825049594297 | 0.00967972688710889 | 18.7326538214079 | 0.999279047927446 |
| ebi-fl187-GCST90094803 | ebi-met1400-GCST90199782 | Glutamine degradant levels \|\| id:ebi-met1400-GCST90199782 | Mushroom liking \|\| id:ebi-fl187-GCST90094803 | MR Egger | 5 | 2.35832117545552 | 1.64289442170482 | 0.246658109330966 | -0.861751891 | 5.57839424199697 | 10.5731860198807 | 0.422421397426896 | 264.64630648912 | 0.999279047927446 |
| ebi-fl187-GCST90094803 | ebi-met1400-GCST90199783 | 5-methyluridine (ribothymidine) levels \|\| id:ebi-met1400-GCST90199783 | Mushroom liking \|\| id:ebi-fl187-GCST90094803 | MR Egger | 5 | -1.218141675 | 1.30772060150477 | 0.420307352933493 | -3.781274054 | 1.34499070436885 | 0.29577931073373 | 0.0227936326096962 | 3.83815086239939 | 0.999279047927446 |
| ebi-fl187-GCST90094803 | ebi-met1400-GCST90199787 | Homostachydrine levels \|\| id:ebi-met1400-GCST90199787 | Mushroom liking \|\| id:ebi-fl187-GCST90094803 | MR Egger | 5 | -1.316261283 | 1.27811319258989 | 0.378843750692846 | -3.821363141 | 1.1888405742943 | 0.268135914550025 | 0.0218979305806655 | 3.28327228943994 | 0.999279047927446 |
| ebi-fl187-GCST90094803 | ebi-met1400-GCST90199802 | Beta-hydroxyisovaleroylcarnitine levels \|\| id:ebi-met1400-GCST90199802 | Mushroom liking \|\| id:ebi-fl187-GCST90094803 | MR Egger | 5 | -0.69655889 | 1.48411642154624 | 0.670829746145092 | -3.605427076 | 2.21230929633028 | 0.498297051963479 | 0.0271758361833716 | 9.13679160854762 | 0.999279047927446 |
| ebi-fl187-GCST90094803 | ebi-met1400-GCST90199873 | Pregnenolone sulfate levels \|\| id:ebi-met1400-GCST90199873 | Mushroom liking \|\| id:ebi-fl187-GCST90094803 | MR Egger | 5 | 0.105221524260474 | 1.32473519678478 | 0.941693514868546 | -2.491259461 | 2.70170250995863 | 1.11095668695723 | 0.0828056103995555 | 14.9050861933095 | 0.999279047927446 |
| ebi-fl187-GCST90094803 | ebi-met1400-GCST90199911 | Beta-citrylglutamate levels \|\| id:ebi-met1400-GCST90199911 | Mushroom liking \|\| id:ebi-fl187-GCST90094803 | MR Egger | 5 | 0.943240103888901 | 1.34951513408815 | 0.534898983627302 | -1.701809559 | 3.58828976670167 | 2.56828947629542 | 0.182353246370129 | 36.172160163584 | 0.999279047927446 |
| ebi-fl187-GCST90094803 | ebi-met1400-GCST90199934 | 2-hydroxydecanoate levels \|\| id:ebi-met1400-GCST90199934 | Mushroom liking \|\| id:ebi-fl187-GCST90094803 | MR Egger | 5 | -1.258542389 | 1.30602522903919 | 0.406310508658682 | -3.818351838 | 1.30126706001657 | 0.284067785233692 | 0.021963971265582 | 3.67394883337992 | 0.999279047927446 |
| ebi-fl187-GCST90094803 | ebi-met1400-GCST90199945 | Sphingomyelin (d18:1/14:0, d16:1/16:0) levels \|\| id:ebi-met1400-GCST90199945 | Mushroom liking \|\| id:ebi-fl187-GCST90094803 | MR Egger | 5 | 0.526879480950504 | 1.25524772578248 | 0.702921599558004 | -1.933406062 | 2.98716502348416 | 1.69363902118318 | 0.144654655776476 | 19.8293868847656 | 0.999279047927446 |
| ebi-fl187-GCST90094803 | ebi-met1400-GCST90199979 | Carnitine C14:1 levels \|\| id:ebi-met1400-GCST90199979 | Mushroom liking \|\| id:ebi-fl187-GCST90094803 | MR Egger | 5 | 3.23064211403764 | 2.37677491960575 | 0.267220130661309 | -1.427836728 | 7.88912095646492 | 25.2958946062194 | 0.239827172797598 | 2668.0975156597 | 0.999279047927446 |
| ebi-fl187-GCST90094803 | ebi-met1400-GCST90199986 | Eugenol sulfate levels \|\| id:ebi-met1400-GCST90199986 | Mushroom liking \|\| id:ebi-fl187-GCST90094803 | MR Egger | 5 | 0.130870615003176 | 1.42417656944154 | 0.932576056479875 | -2.660515461 | 2.9222566911086 | 1.13982029612562 | 0.0699121754482045 | 18.5831766660217 | 0.999279047927446 |
| ebi-fl187-GCST90094803 | ebi-met1400-GCST90200002 | 6-hydroxyindole sulfate levels \|\| id:ebi-met1400-GCST90200002 | Mushroom liking \|\| id:ebi-fl187-GCST90094803 | MR Egger | 5 | -2.239385716 | 1.29814544700347 | 0.182979068805905 | -4.783750792 | 0.304979360471138 | 0.106523920261734 | 0.00836456630908785 | 1.35659700319427 | 0.999279047927446 |
| ebi-fl187-GCST90094803 | ebi-met1400-GCST90200023 | Trans 3,4-methyleneheptanoate levels \|\| id:ebi-met1400-GCST90200023 | Mushroom liking \|\| id:ebi-fl187-GCST90094803 | MR Egger | 5 | 0.705480643743418 | 1.41752986996006 | 0.652900223457295 | -2.072877901 | 3.48383918886514 | 2.02481966797947 | 0.125823153524301 | 32.5845806037969 | 0.999279047927446 |
| ebi-fl187-GCST90094803 | ebi-met1400-GCST90200032 | 2-hydroxybutyrate/2-hydroxyisobutyrate levels \|\| id:ebi-met1400-GCST90200032 | Mushroom liking \|\| id:ebi-fl187-GCST90094803 | MR Egger | 5 | 1.2082625044084 | 1.47114865269129 | 0.471660001546307 | -1.675188855 | 4.09171386368332 | 3.34766304672051 | 0.187272808760794 | 59.8423655229775 | 0.999279047927446 |
| ebi-fl187-GCST90094803 | ebi-met1400-GCST90200051 | 1-palmitoyl-2-dihomo-linolenoyl-GPC (16:0/20:3n3 or 6) levels \|\| id:ebi-met1400-GCST90200051 | Mushroom liking \|\| id:ebi-fl187-GCST90094803 | MR Egger | 5 | -0.607625479 | 1.28828907861992 | 0.669356653259995 | -3.132672073 | 1.91742111535568 | 0.544642600279771 | 0.0436011360473635 | 6.80339066663946 | 0.999279047927446 |
| ebi-fl187-GCST90094803 | ebi-met1400-GCST90200069 | Phenylacetylglutamate levels \|\| id:ebi-met1400-GCST90200069 | Mushroom liking \|\| id:ebi-fl187-GCST90094803 | MR Egger | 5 | -1.952054253 | 1.4482983107995 | 0.270458417200428 | -4.790718942 | 0.886610436267009 | 0.141982104651997 | 0.00830648335437819 | 2.42688959712238 | 0.999279047927446 |
| ebi-fl187-GCST90094803 | ebi-met1400-GCST90200072 | Thioproline levels \|\| id:ebi-met1400-GCST90200072 | Mushroom liking \|\| id:ebi-fl187-GCST90094803 | MR Egger | 5 | -2.061892502 | 1.30786563755577 | 0.212995591612538 | -4.625309151 | 0.501524147902863 | 0.12721299113758 | 0.00980062469141006 | 1.65123608175244 | 0.999279047927446 |
| ebi-fl187-GCST90094803 | ebi-met1400-GCST90200081 | 1-oleoyl-2-docosahexaenoyl-GPC (18:1/22:6) levels \|\| id:ebi-met1400-GCST90200081 | Mushroom liking \|\| id:ebi-fl187-GCST90094803 | MR Egger | 5 | 0.0488063977711873 | 1.25837593064103 | 0.971498303869438 | -2.417610426 | 2.51522322182761 | 1.0500170454202 | 0.089134356295478 | 12.3693695842497 | 0.999279047927446 |
| ebi-fl187-GCST90094803 | ebi-met1400-GCST90200097 | Furaneol sulfate levels \|\| id:ebi-met1400-GCST90200097 | Mushroom liking \|\| id:ebi-fl187-GCST90094803 | MR Egger | 5 | 0.218771893112857 | 2.71735398217792 | 0.940902376907617 | -5.107241912 | 5.54478569818157 | 1.24454735448352 | 0.00605275395125751 | 255.899732588691 | 0.999279047927446 |
| ebi-fl187-GCST90094803 | ebi-met1400-GCST90200124 | Sphingomyelin (d18:2/24:2) levels \|\| id:ebi-met1400-GCST90200124 | Mushroom liking \|\| id:ebi-fl187-GCST90094803 | MR Egger | 5 | 1.8436179667739 | 1.19610487444193 | 0.220889269993265 | -0.500747587 | 4.18798352068009 | 6.31936018683597 | 0.606077394644769 | 65.8897915081846 | 0.999279047927446 |
| ebi-fl187-GCST90094803 | ebi-met1400-GCST90200137 | Lignoceroylcarnitine (C24) levels \|\| id:ebi-met1400-GCST90200137 | Mushroom liking \|\| id:ebi-fl187-GCST90094803 | MR Egger | 5 | -0.058198656 | 1.31806308495236 | 0.967555718590675 | -2.641602302 | 2.52520499054878 | 0.943462504371576 | 0.0712470187749058 | 12.4934560415404 | 0.999279047927446 |
| ebi-fl187-GCST90094803 | ebi-met1400-GCST90200165 | Octadecenedioate (C18:1-DC) levels \|\| id:ebi-met1400-GCST90200165 | Mushroom liking \|\| id:ebi-fl187-GCST90094803 | MR Egger | 5 | -1.440526342 | 1.40063654271045 | 0.379392811246072 | -4.185773966 | 1.30472128127503 | 0.236803086361159 | 0.0152104290278432 | 3.68666140892687 | 0.999279047927446 |
| ebi-fl187-GCST90094803 | ebi-met1400-GCST90200176 | Glutamine conjugate of C6H10O2 (1) levels \|\| id:ebi-met1400-GCST90200176 | Mushroom liking \|\| id:ebi-fl187-GCST90094803 | MR Egger | 5 | -0.259574149 | 1.29377627541217 | 0.853817247135303 | -2.795375648 | 2.27622735113725 | 0.771380009071194 | 0.0610919209345091 | 9.73986591504544 | 0.999279047927446 |
| ebi-fl187-GCST90094803 | ebi-met1400-GCST90200182 | Glycine conjugate of C10H12O2 levels \|\| id:ebi-met1400-GCST90200182 | Mushroom liking \|\| id:ebi-fl187-GCST90094803 | MR Egger | 5 | -0.788382847 | 1.51246342486218 | 0.63822084150134 | -3.752811159 | 2.17604546615398 | 0.454579325711181 | 0.0234517265647449 | 8.81139232088261 | 0.999279047927446 |
| ebi-fl187-GCST90094803 | ebi-met1400-GCST90200208 | Glucuronide of piperine metabolite C17H21NO3 (4) levels \|\| id:ebi-met1400-GCST90200208 | Mushroom liking \|\| id:ebi-fl187-GCST90094803 | MR Egger | 5 | 1.44009166177481 | 1.38121404442346 | 0.373748845426258 | -1.267087865 | 4.14727118884479 | 4.22108271119751 | 0.281650633247432 | 63.2611368536059 | 0.999279047927446 |
| ebi-fl187-GCST90094803 | ebi-met1400-GCST90200227 | 3-ethylcatechol sulfate (2) levels \|\| id:ebi-met1400-GCST90200227 | Mushroom liking \|\| id:ebi-fl187-GCST90094803 | MR Egger | 4 | -1.135502381 | 1.70630079198139 | 0.574221911778607 | -4.479851934 | 2.20884717080293 | 0.321260685367795 | 0.0113350913747337 | 9.10521358416578 | 0.999279047927446 |
| ebi-fl187-GCST90094803 | ebi-met1400-GCST90200259 | Pentose acid levels \|\| id:ebi-met1400-GCST90200259 | Mushroom liking \|\| id:ebi-fl187-GCST90094803 | MR Egger | 5 | 0.96866111504979 | 1.55129038289723 | 0.576614333416494 | -2.071868035 | 4.00919026552835 | 2.63441491856981 | 0.125950282223376 | 55.1022343155583 | 0.999279047927446 |
| ebi-fl187-GCST90094803 | ebi-met1400-GCST90200291 | Sarcosine levels \|\| id:ebi-met1400-GCST90200291 | Mushroom liking \|\| id:ebi-fl187-GCST90094803 | MR Egger | 5 | -0.824953961 | 1.31484224138231 | 0.574892583607061 | -3.402044754 | 1.75213683226355 | 0.438255168897802 | 0.0333050995541854 | 5.76691244393848 | 0.999279047927446 |
| ebi-fl187-GCST90094803 | ebi-met1400-GCST90200306 | Butyrate/isobutyrate (4:0) levels \|\| id:ebi-met1400-GCST90200306 | Mushroom liking \|\| id:ebi-fl187-GCST90094803 | MR Egger | 5 | -1.013174963 | 1.32930005874193 | 0.501394285175258 | -3.618603078 | 1.59225315177188 | 0.363064431449382 | 0.0268201159332442 | 4.91481027568094 | 0.999279047927446 |
| ebi-fl187-GCST90094803 | ebi-met1400-GCST90200321 | Cholate levels \|\| id:ebi-met1400-GCST90200321 | Mushroom liking \|\| id:ebi-fl187-GCST90094803 | MR Egger | 5 | 0.160247324533162 | 1.35187188430368 | 0.913133512823978 | -2.489421569 | 2.80991621776837 | 1.17380114491459 | 0.0829579381675499 | 16.6085266610658 | 0.999279047927446 |
| ebi-fl187-GCST90094803 | ebi-met1400-GCST90200336 | Ceramide (d18:1/16:0) levels \|\| id:ebi-met1400-GCST90200336 | Mushroom liking \|\| id:ebi-fl187-GCST90094803 | MR Egger | 5 | -0.627729895 | 1.29952708657548 | 0.662123071363411 | -3.174802984 | 1.91934319518235 | 0.533802213943359 | 0.041802338995219 | 6.81647990184044 | 0.999279047927446 |
| ebi-fl187-GCST90094803 | ebi-met1400-GCST90200358 | Arachidonate (20:4n6) levels \|\| id:ebi-met1400-GCST90200358 | Mushroom liking \|\| id:ebi-fl187-GCST90094803 | MR Egger | 5 | -0.171101335 | 1.29017751752491 | 0.902890514444569 | -2.69984927 | 2.35764659910951 | 0.842736170273034 | 0.0672156434178474 | 10.5660560038303 | 0.999279047927446 |
| ebi-fl187-GCST90094803 | ebi-met1400-GCST90200413 | 2'-deoxyuridine levels \|\| id:ebi-met1400-GCST90200413 | Mushroom liking \|\| id:ebi-fl187-GCST90094803 | MR Egger | 5 | 0.101274288214199 | 1.31130404676687 | 0.943301668278725 | -2.468881643 | 2.67143021987726 | 1.10658012202653 | 0.084679507948526 | 14.4606363 | 0.999279047927446 |
| ebi-fl187-GCST90094803 | ebi-met1400-GCST90200432 | Threonine levels \|\| id:ebi-met1400-GCST90200432 | Mushroom liking \|\| id:ebi-fl187-GCST90094803 | MR Egger | 5 | -1.893717214 | 1.61918005407718 | 0.326649661029819 | -5.06731012 | 1.27987569201151 | 0.150511285035499 | 0.00629934184574651 | 3.59619266230703 | 0.999279047927446 |
| ebi-fl187-GCST90094803 | ebi-met1400-GCST90200436 | Caffeine levels \|\| id:ebi-met1400-GCST90200436 | Mushroom liking \|\| id:ebi-fl187-GCST90094803 | MR Egger | 5 | 0.403890661996528 | 1.34130919194244 | 0.782989657304916 | -2.225075354 | 3.03285667820371 | 1.49764018901763 | 0.108059275606611 | 20.7564424541039 | 0.999279047927446 |
| ebi-fl187-GCST90094803 | ebi-met1400-GCST90200442 | Valine levels \|\| id:ebi-met1400-GCST90200442 | Mushroom liking \|\| id:ebi-fl187-GCST90094803 | MR Egger | 5 | -3.48499481 | 1.28598955944173 | 0.0731662485933854 | -6.005534347 | -0.964455274 | 0.0306539175093866 | 0.00246507179306081 | 0.381190787756136 | 0.999279047927446 |
| ebi-fl187-GCST90094803 | ebi-met1400-GCST90200459 | Margarate (17:0) levels \|\| id:ebi-met1400-GCST90200459 | Mushroom liking \|\| id:ebi-fl187-GCST90094803 | MR Egger | 5 | 0.537824302731545 | 1.2861950436445 | 0.703964782263884 | -1.983117983 | 3.05876658827477 | 1.71227740929355 | 0.137639410247059 | 21.3012677191392 | 0.999279047927446 |
| ebi-fl187-GCST90094803 | ebi-met1400-GCST90200462 | X-11299 levels \|\| id:ebi-met1400-GCST90200462 | Mushroom liking \|\| id:ebi-fl187-GCST90094803 | MR Egger | 5 | 0.61421200948626 | 1.30333839271857 | 0.669606731979566 | -1.94033124 | 3.16875525921466 | 1.84819966180615 | 0.143656357129317 | 23.7778686454195 | 0.999279047927446 |
| ebi-fl187-GCST90094803 | ebi-met1400-GCST90200497 | X-12740 levels \|\| id:ebi-met1400-GCST90200497 | Mushroom liking \|\| id:ebi-fl187-GCST90094803 | MR Egger | 5 | 0.303965033537153 | 1.69921673565782 | 0.869426647862632 | -3.026499768 | 3.63442983542649 | 1.35522166795308 | 0.0484850503698053 | 37.8802487628909 | 0.999279047927446 |
| ebi-fl187-GCST90094803 | ebi-met1400-GCST90200527 | X-13866 levels \|\| id:ebi-met1400-GCST90200527 | Mushroom liking \|\| id:ebi-fl187-GCST90094803 | MR Egger | 5 | 0.591594079123299 | 1.43199832112857 | 0.707268418912045 | -2.21512263 | 3.39831078853531 | 1.80686640893479 | 0.1091401295422 | 29.9135270723179 | 0.999279047927446 |
| ebi-fl187-GCST90094803 | ebi-met1400-GCST90200547 | X-17654 levels \|\| id:ebi-met1400-GCST90200547 | Mushroom liking \|\| id:ebi-fl187-GCST90094803 | MR Egger | 5 | -1.746811204 | 1.32865309909245 | 0.280075044867911 | -4.350971278 | 0.857348870684987 | 0.174328957630144 | 0.0128942825665483 | 2.35690394650221 | 0.999279047927446 |
| ebi-fl187-GCST90094803 | ebi-met1400-GCST90200553 | X-17653 levels \|\| id:ebi-met1400-GCST90200553 | Mushroom liking \|\| id:ebi-fl187-GCST90094803 | MR Egger | 5 | -3.01252007 | 1.32539027542179 | 0.107633866974608 | -5.61028501 | -0.41475513 | 0.0491676166823284 | 0.00366002606675027 | 0.660501998109218 | 0.999279047927446 |
| ebi-fl187-GCST90094803 | ebi-met1400-GCST90200557 | X-17685 levels \|\| id:ebi-met1400-GCST90200557 | Mushroom liking \|\| id:ebi-fl187-GCST90094803 | MR Egger | 5 | 0.226639072300382 | 1.39175071122058 | 0.880991966665745 | -2.501192322 | 2.95447046629272 | 1.25437704680539 | 0.081987185223563 | 19.1915574520786 | 0.999279047927446 |
| ebi-fl187-GCST90094803 | ebi-met1400-GCST90200561 | X-18779 levels \|\| id:ebi-met1400-GCST90200561 | Mushroom liking \|\| id:ebi-fl187-GCST90094803 | MR Egger | 5 | -0.572204561 | 1.30891676241344 | 0.691552062074348 | -3.137681415 | 1.99327229344426 | 0.564280076679879 | 0.0433832691691385 | 7.33951154525623 | 0.999279047927446 |
| ebi-fl187-GCST90094803 | ebi-met1400-GCST90200570 | X-18921 levels \|\| id:ebi-met1400-GCST90200570 | Mushroom liking \|\| id:ebi-fl187-GCST90094803 | MR Egger | 5 | 2.21113664015257 | 1.32259152982613 | 0.193152815274754 | -0.381142758 | 4.80341603861179 | 9.1260835730725 | 0.683080367262469 | 121.926211576656 | 0.999279047927446 |
| ebi-fl187-GCST90094803 | ebi-met1400-GCST90200575 | X-21283 levels \|\| id:ebi-met1400-GCST90200575 | Mushroom liking \|\| id:ebi-fl187-GCST90094803 | MR Egger | 5 | 1.33117511278522 | 1.38411402298397 | 0.407121752882305 | -1.381688372 | 4.04403859783379 | 3.78548915162048 | 0.251154153182023 | 57.0563056014876 | 0.999279047927446 |
| ebi-fl187-GCST90094803 | ebi-met1400-GCST90200578 | X-18922 levels \|\| id:ebi-met1400-GCST90200578 | Mushroom liking \|\| id:ebi-fl187-GCST90094803 | MR Egger | 5 | 0.630337234711636 | 1.50634952180747 | 0.703766530700955 | -2.322107828 | 3.58278229745427 | 1.87824388150635 | 0.0980666599443839 | 35.9734906890553 | 0.999279047927446 |
| ebi-fl187-GCST90094803 | ebi-met1400-GCST90200623 | X-24241 levels \|\| id:ebi-met1400-GCST90200623 | Mushroom liking \|\| id:ebi-fl187-GCST90094803 | MR Egger | 5 | -2.548529898 | 1.32490096141551 | 0.150111528915928 | -5.145335782 | 0.0482759866866558 | 0.0781965384553287 | 0.00582651759540885 | 1.049460252 | 0.999279047927446 |
| ebi-fl187-GCST90094803 | ebi-met1400-GCST90200636 | X-24544 levels \|\| id:ebi-met1400-GCST90200636 | Mushroom liking \|\| id:ebi-fl187-GCST90094803 | MR Egger | 5 | 0.0500210053829277 | 1.23036647034412 | 0.970124997318441 | -2.361497276 | 2.46153928725739 | 1.05129317895989 | 0.0942789558003517 | 11.7228424810733 | 0.999279047927446 |
| ebi-fl187-GCST90094803 | ebi-met1400-GCST90200659 | X-25172 levels \|\| id:ebi-met1400-GCST90200659 | Mushroom liking \|\| id:ebi-fl187-GCST90094803 | MR Egger | 5 | -0.446767886 | 1.33079479035196 | 0.759193196737647 | -3.055125675 | 2.16158990321627 | 0.639692372680063 | 0.047116798996237 | 8.68493489334304 | 0.999279047927446 |
| ebi-fl187-GCST90094803 | ebi-met1400-GCST90200670 | X-26111 levels \|\| id:ebi-met1400-GCST90200670 | Mushroom liking \|\| id:ebi-fl187-GCST90094803 | MR Egger | 5 | 0.999341389666904 | 1.3233654914834 | 0.505034979980526 | -1.594454974 | 3.59313775297437 | 2.7164921293804 | 0.203019149141976 | 36.34794806 | 0.999279047927446 |
| ebi-fl187-GCST90094803 | ebi-met1400-GCST90200673 | Carnitine C4 levels \|\| id:ebi-met1400-GCST90200673 | Mushroom liking \|\| id:ebi-fl187-GCST90094803 | MR Egger | 5 | -1.145048492 | 2.28610022244762 | 0.650900581802384 | -5.625804928 | 3.33570794423185 | 0.318208486924963 | 0.00360366128126757 | 28.0982681911375 | 0.999279047927446 |
| ebi-fl187-GCST90094803 | ebi-met1400-GCST90200710 | X-13431 levels \|\| id:ebi-met1400-GCST90200710 | Mushroom liking \|\| id:ebi-fl187-GCST90094803 | MR Egger | 5 | 0.569442318036137 | 1.29656726594879 | 0.690231705956861 | -1.971829523 | 3.11071415929577 | 1.76728119571438 | 0.139201949907536 | 22.437062317017 | 0.999279047927446 |
| ebi-fl187-GCST90094803 | ebi-met1400-GCST90200730 | Adenosine 5'-diphosphate (ADP) to fructose ratio \|\| id:ebi-met1400-GCST90200730 | Mushroom liking \|\| id:ebi-fl187-GCST90094803 | MR Egger | 5 | -0.813611992 | 1.75246736984491 | 0.674072846009505 | -4.248448037 | 2.62122405305145 | 0.443254140942067 | 0.0142863886631851 | 13.7525471338036 | 0.999279047927446 |
| ebi-fl187-GCST90094803 | ebi-met1400-GCST90200731 | Adenosine 5'-diphosphate (ADP) to mannose ratio \|\| id:ebi-met1400-GCST90200731 | Mushroom liking \|\| id:ebi-fl187-GCST90094803 | MR Egger | 5 | 1.73324035710632 | 1.94784717373488 | 0.439111418706921 | -2.084540103 | 5.55102081762668 | 5.65896128660745 | 0.12436430173375 | 257.500282612299 | 0.999279047927446 |
| ebi-fl187-GCST90094803 | ebi-met1400-GCST90200748 | Aspartate to glutamate ratio \|\| id:ebi-met1400-GCST90200748 | Mushroom liking \|\| id:ebi-fl187-GCST90094803 | MR Egger | 5 | 1.59782639391276 | 1.2717716854007 | 0.297909354003818 | -0.894846109 | 4.09049889729813 | 4.94227817495226 | 0.408670484318329 | 59.7697032105285 | 0.999279047927446 |
| ebi-fl187-GCST90094803 | ebi-met1400-GCST90200774 | Uridine to pseudouridine ratio \|\| id:ebi-met1400-GCST90200774 | Mushroom liking \|\| id:ebi-fl187-GCST90094803 | MR Egger | 5 | -1.759075089 | 1.25060646091839 | 0.254245000341599 | -4.210263752 | 0.692113574548368 | 0.172204063647109 | 0.014842453091358 | 1.99793385595025 | 0.999279047927446 |
| ebi-fl187-GCST90094803 | ebi-met1400-GCST90200799 | Spermidine to adenosine 5'-diphosphate (ADP) ratio \|\| id:ebi-met1400-GCST90200799 | Mushroom liking \|\| id:ebi-fl187-GCST90094803 | MR Egger | 5 | -2.297145346 | 2.95926161672538 | 0.494178748604819 | -8.097298115 | 3.50300742260169 | 0.100545456910099 | 0.000304360374878661 | 33.2151940254738 | 0.999279047927446 |
| ebi-fl187-GCST90094803 | ebi-met1400-GCST90200811 | Choline to taurocholate ratio \|\| id:ebi-met1400-GCST90200811 | Mushroom liking \|\| id:ebi-fl187-GCST90094803 | MR Egger | 5 | 0.0721669744869278 | 1.37100983963807 | 0.96132946736051 | -2.615012311 | 2.75934626017755 | 1.0748347990703 | 0.0731668879179763 | 15.789517337236 | 0.999279047927446 |
| ebi-fl187-GCST90094803 | ebi-met1400-GCST90200812 | Glutamate to kynurenine ratio \|\| id:ebi-met1400-GCST90200812 | Mushroom liking \|\| id:ebi-fl187-GCST90094803 | MR Egger | 5 | -1.656812264 | 1.43092351400958 | 0.330729334151289 | -4.461422351 | 1.14779782384335 | 0.190746060137996 | 0.0115459292378907 | 3.15124566490195 | 0.999279047927446 |
| ebi-fl187-GCST90094803 | ebi-met1400-GCST90200876 | Cortisone to 4-cholesten-3-one ratio \|\| id:ebi-met1400-GCST90200876 | Mushroom liking \|\| id:ebi-fl187-GCST90094803 | MR Egger | 5 | -0.413200242 | 2.08296110025801 | 0.855436358560787 | -4.495803999 | 3.6694035141038 | 0.66152980324755 | 0.0111557078316366 | 39.2284996334958 | 0.999279047927446 |
| ebi-fl187-GCST90094803 | ebi-met1400-GCST90200906 | 2'-deoxyuridine to cytidine ratio \|\| id:ebi-met1400-GCST90200906 | Mushroom liking \|\| id:ebi-fl187-GCST90094803 | MR Egger | 5 | 2.55303332406007 | 1.35618639048033 | 0.156306857704796 | -0.105092001 | 5.21115864940151 | 12.8460108577581 | 0.900241695386695 | 183.30632296114 | 0.999279047927446 |
| ebi-fl187-GCST90094803 | ebi-met1400-GCST90200907 | Retinol (Vitamin A) to linoleoyl-arachidonoyl-glycerol (18:2 to 20:4) [1] ratio \|\| id:ebi-met1400-GCST90200907 | Mushroom liking \|\| id:ebi-fl187-GCST90094803 | MR Egger | 5 | 0.849192098309427 | 1.36397888719665 | 0.577673428231879 | -1.824206521 | 3.52259071721486 | 2.33775741062316 | 0.161345617775466 | 33.8720678396666 | 0.999279047927446 |
| ebi-fl187-GCST90094803 | ebi-met1400-GCST90200916 | Fructose to sucrose ratio \|\| id:ebi-met1400-GCST90200916 | Mushroom liking \|\| id:ebi-fl187-GCST90094803 | MR Egger | 5 | 1.8984978693686 | 1.6307400410967 | 0.328513451317145 | -1.297752611 | 5.09474834991813 | 6.67585889467799 | 0.273144966700259 | 163.16277953076 | 0.999279047927446 |
| ebi-fl187-GCST90094803 | ebi-met1400-GCST90200917 | Alpha-ketobutyrate to 3-methyl-2-oxobutyrate ratio \|\| id:ebi-met1400-GCST90200917 | Mushroom liking \|\| id:ebi-fl187-GCST90094803 | MR Egger | 5 | 0.393516608841484 | 1.96531483487479 | 0.854105090217094 | -3.458500468 | 4.24553368519606 | 1.48218390088512 | 0.0314769273216842 | 69.7929976961135 | 0.999279047927446 |
| ebi-fl187-GCST90094803 | ebi-met1400-GCST90200924 | Alpha-tocopherol to glycerol ratio \|\| id:ebi-met1400-GCST90200924 | Mushroom liking \|\| id:ebi-fl187-GCST90094803 | MR Egger | 5 | -0.320859078 | 1.2263188408825 | 0.810531225413075 | -2.724444006 | 2.08272585007196 | 0.725525486247067 | 0.0655826561341717 | 8.02631766113802 | 0.999279047927446 |
| ebi-fl187-GCST90094803 | ebi-met1400-GCST90200938 | Alpha-ketoglutarate to alanine ratio \|\| id:ebi-met1400-GCST90200938 | Mushroom liking \|\| id:ebi-fl187-GCST90094803 | MR Egger | 5 | 0.0752352894742666 | 1.30224642330944 | 0.957561894405058 | -2.4771677 | 2.62763827916077 | 1.0781377955186 | 0.0839807477204218 | 13.8410425922303 | 0.999279047927446 |
| ebi-fl187-GCST90094803 | ebi-met1400-GCST90200943 | Phosphate to EDTA ratio \|\| id:ebi-met1400-GCST90200943 | Mushroom liking \|\| id:ebi-fl187-GCST90094803 | MR Egger | 5 | -1.495872207 | 1.65556164862738 | 0.432838661812003 | -4.740773038 | 1.74902862426043 | 0.22405309878993 | 0.00873189348715066 | 5.74901550863411 | 0.999279047927446 |
| ebi-fl187-GCST90094803 | ebi-met1400-GCST90200947 | Adenosine 5'-diphosphate (ADP) to glucose ratio \|\| id:ebi-met1400-GCST90200947 | Mushroom liking \|\| id:ebi-fl187-GCST90094803 | MR Egger | 5 | 1.78962861507428 | 1.74147147256215 | 0.379725096579277 | -1.623655471 | 5.2029127012961 | 5.98722848702555 | 0.197176606696999 | 181.801003457455 | 0.999279047927446 |
| ebi-fl187-GCST90094803 | ebi-met1400-GCST90200975 | Arachidonate (20:4n6) to caffeine ratio \|\| id:ebi-met1400-GCST90200975 | Mushroom liking \|\| id:ebi-fl187-GCST90094803 | MR Egger | 5 | -0.527924335 | 1.34177716234032 | 0.720280971412184 | -3.157807573 | 2.10195890316262 | 0.589827985244568 | 0.0425188584509912 | 8.18218232690007 | 0.999279047927446 |
| ebi-fl187-GCST90094803 | ebi-met1400-GCST90200985 | Cholesterol to oleoyl-linoleoyl-glycerol (18:1 to 18:2) [2] ratio \|\| id:ebi-met1400-GCST90200985 | Mushroom liking \|\| id:ebi-fl187-GCST90094803 | MR Egger | 5 | 0.986004102222273 | 1.30880235830943 | 0.505963622086513 | -1.57924852 | 3.55125672450875 | 2.68050203165041 | 0.206129942532374 | 34.8570957397589 | 0.999279047927446 |
| ebi-fl187-GCST90094803 | ebi-met1400-GCST90201016 | Bilirubin (Z,Z) to etiocholanolone glucuronide ratio \|\| id:ebi-met1400-GCST90201016 | Mushroom liking \|\| id:ebi-fl187-GCST90094803 | MR Egger | 5 | 1.19549197618766 | 1.367761476 | 0.446423509709988 | -1.485320517 | 3.8763044697533 | 3.30518344212238 | 0.226429754373334 | 48.2455921763188 | 0.999279047927446 |
| ebi-fl187-GCST90094803 | ebi-pw11-GCST90275189 | Placental weight (fetal genotype, adjusted for sex and gestational age) \|\| id:ebi-pw11-GCST90275189 | Mushroom liking \|\| id:ebi-fl187-GCST90094803 | MR Egger | 6 | 0.762575036126806 | 0.38924852103985 | 0.12168077642238 | -0.000352065 | 1.52550213736491 | 2.14378945435543 | 0.999647996856347 | 4.59745154200114 | 0.999279047927446 |
| ebi-fl187-GCST90094803 | ebi-pw11-GCST90275192 | Placental weight (fetal effect, conditional on maternal and paternal genotype) \|\| id:ebi-pw11-GCST90275192 | Mushroom liking \|\| id:ebi-fl187-GCST90094803 | MR Egger | 6 | 1.13887800747267 | 0.852686560522887 | 0.252600696299404 | -0.532387651 | 2.81014366609753 | 3.123262122 | 0.587201262792366 | 16.6123046723396 | 0.999279047927446 |
| ebi-fl187-GCST90094803 | ebi-pw11-GCST90275193 | Placental weight (maternal effect, conditional on fetal and paternal genotype) \|\| id:ebi-pw11-GCST90275193 | Mushroom liking \|\| id:ebi-fl187-GCST90094803 | MR Egger | 6 | -0.776257897 | 0.700140052158522 | 0.329722131784072 | -2.1485324 | 0.596016604769167 | 0.46012462721673 | 0.116655235465425 | 1.81487501805338 | 0.999279047927446 |
| ebi-fl187-GCST90094803 | ebi-pw11-GCST90275195 | Placental weight (fetal effect, conditional on maternal genotype) \|\| id:ebi-pw11-GCST90275195 | Mushroom liking \|\| id:ebi-fl187-GCST90094803 | MR Egger | 6 | 1.19139959500665 | 0.526000187387956 | 0.0862009139139505 | 0.160439227726256 | 2.22235996228704 | 3.29168501080605 | 1.17402642271743 | 9.22908547942712 | 0.999279047927446 |
| ebi-fl187-GCST90094803 | ebi-pw11-GCST90275197 | Placental weight (fetal genotype, adjusted for sex) \|\| id:ebi-pw11-GCST90275197 | Mushroom liking \|\| id:ebi-fl187-GCST90094803 | MR Egger | 6 | 0.570243481095336 | 0.3937895187048 | 0.221161189600312 | -0.201583976 | 1.34207093775674 | 1.76869764345065 | 0.817434930116802 | 3.8269607019373 | 0.999279047927446 |
| ebi-fl187-GCST90094803 | prot-a-1030 | Protein FAM171B \|\| id:prot-a-1030 | Mushroom liking \|\| id:ebi-fl187-GCST90094803 | MR Egger | 7 | 0.823121575481405 | 1.60848946504917 | 0.630628058256236 | -2.329517776 | 3.97576092697778 | 2.27759844781548 | 0.0973426767468147 | 53.2906517763414 | 0.999279047927446 |
| ebi-fl187-GCST90094803 | prot-a-1036 | BRISC complex subunit Abro1 \|\| id:prot-a-1036 | Mushroom liking \|\| id:ebi-fl187-GCST90094803 | MR Egger | 7 | -0.213301628 | 0.952749657120869 | 0.831710909607828 | -2.080690956 | 1.65408769975644 | 0.807912411294283 | 0.124843920714472 | 5.228307959 | 0.999279047927446 |
| ebi-fl187-GCST90094803 | prot-a-1055 | Phenylalanine--tRNA ligase, mitochondrial \|\| id:prot-a-1055 | Mushroom liking \|\| id:ebi-fl187-GCST90094803 | MR Egger | 7 | -0.082759734 | 0.953192043146629 | 0.934181429347849 | -1.951016139 | 1.78549667055623 | 0.920572302865617 | 0.142129574840439 | 5.96254063065125 | 0.999279047927446 |
| ebi-fl187-GCST90094803 | prot-a-1083 | Fc receptor-like protein 5 \|\| id:prot-a-1083 | Mushroom liking \|\| id:ebi-fl187-GCST90094803 | MR Egger | 7 | -0.592828312 | 0.953192043146629 | 0.561239197729222 | -2.461084716 | 1.27542809295294 | 0.552761689476158 | 0.0853423285371184 | 3.58023375492555 | 0.999279047927446 |
| ebi-fl187-GCST90094803 | prot-a-1103 | Fibroblast growth factor receptor 3 \|\| id:prot-a-1103 | Mushroom liking \|\| id:ebi-fl187-GCST90094803 | MR Egger | 7 | -0.369243613 | 0.952749657120869 | 0.71429373459423 | -2.236632941 | 1.49814571465817 | 0.691256990540739 | 0.10681756055974 | 4.47338644009004 | 0.999279047927446 |
| ebi-fl187-GCST90094803 | prot-a-1105 | Fibroblast growth factor receptor 4 \|\| id:prot-a-1105 | Mushroom liking \|\| id:ebi-fl187-GCST90094803 | MR Egger | 7 | 0.779112284284783 | 0.952749657120869 | 0.450695008445664 | -1.088277044 | 2.64650161224169 | 2.17953659757434 | 0.336796279371365 | 14.1046088425697 | 0.999279047927446 |
| ebi-fl187-GCST90094803 | prot-a-1120 | Filamin-A \|\| id:prot-a-1120 | Mushroom liking \|\| id:ebi-fl187-GCST90094803 | MR Egger | 7 | 0.336892462564698 | 0.952749657120869 | 0.738059606332931 | -1.530496865 | 2.2042817905216 | 1.40058843995109 | 0.216428104960958 | 9.06373956597957 | 0.999279047927446 |
| ebi-fl187-GCST90094803 | prot-a-1166 | Galanin peptides \|\| id:prot-a-1166 | Mushroom liking \|\| id:ebi-fl187-GCST90094803 | MR Egger | 7 | 0.149412308224828 | 0.953192043146629 | 0.881575113889656 | -1.718844096 | 2.01766871279222 | 1.16115164289852 | 0.17927325079922 | 7.52077140228771 | 0.999279047927446 |
| ebi-fl187-GCST90094803 | prot-a-1205 | ADP-ribosylation factor-binding protein GGA1 \|\| id:prot-a-1205 | Mushroom liking \|\| id:ebi-fl187-GCST90094803 | MR Egger | 7 | 0.0622769162632747 | 0.953888277136697 | 0.950475120172159 | -1.807344107 | 1.9318979394512 | 1.06425701400227 | 0.164089362391873 | 6.90259853133012 | 0.999279047927446 |
| ebi-fl187-GCST90094803 | prot-a-1227 | Guanine nucleotide-binding protein G(I)/G(S)/G(O) subunit gamma-T2 \|\| id:prot-a-1227 | Mushroom liking \|\| id:ebi-fl187-GCST90094803 | MR Egger | 7 | -0.625208505 | 0.95262509875784 | 0.540628098337543 | -2.492353698 | 1.24193668898647 | 0.535149835694451 | 0.0827150510168822 | 3.46231239808274 | 0.999279047927446 |
| ebi-fl187-GCST90094803 | prot-a-1240 | Golgi SNAP receptor complex member 1 \|\| id:prot-a-1240 | Mushroom liking \|\| id:ebi-fl187-GCST90094803 | MR Egger | 7 | 1.27727393731465 | 0.952749657120869 | 0.23772800428363 | -0.590115391 | 3.14466326527155 | 3.58684841215145 | 0.554263324243396 | 23.2118579184642 | 0.999279047927446 |
| ebi-fl187-GCST90094803 | prot-a-1283 | Glutathione S-transferase A1 \|\| id:prot-a-1283 | Mushroom liking \|\| id:ebi-fl187-GCST90094803 | MR Egger | 7 | -0.798623166 | 1.17042496190398 | 0.525348040042504 | -3.092656092 | 1.49540975889147 | 0.449948041399747 | 0.0453812574000308 | 4.46116418006811 | 0.999279047927446 |
| ebi-fl187-GCST90094803 | prot-a-1324 | E3 ubiquitin-protein ligase HECW1 \|\| id:prot-a-1324 | Mushroom liking \|\| id:ebi-fl187-GCST90094803 | MR Egger | 7 | 1.29820000271583 | 0.952749657120869 | 0.231176587012513 | -0.569189325 | 3.16558933067273 | 3.66269788426044 | 0.565984081778286 | 23.7027086507728 | 0.999279047927446 |
| ebi-fl187-GCST90094803 | prot-a-1339 | Histidine triad nucleotide-binding protein 2, mitochondrial \|\| id:prot-a-1339 | Mushroom liking \|\| id:ebi-fl187-GCST90094803 | MR Egger | 7 | -1.333061867 | 0.953192043146629 | 0.220819737583115 | -3.201318271 | 0.535194537749121 | 0.263668705625843 | 0.0407085037347691 | 1.70778043770323 | 0.999279047927446 |
| ebi-fl187-GCST90094803 | prot-a-1466 | Interleukin-11 \|\| id:prot-a-1466 | Mushroom liking \|\| id:ebi-fl187-GCST90094803 | MR Egger | 7 | -1.668261291 | 0.952749657120869 | 0.140342036746657 | -3.535650619 | 0.199128036803703 | 0.18857465718779 | 0.0291397919150619 | 1.22033820410063 | 0.999279047927446 |
| ebi-fl187-GCST90094803 | prot-a-147 | Rho GTPase-activating protein 1 \|\| id:prot-a-147 | Mushroom liking \|\| id:ebi-fl187-GCST90094803 | MR Egger | 7 | -0.260361033 | 0.953192043146629 | 0.795663539711704 | -2.128617438 | 1.60789537128957 | 0.770773260767499 | 0.119001707427271 | 4.99229323980285 | 0.999279047927446 |
| ebi-fl187-GCST90094803 | prot-a-1473 | Interleukin-12 receptor subunit beta-1 \|\| id:prot-a-1473 | Mushroom liking \|\| id:ebi-fl187-GCST90094803 | MR Egger | 7 | -1.698727392 | 0.952749657120869 | 0.134678163771937 | -3.56611672 | 0.16866193564335 | 0.182916156503158 | 0.0282654033044161 | 1.18371989776845 | 0.999279047927446 |
| ebi-fl187-GCST90094803 | prot-a-1501 | X-linked interleukin-1 receptor accessory protein-like 2 \|\| id:prot-a-1501 | Mushroom liking \|\| id:ebi-fl187-GCST90094803 | MR Egger | 7 | 0.141394927074467 | 0.953192043146629 | 0.887872568470861 | -1.726861477 | 2.00965133164186 | 1.15187946655661 | 0.177841695149308 | 7.46071557831704 | 0.999279047927446 |
| ebi-fl187-GCST90094803 | prot-a-1503 | Interleukin-1 receptor-like 2 \|\| id:prot-a-1503 | Mushroom liking \|\| id:ebi-fl187-GCST90094803 | MR Egger | 7 | -1.36162657 | 0.952749657120869 | 0.212340585299939 | -3.229015898 | 0.5057627574865 | 0.256243639456589 | 0.0395964465462999 | 1.65824988071043 | 0.999279047927446 |
| ebi-fl187-GCST90094803 | prot-a-1536 | Interleukin-5 receptor subunit alpha \|\| id:prot-a-1536 | Mushroom liking \|\| id:ebi-fl187-GCST90094803 | MR Egger | 7 | 0.29481652606012 | 0.952749657120869 | 0.769468752419314 | -1.572572802 | 2.16220585401702 | 1.34287995260673 | 0.207510611284844 | 8.69028603379553 | 0.999279047927446 |
| ebi-fl187-GCST90094803 | prot-a-1559 | Insulin-induced gene 1 protein \|\| id:prot-a-1559 | Mushroom liking \|\| id:ebi-fl187-GCST90094803 | MR Egger | 7 | -0.187541655 | 0.953192043146629 | 0.851769729276347 | -2.055798059 | 1.68071474971122 | 0.828994585810949 | 0.127990650663249 | 5.36939237157262 | 0.999279047927446 |
| ebi-fl187-GCST90094803 | prot-a-1567 | Interferon regulatory factor 1 \|\| id:prot-a-1567 | Mushroom liking \|\| id:ebi-fl187-GCST90094803 | MR Egger | 7 | -0.112515579 | 1.44193265172823 | 0.940829789292946 | -2.938703576 | 2.71367241834608 | 0.893583425827034 | 0.0529343095604998 | 15.08457077 | 0.999279047927446 |
| ebi-fl187-GCST90094803 | prot-a-1613 | Potassium voltage-gated channel subfamily E regulatory beta subunit 5 \|\| id:prot-a-1613 | Mushroom liking \|\| id:ebi-fl187-GCST90094803 | MR Egger | 7 | -0.165290416 | 0.952749657120869 | 0.869072942056489 | -2.032679744 | 1.70209891150648 | 0.847647497560986 | 0.130984046661437 | 5.48544878887861 | 0.999279047927446 |
| ebi-fl187-GCST90094803 | prot-a-1649 | Kin of IRRE-like protein 2 \|\| id:prot-a-1649 | Mushroom liking \|\| id:ebi-fl187-GCST90094803 | MR Egger | 7 | 0.655901607030794 | 1.31496697400339 | 0.63908810204707 | -1.921433662 | 3.23323687607744 | 1.92687902211012 | 0.146396927892513 | 25.3616166629813 | 0.999279047927446 |
| ebi-fl187-GCST90094803 | prot-a-165 | Armadillo repeat-containing protein 5 \|\| id:prot-a-165 | Mushroom liking \|\| id:ebi-fl187-GCST90094803 | MR Egger | 7 | 0.631523273761078 | 1.36108634913757 | 0.662158364463055 | -2.036205971 | 3.29925251807072 | 1.88047287366878 | 0.130522980681221 | 27.0923802854348 | 0.999279047927446 |
| ebi-fl187-GCST90094803 | prot-a-1656 | Kallikrein-11 \|\| id:prot-a-1656 | Mushroom liking \|\| id:ebi-fl187-GCST90094803 | MR Egger | 7 | -0.333377244 | 1.33973578430791 | 0.813384171572611 | -2.959259381 | 2.29250489335964 | 0.71649984798024 | 0.0518573094560125 | 9.89970435298365 | 0.999279047927446 |
| ebi-fl187-GCST90094803 | prot-a-1697 | Laminin subunit gamma-2 \|\| id:prot-a-1697 | Mushroom liking \|\| id:ebi-fl187-GCST90094803 | MR Egger | 7 | -1.04379032 | 0.953192043146629 | 0.323418654938701 | -2.912046725 | 0.824466084489703 | 0.352117511334405 | 0.0543643470741462 | 2.28066275898122 | 0.999279047927446 |
| ebi-fl187-GCST90094803 | prot-a-1698 | Lysosome-associated membrane glycoprotein 3 \|\| id:prot-a-1698 | Mushroom liking \|\| id:ebi-fl187-GCST90094803 | MR Egger | 7 | -0.800024319 | 0.953192043146629 | 0.439567858272295 | -2.668280724 | 1.06823208528602 | 0.449318036892588 | 0.0693713914191121 | 2.91022991102047 | 0.999279047927446 |
| ebi-fl187-GCST90094803 | prot-a-1724 | Leptin receptor \|\| id:prot-a-1724 | Mushroom liking \|\| id:ebi-fl187-GCST90094803 | MR Egger | 7 | -0.044127289 | 0.952749657120869 | 0.964851562060344 | -1.911516617 | 1.82326203904592 | 0.95683215559812 | 0.147855975599115 | 6.19202416592777 | 0.999279047927446 |
| ebi-fl187-GCST90094803 | prot-a-1743 | Leukocyte immunoglobulin-like receptor subfamily B member 2 \|\| id:prot-a-1743 | Mushroom liking \|\| id:ebi-fl187-GCST90094803 | MR Egger | 7 | -1.9239274 | 0.958602028262535 | 0.101030687994067 | -3.802787375 | -0.045067425 | 0.146032307141322 | 0.0223085029416962 | 0.955933026288311 | 0.999279047927446 |
| ebi-fl187-GCST90094803 | prot-a-1746 | Leukocyte immunoglobulin-like receptor subfamily B member 4 \|\| id:prot-a-1746 | Mushroom liking \|\| id:ebi-fl187-GCST90094803 | MR Egger | 7 | -0.893366671 | 1.250517788 | 0.50693714931889 | -3.344381535 | 1.55764819328061 | 0.409275534591792 | 0.0352820290980105 | 4.74764256755411 | 0.999279047927446 |
| ebi-fl187-GCST90094803 | prot-a-1750 | Leucine-rich repeat and immunoglobulin-like domain-containing nogo receptor-interacting protein 3 \|\| id:prot-a-1750 | Mushroom liking \|\| id:ebi-fl187-GCST90094803 | MR Egger | 7 | 0.305650822115794 | 0.952749657120869 | 0.761329939752235 | -1.561738506 | 2.1730401500727 | 1.3575082119358 | 0.209771065787509 | 8.78495105392585 | 0.999279047927446 |
| ebi-fl187-GCST90094803 | prot-a-1753 | Lipase member N \|\| id:prot-a-1753 | Mushroom liking \|\| id:ebi-fl187-GCST90094803 | MR Egger | 7 | -0.183628281 | 0.952749657120869 | 0.854750458762565 | -2.051017609 | 1.68376104718223 | 0.832245107842754 | 0.128603968457519 | 5.38577407708059 | 0.999279047927446 |
| ebi-fl187-GCST90094803 | prot-a-1760 | Ig Kappa chain V-I region HK102- like \|\| id:prot-a-1760 | Mushroom liking \|\| id:ebi-fl187-GCST90094803 | MR Egger | 7 | -0.427644972 | 0.952749657120869 | 0.672322103293175 | -2.2950343 | 1.4397443560715 | 0.652042867260035 | 0.100757937227675 | 4.21961695964443 | 0.999279047927446 |
| ebi-fl187-GCST90094803 | prot-a-1792 | Leucine-rich repeat neuronal protein 1 \|\| id:prot-a-1792 | Mushroom liking \|\| id:ebi-fl187-GCST90094803 | MR Egger | 7 | 1.29451822417506 | 0.998526097632575 | 0.251434822606287 | -0.662592927 | 3.25162937553491 | 3.64923743618854 | 0.515512912581164 | 25.8323963196233 | 0.999279047927446 |
| ebi-fl187-GCST90094803 | prot-a-1795 | Leucine-rich repeat transmembrane neuronal protein 1 \|\| id:prot-a-1795 | Mushroom liking \|\| id:ebi-fl187-GCST90094803 | MR Egger | 7 | -1.533489663 | 0.953192043146629 | 0.16857447641386 | -3.401746067 | 0.334766741979713 | 0.215781347824623 | 0.0333150488335814 | 1.39761434244326 | 0.999279047927446 |
| ebi-fl187-GCST90094803 | prot-a-1820 | LysM and putative peptidoglycan-binding domain-containing protein 4 \|\| id:prot-a-1820 | Mushroom liking \|\| id:ebi-fl187-GCST90094803 | MR Egger | 7 | -1.809466448 | 1.36087267850195 | 0.241068161524385 | -4.476776898 | 0.85784400180544 | 0.163741478083633 | 0.0113700008333927 | 2.35807121194492 | 0.999279047927446 |
| ebi-fl187-GCST90094803 | prot-a-1844 | Mitogen-activated protein kinase kinase kinase 7:TGF-beta-activated kinase 1 and MAP3K7-binding protein 1 fusion \|\| id:prot-a-1844 | Mushroom liking \|\| id:ebi-fl187-GCST90094803 | MR Egger | 7 | 0.0440457853040867 | 1.10433168471874 | 0.969728737416353 | -2.120444317 | 2.20853588735281 | 1.04503020081864 | 0.119978308295433 | 9.10237972295711 | 0.999279047927446 |
| ebi-fl187-GCST90094803 | prot-a-1856 | Megakaryocyte-associated tyrosine-protein kinase \|\| id:prot-a-1856 | Mushroom liking \|\| id:ebi-fl187-GCST90094803 | MR Egger | 7 | -0.758708443 | 0.952749657120869 | 0.461955486005581 | -2.626097771 | 1.10868088451645 | 0.46827083488085 | 0.0723602783736297 | 3.03035836412592 | 0.999279047927446 |
| ebi-fl187-GCST90094803 | prot-a-1910 | Stromelysin-2 \|\| id:prot-a-1910 | Mushroom liking \|\| id:ebi-fl187-GCST90094803 | MR Egger | 7 | -1.272311816 | 1.39347428655101 | 0.403094685641618 | -4.003521418 | 1.45889778520387 | 0.280183140493039 | 0.018251255294185 | 4.30121605068739 | 0.999279047927446 |
| ebi-fl187-GCST90094803 | prot-a-1940 | 39S ribosomal protein L14, mitochondrial \|\| id:prot-a-1940 | Mushroom liking \|\| id:ebi-fl187-GCST90094803 | MR Egger | 7 | 0.908239077221816 | 1.03011329612274 | 0.418307369492179 | -1.110782983 | 2.92726113762238 | 2.4799516827421 | 0.329301022946046 | 18.6764082714162 | 0.999279047927446 |
| ebi-fl187-GCST90094803 | prot-a-1942 | 39S ribosomal protein L33, mitochondrial \|\| id:prot-a-1942 | Mushroom liking \|\| id:ebi-fl187-GCST90094803 | MR Egger | 7 | -1.117664397 | 1.37379893753168 | 0.452883871122443 | -3.810310315 | 1.57498152022192 | 0.327042745207086 | 0.0221413071133215 | 4.83065234790204 | 0.999279047927446 |
| ebi-fl187-GCST90094803 | prot-a-1948 | Mesothelin \|\| id:prot-a-1948 | Mushroom liking \|\| id:ebi-fl187-GCST90094803 | MR Egger | 7 | -0.519236296 | 0.952749657120869 | 0.609180673941045 | -2.386625624 | 1.34815303215441 | 0.59497475922763 | 0.0919393991597815 | 3.85030756512525 | 0.999279047927446 |
| ebi-fl187-GCST90094803 | prot-a-1980 | Unconventional myosin-VI \|\| id:prot-a-1980 | Mushroom liking \|\| id:ebi-fl187-GCST90094803 | MR Egger | 7 | 0.595945363979489 | 1.15240684432339 | 0.627119067843116 | -1.662772051 | 2.85466277885334 | 1.81474572952929 | 0.189612635036379 | 17.3685791678015 | 0.999279047927446 |
| ebi-fl187-GCST90094803 | prot-a-2039 | Neurofascin \|\| id:prot-a-2039 | Mushroom liking \|\| id:ebi-fl187-GCST90094803 | MR Egger | 7 | -1.742784538 | 0.953192043146629 | 0.127034310984677 | -3.611040942 | 0.125471866719243 | 0.175032337249172 | 0.027023702102609 | 1.13368327428203 | 0.999279047927446 |
| ebi-fl187-GCST90094803 | prot-a-2041 | NFU1 iron-sulfur cluster scaffold homolog, mitochondrial \|\| id:prot-a-2041 | Mushroom liking \|\| id:ebi-fl187-GCST90094803 | MR Egger | 7 | -1.879786895 | 1.14259777615498 | 0.160850631146301 | -4.119278536 | 0.359704746688806 | 0.152622627001332 | 0.0162562385056744 | 1.43290628177341 | 0.999279047927446 |
| ebi-fl187-GCST90094803 | prot-a-2088 | Glucocorticoid receptor \|\| id:prot-a-2088 | Mushroom liking \|\| id:ebi-fl187-GCST90094803 | MR Egger | 7 | -0.101254792 | 0.953192043146629 | 0.919532511896427 | -1.969511197 | 1.76700161244315 | 0.903702747206978 | 0.139525039850579 | 5.85327663180775 | 0.999279047927446 |
| ebi-fl187-GCST90094803 | prot-a-2111 | Neuron-specific protein family member 2 \|\| id:prot-a-2111 | Mushroom liking \|\| id:ebi-fl187-GCST90094803 | MR Egger | 7 | -0.506263063 | 0.952749657120869 | 0.617911667196028 | -2.373652391 | 1.36112626450456 | 0.602743790783878 | 0.0931399208327715 | 3.90058391804743 | 0.999279047927446 |
| ebi-fl187-GCST90094803 | prot-a-2125 | Nuclear migration protein nudC \|\| id:prot-a-2125 | Mushroom liking \|\| id:ebi-fl187-GCST90094803 | MR Egger | 7 | 0.364017872169218 | 0.952749657120869 | 0.718108837735471 | -1.503371456 | 2.23140720012612 | 1.43909993376575 | 0.222379153383016 | 9.31296206437827 | 0.999279047927446 |
| ebi-fl187-GCST90094803 | prot-a-2142 | Ornithine decarboxylase \|\| id:prot-a-2142 | Mushroom liking \|\| id:ebi-fl187-GCST90094803 | MR Egger | 7 | 0.519018286189889 | 0.952749657120869 | 0.609326793637649 | -1.348371042 | 2.38640761414679 | 1.68037719019818 | 0.259662896337281 | 10.8743587981497 | 0.999279047927446 |
| ebi-fl187-GCST90094803 | prot-a-2186 | Prostate and testis expressed protein 1 \|\| id:prot-a-2186 | Mushroom liking \|\| id:ebi-fl187-GCST90094803 | MR Egger | 7 | 0.370205877751721 | 1.17379960987256 | 0.765203484727239 | -1.930441358 | 2.67085311310194 | 1.44803270169457 | 0.145084150358417 | 14.4522933759265 | 0.999279047927446 |
| ebi-fl187-GCST90094803 | prot-a-2227 | Platelet-derived growth factor C \|\| id:prot-a-2227 | Mushroom liking \|\| id:ebi-fl187-GCST90094803 | MR Egger | 7 | 0.35490597937249 | 0.953192043146629 | 0.724904239215818 | -1.513350425 | 2.22316238393988 | 1.42604657018486 | 0.22017107411565 | 9.23649406946072 | 0.999279047927446 |
| ebi-fl187-GCST90094803 | prot-a-2229 | Platelet-derived growth factor receptor alpha \|\| id:prot-a-2229 | Mushroom liking \|\| id:ebi-fl187-GCST90094803 | MR Egger | 7 | 0.910267235940058 | 1.20192580423749 | 0.482988682462795 | -1.44550734 | 3.26604181224555 | 2.48498652236934 | 0.235626503389946 | 26.2073999635678 | 0.999279047927446 |
| ebi-fl187-GCST90094803 | prot-a-2243 | Platelet endothelial aggregation receptor 1 \|\| id:prot-a-2243 | Mushroom liking \|\| id:ebi-fl187-GCST90094803 | MR Egger | 7 | -0.528103099 | 0.952749657120869 | 0.603255292775895 | -2.395492427 | 1.33928622919474 | 0.58972255481314 | 0.0911277941115951 | 3.81631855621847 | 0.999279047927446 |
| ebi-fl187-GCST90094803 | prot-a-2246 | E3 ubiquitin-protein ligase pellino homolog 2 \|\| id:prot-a-2246 | Mushroom liking \|\| id:ebi-fl187-GCST90094803 | MR Egger | 7 | -0.947958201 | 0.952749657120869 | 0.36543260224561 | -2.815347529 | 0.919431127020508 | 0.387531477613716 | 0.0598839037366222 | 2.50786332838263 | 0.999279047927446 |
| ebi-fl187-GCST90094803 | prot-a-2251 | Platelet factor 4 variant \|\| id:prot-a-2251 | Mushroom liking \|\| id:ebi-fl187-GCST90094803 | MR Egger | 7 | 0.057416274707218 | 0.953192043146629 | 0.954301340457887 | -1.81084013 | 1.92567267927461 | 1.05909659377014 | 0.163516703814497 | 6.85976153364742 | 0.999279047927446 |
| ebi-fl187-GCST90094803 | prot-a-2346 | Protein phosphatase 1L \|\| id:prot-a-2346 | Mushroom liking \|\| id:ebi-fl187-GCST90094803 | MR Egger | 7 | -0.669811438 | 1.20483709267791 | 0.602213663168769 | -3.031292139 | 1.69166926385226 | 0.511805075781207 | 0.0482532479013446 | 5.42853480310717 | 0.999279047927446 |
| ebi-fl187-GCST90094803 | prot-a-2358 | PR domain zinc finger protein 4 \|\| id:prot-a-2358 | Mushroom liking \|\| id:ebi-fl187-GCST90094803 | MR Egger | 7 | -0.155828652 | 0.953192043146629 | 0.876542346641949 | -2.024085057 | 1.71242775233994 | 0.855705801092925 | 0.132114665322044 | 5.54240073378066 | 0.999279047927446 |
| ebi-fl187-GCST90094803 | prot-a-2390 | Trypsin-1 \|\| id:prot-a-2390 | Mushroom liking \|\| id:ebi-fl187-GCST90094803 | MR Egger | 7 | -0.223414164 | 1.30848397821193 | 0.871120385588142 | -2.788042761 | 2.34121443353604 | 0.799783539333687 | 0.061541547629442 | 10.3938515430363 | 0.999279047927446 |
| ebi-fl187-GCST90094803 | prot-a-242 | Brain-derived neurotrophic factor \|\| id:prot-a-242 | Mushroom liking \|\| id:ebi-fl187-GCST90094803 | MR Egger | 7 | 0.198228203357969 | 0.953192043146629 | 0.843463734377265 | -1.670028201 | 2.06648460792536 | 1.21924059687538 | 0.188241756918682 | 7.8970131675471 | 0.999279047927446 |
| ebi-fl187-GCST90094803 | prot-a-2464 | Dihydropteridine reductase \|\| id:prot-a-2464 | Mushroom liking \|\| id:ebi-fl187-GCST90094803 | MR Egger | 7 | -0.164860626 | 1.37281024600188 | 0.909088218748522 | -2.855568708 | 2.52584745593619 | 0.848011886467709 | 0.0575230979483106 | 12.501485233579 | 0.999279047927446 |
| ebi-fl187-GCST90094803 | prot-a-2474 | Ras-related protein Rab-27A \|\| id:prot-a-2474 | Mushroom liking \|\| id:ebi-fl187-GCST90094803 | MR Egger | 7 | -0.282539821 | 0.953192043146629 | 0.778831064615701 | -2.150796226 | 1.58571658318806 | 0.753866621352897 | 0.116391446978963 | 4.88278904972073 | 0.999279047927446 |
| ebi-fl187-GCST90094803 | prot-a-2478 | Ras-related protein Rab-7b \|\| id:prot-a-2478 | Mushroom liking \|\| id:ebi-fl187-GCST90094803 | MR Egger | 7 | -0.121461594 | 1.52821435854273 | 0.939734279498342 | -3.116761737 | 2.87383854857268 | 0.885625065860495 | 0.0443003927219593 | 17.7048488532161 | 0.999279047927446 |
| ebi-fl187-GCST90094803 | prot-a-2528 | E3 ubiquitin-protein ligase rififylin \|\| id:prot-a-2528 | Mushroom liking \|\| id:ebi-fl187-GCST90094803 | MR Egger | 7 | 1.58545182405282 | 1.12419948697568 | 0.217524269965746 | -0.61797917 | 3.78888281852515 | 4.88149645783469 | 0.539032630782993 | 44.2069854532534 | 0.999279047927446 |
| ebi-fl187-GCST90094803 | prot-a-2543 | Protein RIC-3 \|\| id:prot-a-2543 | Mushroom liking \|\| id:ebi-fl187-GCST90094803 | MR Egger | 7 | 0.874894191912386 | 0.952749657120869 | 0.400599178340553 | -0.992495136 | 2.74228351986929 | 2.39862148698747 | 0.370650712328787 | 15.5223903434305 | 0.999279047927446 |
| ebi-fl187-GCST90094803 | prot-a-255 | Bone morphogenetic protein 6 \|\| id:prot-a-255 | Mushroom liking \|\| id:ebi-fl187-GCST90094803 | MR Egger | 7 | -0.616196391 | 0.952749657120869 | 0.546303102639505 | -2.483585719 | 1.25119293695474 | 0.53999446417607 | 0.083443483636861 | 3.49450920109944 | 0.999279047927446 |
| ebi-fl187-GCST90094803 | prot-a-2568 | RING finger protein 165 \|\| id:prot-a-2568 | Mushroom liking \|\| id:ebi-fl187-GCST90094803 | MR Egger | 7 | 0.794408598273946 | 1.05229920998887 | 0.484313189733937 | -1.268097853 | 2.85691504985212 | 2.21313176 | 0.281366313089251 | 17.4077420010528 | 0.999279047927446 |
| ebi-fl187-GCST90094803 | prot-a-2573 | E3 ubiquitin-protein ligase RNF8 \|\| id:prot-a-2573 | Mushroom liking \|\| id:ebi-fl187-GCST90094803 | MR Egger | 7 | 1.38140104927764 | 1.58821371713406 | 0.424198492839536 | -1.731497836 | 4.49429993486039 | 3.98047456361608 | 0.177019065715031 | 89.5054873755854 | 0.999279047927446 |
| ebi-fl187-GCST90094803 | prot-a-260 | Cytoplasmic tyrosine-protein kinase BMX \|\| id:prot-a-260 | Mushroom liking \|\| id:ebi-fl187-GCST90094803 | MR Egger | 7 | -0.006914148 | 1.32954725301846 | 0.99605182481529 | -2.612826764 | 2.59899846766191 | 0.993109699474862 | 0.073326972465228 | 13.4502604162301 | 0.999279047927446 |
| ebi-fl187-GCST90094803 | prot-a-2623 | Serum amyloid A-1 protein \|\| id:prot-a-2623 | Mushroom liking \|\| id:ebi-fl187-GCST90094803 | MR Egger | 7 | -0.810065117 | 0.974213597461198 | 0.443571433919132 | -2.719523768 | 1.09939353381377 | 0.444829099249879 | 0.0659061335483044 | 3.00234465119142 | 0.999279047927446 |
| ebi-fl187-GCST90094803 | prot-a-2640 | Lysosome membrane protein 2 \|\| id:prot-a-2640 | Mushroom liking \|\| id:ebi-fl187-GCST90094803 | MR Egger | 7 | -1.428646068 | 1.17383121197947 | 0.277880090138087 | -3.729355243 | 0.87206310770302 | 0.239633149746396 | 0.0240083103675284 | 2.39184039102749 | 0.999279047927446 |
| ebi-fl187-GCST90094803 | prot-a-2641 | Scavenger receptor class F member 2 \|\| id:prot-a-2641 | Mushroom liking \|\| id:ebi-fl187-GCST90094803 | MR Egger | 7 | -0.954937178 | 1.04628056712525 | 0.403263477547708 | -3.005647089 | 1.09577273371887 | 0.384836320039914 | 0.0495067086943418 | 2.99149341832109 | 0.999279047927446 |
| ebi-fl187-GCST90094803 | prot-a-2669 | Semaphorin-3A \|\| id:prot-a-2669 | Mushroom liking \|\| id:ebi-fl187-GCST90094803 | MR Egger | 7 | -0.080237695 | 0.953192043146629 | 0.936181436367976 | -1.9484941 | 1.78801870928911 | 0.922896952065323 | 0.142488483533845 | 5.97759736792448 | 0.999279047927446 |
| ebi-fl187-GCST90094803 | prot-a-2683 | Sentrin-specific protease 7 \|\| id:prot-a-2683 | Mushroom liking \|\| id:ebi-fl187-GCST90094803 | MR Egger | 7 | -1.31482402 | 0.953192043146629 | 0.226277622632929 | -3.183080424 | 0.553432384752641 | 0.268521573557813 | 0.0414577507561932 | 1.73921243074656 | 0.999279047927446 |
| ebi-fl187-GCST90094803 | prot-a-270 | Bromodomain testis-specific protein \|\| id:prot-a-270 | Mushroom liking \|\| id:ebi-fl187-GCST90094803 | MR Egger | 7 | 0.466999428584433 | 0.953192043146629 | 0.644920653566594 | -1.401256976 | 2.33525583315183 | 1.59520049189435 | 0.246287192209062 | 10.3321028857234 | 0.999279047927446 |
| ebi-fl187-GCST90094803 | prot-a-2708 | 14-3-3 protein sigma \|\| id:prot-a-2708 | Mushroom liking \|\| id:ebi-fl187-GCST90094803 | MR Egger | 7 | 0.433586744050017 | 0.976538655606982 | 0.675596729329736 | -1.480429021 | 2.3476025090397 | 1.54278117286068 | 0.227540047995222 | 10.4604607773632 | 0.999279047927446 |
| ebi-fl187-GCST90094803 | prot-a-2713 | Beta-sarcoglycan \|\| id:prot-a-2713 | Mushroom liking \|\| id:ebi-fl187-GCST90094803 | MR Egger | 7 | -0.072590276 | 0.952749657120869 | 0.942222373910443 | -1.939979604 | 1.79479905237232 | 0.929981788221053 | 0.143706880859244 | 6.01826524416692 | 0.999279047927446 |
| ebi-fl187-GCST90094803 | prot-a-2720 | Protein shisa-3 homolog \|\| id:prot-a-2720 | Mushroom liking \|\| id:ebi-fl187-GCST90094803 | MR Egger | 7 | -0.338728913 | 1.18979015654343 | 0.787291658444543 | -2.670717619 | 1.99325979418646 | 0.71267562025826 | 0.0692025463919641 | 7.33941980738263 | 0.999279047927446 |
| ebi-fl187-GCST90094803 | prot-a-2730 | Sialic acid-binding Ig-like lectin 8 \|\| id:prot-a-2730 | Mushroom liking \|\| id:ebi-fl187-GCST90094803 | MR Egger | 7 | 0.930199911164075 | 0.952749657120869 | 0.373739634433775 | -0.937189417 | 2.79758923912098 | 2.53501590494645 | 0.391727271697752 | 16.4050504078508 | 0.999279047927446 |
| ebi-fl187-GCST90094803 | prot-a-2761 | Sodium- and chloride-dependent neutral and basic amino acid transporter B(0+) \|\| id:prot-a-2761 | Mushroom liking \|\| id:ebi-fl187-GCST90094803 | MR Egger | 7 | -0.354424671 | 0.953192043146629 | 0.725257439750962 | -2.222681076 | 1.51383173308989 | 0.70157696435715 | 0.108318309546712 | 4.54410929210752 | 0.999279047927446 |
| ebi-fl187-GCST90094803 | prot-a-2770 | Secreted Ly-6/uPAR-related protein 1 \|\| id:prot-a-2770 | Mushroom liking \|\| id:ebi-fl187-GCST90094803 | MR Egger | 7 | 0.984316554164528 | 0.952749657120869 | 0.348900651947667 | -0.883072774 | 2.85170588212143 | 2.67598237029858 | 0.413510333794332 | 17.3172979268529 | 0.999279047927446 |
| ebi-fl187-GCST90094803 | prot-a-2784 | N-lysine methyltransferase SMYD2 \|\| id:prot-a-2784 | Mushroom liking \|\| id:ebi-fl187-GCST90094803 | MR Egger | 7 | 0.072193943819285 | 1.03738020926883 | 0.94721551625044 | -1.961071266 | 2.10545915398619 | 1.07486378703812 | 0.140707604831457 | 8.21087219891068 | 0.999279047927446 |
| ebi-fl187-GCST90094803 | prot-a-2837 | Serine palmitoyltransferase 2 \|\| id:prot-a-2837 | Mushroom liking \|\| id:ebi-fl187-GCST90094803 | MR Egger | 7 | 1.18863561478769 | 1.25781872162999 | 0.388051665408849 | -1.27668908 | 3.65396030918246 | 3.28259942052921 | 0.278959385453934 | 38.6273397402437 | 0.999279047927446 |
| ebi-fl187-GCST90094803 | prot-a-2858 | Alpha-2,8-sialyltransferase 8B \|\| id:prot-a-2858 | Mushroom liking \|\| id:ebi-fl187-GCST90094803 | MR Egger | 7 | -0.675074296 | 0.952749657120869 | 0.510259205848654 | -2.542463624 | 1.19231503183665 | 0.50911859366611 | 0.0786723417704294 | 3.29469972017515 | 0.999279047927446 |
| ebi-fl187-GCST90094803 | prot-a-2911 | Synaptotagmin-11 \|\| id:prot-a-2911 | Mushroom liking \|\| id:ebi-fl187-GCST90094803 | MR Egger | 7 | 0.135653443496945 | 0.953192043146629 | 0.89238816435768 | -1.732602961 | 2.00390984806434 | 1.14528491887526 | 0.176823545618524 | 7.41800273721979 | 0.999279047927446 |
| ebi-fl187-GCST90094803 | prot-a-2962 | Transforming growth factor beta-1 \|\| id:prot-a-2962 | Mushroom liking \|\| id:ebi-fl187-GCST90094803 | MR Egger | 7 | -0.153545133 | 0.952749657120869 | 0.878276808712147 | -2.020934461 | 1.71384419467081 | 0.857662054218418 | 0.132531561589854 | 5.55025678730454 | 0.999279047927446 |
| ebi-fl187-GCST90094803 | prot-a-2978 | Tyrosine-protein kinase receptor Tie-1, soluble \|\| id:prot-a-2978 | Mushroom liking \|\| id:ebi-fl187-GCST90094803 | MR Egger | 7 | 0.456000232379662 | 0.952749657120869 | 0.652408902791609 | -1.411389096 | 2.32338956033656 | 1.57775071140361 | 0.243804380237362 | 10.2102238889682 | 0.999279047927446 |
| ebi-fl187-GCST90094803 | prot-a-299 | Uncharacterized protein C1orf226 \|\| id:prot-a-299 | Mushroom liking \|\| id:ebi-fl187-GCST90094803 | MR Egger | 7 | -1.91762275 | 0.952749657120869 | 0.100296948359561 | -3.785012078 | -0.050233422 | 0.146955898131075 | 0.0227085885033114 | 0.951007412563822 | 0.999279047927446 |
| ebi-fl187-GCST90094803 | prot-a-3015 | Transmembrane protein 70, mitochondrial \|\| id:prot-a-3015 | Mushroom liking \|\| id:ebi-fl187-GCST90094803 | MR Egger | 7 | 0.177259915057432 | 0.952749657120869 | 0.859717510669619 | -1.690129413 | 2.04464924301433 | 1.19394137614313 | 0.184495646331452 | 7.72644795696479 | 0.999279047927446 |
| ebi-fl187-GCST90094803 | prot-a-3016 | Transmembrane protein 87B \|\| id:prot-a-3016 | Mushroom liking \|\| id:ebi-fl187-GCST90094803 | MR Egger | 7 | -0.144833576 | 0.952749657120869 | 0.885117585909579 | -2.012222904 | 1.72255575241349 | 0.865166265955779 | 0.133691161568845 | 5.59881939063274 | 0.999279047927446 |
| ebi-fl187-GCST90094803 | prot-a-3123 | Thioredoxin domain-containing protein 12 \|\| id:prot-a-3123 | Mushroom liking \|\| id:ebi-fl187-GCST90094803 | MR Egger | 7 | 0.195844945557536 | 0.952749657120869 | 0.845243516003413 | -1.671544382 | 2.06323427351444 | 1.21633829205631 | 0.187956564563955 | 7.87138690343024 | 0.999279047927446 |
| ebi-fl187-GCST90094803 | prot-a-3133 | PolyUbiquitin K48-linked \|\| id:prot-a-3133 | Mushroom liking \|\| id:ebi-fl187-GCST90094803 | MR Egger | 7 | 0.69505974322991 | 1.05181072307916 | 0.537953504215233 | -1.366489274 | 2.75660876046506 | 2.003828786 | 0.255000627254482 | 15.7463526466849 | 0.999279047927446 |
| ebi-fl187-GCST90094803 | prot-a-3135 | Ubiquitin-conjugating enzyme E2 B \|\| id:prot-a-3135 | Mushroom liking \|\| id:ebi-fl187-GCST90094803 | MR Egger | 7 | -0.618167878 | 0.953192043146629 | 0.545248894210446 | -2.486424283 | 1.25008852623064 | 0.538930920654968 | 0.0832069598255539 | 3.49065195804465 | 0.999279047927446 |
| ebi-fl187-GCST90094803 | prot-a-3162 | NKG2D ligand 3 \|\| id:prot-a-3162 | Mushroom liking \|\| id:ebi-fl187-GCST90094803 | MR Egger | 7 | -0.323686456 | 0.952749657120869 | 0.747859189686443 | -2.191075784 | 1.54370287216442 | 0.723477048858994 | 0.111796415135889 | 4.68189467067885 | 0.999279047927446 |
| ebi-fl187-GCST90094803 | prot-a-3188 | Vesicle-associated membrane protein 8 \|\| id:prot-a-3188 | Mushroom liking \|\| id:ebi-fl187-GCST90094803 | MR Egger | 7 | 1.89177855927755 | 1.17555556006995 | 0.168472555583776 | -0.412310338 | 4.19586745701466 | 6.63115209626732 | 0.662118763247477 | 66.411315559404 | 0.999279047927446 |
| ebi-fl187-GCST90094803 | prot-a-3201 | Vitrin \|\| id:prot-a-3201 | Mushroom liking \|\| id:ebi-fl187-GCST90094803 | MR Egger | 7 | 0.110513026581396 | 0.952749657120869 | 0.91217218596866 | -1.756876301 | 1.9779023545383 | 1.11685089770648 | 0.172583120365467 | 7.22756620152854 | 0.999279047927446 |
| ebi-fl187-GCST90094803 | prot-a-3232 | WNT1-inducible-signaling pathway protein 3 \|\| id:prot-a-3232 | Mushroom liking \|\| id:ebi-fl187-GCST90094803 | MR Egger | 7 | -0.013782411 | 1.3378879559335 | 0.992179039521477 | -2.636042805 | 2.60847798252704 | 0.986312131485275 | 0.0716442195094451 | 13.578368602184 | 0.999279047927446 |
| ebi-fl187-GCST90094803 | prot-a-327 | Carbonic anhydrase-related protein 10 \|\| id:prot-a-327 | Mushroom liking \|\| id:ebi-fl187-GCST90094803 | MR Egger | 7 | -0.846918007 | 0.952749657120869 | 0.414760418011643 | -2.714307335 | 1.02047132101119 | 0.428734253826407 | 0.0662508267530327 | 2.77450213699668 | 0.999279047927446 |
| ebi-fl187-GCST90094803 | prot-a-332 | Carbonic anhydrase 5A, mitochondrial \|\| id:prot-a-332 | Mushroom liking \|\| id:ebi-fl187-GCST90094803 | MR Egger | 7 | 0.365150460348204 | 1.38149276168363 | 0.802085518370066 | -2.342575353 | 3.07287627324812 | 1.4407307646947 | 0.0960798797712032 | 21.6039522663922 | 0.999279047927446 |
| ebi-fl187-GCST90094803 | prot-a-338 | Cell adhesion molecule 3 \|\| id:prot-a-338 | Mushroom liking \|\| id:ebi-fl187-GCST90094803 | MR Egger | 7 | 0.696531969938941 | 0.953192043146629 | 0.497728837061585 | -1.171724435 | 2.56478837450633 | 2.00678104849436 | 0.309832194964473 | 12.9979073900241 | 0.999279047927446 |
| ebi-fl187-GCST90094803 | prot-a-34 | A disintegrin and metalloproteinase with thrombospondin motifs 3 \|\| id:prot-a-34 | Mushroom liking \|\| id:ebi-fl187-GCST90094803 | MR Egger | 7 | -0.466816056 | 1.28944627009468 | 0.732128286269241 | -2.994130745 | 2.06049863355663 | 0.626995411956995 | 0.0500801405735437 | 7.84988304970536 | 0.999279047927446 |
| ebi-fl187-GCST90094803 | prot-a-358 | Cysteine--tRNA ligase, cytoplasmic \|\| id:prot-a-358 | Mushroom liking \|\| id:ebi-fl187-GCST90094803 | MR Egger | 7 | -0.619860261 | 1.01493546389192 | 0.568066214450656 | -2.60913377 | 1.36941324810861 | 0.538019614600585 | 0.0735982691617634 | 3.93304229830106 | 0.999279047927446 |
| ebi-fl187-GCST90094803 | prot-a-382 | Coiled-coil domain-containing protein 167 \|\| id:prot-a-382 | Mushroom liking \|\| id:ebi-fl187-GCST90094803 | MR Egger | 7 | 1.51266578970228 | 1.43563699688721 | 0.340272113563156 | -1.301182724 | 4.32651430360122 | 4.53881420541134 | 0.272209653626621 | 75.6800286719481 | 0.999279047927446 |
| ebi-fl187-GCST90094803 | prot-a-40 | Pituitary adenylate cyclase-activating polypeptide 27 \|\| id:prot-a-40 | Mushroom liking \|\| id:ebi-fl187-GCST90094803 | MR Egger | 7 | -0.33925424 | 1.01478911815971 | 0.751712461355913 | -2.328240912 | 1.64973243119233 | 0.712301330290674 | 0.0974670494951287 | 5.20558678817112 | 0.999279047927446 |
| ebi-fl187-GCST90094803 | prot-a-403 | C-C motif chemokine 25 \|\| id:prot-a-403 | Mushroom liking \|\| id:ebi-fl187-GCST90094803 | MR Egger | 7 | -0.244045501 | 1.4802182042997 | 0.875503592936949 | -3.145273181 | 2.6571821794292 | 0.783451985604449 | 0.0430551605699775 | 14.2560614249702 | 0.999279047927446 |
| ebi-fl187-GCST90094803 | prot-a-415 | Cyclin-C \|\| id:prot-a-415 | Mushroom liking \|\| id:ebi-fl187-GCST90094803 | MR Egger | 7 | -0.748174714 | 1.05838755672302 | 0.51120174293369 | -2.822614325 | 1.3262648970824 | 0.47322954411525 | 0.0594503169034386 | 3.76694714323002 | 0.999279047927446 |
| ebi-fl187-GCST90094803 | prot-a-44 | Adhesion G protein-coupled receptor F5 \|\| id:prot-a-44 | Mushroom liking \|\| id:ebi-fl187-GCST90094803 | MR Egger | 7 | -0.49139144 | 0.953192043146629 | 0.628164420621823 | -2.359647844 | 1.37686496504661 | 0.611774554387292 | 0.094453479691489 | 3.96245968510882 | 0.999279047927446 |
| ebi-fl187-GCST90094803 | prot-a-455 | CD59 glycoprotein \|\| id:prot-a-455 | Mushroom liking \|\| id:ebi-fl187-GCST90094803 | MR Egger | 7 | 0.571257997285149 | 0.952749657120869 | 0.574913754572582 | -1.296131331 | 2.43864732524205 | 1.77049292636262 | 0.2735881704927 | 11.4575319417317 | 0.999279047927446 |
| ebi-fl187-GCST90094803 | prot-a-478 | Cadherin-12 \|\| id:prot-a-478 | Mushroom liking \|\| id:ebi-fl187-GCST90094803 | MR Egger | 7 | 1.32427542024837 | 0.953192043146629 | 0.223433505884496 | -0.543980984 | 3.19253182481576 | 3.75946033903471 | 0.580432952363652 | 24.3499993982424 | 0.999279047927446 |
| ebi-fl187-GCST90094803 | prot-a-516 | Centrin-2 \|\| id:prot-a-516 | Mushroom liking \|\| id:ebi-fl187-GCST90094803 | MR Egger | 7 | -0.522384167 | 1.15336409910822 | 0.669581195591353 | -2.782977801 | 1.73820946717267 | 0.593104800012125 | 0.0618540438117148 | 5.68715126966039 | 0.999279047927446 |
| ebi-fl187-GCST90094803 | prot-a-519 | Cryptic protein \|\| id:prot-a-519 | Mushroom liking \|\| id:ebi-fl187-GCST90094803 | MR Egger | 7 | -0.370253159 | 0.953192043146629 | 0.713683032955432 | -2.238509564 | 1.49800324549594 | 0.690559487108135 | 0.10661729230739 | 4.47274916586862 | 0.999279047927446 |
| ebi-fl187-GCST90094803 | prot-a-583 | Chloride intracellular channel protein 4 \|\| id:prot-a-583 | Mushroom liking \|\| id:ebi-fl187-GCST90094803 | MR Egger | 7 | -1.312447081 | 1.08076670591009 | 0.278827192956136 | -3.430749825 | 0.805855662320338 | 0.269160591990772 | 0.0323626653422878 | 2.23861117477727 | 0.999279047927446 |
| ebi-fl187-GCST90094803 | prot-a-602 | CCR4-NOT transcription complex subunit 1 \|\| id:prot-a-602 | Mushroom liking \|\| id:ebi-fl187-GCST90094803 | MR Egger | 7 | -0.315203694 | 0.953192043146629 | 0.754291852783043 | -2.183460098 | 1.55305271093408 | 0.729640236060551 | 0.112651071746292 | 4.72587491468781 | 0.999279047927446 |
| ebi-fl187-GCST90094803 | prot-a-606 | Ciliary neurotrophic factor receptor subunit alpha \|\| id:prot-a-606 | Mushroom liking \|\| id:ebi-fl187-GCST90094803 | MR Egger | 7 | -1.19840021 | 1.03334855785027 | 0.298526423145085 | -3.223763383 | 0.826962963747987 | 0.301676445142536 | 0.0398049746608034 | 2.2863644137288 | 0.999279047927446 |
| ebi-fl187-GCST90094803 | prot-a-626 | Collagen alpha-2(IX) chain \|\| id:prot-a-626 | Mushroom liking \|\| id:ebi-fl187-GCST90094803 | MR Egger | 7 | -2.227261427 | 0.952749657120869 | 0.0665687313871531 | -4.094650755 | -0.359872099 | 0.107823308218701 | 0.0166615642416768 | 0.697765565501014 | 0.999279047927446 |
| ebi-fl187-GCST90094803 | prot-a-647 | Probable serine carboxypeptidase CPVL \|\| id:prot-a-647 | Mushroom liking \|\| id:ebi-fl187-GCST90094803 | MR Egger | 7 | 0.567736957190383 | 1.04449705380528 | 0.610098370562322 | -1.479477268 | 2.61495118264872 | 1.76426991195229 | 0.227756712933184 | 13.666549196876 | 0.999279047927446 |
| ebi-fl187-GCST90094803 | prot-a-651 | Cellular retinoic acid-binding protein 2 \|\| id:prot-a-651 | Mushroom liking \|\| id:ebi-fl187-GCST90094803 | MR Egger | 7 | 1.25479903586265 | 0.952749657120869 | 0.244956539998275 | -0.612590292 | 3.12218836381955 | 3.50713349545977 | 0.541945252878118 | 22.6959924266411 | 0.999279047927446 |
| ebi-fl187-GCST90094803 | prot-a-671 | Cytotoxic and regulatory T-cell molecule \|\| id:prot-a-671 | Mushroom liking \|\| id:ebi-fl187-GCST90094803 | MR Egger | 7 | -1.665305188 | 1.01664126373039 | 0.162337992508729 | -3.657922065 | 0.32731168861903 | 0.18913292801781 | 0.0257860388025281 | 1.38723379478833 | 0.999279047927446 |
| ebi-fl187-GCST90094803 | prot-a-688 | Chorionic somatomammotropin hormone \|\| id:prot-a-688 | Mushroom liking \|\| id:ebi-fl187-GCST90094803 | MR Egger | 7 | 0.0468645870043668 | 0.953192043146629 | 0.962690592969886 | -1.821391818 | 1.91512099157176 | 1.04798008934772 | 0.161800397509874 | 6.78776000907066 | 0.999279047927446 |
| ebi-fl187-GCST90094803 | prot-a-69 | Aldo-keto reductase family 1 member C1 \|\| id:prot-a-69 | Mushroom liking \|\| id:ebi-fl187-GCST90094803 | MR Egger | 7 | -0.273664518 | 0.952749657120869 | 0.785454722133944 | -2.141053846 | 1.5937248096369 | 0.760587195534539 | 0.117530918213818 | 4.9220485196812 | 0.999279047927446 |
| ebi-fl187-GCST90094803 | prot-a-741 | Stromal cell-derived factor 1 \|\| id:prot-a-741 | Mushroom liking \|\| id:ebi-fl187-GCST90094803 | MR Egger | 7 | -0.152975024 | 1.24747993638779 | 0.90717874790715 | -2.5980357 | 2.29208565089487 | 0.858151154362099 | 0.0744196172082001 | 9.89555484641567 | 0.999279047927446 |
| ebi-fl187-GCST90094803 | prot-a-742 | Growth-regulated alpha protein \|\| id:prot-a-742 | Mushroom liking \|\| id:ebi-fl187-GCST90094803 | MR Egger | 7 | -0.992106152 | 0.953192043146629 | 0.345644979628096 | -2.860362557 | 0.876150252352696 | 0.370794917507317 | 0.0572480008514918 | 2.40163619347898 | 0.999279047927446 |
| ebi-fl187-GCST90094803 | prot-a-788 | Beta-defensin 112 \|\| id:prot-a-788 | Mushroom liking \|\| id:ebi-fl187-GCST90094803 | MR Egger | 7 | 0.478364109693922 | 0.952749657120869 | 0.636930342850513 | -1.389025218 | 2.34575343765082 | 1.61343284298409 | 0.249318217063832 | 10.4411365101061 | 0.999279047927446 |
| ebi-fl187-GCST90094803 | prot-a-838 | DnaJ homolog subfamily B member 1 \|\| id:prot-a-838 | Mushroom liking \|\| id:ebi-fl187-GCST90094803 | MR Egger | 7 | -0.46727432 | 1.00291122325103 | 0.660865675120385 | -2.432980318 | 1.49843167728251 | 0.626708148069199 | 0.087774845434163 | 4.47466584433832 | 0.999279047927446 |
| ebi-fl187-GCST90094803 | prot-a-85 | Junctional adhesion molecule-like \|\| id:prot-a-85 | Mushroom liking \|\| id:ebi-fl187-GCST90094803 | MR Egger | 7 | 1.53387950717766 | 0.952749657120869 | 0.168323541257414 | -0.333509821 | 3.40126883513456 | 4.63612787052534 | 0.716404862951407 | 30.0021436807579 | 0.999279047927446 |
| ebi-fl187-GCST90094803 | prot-a-856 | Dipeptidase 1 \|\| id:prot-a-856 | Mushroom liking \|\| id:ebi-fl187-GCST90094803 | MR Egger | 7 | 0.191689529282889 | 1.33555162077271 | 0.89147810176345 | -2.425991647 | 2.8093707059974 | 1.21129438714226 | 0.0883904234313345 | 16.5994689850328 | 0.999279047927446 |
| ebi-fl187-GCST90094803 | prot-a-860 | Dipeptidyl peptidase 2 \|\| id:prot-a-860 | Mushroom liking \|\| id:ebi-fl187-GCST90094803 | MR Egger | 7 | -0.179201019 | 0.953192043146629 | 0.858267628765608 | -2.047457424 | 1.68905538535146 | 0.835937842936555 | 0.129062638360683 | 5.41436380140124 | 0.999279047927446 |
| ebi-fl187-GCST90094803 | prot-a-861 | Dermatopontin \|\| id:prot-a-861 | Mushroom liking \|\| id:ebi-fl187-GCST90094803 | MR Egger | 7 | -1.379107311 | 0.953192043146629 | 0.207589374602763 | -3.247363716 | 0.489149093190015 | 0.251803234642194 | 0.0388765625163141 | 1.63092786173327 | 0.999279047927446 |
| ebi-fl187-GCST90094803 | prot-a-877 | Dual specificity protein phosphatase 6 \|\| id:prot-a-877 | Mushroom liking \|\| id:ebi-fl187-GCST90094803 | MR Egger | 7 | -0.075401303 | 0.952749657120869 | 0.939990451159627 | -1.942790631 | 1.79198802465635 | 0.927371254499352 | 0.143303484079571 | 6.00137148929448 | 0.999279047927446 |
| ebi-fl187-GCST90094803 | prot-a-879 | Dynactin-associated protein \|\| id:prot-a-879 | Mushroom liking \|\| id:ebi-fl187-GCST90094803 | MR Egger | 7 | -0.647815469 | 0.953192043146629 | 0.526924031710502 | -2.516071873 | 1.22044093576418 | 0.523187448601353 | 0.0807762838400531 | 3.38868159515735 | 0.999279047927446 |
| ebi-fl187-GCST90094803 | prot-a-937 | EMI domain-containing protein 1 \|\| id:prot-a-937 | Mushroom liking \|\| id:ebi-fl187-GCST90094803 | MR Egger | 7 | 1.5754778141208 | 1.18035790241717 | 0.239508844092543 | -0.738023675 | 3.88897930285844 | 4.83305036620164 | 0.478057780247627 | 48.8609887912346 | 0.999279047927446 |
| ebi-fl187-GCST90094803 | prot-b-1 | SRC proto-oncogene, non-receptor tyrosine kinase \|\| id:prot-b-1 | Mushroom liking \|\| id:ebi-fl187-GCST90094803 | MR Egger | 7 | 1.22179033352653 | 1.34627111523935 | 0.405737085176754 | -1.416901052 | 3.86048171939566 | 3.39325736108901 | 0.242464237830767 | 47.4882218573666 | 0.999279047927446 |
| ebi-fl187-GCST90094803 | prot-b-10 | coagulation factor III, tissue factor \|\| id:prot-b-10 | Mushroom liking \|\| id:ebi-fl187-GCST90094803 | MR Egger | 7 | 2.46133695431733 | 2.04787459246491 | 0.283217279870861 | -1.552497247 | 6.47517115554856 | 11.7204708038304 | 0.211718599493442 | 648.830269008543 | 0.999279047927446 |
| ebi-fl187-GCST90094803 | prot-b-29 | myeloperoxidase \|\| id:prot-b-29 | Mushroom liking \|\| id:ebi-fl187-GCST90094803 | MR Egger | 7 | 2.17810743246794 | 1.76220587904867 | 0.27135093861629 | -1.27581609 | 5.63203095540333 | 8.8295798596295 | 0.279203020297649 | 279.228643065762 | 0.999279047927446 |
| ebi-fl187-GCST90094803 | prot-b-33 | adrenomedullin \|\| id:prot-b-33 | Mushroom liking \|\| id:ebi-fl187-GCST90094803 | MR Egger | 7 | 1.89027115218739 | 1.34627111523935 | 0.219258517987151 | -0.748420234 | 4.52896253805652 | 6.62116378070166 | 0.473113371254953 | 92.6623775070835 | 0.999279047927446 |
| ebi-fl187-GCST90094803 | ukb-rsfMRI191-edge-pheno102 | Net25_Pair11_15 (Postcentral\|Precentral)&(Cerebellum) (Motor)&(Subcortical-cerebellum) \|\| id:ukb-rsfMRI191-edge-pheno102 | Mushroom liking \|\| id:ebi-fl187-GCST90094803 | MR Egger | 6 | -0.251887745 | 0.408837393407645 | 0.571156188541729 | -1.053209036 | 0.549433546322337 | 0.77733199276347 | 0.348816586222098 | 1.73227148834285 | 0.999279047927446 |
| ebi-fl187-GCST90094803 | ukb-rsfMRI191-edge-pheno1167 | Net100_Pair11_45 (Angular\|Temporal)&(Frontal) (Default_mode\|Central_executive)&(Salience\|Default_mode) \|\| id:ukb-rsfMRI191-edge-pheno1167 | Mushroom liking \|\| id:ebi-fl187-GCST90094803 | MR Egger | 6 | 0.571943793674909 | 0.301948354857667 | 0.131116732461559 | -0.019874982 | 1.16376256919594 | 1.77170754046099 | 0.980321223595674 | 3.20195822897023 | 0.999279047927446 |
| ebi-fl187-GCST90094803 | ukb-rsfMRI191-edge-pheno1317 | Net100_Pair26_48 (Parietal\|Frontal)&(Temporal) (Attention\|Central_executive)&(Default_mode\|Central_executive) \|\| id:ukb-rsfMRI191-edge-pheno1317 | Mushroom liking \|\| id:ebi-fl187-GCST90094803 | MR Egger | 6 | -0.069689922 | 0.3007532524199 | 0.828128550977929 | -0.659166297 | 0.51978645272875 | 0.932682979532248 | 0.517282414796837 | 1.68166849563368 | 0.999279047927446 |
| ebi-fl187-GCST90094803 | ukb-rsfMRI191-edge-pheno286 | Net100_Pair10_13 (OccipitalPrecuneus)&(Frontal_Sup) (Default_mode\|Central_executive)&(Default_mode) \|\| id:ukb-rsfMRI191-edge-pheno286 | Mushroom liking \|\| id:ebi-fl187-GCST90094803 | MR Egger | 6 | -0.065212578 | 0.297685506502833 | 0.837323197629372 | -0.64867617 | 0.518251015179299 | 0.936868285027797 | 0.522737334109965 | 1.67908837998987 | 0.999279047927446 |
| ebi-fl187-GCST90094803 | ukb-rsfMRI191-edge-pheno599 | Net100_Pair11_29 (Angular\|Temporal)&(Parietal) (Default_mode\|Central_executive)&(Central_executive\|Salience) \|\| id:ukb-rsfMRI191-edge-pheno599 | Mushroom liking \|\| id:ebi-fl187-GCST90094803 | MR Egger | 6 | 0.638018685438392 | 0.448915123583945 | 0.228293650988987 | -0.241854957 | 1.51789232766293 | 1.89272707408544 | 0.785170052881104 | 4.56259859100677 | 0.999279047927446 |
| ebi-fl187-GCST90094803 | ukb-rsfMRI191-edge-pheno621 | Net100_Pair5_30 (Precuneus\|Angular\|Cingulate)&(Paracentral) (Default_mode\|Central_executive)&(Motor) \|\| id:ukb-rsfMRI191-edge-pheno621 | Mushroom liking \|\| id:ebi-fl187-GCST90094803 | MR Egger | 6 | -0.126642027 | 0.354268656725191 | 0.738800240531825 | -0.821008594 | 0.567724540488315 | 0.881049008179964 | 0.439987661778968 | 1.76424800567442 | 0.999279047927446 |
| ebi-fl187-GCST90094803 | ukb-rsfMRI191-edge-pheno624 | Net100_Pair8_30 (Calcarine\|Lingual)&(Paracentral) (Visual)&(Motor) \|\| id:ukb-rsfMRI191-edge-pheno624 | Mushroom liking \|\| id:ebi-fl187-GCST90094803 | MR Egger | 6 | 0.176939324829486 | 0.378954715256945 | 0.664859311628874 | -0.565811917 | 0.919690566733098 | 1.19355867155415 | 0.567898872733603 | 2.5085140521319 | 0.999279047927446 |
| ebi-fl187-GCST90094803 | ukb-rsfMRI191-edge-pheno882 | Net100_Pair6_38 (Postcentral\|Precentral)&(Subcortical) (Motor)&(Subcortical-cerebellum) \|\| id:ukb-rsfMRI191-edge-pheno882 | Mushroom liking \|\| id:ebi-fl187-GCST90094803 | MR Egger | 6 | -0.190717165 | 0.30510838736849 | 0.565808411680731 | -0.788729605 | 0.407295273780112 | 0.826366280026493 | 0.454421723961377 | 1.50274776217095 | 0.999279047927446 |
| ebi-fl187-GCST90094803 | ukb-rsfMRI191-edge-pheno899 | Net100_Pair23_38 (Cerebellum)&(Subcortical) (Subcortical-cerebellum)&(Subcortical-cerebellum) \|\| id:ukb-rsfMRI191-edge-pheno899 | Mushroom liking \|\| id:ebi-fl187-GCST90094803 | MR Egger | 6 | -0.028281604 | 0.303221587500652 | 0.930173677390809 | -0.622595916 | 0.566032707054912 | 0.972114576465659 | 0.536549790027292 | 1.76126571539416 | 0.999279047927446 |
| ebi-fl187-GCST90094803 | ebi-a-GCST90001392 | IgD+ CD38+ B cell Absolute Count \|\| id:ebi-a-GCST90001392 | Mushroom liking \|\| id:ebi-fl187-GCST90094803 | Weighted median | 6 | 0.201262994945156 | 0.29943069500353 | 0.501486819392491 | -0.385621167 | 0.788147157152075 | 1.22294635824409 | 0.680028093794473 | 2.19931765877086 | 0.999489402457283 |
| ebi-fl187-GCST90094803 | ebi-a-GCST90001402 | Switched memory B cell %B cell \|\| id:ebi-a-GCST90001402 | Mushroom liking \|\| id:ebi-fl187-GCST90094803 | Weighted median | 6 | 0.57119760251926 | 0.328775883177426 | 0.0823260431453049 | -0.073203129 | 1.21559833354701 | 1.77038600108572 | 0.929412020772615 | 3.37231122773168 | 0.999489402457283 |
| ebi-fl187-GCST90094803 | ebi-a-GCST90001407 | Memory B cell Absolute Count \|\| id:ebi-a-GCST90001407 | Mushroom liking \|\| id:ebi-fl187-GCST90094803 | Weighted median | 6 | 0.262329531531188 | 0.320492342733124 | 0.413060078828475 | -0.36583546 | 0.890494523288111 | 1.29995484796694 | 0.693616919392352 | 2.43633417742057 | 0.999489402457283 |
| ebi-fl187-GCST90094803 | ebi-a-GCST90001411 | IgD+ CD24+ B cell %B cell \|\| id:ebi-a-GCST90001411 | Mushroom liking \|\| id:ebi-fl187-GCST90094803 | Weighted median | 6 | 0.000675734761044225 | 0.339168558108215 | 0.998410353897908 | -0.664094639 | 0.665446108653145 | 1.00067596312121 | 0.514739341667599 | 1.94535816890251 | 0.999911036302558 |
| ebi-fl187-GCST90094803 | ebi-a-GCST90001418 | CD24+ CD27+ B cell Absolute Count \|\| id:ebi-a-GCST90001418 | Mushroom liking \|\| id:ebi-fl187-GCST90094803 | Weighted median | 6 | 0.148890951278307 | 0.341167324250181 | 0.662534778669161 | -0.519797004 | 0.817578906808662 | 1.1605464262042 | 0.594641245363617 | 2.26500939495334 | 0.999489402457283 |
| ebi-fl187-GCST90094803 | ebi-a-GCST90001436 | Memory B cell %lymphocyte \|\| id:ebi-a-GCST90001436 | Mushroom liking \|\| id:ebi-fl187-GCST90094803 | Weighted median | 6 | 0.122520130111695 | 0.344753815934032 | 0.722301629578424 | -0.553197349 | 0.798237609342397 | 1.13034187375317 | 0.57510804636287 | 2.22162211021067 | 0.999489402457283 |
| ebi-fl187-GCST90094803 | ebi-a-GCST90001439 | IgD+ CD24+ B cell %lymphocyte \|\| id:ebi-a-GCST90001439 | Mushroom liking \|\| id:ebi-fl187-GCST90094803 | Weighted median | 6 | -0.091173597 | 0.340446725012023 | 0.788848829425536 | -0.758449178 | 0.576101983834387 | 0.912859227347532 | 0.468392256965068 | 1.77908997546832 | 0.999489402457283 |
| ebi-fl187-GCST90094803 | ebi-a-GCST90001457 | CD11c+ HLA DR++ monocyte %monocyte \|\| id:ebi-a-GCST90001457 | Mushroom liking \|\| id:ebi-fl187-GCST90094803 | Weighted median | 6 | 0.135942857335409 | 0.38294802932965 | 0.722596815990591 | -0.61463528 | 0.886520994821522 | 1.14561642814914 | 0.540838113737729 | 2.42667254231576 | 0.999489402457283 |
| ebi-fl187-GCST90094803 | ebi-a-GCST90001483 | CD39+ resting CD4 regulatory T cell Absolute Count \|\| id:ebi-a-GCST90001483 | Mushroom liking \|\| id:ebi-fl187-GCST90094803 | Weighted median | 6 | 0.0795423663567086 | 0.365714082823882 | 0.827819658698333 | -0.637257236 | 0.796341968691518 | 1.08279143248577 | 0.528740647892984 | 2.21741470215447 | 0.999489402457283 |
| ebi-fl187-GCST90094803 | ebi-a-GCST90001525 | CD33dim HLA DR+ CD11b+ Absolute Count \|\| id:ebi-a-GCST90001525 | Mushroom liking \|\| id:ebi-fl187-GCST90094803 | Weighted median | 6 | 0.418674841302263 | 0.437397956050139 | 0.338469180915218 | -0.438625153 | 1.27597483516054 | 1.51994605075243 | 0.644922481870139 | 3.58219175504429 | 0.999489402457283 |
| ebi-fl187-GCST90094803 | ebi-a-GCST90001540 | Naive CD4+ T cell Absolute Count \|\| id:ebi-a-GCST90001540 | Mushroom liking \|\| id:ebi-fl187-GCST90094803 | Weighted median | 6 | -0.189666285 | 0.317152220190619 | 0.54982045680367 | -0.811284637 | 0.431952066475423 | 0.827235148582084 | 0.444286952152905 | 1.54026128323955 | 0.999489402457283 |
| ebi-fl187-GCST90094803 | ebi-a-GCST90001624 | HLA DR+ CD4+ T cell Absolute Count \|\| id:ebi-a-GCST90001624 | Mushroom liking \|\| id:ebi-fl187-GCST90094803 | Weighted median | 6 | -0.048400263 | 0.31277422671637 | 0.877022313809322 | -0.661437747 | 0.564637221344765 | 0.952752359210901 | 0.516108766778233 | 1.75880960838626 | 0.999489402457283 |
| ebi-fl187-GCST90094803 | ebi-a-GCST90001672 | CD39+ CD8+ T cell Absolute Count \|\| id:ebi-a-GCST90001672 | Mushroom liking \|\| id:ebi-fl187-GCST90094803 | Weighted median | 6 | 0.365473554466717 | 0.33729713685197 | 0.278570628056215 | -0.295628834 | 1.02657594269658 | 1.44119633153804 | 0.744063548040903 | 2.79149122612914 | 0.999489402457283 |
| ebi-fl187-GCST90094803 | ebi-a-GCST90001722 | CD19 on CD20- CD38- B cell \|\| id:ebi-a-GCST90001722 | Mushroom liking \|\| id:ebi-fl187-GCST90094803 | Weighted median | 6 | 0.128824267692026 | 0.352120550191959 | 0.714474963955107 | -0.561332011 | 0.818980546068266 | 1.13749021282041 | 0.570448713782475 | 2.26818634699491 | 0.999489402457283 |
| ebi-fl187-GCST90094803 | ebi-a-GCST90001742 | CD19 on transitional B cell \|\| id:ebi-a-GCST90001742 | Mushroom liking \|\| id:ebi-fl187-GCST90094803 | Weighted median | 6 | -0.175035677 | 0.439676211367959 | 0.69055550596426 | -1.036801052 | 0.686729696875721 | 0.839427071654952 | 0.354587175646094 | 1.98720612877013 | 0.999489402457283 |
| ebi-fl187-GCST90094803 | ebi-a-GCST90001784 | CD25 on IgD+ CD38dim B cell \|\| id:ebi-a-GCST90001784 | Mushroom liking \|\| id:ebi-fl187-GCST90094803 | Weighted median | 6 | 0.20981693956147 | 0.333553128641358 | 0.529325461119684 | -0.443947193 | 0.863581071698532 | 1.23345224297979 | 0.64149929585846 | 2.37163851236332 | 0.999489402457283 |
| ebi-fl187-GCST90094803 | ebi-a-GCST90001794 | CD25 on IgD+ B cell \|\| id:ebi-a-GCST90001794 | Mushroom liking \|\| id:ebi-fl187-GCST90094803 | Weighted median | 6 | 0.00258087691530218 | 0.340155258527238 | 0.993946228861956 | -0.66412343 | 0.669285183628688 | 1.00258421024515 | 0.51472452219194 | 1.95284089895772 | 0.999911036302558 |
| ebi-fl187-GCST90094803 | ebi-a-GCST90001802 | CD27 on IgD- CD38- B cell \|\| id:ebi-a-GCST90001802 | Mushroom liking \|\| id:ebi-fl187-GCST90094803 | Weighted median | 6 | 0.607672293869631 | 0.388363462413483 | 0.117653278022151 | -0.153520092 | 1.36886468020006 | 1.8361523973931 | 0.857683531052977 | 3.93088534918385 | 0.999489402457283 |
| ebi-fl187-GCST90094803 | ebi-a-GCST90001804 | CD27 on IgD- CD38dim B cell \|\| id:ebi-a-GCST90001804 | Mushroom liking \|\| id:ebi-fl187-GCST90094803 | Weighted median | 6 | 0.829480228939871 | 0.339813343910585 | 0.0146471493172311 | 0.163446074875125 | 1.49551438300462 | 2.29212704928142 | 1.17756185330528 | 4.4616309498313 | 0.999489402457283 |
| ebi-fl187-GCST90094803 | ebi-a-GCST90001805 | CD27 on memory B cell \|\| id:ebi-a-GCST90001805 | Mushroom liking \|\| id:ebi-fl187-GCST90094803 | Weighted median | 6 | 0.638634858241001 | 0.333541021277577 | 0.0555292950483531 | -0.015105543 | 1.29237525994505 | 1.89389368040989 | 0.985007972964062 | 3.64142562410242 | 0.999489402457283 |
| ebi-fl187-GCST90094803 | ebi-a-GCST90001806 | CD27 on unswitched memory B cell \|\| id:ebi-a-GCST90001806 | Mushroom liking \|\| id:ebi-fl187-GCST90094803 | Weighted median | 6 | 0.220874132852217 | 0.317397824613872 | 0.48649740325393 | -0.401225603 | 0.842973869095406 | 1.24716644340147 | 0.669499002753671 | 2.32326580196409 | 0.999489402457283 |
| ebi-fl187-GCST90094803 | ebi-a-GCST90001839 | CD3 on Effector Memory CD8+ T cell \|\| id:ebi-a-GCST90001839 | Mushroom liking \|\| id:ebi-fl187-GCST90094803 | Weighted median | 6 | 0.272198976997209 | 0.3852523535374 | 0.479847902299937 | -0.482895636 | 1.02729358993051 | 1.3128482019168 | 0.616994212036932 | 2.7934952510916 | 0.999489402457283 |
| ebi-fl187-GCST90094803 | ebi-a-GCST90001859 | CD3 on CD8+ T cell \|\| id:ebi-a-GCST90001859 | Mushroom liking \|\| id:ebi-fl187-GCST90094803 | Weighted median | 6 | 0.209144706149731 | 0.39415418846082 | 0.59568457655937 | -0.563397503 | 0.981686915532938 | 1.23262335380499 | 0.569271672218176 | 2.66895474778369 | 0.999489402457283 |
| ebi-fl187-GCST90094803 | ebi-a-GCST90001905 | CD86 on monocyte \|\| id:ebi-a-GCST90001905 | Mushroom liking \|\| id:ebi-fl187-GCST90094803 | Weighted median | 6 | -0.066885729 | 0.36478446871339 | 0.854518035579422 | -0.781863287 | 0.648091829906259 | 0.935302073346949 | 0.45755266439943 | 1.91188913642395 | 0.999489402457283 |
| ebi-fl187-GCST90094803 | ebi-a-GCST90001943 | CD25 on activated & secreting CD4 regulatory T cell \|\| id:ebi-a-GCST90001943 | Mushroom liking \|\| id:ebi-fl187-GCST90094803 | Weighted median | 6 | -0.218573802 | 0.332973078602913 | 0.511546945285531 | -0.871201036 | 0.43405343247815 | 0.803664165567886 | 0.41844867554842 | 1.54350133901473 | 0.999489402457283 |
| ebi-fl187-GCST90094803 | ebi-a-GCST90001949 | CD33 on CD33dim HLA DR+ CD11b- \|\| id:ebi-a-GCST90001949 | Mushroom liking \|\| id:ebi-fl187-GCST90094803 | Weighted median | 6 | -0.388659054 | 0.513549659392208 | 0.449164242311359 | -1.395216386 | 0.617898278725196 | 0.677965380412757 | 0.247779414546674 | 1.85502519601617 | 0.999489402457283 |
| ebi-fl187-GCST90094803 | ebi-a-GCST90001955 | CD33 on Immature Myeloid-Derived Suppressor Cells \|\| id:ebi-a-GCST90001955 | Mushroom liking \|\| id:ebi-fl187-GCST90094803 | Weighted median | 6 | -0.059081976 | 0.526057500429238 | 0.9105769223022 | -1.090154677 | 0.971990724621979 | 0.942629492787686 | 0.336164492749532 | 2.64320111087761 | 0.999489402457283 |
| ebi-fl187-GCST90094803 | ebi-a-GCST90001956 | CD33 on CD33+ HLA DR+ \|\| id:ebi-a-GCST90001956 | Mushroom liking \|\| id:ebi-fl187-GCST90094803 | Weighted median | 6 | -0.282912039 | 0.4966937979054 | 0.568955513340723 | -1.256431883 | 0.690607804503773 | 0.753586070834397 | 0.284667944986919 | 1.99492769086354 | 0.999489402457283 |
| ebi-fl187-GCST90094803 | ebi-a-GCST90001957 | CD33 on CD33+ HLA DR+ CD14- \|\| id:ebi-a-GCST90001957 | Mushroom liking \|\| id:ebi-fl187-GCST90094803 | Weighted median | 6 | -0.243473198 | 0.510720513160631 | 0.63355801238346 | -1.244485404 | 0.757539007961459 | 0.783900485982156 | 0.288089119608657 | 2.13302040964894 | 0.999489402457283 |
| ebi-fl187-GCST90094803 | ebi-a-GCST90002064 | CD4 on resting CD4 regulatory T cell \|\| id:ebi-a-GCST90002064 | Mushroom liking \|\| id:ebi-fl187-GCST90094803 | Weighted median | 6 | -0.575486717 | 0.359955556754073 | 0.109871364169323 | -1.280999609 | 0.130026173975482 | 0.562431057273419 | 0.277759510867756 | 1.1388581913809 | 0.999489402457283 |
| ebi-fl187-GCST90094803 | ebi-a-GCST90002080 | SSC-A on T cell \|\| id:ebi-a-GCST90002080 | Mushroom liking \|\| id:ebi-fl187-GCST90094803 | Weighted median | 6 | 0.348384454867581 | 0.323303134390783 | 0.281221972700018 | -0.285289689 | 0.982058598273517 | 1.41677683179897 | 0.751796435960492 | 2.66994693657712 | 0.999489402457283 |
| ebi-fl187-GCST90094803 | ebi-a-GCST90002121 | CD8 on CD39+ CD8+ T cell \|\| id:ebi-a-GCST90002121 | Mushroom liking \|\| id:ebi-fl187-GCST90094803 | Weighted median | 6 | -0.036578097 | 0.346676707252845 | 0.915970591824454 | -0.716064443 | 0.642908249321727 | 0.964082799084195 | 0.488671671662343 | 1.90200434645257 | 0.999489402457283 |
| ebi-fl187-GCST90094803 | ebi-cfmet338-GCST90026053 | 1-palmitoyl-2-docosahexaenoyl-gpc (16:0/22:6) levels \|\| id:ebi-cfmet338-GCST90026053 | Mushroom liking \|\| id:ebi-fl187-GCST90094803 | Weighted median | 6 | 0.177229923772791 | 0.186749055253866 | 0.342606803471476 | -0.188798225 | 0.543258072070369 | 1.19390556884443 | 0.827953550562608 | 1.72160685384965 | 0.999489402457283 |
| ebi-fl187-GCST90094803 | ebi-cfmet338-GCST90026063 | 1-stearoyl-2-oleoyl-gpc (18:0/18:1) levels \|\| id:ebi-cfmet338-GCST90026063 | Mushroom liking \|\| id:ebi-fl187-GCST90094803 | Weighted median | 6 | 0.181189617279676 | 0.161326174091187 | 0.261383993906092 | -0.135009684 | 0.497388918498402 | 1.19864244106512 | 0.873707450717366 | 1.64442194048236 | 0.999489402457283 |
| ebi-fl187-GCST90094803 | ebi-cfmet338-GCST90026113 | Adenosine 3',5'-cyclic monophosphate (camp) levels \|\| id:ebi-cfmet338-GCST90026113 | Mushroom liking \|\| id:ebi-fl187-GCST90094803 | Weighted median | 6 | 0.448738596878516 | 0.344423966093325 | 0.192620194268984 | -0.226332377 | 1.12380957042143 | 1.56633515877991 | 0.797453002842682 | 3.07655224932932 | 0.999489402457283 |
| ebi-fl187-GCST90094803 | ebi-cfmet338-GCST90026117 | Alpha-ketoglutaramate levels \|\| id:ebi-cfmet338-GCST90026117 | Mushroom liking \|\| id:ebi-fl187-GCST90094803 | Weighted median | 6 | 0.0485381584330017 | 0.129378121763096 | 0.707537669856318 | -0.20504296 | 0.302119277088669 | 1.04973542731507 | 0.814612319733046 | 1.35272256589669 | 0.999489402457283 |
| ebi-fl187-GCST90094803 | ebi-cfmet338-GCST90026124 | Arginine levels \|\| id:ebi-cfmet338-GCST90026124 | Mushroom liking \|\| id:ebi-fl187-GCST90094803 | Weighted median | 6 | 0.0777223077137136 | 0.091149452804299 | 0.393830746126068 | -0.10093062 | 0.25637523521014 | 1.08082248092769 | 0.903995750131742 | 1.29223752999773 | 0.999489402457283 |
| ebi-fl187-GCST90094803 | ebi-cfmet338-GCST90026169 | Glutamate levels \|\| id:ebi-cfmet338-GCST90026169 | Mushroom liking \|\| id:ebi-fl187-GCST90094803 | Weighted median | 6 | 0.0185536315689017 | 0.0998403580997813 | 0.852575738338739 | -0.17713347 | 0.214240733444473 | 1.01872681962211 | 0.83766797326014 | 1.23892086858512 | 0.999489402457283 |
| ebi-fl187-GCST90094803 | ebi-cfmet338-GCST90026170 | Glutamine levels \|\| id:ebi-cfmet338-GCST90026170 | Mushroom liking \|\| id:ebi-fl187-GCST90094803 | Weighted median | 6 | -0.012506956 | 0.0567772523422123 | 0.825652234538193 | -0.12379037 | 0.0987764586876723 | 0.987570931022325 | 0.883565042773795 | 1.10381952271282 | 0.999489402457283 |
| ebi-fl187-GCST90094803 | ebi-cfmet338-GCST90026191 | Kynurenate levels \|\| id:ebi-cfmet338-GCST90026191 | Mushroom liking \|\| id:ebi-fl187-GCST90094803 | Weighted median | 6 | 0.22945210755388 | 0.475229061841916 | 0.62922054247982 | -0.701996854 | 1.16090106876404 | 1.25791062136399 | 0.495594685001426 | 3.19280892073285 | 0.999489402457283 |
| ebi-fl187-GCST90094803 | ebi-cfmet338-GCST90026202 | Methylsuccinoylcarnitine levels \|\| id:ebi-cfmet338-GCST90026202 | Mushroom liking \|\| id:ebi-fl187-GCST90094803 | Weighted median | 6 | 0.594808568248455 | 0.319297206185171 | 0.0624808310024423 | -0.031013956 | 1.22063109237139 | 1.81268390648891 | 0.969462043291027 | 3.38932603662288 | 0.999489402457283 |
| ebi-fl187-GCST90094803 | ebi-cfmet338-GCST90026222 | N-acetylputrescine levels \|\| id:ebi-cfmet338-GCST90026222 | Mushroom liking \|\| id:ebi-fl187-GCST90094803 | Weighted median | 6 | 0.122409919676685 | 0.118093497338401 | 0.299945211153899 | -0.109053335 | 0.35387317445995 | 1.13021730514806 | 0.896682591561257 | 1.42457450259183 | 0.999489402457283 |
| ebi-fl187-GCST90094803 | ebi-cfmet338-GCST90026252 | Propionylcarnitine (c3) levels \|\| id:ebi-cfmet338-GCST90026252 | Mushroom liking \|\| id:ebi-fl187-GCST90094803 | Weighted median | 6 | 0.207536740187259 | 0.182557551321113 | 0.255609727970121 | -0.15027606 | 0.565349540776642 | 1.23064293006122 | 0.860470401828954 | 1.76006288896234 | 0.999489402457283 |
| ebi-fl187-GCST90094803 | ebi-cfmet338-GCST90026316 | Ethyl beta-glucopyranoside levels \|\| id:ebi-cfmet338-GCST90026316 | Mushroom liking \|\| id:ebi-fl187-GCST90094803 | Weighted median | 6 | 0.0822200049376273 | 0.582230972658316 | 0.88769974630075 | -1.058952701 | 1.22339271134793 | 1.08569464173842 | 0.346818843058639 | 3.39869900004316 | 0.999489402457283 |
| ebi-fl187-GCST90094803 | ebi-gm430-GCST90011315 | F_Prevotellaceae abundance \|\| id:ebi-gm430-GCST90011315 | Mushroom liking \|\| id:ebi-fl187-GCST90094803 | Weighted median | 6 | 0.0392386765410166 | 0.256247662297505 | 0.878297383131747 | -0.463006742 | 0.541484094644126 | 1.04001868209104 | 0.629388389432953 | 1.71855546949776 | 0.999489402457283 |
| ebi-fl187-GCST90094803 | ebi-gm430-GCST90011354 | OTU97_1 (Bacteroides) abundance \|\| id:ebi-gm430-GCST90011354 | Mushroom liking \|\| id:ebi-fl187-GCST90094803 | Weighted median | 6 | 0.0228506822132988 | 0.265401091032864 | 0.931387977115939 | -0.497335456 | 0.543036820637713 | 1.02311375905903 | 0.608148942249625 | 1.72122598800187 | 0.999489402457283 |
| ebi-fl187-GCST90094803 | ebi-gm430-GCST90011419 | OTU99_1 (Bacteroides) abundance \|\| id:ebi-gm430-GCST90011419 | Mushroom liking \|\| id:ebi-fl187-GCST90094803 | Weighted median | 6 | 0.0307892685388812 | 0.262451941764402 | 0.906611287270773 | -0.483616537 | 0.545195074397108 | 1.03126816034053 | 0.616549580343074 | 1.72494484213315 | 0.999489402457283 |
| ebi-fl187-GCST90094803 | ebi-gm430-GCST90011435 | OTU99_155 (Butyrivibrio) abundance \|\| id:ebi-gm430-GCST90011435 | Mushroom liking \|\| id:ebi-fl187-GCST90094803 | Weighted median | 6 | -0.919144886 | 0.72882657242529 | 0.207262097223914 | -2.347644968 | 0.509355195940801 | 0.398859966034797 | 0.0955940243223963 | 1.66421775454019 | 0.999489402457283 |
| ebi-fl187-GCST90094803 | ebi-gm430-GCST90011466 | OTU99_58 (Bacteroides) abundance \|\| id:ebi-gm430-GCST90011466 | Mushroom liking \|\| id:ebi-fl187-GCST90094803 | Weighted median | 6 | 0.0219637426053131 | 0.409487757652635 | 0.957224284287943 | -0.780632262 | 0.824559747604478 | 1.02220672124599 | 0.458116270029068 | 2.28087638296317 | 0.999489402457283 |
| ebi-fl187-GCST90094803 | ebi-gm430-GCST90011472 | OTU99_72 (Parabacteroides) abundance \|\| id:ebi-gm430-GCST90011472 | Mushroom liking \|\| id:ebi-fl187-GCST90094803 | Weighted median | 6 | -0.175351568 | 0.683332735101374 | 0.797478098166813 | -1.514683729 | 1.16398059305832 | 0.839161946633588 | 0.219877714898115 | 3.20265640837761 | 0.999489402457283 |
| ebi-fl187-GCST90094803 | ebi-gm430-GCST90011505 | TestASV_28 (Holdemanella) abundance \|\| id:ebi-gm430-GCST90011505 | Mushroom liking \|\| id:ebi-fl187-GCST90094803 | Weighted median | 6 | 0.0628920797544032 | 0.564779794954407 | 0.911333402788989 | -1.044076318 | 1.16986047786504 | 1.06491190747509 | 0.352016820731674 | 3.22154313059563 | 0.999489402457283 |
| ebi-fl187-GCST90094803 | ebi-gm430-GCST90011513 | TestASV_35 (Alphaproteobacteria) abundance \|\| id:ebi-gm430-GCST90011513 | Mushroom liking \|\| id:ebi-fl187-GCST90094803 | Weighted median | 6 | -0.426637542 | 0.671093938071323 | 0.524949512241847 | -1.741981661 | 0.888706576708749 | 0.652700085784315 | 0.175172923171618 | 2.4319820339213 | 0.999489402457283 |
| ebi-fl187-GCST90094803 | ebi-gm430-GCST90011543 | G_Coprococcus prevalence \|\| id:ebi-gm430-GCST90011543 | Mushroom liking \|\| id:ebi-fl187-GCST90094803 | Weighted median | 6 | 0.570289471588991 | 0.652438771649272 | 0.382069855799077 | -0.708490521 | 1.84906946402156 | 1.76877898859893 | 0.492386884523707 | 6.35390423434081 | 0.999489402457283 |
| ebi-fl187-GCST90094803 | ebi-gm430-GCST90011547 | G_Parasutterella prevalence \|\| id:ebi-gm430-GCST90011547 | Mushroom liking \|\| id:ebi-fl187-GCST90094803 | Weighted median | 6 | -0.086481719 | 0.648444614160275 | 0.89390240303895 | -1.357433163 | 1.1844697246349 | 0.917152314983567 | 0.257320429613072 | 3.26895291658173 | 0.999489402457283 |
| ebi-fl187-GCST90094803 | ebi-gm430-GCST90011569 | OTU97_13 (Subdoligranulum) prevalence \|\| id:ebi-gm430-GCST90011569 | Mushroom liking \|\| id:ebi-fl187-GCST90094803 | Weighted median | 6 | 0.510185077463068 | 0.693182253047026 | 0.461728117333766 | -0.848452139 | 1.86882229343524 | 1.66559943133844 | 0.42807702334322 | 6.48065958786731 | 0.999489402457283 |
| ebi-fl187-GCST90094803 | ebi-gm430-GCST90011590 | OTU97_277 (Prevotella) prevalence \|\| id:ebi-gm430-GCST90011590 | Mushroom liking \|\| id:ebi-fl187-GCST90094803 | Weighted median | 6 | 0.745592376138564 | 0.565067362281303 | 0.187010292680251 | -0.361939654 | 1.85312440620992 | 2.10768961028222 | 0.696324387012171 | 6.37972125657289 | 0.999489402457283 |
| ebi-fl187-GCST90094803 | ebi-gm430-GCST90011616 | OTU97_86 (Phascolarctobacterium) prevalence \|\| id:ebi-gm430-GCST90011616 | Mushroom liking \|\| id:ebi-fl187-GCST90094803 | Weighted median | 6 | 1.01917983255195 | 0.70256239644465 | 0.146874294619903 | -0.357842464 | 2.39620212958346 | 2.77092121236574 | 0.699183212522276 | 10.9813911827779 | 0.999489402457283 |
| ebi-fl187-GCST90094803 | ebi-gm430-GCST90011665 | OTU99_55 (Barnesiella) prevalence \|\| id:ebi-gm430-GCST90011665 | Mushroom liking \|\| id:ebi-fl187-GCST90094803 | Weighted median | 6 | 0.368527013732532 | 0.490488044782269 | 0.452443491302055 | -0.592829554 | 1.32988358150578 | 1.44560369125273 | 0.552761002710943 | 3.78060322981276 | 0.999489402457283 |
| ebi-fl187-GCST90094803 | ebi-gm430-GCST90011687 | TestASV_14 (Lachnospiraceae) prevalence \|\| id:ebi-gm430-GCST90011687 | Mushroom liking \|\| id:ebi-fl187-GCST90094803 | Weighted median | 6 | 0.491346842426203 | 0.497494652668504 | 0.323327783961568 | -0.483742677 | 1.46643636165647 | 1.63451617385646 | 0.616471814001512 | 4.33376362376856 | 0.999489402457283 |
| ebi-fl187-GCST90094803 | ebi-gm430-GCST90011697 | TestASV_23 (Barnesiella) prevalence \|\| id:ebi-gm430-GCST90011697 | Mushroom liking \|\| id:ebi-fl187-GCST90094803 | Weighted median | 6 | 0.219513427104884 | 0.65617419969819 | 0.737975664664074 | -1.066588004 | 1.50561485851334 | 1.24547057090744 | 0.344180859880357 | 4.50692389906784 | 0.999489402457283 |
| ebi-fl187-GCST90094803 | ebi-gm430-GCST90011727 | TestASV_6 (Subdoligranulum) prevalence \|\| id:ebi-gm430-GCST90011727 | Mushroom liking \|\| id:ebi-fl187-GCST90094803 | Weighted median | 6 | 0.100101476356196 | 0.884843170233527 | 0.909928236095742 | -1.634191137 | 1.83439409001391 | 1.1052830724838 | 0.195110124792164 | 6.26133918791022 | 0.999489402457283 |
| ebi-fl187-GCST90094803 | ebi-hbsm44-GCST90104908 | Sulcal depth of the left posterior inferior temporal sulcus \|\| id:ebi-hbsm44-GCST90104908 | Mushroom liking \|\| id:ebi-fl187-GCST90094803 | Weighted median | 5 | -0.205630074 | 0.298475547977624 | 0.490864547199169 | -0.790642148 | 0.379381999964092 | 0.814134189930299 | 0.453553453258027 | 1.46138117668875 | 0.999489402457283 |
| ebi-fl187-GCST90094803 | ebi-hbsm44-GCST90104918 | Sulcal depth of the left inferior frontal sulcus \|\| id:ebi-hbsm44-GCST90104918 | Mushroom liking \|\| id:ebi-fl187-GCST90094803 | Weighted median | 5 | -0.281055039 | 0.462982980912614 | 0.543815981002456 | -1.188501682 | 0.62639160335476 | 0.754986780442592 | 0.304677425969077 | 1.87084762459862 | 0.999489402457283 |
| ebi-fl187-GCST90094803 | ebi-hbsm44-GCST90104925 | Sulcal depth of the right anterior inferior temporal sulcus \|\| id:ebi-hbsm44-GCST90104925 | Mushroom liking \|\| id:ebi-fl187-GCST90094803 | Weighted median | 4 | -0.233473812 | 0.155267806940602 | 0.132662593299902 | -0.537798714 | 0.0708510896108247 | 0.791778330565401 | 0.584032461115751 | 1.07342137037257 | 0.999489402457283 |
| ebi-fl187-GCST90094803 | ebi-IPs91-GCST90274770 | C-C motif chemokine 4 levels \|\| id:ebi-IPs91-GCST90274770 | Mushroom liking \|\| id:ebi-fl187-GCST90094803 | Weighted median | 7 | 0.0373917429119456 | 0.139113995280699 | 0.788095205073194 | -0.235271688 | 0.310055173662116 | 1.03809960935773 | 0.790356090318704 | 1.36350034136401 | 0.999489402457283 |
| ebi-fl187-GCST90094803 | ebi-IPs91-GCST90274779 | C-X-C motif chemokine 1 levels \|\| id:ebi-IPs91-GCST90274779 | Mushroom liking \|\| id:ebi-fl187-GCST90094803 | Weighted median | 7 | -0.09484426 | 0.143120200439291 | 0.507529411416043 | -0.375359852 | 0.185671333314127 | 0.909514571650131 | 0.687042000583578 | 1.20402647194972 | 0.999489402457283 |
| ebi-fl187-GCST90094803 | ebi-IPs91-GCST90274780 | C-X-C motif chemokine 10 levels \|\| id:ebi-IPs91-GCST90274780 | Mushroom liking \|\| id:ebi-fl187-GCST90094803 | Weighted median | 7 | 0.109262486972842 | 0.133719014767201 | 0.413868377059263 | -0.152826782 | 0.371351755916557 | 1.11545510435139 | 0.858278378225156 | 1.44969292177272 | 0.999489402457283 |
| ebi-fl187-GCST90094803 | ebi-IPs91-GCST90274796 | Interleukin-10 receptor subunit alpha levels \|\| id:ebi-IPs91-GCST90274796 | Mushroom liking \|\| id:ebi-fl187-GCST90094803 | Weighted median | 7 | 0.0421985660510841 | 0.145650049942644 | 0.772026075703579 | -0.243275532 | 0.327672663938666 | 1.04310158275034 | 0.784055451768401 | 1.38773464234218 | 0.999489402457283 |
| ebi-fl187-GCST90094803 | ebi-IPs91-GCST90274807 | Interleukin-20 levels \|\| id:ebi-IPs91-GCST90274807 | Mushroom liking \|\| id:ebi-fl187-GCST90094803 | Weighted median | 7 | 0.168226500440939 | 0.152422368174579 | 0.269729181123779 | -0.130521341 | 0.466974342063113 | 1.18320457665779 | 0.877637762922321 | 1.59516047436515 | 0.999489402457283 |
| ebi-fl187-GCST90094803 | ebi-IPs91-GCST90274808 | Interleukin-20 receptor subunit alpha levels \|\| id:ebi-IPs91-GCST90274808 | Mushroom liking \|\| id:ebi-fl187-GCST90094803 | Weighted median | 7 | 0.263677864520442 | 0.152993559850167 | 0.084805864183289 | -0.036189513 | 0.56354524182677 | 1.30170880216443 | 0.964457499135208 | 1.75689007255549 | 0.999489402457283 |
| ebi-fl187-GCST90094803 | ebi-IPs91-GCST90274810 | Interleukin-24 levels \|\| id:ebi-IPs91-GCST90274810 | Mushroom liking \|\| id:ebi-fl187-GCST90094803 | Weighted median | 7 | 0.0167070386801477 | 0.152409480595365 | 0.912711209505733 | -0.282015543 | 0.315429620647064 | 1.0168473817338 | 0.754261960732022 | 1.37084812912398 | 0.999489402457283 |
| ebi-fl187-GCST90094803 | ebi-IPs91-GCST90274823 | Monocyte chemoattractant protein-3 levels \|\| id:ebi-IPs91-GCST90274823 | Mushroom liking \|\| id:ebi-fl187-GCST90094803 | Weighted median | 7 | 0.269784763693084 | 0.173699400287089 | 0.120382435430652 | -0.070666061 | 0.610235588255779 | 1.30968252916365 | 0.931772995643767 | 1.84086503388242 | 0.999489402457283 |
| ebi-fl187-GCST90094803 | ebi-IPs91-GCST90274824 | Monocyte chemoattractant protein-4 levels \|\| id:ebi-IPs91-GCST90274824 | Mushroom liking \|\| id:ebi-fl187-GCST90094803 | Weighted median | 7 | -0.08433822 | 0.140848907579661 | 0.549316232151574 | -0.360402079 | 0.191725638517971 | 0.91912033832709 | 0.697395861324262 | 1.21133812684574 | 0.999489402457283 |
| ebi-fl187-GCST90094803 | ebi-IPs91-GCST90274834 | SIR2-like protein 2 levels \|\| id:ebi-IPs91-GCST90274834 | Mushroom liking \|\| id:ebi-fl187-GCST90094803 | Weighted median | 7 | -0.159055206 | 0.150357257626358 | 0.290124497729171 | -0.453755431 | 0.135645019026449 | 0.852949269819156 | 0.635238073873178 | 1.1452752704969 | 0.999489402457283 |
| ebi-fl187-GCST90094803 | ebi-met1400-GCST90199652 | 1-stearoyl-2-oleoyl-GPS (18:0/18:1) levels \|\| id:ebi-met1400-GCST90199652 | Mushroom liking \|\| id:ebi-fl187-GCST90094803 | Weighted median | 5 | -0.105045432 | 0.235261350325311 | 0.655232309284273 | -0.566157679 | 0.356066814736107 | 0.900283620060381 | 0.567702549129926 | 1.42770293667209 | 0.999489402457283 |
| ebi-fl187-GCST90094803 | ebi-met1400-GCST90199665 | N-acetylaspartate (naa) levels \|\| id:ebi-met1400-GCST90199665 | Mushroom liking \|\| id:ebi-fl187-GCST90094803 | Weighted median | 5 | -0.348630799 | 0.269728420441387 | 0.196175301327505 | -0.877298503 | 0.180036905257227 | 0.705653610336372 | 0.415904961450526 | 1.19726154755187 | 0.999489402457283 |
| ebi-fl187-GCST90094803 | ebi-met1400-GCST90199669 | Acetylcarnitine levels (Biocrates platform) \|\| id:ebi-met1400-GCST90199669 | Mushroom liking \|\| id:ebi-fl187-GCST90094803 | Weighted median | 5 | 0.345030588809095 | 0.274123956567351 | 0.208150885120826 | -0.192252366 | 0.882313543681103 | 1.41203311141834 | 0.825098615325707 | 2.41648388532891 | 0.999489402457283 |
| ebi-fl187-GCST90094803 | ebi-met1400-GCST90199670 | Iminodiacetate (IDA) levels \|\| id:ebi-met1400-GCST90199670 | Mushroom liking \|\| id:ebi-fl187-GCST90094803 | Weighted median | 5 | -0.018871466 | 0.284741310376239 | 0.947158228579568 | -0.576964434 | 0.539221502660693 | 0.981305485573547 | 0.561600557251454 | 1.71467147527344 | 0.999489402457283 |
| ebi-fl187-GCST90094803 | ebi-met1400-GCST90199686 | Indolepropionate levels \|\| id:ebi-met1400-GCST90199686 | Mushroom liking \|\| id:ebi-fl187-GCST90094803 | Weighted median | 5 | 0.341747990106748 | 0.258923297747127 | 0.186874688990076 | -0.165741673 | 0.849237653691117 | 1.40740557269076 | 0.847265076962601 | 2.3378639104801 | 0.999489402457283 |
| ebi-fl187-GCST90094803 | ebi-met1400-GCST90199704 | 3-hydroxysebacate levels \|\| id:ebi-met1400-GCST90199704 | Mushroom liking \|\| id:ebi-fl187-GCST90094803 | Weighted median | 5 | 0.063888310983136 | 0.247659814522894 | 0.796431591541505 | -0.421524925 | 0.549301547448009 | 1.0659733345988 | 0.656045635936244 | 1.73204284554699 | 0.999489402457283 |
| ebi-fl187-GCST90094803 | ebi-met1400-GCST90199713 | Docosapentaenoate n3 DPA; 22:5n3 levels \|\| id:ebi-met1400-GCST90199713 | Mushroom liking \|\| id:ebi-fl187-GCST90094803 | Weighted median | 5 | 0.182815751780641 | 0.249044050333762 | 0.462906111307631 | -0.305310587 | 0.670942090434815 | 1.20059318054538 | 0.736894469096698 | 1.95607925642188 | 0.999489402457283 |
| ebi-fl187-GCST90094803 | ebi-met1400-GCST90199715 | Myristoleate (14:1n5) levels \|\| id:ebi-met1400-GCST90199715 | Mushroom liking \|\| id:ebi-fl187-GCST90094803 | Weighted median | 5 | -0.013246974 | 0.233121387769396 | 0.9546851080484 | -0.470164894 | 0.443670946141851 | 0.986840381117153 | 0.62489921770875 | 1.5584175979195 | 0.999489402457283 |
| ebi-fl187-GCST90094803 | ebi-met1400-GCST90199725 | Campesterol levels \|\| id:ebi-met1400-GCST90199725 | Mushroom liking \|\| id:ebi-fl187-GCST90094803 | Weighted median | 5 | -0.272498847 | 0.34520751757055 | 0.429891741300193 | -0.949105581 | 0.404107887454121 | 0.761474307166639 | 0.387087086550146 | 1.49796554993006 | 0.999489402457283 |
| ebi-fl187-GCST90094803 | ebi-met1400-GCST90199737 | N-acetylproline levels \|\| id:ebi-met1400-GCST90199737 | Mushroom liking \|\| id:ebi-fl187-GCST90094803 | Weighted median | 5 | -0.208515258 | 0.258199121396976 | 0.419335053517088 | -0.714585536 | 0.297555019472079 | 0.811788647958564 | 0.489394906140623 | 1.34656246047039 | 0.999489402457283 |
| ebi-fl187-GCST90094803 | ebi-met1400-GCST90199775 | Gamma-glutamylisoleucine levels \|\| id:ebi-met1400-GCST90199775 | Mushroom liking \|\| id:ebi-fl187-GCST90094803 | Weighted median | 5 | -0.158778585 | 0.234901936306579 | 0.499081524182858 | -0.61918638 | 0.301629210325357 | 0.853185246208717 | 0.538382298051769 | 1.35205980393923 | 0.999489402457283 |
| ebi-fl187-GCST90094803 | ebi-met1400-GCST90199777 | 1-palmitoleoyl-GPC (16:1) levels \|\| id:ebi-met1400-GCST90199777 | Mushroom liking \|\| id:ebi-fl187-GCST90094803 | Weighted median | 5 | 0.0325552575243093 | 0.257267282015363 | 0.89930247085254 | -0.471688615 | 0.536799130274421 | 1.03309097761659 | 0.623947770507385 | 1.71052292913062 | 0.999489402457283 |
| ebi-fl187-GCST90094803 | ebi-met1400-GCST90199782 | Glutamine degradant levels \|\| id:ebi-met1400-GCST90199782 | Mushroom liking \|\| id:ebi-fl187-GCST90094803 | Weighted median | 5 | -0.243119828 | 0.228662484204939 | 0.287679614213596 | -0.691298297 | 0.205058641189487 | 0.784177541830864 | 0.500925296952662 | 1.22759705060375 | 0.999489402457283 |
| ebi-fl187-GCST90094803 | ebi-met1400-GCST90199783 | 5-methyluridine (ribothymidine) levels \|\| id:ebi-met1400-GCST90199783 | Mushroom liking \|\| id:ebi-fl187-GCST90094803 | Weighted median | 5 | -0.270687575 | 0.253798138125396 | 0.286176564736222 | -0.768131926 | 0.226756775534998 | 0.762854793941557 | 0.46387881941627 | 1.25452469973067 | 0.999489402457283 |
| ebi-fl187-GCST90094803 | ebi-met1400-GCST90199787 | Homostachydrine levels \|\| id:ebi-met1400-GCST90199787 | Mushroom liking \|\| id:ebi-fl187-GCST90094803 | Weighted median | 5 | 0.00172916480552054 | 0.246909076506421 | 0.994412264452865 | -0.482212625 | 0.485670954758105 | 1.00173066067306 | 0.617415769688347 | 1.62526512246197 | 0.999911036302558 |
| ebi-fl187-GCST90094803 | ebi-met1400-GCST90199802 | Beta-hydroxyisovaleroylcarnitine levels \|\| id:ebi-met1400-GCST90199802 | Mushroom liking \|\| id:ebi-fl187-GCST90094803 | Weighted median | 5 | -0.268973079 | 0.264113982263086 | 0.308488982312539 | -0.786636484 | 0.248690326222583 | 0.764163827415706 | 0.455373879507792 | 1.28234486299873 | 0.999489402457283 |
| ebi-fl187-GCST90094803 | ebi-met1400-GCST90199873 | Pregnenolone sulfate levels \|\| id:ebi-met1400-GCST90199873 | Mushroom liking \|\| id:ebi-fl187-GCST90094803 | Weighted median | 5 | -0.660212851 | 0.245057933357139 | 0.00705770220410161 | -1.1405264 | -0.179899301 | 0.516741334037583 | 0.319650713364629 | 0.835354326265326 | 0.999489402457283 |
| ebi-fl187-GCST90094803 | ebi-met1400-GCST90199911 | Beta-citrylglutamate levels \|\| id:ebi-met1400-GCST90199911 | Mushroom liking \|\| id:ebi-fl187-GCST90094803 | Weighted median | 5 | 0.0728487203685057 | 0.256691339661942 | 0.776564495965166 | -0.430266305 | 0.575963746105912 | 1.0755678131042 | 0.650335883723407 | 1.77884405510948 | 0.999489402457283 |
| ebi-fl187-GCST90094803 | ebi-met1400-GCST90199934 | 2-hydroxydecanoate levels \|\| id:ebi-met1400-GCST90199934 | Mushroom liking \|\| id:ebi-fl187-GCST90094803 | Weighted median | 5 | -0.197900915 | 0.254632872034723 | 0.437040210248048 | -0.696981344 | 0.301179514143492 | 0.820451143475428 | 0.498086588673818 | 1.35145192449852 | 0.999489402457283 |
| ebi-fl187-GCST90094803 | ebi-met1400-GCST90199945 | Sphingomyelin (d18:1/14:0, d16:1/16:0) levels \|\| id:ebi-met1400-GCST90199945 | Mushroom liking \|\| id:ebi-fl187-GCST90094803 | Weighted median | 5 | 0.125723695601944 | 0.242181991025255 | 0.60367078421867 | -0.348953007 | 0.600400398011444 | 1.13396880442519 | 0.705426279723967 | 1.82284851921403 | 0.999489402457283 |
| ebi-fl187-GCST90094803 | ebi-met1400-GCST90199979 | Carnitine C14:1 levels \|\| id:ebi-met1400-GCST90199979 | Mushroom liking \|\| id:ebi-fl187-GCST90094803 | Weighted median | 5 | 0.3454219433453 | 0.31386530736384 | 0.271096054056146 | -0.269754059 | 0.960597945778425 | 1.41258582512822 | 0.76356726367516 | 2.61325859328888 | 0.999489402457283 |
| ebi-fl187-GCST90094803 | ebi-met1400-GCST90199986 | Eugenol sulfate levels \|\| id:ebi-met1400-GCST90199986 | Mushroom liking \|\| id:ebi-fl187-GCST90094803 | Weighted median | 5 | -0.098957482 | 0.252901366144526 | 0.69558374702056 | -0.59464416 | 0.396729195451578 | 0.90578121903708 | 0.551758868906478 | 1.4869532018331 | 0.999489402457283 |
| ebi-fl187-GCST90094803 | ebi-met1400-GCST90200002 | 6-hydroxyindole sulfate levels \|\| id:ebi-met1400-GCST90200002 | Mushroom liking \|\| id:ebi-fl187-GCST90094803 | Weighted median | 5 | -0.09374632 | 0.252397104052529 | 0.710321817393758 | -0.588444644 | 0.400952003924311 | 0.910513712047352 | 0.555190131867371 | 1.49324559685131 | 0.999489402457283 |
| ebi-fl187-GCST90094803 | ebi-met1400-GCST90200023 | Trans 3,4-methyleneheptanoate levels \|\| id:ebi-met1400-GCST90200023 | Mushroom liking \|\| id:ebi-fl187-GCST90094803 | Weighted median | 5 | -0.190827175 | 0.264740010182426 | 0.471026589148428 | -0.709717595 | 0.328063245143676 | 0.826275377007903 | 0.491783059959748 | 1.38827677127682 | 0.999489402457283 |
| ebi-fl187-GCST90094803 | ebi-met1400-GCST90200032 | 2-hydroxybutyrate/2-hydroxyisobutyrate levels \|\| id:ebi-met1400-GCST90200032 | Mushroom liking \|\| id:ebi-fl187-GCST90094803 | Weighted median | 5 | 0.512159079087817 | 0.255129100320822 | 0.0447016999774347 | 0.0121060424590058 | 1.01221211571663 | 1.66889057461446 | 1.01217961719086 | 2.75168132486884 | 0.999489402457283 |
| ebi-fl187-GCST90094803 | ebi-met1400-GCST90200051 | 1-palmitoyl-2-dihomo-linolenoyl-GPC (16:0/20:3n3 or 6) levels \|\| id:ebi-met1400-GCST90200051 | Mushroom liking \|\| id:ebi-fl187-GCST90094803 | Weighted median | 5 | -0.105776142 | 0.251134744128269 | 0.673614314155446 | -0.59800024 | 0.386447956786597 | 0.8996260142822 | 0.549910225635229 | 1.47174380079655 | 0.999489402457283 |
| ebi-fl187-GCST90094803 | ebi-met1400-GCST90200069 | Phenylacetylglutamate levels \|\| id:ebi-met1400-GCST90200069 | Mushroom liking \|\| id:ebi-fl187-GCST90094803 | Weighted median | 5 | 0.0777443715031199 | 0.274219544003967 | 0.776784831005471 | -0.459725935 | 0.615214677750894 | 1.08084632823038 | 0.631456682131008 | 1.85005372230223 | 0.999489402457283 |
| ebi-fl187-GCST90094803 | ebi-met1400-GCST90200072 | Thioproline levels \|\| id:ebi-met1400-GCST90200072 | Mushroom liking \|\| id:ebi-fl187-GCST90094803 | Weighted median | 5 | 0.0185715002295893 | 0.257399055719142 | 0.942482046023522 | -0.485930649 | 0.523073649439107 | 1.01874502306862 | 0.615124465343465 | 1.68720556651505 | 0.999489402457283 |
| ebi-fl187-GCST90094803 | ebi-met1400-GCST90200081 | 1-oleoyl-2-docosahexaenoyl-GPC (18:1/22:6) levels \|\| id:ebi-met1400-GCST90200081 | Mushroom liking \|\| id:ebi-fl187-GCST90094803 | Weighted median | 5 | 0.357311624505413 | 0.240648551445597 | 0.137600494793773 | -0.114359536 | 0.828982785338783 | 1.42948126184396 | 0.891937214395924 | 2.29098712889443 | 0.999489402457283 |
| ebi-fl187-GCST90094803 | ebi-met1400-GCST90200097 | Furaneol sulfate levels \|\| id:ebi-met1400-GCST90200097 | Mushroom liking \|\| id:ebi-fl187-GCST90094803 | Weighted median | 5 | -0.099868006 | 0.351765049358445 | 0.776482857746115 | -0.789327503 | 0.589591490353769 | 0.904956858676858 | 0.45415010713517 | 1.80325161923289 | 0.999489402457283 |
| ebi-fl187-GCST90094803 | ebi-met1400-GCST90200124 | Sphingomyelin (d18:2/24:2) levels \|\| id:ebi-met1400-GCST90200124 | Mushroom liking \|\| id:ebi-fl187-GCST90094803 | Weighted median | 5 | 0.0877618647099081 | 0.234215571225839 | 0.707879495089565 | -0.371300655 | 0.546824384312552 | 1.09172811207998 | 0.689836507656128 | 1.72775760267511 | 0.999489402457283 |
| ebi-fl187-GCST90094803 | ebi-met1400-GCST90200137 | Lignoceroylcarnitine (C24) levels \|\| id:ebi-met1400-GCST90200137 | Mushroom liking \|\| id:ebi-fl187-GCST90094803 | Weighted median | 5 | 0.33006883605407 | 0.247353049764893 | 0.182071573217827 | -0.154743141 | 0.81488081359326 | 1.39106388051664 | 0.856635183268193 | 2.25890642536473 | 0.999489402457283 |
| ebi-fl187-GCST90094803 | ebi-met1400-GCST90200165 | Octadecenedioate (C18:1-DC) levels \|\| id:ebi-met1400-GCST90200165 | Mushroom liking \|\| id:ebi-fl187-GCST90094803 | Weighted median | 5 | -0.186640958 | 0.260983559336171 | 0.474519757043295 | -0.698168734 | 0.324886818368379 | 0.829741595049731 | 0.497495516626855 | 1.38387400799846 | 0.999489402457283 |
| ebi-fl187-GCST90094803 | ebi-met1400-GCST90200176 | Glutamine conjugate of C6H10O2 (1) levels \|\| id:ebi-met1400-GCST90200176 | Mushroom liking \|\| id:ebi-fl187-GCST90094803 | Weighted median | 5 | 0.0527437485292862 | 0.248902096545145 | 0.832180680325038 | -0.435104361 | 0.54059185775777 | 1.05415948058854 | 0.64719712160942 | 1.71702279477276 | 0.999489402457283 |
| ebi-fl187-GCST90094803 | ebi-met1400-GCST90200182 | Glycine conjugate of C10H12O2 levels \|\| id:ebi-met1400-GCST90200182 | Mushroom liking \|\| id:ebi-fl187-GCST90094803 | Weighted median | 5 | -0.158693607 | 0.270876339797918 | 0.55797457613121 | -0.689611233 | 0.372224019449278 | 0.853257751504869 | 0.50177110342016 | 1.45095798769725 | 0.999489402457283 |
| ebi-fl187-GCST90094803 | ebi-met1400-GCST90200208 | Glucuronide of piperine metabolite C17H21NO3 (4) levels \|\| id:ebi-met1400-GCST90200208 | Mushroom liking \|\| id:ebi-fl187-GCST90094803 | Weighted median | 5 | 0.0523016673444334 | 0.276608741543421 | 0.850028737988354 | -0.489851466 | 0.594454800769538 | 1.05369355951139 | 0.612717396742144 | 1.81204275128985 | 0.999489402457283 |
| ebi-fl187-GCST90094803 | ebi-met1400-GCST90200227 | 3-ethylcatechol sulfate (2) levels \|\| id:ebi-met1400-GCST90200227 | Mushroom liking \|\| id:ebi-fl187-GCST90094803 | Weighted median | 4 | 0.111450553563841 | 0.325616713639279 | 0.732143733049106 | -0.526758205 | 0.749659312296829 | 1.11789846654375 | 0.590516202464958 | 2.11627890358355 | 0.999489402457283 |
| ebi-fl187-GCST90094803 | ebi-met1400-GCST90200259 | Pentose acid levels \|\| id:ebi-met1400-GCST90200259 | Mushroom liking \|\| id:ebi-fl187-GCST90094803 | Weighted median | 5 | -0.225418635 | 0.269640244532235 | 0.40315617532784 | -0.753913514 | 0.303076244654241 | 0.798182002180633 | 0.470521552237585 | 1.35401769711792 | 0.999489402457283 |
| ebi-fl187-GCST90094803 | ebi-met1400-GCST90200291 | Sarcosine levels \|\| id:ebi-met1400-GCST90200291 | Mushroom liking \|\| id:ebi-fl187-GCST90094803 | Weighted median | 5 | 0.227675240615202 | 0.248251474706416 | 0.359082192961793 | -0.25889765 | 0.714248131039777 | 1.25567746616645 | 0.77190202332002 | 2.04265029939494 | 0.999489402457283 |
| ebi-fl187-GCST90094803 | ebi-met1400-GCST90200306 | Butyrate/isobutyrate (4:0) levels \|\| id:ebi-met1400-GCST90200306 | Mushroom liking \|\| id:ebi-fl187-GCST90094803 | Weighted median | 5 | -0.094655727 | 0.254110716173279 | 0.709522119420502 | -0.592712731 | 0.403401276501115 | 0.909686060733314 | 0.552825581760463 | 1.49690744494356 | 0.999489402457283 |
| ebi-fl187-GCST90094803 | ebi-met1400-GCST90200321 | Cholate levels \|\| id:ebi-met1400-GCST90200321 | Mushroom liking \|\| id:ebi-fl187-GCST90094803 | Weighted median | 5 | -0.028550087 | 0.256874221615055 | 0.911501977300144 | -0.532023561 | 0.474923387373015 | 0.971853615702314 | 0.587415095704109 | 1.60789100801288 | 0.999489402457283 |
| ebi-fl187-GCST90094803 | ebi-met1400-GCST90200336 | Ceramide (d18:1/16:0) levels \|\| id:ebi-met1400-GCST90200336 | Mushroom liking \|\| id:ebi-fl187-GCST90094803 | Weighted median | 5 | -0.014583699 | 0.239510756235361 | 0.951447182237114 | -0.484024781 | 0.454857383163841 | 0.985522128007005 | 0.616297929072261 | 1.57594861020144 | 0.999489402457283 |
| ebi-fl187-GCST90094803 | ebi-met1400-GCST90200358 | Arachidonate (20:4n6) levels \|\| id:ebi-met1400-GCST90200358 | Mushroom liking \|\| id:ebi-fl187-GCST90094803 | Weighted median | 5 | 0.122995169807521 | 0.252914652536619 | 0.626746703128273 | -0.372717549 | 0.618707888779295 | 1.13087895857126 | 0.68885977438744 | 1.85652765118481 | 0.999489402457283 |
| ebi-fl187-GCST90094803 | ebi-met1400-GCST90200413 | 2'-deoxyuridine levels \|\| id:ebi-met1400-GCST90200413 | Mushroom liking \|\| id:ebi-fl187-GCST90094803 | Weighted median | 5 | -0.216823335 | 0.256362401541441 | 0.397681702566836 | -0.719293642 | 0.285646971933381 | 0.80507218474919 | 0.487096198715937 | 1.3306226251928 | 0.999489402457283 |
| ebi-fl187-GCST90094803 | ebi-met1400-GCST90200432 | Threonine levels \|\| id:ebi-met1400-GCST90200432 | Mushroom liking \|\| id:ebi-fl187-GCST90094803 | Weighted median | 5 | 0.0378296200292897 | 0.291609926306925 | 0.896782585342942 | -0.533725836 | 0.609385075590863 | 1.03855426895745 | 0.586416004761721 | 1.8393000204829 | 0.999489402457283 |
| ebi-fl187-GCST90094803 | ebi-met1400-GCST90200436 | Caffeine levels \|\| id:ebi-met1400-GCST90200436 | Mushroom liking \|\| id:ebi-fl187-GCST90094803 | Weighted median | 5 | -0.009928118 | 0.244106171639273 | 0.967557932743988 | -0.488376215 | 0.468519978258961 | 0.990121002916691 | 0.613621976086113 | 1.59762791852679 | 0.999489402457283 |
| ebi-fl187-GCST90094803 | ebi-met1400-GCST90200442 | Valine levels \|\| id:ebi-met1400-GCST90200442 | Mushroom liking \|\| id:ebi-fl187-GCST90094803 | Weighted median | 5 | -0.033640347 | 0.293336486566117 | 0.908697364078107 | -0.608579861 | 0.541299166208627 | 0.966919197053793 | 0.544123050936381 | 1.71823768910769 | 0.999489402457283 |
| ebi-fl187-GCST90094803 | ebi-met1400-GCST90200459 | Margarate (17:0) levels \|\| id:ebi-met1400-GCST90200459 | Mushroom liking \|\| id:ebi-fl187-GCST90094803 | Weighted median | 5 | 0.118464639053865 | 0.238649266480272 | 0.619614757668679 | -0.349287923 | 0.586217201355197 | 1.1257670652096 | 0.705190060424813 | 1.79717718135046 | 0.999489402457283 |
| ebi-fl187-GCST90094803 | ebi-met1400-GCST90200462 | X-11299 levels \|\| id:ebi-met1400-GCST90200462 | Mushroom liking \|\| id:ebi-fl187-GCST90094803 | Weighted median | 5 | 0.191719940347326 | 0.238737123545366 | 0.4219407678625 | -0.276204822 | 0.659644702496243 | 1.21133122445405 | 0.758657525245454 | 1.9341050296214 | 0.999489402457283 |
| ebi-fl187-GCST90094803 | ebi-met1400-GCST90200497 | X-12740 levels \|\| id:ebi-met1400-GCST90200497 | Mushroom liking \|\| id:ebi-fl187-GCST90094803 | Weighted median | 5 | -0.183761766 | 0.286707658957547 | 0.521563118545994 | -0.745708778 | 0.378185245497706 | 0.832134022782109 | 0.474397938111574 | 1.4596333083317 | 0.999489402457283 |
| ebi-fl187-GCST90094803 | ebi-met1400-GCST90200527 | X-13866 levels \|\| id:ebi-met1400-GCST90200527 | Mushroom liking \|\| id:ebi-fl187-GCST90094803 | Weighted median | 5 | 0.0876538656904779 | 0.265582709444874 | 0.741367040420291 | -0.432888245 | 0.60819597620243 | 1.09161021288101 | 0.64863297584868 | 1.83711420978404 | 0.999489402457283 |
| ebi-fl187-GCST90094803 | ebi-met1400-GCST90200547 | X-17654 levels \|\| id:ebi-met1400-GCST90200547 | Mushroom liking \|\| id:ebi-fl187-GCST90094803 | Weighted median | 5 | 0.25993313795327 | 0.263484935178564 | 0.323878043643713 | -0.256497335 | 0.776363610903256 | 1.29684337416463 | 0.773757056621822 | 2.17355398922933 | 0.999489402457283 |
| ebi-fl187-GCST90094803 | ebi-met1400-GCST90200553 | X-17653 levels \|\| id:ebi-met1400-GCST90200553 | Mushroom liking \|\| id:ebi-fl187-GCST90094803 | Weighted median | 5 | 0.156861835206418 | 0.282445298473967 | 0.578641148550854 | -0.39673095 | 0.710454620215392 | 1.16983397286523 | 0.672514941571645 | 2.03491616241491 | 0.999489402457283 |
| ebi-fl187-GCST90094803 | ebi-met1400-GCST90200557 | X-17685 levels \|\| id:ebi-met1400-GCST90200557 | Mushroom liking \|\| id:ebi-fl187-GCST90094803 | Weighted median | 5 | 0.213990145217576 | 0.260770824645042 | 0.411870637417979 | -0.297120671 | 0.725100961521858 | 1.23861044849683 | 0.742954353843201 | 2.06493956888406 | 0.999489402457283 |
| ebi-fl187-GCST90094803 | ebi-met1400-GCST90200561 | X-18779 levels \|\| id:ebi-met1400-GCST90200561 | Mushroom liking \|\| id:ebi-fl187-GCST90094803 | Weighted median | 5 | -0.136776696 | 0.24763766212616 | 0.580725084058456 | -0.622146514 | 0.348593121464337 | 0.87216496204915 | 0.536790970699731 | 1.41707249664544 | 0.999489402457283 |
| ebi-fl187-GCST90094803 | ebi-met1400-GCST90200570 | X-18921 levels \|\| id:ebi-met1400-GCST90200570 | Mushroom liking \|\| id:ebi-fl187-GCST90094803 | Weighted median | 5 | -0.090309675 | 0.279801131818108 | 0.746874135803996 | -0.638719893 | 0.45810054384975 | 0.913648207893537 | 0.527967847045511 | 1.58106796172972 | 0.999489402457283 |
| ebi-fl187-GCST90094803 | ebi-met1400-GCST90200575 | X-21283 levels \|\| id:ebi-met1400-GCST90200575 | Mushroom liking \|\| id:ebi-fl187-GCST90094803 | Weighted median | 5 | -0.103765765 | 0.256762576121829 | 0.686116251526362 | -0.607020414 | 0.399488884203813 | 0.901436420658539 | 0.544972243925012 | 1.49106239730163 | 0.999489402457283 |
| ebi-fl187-GCST90094803 | ebi-met1400-GCST90200578 | X-18922 levels \|\| id:ebi-met1400-GCST90200578 | Mushroom liking \|\| id:ebi-fl187-GCST90094803 | Weighted median | 5 | -0.047071676 | 0.259698227868577 | 0.856167202440427 | -0.556080203 | 0.461936850455869 | 0.954019014713907 | 0.573452481542953 | 1.58714507257307 | 0.999489402457283 |
| ebi-fl187-GCST90094803 | ebi-met1400-GCST90200623 | X-24241 levels \|\| id:ebi-met1400-GCST90200623 | Mushroom liking \|\| id:ebi-fl187-GCST90094803 | Weighted median | 5 | -0.272374521 | 0.265206516262655 | 0.304407274665742 | -0.792179293 | 0.247430251064955 | 0.76156898423938 | 0.452856811583873 | 1.28073002971268 | 0.999489402457283 |
| ebi-fl187-GCST90094803 | ebi-met1400-GCST90200636 | X-24544 levels \|\| id:ebi-met1400-GCST90200636 | Mushroom liking \|\| id:ebi-fl187-GCST90094803 | Weighted median | 5 | -0.248413913 | 0.234774097362808 | 0.290010939777691 | -0.708571144 | 0.211743317384883 | 0.780037008642724 | 0.492347188202596 | 1.2358306281256 | 0.999489402457283 |
| ebi-fl187-GCST90094803 | ebi-met1400-GCST90200659 | X-25172 levels \|\| id:ebi-met1400-GCST90200659 | Mushroom liking \|\| id:ebi-fl187-GCST90094803 | Weighted median | 5 | -0.094004046 | 0.240477904309422 | 0.695867362487021 | -0.565340739 | 0.377332646191134 | 0.910279079011737 | 0.568166517449352 | 1.45838935635682 | 0.999489402457283 |
| ebi-fl187-GCST90094803 | ebi-met1400-GCST90200670 | X-26111 levels \|\| id:ebi-met1400-GCST90200670 | Mushroom liking \|\| id:ebi-fl187-GCST90094803 | Weighted median | 5 | 0.132444417503082 | 0.253279785486621 | 0.60103170631688 | -0.363983962 | 0.62887279705686 | 1.14161556048232 | 0.694902339462033 | 1.87549532347857 | 0.999489402457283 |
| ebi-fl187-GCST90094803 | ebi-met1400-GCST90200673 | Carnitine C4 levels \|\| id:ebi-met1400-GCST90200673 | Mushroom liking \|\| id:ebi-fl187-GCST90094803 | Weighted median | 5 | -0.311635929 | 0.285185120672698 | 0.274503752208221 | -0.870598765 | 0.247326907654674 | 0.732248070092117 | 0.418700770649352 | 1.28059768154253 | 0.999489402457283 |
| ebi-fl187-GCST90094803 | ebi-met1400-GCST90200710 | X-13431 levels \|\| id:ebi-met1400-GCST90200710 | Mushroom liking \|\| id:ebi-fl187-GCST90094803 | Weighted median | 5 | -0.01967764 | 0.258929043310009 | 0.939422070381214 | -0.527178565 | 0.487823284916233 | 0.98051470111376 | 0.590268025422701 | 1.62876699684304 | 0.999489402457283 |
| ebi-fl187-GCST90094803 | ebi-met1400-GCST90200730 | Adenosine 5'-diphosphate (ADP) to fructose ratio \|\| id:ebi-met1400-GCST90200730 | Mushroom liking \|\| id:ebi-fl187-GCST90094803 | Weighted median | 5 | 0.315596252208945 | 0.345186514118208 | 0.360571066898391 | -0.360969315 | 0.992161819880632 | 1.37107657472143 | 0.697000385273039 | 2.69705872976448 | 0.999489402457283 |
| ebi-fl187-GCST90094803 | ebi-met1400-GCST90200731 | Adenosine 5'-diphosphate (ADP) to mannose ratio \|\| id:ebi-met1400-GCST90200731 | Mushroom liking \|\| id:ebi-fl187-GCST90094803 | Weighted median | 5 | 0.228750597567313 | 0.35830485707553 | 0.523197389738624 | -0.473526922 | 0.931028117435351 | 1.25702849394767 | 0.622801816536842 | 2.53711629067297 | 0.999489402457283 |
| ebi-fl187-GCST90094803 | ebi-met1400-GCST90200748 | Aspartate to glutamate ratio \|\| id:ebi-met1400-GCST90200748 | Mushroom liking \|\| id:ebi-fl187-GCST90094803 | Weighted median | 5 | 0.0519095058092038 | 0.267689437667777 | 0.846240950898736 | -0.472761792 | 0.576580803638047 | 1.05328042244096 | 0.623278523413966 | 1.77994204295818 | 0.999489402457283 |
| ebi-fl187-GCST90094803 | ebi-met1400-GCST90200774 | Uridine to pseudouridine ratio \|\| id:ebi-met1400-GCST90200774 | Mushroom liking \|\| id:ebi-fl187-GCST90094803 | Weighted median | 5 | 0.153032084912047 | 0.230435614279361 | 0.506626883752628 | -0.298621719 | 0.604685888899595 | 1.16536236889202 | 0.741839980277935 | 1.83067708257083 | 0.999489402457283 |
| ebi-fl187-GCST90094803 | ebi-met1400-GCST90200799 | Spermidine to adenosine 5'-diphosphate (ADP) ratio \|\| id:ebi-met1400-GCST90200799 | Mushroom liking \|\| id:ebi-fl187-GCST90094803 | Weighted median | 5 | -0.781345473 | 0.414752099621748 | 0.0595804629957934 | -1.594259588 | 0.0315686425917806 | 0.457789653286111 | 0.203058820059048 | 1.03207221727614 | 0.999489402457283 |
| ebi-fl187-GCST90094803 | ebi-met1400-GCST90200811 | Choline to taurocholate ratio \|\| id:ebi-met1400-GCST90200811 | Mushroom liking \|\| id:ebi-fl187-GCST90094803 | Weighted median | 5 | 0.0134247743977286 | 0.263474886868341 | 0.959363158469785 | -0.502986004 | 0.529835552659676 | 1.01351529128425 | 0.604722258109726 | 1.6986529466898 | 0.999489402457283 |
| ebi-fl187-GCST90094803 | ebi-met1400-GCST90200812 | Glutamate to kynurenine ratio \|\| id:ebi-met1400-GCST90200812 | Mushroom liking \|\| id:ebi-fl187-GCST90094803 | Weighted median | 5 | -0.063946869 | 0.255136748234107 | 0.802094288089714 | -0.564014896 | 0.436121157462043 | 0.938054837926283 | 0.568920316697629 | 1.54669617718114 | 0.999489402457283 |
| ebi-fl187-GCST90094803 | ebi-met1400-GCST90200876 | Cortisone to 4-cholesten-3-one ratio \|\| id:ebi-met1400-GCST90200876 | Mushroom liking \|\| id:ebi-fl187-GCST90094803 | Weighted median | 5 | -0.274588043 | 0.305865589610506 | 0.36932371077663 | -0.874084599 | 0.324908512665985 | 0.759885098762273 | 0.417243790462023 | 1.3839040304987 | 0.999489402457283 |
| ebi-fl187-GCST90094803 | ebi-met1400-GCST90200906 | 2'-deoxyuridine to cytidine ratio \|\| id:ebi-met1400-GCST90200906 | Mushroom liking \|\| id:ebi-fl187-GCST90094803 | Weighted median | 5 | 0.0473663477180386 | 0.259132714285451 | 0.854964373255992 | -0.460533772 | 0.555266467717522 | 1.04850605652907 | 0.630946773709637 | 1.74240521766114 | 0.999489402457283 |
| ebi-fl187-GCST90094803 | ebi-met1400-GCST90200907 | Retinol (Vitamin A) to linoleoyl-arachidonoyl-glycerol (18:2 to 20:4) [1] ratio \|\| id:ebi-met1400-GCST90200907 | Mushroom liking \|\| id:ebi-fl187-GCST90094803 | Weighted median | 5 | -0.133054841 | 0.259348858938699 | 0.607927378680499 | -0.641378605 | 0.375268922110355 | 0.875417081685648 | 0.526565996951093 | 1.45538274659652 | 0.999489402457283 |
| ebi-fl187-GCST90094803 | ebi-met1400-GCST90200916 | Fructose to sucrose ratio \|\| id:ebi-met1400-GCST90200916 | Mushroom liking \|\| id:ebi-fl187-GCST90094803 | Weighted median | 5 | -0.281415125 | 0.285414612835635 | 0.324139446350583 | -0.840827766 | 0.277997516619538 | 0.754714969738439 | 0.431353316129489 | 1.32048291794355 | 0.999489402457283 |
| ebi-fl187-GCST90094803 | ebi-met1400-GCST90200917 | Alpha-ketobutyrate to 3-methyl-2-oxobutyrate ratio \|\| id:ebi-met1400-GCST90200917 | Mushroom liking \|\| id:ebi-fl187-GCST90094803 | Weighted median | 5 | 0.499448704509691 | 0.283235053424586 | 0.0778378036407036 | -0.055692 | 1.05458940922188 | 1.647812589 | 0.945830406606139 | 2.8707961894407 | 0.999489402457283 |
| ebi-fl187-GCST90094803 | ebi-met1400-GCST90200924 | Alpha-tocopherol to glycerol ratio \|\| id:ebi-met1400-GCST90200924 | Mushroom liking \|\| id:ebi-fl187-GCST90094803 | Weighted median | 5 | -0.012264898 | 0.217486841007778 | 0.955028136112067 | -0.438539107 | 0.41400930991729 | 0.987810008852868 | 0.644977977078775 | 1.51287121152468 | 0.999489402457283 |
| ebi-fl187-GCST90094803 | ebi-met1400-GCST90200938 | Alpha-ketoglutarate to alanine ratio \|\| id:ebi-met1400-GCST90200938 | Mushroom liking \|\| id:ebi-fl187-GCST90094803 | Weighted median | 5 | -0.156294004 | 0.23902033881155 | 0.513179995522516 | -0.624773868 | 0.312185859965537 | 0.855307689429859 | 0.535382481848767 | 1.36640863009128 | 0.999489402457283 |
| ebi-fl187-GCST90094803 | ebi-met1400-GCST90200943 | Phosphate to EDTA ratio \|\| id:ebi-met1400-GCST90200943 | Mushroom liking \|\| id:ebi-fl187-GCST90094803 | Weighted median | 5 | 0.163044810056026 | 0.267777673196543 | 0.542603139134682 | -0.361799429 | 0.68788904952125 | 1.1770894339502 | 0.696422035613809 | 1.98951133746943 | 0.999489402457283 |
| ebi-fl187-GCST90094803 | ebi-met1400-GCST90200947 | Adenosine 5'-diphosphate (ADP) to glucose ratio \|\| id:ebi-met1400-GCST90200947 | Mushroom liking \|\| id:ebi-fl187-GCST90094803 | Weighted median | 5 | 0.479837307454293 | 0.334855113750245 | 0.151866432131265 | -0.176478715 | 1.13615333040477 | 1.61581150032102 | 0.83821661999016 | 3.11476382393885 | 0.999489402457283 |
| ebi-fl187-GCST90094803 | ebi-met1400-GCST90200975 | Arachidonate (20:4n6) to caffeine ratio \|\| id:ebi-met1400-GCST90200975 | Mushroom liking \|\| id:ebi-fl187-GCST90094803 | Weighted median | 5 | -0.013987243 | 0.243275076150855 | 0.954150421468059 | -0.490806393 | 0.462831905983132 | 0.986110123720716 | 0.612132575969975 | 1.58856629148288 | 0.999489402457283 |
| ebi-fl187-GCST90094803 | ebi-met1400-GCST90200985 | Cholesterol to oleoyl-linoleoyl-glycerol (18:1 to 18:2) [2] ratio \|\| id:ebi-met1400-GCST90200985 | Mushroom liking \|\| id:ebi-fl187-GCST90094803 | Weighted median | 5 | 0.0632971797252513 | 0.246637539424011 | 0.797456312720668 | -0.420112398 | 0.546706756996312 | 1.06534339064878 | 0.656972973515177 | 1.72755438313751 | 0.999489402457283 |
| ebi-fl187-GCST90094803 | ebi-met1400-GCST90201016 | Bilirubin (Z,Z) to etiocholanolone glucuronide ratio \|\| id:ebi-met1400-GCST90201016 | Mushroom liking \|\| id:ebi-fl187-GCST90094803 | Weighted median | 5 | 0.131169938909378 | 0.267683845043737 | 0.624121065082415 | -0.393490397 | 0.655830275195103 | 1.1401615226551 | 0.674697796414784 | 1.9267415791944 | 0.999489402457283 |
| ebi-fl187-GCST90094803 | ebi-pw11-GCST90275189 | Placental weight (fetal genotype, adjusted for sex and gestational age) \|\| id:ebi-pw11-GCST90275189 | Mushroom liking \|\| id:ebi-fl187-GCST90094803 | Weighted median | 6 | 0.0406625124708249 | 0.0795126533378301 | 0.609073270459704 | -0.115182288 | 0.196507313012972 | 1.04150055277829 | 0.891203673300293 | 1.21714422183713 | 0.999489402457283 |
| ebi-fl187-GCST90094803 | ebi-pw11-GCST90275192 | Placental weight (fetal effect, conditional on maternal and paternal genotype) \|\| id:ebi-pw11-GCST90275192 | Mushroom liking \|\| id:ebi-fl187-GCST90094803 | Weighted median | 6 | 0.0823485433040171 | 0.154510448029752 | 0.594058335622305 | -0.220491935 | 0.385189021442332 | 1.08583420412344 | 0.802124108099388 | 1.46989213631551 | 0.999489402457283 |
| ebi-fl187-GCST90094803 | ebi-pw11-GCST90275193 | Placental weight (maternal effect, conditional on fetal and paternal genotype) \|\| id:ebi-pw11-GCST90275193 | Mushroom liking \|\| id:ebi-fl187-GCST90094803 | Weighted median | 6 | 0.0129441260677166 | 0.127721010690669 | 0.919275102113033 | -0.237389055 | 0.263277307021428 | 1.01302826390588 | 0.788684386806578 | 1.30118749735543 | 0.999489402457283 |
| ebi-fl187-GCST90094803 | ebi-pw11-GCST90275195 | Placental weight (fetal effect, conditional on maternal genotype) \|\| id:ebi-pw11-GCST90275195 | Mushroom liking \|\| id:ebi-fl187-GCST90094803 | Weighted median | 6 | 0.0321057396102249 | 0.103839465766897 | 0.757179819132716 | -0.171419613 | 0.235631092513344 | 1.0326266890761 | 0.842467988525513 | 1.26570729513241 | 0.999489402457283 |
| ebi-fl187-GCST90094803 | ebi-pw11-GCST90275197 | Placental weight (fetal genotype, adjusted for sex) \|\| id:ebi-pw11-GCST90275197 | Mushroom liking \|\| id:ebi-fl187-GCST90094803 | Weighted median | 6 | 0.0701751164748773 | 0.0753331556709214 | 0.351579322848901 | -0.077477869 | 0.217828101589883 | 1.0726960115518 | 0.925447505581786 | 1.24337331535166 | 0.999489402457283 |
| ebi-fl187-GCST90094803 | prot-a-1030 | Protein FAM171B \|\| id:prot-a-1030 | Mushroom liking \|\| id:ebi-fl187-GCST90094803 | Weighted median | 7 | -0.032895504 | 0.308442024130327 | 0.915066233813545 | -0.637441871 | 0.571650863426254 | 0.967639668907596 | 0.528643032776705 | 1.77118862973665 | 0.999489402457283 |
| ebi-fl187-GCST90094803 | prot-a-1036 | BRISC complex subunit Abro1 \|\| id:prot-a-1036 | Mushroom liking \|\| id:ebi-fl187-GCST90094803 | Weighted median | 7 | 0.270923936633619 | 0.275042931537475 | 0.324612196511864 | -0.268160209 | 0.81000808244707 | 1.31117533418167 | 0.764785245670519 | 2.24792615534722 | 0.999489402457283 |
| ebi-fl187-GCST90094803 | prot-a-1055 | Phenylalanine--tRNA ligase, mitochondrial \|\| id:prot-a-1055 | Mushroom liking \|\| id:ebi-fl187-GCST90094803 | Weighted median | 7 | -0.112519716 | 0.290046454260072 | 0.698062745672269 | -0.681010767 | 0.455971334121991 | 0.893579728913385 | 0.506105179547336 | 1.5777051178158 | 0.999489402457283 |
| ebi-fl187-GCST90094803 | prot-a-1083 | Fc receptor-like protein 5 \|\| id:prot-a-1083 | Mushroom liking \|\| id:ebi-fl187-GCST90094803 | Weighted median | 7 | -0.098630608 | 0.277997848384476 | 0.72274759160109 | -0.64350639 | 0.446245175213337 | 0.906077344280156 | 0.525446768606314 | 1.56243448978728 | 0.999489402457283 |
| ebi-fl187-GCST90094803 | prot-a-1103 | Fibroblast growth factor receptor 3 \|\| id:prot-a-1103 | Mushroom liking \|\| id:ebi-fl187-GCST90094803 | Weighted median | 7 | -0.200338022 | 0.273581444883225 | 0.463998127263037 | -0.736557654 | 0.335881610244945 | 0.818454051063834 | 0.478759136930393 | 1.39917336721282 | 0.999489402457283 |
| ebi-fl187-GCST90094803 | prot-a-1105 | Fibroblast growth factor receptor 4 \|\| id:prot-a-1105 | Mushroom liking \|\| id:ebi-fl187-GCST90094803 | Weighted median | 7 | -0.074283083 | 0.289982063758871 | 0.7978238928967 | -0.642647928 | 0.494081762294407 | 0.928408840182288 | 0.525898038789227 | 1.63899256310799 | 0.999489402457283 |
| ebi-fl187-GCST90094803 | prot-a-1120 | Filamin-A \|\| id:prot-a-1120 | Mushroom liking \|\| id:ebi-fl187-GCST90094803 | Weighted median | 7 | 0.14764041927049 | 0.275900552985688 | 0.592565589361865 | -0.393124665 | 0.688405503122439 | 1.15909603282236 | 0.674944600655023 | 1.99053909313549 | 0.999489402457283 |
| ebi-fl187-GCST90094803 | prot-a-1166 | Galanin peptides \|\| id:prot-a-1166 | Mushroom liking \|\| id:ebi-fl187-GCST90094803 | Weighted median | 7 | -0.009671606 | 0.27608914169012 | 0.972055231939472 | -0.550806324 | 0.531463111813721 | 0.990375013665014 | 0.5764847896295 | 1.70141985588614 | 0.999489402457283 |
| ebi-fl187-GCST90094803 | prot-a-1205 | ADP-ribosylation factor-binding protein GGA1 \|\| id:prot-a-1205 | Mushroom liking \|\| id:ebi-fl187-GCST90094803 | Weighted median | 7 | -0.073050413 | 0.279286315881738 | 0.793659902541734 | -0.620451592 | 0.474350766575413 | 0.929553967656108 | 0.537701561206422 | 1.60697055974048 | 0.999489402457283 |
| ebi-fl187-GCST90094803 | prot-a-1227 | Guanine nucleotide-binding protein G(I)/G(S)/G(O) subunit gamma-T2 \|\| id:prot-a-1227 | Mushroom liking \|\| id:ebi-fl187-GCST90094803 | Weighted median | 7 | -0.159506747 | 0.263239339254127 | 0.544555853778579 | -0.675455852 | 0.356442358109682 | 0.852564215272554 | 0.50892437364732 | 1.42823920173848 | 0.999489402457283 |
| ebi-fl187-GCST90094803 | prot-a-1240 | Golgi SNAP receptor complex member 1 \|\| id:prot-a-1240 | Mushroom liking \|\| id:ebi-fl187-GCST90094803 | Weighted median | 7 | 0.16620695997757 | 0.299149731170185 | 0.578485772961191 | -0.420126513 | 0.752540433071133 | 1.18081745839108 | 0.65696370003251 | 2.12238495060256 | 0.999489402457283 |
| ebi-fl187-GCST90094803 | prot-a-1283 | Glutathione S-transferase A1 \|\| id:prot-a-1283 | Mushroom liking \|\| id:ebi-fl187-GCST90094803 | Weighted median | 7 | 0.018595779849639 | 0.287764016890744 | 0.94847526239057 | -0.545421693 | 0.582613252955497 | 1.01876975811099 | 0.579597319518133 | 1.79071190478315 | 0.999489402457283 |
| ebi-fl187-GCST90094803 | prot-a-1324 | E3 ubiquitin-protein ligase HECW1 \|\| id:prot-a-1324 | Mushroom liking \|\| id:ebi-fl187-GCST90094803 | Weighted median | 7 | 0.220222689970912 | 0.279304875885059 | 0.430423567957281 | -0.327214867 | 0.767660246705627 | 1.24635425027755 | 0.720928822740518 | 2.15471884073086 | 0.999489402457283 |
| ebi-fl187-GCST90094803 | prot-a-1339 | Histidine triad nucleotide-binding protein 2, mitochondrial \|\| id:prot-a-1339 | Mushroom liking \|\| id:ebi-fl187-GCST90094803 | Weighted median | 7 | -0.21905036 | 0.274633979238727 | 0.42509779352294 | -0.757332959 | 0.319232239354348 | 0.803281263927855 | 0.468915377182962 | 1.37607086560901 | 0.999489402457283 |
| ebi-fl187-GCST90094803 | prot-a-1466 | Interleukin-11 \|\| id:prot-a-1466 | Mushroom liking \|\| id:ebi-fl187-GCST90094803 | Weighted median | 7 | -0.3411895 | 0.275067750518013 | 0.214833580470879 | -0.880322291 | 0.197943291512036 | 0.710924175662788 | 0.414649252621561 | 1.21889327026737 | 0.999489402457283 |
| ebi-fl187-GCST90094803 | prot-a-147 | Rho GTPase-activating protein 1 \|\| id:prot-a-147 | Mushroom liking \|\| id:ebi-fl187-GCST90094803 | Weighted median | 7 | -0.087608052 | 0.271442040722992 | 0.746884209438956 | -0.619634452 | 0.444418347554451 | 0.916119877477114 | 0.538141118010954 | 1.55958279681502 | 0.999489402457283 |
| ebi-fl187-GCST90094803 | prot-a-1473 | Interleukin-12 receptor subunit beta-1 \|\| id:prot-a-1473 | Mushroom liking \|\| id:ebi-fl187-GCST90094803 | Weighted median | 7 | -0.355745535 | 0.293239725011478 | 0.225070178318472 | -0.930495396 | 0.219004325629431 | 0.700650888405737 | 0.394358298284956 | 1.24483666137798 | 0.999489402457283 |
| ebi-fl187-GCST90094803 | prot-a-1501 | X-linked interleukin-1 receptor accessory protein-like 2 \|\| id:prot-a-1501 | Mushroom liking \|\| id:ebi-fl187-GCST90094803 | Weighted median | 7 | -0.281928629 | 0.294348818994976 | 0.338161339435213 | -0.858852315 | 0.294995055879073 | 0.754327519456511 | 0.423648018066747 | 1.34311971812356 | 0.999489402457283 |
| ebi-fl187-GCST90094803 | prot-a-1503 | Interleukin-1 receptor-like 2 \|\| id:prot-a-1503 | Mushroom liking \|\| id:ebi-fl187-GCST90094803 | Weighted median | 7 | -0.094180969 | 0.285591801606024 | 0.741570156286756 | -0.6539409 | 0.465578961877339 | 0.910118043938289 | 0.519992494888123 | 1.59293617128123 | 0.999489402457283 |
| ebi-fl187-GCST90094803 | prot-a-1536 | Interleukin-5 receptor subunit alpha \|\| id:prot-a-1536 | Mushroom liking \|\| id:ebi-fl187-GCST90094803 | Weighted median | 7 | 0.198523649043141 | 0.272128852416393 | 0.465683169530005 | -0.334848902 | 0.731896199779271 | 1.21960086946678 | 0.715446180892594 | 2.07901910797597 | 0.999489402457283 |
| ebi-fl187-GCST90094803 | prot-a-1559 | Insulin-induced gene 1 protein \|\| id:prot-a-1559 | Mushroom liking \|\| id:ebi-fl187-GCST90094803 | Weighted median | 7 | -0.028265338 | 0.26730730576141 | 0.915787861708605 | -0.552187657 | 0.495656981623773 | 0.972130389766062 | 0.575689021701081 | 1.64157637037139 | 0.999489402457283 |
| ebi-fl187-GCST90094803 | prot-a-1567 | Interferon regulatory factor 1 \|\| id:prot-a-1567 | Mushroom liking \|\| id:ebi-fl187-GCST90094803 | Weighted median | 7 | -0.22921473 | 0.322710604576097 | 0.477530823584929 | -0.861727515 | 0.403298054933909 | 0.795157770915651 | 0.422431694530828 | 1.49675293978541 | 0.999489402457283 |
| ebi-fl187-GCST90094803 | prot-a-1613 | Potassium voltage-gated channel subfamily E regulatory beta subunit 5 \|\| id:prot-a-1613 | Mushroom liking \|\| id:ebi-fl187-GCST90094803 | Weighted median | 7 | 0.113009243613042 | 0.267929311183493 | 0.673180105121881 | -0.412132206 | 0.638150693532688 | 1.11964228244076 | 0.662236718393776 | 1.8929769458717 | 0.999489402457283 |
| ebi-fl187-GCST90094803 | prot-a-1649 | Kin of IRRE-like protein 2 \|\| id:prot-a-1649 | Mushroom liking \|\| id:ebi-fl187-GCST90094803 | Weighted median | 7 | -0.12222612 | 0.297508175572719 | 0.681195133094485 | -0.705342144 | 0.460889904379953 | 0.884948241608573 | 0.49393954695624 | 1.5854842867957 | 0.999489402457283 |
| ebi-fl187-GCST90094803 | prot-a-165 | Armadillo repeat-containing protein 5 \|\| id:prot-a-165 | Mushroom liking \|\| id:ebi-fl187-GCST90094803 | Weighted median | 7 | 0.431053536353783 | 0.299636521931702 | 0.150267258441219 | -0.156234047 | 1.01834111933992 | 1.53887793366507 | 0.855358973054743 | 2.76859817845123 | 0.999489402457283 |
| ebi-fl187-GCST90094803 | prot-a-1656 | Kallikrein-11 \|\| id:prot-a-1656 | Mushroom liking \|\| id:ebi-fl187-GCST90094803 | Weighted median | 7 | -0.411760999 | 0.320202921539563 | 0.198464814890083 | -1.039358726 | 0.215836726723183 | 0.662482590806896 | 0.353681416049929 | 1.24089975669024 | 0.999489402457283 |
| ebi-fl187-GCST90094803 | prot-a-1697 | Laminin subunit gamma-2 \|\| id:prot-a-1697 | Mushroom liking \|\| id:ebi-fl187-GCST90094803 | Weighted median | 7 | -0.269677195 | 0.288439565528743 | 0.349812954821632 | -0.835018743 | 0.295664353824501 | 0.763625957128458 | 0.433866349378895 | 1.34401896629027 | 0.999489402457283 |
| ebi-fl187-GCST90094803 | prot-a-1698 | Lysosome-associated membrane glycoprotein 3 \|\| id:prot-a-1698 | Mushroom liking \|\| id:ebi-fl187-GCST90094803 | Weighted median | 7 | -0.215941086 | 0.265914867485532 | 0.416752330239475 | -0.737134226 | 0.305252054274356 | 0.805782772363565 | 0.478483177106698 | 1.35696698923467 | 0.999489402457283 |
| ebi-fl187-GCST90094803 | prot-a-1724 | Leptin receptor \|\| id:prot-a-1724 | Mushroom liking \|\| id:ebi-fl187-GCST90094803 | Weighted median | 7 | -0.091390771 | 0.276397751493481 | 0.74090927100131 | -0.633130364 | 0.45034882166968 | 0.912660999521035 | 0.530927201468623 | 1.5688593421898 | 0.999489402457283 |
| ebi-fl187-GCST90094803 | prot-a-1743 | Leukocyte immunoglobulin-like receptor subfamily B member 2 \|\| id:prot-a-1743 | Mushroom liking \|\| id:ebi-fl187-GCST90094803 | Weighted median | 7 | -0.127652957 | 0.289557085849628 | 0.659317302532403 | -0.695184846 | 0.43987893094896 | 0.88015877881289 | 0.498982204804181 | 1.55251924510096 | 0.999489402457283 |
| ebi-fl187-GCST90094803 | prot-a-1746 | Leukocyte immunoglobulin-like receptor subfamily B member 4 \|\| id:prot-a-1746 | Mushroom liking \|\| id:ebi-fl187-GCST90094803 | Weighted median | 7 | -0.096827302 | 0.320031413211709 | 0.762228542854971 | -0.724088871 | 0.530434268378886 | 0.90771275321422 | 0.48476605200986 | 1.69967026142124 | 0.999489402457283 |
| ebi-fl187-GCST90094803 | prot-a-1750 | Leucine-rich repeat and immunoglobulin-like domain-containing nogo receptor-interacting protein 3 \|\| id:prot-a-1750 | Mushroom liking \|\| id:ebi-fl187-GCST90094803 | Weighted median | 7 | -0.014792932 | 0.27233481254563 | 0.956681078498392 | -0.548569165 | 0.518983300607173 | 0.985315945900506 | 0.577775921483107 | 1.68031840225137 | 0.999489402457283 |
| ebi-fl187-GCST90094803 | prot-a-1753 | Lipase member N \|\| id:prot-a-1753 | Mushroom liking \|\| id:ebi-fl187-GCST90094803 | Weighted median | 7 | 0.334095607347031 | 0.286814617704879 | 0.244080280355619 | -0.228061043 | 0.896252258048594 | 1.39667666974242 | 0.796075663223274 | 2.45040240509859 | 0.999489402457283 |
| ebi-fl187-GCST90094803 | prot-a-1760 | Ig Kappa chain V-I region HK102- like \|\| id:prot-a-1760 | Mushroom liking \|\| id:ebi-fl187-GCST90094803 | Weighted median | 7 | -0.144949099 | 0.286496896718323 | 0.612901565479641 | -0.706483017 | 0.416584818130201 | 0.865066324352451 | 0.49337634592563 | 1.51677264568631 | 0.999489402457283 |
| ebi-fl187-GCST90094803 | prot-a-1792 | Leucine-rich repeat neuronal protein 1 \|\| id:prot-a-1792 | Mushroom liking \|\| id:ebi-fl187-GCST90094803 | Weighted median | 7 | 0.071271348081559 | 0.293780635786068 | 0.808314843867541 | -0.504538698 | 0.647081394222253 | 1.07387257960175 | 0.60378403794302 | 1.90995827108854 | 0.999489402457283 |
| ebi-fl187-GCST90094803 | prot-a-1795 | Leucine-rich repeat transmembrane neuronal protein 1 \|\| id:prot-a-1795 | Mushroom liking \|\| id:ebi-fl187-GCST90094803 | Weighted median | 7 | -0.459219463 | 0.271804202268813 | 0.0911192396045346 | -0.9919557 | 0.0735167732771569 | 0.631776577993554 | 0.370850708762886 | 1.07628658937375 | 0.999489402457283 |
| ebi-fl187-GCST90094803 | prot-a-1820 | LysM and putative peptidoglycan-binding domain-containing protein 4 \|\| id:prot-a-1820 | Mushroom liking \|\| id:ebi-fl187-GCST90094803 | Weighted median | 7 | -0.285123315 | 0.303830825056603 | 0.348024567150352 | -0.880631732 | 0.310385102060406 | 0.751921525361163 | 0.414520962725743 | 1.36395027306624 | 0.999489402457283 |
| ebi-fl187-GCST90094803 | prot-a-1844 | Mitogen-activated protein kinase kinase kinase 7:TGF-beta-activated kinase 1 and MAP3K7-binding protein 1 fusion \|\| id:prot-a-1844 | Mushroom liking \|\| id:ebi-fl187-GCST90094803 | Weighted median | 7 | 0.206962007875209 | 0.28900670077613 | 0.473920397201221 | -0.359491126 | 0.773415141396423 | 1.22993584301706 | 0.698031446008809 | 2.16715477015774 | 0.999489402457283 |
| ebi-fl187-GCST90094803 | prot-a-1856 | Megakaryocyte-associated tyrosine-protein kinase \|\| id:prot-a-1856 | Mushroom liking \|\| id:ebi-fl187-GCST90094803 | Weighted median | 7 | 0.104722832212057 | 0.257479069197236 | 0.68421095304209 | -0.399936143 | 0.60938180783864 | 1.11040279981234 | 0.670362851751653 | 1.83929401011599 | 0.999489402457283 |
| ebi-fl187-GCST90094803 | prot-a-1910 | Stromelysin-2 \|\| id:prot-a-1910 | Mushroom liking \|\| id:ebi-fl187-GCST90094803 | Weighted median | 7 | -0.296088739 | 0.303698190634418 | 0.329588022483076 | -0.891337192 | 0.299159714874242 | 0.743721428167674 | 0.410106993975348 | 1.34872501771821 | 0.999489402457283 |
| ebi-fl187-GCST90094803 | prot-a-1940 | 39S ribosomal protein L14, mitochondrial \|\| id:prot-a-1940 | Mushroom liking \|\| id:ebi-fl187-GCST90094803 | Weighted median | 7 | -0.122702505 | 0.284769309112809 | 0.666552805570051 | -0.68085035 | 0.435445341234045 | 0.884526766042986 | 0.50618637347326 | 1.54565124793466 | 0.999489402457283 |
| ebi-fl187-GCST90094803 | prot-a-1942 | 39S ribosomal protein L33, mitochondrial \|\| id:prot-a-1942 | Mushroom liking \|\| id:ebi-fl187-GCST90094803 | Weighted median | 7 | -0.144068157 | 0.291678298438293 | 0.621356839751357 | -0.715757622 | 0.42762130810669 | 0.865828733904246 | 0.488821629562453 | 1.53360520712894 | 0.999489402457283 |
| ebi-fl187-GCST90094803 | prot-a-1948 | Mesothelin \|\| id:prot-a-1948 | Mushroom liking \|\| id:ebi-fl187-GCST90094803 | Weighted median | 7 | -0.194833522 | 0.285805259431909 | 0.495428497209477 | -0.755011831 | 0.365344786192241 | 0.822971653085055 | 0.470005054170856 | 1.44101076312116 | 0.999489402457283 |
| ebi-fl187-GCST90094803 | prot-a-1980 | Unconventional myosin-VI \|\| id:prot-a-1980 | Mushroom liking \|\| id:ebi-fl187-GCST90094803 | Weighted median | 7 | 0.084007550483096 | 0.300505849355283 | 0.779819860670575 | -0.504983914 | 0.672999015219452 | 1.08763710596341 | 0.603515283342963 | 1.96010690519035 | 0.999489402457283 |
| ebi-fl187-GCST90094803 | prot-a-2039 | Neurofascin \|\| id:prot-a-2039 | Mushroom liking \|\| id:ebi-fl187-GCST90094803 | Weighted median | 7 | -0.510977759 | 0.297517551419799 | 0.0858931534424272 | -1.09411216 | 0.072156641930873 | 0.599908725891608 | 0.334836756606647 | 1.07482369333686 | 0.999489402457283 |
| ebi-fl187-GCST90094803 | prot-a-2041 | NFU1 iron-sulfur cluster scaffold homolog, mitochondrial \|\| id:prot-a-2041 | Mushroom liking \|\| id:ebi-fl187-GCST90094803 | Weighted median | 7 | -0.058543534 | 0.293255691032417 | 0.841767561268925 | -0.633324689 | 0.516237620164861 | 0.943137180727929 | 0.530824039330806 | 1.6757111128441 | 0.999489402457283 |
| ebi-fl187-GCST90094803 | prot-a-2088 | Glucocorticoid receptor \|\| id:prot-a-2088 | Mushroom liking \|\| id:ebi-fl187-GCST90094803 | Weighted median | 7 | 0.134075469256079 | 0.288341797029637 | 0.641940065639989 | -0.431074453 | 0.699225391434167 | 1.14347911390639 | 0.649810528681065 | 2.01219344136221 | 0.999489402457283 |
| ebi-fl187-GCST90094803 | prot-a-2111 | Neuron-specific protein family member 2 \|\| id:prot-a-2111 | Mushroom liking \|\| id:ebi-fl187-GCST90094803 | Weighted median | 7 | 0.0605112908979089 | 0.267783759169253 | 0.8212241874887 | -0.464344877 | 0.585367458869645 | 1.06237959272213 | 0.628546745720637 | 1.7956506921986 | 0.999489402457283 |
| ebi-fl187-GCST90094803 | prot-a-2125 | Nuclear migration protein nudC \|\| id:prot-a-2125 | Mushroom liking \|\| id:ebi-fl187-GCST90094803 | Weighted median | 7 | 0.0841069192658167 | 0.274006927627464 | 0.758880061312248 | -0.452946659 | 0.621160497415646 | 1.08774518850859 | 0.635752044445528 | 1.86108657527876 | 0.999489402457283 |
| ebi-fl187-GCST90094803 | prot-a-2142 | Ornithine decarboxylase \|\| id:prot-a-2142 | Mushroom liking \|\| id:ebi-fl187-GCST90094803 | Weighted median | 7 | 0.0190636825539885 | 0.282328335559014 | 0.946165278151883 | -0.534299855 | 0.572427220249657 | 1.01924655477444 | 0.586079487068842 | 1.77256423802723 | 0.999489402457283 |
| ebi-fl187-GCST90094803 | prot-a-2186 | Prostate and testis expressed protein 1 \|\| id:prot-a-2186 | Mushroom liking \|\| id:ebi-fl187-GCST90094803 | Weighted median | 7 | 0.351795034464291 | 0.307254449638929 | 0.252224406414635 | -0.250423687 | 0.954013755756591 | 1.42161711134205 | 0.778470885329573 | 2.59610892243569 | 0.999489402457283 |
| ebi-fl187-GCST90094803 | prot-a-2227 | Platelet-derived growth factor C \|\| id:prot-a-2227 | Mushroom liking \|\| id:ebi-fl187-GCST90094803 | Weighted median | 7 | -0.125412729 | 0.285387752747601 | 0.660337837759156 | -0.684772724 | 0.433947266849433 | 0.882132746084145 | 0.504204810245813 | 1.54333748092285 | 0.999489402457283 |
| ebi-fl187-GCST90094803 | prot-a-2229 | Platelet-derived growth factor receptor alpha \|\| id:prot-a-2229 | Mushroom liking \|\| id:ebi-fl187-GCST90094803 | Weighted median | 7 | 0.308629163293043 | 0.296326091454844 | 0.297634882460075 | -0.272169976 | 0.889428302544538 | 1.36155736142301 | 0.761724775186665 | 2.43373789173512 | 0.999489402457283 |
| ebi-fl187-GCST90094803 | prot-a-2243 | Platelet endothelial aggregation receptor 1 \|\| id:prot-a-2243 | Mushroom liking \|\| id:ebi-fl187-GCST90094803 | Weighted median | 7 | -0.039465441 | 0.288020068336623 | 0.891012431556552 | -0.603984775 | 0.525053893196427 | 0.961303175339693 | 0.546629096732446 | 1.69054995506485 | 0.999489402457283 |
| ebi-fl187-GCST90094803 | prot-a-2246 | E3 ubiquitin-protein ligase pellino homolog 2 \|\| id:prot-a-2246 | Mushroom liking \|\| id:ebi-fl187-GCST90094803 | Weighted median | 7 | -0.062945424 | 0.28114916960204 | 0.822845839941749 | -0.613997797 | 0.488106948128535 | 0.938994718592837 | 0.541182998996189 | 1.62922908365688 | 0.999489402457283 |
| ebi-fl187-GCST90094803 | prot-a-2251 | Platelet factor 4 variant \|\| id:prot-a-2251 | Mushroom liking \|\| id:ebi-fl187-GCST90094803 | Weighted median | 7 | 0.253468505881849 | 0.284011656178064 | 0.372147436171246 | -0.30319434 | 0.810131351990854 | 1.28848679909079 | 0.738455570801165 | 2.24820327325859 | 0.999489402457283 |
| ebi-fl187-GCST90094803 | prot-a-2346 | Protein phosphatase 1L \|\| id:prot-a-2346 | Mushroom liking \|\| id:ebi-fl187-GCST90094803 | Weighted median | 7 | -0.004354347 | 0.29847253572278 | 0.988360259300933 | -0.589360517 | 0.580651823125351 | 0.995655119532112 | 0.554681881027641 | 1.78720299140455 | 0.999911036302558 |
| ebi-fl187-GCST90094803 | prot-a-2358 | PR domain zinc finger protein 4 \|\| id:prot-a-2358 | Mushroom liking \|\| id:ebi-fl187-GCST90094803 | Weighted median | 7 | 0.276411840649878 | 0.280046280027594 | 0.323631851425373 | -0.272478868 | 0.825302549503962 | 1.31839071912529 | 0.761489520646233 | 2.28257125167084 | 0.999489402457283 |
| ebi-fl187-GCST90094803 | prot-a-2390 | Trypsin-1 \|\| id:prot-a-2390 | Mushroom liking \|\| id:ebi-fl187-GCST90094803 | Weighted median | 7 | -0.455170071 | 0.312780877166392 | 0.145603868397581 | -1.06822059 | 0.157880448344679 | 0.634340075985298 | 0.343619413510138 | 1.17102618821961 | 0.999489402457283 |
| ebi-fl187-GCST90094803 | prot-a-242 | Brain-derived neurotrophic factor \|\| id:prot-a-242 | Mushroom liking \|\| id:ebi-fl187-GCST90094803 | Weighted median | 7 | 0.157830935464188 | 0.265735669770881 | 0.552552516574006 | -0.363010977 | 0.678672848215115 | 1.17096820877527 | 0.695578797889272 | 1.97125983443309 | 0.999489402457283 |
| ebi-fl187-GCST90094803 | prot-a-2464 | Dihydropteridine reductase \|\| id:prot-a-2464 | Mushroom liking \|\| id:ebi-fl187-GCST90094803 | Weighted median | 7 | -0.093173528 | 0.307836661994457 | 0.762139863997041 | -0.696533385 | 0.510186329758482 | 0.911035396655705 | 0.498309761012756 | 1.66560151716228 | 0.999489402457283 |
| ebi-fl187-GCST90094803 | prot-a-2474 | Ras-related protein Rab-27A \|\| id:prot-a-2474 | Mushroom liking \|\| id:ebi-fl187-GCST90094803 | Weighted median | 7 | 0.185589095458484 | 0.284443704974147 | 0.514102302097976 | -0.371920566 | 0.743098757207813 | 1.20392745946587 | 0.6894090026635 | 2.10244038307608 | 0.999489402457283 |
| ebi-fl187-GCST90094803 | prot-a-2478 | Ras-related protein Rab-7b \|\| id:prot-a-2478 | Mushroom liking \|\| id:ebi-fl187-GCST90094803 | Weighted median | 7 | -0.502094256 | 0.282207075330377 | 0.075211956847588 | -1.055220124 | 0.0510316116149745 | 0.605261758385499 | 0.34811579034283 | 1.05235615943513 | 0.999489402457283 |
| ebi-fl187-GCST90094803 | prot-a-2528 | E3 ubiquitin-protein ligase rififylin \|\| id:prot-a-2528 | Mushroom liking \|\| id:ebi-fl187-GCST90094803 | Weighted median | 7 | 0.0914964531496507 | 0.290228051705845 | 0.752566456643235 | -0.477350528 | 0.660343434493107 | 1.09581289000132 | 0.620425014713369 | 1.93545692294145 | 0.999489402457283 |
| ebi-fl187-GCST90094803 | prot-a-2543 | Protein RIC-3 \|\| id:prot-a-2543 | Mushroom liking \|\| id:ebi-fl187-GCST90094803 | Weighted median | 7 | 0.515097749030254 | 0.275532835764325 | 0.0615587761620646 | -0.024946609 | 1.05514210712833 | 1.67380210633404 | 0.975361986123828 | 2.87238331104344 | 0.999489402457283 |
| ebi-fl187-GCST90094803 | prot-a-255 | Bone morphogenetic protein 6 \|\| id:prot-a-255 | Mushroom liking \|\| id:ebi-fl187-GCST90094803 | Weighted median | 7 | -0.040246173 | 0.288306816093334 | 0.888980038642411 | -0.605327532 | 0.524835186802112 | 0.960552948092755 | 0.545895598923175 | 1.69018026140843 | 0.999489402457283 |
| ebi-fl187-GCST90094803 | prot-a-2568 | RING finger protein 165 \|\| id:prot-a-2568 | Mushroom liking \|\| id:ebi-fl187-GCST90094803 | Weighted median | 7 | 0.104625072934021 | 0.284178203297355 | 0.712748619614191 | -0.452364206 | 0.661614351396838 | 1.11029425294212 | 0.63612244821789 | 1.93791829162748 | 0.999489402457283 |
| ebi-fl187-GCST90094803 | prot-a-2573 | E3 ubiquitin-protein ligase RNF8 \|\| id:prot-a-2573 | Mushroom liking \|\| id:ebi-fl187-GCST90094803 | Weighted median | 7 | 0.538490569163218 | 0.304847172232696 | 0.0773240773923659 | -0.059009888 | 1.1359910267393 | 1.71341862238685 | 0.942697447329443 | 3.11425832737623 | 0.999489402457283 |
| ebi-fl187-GCST90094803 | prot-a-260 | Cytoplasmic tyrosine-protein kinase BMX \|\| id:prot-a-260 | Mushroom liking \|\| id:ebi-fl187-GCST90094803 | Weighted median | 7 | -0.110228326 | 0.299341777320391 | 0.712697499470747 | -0.69693821 | 0.476481557282989 | 0.895629616183807 | 0.498108073813141 | 1.61039832830831 | 0.999489402457283 |
| ebi-fl187-GCST90094803 | prot-a-2623 | Serum amyloid A-1 protein \|\| id:prot-a-2623 | Mushroom liking \|\| id:ebi-fl187-GCST90094803 | Weighted median | 7 | -0.088765739 | 0.295490037670095 | 0.76387069236642 | -0.667926212 | 0.490394735221636 | 0.915059911673141 | 0.512770853753511 | 1.63296067984738 | 0.999489402457283 |
| ebi-fl187-GCST90094803 | prot-a-2640 | Lysosome membrane protein 2 \|\| id:prot-a-2640 | Mushroom liking \|\| id:ebi-fl187-GCST90094803 | Weighted median | 7 | -0.323320656 | 0.304682175974616 | 0.288611052811478 | -0.920497721 | 0.27385640893678 | 0.723741745042488 | 0.3983207391888 | 1.31502596270507 | 0.999489402457283 |
| ebi-fl187-GCST90094803 | prot-a-2641 | Scavenger receptor class F member 2 \|\| id:prot-a-2641 | Mushroom liking \|\| id:ebi-fl187-GCST90094803 | Weighted median | 7 | 0.039055171118407 | 0.280625232205962 | 0.889314244328895 | -0.510970284 | 0.589080626242093 | 1.03982785053311 | 0.5999132101341 | 1.80233063796448 | 0.999489402457283 |
| ebi-fl187-GCST90094803 | prot-a-2669 | Semaphorin-3A \|\| id:prot-a-2669 | Mushroom liking \|\| id:ebi-fl187-GCST90094803 | Weighted median | 7 | 0.223795238050292 | 0.276717042021554 | 0.418658274867555 | -0.318570164 | 0.766160640412537 | 1.25081487391252 | 0.727188053516118 | 2.15149003237321 | 0.999489402457283 |
| ebi-fl187-GCST90094803 | prot-a-2683 | Sentrin-specific protease 7 \|\| id:prot-a-2683 | Mushroom liking \|\| id:ebi-fl187-GCST90094803 | Weighted median | 7 | -0.190794374 | 0.273875974639399 | 0.486024882613066 | -0.727591284 | 0.346002536424321 | 0.82630248006558 | 0.483071171026956 | 1.41340620081099 | 0.999489402457283 |
| ebi-fl187-GCST90094803 | prot-a-270 | Bromodomain testis-specific protein \|\| id:prot-a-270 | Mushroom liking \|\| id:ebi-fl187-GCST90094803 | Weighted median | 7 | -0.024878275 | 0.264366477922766 | 0.925025545079532 | -0.543036572 | 0.493280021596296 | 0.975428638724166 | 0.580981379404846 | 1.637679043 | 0.999489402457283 |
| ebi-fl187-GCST90094803 | prot-a-2708 | 14-3-3 protein sigma \|\| id:prot-a-2708 | Mushroom liking \|\| id:ebi-fl187-GCST90094803 | Weighted median | 7 | 0.514070070224831 | 0.264519632220517 | 0.0519666528657939 | -0.004388409 | 1.03252854937704 | 1.67208285895324 | 0.995621206069087 | 2.80815742991642 | 0.999489402457283 |
| ebi-fl187-GCST90094803 | prot-a-2713 | Beta-sarcoglycan \|\| id:prot-a-2713 | Mushroom liking \|\| id:ebi-fl187-GCST90094803 | Weighted median | 7 | -0.108604934 | 0.256581203692802 | 0.672093140488078 | -0.611504093 | 0.394294225613517 | 0.897084755523101 | 0.54253423321266 | 1.48333692019116 | 0.999489402457283 |
| ebi-fl187-GCST90094803 | prot-a-2720 | Protein shisa-3 homolog \|\| id:prot-a-2720 | Mushroom liking \|\| id:ebi-fl187-GCST90094803 | Weighted median | 7 | -0.032910157 | 0.280740164033596 | 0.906680651820304 | -0.583160879 | 0.517340564458754 | 0.96762549001526 | 0.558131389897202 | 1.6775603484687 | 0.999489402457283 |
| ebi-fl187-GCST90094803 | prot-a-2730 | Sialic acid-binding Ig-like lectin 8 \|\| id:prot-a-2730 | Mushroom liking \|\| id:ebi-fl187-GCST90094803 | Weighted median | 7 | 0.18087082939575 | 0.289096531887461 | 0.531550017725369 | -0.385758373 | 0.747500031895174 | 1.19826038927772 | 0.679934796367964 | 2.11171419404011 | 0.999489402457283 |
| ebi-fl187-GCST90094803 | prot-a-2761 | Sodium- and chloride-dependent neutral and basic amino acid transporter B(0+) \|\| id:prot-a-2761 | Mushroom liking \|\| id:ebi-fl187-GCST90094803 | Weighted median | 7 | 0.00852722371904024 | 0.283284730890893 | 0.97598630604295 | -0.546710849 | 0.563765296265191 | 1.00856368405267 | 0.5788506098207 | 1.75727672655468 | 0.999489402457283 |
| ebi-fl187-GCST90094803 | prot-a-2770 | Secreted Ly-6/uPAR-related protein 1 \|\| id:prot-a-2770 | Mushroom liking \|\| id:ebi-fl187-GCST90094803 | Weighted median | 7 | 0.64429122713271 | 0.285393097902379 | 0.0239732830323021 | 0.0849207552440471 | 1.20366169902137 | 1.90463659601193 | 1.08863079499848 | 3.33229647694558 | 0.999489402457283 |
| ebi-fl187-GCST90094803 | prot-a-2784 | N-lysine methyltransferase SMYD2 \|\| id:prot-a-2784 | Mushroom liking \|\| id:ebi-fl187-GCST90094803 | Weighted median | 7 | -0.009064393 | 0.293064020974094 | 0.97532557447961 | -0.583469874 | 0.565341087984366 | 0.990976564640651 | 0.557958956350198 | 1.76004801157923 | 0.999489402457283 |
| ebi-fl187-GCST90094803 | prot-a-2837 | Serine palmitoyltransferase 2 \|\| id:prot-a-2837 | Mushroom liking \|\| id:ebi-fl187-GCST90094803 | Weighted median | 7 | 0.357191936175315 | 0.304394477285917 | 0.2406150846557 | -0.239421239 | 0.953805111655712 | 1.42931017985729 | 0.787083262125471 | 2.59556731612723 | 0.999489402457283 |
| ebi-fl187-GCST90094803 | prot-a-2858 | Alpha-2,8-sialyltransferase 8B \|\| id:prot-a-2858 | Mushroom liking \|\| id:ebi-fl187-GCST90094803 | Weighted median | 7 | 0.0361915094647406 | 0.273143301565958 | 0.894588759300866 | -0.499169362 | 0.571552380534019 | 1.03685439490855 | 0.607034676665553 | 1.77101420654669 | 0.999489402457283 |
| ebi-fl187-GCST90094803 | prot-a-2911 | Synaptotagmin-11 \|\| id:prot-a-2911 | Mushroom liking \|\| id:ebi-fl187-GCST90094803 | Weighted median | 7 | 0.191424340912354 | 0.278721345074876 | 0.492212113887738 | -0.354869495 | 0.737718177259111 | 1.2109732085458 | 0.701264955515444 | 2.09115841349555 | 0.999489402457283 |
| ebi-fl187-GCST90094803 | prot-a-2962 | Transforming growth factor beta-1 \|\| id:prot-a-2962 | Mushroom liking \|\| id:ebi-fl187-GCST90094803 | Weighted median | 7 | 0.335180813845649 | 0.279574412243536 | 0.230568151801851 | -0.212785034 | 0.88314666184298 | 1.39819317505277 | 0.808329881860012 | 2.41849794079827 | 0.999489402457283 |
| ebi-fl187-GCST90094803 | prot-a-2978 | Tyrosine-protein kinase receptor Tie-1, soluble \|\| id:prot-a-2978 | Mushroom liking \|\| id:ebi-fl187-GCST90094803 | Weighted median | 7 | 0.235552948878916 | 0.274894773930841 | 0.391508965148809 | -0.303240808 | 0.774346705783365 | 1.26560839202863 | 0.738421257193835 | 2.16917455499635 | 0.999489402457283 |
| ebi-fl187-GCST90094803 | prot-a-299 | Uncharacterized protein C1orf226 \|\| id:prot-a-299 | Mushroom liking \|\| id:ebi-fl187-GCST90094803 | Weighted median | 7 | 0.178985458749902 | 0.291183428012203 | 0.538764189388617 | -0.39173406 | 0.74970497765382 | 1.19600335265757 | 0.675883834504992 | 2.11637554642177 | 0.999489402457283 |
| ebi-fl187-GCST90094803 | prot-a-3015 | Transmembrane protein 70, mitochondrial \|\| id:prot-a-3015 | Mushroom liking \|\| id:ebi-fl187-GCST90094803 | Weighted median | 7 | -0.018511163 | 0.252821830750432 | 0.941632470790308 | -0.514041952 | 0.477019624784138 | 0.981659115792132 | 0.598073303310245 | 1.61126506447305 | 0.999489402457283 |
| ebi-fl187-GCST90094803 | prot-a-3016 | Transmembrane protein 87B \|\| id:prot-a-3016 | Mushroom liking \|\| id:ebi-fl187-GCST90094803 | Weighted median | 7 | 0.178469060618244 | 0.267422939032163 | 0.504538178624249 | -0.3456799 | 0.702618021121283 | 1.19538589820068 | 0.707738998186311 | 2.01903166178344 | 0.999489402457283 |
| ebi-fl187-GCST90094803 | prot-a-3123 | Thioredoxin domain-containing protein 12 \|\| id:prot-a-3123 | Mushroom liking \|\| id:ebi-fl187-GCST90094803 | Weighted median | 7 | 0.0547693666062089 | 0.259945759901035 | 0.833124992085743 | -0.454724323 | 0.564263056012237 | 1.05629696922532 | 0.634622894898271 | 1.75815164590533 | 0.999489402457283 |
| ebi-fl187-GCST90094803 | prot-a-3133 | PolyUbiquitin K48-linked \|\| id:prot-a-3133 | Mushroom liking \|\| id:ebi-fl187-GCST90094803 | Weighted median | 7 | -0.052390099 | 0.297766470006268 | 0.860338265809074 | -0.636012381 | 0.531232181846917 | 0.948958606478271 | 0.529399263399432 | 1.70102699241902 | 0.999489402457283 |
| ebi-fl187-GCST90094803 | prot-a-3135 | Ubiquitin-conjugating enzyme E2 B \|\| id:prot-a-3135 | Mushroom liking \|\| id:ebi-fl187-GCST90094803 | Weighted median | 7 | -0.244505721 | 0.286267009371109 | 0.393039774249874 | -0.80558906 | 0.316577616979388 | 0.783091507982202 | 0.446824642607316 | 1.3724227614115 | 0.999489402457283 |
| ebi-fl187-GCST90094803 | prot-a-3162 | NKG2D ligand 3 \|\| id:prot-a-3162 | Mushroom liking \|\| id:ebi-fl187-GCST90094803 | Weighted median | 7 | 0.22579423944765 | 0.276349393439573 | 0.413893718502227 | -0.315850572 | 0.767439050589213 | 1.25331775539164 | 0.729168400426223 | 2.15424227800018 | 0.999489402457283 |
| ebi-fl187-GCST90094803 | prot-a-3188 | Vesicle-associated membrane protein 8 \|\| id:prot-a-3188 | Mushroom liking \|\| id:ebi-fl187-GCST90094803 | Weighted median | 7 | 0.702018387618073 | 0.302273799755852 | 0.020208254827078 | 0.109561740096604 | 1.29447503513954 | 2.01782134566431 | 1.11578895772663 | 3.64907983254668 | 0.999489402457283 |
| ebi-fl187-GCST90094803 | prot-a-3201 | Vitrin \|\| id:prot-a-3201 | Mushroom liking \|\| id:ebi-fl187-GCST90094803 | Weighted median | 7 | -0.234597092 | 0.284020871459184 | 0.408812375187656 | -0.791278001 | 0.322083815573539 | 0.790889440741103 | 0.453265151857177 | 1.38000043663818 | 0.999489402457283 |
| ebi-fl187-GCST90094803 | prot-a-3232 | WNT1-inducible-signaling pathway protein 3 \|\| id:prot-a-3232 | Mushroom liking \|\| id:ebi-fl187-GCST90094803 | Weighted median | 7 | 0.0891659254739094 | 0.286582474506909 | 0.755697763055021 | -0.472535725 | 0.650867575507451 | 1.09326204130003 | 0.623419442334623 | 1.91720342643079 | 0.999489402457283 |
| ebi-fl187-GCST90094803 | prot-a-327 | Carbonic anhydrase-related protein 10 \|\| id:prot-a-327 | Mushroom liking \|\| id:ebi-fl187-GCST90094803 | Weighted median | 7 | -0.10577438 | 0.258863086458005 | 0.682824941819728 | -0.61314603 | 0.401597268994901 | 0.899627598742737 | 0.541644157074265 | 1.49420944701274 | 0.999489402457283 |
| ebi-fl187-GCST90094803 | prot-a-332 | Carbonic anhydrase 5A, mitochondrial \|\| id:prot-a-332 | Mushroom liking \|\| id:ebi-fl187-GCST90094803 | Weighted median | 7 | -0.269902042 | 0.3058705632075 | 0.377557253399624 | -0.869408346 | 0.329604261716939 | 0.763454276998481 | 0.419199496926281 | 1.39041777802938 | 0.999489402457283 |
| ebi-fl187-GCST90094803 | prot-a-338 | Cell adhesion molecule 3 \|\| id:prot-a-338 | Mushroom liking \|\| id:ebi-fl187-GCST90094803 | Weighted median | 7 | 0.157973427950846 | 0.277732594865309 | 0.569494005648406 | -0.386382458 | 0.702329313886853 | 1.17113507483544 | 0.679510591724775 | 2.01844883687307 | 0.999489402457283 |
| ebi-fl187-GCST90094803 | prot-a-34 | A disintegrin and metalloproteinase with thrombospondin motifs 3 \|\| id:prot-a-34 | Mushroom liking \|\| id:ebi-fl187-GCST90094803 | Weighted median | 7 | -0.094890473 | 0.314852032602247 | 0.763123853103962 | -0.712000457 | 0.522219510652748 | 0.909472540587081 | 0.49066166739416 | 1.68576507407794 | 0.999489402457283 |
| ebi-fl187-GCST90094803 | prot-a-358 | Cysteine--tRNA ligase, cytoplasmic \|\| id:prot-a-358 | Mushroom liking \|\| id:ebi-fl187-GCST90094803 | Weighted median | 7 | 0.0089111718572549 | 0.288033851468453 | 0.97531903862378 | -0.555635177 | 0.573457520735423 | 1.00895099455025 | 0.573707739467949 | 1.7743914529513 | 0.999489402457283 |
| ebi-fl187-GCST90094803 | prot-a-382 | Coiled-coil domain-containing protein 167 \|\| id:prot-a-382 | Mushroom liking \|\| id:ebi-fl187-GCST90094803 | Weighted median | 7 | 0.185796882506333 | 0.323047261623666 | 0.565197705012342 | -0.44737575 | 0.818969515288718 | 1.20417764599036 | 0.639303644617114 | 2.26816132726933 | 0.999489402457283 |
| ebi-fl187-GCST90094803 | prot-a-40 | Pituitary adenylate cyclase-activating polypeptide 27 \|\| id:prot-a-40 | Mushroom liking \|\| id:ebi-fl187-GCST90094803 | Weighted median | 7 | 0.0322005268939149 | 0.283736453710509 | 0.909644111438908 | -0.523922922 | 0.588323976166513 | 1.03272457359406 | 0.592192858665336 | 1.80096742015551 | 0.999489402457283 |
| ebi-fl187-GCST90094803 | prot-a-403 | C-C motif chemokine 25 \|\| id:prot-a-403 | Mushroom liking \|\| id:ebi-fl187-GCST90094803 | Weighted median | 7 | -0.096858027 | 0.314991859064713 | 0.758467582584682 | -0.71424207 | 0.520526017067403 | 0.907684864001884 | 0.489563025490995 | 1.68291265769472 | 0.999489402457283 |
| ebi-fl187-GCST90094803 | prot-a-415 | Cyclin-C \|\| id:prot-a-415 | Mushroom liking \|\| id:ebi-fl187-GCST90094803 | Weighted median | 7 | -0.214481371 | 0.301849193471918 | 0.47735815248356 | -0.806105791 | 0.377143047829787 | 0.806959844144071 | 0.44659381418443 | 1.45811287433575 | 0.999489402457283 |
| ebi-fl187-GCST90094803 | prot-a-44 | Adhesion G protein-coupled receptor F5 \|\| id:prot-a-44 | Mushroom liking \|\| id:ebi-fl187-GCST90094803 | Weighted median | 7 | -0.204396657 | 0.266944848088235 | 0.443861545947345 | -0.727608559 | 0.318815245694713 | 0.81513897683226 | 0.483062826214091 | 1.37549717240437 | 0.999489402457283 |
| ebi-fl187-GCST90094803 | prot-a-455 | CD59 glycoprotein \|\| id:prot-a-455 | Mushroom liking \|\| id:ebi-fl187-GCST90094803 | Weighted median | 7 | 0.160801736793494 | 0.293668089452497 | 0.583992102173039 | -0.414787719 | 0.736391192120388 | 1.17445209509123 | 0.660480473587875 | 2.08838531769351 | 0.999489402457283 |
| ebi-fl187-GCST90094803 | prot-a-478 | Cadherin-12 \|\| id:prot-a-478 | Mushroom liking \|\| id:ebi-fl187-GCST90094803 | Weighted median | 7 | 0.336589413946689 | 0.261676743197552 | 0.198345556232007 | -0.176297003 | 0.849475830613891 | 1.40016405786741 | 0.838368948498394 | 2.33842080202889 | 0.999489402457283 |
| ebi-fl187-GCST90094803 | prot-a-516 | Centrin-2 \|\| id:prot-a-516 | Mushroom liking \|\| id:ebi-fl187-GCST90094803 | Weighted median | 7 | 0.113515847165576 | 0.286102621696322 | 0.691539885075471 | -0.447245291 | 0.674276985690367 | 1.12020964089938 | 0.639387052918681 | 1.96261346524875 | 0.999489402457283 |
| ebi-fl187-GCST90094803 | prot-a-519 | Cryptic protein \|\| id:prot-a-519 | Mushroom liking \|\| id:ebi-fl187-GCST90094803 | Weighted median | 7 | -0.146357303 | 0.271832007929698 | 0.590293436229017 | -0.679148038 | 0.386433432637659 | 0.863848992282243 | 0.507048794478587 | 1.47172242512562 | 0.999489402457283 |
| ebi-fl187-GCST90094803 | prot-a-583 | Chloride intracellular channel protein 4 \|\| id:prot-a-583 | Mushroom liking \|\| id:ebi-fl187-GCST90094803 | Weighted median | 7 | 0.0405875467434903 | 0.280176193793007 | 0.884817961237769 | -0.508557793 | 0.589732886577784 | 1.0414224788583 | 0.601362242488923 | 1.80350661022976 | 0.999489402457283 |
| ebi-fl187-GCST90094803 | prot-a-602 | CCR4-NOT transcription complex subunit 1 \|\| id:prot-a-602 | Mushroom liking \|\| id:ebi-fl187-GCST90094803 | Weighted median | 7 | 0.114678757854687 | 0.285131196988169 | 0.68753944782559 | -0.444178388 | 0.673535903951497 | 1.1215131024226 | 0.641351001144382 | 1.96115954705185 | 0.999489402457283 |
| ebi-fl187-GCST90094803 | prot-a-606 | Ciliary neurotrophic factor receptor subunit alpha \|\| id:prot-a-606 | Mushroom liking \|\| id:ebi-fl187-GCST90094803 | Weighted median | 7 | -0.442471022 | 0.29123927241187 | 0.128694606258086 | -1.013299996 | 0.128357952174766 | 0.642446957685323 | 0.363019039499918 | 1.13695990713793 | 0.999489402457283 |
| ebi-fl187-GCST90094803 | prot-a-626 | Collagen alpha-2(IX) chain \|\| id:prot-a-626 | Mushroom liking \|\| id:ebi-fl187-GCST90094803 | Weighted median | 7 | 0.0226485509141464 | 0.333452850103908 | 0.945848283324276 | -0.630919035 | 0.676216137117806 | 1.02290697664504 | 0.532102555198384 | 1.96642296235353 | 0.999489402457283 |
| ebi-fl187-GCST90094803 | prot-a-647 | Probable serine carboxypeptidase CPVL \|\| id:prot-a-647 | Mushroom liking \|\| id:ebi-fl187-GCST90094803 | Weighted median | 7 | 0.115682117038975 | 0.276244327732248 | 0.675386137021284 | -0.425756765 | 0.657120999394182 | 1.12263894761332 | 0.65327522198938 | 1.9292300768126 | 0.999489402457283 |
| ebi-fl187-GCST90094803 | prot-a-651 | Cellular retinoic acid-binding protein 2 \|\| id:prot-a-651 | Mushroom liking \|\| id:ebi-fl187-GCST90094803 | Weighted median | 7 | 0.196349391791751 | 0.283366518148951 | 0.488361830662753 | -0.359048984 | 0.751747767363695 | 1.21695202411166 | 0.698340143173684 | 2.12070327542541 | 0.999489402457283 |
| ebi-fl187-GCST90094803 | prot-a-671 | Cytotoxic and regulatory T-cell molecule \|\| id:prot-a-671 | Mushroom liking \|\| id:ebi-fl187-GCST90094803 | Weighted median | 7 | -0.08399309 | 0.307383647788754 | 0.78465977623089 | -0.68646504 | 0.518478859566588 | 0.91943760929569 | 0.503352258053992 | 1.67947099443964 | 0.999489402457283 |
| ebi-fl187-GCST90094803 | prot-a-688 | Chorionic somatomammotropin hormone \|\| id:prot-a-688 | Mushroom liking \|\| id:ebi-fl187-GCST90094803 | Weighted median | 7 | -0.071857576 | 0.278129978251751 | 0.796129321481523 | -0.616992333 | 0.473277181702881 | 0.930663435488286 | 0.539564830883962 | 1.60524626621028 | 0.999489402457283 |
| ebi-fl187-GCST90094803 | prot-a-69 | Aldo-keto reductase family 1 member C1 \|\| id:prot-a-69 | Mushroom liking \|\| id:ebi-fl187-GCST90094803 | Weighted median | 7 | -0.048253207 | 0.273234838179687 | 0.859822783449956 | -0.583793489 | 0.487287076276819 | 0.952892477906295 | 0.557778421590375 | 1.6278938720172 | 0.999489402457283 |
| ebi-fl187-GCST90094803 | prot-a-741 | Stromal cell-derived factor 1 \|\| id:prot-a-741 | Mushroom liking \|\| id:ebi-fl187-GCST90094803 | Weighted median | 7 | -0.189869684 | 0.285119041957296 | 0.505455036348989 | -0.748703006 | 0.368963638357046 | 0.827066907071849 | 0.472979606748847 | 1.44623501523739 | 0.999489402457283 |
| ebi-fl187-GCST90094803 | prot-a-742 | Growth-regulated alpha protein \|\| id:prot-a-742 | Mushroom liking \|\| id:ebi-fl187-GCST90094803 | Weighted median | 7 | -0.166909525 | 0.278359626738973 | 0.548760650770804 | -0.712494394 | 0.378675343271502 | 0.846276174594665 | 0.490419371582204 | 1.46034884669423 | 0.999489402457283 |
| ebi-fl187-GCST90094803 | prot-a-788 | Beta-defensin 112 \|\| id:prot-a-788 | Mushroom liking \|\| id:ebi-fl187-GCST90094803 | Weighted median | 7 | 0.114476128526592 | 0.269468341591466 | 0.670966306956428 | -0.413681821 | 0.642634078045866 | 1.12128587399856 | 0.661211301355139 | 1.9014829429744 | 0.999489402457283 |
| ebi-fl187-GCST90094803 | prot-a-838 | DnaJ homolog subfamily B member 1 \|\| id:prot-a-838 | Mushroom liking \|\| id:ebi-fl187-GCST90094803 | Weighted median | 7 | -0.175490024 | 0.295168041320887 | 0.552149146888603 | -0.754019385 | 0.403039336992705 | 0.839045767455448 | 0.470471740252824 | 1.49636575303457 | 0.999489402457283 |
| ebi-fl187-GCST90094803 | prot-a-85 | Junctional adhesion molecule-like \|\| id:prot-a-85 | Mushroom liking \|\| id:ebi-fl187-GCST90094803 | Weighted median | 7 | 0.42047076615588 | 0.279565154653045 | 0.132576970278166 | -0.127476937 | 0.968418469275848 | 1.52267821228519 | 0.880313718307002 | 2.63377576646992 | 0.999489402457283 |
| ebi-fl187-GCST90094803 | prot-a-856 | Dipeptidase 1 \|\| id:prot-a-856 | Mushroom liking \|\| id:ebi-fl187-GCST90094803 | Weighted median | 7 | 0.371387702547821 | 0.302824745695134 | 0.220043920255866 | -0.222148799 | 0.964924204110283 | 1.44974503428625 | 0.800796197783788 | 2.62458871589837 | 0.999489402457283 |
| ebi-fl187-GCST90094803 | prot-a-860 | Dipeptidyl peptidase 2 \|\| id:prot-a-860 | Mushroom liking \|\| id:ebi-fl187-GCST90094803 | Weighted median | 7 | 0.0315741634181491 | 0.27331870713369 | 0.908031848481726 | -0.504130503 | 0.567278829400182 | 1.03207791518338 | 0.604030550176581 | 1.76346183599137 | 0.999489402457283 |
| ebi-fl187-GCST90094803 | prot-a-861 | Dermatopontin \|\| id:prot-a-861 | Mushroom liking \|\| id:ebi-fl187-GCST90094803 | Weighted median | 7 | -0.504202239 | 0.277097416028905 | 0.0688222620179484 | -1.047313175 | 0.0389086961047412 | 0.603987220543384 | 0.350879234936466 | 1.03967555288867 | 0.999489402457283 |
| ebi-fl187-GCST90094803 | prot-a-877 | Dual specificity protein phosphatase 6 \|\| id:prot-a-877 | Mushroom liking \|\| id:ebi-fl187-GCST90094803 | Weighted median | 7 | -0.159148946 | 0.279859874517077 | 0.569577610051892 | -0.7076743 | 0.389376408246813 | 0.852869318199699 | 0.492788945093649 | 1.47606004795458 | 0.999489402457283 |
| ebi-fl187-GCST90094803 | prot-a-879 | Dynactin-associated protein \|\| id:prot-a-879 | Mushroom liking \|\| id:ebi-fl187-GCST90094803 | Weighted median | 7 | -0.120435597 | 0.273104963349415 | 0.65922278352415 | -0.655721325 | 0.414850131211633 | 0.886534181009186 | 0.519067511082863 | 1.51414380079003 | 0.999489402457283 |
| ebi-fl187-GCST90094803 | prot-a-937 | EMI domain-containing protein 1 \|\| id:prot-a-937 | Mushroom liking \|\| id:ebi-fl187-GCST90094803 | Weighted median | 7 | 0.690641220014681 | 0.299688931027808 | 0.0211930809602957 | 0.103250915200178 | 1.27803152482918 | 1.99499435350534 | 1.10876958131717 | 3.58956679330081 | 0.999489402457283 |
| ebi-fl187-GCST90094803 | prot-b-1 | SRC proto-oncogene, non-receptor tyrosine kinase \|\| id:prot-b-1 | Mushroom liking \|\| id:ebi-fl187-GCST90094803 | Weighted median | 7 | -0.177436567 | 0.358636714629318 | 0.620774531264311 | -0.880364527 | 0.52549139390469 | 0.837414117534356 | 0.414631739482584 | 1.69128973368258 | 0.999489402457283 |
| ebi-fl187-GCST90094803 | prot-b-10 | coagulation factor III, tissue factor \|\| id:prot-b-10 | Mushroom liking \|\| id:ebi-fl187-GCST90094803 | Weighted median | 7 | 0.480637447808126 | 0.41779148467968 | 0.249969184473977 | -0.338233862 | 1.2995087577803 | 1.61710489368572 | 0.71302851800621 | 3.66749459684237 | 0.999489402457283 |
| ebi-fl187-GCST90094803 | prot-b-29 | myeloperoxidase \|\| id:prot-b-29 | Mushroom liking \|\| id:ebi-fl187-GCST90094803 | Weighted median | 7 | -0.234461184 | 0.389079518166244 | 0.546771628965696 | -0.997057039 | 0.52813467186732 | 0.790996936839418 | 0.368963690561919 | 1.69576619622505 | 0.999489402457283 |
| ebi-fl187-GCST90094803 | prot-b-33 | adrenomedullin \|\| id:prot-b-33 | Mushroom liking \|\| id:ebi-fl187-GCST90094803 | Weighted median | 7 | -0.150270039 | 0.356981893015743 | 0.67379463925064 | -0.849954549 | 0.549414471557348 | 0.860475583294957 | 0.427434358798769 | 1.73223844598645 | 0.999489402457283 |
| ebi-fl187-GCST90094803 | ukb-rsfMRI191-edge-pheno102 | Net25_Pair11_15 (Postcentral\|Precentral)&(Cerebellum) (Motor)&(Subcortical-cerebellum) \|\| id:ukb-rsfMRI191-edge-pheno102 | Mushroom liking \|\| id:ebi-fl187-GCST90094803 | Weighted median | 6 | -0.065752942 | 0.105094510822531 | 0.531541029602639 | -0.271738184 | 0.140232298898073 | 0.93636217118856 | 0.762053753200011 | 1.15054103723157 | 0.999489402457283 |
| ebi-fl187-GCST90094803 | ukb-rsfMRI191-edge-pheno1167 | Net100_Pair11_45 (Angular\|Temporal)&(Frontal) (Default_mode\|Central_executive)&(Salience\|Default_mode) \|\| id:ukb-rsfMRI191-edge-pheno1167 | Mushroom liking \|\| id:ebi-fl187-GCST90094803 | Weighted median | 6 | 0.06617308921768 | 0.0988771277073496 | 0.503338779990174 | -0.127626081 | 0.259972259524085 | 1.06841163169567 | 0.880182434478427 | 1.29689410970695 | 0.999489402457283 |
| ebi-fl187-GCST90094803 | ukb-rsfMRI191-edge-pheno1317 | Net100_Pair26_48 (Parietal\|Frontal)&(Temporal) (Attention\|Central_executive)&(Default_mode\|Central_executive) \|\| id:ukb-rsfMRI191-edge-pheno1317 | Mushroom liking \|\| id:ebi-fl187-GCST90094803 | Weighted median | 6 | 0.00364498061598841 | 0.0928303018866594 | 0.968679121238778 | -0.178302411 | 0.185592372313841 | 1.00365163163633 | 0.836689361091388 | 1.20393140456848 | 0.999489402457283 |
| ebi-fl187-GCST90094803 | ukb-rsfMRI191-edge-pheno286 | Net100_Pair10_13 (OccipitalPrecuneus)&(Frontal_Sup) (Default_mode\|Central_executive)&(Default_mode) \|\| id:ukb-rsfMRI191-edge-pheno286 | Mushroom liking \|\| id:ebi-fl187-GCST90094803 | Weighted median | 6 | -0.003087513 | 0.0953096696570519 | 0.974157419059486 | -0.189894465 | 0.183719439909301 | 0.996917248846942 | 0.827046411559878 | 1.20167863273124 | 0.999489402457283 |
| ebi-fl187-GCST90094803 | ukb-rsfMRI191-edge-pheno599 | Net100_Pair11_29 (Angular\|Temporal)&(Parietal) (Default_mode\|Central_executive)&(Central_executive\|Salience) \|\| id:ukb-rsfMRI191-edge-pheno599 | Mushroom liking \|\| id:ebi-fl187-GCST90094803 | Weighted median | 6 | 0.310108142777311 | 0.111505858827647 | 0.00541762457036485 | 0.0915566594751231 | 0.5286596260795 | 1.3635725666835 | 1.09587886685492 | 1.69665662953054 | 0.999489402457283 |
| ebi-fl187-GCST90094803 | ukb-rsfMRI191-edge-pheno621 | Net100_Pair5_30 (Precuneus\|Angular\|Cingulate)&(Paracentral) (Default_mode\|Central_executive)&(Motor) \|\| id:ukb-rsfMRI191-edge-pheno621 | Mushroom liking \|\| id:ebi-fl187-GCST90094803 | Weighted median | 6 | -0.036361567 | 0.0977754423633231 | 0.709975836907735 | -0.228001434 | 0.155278300387881 | 0.964291574775643 | 0.796123118451753 | 1.16798296599101 | 0.999489402457283 |
| ebi-fl187-GCST90094803 | ukb-rsfMRI191-edge-pheno624 | Net100_Pair8_30 (Calcarine\|Lingual)&(Paracentral) (Visual)&(Motor) \|\| id:ukb-rsfMRI191-edge-pheno624 | Mushroom liking \|\| id:ebi-fl187-GCST90094803 | Weighted median | 6 | 0.0496764643130115 | 0.0980085376457981 | 0.612254077904572 | -0.142420269 | 0.241773198098776 | 1.05093102767479 | 0.867256698369591 | 1.27350532663043 | 0.999489402457283 |
| ebi-fl187-GCST90094803 | ukb-rsfMRI191-edge-pheno882 | Net100_Pair6_38 (Postcentral\|Precentral)&(Subcortical) (Motor)&(Subcortical-cerebellum) \|\| id:ukb-rsfMRI191-edge-pheno882 | Mushroom liking \|\| id:ebi-fl187-GCST90094803 | Weighted median | 6 | -0.137542226 | 0.0939677712409674 | 0.143271284611827 | -0.321719057 | 0.0466346060401652 | 0.871497549720129 | 0.72490181765342 | 1.04773910158867 | 0.999489402457283 |
| ebi-fl187-GCST90094803 | ukb-rsfMRI191-edge-pheno899 | Net100_Pair23_38 (Cerebellum)&(Subcortical) (Subcortical-cerebellum)&(Subcortical-cerebellum) \|\| id:ukb-rsfMRI191-edge-pheno899 | Mushroom liking \|\| id:ebi-fl187-GCST90094803 | Weighted median | 6 | 0.0796107869799039 | 0.0920822064893408 | 0.387279175082235 | -0.100870338 | 0.260091911699012 | 1.08286552028491 | 0.904050246485442 | 1.29704929519178 | 0.999489402457283 |
| ebi-fl187-GCST90094803 | ebi-a-GCST90001392 | IgD+ CD38+ B cell Absolute Count \|\| id:ebi-a-GCST90001392 | Mushroom liking \|\| id:ebi-fl187-GCST90094803 | Weighted mode | 6 | 0.198786068225182 | 0.421748372191056 | 0.657248494779028 | -0.627840741 | 1.02541287771965 | 1.21992095812623 | 0.533743046974784 | 2.78824642777207 | 0.999228396881742 |
| ebi-fl187-GCST90094803 | ebi-a-GCST90001402 | Switched memory B cell %B cell \|\| id:ebi-a-GCST90001402 | Mushroom liking \|\| id:ebi-fl187-GCST90094803 | Weighted mode | 6 | 0.616062807859269 | 0.441993396672033 | 0.222147628374771 | -0.25024425 | 1.48236986533645 | 1.85162347414903 | 0.778610584506615 | 4.40336871632985 | 0.999228396881742 |
| ebi-fl187-GCST90094803 | ebi-a-GCST90001407 | Memory B cell Absolute Count \|\| id:ebi-a-GCST90001407 | Mushroom liking \|\| id:ebi-fl187-GCST90094803 | Weighted mode | 6 | 0.300349500151925 | 0.44059200492001 | 0.525720575596979 | -0.563210829 | 1.16390982979514 | 1.3503306658868 | 0.569377950210893 | 3.20242978597769 | 0.999228396881742 |
| ebi-fl187-GCST90094803 | ebi-a-GCST90001411 | IgD+ CD24+ B cell %B cell \|\| id:ebi-a-GCST90001411 | Mushroom liking \|\| id:ebi-fl187-GCST90094803 | Weighted mode | 6 | -0.007234944 | 0.455448186179151 | 0.987940255455461 | -0.899913389 | 0.885443501314221 | 0.992791165603369 | 0.406604874870406 | 2.42405922657528 | 0.999228396881742 |
| ebi-fl187-GCST90094803 | ebi-a-GCST90001418 | CD24+ CD27+ B cell Absolute Count \|\| id:ebi-a-GCST90001418 | Mushroom liking \|\| id:ebi-fl187-GCST90094803 | Weighted mode | 6 | 0.172868138492104 | 0.421446532462287 | 0.698659275671208 | -0.653167065 | 0.998903342118186 | 1.18870934974195 | 0.52039503915963 | 2.7153024372519 | 0.999228396881742 |
| ebi-fl187-GCST90094803 | ebi-a-GCST90001436 | Memory B cell %lymphocyte \|\| id:ebi-a-GCST90001436 | Mushroom liking \|\| id:ebi-fl187-GCST90094803 | Weighted mode | 6 | 0.32252992914317 | 0.502198039279782 | 0.549003689711487 | -0.661778228 | 1.30683808613154 | 1.38061621090147 | 0.515933071739044 | 3.69447361724475 | 0.999228396881742 |
| ebi-fl187-GCST90094803 | ebi-a-GCST90001439 | IgD+ CD24+ B cell %lymphocyte \|\| id:ebi-a-GCST90001439 | Mushroom liking \|\| id:ebi-fl187-GCST90094803 | Weighted mode | 6 | -0.108382887 | 0.475858601631166 | 0.828851452523613 | -1.041065747 | 0.824299971706663 | 0.89728397184168 | 0.35307818946075 | 2.28028394320709 | 0.999228396881742 |
| ebi-fl187-GCST90094803 | ebi-a-GCST90001457 | CD11c+ HLA DR++ monocyte %monocyte \|\| id:ebi-a-GCST90001457 | Mushroom liking \|\| id:ebi-fl187-GCST90094803 | Weighted mode | 6 | -0.082386876 | 0.574730538137075 | 0.891612828119044 | -1.208858731 | 1.04408497825763 | 0.920915609169749 | 0.298537796943346 | 2.84079794215616 | 0.999228396881742 |
| ebi-fl187-GCST90094803 | ebi-a-GCST90001483 | CD39+ resting CD4 regulatory T cell Absolute Count \|\| id:ebi-a-GCST90001483 | Mushroom liking \|\| id:ebi-fl187-GCST90094803 | Weighted mode | 6 | 0.0159631497150459 | 0.534049141216904 | 0.977310568017941 | -1.030773167 | 1.06269946650018 | 1.0160912414644 | 0.356731041223065 | 2.89417317719509 | 0.999228396881742 |
| ebi-fl187-GCST90094803 | ebi-a-GCST90001525 | CD33dim HLA DR+ CD11b+ Absolute Count \|\| id:ebi-a-GCST90001525 | Mushroom liking \|\| id:ebi-fl187-GCST90094803 | Weighted mode | 6 | 0.43474302659966 | 0.583116862161483 | 0.489482139021473 | -0.708166023 | 1.57765207643617 | 1.54456609554738 | 0.492546688815976 | 4.84357011768649 | 0.999228396881742 |
| ebi-fl187-GCST90094803 | ebi-a-GCST90001540 | Naive CD4+ T cell Absolute Count \|\| id:ebi-a-GCST90001540 | Mushroom liking \|\| id:ebi-fl187-GCST90094803 | Weighted mode | 6 | -0.130118166 | 0.452782028914796 | 0.785354754504365 | -1.017570942 | 0.757334610980037 | 0.877991676295718 | 0.361471910740126 | 2.13258447126966 | 0.999228396881742 |
| ebi-fl187-GCST90094803 | ebi-a-GCST90001624 | HLA DR+ CD4+ T cell Absolute Count \|\| id:ebi-a-GCST90001624 | Mushroom liking \|\| id:ebi-fl187-GCST90094803 | Weighted mode | 6 | -0.122472156 | 0.380794349069905 | 0.760748912538428 | -0.86882908 | 0.623884768221004 | 0.884730539076575 | 0.419442395254543 | 1.86616359154565 | 0.999228396881742 |
| ebi-fl187-GCST90094803 | ebi-a-GCST90001672 | CD39+ CD8+ T cell Absolute Count \|\| id:ebi-a-GCST90001672 | Mushroom liking \|\| id:ebi-fl187-GCST90094803 | Weighted mode | 6 | 0.537815838285057 | 0.46542587335324 | 0.30008674578111 | -0.374418873 | 1.45005055005741 | 1.71226291587439 | 0.687688796886811 | 4.26333002129917 | 0.999228396881742 |
| ebi-fl187-GCST90094803 | ebi-a-GCST90001722 | CD19 on CD20- CD38- B cell \|\| id:ebi-a-GCST90001722 | Mushroom liking \|\| id:ebi-fl187-GCST90094803 | Weighted mode | 6 | 0.167058089188175 | 0.461963445549266 | 0.732410791072587 | -0.738390264 | 1.07250644246474 | 1.18182291444812 | 0.477882561417251 | 2.92269589618935 | 0.999228396881742 |
| ebi-fl187-GCST90094803 | ebi-a-GCST90001742 | CD19 on transitional B cell \|\| id:ebi-a-GCST90001742 | Mushroom liking \|\| id:ebi-fl187-GCST90094803 | Weighted mode | 6 | 0.574133321341499 | 0.886183461285763 | 0.545637541868451 | -1.162786263 | 2.31105290546159 | 1.77559099304993 | 0.312613941713258 | 10.0850376580192 | 0.999228396881742 |
| ebi-fl187-GCST90094803 | ebi-a-GCST90001784 | CD25 on IgD+ CD38dim B cell \|\| id:ebi-a-GCST90001784 | Mushroom liking \|\| id:ebi-fl187-GCST90094803 | Weighted mode | 6 | 0.314129777622118 | 0.440829425809488 | 0.507964138189765 | -0.549895897 | 1.17815545220871 | 1.3690673993304 | 0.577009875733529 | 3.24837688701001 | 0.999228396881742 |
| ebi-fl187-GCST90094803 | ebi-a-GCST90001794 | CD25 on IgD+ B cell \|\| id:ebi-a-GCST90001794 | Mushroom liking \|\| id:ebi-fl187-GCST90094803 | Weighted mode | 6 | -0.060122901 | 0.455362051027587 | 0.900106680855364 | -0.952632521 | 0.832386718640945 | 0.941648796542178 | 0.385724258354084 | 2.29879878391098 | 0.999228396881742 |
| ebi-fl187-GCST90094803 | ebi-a-GCST90001802 | CD27 on IgD- CD38- B cell \|\| id:ebi-a-GCST90001802 | Mushroom liking \|\| id:ebi-fl187-GCST90094803 | Weighted mode | 6 | 0.233488100019799 | 0.47274402024398 | 0.642306671429338 | -0.69309018 | 1.16006638 | 1.26299779815269 | 0.500028501263063 | 3.19014502995169 | 0.999228396881742 |
| ebi-fl187-GCST90094803 | ebi-a-GCST90001804 | CD27 on IgD- CD38dim B cell \|\| id:ebi-a-GCST90001804 | Mushroom liking \|\| id:ebi-fl187-GCST90094803 | Weighted mode | 6 | 0.766387928922177 | 0.397146222290695 | 0.111527805630809 | -0.012018667 | 1.54479452461194 | 2.15197909691344 | 0.988053268929054 | 4.68700846318935 | 0.999228396881742 |
| ebi-fl187-GCST90094803 | ebi-a-GCST90001805 | CD27 on memory B cell \|\| id:ebi-a-GCST90001805 | Mushroom liking \|\| id:ebi-fl187-GCST90094803 | Weighted mode | 6 | 0.392292364162899 | 0.459928375089782 | 0.432640644830676 | -0.509167251 | 1.29375197933887 | 1.48037045540876 | 0.600995849168082 | 3.64644229786394 | 0.999228396881742 |
| ebi-fl187-GCST90094803 | ebi-a-GCST90001806 | CD27 on unswitched memory B cell \|\| id:ebi-a-GCST90001806 | Mushroom liking \|\| id:ebi-fl187-GCST90094803 | Weighted mode | 6 | 0.151743894146983 | 0.464700582682925 | 0.757240573362787 | -0.759069248 | 1.06255703620552 | 1.16386212635721 | 0.468101911145994 | 2.89376098861142 | 0.999228396881742 |
| ebi-fl187-GCST90094803 | ebi-a-GCST90001839 | CD3 on Effector Memory CD8+ T cell \|\| id:ebi-a-GCST90001839 | Mushroom liking \|\| id:ebi-fl187-GCST90094803 | Weighted mode | 6 | 0.504757730246933 | 0.511050511496395 | 0.368660841051311 | -0.496901272 | 1.50641673277987 | 1.65658413161212 | 0.608413048075527 | 4.51053933473239 | 0.999228396881742 |
| ebi-fl187-GCST90094803 | ebi-a-GCST90001859 | CD3 on CD8+ T cell \|\| id:ebi-a-GCST90001859 | Mushroom liking \|\| id:ebi-fl187-GCST90094803 | Weighted mode | 6 | 0.507311328678645 | 0.521844151951998 | 0.375624064122861 | -0.515503209 | 1.53012586650456 | 1.66081978803373 | 0.597200002490224 | 4.61875813265683 | 0.999228396881742 |
| ebi-fl187-GCST90094803 | ebi-a-GCST90001905 | CD86 on monocyte \|\| id:ebi-a-GCST90001905 | Mushroom liking \|\| id:ebi-fl187-GCST90094803 | Weighted mode | 6 | -0.097779834 | 0.469387821407161 | 0.843204039708181 | -1.017779964 | 0.822220296363912 | 0.906848539360173 | 0.361396363348344 | 2.2755466206698 | 0.999228396881742 |
| ebi-fl187-GCST90094803 | ebi-a-GCST90001943 | CD25 on activated & secreting CD4 regulatory T cell \|\| id:ebi-a-GCST90001943 | Mushroom liking \|\| id:ebi-fl187-GCST90094803 | Weighted mode | 6 | -0.248100837 | 0.452506902431344 | 0.607079717023216 | -1.135014366 | 0.638812691621459 | 0.780281257977349 | 0.321417503846482 | 1.894230508 | 0.999228396881742 |
| ebi-fl187-GCST90094803 | ebi-a-GCST90001949 | CD33 on CD33dim HLA DR+ CD11b- \|\| id:ebi-a-GCST90001949 | Mushroom liking \|\| id:ebi-fl187-GCST90094803 | Weighted mode | 6 | -0.383443678 | 0.643164201335522 | 0.577015537525811 | -1.644045513 | 0.877158156381738 | 0.681510460839271 | 0.193196878753674 | 2.40405803256034 | 0.999228396881742 |
| ebi-fl187-GCST90094803 | ebi-a-GCST90001955 | CD33 on Immature Myeloid-Derived Suppressor Cells \|\| id:ebi-a-GCST90001955 | Mushroom liking \|\| id:ebi-fl187-GCST90094803 | Weighted mode | 6 | -0.022975593 | 0.742675561703274 | 0.976517283826539 | -1.478619694 | 1.43266850808592 | 0.977286336253947 | 0.227952115051129 | 4.18986497587198 | 0.999228396881742 |
| ebi-fl187-GCST90094803 | ebi-a-GCST90001956 | CD33 on CD33+ HLA DR+ \|\| id:ebi-a-GCST90001956 | Mushroom liking \|\| id:ebi-fl187-GCST90094803 | Weighted mode | 6 | -0.240078409 | 0.595424840586911 | 0.703460737183064 | -1.407111096 | 0.92695427864802 | 0.786566184857442 | 0.244849609313034 | 2.52680151255711 | 0.999228396881742 |
| ebi-fl187-GCST90094803 | ebi-a-GCST90001957 | CD33 on CD33+ HLA DR+ CD14- \|\| id:ebi-a-GCST90001957 | Mushroom liking \|\| id:ebi-fl187-GCST90094803 | Weighted mode | 6 | -0.226576805 | 0.624537134928272 | 0.731592180240532 | -1.45066959 | 0.997515979214595 | 0.797258106357354 | 0.234413274817075 | 2.71153793934462 | 0.999228396881742 |
| ebi-fl187-GCST90094803 | ebi-a-GCST90002064 | CD4 on resting CD4 regulatory T cell \|\| id:ebi-a-GCST90002064 | Mushroom liking \|\| id:ebi-fl187-GCST90094803 | Weighted mode | 6 | -0.665341171 | 0.539334089284018 | 0.272163144224482 | -1.722435986 | 0.391753644484951 | 0.514098102670644 | 0.178630476230527 | 1.47957316549095 | 0.999228396881742 |
| ebi-fl187-GCST90094803 | ebi-a-GCST90002080 | SSC-A on T cell \|\| id:ebi-a-GCST90002080 | Mushroom liking \|\| id:ebi-fl187-GCST90094803 | Weighted mode | 6 | 0.368480270155432 | 0.459462281121198 | 0.458966759570733 | -0.532065801 | 1.26902634115298 | 1.44553612014441 | 0.587390284117524 | 3.55738719407226 | 0.999228396881742 |
| ebi-fl187-GCST90094803 | ebi-a-GCST90002121 | CD8 on CD39+ CD8+ T cell \|\| id:ebi-a-GCST90002121 | Mushroom liking \|\| id:ebi-fl187-GCST90094803 | Weighted mode | 6 | -0.167131384 | 0.517039139195397 | 0.759589601457148 | -1.180528097 | 0.846265328553703 | 0.846088441322747 | 0.307116508433175 | 2.3309253357695 | 0.999228396881742 |
| ebi-fl187-GCST90094803 | ebi-cfmet338-GCST90026053 | 1-palmitoyl-2-docosahexaenoyl-gpc (16:0/22:6) levels \|\| id:ebi-cfmet338-GCST90026053 | Mushroom liking \|\| id:ebi-fl187-GCST90094803 | Weighted mode | 6 | 0.100307613633714 | 0.277798805964652 | 0.732794563960368 | -0.444178046 | 0.644793273324433 | 1.10551093601203 | 0.641351220605188 | 1.90559305163393 | 0.999228396881742 |
| ebi-fl187-GCST90094803 | ebi-cfmet338-GCST90026063 | 1-stearoyl-2-oleoyl-gpc (18:0/18:1) levels \|\| id:ebi-cfmet338-GCST90026063 | Mushroom liking \|\| id:ebi-fl187-GCST90094803 | Weighted mode | 6 | 0.0689124361704503 | 0.228555946675783 | 0.775160986979213 | -0.379057219 | 0.516882091654984 | 1.07134239419821 | 0.684506444556459 | 1.67679140895464 | 0.999228396881742 |
| ebi-fl187-GCST90094803 | ebi-cfmet338-GCST90026113 | Adenosine 3',5'-cyclic monophosphate (camp) levels \|\| id:ebi-cfmet338-GCST90026113 | Mushroom liking \|\| id:ebi-fl187-GCST90094803 | Weighted mode | 6 | 0.451363766948974 | 0.643795423727273 | 0.514519402051279 | -0.810475264 | 1.71320279745443 | 1.5704524568984 | 0.444646691629558 | 5.54669800946805 | 0.999228396881742 |
| ebi-fl187-GCST90094803 | ebi-cfmet338-GCST90026117 | Alpha-ketoglutaramate levels \|\| id:ebi-cfmet338-GCST90026117 | Mushroom liking \|\| id:ebi-fl187-GCST90094803 | Weighted mode | 6 | 0.0764326578278529 | 0.180622041453165 | 0.689760201348724 | -0.277586543 | 0.430451859076056 | 1.07942949676253 | 0.757609995605422 | 1.5379523042722 | 0.999228396881742 |
| ebi-fl187-GCST90094803 | ebi-cfmet338-GCST90026124 | Arginine levels \|\| id:ebi-cfmet338-GCST90026124 | Mushroom liking \|\| id:ebi-fl187-GCST90094803 | Weighted mode | 6 | 0.0788159170332663 | 0.128784413883096 | 0.567295480892592 | -0.173601534 | 0.331233368244135 | 1.08200512502305 | 0.840631793970752 | 1.39268476278555 | 0.999228396881742 |
| ebi-fl187-GCST90094803 | ebi-cfmet338-GCST90026169 | Glutamate levels \|\| id:ebi-cfmet338-GCST90026169 | Mushroom liking \|\| id:ebi-fl187-GCST90094803 | Weighted mode | 6 | 0.00596977286175865 | 0.145245950834762 | 0.968806009885689 | -0.278712291 | 0.290651836497891 | 1.0059876274674 | 0.75675759803966 | 1.33729890421854 | 0.999228396881742 |
| ebi-fl187-GCST90094803 | ebi-cfmet338-GCST90026170 | Glutamine levels \|\| id:ebi-cfmet338-GCST90026170 | Mushroom liking \|\| id:ebi-fl187-GCST90094803 | Weighted mode | 6 | 0.000118857154672858 | 0.0809303529435245 | 0.998884993084974 | -0.158504635 | 0.158742348923981 | 1.00011886421846 | 0.853419008513542 | 1.17203593145624 | 0.999700494083162 |
| ebi-fl187-GCST90094803 | ebi-cfmet338-GCST90026191 | Kynurenate levels \|\| id:ebi-cfmet338-GCST90026191 | Mushroom liking \|\| id:ebi-fl187-GCST90094803 | Weighted mode | 6 | 0.462488985863121 | 0.712642775712771 | 0.544978814982435 | -0.934290855 | 1.85926882626015 | 1.58802163353198 | 0.392864364754671 | 6.41904162048488 | 0.999228396881742 |
| ebi-fl187-GCST90094803 | ebi-cfmet338-GCST90026202 | Methylsuccinoylcarnitine levels \|\| id:ebi-cfmet338-GCST90026202 | Mushroom liking \|\| id:ebi-fl187-GCST90094803 | Weighted mode | 6 | 0.175771651578404 | 0.500247266764636 | 0.739632923004783 | -0.804712991 | 1.15625629443709 | 1.19216579838841 | 0.447216263108733 | 3.1780134312815 | 0.999228396881742 |
| ebi-fl187-GCST90094803 | ebi-cfmet338-GCST90026222 | N-acetylputrescine levels \|\| id:ebi-cfmet338-GCST90026222 | Mushroom liking \|\| id:ebi-fl187-GCST90094803 | Weighted mode | 6 | 0.151209289335439 | 0.179321694093031 | 0.437568004507678 | -0.200261231 | 0.50267980975778 | 1.16324008635209 | 0.818516903086824 | 1.65314545538821 | 0.999228396881742 |
| ebi-fl187-GCST90094803 | ebi-cfmet338-GCST90026252 | Propionylcarnitine (c3) levels \|\| id:ebi-cfmet338-GCST90026252 | Mushroom liking \|\| id:ebi-fl187-GCST90094803 | Weighted mode | 6 | 0.266360634294748 | 0.254405240236078 | 0.343052870908185 | -0.232273637 | 0.764994905157461 | 1.30520567574988 | 0.792729173934915 | 2.14898342589516 | 0.999228396881742 |
| ebi-fl187-GCST90094803 | ebi-cfmet338-GCST90026316 | Ethyl beta-glucopyranoside levels \|\| id:ebi-cfmet338-GCST90026316 | Mushroom liking \|\| id:ebi-fl187-GCST90094803 | Weighted mode | 6 | 0.182564239423122 | 0.741153216966485 | 0.815223952889853 | -1.270096066 | 1.63522454467743 | 1.20029125449477 | 0.280804644751016 | 5.13060991884262 | 0.999228396881742 |
| ebi-fl187-GCST90094803 | ebi-gm430-GCST90011315 | F_Prevotellaceae abundance \|\| id:ebi-gm430-GCST90011315 | Mushroom liking \|\| id:ebi-fl187-GCST90094803 | Weighted mode | 6 | -0.071114248 | 0.329499952605025 | 0.837652581064135 | -0.716934155 | 0.57470565960055 | 0.931355481008854 | 0.488246853050525 | 1.7766075225793 | 0.999228396881742 |
| ebi-fl187-GCST90094803 | ebi-gm430-GCST90011354 | OTU97_1 (Bacteroides) abundance \|\| id:ebi-gm430-GCST90011354 | Mushroom liking \|\| id:ebi-fl187-GCST90094803 | Weighted mode | 6 | 0.0201887148835929 | 0.350937818450079 | 0.956352936249212 | -0.667649409 | 0.708026839045747 | 1.0203938853714 | 0.51291280999562 | 2.02998182344524 | 0.999228396881742 |
| ebi-fl187-GCST90094803 | ebi-gm430-GCST90011419 | OTU99_1 (Bacteroides) abundance \|\| id:ebi-gm430-GCST90011419 | Mushroom liking \|\| id:ebi-fl187-GCST90094803 | Weighted mode | 6 | 0.0422052722677271 | 0.333107915911894 | 0.904114185332706 | -0.610686243 | 0.695096787455039 | 1.04310857803899 | 0.54297812629842 | 2.00390301722857 | 0.999228396881742 |
| ebi-fl187-GCST90094803 | ebi-gm430-GCST90011435 | OTU99_155 (Butyrivibrio) abundance \|\| id:ebi-gm430-GCST90011435 | Mushroom liking \|\| id:ebi-fl187-GCST90094803 | Weighted mode | 6 | -0.667879211 | 0.992541883609792 | 0.5308534390246 | -2.613261303 | 1.27750288063255 | 0.512794955166634 | 0.0732951159617461 | 3.5876696911373 | 0.999228396881742 |
| ebi-fl187-GCST90094803 | ebi-gm430-GCST90011466 | OTU99_58 (Bacteroides) abundance \|\| id:ebi-gm430-GCST90011466 | Mushroom liking \|\| id:ebi-fl187-GCST90094803 | Weighted mode | 6 | 0.269666153969597 | 0.552416385769733 | 0.646091829002578 | -0.813069962 | 1.35240227007827 | 1.30952719729312 | 0.443494462978497 | 3.86670324795809 | 0.999228396881742 |
| ebi-fl187-GCST90094803 | ebi-gm430-GCST90011472 | OTU99_72 (Parabacteroides) abundance \|\| id:ebi-gm430-GCST90011472 | Mushroom liking \|\| id:ebi-fl187-GCST90094803 | Weighted mode | 6 | -0.076114143 | 0.942571687300256 | 0.938772158304118 | -1.92355465 | 1.77132636402021 | 0.926710422932637 | 0.146086750795986 | 5.87864541645748 | 0.999228396881742 |
| ebi-fl187-GCST90094803 | ebi-gm430-GCST90011505 | TestASV_28 (Holdemanella) abundance \|\| id:ebi-gm430-GCST90011505 | Mushroom liking \|\| id:ebi-fl187-GCST90094803 | Weighted mode | 6 | 0.0450772329161548 | 0.892303749703379 | 0.96166576552799 | -1.703838117 | 1.79399258233478 | 1.04610865081054 | 0.181983707252635 | 6.01341365016507 | 0.999228396881742 |
| ebi-fl187-GCST90094803 | ebi-gm430-GCST90011513 | TestASV_35 (Alphaproteobacteria) abundance \|\| id:ebi-gm430-GCST90011513 | Mushroom liking \|\| id:ebi-fl187-GCST90094803 | Weighted mode | 6 | -0.444334179 | 0.959516198500159 | 0.662762802057723 | -2.324985928 | 1.43631757041557 | 0.641251092596294 | 0.0977848200984979 | 4.20518198368356 | 0.999228396881742 |
| ebi-fl187-GCST90094803 | ebi-gm430-GCST90011543 | G_Coprococcus prevalence \|\| id:ebi-gm430-GCST90011543 | Mushroom liking \|\| id:ebi-fl187-GCST90094803 | Weighted mode | 6 | 0.447266846746126 | 0.893437212526939 | 0.637896500474559 | -1.30387009 | 2.19840378329893 | 1.56403160034962 | 0.2714791088249 | 9.01061911349482 | 0.999228396881742 |
| ebi-fl187-GCST90094803 | ebi-gm430-GCST90011547 | G_Parasutterella prevalence \|\| id:ebi-gm430-GCST90011547 | Mushroom liking \|\| id:ebi-fl187-GCST90094803 | Weighted mode | 6 | -0.537310061 | 0.922204607066872 | 0.58542321526678 | -2.344831091 | 1.27021096866183 | 0.584317919723034 | 0.0958633929490484 | 3.56160386990395 | 0.999228396881742 |
| ebi-fl187-GCST90094803 | ebi-gm430-GCST90011569 | OTU97_13 (Subdoligranulum) prevalence \|\| id:ebi-gm430-GCST90011569 | Mushroom liking \|\| id:ebi-fl187-GCST90094803 | Weighted mode | 6 | 0.999897545402591 | 0.952812538360123 | 0.34203902402895 | -0.86761503 | 2.86741012058843 | 2.71800334225501 | 0.419951928678243 | 17.591399548422 | 0.999228396881742 |
| ebi-fl187-GCST90094803 | ebi-gm430-GCST90011590 | OTU97_277 (Prevotella) prevalence \|\| id:ebi-gm430-GCST90011590 | Mushroom liking \|\| id:ebi-fl187-GCST90094803 | Weighted mode | 6 | 1.14739071175375 | 0.846983635688016 | 0.233514465177538 | -0.512697214 | 2.80747863770226 | 3.14996301580317 | 0.598878095942673 | 16.5680913497254 | 0.999228396881742 |
| ebi-fl187-GCST90094803 | ebi-gm430-GCST90011616 | OTU97_86 (Phascolarctobacterium) prevalence \|\| id:ebi-gm430-GCST90011616 | Mushroom liking \|\| id:ebi-fl187-GCST90094803 | Weighted mode | 6 | 1.40498478699601 | 1.01410254881101 | 0.224536529947132 | -0.582656209 | 3.39262578266558 | 4.07546474073456 | 0.558413133086092 | 29.7439509726035 | 0.999228396881742 |
| ebi-fl187-GCST90094803 | ebi-gm430-GCST90011665 | OTU99_55 (Barnesiella) prevalence \|\| id:ebi-gm430-GCST90011665 | Mushroom liking \|\| id:ebi-fl187-GCST90094803 | Weighted mode | 6 | 0.57000323803833 | 0.715729545010071 | 0.461924095951257 | -0.83282667 | 1.97283314625807 | 1.7682727771595 | 0.434818459196964 | 7.19102086930257 | 0.999228396881742 |
| ebi-fl187-GCST90094803 | ebi-gm430-GCST90011687 | TestASV_14 (Lachnospiraceae) prevalence \|\| id:ebi-gm430-GCST90011687 | Mushroom liking \|\| id:ebi-fl187-GCST90094803 | Weighted mode | 6 | 0.605485242762097 | 0.650148971596629 | 0.394444366377971 | -0.668806742 | 1.87977722709149 | 1.8321410263944 | 0.512319542809162 | 6.55204508145789 | 0.999228396881742 |
| ebi-fl187-GCST90094803 | ebi-gm430-GCST90011697 | TestASV_23 (Barnesiella) prevalence \|\| id:ebi-gm430-GCST90011697 | Mushroom liking \|\| id:ebi-fl187-GCST90094803 | Weighted mode | 6 | 0.396958480474631 | 0.99313020760555 | 0.70587733953493 | -1.549576726 | 2.34349368738151 | 1.48729417702102 | 0.212337831799784 | 10.417568787678 | 0.999228396881742 |
| ebi-fl187-GCST90094803 | ebi-gm430-GCST90011727 | TestASV_6 (Subdoligranulum) prevalence \|\| id:ebi-gm430-GCST90011727 | Mushroom liking \|\| id:ebi-fl187-GCST90094803 | Weighted mode | 6 | -0.194709278 | 1.75581325830324 | 0.916014199803014 | -3.636103265 | 3.24668470802946 | 0.823073908767979 | 0.0263548419799977 | 25.7049789867365 | 0.999228396881742 |
| ebi-fl187-GCST90094803 | ebi-hbsm44-GCST90104908 | Sulcal depth of the left posterior inferior temporal sulcus \|\| id:ebi-hbsm44-GCST90104908 | Mushroom liking \|\| id:ebi-fl187-GCST90094803 | Weighted mode | 5 | -0.278969779 | 0.390290037412364 | 0.514246878516748 | -1.043938252 | 0.485998694436152 | 0.756562767034591 | 0.352065425689163 | 1.62579787362703 | 0.999228396881742 |
| ebi-fl187-GCST90094803 | ebi-hbsm44-GCST90104918 | Sulcal depth of the left inferior frontal sulcus \|\| id:ebi-hbsm44-GCST90104918 | Mushroom liking \|\| id:ebi-fl187-GCST90094803 | Weighted mode | 5 | -0.548779799 | 0.653385342748863 | 0.448232694253286 | -1.829415071 | 0.731855472799897 | 0.577654234805355 | 0.160507425806424 | 2.07893443753184 | 0.999228396881742 |
| ebi-fl187-GCST90094803 | ebi-hbsm44-GCST90104925 | Sulcal depth of the right anterior inferior temporal sulcus \|\| id:ebi-hbsm44-GCST90104925 | Mushroom liking \|\| id:ebi-fl187-GCST90094803 | Weighted mode | 4 | -0.306476786 | 0.229878075387693 | 0.274659437776621 | -0.757037813 | 0.144084242198141 | 0.736035604634136 | 0.469053796078551 | 1.15498140259889 | 0.999228396881742 |
| ebi-fl187-GCST90094803 | ebi-IPs91-GCST90274770 | C-C motif chemokine 4 levels \|\| id:ebi-IPs91-GCST90274770 | Mushroom liking \|\| id:ebi-fl187-GCST90094803 | Weighted mode | 7 | 0.0936798235799658 | 0.176349807495825 | 0.614350021381533 | -0.251965799 | 0.439325446271783 | 1.09820806929282 | 0.777271320983874 | 1.55166018724738 | 0.999228396881742 |
| ebi-fl187-GCST90094803 | ebi-IPs91-GCST90274779 | C-X-C motif chemokine 1 levels \|\| id:ebi-IPs91-GCST90274779 | Mushroom liking \|\| id:ebi-fl187-GCST90094803 | Weighted mode | 7 | -0.114645474 | 0.205661960711367 | 0.597389010648124 | -0.517742917 | 0.288451968840276 | 0.891682212266984 | 0.595863945613584 | 1.33436025711306 | 0.999228396881742 |
| ebi-fl187-GCST90094803 | ebi-IPs91-GCST90274780 | C-X-C motif chemokine 10 levels \|\| id:ebi-IPs91-GCST90274780 | Mushroom liking \|\| id:ebi-fl187-GCST90094803 | Weighted mode | 7 | 0.187631581026738 | 0.207902777668047 | 0.401569734207598 | -0.219857863 | 0.59512102525611 | 1.20638897687821 | 0.802632873521221 | 1.81325038077298 | 0.999228396881742 |
| ebi-fl187-GCST90094803 | ebi-IPs91-GCST90274796 | Interleukin-10 receptor subunit alpha levels \|\| id:ebi-IPs91-GCST90274796 | Mushroom liking \|\| id:ebi-fl187-GCST90094803 | Weighted mode | 7 | -0.015359751 | 0.191562119663904 | 0.938700390301763 | -0.390821505 | 0.360102004008026 | 0.984757608796672 | 0.67650089724039 | 1.43347562736247 | 0.999228396881742 |
| ebi-fl187-GCST90094803 | ebi-IPs91-GCST90274807 | Interleukin-20 levels \|\| id:ebi-IPs91-GCST90274807 | Mushroom liking \|\| id:ebi-fl187-GCST90094803 | Weighted mode | 7 | 0.166259945673721 | 0.197734140897733 | 0.432670442864879 | -0.22129897 | 0.553818861833278 | 1.18088002648373 | 0.801477026491278 | 1.73988472639446 | 0.999228396881742 |
| ebi-fl187-GCST90094803 | ebi-IPs91-GCST90274808 | Interleukin-20 receptor subunit alpha levels \|\| id:ebi-IPs91-GCST90274808 | Mushroom liking \|\| id:ebi-fl187-GCST90094803 | Weighted mode | 7 | 0.275185162492179 | 0.214356895708843 | 0.246567433057835 | -0.144954353 | 0.695324678081512 | 1.31677446953853 | 0.865061779600526 | 2.00435973998199 | 0.999228396881742 |
| ebi-fl187-GCST90094803 | ebi-IPs91-GCST90274810 | Interleukin-24 levels \|\| id:ebi-IPs91-GCST90274810 | Mushroom liking \|\| id:ebi-fl187-GCST90094803 | Weighted mode | 7 | 0.0403853273777322 | 0.218654011789597 | 0.859549429266129 | -0.388176536 | 0.468947190485342 | 1.04121190435698 | 0.67829258981662 | 1.59831059051932 | 0.999228396881742 |
| ebi-fl187-GCST90094803 | ebi-IPs91-GCST90274823 | Monocyte chemoattractant protein-3 levels \|\| id:ebi-IPs91-GCST90274823 | Mushroom liking \|\| id:ebi-fl187-GCST90094803 | Weighted mode | 7 | 0.349106999906863 | 0.223389219587008 | 0.169134264075535 | -0.08873587 | 0.786949870297398 | 1.41780088678892 | 0.915087243207951 | 2.19668602037616 | 0.999228396881742 |
| ebi-fl187-GCST90094803 | ebi-IPs91-GCST90274824 | Monocyte chemoattractant protein-4 levels \|\| id:ebi-IPs91-GCST90274824 | Mushroom liking \|\| id:ebi-fl187-GCST90094803 | Weighted mode | 7 | -0.023582881 | 0.205900119724559 | 0.912549968541965 | -0.427147116 | 0.379981353292229 | 0.976693021660481 | 0.652367571441982 | 1.46225732289502 | 0.999228396881742 |
| ebi-fl187-GCST90094803 | ebi-IPs91-GCST90274834 | SIR2-like protein 2 levels \|\| id:ebi-IPs91-GCST90274834 | Mushroom liking \|\| id:ebi-fl187-GCST90094803 | Weighted mode | 7 | -0.30206347 | 0.224518463376557 | 0.22711693131655 | -0.742119658 | 0.137992717973661 | 0.739291140411035 | 0.47610366804051 | 1.14796719071642 | 0.999228396881742 |
| ebi-fl187-GCST90094803 | ebi-met1400-GCST90199652 | 1-stearoyl-2-oleoyl-GPS (18:0/18:1) levels \|\| id:ebi-met1400-GCST90199652 | Mushroom liking \|\| id:ebi-fl187-GCST90094803 | Weighted mode | 5 | -0.101434392 | 0.306276248393375 | 0.757127689539825 | -0.701735839 | 0.49886705474531 | 0.903540456784445 | 0.495724059382805 | 1.64685441748112 | 0.999228396881742 |
| ebi-fl187-GCST90094803 | ebi-met1400-GCST90199665 | N-acetylaspartate (naa) levels \|\| id:ebi-met1400-GCST90199665 | Mushroom liking \|\| id:ebi-fl187-GCST90094803 | Weighted mode | 5 | -0.504394091 | 0.430768972659909 | 0.306627581933428 | -1.348701278 | 0.339913095158235 | 0.603871355536228 | 0.259577160483906 | 1.40482549912079 | 0.999228396881742 |
| ebi-fl187-GCST90094803 | ebi-met1400-GCST90199669 | Acetylcarnitine levels (Biocrates platform) \|\| id:ebi-met1400-GCST90199669 | Mushroom liking \|\| id:ebi-fl187-GCST90094803 | Weighted mode | 5 | 0.475899470967795 | 0.449055334758787 | 0.348996911757332 | -0.404248985 | 1.35604792709502 | 1.60946121024765 | 0.667477908484071 | 3.88082564885913 | 0.999228396881742 |
| ebi-fl187-GCST90094803 | ebi-met1400-GCST90199670 | Iminodiacetate (IDA) levels \|\| id:ebi-met1400-GCST90199670 | Mushroom liking \|\| id:ebi-fl187-GCST90094803 | Weighted mode | 5 | 0.0123126671768103 | 0.435795292850724 | 0.978813527502136 | -0.841846107 | 0.86647144116423 | 1.01238878 | 0.430914274897626 | 2.37850334006813 | 0.999228396881742 |
| ebi-fl187-GCST90094803 | ebi-met1400-GCST90199686 | Indolepropionate levels \|\| id:ebi-met1400-GCST90199686 | Mushroom liking \|\| id:ebi-fl187-GCST90094803 | Weighted mode | 5 | 0.56963336834472 | 0.400312634909259 | 0.227828368302964 | -0.214979396 | 1.35424613276687 | 1.76761886758731 | 0.80655805826591 | 3.87383949491278 | 0.999228396881742 |
| ebi-fl187-GCST90094803 | ebi-met1400-GCST90199704 | 3-hydroxysebacate levels \|\| id:ebi-met1400-GCST90199704 | Mushroom liking \|\| id:ebi-fl187-GCST90094803 | Weighted mode | 5 | 0.0732767885352426 | 0.310023069891593 | 0.82476401011871 | -0.534368428 | 0.680922005522764 | 1.07602832800501 | 0.586039299036068 | 1.97569849764973 | 0.999228396881742 |
| ebi-fl187-GCST90094803 | ebi-met1400-GCST90199713 | Docosapentaenoate n3 DPA; 22:5n3 levels \|\| id:ebi-met1400-GCST90199713 | Mushroom liking \|\| id:ebi-fl187-GCST90094803 | Weighted mode | 5 | 0.264471518306948 | 0.31301716266127 | 0.445735586423706 | -0.349042121 | 0.877985157123038 | 1.30274231835696 | 0.705363419377886 | 2.40604701266605 | 0.999228396881742 |
| ebi-fl187-GCST90094803 | ebi-met1400-GCST90199715 | Myristoleate (14:1n5) levels \|\| id:ebi-met1400-GCST90199715 | Mushroom liking \|\| id:ebi-fl187-GCST90094803 | Weighted mode | 5 | -0.06691108 | 0.304692595874195 | 0.83693261158278 | -0.664108568 | 0.530286407569971 | 0.935278362270152 | 0.514732171848482 | 1.69941896538037 | 0.999228396881742 |
| ebi-fl187-GCST90094803 | ebi-met1400-GCST90199725 | Campesterol levels \|\| id:ebi-met1400-GCST90199725 | Mushroom liking \|\| id:ebi-fl187-GCST90094803 | Weighted mode | 5 | -0.383108714 | 0.456623304753528 | 0.44868178800737 | -1.278090391 | 0.511872963555481 | 0.681738780870035 | 0.278568750231812 | 1.66841314740222 | 0.999228396881742 |
| ebi-fl187-GCST90094803 | ebi-met1400-GCST90199737 | N-acetylproline levels \|\| id:ebi-met1400-GCST90199737 | Mushroom liking \|\| id:ebi-fl187-GCST90094803 | Weighted mode | 5 | -0.239377385 | 0.341585075601576 | 0.522059786753144 | -0.908884133 | 0.430129363115945 | 0.787117779822142 | 0.402973638130925 | 1.53745640083495 | 0.999228396881742 |
| ebi-fl187-GCST90094803 | ebi-met1400-GCST90199775 | Gamma-glutamylisoleucine levels \|\| id:ebi-met1400-GCST90199775 | Mushroom liking \|\| id:ebi-fl187-GCST90094803 | Weighted mode | 5 | -0.261561697 | 0.31714221748464 | 0.455857657499555 | -0.883160443 | 0.360037049637367 | 0.769848376906695 | 0.413474083300421 | 1.43338251987914 | 0.999228396881742 |
| ebi-fl187-GCST90094803 | ebi-met1400-GCST90199777 | 1-palmitoleoyl-GPC (16:1) levels \|\| id:ebi-met1400-GCST90199777 | Mushroom liking \|\| id:ebi-fl187-GCST90094803 | Weighted mode | 5 | 0.0824172180252942 | 0.315444484051117 | 0.806782787605114 | -0.535853971 | 0.700688406765484 | 1.08590877604533 | 0.585169359215965 | 2.01513946573043 | 0.999228396881742 |
| ebi-fl187-GCST90094803 | ebi-met1400-GCST90199782 | Glutamine degradant levels \|\| id:ebi-met1400-GCST90199782 | Mushroom liking \|\| id:ebi-fl187-GCST90094803 | Weighted mode | 5 | -0.269473903 | 0.273153042843011 | 0.379722622850159 | -0.804853867 | 0.265906060714432 | 0.763781211463604 | 0.447153265530389 | 1.30461249856435 | 0.999228396881742 |
| ebi-fl187-GCST90094803 | ebi-met1400-GCST90199783 | 5-methyluridine (ribothymidine) levels \|\| id:ebi-met1400-GCST90199783 | Mushroom liking \|\| id:ebi-fl187-GCST90094803 | Weighted mode | 5 | -0.38504206 | 0.349864450607146 | 0.332872057942477 | -1.070776383 | 0.300692263317821 | 0.680422017139661 | 0.342742314766582 | 1.35079358883278 | 0.999228396881742 |
| ebi-fl187-GCST90094803 | ebi-met1400-GCST90199787 | Homostachydrine levels \|\| id:ebi-met1400-GCST90199787 | Mushroom liking \|\| id:ebi-fl187-GCST90094803 | Weighted mode | 5 | 0.0556214996283068 | 0.311291973983109 | 0.866874297306053 | -0.554510769 | 0.665753768635201 | 1.05719745836772 | 0.574353183637844 | 1.9459567698399 | 0.999228396881742 |
| ebi-fl187-GCST90094803 | ebi-met1400-GCST90199802 | Beta-hydroxyisovaleroylcarnitine levels \|\| id:ebi-met1400-GCST90199802 | Mushroom liking \|\| id:ebi-fl187-GCST90094803 | Weighted mode | 5 | -0.355796935 | 0.325833552890663 | 0.336211601902316 | -0.994430698 | 0.282836828876787 | 0.700614876298881 | 0.369933988598245 | 1.32688863424329 | 0.999228396881742 |
| ebi-fl187-GCST90094803 | ebi-met1400-GCST90199873 | Pregnenolone sulfate levels \|\| id:ebi-met1400-GCST90199873 | Mushroom liking \|\| id:ebi-fl187-GCST90094803 | Weighted mode | 5 | -0.718116398 | 0.366452936683298 | 0.121604612688097 | -1.436364154 | 0.000131357661960263 | 0.487669967397402 | 0.237790759411581 | 1.00013136628976 | 0.999228396881742 |
| ebi-fl187-GCST90094803 | ebi-met1400-GCST90199911 | Beta-citrylglutamate levels \|\| id:ebi-met1400-GCST90199911 | Mushroom liking \|\| id:ebi-fl187-GCST90094803 | Weighted mode | 5 | 0.0740761623714006 | 0.340723028677676 | 0.838529426768707 | -0.593740974 | 0.741893298579645 | 1.07688882077927 | 0.552257434906235 | 2.0999075051226 | 0.999228396881742 |
| ebi-fl187-GCST90094803 | ebi-met1400-GCST90199934 | 2-hydroxydecanoate levels \|\| id:ebi-met1400-GCST90199934 | Mushroom liking \|\| id:ebi-fl187-GCST90094803 | Weighted mode | 5 | -0.2494823 | 0.329200697678729 | 0.490739949609941 | -0.894715667 | 0.395751067693769 | 0.779204072808999 | 0.408723795699207 | 1.48549948270924 | 0.999228396881742 |
| ebi-fl187-GCST90094803 | ebi-met1400-GCST90199945 | Sphingomyelin (d18:1/14:0, d16:1/16:0) levels \|\| id:ebi-met1400-GCST90199945 | Mushroom liking \|\| id:ebi-fl187-GCST90094803 | Weighted mode | 5 | 0.132960775403977 | 0.303124116205898 | 0.683577072669704 | -0.461162492 | 0.727084043167536 | 1.14220519491486 | 0.630550209481779 | 2.06903857563175 | 0.999228396881742 |
| ebi-fl187-GCST90094803 | ebi-met1400-GCST90199979 | Carnitine C14:1 levels \|\| id:ebi-met1400-GCST90199979 | Mushroom liking \|\| id:ebi-fl187-GCST90094803 | Weighted mode | 5 | 0.361926230420302 | 0.503046121517154 | 0.511645155811404 | -0.624044168 | 1.34789662859392 | 1.43609299807581 | 0.535773293242206 | 3.84932045911073 | 0.999228396881742 |
| ebi-fl187-GCST90094803 | ebi-met1400-GCST90199986 | Eugenol sulfate levels \|\| id:ebi-met1400-GCST90199986 | Mushroom liking \|\| id:ebi-fl187-GCST90094803 | Weighted mode | 5 | -0.053456336 | 0.322209798439276 | 0.876279366398965 | -0.684987541 | 0.578074868814962 | 0.947947331152977 | 0.504096510040345 | 1.78260337999192 | 0.999228396881742 |
| ebi-fl187-GCST90094803 | ebi-met1400-GCST90200002 | 6-hydroxyindole sulfate levels \|\| id:ebi-met1400-GCST90200002 | Mushroom liking \|\| id:ebi-fl187-GCST90094803 | Weighted mode | 5 | 0.102778107613409 | 0.364545333231494 | 0.791978777179119 | -0.611730746 | 0.817286960747136 | 1.10824547055868 | 0.542411280320944 | 2.26434823089798 | 0.999228396881742 |
| ebi-fl187-GCST90094803 | ebi-met1400-GCST90200023 | Trans 3,4-methyleneheptanoate levels \|\| id:ebi-met1400-GCST90200023 | Mushroom liking \|\| id:ebi-fl187-GCST90094803 | Weighted mode | 5 | -0.111362362 | 0.353480282549542 | 0.768477742408354 | -0.804183716 | 0.581458991393441 | 0.894614515521385 | 0.447453026182821 | 1.78864614730432 | 0.999228396881742 |
| ebi-fl187-GCST90094803 | ebi-met1400-GCST90200032 | 2-hydroxybutyrate/2-hydroxyisobutyrate levels \|\| id:ebi-met1400-GCST90200032 | Mushroom liking \|\| id:ebi-fl187-GCST90094803 | Weighted mode | 5 | 0.592110558697537 | 0.355705672261046 | 0.171322345300492 | -0.105072559 | 1.28929367632919 | 1.80779985956172 | 0.900259198368508 | 3.63022153859028 | 0.999228396881742 |
| ebi-fl187-GCST90094803 | ebi-met1400-GCST90200051 | 1-palmitoyl-2-dihomo-linolenoyl-GPC (16:0/20:3n3 or 6) levels \|\| id:ebi-met1400-GCST90200051 | Mushroom liking \|\| id:ebi-fl187-GCST90094803 | Weighted mode | 5 | -0.22184609 | 0.330960176756654 | 0.539372563043015 | -0.870528036 | 0.426835856398799 | 0.801038642669587 | 0.418730385939518 | 1.53240110724283 | 0.999228396881742 |
| ebi-fl187-GCST90094803 | ebi-met1400-GCST90200069 | Phenylacetylglutamate levels \|\| id:ebi-met1400-GCST90200069 | Mushroom liking \|\| id:ebi-fl187-GCST90094803 | Weighted mode | 5 | 0.114827719789189 | 0.370075884274888 | 0.771841381285612 | -0.610521013 | 0.840176452967969 | 1.12168017762752 | 0.543067849731302 | 2.31677574267197 | 0.999228396881742 |
| ebi-fl187-GCST90094803 | ebi-met1400-GCST90200072 | Thioproline levels \|\| id:ebi-met1400-GCST90200072 | Mushroom liking \|\| id:ebi-fl187-GCST90094803 | Weighted mode | 5 | -0.001497124 | 0.328185267721354 | 0.99657864448989 | -0.644740249 | 0.64174600027774 | 0.998503995675643 | 0.52479884132805 | 1.89979502785716 | 0.999228396881742 |
| ebi-fl187-GCST90094803 | ebi-met1400-GCST90200081 | 1-oleoyl-2-docosahexaenoyl-GPC (18:1/22:6) levels \|\| id:ebi-met1400-GCST90200081 | Mushroom liking \|\| id:ebi-fl187-GCST90094803 | Weighted mode | 5 | 0.373279025391583 | 0.306379441338673 | 0.29003343016889 | -0.22722468 | 0.973782730415381 | 1.4524895647657 | 0.796741750535017 | 2.6479419891534 | 0.999228396881742 |
| ebi-fl187-GCST90094803 | ebi-met1400-GCST90200097 | Furaneol sulfate levels \|\| id:ebi-met1400-GCST90200097 | Mushroom liking \|\| id:ebi-fl187-GCST90094803 | Weighted mode | 5 | -0.426878581 | 0.569115329029942 | 0.494919520504208 | -1.542344626 | 0.68858746364973 | 0.652542778347184 | 0.213879046686342 | 1.99090132563347 | 0.999228396881742 |
| ebi-fl187-GCST90094803 | ebi-met1400-GCST90200124 | Sphingomyelin (d18:2/24:2) levels \|\| id:ebi-met1400-GCST90200124 | Mushroom liking \|\| id:ebi-fl187-GCST90094803 | Weighted mode | 5 | 0.0472596407690862 | 0.324818775597657 | 0.891357030590573 | -0.589385159 | 0.683904440940494 | 1.04839417961594 | 0.554668212450923 | 1.98159968640683 | 0.999228396881742 |
| ebi-fl187-GCST90094803 | ebi-met1400-GCST90200137 | Lignoceroylcarnitine (C24) levels \|\| id:ebi-met1400-GCST90200137 | Mushroom liking \|\| id:ebi-fl187-GCST90094803 | Weighted mode | 5 | 0.407868154371568 | 0.320267636982635 | 0.271805082525865 | -0.219856414 | 1.03559272285753 | 1.50360890383987 | 0.802634036607921 | 2.81677530803612 | 0.999228396881742 |
| ebi-fl187-GCST90094803 | ebi-met1400-GCST90200165 | Octadecenedioate (C18:1-DC) levels \|\| id:ebi-met1400-GCST90200165 | Mushroom liking \|\| id:ebi-fl187-GCST90094803 | Weighted mode | 5 | -0.377782425 | 0.399790559145238 | 0.398189953826958 | -1.161371921 | 0.405807070921796 | 0.685379605910285 | 0.31305639751237 | 1.50051303193437 | 0.999228396881742 |
| ebi-fl187-GCST90094803 | ebi-met1400-GCST90200176 | Glutamine conjugate of C6H10O2 (1) levels \|\| id:ebi-met1400-GCST90200176 | Mushroom liking \|\| id:ebi-fl187-GCST90094803 | Weighted mode | 5 | -0.027066324 | 0.332416839820054 | 0.9390170670346 | -0.67860333 | 0.624470682293927 | 0.973296686694189 | 0.507325063577171 | 1.86725732344148 | 0.999228396881742 |
| ebi-fl187-GCST90094803 | ebi-met1400-GCST90200182 | Glycine conjugate of C10H12O2 levels \|\| id:ebi-met1400-GCST90200182 | Mushroom liking \|\| id:ebi-fl187-GCST90094803 | Weighted mode | 5 | -0.146824111 | 0.34927079454961 | 0.695813091021242 | -0.831394868 | 0.537746646140256 | 0.863445834532521 | 0.43544147891437 | 1.71214444482945 | 0.999228396881742 |
| ebi-fl187-GCST90094803 | ebi-met1400-GCST90200208 | Glucuronide of piperine metabolite C17H21NO3 (4) levels \|\| id:ebi-met1400-GCST90200208 | Mushroom liking \|\| id:ebi-fl187-GCST90094803 | Weighted mode | 5 | 0.282515469626995 | 0.424203128667392 | 0.541859236477791 | -0.548922663 | 1.11395360181508 | 1.32646229481496 | 0.577571714951968 | 3.04637878555407 | 0.999228396881742 |
| ebi-fl187-GCST90094803 | ebi-met1400-GCST90200227 | 3-ethylcatechol sulfate (2) levels \|\| id:ebi-met1400-GCST90200227 | Mushroom liking \|\| id:ebi-fl187-GCST90094803 | Weighted mode | 4 | 0.140642681540005 | 0.441131951007802 | 0.770769701166329 | -0.723975942 | 1.0052613055153 | 1.1510132961989 | 0.484820799234798 | 2.73262122853981 | 0.999228396881742 |
| ebi-fl187-GCST90094803 | ebi-met1400-GCST90200259 | Pentose acid levels \|\| id:ebi-met1400-GCST90200259 | Mushroom liking \|\| id:ebi-fl187-GCST90094803 | Weighted mode | 5 | -0.152046828 | 0.411708102966474 | 0.730617818609554 | -0.95899471 | 0.654901053496403 | 0.858948056706672 | 0.383277997855948 | 1.924952041 | 0.999228396881742 |
| ebi-fl187-GCST90094803 | ebi-met1400-GCST90200291 | Sarcosine levels \|\| id:ebi-met1400-GCST90200291 | Mushroom liking \|\| id:ebi-fl187-GCST90094803 | Weighted mode | 5 | 0.277267225613389 | 0.307513361750797 | 0.418229068138922 | -0.325458963 | 0.87999341464495 | 1.31951893318234 | 0.722195816105742 | 2.41088382983893 | 0.999228396881742 |
| ebi-fl187-GCST90094803 | ebi-met1400-GCST90200306 | Butyrate/isobutyrate (4:0) levels \|\| id:ebi-met1400-GCST90200306 | Mushroom liking \|\| id:ebi-fl187-GCST90094803 | Weighted mode | 5 | -0.173841264 | 0.341556024207821 | 0.637564840139537 | -0.843291071 | 0.495608543400376 | 0.840430293574408 | 0.43029206863001 | 1.64149685725419 | 0.999228396881742 |
| ebi-fl187-GCST90094803 | ebi-met1400-GCST90200321 | Cholate levels \|\| id:ebi-met1400-GCST90200321 | Mushroom liking \|\| id:ebi-fl187-GCST90094803 | Weighted mode | 5 | 0.0493625404724931 | 0.352834108030579 | 0.895498443067059 | -0.642192311 | 0.740917392212428 | 1.05060116714872 | 0.526137701139088 | 2.09785919166142 | 0.999228396881742 |
| ebi-fl187-GCST90094803 | ebi-met1400-GCST90200336 | Ceramide (d18:1/16:0) levels \|\| id:ebi-met1400-GCST90200336 | Mushroom liking \|\| id:ebi-fl187-GCST90094803 | Weighted mode | 5 | -0.053024698 | 0.289354584095051 | 0.863514411694901 | -0.620159683 | 0.514110286816288 | 0.948356589672505 | 0.537858543959154 | 1.67215010577868 | 0.999228396881742 |
| ebi-fl187-GCST90094803 | ebi-met1400-GCST90200358 | Arachidonate (20:4n6) levels \|\| id:ebi-met1400-GCST90200358 | Mushroom liking \|\| id:ebi-fl187-GCST90094803 | Weighted mode | 5 | 0.134619850544796 | 0.310729624002563 | 0.687181253714858 | -0.474410213 | 0.743649913589819 | 1.14410177200637 | 0.622251944680025 | 2.10359947590236 | 0.999228396881742 |
| ebi-fl187-GCST90094803 | ebi-met1400-GCST90200413 | 2'-deoxyuridine levels \|\| id:ebi-met1400-GCST90200413 | Mushroom liking \|\| id:ebi-fl187-GCST90094803 | Weighted mode | 5 | -0.209792485 | 0.310035883205988 | 0.535725296477379 | -0.817462816 | 0.397877846376009 | 0.81075247205104 | 0.441550529540575 | 1.48866217332091 | 0.999228396881742 |
| ebi-fl187-GCST90094803 | ebi-met1400-GCST90200432 | Threonine levels \|\| id:ebi-met1400-GCST90200432 | Mushroom liking \|\| id:ebi-fl187-GCST90094803 | Weighted mode | 5 | -0.152118173 | 0.436538026517192 | 0.745058691890353 | -1.007732705 | 0.703496358767159 | 0.85888677733922 | 0.365045708241824 | 2.02080583234654 | 0.999228396881742 |
| ebi-fl187-GCST90094803 | ebi-met1400-GCST90200436 | Caffeine levels \|\| id:ebi-met1400-GCST90200436 | Mushroom liking \|\| id:ebi-fl187-GCST90094803 | Weighted mode | 5 | 0.0117606280773305 | 0.33537512701922 | 0.973706421909397 | -0.645574621 | 0.669095877035001 | 1.01183005616942 | 0.524361146657119 | 1.95247124828888 | 0.999228396881742 |
| ebi-fl187-GCST90094803 | ebi-met1400-GCST90200442 | Valine levels \|\| id:ebi-met1400-GCST90200442 | Mushroom liking \|\| id:ebi-fl187-GCST90094803 | Weighted mode | 5 | 0.0724719347034624 | 0.436772685899641 | 0.876264174453659 | -0.78360253 | 0.928546399066759 | 1.07516263090866 | 0.456757561131401 | 2.53082768906779 | 0.999228396881742 |
| ebi-fl187-GCST90094803 | ebi-met1400-GCST90200459 | Margarate (17:0) levels \|\| id:ebi-met1400-GCST90200459 | Mushroom liking \|\| id:ebi-fl187-GCST90094803 | Weighted mode | 5 | 0.0911774893175824 | 0.307322089400448 | 0.781475421289266 | -0.511173806 | 0.693528784542461 | 1.09546342105963 | 0.599791127080237 | 2.00076335360516 | 0.999228396881742 |
| ebi-fl187-GCST90094803 | ebi-met1400-GCST90200462 | X-11299 levels \|\| id:ebi-met1400-GCST90200462 | Mushroom liking \|\| id:ebi-fl187-GCST90094803 | Weighted mode | 5 | 0.204638266163152 | 0.311941032794997 | 0.547632541039885 | -0.406766158 | 0.816042690441346 | 1.22708110791359 | 0.665799863994499 | 2.26153252174722 | 0.999228396881742 |
| ebi-fl187-GCST90094803 | ebi-met1400-GCST90200497 | X-12740 levels \|\| id:ebi-met1400-GCST90200497 | Mushroom liking \|\| id:ebi-fl187-GCST90094803 | Weighted mode | 5 | -0.234041586 | 0.368046933179247 | 0.559404054616302 | -0.955413575 | 0.487330402602152 | 0.791328906667771 | 0.384653028598586 | 1.62796440420465 | 0.999228396881742 |
| ebi-fl187-GCST90094803 | ebi-met1400-GCST90200527 | X-13866 levels \|\| id:ebi-met1400-GCST90200527 | Mushroom liking \|\| id:ebi-fl187-GCST90094803 | Weighted mode | 5 | -0.116099053 | 0.365628053328931 | 0.766723669607361 | -0.832730038 | 0.600531931367299 | 0.89038702328333 | 0.434860478821615 | 1.82308830036624 | 0.999228396881742 |
| ebi-fl187-GCST90094803 | ebi-met1400-GCST90200547 | X-17654 levels \|\| id:ebi-met1400-GCST90200547 | Mushroom liking \|\| id:ebi-fl187-GCST90094803 | Weighted mode | 5 | 0.25333958145957 | 0.343411564459137 | 0.501624333138916 | -0.419747085 | 0.926426247799478 | 1.28832069238244 | 0.657213017906233 | 2.52546763560542 | 0.999228396881742 |
| ebi-fl187-GCST90094803 | ebi-met1400-GCST90200553 | X-17653 levels \|\| id:ebi-met1400-GCST90200553 | Mushroom liking \|\| id:ebi-fl187-GCST90094803 | Weighted mode | 5 | 0.248904059242327 | 0.359939128581903 | 0.527281755327537 | -0.456576633 | 0.954384751262856 | 1.28261897173065 | 0.633448464617798 | 2.59707224586297 | 0.999228396881742 |
| ebi-fl187-GCST90094803 | ebi-met1400-GCST90200557 | X-17685 levels \|\| id:ebi-met1400-GCST90200557 | Mushroom liking \|\| id:ebi-fl187-GCST90094803 | Weighted mode | 5 | 0.270487173307404 | 0.291480445398498 | 0.405945575181526 | -0.3008145 | 0.841788846288461 | 1.3106027859246 | 0.740215070149037 | 2.32051430961475 | 0.999228396881742 |
| ebi-fl187-GCST90094803 | ebi-met1400-GCST90200561 | X-18779 levels \|\| id:ebi-met1400-GCST90200561 | Mushroom liking \|\| id:ebi-fl187-GCST90094803 | Weighted mode | 5 | -0.007544332 | 0.313275027707475 | 0.981940579331743 | -0.621563386 | 0.606474722237968 | 0.992484054972552 | 0.537104079663488 | 1.83395478952963 | 0.999228396881742 |
| ebi-fl187-GCST90094803 | ebi-met1400-GCST90200570 | X-18921 levels \|\| id:ebi-met1400-GCST90200570 | Mushroom liking \|\| id:ebi-fl187-GCST90094803 | Weighted mode | 5 | -0.207061617 | 0.380486836572615 | 0.615217408395277 | -0.952815817 | 0.538692582296788 | 0.812969555385713 | 0.385653563242851 | 1.71376479041594 | 0.999228396881742 |
| ebi-fl187-GCST90094803 | ebi-met1400-GCST90200575 | X-21283 levels \|\| id:ebi-met1400-GCST90200575 | Mushroom liking \|\| id:ebi-fl187-GCST90094803 | Weighted mode | 5 | -0.169870616 | 0.344196206376083 | 0.64750895765819 | -0.84449518 | 0.504753948497384 | 0.843773980387107 | 0.429774261886878 | 1.65657786683768 | 0.999228396881742 |
| ebi-fl187-GCST90094803 | ebi-met1400-GCST90200578 | X-18922 levels \|\| id:ebi-met1400-GCST90200578 | Mushroom liking \|\| id:ebi-fl187-GCST90094803 | Weighted mode | 5 | -0.159415183 | 0.334321850367045 | 0.65836343516227 | -0.81468601 | 0.495855643356034 | 0.852642282580268 | 0.442778333469222 | 1.64190252117253 | 0.999228396881742 |
| ebi-fl187-GCST90094803 | ebi-met1400-GCST90200623 | X-24241 levels \|\| id:ebi-met1400-GCST90200623 | Mushroom liking \|\| id:ebi-fl187-GCST90094803 | Weighted mode | 5 | -0.477257458 | 0.393744915490428 | 0.292172784025536 | -1.248997492 | 0.294482576756513 | 0.620482760722154 | 0.286792164240818 | 1.34243157365382 | 0.999228396881742 |
| ebi-fl187-GCST90094803 | ebi-met1400-GCST90200636 | X-24544 levels \|\| id:ebi-met1400-GCST90200636 | Mushroom liking \|\| id:ebi-fl187-GCST90094803 | Weighted mode | 5 | -0.234658698 | 0.299847922792682 | 0.477603277803275 | -0.822360626 | 0.353043231068634 | 0.790840719404094 | 0.439393186168478 | 1.42339267689002 | 0.999228396881742 |
| ebi-fl187-GCST90094803 | ebi-met1400-GCST90200659 | X-25172 levels \|\| id:ebi-met1400-GCST90200659 | Mushroom liking \|\| id:ebi-fl187-GCST90094803 | Weighted mode | 5 | -0.11458904 | 0.310680634303065 | 0.730945521172321 | -0.723523084 | 0.494345002807999 | 0.891732534638335 | 0.485040404309455 | 1.63942406914452 | 0.999228396881742 |
| ebi-fl187-GCST90094803 | ebi-met1400-GCST90200670 | X-26111 levels \|\| id:ebi-met1400-GCST90200670 | Mushroom liking \|\| id:ebi-fl187-GCST90094803 | Weighted mode | 5 | 0.248410607569532 | 0.35025256400463 | 0.517331699121813 | -0.438084418 | 0.934905633018606 | 1.28198621738318 | 0.645271308122445 | 2.54697309623531 | 0.999228396881742 |
| ebi-fl187-GCST90094803 | ebi-met1400-GCST90200673 | Carnitine C4 levels \|\| id:ebi-met1400-GCST90200673 | Mushroom liking \|\| id:ebi-fl187-GCST90094803 | Weighted mode | 5 | -0.435593305 | 0.351017290429352 | 0.282432682436826 | -1.123587194 | 0.252400584423087 | 0.646880755731737 | 0.325111462451828 | 1.28711153085864 | 0.999228396881742 |
| ebi-fl187-GCST90094803 | ebi-met1400-GCST90200710 | X-13431 levels \|\| id:ebi-met1400-GCST90200710 | Mushroom liking \|\| id:ebi-fl187-GCST90094803 | Weighted mode | 5 | -0.058362056 | 0.341382831599017 | 0.872556415834602 | -0.727472406 | 0.610748294017792 | 0.943308355231902 | 0.483128601125762 | 1.84180909798525 | 0.999228396881742 |
| ebi-fl187-GCST90094803 | ebi-met1400-GCST90200730 | Adenosine 5'-diphosphate (ADP) to fructose ratio \|\| id:ebi-met1400-GCST90200730 | Mushroom liking \|\| id:ebi-fl187-GCST90094803 | Weighted mode | 5 | 0.166611018386697 | 0.454803711593271 | 0.732668435014472 | -0.724804256 | 1.05802629310951 | 1.18129467401979 | 0.484419381700194 | 2.88067975721741 | 0.999228396881742 |
| ebi-fl187-GCST90094803 | ebi-met1400-GCST90200731 | Adenosine 5'-diphosphate (ADP) to mannose ratio \|\| id:ebi-met1400-GCST90200731 | Mushroom liking \|\| id:ebi-fl187-GCST90094803 | Weighted mode | 5 | 0.358969268616497 | 0.540727429385975 | 0.543086902898565 | -0.700856493 | 1.41879503021301 | 1.43185279807806 | 0.496160164055324 | 4.13213833736029 | 0.999228396881742 |
| ebi-fl187-GCST90094803 | ebi-met1400-GCST90200748 | Aspartate to glutamate ratio \|\| id:ebi-met1400-GCST90200748 | Mushroom liking \|\| id:ebi-fl187-GCST90094803 | Weighted mode | 5 | -0.096270206 | 0.381743109519559 | 0.813325492052903 | -0.844486701 | 0.651946288697706 | 0.908218576837612 | 0.429777906335594 | 1.91927265490665 | 0.999228396881742 |
| ebi-fl187-GCST90094803 | ebi-met1400-GCST90200774 | Uridine to pseudouridine ratio \|\| id:ebi-met1400-GCST90200774 | Mushroom liking \|\| id:ebi-fl187-GCST90094803 | Weighted mode | 5 | 0.159707201130539 | 0.335226086171063 | 0.658634599952093 | -0.497335928 | 0.816750330025822 | 1.17316731863381 | 0.608148655474851 | 2.26313343805026 | 0.999228396881742 |
| ebi-fl187-GCST90094803 | ebi-met1400-GCST90200799 | Spermidine to adenosine 5'-diphosphate (ADP) ratio \|\| id:ebi-met1400-GCST90200799 | Mushroom liking \|\| id:ebi-fl187-GCST90094803 | Weighted mode | 5 | -0.845790978 | 0.563084036602412 | 0.207492195707534 | -1.94943569 | 0.257853733272531 | 0.42921772192974 | 0.14235438089566 | 1.29414951376584 | 0.999228396881742 |
| ebi-fl187-GCST90094803 | ebi-met1400-GCST90200811 | Choline to taurocholate ratio \|\| id:ebi-met1400-GCST90200811 | Mushroom liking \|\| id:ebi-fl187-GCST90094803 | Weighted mode | 5 | -0.047593682 | 0.309902767801917 | 0.885380354350577 | -0.655003107 | 0.559815743208816 | 0.953521141483067 | 0.519440448903985 | 1.75034995671512 | 0.999228396881742 |
| ebi-fl187-GCST90094803 | ebi-met1400-GCST90200812 | Glutamate to kynurenine ratio \|\| id:ebi-met1400-GCST90200812 | Mushroom liking \|\| id:ebi-fl187-GCST90094803 | Weighted mode | 5 | -0.155138118 | 0.317168500204764 | 0.650355191387553 | -0.776788379 | 0.466512141963676 | 0.856296898925723 | 0.459880604401182 | 1.59442336139525 | 0.999228396881742 |
| ebi-fl187-GCST90094803 | ebi-met1400-GCST90200876 | Cortisone to 4-cholesten-3-one ratio \|\| id:ebi-met1400-GCST90200876 | Mushroom liking \|\| id:ebi-fl187-GCST90094803 | Weighted mode | 5 | -0.313288244 | 0.453538460600528 | 0.527707519623632 | -1.202223627 | 0.575647138674472 | 0.731039164468992 | 0.30052521244487 | 1.77828094900875 | 0.999228396881742 |
| ebi-fl187-GCST90094803 | ebi-met1400-GCST90200906 | 2'-deoxyuridine to cytidine ratio \|\| id:ebi-met1400-GCST90200906 | Mushroom liking \|\| id:ebi-fl187-GCST90094803 | Weighted mode | 5 | 0.0357848928734961 | 0.352279768199822 | 0.923977667256219 | -0.654683453 | 0.726253238545147 | 1.03643287841248 | 0.519606516545828 | 2.0673203226843 | 0.999228396881742 |
| ebi-fl187-GCST90094803 | ebi-met1400-GCST90200907 | Retinol (Vitamin A) to linoleoyl-arachidonoyl-glycerol (18:2 to 20:4) [1] ratio \|\| id:ebi-met1400-GCST90200907 | Mushroom liking \|\| id:ebi-fl187-GCST90094803 | Weighted mode | 5 | -0.076036418 | 0.321296110994961 | 0.824549495263751 | -0.705776796 | 0.553703959102527 | 0.926782453966544 | 0.493724901730154 | 1.7396848203733 | 0.999228396881742 |
| ebi-fl187-GCST90094803 | ebi-met1400-GCST90200916 | Fructose to sucrose ratio \|\| id:ebi-met1400-GCST90200916 | Mushroom liking \|\| id:ebi-fl187-GCST90094803 | Weighted mode | 5 | -0.461524047 | 0.441447327545225 | 0.354816743409182 | -1.326760809 | 0.403712715049514 | 0.630322272374244 | 0.26533534267793 | 1.49737371222834 | 0.999228396881742 |
| ebi-fl187-GCST90094803 | ebi-met1400-GCST90200917 | Alpha-ketobutyrate to 3-methyl-2-oxobutyrate ratio \|\| id:ebi-met1400-GCST90200917 | Mushroom liking \|\| id:ebi-fl187-GCST90094803 | Weighted mode | 5 | 0.604901520228302 | 0.429509431484867 | 0.231799804483474 | -0.236936965 | 1.44674000593864 | 1.83107187646601 | 0.789041023270629 | 4.24923941582541 | 0.999228396881742 |
| ebi-fl187-GCST90094803 | ebi-met1400-GCST90200924 | Alpha-tocopherol to glycerol ratio \|\| id:ebi-met1400-GCST90200924 | Mushroom liking \|\| id:ebi-fl187-GCST90094803 | Weighted mode | 5 | -0.041862112 | 0.288907054927487 | 0.891799090455651 | -0.60811994 | 0.524395715629781 | 0.95900200629781 | 0.544373362353382 | 1.689437639 | 0.999228396881742 |
| ebi-fl187-GCST90094803 | ebi-met1400-GCST90200938 | Alpha-ketoglutarate to alanine ratio \|\| id:ebi-met1400-GCST90200938 | Mushroom liking \|\| id:ebi-fl187-GCST90094803 | Weighted mode | 5 | -0.156646904 | 0.318463957972166 | 0.648574221568964 | -0.780836262 | 0.467542453648825 | 0.855005904709175 | 0.458022824204473 | 1.5960669609801 | 0.999228396881742 |
| ebi-fl187-GCST90094803 | ebi-met1400-GCST90200943 | Phosphate to EDTA ratio \|\| id:ebi-met1400-GCST90200943 | Mushroom liking \|\| id:ebi-fl187-GCST90094803 | Weighted mode | 5 | 0.39026487540277 | 0.408620810184406 | 0.393622619002098 | -0.410631913 | 1.19116166336421 | 1.47737206158161 | 0.663231013683037 | 3.29090190795118 | 0.999228396881742 |
| ebi-fl187-GCST90094803 | ebi-met1400-GCST90200947 | Adenosine 5'-diphosphate (ADP) to glucose ratio \|\| id:ebi-met1400-GCST90200947 | Mushroom liking \|\| id:ebi-fl187-GCST90094803 | Weighted mode | 5 | 0.575069955808021 | 0.431001021062122 | 0.253009427945029 | -0.269692045 | 1.41983195708978 | 1.77725485186458 | 0.763614616709031 | 4.13642528490229 | 0.999228396881742 |
| ebi-fl187-GCST90094803 | ebi-met1400-GCST90200975 | Arachidonate (20:4n6) to caffeine ratio \|\| id:ebi-met1400-GCST90200975 | Mushroom liking \|\| id:ebi-fl187-GCST90094803 | Weighted mode | 5 | -0.024528536 | 0.310893367781053 | 0.940903903409234 | -0.633879537 | 0.584822464363353 | 0.975769843477151 | 0.530529594019771 | 1.79467233905889 | 0.999228396881742 |
| ebi-fl187-GCST90094803 | ebi-met1400-GCST90200985 | Cholesterol to oleoyl-linoleoyl-glycerol (18:1 to 18:2) [2] ratio \|\| id:ebi-met1400-GCST90200985 | Mushroom liking \|\| id:ebi-fl187-GCST90094803 | Weighted mode | 5 | 0.139410716351987 | 0.289939212514681 | 0.655756311669126 | -0.42887014 | 0.707691572880762 | 1.14959616099689 | 0.651244494184999 | 2.02930135329999 | 0.999228396881742 |
| ebi-fl187-GCST90094803 | ebi-met1400-GCST90201016 | Bilirubin (Z,Z) to etiocholanolone glucuronide ratio \|\| id:ebi-met1400-GCST90201016 | Mushroom liking \|\| id:ebi-fl187-GCST90094803 | Weighted mode | 5 | 0.218802164246916 | 0.339860953513839 | 0.554761523929864 | -0.447325305 | 0.88492963313404 | 1.24458502891355 | 0.639335895509415 | 2.42281389966621 | 0.999228396881742 |
| ebi-fl187-GCST90094803 | ebi-pw11-GCST90275189 | Placental weight (fetal genotype, adjusted for sex and gestational age) \|\| id:ebi-pw11-GCST90275189 | Mushroom liking \|\| id:ebi-fl187-GCST90094803 | Weighted mode | 6 | 0.00537310019666604 | 0.12933106916553 | 0.968469123672616 | -0.248115795 | 0.258861995761105 | 1.00538756118805 | 0.78026958644296 | 1.29545501420814 | 0.999228396881742 |
| ebi-fl187-GCST90094803 | ebi-pw11-GCST90275192 | Placental weight (fetal effect, conditional on maternal and paternal genotype) \|\| id:ebi-pw11-GCST90275192 | Mushroom liking \|\| id:ebi-fl187-GCST90094803 | Weighted mode | 6 | 0.0368587954689528 | 0.210778395197209 | 0.868042489457393 | -0.376266859 | 0.449984450055482 | 1.03754650422643 | 0.686419131395182 | 1.56828779851231 | 0.999228396881742 |
| ebi-fl187-GCST90094803 | ebi-pw11-GCST90275193 | Placental weight (maternal effect, conditional on fetal and paternal genotype) \|\| id:ebi-pw11-GCST90275193 | Mushroom liking \|\| id:ebi-fl187-GCST90094803 | Weighted mode | 6 | 0.00830036242039367 | 0.169301578772081 | 0.96279586668195 | -0.323530732 | 0.340131456813672 | 1.00833490593697 | 0.723589720241018 | 1.40513229263713 | 0.999228396881742 |
| ebi-fl187-GCST90094803 | ebi-pw11-GCST90275195 | Placental weight (fetal effect, conditional on maternal genotype) \|\| id:ebi-pw11-GCST90275195 | Mushroom liking \|\| id:ebi-fl187-GCST90094803 | Weighted mode | 6 | -0.047498681 | 0.17308590149689 | 0.794736985946394 | -0.386747048 | 0.291749685769188 | 0.953611730788594 | 0.679262894037848 | 1.33876786304619 | 0.999228396881742 |
| ebi-fl187-GCST90094803 | ebi-pw11-GCST90275197 | Placental weight (fetal genotype, adjusted for sex) \|\| id:ebi-pw11-GCST90275197 | Mushroom liking \|\| id:ebi-fl187-GCST90094803 | Weighted mode | 6 | 0.0536911132696116 | 0.10710167206204 | 0.637440205625673 | -0.156228164 | 0.26361039051121 | 1.05515862731459 | 0.855364004855872 | 1.3016209736158 | 0.999228396881742 |
| ebi-fl187-GCST90094803 | prot-a-1030 | Protein FAM171B \|\| id:prot-a-1030 | Mushroom liking \|\| id:ebi-fl187-GCST90094803 | Weighted mode | 7 | -0.1359011 | 0.391477098679357 | 0.740331351321323 | -0.903196214 | 0.63139401302108 | 0.872928960553097 | 0.40527225068553 | 1.88022981806269 | 0.999228396881742 |
| ebi-fl187-GCST90094803 | prot-a-1036 | BRISC complex subunit Abro1 \|\| id:prot-a-1036 | Mushroom liking \|\| id:ebi-fl187-GCST90094803 | Weighted mode | 7 | 0.320530018212358 | 0.342216129018351 | 0.385099489266765 | -0.350213595 | 0.991273631088326 | 1.37785786059686 | 0.704537588176975 | 2.69466429594056 | 0.999228396881742 |
| ebi-fl187-GCST90094803 | prot-a-1055 | Phenylalanine--tRNA ligase, mitochondrial \|\| id:prot-a-1055 | Mushroom liking \|\| id:ebi-fl187-GCST90094803 | Weighted mode | 7 | -0.151435904 | 0.360065301755029 | 0.688709937095638 | -0.857163895 | 0.554292087799392 | 0.85947296959593 | 0.424363917841473 | 1.74070827987311 | 0.999228396881742 |
| ebi-fl187-GCST90094803 | prot-a-1083 | Fc receptor-like protein 5 \|\| id:prot-a-1083 | Mushroom liking \|\| id:ebi-fl187-GCST90094803 | Weighted mode | 7 | -0.058486944 | 0.355104252672349 | 0.874586057859608 | -0.754491279 | 0.637517391320629 | 0.943190554693279 | 0.470249779756591 | 1.89177849891411 | 0.999228396881742 |
| ebi-fl187-GCST90094803 | prot-a-1103 | Fibroblast growth factor receptor 3 \|\| id:prot-a-1103 | Mushroom liking \|\| id:ebi-fl187-GCST90094803 | Weighted mode | 7 | -0.195870317 | 0.370195279624476 | 0.615729891013676 | -0.921453065 | 0.529712430782717 | 0.82211884237206 | 0.397940387389131 | 1.69844381822511 | 0.999228396881742 |
| ebi-fl187-GCST90094803 | prot-a-1105 | Fibroblast growth factor receptor 4 \|\| id:prot-a-1105 | Mushroom liking \|\| id:ebi-fl187-GCST90094803 | Weighted mode | 7 | 0.0883739210097727 | 0.386855070685525 | 0.826889358199609 | -0.669862018 | 0.846609859553402 | 1.09239651567817 | 0.511779189469528 | 2.33172855016384 | 0.999228396881742 |
| ebi-fl187-GCST90094803 | prot-a-1120 | Filamin-A \|\| id:prot-a-1120 | Mushroom liking \|\| id:ebi-fl187-GCST90094803 | Weighted mode | 7 | 0.142381245417571 | 0.351865761613831 | 0.699767915839942 | -0.547275647 | 0.832038138180681 | 1.1530161468757 | 0.578523768162413 | 2.29799760721821 | 0.999228396881742 |
| ebi-fl187-GCST90094803 | prot-a-1166 | Galanin peptides \|\| id:prot-a-1166 | Mushroom liking \|\| id:ebi-fl187-GCST90094803 | Weighted mode | 7 | 0.0355563104181142 | 0.370399001754219 | 0.92665081797788 | -0.690425733 | 0.761538353856384 | 1.03619599511501 | 0.501362577019866 | 2.14156817741473 | 0.999228396881742 |
| ebi-fl187-GCST90094803 | prot-a-1205 | ADP-ribosylation factor-binding protein GGA1 \|\| id:prot-a-1205 | Mushroom liking \|\| id:ebi-fl187-GCST90094803 | Weighted mode | 7 | -0.037051441 | 0.368547012364491 | 0.923195714701367 | -0.759403585 | 0.685300703419577 | 0.963626564338421 | 0.467945433452489 | 1.98436845220961 | 0.999228396881742 |
| ebi-fl187-GCST90094803 | prot-a-1227 | Guanine nucleotide-binding protein G(I)/G(S)/G(O) subunit gamma-T2 \|\| id:prot-a-1227 | Mushroom liking \|\| id:ebi-fl187-GCST90094803 | Weighted mode | 7 | -0.168572537 | 0.359174669592507 | 0.65540152391476 | -0.872554889 | 0.535409815545663 | 0.844869976984637 | 0.417882540616105 | 1.70814812448882 | 0.999228396881742 |
| ebi-fl187-GCST90094803 | prot-a-1240 | Golgi SNAP receptor complex member 1 \|\| id:prot-a-1240 | Mushroom liking \|\| id:ebi-fl187-GCST90094803 | Weighted mode | 7 | 0.461679019490442 | 0.390774089510679 | 0.282126392080769 | -0.304238196 | 1.22759623493137 | 1.58673591017637 | 0.737685131910637 | 3.41301558040378 | 0.999228396881742 |
| ebi-fl187-GCST90094803 | prot-a-1283 | Glutathione S-transferase A1 \|\| id:prot-a-1283 | Mushroom liking \|\| id:ebi-fl187-GCST90094803 | Weighted mode | 7 | -0.015938124 | 0.338088118778979 | 0.963930068719188 | -0.678590837 | 0.646714589095912 | 0.984188216085857 | 0.507331401772357 | 1.90925781707652 | 0.999228396881742 |
| ebi-fl187-GCST90094803 | prot-a-1324 | E3 ubiquitin-protein ligase HECW1 \|\| id:prot-a-1324 | Mushroom liking \|\| id:ebi-fl187-GCST90094803 | Weighted mode | 7 | 0.27561458108034 | 0.346953732227585 | 0.457232411757653 | -0.404414734 | 0.955643896246406 | 1.31734003839636 | 0.667367283905617 | 2.60034439597665 | 0.999228396881742 |
| ebi-fl187-GCST90094803 | prot-a-1339 | Histidine triad nucleotide-binding protein 2, mitochondrial \|\| id:prot-a-1339 | Mushroom liking \|\| id:ebi-fl187-GCST90094803 | Weighted mode | 7 | -0.286449624 | 0.359257417732792 | 0.455641059403563 | -0.990594163 | 0.417694915001932 | 0.750924906355824 | 0.371355979658125 | 1.51845734517222 | 0.999228396881742 |
| ebi-fl187-GCST90094803 | prot-a-1466 | Interleukin-11 \|\| id:prot-a-1466 | Mushroom liking \|\| id:ebi-fl187-GCST90094803 | Weighted mode | 7 | -0.465105378 | 0.370467350435623 | 0.255991911598959 | -1.191221385 | 0.261010629015728 | 0.628068917157663 | 0.303849919695147 | 1.2982414643893 | 0.999228396881742 |
| ebi-fl187-GCST90094803 | prot-a-147 | Rho GTPase-activating protein 1 \|\| id:prot-a-147 | Mushroom liking \|\| id:ebi-fl187-GCST90094803 | Weighted mode | 7 | -0.049771821 | 0.353365296221097 | 0.892597549273477 | -0.742367802 | 0.64282415965946 | 0.951446499907542 | 0.475985540873986 | 1.90184441427386 | 0.999228396881742 |
| ebi-fl187-GCST90094803 | prot-a-1473 | Interleukin-12 receptor subunit beta-1 \|\| id:prot-a-1473 | Mushroom liking \|\| id:ebi-fl187-GCST90094803 | Weighted mode | 7 | -0.403500015 | 0.324432076505767 | 0.259992751774345 | -1.039386885 | 0.23238685491844 | 0.667978016757367 | 0.353671456778864 | 1.26160769357786 | 0.999228396881742 |
| ebi-fl187-GCST90094803 | prot-a-1501 | X-linked interleukin-1 receptor accessory protein-like 2 \|\| id:prot-a-1501 | Mushroom liking \|\| id:ebi-fl187-GCST90094803 | Weighted mode | 7 | -0.261438737 | 0.34860246143716 | 0.481638850697748 | -0.944699561 | 0.421822087733184 | 0.769943043243728 | 0.388796362907852 | 1.52473723109368 | 0.999228396881742 |
| ebi-fl187-GCST90094803 | prot-a-1503 | Interleukin-1 receptor-like 2 \|\| id:prot-a-1503 | Mushroom liking \|\| id:ebi-fl187-GCST90094803 | Weighted mode | 7 | -0.172168017 | 0.383261629652673 | 0.669044878513955 | -0.923360811 | 0.579024777301999 | 0.841837718391365 | 0.397181942059904 | 1.78429749457112 | 0.999228396881742 |
| ebi-fl187-GCST90094803 | prot-a-1536 | Interleukin-5 receptor subunit alpha \|\| id:prot-a-1536 | Mushroom liking \|\| id:ebi-fl187-GCST90094803 | Weighted mode | 7 | 0.246765058766782 | 0.339630856522629 | 0.494847672064672 | -0.41891142 | 0.912441537551135 | 1.27987838124728 | 0.657762457274264 | 2.49039551082363 | 0.999228396881742 |
| ebi-fl187-GCST90094803 | prot-a-1559 | Insulin-induced gene 1 protein \|\| id:prot-a-1559 | Mushroom liking \|\| id:ebi-fl187-GCST90094803 | Weighted mode | 7 | -0.041149024 | 0.326867791190954 | 0.903932284699583 | -0.681809895 | 0.599511846295591 | 0.95968610260803 | 0.505700897984045 | 1.82122954341293 | 0.999228396881742 |
| ebi-fl187-GCST90094803 | prot-a-1567 | Interferon regulatory factor 1 \|\| id:prot-a-1567 | Mushroom liking \|\| id:ebi-fl187-GCST90094803 | Weighted mode | 7 | -0.501540191 | 0.4260795099609 | 0.283728952993425 | -1.336656031 | 0.33357564805911 | 0.605597205401445 | 0.262722738011293 | 1.39595064350416 | 0.999228396881742 |
| ebi-fl187-GCST90094803 | prot-a-1613 | Potassium voltage-gated channel subfamily E regulatory beta subunit 5 \|\| id:prot-a-1613 | Mushroom liking \|\| id:ebi-fl187-GCST90094803 | Weighted mode | 7 | 0.103996093198896 | 0.354764340323679 | 0.779288568956653 | -0.591342014 | 0.799334200233307 | 1.10959611993577 | 0.553583868797801 | 2.22405965703133 | 0.999228396881742 |
| ebi-fl187-GCST90094803 | prot-a-1649 | Kin of IRRE-like protein 2 \|\| id:prot-a-1649 | Mushroom liking \|\| id:ebi-fl187-GCST90094803 | Weighted mode | 7 | 0.0703926333103881 | 0.333028413546325 | 0.839594185867198 | -0.582343057 | 0.723128323861184 | 1.07292936637208 | 0.55858802834193 | 2.06087020633197 | 0.999228396881742 |
| ebi-fl187-GCST90094803 | prot-a-165 | Armadillo repeat-containing protein 5 \|\| id:prot-a-165 | Mushroom liking \|\| id:ebi-fl187-GCST90094803 | Weighted mode | 7 | 0.610772030227748 | 0.405439229434218 | 0.182668867872761 | -0.183888859 | 1.40543291991882 | 1.84185281607155 | 0.832028270756709 | 4.07729149994552 | 0.999228396881742 |
| ebi-fl187-GCST90094803 | prot-a-1656 | Kallikrein-11 \|\| id:prot-a-1656 | Mushroom liking \|\| id:ebi-fl187-GCST90094803 | Weighted mode | 7 | -0.470690369 | 0.385212317942041 | 0.267569087797862 | -1.225706512 | 0.284325774612933 | 0.624570935277335 | 0.293550230364062 | 1.32886577097697 | 0.999228396881742 |
| ebi-fl187-GCST90094803 | prot-a-1697 | Laminin subunit gamma-2 \|\| id:prot-a-1697 | Mushroom liking \|\| id:ebi-fl187-GCST90094803 | Weighted mode | 7 | -0.185265333 | 0.357117635902825 | 0.622484821591949 | -0.8852159 | 0.514685232954058 | 0.830883793366913 | 0.412625077995939 | 1.67311177845227 | 0.999228396881742 |
| ebi-fl187-GCST90094803 | prot-a-1698 | Lysosome-associated membrane glycoprotein 3 \|\| id:prot-a-1698 | Mushroom liking \|\| id:ebi-fl187-GCST90094803 | Weighted mode | 7 | -0.197528371 | 0.352824653205426 | 0.595849119147523 | -0.889064692 | 0.494007948874141 | 0.820756854269406 | 0.411040022167155 | 1.63887158792597 | 0.999228396881742 |
| ebi-fl187-GCST90094803 | prot-a-1724 | Leptin receptor \|\| id:prot-a-1724 | Mushroom liking \|\| id:ebi-fl187-GCST90094803 | Weighted mode | 7 | -0.145703405 | 0.325639972991631 | 0.670259595492811 | -0.783957752 | 0.492550941681179 | 0.864414045721178 | 0.456595339252086 | 1.63648547894511 | 0.999228396881742 |
| ebi-fl187-GCST90094803 | prot-a-1743 | Leukocyte immunoglobulin-like receptor subfamily B member 2 \|\| id:prot-a-1743 | Mushroom liking \|\| id:ebi-fl187-GCST90094803 | Weighted mode | 7 | -0.740695821 | 0.432091555247714 | 0.137318357139162 | -1.587595269 | 0.106203627350892 | 0.476782045144981 | 0.204416588025853 | 1.11204829690182 | 0.999228396881742 |
| ebi-fl187-GCST90094803 | prot-a-1746 | Leukocyte immunoglobulin-like receptor subfamily B member 4 \|\| id:prot-a-1746 | Mushroom liking \|\| id:ebi-fl187-GCST90094803 | Weighted mode | 7 | -0.306357096 | 0.390297816988027 | 0.462351441639551 | -1.071340817 | 0.458626625480021 | 0.736123705820431 | 0.342548913919606 | 1.58189995136864 | 0.999228396881742 |
| ebi-fl187-GCST90094803 | prot-a-1750 | Leucine-rich repeat and immunoglobulin-like domain-containing nogo receptor-interacting protein 3 \|\| id:prot-a-1750 | Mushroom liking \|\| id:ebi-fl187-GCST90094803 | Weighted mode | 7 | 0.0128378949446681 | 0.373869661762793 | 0.973721553678209 | -0.719946642 | 0.745622431999742 | 1.01292065449156 | 0.486778228726025 | 2.10775295966056 | 0.999228396881742 |
| ebi-fl187-GCST90094803 | prot-a-1753 | Lipase member N \|\| id:prot-a-1753 | Mushroom liking \|\| id:ebi-fl187-GCST90094803 | Weighted mode | 7 | 0.266988455744922 | 0.348403229748779 | 0.472548161049532 | -0.415881875 | 0.949858786052528 | 1.30602536915363 | 0.659758200107473 | 2.58534454682793 | 0.999228396881742 |
| ebi-fl187-GCST90094803 | prot-a-1760 | Ig Kappa chain V-I region HK102- like \|\| id:prot-a-1760 | Mushroom liking \|\| id:ebi-fl187-GCST90094803 | Weighted mode | 7 | -0.235101412 | 0.365994345429845 | 0.544385058363509 | -0.952450329 | 0.482247504728627 | 0.790490680074757 | 0.385794540642297 | 1.61971062173331 | 0.999228396881742 |
| ebi-fl187-GCST90094803 | prot-a-1792 | Leucine-rich repeat neuronal protein 1 \|\| id:prot-a-1792 | Mushroom liking \|\| id:ebi-fl187-GCST90094803 | Weighted mode | 7 | -0.115338674 | 0.455899786445323 | 0.808719772520131 | -1.008902255 | 0.778224907678495 | 0.891064312702423 | 0.364619018597292 | 2.17760338565548 | 0.999228396881742 |
| ebi-fl187-GCST90094803 | prot-a-1795 | Leucine-rich repeat transmembrane neuronal protein 1 \|\| id:prot-a-1795 | Mushroom liking \|\| id:ebi-fl187-GCST90094803 | Weighted mode | 7 | -0.410819563 | 0.349058449056056 | 0.283791248161866 | -1.094974123 | 0.273334997315317 | 0.663106569876666 | 0.334548263947237 | 1.31434047161263 | 0.999228396881742 |
| ebi-fl187-GCST90094803 | prot-a-1820 | LysM and putative peptidoglycan-binding domain-containing protein 4 \|\| id:prot-a-1820 | Mushroom liking \|\| id:ebi-fl187-GCST90094803 | Weighted mode | 7 | -0.42055558 | 0.310153491659731 | 0.223920162816019 | -1.028456424 | 0.187345263533251 | 0.656681879050479 | 0.357558453552622 | 1.20604361605397 | 0.999228396881742 |
| ebi-fl187-GCST90094803 | prot-a-1844 | Mitogen-activated protein kinase kinase kinase 7:TGF-beta-activated kinase 1 and MAP3K7-binding protein 1 fusion \|\| id:prot-a-1844 | Mushroom liking \|\| id:ebi-fl187-GCST90094803 | Weighted mode | 7 | 0.0701969716199531 | 0.435459218933616 | 0.877225163432864 | -0.783303097 | 0.923697040729841 | 1.07271945573495 | 0.456894349517448 | 2.51858450849223 | 0.999228396881742 |
| ebi-fl187-GCST90094803 | prot-a-1856 | Megakaryocyte-associated tyrosine-protein kinase \|\| id:prot-a-1856 | Mushroom liking \|\| id:ebi-fl187-GCST90094803 | Weighted mode | 7 | 0.12618527617418 | 0.342944820863977 | 0.725544798362375 | -0.545986573 | 0.798357125067575 | 1.13449234321321 | 0.579270009349629 | 2.22188764485571 | 0.999228396881742 |
| ebi-fl187-GCST90094803 | prot-a-1910 | Stromelysin-2 \|\| id:prot-a-1910 | Mushroom liking \|\| id:ebi-fl187-GCST90094803 | Weighted mode | 7 | -0.294448167 | 0.391833651156829 | 0.480800371900115 | -1.062442123 | 0.473545789171533 | 0.744942557877119 | 0.345610754786254 | 1.60567750526083 | 0.999228396881742 |
| ebi-fl187-GCST90094803 | prot-a-1940 | 39S ribosomal protein L14, mitochondrial \|\| id:prot-a-1940 | Mushroom liking \|\| id:ebi-fl187-GCST90094803 | Weighted mode | 7 | -0.135136608 | 0.334284675137088 | 0.700039919673892 | -0.790334571 | 0.520061355345908 | 0.873596563324387 | 0.453692977286833 | 1.6821308542532 | 0.999228396881742 |
| ebi-fl187-GCST90094803 | prot-a-1942 | 39S ribosomal protein L33, mitochondrial \|\| id:prot-a-1942 | Mushroom liking \|\| id:ebi-fl187-GCST90094803 | Weighted mode | 7 | -0.078888196 | 0.350429840787087 | 0.829358554917343 | -0.765730684 | 0.607954292122139 | 0.924143241746163 | 0.46499404321682 | 1.83667026217555 | 0.999228396881742 |
| ebi-fl187-GCST90094803 | prot-a-1948 | Mesothelin \|\| id:prot-a-1948 | Mushroom liking \|\| id:ebi-fl187-GCST90094803 | Weighted mode | 7 | -0.202957799 | 0.35738375022437 | 0.590705776125453 | -0.90342995 | 0.497514351254901 | 0.816312689759644 | 0.405177535112269 | 1.6446282177959 | 0.999228396881742 |
| ebi-fl187-GCST90094803 | prot-a-1980 | Unconventional myosin-VI \|\| id:prot-a-1980 | Mushroom liking \|\| id:ebi-fl187-GCST90094803 | Weighted mode | 7 | 0.0457082326411644 | 0.373554669138825 | 0.906609222545734 | -0.686458919 | 0.77787538415326 | 1.04676895338499 | 0.503355339029437 | 2.17684239504337 | 0.999228396881742 |
| ebi-fl187-GCST90094803 | prot-a-2039 | Neurofascin \|\| id:prot-a-2039 | Mushroom liking \|\| id:ebi-fl187-GCST90094803 | Weighted mode | 7 | -0.589021756 | 0.370317644383062 | 0.16280668402472 | -1.314844339 | 0.136800826577944 | 0.554869817167147 | 0.268516117365259 | 1.1465997535794 | 0.999228396881742 |
| ebi-fl187-GCST90094803 | prot-a-2041 | NFU1 iron-sulfur cluster scaffold homolog, mitochondrial \|\| id:prot-a-2041 | Mushroom liking \|\| id:ebi-fl187-GCST90094803 | Weighted mode | 7 | -0.063841865 | 0.359828721489182 | 0.865014300064868 | -0.769106159 | 0.64142242876616 | 0.938153342349316 | 0.463427113174414 | 1.89918040775044 | 0.999228396881742 |
| ebi-fl187-GCST90094803 | prot-a-2088 | Glucocorticoid receptor \|\| id:prot-a-2088 | Mushroom liking \|\| id:ebi-fl187-GCST90094803 | Weighted mode | 7 | 0.121428740717572 | 0.346802328073987 | 0.738198734525236 | -0.558303822 | 0.801161303742587 | 1.1291089035681 | 0.572178758075074 | 2.22812695879471 | 0.999228396881742 |
| ebi-fl187-GCST90094803 | prot-a-2111 | Neuron-specific protein family member 2 \|\| id:prot-a-2111 | Mushroom liking \|\| id:ebi-fl187-GCST90094803 | Weighted mode | 7 | -0.042291567 | 0.369118689677196 | 0.912520418527946 | -0.765764199 | 0.681181064549003 | 0.95859024633134 | 0.464978459099218 | 1.97621038648052 | 0.999228396881742 |
| ebi-fl187-GCST90094803 | prot-a-2125 | Nuclear migration protein nudC \|\| id:prot-a-2125 | Mushroom liking \|\| id:ebi-fl187-GCST90094803 | Weighted mode | 7 | 0.103531307947898 | 0.360087660396234 | 0.783388667572912 | -0.602240506 | 0.809303122324516 | 1.10908051585653 | 0.547583396548301 | 2.24634201549257 | 0.999228396881742 |
| ebi-fl187-GCST90094803 | prot-a-2142 | Ornithine decarboxylase \|\| id:prot-a-2142 | Mushroom liking \|\| id:ebi-fl187-GCST90094803 | Weighted mode | 7 | 0.0652583693475242 | 0.353715867517978 | 0.859703967218987 | -0.628024731 | 0.758541469682762 | 1.06743478118705 | 0.533644852775498 | 2.13515975308617 | 0.999228396881742 |
| ebi-fl187-GCST90094803 | prot-a-2186 | Prostate and testis expressed protein 1 \|\| id:prot-a-2186 | Mushroom liking \|\| id:ebi-fl187-GCST90094803 | Weighted mode | 7 | 0.711000738746494 | 0.489340371848288 | 0.196447213480251 | -0.24810639 | 1.67010786756914 | 2.03602777134793 | 0.780276925140475 | 5.31274083871405 | 0.999228396881742 |
| ebi-fl187-GCST90094803 | prot-a-2227 | Platelet-derived growth factor C \|\| id:prot-a-2227 | Mushroom liking \|\| id:ebi-fl187-GCST90094803 | Weighted mode | 7 | 0.217536879729437 | 0.411698079597023 | 0.616193710247835 | -0.589391356 | 1.0244651157396 | 1.24301127058177 | 0.554664775250105 | 2.78560508569633 | 0.999228396881742 |
| ebi-fl187-GCST90094803 | prot-a-2229 | Platelet-derived growth factor receptor alpha \|\| id:prot-a-2229 | Mushroom liking \|\| id:ebi-fl187-GCST90094803 | Weighted mode | 7 | 0.491734278269736 | 0.373619840676549 | 0.23617252026853 | -0.240560609 | 1.22402916599577 | 1.63514956670067 | 0.786186993637821 | 3.40086280632762 | 0.999228396881742 |
| ebi-fl187-GCST90094803 | prot-a-2243 | Platelet endothelial aggregation receptor 1 \|\| id:prot-a-2243 | Mushroom liking \|\| id:ebi-fl187-GCST90094803 | Weighted mode | 7 | -0.142165179 | 0.364662748534199 | 0.710107561391742 | -0.856904166 | 0.572573808530309 | 0.867477955859706 | 0.4244741519237 | 1.7728240942167 | 0.999228396881742 |
| ebi-fl187-GCST90094803 | prot-a-2246 | E3 ubiquitin-protein ligase pellino homolog 2 \|\| id:prot-a-2246 | Mushroom liking \|\| id:ebi-fl187-GCST90094803 | Weighted mode | 7 | -0.100037802 | 0.363238550223455 | 0.792243700126865 | -0.811985361 | 0.611909755938935 | 0.904803213566847 | 0.443975738555011 | 1.8439495318942 | 0.999228396881742 |
| ebi-fl187-GCST90094803 | prot-a-2251 | Platelet factor 4 variant \|\| id:prot-a-2251 | Mushroom liking \|\| id:ebi-fl187-GCST90094803 | Weighted mode | 7 | 0.133635837598089 | 0.377150653836901 | 0.735211781124603 | -0.605579444 | 0.872851119118414 | 1.14297651477501 | 0.545758098788081 | 2.39372593137551 | 0.999228396881742 |
| ebi-fl187-GCST90094803 | prot-a-2346 | Protein phosphatase 1L \|\| id:prot-a-2346 | Mushroom liking \|\| id:ebi-fl187-GCST90094803 | Weighted mode | 7 | -0.069385183 | 0.363432910744458 | 0.854887634145014 | -0.781713688 | 0.642943321878036 | 0.932967247566685 | 0.457621119036804 | 1.90207105577693 | 0.999228396881742 |
| ebi-fl187-GCST90094803 | prot-a-2358 | PR domain zinc finger protein 4 \|\| id:prot-a-2358 | Mushroom liking \|\| id:ebi-fl187-GCST90094803 | Weighted mode | 7 | 0.367606086280106 | 0.354564507096266 | 0.339803233178748 | -0.327340348 | 1.06255252018879 | 1.44427300795357 | 0.720838365643837 | 2.8937479203679 | 0.999228396881742 |
| ebi-fl187-GCST90094803 | prot-a-2390 | Trypsin-1 \|\| id:prot-a-2390 | Mushroom liking \|\| id:ebi-fl187-GCST90094803 | Weighted mode | 7 | -0.484678199 | 0.393813813026639 | 0.264479072352901 | -1.256553273 | 0.287196874255788 | 0.615895360450606 | 0.28463339137806 | 1.33268655932482 | 0.999228396881742 |
| ebi-fl187-GCST90094803 | prot-a-242 | Brain-derived neurotrophic factor \|\| id:prot-a-242 | Mushroom liking \|\| id:ebi-fl187-GCST90094803 | Weighted mode | 7 | 0.191905466351585 | 0.365560173033961 | 0.618432917215357 | -0.524592473 | 0.908403405498149 | 1.2115559787442 | 0.591796488400036 | 2.48035924241338 | 0.999228396881742 |
| ebi-fl187-GCST90094803 | prot-a-2464 | Dihydropteridine reductase \|\| id:prot-a-2464 | Mushroom liking \|\| id:ebi-fl187-GCST90094803 | Weighted mode | 7 | -0.166676804 | 0.407608791574628 | 0.696798210263618 | -0.965590036 | 0.63223642712464 | 0.846473143560591 | 0.380758472299185 | 1.88181441753012 | 0.999228396881742 |
| ebi-fl187-GCST90094803 | prot-a-2474 | Ras-related protein Rab-27A \|\| id:prot-a-2474 | Mushroom liking \|\| id:ebi-fl187-GCST90094803 | Weighted mode | 7 | 0.1867480672741 | 0.341569960019192 | 0.604282870281772 | -0.482729054 | 0.856225188911715 | 1.20532358633901 | 0.617097000464137 | 2.35425702392402 | 0.999228396881742 |
| ebi-fl187-GCST90094803 | prot-a-2478 | Ras-related protein Rab-7b \|\| id:prot-a-2478 | Mushroom liking \|\| id:ebi-fl187-GCST90094803 | Weighted mode | 7 | -0.58937866 | 0.37327691344612 | 0.165430352891489 | -1.321001411 | 0.14224409006121 | 0.554671817311884 | 0.266867923730167 | 1.15285801537972 | 0.999228396881742 |
| ebi-fl187-GCST90094803 | prot-a-2528 | E3 ubiquitin-protein ligase rififylin \|\| id:prot-a-2528 | Mushroom liking \|\| id:ebi-fl187-GCST90094803 | Weighted mode | 7 | 0.0980836487485699 | 0.367056774525983 | 0.798249957680111 | -0.621347629 | 0.817514926819496 | 1.10305505042409 | 0.537219976159152 | 2.26486448431253 | 0.999228396881742 |
| ebi-fl187-GCST90094803 | prot-a-2543 | Protein RIC-3 \|\| id:prot-a-2543 | Mushroom liking \|\| id:ebi-fl187-GCST90094803 | Weighted mode | 7 | 0.61832673049754 | 0.355802011895345 | 0.132906817571721 | -0.079045213 | 1.31569867381242 | 1.8558201551378 | 0.923998146941217 | 3.72735428054361 | 0.999228396881742 |
| ebi-fl187-GCST90094803 | prot-a-255 | Bone morphogenetic protein 6 \|\| id:prot-a-255 | Mushroom liking \|\| id:ebi-fl187-GCST90094803 | Weighted mode | 7 | -0.404414426 | 0.439034026901304 | 0.392505557465909 | -1.264921119 | 0.456092266411426 | 0.667367489301677 | 0.282261560324776 | 1.57789592484489 | 0.999228396881742 |
| ebi-fl187-GCST90094803 | prot-a-2568 | RING finger protein 165 \|\| id:prot-a-2568 | Mushroom liking \|\| id:ebi-fl187-GCST90094803 | Weighted mode | 7 | 0.207601436139803 | 0.398138107431749 | 0.62074570838953 | -0.572749254 | 0.987952126706032 | 1.23072255025334 | 0.563972800660392 | 2.68572880452471 | 0.999228396881742 |
| ebi-fl187-GCST90094803 | prot-a-2573 | E3 ubiquitin-protein ligase RNF8 \|\| id:prot-a-2573 | Mushroom liking \|\| id:ebi-fl187-GCST90094803 | Weighted mode | 7 | 0.659845849697525 | 0.343753910906059 | 0.103333486081381 | -0.013911816 | 1.3336035150734 | 1.93449410856485 | 0.986184506440176 | 3.79469301295409 | 0.999228396881742 |
| ebi-fl187-GCST90094803 | prot-a-260 | Cytoplasmic tyrosine-protein kinase BMX \|\| id:prot-a-260 | Mushroom liking \|\| id:ebi-fl187-GCST90094803 | Weighted mode | 7 | -0.072667106 | 0.348086977264734 | 0.841541392794565 | -0.754917582 | 0.609583369131894 | 0.929910339793197 | 0.470049353781211 | 1.83966477796031 | 0.999228396881742 |
| ebi-fl187-GCST90094803 | prot-a-2623 | Serum amyloid A-1 protein \|\| id:prot-a-2623 | Mushroom liking \|\| id:ebi-fl187-GCST90094803 | Weighted mode | 7 | -0.260523382 | 0.374441792160584 | 0.512610418696812 | -0.994429295 | 0.473382530238279 | 0.770648136565114 | 0.369934507772626 | 1.60541538546145 | 0.999228396881742 |
| ebi-fl187-GCST90094803 | prot-a-2640 | Lysosome membrane protein 2 \|\| id:prot-a-2640 | Mushroom liking \|\| id:ebi-fl187-GCST90094803 | Weighted mode | 7 | -0.388427241 | 0.366916398887486 | 0.330516070018923 | -1.107583383 | 0.330728900688311 | 0.67812255951535 | 0.33035634197337 | 1.39198237568788 | 0.999228396881742 |
| ebi-fl187-GCST90094803 | prot-a-2641 | Scavenger receptor class F member 2 \|\| id:prot-a-2641 | Mushroom liking \|\| id:ebi-fl187-GCST90094803 | Weighted mode | 7 | 0.076391460703404 | 0.358854711822332 | 0.838472281778351 | -0.626963774 | 0.779746695875176 | 1.07938502828721 | 0.534211327210301 | 2.18091975955413 | 0.999228396881742 |
| ebi-fl187-GCST90094803 | prot-a-2669 | Semaphorin-3A \|\| id:prot-a-2669 | Mushroom liking \|\| id:ebi-fl187-GCST90094803 | Weighted mode | 7 | 0.193033276265034 | 0.338095524735248 | 0.588767562471028 | -0.469633952 | 0.855700504746119 | 1.21292315439994 | 0.625231090855411 | 2.35302210654095 | 0.999228396881742 |
| ebi-fl187-GCST90094803 | prot-a-2683 | Sentrin-specific protease 7 \|\| id:prot-a-2683 | Mushroom liking \|\| id:ebi-fl187-GCST90094803 | Weighted mode | 7 | 0.0349457019467947 | 0.366708692605357 | 0.927183093689431 | -0.683803336 | 0.753694739453294 | 1.03556347819219 | 0.504693817501334 | 2.12483624759814 | 0.999228396881742 |
| ebi-fl187-GCST90094803 | prot-a-270 | Bromodomain testis-specific protein \|\| id:prot-a-270 | Mushroom liking \|\| id:ebi-fl187-GCST90094803 | Weighted mode | 7 | 0.0568916156146888 | 0.339312860531194 | 0.872353734091897 | -0.608161591 | 0.721944822255829 | 1.05854107485412 | 0.544350688945308 | 2.0584326058709 | 0.999228396881742 |
| ebi-fl187-GCST90094803 | prot-a-2708 | 14-3-3 protein sigma \|\| id:prot-a-2708 | Mushroom liking \|\| id:ebi-fl187-GCST90094803 | Weighted mode | 7 | 0.753110599767668 | 0.395797347457477 | 0.105763115371534 | -0.022652201 | 1.52887340078432 | 2.12359540886732 | 0.977602433558174 | 4.6129769175682 | 0.999228396881742 |
| ebi-fl187-GCST90094803 | prot-a-2713 | Beta-sarcoglycan \|\| id:prot-a-2713 | Mushroom liking \|\| id:ebi-fl187-GCST90094803 | Weighted mode | 7 | -0.157805407 | 0.354910533901333 | 0.672173541115946 | -0.853430054 | 0.537819239136161 | 0.854015951061145 | 0.425951387209052 | 1.71226873903552 | 0.999228396881742 |
| ebi-fl187-GCST90094803 | prot-a-2720 | Protein shisa-3 homolog \|\| id:prot-a-2720 | Mushroom liking \|\| id:ebi-fl187-GCST90094803 | Weighted mode | 7 | 0.0441732495674905 | 0.359348157090524 | 0.906179880354364 | -0.660149138 | 0.748495637464917 | 1.04516341331316 | 0.516774257894528 | 2.11381767539853 | 0.999228396881742 |
| ebi-fl187-GCST90094803 | prot-a-2730 | Sialic acid-binding Ig-like lectin 8 \|\| id:prot-a-2730 | Mushroom liking \|\| id:ebi-fl187-GCST90094803 | Weighted mode | 7 | 0.326393980646798 | 0.365820507692572 | 0.40662995617758 | -0.390614214 | 1.04340217572424 | 1.38596130325263 | 0.676641144082362 | 2.83885890019112 | 0.999228396881742 |
| ebi-fl187-GCST90094803 | prot-a-2761 | Sodium- and chloride-dependent neutral and basic amino acid transporter B(0+) \|\| id:prot-a-2761 | Mushroom liking \|\| id:ebi-fl187-GCST90094803 | Weighted mode | 7 | -0.122285097 | 0.414342009002911 | 0.777840116048279 | -0.934395435 | 0.689825240789508 | 0.884896051454607 | 0.392823281160278 | 1.99336714353359 | 0.999228396881742 |
| ebi-fl187-GCST90094803 | prot-a-2770 | Secreted Ly-6/uPAR-related protein 1 \|\| id:prot-a-2770 | Mushroom liking \|\| id:ebi-fl187-GCST90094803 | Weighted mode | 7 | 0.644970823550504 | 0.374801900398466 | 0.136068061181138 | -0.089640901 | 1.3795825483315 | 1.90593142014886 | 0.914259435768923 | 3.97324264447492 | 0.999228396881742 |
| ebi-fl187-GCST90094803 | prot-a-2784 | N-lysine methyltransferase SMYD2 \|\| id:prot-a-2784 | Mushroom liking \|\| id:ebi-fl187-GCST90094803 | Weighted mode | 7 | -0.13637302 | 0.377877199438361 | 0.730546011385804 | -0.87701233 | 0.60426629134587 | 0.87251710583751 | 0.416023999011866 | 1.82990909607922 | 0.999228396881742 |
| ebi-fl187-GCST90094803 | prot-a-2837 | Serine palmitoyltransferase 2 \|\| id:prot-a-2837 | Mushroom liking \|\| id:ebi-fl187-GCST90094803 | Weighted mode | 7 | 0.483214882950287 | 0.427793995956992 | 0.301791184916857 | -0.355261349 | 1.32169111502599 | 1.62127825263791 | 0.700990216086591 | 3.74975728926857 | 0.999228396881742 |
| ebi-fl187-GCST90094803 | prot-a-2858 | Alpha-2,8-sialyltransferase 8B \|\| id:prot-a-2858 | Mushroom liking \|\| id:ebi-fl187-GCST90094803 | Weighted mode | 7 | 0.0390634277969077 | 0.347013914192245 | 0.914043102598893 | -0.641083844 | 0.719210699613708 | 1.03983643609281 | 0.526721230900567 | 2.05281228549969 | 0.999228396881742 |
| ebi-fl187-GCST90094803 | prot-a-2911 | Synaptotagmin-11 \|\| id:prot-a-2911 | Mushroom liking \|\| id:ebi-fl187-GCST90094803 | Weighted mode | 7 | 0.244325602240354 | 0.359972809525473 | 0.522610204532457 | -0.461221104 | 0.94987230891028 | 1.27675997872725 | 0.630513252711835 | 2.58537950831087 | 0.999228396881742 |
| ebi-fl187-GCST90094803 | prot-a-2962 | Transforming growth factor beta-1 \|\| id:prot-a-2962 | Mushroom liking \|\| id:ebi-fl187-GCST90094803 | Weighted mode | 7 | 0.193786691140704 | 0.359149005393613 | 0.608917736981191 | -0.510145359 | 0.897718741712187 | 1.21383733308217 | 0.600408297460483 | 2.45399851636964 | 0.999228396881742 |
| ebi-fl187-GCST90094803 | prot-a-2978 | Tyrosine-protein kinase receptor Tie-1, soluble \|\| id:prot-a-2978 | Mushroom liking \|\| id:ebi-fl187-GCST90094803 | Weighted mode | 7 | 0.207757941003897 | 0.355195911656904 | 0.579922334772495 | -0.488426046 | 0.903941927851428 | 1.23091517939211 | 0.613591399281514 | 2.46931782393314 | 0.999228396881742 |
| ebi-fl187-GCST90094803 | prot-a-299 | Uncharacterized protein C1orf226 \|\| id:prot-a-299 | Mushroom liking \|\| id:ebi-fl187-GCST90094803 | Weighted mode | 7 | 0.202522406755101 | 0.635852111084864 | 0.760896799162257 | -1.043747731 | 1.44879254448144 | 1.22448752160655 | 0.352132508023926 | 4.25797010047218 | 0.999228396881742 |
| ebi-fl187-GCST90094803 | prot-a-3015 | Transmembrane protein 70, mitochondrial \|\| id:prot-a-3015 | Mushroom liking \|\| id:ebi-fl187-GCST90094803 | Weighted mode | 7 | 0.140915860493559 | 0.358847362357969 | 0.708119663275317 | -0.56242497 | 0.844256690715177 | 1.15132777175878 | 0.569825577295058 | 2.32624805000048 | 0.999228396881742 |
| ebi-fl187-GCST90094803 | prot-a-3016 | Transmembrane protein 87B \|\| id:prot-a-3016 | Mushroom liking \|\| id:ebi-fl187-GCST90094803 | Weighted mode | 7 | 0.169303629204759 | 0.352557804010948 | 0.648080323631981 | -0.521709667 | 0.860316925066217 | 1.18447972696804 | 0.593504984397567 | 2.36390975725737 | 0.999228396881742 |
| ebi-fl187-GCST90094803 | prot-a-3123 | Thioredoxin domain-containing protein 12 \|\| id:prot-a-3123 | Mushroom liking \|\| id:ebi-fl187-GCST90094803 | Weighted mode | 7 | 0.0322387118686431 | 0.351622975911185 | 0.929932443732492 | -0.656942321 | 0.721419744654566 | 1.03276400890871 | 0.518434118595801 | 2.0573520527278 | 0.999228396881742 |
| ebi-fl187-GCST90094803 | prot-a-3133 | PolyUbiquitin K48-linked \|\| id:prot-a-3133 | Mushroom liking \|\| id:ebi-fl187-GCST90094803 | Weighted mode | 7 | 0.0238546907389616 | 0.390989581607619 | 0.953331906048881 | -0.742484889 | 0.790194270689895 | 1.02414148983475 | 0.475929812091681 | 2.20382452318177 | 0.999228396881742 |
| ebi-fl187-GCST90094803 | prot-a-3135 | Ubiquitin-conjugating enzyme E2 B \|\| id:prot-a-3135 | Mushroom liking \|\| id:ebi-fl187-GCST90094803 | Weighted mode | 7 | -0.362984823 | 0.388171847779839 | 0.385820087910458 | -1.123801645 | 0.397831998315725 | 0.695596990263042 | 0.325041749474464 | 1.48859392261244 | 0.999228396881742 |
| ebi-fl187-GCST90094803 | prot-a-3162 | NKG2D ligand 3 \|\| id:prot-a-3162 | Mushroom liking \|\| id:ebi-fl187-GCST90094803 | Weighted mode | 7 | 0.186930561376381 | 0.353362791772494 | 0.615792101059461 | -0.50566051 | 0.879521633250469 | 1.20554357085719 | 0.603107085276997 | 2.40974668796604 | 0.999228396881742 |
| ebi-fl187-GCST90094803 | prot-a-3188 | Vesicle-associated membrane protein 8 \|\| id:prot-a-3188 | Mushroom liking \|\| id:ebi-fl187-GCST90094803 | Weighted mode | 7 | 0.888359008899058 | 0.390755572939237 | 0.0633678093870101 | 0.122478085938154 | 1.65423993185996 | 2.43113690213138 | 1.13029435046231 | 5.22910393605655 | 0.999228396881742 |
| ebi-fl187-GCST90094803 | prot-a-3201 | Vitrin \|\| id:prot-a-3201 | Mushroom liking \|\| id:ebi-fl187-GCST90094803 | Weighted mode | 7 | -0.343750826 | 0.385903967825754 | 0.407351370812534 | -1.100122603 | 0.41262095126315 | 0.709105596946161 | 0.332830275334815 | 1.51077226107072 | 0.999228396881742 |
| ebi-fl187-GCST90094803 | prot-a-3232 | WNT1-inducible-signaling pathway protein 3 \|\| id:prot-a-3232 | Mushroom liking \|\| id:ebi-fl187-GCST90094803 | Weighted mode | 7 | -0.05947621 | 0.368063947553624 | 0.876931210431601 | -0.780881548 | 0.661929126837578 | 0.942257949294909 | 0.458002082665994 | 1.93852839672986 | 0.999228396881742 |
| ebi-fl187-GCST90094803 | prot-a-327 | Carbonic anhydrase-related protein 10 \|\| id:prot-a-327 | Mushroom liking \|\| id:ebi-fl187-GCST90094803 | Weighted mode | 7 | -0.207201198 | 0.36593113523848 | 0.591769828001109 | -0.924426224 | 0.510023826598205 | 0.8128560881333 | 0.396759004755652 | 1.66533087364278 | 0.999228396881742 |
| ebi-fl187-GCST90094803 | prot-a-332 | Carbonic anhydrase 5A, mitochondrial \|\| id:prot-a-332 | Mushroom liking \|\| id:ebi-fl187-GCST90094803 | Weighted mode | 7 | 0.236172089087514 | 0.431745540980222 | 0.604100543962292 | -0.610049171 | 1.08239334940875 | 1.26639222369828 | 0.543324152498773 | 2.95173563860131 | 0.999228396881742 |
| ebi-fl187-GCST90094803 | prot-a-338 | Cell adhesion molecule 3 \|\| id:prot-a-338 | Mushroom liking \|\| id:ebi-fl187-GCST90094803 | Weighted mode | 7 | 0.252545078168622 | 0.371371749817366 | 0.52184269766255 | -0.475343551 | 0.98043370781066 | 1.28729752386174 | 0.621671443633179 | 2.66561208804433 | 0.999228396881742 |
| ebi-fl187-GCST90094803 | prot-a-34 | A disintegrin and metalloproteinase with thrombospondin motifs 3 \|\| id:prot-a-34 | Mushroom liking \|\| id:ebi-fl187-GCST90094803 | Weighted mode | 7 | -0.585350854 | 0.524782598415324 | 0.307343398347622 | -1.613924747 | 0.443223038873663 | 0.556910433266234 | 0.19910464326476 | 1.55771972765277 | 0.999228396881742 |
| ebi-fl187-GCST90094803 | prot-a-358 | Cysteine--tRNA ligase, cytoplasmic \|\| id:prot-a-358 | Mushroom liking \|\| id:ebi-fl187-GCST90094803 | Weighted mode | 7 | 0.19369638270702 | 0.406702834438683 | 0.650736207228014 | -0.603441173 | 0.99083393820684 | 1.21372771828352 | 0.546926326122653 | 2.69347973167296 | 0.999228396881742 |
| ebi-fl187-GCST90094803 | prot-a-382 | Coiled-coil domain-containing protein 167 \|\| id:prot-a-382 | Mushroom liking \|\| id:ebi-fl187-GCST90094803 | Weighted mode | 7 | 0.222881739187973 | 0.479885542418791 | 0.658701980795207 | -0.717693924 | 1.1634574023288 | 1.24967277767946 | 0.487876038944824 | 3.20098124648772 | 0.999228396881742 |
| ebi-fl187-GCST90094803 | prot-a-40 | Pituitary adenylate cyclase-activating polypeptide 27 \|\| id:prot-a-40 | Mushroom liking \|\| id:ebi-fl187-GCST90094803 | Weighted mode | 7 | 0.0492162724935323 | 0.331399782983982 | 0.88680561275945 | -0.600327302 | 0.698759847142136 | 1.05044750907721 | 0.548632038255788 | 2.01125689421013 | 0.999228396881742 |
| ebi-fl187-GCST90094803 | prot-a-403 | C-C motif chemokine 25 \|\| id:prot-a-403 | Mushroom liking \|\| id:ebi-fl187-GCST90094803 | Weighted mode | 7 | -0.211609186 | 0.369402903359221 | 0.587560922167334 | -0.935638877 | 0.512420504398112 | 0.809280913932216 | 0.392335131644343 | 1.6693269218844 | 0.999228396881742 |
| ebi-fl187-GCST90094803 | prot-a-415 | Cyclin-C \|\| id:prot-a-415 | Mushroom liking \|\| id:ebi-fl187-GCST90094803 | Weighted mode | 7 | -0.343354601 | 0.381044103915946 | 0.402260426891443 | -1.090201045 | 0.403491842812792 | 0.709386617848663 | 0.336148906011479 | 1.49704302046897 | 0.999228396881742 |
| ebi-fl187-GCST90094803 | prot-a-44 | Adhesion G protein-coupled receptor F5 \|\| id:prot-a-44 | Mushroom liking \|\| id:ebi-fl187-GCST90094803 | Weighted mode | 7 | -0.260455601 | 0.338523465408021 | 0.470857157387298 | -0.923961593 | 0.403050390914209 | 0.770700373722292 | 0.396943393745442 | 1.49638229383557 | 0.999228396881742 |
| ebi-fl187-GCST90094803 | prot-a-455 | CD59 glycoprotein \|\| id:prot-a-455 | Mushroom liking \|\| id:ebi-fl187-GCST90094803 | Weighted mode | 7 | 0.12097520225617 | 0.36341803618956 | 0.750547136755057 | -0.591324149 | 0.833274553187708 | 1.1285969253629 | 0.553593758750614 | 2.30084064316986 | 0.999228396881742 |
| ebi-fl187-GCST90094803 | prot-a-478 | Cadherin-12 \|\| id:prot-a-478 | Mushroom liking \|\| id:ebi-fl187-GCST90094803 | Weighted mode | 7 | 0.451292882569627 | 0.352740686557779 | 0.248005303734201 | -0.240078863 | 1.14266462822287 | 1.57034114029605 | 0.786565827613875 | 3.13511115069296 | 0.999228396881742 |
| ebi-fl187-GCST90094803 | prot-a-516 | Centrin-2 \|\| id:prot-a-516 | Mushroom liking \|\| id:ebi-fl187-GCST90094803 | Weighted mode | 7 | 0.104142674761013 | 0.329561062802651 | 0.762703183840469 | -0.541797008 | 0.750082357854209 | 1.10975877818941 | 0.581701989262226 | 2.11717437537121 | 0.999228396881742 |
| ebi-fl187-GCST90094803 | prot-a-519 | Cryptic protein \|\| id:prot-a-519 | Mushroom liking \|\| id:ebi-fl187-GCST90094803 | Weighted mode | 7 | -0.181249243 | 0.343595546044477 | 0.616769515032286 | -0.854696513 | 0.4921980273153 | 0.834227407496789 | 0.425412278513116 | 1.635908042 | 0.999228396881742 |
| ebi-fl187-GCST90094803 | prot-a-583 | Chloride intracellular channel protein 4 \|\| id:prot-a-583 | Mushroom liking \|\| id:ebi-fl187-GCST90094803 | Weighted mode | 7 | 0.0982564945451498 | 0.364831952993924 | 0.796705751608714 | -0.616814133 | 0.813327122413242 | 1.10324572533115 | 0.539660989753797 | 2.25539950741436 | 0.999228396881742 |
| ebi-fl187-GCST90094803 | prot-a-602 | CCR4-NOT transcription complex subunit 1 \|\| id:prot-a-602 | Mushroom liking \|\| id:ebi-fl187-GCST90094803 | Weighted mode | 7 | 0.0775562328181068 | 0.362576094926496 | 0.837706237801107 | -0.633092913 | 0.788205378874038 | 1.08064299835119 | 0.530947085567411 | 2.19944571055976 | 0.999228396881742 |
| ebi-fl187-GCST90094803 | prot-a-606 | Ciliary neurotrophic factor receptor subunit alpha \|\| id:prot-a-606 | Mushroom liking \|\| id:ebi-fl187-GCST90094803 | Weighted mode | 7 | -0.59184053 | 0.345229701217444 | 0.137294074551147 | -1.268490744 | 0.0848096843925698 | 0.553307967070787 | 0.281255788489356 | 1.08850988656396 | 0.999228396881742 |
| ebi-fl187-GCST90094803 | prot-a-626 | Collagen alpha-2(IX) chain \|\| id:prot-a-626 | Mushroom liking \|\| id:ebi-fl187-GCST90094803 | Weighted mode | 7 | 0.1908739321533 | 0.635860249711068 | 0.774166834828082 | -1.055412157 | 1.43716002158699 | 1.21030686168323 | 0.348048946832597 | 4.20872613685013 | 0.999228396881742 |
| ebi-fl187-GCST90094803 | prot-a-647 | Probable serine carboxypeptidase CPVL \|\| id:prot-a-647 | Mushroom liking \|\| id:ebi-fl187-GCST90094803 | Weighted mode | 7 | 0.112830615493532 | 0.337252269217617 | 0.749343594696489 | -0.548183832 | 0.773845063160062 | 1.11944230070703 | 0.577998600164744 | 2.16808667746786 | 0.999228396881742 |
| ebi-fl187-GCST90094803 | prot-a-651 | Cellular retinoic acid-binding protein 2 \|\| id:prot-a-651 | Mushroom liking \|\| id:ebi-fl187-GCST90094803 | Weighted mode | 7 | 0.242133241158046 | 0.349477187320385 | 0.514316178117084 | -0.442842046 | 0.927108528 | 1.27396392593703 | 0.642208638506602 | 2.52719130088779 | 0.999228396881742 |
| ebi-fl187-GCST90094803 | prot-a-671 | Cytotoxic and regulatory T-cell molecule \|\| id:prot-a-671 | Mushroom liking \|\| id:ebi-fl187-GCST90094803 | Weighted mode | 7 | -0.158894035 | 0.390666962891116 | 0.69832063655307 | -0.924601282 | 0.606813212300287 | 0.853086751546197 | 0.396689554720647 | 1.83457567007565 | 0.999228396881742 |
| ebi-fl187-GCST90094803 | prot-a-688 | Chorionic somatomammotropin hormone \|\| id:prot-a-688 | Mushroom liking \|\| id:ebi-fl187-GCST90094803 | Weighted mode | 7 | -0.110450638 | 0.369708031250978 | 0.775206949685376 | -0.835078379 | 0.614177103332156 | 0.895430529412236 | 0.433840476043199 | 1.84813514938989 | 0.999228396881742 |
| ebi-fl187-GCST90094803 | prot-a-69 | Aldo-keto reductase family 1 member C1 \|\| id:prot-a-69 | Mushroom liking \|\| id:ebi-fl187-GCST90094803 | Weighted mode | 7 | -0.018694187 | 0.335847081161262 | 0.957417700878136 | -0.676954466 | 0.639566092294914 | 0.981479465747304 | 0.508162263606906 | 1.89565816014387 | 0.999228396881742 |
| ebi-fl187-GCST90094803 | prot-a-741 | Stromal cell-derived factor 1 \|\| id:prot-a-741 | Mushroom liking \|\| id:ebi-fl187-GCST90094803 | Weighted mode | 7 | -0.232851635 | 0.333324073740108 | 0.510972958118404 | -0.88616682 | 0.420463549140038 | 0.792271109798753 | 0.412232890999393 | 1.52266722313206 | 0.999228396881742 |
| ebi-fl187-GCST90094803 | prot-a-742 | Growth-regulated alpha protein \|\| id:prot-a-742 | Mushroom liking \|\| id:ebi-fl187-GCST90094803 | Weighted mode | 7 | -0.207843678 | 0.383204823497255 | 0.60709644753613 | -0.958925132 | 0.543237776007535 | 0.812334012426377 | 0.383304666511928 | 1.72157191236336 | 0.999228396881742 |
| ebi-fl187-GCST90094803 | prot-a-788 | Beta-defensin 112 \|\| id:prot-a-788 | Mushroom liking \|\| id:ebi-fl187-GCST90094803 | Weighted mode | 7 | 0.139073590985161 | 0.346007215483284 | 0.701655607227172 | -0.539100551 | 0.817247733332398 | 1.14920866828989 | 0.583272640290486 | 2.26425940811297 | 0.999228396881742 |
| ebi-fl187-GCST90094803 | prot-a-838 | DnaJ homolog subfamily B member 1 \|\| id:prot-a-838 | Mushroom liking \|\| id:ebi-fl187-GCST90094803 | Weighted mode | 7 | -0.251083655 | 0.370167295281898 | 0.522867688787633 | -0.976611554 | 0.474444243987216 | 0.777957289002335 | 0.376584977439892 | 1.60712078221034 | 0.999228396881742 |
| ebi-fl187-GCST90094803 | prot-a-85 | Junctional adhesion molecule-like \|\| id:prot-a-85 | Mushroom liking \|\| id:ebi-fl187-GCST90094803 | Weighted mode | 7 | 0.480690676829308 | 0.343139483676942 | 0.210796518845439 | -0.191862711 | 1.15324406483611 | 1.61719097288729 | 0.825420181677999 | 3.16845492858132 | 0.999228396881742 |
| ebi-fl187-GCST90094803 | prot-a-856 | Dipeptidase 1 \|\| id:prot-a-856 | Mushroom liking \|\| id:ebi-fl187-GCST90094803 | Weighted mode | 7 | 0.498499002333613 | 0.409817943096811 | 0.269511972178267 | -0.304744166 | 1.30174217080336 | 1.64624840027094 | 0.737311979637418 | 3.67569478082718 | 0.999228396881742 |
| ebi-fl187-GCST90094803 | prot-a-860 | Dipeptidyl peptidase 2 \|\| id:prot-a-860 | Mushroom liking \|\| id:ebi-fl187-GCST90094803 | Weighted mode | 7 | 0.0218174443679073 | 0.341257333071763 | 0.951100710455638 | -0.647046928 | 0.690681817188563 | 1.02205718514311 | 0.523589693817503 | 1.99507534628203 | 0.999228396881742 |
| ebi-fl187-GCST90094803 | prot-a-861 | Dermatopontin \|\| id:prot-a-861 | Mushroom liking \|\| id:ebi-fl187-GCST90094803 | Weighted mode | 7 | -0.518598507 | 0.345210921587243 | 0.183712627344483 | -1.195211913 | 0.158014899260136 | 0.595354348472577 | 0.302639813965889 | 1.17118364434746 | 0.999228396881742 |
| ebi-fl187-GCST90094803 | prot-a-877 | Dual specificity protein phosphatase 6 \|\| id:prot-a-877 | Mushroom liking \|\| id:ebi-fl187-GCST90094803 | Weighted mode | 7 | -0.118240114 | 0.328779339309087 | 0.731440025625969 | -0.762647619 | 0.526167391346366 | 0.888482690134395 | 0.46642986230952 | 1.69243342773926 | 0.999228396881742 |
| ebi-fl187-GCST90094803 | prot-a-879 | Dynactin-associated protein \|\| id:prot-a-879 | Mushroom liking \|\| id:ebi-fl187-GCST90094803 | Weighted mode | 7 | -0.142988909 | 0.335836346194956 | 0.68512533925274 | -0.801228148 | 0.515250329546635 | 0.866763682122097 | 0.448777460589544 | 1.67405751540847 | 0.999228396881742 |
| ebi-fl187-GCST90094803 | prot-a-937 | EMI domain-containing protein 1 \|\| id:prot-a-937 | Mushroom liking \|\| id:ebi-fl187-GCST90094803 | Weighted mode | 7 | 1.03732288674659 | 0.362110987706326 | 0.0286266736398906 | 0.327585350842191 | 1.74706042265099 | 2.82165300935224 | 1.38761348022307 | 5.73771141507408 | 0.999228396881742 |
| ebi-fl187-GCST90094803 | prot-b-1 | SRC proto-oncogene, non-receptor tyrosine kinase \|\| id:prot-b-1 | Mushroom liking \|\| id:ebi-fl187-GCST90094803 | Weighted mode | 7 | -0.227627548 | 0.43191589752468 | 0.617089165133834 | -1.074182707 | 0.618927610959219 | 0.796420832983863 | 0.34157680947105 | 1.85693561630527 | 0.999228396881742 |
| ebi-fl187-GCST90094803 | prot-b-10 | coagulation factor III, tissue factor \|\| id:prot-b-10 | Mushroom liking \|\| id:ebi-fl187-GCST90094803 | Weighted mode | 7 | 0.149423864234916 | 0.664027269102374 | 0.829426694002849 | -1.152069583 | 1.45091731167557 | 1.16116506125615 | 0.315982140876562 | 4.26702691405812 | 0.999228396881742 |
| ebi-fl187-GCST90094803 | prot-b-29 | myeloperoxidase \|\| id:prot-b-29 | Mushroom liking \|\| id:ebi-fl187-GCST90094803 | Weighted mode | 7 | -0.280834916 | 0.464068661946845 | 0.567241835717027 | -1.190409493 | 0.628739661453367 | 0.755152988895156 | 0.304096712976705 | 1.87524564489775 | 0.999228396881742 |
| ebi-fl187-GCST90094803 | prot-b-33 | adrenomedullin \|\| id:prot-b-33 | Mushroom liking \|\| id:ebi-fl187-GCST90094803 | Weighted mode | 7 | -0.148094665 | 0.479997871665734 | 0.768109135230123 | -1.088890493 | 0.792701163939378 | 0.862349477173968 | 0.336589735282061 | 2.20935620677512 | 0.999228396881742 |
| ebi-fl187-GCST90094803 | ukb-rsfMRI191-edge-pheno102 | Net25_Pair11_15 (Postcentral\|Precentral)&(Cerebellum) (Motor)&(Subcortical-cerebellum) \|\| id:ukb-rsfMRI191-edge-pheno102 | Mushroom liking \|\| id:ebi-fl187-GCST90094803 | Weighted mode | 6 | -0.040852741 | 0.132368713045339 | 0.770049191604271 | -0.300295419 | 0.218589936163642 | 0.959970483444206 | 0.740599401246279 | 1.24432092104494 | 0.999228396881742 |
| ebi-fl187-GCST90094803 | ukb-rsfMRI191-edge-pheno1167 | Net100_Pair11_45 (Angular\|Temporal)&(Frontal) (Default_mode\|Central_executive)&(Salience\|Default_mode) \|\| id:ukb-rsfMRI191-edge-pheno1167 | Mushroom liking \|\| id:ebi-fl187-GCST90094803 | Weighted mode | 6 | 0.113804713642295 | 0.113029012726965 | 0.360212548272607 | -0.107732151 | 0.335341578587147 | 1.12053327865333 | 0.897868057015017 | 1.39841797328645 | 0.999228396881742 |
| ebi-fl187-GCST90094803 | ukb-rsfMRI191-edge-pheno1317 | Net100_Pair26_48 (Parietal\|Frontal)&(Temporal) (Attention\|Central_executive)&(Default_mode\|Central_executive) \|\| id:ukb-rsfMRI191-edge-pheno1317 | Mushroom liking \|\| id:ebi-fl187-GCST90094803 | Weighted mode | 6 | -0.017917933 | 0.113032514263071 | 0.880250589585021 | -0.239461661 | 0.203625794581082 | 0.982241638307372 | 0.787051447269129 | 1.22583935189035 | 0.999228396881742 |
| ebi-fl187-GCST90094803 | ukb-rsfMRI191-edge-pheno286 | Net100_Pair10_13 (OccipitalPrecuneus)&(Frontal_Sup) (Default_mode\|Central_executive)&(Default_mode) \|\| id:ukb-rsfMRI191-edge-pheno286 | Mushroom liking \|\| id:ebi-fl187-GCST90094803 | Weighted mode | 6 | -0.05567629 | 0.121330498321816 | 0.665577347710143 | -0.293484066 | 0.182131487207423 | 0.945845266379844 | 0.745661103973834 | 1.19977193817066 | 0.999228396881742 |
| ebi-fl187-GCST90094803 | ukb-rsfMRI191-edge-pheno599 | Net100_Pair11_29 (Angular\|Temporal)&(Parietal) (Default_mode\|Central_executive)&(Central_executive\|Salience) \|\| id:ukb-rsfMRI191-edge-pheno599 | Mushroom liking \|\| id:ebi-fl187-GCST90094803 | Weighted mode | 6 | 0.352687836971341 | 0.130472711086241 | 0.0426231529042521 | 0.0969613232423083 | 0.608414350700374 | 1.42288690141466 | 1.10181775805845 | 1.83751543248407 | 0.999228396881742 |
| ebi-fl187-GCST90094803 | ukb-rsfMRI191-edge-pheno621 | Net100_Pair5_30 (Precuneus\|Angular\|Cingulate)&(Paracentral) (Default_mode\|Central_executive)&(Motor) \|\| id:ukb-rsfMRI191-edge-pheno621 | Mushroom liking \|\| id:ebi-fl187-GCST90094803 | Weighted mode | 6 | -0.024225132 | 0.122467173331106 | 0.850984938907121 | -0.264260792 | 0.215810527249181 | 0.976065940874576 | 0.767773284254694 | 1.24086724619521 | 0.999228396881742 |
| ebi-fl187-GCST90094803 | ukb-rsfMRI191-edge-pheno624 | Net100_Pair8_30 (Calcarine\|Lingual)&(Paracentral) (Visual)&(Motor) \|\| id:ukb-rsfMRI191-edge-pheno624 | Mushroom liking \|\| id:ebi-fl187-GCST90094803 | Weighted mode | 6 | 0.0447502086112259 | 0.12704661215673 | 0.739022631718271 | -0.204261151 | 0.293761568438417 | 1.04576660378802 | 0.815249440002313 | 1.34146401817243 | 0.999228396881742 |
| ebi-fl187-GCST90094803 | ukb-rsfMRI191-edge-pheno882 | Net100_Pair6_38 (Postcentral\|Precentral)&(Subcortical) (Motor)&(Subcortical-cerebellum) \|\| id:ukb-rsfMRI191-edge-pheno882 | Mushroom liking \|\| id:ebi-fl187-GCST90094803 | Weighted mode | 6 | -0.141595718 | 0.121372545228872 | 0.295974216508994 | -0.379485906 | 0.0962944708972973 | 0.867972091271873 | 0.684213068371317 | 1.10108325323307 | 0.999228396881742 |
| ebi-fl187-GCST90094803 | ukb-rsfMRI191-edge-pheno899 | Net100_Pair23_38 (Cerebellum)&(Subcortical) (Subcortical-cerebellum)&(Subcortical-cerebellum) \|\| id:ukb-rsfMRI191-edge-pheno899 | Mushroom liking \|\| id:ebi-fl187-GCST90094803 | Weighted mode | 6 | 0.0851550005897347 | 0.12427145351497 | 0.523665554912171 | -0.158417048 | 0.328727049479075 | 1.08888583156478 | 0.853493759613212 | 1.38919862134651 | 0.999228396881742 |
| ebi-fl187-GCST90094838 | ebi-a-GCST90001392 | IgD+ CD38+ B cell Absolute Count \|\| id:ebi-a-GCST90001392 | Shellfish liking \|\| id:ebi-fl187-GCST90094838 | Inverse variance weighted | 30 | -0.026162099 | 0.0662484888333405 | 0.692910327641183 | -0.156009137 | 0.103684938766109 | 0.974177163326951 | 0.855551372768354 | 1.1092509178928 | 0.998074519377826 |
| ebi-fl187-GCST90094838 | ebi-a-GCST90001402 | Switched memory B cell %B cell \|\| id:ebi-a-GCST90001402 | Shellfish liking \|\| id:ebi-fl187-GCST90094838 | Inverse variance weighted | 30 | -0.055668316 | 0.0712047187825808 | 0.434328270091959 | -0.195229564 | 0.0838929331584485 | 0.945852808436229 | 0.822645786134417 | 1.08751245105208 | 0.998074519377826 |
| ebi-fl187-GCST90094838 | ebi-a-GCST90001407 | Memory B cell Absolute Count \|\| id:ebi-a-GCST90001407 | Shellfish liking \|\| id:ebi-fl187-GCST90094838 | Inverse variance weighted | 30 | -0.022767021 | 0.0692063905183123 | 0.742176232304432 | -0.158411547 | 0.112877503980255 | 0.977490191508233 | 0.85349845507773 | 1.11949479089307 | 0.998074519377826 |
| ebi-fl187-GCST90094838 | ebi-a-GCST90001411 | IgD+ CD24+ B cell %B cell \|\| id:ebi-a-GCST90001411 | Shellfish liking \|\| id:ebi-fl187-GCST90094838 | Inverse variance weighted | 30 | -0.029215239 | 0.070212101323934 | 0.677336997063093 | -0.166830958 | 0.108400479268694 | 0.971207399941579 | 0.846342666769484 | 1.11449398776228 | 0.998074519377826 |
| ebi-fl187-GCST90094838 | ebi-a-GCST90001418 | CD24+ CD27+ B cell Absolute Count \|\| id:ebi-a-GCST90001418 | Shellfish liking \|\| id:ebi-fl187-GCST90094838 | Inverse variance weighted | 30 | -0.024080826 | 0.0689722678318862 | 0.726985854282427 | -0.159266471 | 0.111104818604135 | 0.976206803339886 | 0.852769090204787 | 1.11751203676745 | 0.998074519377826 |
| ebi-fl187-GCST90094838 | ebi-a-GCST90001436 | Memory B cell %lymphocyte \|\| id:ebi-a-GCST90001436 | Shellfish liking \|\| id:ebi-fl187-GCST90094838 | Inverse variance weighted | 30 | -0.034573846 | 0.0682451464805417 | 0.612426718355921 | -0.168334333 | 0.0991866411212792 | 0.966017000579804 | 0.845071252172326 | 1.10427238296222 | 0.998074519377826 |
| ebi-fl187-GCST90094838 | ebi-a-GCST90001439 | IgD+ CD24+ B cell %lymphocyte \|\| id:ebi-a-GCST90001439 | Shellfish liking \|\| id:ebi-fl187-GCST90094838 | Inverse variance weighted | 30 | -0.035215797 | 0.0689540229906885 | 0.609551210689131 | -0.170365682 | 0.0999340878163511 | 0.965397063750354 | 0.843356359701843 | 1.10509807624774 | 0.998074519377826 |
| ebi-fl187-GCST90094838 | ebi-a-GCST90001457 | CD11c+ HLA DR++ monocyte %monocyte \|\| id:ebi-a-GCST90001457 | Shellfish liking \|\| id:ebi-fl187-GCST90094838 | Inverse variance weighted | 30 | 0.084452151449882 | 0.0757146227558833 | 0.264678703166815 | -0.063948509 | 0.232852812051413 | 1.08812077798479 | 0.938053299447404 | 1.26219568566062 | 0.998074519377826 |
| ebi-fl187-GCST90094838 | ebi-a-GCST90001483 | CD39+ resting CD4 regulatory T cell Absolute Count \|\| id:ebi-a-GCST90001483 | Shellfish liking \|\| id:ebi-fl187-GCST90094838 | Inverse variance weighted | 30 | 0.0384589753461958 | 0.0696320183584787 | 0.580730911242554 | -0.098019781 | 0.174937731328814 | 1.03920809433117 | 0.906630969838966 | 1.19117204160281 | 0.998074519377826 |
| ebi-fl187-GCST90094838 | ebi-a-GCST90001525 | CD33dim HLA DR+ CD11b+ Absolute Count \|\| id:ebi-a-GCST90001525 | Shellfish liking \|\| id:ebi-fl187-GCST90094838 | Inverse variance weighted | 30 | 0.0273040145502581 | 0.0893766044185753 | 0.759989954689244 | -0.14787413 | 0.202482159210666 | 1.02768018500596 | 0.86253967588362 | 1.22443823998236 | 0.998074519377826 |
| ebi-fl187-GCST90094838 | ebi-a-GCST90001540 | Naive CD4+ T cell Absolute Count \|\| id:ebi-a-GCST90001540 | Shellfish liking \|\| id:ebi-fl187-GCST90094838 | Inverse variance weighted | 30 | -0.087552759 | 0.0676556506124073 | 0.195633019249467 | -0.220157834 | 0.0450523164988501 | 0.916170534408065 | 0.802392143284803 | 1.04608258585557 | 0.998074519377826 |
| ebi-fl187-GCST90094838 | ebi-a-GCST90001624 | HLA DR+ CD4+ T cell Absolute Count \|\| id:ebi-a-GCST90001624 | Shellfish liking \|\| id:ebi-fl187-GCST90094838 | Inverse variance weighted | 30 | 0.0510881603344187 | 0.0666007777854716 | 0.443033633513523 | -0.079449364 | 0.181625684793943 | 1.05241567051097 | 0.923624787333724 | 1.19916524407531 | 0.998074519377826 |
| ebi-fl187-GCST90094838 | ebi-a-GCST90001672 | CD39+ CD8+ T cell Absolute Count \|\| id:ebi-a-GCST90001672 | Shellfish liking \|\| id:ebi-fl187-GCST90094838 | Inverse variance weighted | 30 | 0.217905250323849 | 0.0723376692546181 | 0.00259246831122038 | 0.076123418584798 | 0.359687082062901 | 1.24346924372912 | 1.07909574640903 | 1.43288097024355 | 0.637747204560213 |
| ebi-fl187-GCST90094838 | ebi-a-GCST90001722 | CD19 on CD20- CD38- B cell \|\| id:ebi-a-GCST90001722 | Shellfish liking \|\| id:ebi-fl187-GCST90094838 | Inverse variance weighted (multiplicative random effects) | 30 | -0.07930241 | 0.0840717101176395 | 0.345542283598381 | -0.244082961 | 0.0854781421938963 | 0.923760528115461 | 0.783422637675295 | 1.08923775273626 | 0.998074519377826 |
| ebi-fl187-GCST90094838 | ebi-a-GCST90001742 | CD19 on transitional B cell \|\| id:ebi-a-GCST90001742 | Shellfish liking \|\| id:ebi-fl187-GCST90094838 | Inverse variance weighted | 30 | -0.095660066 | 0.0803611302453968 | 0.23389812964276 | -0.253167881 | 0.0618477497635498 | 0.908772886808877 | 0.776337538717159 | 1.06380036854024 | 0.998074519377826 |
| ebi-fl187-GCST90094838 | ebi-a-GCST90001784 | CD25 on IgD+ CD38dim B cell \|\| id:ebi-a-GCST90001784 | Shellfish liking \|\| id:ebi-fl187-GCST90094838 | Inverse variance weighted | 30 | -0.071736761 | 0.0702927846547137 | 0.30747133018299 | -0.209510619 | 0.0660370965556569 | 0.930775879734844 | 0.810981027343986 | 1.06826634543289 | 0.998074519377826 |
| ebi-fl187-GCST90094838 | ebi-a-GCST90001794 | CD25 on IgD+ B cell \|\| id:ebi-a-GCST90001794 | Shellfish liking \|\| id:ebi-fl187-GCST90094838 | Inverse variance weighted | 30 | -0.083245374 | 0.0702782418116724 | 0.236210711262344 | -0.220990728 | 0.0544999797881153 | 0.920125344532134 | 0.801724113750609 | 1.05601245506972 | 0.998074519377826 |
| ebi-fl187-GCST90094838 | ebi-a-GCST90001802 | CD27 on IgD- CD38- B cell \|\| id:ebi-a-GCST90001802 | Shellfish liking \|\| id:ebi-fl187-GCST90094838 | Inverse variance weighted | 30 | 0.0255468587284144 | 0.0684109364158959 | 0.708826664706991 | -0.108538577 | 0.15963229410357 | 1.02587597638795 | 0.897144285331245 | 1.17307944344911 | 0.998074519377826 |
| ebi-fl187-GCST90094838 | ebi-a-GCST90001804 | CD27 on IgD- CD38dim B cell \|\| id:ebi-a-GCST90001804 | Shellfish liking \|\| id:ebi-fl187-GCST90094838 | Inverse variance weighted | 30 | 0.0218020171756774 | 0.0675249418305766 | 0.746791014113325 | -0.110546869 | 0.154150903163608 | 1.02204141779207 | 0.895344365479108 | 1.16666692722577 | 0.998074519377826 |
| ebi-fl187-GCST90094838 | ebi-a-GCST90001805 | CD27 on memory B cell \|\| id:ebi-a-GCST90001805 | Shellfish liking \|\| id:ebi-fl187-GCST90094838 | Inverse variance weighted | 30 | -0.056821546 | 0.0684941938162484 | 0.406775190427422 | -0.191070166 | 0.0774270736047703 | 0.944762650738282 | 0.826074623637624 | 1.08050343236494 | 0.998074519377826 |
| ebi-fl187-GCST90094838 | ebi-a-GCST90001806 | CD27 on unswitched memory B cell \|\| id:ebi-a-GCST90001806 | Shellfish liking \|\| id:ebi-fl187-GCST90094838 | Inverse variance weighted | 30 | -0.045567099 | 0.068344809130877 | 0.504948682990603 | -0.179522925 | 0.0883887267522847 | 0.95545549017529 | 0.835668792982425 | 1.09241268953944 | 0.998074519377826 |
| ebi-fl187-GCST90094838 | ebi-a-GCST90001839 | CD3 on Effector Memory CD8+ T cell \|\| id:ebi-a-GCST90001839 | Shellfish liking \|\| id:ebi-fl187-GCST90094838 | Inverse variance weighted | 30 | 0.16135391972078 | 0.0821607759376533 | 0.0495439884525481 | 0.000318798882979948 | 0.322389040558581 | 1.17510078656875 | 1.00031884970474 | 1.38042171153935 | 0.998074519377826 |
| ebi-fl187-GCST90094838 | ebi-a-GCST90001859 | CD3 on CD8+ T cell \|\| id:ebi-a-GCST90001859 | Shellfish liking \|\| id:ebi-fl187-GCST90094838 | Inverse variance weighted | 30 | 0.0458268691755328 | 0.0739324190689302 | 0.535357800519475 | -0.099080672 | 0.190734410550636 | 1.04689314579264 | 0.905669642714257 | 1.21013800950971 | 0.998074519377826 |
| ebi-fl187-GCST90094838 | ebi-a-GCST90001905 | CD86 on monocyte \|\| id:ebi-a-GCST90001905 | Shellfish liking \|\| id:ebi-fl187-GCST90094838 | Inverse variance weighted | 30 | 0.142124009596703 | 0.0752326212185579 | 0.0588747535860573 | -0.005331928 | 0.289579947185076 | 1.15271958796511 | 0.994682261506055 | 1.33586623578323 | 0.998074519377826 |
| ebi-fl187-GCST90094838 | ebi-a-GCST90001943 | CD25 on activated & secreting CD4 regulatory T cell \|\| id:ebi-a-GCST90001943 | Shellfish liking \|\| id:ebi-fl187-GCST90094838 | Inverse variance weighted | 30 | -0.032800655 | 0.0688830488161059 | 0.633946922100655 | -0.167811431 | 0.102210120469506 | 0.967731452585419 | 0.845513257335835 | 1.10761618011048 | 0.998074519377826 |
| ebi-fl187-GCST90094838 | ebi-a-GCST90001949 | CD33 on CD33dim HLA DR+ CD11b- \|\| id:ebi-a-GCST90001949 | Shellfish liking \|\| id:ebi-fl187-GCST90094838 | Inverse variance weighted | 30 | 0.177278661119307 | 0.111593135029183 | 0.112147070521038 | -0.041443884 | 0.396001205776506 | 1.19396375805183 | 0.959403172142649 | 1.48587110917878 | 0.998074519377826 |
| ebi-fl187-GCST90094838 | ebi-a-GCST90001955 | CD33 on Immature Myeloid-Derived Suppressor Cells \|\| id:ebi-a-GCST90001955 | Shellfish liking \|\| id:ebi-fl187-GCST90094838 | Inverse variance weighted | 30 | -0.065551257 | 0.110977958151883 | 0.554742115186657 | -0.283068055 | 0.151965540595995 | 0.936551040375144 | 0.753468508544686 | 1.16412012085538 | 0.998074519377826 |
| ebi-fl187-GCST90094838 | ebi-a-GCST90001956 | CD33 on CD33+ HLA DR+ \|\| id:ebi-a-GCST90001956 | Shellfish liking \|\| id:ebi-fl187-GCST90094838 | Inverse variance weighted (multiplicative random effects) | 30 | 0.0748378653790401 | 0.124988985782176 | 0.549335819912922 | -0.170140547 | 0.319816277512105 | 1.07770940271314 | 0.843546250577156 | 1.37687477823727 | 0.998074519377826 |
| ebi-fl187-GCST90094838 | ebi-a-GCST90001957 | CD33 on CD33+ HLA DR+ CD14- \|\| id:ebi-a-GCST90001957 | Shellfish liking \|\| id:ebi-fl187-GCST90094838 | Inverse variance weighted (multiplicative random effects) | 30 | 0.0690266789597156 | 0.124504009974444 | 0.579296026362915 | -0.175001181 | 0.313054538509626 | 1.07146479433312 | 0.839456029715065 | 1.36759611564764 | 0.998074519377826 |
| ebi-fl187-GCST90094838 | ebi-a-GCST90002064 | CD4 on resting CD4 regulatory T cell \|\| id:ebi-a-GCST90002064 | Shellfish liking \|\| id:ebi-fl187-GCST90094838 | Inverse variance weighted | 30 | -0.127318323 | 0.0732649255573387 | 0.082249583696794 | -0.270917577 | 0.0162809307800245 | 0.880453359154708 | 0.762679355826367 | 1.01641418733155 | 0.998074519377826 |
| ebi-fl187-GCST90094838 | ebi-a-GCST90002080 | SSC-A on T cell \|\| id:ebi-a-GCST90002080 | Shellfish liking \|\| id:ebi-fl187-GCST90094838 | Inverse variance weighted | 30 | 0.125798695997277 | 0.0697901150899504 | 0.0714622396323821 | -0.01098993 | 0.26258732157358 | 1.13405385572322 | 0.989070239078897 | 1.30029000658073 | 0.998074519377826 |
| ebi-fl187-GCST90094838 | ebi-a-GCST90002121 | CD8 on CD39+ CD8+ T cell \|\| id:ebi-a-GCST90002121 | Shellfish liking \|\| id:ebi-fl187-GCST90094838 | Inverse variance weighted | 30 | -0.034265929 | 0.0818533406912427 | 0.675489549021795 | -0.194698477 | 0.126166618337362 | 0.966314499014524 | 0.823082798897021 | 1.13447117623766 | 0.998074519377826 |
| ebi-fl187-GCST90094838 | ebi-cfmet338-GCST90026053 | 1-palmitoyl-2-docosahexaenoyl-gpc (16:0/22:6) levels \|\| id:ebi-cfmet338-GCST90026053 | Shellfish liking \|\| id:ebi-fl187-GCST90094838 | Inverse variance weighted | 27 | 0.035548290336599 | 0.0421801951793855 | 0.399355932250703 | -0.047124892 | 0.118221472888195 | 1.03618768477199 | 0.953968246942635 | 1.1254933500294 | 0.998074519377826 |
| ebi-fl187-GCST90094838 | ebi-cfmet338-GCST90026063 | 1-stearoyl-2-oleoyl-gpc (18:0/18:1) levels \|\| id:ebi-cfmet338-GCST90026063 | Shellfish liking \|\| id:ebi-fl187-GCST90094838 | Inverse variance weighted | 26 | 0.0319914872781996 | 0.0321152034959042 | 0.319178368535045 | -0.030954312 | 0.0949372861301718 | 1.03250871580825 | 0.969519867901098 | 1.09958989342626 | 0.998074519377826 |
| ebi-fl187-GCST90094838 | ebi-cfmet338-GCST90026113 | Adenosine 3',5'-cyclic monophosphate (camp) levels \|\| id:ebi-cfmet338-GCST90026113 | Shellfish liking \|\| id:ebi-fl187-GCST90094838 | Inverse variance weighted | 27 | 0.0329996630454729 | 0.0629648359025794 | 0.60021128001946 | -0.090411415 | 0.156410741414528 | 1.03355019098183 | 0.913555257313471 | 1.16930638702683 | 0.998074519377826 |
| ebi-fl187-GCST90094838 | ebi-cfmet338-GCST90026117 | Alpha-ketoglutaramate levels \|\| id:ebi-cfmet338-GCST90026117 | Shellfish liking \|\| id:ebi-fl187-GCST90094838 | Inverse variance weighted | 27 | -0.012403446 | 0.0248195924689663 | 0.617255249224507 | -0.061049847 | 0.0362429553942966 | 0.987673159837622 | 0.940776343649727 | 1.03690773821885 | 0.998074519377826 |
| ebi-fl187-GCST90094838 | ebi-cfmet338-GCST90026124 | Arginine levels \|\| id:ebi-cfmet338-GCST90026124 | Shellfish liking \|\| id:ebi-fl187-GCST90094838 | Inverse variance weighted | 27 | 0.0278895563727173 | 0.0219895944939716 | 0.204688342324412 | -0.015210049 | 0.0709891615809016 | 1.0282821109436 | 0.984905039717645 | 1.07356959000815 | 0.998074519377826 |
| ebi-fl187-GCST90094838 | ebi-cfmet338-GCST90026169 | Glutamate levels \|\| id:ebi-cfmet338-GCST90026169 | Shellfish liking \|\| id:ebi-fl187-GCST90094838 | Inverse variance weighted | 27 | 0.0271655160962706 | 0.0239021807327158 | 0.255735331395032 | -0.019682758 | 0.0740137903323935 | 1.02753786274508 | 0.980509682687178 | 1.07682165512238 | 0.998074519377826 |
| ebi-fl187-GCST90094838 | ebi-cfmet338-GCST90026170 | Glutamine levels \|\| id:ebi-cfmet338-GCST90026170 | Shellfish liking \|\| id:ebi-fl187-GCST90094838 | Inverse variance weighted | 26 | 0.0107507754644556 | 0.0128225452479475 | 0.401790610223384 | -0.014381413 | 0.0358829641504327 | 1.01080877270309 | 0.985721505339438 | 1.03653452769268 | 0.998074519377826 |
| ebi-fl187-GCST90094838 | ebi-cfmet338-GCST90026191 | Kynurenate levels \|\| id:ebi-cfmet338-GCST90026191 | Shellfish liking \|\| id:ebi-fl187-GCST90094838 | Inverse variance weighted | 27 | -0.172926221 | 0.0958013479121123 | 0.0710668614760913 | -0.360696863 | 0.0148444208620932 | 0.841199675387666 | 0.697190310648676 | 1.01495514648495 | 0.998074519377826 |
| ebi-fl187-GCST90094838 | ebi-cfmet338-GCST90026202 | Methylsuccinoylcarnitine levels \|\| id:ebi-cfmet338-GCST90026202 | Shellfish liking \|\| id:ebi-fl187-GCST90094838 | Inverse variance weighted | 27 | -0.13283443 | 0.0643786892098708 | 0.0390813603694026 | -0.259016661 | -0.006652199 | 0.875610054748143 | 0.771810163819625 | 0.993369877615689 | 0.998074519377826 |
| ebi-fl187-GCST90094838 | ebi-cfmet338-GCST90026222 | N-acetylputrescine levels \|\| id:ebi-cfmet338-GCST90026222 | Shellfish liking \|\| id:ebi-fl187-GCST90094838 | Inverse variance weighted | 27 | 0.0113384333261124 | 0.0231071786950817 | 0.623646583252613 | -0.033951637 | 0.0566285035684726 | 1.01140295699644 | 0.966618252146556 | 1.05826259658301 | 0.998074519377826 |
| ebi-fl187-GCST90094838 | ebi-cfmet338-GCST90026252 | Propionylcarnitine (c3) levels \|\| id:ebi-cfmet338-GCST90026252 | Shellfish liking \|\| id:ebi-fl187-GCST90094838 | Inverse variance weighted | 27 | -0.00429699 | 0.041987099250435 | 0.918486231082603 | -0.086591705 | 0.0779977243513463 | 0.995712228673628 | 0.917051446991169 | 1.08112019841756 | 0.998074519377826 |
| ebi-fl187-GCST90094838 | ebi-cfmet338-GCST90026316 | Ethyl beta-glucopyranoside levels \|\| id:ebi-cfmet338-GCST90026316 | Shellfish liking \|\| id:ebi-fl187-GCST90094838 | Inverse variance weighted | 27 | -0.053147282 | 0.124709113121566 | 0.669983982243445 | -0.297577143 | 0.191282580051345 | 0.948240343778755 | 0.742615293153426 | 1.21080155210846 | 0.998074519377826 |
| ebi-fl187-GCST90094838 | ebi-gm430-GCST90011315 | F_Prevotellaceae abundance \|\| id:ebi-gm430-GCST90011315 | Shellfish liking \|\| id:ebi-fl187-GCST90094838 | Inverse variance weighted | 23 | 0.0225869683626133 | 0.0656716957945604 | 0.730893180717436 | -0.106129555 | 0.151303492119952 | 1.02284398536304 | 0.899308130308533 | 1.16334967196884 | 0.998074519377826 |
| ebi-fl187-GCST90094838 | ebi-gm430-GCST90011354 | OTU97_1 (Bacteroides) abundance \|\| id:ebi-gm430-GCST90011354 | Shellfish liking \|\| id:ebi-fl187-GCST90094838 | Inverse variance weighted | 23 | -0.011687164 | 0.0528471059947405 | 0.824975242353478 | -0.115267492 | 0.0918931633002418 | 0.988380865174603 | 0.891127742303464 | 1.09624769633827 | 0.998074519377826 |
| ebi-fl187-GCST90094838 | ebi-gm430-GCST90011419 | OTU99_1 (Bacteroides) abundance \|\| id:ebi-gm430-GCST90011419 | Shellfish liking \|\| id:ebi-fl187-GCST90094838 | Inverse variance weighted | 23 | -0.01386506 | 0.0528623144845098 | 0.793100733472061 | -0.117475197 | 0.089745076203167 | 0.986230617059812 | 0.889162565748964 | 1.09389538819247 | 0.998074519377826 |
| ebi-fl187-GCST90094838 | ebi-gm430-GCST90011435 | OTU99_155 (Butyrivibrio) abundance \|\| id:ebi-gm430-GCST90011435 | Shellfish liking \|\| id:ebi-fl187-GCST90094838 | Inverse variance weighted | 23 | -0.049829614 | 0.165467085558892 | 0.76330379609501 | -0.374145102 | 0.274485873655999 | 0.951391514448464 | 0.687877092427689 | 1.31585398573175 | 0.998074519377826 |
| ebi-fl187-GCST90094838 | ebi-gm430-GCST90011466 | OTU99_58 (Bacteroides) abundance \|\| id:ebi-gm430-GCST90011466 | Shellfish liking \|\| id:ebi-fl187-GCST90094838 | Inverse variance weighted | 23 | 0.0297734163093803 | 0.0973187158115408 | 0.759652454604107 | -0.160971267 | 0.220518099 | 1.03022107621206 | 0.851316531905007 | 1.24672248933838 | 0.998074519377826 |
| ebi-fl187-GCST90094838 | ebi-gm430-GCST90011472 | OTU99_72 (Parabacteroides) abundance \|\| id:ebi-gm430-GCST90011472 | Shellfish liking \|\| id:ebi-fl187-GCST90094838 | Inverse variance weighted | 23 | -0.178614395 | 0.145967405409539 | 0.221080721767166 | -0.46471051 | 0.107481719235363 | 0.836428367873917 | 0.628316970362875 | 1.11347050546824 | 0.998074519377826 |
| ebi-fl187-GCST90094838 | ebi-gm430-GCST90011505 | TestASV_28 (Holdemanella) abundance \|\| id:ebi-gm430-GCST90011505 | Shellfish liking \|\| id:ebi-fl187-GCST90094838 | Inverse variance weighted | 23 | 0.0688414614729291 | 0.12403476627479 | 0.578882714943593 | -0.17426668 | 0.311949603371518 | 1.07126635869417 | 0.840072836801733 | 1.36608584517383 | 0.998074519377826 |
| ebi-fl187-GCST90094838 | ebi-gm430-GCST90011513 | TestASV_35 (Alphaproteobacteria) abundance \|\| id:ebi-gm430-GCST90011513 | Shellfish liking \|\| id:ebi-fl187-GCST90094838 | Inverse variance weighted | 23 | -0.184425083 | 0.156692413583961 | 0.239200314392902 | -0.491542214 | 0.122692047402968 | 0.831582237028002 | 0.611682321444925 | 1.13053621577121 | 0.998074519377826 |
| ebi-fl187-GCST90094838 | ebi-gm430-GCST90011543 | G_Coprococcus prevalence \|\| id:ebi-gm430-GCST90011543 | Shellfish liking \|\| id:ebi-fl187-GCST90094838 | Inverse variance weighted | 23 | 0.291387489075508 | 0.170447856139436 | 0.0873509025821816 | -0.042690309 | 0.625465287108803 | 1.33828305355608 | 0.958208092584454 | 1.8691154304539 | 0.998074519377826 |
| ebi-fl187-GCST90094838 | ebi-gm430-GCST90011547 | G_Parasutterella prevalence \|\| id:ebi-gm430-GCST90011547 | Shellfish liking \|\| id:ebi-fl187-GCST90094838 | Inverse variance weighted | 23 | 0.13309312982555 | 0.139819268084586 | 0.341150715737319 | -0.140952636 | 0.407138895271339 | 1.14235638082759 | 0.868530448129438 | 1.50251278309016 | 0.998074519377826 |
| ebi-fl187-GCST90094838 | ebi-gm430-GCST90011569 | OTU97_13 (Subdoligranulum) prevalence \|\| id:ebi-gm430-GCST90011569 | Shellfish liking \|\| id:ebi-fl187-GCST90094838 | Inverse variance weighted | 23 | -0.054588034 | 0.166849440360182 | 0.743539752591836 | -0.381612937 | 0.272436869364933 | 0.946875148227025 | 0.682759273024185 | 1.31316055563582 | 0.998074519377826 |
| ebi-fl187-GCST90094838 | ebi-gm430-GCST90011590 | OTU97_277 (Prevotella) prevalence \|\| id:ebi-gm430-GCST90011590 | Shellfish liking \|\| id:ebi-fl187-GCST90094838 | Inverse variance weighted | 23 | 0.249161022861245 | 0.124221039621368 | 0.0448789125427309 | 0.00568778520336358 | 0.492634260519127 | 1.28294860049281 | 1.00570399136478 | 1.63662183469396 | 0.998074519377826 |
| ebi-fl187-GCST90094838 | ebi-gm430-GCST90011616 | OTU97_86 (Phascolarctobacterium) prevalence \|\| id:ebi-gm430-GCST90011616 | Shellfish liking \|\| id:ebi-fl187-GCST90094838 | Inverse variance weighted | 23 | 0.201019058853264 | 0.158721274637376 | 0.205337365321821 | -0.110074639 | 0.51211275714252 | 1.2226480738715 | 0.895767273237231 | 1.66881327014702 | 0.998074519377826 |
| ebi-fl187-GCST90094838 | ebi-gm430-GCST90011665 | OTU99_55 (Barnesiella) prevalence \|\| id:ebi-gm430-GCST90011665 | Shellfish liking \|\| id:ebi-fl187-GCST90094838 | Inverse variance weighted | 23 | 0.158803428591922 | 0.102061413110676 | 0.119717715602966 | -0.041236941 | 0.358843798288847 | 1.17210752120807 | 0.959601733913932 | 1.43167315430856 | 0.998074519377826 |
| ebi-fl187-GCST90094838 | ebi-gm430-GCST90011687 | TestASV_14 (Lachnospiraceae) prevalence \|\| id:ebi-gm430-GCST90011687 | Shellfish liking \|\| id:ebi-fl187-GCST90094838 | Inverse variance weighted | 23 | 0.0367802654058419 | 0.114869647084951 | 0.748823293001517 | -0.188364243 | 0.261924773692346 | 1.03746502883315 | 0.828312945185434 | 1.29942878752281 | 0.998074519377826 |
| ebi-fl187-GCST90094838 | ebi-gm430-GCST90011697 | TestASV_23 (Barnesiella) prevalence \|\| id:ebi-gm430-GCST90011697 | Shellfish liking \|\| id:ebi-fl187-GCST90094838 | Inverse variance weighted | 23 | -0.079210604 | 0.146316623506989 | 0.588256475255257 | -0.365991186 | 0.207569978484114 | 0.923845338811015 | 0.6935089140039 | 1.23068383521605 | 0.998074519377826 |
| ebi-fl187-GCST90094838 | ebi-gm430-GCST90011727 | TestASV_6 (Subdoligranulum) prevalence \|\| id:ebi-gm430-GCST90011727 | Shellfish liking \|\| id:ebi-fl187-GCST90094838 | Inverse variance weighted | 23 | -0.088623612 | 0.170072327398947 | 0.602301523860385 | -0.421965374 | 0.244718149309967 | 0.91518997492174 | 0.655756745171814 | 1.27726126549839 | 0.998074519377826 |
| ebi-fl187-GCST90094838 | ebi-hbsm44-GCST90104908 | Sulcal depth of the left posterior inferior temporal sulcus \|\| id:ebi-hbsm44-GCST90104908 | Shellfish liking \|\| id:ebi-fl187-GCST90094838 | Inverse variance weighted | 26 | 0.0356803462050722 | 0.0464431730452435 | 0.442333832512948 | -0.055348273 | 0.126708965373749 | 1.03632452847191 | 0.946155570160846 | 1.13508662019498 | 0.998074519377826 |
| ebi-fl187-GCST90094838 | ebi-hbsm44-GCST90104918 | Sulcal depth of the left inferior frontal sulcus \|\| id:ebi-hbsm44-GCST90104918 | Shellfish liking \|\| id:ebi-fl187-GCST90094838 | Inverse variance weighted | 24 | 0.0976909244240683 | 0.0814780873065091 | 0.230534160428883 | -0.062006127 | 0.257387975544826 | 1.1026219389268 | 0.939877128431358 | 1.2935468939773 | 0.998074519377826 |
| ebi-fl187-GCST90094838 | ebi-hbsm44-GCST90104925 | Sulcal depth of the right anterior inferior temporal sulcus \|\| id:ebi-hbsm44-GCST90104925 | Shellfish liking \|\| id:ebi-fl187-GCST90094838 | Inverse variance weighted | 26 | -0.044406237 | 0.0261561935586059 | 0.0895581353491254 | -0.095672377 | 0.00685990211324582 | 0.956565286069613 | 0.908761698866536 | 1.00688348513665 | 0.998074519377826 |
| ebi-fl187-GCST90094838 | ebi-IPs91-GCST90274770 | C-C motif chemokine 4 levels \|\| id:ebi-IPs91-GCST90274770 | Shellfish liking \|\| id:ebi-fl187-GCST90094838 | Inverse variance weighted | 37 | 0.044839685295513 | 0.0319195423821801 | 0.16008903272633 | -0.017722618 | 0.107401988364586 | 1.04586017970263 | 0.982433504159849 | 1.11338173103433 | 0.998074519377826 |
| ebi-fl187-GCST90094838 | ebi-IPs91-GCST90274779 | C-X-C motif chemokine 1 levels \|\| id:ebi-IPs91-GCST90274779 | Shellfish liking \|\| id:ebi-fl187-GCST90094838 | Inverse variance weighted | 37 | -0.000966909 | 0.0334505828345555 | 0.976939874282054 | -0.066530052 | 0.0645962329766036 | 0.999033557927122 | 0.935634797984968 | 1.06672822773802 | 0.998074519377826 |
| ebi-fl187-GCST90094838 | ebi-IPs91-GCST90274780 | C-X-C motif chemokine 10 levels \|\| id:ebi-IPs91-GCST90274780 | Shellfish liking \|\| id:ebi-fl187-GCST90094838 | Inverse variance weighted | 37 | -0.017411276 | 0.0288839715924304 | 0.546641324308656 | -0.074023861 | 0.0392013079048575 | 0.982739423960718 | 0.928649535314165 | 1.03997981873745 | 0.998074519377826 |
| ebi-fl187-GCST90094838 | ebi-IPs91-GCST90274796 | Interleukin-10 receptor subunit alpha levels \|\| id:ebi-IPs91-GCST90274796 | Shellfish liking \|\| id:ebi-fl187-GCST90094838 | Inverse variance weighted | 37 | -0.036319495 | 0.0374385903447293 | 0.331992404570874 | -0.109699132 | 0.0370601417531758 | 0.964332144650143 | 0.896103703314567 | 1.03775543139241 | 0.998074519377826 |
| ebi-fl187-GCST90094838 | ebi-IPs91-GCST90274807 | Interleukin-20 levels \|\| id:ebi-IPs91-GCST90274807 | Shellfish liking \|\| id:ebi-fl187-GCST90094838 | Inverse variance weighted | 37 | -0.001885739 | 0.0328686583979665 | 0.954248913532821 | -0.066308309 | 0.0625368317098098 | 0.998116038138021 | 0.935842291011373 | 1.06453366679091 | 0.998074519377826 |
| ebi-fl187-GCST90094838 | ebi-IPs91-GCST90274808 | Interleukin-20 receptor subunit alpha levels \|\| id:ebi-IPs91-GCST90274808 | Shellfish liking \|\| id:ebi-fl187-GCST90094838 | Inverse variance weighted | 37 | -0.047466261 | 0.0325458568746139 | 0.144718610437608 | -0.111256141 | 0.0163236182673627 | 0.953642647341852 | 0.894709548063402 | 1.01645757642538 | 0.998074519377826 |
| ebi-fl187-GCST90094838 | ebi-IPs91-GCST90274810 | Interleukin-24 levels \|\| id:ebi-IPs91-GCST90274810 | Shellfish liking \|\| id:ebi-fl187-GCST90094838 | Inverse variance weighted | 37 | -0.046762942 | 0.0327158099883079 | 0.152898403941899 | -0.110885929 | 0.0173600457933104 | 0.95431359865732 | 0.895040840986858 | 1.01751160715543 | 0.998074519377826 |
| ebi-fl187-GCST90094838 | ebi-IPs91-GCST90274823 | Monocyte chemoattractant protein-3 levels \|\| id:ebi-IPs91-GCST90274823 | Shellfish liking \|\| id:ebi-fl187-GCST90094838 | Inverse variance weighted | 37 | -0.000747282 | 0.0368733407524931 | 0.983831022971918 | -0.07301903 | 0.0715244655721866 | 0.999252996843182 | 0.929583139725238 | 1.07414442993793 | 0.999355215041207 |
| ebi-fl187-GCST90094838 | ebi-IPs91-GCST90274824 | Monocyte chemoattractant protein-4 levels \|\| id:ebi-IPs91-GCST90274824 | Shellfish liking \|\| id:ebi-fl187-GCST90094838 | Inverse variance weighted | 37 | 0.0219951838381321 | 0.0288752219238293 | 0.446219904418357 | -0.034600251 | 0.0785906188088375 | 1.02223886119076 | 0.965991493090844 | 1.0817613786484 | 0.998074519377826 |
| ebi-fl187-GCST90094838 | ebi-IPs91-GCST90274834 | SIR2-like protein 2 levels \|\| id:ebi-IPs91-GCST90274834 | Shellfish liking \|\| id:ebi-fl187-GCST90094838 | Inverse variance weighted | 37 | 0.00334156821444668 | 0.0289307540907175 | 0.908047009152363 | -0.05336271 | 0.0600458462322531 | 1.00334715747741 | 0.948036088130598 | 1.06188522886623 | 0.998074519377826 |
| ebi-fl187-GCST90094838 | ebi-met1400-GCST90199652 | 1-stearoyl-2-oleoyl-GPS (18:0/18:1) levels \|\| id:ebi-met1400-GCST90199652 | Shellfish liking \|\| id:ebi-fl187-GCST90094838 | Inverse variance weighted | 30 | 0.0383885045934809 | 0.0499759813782863 | 0.442404558727616 | -0.059564419 | 0.136341428094922 | 1.03913486313489 | 0.942174837762115 | 1.14607312836665 | 0.998074519377826 |
| ebi-fl187-GCST90094838 | ebi-met1400-GCST90199665 | N-acetylaspartate (naa) levels \|\| id:ebi-met1400-GCST90199665 | Shellfish liking \|\| id:ebi-fl187-GCST90094838 | Inverse variance weighted | 30 | 0.0175559694341087 | 0.0424366162831113 | 0.679094840628934 | -0.065619798 | 0.100731737349007 | 1.01771098126447 | 0.936486850337332 | 1.10597990886173 | 0.998074519377826 |
| ebi-fl187-GCST90094838 | ebi-met1400-GCST90199669 | Acetylcarnitine levels (Biocrates platform) \|\| id:ebi-met1400-GCST90199669 | Shellfish liking \|\| id:ebi-fl187-GCST90094838 | Inverse variance weighted | 30 | 0.0401648434519712 | 0.0452237629899148 | 0.374467701187117 | -0.048473732 | 0.128803418912204 | 1.0409823591752 | 0.952682364029626 | 1.13746649778463 | 0.998074519377826 |
| ebi-fl187-GCST90094838 | ebi-met1400-GCST90199670 | Iminodiacetate (IDA) levels \|\| id:ebi-met1400-GCST90199670 | Shellfish liking \|\| id:ebi-fl187-GCST90094838 | Inverse variance weighted (multiplicative random effects) | 30 | 0.0329113808225479 | 0.0515630403722631 | 0.523294919849682 | -0.068152178 | 0.133974939952184 | 1.03345895090096 | 0.93411831021733 | 1.14336416652494 | 0.998074519377826 |
| ebi-fl187-GCST90094838 | ebi-met1400-GCST90199686 | Indolepropionate levels \|\| id:ebi-met1400-GCST90199686 | Shellfish liking \|\| id:ebi-fl187-GCST90094838 | Inverse variance weighted | 30 | -0.014865093 | 0.0494524813581044 | 0.763724510596896 | -0.111791956 | 0.0820617704323784 | 0.9852448470353 | 0.894230276953939 | 1.08552286097512 | 0.998074519377826 |
| ebi-fl187-GCST90094838 | ebi-met1400-GCST90199704 | 3-hydroxysebacate levels \|\| id:ebi-met1400-GCST90199704 | Shellfish liking \|\| id:ebi-fl187-GCST90094838 | Inverse variance weighted | 30 | 0.0792588440314554 | 0.05252683628052 | 0.131318983715546 | -0.023693755 | 0.182211443141275 | 1.08248448 | 0.976584738084205 | 1.19986787089153 | 0.998074519377826 |
| ebi-fl187-GCST90094838 | ebi-met1400-GCST90199713 | Docosapentaenoate n3 DPA; 22:5n3 levels \|\| id:ebi-met1400-GCST90199713 | Shellfish liking \|\| id:ebi-fl187-GCST90094838 | Inverse variance weighted | 30 | 0.0268070358515071 | 0.0405203849899766 | 0.508247396846966 | -0.052612919 | 0.106226990431861 | 1.02716957673611 | 0.948747183681028 | 1.11207427807972 | 0.998074519377826 |
| ebi-fl187-GCST90094838 | ebi-met1400-GCST90199715 | Myristoleate (14:1n5) levels \|\| id:ebi-met1400-GCST90199715 | Shellfish liking \|\| id:ebi-fl187-GCST90094838 | Inverse variance weighted | 30 | 0.0269206199226449 | 0.0388268863152405 | 0.488089959650113 | -0.049180077 | 0.103021317100516 | 1.02728625346457 | 0.952009678971842 | 1.10851503915066 | 0.998074519377826 |
| ebi-fl187-GCST90094838 | ebi-met1400-GCST90199725 | Campesterol levels \|\| id:ebi-met1400-GCST90199725 | Shellfish liking \|\| id:ebi-fl187-GCST90094838 | Inverse variance weighted | 30 | 0.0674477263169 | 0.0595883425819576 | 0.257678727172156 | -0.049345425 | 0.184240877777537 | 1.06977433709159 | 0.951852279194801 | 1.20230539687088 | 0.998074519377826 |
| ebi-fl187-GCST90094838 | ebi-met1400-GCST90199737 | N-acetylproline levels \|\| id:ebi-met1400-GCST90199737 | Shellfish liking \|\| id:ebi-fl187-GCST90094838 | Inverse variance weighted | 30 | 0.00178892169025005 | 0.0455166926125106 | 0.968649180536438 | -0.087423796 | 0.0910016392107708 | 1.00179052276525 | 0.916288694009626 | 1.09527080063669 | 0.998074519377826 |
| ebi-fl187-GCST90094838 | ebi-met1400-GCST90199775 | Gamma-glutamylisoleucine levels \|\| id:ebi-met1400-GCST90199775 | Shellfish liking \|\| id:ebi-fl187-GCST90094838 | Inverse variance weighted | 30 | 0.0223802156672941 | 0.0460748469135695 | 0.627154296998325 | -0.067926484 | 0.11268691561789 | 1.02263253147226 | 0.934329158930207 | 1.11928144854513 | 0.998074519377826 |
| ebi-fl187-GCST90094838 | ebi-met1400-GCST90199777 | 1-palmitoleoyl-GPC (16:1) levels \|\| id:ebi-met1400-GCST90199777 | Shellfish liking \|\| id:ebi-fl187-GCST90094838 | Inverse variance weighted | 30 | -0.091162403 | 0.0484929928411922 | 0.0601205109906197 | -0.186208669 | 0.00388386291895629 | 0.912869446078168 | 0.830100360680711 | 1.00389141488832 | 0.998074519377826 |
| ebi-fl187-GCST90094838 | ebi-met1400-GCST90199782 | Glutamine degradant levels \|\| id:ebi-met1400-GCST90199782 | Shellfish liking \|\| id:ebi-fl187-GCST90094838 | Inverse variance weighted | 30 | -0.075522669 | 0.0407324377866315 | 0.0637225494693638 | -0.155358247 | 0.00431290869969746 | 0.927258709932282 | 0.856108423902614 | 1.00432222267572 | 0.998074519377826 |
| ebi-fl187-GCST90094838 | ebi-met1400-GCST90199783 | 5-methyluridine (ribothymidine) levels \|\| id:ebi-met1400-GCST90199783 | Shellfish liking \|\| id:ebi-fl187-GCST90094838 | Inverse variance weighted | 30 | -0.016066826 | 0.042080848629279 | 0.70260340405487 | -0.09854529 | 0.0664116370844792 | 0.984061556735103 | 0.906154652355477 | 1.06866652941272 | 0.998074519377826 |
| ebi-fl187-GCST90094838 | ebi-met1400-GCST90199787 | Homostachydrine levels \|\| id:ebi-met1400-GCST90199787 | Shellfish liking \|\| id:ebi-fl187-GCST90094838 | Inverse variance weighted | 30 | -0.034627199 | 0.0468785503233697 | 0.460114917828597 | -0.126509158 | 0.0572547595773804 | 0.965965461976391 | 0.881166080060565 | 1.05892554745994 | 0.998074519377826 |
| ebi-fl187-GCST90094838 | ebi-met1400-GCST90199802 | Beta-hydroxyisovaleroylcarnitine levels \|\| id:ebi-met1400-GCST90199802 | Shellfish liking \|\| id:ebi-fl187-GCST90094838 | Inverse variance weighted | 30 | 0.036804267207915 | 0.0472473278197101 | 0.435997184345501 | -0.055800495 | 0.129409029734547 | 1.03748993016226 | 0.945727794192824 | 1.138155568 | 0.998074519377826 |
| ebi-fl187-GCST90094838 | ebi-met1400-GCST90199873 | Pregnenolone sulfate levels \|\| id:ebi-met1400-GCST90199873 | Shellfish liking \|\| id:ebi-fl187-GCST90094838 | Inverse variance weighted | 30 | 0.0159800667542812 | 0.0402620693709049 | 0.691440262364812 | -0.062933589 | 0.0948937227212547 | 1.0161084308652 | 0.939005831735059 | 1.09954199258546 | 0.998074519377826 |
| ebi-fl187-GCST90094838 | ebi-met1400-GCST90199911 | Beta-citrylglutamate levels \|\| id:ebi-met1400-GCST90199911 | Shellfish liking \|\| id:ebi-fl187-GCST90094838 | Inverse variance weighted | 30 | 0.0749204344499549 | 0.0419219040491855 | 0.0739143903823081 | -0.007246497 | 0.157087366386359 | 1.07779839185106 | 0.992779695070176 | 1.17009783665511 | 0.998074519377826 |
| ebi-fl187-GCST90094838 | ebi-met1400-GCST90199934 | 2-hydroxydecanoate levels \|\| id:ebi-met1400-GCST90199934 | Shellfish liking \|\| id:ebi-fl187-GCST90094838 | Inverse variance weighted | 29 | 0.010861289964553 | 0.0466986105461056 | 0.816085445092164 | -0.080667987 | 0.10239056663492 | 1.01092048790226 | 0.922499922843617 | 1.10781606323646 | 0.998074519377826 |
| ebi-fl187-GCST90094838 | ebi-met1400-GCST90199945 | Sphingomyelin (d18:1/14:0, d16:1/16:0) levels \|\| id:ebi-met1400-GCST90199945 | Shellfish liking \|\| id:ebi-fl187-GCST90094838 | Inverse variance weighted | 30 | 0.0372064486843991 | 0.040423037203413 | 0.357349744413705 | -0.042022704 | 0.116435601603089 | 1.03790727331243 | 0.958848010415471 | 1.12348515749442 | 0.998074519377826 |
| ebi-fl187-GCST90094838 | ebi-met1400-GCST90199979 | Carnitine C14:1 levels \|\| id:ebi-met1400-GCST90199979 | Shellfish liking \|\| id:ebi-fl187-GCST90094838 | Inverse variance weighted | 30 | 0.0530204849341916 | 0.0408303132921487 | 0.194096038279248 | -0.027006929 | 0.133047898986803 | 1.05445124526248 | 0.973354497012396 | 1.14230471225885 | 0.998074519377826 |
| ebi-fl187-GCST90094838 | ebi-met1400-GCST90199986 | Eugenol sulfate levels \|\| id:ebi-met1400-GCST90199986 | Shellfish liking \|\| id:ebi-fl187-GCST90094838 | Inverse variance weighted | 30 | -0.041987065 | 0.0536250187051901 | 0.433641615263535 | -0.147092102 | 0.0631179714127221 | 0.958882183394119 | 0.863214470052153 | 1.06515248936353 | 0.998074519377826 |
| ebi-fl187-GCST90094838 | ebi-met1400-GCST90200002 | 6-hydroxyindole sulfate levels \|\| id:ebi-met1400-GCST90200002 | Shellfish liking \|\| id:ebi-fl187-GCST90094838 | Inverse variance weighted | 30 | -0.011373636 | 0.0487307295953016 | 0.815452672911725 | -0.106885866 | 0.0841385944388304 | 0.98869079970592 | 0.898628231554183 | 1.08777964357132 | 0.998074519377826 |
| ebi-fl187-GCST90094838 | ebi-met1400-GCST90200023 | Trans 3,4-methyleneheptanoate levels \|\| id:ebi-met1400-GCST90200023 | Shellfish liking \|\| id:ebi-fl187-GCST90094838 | Inverse variance weighted | 30 | 0.0288029825382102 | 0.0454144862667494 | 0.525934281992161 | -0.060209411 | 0.117815375621039 | 1.02922179983212 | 0.941567338808422 | 1.12503638304855 | 0.998074519377826 |
| ebi-fl187-GCST90094838 | ebi-met1400-GCST90200032 | 2-hydroxybutyrate/2-hydroxyisobutyrate levels \|\| id:ebi-met1400-GCST90200032 | Shellfish liking \|\| id:ebi-fl187-GCST90094838 | Inverse variance weighted | 30 | 0.136570404354027 | 0.0434710974537906 | 0.00168006238804751 | 0.0513670533445975 | 0.221773755363457 | 1.14633558195092 | 1.05270922281822 | 1.24828892723937 | 0.598008988154769 |
| ebi-fl187-GCST90094838 | ebi-met1400-GCST90200051 | 1-palmitoyl-2-dihomo-linolenoyl-GPC (16:0/20:3n3 or 6) levels \|\| id:ebi-met1400-GCST90200051 | Shellfish liking \|\| id:ebi-fl187-GCST90094838 | Inverse variance weighted | 30 | 0.00534533992062729 | 0.0428239273254927 | 0.90066499397553 | -0.078589558 | 0.0892802374785929 | 1.00535965173921 | 0.924419267418758 | 1.09338702141884 | 0.998074519377826 |
| ebi-fl187-GCST90094838 | ebi-met1400-GCST90200069 | Phenylacetylglutamate levels \|\| id:ebi-met1400-GCST90200069 | Shellfish liking \|\| id:ebi-fl187-GCST90094838 | Inverse variance weighted | 30 | -0.019109801 | 0.0464219807658245 | 0.680592934896846 | -0.110096883 | 0.0718772812164435 | 0.981071633599141 | 0.895747348056764 | 1.07452347175902 | 0.998074519377826 |
| ebi-fl187-GCST90094838 | ebi-met1400-GCST90200072 | Thioproline levels \|\| id:ebi-met1400-GCST90200072 | Shellfish liking \|\| id:ebi-fl187-GCST90094838 | Inverse variance weighted | 30 | 0.00649214758279125 | 0.0435239257866346 | 0.881425228226098 | -0.078814747 | 0.091799042124595 | 1.00651326725218 | 0.924211121507983 | 1.09614452107186 | 0.998074519377826 |
| ebi-fl187-GCST90094838 | ebi-met1400-GCST90200081 | 1-oleoyl-2-docosahexaenoyl-GPC (18:1/22:6) levels \|\| id:ebi-met1400-GCST90200081 | Shellfish liking \|\| id:ebi-fl187-GCST90094838 | Inverse variance weighted | 30 | -0.073393187 | 0.0405281539223357 | 0.0701534569246477 | -0.152828368 | 0.00604199497112005 | 0.929235395174405 | 0.85827701662461 | 1.00606028463954 | 0.998074519377826 |
| ebi-fl187-GCST90094838 | ebi-met1400-GCST90200097 | Furaneol sulfate levels \|\| id:ebi-met1400-GCST90200097 | Shellfish liking \|\| id:ebi-fl187-GCST90094838 | Inverse variance weighted | 30 | -0.084798139 | 0.0500626454016712 | 0.0902955871734709 | -0.182920924 | 0.0133246463879426 | 0.918697715292757 | 0.832834010659784 | 1.01341381509563 | 0.998074519377826 |
| ebi-fl187-GCST90094838 | ebi-met1400-GCST90200124 | Sphingomyelin (d18:2/24:2) levels \|\| id:ebi-met1400-GCST90200124 | Shellfish liking \|\| id:ebi-fl187-GCST90094838 | Inverse variance weighted | 30 | -0.088601566 | 0.0384769115610022 | 0.0212945844117522 | -0.164016313 | -0.013186819 | 0.915210151851524 | 0.84872817681881 | 0.986899745913473 | 0.998074519377826 |
| ebi-fl187-GCST90094838 | ebi-met1400-GCST90200137 | Lignoceroylcarnitine (C24) levels \|\| id:ebi-met1400-GCST90200137 | Shellfish liking \|\| id:ebi-fl187-GCST90094838 | Inverse variance weighted | 30 | 0.00917650712432827 | 0.0424119573858492 | 0.828702438097594 | -0.073950929 | 0.0923039436005927 | 1.00921874035148 | 0.928717265481248 | 1.09669810579956 | 0.998074519377826 |
| ebi-fl187-GCST90094838 | ebi-met1400-GCST90200165 | Octadecenedioate (C18:1-DC) levels \|\| id:ebi-met1400-GCST90200165 | Shellfish liking \|\| id:ebi-fl187-GCST90094838 | Inverse variance weighted | 30 | -0.044855805 | 0.0413860054615835 | 0.278435782937286 | -0.125972375 | 0.0362607659539416 | 0.95613534206742 | 0.881639201328403 | 1.03692620629043 | 0.998074519377826 |
| ebi-fl187-GCST90094838 | ebi-met1400-GCST90200176 | Glutamine conjugate of C6H10O2 (1) levels \|\| id:ebi-met1400-GCST90200176 | Shellfish liking \|\| id:ebi-fl187-GCST90094838 | Inverse variance weighted | 30 | 0.0477632797683425 | 0.0417262252982984 | 0.252340704468781 | -0.034020122 | 0.129546681353007 | 1.04892232479748 | 0.966552055658903 | 1.13831224817842 | 0.998074519377826 |
| ebi-fl187-GCST90094838 | ebi-met1400-GCST90200182 | Glycine conjugate of C10H12O2 levels \|\| id:ebi-met1400-GCST90200182 | Shellfish liking \|\| id:ebi-fl187-GCST90094838 | Inverse variance weighted (multiplicative random effects) | 30 | -0.043983395 | 0.061478865507503 | 0.474347700531858 | -0.164481971 | 0.0765151815889508 | 0.956969848011326 | 0.848333051232993 | 1.07951857902009 | 0.998074519377826 |
| ebi-fl187-GCST90094838 | ebi-met1400-GCST90200208 | Glucuronide of piperine metabolite C17H21NO3 (4) levels \|\| id:ebi-met1400-GCST90200208 | Shellfish liking \|\| id:ebi-fl187-GCST90094838 | Inverse variance weighted | 30 | -0.03227616 | 0.0431717126384091 | 0.454687659248507 | -0.116892717 | 0.0523403965791283 | 0.968239156043301 | 0.889680635683807 | 1.05373436904681 | 0.998074519377826 |
| ebi-fl187-GCST90094838 | ebi-met1400-GCST90200227 | 3-ethylcatechol sulfate (2) levels \|\| id:ebi-met1400-GCST90200227 | Shellfish liking \|\| id:ebi-fl187-GCST90094838 | Inverse variance weighted | 29 | -0.090285478 | 0.0573306810273887 | 0.115298099503547 | -0.202653613 | 0.0220826567748754 | 0.913670315226959 | 0.816561038693075 | 1.02232828333695 | 0.998074519377826 |
| ebi-fl187-GCST90094838 | ebi-met1400-GCST90200259 | Pentose acid levels \|\| id:ebi-met1400-GCST90200259 | Shellfish liking \|\| id:ebi-fl187-GCST90094838 | Inverse variance weighted | 30 | -0.059804867 | 0.0432413399743287 | 0.166650019340544 | -0.144557893 | 0.0249481593691859 | 0.941948320872248 | 0.865404809786423 | 1.02526196891952 | 0.998074519377826 |
| ebi-fl187-GCST90094838 | ebi-met1400-GCST90200291 | Sarcosine levels \|\| id:ebi-met1400-GCST90200291 | Shellfish liking \|\| id:ebi-fl187-GCST90094838 | Inverse variance weighted | 30 | 0.0577269372428781 | 0.0430459340569874 | 0.179902806228199 | -0.026643094 | 0.142096967994573 | 1.05942566651611 | 0.973708702471548 | 1.1526884170021 | 0.998074519377826 |
| ebi-fl187-GCST90094838 | ebi-met1400-GCST90200306 | Butyrate/isobutyrate (4:0) levels \|\| id:ebi-met1400-GCST90200306 | Shellfish liking \|\| id:ebi-fl187-GCST90094838 | Inverse variance weighted | 30 | 0.0187776466193251 | 0.0437260300130675 | 0.667604043019722 | -0.066925372 | 0.104480665444937 | 1.01895505532514 | 0.935264995495603 | 1.11013392971313 | 0.998074519377826 |
| ebi-fl187-GCST90094838 | ebi-met1400-GCST90200321 | Cholate levels \|\| id:ebi-met1400-GCST90200321 | Shellfish liking \|\| id:ebi-fl187-GCST90094838 | Inverse variance weighted | 30 | -0.059761991 | 0.0436828353625175 | 0.171284328848656 | -0.145380349 | 0.0258563660318352 | 0.94198870843338 | 0.864693345663216 | 1.02619354163689 | 0.998074519377826 |
| ebi-fl187-GCST90094838 | ebi-met1400-GCST90200336 | Ceramide (d18:1/16:0) levels \|\| id:ebi-met1400-GCST90200336 | Shellfish liking \|\| id:ebi-fl187-GCST90094838 | Inverse variance weighted | 30 | 0.0473499619666656 | 0.0418497662372899 | 0.257875347295133 | -0.03467558 | 0.129375503791754 | 1.04848887611027 | 0.965918728923138 | 1.13811741134015 | 0.998074519377826 |
| ebi-fl187-GCST90094838 | ebi-met1400-GCST90200358 | Arachidonate (20:4n6) levels \|\| id:ebi-met1400-GCST90200358 | Shellfish liking \|\| id:ebi-fl187-GCST90094838 | Inverse variance weighted | 30 | 0.0221751847540515 | 0.0415285621778592 | 0.59335931778068 | -0.059220797 | 0.103571166622656 | 1.02242288168349 | 0.942498645200031 | 1.1091247232171 | 0.998074519377826 |
| ebi-fl187-GCST90094838 | ebi-met1400-GCST90200413 | 2'-deoxyuridine levels \|\| id:ebi-met1400-GCST90200413 | Shellfish liking \|\| id:ebi-fl187-GCST90094838 | Inverse variance weighted | 30 | -0.041924051 | 0.0420700368643703 | 0.318992725388958 | -0.124381324 | 0.0405332207704899 | 0.958942608075203 | 0.883043051396885 | 1.04136590410558 | 0.998074519377826 |
| ebi-fl187-GCST90094838 | ebi-met1400-GCST90200432 | Threonine levels \|\| id:ebi-met1400-GCST90200432 | Shellfish liking \|\| id:ebi-fl187-GCST90094838 | Inverse variance weighted | 30 | -0.072726771 | 0.0422558152330185 | 0.0852314451673366 | -0.155548169 | 0.0100946268222772 | 0.929854858601367 | 0.855945845973623 | 1.01014574944408 | 0.998074519377826 |
| ebi-fl187-GCST90094838 | ebi-met1400-GCST90200436 | Caffeine levels \|\| id:ebi-met1400-GCST90200436 | Shellfish liking \|\| id:ebi-fl187-GCST90094838 | Inverse variance weighted | 30 | -0.011061006 | 0.0432432570296086 | 0.798116323367285 | -0.09581779 | 0.0736957773971203 | 0.98899994162775 | 0.90862956223429 | 1.07647926635198 | 0.998074519377826 |
| ebi-fl187-GCST90094838 | ebi-met1400-GCST90200442 | Valine levels \|\| id:ebi-met1400-GCST90200442 | Shellfish liking \|\| id:ebi-fl187-GCST90094838 | Inverse variance weighted | 30 | -0.019308133 | 0.0457749074043091 | 0.673166581731797 | -0.109026952 | 0.0704106851513021 | 0.98087707472286 | 0.896706249259071 | 1.07294873489714 | 0.998074519377826 |
| ebi-fl187-GCST90094838 | ebi-met1400-GCST90200459 | Margarate (17:0) levels \|\| id:ebi-met1400-GCST90200459 | Shellfish liking \|\| id:ebi-fl187-GCST90094838 | Inverse variance weighted | 30 | 0.0885824377167189 | 0.0432399919030294 | 0.040499003365073 | 0.00383205358678129 | 0.173332821846657 | 1.09262432235222 | 1.00383940529184 | 1.18926185154944 | 0.998074519377826 |
| ebi-fl187-GCST90094838 | ebi-met1400-GCST90200462 | X-11299 levels \|\| id:ebi-met1400-GCST90200462 | Shellfish liking \|\| id:ebi-fl187-GCST90094838 | Inverse variance weighted | 30 | 0.00232298993580274 | 0.0419764763378886 | 0.955867372862129 | -0.079950904 | 0.0845968835580644 | 1.00232569016739 | 0.923161669108793 | 1.08827827539613 | 0.998074519377826 |
| ebi-fl187-GCST90094838 | ebi-met1400-GCST90200497 | X-12740 levels \|\| id:ebi-met1400-GCST90200497 | Shellfish liking \|\| id:ebi-fl187-GCST90094838 | Inverse variance weighted | 30 | -0.008631774 | 0.0482271652261854 | 0.857952174562687 | -0.103157017 | 0.085893470188924 | 0.99140537314605 | 0.901985334881672 | 1.08969023762653 | 0.998074519377826 |
| ebi-fl187-GCST90094838 | ebi-met1400-GCST90200527 | X-13866 levels \|\| id:ebi-met1400-GCST90200527 | Shellfish liking \|\| id:ebi-fl187-GCST90094838 | Inverse variance weighted | 30 | 0.0247095934941651 | 0.0414516423088588 | 0.55110408089655 | -0.056535625 | 0.105954812419528 | 1.02501740557417 | 0.945032816693844 | 1.11177163710112 | 0.998074519377826 |
| ebi-fl187-GCST90094838 | ebi-met1400-GCST90200547 | X-17654 levels \|\| id:ebi-met1400-GCST90200547 | Shellfish liking \|\| id:ebi-fl187-GCST90094838 | Inverse variance weighted | 30 | 0.00883202953831757 | 0.0507845337747279 | 0.861934781097544 | -0.090705657 | 0.108369715736784 | 1.00887114698855 | 0.913286491136531 | 1.11445970251829 | 0.998074519377826 |
| ebi-fl187-GCST90094838 | ebi-met1400-GCST90200553 | X-17653 levels \|\| id:ebi-met1400-GCST90200553 | Shellfish liking \|\| id:ebi-fl187-GCST90094838 | Inverse variance weighted | 30 | -0.025433567 | 0.0457366168777494 | 0.578150952278429 | -0.115077336 | 0.0642102022829941 | 0.974887141689521 | 0.891297211989155 | 1.0663165173719 | 0.998074519377826 |
| ebi-fl187-GCST90094838 | ebi-met1400-GCST90200557 | X-17685 levels \|\| id:ebi-met1400-GCST90200557 | Shellfish liking \|\| id:ebi-fl187-GCST90094838 | Inverse variance weighted (multiplicative random effects) | 30 | -0.023304982 | 0.0552754477119011 | 0.673305732563795 | -0.13164486 | 0.0850348951527522 | 0.976964481397165 | 0.876652274197948 | 1.0887550583096 | 0.998074519377826 |
| ebi-fl187-GCST90094838 | ebi-met1400-GCST90200561 | X-18779 levels \|\| id:ebi-met1400-GCST90200561 | Shellfish liking \|\| id:ebi-fl187-GCST90094838 | Inverse variance weighted | 30 | 0.010462257049046 | 0.0421095100010922 | 0.803783508850473 | -0.072072383 | 0.0929968966511867 | 1.01051717782498 | 0.930463544046809 | 1.09745832946679 | 0.998074519377826 |
| ebi-fl187-GCST90094838 | ebi-met1400-GCST90200570 | X-18921 levels \|\| id:ebi-met1400-GCST90200570 | Shellfish liking \|\| id:ebi-fl187-GCST90094838 | Inverse variance weighted | 30 | -0.009799269 | 0.0422923140469994 | 0.816768464708794 | -0.092692204 | 0.0730936666180534 | 0.990248587474784 | 0.911474004770998 | 1.07583130167511 | 0.998074519377826 |
| ebi-fl187-GCST90094838 | ebi-met1400-GCST90200575 | X-21283 levels \|\| id:ebi-met1400-GCST90200575 | Shellfish liking \|\| id:ebi-fl187-GCST90094838 | Inverse variance weighted | 30 | -0.021480118 | 0.051007756660834 | 0.673671297358207 | -0.121455321 | 0.0784950847810218 | 0.978748936493677 | 0.885630621263699 | 1.08165803856309 | 0.998074519377826 |
| ebi-fl187-GCST90094838 | ebi-met1400-GCST90200578 | X-18922 levels \|\| id:ebi-met1400-GCST90200578 | Shellfish liking \|\| id:ebi-fl187-GCST90094838 | Inverse variance weighted (multiplicative random effects) | 30 | 0.117790170651438 | 0.0527284077355372 | 0.025489428145737 | 0.0144424914897851 | 0.221137849813091 | 1.12500802689807 | 1.01454728817061 | 1.24749538571755 | 0.998074519377826 |
| ebi-fl187-GCST90094838 | ebi-met1400-GCST90200623 | X-24241 levels \|\| id:ebi-met1400-GCST90200623 | Shellfish liking \|\| id:ebi-fl187-GCST90094838 | Inverse variance weighted | 30 | -0.034058014 | 0.042650475687613 | 0.424558504748601 | -0.117652947 | 0.04953691813331 | 0.966515431377531 | 0.88900453116107 | 1.05078438449674 | 0.998074519377826 |
| ebi-fl187-GCST90094838 | ebi-met1400-GCST90200636 | X-24544 levels \|\| id:ebi-met1400-GCST90200636 | Shellfish liking \|\| id:ebi-fl187-GCST90094838 | Inverse variance weighted | 30 | 0.00706639768861513 | 0.0395656361143202 | 0.858252289104228 | -0.070482249 | 0.0846150444726827 | 1.00709142358969 | 0.931944282232973 | 1.08829803970444 | 0.998074519377826 |
| ebi-fl187-GCST90094838 | ebi-met1400-GCST90200659 | X-25172 levels \|\| id:ebi-met1400-GCST90200659 | Shellfish liking \|\| id:ebi-fl187-GCST90094838 | Inverse variance weighted | 30 | -0.039036293 | 0.0428419363164131 | 0.362205729760253 | -0.123006489 | 0.0449339016825237 | 0.961715804482678 | 0.884257924877426 | 1.04595872151213 | 0.998074519377826 |
| ebi-fl187-GCST90094838 | ebi-met1400-GCST90200670 | X-26111 levels \|\| id:ebi-met1400-GCST90200670 | Shellfish liking \|\| id:ebi-fl187-GCST90094838 | Inverse variance weighted | 30 | -0.00382077 | 0.0425868304451861 | 0.928511961859914 | -0.087290958 | 0.0796494172609456 | 0.996186519444406 | 0.916410419819215 | 1.08290735249227 | 0.998074519377826 |
| ebi-fl187-GCST90094838 | ebi-met1400-GCST90200673 | Carnitine C4 levels \|\| id:ebi-met1400-GCST90200673 | Shellfish liking \|\| id:ebi-fl187-GCST90094838 | Inverse variance weighted | 30 | 0.0179616664028002 | 0.0453720615307547 | 0.692197219617245 | -0.070967574 | 0.106890907003079 | 1.01812394728844 | 0.931492096016652 | 1.11281284776856 | 0.998074519377826 |
| ebi-fl187-GCST90094838 | ebi-met1400-GCST90200710 | X-13431 levels \|\| id:ebi-met1400-GCST90200710 | Shellfish liking \|\| id:ebi-fl187-GCST90094838 | Inverse variance weighted | 30 | 0.0827695030414809 | 0.0435322055161315 | 0.0572575548948175 | -0.00255362 | 0.168092625853099 | 1.0862913928272 | 0.997449637943251 | 1.18304618623522 | 0.998074519377826 |
| ebi-fl187-GCST90094838 | ebi-met1400-GCST90200730 | Adenosine 5'-diphosphate (ADP) to fructose ratio \|\| id:ebi-met1400-GCST90200730 | Shellfish liking \|\| id:ebi-fl187-GCST90094838 | Inverse variance weighted | 30 | 0.127352653304316 | 0.0565894459544038 | 0.0244193467093371 | 0.016437339233685 | 0.238267967374948 | 1.13581749695559 | 1.0165731755361 | 1.26904921105175 | 0.998074519377826 |
| ebi-fl187-GCST90094838 | ebi-met1400-GCST90200731 | Adenosine 5'-diphosphate (ADP) to mannose ratio \|\| id:ebi-met1400-GCST90200731 | Shellfish liking \|\| id:ebi-fl187-GCST90094838 | Inverse variance weighted | 30 | 0.0104502259572972 | 0.0570527253019575 | 0.854666306802762 | -0.101373116 | 0.122273567549134 | 1.01050502027323 | 0.903595824251537 | 1.13006320811987 | 0.998074519377826 |
| ebi-fl187-GCST90094838 | ebi-met1400-GCST90200748 | Aspartate to glutamate ratio \|\| id:ebi-met1400-GCST90200748 | Shellfish liking \|\| id:ebi-fl187-GCST90094838 | Inverse variance weighted | 30 | -0.024888021 | 0.0475805106072462 | 0.600924320367633 | -0.118145822 | 0.0683697796140799 | 0.975419132200257 | 0.888566470656937 | 1.07076118093774 | 0.998074519377826 |
| ebi-fl187-GCST90094838 | ebi-met1400-GCST90200774 | Uridine to pseudouridine ratio \|\| id:ebi-met1400-GCST90200774 | Shellfish liking \|\| id:ebi-fl187-GCST90094838 | Inverse variance weighted | 30 | -0.013732156 | 0.0424169875327931 | 0.746133452611993 | -0.096869451 | 0.0694051397921244 | 0.986361700172928 | 0.907674494091047 | 1.0718703785351 | 0.998074519377826 |
| ebi-fl187-GCST90094838 | ebi-met1400-GCST90200799 | Spermidine to adenosine 5'-diphosphate (ADP) ratio \|\| id:ebi-met1400-GCST90200799 | Shellfish liking \|\| id:ebi-fl187-GCST90094838 | Inverse variance weighted | 30 | -0.105918753 | 0.0587132247160731 | 0.0712310525361801 | -0.220996673 | 0.00915916776994084 | 0.899497726892601 | 0.801719347512174 | 1.0092012413018 | 0.998074519377826 |
| ebi-fl187-GCST90094838 | ebi-met1400-GCST90200811 | Choline to taurocholate ratio \|\| id:ebi-met1400-GCST90200811 | Shellfish liking \|\| id:ebi-fl187-GCST90094838 | Inverse variance weighted | 30 | -0.040582159 | 0.0441619343903899 | 0.358127064444417 | -0.12713955 | 0.0459752325495546 | 0.960230269850417 | 0.8806107745589 | 1.0470484779145 | 0.998074519377826 |
| ebi-fl187-GCST90094838 | ebi-met1400-GCST90200812 | Glutamate to kynurenine ratio \|\| id:ebi-met1400-GCST90200812 | Shellfish liking \|\| id:ebi-fl187-GCST90094838 | Inverse variance weighted | 30 | 0.0734204056399918 | 0.0410688154279223 | 0.0738178017654132 | -0.007074473 | 0.15391528387872 | 1.07618287517565 | 0.992950492576033 | 1.16639207038073 | 0.998074519377826 |
| ebi-fl187-GCST90094838 | ebi-met1400-GCST90200876 | Cortisone to 4-cholesten-3-one ratio \|\| id:ebi-met1400-GCST90200876 | Shellfish liking \|\| id:ebi-fl187-GCST90094838 | Inverse variance weighted | 30 | -0.009037087 | 0.0488225011886013 | 0.853149926114531 | -0.104729189 | 0.0866550153121513 | 0.991003624722559 | 0.900568373075155 | 1.09052040197651 | 0.998074519377826 |
| ebi-fl187-GCST90094838 | ebi-met1400-GCST90200906 | 2'-deoxyuridine to cytidine ratio \|\| id:ebi-met1400-GCST90200906 | Shellfish liking \|\| id:ebi-fl187-GCST90094838 | Inverse variance weighted | 30 | 0.0204331005649432 | 0.0523521672531869 | 0.696314021583031 | -0.082177147 | 0.12304334838119 | 1.02064328549999 | 0.921108772355596 | 1.13093344401898 | 0.998074519377826 |
| ebi-fl187-GCST90094838 | ebi-met1400-GCST90200907 | Retinol (Vitamin A) to linoleoyl-arachidonoyl-glycerol (18:2 to 20:4) [1] ratio \|\| id:ebi-met1400-GCST90200907 | Shellfish liking \|\| id:ebi-fl187-GCST90094838 | Inverse variance weighted (multiplicative random effects) | 30 | 0.0229519254660866 | 0.0593636938549205 | 0.699028392563873 | -0.093400914 | 0.139304765421731 | 1.02321734766771 | 0.910828262838468 | 1.14947436666644 | 0.998074519377826 |
| ebi-fl187-GCST90094838 | ebi-met1400-GCST90200916 | Fructose to sucrose ratio \|\| id:ebi-met1400-GCST90200916 | Shellfish liking \|\| id:ebi-fl187-GCST90094838 | Inverse variance weighted | 30 | -0.000817572 | 0.0434425713933366 | 0.98498500784518 | -0.085965012 | 0.0843298675584883 | 0.999182761748778 | 0.917626336290361 | 1.08798772647694 | 0.999355215041207 |
| ebi-fl187-GCST90094838 | ebi-met1400-GCST90200917 | Alpha-ketobutyrate to 3-methyl-2-oxobutyrate ratio \|\| id:ebi-met1400-GCST90200917 | Shellfish liking \|\| id:ebi-fl187-GCST90094838 | Inverse variance weighted | 30 | 0.0939533458286851 | 0.0439061202007823 | 0.0323653544537779 | 0.00789735023515176 | 0.180009341422218 | 1.09850849471814 | 1.00792861655836 | 1.19722854688692 | 0.998074519377826 |
| ebi-fl187-GCST90094838 | ebi-met1400-GCST90200924 | Alpha-tocopherol to glycerol ratio \|\| id:ebi-met1400-GCST90200924 | Shellfish liking \|\| id:ebi-fl187-GCST90094838 | Inverse variance weighted | 30 | -0.007251069 | 0.0393970505882312 | 0.85397340370839 | -0.084469288 | 0.069967150469424 | 0.992775156888924 | 0.918999879418025 | 1.07247295044258 | 0.998074519377826 |
| ebi-fl187-GCST90094838 | ebi-met1400-GCST90200938 | Alpha-ketoglutarate to alanine ratio \|\| id:ebi-met1400-GCST90200938 | Shellfish liking \|\| id:ebi-fl187-GCST90094838 | Inverse variance weighted | 30 | 0.044676835695608 | 0.0432340475045604 | 0.301430044161978 | -0.040061897 | 0.129415568804546 | 1.04568987565816 | 0.960729970611778 | 1.13816301094227 | 0.998074519377826 |
| ebi-fl187-GCST90094838 | ebi-met1400-GCST90200943 | Phosphate to EDTA ratio \|\| id:ebi-met1400-GCST90200943 | Shellfish liking \|\| id:ebi-fl187-GCST90094838 | Inverse variance weighted | 30 | -0.008047965 | 0.0448133431065549 | 0.857475570918323 | -0.095882117 | 0.0797861878446346 | 0.991984333520337 | 0.908571114723856 | 1.08305547249195 | 0.998074519377826 |
| ebi-fl187-GCST90094838 | ebi-met1400-GCST90200947 | Adenosine 5'-diphosphate (ADP) to glucose ratio \|\| id:ebi-met1400-GCST90200947 | Shellfish liking \|\| id:ebi-fl187-GCST90094838 | Inverse variance weighted | 30 | 0.0808354229173586 | 0.0562175398561946 | 0.150461307297322 | -0.029350955 | 0.1910218010355 | 1.08419244865272 | 0.971075600623742 | 1.21048584163844 | 0.998074519377826 |
| ebi-fl187-GCST90094838 | ebi-met1400-GCST90200975 | Arachidonate (20:4n6) to caffeine ratio \|\| id:ebi-met1400-GCST90200975 | Shellfish liking \|\| id:ebi-fl187-GCST90094838 | Inverse variance weighted | 30 | 0.00355630896530385 | 0.0432466478913757 | 0.934461296595343 | -0.081207121 | 0.0883197388324003 | 1.00356264013501 | 0.922002705634868 | 1.09233732885985 | 0.998074519377826 |
| ebi-fl187-GCST90094838 | ebi-met1400-GCST90200985 | Cholesterol to oleoyl-linoleoyl-glycerol (18:1 to 18:2) [2] ratio \|\| id:ebi-met1400-GCST90200985 | Shellfish liking \|\| id:ebi-fl187-GCST90094838 | Inverse variance weighted | 30 | 0.020330100459541 | 0.0422308193671531 | 0.630229140386946 | -0.062442306 | 0.103102506419161 | 1.02053816454783 | 0.939467263343875 | 1.10860504238499 | 0.998074519377826 |
| ebi-fl187-GCST90094838 | ebi-met1400-GCST90201016 | Bilirubin (Z,Z) to etiocholanolone glucuronide ratio \|\| id:ebi-met1400-GCST90201016 | Shellfish liking \|\| id:ebi-fl187-GCST90094838 | Inverse variance weighted | 30 | 0.0195857798292136 | 0.0514882604537238 | 0.703653623929023 | -0.081331211 | 0.120502770318512 | 1.0197788395636 | 0.92188830164031 | 1.12806386605765 | 0.998074519377826 |
| ebi-fl187-GCST90094838 | ebi-pw11-GCST90275189 | Placental weight (fetal genotype, adjusted for sex and gestational age) \|\| id:ebi-pw11-GCST90275189 | Shellfish liking \|\| id:ebi-fl187-GCST90094838 | Inverse variance weighted (multiplicative random effects) | 33 | 0.00865182421546147 | 0.0182543744380658 | 0.63552917750323 | -0.02712675 | 0.0444303981140705 | 1.00868935941782 | 0.973237876113814 | 1.0454322101247 | 0.998074519377826 |
| ebi-fl187-GCST90094838 | ebi-pw11-GCST90275192 | Placental weight (fetal effect, conditional on maternal and paternal genotype) \|\| id:ebi-pw11-GCST90275192 | Shellfish liking \|\| id:ebi-fl187-GCST90094838 | Inverse variance weighted | 33 | -0.008454158 | 0.0313654546075457 | 0.787516286868114 | -0.069930449 | 0.053022132884979 | 0.991581477754611 | 0.93245867091905 | 1.05445298294768 | 0.998074519377826 |
| ebi-fl187-GCST90094838 | ebi-pw11-GCST90275193 | Placental weight (maternal effect, conditional on fetal and paternal genotype) \|\| id:ebi-pw11-GCST90275193 | Shellfish liking \|\| id:ebi-fl187-GCST90094838 | Inverse variance weighted | 33 | 0.0253367660336189 | 0.027973229960333 | 0.365067945195836 | -0.029490765 | 0.0801642967558716 | 1.0256604699785 | 0.97093984453157 | 1.08346506284747 | 0.998074519377826 |
| ebi-fl187-GCST90094838 | ebi-pw11-GCST90275195 | Placental weight (fetal effect, conditional on maternal genotype) \|\| id:ebi-pw11-GCST90275195 | Shellfish liking \|\| id:ebi-fl187-GCST90094838 | Inverse variance weighted (multiplicative random effects) | 33 | -0.007856416 | 0.024394674624987 | 0.747411367854168 | -0.055669978 | 0.039957146263316 | 0.99217436497241 | 0.945851235852041 | 1.04076617251721 | 0.998074519377826 |
| ebi-fl187-GCST90094838 | ebi-pw11-GCST90275197 | Placental weight (fetal genotype, adjusted for sex) \|\| id:ebi-pw11-GCST90275197 | Shellfish liking \|\| id:ebi-fl187-GCST90094838 | Inverse variance weighted | 33 | 0.0127434166924187 | 0.0177115579437841 | 0.471834614844684 | -0.021971237 | 0.0474580702622356 | 1.01282496003905 | 0.978268372699686 | 1.04860223258287 | 0.998074519377826 |
| ebi-fl187-GCST90094838 | prot-a-1030 | Protein FAM171B \|\| id:prot-a-1030 | Shellfish liking \|\| id:ebi-fl187-GCST90094838 | Inverse variance weighted | 34 | -0.026612838 | 0.0636538025094573 | 0.675883388684161 | -0.151374291 | 0.0981486150791302 | 0.973738163125826 | 0.859525925834413 | 1.10312671419096 | 0.998074519377826 |
| ebi-fl187-GCST90094838 | prot-a-1036 | BRISC complex subunit Abro1 \|\| id:prot-a-1036 | Shellfish liking \|\| id:ebi-fl187-GCST90094838 | Inverse variance weighted | 34 | 0.0144478863235029 | 0.0614221847654225 | 0.814035961668631 | -0.105939596 | 0.134835368463731 | 1.01455276149929 | 0.899478978728091 | 1.14434837301184 | 0.998074519377826 |
| ebi-fl187-GCST90094838 | prot-a-1055 | Phenylalanine--tRNA ligase, mitochondrial \|\| id:prot-a-1055 | Shellfish liking \|\| id:ebi-fl187-GCST90094838 | Inverse variance weighted | 34 | -0.036077141 | 0.0663512699363065 | 0.586627651263127 | -0.16612563 | 0.0939713483863774 | 0.964565883336518 | 0.846939826655065 | 1.0985282708587 | 0.998074519377826 |
| ebi-fl187-GCST90094838 | prot-a-1083 | Fc receptor-like protein 5 \|\| id:prot-a-1083 | Shellfish liking \|\| id:ebi-fl187-GCST90094838 | Inverse variance weighted (multiplicative random effects) | 34 | 0.0267238648989904 | 0.0747177499867722 | 0.720594621127359 | -0.119722925 | 0.173170654873064 | 1.0270841496166 | 0.887166214178382 | 1.189069008 | 0.998074519377826 |
| ebi-fl187-GCST90094838 | prot-a-1103 | Fibroblast growth factor receptor 3 \|\| id:prot-a-1103 | Shellfish liking \|\| id:ebi-fl187-GCST90094838 | Inverse variance weighted | 34 | -0.084679513 | 0.0614209465876551 | 0.167995035782823 | -0.205064568 | 0.0357055427077949 | 0.918806703187906 | 0.814594718030173 | 1.03635064055468 | 0.998074519377826 |
| ebi-fl187-GCST90094838 | prot-a-1105 | Fibroblast growth factor receptor 4 \|\| id:prot-a-1105 | Shellfish liking \|\| id:ebi-fl187-GCST90094838 | Inverse variance weighted | 34 | 0.0167624041042455 | 0.0621591445473435 | 0.787414790935693 | -0.105069519 | 0.138594327417039 | 1.01690368147884 | 0.900261934913567 | 1.14865802640485 | 0.998074519377826 |
| ebi-fl187-GCST90094838 | prot-a-1120 | Filamin-A \|\| id:prot-a-1120 | Shellfish liking \|\| id:ebi-fl187-GCST90094838 | Inverse variance weighted | 34 | -0.123478221 | 0.0634584706752719 | 0.0516773893460893 | -0.247856823 | 0.000900381899358482 | 0.883840890537384 | 0.780471680757288 | 1.00090078736482 | 0.998074519377826 |
| ebi-fl187-GCST90094838 | prot-a-1166 | Galanin peptides \|\| id:prot-a-1166 | Shellfish liking \|\| id:ebi-fl187-GCST90094838 | Inverse variance weighted | 34 | -0.074386796 | 0.0711475901867176 | 0.295779016790961 | -0.213836073 | 0.0650624806726398 | 0.928312556719022 | 0.807480742183161 | 1.0672257032809 | 0.998074519377826 |
| ebi-fl187-GCST90094838 | prot-a-1205 | ADP-ribosylation factor-binding protein GGA1 \|\| id:prot-a-1205 | Shellfish liking \|\| id:ebi-fl187-GCST90094838 | Inverse variance weighted | 34 | -0.042055095 | 0.0618081002096695 | 0.496242373225153 | -0.163198972 | 0.0790887811153588 | 0.958816952813782 | 0.849422160622397 | 1.08230040564221 | 0.998074519377826 |
| ebi-fl187-GCST90094838 | prot-a-1227 | Guanine nucleotide-binding protein G(I)/G(S)/G(O) subunit gamma-T2 \|\| id:prot-a-1227 | Shellfish liking \|\| id:ebi-fl187-GCST90094838 | Inverse variance weighted | 34 | 0.111353933399812 | 0.0637439304705191 | 0.080655607794095 | -0.01358417 | 0.23629203712203 | 1.11779046022842 | 0.986507678153782 | 1.26654413406693 | 0.998074519377826 |
| ebi-fl187-GCST90094838 | prot-a-1240 | Golgi SNAP receptor complex member 1 \|\| id:prot-a-1240 | Shellfish liking \|\| id:ebi-fl187-GCST90094838 | Inverse variance weighted | 34 | -0.010398951 | 0.0614364571658483 | 0.865589364685951 | -0.130814407 | 0.110016504732415 | 0.989654930847161 | 0.877380594664311 | 1.11629649448177 | 0.998074519377826 |
| ebi-fl187-GCST90094838 | prot-a-1283 | Glutathione S-transferase A1 \|\| id:prot-a-1283 | Shellfish liking \|\| id:ebi-fl187-GCST90094838 | Inverse variance weighted (multiplicative random effects) | 34 | -0.012943302 | 0.085741975869096 | 0.880010093185734 | -0.180997574 | 0.155110970931445 | 0.987140102528261 | 0.834437382641725 | 1.16778754438653 | 0.998074519377826 |
| ebi-fl187-GCST90094838 | prot-a-1324 | E3 ubiquitin-protein ligase HECW1 \|\| id:prot-a-1324 | Shellfish liking \|\| id:ebi-fl187-GCST90094838 | Inverse variance weighted | 34 | 0.00655756905430445 | 0.0614112311722676 | 0.914962509498356 | -0.113808444 | 0.126923582151949 | 1.00657911698519 | 0.892428889579896 | 1.1353302549715 | 0.998074519377826 |
| ebi-fl187-GCST90094838 | prot-a-1339 | Histidine triad nucleotide-binding protein 2, mitochondrial \|\| id:prot-a-1339 | Shellfish liking \|\| id:ebi-fl187-GCST90094838 | Inverse variance weighted | 34 | -0.134817826 | 0.0614209465876551 | 0.0281649433012993 | -0.255202881 | -0.01443277 | 0.873875094809499 | 0.774759298098365 | 0.985670882818311 | 0.998074519377826 |
| ebi-fl187-GCST90094838 | prot-a-1466 | Interleukin-11 \|\| id:prot-a-1466 | Shellfish liking \|\| id:ebi-fl187-GCST90094838 | Inverse variance weighted | 34 | 0.0370839463863802 | 0.0678589252171329 | 0.584731971617868 | -0.095919547 | 0.170087439811961 | 1.03778013507384 | 0.908537107628756 | 1.18540849868507 | 0.998074519377826 |
| ebi-fl187-GCST90094838 | prot-a-147 | Rho GTPase-activating protein 1 \|\| id:prot-a-147 | Shellfish liking \|\| id:ebi-fl187-GCST90094838 | Inverse variance weighted | 34 | 0.011058764322096 | 0.0614283019256099 | 0.857131225711759 | -0.109340707 | 0.131458236096291 | 1.01112013848843 | 0.896424946803496 | 1.14049027540168 | 0.998074519377826 |
| ebi-fl187-GCST90094838 | prot-a-1473 | Interleukin-12 receptor subunit beta-1 \|\| id:prot-a-1473 | Shellfish liking \|\| id:ebi-fl187-GCST90094838 | Inverse variance weighted | 34 | -0.058309316 | 0.0726129506743682 | 0.421965844224291 | -0.2006307 | 0.0840120670320921 | 0.94335810627425 | 0.818214542714098 | 1.08764201834078 | 0.998074519377826 |
| ebi-fl187-GCST90094838 | prot-a-1501 | X-linked interleukin-1 receptor accessory protein-like 2 \|\| id:prot-a-1501 | Shellfish liking \|\| id:ebi-fl187-GCST90094838 | Inverse variance weighted | 34 | -0.097298447 | 0.0649015006450734 | 0.133829279933952 | -0.224505388 | 0.0299084945609444 | 0.907285189449275 | 0.798911272180161 | 1.03036024607295 | 0.998074519377826 |
| ebi-fl187-GCST90094838 | prot-a-1503 | Interleukin-1 receptor-like 2 \|\| id:prot-a-1503 | Shellfish liking \|\| id:ebi-fl187-GCST90094838 | Inverse variance weighted | 34 | -0.125644045 | 0.0620225328045061 | 0.0427872699966584 | -0.24720821 | -0.004079881 | 0.881928717481538 | 0.780978069376729 | 0.995928430281486 | 0.998074519377826 |
| ebi-fl187-GCST90094838 | prot-a-1536 | Interleukin-5 receptor subunit alpha \|\| id:prot-a-1536 | Shellfish liking \|\| id:ebi-fl187-GCST90094838 | Inverse variance weighted | 34 | 0.0262897178252269 | 0.0701661190019457 | 0.707899778868392 | -0.111235875 | 0.16381531106904 | 1.02663834081883 | 0.89472767977101 | 1.17799673204364 | 0.998074519377826 |
| ebi-fl187-GCST90094838 | prot-a-1559 | Insulin-induced gene 1 protein \|\| id:prot-a-1559 | Shellfish liking \|\| id:ebi-fl187-GCST90094838 | Inverse variance weighted | 34 | -0.0508465 | 0.0614280233265093 | 0.407817131715958 | -0.171245426 | 0.069552425470749 | 0.950424549267424 | 0.842614748551257 | 1.07202826131786 | 0.998074519377826 |
| ebi-fl187-GCST90094838 | prot-a-1567 | Interferon regulatory factor 1 \|\| id:prot-a-1567 | Shellfish liking \|\| id:ebi-fl187-GCST90094838 | Inverse variance weighted | 34 | -0.074431367 | 0.0614280233265093 | 0.225633278703572 | -0.194830293 | 0.0459675586855755 | 0.928271181876839 | 0.822974310909185 | 1.04704044303773 | 0.998074519377826 |
| ebi-fl187-GCST90094838 | prot-a-1613 | Potassium voltage-gated channel subfamily E regulatory beta subunit 5 \|\| id:prot-a-1613 | Shellfish liking \|\| id:ebi-fl187-GCST90094838 | Inverse variance weighted | 34 | -0.055503975 | 0.0614221847654225 | 0.36618259206261 | -0.175891457 | 0.0648835069648584 | 0.946008263114213 | 0.838709014124447 | 1.06703471503119 | 0.998074519377826 |
| ebi-fl187-GCST90094838 | prot-a-1649 | Kin of IRRE-like protein 2 \|\| id:prot-a-1649 | Shellfish liking \|\| id:ebi-fl187-GCST90094838 | Inverse variance weighted | 34 | -0.026110314 | 0.0614221847654225 | 0.670766822074645 | -0.146497796 | 0.0942771678996738 | 0.974227612501531 | 0.863727635659482 | 1.09886427361532 | 0.998074519377826 |
| ebi-fl187-GCST90094838 | prot-a-165 | Armadillo repeat-containing protein 5 \|\| id:prot-a-165 | Shellfish liking \|\| id:ebi-fl187-GCST90094838 | Inverse variance weighted (multiplicative random effects) | 34 | -0.024461518 | 0.0753454449586639 | 0.745439763922585 | -0.17213859 | 0.123215554591892 | 0.975835240749077 | 0.841862491658504 | 1.1311282145518 | 0.998074519377826 |
| ebi-fl187-GCST90094838 | prot-a-1656 | Kallikrein-11 \|\| id:prot-a-1656 | Shellfish liking \|\| id:ebi-fl187-GCST90094838 | Inverse variance weighted | 34 | -0.109378694 | 0.0623089807463482 | 0.0791867710708937 | -0.231504297 | 0.0127469079038265 | 0.896390895039248 | 0.79333928681688 | 1.01282849603127 | 0.998074519377826 |
| ebi-fl187-GCST90094838 | prot-a-1697 | Laminin subunit gamma-2 \|\| id:prot-a-1697 | Shellfish liking \|\| id:ebi-fl187-GCST90094838 | Inverse variance weighted | 34 | -0.071963689 | 0.0614280233265093 | 0.241392889767433 | -0.192362615 | 0.0484352367110828 | 0.930564684923759 | 0.825007654318372 | 1.049627392 | 0.998074519377826 |
| ebi-fl187-GCST90094838 | prot-a-1698 | Lysosome-associated membrane glycoprotein 3 \|\| id:prot-a-1698 | Shellfish liking \|\| id:ebi-fl187-GCST90094838 | Inverse variance weighted | 34 | -0.046074155 | 0.0614041912852119 | 0.453048642696831 | -0.16642637 | 0.0742780599557853 | 0.954971143714929 | 0.846685156168139 | 1.07710626398072 | 0.998074519377826 |
| ebi-fl187-GCST90094838 | prot-a-1724 | Leptin receptor \|\| id:prot-a-1724 | Shellfish liking \|\| id:ebi-fl187-GCST90094838 | Inverse variance weighted | 34 | 0.0338501218126555 | 0.0659183037898102 | 0.607590229282845 | -0.095349754 | 0.163049997240684 | 1.03442955668221 | 0.909054933610896 | 1.17709553974629 | 0.998074519377826 |
| ebi-fl187-GCST90094838 | prot-a-1743 | Leukocyte immunoglobulin-like receptor subfamily B member 2 \|\| id:prot-a-1743 | Shellfish liking \|\| id:ebi-fl187-GCST90094838 | Inverse variance weighted | 34 | -0.063696138 | 0.0623632094266533 | 0.307077448296219 | -0.185928029 | 0.0585357521354247 | 0.938290066594492 | 0.830333352905129 | 1.06028289239453 | 0.998074519377826 |
| ebi-fl187-GCST90094838 | prot-a-1746 | Leukocyte immunoglobulin-like receptor subfamily B member 4 \|\| id:prot-a-1746 | Shellfish liking \|\| id:ebi-fl187-GCST90094838 | Inverse variance weighted | 34 | -0.026052051 | 0.0623279755818732 | 0.675959348316571 | -0.148214883 | 0.0961107810186844 | 0.974284375694208 | 0.862245812840467 | 1.10088101395916 | 0.998074519377826 |
| ebi-fl187-GCST90094838 | prot-a-1750 | Leucine-rich repeat and immunoglobulin-like domain-containing nogo receptor-interacting protein 3 \|\| id:prot-a-1750 | Shellfish liking \|\| id:ebi-fl187-GCST90094838 | Inverse variance weighted | 34 | 0.044890762755163 | 0.0614058053101225 | 0.464748127839845 | -0.075464616 | 0.165246141163003 | 1.04591360094806 | 0.927312542302558 | 1.17968345163524 | 0.998074519377826 |
| ebi-fl187-GCST90094838 | prot-a-1753 | Lipase member N \|\| id:prot-a-1753 | Shellfish liking \|\| id:ebi-fl187-GCST90094838 | Inverse variance weighted | 34 | 0.0604725490993528 | 0.0649863496252929 | 0.352090358720652 | -0.066900696 | 0.187845794364927 | 1.06233843502322 | 0.935288074416851 | 1.20664742916908 | 0.998074519377826 |
| ebi-fl187-GCST90094838 | prot-a-1760 | Ig Kappa chain V-I region HK102- like \|\| id:prot-a-1760 | Shellfish liking \|\| id:ebi-fl187-GCST90094838 | Inverse variance weighted | 34 | -0.071545882 | 0.0618704607403171 | 0.247524974657253 | -0.192811985 | 0.0497202210329176 | 0.930953562586731 | 0.824637003633957 | 1.05097701397551 | 0.998074519377826 |
| ebi-fl187-GCST90094838 | prot-a-1792 | Leucine-rich repeat neuronal protein 1 \|\| id:prot-a-1792 | Shellfish liking \|\| id:ebi-fl187-GCST90094838 | Inverse variance weighted | 34 | -0.010255234 | 0.0618703546862457 | 0.86835086402066 | -0.131521129 | 0.111010661264049 | 0.989797171693457 | 0.876760749771237 | 1.11740681976011 | 0.998074519377826 |
| ebi-fl187-GCST90094838 | prot-a-1795 | Leucine-rich repeat transmembrane neuronal protein 1 \|\| id:prot-a-1795 | Shellfish liking \|\| id:ebi-fl187-GCST90094838 | Inverse variance weighted | 34 | 0.00458382030316011 | 0.0614179996832985 | 0.940506578088199 | -0.115795459 | 0.124963099682425 | 1.00459434207794 | 0.890657380551023 | 1.13310664030051 | 0.998074519377826 |
| ebi-fl187-GCST90094838 | prot-a-1820 | LysM and putative peptidoglycan-binding domain-containing protein 4 \|\| id:prot-a-1820 | Shellfish liking \|\| id:ebi-fl187-GCST90094838 | Inverse variance weighted | 34 | 0.0194492636427646 | 0.0655544605837796 | 0.766704232043775 | -0.109037479 | 0.147936006386973 | 1.01963963274761 | 0.896696809477754 | 1.15943869731754 | 0.998074519377826 |
| ebi-fl187-GCST90094838 | prot-a-1844 | Mitogen-activated protein kinase kinase kinase 7:TGF-beta-activated kinase 1 and MAP3K7-binding protein 1 fusion \|\| id:prot-a-1844 | Shellfish liking \|\| id:ebi-fl187-GCST90094838 | Inverse variance weighted | 34 | -0.025621331 | 0.0614232793678283 | 0.67658524677644 | -0.146010958 | 0.0947682966229927 | 0.974704110027151 | 0.864148233365296 | 1.09940408997193 | 0.998074519377826 |
| ebi-fl187-GCST90094838 | prot-a-1856 | Megakaryocyte-associated tyrosine-protein kinase \|\| id:prot-a-1856 | Shellfish liking \|\| id:ebi-fl187-GCST90094838 | Inverse variance weighted | 34 | 0.0293993254314258 | 0.0630861281192889 | 0.641202001694566 | -0.094249486 | 0.153048136545232 | 1.0298357519828 | 0.910055688051728 | 1.16538107501142 | 0.998074519377826 |
| ebi-fl187-GCST90094838 | prot-a-1910 | Stromelysin-2 \|\| id:prot-a-1910 | Shellfish liking \|\| id:ebi-fl187-GCST90094838 | Inverse variance weighted | 34 | -0.063112786 | 0.0687687636297791 | 0.35874783981512 | -0.197899562 | 0.0716739911507207 | 0.938837580391951 | 0.820452253355042 | 1.07430505401365 | 0.998074519377826 |
| ebi-fl187-GCST90094838 | prot-a-1940 | 39S ribosomal protein L14, mitochondrial \|\| id:prot-a-1940 | Shellfish liking \|\| id:ebi-fl187-GCST90094838 | Inverse variance weighted | 34 | 0.0059456798977659 | 0.0614209465876551 | 0.922883513747803 | -0.114439375 | 0.12633073520957 | 1.00596339053569 | 0.891866005786537 | 1.13465737737767 | 0.998074519377826 |
| ebi-fl187-GCST90094838 | prot-a-1942 | 39S ribosomal protein L33, mitochondrial \|\| id:prot-a-1942 | Shellfish liking \|\| id:ebi-fl187-GCST90094838 | Inverse variance weighted | 34 | -0.035491286 | 0.0726553086552767 | 0.625203834769024 | -0.177895691 | 0.106913118668839 | 0.96513114406095 | 0.8370297284518 | 1.1128375654701 | 0.998074519377826 |
| ebi-fl187-GCST90094838 | prot-a-1948 | Mesothelin \|\| id:prot-a-1948 | Shellfish liking \|\| id:ebi-fl187-GCST90094838 | Inverse variance weighted | 34 | 0.035282163211407 | 0.0614209465876551 | 0.565675437422381 | -0.085102892 | 0.155667218523211 | 1.03591196381232 | 0.918417781605154 | 1.16843730409268 | 0.998074519377826 |
| ebi-fl187-GCST90094838 | prot-a-1980 | Unconventional myosin-VI \|\| id:prot-a-1980 | Shellfish liking \|\| id:ebi-fl187-GCST90094838 | Inverse variance weighted | 34 | -0.021126609 | 0.0729495535376976 | 0.772117874740053 | -0.164107734 | 0.121854515452477 | 0.979094994012258 | 0.848650588080113 | 1.12958975197148 | 0.998074519377826 |
| ebi-fl187-GCST90094838 | prot-a-2039 | Neurofascin \|\| id:prot-a-2039 | Shellfish liking \|\| id:ebi-fl187-GCST90094838 | Inverse variance weighted | 34 | 0.0386329339103849 | 0.0614067700185806 | 0.529262963457829 | -0.081724335 | 0.158990203146803 | 1.0393888892041 | 0.921525955837889 | 1.17232646151422 | 0.998074519377826 |
| ebi-fl187-GCST90094838 | prot-a-2041 | NFU1 iron-sulfur cluster scaffold homolog, mitochondrial \|\| id:prot-a-2041 | Shellfish liking \|\| id:ebi-fl187-GCST90094838 | Inverse variance weighted | 34 | -0.098533395 | 0.0614164833164889 | 0.108637428394694 | -0.218909702 | 0.0218429124552108 | 0.906165430854775 | 0.803394259656431 | 1.0220832153162 | 0.998074519377826 |
| ebi-fl187-GCST90094838 | prot-a-2088 | Glucocorticoid receptor \|\| id:prot-a-2088 | Shellfish liking \|\| id:ebi-fl187-GCST90094838 | Inverse variance weighted | 34 | 0.0223968203255728 | 0.0727015158476289 | 0.758032581409763 | -0.120098151 | 0.164891791386925 | 1.02264951207696 | 0.886833389095679 | 1.17926550512231 | 0.998074519377826 |
| ebi-fl187-GCST90094838 | prot-a-2111 | Neuron-specific protein family member 2 \|\| id:prot-a-2111 | Shellfish liking \|\| id:ebi-fl187-GCST90094838 | Inverse variance weighted | 34 | -0.078911728 | 0.0614213160132143 | 0.198875722893108 | -0.199297507 | 0.0414740518807944 | 0.924121495354781 | 0.819306107857864 | 1.04234611457933 | 0.998074519377826 |
| ebi-fl187-GCST90094838 | prot-a-2125 | Nuclear migration protein nudC \|\| id:prot-a-2125 | Shellfish liking \|\| id:ebi-fl187-GCST90094838 | Inverse variance weighted | 34 | -0.068421611 | 0.0645366910534393 | 0.289054950298605 | -0.194913526 | 0.0580703032040243 | 0.933866661864671 | 0.822905815162727 | 1.05978949968874 | 0.998074519377826 |
| ebi-fl187-GCST90094838 | prot-a-2142 | Ornithine decarboxylase \|\| id:prot-a-2142 | Shellfish liking \|\| id:ebi-fl187-GCST90094838 | Inverse variance weighted | 34 | 0.0130579595996395 | 0.0614067700185806 | 0.831602383025954 | -0.10729931 | 0.133415228836058 | 1.01314358705479 | 0.898256775841248 | 1.14272439195233 | 0.998074519377826 |
| ebi-fl187-GCST90094838 | prot-a-2186 | Prostate and testis expressed protein 1 \|\| id:prot-a-2186 | Shellfish liking \|\| id:ebi-fl187-GCST90094838 | Inverse variance weighted | 34 | 0.0483940834033355 | 0.0700193285167544 | 0.489469266861902 | -0.0888438 | 0.185631967296174 | 1.04958419754671 | 0.914988483166138 | 1.20397907515493 | 0.998074519377826 |
| ebi-fl187-GCST90094838 | prot-a-2227 | Platelet-derived growth factor C \|\| id:prot-a-2227 | Shellfish liking \|\| id:ebi-fl187-GCST90094838 | Inverse variance weighted | 34 | -0.032236759 | 0.0689591568623863 | 0.640159275215576 | -0.167396706 | 0.102923188549188 | 0.968277306667695 | 0.845863985153768 | 1.10840626751263 | 0.998074519377826 |
| ebi-fl187-GCST90094838 | prot-a-2229 | Platelet-derived growth factor receptor alpha \|\| id:prot-a-2229 | Shellfish liking \|\| id:ebi-fl187-GCST90094838 | Inverse variance weighted | 34 | -0.043775663 | 0.0720402313191569 | 0.543415797117211 | -0.184974516 | 0.097423190432045 | 0.957168661779665 | 0.831125463701778 | 1.10232676906866 | 0.998074519377826 |
| ebi-fl187-GCST90094838 | prot-a-2243 | Platelet endothelial aggregation receptor 1 \|\| id:prot-a-2243 | Shellfish liking \|\| id:ebi-fl187-GCST90094838 | Inverse variance weighted | 34 | -0.038879044 | 0.0614864283470625 | 0.527178377255997 | -0.159392443 | 0.0816343557690215 | 0.96186704590162 | 0.852661671896385 | 1.08505899172625 | 0.998074519377826 |
| ebi-fl187-GCST90094838 | prot-a-2246 | E3 ubiquitin-protein ligase pellino homolog 2 \|\| id:prot-a-2246 | Shellfish liking \|\| id:ebi-fl187-GCST90094838 | Inverse variance weighted | 34 | 0.0331045566061092 | 0.0623143125547268 | 0.595244680764676 | -0.089031496 | 0.155240609213374 | 1.03365860942756 | 0.914816760050968 | 1.16793894417084 | 0.998074519377826 |
| ebi-fl187-GCST90094838 | prot-a-2251 | Platelet factor 4 variant \|\| id:prot-a-2251 | Shellfish liking \|\| id:ebi-fl187-GCST90094838 | Inverse variance weighted | 34 | 0.0332835820686388 | 0.0614082856660253 | 0.587815238726189 | -0.087076658 | 0.153643821974048 | 1.03384367720364 | 0.916606827843307 | 1.16607548234045 | 0.998074519377826 |
| ebi-fl187-GCST90094838 | prot-a-2346 | Protein phosphatase 1L \|\| id:prot-a-2346 | Shellfish liking \|\| id:ebi-fl187-GCST90094838 | Inverse variance weighted | 34 | 0.085925034494211 | 0.0682283491597491 | 0.207895141079061 | -0.04780253 | 0.219652598847319 | 1.0897246334847 | 0.953322021125619 | 1.24564391727912 | 0.998074519377826 |
| ebi-fl187-GCST90094838 | prot-a-2358 | PR domain zinc finger protein 4 \|\| id:prot-a-2358 | Shellfish liking \|\| id:ebi-fl187-GCST90094838 | Inverse variance weighted | 34 | 0.0277350376071411 | 0.0614221847654225 | 0.651594955895554 | -0.092652445 | 0.148122519747369 | 1.02812323433618 | 0.91151024561868 | 1.15965496829324 | 0.998074519377826 |
| ebi-fl187-GCST90094838 | prot-a-2390 | Trypsin-1 \|\| id:prot-a-2390 | Shellfish liking \|\| id:ebi-fl187-GCST90094838 | Inverse variance weighted | 34 | -0.07592591 | 0.0614232793678283 | 0.216417798725976 | -0.196315538 | 0.0444637176096126 | 0.926884876961099 | 0.821752899899675 | 1.04546704397889 | 0.998074519377826 |
| ebi-fl187-GCST90094838 | prot-a-242 | Brain-derived neurotrophic factor \|\| id:prot-a-242 | Shellfish liking \|\| id:ebi-fl187-GCST90094838 | Inverse variance weighted | 34 | -0.039711587 | 0.0614235709359605 | 0.51794244068857 | -0.160101786 | 0.0806786121919749 | 0.961066583432262 | 0.85205705717744 | 1.08402244897763 | 0.998074519377826 |
| ebi-fl187-GCST90094838 | prot-a-2464 | Dihydropteridine reductase \|\| id:prot-a-2464 | Shellfish liking \|\| id:ebi-fl187-GCST90094838 | Inverse variance weighted | 34 | 0.0112577072468722 | 0.0731315382268189 | 0.877658788476094 | -0.132080108 | 0.154595522171437 | 1.01132131369661 | 0.876270796249161 | 1.16718576485141 | 0.998074519377826 |
| ebi-fl187-GCST90094838 | prot-a-2474 | Ras-related protein Rab-27A \|\| id:prot-a-2474 | Shellfish liking \|\| id:ebi-fl187-GCST90094838 | Inverse variance weighted | 34 | -0.075064548 | 0.0712930158672526 | 0.292385978541817 | -0.214798859 | 0.0646697628632443 | 0.927683604055188 | 0.806703684774282 | 1.06680666702746 | 0.998074519377826 |
| ebi-fl187-GCST90094838 | prot-a-2478 | Ras-related protein Rab-7b \|\| id:prot-a-2478 | Shellfish liking \|\| id:ebi-fl187-GCST90094838 | Inverse variance weighted (multiplicative random effects) | 34 | -0.009094384 | 0.0772355916035586 | 0.906266846139368 | -0.160476143 | 0.142287375666843 | 0.990946844954643 | 0.851738142889481 | 1.15290791861717 | 0.998074519377826 |
| ebi-fl187-GCST90094838 | prot-a-2528 | E3 ubiquitin-protein ligase rififylin \|\| id:prot-a-2528 | Shellfish liking \|\| id:ebi-fl187-GCST90094838 | Inverse variance weighted | 34 | 0.0129670507085672 | 0.0629652367756494 | 0.836837905555753 | -0.110444813 | 0.13637891478884 | 1.01305148748119 | 0.895435744905573 | 1.14611609166449 | 0.998074519377826 |
| ebi-fl187-GCST90094838 | prot-a-2543 | Protein RIC-3 \|\| id:prot-a-2543 | Shellfish liking \|\| id:ebi-fl187-GCST90094838 | Inverse variance weighted | 34 | -0.004510269 | 0.0698459090417213 | 0.94851273733995 | -0.141408251 | 0.132387712591471 | 0.995499886859026 | 0.868134822561318 | 1.14155082710824 | 0.998074519377826 |
| ebi-fl187-GCST90094838 | prot-a-255 | Bone morphogenetic protein 6 \|\| id:prot-a-255 | Shellfish liking \|\| id:ebi-fl187-GCST90094838 | Inverse variance weighted (multiplicative random effects) | 34 | -0.010773173 | 0.0740352972145717 | 0.884305059613905 | -0.155882355 | 0.134336009984733 | 0.989284650236377 | 0.855659848470344 | 1.14377707560182 | 0.998074519377826 |
| ebi-fl187-GCST90094838 | prot-a-2568 | RING finger protein 165 \|\| id:prot-a-2568 | Shellfish liking \|\| id:ebi-fl187-GCST90094838 | Inverse variance weighted | 34 | -0.053842927 | 0.0670842554879463 | 0.422196459243859 | -0.185328068 | 0.0776422139797239 | 0.947580934404804 | 0.830831670240318 | 1.08073591728606 | 0.998074519377826 |
| ebi-fl187-GCST90094838 | prot-a-2573 | E3 ubiquitin-protein ligase RNF8 \|\| id:prot-a-2573 | Shellfish liking \|\| id:ebi-fl187-GCST90094838 | Inverse variance weighted (multiplicative random effects) | 34 | -0.036008234 | 0.0774577683743424 | 0.64202047953332 | -0.18782546 | 0.115808992404188 | 0.964632351044313 | 0.82875934657821 | 1.12278139187594 | 0.998074519377826 |
| ebi-fl187-GCST90094838 | prot-a-260 | Cytoplasmic tyrosine-protein kinase BMX \|\| id:prot-a-260 | Shellfish liking \|\| id:ebi-fl187-GCST90094838 | Inverse variance weighted | 34 | 0.0796143276238341 | 0.0727436215447607 | 0.27375723705792 | -0.062963171 | 0.222191825851565 | 1.08286935433292 | 0.938978055047046 | 1.24881090910549 | 0.998074519377826 |
| ebi-fl187-GCST90094838 | prot-a-2623 | Serum amyloid A-1 protein \|\| id:prot-a-2623 | Shellfish liking \|\| id:ebi-fl187-GCST90094838 | Inverse variance weighted | 34 | 0.0507813962386596 | 0.0614042161481257 | 0.408236050880487 | -0.069570867 | 0.171133659888986 | 1.05209287668275 | 0.932794026343901 | 1.18664934584229 | 0.998074519377826 |
| ebi-fl187-GCST90094838 | prot-a-2640 | Lysosome membrane protein 2 \|\| id:prot-a-2640 | Shellfish liking \|\| id:ebi-fl187-GCST90094838 | Inverse variance weighted | 34 | -0.002695498 | 0.0657907378571297 | 0.967319183420847 | -0.131645345 | 0.126254347770879 | 0.997308131164874 | 0.876651849239835 | 1.13457070711712 | 0.998074519377826 |
| ebi-fl187-GCST90094838 | prot-a-2641 | Scavenger receptor class F member 2 \|\| id:prot-a-2641 | Shellfish liking \|\| id:ebi-fl187-GCST90094838 | Inverse variance weighted | 34 | -0.03724947 | 0.0614125676285508 | 0.544153078789203 | -0.157618103 | 0.0831191623434205 | 0.963435756847488 | 0.854175927116166 | 1.08667129113094 | 0.998074519377826 |
| ebi-fl187-GCST90094838 | prot-a-2669 | Semaphorin-3A \|\| id:prot-a-2669 | Shellfish liking \|\| id:ebi-fl187-GCST90094838 | Inverse variance weighted | 34 | 0.00720350644882789 | 0.0614221847654225 | 0.906639300642423 | -0.113183976 | 0.127590988589056 | 1.00722951411269 | 0.892986357220129 | 1.13608823460402 | 0.998074519377826 |
| ebi-fl187-GCST90094838 | prot-a-2683 | Sentrin-specific protease 7 \|\| id:prot-a-2683 | Shellfish liking \|\| id:ebi-fl187-GCST90094838 | Inverse variance weighted | 34 | 0.0212672600410368 | 0.0723612478835124 | 0.768831263130344 | -0.120560786 | 0.163095305892721 | 1.02149501996009 | 0.886423203754699 | 1.17714887356675 | 0.998074519377826 |
| ebi-fl187-GCST90094838 | prot-a-270 | Bromodomain testis-specific protein \|\| id:prot-a-270 | Shellfish liking \|\| id:ebi-fl187-GCST90094838 | Inverse variance weighted | 34 | -0.090078109 | 0.0614153884674535 | 0.142456981693934 | -0.21045227 | 0.0302960528958528 | 0.913859802264764 | 0.810217726006326 | 1.03075964816508 | 0.998074519377826 |
| ebi-fl187-GCST90094838 | prot-a-2708 | 14-3-3 protein sigma \|\| id:prot-a-2708 | Shellfish liking \|\| id:ebi-fl187-GCST90094838 | Inverse variance weighted | 34 | 0.0612597130302255 | 0.0614209465876551 | 0.318582549717284 | -0.059125342 | 0.181644768342029 | 1.06317499873485 | 0.94258861554477 | 1.19918812862127 | 0.998074519377826 |
| ebi-fl187-GCST90094838 | prot-a-2713 | Beta-sarcoglycan \|\| id:prot-a-2713 | Shellfish liking \|\| id:ebi-fl187-GCST90094838 | Inverse variance weighted | 34 | -0.076367034 | 0.0614283019256099 | 0.213797926698069 | -0.196766506 | 0.0440324377561029 | 0.926476095902964 | 0.821382398956176 | 1.04501625232098 | 0.998074519377826 |
| ebi-fl187-GCST90094838 | prot-a-2720 | Protein shisa-3 homolog \|\| id:prot-a-2720 | Shellfish liking \|\| id:ebi-fl187-GCST90094838 | Inverse variance weighted | 34 | -0.043038152 | 0.0685204085941189 | 0.529933810598124 | -0.177338153 | 0.0912618486761355 | 0.957874844367578 | 0.837496534658382 | 1.09555583754919 | 0.998074519377826 |
| ebi-fl187-GCST90094838 | prot-a-2730 | Sialic acid-binding Ig-like lectin 8 \|\| id:prot-a-2730 | Shellfish liking \|\| id:ebi-fl187-GCST90094838 | Inverse variance weighted | 34 | -0.056390619 | 0.0614018499465706 | 0.358416648012363 | -0.176738245 | 0.0639570066391934 | 0.945169862223933 | 0.83799910614668 | 1.06604656485164 | 0.998074519377826 |
| ebi-fl187-GCST90094838 | prot-a-2761 | Sodium- and chloride-dependent neutral and basic amino acid transporter B(0+) \|\| id:prot-a-2761 | Shellfish liking \|\| id:ebi-fl187-GCST90094838 | Inverse variance weighted (multiplicative random effects) | 34 | 0.0164698347895045 | 0.0738414465917965 | 0.823501860825135 | -0.128259401 | 0.161199070109426 | 1.0166062101832 | 0.879625174310912 | 1.17491883675644 | 0.998074519377826 |
| ebi-fl187-GCST90094838 | prot-a-2770 | Secreted Ly-6/uPAR-related protein 1 \|\| id:prot-a-2770 | Shellfish liking \|\| id:ebi-fl187-GCST90094838 | Inverse variance weighted | 34 | 0.033564986105908 | 0.0614209465876551 | 0.584739755295916 | -0.086820069 | 0.153950041417712 | 1.03413464592627 | 0.916842048910549 | 1.16643261200316 | 0.998074519377826 |
| ebi-fl187-GCST90094838 | prot-a-2784 | N-lysine methyltransferase SMYD2 \|\| id:prot-a-2784 | Shellfish liking \|\| id:ebi-fl187-GCST90094838 | Inverse variance weighted | 34 | -0.036820562 | 0.0614280233265093 | 0.548899615139462 | -0.157219488 | 0.0835783639347852 | 0.963849071189637 | 0.854516482542267 | 1.08717040690573 | 0.998074519377826 |
| ebi-fl187-GCST90094838 | prot-a-2837 | Serine palmitoyltransferase 2 \|\| id:prot-a-2837 | Shellfish liking \|\| id:ebi-fl187-GCST90094838 | Inverse variance weighted (multiplicative random effects) | 34 | 0.055370881672234 | 0.0856207465107629 | 0.517826539656467 | -0.112445781 | 0.223187544833329 | 1.05693253889971 | 0.893645797939687 | 1.25005499310856 | 0.998074519377826 |
| ebi-fl187-GCST90094838 | prot-a-2858 | Alpha-2,8-sialyltransferase 8B \|\| id:prot-a-2858 | Shellfish liking \|\| id:ebi-fl187-GCST90094838 | Inverse variance weighted | 34 | -0.026972529 | 0.0710332031483527 | 0.704155348346071 | -0.166197607 | 0.112252548904275 | 0.973387980838878 | 0.846878868090752 | 1.11879537551533 | 0.998074519377826 |
| ebi-fl187-GCST90094838 | prot-a-2911 | Synaptotagmin-11 \|\| id:prot-a-2911 | Shellfish liking \|\| id:ebi-fl187-GCST90094838 | Inverse variance weighted | 34 | -0.093354923 | 0.0614162166871798 | 0.128501673395053 | -0.213730707 | 0.0270208621307124 | 0.910870154536384 | 0.807565827339326 | 1.02738923606845 | 0.998074519377826 |
| ebi-fl187-GCST90094838 | prot-a-2962 | Transforming growth factor beta-1 \|\| id:prot-a-2962 | Shellfish liking \|\| id:ebi-fl187-GCST90094838 | Inverse variance weighted | 34 | -0.117470238 | 0.0614191070927015 | 0.0557991671832476 | -0.237851688 | 0.002911211742904 | 0.889166974598961 | 0.788319599632869 | 1.00291545343497 | 0.998074519377826 |
| ebi-fl187-GCST90094838 | prot-a-2978 | Tyrosine-protein kinase receptor Tie-1, soluble \|\| id:prot-a-2978 | Shellfish liking \|\| id:ebi-fl187-GCST90094838 | Inverse variance weighted | 34 | -0.074265771 | 0.0636928817743446 | 0.243615263706844 | -0.19910382 | 0.050572277027846 | 0.928424912399655 | 0.819464812466772 | 1.05187288685353 | 0.998074519377826 |
| ebi-fl187-GCST90094838 | prot-a-299 | Uncharacterized protein C1orf226 \|\| id:prot-a-299 | Shellfish liking \|\| id:ebi-fl187-GCST90094838 | Inverse variance weighted | 34 | 0.0815547600482209 | 0.0614221847654225 | 0.184252885753011 | -0.038832722 | 0.201942242188449 | 1.08497262911078 | 0.961911602249559 | 1.22377732337006 | 0.998074519377826 |
| ebi-fl187-GCST90094838 | prot-a-3015 | Transmembrane protein 70, mitochondrial \|\| id:prot-a-3015 | Shellfish liking \|\| id:ebi-fl187-GCST90094838 | Inverse variance weighted | 34 | 0.0298916382867319 | 0.0654939387808543 | 0.648100119438452 | -0.098476482 | 0.158259758297206 | 1.03034287818449 | 0.906217005025528 | 1.17147045435943 | 0.998074519377826 |
| ebi-fl187-GCST90094838 | prot-a-3016 | Transmembrane protein 87B \|\| id:prot-a-3016 | Shellfish liking \|\| id:ebi-fl187-GCST90094838 | Inverse variance weighted | 34 | -0.097515126 | 0.069798876738945 | 0.162387314070471 | -0.234320924 | 0.0392906726941508 | 0.907088621088816 | 0.791107889546952 | 1.04007276046762 | 0.998074519377826 |
| ebi-fl187-GCST90094838 | prot-a-3123 | Thioredoxin domain-containing protein 12 \|\| id:prot-a-3123 | Shellfish liking \|\| id:ebi-fl187-GCST90094838 | Inverse variance weighted | 33 | -0.092510546 | 0.0620984247459224 | 0.136292448216087 | -0.214223459 | 0.0292023663753334 | 0.911639596647097 | 0.807167996215164 | 1.02963293647899 | 0.998074519377826 |
| ebi-fl187-GCST90094838 | prot-a-3133 | PolyUbiquitin K48-linked \|\| id:prot-a-3133 | Shellfish liking \|\| id:ebi-fl187-GCST90094838 | Inverse variance weighted | 34 | 0.107476227229841 | 0.0664134112399106 | 0.105599894357561 | -0.022694059 | 0.237646513260065 | 1.11346439029887 | 0.97756151437046 | 1.26826080020351 | 0.998074519377826 |
| ebi-fl187-GCST90094838 | prot-a-3135 | Ubiquitin-conjugating enzyme E2 B \|\| id:prot-a-3135 | Shellfish liking \|\| id:ebi-fl187-GCST90094838 | Inverse variance weighted | 34 | 0.00633905170331608 | 0.0614170300460244 | 0.917793718580276 | -0.114038327 | 0.126716430593524 | 1.00635918601323 | 0.892223758800321 | 1.13509509389769 | 0.998074519377826 |
| ebi-fl187-GCST90094838 | prot-a-3162 | NKG2D ligand 3 \|\| id:prot-a-3162 | Shellfish liking \|\| id:ebi-fl187-GCST90094838 | Inverse variance weighted | 34 | 0.0107674970258 | 0.0711920045673838 | 0.879781763459964 | -0.128768832 | 0.150303825977872 | 1.01082567514531 | 0.879177179751468 | 1.16218729178323 | 0.998074519377826 |
| ebi-fl187-GCST90094838 | prot-a-3188 | Vesicle-associated membrane protein 8 \|\| id:prot-a-3188 | Shellfish liking \|\| id:ebi-fl187-GCST90094838 | Inverse variance weighted | 34 | 0.0105820691121159 | 0.068754377572651 | 0.877679748942385 | -0.124176511 | 0.145340649154512 | 1.01063825722606 | 0.883223928445776 | 1.15643344125232 | 0.998074519377826 |
| ebi-fl187-GCST90094838 | prot-a-3201 | Vitrin \|\| id:prot-a-3201 | Shellfish liking \|\| id:ebi-fl187-GCST90094838 | Inverse variance weighted | 34 | -0.09527246 | 0.0614151100440166 | 0.120832929807957 | -0.215646075 | 0.0251011561746881 | 0.909125200912872 | 0.806020522051045 | 1.02541884272585 | 0.998074519377826 |
| ebi-fl187-GCST90094838 | prot-a-3232 | WNT1-inducible-signaling pathway protein 3 \|\| id:prot-a-3232 | Shellfish liking \|\| id:ebi-fl187-GCST90094838 | Inverse variance weighted | 34 | 0.0316199641584045 | 0.0729100176547154 | 0.664517368470263 | -0.11128367 | 0.174523598761647 | 1.03212518619841 | 0.894684917259993 | 1.19067884059963 | 0.998074519377826 |
| ebi-fl187-GCST90094838 | prot-a-327 | Carbonic anhydrase-related protein 10 \|\| id:prot-a-327 | Shellfish liking \|\| id:ebi-fl187-GCST90094838 | Inverse variance weighted (multiplicative random effects) | 34 | -0.097993026 | 0.0767081185598865 | 0.201433117030778 | -0.248340939 | 0.0523548859749299 | 0.906655226380543 | 0.780093933660223 | 1.05374963713177 | 0.998074519377826 |
| ebi-fl187-GCST90094838 | prot-a-332 | Carbonic anhydrase 5A, mitochondrial \|\| id:prot-a-332 | Shellfish liking \|\| id:ebi-fl187-GCST90094838 | Inverse variance weighted | 34 | -0.037102455 | 0.0614328463190636 | 0.545875936971237 | -0.157510834 | 0.083305923655163 | 0.963577406842841 | 0.854267558495745 | 1.08687425823932 | 0.998074519377826 |
| ebi-fl187-GCST90094838 | prot-a-338 | Cell adhesion molecule 3 \|\| id:prot-a-338 | Shellfish liking \|\| id:ebi-fl187-GCST90094838 | Inverse variance weighted | 34 | 0.00404804056297616 | 0.0614242408331277 | 0.947455067426243 | -0.116343471 | 0.124439552595906 | 1.004056245 | 0.890169422983417 | 1.13251356088631 | 0.998074519377826 |
| ebi-fl187-GCST90094838 | prot-a-34 | A disintegrin and metalloproteinase with thrombospondin motifs 3 \|\| id:prot-a-34 | Shellfish liking \|\| id:ebi-fl187-GCST90094838 | Inverse variance weighted | 34 | -0.069449417 | 0.0690310370228358 | 0.314386346530832 | -0.20475025 | 0.0658514154867801 | 0.932907321369383 | 0.814850800278759 | 1.06806800701044 | 0.998074519377826 |
| ebi-fl187-GCST90094838 | prot-a-358 | Cysteine--tRNA ligase, cytoplasmic \|\| id:prot-a-358 | Shellfish liking \|\| id:ebi-fl187-GCST90094838 | Inverse variance weighted | 34 | 0.0360737189729671 | 0.0698912692940602 | 0.605756240360116 | -0.100913169 | 0.173060606789325 | 1.03673227051216 | 0.904011525844375 | 1.1889381606251 | 0.998074519377826 |
| ebi-fl187-GCST90094838 | prot-a-382 | Coiled-coil domain-containing protein 167 \|\| id:prot-a-382 | Shellfish liking \|\| id:ebi-fl187-GCST90094838 | Inverse variance weighted | 34 | -0.06048327 | 0.0618189126374818 | 0.327879357648271 | -0.181648339 | 0.0606817984373726 | 0.941309516682292 | 0.833894536961909 | 1.06256075189652 | 0.998074519377826 |
| ebi-fl187-GCST90094838 | prot-a-40 | Pituitary adenylate cyclase-activating polypeptide 27 \|\| id:prot-a-40 | Shellfish liking \|\| id:ebi-fl187-GCST90094838 | Inverse variance weighted | 34 | -0.075523576 | 0.0678860618160615 | 0.265921129786435 | -0.208580257 | 0.0575331055779044 | 0.92725786963276 | 0.811735884811506 | 1.05922033617565 | 0.998074519377826 |
| ebi-fl187-GCST90094838 | prot-a-403 | C-C motif chemokine 25 \|\| id:prot-a-403 | Shellfish liking \|\| id:ebi-fl187-GCST90094838 | Inverse variance weighted (multiplicative random effects) | 34 | 0.0461134143073303 | 0.0771740844394578 | 0.550157184685863 | -0.105147791 | 0.197374619808668 | 1.04719317091039 | 0.900191472382145 | 1.21820031720522 | 0.998074519377826 |
| ebi-fl187-GCST90094838 | prot-a-415 | Cyclin-C \|\| id:prot-a-415 | Shellfish liking \|\| id:ebi-fl187-GCST90094838 | Inverse variance weighted | 34 | -0.001664275 | 0.0614070117825261 | 0.978378092431175 | -0.122022018 | 0.118693468152789 | 0.998337109196609 | 0.885128879489678 | 1.12602470294911 | 0.998074519377826 |
| ebi-fl187-GCST90094838 | prot-a-44 | Adhesion G protein-coupled receptor F5 \|\| id:prot-a-44 | Shellfish liking \|\| id:ebi-fl187-GCST90094838 | Inverse variance weighted | 34 | 0.0103886592029025 | 0.0614067700185806 | 0.865656830233843 | -0.10996861 | 0.13074592843932 | 1.01044280867394 | 0.895862255941362 | 1.13967818470936 | 0.998074519377826 |
| ebi-fl187-GCST90094838 | prot-a-455 | CD59 glycoprotein \|\| id:prot-a-455 | Shellfish liking \|\| id:ebi-fl187-GCST90094838 | Inverse variance weighted | 34 | -0.00564614 | 0.062059459342084 | 0.927508856650402 | -0.127282681 | 0.11599039989611 | 0.994369769079919 | 0.88048474134987 | 1.12298509130796 | 0.998074519377826 |
| ebi-fl187-GCST90094838 | prot-a-478 | Cadherin-12 \|\| id:prot-a-478 | Shellfish liking \|\| id:ebi-fl187-GCST90094838 | Inverse variance weighted | 34 | -0.064026866 | 0.0634940332798658 | 0.313266384042026 | -0.188475171 | 0.0604214395571522 | 0.937979799735161 | 0.828221067167115 | 1.06228414077964 | 0.998074519377826 |
| ebi-fl187-GCST90094838 | prot-a-516 | Centrin-2 \|\| id:prot-a-516 | Shellfish liking \|\| id:ebi-fl187-GCST90094838 | Inverse variance weighted | 34 | -0.057153416 | 0.0678940492414175 | 0.399898217444264 | -0.190225753 | 0.0759189203623525 | 0.944449164495966 | 0.826772466786949 | 1.07887509580914 | 0.998074519377826 |
| ebi-fl187-GCST90094838 | prot-a-519 | Cryptic protein \|\| id:prot-a-519 | Shellfish liking \|\| id:ebi-fl187-GCST90094838 | Inverse variance weighted | 34 | 0.040879421374601 | 0.0614209465876551 | 0.505691379728667 | -0.079505634 | 0.161264476686405 | 1.04172648802427 | 0.923572816602698 | 1.174995687 | 0.998074519377826 |
| ebi-fl187-GCST90094838 | prot-a-583 | Chloride intracellular channel protein 4 \|\| id:prot-a-583 | Shellfish liking \|\| id:ebi-fl187-GCST90094838 | Inverse variance weighted | 34 | -0.072777803 | 0.0627119999294527 | 0.245841872324683 | -0.195693323 | 0.0501377166352022 | 0.929807407280394 | 0.822264365511113 | 1.0514158838637 | 0.998074519377826 |
| ebi-fl187-GCST90094838 | prot-a-602 | CCR4-NOT transcription complex subunit 1 \|\| id:prot-a-602 | Shellfish liking \|\| id:ebi-fl187-GCST90094838 | Inverse variance weighted | 34 | -0.067436844 | 0.0623264110683042 | 0.279254897749454 | -0.18959661 | 0.054722921319163 | 0.934786755793878 | 0.827292788223547 | 1.05624791034867 | 0.998074519377826 |
| ebi-fl187-GCST90094838 | prot-a-606 | Ciliary neurotrophic factor receptor subunit alpha \|\| id:prot-a-606 | Shellfish liking \|\| id:ebi-fl187-GCST90094838 | Inverse variance weighted | 34 | -0.061410813 | 0.0614209465876551 | 0.317390359484885 | -0.181795868 | 0.058974242507175 | 0.94043681692138 | 0.833771522396692 | 1.06074791818139 | 0.998074519377826 |
| ebi-fl187-GCST90094838 | prot-a-626 | Collagen alpha-2(IX) chain \|\| id:prot-a-626 | Shellfish liking \|\| id:ebi-fl187-GCST90094838 | Inverse variance weighted | 34 | 0.010095550021425 | 0.0688976154835273 | 0.883503210288689 | -0.124943776 | 0.145134876369139 | 1.0101466820102 | 0.882546521197355 | 1.15619550320344 | 0.998074519377826 |
| ebi-fl187-GCST90094838 | prot-a-647 | Probable serine carboxypeptidase CPVL \|\| id:prot-a-647 | Shellfish liking \|\| id:ebi-fl187-GCST90094838 | Inverse variance weighted | 34 | -0.139456165 | 0.061403884385884 | 0.0231391473344537 | -0.259807779 | -0.019104552 | 0.869831151030454 | 0.771199812478733 | 0.981076783293212 | 0.998074519377826 |
| ebi-fl187-GCST90094838 | prot-a-651 | Cellular retinoic acid-binding protein 2 \|\| id:prot-a-651 | Shellfish liking \|\| id:ebi-fl187-GCST90094838 | Inverse variance weighted | 34 | -0.046508834 | 0.063271593744274 | 0.462298951396763 | -0.170521157 | 0.0775034902176888 | 0.954556128441037 | 0.843225249104166 | 1.08058600393236 | 0.998074519377826 |
| ebi-fl187-GCST90094838 | prot-a-671 | Cytotoxic and regulatory T-cell molecule \|\| id:prot-a-671 | Shellfish liking \|\| id:ebi-fl187-GCST90094838 | Inverse variance weighted | 34 | -0.086779276 | 0.071073915682277 | 0.222096543042108 | -0.22608415 | 0.0525255990423356 | 0.916879450879601 | 0.797650976167013 | 1.05392954132016 | 0.998074519377826 |
| ebi-fl187-GCST90094838 | prot-a-688 | Chorionic somatomammotropin hormone \|\| id:prot-a-688 | Shellfish liking \|\| id:ebi-fl187-GCST90094838 | Inverse variance weighted | 34 | -0.042773843 | 0.0702251619002383 | 0.54246102250931 | -0.18041516 | 0.0948674747582786 | 0.958128053347909 | 0.834923512694315 | 1.09951313222671 | 0.998074519377826 |
| ebi-fl187-GCST90094838 | prot-a-69 | Aldo-keto reductase family 1 member C1 \|\| id:prot-a-69 | Shellfish liking \|\| id:ebi-fl187-GCST90094838 | Inverse variance weighted | 34 | 0.00413937610032825 | 0.0614179996832985 | 0.946265823637279 | -0.116239903 | 0.124518655479593 | 1.0041479551508 | 0.890261620994562 | 1.1326031495181 | 0.998074519377826 |
| ebi-fl187-GCST90094838 | prot-a-741 | Stromal cell-derived factor 1 \|\| id:prot-a-741 | Shellfish liking \|\| id:ebi-fl187-GCST90094838 | Inverse variance weighted | 34 | -0.082024974 | 0.0621638685133733 | 0.187003356962102 | -0.203866156 | 0.0398162083840944 | 0.921248951227923 | 0.815571523083817 | 1.04061949947623 | 0.998074519377826 |
| ebi-fl187-GCST90094838 | prot-a-742 | Growth-regulated alpha protein \|\| id:prot-a-742 | Shellfish liking \|\| id:ebi-fl187-GCST90094838 | Inverse variance weighted | 34 | -0.107584354 | 0.0618147788329897 | 0.0817833769399096 | -0.22874132 | 0.0135726129862103 | 0.898000769724162 | 0.795534295671163 | 1.01366513903068 | 0.998074519377826 |
| ebi-fl187-GCST90094838 | prot-a-788 | Beta-defensin 112 \|\| id:prot-a-788 | Shellfish liking \|\| id:ebi-fl187-GCST90094838 | Inverse variance weighted | 34 | -0.068545303 | 0.0685222154841822 | 0.317147477673041 | -0.202848845 | 0.065758239212099 | 0.933751157288798 | 0.816401634892072 | 1.06796849304869 | 0.998074519377826 |
| ebi-fl187-GCST90094838 | prot-a-838 | DnaJ homolog subfamily B member 1 \|\| id:prot-a-838 | Shellfish liking \|\| id:ebi-fl187-GCST90094838 | Inverse variance weighted | 34 | 0.00457382728498529 | 0.0614209465876551 | 0.940638880613333 | -0.115811228 | 0.124958882596789 | 1.00458430319859 | 0.890643335929421 | 1.13310186190284 | 0.998074519377826 |
| ebi-fl187-GCST90094838 | prot-a-85 | Junctional adhesion molecule-like \|\| id:prot-a-85 | Shellfish liking \|\| id:ebi-fl187-GCST90094838 | Inverse variance weighted | 34 | -0.027300072 | 0.0614212250904793 | 0.656700695115138 | -0.147685673 | 0.0930855292239549 | 0.97306920693313 | 0.862702242823528 | 1.09755560433284 | 0.998074519377826 |
| ebi-fl187-GCST90094838 | prot-a-856 | Dipeptidase 1 \|\| id:prot-a-856 | Shellfish liking \|\| id:ebi-fl187-GCST90094838 | Inverse variance weighted | 34 | 0.0346094837535599 | 0.0646357301435934 | 0.592335706694444 | -0.092076547 | 0.161295514835003 | 1.03521536143545 | 0.912035333005313 | 1.17503215694571 | 0.998074519377826 |
| ebi-fl187-GCST90094838 | prot-a-860 | Dipeptidyl peptidase 2 \|\| id:prot-a-860 | Shellfish liking \|\| id:ebi-fl187-GCST90094838 | Inverse variance weighted | 34 | 0.0191039974660858 | 0.0628602310757504 | 0.761194705966109 | -0.104102055 | 0.142310050374557 | 1.019287646 | 0.9011333271679 | 1.15293406076362 | 0.998074519377826 |
| ebi-fl187-GCST90094838 | prot-a-861 | Dermatopontin \|\| id:prot-a-861 | Shellfish liking \|\| id:ebi-fl187-GCST90094838 | Inverse variance weighted | 34 | -0.009558501 | 0.0614148310144699 | 0.876318115505042 | -0.12993157 | 0.110814567472188 | 0.990487035952689 | 0.878155520955063 | 1.11718772470206 | 0.998074519377826 |
| ebi-fl187-GCST90094838 | prot-a-877 | Dual specificity protein phosphatase 6 \|\| id:prot-a-877 | Shellfish liking \|\| id:ebi-fl187-GCST90094838 | Inverse variance weighted | 34 | -0.005814242 | 0.0614209465876551 | 0.924583141645139 | -0.126199297 | 0.11457081326668 | 0.99420262794891 | 0.881439160782083 | 1.12139204768653 | 0.998074519377826 |
| ebi-fl187-GCST90094838 | prot-a-879 | Dynactin-associated protein \|\| id:prot-a-879 | Shellfish liking \|\| id:ebi-fl187-GCST90094838 | Inverse variance weighted | 34 | 0.0385865797477608 | 0.0632560642772528 | 0.541857822501402 | -0.085395306 | 0.162568465731176 | 1.03934071031916 | 0.918149262525041 | 1.1765288676004 | 0.998074519377826 |
| ebi-fl187-GCST90094838 | prot-a-937 | EMI domain-containing protein 1 \|\| id:prot-a-937 | Shellfish liking \|\| id:ebi-fl187-GCST90094838 | Inverse variance weighted | 34 | 0.000198795102808171 | 0.0696516378802263 | 0.99772273369626 | -0.136318415 | 0.136716005348052 | 1.00019881486386 | 0.872564750420849 | 1.14650250170268 | 0.999355215041207 |
| ebi-fl187-GCST90094838 | prot-b-1 | SRC proto-oncogene, non-receptor tyrosine kinase \|\| id:prot-b-1 | Shellfish liking \|\| id:ebi-fl187-GCST90094838 | Inverse variance weighted | 23 | 0.0212949037915949 | 0.0881768236512844 | 0.809165851025353 | -0.151531671 | 0.194121478148112 | 1.02152325830393 | 0.859390664454095 | 1.21424377808285 | 0.998074519377826 |
| ebi-fl187-GCST90094838 | prot-b-10 | coagulation factor III, tissue factor \|\| id:prot-b-10 | Shellfish liking \|\| id:ebi-fl187-GCST90094838 | Inverse variance weighted | 23 | -0.096269532 | 0.0989881284486318 | 0.330783898569391 | -0.290286263 | 0.0977472001801755 | 0.908219189323612 | 0.748049397807601 | 1.10268399155612 | 0.998074519377826 |
| ebi-fl187-GCST90094838 | prot-b-29 | myeloperoxidase \|\| id:prot-b-29 | Shellfish liking \|\| id:ebi-fl187-GCST90094838 | Inverse variance weighted | 23 | -0.071032362 | 0.0920563165248588 | 0.440340378667896 | -0.251462743 | 0.109398018045033 | 0.931431748325469 | 0.777662430647306 | 1.11560629342284 | 0.998074519377826 |
| ebi-fl187-GCST90094838 | prot-b-33 | adrenomedullin \|\| id:prot-b-33 | Shellfish liking \|\| id:ebi-fl187-GCST90094838 | Inverse variance weighted | 23 | -0.043319358 | 0.0881062029208048 | 0.622951196695239 | -0.216007516 | 0.129368799905276 | 0.957605522417436 | 0.805729246356897 | 1.1381097815558 | 0.998074519377826 |
| ebi-fl187-GCST90094838 | ukb-rsfMRI191-edge-pheno102 | Net25_Pair11_15 (Postcentral\|Precentral)&(Cerebellum) (Motor)&(Subcortical-cerebellum) \|\| id:ukb-rsfMRI191-edge-pheno102 | Shellfish liking \|\| id:ebi-fl187-GCST90094838 | Inverse variance weighted | 30 | -0.023530048 | 0.0199587128510812 | 0.238423602815874 | -0.062649125 | 0.0155890291623591 | 0.976744624980244 | 0.939272983084532 | 1.01571117194791 | 0.998074519377826 |
| ebi-fl187-GCST90094838 | ukb-rsfMRI191-edge-pheno1167 | Net100_Pair11_45 (Angular\|Temporal)&(Frontal) (Default_mode\|Central_executive)&(Salience\|Default_mode) \|\| id:ukb-rsfMRI191-edge-pheno1167 | Shellfish liking \|\| id:ebi-fl187-GCST90094838 | Inverse variance weighted | 30 | -0.042364972 | 0.0199841296929589 | 0.0340118809676034 | -0.081533866 | -0.003196078 | 0.958519883636977 | 0.921701494637217 | 0.996809024042073 | 0.998074519377826 |
| ebi-fl187-GCST90094838 | ukb-rsfMRI191-edge-pheno1317 | Net100_Pair26_48 (Parietal\|Frontal)&(Temporal) (Attention\|Central_executive)&(Default_mode\|Central_executive) \|\| id:ukb-rsfMRI191-edge-pheno1317 | Shellfish liking \|\| id:ebi-fl187-GCST90094838 | Inverse variance weighted | 30 | 0.0310285887738489 | 0.0237338079044518 | 0.191091080988617 | -0.015489675 | 0.0775468522665743 | 1.03151499321385 | 0.984629673277426 | 1.0806328613714 | 0.998074519377826 |
| ebi-fl187-GCST90094838 | ukb-rsfMRI191-edge-pheno286 | Net100_Pair10_13 (OccipitalPrecuneus)&(Frontal_Sup) (Default_mode\|Central_executive)&(Default_mode) \|\| id:ukb-rsfMRI191-edge-pheno286 | Shellfish liking \|\| id:ebi-fl187-GCST90094838 | Inverse variance weighted | 30 | -0.000133877 | 0.0231917484909026 | 0.995394143501107 | -0.045589704 | 0.0453219499576809 | 0.999866131876649 | 0.955433892364839 | 1.04636468275109 | 0.999355215041207 |
| ebi-fl187-GCST90094838 | ukb-rsfMRI191-edge-pheno599 | Net100_Pair11_29 (Angular\|Temporal)&(Parietal) (Default_mode\|Central_executive)&(Central_executive\|Salience) \|\| id:ukb-rsfMRI191-edge-pheno599 | Shellfish liking \|\| id:ebi-fl187-GCST90094838 | Inverse variance weighted | 30 | -0.018718288 | 0.0205375475078726 | 0.362075238969888 | -0.058971881 | 0.0215353052123188 | 0.981455811276057 | 0.942733277483954 | 1.02176886346726 | 0.998074519377826 |
| ebi-fl187-GCST90094838 | ukb-rsfMRI191-edge-pheno621 | Net100_Pair5_30 (Precuneus\|Angular\|Cingulate)&(Paracentral) (Default_mode\|Central_executive)&(Motor) \|\| id:ukb-rsfMRI191-edge-pheno621 | Shellfish liking \|\| id:ebi-fl187-GCST90094838 | Inverse variance weighted | 30 | -0.002902692 | 0.0201481106646914 | 0.8854470147482 | -0.042392989 | 0.0365876050543761 | 0.997101516888358 | 0.958493029569142 | 1.03726516970914 | 0.998074519377826 |
| ebi-fl187-GCST90094838 | ukb-rsfMRI191-edge-pheno624 | Net100_Pair8_30 (Calcarine\|Lingual)&(Paracentral) (Visual)&(Motor) \|\| id:ukb-rsfMRI191-edge-pheno624 | Shellfish liking \|\| id:ebi-fl187-GCST90094838 | Inverse variance weighted | 30 | -0.029390934 | 0.0200788495938117 | 0.143255216895288 | -0.068745479 | 0.00996361109176387 | 0.971036778854447 | 0.933564261256611 | 1.01001341313005 | 0.998074519377826 |
| ebi-fl187-GCST90094838 | ukb-rsfMRI191-edge-pheno882 | Net100_Pair6_38 (Postcentral\|Precentral)&(Subcortical) (Motor)&(Subcortical-cerebellum) \|\| id:ukb-rsfMRI191-edge-pheno882 | Shellfish liking \|\| id:ebi-fl187-GCST90094838 | Inverse variance weighted | 29 | -0.042966983 | 0.021422743262699 | 0.044891321269429 | -0.08495556 | -0.000978406 | 0.957943018023112 | 0.918553104253349 | 0.999022072354923 | 0.998074519377826 |
| ebi-fl187-GCST90094838 | ukb-rsfMRI191-edge-pheno899 | Net100_Pair23_38 (Cerebellum)&(Subcortical) (Subcortical-cerebellum)&(Subcortical-cerebellum) \|\| id:ukb-rsfMRI191-edge-pheno899 | Shellfish liking \|\| id:ebi-fl187-GCST90094838 | Inverse variance weighted | 30 | 0.0267923892444845 | 0.0200683800008728 | 0.18185837571541 | -0.012541636 | 0.0661264140461953 | 1.02715453229715 | 0.987536682997835 | 1.06836176456333 | 0.998074519377826 |
| ebi-fl187-GCST90094838 | ebi-a-GCST90001392 | IgD+ CD38+ B cell Absolute Count \|\| id:ebi-a-GCST90001392 | Shellfish liking \|\| id:ebi-fl187-GCST90094838 | MR Egger | 30 | -0.345056782 | 0.268329867940129 | 0.208996284433517 | -0.870983324 | 0.180869758707757 | 0.708180140119239 | 0.418539786775715 | 1.1982591063154 | 0.999279047927446 |
| ebi-fl187-GCST90094838 | ebi-a-GCST90001402 | Switched memory B cell %B cell \|\| id:ebi-a-GCST90001402 | Shellfish liking \|\| id:ebi-fl187-GCST90094838 | MR Egger | 30 | -0.171456744 | 0.292904902212571 | 0.562989705930949 | -0.745550353 | 0.40263686398515 | 0.842436707378021 | 0.474473100524286 | 1.49576362738733 | 0.999279047927446 |
| ebi-fl187-GCST90094838 | ebi-a-GCST90001407 | Memory B cell Absolute Count \|\| id:ebi-a-GCST90001407 | Shellfish liking \|\| id:ebi-fl187-GCST90094838 | MR Egger | 30 | -0.009216828 | 0.280649768876166 | 0.974034349555654 | -0.559290375 | 0.5408567188933 | 0.990825516661495 | 0.571614551878087 | 1.71747762761103 | 0.999279047927446 |
| ebi-fl187-GCST90094838 | ebi-a-GCST90001411 | IgD+ CD24+ B cell %B cell \|\| id:ebi-a-GCST90001411 | Shellfish liking \|\| id:ebi-fl187-GCST90094838 | MR Egger | 30 | 0.0432759056950881 | 0.284828105063109 | 0.880326180845248 | -0.51498718 | 0.601538991618782 | 1.04422596299911 | 0.597508254488367 | 1.82492518489995 | 0.999279047927446 |
| ebi-fl187-GCST90094838 | ebi-a-GCST90001418 | CD24+ CD27+ B cell Absolute Count \|\| id:ebi-a-GCST90001418 | Shellfish liking \|\| id:ebi-fl187-GCST90094838 | MR Egger | 30 | -0.003461872 | 0.279691717928173 | 0.990212244572101 | -0.551657639 | 0.544733895626005 | 0.996544113855122 | 0.575994228297994 | 1.72414951759813 | 0.999279047927446 |
| ebi-fl187-GCST90094838 | ebi-a-GCST90001436 | Memory B cell %lymphocyte \|\| id:ebi-a-GCST90001436 | Shellfish liking \|\| id:ebi-fl187-GCST90094838 | MR Egger | 30 | -0.021322877 | 0.276591439452785 | 0.939098919644226 | -0.563442099 | 0.520796344142587 | 0.978902848142943 | 0.569246285955267 | 1.68336765604768 | 0.999279047927446 |
| ebi-fl187-GCST90094838 | ebi-a-GCST90001439 | IgD+ CD24+ B cell %lymphocyte \|\| id:ebi-a-GCST90001439 | Shellfish liking \|\| id:ebi-fl187-GCST90094838 | MR Egger | 30 | 0.0674230979803254 | 0.279727770470002 | 0.811287278415959 | -0.480843332 | 0.615689528101529 | 1.06974799065359 | 0.618261771863936 | 1.85093242957168 | 0.999279047927446 |
| ebi-fl187-GCST90094838 | ebi-a-GCST90001457 | CD11c+ HLA DR++ monocyte %monocyte \|\| id:ebi-a-GCST90001457 | Shellfish liking \|\| id:ebi-fl187-GCST90094838 | MR Egger | 30 | -0.116804156 | 0.302251918067424 | 0.702087309818519 | -0.709217916 | 0.475609603228093 | 0.889759429983291 | 0.492028855117785 | 1.60899474697409 | 0.999279047927446 |
| ebi-fl187-GCST90094838 | ebi-a-GCST90001483 | CD39+ resting CD4 regulatory T cell Absolute Count \|\| id:ebi-a-GCST90001483 | Shellfish liking \|\| id:ebi-fl187-GCST90094838 | MR Egger | 30 | -0.142185391 | 0.278461480066236 | 0.613626108231453 | -0.687969892 | 0.403599110019687 | 0.86746042230058 | 0.502595357019075 | 1.49720361270536 | 0.999279047927446 |
| ebi-fl187-GCST90094838 | ebi-a-GCST90001525 | CD33dim HLA DR+ CD11b+ Absolute Count \|\| id:ebi-a-GCST90001525 | Shellfish liking \|\| id:ebi-fl187-GCST90094838 | MR Egger | 30 | 0.0625671197713781 | 0.359978477313387 | 0.863266912083187 | -0.642990696 | 0.768124935305617 | 1.06456590994044 | 0.525717808596194 | 2.15572034668851 | 0.999279047927446 |
| ebi-fl187-GCST90094838 | ebi-a-GCST90001540 | Naive CD4+ T cell Absolute Count \|\| id:ebi-a-GCST90001540 | Shellfish liking \|\| id:ebi-fl187-GCST90094838 | MR Egger | 30 | -0.463571437 | 0.265344050927747 | 0.0915891964675162 | -0.983645776 | 0.0565029031534597 | 0.629033077222635 | 0.373945289703544 | 1.05812968670863 | 0.999279047927446 |
| ebi-fl187-GCST90094838 | ebi-a-GCST90001624 | HLA DR+ CD4+ T cell Absolute Count \|\| id:ebi-a-GCST90001624 | Shellfish liking \|\| id:ebi-fl187-GCST90094838 | MR Egger | 30 | 0.195551809326616 | 0.262860113269829 | 0.463111339677136 | -0.319654013 | 0.710757631335482 | 1.21598179148802 | 0.726400318898971 | 2.03553285806867 | 0.999279047927446 |
| ebi-fl187-GCST90094838 | ebi-a-GCST90001672 | CD39+ CD8+ T cell Absolute Count \|\| id:ebi-a-GCST90001672 | Shellfish liking \|\| id:ebi-fl187-GCST90094838 | MR Egger | 30 | 0.230358297902048 | 0.288837525047656 | 0.431851697781481 | -0.335763251 | 0.796479846995454 | 1.25905104447009 | 0.714792312013881 | 2.21772045661069 | 0.999279047927446 |
| ebi-fl187-GCST90094838 | ebi-a-GCST90001722 | CD19 on CD20- CD38- B cell \|\| id:ebi-a-GCST90001722 | Shellfish liking \|\| id:ebi-fl187-GCST90094838 | MR Egger | 30 | -0.367525316 | 0.340100226689902 | 0.289078354223157 | -1.03412176 | 0.299071128750611 | 0.69244579693253 | 0.355538492020746 | 1.34860554468895 | 0.999279047927446 |
| ebi-fl187-GCST90094838 | ebi-a-GCST90001742 | CD19 on transitional B cell \|\| id:ebi-a-GCST90001742 | Shellfish liking \|\| id:ebi-fl187-GCST90094838 | MR Egger | 30 | -0.681239224 | 0.311087080671036 | 0.0370272351637367 | -1.290969903 | -0.071508546 | 0.505989569055675 | 0.275003926692178 | 0.930988321049418 | 0.999279047927446 |
| ebi-fl187-GCST90094838 | ebi-a-GCST90001784 | CD25 on IgD+ CD38dim B cell \|\| id:ebi-a-GCST90001784 | Shellfish liking \|\| id:ebi-fl187-GCST90094838 | MR Egger | 30 | -0.166889059 | 0.289915330245389 | 0.56945141029374 | -0.735123106 | 0.401344988521543 | 0.846293494979537 | 0.479446432589826 | 1.49383253469196 | 0.999279047927446 |
| ebi-fl187-GCST90094838 | ebi-a-GCST90001794 | CD25 on IgD+ B cell \|\| id:ebi-a-GCST90001794 | Shellfish liking \|\| id:ebi-fl187-GCST90094838 | MR Egger | 30 | -0.107564649 | 0.290437143086941 | 0.713905464374122 | -0.676821449 | 0.461692151437259 | 0.89801846456661 | 0.508229862014366 | 1.58675674724477 | 0.999279047927446 |
| ebi-fl187-GCST90094838 | ebi-a-GCST90001802 | CD27 on IgD- CD38- B cell \|\| id:ebi-a-GCST90001802 | Shellfish liking \|\| id:ebi-fl187-GCST90094838 | MR Egger | 30 | 0.0502008080836718 | 0.277800146608678 | 0.857898142362749 | -0.494287479 | 0.594689095436681 | 1.05148222130739 | 0.610005393976568 | 1.81246735298213 | 0.999279047927446 |
| ebi-fl187-GCST90094838 | ebi-a-GCST90001804 | CD27 on IgD- CD38dim B cell \|\| id:ebi-a-GCST90001804 | Shellfish liking \|\| id:ebi-fl187-GCST90094838 | MR Egger | 30 | 0.0484664026577185 | 0.274767877788557 | 0.86125691254798 | -0.490078638 | 0.587011443123289 | 1.04966010543806 | 0.612578220481907 | 1.79860514153029 | 0.999279047927446 |
| ebi-fl187-GCST90094838 | ebi-a-GCST90001805 | CD27 on memory B cell \|\| id:ebi-a-GCST90001805 | Shellfish liking \|\| id:ebi-fl187-GCST90094838 | MR Egger | 30 | 0.178360733312916 | 0.279578936436943 | 0.528681783304019 | -0.369613982 | 0.726335448729324 | 1.19525641228106 | 0.691001017920434 | 2.06749028445498 | 0.999279047927446 |
| ebi-fl187-GCST90094838 | ebi-a-GCST90001806 | CD27 on unswitched memory B cell \|\| id:ebi-a-GCST90001806 | Shellfish liking \|\| id:ebi-fl187-GCST90094838 | MR Egger | 30 | 0.185138765512452 | 0.277367272369093 | 0.509926136597473 | -0.358501088 | 0.728778619355875 | 1.20338541693617 | 0.698722865396447 | 2.07254769152716 | 0.999279047927446 |
| ebi-fl187-GCST90094838 | ebi-a-GCST90001839 | CD3 on Effector Memory CD8+ T cell \|\| id:ebi-a-GCST90001839 | Shellfish liking \|\| id:ebi-fl187-GCST90094838 | MR Egger | 30 | 0.207866025919154 | 0.335910732898183 | 0.541041584236487 | -0.450519011 | 0.866251062399592 | 1.23104822994521 | 0.637297301741628 | 2.37797922619426 | 0.999279047927446 |
| ebi-fl187-GCST90094838 | ebi-a-GCST90001859 | CD3 on CD8+ T cell \|\| id:ebi-a-GCST90001859 | Shellfish liking \|\| id:ebi-fl187-GCST90094838 | MR Egger | 30 | 0.14479331020079 | 0.299584429516666 | 0.632630905301097 | -0.442392172 | 0.731978792053455 | 1.15580065337306 | 0.64249761668979 | 2.07919082598336 | 0.999279047927446 |
| ebi-fl187-GCST90094838 | ebi-a-GCST90001905 | CD86 on monocyte \|\| id:ebi-a-GCST90001905 | Shellfish liking \|\| id:ebi-fl187-GCST90094838 | MR Egger | 30 | 0.0693356733420626 | 0.300830823903279 | 0.81939402675451 | -0.520292742 | 0.658964088192489 | 1.07179592209112 | 0.594346532600232 | 1.93278909794503 | 0.999279047927446 |
| ebi-fl187-GCST90094838 | ebi-a-GCST90001943 | CD25 on activated & secreting CD4 regulatory T cell \|\| id:ebi-a-GCST90001943 | Shellfish liking \|\| id:ebi-fl187-GCST90094838 | MR Egger | 30 | -0.184285617 | 0.276139507569463 | 0.510002749531191 | -0.725519052 | 0.356947817914539 | 0.8316982228136 | 0.48407324466732 | 1.42896130172757 | 0.999279047927446 |
| ebi-fl187-GCST90094838 | ebi-a-GCST90001949 | CD33 on CD33dim HLA DR+ CD11b- \|\| id:ebi-a-GCST90001949 | Shellfish liking \|\| id:ebi-fl187-GCST90094838 | MR Egger | 30 | 0.547498924663063 | 0.454217325288033 | 0.238149692330676 | -0.342767033 | 1.43776488222761 | 1.72892343805131 | 0.709803553174183 | 4.21127259968737 | 0.999279047927446 |
| ebi-fl187-GCST90094838 | ebi-a-GCST90001955 | CD33 on Immature Myeloid-Derived Suppressor Cells \|\| id:ebi-a-GCST90001955 | Shellfish liking \|\| id:ebi-fl187-GCST90094838 | MR Egger | 30 | 0.307619961534399 | 0.449518519542035 | 0.499390934538108 | -0.573436337 | 1.18867625983679 | 1.36018396847123 | 0.563585437998197 | 3.28273284465532 | 0.999279047927446 |
| ebi-fl187-GCST90094838 | ebi-a-GCST90001956 | CD33 on CD33+ HLA DR+ \|\| id:ebi-a-GCST90001956 | Shellfish liking \|\| id:ebi-fl187-GCST90094838 | MR Egger | 30 | 0.491421636582164 | 0.507732319086966 | 0.341393047546166 | -0.503733709 | 1.48657698199262 | 1.63463843068608 | 0.604270273272165 | 4.42193322634681 | 0.999279047927446 |
| ebi-fl187-GCST90094838 | ebi-a-GCST90001957 | CD33 on CD33+ HLA DR+ CD14- \|\| id:ebi-a-GCST90001957 | Shellfish liking \|\| id:ebi-fl187-GCST90094838 | MR Egger | 30 | 0.497977082388011 | 0.505318223423177 | 0.332831779599669 | -0.492446636 | 1.48840080029744 | 1.6453894145758 | 0.611129352791149 | 4.43000538794822 | 0.999279047927446 |
| ebi-fl187-GCST90094838 | ebi-a-GCST90002064 | CD4 on resting CD4 regulatory T cell \|\| id:ebi-a-GCST90002064 | Shellfish liking \|\| id:ebi-fl187-GCST90094838 | MR Egger | 30 | -0.289932233 | 0.297907814350122 | 0.338773287609355 | -0.873831549 | 0.293967083001851 | 0.748314276780857 | 0.417349387094749 | 1.34173973689562 | 0.999279047927446 |
| ebi-fl187-GCST90094838 | ebi-a-GCST90002080 | SSC-A on T cell \|\| id:ebi-a-GCST90002080 | Shellfish liking \|\| id:ebi-fl187-GCST90094838 | MR Egger | 30 | 0.422259381297694 | 0.276598084308486 | 0.138074385138923 | -0.119872864 | 0.964391626542326 | 1.5254041346781 | 0.887033203449068 | 2.62319129097478 | 0.999279047927446 |
| ebi-fl187-GCST90094838 | ebi-a-GCST90002121 | CD8 on CD39+ CD8+ T cell \|\| id:ebi-a-GCST90002121 | Shellfish liking \|\| id:ebi-fl187-GCST90094838 | MR Egger | 30 | 0.0182500904743049 | 0.334046152082195 | 0.956818463355957 | -0.636480368 | 0.672980548555406 | 1.01841764109476 | 0.529151569374239 | 1.96007070888886 | 0.999279047927446 |
| ebi-fl187-GCST90094838 | ebi-cfmet338-GCST90026053 | 1-palmitoyl-2-docosahexaenoyl-gpc (16:0/22:6) levels \|\| id:ebi-cfmet338-GCST90026053 | Shellfish liking \|\| id:ebi-fl187-GCST90094838 | MR Egger | 27 | 0.209358643636044 | 0.229396300103518 | 0.370144068863169 | -0.240258105 | 0.658975391838939 | 1.23288708635701 | 0.786424855022721 | 1.93281094563314 | 0.999279047927446 |
| ebi-fl187-GCST90094838 | ebi-cfmet338-GCST90026063 | 1-stearoyl-2-oleoyl-gpc (18:0/18:1) levels \|\| id:ebi-cfmet338-GCST90026063 | Shellfish liking \|\| id:ebi-fl187-GCST90094838 | MR Egger | 26 | 0.119288927174904 | 0.173089803242091 | 0.49732343169657 | -0.219967087 | 0.458544941529403 | 1.12669540418534 | 0.802545211554218 | 1.58177074080841 | 0.999279047927446 |
| ebi-fl187-GCST90094838 | ebi-cfmet338-GCST90026113 | Adenosine 3',5'-cyclic monophosphate (camp) levels \|\| id:ebi-cfmet338-GCST90026113 | Shellfish liking \|\| id:ebi-fl187-GCST90094838 | MR Egger | 27 | 0.61374757552401 | 0.335371244642646 | 0.0791911558110854 | -0.043580064 | 1.2710752150236 | 1.84734149441066 | 0.957355901302945 | 3.56468330359358 | 0.999279047927446 |
| ebi-fl187-GCST90094838 | ebi-cfmet338-GCST90026117 | Alpha-ketoglutaramate levels \|\| id:ebi-cfmet338-GCST90026117 | Shellfish liking \|\| id:ebi-fl187-GCST90094838 | MR Egger | 27 | 0.144885015593493 | 0.133976126931404 | 0.289830224264176 | -0.117708193 | 0.407478224379044 | 1.15590665138608 | 0.888955418013415 | 1.50302271592479 | 0.999279047927446 |
| ebi-fl187-GCST90094838 | ebi-cfmet338-GCST90026124 | Arginine levels \|\| id:ebi-cfmet338-GCST90026124 | Shellfish liking \|\| id:ebi-fl187-GCST90094838 | MR Egger | 27 | 0.0513383190513597 | 0.12120880734504 | 0.675513914127768 | -0.186230943 | 0.288907581447638 | 1.0526789743973 | 0.830081870960256 | 1.33496834698515 | 0.999279047927446 |
| ebi-fl187-GCST90094838 | ebi-cfmet338-GCST90026169 | Glutamate levels \|\| id:ebi-cfmet338-GCST90026169 | Shellfish liking \|\| id:ebi-fl187-GCST90094838 | MR Egger | 27 | 0.00692080242538711 | 0.131678374381183 | 0.958501502946207 | -0.251168811 | 0.265010416212505 | 1.00694480652241 | 0.777891043628093 | 1.30344455266311 | 0.999279047927446 |
| ebi-fl187-GCST90094838 | ebi-cfmet338-GCST90026170 | Glutamine levels \|\| id:ebi-cfmet338-GCST90026170 | Shellfish liking \|\| id:ebi-fl187-GCST90094838 | MR Egger | 26 | 0.0293964804340941 | 0.0688919720542191 | 0.673398265843905 | -0.105631785 | 0.164424745660363 | 1.029832822 | 0.899755890890216 | 1.17871486280525 | 0.999279047927446 |
| ebi-fl187-GCST90094838 | ebi-cfmet338-GCST90026191 | Kynurenate levels \|\| id:ebi-cfmet338-GCST90026191 | Shellfish liking \|\| id:ebi-fl187-GCST90094838 | MR Egger | 27 | 0.0615144657643369 | 0.520644807878059 | 0.906892570562323 | -0.958949358 | 1.08197828920533 | 1.06344587997502 | 0.383295380848474 | 2.95051074482664 | 0.999279047927446 |
| ebi-fl187-GCST90094838 | ebi-cfmet338-GCST90026202 | Methylsuccinoylcarnitine levels \|\| id:ebi-cfmet338-GCST90026202 | Shellfish liking \|\| id:ebi-fl187-GCST90094838 | MR Egger | 27 | 0.187404044087936 | 0.348225099578256 | 0.595219609111167 | -0.495117151 | 0.869925239261319 | 1.20611451005027 | 0.60949949958569 | 2.38673241297596 | 0.999279047927446 |
| ebi-fl187-GCST90094838 | ebi-cfmet338-GCST90026222 | N-acetylputrescine levels \|\| id:ebi-cfmet338-GCST90026222 | Shellfish liking \|\| id:ebi-fl187-GCST90094838 | MR Egger | 27 | 0.0120285206986388 | 0.126017208806352 | 0.924717281867314 | -0.234965209 | 0.259022249959088 | 1.01210115428646 | 0.790598355204061 | 1.29566263294791 | 0.999279047927446 |
| ebi-fl187-GCST90094838 | ebi-cfmet338-GCST90026252 | Propionylcarnitine (c3) levels \|\| id:ebi-cfmet338-GCST90026252 | Shellfish liking \|\| id:ebi-fl187-GCST90094838 | MR Egger | 27 | -0.188770699 | 0.2277810140505 | 0.415095411928488 | -0.635221487 | 0.257680088126459 | 0.827976340390683 | 0.529818127519191 | 1.29392481049436 | 0.999279047927446 |
| ebi-fl187-GCST90094838 | ebi-cfmet338-GCST90026316 | Ethyl beta-glucopyranoside levels \|\| id:ebi-cfmet338-GCST90026316 | Shellfish liking \|\| id:ebi-fl187-GCST90094838 | MR Egger | 27 | -0.855681208 | 0.678867788408908 | 0.219143900589725 | -2.186262073 | 0.474899657328689 | 0.424993583443042 | 0.112335868058414 | 1.6078528531407 | 0.999279047927446 |
| ebi-fl187-GCST90094838 | ebi-gm430-GCST90011315 | F_Prevotellaceae abundance \|\| id:ebi-gm430-GCST90011315 | Shellfish liking \|\| id:ebi-fl187-GCST90094838 | MR Egger | 23 | -0.557504762 | 0.375150526676481 | 0.152116739889543 | -1.292799794 | 0.177790270180048 | 0.572636146064778 | 0.274501159391846 | 1.19457475701162 | 0.999279047927446 |
| ebi-fl187-GCST90094838 | ebi-gm430-GCST90011354 | OTU97_1 (Bacteroides) abundance \|\| id:ebi-gm430-GCST90011354 | Shellfish liking \|\| id:ebi-fl187-GCST90094838 | MR Egger | 23 | -0.595834245 | 0.312152310733146 | 0.0700502527176245 | -1.207652774 | 0.0159842841462391 | 0.551102619495969 | 0.298898037956464 | 1.01611271620176 | 0.999279047927446 |
| ebi-fl187-GCST90094838 | ebi-gm430-GCST90011419 | OTU99_1 (Bacteroides) abundance \|\| id:ebi-gm430-GCST90011419 | Shellfish liking \|\| id:ebi-fl187-GCST90094838 | MR Egger | 23 | -0.608633841 | 0.312220039597592 | 0.0647318270009081 | -1.220585119 | 0.0033174365087405 | 0.544093679982062 | 0.295057472756684 | 1.00332294529123 | 0.999279047927446 |
| ebi-fl187-GCST90094838 | ebi-gm430-GCST90011435 | OTU99_155 (Butyrivibrio) abundance \|\| id:ebi-gm430-GCST90011435 | Shellfish liking \|\| id:ebi-fl187-GCST90094838 | MR Egger | 23 | 0.289558275033136 | 0.96590610006154 | 0.76729294177412 | -1.603617681 | 2.18273423115375 | 1.33583728500091 | 0.20116744036022 | 8.87052720262915 | 0.999279047927446 |
| ebi-fl187-GCST90094838 | ebi-gm430-GCST90011466 | OTU99_58 (Bacteroides) abundance \|\| id:ebi-gm430-GCST90011466 | Shellfish liking \|\| id:ebi-fl187-GCST90094838 | MR Egger | 23 | -0.821350357 | 0.570609651575491 | 0.16477052779322 | -1.939745274 | 0.297044560233907 | 0.43983731597746 | 0.143740559580884 | 1.34587527062879 | 0.999279047927446 |
| ebi-fl187-GCST90094838 | ebi-gm430-GCST90011472 | OTU99_72 (Parabacteroides) abundance \|\| id:ebi-gm430-GCST90011472 | Shellfish liking \|\| id:ebi-fl187-GCST90094838 | MR Egger | 23 | -0.446013134 | 0.854010887598216 | 0.606957340304545 | -2.119874473 | 1.22784820615746 | 0.640175364240856 | 0.120046696640083 | 3.41387567047866 | 0.999279047927446 |
| ebi-fl187-GCST90094838 | ebi-gm430-GCST90011505 | TestASV_28 (Holdemanella) abundance \|\| id:ebi-gm430-GCST90011505 | Shellfish liking \|\| id:ebi-fl187-GCST90094838 | MR Egger | 23 | 0.702782106143525 | 0.727485095326826 | 0.345016848506693 | -0.723088681 | 2.1286528929841 | 2.01936298182016 | 0.485251153069871 | 8.40353871711138 | 0.999279047927446 |
| ebi-fl187-GCST90094838 | ebi-gm430-GCST90011513 | TestASV_35 (Alphaproteobacteria) abundance \|\| id:ebi-gm430-GCST90011513 | Shellfish liking \|\| id:ebi-fl187-GCST90094838 | MR Egger | 23 | 0.485525564989905 | 0.93094276404698 | 0.607446682952704 | -1.339122253 | 2.31017338252199 | 1.6250288427193 | 0.262075603848051 | 10.0761715356027 | 0.999279047927446 |
| ebi-fl187-GCST90094838 | ebi-gm430-GCST90011543 | G_Coprococcus prevalence \|\| id:ebi-gm430-GCST90011543 | Shellfish liking \|\| id:ebi-fl187-GCST90094838 | MR Egger | 23 | 1.01717434754054 | 1.02099629123396 | 0.330467267537953 | -0.983978383 | 3.0183270783591 | 2.76536973996455 | 0.373820933641321 | 20.4570400170503 | 0.999279047927446 |
| ebi-fl187-GCST90094838 | ebi-gm430-GCST90011547 | G_Parasutterella prevalence \|\| id:ebi-gm430-GCST90011547 | Shellfish liking \|\| id:ebi-fl187-GCST90094838 | MR Egger | 23 | 0.555024658864033 | 0.827531071667403 | 0.50972188761736 | -1.066936242 | 2.17698555933214 | 1.74198393958958 | 0.344061024133674 | 8.81967974555893 | 0.999279047927446 |
| ebi-fl187-GCST90094838 | ebi-gm430-GCST90011569 | OTU97_13 (Subdoligranulum) prevalence \|\| id:ebi-gm430-GCST90011569 | Shellfish liking \|\| id:ebi-fl187-GCST90094838 | MR Egger | 23 | 0.24408440479932 | 1.0134540703314 | 0.812011776667859 | -1.742285573 | 2.23045438264886 | 1.27645206462311 | 0.175119694016122 | 9.30409273745416 | 0.999279047927446 |
| ebi-fl187-GCST90094838 | ebi-gm430-GCST90011590 | OTU97_277 (Prevotella) prevalence \|\| id:ebi-gm430-GCST90011590 | Shellfish liking \|\| id:ebi-fl187-GCST90094838 | MR Egger | 23 | -0.119338676 | 0.733510193369151 | 0.872313736099442 | -1.557018655 | 1.31834130317049 | 0.887507172626057 | 0.210763494177794 | 3.737217323 | 0.999279047927446 |
| ebi-fl187-GCST90094838 | ebi-gm430-GCST90011616 | OTU97_86 (Phascolarctobacterium) prevalence \|\| id:ebi-gm430-GCST90011616 | Shellfish liking \|\| id:ebi-fl187-GCST90094838 | MR Egger | 23 | 0.198216736022064 | 0.95239137091973 | 0.837136406158972 | -1.668470351 | 2.06490382302474 | 1.21922661551407 | 0.188535237923141 | 7.88453955002242 | 0.999279047927446 |
| ebi-fl187-GCST90094838 | ebi-gm430-GCST90011665 | OTU99_55 (Barnesiella) prevalence \|\| id:ebi-gm430-GCST90011665 | Shellfish liking \|\| id:ebi-fl187-GCST90094838 | MR Egger | 23 | 0.0125520650991233 | 0.602869263831743 | 0.983585373408272 | -1.169071692 | 1.19417582220934 | 1.01263117291054 | 0.310655191148189 | 3.30083617325112 | 0.999279047927446 |
| ebi-fl187-GCST90094838 | ebi-gm430-GCST90011687 | TestASV_14 (Lachnospiraceae) prevalence \|\| id:ebi-gm430-GCST90011687 | Shellfish liking \|\| id:ebi-fl187-GCST90094838 | MR Egger | 23 | 0.537213427365176 | 0.68471728270392 | 0.441462901015189 | -0.804832447 | 1.87925930146486 | 1.71123174062296 | 0.447162843877559 | 6.54865248803474 | 0.999279047927446 |
| ebi-fl187-GCST90094838 | ebi-gm430-GCST90011697 | TestASV_23 (Barnesiella) prevalence \|\| id:ebi-gm430-GCST90011697 | Shellfish liking \|\| id:ebi-fl187-GCST90094838 | MR Egger | 23 | 1.20408314760905 | 0.860522332011775 | 0.176336849212629 | -0.482540623 | 2.89070691835213 | 3.33370116457052 | 0.617213291766758 | 18.0060338993131 | 0.999279047927446 |
| ebi-fl187-GCST90094838 | ebi-gm430-GCST90011727 | TestASV_6 (Subdoligranulum) prevalence \|\| id:ebi-gm430-GCST90011727 | Shellfish liking \|\| id:ebi-fl187-GCST90094838 | MR Egger | 23 | -0.908835052 | 1.00563302113061 | 0.376381946375074 | -2.879875774 | 1.06220566891875 | 0.402993416862669 | 0.0561417366692094 | 2.89274439427376 | 0.999279047927446 |
| ebi-fl187-GCST90094838 | ebi-hbsm44-GCST90104908 | Sulcal depth of the left posterior inferior temporal sulcus \|\| id:ebi-hbsm44-GCST90104908 | Shellfish liking \|\| id:ebi-fl187-GCST90094838 | MR Egger | 26 | 0.0149648048684233 | 0.205598270358419 | 0.94257912721024 | -0.388007805 | 0.417937414770924 | 1.01507733820655 | 0.67840704825331 | 1.51882561537856 | 0.999279047927446 |
| ebi-fl187-GCST90094838 | ebi-hbsm44-GCST90104918 | Sulcal depth of the left inferior frontal sulcus \|\| id:ebi-hbsm44-GCST90104918 | Shellfish liking \|\| id:ebi-fl187-GCST90094838 | MR Egger | 24 | 0.0823075391683807 | 0.352475158405165 | 0.817522180173277 | -0.608543771 | 0.773158849642504 | 1.08578968134327 | 0.544142688596322 | 2.16659941742988 | 0.999279047927446 |
| ebi-fl187-GCST90094838 | ebi-hbsm44-GCST90104925 | Sulcal depth of the right anterior inferior temporal sulcus \|\| id:ebi-hbsm44-GCST90104925 | Shellfish liking \|\| id:ebi-fl187-GCST90094838 | MR Egger | 26 | -0.238826144 | 0.115154670207836 | 0.0489787612140981 | -0.464529298 | -0.01312299 | 0.787551791032897 | 0.628430839448654 | 0.986962740567096 | 0.999279047927446 |
| ebi-fl187-GCST90094838 | ebi-IPs91-GCST90274770 | C-C motif chemokine 4 levels \|\| id:ebi-IPs91-GCST90274770 | Shellfish liking \|\| id:ebi-fl187-GCST90094838 | MR Egger | 37 | -0.183311186 | 0.120614262193605 | 0.13754097240662 | -0.41971514 | 0.053092767798723 | 0.83250905 | 0.657234012832648 | 1.05452746677372 | 0.999279047927446 |
| ebi-fl187-GCST90094838 | ebi-IPs91-GCST90274779 | C-X-C motif chemokine 1 levels \|\| id:ebi-IPs91-GCST90274779 | Shellfish liking \|\| id:ebi-fl187-GCST90094838 | MR Egger | 37 | 0.0264764399824405 | 0.133461748040363 | 0.843893854356158 | -0.235108586 | 0.288061466141552 | 1.02683005484257 | 0.790485009223412 | 1.33383928755821 | 0.999279047927446 |
| ebi-fl187-GCST90094838 | ebi-IPs91-GCST90274780 | C-X-C motif chemokine 10 levels \|\| id:ebi-IPs91-GCST90274780 | Shellfish liking \|\| id:ebi-fl187-GCST90094838 | MR Egger | 37 | 0.156365922562062 | 0.11375661735867 | 0.178003749805613 | -0.066597047 | 0.379328892585056 | 1.16925398123077 | 0.935572116552051 | 1.46130356862549 | 0.999279047927446 |
| ebi-fl187-GCST90094838 | ebi-IPs91-GCST90274796 | Interleukin-10 receptor subunit alpha levels \|\| id:ebi-IPs91-GCST90274796 | Shellfish liking \|\| id:ebi-fl187-GCST90094838 | MR Egger | 37 | -0.185958577 | 0.146973154985585 | 0.214138755732961 | -0.474025961 | 0.102108806742029 | 0.830307988092712 | 0.622491091990265 | 1.10750396907101 | 0.999279047927446 |
| ebi-fl187-GCST90094838 | ebi-IPs91-GCST90274807 | Interleukin-20 levels \|\| id:ebi-IPs91-GCST90274807 | Shellfish liking \|\| id:ebi-fl187-GCST90094838 | MR Egger | 37 | -0.230500039 | 0.125899824656667 | 0.0756521896771838 | -0.477263696 | 0.0162636169623582 | 0.794136403741283 | 0.620478890108752 | 1.01639658947398 | 0.999279047927446 |
| ebi-fl187-GCST90094838 | ebi-IPs91-GCST90274808 | Interleukin-20 receptor subunit alpha levels \|\| id:ebi-IPs91-GCST90274808 | Shellfish liking \|\| id:ebi-fl187-GCST90094838 | MR Egger | 37 | 0.0221472285265383 | 0.12786713831532 | 0.86348811322248 | -0.228472363 | 0.272766819624565 | 1.02239429899633 | 0.79574828933704 | 1.313593905 | 0.999279047927446 |
| ebi-fl187-GCST90094838 | ebi-IPs91-GCST90274810 | Interleukin-24 levels \|\| id:ebi-IPs91-GCST90274810 | Shellfish liking \|\| id:ebi-fl187-GCST90094838 | MR Egger | 37 | -0.040951019 | 0.128339298735142 | 0.75155943986009 | -0.292496044 | 0.210594006902977 | 0.959876144856581 | 0.746398197677727 | 1.23441109093158 | 0.999279047927446 |
| ebi-fl187-GCST90094838 | ebi-IPs91-GCST90274823 | Monocyte chemoattractant protein-3 levels \|\| id:ebi-IPs91-GCST90274823 | Shellfish liking \|\| id:ebi-fl187-GCST90094838 | MR Egger | 37 | -0.151535907 | 0.143612795280022 | 0.298577217113991 | -0.433016986 | 0.129945171795985 | 0.859387023749564 | 0.648549475643486 | 1.13876594512139 | 0.999279047927446 |
| ebi-fl187-GCST90094838 | ebi-IPs91-GCST90274824 | Monocyte chemoattractant protein-4 levels \|\| id:ebi-IPs91-GCST90274824 | Shellfish liking \|\| id:ebi-fl187-GCST90094838 | MR Egger | 37 | -0.130114835 | 0.113333922362606 | 0.258727907365868 | -0.352249322 | 0.0920196532479679 | 0.877994600987638 | 0.703104800335716 | 1.09638636942227 | 0.999279047927446 |
| ebi-fl187-GCST90094838 | ebi-IPs91-GCST90274834 | SIR2-like protein 2 levels \|\| id:ebi-IPs91-GCST90274834 | Shellfish liking \|\| id:ebi-fl187-GCST90094838 | MR Egger | 37 | -0.10998277 | 0.11378296439684 | 0.340375360178955 | -0.332997381 | 0.11303183985075 | 0.895849570322827 | 0.716772071676854 | 1.11966758242976 | 0.999279047927446 |
| ebi-fl187-GCST90094838 | ebi-met1400-GCST90199652 | 1-stearoyl-2-oleoyl-GPS (18:0/18:1) levels \|\| id:ebi-met1400-GCST90199652 | Shellfish liking \|\| id:ebi-fl187-GCST90094838 | MR Egger | 30 | 1.42468153317127e-05 | 0.215072866205609 | 0.999947616389678 | -0.421528571 | 0.421557064578326 | 1.00001424691682 | 0.656043244348591 | 1.52433319396433 | 0.999947616389678 |
| ebi-fl187-GCST90094838 | ebi-met1400-GCST90199665 | N-acetylaspartate (naa) levels \|\| id:ebi-met1400-GCST90199665 | Shellfish liking \|\| id:ebi-fl187-GCST90094838 | MR Egger | 30 | -0.051125043 | 0.180465106577361 | 0.779034029354595 | -0.404836652 | 0.302586566231006 | 0.950159852587942 | 0.667085769384074 | 1.35335482617701 | 0.999279047927446 |
| ebi-fl187-GCST90094838 | ebi-met1400-GCST90199669 | Acetylcarnitine levels (Biocrates platform) \|\| id:ebi-met1400-GCST90199669 | Shellfish liking \|\| id:ebi-fl187-GCST90094838 | MR Egger | 30 | 0.212330806821703 | 0.19285674800686 | 0.280281805511014 | -0.165668419 | 0.590330032915148 | 1.23655687887676 | 0.847327144966326 | 1.80458388921106 | 0.999279047927446 |
| ebi-fl187-GCST90094838 | ebi-met1400-GCST90199670 | Iminodiacetate (IDA) levels \|\| id:ebi-met1400-GCST90199670 | Shellfish liking \|\| id:ebi-fl187-GCST90094838 | MR Egger | 30 | 0.0836126605310181 | 0.223145342111969 | 0.710706331395879 | -0.35375221 | 0.520977531070477 | 1.08720769378961 | 0.70204890649693 | 1.68367268789485 | 0.999279047927446 |
| ebi-fl187-GCST90094838 | ebi-met1400-GCST90199686 | Indolepropionate levels \|\| id:ebi-met1400-GCST90199686 | Shellfish liking \|\| id:ebi-fl187-GCST90094838 | MR Egger | 30 | -0.170548696 | 0.211689370946794 | 0.427230916863084 | -0.585459863 | 0.24436247117355 | 0.843202028162254 | 0.556849728371502 | 1.27680705237339 | 0.999279047927446 |
| ebi-fl187-GCST90094838 | ebi-met1400-GCST90199704 | 3-hydroxysebacate levels \|\| id:ebi-met1400-GCST90199704 | Shellfish liking \|\| id:ebi-fl187-GCST90094838 | MR Egger | 30 | 0.286652836607019 | 0.225613458530712 | 0.214345248459399 | -0.155549542 | 0.728855215327213 | 1.33196172484904 | 0.855944670570746 | 2.07270644641066 | 0.999279047927446 |
| ebi-fl187-GCST90094838 | ebi-met1400-GCST90199713 | Docosapentaenoate n3 DPA; 22:5n3 levels \|\| id:ebi-met1400-GCST90199713 | Shellfish liking \|\| id:ebi-fl187-GCST90094838 | MR Egger | 30 | -0.182484748 | 0.172408743801355 | 0.29889992877585 | -0.520405885 | 0.15543639026517 | 0.833197352102803 | 0.594279289703208 | 1.1681676268709 | 0.999279047927446 |
| ebi-fl187-GCST90094838 | ebi-met1400-GCST90199715 | Myristoleate (14:1n5) levels \|\| id:ebi-met1400-GCST90199715 | Shellfish liking \|\| id:ebi-fl187-GCST90094838 | MR Egger | 30 | -0.056999772 | 0.165346874699876 | 0.732874537320704 | -0.381079646 | 0.267080102779657 | 0.944594285081513 | 0.683123479370551 | 1.30614506799094 | 0.999279047927446 |
| ebi-fl187-GCST90094838 | ebi-met1400-GCST90199725 | Campesterol levels \|\| id:ebi-met1400-GCST90199725 | Shellfish liking \|\| id:ebi-fl187-GCST90094838 | MR Egger | 30 | -0.022111656 | 0.254634216940706 | 0.931419158813427 | -0.521194722 | 0.476971408709824 | 0.978131014274032 | 0.593810685500264 | 1.61118737746985 | 0.999279047927446 |
| ebi-fl187-GCST90094838 | ebi-met1400-GCST90199737 | N-acetylproline levels \|\| id:ebi-met1400-GCST90199737 | Shellfish liking \|\| id:ebi-fl187-GCST90094838 | MR Egger | 30 | 0.318290985906611 | 0.193540813380206 | 0.11124149274566 | -0.061049008 | 0.697630980131814 | 1.37477624354175 | 0.94077713274076 | 2.00898773368417 | 0.999279047927446 |
| ebi-fl187-GCST90094838 | ebi-met1400-GCST90199775 | Gamma-glutamylisoleucine levels \|\| id:ebi-met1400-GCST90199775 | Shellfish liking \|\| id:ebi-fl187-GCST90094838 | MR Egger | 30 | 0.0792233738057555 | 0.199381870658816 | 0.69412601652538 | -0.311565093 | 0.470011840297035 | 1.08244608516911 | 0.732299941584131 | 1.60001313773602 | 0.999279047927446 |
| ebi-fl187-GCST90094838 | ebi-met1400-GCST90199777 | 1-palmitoleoyl-GPC (16:1) levels \|\| id:ebi-met1400-GCST90199777 | Shellfish liking \|\| id:ebi-fl187-GCST90094838 | MR Egger | 30 | -0.359164148 | 0.203153654538173 | 0.0879671582808956 | -0.757345311 | 0.0390170145300708 | 0.698259723752042 | 0.468909585176883 | 1.03978817500682 | 0.999279047927446 |
| ebi-fl187-GCST90094838 | ebi-met1400-GCST90199782 | Glutamine degradant levels \|\| id:ebi-met1400-GCST90199782 | Shellfish liking \|\| id:ebi-fl187-GCST90094838 | MR Egger | 30 | -0.191154658 | 0.174990432755444 | 0.283979757549973 | -0.534135906 | 0.151826590496057 | 0.82600483026103 | 0.586175582230363 | 1.16395837748564 | 0.999279047927446 |
| ebi-fl187-GCST90094838 | ebi-met1400-GCST90199783 | 5-methyluridine (ribothymidine) levels \|\| id:ebi-met1400-GCST90199783 | Shellfish liking \|\| id:ebi-fl187-GCST90094838 | MR Egger | 30 | -0.378398753 | 0.178979065464345 | 0.0435336547438977 | -0.729197722 | -0.027599785 | 0.684957317229739 | 0.48229577040298 | 0.972777609130911 | 0.999279047927446 |
| ebi-fl187-GCST90094838 | ebi-met1400-GCST90199787 | Homostachydrine levels \|\| id:ebi-met1400-GCST90199787 | Shellfish liking \|\| id:ebi-fl187-GCST90094838 | MR Egger | 30 | -0.156185797 | 0.201513827393026 | 0.444799432518865 | -0.551152898 | 0.238781305092072 | 0.855400245150037 | 0.576285029217664 | 1.26970082911242 | 0.999279047927446 |
| ebi-fl187-GCST90094838 | ebi-met1400-GCST90199802 | Beta-hydroxyisovaleroylcarnitine levels \|\| id:ebi-met1400-GCST90199802 | Shellfish liking \|\| id:ebi-fl187-GCST90094838 | MR Egger | 30 | 0.290552150695508 | 0.198595743124877 | 0.154594095162371 | -0.098695506 | 0.679799807220267 | 1.33716560114857 | 0.906018543391341 | 1.97348261571144 | 0.999279047927446 |
| ebi-fl187-GCST90094838 | ebi-met1400-GCST90199873 | Pregnenolone sulfate levels \|\| id:ebi-met1400-GCST90199873 | Shellfish liking \|\| id:ebi-fl187-GCST90094838 | MR Egger | 30 | 0.0442139163910529 | 0.171209228035616 | 0.79810668 | -0.291356171 | 0.37978400334086 | 1.04520591765353 | 0.747249482349718 | 1.46196877495686 | 0.999279047927446 |
| ebi-fl187-GCST90094838 | ebi-met1400-GCST90199911 | Beta-citrylglutamate levels \|\| id:ebi-met1400-GCST90199911 | Shellfish liking \|\| id:ebi-fl187-GCST90094838 | MR Egger | 30 | -0.030518345 | 0.178794803403876 | 0.865695600033553 | -0.380956159 | 0.319919469956078 | 0.969942638582228 | 0.683207841213832 | 1.37701686864189 | 0.999279047927446 |
| ebi-fl187-GCST90094838 | ebi-met1400-GCST90199934 | 2-hydroxydecanoate levels \|\| id:ebi-met1400-GCST90199934 | Shellfish liking \|\| id:ebi-fl187-GCST90094838 | MR Egger | 29 | -0.079522298 | 0.200137101787056 | 0.694241207714652 | -0.471791017 | 0.312746421583801 | 0.923557426330482 | 0.623883880157021 | 1.36717480104713 | 0.999279047927446 |
| ebi-fl187-GCST90094838 | ebi-met1400-GCST90199945 | Sphingomyelin (d18:1/14:0, d16:1/16:0) levels \|\| id:ebi-met1400-GCST90199945 | Shellfish liking \|\| id:ebi-fl187-GCST90094838 | MR Egger | 30 | -0.268687096 | 0.172030864746712 | 0.12955529395625 | -0.605867591 | 0.0684933991915119 | 0.764382396761693 | 0.545600863051105 | 1.07089355616437 | 0.999279047927446 |
| ebi-fl187-GCST90094838 | ebi-met1400-GCST90199979 | Carnitine C14:1 levels \|\| id:ebi-met1400-GCST90199979 | Shellfish liking \|\| id:ebi-fl187-GCST90094838 | MR Egger | 30 | 0.0164730125103912 | 0.173865704058943 | 0.925191684004353 | -0.324303767 | 0.357249792465919 | 1.01660944067912 | 0.723030575866977 | 1.42939287683466 | 0.999279047927446 |
| ebi-fl187-GCST90094838 | ebi-met1400-GCST90199986 | Eugenol sulfate levels \|\| id:ebi-met1400-GCST90199986 | Shellfish liking \|\| id:ebi-fl187-GCST90094838 | MR Egger | 30 | -0.320694441 | 0.224178671562693 | 0.163630733871948 | -0.760084637 | 0.118695755590861 | 0.725644944699783 | 0.467626846831971 | 1.12602727866383 | 0.999279047927446 |
| ebi-fl187-GCST90094838 | ebi-met1400-GCST90200002 | 6-hydroxyindole sulfate levels \|\| id:ebi-met1400-GCST90200002 | Shellfish liking \|\| id:ebi-fl187-GCST90094838 | MR Egger | 30 | 0.304428198237289 | 0.202041059920733 | 0.143071304423572 | -0.091572279 | 0.700428675681926 | 1.3558495041751 | 0.912495359327106 | 2.01461613933848 | 0.999279047927446 |
| ebi-fl187-GCST90094838 | ebi-met1400-GCST90200023 | Trans 3,4-methyleneheptanoate levels \|\| id:ebi-met1400-GCST90200023 | Shellfish liking \|\| id:ebi-fl187-GCST90094838 | MR Egger | 30 | -0.035254146 | 0.190805109648616 | 0.854744977517665 | -0.409232161 | 0.338723868604144 | 0.965360042388619 | 0.664160022222114 | 1.40315583633381 | 0.999279047927446 |
| ebi-fl187-GCST90094838 | ebi-met1400-GCST90200032 | 2-hydroxybutyrate/2-hydroxyisobutyrate levels \|\| id:ebi-met1400-GCST90200032 | Shellfish liking \|\| id:ebi-fl187-GCST90094838 | MR Egger | 30 | 0.385121504999802 | 0.181916302691674 | 0.0432740405765681 | 0.0285655517241201 | 0.741677458275483 | 1.46979289777772 | 1.02897745987037 | 2.0994543093587 | 0.999279047927446 |
| ebi-fl187-GCST90094838 | ebi-met1400-GCST90200051 | 1-palmitoyl-2-dihomo-linolenoyl-GPC (16:0/20:3n3 or 6) levels \|\| id:ebi-met1400-GCST90200051 | Shellfish liking \|\| id:ebi-fl187-GCST90094838 | MR Egger | 30 | -0.310392362 | 0.176686645280201 | 0.0898948004448518 | -0.656698187 | 0.0359134623681027 | 0.733159235679191 | 0.518560701331414 | 1.0365661406304 | 0.999279047927446 |
| ebi-fl187-GCST90094838 | ebi-met1400-GCST90200069 | Phenylacetylglutamate levels \|\| id:ebi-met1400-GCST90200069 | Shellfish liking \|\| id:ebi-fl187-GCST90094838 | MR Egger | 30 | 0.258393528510055 | 0.194977999341083 | 0.195803009365684 | -0.12376335 | 0.640550407218578 | 1.29484827808787 | 0.883588917284741 | 1.89752500339117 | 0.999279047927446 |
| ebi-fl187-GCST90094838 | ebi-met1400-GCST90200072 | Thioproline levels \|\| id:ebi-met1400-GCST90200072 | Shellfish liking \|\| id:ebi-fl187-GCST90094838 | MR Egger | 30 | 0.136426945842756 | 0.186990219415402 | 0.471698272605757 | -0.230073884 | 0.502927775896944 | 1.14617114215034 | 0.794474901183234 | 1.65355543031211 | 0.999279047927446 |
| ebi-fl187-GCST90094838 | ebi-met1400-GCST90200081 | 1-oleoyl-2-docosahexaenoyl-GPC (18:1/22:6) levels \|\| id:ebi-met1400-GCST90200081 | Shellfish liking \|\| id:ebi-fl187-GCST90094838 | MR Egger | 30 | -0.125207903 | 0.172368258929939 | 0.473624188243453 | -0.46304969 | 0.212633884760852 | 0.882313448129897 | 0.629361358611372 | 1.23693170878572 | 0.999279047927446 |
| ebi-fl187-GCST90094838 | ebi-met1400-GCST90200097 | Furaneol sulfate levels \|\| id:ebi-met1400-GCST90200097 | Shellfish liking \|\| id:ebi-fl187-GCST90094838 | MR Egger | 30 | -0.182378536 | 0.214729474831029 | 0.402895336732433 | -0.603248307 | 0.238491234418891 | 0.833285851806106 | 0.547031819719316 | 1.26933257954996 | 0.999279047927446 |
| ebi-fl187-GCST90094838 | ebi-met1400-GCST90200124 | Sphingomyelin (d18:2/24:2) levels \|\| id:ebi-met1400-GCST90200124 | Shellfish liking \|\| id:ebi-fl187-GCST90094838 | MR Egger | 30 | -0.262056843 | 0.163423624120306 | 0.120037023804074 | -0.582367146 | 0.0582534601230171 | 0.769467283518064 | 0.558574572571873 | 1.05998362524546 | 0.999279047927446 |
| ebi-fl187-GCST90094838 | ebi-met1400-GCST90200137 | Lignoceroylcarnitine (C24) levels \|\| id:ebi-met1400-GCST90200137 | Shellfish liking \|\| id:ebi-fl187-GCST90094838 | MR Egger | 30 | 0.311652099537822 | 0.18043010337138 | 0.0951360940971905 | -0.041990903 | 0.665295102145728 | 1.36567948984687 | 0.958878503383353 | 1.9450644293386 | 0.999279047927446 |
| ebi-fl187-GCST90094838 | ebi-met1400-GCST90200165 | Octadecenedioate (C18:1-DC) levels \|\| id:ebi-met1400-GCST90200165 | Shellfish liking \|\| id:ebi-fl187-GCST90094838 | MR Egger | 30 | -0.225334397 | 0.176136561290734 | 0.211286023143093 | -0.570562057 | 0.119893262846534 | 0.798249241745831 | 0.565207670244818 | 1.12737651219735 | 0.999279047927446 |
| ebi-fl187-GCST90094838 | ebi-met1400-GCST90200176 | Glutamine conjugate of C6H10O2 (1) levels \|\| id:ebi-met1400-GCST90200176 | Shellfish liking \|\| id:ebi-fl187-GCST90094838 | MR Egger | 30 | 0.0598199032227277 | 0.179972761583426 | 0.742075614212222 | -0.292926709 | 0.412566515926243 | 1.06164533042456 | 0.746076819050935 | 1.51069002391203 | 0.999279047927446 |
| ebi-fl187-GCST90094838 | ebi-met1400-GCST90200182 | Glycine conjugate of C10H12O2 levels \|\| id:ebi-met1400-GCST90200182 | Shellfish liking \|\| id:ebi-fl187-GCST90094838 | MR Egger | 30 | -0.115164524 | 0.261885672342061 | 0.663493013282375 | -0.628460442 | 0.398131393573444 | 0.891219504653133 | 0.53341238847983 | 1.48903966729713 | 0.999279047927446 |
| ebi-fl187-GCST90094838 | ebi-met1400-GCST90200208 | Glucuronide of piperine metabolite C17H21NO3 (4) levels \|\| id:ebi-met1400-GCST90200208 | Shellfish liking \|\| id:ebi-fl187-GCST90094838 | MR Egger | 30 | -0.020127456 | 0.183539920502319 | 0.913459195895568 | -0.379865701 | 0.339610787724109 | 0.980073748614731 | 0.683953257525983 | 1.40440087411564 | 0.999279047927446 |
| ebi-fl187-GCST90094838 | ebi-met1400-GCST90200227 | 3-ethylcatechol sulfate (2) levels \|\| id:ebi-met1400-GCST90200227 | Shellfish liking \|\| id:ebi-fl187-GCST90094838 | MR Egger | 29 | -0.058399605 | 0.26612678200573 | 0.827955692003631 | -0.580008098 | 0.463208887811479 | 0.943272935608187 | 0.559893832722201 | 1.58916526500187 | 0.999279047927446 |
| ebi-fl187-GCST90094838 | ebi-met1400-GCST90200259 | Pentose acid levels \|\| id:ebi-met1400-GCST90200259 | Shellfish liking \|\| id:ebi-fl187-GCST90094838 | MR Egger | 30 | 0.0545965374993741 | 0.185741071279399 | 0.770972503259185 | -0.309455962 | 0.418649037206996 | 1.05611442613841 | 0.733846087648165 | 1.51990683042576 | 0.999279047927446 |
| ebi-fl187-GCST90094838 | ebi-met1400-GCST90200291 | Sarcosine levels \|\| id:ebi-met1400-GCST90200291 | Shellfish liking \|\| id:ebi-fl187-GCST90094838 | MR Egger | 30 | 0.146347564653519 | 0.185688725239073 | 0.437240344025073 | -0.217602337 | 0.510297466122102 | 1.15759845844639 | 0.804445276339803 | 1.66578663634468 | 0.999279047927446 |
| ebi-fl187-GCST90094838 | ebi-met1400-GCST90200306 | Butyrate/isobutyrate (4:0) levels \|\| id:ebi-met1400-GCST90200306 | Shellfish liking \|\| id:ebi-fl187-GCST90094838 | MR Egger | 30 | -0.045110892 | 0.189387645357539 | 0.813465639411936 | -0.416310676 | 0.326088893336334 | 0.955891475654439 | 0.659475355182608 | 1.38553852854109 | 0.999279047927446 |
| ebi-fl187-GCST90094838 | ebi-met1400-GCST90200321 | Cholate levels \|\| id:ebi-met1400-GCST90200321 | Shellfish liking \|\| id:ebi-fl187-GCST90094838 | MR Egger | 30 | 0.152369671273385 | 0.18479157138163 | 0.416597016029897 | -0.209821809 | 0.514561151181379 | 1.16459067258418 | 0.810728697953407 | 1.67290418865623 | 0.999279047927446 |
| ebi-fl187-GCST90094838 | ebi-met1400-GCST90200336 | Ceramide (d18:1/16:0) levels \|\| id:ebi-met1400-GCST90200336 | Shellfish liking \|\| id:ebi-fl187-GCST90094838 | MR Egger | 30 | -0.001943448 | 0.178002475393448 | 0.991366217615989 | -0.350828299 | 0.346941404236037 | 0.998058439736238 | 0.704104638732238 | 1.41473382553235 | 0.999279047927446 |
| ebi-fl187-GCST90094838 | ebi-met1400-GCST90200358 | Arachidonate (20:4n6) levels \|\| id:ebi-met1400-GCST90200358 | Shellfish liking \|\| id:ebi-fl187-GCST90094838 | MR Egger | 30 | -0.197953045 | 0.176612319376432 | 0.271880136093435 | -0.544113191 | 0.148207100556568 | 0.820408374163076 | 0.580356220090258 | 1.159753057 | 0.999279047927446 |
| ebi-fl187-GCST90094838 | ebi-met1400-GCST90200413 | 2'-deoxyuridine levels \|\| id:ebi-met1400-GCST90200413 | Shellfish liking \|\| id:ebi-fl187-GCST90094838 | MR Egger | 30 | -0.213841877 | 0.178888374127323 | 0.241960116234231 | -0.56446309 | 0.136779336750002 | 0.80747605583672 | 0.56866538703704 | 1.14657511361274 | 0.999279047927446 |
| ebi-fl187-GCST90094838 | ebi-met1400-GCST90200432 | Threonine levels \|\| id:ebi-met1400-GCST90200432 | Shellfish liking \|\| id:ebi-fl187-GCST90094838 | MR Egger | 30 | -0.151460448 | 0.1803897703027 | 0.408228637691575 | -0.505024398 | 0.202103501749548 | 0.85945187460364 | 0.603490851375979 | 1.22397468507691 | 0.999279047927446 |
| ebi-fl187-GCST90094838 | ebi-met1400-GCST90200436 | Caffeine levels \|\| id:ebi-met1400-GCST90200436 | Shellfish liking \|\| id:ebi-fl187-GCST90094838 | MR Egger | 30 | 0.0548157268848097 | 0.185470914336336 | 0.769755286646423 | -0.308707265 | 0.418338718984029 | 1.05634594058225 | 0.734395721736949 | 1.51943524881303 | 0.999279047927446 |
| ebi-fl187-GCST90094838 | ebi-met1400-GCST90200442 | Valine levels \|\| id:ebi-met1400-GCST90200442 | Shellfish liking \|\| id:ebi-fl187-GCST90094838 | MR Egger | 30 | -0.040931205 | 0.1980337773716 | 0.837749220492159 | -0.429077408 | 0.347214998826768 | 0.959895163835438 | 0.6511095258381 | 1.41512094200841 | 0.999279047927446 |
| ebi-fl187-GCST90094838 | ebi-met1400-GCST90200459 | Margarate (17:0) levels \|\| id:ebi-met1400-GCST90200459 | Shellfish liking \|\| id:ebi-fl187-GCST90094838 | MR Egger | 30 | 0.0301043815657982 | 0.186843363407844 | 0.873155056630779 | -0.336108611 | 0.396317373845172 | 1.03056210002511 | 0.714545494305394 | 1.48634096845099 | 0.999279047927446 |
| ebi-fl187-GCST90094838 | ebi-met1400-GCST90200462 | X-11299 levels \|\| id:ebi-met1400-GCST90200462 | Shellfish liking \|\| id:ebi-fl187-GCST90094838 | MR Egger | 30 | 0.138807308676097 | 0.178779191510369 | 0.444012120050996 | -0.211599907 | 0.489214524036421 | 1.14890269509153 | 0.809288423690704 | 1.63103457821493 | 0.999279047927446 |
| ebi-fl187-GCST90094838 | ebi-met1400-GCST90200497 | X-12740 levels \|\| id:ebi-met1400-GCST90200497 | Shellfish liking \|\| id:ebi-fl187-GCST90094838 | MR Egger | 30 | 0.072907696690071 | 0.205527251562147 | 0.725446143625322 | -0.329925716 | 0.47574110975188 | 1.07563124800797 | 0.71897713967887 | 1.60920635419362 | 0.999279047927446 |
| ebi-fl187-GCST90094838 | ebi-met1400-GCST90200527 | X-13866 levels \|\| id:ebi-met1400-GCST90200527 | Shellfish liking \|\| id:ebi-fl187-GCST90094838 | MR Egger | 30 | -0.039678651 | 0.17951192796292 | 0.826668145871073 | -0.39152203 | 0.312164728072732 | 0.961098237746251 | 0.676027157762054 | 1.36637975559567 | 0.999279047927446 |
| ebi-fl187-GCST90094838 | ebi-met1400-GCST90200547 | X-17654 levels \|\| id:ebi-met1400-GCST90200547 | Shellfish liking \|\| id:ebi-fl187-GCST90094838 | MR Egger | 30 | -0.047119059 | 0.21866573094429 | 0.830951630826409 | -0.475703891 | 0.381465774119625 | 0.953973812107989 | 0.621447471081877 | 1.4644295399634 | 0.999279047927446 |
| ebi-fl187-GCST90094838 | ebi-met1400-GCST90200553 | X-17653 levels \|\| id:ebi-met1400-GCST90200553 | Shellfish liking \|\| id:ebi-fl187-GCST90094838 | MR Egger | 30 | -0.125252807 | 0.197122628104132 | 0.530323084975239 | -0.511613158 | 0.261107544575937 | 0.8822738298225 | 0.599527665718261 | 1.29836729028525 | 0.999279047927446 |
| ebi-fl187-GCST90094838 | ebi-met1400-GCST90200557 | X-17685 levels \|\| id:ebi-met1400-GCST90200557 | Shellfish liking \|\| id:ebi-fl187-GCST90094838 | MR Egger | 30 | 0.362825935651147 | 0.230439522507447 | 0.126605874170265 | -0.088835528 | 0.814487399765744 | 1.43738563986925 | 0.914996052006015 | 2.25801791512949 | 0.999279047927446 |
| ebi-fl187-GCST90094838 | ebi-met1400-GCST90200561 | X-18779 levels \|\| id:ebi-met1400-GCST90200561 | Shellfish liking \|\| id:ebi-fl187-GCST90094838 | MR Egger | 30 | -0.054944559 | 0.178801506181267 | 0.760893891775514 | -0.405395511 | 0.295506393385036 | 0.946537623746538 | 0.666713066456387 | 1.34380668123046 | 0.999279047927446 |
| ebi-fl187-GCST90094838 | ebi-met1400-GCST90200570 | X-18921 levels \|\| id:ebi-met1400-GCST90200570 | Shellfish liking \|\| id:ebi-fl187-GCST90094838 | MR Egger | 30 | 0.0123403548701252 | 0.18110895142479 | 0.946160246689105 | -0.34263319 | 0.367313899662714 | 1.01241681122512 | 0.709898561754232 | 1.44385107235375 | 0.999279047927446 |
| ebi-fl187-GCST90094838 | ebi-met1400-GCST90200575 | X-21283 levels \|\| id:ebi-met1400-GCST90200575 | Shellfish liking \|\| id:ebi-fl187-GCST90094838 | MR Egger | 30 | -0.12057402 | 0.222215847196428 | 0.591699267841136 | -0.55611708 | 0.314969040921229 | 0.886411473108751 | 0.573431334553784 | 1.37021688964772 | 0.999279047927446 |
| ebi-fl187-GCST90094838 | ebi-met1400-GCST90200578 | X-18922 levels \|\| id:ebi-met1400-GCST90200578 | Shellfish liking \|\| id:ebi-fl187-GCST90094838 | MR Egger | 30 | 0.489054481856535 | 0.217112681963502 | 0.0323136215834122 | 0.0635136252080713 | 0.914595338504998 | 1.6307735647727 | 1.06557400437008 | 2.49576510749583 | 0.999279047927446 |
| ebi-fl187-GCST90094838 | ebi-met1400-GCST90200623 | X-24241 levels \|\| id:ebi-met1400-GCST90200623 | Shellfish liking \|\| id:ebi-fl187-GCST90094838 | MR Egger | 30 | -0.109128178 | 0.181741525714415 | 0.553030535428035 | -0.465341568 | 0.247085212743108 | 0.896615484060336 | 0.62792059093967 | 1.2802882050001 | 0.999279047927446 |
| ebi-fl187-GCST90094838 | ebi-met1400-GCST90200636 | X-24544 levels \|\| id:ebi-met1400-GCST90200636 | Shellfish liking \|\| id:ebi-fl187-GCST90094838 | MR Egger | 30 | 0.106753008577651 | 0.167698497155681 | 0.529570624765553 | -0.221936046 | 0.435442063002786 | 1.11265940320918 | 0.800966587835983 | 1.54564618094073 | 0.999279047927446 |
| ebi-fl187-GCST90094838 | ebi-met1400-GCST90200659 | X-25172 levels \|\| id:ebi-met1400-GCST90200659 | Shellfish liking \|\| id:ebi-fl187-GCST90094838 | MR Egger | 30 | 0.157814859850965 | 0.182405433192691 | 0.394288542154555 | -0.199699789 | 0.51532950890864 | 1.17094938489456 | 0.818976581785223 | 1.67419007146228 | 0.999279047927446 |
| ebi-fl187-GCST90094838 | ebi-met1400-GCST90200670 | X-26111 levels \|\| id:ebi-met1400-GCST90200670 | Shellfish liking \|\| id:ebi-fl187-GCST90094838 | MR Egger | 30 | 0.180967260398413 | 0.181421582586923 | 0.327066778124513 | -0.174619041 | 0.536553562268782 | 1.19837594429997 | 0.839776880002879 | 1.71010293099747 | 0.999279047927446 |
| ebi-fl187-GCST90094838 | ebi-met1400-GCST90200673 | Carnitine C4 levels \|\| id:ebi-met1400-GCST90200673 | Shellfish liking \|\| id:ebi-fl187-GCST90094838 | MR Egger | 30 | 0.141746404677494 | 0.194589792207436 | 0.472394893247262 | -0.239649588 | 0.523142397404069 | 1.15228439754841 | 0.78690355317008 | 1.68732156245142 | 0.999279047927446 |
| ebi-fl187-GCST90094838 | ebi-met1400-GCST90200710 | X-13431 levels \|\| id:ebi-met1400-GCST90200710 | Shellfish liking \|\| id:ebi-fl187-GCST90094838 | MR Egger | 30 | 0.180612443392868 | 0.18752242050048 | 0.343716595103787 | -0.186931501 | 0.54815638757381 | 1.19795081556181 | 0.829500554573662 | 1.7300605148393 | 0.999279047927446 |
| ebi-fl187-GCST90094838 | ebi-met1400-GCST90200730 | Adenosine 5'-diphosphate (ADP) to fructose ratio \|\| id:ebi-met1400-GCST90200730 | Shellfish liking \|\| id:ebi-fl187-GCST90094838 | MR Egger | 30 | 0.227135741428144 | 0.236215291537033 | 0.344500410902663 | -0.23584623 | 0.690117712840728 | 1.25500021189974 | 0.789902127857069 | 1.99395023297542 | 0.999279047927446 |
| ebi-fl187-GCST90094838 | ebi-met1400-GCST90200731 | Adenosine 5'-diphosphate (ADP) to mannose ratio \|\| id:ebi-met1400-GCST90200731 | Shellfish liking \|\| id:ebi-fl187-GCST90094838 | MR Egger | 30 | -0.019909998 | 0.242877117472422 | 0.935249314777294 | -0.495949149 | 0.456129151851033 | 0.980286896730722 | 0.608992608387367 | 1.57795412730314 | 0.999279047927446 |
| ebi-fl187-GCST90094838 | ebi-met1400-GCST90200748 | Aspartate to glutamate ratio \|\| id:ebi-met1400-GCST90200748 | Shellfish liking \|\| id:ebi-fl187-GCST90094838 | MR Egger | 30 | -0.389303758 | 0.193306100561213 | 0.0537168261419733 | -0.768183715 | -0.010423801 | 0.677528433838431 | 0.463854795973642 | 0.98963033829587 | 0.999279047927446 |
| ebi-fl187-GCST90094838 | ebi-met1400-GCST90200774 | Uridine to pseudouridine ratio \|\| id:ebi-met1400-GCST90200774 | Shellfish liking \|\| id:ebi-fl187-GCST90094838 | MR Egger | 30 | -0.304974727 | 0.174801387281816 | 0.0920072249566996 | -0.647585446 | 0.0376359917753893 | 0.737142003727171 | 0.523307807293729 | 1.0383531949752 | 0.999279047927446 |
| ebi-fl187-GCST90094838 | ebi-met1400-GCST90200799 | Spermidine to adenosine 5'-diphosphate (ADP) ratio \|\| id:ebi-met1400-GCST90200799 | Shellfish liking \|\| id:ebi-fl187-GCST90094838 | MR Egger | 30 | -0.131829588 | 0.245336883613639 | 0.595277038040575 | -0.61268988 | 0.349030703885563 | 0.876490346828812 | 0.541891284437786 | 1.41769271834873 | 0.999279047927446 |
| ebi-fl187-GCST90094838 | ebi-met1400-GCST90200811 | Choline to taurocholate ratio \|\| id:ebi-met1400-GCST90200811 | Shellfish liking \|\| id:ebi-fl187-GCST90094838 | MR Egger | 30 | 0.127623259176249 | 0.186961123735485 | 0.500456542916539 | -0.238820543 | 0.4940670616978 | 1.13612489743002 | 0.787556201893323 | 1.63896846911658 | 0.999279047927446 |
| ebi-fl187-GCST90094838 | ebi-met1400-GCST90200812 | Glutamate to kynurenine ratio \|\| id:ebi-met1400-GCST90200812 | Shellfish liking \|\| id:ebi-fl187-GCST90094838 | MR Egger | 30 | 0.299253451860904 | 0.176179928271706 | 0.100488054848955 | -0.046059208 | 0.644566111273447 | 1.34885144906276 | 0.954985418168354 | 1.90516022237103 | 0.999279047927446 |
| ebi-fl187-GCST90094838 | ebi-met1400-GCST90200876 | Cortisone to 4-cholesten-3-one ratio \|\| id:ebi-met1400-GCST90200876 | Shellfish liking \|\| id:ebi-fl187-GCST90094838 | MR Egger | 30 | -0.274101204 | 0.202486296067593 | 0.186666234631264 | -0.670974344 | 0.12277193674648 | 0.760255130852316 | 0.511210240502032 | 1.13062653717512 | 0.999279047927446 |
| ebi-fl187-GCST90094838 | ebi-met1400-GCST90200906 | 2'-deoxyuridine to cytidine ratio \|\| id:ebi-met1400-GCST90200906 | Shellfish liking \|\| id:ebi-fl187-GCST90094838 | MR Egger | 30 | -0.179790788 | 0.224108874523483 | 0.429166643205896 | -0.619044182 | 0.259462606122143 | 0.835444978290646 | 0.538458860373906 | 1.29623331161528 | 0.999279047927446 |
| ebi-fl187-GCST90094838 | ebi-met1400-GCST90200907 | Retinol (Vitamin A) to linoleoyl-arachidonoyl-glycerol (18:2 to 20:4) [1] ratio \|\| id:ebi-met1400-GCST90200907 | Shellfish liking \|\| id:ebi-fl187-GCST90094838 | MR Egger | 30 | -0.030056475 | 0.254855761278592 | 0.906960944134767 | -0.529573767 | 0.469460817401764 | 0.970390729471522 | 0.588855906126967 | 1.59913173672276 | 0.999279047927446 |
| ebi-fl187-GCST90094838 | ebi-met1400-GCST90200916 | Fructose to sucrose ratio \|\| id:ebi-met1400-GCST90200916 | Shellfish liking \|\| id:ebi-fl187-GCST90094838 | MR Egger | 30 | -0.041392942 | 0.184778577680747 | 0.824373148143713 | -0.403558954 | 0.320773070010209 | 0.959452046626404 | 0.667938647650333 | 1.37819279212827 | 0.999279047927446 |
| ebi-fl187-GCST90094838 | ebi-met1400-GCST90200917 | Alpha-ketobutyrate to 3-methyl-2-oxobutyrate ratio \|\| id:ebi-met1400-GCST90200917 | Shellfish liking \|\| id:ebi-fl187-GCST90094838 | MR Egger | 30 | 0.201863784779265 | 0.18928026841699 | 0.29531617647283 | -0.169125541 | 0.572853110876567 | 1.22368131273827 | 0.844402889279925 | 1.77331931730098 | 0.999279047927446 |
| ebi-fl187-GCST90094838 | ebi-met1400-GCST90200924 | Alpha-tocopherol to glycerol ratio \|\| id:ebi-met1400-GCST90200924 | Shellfish liking \|\| id:ebi-fl187-GCST90094838 | MR Egger | 30 | -0.059500178 | 0.167116351351633 | 0.724478984545665 | -0.387048227 | 0.268047870233758 | 0.942235365481875 | 0.679058345146222 | 1.30740972452609 | 0.999279047927446 |
| ebi-fl187-GCST90094838 | ebi-met1400-GCST90200938 | Alpha-ketoglutarate to alanine ratio \|\| id:ebi-met1400-GCST90200938 | Shellfish liking \|\| id:ebi-fl187-GCST90094838 | MR Egger | 30 | -0.06873427 | 0.185853570048889 | 0.714291590911838 | -0.433007267 | 0.295538727517025 | 0.933574726138561 | 0.648555778684612 | 1.34385013275554 | 0.999279047927446 |
| ebi-fl187-GCST90094838 | ebi-met1400-GCST90200943 | Phosphate to EDTA ratio \|\| id:ebi-met1400-GCST90200943 | Shellfish liking \|\| id:ebi-fl187-GCST90094838 | MR Egger | 30 | 0.249987031540329 | 0.187807863746957 | 0.193900613045182 | -0.118116381 | 0.618090444484364 | 1.28400876496388 | 0.888592630938842 | 1.85538170259432 | 0.999279047927446 |
| ebi-fl187-GCST90094838 | ebi-met1400-GCST90200947 | Adenosine 5'-diphosphate (ADP) to glucose ratio \|\| id:ebi-met1400-GCST90200947 | Shellfish liking \|\| id:ebi-fl187-GCST90094838 | MR Egger | 30 | 0.166293937368441 | 0.235741337651855 | 0.486383622378275 | -0.295759084 | 0.628346959166077 | 1.18092016727932 | 0.743966639579519 | 1.87450937622043 | 0.999279047927446 |
| ebi-fl187-GCST90094838 | ebi-met1400-GCST90200975 | Arachidonate (20:4n6) to caffeine ratio \|\| id:ebi-met1400-GCST90200975 | Shellfish liking \|\| id:ebi-fl187-GCST90094838 | MR Egger | 30 | -0.104571535 | 0.184994286056821 | 0.57639259628816 | -0.467160336 | 0.258017265730454 | 0.900710362839662 | 0.626779587266584 | 1.29436116652232 | 0.999279047927446 |
| ebi-fl187-GCST90094838 | ebi-met1400-GCST90200985 | Cholesterol to oleoyl-linoleoyl-glycerol (18:1 to 18:2) [2] ratio \|\| id:ebi-met1400-GCST90200985 | Shellfish liking \|\| id:ebi-fl187-GCST90094838 | MR Egger | 30 | 0.177336494651748 | 0.179074602618642 | 0.330511625501388 | -0.173649726 | 0.528322715784286 | 1.19403281119034 | 0.840591282964634 | 1.69608510472631 | 0.999279047927446 |
| ebi-fl187-GCST90094838 | ebi-met1400-GCST90201016 | Bilirubin (Z,Z) to etiocholanolone glucuronide ratio \|\| id:ebi-met1400-GCST90201016 | Shellfish liking \|\| id:ebi-fl187-GCST90094838 | MR Egger | 30 | -0.212436859 | 0.216927281717471 | 0.335818004547038 | -0.637614331 | 0.212740612922647 | 0.808611371043223 | 0.528551870730784 | 1.2370637312784 | 0.999279047927446 |
| ebi-fl187-GCST90094838 | ebi-pw11-GCST90275189 | Placental weight (fetal genotype, adjusted for sex and gestational age) \|\| id:ebi-pw11-GCST90275189 | Shellfish liking \|\| id:ebi-fl187-GCST90094838 | MR Egger | 33 | 0.0157678211760301 | 0.0679628777162123 | 0.818057836232726 | -0.117439419 | 0.148975061499806 | 1.01589278922902 | 0.889194378268047 | 1.16064404412644 | 0.999279047927446 |
| ebi-fl187-GCST90094838 | ebi-pw11-GCST90275192 | Placental weight (fetal effect, conditional on maternal and paternal genotype) \|\| id:ebi-pw11-GCST90275192 | Shellfish liking \|\| id:ebi-fl187-GCST90094838 | MR Egger | 33 | 0.0488807369483228 | 0.120448216730881 | 0.687659789769154 | -0.187197768 | 0.28495924174085 | 1.05009510572477 | 0.829279715305353 | 1.32970783044063 | 0.999279047927446 |
| ebi-fl187-GCST90094838 | ebi-pw11-GCST90275193 | Placental weight (maternal effect, conditional on fetal and paternal genotype) \|\| id:ebi-pw11-GCST90275193 | Shellfish liking \|\| id:ebi-fl187-GCST90094838 | MR Egger | 33 | -0.043599267 | 0.112312748477855 | 0.700524738784607 | -0.263732254 | 0.176533719695589 | 0.957337517043391 | 0.768179188770385 | 1.19307465619555 | 0.999279047927446 |
| ebi-fl187-GCST90094838 | ebi-pw11-GCST90275195 | Placental weight (fetal effect, conditional on maternal genotype) \|\| id:ebi-pw11-GCST90275195 | Shellfish liking \|\| id:ebi-fl187-GCST90094838 | MR Egger | 33 | 0.0333994667136126 | 0.0919839433536795 | 0.718993670616953 | -0.146889062 | 0.213687995686824 | 1.03396349075327 | 0.86338975461203 | 1.2382362594644 | 0.999279047927446 |
| ebi-fl187-GCST90094838 | ebi-pw11-GCST90275197 | Placental weight (fetal genotype, adjusted for sex) \|\| id:ebi-pw11-GCST90275197 | Shellfish liking \|\| id:ebi-fl187-GCST90094838 | MR Egger | 33 | 0.0204137120917909 | 0.0660981378024253 | 0.759509758117337 | -0.109138638 | 0.149966062184545 | 1.02062349697688 | 0.896606105203154 | 1.16179481328124 | 0.999279047927446 |
| ebi-fl187-GCST90094838 | prot-a-1030 | Protein FAM171B \|\| id:prot-a-1030 | Shellfish liking \|\| id:ebi-fl187-GCST90094838 | MR Egger | 34 | -0.104423245 | 0.307511162734885 | 0.736394970205793 | -0.707145124 | 0.49829863366153 | 0.900843938760694 | 0.493049785989021 | 1.64591857670931 | 0.999279047927446 |
| ebi-fl187-GCST90094838 | prot-a-1036 | BRISC complex subunit Abro1 \|\| id:prot-a-1036 | Shellfish liking \|\| id:ebi-fl187-GCST90094838 | MR Egger | 34 | 0.428881566793421 | 0.29247797913658 | 0.152305930130878 | -0.144375272 | 1.00213840590112 | 1.53553916480075 | 0.865562865323649 | 2.72410083784651 | 0.999279047927446 |
| ebi-fl187-GCST90094838 | prot-a-1055 | Phenylalanine--tRNA ligase, mitochondrial \|\| id:prot-a-1055 | Shellfish liking \|\| id:ebi-fl187-GCST90094838 | MR Egger | 34 | -0.615731492 | 0.303304579011705 | 0.0507308177736971 | -1.210208466 | -0.021254517 | 0.540245565650534 | 0.298135121780745 | 0.978969768679952 | 0.999279047927446 |
| ebi-fl187-GCST90094838 | prot-a-1083 | Fc receptor-like protein 5 \|\| id:prot-a-1083 | Shellfish liking \|\| id:ebi-fl187-GCST90094838 | MR Egger | 34 | -0.207321739 | 0.358898071856008 | 0.567535204516914 | -0.91076196 | 0.496118481646238 | 0.812758111778481 | 0.402217633483797 | 1.64233413274342 | 0.999279047927446 |
| ebi-fl187-GCST90094838 | prot-a-1103 | Fibroblast growth factor receptor 3 \|\| id:prot-a-1103 | Shellfish liking \|\| id:ebi-fl187-GCST90094838 | MR Egger | 34 | -0.539955275 | 0.292504920392947 | 0.074165499322877 | -1.113264919 | 0.0333543685022697 | 0.582774316099745 | 0.328484732206783 | 1.03391686190069 | 0.999279047927446 |
| ebi-fl187-GCST90094838 | prot-a-1105 | Fibroblast growth factor receptor 4 \|\| id:prot-a-1105 | Shellfish liking \|\| id:ebi-fl187-GCST90094838 | MR Egger | 34 | -0.087013471 | 0.299993174782848 | 0.773648138440898 | -0.675000093 | 0.500973152024359 | 0.916664747571647 | 0.509156373192662 | 1.65032650808564 | 0.999279047927446 |
| ebi-fl187-GCST90094838 | prot-a-1120 | Filamin-A \|\| id:prot-a-1120 | Shellfish liking \|\| id:ebi-fl187-GCST90094838 | MR Egger | 34 | -0.03135063 | 0.306457988992719 | 0.919156989059647 | -0.632008289 | 0.569307028206539 | 0.969135705230788 | 0.531523276252755 | 1.76704211671541 | 0.999279047927446 |
| ebi-fl187-GCST90094838 | prot-a-1166 | Galanin peptides \|\| id:prot-a-1166 | Shellfish liking \|\| id:ebi-fl187-GCST90094838 | MR Egger | 34 | -0.253757551 | 0.342510613325727 | 0.464171468804326 | -0.925078353 | 0.417563251329633 | 0.775879890712076 | 0.396500350902663 | 1.51825743266282 | 0.999279047927446 |
| ebi-fl187-GCST90094838 | prot-a-1205 | ADP-ribosylation factor-binding protein GGA1 \|\| id:prot-a-1205 | Shellfish liking \|\| id:ebi-fl187-GCST90094838 | MR Egger | 34 | 0.287842253648619 | 0.292904243807459 | 0.333118403305188 | -0.286250064 | 0.861934571511238 | 1.33354692536871 | 0.751074775538383 | 2.36773682205688 | 0.999279047927446 |
| ebi-fl187-GCST90094838 | prot-a-1227 | Guanine nucleotide-binding protein G(I)/G(S)/G(O) subunit gamma-T2 \|\| id:prot-a-1227 | Shellfish liking \|\| id:ebi-fl187-GCST90094838 | MR Egger | 34 | 0.233594768973531 | 0.307469306769114 | 0.452974862056643 | -0.369045072 | 0.836234610240994 | 1.26313252799201 | 0.691394247023286 | 2.30766135260848 | 0.999279047927446 |
| ebi-fl187-GCST90094838 | prot-a-1240 | Golgi SNAP receptor complex member 1 \|\| id:prot-a-1240 | Shellfish liking \|\| id:ebi-fl187-GCST90094838 | MR Egger | 34 | 0.0289950727088312 | 0.296997905188892 | 0.922837268610646 | -0.553120821 | 0.611110966879059 | 1.02941952221291 | 0.57515205971863 | 1.84247719330342 | 0.999279047927446 |
| ebi-fl187-GCST90094838 | prot-a-1283 | Glutathione S-transferase A1 \|\| id:prot-a-1283 | Shellfish liking \|\| id:ebi-fl187-GCST90094838 | MR Egger | 34 | 0.0210385613652019 | 0.414619362334351 | 0.959846630419959 | -0.791615389 | 0.83369251154053 | 1.02126143211329 | 0.453112251309488 | 2.30180249972917 | 0.999279047927446 |
| ebi-fl187-GCST90094838 | prot-a-1324 | E3 ubiquitin-protein ligase HECW1 \|\| id:prot-a-1324 | Shellfish liking \|\| id:ebi-fl187-GCST90094838 | MR Egger | 34 | 0.236942275745289 | 0.292504270805686 | 0.423896228775409 | -0.336366095 | 0.810250646524433 | 1.26736795779294 | 0.714361533728982 | 2.2484714876174 | 0.999279047927446 |
| ebi-fl187-GCST90094838 | prot-a-1339 | Histidine triad nucleotide-binding protein 2, mitochondrial \|\| id:prot-a-1339 | Shellfish liking \|\| id:ebi-fl187-GCST90094838 | MR Egger | 34 | 0.465402542583048 | 0.292504920392947 | 0.121420666985343 | -0.107907101 | 1.03871218655322 | 1.59265517139367 | 0.897710988662469 | 2.82557585570639 | 0.999279047927446 |
| ebi-fl187-GCST90094838 | prot-a-1466 | Interleukin-11 \|\| id:prot-a-1466 | Shellfish liking \|\| id:ebi-fl187-GCST90094838 | MR Egger | 34 | -0.145553701 | 0.326474071111365 | 0.65871816602855 | -0.78544288 | 0.494335478337519 | 0.864543461943659 | 0.455917740026739 | 1.63940845457273 | 0.999279047927446 |
| ebi-fl187-GCST90094838 | prot-a-147 | Rho GTPase-activating protein 1 \|\| id:prot-a-147 | Shellfish liking \|\| id:ebi-fl187-GCST90094838 | MR Egger | 34 | -0.025577455 | 0.292518233422323 | 0.930867586224442 | -0.598913193 | 0.547758282362812 | 0.974746876881137 | 0.549408412844359 | 1.72937190581152 | 0.999279047927446 |
| ebi-fl187-GCST90094838 | prot-a-1473 | Interleukin-12 receptor subunit beta-1 \|\| id:prot-a-1473 | Shellfish liking \|\| id:ebi-fl187-GCST90094838 | MR Egger | 34 | -0.642113707 | 0.33488293191449 | 0.0641473678119238 | -1.298484254 | 0.0142568394315652 | 0.526179059369526 | 0.272945195325585 | 1.01435895286136 | 0.999279047927446 |
| ebi-fl187-GCST90094838 | prot-a-1501 | X-linked interleukin-1 receptor accessory protein-like 2 \|\| id:prot-a-1501 | Shellfish liking \|\| id:ebi-fl187-GCST90094838 | MR Egger | 34 | -0.075841733 | 0.313864487964992 | 0.810602103368968 | -0.691016129 | 0.539332663680277 | 0.926962902837463 | 0.501066661860686 | 1.71486209049717 | 0.999279047927446 |
| ebi-fl187-GCST90094838 | prot-a-1503 | Interleukin-1 receptor-like 2 \|\| id:prot-a-1503 | Shellfish liking \|\| id:ebi-fl187-GCST90094838 | MR Egger | 34 | -0.313242041 | 0.297978622950809 | 0.301030195683944 | -0.897280142 | 0.270796060122996 | 0.7310729416287 | 0.407676976735508 | 1.31100767637509 | 0.999279047927446 |
| ebi-fl187-GCST90094838 | prot-a-1536 | Interleukin-5 receptor subunit alpha \|\| id:prot-a-1536 | Shellfish liking \|\| id:ebi-fl187-GCST90094838 | MR Egger | 34 | 0.231907717481958 | 0.33730995234048 | 0.496707708101093 | -0.429219789 | 0.893035224069299 | 1.26100335489392 | 0.651016827049457 | 2.4425320436962 | 0.999279047927446 |
| ebi-fl187-GCST90094838 | prot-a-1559 | Insulin-induced gene 1 protein \|\| id:prot-a-1559 | Shellfish liking \|\| id:ebi-fl187-GCST90094838 | MR Egger | 34 | -0.041176495 | 0.292508672078529 | 0.888933922987659 | -0.614493492 | 0.532140502447641 | 0.959659740020916 | 0.540914803556238 | 1.70257277220416 | 0.999279047927446 |
| ebi-fl187-GCST90094838 | prot-a-1567 | Interferon regulatory factor 1 \|\| id:prot-a-1567 | Shellfish liking \|\| id:ebi-fl187-GCST90094838 | MR Egger | 34 | 0.0877331682508756 | 0.292508672078529 | 0.7661675925629 | -0.485583829 | 0.661050165524793 | 1.09169678379845 | 0.615337839783134 | 1.93682525387339 | 0.999279047927446 |
| ebi-fl187-GCST90094838 | prot-a-1613 | Potassium voltage-gated channel subfamily E regulatory beta subunit 5 \|\| id:prot-a-1613 | Shellfish liking \|\| id:ebi-fl187-GCST90094838 | MR Egger | 34 | 0.086817433275225 | 0.29247797913658 | 0.768511424070956 | -0.486439406 | 0.660074272382921 | 1.09069753646341 | 0.614811596150476 | 1.93493604137583 | 0.999279047927446 |
| ebi-fl187-GCST90094838 | prot-a-1649 | Kin of IRRE-like protein 2 \|\| id:prot-a-1649 | Shellfish liking \|\| id:ebi-fl187-GCST90094838 | MR Egger | 34 | 0.0279856173548727 | 0.29247797913658 | 0.924367843115143 | -0.545271222 | 0.601242456462569 | 1.02838089347761 | 0.579684538960021 | 1.8243841106529 | 0.999279047927446 |
| ebi-fl187-GCST90094838 | prot-a-165 | Armadillo repeat-containing protein 5 \|\| id:prot-a-165 | Shellfish liking \|\| id:ebi-fl187-GCST90094838 | MR Egger | 34 | -0.18761949 | 0.363201553476696 | 0.609007280682649 | -0.899494535 | 0.524255554427543 | 0.8289300630886 | 0.406775218338838 | 1.68920086208326 | 0.999279047927446 |
| ebi-fl187-GCST90094838 | prot-a-1656 | Kallikrein-11 \|\| id:prot-a-1656 | Shellfish liking \|\| id:ebi-fl187-GCST90094838 | MR Egger | 34 | 0.0615498639090947 | 0.299723283374633 | 0.838595058237231 | -0.525907772 | 0.649007499323376 | 1.06348352465249 | 0.591018610924902 | 1.91364059659197 | 0.999279047927446 |
| ebi-fl187-GCST90094838 | prot-a-1697 | Laminin subunit gamma-2 \|\| id:prot-a-1697 | Shellfish liking \|\| id:ebi-fl187-GCST90094838 | MR Egger | 34 | -0.212645141 | 0.292508672078529 | 0.472529217805285 | -0.785962138 | 0.360671856352836 | 0.808442969648499 | 0.455681062648631 | 1.43429272960165 | 0.999279047927446 |
| ebi-fl187-GCST90094838 | prot-a-1698 | Lysosome-associated membrane glycoprotein 3 \|\| id:prot-a-1698 | Shellfish liking \|\| id:ebi-fl187-GCST90094838 | MR Egger | 34 | 0.0570982253684979 | 0.292389271158579 | 0.846407229349158 | -0.515984746 | 0.630181196839313 | 1.05875980235983 | 0.596912497847081 | 1.87795082719176 | 0.999279047927446 |
| ebi-fl187-GCST90094838 | prot-a-1724 | Leptin receptor \|\| id:prot-a-1724 | Shellfish liking \|\| id:ebi-fl187-GCST90094838 | MR Egger | 34 | -0.331447985 | 0.311846944973773 | 0.295803288054483 | -0.942667997 | 0.279772027381748 | 0.717883496123032 | 0.389587030548099 | 1.32282820934975 | 0.999279047927446 |
| ebi-fl187-GCST90094838 | prot-a-1743 | Leukocyte immunoglobulin-like receptor subfamily B member 2 \|\| id:prot-a-1743 | Shellfish liking \|\| id:ebi-fl187-GCST90094838 | MR Egger | 34 | -0.014461826 | 0.301490584205232 | 0.962040075166989 | -0.605383371 | 0.576459719159687 | 0.985642244036991 | 0.54586511770579 | 1.77972653265214 | 0.999279047927446 |
| ebi-fl187-GCST90094838 | prot-a-1746 | Leukocyte immunoglobulin-like receptor subfamily B member 4 \|\| id:prot-a-1746 | Shellfish liking \|\| id:ebi-fl187-GCST90094838 | MR Egger | 34 | 0.108107440532489 | 0.300444537725712 | 0.721341990920685 | -0.480763853 | 0.696978734474884 | 1.11416744569967 | 0.618310912477763 | 2.00767780740335 | 0.999279047927446 |
| ebi-fl187-GCST90094838 | prot-a-1750 | Leucine-rich repeat and immunoglobulin-like domain-containing nogo receptor-interacting protein 3 \|\| id:prot-a-1750 | Shellfish liking \|\| id:ebi-fl187-GCST90094838 | MR Egger | 34 | -0.083716917 | 0.292452692194069 | 0.77652566882957 | -0.656924194 | 0.489490359287211 | 0.919691567916651 | 0.518443516234495 | 1.63148453710127 | 0.999279047927446 |
| ebi-fl187-GCST90094838 | prot-a-1753 | Lipase member N \|\| id:prot-a-1753 | Shellfish liking \|\| id:ebi-fl187-GCST90094838 | MR Egger | 34 | 0.34467106388209 | 0.310030046222623 | 0.274535412449685 | -0.262987827 | 0.952329954478431 | 1.41152554156451 | 0.768751255482904 | 2.59174126907593 | 0.999279047927446 |
| ebi-fl187-GCST90094838 | prot-a-1760 | Ig Kappa chain V-I region HK102- like \|\| id:prot-a-1760 | Shellfish liking \|\| id:ebi-fl187-GCST90094838 | MR Egger | 34 | -0.234023409 | 0.29776050509534 | 0.437681016656922 | -0.817633999 | 0.349587180990924 | 0.791343291126871 | 0.441474949980947 | 1.41848185143581 | 0.999279047927446 |
| ebi-fl187-GCST90094838 | prot-a-1792 | Leucine-rich repeat neuronal protein 1 \|\| id:prot-a-1792 | Shellfish liking \|\| id:ebi-fl187-GCST90094838 | MR Egger | 34 | -0.023760889 | 0.299199193858616 | 0.937197138799191 | -0.610191309 | 0.562669530611783 | 0.976519177978733 | 0.543246930934964 | 1.75535221767212 | 0.999279047927446 |
| ebi-fl187-GCST90094838 | prot-a-1795 | Leucine-rich repeat transmembrane neuronal protein 1 \|\| id:prot-a-1795 | Shellfish liking \|\| id:ebi-fl187-GCST90094838 | MR Egger | 34 | -0.305705319 | 0.292464879637777 | 0.30372934874645 | -0.878936483 | 0.267525845551762 | 0.736603650917516 | 0.415224275170898 | 1.30672740249037 | 0.999279047927446 |
| ebi-fl187-GCST90094838 | prot-a-1820 | LysM and putative peptidoglycan-binding domain-containing protein 4 \|\| id:prot-a-1820 | Shellfish liking \|\| id:ebi-fl187-GCST90094838 | MR Egger | 34 | -0.016242998 | 0.316941165293919 | 0.959445485748615 | -0.637447682 | 0.60496168641889 | 0.983888208573477 | 0.528639961174728 | 1.83118204839979 | 0.999279047927446 |
| ebi-fl187-GCST90094838 | prot-a-1844 | Mitogen-activated protein kinase kinase kinase 7:TGF-beta-activated kinase 1 and MAP3K7-binding protein 1 fusion \|\| id:prot-a-1844 | Shellfish liking \|\| id:ebi-fl187-GCST90094838 | MR Egger | 34 | -0.409530539 | 0.292476414643879 | 0.17106990597755 | -0.982784312 | 0.163723233393863 | 0.663961880985395 | 0.374267569081717 | 1.17788826983673 | 0.999279047927446 |
| ebi-fl187-GCST90094838 | prot-a-1856 | Megakaryocyte-associated tyrosine-protein kinase \|\| id:prot-a-1856 | Shellfish liking \|\| id:ebi-fl187-GCST90094838 | MR Egger | 34 | -0.225976968 | 0.301600846673376 | 0.459174316286223 | -0.817114628 | 0.365160691357568 | 0.797736474823055 | 0.441704298988782 | 1.44074550490005 | 0.999279047927446 |
| ebi-fl187-GCST90094838 | prot-a-1910 | Stromelysin-2 \|\| id:prot-a-1910 | Shellfish liking \|\| id:ebi-fl187-GCST90094838 | MR Egger | 34 | -0.163176081 | 0.332131382921661 | 0.62656734965639 | -0.814153592 | 0.487801429287707 | 0.84944160451555 | 0.443014139532752 | 1.62873139950561 | 0.999279047927446 |
| ebi-fl187-GCST90094838 | prot-a-1940 | 39S ribosomal protein L14, mitochondrial \|\| id:prot-a-1940 | Shellfish liking \|\| id:ebi-fl187-GCST90094838 | MR Egger | 34 | -0.087932579 | 0.292504920392947 | 0.765649456668881 | -0.661242223 | 0.485377065178396 | 0.915822620509471 | 0.516209688615513 | 1.6247875441593 | 0.999279047927446 |
| ebi-fl187-GCST90094838 | prot-a-1942 | 39S ribosomal protein L33, mitochondrial \|\| id:prot-a-1942 | Shellfish liking \|\| id:ebi-fl187-GCST90094838 | MR Egger | 34 | 0.185276345810438 | 0.349127707105846 | 0.599302054418664 | -0.49901396 | 0.869566651737896 | 1.20355099044999 | 0.60712901808749 | 2.38587671394158 | 0.999279047927446 |
| ebi-fl187-GCST90094838 | prot-a-1948 | Mesothelin \|\| id:prot-a-1948 | Shellfish liking \|\| id:ebi-fl187-GCST90094838 | MR Egger | 34 | 0.246976239933266 | 0.292504920392947 | 0.404742820729272 | -0.326333404 | 0.820285883903443 | 1.28014869599847 | 0.721564574781207 | 2.2711490296811 | 0.999279047927446 |
| ebi-fl187-GCST90094838 | prot-a-1980 | Unconventional myosin-VI \|\| id:prot-a-1980 | Shellfish liking \|\| id:ebi-fl187-GCST90094838 | MR Egger | 34 | 7.88402991835108e-05 | 0.352767985815728 | 0.999823067838974 | -0.691346412 | 0.691504092498011 | 1.00007884340716 | 0.500901195508935 | 1.99671652213648 | 0.999947616389678 |
| ebi-fl187-GCST90094838 | prot-a-2039 | Neurofascin \|\| id:prot-a-2039 | Shellfish liking \|\| id:ebi-fl187-GCST90094838 | MR Egger | 34 | -0.239453671 | 0.292481596626919 | 0.419014947825194 | -0.812717601 | 0.33381025796564 | 0.787057735762146 | 0.443650760811116 | 1.39627818577498 | 0.999279047927446 |
| ebi-fl187-GCST90094838 | prot-a-2041 | NFU1 iron-sulfur cluster scaffold homolog, mitochondrial \|\| id:prot-a-2041 | Shellfish liking \|\| id:ebi-fl187-GCST90094838 | MR Egger | 34 | 0.0728698585712179 | 0.292482248107197 | 0.804842539295767 | -0.500395348 | 0.646135064861325 | 1.07559054891496 | 0.60629091659395 | 1.90815167645006 | 0.999279047927446 |
| ebi-fl187-GCST90094838 | prot-a-2088 | Glucocorticoid receptor \|\| id:prot-a-2088 | Shellfish liking \|\| id:ebi-fl187-GCST90094838 | MR Egger | 34 | 0.391320630055159 | 0.345238630207757 | 0.265434171281494 | -0.285347085 | 1.06798834526236 | 1.47893262765143 | 0.751753286629208 | 2.90952065795329 | 0.999279047927446 |
| ebi-fl187-GCST90094838 | prot-a-2111 | Neuron-specific protein family member 2 \|\| id:prot-a-2111 | Shellfish liking \|\| id:ebi-fl187-GCST90094838 | MR Egger | 34 | 0.0698498485081994 | 0.292527816888034 | 0.812798655624709 | -0.503504673 | 0.643204369608745 | 1.07234715464033 | 0.604408688911541 | 1.90256765192452 | 0.999279047927446 |
| ebi-fl187-GCST90094838 | prot-a-2125 | Nuclear migration protein nudC \|\| id:prot-a-2125 | Shellfish liking \|\| id:ebi-fl187-GCST90094838 | MR Egger | 34 | 0.228643890226853 | 0.30744873213187 | 0.462495142208605 | -0.373955625 | 0.831243405205319 | 1.25689436693649 | 0.688007441652706 | 2.29617203825846 | 0.999279047927446 |
| ebi-fl187-GCST90094838 | prot-a-2142 | Ornithine decarboxylase \|\| id:prot-a-2142 | Shellfish liking \|\| id:ebi-fl187-GCST90094838 | MR Egger | 34 | -0.437812769 | 0.292481596626919 | 0.144221217487338 | -1.011076698 | 0.135451160691543 | 0.645446619356172 | 0.363827036733257 | 1.14505327085888 | 0.999279047927446 |
| ebi-fl187-GCST90094838 | prot-a-2186 | Prostate and testis expressed protein 1 \|\| id:prot-a-2186 | Shellfish liking \|\| id:ebi-fl187-GCST90094838 | MR Egger | 34 | 0.11642720259311 | 0.338475786918213 | 0.733115206747533 | -0.54698534 | 0.779839744952808 | 1.123475721 | 0.578691742377827 | 2.18112270156779 | 0.999279047927446 |
| ebi-fl187-GCST90094838 | prot-a-2227 | Platelet-derived growth factor C \|\| id:prot-a-2227 | Shellfish liking \|\| id:ebi-fl187-GCST90094838 | MR Egger | 34 | -0.241452552 | 0.331308308603179 | 0.471433541090208 | -0.890816837 | 0.407911732747876 | 0.785486072558163 | 0.410320450910965 | 1.50367443010226 | 0.999279047927446 |
| ebi-fl187-GCST90094838 | prot-a-2229 | Platelet-derived growth factor receptor alpha \|\| id:prot-a-2229 | Shellfish liking \|\| id:ebi-fl187-GCST90094838 | MR Egger | 34 | -0.616460246 | 0.332717345273979 | 0.0731525225953115 | -1.268586243 | 0.0356657505852437 | 0.539852002651375 | 0.281228930263405 | 1.03630940278346 | 0.999279047927446 |
| ebi-fl187-GCST90094838 | prot-a-2243 | Platelet endothelial aggregation receptor 1 \|\| id:prot-a-2243 | Shellfish liking \|\| id:ebi-fl187-GCST90094838 | MR Egger | 34 | 0.0754821041769563 | 0.296603563544442 | 0.80074675175172 | -0.50586088 | 0.656825088724063 | 1.07840392861949 | 0.60298625289324 | 1.92865928150414 | 0.999279047927446 |
| ebi-fl187-GCST90094838 | prot-a-2246 | E3 ubiquitin-protein ligase pellino homolog 2 \|\| id:prot-a-2246 | Shellfish liking \|\| id:ebi-fl187-GCST90094838 | MR Egger | 34 | 0.617880543182071 | 0.292503615622521 | 0.0425492360420572 | 0.0445734565619305 | 1.19118762980221 | 1.85499229642855 | 1.0455817787323 | 3.29098736206103 | 0.999279047927446 |
| ebi-fl187-GCST90094838 | prot-a-2251 | Platelet factor 4 variant \|\| id:prot-a-2251 | Shellfish liking \|\| id:ebi-fl187-GCST90094838 | MR Egger | 34 | -0.41509737 | 0.292464226344514 | 0.16547696084408 | -0.988327253 | 0.158132513849639 | 0.660275986643623 | 0.372198764801686 | 1.17132140073194 | 0.999279047927446 |
| ebi-fl187-GCST90094838 | prot-a-2346 | Protein phosphatase 1L \|\| id:prot-a-2346 | Shellfish liking \|\| id:ebi-fl187-GCST90094838 | MR Egger | 34 | 0.0100453997540779 | 0.329697136584281 | 0.97588260222782 | -0.636160988 | 0.656251787459268 | 1.0100960241543 | 0.529320596610764 | 1.92755389558854 | 0.999279047927446 |
| ebi-fl187-GCST90094838 | prot-a-2358 | PR domain zinc finger protein 4 \|\| id:prot-a-2358 | Shellfish liking \|\| id:ebi-fl187-GCST90094838 | MR Egger | 34 | 0.249387701798462 | 0.29247797913658 | 0.400178521692825 | -0.323869137 | 0.822644540906158 | 1.28323945088165 | 0.723344895045773 | 2.27651221371355 | 0.999279047927446 |
| ebi-fl187-GCST90094838 | prot-a-2390 | Trypsin-1 \|\| id:prot-a-2390 | Shellfish liking \|\| id:ebi-fl187-GCST90094838 | MR Egger | 34 | -0.032005284 | 0.292476414643879 | 0.913545913343299 | -0.605259057 | 0.541248488208152 | 0.968501464025643 | 0.545932980452134 | 1.71815061446367 | 0.999279047927446 |
| ebi-fl187-GCST90094838 | prot-a-242 | Brain-derived neurotrophic factor \|\| id:prot-a-242 | Shellfish liking \|\| id:ebi-fl187-GCST90094838 | MR Egger | 34 | -0.154981378 | 0.292505155600349 | 0.599879092462385 | -0.728291483 | 0.418328727286199 | 0.856431126060621 | 0.482733043707109 | 1.519420067 | 0.999279047927446 |
| ebi-fl187-GCST90094838 | prot-a-2464 | Dihydropteridine reductase \|\| id:prot-a-2464 | Shellfish liking \|\| id:ebi-fl187-GCST90094838 | MR Egger | 34 | 0.299068642343367 | 0.349823118 | 0.398954445357682 | -0.38658467 | 0.984721954268207 | 1.34860219151053 | 0.679373200694808 | 2.6770674337565 | 0.999279047927446 |
| ebi-fl187-GCST90094838 | prot-a-2474 | Ras-related protein Rab-27A \|\| id:prot-a-2474 | Shellfish liking \|\| id:ebi-fl187-GCST90094838 | MR Egger | 34 | -0.248718887 | 0.343360979645978 | 0.474104485302544 | -0.921706407 | 0.424268632857292 | 0.779799154061577 | 0.397839585140904 | 1.52847213647627 | 0.999279047927446 |
| ebi-fl187-GCST90094838 | prot-a-2478 | Ras-related protein Rab-7b \|\| id:prot-a-2478 | Shellfish liking \|\| id:ebi-fl187-GCST90094838 | MR Egger | 34 | 0.0860771271874842 | 0.373088822920395 | 0.819005066362403 | -0.645176966 | 0.817331220111458 | 1.08989038524363 | 0.524569703028331 | 2.26444845172912 | 0.999279047927446 |
| ebi-fl187-GCST90094838 | prot-a-2528 | E3 ubiquitin-protein ligase rififylin \|\| id:prot-a-2528 | Shellfish liking \|\| id:ebi-fl187-GCST90094838 | MR Egger | 34 | -0.22438163 | 0.3014663660001 | 0.46212566010302 | -0.815255707 | 0.366492447330069 | 0.799010149909871 | 0.442526155684131 | 1.44266550453277 | 0.999279047927446 |
| ebi-fl187-GCST90094838 | prot-a-2543 | Protein RIC-3 \|\| id:prot-a-2543 | Shellfish liking \|\| id:ebi-fl187-GCST90094838 | MR Egger | 34 | -0.364281868 | 0.331424763016561 | 0.279908366411985 | -1.013874403 | 0.285310667932392 | 0.694695355045165 | 0.362810578549144 | 1.33017520671867 | 0.999279047927446 |
| ebi-fl187-GCST90094838 | prot-a-255 | Bone morphogenetic protein 6 \|\| id:prot-a-255 | Shellfish liking \|\| id:ebi-fl187-GCST90094838 | MR Egger | 34 | -0.689714782 | 0.336432504003166 | 0.0486242391691176 | -1.34912249 | -0.030307074 | 0.501719148090365 | 0.259467846497213 | 0.970147580745522 | 0.999279047927446 |
| ebi-fl187-GCST90094838 | prot-a-2568 | RING finger protein 165 \|\| id:prot-a-2568 | Shellfish liking \|\| id:ebi-fl187-GCST90094838 | MR Egger | 34 | -0.323110105 | 0.320771106239839 | 0.321351001135667 | -0.951821473 | 0.305601263411654 | 0.723894145746363 | 0.386037226271909 | 1.35744093725499 | 0.999279047927446 |
| ebi-fl187-GCST90094838 | prot-a-2573 | E3 ubiquitin-protein ligase RNF8 \|\| id:prot-a-2573 | Shellfish liking \|\| id:ebi-fl187-GCST90094838 | MR Egger | 34 | -0.199341396 | 0.373424999414527 | 0.597155726807879 | -0.931254395 | 0.532571602358102 | 0.819270149626857 | 0.394059094320125 | 1.70330690940568 | 0.999279047927446 |
| ebi-fl187-GCST90094838 | prot-a-260 | Cytoplasmic tyrosine-protein kinase BMX \|\| id:prot-a-260 | Shellfish liking \|\| id:ebi-fl187-GCST90094838 | MR Egger | 34 | -0.305656611 | 0.344817056127718 | 0.381998719253298 | -0.981498041 | 0.370184819292935 | 0.736639530150003 | 0.374749288452249 | 1.44800220867867 | 0.999279047927446 |
| ebi-fl187-GCST90094838 | prot-a-2623 | Serum amyloid A-1 protein \|\| id:prot-a-2623 | Shellfish liking \|\| id:ebi-fl187-GCST90094838 | MR Egger | 34 | 0.0793518790408953 | 0.292371911153034 | 0.787821036202183 | -0.493697067 | 0.652400824900842 | 1.08258519409573 | 0.610365655096774 | 1.92014523210594 | 0.999279047927446 |
| ebi-fl187-GCST90094838 | prot-a-2640 | Lysosome membrane protein 2 \|\| id:prot-a-2640 | Shellfish liking \|\| id:ebi-fl187-GCST90094838 | MR Egger | 34 | -0.089123369 | 0.317811278381003 | 0.780953739842474 | -0.712033475 | 0.533786736207255 | 0.914732716568793 | 0.49064546704462 | 1.70537791330544 | 0.999279047927446 |
| ebi-fl187-GCST90094838 | prot-a-2641 | Scavenger receptor class F member 2 \|\| id:prot-a-2641 | Shellfish liking \|\| id:ebi-fl187-GCST90094838 | MR Egger | 34 | -0.16908186 | 0.292480941859396 | 0.567246098377539 | -0.742344506 | 0.404180786420201 | 0.844439775034006 | 0.475996629495006 | 1.49807475405026 | 0.999279047927446 |
| ebi-fl187-GCST90094838 | prot-a-2669 | Semaphorin-3A \|\| id:prot-a-2669 | Shellfish liking \|\| id:ebi-fl187-GCST90094838 | MR Egger | 34 | 0.135936989497782 | 0.29247797913658 | 0.645238386845945 | -0.43731985 | 0.709193828605478 | 1.14560970587768 | 0.645764850739407 | 2.03235217385773 | 0.999279047927446 |
| ebi-fl187-GCST90094838 | prot-a-2683 | Sentrin-specific protease 7 \|\| id:prot-a-2683 | Shellfish liking \|\| id:ebi-fl187-GCST90094838 | MR Egger | 34 | -0.594371095 | 0.331551336452532 | 0.0824798954310199 | -1.244211714 | 0.0554695246697674 | 0.551909555546581 | 0.288167977338888 | 1.05703680303592 | 0.999279047927446 |
| ebi-fl187-GCST90094838 | prot-a-270 | Bromodomain testis-specific protein \|\| id:prot-a-270 | Shellfish liking \|\| id:ebi-fl187-GCST90094838 | MR Egger | 34 | -0.263127008 | 0.292483793790732 | 0.375037890484643 | -0.836395243 | 0.310141228225025 | 0.768644267444065 | 0.433269543027617 | 1.36361768183868 | 0.999279047927446 |
| ebi-fl187-GCST90094838 | prot-a-2708 | 14-3-3 protein sigma \|\| id:prot-a-2708 | Shellfish liking \|\| id:ebi-fl187-GCST90094838 | MR Egger | 34 | -0.172396681 | 0.292504920392947 | 0.55974454155415 | -0.745706325 | 0.400912963296153 | 0.841645242538866 | 0.474399101797707 | 1.49318730074318 | 0.999279047927446 |
| ebi-fl187-GCST90094838 | prot-a-2713 | Beta-sarcoglycan \|\| id:prot-a-2713 | Shellfish liking \|\| id:ebi-fl187-GCST90094838 | MR Egger | 34 | -0.048600012 | 0.292518233422323 | 0.869089313800936 | -0.621935749 | 0.524735725604892 | 0.952562066996819 | 0.536904119189378 | 1.69001216241591 | 0.999279047927446 |
| ebi-fl187-GCST90094838 | prot-a-2720 | Protein shisa-3 homolog \|\| id:prot-a-2720 | Shellfish liking \|\| id:ebi-fl187-GCST90094838 | MR Egger | 34 | 0.138634998081455 | 0.329707950131342 | 0.676946514177716 | -0.507592584 | 0.784862580338885 | 1.14870474403998 | 0.601942962899589 | 2.19210568161433 | 0.999279047927446 |
| ebi-fl187-GCST90094838 | prot-a-2730 | Sialic acid-binding Ig-like lectin 8 \|\| id:prot-a-2730 | Shellfish liking \|\| id:ebi-fl187-GCST90094838 | MR Egger | 34 | -0.586727457 | 0.292397024707107 | 0.0533030253362898 | -1.159825626 | -0.013629289 | 0.556144316091931 | 0.313540849638264 | 0.986463169562118 | 0.999279047927446 |
| ebi-fl187-GCST90094838 | prot-a-2761 | Sodium- and chloride-dependent neutral and basic amino acid transporter B(0+) \|\| id:prot-a-2761 | Shellfish liking \|\| id:ebi-fl187-GCST90094838 | MR Egger | 34 | -0.307408622 | 0.352278142707869 | 0.389365603716661 | -0.997873782 | 0.383056537305658 | 0.73535005900011 | 0.368662465165744 | 1.46676095443662 | 0.999279047927446 |
| ebi-fl187-GCST90094838 | prot-a-2770 | Secreted Ly-6/uPAR-related protein 1 \|\| id:prot-a-2770 | Shellfish liking \|\| id:ebi-fl187-GCST90094838 | MR Egger | 34 | 0.204772927497944 | 0.292504920392947 | 0.488944410874742 | -0.368536716 | 0.77808257146812 | 1.22724635941972 | 0.69174581066599 | 2.1772934558995 | 0.999279047927446 |
| ebi-fl187-GCST90094838 | prot-a-2784 | N-lysine methyltransferase SMYD2 \|\| id:prot-a-2784 | Shellfish liking \|\| id:ebi-fl187-GCST90094838 | MR Egger | 34 | -0.350225926 | 0.292508672078529 | 0.239977059968685 | -0.923542923 | 0.223091071590232 | 0.704528900563395 | 0.39710961703951 | 1.24993440206632 | 0.999279047927446 |
| ebi-fl187-GCST90094838 | prot-a-2837 | Serine palmitoyltransferase 2 \|\| id:prot-a-2837 | Shellfish liking \|\| id:ebi-fl187-GCST90094838 | MR Egger | 34 | -0.3005189 | 0.409079121502736 | 0.467918745403711 | -1.102313978 | 0.501276177968461 | 0.740433909694097 | 0.332101717717691 | 1.65082667561187 | 0.999279047927446 |
| ebi-fl187-GCST90094838 | prot-a-2858 | Alpha-2,8-sialyltransferase 8B \|\| id:prot-a-2858 | Shellfish liking \|\| id:ebi-fl187-GCST90094838 | MR Egger | 34 | 0.151225151379542 | 0.342060817195614 | 0.661390313222394 | -0.51921405 | 0.821664353082945 | 1.16325853786398 | 0.594987994873138 | 2.27428189740529 | 0.999279047927446 |
| ebi-fl187-GCST90094838 | prot-a-2911 | Synaptotagmin-11 \|\| id:prot-a-2911 | Shellfish liking \|\| id:ebi-fl187-GCST90094838 | MR Egger | 34 | -0.375299358 | 0.292491824586775 | 0.208668749793397 | -0.948583334 | 0.197984617908594 | 0.687083563846363 | 0.387289294397438 | 1.21894364377489 | 0.999279047927446 |
| ebi-fl187-GCST90094838 | prot-a-2962 | Transforming growth factor beta-1 \|\| id:prot-a-2962 | Shellfish liking \|\| id:ebi-fl187-GCST90094838 | MR Egger | 34 | -0.347746617 | 0.292482482244523 | 0.243208192354618 | -0.921012282 | 0.225519048695019 | 0.706277812684804 | 0.398115831665913 | 1.25297290138774 | 0.999279047927446 |
| ebi-fl187-GCST90094838 | prot-a-2978 | Tyrosine-protein kinase receptor Tie-1, soluble \|\| id:prot-a-2978 | Shellfish liking \|\| id:ebi-fl187-GCST90094838 | MR Egger | 34 | 0.038548121643353 | 0.307348381783465 | 0.900974459752081 | -0.563854707 | 0.640950949938944 | 1.0393007400142 | 0.569011458753209 | 1.89828519545253 | 0.999279047927446 |
| ebi-fl187-GCST90094838 | prot-a-299 | Uncharacterized protein C1orf226 \|\| id:prot-a-299 | Shellfish liking \|\| id:ebi-fl187-GCST90094838 | MR Egger | 34 | 0.0595666083942761 | 0.29247797913658 | 0.83990738922539 | -0.513690231 | 0.632823447501972 | 1.0613764552065 | 0.598283695274425 | 1.88291940523299 | 0.999279047927446 |
| ebi-fl187-GCST90094838 | prot-a-3015 | Transmembrane protein 70, mitochondrial \|\| id:prot-a-3015 | Shellfish liking \|\| id:ebi-fl187-GCST90094838 | MR Egger | 34 | 0.00885830392023567 | 0.316703461342016 | 0.977859518561631 | -0.61188048 | 0.629597088150587 | 1.00889765480261 | 0.542330068562167 | 1.87685422009664 | 0.999279047927446 |
| ebi-fl187-GCST90094838 | prot-a-3016 | Transmembrane protein 87B \|\| id:prot-a-3016 | Shellfish liking \|\| id:ebi-fl187-GCST90094838 | MR Egger | 34 | -0.301393382 | 0.335507933724994 | 0.375724934841624 | -0.958988932 | 0.356202168240862 | 0.739786696831573 | 0.383280212508182 | 1.42789619434707 | 0.999279047927446 |
| ebi-fl187-GCST90094838 | prot-a-3123 | Thioredoxin domain-containing protein 12 \|\| id:prot-a-3123 | Shellfish liking \|\| id:ebi-fl187-GCST90094838 | MR Egger | 33 | -0.451543641 | 0.299321954157244 | 0.141537784775643 | -1.038214671 | 0.135127389048282 | 0.636644641888784 | 0.354086278400851 | 1.14468259509015 | 0.999279047927446 |
| ebi-fl187-GCST90094838 | prot-a-3133 | PolyUbiquitin K48-linked \|\| id:prot-a-3133 | Shellfish liking \|\| id:ebi-fl187-GCST90094838 | MR Egger | 34 | 0.0091490058649624 | 0.320720121965835 | 0.97741944321513 | -0.619462433 | 0.637760444917999 | 1.00919098594679 | 0.53823369641199 | 1.89223835836668 | 0.999279047927446 |
| ebi-fl187-GCST90094838 | prot-a-3135 | Ubiquitin-conjugating enzyme E2 B \|\| id:prot-a-3135 | Shellfish liking \|\| id:ebi-fl187-GCST90094838 | MR Egger | 34 | 0.277465695903537 | 0.292503615622521 | 0.349938982747353 | -0.295841391 | 0.850772782523678 | 1.31978084447781 | 0.743905408967326 | 2.34145558891489 | 0.999279047927446 |
| ebi-fl187-GCST90094838 | prot-a-3162 | NKG2D ligand 3 \|\| id:prot-a-3162 | Shellfish liking \|\| id:ebi-fl187-GCST90094838 | MR Egger | 34 | 0.291768054927945 | 0.340521484971279 | 0.397911796481065 | -0.375654056 | 0.959190165471652 | 1.33879245531147 | 0.686839900353791 | 2.60958228762725 | 0.999279047927446 |
| ebi-fl187-GCST90094838 | prot-a-3188 | Vesicle-associated membrane protein 8 \|\| id:prot-a-3188 | Shellfish liking \|\| id:ebi-fl187-GCST90094838 | MR Egger | 34 | -0.448087326 | 0.321960664160313 | 0.173598628115953 | -1.079130228 | 0.182955575276966 | 0.638848893172608 | 0.339891024735622 | 1.20076106341826 | 0.999279047927446 |
| ebi-fl187-GCST90094838 | prot-a-3201 | Vitrin \|\| id:prot-a-3201 | Shellfish liking \|\| id:ebi-fl187-GCST90094838 | MR Egger | 34 | -0.123534317 | 0.292474234764274 | 0.675574640049035 | -0.696783817 | 0.449715182983416 | 0.88379131152063 | 0.498184983911111 | 1.5678655670977 | 0.999279047927446 |
| ebi-fl187-GCST90094838 | prot-a-3232 | WNT1-inducible-signaling pathway protein 3 \|\| id:prot-a-3232 | Shellfish liking \|\| id:ebi-fl187-GCST90094838 | MR Egger | 34 | -0.12399295 | 0.351476812795071 | 0.72656877935533 | -0.812887503 | 0.564901602754969 | 0.88338606844692 | 0.443575389800921 | 1.75927466642444 | 0.999279047927446 |
| ebi-fl187-GCST90094838 | prot-a-327 | Carbonic anhydrase-related protein 10 \|\| id:prot-a-327 | Shellfish liking \|\| id:ebi-fl187-GCST90094838 | MR Egger | 34 | -0.512642876 | 0.363310694069098 | 0.167884041715266 | -1.224731836 | 0.199446084794735 | 0.598910639032566 | 0.293836486139659 | 1.22072639194273 | 0.999279047927446 |
| ebi-fl187-GCST90094838 | prot-a-332 | Carbonic anhydrase 5A, mitochondrial \|\| id:prot-a-332 | Shellfish liking \|\| id:ebi-fl187-GCST90094838 | MR Egger | 34 | 0.170779543537142 | 0.292535079430641 | 0.563453257067092 | -0.402589212 | 0.744148299221197 | 1.1862292082982 | 0.668586690211472 | 2.10464814095342 | 0.999279047927446 |
| ebi-fl187-GCST90094838 | prot-a-338 | Cell adhesion molecule 3 \|\| id:prot-a-338 | Shellfish liking \|\| id:ebi-fl187-GCST90094838 | MR Egger | 34 | -0.231889107 | 0.292508299429514 | 0.433755743031685 | -0.805205374 | 0.341427159618514 | 0.793034060148014 | 0.446996115686731 | 1.40695410649966 | 0.999279047927446 |
| ebi-fl187-GCST90094838 | prot-a-34 | A disintegrin and metalloproteinase with thrombospondin motifs 3 \|\| id:prot-a-34 | Shellfish liking \|\| id:ebi-fl187-GCST90094838 | MR Egger | 34 | -0.387372803 | 0.328863688892346 | 0.247517346218564 | -1.031945633 | 0.257200026965811 | 0.678837974735396 | 0.356313031154781 | 1.29330379652232 | 0.999279047927446 |
| ebi-fl187-GCST90094838 | prot-a-358 | Cysteine--tRNA ligase, cytoplasmic \|\| id:prot-a-358 | Shellfish liking \|\| id:ebi-fl187-GCST90094838 | MR Egger | 34 | 0.331831685461928 | 0.333741812209041 | 0.327547573421167 | -0.322302266 | 0.985965637391648 | 1.39351827938688 | 0.724479171470392 | 2.68039892857671 | 0.999279047927446 |
| ebi-fl187-GCST90094838 | prot-a-382 | Coiled-coil domain-containing protein 167 \|\| id:prot-a-382 | Shellfish liking \|\| id:ebi-fl187-GCST90094838 | MR Egger | 34 | -0.490089124 | 0.292530678789621 | 0.103613449948368 | -1.063449254 | 0.0832710065649148 | 0.612571796986742 | 0.345262854726212 | 1.08683630841531 | 0.999279047927446 |
| ebi-fl187-GCST90094838 | prot-a-40 | Pituitary adenylate cyclase-activating polypeptide 27 \|\| id:prot-a-40 | Shellfish liking \|\| id:ebi-fl187-GCST90094838 | MR Egger | 34 | -0.310346947 | 0.325469281724914 | 0.34746597718323 | -0.948266739 | 0.327572845095689 | 0.733192533078957 | 0.387411927742761 | 1.38759612718915 | 0.999279047927446 |
| ebi-fl187-GCST90094838 | prot-a-403 | C-C motif chemokine 25 \|\| id:prot-a-403 | Shellfish liking \|\| id:ebi-fl187-GCST90094838 | MR Egger | 34 | 0.0487453952084397 | 0.373280586993876 | 0.896919936904958 | -0.682884555 | 0.780375345716436 | 1.04995299364318 | 0.505157733303979 | 2.18229122545552 | 0.999279047927446 |
| ebi-fl187-GCST90094838 | prot-a-415 | Cyclin-C \|\| id:prot-a-415 | Shellfish liking \|\| id:ebi-fl187-GCST90094838 | MR Egger | 34 | -0.084819406 | 0.292459832178663 | 0.773671487046388 | -0.658040677 | 0.488401865098212 | 0.91867817721387 | 0.517865005907719 | 1.62970964182003 | 0.999279047927446 |
| ebi-fl187-GCST90094838 | prot-a-44 | Adhesion G protein-coupled receptor F5 \|\| id:prot-a-44 | Shellfish liking \|\| id:ebi-fl187-GCST90094838 | MR Egger | 34 | 0.11163363288533 | 0.292481596626919 | 0.705223426578875 | -0.461630297 | 0.684897562274091 | 1.11810314937252 | 0.630255304465151 | 1.98356863286959 | 0.999279047927446 |
| ebi-fl187-GCST90094838 | prot-a-455 | CD59 glycoprotein \|\| id:prot-a-455 | Shellfish liking \|\| id:ebi-fl187-GCST90094838 | MR Egger | 34 | -0.227130736 | 0.297475986094913 | 0.450742539188433 | -0.810183669 | 0.355922196737697 | 0.796816602858256 | 0.444776367199059 | 1.42749648006009 | 0.999279047927446 |
| ebi-fl187-GCST90094838 | prot-a-478 | Cadherin-12 \|\| id:prot-a-478 | Shellfish liking \|\| id:ebi-fl187-GCST90094838 | MR Egger | 34 | -0.138473624 | 0.306754432953898 | 0.654734901941012 | -0.739712313 | 0.462765064574833 | 0.870686216172999 | 0.477251194926603 | 1.58846011302338 | 0.999279047927446 |
| ebi-fl187-GCST90094838 | prot-a-516 | Centrin-2 \|\| id:prot-a-516 | Shellfish liking \|\| id:ebi-fl187-GCST90094838 | MR Egger | 34 | 0.0878247904345538 | 0.327329609487983 | 0.790185219766866 | -0.553741244 | 0.729390825031001 | 1.09179681202403 | 0.574795332996664 | 2.07381690545623 | 0.999279047927446 |
| ebi-fl187-GCST90094838 | prot-a-519 | Cryptic protein \|\| id:prot-a-519 | Shellfish liking \|\| id:ebi-fl187-GCST90094838 | MR Egger | 34 | 0.194327673769958 | 0.292504920392947 | 0.511221123584341 | -0.37898197 | 0.767637317740135 | 1.2144941756483 | 0.68455795499788 | 2.15466943582331 | 0.999279047927446 |
| ebi-fl187-GCST90094838 | prot-a-583 | Chloride intracellular channel protein 4 \|\| id:prot-a-583 | Shellfish liking \|\| id:ebi-fl187-GCST90094838 | MR Egger | 34 | 0.341544449752451 | 0.293893671280149 | 0.253774704754713 | -0.234487146 | 0.917576045461543 | 1.40711913801335 | 0.790976401070989 | 2.50321534989227 | 0.999279047927446 |
| ebi-fl187-GCST90094838 | prot-a-602 | CCR4-NOT transcription complex subunit 1 \|\| id:prot-a-602 | Shellfish liking \|\| id:ebi-fl187-GCST90094838 | MR Egger | 34 | -0.088473663 | 0.301403588307856 | 0.771005719949864 | -0.679224696 | 0.502277370377442 | 0.915327217660618 | 0.507009926955193 | 1.6524802984072 | 0.999279047927446 |
| ebi-fl187-GCST90094838 | prot-a-606 | Ciliary neurotrophic factor receptor subunit alpha \|\| id:prot-a-606 | Shellfish liking \|\| id:ebi-fl187-GCST90094838 | MR Egger | 34 | -0.120348136 | 0.292504920392947 | 0.683492890243162 | -0.69365778 | 0.452961508139946 | 0.886611721675074 | 0.499744765546573 | 1.57296363905263 | 0.999279047927446 |
| ebi-fl187-GCST90094838 | prot-a-626 | Collagen alpha-2(IX) chain \|\| id:prot-a-626 | Shellfish liking \|\| id:ebi-fl187-GCST90094838 | MR Egger | 34 | 0.709830999696072 | 0.308235116991082 | 0.027936570620273 | 0.105690170393551 | 1.31397182899859 | 2.03364754255073 | 1.11147745453091 | 3.72092327240959 | 0.999279047927446 |
| ebi-fl187-GCST90094838 | prot-a-647 | Probable serine carboxypeptidase CPVL \|\| id:prot-a-647 | Shellfish liking \|\| id:ebi-fl187-GCST90094838 | MR Egger | 34 | -0.446609866 | 0.292242321134573 | 0.13628405415468 | -1.019404815 | 0.126185083518137 | 0.639793464835365 | 0.360809624635883 | 1.13449212464642 | 0.999279047927446 |
| ebi-fl187-GCST90094838 | prot-a-651 | Cellular retinoic acid-binding protein 2 \|\| id:prot-a-651 | Shellfish liking \|\| id:ebi-fl187-GCST90094838 | MR Egger | 34 | -0.569118528 | 0.292504920392947 | 0.0605227205851287 | -1.142428172 | 0.00419111601615818 | 0.566024153334255 | 0.319043388313549 | 1.00419991102556 | 0.999279047927446 |
| ebi-fl187-GCST90094838 | prot-a-671 | Cytotoxic and regulatory T-cell molecule \|\| id:prot-a-671 | Shellfish liking \|\| id:ebi-fl187-GCST90094838 | MR Egger | 34 | -0.104066157 | 0.343709026473393 | 0.764021613905614 | -0.777735849 | 0.56960353470362 | 0.901165676865355 | 0.459445087569937 | 1.76756613386704 | 0.999279047927446 |
| ebi-fl187-GCST90094838 | prot-a-688 | Chorionic somatomammotropin hormone \|\| id:prot-a-688 | Shellfish liking \|\| id:ebi-fl187-GCST90094838 | MR Egger | 34 | 0.323270196304048 | 0.333153970204548 | 0.339158285221139 | -0.329711585 | 0.976251977904961 | 1.38163861412325 | 0.719131111511086 | 2.65448849240476 | 0.999279047927446 |
| ebi-fl187-GCST90094838 | prot-a-69 | Aldo-keto reductase family 1 member C1 \|\| id:prot-a-69 | Shellfish liking \|\| id:ebi-fl187-GCST90094838 | MR Egger | 34 | -0.137874159 | 0.292464879637777 | 0.640538326159806 | -0.711105323 | 0.435357005326191 | 0.871208318779631 | 0.491101072113253 | 1.54551471745881 | 0.999279047927446 |
| ebi-fl187-GCST90094838 | prot-a-741 | Stromal cell-derived factor 1 \|\| id:prot-a-741 | Shellfish liking \|\| id:ebi-fl187-GCST90094838 | MR Egger | 34 | -0.514576929 | 0.292474234764274 | 0.0880699546752117 | -1.087826429 | 0.0586725716181022 | 0.59775343355988 | 0.336948079030633 | 1.06042796967583 | 0.999279047927446 |
| ebi-fl187-GCST90094838 | prot-a-742 | Growth-regulated alpha protein \|\| id:prot-a-742 | Shellfish liking \|\| id:ebi-fl187-GCST90094838 | MR Egger | 34 | -0.378715717 | 0.294862360750516 | 0.208229454822873 | -0.956645944 | 0.199214510427408 | 0.684740245254746 | 0.384179286389677 | 1.22044373573007 | 0.999279047927446 |
| ebi-fl187-GCST90094838 | prot-a-788 | Beta-defensin 112 \|\| id:prot-a-788 | Shellfish liking \|\| id:ebi-fl187-GCST90094838 | MR Egger | 34 | -0.235051436 | 0.330062809337194 | 0.481537406696677 | -0.881974542 | 0.411871670590787 | 0.790530187101451 | 0.413964713446243 | 1.5096406925993 | 0.999279047927446 |
| ebi-fl187-GCST90094838 | prot-a-838 | DnaJ homolog subfamily B member 1 \|\| id:prot-a-838 | Shellfish liking \|\| id:ebi-fl187-GCST90094838 | MR Egger | 34 | 0.00066904565503277 | 0.293554235412043 | 0.998195675261868 | -0.574697256 | 0.576035347062637 | 1.00066927 | 0.562875250258688 | 1.7789714266056 | 0.999739542784661 |
| ebi-fl187-GCST90094838 | prot-a-85 | Junctional adhesion molecule-like \|\| id:prot-a-85 | Shellfish liking \|\| id:ebi-fl187-GCST90094838 | MR Egger | 34 | 0.129468096700942 | 0.292514481636248 | 0.661029583229053 | -0.443860287 | 0.702796480707988 | 1.13822279782121 | 0.641555047950182 | 2.01939200949214 | 0.999279047927446 |
| ebi-fl187-GCST90094838 | prot-a-856 | Dipeptidase 1 \|\| id:prot-a-856 | Shellfish liking \|\| id:ebi-fl187-GCST90094838 | MR Egger | 34 | -0.49639192 | 0.297444036169679 | 0.104902231755883 | -1.079382231 | 0.0865983904730006 | 0.608723023333662 | 0.33980538194187 | 1.09045865318243 | 0.999279047927446 |
| ebi-fl187-GCST90094838 | prot-a-860 | Dipeptidyl peptidase 2 \|\| id:prot-a-860 | Shellfish liking \|\| id:ebi-fl187-GCST90094838 | MR Egger | 34 | 0.312635644565717 | 0.299426972198024 | 0.304256829760714 | -0.274241221 | 0.899512510073844 | 1.36702335788783 | 0.760148689360292 | 2.45840437162838 | 0.999279047927446 |
| ebi-fl187-GCST90094838 | prot-a-861 | Dermatopontin \|\| id:prot-a-861 | Shellfish liking \|\| id:ebi-fl187-GCST90094838 | MR Egger | 34 | 0.0464115833712598 | 0.292464655772865 | 0.874909417826389 | -0.526819142 | 0.619642308686076 | 1.04750545807279 | 0.5904802194087 | 1.85826323833687 | 0.999279047927446 |
| ebi-fl187-GCST90094838 | prot-a-877 | Dual specificity protein phosphatase 6 \|\| id:prot-a-877 | Shellfish liking \|\| id:ebi-fl187-GCST90094838 | MR Egger | 34 | 0.102759485967193 | 0.292504920392947 | 0.727659499356677 | -0.470550158 | 0.67606912993737 | 1.10822483339576 | 0.624658512851493 | 1.96613390530554 | 0.999279047927446 |
| ebi-fl187-GCST90094838 | prot-a-879 | Dynactin-associated protein \|\| id:prot-a-879 | Shellfish liking \|\| id:ebi-fl187-GCST90094838 | MR Egger | 34 | -0.028108784 | 0.305712137244499 | 0.927314723923792 | -0.627304573 | 0.57108700488836 | 0.972282592150761 | 0.534029299734723 | 1.77019021141536 | 0.999279047927446 |
| ebi-fl187-GCST90094838 | prot-a-937 | EMI domain-containing protein 1 \|\| id:prot-a-937 | Shellfish liking \|\| id:ebi-fl187-GCST90094838 | MR Egger | 34 | -0.676343784 | 0.313854051114434 | 0.0387938964468193 | -1.291497724 | -0.061189844 | 0.508472683699547 | 0.274858811882587 | 0.940644646965378 | 0.999279047927446 |
| ebi-fl187-GCST90094838 | prot-b-1 | SRC proto-oncogene, non-receptor tyrosine kinase \|\| id:prot-b-1 | Shellfish liking \|\| id:ebi-fl187-GCST90094838 | MR Egger | 23 | 0.625543908270009 | 0.51347727489156 | 0.236639361533027 | -0.380871551 | 1.63195936705747 | 1.86926238825639 | 0.683265649102522 | 5.11388488611944 | 0.999279047927446 |
| ebi-fl187-GCST90094838 | prot-b-10 | coagulation factor III, tissue factor \|\| id:prot-b-10 | Shellfish liking \|\| id:ebi-fl187-GCST90094838 | MR Egger | 23 | -0.139223133 | 0.595535170289446 | 0.817421104564491 | -1.306472066 | 1.02802580116058 | 0.870033873871726 | 0.270773644743727 | 2.79554142871129 | 0.999279047927446 |
| ebi-fl187-GCST90094838 | prot-b-29 | myeloperoxidase \|\| id:prot-b-29 | Shellfish liking \|\| id:ebi-fl187-GCST90094838 | MR Egger | 23 | 0.698625465615896 | 0.527141681824924 | 0.199307955238529 | -0.334572231 | 1.73182316199275 | 2.01098663659824 | 0.715644151439367 | 5.65094711448829 | 0.999279047927446 |
| ebi-fl187-GCST90094838 | prot-b-33 | adrenomedullin \|\| id:prot-b-33 | Shellfish liking \|\| id:ebi-fl187-GCST90094838 | MR Egger | 23 | 0.871680644379367 | 0.508972201441388 | 0.101508835524484 | -0.12590487 | 1.86926615920449 | 2.39092577471664 | 0.88169871840008 | 6.48353676931445 | 0.999279047927446 |
| ebi-fl187-GCST90094838 | ukb-rsfMRI191-edge-pheno102 | Net25_Pair11_15 (Postcentral\|Precentral)&(Cerebellum) (Motor)&(Subcortical-cerebellum) \|\| id:ukb-rsfMRI191-edge-pheno102 | Shellfish liking \|\| id:ebi-fl187-GCST90094838 | MR Egger | 30 | 0.0177567883810027 | 0.0879332202974183 | 0.841427595212649 | -0.154592323 | 0.190105900163943 | 1.01791537743459 | 0.856764389087562 | 1.2093776641689 | 0.999279047927446 |
| ebi-fl187-GCST90094838 | ukb-rsfMRI191-edge-pheno1167 | Net100_Pair11_45 (Angular\|Temporal)&(Frontal) (Default_mode\|Central_executive)&(Salience\|Default_mode) \|\| id:ukb-rsfMRI191-edge-pheno1167 | Shellfish liking \|\| id:ebi-fl187-GCST90094838 | MR Egger | 30 | -0.259431259 | 0.0880505455512685 | 0.00641126550305674 | -0.432010328 | -0.08685219 | 0.771490239292641 | 0.649202671596523 | 0.91681260007773 | 0.999279047927446 |
| ebi-fl187-GCST90094838 | ukb-rsfMRI191-edge-pheno1317 | Net100_Pair26_48 (Parietal\|Frontal)&(Temporal) (Attention\|Central_executive)&(Default_mode\|Central_executive) \|\| id:ukb-rsfMRI191-edge-pheno1317 | Shellfish liking \|\| id:ebi-fl187-GCST90094838 | MR Egger | 30 | 0.0904263108958726 | 0.105796539741952 | 0.399961079807408 | -0.116934907 | 0.297787528790098 | 1.09464084156662 | 0.889643100818526 | 1.34687558519054 | 0.999279047927446 |
| ebi-fl187-GCST90094838 | ukb-rsfMRI191-edge-pheno286 | Net100_Pair10_13 (OccipitalPrecuneus)&(Frontal_Sup) (Default_mode\|Central_executive)&(Default_mode) \|\| id:ukb-rsfMRI191-edge-pheno286 | Shellfish liking \|\| id:ebi-fl187-GCST90094838 | MR Egger | 30 | 0.0380923811603222 | 0.103727104507767 | 0.71620370376796 | -0.165212744 | 0.241397505995546 | 1.0388271965076 | 0.847713339251802 | 1.27302697059872 | 0.999279047927446 |
| ebi-fl187-GCST90094838 | ukb-rsfMRI191-edge-pheno599 | Net100_Pair11_29 (Angular\|Temporal)&(Parietal) (Default_mode\|Central_executive)&(Central_executive\|Salience) \|\| id:ukb-rsfMRI191-edge-pheno599 | Shellfish liking \|\| id:ebi-fl187-GCST90094838 | MR Egger | 30 | -0.114185574 | 0.0902080079951387 | 0.216014953004724 | -0.290993269 | 0.0626221221258228 | 0.892092391773429 | 0.747520709402246 | 1.06462446518227 | 0.999279047927446 |
| ebi-fl187-GCST90094838 | ukb-rsfMRI191-edge-pheno621 | Net100_Pair5_30 (Precuneus\|Angular\|Cingulate)&(Paracentral) (Default_mode\|Central_executive)&(Motor) \|\| id:ukb-rsfMRI191-edge-pheno621 | Shellfish liking \|\| id:ebi-fl187-GCST90094838 | MR Egger | 30 | 0.0338923632813351 | 0.0887713590050026 | 0.705496487353332 | -0.1400995 | 0.20788422693114 | 1.03447325342883 | 0.869271738237372 | 1.23107063647271 | 0.999279047927446 |
| ebi-fl187-GCST90094838 | ukb-rsfMRI191-edge-pheno624 | Net100_Pair8_30 (Calcarine\|Lingual)&(Paracentral) (Visual)&(Motor) \|\| id:ukb-rsfMRI191-edge-pheno624 | Shellfish liking \|\| id:ebi-fl187-GCST90094838 | MR Egger | 30 | -0.006109349 | 0.088465281218605 | 0.945433266866669 | -0.179501301 | 0.167282601856125 | 0.993909274795888 | 0.835686864114296 | 1.18208827845137 | 0.999279047927446 |
| ebi-fl187-GCST90094838 | ukb-rsfMRI191-edge-pheno882 | Net100_Pair6_38 (Postcentral\|Precentral)&(Subcortical) (Motor)&(Subcortical-cerebellum) \|\| id:ukb-rsfMRI191-edge-pheno882 | Shellfish liking \|\| id:ebi-fl187-GCST90094838 | MR Egger | 29 | -0.076154266 | 0.0945806575650509 | 0.427751033241583 | -0.261532355 | 0.109223822559659 | 0.926673241109878 | 0.769870965772868 | 1.11541197676811 | 0.999279047927446 |
| ebi-fl187-GCST90094838 | ukb-rsfMRI191-edge-pheno899 | Net100_Pair23_38 (Cerebellum)&(Subcortical) (Subcortical-cerebellum)&(Subcortical-cerebellum) \|\| id:ukb-rsfMRI191-edge-pheno899 | Shellfish liking \|\| id:ebi-fl187-GCST90094838 | MR Egger | 30 | 0.0710307582739319 | 0.0884198913642196 | 0.428548589159587 | -0.102272229 | 0.244333745347802 | 1.07361424788164 | 0.902783754475772 | 1.27677037556328 | 0.999279047927446 |
| ebi-fl187-GCST90094838 | ebi-a-GCST90001392 | IgD+ CD38+ B cell Absolute Count \|\| id:ebi-a-GCST90001392 | Shellfish liking \|\| id:ebi-fl187-GCST90094838 | Weighted median | 30 | -0.043896248 | 0.0991612424730995 | 0.658000498144095 | -0.238252283 | 0.150459787057331 | 0.957053248328989 | 0.788003865690869 | 1.16236856190296 | 0.999489402457283 |
| ebi-fl187-GCST90094838 | ebi-a-GCST90001402 | Switched memory B cell %B cell \|\| id:ebi-a-GCST90001402 | Shellfish liking \|\| id:ebi-fl187-GCST90094838 | Weighted median | 30 | -0.026524825 | 0.102645686114438 | 0.796089344647934 | -0.22771037 | 0.174660719390733 | 0.973823867974662 | 0.796354874558066 | 1.19084211842541 | 0.999489402457283 |
| ebi-fl187-GCST90094838 | ebi-a-GCST90001407 | Memory B cell Absolute Count \|\| id:ebi-a-GCST90001407 | Shellfish liking \|\| id:ebi-fl187-GCST90094838 | Weighted median | 30 | -0.045578199 | 0.0945023103273007 | 0.629594842571858 | -0.230802727 | 0.139646329443584 | 0.955444885009089 | 0.793896064815469 | 1.14986705281404 | 0.999489402457283 |
| ebi-fl187-GCST90094838 | ebi-a-GCST90001411 | IgD+ CD24+ B cell %B cell \|\| id:ebi-a-GCST90001411 | Shellfish liking \|\| id:ebi-fl187-GCST90094838 | Weighted median | 30 | -0.140783906 | 0.0956109975020079 | 0.140895185737602 | -0.328181461 | 0.046613648860481 | 0.868677007094724 | 0.720232313520555 | 1.04771714416214 | 0.999489402457283 |
| ebi-fl187-GCST90094838 | ebi-a-GCST90001418 | CD24+ CD27+ B cell Absolute Count \|\| id:ebi-a-GCST90001418 | Shellfish liking \|\| id:ebi-fl187-GCST90094838 | Weighted median | 30 | 0.011869403547708 | 0.0944246438454068 | 0.899967508744043 | -0.173202898 | 0.196941705484705 | 1.01194012444598 | 0.840966966690104 | 1.21767305497636 | 0.999489402457283 |
| ebi-fl187-GCST90094838 | ebi-a-GCST90001436 | Memory B cell %lymphocyte \|\| id:ebi-a-GCST90001436 | Shellfish liking \|\| id:ebi-fl187-GCST90094838 | Weighted median | 30 | -0.03999893 | 0.0955287937799133 | 0.67542755687433 | -0.227235366 | 0.147237505349156 | 0.960790466756115 | 0.796733236091528 | 1.15862911096555 | 0.999489402457283 |
| ebi-fl187-GCST90094838 | ebi-a-GCST90001439 | IgD+ CD24+ B cell %lymphocyte \|\| id:ebi-a-GCST90001439 | Shellfish liking \|\| id:ebi-fl187-GCST90094838 | Weighted median | 30 | 0.0471134745899945 | 0.0979585688033414 | 0.630549851963811 | -0.14488532 | 0.239112269444544 | 1.04824095104322 | 0.865121499326832 | 1.2701211243727 | 0.999489402457283 |
| ebi-fl187-GCST90094838 | ebi-a-GCST90001457 | CD11c+ HLA DR++ monocyte %monocyte \|\| id:ebi-a-GCST90001457 | Shellfish liking \|\| id:ebi-fl187-GCST90094838 | Weighted median | 30 | 0.046825587746759 | 0.105300462545921 | 0.656547056941406 | -0.159563319 | 0.253214494336763 | 1.0479392196992 | 0.85251598536136 | 1.28815955013239 | 0.999489402457283 |
| ebi-fl187-GCST90094838 | ebi-a-GCST90001483 | CD39+ resting CD4 regulatory T cell Absolute Count \|\| id:ebi-a-GCST90001483 | Shellfish liking \|\| id:ebi-fl187-GCST90094838 | Weighted median | 30 | 0.131418473666132 | 0.100958931038872 | 0.193018169902187 | -0.066461031 | 0.329297978502321 | 1.14044492763833 | 0.935699378255828 | 1.38999198161314 | 0.999489402457283 |
| ebi-fl187-GCST90094838 | ebi-a-GCST90001525 | CD33dim HLA DR+ CD11b+ Absolute Count \|\| id:ebi-a-GCST90001525 | Shellfish liking \|\| id:ebi-fl187-GCST90094838 | Weighted median | 30 | 0.0588172801652104 | 0.129600821892637 | 0.649948194719962 | -0.195200331 | 0.312834891074779 | 1.06058143377015 | 0.822669835486552 | 1.36729575965635 | 0.999489402457283 |
| ebi-fl187-GCST90094838 | ebi-a-GCST90001540 | Naive CD4+ T cell Absolute Count \|\| id:ebi-a-GCST90001540 | Shellfish liking \|\| id:ebi-fl187-GCST90094838 | Weighted median | 30 | -0.101333735 | 0.0945467125350697 | 0.283816675747822 | -0.286645291 | 0.0839778216966339 | 0.903631409244708 | 0.750777988990623 | 1.08760477231276 | 0.999489402457283 |
| ebi-fl187-GCST90094838 | ebi-a-GCST90001624 | HLA DR+ CD4+ T cell Absolute Count \|\| id:ebi-a-GCST90001624 | Shellfish liking \|\| id:ebi-fl187-GCST90094838 | Weighted median | 30 | 0.0204723103570165 | 0.0937085271710424 | 0.827064523003329 | -0.163196403 | 0.20414102361226 | 1.02068330549557 | 0.849424342627912 | 1.22647110264619 | 0.999489402457283 |
| ebi-fl187-GCST90094838 | ebi-a-GCST90001672 | CD39+ CD8+ T cell Absolute Count \|\| id:ebi-a-GCST90001672 | Shellfish liking \|\| id:ebi-fl187-GCST90094838 | Weighted median | 30 | 0.247837494856485 | 0.105195401635044 | 0.0184742751450073 | 0.0416545076517995 | 0.454020482061171 | 1.28125170528326 | 1.04253422892374 | 1.57463024881781 | 0.999489402457283 |
| ebi-fl187-GCST90094838 | ebi-a-GCST90001722 | CD19 on CD20- CD38- B cell \|\| id:ebi-a-GCST90001722 | Shellfish liking \|\| id:ebi-fl187-GCST90094838 | Weighted median | 30 | -0.154383385 | 0.0985383727411416 | 0.117176961 | -0.347518595 | 0.0387518256603124 | 0.856943418847424 | 0.706438877233904 | 1.0395124713143 | 0.999489402457283 |
| ebi-fl187-GCST90094838 | ebi-a-GCST90001742 | CD19 on transitional B cell \|\| id:ebi-a-GCST90001742 | Shellfish liking \|\| id:ebi-fl187-GCST90094838 | Weighted median | 30 | -0.13390742 | 0.0992627462823971 | 0.177330575418176 | -0.328462402 | 0.0606475629953753 | 0.874671038146261 | 0.720029999094044 | 1.06252437528222 | 0.999489402457283 |
| ebi-fl187-GCST90094838 | ebi-a-GCST90001784 | CD25 on IgD+ CD38dim B cell \|\| id:ebi-a-GCST90001784 | Shellfish liking \|\| id:ebi-fl187-GCST90094838 | Weighted median | 30 | -0.044608969 | 0.0995394520222577 | 0.654042308876611 | -0.239706295 | 0.150488356934286 | 0.9563713795544 | 0.786858931539639 | 1.16240177110414 | 0.999489402457283 |
| ebi-fl187-GCST90094838 | ebi-a-GCST90001794 | CD25 on IgD+ B cell \|\| id:ebi-a-GCST90001794 | Shellfish liking \|\| id:ebi-fl187-GCST90094838 | Weighted median | 30 | -0.108712482 | 0.0971773657562018 | 0.263267431224509 | -0.299180119 | 0.0817551552300969 | 0.896988281014893 | 0.741425852869518 | 1.08519007418487 | 0.999489402457283 |
| ebi-fl187-GCST90094838 | ebi-a-GCST90001802 | CD27 on IgD- CD38- B cell \|\| id:ebi-a-GCST90001802 | Shellfish liking \|\| id:ebi-fl187-GCST90094838 | Weighted median | 30 | -0.018544023 | 0.0985417898668839 | 0.850732140279698 | -0.211685931 | 0.174597885474171 | 0.981626859810256 | 0.809218808360744 | 1.19076729550184 | 0.999489402457283 |
| ebi-fl187-GCST90094838 | ebi-a-GCST90001804 | CD27 on IgD- CD38dim B cell \|\| id:ebi-a-GCST90001804 | Shellfish liking \|\| id:ebi-fl187-GCST90094838 | Weighted median | 30 | -0.003991187 | 0.100483151389535 | 0.968316382556515 | -0.200938164 | 0.192955789226316 | 0.996016766705888 | 0.817963009370237 | 1.21282917221776 | 0.999489402457283 |
| ebi-fl187-GCST90094838 | ebi-a-GCST90001805 | CD27 on memory B cell \|\| id:ebi-a-GCST90001805 | Shellfish liking \|\| id:ebi-fl187-GCST90094838 | Weighted median | 30 | -0.035638109 | 0.0983185524054382 | 0.716996403606258 | -0.228342472 | 0.157066253493936 | 0.964989451085301 | 0.795851656301578 | 1.17007313276614 | 0.999489402457283 |
| ebi-fl187-GCST90094838 | ebi-a-GCST90001806 | CD27 on unswitched memory B cell \|\| id:ebi-a-GCST90001806 | Shellfish liking \|\| id:ebi-fl187-GCST90094838 | Weighted median | 30 | -0.07643673 | 0.0997488538587152 | 0.443502691177625 | -0.271944483 | 0.119071023927316 | 0.926411526829317 | 0.76189655797544 | 1.12644992034456 | 0.999489402457283 |
| ebi-fl187-GCST90094838 | ebi-a-GCST90001839 | CD3 on Effector Memory CD8+ T cell \|\| id:ebi-a-GCST90001839 | Shellfish liking \|\| id:ebi-fl187-GCST90094838 | Weighted median | 30 | 0.158284858007634 | 0.109002942352365 | 0.146469503333964 | -0.055360909 | 0.371930625018269 | 1.17149985829762 | 0.946143614577323 | 1.45053234714737 | 0.999489402457283 |
| ebi-fl187-GCST90094838 | ebi-a-GCST90001859 | CD3 on CD8+ T cell \|\| id:ebi-a-GCST90001859 | Shellfish liking \|\| id:ebi-fl187-GCST90094838 | Weighted median | 30 | 0.0711617938908353 | 0.101221039024496 | 0.482034795412737 | -0.127231443 | 0.269555030378848 | 1.07375493880449 | 0.880529856895277 | 1.3093816860138 | 0.999489402457283 |
| ebi-fl187-GCST90094838 | ebi-a-GCST90001905 | CD86 on monocyte \|\| id:ebi-a-GCST90001905 | Shellfish liking \|\| id:ebi-fl187-GCST90094838 | Weighted median | 30 | 0.262559019699707 | 0.107875660967052 | 0.0149369495938807 | 0.0511227242042857 | 0.473995315195128 | 1.30025320645772 | 1.0524520466979 | 1.60639946134181 | 0.999489402457283 |
| ebi-fl187-GCST90094838 | ebi-a-GCST90001943 | CD25 on activated & secreting CD4 regulatory T cell \|\| id:ebi-a-GCST90001943 | Shellfish liking \|\| id:ebi-fl187-GCST90094838 | Weighted median | 30 | 0.0243988539099952 | 0.0929245621675446 | 0.792884915385079 | -0.157733288 | 0.206530995758383 | 1.02469894157404 | 0.854077544376274 | 1.22940583999287 | 0.999489402457283 |
| ebi-fl187-GCST90094838 | ebi-a-GCST90001949 | CD33 on CD33dim HLA DR+ CD11b- \|\| id:ebi-a-GCST90001949 | Shellfish liking \|\| id:ebi-fl187-GCST90094838 | Weighted median | 30 | 0.297608669689643 | 0.147525048363753 | 0.0436596986297888 | 0.00845957489668786 | 0.586757764482598 | 1.34663470577733 | 1.0084954582149 | 1.79814893169064 | 0.999489402457283 |
| ebi-fl187-GCST90094838 | ebi-a-GCST90001955 | CD33 on Immature Myeloid-Derived Suppressor Cells \|\| id:ebi-a-GCST90001955 | Shellfish liking \|\| id:ebi-fl187-GCST90094838 | Weighted median | 30 | -0.102590887 | 0.152754095665176 | 0.501833226029499 | -0.401988914 | 0.19680714092213 | 0.902496121237956 | 0.668988161994897 | 1.21750921035845 | 0.999489402457283 |
| ebi-fl187-GCST90094838 | ebi-a-GCST90001956 | CD33 on CD33+ HLA DR+ \|\| id:ebi-a-GCST90001956 | Shellfish liking \|\| id:ebi-fl187-GCST90094838 | Weighted median | 30 | 0.137404199584332 | 0.160318033530813 | 0.391404743044972 | -0.176819146 | 0.451627545304725 | 1.1472917896768 | 0.837931313936081 | 1.57086676290534 | 0.999489402457283 |
| ebi-fl187-GCST90094838 | ebi-a-GCST90001957 | CD33 on CD33+ HLA DR+ CD14- \|\| id:ebi-a-GCST90001957 | Shellfish liking \|\| id:ebi-fl187-GCST90094838 | Weighted median | 30 | 0.106163516907669 | 0.159001491899478 | 0.504332254387653 | -0.205479407 | 0.417806441030645 | 1.11200369304629 | 0.814256862210754 | 1.51862670213337 | 0.999489402457283 |
| ebi-fl187-GCST90094838 | ebi-a-GCST90002064 | CD4 on resting CD4 regulatory T cell \|\| id:ebi-a-GCST90002064 | Shellfish liking \|\| id:ebi-fl187-GCST90094838 | Weighted median | 30 | -0.082536694 | 0.101601324779585 | 0.416585982654617 | -0.281675291 | 0.116601902224776 | 0.920777649905787 | 0.754518643822001 | 1.12367200931093 | 0.999489402457283 |
| ebi-fl187-GCST90094838 | ebi-a-GCST90002080 | SSC-A on T cell \|\| id:ebi-a-GCST90002080 | Shellfish liking \|\| id:ebi-fl187-GCST90094838 | Weighted median | 30 | 0.133426447855523 | 0.0930443538179276 | 0.151569672819803 | -0.048940486 | 0.315793381338661 | 1.14273721227144 | 0.952237799847163 | 1.37134688049508 | 0.999489402457283 |
| ebi-fl187-GCST90094838 | ebi-a-GCST90002121 | CD8 on CD39+ CD8+ T cell \|\| id:ebi-a-GCST90002121 | Shellfish liking \|\| id:ebi-fl187-GCST90094838 | Weighted median | 30 | -0.063199022 | 0.10982696501654 | 0.564992569609683 | -0.278459873 | 0.152061829604208 | 0.938756622036808 | 0.756948641021346 | 1.16423221822405 | 0.999489402457283 |
| ebi-fl187-GCST90094838 | ebi-cfmet338-GCST90026053 | 1-palmitoyl-2-docosahexaenoyl-gpc (16:0/22:6) levels \|\| id:ebi-cfmet338-GCST90026053 | Shellfish liking \|\| id:ebi-fl187-GCST90094838 | Weighted median | 27 | 0.00524192845246972 | 0.0584275200898387 | 0.928512288187372 | -0.109276011 | 0.119759867828554 | 1.00525569139704 | 0.896482944261698 | 1.12722613581719 | 0.999489402457283 |
| ebi-fl187-GCST90094838 | ebi-cfmet338-GCST90026063 | 1-stearoyl-2-oleoyl-gpc (18:0/18:1) levels \|\| id:ebi-cfmet338-GCST90026063 | Shellfish liking \|\| id:ebi-fl187-GCST90094838 | Weighted median | 26 | 0.0438346996606725 | 0.0445605738062869 | 0.325257927288845 | -0.043504025 | 0.131173424320995 | 1.04480963322646 | 0.957428700433027 | 1.14016549659424 | 0.999489402457283 |
| ebi-fl187-GCST90094838 | ebi-cfmet338-GCST90026113 | Adenosine 3',5'-cyclic monophosphate (camp) levels \|\| id:ebi-cfmet338-GCST90026113 | Shellfish liking \|\| id:ebi-fl187-GCST90094838 | Weighted median | 27 | 0.0677386704003269 | 0.0879051740814055 | 0.440951188934328 | -0.104555471 | 0.240032811599882 | 1.07008562688734 | 0.900724832094767 | 1.2712908627242 | 0.999489402457283 |
| ebi-fl187-GCST90094838 | ebi-cfmet338-GCST90026117 | Alpha-ketoglutaramate levels \|\| id:ebi-cfmet338-GCST90026117 | Shellfish liking \|\| id:ebi-fl187-GCST90094838 | Weighted median | 27 | 0.00991550143650388 | 0.0334726989457482 | 0.767057090043819 | -0.055690988 | 0.0755219913701703 | 1.00996482290177 | 0.945831363508292 | 1.07844694398323 | 0.999489402457283 |
| ebi-fl187-GCST90094838 | ebi-cfmet338-GCST90026124 | Arginine levels \|\| id:ebi-cfmet338-GCST90026124 | Shellfish liking \|\| id:ebi-fl187-GCST90094838 | Weighted median | 27 | 0.0301311704039668 | 0.0297711906575718 | 0.311494283754828 | -0.028220363 | 0.0884827040928075 | 1.03058970795623 | 0.97217411171441 | 1.09251535640286 | 0.999489402457283 |
| ebi-fl187-GCST90094838 | ebi-cfmet338-GCST90026169 | Glutamate levels \|\| id:ebi-cfmet338-GCST90026169 | Shellfish liking \|\| id:ebi-fl187-GCST90094838 | Weighted median | 27 | 0.0106641084166091 | 0.0304575275766481 | 0.726240785379114 | -0.049032646 | 0.0703608624668394 | 1.0107211726869 | 0.952150045649586 | 1.07289527904254 | 0.999489402457283 |
| ebi-fl187-GCST90094838 | ebi-cfmet338-GCST90026170 | Glutamine levels \|\| id:ebi-cfmet338-GCST90026170 | Shellfish liking \|\| id:ebi-fl187-GCST90094838 | Weighted median | 26 | -0.006068133 | 0.0175810468107891 | 0.729980140496551 | -0.040526984 | 0.0283907191738789 | 0.993950241357269 | 0.960283251567003 | 1.02879757684209 | 0.999489402457283 |
| ebi-fl187-GCST90094838 | ebi-cfmet338-GCST90026191 | Kynurenate levels \|\| id:ebi-cfmet338-GCST90026191 | Shellfish liking \|\| id:ebi-fl187-GCST90094838 | Weighted median | 27 | -0.194930888 | 0.13412941844037 | 0.146139226369554 | -0.457824549 | 0.0679627717397769 | 0.822891527438162 | 0.63265846731798 | 1.07032546138226 | 0.999489402457283 |
| ebi-fl187-GCST90094838 | ebi-cfmet338-GCST90026202 | Methylsuccinoylcarnitine levels \|\| id:ebi-cfmet338-GCST90026202 | Shellfish liking \|\| id:ebi-fl187-GCST90094838 | Weighted median | 27 | -0.068600153 | 0.0885679165983235 | 0.43860645862272 | -0.24219327 | 0.104992963556737 | 0.933699942592652 | 0.784904464791297 | 1.11070279493101 | 0.999489402457283 |
| ebi-fl187-GCST90094838 | ebi-cfmet338-GCST90026222 | N-acetylputrescine levels \|\| id:ebi-cfmet338-GCST90026222 | Shellfish liking \|\| id:ebi-fl187-GCST90094838 | Weighted median | 27 | 0.0211961378181082 | 0.0319626355059472 | 0.50723216476969 | -0.041450628 | 0.0838429034097648 | 1.02142237154705 | 0.959396701723385 | 1.08745804443845 | 0.999489402457283 |
| ebi-fl187-GCST90094838 | ebi-cfmet338-GCST90026252 | Propionylcarnitine (c3) levels \|\| id:ebi-cfmet338-GCST90026252 | Shellfish liking \|\| id:ebi-fl187-GCST90094838 | Weighted median | 27 | -0.027331867 | 0.0598573281667118 | 0.647946433923287 | -0.14465223 | 0.0899884959168976 | 0.973038268362138 | 0.865323173799567 | 1.09416169630573 | 0.999489402457283 |
| ebi-fl187-GCST90094838 | ebi-cfmet338-GCST90026316 | Ethyl beta-glucopyranoside levels \|\| id:ebi-cfmet338-GCST90026316 | Shellfish liking \|\| id:ebi-fl187-GCST90094838 | Weighted median | 27 | -0.165599058 | 0.176261229670337 | 0.347469339280659 | -0.511071068 | 0.179872952390311 | 0.847385918893335 | 0.59985275158052 | 1.19706526917934 | 0.999489402457283 |
| ebi-fl187-GCST90094838 | ebi-gm430-GCST90011315 | F_Prevotellaceae abundance \|\| id:ebi-gm430-GCST90011315 | Shellfish liking \|\| id:ebi-fl187-GCST90094838 | Weighted median | 23 | -0.05765342 | 0.0819113293414937 | 0.481525200550187 | -0.218199625 | 0.102892785954189 | 0.943977054736597 | 0.803964934094628 | 1.10837256959804 | 0.999489402457283 |
| ebi-fl187-GCST90094838 | ebi-gm430-GCST90011354 | OTU97_1 (Bacteroides) abundance \|\| id:ebi-gm430-GCST90011354 | Shellfish liking \|\| id:ebi-fl187-GCST90094838 | Weighted median | 23 | -0.002043602 | 0.0762081007455856 | 0.978606430676469 | -0.15141148 | 0.147324275205669 | 0.997958484477684 | 0.859493961554288 | 1.15872964940905 | 0.999489402457283 |
| ebi-fl187-GCST90094838 | ebi-gm430-GCST90011419 | OTU99_1 (Bacteroides) abundance \|\| id:ebi-gm430-GCST90011419 | Shellfish liking \|\| id:ebi-fl187-GCST90094838 | Weighted median | 23 | -0.003757285 | 0.0704043188432852 | 0.957439296349967 | -0.14174975 | 0.13423517983805 | 0.996249764668765 | 0.867838405851057 | 1.14366175420555 | 0.999489402457283 |
| ebi-fl187-GCST90094838 | ebi-gm430-GCST90011435 | OTU99_155 (Butyrivibrio) abundance \|\| id:ebi-gm430-GCST90011435 | Shellfish liking \|\| id:ebi-fl187-GCST90094838 | Weighted median | 23 | -0.095500152 | 0.237316950944922 | 0.687377853503147 | -0.560641376 | 0.369641071769582 | 0.908918223423137 | 0.570842821563736 | 1.44721507508426 | 0.999489402457283 |
| ebi-fl187-GCST90094838 | ebi-gm430-GCST90011466 | OTU99_58 (Bacteroides) abundance \|\| id:ebi-gm430-GCST90011466 | Shellfish liking \|\| id:ebi-fl187-GCST90094838 | Weighted median | 23 | 0.06370645332959 | 0.137045759339548 | 0.642035052934569 | -0.204903235 | 0.332316141635104 | 1.06577949681541 | 0.814726149592686 | 1.394193541 | 0.999489402457283 |
| ebi-fl187-GCST90094838 | ebi-gm430-GCST90011472 | OTU99_72 (Parabacteroides) abundance \|\| id:ebi-gm430-GCST90011472 | Shellfish liking \|\| id:ebi-fl187-GCST90094838 | Weighted median | 23 | -0.259847584 | 0.205121876382249 | 0.205228221964165 | -0.661886462 | 0.142191293278513 | 0.771169115026394 | 0.515877233108792 | 1.15279714979237 | 0.999489402457283 |
| ebi-fl187-GCST90094838 | ebi-gm430-GCST90011505 | TestASV_28 (Holdemanella) abundance \|\| id:ebi-gm430-GCST90011505 | Shellfish liking \|\| id:ebi-fl187-GCST90094838 | Weighted median | 23 | 0.0336421421494025 | 0.167534535946521 | 0.840849327767563 | -0.294725548 | 0.362009832604583 | 1.0342144387422 | 0.744735953464506 | 1.43621306360608 | 0.999489402457283 |
| ebi-fl187-GCST90094838 | ebi-gm430-GCST90011513 | TestASV_35 (Alphaproteobacteria) abundance \|\| id:ebi-gm430-GCST90011513 | Shellfish liking \|\| id:ebi-fl187-GCST90094838 | Weighted median | 23 | -0.151991434 | 0.214500844428029 | 0.478583918920318 | -0.572413089 | 0.268430220936571 | 0.85899563874396 | 0.564162420562631 | 1.30790970913177 | 0.999489402457283 |
| ebi-fl187-GCST90094838 | ebi-gm430-GCST90011543 | G_Coprococcus prevalence \|\| id:ebi-gm430-GCST90011543 | Shellfish liking \|\| id:ebi-fl187-GCST90094838 | Weighted median | 23 | 0.310133188811493 | 0.220887255651506 | 0.160308763414579 | -0.122805832 | 0.743072209888445 | 1.3636067191963 | 0.884435374702857 | 2.10238456966063 | 0.999489402457283 |
| ebi-fl187-GCST90094838 | ebi-gm430-GCST90011547 | G_Parasutterella prevalence \|\| id:ebi-gm430-GCST90011547 | Shellfish liking \|\| id:ebi-fl187-GCST90094838 | Weighted median | 23 | 0.106496749118356 | 0.180821557745225 | 0.555887793420106 | -0.247913504 | 0.460907002298997 | 1.11237431024258 | 0.780427444162317 | 1.58551139550943 | 0.999489402457283 |
| ebi-fl187-GCST90094838 | ebi-gm430-GCST90011569 | OTU97_13 (Subdoligranulum) prevalence \|\| id:ebi-gm430-GCST90011569 | Shellfish liking \|\| id:ebi-fl187-GCST90094838 | Weighted median | 23 | 0.12767995071319 | 0.213045295748238 | 0.548966886262623 | -0.289888829 | 0.545248730379737 | 1.13618930792236 | 0.748346757446605 | 1.72503739822671 | 0.999489402457283 |
| ebi-fl187-GCST90094838 | ebi-gm430-GCST90011590 | OTU97_277 (Prevotella) prevalence \|\| id:ebi-gm430-GCST90011590 | Shellfish liking \|\| id:ebi-fl187-GCST90094838 | Weighted median | 23 | 0.225385022870308 | 0.16797979486665 | 0.17968067385321 | -0.103855375 | 0.554625420808941 | 1.25280498191431 | 0.901355646493903 | 1.74128861211937 | 0.999489402457283 |
| ebi-fl187-GCST90094838 | ebi-gm430-GCST90011616 | OTU97_86 (Phascolarctobacterium) prevalence \|\| id:ebi-gm430-GCST90011616 | Shellfish liking \|\| id:ebi-fl187-GCST90094838 | Weighted median | 23 | 0.180674821105716 | 0.216788141859666 | 0.404609854849726 | -0.244229937 | 0.605579579150661 | 1.19802554332443 | 0.783307502224614 | 1.83231387211486 | 0.999489402457283 |
| ebi-fl187-GCST90094838 | ebi-gm430-GCST90011665 | OTU99_55 (Barnesiella) prevalence \|\| id:ebi-gm430-GCST90011665 | Shellfish liking \|\| id:ebi-fl187-GCST90094838 | Weighted median | 23 | 0.208425594309695 | 0.142635663290202 | 0.143948324361735 | -0.071140306 | 0.487991494358491 | 1.23173727838881 | 0.931331211846184 | 1.62904099387495 | 0.999489402457283 |
| ebi-fl187-GCST90094838 | ebi-gm430-GCST90011687 | TestASV_14 (Lachnospiraceae) prevalence \|\| id:ebi-gm430-GCST90011687 | Shellfish liking \|\| id:ebi-fl187-GCST90094838 | Weighted median | 23 | 0.171586447095262 | 0.159512790758706 | 0.282065547636232 | -0.141058623 | 0.484231516982326 | 1.18718676714399 | 0.868438399921876 | 1.62292733740076 | 0.999489402457283 |
| ebi-fl187-GCST90094838 | ebi-gm430-GCST90011697 | TestASV_23 (Barnesiella) prevalence \|\| id:ebi-gm430-GCST90011697 | Shellfish liking \|\| id:ebi-fl187-GCST90094838 | Weighted median | 23 | 0.00939444951010177 | 0.20922958479983 | 0.964186860071246 | -0.400695537 | 0.419484435717769 | 1.00943871586165 | 0.669853975947722 | 1.52117708884053 | 0.999489402457283 |
| ebi-fl187-GCST90094838 | ebi-gm430-GCST90011727 | TestASV_6 (Subdoligranulum) prevalence \|\| id:ebi-gm430-GCST90011727 | Shellfish liking \|\| id:ebi-fl187-GCST90094838 | Weighted median | 23 | -0.262829309 | 0.240189159131382 | 0.273841110581069 | -0.73360006 | 0.207941443358596 | 0.768873126188316 | 0.480177207731985 | 1.23114107595161 | 0.999489402457283 |
| ebi-fl187-GCST90094838 | ebi-hbsm44-GCST90104908 | Sulcal depth of the left posterior inferior temporal sulcus \|\| id:ebi-hbsm44-GCST90104908 | Shellfish liking \|\| id:ebi-fl187-GCST90094838 | Weighted median | 26 | 0.0662844194640605 | 0.0668501089294401 | 0.321422974678894 | -0.064741794 | 0.197310632965763 | 1.06853058484728 | 0.937309451023561 | 1.21812237090669 | 0.999489402457283 |
| ebi-fl187-GCST90094838 | ebi-hbsm44-GCST90104918 | Sulcal depth of the left inferior frontal sulcus \|\| id:ebi-hbsm44-GCST90104918 | Shellfish liking \|\| id:ebi-fl187-GCST90094838 | Weighted median | 24 | 0.0134791352908052 | 0.107501726000927 | 0.900218531609794 | -0.197224248 | 0.224182518252622 | 1.01357038837818 | 0.821006503871752 | 1.25129938356441 | 0.999489402457283 |
| ebi-fl187-GCST90094838 | ebi-hbsm44-GCST90104925 | Sulcal depth of the right anterior inferior temporal sulcus \|\| id:ebi-hbsm44-GCST90104925 | Shellfish liking \|\| id:ebi-fl187-GCST90094838 | Weighted median | 26 | -0.025563538 | 0.0369987284326812 | 0.489609416433338 | -0.098081046 | 0.0469539694791385 | 0.974760442426467 | 0.906575426485304 | 1.04807376458802 | 0.999489402457283 |
| ebi-fl187-GCST90094838 | ebi-IPs91-GCST90274770 | C-C motif chemokine 4 levels \|\| id:ebi-IPs91-GCST90274770 | Shellfish liking \|\| id:ebi-fl187-GCST90094838 | Weighted median | 37 | -0.004677871 | 0.0416545665936802 | 0.910584346386384 | -0.086320821 | 0.0769650798920751 | 0.995333053564654 | 0.917299894796131 | 1.08000436186523 | 0.999489402457283 |
| ebi-fl187-GCST90094838 | ebi-IPs91-GCST90274779 | C-X-C motif chemokine 1 levels \|\| id:ebi-IPs91-GCST90274779 | Shellfish liking \|\| id:ebi-fl187-GCST90094838 | Weighted median | 37 | 0.0146789499471916 | 0.0447373569673624 | 0.742825523009608 | -0.07300627 | 0.102364169603222 | 1.01478721482256 | 0.929595001717523 | 1.10778682056668 | 0.999489402457283 |
| ebi-fl187-GCST90094838 | ebi-IPs91-GCST90274780 | C-X-C motif chemokine 10 levels \|\| id:ebi-IPs91-GCST90274780 | Shellfish liking \|\| id:ebi-fl187-GCST90094838 | Weighted median | 37 | -0.022582856 | 0.0408815292744008 | 0.580676025027756 | -0.102710653 | 0.0575449417334722 | 0.977670228340628 | 0.902388038962337 | 1.05923287334653 | 0.999489402457283 |
| ebi-fl187-GCST90094838 | ebi-IPs91-GCST90274796 | Interleukin-10 receptor subunit alpha levels \|\| id:ebi-IPs91-GCST90274796 | Shellfish liking \|\| id:ebi-fl187-GCST90094838 | Weighted median | 37 | -0.080097867 | 0.0482995904557103 | 0.0972458355509184 | -0.174765064 | 0.0145693303866293 | 0.923026008266046 | 0.839654262444812 | 1.01467598039086 | 0.999489402457283 |
| ebi-fl187-GCST90094838 | ebi-IPs91-GCST90274807 | Interleukin-20 levels \|\| id:ebi-IPs91-GCST90274807 | Shellfish liking \|\| id:ebi-fl187-GCST90094838 | Weighted median | 37 | -0.021968348 | 0.0478374695614654 | 0.646069813064034 | -0.115729788 | 0.0717930927369237 | 0.978271199188997 | 0.890715872950113 | 1.07443301306957 | 0.999489402457283 |
| ebi-fl187-GCST90094838 | ebi-IPs91-GCST90274808 | Interleukin-20 receptor subunit alpha levels \|\| id:ebi-IPs91-GCST90274808 | Shellfish liking \|\| id:ebi-fl187-GCST90094838 | Weighted median | 37 | -0.032470142 | 0.0465205138807762 | 0.485193014344007 | -0.123650349 | 0.0587100653367368 | 0.968051353603362 | 0.883688769465877 | 1.06046772980913 | 0.999489402457283 |
| ebi-fl187-GCST90094838 | ebi-IPs91-GCST90274810 | Interleukin-24 levels \|\| id:ebi-IPs91-GCST90274810 | Shellfish liking \|\| id:ebi-fl187-GCST90094838 | Weighted median | 37 | -0.044106476 | 0.0463379118469364 | 0.341175969630376 | -0.134928783 | 0.0467158316208044 | 0.956852070651353 | 0.873778137487844 | 1.04782420826193 | 0.999489402457283 |
| ebi-fl187-GCST90094838 | ebi-IPs91-GCST90274823 | Monocyte chemoattractant protein-3 levels \|\| id:ebi-IPs91-GCST90274823 | Shellfish liking \|\| id:ebi-fl187-GCST90094838 | Weighted median | 37 | -0.015433685 | 0.0476926763268823 | 0.746235567568836 | -0.108911331 | 0.078043960425261 | 0.98468480378665 | 0.896809933413642 | 1.08117018632658 | 0.999489402457283 |
| ebi-fl187-GCST90094838 | ebi-IPs91-GCST90274824 | Monocyte chemoattractant protein-4 levels \|\| id:ebi-IPs91-GCST90274824 | Shellfish liking \|\| id:ebi-fl187-GCST90094838 | Weighted median | 37 | 0.0657246277509131 | 0.0424566378019769 | 0.121612359144585 | -0.017490382 | 0.148939637842788 | 1.06793259767038 | 0.982661686524677 | 1.1606029305981 | 0.999489402457283 |
| ebi-fl187-GCST90094838 | ebi-IPs91-GCST90274834 | SIR2-like protein 2 levels \|\| id:ebi-IPs91-GCST90274834 | Shellfish liking \|\| id:ebi-fl187-GCST90094838 | Weighted median | 37 | 0.0344582053923781 | 0.0408130317779712 | 0.398505034865344 | -0.045535337 | 0.114451747677202 | 1.03505876759703 | 0.955485838075099 | 1.1212585364298 | 0.999489402457283 |
| ebi-fl187-GCST90094838 | ebi-met1400-GCST90199652 | 1-stearoyl-2-oleoyl-GPS (18:0/18:1) levels \|\| id:ebi-met1400-GCST90199652 | Shellfish liking \|\| id:ebi-fl187-GCST90094838 | Weighted median | 30 | 0.0130211705008823 | 0.0639011472352188 | 0.838532824988614 | -0.112225078 | 0.138267419081911 | 1.01310631510092 | 0.893843050379819 | 1.14828258189312 | 0.999489402457283 |
| ebi-fl187-GCST90094838 | ebi-met1400-GCST90199665 | N-acetylaspartate (naa) levels \|\| id:ebi-met1400-GCST90199665 | Shellfish liking \|\| id:ebi-fl187-GCST90094838 | Weighted median | 30 | 0.0391996365590128 | 0.0604973335849198 | 0.51701312821287 | -0.079375137 | 0.157774410385455 | 1.03997808057296 | 0.923693347643836 | 1.17090202157572 | 0.999489402457283 |
| ebi-fl187-GCST90094838 | ebi-met1400-GCST90199669 | Acetylcarnitine levels (Biocrates platform) \|\| id:ebi-met1400-GCST90199669 | Shellfish liking \|\| id:ebi-fl187-GCST90094838 | Weighted median | 30 | 0.0674717607914353 | 0.0630762180801296 | 0.284760574594478 | -0.056157627 | 0.191101148228489 | 1.06980004886464 | 0.945390105473996 | 1.21058189410283 | 0.999489402457283 |
| ebi-fl187-GCST90094838 | ebi-met1400-GCST90199670 | Iminodiacetate (IDA) levels \|\| id:ebi-met1400-GCST90199670 | Shellfish liking \|\| id:ebi-fl187-GCST90094838 | Weighted median | 30 | -0.006836746 | 0.0621058455705529 | 0.912344215824924 | -0.128564203 | 0.114890711642655 | 0.993186571701501 | 0.879357103247264 | 1.12175083656635 | 0.999489402457283 |
| ebi-fl187-GCST90094838 | ebi-met1400-GCST90199686 | Indolepropionate levels \|\| id:ebi-met1400-GCST90199686 | Shellfish liking \|\| id:ebi-fl187-GCST90094838 | Weighted median | 30 | -0.06084249 | 0.0641817603273026 | 0.34314387970808 | -0.18663874 | 0.0649537600962032 | 0.940971440379017 | 0.829743435040013 | 1.06710968019441 | 0.999489402457283 |
| ebi-fl187-GCST90094838 | ebi-met1400-GCST90199704 | 3-hydroxysebacate levels \|\| id:ebi-met1400-GCST90199704 | Shellfish liking \|\| id:ebi-fl187-GCST90094838 | Weighted median | 30 | 0.0892712175936497 | 0.0623272394986932 | 0.152058384889162 | -0.032890172 | 0.211432607011088 | 1.09337715923819 | 0.967644828419995 | 1.23544670237714 | 0.999489402457283 |
| ebi-fl187-GCST90094838 | ebi-met1400-GCST90199713 | Docosapentaenoate n3 DPA; 22:5n3 levels \|\| id:ebi-met1400-GCST90199713 | Shellfish liking \|\| id:ebi-fl187-GCST90094838 | Weighted median | 30 | 0.0120345948527102 | 0.0564506241631334 | 0.831180338955127 | -0.098608629 | 0.122677818212452 | 1.01210730196348 | 0.906097259275534 | 1.13052012927046 | 0.999489402457283 |
| ebi-fl187-GCST90094838 | ebi-met1400-GCST90199715 | Myristoleate (14:1n5) levels \|\| id:ebi-met1400-GCST90199715 | Shellfish liking \|\| id:ebi-fl187-GCST90094838 | Weighted median | 30 | 0.0247144156841501 | 0.0571256035328388 | 0.665281482004843 | -0.087251767 | 0.136680598608514 | 1.02502234841475 | 0.916446335420767 | 1.14646190850586 | 0.999489402457283 |
| ebi-fl187-GCST90094838 | ebi-met1400-GCST90199725 | Campesterol levels \|\| id:ebi-met1400-GCST90199725 | Shellfish liking \|\| id:ebi-fl187-GCST90094838 | Weighted median | 30 | 0.0432502349345492 | 0.08026206306746 | 0.589981571054514 | -0.114063409 | 0.200563878546771 | 1.04419915726853 | 0.892201380778978 | 1.22209167518698 | 0.999489402457283 |
| ebi-fl187-GCST90094838 | ebi-met1400-GCST90199737 | N-acetylproline levels \|\| id:ebi-met1400-GCST90199737 | Shellfish liking \|\| id:ebi-fl187-GCST90094838 | Weighted median | 30 | 0.0238477402415173 | 0.0639239559019936 | 0.709100653621141 | -0.101443213 | 0.149138693809425 | 1.02413437156668 | 0.903532486489832 | 1.16083397853131 | 0.999489402457283 |
| ebi-fl187-GCST90094838 | ebi-met1400-GCST90199775 | Gamma-glutamylisoleucine levels \|\| id:ebi-met1400-GCST90199775 | Shellfish liking \|\| id:ebi-fl187-GCST90094838 | Weighted median | 30 | 0.0156869694978695 | 0.0602597097556138 | 0.794614874836843 | -0.102422062 | 0.133796000618873 | 1.01581065591254 | 0.90264849797029 | 1.14315959200701 | 0.999489402457283 |
| ebi-fl187-GCST90094838 | ebi-met1400-GCST90199777 | 1-palmitoleoyl-GPC (16:1) levels \|\| id:ebi-met1400-GCST90199777 | Shellfish liking \|\| id:ebi-fl187-GCST90094838 | Weighted median | 30 | -0.072366146 | 0.0620721241036196 | 0.243679263938571 | -0.19402751 | 0.0492952168918718 | 0.930190247686344 | 0.823635246085435 | 1.05053043929719 | 0.999489402457283 |
| ebi-fl187-GCST90094838 | ebi-met1400-GCST90199782 | Glutamine degradant levels \|\| id:ebi-met1400-GCST90199782 | Shellfish liking \|\| id:ebi-fl187-GCST90094838 | Weighted median | 30 | -0.154060732 | 0.0499562165451662 | 0.00204295860439424 | -0.251974917 | -0.056146548 | 0.857219958344258 | 0.777264234021306 | 0.9454005791338 | 0.999489402457283 |
| ebi-fl187-GCST90094838 | ebi-met1400-GCST90199783 | 5-methyluridine (ribothymidine) levels \|\| id:ebi-met1400-GCST90199783 | Shellfish liking \|\| id:ebi-fl187-GCST90094838 | Weighted median | 30 | -0.001526979 | 0.058507725475149 | 0.9791785722533 | -0.116202121 | 0.113148163297072 | 0.998474186604481 | 0.890295258129708 | 1.11979783359717 | 0.999489402457283 |
| ebi-fl187-GCST90094838 | ebi-met1400-GCST90199787 | Homostachydrine levels \|\| id:ebi-met1400-GCST90199787 | Shellfish liking \|\| id:ebi-fl187-GCST90094838 | Weighted median | 30 | -0.060257625 | 0.0592418587404373 | 0.309083934186339 | -0.176371669 | 0.0558564177567748 | 0.941521942393775 | 0.838306353359432 | 1.05744584238987 | 0.999489402457283 |
| ebi-fl187-GCST90094838 | ebi-met1400-GCST90199802 | Beta-hydroxyisovaleroylcarnitine levels \|\| id:ebi-met1400-GCST90199802 | Shellfish liking \|\| id:ebi-fl187-GCST90094838 | Weighted median | 30 | 0.0813597873232749 | 0.0580616910766079 | 0.161134988623455 | -0.032441127 | 0.195160701833426 | 1.08476110966173 | 0.968079441713714 | 1.21550630488704 | 0.999489402457283 |
| ebi-fl187-GCST90094838 | ebi-met1400-GCST90199873 | Pregnenolone sulfate levels \|\| id:ebi-met1400-GCST90199873 | Shellfish liking \|\| id:ebi-fl187-GCST90094838 | Weighted median | 30 | 0.00616166868059798 | 0.0538667557917163 | 0.908930840999991 | -0.099417173 | 0.111740510032362 | 1.00618069081035 | 0.905364935722272 | 1.11822265543333 | 0.999489402457283 |
| ebi-fl187-GCST90094838 | ebi-met1400-GCST90199911 | Beta-citrylglutamate levels \|\| id:ebi-met1400-GCST90199911 | Shellfish liking \|\| id:ebi-fl187-GCST90094838 | Weighted median | 30 | 0.123605420292648 | 0.0594629965191721 | 0.0376454133495569 | 0.00705794711507106 | 0.240152893470226 | 1.13156928862005 | 1.00708291312551 | 1.2714435308749 | 0.999489402457283 |
| ebi-fl187-GCST90094838 | ebi-met1400-GCST90199934 | 2-hydroxydecanoate levels \|\| id:ebi-met1400-GCST90199934 | Shellfish liking \|\| id:ebi-fl187-GCST90094838 | Weighted median | 29 | -0.037974356 | 0.0639437890118859 | 0.552598454487578 | -0.163304183 | 0.0873554700603419 | 0.962737628630466 | 0.849332796632926 | 1.0912845297573 | 0.999489402457283 |
| ebi-fl187-GCST90094838 | ebi-met1400-GCST90199945 | Sphingomyelin (d18:1/14:0, d16:1/16:0) levels \|\| id:ebi-met1400-GCST90199945 | Shellfish liking \|\| id:ebi-fl187-GCST90094838 | Weighted median | 30 | 0.0700374913817925 | 0.0586223272993822 | 0.232194948858762 | -0.04486227 | 0.184937252888582 | 1.07254839182169 | 0.956129160314599 | 1.20314294401478 | 0.999489402457283 |
| ebi-fl187-GCST90094838 | ebi-met1400-GCST90199979 | Carnitine C14:1 levels \|\| id:ebi-met1400-GCST90199979 | Shellfish liking \|\| id:ebi-fl187-GCST90094838 | Weighted median | 30 | 0.0317550443051073 | 0.0583814787851515 | 0.586494281525405 | -0.082672654 | 0.146182742724004 | 1.03226461523681 | 0.920652469697682 | 1.15740767655781 | 0.999489402457283 |
| ebi-fl187-GCST90094838 | ebi-met1400-GCST90199986 | Eugenol sulfate levels \|\| id:ebi-met1400-GCST90199986 | Shellfish liking \|\| id:ebi-fl187-GCST90094838 | Weighted median | 30 | -0.027932292 | 0.0691296684400054 | 0.686171175358785 | -0.163426442 | 0.107561858583363 | 0.972454207930538 | 0.84922896454183 | 1.11355974184419 | 0.999489402457283 |
| ebi-fl187-GCST90094838 | ebi-met1400-GCST90200002 | 6-hydroxyindole sulfate levels \|\| id:ebi-met1400-GCST90200002 | Shellfish liking \|\| id:ebi-fl187-GCST90094838 | Weighted median | 30 | 0.00355543713970604 | 0.0627359618538724 | 0.95480565725599 | -0.119407048 | 0.126517922373296 | 1.00356176520379 | 0.887446493828553 | 1.13486979055383 | 0.999489402457283 |
| ebi-fl187-GCST90094838 | ebi-met1400-GCST90200023 | Trans 3,4-methyleneheptanoate levels \|\| id:ebi-met1400-GCST90200023 | Shellfish liking \|\| id:ebi-fl187-GCST90094838 | Weighted median | 30 | -0.035367234 | 0.0635189003238118 | 0.57766504749676 | -0.159864279 | 0.0891298105099976 | 0.965250878100986 | 0.852259450827175 | 1.09322255889382 | 0.999489402457283 |
| ebi-fl187-GCST90094838 | ebi-met1400-GCST90200032 | 2-hydroxybutyrate/2-hydroxyisobutyrate levels \|\| id:ebi-met1400-GCST90200032 | Shellfish liking \|\| id:ebi-fl187-GCST90094838 | Weighted median | 30 | 0.156116934203889 | 0.061704709544602 | 0.0114041354153535 | 0.0351757034964694 | 0.277058164911309 | 1.16896288684276 | 1.03580168679009 | 1.31924310246145 | 0.999489402457283 |
| ebi-fl187-GCST90094838 | ebi-met1400-GCST90200051 | 1-palmitoyl-2-dihomo-linolenoyl-GPC (16:0/20:3n3 or 6) levels \|\| id:ebi-met1400-GCST90200051 | Shellfish liking \|\| id:ebi-fl187-GCST90094838 | Weighted median | 30 | 0.0107660190759069 | 0.0584248120383877 | 0.853800587247159 | -0.103746613 | 0.125278650671147 | 1.01082418119672 | 0.901453685562958 | 1.1334642496402 | 0.999489402457283 |
| ebi-fl187-GCST90094838 | ebi-met1400-GCST90200069 | Phenylacetylglutamate levels \|\| id:ebi-met1400-GCST90200069 | Shellfish liking \|\| id:ebi-fl187-GCST90094838 | Weighted median | 30 | -0.023268156 | 0.0626183168022741 | 0.710200330451488 | -0.146000057 | 0.0994637451524732 | 0.977000460322813 | 0.864157654176151 | 1.10457842369168 | 0.999489402457283 |
| ebi-fl187-GCST90094838 | ebi-met1400-GCST90200072 | Thioproline levels \|\| id:ebi-met1400-GCST90200072 | Shellfish liking \|\| id:ebi-fl187-GCST90094838 | Weighted median | 30 | 0.0454596895947225 | 0.0622333619511122 | 0.465102102766773 | -0.0765177 | 0.167437079018902 | 1.04650881856908 | 0.926336518145327 | 1.1822708981997 | 0.999489402457283 |
| ebi-fl187-GCST90094838 | ebi-met1400-GCST90200081 | 1-oleoyl-2-docosahexaenoyl-GPC (18:1/22:6) levels \|\| id:ebi-met1400-GCST90200081 | Shellfish liking \|\| id:ebi-fl187-GCST90094838 | Weighted median | 30 | -0.078952596 | 0.0551923204876542 | 0.152573661235924 | -0.187129544 | 0.0292243520600569 | 0.924083728583428 | 0.829336293676816 | 1.02965557391296 | 0.999489402457283 |
| ebi-fl187-GCST90094838 | ebi-met1400-GCST90200097 | Furaneol sulfate levels \|\| id:ebi-met1400-GCST90200097 | Shellfish liking \|\| id:ebi-fl187-GCST90094838 | Weighted median | 30 | -0.115100227 | 0.0699553905611989 | 0.0999002324905189 | -0.252212792 | 0.022012338724479 | 0.891276809629382 | 0.777079363988361 | 1.02225639773266 | 0.999489402457283 |
| ebi-fl187-GCST90094838 | ebi-met1400-GCST90200124 | Sphingomyelin (d18:2/24:2) levels \|\| id:ebi-met1400-GCST90200124 | Shellfish liking \|\| id:ebi-fl187-GCST90094838 | Weighted median | 30 | -0.086913305 | 0.0526569915012717 | 0.0988293007648869 | -0.190121009 | 0.016294398102868 | 0.916756570179263 | 0.826859070845464 | 1.01642787580173 | 0.999489402457283 |
| ebi-fl187-GCST90094838 | ebi-met1400-GCST90200137 | Lignoceroylcarnitine (C24) levels \|\| id:ebi-met1400-GCST90200137 | Shellfish liking \|\| id:ebi-fl187-GCST90094838 | Weighted median | 30 | -0.000334378 | 0.0595146880121639 | 0.995517180328829 | -0.116983166 | 0.116314410536304 | 0.999665677930545 | 0.889600168147263 | 1.12334900937981 | 0.999911036302558 |
| ebi-fl187-GCST90094838 | ebi-met1400-GCST90200165 | Octadecenedioate (C18:1-DC) levels \|\| id:ebi-met1400-GCST90200165 | Shellfish liking \|\| id:ebi-fl187-GCST90094838 | Weighted median | 30 | -0.084849698 | 0.0565137683552007 | 0.133252471250133 | -0.195616684 | 0.0259172879214191 | 0.918650348959943 | 0.82232738549179 | 1.02625606119091 | 0.999489402457283 |
| ebi-fl187-GCST90094838 | ebi-met1400-GCST90200176 | Glutamine conjugate of C6H10O2 (1) levels \|\| id:ebi-met1400-GCST90200176 | Shellfish liking \|\| id:ebi-fl187-GCST90094838 | Weighted median | 30 | 0.0188796863538605 | 0.0545992721342095 | 0.729503238382623 | -0.088134887 | 0.125894259736911 | 1.01905903453341 | 0.915637360789752 | 1.13416223532912 | 0.999489402457283 |
| ebi-fl187-GCST90094838 | ebi-met1400-GCST90200182 | Glycine conjugate of C10H12O2 levels \|\| id:ebi-met1400-GCST90200182 | Shellfish liking \|\| id:ebi-fl187-GCST90094838 | Weighted median | 30 | -0.032214845 | 0.0734321118448099 | 0.660877176673188 | -0.176141784 | 0.111712094380362 | 0.968298525792634 | 0.838499089110762 | 1.11819088085895 | 0.999489402457283 |
| ebi-fl187-GCST90094838 | ebi-met1400-GCST90200208 | Glucuronide of piperine metabolite C17H21NO3 (4) levels \|\| id:ebi-met1400-GCST90200208 | Shellfish liking \|\| id:ebi-fl187-GCST90094838 | Weighted median | 30 | -0.051519341 | 0.0604344523355287 | 0.393946092512705 | -0.169970867 | 0.066932185748271 | 0.94978528014952 | 0.843689395098129 | 1.06922296716054 | 0.999489402457283 |
| ebi-fl187-GCST90094838 | ebi-met1400-GCST90200227 | 3-ethylcatechol sulfate (2) levels \|\| id:ebi-met1400-GCST90200227 | Shellfish liking \|\| id:ebi-fl187-GCST90094838 | Weighted median | 29 | -0.087704494 | 0.0814373443552822 | 0.281499609566743 | -0.247321689 | 0.0719127011464925 | 0.916031529737254 | 0.780889449789987 | 1.07456153197926 | 0.999489402457283 |
| ebi-fl187-GCST90094838 | ebi-met1400-GCST90200259 | Pentose acid levels \|\| id:ebi-met1400-GCST90200259 | Shellfish liking \|\| id:ebi-fl187-GCST90094838 | Weighted median | 30 | -0.039024215 | 0.0603952917170747 | 0.518184388077219 | -0.157398987 | 0.0793505565158229 | 0.961727420394823 | 0.854363111017851 | 1.08258376235061 | 0.999489402457283 |
| ebi-fl187-GCST90094838 | ebi-met1400-GCST90200291 | Sarcosine levels \|\| id:ebi-met1400-GCST90200291 | Shellfish liking \|\| id:ebi-fl187-GCST90094838 | Weighted median | 30 | 0.110795638764262 | 0.0622996199865978 | 0.0753329283237635 | -0.011311616 | 0.232902893937993 | 1.11716657798207 | 0.988752119378803 | 1.26225890038473 | 0.999489402457283 |
| ebi-fl187-GCST90094838 | ebi-met1400-GCST90200306 | Butyrate/isobutyrate (4:0) levels \|\| id:ebi-met1400-GCST90200306 | Shellfish liking \|\| id:ebi-fl187-GCST90094838 | Weighted median | 30 | -0.049352865 | 0.0619990238525119 | 0.426016642393866 | -0.170870952 | 0.072165221810661 | 0.951845197633865 | 0.842930345188561 | 1.07483291523451 | 0.999489402457283 |
| ebi-fl187-GCST90094838 | ebi-met1400-GCST90200321 | Cholate levels \|\| id:ebi-met1400-GCST90200321 | Shellfish liking \|\| id:ebi-fl187-GCST90094838 | Weighted median | 30 | -0.071456403 | 0.0623952878680006 | 0.252117559224504 | -0.193751168 | 0.050838360889665 | 0.931036866815614 | 0.823862882582042 | 1.05215281049333 | 0.999489402457283 |
| ebi-fl187-GCST90094838 | ebi-met1400-GCST90200336 | Ceramide (d18:1/16:0) levels \|\| id:ebi-met1400-GCST90200336 | Shellfish liking \|\| id:ebi-fl187-GCST90094838 | Weighted median | 30 | 0.0418762678174126 | 0.0586904072037985 | 0.475529432965921 | -0.07315693 | 0.156909465936858 | 1.04276544712355 | 0.929454958932864 | 1.16988969423887 | 0.999489402457283 |
| ebi-fl187-GCST90094838 | ebi-met1400-GCST90200358 | Arachidonate (20:4n6) levels \|\| id:ebi-met1400-GCST90200358 | Shellfish liking \|\| id:ebi-fl187-GCST90094838 | Weighted median | 30 | -0.021702706 | 0.0574516561087091 | 0.705611891233261 | -0.134307952 | 0.0909025403691706 | 0.978531103625787 | 0.874320774679994 | 1.09516226594703 | 0.999489402457283 |
| ebi-fl187-GCST90094838 | ebi-met1400-GCST90200413 | 2'-deoxyuridine levels \|\| id:ebi-met1400-GCST90200413 | Shellfish liking \|\| id:ebi-fl187-GCST90094838 | Weighted median | 30 | 0.0162967177813501 | 0.058766262282952 | 0.781538873043896 | -0.098885156 | 0.131478591855936 | 1.01643023359034 | 0.905846732846696 | 1.14051349118389 | 0.999489402457283 |
| ebi-fl187-GCST90094838 | ebi-met1400-GCST90200432 | Threonine levels \|\| id:ebi-met1400-GCST90200432 | Shellfish liking \|\| id:ebi-fl187-GCST90094838 | Weighted median | 30 | -0.10434429 | 0.0588101638489083 | 0.0760208028137955 | -0.219612212 | 0.0109236307318945 | 0.900915067599923 | 0.802830065827701 | 1.01098351142565 | 0.999489402457283 |
| ebi-fl187-GCST90094838 | ebi-met1400-GCST90200436 | Caffeine levels \|\| id:ebi-met1400-GCST90200436 | Shellfish liking \|\| id:ebi-fl187-GCST90094838 | Weighted median | 30 | 0.000656205958976162 | 0.0598365390193793 | 0.991250060292759 | -0.116623411 | 0.11793582243696 | 1.00065642130921 | 0.889920264677848 | 1.1251718982597 | 0.999911036302558 |
| ebi-fl187-GCST90094838 | ebi-met1400-GCST90200442 | Valine levels \|\| id:ebi-met1400-GCST90200442 | Shellfish liking \|\| id:ebi-fl187-GCST90094838 | Weighted median | 30 | 0.047383538550878 | 0.0640927471136563 | 0.459727022129982 | -0.078238246 | 0.173005322893644 | 1.04852408137635 | 0.924744083910164 | 1.18887243330871 | 0.999489402457283 |
| ebi-fl187-GCST90094838 | ebi-met1400-GCST90200459 | Margarate (17:0) levels \|\| id:ebi-met1400-GCST90200459 | Shellfish liking \|\| id:ebi-fl187-GCST90094838 | Weighted median | 30 | 0.0430531310059318 | 0.057138112104435 | 0.451154099866808 | -0.068937569 | 0.155043830730624 | 1.04399336179459 | 0.933384950677589 | 1.16770914152831 | 0.999489402457283 |
| ebi-fl187-GCST90094838 | ebi-met1400-GCST90200462 | X-11299 levels \|\| id:ebi-met1400-GCST90200462 | Shellfish liking \|\| id:ebi-fl187-GCST90094838 | Weighted median | 30 | 0.0553265472262561 | 0.0586659864137881 | 0.345641477701237 | -0.059658786 | 0.170311880597281 | 1.05688568141987 | 0.942085931521069 | 1.18567458255836 | 0.999489402457283 |
| ebi-fl187-GCST90094838 | ebi-met1400-GCST90200497 | X-12740 levels \|\| id:ebi-met1400-GCST90200497 | Shellfish liking \|\| id:ebi-fl187-GCST90094838 | Weighted median | 30 | 0.0369516970598393 | 0.0656746273464395 | 0.573674606742261 | -0.091770573 | 0.165673966658861 | 1.03764289842481 | 0.912314435520606 | 1.18018825826977 | 0.999489402457283 |
| ebi-fl187-GCST90094838 | ebi-met1400-GCST90200527 | X-13866 levels \|\| id:ebi-met1400-GCST90200527 | Shellfish liking \|\| id:ebi-fl187-GCST90094838 | Weighted median | 30 | -0.009794985 | 0.0580383698714213 | 0.865979604551356 | -0.12355019 | 0.103960220390605 | 0.990252830062028 | 0.883777283786221 | 1.10955631632082 | 0.999489402457283 |
| ebi-fl187-GCST90094838 | ebi-met1400-GCST90200547 | X-17654 levels \|\| id:ebi-met1400-GCST90200547 | Shellfish liking \|\| id:ebi-fl187-GCST90094838 | Weighted median | 30 | -0.005318582 | 0.0653382040132207 | 0.935123211185595 | -0.133381462 | 0.122744297646515 | 0.994695536397583 | 0.875131199056938 | 1.13059528810708 | 0.999489402457283 |
| ebi-fl187-GCST90094838 | ebi-met1400-GCST90200553 | X-17653 levels \|\| id:ebi-met1400-GCST90200553 | Shellfish liking \|\| id:ebi-fl187-GCST90094838 | Weighted median | 30 | -0.022289043 | 0.0602890409464045 | 0.711603747169541 | -0.140455564 | 0.0958774769785169 | 0.97795752214863 | 0.86896227768984 | 1.10062420392943 | 0.999489402457283 |
| ebi-fl187-GCST90094838 | ebi-met1400-GCST90200557 | X-17685 levels \|\| id:ebi-met1400-GCST90200557 | Shellfish liking \|\| id:ebi-fl187-GCST90094838 | Weighted median | 30 | -0.113200201 | 0.0679455357475692 | 0.0957048009278718 | -0.246373451 | 0.0199730490425509 | 0.892971868338194 | 0.781630269738794 | 1.02017384499436 | 0.999489402457283 |
| ebi-fl187-GCST90094838 | ebi-met1400-GCST90200561 | X-18779 levels \|\| id:ebi-met1400-GCST90200561 | Shellfish liking \|\| id:ebi-fl187-GCST90094838 | Weighted median | 30 | 0.00243848910123607 | 0.0615140995053885 | 0.968379227306404 | -0.118129146 | 0.123006124131798 | 1.00244146463389 | 0.88858128854783 | 1.13089134665394 | 0.999489402457283 |
| ebi-fl187-GCST90094838 | ebi-met1400-GCST90200570 | X-18921 levels \|\| id:ebi-met1400-GCST90200570 | Shellfish liking \|\| id:ebi-fl187-GCST90094838 | Weighted median | 30 | -0.033637278 | 0.0606721487979588 | 0.579297928452557 | -0.152554689 | 0.0852801341117909 | 0.966922165431395 | 0.85851194136167 | 1.08902209620946 | 0.999489402457283 |
| ebi-fl187-GCST90094838 | ebi-met1400-GCST90200575 | X-21283 levels \|\| id:ebi-met1400-GCST90200575 | Shellfish liking \|\| id:ebi-fl187-GCST90094838 | Weighted median | 30 | 0.0273475371654755 | 0.0681859719493534 | 0.688366542495984 | -0.106296968 | 0.160992042186208 | 1.02772491330856 | 0.899157587523382 | 1.17467562092682 | 0.999489402457283 |
| ebi-fl187-GCST90094838 | ebi-met1400-GCST90200578 | X-18922 levels \|\| id:ebi-met1400-GCST90200578 | Shellfish liking \|\| id:ebi-fl187-GCST90094838 | Weighted median | 30 | 0.105076152013343 | 0.0632073886025796 | 0.0964319991394062 | -0.01881033 | 0.228962633674399 | 1.11079519642558 | 0.981365480528102 | 1.25729505763558 | 0.999489402457283 |
| ebi-fl187-GCST90094838 | ebi-met1400-GCST90200623 | X-24241 levels \|\| id:ebi-met1400-GCST90200623 | Shellfish liking \|\| id:ebi-fl187-GCST90094838 | Weighted median | 30 | -0.035839334 | 0.0589598511426155 | 0.543280348905685 | -0.151400642 | 0.0797219747170569 | 0.964795291292304 | 0.859503276761672 | 1.08298592834557 | 0.999489402457283 |
| ebi-fl187-GCST90094838 | ebi-met1400-GCST90200636 | X-24544 levels \|\| id:ebi-met1400-GCST90200636 | Shellfish liking \|\| id:ebi-fl187-GCST90094838 | Weighted median | 30 | 0.0656716031988093 | 0.0546671269629064 | 0.229634966930219 | -0.041475966 | 0.172819172046106 | 1.06787597252399 | 0.959372392957708 | 1.18865114429484 | 0.999489402457283 |
| ebi-fl187-GCST90094838 | ebi-met1400-GCST90200659 | X-25172 levels \|\| id:ebi-met1400-GCST90200659 | Shellfish liking \|\| id:ebi-fl187-GCST90094838 | Weighted median | 30 | -0.002289078 | 0.0584025788043746 | 0.968735065341655 | -0.116758133 | 0.112179976027264 | 0.997713539512778 | 0.889800380589173 | 1.11871418426232 | 0.999489402457283 |
| ebi-fl187-GCST90094838 | ebi-met1400-GCST90200670 | X-26111 levels \|\| id:ebi-met1400-GCST90200670 | Shellfish liking \|\| id:ebi-fl187-GCST90094838 | Weighted median | 30 | 0.0267045671205327 | 0.0584858879758544 | 0.647959677224867 | -0.087927773 | 0.141336907553207 | 1.02706432936546 | 0.915827021487001 | 1.15181263700014 | 0.999489402457283 |
| ebi-fl187-GCST90094838 | ebi-met1400-GCST90200673 | Carnitine C4 levels \|\| id:ebi-met1400-GCST90200673 | Shellfish liking \|\| id:ebi-fl187-GCST90094838 | Weighted median | 30 | 0.117526100802277 | 0.0607234197221182 | 0.0529372024282089 | -0.001491802 | 0.236544003457629 | 1.12471098541968 | 0.998509310330189 | 1.26686330075921 | 0.999489402457283 |
| ebi-fl187-GCST90094838 | ebi-met1400-GCST90200710 | X-13431 levels \|\| id:ebi-met1400-GCST90200710 | Shellfish liking \|\| id:ebi-fl187-GCST90094838 | Weighted median | 30 | 0.0988499449426691 | 0.0617429770691072 | 0.109378932839368 | -0.02216629 | 0.219866179998119 | 1.10390064125625 | 0.978077576896793 | 1.24590999175369 | 0.999489402457283 |
| ebi-fl187-GCST90094838 | ebi-met1400-GCST90200730 | Adenosine 5'-diphosphate (ADP) to fructose ratio \|\| id:ebi-met1400-GCST90200730 | Shellfish liking \|\| id:ebi-fl187-GCST90094838 | Weighted median | 30 | 0.148677687933377 | 0.0784269812879382 | 0.0579935519858241 | -0.005039195 | 0.302394571257736 | 1.16029895058108 | 0.994973480053826 | 1.35309501379547 | 0.999489402457283 |
| ebi-fl187-GCST90094838 | ebi-met1400-GCST90200731 | Adenosine 5'-diphosphate (ADP) to mannose ratio \|\| id:ebi-met1400-GCST90200731 | Shellfish liking \|\| id:ebi-fl187-GCST90094838 | Weighted median | 30 | 0.0117327416210156 | 0.0807766480328511 | 0.884514140214641 | -0.146589489 | 0.170054971765404 | 1.01180184020819 | 0.863648442252716 | 1.18537001141155 | 0.999489402457283 |
| ebi-fl187-GCST90094838 | ebi-met1400-GCST90200748 | Aspartate to glutamate ratio \|\| id:ebi-met1400-GCST90200748 | Shellfish liking \|\| id:ebi-fl187-GCST90094838 | Weighted median | 30 | -0.102514293 | 0.0632576847124935 | 0.105107220525349 | -0.226499355 | 0.0214707689346775 | 0.902565249203735 | 0.797319856473862 | 1.02170292443595 | 0.999489402457283 |
| ebi-fl187-GCST90094838 | ebi-met1400-GCST90200774 | Uridine to pseudouridine ratio \|\| id:ebi-met1400-GCST90200774 | Shellfish liking \|\| id:ebi-fl187-GCST90094838 | Weighted median | 30 | 0.00466284640547283 | 0.0578734918033906 | 0.935784211382707 | -0.108769198 | 0.118094890340118 | 1.0046737343902 | 0.896937408980414 | 1.12535089122989 | 0.999489402457283 |
| ebi-fl187-GCST90094838 | ebi-met1400-GCST90200799 | Spermidine to adenosine 5'-diphosphate (ADP) ratio \|\| id:ebi-met1400-GCST90200799 | Shellfish liking \|\| id:ebi-fl187-GCST90094838 | Weighted median | 30 | -0.127073887 | 0.0819415876221932 | 0.120952916870671 | -0.287679399 | 0.033531624370847 | 0.88066859990766 | 0.750002005010403 | 1.03410014597569 | 0.999489402457283 |
| ebi-fl187-GCST90094838 | ebi-met1400-GCST90200811 | Choline to taurocholate ratio \|\| id:ebi-met1400-GCST90200811 | Shellfish liking \|\| id:ebi-fl187-GCST90094838 | Weighted median | 30 | -0.034101374 | 0.0611080989215649 | 0.576810251388067 | -0.153873248 | 0.0856705001306334 | 0.966473524620378 | 0.857380689148479 | 1.08944729641605 | 0.999489402457283 |
| ebi-fl187-GCST90094838 | ebi-met1400-GCST90200812 | Glutamate to kynurenine ratio \|\| id:ebi-met1400-GCST90200812 | Shellfish liking \|\| id:ebi-fl187-GCST90094838 | Weighted median | 30 | 0.123076305124512 | 0.0564879109013332 | 0.0293459436864658 | 0.0123599997578986 | 0.233792610491125 | 1.13097071651639 | 1.01243670023511 | 1.2633824527702 | 0.999489402457283 |
| ebi-fl187-GCST90094838 | ebi-met1400-GCST90200876 | Cortisone to 4-cholesten-3-one ratio \|\| id:ebi-met1400-GCST90200876 | Shellfish liking \|\| id:ebi-fl187-GCST90094838 | Weighted median | 30 | -0.031114404 | 0.0631199858819469 | 0.6220545589637 | -0.154829576 | 0.0926007686187056 | 0.969364667817906 | 0.856561143588534 | 1.09702368155181 | 0.999489402457283 |
| ebi-fl187-GCST90094838 | ebi-met1400-GCST90200906 | 2'-deoxyuridine to cytidine ratio \|\| id:ebi-met1400-GCST90200906 | Shellfish liking \|\| id:ebi-fl187-GCST90094838 | Weighted median | 30 | 0.0869759899002887 | 0.066065932829501 | 0.188005317187101 | -0.042513238 | 0.216465218246111 | 1.09087048749466 | 0.958377778004951 | 1.24167989679817 | 0.999489402457283 |
| ebi-fl187-GCST90094838 | ebi-met1400-GCST90200907 | Retinol (Vitamin A) to linoleoyl-arachidonoyl-glycerol (18:2 to 20:4) [1] ratio \|\| id:ebi-met1400-GCST90200907 | Shellfish liking \|\| id:ebi-fl187-GCST90094838 | Weighted median | 30 | 0.000986485528998785 | 0.0687588312330493 | 0.988553113000012 | -0.133780824 | 0.135753794745775 | 1.00098697226589 | 0.874781775036825 | 1.14539985541405 | 0.999911036302558 |
| ebi-fl187-GCST90094838 | ebi-met1400-GCST90200916 | Fructose to sucrose ratio \|\| id:ebi-met1400-GCST90200916 | Shellfish liking \|\| id:ebi-fl187-GCST90094838 | Weighted median | 30 | -0.044425209 | 0.0603423027585727 | 0.461596831346007 | -0.162696123 | 0.0738457041277567 | 0.956547138268489 | 0.849849399133256 | 1.07664067146816 | 0.999489402457283 |
| ebi-fl187-GCST90094838 | ebi-met1400-GCST90200917 | Alpha-ketobutyrate to 3-methyl-2-oxobutyrate ratio \|\| id:ebi-met1400-GCST90200917 | Shellfish liking \|\| id:ebi-fl187-GCST90094838 | Weighted median | 30 | 0.0647333160725665 | 0.0614428808612023 | 0.292087731700596 | -0.05569473 | 0.185161362560523 | 1.06687446816934 | 0.945827824291294 | 1.20341261020153 | 0.999489402457283 |
| ebi-fl187-GCST90094838 | ebi-met1400-GCST90200924 | Alpha-tocopherol to glycerol ratio \|\| id:ebi-met1400-GCST90200924 | Shellfish liking \|\| id:ebi-fl187-GCST90094838 | Weighted median | 30 | 0.00528081887144252 | 0.0547060243370317 | 0.923098975860685 | -0.101942989 | 0.112504626572025 | 1.00529478697227 | 0.903081035908513 | 1.11907743439316 | 0.999489402457283 |
| ebi-fl187-GCST90094838 | ebi-met1400-GCST90200938 | Alpha-ketoglutarate to alanine ratio \|\| id:ebi-met1400-GCST90200938 | Shellfish liking \|\| id:ebi-fl187-GCST90094838 | Weighted median | 30 | 0.0355116622749091 | 0.0598857462449792 | 0.553187692567067 | -0.0818644 | 0.152887724915068 | 1.03614973192062 | 0.92139689130767 | 1.16519414932634 | 0.999489402457283 |
| ebi-fl187-GCST90094838 | ebi-met1400-GCST90200943 | Phosphate to EDTA ratio \|\| id:ebi-met1400-GCST90200943 | Shellfish liking \|\| id:ebi-fl187-GCST90094838 | Weighted median | 30 | -0.026871448 | 0.0597397083498782 | 0.652848291508836 | -0.143961277 | 0.0902183801416202 | 0.973486376883501 | 0.86592127883478 | 1.09441325573267 | 0.999489402457283 |
| ebi-fl187-GCST90094838 | ebi-met1400-GCST90200947 | Adenosine 5'-diphosphate (ADP) to glucose ratio \|\| id:ebi-met1400-GCST90200947 | Shellfish liking \|\| id:ebi-fl187-GCST90094838 | Weighted median | 30 | 0.0899678385266303 | 0.0806655237491698 | 0.264713495350768 | -0.068136588 | 0.248072265075003 | 1.094139094 | 0.934132873501855 | 1.28155254033826 | 0.999489402457283 |
| ebi-fl187-GCST90094838 | ebi-met1400-GCST90200975 | Arachidonate (20:4n6) to caffeine ratio \|\| id:ebi-met1400-GCST90200975 | Shellfish liking \|\| id:ebi-fl187-GCST90094838 | Weighted median | 30 | -0.050442574 | 0.0609455797382195 | 0.407859775720819 | -0.16989591 | 0.0690107624587225 | 0.950808528398498 | 0.843752638140627 | 1.07144774049837 | 0.999489402457283 |
| ebi-fl187-GCST90094838 | ebi-met1400-GCST90200985 | Cholesterol to oleoyl-linoleoyl-glycerol (18:1 to 18:2) [2] ratio \|\| id:ebi-met1400-GCST90200985 | Shellfish liking \|\| id:ebi-fl187-GCST90094838 | Weighted median | 30 | 0.0346470294094663 | 0.0603611209595427 | 0.565970649068273 | -0.083660768 | 0.15295482649017 | 1.03525423000487 | 0.919743209810668 | 1.16527233831234 | 0.999489402457283 |
| ebi-fl187-GCST90094838 | ebi-met1400-GCST90201016 | Bilirubin (Z,Z) to etiocholanolone glucuronide ratio \|\| id:ebi-met1400-GCST90201016 | Shellfish liking \|\| id:ebi-fl187-GCST90094838 | Weighted median | 30 | 0.0450372672409258 | 0.0641656224595268 | 0.48274719717549 | -0.080727353 | 0.170801887261598 | 1.04606684320738 | 0.92244515927056 | 1.18625571337288 | 0.999489402457283 |
| ebi-fl187-GCST90094838 | ebi-pw11-GCST90275189 | Placental weight (fetal genotype, adjusted for sex and gestational age) \|\| id:ebi-pw11-GCST90275189 | Shellfish liking \|\| id:ebi-fl187-GCST90094838 | Weighted median | 33 | 0.0194999003382683 | 0.0223297390748318 | 0.382515706355545 | -0.024266388 | 0.0632661889249386 | 1.01969126523646 | 0.976025673354606 | 1.06531037531608 | 0.999489402457283 |
| ebi-fl187-GCST90094838 | ebi-pw11-GCST90275192 | Placental weight (fetal effect, conditional on maternal and paternal genotype) \|\| id:ebi-pw11-GCST90275192 | Shellfish liking \|\| id:ebi-fl187-GCST90094838 | Weighted median | 33 | -0.013827902 | 0.0453174730247925 | 0.760264101755014 | -0.102650149 | 0.0749943456005833 | 0.986267264747362 | 0.902442639029892 | 1.07787805611435 | 0.999489402457283 |
| ebi-fl187-GCST90094838 | ebi-pw11-GCST90275193 | Placental weight (maternal effect, conditional on fetal and paternal genotype) \|\| id:ebi-pw11-GCST90275193 | Shellfish liking \|\| id:ebi-fl187-GCST90094838 | Weighted median | 33 | -0.001897363 | 0.0361655123986965 | 0.958159520890694 | -0.072781767 | 0.0689870414254325 | 0.998104435979056 | 0.92980372157676 | 1.0714223249523 | 0.999489402457283 |
| ebi-fl187-GCST90094838 | ebi-pw11-GCST90275195 | Placental weight (fetal effect, conditional on maternal genotype) \|\| id:ebi-pw11-GCST90275195 | Shellfish liking \|\| id:ebi-fl187-GCST90094838 | Weighted median | 33 | -0.005974795 | 0.0300629241060846 | 0.842463815668957 | -0.064898126 | 0.0529485366709682 | 0.99404301901299 | 0.937162931215035 | 1.05437538205588 | 0.999489402457283 |
| ebi-fl187-GCST90094838 | ebi-pw11-GCST90275197 | Placental weight (fetal genotype, adjusted for sex) \|\| id:ebi-pw11-GCST90275197 | Shellfish liking \|\| id:ebi-fl187-GCST90094838 | Weighted median | 33 | 0.0214740373224501 | 0.0214059293657389 | 0.315773184843408 | -0.020481584 | 0.0634296588792984 | 1.02170626376276 | 0.979726738726361 | 1.06548453578913 | 0.999489402457283 |
| ebi-fl187-GCST90094838 | prot-a-1030 | Protein FAM171B \|\| id:prot-a-1030 | Shellfish liking \|\| id:ebi-fl187-GCST90094838 | Weighted median | 34 | -0.102116722 | 0.088914370009397 | 0.250768856554144 | -0.276388887 | 0.072155442975488 | 0.902924154185409 | 0.758517895298474 | 1.07482240467198 | 0.999489402457283 |
| ebi-fl187-GCST90094838 | prot-a-1036 | BRISC complex subunit Abro1 \|\| id:prot-a-1036 | Shellfish liking \|\| id:ebi-fl187-GCST90094838 | Weighted median | 34 | 0.0941043915077694 | 0.0844201619686943 | 0.264972720029408 | -0.071359126 | 0.25956790896641 | 1.09867443221146 | 0.931127440048686 | 1.29636981585684 | 0.999489402457283 |
| ebi-fl187-GCST90094838 | prot-a-1055 | Phenylalanine--tRNA ligase, mitochondrial \|\| id:prot-a-1055 | Shellfish liking \|\| id:ebi-fl187-GCST90094838 | Weighted median | 34 | -0.063751916 | 0.084313561805844 | 0.44957195173509 | -0.229006497 | 0.101502665394267 | 0.938237732669569 | 0.795323366364145 | 1.10683286853399 | 0.999489402457283 |
| ebi-fl187-GCST90094838 | prot-a-1083 | Fc receptor-like protein 5 \|\| id:prot-a-1083 | Shellfish liking \|\| id:ebi-fl187-GCST90094838 | Weighted median | 34 | 0.0123607148020541 | 0.0927969316348068 | 0.894033817390495 | -0.169521271 | 0.194242700806276 | 1.01243742417232 | 0.844068799931319 | 1.21439098086327 | 0.999489402457283 |
| ebi-fl187-GCST90094838 | prot-a-1103 | Fibroblast growth factor receptor 3 \|\| id:prot-a-1103 | Shellfish liking \|\| id:ebi-fl187-GCST90094838 | Weighted median | 34 | -0.089406161 | 0.0908211147893661 | 0.324908834133982 | -0.267415546 | 0.0886032235795737 | 0.914474074058057 | 0.765354964879576 | 1.09264703372758 | 0.999489402457283 |
| ebi-fl187-GCST90094838 | prot-a-1105 | Fibroblast growth factor receptor 4 \|\| id:prot-a-1105 | Shellfish liking \|\| id:ebi-fl187-GCST90094838 | Weighted median | 34 | 0.0603551492451839 | 0.0913474412153351 | 0.508791418592556 | -0.118685836 | 0.239396134027241 | 1.06221372396655 | 0.888086762240634 | 1.27048171795309 | 0.999489402457283 |
| ebi-fl187-GCST90094838 | prot-a-1120 | Filamin-A \|\| id:prot-a-1120 | Shellfish liking \|\| id:ebi-fl187-GCST90094838 | Weighted median | 34 | -0.191177088 | 0.0879391411304038 | 0.0297073598199261 | -0.363537805 | -0.018816371 | 0.825986302957941 | 0.695212444498201 | 0.981359551419671 | 0.999489402457283 |
| ebi-fl187-GCST90094838 | prot-a-1166 | Galanin peptides \|\| id:prot-a-1166 | Shellfish liking \|\| id:ebi-fl187-GCST90094838 | Weighted median | 34 | -0.057541432 | 0.0915643076618635 | 0.529724616997466 | -0.237007475 | 0.121924610560347 | 0.944082773907236 | 0.788985389955671 | 1.12966893346208 | 0.999489402457283 |
| ebi-fl187-GCST90094838 | prot-a-1205 | ADP-ribosylation factor-binding protein GGA1 \|\| id:prot-a-1205 | Shellfish liking \|\| id:ebi-fl187-GCST90094838 | Weighted median | 34 | 0.0247354094304096 | 0.0877654577708307 | 0.778069755758246 | -0.147284888 | 0.196755706661238 | 1.02504386769973 | 0.863048070523579 | 1.2174465902825 | 0.999489402457283 |
| ebi-fl187-GCST90094838 | prot-a-1227 | Guanine nucleotide-binding protein G(I)/G(S)/G(O) subunit gamma-T2 \|\| id:prot-a-1227 | Shellfish liking \|\| id:ebi-fl187-GCST90094838 | Weighted median | 34 | 0.0426442577473844 | 0.0883152186131937 | 0.629192159708172 | -0.130453571 | 0.215742086229244 | 1.04356658808098 | 0.877697242841076 | 1.24078232288142 | 0.999489402457283 |
| ebi-fl187-GCST90094838 | prot-a-1240 | Golgi SNAP receptor complex member 1 \|\| id:prot-a-1240 | Shellfish liking \|\| id:ebi-fl187-GCST90094838 | Weighted median | 34 | -0.063037855 | 0.0890682746161138 | 0.479102027826728 | -0.237611674 | 0.111535962919171 | 0.938907930348333 | 0.78850883046335 | 1.11799394960861 | 0.999489402457283 |
| ebi-fl187-GCST90094838 | prot-a-1283 | Glutathione S-transferase A1 \|\| id:prot-a-1283 | Shellfish liking \|\| id:ebi-fl187-GCST90094838 | Weighted median | 34 | -0.009764246 | 0.101093268159254 | 0.923054772235483 | -0.207907051 | 0.188378559938177 | 0.990283269815972 | 0.812282533852538 | 1.2072904606557 | 0.999489402457283 |
| ebi-fl187-GCST90094838 | prot-a-1324 | E3 ubiquitin-protein ligase HECW1 \|\| id:prot-a-1324 | Shellfish liking \|\| id:ebi-fl187-GCST90094838 | Weighted median | 34 | 0.00407189826653292 | 0.0859951945249617 | 0.962234039341805 | -0.164478683 | 0.172622479535458 | 1.0040802 | 0.84833584072468 | 1.18841736850871 | 0.999489402457283 |
| ebi-fl187-GCST90094838 | prot-a-1339 | Histidine triad nucleotide-binding protein 2, mitochondrial \|\| id:prot-a-1339 | Shellfish liking \|\| id:ebi-fl187-GCST90094838 | Weighted median | 34 | -0.164122329 | 0.0904176081716031 | 0.0694994884949772 | -0.341340841 | 0.0130961827221588 | 0.848638202217949 | 0.710816591254436 | 1.01318231330636 | 0.999489402457283 |
| ebi-fl187-GCST90094838 | prot-a-1466 | Interleukin-11 \|\| id:prot-a-1466 | Shellfish liking \|\| id:ebi-fl187-GCST90094838 | Weighted median | 34 | 0.00034083399267437 | 0.0899200832245265 | 0.996975697534783 | -0.175902529 | 0.176584197112746 | 1.00034089208318 | 0.838699728147494 | 1.19313488104266 | 0.999911036302558 |
| ebi-fl187-GCST90094838 | prot-a-147 | Rho GTPase-activating protein 1 \|\| id:prot-a-147 | Shellfish liking \|\| id:ebi-fl187-GCST90094838 | Weighted median | 34 | 0.012541234946535 | 0.0803382406780987 | 0.875949924792339 | -0.144921717 | 0.170004186675608 | 1.01262020601981 | 0.865090012489633 | 1.18530981381766 | 0.999489402457283 |
| ebi-fl187-GCST90094838 | prot-a-1473 | Interleukin-12 receptor subunit beta-1 \|\| id:prot-a-1473 | Shellfish liking \|\| id:ebi-fl187-GCST90094838 | Weighted median | 34 | -0.079502399 | 0.088283069794369 | 0.367833170321173 | -0.252537216 | 0.0935324179477097 | 0.92357580444682 | 0.776827302171303 | 1.09804619916859 | 0.999489402457283 |
| ebi-fl187-GCST90094838 | prot-a-1501 | X-linked interleukin-1 receptor accessory protein-like 2 \|\| id:prot-a-1501 | Shellfish liking \|\| id:ebi-fl187-GCST90094838 | Weighted median | 34 | -0.179446021 | 0.0903344664833124 | 0.0469820035737553 | -0.356501576 | -0.002390467 | 0.835733061496534 | 0.700121368341264 | 0.997612387882326 | 0.999489402457283 |
| ebi-fl187-GCST90094838 | prot-a-1503 | Interleukin-1 receptor-like 2 \|\| id:prot-a-1503 | Shellfish liking \|\| id:ebi-fl187-GCST90094838 | Weighted median | 34 | 0.0498477181252614 | 0.0911557619688234 | 0.584487896532102 | -0.128817575 | 0.228513011584155 | 1.05111101903136 | 0.879134326704464 | 1.25672987707207 | 0.999489402457283 |
| ebi-fl187-GCST90094838 | prot-a-1536 | Interleukin-5 receptor subunit alpha \|\| id:prot-a-1536 | Shellfish liking \|\| id:ebi-fl187-GCST90094838 | Weighted median | 34 | 0.069280599908418 | 0.0911204189483462 | 0.447063834962359 | -0.109315421 | 0.247876621047177 | 1.07173689623492 | 0.896447614290077 | 1.28130183676253 | 0.999489402457283 |
| ebi-fl187-GCST90094838 | prot-a-1559 | Insulin-induced gene 1 protein \|\| id:prot-a-1559 | Shellfish liking \|\| id:ebi-fl187-GCST90094838 | Weighted median | 34 | -0.021245562 | 0.0813990062588705 | 0.794088513407646 | -0.180787614 | 0.138296490070176 | 0.978978534930393 | 0.834612599517233 | 1.1483159640878 | 0.999489402457283 |
| ebi-fl187-GCST90094838 | prot-a-1567 | Interferon regulatory factor 1 \|\| id:prot-a-1567 | Shellfish liking \|\| id:ebi-fl187-GCST90094838 | Weighted median | 34 | -0.1094654 | 0.0846644513342791 | 0.196034491461375 | -0.275407724 | 0.0564769248594075 | 0.896313176480397 | 0.75926249028556 | 1.05810219866153 | 0.999489402457283 |
| ebi-fl187-GCST90094838 | prot-a-1613 | Potassium voltage-gated channel subfamily E regulatory beta subunit 5 \|\| id:prot-a-1613 | Shellfish liking \|\| id:ebi-fl187-GCST90094838 | Weighted median | 34 | -0.018703752 | 0.084255302340095 | 0.824322428850599 | -0.183844145 | 0.146436640371796 | 0.981470077515516 | 0.832065475451356 | 1.15770157695316 | 0.999489402457283 |
| ebi-fl187-GCST90094838 | prot-a-1649 | Kin of IRRE-like protein 2 \|\| id:prot-a-1649 | Shellfish liking \|\| id:ebi-fl187-GCST90094838 | Weighted median | 34 | 0.0370985334264574 | 0.0884799540238149 | 0.675006074589855 | -0.136322176 | 0.210519243313135 | 1.03779527332467 | 0.872561468433707 | 1.23431880537693 | 0.999489402457283 |
| ebi-fl187-GCST90094838 | prot-a-165 | Armadillo repeat-containing protein 5 \|\| id:prot-a-165 | Shellfish liking \|\| id:ebi-fl187-GCST90094838 | Weighted median | 34 | -0.034774106 | 0.0942935000474476 | 0.712287373672722 | -0.219589366 | 0.150041154207615 | 0.965823565476526 | 0.802848407153654 | 1.16188205807982 | 0.999489402457283 |
| ebi-fl187-GCST90094838 | prot-a-1656 | Kallikrein-11 \|\| id:prot-a-1656 | Shellfish liking \|\| id:ebi-fl187-GCST90094838 | Weighted median | 34 | -0.093853821 | 0.0886116686513639 | 0.289527503617264 | -0.267532692 | 0.0798250494637649 | 0.910415836106155 | 0.765265312428454 | 1.08309756259906 | 0.999489402457283 |
| ebi-fl187-GCST90094838 | prot-a-1697 | Laminin subunit gamma-2 \|\| id:prot-a-1697 | Shellfish liking \|\| id:ebi-fl187-GCST90094838 | Weighted median | 34 | -0.159665556 | 0.0875032737080892 | 0.0680491907241932 | -0.331171972 | 0.011840860496415 | 0.852428831030557 | 0.718081668165414 | 1.01191124099931 | 0.999489402457283 |
| ebi-fl187-GCST90094838 | prot-a-1698 | Lysosome-associated membrane glycoprotein 3 \|\| id:prot-a-1698 | Shellfish liking \|\| id:ebi-fl187-GCST90094838 | Weighted median | 34 | -0.017893485 | 0.0882470746139102 | 0.839318120592522 | -0.190857752 | 0.155070780984871 | 0.982265652558576 | 0.826250112629933 | 1.16774061201063 | 0.999489402457283 |
| ebi-fl187-GCST90094838 | prot-a-1724 | Leptin receptor \|\| id:prot-a-1724 | Shellfish liking \|\| id:ebi-fl187-GCST90094838 | Weighted median | 34 | 0.0398253880880853 | 0.0894887525278858 | 0.656296390063972 | -0.135572567 | 0.215223343042742 | 1.04062905209905 | 0.87321579409525 | 1.24013884242048 | 0.999489402457283 |
| ebi-fl187-GCST90094838 | prot-a-1743 | Leukocyte immunoglobulin-like receptor subfamily B member 2 \|\| id:prot-a-1743 | Shellfish liking \|\| id:ebi-fl187-GCST90094838 | Weighted median | 34 | -0.0461581 | 0.091507215692879 | 0.613966035889633 | -0.225512243 | 0.133196042969957 | 0.954890982194146 | 0.798107289522773 | 1.14247395036439 | 0.999489402457283 |
| ebi-fl187-GCST90094838 | prot-a-1746 | Leukocyte immunoglobulin-like receptor subfamily B member 4 \|\| id:prot-a-1746 | Shellfish liking \|\| id:ebi-fl187-GCST90094838 | Weighted median | 34 | 0.0215647627761942 | 0.0892168704542159 | 0.809003551332964 | -0.153300303 | 0.196429828866457 | 1.02179896273214 | 0.857872061302122 | 1.21704991610956 | 0.999489402457283 |
| ebi-fl187-GCST90094838 | prot-a-1750 | Leucine-rich repeat and immunoglobulin-like domain-containing nogo receptor-interacting protein 3 \|\| id:prot-a-1750 | Shellfish liking \|\| id:ebi-fl187-GCST90094838 | Weighted median | 34 | -0.054395184 | 0.0918419343113083 | 0.553670082453411 | -0.234405375 | 0.125615007348188 | 0.947057770555735 | 0.791041082492135 | 1.13384556203365 | 0.999489402457283 |
| ebi-fl187-GCST90094838 | prot-a-1753 | Lipase member N \|\| id:prot-a-1753 | Shellfish liking \|\| id:ebi-fl187-GCST90094838 | Weighted median | 34 | 0.0922954628962481 | 0.092184836278011 | 0.316730101316477 | -0.088386816 | 0.27297774200115 | 1.09668880506661 | 0.915406714075301 | 1.31387100036006 | 0.999489402457283 |
| ebi-fl187-GCST90094838 | prot-a-1760 | Ig Kappa chain V-I region HK102- like \|\| id:prot-a-1760 | Shellfish liking \|\| id:ebi-fl187-GCST90094838 | Weighted median | 34 | -0.109067806 | 0.0907245504911065 | 0.229291381345354 | -0.286887925 | 0.0687523132175904 | 0.896669616085598 | 0.750595847372284 | 1.07117086142406 | 0.999489402457283 |
| ebi-fl187-GCST90094838 | prot-a-1792 | Leucine-rich repeat neuronal protein 1 \|\| id:prot-a-1792 | Shellfish liking \|\| id:ebi-fl187-GCST90094838 | Weighted median | 34 | -0.025802427 | 0.0894728685941712 | 0.773053813240764 | -0.201169249 | 0.149564395531083 | 0.974527611017594 | 0.817774012113894 | 1.16132825275376 | 0.999489402457283 |
| ebi-fl187-GCST90094838 | prot-a-1795 | Leucine-rich repeat transmembrane neuronal protein 1 \|\| id:prot-a-1795 | Shellfish liking \|\| id:ebi-fl187-GCST90094838 | Weighted median | 34 | 0.0242634476902168 | 0.085915119175185 | 0.777627730200824 | -0.144130186 | 0.192657081273579 | 1.02456020035738 | 0.865775029026733 | 1.21246694460153 | 0.999489402457283 |
| ebi-fl187-GCST90094838 | prot-a-1820 | LysM and putative peptidoglycan-binding domain-containing protein 4 \|\| id:prot-a-1820 | Shellfish liking \|\| id:ebi-fl187-GCST90094838 | Weighted median | 34 | -0.025914427 | 0.0918063624655525 | 0.777734438824794 | -0.205854898 | 0.154026043233045 | 0.974418469758539 | 0.813951173957429 | 1.16652126636804 | 0.999489402457283 |
| ebi-fl187-GCST90094838 | prot-a-1844 | Mitogen-activated protein kinase kinase kinase 7:TGF-beta-activated kinase 1 and MAP3K7-binding protein 1 fusion \|\| id:prot-a-1844 | Shellfish liking \|\| id:ebi-fl187-GCST90094838 | Weighted median | 34 | -0.024379065 | 0.0885458860415187 | 0.783065096542391 | -0.197929001 | 0.149170871979989 | 0.975915704478316 | 0.82042810039637 | 1.16087133264606 | 0.999489402457283 |
| ebi-fl187-GCST90094838 | prot-a-1856 | Megakaryocyte-associated tyrosine-protein kinase \|\| id:prot-a-1856 | Shellfish liking \|\| id:ebi-fl187-GCST90094838 | Weighted median | 34 | 0.0212353593758708 | 0.0872731078749167 | 0.807756919513106 | -0.149819932 | 0.192290650810708 | 1.02146243410925 | 0.860862976293039 | 1.21202274116761 | 0.999489402457283 |
| ebi-fl187-GCST90094838 | prot-a-1910 | Stromelysin-2 \|\| id:prot-a-1910 | Shellfish liking \|\| id:ebi-fl187-GCST90094838 | Weighted median | 34 | -0.132587133 | 0.0880543214728611 | 0.132133293043058 | -0.305173603 | 0.039999337241498 | 0.875826617516229 | 0.736995418719562 | 1.04081008438643 | 0.999489402457283 |
| ebi-fl187-GCST90094838 | prot-a-1940 | 39S ribosomal protein L14, mitochondrial \|\| id:prot-a-1940 | Shellfish liking \|\| id:ebi-fl187-GCST90094838 | Weighted median | 34 | 0.0295086699085497 | 0.0865451051244047 | 0.733131512464782 | -0.140119736 | 0.199137075952383 | 1.02994836499132 | 0.869254148035156 | 1.22034923496895 | 0.999489402457283 |
| ebi-fl187-GCST90094838 | prot-a-1942 | 39S ribosomal protein L33, mitochondrial \|\| id:prot-a-1942 | Shellfish liking \|\| id:ebi-fl187-GCST90094838 | Weighted median | 34 | 0.0450996859172026 | 0.0960237026876574 | 0.638589067147895 | -0.143106771 | 0.233306143185011 | 1.04613213935286 | 0.866661529333285 | 1.26276800797758 | 0.999489402457283 |
| ebi-fl187-GCST90094838 | prot-a-1948 | Mesothelin \|\| id:prot-a-1948 | Shellfish liking \|\| id:ebi-fl187-GCST90094838 | Weighted median | 34 | 0.0386006212319458 | 0.0822291911675505 | 0.638764290291636 | -0.122568593 | 0.199769835920345 | 1.03935530430776 | 0.88464522198877 | 1.22112166746818 | 0.999489402457283 |
| ebi-fl187-GCST90094838 | prot-a-1980 | Unconventional myosin-VI \|\| id:prot-a-1980 | Shellfish liking \|\| id:ebi-fl187-GCST90094838 | Weighted median | 34 | 0.019576007735458 | 0.090529635444578 | 0.82880171418422 | -0.157862078 | 0.197014093206831 | 1.01976887423786 | 0.853967554985173 | 1.21776120274548 | 0.999489402457283 |
| ebi-fl187-GCST90094838 | prot-a-2039 | Neurofascin \|\| id:prot-a-2039 | Shellfish liking \|\| id:ebi-fl187-GCST90094838 | Weighted median | 34 | 0.0158571618828669 | 0.0888477398708088 | 0.858349409442373 | -0.158284408 | 0.189998732029652 | 1.01598355386331 | 0.853606974564198 | 1.20924806436559 | 0.999489402457283 |
| ebi-fl187-GCST90094838 | prot-a-2041 | NFU1 iron-sulfur cluster scaffold homolog, mitochondrial \|\| id:prot-a-2041 | Shellfish liking \|\| id:ebi-fl187-GCST90094838 | Weighted median | 34 | -0.078160558 | 0.0914696588014374 | 0.392829619177678 | -0.257441089 | 0.101119973665701 | 0.924815928410464 | 0.773027164902244 | 1.10640937378943 | 0.999489402457283 |
| ebi-fl187-GCST90094838 | prot-a-2088 | Glucocorticoid receptor \|\| id:prot-a-2088 | Shellfish liking \|\| id:ebi-fl187-GCST90094838 | Weighted median | 34 | 0.118531568452409 | 0.0870003980519749 | 0.173063613060311 | -0.051989212 | 0.289052348634279 | 1.12584241464369 | 0.949339108514779 | 1.3351616205865 | 0.999489402457283 |
| ebi-fl187-GCST90094838 | prot-a-2111 | Neuron-specific protein family member 2 \|\| id:prot-a-2111 | Shellfish liking \|\| id:ebi-fl187-GCST90094838 | Weighted median | 34 | -0.089206518 | 0.0896093358652224 | 0.319490845151612 | -0.264840816 | 0.0864277806296044 | 0.914656661309167 | 0.767328086637745 | 1.09027262607186 | 0.999489402457283 |
| ebi-fl187-GCST90094838 | prot-a-2125 | Nuclear migration protein nudC \|\| id:prot-a-2125 | Shellfish liking \|\| id:ebi-fl187-GCST90094838 | Weighted median | 34 | -0.113911009 | 0.0900724193341037 | 0.205993142866554 | -0.290452951 | 0.0626309330031311 | 0.892337362439967 | 0.747924717754021 | 1.06463384549913 | 0.999489402457283 |
| ebi-fl187-GCST90094838 | prot-a-2142 | Ornithine decarboxylase \|\| id:prot-a-2142 | Shellfish liking \|\| id:ebi-fl187-GCST90094838 | Weighted median | 34 | -0.026239757 | 0.0888347102348159 | 0.767705667224007 | -0.200355789 | 0.147876274638413 | 0.974101513541587 | 0.818439509101238 | 1.15936944408513 | 0.999489402457283 |
| ebi-fl187-GCST90094838 | prot-a-2186 | Prostate and testis expressed protein 1 \|\| id:prot-a-2186 | Shellfish liking \|\| id:ebi-fl187-GCST90094838 | Weighted median | 34 | -0.049891612 | 0.0897938760728362 | 0.578468079788022 | -0.225887609 | 0.126104385337751 | 0.951332532166836 | 0.797807763144807 | 1.13440057689022 | 0.999489402457283 |
| ebi-fl187-GCST90094838 | prot-a-2227 | Platelet-derived growth factor C \|\| id:prot-a-2227 | Shellfish liking \|\| id:ebi-fl187-GCST90094838 | Weighted median | 34 | -0.10354525 | 0.0910315948160711 | 0.255343962500764 | -0.281967176 | 0.0748766760013706 | 0.90163522297088 | 0.754298443462012 | 1.07775123009742 | 0.999489402457283 |
| ebi-fl187-GCST90094838 | prot-a-2229 | Platelet-derived growth factor receptor alpha \|\| id:prot-a-2229 | Shellfish liking \|\| id:ebi-fl187-GCST90094838 | Weighted median | 34 | -0.147765314 | 0.0939592144496245 | 0.115799007659131 | -0.331925374 | 0.0363947465857491 | 0.862633539430981 | 0.717540868020565 | 1.03706514362593 | 0.999489402457283 |
| ebi-fl187-GCST90094838 | prot-a-2243 | Platelet endothelial aggregation receptor 1 \|\| id:prot-a-2243 | Shellfish liking \|\| id:ebi-fl187-GCST90094838 | Weighted median | 34 | -0.013710459 | 0.0906064665950911 | 0.879724267325486 | -0.191299133 | 0.163878215892452 | 0.98638310163125 | 0.825885501457023 | 1.17807083605077 | 0.999489402457283 |
| ebi-fl187-GCST90094838 | prot-a-2246 | E3 ubiquitin-protein ligase pellino homolog 2 \|\| id:prot-a-2246 | Shellfish liking \|\| id:ebi-fl187-GCST90094838 | Weighted median | 34 | 0.0982890229370094 | 0.0894949481420444 | 0.272089503930676 | -0.077121075 | 0.273699121295416 | 1.1032816127241 | 0.925777757888457 | 1.31481914163869 | 0.999489402457283 |
| ebi-fl187-GCST90094838 | prot-a-2251 | Platelet factor 4 variant \|\| id:prot-a-2251 | Shellfish liking \|\| id:ebi-fl187-GCST90094838 | Weighted median | 34 | 0.0268654851706856 | 0.0888245142507744 | 0.762304660278729 | -0.147230563 | 0.200961533102203 | 1.02722961585316 | 0.863094956917716 | 1.22257774214573 | 0.999489402457283 |
| ebi-fl187-GCST90094838 | prot-a-2346 | Protein phosphatase 1L \|\| id:prot-a-2346 | Shellfish liking \|\| id:ebi-fl187-GCST90094838 | Weighted median | 34 | 0.154180174080734 | 0.0933813578734378 | 0.098722061895075 | -0.028847287 | 0.337207635512672 | 1.16670107713651 | 0.97156482337583 | 1.40102993710893 | 0.999489402457283 |
| ebi-fl187-GCST90094838 | prot-a-2358 | PR domain zinc finger protein 4 \|\| id:prot-a-2358 | Shellfish liking \|\| id:ebi-fl187-GCST90094838 | Weighted median | 34 | 0.0330775666587468 | 0.0850974589192354 | 0.697496479409384 | -0.133713453 | 0.199868586140448 | 1.03363071141258 | 0.87484071182683 | 1.22124225945576 | 0.999489402457283 |
| ebi-fl187-GCST90094838 | prot-a-2390 | Trypsin-1 \|\| id:prot-a-2390 | Shellfish liking \|\| id:ebi-fl187-GCST90094838 | Weighted median | 34 | -0.128023256 | 0.0866275113892836 | 0.1394454970822 | -0.297813178 | 0.0417666667758466 | 0.87983291791082 | 0.742440031019956 | 1.04265116520726 | 0.999489402457283 |
| ebi-fl187-GCST90094838 | prot-a-242 | Brain-derived neurotrophic factor \|\| id:prot-a-242 | Shellfish liking \|\| id:ebi-fl187-GCST90094838 | Weighted median | 34 | -0.003452421 | 0.0879937001247767 | 0.968703136846573 | -0.175920073 | 0.169015231307018 | 0.996553531815181 | 0.838685014082652 | 1.18413817475871 | 0.999489402457283 |
| ebi-fl187-GCST90094838 | prot-a-2464 | Dihydropteridine reductase \|\| id:prot-a-2464 | Shellfish liking \|\| id:ebi-fl187-GCST90094838 | Weighted median | 34 | 0.0837448648058564 | 0.0909131107966193 | 0.356970667122485 | -0.094444832 | 0.26193456196723 | 1.08735143679588 | 0.909877929063576 | 1.29944150675123 | 0.999489402457283 |
| ebi-fl187-GCST90094838 | prot-a-2474 | Ras-related protein Rab-27A \|\| id:prot-a-2474 | Shellfish liking \|\| id:ebi-fl187-GCST90094838 | Weighted median | 34 | -0.127826918 | 0.0973307708333041 | 0.189073516511242 | -0.318595228 | 0.0629413931730207 | 0.880005679406001 | 0.727169827371116 | 1.06496442321663 | 0.999489402457283 |
| ebi-fl187-GCST90094838 | prot-a-2478 | Ras-related protein Rab-7b \|\| id:prot-a-2478 | Shellfish liking \|\| id:ebi-fl187-GCST90094838 | Weighted median | 34 | 0.0212968179617541 | 0.0905528667927585 | 0.814063930843456 | -0.156186801 | 0.198780436875561 | 1.02152521367514 | 0.855399386025986 | 1.21991408834415 | 0.999489402457283 |
| ebi-fl187-GCST90094838 | prot-a-2528 | E3 ubiquitin-protein ligase rififylin \|\| id:prot-a-2528 | Shellfish liking \|\| id:ebi-fl187-GCST90094838 | Weighted median | 34 | 0.0724614781304937 | 0.0897111783312247 | 0.419252011106452 | -0.103372431 | 0.248295387659694 | 1.07515138845093 | 0.901791055628033 | 1.28183851555606 | 0.999489402457283 |
| ebi-fl187-GCST90094838 | prot-a-2543 | Protein RIC-3 \|\| id:prot-a-2543 | Shellfish liking \|\| id:ebi-fl187-GCST90094838 | Weighted median | 34 | 0.0684966196373043 | 0.0885530599898084 | 0.439220998984405 | -0.105067378 | 0.242060617217329 | 1.07089700492457 | 0.900263862615748 | 1.27387140901591 | 0.999489402457283 |
| ebi-fl187-GCST90094838 | prot-a-255 | Bone morphogenetic protein 6 \|\| id:prot-a-255 | Shellfish liking \|\| id:ebi-fl187-GCST90094838 | Weighted median | 34 | 0.0134617505307929 | 0.0945989602138712 | 0.886840387298323 | -0.171952211 | 0.19887571254998 | 1.01355276785339 | 0.842019411061435 | 1.22003032201868 | 0.999489402457283 |
| ebi-fl187-GCST90094838 | prot-a-2568 | RING finger protein 165 \|\| id:prot-a-2568 | Shellfish liking \|\| id:ebi-fl187-GCST90094838 | Weighted median | 34 | -0.101204199 | 0.0869133249270541 | 0.244251117637663 | -0.271554316 | 0.0691459175362595 | 0.903748469219059 | 0.762193882885233 | 1.07159256188727 | 0.999489402457283 |
| ebi-fl187-GCST90094838 | prot-a-2573 | E3 ubiquitin-protein ligase RNF8 \|\| id:prot-a-2573 | Shellfish liking \|\| id:ebi-fl187-GCST90094838 | Weighted median | 34 | -0.162401712 | 0.0942444931057573 | 0.0848531273643315 | -0.347120919 | 0.0223174940664907 | 0.850099640355096 | 0.70671986729626 | 1.02256839233432 | 0.999489402457283 |
| ebi-fl187-GCST90094838 | prot-a-260 | Cytoplasmic tyrosine-protein kinase BMX \|\| id:prot-a-260 | Shellfish liking \|\| id:ebi-fl187-GCST90094838 | Weighted median | 34 | 0.0669651869584247 | 0.0930415310098629 | 0.471688565200745 | -0.115396214 | 0.249326587737756 | 1.06925825339462 | 0.891013042277619 | 1.28316102930431 | 0.999489402457283 |
| ebi-fl187-GCST90094838 | prot-a-2623 | Serum amyloid A-1 protein \|\| id:prot-a-2623 | Shellfish liking \|\| id:ebi-fl187-GCST90094838 | Weighted median | 34 | 0.0222480233955142 | 0.0836395959982931 | 0.790240213980409 | -0.141685585 | 0.186181631552169 | 1.02249735628948 | 0.867894092720199 | 1.20464104133041 | 0.999489402457283 |
| ebi-fl187-GCST90094838 | prot-a-2640 | Lysosome membrane protein 2 \|\| id:prot-a-2640 | Shellfish liking \|\| id:ebi-fl187-GCST90094838 | Weighted median | 34 | 0.00704275800427583 | 0.0878256307400823 | 0.936085956164457 | -0.165095478 | 0.179180994254837 | 1.00706761654773 | 0.84781275254863 | 1.1962372366426 | 0.999489402457283 |
| ebi-fl187-GCST90094838 | prot-a-2641 | Scavenger receptor class F member 2 \|\| id:prot-a-2641 | Shellfish liking \|\| id:ebi-fl187-GCST90094838 | Weighted median | 34 | -0.036401569 | 0.0890905033169281 | 0.682839907776498 | -0.211018956 | 0.138215817307962 | 0.964253001426208 | 0.809758717197017 | 1.14822333000366 | 0.999489402457283 |
| ebi-fl187-GCST90094838 | prot-a-2669 | Semaphorin-3A \|\| id:prot-a-2669 | Shellfish liking \|\| id:ebi-fl187-GCST90094838 | Weighted median | 34 | -0.066134356 | 0.0898306943041172 | 0.461602225094623 | -0.242202517 | 0.109933804644125 | 0.936005097762476 | 0.784897206405653 | 1.11620418048036 | 0.999489402457283 |
| ebi-fl187-GCST90094838 | prot-a-2683 | Sentrin-specific protease 7 \|\| id:prot-a-2683 | Shellfish liking \|\| id:ebi-fl187-GCST90094838 | Weighted median | 34 | 0.0462686386313958 | 0.0908406502148935 | 0.610514990665201 | -0.131779036 | 0.224316313052587 | 1.04735573337901 | 0.876534656470551 | 1.25146681211542 | 0.999489402457283 |
| ebi-fl187-GCST90094838 | prot-a-270 | Bromodomain testis-specific protein \|\| id:prot-a-270 | Shellfish liking \|\| id:ebi-fl187-GCST90094838 | Weighted median | 34 | -0.068692263 | 0.0877675141572676 | 0.433825861123699 | -0.24071659 | 0.103332065220355 | 0.933613943870032 | 0.786064373109485 | 1.10885956164197 | 0.999489402457283 |
| ebi-fl187-GCST90094838 | prot-a-2708 | 14-3-3 protein sigma \|\| id:prot-a-2708 | Shellfish liking \|\| id:ebi-fl187-GCST90094838 | Weighted median | 34 | 0.118126975299932 | 0.088234369674032 | 0.180640215142475 | -0.054812389 | 0.291066339861035 | 1.12538699864727 | 0.946662735389516 | 1.33785333401043 | 0.999489402457283 |
| ebi-fl187-GCST90094838 | prot-a-2713 | Beta-sarcoglycan \|\| id:prot-a-2713 | Shellfish liking \|\| id:ebi-fl187-GCST90094838 | Weighted median | 34 | -0.072866056 | 0.0871559502285624 | 0.40313113816813 | -0.243691719 | 0.0979596062266607 | 0.929725352612956 | 0.783729206107469 | 1.10291823317958 | 0.999489402457283 |
| ebi-fl187-GCST90094838 | prot-a-2720 | Protein shisa-3 homolog \|\| id:prot-a-2720 | Shellfish liking \|\| id:ebi-fl187-GCST90094838 | Weighted median | 34 | -0.060660225 | 0.081663792703765 | 0.45760010652177 | -0.220721259 | 0.0994008085273233 | 0.941142962144161 | 0.801940182850679 | 1.10450890744106 | 0.999489402457283 |
| ebi-fl187-GCST90094838 | prot-a-2730 | Sialic acid-binding Ig-like lectin 8 \|\| id:prot-a-2730 | Shellfish liking \|\| id:ebi-fl187-GCST90094838 | Weighted median | 34 | -0.056653012 | 0.0891167569744687 | 0.524961078575161 | -0.231321856 | 0.118015831508283 | 0.944921888892152 | 0.793484037467093 | 1.12526192581983 | 0.999489402457283 |
| ebi-fl187-GCST90094838 | prot-a-2761 | Sodium- and chloride-dependent neutral and basic amino acid transporter B(0+) \|\| id:prot-a-2761 | Shellfish liking \|\| id:ebi-fl187-GCST90094838 | Weighted median | 34 | 0.0883012034341622 | 0.0966343405421294 | 0.360839659862094 | -0.101102104 | 0.277704510896736 | 1.09231708214009 | 0.903840742393488 | 1.32009606556947 | 0.999489402457283 |
| ebi-fl187-GCST90094838 | prot-a-2770 | Secreted Ly-6/uPAR-related protein 1 \|\| id:prot-a-2770 | Shellfish liking \|\| id:ebi-fl187-GCST90094838 | Weighted median | 34 | 0.0657916756114873 | 0.0806360309745423 | 0.414552097634366 | -0.092254945 | 0.22383829632159 | 1.06800420266675 | 0.911872642447324 | 1.25086873299824 | 0.999489402457283 |
| ebi-fl187-GCST90094838 | prot-a-2784 | N-lysine methyltransferase SMYD2 \|\| id:prot-a-2784 | Shellfish liking \|\| id:ebi-fl187-GCST90094838 | Weighted median | 34 | -0.076066046 | 0.0902167513547574 | 0.399145844699467 | -0.252890879 | 0.100758786333804 | 0.926754995779609 | 0.776552615416474 | 1.10600982490007 | 0.999489402457283 |
| ebi-fl187-GCST90094838 | prot-a-2837 | Serine palmitoyltransferase 2 \|\| id:prot-a-2837 | Shellfish liking \|\| id:ebi-fl187-GCST90094838 | Weighted median | 34 | 0.0493852016054065 | 0.0933563721112851 | 0.596807822071042 | -0.133593288 | 0.232363690943525 | 1.05062497523116 | 0.874945843456364 | 1.26157847006737 | 0.999489402457283 |
| ebi-fl187-GCST90094838 | prot-a-2858 | Alpha-2,8-sialyltransferase 8B \|\| id:prot-a-2858 | Shellfish liking \|\| id:ebi-fl187-GCST90094838 | Weighted median | 34 | -0.025967989 | 0.0856218919050711 | 0.76167130773504 | -0.193786897 | 0.141850918854644 | 0.974366279276375 | 0.823833446602185 | 1.15240483389761 | 0.999489402457283 |
| ebi-fl187-GCST90094838 | prot-a-2911 | Synaptotagmin-11 \|\| id:prot-a-2911 | Shellfish liking \|\| id:ebi-fl187-GCST90094838 | Weighted median | 34 | -0.067290596 | 0.0856127447930593 | 0.431874475613952 | -0.235091576 | 0.100510383635709 | 0.934923476686627 | 0.790498455664525 | 1.10573512319518 | 0.999489402457283 |
| ebi-fl187-GCST90094838 | prot-a-2962 | Transforming growth factor beta-1 \|\| id:prot-a-2962 | Shellfish liking \|\| id:ebi-fl187-GCST90094838 | Weighted median | 34 | -0.181430123 | 0.0842973164196924 | 0.0313765268927942 | -0.346652863 | -0.016207383 | 0.834076526112829 | 0.707050729069267 | 0.983923250214614 | 0.999489402457283 |
| ebi-fl187-GCST90094838 | prot-a-2978 | Tyrosine-protein kinase receptor Tie-1, soluble \|\| id:prot-a-2978 | Shellfish liking \|\| id:ebi-fl187-GCST90094838 | Weighted median | 34 | -0.164360787 | 0.0896875779197931 | 0.06686318709717 | -0.34014844 | 0.0114268657195959 | 0.848435862022214 | 0.71166467561224 | 1.01149240173555 | 0.999489402457283 |
| ebi-fl187-GCST90094838 | prot-a-299 | Uncharacterized protein C1orf226 \|\| id:prot-a-299 | Shellfish liking \|\| id:ebi-fl187-GCST90094838 | Weighted median | 34 | 0.0572158488821082 | 0.0809170204345628 | 0.479508761390755 | -0.101381511 | 0.215813208933851 | 1.05888434473226 | 0.903588238112927 | 1.24087057381434 | 0.999489402457283 |
| ebi-fl187-GCST90094838 | prot-a-3015 | Transmembrane protein 70, mitochondrial \|\| id:prot-a-3015 | Shellfish liking \|\| id:ebi-fl187-GCST90094838 | Weighted median | 34 | -0.030762983 | 0.0899647276276743 | 0.732392331804549 | -0.207093849 | 0.145567883136336 | 0.96970538248774 | 0.812943352353275 | 1.15669625208653 | 0.999489402457283 |
| ebi-fl187-GCST90094838 | prot-a-3016 | Transmembrane protein 87B \|\| id:prot-a-3016 | Shellfish liking \|\| id:ebi-fl187-GCST90094838 | Weighted median | 34 | -0.070787347 | 0.0904932767881478 | 0.434074281467666 | -0.248154169 | 0.106579475724909 | 0.931659991560885 | 0.780239645017212 | 1.11246633700096 | 0.999489402457283 |
| ebi-fl187-GCST90094838 | prot-a-3123 | Thioredoxin domain-containing protein 12 \|\| id:prot-a-3123 | Shellfish liking \|\| id:ebi-fl187-GCST90094838 | Weighted median | 33 | -0.059822059 | 0.0888402585214497 | 0.500714122253048 | -0.233948965 | 0.114304848112838 | 0.941932127404491 | 0.791402203845963 | 1.12109383613675 | 0.999489402457283 |
| ebi-fl187-GCST90094838 | prot-a-3133 | PolyUbiquitin K48-linked \|\| id:prot-a-3133 | Shellfish liking \|\| id:ebi-fl187-GCST90094838 | Weighted median | 34 | 0.155083962879422 | 0.093113468298438 | 0.0958055906671765 | -0.027418435 | 0.337586360744361 | 1.16775600514569 | 0.972954038327311 | 1.40156064298597 | 0.999489402457283 |
| ebi-fl187-GCST90094838 | prot-a-3135 | Ubiquitin-conjugating enzyme E2 B \|\| id:prot-a-3135 | Shellfish liking \|\| id:ebi-fl187-GCST90094838 | Weighted median | 34 | 0.0309468982289977 | 0.0808167711189079 | 0.70177412513952 | -0.127453973 | 0.189347769622057 | 1.03143073163377 | 0.880333933887301 | 1.20846114548932 | 0.999489402457283 |
| ebi-fl187-GCST90094838 | prot-a-3162 | NKG2D ligand 3 \|\| id:prot-a-3162 | Shellfish liking \|\| id:ebi-fl187-GCST90094838 | Weighted median | 34 | 0.0798218909854315 | 0.0878650739077335 | 0.363635322466461 | -0.092393654 | 0.252037435844589 | 1.08309414166428 | 0.911746166481899 | 1.2866442029957 | 0.999489402457283 |
| ebi-fl187-GCST90094838 | prot-a-3188 | Vesicle-associated membrane protein 8 \|\| id:prot-a-3188 | Shellfish liking \|\| id:ebi-fl187-GCST90094838 | Weighted median | 34 | -0.061478845 | 0.0902195489518735 | 0.49559567850107 | -0.23830916 | 0.115351471425583 | 0.940372839567713 | 0.787959047647079 | 1.12226781333021 | 0.999489402457283 |
| ebi-fl187-GCST90094838 | prot-a-3201 | Vitrin \|\| id:prot-a-3201 | Shellfish liking \|\| id:ebi-fl187-GCST90094838 | Weighted median | 34 | -0.07320894 | 0.0861293742729362 | 0.39533172048679 | -0.242022514 | 0.0956046331969697 | 0.929406619166943 | 0.785038503032955 | 1.10032394642313 | 0.999489402457283 |
| ebi-fl187-GCST90094838 | prot-a-3232 | WNT1-inducible-signaling pathway protein 3 \|\| id:prot-a-3232 | Shellfish liking \|\| id:ebi-fl187-GCST90094838 | Weighted median | 34 | 0.0691253611522294 | 0.0943482722307008 | 0.463764817456017 | -0.115797252 | 0.254047974724403 | 1.07157053404545 | 0.890655783297529 | 1.28923365341341 | 0.999489402457283 |
| ebi-fl187-GCST90094838 | prot-a-327 | Carbonic anhydrase-related protein 10 \|\| id:prot-a-327 | Shellfish liking \|\| id:ebi-fl187-GCST90094838 | Weighted median | 34 | -0.057276018 | 0.0954311671585961 | 0.548385303520584 | -0.244321106 | 0.129769069724375 | 0.944333380468087 | 0.783236092432977 | 1.13856542373601 | 0.999489402457283 |
| ebi-fl187-GCST90094838 | prot-a-332 | Carbonic anhydrase 5A, mitochondrial \|\| id:prot-a-332 | Shellfish liking \|\| id:ebi-fl187-GCST90094838 | Weighted median | 34 | -0.073954286 | 0.0869339291572378 | 0.394938625789006 | -0.244344787 | 0.0964362148090097 | 0.928714147794471 | 0.783217544094596 | 1.10123933614214 | 0.999489402457283 |
| ebi-fl187-GCST90094838 | prot-a-338 | Cell adhesion molecule 3 \|\| id:prot-a-338 | Shellfish liking \|\| id:ebi-fl187-GCST90094838 | Weighted median | 34 | 0.0073177552476734 | 0.0847442563297179 | 0.931187360470288 | -0.158780987 | 0.173416497653921 | 1.00734459544867 | 0.853183196584607 | 1.18936136815842 | 0.999489402457283 |
| ebi-fl187-GCST90094838 | prot-a-34 | A disintegrin and metalloproteinase with thrombospondin motifs 3 \|\| id:prot-a-34 | Shellfish liking \|\| id:ebi-fl187-GCST90094838 | Weighted median | 34 | -0.071856835 | 0.0950800109302965 | 0.449798363866779 | -0.258213656 | 0.114499986470399 | 0.930664124847299 | 0.772430179842103 | 1.12131262589305 | 0.999489402457283 |
| ebi-fl187-GCST90094838 | prot-a-358 | Cysteine--tRNA ligase, cytoplasmic \|\| id:prot-a-358 | Shellfish liking \|\| id:ebi-fl187-GCST90094838 | Weighted median | 34 | 0.0715538432686402 | 0.0905398386955591 | 0.429351240661724 | -0.105904241 | 0.249011927111936 | 1.07417598629047 | 0.899510780587298 | 1.28275733256886 | 0.999489402457283 |
| ebi-fl187-GCST90094838 | prot-a-382 | Coiled-coil domain-containing protein 167 \|\| id:prot-a-382 | Shellfish liking \|\| id:ebi-fl187-GCST90094838 | Weighted median | 34 | -0.114504847 | 0.0912636366497555 | 0.209602163079437 | -0.293381575 | 0.0643718811062516 | 0.891807616059359 | 0.745737531929731 | 1.06648893210918 | 0.999489402457283 |
| ebi-fl187-GCST90094838 | prot-a-40 | Pituitary adenylate cyclase-activating polypeptide 27 \|\| id:prot-a-40 | Shellfish liking \|\| id:ebi-fl187-GCST90094838 | Weighted median | 34 | -0.024650789 | 0.0898741865676324 | 0.783868619910948 | -0.200804195 | 0.151502616336678 | 0.975650560125946 | 0.818072598570855 | 1.16358134612625 | 0.999489402457283 |
| ebi-fl187-GCST90094838 | prot-a-403 | C-C motif chemokine 25 \|\| id:prot-a-403 | Shellfish liking \|\| id:ebi-fl187-GCST90094838 | Weighted median | 34 | 0.00925576935231683 | 0.0918709667972258 | 0.919750911183659 | -0.170811326 | 0.189322864274879 | 1.00929873644766 | 0.842980607353721 | 1.20843104871973 | 0.999489402457283 |
| ebi-fl187-GCST90094838 | prot-a-415 | Cyclin-C \|\| id:prot-a-415 | Shellfish liking \|\| id:ebi-fl187-GCST90094838 | Weighted median | 34 | 0.00563176726672463 | 0.0856698871005291 | 0.947586416082366 | -0.162281211 | 0.173544745983762 | 1.00564765548023 | 0.850202084358941 | 1.18951391154897 | 0.999489402457283 |
| ebi-fl187-GCST90094838 | prot-a-44 | Adhesion G protein-coupled receptor F5 \|\| id:prot-a-44 | Shellfish liking \|\| id:ebi-fl187-GCST90094838 | Weighted median | 34 | -0.006628754 | 0.0877212851524795 | 0.939764319016479 | -0.178562473 | 0.165304964751409 | 0.993393167578667 | 0.836471798303641 | 1.17975284689007 | 0.999489402457283 |
| ebi-fl187-GCST90094838 | prot-a-455 | CD59 glycoprotein \|\| id:prot-a-455 | Shellfish liking \|\| id:ebi-fl187-GCST90094838 | Weighted median | 34 | 0.00454331001280296 | 0.0859653240751309 | 0.957851027730262 | -0.163948725 | 0.17303534520006 | 1.00455364649376 | 0.848785542095049 | 1.18890812653698 | 0.999489402457283 |
| ebi-fl187-GCST90094838 | prot-a-478 | Cadherin-12 \|\| id:prot-a-478 | Shellfish liking \|\| id:ebi-fl187-GCST90094838 | Weighted median | 34 | -0.067428336 | 0.0919196999779719 | 0.463218801345924 | -0.247590948 | 0.112734276397509 | 0.934794709755658 | 0.780679216746487 | 1.11933445984246 | 0.999489402457283 |
| ebi-fl187-GCST90094838 | prot-a-516 | Centrin-2 \|\| id:prot-a-516 | Shellfish liking \|\| id:ebi-fl187-GCST90094838 | Weighted median | 34 | -0.024102176 | 0.0903257668341465 | 0.789595352784472 | -0.201140679 | 0.152936327045669 | 0.976185961934768 | 0.817797376589647 | 1.16525078162078 | 0.999489402457283 |
| ebi-fl187-GCST90094838 | prot-a-519 | Cryptic protein \|\| id:prot-a-519 | Shellfish liking \|\| id:ebi-fl187-GCST90094838 | Weighted median | 34 | 0.0515765495129147 | 0.0876801166213315 | 0.556374291405437 | -0.120276479 | 0.223429578090725 | 1.05292978446935 | 0.886675255679451 | 1.25035758460759 | 0.999489402457283 |
| ebi-fl187-GCST90094838 | prot-a-583 | Chloride intracellular channel protein 4 \|\| id:prot-a-583 | Shellfish liking \|\| id:ebi-fl187-GCST90094838 | Weighted median | 34 | -0.038274266 | 0.0912487372451104 | 0.674887425852126 | -0.217121791 | 0.140573259249699 | 0.962448937908884 | 0.804831942248945 | 1.15093339299327 | 0.999489402457283 |
| ebi-fl187-GCST90094838 | prot-a-602 | CCR4-NOT transcription complex subunit 1 \|\| id:prot-a-602 | Shellfish liking \|\| id:ebi-fl187-GCST90094838 | Weighted median | 34 | -0.063819938 | 0.0914228716064993 | 0.485130860603733 | -0.243008766 | 0.115368890426178 | 0.93817391386667 | 0.784264638664829 | 1.12228736228418 | 0.999489402457283 |
| ebi-fl187-GCST90094838 | prot-a-606 | Ciliary neurotrophic factor receptor subunit alpha \|\| id:prot-a-606 | Shellfish liking \|\| id:ebi-fl187-GCST90094838 | Weighted median | 34 | -0.068399624 | 0.086987601515282 | 0.431683181916173 | -0.238895323 | 0.102096075027132 | 0.933887195313574 | 0.787497310983131 | 1.10748986873599 | 0.999489402457283 |
| ebi-fl187-GCST90094838 | prot-a-626 | Collagen alpha-2(IX) chain \|\| id:prot-a-626 | Shellfish liking \|\| id:ebi-fl187-GCST90094838 | Weighted median | 34 | 0.100344133622405 | 0.0910991813934248 | 0.2706868144469 | -0.078210262 | 0.278898529153518 | 1.10555130999613 | 0.924769962202666 | 1.32167322576412 | 0.999489402457283 |
| ebi-fl187-GCST90094838 | prot-a-647 | Probable serine carboxypeptidase CPVL \|\| id:prot-a-647 | Shellfish liking \|\| id:ebi-fl187-GCST90094838 | Weighted median | 34 | -0.139834675 | 0.0897027952493718 | 0.119027999570869 | -0.315652154 | 0.0359828033743814 | 0.869501973657172 | 0.729313094690884 | 1.03663801966172 | 0.999489402457283 |
| ebi-fl187-GCST90094838 | prot-a-651 | Cellular retinoic acid-binding protein 2 \|\| id:prot-a-651 | Shellfish liking \|\| id:ebi-fl187-GCST90094838 | Weighted median | 34 | -0.007099288 | 0.0910634575183743 | 0.937860041795795 | -0.185583664 | 0.17138508908458 | 0.992925852762954 | 0.830619339415552 | 1.1869477416439 | 0.999489402457283 |
| ebi-fl187-GCST90094838 | prot-a-671 | Cytotoxic and regulatory T-cell molecule \|\| id:prot-a-671 | Shellfish liking \|\| id:ebi-fl187-GCST90094838 | Weighted median | 34 | -0.010183013 | 0.0944113367413339 | 0.914108372932917 | -0.195229233 | 0.174863206707349 | 0.989868658035609 | 0.822646058554098 | 1.19108327326506 | 0.999489402457283 |
| ebi-fl187-GCST90094838 | prot-a-688 | Chorionic somatomammotropin hormone \|\| id:prot-a-688 | Shellfish liking \|\| id:ebi-fl187-GCST90094838 | Weighted median | 34 | 0.0226401432839445 | 0.0939178092663039 | 0.809506004830064 | -0.161438763 | 0.2067190494459 | 1.0228983764576 | 0.850918637678358 | 1.22963705603437 | 0.999489402457283 |
| ebi-fl187-GCST90094838 | prot-a-69 | Aldo-keto reductase family 1 member C1 \|\| id:prot-a-69 | Shellfish liking \|\| id:ebi-fl187-GCST90094838 | Weighted median | 34 | -0.014795876 | 0.0948758883363262 | 0.876072588979092 | -0.200752617 | 0.17116086511401 | 0.985313045092336 | 0.818114794079935 | 1.18668162934391 | 0.999489402457283 |
| ebi-fl187-GCST90094838 | prot-a-741 | Stromal cell-derived factor 1 \|\| id:prot-a-741 | Shellfish liking \|\| id:ebi-fl187-GCST90094838 | Weighted median | 34 | -0.060528918 | 0.0895869039657239 | 0.499266244129652 | -0.23611925 | 0.115061413524543 | 0.941266548845075 | 0.789686498186118 | 1.12194233788952 | 0.999489402457283 |
| ebi-fl187-GCST90094838 | prot-a-742 | Growth-regulated alpha protein \|\| id:prot-a-742 | Shellfish liking \|\| id:ebi-fl187-GCST90094838 | Weighted median | 34 | -0.180269304 | 0.0886616619115664 | 0.0420295725303494 | -0.354046161 | -0.006492447 | 0.835045300005508 | 0.701842568539847 | 0.993528583642328 | 0.999489402457283 |
| ebi-fl187-GCST90094838 | prot-a-788 | Beta-defensin 112 \|\| id:prot-a-788 | Shellfish liking \|\| id:ebi-fl187-GCST90094838 | Weighted median | 34 | -0.089938424 | 0.0844979231338634 | 0.287153779664957 | -0.255554353 | 0.0756775054590816 | 0.913987463337231 | 0.774487039547643 | 1.07861467071876 | 0.999489402457283 |
| ebi-fl187-GCST90094838 | prot-a-838 | DnaJ homolog subfamily B member 1 \|\| id:prot-a-838 | Shellfish liking \|\| id:ebi-fl187-GCST90094838 | Weighted median | 34 | 0.0484991725847874 | 0.0871190103833776 | 0.577732263937859 | -0.122254088 | 0.219252432936207 | 1.04969450328677 | 0.884923491700968 | 1.24514555276694 | 0.999489402457283 |
| ebi-fl187-GCST90094838 | prot-a-85 | Junctional adhesion molecule-like \|\| id:prot-a-85 | Shellfish liking \|\| id:ebi-fl187-GCST90094838 | Weighted median | 34 | 0.0159416479003644 | 0.0831127390948604 | 0.847892984621305 | -0.146959321 | 0.178842616526291 | 1.01606939389371 | 0.863329096303248 | 1.19583252508021 | 0.999489402457283 |
| ebi-fl187-GCST90094838 | prot-a-856 | Dipeptidase 1 \|\| id:prot-a-856 | Shellfish liking \|\| id:ebi-fl187-GCST90094838 | Weighted median | 34 | 0.0461176379749956 | 0.0914017302587479 | 0.613868056729025 | -0.133029753 | 0.225265029282142 | 1.04719759391567 | 0.875439044492603 | 1.25265466236813 | 0.999489402457283 |
| ebi-fl187-GCST90094838 | prot-a-860 | Dipeptidyl peptidase 2 \|\| id:prot-a-860 | Shellfish liking \|\| id:ebi-fl187-GCST90094838 | Weighted median | 34 | 0.0156235326135683 | 0.0857522146691594 | 0.85543053993708 | -0.152450808 | 0.183697873365121 | 1.01574621809337 | 0.858601129105843 | 1.20165271695538 | 0.999489402457283 |
| ebi-fl187-GCST90094838 | prot-a-861 | Dermatopontin \|\| id:prot-a-861 | Shellfish liking \|\| id:ebi-fl187-GCST90094838 | Weighted median | 34 | -0.03615691 | 0.0854739434729491 | 0.672283041303901 | -0.203685839 | 0.131372019551079 | 0.964488943981014 | 0.815718598019251 | 1.14039195050896 | 0.999489402457283 |
| ebi-fl187-GCST90094838 | prot-a-877 | Dual specificity protein phosphatase 6 \|\| id:prot-a-877 | Shellfish liking \|\| id:ebi-fl187-GCST90094838 | Weighted median | 34 | 0.062746478619952 | 0.0848199510643912 | 0.459445121672244 | -0.103500625 | 0.228993582706159 | 1.06475686638063 | 0.901675458774107 | 1.25733397030241 | 0.999489402457283 |
| ebi-fl187-GCST90094838 | prot-a-879 | Dynactin-associated protein \|\| id:prot-a-879 | Shellfish liking \|\| id:ebi-fl187-GCST90094838 | Weighted median | 34 | -0.044442768 | 0.0872492827305561 | 0.610488075340737 | -0.215451362 | 0.126565826290431 | 0.956530342804183 | 0.806177480154083 | 1.1349241565644 | 0.999489402457283 |
| ebi-fl187-GCST90094838 | prot-a-937 | EMI domain-containing protein 1 \|\| id:prot-a-937 | Shellfish liking \|\| id:ebi-fl187-GCST90094838 | Weighted median | 34 | -0.106229917 | 0.0908808698219443 | 0.242447165809441 | -0.284356422 | 0.0718965873821811 | 0.899217878408326 | 0.752498389681271 | 1.07454421688751 | 0.999489402457283 |
| ebi-fl187-GCST90094838 | prot-b-1 | SRC proto-oncogene, non-receptor tyrosine kinase \|\| id:prot-b-1 | Shellfish liking \|\| id:ebi-fl187-GCST90094838 | Weighted median | 23 | -0.031893886 | 0.126510145374709 | 0.800959625359945 | -0.279853771 | 0.216065998911342 | 0.968609359617257 | 0.755894267069392 | 1.24118429310964 | 0.999489402457283 |
| ebi-fl187-GCST90094838 | prot-b-10 | coagulation factor III, tissue factor \|\| id:prot-b-10 | Shellfish liking \|\| id:ebi-fl187-GCST90094838 | Weighted median | 23 | -0.191631759 | 0.119878335972052 | 0.109920172677646 | -0.426593297 | 0.0433297795277885 | 0.825610836300141 | 0.652728964765383 | 1.04428222096935 | 0.999489402457283 |
| ebi-fl187-GCST90094838 | prot-b-29 | myeloperoxidase \|\| id:prot-b-29 | Shellfish liking \|\| id:ebi-fl187-GCST90094838 | Weighted median | 23 | -0.08719136 | 0.122091145749775 | 0.475134590230931 | -0.326490006 | 0.152107285399685 | 0.91650169683946 | 0.721451585243423 | 1.16428514052845 | 0.999489402457283 |
| ebi-fl187-GCST90094838 | prot-b-33 | adrenomedullin \|\| id:prot-b-33 | Shellfish liking \|\| id:ebi-fl187-GCST90094838 | Weighted median | 23 | 0.0698900088768758 | 0.119531541257465 | 0.558749922712315 | -0.164391812 | 0.304171829741506 | 1.07239022136219 | 0.848409539721024 | 1.35550195162987 | 0.999489402457283 |
| ebi-fl187-GCST90094838 | ukb-rsfMRI191-edge-pheno102 | Net25_Pair11_15 (Postcentral\|Precentral)&(Cerebellum) (Motor)&(Subcortical-cerebellum) \|\| id:ukb-rsfMRI191-edge-pheno102 | Shellfish liking \|\| id:ebi-fl187-GCST90094838 | Weighted median | 30 | -0.03790921 | 0.0289940682176111 | 0.191049211526958 | -0.094737583 | 0.0189191639884385 | 0.962800349838393 | 0.909611600313026 | 1.01909926536769 | 0.999489402457283 |
| ebi-fl187-GCST90094838 | ukb-rsfMRI191-edge-pheno1167 | Net100_Pair11_45 (Angular\|Temporal)&(Frontal) (Default_mode\|Central_executive)&(Salience\|Default_mode) \|\| id:ukb-rsfMRI191-edge-pheno1167 | Shellfish liking \|\| id:ebi-fl187-GCST90094838 | Weighted median | 30 | -0.054539056 | 0.0281352363420106 | 0.0525670150506474 | -0.109684119 | 0.000606007293902151 | 0.946921525228713 | 0.896117156827799 | 1.00060619095342 | 0.999489402457283 |
| ebi-fl187-GCST90094838 | ukb-rsfMRI191-edge-pheno1317 | Net100_Pair26_48 (Parietal\|Frontal)&(Temporal) (Attention\|Central_executive)&(Default_mode\|Central_executive) \|\| id:ukb-rsfMRI191-edge-pheno1317 | Shellfish liking \|\| id:ebi-fl187-GCST90094838 | Weighted median | 30 | 0.0636682153335587 | 0.0285788101045773 | 0.0258930560869594 | 0.0076537475285872 | 0.11968268313853 | 1.06573874432239 | 1.0076831123233 | 1.12713913457494 | 0.999489402457283 |
| ebi-fl187-GCST90094838 | ukb-rsfMRI191-edge-pheno286 | Net100_Pair10_13 (OccipitalPrecuneus)&(Frontal_Sup) (Default_mode\|Central_executive)&(Default_mode) \|\| id:ukb-rsfMRI191-edge-pheno286 | Shellfish liking \|\| id:ebi-fl187-GCST90094838 | Weighted median | 30 | 0.00996317606782974 | 0.0295523721055113 | 0.736014187815828 | -0.047959473 | 0.0678858253946319 | 1.01001297375013 | 0.95317241526639 | 1.07024310691837 | 0.999489402457283 |
| ebi-fl187-GCST90094838 | ukb-rsfMRI191-edge-pheno599 | Net100_Pair11_29 (Angular\|Temporal)&(Parietal) (Default_mode\|Central_executive)&(Central_executive\|Salience) \|\| id:ukb-rsfMRI191-edge-pheno599 | Shellfish liking \|\| id:ebi-fl187-GCST90094838 | Weighted median | 30 | -0.015856469 | 0.0282206334200776 | 0.574201182016886 | -0.07116891 | 0.0394559728551923 | 0.984268583317527 | 0.931304572045119 | 1.04024469887275 | 0.999489402457283 |
| ebi-fl187-GCST90094838 | ukb-rsfMRI191-edge-pheno621 | Net100_Pair5_30 (Precuneus\|Angular\|Cingulate)&(Paracentral) (Default_mode\|Central_executive)&(Motor) \|\| id:ukb-rsfMRI191-edge-pheno621 | Shellfish liking \|\| id:ebi-fl187-GCST90094838 | Weighted median | 30 | -0.01729126 | 0.0284360437743684 | 0.543137430500248 | -0.073025906 | 0.0384433855404694 | 0.982857375649606 | 0.929576748047447 | 1.03919189340515 | 0.999489402457283 |
| ebi-fl187-GCST90094838 | ukb-rsfMRI191-edge-pheno624 | Net100_Pair8_30 (Calcarine\|Lingual)&(Paracentral) (Visual)&(Motor) \|\| id:ukb-rsfMRI191-edge-pheno624 | Shellfish liking \|\| id:ebi-fl187-GCST90094838 | Weighted median | 30 | -0.033191784 | 0.029751876412772 | 0.264584894665462 | -0.091505461 | 0.0251218940764981 | 0.967353019263777 | 0.912556332247064 | 1.02544010798163 | 0.999489402457283 |
| ebi-fl187-GCST90094838 | ukb-rsfMRI191-edge-pheno882 | Net100_Pair6_38 (Postcentral\|Precentral)&(Subcortical) (Motor)&(Subcortical-cerebellum) \|\| id:ukb-rsfMRI191-edge-pheno882 | Shellfish liking \|\| id:ebi-fl187-GCST90094838 | Weighted median | 29 | -0.031475313 | 0.0304440383339113 | 0.301194870652332 | -0.091145628 | 0.0281950023824992 | 0.969014878469107 | 0.912884759740685 | 1.02859624358416 | 0.999489402457283 |
| ebi-fl187-GCST90094838 | ukb-rsfMRI191-edge-pheno899 | Net100_Pair23_38 (Cerebellum)&(Subcortical) (Subcortical-cerebellum)&(Subcortical-cerebellum) \|\| id:ukb-rsfMRI191-edge-pheno899 | Shellfish liking \|\| id:ebi-fl187-GCST90094838 | Weighted median | 30 | 0.0213949879511225 | 0.0275899566078211 | 0.438066175729094 | -0.032681327 | 0.0754713029024518 | 1.02162550171706 | 0.967846937137421 | 1.07839228054554 | 0.999489402457283 |
| ebi-fl187-GCST90094838 | ebi-a-GCST90001392 | IgD+ CD38+ B cell Absolute Count \|\| id:ebi-a-GCST90001392 | Shellfish liking \|\| id:ebi-fl187-GCST90094838 | Weighted mode | 30 | 0.0870660696474273 | 0.209383482379972 | 0.680601979504829 | -0.323325556 | 0.497457695112172 | 1.09096875725833 | 0.723738198829637 | 1.64453504214436 | 0.999228396881742 |
| ebi-fl187-GCST90094838 | ebi-a-GCST90001402 | Switched memory B cell %B cell \|\| id:ebi-a-GCST90001402 | Shellfish liking \|\| id:ebi-fl187-GCST90094838 | Weighted mode | 30 | 0.00795051747454179 | 0.176524043858145 | 0.964384705163131 | -0.338036608 | 0.353937643436506 | 1.00798220676501 | 0.713169179375455 | 1.42466634641255 | 0.999228396881742 |
| ebi-fl187-GCST90094838 | ebi-a-GCST90001407 | Memory B cell Absolute Count \|\| id:ebi-a-GCST90001407 | Shellfish liking \|\| id:ebi-fl187-GCST90094838 | Weighted mode | 30 | -0.159212796 | 0.156323899685192 | 0.316871853571882 | -0.465607639 | 0.147182047794036 | 0.852814864417865 | 0.627753541758244 | 1.15856485800944 | 0.999228396881742 |
| ebi-fl187-GCST90094838 | ebi-a-GCST90001411 | IgD+ CD24+ B cell %B cell \|\| id:ebi-a-GCST90001411 | Shellfish liking \|\| id:ebi-fl187-GCST90094838 | Weighted mode | 30 | -0.20683333 | 0.203023375705858 | 0.316738379596379 | -0.604759147 | 0.191092486038351 | 0.813155166985009 | 0.546205966292029 | 1.2105714078577 | 0.999228396881742 |
| ebi-fl187-GCST90094838 | ebi-a-GCST90001418 | CD24+ CD27+ B cell Absolute Count \|\| id:ebi-a-GCST90001418 | Shellfish liking \|\| id:ebi-fl187-GCST90094838 | Weighted mode | 30 | 0.0236581942626488 | 0.162535104937105 | 0.88527778616914 | -0.294910611 | 0.342226999939374 | 1.02394026941095 | 0.744598143066299 | 1.40807989528926 | 0.999228396881742 |
| ebi-fl187-GCST90094838 | ebi-a-GCST90001436 | Memory B cell %lymphocyte \|\| id:ebi-a-GCST90001436 | Shellfish liking \|\| id:ebi-fl187-GCST90094838 | Weighted mode | 30 | -0.055481175 | 0.194606405950025 | 0.777598131139757 | -0.43690973 | 0.325947381044174 | 0.946029832875907 | 0.646029745702882 | 1.38534247168059 | 0.999228396881742 |
| ebi-fl187-GCST90094838 | ebi-a-GCST90001439 | IgD+ CD24+ B cell %lymphocyte \|\| id:ebi-a-GCST90001439 | Shellfish liking \|\| id:ebi-fl187-GCST90094838 | Weighted mode | 30 | 0.170716913005804 | 0.192513675995224 | 0.382493641209079 | -0.206609892 | 0.548043717956443 | 1.18615491645909 | 0.813336877374495 | 1.72986560056374 | 0.999228396881742 |
| ebi-fl187-GCST90094838 | ebi-a-GCST90001457 | CD11c+ HLA DR++ monocyte %monocyte \|\| id:ebi-a-GCST90001457 | Shellfish liking \|\| id:ebi-fl187-GCST90094838 | Weighted mode | 30 | 0.0476739941120914 | 0.164626229634386 | 0.77419086183018 | -0.274993416 | 0.370341404195487 | 1.0488286752602 | 0.759577124286088 | 1.448228962 | 0.999228396881742 |
| ebi-fl187-GCST90094838 | ebi-a-GCST90001483 | CD39+ resting CD4 regulatory T cell Absolute Count \|\| id:ebi-a-GCST90001483 | Shellfish liking \|\| id:ebi-fl187-GCST90094838 | Weighted mode | 30 | 0.203619214528777 | 0.163537408948083 | 0.223065242149231 | -0.116914107 | 0.52415253606702 | 1.22583128582983 | 0.889661605577584 | 1.6890268523431 | 0.999228396881742 |
| ebi-fl187-GCST90094838 | ebi-a-GCST90001525 | CD33dim HLA DR+ CD11b+ Absolute Count \|\| id:ebi-a-GCST90001525 | Shellfish liking \|\| id:ebi-fl187-GCST90094838 | Weighted mode | 30 | 0.243388779387023 | 0.254547929194806 | 0.346894387510237 | -0.255525162 | 0.742302720608843 | 1.27556444089198 | 0.774509648231523 | 2.10076742953846 | 0.999228396881742 |
| ebi-fl187-GCST90094838 | ebi-a-GCST90001540 | Naive CD4+ T cell Absolute Count \|\| id:ebi-a-GCST90001540 | Shellfish liking \|\| id:ebi-fl187-GCST90094838 | Weighted mode | 30 | -0.118119698 | 0.162310521464834 | 0.47260854204076 | -0.43624832 | 0.200008923970292 | 0.88858968375115 | 0.646457177645125 | 1.22141365797073 | 0.999228396881742 |
| ebi-fl187-GCST90094838 | ebi-a-GCST90001624 | HLA DR+ CD4+ T cell Absolute Count \|\| id:ebi-a-GCST90001624 | Shellfish liking \|\| id:ebi-fl187-GCST90094838 | Weighted mode | 30 | 0.0675610410583583 | 0.190160366705654 | 0.724947572678735 | -0.305153278 | 0.440275359801439 | 1.06989556516235 | 0.73701039848601 | 1.5531348305336 | 0.999228396881742 |
| ebi-fl187-GCST90094838 | ebi-a-GCST90001672 | CD39+ CD8+ T cell Absolute Count \|\| id:ebi-a-GCST90001672 | Shellfish liking \|\| id:ebi-fl187-GCST90094838 | Weighted mode | 30 | 0.315308637838339 | 0.169887644329743 | 0.0736441261672497 | -0.017671145 | 0.648288420724635 | 1.37068229009902 | 0.982484073991504 | 1.91226503322164 | 0.999228396881742 |
| ebi-fl187-GCST90094838 | ebi-a-GCST90001722 | CD19 on CD20- CD38- B cell \|\| id:ebi-a-GCST90001722 | Shellfish liking \|\| id:ebi-fl187-GCST90094838 | Weighted mode | 30 | -0.322414739 | 0.152377250140064 | 0.043059916446771 | -0.621074149 | -0.023755329 | 0.724397691914431 | 0.537366915098134 | 0.976524607874538 | 0.999228396881742 |
| ebi-fl187-GCST90094838 | ebi-a-GCST90001742 | CD19 on transitional B cell \|\| id:ebi-a-GCST90001742 | Shellfish liking \|\| id:ebi-fl187-GCST90094838 | Weighted mode | 30 | -0.10125699 | 0.178977226601971 | 0.57591317921842 | -0.452052354 | 0.249538373837058 | 0.903700760709154 | 0.636320854629295 | 1.28343281375255 | 0.999228396881742 |
| ebi-fl187-GCST90094838 | ebi-a-GCST90001784 | CD25 on IgD+ CD38dim B cell \|\| id:ebi-a-GCST90001784 | Shellfish liking \|\| id:ebi-fl187-GCST90094838 | Weighted mode | 30 | -0.002958463 | 0.198118322326307 | 0.988188048044307 | -0.391270375 | 0.38535344887068 | 0.997045909049982 | 0.676197304712683 | 1.4701338467708 | 0.999228396881742 |
| ebi-fl187-GCST90094838 | ebi-a-GCST90001794 | CD25 on IgD+ B cell \|\| id:ebi-a-GCST90001794 | Shellfish liking \|\| id:ebi-fl187-GCST90094838 | Weighted mode | 30 | -0.139619454 | 0.195088583934034 | 0.479917026992291 | -0.521993079 | 0.242754170420284 | 0.869689129075368 | 0.59333680182951 | 1.27475521305892 | 0.999228396881742 |
| ebi-fl187-GCST90094838 | ebi-a-GCST90001802 | CD27 on IgD- CD38- B cell \|\| id:ebi-a-GCST90001802 | Shellfish liking \|\| id:ebi-fl187-GCST90094838 | Weighted mode | 30 | 0.0328323897227796 | 0.163046799611688 | 0.841816712337239 | -0.286739338 | 0.352404116961689 | 1.03337732006596 | 0.75070738458743 | 1.42248325719293 | 0.999228396881742 |
| ebi-fl187-GCST90094838 | ebi-a-GCST90001804 | CD27 on IgD- CD38dim B cell \|\| id:ebi-a-GCST90001804 | Shellfish liking \|\| id:ebi-fl187-GCST90094838 | Weighted mode | 30 | -0.036273918 | 0.17884069418506 | 0.840685763056402 | -0.386801678 | 0.314253843064829 | 0.964376097774554 | 0.679225786891018 | 1.36923726382034 | 0.999228396881742 |
| ebi-fl187-GCST90094838 | ebi-a-GCST90001805 | CD27 on memory B cell \|\| id:ebi-a-GCST90001805 | Shellfish liking \|\| id:ebi-fl187-GCST90094838 | Weighted mode | 30 | 0.00591579472676007 | 0.1820017183297 | 0.974292763754767 | -0.350807573 | 0.362639162652973 | 1.00593332759695 | 0.70411923223143 | 1.43711720011319 | 0.999228396881742 |
| ebi-fl187-GCST90094838 | ebi-a-GCST90001806 | CD27 on unswitched memory B cell \|\| id:ebi-a-GCST90001806 | Shellfish liking \|\| id:ebi-fl187-GCST90094838 | Weighted mode | 30 | -0.053431889 | 0.171268101897311 | 0.757289289723388 | -0.389117368 | 0.282253591074044 | 0.947970506360947 | 0.677654730119694 | 1.32611496826932 | 0.999228396881742 |
| ebi-fl187-GCST90094838 | ebi-a-GCST90001839 | CD3 on Effector Memory CD8+ T cell \|\| id:ebi-a-GCST90001839 | Shellfish liking \|\| id:ebi-fl187-GCST90094838 | Weighted mode | 30 | 0.113653719531009 | 0.190885520584877 | 0.556192775295139 | -0.260481901 | 0.487789339877367 | 1.12036409749976 | 0.770680104931348 | 1.62871170922241 | 0.999228396881742 |
| ebi-fl187-GCST90094838 | ebi-a-GCST90001859 | CD3 on CD8+ T cell \|\| id:ebi-a-GCST90001859 | Shellfish liking \|\| id:ebi-fl187-GCST90094838 | Weighted mode | 30 | -0.071578405 | 0.185787959883002 | 0.702849337281315 | -0.435722806 | 0.292565996613338 | 0.930923285919111 | 0.646796989250774 | 1.33986115994494 | 0.999228396881742 |
| ebi-fl187-GCST90094838 | ebi-a-GCST90001905 | CD86 on monocyte \|\| id:ebi-a-GCST90001905 | Shellfish liking \|\| id:ebi-fl187-GCST90094838 | Weighted mode | 30 | 0.320018415291836 | 0.174306932355051 | 0.076638353434822 | -0.021623172 | 0.661660002707736 | 1.37715312477914 | 0.978608932704553 | 1.93800676215729 | 0.999228396881742 |
| ebi-fl187-GCST90094838 | ebi-a-GCST90001943 | CD25 on activated & secreting CD4 regulatory T cell \|\| id:ebi-a-GCST90001943 | Shellfish liking \|\| id:ebi-fl187-GCST90094838 | Weighted mode | 30 | 0.072346339882436 | 0.190479469214203 | 0.706852542089222 | -0.30099342 | 0.445686099542273 | 1.07502760452994 | 0.740082642639215 | 1.56156121481255 | 0.999228396881742 |
| ebi-fl187-GCST90094838 | ebi-a-GCST90001949 | CD33 on CD33dim HLA DR+ CD11b- \|\| id:ebi-a-GCST90001949 | Shellfish liking \|\| id:ebi-fl187-GCST90094838 | Weighted mode | 30 | 0.589164740731963 | 0.331411305600822 | 0.085934985751253 | -0.060401418 | 1.23873089970957 | 1.80248224646281 | 0.941386567983583 | 3.45123072636647 | 0.999228396881742 |
| ebi-fl187-GCST90094838 | ebi-a-GCST90001955 | CD33 on Immature Myeloid-Derived Suppressor Cells \|\| id:ebi-a-GCST90001955 | Shellfish liking \|\| id:ebi-fl187-GCST90094838 | Weighted mode | 30 | -0.202043624 | 0.307411279970775 | 0.516208094612102 | -0.804569733 | 0.400482484304062 | 0.817059283412168 | 0.447280335049689 | 1.49254465331198 | 0.999228396881742 |
| ebi-fl187-GCST90094838 | ebi-a-GCST90001956 | CD33 on CD33+ HLA DR+ \|\| id:ebi-a-GCST90001956 | Shellfish liking \|\| id:ebi-fl187-GCST90094838 | Weighted mode | 30 | -0.069605954 | 0.32237678214888 | 0.830565589283811 | -0.701464447 | 0.562252539350803 | 0.932761298674263 | 0.495858613325097 | 1.75462040372802 | 0.999228396881742 |
| ebi-fl187-GCST90094838 | ebi-a-GCST90001957 | CD33 on CD33+ HLA DR+ CD14- \|\| id:ebi-a-GCST90001957 | Shellfish liking \|\| id:ebi-fl187-GCST90094838 | Weighted mode | 30 | -0.077125433 | 0.340859995414823 | 0.822580867881308 | -0.745211024 | 0.590960158181603 | 0.925773723903938 | 0.47463413025229 | 1.80572136145245 | 0.999228396881742 |
| ebi-fl187-GCST90094838 | ebi-a-GCST90002064 | CD4 on resting CD4 regulatory T cell \|\| id:ebi-a-GCST90002064 | Shellfish liking \|\| id:ebi-fl187-GCST90094838 | Weighted mode | 30 | -0.051378231 | 0.198895021254276 | 0.797985774291406 | -0.441212473 | 0.338456010573584 | 0.949919313564285 | 0.643256017681497 | 1.40278004010719 | 0.999228396881742 |
| ebi-fl187-GCST90094838 | ebi-a-GCST90002080 | SSC-A on T cell \|\| id:ebi-a-GCST90002080 | Shellfish liking \|\| id:ebi-fl187-GCST90094838 | Weighted mode | 30 | 0.124851493093429 | 0.157963524940436 | 0.435720880052026 | -0.184757016 | 0.434460001976684 | 1.13298018519016 | 0.831306253606847 | 1.54412900716683 | 0.999228396881742 |
| ebi-fl187-GCST90094838 | ebi-a-GCST90002121 | CD8 on CD39+ CD8+ T cell \|\| id:ebi-a-GCST90002121 | Shellfish liking \|\| id:ebi-fl187-GCST90094838 | Weighted mode | 30 | -0.056171117 | 0.186567045590688 | 0.765503687473843 | -0.421842527 | 0.309500292162387 | 0.945377351727779 | 0.655837308223705 | 1.36274397011732 | 0.999228396881742 |
| ebi-fl187-GCST90094838 | ebi-cfmet338-GCST90026053 | 1-palmitoyl-2-docosahexaenoyl-gpc (16:0/22:6) levels \|\| id:ebi-cfmet338-GCST90026053 | Shellfish liking \|\| id:ebi-fl187-GCST90094838 | Weighted mode | 27 | -0.146447124 | 0.149693702999973 | 0.336935885117513 | -0.439846782 | 0.146952533747672 | 0.863771403789784 | 0.64413510660764 | 1.15829898161325 | 0.999228396881742 |
| ebi-fl187-GCST90094838 | ebi-cfmet338-GCST90026063 | 1-stearoyl-2-oleoyl-gpc (18:0/18:1) levels \|\| id:ebi-cfmet338-GCST90026063 | Shellfish liking \|\| id:ebi-fl187-GCST90094838 | Weighted mode | 26 | 0.0971298535371961 | 0.0835233709901609 | 0.255848099018029 | -0.066575954 | 0.260835660677911 | 1.10200346337818 | 0.93559185158502 | 1.29801433310916 | 0.999228396881742 |
| ebi-fl187-GCST90094838 | ebi-cfmet338-GCST90026113 | Adenosine 3',5'-cyclic monophosphate (camp) levels \|\| id:ebi-cfmet338-GCST90026113 | Shellfish liking \|\| id:ebi-fl187-GCST90094838 | Weighted mode | 27 | 0.0617381447439211 | 0.177281592869321 | 0.730457167611722 | -0.285733777 | 0.409210066767791 | 1.06368377706959 | 0.751462645749034 | 1.50562796966876 | 0.999228396881742 |
| ebi-fl187-GCST90094838 | ebi-cfmet338-GCST90026117 | Alpha-ketoglutaramate levels \|\| id:ebi-cfmet338-GCST90026117 | Shellfish liking \|\| id:ebi-fl187-GCST90094838 | Weighted mode | 27 | 0.0225562138750406 | 0.0548219190840097 | 0.684118125179211 | -0.084894748 | 0.1300071752797 | 1.02281252880412 | 0.918608965176322 | 1.13883655476612 | 0.999228396881742 |
| ebi-fl187-GCST90094838 | ebi-cfmet338-GCST90026124 | Arginine levels \|\| id:ebi-cfmet338-GCST90026124 | Shellfish liking \|\| id:ebi-fl187-GCST90094838 | Weighted mode | 27 | 0.0215726153861341 | 0.0510304097629769 | 0.675960582388071 | -0.078446988 | 0.121592218521569 | 1.02180698655234 | 0.924551071165613 | 1.12929350290066 | 0.999228396881742 |
| ebi-fl187-GCST90094838 | ebi-cfmet338-GCST90026169 | Glutamate levels \|\| id:ebi-cfmet338-GCST90026169 | Shellfish liking \|\| id:ebi-fl187-GCST90094838 | Weighted mode | 27 | 0.0172422748253782 | 0.0567198849075759 | 0.763555154464901 | -0.0939287 | 0.128413249244227 | 1.01739178088474 | 0.91034766808565 | 1.1370227794272 | 0.999228396881742 |
| ebi-fl187-GCST90094838 | ebi-cfmet338-GCST90026170 | Glutamine levels \|\| id:ebi-cfmet338-GCST90026170 | Shellfish liking \|\| id:ebi-fl187-GCST90094838 | Weighted mode | 26 | -0.015101644 | 0.0295215657379919 | 0.61345756688939 | -0.072963913 | 0.0427606247943914 | 0.985011813922452 | 0.929634377230573 | 1.04368803190908 | 0.999228396881742 |
| ebi-fl187-GCST90094838 | ebi-cfmet338-GCST90026191 | Kynurenate levels \|\| id:ebi-cfmet338-GCST90026191 | Shellfish liking \|\| id:ebi-fl187-GCST90094838 | Weighted mode | 27 | -0.196749381 | 0.219935166458573 | 0.379219194347387 | -0.627822308 | 0.23432354489774 | 0.821396464783011 | 0.533752885897676 | 1.2640534040829 | 0.999228396881742 |
| ebi-fl187-GCST90094838 | ebi-cfmet338-GCST90026202 | Methylsuccinoylcarnitine levels \|\| id:ebi-cfmet338-GCST90026202 | Shellfish liking \|\| id:ebi-fl187-GCST90094838 | Weighted mode | 27 | -0.047867925 | 0.138040277584873 | 0.731556727879588 | -0.318426869 | 0.222691019188762 | 0.95325968065263 | 0.7272922636621 | 1.24943446281471 | 0.999228396881742 |
| ebi-fl187-GCST90094838 | ebi-cfmet338-GCST90026222 | N-acetylputrescine levels \|\| id:ebi-cfmet338-GCST90026222 | Shellfish liking \|\| id:ebi-fl187-GCST90094838 | Weighted mode | 27 | -0.058506836 | 0.0618555088352983 | 0.352926657950841 | -0.179743634 | 0.062729961034638 | 0.943171792588763 | 0.835484374079507 | 1.0647392793135 | 0.999228396881742 |
| ebi-fl187-GCST90094838 | ebi-cfmet338-GCST90026252 | Propionylcarnitine (c3) levels \|\| id:ebi-cfmet338-GCST90026252 | Shellfish liking \|\| id:ebi-fl187-GCST90094838 | Weighted mode | 27 | -0.063913581 | 0.0970576950139769 | 0.5159991682774 | -0.254146663 | 0.126319500997386 | 0.938086064271742 | 0.775578044747132 | 1.13464463046754 | 0.999228396881742 |
| ebi-fl187-GCST90094838 | ebi-cfmet338-GCST90026316 | Ethyl beta-glucopyranoside levels \|\| id:ebi-cfmet338-GCST90026316 | Shellfish liking \|\| id:ebi-fl187-GCST90094838 | Weighted mode | 27 | -0.207044112 | 0.284242827498674 | 0.472875338440401 | -0.764160054 | 0.35007182989165 | 0.812983786851098 | 0.465724950588076 | 1.41916948372247 | 0.999228396881742 |
| ebi-fl187-GCST90094838 | ebi-gm430-GCST90011315 | F_Prevotellaceae abundance \|\| id:ebi-gm430-GCST90011315 | Shellfish liking \|\| id:ebi-fl187-GCST90094838 | Weighted mode | 23 | -0.156897759 | 0.157520734525671 | 0.330058205733393 | -0.465638399 | 0.151842880188188 | 0.854791448670602 | 0.627734232242958 | 1.16397733816369 | 0.999228396881742 |
| ebi-fl187-GCST90094838 | ebi-gm430-GCST90011354 | OTU97_1 (Bacteroides) abundance \|\| id:ebi-gm430-GCST90011354 | Shellfish liking \|\| id:ebi-fl187-GCST90094838 | Weighted mode | 23 | 0.0210085444828729 | 0.161994021931073 | 0.897992375836144 | -0.296499739 | 0.338516827467776 | 1.02123077748914 | 0.743415821665807 | 1.40286535542675 | 0.999228396881742 |
| ebi-fl187-GCST90094838 | ebi-gm430-GCST90011419 | OTU99_1 (Bacteroides) abundance \|\| id:ebi-gm430-GCST90011419 | Shellfish liking \|\| id:ebi-fl187-GCST90094838 | Weighted mode | 23 | 0.0441575437629846 | 0.156591968429786 | 0.78058602417811 | -0.262762714 | 0.351077801885365 | 1.04514699830982 | 0.76892433036824 | 1.4205978468036 | 0.999228396881742 |
| ebi-fl187-GCST90094838 | ebi-gm430-GCST90011435 | OTU99_155 (Butyrivibrio) abundance \|\| id:ebi-gm430-GCST90011435 | Shellfish liking \|\| id:ebi-fl187-GCST90094838 | Weighted mode | 23 | -0.151393194 | 0.410677052030597 | 0.715917653720267 | -0.956320216 | 0.653533828242567 | 0.859509678387054 | 0.384304444721893 | 1.92232199597802 | 0.999228396881742 |
| ebi-fl187-GCST90094838 | ebi-gm430-GCST90011466 | OTU99_58 (Bacteroides) abundance \|\| id:ebi-gm430-GCST90011466 | Shellfish liking \|\| id:ebi-fl187-GCST90094838 | Weighted mode | 23 | -0.103473047 | 0.243285118550329 | 0.67473780908746 | -0.580311879 | 0.373365785576387 | 0.901700326139556 | 0.55972377317128 | 1.45261558849561 | 0.999228396881742 |
| ebi-fl187-GCST90094838 | ebi-gm430-GCST90011472 | OTU99_72 (Parabacteroides) abundance \|\| id:ebi-gm430-GCST90011472 | Shellfish liking \|\| id:ebi-fl187-GCST90094838 | Weighted mode | 23 | -0.270093556 | 0.305989765093687 | 0.386948313582994 | -0.869833496 | 0.329646383165825 | 0.763308078626691 | 0.419021312163538 | 1.39047634567422 | 0.999228396881742 |
| ebi-fl187-GCST90094838 | ebi-gm430-GCST90011505 | TestASV_28 (Holdemanella) abundance \|\| id:ebi-gm430-GCST90011505 | Shellfish liking \|\| id:ebi-fl187-GCST90094838 | Weighted mode | 23 | 0.0474950341296705 | 0.256431917250466 | 0.854757335815303 | -0.455111524 | 0.550101591940584 | 1.04864099369316 | 0.634377215920622 | 1.73342911134969 | 0.999228396881742 |
| ebi-fl187-GCST90094838 | ebi-gm430-GCST90011513 | TestASV_35 (Alphaproteobacteria) abundance \|\| id:ebi-gm430-GCST90011513 | Shellfish liking \|\| id:ebi-fl187-GCST90094838 | Weighted mode | 23 | -0.582272578 | 0.477639119237035 | 0.235730186759508 | -1.518445251 | 0.35390009594594 | 0.558627398724071 | 0.219052193415364 | 1.42461285477059 | 0.999228396881742 |
| ebi-fl187-GCST90094838 | ebi-gm430-GCST90011543 | G_Coprococcus prevalence \|\| id:ebi-gm430-GCST90011543 | Shellfish liking \|\| id:ebi-fl187-GCST90094838 | Weighted mode | 23 | 0.325299281543979 | 0.354989960278067 | 0.36941344681381 | -0.370481041 | 1.02107960368899 | 1.38444492279901 | 0.690402139284961 | 2.77619033198395 | 0.999228396881742 |
| ebi-fl187-GCST90094838 | ebi-gm430-GCST90011547 | G_Parasutterella prevalence \|\| id:ebi-gm430-GCST90011547 | Shellfish liking \|\| id:ebi-fl187-GCST90094838 | Weighted mode | 23 | 0.30326013272155 | 0.3353415954462 | 0.375616274885115 | -0.354009394 | 0.960529659796102 | 1.35426670770968 | 0.701868373725159 | 2.61308015045141 | 0.999228396881742 |
| ebi-fl187-GCST90094838 | ebi-gm430-GCST90011569 | OTU97_13 (Subdoligranulum) prevalence \|\| id:ebi-gm430-GCST90011569 | Shellfish liking \|\| id:ebi-fl187-GCST90094838 | Weighted mode | 23 | 0.164100920629233 | 0.325708951117175 | 0.619391146229211 | -0.474288624 | 0.802490464818897 | 1.17833322722302 | 0.622327608234097 | 2.23109046747531 | 0.999228396881742 |
| ebi-fl187-GCST90094838 | ebi-gm430-GCST90011590 | OTU97_277 (Prevotella) prevalence \|\| id:ebi-gm430-GCST90011590 | Shellfish liking \|\| id:ebi-fl187-GCST90094838 | Weighted mode | 23 | 0.221902165789103 | 0.287807232586031 | 0.448903172776891 | -0.34220001 | 0.786004341657724 | 1.24844923084396 | 0.710206142115716 | 2.19460997246757 | 0.999228396881742 |
| ebi-fl187-GCST90094838 | ebi-gm430-GCST90011616 | OTU97_86 (Phascolarctobacterium) prevalence \|\| id:ebi-gm430-GCST90011616 | Shellfish liking \|\| id:ebi-fl187-GCST90094838 | Weighted mode | 23 | 0.109258274098083 | 0.36045653570648 | 0.764653121986086 | -0.597236536 | 0.815753084082784 | 1.11545040508864 | 0.550330354851267 | 2.26087766237912 | 0.999228396881742 |
| ebi-fl187-GCST90094838 | ebi-gm430-GCST90011665 | OTU99_55 (Barnesiella) prevalence \|\| id:ebi-gm430-GCST90011665 | Shellfish liking \|\| id:ebi-fl187-GCST90094838 | Weighted mode | 23 | 0.322689605070171 | 0.228376674985455 | 0.171656650102117 | -0.124928678 | 0.770307888041663 | 1.38083667967609 | 0.882559846360381 | 2.16043132236532 | 0.999228396881742 |
| ebi-fl187-GCST90094838 | ebi-gm430-GCST90011687 | TestASV_14 (Lachnospiraceae) prevalence \|\| id:ebi-gm430-GCST90011687 | Shellfish liking \|\| id:ebi-fl187-GCST90094838 | Weighted mode | 23 | 0.252555755763367 | 0.263292621979332 | 0.347871644005648 | -0.263497783 | 0.768609294842857 | 1.2873112691764 | 0.768359325647069 | 2.15676474330936 | 0.999228396881742 |
| ebi-fl187-GCST90094838 | ebi-gm430-GCST90011697 | TestASV_23 (Barnesiella) prevalence \|\| id:ebi-gm430-GCST90011697 | Shellfish liking \|\| id:ebi-fl187-GCST90094838 | Weighted mode | 23 | 0.423313121752759 | 0.46112084638411 | 0.368569474813815 | -0.480483737 | 1.32710998066561 | 1.52701236190337 | 0.618484135671966 | 3.77013187391188 | 0.999228396881742 |
| ebi-fl187-GCST90094838 | ebi-gm430-GCST90011727 | TestASV_6 (Subdoligranulum) prevalence \|\| id:ebi-gm430-GCST90011727 | Shellfish liking \|\| id:ebi-fl187-GCST90094838 | Weighted mode | 23 | -0.302938771 | 0.446098613149742 | 0.504166461146529 | -1.177292052 | 0.57141451121912 | 0.738644321768242 | 0.30811196099818 | 1.77077005486226 | 0.999228396881742 |
| ebi-fl187-GCST90094838 | ebi-hbsm44-GCST90104908 | Sulcal depth of the left posterior inferior temporal sulcus \|\| id:ebi-hbsm44-GCST90104908 | Shellfish liking \|\| id:ebi-fl187-GCST90094838 | Weighted mode | 26 | 0.147688796521998 | 0.11075171921665 | 0.194387320723345 | -0.069384573 | 0.364762166186631 | 1.15915210805904 | 0.932967816712768 | 1.4401714459475 | 0.999228396881742 |
| ebi-fl187-GCST90094838 | ebi-hbsm44-GCST90104918 | Sulcal depth of the left inferior frontal sulcus \|\| id:ebi-hbsm44-GCST90104918 | Shellfish liking \|\| id:ebi-fl187-GCST90094838 | Weighted mode | 24 | -0.05474199 | 0.175199689997643 | 0.757510179703169 | -0.398133382 | 0.288649402761513 | 0.946729382439206 | 0.671572445992217 | 1.33462373109946 | 0.999228396881742 |
| ebi-fl187-GCST90094838 | ebi-hbsm44-GCST90104925 | Sulcal depth of the right anterior inferior temporal sulcus \|\| id:ebi-hbsm44-GCST90104925 | Shellfish liking \|\| id:ebi-fl187-GCST90094838 | Weighted mode | 26 | -0.007039489 | 0.0681776388431113 | 0.91858665541935 | -0.140667661 | 0.126588683346469 | 0.99298523037772 | 0.868777992605103 | 1.13495009788592 | 0.999228396881742 |
| ebi-fl187-GCST90094838 | ebi-IPs91-GCST90274770 | C-C motif chemokine 4 levels \|\| id:ebi-IPs91-GCST90274770 | Shellfish liking \|\| id:ebi-fl187-GCST90094838 | Weighted mode | 37 | -0.01747315 | 0.0754254451645882 | 0.818112933140777 | -0.165307023 | 0.130360722297286 | 0.982678620010407 | 0.847633421391359 | 1.13923925821668 | 0.999228396881742 |
| ebi-fl187-GCST90094838 | ebi-IPs91-GCST90274779 | C-X-C motif chemokine 1 levels \|\| id:ebi-IPs91-GCST90274779 | Shellfish liking \|\| id:ebi-fl187-GCST90094838 | Weighted mode | 37 | 0.0614719593220124 | 0.0904069323169707 | 0.500887135941434 | -0.115725628 | 0.238669546663275 | 1.06340067763476 | 0.890719578268843 | 1.26955893727167 | 0.999228396881742 |
| ebi-fl187-GCST90094838 | ebi-IPs91-GCST90274780 | C-X-C motif chemokine 10 levels \|\| id:ebi-IPs91-GCST90274780 | Shellfish liking \|\| id:ebi-fl187-GCST90094838 | Weighted mode | 37 | -0.036605275 | 0.0670760898589599 | 0.58861910039474 | -0.168074411 | 0.0948638609124743 | 0.9640565972921 | 0.845290933117893 | 1.09950915876297 | 0.999228396881742 |
| ebi-fl187-GCST90094838 | ebi-IPs91-GCST90274796 | Interleukin-10 receptor subunit alpha levels \|\| id:ebi-IPs91-GCST90274796 | Shellfish liking \|\| id:ebi-fl187-GCST90094838 | Weighted mode | 37 | -0.135420436 | 0.0995261693039495 | 0.182082285635533 | -0.330491727 | 0.059650856313188 | 0.873348647692966 | 0.718570305865794 | 1.06146587773081 | 0.999228396881742 |
| ebi-fl187-GCST90094838 | ebi-IPs91-GCST90274807 | Interleukin-20 levels \|\| id:ebi-IPs91-GCST90274807 | Shellfish liking \|\| id:ebi-fl187-GCST90094838 | Weighted mode | 37 | -0.024648305 | 0.0838341764326998 | 0.770436827537339 | -0.188963291 | 0.139666680637483 | 0.975652983806196 | 0.827816894484989 | 1.14989045421952 | 0.999228396881742 |
| ebi-fl187-GCST90094838 | ebi-IPs91-GCST90274808 | Interleukin-20 receptor subunit alpha levels \|\| id:ebi-IPs91-GCST90274808 | Shellfish liking \|\| id:ebi-fl187-GCST90094838 | Weighted mode | 37 | -0.005129679 | 0.0888937240130847 | 0.954302129230103 | -0.179361378 | 0.169102019789078 | 0.994883455060264 | 0.835803803421955 | 1.18424094877318 | 0.999228396881742 |
| ebi-fl187-GCST90094838 | ebi-IPs91-GCST90274810 | Interleukin-24 levels \|\| id:ebi-IPs91-GCST90274810 | Shellfish liking \|\| id:ebi-fl187-GCST90094838 | Weighted mode | 37 | -0.039496844 | 0.0922441444799642 | 0.671072983698295 | -0.220295367 | 0.141301679279276 | 0.96127298785809 | 0.802281795329838 | 1.15177206134376 | 0.999228396881742 |
| ebi-fl187-GCST90094838 | ebi-IPs91-GCST90274823 | Monocyte chemoattractant protein-3 levels \|\| id:ebi-IPs91-GCST90274823 | Shellfish liking \|\| id:ebi-fl187-GCST90094838 | Weighted mode | 37 | -0.057535218 | 0.0989958490039492 | 0.564734966773508 | -0.251567082 | 0.136496646021782 | 0.944088640862673 | 0.777581294094491 | 1.14625103326832 | 0.999228396881742 |
| ebi-fl187-GCST90094838 | ebi-IPs91-GCST90274824 | Monocyte chemoattractant protein-4 levels \|\| id:ebi-IPs91-GCST90274824 | Shellfish liking \|\| id:ebi-fl187-GCST90094838 | Weighted mode | 37 | 0.0584764768949833 | 0.0889002348539678 | 0.514866113912203 | -0.115767983 | 0.23272093720876 | 1.06022004573379 | 0.89068185228418 | 1.2620292447781 | 0.999228396881742 |
| ebi-fl187-GCST90094838 | ebi-IPs91-GCST90274834 | SIR2-like protein 2 levels \|\| id:ebi-IPs91-GCST90274834 | Shellfish liking \|\| id:ebi-fl187-GCST90094838 | Weighted mode | 37 | 0.0672800651954454 | 0.0851521780505204 | 0.434634193681723 | -0.099618204 | 0.234178334174465 | 1.0695949925615 | 0.905182947495369 | 1.2638698633002 | 0.999228396881742 |
| ebi-fl187-GCST90094838 | ebi-met1400-GCST90199652 | 1-stearoyl-2-oleoyl-GPS (18:0/18:1) levels \|\| id:ebi-met1400-GCST90199652 | Shellfish liking \|\| id:ebi-fl187-GCST90094838 | Weighted mode | 30 | 0.000152409924250252 | 0.12626497349782 | 0.999045167636696 | -0.247326938 | 0.247631757979977 | 1.00015242153923 | 0.780885350595559 | 1.28098813167373 | 0.999700494083162 |
| ebi-fl187-GCST90094838 | ebi-met1400-GCST90199665 | N-acetylaspartate (naa) levels \|\| id:ebi-met1400-GCST90199665 | Shellfish liking \|\| id:ebi-fl187-GCST90094838 | Weighted mode | 30 | 0.0262570495750005 | 0.129888039676091 | 0.841209826819413 | -0.228323508 | 0.280837607340138 | 1.02660480288843 | 0.795866748772745 | 1.32423853985454 | 0.999228396881742 |
| ebi-fl187-GCST90094838 | ebi-met1400-GCST90199669 | Acetylcarnitine levels (Biocrates platform) \|\| id:ebi-met1400-GCST90199669 | Shellfish liking \|\| id:ebi-fl187-GCST90094838 | Weighted mode | 30 | 0.110125855213634 | 0.104774817047299 | 0.301905745011384 | -0.095232786 | 0.315484496626341 | 1.11641856871494 | 0.909161269636558 | 1.37092335782161 | 0.999228396881742 |
| ebi-fl187-GCST90094838 | ebi-met1400-GCST90199670 | Iminodiacetate (IDA) levels \|\| id:ebi-met1400-GCST90199670 | Shellfish liking \|\| id:ebi-fl187-GCST90094838 | Weighted mode | 30 | -0.06440996 | 0.108976904150238 | 0.559071026752424 | -0.278004692 | 0.149184772131226 | 0.937620533811252 | 0.757293268181702 | 1.1608874690453 | 0.999228396881742 |
| ebi-fl187-GCST90094838 | ebi-met1400-GCST90199686 | Indolepropionate levels \|\| id:ebi-met1400-GCST90199686 | Shellfish liking \|\| id:ebi-fl187-GCST90094838 | Weighted mode | 30 | -0.156922541 | 0.135422570596265 | 0.256006450645775 | -0.42235078 | 0.108505697033867 | 0.854770265617324 | 0.655504061539673 | 1.11461125849834 | 0.999228396881742 |
| ebi-fl187-GCST90094838 | ebi-met1400-GCST90199704 | 3-hydroxysebacate levels \|\| id:ebi-met1400-GCST90199704 | Shellfish liking \|\| id:ebi-fl187-GCST90094838 | Weighted mode | 30 | 0.0902524624828711 | 0.112614340594027 | 0.429401062541524 | -0.130471645 | 0.310976570047164 | 1.09445055653436 | 0.877681379179958 | 1.36475724461368 | 0.999228396881742 |
| ebi-fl187-GCST90094838 | ebi-met1400-GCST90199713 | Docosapentaenoate n3 DPA; 22:5n3 levels \|\| id:ebi-met1400-GCST90199713 | Shellfish liking \|\| id:ebi-fl187-GCST90094838 | Weighted mode | 30 | 0.0157663145214098 | 0.102852983433191 | 0.87923141517959 | -0.185825533 | 0.217358162050464 | 1.01589125863061 | 0.830418462955854 | 1.24278914234227 | 0.999228396881742 |
| ebi-fl187-GCST90094838 | ebi-met1400-GCST90199715 | Myristoleate (14:1n5) levels \|\| id:ebi-met1400-GCST90199715 | Shellfish liking \|\| id:ebi-fl187-GCST90094838 | Weighted mode | 30 | 0.0645957571263538 | 0.106099278481497 | 0.547381235454453 | -0.143358829 | 0.272550342950087 | 1.06672772013525 | 0.8664431084561 | 1.31330957312657 | 0.999228396881742 |
| ebi-fl187-GCST90094838 | ebi-met1400-GCST90199725 | Campesterol levels \|\| id:ebi-met1400-GCST90199725 | Shellfish liking \|\| id:ebi-fl187-GCST90094838 | Weighted mode | 30 | 0.315461564609047 | 0.19252746099234 | 0.112117643128794 | -0.061892259 | 0.692815388154033 | 1.37089192014393 | 0.939984156228745 | 1.9993365252622 | 0.999228396881742 |
| ebi-fl187-GCST90094838 | ebi-met1400-GCST90199737 | N-acetylproline levels \|\| id:ebi-met1400-GCST90199737 | Shellfish liking \|\| id:ebi-fl187-GCST90094838 | Weighted mode | 30 | 0.0433377541555041 | 0.104097335120686 | 0.680241371984601 | -0.160693023 | 0.247368530992048 | 1.0442905487645 | 0.851553438579682 | 1.2806509854012 | 0.999228396881742 |
| ebi-fl187-GCST90094838 | ebi-met1400-GCST90199775 | Gamma-glutamylisoleucine levels \|\| id:ebi-met1400-GCST90199775 | Shellfish liking \|\| id:ebi-fl187-GCST90094838 | Weighted mode | 30 | -0.001130004 | 0.103006040774211 | 0.991322285977378 | -0.203021844 | 0.200761835463877 | 0.998870633761038 | 0.816260410535593 | 1.22233362063401 | 0.999228396881742 |
| ebi-fl187-GCST90094838 | ebi-met1400-GCST90199777 | 1-palmitoleoyl-GPC (16:1) levels \|\| id:ebi-met1400-GCST90199777 | Shellfish liking \|\| id:ebi-fl187-GCST90094838 | Weighted mode | 30 | -0.067432402 | 0.121157229056005 | 0.582091589045496 | -0.304900571 | 0.170035766665691 | 0.934790908210595 | 0.737196669302997 | 1.18534724648089 | 0.999228396881742 |
| ebi-fl187-GCST90094838 | ebi-met1400-GCST90199782 | Glutamine degradant levels \|\| id:ebi-met1400-GCST90199782 | Shellfish liking \|\| id:ebi-fl187-GCST90094838 | Weighted mode | 30 | -0.170916621 | 0.0962158257980207 | 0.0861627189963453 | -0.35949964 | 0.0176663973407882 | 0.842891849833103 | 0.698025502895643 | 1.01782337116311 | 0.999228396881742 |
| ebi-fl187-GCST90094838 | ebi-met1400-GCST90199783 | 5-methyluridine (ribothymidine) levels \|\| id:ebi-met1400-GCST90199783 | Shellfish liking \|\| id:ebi-fl187-GCST90094838 | Weighted mode | 30 | 0.0517824323754172 | 0.109413502105276 | 0.639561795087492 | -0.162668032 | 0.266232896501759 | 1.05314658698459 | 0.849873272532733 | 1.30503896230549 | 0.999228396881742 |
| ebi-fl187-GCST90094838 | ebi-met1400-GCST90199787 | Homostachydrine levels \|\| id:ebi-met1400-GCST90199787 | Shellfish liking \|\| id:ebi-fl187-GCST90094838 | Weighted mode | 30 | -0.079370546 | 0.120822285748159 | 0.516412361948817 | -0.316182226 | 0.157441133787676 | 0.923697588319305 | 0.728926608432654 | 1.17051185235438 | 0.999228396881742 |
| ebi-fl187-GCST90094838 | ebi-met1400-GCST90199802 | Beta-hydroxyisovaleroylcarnitine levels \|\| id:ebi-met1400-GCST90199802 | Shellfish liking \|\| id:ebi-fl187-GCST90094838 | Weighted mode | 30 | 0.104665485378193 | 0.120579067427392 | 0.392508014758564 | -0.131669487 | 0.341000457535881 | 1.11033912355329 | 0.876630685234493 | 1.40635388431939 | 0.999228396881742 |
| ebi-fl187-GCST90094838 | ebi-met1400-GCST90199873 | Pregnenolone sulfate levels \|\| id:ebi-met1400-GCST90199873 | Shellfish liking \|\| id:ebi-fl187-GCST90094838 | Weighted mode | 30 | 0.0396868561400005 | 0.119077769956803 | 0.741316760845775 | -0.193705573 | 0.273079285255335 | 1.04048490171417 | 0.823900447118506 | 1.31400442187093 | 0.999228396881742 |
| ebi-fl187-GCST90094838 | ebi-met1400-GCST90199911 | Beta-citrylglutamate levels \|\| id:ebi-met1400-GCST90199911 | Shellfish liking \|\| id:ebi-fl187-GCST90094838 | Weighted mode | 30 | 0.145810502632151 | 0.098695877856066 | 0.150354744306872 | -0.047633418 | 0.33925442323004 | 1.15697692319479 | 0.953483252850118 | 1.40390048467448 | 0.999228396881742 |
| ebi-fl187-GCST90094838 | ebi-met1400-GCST90199934 | 2-hydroxydecanoate levels \|\| id:ebi-met1400-GCST90199934 | Shellfish liking \|\| id:ebi-fl187-GCST90094838 | Weighted mode | 29 | -0.098874443 | 0.126149627216351 | 0.439745364029257 | -0.346127713 | 0.148378826122827 | 0.905856437299961 | 0.707422134641508 | 1.15995223334878 | 0.999228396881742 |
| ebi-fl187-GCST90094838 | ebi-met1400-GCST90199945 | Sphingomyelin (d18:1/14:0, d16:1/16:0) levels \|\| id:ebi-met1400-GCST90199945 | Shellfish liking \|\| id:ebi-fl187-GCST90094838 | Weighted mode | 30 | 0.107470078448056 | 0.108705346484947 | 0.331018488827697 | -0.105592401 | 0.320532557558551 | 1.11345754387036 | 0.899791327690773 | 1.37786135945942 | 0.999228396881742 |
| ebi-fl187-GCST90094838 | ebi-met1400-GCST90199979 | Carnitine C14:1 levels \|\| id:ebi-met1400-GCST90199979 | Shellfish liking \|\| id:ebi-fl187-GCST90094838 | Weighted mode | 30 | 0.00231710143521824 | 0.108651795413581 | 0.983131713436444 | -0.210640418 | 0.215274620445837 | 1.00231978798935 | 0.810065299761598 | 1.24020243515022 | 0.999228396881742 |
| ebi-fl187-GCST90094838 | ebi-met1400-GCST90199986 | Eugenol sulfate levels \|\| id:ebi-met1400-GCST90199986 | Shellfish liking \|\| id:ebi-fl187-GCST90094838 | Weighted mode | 30 | 0.0078133241915721 | 0.146798634889672 | 0.957917780391212 | -0.279912 | 0.295538648575329 | 1.00784392786258 | 0.755850253206191 | 1.34385002666974 | 0.999228396881742 |
| ebi-fl187-GCST90094838 | ebi-met1400-GCST90200002 | 6-hydroxyindole sulfate levels \|\| id:ebi-met1400-GCST90200002 | Shellfish liking \|\| id:ebi-fl187-GCST90094838 | Weighted mode | 30 | 0.0265228174824937 | 0.119465157744462 | 0.825859843321348 | -0.207628892 | 0.26067452666164 | 1.0268776777578 | 0.812508509423353 | 1.29780519569652 | 0.999228396881742 |
| ebi-fl187-GCST90094838 | ebi-met1400-GCST90200023 | Trans 3,4-methyleneheptanoate levels \|\| id:ebi-met1400-GCST90200023 | Shellfish liking \|\| id:ebi-fl187-GCST90094838 | Weighted mode | 30 | -0.08946277 | 0.110845866565432 | 0.426183955364975 | -0.306720669 | 0.127795128205768 | 0.914422308193111 | 0.735856119825942 | 1.13632017889449 | 0.999228396881742 |
| ebi-fl187-GCST90094838 | ebi-met1400-GCST90200032 | 2-hydroxybutyrate/2-hydroxyisobutyrate levels \|\| id:ebi-met1400-GCST90200032 | Shellfish liking \|\| id:ebi-fl187-GCST90094838 | Weighted mode | 30 | 0.168835565637288 | 0.120473375013394 | 0.171696080583092 | -0.067292249 | 0.404963380663541 | 1.18392544489118 | 0.934921931044106 | 1.49924759759932 | 0.999228396881742 |
| ebi-fl187-GCST90094838 | ebi-met1400-GCST90200051 | 1-palmitoyl-2-dihomo-linolenoyl-GPC (16:0/20:3n3 or 6) levels \|\| id:ebi-met1400-GCST90200051 | Shellfish liking \|\| id:ebi-fl187-GCST90094838 | Weighted mode | 30 | -0.009776009 | 0.107022520233945 | 0.927846245200336 | -0.219540149 | 0.199988130601715 | 0.990271620783443 | 0.802887922127283 | 1.2213882609304 | 0.999228396881742 |
| ebi-fl187-GCST90094838 | ebi-met1400-GCST90200069 | Phenylacetylglutamate levels \|\| id:ebi-met1400-GCST90200069 | Shellfish liking \|\| id:ebi-fl187-GCST90094838 | Weighted mode | 30 | -0.007708683 | 0.104049972384834 | 0.941450351489612 | -0.211646629 | 0.196229262823568 | 0.992320952646855 | 0.809250612805444 | 1.21680584170123 | 0.999228396881742 |
| ebi-fl187-GCST90094838 | ebi-met1400-GCST90200072 | Thioproline levels \|\| id:ebi-met1400-GCST90200072 | Shellfish liking \|\| id:ebi-fl187-GCST90094838 | Weighted mode | 30 | 0.0886153065925044 | 0.102127704291804 | 0.392687064870391 | -0.111554994 | 0.28878560700444 | 1.09266023627558 | 0.894442201257795 | 1.33480552489461 | 0.999228396881742 |
| ebi-fl187-GCST90094838 | ebi-met1400-GCST90200081 | 1-oleoyl-2-docosahexaenoyl-GPC (18:1/22:6) levels \|\| id:ebi-met1400-GCST90200081 | Shellfish liking \|\| id:ebi-fl187-GCST90094838 | Weighted mode | 30 | -0.060512998 | 0.0988463762100944 | 0.545181041974145 | -0.254251895 | 0.133225899746581 | 0.941281534514299 | 0.775496433769514 | 1.14250806146315 | 0.999228396881742 |
| ebi-fl187-GCST90094838 | ebi-met1400-GCST90200097 | Furaneol sulfate levels \|\| id:ebi-met1400-GCST90200097 | Shellfish liking \|\| id:ebi-fl187-GCST90094838 | Weighted mode | 30 | -0.21266662 | 0.143697011594086 | 0.149665134812987 | -0.494312763 | 0.0689795222931053 | 0.808425604875963 | 0.609989970864478 | 1.07141426881634 | 0.999228396881742 |
| ebi-fl187-GCST90094838 | ebi-met1400-GCST90200124 | Sphingomyelin (d18:2/24:2) levels \|\| id:ebi-met1400-GCST90200124 | Shellfish liking \|\| id:ebi-fl187-GCST90094838 | Weighted mode | 30 | -0.078100297 | 0.103333706919622 | 0.455860895446595 | -0.280634363 | 0.124433768219131 | 0.924871659721094 | 0.755304452323044 | 1.13250701002012 | 0.999228396881742 |
| ebi-fl187-GCST90094838 | ebi-met1400-GCST90200137 | Lignoceroylcarnitine (C24) levels \|\| id:ebi-met1400-GCST90200137 | Shellfish liking \|\| id:ebi-fl187-GCST90094838 | Weighted mode | 30 | -0.01261591 | 0.104783997102942 | 0.904997218094429 | -0.217992544 | 0.192760724187392 | 0.987463336852198 | 0.804131436881408 | 1.21259261472087 | 0.999228396881742 |
| ebi-fl187-GCST90094838 | ebi-met1400-GCST90200165 | Octadecenedioate (C18:1-DC) levels \|\| id:ebi-met1400-GCST90200165 | Shellfish liking \|\| id:ebi-fl187-GCST90094838 | Weighted mode | 30 | -0.132605739 | 0.100454058143397 | 0.197139238974518 | -0.329495693 | 0.0642842146300688 | 0.875810321612418 | 0.719286382928804 | 1.0663954408807 | 0.999228396881742 |
| ebi-fl187-GCST90094838 | ebi-met1400-GCST90200176 | Glutamine conjugate of C6H10O2 (1) levels \|\| id:ebi-met1400-GCST90200176 | Shellfish liking \|\| id:ebi-fl187-GCST90094838 | Weighted mode | 30 | 0.015889638490471 | 0.0960948427110452 | 0.869812903652064 | -0.172456253 | 0.20423553020412 | 1.01601655009832 | 0.841595105079708 | 1.2265870177274 | 0.999228396881742 |
| ebi-fl187-GCST90094838 | ebi-met1400-GCST90200182 | Glycine conjugate of C10H12O2 levels \|\| id:ebi-met1400-GCST90200182 | Shellfish liking \|\| id:ebi-fl187-GCST90094838 | Weighted mode | 30 | 0.0343893234779118 | 0.145692341926528 | 0.815060233433047 | -0.251167667 | 0.319946313653906 | 1.03498747322301 | 0.777891934052202 | 1.37705383336275 | 0.999228396881742 |
| ebi-fl187-GCST90094838 | ebi-met1400-GCST90200208 | Glucuronide of piperine metabolite C17H21NO3 (4) levels \|\| id:ebi-met1400-GCST90200208 | Shellfish liking \|\| id:ebi-fl187-GCST90094838 | Weighted mode | 30 | -0.092469093 | 0.117758968535315 | 0.438681168239918 | -0.323276671 | 0.138338485698441 | 0.911677388078666 | 0.723773579533011 | 1.14836418935078 | 0.999228396881742 |
| ebi-fl187-GCST90094838 | ebi-met1400-GCST90200227 | 3-ethylcatechol sulfate (2) levels \|\| id:ebi-met1400-GCST90200227 | Shellfish liking \|\| id:ebi-fl187-GCST90094838 | Weighted mode | 29 | -0.227013476 | 0.151665399448047 | 0.145632574817482 | -0.524277659 | 0.0702507071497891 | 0.796910043242617 | 0.591982823612837 | 1.07277710043204 | 0.999228396881742 |
| ebi-fl187-GCST90094838 | ebi-met1400-GCST90200259 | Pentose acid levels \|\| id:ebi-met1400-GCST90200259 | Shellfish liking \|\| id:ebi-fl187-GCST90094838 | Weighted mode | 30 | 0.00225853906955331 | 0.124299267562953 | 0.985627536920035 | -0.241368025 | 0.245885103492942 | 1.00226109149014 | 0.785552469957448 | 1.27875264088905 | 0.999228396881742 |
| ebi-fl187-GCST90094838 | ebi-met1400-GCST90200291 | Sarcosine levels \|\| id:ebi-met1400-GCST90200291 | Shellfish liking \|\| id:ebi-fl187-GCST90094838 | Weighted mode | 30 | 0.133877671411628 | 0.11540134265256 | 0.255468118927134 | -0.09230896 | 0.360064303010645 | 1.14325295856974 | 0.91182338889582 | 1.43342158492033 | 0.999228396881742 |
| ebi-fl187-GCST90094838 | ebi-met1400-GCST90200306 | Butyrate/isobutyrate (4:0) levels \|\| id:ebi-met1400-GCST90200306 | Shellfish liking \|\| id:ebi-fl187-GCST90094838 | Weighted mode | 30 | -0.119882368 | 0.116710538443489 | 0.31282904848137 | -0.348635023 | 0.108870287293576 | 0.887024773029123 | 0.705650629240538 | 1.115017709 | 0.999228396881742 |
| ebi-fl187-GCST90094838 | ebi-met1400-GCST90200321 | Cholate levels \|\| id:ebi-met1400-GCST90200321 | Shellfish liking \|\| id:ebi-fl187-GCST90094838 | Weighted mode | 30 | -0.085173247 | 0.117063211643453 | 0.472702389954764 | -0.314617142 | 0.144270647854597 | 0.918353168717979 | 0.730068333425991 | 1.15519671773279 | 0.999228396881742 |
| ebi-fl187-GCST90094838 | ebi-met1400-GCST90200336 | Ceramide (d18:1/16:0) levels \|\| id:ebi-met1400-GCST90200336 | Shellfish liking \|\| id:ebi-fl187-GCST90094838 | Weighted mode | 30 | 0.0464013452615378 | 0.103986065663886 | 0.65874777662952 | -0.157411343 | 0.250214033962755 | 1.04749473365187 | 0.854352554209737 | 1.284300271 | 0.999228396881742 |
| ebi-fl187-GCST90094838 | ebi-met1400-GCST90200358 | Arachidonate (20:4n6) levels \|\| id:ebi-met1400-GCST90200358 | Shellfish liking \|\| id:ebi-fl187-GCST90094838 | Weighted mode | 30 | -0.03612092 | 0.104557917470853 | 0.732239550401981 | -0.241054438 | 0.16881259854211 | 0.964523656519488 | 0.78579884795058 | 1.18389825387506 | 0.999228396881742 |
| ebi-fl187-GCST90094838 | ebi-met1400-GCST90200413 | 2'-deoxyuridine levels \|\| id:ebi-met1400-GCST90200413 | Shellfish liking \|\| id:ebi-fl187-GCST90094838 | Weighted mode | 30 | 0.04542084992868 | 0.104260693293199 | 0.666318502353128 | -0.158930109 | 0.249771808783351 | 1.04646817330539 | 0.853055977884177 | 1.28373244679355 | 0.999228396881742 |
| ebi-fl187-GCST90094838 | ebi-met1400-GCST90200432 | Threonine levels \|\| id:ebi-met1400-GCST90200432 | Shellfish liking \|\| id:ebi-fl187-GCST90094838 | Weighted mode | 30 | -0.121707745 | 0.110426074533606 | 0.279453339672207 | -0.338142851 | 0.0947273609473836 | 0.885407095221714 | 0.713093414354757 | 1.09935908604388 | 0.999228396881742 |
| ebi-fl187-GCST90094838 | ebi-met1400-GCST90200436 | Caffeine levels \|\| id:ebi-met1400-GCST90200436 | Shellfish liking \|\| id:ebi-fl187-GCST90094838 | Weighted mode | 30 | 0.0565489265959362 | 0.121356098987218 | 0.644714352582675 | -0.181309027 | 0.294406880610884 | 1.05817838660006 | 0.834177535129887 | 1.34232996060383 | 0.999228396881742 |
| ebi-fl187-GCST90094838 | ebi-met1400-GCST90200442 | Valine levels \|\| id:ebi-met1400-GCST90200442 | Shellfish liking \|\| id:ebi-fl187-GCST90094838 | Weighted mode | 30 | 0.0883407758858702 | 0.113753871196016 | 0.443685777346217 | -0.134616812 | 0.311298363430062 | 1.09236030866035 | 0.874050773592824 | 1.36519648513282 | 0.999228396881742 |
| ebi-fl187-GCST90094838 | ebi-met1400-GCST90200459 | Margarate (17:0) levels \|\| id:ebi-met1400-GCST90200459 | Shellfish liking \|\| id:ebi-fl187-GCST90094838 | Weighted mode | 30 | 0.003676160675955 | 0.101758265243343 | 0.971429097443577 | -0.195770039 | 0.203122360552906 | 1.00368292604226 | 0.822201287004943 | 1.2252223779634 | 0.999228396881742 |
| ebi-fl187-GCST90094838 | ebi-met1400-GCST90200462 | X-11299 levels \|\| id:ebi-met1400-GCST90200462 | Shellfish liking \|\| id:ebi-fl187-GCST90094838 | Weighted mode | 30 | 0.0832082688494329 | 0.103130087829042 | 0.426333268193773 | -0.118926703 | 0.285343240994355 | 1.08676812492707 | 0.887872876532875 | 1.33021853530377 | 0.999228396881742 |
| ebi-fl187-GCST90094838 | ebi-met1400-GCST90200497 | X-12740 levels \|\| id:ebi-met1400-GCST90200497 | Shellfish liking \|\| id:ebi-fl187-GCST90094838 | Weighted mode | 30 | 0.0881092345455521 | 0.126438135047306 | 0.491441330139554 | -0.15970951 | 0.335927979238273 | 1.0921074113696 | 0.852391364047343 | 1.39923824697754 | 0.999228396881742 |
| ebi-fl187-GCST90094838 | ebi-met1400-GCST90200527 | X-13866 levels \|\| id:ebi-met1400-GCST90200527 | Shellfish liking \|\| id:ebi-fl187-GCST90094838 | Weighted mode | 30 | -0.052598877 | 0.128804326719808 | 0.686007133414372 | -0.305055357 | 0.199857603592345 | 0.94876050603543 | 0.737082570472343 | 1.22122884717755 | 0.999228396881742 |
| ebi-fl187-GCST90094838 | ebi-met1400-GCST90200547 | X-17654 levels \|\| id:ebi-met1400-GCST90200547 | Shellfish liking \|\| id:ebi-fl187-GCST90094838 | Weighted mode | 30 | -0.002998373 | 0.121075820917016 | 0.980412493210152 | -0.240306982 | 0.234310236284793 | 0.997006117917588 | 0.786386417761937 | 1.26403658139734 | 0.999228396881742 |
| ebi-fl187-GCST90094838 | ebi-met1400-GCST90200553 | X-17653 levels \|\| id:ebi-met1400-GCST90200553 | Shellfish liking \|\| id:ebi-fl187-GCST90094838 | Weighted mode | 30 | -0.045000711 | 0.119803337345321 | 0.70993192973616 | -0.279815252 | 0.189813829901711 | 0.955996801836999 | 0.75592338351745 | 1.20902449249538 | 0.999228396881742 |
| ebi-fl187-GCST90094838 | ebi-met1400-GCST90200557 | X-17685 levels \|\| id:ebi-met1400-GCST90200557 | Shellfish liking \|\| id:ebi-fl187-GCST90094838 | Weighted mode | 30 | -0.193170408 | 0.153296215720475 | 0.217675069672471 | -0.49363099 | 0.107290175232222 | 0.824341488131497 | 0.610405987210919 | 1.11325724729506 | 0.999228396881742 |
| ebi-fl187-GCST90094838 | ebi-met1400-GCST90200561 | X-18779 levels \|\| id:ebi-met1400-GCST90200561 | Shellfish liking \|\| id:ebi-fl187-GCST90094838 | Weighted mode | 30 | -0.060833395 | 0.130265310634552 | 0.643992394624681 | -0.316153404 | 0.194486613369442 | 0.940979998243632 | 0.728947617877886 | 1.21468722220712 | 0.999228396881742 |
| ebi-fl187-GCST90094838 | ebi-met1400-GCST90200570 | X-18921 levels \|\| id:ebi-met1400-GCST90200570 | Shellfish liking \|\| id:ebi-fl187-GCST90094838 | Weighted mode | 30 | -0.144244487 | 0.110909778415502 | 0.203650862223111 | -0.361627652 | 0.0731386791770857 | 0.865676076055841 | 0.696541675314768 | 1.07587972868499 | 0.999228396881742 |
| ebi-fl187-GCST90094838 | ebi-met1400-GCST90200575 | X-21283 levels \|\| id:ebi-met1400-GCST90200575 | Shellfish liking \|\| id:ebi-fl187-GCST90094838 | Weighted mode | 30 | 0.144054574004171 | 0.143045548436561 | 0.322238177399971 | -0.136314701 | 0.424423848939831 | 1.15494713689491 | 0.872567991316415 | 1.52870939834652 | 0.999228396881742 |
| ebi-fl187-GCST90094838 | ebi-met1400-GCST90200578 | X-18922 levels \|\| id:ebi-met1400-GCST90200578 | Shellfish liking \|\| id:ebi-fl187-GCST90094838 | Weighted mode | 30 | 0.110160060681437 | 0.108610469 | 0.31884314375034 | -0.102716459 | 0.323036580327157 | 1.11645675698746 | 0.902382799764836 | 1.38131587896822 | 0.999228396881742 |
| ebi-fl187-GCST90094838 | ebi-met1400-GCST90200623 | X-24241 levels \|\| id:ebi-met1400-GCST90200623 | Shellfish liking \|\| id:ebi-fl187-GCST90094838 | Weighted mode | 30 | -0.013708185 | 0.104985771044906 | 0.897015583278161 | -0.219480296 | 0.192063926519707 | 0.986385344575877 | 0.802935978606696 | 1.21174797731994 | 0.999228396881742 |
| ebi-fl187-GCST90094838 | ebi-met1400-GCST90200636 | X-24544 levels \|\| id:ebi-met1400-GCST90200636 | Shellfish liking \|\| id:ebi-fl187-GCST90094838 | Weighted mode | 30 | 0.100736057250076 | 0.112934562941041 | 0.379742210256476 | -0.120615686 | 0.322087800614516 | 1.10598468659584 | 0.886374540187425 | 1.38000593600742 | 0.999228396881742 |
| ebi-fl187-GCST90094838 | ebi-met1400-GCST90200659 | X-25172 levels \|\| id:ebi-met1400-GCST90200659 | Shellfish liking \|\| id:ebi-fl187-GCST90094838 | Weighted mode | 30 | 0.0454457067608634 | 0.10876599331812 | 0.679148976824322 | -0.16773564 | 0.258627053664378 | 1.04649418551245 | 0.845577341845664 | 1.29515069304122 | 0.999228396881742 |
| ebi-fl187-GCST90094838 | ebi-met1400-GCST90200670 | X-26111 levels \|\| id:ebi-met1400-GCST90200670 | Shellfish liking \|\| id:ebi-fl187-GCST90094838 | Weighted mode | 30 | 0.0611462258576547 | 0.11372882757933 | 0.594921385258553 | -0.161762276 | 0.284054727913141 | 1.06305434885653 | 0.850643398689126 | 1.3285056351043 | 0.999228396881742 |
| ebi-fl187-GCST90094838 | ebi-met1400-GCST90200673 | Carnitine C4 levels \|\| id:ebi-met1400-GCST90200673 | Shellfish liking \|\| id:ebi-fl187-GCST90094838 | Weighted mode | 30 | 0.19186692442303 | 0.110309512813864 | 0.0925790368638141 | -0.024339721 | 0.408073569538203 | 1.21150928394008 | 0.975954101631121 | 1.50391779963827 | 0.999228396881742 |
| ebi-fl187-GCST90094838 | ebi-met1400-GCST90200710 | X-13431 levels \|\| id:ebi-met1400-GCST90200710 | Shellfish liking \|\| id:ebi-fl187-GCST90094838 | Weighted mode | 30 | 0.0995628542946444 | 0.107124122484481 | 0.360345933132555 | -0.110400426 | 0.309526134364228 | 1.10468790293682 | 0.895475492028482 | 1.36277918687709 | 0.999228396881742 |
| ebi-fl187-GCST90094838 | ebi-met1400-GCST90200730 | Adenosine 5'-diphosphate (ADP) to fructose ratio \|\| id:ebi-met1400-GCST90200730 | Shellfish liking \|\| id:ebi-fl187-GCST90094838 | Weighted mode | 30 | 0.208728378118286 | 0.144255308819132 | 0.158638241643386 | -0.074012027 | 0.491468783403784 | 1.23211028496047 | 0.928660524618707 | 1.63471550050937 | 0.999228396881742 |
| ebi-fl187-GCST90094838 | ebi-met1400-GCST90200731 | Adenosine 5'-diphosphate (ADP) to mannose ratio \|\| id:ebi-met1400-GCST90200731 | Shellfish liking \|\| id:ebi-fl187-GCST90094838 | Weighted mode | 30 | 0.0277933635261152 | 0.16379646907164 | 0.86643813719276 | -0.293247716 | 0.348834442906529 | 1.02818320231747 | 0.745837362072555 | 1.41741450788968 | 0.999228396881742 |
| ebi-fl187-GCST90094838 | ebi-met1400-GCST90200748 | Aspartate to glutamate ratio \|\| id:ebi-met1400-GCST90200748 | Shellfish liking \|\| id:ebi-fl187-GCST90094838 | Weighted mode | 30 | -0.171981649 | 0.117347366400073 | 0.153522759525069 | -0.401982487 | 0.0580191890976002 | 0.841994624430891 | 0.66899246152517 | 1.05973533087987 | 0.999228396881742 |
| ebi-fl187-GCST90094838 | ebi-met1400-GCST90200774 | Uridine to pseudouridine ratio \|\| id:ebi-met1400-GCST90200774 | Shellfish liking \|\| id:ebi-fl187-GCST90094838 | Weighted mode | 30 | 0.0696508971056482 | 0.118268994837135 | 0.560475184355817 | -0.162156333 | 0.301458126986432 | 1.07213383089112 | 0.850308263098461 | 1.35182850881948 | 0.999228396881742 |
| ebi-fl187-GCST90094838 | ebi-met1400-GCST90200799 | Spermidine to adenosine 5'-diphosphate (ADP) ratio \|\| id:ebi-met1400-GCST90200799 | Shellfish liking \|\| id:ebi-fl187-GCST90094838 | Weighted mode | 30 | -0.163165874 | 0.150342572309531 | 0.286731406088896 | -0.457837316 | 0.131505567717234 | 0.849450275005037 | 0.632650390098903 | 1.14054425793261 | 0.999228396881742 |
| ebi-fl187-GCST90094838 | ebi-met1400-GCST90200811 | Choline to taurocholate ratio \|\| id:ebi-met1400-GCST90200811 | Shellfish liking \|\| id:ebi-fl187-GCST90094838 | Weighted mode | 30 | -0.049240878 | 0.120495236078606 | 0.685795565625595 | -0.285411541 | 0.186929784406258 | 0.951951797540935 | 0.751704833278788 | 1.20554263418621 | 0.999228396881742 |
| ebi-fl187-GCST90094838 | ebi-met1400-GCST90200812 | Glutamate to kynurenine ratio \|\| id:ebi-met1400-GCST90200812 | Shellfish liking \|\| id:ebi-fl187-GCST90094838 | Weighted mode | 30 | 0.156526001538197 | 0.105720019867018 | 0.149504500761311 | -0.050685237 | 0.363737240477552 | 1.16944116919296 | 0.95057782979599 | 1.43869613337911 | 0.999228396881742 |
| ebi-fl187-GCST90094838 | ebi-met1400-GCST90200876 | Cortisone to 4-cholesten-3-one ratio \|\| id:ebi-met1400-GCST90200876 | Shellfish liking \|\| id:ebi-fl187-GCST90094838 | Weighted mode | 30 | -0.046388425 | 0.117639619795591 | 0.696224927339029 | -0.276962079 | 0.184185230197771 | 0.954671072433068 | 0.758083243536192 | 1.20223849334693 | 0.999228396881742 |
| ebi-fl187-GCST90094838 | ebi-met1400-GCST90200906 | 2'-deoxyuridine to cytidine ratio \|\| id:ebi-met1400-GCST90200906 | Shellfish liking \|\| id:ebi-fl187-GCST90094838 | Weighted mode | 30 | 0.162672140865331 | 0.124975659189759 | 0.203288239027988 | -0.082280151 | 0.40762443287726 | 1.17665085071179 | 0.921013899450277 | 1.50324248668466 | 0.999228396881742 |
| ebi-fl187-GCST90094838 | ebi-met1400-GCST90200907 | Retinol (Vitamin A) to linoleoyl-arachidonoyl-glycerol (18:2 to 20:4) [1] ratio \|\| id:ebi-met1400-GCST90200907 | Shellfish liking \|\| id:ebi-fl187-GCST90094838 | Weighted mode | 30 | -0.031411789 | 0.125899236378156 | 0.804733287386128 | -0.278174292 | 0.215350714402637 | 0.969076435983371 | 0.757164842087288 | 1.2402968106514 | 0.999228396881742 |
| ebi-fl187-GCST90094838 | ebi-met1400-GCST90200916 | Fructose to sucrose ratio \|\| id:ebi-met1400-GCST90200916 | Shellfish liking \|\| id:ebi-fl187-GCST90094838 | Weighted mode | 30 | -0.081305932 | 0.116699179312348 | 0.491529302393716 | -0.310036323 | 0.147424459924998 | 0.921911606471751 | 0.733420315729438 | 1.15884574222902 | 0.999228396881742 |
| ebi-fl187-GCST90094838 | ebi-met1400-GCST90200917 | Alpha-ketobutyrate to 3-methyl-2-oxobutyrate ratio \|\| id:ebi-met1400-GCST90200917 | Shellfish liking \|\| id:ebi-fl187-GCST90094838 | Weighted mode | 30 | 0.0287046371960432 | 0.117581839373811 | 0.808853125494299 | -0.201755768 | 0.259165042368712 | 1.02912058563911 | 0.817294513061323 | 1.29584765694703 | 0.999228396881742 |
| ebi-fl187-GCST90094838 | ebi-met1400-GCST90200924 | Alpha-tocopherol to glycerol ratio \|\| id:ebi-met1400-GCST90200924 | Shellfish liking \|\| id:ebi-fl187-GCST90094838 | Weighted mode | 30 | -0.007062001 | 0.0985766358697269 | 0.943380436992726 | -0.200272207 | 0.18614820555361 | 0.992962876580557 | 0.818507919120123 | 1.20460077567366 | 0.999228396881742 |
| ebi-fl187-GCST90094838 | ebi-met1400-GCST90200938 | Alpha-ketoglutarate to alanine ratio \|\| id:ebi-met1400-GCST90200938 | Shellfish liking \|\| id:ebi-fl187-GCST90094838 | Weighted mode | 30 | 0.0200312377390933 | 0.106724481232688 | 0.852425894001643 | -0.189148745 | 0.229211220955162 | 1.0202332093078 | 0.827663386352985 | 1.25760764404597 | 0.999228396881742 |
| ebi-fl187-GCST90094838 | ebi-met1400-GCST90200943 | Phosphate to EDTA ratio \|\| id:ebi-met1400-GCST90200943 | Shellfish liking \|\| id:ebi-fl187-GCST90094838 | Weighted mode | 30 | -0.085484541 | 0.139958646391138 | 0.546100159134431 | -0.359803488 | 0.188834406240552 | 0.918067335635597 | 0.697813441583431 | 1.20784092500212 | 0.999228396881742 |
| ebi-fl187-GCST90094838 | ebi-met1400-GCST90200947 | Adenosine 5'-diphosphate (ADP) to glucose ratio \|\| id:ebi-met1400-GCST90200947 | Shellfish liking \|\| id:ebi-fl187-GCST90094838 | Weighted mode | 30 | 0.176144675533343 | 0.14189295549094 | 0.224408880623596 | -0.101965517 | 0.454254868295584 | 1.19261058774285 | 0.903060691167089 | 1.57499936372846 | 0.999228396881742 |
| ebi-fl187-GCST90094838 | ebi-met1400-GCST90200975 | Arachidonate (20:4n6) to caffeine ratio \|\| id:ebi-met1400-GCST90200975 | Shellfish liking \|\| id:ebi-fl187-GCST90094838 | Weighted mode | 30 | -0.066607538 | 0.111912630444491 | 0.556343136611606 | -0.285956294 | 0.152741217208046 | 0.93556230151438 | 0.751295451247382 | 1.16502345190784 | 0.999228396881742 |
| ebi-fl187-GCST90094838 | ebi-met1400-GCST90200985 | Cholesterol to oleoyl-linoleoyl-glycerol (18:1 to 18:2) [2] ratio \|\| id:ebi-met1400-GCST90200985 | Shellfish liking \|\| id:ebi-fl187-GCST90094838 | Weighted mode | 30 | 0.0152308527514348 | 0.109160156777294 | 0.889997774004188 | -0.198723055 | 0.229184760034932 | 1.01534743331099 | 0.819776895393778 | 1.25757436703069 | 0.999228396881742 |
| ebi-fl187-GCST90094838 | ebi-met1400-GCST90201016 | Bilirubin (Z,Z) to etiocholanolone glucuronide ratio \|\| id:ebi-met1400-GCST90201016 | Shellfish liking \|\| id:ebi-fl187-GCST90094838 | Weighted mode | 30 | 0.0803928905022204 | 0.128486827837753 | 0.536411991902028 | -0.171441292 | 0.332227073064217 | 1.08371276449567 | 0.84244972505635 | 1.39406936817778 | 0.999228396881742 |
| ebi-fl187-GCST90094838 | ebi-pw11-GCST90275189 | Placental weight (fetal genotype, adjusted for sex and gestational age) \|\| id:ebi-pw11-GCST90275189 | Shellfish liking \|\| id:ebi-fl187-GCST90094838 | Weighted mode | 33 | 0.0229583986955882 | 0.0340747344779454 | 0.505298049435074 | -0.043828081 | 0.0897448782723612 | 1.02322397120987 | 0.957118490296926 | 1.0938951716769 | 0.999228396881742 |
| ebi-fl187-GCST90094838 | ebi-pw11-GCST90275192 | Placental weight (fetal effect, conditional on maternal and paternal genotype) \|\| id:ebi-pw11-GCST90275192 | Shellfish liking \|\| id:ebi-fl187-GCST90094838 | Weighted mode | 33 | -0.032403208 | 0.0834088846937954 | 0.700228955753863 | -0.195884622 | 0.131078205936313 | 0.968116151133468 | 0.822107082225265 | 1.1400569370459 | 0.999228396881742 |
| ebi-fl187-GCST90094838 | ebi-pw11-GCST90275193 | Placental weight (maternal effect, conditional on fetal and paternal genotype) \|\| id:ebi-pw11-GCST90275193 | Shellfish liking \|\| id:ebi-fl187-GCST90094838 | Weighted mode | 33 | -0.009715156 | 0.0616789421821578 | 0.875831276282078 | -0.130605883 | 0.111175570554126 | 0.990331883550505 | 0.87756356914149 | 1.1175911057203 | 0.999228396881742 |
| ebi-fl187-GCST90094838 | ebi-pw11-GCST90275195 | Placental weight (fetal effect, conditional on maternal genotype) \|\| id:ebi-pw11-GCST90275195 | Shellfish liking \|\| id:ebi-fl187-GCST90094838 | Weighted mode | 33 | -0.029976891 | 0.0441694550274293 | 0.502220290871033 | -0.116549022 | 0.0565952412863268 | 0.970467960253257 | 0.889986466615828 | 1.05822739693935 | 0.999228396881742 |
| ebi-fl187-GCST90094838 | ebi-pw11-GCST90275197 | Placental weight (fetal genotype, adjusted for sex) \|\| id:ebi-pw11-GCST90275197 | Shellfish liking \|\| id:ebi-fl187-GCST90094838 | Weighted mode | 33 | 0.0368311796379631 | 0.0390491595885799 | 0.352645551731517 | -0.039705173 | 0.11336753243158 | 1.03751785191315 | 0.961072747432141 | 1.12004350962463 | 0.999228396881742 |
| ebi-fl187-GCST90094838 | prot-a-1030 | Protein FAM171B \|\| id:prot-a-1030 | Shellfish liking \|\| id:ebi-fl187-GCST90094838 | Weighted mode | 34 | -0.124118125 | 0.140065849577566 | 0.381953678769599 | -0.39864719 | 0.15041094006895 | 0.883275497710885 | 0.671227475163791 | 1.16231178508602 | 0.999228396881742 |
| ebi-fl187-GCST90094838 | prot-a-1036 | BRISC complex subunit Abro1 \|\| id:prot-a-1036 | Shellfish liking \|\| id:ebi-fl187-GCST90094838 | Weighted mode | 34 | 0.125223331376211 | 0.14003413111427 | 0.37767241283928 | -0.149243566 | 0.399690228360181 | 1.13340154893137 | 0.861359291847738 | 1.49136264422758 | 0.999228396881742 |
| ebi-fl187-GCST90094838 | prot-a-1055 | Phenylalanine--tRNA ligase, mitochondrial \|\| id:prot-a-1055 | Shellfish liking \|\| id:ebi-fl187-GCST90094838 | Weighted mode | 34 | -0.071373832 | 0.158369348177709 | 0.655167823346621 | -0.381777754 | 0.239030090823823 | 0.931113747311761 | 0.682646751835181 | 1.2700167518592 | 0.999228396881742 |
| ebi-fl187-GCST90094838 | prot-a-1083 | Fc receptor-like protein 5 \|\| id:prot-a-1083 | Shellfish liking \|\| id:ebi-fl187-GCST90094838 | Weighted mode | 34 | 0.00853753979611893 | 0.148286739916637 | 0.954434752216933 | -0.28210447 | 0.299179550032728 | 1.00857408852704 | 0.754194889344972 | 1.34875177015801 | 0.999228396881742 |
| ebi-fl187-GCST90094838 | prot-a-1103 | Fibroblast growth factor receptor 3 \|\| id:prot-a-1103 | Shellfish liking \|\| id:ebi-fl187-GCST90094838 | Weighted mode | 34 | -0.052746098 | 0.170204553385502 | 0.758586914994201 | -0.386347023 | 0.280854826711221 | 0.948620838707805 | 0.679534670898154 | 1.32426134260568 | 0.999228396881742 |
| ebi-fl187-GCST90094838 | prot-a-1105 | Fibroblast growth factor receptor 4 \|\| id:prot-a-1105 | Shellfish liking \|\| id:ebi-fl187-GCST90094838 | Weighted mode | 34 | 0.0582907128776762 | 0.185572897945628 | 0.755413035638516 | -0.305432167 | 0.422013592851108 | 1.06002311329091 | 0.736804882749464 | 1.52502925403798 | 0.999228396881742 |
| ebi-fl187-GCST90094838 | prot-a-1120 | Filamin-A \|\| id:prot-a-1120 | Shellfish liking \|\| id:ebi-fl187-GCST90094838 | Weighted mode | 34 | -0.231606367 | 0.14028235756189 | 0.108224874856589 | -0.506559788 | 0.0433470540498961 | 0.79325831468972 | 0.602564968682424 | 1.04430026060147 | 0.999228396881742 |
| ebi-fl187-GCST90094838 | prot-a-1166 | Galanin peptides \|\| id:prot-a-1166 | Shellfish liking \|\| id:ebi-fl187-GCST90094838 | Weighted mode | 34 | -0.106542701 | 0.160487697461477 | 0.511386467526314 | -0.421098588 | 0.208013185832451 | 0.898936661674638 | 0.656325392269552 | 1.23122940422647 | 0.999228396881742 |
| ebi-fl187-GCST90094838 | prot-a-1205 | ADP-ribosylation factor-binding protein GGA1 \|\| id:prot-a-1205 | Shellfish liking \|\| id:ebi-fl187-GCST90094838 | Weighted mode | 34 | 0.0973733265541163 | 0.174658564800196 | 0.580943630785013 | -0.24495746 | 0.4397041135625 | 1.1022718041516 | 0.782737834845574 | 1.55224786146608 | 0.999228396881742 |
| ebi-fl187-GCST90094838 | prot-a-1227 | Guanine nucleotide-binding protein G(I)/G(S)/G(O) subunit gamma-T2 \|\| id:prot-a-1227 | Shellfish liking \|\| id:ebi-fl187-GCST90094838 | Weighted mode | 34 | 0.0168783640636634 | 0.15898846861249 | 0.916097396935162 | -0.294739034 | 0.328495762544144 | 1.01702160842575 | 0.744725909940466 | 1.38887735500915 | 0.999228396881742 |
| ebi-fl187-GCST90094838 | prot-a-1240 | Golgi SNAP receptor complex member 1 \|\| id:prot-a-1240 | Shellfish liking \|\| id:ebi-fl187-GCST90094838 | Weighted mode | 34 | -0.100632088 | 0.16835047853127 | 0.554084682140627 | -0.430599026 | 0.229334850099525 | 0.90426566204238 | 0.650119539717875 | 1.25776313061409 | 0.999228396881742 |
| ebi-fl187-GCST90094838 | prot-a-1283 | Glutathione S-transferase A1 \|\| id:prot-a-1283 | Shellfish liking \|\| id:ebi-fl187-GCST90094838 | Weighted mode | 34 | 0.0119948266939156 | 0.212096485245474 | 0.955241811287461 | -0.403714284 | 0.427703937775045 | 1.01206705311989 | 0.66783490487179 | 1.5337319336542 | 0.999228396881742 |
| ebi-fl187-GCST90094838 | prot-a-1324 | E3 ubiquitin-protein ligase HECW1 \|\| id:prot-a-1324 | Shellfish liking \|\| id:ebi-fl187-GCST90094838 | Weighted mode | 34 | 0.044758032495732 | 0.136503683114747 | 0.745067030857624 | -0.222789186 | 0.312305251400637 | 1.04577478577715 | 0.800283542159758 | 1.36657177731756 | 0.999228396881742 |
| ebi-fl187-GCST90094838 | prot-a-1339 | Histidine triad nucleotide-binding protein 2, mitochondrial \|\| id:prot-a-1339 | Shellfish liking \|\| id:ebi-fl187-GCST90094838 | Weighted mode | 34 | -0.195974112 | 0.16625211617754 | 0.246916904718517 | -0.521828259 | 0.129880036159114 | 0.82203351557722 | 0.593434603271692 | 1.13869177329194 | 0.999228396881742 |
| ebi-fl187-GCST90094838 | prot-a-1466 | Interleukin-11 \|\| id:prot-a-1466 | Shellfish liking \|\| id:ebi-fl187-GCST90094838 | Weighted mode | 34 | -0.03806588 | 0.159188978934266 | 0.812488470040024 | -0.350076279 | 0.273944518711908 | 0.962649519452497 | 0.704634339070026 | 1.31514183445157 | 0.999228396881742 |
| ebi-fl187-GCST90094838 | prot-a-147 | Rho GTPase-activating protein 1 \|\| id:prot-a-147 | Shellfish liking \|\| id:ebi-fl187-GCST90094838 | Weighted mode | 34 | 0.0128126013509748 | 0.1340488323223 | 0.924430960147987 | -0.24992311 | 0.275548312702683 | 1.01289503441209 | 0.778860667365266 | 1.31725274330168 | 0.999228396881742 |
| ebi-fl187-GCST90094838 | prot-a-1473 | Interleukin-12 receptor subunit beta-1 \|\| id:prot-a-1473 | Shellfish liking \|\| id:ebi-fl187-GCST90094838 | Weighted mode | 34 | -0.03093465 | 0.142869356915605 | 0.829912273416179 | -0.31095859 | 0.24908928952467 | 0.969538930345899 | 0.732744218483619 | 1.28285657360978 | 0.999228396881742 |
| ebi-fl187-GCST90094838 | prot-a-1501 | X-linked interleukin-1 receptor accessory protein-like 2 \|\| id:prot-a-1501 | Shellfish liking \|\| id:ebi-fl187-GCST90094838 | Weighted mode | 34 | -0.228252204 | 0.159078826018571 | 0.160743653098019 | -0.540046703 | 0.0835422952744158 | 0.795923499651259 | 0.58272103708199 | 1.08713119483272 | 0.999228396881742 |
| ebi-fl187-GCST90094838 | prot-a-1503 | Interleukin-1 receptor-like 2 \|\| id:prot-a-1503 | Shellfish liking \|\| id:ebi-fl187-GCST90094838 | Weighted mode | 34 | 0.120847905791579 | 0.18170155359627 | 0.510614656021778 | -0.235287139 | 0.476982950840269 | 1.12845326810807 | 0.79034387829003 | 1.61120597411206 | 0.999228396881742 |
| ebi-fl187-GCST90094838 | prot-a-1536 | Interleukin-5 receptor subunit alpha \|\| id:prot-a-1536 | Shellfish liking \|\| id:ebi-fl187-GCST90094838 | Weighted mode | 34 | 0.173197357397829 | 0.161404819488352 | 0.291027872556495 | -0.143156089 | 0.489550803594999 | 1.18910075975956 | 0.866618788851676 | 1.63158315403516 | 0.999228396881742 |
| ebi-fl187-GCST90094838 | prot-a-1559 | Insulin-induced gene 1 protein \|\| id:prot-a-1559 | Shellfish liking \|\| id:ebi-fl187-GCST90094838 | Weighted mode | 34 | -0.014842042 | 0.14801438300124 | 0.920733273402239 | -0.304950233 | 0.275266148635963 | 0.985267558159314 | 0.737160059923299 | 1.31688111434337 | 0.999228396881742 |
| ebi-fl187-GCST90094838 | prot-a-1567 | Interferon regulatory factor 1 \|\| id:prot-a-1567 | Shellfish liking \|\| id:ebi-fl187-GCST90094838 | Weighted mode | 34 | -0.127987008 | 0.134379827268015 | 0.347804821562712 | -0.39137147 | 0.135397453034807 | 0.879864809912815 | 0.676128947861272 | 1.1449917743823 | 0.999228396881742 |
| ebi-fl187-GCST90094838 | prot-a-1613 | Potassium voltage-gated channel subfamily E regulatory beta subunit 5 \|\| id:prot-a-1613 | Shellfish liking \|\| id:ebi-fl187-GCST90094838 | Weighted mode | 34 | 0.0295355456767608 | 0.158809466842451 | 0.853598173742466 | -0.281731009 | 0.340802100687965 | 1.02997604601682 | 0.754476604404055 | 1.40607495206081 | 0.999228396881742 |
| ebi-fl187-GCST90094838 | prot-a-1649 | Kin of IRRE-like protein 2 \|\| id:prot-a-1649 | Shellfish liking \|\| id:ebi-fl187-GCST90094838 | Weighted mode | 34 | 0.0691126081971485 | 0.160651801941256 | 0.669845769104259 | -0.245764924 | 0.38399014000201 | 1.0715568684417 | 0.782106057987722 | 1.46813096584225 | 0.999228396881742 |
| ebi-fl187-GCST90094838 | prot-a-165 | Armadillo repeat-containing protein 5 \|\| id:prot-a-165 | Shellfish liking \|\| id:ebi-fl187-GCST90094838 | Weighted mode | 34 | -0.163850953 | 0.167852762488296 | 0.336087769860478 | -0.492842367 | 0.165140461465833 | 0.848868533750686 | 0.610887557216632 | 1.17955878963256 | 0.999228396881742 |
| ebi-fl187-GCST90094838 | prot-a-1656 | Kallikrein-11 \|\| id:prot-a-1656 | Shellfish liking \|\| id:ebi-fl187-GCST90094838 | Weighted mode | 34 | -0.111760346 | 0.177282214746155 | 0.532768472578614 | -0.459233487 | 0.235712794866543 | 0.894258544427059 | 0.631767718167098 | 1.26581071062148 | 0.999228396881742 |
| ebi-fl187-GCST90094838 | prot-a-1697 | Laminin subunit gamma-2 \|\| id:prot-a-1697 | Shellfish liking \|\| id:ebi-fl187-GCST90094838 | Weighted mode | 34 | -0.21023694 | 0.17336814671917 | 0.233869224183482 | -0.550038508 | 0.129564627309972 | 0.8103922086801 | 0.576927593723508 | 1.13833267646434 | 0.999228396881742 |
| ebi-fl187-GCST90094838 | prot-a-1698 | Lysosome-associated membrane glycoprotein 3 \|\| id:prot-a-1698 | Shellfish liking \|\| id:ebi-fl187-GCST90094838 | Weighted mode | 34 | 0.0241145401414111 | 0.157909523251672 | 0.879556017204935 | -0.285388125 | 0.333617205714687 | 1.0244076469678 | 0.75172243509705 | 1.39600865714565 | 0.999228396881742 |
| ebi-fl187-GCST90094838 | prot-a-1724 | Leptin receptor \|\| id:prot-a-1724 | Shellfish liking \|\| id:ebi-fl187-GCST90094838 | Weighted mode | 34 | -0.105755576 | 0.196750308136595 | 0.594520710973526 | -0.491386179 | 0.27987502842473 | 0.899644516344634 | 0.611777772360536 | 1.32296446905232 | 0.999228396881742 |
| ebi-fl187-GCST90094838 | prot-a-1743 | Leukocyte immunoglobulin-like receptor subfamily B member 2 \|\| id:prot-a-1743 | Shellfish liking \|\| id:ebi-fl187-GCST90094838 | Weighted mode | 34 | -0.166261637 | 0.174564811269807 | 0.347800945297081 | -0.508408667 | 0.175885393183584 | 0.846824644623361 | 0.601451927979932 | 1.19230140495187 | 0.999228396881742 |
| ebi-fl187-GCST90094838 | prot-a-1746 | Leukocyte immunoglobulin-like receptor subfamily B member 4 \|\| id:prot-a-1746 | Shellfish liking \|\| id:ebi-fl187-GCST90094838 | Weighted mode | 34 | -0.000560574 | 0.144867316590981 | 0.996935837465757 | -0.284500514 | 0.283379366615359 | 0.999439583189232 | 0.752389968418479 | 1.32760871672054 | 0.999228396881742 |
| ebi-fl187-GCST90094838 | prot-a-1750 | Leucine-rich repeat and immunoglobulin-like domain-containing nogo receptor-interacting protein 3 \|\| id:prot-a-1750 | Shellfish liking \|\| id:ebi-fl187-GCST90094838 | Weighted mode | 34 | -0.116695782 | 0.15718284300867 | 0.463084709029509 | -0.424774155 | 0.19138259000651 | 0.88985586190232 | 0.653917452711061 | 1.21092265037282 | 0.999228396881742 |
| ebi-fl187-GCST90094838 | prot-a-1753 | Lipase member N \|\| id:prot-a-1753 | Shellfish liking \|\| id:ebi-fl187-GCST90094838 | Weighted mode | 34 | 0.0769139796818179 | 0.171237161360131 | 0.65624773927666 | -0.258710857 | 0.412538815947675 | 1.07994917482531 | 0.772046222855926 | 1.51064817841031 | 0.999228396881742 |
| ebi-fl187-GCST90094838 | prot-a-1760 | Ig Kappa chain V-I region HK102- like \|\| id:prot-a-1760 | Shellfish liking \|\| id:ebi-fl187-GCST90094838 | Weighted mode | 34 | -0.097251264 | 0.140046924956739 | 0.492279684603465 | -0.371743237 | 0.177240708954574 | 0.907327998662904 | 0.689531265998763 | 1.19391844540246 | 0.999228396881742 |
| ebi-fl187-GCST90094838 | prot-a-1792 | Leucine-rich repeat neuronal protein 1 \|\| id:prot-a-1792 | Shellfish liking \|\| id:ebi-fl187-GCST90094838 | Weighted mode | 34 | -0.056004415 | 0.157993155960635 | 0.725239156239948 | -0.365671001 | 0.253662170259826 | 0.945534960944348 | 0.693731000401018 | 1.28873635724975 | 0.999228396881742 |
| ebi-fl187-GCST90094838 | prot-a-1795 | Leucine-rich repeat transmembrane neuronal protein 1 \|\| id:prot-a-1795 | Shellfish liking \|\| id:ebi-fl187-GCST90094838 | Weighted mode | 34 | 0.0246356266530772 | 0.141036308047174 | 0.862401690184642 | -0.251795537 | 0.301066790425539 | 1.02494159107853 | 0.777403672014528 | 1.35129959239884 | 0.999228396881742 |
| ebi-fl187-GCST90094838 | prot-a-1820 | LysM and putative peptidoglycan-binding domain-containing protein 4 \|\| id:prot-a-1820 | Shellfish liking \|\| id:ebi-fl187-GCST90094838 | Weighted mode | 34 | -0.061590161 | 0.163908533684818 | 0.709500589472756 | -0.382850887 | 0.25967056484532 | 0.940268166233084 | 0.681914573897351 | 1.29650290267064 | 0.999228396881742 |
| ebi-fl187-GCST90094838 | prot-a-1844 | Mitogen-activated protein kinase kinase kinase 7:TGF-beta-activated kinase 1 and MAP3K7-binding protein 1 fusion \|\| id:prot-a-1844 | Shellfish liking \|\| id:ebi-fl187-GCST90094838 | Weighted mode | 34 | -0.004263463 | 0.154120990929113 | 0.978097478744085 | -0.306340605 | 0.297813679068559 | 0.995745612504034 | 0.736135844926495 | 1.34691080682267 | 0.999228396881742 |
| ebi-fl187-GCST90094838 | prot-a-1856 | Megakaryocyte-associated tyrosine-protein kinase \|\| id:prot-a-1856 | Shellfish liking \|\| id:ebi-fl187-GCST90094838 | Weighted mode | 34 | 0.0308703443547309 | 0.139056623959084 | 0.825683315284883 | -0.241680639 | 0.303421327314536 | 1.03135177463749 | 0.785306934226657 | 1.3544850257758 | 0.999228396881742 |
| ebi-fl187-GCST90094838 | prot-a-1910 | Stromelysin-2 \|\| id:prot-a-1910 | Shellfish liking \|\| id:ebi-fl187-GCST90094838 | Weighted mode | 34 | -0.167656445 | 0.13013364435101 | 0.206587639834228 | -0.422718388 | 0.0874054977352959 | 0.845644309952679 | 0.655263137014817 | 1.09133912555067 | 0.999228396881742 |
| ebi-fl187-GCST90094838 | prot-a-1940 | 39S ribosomal protein L14, mitochondrial \|\| id:prot-a-1940 | Shellfish liking \|\| id:ebi-fl187-GCST90094838 | Weighted mode | 34 | -0.018401227 | 0.177333815201116 | 0.917983099290069 | -0.365975504 | 0.329173051267553 | 0.9817670423436 | 0.693519789239949 | 1.38981834460475 | 0.999228396881742 |
| ebi-fl187-GCST90094838 | prot-a-1942 | 39S ribosomal protein L33, mitochondrial \|\| id:prot-a-1942 | Shellfish liking \|\| id:ebi-fl187-GCST90094838 | Weighted mode | 34 | 0.0449208022992886 | 0.186024038053182 | 0.81067851676382 | -0.319686312 | 0.409527916883525 | 1.04594502018773 | 0.726376856836181 | 1.50610660975704 | 0.999228396881742 |
| ebi-fl187-GCST90094838 | prot-a-1948 | Mesothelin \|\| id:prot-a-1948 | Shellfish liking \|\| id:ebi-fl187-GCST90094838 | Weighted mode | 34 | 0.141442994385395 | 0.165247166589723 | 0.398199122355265 | -0.182441452 | 0.465327440901253 | 1.1519348356358 | 0.833233426542209 | 1.59253556480316 | 0.999228396881742 |
| ebi-fl187-GCST90094838 | prot-a-1980 | Unconventional myosin-VI \|\| id:prot-a-1980 | Shellfish liking \|\| id:ebi-fl187-GCST90094838 | Weighted mode | 34 | 0.0508922867865387 | 0.208173433415595 | 0.808380069498161 | -0.357127643 | 0.458912216281106 | 1.05220955030715 | 0.699683182578332 | 1.58235179196067 | 0.999228396881742 |
| ebi-fl187-GCST90094838 | prot-a-2039 | Neurofascin \|\| id:prot-a-2039 | Shellfish liking \|\| id:ebi-fl187-GCST90094838 | Weighted mode | 34 | -0.10042704 | 0.18863159487938 | 0.598017328502153 | -0.470144966 | 0.269290885782357 | 0.904451098593813 | 0.62491167068032 | 1.309035866 | 0.999228396881742 |
| ebi-fl187-GCST90094838 | prot-a-2041 | NFU1 iron-sulfur cluster scaffold homolog, mitochondrial \|\| id:prot-a-2041 | Shellfish liking \|\| id:ebi-fl187-GCST90094838 | Weighted mode | 34 | -0.05538187 | 0.166389574686002 | 0.741356952134435 | -0.381505437 | 0.27074169600106 | 0.946123782308865 | 0.682832673645129 | 1.31093640653119 | 0.999228396881742 |
| ebi-fl187-GCST90094838 | prot-a-2088 | Glucocorticoid receptor \|\| id:prot-a-2088 | Shellfish liking \|\| id:ebi-fl187-GCST90094838 | Weighted mode | 34 | 0.203657497487732 | 0.165852512191911 | 0.22815500662225 | -0.121413426 | 0.528728421383878 | 1.22587821517693 | 0.885667725465875 | 1.69677335555485 | 0.999228396881742 |
| ebi-fl187-GCST90094838 | prot-a-2111 | Neuron-specific protein family member 2 \|\| id:prot-a-2111 | Shellfish liking \|\| id:ebi-fl187-GCST90094838 | Weighted mode | 34 | -0.083969624 | 0.182125112996736 | 0.647783847509577 | -0.440934845 | 0.272995597804868 | 0.919459185467729 | 0.643434628098855 | 1.31389446079221 | 0.999228396881742 |
| ebi-fl187-GCST90094838 | prot-a-2125 | Nuclear migration protein nudC \|\| id:prot-a-2125 | Shellfish liking \|\| id:ebi-fl187-GCST90094838 | Weighted mode | 34 | -0.126286027 | 0.172213500154729 | 0.468546606017187 | -0.463824487 | 0.211252433223951 | 0.881362717123381 | 0.628873920089273 | 1.23522412731766 | 0.999228396881742 |
| ebi-fl187-GCST90094838 | prot-a-2142 | Ornithine decarboxylase \|\| id:prot-a-2142 | Shellfish liking \|\| id:ebi-fl187-GCST90094838 | Weighted mode | 34 | -0.09023624 | 0.142797064804923 | 0.531793278809525 | -0.370118487 | 0.189646006814006 | 0.913715303482887 | 0.690652492294361 | 1.20882160729682 | 0.999228396881742 |
| ebi-fl187-GCST90094838 | prot-a-2186 | Prostate and testis expressed protein 1 \|\| id:prot-a-2186 | Shellfish liking \|\| id:ebi-fl187-GCST90094838 | Weighted mode | 34 | -0.097422256 | 0.159286754807894 | 0.544983010020769 | -0.409624296 | 0.214779783229213 | 0.90717286588541 | 0.663899633287827 | 1.2395888886445 | 0.999228396881742 |
| ebi-fl187-GCST90094838 | prot-a-2227 | Platelet-derived growth factor C \|\| id:prot-a-2227 | Shellfish liking \|\| id:ebi-fl187-GCST90094838 | Weighted mode | 34 | -0.182806312 | 0.136730826728877 | 0.190373434463643 | -0.450798733 | 0.0851861080656239 | 0.832929468288298 | 0.637119060500183 | 1.08891970458138 | 0.999228396881742 |
| ebi-fl187-GCST90094838 | prot-a-2229 | Platelet-derived growth factor receptor alpha \|\| id:prot-a-2229 | Shellfish liking \|\| id:ebi-fl187-GCST90094838 | Weighted mode | 34 | -0.144664325 | 0.152709250895003 | 0.350362228923546 | -0.443974457 | 0.154645806660579 | 0.865312708127782 | 0.641481806085439 | 1.16724445766698 | 0.999228396881742 |
| ebi-fl187-GCST90094838 | prot-a-2243 | Platelet endothelial aggregation receptor 1 \|\| id:prot-a-2243 | Shellfish liking \|\| id:ebi-fl187-GCST90094838 | Weighted mode | 34 | 0.0273725694667328 | 0.156140001645718 | 0.861908996998487 | -0.278661834 | 0.33340697269234 | 1.0277506399502 | 0.756795782732848 | 1.39571520087462 | 0.999228396881742 |
| ebi-fl187-GCST90094838 | prot-a-2246 | E3 ubiquitin-protein ligase pellino homolog 2 \|\| id:prot-a-2246 | Shellfish liking \|\| id:ebi-fl187-GCST90094838 | Weighted mode | 34 | 0.240141940632948 | 0.166019092944003 | 0.157477150193377 | -0.085255482 | 0.565539362803194 | 1.27142960503706 | 0.918277651444482 | 1.76039701937844 | 0.999228396881742 |
| ebi-fl187-GCST90094838 | prot-a-2251 | Platelet factor 4 variant \|\| id:prot-a-2251 | Shellfish liking \|\| id:ebi-fl187-GCST90094838 | Weighted mode | 34 | 0.0439007151631672 | 0.200105455830964 | 0.827699272894382 | -0.348305978 | 0.436107408591857 | 1.04487860913612 | 0.705882858355023 | 1.54667491200237 | 0.999228396881742 |
| ebi-fl187-GCST90094838 | prot-a-2346 | Protein phosphatase 1L \|\| id:prot-a-2346 | Shellfish liking \|\| id:ebi-fl187-GCST90094838 | Weighted mode | 34 | 0.29182173918906 | 0.157858502715626 | 0.0734885334793293 | -0.017580926 | 0.601224404511686 | 1.33886432932446 | 0.982572716636627 | 1.8243511772578 | 0.999228396881742 |
| ebi-fl187-GCST90094838 | prot-a-2358 | PR domain zinc finger protein 4 \|\| id:prot-a-2358 | Shellfish liking \|\| id:ebi-fl187-GCST90094838 | Weighted mode | 34 | 0.0535538656339778 | 0.149241884056482 | 0.722000983121639 | -0.238960227 | 0.346067958384683 | 1.05501381922528 | 0.787446200755699 | 1.41349867164022 | 0.999228396881742 |
| ebi-fl187-GCST90094838 | prot-a-2390 | Trypsin-1 \|\| id:prot-a-2390 | Shellfish liking \|\| id:ebi-fl187-GCST90094838 | Weighted mode | 34 | -0.134508304 | 0.1698623373716 | 0.434093976615556 | -0.467438485 | 0.198421877004988 | 0.874145619728483 | 0.626605272843867 | 1.21947675451639 | 0.999228396881742 |
| ebi-fl187-GCST90094838 | prot-a-242 | Brain-derived neurotrophic factor \|\| id:prot-a-242 | Shellfish liking \|\| id:ebi-fl187-GCST90094838 | Weighted mode | 34 | 0.156762491204206 | 0.183263923114492 | 0.398502357579841 | -0.202434798 | 0.515959780508609 | 1.16971776264894 | 0.816739733844342 | 1.6752455985167 | 0.999228396881742 |
| ebi-fl187-GCST90094838 | prot-a-2464 | Dihydropteridine reductase \|\| id:prot-a-2464 | Shellfish liking \|\| id:ebi-fl187-GCST90094838 | Weighted mode | 34 | 0.0361216254811008 | 0.15702583595792 | 0.819483660029015 | -0.271649013 | 0.343892263958624 | 1.0367819379248 | 0.762121708966719 | 1.41042667353548 | 0.999228396881742 |
| ebi-fl187-GCST90094838 | prot-a-2474 | Ras-related protein Rab-27A \|\| id:prot-a-2474 | Shellfish liking \|\| id:ebi-fl187-GCST90094838 | Weighted mode | 34 | -0.170495205 | 0.260116994444231 | 0.516718034 | -0.680324514 | 0.339334104142659 | 0.843247133015878 | 0.506452614691922 | 1.40401235320316 | 0.999228396881742 |
| ebi-fl187-GCST90094838 | prot-a-2478 | Ras-related protein Rab-7b \|\| id:prot-a-2478 | Shellfish liking \|\| id:ebi-fl187-GCST90094838 | Weighted mode | 34 | 0.0934378320383411 | 0.157999446414989 | 0.55829492559869 | -0.216241083 | 0.403116747011719 | 1.09794234438199 | 0.805541076255223 | 1.49648159121943 | 0.999228396881742 |
| ebi-fl187-GCST90094838 | prot-a-2528 | E3 ubiquitin-protein ligase rififylin \|\| id:prot-a-2528 | Shellfish liking \|\| id:ebi-fl187-GCST90094838 | Weighted mode | 34 | 0.234459076335484 | 0.209960490953994 | 0.272195309243802 | -0.177063486 | 0.645981638605312 | 1.26422473466822 | 0.837726598978883 | 1.90785893833987 | 0.999228396881742 |
| ebi-fl187-GCST90094838 | prot-a-2543 | Protein RIC-3 \|\| id:prot-a-2543 | Shellfish liking \|\| id:ebi-fl187-GCST90094838 | Weighted mode | 34 | 0.14224961567734 | 0.183641855961217 | 0.444088920907216 | -0.217688422 | 0.502187653361326 | 1.15286438564817 | 0.804376028494722 | 1.65233204945596 | 0.999228396881742 |
| ebi-fl187-GCST90094838 | prot-a-255 | Bone morphogenetic protein 6 \|\| id:prot-a-255 | Shellfish liking \|\| id:ebi-fl187-GCST90094838 | Weighted mode | 34 | -0.00570655 | 0.205635648324599 | 0.978028068776112 | -0.40875242 | 0.39733932095425 | 0.994309701665242 | 0.664478723283652 | 1.48786070672061 | 0.999228396881742 |
| ebi-fl187-GCST90094838 | prot-a-2568 | RING finger protein 165 \|\| id:prot-a-2568 | Shellfish liking \|\| id:ebi-fl187-GCST90094838 | Weighted mode | 34 | -0.18961881 | 0.150281291462533 | 0.215881259828219 | -0.484170141 | 0.104932521644061 | 0.827274422896544 | 0.616208350756159 | 1.11063566395845 | 0.999228396881742 |
| ebi-fl187-GCST90094838 | prot-a-2573 | E3 ubiquitin-protein ligase RNF8 \|\| id:prot-a-2573 | Shellfish liking \|\| id:ebi-fl187-GCST90094838 | Weighted mode | 34 | -0.261610023 | 0.177827159343846 | 0.150723500165357 | -0.610151256 | 0.0869312089654066 | 0.769811173561783 | 0.543268690393996 | 1.09082163838816 | 0.999228396881742 |
| ebi-fl187-GCST90094838 | prot-a-260 | Cytoplasmic tyrosine-protein kinase BMX \|\| id:prot-a-260 | Shellfish liking \|\| id:ebi-fl187-GCST90094838 | Weighted mode | 34 | 0.04639785665866 | 0.15783908113912 | 0.770632013520699 | -0.262966742 | 0.355762455691336 | 1.0474910793651 | 0.768767464266805 | 1.42726846849058 | 0.999228396881742 |
| ebi-fl187-GCST90094838 | prot-a-2623 | Serum amyloid A-1 protein \|\| id:prot-a-2623 | Shellfish liking \|\| id:ebi-fl187-GCST90094838 | Weighted mode | 34 | 0.0480831082023361 | 0.152975813395702 | 0.755257868486428 | -0.251749486 | 0.347915702457911 | 1.04925785363498 | 0.777439473106782 | 1.41611286987413 | 0.999228396881742 |
| ebi-fl187-GCST90094838 | prot-a-2640 | Lysosome membrane protein 2 \|\| id:prot-a-2640 | Shellfish liking \|\| id:ebi-fl187-GCST90094838 | Weighted mode | 34 | 0.0514675581802544 | 0.156942687886541 | 0.745029771902275 | -0.25614011 | 0.359075226437875 | 1.05281503050265 | 0.77403351129928 | 1.43200452211911 | 0.999228396881742 |
| ebi-fl187-GCST90094838 | prot-a-2641 | Scavenger receptor class F member 2 \|\| id:prot-a-2641 | Shellfish liking \|\| id:ebi-fl187-GCST90094838 | Weighted mode | 34 | -0.070550777 | 0.152417860485068 | 0.646490293 | -0.369289784 | 0.228188229328339 | 0.931880420025101 | 0.691225075614325 | 1.25632177978261 | 0.999228396881742 |
| ebi-fl187-GCST90094838 | prot-a-2669 | Semaphorin-3A \|\| id:prot-a-2669 | Shellfish liking \|\| id:ebi-fl187-GCST90094838 | Weighted mode | 34 | -0.121242186 | 0.17851059216482 | 0.501755271997332 | -0.471122947 | 0.228638574308589 | 0.885819400258714 | 0.624300817794162 | 1.25688768540653 | 0.999228396881742 |
| ebi-fl187-GCST90094838 | prot-a-2683 | Sentrin-specific protease 7 \|\| id:prot-a-2683 | Shellfish liking \|\| id:ebi-fl187-GCST90094838 | Weighted mode | 34 | 0.0659190263566534 | 0.189606969986484 | 0.73030228454868 | -0.305710635 | 0.437548687530162 | 1.06814022245873 | 0.736599734937718 | 1.54890570919178 | 0.999228396881742 |
| ebi-fl187-GCST90094838 | prot-a-270 | Bromodomain testis-specific protein \|\| id:prot-a-270 | Shellfish liking \|\| id:ebi-fl187-GCST90094838 | Weighted mode | 34 | -0.064820309 | 0.140632185727535 | 0.647878806276509 | -0.340459393 | 0.210818774725443 | 0.937235860814625 | 0.711443415321632 | 1.23468857800844 | 0.999228396881742 |
| ebi-fl187-GCST90094838 | prot-a-2708 | 14-3-3 protein sigma \|\| id:prot-a-2708 | Shellfish liking \|\| id:ebi-fl187-GCST90094838 | Weighted mode | 34 | 0.154423971012447 | 0.138183114031353 | 0.27183739039397 | -0.116414932 | 0.425262874513898 | 1.16698554995473 | 0.890105812842173 | 1.52999256285569 | 0.999228396881742 |
| ebi-fl187-GCST90094838 | prot-a-2713 | Beta-sarcoglycan \|\| id:prot-a-2713 | Shellfish liking \|\| id:ebi-fl187-GCST90094838 | Weighted mode | 34 | -0.054264607 | 0.154764034946918 | 0.728095866916074 | -0.357602115 | 0.249072901991945 | 0.94718144296937 | 0.699351281040328 | 1.28283555092795 | 0.999228396881742 |
| ebi-fl187-GCST90094838 | prot-a-2720 | Protein shisa-3 homolog \|\| id:prot-a-2720 | Shellfish liking \|\| id:ebi-fl187-GCST90094838 | Weighted mode | 34 | -0.05044236 | 0.134124709508639 | 0.709260395306369 | -0.313326791 | 0.21244207016417 | 0.950808731258678 | 0.731010985640215 | 1.23669446998254 | 0.999228396881742 |
| ebi-fl187-GCST90094838 | prot-a-2730 | Sialic acid-binding Ig-like lectin 8 \|\| id:prot-a-2730 | Shellfish liking \|\| id:ebi-fl187-GCST90094838 | Weighted mode | 34 | -0.071289757 | 0.171760064775541 | 0.680788325508494 | -0.407939484 | 0.265359969571809 | 0.931192033261162 | 0.665019121670262 | 1.30390025572679 | 0.999228396881742 |
| ebi-fl187-GCST90094838 | prot-a-2761 | Sodium- and chloride-dependent neutral and basic amino acid transporter B(0+) \|\| id:prot-a-2761 | Shellfish liking \|\| id:ebi-fl187-GCST90094838 | Weighted mode | 34 | 0.156954594757363 | 0.234512461343869 | 0.507972420554824 | -0.302689829 | 0.616599018991347 | 1.16994249117222 | 0.738828223571186 | 1.85261660150746 | 0.999228396881742 |
| ebi-fl187-GCST90094838 | prot-a-2770 | Secreted Ly-6/uPAR-related protein 1 \|\| id:prot-a-2770 | Shellfish liking \|\| id:ebi-fl187-GCST90094838 | Weighted mode | 34 | 0.0926639253463972 | 0.143639698625852 | 0.523313674540428 | -0.188869884 | 0.374197734653067 | 1.09709296816567 | 0.827894222004317 | 1.45382459353881 | 0.999228396881742 |
| ebi-fl187-GCST90094838 | prot-a-2784 | N-lysine methyltransferase SMYD2 \|\| id:prot-a-2784 | Shellfish liking \|\| id:ebi-fl187-GCST90094838 | Weighted mode | 34 | -0.173918011 | 0.155525478065585 | 0.27153045430759 | -0.478747948 | 0.130911926162164 | 0.840365795714266 | 0.61955862609479 | 1.13986738439572 | 0.999228396881742 |
| ebi-fl187-GCST90094838 | prot-a-2837 | Serine palmitoyltransferase 2 \|\| id:prot-a-2837 | Shellfish liking \|\| id:ebi-fl187-GCST90094838 | Weighted mode | 34 | 0.00841130196340556 | 0.1597709905796 | 0.958331381545742 | -0.30473984 | 0.321562443499422 | 1.00844677635595 | 0.737315169671437 | 1.37928113047761 | 0.999228396881742 |
| ebi-fl187-GCST90094838 | prot-a-2858 | Alpha-2,8-sialyltransferase 8B \|\| id:prot-a-2858 | Shellfish liking \|\| id:ebi-fl187-GCST90094838 | Weighted mode | 34 | 0.0138460515999586 | 0.146585016477772 | 0.925316975039299 | -0.273460581 | 0.301152683896392 | 1.01394235211976 | 0.760742323697382 | 1.35141566519587 | 0.999228396881742 |
| ebi-fl187-GCST90094838 | prot-a-2911 | Synaptotagmin-11 \|\| id:prot-a-2911 | Shellfish liking \|\| id:ebi-fl187-GCST90094838 | Weighted mode | 34 | -0.073982825 | 0.15352965562456 | 0.633071990058403 | -0.37490095 | 0.226935300378977 | 0.928687644244136 | 0.687357358392923 | 1.2547486835497 | 0.999228396881742 |
| ebi-fl187-GCST90094838 | prot-a-2962 | Transforming growth factor beta-1 \|\| id:prot-a-2962 | Shellfish liking \|\| id:ebi-fl187-GCST90094838 | Weighted mode | 34 | -0.197979085 | 0.159669092796024 | 0.223744812779274 | -0.510930506 | 0.114972337288869 | 0.820387011688002 | 0.599937073676668 | 1.12184240394035 | 0.999228396881742 |
| ebi-fl187-GCST90094838 | prot-a-2978 | Tyrosine-protein kinase receptor Tie-1, soluble \|\| id:prot-a-2978 | Shellfish liking \|\| id:ebi-fl187-GCST90094838 | Weighted mode | 34 | -0.281599754 | 0.190444180046899 | 0.148717312914554 | -0.654870347 | 0.0916708384412013 | 0.754575639642325 | 0.519509413996856 | 1.09600400031455 | 0.999228396881742 |
| ebi-fl187-GCST90094838 | prot-a-299 | Uncharacterized protein C1orf226 \|\| id:prot-a-299 | Shellfish liking \|\| id:ebi-fl187-GCST90094838 | Weighted mode | 34 | 0.0588981658098697 | 0.132983329569653 | 0.66072869758995 | -0.20174916 | 0.31954549176639 | 1.06066722305265 | 0.817299913622348 | 1.37650199064875 | 0.999228396881742 |
| ebi-fl187-GCST90094838 | prot-a-3015 | Transmembrane protein 70, mitochondrial \|\| id:prot-a-3015 | Shellfish liking \|\| id:ebi-fl187-GCST90094838 | Weighted mode | 34 | -0.069614164 | 0.172125838171822 | 0.688500408068907 | -0.406980807 | 0.26775247861261 | 0.932753640228803 | 0.665656966119134 | 1.30702358368225 | 0.999228396881742 |
| ebi-fl187-GCST90094838 | prot-a-3016 | Transmembrane protein 87B \|\| id:prot-a-3016 | Shellfish liking \|\| id:ebi-fl187-GCST90094838 | Weighted mode | 34 | -0.046891849 | 0.18094728914382 | 0.797132013128692 | -0.401548536 | 0.307764837487023 | 0.954190588452411 | 0.669282834628667 | 1.36038104069457 | 0.999228396881742 |
| ebi-fl187-GCST90094838 | prot-a-3123 | Thioredoxin domain-containing protein 12 \|\| id:prot-a-3123 | Shellfish liking \|\| id:ebi-fl187-GCST90094838 | Weighted mode | 33 | -0.04795941 | 0.161040624911524 | 0.76777365664231 | -0.363599035 | 0.267680214654476 | 0.953172475399048 | 0.69516987765903 | 1.30692913639732 | 0.999228396881742 |
| ebi-fl187-GCST90094838 | prot-a-3133 | PolyUbiquitin K48-linked \|\| id:prot-a-3133 | Shellfish liking \|\| id:ebi-fl187-GCST90094838 | Weighted mode | 34 | 0.34878862434611 | 0.165688752588168 | 0.0429796837974851 | 0.0240386692733002 | 0.673538579418921 | 1.41734956548521 | 1.02432992721869 | 1.96116479407735 | 0.999228396881742 |
| ebi-fl187-GCST90094838 | prot-a-3135 | Ubiquitin-conjugating enzyme E2 B \|\| id:prot-a-3135 | Shellfish liking \|\| id:ebi-fl187-GCST90094838 | Weighted mode | 34 | 0.0488600090842561 | 0.145830010232063 | 0.739709635024842 | -0.236966811 | 0.334686829139099 | 1.05007333972174 | 0.789017474307221 | 1.39750265957358 | 0.999228396881742 |
| ebi-fl187-GCST90094838 | prot-a-3162 | NKG2D ligand 3 \|\| id:prot-a-3162 | Shellfish liking \|\| id:ebi-fl187-GCST90094838 | Weighted mode | 34 | 0.11911150307792 | 0.149026656018972 | 0.429853488754974 | -0.172980743 | 0.411203748875106 | 1.12649551900342 | 0.841153813023774 | 1.50863270746289 | 0.999228396881742 |
| ebi-fl187-GCST90094838 | prot-a-3188 | Vesicle-associated membrane protein 8 \|\| id:prot-a-3188 | Shellfish liking \|\| id:ebi-fl187-GCST90094838 | Weighted mode | 34 | -0.146298154 | 0.199942386517742 | 0.469515344579371 | -0.538185232 | 0.245588923287734 | 0.863900089267015 | 0.583806765522442 | 1.27837395575175 | 0.999228396881742 |
| ebi-fl187-GCST90094838 | prot-a-3201 | Vitrin \|\| id:prot-a-3201 | Shellfish liking \|\| id:ebi-fl187-GCST90094838 | Weighted mode | 34 | -0.038798716 | 0.178121276216913 | 0.828909191597366 | -0.387916417 | 0.310318985662323 | 0.961944313926824 | 0.678469049299534 | 1.36386009656813 | 0.999228396881742 |
| ebi-fl187-GCST90094838 | prot-a-3232 | WNT1-inducible-signaling pathway protein 3 \|\| id:prot-a-3232 | Shellfish liking \|\| id:ebi-fl187-GCST90094838 | Weighted mode | 34 | 0.0623597177713731 | 0.188152899706988 | 0.742414530596116 | -0.306419966 | 0.43113940119707 | 1.06434513973646 | 0.736077427297213 | 1.53901007485074 | 0.999228396881742 |
| ebi-fl187-GCST90094838 | prot-a-327 | Carbonic anhydrase-related protein 10 \|\| id:prot-a-327 | Shellfish liking \|\| id:ebi-fl187-GCST90094838 | Weighted mode | 34 | -0.077381188 | 0.15187527259462 | 0.613788924122027 | -0.375056722 | 0.220294346374902 | 0.925536982847103 | 0.68725029533941 | 1.24644356274109 | 0.999228396881742 |
| ebi-fl187-GCST90094838 | prot-a-332 | Carbonic anhydrase 5A, mitochondrial \|\| id:prot-a-332 | Shellfish liking \|\| id:ebi-fl187-GCST90094838 | Weighted mode | 34 | -0.14502736 | 0.18786859799886 | 0.445630482038436 | -0.513249812 | 0.223195092036778 | 0.86499862638911 | 0.598547248571336 | 1.25006442756352 | 0.999228396881742 |
| ebi-fl187-GCST90094838 | prot-a-338 | Cell adhesion molecule 3 \|\| id:prot-a-338 | Shellfish liking \|\| id:ebi-fl187-GCST90094838 | Weighted mode | 34 | 0.00316840642545513 | 0.149747153994332 | 0.983246738083082 | -0.290336015 | 0.296672828254345 | 1.00317343113046 | 0.748012181731153 | 1.34537505872834 | 0.999228396881742 |
| ebi-fl187-GCST90094838 | prot-a-34 | A disintegrin and metalloproteinase with thrombospondin motifs 3 \|\| id:prot-a-34 | Shellfish liking \|\| id:ebi-fl187-GCST90094838 | Weighted mode | 34 | -0.017384228 | 0.228870382182579 | 0.93991221415785 | -0.465970177 | 0.431201721173639 | 0.98276600595941 | 0.627525998487384 | 1.5391059889112 | 0.999228396881742 |
| ebi-fl187-GCST90094838 | prot-a-358 | Cysteine--tRNA ligase, cytoplasmic \|\| id:prot-a-358 | Shellfish liking \|\| id:ebi-fl187-GCST90094838 | Weighted mode | 34 | 0.166456869821777 | 0.144438342011522 | 0.257419603508835 | -0.116642281 | 0.449556020164361 | 1.18111259317515 | 0.889903472039291 | 1.56761604105231 | 0.999228396881742 |
| ebi-fl187-GCST90094838 | prot-a-382 | Coiled-coil domain-containing protein 167 \|\| id:prot-a-382 | Shellfish liking \|\| id:ebi-fl187-GCST90094838 | Weighted mode | 34 | -0.127324797 | 0.176764497837505 | 0.476407195839957 | -0.473783212 | 0.219133619160669 | 0.880447659744567 | 0.622642219073398 | 1.24499762111105 | 0.999228396881742 |
| ebi-fl187-GCST90094838 | prot-a-40 | Pituitary adenylate cyclase-activating polypeptide 27 \|\| id:prot-a-40 | Shellfish liking \|\| id:ebi-fl187-GCST90094838 | Weighted mode | 34 | 0.0507167776993998 | 0.153782426272854 | 0.743638759296391 | -0.250696778 | 0.352130333194194 | 1.05202489417438 | 0.778258320988439 | 1.42209385767565 | 0.999228396881742 |
| ebi-fl187-GCST90094838 | prot-a-403 | C-C motif chemokine 25 \|\| id:prot-a-403 | Shellfish liking \|\| id:ebi-fl187-GCST90094838 | Weighted mode | 34 | -0.074398111 | 0.180192737193321 | 0.682364430411879 | -0.427575876 | 0.2787796535902 | 0.928302052721929 | 0.652087922160425 | 1.32151612045307 | 0.999228396881742 |
| ebi-fl187-GCST90094838 | prot-a-415 | Cyclin-C \|\| id:prot-a-415 | Shellfish liking \|\| id:ebi-fl187-GCST90094838 | Weighted mode | 34 | -0.021169215 | 0.149758626912139 | 0.888448467595083 | -0.314696124 | 0.272357693883346 | 0.979053280183632 | 0.730010673574076 | 1.31305658963227 | 0.999228396881742 |
| ebi-fl187-GCST90094838 | prot-a-44 | Adhesion G protein-coupled receptor F5 \|\| id:prot-a-44 | Shellfish liking \|\| id:ebi-fl187-GCST90094838 | Weighted mode | 34 | 0.0145816886101628 | 0.188658802641236 | 0.938858527267976 | -0.355189565 | 0.384352941786986 | 1.01468852006092 | 0.701040538166099 | 1.46866370301039 | 0.999228396881742 |
| ebi-fl187-GCST90094838 | prot-a-455 | CD59 glycoprotein \|\| id:prot-a-455 | Shellfish liking \|\| id:ebi-fl187-GCST90094838 | Weighted mode | 34 | -0.009259796 | 0.151691204302745 | 0.95169260258374 | -0.306574556 | 0.288054964862129 | 0.990782944313162 | 0.735963645624875 | 1.33383061592449 | 0.999228396881742 |
| ebi-fl187-GCST90094838 | prot-a-478 | Cadherin-12 \|\| id:prot-a-478 | Shellfish liking \|\| id:ebi-fl187-GCST90094838 | Weighted mode | 34 | -0.069390924 | 0.157598353389654 | 0.662588821598323 | -0.378283697 | 0.239501848737406 | 0.932961891673457 | 0.685036130709197 | 1.27061603365913 | 0.999228396881742 |
| ebi-fl187-GCST90094838 | prot-a-516 | Centrin-2 \|\| id:prot-a-516 | Shellfish liking \|\| id:ebi-fl187-GCST90094838 | Weighted mode | 34 | 0.0517018513697629 | 0.193800968799908 | 0.791300084923423 | -0.328148047 | 0.431551750217583 | 1.05306172679261 | 0.720256379671037 | 1.53964481500591 | 0.999228396881742 |
| ebi-fl187-GCST90094838 | prot-a-519 | Cryptic protein \|\| id:prot-a-519 | Shellfish liking \|\| id:ebi-fl187-GCST90094838 | Weighted mode | 34 | 0.034942789276686 | 0.146971170186806 | 0.813542931701738 | -0.253120704 | 0.323006282842827 | 1.0355604619418 | 0.776374164475935 | 1.38127402920599 | 0.999228396881742 |
| ebi-fl187-GCST90094838 | prot-a-583 | Chloride intracellular channel protein 4 \|\| id:prot-a-583 | Shellfish liking \|\| id:ebi-fl187-GCST90094838 | Weighted mode | 34 | 0.0112904131241138 | 0.176179715945412 | 0.949289463008249 | -0.33402183 | 0.356602656377122 | 1.01135439038824 | 0.716038150851465 | 1.42846816435868 | 0.999228396881742 |
| ebi-fl187-GCST90094838 | prot-a-602 | CCR4-NOT transcription complex subunit 1 \|\| id:prot-a-602 | Shellfish liking \|\| id:ebi-fl187-GCST90094838 | Weighted mode | 34 | -0.255157187 | 0.226298487393055 | 0.267652285664789 | -0.698702222 | 0.188387848399133 | 0.774794700818492 | 0.497230179545808 | 1.20730167457809 | 0.999228396881742 |
| ebi-fl187-GCST90094838 | prot-a-606 | Ciliary neurotrophic factor receptor subunit alpha \|\| id:prot-a-606 | Shellfish liking \|\| id:ebi-fl187-GCST90094838 | Weighted mode | 34 | -0.080218329 | 0.139792980323767 | 0.569969115793803 | -0.35421257 | 0.193775912836993 | 0.922914825688983 | 0.701725785627346 | 1.21382425004525 | 0.999228396881742 |
| ebi-fl187-GCST90094838 | prot-a-626 | Collagen alpha-2(IX) chain \|\| id:prot-a-626 | Shellfish liking \|\| id:ebi-fl187-GCST90094838 | Weighted mode | 34 | 0.172753699741312 | 0.150733305289526 | 0.260001588147017 | -0.122683579 | 0.468190978108783 | 1.18857332311242 | 0.884543506755773 | 1.59710238515669 | 0.999228396881742 |
| ebi-fl187-GCST90094838 | prot-a-647 | Probable serine carboxypeptidase CPVL \|\| id:prot-a-647 | Shellfish liking \|\| id:ebi-fl187-GCST90094838 | Weighted mode | 34 | -0.167419444 | 0.169251454880762 | 0.329774960348514 | -0.499152296 | 0.164313407303128 | 0.845844752191739 | 0.607045036271207 | 1.17858363393431 | 0.999228396881742 |
| ebi-fl187-GCST90094838 | prot-a-651 | Cellular retinoic acid-binding protein 2 \|\| id:prot-a-651 | Shellfish liking \|\| id:ebi-fl187-GCST90094838 | Weighted mode | 34 | 0.0639172039580558 | 0.153420171710377 | 0.679657267044286 | -0.236786333 | 0.364620740510394 | 1.06600413418457 | 0.789159887750746 | 1.43996778312878 | 0.999228396881742 |
| ebi-fl187-GCST90094838 | prot-a-671 | Cytotoxic and regulatory T-cell molecule \|\| id:prot-a-671 | Shellfish liking \|\| id:ebi-fl187-GCST90094838 | Weighted mode | 34 | 0.0958143231252975 | 0.194762573138574 | 0.626008299819636 | -0.28592032 | 0.477548966476902 | 1.10055469746483 | 0.751322478767005 | 1.61211820002996 | 0.999228396881742 |
| ebi-fl187-GCST90094838 | prot-a-688 | Chorionic somatomammotropin hormone \|\| id:prot-a-688 | Shellfish liking \|\| id:ebi-fl187-GCST90094838 | Weighted mode | 34 | 0.0974136049566083 | 0.161378844093434 | 0.550214738217235 | -0.218888929 | 0.413716139379738 | 1.10231620279313 | 0.803410948480755 | 1.51242774726686 | 0.999228396881742 |
| ebi-fl187-GCST90094838 | prot-a-69 | Aldo-keto reductase family 1 member C1 \|\| id:prot-a-69 | Shellfish liking \|\| id:ebi-fl187-GCST90094838 | Weighted mode | 34 | 0.0430742462108099 | 0.150779969322311 | 0.776911719790038 | -0.252454494 | 0.338602986082539 | 1.04401540616105 | 0.776891565525932 | 1.40298622956957 | 0.999228396881742 |
| ebi-fl187-GCST90094838 | prot-a-741 | Stromal cell-derived factor 1 \|\| id:prot-a-741 | Shellfish liking \|\| id:ebi-fl187-GCST90094838 | Weighted mode | 34 | -0.086107858 | 0.144672051106668 | 0.555774058240998 | -0.369665078 | 0.197449362384707 | 0.917495266876561 | 0.690965711538126 | 1.21829137203785 | 0.999228396881742 |
| ebi-fl187-GCST90094838 | prot-a-742 | Growth-regulated alpha protein \|\| id:prot-a-742 | Shellfish liking \|\| id:ebi-fl187-GCST90094838 | Weighted mode | 34 | -0.234776674 | 0.160609446526839 | 0.153258015957772 | -0.54957119 | 0.0800178407309827 | 0.790747424005406 | 0.577197265480033 | 1.08330639448051 | 0.999228396881742 |
| ebi-fl187-GCST90094838 | prot-a-788 | Beta-defensin 112 \|\| id:prot-a-788 | Shellfish liking \|\| id:ebi-fl187-GCST90094838 | Weighted mode | 34 | -0.150287828 | 0.138211995560579 | 0.284751073234916 | -0.42118334 | 0.120607682949895 | 0.860460275918693 | 0.656269770110392 | 1.12818222041453 | 0.999228396881742 |
| ebi-fl187-GCST90094838 | prot-a-838 | DnaJ homolog subfamily B member 1 \|\| id:prot-a-838 | Shellfish liking \|\| id:ebi-fl187-GCST90094838 | Weighted mode | 34 | 0.0806454100011118 | 0.139600944109972 | 0.56739773807403 | -0.19297244 | 0.354263260456657 | 1.08398645765488 | 0.824504696800755 | 1.42513031755733 | 0.999228396881742 |
| ebi-fl187-GCST90094838 | prot-a-85 | Junctional adhesion molecule-like \|\| id:prot-a-85 | Shellfish liking \|\| id:ebi-fl187-GCST90094838 | Weighted mode | 34 | 0.0310996209284615 | 0.132498451531663 | 0.815878811842245 | -0.228597344 | 0.290796585930521 | 1.03158826654869 | 0.795648841735222 | 1.33749249148667 | 0.999228396881742 |
| ebi-fl187-GCST90094838 | prot-a-856 | Dipeptidase 1 \|\| id:prot-a-856 | Shellfish liking \|\| id:ebi-fl187-GCST90094838 | Weighted mode | 34 | -0.225294262 | 0.206069562394176 | 0.282182376588796 | -0.629190604 | 0.178602080292586 | 0.798281280348232 | 0.533023053027661 | 1.19554491861976 | 0.999228396881742 |
| ebi-fl187-GCST90094838 | prot-a-860 | Dipeptidyl peptidase 2 \|\| id:prot-a-860 | Shellfish liking \|\| id:ebi-fl187-GCST90094838 | Weighted mode | 34 | 0.136625729711885 | 0.163992872239052 | 0.410766596980499 | -0.1848003 | 0.458051759300428 | 1.14639900513165 | 0.831270272053502 | 1.58099083192314 | 0.999228396881742 |
| ebi-fl187-GCST90094838 | prot-a-861 | Dermatopontin \|\| id:prot-a-861 | Shellfish liking \|\| id:ebi-fl187-GCST90094838 | Weighted mode | 34 | -0.073054131 | 0.145963356993079 | 0.620047331400329 | -0.35914231 | 0.213034048938698 | 0.929550511381081 | 0.698274972437982 | 1.23742678359501 | 0.999228396881742 |
| ebi-fl187-GCST90094838 | prot-a-877 | Dual specificity protein phosphatase 6 \|\| id:prot-a-877 | Shellfish liking \|\| id:ebi-fl187-GCST90094838 | Weighted mode | 34 | 0.127273101602848 | 0.159219855949295 | 0.429801255065804 | -0.184797816 | 0.439344019263466 | 1.13572714433505 | 0.831272336780868 | 1.55168900648677 | 0.999228396881742 |
| ebi-fl187-GCST90094838 | prot-a-879 | Dynactin-associated protein \|\| id:prot-a-879 | Shellfish liking \|\| id:ebi-fl187-GCST90094838 | Weighted mode | 34 | -0.085392011 | 0.156503006145997 | 0.588993027869854 | -0.392137903 | 0.221353881094884 | 0.918152288092955 | 0.675610938762658 | 1.24776491285686 | 0.999228396881742 |
| ebi-fl187-GCST90094838 | prot-a-937 | EMI domain-containing protein 1 \|\| id:prot-a-937 | Shellfish liking \|\| id:ebi-fl187-GCST90094838 | Weighted mode | 34 | -0.170184763 | 0.135434915044061 | 0.217728317537233 | -0.435637196 | 0.0952676707226259 | 0.843508953152528 | 0.646852363832346 | 1.09995324100398 | 0.999228396881742 |
| ebi-fl187-GCST90094838 | prot-b-1 | SRC proto-oncogene, non-receptor tyrosine kinase \|\| id:prot-b-1 | Shellfish liking \|\| id:ebi-fl187-GCST90094838 | Weighted mode | 23 | -0.069600221 | 0.217537849236025 | 0.752028475667386 | -0.495974406 | 0.35677396301552 | 0.93276664543957 | 0.608977227042864 | 1.42871289139905 | 0.999228396881742 |
| ebi-fl187-GCST90094838 | prot-b-10 | coagulation factor III, tissue factor \|\| id:prot-b-10 | Shellfish liking \|\| id:ebi-fl187-GCST90094838 | Weighted mode | 23 | -0.201474021 | 0.193930263155147 | 0.310135588564493 | -0.581577337 | 0.17862929434732 | 0.817524815404345 | 0.559015914177383 | 1.19557745468733 | 0.999228396881742 |
| ebi-fl187-GCST90094838 | prot-b-29 | myeloperoxidase \|\| id:prot-b-29 | Shellfish liking \|\| id:ebi-fl187-GCST90094838 | Weighted mode | 23 | -0.146693799 | 0.216670150760222 | 0.50543966478746 | -0.571367295 | 0.277979696021751 | 0.863558358966052 | 0.564752727001189 | 1.32045938635825 | 0.999228396881742 |
| ebi-fl187-GCST90094838 | prot-b-33 | adrenomedullin \|\| id:prot-b-33 | Shellfish liking \|\| id:ebi-fl187-GCST90094838 | Weighted mode | 23 | 0.20998370677324 | 0.170595547195301 | 0.231363285824249 | -0.124383566 | 0.544350979276029 | 1.23365795952411 | 0.883041071623906 | 1.72348943894356 | 0.999228396881742 |
| ebi-fl187-GCST90094838 | ukb-rsfMRI191-edge-pheno102 | Net25_Pair11_15 (Postcentral\|Precentral)&(Cerebellum) (Motor)&(Subcortical-cerebellum) \|\| id:ukb-rsfMRI191-edge-pheno102 | Shellfish liking \|\| id:ebi-fl187-GCST90094838 | Weighted mode | 30 | -0.071981641 | 0.0570694239147998 | 0.217253125314322 | -0.183837711 | 0.0398744302848691 | 0.930547979967999 | 0.832070828428871 | 1.04068008808525 | 0.999228396881742 |
| ebi-fl187-GCST90094838 | ukb-rsfMRI191-edge-pheno1167 | Net100_Pair11_45 (Angular\|Temporal)&(Frontal) (Default_mode\|Central_executive)&(Salience\|Default_mode) \|\| id:ukb-rsfMRI191-edge-pheno1167 | Shellfish liking \|\| id:ebi-fl187-GCST90094838 | Weighted mode | 30 | -0.073980131 | 0.048113214854681 | 0.134980699290675 | -0.168282033 | 0.0203217696550643 | 0.928690145375182 | 0.8451154509832 | 1.02052966267933 | 0.999228396881742 |
| ebi-fl187-GCST90094838 | ukb-rsfMRI191-edge-pheno1317 | Net100_Pair26_48 (Parietal\|Frontal)&(Temporal) (Attention\|Central_executive)&(Default_mode\|Central_executive) \|\| id:ukb-rsfMRI191-edge-pheno1317 | Shellfish liking \|\| id:ebi-fl187-GCST90094838 | Weighted mode | 30 | 0.0837722426856352 | 0.0557143395155751 | 0.143496989292411 | -0.025427863 | 0.192972348136162 | 1.08738120658031 | 0.974892702493323 | 1.21284925551296 | 0.999228396881742 |
| ebi-fl187-GCST90094838 | ukb-rsfMRI191-edge-pheno286 | Net100_Pair10_13 (OccipitalPrecuneus)&(Frontal_Sup) (Default_mode\|Central_executive)&(Default_mode) \|\| id:ukb-rsfMRI191-edge-pheno286 | Shellfish liking \|\| id:ebi-fl187-GCST90094838 | Weighted mode | 30 | 0.0127094717405824 | 0.0576197122722202 | 0.826968873832617 | -0.100225164 | 0.125644107794134 | 1.01279058032807 | 0.904633703875819 | 1.13387855792523 | 0.999228396881742 |
| ebi-fl187-GCST90094838 | ukb-rsfMRI191-edge-pheno599 | Net100_Pair11_29 (Angular\|Temporal)&(Parietal) (Default_mode\|Central_executive)&(Central_executive\|Salience) \|\| id:ukb-rsfMRI191-edge-pheno599 | Shellfish liking \|\| id:ebi-fl187-GCST90094838 | Weighted mode | 30 | -0.027608701 | 0.0555602318118393 | 0.622995455073995 | -0.136506755 | 0.0812893536728364 | 0.972768936162949 | 0.872400427149033 | 1.08468470866755 | 0.999228396881742 |
| ebi-fl187-GCST90094838 | ukb-rsfMRI191-edge-pheno621 | Net100_Pair5_30 (Precuneus\|Angular\|Cingulate)&(Paracentral) (Default_mode\|Central_executive)&(Motor) \|\| id:ukb-rsfMRI191-edge-pheno621 | Shellfish liking \|\| id:ebi-fl187-GCST90094838 | Weighted mode | 30 | -0.023403728 | 0.0546894920089882 | 0.671857424705423 | -0.130595132 | 0.0837876767013601 | 0.976868015534924 | 0.877573003725492 | 1.08739798936846 | 0.999228396881742 |
| ebi-fl187-GCST90094838 | ukb-rsfMRI191-edge-pheno624 | Net100_Pair8_30 (Calcarine\|Lingual)&(Paracentral) (Visual)&(Motor) \|\| id:ukb-rsfMRI191-edge-pheno624 | Shellfish liking \|\| id:ebi-fl187-GCST90094838 | Weighted mode | 30 | -0.063187051 | 0.0582140474726055 | 0.28667381851138 | -0.177286584 | 0.0509124824331598 | 0.93876786016149 | 0.837539724926751 | 1.05223080057398 | 0.999228396881742 |
| ebi-fl187-GCST90094838 | ukb-rsfMRI191-edge-pheno882 | Net100_Pair6_38 (Postcentral\|Precentral)&(Subcortical) (Motor)&(Subcortical-cerebellum) \|\| id:ukb-rsfMRI191-edge-pheno882 | Shellfish liking \|\| id:ebi-fl187-GCST90094838 | Weighted mode | 29 | -0.032833126 | 0.0498369351932446 | 0.515398687917158 | -0.130513519 | 0.0648472665536363 | 0.967700029679473 | 0.877644627636346 | 1.06699604595503 | 0.999228396881742 |
| ebi-fl187-GCST90094838 | ukb-rsfMRI191-edge-pheno899 | Net100_Pair23_38 (Cerebellum)&(Subcortical) (Subcortical-cerebellum)&(Subcortical-cerebellum) \|\| id:ukb-rsfMRI191-edge-pheno899 | Shellfish liking \|\| id:ebi-fl187-GCST90094838 | Weighted mode | 30 | 0.0304537964233244 | 0.0481974965490067 | 0.532431936097937 | -0.064013297 | 0.124920889659377 | 1.03092225665284 | 0.937992527136834 | 1.13305881285251 | 0.999228396881742 |
| ebi-fl187-GCST90094852 | ebi-a-GCST90001392 | IgD+ CD38+ B cell Absolute Count \|\| id:ebi-a-GCST90001392 | F-strong vegetable liking (derived food-liking factor) \|\| id:ebi-fl187-GCST90094852 | Inverse variance weighted | 23 | -0.176816504 | 0.0825899278182324 | 0.0322823694668474 | -0.338692762 | -0.014940245 | 0.837933528050767 | 0.712701384185356 | 0.985170806472011 | 0.998074519377826 |
| ebi-fl187-GCST90094852 | ebi-a-GCST90001402 | Switched memory B cell %B cell \|\| id:ebi-a-GCST90001402 | F-strong vegetable liking (derived food-liking factor) \|\| id:ebi-fl187-GCST90094852 | Inverse variance weighted | 23 | 0.0779084319255651 | 0.0987685180887436 | 0.430229946101153 | -0.115677864 | 0.271494727379503 | 1.08102366688231 | 0.890762124052095 | 1.31192395456112 | 0.998074519377826 |
| ebi-fl187-GCST90094852 | ebi-a-GCST90001407 | Memory B cell Absolute Count \|\| id:ebi-a-GCST90001407 | F-strong vegetable liking (derived food-liking factor) \|\| id:ebi-fl187-GCST90094852 | Inverse variance weighted | 23 | -0.055691145 | 0.0863984078599813 | 0.519195951490883 | -0.225032025 | 0.113649734146023 | 0.94583121523753 | 0.798490646954358 | 1.1203596324264 | 0.998074519377826 |
| ebi-fl187-GCST90094852 | ebi-a-GCST90001411 | IgD+ CD24+ B cell %B cell \|\| id:ebi-a-GCST90001411 | F-strong vegetable liking (derived food-liking factor) \|\| id:ebi-fl187-GCST90094852 | Inverse variance weighted (multiplicative random effects) | 23 | 0.0290665828094375 | 0.110880860445295 | 0.793211603743426 | -0.188259904 | 0.246393069282216 | 1.02949313873864 | 0.828399375218774 | 1.27940236848929 | 0.998074519377826 |
| ebi-fl187-GCST90094852 | ebi-a-GCST90001418 | CD24+ CD27+ B cell Absolute Count \|\| id:ebi-a-GCST90001418 | F-strong vegetable liking (derived food-liking factor) \|\| id:ebi-fl187-GCST90094852 | Inverse variance weighted | 23 | -0.071797044 | 0.0880889714736011 | 0.415042860039658 | -0.244451428 | 0.100857340115588 | 0.930719771831243 | 0.783134025779767 | 1.10611883172243 | 0.998074519377826 |
| ebi-fl187-GCST90094852 | ebi-a-GCST90001436 | Memory B cell %lymphocyte \|\| id:ebi-a-GCST90001436 | F-strong vegetable liking (derived food-liking factor) \|\| id:ebi-fl187-GCST90094852 | Inverse variance weighted | 23 | 0.0189837375695871 | 0.0893961115560584 | 0.831829860414245 | -0.156232641 | 0.194200116219462 | 1.01916507438154 | 0.855360175307158 | 1.21433926762622 | 0.998074519377826 |
| ebi-fl187-GCST90094852 | ebi-a-GCST90001439 | IgD+ CD24+ B cell %lymphocyte \|\| id:ebi-a-GCST90001439 | F-strong vegetable liking (derived food-liking factor) \|\| id:ebi-fl187-GCST90094852 | Inverse variance weighted | 23 | -0.029852019 | 0.0955893318604208 | 0.754816746310323 | -0.21720711 | 0.157503071213708 | 0.970589151449251 | 0.804763277779637 | 1.17058435309088 | 0.998074519377826 |
| ebi-fl187-GCST90094852 | ebi-a-GCST90001457 | CD11c+ HLA DR++ monocyte %monocyte \|\| id:ebi-a-GCST90001457 | F-strong vegetable liking (derived food-liking factor) \|\| id:ebi-fl187-GCST90094852 | Inverse variance weighted | 23 | -0.018304841 | 0.0919354046931266 | 0.842180253589705 | -0.198498234 | 0.161888552546072 | 0.981861675378786 | 0.819961218912826 | 1.17572920199306 | 0.998074519377826 |
| ebi-fl187-GCST90094852 | ebi-a-GCST90001483 | CD39+ resting CD4 regulatory T cell Absolute Count \|\| id:ebi-a-GCST90001483 | F-strong vegetable liking (derived food-liking factor) \|\| id:ebi-fl187-GCST90094852 | Inverse variance weighted | 23 | -0.023695854 | 0.0870504764906301 | 0.785461933095266 | -0.194314788 | 0.14692307994934 | 0.976582688338576 | 0.823398667536007 | 1.15826486581106 | 0.998074519377826 |
| ebi-fl187-GCST90094852 | ebi-a-GCST90001525 | CD33dim HLA DR+ CD11b+ Absolute Count \|\| id:ebi-a-GCST90001525 | F-strong vegetable liking (derived food-liking factor) \|\| id:ebi-fl187-GCST90094852 | Inverse variance weighted | 23 | -0.266643888 | 0.111161359026434 | 0.0164529295385144 | -0.484520151 | -0.048767624 | 0.765945785735791 | 0.615992709195704 | 0.952402419587778 | 0.998074519377826 |
| ebi-fl187-GCST90094852 | ebi-a-GCST90001540 | Naive CD4+ T cell Absolute Count \|\| id:ebi-a-GCST90001540 | F-strong vegetable liking (derived food-liking factor) \|\| id:ebi-fl187-GCST90094852 | Inverse variance weighted | 23 | -0.079502545 | 0.0918798812010343 | 0.386880817344535 | -0.259587112 | 0.100582022208791 | 0.923575669516115 | 0.771370009406291 | 1.10581433931904 | 0.998074519377826 |
| ebi-fl187-GCST90094852 | ebi-a-GCST90001624 | HLA DR+ CD4+ T cell Absolute Count \|\| id:ebi-a-GCST90001624 | F-strong vegetable liking (derived food-liking factor) \|\| id:ebi-fl187-GCST90094852 | Inverse variance weighted | 23 | -0.133123105 | 0.085986858006317 | 0.121579044914424 | -0.301657347 | 0.0354111363210784 | 0.875357324287079 | 0.739591444656578 | 1.03604557721572 | 0.998074519377826 |
| ebi-fl187-GCST90094852 | ebi-a-GCST90001672 | CD39+ CD8+ T cell Absolute Count \|\| id:ebi-a-GCST90001672 | F-strong vegetable liking (derived food-liking factor) \|\| id:ebi-fl187-GCST90094852 | Inverse variance weighted | 23 | 0.00384100501449178 | 0.106078478656309 | 0.97111563922155 | -0.204072813 | 0.211754823180857 | 1.00384839112792 | 0.815402996963451 | 1.23584484742247 | 0.998074519377826 |
| ebi-fl187-GCST90094852 | ebi-a-GCST90001722 | CD19 on CD20- CD38- B cell \|\| id:ebi-a-GCST90001722 | F-strong vegetable liking (derived food-liking factor) \|\| id:ebi-fl187-GCST90094852 | Inverse variance weighted | 23 | -0.058416295 | 0.082071009363544 | 0.476602590899964 | -0.219275473 | 0.102442883559965 | 0.943257192634222 | 0.803100455070795 | 1.10787402228251 | 0.998074519377826 |
| ebi-fl187-GCST90094852 | ebi-a-GCST90001742 | CD19 on transitional B cell \|\| id:ebi-a-GCST90001742 | F-strong vegetable liking (derived food-liking factor) \|\| id:ebi-fl187-GCST90094852 | Inverse variance weighted | 23 | -0.014087742 | 0.0841796114941453 | 0.867092006792585 | -0.179079781 | 0.150904296194235 | 0.986011025558223 | 0.836039196807807 | 1.16288536020145 | 0.998074519377826 |
| ebi-fl187-GCST90094852 | ebi-a-GCST90001784 | CD25 on IgD+ CD38dim B cell \|\| id:ebi-a-GCST90001784 | F-strong vegetable liking (derived food-liking factor) \|\| id:ebi-fl187-GCST90094852 | Inverse variance weighted | 23 | 0.0485871135203319 | 0.0973426684643028 | 0.61768442181575 | -0.142204517 | 0.239378743710365 | 1.0497868184625 | 0.867443831619765 | 1.27045962406554 | 0.998074519377826 |
| ebi-fl187-GCST90094852 | ebi-a-GCST90001794 | CD25 on IgD+ B cell \|\| id:ebi-a-GCST90001794 | F-strong vegetable liking (derived food-liking factor) \|\| id:ebi-fl187-GCST90094852 | Inverse variance weighted | 23 | 0.0668343905120591 | 0.093258161032718 | 0.473583933805695 | -0.115951605 | 0.249620386136186 | 1.06911840736071 | 0.890518318788902 | 1.2835380753447 | 0.998074519377826 |
| ebi-fl187-GCST90094852 | ebi-a-GCST90001802 | CD27 on IgD- CD38- B cell \|\| id:ebi-a-GCST90001802 | F-strong vegetable liking (derived food-liking factor) \|\| id:ebi-fl187-GCST90094852 | Inverse variance weighted | 23 | 0.000439806378346572 | 0.0902884338407546 | 0.996113418780683 | -0.176525524 | 0.177405136706226 | 1.00043990310735 | 0.838177385284876 | 1.19411477486866 | 0.999355215041207 |
| ebi-fl187-GCST90094852 | ebi-a-GCST90001804 | CD27 on IgD- CD38dim B cell \|\| id:ebi-a-GCST90001804 | F-strong vegetable liking (derived food-liking factor) \|\| id:ebi-fl187-GCST90094852 | Inverse variance weighted (multiplicative random effects) | 23 | 0.0895006170562816 | 0.105374352373065 | 0.395681757916602 | -0.117033114 | 0.296034347707489 | 1.09362800814217 | 0.889555736287388 | 1.34451633709282 | 0.998074519377826 |
| ebi-fl187-GCST90094852 | ebi-a-GCST90001805 | CD27 on memory B cell \|\| id:ebi-a-GCST90001805 | F-strong vegetable liking (derived food-liking factor) \|\| id:ebi-fl187-GCST90094852 | Inverse variance weighted | 23 | 0.143984687915062 | 0.0954624739917145 | 0.131481412124026 | -0.043121761 | 0.331091136938823 | 1.15486642497674 | 0.957794760814739 | 1.39248669350001 | 0.998074519377826 |
| ebi-fl187-GCST90094852 | ebi-a-GCST90001806 | CD27 on unswitched memory B cell \|\| id:ebi-a-GCST90001806 | F-strong vegetable liking (derived food-liking factor) \|\| id:ebi-fl187-GCST90094852 | Inverse variance weighted (multiplicative random effects) | 23 | 0.133227610178789 | 0.116817184643644 | 0.254086567717866 | -0.095734072 | 0.362189292080331 | 1.14251001564741 | 0.908705634464598 | 1.43647082877804 | 0.998074519377826 |
| ebi-fl187-GCST90094852 | ebi-a-GCST90001839 | CD3 on Effector Memory CD8+ T cell \|\| id:ebi-a-GCST90001839 | F-strong vegetable liking (derived food-liking factor) \|\| id:ebi-fl187-GCST90094852 | Inverse variance weighted | 23 | -0.167484921 | 0.110206623251206 | 0.128577457965429 | -0.383489903 | 0.0485200605382519 | 0.84578937082176 | 0.681478959175245 | 1.0497164294857 | 0.998074519377826 |
| ebi-fl187-GCST90094852 | ebi-a-GCST90001859 | CD3 on CD8+ T cell \|\| id:ebi-a-GCST90001859 | F-strong vegetable liking (derived food-liking factor) \|\| id:ebi-fl187-GCST90094852 | Inverse variance weighted | 23 | -0.13110329 | 0.110432679731307 | 0.235157266363594 | -0.347551342 | 0.0853447623070195 | 0.87712717127593 | 0.706415744032023 | 1.08909248001647 | 0.998074519377826 |
| ebi-fl187-GCST90094852 | ebi-a-GCST90001905 | CD86 on monocyte \|\| id:ebi-a-GCST90001905 | F-strong vegetable liking (derived food-liking factor) \|\| id:ebi-fl187-GCST90094852 | Inverse variance weighted | 23 | 0.0231118656259735 | 0.0930622707459629 | 0.803864857342635 | -0.159290185 | 0.205513916288061 | 1.02338101430198 | 0.852748868100709 | 1.22815607221663 | 0.998074519377826 |
| ebi-fl187-GCST90094852 | ebi-a-GCST90001943 | CD25 on activated & secreting CD4 regulatory T cell \|\| id:ebi-a-GCST90001943 | F-strong vegetable liking (derived food-liking factor) \|\| id:ebi-fl187-GCST90094852 | Inverse variance weighted | 23 | -0.003786338 | 0.0851259487113131 | 0.9645223922439 | -0.170633198 | 0.163060521177375 | 0.996220820843558 | 0.843130779008616 | 1.17710792749042 | 0.998074519377826 |
| ebi-fl187-GCST90094852 | ebi-a-GCST90001949 | CD33 on CD33dim HLA DR+ CD11b- \|\| id:ebi-a-GCST90001949 | F-strong vegetable liking (derived food-liking factor) \|\| id:ebi-fl187-GCST90094852 | Inverse variance weighted | 23 | 0.181912304693296 | 0.12106018137733 | 0.132926715799037 | -0.055365651 | 0.419190260192863 | 1.19950899795787 | 0.946139128161075 | 1.52072966158625 | 0.998074519377826 |
| ebi-fl187-GCST90094852 | ebi-a-GCST90001955 | CD33 on Immature Myeloid-Derived Suppressor Cells \|\| id:ebi-a-GCST90001955 | F-strong vegetable liking (derived food-liking factor) \|\| id:ebi-fl187-GCST90094852 | Inverse variance weighted | 23 | 0.207432591066588 | 0.129104191758043 | 0.108118687192899 | -0.045611625 | 0.460476806912353 | 1.23051476635637 | 0.955412948860027 | 1.58482946251435 | 0.998074519377826 |
| ebi-fl187-GCST90094852 | ebi-a-GCST90001956 | CD33 on CD33+ HLA DR+ \|\| id:ebi-a-GCST90001956 | F-strong vegetable liking (derived food-liking factor) \|\| id:ebi-fl187-GCST90094852 | Inverse variance weighted | 23 | 0.195962023108565 | 0.124542177137531 | 0.115612549454265 | -0.048140644 | 0.440064690298126 | 1.21648070630136 | 0.952999743878367 | 1.55280766685318 | 0.998074519377826 |
| ebi-fl187-GCST90094852 | ebi-a-GCST90001957 | CD33 on CD33+ HLA DR+ CD14- \|\| id:ebi-a-GCST90001957 | F-strong vegetable liking (derived food-liking factor) \|\| id:ebi-fl187-GCST90094852 | Inverse variance weighted | 23 | 0.191530065832285 | 0.124638374066312 | 0.124370179153386 | -0.052761147 | 0.435821279002257 | 1.21110124535953 | 0.948606562628139 | 1.54623242585176 | 0.998074519377826 |
| ebi-fl187-GCST90094852 | ebi-a-GCST90002064 | CD4 on resting CD4 regulatory T cell \|\| id:ebi-a-GCST90002064 | F-strong vegetable liking (derived food-liking factor) \|\| id:ebi-fl187-GCST90094852 | Inverse variance weighted | 23 | 0.0395879051559108 | 0.0900760122040232 | 0.660303320996421 | -0.136961079 | 0.216136889075796 | 1.0403819498029 | 0.872004164951554 | 1.2412722839871 | 0.998074519377826 |
| ebi-fl187-GCST90094852 | ebi-a-GCST90002080 | SSC-A on T cell \|\| id:ebi-a-GCST90002080 | F-strong vegetable liking (derived food-liking factor) \|\| id:ebi-fl187-GCST90094852 | Inverse variance weighted | 23 | -0.118501064 | 0.0917487984930559 | 0.196502063990685 | -0.298328709 | 0.0613265809803825 | 0.888250870498745 | 0.742057378631573 | 1.06324609344462 | 0.998074519377826 |
| ebi-fl187-GCST90094852 | ebi-a-GCST90002121 | CD8 on CD39+ CD8+ T cell \|\| id:ebi-a-GCST90002121 | F-strong vegetable liking (derived food-liking factor) \|\| id:ebi-fl187-GCST90094852 | Inverse variance weighted | 23 | 0.0102475502420912 | 0.0967214178580956 | 0.915622711922036 | -0.179326429 | 0.199821529243959 | 1.01030023619861 | 0.835833014926347 | 1.22118479293728 | 0.998074519377826 |
| ebi-fl187-GCST90094852 | ebi-cfmet338-GCST90026053 | 1-palmitoyl-2-docosahexaenoyl-gpc (16:0/22:6) levels \|\| id:ebi-cfmet338-GCST90026053 | F-strong vegetable liking (derived food-liking factor) \|\| id:ebi-fl187-GCST90094852 | Inverse variance weighted | 20 | 0.121868456469059 | 0.0528389118459356 | 0.021087457165368 | 0.0183041892510256 | 0.225432723687093 | 1.12960549971071 | 1.01847273773365 | 1.25286474316054 | 0.998074519377826 |
| ebi-fl187-GCST90094852 | ebi-cfmet338-GCST90026063 | 1-stearoyl-2-oleoyl-gpc (18:0/18:1) levels \|\| id:ebi-cfmet338-GCST90026063 | F-strong vegetable liking (derived food-liking factor) \|\| id:ebi-fl187-GCST90094852 | Inverse variance weighted | 20 | 0.0817950682597034 | 0.0431902560648277 | 0.0582473765361689 | -0.002857834 | 0.166447970146766 | 1.08523338827282 | 0.99714624609185 | 1.1811020817037 | 0.998074519377826 |
| ebi-fl187-GCST90094852 | ebi-cfmet338-GCST90026113 | Adenosine 3',5'-cyclic monophosphate (camp) levels \|\| id:ebi-cfmet338-GCST90026113 | F-strong vegetable liking (derived food-liking factor) \|\| id:ebi-fl187-GCST90094852 | Inverse variance weighted | 20 | 0.0773103396582287 | 0.0835989088728076 | 0.355081969678876 | -0.086543522 | 0.241164201048932 | 1.08037730829665 | 0.917095634325288 | 1.2727300017528 | 0.998074519377826 |
| ebi-fl187-GCST90094852 | ebi-cfmet338-GCST90026117 | Alpha-ketoglutaramate levels \|\| id:ebi-cfmet338-GCST90026117 | F-strong vegetable liking (derived food-liking factor) \|\| id:ebi-fl187-GCST90094852 | Inverse variance weighted | 19 | 0.0190835185116179 | 0.0408608779536797 | 0.640473815203709 | -0.061003802 | 0.0991708393008301 | 1.01926677270644 | 0.940819662511686 | 1.10425493358616 | 0.998074519377826 |
| ebi-fl187-GCST90094852 | ebi-cfmet338-GCST90026124 | Arginine levels \|\| id:ebi-cfmet338-GCST90026124 | F-strong vegetable liking (derived food-liking factor) \|\| id:ebi-fl187-GCST90094852 | Inverse variance weighted | 19 | -0.004781533 | 0.0278944234080281 | 0.86389715634938 | -0.059454603 | 0.0498915364813866 | 0.995229879934148 | 0.942278308966652 | 1.05115707799742 | 0.998074519377826 |
| ebi-fl187-GCST90094852 | ebi-cfmet338-GCST90026169 | Glutamate levels \|\| id:ebi-cfmet338-GCST90026169 | F-strong vegetable liking (derived food-liking factor) \|\| id:ebi-fl187-GCST90094852 | Inverse variance weighted | 20 | 0.0171824436359905 | 0.0333424900346505 | 0.606321218116749 | -0.048168837 | 0.0825337241039055 | 1.01733091094539 | 0.952972876572687 | 1.08603529838873 | 0.998074519377826 |
| ebi-fl187-GCST90094852 | ebi-cfmet338-GCST90026170 | Glutamine levels \|\| id:ebi-cfmet338-GCST90026170 | F-strong vegetable liking (derived food-liking factor) \|\| id:ebi-fl187-GCST90094852 | Inverse variance weighted (multiplicative random effects) | 20 | 0.00152815081087964 | 0.0225372097829365 | 0.945940323024098 | -0.04264478 | 0.0457010819854353 | 1.00152931902833 | 0.958251719444901 | 1.04676146832734 | 0.998074519377826 |
| ebi-fl187-GCST90094852 | ebi-cfmet338-GCST90026191 | Kynurenate levels \|\| id:ebi-cfmet338-GCST90026191 | F-strong vegetable liking (derived food-liking factor) \|\| id:ebi-fl187-GCST90094852 | Inverse variance weighted | 20 | 0.0417960531119424 | 0.143420477369082 | 0.770727680341858 | -0.239308083 | 0.322900188755343 | 1.04268180535503 | 0.787172330967263 | 1.3811274919718 | 0.998074519377826 |
| ebi-fl187-GCST90094852 | ebi-cfmet338-GCST90026202 | Methylsuccinoylcarnitine levels \|\| id:ebi-cfmet338-GCST90026202 | F-strong vegetable liking (derived food-liking factor) \|\| id:ebi-fl187-GCST90094852 | Inverse variance weighted | 20 | -0.016163194 | 0.0879215506523333 | 0.854141687090225 | -0.188489433 | 0.156163045305384 | 0.983966729511862 | 0.828209254871089 | 1.16901679025184 | 0.998074519377826 |
| ebi-fl187-GCST90094852 | ebi-cfmet338-GCST90026222 | N-acetylputrescine levels \|\| id:ebi-cfmet338-GCST90026222 | F-strong vegetable liking (derived food-liking factor) \|\| id:ebi-fl187-GCST90094852 | Inverse variance weighted | 19 | -0.027141526 | 0.0324191082614524 | 0.402475844328163 | -0.090682978 | 0.0363999259520514 | 0.973223495114873 | 0.913307203090028 | 1.0370705149801 | 0.998074519377826 |
| ebi-fl187-GCST90094852 | ebi-cfmet338-GCST90026252 | Propionylcarnitine (c3) levels \|\| id:ebi-cfmet338-GCST90026252 | F-strong vegetable liking (derived food-liking factor) \|\| id:ebi-fl187-GCST90094852 | Inverse variance weighted | 20 | 0.046888547684474 | 0.0528520321015733 | 0.374989312897757 | -0.056701435 | 0.150478530603558 | 1.04800519996423 | 0.944876133978415 | 1.16239034901602 | 0.998074519377826 |
| ebi-fl187-GCST90094852 | ebi-cfmet338-GCST90026316 | Ethyl beta-glucopyranoside levels \|\| id:ebi-cfmet338-GCST90026316 | F-strong vegetable liking (derived food-liking factor) \|\| id:ebi-fl187-GCST90094852 | Inverse variance weighted | 20 | -0.082528993 | 0.170251023683749 | 0.627854488547079 | -0.416221 | 0.251163013320948 | 0.920784741066456 | 0.659534497570154 | 1.28551962407491 | 0.998074519377826 |
| ebi-fl187-GCST90094852 | ebi-gm430-GCST90011315 | F_Prevotellaceae abundance \|\| id:ebi-gm430-GCST90011315 | F-strong vegetable liking (derived food-liking factor) \|\| id:ebi-fl187-GCST90094852 | Inverse variance weighted | 19 | 0.00358346213307573 | 0.0734403839648377 | 0.961083333158493 | -0.14035969 | 0.147526614704158 | 1.00358989040971 | 0.869045591785161 | 1.15896412990673 | 0.998074519377826 |
| ebi-fl187-GCST90094852 | ebi-gm430-GCST90011354 | OTU97_1 (Bacteroides) abundance \|\| id:ebi-gm430-GCST90011354 | F-strong vegetable liking (derived food-liking factor) \|\| id:ebi-fl187-GCST90094852 | Inverse variance weighted | 19 | -0.052422495 | 0.0744838955312175 | 0.481551238804271 | -0.19841093 | 0.093565940693209 | 0.948927865288861 | 0.820032807982735 | 1.09808300930886 | 0.998074519377826 |
| ebi-fl187-GCST90094852 | ebi-gm430-GCST90011419 | OTU99_1 (Bacteroides) abundance \|\| id:ebi-gm430-GCST90011419 | F-strong vegetable liking (derived food-liking factor) \|\| id:ebi-fl187-GCST90094852 | Inverse variance weighted | 19 | -0.050932542 | 0.0739293736558951 | 0.490864097264898 | -0.195834114 | 0.0939690304536083 | 0.950342776676874 | 0.822148606082361 | 1.09852572454697 | 0.998074519377826 |
| ebi-fl187-GCST90094852 | ebi-gm430-GCST90011435 | OTU99_155 (Butyrivibrio) abundance \|\| id:ebi-gm430-GCST90011435 | F-strong vegetable liking (derived food-liking factor) \|\| id:ebi-fl187-GCST90094852 | Inverse variance weighted | 19 | -0.249678131 | 0.263780789515098 | 0.343875042276451 | -0.766688478 | 0.267332216485377 | 0.779051495274603 | 0.464548887627533 | 1.30647440657788 | 0.998074519377826 |
| ebi-fl187-GCST90094852 | ebi-gm430-GCST90011466 | OTU99_58 (Bacteroides) abundance \|\| id:ebi-gm430-GCST90011466 | F-strong vegetable liking (derived food-liking factor) \|\| id:ebi-fl187-GCST90094852 | Inverse variance weighted | 19 | -0.056233431 | 0.123115274364452 | 0.647847658082961 | -0.297539369 | 0.185072507003199 | 0.945318443738855 | 0.742643345886659 | 1.20330568505388 | 0.998074519377826 |
| ebi-fl187-GCST90094852 | ebi-gm430-GCST90011472 | OTU99_72 (Parabacteroides) abundance \|\| id:ebi-gm430-GCST90011472 | F-strong vegetable liking (derived food-liking factor) \|\| id:ebi-fl187-GCST90094852 | Inverse variance weighted | 19 | -0.269783359 | 0.1833757120137 | 0.141235546007592 | -0.629199754 | 0.0896330366007704 | 0.763544891590317 | 0.533018175782249 | 1.09377283545362 | 0.998074519377826 |
| ebi-fl187-GCST90094852 | ebi-gm430-GCST90011505 | TestASV_28 (Holdemanella) abundance \|\| id:ebi-gm430-GCST90011505 | F-strong vegetable liking (derived food-liking factor) \|\| id:ebi-fl187-GCST90094852 | Inverse variance weighted | 19 | -0.028664591 | 0.156868629559071 | 0.855009944276283 | -0.336127105 | 0.278797923012906 | 0.971742341014379 | 0.714532279519558 | 1.32154026401023 | 0.998074519377826 |
| ebi-fl187-GCST90094852 | ebi-gm430-GCST90011513 | TestASV_35 (Alphaproteobacteria) abundance \|\| id:ebi-gm430-GCST90011513 | F-strong vegetable liking (derived food-liking factor) \|\| id:ebi-fl187-GCST90094852 | Inverse variance weighted | 19 | -0.060665985 | 0.187390871337471 | 0.746134626396398 | -0.427952092 | 0.30662012320634 | 0.941137541700481 | 0.651842642243456 | 1.35882468405191 | 0.998074519377826 |
| ebi-fl187-GCST90094852 | ebi-gm430-GCST90011543 | G_Coprococcus prevalence \|\| id:ebi-gm430-GCST90011543 | F-strong vegetable liking (derived food-liking factor) \|\| id:ebi-fl187-GCST90094852 | Inverse variance weighted | 19 | 0.125722473777217 | 0.196367711766174 | 0.522016548393623 | -0.259158241 | 0.510603188838918 | 1.13396741891491 | 0.771700898439975 | 1.66629598301624 | 0.998074519377826 |
| ebi-fl187-GCST90094852 | ebi-gm430-GCST90011547 | G_Parasutterella prevalence \|\| id:ebi-gm430-GCST90011547 | F-strong vegetable liking (derived food-liking factor) \|\| id:ebi-fl187-GCST90094852 | Inverse variance weighted | 19 | 0.243819567497868 | 0.176474724520184 | 0.167090918937472 | -0.102070893 | 0.589710027557428 | 1.27611405726333 | 0.902965535859824 | 1.80346538430664 | 0.998074519377826 |
| ebi-fl187-GCST90094852 | ebi-gm430-GCST90011569 | OTU97_13 (Subdoligranulum) prevalence \|\| id:ebi-gm430-GCST90011569 | F-strong vegetable liking (derived food-liking factor) \|\| id:ebi-fl187-GCST90094852 | Inverse variance weighted | 19 | 0.195412125471957 | 0.181774201171688 | 0.282362760189775 | -0.160865309 | 0.551689559768466 | 1.2158119503265 | 0.851406740359153 | 1.73618392771142 | 0.998074519377826 |
| ebi-fl187-GCST90094852 | ebi-gm430-GCST90011590 | OTU97_277 (Prevotella) prevalence \|\| id:ebi-gm430-GCST90011590 | F-strong vegetable liking (derived food-liking factor) \|\| id:ebi-fl187-GCST90094852 | Inverse variance weighted | 19 | -0.111384666 | 0.179427989352207 | 0.534746735686194 | -0.463063525 | 0.240294193081271 | 0.894594562578981 | 0.629352651498197 | 1.27162319804443 | 0.998074519377826 |
| ebi-fl187-GCST90094852 | ebi-gm430-GCST90011616 | OTU97_86 (Phascolarctobacterium) prevalence \|\| id:ebi-gm430-GCST90011616 | F-strong vegetable liking (derived food-liking factor) \|\| id:ebi-fl187-GCST90094852 | Inverse variance weighted | 19 | 0.0231181219727264 | 0.194003473323319 | 0.905145867930915 | -0.357128686 | 0.403364929686431 | 1.02338741694849 | 0.699682452786098 | 1.49685303811483 | 0.998074519377826 |
| ebi-fl187-GCST90094852 | ebi-gm430-GCST90011665 | OTU99_55 (Barnesiella) prevalence \|\| id:ebi-gm430-GCST90011665 | F-strong vegetable liking (derived food-liking factor) \|\| id:ebi-fl187-GCST90094852 | Inverse variance weighted | 19 | 0.0269551310507088 | 0.129802655757031 | 0.835492577902537 | -0.227458074 | 0.281368336334489 | 1.02732170688379 | 0.796555817010951 | 1.32494153817737 | 0.998074519377826 |
| ebi-fl187-GCST90094852 | ebi-gm430-GCST90011687 | TestASV_14 (Lachnospiraceae) prevalence \|\| id:ebi-gm430-GCST90011687 | F-strong vegetable liking (derived food-liking factor) \|\| id:ebi-fl187-GCST90094852 | Inverse variance weighted | 19 | -0.141945825 | 0.140193200232308 | 0.311298330231206 | -0.416724498 | 0.132832847040575 | 0.867668260780848 | 0.659202506623843 | 1.14205908381968 | 0.998074519377826 |
| ebi-fl187-GCST90094852 | ebi-gm430-GCST90011697 | TestASV_23 (Barnesiella) prevalence \|\| id:ebi-gm430-GCST90011697 | F-strong vegetable liking (derived food-liking factor) \|\| id:ebi-fl187-GCST90094852 | Inverse variance weighted | 19 | 0.115121681540549 | 0.22506396986001 | 0.608996444203391 | -0.326003699 | 0.556247062466168 | 1.12200995716591 | 0.721802517201321 | 1.74411464906034 | 0.998074519377826 |
| ebi-fl187-GCST90094852 | ebi-gm430-GCST90011727 | TestASV_6 (Subdoligranulum) prevalence \|\| id:ebi-gm430-GCST90011727 | F-strong vegetable liking (derived food-liking factor) \|\| id:ebi-fl187-GCST90094852 | Inverse variance weighted | 19 | 0.44440911416794 | 0.216032700948852 | 0.0396729374087464 | 0.0209850203081889 | 0.867833208027691 | 1.55956839665074 | 1.0212067541605 | 2.38174451346165 | 0.998074519377826 |
| ebi-fl187-GCST90094852 | ebi-hbsm44-GCST90104908 | Sulcal depth of the left posterior inferior temporal sulcus \|\| id:ebi-hbsm44-GCST90104908 | F-strong vegetable liking (derived food-liking factor) \|\| id:ebi-fl187-GCST90094852 | Inverse variance weighted | 22 | 0.0286650470643981 | 0.0663590099034949 | 0.665763813625231 | -0.101398612 | 0.158728706475248 | 1.02907984342614 | 0.903572785822824 | 1.1720199421252 | 0.998074519377826 |
| ebi-fl187-GCST90094852 | ebi-hbsm44-GCST90104918 | Sulcal depth of the left inferior frontal sulcus \|\| id:ebi-hbsm44-GCST90104918 | F-strong vegetable liking (derived food-liking factor) \|\| id:ebi-fl187-GCST90094852 | Inverse variance weighted | 23 | -0.119843159 | 0.0896342336238964 | 0.181214664984933 | -0.295526257 | 0.0558399384466148 | 0.887059552709969 | 0.744139875318694 | 1.05742841655544 | 0.998074519377826 |
| ebi-fl187-GCST90094852 | ebi-hbsm44-GCST90104925 | Sulcal depth of the right anterior inferior temporal sulcus \|\| id:ebi-hbsm44-GCST90104925 | F-strong vegetable liking (derived food-liking factor) \|\| id:ebi-fl187-GCST90094852 | Inverse variance weighted | 24 | -0.006200503 | 0.0299613466582231 | 0.836048835813189 | -0.064924743 | 0.0525237360164699 | 0.99381868001827 | 0.937137987026085 | 1.05392757782399 | 0.998074519377826 |
| ebi-fl187-GCST90094852 | ebi-IPs91-GCST90274770 | C-C motif chemokine 4 levels \|\| id:ebi-IPs91-GCST90274770 | F-strong vegetable liking (derived food-liking factor) \|\| id:ebi-fl187-GCST90094852 | Inverse variance weighted | 27 | -0.024558193 | 0.0373937679761749 | 0.511344504013845 | -0.097849978 | 0.0487335923886872 | 0.975740906127312 | 0.906784931168407 | 1.04994060131038 | 0.998074519377826 |
| ebi-fl187-GCST90094852 | ebi-IPs91-GCST90274779 | C-X-C motif chemokine 1 levels \|\| id:ebi-IPs91-GCST90274779 | F-strong vegetable liking (derived food-liking factor) \|\| id:ebi-fl187-GCST90094852 | Inverse variance weighted | 27 | -0.0378073 | 0.0410044181538364 | 0.356512970565551 | -0.11817596 | 0.0425613592925123 | 0.962898473272123 | 0.888539691529303 | 1.04348008160894 | 0.998074519377826 |
| ebi-fl187-GCST90094852 | ebi-IPs91-GCST90274780 | C-X-C motif chemokine 10 levels \|\| id:ebi-IPs91-GCST90274780 | F-strong vegetable liking (derived food-liking factor) \|\| id:ebi-fl187-GCST90094852 | Inverse variance weighted | 27 | -0.070168418 | 0.0375197548807435 | 0.0614598513275416 | -0.143707138 | 0.00337030148362638 | 0.932236801149313 | 0.866141371117411 | 1.00337598733556 | 0.998074519377826 |
| ebi-fl187-GCST90094852 | ebi-IPs91-GCST90274796 | Interleukin-10 receptor subunit alpha levels \|\| id:ebi-IPs91-GCST90274796 | F-strong vegetable liking (derived food-liking factor) \|\| id:ebi-fl187-GCST90094852 | Inverse variance weighted | 27 | -0.030836948 | 0.0416542349525788 | 0.459113346669001 | -0.112479249 | 0.0508053521641095 | 0.969633660562553 | 0.893615890473503 | 1.05211808084316 | 0.998074519377826 |
| ebi-fl187-GCST90094852 | ebi-IPs91-GCST90274807 | Interleukin-20 levels \|\| id:ebi-IPs91-GCST90274807 | F-strong vegetable liking (derived food-liking factor) \|\| id:ebi-fl187-GCST90094852 | Inverse variance weighted | 27 | -0.016266136 | 0.0444997572749692 | 0.714713476997502 | -0.10348566 | 0.0709533884578708 | 0.983865443391532 | 0.901688952814621 | 1.07353118575805 | 0.998074519377826 |
| ebi-fl187-GCST90094852 | ebi-IPs91-GCST90274808 | Interleukin-20 receptor subunit alpha levels \|\| id:ebi-IPs91-GCST90274808 | F-strong vegetable liking (derived food-liking factor) \|\| id:ebi-fl187-GCST90094852 | Inverse variance weighted | 27 | -0.037269785 | 0.0492941143172075 | 0.449607424828806 | -0.133886249 | 0.0593466792646579 | 0.963416185245308 | 0.874689555879785 | 1.06114305327339 | 0.998074519377826 |
| ebi-fl187-GCST90094852 | ebi-IPs91-GCST90274810 | Interleukin-24 levels \|\| id:ebi-IPs91-GCST90274810 | F-strong vegetable liking (derived food-liking factor) \|\| id:ebi-fl187-GCST90094852 | Inverse variance weighted | 27 | 0.00311105171657712 | 0.0493228933144681 | 0.949706616748816 | -0.093561819 | 0.0997839226129346 | 1.00311589606033 | 0.910681718089174 | 1.10493214142946 | 0.998074519377826 |
| ebi-fl187-GCST90094852 | ebi-IPs91-GCST90274823 | Monocyte chemoattractant protein-3 levels \|\| id:ebi-IPs91-GCST90274823 | F-strong vegetable liking (derived food-liking factor) \|\| id:ebi-fl187-GCST90094852 | Inverse variance weighted | 27 | -0.071851925 | 0.0440586955658003 | 0.102927714966316 | -0.158206969 | 0.0145031180226081 | 0.930668694109106 | 0.853673080164979 | 1.01460879851964 | 0.998074519377826 |
| ebi-fl187-GCST90094852 | ebi-IPs91-GCST90274824 | Monocyte chemoattractant protein-4 levels \|\| id:ebi-IPs91-GCST90274824 | F-strong vegetable liking (derived food-liking factor) \|\| id:ebi-fl187-GCST90094852 | Inverse variance weighted | 27 | -0.074742817 | 0.0371158182436026 | 0.0440334160463505 | -0.147489821 | -0.001995813 | 0.927982116886999 | 0.862871221671511 | 0.998006177090826 | 0.998074519377826 |
| ebi-fl187-GCST90094852 | ebi-IPs91-GCST90274834 | SIR2-like protein 2 levels \|\| id:ebi-IPs91-GCST90274834 | F-strong vegetable liking (derived food-liking factor) \|\| id:ebi-fl187-GCST90094852 | Inverse variance weighted | 27 | -0.002998518 | 0.0412382787549697 | 0.942035299952797 | -0.083825544 | 0.0778285086734438 | 0.997005973377894 | 0.919591670344213 | 1.08093727140779 | 0.998074519377826 |
| ebi-fl187-GCST90094852 | ebi-met1400-GCST90199652 | 1-stearoyl-2-oleoyl-GPS (18:0/18:1) levels \|\| id:ebi-met1400-GCST90199652 | F-strong vegetable liking (derived food-liking factor) \|\| id:ebi-fl187-GCST90094852 | Inverse variance weighted | 26 | 0.045349839934331 | 0.0490023838639748 | 0.354725686281718 | -0.050694832 | 0.141394512307722 | 1.04639386624462 | 0.950568709009444 | 1.15187898879542 | 0.998074519377826 |
| ebi-fl187-GCST90094852 | ebi-met1400-GCST90199665 | N-acetylaspartate (naa) levels \|\| id:ebi-met1400-GCST90199665 | F-strong vegetable liking (derived food-liking factor) \|\| id:ebi-fl187-GCST90094852 | Inverse variance weighted | 26 | -0.07570146 | 0.0486465682276229 | 0.119671925597354 | -0.171048734 | 0.0196458138271599 | 0.927092939669224 | 0.842780500721834 | 1.01984006280208 | 0.998074519377826 |
| ebi-fl187-GCST90094852 | ebi-met1400-GCST90199669 | Acetylcarnitine levels (Biocrates platform) \|\| id:ebi-met1400-GCST90199669 | F-strong vegetable liking (derived food-liking factor) \|\| id:ebi-fl187-GCST90094852 | Inverse variance weighted | 26 | -0.049525994 | 0.0570724714831257 | 0.385518194054266 | -0.161388038 | 0.0623360505749653 | 0.95168042027953 | 0.850961801824476 | 1.06431994996908 | 0.998074519377826 |
| ebi-fl187-GCST90094852 | ebi-met1400-GCST90199670 | Iminodiacetate (IDA) levels \|\| id:ebi-met1400-GCST90199670 | F-strong vegetable liking (derived food-liking factor) \|\| id:ebi-fl187-GCST90094852 | Inverse variance weighted | 26 | -0.049964254 | 0.0562042206438629 | 0.374015489586795 | -0.160124527 | 0.0601960181001338 | 0.951263427411256 | 0.852037680813536 | 1.06204470612872 | 0.998074519377826 |
| ebi-fl187-GCST90094852 | ebi-met1400-GCST90199686 | Indolepropionate levels \|\| id:ebi-met1400-GCST90199686 | F-strong vegetable liking (derived food-liking factor) \|\| id:ebi-fl187-GCST90094852 | Inverse variance weighted | 26 | 0.179633707659393 | 0.0575233569394113 | 0.00179141882162268 | 0.0668879280581465 | 0.292379487260639 | 1.19677891187723 | 1.06917564686894 | 1.33961128660987 | 0.598008988154769 |
| ebi-fl187-GCST90094852 | ebi-met1400-GCST90199704 | 3-hydroxysebacate levels \|\| id:ebi-met1400-GCST90199704 | F-strong vegetable liking (derived food-liking factor) \|\| id:ebi-fl187-GCST90094852 | Inverse variance weighted | 26 | 0.0406191590723905 | 0.0498215664025919 | 0.414904790000971 | -0.057031111 | 0.138269429221471 | 1.0414554011686 | 0.944564682485122 | 1.14828489010368 | 0.998074519377826 |
| ebi-fl187-GCST90094852 | ebi-met1400-GCST90199713 | Docosapentaenoate n3 DPA; 22:5n3 levels \|\| id:ebi-met1400-GCST90199713 | F-strong vegetable liking (derived food-liking factor) \|\| id:ebi-fl187-GCST90094852 | Inverse variance weighted | 26 | 0.0103097755422537 | 0.0516182761396072 | 0.841690882432108 | -0.090862046 | 0.111481596775884 | 1.01036310439004 | 0.913143674314732 | 1.11793317024154 | 0.998074519377826 |
| ebi-fl187-GCST90094852 | ebi-met1400-GCST90199715 | Myristoleate (14:1n5) levels \|\| id:ebi-met1400-GCST90199715 | F-strong vegetable liking (derived food-liking factor) \|\| id:ebi-fl187-GCST90094852 | Inverse variance weighted | 26 | -0.066568737 | 0.0495433961849589 | 0.179063208366997 | -0.163673793 | 0.0305363196294179 | 0.935598603504845 | 0.849018932278725 | 1.03100733517312 | 0.998074519377826 |
| ebi-fl187-GCST90094852 | ebi-met1400-GCST90199725 | Campesterol levels \|\| id:ebi-met1400-GCST90199725 | F-strong vegetable liking (derived food-liking factor) \|\| id:ebi-fl187-GCST90094852 | Inverse variance weighted | 26 | -0.031919621 | 0.0608113977740088 | 0.599655927783916 | -0.151109961 | 0.0872707184650787 | 0.968584432631922 | 0.859753154308795 | 1.09119204557166 | 0.998074519377826 |
| ebi-fl187-GCST90094852 | ebi-met1400-GCST90199737 | N-acetylproline levels \|\| id:ebi-met1400-GCST90199737 | F-strong vegetable liking (derived food-liking factor) \|\| id:ebi-fl187-GCST90094852 | Inverse variance weighted | 26 | -0.017482519 | 0.057637043976463 | 0.761645336658333 | -0.130451125 | 0.0954860873948195 | 0.982669413756417 | 0.877699389464325 | 1.10019351536946 | 0.998074519377826 |
| ebi-fl187-GCST90094852 | ebi-met1400-GCST90199775 | Gamma-glutamylisoleucine levels \|\| id:ebi-met1400-GCST90199775 | F-strong vegetable liking (derived food-liking factor) \|\| id:ebi-fl187-GCST90094852 | Inverse variance weighted | 26 | -0.048592734 | 0.0500476280079446 | 0.331583230399835 | -0.146686085 | 0.0495006167409815 | 0.952568999528981 | 0.863565020842002 | 1.05074624025289 | 0.998074519377826 |
| ebi-fl187-GCST90094852 | ebi-met1400-GCST90199777 | 1-palmitoleoyl-GPC (16:1) levels \|\| id:ebi-met1400-GCST90199777 | F-strong vegetable liking (derived food-liking factor) \|\| id:ebi-fl187-GCST90094852 | Inverse variance weighted | 26 | -0.070675852 | 0.0476548585816624 | 0.138053799191693 | -0.164079375 | 0.0227276703437751 | 0.931763872133753 | 0.848674655404355 | 1.02298791166332 | 0.998074519377826 |
| ebi-fl187-GCST90094852 | ebi-met1400-GCST90199782 | Glutamine degradant levels \|\| id:ebi-met1400-GCST90199782 | F-strong vegetable liking (derived food-liking factor) \|\| id:ebi-fl187-GCST90094852 | Inverse variance weighted | 26 | 0.0355355830649518 | 0.0430211005629169 | 0.408802061208277 | -0.048785774 | 0.119856940168269 | 1.03617451773726 | 0.952385133467337 | 1.1273355636067 | 0.998074519377826 |
| ebi-fl187-GCST90094852 | ebi-met1400-GCST90199783 | 5-methyluridine (ribothymidine) levels \|\| id:ebi-met1400-GCST90199783 | F-strong vegetable liking (derived food-liking factor) \|\| id:ebi-fl187-GCST90094852 | Inverse variance weighted | 26 | -0.022177526 | 0.0521576482909381 | 0.670689573230152 | -0.124406517 | 0.0800514647918175 | 0.978066587527659 | 0.883020805375889 | 1.08334282025301 | 0.998074519377826 |
| ebi-fl187-GCST90094852 | ebi-met1400-GCST90199787 | Homostachydrine levels \|\| id:ebi-met1400-GCST90199787 | F-strong vegetable liking (derived food-liking factor) \|\| id:ebi-fl187-GCST90094852 | Inverse variance weighted | 26 | -0.071584393 | 0.047209673787049 | 0.129441559457157 | -0.164115354 | 0.0209465676098166 | 0.930917711329359 | 0.848644122049113 | 1.02116748676018 | 0.998074519377826 |
| ebi-fl187-GCST90094852 | ebi-met1400-GCST90199802 | Beta-hydroxyisovaleroylcarnitine levels \|\| id:ebi-met1400-GCST90199802 | F-strong vegetable liking (derived food-liking factor) \|\| id:ebi-fl187-GCST90094852 | Inverse variance weighted | 26 | 0.00356490324284376 | 0.0476755584208233 | 0.940394344679985 | -0.089879191 | 0.0970089977476574 | 1.00357126506793 | 0.914041602813935 | 1.10187028792721 | 0.998074519377826 |
| ebi-fl187-GCST90094852 | ebi-met1400-GCST90199873 | Pregnenolone sulfate levels \|\| id:ebi-met1400-GCST90199873 | F-strong vegetable liking (derived food-liking factor) \|\| id:ebi-fl187-GCST90094852 | Inverse variance weighted (multiplicative random effects) | 26 | 0.0669125016014877 | 0.0665960812115748 | 0.31501660171973 | -0.063615818 | 0.197440820776174 | 1.06920192062585 | 0.938365433799624 | 1.21828096591431 | 0.998074519377826 |
| ebi-fl187-GCST90094852 | ebi-met1400-GCST90199911 | Beta-citrylglutamate levels \|\| id:ebi-met1400-GCST90199911 | F-strong vegetable liking (derived food-liking factor) \|\| id:ebi-fl187-GCST90094852 | Inverse variance weighted | 26 | -0.038478014 | 0.0480254825802471 | 0.423015907229782 | -0.13260796 | 0.0556519322013369 | 0.962252860929709 | 0.875808377156052 | 1.05722963209614 | 0.998074519377826 |
| ebi-fl187-GCST90094852 | ebi-met1400-GCST90199934 | 2-hydroxydecanoate levels \|\| id:ebi-met1400-GCST90199934 | F-strong vegetable liking (derived food-liking factor) \|\| id:ebi-fl187-GCST90094852 | Inverse variance weighted | 26 | 0.0926965894718206 | 0.0481815465015 | 0.0543668274121828 | -0.001739242 | 0.187132420614761 | 1.09712880433326 | 0.9982622699332 | 1.20578694552712 | 0.998074519377826 |
| ebi-fl187-GCST90094852 | ebi-met1400-GCST90199945 | Sphingomyelin (d18:1/14:0, d16:1/16:0) levels \|\| id:ebi-met1400-GCST90199945 | F-strong vegetable liking (derived food-liking factor) \|\| id:ebi-fl187-GCST90094852 | Inverse variance weighted | 26 | -0.046813707 | 0.0463059888269863 | 0.312033459364988 | -0.137573445 | 0.0439460309665353 | 0.954265153822586 | 0.871470342302352 | 1.04492595972257 | 0.998074519377826 |
| ebi-fl187-GCST90094852 | ebi-met1400-GCST90199979 | Carnitine C14:1 levels \|\| id:ebi-met1400-GCST90199979 | F-strong vegetable liking (derived food-liking factor) \|\| id:ebi-fl187-GCST90094852 | Inverse variance weighted | 26 | -0.014002759 | 0.0573558748405749 | 0.80712380420029 | -0.126420274 | 0.0984147558332443 | 0.986094823767182 | 0.881244405238089 | 1.10342034023775 | 0.998074519377826 |
| ebi-fl187-GCST90094852 | ebi-met1400-GCST90199986 | Eugenol sulfate levels \|\| id:ebi-met1400-GCST90199986 | F-strong vegetable liking (derived food-liking factor) \|\| id:ebi-fl187-GCST90094852 | Inverse variance weighted | 26 | -0.060117653 | 0.0522521158974374 | 0.249925440791385 | -0.1625318 | 0.0422964942882841 | 0.941653738801145 | 0.84998906011829 | 1.04320373685136 | 0.998074519377826 |
| ebi-fl187-GCST90094852 | ebi-met1400-GCST90200002 | 6-hydroxyindole sulfate levels \|\| id:ebi-met1400-GCST90200002 | F-strong vegetable liking (derived food-liking factor) \|\| id:ebi-fl187-GCST90094852 | Inverse variance weighted | 26 | -0.087006974 | 0.0479121640823514 | 0.0693752248606151 | -0.180914816 | 0.00690086728060034 | 0.916670702455304 | 0.834506442329843 | 1.00692473313199 | 0.998074519377826 |
| ebi-fl187-GCST90094852 | ebi-met1400-GCST90200023 | Trans 3,4-methyleneheptanoate levels \|\| id:ebi-met1400-GCST90200023 | F-strong vegetable liking (derived food-liking factor) \|\| id:ebi-fl187-GCST90094852 | Inverse variance weighted | 26 | -0.038609016 | 0.0520599323134732 | 0.458313767980509 | -0.140646484 | 0.0634284509498899 | 0.962126811435947 | 0.868796391084876 | 1.0654832487598 | 0.998074519377826 |
| ebi-fl187-GCST90094852 | ebi-met1400-GCST90200032 | 2-hydroxybutyrate/2-hydroxyisobutyrate levels \|\| id:ebi-met1400-GCST90200032 | F-strong vegetable liking (derived food-liking factor) \|\| id:ebi-fl187-GCST90094852 | Inverse variance weighted | 26 | -0.039884162 | 0.0591635029915305 | 0.500225769705003 | -0.155844628 | 0.0760763038452796 | 0.960900741508362 | 0.855692130742258 | 1.07904490629169 | 0.998074519377826 |
| ebi-fl187-GCST90094852 | ebi-met1400-GCST90200051 | 1-palmitoyl-2-dihomo-linolenoyl-GPC (16:0/20:3n3 or 6) levels \|\| id:ebi-met1400-GCST90200051 | F-strong vegetable liking (derived food-liking factor) \|\| id:ebi-fl187-GCST90094852 | Inverse variance weighted | 26 | -0.049681535 | 0.0475129093256114 | 0.295725944169908 | -0.142806838 | 0.0434437669574954 | 0.951532405716173 | 0.86692150936368 | 1.04440126280011 | 0.998074519377826 |
| ebi-fl187-GCST90094852 | ebi-met1400-GCST90200069 | Phenylacetylglutamate levels \|\| id:ebi-met1400-GCST90200069 | F-strong vegetable liking (derived food-liking factor) \|\| id:ebi-fl187-GCST90094852 | Inverse variance weighted | 26 | 0.0231605869221336 | 0.0532548740146534 | 0.663634439216691 | -0.081218966 | 0.127540139990854 | 1.02343087596611 | 0.921991784351801 | 1.13603046757856 | 0.998074519377826 |
| ebi-fl187-GCST90094852 | ebi-met1400-GCST90200072 | Thioproline levels \|\| id:ebi-met1400-GCST90200072 | F-strong vegetable liking (derived food-liking factor) \|\| id:ebi-fl187-GCST90094852 | Inverse variance weighted | 26 | 0.0230754641583522 | 0.0482525910744747 | 0.632492005841363 | -0.071499614 | 0.117650542664323 | 1.02334376240914 | 0.930996636635863 | 1.12485095525784 | 0.998074519377826 |
| ebi-fl187-GCST90094852 | ebi-met1400-GCST90200081 | 1-oleoyl-2-docosahexaenoyl-GPC (18:1/22:6) levels \|\| id:ebi-met1400-GCST90200081 | F-strong vegetable liking (derived food-liking factor) \|\| id:ebi-fl187-GCST90094852 | Inverse variance weighted | 26 | 0.0450042249575226 | 0.0464331842347066 | 0.332432704190201 | -0.046004816 | 0.136013266057548 | 1.04603227934133 | 0.955037362583605 | 1.14569709237761 | 0.998074519377826 |
| ebi-fl187-GCST90094852 | ebi-met1400-GCST90200097 | Furaneol sulfate levels \|\| id:ebi-met1400-GCST90200097 | F-strong vegetable liking (derived food-liking factor) \|\| id:ebi-fl187-GCST90094852 | Inverse variance weighted | 26 | 0.0225047267995718 | 0.0572228765544738 | 0.694111218223874 | -0.089652111 | 0.13466156484634 | 1.02275986853393 | 0.914249186962819 | 1.14414949840812 | 0.998074519377826 |
| ebi-fl187-GCST90094852 | ebi-met1400-GCST90200124 | Sphingomyelin (d18:2/24:2) levels \|\| id:ebi-met1400-GCST90200124 | F-strong vegetable liking (derived food-liking factor) \|\| id:ebi-fl187-GCST90094852 | Inverse variance weighted | 26 | -0.028536374 | 0.0445500799927203 | 0.521817713050733 | -0.115854531 | 0.0587817825921324 | 0.971866942626875 | 0.89060476927837 | 1.0605437863714 | 0.998074519377826 |
| ebi-fl187-GCST90094852 | ebi-met1400-GCST90200137 | Lignoceroylcarnitine (C24) levels \|\| id:ebi-met1400-GCST90200137 | F-strong vegetable liking (derived food-liking factor) \|\| id:ebi-fl187-GCST90094852 | Inverse variance weighted | 26 | -0.013486383 | 0.048599485190016 | 0.781395804044926 | -0.108741374 | 0.081768608399412 | 0.986604151236246 | 0.896962365699477 | 1.08520467352888 | 0.998074519377826 |
| ebi-fl187-GCST90094852 | ebi-met1400-GCST90200165 | Octadecenedioate (C18:1-DC) levels \|\| id:ebi-met1400-GCST90200165 | F-strong vegetable liking (derived food-liking factor) \|\| id:ebi-fl187-GCST90094852 | Inverse variance weighted | 26 | 0.0343606816248258 | 0.0474213804710246 | 0.468708467091328 | -0.058585224 | 0.127306587348034 | 1.03495782968838 | 0.943097862309638 | 1.13576517564155 | 0.998074519377826 |
| ebi-fl187-GCST90094852 | ebi-met1400-GCST90200176 | Glutamine conjugate of C6H10O2 (1) levels \|\| id:ebi-met1400-GCST90200176 | F-strong vegetable liking (derived food-liking factor) \|\| id:ebi-fl187-GCST90094852 | Inverse variance weighted | 26 | -0.013964637 | 0.0476232782540707 | 0.769345338315506 | -0.107306263 | 0.0793769881643315 | 0.986132416036221 | 0.898250530324157 | 1.08261237720227 | 0.998074519377826 |
| ebi-fl187-GCST90094852 | ebi-met1400-GCST90200182 | Glycine conjugate of C10H12O2 levels \|\| id:ebi-met1400-GCST90200182 | F-strong vegetable liking (derived food-liking factor) \|\| id:ebi-fl187-GCST90094852 | Inverse variance weighted | 26 | -0.14316594 | 0.0681713508605311 | 0.0357209663383245 | -0.276781788 | -0.009550092 | 0.866610251612671 | 0.758219931945498 | 0.990495364944009 | 0.998074519377826 |
| ebi-fl187-GCST90094852 | ebi-met1400-GCST90200208 | Glucuronide of piperine metabolite C17H21NO3 (4) levels \|\| id:ebi-met1400-GCST90200208 | F-strong vegetable liking (derived food-liking factor) \|\| id:ebi-fl187-GCST90094852 | Inverse variance weighted (multiplicative random effects) | 26 | -0.046853444 | 0.0661098142851157 | 0.478497349684362 | -0.17642868 | 0.0827217924094834 | 0.954227235461633 | 0.838258561969151 | 1.08623956641466 | 0.998074519377826 |
| ebi-fl187-GCST90094852 | ebi-met1400-GCST90200227 | 3-ethylcatechol sulfate (2) levels \|\| id:ebi-met1400-GCST90200227 | F-strong vegetable liking (derived food-liking factor) \|\| id:ebi-fl187-GCST90094852 | Inverse variance weighted (multiplicative random effects) | 26 | 0.0626820807848701 | 0.0852472333067264 | 0.462158307192445 | -0.104402496 | 0.229766658066054 | 1.06468830055131 | 0.900862630387605 | 1.25830636003085 | 0.998074519377826 |
| ebi-fl187-GCST90094852 | ebi-met1400-GCST90200259 | Pentose acid levels \|\| id:ebi-met1400-GCST90200259 | F-strong vegetable liking (derived food-liking factor) \|\| id:ebi-fl187-GCST90094852 | Inverse variance weighted | 26 | -0.007641272 | 0.0476793664601643 | 0.872673354472488 | -0.10109283 | 0.0858102867031255 | 0.992387848737484 | 0.903849124839124 | 1.08959959716407 | 0.998074519377826 |
| ebi-fl187-GCST90094852 | ebi-met1400-GCST90200291 | Sarcosine levels \|\| id:ebi-met1400-GCST90200291 | F-strong vegetable liking (derived food-liking factor) \|\| id:ebi-fl187-GCST90094852 | Inverse variance weighted | 26 | 0.051924008804468 | 0.0484867327403258 | 0.284218414387034 | -0.043109987 | 0.146958004975507 | 1.05329569827271 | 0.95780603770965 | 1.15830531894822 | 0.998074519377826 |
| ebi-fl187-GCST90094852 | ebi-met1400-GCST90200306 | Butyrate/isobutyrate (4:0) levels \|\| id:ebi-met1400-GCST90200306 | F-strong vegetable liking (derived food-liking factor) \|\| id:ebi-fl187-GCST90094852 | Inverse variance weighted | 26 | -0.076264294 | 0.0489572701754013 | 0.119287163830912 | -0.172220544 | 0.0196919555771658 | 0.92657128699463 | 0.841793500601152 | 1.01988712109297 | 0.998074519377826 |
| ebi-fl187-GCST90094852 | ebi-met1400-GCST90200321 | Cholate levels \|\| id:ebi-met1400-GCST90200321 | F-strong vegetable liking (derived food-liking factor) \|\| id:ebi-fl187-GCST90094852 | Inverse variance weighted | 26 | 0.0351436198508905 | 0.0513480298617158 | 0.493709974939099 | -0.065498519 | 0.135785758379854 | 1.03576845502898 | 0.936600434165361 | 1.14543646714102 | 0.998074519377826 |
| ebi-fl187-GCST90094852 | ebi-met1400-GCST90200336 | Ceramide (d18:1/16:0) levels \|\| id:ebi-met1400-GCST90200336 | F-strong vegetable liking (derived food-liking factor) \|\| id:ebi-fl187-GCST90094852 | Inverse variance weighted | 26 | -0.027660548 | 0.0479547711195812 | 0.564071246525705 | -0.121651899 | 0.0663308035425674 | 0.972718502150638 | 0.885456542951703 | 1.06858014880329 | 0.998074519377826 |
| ebi-fl187-GCST90094852 | ebi-met1400-GCST90200358 | Arachidonate (20:4n6) levels \|\| id:ebi-met1400-GCST90200358 | F-strong vegetable liking (derived food-liking factor) \|\| id:ebi-fl187-GCST90094852 | Inverse variance weighted | 26 | 0.00399370697970981 | 0.055427949532365 | 0.94256034787504 | -0.104645074 | 0.112632488063145 | 1.00400169245444 | 0.900644127789109 | 1.11922053045064 | 0.998074519377826 |
| ebi-fl187-GCST90094852 | ebi-met1400-GCST90200413 | 2'-deoxyuridine levels \|\| id:ebi-met1400-GCST90200413 | F-strong vegetable liking (derived food-liking factor) \|\| id:ebi-fl187-GCST90094852 | Inverse variance weighted | 26 | -0.006112866 | 0.0482182448050994 | 0.899118514993571 | -0.100620626 | 0.0883948937715649 | 0.993905779507284 | 0.904276026756221 | 1.09241942649033 | 0.998074519377826 |
| ebi-fl187-GCST90094852 | ebi-met1400-GCST90200432 | Threonine levels \|\| id:ebi-met1400-GCST90200432 | F-strong vegetable liking (derived food-liking factor) \|\| id:ebi-fl187-GCST90094852 | Inverse variance weighted (multiplicative random effects) | 26 | 0.0340195714067807 | 0.0597163630599275 | 0.568890739465987 | -0.0830245 | 0.151063643004239 | 1.03460485520245 | 0.92032859871775 | 1.16307067703843 | 0.998074519377826 |
| ebi-fl187-GCST90094852 | ebi-met1400-GCST90200436 | Caffeine levels \|\| id:ebi-met1400-GCST90200436 | F-strong vegetable liking (derived food-liking factor) \|\| id:ebi-fl187-GCST90094852 | Inverse variance weighted | 26 | 0.0252766132239408 | 0.04968657196068 | 0.610947650223825 | -0.072109068 | 0.122662294266874 | 1.02559877547502 | 0.930429410370393 | 1.13050257927372 | 0.998074519377826 |
| ebi-fl187-GCST90094852 | ebi-met1400-GCST90200442 | Valine levels \|\| id:ebi-met1400-GCST90200442 | F-strong vegetable liking (derived food-liking factor) \|\| id:ebi-fl187-GCST90094852 | Inverse variance weighted | 26 | -0.007790888 | 0.0474580829070106 | 0.869602389652599 | -0.10080873 | 0.0852269548299658 | 0.992239382635696 | 0.904105944543314 | 1.08896418433636 | 0.998074519377826 |
| ebi-fl187-GCST90094852 | ebi-met1400-GCST90200459 | Margarate (17:0) levels \|\| id:ebi-met1400-GCST90200459 | F-strong vegetable liking (derived food-liking factor) \|\| id:ebi-fl187-GCST90094852 | Inverse variance weighted | 26 | 0.00470231569825295 | 0.0509909411051358 | 0.926524321447243 | -0.095239929 | 0.104644560264319 | 1.00471338893453 | 0.90915477582197 | 1.11031588982378 | 0.998074519377826 |
| ebi-fl187-GCST90094852 | ebi-met1400-GCST90200462 | X-11299 levels \|\| id:ebi-met1400-GCST90200462 | F-strong vegetable liking (derived food-liking factor) \|\| id:ebi-fl187-GCST90094852 | Inverse variance weighted | 26 | 0.0639463220777423 | 0.0516087241297973 | 0.215323161524968 | -0.037206777 | 0.165099421372145 | 1.06603517467245 | 0.963476889680466 | 1.17951038142266 | 0.998074519377826 |
| ebi-fl187-GCST90094852 | ebi-met1400-GCST90200497 | X-12740 levels \|\| id:ebi-met1400-GCST90200497 | F-strong vegetable liking (derived food-liking factor) \|\| id:ebi-fl187-GCST90094852 | Inverse variance weighted | 26 | 0.0194482073186198 | 0.0645580287077223 | 0.763222670170471 | -0.107085529 | 0.145981943585756 | 1.01963855567822 | 0.898448826320635 | 1.1571752934257 | 0.998074519377826 |
| ebi-fl187-GCST90094852 | ebi-met1400-GCST90200527 | X-13866 levels \|\| id:ebi-met1400-GCST90200527 | F-strong vegetable liking (derived food-liking factor) \|\| id:ebi-fl187-GCST90094852 | Inverse variance weighted | 26 | 0.00133975560829583 | 0.0514363320229704 | 0.979219951862698 | -0.099475455 | 0.102154966373318 | 1.00134065348177 | 0.905312170341143 | 1.10755509222578 | 0.998352135646871 |
| ebi-fl187-GCST90094852 | ebi-met1400-GCST90200547 | X-17654 levels \|\| id:ebi-met1400-GCST90200547 | F-strong vegetable liking (derived food-liking factor) \|\| id:ebi-fl187-GCST90094852 | Inverse variance weighted | 25 | -0.022724633 | 0.0497725842408117 | 0.64798065703467 | -0.120278899 | 0.0748296316817629 | 0.97753162624592 | 0.886673110391372 | 1.07770052921669 | 0.998074519377826 |
| ebi-fl187-GCST90094852 | ebi-met1400-GCST90200553 | X-17653 levels \|\| id:ebi-met1400-GCST90200553 | F-strong vegetable liking (derived food-liking factor) \|\| id:ebi-fl187-GCST90094852 | Inverse variance weighted | 26 | -0.012604701 | 0.0487426686487266 | 0.795946312033434 | -0.108140331 | 0.0829309298583038 | 0.987474405826422 | 0.89750164007056 | 1.08646676354328 | 0.998074519377826 |
| ebi-fl187-GCST90094852 | ebi-met1400-GCST90200557 | X-17685 levels \|\| id:ebi-met1400-GCST90200557 | F-strong vegetable liking (derived food-liking factor) \|\| id:ebi-fl187-GCST90094852 | Inverse variance weighted | 26 | -0.07029522 | 0.0513652149224988 | 0.17114390804277 | -0.170971041 | 0.0303806010234777 | 0.932118599020386 | 0.842845980696547 | 1.0308468006476 | 0.998074519377826 |
| ebi-fl187-GCST90094852 | ebi-met1400-GCST90200561 | X-18779 levels \|\| id:ebi-met1400-GCST90200561 | F-strong vegetable liking (derived food-liking factor) \|\| id:ebi-fl187-GCST90094852 | Inverse variance weighted | 26 | 9.77301927131873e-05 | 0.0482503012274293 | 0.99838389903092 | -0.09447286 | 0.0946683205984747 | 1.00009773496846 | 0.909852427491989 | 1.09929418141587 | 0.999355215041207 |
| ebi-fl187-GCST90094852 | ebi-met1400-GCST90200570 | X-18921 levels \|\| id:ebi-met1400-GCST90200570 | F-strong vegetable liking (derived food-liking factor) \|\| id:ebi-fl187-GCST90094852 | Inverse variance weighted | 26 | -0.007202606 | 0.0484676002159257 | 0.881864059789645 | -0.102199102 | 0.0877938908374103 | 0.992823271014222 | 0.902849774568521 | 1.09176307646358 | 0.998074519377826 |
| ebi-fl187-GCST90094852 | ebi-met1400-GCST90200575 | X-21283 levels \|\| id:ebi-met1400-GCST90200575 | F-strong vegetable liking (derived food-liking factor) \|\| id:ebi-fl187-GCST90094852 | Inverse variance weighted (multiplicative random effects) | 26 | -0.054994593 | 0.0677883186579785 | 0.417210937981589 | -0.187859698 | 0.0778705111535434 | 0.946490265218685 | 0.828730971701036 | 1.08098267440753 | 0.998074519377826 |
| ebi-fl187-GCST90094852 | ebi-met1400-GCST90200578 | X-18922 levels \|\| id:ebi-met1400-GCST90200578 | F-strong vegetable liking (derived food-liking factor) \|\| id:ebi-fl187-GCST90094852 | Inverse variance weighted | 26 | -0.053872444 | 0.048613475249899 | 0.267784418648378 | -0.149154855 | 0.0414099678847272 | 0.947552965233728 | 0.861435706861651 | 1.04227931901531 | 0.998074519377826 |
| ebi-fl187-GCST90094852 | ebi-met1400-GCST90200623 | X-24241 levels \|\| id:ebi-met1400-GCST90200623 | F-strong vegetable liking (derived food-liking factor) \|\| id:ebi-fl187-GCST90094852 | Inverse variance weighted | 26 | -0.087514246 | 0.0488287548845301 | 0.0730899449989829 | -0.183218605 | 0.00819011371977285 | 0.916205819423656 | 0.832586127995178 | 1.00822374445162 | 0.998074519377826 |
| ebi-fl187-GCST90094852 | ebi-met1400-GCST90200636 | X-24544 levels \|\| id:ebi-met1400-GCST90200636 | F-strong vegetable liking (derived food-liking factor) \|\| id:ebi-fl187-GCST90094852 | Inverse variance weighted (multiplicative random effects) | 26 | 0.0171492287898033 | 0.0640680665471993 | 0.788951788934941 | -0.108424182 | 0.142722639222314 | 1.01729712101683 | 0.897246920025721 | 1.15340984664452 | 0.998074519377826 |
| ebi-fl187-GCST90094852 | ebi-met1400-GCST90200659 | X-25172 levels \|\| id:ebi-met1400-GCST90200659 | F-strong vegetable liking (derived food-liking factor) \|\| id:ebi-fl187-GCST90094852 | Inverse variance weighted | 26 | -0.061249265 | 0.0506035598967286 | 0.226135175732605 | -0.160432242 | 0.0379337127600015 | 0.940588755037705 | 0.851775536193505 | 1.03866238053401 | 0.998074519377826 |
| ebi-fl187-GCST90094852 | ebi-met1400-GCST90200670 | X-26111 levels \|\| id:ebi-met1400-GCST90200670 | F-strong vegetable liking (derived food-liking factor) \|\| id:ebi-fl187-GCST90094852 | Inverse variance weighted | 26 | 0.0338569419242611 | 0.0543374118738218 | 0.533227267762338 | -0.072644385 | 0.140358269196952 | 1.03443661163129 | 0.929931468487518 | 1.15068598035898 | 0.998074519377826 |
| ebi-fl187-GCST90094852 | ebi-met1400-GCST90200673 | Carnitine C4 levels \|\| id:ebi-met1400-GCST90200673 | F-strong vegetable liking (derived food-liking factor) \|\| id:ebi-fl187-GCST90094852 | Inverse variance weighted (multiplicative random effects) | 26 | -0.147923082 | 0.0598592160359742 | 0.0134666851772483 | -0.265247146 | -0.030599019 | 0.862497453556722 | 0.767016361592542 | 0.969864392263132 | 0.998074519377826 |
| ebi-fl187-GCST90094852 | ebi-met1400-GCST90200710 | X-13431 levels \|\| id:ebi-met1400-GCST90200710 | F-strong vegetable liking (derived food-liking factor) \|\| id:ebi-fl187-GCST90094852 | Inverse variance weighted | 26 | -0.037438841 | 0.0522659368800906 | 0.473797268768102 | -0.139880077 | 0.0650023957458689 | 0.963253327973584 | 0.869462497851019 | 1.06716158102908 | 0.998074519377826 |
| ebi-fl187-GCST90094852 | ebi-met1400-GCST90200730 | Adenosine 5'-diphosphate (ADP) to fructose ratio \|\| id:ebi-met1400-GCST90200730 | F-strong vegetable liking (derived food-liking factor) \|\| id:ebi-fl187-GCST90094852 | Inverse variance weighted | 26 | 0.0545009521034984 | 0.0650270688525348 | 0.401959294302877 | -0.072952103 | 0.181954007054467 | 1.05601348184738 | 0.929645356324874 | 1.19955902135837 | 0.998074519377826 |
| ebi-fl187-GCST90094852 | ebi-met1400-GCST90200731 | Adenosine 5'-diphosphate (ADP) to mannose ratio \|\| id:ebi-met1400-GCST90200731 | F-strong vegetable liking (derived food-liking factor) \|\| id:ebi-fl187-GCST90094852 | Inverse variance weighted | 26 | 0.068555911516783 | 0.0715486270351805 | 0.337975889734237 | -0.071679397 | 0.208791220505737 | 1.07096050230315 | 0.930829274196502 | 1.23218771614533 | 0.998074519377826 |
| ebi-fl187-GCST90094852 | ebi-met1400-GCST90200748 | Aspartate to glutamate ratio \|\| id:ebi-met1400-GCST90200748 | F-strong vegetable liking (derived food-liking factor) \|\| id:ebi-fl187-GCST90094852 | Inverse variance weighted | 26 | 0.110571612190868 | 0.0469047742757395 | 0.0184052106322389 | 0.018638254610419 | 0.202504969771318 | 1.11691633101372 | 1.01881303103175 | 1.22446617042365 | 0.998074519377826 |
| ebi-fl187-GCST90094852 | ebi-met1400-GCST90200774 | Uridine to pseudouridine ratio \|\| id:ebi-met1400-GCST90200774 | F-strong vegetable liking (derived food-liking factor) \|\| id:ebi-fl187-GCST90094852 | Inverse variance weighted | 26 | 0.0280677807330059 | 0.0461540959303397 | 0.543099957944797 | -0.062394247 | 0.118529808756472 | 1.02846539219713 | 0.939512413543458 | 1.12584043350511 | 0.998074519377826 |
| ebi-fl187-GCST90094852 | ebi-met1400-GCST90200799 | Spermidine to adenosine 5'-diphosphate (ADP) ratio \|\| id:ebi-met1400-GCST90200799 | F-strong vegetable liking (derived food-liking factor) \|\| id:ebi-fl187-GCST90094852 | Inverse variance weighted | 26 | -0.047522049 | 0.0759257835786312 | 0.531379659618458 | -0.196336585 | 0.101292486601701 | 0.953589447004547 | 0.821735604225812 | 1.10660026018363 | 0.998074519377826 |
| ebi-fl187-GCST90094852 | ebi-met1400-GCST90200811 | Choline to taurocholate ratio \|\| id:ebi-met1400-GCST90200811 | F-strong vegetable liking (derived food-liking factor) \|\| id:ebi-fl187-GCST90094852 | Inverse variance weighted | 26 | -0.018217925 | 0.0506296718170844 | 0.718976479099007 | -0.117452082 | 0.0810162317038137 | 0.981947018179054 | 0.889183118763102 | 1.08438849789683 | 0.998074519377826 |
| ebi-fl187-GCST90094852 | ebi-met1400-GCST90200812 | Glutamate to kynurenine ratio \|\| id:ebi-met1400-GCST90200812 | F-strong vegetable liking (derived food-liking factor) \|\| id:ebi-fl187-GCST90094852 | Inverse variance weighted | 26 | -0.050823872 | 0.047011208700465 | 0.279652122460484 | -0.142965841 | 0.0413180973197035 | 0.950446056207879 | 0.866783677038803 | 1.04218356862374 | 0.998074519377826 |
| ebi-fl187-GCST90094852 | ebi-met1400-GCST90200876 | Cortisone to 4-cholesten-3-one ratio \|\| id:ebi-met1400-GCST90200876 | F-strong vegetable liking (derived food-liking factor) \|\| id:ebi-fl187-GCST90094852 | Inverse variance weighted | 26 | 0.040013935474611 | 0.0612691877409943 | 0.513702029078575 | -0.080073672 | 0.16010154344696 | 1.04082527848557 | 0.923048340604804 | 1.17363003938098 | 0.998074519377826 |
| ebi-fl187-GCST90094852 | ebi-met1400-GCST90200906 | 2'-deoxyuridine to cytidine ratio \|\| id:ebi-met1400-GCST90200906 | F-strong vegetable liking (derived food-liking factor) \|\| id:ebi-fl187-GCST90094852 | Inverse variance weighted | 26 | 0.0358630174986257 | 0.056658633963848 | 0.526755516602567 | -0.075187905 | 0.146913940067768 | 1.03651385250557 | 0.927569175000837 | 1.15825427945574 | 0.998074519377826 |
| ebi-fl187-GCST90094852 | ebi-met1400-GCST90200907 | Retinol (Vitamin A) to linoleoyl-arachidonoyl-glycerol (18:2 to 20:4) [1] ratio \|\| id:ebi-met1400-GCST90200907 | F-strong vegetable liking (derived food-liking factor) \|\| id:ebi-fl187-GCST90094852 | Inverse variance weighted | 26 | 0.0432397719888207 | 0.0504347541417647 | 0.391256571472163 | -0.055612346 | 0.14209189010668 | 1.04418823192658 | 0.945905748851446 | 1.1526825637944 | 0.998074519377826 |
| ebi-fl187-GCST90094852 | ebi-met1400-GCST90200916 | Fructose to sucrose ratio \|\| id:ebi-met1400-GCST90200916 | F-strong vegetable liking (derived food-liking factor) \|\| id:ebi-fl187-GCST90094852 | Inverse variance weighted | 26 | 0.0336400597638683 | 0.0502667263840698 | 0.503348030136823 | -0.064882724 | 0.132162843476645 | 1.03421228511126 | 0.937177365393426 | 1.14129415644395 | 0.998074519377826 |
| ebi-fl187-GCST90094852 | ebi-met1400-GCST90200917 | Alpha-ketobutyrate to 3-methyl-2-oxobutyrate ratio \|\| id:ebi-met1400-GCST90200917 | F-strong vegetable liking (derived food-liking factor) \|\| id:ebi-fl187-GCST90094852 | Inverse variance weighted | 26 | -0.067649337 | 0.0534370804893069 | 0.20552652934485 | -0.172386015 | 0.0370873408934866 | 0.93458814173048 | 0.841654219616498 | 1.03778365783186 | 0.998074519377826 |
| ebi-fl187-GCST90094852 | ebi-met1400-GCST90200924 | Alpha-tocopherol to glycerol ratio \|\| id:ebi-met1400-GCST90200924 | F-strong vegetable liking (derived food-liking factor) \|\| id:ebi-fl187-GCST90094852 | Inverse variance weighted | 26 | 0.097141237190568 | 0.0540540189259388 | 0.0723175147176087 | -0.00880464 | 0.203087114285408 | 1.10201600827502 | 0.991234007429192 | 1.22517919420875 | 0.998074519377826 |
| ebi-fl187-GCST90094852 | ebi-met1400-GCST90200938 | Alpha-ketoglutarate to alanine ratio \|\| id:ebi-met1400-GCST90200938 | F-strong vegetable liking (derived food-liking factor) \|\| id:ebi-fl187-GCST90094852 | Inverse variance weighted (multiplicative random effects) | 26 | -0.03306714 | 0.0707816812043052 | 0.640377629746407 | -0.171799235 | 0.105664955243772 | 0.967473601311441 | 0.842148230022056 | 1.11144942881376 | 0.998074519377826 |
| ebi-fl187-GCST90094852 | ebi-met1400-GCST90200943 | Phosphate to EDTA ratio \|\| id:ebi-met1400-GCST90200943 | F-strong vegetable liking (derived food-liking factor) \|\| id:ebi-fl187-GCST90094852 | Inverse variance weighted | 26 | -0.029022152 | 0.0526350016889209 | 0.581369766870349 | -0.132186755 | 0.0741424518093893 | 0.971394946372252 | 0.876177349463586 | 1.07696020950011 | 0.998074519377826 |
| ebi-fl187-GCST90094852 | ebi-met1400-GCST90200947 | Adenosine 5'-diphosphate (ADP) to glucose ratio \|\| id:ebi-met1400-GCST90200947 | F-strong vegetable liking (derived food-liking factor) \|\| id:ebi-fl187-GCST90094852 | Inverse variance weighted | 26 | 0.0652835554871462 | 0.0787454121562186 | 0.407078475106368 | -0.089057452 | 0.219624563313335 | 1.06746166608704 | 0.914793015066207 | 1.24560899547627 | 0.998074519377826 |
| ebi-fl187-GCST90094852 | ebi-met1400-GCST90200975 | Arachidonate (20:4n6) to caffeine ratio \|\| id:ebi-met1400-GCST90200975 | F-strong vegetable liking (derived food-liking factor) \|\| id:ebi-fl187-GCST90094852 | Inverse variance weighted | 26 | -0.029181592 | 0.0516642899103648 | 0.572188730378205 | -0.1304436 | 0.0720804165242827 | 0.971240079314907 | 0.877705994237263 | 1.07474176758628 | 0.998074519377826 |
| ebi-fl187-GCST90094852 | ebi-met1400-GCST90200985 | Cholesterol to oleoyl-linoleoyl-glycerol (18:1 to 18:2) [2] ratio \|\| id:ebi-met1400-GCST90200985 | F-strong vegetable liking (derived food-liking factor) \|\| id:ebi-fl187-GCST90094852 | Inverse variance weighted | 26 | 0.00866179514914973 | 0.0483906998091151 | 0.857939975274848 | -0.086183976 | 0.103507566775015 | 1.00869941704268 | 0.917425430994548 | 1.10905418529682 | 0.998074519377826 |
| ebi-fl187-GCST90094852 | ebi-met1400-GCST90201016 | Bilirubin (Z,Z) to etiocholanolone glucuronide ratio \|\| id:ebi-met1400-GCST90201016 | F-strong vegetable liking (derived food-liking factor) \|\| id:ebi-fl187-GCST90094852 | Inverse variance weighted (multiplicative random effects) | 26 | -0.091510397 | 0.0686565519382013 | 0.182573805024395 | -0.226077239 | 0.0430564445137852 | 0.912551828041171 | 0.797656489029723 | 1.04399682108052 | 0.998074519377826 |
| ebi-fl187-GCST90094852 | ebi-pw11-GCST90275189 | Placental weight (fetal genotype, adjusted for sex and gestational age) \|\| id:ebi-pw11-GCST90275189 | F-strong vegetable liking (derived food-liking factor) \|\| id:ebi-fl187-GCST90094852 | Inverse variance weighted (multiplicative random effects) | 20 | 0.00917891094509922 | 0.0312970702080279 | 0.769305546210557 | -0.052163347 | 0.0705211685528339 | 1.00922116633537 | 0.949173809805117 | 1.0730672844718 | 0.998074519377826 |
| ebi-fl187-GCST90094852 | ebi-pw11-GCST90275192 | Placental weight (fetal effect, conditional on maternal and paternal genotype) \|\| id:ebi-pw11-GCST90275192 | F-strong vegetable liking (derived food-liking factor) \|\| id:ebi-fl187-GCST90094852 | Inverse variance weighted (multiplicative random effects) | 20 | 0.0280804840560452 | 0.05824374332669 | 0.629720574631322 | -0.086077253 | 0.142238220976358 | 1.02847845720823 | 0.917523347175587 | 1.15285124917806 | 0.998074519377826 |
| ebi-fl187-GCST90094852 | ebi-pw11-GCST90275193 | Placental weight (maternal effect, conditional on fetal and paternal genotype) \|\| id:ebi-pw11-GCST90275193 | F-strong vegetable liking (derived food-liking factor) \|\| id:ebi-fl187-GCST90094852 | Inverse variance weighted (multiplicative random effects) | 20 | -0.032208946 | 0.046642755908435 | 0.489851191347401 | -0.123628748 | 0.0592108552204654 | 0.96830423729451 | 0.883707858352724 | 1.06099893432006 | 0.998074519377826 |
| ebi-fl187-GCST90094852 | ebi-pw11-GCST90275195 | Placental weight (fetal effect, conditional on maternal genotype) \|\| id:ebi-pw11-GCST90275195 | F-strong vegetable liking (derived food-liking factor) \|\| id:ebi-fl187-GCST90094852 | Inverse variance weighted (multiplicative random effects) | 20 | 0.0280794398235971 | 0.0428278145607374 | 0.512058615163932 | -0.055863077 | 0.112021956362642 | 1.02847738323821 | 0.945668611078436 | 1.11853741938864 | 0.998074519377826 |
| ebi-fl187-GCST90094852 | ebi-pw11-GCST90275197 | Placental weight (fetal genotype, adjusted for sex) \|\| id:ebi-pw11-GCST90275197 | F-strong vegetable liking (derived food-liking factor) \|\| id:ebi-fl187-GCST90094852 | Inverse variance weighted (multiplicative random effects) | 20 | 0.0121419289610167 | 0.0311505095810593 | 0.69669725566391 | -0.04891307 | 0.073196927739893 | 1.0122159414285 | 0.952263906575393 | 1.07594239895814 | 0.998074519377826 |
| ebi-fl187-GCST90094852 | prot-a-1030 | Protein FAM171B \|\| id:prot-a-1030 | F-strong vegetable liking (derived food-liking factor) \|\| id:ebi-fl187-GCST90094852 | Inverse variance weighted | 27 | 0.0236039627666445 | 0.0761652331449943 | 0.756633438170574 | -0.125679894 | 0.172887819730833 | 1.02388474110403 | 0.881897101984756 | 1.18873274524467 | 0.998074519377826 |
| ebi-fl187-GCST90094852 | prot-a-1036 | BRISC complex subunit Abro1 \|\| id:prot-a-1036 | F-strong vegetable liking (derived food-liking factor) \|\| id:ebi-fl187-GCST90094852 | Inverse variance weighted | 27 | -0.030082752 | 0.0761752829132372 | 0.692905712951463 | -0.179386307 | 0.119220802303536 | 0.970365230362086 | 0.835782968451728 | 1.12661865082029 | 0.998074519377826 |
| ebi-fl187-GCST90094852 | prot-a-1055 | Phenylalanine--tRNA ligase, mitochondrial \|\| id:prot-a-1055 | F-strong vegetable liking (derived food-liking factor) \|\| id:ebi-fl187-GCST90094852 | Inverse variance weighted | 26 | 0.0079456940232474 | 0.0807695952926193 | 0.921634672097221 | -0.150362713 | 0.166254100796781 | 1.00797734482366 | 0.860395843278493 | 1.18087312440547 | 0.998074519377826 |
| ebi-fl187-GCST90094852 | prot-a-1083 | Fc receptor-like protein 5 \|\| id:prot-a-1083 | F-strong vegetable liking (derived food-liking factor) \|\| id:ebi-fl187-GCST90094852 | Inverse variance weighted | 27 | 0.0518311646933146 | 0.0761752829132372 | 0.496238737898675 | -0.09747239 | 0.20113471920326 | 1.05319791050941 | 0.907127387163542 | 1.22278949395384 | 0.998074519377826 |
| ebi-fl187-GCST90094852 | prot-a-1103 | Fibroblast growth factor receptor 3 \|\| id:prot-a-1103 | F-strong vegetable liking (derived food-liking factor) \|\| id:ebi-fl187-GCST90094852 | Inverse variance weighted | 27 | -0.063253959 | 0.0761752829132372 | 0.406327476763692 | -0.212557513 | 0.0860495957372335 | 0.938705051032954 | 0.808513814700678 | 1.08986037939376 | 0.998074519377826 |
| ebi-fl187-GCST90094852 | prot-a-1105 | Fibroblast growth factor receptor 4 \|\| id:prot-a-1105 | F-strong vegetable liking (derived food-liking factor) \|\| id:ebi-fl187-GCST90094852 | Inverse variance weighted | 27 | 0.0291661832987937 | 0.0847596862960734 | 0.730767753600823 | -0.136962802 | 0.195295168439097 | 1.02959568186564 | 0.872002662422061 | 1.21566976088347 | 0.998074519377826 |
| ebi-fl187-GCST90094852 | prot-a-1120 | Filamin-A \|\| id:prot-a-1120 | F-strong vegetable liking (derived food-liking factor) \|\| id:ebi-fl187-GCST90094852 | Inverse variance weighted | 27 | 0.0140780244127086 | 0.0761752829132372 | 0.853377339431925 | -0.13522553 | 0.163381578922653 | 1.01417758646208 | 0.873518884672111 | 1.17748590778107 | 0.998074519377826 |
| ebi-fl187-GCST90094852 | prot-a-1166 | Galanin peptides \|\| id:prot-a-1166 | F-strong vegetable liking (derived food-liking factor) \|\| id:ebi-fl187-GCST90094852 | Inverse variance weighted | 27 | 0.0808328255164062 | 0.0761652331449943 | 0.288561557341825 | -0.068451031 | 0.230116682480595 | 1.08418963257388 | 0.933839187736933 | 1.25874687506884 | 0.998074519377826 |
| ebi-fl187-GCST90094852 | prot-a-1205 | ADP-ribosylation factor-binding protein GGA1 \|\| id:prot-a-1205 | F-strong vegetable liking (derived food-liking factor) \|\| id:ebi-fl187-GCST90094852 | Inverse variance weighted | 27 | -0.242188583 | 0.0761752829132372 | 0.00147601083270068 | -0.391492137 | -0.092885028 | 0.784908143433306 | 0.676047366037462 | 0.911298267810691 | 0.598008988154769 |
| ebi-fl187-GCST90094852 | prot-a-1227 | Guanine nucleotide-binding protein G(I)/G(S)/G(O) subunit gamma-T2 \|\| id:prot-a-1227 | F-strong vegetable liking (derived food-liking factor) \|\| id:ebi-fl187-GCST90094852 | Inverse variance weighted | 27 | 0.123763110417568 | 0.0761752829132372 | 0.10422333989517 | -0.025540444 | 0.273066664927513 | 1.13174773999216 | 0.974782953956655 | 1.31398783880901 | 0.998074519377826 |
| ebi-fl187-GCST90094852 | prot-a-1240 | Golgi SNAP receptor complex member 1 \|\| id:prot-a-1240 | F-strong vegetable liking (derived food-liking factor) \|\| id:ebi-fl187-GCST90094852 | Inverse variance weighted | 27 | 0.100415054520258 | 0.0780907991230182 | 0.19848647448653 | -0.052642912 | 0.253473020801374 | 1.10562971946806 | 0.948718728303112 | 1.28849261651813 | 0.998074519377826 |
| ebi-fl187-GCST90094852 | prot-a-1283 | Glutathione S-transferase A1 \|\| id:prot-a-1283 | F-strong vegetable liking (derived food-liking factor) \|\| id:ebi-fl187-GCST90094852 | Inverse variance weighted | 27 | -0.176413934 | 0.0849892208893421 | 0.0379197366577386 | -0.342992807 | -0.009835061 | 0.838270922385431 | 0.709643315800765 | 0.990213144647173 | 0.998074519377826 |
| ebi-fl187-GCST90094852 | prot-a-1324 | E3 ubiquitin-protein ligase HECW1 \|\| id:prot-a-1324 | F-strong vegetable liking (derived food-liking factor) \|\| id:ebi-fl187-GCST90094852 | Inverse variance weighted | 27 | 0.0427052562175988 | 0.0761752829132372 | 0.575057785960279 | -0.106598298 | 0.192008810727544 | 1.04363024598791 | 0.898886684792191 | 1.21168119271073 | 0.998074519377826 |
| ebi-fl187-GCST90094852 | prot-a-1339 | Histidine triad nucleotide-binding protein 2, mitochondrial \|\| id:prot-a-1339 | F-strong vegetable liking (derived food-liking factor) \|\| id:ebi-fl187-GCST90094852 | Inverse variance weighted (multiplicative random effects) | 27 | 0.0293430157527557 | 0.103432133916242 | 0.776645338145818 | -0.173383967 | 0.23206999822859 | 1.02977776389518 | 0.840814707987704 | 1.26120800806505 | 0.998074519377826 |
| ebi-fl187-GCST90094852 | prot-a-1466 | Interleukin-11 \|\| id:prot-a-1466 | F-strong vegetable liking (derived food-liking factor) \|\| id:ebi-fl187-GCST90094852 | Inverse variance weighted | 27 | 0.0148385351331316 | 0.0794138979607021 | 0.851777766002159 | -0.140812705 | 0.170489775136108 | 1.01494917275163 | 0.868651990750363 | 1.18588552635374 | 0.998074519377826 |
| ebi-fl187-GCST90094852 | prot-a-147 | Rho GTPase-activating protein 1 \|\| id:prot-a-147 | F-strong vegetable liking (derived food-liking factor) \|\| id:ebi-fl187-GCST90094852 | Inverse variance weighted | 27 | -0.008288948 | 0.0791589576701306 | 0.916603787013872 | -0.163440505 | 0.146862609124919 | 0.991745310698893 | 0.84921702171461 | 1.15819482669741 | 0.998074519377826 |
| ebi-fl187-GCST90094852 | prot-a-1473 | Interleukin-12 receptor subunit beta-1 \|\| id:prot-a-1473 | F-strong vegetable liking (derived food-liking factor) \|\| id:ebi-fl187-GCST90094852 | Inverse variance weighted | 27 | 0.157921424590954 | 0.0865299281283095 | 0.0679935205927662 | -0.011677235 | 0.327520083722441 | 1.17107417346021 | 0.988390679755298 | 1.3875229176433 | 0.998074519377826 |
| ebi-fl187-GCST90094852 | prot-a-1501 | X-linked interleukin-1 receptor accessory protein-like 2 \|\| id:prot-a-1501 | F-strong vegetable liking (derived food-liking factor) \|\| id:ebi-fl187-GCST90094852 | Inverse variance weighted (multiplicative random effects) | 27 | 0.0140263283854814 | 0.0932828860246057 | 0.880477945263138 | -0.168808128 | 0.196860784993709 | 1.01412515886512 | 0.844670956356433 | 1.21757452426151 | 0.998074519377826 |
| ebi-fl187-GCST90094852 | prot-a-1503 | Interleukin-1 receptor-like 2 \|\| id:prot-a-1503 | F-strong vegetable liking (derived food-liking factor) \|\| id:ebi-fl187-GCST90094852 | Inverse variance weighted | 27 | -0.091950799 | 0.0761752829132372 | 0.227395582387914 | -0.241254353 | 0.0573527557634013 | 0.912150027365746 | 0.785641770429674 | 1.0590293232096 | 0.998074519377826 |
| ebi-fl187-GCST90094852 | prot-a-1536 | Interleukin-5 receptor subunit alpha \|\| id:prot-a-1536 | F-strong vegetable liking (derived food-liking factor) \|\| id:ebi-fl187-GCST90094852 | Inverse variance weighted | 27 | 0.0562400214087608 | 0.0761752829132372 | 0.460333681587188 | -0.093063533 | 0.205543575918706 | 1.05785156028925 | 0.911135611186245 | 1.22819249941231 | 0.998074519377826 |
| ebi-fl187-GCST90094852 | prot-a-1559 | Insulin-induced gene 1 protein \|\| id:prot-a-1559 | F-strong vegetable liking (derived food-liking factor) \|\| id:ebi-fl187-GCST90094852 | Inverse variance weighted | 27 | 0.0246738240609088 | 0.0761752829132372 | 0.746007284356879 | -0.12462973 | 0.173977378570854 | 1.02498074193822 | 0.882823724818869 | 1.19002864536719 | 0.998074519377826 |
| ebi-fl187-GCST90094852 | prot-a-1567 | Interferon regulatory factor 1 \|\| id:prot-a-1567 | F-strong vegetable liking (derived food-liking factor) \|\| id:ebi-fl187-GCST90094852 | Inverse variance weighted (multiplicative random effects) | 27 | -0.108493712 | 0.105773811964573 | 0.305026268357515 | -0.315810384 | 0.098822959165909 | 0.897184536040211 | 0.729197704804618 | 1.10387085204192 | 0.998074519377826 |
| ebi-fl187-GCST90094852 | prot-a-1613 | Potassium voltage-gated channel subfamily E regulatory beta subunit 5 \|\| id:prot-a-1613 | F-strong vegetable liking (derived food-liking factor) \|\| id:ebi-fl187-GCST90094852 | Inverse variance weighted | 27 | 0.0785414098062875 | 0.0905936696298092 | 0.38596197922048 | -0.099022183 | 0.256105002280714 | 1.0817081475597 | 0.905722616456481 | 1.2918883720436 | 0.998074519377826 |
| ebi-fl187-GCST90094852 | prot-a-1649 | Kin of IRRE-like protein 2 \|\| id:prot-a-1649 | F-strong vegetable liking (derived food-liking factor) \|\| id:ebi-fl187-GCST90094852 | Inverse variance weighted | 26 | 0.0433122994716893 | 0.0924382774411328 | 0.639388658700911 | -0.137866724 | 0.22449132325631 | 1.04426396701708 | 0.871214795759195 | 1.2516858507436 | 0.998074519377826 |
| ebi-fl187-GCST90094852 | prot-a-165 | Armadillo repeat-containing protein 5 \|\| id:prot-a-165 | F-strong vegetable liking (derived food-liking factor) \|\| id:ebi-fl187-GCST90094852 | Inverse variance weighted | 27 | 0.084504748091608 | 0.0829478247683426 | 0.30831221334904 | -0.078072988 | 0.247082484637559 | 1.08817801098862 | 0.924896917283452 | 1.28028471224351 | 0.998074519377826 |
| ebi-fl187-GCST90094852 | prot-a-1656 | Kallikrein-11 \|\| id:prot-a-1656 | F-strong vegetable liking (derived food-liking factor) \|\| id:ebi-fl187-GCST90094852 | Inverse variance weighted | 27 | 0.113334523883704 | 0.0796082176890405 | 0.154546778260747 | -0.042697583 | 0.269366630554223 | 1.12000653922499 | 0.958201122767962 | 1.30913502197025 | 0.998074519377826 |
| ebi-fl187-GCST90094852 | prot-a-1697 | Laminin subunit gamma-2 \|\| id:prot-a-1697 | F-strong vegetable liking (derived food-liking factor) \|\| id:ebi-fl187-GCST90094852 | Inverse variance weighted | 26 | -0.026529588 | 0.0773032760544113 | 0.731456723933141 | -0.178044009 | 0.124984832735711 | 0.973819229723616 | 0.836905590967394 | 1.1331312664351 | 0.998074519377826 |
| ebi-fl187-GCST90094852 | prot-a-1698 | Lysosome-associated membrane glycoprotein 3 \|\| id:prot-a-1698 | F-strong vegetable liking (derived food-liking factor) \|\| id:ebi-fl187-GCST90094852 | Inverse variance weighted | 27 | -0.026580568 | 0.0774516429714217 | 0.73145612645528 | -0.178385788 | 0.12522465257089 | 0.973769586344781 | 0.836619603521988 | 1.13340304637646 | 0.998074519377826 |
| ebi-fl187-GCST90094852 | prot-a-1724 | Leptin receptor \|\| id:prot-a-1724 | F-strong vegetable liking (derived food-liking factor) \|\| id:ebi-fl187-GCST90094852 | Inverse variance weighted | 27 | 0.161525332899304 | 0.0761752829132372 | 0.0339687175188338 | 0.0122217783893587 | 0.310828887409249 | 1.17530223159433 | 1.01229676951978 | 1.36455570854574 | 0.998074519377826 |
| ebi-fl187-GCST90094852 | prot-a-1743 | Leukocyte immunoglobulin-like receptor subfamily B member 2 \|\| id:prot-a-1743 | F-strong vegetable liking (derived food-liking factor) \|\| id:ebi-fl187-GCST90094852 | Inverse variance weighted | 27 | 0.0413433346789078 | 0.0761752829132372 | 0.587309176238318 | -0.10796022 | 0.190646889188853 | 1.04220987091672 | 0.897663304918127 | 1.21003210121786 | 0.998074519377826 |
| ebi-fl187-GCST90094852 | prot-a-1746 | Leukocyte immunoglobulin-like receptor subfamily B member 4 \|\| id:prot-a-1746 | F-strong vegetable liking (derived food-liking factor) \|\| id:ebi-fl187-GCST90094852 | Inverse variance weighted | 27 | 0.0393919116361225 | 0.076166166324255 | 0.605027820195972 | -0.109893774 | 0.188677597631662 | 1.04017806166366 | 0.89592930090583 | 1.2076515399959 | 0.998074519377826 |
| ebi-fl187-GCST90094852 | prot-a-1750 | Leucine-rich repeat and immunoglobulin-like domain-containing nogo receptor-interacting protein 3 \|\| id:prot-a-1750 | F-strong vegetable liking (derived food-liking factor) \|\| id:ebi-fl187-GCST90094852 | Inverse variance weighted | 27 | -0.027892791 | 0.0761752829132372 | 0.714241286785797 | -0.177196345 | 0.1214107635943 | 0.972492621256102 | 0.837615306442439 | 1.1290886056206 | 0.998074519377826 |
| ebi-fl187-GCST90094852 | prot-a-1753 | Lipase member N \|\| id:prot-a-1753 | F-strong vegetable liking (derived food-liking factor) \|\| id:ebi-fl187-GCST90094852 | Inverse variance weighted | 27 | 0.0404023199761031 | 0.078895649956927 | 0.608582287390472 | -0.114233154 | 0.19503779389168 | 1.04122959740301 | 0.892049946674993 | 1.21535691868948 | 0.998074519377826 |
| ebi-fl187-GCST90094852 | prot-a-1760 | Ig Kappa chain V-I region HK102- like \|\| id:prot-a-1760 | F-strong vegetable liking (derived food-liking factor) \|\| id:ebi-fl187-GCST90094852 | Inverse variance weighted | 26 | -0.036648251 | 0.0772993557639286 | 0.635422818982676 | -0.188154988 | 0.114858486195193 | 0.964015166991096 | 0.828486291517212 | 1.12171468822615 | 0.998074519377826 |
| ebi-fl187-GCST90094852 | prot-a-1792 | Leucine-rich repeat neuronal protein 1 \|\| id:prot-a-1792 | F-strong vegetable liking (derived food-liking factor) \|\| id:ebi-fl187-GCST90094852 | Inverse variance weighted | 27 | 0.00998779166668036 | 0.0761752829132372 | 0.895683614126404 | -0.139315763 | 0.159291346176625 | 1.01003783613033 | 0.869953286160684 | 1.17267955261957 | 0.998074519377826 |
| ebi-fl187-GCST90094852 | prot-a-1795 | Leucine-rich repeat transmembrane neuronal protein 1 \|\| id:prot-a-1795 | F-strong vegetable liking (derived food-liking factor) \|\| id:ebi-fl187-GCST90094852 | Inverse variance weighted | 27 | 0.066565874112414 | 0.0761752829132372 | 0.382199209540776 | -0.08273768 | 0.215869428622359 | 1.06883137007401 | 0.92059260503537 | 1.2409403371325 | 0.998074519377826 |
| ebi-fl187-GCST90094852 | prot-a-1820 | LysM and putative peptidoglycan-binding domain-containing protein 4 \|\| id:prot-a-1820 | F-strong vegetable liking (derived food-liking factor) \|\| id:ebi-fl187-GCST90094852 | Inverse variance weighted | 27 | 0.0432837281654646 | 0.0761752829132372 | 0.569890860970839 | -0.106019826 | 0.19258728267541 | 1.04423413145772 | 0.899406815949791 | 1.21238231906188 | 0.998074519377826 |
| ebi-fl187-GCST90094852 | prot-a-1844 | Mitogen-activated protein kinase kinase kinase 7:TGF-beta-activated kinase 1 and MAP3K7-binding protein 1 fusion \|\| id:prot-a-1844 | F-strong vegetable liking (derived food-liking factor) \|\| id:ebi-fl187-GCST90094852 | Inverse variance weighted | 27 | -0.06116146 | 0.076542793345787 | 0.424261864156988 | -0.211185335 | 0.0888624145359015 | 0.940671346321558 | 0.809624001003902 | 1.09293027466234 | 0.998074519377826 |
| ebi-fl187-GCST90094852 | prot-a-1856 | Megakaryocyte-associated tyrosine-protein kinase \|\| id:prot-a-1856 | F-strong vegetable liking (derived food-liking factor) \|\| id:ebi-fl187-GCST90094852 | Inverse variance weighted | 27 | -0.05693165 | 0.080206798265176 | 0.477821482089316 | -0.214136975 | 0.10027367440839 | 0.944658634396891 | 0.807237806219726 | 1.10547341646397 | 0.998074519377826 |
| ebi-fl187-GCST90094852 | prot-a-1910 | Stromelysin-2 \|\| id:prot-a-1910 | F-strong vegetable liking (derived food-liking factor) \|\| id:ebi-fl187-GCST90094852 | Inverse variance weighted | 27 | 0.0343004150938868 | 0.0761752829132372 | 0.652506582917559 | -0.115003139 | 0.183603969603832 | 1.03489545824979 | 0.891363345542038 | 1.20153988254336 | 0.998074519377826 |
| ebi-fl187-GCST90094852 | prot-a-1940 | 39S ribosomal protein L14, mitochondrial \|\| id:prot-a-1940 | F-strong vegetable liking (derived food-liking factor) \|\| id:ebi-fl187-GCST90094852 | Inverse variance weighted | 27 | -0.157546384 | 0.0761752829132372 | 0.0386204014349768 | -0.306849938 | -0.008242829 | 0.854237189982963 | 0.735761002215027 | 0.99179104974733 | 0.998074519377826 |
| ebi-fl187-GCST90094852 | prot-a-1942 | 39S ribosomal protein L33, mitochondrial \|\| id:prot-a-1942 | F-strong vegetable liking (derived food-liking factor) \|\| id:ebi-fl187-GCST90094852 | Inverse variance weighted | 27 | 0.0621411375662011 | 0.0923442709628512 | 0.500992463990361 | -0.118853634 | 0.243135908653389 | 1.06411252038136 | 0.88793775557407 | 1.27524192875468 | 0.998074519377826 |
| ebi-fl187-GCST90094852 | prot-a-1948 | Mesothelin \|\| id:prot-a-1948 | F-strong vegetable liking (derived food-liking factor) \|\| id:ebi-fl187-GCST90094852 | Inverse variance weighted | 27 | -0.033499524 | 0.0796035074571512 | 0.673879407342495 | -0.189522399 | 0.12252335062339 | 0.967055371556781 | 0.827354185107313 | 1.13034551403825 | 0.998074519377826 |
| ebi-fl187-GCST90094852 | prot-a-1980 | Unconventional myosin-VI \|\| id:prot-a-1980 | F-strong vegetable liking (derived food-liking factor) \|\| id:ebi-fl187-GCST90094852 | Inverse variance weighted | 27 | -0.207361848 | 0.0761699167101254 | 0.00648177137448042 | -0.356654885 | -0.058068811 | 0.812725513859565 | 0.700014041732524 | 0.943585016156977 | 0.877504772527479 |
| ebi-fl187-GCST90094852 | prot-a-2039 | Neurofascin \|\| id:prot-a-2039 | F-strong vegetable liking (derived food-liking factor) \|\| id:ebi-fl187-GCST90094852 | Inverse variance weighted | 27 | 0.0363354949084655 | 0.081359219970353 | 0.655159748075478 | -0.123128576 | 0.195799566050357 | 1.03700369759713 | 0.884149974578676 | 1.21628309647644 | 0.998074519377826 |
| ebi-fl187-GCST90094852 | prot-a-2041 | NFU1 iron-sulfur cluster scaffold homolog, mitochondrial \|\| id:prot-a-2041 | F-strong vegetable liking (derived food-liking factor) \|\| id:ebi-fl187-GCST90094852 | Inverse variance weighted | 27 | -0.127557226 | 0.0875764697871544 | 0.145247833470033 | -0.299207107 | 0.044092654975488 | 0.880243041774171 | 0.741405843497131 | 1.04507918218862 | 0.998074519377826 |
| ebi-fl187-GCST90094852 | prot-a-2088 | Glucocorticoid receptor \|\| id:prot-a-2088 | F-strong vegetable liking (derived food-liking factor) \|\| id:ebi-fl187-GCST90094852 | Inverse variance weighted | 27 | 0.116631582081657 | 0.0761652331449943 | 0.12569604214436 | -0.032652275 | 0.265915439045845 | 1.12370536023027 | 0.967875055548978 | 1.3046247337101 | 0.998074519377826 |
| ebi-fl187-GCST90094852 | prot-a-2111 | Neuron-specific protein family member 2 \|\| id:prot-a-2111 | F-strong vegetable liking (derived food-liking factor) \|\| id:ebi-fl187-GCST90094852 | Inverse variance weighted | 27 | 0.0416558366485701 | 0.0801008507072603 | 0.603034040615833 | -0.115341831 | 0.1986535040348 | 1.04253561444928 | 0.89106149963169 | 1.21975925101061 | 0.998074519377826 |
| ebi-fl187-GCST90094852 | prot-a-2125 | Nuclear migration protein nudC \|\| id:prot-a-2125 | F-strong vegetable liking (derived food-liking factor) \|\| id:ebi-fl187-GCST90094852 | Inverse variance weighted | 27 | -0.096861987 | 0.0792502267142356 | 0.221620744394014 | -0.252192432 | 0.0584684570318946 | 0.907681269006395 | 0.777095185941827 | 1.06021154294827 | 0.998074519377826 |
| ebi-fl187-GCST90094852 | prot-a-2142 | Ornithine decarboxylase \|\| id:prot-a-2142 | F-strong vegetable liking (derived food-liking factor) \|\| id:ebi-fl187-GCST90094852 | Inverse variance weighted (multiplicative random effects) | 27 | -0.003098878 | 0.106079574437577 | 0.976694899335929 | -0.211014844 | 0.20481708805264 | 0.996905918721002 | 0.809762046892625 | 1.22730055649638 | 0.998074519377826 |
| ebi-fl187-GCST90094852 | prot-a-2186 | Prostate and testis expressed protein 1 \|\| id:prot-a-2186 | F-strong vegetable liking (derived food-liking factor) \|\| id:ebi-fl187-GCST90094852 | Inverse variance weighted | 27 | 0.0796087239826994 | 0.0761752829132372 | 0.295989277012987 | -0.069694831 | 0.228912278492644 | 1.08286328633867 | 0.932678401456961 | 1.25723174790844 | 0.998074519377826 |
| ebi-fl187-GCST90094852 | prot-a-2227 | Platelet-derived growth factor C \|\| id:prot-a-2227 | F-strong vegetable liking (derived food-liking factor) \|\| id:ebi-fl187-GCST90094852 | Inverse variance weighted | 27 | 0.0278716185027913 | 0.0761752829132372 | 0.714448684460252 | -0.121431936 | 0.177175173012736 | 1.02826366591828 | 0.885651332263376 | 1.19384020339652 | 0.998074519377826 |
| ebi-fl187-GCST90094852 | prot-a-2229 | Platelet-derived growth factor receptor alpha \|\| id:prot-a-2229 | F-strong vegetable liking (derived food-liking factor) \|\| id:ebi-fl187-GCST90094852 | Inverse variance weighted | 27 | -0.03321594 | 0.0761592289930772 | 0.662736545777366 | -0.182488029 | 0.116056148470453 | 0.967329651524705 | 0.833194617889421 | 1.12305892840404 | 0.998074519377826 |
| ebi-fl187-GCST90094852 | prot-a-2243 | Platelet endothelial aggregation receptor 1 \|\| id:prot-a-2243 | F-strong vegetable liking (derived food-liking factor) \|\| id:ebi-fl187-GCST90094852 | Inverse variance weighted | 27 | 0.144101123108594 | 0.0915618395396072 | 0.1155309157019 | -0.035360082 | 0.323562328606224 | 1.15500089990109 | 0.965257781344775 | 1.38204229435352 | 0.998074519377826 |
| ebi-fl187-GCST90094852 | prot-a-2246 | E3 ubiquitin-protein ligase pellino homolog 2 \|\| id:prot-a-2246 | F-strong vegetable liking (derived food-liking factor) \|\| id:ebi-fl187-GCST90094852 | Inverse variance weighted | 27 | -0.018922301 | 0.0903223983374813 | 0.834060019983721 | -0.195954202 | 0.158109599507046 | 0.981255601629882 | 0.822049882076418 | 1.17129456097959 | 0.998074519377826 |
| ebi-fl187-GCST90094852 | prot-a-2251 | Platelet factor 4 variant \|\| id:prot-a-2251 | F-strong vegetable liking (derived food-liking factor) \|\| id:ebi-fl187-GCST90094852 | Inverse variance weighted | 27 | -0.015875418 | 0.0761752829132372 | 0.834911640293462 | -0.165178973 | 0.133428136148935 | 0.984249931887225 | 0.847741967696184 | 1.14273914154878 | 0.998074519377826 |
| ebi-fl187-GCST90094852 | prot-a-2346 | Protein phosphatase 1L \|\| id:prot-a-2346 | F-strong vegetable liking (derived food-liking factor) \|\| id:ebi-fl187-GCST90094852 | Inverse variance weighted | 27 | -0.15210616 | 0.0829142269582446 | 0.0665797889781522 | -0.314618045 | 0.0104057249627525 | 0.858897095492457 | 0.730067674228734 | 1.0104600527954 | 0.998074519377826 |
| ebi-fl187-GCST90094852 | prot-a-2358 | PR domain zinc finger protein 4 \|\| id:prot-a-2358 | F-strong vegetable liking (derived food-liking factor) \|\| id:ebi-fl187-GCST90094852 | Inverse variance weighted | 27 | 0.0791642160774394 | 0.0776011098273303 | 0.307660724812345 | -0.072933959 | 0.231262391339007 | 1.08238205201173 | 0.929662223650263 | 1.26018985897598 | 0.998074519377826 |
| ebi-fl187-GCST90094852 | prot-a-2390 | Trypsin-1 \|\| id:prot-a-2390 | F-strong vegetable liking (derived food-liking factor) \|\| id:ebi-fl187-GCST90094852 | Inverse variance weighted | 27 | 0.0339884622115334 | 0.0762294261699254 | 0.65569069924363 | -0.115421213 | 0.183398137504587 | 1.03457266997864 | 0.890990767888788 | 1.201292593 | 0.998074519377826 |
| ebi-fl187-GCST90094852 | prot-a-242 | Brain-derived neurotrophic factor \|\| id:prot-a-242 | F-strong vegetable liking (derived food-liking factor) \|\| id:ebi-fl187-GCST90094852 | Inverse variance weighted | 27 | 0.0609769392325009 | 0.0761752829132372 | 0.423431615608212 | -0.088326615 | 0.210280493742446 | 1.06287440320508 | 0.915461824070743 | 1.23402414746818 | 0.998074519377826 |
| ebi-fl187-GCST90094852 | prot-a-2464 | Dihydropteridine reductase \|\| id:prot-a-2464 | F-strong vegetable liking (derived food-liking factor) \|\| id:ebi-fl187-GCST90094852 | Inverse variance weighted | 26 | -0.255816424 | 0.0930573891521746 | 0.00597741305381885 | -0.438208907 | -0.073423941 | 0.774284095668912 | 0.64519098398811 | 0.929206817336543 | 0.877504772527479 |
| ebi-fl187-GCST90094852 | prot-a-2474 | Ras-related protein Rab-27A \|\| id:prot-a-2474 | F-strong vegetable liking (derived food-liking factor) \|\| id:ebi-fl187-GCST90094852 | Inverse variance weighted | 27 | -0.146414426 | 0.0808455213677525 | 0.0701347510510435 | -0.304871647 | 0.0120427963770094 | 0.863799648391794 | 0.737217992176708 | 1.01211560282015 | 0.998074519377826 |
| ebi-fl187-GCST90094852 | prot-a-2478 | Ras-related protein Rab-7b \|\| id:prot-a-2478 | F-strong vegetable liking (derived food-liking factor) \|\| id:ebi-fl187-GCST90094852 | Inverse variance weighted | 27 | 0.0578728105773011 | 0.0761683430909296 | 0.447373330589957 | -0.091417142 | 0.207162763035523 | 1.05958021974298 | 0.912636932398879 | 1.23018278377089 | 0.998074519377826 |
| ebi-fl187-GCST90094852 | prot-a-2528 | E3 ubiquitin-protein ligase rififylin \|\| id:prot-a-2528 | F-strong vegetable liking (derived food-liking factor) \|\| id:ebi-fl187-GCST90094852 | Inverse variance weighted | 27 | -0.065386156 | 0.0856629816266822 | 0.445287268398084 | -0.2332856 | 0.102513287549828 | 0.936705678600396 | 0.79192736642961 | 1.10795202377972 | 0.998074519377826 |
| ebi-fl187-GCST90094852 | prot-a-2543 | Protein RIC-3 \|\| id:prot-a-2543 | F-strong vegetable liking (derived food-liking factor) \|\| id:ebi-fl187-GCST90094852 | Inverse variance weighted (multiplicative random effects) | 27 | 0.0204632866606627 | 0.0953340827532875 | 0.830041632804869 | -0.166391516 | 0.207318088857106 | 1.02067409520091 | 0.846714667340212 | 1.23037387776301 | 0.998074519377826 |
| ebi-fl187-GCST90094852 | prot-a-255 | Bone morphogenetic protein 6 \|\| id:prot-a-255 | F-strong vegetable liking (derived food-liking factor) \|\| id:ebi-fl187-GCST90094852 | Inverse variance weighted | 27 | 0.00144544599071454 | 0.0893007247496372 | 0.987085785867638 | -0.173583975 | 0.176474866500004 | 1.00144649115128 | 0.840646555308023 | 1.19300444200565 | 0.999355215041207 |
| ebi-fl187-GCST90094852 | prot-a-2568 | RING finger protein 165 \|\| id:prot-a-2568 | F-strong vegetable liking (derived food-liking factor) \|\| id:ebi-fl187-GCST90094852 | Inverse variance weighted | 27 | -0.034511624 | 0.0761752829132372 | 0.650508839647714 | -0.183815179 | 0.114791930175741 | 0.966077109618032 | 0.832089577373394 | 1.12164003384592 | 0.998074519377826 |
| ebi-fl187-GCST90094852 | prot-a-2573 | E3 ubiquitin-protein ligase RNF8 \|\| id:prot-a-2573 | F-strong vegetable liking (derived food-liking factor) \|\| id:ebi-fl187-GCST90094852 | Inverse variance weighted | 27 | 0.135539497382667 | 0.0923152666682979 | 0.142043365661328 | -0.045398425 | 0.31647742005253 | 1.14515442554376 | 0.95561666413058 | 1.37228525575739 | 0.998074519377826 |
| ebi-fl187-GCST90094852 | prot-a-260 | Cytoplasmic tyrosine-protein kinase BMX \|\| id:prot-a-260 | F-strong vegetable liking (derived food-liking factor) \|\| id:ebi-fl187-GCST90094852 | Inverse variance weighted | 27 | -0.0803252 | 0.0893857659449014 | 0.368846978184957 | -0.255521301 | 0.0948709014291833 | 0.922816197921105 | 0.774512638433139 | 1.09951689990283 | 0.998074519377826 |
| ebi-fl187-GCST90094852 | prot-a-2623 | Serum amyloid A-1 protein \|\| id:prot-a-2623 | F-strong vegetable liking (derived food-liking factor) \|\| id:ebi-fl187-GCST90094852 | Inverse variance weighted | 27 | 0.0257406169984988 | 0.0761652331449943 | 0.735395163746212 | -0.12354324 | 0.175024473962688 | 1.02607476760055 | 0.883783425652861 | 1.19127537148458 | 0.998074519377826 |
| ebi-fl187-GCST90094852 | prot-a-2640 | Lysosome membrane protein 2 \|\| id:prot-a-2640 | F-strong vegetable liking (derived food-liking factor) \|\| id:ebi-fl187-GCST90094852 | Inverse variance weighted | 27 | -0.025808747 | 0.08070916882838 | 0.749139160829003 | -0.183998718 | 0.13238122416376 | 0.974521452191759 | 0.83193687066565 | 1.14154342026224 | 0.998074519377826 |
| ebi-fl187-GCST90094852 | prot-a-2641 | Scavenger receptor class F member 2 \|\| id:prot-a-2641 | F-strong vegetable liking (derived food-liking factor) \|\| id:ebi-fl187-GCST90094852 | Inverse variance weighted | 27 | 0.00366187222132084 | 0.0903936085564134 | 0.967686295389042 | -0.173509601 | 0.180833344991891 | 1.00366858506676 | 0.840709079854211 | 1.19821547404318 | 0.998074519377826 |
| ebi-fl187-GCST90094852 | prot-a-2669 | Semaphorin-3A \|\| id:prot-a-2669 | F-strong vegetable liking (derived food-liking factor) \|\| id:ebi-fl187-GCST90094852 | Inverse variance weighted | 27 | -0.070570248 | 0.0911992427541921 | 0.439047297726847 | -0.249320764 | 0.108180268088995 | 0.931862276036296 | 0.779329952679012 | 1.11424859074704 | 0.998074519377826 |
| ebi-fl187-GCST90094852 | prot-a-2683 | Sentrin-specific protease 7 \|\| id:prot-a-2683 | F-strong vegetable liking (derived food-liking factor) \|\| id:ebi-fl187-GCST90094852 | Inverse variance weighted | 27 | -0.173135755 | 0.0910909959007751 | 0.0573426149780237 | -0.351674107 | 0.00540259712084148 | 0.841023434088838 | 0.703509353529089 | 1.00541721746609 | 0.998074519377826 |
| ebi-fl187-GCST90094852 | prot-a-270 | Bromodomain testis-specific protein \|\| id:prot-a-270 | F-strong vegetable liking (derived food-liking factor) \|\| id:ebi-fl187-GCST90094852 | Inverse variance weighted | 27 | 0.0650191409179252 | 0.0761752829132372 | 0.393356350360023 | -0.084284414 | 0.21432269542787 | 1.06717945098298 | 0.919169794532337 | 1.23902241715829 | 0.998074519377826 |
| ebi-fl187-GCST90094852 | prot-a-2708 | 14-3-3 protein sigma \|\| id:prot-a-2708 | F-strong vegetable liking (derived food-liking factor) \|\| id:ebi-fl187-GCST90094852 | Inverse variance weighted | 27 | 0.0208457487139756 | 0.0761752829132372 | 0.7843497459708 | -0.128457806 | 0.170149303223921 | 1.02106453897133 | 0.879450669356539 | 1.18548183436774 | 0.998074519377826 |
| ebi-fl187-GCST90094852 | prot-a-2713 | Beta-sarcoglycan \|\| id:prot-a-2713 | F-strong vegetable liking (derived food-liking factor) \|\| id:ebi-fl187-GCST90094852 | Inverse variance weighted | 27 | 0.0588372445523055 | 0.0761707582580822 | 0.439854616404518 | -0.090457442 | 0.208131930738147 | 1.0606026078398 | 0.913513210703672 | 1.23137561512669 | 0.998074519377826 |
| ebi-fl187-GCST90094852 | prot-a-2720 | Protein shisa-3 homolog \|\| id:prot-a-2720 | F-strong vegetable liking (derived food-liking factor) \|\| id:ebi-fl187-GCST90094852 | Inverse variance weighted | 26 | 0.0526149109238193 | 0.083920877202168 | 0.53068641040333 | -0.111870008 | 0.217099830240069 | 1.05402367395397 | 0.894160483304654 | 1.24246813183858 | 0.998074519377826 |
| ebi-fl187-GCST90094852 | prot-a-2730 | Sialic acid-binding Ig-like lectin 8 \|\| id:prot-a-2730 | F-strong vegetable liking (derived food-liking factor) \|\| id:ebi-fl187-GCST90094852 | Inverse variance weighted | 27 | -0.050044866 | 0.0761752829132372 | 0.511200291185563 | -0.19934842 | 0.0992586889208639 | 0.951186747989593 | 0.819264395417428 | 1.1043519462237 | 0.998074519377826 |
| ebi-fl187-GCST90094852 | prot-a-2761 | Sodium- and chloride-dependent neutral and basic amino acid transporter B(0+) \|\| id:prot-a-2761 | F-strong vegetable liking (derived food-liking factor) \|\| id:ebi-fl187-GCST90094852 | Inverse variance weighted (multiplicative random effects) | 27 | -0.068526845 | 0.0998409743735835 | 0.492486162920361 | -0.264215154 | 0.127161465119586 | 0.93376839307891 | 0.767808324525493 | 1.13560036282756 | 0.998074519377826 |
| ebi-fl187-GCST90094852 | prot-a-2770 | Secreted Ly-6/uPAR-related protein 1 \|\| id:prot-a-2770 | F-strong vegetable liking (derived food-liking factor) \|\| id:ebi-fl187-GCST90094852 | Inverse variance weighted | 27 | 0.0944665881587654 | 0.0761752829132372 | 0.214930544922087 | -0.054836966 | 0.24377014266871 | 1.09907244048562 | 0.946639469460166 | 1.2760509871027 | 0.998074519377826 |
| ebi-fl187-GCST90094852 | prot-a-2784 | N-lysine methyltransferase SMYD2 \|\| id:prot-a-2784 | F-strong vegetable liking (derived food-liking factor) \|\| id:ebi-fl187-GCST90094852 | Inverse variance weighted | 27 | -0.071660001 | 0.0761752829132372 | 0.346845748077093 | -0.220963556 | 0.0776435531773397 | 0.930847328866111 | 0.801745898711539 | 1.080737365 | 0.998074519377826 |
| ebi-fl187-GCST90094852 | prot-a-2837 | Serine palmitoyltransferase 2 \|\| id:prot-a-2837 | F-strong vegetable liking (derived food-liking factor) \|\| id:ebi-fl187-GCST90094852 | Inverse variance weighted | 27 | 0.100125515168815 | 0.0761652331449943 | 0.188650110918958 | -0.049158342 | 0.249409372133004 | 1.10530964249583 | 0.952030371564879 | 1.28326725941116 | 0.998074519377826 |
| ebi-fl187-GCST90094852 | prot-a-2858 | Alpha-2,8-sialyltransferase 8B \|\| id:prot-a-2858 | F-strong vegetable liking (derived food-liking factor) \|\| id:ebi-fl187-GCST90094852 | Inverse variance weighted (multiplicative random effects) | 27 | 0.091130819200004 | 0.0931960615886719 | 0.328153557860817 | -0.091533462 | 0.273795099913801 | 1.09541229684596 | 0.912530780979823 | 1.31494534221951 | 0.998074519377826 |
| ebi-fl187-GCST90094852 | prot-a-2911 | Synaptotagmin-11 \|\| id:prot-a-2911 | F-strong vegetable liking (derived food-liking factor) \|\| id:ebi-fl187-GCST90094852 | Inverse variance weighted | 27 | -0.07373213 | 0.0761752829132372 | 0.33308071700792 | -0.223035685 | 0.0755714243935802 | 0.928920490340909 | 0.800086298004576 | 1.07850025619394 | 0.998074519377826 |
| ebi-fl187-GCST90094852 | prot-a-2962 | Transforming growth factor beta-1 \|\| id:prot-a-2962 | F-strong vegetable liking (derived food-liking factor) \|\| id:ebi-fl187-GCST90094852 | Inverse variance weighted | 27 | 0.0216331504609948 | 0.0783097302784628 | 0.782355198443076 | -0.131853921 | 0.175120221806782 | 1.021868844 | 0.876469019547178 | 1.1913894389939 | 0.998074519377826 |
| ebi-fl187-GCST90094852 | prot-a-2978 | Tyrosine-protein kinase receptor Tie-1, soluble \|\| id:prot-a-2978 | F-strong vegetable liking (derived food-liking factor) \|\| id:ebi-fl187-GCST90094852 | Inverse variance weighted | 27 | 0.123586290224693 | 0.0761752829132372 | 0.104719102154219 | -0.025717264 | 0.272889844734638 | 1.13154764182971 | 0.974610607884307 | 1.31375551976587 | 0.998074519377826 |
| ebi-fl187-GCST90094852 | prot-a-299 | Uncharacterized protein C1orf226 \|\| id:prot-a-299 | F-strong vegetable liking (derived food-liking factor) \|\| id:ebi-fl187-GCST90094852 | Inverse variance weighted | 27 | 0.0493430247329331 | 0.0814660551137528 | 0.544721850120344 | -0.110330443 | 0.209016492755889 | 1.05058066409002 | 0.895538161821464 | 1.23246532511238 | 0.998074519377826 |
| ebi-fl187-GCST90094852 | prot-a-3015 | Transmembrane protein 70, mitochondrial \|\| id:prot-a-3015 | F-strong vegetable liking (derived food-liking factor) \|\| id:ebi-fl187-GCST90094852 | Inverse variance weighted | 27 | -0.060336396 | 0.0761752829132372 | 0.428318029212518 | -0.20963995 | 0.0889671587912607 | 0.941447781307184 | 0.810876149189316 | 1.09304475882579 | 0.998074519377826 |
| ebi-fl187-GCST90094852 | prot-a-3016 | Transmembrane protein 87B \|\| id:prot-a-3016 | F-strong vegetable liking (derived food-liking factor) \|\| id:ebi-fl187-GCST90094852 | Inverse variance weighted | 27 | -0.180327156 | 0.080343167298005 | 0.0248027150866049 | -0.337799764 | -0.022854548 | 0.834996992318572 | 0.713338109528871 | 0.977404638652414 | 0.998074519377826 |
| ebi-fl187-GCST90094852 | prot-a-3123 | Thioredoxin domain-containing protein 12 \|\| id:prot-a-3123 | F-strong vegetable liking (derived food-liking factor) \|\| id:ebi-fl187-GCST90094852 | Inverse variance weighted | 26 | -0.029011027 | 0.0868606262744067 | 0.738383228368098 | -0.199257854 | 0.141235800735065 | 0.971405752946755 | 0.819338596144258 | 1.15169618677638 | 0.998074519377826 |
| ebi-fl187-GCST90094852 | prot-a-3133 | PolyUbiquitin K48-linked \|\| id:prot-a-3133 | F-strong vegetable liking (derived food-liking factor) \|\| id:ebi-fl187-GCST90094852 | Inverse variance weighted | 27 | -0.140384661 | 0.0835464205553722 | 0.09289521286493 | -0.304135645 | 0.0233663235032637 | 0.86902389168588 | 0.737760786046771 | 1.0236414547967 | 0.998074519377826 |
| ebi-fl187-GCST90094852 | prot-a-3135 | Ubiquitin-conjugating enzyme E2 B \|\| id:prot-a-3135 | F-strong vegetable liking (derived food-liking factor) \|\| id:ebi-fl187-GCST90094852 | Inverse variance weighted | 27 | 0.00062283962807467 | 0.076166166324255 | 0.993475468063838 | -0.148662846 | 0.149908525623614 | 1.00062303363295 | 0.861859645029619 | 1.16172796952618 | 0.999355215041207 |
| ebi-fl187-GCST90094852 | prot-a-3162 | NKG2D ligand 3 \|\| id:prot-a-3162 | F-strong vegetable liking (derived food-liking factor) \|\| id:ebi-fl187-GCST90094852 | Inverse variance weighted | 27 | -0.029244064 | 0.0761752829132372 | 0.70104893977135 | -0.178547618 | 0.120059490685634 | 0.971179405779191 | 0.836484223943636 | 1.12756392913535 | 0.998074519377826 |
| ebi-fl187-GCST90094852 | prot-a-3188 | Vesicle-associated membrane protein 8 \|\| id:prot-a-3188 | F-strong vegetable liking (derived food-liking factor) \|\| id:ebi-fl187-GCST90094852 | Inverse variance weighted (multiplicative random effects) | 27 | -0.042242009 | 0.100702168999262 | 0.674869265283638 | -0.23961826 | 0.155134242458024 | 0.958637753743599 | 0.786928205694355 | 1.16781472090163 | 0.998074519377826 |
| ebi-fl187-GCST90094852 | prot-a-3201 | Vitrin \|\| id:prot-a-3201 | F-strong vegetable liking (derived food-liking factor) \|\| id:ebi-fl187-GCST90094852 | Inverse variance weighted | 27 | 0.0406134233140951 | 0.0886372983350616 | 0.646810273699249 | -0.133115681 | 0.214342528050816 | 1.04144942764927 | 0.875363822919052 | 1.23904699046638 | 0.998074519377826 |
| ebi-fl187-GCST90094852 | prot-a-3232 | WNT1-inducible-signaling pathway protein 3 \|\| id:prot-a-3232 | F-strong vegetable liking (derived food-liking factor) \|\| id:ebi-fl187-GCST90094852 | Inverse variance weighted | 27 | -0.12483517 | 0.0761683430909296 | 0.10122623257229 | -0.274125122 | 0.0244547824724491 | 0.882642376550512 | 0.760236946604867 | 1.02475625311545 | 0.998074519377826 |
| ebi-fl187-GCST90094852 | prot-a-327 | Carbonic anhydrase-related protein 10 \|\| id:prot-a-327 | F-strong vegetable liking (derived food-liking factor) \|\| id:ebi-fl187-GCST90094852 | Inverse variance weighted (multiplicative random effects) | 25 | 0.0423450974229381 | 0.102103501398452 | 0.678341631253395 | -0.157777765 | 0.242467960163904 | 1.04325444105524 | 0.854039558089867 | 1.27439041724921 | 0.998074519377826 |
| ebi-fl187-GCST90094852 | prot-a-332 | Carbonic anhydrase 5A, mitochondrial \|\| id:prot-a-332 | F-strong vegetable liking (derived food-liking factor) \|\| id:ebi-fl187-GCST90094852 | Inverse variance weighted | 27 | 0.0422771124458523 | 0.0810213271000586 | 0.601808008608978 | -0.116524689 | 0.201078913561967 | 1.04318351783685 | 0.890008123588338 | 1.22272125730598 | 0.998074519377826 |
| ebi-fl187-GCST90094852 | prot-a-338 | Cell adhesion molecule 3 \|\| id:prot-a-338 | F-strong vegetable liking (derived food-liking factor) \|\| id:ebi-fl187-GCST90094852 | Inverse variance weighted | 27 | 0.103794477240949 | 0.0761752829132372 | 0.173016735996271 | -0.045509077 | 0.253098031750893 | 1.10937243020158 | 0.955510929102856 | 1.28800953647583 | 0.998074519377826 |
| ebi-fl187-GCST90094852 | prot-a-34 | A disintegrin and metalloproteinase with thrombospondin motifs 3 \|\| id:prot-a-34 | F-strong vegetable liking (derived food-liking factor) \|\| id:ebi-fl187-GCST90094852 | Inverse variance weighted | 27 | -0.023861682 | 0.0885491085730155 | 0.787564954667618 | -0.197417934 | 0.149694571151103 | 0.976420757324014 | 0.820847501161145 | 1.16147943921929 | 0.998074519377826 |
| ebi-fl187-GCST90094852 | prot-a-358 | Cysteine--tRNA ligase, cytoplasmic \|\| id:prot-a-358 | F-strong vegetable liking (derived food-liking factor) \|\| id:ebi-fl187-GCST90094852 | Inverse variance weighted | 27 | -0.098468443 | 0.0823277466614351 | 0.23167542891201 | -0.259830826 | 0.0628939405128846 | 0.906224289934138 | 0.771182038410425 | 1.0649138890208 | 0.998074519377826 |
| ebi-fl187-GCST90094852 | prot-a-382 | Coiled-coil domain-containing protein 167 \|\| id:prot-a-382 | F-strong vegetable liking (derived food-liking factor) \|\| id:ebi-fl187-GCST90094852 | Inverse variance weighted | 27 | -0.070006477 | 0.0761752829132372 | 0.358086014662314 | -0.219310031 | 0.0792970775812981 | 0.932387780877263 | 0.803072701769144 | 1.08252586847253 | 0.998074519377826 |
| ebi-fl187-GCST90094852 | prot-a-40 | Pituitary adenylate cyclase-activating polypeptide 27 \|\| id:prot-a-40 | F-strong vegetable liking (derived food-liking factor) \|\| id:ebi-fl187-GCST90094852 | Inverse variance weighted | 27 | 0.0877124725346963 | 0.0764956667391043 | 0.25153324154291 | -0.062219034 | 0.237643979343341 | 1.09167419058545 | 0.939677042769655 | 1.26825758654032 | 0.998074519377826 |
| ebi-fl187-GCST90094852 | prot-a-403 | C-C motif chemokine 25 \|\| id:prot-a-403 | F-strong vegetable liking (derived food-liking factor) \|\| id:ebi-fl187-GCST90094852 | Inverse variance weighted | 27 | -0.021528133 | 0.0761752829132372 | 0.777473437139719 | -0.170831687 | 0.127775421655376 | 0.978701943402403 | 0.842963442930772 | 1.13629778616426 | 0.998074519377826 |
| ebi-fl187-GCST90094852 | prot-a-415 | Cyclin-C \|\| id:prot-a-415 | F-strong vegetable liking (derived food-liking factor) \|\| id:ebi-fl187-GCST90094852 | Inverse variance weighted | 27 | -0.045023251 | 0.0761752829132372 | 0.554488239120007 | -0.194326805 | 0.104280303527277 | 0.955975254210627 | 0.823388772320723 | 1.10991152343173 | 0.998074519377826 |
| ebi-fl187-GCST90094852 | prot-a-44 | Adhesion G protein-coupled receptor F5 \|\| id:prot-a-44 | F-strong vegetable liking (derived food-liking factor) \|\| id:ebi-fl187-GCST90094852 | Inverse variance weighted | 27 | 0.0265203892612888 | 0.0761752829132372 | 0.727727885552555 | -0.122783165 | 0.175823943771234 | 1.02687518427468 | 0.884455422441566 | 1.19222814098222 | 0.998074519377826 |
| ebi-fl187-GCST90094852 | prot-a-455 | CD59 glycoprotein \|\| id:prot-a-455 | F-strong vegetable liking (derived food-liking factor) \|\| id:ebi-fl187-GCST90094852 | Inverse variance weighted | 27 | 0.154107384234894 | 0.0835721467252037 | 0.0651825373846698 | -0.009694023 | 0.317908791816294 | 1.16661615623569 | 0.9903528122339 | 1.37425091258154 | 0.998074519377826 |
| ebi-fl187-GCST90094852 | prot-a-478 | Cadherin-12 \|\| id:prot-a-478 | F-strong vegetable liking (derived food-liking factor) \|\| id:ebi-fl187-GCST90094852 | Inverse variance weighted | 27 | -0.005214677 | 0.0816895812444061 | 0.949101387384616 | -0.165326256 | 0.154896902073178 | 0.99479889566023 | 0.847617118457658 | 1.16753758419551 | 0.998074519377826 |
| ebi-fl187-GCST90094852 | prot-a-516 | Centrin-2 \|\| id:prot-a-516 | F-strong vegetable liking (derived food-liking factor) \|\| id:ebi-fl187-GCST90094852 | Inverse variance weighted | 27 | -0.025780178 | 0.0914799333950349 | 0.778087372277405 | -0.205080848 | 0.15352049106017 | 0.974549293055244 | 0.814581456591053 | 1.16593167805351 | 0.998074519377826 |
| ebi-fl187-GCST90094852 | prot-a-519 | Cryptic protein \|\| id:prot-a-519 | F-strong vegetable liking (derived food-liking factor) \|\| id:ebi-fl187-GCST90094852 | Inverse variance weighted | 27 | -0.200631289 | 0.0761752829132372 | 0.00844325030530582 | -0.349934843 | -0.051327734 | 0.81821406079681 | 0.704734006500342 | 0.949967282847845 | 0.998074519377826 |
| ebi-fl187-GCST90094852 | prot-a-583 | Chloride intracellular channel protein 4 \|\| id:prot-a-583 | F-strong vegetable liking (derived food-liking factor) \|\| id:ebi-fl187-GCST90094852 | Inverse variance weighted | 27 | -0.06955589 | 0.0857440202472664 | 0.417248543384139 | -0.23761417 | 0.0985023896436599 | 0.932807997250429 | 0.788506862229782 | 1.10351704140374 | 0.998074519377826 |
| ebi-fl187-GCST90094852 | prot-a-602 | CCR4-NOT transcription complex subunit 1 \|\| id:prot-a-602 | F-strong vegetable liking (derived food-liking factor) \|\| id:ebi-fl187-GCST90094852 | Inverse variance weighted | 27 | -0.08274539 | 0.0761752829132372 | 0.277368463921876 | -0.232048945 | 0.0665581641845453 | 0.92058550736015 | 0.792907313639016 | 1.06882312949301 | 0.998074519377826 |
| ebi-fl187-GCST90094852 | prot-a-606 | Ciliary neurotrophic factor receptor subunit alpha \|\| id:prot-a-606 | F-strong vegetable liking (derived food-liking factor) \|\| id:ebi-fl187-GCST90094852 | Inverse variance weighted | 27 | -0.065268934 | 0.0761683430909296 | 0.391498118604405 | -0.214558886 | 0.0840210189314898 | 0.936815488403469 | 0.806897295390895 | 1.08765175484629 | 0.998074519377826 |
| ebi-fl187-GCST90094852 | prot-a-626 | Collagen alpha-2(IX) chain \|\| id:prot-a-626 | F-strong vegetable liking (derived food-liking factor) \|\| id:ebi-fl187-GCST90094852 | Inverse variance weighted | 27 | -0.006143512 | 0.076166166324255 | 0.935712878012878 | -0.155429198 | 0.143142173732225 | 0.993875320521931 | 0.856047684449907 | 1.15389384339883 | 0.998074519377826 |
| ebi-fl187-GCST90094852 | prot-a-647 | Probable serine carboxypeptidase CPVL \|\| id:prot-a-647 | F-strong vegetable liking (derived food-liking factor) \|\| id:ebi-fl187-GCST90094852 | Inverse variance weighted | 27 | -0.172533466 | 0.0887846183059037 | 0.0519822103878723 | -0.346551318 | 0.00148438599016648 | 0.841530125786405 | 0.707122530405494 | 1.00148548823637 | 0.998074519377826 |
| ebi-fl187-GCST90094852 | prot-a-651 | Cellular retinoic acid-binding protein 2 \|\| id:prot-a-651 | F-strong vegetable liking (derived food-liking factor) \|\| id:ebi-fl187-GCST90094852 | Inverse variance weighted | 27 | -0.04972476 | 0.0797646037563226 | 0.533025763329315 | -0.206063384 | 0.106613862959478 | 0.951491276538622 | 0.813781494112968 | 1.11250459229959 | 0.998074519377826 |
| ebi-fl187-GCST90094852 | prot-a-671 | Cytotoxic and regulatory T-cell molecule \|\| id:prot-a-671 | F-strong vegetable liking (derived food-liking factor) \|\| id:ebi-fl187-GCST90094852 | Inverse variance weighted | 27 | -0.075203947 | 0.0823547121999523 | 0.361152535605894 | -0.236619183 | 0.0862112888161889 | 0.927554295032109 | 0.789291806524568 | 1.09003661652192 | 0.998074519377826 |
| ebi-fl187-GCST90094852 | prot-a-688 | Chorionic somatomammotropin hormone \|\| id:prot-a-688 | F-strong vegetable liking (derived food-liking factor) \|\| id:ebi-fl187-GCST90094852 | Inverse variance weighted | 27 | -0.061018492 | 0.0886324305404317 | 0.49117303335943 | -0.234738056 | 0.112701072044291 | 0.940805842407609 | 0.790777962301748 | 1.11929729368273 | 0.998074519377826 |
| ebi-fl187-GCST90094852 | prot-a-69 | Aldo-keto reductase family 1 member C1 \|\| id:prot-a-69 | F-strong vegetable liking (derived food-liking factor) \|\| id:ebi-fl187-GCST90094852 | Inverse variance weighted | 27 | -0.125165451 | 0.0846326307186204 | 0.139159925794925 | -0.291045407 | 0.0407145050719501 | 0.882350904547168 | 0.747481736086387 | 1.04155470450883 | 0.998074519377826 |
| ebi-fl187-GCST90094852 | prot-a-741 | Stromal cell-derived factor 1 \|\| id:prot-a-741 | F-strong vegetable liking (derived food-liking factor) \|\| id:ebi-fl187-GCST90094852 | Inverse variance weighted | 27 | 0.0518954985877372 | 0.0804576520506871 | 0.518924664559522 | -0.105801499 | 0.209592496607084 | 1.05326566901215 | 0.899603202100743 | 1.23317543437931 | 0.998074519377826 |
| ebi-fl187-GCST90094852 | prot-a-742 | Growth-regulated alpha protein \|\| id:prot-a-742 | F-strong vegetable liking (derived food-liking factor) \|\| id:ebi-fl187-GCST90094852 | Inverse variance weighted | 27 | -0.013359453 | 0.0820621041711901 | 0.870678382922416 | -0.174201177 | 0.147482271528876 | 0.98672938877654 | 0.840127866402021 | 1.1589127388966 | 0.998074519377826 |
| ebi-fl187-GCST90094852 | prot-a-788 | Beta-defensin 112 \|\| id:prot-a-788 | F-strong vegetable liking (derived food-liking factor) \|\| id:ebi-fl187-GCST90094852 | Inverse variance weighted | 27 | -0.022571436 | 0.0761652331449943 | 0.766964119540272 | -0.171855293 | 0.126712421203767 | 0.977681393284911 | 0.842101022496614 | 1.13509054286817 | 0.998074519377826 |
| ebi-fl187-GCST90094852 | prot-a-838 | DnaJ homolog subfamily B member 1 \|\| id:prot-a-838 | F-strong vegetable liking (derived food-liking factor) \|\| id:ebi-fl187-GCST90094852 | Inverse variance weighted | 27 | -0.111697684 | 0.0843151928388647 | 0.185249626536612 | -0.276955462 | 0.0535600936172026 | 0.894314581933494 | 0.758088259857686 | 1.05502038985411 | 0.998074519377826 |
| ebi-fl187-GCST90094852 | prot-a-85 | Junctional adhesion molecule-like \|\| id:prot-a-85 | F-strong vegetable liking (derived food-liking factor) \|\| id:ebi-fl187-GCST90094852 | Inverse variance weighted | 26 | -0.02202218 | 0.0773088853940751 | 0.775751694646232 | -0.173547595 | 0.129503235786396 | 0.97821853832841 | 0.840677138216278 | 1.13826279463209 | 0.998074519377826 |
| ebi-fl187-GCST90094852 | prot-a-856 | Dipeptidase 1 \|\| id:prot-a-856 | F-strong vegetable liking (derived food-liking factor) \|\| id:ebi-fl187-GCST90094852 | Inverse variance weighted | 27 | -0.088579144 | 0.0793413452424954 | 0.264237657331799 | -0.24408818 | 0.0669298931737599 | 0.915230673309365 | 0.783418549230745 | 1.06922051589003 | 0.998074519377826 |
| ebi-fl187-GCST90094852 | prot-a-860 | Dipeptidyl peptidase 2 \|\| id:prot-a-860 | F-strong vegetable liking (derived food-liking factor) \|\| id:ebi-fl187-GCST90094852 | Inverse variance weighted | 27 | 0.0247596315650119 | 0.0820354949565367 | 0.762792271584947 | -0.136029939 | 0.185549201679824 | 1.02506869675097 | 0.872816501237267 | 1.2038794312083 | 0.998074519377826 |
| ebi-fl187-GCST90094852 | prot-a-861 | Dermatopontin \|\| id:prot-a-861 | F-strong vegetable liking (derived food-liking factor) \|\| id:ebi-fl187-GCST90094852 | Inverse variance weighted (multiplicative random effects) | 26 | 0.0423139735633762 | 0.0995451532741201 | 0.670783338004363 | -0.152794527 | 0.237422473980652 | 1.04322197145582 | 0.858306062541119 | 1.26797669179463 | 0.998074519377826 |
| ebi-fl187-GCST90094852 | prot-a-877 | Dual specificity protein phosphatase 6 \|\| id:prot-a-877 | F-strong vegetable liking (derived food-liking factor) \|\| id:ebi-fl187-GCST90094852 | Inverse variance weighted | 27 | -0.029606835 | 0.078943331302175 | 0.707631397902944 | -0.184335764 | 0.12512209450218 | 0.970827153927192 | 0.831656516455071 | 1.1332868127094 | 0.998074519377826 |
| ebi-fl187-GCST90094852 | prot-a-879 | Dynactin-associated protein \|\| id:prot-a-879 | F-strong vegetable liking (derived food-liking factor) \|\| id:ebi-fl187-GCST90094852 | Inverse variance weighted | 27 | 0.0165229742365278 | 0.0772917111105429 | 0.830723139962675 | -0.13496878 | 0.168014728013192 | 1.01666023351043 | 0.873743189926405 | 1.18295403308212 | 0.998074519377826 |
| ebi-fl187-GCST90094852 | prot-a-937 | EMI domain-containing protein 1 \|\| id:prot-a-937 | F-strong vegetable liking (derived food-liking factor) \|\| id:ebi-fl187-GCST90094852 | Inverse variance weighted (multiplicative random effects) | 27 | 0.103715021689062 | 0.0991882244276887 | 0.295727987969998 | -0.090693898 | 0.298123941567332 | 1.10928428790463 | 0.913297230052334 | 1.34732876757064 | 0.998074519377826 |
| ebi-fl187-GCST90094852 | prot-b-1 | SRC proto-oncogene, non-receptor tyrosine kinase \|\| id:prot-b-1 | F-strong vegetable liking (derived food-liking factor) \|\| id:ebi-fl187-GCST90094852 | Inverse variance weighted | 20 | 0.061226769851842 | 0.107944255432207 | 0.570573347835833 | -0.150343971 | 0.272797510498968 | 1.06313997494811 | 0.860411968929785 | 1.31363422075421 | 0.998074519377826 |
| ebi-fl187-GCST90094852 | prot-b-10 | coagulation factor III, tissue factor \|\| id:prot-b-10 | F-strong vegetable liking (derived food-liking factor) \|\| id:ebi-fl187-GCST90094852 | Inverse variance weighted | 20 | -0.18482571 | 0.105703307278918 | 0.08037179443902 | -0.392004192 | 0.0223527722399284 | 0.831249149619484 | 0.675701281216646 | 1.02260446731575 | 0.998074519377826 |
| ebi-fl187-GCST90094852 | prot-b-29 | myeloperoxidase \|\| id:prot-b-29 | F-strong vegetable liking (derived food-liking factor) \|\| id:ebi-fl187-GCST90094852 | Inverse variance weighted | 20 | 0.0161964002378442 | 0.126054538094384 | 0.897763392086137 | -0.230870494 | 0.263263294902837 | 1.01632827292047 | 0.793842266375952 | 1.30116926508965 | 0.998074519377826 |
| ebi-fl187-GCST90094852 | prot-b-33 | adrenomedullin \|\| id:prot-b-33 | F-strong vegetable liking (derived food-liking factor) \|\| id:ebi-fl187-GCST90094852 | Inverse variance weighted | 20 | -0.142042183 | 0.120423803840805 | 0.23819151387583 | -0.378072839 | 0.0939884721898987 | 0.867584658097053 | 0.685180591070915 | 1.09854708200203 | 0.998074519377826 |
| ebi-fl187-GCST90094852 | prot-c-2201_17_6 | Endostatin \|\| id:prot-c-2201_17_6 | F-strong vegetable liking (derived food-liking factor) \|\| id:ebi-fl187-GCST90094852 | Inverse variance weighted | 2 | -0.391843078 | 0.503569381202967 | 0.436491634576425 | -1.378839065 | 0.595152909068665 | 0.675810155061226 | 0.251870788945697 | 1.8133081950299 | 0.998074519377826 |
| ebi-fl187-GCST90094852 | prot-c-2515_14_3 | GFRa-2 \|\| id:prot-c-2515_14_3 | F-strong vegetable liking (derived food-liking factor) \|\| id:ebi-fl187-GCST90094852 | Inverse variance weighted | 2 | 1.02174956972978 | 0.514913655954721 | 0.0472210209527439 | 0.0125188040585253 | 2.03098033540103 | 2.77805090842106 | 1.01259749230412 | 7.6215543771673 | 0.998074519377826 |
| ebi-fl187-GCST90094852 | prot-c-2570_72_5 | IGFBP-2 \|\| id:prot-c-2570_72_5 | F-strong vegetable liking (derived food-liking factor) \|\| id:ebi-fl187-GCST90094852 | Inverse variance weighted | 2 | 0.341638305425209 | 0.445215434355724 | 0.442870454842367 | -0.530983946 | 1.21426055676243 | 1.40725121032447 | 0.588026099060043 | 3.36780284433851 | 0.998074519377826 |
| ebi-fl187-GCST90094852 | prot-c-2585_2_5 | PRL \|\| id:prot-c-2585_2_5 | F-strong vegetable liking (derived food-liking factor) \|\| id:ebi-fl187-GCST90094852 | Inverse variance weighted | 2 | 0.6091510909378 | 0.507725008490627 | 0.23023031766588 | -0.385989926 | 1.60429210757943 | 1.83886970285155 | 0.67977737392442 | 4.97433706059378 | 0.998074519377826 |
| ebi-fl187-GCST90094852 | prot-c-2614_28_2 | Ephrin-A4 \|\| id:prot-c-2614_28_2 | F-strong vegetable liking (derived food-liking factor) \|\| id:ebi-fl187-GCST90094852 | Inverse variance weighted | 2 | 0.100474909946439 | 0.499355461099454 | 0.840535002581818 | -0.878261794 | 1.07921161370137 | 1.10569589938671 | 0.415504516874419 | 2.9423589209503 | 0.998074519377826 |
| ebi-fl187-GCST90094852 | prot-c-2642_4_1 | PAFAH beta subunit \|\| id:prot-c-2642_4_1 | F-strong vegetable liking (derived food-liking factor) \|\| id:ebi-fl187-GCST90094852 | Inverse variance weighted | 2 | -0.720626458 | 0.514931201472279 | 0.161674596937333 | -1.729891613 | 0.288638696601197 | 0.486447421469757 | 0.177303626306439 | 1.33460944248032 | 0.998074519377826 |
| ebi-fl187-GCST90094852 | prot-c-2658_27_1 | TrkC \|\| id:prot-c-2658_27_1 | F-strong vegetable liking (derived food-liking factor) \|\| id:ebi-fl187-GCST90094852 | Inverse variance weighted | 2 | -0.289871414 | 0.486759072681693 | 0.551500531366646 | -1.243919197 | 0.664176368095186 | 0.748359789713853 | 0.288252283818386 | 1.94288963626536 | 0.998074519377826 |
| ebi-fl187-GCST90094852 | prot-c-2685_21_2 | IGFBP-5 \|\| id:prot-c-2685_21_2 | F-strong vegetable liking (derived food-liking factor) \|\| id:ebi-fl187-GCST90094852 | Inverse variance weighted | 2 | 0.705930054303611 | 0.504672893247843 | 0.16187676359288 | -0.283228816 | 1.69508892506938 | 2.02572984782765 | 0.753347389852195 | 5.44713033011893 | 0.998074519377826 |
| ebi-fl187-GCST90094852 | prot-c-2752_62_2 | BMP-14 \|\| id:prot-c-2752_62_2 | F-strong vegetable liking (derived food-liking factor) \|\| id:ebi-fl187-GCST90094852 | Inverse variance weighted | 2 | 0.183607718904185 | 0.519864979878098 | 0.723950897426385 | -0.835327642 | 1.20254307946526 | 1.20154438748571 | 0.433732349364478 | 3.32857098902999 | 0.998074519377826 |
| ebi-fl187-GCST90094852 | prot-c-2768_56_2 | Hemopexin \|\| id:prot-c-2768_56_2 | F-strong vegetable liking (derived food-liking factor) \|\| id:ebi-fl187-GCST90094852 | Inverse variance weighted | 2 | -0.727767615 | 0.978086112302598 | 0.456832221845729 | -2.644816395 | 1.18928116531316 | 0.482985998288763 | 0.0710183918806838 | 3.28471918844507 | 0.998074519377826 |
| ebi-fl187-GCST90094852 | prot-c-2864_2_3 | MEK1 \|\| id:prot-c-2864_2_3 | F-strong vegetable liking (derived food-liking factor) \|\| id:ebi-fl187-GCST90094852 | Inverse variance weighted | 2 | -1.301395239 | 1.06208592822845 | 0.220454637058663 | -3.383083658 | 0.780293180652817 | 0.272151811280479 | 0.0339426257397869 | 2.18211192472438 | 0.998074519377826 |
| ebi-fl187-GCST90094852 | prot-c-2888_49_2 | C7 \|\| id:prot-c-2888_49_2 | F-strong vegetable liking (derived food-liking factor) \|\| id:ebi-fl187-GCST90094852 | Inverse variance weighted | 2 | 1.77876214302243 | 0.501129043931169 | 0.000385950004855397 | 0.796549216917342 | 2.76097506912753 | 5.92252064570296 | 2.2178743050417 | 15.8152564007086 | 0.443070605573995 |
| ebi-fl187-GCST90094852 | prot-c-2911_27_2 | Midkine \|\| id:prot-c-2911_27_2 | F-strong vegetable liking (derived food-liking factor) \|\| id:ebi-fl187-GCST90094852 | Inverse variance weighted | 2 | -0.530731517 | 1.18317441137549 | 0.653744945736493 | -2.849753363 | 1.78829032927019 | 0.588174552569504 | 0.0578585891654664 | 5.9792212233343 | 0.998074519377826 |
| ebi-fl187-GCST90094852 | prot-c-2957_21_2 | NG36 \|\| id:prot-c-2957_21_2 | F-strong vegetable liking (derived food-liking factor) \|\| id:ebi-fl187-GCST90094852 | Inverse variance weighted | 2 | -0.922562178 | 0.512601146933101 | 0.0718975754730993 | -1.927260426 | 0.0821360698446705 | 0.397499271281226 | 0.145546387880609 | 1.08560351768205 | 0.998074519377826 |
| ebi-fl187-GCST90094852 | prot-c-2985_35_1 | Gro-a \|\| id:prot-c-2985_35_1 | F-strong vegetable liking (derived food-liking factor) \|\| id:ebi-fl187-GCST90094852 | Inverse variance weighted | 2 | -0.678570274 | 0.514267499768053 | 0.187005707833796 | -1.686534574 | 0.329394025400395 | 0.507341833816973 | 0.185160072051083 | 1.39012549244287 | 0.998074519377826 |
| ebi-fl187-GCST90094852 | prot-c-2994_71_2 | IL-1Rrp2 \|\| id:prot-c-2994_71_2 | F-strong vegetable liking (derived food-liking factor) \|\| id:ebi-fl187-GCST90094852 | Inverse variance weighted | 2 | 0.0800860460302262 | 0.51484652163672 | 0.876385182952786 | -0.929013136 | 1.0891852284382 | 1.08338028423713 | 0.394943273266501 | 2.9718517056036 | 0.998074519377826 |
| ebi-fl187-GCST90094852 | prot-c-3060_43_2 | C9 \|\| id:prot-c-3060_43_2 | F-strong vegetable liking (derived food-liking factor) \|\| id:ebi-fl187-GCST90094852 | Inverse variance weighted | 2 | -0.375770358 | 0.508424454393901 | 0.459853629036174 | -1.372282289 | 0.620741572689503 | 0.686760023933987 | 0.253527675450636 | 1.8603070833805 | 0.998074519377826 |
| ebi-fl187-GCST90094852 | prot-c-3173_49_2 | ASAHL \|\| id:prot-c-3173_49_2 | F-strong vegetable liking (derived food-liking factor) \|\| id:ebi-fl187-GCST90094852 | Inverse variance weighted | 2 | 0.339998367186767 | 0.51470915108744 | 0.508891923077436 | -0.668831569 | 1.34882830331815 | 1.40494529654845 | 0.51230682341646 | 3.8529084448462 | 0.998074519377826 |
| ebi-fl187-GCST90094852 | prot-c-3197_70_2 | IDE \|\| id:prot-c-3197_70_2 | F-strong vegetable liking (derived food-liking factor) \|\| id:ebi-fl187-GCST90094852 | Inverse variance weighted | 2 | 0.22136790534323 | 0.51753493699554 | 0.66884392562166 | -0.793000571 | 1.23573638185449 | 1.24778241194458 | 0.452485042712254 | 3.44091141273004 | 0.998074519377826 |
| ebi-fl187-GCST90094852 | prot-c-3389_7_2 | PCI \|\| id:prot-c-3389_7_2 | F-strong vegetable liking (derived food-liking factor) \|\| id:ebi-fl187-GCST90094852 | Inverse variance weighted | 2 | -0.191961292 | 0.5009042819569 | 0.701549614468677 | -1.173733685 | 0.78981110030027 | 0.82533881481154 | 0.309210289511773 | 2.20298024464216 | 0.998074519377826 |
| ebi-fl187-GCST90094852 | prot-c-3404_51_2 | TPSG1 \|\| id:prot-c-3404_51_2 | F-strong vegetable liking (derived food-liking factor) \|\| id:ebi-fl187-GCST90094852 | Inverse variance weighted | 2 | 0.164833255301424 | 0.515731915006243 | 0.749263731213945 | -0.846001298 | 1.17566780871366 | 1.1791964775562 | 0.429127458504277 | 3.24030612612753 | 0.998074519377826 |
| ebi-fl187-GCST90094852 | prot-c-3452_17_2 | LCK \|\| id:prot-c-3452_17_2 | F-strong vegetable liking (derived food-liking factor) \|\| id:ebi-fl187-GCST90094852 | Inverse variance weighted | 2 | 0.304780278652638 | 0.516719087381703 | 0.555299619846448 | -0.707989133 | 1.31754968992078 | 1.35632695627751 | 0.492633823412222 | 3.73426006274376 | 0.998074519377826 |
| ebi-fl187-GCST90094852 | prot-c-3580_25_8 | a1-Antitrypsin \|\| id:prot-c-3580_25_8 | F-strong vegetable liking (derived food-liking factor) \|\| id:ebi-fl187-GCST90094852 | Inverse variance weighted | 2 | -0.00074561 | 0.512086507015993 | 0.998838262181667 | -1.004435163 | 1.00294394404678 | 0.999254668193275 | 0.366251448593014 | 2.7262960890446 | 0.999355215041207 |
| ebi-fl187-GCST90094852 | prot-c-3620_67_5 | IL22RA1 \|\| id:prot-c-3620_67_5 | F-strong vegetable liking (derived food-liking factor) \|\| id:ebi-fl187-GCST90094852 | Inverse variance weighted | 2 | -0.154227062 | 0.828876632445643 | 0.852391771872226 | -1.778825262 | 1.47037113739252 | 0.857077389037185 | 0.168836369349845 | 4.3508496044278 | 0.998074519377826 |
| ebi-fl187-GCST90094852 | prot-c-3623_84_4 | LY86 \|\| id:prot-c-3623_84_4 | F-strong vegetable liking (derived food-liking factor) \|\| id:ebi-fl187-GCST90094852 | Inverse variance weighted | 2 | -0.447304996 | 0.688422394803012 | 0.515851332297908 | -1.79661289 | 0.902002897511198 | 0.6393488794904 | 0.165859722987904 | 2.46453438087222 | 0.998074519377826 |
| ebi-fl187-GCST90094852 | prot-c-3635_76_4 | OCAD1 \|\| id:prot-c-3635_76_4 | F-strong vegetable liking (derived food-liking factor) \|\| id:ebi-fl187-GCST90094852 | Inverse variance weighted | 2 | 0.90450534113548 | 0.512326067953797 | 0.0774818455396711 | -0.099653752 | 1.90866443432492 | 2.47070946239467 | 0.905150770379125 | 6.74407562511349 | 0.998074519377826 |
| ebi-fl187-GCST90094852 | prot-c-3707_12_2 | Albumin \|\| id:prot-c-3707_12_2 | F-strong vegetable liking (derived food-liking factor) \|\| id:ebi-fl187-GCST90094852 | Inverse variance weighted | 2 | -0.270207996 | 0.472337493986671 | 0.56727758471044 | -1.195989484 | 0.655573492256967 | 0.763220731000084 | 0.302404581547714 | 1.92624688834747 | 0.998074519377826 |
| ebi-fl187-GCST90094852 | prot-c-4336_2_1 | SAA \|\| id:prot-c-4336_2_1 | F-strong vegetable liking (derived food-liking factor) \|\| id:ebi-fl187-GCST90094852 | Inverse variance weighted | 2 | 0.32430217456318 | 0.511549434604244 | 0.526106550646051 | -0.678334717 | 1.3269390663875 | 1.38306517109629 | 0.507461355754949 | 3.76948755960707 | 0.998074519377826 |
| ebi-fl187-GCST90094852 | prot-c-4452_9_2 | LRRT1 \|\| id:prot-c-4452_9_2 | F-strong vegetable liking (derived food-liking factor) \|\| id:ebi-fl187-GCST90094852 | Inverse variance weighted | 2 | 0.0118668892168974 | 0.64677120072282 | 0.985361320265712 | -1.255804664 | 1.27953844263363 | 1.01193758009694 | 0.284846550161495 | 3.59498005305625 | 0.999355215041207 |
| ebi-fl187-GCST90094852 | prot-c-4455_89_2 | MFGM \|\| id:prot-c-4455_89_2 | F-strong vegetable liking (derived food-liking factor) \|\| id:ebi-fl187-GCST90094852 | Inverse variance weighted | 2 | -0.040156848 | 0.719452447760402 | 0.955488510103699 | -1.450283646 | 1.36996994946497 | 0.960638752928393 | 0.234503762662416 | 3.93523243785341 | 0.998074519377826 |
| ebi-fl187-GCST90094852 | prot-c-4462_8_2 | RASA1 \|\| id:prot-c-4462_8_2 | F-strong vegetable liking (derived food-liking factor) \|\| id:ebi-fl187-GCST90094852 | Inverse variance weighted | 2 | 0.868986141027973 | 0.516293267806579 | 0.0923508827879038 | -0.142948664 | 1.88092094592887 | 2.3844920888903 | 0.866798565834655 | 6.55954306581655 | 0.998074519377826 |
| ebi-fl187-GCST90094852 | prot-c-4467_49_2 | SPARCL1 \|\| id:prot-c-4467_49_2 | F-strong vegetable liking (derived food-liking factor) \|\| id:ebi-fl187-GCST90094852 | Inverse variance weighted | 2 | -0.464161508 | 0.79619727815076 | 0.559911482684575 | -2.024708174 | 1.09638515683434 | 0.628662012108631 | 0.132032368107936 | 2.99332603915265 | 0.998074519377826 |
| ebi-fl187-GCST90094852 | prot-c-4811_33_3 | ITI heavy chain H4 \|\| id:prot-c-4811_33_3 | F-strong vegetable liking (derived food-liking factor) \|\| id:ebi-fl187-GCST90094852 | Inverse variance weighted | 2 | -0.640914852 | 0.745633320567581 | 0.390032908191446 | -2.10235616 | 0.820526456272386 | 0.526810250085741 | 0.122168240912934 | 2.27169547111011 | 0.998074519377826 |
| ebi-fl187-GCST90094852 | prot-c-4962_52_1 | ARMEL \|\| id:prot-c-4962_52_1 | F-strong vegetable liking (derived food-liking factor) \|\| id:ebi-fl187-GCST90094852 | Inverse variance weighted | 2 | -0.056502893 | 0.495555258227958 | 0.909222446863206 | -1.027791199 | 0.914785412987381 | 0.945063750290004 | 0.357796389330077 | 2.49623953384353 | 0.998074519377826 |
| ebi-fl187-GCST90094852 | prot-c-4984_83_1 | Esterase D \|\| id:prot-c-4984_83_1 | F-strong vegetable liking (derived food-liking factor) \|\| id:ebi-fl187-GCST90094852 | Inverse variance weighted | 2 | 0.519192648800062 | 0.516250409878721 | 0.314560265220913 | -0.492658155 | 1.53104345216236 | 1.68067021069631 | 0.611000100966726 | 4.62299818388378 | 0.998074519377826 |
| ebi-fl187-GCST90094852 | prot-c-4988_49_2 | FGFR4 \|\| id:prot-c-4988_49_2 | F-strong vegetable liking (derived food-liking factor) \|\| id:ebi-fl187-GCST90094852 | Inverse variance weighted | 2 | 0.410230070884243 | 0.518828402286703 | 0.429127566324064 | -0.606673598 | 1.42713373936618 | 1.50716449989603 | 0.545161282122495 | 4.16673909948806 | 0.998074519377826 |
| ebi-fl187-GCST90094852 | prot-c-5066_134_3 | CLM6 \|\| id:prot-c-5066_134_3 | F-strong vegetable liking (derived food-liking factor) \|\| id:ebi-fl187-GCST90094852 | Inverse variance weighted | 2 | 0.55449413714776 | 0.515841065120004 | 0.282405113714135 | -0.45655435 | 1.56554262478297 | 1.74106002438044 | 0.633462579457919 | 4.7852708380811 | 0.998074519377826 |
| ebi-fl187-GCST90094852 | prot-c-5085_18_3 | IL-20 Ra \|\| id:prot-c-5085_18_3 | F-strong vegetable liking (derived food-liking factor) \|\| id:ebi-fl187-GCST90094852 | Inverse variance weighted | 2 | 0.181724631746377 | 0.516838097954282 | 0.725131942848274 | -0.83127804 | 1.19472730373677 | 1.19928390369208 | 0.435492353752168 | 3.30265702546278 | 0.998074519377826 |
| ebi-fl187-GCST90094852 | prot-c-5265_12_3 | GRB2-related adapter protein 2 \|\| id:prot-c-5265_12_3 | F-strong vegetable liking (derived food-liking factor) \|\| id:ebi-fl187-GCST90094852 | Inverse variance weighted | 2 | 0.568104890816961 | 0.599337562729673 | 0.343186468493142 | -0.606596732 | 1.74280651376712 | 1.76491916561306 | 0.5452031878082 | 5.71335555404732 | 0.998074519377826 |
| ebi-fl187-GCST90094852 | prot-c-5340_24_3 | Caspase-10 \|\| id:prot-c-5340_24_3 | F-strong vegetable liking (derived food-liking factor) \|\| id:ebi-fl187-GCST90094852 | Inverse variance weighted | 2 | -0.6505727 | 0.571875569041401 | 0.255282394449022 | -1.771448816 | 0.47030341498155 | 0.521746886562809 | 0.170086386407517 | 1.60047972908168 | 0.998074519377826 |
| ebi-fl187-GCST90094852 | prot-c-5400_52_3 | sLeptin R \|\| id:prot-c-5400_52_3 | F-strong vegetable liking (derived food-liking factor) \|\| id:ebi-fl187-GCST90094852 | Inverse variance weighted | 2 | -0.426786846 | 0.846435376385218 | 0.614109111255108 | -2.085800184 | 1.23222649147532 | 0.652602642110749 | 0.124207691388725 | 3.42885536095386 | 0.998074519377826 |
| ebi-fl187-GCST90094852 | prot-c-5468_67_3 | IL-17 RC \|\| id:prot-c-5468_67_3 | F-strong vegetable liking (derived food-liking factor) \|\| id:ebi-fl187-GCST90094852 | Inverse variance weighted | 2 | -0.413454889 | 1.00384078255594 | 0.680432530246036 | -2.380982823 | 1.55407304463069 | 0.661361368261851 | 0.0924596613597187 | 4.73069934495506 | 0.998074519377826 |
| ebi-fl187-GCST90094852 | ukb-rsfMRI191-edge-pheno102 | Net25_Pair11_15 (Postcentral\|Precentral)&(Cerebellum) (Motor)&(Subcortical-cerebellum) \|\| id:ukb-rsfMRI191-edge-pheno102 | F-strong vegetable liking (derived food-liking factor) \|\| id:ebi-fl187-GCST90094852 | Inverse variance weighted | 27 | 0.0150642731517023 | 0.0236459382311659 | 0.524074971291041 | -0.031281766 | 0.0614103120847875 | 1.0151783112285 | 0.969202446514313 | 1.06333512394154 | 0.998074519377826 |
| ebi-fl187-GCST90094852 | ukb-rsfMRI191-edge-pheno1167 | Net100_Pair11_45 (Angular\|Temporal)&(Frontal) (Default_mode\|Central_executive)&(Salience\|Default_mode) \|\| id:ukb-rsfMRI191-edge-pheno1167 | F-strong vegetable liking (derived food-liking factor) \|\| id:ebi-fl187-GCST90094852 | Inverse variance weighted (multiplicative random effects) | 27 | -0.030198625 | 0.0355913661164465 | 0.396169730552217 | -0.099957702 | 0.0395604528931958 | 0.970252798241984 | 0.904875691402183 | 1.04035338935632 | 0.998074519377826 |
| ebi-fl187-GCST90094852 | ukb-rsfMRI191-edge-pheno1317 | Net100_Pair26_48 (Parietal\|Frontal)&(Temporal) (Attention\|Central_executive)&(Default_mode\|Central_executive) \|\| id:ukb-rsfMRI191-edge-pheno1317 | F-strong vegetable liking (derived food-liking factor) \|\| id:ebi-fl187-GCST90094852 | Inverse variance weighted | 27 | -0.036070893 | 0.0271788733578401 | 0.184453576911564 | -0.089341485 | 0.0171996987229015 | 0.964571909606399 | 0.914533221015156 | 1.01734846523013 | 0.998074519377826 |
| ebi-fl187-GCST90094852 | ukb-rsfMRI191-edge-pheno286 | Net100_Pair10_13 (OccipitalPrecuneus)&(Frontal_Sup) (Default_mode\|Central_executive)&(Default_mode) \|\| id:ukb-rsfMRI191-edge-pheno286 | F-strong vegetable liking (derived food-liking factor) \|\| id:ebi-fl187-GCST90094852 | Inverse variance weighted (multiplicative random effects) | 26 | -0.013130411 | 0.0298167237036646 | 0.65966866168288 | -0.071571189 | 0.045310367763216 | 0.986955417084542 | 0.930930003115453 | 1.04635256362203 | 0.998074519377826 |
| ebi-fl187-GCST90094852 | ukb-rsfMRI191-edge-pheno599 | Net100_Pair11_29 (Angular\|Temporal)&(Parietal) (Default_mode\|Central_executive)&(Central_executive\|Salience) \|\| id:ukb-rsfMRI191-edge-pheno599 | F-strong vegetable liking (derived food-liking factor) \|\| id:ebi-fl187-GCST90094852 | Inverse variance weighted (multiplicative random effects) | 27 | 0.0233711262099342 | 0.0299429101441463 | 0.435083170450019 | -0.035316978 | 0.082059230092461 | 1.02364637105815 | 0.96529938940448 | 1.08552010338157 | 0.998074519377826 |
| ebi-fl187-GCST90094852 | ukb-rsfMRI191-edge-pheno621 | Net100_Pair5_30 (Precuneus\|Angular\|Cingulate)&(Paracentral) (Default_mode\|Central_executive)&(Motor) \|\| id:ukb-rsfMRI191-edge-pheno621 | F-strong vegetable liking (derived food-liking factor) \|\| id:ebi-fl187-GCST90094852 | Inverse variance weighted | 27 | -0.012302348 | 0.0243696235582124 | 0.613683129814014 | -0.06006681 | 0.0354621146562139 | 0.98777301698931 | 0.941701616687596 | 1.03609839444063 | 0.998074519377826 |
| ebi-fl187-GCST90094852 | ukb-rsfMRI191-edge-pheno624 | Net100_Pair8_30 (Calcarine\|Lingual)&(Paracentral) (Visual)&(Motor) \|\| id:ukb-rsfMRI191-edge-pheno624 | F-strong vegetable liking (derived food-liking factor) \|\| id:ebi-fl187-GCST90094852 | Inverse variance weighted | 27 | -0.020433507 | 0.0243615048489033 | 0.401602811777565 | -0.068182057 | 0.02731504207059 | 0.979773841986226 | 0.934090400458896 | 1.02769151783259 | 0.998074519377826 |
| ebi-fl187-GCST90094852 | ukb-rsfMRI191-edge-pheno882 | Net100_Pair6_38 (Postcentral\|Precentral)&(Subcortical) (Motor)&(Subcortical-cerebellum) \|\| id:ukb-rsfMRI191-edge-pheno882 | F-strong vegetable liking (derived food-liking factor) \|\| id:ebi-fl187-GCST90094852 | Inverse variance weighted | 27 | -0.00603149 | 0.0239233069130847 | 0.800950052735939 | -0.052921172 | 0.0408581913070191 | 0.993986662679918 | 0.948454774525566 | 1.04170437233529 | 0.998074519377826 |
| ebi-fl187-GCST90094852 | ukb-rsfMRI191-edge-pheno899 | Net100_Pair23_38 (Cerebellum)&(Subcortical) (Subcortical-cerebellum)&(Subcortical-cerebellum) \|\| id:ukb-rsfMRI191-edge-pheno899 | F-strong vegetable liking (derived food-liking factor) \|\| id:ebi-fl187-GCST90094852 | Inverse variance weighted | 27 | -0.006977251 | 0.0274723461716221 | 0.799515948905689 | -0.060823049 | 0.0468685476691153 | 0.993047033674778 | 0.940989733814802 | 1.04798424005374 | 0.998074519377826 |
| ebi-fl187-GCST90094852 | ebi-a-GCST90001392 | IgD+ CD38+ B cell Absolute Count \|\| id:ebi-a-GCST90001392 | F-strong vegetable liking (derived food-liking factor) \|\| id:ebi-fl187-GCST90094852 | MR Egger | 23 | -0.716731789 | 0.339368390117046 | 0.0468427737116556 | -1.381893833 | -0.051569744 | 0.488345667620633 | 0.251102556109903 | 0.94973740920209 | 0.999279047927446 |
| ebi-fl187-GCST90094852 | ebi-a-GCST90001402 | Switched memory B cell %B cell \|\| id:ebi-a-GCST90001402 | F-strong vegetable liking (derived food-liking factor) \|\| id:ebi-fl187-GCST90094852 | MR Egger | 23 | -0.436464319 | 0.39835517726877 | 0.285631941201087 | -1.217240466 | 0.344311828605104 | 0.646317558833933 | 0.296045989649748 | 1.41101856286338 | 0.999279047927446 |
| ebi-fl187-GCST90094852 | ebi-a-GCST90001407 | Memory B cell Absolute Count \|\| id:ebi-a-GCST90001407 | F-strong vegetable liking (derived food-liking factor) \|\| id:ebi-fl187-GCST90094852 | MR Egger | 23 | -0.090392774 | 0.353855605615601 | 0.800860106377392 | -0.783949761 | 0.603164212890514 | 0.913572287245332 | 0.456598988067673 | 1.82789350356372 | 0.999279047927446 |
| ebi-fl187-GCST90094852 | ebi-a-GCST90001411 | IgD+ CD24+ B cell %B cell \|\| id:ebi-a-GCST90001411 | F-strong vegetable liking (derived food-liking factor) \|\| id:ebi-fl187-GCST90094852 | MR Egger | 23 | 0.119619629147026 | 0.463709764948397 | 0.798947186420788 | -0.78925151 | 1.02849076844588 | 1.12706806619412 | 0.454184620666328 | 2.79684156625764 | 0.999279047927446 |
| ebi-fl187-GCST90094852 | ebi-a-GCST90001418 | CD24+ CD27+ B cell Absolute Count \|\| id:ebi-a-GCST90001418 | F-strong vegetable liking (derived food-liking factor) \|\| id:ebi-fl187-GCST90094852 | MR Egger | 23 | -0.197832119 | 0.3682108895412 | 0.596722377813481 | -0.919525462 | 0.523861224676602 | 0.820507589354743 | 0.398708198261306 | 1.68853489124271 | 0.999279047927446 |
| ebi-fl187-GCST90094852 | ebi-a-GCST90001436 | Memory B cell %lymphocyte \|\| id:ebi-a-GCST90001436 | F-strong vegetable liking (derived food-liking factor) \|\| id:ebi-fl187-GCST90094852 | MR Egger | 23 | 0.34099910273509 | 0.368345530210865 | 0.365088664714074 | -0.380958136 | 1.06295634194839 | 1.40635197899132 | 0.683206490451032 | 2.89491671472135 | 0.999279047927446 |
| ebi-fl187-GCST90094852 | ebi-a-GCST90001439 | IgD+ CD24+ B cell %lymphocyte \|\| id:ebi-a-GCST90001439 | F-strong vegetable liking (derived food-liking factor) \|\| id:ebi-fl187-GCST90094852 | MR Egger | 23 | 0.240693002647042 | 0.395417630568406 | 0.549247961677011 | -0.534325553 | 1.01571155856112 | 1.27213043467855 | 0.586064426118236 | 2.76132754474498 | 0.999279047927446 |
| ebi-fl187-GCST90094852 | ebi-a-GCST90001457 | CD11c+ HLA DR++ monocyte %monocyte \|\| id:ebi-a-GCST90001457 | F-strong vegetable liking (derived food-liking factor) \|\| id:ebi-fl187-GCST90094852 | MR Egger | 23 | -0.459061272 | 0.37934573179517 | 0.239671235884864 | -1.202578906 | 0.284456362368792 | 0.63187652740653 | 0.300418460995529 | 1.32903931590701 | 0.999279047927446 |
| ebi-fl187-GCST90094852 | ebi-a-GCST90001483 | CD39+ resting CD4 regulatory T cell Absolute Count \|\| id:ebi-a-GCST90001483 | F-strong vegetable liking (derived food-liking factor) \|\| id:ebi-fl187-GCST90094852 | MR Egger | 23 | 0.0980660115349749 | 0.35525540938516 | 0.785212840667183 | -0.598234591 | 0.794366613929889 | 1.10303559577812 | 0.549781368908284 | 2.21303884482226 | 0.999279047927446 |
| ebi-fl187-GCST90094852 | ebi-a-GCST90001525 | CD33dim HLA DR+ CD11b+ Absolute Count \|\| id:ebi-a-GCST90001525 | F-strong vegetable liking (derived food-liking factor) \|\| id:ebi-fl187-GCST90094852 | MR Egger | 23 | -0.594147726 | 0.458380314555096 | 0.208974969496393 | -1.492573142 | 0.30427769087116 | 0.552032848867974 | 0.224793485115651 | 1.35564545419325 | 0.999279047927446 |
| ebi-fl187-GCST90094852 | ebi-a-GCST90001540 | Naive CD4+ T cell Absolute Count \|\| id:ebi-a-GCST90001540 | F-strong vegetable liking (derived food-liking factor) \|\| id:ebi-fl187-GCST90094852 | MR Egger | 23 | -0.51331889 | 0.371265302318146 | 0.181309312338252 | -1.240998883 | 0.214361102121552 | 0.598505903371028 | 0.289095301294224 | 1.23907000482656 | 0.999279047927446 |
| ebi-fl187-GCST90094852 | ebi-a-GCST90001624 | HLA DR+ CD4+ T cell Absolute Count \|\| id:ebi-a-GCST90001624 | F-strong vegetable liking (derived food-liking factor) \|\| id:ebi-fl187-GCST90094852 | MR Egger | 23 | -0.082825018 | 0.360953893062022 | 0.82073039275635 | -0.790294648 | 0.624644612701173 | 0.920512206471161 | 0.453711090473745 | 1.86758212451375 | 0.999279047927446 |
| ebi-fl187-GCST90094852 | ebi-a-GCST90001672 | CD39+ CD8+ T cell Absolute Count \|\| id:ebi-a-GCST90001672 | F-strong vegetable liking (derived food-liking factor) \|\| id:ebi-fl187-GCST90094852 | MR Egger | 23 | 0.133069141240975 | 0.440318921186336 | 0.765464194114632 | -0.729955944 | 0.996094226766193 | 1.14232897764361 | 0.481930221403367 | 2.70768554286641 | 0.999279047927446 |
| ebi-fl187-GCST90094852 | ebi-a-GCST90001722 | CD19 on CD20- CD38- B cell \|\| id:ebi-a-GCST90001722 | F-strong vegetable liking (derived food-liking factor) \|\| id:ebi-fl187-GCST90094852 | MR Egger | 23 | -0.283679845 | 0.345162825497953 | 0.420383286766382 | -0.960198983 | 0.392839292598668 | 0.753007685010324 | 0.382816704244403 | 1.4811803335588 | 0.999279047927446 |
| ebi-fl187-GCST90094852 | ebi-a-GCST90001742 | CD19 on transitional B cell \|\| id:ebi-a-GCST90001742 | F-strong vegetable liking (derived food-liking factor) \|\| id:ebi-fl187-GCST90094852 | MR Egger | 23 | -0.017801614 | 0.350289813705718 | 0.959949502985982 | -0.704369649 | 0.668766420960136 | 0.982355898780821 | 0.494420134391358 | 1.95182810072539 | 0.999279047927446 |
| ebi-fl187-GCST90094852 | ebi-a-GCST90001784 | CD25 on IgD+ CD38dim B cell \|\| id:ebi-a-GCST90001784 | F-strong vegetable liking (derived food-liking factor) \|\| id:ebi-fl187-GCST90094852 | MR Egger | 23 | 0.322926942113123 | 0.40928496060614 | 0.438927963790438 | -0.479271581 | 1.12512546490116 | 1.38116444226401 | 0.619234289788241 | 3.08060333226507 | 0.999279047927446 |
| ebi-fl187-GCST90094852 | ebi-a-GCST90001794 | CD25 on IgD+ B cell \|\| id:ebi-a-GCST90001794 | F-strong vegetable liking (derived food-liking factor) \|\| id:ebi-fl187-GCST90094852 | MR Egger | 23 | 0.364692521674867 | 0.390745403434321 | 0.361260086238227 | -0.401168469 | 1.13055351240614 | 1.44007114940288 | 0.669537255226443 | 3.09737045870757 | 0.999279047927446 |
| ebi-fl187-GCST90094852 | ebi-a-GCST90001802 | CD27 on IgD- CD38- B cell \|\| id:ebi-a-GCST90001802 | F-strong vegetable liking (derived food-liking factor) \|\| id:ebi-fl187-GCST90094852 | MR Egger | 23 | -0.396779745 | 0.373397837429896 | 0.300022333959642 | -1.128639507 | 0.335080016161721 | 0.672482126737808 | 0.323473040140992 | 1.39805224752174 | 0.999279047927446 |
| ebi-fl187-GCST90094852 | ebi-a-GCST90001804 | CD27 on IgD- CD38dim B cell \|\| id:ebi-a-GCST90001804 | F-strong vegetable liking (derived food-liking factor) \|\| id:ebi-fl187-GCST90094852 | MR Egger | 23 | -0.402751473 | 0.433461817391958 | 0.363368863513364 | -1.252336635 | 0.44683368929875 | 0.668478213706475 | 0.285836121288782 | 1.56335427511882 | 0.999279047927446 |
| ebi-fl187-GCST90094852 | ebi-a-GCST90001805 | CD27 on memory B cell \|\| id:ebi-a-GCST90001805 | F-strong vegetable liking (derived food-liking factor) \|\| id:ebi-fl187-GCST90094852 | MR Egger | 23 | -0.259063918 | 0.395427275060799 | 0.519484407093301 | -1.034101377 | 0.515973541595819 | 0.771773691604198 | 0.355545739117922 | 1.6752686518761 | 0.999279047927446 |
| ebi-fl187-GCST90094852 | ebi-a-GCST90001806 | CD27 on unswitched memory B cell \|\| id:ebi-a-GCST90001806 | F-strong vegetable liking (derived food-liking factor) \|\| id:ebi-fl187-GCST90094852 | MR Egger | 23 | -0.103670007 | 0.494050146505179 | 0.83581730299604 | -1.072008294 | 0.864668280523852 | 0.90152274487271 | 0.342320346803584 | 2.37421838085821 | 0.999279047927446 |
| ebi-fl187-GCST90094852 | ebi-a-GCST90001839 | CD3 on Effector Memory CD8+ T cell \|\| id:ebi-a-GCST90001839 | F-strong vegetable liking (derived food-liking factor) \|\| id:ebi-fl187-GCST90094852 | MR Egger | 23 | -0.032753328 | 0.468324233559683 | 0.944905290970622 | -0.950668826 | 0.885162169946405 | 0.967777253862937 | 0.386482447635228 | 2.42337735859732 | 0.999279047927446 |
| ebi-fl187-GCST90094852 | ebi-a-GCST90001859 | CD3 on CD8+ T cell \|\| id:ebi-a-GCST90001859 | F-strong vegetable liking (derived food-liking factor) \|\| id:ebi-fl187-GCST90094852 | MR Egger | 23 | -0.17873224 | 0.470211576459063 | 0.707680862086869 | -1.10034693 | 0.742882449634687 | 0.836329804899575 | 0.332755620734717 | 2.10198565848113 | 0.999279047927446 |
| ebi-fl187-GCST90094852 | ebi-a-GCST90001905 | CD86 on monocyte \|\| id:ebi-a-GCST90001905 | F-strong vegetable liking (derived food-liking factor) \|\| id:ebi-fl187-GCST90094852 | MR Egger | 23 | 0.209763294997786 | 0.388166481513326 | 0.594610137507412 | -0.551043009 | 0.970569598763904 | 1.23338607674714 | 0.576348360382708 | 2.63944745726969 | 0.999279047927446 |
| ebi-fl187-GCST90094852 | ebi-a-GCST90001943 | CD25 on activated & secreting CD4 regulatory T cell \|\| id:ebi-a-GCST90001943 | F-strong vegetable liking (derived food-liking factor) \|\| id:ebi-fl187-GCST90094852 | MR Egger | 23 | 0.497100231564336 | 0.354859575469163 | 0.175867209858141 | -0.198424536 | 1.1926249994839 | 1.64394728587064 | 0.820021650228071 | 3.29572112878944 | 0.999279047927446 |
| ebi-fl187-GCST90094852 | ebi-a-GCST90001949 | CD33 on CD33dim HLA DR+ CD11b- \|\| id:ebi-a-GCST90001949 | F-strong vegetable liking (derived food-liking factor) \|\| id:ebi-fl187-GCST90094852 | MR Egger | 23 | 0.0456442385496252 | 0.50215630567778 | 0.928436296909684 | -0.938582121 | 1.02987059767807 | 1.04670196850011 | 0.391182091368854 | 2.80070339372706 | 0.999279047927446 |
| ebi-fl187-GCST90094852 | ebi-a-GCST90001955 | CD33 on Immature Myeloid-Derived Suppressor Cells \|\| id:ebi-a-GCST90001955 | F-strong vegetable liking (derived food-liking factor) \|\| id:ebi-fl187-GCST90094852 | MR Egger | 23 | 0.532411882008305 | 0.542245782099774 | 0.337344092726366 | -0.530389851 | 1.59521361492386 | 1.70303487835532 | 0.588375546220291 | 4.92938194920976 | 0.999279047927446 |
| ebi-fl187-GCST90094852 | ebi-a-GCST90001956 | CD33 on CD33+ HLA DR+ \|\| id:ebi-a-GCST90001956 | F-strong vegetable liking (derived food-liking factor) \|\| id:ebi-fl187-GCST90094852 | MR Egger | 23 | 0.357860718945754 | 0.516503212751872 | 0.495993488664737 | -0.654485578 | 1.37020701593942 | 1.43026639759452 | 0.519709343728628 | 3.93616546012338 | 0.999279047927446 |
| ebi-fl187-GCST90094852 | ebi-a-GCST90001957 | CD33 on CD33+ HLA DR+ CD14- \|\| id:ebi-a-GCST90001957 | F-strong vegetable liking (derived food-liking factor) \|\| id:ebi-fl187-GCST90094852 | MR Egger | 23 | 0.382295434675104 | 0.516914359377939 | 0.467747242534564 | -0.63085671 | 1.39544757905586 | 1.46564502353865 | 0.5321357198343 | 4.03678094695936 | 0.999279047927446 |
| ebi-fl187-GCST90094852 | ebi-a-GCST90002064 | CD4 on resting CD4 regulatory T cell \|\| id:ebi-a-GCST90002064 | F-strong vegetable liking (derived food-liking factor) \|\| id:ebi-fl187-GCST90094852 | MR Egger | 23 | 0.436096861009634 | 0.375447937819621 | 0.258456044289348 | -0.299781097 | 1.17197481913609 | 1.54665859840759 | 0.740980405676868 | 3.22836177812687 | 0.999279047927446 |
| ebi-fl187-GCST90094852 | ebi-a-GCST90002080 | SSC-A on T cell \|\| id:ebi-a-GCST90002080 | F-strong vegetable liking (derived food-liking factor) \|\| id:ebi-fl187-GCST90094852 | MR Egger | 23 | 0.188813223780159 | 0.383767498601855 | 0.627823849673197 | -0.563371073 | 0.940997521039795 | 1.20781534023054 | 0.56928671812726 | 2.56253632773163 | 0.999279047927446 |
| ebi-fl187-GCST90094852 | ebi-a-GCST90002121 | CD8 on CD39+ CD8+ T cell \|\| id:ebi-a-GCST90002121 | F-strong vegetable liking (derived food-liking factor) \|\| id:ebi-fl187-GCST90094852 | MR Egger | 23 | 0.098020060533214 | 0.413327212352138 | 0.814839516011819 | -0.712101276 | 0.908141396743405 | 1.10298491135202 | 0.490612202100208 | 2.4797094517061 | 0.999279047927446 |
| ebi-fl187-GCST90094852 | ebi-cfmet338-GCST90026053 | 1-palmitoyl-2-docosahexaenoyl-gpc (16:0/22:6) levels \|\| id:ebi-cfmet338-GCST90026053 | F-strong vegetable liking (derived food-liking factor) \|\| id:ebi-fl187-GCST90094852 | MR Egger | 20 | 0.344741771037364 | 0.307911749966705 | 0.277597044358666 | -0.258765259 | 0.948248800972106 | 1.41162535004869 | 0.772004222897856 | 2.58118552955603 | 0.999279047927446 |
| ebi-fl187-GCST90094852 | ebi-cfmet338-GCST90026063 | 1-stearoyl-2-oleoyl-gpc (18:0/18:1) levels \|\| id:ebi-cfmet338-GCST90026063 | F-strong vegetable liking (derived food-liking factor) \|\| id:ebi-fl187-GCST90094852 | MR Egger | 20 | -0.10076717 | 0.251363174962553 | 0.693225046368886 | -0.593438993 | 0.39190465320198 | 0.90414352036581 | 0.552424231444634 | 1.47979661080709 | 0.999279047927446 |
| ebi-fl187-GCST90094852 | ebi-cfmet338-GCST90026113 | Adenosine 3',5'-cyclic monophosphate (camp) levels \|\| id:ebi-cfmet338-GCST90026113 | F-strong vegetable liking (derived food-liking factor) \|\| id:ebi-fl187-GCST90094852 | MR Egger | 20 | 0.279532354071587 | 0.483947456811101 | 0.570683615542285 | -0.669004661 | 1.22806936942134 | 1.32251120072481 | 0.512218154707056 | 3.41463077786236 | 0.999279047927446 |
| ebi-fl187-GCST90094852 | ebi-cfmet338-GCST90026117 | Alpha-ketoglutaramate levels \|\| id:ebi-cfmet338-GCST90026117 | F-strong vegetable liking (derived food-liking factor) \|\| id:ebi-fl187-GCST90094852 | MR Egger | 19 | -0.13061151 | 0.244836411751152 | 0.600618107307574 | -0.610490877 | 0.349267856734204 | 0.877558630668048 | 0.543084215902819 | 1.4180289680851 | 0.999279047927446 |
| ebi-fl187-GCST90094852 | ebi-cfmet338-GCST90026124 | Arginine levels \|\| id:ebi-cfmet338-GCST90026124 | F-strong vegetable liking (derived food-liking factor) \|\| id:ebi-fl187-GCST90094852 | MR Egger | 19 | -0.04731597 | 0.163140664924403 | 0.775299894727279 | -0.367071674 | 0.272439732802539 | 0.953785981788442 | 0.692759990592439 | 1.31316431579453 | 0.999279047927446 |
| ebi-fl187-GCST90094852 | ebi-cfmet338-GCST90026169 | Glutamate levels \|\| id:ebi-cfmet338-GCST90026169 | F-strong vegetable liking (derived food-liking factor) \|\| id:ebi-fl187-GCST90094852 | MR Egger | 20 | 0.255544060467451 | 0.191827565659775 | 0.199431167594003 | -0.120437968 | 0.63152608916061 | 1.29116390104969 | 0.886532078797566 | 1.88047816795868 | 0.999279047927446 |
| ebi-fl187-GCST90094852 | ebi-cfmet338-GCST90026170 | Glutamine levels \|\| id:ebi-cfmet338-GCST90026170 | F-strong vegetable liking (derived food-liking factor) \|\| id:ebi-fl187-GCST90094852 | MR Egger | 20 | 0.101121388684895 | 0.132895789058942 | 0.456565485072002 | -0.159354358 | 0.361597135240422 | 1.10641093938104 | 0.852694146544549 | 1.4356204645508 | 0.999279047927446 |
| ebi-fl187-GCST90094852 | ebi-cfmet338-GCST90026191 | Kynurenate levels \|\| id:ebi-cfmet338-GCST90026191 | F-strong vegetable liking (derived food-liking factor) \|\| id:ebi-fl187-GCST90094852 | MR Egger | 20 | -0.087166282 | 0.853370435442935 | 0.919772031424721 | -1.759772336 | 1.58543977130358 | 0.916524681253726 | 0.172084036767089 | 4.88143762273654 | 0.999279047927446 |
| ebi-fl187-GCST90094852 | ebi-cfmet338-GCST90026202 | Methylsuccinoylcarnitine levels \|\| id:ebi-cfmet338-GCST90026202 | F-strong vegetable liking (derived food-liking factor) \|\| id:ebi-fl187-GCST90094852 | MR Egger | 20 | -0.623970957 | 0.513269145555984 | 0.23981107960296 | -1.629978482 | 0.38203656874141 | 0.535812519286487 | 0.195933790216578 | 1.46526566707452 | 0.999279047927446 |
| ebi-fl187-GCST90094852 | ebi-cfmet338-GCST90026222 | N-acetylputrescine levels \|\| id:ebi-cfmet338-GCST90026222 | F-strong vegetable liking (derived food-liking factor) \|\| id:ebi-fl187-GCST90094852 | MR Egger | 19 | 0.100841302492534 | 0.190816285222246 | 0.604002402139056 | -0.273158617 | 0.474841221528135 | 1.1061010923478 | 0.760972075295746 | 1.6077588997172 | 0.999279047927446 |
| ebi-fl187-GCST90094852 | ebi-cfmet338-GCST90026252 | Propionylcarnitine (c3) levels \|\| id:ebi-cfmet338-GCST90026252 | F-strong vegetable liking (derived food-liking factor) \|\| id:ebi-fl187-GCST90094852 | MR Egger | 20 | 0.754258344079694 | 0.306057494671224 | 0.0240159092518889 | 0.154385654524094 | 1.35413103363529 | 2.12603415267834 | 1.16694083602314 | 3.87339364501002 | 0.999279047927446 |
| ebi-fl187-GCST90094852 | ebi-cfmet338-GCST90026316 | Ethyl beta-glucopyranoside levels \|\| id:ebi-cfmet338-GCST90026316 | F-strong vegetable liking (derived food-liking factor) \|\| id:ebi-fl187-GCST90094852 | MR Egger | 20 | -0.005327764 | 0.990660585032107 | 0.995768160583577 | -1.947022511 | 1.93636698254813 | 0.994686403249138 | 0.142698322388936 | 6.93351557499055 | 0.999279047927446 |
| ebi-fl187-GCST90094852 | ebi-gm430-GCST90011315 | F_Prevotellaceae abundance \|\| id:ebi-gm430-GCST90011315 | F-strong vegetable liking (derived food-liking factor) \|\| id:ebi-fl187-GCST90094852 | MR Egger | 19 | 0.0165046006427722 | 0.435165772443981 | 0.970187588531355 | -0.836420313 | 0.869429514632974 | 1.01664155397991 | 0.433258681134115 | 2.3855495441504 | 0.999279047927446 |
| ebi-fl187-GCST90094852 | ebi-gm430-GCST90011354 | OTU97_1 (Bacteroides) abundance \|\| id:ebi-gm430-GCST90011354 | F-strong vegetable liking (derived food-liking factor) \|\| id:ebi-fl187-GCST90094852 | MR Egger | 19 | 0.244842896760503 | 0.45843856001005 | 0.600201299329668 | -0.653696681 | 1.1433824743802 | 1.27742061052367 | 0.520119502734219 | 3.13736248614486 | 0.999279047927446 |
| ebi-fl187-GCST90094852 | ebi-gm430-GCST90011419 | OTU99_1 (Bacteroides) abundance \|\| id:ebi-gm430-GCST90011419 | F-strong vegetable liking (derived food-liking factor) \|\| id:ebi-fl187-GCST90094852 | MR Egger | 19 | 0.274737043684744 | 0.453700702982719 | 0.552814972977522 | -0.614516334 | 1.16399042153087 | 1.31618453032488 | 0.540902448088309 | 3.20268788575291 | 0.999279047927446 |
| ebi-fl187-GCST90094852 | ebi-gm430-GCST90011435 | OTU99_155 (Butyrivibrio) abundance \|\| id:ebi-gm430-GCST90011435 | F-strong vegetable liking (derived food-liking factor) \|\| id:ebi-fl187-GCST90094852 | MR Egger | 19 | -1.391130935 | 1.52046019279675 | 0.373018655761499 | -4.371232913 | 1.5889710431524 | 0.24879377594476 | 0.0126356523054235 | 4.89870577732525 | 0.999279047927446 |
| ebi-fl187-GCST90094852 | ebi-gm430-GCST90011466 | OTU99_58 (Bacteroides) abundance \|\| id:ebi-gm430-GCST90011466 | F-strong vegetable liking (derived food-liking factor) \|\| id:ebi-fl187-GCST90094852 | MR Egger | 19 | 0.218804806115277 | 0.72966096135928 | 0.767910555471572 | -1.211330678 | 1.64894029037947 | 1.24458831694771 | 0.297800738718628 | 5.20146486320935 | 0.999279047927446 |
| ebi-fl187-GCST90094852 | ebi-gm430-GCST90011472 | OTU99_72 (Parabacteroides) abundance \|\| id:ebi-gm430-GCST90011472 | F-strong vegetable liking (derived food-liking factor) \|\| id:ebi-fl187-GCST90094852 | MR Egger | 19 | -1.336683459 | 1.01053079883773 | 0.203436156107396 | -3.317323825 | 0.643956906343479 | 0.262715532048174 | 0.0362497126322016 | 1.90399994283108 | 0.999279047927446 |
| ebi-fl187-GCST90094852 | ebi-gm430-GCST90011505 | TestASV_28 (Holdemanella) abundance \|\| id:ebi-gm430-GCST90011505 | F-strong vegetable liking (derived food-liking factor) \|\| id:ebi-fl187-GCST90094852 | MR Egger | 19 | -0.292872195 | 0.888926069109349 | 0.745827010358807 | -2.03516729 | 1.44942290044123 | 0.746117492140207 | 0.130658622733541 | 4.26065498342871 | 0.999279047927446 |
| ebi-fl187-GCST90094852 | ebi-gm430-GCST90011513 | TestASV_35 (Alphaproteobacteria) abundance \|\| id:ebi-gm430-GCST90011513 | F-strong vegetable liking (derived food-liking factor) \|\| id:ebi-fl187-GCST90094852 | MR Egger | 19 | -1.793948656 | 1.04959979157806 | 0.105608883543368 | -3.851164247 | 0.263266935846865 | 0.166302201350472 | 0.0212549759825488 | 1.30117400258274 | 0.999279047927446 |
| ebi-fl187-GCST90094852 | ebi-gm430-GCST90011543 | G_Coprococcus prevalence \|\| id:ebi-gm430-GCST90011543 | F-strong vegetable liking (derived food-liking factor) \|\| id:ebi-fl187-GCST90094852 | MR Egger | 19 | -0.16228137 | 1.11436532923391 | 0.885928845868761 | -2.346437415 | 2.02187467573638 | 0.850201949931994 | 0.0957095289082871 | 7.55247010316835 | 0.999279047927446 |
| ebi-fl187-GCST90094852 | ebi-gm430-GCST90011547 | G_Parasutterella prevalence \|\| id:ebi-gm430-GCST90011547 | F-strong vegetable liking (derived food-liking factor) \|\| id:ebi-fl187-GCST90094852 | MR Egger | 19 | 1.47822529301561 | 1.01463909252774 | 0.16336788143604 | -0.510467328 | 3.46691791436998 | 4.3851564034238 | 0.600215015773626 | 32.03784673 | 0.999279047927446 |
| ebi-fl187-GCST90094852 | ebi-gm430-GCST90011569 | OTU97_13 (Subdoligranulum) prevalence \|\| id:ebi-gm430-GCST90011569 | F-strong vegetable liking (derived food-liking factor) \|\| id:ebi-fl187-GCST90094852 | MR Egger | 19 | 1.41045777074084 | 1.05507253509855 | 0.19889254802585 | -0.657484398 | 3.47839993953401 | 4.09783084183846 | 0.518153163470636 | 32.4078260872649 | 0.999279047927446 |
| ebi-fl187-GCST90094852 | ebi-gm430-GCST90011590 | OTU97_277 (Prevotella) prevalence \|\| id:ebi-gm430-GCST90011590 | F-strong vegetable liking (derived food-liking factor) \|\| id:ebi-fl187-GCST90094852 | MR Egger | 19 | 0.51965889822775 | 1.07644852135522 | 0.635428914237101 | -1.590180204 | 2.62949800008398 | 1.68145400492779 | 0.203888866909914 | 13.8668070186338 | 0.999279047927446 |
| ebi-fl187-GCST90094852 | ebi-gm430-GCST90011616 | OTU97_86 (Phascolarctobacterium) prevalence \|\| id:ebi-gm430-GCST90011616 | F-strong vegetable liking (derived food-liking factor) \|\| id:ebi-fl187-GCST90094852 | MR Egger | 19 | -1.212475274 | 1.1194963502616 | 0.293907474985131 | -3.40668812 | 0.981737572772139 | 0.297460072305722 | 0.0331508102654162 | 2.6690899530873 | 0.999279047927446 |
| ebi-fl187-GCST90094852 | ebi-gm430-GCST90011665 | OTU99_55 (Barnesiella) prevalence \|\| id:ebi-gm430-GCST90011665 | F-strong vegetable liking (derived food-liking factor) \|\| id:ebi-fl187-GCST90094852 | MR Egger | 19 | -0.087533151 | 0.761611522641204 | 0.90984601630179 | -1.580291735 | 1.40522543332427 | 0.916188498534419 | 0.205915016735565 | 4.07644560437623 | 0.999279047927446 |
| ebi-fl187-GCST90094852 | ebi-gm430-GCST90011687 | TestASV_14 (Lachnospiraceae) prevalence \|\| id:ebi-gm430-GCST90011687 | F-strong vegetable liking (derived food-liking factor) \|\| id:ebi-fl187-GCST90094852 | MR Egger | 19 | 0.529046193722811 | 0.807859451668053 | 0.521311934244462 | -1.054358332 | 2.11245071899219 | 1.69731262887122 | 0.348415923099954 | 8.26848019600774 | 0.999279047927446 |
| ebi-fl187-GCST90094852 | ebi-gm430-GCST90011697 | TestASV_23 (Barnesiella) prevalence \|\| id:ebi-gm430-GCST90011697 | F-strong vegetable liking (derived food-liking factor) \|\| id:ebi-fl187-GCST90094852 | MR Egger | 19 | 0.130578333723991 | 1.33365974314423 | 0.92314908380474 | -2.483394763 | 2.74455143028669 | 1.13948719667316 | 0.0834594192022138 | 15.5576336834563 | 0.999279047927446 |
| ebi-fl187-GCST90094852 | ebi-gm430-GCST90011727 | TestASV_6 (Subdoligranulum) prevalence \|\| id:ebi-gm430-GCST90011727 | F-strong vegetable liking (derived food-liking factor) \|\| id:ebi-fl187-GCST90094852 | MR Egger | 19 | 1.16682454388903 | 1.42176748609425 | 0.423184520994099 | -1.619839729 | 3.95348881663377 | 3.21177756965009 | 0.197930419076403 | 52.1168762489486 | 0.999279047927446 |
| ebi-fl187-GCST90094852 | ebi-hbsm44-GCST90104908 | Sulcal depth of the left posterior inferior temporal sulcus \|\| id:ebi-hbsm44-GCST90104908 | F-strong vegetable liking (derived food-liking factor) \|\| id:ebi-fl187-GCST90094852 | MR Egger | 22 | -0.313798815 | 0.279603271112545 | 0.27502820952034 | -0.861821226 | 0.234223596817277 | 0.730666012734197 | 0.42239210991464 | 1.26392707068505 | 0.999279047927446 |
| ebi-fl187-GCST90094852 | ebi-hbsm44-GCST90104918 | Sulcal depth of the left inferior frontal sulcus \|\| id:ebi-hbsm44-GCST90104918 | F-strong vegetable liking (derived food-liking factor) \|\| id:ebi-fl187-GCST90094852 | MR Egger | 23 | 0.0188079673454784 | 0.392372334203767 | 0.962221811849811 | -0.750241808 | 0.787857742384862 | 1.01898595125073 | 0.472252344682969 | 2.19868123586216 | 0.999279047927446 |
| ebi-fl187-GCST90094852 | ebi-hbsm44-GCST90104925 | Sulcal depth of the right anterior inferior temporal sulcus \|\| id:ebi-hbsm44-GCST90104925 | F-strong vegetable liking (derived food-liking factor) \|\| id:ebi-fl187-GCST90094852 | MR Egger | 24 | -0.146586056 | 0.129383390227752 | 0.269428306867197 | -0.400177501 | 0.107005389013313 | 0.863651406895427 | 0.670201074331094 | 1.112940252 | 0.999279047927446 |
| ebi-fl187-GCST90094852 | ebi-IPs91-GCST90274770 | C-C motif chemokine 4 levels \|\| id:ebi-IPs91-GCST90274770 | F-strong vegetable liking (derived food-liking factor) \|\| id:ebi-fl187-GCST90094852 | MR Egger | 27 | 0.0872033958374797 | 0.170507673284569 | 0.613535066666237 | -0.246991644 | 0.421398435475235 | 1.09111858612871 | 0.781147220926296 | 1.52409140953449 | 0.999279047927446 |
| ebi-fl187-GCST90094852 | ebi-IPs91-GCST90274779 | C-X-C motif chemokine 1 levels \|\| id:ebi-IPs91-GCST90274779 | F-strong vegetable liking (derived food-liking factor) \|\| id:ebi-fl187-GCST90094852 | MR Egger | 27 | -0.29683108 | 0.183211856195444 | 0.117746749165184 | -0.655926318 | 0.0622641578818237 | 0.743169537763951 | 0.518961116633433 | 1.06424343589195 | 0.999279047927446 |
| ebi-fl187-GCST90094852 | ebi-IPs91-GCST90274780 | C-X-C motif chemokine 10 levels \|\| id:ebi-IPs91-GCST90274780 | F-strong vegetable liking (derived food-liking factor) \|\| id:ebi-fl187-GCST90094852 | MR Egger | 27 | -0.047615397 | 0.174621404828125 | 0.787340515878232 | -0.38987335 | 0.294642556934134 | 0.953500436143083 | 0.677142629186959 | 1.3426463532752 | 0.999279047927446 |
[truncated: 331,378 more chars]
