# Supplementary material for: Mendelian Randomization Mediation Analysis Reveals the Impact of Dietary Preferences on Preeclampsia and Fetal Growth Restriction via Immune Modulation
Source: Food Sci Nutr. 2025 Nov 26;13(12):e71280. doi: 10.1002/fsn3.71280 (PMC12648301; doi:10.1002/fsn3.71280)
Supplement: Supplementary file 1 — Data S1: fsn371280‐sup‐0001‐Supinfo1.zip. [file FSN3-13-e71280-s001.zip › fsn371280-sup-0014-Supinfo14@Supplementary Material 4_FGR - MR (Dietary Preferences to Immune Cells) MR Analysis.docx]

| id.exposure | id.outcome | outcome | exposure | method | nsnp | b | se | pval | lo_ci | up_ci | or | or_lci95 | or_uci95 | p_fdr |
| --- | --- | --- | --- | --- | --- | --- | --- | --- | --- | --- | --- | --- | --- | --- |
| ebi-fl187-GCST90094697 | ebi-a-GCST90001392 | IgD+ CD38+ B cell Absolute Count \|\| id:ebi-a-GCST90001392 | Banana liking \|\| id:ebi-fl187-GCST90094697 | Inverse variance weighted | 2 | -0.624761907 | 0.327898727603401 | 0.0567345636192079 | -1.267443413 | 0.0179195987572185 | 0.53538888550607 | 0.281550510685255 | 1.01808111811194 | 0.998074519377826 |
| ebi-fl187-GCST90094697 | ebi-a-GCST90001402 | Switched memory B cell %B cell \|\| id:ebi-a-GCST90001402 | Banana liking \|\| id:ebi-fl187-GCST90094697 | Inverse variance weighted | 2 | 0.084490517512682 | 0.344819712503957 | 0.806434677111133 | -0.591356119 | 0.760337154020437 | 1.08816252569573 | 0.553576060464072 | 2.13899727046698 | 0.998074519377826 |
| ebi-fl187-GCST90094697 | ebi-a-GCST90001407 | Memory B cell Absolute Count \|\| id:ebi-a-GCST90001407 | Banana liking \|\| id:ebi-fl187-GCST90094697 | Inverse variance weighted | 2 | -0.127875622 | 0.341156560793706 | 0.707787101578816 | -0.796542481 | 0.540791237286714 | 0.879962820469454 | 0.450885216372357 | 1.71736516809875 | 0.998074519377826 |
| ebi-fl187-GCST90094697 | ebi-a-GCST90001411 | IgD+ CD24+ B cell %B cell \|\| id:ebi-a-GCST90001411 | Banana liking \|\| id:ebi-fl187-GCST90094697 | Inverse variance weighted | 2 | 0.0295580097908073 | 0.345449246750365 | 0.931813039302702 | -0.647522514 | 0.706638533421524 | 1.02999918377606 | 0.523340741414011 | 2.02716554364356 | 0.998074519377826 |
| ebi-fl187-GCST90094697 | ebi-a-GCST90001418 | CD24+ CD27+ B cell Absolute Count \|\| id:ebi-a-GCST90001418 | Banana liking \|\| id:ebi-fl187-GCST90094697 | Inverse variance weighted | 2 | -0.150786382 | 0.425809728611383 | 0.723251345834984 | -0.98537345 | 0.683800685937878 | 0.860031397104083 | 0.373299792013296 | 1.98139409619188 | 0.998074519377826 |
| ebi-fl187-GCST90094697 | ebi-a-GCST90001436 | Memory B cell %lymphocyte \|\| id:ebi-a-GCST90001436 | Banana liking \|\| id:ebi-fl187-GCST90094697 | Inverse variance weighted | 2 | -0.04674075 | 0.337173281067245 | 0.889746243873504 | -0.707600381 | 0.614118881111033 | 0.954334777022564 | 0.492825372998453 | 1.84802754998892 | 0.998074519377826 |
| ebi-fl187-GCST90094697 | ebi-a-GCST90001439 | IgD+ CD24+ B cell %lymphocyte \|\| id:ebi-a-GCST90001439 | Banana liking \|\| id:ebi-fl187-GCST90094697 | Inverse variance weighted | 2 | -0.10458135 | 0.3392952357335 | 0.757906474777796 | -0.769600012 | 0.560437311810783 | 0.90070152215327 | 0.463198304903625 | 1.75143825748242 | 0.998074519377826 |
| ebi-fl187-GCST90094697 | ebi-a-GCST90001457 | CD11c+ HLA DR++ monocyte %monocyte \|\| id:ebi-a-GCST90001457 | Banana liking \|\| id:ebi-fl187-GCST90094697 | Inverse variance weighted | 2 | 0.0981633470096518 | 0.366993695686486 | 0.78909987686548 | -0.621144297 | 0.817470990555165 | 1.10314296549678 | 0.537329221700089 | 2.26476497681388 | 0.998074519377826 |
| ebi-fl187-GCST90094697 | ebi-a-GCST90001483 | CD39+ resting CD4 regulatory T cell Absolute Count \|\| id:ebi-a-GCST90001483 | Banana liking \|\| id:ebi-fl187-GCST90094697 | Inverse variance weighted | 2 | -0.18660215 | 0.776697903942703 | 0.810135976822946 | -1.708930041 | 1.33572574208491 | 0.829773796525131 | 0.181059415098267 | 3.80275476437413 | 0.998074519377826 |
| ebi-fl187-GCST90094697 | ebi-a-GCST90001525 | CD33dim HLA DR+ CD11b+ Absolute Count \|\| id:ebi-a-GCST90001525 | Banana liking \|\| id:ebi-fl187-GCST90094697 | Inverse variance weighted | 2 | 0.352799814248368 | 0.446989729849181 | 0.429948686731995 | -0.523300056 | 1.22889968475276 | 1.42304624133645 | 0.592561830433156 | 3.41746717553764 | 0.998074519377826 |
| ebi-fl187-GCST90094697 | ebi-a-GCST90001540 | Naive CD4+ T cell Absolute Count \|\| id:ebi-a-GCST90001540 | Banana liking \|\| id:ebi-fl187-GCST90094697 | Inverse variance weighted | 2 | -0.119191574 | 0.37312481172633 | 0.749391696116703 | -0.850516205 | 0.612133057153518 | 0.887637736311644 | 0.427194355239687 | 1.84436133404055 | 0.998074519377826 |
| ebi-fl187-GCST90094697 | ebi-a-GCST90001624 | HLA DR+ CD4+ T cell Absolute Count \|\| id:ebi-a-GCST90001624 | Banana liking \|\| id:ebi-fl187-GCST90094697 | Inverse variance weighted | 2 | 0.0753379922572025 | 0.330183412312518 | 0.819513981445707 | -0.571821496 | 0.722497480389739 | 1.07824852895681 | 0.564496274039692 | 2.05957052980613 | 0.998074519377826 |
| ebi-fl187-GCST90094697 | ebi-a-GCST90001672 | CD39+ CD8+ T cell Absolute Count \|\| id:ebi-a-GCST90001672 | Banana liking \|\| id:ebi-fl187-GCST90094697 | Inverse variance weighted | 2 | -0.696467278 | 0.355859697658888 | 0.0503310009470296 | -1.393952286 | 0.00101772914759557 | 0.498342703862981 | 0.248092830660915 | 1.00101824720964 | 0.998074519377826 |
| ebi-fl187-GCST90094697 | ebi-a-GCST90001722 | CD19 on CD20- CD38- B cell \|\| id:ebi-a-GCST90001722 | Banana liking \|\| id:ebi-fl187-GCST90094697 | Inverse variance weighted | 2 | 0.341465564774271 | 0.333423138271048 | 0.30577823005207 | -0.312043786 | 0.994974915785525 | 1.40700814182888 | 0.73194947821333 | 2.7046564962449 | 0.998074519377826 |
| ebi-fl187-GCST90094697 | ebi-a-GCST90001742 | CD19 on transitional B cell \|\| id:ebi-a-GCST90001742 | Banana liking \|\| id:ebi-fl187-GCST90094697 | Inverse variance weighted | 2 | 0.329111955034344 | 0.340224927290815 | 0.333375894688736 | -0.337728902 | 0.995952812524342 | 1.38973343453292 | 0.713388659599583 | 2.70730266464105 | 0.998074519377826 |
| ebi-fl187-GCST90094697 | ebi-a-GCST90001784 | CD25 on IgD+ CD38dim B cell \|\| id:ebi-a-GCST90001784 | Banana liking \|\| id:ebi-fl187-GCST90094697 | Inverse variance weighted | 2 | -0.074016507 | 0.511717764342443 | 0.884992569043634 | -1.076983325 | 0.928950311409559 | 0.928656364661243 | 0.340621521811344 | 2.53185012808288 | 0.998074519377826 |
| ebi-fl187-GCST90094697 | ebi-a-GCST90001794 | CD25 on IgD+ B cell \|\| id:ebi-a-GCST90001794 | Banana liking \|\| id:ebi-fl187-GCST90094697 | Inverse variance weighted | 2 | -0.060468352 | 0.580085699462761 | 0.916978591948132 | -1.197436323 | 1.07649961870163 | 0.941323559324027 | 0.301967367164502 | 2.93439006889689 | 0.998074519377826 |
| ebi-fl187-GCST90094697 | ebi-a-GCST90001802 | CD27 on IgD- CD38- B cell \|\| id:ebi-a-GCST90001802 | Banana liking \|\| id:ebi-fl187-GCST90094697 | Inverse variance weighted | 2 | 0.454976496921265 | 0.341007474074175 | 0.18213416409631 | -0.213398152 | 1.12335114610665 | 1.5761363385422 | 0.807834432068762 | 3.07514220619624 | 0.998074519377826 |
| ebi-fl187-GCST90094697 | ebi-a-GCST90001804 | CD27 on IgD- CD38dim B cell \|\| id:ebi-a-GCST90001804 | Banana liking \|\| id:ebi-fl187-GCST90094697 | Inverse variance weighted | 2 | 0.534380638252262 | 0.512497184450483 | 0.297087416970951 | -0.470113843 | 1.53887511977521 | 1.70639104155535 | 0.624931120030246 | 4.65934611571179 | 0.998074519377826 |
| ebi-fl187-GCST90094697 | ebi-a-GCST90001805 | CD27 on memory B cell \|\| id:ebi-a-GCST90001805 | Banana liking \|\| id:ebi-fl187-GCST90094697 | Inverse variance weighted | 2 | -0.06148101 | 0.340871765518552 | 0.856866781004264 | -0.72958967 | 0.606627650692204 | 0.940370803470818 | 0.482106772313589 | 1.83423527484728 | 0.998074519377826 |
| ebi-fl187-GCST90094697 | ebi-a-GCST90001806 | CD27 on unswitched memory B cell \|\| id:ebi-a-GCST90001806 | Banana liking \|\| id:ebi-fl187-GCST90094697 | Inverse variance weighted | 2 | -0.037671811 | 0.341095303334094 | 0.912057535734606 | -0.706218605 | 0.630874983628221 | 0.963028944630126 | 0.493506817585341 | 1.87925417673695 | 0.998074519377826 |
| ebi-fl187-GCST90094697 | ebi-a-GCST90001839 | CD3 on Effector Memory CD8+ T cell \|\| id:ebi-a-GCST90001839 | Banana liking \|\| id:ebi-fl187-GCST90094697 | Inverse variance weighted | 2 | -0.090927834 | 0.378587092458694 | 0.810193203384586 | -0.832958535 | 0.651102867324993 | 0.913083602209658 | 0.434761125670773 | 1.91765458178409 | 0.998074519377826 |
| ebi-fl187-GCST90094697 | ebi-a-GCST90001859 | CD3 on CD8+ T cell \|\| id:ebi-a-GCST90001859 | Banana liking \|\| id:ebi-fl187-GCST90094697 | Inverse variance weighted | 2 | 0.162949102809759 | 0.370882357207917 | 0.660404162016403 | -0.563980317 | 0.889878522937277 | 1.1769767833527 | 0.568939989333941 | 2.43483385685897 | 0.998074519377826 |
| ebi-fl187-GCST90094697 | ebi-a-GCST90001905 | CD86 on monocyte \|\| id:ebi-a-GCST90001905 | Banana liking \|\| id:ebi-fl187-GCST90094697 | Inverse variance weighted | 2 | 0.338547780493663 | 0.379012320283174 | 0.371730334720575 | -0.404316367 | 1.08141192824868 | 1.40290877902645 | 0.667432933934862 | 2.94884016385917 | 0.998074519377826 |
| ebi-fl187-GCST90094697 | ebi-a-GCST90001943 | CD25 on activated & secreting CD4 regulatory T cell \|\| id:ebi-a-GCST90001943 | Banana liking \|\| id:ebi-fl187-GCST90094697 | Inverse variance weighted | 2 | 0.124557592101445 | 0.345577302957886 | 0.718523075140871 | -0.552773922 | 0.801889105898902 | 1.1326472501172 | 0.575351614443949 | 2.229749185 | 0.998074519377826 |
| ebi-fl187-GCST90094697 | ebi-a-GCST90001949 | CD33 on CD33dim HLA DR+ CD11b- \|\| id:ebi-a-GCST90001949 | Banana liking \|\| id:ebi-fl187-GCST90094697 | Inverse variance weighted | 2 | -0.330453847 | 0.491540981503853 | 0.501404584546375 | -1.293874171 | 0.63296647688151 | 0.718597526178254 | 0.274206400242793 | 1.88318873728798 | 0.998074519377826 |
| ebi-fl187-GCST90094697 | ebi-a-GCST90001955 | CD33 on Immature Myeloid-Derived Suppressor Cells \|\| id:ebi-a-GCST90001955 | Banana liking \|\| id:ebi-fl187-GCST90094697 | Inverse variance weighted | 2 | -0.089130969 | 0.838593934947703 | 0.915355274652931 | -1.732775081 | 1.55451314376354 | 0.914725765253658 | 0.176793113340461 | 4.73278177983981 | 0.998074519377826 |
| ebi-fl187-GCST90094697 | ebi-a-GCST90001956 | CD33 on CD33+ HLA DR+ \|\| id:ebi-a-GCST90001956 | Banana liking \|\| id:ebi-fl187-GCST90094697 | Inverse variance weighted | 2 | -0.184707726 | 0.507757781223083 | 0.716028769114391 | -1.179912977 | 0.810497524939441 | 0.831347229312885 | 0.307305479942509 | 2.24902665522109 | 0.998074519377826 |
| ebi-fl187-GCST90094697 | ebi-a-GCST90001957 | CD33 on CD33+ HLA DR+ CD14- \|\| id:ebi-a-GCST90001957 | Banana liking \|\| id:ebi-fl187-GCST90094697 | Inverse variance weighted | 2 | -0.148511038 | 0.508004699367232 | 0.770025256749758 | -1.144200249 | 0.847178172339281 | 0.861990492104469 | 0.318478519424892 | 2.33305407793361 | 0.998074519377826 |
| ebi-fl187-GCST90094697 | ebi-a-GCST90002064 | CD4 on resting CD4 regulatory T cell \|\| id:ebi-a-GCST90002064 | Banana liking \|\| id:ebi-fl187-GCST90094697 | Inverse variance weighted | 2 | 0.233816680247785 | 0.479406966624572 | 0.62574771870591 | -0.705820974 | 1.17345433483195 | 1.26341286244438 | 0.493703090266043 | 3.23314172518092 | 0.998074519377826 |
| ebi-fl187-GCST90094697 | ebi-a-GCST90002080 | SSC-A on T cell \|\| id:ebi-a-GCST90002080 | Banana liking \|\| id:ebi-fl187-GCST90094697 | Inverse variance weighted | 2 | -0.133167198 | 0.350170285116958 | 0.703728163820554 | -0.819500957 | 0.553166560390365 | 0.875318727948354 | 0.440651503574552 | 1.73875016715448 | 0.998074519377826 |
| ebi-fl187-GCST90094697 | ebi-a-GCST90002121 | CD8 on CD39+ CD8+ T cell \|\| id:ebi-a-GCST90002121 | Banana liking \|\| id:ebi-fl187-GCST90094697 | Inverse variance weighted | 2 | -0.26927894 | 0.37201627053122 | 0.469166142616569 | -0.998430831 | 0.459872949910871 | 0.763930135001411 | 0.368457159494321 | 1.5838727410378 | 0.998074519377826 |
| ebi-fl187-GCST90094697 | ebi-cfmet338-GCST90026053 | 1-palmitoyl-2-docosahexaenoyl-gpc (16:0/22:6) levels \|\| id:ebi-cfmet338-GCST90026053 | Banana liking \|\| id:ebi-fl187-GCST90094697 | Inverse variance weighted | 2 | -0.164653 | 0.196545675582709 | 0.402180099000811 | -0.549882524 | 0.220576524070802 | 0.848187974195673 | 0.577017591994551 | 1.24679533094193 | 0.998074519377826 |
| ebi-fl187-GCST90094697 | ebi-cfmet338-GCST90026063 | 1-stearoyl-2-oleoyl-gpc (18:0/18:1) levels \|\| id:ebi-cfmet338-GCST90026063 | Banana liking \|\| id:ebi-fl187-GCST90094697 | Inverse variance weighted | 2 | -0.18341368 | 0.16122068490005 | 0.255264152744037 | -0.499406222 | 0.132578862782643 | 0.832423727767993 | 0.606890911203313 | 1.14176905562362 | 0.998074519377826 |
| ebi-fl187-GCST90094697 | ebi-cfmet338-GCST90026113 | Adenosine 3',5'-cyclic monophosphate (camp) levels \|\| id:ebi-cfmet338-GCST90026113 | Banana liking \|\| id:ebi-fl187-GCST90094697 | Inverse variance weighted | 2 | -0.545131144 | 0.312028432315092 | 0.080627625098044 | -1.156706871 | 0.0664445831986384 | 0.579765745474516 | 0.314520232865126 | 1.06870173840219 | 0.998074519377826 |
| ebi-fl187-GCST90094697 | ebi-cfmet338-GCST90026117 | Alpha-ketoglutaramate levels \|\| id:ebi-cfmet338-GCST90026117 | Banana liking \|\| id:ebi-fl187-GCST90094697 | Inverse variance weighted | 2 | -0.174817433 | 0.194601880714744 | 0.369007656525828 | -0.556237119 | 0.206602253299088 | 0.83961029199226 | 0.573362504553048 | 1.22949344755087 | 0.998074519377826 |
| ebi-fl187-GCST90094697 | ebi-cfmet338-GCST90026124 | Arginine levels \|\| id:ebi-cfmet338-GCST90026124 | Banana liking \|\| id:ebi-fl187-GCST90094697 | Inverse variance weighted | 2 | -0.043619107 | 0.101894470398391 | 0.668592002197331 | -0.243332269 | 0.156094054619386 | 0.957318523616743 | 0.784010967679623 | 1.16893614176357 | 0.998074519377826 |
| ebi-fl187-GCST90094697 | ebi-cfmet338-GCST90026169 | Glutamate levels \|\| id:ebi-cfmet338-GCST90026169 | Banana liking \|\| id:ebi-fl187-GCST90094697 | Inverse variance weighted | 2 | 0.0111689932321576 | 0.106415554043654 | 0.916410406998945 | -0.197405493 | 0.21974347915772 | 1.01123159930222 | 0.820857714013691 | 1.24575712692916 | 0.998074519377826 |
| ebi-fl187-GCST90094697 | ebi-cfmet338-GCST90026170 | Glutamine levels \|\| id:ebi-cfmet338-GCST90026170 | Banana liking \|\| id:ebi-fl187-GCST90094697 | Inverse variance weighted | 2 | -0.021293395 | 0.0638531617957579 | 0.738776209182561 | -0.146445592 | 0.103858802192046 | 0.978931708833955 | 0.863772727161966 | 1.10944379282408 | 0.998074519377826 |
| ebi-fl187-GCST90094697 | ebi-cfmet338-GCST90026191 | Kynurenate levels \|\| id:ebi-cfmet338-GCST90026191 | Banana liking \|\| id:ebi-fl187-GCST90094697 | Inverse variance weighted | 2 | -0.038117474 | 0.706100939576134 | 0.956948678596194 | -1.422075315 | 1.34584036802347 | 0.962599854230964 | 0.241212904307388 | 3.8414133855155 | 0.998074519377826 |
| ebi-fl187-GCST90094697 | ebi-cfmet338-GCST90026202 | Methylsuccinoylcarnitine levels \|\| id:ebi-cfmet338-GCST90026202 | Banana liking \|\| id:ebi-fl187-GCST90094697 | Inverse variance weighted | 2 | -0.720826505 | 0.326067123241545 | 0.0270587617132203 | -1.359918067 | -0.081734943 | 0.486350119017972 | 0.256681806929961 | 0.921516180277388 | 0.998074519377826 |
| ebi-fl187-GCST90094697 | ebi-cfmet338-GCST90026222 | N-acetylputrescine levels \|\| id:ebi-cfmet338-GCST90026222 | Banana liking \|\| id:ebi-fl187-GCST90094697 | Inverse variance weighted | 2 | 0.254588450150266 | 0.117291232778488 | 0.0299639376487112 | 0.0246976339044299 | 0.484479266396103 | 1.2899306408566 | 1.02500514685983 | 1.62332946650878 | 0.998074519377826 |
| ebi-fl187-GCST90094697 | ebi-cfmet338-GCST90026252 | Propionylcarnitine (c3) levels \|\| id:ebi-cfmet338-GCST90026252 | Banana liking \|\| id:ebi-fl187-GCST90094697 | Inverse variance weighted | 2 | -0.392837931 | 0.193827968154481 | 0.0426895188808133 | -0.772740749 | -0.012935114 | 0.675138157592482 | 0.461745803257139 | 0.98714818547801 | 0.998074519377826 |
| ebi-fl187-GCST90094697 | ebi-cfmet338-GCST90026316 | Ethyl beta-glucopyranoside levels \|\| id:ebi-cfmet338-GCST90026316 | Banana liking \|\| id:ebi-fl187-GCST90094697 | Inverse variance weighted | 2 | 0.284760229309031 | 0.633567400621541 | 0.653103243134523 | -0.957031876 | 1.52655233452725 | 1.32944322838211 | 0.384031047841424 | 4.60228230874931 | 0.998074519377826 |
| ebi-fl187-GCST90094697 | ebi-gm430-GCST90011315 | F_Prevotellaceae abundance \|\| id:ebi-gm430-GCST90011315 | Banana liking \|\| id:ebi-fl187-GCST90094697 | Inverse variance weighted | 2 | -0.131768593 | 0.271638884739168 | 0.627614369720664 | -0.664180807 | 0.4006436214988 | 0.876543810344921 | 0.514694989751707 | 1.49278517714859 | 0.998074519377826 |
| ebi-fl187-GCST90094697 | ebi-gm430-GCST90011354 | OTU97_1 (Bacteroides) abundance \|\| id:ebi-gm430-GCST90011354 | Banana liking \|\| id:ebi-fl187-GCST90094697 | Inverse variance weighted | 2 | -0.322199949 | 0.340958705263232 | 0.344667901178733 | -0.990479011 | 0.346079113250061 | 0.724553302089323 | 0.371398744180515 | 1.41351443911555 | 0.998074519377826 |
| ebi-fl187-GCST90094697 | ebi-gm430-GCST90011419 | OTU99_1 (Bacteroides) abundance \|\| id:ebi-gm430-GCST90011419 | Banana liking \|\| id:ebi-fl187-GCST90094697 | Inverse variance weighted | 2 | -0.331958565 | 0.336780775555387 | 0.324289453119231 | -0.992048885 | 0.328131755584021 | 0.71751705291309 | 0.370816152658383 | 1.38837188598787 | 0.998074519377826 |
| ebi-fl187-GCST90094697 | ebi-gm430-GCST90011435 | OTU99_155 (Butyrivibrio) abundance \|\| id:ebi-gm430-GCST90011435 | Banana liking \|\| id:ebi-fl187-GCST90094697 | Inverse variance weighted | 2 | -0.102377599 | 0.782925205539641 | 0.895962843302996 | -1.636911002 | 1.43215580365792 | 0.902688632802188 | 0.194580172665968 | 4.18771736413821 | 0.998074519377826 |
| ebi-fl187-GCST90094697 | ebi-gm430-GCST90011466 | OTU99_58 (Bacteroides) abundance \|\| id:ebi-gm430-GCST90011466 | Banana liking \|\| id:ebi-fl187-GCST90094697 | Inverse variance weighted | 2 | -0.20730903 | 0.85145480240838 | 0.807636474246161 | -1.876160443 | 1.46154238254495 | 0.812768441200015 | 0.153177110379197 | 4.31260608961336 | 0.998074519377826 |
| ebi-fl187-GCST90094697 | ebi-gm430-GCST90011472 | OTU99_72 (Parabacteroides) abundance \|\| id:ebi-gm430-GCST90011472 | Banana liking \|\| id:ebi-fl187-GCST90094697 | Inverse variance weighted | 2 | -0.53937071 | 0.697291090499857 | 0.439212910504219 | -1.906061247 | 0.827319827262727 | 0.583115085363588 | 0.148664788741037 | 2.28718047937282 | 0.998074519377826 |
| ebi-fl187-GCST90094697 | ebi-gm430-GCST90011505 | TestASV_28 (Holdemanella) abundance \|\| id:ebi-gm430-GCST90011505 | Banana liking \|\| id:ebi-fl187-GCST90094697 | Inverse variance weighted | 2 | 0.40668438247001 | 0.580003519370901 | 0.483193363328973 | -0.730122515 | 1.54349128043698 | 1.50183002696854 | 0.481849952387384 | 4.68090412529709 | 0.998074519377826 |
| ebi-fl187-GCST90094697 | ebi-gm430-GCST90011513 | TestASV_35 (Alphaproteobacteria) abundance \|\| id:ebi-gm430-GCST90011513 | Banana liking \|\| id:ebi-fl187-GCST90094697 | Inverse variance weighted | 2 | 1.87686231323167 | 0.690096400863368 | 0.00653391255046738 | 0.524273367539466 | 3.22945125892387 | 6.53297424286894 | 1.68923095227527 | 25.2657887901606 | 0.877504772527479 |
| ebi-fl187-GCST90094697 | ebi-gm430-GCST90011543 | G_Coprococcus prevalence \|\| id:ebi-gm430-GCST90011543 | Banana liking \|\| id:ebi-fl187-GCST90094697 | Inverse variance weighted | 2 | 0.0800866180619845 | 0.727867875066309 | 0.912386328320821 | -1.346534417 | 1.50670765319195 | 1.08338090396523 | 0.260140237840534 | 4.51185173358692 | 0.998074519377826 |
| ebi-fl187-GCST90094697 | ebi-gm430-GCST90011547 | G_Parasutterella prevalence \|\| id:ebi-gm430-GCST90011547 | Banana liking \|\| id:ebi-fl187-GCST90094697 | Inverse variance weighted | 2 | -0.237950767 | 0.893299923518365 | 0.789952141539731 | -1.988818617 | 1.51291708346387 | 0.788241497922108 | 0.136857010562672 | 4.53995492442795 | 0.998074519377826 |
| ebi-fl187-GCST90094697 | ebi-gm430-GCST90011569 | OTU97_13 (Subdoligranulum) prevalence \|\| id:ebi-gm430-GCST90011569 | Banana liking \|\| id:ebi-fl187-GCST90094697 | Inverse variance weighted | 2 | 0.229186614944601 | 0.667953353312262 | 0.731509919631064 | -1.080001958 | 1.53837518743664 | 1.25757669971971 | 0.33959486087124 | 4.65701734007558 | 0.998074519377826 |
| ebi-fl187-GCST90094697 | ebi-gm430-GCST90011590 | OTU97_277 (Prevotella) prevalence \|\| id:ebi-gm430-GCST90011590 | Banana liking \|\| id:ebi-fl187-GCST90094697 | Inverse variance weighted | 2 | -0.229049784 | 0.581264068626464 | 0.693540723803326 | -1.368327358 | 0.910227790625014 | 0.795288939948119 | 0.25453234508615 | 2.4848885032263 | 0.998074519377826 |
| ebi-fl187-GCST90094697 | ebi-gm430-GCST90011616 | OTU97_86 (Phascolarctobacterium) prevalence \|\| id:ebi-gm430-GCST90011616 | Banana liking \|\| id:ebi-fl187-GCST90094697 | Inverse variance weighted | 2 | -0.512672367 | 0.70906782057798 | 0.469665732438871 | -1.902445296 | 0.877100560863342 | 0.598892976287053 | 0.149203326512275 | 2.40391957357905 | 0.998074519377826 |
| ebi-fl187-GCST90094697 | ebi-gm430-GCST90011665 | OTU99_55 (Barnesiella) prevalence \|\| id:ebi-gm430-GCST90011665 | Banana liking \|\| id:ebi-fl187-GCST90094697 | Inverse variance weighted | 2 | -0.566664018 | 0.476849356940027 | 0.234695476312974 | -1.501288758 | 0.367960721300417 | 0.567415171517285 | 0.222842784609332 | 1.44478528857203 | 0.998074519377826 |
| ebi-fl187-GCST90094697 | ebi-gm430-GCST90011687 | TestASV_14 (Lachnospiraceae) prevalence \|\| id:ebi-gm430-GCST90011687 | Banana liking \|\| id:ebi-fl187-GCST90094697 | Inverse variance weighted | 2 | -0.606758742 | 0.582793153639868 | 0.297818955713049 | -1.749033323 | 0.535515839337581 | 0.545114866777904 | 0.173942007955785 | 1.70832923843116 | 0.998074519377826 |
| ebi-fl187-GCST90094697 | ebi-gm430-GCST90011697 | TestASV_23 (Barnesiella) prevalence \|\| id:ebi-gm430-GCST90011697 | Banana liking \|\| id:ebi-fl187-GCST90094697 | Inverse variance weighted | 2 | -0.04086597 | 0.680160696836313 | 0.952089676447721 | -1.373980935 | 1.29224899611419 | 0.959957784770082 | 0.25309738699565 | 3.64096587277892 | 0.998074519377826 |
| ebi-fl187-GCST90094697 | ebi-gm430-GCST90011727 | TestASV_6 (Subdoligranulum) prevalence \|\| id:ebi-gm430-GCST90011727 | Banana liking \|\| id:ebi-fl187-GCST90094697 | Inverse variance weighted | 2 | 0.938028033425521 | 0.774071043624449 | 0.225584623854797 | -0.579151212 | 2.45520727892944 | 2.55493819470952 | 0.560373803280194 | 11.6488478593665 | 0.998074519377826 |
| ebi-fl187-GCST90094697 | ebi-hbsm44-GCST90104908 | Sulcal depth of the left posterior inferior temporal sulcus \|\| id:ebi-hbsm44-GCST90104908 | Banana liking \|\| id:ebi-fl187-GCST90094697 | Inverse variance weighted | 2 | -0.237400035 | 0.234888491818379 | 0.312163632587376 | -0.697781479 | 0.222981409296292 | 0.788675727271692 | 0.497688211859225 | 1.24979733890799 | 0.998074519377826 |
| ebi-fl187-GCST90094697 | ebi-hbsm44-GCST90104918 | Sulcal depth of the left inferior frontal sulcus \|\| id:ebi-hbsm44-GCST90104918 | Banana liking \|\| id:ebi-fl187-GCST90094697 | Inverse variance weighted | 2 | -0.083606108 | 0.384138341740005 | 0.827705073263748 | -0.836517258 | 0.669305041632892 | 0.919793483882833 | 0.433216681062903 | 1.95287967886554 | 0.998074519377826 |
| ebi-fl187-GCST90094697 | ebi-hbsm44-GCST90104925 | Sulcal depth of the right anterior inferior temporal sulcus \|\| id:ebi-hbsm44-GCST90104925 | Banana liking \|\| id:ebi-fl187-GCST90094697 | Inverse variance weighted | 2 | -0.027223408 | 0.130862307908965 | 0.835204817051084 | -0.283713531 | 0.229266715897931 | 0.973143809510778 | 0.75298231982557 | 1.25767743684676 | 0.998074519377826 |
| ebi-fl187-GCST90094697 | ebi-IPs91-GCST90274770 | C-C motif chemokine 4 levels \|\| id:ebi-IPs91-GCST90274770 | Banana liking \|\| id:ebi-fl187-GCST90094697 | Inverse variance weighted | 2 | 0.0312581255139496 | 0.184539697113322 | 0.865494379969841 | -0.330439681 | 0.39295593185606 | 1.03175179097861 | 0.718607705930134 | 1.48135310740889 | 0.998074519377826 |
| ebi-fl187-GCST90094697 | ebi-IPs91-GCST90274779 | C-X-C motif chemokine 1 levels \|\| id:ebi-IPs91-GCST90274779 | Banana liking \|\| id:ebi-fl187-GCST90094697 | Inverse variance weighted | 2 | -0.124043706 | 0.167956744096091 | 0.460182999794813 | -0.453238924 | 0.205151512922975 | 0.883341233164068 | 0.63556626349263 | 1.22771106496414 | 0.998074519377826 |
| ebi-fl187-GCST90094697 | ebi-IPs91-GCST90274780 | C-X-C motif chemokine 10 levels \|\| id:ebi-IPs91-GCST90274780 | Banana liking \|\| id:ebi-fl187-GCST90094697 | Inverse variance weighted | 2 | 0.281027192532473 | 0.166114758402327 | 0.0906907225091293 | -0.044557734 | 0.606612119001035 | 1.32448961967257 | 0.956420380586489 | 1.8342067862927 | 0.998074519377826 |
| ebi-fl187-GCST90094697 | ebi-IPs91-GCST90274796 | Interleukin-10 receptor subunit alpha levels \|\| id:ebi-IPs91-GCST90274796 | Banana liking \|\| id:ebi-fl187-GCST90094697 | Inverse variance weighted | 2 | 0.21569249521771 | 0.190149296623106 | 0.256655171485678 | -0.157000126 | 0.588385116598997 | 1.24072079275662 | 0.85470395098517 | 1.80107753544868 | 0.998074519377826 |
| ebi-fl187-GCST90094697 | ebi-IPs91-GCST90274807 | Interleukin-20 levels \|\| id:ebi-IPs91-GCST90274807 | Banana liking \|\| id:ebi-fl187-GCST90094697 | Inverse variance weighted | 2 | 0.105109239138155 | 0.190149296623106 | 0.580419428112694 | -0.267583382 | 0.477801860519442 | 1.11083195005292 | 0.765226521658715 | 1.61252594667479 | 0.998074519377826 |
| ebi-fl187-GCST90094697 | ebi-IPs91-GCST90274808 | Interleukin-20 receptor subunit alpha levels \|\| id:ebi-IPs91-GCST90274808 | Banana liking \|\| id:ebi-fl187-GCST90094697 | Inverse variance weighted | 2 | 0.192340579061211 | 0.190149296623106 | 0.311765695339992 | -0.180352042 | 0.565033200442499 | 1.21208325685336 | 0.834976212701186 | 1.75950619813646 | 0.998074519377826 |
| ebi-fl187-GCST90094697 | ebi-IPs91-GCST90274810 | Interleukin-24 levels \|\| id:ebi-IPs91-GCST90274810 | Banana liking \|\| id:ebi-fl187-GCST90094697 | Inverse variance weighted | 2 | 0.35764668248865 | 0.190149296623106 | 0.0599891936330324 | -0.015045939 | 0.730339303869938 | 1.42996030120113 | 0.985066685690899 | 2.07578481001727 | 0.998074519377826 |
| ebi-fl187-GCST90094697 | ebi-IPs91-GCST90274823 | Monocyte chemoattractant protein-3 levels \|\| id:ebi-IPs91-GCST90274823 | Banana liking \|\| id:ebi-fl187-GCST90094697 | Inverse variance weighted | 2 | 0.131676302670852 | 0.187435177720093 | 0.482357123289257 | -0.235696646 | 0.499049251002235 | 1.14073900532821 | 0.790020293670476 | 1.6471544955274 | 0.998074519377826 |
| ebi-fl187-GCST90094697 | ebi-IPs91-GCST90274824 | Monocyte chemoattractant protein-4 levels \|\| id:ebi-IPs91-GCST90274824 | Banana liking \|\| id:ebi-fl187-GCST90094697 | Inverse variance weighted | 2 | 0.121568625419406 | 0.166599783450417 | 0.46557077947123 | -0.20496695 | 0.448104200982224 | 1.12926685967797 | 0.814674240833313 | 1.56534179730849 | 0.998074519377826 |
| ebi-fl187-GCST90094697 | ebi-IPs91-GCST90274834 | SIR2-like protein 2 levels \|\| id:ebi-IPs91-GCST90274834 | Banana liking \|\| id:ebi-fl187-GCST90094697 | Inverse variance weighted | 2 | -0.156934875 | 0.166821714496431 | 0.346841140895916 | -0.483905435 | 0.170035685437293 | 0.854759723252828 | 0.616371486086423 | 1.18534715019703 | 0.998074519377826 |
| ebi-fl187-GCST90094697 | ebi-met1400-GCST90199652 | 1-stearoyl-2-oleoyl-GPS (18:0/18:1) levels \|\| id:ebi-met1400-GCST90199652 | Banana liking \|\| id:ebi-fl187-GCST90094697 | Inverse variance weighted | 2 | -0.125814119 | 0.286065279990341 | 0.660075360805017 | -0.686502068 | 0.434873829357186 | 0.88177873709059 | 0.503333620050366 | 1.54476814226571 | 0.998074519377826 |
| ebi-fl187-GCST90094697 | ebi-met1400-GCST90199665 | N-acetylaspartate (naa) levels \|\| id:ebi-met1400-GCST90199665 | Banana liking \|\| id:ebi-fl187-GCST90094697 | Inverse variance weighted | 2 | -0.102753456 | 0.279864345898833 | 0.713503771501534 | -0.651287574 | 0.445780662096667 | 0.902349415015514 | 0.521374036832668 | 1.56170888701189 | 0.998074519377826 |
| ebi-fl187-GCST90094697 | ebi-met1400-GCST90199669 | Acetylcarnitine levels (Biocrates platform) \|\| id:ebi-met1400-GCST90199669 | Banana liking \|\| id:ebi-fl187-GCST90094697 | Inverse variance weighted | 2 | 0.378713702131714 | 0.214079035448377 | 0.0768877255097341 | -0.040881207 | 0.798308611610533 | 1.4604048650859 | 0.959943157369147 | 2.22177985601953 | 0.998074519377826 |
| ebi-fl187-GCST90094697 | ebi-met1400-GCST90199670 | Iminodiacetate (IDA) levels \|\| id:ebi-met1400-GCST90199670 | Banana liking \|\| id:ebi-fl187-GCST90094697 | Inverse variance weighted | 2 | -0.035244601 | 0.220214144606391 | 0.872844094243668 | -0.466864324 | 0.396375122391071 | 0.965369257054548 | 0.626965148473428 | 1.48642680495908 | 0.998074519377826 |
| ebi-fl187-GCST90094697 | ebi-met1400-GCST90199686 | Indolepropionate levels \|\| id:ebi-met1400-GCST90199686 | Banana liking \|\| id:ebi-fl187-GCST90094697 | Inverse variance weighted | 2 | -0.013168066 | 0.222078260050118 | 0.952717374483004 | -0.448441456 | 0.422105323663229 | 0.986918253643403 | 0.638622698142883 | 1.52516915262637 | 0.998074519377826 |
| ebi-fl187-GCST90094697 | ebi-met1400-GCST90199704 | 3-hydroxysebacate levels \|\| id:ebi-met1400-GCST90199704 | Banana liking \|\| id:ebi-fl187-GCST90094697 | Inverse variance weighted | 2 | 0.226028800900808 | 0.226792827506237 | 0.318943570725219 | -0.218485141 | 0.670542742813033 | 1.2536117699065 | 0.803735422051389 | 1.95529825677836 | 0.998074519377826 |
| ebi-fl187-GCST90094697 | ebi-met1400-GCST90199713 | Docosapentaenoate n3 DPA; 22:5n3 levels \|\| id:ebi-met1400-GCST90199713 | Banana liking \|\| id:ebi-fl187-GCST90094697 | Inverse variance weighted | 2 | 0.219614112881142 | 0.211364812088647 | 0.298791373455763 | -0.194660919 | 0.63388914457489 | 1.24559597839194 | 0.823113713117389 | 1.88492709653705 | 0.998074519377826 |
| ebi-fl187-GCST90094697 | ebi-met1400-GCST90199715 | Myristoleate (14:1n5) levels \|\| id:ebi-met1400-GCST90199715 | Banana liking \|\| id:ebi-fl187-GCST90094697 | Inverse variance weighted | 2 | 0.0370198812532267 | 0.202447240577079 | 0.85490640067753 | -0.35977671 | 0.433816472784301 | 1.03771365168096 | 0.697832127417804 | 1.54313563474028 | 0.998074519377826 |
| ebi-fl187-GCST90094697 | ebi-met1400-GCST90199725 | Campesterol levels \|\| id:ebi-met1400-GCST90199725 | Banana liking \|\| id:ebi-fl187-GCST90094697 | Inverse variance weighted | 2 | 0.507879371346872 | 0.29654205918021 | 0.0867728433912223 | -0.073343065 | 1.08910180734008 | 1.66176347253872 | 0.929281971543467 | 2.97160380081127 | 0.998074519377826 |
| ebi-fl187-GCST90094697 | ebi-met1400-GCST90199737 | N-acetylproline levels \|\| id:ebi-met1400-GCST90199737 | Banana liking \|\| id:ebi-fl187-GCST90094697 | Inverse variance weighted | 2 | 0.0035935949238185 | 0.23787500028245 | 0.987946758404459 | -0.462641406 | 0.469828595477421 | 1.00360005962758 | 0.629618369634448 | 1.59971997047872 | 0.999355215041207 |
| ebi-fl187-GCST90094697 | ebi-met1400-GCST90199775 | Gamma-glutamylisoleucine levels \|\| id:ebi-met1400-GCST90199775 | Banana liking \|\| id:ebi-fl187-GCST90094697 | Inverse variance weighted | 2 | 0.177395810184592 | 0.346225821429995 | 0.608391908964594 | -0.5012068 | 0.855998420187381 | 1.19410363798331 | 0.605799140110402 | 2.35372321259009 | 0.998074519377826 |
| ebi-fl187-GCST90094697 | ebi-met1400-GCST90199777 | 1-palmitoleoyl-GPC (16:1) levels \|\| id:ebi-met1400-GCST90199777 | Banana liking \|\| id:ebi-fl187-GCST90094697 | Inverse variance weighted | 2 | 0.365081532531216 | 0.362837879410583 | 0.314327247995132 | -0.346080711 | 1.07624377617596 | 1.44063146169063 | 0.70745538529024 | 2.933639423 | 0.998074519377826 |
| ebi-fl187-GCST90094697 | ebi-met1400-GCST90199782 | Glutamine degradant levels \|\| id:ebi-met1400-GCST90199782 | Banana liking \|\| id:ebi-fl187-GCST90094697 | Inverse variance weighted | 2 | -0.27119375 | 0.183930819306159 | 0.14036492754616 | -0.631698156 | 0.0893106559911055 | 0.762468753887075 | 0.531688144701788 | 1.09342028113149 | 0.998074519377826 |
| ebi-fl187-GCST90094697 | ebi-met1400-GCST90199783 | 5-methyluridine (ribothymidine) levels \|\| id:ebi-met1400-GCST90199783 | Banana liking \|\| id:ebi-fl187-GCST90094697 | Inverse variance weighted | 2 | 0.161951931708777 | 0.219670413506634 | 0.460970555501857 | -0.268602079 | 0.592505942181781 | 1.17580372108691 | 0.764447384982542 | 1.80851477509259 | 0.998074519377826 |
| ebi-fl187-GCST90094697 | ebi-met1400-GCST90199787 | Homostachydrine levels \|\| id:ebi-met1400-GCST90199787 | Banana liking \|\| id:ebi-fl187-GCST90094697 | Inverse variance weighted | 2 | -0.369950803 | 0.214822087669782 | 0.0850466118851131 | -0.791002094 | 0.0511004893321736 | 0.690768313875111 | 0.453390227782571 | 1.05242864582139 | 0.998074519377826 |
| ebi-fl187-GCST90094697 | ebi-met1400-GCST90199802 | Beta-hydroxyisovaleroylcarnitine levels \|\| id:ebi-met1400-GCST90199802 | Banana liking \|\| id:ebi-fl187-GCST90094697 | Inverse variance weighted | 2 | 0.19912415200231 | 0.217162836093015 | 0.359176830245894 | -0.226515007 | 0.624763310744619 | 1.22033346333828 | 0.7973073772387 | 1.86780381601481 | 0.998074519377826 |
| ebi-fl187-GCST90094697 | ebi-met1400-GCST90199873 | Pregnenolone sulfate levels \|\| id:ebi-met1400-GCST90199873 | Banana liking \|\| id:ebi-fl187-GCST90094697 | Inverse variance weighted | 2 | 0.292381956745895 | 0.210216549555179 | 0.164267644022785 | -0.11964248 | 0.704406393874046 | 1.33961459476428 | 0.887237584862625 | 2.02264567363128 | 0.998074519377826 |
| ebi-fl187-GCST90094697 | ebi-met1400-GCST90199911 | Beta-citrylglutamate levels \|\| id:ebi-met1400-GCST90199911 | Banana liking \|\| id:ebi-fl187-GCST90094697 | Inverse variance weighted | 2 | 0.0414305483567622 | 0.218531149268718 | 0.849633193232251 | -0.386890504 | 0.46975160092345 | 1.04230076983642 | 0.67916545661368 | 1.59959680549469 | 0.998074519377826 |
| ebi-fl187-GCST90094697 | ebi-met1400-GCST90199934 | 2-hydroxydecanoate levels \|\| id:ebi-met1400-GCST90199934 | Banana liking \|\| id:ebi-fl187-GCST90094697 | Inverse variance weighted | 2 | -0.134311329 | 0.2193252541826 | 0.540283567507861 | -0.564188827 | 0.295566169453888 | 0.874317821957678 | 0.568821372237436 | 1.34388701111204 | 0.998074519377826 |
| ebi-fl187-GCST90094697 | ebi-met1400-GCST90199945 | Sphingomyelin (d18:1/14:0, d16:1/16:0) levels \|\| id:ebi-met1400-GCST90199945 | Banana liking \|\| id:ebi-fl187-GCST90094697 | Inverse variance weighted | 2 | -0.006388913 | 0.210724573615981 | 0.975812815295656 | -0.419409077 | 0.406631251272117 | 0.993631452694888 | 0.657435198433757 | 1.50175023505991 | 0.998074519377826 |
| ebi-fl187-GCST90094697 | ebi-met1400-GCST90199979 | Carnitine C14:1 levels \|\| id:ebi-met1400-GCST90199979 | Banana liking \|\| id:ebi-fl187-GCST90094697 | Inverse variance weighted | 2 | 0.190929226692755 | 0.212831675694315 | 0.369670747072264 | -0.226220858 | 0.608079311053611 | 1.21037378689403 | 0.797541938960096 | 1.83689989508313 | 0.998074519377826 |
| ebi-fl187-GCST90094697 | ebi-met1400-GCST90199986 | Eugenol sulfate levels \|\| id:ebi-met1400-GCST90199986 | Banana liking \|\| id:ebi-fl187-GCST90094697 | Inverse variance weighted | 2 | 0.108460054806059 | 0.237230496899137 | 0.647532534579374 | -0.356511719 | 0.573431828728368 | 1.11456038631833 | 0.700114266701065 | 1.77434586585919 | 0.998074519377826 |
| ebi-fl187-GCST90094697 | ebi-met1400-GCST90200002 | 6-hydroxyindole sulfate levels \|\| id:ebi-met1400-GCST90200002 | Banana liking \|\| id:ebi-fl187-GCST90094697 | Inverse variance weighted | 2 | -0.13772951 | 0.297818855455522 | 0.643750993325884 | -0.721454467 | 0.445995446338453 | 0.871334346791926 | 0.486044805449974 | 1.56204435349624 | 0.998074519377826 |
| ebi-fl187-GCST90094697 | ebi-met1400-GCST90200023 | Trans 3,4-methyleneheptanoate levels \|\| id:ebi-met1400-GCST90200023 | Banana liking \|\| id:ebi-fl187-GCST90094697 | Inverse variance weighted | 2 | -0.002188004 | 0.237736603617159 | 0.992656790943211 | -0.468151747 | 0.463775739571404 | 0.997814388416632 | 0.626158499019698 | 1.59006634149341 | 0.999355215041207 |
| ebi-fl187-GCST90094697 | ebi-met1400-GCST90200032 | 2-hydroxybutyrate/2-hydroxyisobutyrate levels \|\| id:ebi-met1400-GCST90200032 | Banana liking \|\| id:ebi-fl187-GCST90094697 | Inverse variance weighted | 2 | 0.121724683239787 | 0.222130109503611 | 0.583699991727867 | -0.313650331 | 0.557099697866863 | 1.12944310435455 | 0.730774512399366 | 1.74560237710767 | 0.998074519377826 |
| ebi-fl187-GCST90094697 | ebi-met1400-GCST90200051 | 1-palmitoyl-2-dihomo-linolenoyl-GPC (16:0/20:3n3 or 6) levels \|\| id:ebi-met1400-GCST90200051 | Banana liking \|\| id:ebi-fl187-GCST90094697 | Inverse variance weighted | 2 | 0.44137615918202 | 0.216173723241685 | 0.0411747479274858 | 0.0176756616283184 | 0.865076656735722 | 1.55484546175116 | 1.01783280061516 | 2.37518815316923 | 0.998074519377826 |
| ebi-fl187-GCST90094697 | ebi-met1400-GCST90200069 | Phenylacetylglutamate levels \|\| id:ebi-met1400-GCST90200069 | Banana liking \|\| id:ebi-fl187-GCST90094697 | Inverse variance weighted | 2 | -0.307981908 | 0.240864809569458 | 0.201020159165056 | -0.780076935 | 0.164113118276619 | 0.734928613864868 | 0.458370745087343 | 1.17834760020388 | 0.998074519377826 |
| ebi-fl187-GCST90094697 | ebi-met1400-GCST90200072 | Thioproline levels \|\| id:ebi-met1400-GCST90200072 | Banana liking \|\| id:ebi-fl187-GCST90094697 | Inverse variance weighted | 2 | 0.242512744802298 | 0.219561650009807 | 0.269362674309515 | -0.187828089 | 0.672853578821519 | 1.27444749164123 | 0.828757167280728 | 1.95982185503134 | 0.998074519377826 |
| ebi-fl187-GCST90094697 | ebi-met1400-GCST90200081 | 1-oleoyl-2-docosahexaenoyl-GPC (18:1/22:6) levels \|\| id:ebi-met1400-GCST90200081 | Banana liking \|\| id:ebi-fl187-GCST90094697 | Inverse variance weighted | 2 | 0.248142254866458 | 0.281419524035455 | 0.377910882617867 | -0.303440012 | 0.79972452197595 | 1.28164223907226 | 0.738274175215271 | 2.22492792531337 | 0.998074519377826 |
| ebi-fl187-GCST90094697 | ebi-met1400-GCST90200097 | Furaneol sulfate levels \|\| id:ebi-met1400-GCST90200097 | Banana liking \|\| id:ebi-fl187-GCST90094697 | Inverse variance weighted | 2 | 0.618332823453047 | 0.260032961496597 | 0.0174114664061826 | 0.108668218919717 | 1.12799742798638 | 1.85583146260189 | 1.11479242194318 | 3.08946342815974 | 0.998074519377826 |
| ebi-fl187-GCST90094697 | ebi-met1400-GCST90200124 | Sphingomyelin (d18:2/24:2) levels \|\| id:ebi-met1400-GCST90200124 | Banana liking \|\| id:ebi-fl187-GCST90094697 | Inverse variance weighted | 2 | -0.141320792 | 0.200799291960917 | 0.481562759651171 | -0.534887405 | 0.252245819826626 | 0.868210751594802 | 0.585735237490159 | 1.28691234697567 | 0.998074519377826 |
| ebi-fl187-GCST90094697 | ebi-met1400-GCST90200137 | Lignoceroylcarnitine (C24) levels \|\| id:ebi-met1400-GCST90200137 | Banana liking \|\| id:ebi-fl187-GCST90094697 | Inverse variance weighted | 2 | 0.309141359023013 | 0.221090524848958 | 0.162035925837521 | -0.12419607 | 0.742478787726971 | 1.36225492391866 | 0.883206653858112 | 2.10113733816906 | 0.998074519377826 |
| ebi-fl187-GCST90094697 | ebi-met1400-GCST90200165 | Octadecenedioate (C18:1-DC) levels \|\| id:ebi-met1400-GCST90200165 | Banana liking \|\| id:ebi-fl187-GCST90094697 | Inverse variance weighted | 2 | 0.472122797380413 | 0.215778026377768 | 0.0286694417441918 | 0.0491978656799886 | 0.895047729080838 | 1.60339426425354 | 1.05042817386372 | 2.44745260133768 | 0.998074519377826 |
| ebi-fl187-GCST90094697 | ebi-met1400-GCST90200176 | Glutamine conjugate of C6H10O2 (1) levels \|\| id:ebi-met1400-GCST90200176 | Banana liking \|\| id:ebi-fl187-GCST90094697 | Inverse variance weighted | 2 | -0.074519149 | 0.216299197624926 | 0.730456130021308 | -0.498465576 | 0.349427278694504 | 0.928189700308782 | 0.607462049307073 | 1.41825505106377 | 0.998074519377826 |
| ebi-fl187-GCST90094697 | ebi-met1400-GCST90200182 | Glycine conjugate of C10H12O2 levels \|\| id:ebi-met1400-GCST90200182 | Banana liking \|\| id:ebi-fl187-GCST90094697 | Inverse variance weighted | 2 | 0.0497535690724152 | 0.252330620299405 | 0.843689728396343 | -0.444814447 | 0.544321584859249 | 1.05101206258288 | 0.640943194114696 | 1.72343877872124 | 0.998074519377826 |
| ebi-fl187-GCST90094697 | ebi-met1400-GCST90200208 | Glucuronide of piperine metabolite C17H21NO3 (4) levels \|\| id:ebi-met1400-GCST90200208 | Banana liking \|\| id:ebi-fl187-GCST90094697 | Inverse variance weighted | 2 | 0.214427219125414 | 0.312556494739032 | 0.492685945135628 | -0.398183511 | 0.827037948813917 | 1.23915193113119 | 0.671538781893904 | 2.2865358633431 | 0.998074519377826 |
| ebi-fl187-GCST90094697 | ebi-met1400-GCST90200227 | 3-ethylcatechol sulfate (2) levels \|\| id:ebi-met1400-GCST90200227 | Banana liking \|\| id:ebi-fl187-GCST90094697 | Inverse variance weighted | 2 | -0.257995862 | 0.340321626476621 | 0.448394573450522 | -0.925026249 | 0.409034526383556 | 0.772598429490706 | 0.396521010497868 | 1.50536369435262 | 0.998074519377826 |
| ebi-fl187-GCST90094697 | ebi-met1400-GCST90200259 | Pentose acid levels \|\| id:ebi-met1400-GCST90200259 | Banana liking \|\| id:ebi-fl187-GCST90094697 | Inverse variance weighted | 2 | 0.483253989620707 | 0.217000947376734 | 0.0259494667860568 | 0.0579321327623079 | 0.908575846479106 | 1.62134165667195 | 1.05964307822124 | 2.48078699487424 | 0.998074519377826 |
| ebi-fl187-GCST90094697 | ebi-met1400-GCST90200291 | Sarcosine levels \|\| id:ebi-met1400-GCST90200291 | Banana liking \|\| id:ebi-fl187-GCST90094697 | Inverse variance weighted | 2 | 0.031623142675132 | 0.226093868659864 | 0.888764825096807 | -0.41152084 | 0.474767125248466 | 1.03212846683079 | 0.662641711464778 | 1.60763977517752 | 0.998074519377826 |
| ebi-fl187-GCST90094697 | ebi-met1400-GCST90200306 | Butyrate/isobutyrate (4:0) levels \|\| id:ebi-met1400-GCST90200306 | Banana liking \|\| id:ebi-fl187-GCST90094697 | Inverse variance weighted | 2 | -0.21569707 | 0.264011545668891 | 0.4139292124423 | -0.7331597 | 0.301765559076935 | 0.80597941989213 | 0.48038870536728 | 1.35224416817419 | 0.998074519377826 |
| ebi-fl187-GCST90094697 | ebi-met1400-GCST90200321 | Cholate levels \|\| id:ebi-met1400-GCST90200321 | Banana liking \|\| id:ebi-fl187-GCST90094697 | Inverse variance weighted | 2 | 0.16125221834044 | 0.233502532860714 | 0.489829414098341 | -0.296412746 | 0.61891718274744 | 1.17498128327365 | 0.74348049603175 | 1.85691625188837 | 0.998074519377826 |
| ebi-fl187-GCST90094697 | ebi-met1400-GCST90200336 | Ceramide (d18:1/16:0) levels \|\| id:ebi-met1400-GCST90200336 | Banana liking \|\| id:ebi-fl187-GCST90094697 | Inverse variance weighted | 2 | 0.161269827280968 | 0.34146671485674 | 0.63672291604968 | -0.508004934 | 0.830544588400178 | 1.17500197363135 | 0.601694803089956 | 2.29456799518204 | 0.998074519377826 |
| ebi-fl187-GCST90094697 | ebi-met1400-GCST90200358 | Arachidonate (20:4n6) levels \|\| id:ebi-met1400-GCST90200358 | Banana liking \|\| id:ebi-fl187-GCST90094697 | Inverse variance weighted | 2 | 0.314078334395703 | 0.216572345556739 | 0.146996165986352 | -0.110403463 | 0.73856013168691 | 1.36899697189773 | 0.895472772365579 | 2.09291981498686 | 0.998074519377826 |
| ebi-fl187-GCST90094697 | ebi-met1400-GCST90200413 | 2'-deoxyuridine levels \|\| id:ebi-met1400-GCST90200413 | Banana liking \|\| id:ebi-fl187-GCST90094697 | Inverse variance weighted | 2 | 0.238974586724222 | 0.219691546791265 | 0.276695223652539 | -0.191620845 | 0.669570018435102 | 1.26994626267915 | 0.825619847058363 | 1.95339721524249 | 0.998074519377826 |
| ebi-fl187-GCST90094697 | ebi-met1400-GCST90200432 | Threonine levels \|\| id:ebi-met1400-GCST90200432 | Banana liking \|\| id:ebi-fl187-GCST90094697 | Inverse variance weighted | 2 | -0.17361635 | 0.227240281211168 | 0.444854719806591 | -0.619007301 | 0.271774601058945 | 0.840619339315028 | 0.538478719491153 | 1.31229117893125 | 0.998074519377826 |
| ebi-fl187-GCST90094697 | ebi-met1400-GCST90200436 | Caffeine levels \|\| id:ebi-met1400-GCST90200436 | Banana liking \|\| id:ebi-fl187-GCST90094697 | Inverse variance weighted | 2 | 0.0616331131606653 | 0.225251971365956 | 0.784377792799324 | -0.379860751 | 0.503126977037939 | 1.06357206254528 | 0.683956643053996 | 1.65388485325015 | 0.998074519377826 |
| ebi-fl187-GCST90094697 | ebi-met1400-GCST90200442 | Valine levels \|\| id:ebi-met1400-GCST90200442 | Banana liking \|\| id:ebi-fl187-GCST90094697 | Inverse variance weighted | 2 | 0.00327672208447056 | 0.216030167698639 | 0.987898237807098 | -0.420142407 | 0.426695850773803 | 1.00328209640673 | 0.656953258670232 | 1.5321865774861 | 0.999355215041207 |
| ebi-fl187-GCST90094697 | ebi-met1400-GCST90200459 | Margarate (17:0) levels \|\| id:ebi-met1400-GCST90200459 | Banana liking \|\| id:ebi-fl187-GCST90094697 | Inverse variance weighted | 2 | 0.164793549616711 | 0.21596795832233 | 0.445435789161647 | -0.258503649 | 0.588090747928479 | 1.17914965768216 | 0.772206213499051 | 1.80054743267572 | 0.998074519377826 |
| ebi-fl187-GCST90094697 | ebi-met1400-GCST90200462 | X-11299 levels \|\| id:ebi-met1400-GCST90200462 | Banana liking \|\| id:ebi-fl187-GCST90094697 | Inverse variance weighted | 2 | -0.136267574 | 0.222024059731928 | 0.539379627479791 | -0.571434731 | 0.298899583088447 | 0.872609113749443 | 0.564714643562579 | 1.34837421709998 | 0.998074519377826 |
| ebi-fl187-GCST90094697 | ebi-met1400-GCST90200497 | X-12740 levels \|\| id:ebi-met1400-GCST90200497 | Banana liking \|\| id:ebi-fl187-GCST90094697 | Inverse variance weighted | 2 | 0.0704001554954563 | 0.290332253988055 | 0.80840721000665 | -0.498651062 | 0.639451373312044 | 1.0729374371757 | 0.607349383852681 | 1.89544070464125 | 0.998074519377826 |
| ebi-fl187-GCST90094697 | ebi-met1400-GCST90200547 | X-17654 levels \|\| id:ebi-met1400-GCST90200547 | Banana liking \|\| id:ebi-fl187-GCST90094697 | Inverse variance weighted | 2 | -0.067980333 | 0.223311781714047 | 0.76080875008698 | -0.505671425 | 0.369710759220363 | 0.934278847915464 | 0.603100502639729 | 1.44731593132774 | 0.998074519377826 |
| ebi-fl187-GCST90094697 | ebi-met1400-GCST90200553 | X-17653 levels \|\| id:ebi-met1400-GCST90200553 | Banana liking \|\| id:ebi-fl187-GCST90094697 | Inverse variance weighted | 2 | -0.112850613 | 0.221770427627017 | 0.610848736657523 | -0.547520651 | 0.321819424988597 | 0.893284095036919 | 0.578382044908166 | 1.37963562574392 | 0.998074519377826 |
| ebi-fl187-GCST90094697 | ebi-met1400-GCST90200557 | X-17685 levels \|\| id:ebi-met1400-GCST90200557 | Banana liking \|\| id:ebi-fl187-GCST90094697 | Inverse variance weighted | 2 | -0.005210583 | 0.233766163105605 | 0.982216847458945 | -0.463392262 | 0.452971096885503 | 0.994802968737726 | 0.629145793803857 | 1.57297872187304 | 0.999355215041207 |
| ebi-fl187-GCST90094697 | ebi-met1400-GCST90200561 | X-18779 levels \|\| id:ebi-met1400-GCST90200561 | Banana liking \|\| id:ebi-fl187-GCST90094697 | Inverse variance weighted | 2 | -0.051054327 | 0.219592809126553 | 0.816153226109071 | -0.481456233 | 0.379347578419255 | 0.950227045699949 | 0.61788295457283 | 1.4613308745568 | 0.998074519377826 |
| ebi-fl187-GCST90094697 | ebi-met1400-GCST90200570 | X-18921 levels \|\| id:ebi-met1400-GCST90200570 | Banana liking \|\| id:ebi-fl187-GCST90094697 | Inverse variance weighted | 2 | -0.29767284 | 0.240322952351652 | 0.215480053062252 | -0.768705827 | 0.173360146703043 | 0.74254423085369 | 0.463612675461393 | 1.18929434840272 | 0.998074519377826 |
| ebi-fl187-GCST90094697 | ebi-met1400-GCST90200575 | X-21283 levels \|\| id:ebi-met1400-GCST90200575 | Banana liking \|\| id:ebi-fl187-GCST90094697 | Inverse variance weighted | 2 | 0.0362825727843784 | 0.234258938944914 | 0.876914075751702 | -0.422864948 | 0.49543009311641 | 1.03694881861093 | 0.655167109062001 | 1.64120395780865 | 0.998074519377826 |
| ebi-fl187-GCST90094697 | ebi-met1400-GCST90200578 | X-18922 levels \|\| id:ebi-met1400-GCST90200578 | Banana liking \|\| id:ebi-fl187-GCST90094697 | Inverse variance weighted | 2 | 0.314724572046904 | 0.221319643754181 | 0.155015707052109 | -0.11906193 | 0.748511073805098 | 1.36988195520932 | 0.887752820783632 | 2.11385030525909 | 0.998074519377826 |
| ebi-fl187-GCST90094697 | ebi-met1400-GCST90200623 | X-24241 levels \|\| id:ebi-met1400-GCST90200623 | Banana liking \|\| id:ebi-fl187-GCST90094697 | Inverse variance weighted | 2 | -0.133167532 | 0.222890806857299 | 0.550202729854426 | -0.570033513 | 0.303698449651396 | 0.875318436160872 | 0.565506486433461 | 1.35486043584624 | 0.998074519377826 |
| ebi-fl187-GCST90094697 | ebi-met1400-GCST90200636 | X-24544 levels \|\| id:ebi-met1400-GCST90200636 | Banana liking \|\| id:ebi-fl187-GCST90094697 | Inverse variance weighted | 2 | -0.1810151 | 0.226486883982837 | 0.424157090062998 | -0.624929393 | 0.262899192241789 | 0.834422758512796 | 0.535299223072497 | 1.30069559213578 | 0.998074519377826 |
| ebi-fl187-GCST90094697 | ebi-met1400-GCST90200659 | X-25172 levels \|\| id:ebi-met1400-GCST90200659 | Banana liking \|\| id:ebi-fl187-GCST90094697 | Inverse variance weighted | 2 | 0.231044279492489 | 0.223043421261617 | 0.300262186969232 | -0.206120826 | 0.668209385165258 | 1.25991502661207 | 0.81373474988125 | 1.95074116536689 | 0.998074519377826 |
| ebi-fl187-GCST90094697 | ebi-met1400-GCST90200670 | X-26111 levels \|\| id:ebi-met1400-GCST90200670 | Banana liking \|\| id:ebi-fl187-GCST90094697 | Inverse variance weighted | 2 | -0.178989829 | 0.221942881893019 | 0.419973058659227 | -0.613997878 | 0.256018219011801 | 0.836114403056569 | 0.541182954999434 | 1.29177626261229 | 0.998074519377826 |
| ebi-fl187-GCST90094697 | ebi-met1400-GCST90200673 | Carnitine C4 levels \|\| id:ebi-met1400-GCST90200673 | Banana liking \|\| id:ebi-fl187-GCST90094697 | Inverse variance weighted | 2 | 0.159177824153758 | 0.220467896534042 | 0.4702944695579 | -0.272939253 | 0.591294901360482 | 1.1725464352206 | 0.761139023096511 | 1.80632591554069 | 0.998074519377826 |
| ebi-fl187-GCST90094697 | ebi-met1400-GCST90200710 | X-13431 levels \|\| id:ebi-met1400-GCST90200710 | Banana liking \|\| id:ebi-fl187-GCST90094697 | Inverse variance weighted | 2 | -0.106417487 | 0.257994101891515 | 0.679987410066677 | -0.612085927 | 0.399250952683224 | 0.899049228328045 | 0.542218660234976 | 1.49070766876037 | 0.998074519377826 |
| ebi-fl187-GCST90094697 | ebi-met1400-GCST90200730 | Adenosine 5'-diphosphate (ADP) to fructose ratio \|\| id:ebi-met1400-GCST90200730 | Banana liking \|\| id:ebi-fl187-GCST90094697 | Inverse variance weighted | 2 | -0.323380191 | 0.295337064218063 | 0.273537247997824 | -0.902240837 | 0.2554804550067 | 0.7236986584419 | 0.405659623516617 | 1.2910817785866 | 0.998074519377826 |
| ebi-fl187-GCST90094697 | ebi-met1400-GCST90200731 | Adenosine 5'-diphosphate (ADP) to mannose ratio \|\| id:ebi-met1400-GCST90200731 | Banana liking \|\| id:ebi-fl187-GCST90094697 | Inverse variance weighted | 2 | -0.086577934 | 0.515091310825611 | 0.866518225048887 | -1.096156904 | 0.923001034830621 | 0.917064075172549 | 0.334152800662991 | 2.51683216870679 | 0.998074519377826 |
| ebi-fl187-GCST90094697 | ebi-met1400-GCST90200748 | Aspartate to glutamate ratio \|\| id:ebi-met1400-GCST90200748 | Banana liking \|\| id:ebi-fl187-GCST90094697 | Inverse variance weighted | 2 | 0.263486345408189 | 0.213477489293395 | 0.217106699995434 | -0.154929534 | 0.681902224423244 | 1.30145952392176 | 0.856475528098517 | 1.97763606412327 | 0.998074519377826 |
| ebi-fl187-GCST90094697 | ebi-met1400-GCST90200774 | Uridine to pseudouridine ratio \|\| id:ebi-met1400-GCST90200774 | Banana liking \|\| id:ebi-fl187-GCST90094697 | Inverse variance weighted | 2 | 0.0754535103891302 | 0.210162296340207 | 0.719576415775566 | -0.33646459 | 0.487371611215936 | 1.07837309340721 | 0.714291175866264 | 1.62803149174332 | 0.998074519377826 |
| ebi-fl187-GCST90094697 | ebi-met1400-GCST90200799 | Spermidine to adenosine 5'-diphosphate (ADP) ratio \|\| id:ebi-met1400-GCST90200799 | Banana liking \|\| id:ebi-fl187-GCST90094697 | Inverse variance weighted | 2 | -0.050258885 | 0.306366140905578 | 0.869693018293102 | -0.650736521 | 0.550218751640162 | 0.950983197787211 | 0.521661420795458 | 1.73363221128095 | 0.998074519377826 |
| ebi-fl187-GCST90094697 | ebi-met1400-GCST90200811 | Choline to taurocholate ratio \|\| id:ebi-met1400-GCST90200811 | Banana liking \|\| id:ebi-fl187-GCST90094697 | Inverse variance weighted | 2 | 0.239464741110199 | 0.341780866779188 | 0.483528814638741 | -0.430425758 | 0.909355239997407 | 1.27056888498782 | 0.650232194367646 | 2.48272125785643 | 0.998074519377826 |
| ebi-fl187-GCST90094697 | ebi-met1400-GCST90200812 | Glutamate to kynurenine ratio \|\| id:ebi-met1400-GCST90200812 | Banana liking \|\| id:ebi-fl187-GCST90094697 | Inverse variance weighted | 2 | 0.0615334329609241 | 0.213887292680693 | 0.773583337699348 | -0.357685661 | 0.480752526615082 | 1.06346605075337 | 0.699292855693347 | 1.61729099889578 | 0.998074519377826 |
| ebi-fl187-GCST90094697 | ebi-met1400-GCST90200876 | Cortisone to 4-cholesten-3-one ratio \|\| id:ebi-met1400-GCST90200876 | Banana liking \|\| id:ebi-fl187-GCST90094697 | Inverse variance weighted | 2 | 0.0613960887953466 | 0.290259454338272 | 0.83248042805124 | -0.507512442 | 0.630304619298359 | 1.06331999992586 | 0.601991206027553 | 1.87818262280492 | 0.998074519377826 |
| ebi-fl187-GCST90094697 | ebi-met1400-GCST90200906 | 2'-deoxyuridine to cytidine ratio \|\| id:ebi-met1400-GCST90200906 | Banana liking \|\| id:ebi-fl187-GCST90094697 | Inverse variance weighted | 2 | 0.182191393474715 | 0.377468470303373 | 0.629332538033854 | -0.557646808 | 0.922029595269326 | 1.19984381418196 | 0.572554811045009 | 2.51438840554526 | 0.998074519377826 |
| ebi-fl187-GCST90094697 | ebi-met1400-GCST90200907 | Retinol (Vitamin A) to linoleoyl-arachidonoyl-glycerol (18:2 to 20:4) [1] ratio \|\| id:ebi-met1400-GCST90200907 | Banana liking \|\| id:ebi-fl187-GCST90094697 | Inverse variance weighted | 2 | 0.485039672496565 | 0.347328047105804 | 0.162567398816676 | -0.1957233 | 1.16580264482394 | 1.62423944519986 | 0.822239717073354 | 3.20849713357714 | 0.998074519377826 |
| ebi-fl187-GCST90094697 | ebi-met1400-GCST90200916 | Fructose to sucrose ratio \|\| id:ebi-met1400-GCST90200916 | Banana liking \|\| id:ebi-fl187-GCST90094697 | Inverse variance weighted | 2 | -0.289510454 | 0.22641436360771 | 0.201011421991379 | -0.733282606 | 0.154261698893075 | 0.74862996685848 | 0.480329666099157 | 1.1667961961001 | 0.998074519377826 |
| ebi-fl187-GCST90094697 | ebi-met1400-GCST90200917 | Alpha-ketobutyrate to 3-methyl-2-oxobutyrate ratio \|\| id:ebi-met1400-GCST90200917 | Banana liking \|\| id:ebi-fl187-GCST90094697 | Inverse variance weighted | 2 | -0.17023682 | 0.22131499928881 | 0.441771043259417 | -0.604014219 | 0.263540578537547 | 0.843465043492783 | 0.546613002026942 | 1.30153010805846 | 0.998074519377826 |
| ebi-fl187-GCST90094697 | ebi-met1400-GCST90200924 | Alpha-tocopherol to glycerol ratio \|\| id:ebi-met1400-GCST90200924 | Banana liking \|\| id:ebi-fl187-GCST90094697 | Inverse variance weighted | 2 | -0.046097161 | 0.205476540480113 | 0.822490657134741 | -0.44883118 | 0.35663685854515 | 0.954949174061334 | 0.638373859785026 | 1.42851702190237 | 0.998074519377826 |
| ebi-fl187-GCST90094697 | ebi-met1400-GCST90200938 | Alpha-ketoglutarate to alanine ratio \|\| id:ebi-met1400-GCST90200938 | Banana liking \|\| id:ebi-fl187-GCST90094697 | Inverse variance weighted | 2 | -0.172244495 | 0.218764439554586 | 0.43107616833742 | -0.601022797 | 0.256533806301466 | 0.841773338444499 | 0.548250600296593 | 1.2924424577605 | 0.998074519377826 |
| ebi-fl187-GCST90094697 | ebi-met1400-GCST90200943 | Phosphate to EDTA ratio \|\| id:ebi-met1400-GCST90200943 | Banana liking \|\| id:ebi-fl187-GCST90094697 | Inverse variance weighted | 2 | 0.0983946692946737 | 0.209723635975311 | 0.638952792701801 | -0.312663657 | 0.509452995806284 | 1.10339817656513 | 0.731495904566088 | 1.66438052277195 | 0.998074519377826 |
| ebi-fl187-GCST90094697 | ebi-met1400-GCST90200947 | Adenosine 5'-diphosphate (ADP) to glucose ratio \|\| id:ebi-met1400-GCST90200947 | Banana liking \|\| id:ebi-fl187-GCST90094697 | Inverse variance weighted | 2 | -0.275005341 | 0.293204481668227 | 0.348280277497758 | -0.849686125 | 0.2996754433831 | 0.759568066599123 | 0.427549107970797 | 1.34942077305547 | 0.998074519377826 |
| ebi-fl187-GCST90094697 | ebi-met1400-GCST90200975 | Arachidonate (20:4n6) to caffeine ratio \|\| id:ebi-met1400-GCST90200975 | Banana liking \|\| id:ebi-fl187-GCST90094697 | Inverse variance weighted | 2 | 0.0779664501208172 | 0.225304559068828 | 0.729305781000118 | -0.363630486 | 0.519563385895721 | 1.08108638774395 | 0.695148014454579 | 1.68129341300395 | 0.998074519377826 |
| ebi-fl187-GCST90094697 | ebi-met1400-GCST90200985 | Cholesterol to oleoyl-linoleoyl-glycerol (18:1 to 18:2) [2] ratio \|\| id:ebi-met1400-GCST90200985 | Banana liking \|\| id:ebi-fl187-GCST90094697 | Inverse variance weighted | 2 | 0.130288793096351 | 0.382384702309779 | 0.733309109355714 | -0.619185223 | 0.879762809623518 | 1.1391573165942 | 0.538382920726583 | 2.41032793202056 | 0.998074519377826 |
| ebi-fl187-GCST90094697 | ebi-met1400-GCST90201016 | Bilirubin (Z,Z) to etiocholanolone glucuronide ratio \|\| id:ebi-met1400-GCST90201016 | Banana liking \|\| id:ebi-fl187-GCST90094697 | Inverse variance weighted | 2 | -0.092435286 | 0.226327369924955 | 0.68296969357756 | -0.536036931 | 0.351166359087092 | 0.911708209371652 | 0.585062306245727 | 1.42072365654429 | 0.998074519377826 |
| ebi-fl187-GCST90094697 | ebi-pw11-GCST90275189 | Placental weight (fetal genotype, adjusted for sex and gestational age) \|\| id:ebi-pw11-GCST90275189 | Banana liking \|\| id:ebi-fl187-GCST90094697 | Inverse variance weighted | 2 | 0.00945719545407802 | 0.0807504487081112 | 0.906767876627214 | -0.148813684 | 0.167728074921976 | 1.00950205603391 | 0.861729653953295 | 1.18261498424879 | 0.998074519377826 |
| ebi-fl187-GCST90094697 | ebi-pw11-GCST90275192 | Placental weight (fetal effect, conditional on maternal and paternal genotype) \|\| id:ebi-pw11-GCST90275192 | Banana liking \|\| id:ebi-fl187-GCST90094697 | Inverse variance weighted | 2 | 0.234389437935147 | 0.176812163842068 | 0.184958668318284 | -0.112162403 | 0.580941279065601 | 1.26413669914539 | 0.893899073645674 | 1.78772038280424 | 0.998074519377826 |
| ebi-fl187-GCST90094697 | ebi-pw11-GCST90275193 | Placental weight (maternal effect, conditional on fetal and paternal genotype) \|\| id:ebi-pw11-GCST90275193 | Banana liking \|\| id:ebi-fl187-GCST90094697 | Inverse variance weighted | 2 | -0.162723075 | 0.143075315233419 | 0.255402615414403 | -0.443150693 | 0.11770454246655 | 0.849826493701701 | 0.642010453157169 | 1.12491169862701 | 0.998074519377826 |
| ebi-fl187-GCST90094697 | ebi-pw11-GCST90275195 | Placental weight (fetal effect, conditional on maternal genotype) \|\| id:ebi-pw11-GCST90275195 | Banana liking \|\| id:ebi-fl187-GCST90094697 | Inverse variance weighted | 2 | 0.0289793554653884 | 0.109439932010431 | 0.791165919962888 | -0.185522911 | 0.243481622205833 | 1.02940334270283 | 0.830669803658548 | 1.27568287338797 | 0.998074519377826 |
| ebi-fl187-GCST90094697 | ebi-pw11-GCST90275197 | Placental weight (fetal genotype, adjusted for sex) \|\| id:ebi-pw11-GCST90275197 | Banana liking \|\| id:ebi-fl187-GCST90094697 | Inverse variance weighted | 2 | 0.00575327050624827 | 0.0821205947746307 | 0.9441468571709 | -0.155203095 | 0.166709636264525 | 1.0057698523517 | 0.856241261288676 | 1.18141117653815 | 0.998074519377826 |
| ebi-fl187-GCST90094697 | prot-a-1030 | Protein FAM171B \|\| id:prot-a-1030 | Banana liking \|\| id:ebi-fl187-GCST90094697 | Inverse variance weighted | 2 | -0.459368098 | 0.352196524978385 | 0.192133085448274 | -1.149673287 | 0.230937090613826 | 0.631682680750286 | 0.316740235533372 | 1.25977998497076 | 0.998074519377826 |
| ebi-fl187-GCST90094697 | prot-a-1036 | BRISC complex subunit Abro1 \|\| id:prot-a-1036 | Banana liking \|\| id:ebi-fl187-GCST90094697 | Inverse variance weighted | 2 | -0.614797123 | 0.352196524978385 | 0.0808790660327757 | -1.305102311 | 0.0755080664532545 | 0.540750590307134 | 0.2711447924063 | 1.07843192680369 | 0.998074519377826 |
| ebi-fl187-GCST90094697 | prot-a-1055 | Phenylalanine--tRNA ligase, mitochondrial \|\| id:prot-a-1055 | Banana liking \|\| id:ebi-fl187-GCST90094697 | Inverse variance weighted | 2 | 0.197673202669552 | 0.729799851536209 | 0.786499116597016 | -1.232734506 | 1.62808091168052 | 1.21856410524879 | 0.29149439359074 | 5.09408932470103 | 0.998074519377826 |
| ebi-fl187-GCST90094697 | prot-a-1083 | Fc receptor-like protein 5 \|\| id:prot-a-1083 | Banana liking \|\| id:ebi-fl187-GCST90094697 | Inverse variance weighted | 2 | -0.535396406 | 0.352196524978385 | 0.128469814531591 | -1.225701595 | 0.154908783307948 | 0.585437173538539 | 0.293551673787131 | 1.16755145606606 | 0.998074519377826 |
| ebi-fl187-GCST90094697 | prot-a-1103 | Fibroblast growth factor receptor 3 \|\| id:prot-a-1103 | Banana liking \|\| id:ebi-fl187-GCST90094697 | Inverse variance weighted | 2 | -0.814411035 | 0.352196524978385 | 0.0207569337645241 | -1.504716224 | -0.124105846 | 0.44290010332777 | 0.222080306015623 | 0.883286343787502 | 0.998074519377826 |
| ebi-fl187-GCST90094697 | prot-a-1105 | Fibroblast growth factor receptor 4 \|\| id:prot-a-1105 | Banana liking \|\| id:ebi-fl187-GCST90094697 | Inverse variance weighted | 2 | 0.284912553129239 | 0.352196524978385 | 0.418538722680961 | -0.405392636 | 0.975217742086873 | 1.32964574967743 | 0.666714983270632 | 2.6517445445164 | 0.998074519377826 |
| ebi-fl187-GCST90094697 | prot-a-1120 | Filamin-A \|\| id:prot-a-1120 | Banana liking \|\| id:ebi-fl187-GCST90094697 | Inverse variance weighted | 2 | -0.564161962 | 0.352196524978385 | 0.109191321646212 | -1.254467151 | 0.126143227146805 | 0.568836653903431 | 0.28522779114885 | 1.13444463991656 | 0.998074519377826 |
| ebi-fl187-GCST90094697 | prot-a-1166 | Galanin peptides \|\| id:prot-a-1166 | Banana liking \|\| id:ebi-fl187-GCST90094697 | Inverse variance weighted | 2 | 0.0635545735122403 | 0.352196524978385 | 0.856797720394742 | -0.626750615 | 0.753859762469875 | 1.0656176387119 | 0.534325211312151 | 2.12518692341946 | 0.998074519377826 |
| ebi-fl187-GCST90094697 | prot-a-1205 | ADP-ribosylation factor-binding protein GGA1 \|\| id:prot-a-1205 | Banana liking \|\| id:ebi-fl187-GCST90094697 | Inverse variance weighted | 2 | 0.244798543182325 | 0.38432280836421 | 0.524150412053489 | -0.508474161 | 0.998071247576175 | 1.27736395360523 | 0.601412537646499 | 2.71304398866563 | 0.998074519377826 |
| ebi-fl187-GCST90094697 | prot-a-1227 | Guanine nucleotide-binding protein G(I)/G(S)/G(O) subunit gamma-T2 \|\| id:prot-a-1227 | Banana liking \|\| id:ebi-fl187-GCST90094697 | Inverse variance weighted | 2 | -0.520688273 | 0.597535643367054 | 0.383539701097045 | -1.691858134 | 0.650481587599511 | 0.594111496077241 | 0.18417698026281 | 1.91646355189162 | 0.998074519377826 |
| ebi-fl187-GCST90094697 | prot-a-1240 | Golgi SNAP receptor complex member 1 \|\| id:prot-a-1240 | Banana liking \|\| id:ebi-fl187-GCST90094697 | Inverse variance weighted | 2 | -0.565498141 | 0.352196524978385 | 0.108354708714862 | -1.25580333 | 0.124807047690346 | 0.568077093619836 | 0.284846930139898 | 1.13292983054816 | 0.998074519377826 |
| ebi-fl187-GCST90094697 | prot-a-1283 | Glutathione S-transferase A1 \|\| id:prot-a-1283 | Banana liking \|\| id:ebi-fl187-GCST90094697 | Inverse variance weighted | 2 | -0.119614669 | 0.352196524978385 | 0.73413911173622 | -0.809919858 | 0.570690520355441 | 0.887262260862339 | 0.444893719662556 | 1.76948849749476 | 0.998074519377826 |
| ebi-fl187-GCST90094697 | prot-a-1324 | E3 ubiquitin-protein ligase HECW1 \|\| id:prot-a-1324 | Banana liking \|\| id:ebi-fl187-GCST90094697 | Inverse variance weighted | 2 | -0.021918222 | 0.508465460399347 | 0.965616550013222 | -1.018510525 | 0.974674079975882 | 0.978320236454283 | 0.361132437592646 | 2.65030328329458 | 0.998074519377826 |
| ebi-fl187-GCST90094697 | prot-a-1339 | Histidine triad nucleotide-binding protein 2, mitochondrial \|\| id:prot-a-1339 | Banana liking \|\| id:ebi-fl187-GCST90094697 | Inverse variance weighted | 2 | -0.212673309 | 0.352196524978385 | 0.54594443661408 | -0.902978498 | 0.477631880400454 | 0.808420198041837 | 0.405360494660975 | 1.61225187261673 | 0.998074519377826 |
| ebi-fl187-GCST90094697 | prot-a-1466 | Interleukin-11 \|\| id:prot-a-1466 | Banana liking \|\| id:ebi-fl187-GCST90094697 | Inverse variance weighted | 2 | -0.000419226 | 0.352196524978385 | 0.999050263466659 | -0.690724415 | 0.689885962916539 | 0.999580861821863 | 0.501212851414662 | 1.99348818870152 | 0.999355215041207 |
| ebi-fl187-GCST90094697 | prot-a-147 | Rho GTPase-activating protein 1 \|\| id:prot-a-147 | Banana liking \|\| id:ebi-fl187-GCST90094697 | Inverse variance weighted | 2 | -0.219819477 | 0.352196524978385 | 0.532536508393026 | -0.910124666 | 0.470485712058722 | 0.802663684221833 | 0.402474046134267 | 1.60077151845373 | 0.998074519377826 |
| ebi-fl187-GCST90094697 | prot-a-1473 | Interleukin-12 receptor subunit beta-1 \|\| id:prot-a-1473 | Banana liking \|\| id:ebi-fl187-GCST90094697 | Inverse variance weighted | 2 | 0.102990697713733 | 0.352196524978385 | 0.769962501460497 | -0.587314491 | 0.793295886671367 | 1.10848109761956 | 0.555817936194304 | 2.21067055193108 | 0.998074519377826 |
| ebi-fl187-GCST90094697 | prot-a-1501 | X-linked interleukin-1 receptor accessory protein-like 2 \|\| id:prot-a-1501 | Banana liking \|\| id:ebi-fl187-GCST90094697 | Inverse variance weighted | 2 | 0.00414594900134009 | 0.352196524978385 | 0.990607765649217 | -0.68615924 | 0.694451137958974 | 1.0041545553376 | 0.503506206615902 | 2.00260961584224 | 0.999355215041207 |
| ebi-fl187-GCST90094697 | prot-a-1503 | Interleukin-1 receptor-like 2 \|\| id:prot-a-1503 | Banana liking \|\| id:ebi-fl187-GCST90094697 | Inverse variance weighted | 2 | 0.0091678189162473 | 1.22659626288015 | 0.994036511209336 | -2.394960856 | 2.41329649416134 | 1.00920997208716 | 0.0911762478258097 | 11.170724745177 | 0.999355215041207 |
| ebi-fl187-GCST90094697 | prot-a-1536 | Interleukin-5 receptor subunit alpha \|\| id:prot-a-1536 | Banana liking \|\| id:ebi-fl187-GCST90094697 | Inverse variance weighted | 2 | -0.209294038 | 0.352196524978385 | 0.552342535881831 | -0.899599227 | 0.48101115089884 | 0.811156689635929 | 0.406732634531928 | 1.61770932371415 | 0.998074519377826 |
| ebi-fl187-GCST90094697 | prot-a-1559 | Insulin-induced gene 1 protein \|\| id:prot-a-1559 | Banana liking \|\| id:ebi-fl187-GCST90094697 | Inverse variance weighted | 2 | -0.315662291 | 0.352196524978385 | 0.370109924486307 | -1.00596748 | 0.374642897924114 | 0.72930570165937 | 0.365690665200837 | 1.45447192692423 | 0.998074519377826 |
| ebi-fl187-GCST90094697 | prot-a-1567 | Interferon regulatory factor 1 \|\| id:prot-a-1567 | Banana liking \|\| id:ebi-fl187-GCST90094697 | Inverse variance weighted | 2 | 0.0629355970656073 | 0.352196524978385 | 0.858177554299706 | -0.627369592 | 0.753240786023242 | 1.06495825058633 | 0.533994578928892 | 2.12387188979857 | 0.998074519377826 |
| ebi-fl187-GCST90094697 | prot-a-1613 | Potassium voltage-gated channel subfamily E regulatory beta subunit 5 \|\| id:prot-a-1613 | Banana liking \|\| id:ebi-fl187-GCST90094697 | Inverse variance weighted | 2 | -0.185867196 | 0.409068205449228 | 0.64956451867679 | -0.987640879 | 0.615906486287875 | 0.830383865632473 | 0.372454320179407 | 1.85133404808028 | 0.998074519377826 |
| ebi-fl187-GCST90094697 | prot-a-1649 | Kin of IRRE-like protein 2 \|\| id:prot-a-1649 | Banana liking \|\| id:ebi-fl187-GCST90094697 | Inverse variance weighted | 2 | -0.05480499 | 0.594882801827661 | 0.926596840357442 | -1.220775281 | 1.11116530175946 | 0.946669740188041 | 0.295001369168194 | 3.03789639863243 | 0.998074519377826 |
| ebi-fl187-GCST90094697 | prot-a-165 | Armadillo repeat-containing protein 5 \|\| id:prot-a-165 | Banana liking \|\| id:ebi-fl187-GCST90094697 | Inverse variance weighted | 2 | 0.384523980344585 | 0.352196524978385 | 0.27492624389944 | -0.305781209 | 1.07482916930222 | 1.46891492261526 | 0.736547752132481 | 2.92949241055276 | 0.998074519377826 |
| ebi-fl187-GCST90094697 | prot-a-1656 | Kallikrein-11 \|\| id:prot-a-1656 | Banana liking \|\| id:ebi-fl187-GCST90094697 | Inverse variance weighted | 2 | -0.211313249 | 0.352196524978385 | 0.548515064871197 | -0.901618438 | 0.47899193998478 | 0.809520445711941 | 0.405912184167105 | 1.61444612304586 | 0.998074519377826 |
| ebi-fl187-GCST90094697 | prot-a-1697 | Laminin subunit gamma-2 \|\| id:prot-a-1697 | Banana liking \|\| id:ebi-fl187-GCST90094697 | Inverse variance weighted | 2 | -0.47344782 | 0.65626781070308 | 0.470648243085017 | -1.759732729 | 0.81283708863675 | 0.622851083329396 | 0.172090852516352 | 2.25429455623006 | 0.998074519377826 |
| ebi-fl187-GCST90094697 | prot-a-1698 | Lysosome-associated membrane glycoprotein 3 \|\| id:prot-a-1698 | Banana liking \|\| id:ebi-fl187-GCST90094697 | Inverse variance weighted | 2 | -0.415339488 | 0.352196524978385 | 0.238285376500252 | -1.105644677 | 0.274965700460407 | 0.660116140824036 | 0.330997426865722 | 1.31648551924603 | 0.998074519377826 |
| ebi-fl187-GCST90094697 | prot-a-1724 | Leptin receptor \|\| id:prot-a-1724 | Banana liking \|\| id:ebi-fl187-GCST90094697 | Inverse variance weighted | 2 | -0.278748251 | 0.352196524978385 | 0.428677614591589 | -0.96905344 | 0.411556938125099 | 0.756730385481705 | 0.379442032901781 | 1.50916563442385 | 0.998074519377826 |
| ebi-fl187-GCST90094697 | prot-a-1743 | Leukocyte immunoglobulin-like receptor subfamily B member 2 \|\| id:prot-a-1743 | Banana liking \|\| id:ebi-fl187-GCST90094697 | Inverse variance weighted | 2 | -0.258514518 | 0.352196524978385 | 0.46294481228513 | -0.948819707 | 0.431790670994923 | 0.772197820228646 | 0.387197760696983 | 1.54001271208932 | 0.998074519377826 |
| ebi-fl187-GCST90094697 | prot-a-1746 | Leukocyte immunoglobulin-like receptor subfamily B member 4 \|\| id:prot-a-1746 | Banana liking \|\| id:ebi-fl187-GCST90094697 | Inverse variance weighted | 2 | 0.0657206288639245 | 0.352196524978385 | 0.851972580723394 | -0.62458456 | 0.756025817821559 | 1.06792832713715 | 0.535483843673562 | 2.12979518500134 | 0.998074519377826 |
| ebi-fl187-GCST90094697 | prot-a-1750 | Leucine-rich repeat and immunoglobulin-like domain-containing nogo receptor-interacting protein 3 \|\| id:prot-a-1750 | Banana liking \|\| id:ebi-fl187-GCST90094697 | Inverse variance weighted | 2 | 0.00521746542259672 | 0.352196524978385 | 0.988180511297161 | -0.685087724 | 0.695522654380231 | 1.00523110009782 | 0.504046010937445 | 2.00475659498727 | 0.999355215041207 |
| ebi-fl187-GCST90094697 | prot-a-1753 | Lipase member N \|\| id:prot-a-1753 | Banana liking \|\| id:ebi-fl187-GCST90094697 | Inverse variance weighted | 2 | 0.110433985405499 | 0.532731599475237 | 0.835777443394248 | -0.93371995 | 1.15458792037696 | 1.1167626239869 | 0.393088717008052 | 3.17271573660703 | 0.998074519377826 |
| ebi-fl187-GCST90094697 | prot-a-1760 | Ig Kappa chain V-I region HK102- like \|\| id:prot-a-1760 | Banana liking \|\| id:ebi-fl187-GCST90094697 | Inverse variance weighted | 2 | 0.196322576890887 | 0.352196524978385 | 0.577238196053395 | -0.493982612 | 0.886627765848521 | 1.2169193921013 | 0.61019139296565 | 2.42693165446789 | 0.998074519377826 |
| ebi-fl187-GCST90094697 | prot-a-1792 | Leucine-rich repeat neuronal protein 1 \|\| id:prot-a-1792 | Banana liking \|\| id:ebi-fl187-GCST90094697 | Inverse variance weighted | 2 | -0.044419668 | 0.436562969660503 | 0.918956231084978 | -0.900083088 | 0.811243752868259 | 0.956552439096965 | 0.406535880002394 | 2.25070556807179 | 0.998074519377826 |
| ebi-fl187-GCST90094697 | prot-a-1795 | Leucine-rich repeat transmembrane neuronal protein 1 \|\| id:prot-a-1795 | Banana liking \|\| id:ebi-fl187-GCST90094697 | Inverse variance weighted | 2 | -0.339873821 | 0.352196524978385 | 0.33453887496775 | -1.03017901 | 0.350431367762352 | 0.711860138757488 | 0.356943058418281 | 1.41967982063404 | 0.998074519377826 |
| ebi-fl187-GCST90094697 | prot-a-1820 | LysM and putative peptidoglycan-binding domain-containing protein 4 \|\| id:prot-a-1820 | Banana liking \|\| id:ebi-fl187-GCST90094697 | Inverse variance weighted | 2 | 0.117241144276703 | 0.352196524978385 | 0.739220655229623 | -0.573064045 | 0.807546333234337 | 1.12439053734405 | 0.563795295458857 | 2.24239913076945 | 0.998074519377826 |
| ebi-fl187-GCST90094697 | prot-a-1844 | Mitogen-activated protein kinase kinase kinase 7:TGF-beta-activated kinase 1 and MAP3K7-binding protein 1 fusion \|\| id:prot-a-1844 | Banana liking \|\| id:ebi-fl187-GCST90094697 | Inverse variance weighted | 2 | 0.382540656220144 | 0.352196524978385 | 0.277409619128959 | -0.307764533 | 1.07284584517778 | 1.46600447534666 | 0.735088386882404 | 2.92368803546377 | 0.998074519377826 |
| ebi-fl187-GCST90094697 | prot-a-1856 | Megakaryocyte-associated tyrosine-protein kinase \|\| id:prot-a-1856 | Banana liking \|\| id:ebi-fl187-GCST90094697 | Inverse variance weighted | 2 | -0.205090037 | 0.352196524978385 | 0.560353150855678 | -0.895395226 | 0.485215151580273 | 0.814573971005534 | 0.408446138066024 | 1.62452449025841 | 0.998074519377826 |
| ebi-fl187-GCST90094697 | prot-a-1910 | Stromelysin-2 \|\| id:prot-a-1910 | Banana liking \|\| id:ebi-fl187-GCST90094697 | Inverse variance weighted | 2 | -0.103044461 | 0.352196524978385 | 0.769845803063795 | -0.79334965 | 0.587260727657292 | 0.902086864634854 | 0.452327117211177 | 1.79905356186466 | 0.998074519377826 |
| ebi-fl187-GCST90094697 | prot-a-1940 | 39S ribosomal protein L14, mitochondrial \|\| id:prot-a-1940 | Banana liking \|\| id:ebi-fl187-GCST90094697 | Inverse variance weighted | 2 | -0.145977675 | 0.352196524978385 | 0.678524151858323 | -0.836282864 | 0.544327514359835 | 0.864176996068291 | 0.433318236542563 | 1.72344899788278 | 0.998074519377826 |
| ebi-fl187-GCST90094697 | prot-a-1942 | 39S ribosomal protein L33, mitochondrial \|\| id:prot-a-1942 | Banana liking \|\| id:ebi-fl187-GCST90094697 | Inverse variance weighted | 2 | 0.264528545347005 | 0.352196524978385 | 0.452603179713661 | -0.425776644 | 0.954833734304639 | 1.30281661201369 | 0.653262236121262 | 2.59823854906524 | 0.998074519377826 |
| ebi-fl187-GCST90094697 | prot-a-1948 | Mesothelin \|\| id:prot-a-1948 | Banana liking \|\| id:ebi-fl187-GCST90094697 | Inverse variance weighted | 2 | -0.099250253 | 0.447464823369257 | 0.824465134226652 | -0.976281307 | 0.7777808009494 | 0.905516071684943 | 0.376709363984117 | 2.17663651205204 | 0.998074519377826 |
| ebi-fl187-GCST90094697 | prot-a-1980 | Unconventional myosin-VI \|\| id:prot-a-1980 | Banana liking \|\| id:ebi-fl187-GCST90094697 | Inverse variance weighted | 2 | 0.319724592989507 | 0.352196524978385 | 0.363983016745701 | -0.370580596 | 1.01002978194714 | 1.37674854591742 | 0.690333409467809 | 2.74568278557886 | 0.998074519377826 |
| ebi-fl187-GCST90094697 | prot-a-2039 | Neurofascin \|\| id:prot-a-2039 | Banana liking \|\| id:ebi-fl187-GCST90094697 | Inverse variance weighted | 2 | -0.401712306 | 0.356732156119485 | 0.260127672196633 | -1.100907332 | 0.297482719519819 | 0.66917323480726 | 0.332569195932991 | 1.34646510758813 | 0.998074519377826 |
| ebi-fl187-GCST90094697 | prot-a-2041 | NFU1 iron-sulfur cluster scaffold homolog, mitochondrial \|\| id:prot-a-2041 | Banana liking \|\| id:ebi-fl187-GCST90094697 | Inverse variance weighted | 2 | 0.14005930092061 | 0.352196524978385 | 0.690870714375341 | -0.550245888 | 0.830364489878244 | 1.15034201317502 | 0.576807962764181 | 2.2941547840881 | 0.998074519377826 |
| ebi-fl187-GCST90094697 | prot-a-2088 | Glucocorticoid receptor \|\| id:prot-a-2088 | Banana liking \|\| id:ebi-fl187-GCST90094697 | Inverse variance weighted | 2 | 1.11638380399191 | 0.352196524978385 | 0.00152556924946109 | 0.426078615034274 | 1.80668899294954 | 3.05379110497954 | 1.531241149 | 6.09024915447349 | 0.598008988154769 |
| ebi-fl187-GCST90094697 | prot-a-2111 | Neuron-specific protein family member 2 \|\| id:prot-a-2111 | Banana liking \|\| id:ebi-fl187-GCST90094697 | Inverse variance weighted | 2 | 0.126398804964226 | 0.352196524978385 | 0.719679517466078 | -0.563906384 | 0.816703993921861 | 1.13473461585574 | 0.568982054513699 | 2.26302857569347 | 0.998074519377826 |
| ebi-fl187-GCST90094697 | prot-a-2125 | Nuclear migration protein nudC \|\| id:prot-a-2125 | Banana liking \|\| id:ebi-fl187-GCST90094697 | Inverse variance weighted | 2 | -0.174275387 | 0.352196524978385 | 0.620724174019804 | -0.864580576 | 0.516029802114218 | 0.84006552280851 | 0.421228188877668 | 1.67536290601028 | 0.998074519377826 |
| ebi-fl187-GCST90094697 | prot-a-2142 | Ornithine decarboxylase \|\| id:prot-a-2142 | Banana liking \|\| id:ebi-fl187-GCST90094697 | Inverse variance weighted | 2 | 0.221648238017462 | 0.352196524978385 | 0.529132293479953 | -0.468656951 | 0.911953426975096 | 1.24813225515893 | 0.625842240927483 | 2.48918021905879 | 0.998074519377826 |
| ebi-fl187-GCST90094697 | prot-a-2186 | Prostate and testis expressed protein 1 \|\| id:prot-a-2186 | Banana liking \|\| id:ebi-fl187-GCST90094697 | Inverse variance weighted | 2 | -0.409996486 | 0.495324792338181 | 0.407821943732113 | -1.380833079 | 0.560840107074051 | 0.663652582267931 | 0.25136905555371 | 1.75214387061571 | 0.998074519377826 |
| ebi-fl187-GCST90094697 | prot-a-2227 | Platelet-derived growth factor C \|\| id:prot-a-2227 | Banana liking \|\| id:ebi-fl187-GCST90094697 | Inverse variance weighted | 2 | -0.204912452 | 0.352196524978385 | 0.560692770761491 | -0.895217641 | 0.485392737095336 | 0.814718640388945 | 0.408518678624713 | 1.62481300789427 | 0.998074519377826 |
| ebi-fl187-GCST90094697 | prot-a-2229 | Platelet-derived growth factor receptor alpha \|\| id:prot-a-2229 | Banana liking \|\| id:ebi-fl187-GCST90094697 | Inverse variance weighted | 2 | -0.390266484 | 0.352196524978385 | 0.26782167854336 | -1.080571673 | 0.30003870521353 | 0.67687647388526 | 0.339401443634275 | 1.34991105516051 | 0.998074519377826 |
| ebi-fl187-GCST90094697 | prot-a-2243 | Platelet endothelial aggregation receptor 1 \|\| id:prot-a-2243 | Banana liking \|\| id:ebi-fl187-GCST90094697 | Inverse variance weighted | 2 | -0.029576698 | 0.512621762183569 | 0.953990045793341 | -1.034315352 | 0.975161956239116 | 0.970856412389307 | 0.355469669400578 | 2.65159661882506 | 0.998074519377826 |
| ebi-fl187-GCST90094697 | prot-a-2246 | E3 ubiquitin-protein ligase pellino homolog 2 \|\| id:prot-a-2246 | Banana liking \|\| id:ebi-fl187-GCST90094697 | Inverse variance weighted | 2 | 0.329867550196845 | 0.721935848765592 | 0.647727928567852 | -1.085126713 | 1.74486181377741 | 1.39078390720927 | 0.337858971928667 | 5.72511028939223 | 0.998074519377826 |
| ebi-fl187-GCST90094697 | prot-a-2251 | Platelet factor 4 variant \|\| id:prot-a-2251 | Banana liking \|\| id:ebi-fl187-GCST90094697 | Inverse variance weighted | 2 | 0.0497745437208857 | 0.352196524978385 | 0.88761237117078 | -0.640530645 | 0.74007973267852 | 1.05103410742262 | 0.527012693055379 | 2.09610263570936 | 0.998074519377826 |
| ebi-fl187-GCST90094697 | prot-a-2346 | Protein phosphatase 1L \|\| id:prot-a-2346 | Banana liking \|\| id:ebi-fl187-GCST90094697 | Inverse variance weighted | 2 | 0.599368489965629 | 0.530878067758746 | 0.258892151009678 | -0.441152523 | 1.63988950277277 | 1.82096847734275 | 0.643294581972452 | 5.1545999117679 | 0.998074519377826 |
| ebi-fl187-GCST90094697 | prot-a-2358 | PR domain zinc finger protein 4 \|\| id:prot-a-2358 | Banana liking \|\| id:ebi-fl187-GCST90094697 | Inverse variance weighted | 2 | -0.807352802 | 0.352196524978385 | 0.0218862573647999 | -1.497657991 | -0.117047613 | 0.446037253888729 | 0.223653345514508 | 0.889542838712836 | 0.998074519377826 |
| ebi-fl187-GCST90094697 | prot-a-2390 | Trypsin-1 \|\| id:prot-a-2390 | Banana liking \|\| id:ebi-fl187-GCST90094697 | Inverse variance weighted | 2 | -0.094098558 | 0.352196524978385 | 0.789333437661882 | -0.784403747 | 0.596206631277526 | 0.910193051304402 | 0.456391745786936 | 1.81521992518588 | 0.998074519377826 |
| ebi-fl187-GCST90094697 | prot-a-242 | Brain-derived neurotrophic factor \|\| id:prot-a-242 | Banana liking \|\| id:ebi-fl187-GCST90094697 | Inverse variance weighted | 2 | 0.237952924665413 | 0.352196524978385 | 0.499278498812407 | -0.452352264 | 0.928258113623048 | 1.26864946932093 | 0.636130044351888 | 2.53009819344103 | 0.998074519377826 |
| ebi-fl187-GCST90094697 | prot-a-2464 | Dihydropteridine reductase \|\| id:prot-a-2464 | Banana liking \|\| id:ebi-fl187-GCST90094697 | Inverse variance weighted | 2 | 0.0848043439793181 | 0.480714873326795 | 0.859969506964725 | -0.857396808 | 1.02700549569984 | 1.08850407348686 | 0.424265089621806 | 2.79269057714286 | 0.998074519377826 |
| ebi-fl187-GCST90094697 | prot-a-2474 | Ras-related protein Rab-27A \|\| id:prot-a-2474 | Banana liking \|\| id:ebi-fl187-GCST90094697 | Inverse variance weighted | 2 | -0.538203602 | 0.352196524978385 | 0.126479188297532 | -1.228508791 | 0.152101586619156 | 0.583796040812389 | 0.292728772064379 | 1.16427850554187 | 0.998074519377826 |
| ebi-fl187-GCST90094697 | prot-a-2478 | Ras-related protein Rab-7b \|\| id:prot-a-2478 | Banana liking \|\| id:ebi-fl187-GCST90094697 | Inverse variance weighted | 2 | -0.017302714 | 0.352196524978385 | 0.960817281936955 | -0.707607903 | 0.673002474705055 | 0.98284611807033 | 0.492821665714975 | 1.9601136861637 | 0.998074519377826 |
| ebi-fl187-GCST90094697 | prot-a-2528 | E3 ubiquitin-protein ligase rififylin \|\| id:prot-a-2528 | Banana liking \|\| id:ebi-fl187-GCST90094697 | Inverse variance weighted | 2 | 0.191463712973203 | 0.352196524978385 | 0.586697838556327 | -0.498841476 | 0.881768901930838 | 1.21102088799526 | 0.607233747241344 | 2.41516812565747 | 0.998074519377826 |
| ebi-fl187-GCST90094697 | prot-a-2543 | Protein RIC-3 \|\| id:prot-a-2543 | Banana liking \|\| id:ebi-fl187-GCST90094697 | Inverse variance weighted | 2 | 0.161893876282096 | 0.352196524978385 | 0.645753718287035 | -0.528411313 | 0.85219906523973 | 1.17573546128163 | 0.589540822124422 | 2.34479754927537 | 0.998074519377826 |
| ebi-fl187-GCST90094697 | prot-a-255 | Bone morphogenetic protein 6 \|\| id:prot-a-255 | Banana liking \|\| id:ebi-fl187-GCST90094697 | Inverse variance weighted | 2 | -0.516989719 | 0.352196524978385 | 0.142131049074014 | -1.207294908 | 0.173315470296616 | 0.596312918497658 | 0.299005022636052 | 1.18924121619193 | 0.998074519377826 |
| ebi-fl187-GCST90094697 | prot-a-2568 | RING finger protein 165 \|\| id:prot-a-2568 | Banana liking \|\| id:ebi-fl187-GCST90094697 | Inverse variance weighted | 2 | -0.241594285 | 0.352196524978385 | 0.49273554465085 | -0.931899474 | 0.448710903614722 | 0.785374750970282 | 0.393804977063522 | 1.56629178244778 | 0.998074519377826 |
| ebi-fl187-GCST90094697 | prot-a-2573 | E3 ubiquitin-protein ligase RNF8 \|\| id:prot-a-2573 | Banana liking \|\| id:ebi-fl187-GCST90094697 | Inverse variance weighted | 2 | 0.5164638230654 | 0.352196524978385 | 0.142537151748218 | -0.173841366 | 1.20676901202303 | 1.67609020643271 | 0.840430207980552 | 3.34266706910737 | 0.998074519377826 |
| ebi-fl187-GCST90094697 | prot-a-260 | Cytoplasmic tyrosine-protein kinase BMX \|\| id:prot-a-260 | Banana liking \|\| id:ebi-fl187-GCST90094697 | Inverse variance weighted | 2 | 0.45260612687225 | 0.352196524978385 | 0.198758956061511 | -0.237699062 | 1.14291131582988 | 1.57240473655816 | 0.788439926862751 | 3.13588463916152 | 0.998074519377826 |
| ebi-fl187-GCST90094697 | prot-a-2623 | Serum amyloid A-1 protein \|\| id:prot-a-2623 | Banana liking \|\| id:ebi-fl187-GCST90094697 | Inverse variance weighted | 2 | 0.171256433087336 | 0.352196524978385 | 0.626788168014046 | -0.519048756 | 0.86156162204497 | 1.1867950435215 | 0.595086351217282 | 2.36685394051816 | 0.998074519377826 |
| ebi-fl187-GCST90094697 | prot-a-2640 | Lysosome membrane protein 2 \|\| id:prot-a-2640 | Banana liking \|\| id:ebi-fl187-GCST90094697 | Inverse variance weighted | 2 | 0.186936139957779 | 0.574421800115621 | 0.744852948080775 | -0.938930588 | 1.3128028681844 | 1.20555029609888 | 0.39104580079691 | 3.71657620018507 | 0.998074519377826 |
| ebi-fl187-GCST90094697 | prot-a-2641 | Scavenger receptor class F member 2 \|\| id:prot-a-2641 | Banana liking \|\| id:ebi-fl187-GCST90094697 | Inverse variance weighted | 2 | -0.206594369 | 0.522212660346866 | 0.692390190205813 | -1.230131183 | 0.816942445736635 | 0.813349503227486 | 0.292254236430265 | 2.26356826330642 | 0.998074519377826 |
| ebi-fl187-GCST90094697 | prot-a-2669 | Semaphorin-3A \|\| id:prot-a-2669 | Banana liking \|\| id:ebi-fl187-GCST90094697 | Inverse variance weighted | 2 | 0.0222179146129625 | 0.352196524978385 | 0.949699730308305 | -0.668087274 | 0.712523103570597 | 1.02246657060238 | 0.512688272556301 | 2.03912970894919 | 0.998074519377826 |
| ebi-fl187-GCST90094697 | prot-a-2683 | Sentrin-specific protease 7 \|\| id:prot-a-2683 | Banana liking \|\| id:ebi-fl187-GCST90094697 | Inverse variance weighted | 2 | -0.199313362 | 0.352196524978385 | 0.571451436790961 | -0.889618551 | 0.490991826532017 | 0.819293117424498 | 0.410812426700823 | 1.63393599762742 | 0.998074519377826 |
| ebi-fl187-GCST90094697 | prot-a-270 | Bromodomain testis-specific protein \|\| id:prot-a-270 | Banana liking \|\| id:ebi-fl187-GCST90094697 | Inverse variance weighted | 2 | 0.311932995217186 | 0.352196524978385 | 0.375790636014471 | -0.378372194 | 1.00223818417482 | 1.36606315719769 | 0.684975509618738 | 2.72437265748612 | 0.998074519377826 |
| ebi-fl187-GCST90094697 | prot-a-2708 | 14-3-3 protein sigma \|\| id:prot-a-2708 | Banana liking \|\| id:ebi-fl187-GCST90094697 | Inverse variance weighted | 2 | 0.325416763277202 | 0.352196524978385 | 0.355505276384347 | -0.364888426 | 1.01572195223484 | 1.38460757934249 | 0.694274109718075 | 2.76135624523167 | 0.998074519377826 |
| ebi-fl187-GCST90094697 | prot-a-2713 | Beta-sarcoglycan \|\| id:prot-a-2713 | Banana liking \|\| id:ebi-fl187-GCST90094697 | Inverse variance weighted | 2 | -0.505290382 | 0.352196524978385 | 0.151377438645377 | -1.195595571 | 0.185014807019032 | 0.603330353749927 | 0.302523726191456 | 1.20323625633794 | 0.998074519377826 |
| ebi-fl187-GCST90094697 | prot-a-2720 | Protein shisa-3 homolog \|\| id:prot-a-2720 | Banana liking \|\| id:ebi-fl187-GCST90094697 | Inverse variance weighted | 2 | -0.607418747 | 0.352196524978385 | 0.0845890254325659 | -1.297723936 | 0.0828864415153566 | 0.544755206590199 | 0.27315279918466 | 1.08641842951246 | 0.998074519377826 |
| ebi-fl187-GCST90094697 | prot-a-2730 | Sialic acid-binding Ig-like lectin 8 \|\| id:prot-a-2730 | Banana liking \|\| id:ebi-fl187-GCST90094697 | Inverse variance weighted | 2 | 0.00521832304129317 | 0.352196524978385 | 0.988178568615803 | -0.685086866 | 0.695523511998927 | 1.00523196220317 | 0.504046443216914 | 2.00475831430474 | 0.999355215041207 |
| ebi-fl187-GCST90094697 | prot-a-2761 | Sodium- and chloride-dependent neutral and basic amino acid transporter B(0+) \|\| id:prot-a-2761 | Banana liking \|\| id:ebi-fl187-GCST90094697 | Inverse variance weighted | 2 | 0.0750771823382894 | 0.352196524978385 | 0.831195586987917 | -0.615228007 | 0.765382371295924 | 1.07796734771442 | 0.540517639658584 | 2.14981624553909 | 0.998074519377826 |
| ebi-fl187-GCST90094697 | prot-a-2770 | Secreted Ly-6/uPAR-related protein 1 \|\| id:prot-a-2770 | Banana liking \|\| id:ebi-fl187-GCST90094697 | Inverse variance weighted | 2 | -0.24786783 | 0.483719607919033 | 0.60835676594422 | -1.195958262 | 0.700222601471076 | 0.780463090228854 | 0.302414023552175 | 2.01420102168139 | 0.998074519377826 |
| ebi-fl187-GCST90094697 | prot-a-2784 | N-lysine methyltransferase SMYD2 \|\| id:prot-a-2784 | Banana liking \|\| id:ebi-fl187-GCST90094697 | Inverse variance weighted | 2 | -0.374561182 | 0.441869472450822 | 0.396619652966517 | -1.240625348 | 0.49150298449521 | 0.687590940218049 | 0.28920330880978 | 1.63477141051974 | 0.998074519377826 |
| ebi-fl187-GCST90094697 | prot-a-2837 | Serine palmitoyltransferase 2 \|\| id:prot-a-2837 | Banana liking \|\| id:ebi-fl187-GCST90094697 | Inverse variance weighted | 2 | -0.109386349 | 0.352196524978385 | 0.756117445711923 | -0.799691538 | 0.580918839845524 | 0.896384033414533 | 0.449467586375559 | 1.78768026820321 | 0.998074519377826 |
| ebi-fl187-GCST90094697 | prot-a-2858 | Alpha-2,8-sialyltransferase 8B \|\| id:prot-a-2858 | Banana liking \|\| id:ebi-fl187-GCST90094697 | Inverse variance weighted | 2 | -0.082276257 | 0.352196524978385 | 0.815288587151234 | -0.772581446 | 0.608028932309699 | 0.921017486344661 | 0.461819366661542 | 1.83680735670472 | 0.998074519377826 |
| ebi-fl187-GCST90094697 | prot-a-2911 | Synaptotagmin-11 \|\| id:prot-a-2911 | Banana liking \|\| id:ebi-fl187-GCST90094697 | Inverse variance weighted | 2 | 0.227615362764716 | 0.493727265641086 | 0.644788297640336 | -0.740090078 | 1.19532080342124 | 1.25560228114985 | 0.47707094004097 | 3.30461773314746 | 0.998074519377826 |
| ebi-fl187-GCST90094697 | prot-a-2962 | Transforming growth factor beta-1 \|\| id:prot-a-2962 | Banana liking \|\| id:ebi-fl187-GCST90094697 | Inverse variance weighted | 2 | 0.45474744447737 | 0.352196524978385 | 0.196642907114332 | -0.235557744 | 1.145052633 | 1.5757753620048 | 0.790130036043197 | 3.14260675867487 | 0.998074519377826 |
| ebi-fl187-GCST90094697 | prot-a-2978 | Tyrosine-protein kinase receptor Tie-1, soluble \|\| id:prot-a-2978 | Banana liking \|\| id:ebi-fl187-GCST90094697 | Inverse variance weighted | 2 | -0.211491692 | 0.352196524978385 | 0.548177451798301 | -0.901796881 | 0.47881349685102 | 0.809376005234354 | 0.405839758387065 | 1.61415806192241 | 0.998074519377826 |
| ebi-fl187-GCST90094697 | prot-a-299 | Uncharacterized protein C1orf226 \|\| id:prot-a-299 | Banana liking \|\| id:ebi-fl187-GCST90094697 | Inverse variance weighted | 2 | 0.121830199447005 | 0.352196524978385 | 0.729406299831459 | -0.56847499 | 0.81213538840464 | 1.12956228519483 | 0.56638852886907 | 2.25271327207532 | 0.998074519377826 |
| ebi-fl187-GCST90094697 | prot-a-3015 | Transmembrane protein 70, mitochondrial \|\| id:prot-a-3015 | Banana liking \|\| id:ebi-fl187-GCST90094697 | Inverse variance weighted | 2 | 0.00649130510548076 | 0.352196524978385 | 0.985295088007757 | -0.683813884 | 0.696796494063115 | 1.00651241928794 | 0.504688493871427 | 2.00731196071007 | 0.999355215041207 |
| ebi-fl187-GCST90094697 | prot-a-3016 | Transmembrane protein 87B \|\| id:prot-a-3016 | Banana liking \|\| id:ebi-fl187-GCST90094697 | Inverse variance weighted | 2 | -0.406489239 | 0.836647628544319 | 0.627070140880236 | -2.046318591 | 1.23334011336277 | 0.665984262511136 | 0.129209702909991 | 3.43267593627603 | 0.998074519377826 |
| ebi-fl187-GCST90094697 | prot-a-3123 | Thioredoxin domain-containing protein 12 \|\| id:prot-a-3123 | Banana liking \|\| id:ebi-fl187-GCST90094697 | Inverse variance weighted | 2 | -0.585941058 | 0.352196524978385 | 0.0961771083823664 | -1.276246247 | 0.104364130609322 | 0.556581839296576 | 0.279082945036586 | 1.11000456797584 | 0.998074519377826 |
| ebi-fl187-GCST90094697 | prot-a-3133 | PolyUbiquitin K48-linked \|\| id:prot-a-3133 | Banana liking \|\| id:ebi-fl187-GCST90094697 | Inverse variance weighted | 2 | 0.850051235272918 | 0.352196524978385 | 0.015797060091985 | 0.159746046315284 | 1.54035642423055 | 2.33976672744188 | 1.17321289142017 | 4.66625314031216 | 0.998074519377826 |
| ebi-fl187-GCST90094697 | prot-a-3135 | Ubiquitin-conjugating enzyme E2 B \|\| id:prot-a-3135 | Banana liking \|\| id:ebi-fl187-GCST90094697 | Inverse variance weighted | 2 | -0.198742146 | 0.564446256869816 | 0.724762445729463 | -1.30505681 | 0.907572517230121 | 0.819761244606447 | 0.27115713025293 | 2.47829919697068 | 0.998074519377826 |
| ebi-fl187-GCST90094697 | prot-a-3162 | NKG2D ligand 3 \|\| id:prot-a-3162 | Banana liking \|\| id:ebi-fl187-GCST90094697 | Inverse variance weighted | 2 | 0.589734409175614 | 0.352196524978385 | 0.0940427854118477 | -0.10057078 | 1.28003959813325 | 1.8035093562471 | 0.904321102496983 | 3.59678214860821 | 0.998074519377826 |
| ebi-fl187-GCST90094697 | prot-a-3188 | Vesicle-associated membrane protein 8 \|\| id:prot-a-3188 | Banana liking \|\| id:ebi-fl187-GCST90094697 | Inverse variance weighted | 2 | 0.122544829600742 | 0.352196524978385 | 0.727881894905423 | -0.567760359 | 0.812850018558376 | 1.13036979296469 | 0.566793431851239 | 2.25432370427043 | 0.998074519377826 |
| ebi-fl187-GCST90094697 | prot-a-3201 | Vitrin \|\| id:prot-a-3201 | Banana liking \|\| id:ebi-fl187-GCST90094697 | Inverse variance weighted | 2 | -0.306337336 | 0.367570145191979 | 0.404612460766265 | -1.026774821 | 0.414100148320989 | 0.736138251445569 | 0.358160230733142 | 1.51300864457254 | 0.998074519377826 |
| ebi-fl187-GCST90094697 | prot-a-3232 | WNT1-inducible-signaling pathway protein 3 \|\| id:prot-a-3232 | Banana liking \|\| id:ebi-fl187-GCST90094697 | Inverse variance weighted | 2 | 0.38052487578087 | 0.352196524978385 | 0.279949245935443 | -0.309780313 | 1.0708300647385 | 1.46305230866095 | 0.733608102556329 | 2.91780045833636 | 0.998074519377826 |
| ebi-fl187-GCST90094697 | prot-a-327 | Carbonic anhydrase-related protein 10 \|\| id:prot-a-327 | Banana liking \|\| id:ebi-fl187-GCST90094697 | Inverse variance weighted | 2 | 0.246797171247669 | 0.7720333569801 | 0.749217258700801 | -1.266388208 | 1.75998255092867 | 1.27991948197725 | 0.281847760998476 | 5.81233597365272 | 0.998074519377826 |
| ebi-fl187-GCST90094697 | prot-a-332 | Carbonic anhydrase 5A, mitochondrial \|\| id:prot-a-332 | Banana liking \|\| id:ebi-fl187-GCST90094697 | Inverse variance weighted | 2 | 0.0724099691375503 | 0.352196524978385 | 0.837107007177836 | -0.61789522 | 0.762715158095184 | 1.07509600991191 | 0.539077884794981 | 2.14408986740031 | 0.998074519377826 |
| ebi-fl187-GCST90094697 | prot-a-338 | Cell adhesion molecule 3 \|\| id:prot-a-338 | Banana liking \|\| id:ebi-fl187-GCST90094697 | Inverse variance weighted | 2 | 0.0144938023263946 | 0.381157319232612 | 0.969667131688975 | -0.732574543 | 0.761562148022314 | 1.01459934677632 | 0.480669890237631 | 2.14161913484954 | 0.998074519377826 |
| ebi-fl187-GCST90094697 | prot-a-34 | A disintegrin and metalloproteinase with thrombospondin motifs 3 \|\| id:prot-a-34 | Banana liking \|\| id:ebi-fl187-GCST90094697 | Inverse variance weighted | 2 | -0.125218046 | 0.481642094580336 | 0.794878024824717 | -1.069236552 | 0.818800459245103 | 0.882304498525413 | 0.343270486740768 | 2.2677779132992 | 0.998074519377826 |
| ebi-fl187-GCST90094697 | prot-a-358 | Cysteine--tRNA ligase, cytoplasmic \|\| id:prot-a-358 | Banana liking \|\| id:ebi-fl187-GCST90094697 | Inverse variance weighted | 2 | -0.382771148 | 0.352196524978385 | 0.277120232175642 | -1.073076337 | 0.307534041254513 | 0.681968951589817 | 0.341954929168616 | 1.36006710610446 | 0.998074519377826 |
| ebi-fl187-GCST90094697 | prot-a-382 | Coiled-coil domain-containing protein 167 \|\| id:prot-a-382 | Banana liking \|\| id:ebi-fl187-GCST90094697 | Inverse variance weighted | 2 | -0.50507691 | 0.381316343269848 | 0.185316665508811 | -1.252456942 | 0.242303123174981 | 0.603459161819015 | 0.285801735109513 | 1.27418036788254 | 0.998074519377826 |
| ebi-fl187-GCST90094697 | prot-a-40 | Pituitary adenylate cyclase-activating polypeptide 27 \|\| id:prot-a-40 | Banana liking \|\| id:ebi-fl187-GCST90094697 | Inverse variance weighted | 2 | 0.316762833294823 | 0.352196524978385 | 0.36844375328799 | -0.373542356 | 1.00706802225246 | 1.37267698004058 | 0.688291832622157 | 2.73756276368267 | 0.998074519377826 |
| ebi-fl187-GCST90094697 | prot-a-403 | C-C motif chemokine 25 \|\| id:prot-a-403 | Banana liking \|\| id:ebi-fl187-GCST90094697 | Inverse variance weighted | 2 | -0.279076111 | 0.352196524978385 | 0.428134786685538 | -0.9693813 | 0.411229077697625 | 0.756482324 | 0.379317649266022 | 1.50867091983692 | 0.998074519377826 |
| ebi-fl187-GCST90094697 | prot-a-415 | Cyclin-C \|\| id:prot-a-415 | Banana liking \|\| id:ebi-fl187-GCST90094697 | Inverse variance weighted | 2 | 0.818592012927079 | 0.530814476359663 | 0.123038763539591 | -0.221804361 | 1.85898838659202 | 2.26730525260698 | 0.801072070153979 | 6.41724171897679 | 0.998074519377826 |
| ebi-fl187-GCST90094697 | prot-a-44 | Adhesion G protein-coupled receptor F5 \|\| id:prot-a-44 | Banana liking \|\| id:ebi-fl187-GCST90094697 | Inverse variance weighted | 2 | -0.06038706 | 0.352196524978385 | 0.863863321431616 | -0.750692249 | 0.629918128876492 | 0.941400084663827 | 0.472039670603906 | 1.87745686346924 | 0.998074519377826 |
| ebi-fl187-GCST90094697 | prot-a-455 | CD59 glycoprotein \|\| id:prot-a-455 | Banana liking \|\| id:ebi-fl187-GCST90094697 | Inverse variance weighted | 2 | -0.068858259 | 0.352196524978385 | 0.844993007011579 | -0.759163448 | 0.621446930121355 | 0.933458980263937 | 0.468057818077842 | 1.86161972769458 | 0.998074519377826 |
| ebi-fl187-GCST90094697 | prot-a-478 | Cadherin-12 \|\| id:prot-a-478 | Banana liking \|\| id:ebi-fl187-GCST90094697 | Inverse variance weighted | 2 | -0.202454423 | 0.352196524978385 | 0.565403789433092 | -0.892759612 | 0.487850766084876 | 0.816723705670099 | 0.40952406450716 | 1.62881175787861 | 0.998074519377826 |
| ebi-fl187-GCST90094697 | prot-a-516 | Centrin-2 \|\| id:prot-a-516 | Banana liking \|\| id:ebi-fl187-GCST90094697 | Inverse variance weighted | 2 | 0.189421479549972 | 0.352196524978385 | 0.590695158537008 | -0.500883709 | 0.879726668507607 | 1.2085502243562 | 0.605994899625591 | 2.41024082165352 | 0.998074519377826 |
| ebi-fl187-GCST90094697 | prot-a-519 | Cryptic protein \|\| id:prot-a-519 | Banana liking \|\| id:ebi-fl187-GCST90094697 | Inverse variance weighted | 2 | 0.311578681805755 | 0.352196524978385 | 0.376333136163146 | -0.378726507 | 1.00188387076339 | 1.36557922843652 | 0.684732856599326 | 2.72340754670178 | 0.998074519377826 |
| ebi-fl187-GCST90094697 | prot-a-583 | Chloride intracellular channel protein 4 \|\| id:prot-a-583 | Banana liking \|\| id:ebi-fl187-GCST90094697 | Inverse variance weighted | 2 | -0.383183513 | 0.416836229107337 | 0.357956281631009 | -1.200182522 | 0.433815496255736 | 0.681687789375404 | 0.301139242405654 | 1.54313412782498 | 0.998074519377826 |
| ebi-fl187-GCST90094697 | prot-a-602 | CCR4-NOT transcription complex subunit 1 \|\| id:prot-a-602 | Banana liking \|\| id:ebi-fl187-GCST90094697 | Inverse variance weighted | 2 | 0.378426305914939 | 0.517290074006582 | 0.464440027291709 | -0.635462239 | 1.39231485096784 | 1.45998521055916 | 0.52969058799989 | 4.02415459768736 | 0.998074519377826 |
| ebi-fl187-GCST90094697 | prot-a-606 | Ciliary neurotrophic factor receptor subunit alpha \|\| id:prot-a-606 | Banana liking \|\| id:ebi-fl187-GCST90094697 | Inverse variance weighted | 2 | 0.164873512773772 | 0.77305655734615 | 0.831112597679183 | -1.35031734 | 1.68006436517222 | 1.17924394998134 | 0.259158006490818 | 5.36590133717079 | 0.998074519377826 |
| ebi-fl187-GCST90094697 | prot-a-626 | Collagen alpha-2(IX) chain \|\| id:prot-a-626 | Banana liking \|\| id:ebi-fl187-GCST90094697 | Inverse variance weighted | 2 | -0.188618914 | 0.352196524978385 | 0.59226946453169 | -0.878924103 | 0.501686274700234 | 0.828102024446191 | 0.415229415435624 | 1.6515038130727 | 0.998074519377826 |
| ebi-fl187-GCST90094697 | prot-a-647 | Probable serine carboxypeptidase CPVL \|\| id:prot-a-647 | Banana liking \|\| id:ebi-fl187-GCST90094697 | Inverse variance weighted | 2 | -0.790196074 | 0.352196524978385 | 0.0248566955330146 | -1.480501263 | -0.099890885 | 0.453755816787765 | 0.227523610609818 | 0.904936154612194 | 0.998074519377826 |
| ebi-fl187-GCST90094697 | prot-a-651 | Cellular retinoic acid-binding protein 2 \|\| id:prot-a-651 | Banana liking \|\| id:ebi-fl187-GCST90094697 | Inverse variance weighted | 2 | -0.008139908 | 0.574453459196573 | 0.988694489752329 | -1.134068688 | 1.11778887217241 | 0.991893131490537 | 0.321721605086996 | 3.05808490552589 | 0.999355215041207 |
| ebi-fl187-GCST90094697 | prot-a-671 | Cytotoxic and regulatory T-cell molecule \|\| id:prot-a-671 | Banana liking \|\| id:ebi-fl187-GCST90094697 | Inverse variance weighted | 2 | 0.835976658987619 | 0.352196524978385 | 0.0176152176721344 | 0.145671470029984 | 1.52628184794525 | 2.30706616523817 | 1.15681607686421 | 4.60103762148142 | 0.998074519377826 |
| ebi-fl187-GCST90094697 | prot-a-688 | Chorionic somatomammotropin hormone \|\| id:prot-a-688 | Banana liking \|\| id:ebi-fl187-GCST90094697 | Inverse variance weighted | 2 | 0.0276155384067122 | 0.352196524978385 | 0.937502350810828 | -0.662689651 | 0.717920727364346 | 1.02800038177227 | 0.51546305284831 | 2.05016592185305 | 0.998074519377826 |
| ebi-fl187-GCST90094697 | prot-a-69 | Aldo-keto reductase family 1 member C1 \|\| id:prot-a-69 | Banana liking \|\| id:ebi-fl187-GCST90094697 | Inverse variance weighted | 2 | -0.199105893 | 0.352196524978385 | 0.571851968537661 | -0.889411082 | 0.491199295505823 | 0.819463112960607 | 0.410897666375406 | 1.63427502381961 | 0.998074519377826 |
| ebi-fl187-GCST90094697 | prot-a-741 | Stromal cell-derived factor 1 \|\| id:prot-a-741 | Banana liking \|\| id:ebi-fl187-GCST90094697 | Inverse variance weighted | 2 | 0.12002617607502 | 0.352196524978385 | 0.733259280026904 | -0.570279013 | 0.810331365032654 | 1.12752636540782 | 0.565367671827212 | 2.24865298820679 | 0.998074519377826 |
| ebi-fl187-GCST90094697 | prot-a-742 | Growth-regulated alpha protein \|\| id:prot-a-742 | Banana liking \|\| id:ebi-fl187-GCST90094697 | Inverse variance weighted | 2 | 0.0402386894940643 | 0.352196524978385 | 0.909039084763265 | -0.650066499 | 0.730543878451699 | 1.04105923444074 | 0.522011062151167 | 2.07620950626616 | 0.998074519377826 |
| ebi-fl187-GCST90094697 | prot-a-788 | Beta-defensin 112 \|\| id:prot-a-788 | Banana liking \|\| id:ebi-fl187-GCST90094697 | Inverse variance weighted | 2 | -0.650470637 | 0.352196524978385 | 0.0647620977944062 | -1.340775826 | 0.0398345520016253 | 0.521800140533021 | 0.261642600708111 | 1.0406385883774 | 0.998074519377826 |
| ebi-fl187-GCST90094697 | prot-a-838 | DnaJ homolog subfamily B member 1 \|\| id:prot-a-838 | Banana liking \|\| id:ebi-fl187-GCST90094697 | Inverse variance weighted | 2 | 0.0460486783793371 | 0.395448370457993 | 0.907298484536914 | -0.729030128 | 0.821127484477003 | 1.04712538208289 | 0.482376606989659 | 2.27306123455061 | 0.998074519377826 |
| ebi-fl187-GCST90094697 | prot-a-85 | Junctional adhesion molecule-like \|\| id:prot-a-85 | Banana liking \|\| id:ebi-fl187-GCST90094697 | Inverse variance weighted | 2 | 0.214809480450122 | 0.352196524978385 | 0.541918986555458 | -0.475495709 | 0.905114669407756 | 1.23962570153618 | 0.621576859146163 | 2.47221539427953 | 0.998074519377826 |
| ebi-fl187-GCST90094697 | prot-a-856 | Dipeptidase 1 \|\| id:prot-a-856 | Banana liking \|\| id:ebi-fl187-GCST90094697 | Inverse variance weighted | 2 | -0.006393936 | 0.421920207540395 | 0.987909022624944 | -0.833357543 | 0.820569670618191 | 0.993626461551786 | 0.434587687182682 | 2.27179364306496 | 0.999355215041207 |
| ebi-fl187-GCST90094697 | prot-a-860 | Dipeptidyl peptidase 2 \|\| id:prot-a-860 | Banana liking \|\| id:ebi-fl187-GCST90094697 | Inverse variance weighted | 2 | -0.221255465 | 0.892244053169459 | 0.804152669514323 | -1.970053809 | 1.52754287925182 | 0.801511895929002 | 0.139449352358914 | 4.60684333378792 | 0.998074519377826 |
| ebi-fl187-GCST90094697 | prot-a-861 | Dermatopontin \|\| id:prot-a-861 | Banana liking \|\| id:ebi-fl187-GCST90094697 | Inverse variance weighted | 2 | -0.435724354 | 0.352196524978385 | 0.216026738667545 | -1.126029543 | 0.254580835059476 | 0.64679598815849 | 0.324318395730003 | 1.28992081795506 | 0.998074519377826 |
| ebi-fl187-GCST90094697 | prot-a-877 | Dual specificity protein phosphatase 6 \|\| id:prot-a-877 | Banana liking \|\| id:ebi-fl187-GCST90094697 | Inverse variance weighted | 2 | 0.0536848555270697 | 0.352196524978385 | 0.878848828767324 | -0.636620333 | 0.743990044484704 | 1.05515202442422 | 0.529077511421845 | 2.10431509677006 | 0.998074519377826 |
| ebi-fl187-GCST90094697 | prot-a-879 | Dynactin-associated protein \|\| id:prot-a-879 | Banana liking \|\| id:ebi-fl187-GCST90094697 | Inverse variance weighted | 2 | 0.197569105971118 | 0.352196524978385 | 0.574822979942876 | -0.492736083 | 0.887874294928752 | 1.21843726335063 | 0.610952488546824 | 2.42995878165998 | 0.998074519377826 |
| ebi-fl187-GCST90094697 | prot-a-937 | EMI domain-containing protein 1 \|\| id:prot-a-937 | Banana liking \|\| id:ebi-fl187-GCST90094697 | Inverse variance weighted | 2 | 0.175136003816081 | 0.352196524978385 | 0.619000190125375 | -0.515169185 | 0.865441192773715 | 1.19140824166147 | 0.597399514946389 | 2.3760541526825 | 0.998074519377826 |
| ebi-fl187-GCST90094697 | ukb-rsfMRI191-edge-pheno102 | Net25_Pair11_15 (Postcentral\|Precentral)&(Cerebellum) (Motor)&(Subcortical-cerebellum) \|\| id:ukb-rsfMRI191-edge-pheno102 | Banana liking \|\| id:ebi-fl187-GCST90094697 | Inverse variance weighted | 2 | 0.133052665204595 | 0.108682604828326 | 0.220864920193312 | -0.07996524 | 0.346070570668115 | 1.14231015674487 | 0.923148434229498 | 1.4135023641042 | 0.998074519377826 |
| ebi-fl187-GCST90094697 | ukb-rsfMRI191-edge-pheno1167 | Net100_Pair11_45 (Angular\|Temporal)&(Frontal) (Default_mode\|Central_executive)&(Salience\|Default_mode) \|\| id:ukb-rsfMRI191-edge-pheno1167 | Banana liking \|\| id:ebi-fl187-GCST90094697 | Inverse variance weighted | 2 | -0.126415809 | 0.111091016847269 | 0.255142164808476 | -0.344154202 | 0.0913225841784942 | 0.881248339738466 | 0.708819618316311 | 1.09562237870403 | 0.998074519377826 |
| ebi-fl187-GCST90094697 | ukb-rsfMRI191-edge-pheno1317 | Net100_Pair26_48 (Parietal\|Frontal)&(Temporal) (Attention\|Central_executive)&(Default_mode\|Central_executive) \|\| id:ukb-rsfMRI191-edge-pheno1317 | Banana liking \|\| id:ebi-fl187-GCST90094697 | Inverse variance weighted | 2 | 0.100235142621921 | 0.180045718686897 | 0.577718407993997 | -0.252654466 | 0.453124751248239 | 1.10543082141897 | 0.776736224231445 | 1.57322043548585 | 0.998074519377826 |
| ebi-fl187-GCST90094697 | ukb-rsfMRI191-edge-pheno286 | Net100_Pair10_13 (OccipitalPrecuneus)&(Frontal_Sup) (Default_mode\|Central_executive)&(Default_mode) \|\| id:ukb-rsfMRI191-edge-pheno286 | Banana liking \|\| id:ebi-fl187-GCST90094697 | Inverse variance weighted | 2 | -0.366544105 | 0.191704109657939 | 0.0558722816297663 | -0.74228416 | 0.0091959495702692 | 0.693125565256293 | 0.476025354559091 | 1.00923836222303 | 0.998074519377826 |
| ebi-fl187-GCST90094697 | ukb-rsfMRI191-edge-pheno599 | Net100_Pair11_29 (Angular\|Temporal)&(Parietal) (Default_mode\|Central_executive)&(Central_executive\|Salience) \|\| id:ukb-rsfMRI191-edge-pheno599 | Banana liking \|\| id:ebi-fl187-GCST90094697 | Inverse variance weighted | 2 | 0.101088051658116 | 0.109150932030519 | 0.354377953870643 | -0.112847775 | 0.315023878437933 | 1.10637405554473 | 0.893286630215307 | 1.37029203099956 | 0.998074519377826 |
| ebi-fl187-GCST90094697 | ukb-rsfMRI191-edge-pheno621 | Net100_Pair5_30 (Precuneus\|Angular\|Cingulate)&(Paracentral) (Default_mode\|Central_executive)&(Motor) \|\| id:ukb-rsfMRI191-edge-pheno621 | Banana liking \|\| id:ebi-fl187-GCST90094697 | Inverse variance weighted | 2 | 0.0502769760982427 | 0.111039836708377 | 0.650704839339797 | -0.167361104 | 0.267915056046661 | 1.05156231367075 | 0.845894100563395 | 1.30723609349691 | 0.998074519377826 |
| ebi-fl187-GCST90094697 | ukb-rsfMRI191-edge-pheno624 | Net100_Pair8_30 (Calcarine\|Lingual)&(Paracentral) (Visual)&(Motor) \|\| id:ukb-rsfMRI191-edge-pheno624 | Banana liking \|\| id:ebi-fl187-GCST90094697 | Inverse variance weighted | 2 | -0.161753919 | 0.10933759773517 | 0.139033740135314 | -0.376055611 | 0.0525477722723846 | 0.850650507468545 | 0.686564151564967 | 1.05395291060142 | 0.998074519377826 |
| ebi-fl187-GCST90094697 | ukb-rsfMRI191-edge-pheno882 | Net100_Pair6_38 (Postcentral\|Precentral)&(Subcortical) (Motor)&(Subcortical-cerebellum) \|\| id:ukb-rsfMRI191-edge-pheno882 | Banana liking \|\| id:ebi-fl187-GCST90094697 | Inverse variance weighted | 2 | 0.0695787014416159 | 0.135553665234654 | 0.607746617392278 | -0.196106482 | 0.335263885301537 | 1.0720564302713 | 0.821924709487591 | 1.39830932981994 | 0.998074519377826 |
| ebi-fl187-GCST90094697 | ukb-rsfMRI191-edge-pheno899 | Net100_Pair23_38 (Cerebellum)&(Subcortical) (Subcortical-cerebellum)&(Subcortical-cerebellum) \|\| id:ukb-rsfMRI191-edge-pheno899 | Banana liking \|\| id:ebi-fl187-GCST90094697 | Inverse variance weighted | 2 | -0.135098018 | 0.109284743901344 | 0.216383491193486 | -0.349296116 | 0.0791000801603092 | 0.8736302760981 | 0.705184283048101 | 1.08231263467224 | 0.998074519377826 |
| ebi-fl187-GCST90094727 | ebi-a-GCST90001392 | IgD+ CD38+ B cell Absolute Count \|\| id:ebi-a-GCST90001392 | Cauliflower liking \|\| id:ebi-fl187-GCST90094727 | Inverse variance weighted (multiplicative random effects) | 4 | -0.264314815 | 0.573086153842705 | 0.644645756725654 | -1.387563677 | 0.858934046100005 | 0.767731807787745 | 0.249682872349278 | 2.3606430154509 | 0.998074519377826 |
| ebi-fl187-GCST90094727 | ebi-a-GCST90001402 | Switched memory B cell %B cell \|\| id:ebi-a-GCST90001402 | Cauliflower liking \|\| id:ebi-fl187-GCST90094727 | Inverse variance weighted | 4 | 0.0600746181642989 | 0.35047858092364 | 0.863903445835981 | -0.6268634 | 0.747012636774632 | 1.06191578179541 | 0.534264950841153 | 2.11068520562829 | 0.998074519377826 |
| ebi-fl187-GCST90094727 | ebi-a-GCST90001407 | Memory B cell Absolute Count \|\| id:ebi-a-GCST90001407 | Cauliflower liking \|\| id:ebi-fl187-GCST90094727 | Inverse variance weighted | 4 | 0.187908842918814 | 0.34705183215631 | 0.588202160953937 | -0.492312748 | 0.868130433945182 | 1.20672350894288 | 0.611211180797478 | 2.38245253487584 | 0.998074519377826 |
| ebi-fl187-GCST90094727 | ebi-a-GCST90001411 | IgD+ CD24+ B cell %B cell \|\| id:ebi-a-GCST90001411 | Cauliflower liking \|\| id:ebi-fl187-GCST90094727 | Inverse variance weighted | 4 | 0.517826682642916 | 0.470204781224444 | 0.270775218963167 | -0.403774689 | 1.43942805384283 | 1.67837603930413 | 0.667794566077139 | 4.21828249645391 | 0.998074519377826 |
| ebi-fl187-GCST90094727 | ebi-a-GCST90001418 | CD24+ CD27+ B cell Absolute Count \|\| id:ebi-a-GCST90001418 | Cauliflower liking \|\| id:ebi-fl187-GCST90094727 | Inverse variance weighted | 4 | 0.0513643669044562 | 0.345886363322161 | 0.881947633946791 | -0.626572905 | 0.729301639015891 | 1.0527063947817 | 0.534420174810587 | 2.07363195823784 | 0.998074519377826 |
| ebi-fl187-GCST90094727 | ebi-a-GCST90001436 | Memory B cell %lymphocyte \|\| id:ebi-a-GCST90001436 | Cauliflower liking \|\| id:ebi-fl187-GCST90094727 | Inverse variance weighted | 4 | 0.500698779904912 | 0.342529169429479 | 0.143804314941257 | -0.170658392 | 1.17205595198669 | 1.64987376661649 | 0.843109537097137 | 3.22862371494639 | 0.998074519377826 |
| ebi-fl187-GCST90094727 | ebi-a-GCST90001439 | IgD+ CD24+ B cell %lymphocyte \|\| id:ebi-a-GCST90001439 | Cauliflower liking \|\| id:ebi-fl187-GCST90094727 | Inverse variance weighted | 4 | 0.413784965942011 | 0.345503203402109 | 0.231061131663487 | -0.263401313 | 1.09097124461015 | 1.51253184605173 | 0.768433453300059 | 2.97716422351971 | 0.998074519377826 |
| ebi-fl187-GCST90094727 | ebi-a-GCST90001457 | CD11c+ HLA DR++ monocyte %monocyte \|\| id:ebi-a-GCST90001457 | Cauliflower liking \|\| id:ebi-fl187-GCST90094727 | Inverse variance weighted | 4 | 0.0201104182562414 | 0.374483168250951 | 0.95717275282627 | -0.713876592 | 0.754097428028105 | 1.02031399509921 | 0.489741983172468 | 2.12569206718121 | 0.998074519377826 |
| ebi-fl187-GCST90094727 | ebi-a-GCST90001483 | CD39+ resting CD4 regulatory T cell Absolute Count \|\| id:ebi-a-GCST90001483 | Cauliflower liking \|\| id:ebi-fl187-GCST90094727 | Inverse variance weighted | 4 | -0.011657151 | 0.350696789746385 | 0.973483224687252 | -0.699022858 | 0.675708557316069 | 0.988410530747278 | 0.497070775053553 | 1.96542509904522 | 0.998074519377826 |
| ebi-fl187-GCST90094727 | ebi-a-GCST90001525 | CD33dim HLA DR+ CD11b+ Absolute Count \|\| id:ebi-a-GCST90001525 | Cauliflower liking \|\| id:ebi-fl187-GCST90094727 | Inverse variance weighted | 4 | -0.105609638 | 0.453483881765144 | 0.815850631086932 | -0.994438046 | 0.783218770415563 | 0.899775817957857 | 0.36993127046306 | 2.1885052365763 | 0.998074519377826 |
| ebi-fl187-GCST90094727 | ebi-a-GCST90001540 | Naive CD4+ T cell Absolute Count \|\| id:ebi-a-GCST90001540 | Cauliflower liking \|\| id:ebi-fl187-GCST90094727 | Inverse variance weighted | 4 | -0.529873003 | 0.332069212817736 | 0.110562407690367 | -1.18072866 | 0.12098265404105 | 0.588679725442368 | 0.307054918366983 | 1.12860533545574 | 0.998074519377826 |
| ebi-fl187-GCST90094727 | ebi-a-GCST90001624 | HLA DR+ CD4+ T cell Absolute Count \|\| id:ebi-a-GCST90001624 | Cauliflower liking \|\| id:ebi-fl187-GCST90094727 | Inverse variance weighted | 4 | -0.232578031 | 0.336922382891901 | 0.490004595579807 | -0.892945902 | 0.427789839242563 | 0.792487908131322 | 0.40944778143077 | 1.533863689 | 0.998074519377826 |
| ebi-fl187-GCST90094727 | ebi-a-GCST90001672 | CD39+ CD8+ T cell Absolute Count \|\| id:ebi-a-GCST90001672 | Cauliflower liking \|\| id:ebi-fl187-GCST90094727 | Inverse variance weighted | 4 | 0.265287285805125 | 0.448313088555038 | 0.554020973801163 | -0.613406368 | 1.143980939 | 1.30380548678894 | 0.541503164956721 | 3.13924064971405 | 0.998074519377826 |
| ebi-fl187-GCST90094727 | ebi-a-GCST90001722 | CD19 on CD20- CD38- B cell \|\| id:ebi-a-GCST90001722 | Cauliflower liking \|\| id:ebi-fl187-GCST90094727 | Inverse variance weighted | 4 | -0.71846377 | 0.336221485014156 | 0.0326080427231497 | -1.377457881 | -0.059469659 | 0.48750059405256 | 0.252218909365507 | 0.942264122065465 | 0.998074519377826 |
| ebi-fl187-GCST90094727 | ebi-a-GCST90001742 | CD19 on transitional B cell \|\| id:ebi-a-GCST90001742 | Cauliflower liking \|\| id:ebi-fl187-GCST90094727 | Inverse variance weighted | 4 | -0.337727848 | 0.343606440035301 | 0.325660843129661 | -1.011196471 | 0.3357407742148 | 0.713389411655206 | 0.363783462818971 | 1.3989763270658 | 0.998074519377826 |
| ebi-fl187-GCST90094727 | ebi-a-GCST90001784 | CD25 on IgD+ CD38dim B cell \|\| id:ebi-a-GCST90001784 | Cauliflower liking \|\| id:ebi-fl187-GCST90094727 | Inverse variance weighted | 4 | 0.552731383298637 | 0.345181491396388 | 0.109315452412054 | -0.12382434 | 1.22928710643556 | 1.73799366753109 | 0.883535029158257 | 3.41879143292813 | 0.998074519377826 |
| ebi-fl187-GCST90094727 | ebi-a-GCST90001794 | CD25 on IgD+ B cell \|\| id:ebi-a-GCST90001794 | Cauliflower liking \|\| id:ebi-fl187-GCST90094727 | Inverse variance weighted | 4 | 0.686572846209857 | 0.344242731014818 | 0.0461035551106732 | 0.0118570934208141 | 1.3612885989989 | 1.9868944586091 | 1.01192766741131 | 3.90121716876323 | 0.998074519377826 |
| ebi-fl187-GCST90094727 | ebi-a-GCST90001802 | CD27 on IgD- CD38- B cell \|\| id:ebi-a-GCST90001802 | Cauliflower liking \|\| id:ebi-fl187-GCST90094727 | Inverse variance weighted | 4 | 0.279337668383422 | 0.344848867341139 | 0.417923366515593 | -0.396566112 | 0.955241448372053 | 1.32225375178322 | 0.672625806859469 | 2.59929810345513 | 0.998074519377826 |
| ebi-fl187-GCST90094727 | ebi-a-GCST90001804 | CD27 on IgD- CD38dim B cell \|\| id:ebi-a-GCST90001804 | Cauliflower liking \|\| id:ebi-fl187-GCST90094727 | Inverse variance weighted | 4 | 0.64770110864281 | 0.339898216911056 | 0.0567055348126547 | -0.018499397 | 1.31390161378848 | 1.91114226660345 | 0.981670667027052 | 3.7206620161724 | 0.998074519377826 |
| ebi-fl187-GCST90094727 | ebi-a-GCST90001805 | CD27 on memory B cell \|\| id:ebi-a-GCST90001805 | Cauliflower liking \|\| id:ebi-fl187-GCST90094727 | Inverse variance weighted | 4 | 0.435892089771994 | 0.344696678861449 | 0.206026542408596 | -0.239713401 | 1.11149758034043 | 1.54634191963665 | 0.786853340294568 | 3.03890599426113 | 0.998074519377826 |
| ebi-fl187-GCST90094727 | ebi-a-GCST90001806 | CD27 on unswitched memory B cell \|\| id:ebi-a-GCST90001806 | Cauliflower liking \|\| id:ebi-fl187-GCST90094727 | Inverse variance weighted | 4 | 0.579271149709913 | 0.397216193254858 | 0.144750396606519 | -0.199272589 | 1.35781488848943 | 1.7847371503986 | 0.819326523435484 | 3.88768897979383 | 0.998074519377826 |
| ebi-fl187-GCST90094727 | ebi-a-GCST90001839 | CD3 on Effector Memory CD8+ T cell \|\| id:ebi-a-GCST90001839 | Cauliflower liking \|\| id:ebi-fl187-GCST90094727 | Inverse variance weighted | 4 | 0.38646640123742 | 0.382555727029501 | 0.312388703925001 | -0.363342824 | 1.13627562621524 | 1.47177094655305 | 0.695348010827001 | 3.11514476979868 | 0.998074519377826 |
| ebi-fl187-GCST90094727 | ebi-a-GCST90001859 | CD3 on CD8+ T cell \|\| id:ebi-a-GCST90001859 | Cauliflower liking \|\| id:ebi-fl187-GCST90094727 | Inverse variance weighted | 4 | 0.332742715495718 | 0.381079693262873 | 0.382577060141438 | -0.414173483 | 1.07965891429095 | 1.39478839486086 | 0.660886288586223 | 2.94367533422466 | 0.998074519377826 |
| ebi-fl187-GCST90094727 | ebi-a-GCST90001905 | CD86 on monocyte \|\| id:ebi-a-GCST90001905 | Cauliflower liking \|\| id:ebi-fl187-GCST90094727 | Inverse variance weighted | 4 | 0.12660678689487 | 0.381589568244716 | 0.740049450555084 | -0.621308767 | 0.874522340654513 | 1.13497064469593 | 0.537240854253373 | 2.39772972238255 | 0.998074519377826 |
| ebi-fl187-GCST90094727 | ebi-a-GCST90001943 | CD25 on activated & secreting CD4 regulatory T cell \|\| id:ebi-a-GCST90001943 | Cauliflower liking \|\| id:ebi-fl187-GCST90094727 | Inverse variance weighted | 4 | 0.363479552368297 | 0.350056649622819 | 0.299109491398035 | -0.322631481 | 1.04959058562902 | 1.43832544625558 | 0.724240701732788 | 2.85648139409543 | 0.998074519377826 |
| ebi-fl187-GCST90094727 | ebi-a-GCST90001949 | CD33 on CD33dim HLA DR+ CD11b- \|\| id:ebi-a-GCST90001949 | Cauliflower liking \|\| id:ebi-fl187-GCST90094727 | Inverse variance weighted | 4 | -0.411248662 | 0.675404955902816 | 0.542596038932662 | -1.735042376 | 0.912545051072207 | 0.66282209211018 | 0.176392725298045 | 2.49065331377464 | 0.998074519377826 |
| ebi-fl187-GCST90094727 | ebi-a-GCST90001955 | CD33 on Immature Myeloid-Derived Suppressor Cells \|\| id:ebi-a-GCST90001955 | Cauliflower liking \|\| id:ebi-fl187-GCST90094727 | Inverse variance weighted | 4 | -0.208292092 | 0.51490104992302 | 0.685824205842856 | -1.21749815 | 0.800913965393583 | 0.81196983180875 | 0.295969713158199 | 2.22757592569996 | 0.998074519377826 |
| ebi-fl187-GCST90094727 | ebi-a-GCST90001956 | CD33 on CD33+ HLA DR+ \|\| id:ebi-a-GCST90001956 | Cauliflower liking \|\| id:ebi-fl187-GCST90094727 | Inverse variance weighted | 4 | -0.373551365 | 0.514184689248824 | 0.46753684997919 | -1.381353356 | 0.634250625564922 | 0.688285631347195 | 0.251238307930801 | 1.88560858501518 | 0.998074519377826 |
| ebi-fl187-GCST90094727 | ebi-a-GCST90001957 | CD33 on CD33+ HLA DR+ CD14- \|\| id:ebi-a-GCST90001957 | Cauliflower liking \|\| id:ebi-fl187-GCST90094727 | Inverse variance weighted | 4 | -0.435092078 | 0.514520910540005 | 0.397761612787894 | -1.443553063 | 0.573368906164545 | 0.647205070666174 | 0.236087433141894 | 1.77423422298066 | 0.998074519377826 |
| ebi-fl187-GCST90094727 | ebi-a-GCST90002064 | CD4 on resting CD4 regulatory T cell \|\| id:ebi-a-GCST90002064 | Cauliflower liking \|\| id:ebi-fl187-GCST90094727 | Inverse variance weighted | 4 | 0.272772194944283 | 0.370310485466411 | 0.461363185153293 | -0.453036357 | 0.998580746458449 | 1.31360096579619 | 0.635695021515804 | 2.71442663374357 | 0.998074519377826 |
| ebi-fl187-GCST90094727 | ebi-a-GCST90002080 | SSC-A on T cell \|\| id:ebi-a-GCST90002080 | Cauliflower liking \|\| id:ebi-fl187-GCST90094727 | Inverse variance weighted | 4 | 0.390155459743805 | 0.353881613324519 | 0.270243153936558 | -0.303452502 | 1.08376342185986 | 1.47721042278703 | 0.738264954133011 | 2.95578250189765 | 0.998074519377826 |
| ebi-fl187-GCST90094727 | ebi-a-GCST90002121 | CD8 on CD39+ CD8+ T cell \|\| id:ebi-a-GCST90002121 | Cauliflower liking \|\| id:ebi-fl187-GCST90094727 | Inverse variance weighted | 4 | -0.597520051 | 0.375776293683588 | 0.111813365425634 | -1.334041587 | 0.139001484177673 | 0.550174349750962 | 0.263410510557427 | 1.1491258055092 | 0.998074519377826 |
| ebi-fl187-GCST90094727 | ebi-cfmet338-GCST90026053 | 1-palmitoyl-2-docosahexaenoyl-gpc (16:0/22:6) levels \|\| id:ebi-cfmet338-GCST90026053 | Cauliflower liking \|\| id:ebi-fl187-GCST90094727 | Inverse variance weighted | 4 | 0.679356664710315 | 0.189918034321076 | 0.00034742124257211 | 0.307117317441007 | 1.05159601197962 | 1.97260827541475 | 1.35950045183074 | 2.86221560500018 | 0.443070605573995 |
| ebi-fl187-GCST90094727 | ebi-cfmet338-GCST90026063 | 1-stearoyl-2-oleoyl-gpc (18:0/18:1) levels \|\| id:ebi-cfmet338-GCST90026063 | Cauliflower liking \|\| id:ebi-fl187-GCST90094727 | Inverse variance weighted | 4 | 0.439670342726477 | 0.158353085188757 | 0.00549444310607977 | 0.129298295756514 | 0.750042389696441 | 1.55219544164322 | 1.13802954292307 | 2.11708975750278 | 0.877504772527479 |
| ebi-fl187-GCST90094727 | ebi-cfmet338-GCST90026113 | Adenosine 3',5'-cyclic monophosphate (camp) levels \|\| id:ebi-cfmet338-GCST90026113 | Cauliflower liking \|\| id:ebi-fl187-GCST90094727 | Inverse variance weighted | 4 | 0.373263282637751 | 0.304582251197003 | 0.220389691736164 | -0.22371793 | 0.970244494983877 | 1.45246669876003 | 0.799540629223622 | 2.63858950239389 | 0.998074519377826 |
| ebi-fl187-GCST90094727 | ebi-cfmet338-GCST90026117 | Alpha-ketoglutaramate levels \|\| id:ebi-cfmet338-GCST90026117 | Cauliflower liking \|\| id:ebi-fl187-GCST90094727 | Inverse variance weighted | 4 | 0.3210575710679 | 0.121224207850303 | 0.00808592092085209 | 0.0834581236813056 | 0.558657018454495 | 1.37858494521667 | 1.08703969311911 | 1.74832295748543 | 0.998074519377826 |
| ebi-fl187-GCST90094727 | ebi-cfmet338-GCST90026124 | Arginine levels \|\| id:ebi-cfmet338-GCST90026124 | Cauliflower liking \|\| id:ebi-fl187-GCST90094727 | Inverse variance weighted | 4 | -0.054738992 | 0.098937792363765 | 0.580080759655136 | -0.248657065 | 0.139179081347409 | 0.9467322206892 | 0.779847364708625 | 1.14932990512315 | 0.998074519377826 |
| ebi-fl187-GCST90094727 | ebi-cfmet338-GCST90026169 | Glutamate levels \|\| id:ebi-cfmet338-GCST90026169 | Cauliflower liking \|\| id:ebi-fl187-GCST90094727 | Inverse variance weighted | 4 | 0.165489344280612 | 0.1126107367798 | 0.141678289239316 | -0.0552277 | 0.38620638836902 | 1.17997038921912 | 0.946269658001655 | 1.47138831691408 | 0.998074519377826 |
| ebi-fl187-GCST90094727 | ebi-cfmet338-GCST90026170 | Glutamine levels \|\| id:ebi-cfmet338-GCST90026170 | Cauliflower liking \|\| id:ebi-fl187-GCST90094727 | Inverse variance weighted | 4 | 0.0235559074356002 | 0.0688325836826803 | 0.732185109361173 | -0.111355957 | 0.158467771453654 | 1.02383553916606 | 0.894620246280378 | 1.17171416097253 | 0.998074519377826 |
| ebi-fl187-GCST90094727 | ebi-cfmet338-GCST90026191 | Kynurenate levels \|\| id:ebi-cfmet338-GCST90026191 | Cauliflower liking \|\| id:ebi-fl187-GCST90094727 | Inverse variance weighted | 4 | -0.378913099 | 0.544298526479149 | 0.486335264952629 | -1.445738211 | 0.687912012904394 | 0.684605102953012 | 0.235572110453699 | 1.9895570239051 | 0.998074519377826 |
| ebi-fl187-GCST90094727 | ebi-cfmet338-GCST90026202 | Methylsuccinoylcarnitine levels \|\| id:ebi-cfmet338-GCST90026202 | Cauliflower liking \|\| id:ebi-fl187-GCST90094727 | Inverse variance weighted | 4 | 0.514430337134123 | 0.31695661493924 | 0.104582828461702 | -0.106804628 | 1.13566530241503 | 1.67268536360165 | 0.898701236765749 | 3.11324410287471 | 0.998074519377826 |
| ebi-fl187-GCST90094727 | ebi-cfmet338-GCST90026222 | N-acetylputrescine levels \|\| id:ebi-cfmet338-GCST90026222 | Cauliflower liking \|\| id:ebi-fl187-GCST90094727 | Inverse variance weighted | 4 | -0.060599477 | 0.117304929942468 | 0.60543690285528 | -0.29051714 | 0.169318185731009 | 0.941200136636558 | 0.747876710862105 | 1.18449696900377 | 0.998074519377826 |
| ebi-fl187-GCST90094727 | ebi-cfmet338-GCST90026252 | Propionylcarnitine (c3) levels \|\| id:ebi-cfmet338-GCST90026252 | Cauliflower liking \|\| id:ebi-fl187-GCST90094727 | Inverse variance weighted | 4 | 0.0783678171467502 | 0.188216285674839 | 0.677138491064779 | -0.290536103 | 0.447271737069434 | 1.0815203872629 | 0.747862528911433 | 1.56403924898851 | 0.998074519377826 |
| ebi-fl187-GCST90094727 | ebi-cfmet338-GCST90026316 | Ethyl beta-glucopyranoside levels \|\| id:ebi-cfmet338-GCST90026316 | Cauliflower liking \|\| id:ebi-fl187-GCST90094727 | Inverse variance weighted | 4 | -0.3459828 | 0.615497310756258 | 0.574034870080539 | -1.552357529 | 0.860391929526261 | 0.707524656740073 | 0.211748182517678 | 2.36408706768176 | 0.998074519377826 |
| ebi-fl187-GCST90094727 | ebi-gm430-GCST90011315 | F_Prevotellaceae abundance \|\| id:ebi-gm430-GCST90011315 | Cauliflower liking \|\| id:ebi-fl187-GCST90094727 | Inverse variance weighted | 4 | 0.110464514686364 | 0.263685629802809 | 0.675270911729379 | -0.40635932 | 0.627288349099871 | 1.11679671846715 | 0.666070792046059 | 1.87252605169443 | 0.998074519377826 |
| ebi-fl187-GCST90094727 | ebi-gm430-GCST90011354 | OTU97_1 (Bacteroides) abundance \|\| id:ebi-gm430-GCST90011354 | Cauliflower liking \|\| id:ebi-fl187-GCST90094727 | Inverse variance weighted | 4 | -0.467688016 | 0.239432713387561 | 0.0507820309922921 | -0.936976135 | 0.00160010186002613 | 0.626448934980139 | 0.391810829052053 | 1.00160138270608 | 0.998074519377826 |
| ebi-fl187-GCST90094727 | ebi-gm430-GCST90011419 | OTU99_1 (Bacteroides) abundance \|\| id:ebi-gm430-GCST90011419 | Cauliflower liking \|\| id:ebi-fl187-GCST90094727 | Inverse variance weighted | 4 | -0.463048237 | 0.239471966797105 | 0.053159634019461 | -0.932413292 | 0.00631681820586627 | 0.629362273406424 | 0.393602685213403 | 1.00633681137756 | 0.998074519377826 |
| ebi-fl187-GCST90094727 | ebi-gm430-GCST90011435 | OTU99_155 (Butyrivibrio) abundance \|\| id:ebi-gm430-GCST90011435 | Cauliflower liking \|\| id:ebi-fl187-GCST90094727 | Inverse variance weighted | 4 | -0.022481985 | 0.922098543185281 | 0.980548443958007 | -1.829795129 | 1.7848311598811 | 0.977768851773197 | 0.160446435164883 | 5.95857381633571 | 0.999115041713425 |
| ebi-fl187-GCST90094727 | ebi-gm430-GCST90011466 | OTU99_58 (Bacteroides) abundance \|\| id:ebi-gm430-GCST90011466 | Cauliflower liking \|\| id:ebi-fl187-GCST90094727 | Inverse variance weighted | 4 | -0.653526334 | 0.44086763883538 | 0.138243456755249 | -1.517626906 | 0.21057423774751 | 0.52020811081238 | 0.219231527045719 | 1.23438668790803 | 0.998074519377826 |
| ebi-fl187-GCST90094727 | ebi-gm430-GCST90011472 | OTU99_72 (Parabacteroides) abundance \|\| id:ebi-gm430-GCST90011472 | Cauliflower liking \|\| id:ebi-fl187-GCST90094727 | Inverse variance weighted | 4 | -0.109360872 | 0.693210870189735 | 0.874646004231692 | -1.468054178 | 1.24933243345486 | 0.896406870877061 | 0.230373314882898 | 3.48801369882643 | 0.998074519377826 |
| ebi-fl187-GCST90094727 | ebi-gm430-GCST90011505 | TestASV_28 (Holdemanella) abundance \|\| id:ebi-gm430-GCST90011505 | Cauliflower liking \|\| id:ebi-fl187-GCST90094727 | Inverse variance weighted | 4 | 0.162427669446564 | 0.56106292151589 | 0.772198843075481 | -0.937255657 | 1.26211099561771 | 1.17636322836807 | 0.391701324569386 | 3.5328714973784 | 0.998074519377826 |
| ebi-fl187-GCST90094727 | ebi-gm430-GCST90011513 | TestASV_35 (Alphaproteobacteria) abundance \|\| id:ebi-gm430-GCST90011513 | Cauliflower liking \|\| id:ebi-fl187-GCST90094727 | Inverse variance weighted | 4 | 0.791290077660407 | 0.854251690926584 | 0.354292080827383 | -0.883043237 | 2.46562339187651 | 2.20624081310795 | 0.413522547927315 | 11.7708176974156 | 0.998074519377826 |
| ebi-fl187-GCST90094727 | ebi-gm430-GCST90011543 | G_Coprococcus prevalence \|\| id:ebi-gm430-GCST90011543 | Cauliflower liking \|\| id:ebi-fl187-GCST90094727 | Inverse variance weighted | 4 | -0.286229505 | 1.07867387159587 | 0.790737427269498 | -2.400430293 | 1.82797128369323 | 0.751090217478543 | 0.0906789263896337 | 6.22125269070738 | 0.998074519377826 |
| ebi-fl187-GCST90094727 | ebi-gm430-GCST90011547 | G_Parasutterella prevalence \|\| id:ebi-gm430-GCST90011547 | Cauliflower liking \|\| id:ebi-fl187-GCST90094727 | Inverse variance weighted | 4 | 0.775037014553837 | 0.63447582043055 | 0.221881975642494 | -0.468535593 | 2.01860962259772 | 2.17067247216955 | 0.6259181961548 | 7.52785110000119 | 0.998074519377826 |
| ebi-fl187-GCST90094727 | ebi-gm430-GCST90011569 | OTU97_13 (Subdoligranulum) prevalence \|\| id:ebi-gm430-GCST90011569 | Cauliflower liking \|\| id:ebi-fl187-GCST90094727 | Inverse variance weighted | 4 | 0.0150911511866455 | 0.643061418322898 | 0.981277229423177 | -1.245309229 | 1.27549153109953 | 1.01520559759332 | 0.2878518822959 | 3.58046088552358 | 0.999355215041207 |
| ebi-fl187-GCST90094727 | ebi-gm430-GCST90011590 | OTU97_277 (Prevotella) prevalence \|\| id:ebi-gm430-GCST90011590 | Cauliflower liking \|\| id:ebi-fl187-GCST90094727 | Inverse variance weighted | 4 | 0.136644689631011 | 0.56140700687978 | 0.807697994013385 | -0.963713044 | 1.23700242311538 | 1.14642074097013 | 0.381473824048277 | 3.44527050736821 | 0.998074519377826 |
| ebi-fl187-GCST90094727 | ebi-gm430-GCST90011616 | OTU97_86 (Phascolarctobacterium) prevalence \|\| id:ebi-gm430-GCST90011616 | Cauliflower liking \|\| id:ebi-fl187-GCST90094727 | Inverse variance weighted | 4 | 0.319077826790013 | 0.731923715477063 | 0.662877286373849 | -1.115492656 | 1.75364830912506 | 1.37585839938209 | 0.327753769406679 | 5.77563558941536 | 0.998074519377826 |
| ebi-fl187-GCST90094727 | ebi-gm430-GCST90011665 | OTU99_55 (Barnesiella) prevalence \|\| id:ebi-gm430-GCST90011665 | Cauliflower liking \|\| id:ebi-fl187-GCST90094727 | Inverse variance weighted | 4 | -0.283681888 | 0.495390671067234 | 0.566886592009527 | -1.254647603 | 0.687283827405438 | 0.753006146986905 | 0.285176325750304 | 1.98830760550768 | 0.998074519377826 |
| ebi-fl187-GCST90094727 | ebi-gm430-GCST90011687 | TestASV_14 (Lachnospiraceae) prevalence \|\| id:ebi-gm430-GCST90011687 | Cauliflower liking \|\| id:ebi-fl187-GCST90094727 | Inverse variance weighted | 4 | 0.57702905862391 | 0.501709181771061 | 0.250091745792613 | -0.406320938 | 1.56037905489519 | 1.78074008970434 | 0.666096357718962 | 4.76062544154929 | 0.998074519377826 |
| ebi-fl187-GCST90094727 | ebi-gm430-GCST90011697 | TestASV_23 (Barnesiella) prevalence \|\| id:ebi-gm430-GCST90011697 | Cauliflower liking \|\| id:ebi-fl187-GCST90094727 | Inverse variance weighted | 4 | 0.19430697888176 | 0.813918480598731 | 0.811314742986981 | -1.400973243 | 1.78958720085527 | 1.21446904208719 | 0.246357081900708 | 5.98698053576813 | 0.998074519377826 |
| ebi-fl187-GCST90094727 | ebi-gm430-GCST90011727 | TestASV_6 (Subdoligranulum) prevalence \|\| id:ebi-gm430-GCST90011727 | Cauliflower liking \|\| id:ebi-fl187-GCST90094727 | Inverse variance weighted | 4 | -0.820720487 | 0.756162846436038 | 0.277755461392195 | -2.302799666 | 0.661358692270789 | 0.440114443624197 | 0.0999785450254653 | 1.937422908 | 0.998074519377826 |
| ebi-fl187-GCST90094727 | ebi-hbsm44-GCST90104908 | Sulcal depth of the left posterior inferior temporal sulcus \|\| id:ebi-hbsm44-GCST90104908 | Cauliflower liking \|\| id:ebi-fl187-GCST90094727 | Inverse variance weighted | 4 | -0.073367037 | 0.274987423527135 | 0.789622056063171 | -0.612342387 | 0.465608313316174 | 0.929259694923039 | 0.542079620570318 | 1.59298292693599 | 0.998074519377826 |
| ebi-fl187-GCST90094727 | ebi-hbsm44-GCST90104918 | Sulcal depth of the left inferior frontal sulcus \|\| id:ebi-hbsm44-GCST90104918 | Cauliflower liking \|\| id:ebi-fl187-GCST90094727 | Inverse variance weighted | 4 | -0.295035532 | 0.365606119109293 | 0.419680044372096 | -1.011623526 | 0.421552461008793 | 0.744505132907914 | 0.363628140376198 | 1.52432617660663 | 0.998074519377826 |
| ebi-fl187-GCST90094727 | ebi-hbsm44-GCST90104925 | Sulcal depth of the right anterior inferior temporal sulcus \|\| id:ebi-hbsm44-GCST90104925 | Cauliflower liking \|\| id:ebi-fl187-GCST90094727 | Inverse variance weighted | 4 | -0.028207435 | 0.124485639165114 | 0.820741109918957 | -0.272199287 | 0.215784418235191 | 0.97218668079797 | 0.761702448344882 | 1.24083484879785 | 0.998074519377826 |
| ebi-fl187-GCST90094727 | ebi-IPs91-GCST90274770 | C-C motif chemokine 4 levels \|\| id:ebi-IPs91-GCST90274770 | Cauliflower liking \|\| id:ebi-fl187-GCST90094727 | Inverse variance weighted | 4 | 0.00933070818951167 | 0.200045816482506 | 0.962797875120463 | -0.382759092 | 0.401420508495224 | 1.00937437495545 | 0.681977173175348 | 1.49394535314564 | 0.998074519377826 |
| ebi-fl187-GCST90094727 | ebi-IPs91-GCST90274779 | C-X-C motif chemokine 1 levels \|\| id:ebi-IPs91-GCST90274779 | Cauliflower liking \|\| id:ebi-fl187-GCST90094727 | Inverse variance weighted | 4 | -0.226963599 | 0.18700434263955 | 0.224869599553621 | -0.593492111 | 0.139564912117969 | 0.796949791168465 | 0.552394888344916 | 1.1497734375248 | 0.998074519377826 |
| ebi-fl187-GCST90094727 | ebi-IPs91-GCST90274780 | C-X-C motif chemokine 10 levels \|\| id:ebi-IPs91-GCST90274780 | Cauliflower liking \|\| id:ebi-fl187-GCST90094727 | Inverse variance weighted | 4 | 0.0188757064659154 | 0.178974504583957 | 0.916006130175524 | -0.331914323 | 0.369665735450471 | 1.01905497880071 | 0.717548797994653 | 1.44725076917522 | 0.998074519377826 |
| ebi-fl187-GCST90094727 | ebi-IPs91-GCST90274796 | Interleukin-10 receptor subunit alpha levels \|\| id:ebi-IPs91-GCST90274796 | Cauliflower liking \|\| id:ebi-fl187-GCST90094727 | Inverse variance weighted (multiplicative random effects) | 4 | 0.017501197334243 | 0.299864490623453 | 0.953458938858458 | -0.570233204 | 0.605235598956211 | 1.0176552406235 | 0.565393571119055 | 1.83168370082228 | 0.998074519377826 |
| ebi-fl187-GCST90094727 | ebi-IPs91-GCST90274807 | Interleukin-20 levels \|\| id:ebi-IPs91-GCST90274807 | Cauliflower liking \|\| id:ebi-fl187-GCST90094727 | Inverse variance weighted | 4 | -0.080087165 | 0.255232180414134 | 0.753686909771637 | -0.580342239 | 0.420167908171882 | 0.923035886051029 | 0.559706780265506 | 1.52221712685676 | 0.998074519377826 |
| ebi-fl187-GCST90094727 | ebi-IPs91-GCST90274808 | Interleukin-20 receptor subunit alpha levels \|\| id:ebi-IPs91-GCST90274808 | Cauliflower liking \|\| id:ebi-fl187-GCST90094727 | Inverse variance weighted | 4 | -0.013655863 | 0.251456631745209 | 0.956690547819006 | -0.506510861 | 0.479199135101586 | 0.986436955194643 | 0.602594450649794 | 1.61478066305523 | 0.998074519377826 |
| ebi-fl187-GCST90094727 | ebi-IPs91-GCST90274810 | Interleukin-24 levels \|\| id:ebi-IPs91-GCST90274810 | Cauliflower liking \|\| id:ebi-fl187-GCST90094727 | Inverse variance weighted | 4 | 0.0362909978964189 | 0.183667884807596 | 0.843365601497199 | -0.323698056 | 0.396280052119307 | 1.03695755505771 | 0.723468656187583 | 1.48628549667599 | 0.998074519377826 |
| ebi-fl187-GCST90094727 | ebi-IPs91-GCST90274823 | Monocyte chemoattractant protein-3 levels \|\| id:ebi-IPs91-GCST90274823 | Cauliflower liking \|\| id:ebi-fl187-GCST90094727 | Inverse variance weighted | 4 | -0.204641988 | 0.189066636785755 | 0.279083614235179 | -0.575212596 | 0.165928620394417 | 0.814939022380285 | 0.562585252827356 | 1.18048883588838 | 0.998074519377826 |
| ebi-fl187-GCST90094727 | ebi-IPs91-GCST90274824 | Monocyte chemoattractant protein-4 levels \|\| id:ebi-IPs91-GCST90274824 | Cauliflower liking \|\| id:ebi-fl187-GCST90094727 | Inverse variance weighted (multiplicative random effects) | 4 | -0.146247003 | 0.286259226769462 | 0.609428017663085 | -0.707315088 | 0.414821081059916 | 0.863944279645964 | 0.492965992585073 | 1.51409981532179 | 0.998074519377826 |
| ebi-fl187-GCST90094727 | ebi-IPs91-GCST90274834 | SIR2-like protein 2 levels \|\| id:ebi-IPs91-GCST90274834 | Cauliflower liking \|\| id:ebi-fl187-GCST90094727 | Inverse variance weighted (multiplicative random effects) | 4 | -0.146693991 | 0.297089769596307 | 0.621468680952637 | -0.728989939 | 0.435601957495954 | 0.86355819364255 | 0.482395993321679 | 1.54589334101269 | 0.998074519377826 |
| ebi-fl187-GCST90094727 | ebi-met1400-GCST90199652 | 1-stearoyl-2-oleoyl-GPS (18:0/18:1) levels \|\| id:ebi-met1400-GCST90199652 | Cauliflower liking \|\| id:ebi-fl187-GCST90094727 | Inverse variance weighted | 4 | 0.0538434799247677 | 0.209523279466475 | 0.7971935213403 | -0.356822148 | 0.464509107679058 | 1.05531941055398 | 0.69989696486022 | 1.59123287313359 | 0.998074519377826 |
| ebi-fl187-GCST90094727 | ebi-met1400-GCST90199665 | N-acetylaspartate (naa) levels \|\| id:ebi-met1400-GCST90199665 | Cauliflower liking \|\| id:ebi-fl187-GCST90094727 | Inverse variance weighted | 4 | -0.266554388 | 0.280768990542083 | 0.342431138389084 | -0.816861609 | 0.283752833512205 | 0.766014340643349 | 0.441816072350472 | 1.32810462722552 | 0.998074519377826 |
| ebi-fl187-GCST90094727 | ebi-met1400-GCST90199669 | Acetylcarnitine levels (Biocrates platform) \|\| id:ebi-met1400-GCST90199669 | Cauliflower liking \|\| id:ebi-fl187-GCST90094727 | Inverse variance weighted (multiplicative random effects) | 4 | 0.0968837764033172 | 0.341670365429708 | 0.77674820576396 | -0.57279014 | 0.766557692645544 | 1.10173231888698 | 0.563949742871174 | 2.15234445590901 | 0.998074519377826 |
| ebi-fl187-GCST90094727 | ebi-met1400-GCST90199670 | Iminodiacetate (IDA) levels \|\| id:ebi-met1400-GCST90199670 | Cauliflower liking \|\| id:ebi-fl187-GCST90094727 | Inverse variance weighted | 4 | 0.0669651553010018 | 0.206743520614151 | 0.746010342018851 | -0.338252145 | 0.472182455704738 | 1.06925821954466 | 0.713015481868703 | 1.60348992292197 | 0.998074519377826 |
| ebi-fl187-GCST90094727 | ebi-met1400-GCST90199686 | Indolepropionate levels \|\| id:ebi-met1400-GCST90199686 | Cauliflower liking \|\| id:ebi-fl187-GCST90094727 | Inverse variance weighted | 4 | 0.246779698932558 | 0.208726020438266 | 0.237081127243415 | -0.162323301 | 0.655882698991559 | 1.27989711901611 | 0.850166300381637 | 1.92684258895041 | 0.998074519377826 |
| ebi-fl187-GCST90094727 | ebi-met1400-GCST90199704 | 3-hydroxysebacate levels \|\| id:ebi-met1400-GCST90199704 | Cauliflower liking \|\| id:ebi-fl187-GCST90094727 | Inverse variance weighted | 4 | 0.0243036281260509 | 0.213315148763572 | 0.909290930632878 | -0.393794063 | 0.442401319702651 | 1.02460136845984 | 0.674492944688507 | 1.5564402452491 | 0.998074519377826 |
| ebi-fl187-GCST90094727 | ebi-met1400-GCST90199713 | Docosapentaenoate n3 DPA; 22:5n3 levels \|\| id:ebi-met1400-GCST90199713 | Cauliflower liking \|\| id:ebi-fl187-GCST90094727 | Inverse variance weighted | 4 | 0.411616550125664 | 0.198526772979307 | 0.0381392195319971 | 0.0225040750862233 | 0.800729025165106 | 1.50925560148803 | 1.02275920198789 | 2.22716399539173 | 0.998074519377826 |
| ebi-fl187-GCST90094727 | ebi-met1400-GCST90199715 | Myristoleate (14:1n5) levels \|\| id:ebi-met1400-GCST90199715 | Cauliflower liking \|\| id:ebi-fl187-GCST90094727 | Inverse variance weighted | 4 | 0.188569586069184 | 0.206943655178721 | 0.362183570099471 | -0.217039978 | 0.594179150219477 | 1.20752110671041 | 0.804897790392631 | 1.81154332954483 | 0.998074519377826 |
| ebi-fl187-GCST90094727 | ebi-met1400-GCST90199725 | Campesterol levels \|\| id:ebi-met1400-GCST90199725 | Cauliflower liking \|\| id:ebi-fl187-GCST90094727 | Inverse variance weighted | 4 | -0.211523848 | 0.292065959474721 | 0.468922613212043 | -0.783973129 | 0.360925432218556 | 0.809349979159457 | 0.456588318478635 | 1.43465647773918 | 0.998074519377826 |
| ebi-fl187-GCST90094727 | ebi-met1400-GCST90199737 | N-acetylproline levels \|\| id:ebi-met1400-GCST90199737 | Cauliflower liking \|\| id:ebi-fl187-GCST90094727 | Inverse variance weighted | 4 | 0.0427188507638261 | 0.230726280948674 | 0.853111793615559 | -0.40950466 | 0.494942361423227 | 1.04364443376398 | 0.663979064151185 | 1.64040368579833 | 0.998074519377826 |
| ebi-fl187-GCST90094727 | ebi-met1400-GCST90199775 | Gamma-glutamylisoleucine levels \|\| id:ebi-met1400-GCST90199775 | Cauliflower liking \|\| id:ebi-fl187-GCST90094727 | Inverse variance weighted | 4 | -0.079725531 | 0.205365792157173 | 0.69785891624309 | -0.482242483 | 0.322791421874322 | 0.923369748208355 | 0.617397335018583 | 1.38097727921145 | 0.998074519377826 |
| ebi-fl187-GCST90094727 | ebi-met1400-GCST90199777 | 1-palmitoleoyl-GPC (16:1) levels \|\| id:ebi-met1400-GCST90199777 | Cauliflower liking \|\| id:ebi-fl187-GCST90094727 | Inverse variance weighted | 4 | -0.43367145 | 0.203876369499978 | 0.033409320566795 | -0.833269134 | -0.034073766 | 0.648125161850195 | 0.434626110101655 | 0.966500207097758 | 0.998074519377826 |
| ebi-fl187-GCST90094727 | ebi-met1400-GCST90199782 | Glutamine degradant levels \|\| id:ebi-met1400-GCST90199782 | Cauliflower liking \|\| id:ebi-fl187-GCST90094727 | Inverse variance weighted (multiplicative random effects) | 4 | -0.645259501 | 0.300370096912369 | 0.0316966919136852 | -1.233984891 | -0.056534111 | 0.524526409163115 | 0.291130141178196 | 0.945034247557178 | 0.998074519377826 |
| ebi-fl187-GCST90094727 | ebi-met1400-GCST90199783 | 5-methyluridine (ribothymidine) levels \|\| id:ebi-met1400-GCST90199783 | Cauliflower liking \|\| id:ebi-fl187-GCST90094727 | Inverse variance weighted | 4 | -0.052521507 | 0.289756872464194 | 0.856163022443922 | -0.620444978 | 0.515401962551804 | 0.948833913811805 | 0.53770511766942 | 1.67431137702677 | 0.998074519377826 |
| ebi-fl187-GCST90094727 | ebi-met1400-GCST90199787 | Homostachydrine levels \|\| id:ebi-met1400-GCST90199787 | Cauliflower liking \|\| id:ebi-fl187-GCST90094727 | Inverse variance weighted | 4 | -0.055988355 | 0.201930430138541 | 0.781576196211164 | -0.451771998 | 0.339795287656586 | 0.945550146365385 | 0.636499275980505 | 1.40466000988662 | 0.998074519377826 |
| ebi-fl187-GCST90094727 | ebi-met1400-GCST90199802 | Beta-hydroxyisovaleroylcarnitine levels \|\| id:ebi-met1400-GCST90199802 | Cauliflower liking \|\| id:ebi-fl187-GCST90094727 | Inverse variance weighted | 4 | -0.113871855 | 0.203787033941681 | 0.576312755640896 | -0.513294442 | 0.2855507310622 | 0.892372301190814 | 0.598520536081273 | 1.33049457107426 | 0.998074519377826 |
| ebi-fl187-GCST90094727 | ebi-met1400-GCST90199873 | Pregnenolone sulfate levels \|\| id:ebi-met1400-GCST90199873 | Cauliflower liking \|\| id:ebi-fl187-GCST90094727 | Inverse variance weighted | 4 | -0.180079414 | 0.273699231746609 | 0.510572596750784 | -0.716529909 | 0.356371079819664 | 0.835203881559321 | 0.488444264778884 | 1.42813740291851 | 0.998074519377826 |
| ebi-fl187-GCST90094727 | ebi-met1400-GCST90199911 | Beta-citrylglutamate levels \|\| id:ebi-met1400-GCST90199911 | Cauliflower liking \|\| id:ebi-fl187-GCST90094727 | Inverse variance weighted | 4 | -0.243053269 | 0.252577577459481 | 0.335903170586262 | -0.738105321 | 0.251998782394745 | 0.784229737191033 | 0.478018750034491 | 1.2865944707197 | 0.998074519377826 |
| ebi-fl187-GCST90094727 | ebi-met1400-GCST90199934 | 2-hydroxydecanoate levels \|\| id:ebi-met1400-GCST90199934 | Cauliflower liking \|\| id:ebi-fl187-GCST90094727 | Inverse variance weighted | 4 | -0.274289422 | 0.206010658754212 | 0.183046525621056 | -0.678070313 | 0.129491469504938 | 0.760112050536086 | 0.507595548535195 | 1.13824940159047 | 0.998074519377826 |
| ebi-fl187-GCST90094727 | ebi-met1400-GCST90199945 | Sphingomyelin (d18:1/14:0, d16:1/16:0) levels \|\| id:ebi-met1400-GCST90199945 | Cauliflower liking \|\| id:ebi-fl187-GCST90094727 | Inverse variance weighted | 4 | -0.302791394 | 0.232350581764705 | 0.192518071746249 | -0.758198534 | 0.152615746171214 | 0.738753188580588 | 0.468509671325285 | 1.16487728437748 | 0.998074519377826 |
| ebi-fl187-GCST90094727 | ebi-met1400-GCST90199979 | Carnitine C14:1 levels \|\| id:ebi-met1400-GCST90199979 | Cauliflower liking \|\| id:ebi-fl187-GCST90094727 | Inverse variance weighted | 4 | 0.125247508507438 | 0.317367654143782 | 0.693105013131065 | -0.496793094 | 0.747288110629249 | 1.13342895166061 | 0.608478868951012 | 2.11126672431069 | 0.998074519377826 |
| ebi-fl187-GCST90094727 | ebi-met1400-GCST90199986 | Eugenol sulfate levels \|\| id:ebi-met1400-GCST90199986 | Cauliflower liking \|\| id:ebi-fl187-GCST90094727 | Inverse variance weighted | 4 | 0.137368443069722 | 0.223665872053154 | 0.539104252608992 | -0.301016666 | 0.575753552293904 | 1.14725076725457 | 0.740065438599016 | 1.77847019238976 | 0.998074519377826 |
| ebi-fl187-GCST90094727 | ebi-met1400-GCST90200002 | 6-hydroxyindole sulfate levels \|\| id:ebi-met1400-GCST90200002 | Cauliflower liking \|\| id:ebi-fl187-GCST90094727 | Inverse variance weighted | 4 | 0.0452689625873804 | 0.204850324186804 | 0.825103680371332 | -0.356237673 | 0.446775597993516 | 1.04630924010707 | 0.700306156715851 | 1.56326346046623 | 0.998074519377826 |
| ebi-fl187-GCST90094727 | ebi-met1400-GCST90200023 | Trans 3,4-methyleneheptanoate levels \|\| id:ebi-met1400-GCST90200023 | Cauliflower liking \|\| id:ebi-fl187-GCST90094727 | Inverse variance weighted | 4 | -0.37842513 | 0.222950523113253 | 0.0896304592774187 | -0.815408155 | 0.0585578956216618 | 0.684939250756058 | 0.442458698779267 | 1.06030637101411 | 0.998074519377826 |
| ebi-fl187-GCST90094727 | ebi-met1400-GCST90200032 | 2-hydroxybutyrate/2-hydroxyisobutyrate levels \|\| id:ebi-met1400-GCST90200032 | Cauliflower liking \|\| id:ebi-fl187-GCST90094727 | Inverse variance weighted | 4 | 0.21363293950796 | 0.208585441857028 | 0.305741430136329 | -0.195194527 | 0.622460405547734 | 1.23816808878405 | 0.822674610450987 | 1.86350738992993 | 0.998074519377826 |
| ebi-fl187-GCST90094727 | ebi-met1400-GCST90200051 | 1-palmitoyl-2-dihomo-linolenoyl-GPC (16:0/20:3n3 or 6) levels \|\| id:ebi-met1400-GCST90200051 | Cauliflower liking \|\| id:ebi-fl187-GCST90094727 | Inverse variance weighted | 4 | 0.0100591283222248 | 0.203285573262236 | 0.96053458730653 | -0.388380595 | 0.408498851916207 | 1.01010989142159 | 0.678154191862674 | 1.50455752539882 | 0.998074519377826 |
| ebi-fl187-GCST90094727 | ebi-met1400-GCST90200069 | Phenylacetylglutamate levels \|\| id:ebi-met1400-GCST90200069 | Cauliflower liking \|\| id:ebi-fl187-GCST90094727 | Inverse variance weighted | 4 | 0.170512109348713 | 0.227094858368966 | 0.452748407559608 | -0.274593813 | 0.615618031751886 | 1.18591201246898 | 0.759880714174183 | 1.85080009939014 | 0.998074519377826 |
| ebi-fl187-GCST90094727 | ebi-met1400-GCST90200072 | Thioproline levels \|\| id:ebi-met1400-GCST90200072 | Cauliflower liking \|\| id:ebi-fl187-GCST90094727 | Inverse variance weighted | 4 | 0.144126958910797 | 0.206225948250219 | 0.484627729876574 | -0.2600759 | 0.548329817481226 | 1.15503074066136 | 0.77099306547151 | 1.73036058509404 | 0.998074519377826 |
| ebi-fl187-GCST90094727 | ebi-met1400-GCST90200081 | 1-oleoyl-2-docosahexaenoyl-GPC (18:1/22:6) levels \|\| id:ebi-met1400-GCST90200081 | Cauliflower liking \|\| id:ebi-fl187-GCST90094727 | Inverse variance weighted | 4 | 0.113582423519101 | 0.198649733610309 | 0.567476216861747 | -0.275771054 | 0.502935901395307 | 1.12028422285512 | 0.758986677564039 | 1.65356886632864 | 0.998074519377826 |
| ebi-fl187-GCST90094727 | ebi-met1400-GCST90200097 | Furaneol sulfate levels \|\| id:ebi-met1400-GCST90200097 | Cauliflower liking \|\| id:ebi-fl187-GCST90094727 | Inverse variance weighted | 4 | -0.56679642 | 0.275733714246161 | 0.0398217187591341 | -1.1072345 | -0.02635834 | 0.567340049993918 | 0.330471617939262 | 0.973986009250151 | 0.998074519377826 |
| ebi-fl187-GCST90094727 | ebi-met1400-GCST90200124 | Sphingomyelin (d18:2/24:2) levels \|\| id:ebi-met1400-GCST90200124 | Cauliflower liking \|\| id:ebi-fl187-GCST90094727 | Inverse variance weighted | 4 | -0.089344144 | 0.188598065585953 | 0.63569403638525 | -0.458996353 | 0.28030806449656 | 0.914530789080605 | 0.631917549751127 | 1.32353748444839 | 0.998074519377826 |
| ebi-fl187-GCST90094727 | ebi-met1400-GCST90200137 | Lignoceroylcarnitine (C24) levels \|\| id:ebi-met1400-GCST90200137 | Cauliflower liking \|\| id:ebi-fl187-GCST90094727 | Inverse variance weighted | 4 | -0.151766991 | 0.256510127014544 | 0.55407774012971 | -0.65452684 | 0.350992857753834 | 0.859188455894591 | 0.51968789987459 | 1.42047718047827 | 0.998074519377826 |
| ebi-fl187-GCST90094727 | ebi-met1400-GCST90200165 | Octadecenedioate (C18:1-DC) levels \|\| id:ebi-met1400-GCST90200165 | Cauliflower liking \|\| id:ebi-fl187-GCST90094727 | Inverse variance weighted | 4 | -0.228927954 | 0.202728819088861 | 0.258799802913735 | -0.626276439 | 0.168420531452056 | 0.79538583583893 | 0.534578635619602 | 1.18343417731226 | 0.998074519377826 |
| ebi-fl187-GCST90094727 | ebi-met1400-GCST90200176 | Glutamine conjugate of C6H10O2 (1) levels \|\| id:ebi-met1400-GCST90200176 | Cauliflower liking \|\| id:ebi-fl187-GCST90094727 | Inverse variance weighted | 4 | 0.166689241178748 | 0.208997996824878 | 0.425123652371584 | -0.242946833 | 0.576325314955509 | 1.18138708180158 | 0.784313212558895 | 1.77948734599807 | 0.998074519377826 |
| ebi-fl187-GCST90094727 | ebi-met1400-GCST90200182 | Glycine conjugate of C10H12O2 levels \|\| id:ebi-met1400-GCST90200182 | Cauliflower liking \|\| id:ebi-fl187-GCST90094727 | Inverse variance weighted | 4 | -0.335681298 | 0.238695132225209 | 0.159629671880846 | -0.803523757 | 0.132161161541896 | 0.714850894197453 | 0.447748424487113 | 1.14129223686326 | 0.998074519377826 |
| ebi-fl187-GCST90094727 | ebi-met1400-GCST90200208 | Glucuronide of piperine metabolite C17H21NO3 (4) levels \|\| id:ebi-met1400-GCST90200208 | Cauliflower liking \|\| id:ebi-fl187-GCST90094727 | Inverse variance weighted | 4 | 0.181335683197476 | 0.21199454035057 | 0.392341351702731 | -0.234173616 | 0.596844982284592 | 1.19881753466068 | 0.791224434836003 | 1.81637904257569 | 0.998074519377826 |
| ebi-fl187-GCST90094727 | ebi-met1400-GCST90200227 | 3-ethylcatechol sulfate (2) levels \|\| id:ebi-met1400-GCST90200227 | Cauliflower liking \|\| id:ebi-fl187-GCST90094727 | Inverse variance weighted | 4 | -0.03377958 | 0.27006059019037 | 0.900459041305471 | -0.563098336 | 0.495539177216159 | 0.966784580239029 | 0.569442004939223 | 1.64138299682988 | 0.998074519377826 |
| ebi-fl187-GCST90094727 | ebi-met1400-GCST90200259 | Pentose acid levels \|\| id:ebi-met1400-GCST90200259 | Cauliflower liking \|\| id:ebi-fl187-GCST90094727 | Inverse variance weighted | 4 | -0.439673037 | 0.203845225005214 | 0.0310137355310456 | -0.839209678 | -0.040136396 | 0.644247031499613 | 0.432051848547281 | 0.960658400124034 | 0.998074519377826 |
| ebi-fl187-GCST90094727 | ebi-met1400-GCST90200291 | Sarcosine levels \|\| id:ebi-met1400-GCST90200291 | Cauliflower liking \|\| id:ebi-fl187-GCST90094727 | Inverse variance weighted | 4 | -0.050522758 | 0.20745894382263 | 0.807593797284294 | -0.457142288 | 0.356096771918487 | 0.950732291685472 | 0.633090252592567 | 1.42774570726999 | 0.998074519377826 |
| ebi-fl187-GCST90094727 | ebi-met1400-GCST90200306 | Butyrate/isobutyrate (4:0) levels \|\| id:ebi-met1400-GCST90200306 | Cauliflower liking \|\| id:ebi-fl187-GCST90094727 | Inverse variance weighted | 4 | 0.120481142773304 | 0.20900974247288 | 0.564319214637645 | -0.289177952 | 0.530140238020149 | 1.12803946906921 | 0.748878928686818 | 1.69917058022886 | 0.998074519377826 |
| ebi-fl187-GCST90094727 | ebi-met1400-GCST90200321 | Cholate levels \|\| id:ebi-met1400-GCST90200321 | Cauliflower liking \|\| id:ebi-fl187-GCST90094727 | Inverse variance weighted | 4 | 0.136483688981111 | 0.325234522436219 | 0.674743354221207 | -0.500975975 | 0.7739433529561 | 1.14623618134328 | 0.605938989730252 | 2.16829978873835 | 0.998074519377826 |
| ebi-fl187-GCST90094727 | ebi-met1400-GCST90200336 | Ceramide (d18:1/16:0) levels \|\| id:ebi-met1400-GCST90200336 | Cauliflower liking \|\| id:ebi-fl187-GCST90094727 | Inverse variance weighted | 4 | 0.0047593779804409 | 0.2820449367849 | 0.986536705672916 | -0.548048698 | 0.557567454078845 | 1.0047707218092 | 0.578076712737104 | 1.74641908445829 | 0.999355215041207 |
| ebi-fl187-GCST90094727 | ebi-met1400-GCST90200358 | Arachidonate (20:4n6) levels \|\| id:ebi-met1400-GCST90200358 | Cauliflower liking \|\| id:ebi-fl187-GCST90094727 | Inverse variance weighted | 4 | 0.498292542485353 | 0.217261907751906 | 0.0218186114882551 | 0.0724592032916161 | 0.924125881679089 | 1.64590855115982 | 1.07514894265754 | 2.51966481228631 | 0.998074519377826 |
| ebi-fl187-GCST90094727 | ebi-met1400-GCST90200413 | 2'-deoxyuridine levels \|\| id:ebi-met1400-GCST90200413 | Cauliflower liking \|\| id:ebi-fl187-GCST90094727 | Inverse variance weighted | 4 | -0.017094013 | 0.308176186964248 | 0.955765375245352 | -0.621119339 | 0.586931313875693 | 0.983051261110831 | 0.537342632267773 | 1.79846102642758 | 0.998074519377826 |
| ebi-fl187-GCST90094727 | ebi-met1400-GCST90200432 | Threonine levels \|\| id:ebi-met1400-GCST90200432 | Cauliflower liking \|\| id:ebi-fl187-GCST90094727 | Inverse variance weighted | 4 | 0.174494558535309 | 0.238671677841647 | 0.464713737153489 | -0.29330193 | 0.642291047104938 | 1.19064426351867 | 0.745796928207595 | 1.90083078735249 | 0.998074519377826 |
| ebi-fl187-GCST90094727 | ebi-met1400-GCST90200436 | Caffeine levels \|\| id:ebi-met1400-GCST90200436 | Cauliflower liking \|\| id:ebi-fl187-GCST90094727 | Inverse variance weighted | 4 | -0.168901887 | 0.211681280528611 | 0.424924702486664 | -0.583797197 | 0.245993422444986 | 0.844591764422582 | 0.557776353441282 | 1.2788911615371 | 0.998074519377826 |
| ebi-fl187-GCST90094727 | ebi-met1400-GCST90200442 | Valine levels \|\| id:ebi-met1400-GCST90200442 | Cauliflower liking \|\| id:ebi-fl187-GCST90094727 | Inverse variance weighted | 4 | 0.403526815929607 | 0.259097396379431 | 0.119367734077582 | -0.104304081 | 0.911357712833292 | 1.49709537764494 | 0.900951293616685 | 2.48769782078745 | 0.998074519377826 |
| ebi-fl187-GCST90094727 | ebi-met1400-GCST90200459 | Margarate (17:0) levels \|\| id:ebi-met1400-GCST90200459 | Cauliflower liking \|\| id:ebi-fl187-GCST90094727 | Inverse variance weighted | 4 | 0.291857786902908 | 0.202958493379096 | 0.150429198367744 | -0.10594086 | 0.689656433925937 | 1.33891259319258 | 0.899477841514483 | 1.9930306778778 | 0.998074519377826 |
| ebi-fl187-GCST90094727 | ebi-met1400-GCST90200462 | X-11299 levels \|\| id:ebi-met1400-GCST90200462 | Cauliflower liking \|\| id:ebi-fl187-GCST90094727 | Inverse variance weighted | 4 | 0.174099169833886 | 0.229942881497511 | 0.448965427870142 | -0.276588878 | 0.624787217569008 | 1.19017358928523 | 0.758366214138827 | 1.8678484698064 | 0.998074519377826 |
| ebi-fl187-GCST90094727 | ebi-met1400-GCST90200497 | X-12740 levels \|\| id:ebi-met1400-GCST90200497 | Cauliflower liking \|\| id:ebi-fl187-GCST90094727 | Inverse variance weighted | 4 | 0.0375145471249991 | 0.236101029978929 | 0.873754022865309 | -0.425243472 | 0.500272565883701 | 1.03822710019138 | 0.653610630107849 | 1.64917071711935 | 0.998074519377826 |
| ebi-fl187-GCST90094727 | ebi-met1400-GCST90200527 | X-13866 levels \|\| id:ebi-met1400-GCST90200527 | Cauliflower liking \|\| id:ebi-fl187-GCST90094727 | Inverse variance weighted | 4 | 0.189639954730652 | 0.278709417312828 | 0.496237441137645 | -0.356630503 | 0.735910412663794 | 1.20881429142983 | 0.700031109206623 | 2.08738150626061 | 0.998074519377826 |
| ebi-fl187-GCST90094727 | ebi-met1400-GCST90200547 | X-17654 levels \|\| id:ebi-met1400-GCST90200547 | Cauliflower liking \|\| id:ebi-fl187-GCST90094727 | Inverse variance weighted | 4 | 0.250301776405806 | 0.209150300975856 | 0.231401861395765 | -0.159632814 | 0.660236366318484 | 1.28441296373629 | 0.85245674210829 | 1.93524970819489 | 0.998074519377826 |
| ebi-fl187-GCST90094727 | ebi-met1400-GCST90200553 | X-17653 levels \|\| id:ebi-met1400-GCST90200553 | Cauliflower liking \|\| id:ebi-fl187-GCST90094727 | Inverse variance weighted | 4 | 0.257444187397638 | 0.208430938618229 | 0.216773409885796 | -0.151080452 | 0.665968827089366 | 1.29361960868861 | 0.859778524721918 | 1.94637530929831 | 0.998074519377826 |
| ebi-fl187-GCST90094727 | ebi-met1400-GCST90200557 | X-17685 levels \|\| id:ebi-met1400-GCST90200557 | Cauliflower liking \|\| id:ebi-fl187-GCST90094727 | Inverse variance weighted | 4 | 0.0767173526168312 | 0.219622704972036 | 0.726853359993051 | -0.353743149 | 0.507177854362022 | 1.07973684846397 | 0.702055267706702 | 1.66059812604091 | 0.998074519377826 |
| ebi-fl187-GCST90094727 | ebi-met1400-GCST90200561 | X-18779 levels \|\| id:ebi-met1400-GCST90200561 | Cauliflower liking \|\| id:ebi-fl187-GCST90094727 | Inverse variance weighted | 4 | 0.20923664639279 | 0.20620543321133 | 0.310248869600743 | -0.194926003 | 0.613399295486996 | 1.23273668670559 | 0.822895547850708 | 1.84669821427392 | 0.998074519377826 |
| ebi-fl187-GCST90094727 | ebi-met1400-GCST90200570 | X-18921 levels \|\| id:ebi-met1400-GCST90200570 | Cauliflower liking \|\| id:ebi-fl187-GCST90094727 | Inverse variance weighted | 4 | -0.131894928 | 0.207263425711179 | 0.52453937283241 | -0.538131242 | 0.274341386893192 | 0.876433079255621 | 0.583838286081443 | 1.31566387598352 | 0.998074519377826 |
| ebi-fl187-GCST90094727 | ebi-met1400-GCST90200575 | X-21283 levels \|\| id:ebi-met1400-GCST90200575 | Cauliflower liking \|\| id:ebi-fl187-GCST90094727 | Inverse variance weighted | 4 | 0.145338370850661 | 0.251692074383925 | 0.563638772811837 | -0.347978095 | 0.638654836643154 | 1.15643080654855 | 0.706114343520746 | 1.89393151775743 | 0.998074519377826 |
| ebi-fl187-GCST90094727 | ebi-met1400-GCST90200578 | X-18922 levels \|\| id:ebi-met1400-GCST90200578 | Cauliflower liking \|\| id:ebi-fl187-GCST90094727 | Inverse variance weighted | 4 | -0.041918675 | 0.207726946559211 | 0.840075427282508 | -0.44906349 | 0.365226140707653 | 0.958947764261401 | 0.638225576590357 | 1.44083980384285 | 0.998074519377826 |
| ebi-fl187-GCST90094727 | ebi-met1400-GCST90200623 | X-24241 levels \|\| id:ebi-met1400-GCST90200623 | Cauliflower liking \|\| id:ebi-fl187-GCST90094727 | Inverse variance weighted | 4 | 0.0588771850185343 | 0.212131564561283 | 0.781357567997842 | -0.356900682 | 0.474655051558649 | 1.06064496964841 | 0.699842001525774 | 1.60745961115203 | 0.998074519377826 |
| ebi-fl187-GCST90094727 | ebi-met1400-GCST90200636 | X-24544 levels \|\| id:ebi-met1400-GCST90200636 | Cauliflower liking \|\| id:ebi-fl187-GCST90094727 | Inverse variance weighted | 4 | 0.0194032533557318 | 0.259136598195522 | 0.940312937923691 | -0.488504479 | 0.527310985818954 | 1.01959271991468 | 0.613543275192684 | 1.69436999236369 | 0.998074519377826 |
| ebi-fl187-GCST90094727 | ebi-met1400-GCST90200659 | X-25172 levels \|\| id:ebi-met1400-GCST90200659 | Cauliflower liking \|\| id:ebi-fl187-GCST90094727 | Inverse variance weighted | 4 | 0.0119344969223172 | 0.277165938600132 | 0.965654476997361 | -0.531310743 | 0.555179736578575 | 1.0120059971875 | 0.587833965395704 | 1.74225410342535 | 0.998074519377826 |
| ebi-fl187-GCST90094727 | ebi-met1400-GCST90200670 | X-26111 levels \|\| id:ebi-met1400-GCST90200670 | Cauliflower liking \|\| id:ebi-fl187-GCST90094727 | Inverse variance weighted | 4 | 0.310532823249098 | 0.231378822859726 | 0.179564547574715 | -0.14296967 | 0.764035316054161 | 1.36415177230438 | 0.866780358329951 | 2.14692227390415 | 0.998074519377826 |
| ebi-fl187-GCST90094727 | ebi-met1400-GCST90200673 | Carnitine C4 levels \|\| id:ebi-met1400-GCST90200673 | Cauliflower liking \|\| id:ebi-fl187-GCST90094727 | Inverse variance weighted | 4 | -0.259645205 | 0.207326384887775 | 0.210442344459358 | -0.66600492 | 0.146714509106571 | 0.77132519937555 | 0.513756983717435 | 1.1580233107234 | 0.998074519377826 |
| ebi-fl187-GCST90094727 | ebi-met1400-GCST90200710 | X-13431 levels \|\| id:ebi-met1400-GCST90200710 | Cauliflower liking \|\| id:ebi-fl187-GCST90094727 | Inverse variance weighted | 4 | -0.36409845 | 0.204484366025427 | 0.0749829463773316 | -0.764887807 | 0.0366909075031989 | 0.694822786137131 | 0.4653861409655 | 1.03737232727597 | 0.998074519377826 |
| ebi-fl187-GCST90094727 | ebi-met1400-GCST90200730 | Adenosine 5'-diphosphate (ADP) to fructose ratio \|\| id:ebi-met1400-GCST90200730 | Cauliflower liking \|\| id:ebi-fl187-GCST90094727 | Inverse variance weighted | 4 | 0.330441178278664 | 0.277648539437258 | 0.233990598731726 | -0.213749959 | 0.874632315575689 | 1.3915819287761 | 0.80755028044541 | 2.39799342701995 | 0.998074519377826 |
| ebi-fl187-GCST90094727 | ebi-met1400-GCST90200731 | Adenosine 5'-diphosphate (ADP) to mannose ratio \|\| id:ebi-met1400-GCST90200731 | Cauliflower liking \|\| id:ebi-fl187-GCST90094727 | Inverse variance weighted | 4 | -0.05745025 | 0.353109486777037 | 0.870756125850684 | -0.749544844 | 0.634644343784674 | 0.944168861337205 | 0.472581601968294 | 1.8863511296375 | 0.998074519377826 |
| ebi-fl187-GCST90094727 | ebi-met1400-GCST90200748 | Aspartate to glutamate ratio \|\| id:ebi-met1400-GCST90200748 | Cauliflower liking \|\| id:ebi-fl187-GCST90094727 | Inverse variance weighted | 4 | -0.029337041 | 0.213089752273031 | 0.890497615887503 | -0.446992955 | 0.388318873820247 | 0.971089112813178 | 0.639548413819923 | 1.47449988874463 | 0.998074519377826 |
| ebi-fl187-GCST90094727 | ebi-met1400-GCST90200774 | Uridine to pseudouridine ratio \|\| id:ebi-met1400-GCST90200774 | Cauliflower liking \|\| id:ebi-fl187-GCST90094727 | Inverse variance weighted | 4 | 0.202783317800925 | 0.197387936813166 | 0.304263267528755 | -0.184097038 | 0.589663673954731 | 1.22480704560827 | 0.831855078063377 | 1.80338178912622 | 0.998074519377826 |
| ebi-fl187-GCST90094727 | ebi-met1400-GCST90200799 | Spermidine to adenosine 5'-diphosphate (ADP) ratio \|\| id:ebi-met1400-GCST90200799 | Cauliflower liking \|\| id:ebi-fl187-GCST90094727 | Inverse variance weighted | 4 | -0.288730533 | 0.441918859188209 | 0.513526514426833 | -1.154891497 | 0.577430431493107 | 0.749214067033659 | 0.31509172359575 | 1.7814549739214 | 0.998074519377826 |
| ebi-fl187-GCST90094727 | ebi-met1400-GCST90200811 | Choline to taurocholate ratio \|\| id:ebi-met1400-GCST90200811 | Cauliflower liking \|\| id:ebi-fl187-GCST90094727 | Inverse variance weighted | 4 | 0.284658040155237 | 0.262840299486686 | 0.278805052681654 | -0.230508947 | 0.799825027149141 | 1.32930738064479 | 0.794129330023452 | 2.22515155331754 | 0.998074519377826 |
| ebi-fl187-GCST90094727 | ebi-met1400-GCST90200812 | Glutamate to kynurenine ratio \|\| id:ebi-met1400-GCST90200812 | Cauliflower liking \|\| id:ebi-fl187-GCST90094727 | Inverse variance weighted (multiplicative random effects) | 4 | 0.295087782364308 | 0.368127306379967 | 0.422789197614922 | -0.426441738 | 1.01661730286904 | 1.34324426666865 | 0.652827899434983 | 2.76382973445194 | 0.998074519377826 |
| ebi-fl187-GCST90094727 | ebi-met1400-GCST90200876 | Cortisone to 4-cholesten-3-one ratio \|\| id:ebi-met1400-GCST90200876 | Cauliflower liking \|\| id:ebi-fl187-GCST90094727 | Inverse variance weighted (multiplicative random effects) | 4 | 0.0144114116013068 | 0.385642782776491 | 0.970190114866656 | -0.741448443 | 0.770271265843229 | 1.01451575664404 | 0.476423343628984 | 2.16035220406948 | 0.998074519377826 |
| ebi-fl187-GCST90094727 | ebi-met1400-GCST90200906 | 2'-deoxyuridine to cytidine ratio \|\| id:ebi-met1400-GCST90200906 | Cauliflower liking \|\| id:ebi-fl187-GCST90094727 | Inverse variance weighted | 4 | 0.189040594746006 | 0.289474574840803 | 0.513725773818742 | -0.378329572 | 0.756410761433979 | 1.20808999359393 | 0.685004705129051 | 2.13061519387203 | 0.998074519377826 |
| ebi-fl187-GCST90094727 | ebi-met1400-GCST90200907 | Retinol (Vitamin A) to linoleoyl-arachidonoyl-glycerol (18:2 to 20:4) [1] ratio \|\| id:ebi-met1400-GCST90200907 | Cauliflower liking \|\| id:ebi-fl187-GCST90094727 | Inverse variance weighted | 4 | -0.470758998 | 0.299381946615341 | 0.115849434128271 | -1.057547613 | 0.116029617356867 | 0.624528072784812 | 0.347306496604842 | 1.1230291327953 | 0.998074519377826 |
| ebi-fl187-GCST90094727 | ebi-met1400-GCST90200916 | Fructose to sucrose ratio \|\| id:ebi-met1400-GCST90200916 | Cauliflower liking \|\| id:ebi-fl187-GCST90094727 | Inverse variance weighted | 4 | -0.270320029 | 0.281027983135702 | 0.33610124744956 | -0.821134876 | 0.280494818093147 | 0.763135229960916 | 0.43993210279846 | 1.32378468291571 | 0.998074519377826 |
| ebi-fl187-GCST90094727 | ebi-met1400-GCST90200917 | Alpha-ketobutyrate to 3-methyl-2-oxobutyrate ratio \|\| id:ebi-met1400-GCST90200917 | Cauliflower liking \|\| id:ebi-fl187-GCST90094727 | Inverse variance weighted (multiplicative random effects) | 4 | 0.0740783173167158 | 0.353912571317056 | 0.83420431144215 | -0.619590322 | 0.767746957098145 | 1.07689114141829 | 0.538164866495291 | 2.15490568534787 | 0.998074519377826 |
| ebi-fl187-GCST90094727 | ebi-met1400-GCST90200924 | Alpha-tocopherol to glycerol ratio \|\| id:ebi-met1400-GCST90200924 | Cauliflower liking \|\| id:ebi-fl187-GCST90094727 | Inverse variance weighted | 4 | -0.218130422 | 0.193278864662862 | 0.259075573848745 | -0.596956997 | 0.160696152844486 | 0.804020572941599 | 0.550484215291405 | 1.17432809834726 | 0.998074519377826 |
| ebi-fl187-GCST90094727 | ebi-met1400-GCST90200938 | Alpha-ketoglutarate to alanine ratio \|\| id:ebi-met1400-GCST90200938 | Cauliflower liking \|\| id:ebi-fl187-GCST90094727 | Inverse variance weighted | 4 | 0.0730994290199543 | 0.205479057770197 | 0.722026870114365 | -0.329639524 | 0.47583838224954 | 1.07583750106531 | 0.719182934748043 | 1.60936289332829 | 0.998074519377826 |
| ebi-fl187-GCST90094727 | ebi-met1400-GCST90200943 | Phosphate to EDTA ratio \|\| id:ebi-met1400-GCST90200943 | Cauliflower liking \|\| id:ebi-fl187-GCST90094727 | Inverse variance weighted | 4 | -0.049598209 | 0.228579566673071 | 0.828220472984669 | -0.497614159 | 0.398417741947087 | 0.951611696968944 | 0.607979472810133 | 1.48946611243718 | 0.998074519377826 |
| ebi-fl187-GCST90094727 | ebi-met1400-GCST90200947 | Adenosine 5'-diphosphate (ADP) to glucose ratio \|\| id:ebi-met1400-GCST90200947 | Cauliflower liking \|\| id:ebi-fl187-GCST90094727 | Inverse variance weighted | 4 | 0.151969453709445 | 0.311202630321279 | 0.625316403697563 | -0.457987702 | 0.761926609139152 | 1.1641246761984 | 0.632555255501009 | 2.14239981400623 | 0.998074519377826 |
| ebi-fl187-GCST90094727 | ebi-met1400-GCST90200975 | Arachidonate (20:4n6) to caffeine ratio \|\| id:ebi-met1400-GCST90200975 | Cauliflower liking \|\| id:ebi-fl187-GCST90094727 | Inverse variance weighted | 4 | 0.264376421621452 | 0.211791285222763 | 0.211925776925916 | -0.150734497 | 0.679487340658068 | 1.30261843777084 | 0.860076020754477 | 1.97286606471375 | 0.998074519377826 |
| ebi-fl187-GCST90094727 | ebi-met1400-GCST90200985 | Cholesterol to oleoyl-linoleoyl-glycerol (18:1 to 18:2) [2] ratio \|\| id:ebi-met1400-GCST90200985 | Cauliflower liking \|\| id:ebi-fl187-GCST90094727 | Inverse variance weighted | 4 | -0.33843628 | 0.317314915092503 | 0.286169313085868 | -0.960373513 | 0.283500954075141 | 0.712884203275137 | 0.382749897176858 | 1.32777014710575 | 0.998074519377826 |
| ebi-fl187-GCST90094727 | ebi-met1400-GCST90201016 | Bilirubin (Z,Z) to etiocholanolone glucuronide ratio \|\| id:ebi-met1400-GCST90201016 | Cauliflower liking \|\| id:ebi-fl187-GCST90094727 | Inverse variance weighted | 4 | -0.059321846 | 0.213046335119023 | 0.780670204910634 | -0.476892663 | 0.358248970498547 | 0.942403411258647 | 0.620709150671723 | 1.43082180855691 | 0.998074519377826 |
| ebi-fl187-GCST90094727 | ebi-pw11-GCST90275189 | Placental weight (fetal genotype, adjusted for sex and gestational age) \|\| id:ebi-pw11-GCST90275189 | Cauliflower liking \|\| id:ebi-fl187-GCST90094727 | Inverse variance weighted | 4 | -0.067385849 | 0.0778663329656541 | 0.386816996483793 | -0.220003861 | 0.0852321638504451 | 0.934834427032526 | 0.802515699142503 | 1.08896985678787 | 0.998074519377826 |
| ebi-fl187-GCST90094727 | ebi-pw11-GCST90275192 | Placental weight (fetal effect, conditional on maternal and paternal genotype) \|\| id:ebi-pw11-GCST90275192 | Cauliflower liking \|\| id:ebi-fl187-GCST90094727 | Inverse variance weighted | 4 | -0.065347444 | 0.161792866433782 | 0.686289415170463 | -0.382461462 | 0.251766574431276 | 0.93674194167033 | 0.682180180337072 | 1.28629574792209 | 0.998074519377826 |
| ebi-fl187-GCST90094727 | ebi-pw11-GCST90275193 | Placental weight (maternal effect, conditional on fetal and paternal genotype) \|\| id:ebi-pw11-GCST90275193 | Cauliflower liking \|\| id:ebi-fl187-GCST90094727 | Inverse variance weighted | 4 | 0.00651312891231174 | 0.125829002113385 | 0.958718536522669 | -0.240111715 | 0.253137973054547 | 1.00653438546025 | 0.78653998766268 | 1.28806098228324 | 0.998074519377826 |
| ebi-fl187-GCST90094727 | ebi-pw11-GCST90275195 | Placental weight (fetal effect, conditional on maternal genotype) \|\| id:ebi-pw11-GCST90275195 | Cauliflower liking \|\| id:ebi-fl187-GCST90094727 | Inverse variance weighted | 4 | -0.072815869 | 0.103219836519806 | 0.480533639089933 | -0.275126749 | 0.12949501029485 | 0.929772013851864 | 0.759475854423252 | 1.1382534318996 | 0.998074519377826 |
| ebi-fl187-GCST90094727 | ebi-pw11-GCST90275197 | Placental weight (fetal genotype, adjusted for sex) \|\| id:ebi-pw11-GCST90275197 | Cauliflower liking \|\| id:ebi-fl187-GCST90094727 | Inverse variance weighted | 4 | -0.066265631 | 0.0785927419108988 | 0.399143413337045 | -0.220307405 | 0.087776142926568 | 0.935882231732933 | 0.802272137293498 | 1.09174370012178 | 0.998074519377826 |
| ebi-fl187-GCST90094727 | prot-a-1030 | Protein FAM171B \|\| id:prot-a-1030 | Cauliflower liking \|\| id:ebi-fl187-GCST90094727 | Inverse variance weighted | 4 | 0.0573746867964048 | 0.335893159052301 | 0.864371348449152 | -0.600975905 | 0.715725278538914 | 1.05905254907132 | 0.548276309360373 | 2.04566982476943 | 0.998074519377826 |
| ebi-fl187-GCST90094727 | prot-a-1036 | BRISC complex subunit Abro1 \|\| id:prot-a-1036 | Cauliflower liking \|\| id:ebi-fl187-GCST90094727 | Inverse variance weighted | 4 | 0.52821516012787 | 0.438683622118008 | 0.228554268150288 | -0.331604739 | 1.38803505947917 | 1.69590269098953 | 0.717770973505152 | 4.00696885701644 | 0.998074519377826 |
| ebi-fl187-GCST90094727 | prot-a-1055 | Phenylalanine--tRNA ligase, mitochondrial \|\| id:prot-a-1055 | Cauliflower liking \|\| id:ebi-fl187-GCST90094727 | Inverse variance weighted | 4 | -0.386337585 | 0.335893159052301 | 0.250069752497088 | -1.044688177 | 0.272013006715388 | 0.67954108405918 | 0.35180150215721 | 1.31260407386768 | 0.998074519377826 |
| ebi-fl187-GCST90094727 | prot-a-1083 | Fc receptor-like protein 5 \|\| id:prot-a-1083 | Cauliflower liking \|\| id:ebi-fl187-GCST90094727 | Inverse variance weighted | 4 | -0.113061634 | 0.33605907840077 | 0.736543761067458 | -0.771737428 | 0.545614159273077 | 0.893095613014145 | 0.462209314849248 | 1.7256678919274 | 0.998074519377826 |
| ebi-fl187-GCST90094727 | prot-a-1103 | Fibroblast growth factor receptor 3 \|\| id:prot-a-1103 | Cauliflower liking \|\| id:ebi-fl187-GCST90094727 | Inverse variance weighted | 4 | -0.228708666 | 0.424828704415743 | 0.590331973035662 | -1.061372927 | 0.603955594796044 | 0.795560273615629 | 0.345980478334854 | 1.82934063795013 | 0.998074519377826 |
| ebi-fl187-GCST90094727 | prot-a-1105 | Fibroblast growth factor receptor 4 \|\| id:prot-a-1105 | Cauliflower liking \|\| id:ebi-fl187-GCST90094727 | Inverse variance weighted | 4 | -0.746923554 | 0.375373667457672 | 0.0466114946969214 | -1.482655942 | -0.011191166 | 0.4738220007106 | 0.227033898066819 | 0.988871222619454 | 0.998074519377826 |
| ebi-fl187-GCST90094727 | prot-a-1120 | Filamin-A \|\| id:prot-a-1120 | Cauliflower liking \|\| id:ebi-fl187-GCST90094727 | Inverse variance weighted | 4 | -0.043897756 | 0.336059078400769 | 0.896071874470359 | -0.702573549 | 0.614778038077968 | 0.95705180567031 | 0.495308960124859 | 1.84924609178462 | 0.998074519377826 |
| ebi-fl187-GCST90094727 | prot-a-1166 | Galanin peptides \|\| id:prot-a-1166 | Cauliflower liking \|\| id:ebi-fl187-GCST90094727 | Inverse variance weighted | 4 | -0.1962498 | 0.342375180834204 | 0.566508600021376 | -0.867305154 | 0.474805554686276 | 0.821806921873094 | 0.420082081698461 | 1.6077015570574 | 0.998074519377826 |
| ebi-fl187-GCST90094727 | prot-a-1205 | ADP-ribosylation factor-binding protein GGA1 \|\| id:prot-a-1205 | Cauliflower liking \|\| id:ebi-fl187-GCST90094727 | Inverse variance weighted | 4 | 0.0464237173226083 | 0.336059078400769 | 0.890128559354134 | -0.612252076 | 0.705099510988116 | 1.04751816853017 | 0.542128578299039 | 2.0240480899266 | 0.998074519377826 |
| ebi-fl187-GCST90094727 | prot-a-1227 | Guanine nucleotide-binding protein G(I)/G(S)/G(O) subunit gamma-T2 \|\| id:prot-a-1227 | Cauliflower liking \|\| id:ebi-fl187-GCST90094727 | Inverse variance weighted | 4 | -0.564323332 | 0.336059078400769 | 0.0931055774912261 | -1.222999126 | 0.0943524614094485 | 0.568744867885302 | 0.294346060912884 | 1.09894701407811 | 0.998074519377826 |
| ebi-fl187-GCST90094727 | prot-a-1240 | Golgi SNAP receptor complex member 1 \|\| id:prot-a-1240 | Cauliflower liking \|\| id:ebi-fl187-GCST90094727 | Inverse variance weighted | 4 | -0.218228985 | 0.336059078400769 | 0.516094824882293 | -0.876904779 | 0.440446808602959 | 0.803941330032214 | 0.41606874551661 | 1.55340113646716 | 0.998074519377826 |
| ebi-fl187-GCST90094727 | prot-a-1283 | Glutathione S-transferase A1 \|\| id:prot-a-1283 | Cauliflower liking \|\| id:ebi-fl187-GCST90094727 | Inverse variance weighted | 4 | -0.442234988 | 0.33605907840077 | 0.188192779183665 | -1.100910782 | 0.21644080577044 | 0.642598614816346 | 0.332568048873211 | 1.24164958468789 | 0.998074519377826 |
| ebi-fl187-GCST90094727 | prot-a-1324 | E3 ubiquitin-protein ligase HECW1 \|\| id:prot-a-1324 | Cauliflower liking \|\| id:ebi-fl187-GCST90094727 | Inverse variance weighted | 4 | -0.04133553 | 0.335893159052301 | 0.902058364396746 | -0.699686122 | 0.617015061479133 | 0.959507132250039 | 0.496741195454672 | 1.85338752908546 | 0.998074519377826 |
| ebi-fl187-GCST90094727 | prot-a-1339 | Histidine triad nucleotide-binding protein 2, mitochondrial \|\| id:prot-a-1339 | Cauliflower liking \|\| id:ebi-fl187-GCST90094727 | Inverse variance weighted | 4 | 0.113726874839488 | 0.33605907840077 | 0.73505172340266 | -0.544948919 | 0.772402668504996 | 1.12044606107891 | 0.579871403095361 | 2.16496169510329 | 0.998074519377826 |
| ebi-fl187-GCST90094727 | prot-a-1466 | Interleukin-11 \|\| id:prot-a-1466 | Cauliflower liking \|\| id:ebi-fl187-GCST90094727 | Inverse variance weighted | 4 | -0.101744609 | 0.3358931590523 | 0.761960237653294 | -0.760095201 | 0.55660598270845 | 0.903260206710704 | 0.46762190692209 | 1.74474075946788 | 0.998074519377826 |
| ebi-fl187-GCST90094727 | prot-a-147 | Rho GTPase-activating protein 1 \|\| id:prot-a-147 | Cauliflower liking \|\| id:ebi-fl187-GCST90094727 | Inverse variance weighted | 4 | -0.041473821 | 0.335893159052301 | 0.90173235287747 | -0.699824413 | 0.61687677059087 | 0.959374450330987 | 0.496672505423228 | 1.85313124019939 | 0.998074519377826 |
| ebi-fl187-GCST90094727 | prot-a-1473 | Interleukin-12 receptor subunit beta-1 \|\| id:prot-a-1473 | Cauliflower liking \|\| id:ebi-fl187-GCST90094727 | Inverse variance weighted | 4 | 0.349724569115879 | 0.437884786808004 | 0.424482943641916 | -0.508529613 | 1.20797875125957 | 1.41867674738555 | 0.601379189153578 | 3.346713272 | 0.998074519377826 |
| ebi-fl187-GCST90094727 | prot-a-1501 | X-linked interleukin-1 receptor accessory protein-like 2 \|\| id:prot-a-1501 | Cauliflower liking \|\| id:ebi-fl187-GCST90094727 | Inverse variance weighted | 4 | -0.010624995 | 0.336059078400769 | 0.974777924226503 | -0.669300789 | 0.64805079839773 | 0.989431250614112 | 0.51206649520239 | 1.91181069033793 | 0.998074519377826 |
| ebi-fl187-GCST90094727 | prot-a-1503 | Interleukin-1 receptor-like 2 \|\| id:prot-a-1503 | Cauliflower liking \|\| id:ebi-fl187-GCST90094727 | Inverse variance weighted | 4 | -0.151666598 | 0.336059078400769 | 0.651766619311812 | -0.810342392 | 0.50700919518487 | 0.85927471648544 | 0.444705776387826 | 1.66031807454468 | 0.998074519377826 |
| ebi-fl187-GCST90094727 | prot-a-1536 | Interleukin-5 receptor subunit alpha \|\| id:prot-a-1536 | Cauliflower liking \|\| id:ebi-fl187-GCST90094727 | Inverse variance weighted | 4 | 0.0298767966268086 | 0.342101758185842 | 0.930406768764316 | -0.640642649 | 0.700396242671058 | 1.03032758629936 | 0.526953668736025 | 2.01455080033092 | 0.998074519377826 |
| ebi-fl187-GCST90094727 | prot-a-1559 | Insulin-induced gene 1 protein \|\| id:prot-a-1559 | Cauliflower liking \|\| id:ebi-fl187-GCST90094727 | Inverse variance weighted | 4 | 0.254242790546103 | 0.336059078400769 | 0.449324330672681 | -0.404433003 | 0.912918584211611 | 1.28948484099382 | 0.667355091861601 | 2.49158382910444 | 0.998074519377826 |
| ebi-fl187-GCST90094727 | prot-a-1567 | Interferon regulatory factor 1 \|\| id:prot-a-1567 | Cauliflower liking \|\| id:ebi-fl187-GCST90094727 | Inverse variance weighted | 4 | -0.327150793 | 0.336059078400769 | 0.330308881169497 | -0.985826586 | 0.331525001001545 | 0.720975017085799 | 0.373130674716878 | 1.39309097451257 | 0.998074519377826 |
| ebi-fl187-GCST90094727 | prot-a-1613 | Potassium voltage-gated channel subfamily E regulatory beta subunit 5 \|\| id:prot-a-1613 | Cauliflower liking \|\| id:ebi-fl187-GCST90094727 | Inverse variance weighted | 3 | -0.451209638 | 0.442657911224513 | 0.308051526646571 | -1.318819144 | 0.4163998679432 | 0.636857318651896 | 0.26745093659763 | 1.51649214424205 | 0.998074519377826 |
| ebi-fl187-GCST90094727 | prot-a-1649 | Kin of IRRE-like protein 2 \|\| id:prot-a-1649 | Cauliflower liking \|\| id:ebi-fl187-GCST90094727 | Inverse variance weighted | 4 | -0.189875646 | 0.336059078400769 | 0.572069176943611 | -0.848551439 | 0.468800147918029 | 0.827061976222634 | 0.428034517018363 | 1.59807558810483 | 0.998074519377826 |
| ebi-fl187-GCST90094727 | prot-a-165 | Armadillo repeat-containing protein 5 \|\| id:prot-a-165 | Cauliflower liking \|\| id:ebi-fl187-GCST90094727 | Inverse variance weighted | 4 | -0.122227773 | 0.335893159052301 | 0.715941015519783 | -0.780578365 | 0.53612281835283 | 0.884946778217687 | 0.458140961906953 | 1.70936647318804 | 0.998074519377826 |
| ebi-fl187-GCST90094727 | prot-a-1656 | Kallikrein-11 \|\| id:prot-a-1656 | Cauliflower liking \|\| id:ebi-fl187-GCST90094727 | Inverse variance weighted | 4 | -0.276385642 | 0.368854909391148 | 0.453672065577497 | -0.999341264 | 0.446569980846561 | 0.758520357376661 | 0.368121856450312 | 1.56294205973743 | 0.998074519377826 |
| ebi-fl187-GCST90094727 | prot-a-1697 | Laminin subunit gamma-2 \|\| id:prot-a-1697 | Cauliflower liking \|\| id:ebi-fl187-GCST90094727 | Inverse variance weighted | 4 | -0.101599424 | 0.336059078400769 | 0.762403497554663 | -0.760275218 | 0.557076369205074 | 0.903391355678861 | 0.467537734442378 | 1.74556165501519 | 0.998074519377826 |
| ebi-fl187-GCST90094727 | prot-a-1698 | Lysosome-associated membrane glycoprotein 3 \|\| id:prot-a-1698 | Cauliflower liking \|\| id:ebi-fl187-GCST90094727 | Inverse variance weighted | 4 | -0.364987729 | 0.335893159052301 | 0.277205435172384 | -1.02333832 | 0.293362863127376 | 0.694205169684524 | 0.359393165813427 | 1.34092927595318 | 0.998074519377826 |
| ebi-fl187-GCST90094727 | prot-a-1724 | Leptin receptor \|\| id:prot-a-1724 | Cauliflower liking \|\| id:ebi-fl187-GCST90094727 | Inverse variance weighted | 4 | -0.183110405 | 0.444416915801273 | 0.68032188173959 | -1.05416756 | 0.687946749653959 | 0.832676218780513 | 0.348482397184919 | 1.98962613584954 | 0.998074519377826 |
| ebi-fl187-GCST90094727 | prot-a-1743 | Leukocyte immunoglobulin-like receptor subfamily B member 2 \|\| id:prot-a-1743 | Cauliflower liking \|\| id:ebi-fl187-GCST90094727 | Inverse variance weighted | 4 | -0.203606323 | 0.33605907840077 | 0.544604620679341 | -0.862282117 | 0.455069470487592 | 0.81578346302117 | 0.422197478090613 | 1.57628288437095 | 0.998074519377826 |
| ebi-fl187-GCST90094727 | prot-a-1746 | Leukocyte immunoglobulin-like receptor subfamily B member 4 \|\| id:prot-a-1746 | Cauliflower liking \|\| id:ebi-fl187-GCST90094727 | Inverse variance weighted | 4 | 0.0798157701251799 | 0.335893159052301 | 0.81217390257464 | -0.578534822 | 0.738166361867689 | 1.08308751221669 | 0.560719318822472 | 2.09209584856687 | 0.998074519377826 |
| ebi-fl187-GCST90094727 | prot-a-1750 | Leucine-rich repeat and immunoglobulin-like domain-containing nogo receptor-interacting protein 3 \|\| id:prot-a-1750 | Cauliflower liking \|\| id:ebi-fl187-GCST90094727 | Inverse variance weighted | 4 | -0.187419075 | 0.356825462485314 | 0.599416226001837 | -0.886796982 | 0.511958831005066 | 0.829096209689975 | 0.411973199322173 | 1.66855641593501 | 0.998074519377826 |
| ebi-fl187-GCST90094727 | prot-a-1753 | Lipase member N \|\| id:prot-a-1753 | Cauliflower liking \|\| id:ebi-fl187-GCST90094727 | Inverse variance weighted | 4 | 0.435494064710536 | 0.33605907840077 | 0.195014958199814 | -0.223181729 | 1.09416985837604 | 1.5457265592716 | 0.799969458470395 | 2.9867022681167 | 0.998074519377826 |
| ebi-fl187-GCST90094727 | prot-a-1760 | Ig Kappa chain V-I region HK102- like \|\| id:prot-a-1760 | Cauliflower liking \|\| id:ebi-fl187-GCST90094727 | Inverse variance weighted | 4 | -0.456872423 | 0.335893159052301 | 0.173775544921024 | -1.115223015 | 0.201478168510664 | 0.633261124317192 | 0.327842157036013 | 1.22320953228545 | 0.998074519377826 |
| ebi-fl187-GCST90094727 | prot-a-1792 | Leucine-rich repeat neuronal protein 1 \|\| id:prot-a-1792 | Cauliflower liking \|\| id:ebi-fl187-GCST90094727 | Inverse variance weighted | 4 | -0.416992507 | 0.335893159052301 | 0.214441816715429 | -1.075343099 | 0.241358084248037 | 0.659025857680504 | 0.341180676387558 | 1.27297678664009 | 0.998074519377826 |
| ebi-fl187-GCST90094727 | prot-a-1795 | Leucine-rich repeat transmembrane neuronal protein 1 \|\| id:prot-a-1795 | Cauliflower liking \|\| id:ebi-fl187-GCST90094727 | Inverse variance weighted | 4 | -0.18810044 | 0.336059078400769 | 0.575667489345833 | -0.846776234 | 0.470575353605929 | 0.828531485301596 | 0.42879504117127 | 1.60091502052115 | 0.998074519377826 |
| ebi-fl187-GCST90094727 | prot-a-1820 | LysM and putative peptidoglycan-binding domain-containing protein 4 \|\| id:prot-a-1820 | Cauliflower liking \|\| id:ebi-fl187-GCST90094727 | Inverse variance weighted | 4 | 0.0741771554175634 | 0.422688398950922 | 0.860695430168179 | -0.754292107 | 0.902646417361371 | 1.07699758455376 | 0.470343449969349 | 2.466120868 | 0.998074519377826 |
| ebi-fl187-GCST90094727 | prot-a-1844 | Mitogen-activated protein kinase kinase kinase 7:TGF-beta-activated kinase 1 and MAP3K7-binding protein 1 fusion \|\| id:prot-a-1844 | Cauliflower liking \|\| id:ebi-fl187-GCST90094727 | Inverse variance weighted | 4 | -0.302106805 | 0.336059078400769 | 0.368668996931137 | -0.960782598 | 0.356568988923105 | 0.73925910429472 | 0.382593351834906 | 1.42842007228201 | 0.998074519377826 |
| ebi-fl187-GCST90094727 | prot-a-1856 | Megakaryocyte-associated tyrosine-protein kinase \|\| id:prot-a-1856 | Cauliflower liking \|\| id:ebi-fl187-GCST90094727 | Inverse variance weighted | 4 | -0.348714504 | 0.481626790367607 | 0.469044438586337 | -1.292703013 | 0.595274005552775 | 0.705594546242393 | 0.274527727367295 | 1.81352779357296 | 0.998074519377826 |
| ebi-fl187-GCST90094727 | prot-a-1910 | Stromelysin-2 \|\| id:prot-a-1910 | Cauliflower liking \|\| id:ebi-fl187-GCST90094727 | Inverse variance weighted | 4 | -0.191722999 | 0.417247769371194 | 0.645879197666531 | -1.009528627 | 0.626082629124237 | 0.825535511114558 | 0.36439070310323 | 1.8702696701845 | 0.998074519377826 |
| ebi-fl187-GCST90094727 | prot-a-1940 | 39S ribosomal protein L14, mitochondrial \|\| id:prot-a-1940 | Cauliflower liking \|\| id:ebi-fl187-GCST90094727 | Inverse variance weighted | 4 | -0.606501016 | 0.336521973380864 | 0.0715040767369259 | -1.266084084 | 0.0530820517698721 | 0.545255375015813 | 0.281933490857635 | 1.05451616648751 | 0.998074519377826 |
| ebi-fl187-GCST90094727 | prot-a-1942 | 39S ribosomal protein L33, mitochondrial \|\| id:prot-a-1942 | Cauliflower liking \|\| id:ebi-fl187-GCST90094727 | Inverse variance weighted | 4 | 0.359256017839953 | 0.527185052884945 | 0.495579878710461 | -0.674026686 | 1.39253872149445 | 1.43226343962877 | 0.509652231024859 | 4.02505558814513 | 0.998074519377826 |
| ebi-fl187-GCST90094727 | prot-a-1948 | Mesothelin \|\| id:prot-a-1948 | Cauliflower liking \|\| id:ebi-fl187-GCST90094727 | Inverse variance weighted | 4 | 0.510238747760196 | 0.336059078400769 | 0.128938600972288 | -0.148437046 | 1.1689145414257 | 1.66568882695374 | 0.862054275308754 | 3.2184971964147 | 0.998074519377826 |
| ebi-fl187-GCST90094727 | prot-a-1980 | Unconventional myosin-VI \|\| id:prot-a-1980 | Cauliflower liking \|\| id:ebi-fl187-GCST90094727 | Inverse variance weighted | 4 | -0.138922113 | 0.370800555960394 | 0.707917402495361 | -0.865691203 | 0.58784697650258 | 0.870295810391869 | 0.420760621146945 | 1.80010856415464 | 0.998074519377826 |
| ebi-fl187-GCST90094727 | prot-a-2039 | Neurofascin \|\| id:prot-a-2039 | Cauliflower liking \|\| id:ebi-fl187-GCST90094727 | Inverse variance weighted | 4 | -0.327627684 | 0.336059078400769 | 0.329604421874118 | -0.986303477 | 0.33104811006242 | 0.720631272603644 | 0.372952774501868 | 1.3924267804361 | 0.998074519377826 |
| ebi-fl187-GCST90094727 | prot-a-2041 | NFU1 iron-sulfur cluster scaffold homolog, mitochondrial \|\| id:prot-a-2041 | Cauliflower liking \|\| id:ebi-fl187-GCST90094727 | Inverse variance weighted | 4 | 0.13568969784795 | 0.336059078400769 | 0.686383776657439 | -0.522986096 | 0.794365491513458 | 1.14532644118939 | 0.592747900613068 | 2.21303636087249 | 0.998074519377826 |
| ebi-fl187-GCST90094727 | prot-a-2088 | Glucocorticoid receptor \|\| id:prot-a-2088 | Cauliflower liking \|\| id:ebi-fl187-GCST90094727 | Inverse variance weighted | 4 | 0.358517285225292 | 0.335893159052301 | 0.285811503670301 | -0.299833307 | 1.0168678769678 | 1.43120577062852 | 0.740941720544057 | 2.76452236537083 | 0.998074519377826 |
| ebi-fl187-GCST90094727 | prot-a-2111 | Neuron-specific protein family member 2 \|\| id:prot-a-2111 | Cauliflower liking \|\| id:ebi-fl187-GCST90094727 | Inverse variance weighted | 4 | 0.224335667545284 | 0.336059078400769 | 0.504421966634951 | -0.434340126 | 0.883011461210792 | 1.25149103385509 | 0.647691921076515 | 2.41817098045085 | 0.998074519377826 |
| ebi-fl187-GCST90094727 | prot-a-2125 | Nuclear migration protein nudC \|\| id:prot-a-2125 | Cauliflower liking \|\| id:ebi-fl187-GCST90094727 | Inverse variance weighted | 4 | 0.124420313149797 | 0.336059078400769 | 0.711208552553494 | -0.534255481 | 0.783096106815305 | 1.13249177216229 | 0.586105494703904 | 2.18823680310864 | 0.998074519377826 |
| ebi-fl187-GCST90094727 | prot-a-2142 | Ornithine decarboxylase \|\| id:prot-a-2142 | Cauliflower liking \|\| id:ebi-fl187-GCST90094727 | Inverse variance weighted | 4 | -0.318782267 | 0.336059078400769 | 0.342829199845618 | -0.977458061 | 0.339893526271609 | 0.727033831044922 | 0.376266330304072 | 1.40479800851885 | 0.998074519377826 |
| ebi-fl187-GCST90094727 | prot-a-2186 | Prostate and testis expressed protein 1 \|\| id:prot-a-2186 | Cauliflower liking \|\| id:ebi-fl187-GCST90094727 | Inverse variance weighted | 4 | 0.508262234424541 | 0.336059078400769 | 0.13042722940403 | -0.150413559 | 1.16693802809005 | 1.6623998222258 | 0.860352096281466 | 3.21214207634391 | 0.998074519377826 |
| ebi-fl187-GCST90094727 | prot-a-2227 | Platelet-derived growth factor C \|\| id:prot-a-2227 | Cauliflower liking \|\| id:ebi-fl187-GCST90094727 | Inverse variance weighted (multiplicative random effects) | 4 | -0.032440564 | 0.655452211324472 | 0.96052608978228 | -1.317126898 | 1.25224576995397 | 0.968079986689237 | 0.267903912438459 | 3.49819027313951 | 0.998074519377826 |
| ebi-fl187-GCST90094727 | prot-a-2229 | Platelet-derived growth factor receptor alpha \|\| id:prot-a-2229 | Cauliflower liking \|\| id:ebi-fl187-GCST90094727 | Inverse variance weighted (multiplicative random effects) | 4 | -0.490794464 | 0.557966623831543 | 0.379069219613883 | -1.584409046 | 0.602820119154786 | 0.61213987812661 | 0.20506894357428 | 1.82726464505891 | 0.998074519377826 |
| ebi-fl187-GCST90094727 | prot-a-2243 | Platelet endothelial aggregation receptor 1 \|\| id:prot-a-2243 | Cauliflower liking \|\| id:ebi-fl187-GCST90094727 | Inverse variance weighted (multiplicative random effects) | 4 | 0.569072144089647 | 0.613887235782034 | 0.353927653639401 | -0.634146838 | 1.77229112622243 | 1.76662711532884 | 0.530387802036785 | 5.88431964805752 | 0.998074519377826 |
| ebi-fl187-GCST90094727 | prot-a-2246 | E3 ubiquitin-protein ligase pellino homolog 2 \|\| id:prot-a-2246 | Cauliflower liking \|\| id:ebi-fl187-GCST90094727 | Inverse variance weighted | 4 | 0.638310303762207 | 0.3358931590523 | 0.0573888675107936 | -0.020040288 | 1.29666089550472 | 1.89327910846998 | 0.980159183877393 | 3.65706493550264 | 0.998074519377826 |
| ebi-fl187-GCST90094727 | prot-a-2251 | Platelet factor 4 variant \|\| id:prot-a-2251 | Cauliflower liking \|\| id:ebi-fl187-GCST90094727 | Inverse variance weighted | 4 | -0.524535531 | 0.335893159052301 | 0.118378899384461 | -1.182886123 | 0.133815060833686 | 0.591830187367738 | 0.306393173013541 | 1.14318138108205 | 0.998074519377826 |
| ebi-fl187-GCST90094727 | prot-a-2346 | Protein phosphatase 1L \|\| id:prot-a-2346 | Cauliflower liking \|\| id:ebi-fl187-GCST90094727 | Inverse variance weighted | 4 | 0.832353049996045 | 0.513817298769085 | 0.105245226878405 | -0.174728856 | 1.83943495558345 | 2.29872138777394 | 0.839684665707612 | 6.29298144221363 | 0.998074519377826 |
| ebi-fl187-GCST90094727 | prot-a-2358 | PR domain zinc finger protein 4 \|\| id:prot-a-2358 | Cauliflower liking \|\| id:ebi-fl187-GCST90094727 | Inverse variance weighted | 4 | 0.223951996269168 | 0.364501006601274 | 0.538946363580888 | -0.490469977 | 0.938373969207666 | 1.25101096479333 | 0.612338541719404 | 2.5558221921466 | 0.998074519377826 |
| ebi-fl187-GCST90094727 | prot-a-2390 | Trypsin-1 \|\| id:prot-a-2390 | Cauliflower liking \|\| id:ebi-fl187-GCST90094727 | Inverse variance weighted | 4 | 0.110014488610899 | 0.350809168409606 | 0.753823271529638 | -0.577571481 | 0.797600458693726 | 1.11629424389465 | 0.561259742516721 | 2.2202070530922 | 0.998074519377826 |
| ebi-fl187-GCST90094727 | prot-a-242 | Brain-derived neurotrophic factor \|\| id:prot-a-242 | Cauliflower liking \|\| id:ebi-fl187-GCST90094727 | Inverse variance weighted | 4 | 0.0314930795547182 | 0.370611172973132 | 0.932280418308961 | -0.694904819 | 0.757890978582058 | 1.03199423371131 | 0.499121952415009 | 2.1337713023046 | 0.998074519377826 |
| ebi-fl187-GCST90094727 | prot-a-2464 | Dihydropteridine reductase \|\| id:prot-a-2464 | Cauliflower liking \|\| id:ebi-fl187-GCST90094727 | Inverse variance weighted | 4 | 0.292760781325173 | 0.336059078400769 | 0.383667520630335 | -0.365915012 | 0.951436574990681 | 1.34012216983442 | 0.693561742894424 | 2.58942689454988 | 0.998074519377826 |
| ebi-fl187-GCST90094727 | prot-a-2474 | Ras-related protein Rab-27A \|\| id:prot-a-2474 | Cauliflower liking \|\| id:ebi-fl187-GCST90094727 | Inverse variance weighted (multiplicative random effects) | 4 | -0.226681011 | 0.70536106685079 | 0.747930955034527 | -1.609188702 | 1.15582668017955 | 0.797175031923974 | 0.200049848322816 | 3.1766484046392 | 0.998074519377826 |
| ebi-fl187-GCST90094727 | prot-a-2478 | Ras-related protein Rab-7b \|\| id:prot-a-2478 | Cauliflower liking \|\| id:ebi-fl187-GCST90094727 | Inverse variance weighted | 4 | -0.312178093 | 0.379947644458763 | 0.411284948300377 | -1.056875476 | 0.432519290537599 | 0.731851179341076 | 0.347540012840657 | 1.54113520433255 | 0.998074519377826 |
| ebi-fl187-GCST90094727 | prot-a-2528 | E3 ubiquitin-protein ligase rififylin \|\| id:prot-a-2528 | Cauliflower liking \|\| id:ebi-fl187-GCST90094727 | Inverse variance weighted | 4 | -0.302727608 | 0.453090599240854 | 0.504044717234304 | -1.190785182 | 0.585329966857375 | 0.738800312514205 | 0.30398248870875 | 1.79558337090283 | 0.998074519377826 |
| ebi-fl187-GCST90094727 | prot-a-2543 | Protein RIC-3 \|\| id:prot-a-2543 | Cauliflower liking \|\| id:ebi-fl187-GCST90094727 | Inverse variance weighted | 4 | -0.329701714 | 0.336059078400769 | 0.326552010289937 | -0.988377508 | 0.328974079314304 | 0.719138210050246 | 0.372180060573172 | 1.38954183724358 | 0.998074519377826 |
| ebi-fl187-GCST90094727 | prot-a-255 | Bone morphogenetic protein 6 \|\| id:prot-a-255 | Cauliflower liking \|\| id:ebi-fl187-GCST90094727 | Inverse variance weighted | 4 | -0.764403676 | 0.502405055558973 | 0.128137214372266 | -1.749117585 | 0.220310232898094 | 0.465611503519894 | 0.173927351878337 | 1.24646336455295 | 0.998074519377826 |
| ebi-fl187-GCST90094727 | prot-a-2568 | RING finger protein 165 \|\| id:prot-a-2568 | Cauliflower liking \|\| id:ebi-fl187-GCST90094727 | Inverse variance weighted | 4 | 0.0582511008308854 | 0.336059078400769 | 0.862387411266118 | -0.600424693 | 0.716926894496394 | 1.05998112443739 | 0.548578609210546 | 2.04812941171813 | 0.998074519377826 |
| ebi-fl187-GCST90094727 | prot-a-2573 | E3 ubiquitin-protein ligase RNF8 \|\| id:prot-a-2573 | Cauliflower liking \|\| id:ebi-fl187-GCST90094727 | Inverse variance weighted (multiplicative random effects) | 4 | 0.0828125483992241 | 0.663707304280806 | 0.900703505437467 | -1.218053768 | 1.3836788647896 | 1.08633815363523 | 0.295805312826996 | 3.98955168440066 | 0.998074519377826 |
| ebi-fl187-GCST90094727 | prot-a-260 | Cytoplasmic tyrosine-protein kinase BMX \|\| id:prot-a-260 | Cauliflower liking \|\| id:ebi-fl187-GCST90094727 | Inverse variance weighted | 4 | 0.273846940820424 | 0.37745905706048 | 0.468144703240672 | -0.465972811 | 1.01366669265896 | 1.31501351194518 | 0.627524345563461 | 2.75568676948412 | 0.998074519377826 |
| ebi-fl187-GCST90094727 | prot-a-2623 | Serum amyloid A-1 protein \|\| id:prot-a-2623 | Cauliflower liking \|\| id:ebi-fl187-GCST90094727 | Inverse variance weighted | 4 | -0.038519281 | 0.336059078400769 | 0.908745878613511 | -0.697195075 | 0.620156512219712 | 0.962213151700224 | 0.497980143565269 | 1.85921900956986 | 0.998074519377826 |
| ebi-fl187-GCST90094727 | prot-a-2640 | Lysosome membrane protein 2 \|\| id:prot-a-2640 | Cauliflower liking \|\| id:ebi-fl187-GCST90094727 | Inverse variance weighted | 4 | 0.163057871844666 | 0.456814319517051 | 0.721132514212245 | -0.732298194 | 1.05841393809809 | 1.17710480894401 | 0.480802741218041 | 2.88179665475487 | 0.998074519377826 |
| ebi-fl187-GCST90094727 | prot-a-2641 | Scavenger receptor class F member 2 \|\| id:prot-a-2641 | Cauliflower liking \|\| id:ebi-fl187-GCST90094727 | Inverse variance weighted | 4 | -0.145802661 | 0.335893159052301 | 0.664234328295036 | -0.804153253 | 0.512547930257176 | 0.864328251609561 | 0.447466657139861 | 1.66953965085478 | 0.998074519377826 |
| ebi-fl187-GCST90094727 | prot-a-2669 | Semaphorin-3A \|\| id:prot-a-2669 | Cauliflower liking \|\| id:ebi-fl187-GCST90094727 | Inverse variance weighted | 4 | 0.00547271273964854 | 0.336059078400769 | 0.987007046423241 | -0.653203081 | 0.664148506405157 | 1.00548771538792 | 0.520376297057711 | 1.94283550483065 | 0.999355215041207 |
| ebi-fl187-GCST90094727 | prot-a-2683 | Sentrin-specific protease 7 \|\| id:prot-a-2683 | Cauliflower liking \|\| id:ebi-fl187-GCST90094727 | Inverse variance weighted (multiplicative random effects) | 4 | -0.373272835 | 0.587711075887519 | 0.525343744564634 | -1.525186543 | 0.778640874117558 | 0.688477366754835 | 0.217580464887948 | 2.17850938400089 | 0.998074519377826 |
| ebi-fl187-GCST90094727 | prot-a-270 | Bromodomain testis-specific protein \|\| id:prot-a-270 | Cauliflower liking \|\| id:ebi-fl187-GCST90094727 | Inverse variance weighted | 4 | 0.812562551105465 | 0.457845329637492 | 0.075938615401151 | -0.084814295 | 1.70993939719495 | 2.25367575271408 | 0.91868287257896 | 5.52862641720234 | 0.998074519377826 |
| ebi-fl187-GCST90094727 | prot-a-2708 | 14-3-3 protein sigma \|\| id:prot-a-2708 | Cauliflower liking \|\| id:ebi-fl187-GCST90094727 | Inverse variance weighted | 4 | 0.0988182705078565 | 0.340234431720569 | 0.771478337428443 | -0.568041216 | 0.765677756680171 | 1.1038656763811 | 0.566634266693132 | 2.15045136363453 | 0.998074519377826 |
| ebi-fl187-GCST90094727 | prot-a-2713 | Beta-sarcoglycan \|\| id:prot-a-2713 | Cauliflower liking \|\| id:ebi-fl187-GCST90094727 | Inverse variance weighted | 4 | -0.009456722 | 0.335893159052301 | 0.97753935905857 | -0.667807314 | 0.648893869939602 | 0.990587852371539 | 0.512831825268483 | 1.91342316314386 | 0.998074519377826 |
| ebi-fl187-GCST90094727 | prot-a-2720 | Protein shisa-3 homolog \|\| id:prot-a-2720 | Cauliflower liking \|\| id:ebi-fl187-GCST90094727 | Inverse variance weighted | 4 | -0.119705627 | 0.407475175150192 | 0.768930576226717 | -0.91835697 | 0.678945716450977 | 0.88718156071783 | 0.399174357974869 | 1.97179780202033 | 0.998074519377826 |
| ebi-fl187-GCST90094727 | prot-a-2730 | Sialic acid-binding Ig-like lectin 8 \|\| id:prot-a-2730 | Cauliflower liking \|\| id:ebi-fl187-GCST90094727 | Inverse variance weighted | 4 | -0.734186824 | 0.335893159052301 | 0.0288320806867119 | -1.392537415 | -0.075836232 | 0.479895540026412 | 0.248444098260194 | 0.926968001856298 | 0.998074519377826 |
| ebi-fl187-GCST90094727 | prot-a-2761 | Sodium- and chloride-dependent neutral and basic amino acid transporter B(0+) \|\| id:prot-a-2761 | Cauliflower liking \|\| id:ebi-fl187-GCST90094727 | Inverse variance weighted | 4 | -0.227789928 | 0.418068479400655 | 0.585847939686478 | -1.047204147 | 0.591624291866031 | 0.796291521010481 | 0.350917492453046 | 1.80692100014942 | 0.998074519377826 |
| ebi-fl187-GCST90094727 | prot-a-2770 | Secreted Ly-6/uPAR-related protein 1 \|\| id:prot-a-2770 | Cauliflower liking \|\| id:ebi-fl187-GCST90094727 | Inverse variance weighted | 4 | 0.0387688738947679 | 0.335893159052301 | 0.908112025497721 | -0.619581718 | 0.697119465637277 | 1.03953019331769 | 0.538169497217758 | 2.00796037011712 | 0.998074519377826 |
| ebi-fl187-GCST90094727 | prot-a-2784 | N-lysine methyltransferase SMYD2 \|\| id:prot-a-2784 | Cauliflower liking \|\| id:ebi-fl187-GCST90094727 | Inverse variance weighted | 4 | 0.29330217814742 | 0.406964113991613 | 0.471089362467506 | -0.504347485 | 1.09095184157098 | 1.34084790415599 | 0.60389950020782 | 2.97710645804611 | 0.998074519377826 |
| ebi-fl187-GCST90094727 | prot-a-2837 | Serine palmitoyltransferase 2 \|\| id:prot-a-2837 | Cauliflower liking \|\| id:ebi-fl187-GCST90094727 | Inverse variance weighted | 4 | 0.191885015805756 | 0.336059078400769 | 0.568009159439102 | -0.466790778 | 0.850560809471264 | 1.21153120201648 | 0.627011261328024 | 2.34095931602672 | 0.998074519377826 |
| ebi-fl187-GCST90094727 | prot-a-2858 | Alpha-2,8-sialyltransferase 8B \|\| id:prot-a-2858 | Cauliflower liking \|\| id:ebi-fl187-GCST90094727 | Inverse variance weighted | 4 | 0.284821589257033 | 0.335893159052301 | 0.396464183780686 | -0.373529002 | 0.943172180999542 | 1.32952480545223 | 0.68830102356642 | 2.56811503657777 | 0.998074519377826 |
| ebi-fl187-GCST90094727 | prot-a-2911 | Synaptotagmin-11 \|\| id:prot-a-2911 | Cauliflower liking \|\| id:ebi-fl187-GCST90094727 | Inverse variance weighted | 4 | -0.200388361 | 0.335893159052301 | 0.550785401712294 | -0.858738953 | 0.457962230830334 | 0.818412851790103 | 0.423696046344492 | 1.58084929456863 | 0.998074519377826 |
| ebi-fl187-GCST90094727 | prot-a-2962 | Transforming growth factor beta-1 \|\| id:prot-a-2962 | Cauliflower liking \|\| id:ebi-fl187-GCST90094727 | Inverse variance weighted | 4 | 0.054617445862707 | 0.434322547677633 | 0.899927296483962 | -0.796654748 | 0.905889639310868 | 1.0561365079934 | 0.450834599881103 | 2.47413202937544 | 0.998074519377826 |
| ebi-fl187-GCST90094727 | prot-a-2978 | Tyrosine-protein kinase receptor Tie-1, soluble \|\| id:prot-a-2978 | Cauliflower liking \|\| id:ebi-fl187-GCST90094727 | Inverse variance weighted | 4 | 0.0451939444025126 | 0.365018602983295 | 0.901463563860266 | -0.670242517 | 0.76063040624977 | 1.04623075083116 | 0.511584494576583 | 2.13962462816761 | 0.998074519377826 |
| ebi-fl187-GCST90094727 | prot-a-299 | Uncharacterized protein C1orf226 \|\| id:prot-a-299 | Cauliflower liking \|\| id:ebi-fl187-GCST90094727 | Inverse variance weighted | 4 | -0.101639462 | 0.367618610935824 | 0.782178858349989 | -0.822171939 | 0.618893015555803 | 0.903355186945612 | 0.439476101757979 | 1.85687137597972 | 0.998074519377826 |
| ebi-fl187-GCST90094727 | prot-a-3015 | Transmembrane protein 70, mitochondrial \|\| id:prot-a-3015 | Cauliflower liking \|\| id:ebi-fl187-GCST90094727 | Inverse variance weighted | 4 | -0.216682465 | 0.336059078400769 | 0.519073056894841 | -0.875358259 | 0.44199332822272 | 0.805185602970505 | 0.416712701811896 | 1.55580536041263 | 0.998074519377826 |
| ebi-fl187-GCST90094727 | prot-a-3016 | Transmembrane protein 87B \|\| id:prot-a-3016 | Cauliflower liking \|\| id:ebi-fl187-GCST90094727 | Inverse variance weighted (multiplicative random effects) | 4 | -0.461172892 | 0.660925073406482 | 0.485321796934563 | -1.756586036 | 0.834240251676924 | 0.630543651894462 | 0.172633222527613 | 2.30306363469991 | 0.998074519377826 |
| ebi-fl187-GCST90094727 | prot-a-3123 | Thioredoxin domain-containing protein 12 \|\| id:prot-a-3123 | Cauliflower liking \|\| id:ebi-fl187-GCST90094727 | Inverse variance weighted | 4 | -0.383001262 | 0.336059078400769 | 0.254417842150343 | -1.041677056 | 0.275674531541773 | 0.681812038754291 | 0.352862415508919 | 1.31741901590687 | 0.998074519377826 |
| ebi-fl187-GCST90094727 | prot-a-3133 | PolyUbiquitin K48-linked \|\| id:prot-a-3133 | Cauliflower liking \|\| id:ebi-fl187-GCST90094727 | Inverse variance weighted | 4 | 0.128249375354963 | 0.477055026482452 | 0.788056194898361 | -0.806778477 | 1.06327722726057 | 1.1368364663485 | 0.446293497811741 | 2.89584580003204 | 0.998074519377826 |
| ebi-fl187-GCST90094727 | prot-a-3135 | Ubiquitin-conjugating enzyme E2 B \|\| id:prot-a-3135 | Cauliflower liking \|\| id:ebi-fl187-GCST90094727 | Inverse variance weighted | 4 | -0.280466343 | 0.409006519445139 | 0.492886003717287 | -1.082119121 | 0.521186435606887 | 0.755431369541441 | 0.338876643732508 | 1.6840244514984 | 0.998074519377826 |
| ebi-fl187-GCST90094727 | prot-a-3162 | NKG2D ligand 3 \|\| id:prot-a-3162 | Cauliflower liking \|\| id:ebi-fl187-GCST90094727 | Inverse variance weighted | 4 | 0.737677134453237 | 0.362278947532516 | 0.0417289625925962 | 0.0276103972895048 | 1.44774387161697 | 2.091072588 | 1.02799509671541 | 4.25350722322823 | 0.998074519377826 |
| ebi-fl187-GCST90094727 | prot-a-3188 | Vesicle-associated membrane protein 8 \|\| id:prot-a-3188 | Cauliflower liking \|\| id:ebi-fl187-GCST90094727 | Inverse variance weighted | 4 | -0.645221182 | 0.335893159052301 | 0.054742809366436 | -1.303571774 | 0.0131294096807839 | 0.524546508996514 | 0.271560107468386 | 1.01321597883247 | 0.998074519377826 |
| ebi-fl187-GCST90094727 | prot-a-3201 | Vitrin \|\| id:prot-a-3201 | Cauliflower liking \|\| id:ebi-fl187-GCST90094727 | Inverse variance weighted (multiplicative random effects) | 4 | -0.133644744 | 0.554929654900304 | 0.809685431627699 | -1.221306867 | 0.95401737984894 | 0.874900823381256 | 0.29484459225745 | 2.59611833099123 | 0.998074519377826 |
| ebi-fl187-GCST90094727 | prot-a-3232 | WNT1-inducible-signaling pathway protein 3 \|\| id:prot-a-3232 | Cauliflower liking \|\| id:ebi-fl187-GCST90094727 | Inverse variance weighted | 4 | -0.335148648 | 0.393207256718046 | 0.39402206020452 | -1.105834871 | 0.435537575324267 | 0.715231760791662 | 0.330934479274511 | 1.54579381624604 | 0.998074519377826 |
| ebi-fl187-GCST90094727 | prot-a-327 | Carbonic anhydrase-related protein 10 \|\| id:prot-a-327 | Cauliflower liking \|\| id:ebi-fl187-GCST90094727 | Inverse variance weighted | 4 | 0.062457728461442 | 0.336059078400769 | 0.852559712498937 | -0.596218065 | 0.72113352212695 | 1.06444946205034 | 0.550891135704356 | 2.0567632764876 | 0.998074519377826 |
| ebi-fl187-GCST90094727 | prot-a-332 | Carbonic anhydrase 5A, mitochondrial \|\| id:prot-a-332 | Cauliflower liking \|\| id:ebi-fl187-GCST90094727 | Inverse variance weighted | 4 | -0.086585244 | 0.336059078400769 | 0.796677905359556 | -0.745261037 | 0.572090549914297 | 0.917057372042245 | 0.474610392697545 | 1.77196756867684 | 0.998074519377826 |
| ebi-fl187-GCST90094727 | prot-a-338 | Cell adhesion molecule 3 \|\| id:prot-a-338 | Cauliflower liking \|\| id:ebi-fl187-GCST90094727 | Inverse variance weighted | 4 | -0.28908606 | 0.34771267126251 | 0.405751360618046 | -0.970602896 | 0.392430775240743 | 0.74894774786103 | 0.378854559296418 | 1.48057536925995 | 0.998074519377826 |
| ebi-fl187-GCST90094727 | prot-a-34 | A disintegrin and metalloproteinase with thrombospondin motifs 3 \|\| id:prot-a-34 | Cauliflower liking \|\| id:ebi-fl187-GCST90094727 | Inverse variance weighted | 4 | -0.720754249 | 0.507821422945069 | 0.155809676482675 | -1.716084238 | 0.274575740026471 | 0.486385262003817 | 0.179768702965138 | 1.31597224206707 | 0.998074519377826 |
| ebi-fl187-GCST90094727 | prot-a-358 | Cysteine--tRNA ligase, cytoplasmic \|\| id:prot-a-358 | Cauliflower liking \|\| id:ebi-fl187-GCST90094727 | Inverse variance weighted | 4 | 0.180211905887186 | 0.449136844484299 | 0.68824304276009 | -0.700096309 | 1.06052012107641 | 1.19747108741118 | 0.496537480310358 | 2.88787264214062 | 0.998074519377826 |
| ebi-fl187-GCST90094727 | prot-a-382 | Coiled-coil domain-containing protein 167 \|\| id:prot-a-382 | Cauliflower liking \|\| id:ebi-fl187-GCST90094727 | Inverse variance weighted | 4 | -0.689801538 | 0.335893159052301 | 0.0400111500997114 | -1.34815213 | -0.031450946 | 0.501675622715298 | 0.259719745880038 | 0.96903849021564 | 0.998074519377826 |
| ebi-fl187-GCST90094727 | prot-a-40 | Pituitary adenylate cyclase-activating polypeptide 27 \|\| id:prot-a-40 | Cauliflower liking \|\| id:ebi-fl187-GCST90094727 | Inverse variance weighted | 4 | -0.330178405 | 0.33605907840077 | 0.325853054391082 | -0.988854199 | 0.328497388399501 | 0.718795485092454 | 0.372002687998932 | 1.38887961312468 | 0.998074519377826 |
| ebi-fl187-GCST90094727 | prot-a-403 | C-C motif chemokine 25 \|\| id:prot-a-403 | Cauliflower liking \|\| id:ebi-fl187-GCST90094727 | Inverse variance weighted | 4 | 0.41811427635772 | 0.335893159052301 | 0.21321132731101 | -0.240236315 | 1.07646486810023 | 1.51909426104281 | 0.786441990763751 | 2.93428809884902 | 0.998074519377826 |
| ebi-fl187-GCST90094727 | prot-a-415 | Cyclin-C \|\| id:prot-a-415 | Cauliflower liking \|\| id:ebi-fl187-GCST90094727 | Inverse variance weighted | 4 | -0.120689782 | 0.398940842528997 | 0.762251489057838 | -0.902613834 | 0.661234269137511 | 0.886308865719604 | 0.405508341971187 | 1.93718186322536 | 0.998074519377826 |
| ebi-fl187-GCST90094727 | prot-a-44 | Adhesion G protein-coupled receptor F5 \|\| id:prot-a-44 | Cauliflower liking \|\| id:ebi-fl187-GCST90094727 | Inverse variance weighted | 4 | -0.361826171 | 0.33605907840077 | 0.28162580000725 | -1.020501965 | 0.296849622383738 | 0.69640341223349 | 0.360413979574684 | 1.34561293416742 | 0.998074519377826 |
| ebi-fl187-GCST90094727 | prot-a-455 | CD59 glycoprotein \|\| id:prot-a-455 | Cauliflower liking \|\| id:ebi-fl187-GCST90094727 | Inverse variance weighted | 4 | -0.373287442 | 0.430131847407039 | 0.385479613491854 | -1.216345862 | 0.469770979347898 | 0.688467310275242 | 0.296310952017794 | 1.59962780346088 | 0.998074519377826 |
| ebi-fl187-GCST90094727 | prot-a-478 | Cadherin-12 \|\| id:prot-a-478 | Cauliflower liking \|\| id:ebi-fl187-GCST90094727 | Inverse variance weighted | 4 | -0.510873409 | 0.336059078400769 | 0.128463410214351 | -1.169549203 | 0.147802384349143 | 0.599971329354802 | 0.310506885297939 | 1.15928378110641 | 0.998074519377826 |
| ebi-fl187-GCST90094727 | prot-a-516 | Centrin-2 \|\| id:prot-a-516 | Cauliflower liking \|\| id:ebi-fl187-GCST90094727 | Inverse variance weighted | 4 | 0.273168179303759 | 0.3419195360715 | 0.424333708531079 | -0.396994111 | 0.943330470003899 | 1.31412123423552 | 0.67233798475289 | 2.56852157312427 | 0.998074519377826 |
| ebi-fl187-GCST90094727 | prot-a-519 | Cryptic protein \|\| id:prot-a-519 | Cauliflower liking \|\| id:ebi-fl187-GCST90094727 | Inverse variance weighted | 4 | -0.078702661 | 0.336059078400769 | 0.814835202798656 | -0.737378454 | 0.579973133153676 | 0.924314718854858 | 0.47836633243009 | 1.78599044617463 | 0.998074519377826 |
| ebi-fl187-GCST90094727 | prot-a-583 | Chloride intracellular channel protein 4 \|\| id:prot-a-583 | Cauliflower liking \|\| id:ebi-fl187-GCST90094727 | Inverse variance weighted | 4 | 0.43174838892197 | 0.335893159052301 | 0.198661564607737 | -0.226602203 | 1.09009898066448 | 1.53994759853606 | 0.797237858191357 | 2.97456848275733 | 0.998074519377826 |
| ebi-fl187-GCST90094727 | prot-a-602 | CCR4-NOT transcription complex subunit 1 \|\| id:prot-a-602 | Cauliflower liking \|\| id:ebi-fl187-GCST90094727 | Inverse variance weighted | 4 | 0.273237778099458 | 0.371362400552339 | 0.461869919724653 | -0.454632527 | 1.00110808318204 | 1.3142126986737 | 0.634681153299098 | 2.72129558027269 | 0.998074519377826 |
| ebi-fl187-GCST90094727 | prot-a-606 | Ciliary neurotrophic factor receptor subunit alpha \|\| id:prot-a-606 | Cauliflower liking \|\| id:ebi-fl187-GCST90094727 | Inverse variance weighted | 4 | -0.05630606 | 0.33605907840077 | 0.866938803879589 | -0.714981854 | 0.602369733518856 | 0.945249788324938 | 0.489200988848824 | 1.82644185660969 | 0.998074519377826 |
| ebi-fl187-GCST90094727 | prot-a-626 | Collagen alpha-2(IX) chain \|\| id:prot-a-626 | Cauliflower liking \|\| id:ebi-fl187-GCST90094727 | Inverse variance weighted | 4 | 0.100304622132733 | 0.440033925832915 | 0.819687074701388 | -0.762161872 | 0.962771116765247 | 1.10550762887992 | 0.466656483899751 | 2.61894382629913 | 0.998074519377826 |
| ebi-fl187-GCST90094727 | prot-a-647 | Probable serine carboxypeptidase CPVL \|\| id:prot-a-647 | Cauliflower liking \|\| id:ebi-fl187-GCST90094727 | Inverse variance weighted (multiplicative random effects) | 4 | -0.403627425 | 0.569017525903228 | 0.478112930007762 | -1.518901776 | 0.711646925499128 | 0.667892914940585 | 0.218952213528516 | 2.0373438506925 | 0.998074519377826 |
| ebi-fl187-GCST90094727 | prot-a-651 | Cellular retinoic acid-binding protein 2 \|\| id:prot-a-651 | Cauliflower liking \|\| id:ebi-fl187-GCST90094727 | Inverse variance weighted | 4 | -0.179218381 | 0.50417137474094 | 0.722236650133562 | -1.167394276 | 0.808957513220649 | 0.835923329463192 | 0.31117672650677 | 2.24556579338406 | 0.998074519377826 |
| ebi-fl187-GCST90094727 | prot-a-671 | Cytotoxic and regulatory T-cell molecule \|\| id:prot-a-671 | Cauliflower liking \|\| id:ebi-fl187-GCST90094727 | Inverse variance weighted | 4 | 0.00163505083691461 | 0.452628878388476 | 0.997117773425551 | -0.885517551 | 0.888787652478327 | 1.00163638826135 | 0.412500627991676 | 2.43217921672957 | 0.999355215041207 |
| ebi-fl187-GCST90094727 | prot-a-688 | Chorionic somatomammotropin hormone \|\| id:prot-a-688 | Cauliflower liking \|\| id:ebi-fl187-GCST90094727 | Inverse variance weighted | 4 | 0.0682634738524635 | 0.336059078400769 | 0.839033904728457 | -0.59041232 | 0.726939267517972 | 1.07064735890497 | 0.554098771725558 | 2.06873905091046 | 0.998074519377826 |
| ebi-fl187-GCST90094727 | prot-a-69 | Aldo-keto reductase family 1 member C1 \|\| id:prot-a-69 | Cauliflower liking \|\| id:ebi-fl187-GCST90094727 | Inverse variance weighted | 4 | -0.278966472 | 0.336059078400769 | 0.406475803052297 | -0.937642265 | 0.379709322095941 | 0.756565269235801 | 0.391549918773006 | 1.46185959738552 | 0.998074519377826 |
| ebi-fl187-GCST90094727 | prot-a-741 | Stromal cell-derived factor 1 \|\| id:prot-a-741 | Cauliflower liking \|\| id:ebi-fl187-GCST90094727 | Inverse variance weighted | 4 | -0.116544291 | 0.351287349950506 | 0.740067910829483 | -0.805067497 | 0.571978914648552 | 0.889990677300173 | 0.447057750413605 | 1.77176976564752 | 0.998074519377826 |
| ebi-fl187-GCST90094727 | prot-a-742 | Growth-regulated alpha protein \|\| id:prot-a-742 | Cauliflower liking \|\| id:ebi-fl187-GCST90094727 | Inverse variance weighted | 4 | -0.161986532 | 0.335893159052301 | 0.629624014346593 | -0.820337123 | 0.496364059988113 | 0.850452658568381 | 0.440283199671697 | 1.64273750396413 | 0.998074519377826 |
| ebi-fl187-GCST90094727 | prot-a-788 | Beta-defensin 112 \|\| id:prot-a-788 | Cauliflower liking \|\| id:ebi-fl187-GCST90094727 | Inverse variance weighted | 4 | -0.289541521 | 0.336059078400769 | 0.388919105590051 | -0.948217315 | 0.369134272304732 | 0.748606709096327 | 0.387431075755921 | 1.44648181308277 | 0.998074519377826 |
| ebi-fl187-GCST90094727 | prot-a-838 | DnaJ homolog subfamily B member 1 \|\| id:prot-a-838 | Cauliflower liking \|\| id:ebi-fl187-GCST90094727 | Inverse variance weighted | 4 | 0.331937486344464 | 0.336059078400769 | 0.32328219701744 | -0.326738307 | 0.990613280009972 | 1.39366572265035 | 0.721272470056235 | 2.69288545886007 | 0.998074519377826 |
| ebi-fl187-GCST90094727 | prot-a-85 | Junctional adhesion molecule-like \|\| id:prot-a-85 | Cauliflower liking \|\| id:ebi-fl187-GCST90094727 | Inverse variance weighted | 4 | -0.184537328 | 0.336059078400769 | 0.582921944187261 | -0.843213121 | 0.474138466124069 | 0.83148890188368 | 0.430325611327712 | 1.60662943537706 | 0.998074519377826 |
| ebi-fl187-GCST90094727 | prot-a-856 | Dipeptidase 1 \|\| id:prot-a-856 | Cauliflower liking \|\| id:ebi-fl187-GCST90094727 | Inverse variance weighted (multiplicative random effects) | 4 | -0.186638282 | 0.67206810558872 | 0.781236984671734 | -1.503891769 | 1.13061520518033 | 0.829743815571445 | 0.222263476728617 | 3.0975615499783 | 0.998074519377826 |
| ebi-fl187-GCST90094727 | prot-a-860 | Dipeptidyl peptidase 2 \|\| id:prot-a-860 | Cauliflower liking \|\| id:ebi-fl187-GCST90094727 | Inverse variance weighted | 4 | 0.0575194400337815 | 0.343394115694699 | 0.866974526708413 | -0.615533027 | 0.730571906795391 | 1.05920586145232 | 0.540352796051084 | 2.07626769979531 | 0.998074519377826 |
| ebi-fl187-GCST90094727 | prot-a-861 | Dermatopontin \|\| id:prot-a-861 | Cauliflower liking \|\| id:ebi-fl187-GCST90094727 | Inverse variance weighted | 4 | -0.350279795 | 0.336059078400769 | 0.297265140413042 | -1.008955589 | 0.308395998487095 | 0.704490948969727 | 0.364599572650276 | 1.36123993117327 | 0.998074519377826 |
| ebi-fl187-GCST90094727 | prot-a-877 | Dual specificity protein phosphatase 6 \|\| id:prot-a-877 | Cauliflower liking \|\| id:ebi-fl187-GCST90094727 | Inverse variance weighted | 4 | -0.102438271 | 0.336059078400769 | 0.760501565089941 | -0.761114065 | 0.556237522776731 | 0.902633866819584 | 0.467145706532319 | 1.74409801082761 | 0.998074519377826 |
| ebi-fl187-GCST90094727 | prot-a-879 | Dynactin-associated protein \|\| id:prot-a-879 | Cauliflower liking \|\| id:ebi-fl187-GCST90094727 | Inverse variance weighted | 4 | -0.30154872 | 0.336059078400769 | 0.369554238558002 | -0.960224514 | 0.357127073502733 | 0.739671788546825 | 0.382806930876875 | 1.42921747398568 | 0.998074519377826 |
| ebi-fl187-GCST90094727 | prot-a-937 | EMI domain-containing protein 1 \|\| id:prot-a-937 | Cauliflower liking \|\| id:ebi-fl187-GCST90094727 | Inverse variance weighted | 4 | -0.305334382 | 0.335893159052301 | 0.3633383398045 | -0.963684973 | 0.353016210242102 | 0.736876935176367 | 0.381484532401681 | 1.42335421616304 | 0.998074519377826 |
| ebi-fl187-GCST90094727 | prot-b-1 | SRC proto-oncogene, non-receptor tyrosine kinase \|\| id:prot-b-1 | Cauliflower liking \|\| id:ebi-fl187-GCST90094727 | Inverse variance weighted | 4 | 0.0430803744990987 | 0.41987797904594 | 0.918278753196886 | -0.779880464 | 0.866041213429142 | 1.04402180420804 | 0.458460810403799 | 2.37748026205728 | 0.998074519377826 |
| ebi-fl187-GCST90094727 | prot-b-10 | coagulation factor III, tissue factor \|\| id:prot-b-10 | Cauliflower liking \|\| id:ebi-fl187-GCST90094727 | Inverse variance weighted | 4 | -0.215184192 | 0.41987797904594 | 0.608306573919715 | -1.038145031 | 0.607776647334675 | 0.806392895703472 | 0.354110938083827 | 1.83634401625599 | 0.998074519377826 |
| ebi-fl187-GCST90094727 | prot-b-29 | myeloperoxidase \|\| id:prot-b-29 | Cauliflower liking \|\| id:ebi-fl187-GCST90094727 | Inverse variance weighted (multiplicative random effects) | 4 | 0.828528280666248 | 0.728324632120688 | 0.255295474139906 | -0.598987998 | 2.2560445596228 | 2.28994610113354 | 0.549367315534904 | 9.54525869634397 | 0.998074519377826 |
| ebi-fl187-GCST90094727 | prot-b-33 | adrenomedullin \|\| id:prot-b-33 | Cauliflower liking \|\| id:ebi-fl187-GCST90094727 | Inverse variance weighted | 4 | 0.0720577313558037 | 0.41987797904594 | 0.863739517585758 | -0.750903108 | 0.895018570285847 | 1.07471738716475 | 0.471940147503298 | 2.44738123760948 | 0.998074519377826 |
| ebi-fl187-GCST90094727 | ukb-rsfMRI191-edge-pheno102 | Net25_Pair11_15 (Postcentral\|Precentral)&(Cerebellum) (Motor)&(Subcortical-cerebellum) \|\| id:ukb-rsfMRI191-edge-pheno102 | Cauliflower liking \|\| id:ebi-fl187-GCST90094727 | Inverse variance weighted | 4 | -0.054715064 | 0.148673038290655 | 0.71285625030884 | -0.34611422 | 0.236684090561796 | 0.946754873609206 | 0.707431679972362 | 1.26704078440169 | 0.998074519377826 |
| ebi-fl187-GCST90094727 | ukb-rsfMRI191-edge-pheno1167 | Net100_Pair11_45 (Angular\|Temporal)&(Frontal) (Default_mode\|Central_executive)&(Salience\|Default_mode) \|\| id:ukb-rsfMRI191-edge-pheno1167 | Cauliflower liking \|\| id:ebi-fl187-GCST90094727 | Inverse variance weighted | 4 | -0.285296314 | 0.103283491295346 | 0.00574019682306084 | -0.487731957 | -0.082860671 | 0.751791455052711 | 0.614017434160303 | 0.920479387793273 | 0.877504772527479 |
| ebi-fl187-GCST90094727 | ukb-rsfMRI191-edge-pheno1317 | Net100_Pair26_48 (Parietal\|Frontal)&(Temporal) (Attention\|Central_executive)&(Default_mode\|Central_executive) \|\| id:ukb-rsfMRI191-edge-pheno1317 | Cauliflower liking \|\| id:ebi-fl187-GCST90094727 | Inverse variance weighted | 4 | -0.147294882 | 0.10287451341845 | 0.152203423086333 | -0.348928928 | 0.0543391645652397 | 0.863039445320789 | 0.705443265727353 | 1.05584264584572 | 0.998074519377826 |
| ebi-fl187-GCST90094727 | ukb-rsfMRI191-edge-pheno286 | Net100_Pair10_13 (OccipitalPrecuneus)&(Frontal_Sup) (Default_mode\|Central_executive)&(Default_mode) \|\| id:ukb-rsfMRI191-edge-pheno286 | Cauliflower liking \|\| id:ebi-fl187-GCST90094727 | Inverse variance weighted | 4 | 0.261031649750516 | 0.103183328337041 | 0.0114132397336047 | 0.0587923262099152 | 0.463270973291116 | 1.29826875466564 | 1.06055496839867 | 1.58926393215243 | 0.998074519377826 |
| ebi-fl187-GCST90094727 | ukb-rsfMRI191-edge-pheno599 | Net100_Pair11_29 (Angular\|Temporal)&(Parietal) (Default_mode\|Central_executive)&(Central_executive\|Salience) \|\| id:ukb-rsfMRI191-edge-pheno599 | Cauliflower liking \|\| id:ebi-fl187-GCST90094727 | Inverse variance weighted | 4 | 0.0522713722707185 | 0.103593812816824 | 0.613853817986402 | -0.150772501 | 0.255315245391693 | 1.05366163827087 | 0.860043335532327 | 1.29086849708158 | 0.998074519377826 |
| ebi-fl187-GCST90094727 | ukb-rsfMRI191-edge-pheno621 | Net100_Pair5_30 (Precuneus\|Angular\|Cingulate)&(Paracentral) (Default_mode\|Central_executive)&(Motor) \|\| id:ukb-rsfMRI191-edge-pheno621 | Cauliflower liking \|\| id:ebi-fl187-GCST90094727 | Inverse variance weighted | 4 | -0.047039988 | 0.104134555597663 | 0.651468449818394 | -0.251143717 | 0.15706374109749 | 0.954049246426595 | 0.777910564672564 | 1.17007019308226 | 0.998074519377826 |
| ebi-fl187-GCST90094727 | ukb-rsfMRI191-edge-pheno624 | Net100_Pair8_30 (Calcarine\|Lingual)&(Paracentral) (Visual)&(Motor) \|\| id:ukb-rsfMRI191-edge-pheno624 | Cauliflower liking \|\| id:ebi-fl187-GCST90094727 | Inverse variance weighted | 4 | -0.018977561 | 0.103775108788484 | 0.854898457321632 | -0.222376774 | 0.184421652082491 | 0.981201379033283 | 0.80061365679555 | 1.2025227624398 | 0.998074519377826 |
| ebi-fl187-GCST90094727 | ukb-rsfMRI191-edge-pheno882 | Net100_Pair6_38 (Postcentral\|Precentral)&(Subcortical) (Motor)&(Subcortical-cerebellum) \|\| id:ukb-rsfMRI191-edge-pheno882 | Cauliflower liking \|\| id:ebi-fl187-GCST90094727 | Inverse variance weighted | 4 | -0.139278926 | 0.127823466106154 | 0.27588084140474 | -0.38981292 | 0.111255067351236 | 0.86998533289515 | 0.677183550292788 | 1.11767995416522 | 0.998074519377826 |
| ebi-fl187-GCST90094727 | ukb-rsfMRI191-edge-pheno899 | Net100_Pair23_38 (Cerebellum)&(Subcortical) (Subcortical-cerebellum)&(Subcortical-cerebellum) \|\| id:ukb-rsfMRI191-edge-pheno899 | Cauliflower liking \|\| id:ebi-fl187-GCST90094727 | Inverse variance weighted (multiplicative random effects) | 4 | 0.0098900447083011 | 0.179523394647954 | 0.95606631513801 | -0.341975809 | 0.361755898218291 | 1.00993911282903 | 0.710365389091322 | 1.4358484060247 | 0.998074519377826 |
| ebi-fl187-GCST90094727 | ebi-a-GCST90001392 | IgD+ CD38+ B cell Absolute Count \|\| id:ebi-a-GCST90001392 | Cauliflower liking \|\| id:ebi-fl187-GCST90094727 | MR Egger | 4 | -1.050275077 | 2.85282003030816 | 0.748072747422411 | -6.641802337 | 4.54125218217752 | 0.34984150244393 | 0.00130467366236795 | 93.8081915519725 | 0.999279047927446 |
| ebi-fl187-GCST90094727 | ebi-a-GCST90001402 | Switched memory B cell %B cell \|\| id:ebi-a-GCST90001402 | Cauliflower liking \|\| id:ebi-fl187-GCST90094727 | MR Egger | 4 | 1.05953002124663 | 1.51045118014762 | 0.555647311781319 | -1.900954292 | 4.02001433433596 | 2.88501477494957 | 0.149425955191772 | 55.7019042708825 | 0.999279047927446 |
| ebi-fl187-GCST90094727 | ebi-a-GCST90001407 | Memory B cell Absolute Count \|\| id:ebi-a-GCST90001407 | Cauliflower liking \|\| id:ebi-fl187-GCST90094727 | MR Egger | 4 | -0.606809208 | 1.43883312953386 | 0.714223480375086 | -3.426922142 | 2.21330372565166 | 0.545087357466349 | 0.032486776735377 | 9.1458820211638 | 0.999279047927446 |
| ebi-fl187-GCST90094727 | ebi-a-GCST90001411 | IgD+ CD24+ B cell %B cell \|\| id:ebi-a-GCST90001411 | Cauliflower liking \|\| id:ebi-fl187-GCST90094727 | MR Egger | 4 | -1.922752233 | 1.59439208851141 | 0.35114498494186 | -5.047760726 | 1.20225626065877 | 0.146204020386104 | 0.00642370177935009 | 3.327616429 | 0.999279047927446 |
| ebi-fl187-GCST90094727 | ebi-a-GCST90001418 | CD24+ CD27+ B cell Absolute Count \|\| id:ebi-a-GCST90001418 | Cauliflower liking \|\| id:ebi-fl187-GCST90094727 | MR Egger | 4 | -0.697593436 | 1.43388849034275 | 0.674699732285394 | -3.508014877 | 2.11282800500744 | 0.497781807228028 | 0.0299563224319092 | 8.27160036651434 | 0.999279047927446 |
| ebi-fl187-GCST90094727 | ebi-a-GCST90001436 | Memory B cell %lymphocyte \|\| id:ebi-a-GCST90001436 | Cauliflower liking \|\| id:ebi-fl187-GCST90094727 | MR Egger | 4 | -0.750277704 | 1.42003911305714 | 0.650026692214026 | -3.533554366 | 2.03299895766441 | 0.472235392906704 | 0.0292009403774413 | 7.6369549552599 | 0.999279047927446 |
| ebi-fl187-GCST90094727 | ebi-a-GCST90001439 | IgD+ CD24+ B cell %lymphocyte \|\| id:ebi-a-GCST90001439 | Cauliflower liking \|\| id:ebi-fl187-GCST90094727 | MR Egger | 4 | -1.229014237 | 1.43254777828148 | 0.481334501273936 | -4.036807882 | 1.578779409 | 0.292580850992549 | 0.0176537353702305 | 4.84903350890133 | 0.999279047927446 |
| ebi-fl187-GCST90094727 | ebi-a-GCST90001457 | CD11c+ HLA DR++ monocyte %monocyte \|\| id:ebi-a-GCST90001457 | Cauliflower liking \|\| id:ebi-fl187-GCST90094727 | MR Egger | 4 | 1.31998764790525 | 1.55702247597787 | 0.485845034494444 | -1.731776405 | 4.37175170082188 | 3.7433751384509 | 0.17696976061066 | 79.1822138359619 | 0.999279047927446 |
| ebi-fl187-GCST90094727 | ebi-a-GCST90001483 | CD39+ resting CD4 regulatory T cell Absolute Count \|\| id:ebi-a-GCST90001483 | Cauliflower liking \|\| id:ebi-fl187-GCST90094727 | MR Egger | 4 | -0.101063691 | 1.46064357178092 | 0.951132880559919 | -2.963925092 | 2.76179770956697 | 0.903875462208708 | 0.0516159218134387 | 15.8282720230387 | 0.999279047927446 |
| ebi-fl187-GCST90094727 | ebi-a-GCST90001525 | CD33dim HLA DR+ CD11b+ Absolute Count \|\| id:ebi-a-GCST90001525 | Cauliflower liking \|\| id:ebi-fl187-GCST90094727 | MR Egger | 4 | -0.656469736 | 1.97390809795798 | 0.771079714907318 | -4.525329608 | 3.21239013641864 | 0.518679180860965 | 0.0108311438209503 | 24.8383824558058 | 0.999279047927446 |
| ebi-fl187-GCST90094727 | ebi-a-GCST90001540 | Naive CD4+ T cell Absolute Count \|\| id:ebi-a-GCST90001540 | Cauliflower liking \|\| id:ebi-fl187-GCST90094727 | MR Egger | 4 | 0.586835555994507 | 1.38269004876206 | 0.71255781470645 | -2.12323694 | 3.29690805156814 | 1.79828881785558 | 0.119643721536801 | 27.0289375061753 | 0.999279047927446 |
| ebi-fl187-GCST90094727 | ebi-a-GCST90001624 | HLA DR+ CD4+ T cell Absolute Count \|\| id:ebi-a-GCST90001624 | Cauliflower liking \|\| id:ebi-fl187-GCST90094727 | MR Egger | 4 | -0.571071531 | 1.59525951335224 | 0.754609323452003 | -3.697780178 | 2.55563711476931 | 0.564919784980302 | 0.0247784692679767 | 12.8795027655173 | 0.999279047927446 |
| ebi-fl187-GCST90094727 | ebi-a-GCST90001672 | CD39+ CD8+ T cell Absolute Count \|\| id:ebi-a-GCST90001672 | Cauliflower liking \|\| id:ebi-fl187-GCST90094727 | MR Egger | 4 | -0.878977646 | 2.12996585744412 | 0.719879127228352 | -5.053710727 | 3.29575543450518 | 0.415207183456097 | 0.00638559423376784 | 26.9978014390403 | 0.999279047927446 |
| ebi-fl187-GCST90094727 | ebi-a-GCST90001722 | CD19 on CD20- CD38- B cell \|\| id:ebi-a-GCST90001722 | Cauliflower liking \|\| id:ebi-fl187-GCST90094727 | MR Egger | 4 | -1.802503592 | 1.39154864721844 | 0.324570225998255 | -4.529938941 | 0.924931756195306 | 0.164885564801671 | 0.0107813343512076 | 2.52169616434544 | 0.999279047927446 |
| ebi-fl187-GCST90094727 | ebi-a-GCST90001742 | CD19 on transitional B cell \|\| id:ebi-a-GCST90001742 | Cauliflower liking \|\| id:ebi-fl187-GCST90094727 | MR Egger | 4 | -0.705160005 | 1.42643258325334 | 0.670019834707812 | -3.500967869 | 2.09064785771951 | 0.494029520512578 | 0.0301681704615227 | 8.09015473607109 | 0.999279047927446 |
| ebi-fl187-GCST90094727 | ebi-a-GCST90001784 | CD25 on IgD+ CD38dim B cell \|\| id:ebi-a-GCST90001784 | Cauliflower liking \|\| id:ebi-fl187-GCST90094727 | MR Egger | 4 | 0.46871026858114 | 1.43399072549217 | 0.774813269665813 | -2.341911553 | 3.27933209054578 | 1.59793196058534 | 0.0961436786879678 | 26.5580284164816 | 0.999279047927446 |
| ebi-fl187-GCST90094727 | ebi-a-GCST90001794 | CD25 on IgD+ B cell \|\| id:ebi-a-GCST90001794 | Cauliflower liking \|\| id:ebi-fl187-GCST90094727 | MR Egger | 4 | -0.041287918 | 1.43021892983307 | 0.979591316293256 | -2.84451702 | 2.76194118494478 | 0.959552818096791 | 0.0581623512094946 | 15.830543153269 | 0.999279047927446 |
| ebi-fl187-GCST90094727 | ebi-a-GCST90001802 | CD27 on IgD- CD38- B cell \|\| id:ebi-a-GCST90001802 | Cauliflower liking \|\| id:ebi-fl187-GCST90094727 | MR Egger | 4 | 0.938927089182202 | 1.4327262534237 | 0.579551812221735 | -1.869216368 | 3.74707054589266 | 2.55723625949083 | 0.154244485455675 | 42.396700715332 | 0.999279047927446 |
| ebi-fl187-GCST90094727 | ebi-a-GCST90001804 | CD27 on IgD- CD38dim B cell \|\| id:ebi-a-GCST90001804 | Cauliflower liking \|\| id:ebi-fl187-GCST90094727 | MR Egger | 4 | 0.874228785751026 | 1.41304961880108 | 0.599200814616068 | -1.895348467 | 3.64380603860114 | 2.39702596036675 | 0.150265963177714 | 38.2370919745604 | 0.999279047927446 |
| ebi-fl187-GCST90094727 | ebi-a-GCST90001805 | CD27 on memory B cell \|\| id:ebi-a-GCST90001805 | Cauliflower liking \|\| id:ebi-fl187-GCST90094727 | MR Egger | 4 | 0.18984009258625 | 1.43240989156237 | 0.906694576584247 | -2.617683295 | 2.9973634800485 | 1.20905624514117 | 0.0729717211146956 | 20.0326507527104 | 0.999279047927446 |
| ebi-fl187-GCST90094727 | ebi-a-GCST90001806 | CD27 on unswitched memory B cell \|\| id:ebi-a-GCST90001806 | Cauliflower liking \|\| id:ebi-fl187-GCST90094727 | MR Egger | 4 | 1.76713369677034 | 1.82587109595891 | 0.435232351670286 | -1.811573651 | 5.3458410448498 | 5.85404980897467 | 0.163396804784619 | 209.734206315289 | 0.999279047927446 |
| ebi-fl187-GCST90094727 | ebi-a-GCST90001839 | CD3 on Effector Memory CD8+ T cell \|\| id:ebi-a-GCST90001839 | Cauliflower liking \|\| id:ebi-fl187-GCST90094727 | MR Egger | 4 | 1.69682915029178 | 1.59934284182751 | 0.399893646680316 | -1.43788282 | 4.83154112027369 | 5.45661781649631 | 0.237429908851317 | 125.404074572384 | 0.999279047927446 |
| ebi-fl187-GCST90094727 | ebi-a-GCST90001859 | CD3 on CD8+ T cell \|\| id:ebi-a-GCST90001859 | Cauliflower liking \|\| id:ebi-fl187-GCST90094727 | MR Egger | 4 | -0.12543537 | 1.92357282130956 | 0.953938806148479 | -3.8956381 | 3.64476735939799 | 0.882112773208036 | 0.0203303974799114 | 38.2738677601183 | 0.999279047927446 |
| ebi-fl187-GCST90094727 | ebi-a-GCST90001905 | CD86 on monocyte \|\| id:ebi-a-GCST90001905 | Cauliflower liking \|\| id:ebi-fl187-GCST90094727 | MR Egger | 4 | -0.958566461 | 1.59184161550139 | 0.608234692707393 | -4.078576027 | 2.1614431058145 | 0.383442171642572 | 0.0169315585778897 | 8.68366006103951 | 0.999279047927446 |
| ebi-fl187-GCST90094727 | ebi-a-GCST90001943 | CD25 on activated & secreting CD4 regulatory T cell \|\| id:ebi-a-GCST90001943 | Cauliflower liking \|\| id:ebi-fl187-GCST90094727 | MR Egger | 4 | 0.677529216826847 | 1.45847517231942 | 0.687921830364647 | -2.181082121 | 3.53614055457292 | 1.96900672841895 | 0.112919272196543 | 34.33415237 | 0.999279047927446 |
| ebi-fl187-GCST90094727 | ebi-a-GCST90001949 | CD33 on CD33dim HLA DR+ CD11b- \|\| id:ebi-a-GCST90001949 | Cauliflower liking \|\| id:ebi-fl187-GCST90094727 | MR Egger | 4 | 3.78816572023031 | 2.08815337372706 | 0.211328665157447 | -0.304614892 | 7.88094633273534 | 44.1752960629266 | 0.73740730096524 | 2646.37572708167 | 0.999279047927446 |
| ebi-fl187-GCST90094727 | ebi-a-GCST90001955 | CD33 on Immature Myeloid-Derived Suppressor Cells \|\| id:ebi-a-GCST90001955 | Cauliflower liking \|\| id:ebi-fl187-GCST90094727 | MR Egger | 4 | 2.36938055486897 | 2.15760400800973 | 0.386682582450105 | -1.859523301 | 6.59828441056804 | 10.6907678884826 | 0.15574685707217 | 733.835148868847 | 0.999279047927446 |
| ebi-fl187-GCST90094727 | ebi-a-GCST90001956 | CD33 on CD33+ HLA DR+ \|\| id:ebi-a-GCST90001956 | Cauliflower liking \|\| id:ebi-fl187-GCST90094727 | MR Egger | 4 | 1.97898783248864 | 2.15303733814738 | 0.455044105853899 | -2.24096535 | 6.1989410152575 | 7.23541582480069 | 0.106355784220663 | 492.227503575732 | 0.999279047927446 |
| ebi-fl187-GCST90094727 | ebi-a-GCST90001957 | CD33 on CD33+ HLA DR+ CD14- \|\| id:ebi-a-GCST90001957 | Cauliflower liking \|\| id:ebi-fl187-GCST90094727 | MR Egger | 4 | 2.19643117311882 | 2.154625037 | 0.415253660055496 | -2.026633899 | 6.41949624572898 | 8.99286219400051 | 0.131778354605867 | 613.693885328596 | 0.999279047927446 |
| ebi-fl187-GCST90094727 | ebi-a-GCST90002064 | CD4 on resting CD4 regulatory T cell \|\| id:ebi-a-GCST90002064 | Cauliflower liking \|\| id:ebi-fl187-GCST90094727 | MR Egger | 4 | 2.26766104367758 | 1.54740017635395 | 0.28042231653718 | -0.765243302 | 5.30056538933131 | 9.65678757434077 | 0.465220728081139 | 200.450110296199 | 0.999279047927446 |
| ebi-fl187-GCST90094727 | ebi-a-GCST90002080 | SSC-A on T cell \|\| id:ebi-a-GCST90002080 | Cauliflower liking \|\| id:ebi-fl187-GCST90094727 | MR Egger | 4 | 0.299683623515901 | 1.54729864749752 | 0.864312853761827 | -2.733021726 | 3.33238897261104 | 1.34943181154175 | 0.0650225123255709 | 28.0051654246021 | 0.999279047927446 |
| ebi-fl187-GCST90094727 | ebi-a-GCST90002121 | CD8 on CD39+ CD8+ T cell \|\| id:ebi-a-GCST90002121 | Cauliflower liking \|\| id:ebi-fl187-GCST90094727 | MR Egger | 4 | -1.463606045 | 1.58106804214978 | 0.45232420637195 | -4.562499408 | 1.63528731760386 | 0.231400328406089 | 0.0104359426447823 | 5.13093199235025 | 0.999279047927446 |
| ebi-fl187-GCST90094727 | ebi-cfmet338-GCST90026053 | 1-palmitoyl-2-docosahexaenoyl-gpc (16:0/22:6) levels \|\| id:ebi-cfmet338-GCST90026053 | Cauliflower liking \|\| id:ebi-fl187-GCST90094727 | MR Egger | 4 | 0.956926632547452 | 0.777106724500827 | 0.343321673634695 | -0.566202547 | 2.48005581256907 | 2.60368209236309 | 0.567677077492565 | 11.9419309090934 | 0.999279047927446 |
| ebi-fl187-GCST90094727 | ebi-cfmet338-GCST90026063 | 1-stearoyl-2-oleoyl-gpc (18:0/18:1) levels \|\| id:ebi-cfmet338-GCST90026063 | Cauliflower liking \|\| id:ebi-fl187-GCST90094727 | MR Egger | 4 | 0.773959926817021 | 0.755410099138459 | 0.413313540603999 | -0.706643867 | 2.2545637211284 | 2.16833572613529 | 0.493296992481449 | 9.53113417047939 | 0.999279047927446 |
| ebi-fl187-GCST90094727 | ebi-cfmet338-GCST90026113 | Adenosine 3',5'-cyclic monophosphate (camp) levels \|\| id:ebi-cfmet338-GCST90026113 | Cauliflower liking \|\| id:ebi-fl187-GCST90094727 | MR Egger | 4 | 0.443638160666111 | 1.24752258207727 | 0.75613378375469 | -2.0015061 | 2.88878242153756 | 1.5583665052947 | 0.135131608154782 | 17.9714146674165 | 0.999279047927446 |
| ebi-fl187-GCST90094727 | ebi-cfmet338-GCST90026117 | Alpha-ketoglutaramate levels \|\| id:ebi-cfmet338-GCST90026117 | Cauliflower liking \|\| id:ebi-fl187-GCST90094727 | MR Egger | 4 | 0.0902055369412737 | 0.496052723106329 | 0.872465001064984 | -0.8820578 | 1.06246887422968 | 1.09439920005422 | 0.413930248867742 | 2.89350588017066 | 0.999279047927446 |
| ebi-fl187-GCST90094727 | ebi-cfmet338-GCST90026124 | Arginine levels \|\| id:ebi-cfmet338-GCST90026124 | Cauliflower liking \|\| id:ebi-fl187-GCST90094727 | MR Egger | 4 | -0.245863312 | 0.405278501741643 | 0.605772617489883 | -1.040209176 | 0.548482551125062 | 0.782029111389794 | 0.353380755559566 | 1.73062488955493 | 0.999279047927446 |
| ebi-fl187-GCST90094727 | ebi-cfmet338-GCST90026169 | Glutamate levels \|\| id:ebi-cfmet338-GCST90026169 | Cauliflower liking \|\| id:ebi-fl187-GCST90094727 | MR Egger | 4 | 0.0351013662171316 | 0.558375508961991 | 0.95559273533012 | -1.059314631 | 1.12951736378263 | 1.03572469097262 | 0.346693341670602 | 3.09416278467081 | 0.999279047927446 |
| ebi-fl187-GCST90094727 | ebi-cfmet338-GCST90026170 | Glutamine levels \|\| id:ebi-cfmet338-GCST90026170 | Cauliflower liking \|\| id:ebi-fl187-GCST90094727 | MR Egger | 4 | 0.0151389194175545 | 0.344980884640254 | 0.968984708805945 | -0.661023614 | 0.691301453312453 | 1.01525409332699 | 0.516322548665664 | 1.9963119501191 | 0.999279047927446 |
| ebi-fl187-GCST90094727 | ebi-cfmet338-GCST90026191 | Kynurenate levels \|\| id:ebi-cfmet338-GCST90026191 | Cauliflower liking \|\| id:ebi-fl187-GCST90094727 | MR Egger | 4 | -2.347763202 | 2.32488917332475 | 0.418881378519177 | -6.904545982 | 2.20901957793028 | 0.0955827225438805 | 0.00100321445278804 | 9.10678352321415 | 0.999279047927446 |
| ebi-fl187-GCST90094727 | ebi-cfmet338-GCST90026202 | Methylsuccinoylcarnitine levels \|\| id:ebi-cfmet338-GCST90026202 | Cauliflower liking \|\| id:ebi-fl187-GCST90094727 | MR Egger | 4 | -0.339791595 | 1.29771237394564 | 0.817946025103479 | -2.883307848 | 2.20372465785025 | 0.711918674655601 | 0.0559493843411999 | 9.05869126695949 | 0.999279047927446 |
| ebi-fl187-GCST90094727 | ebi-cfmet338-GCST90026222 | N-acetylputrescine levels \|\| id:ebi-cfmet338-GCST90026222 | Cauliflower liking \|\| id:ebi-fl187-GCST90094727 | MR Egger | 4 | -0.815509206 | 0.466761826018448 | 0.222721003906993 | -1.730362385 | 0.0993439725275992 | 0.442413989929128 | 0.177220176315958 | 1.10444613335703 | 0.999279047927446 |
| ebi-fl187-GCST90094727 | ebi-cfmet338-GCST90026252 | Propionylcarnitine (c3) levels \|\| id:ebi-cfmet338-GCST90026252 | Cauliflower liking \|\| id:ebi-fl187-GCST90094727 | MR Egger | 4 | -0.460743362 | 0.770433381389643 | 0.610520050490096 | -1.970792789 | 1.04930606583045 | 0.630814547803513 | 0.13934634013698 | 2.85566878419184 | 0.999279047927446 |
| ebi-fl187-GCST90094727 | ebi-cfmet338-GCST90026316 | Ethyl beta-glucopyranoside levels \|\| id:ebi-cfmet338-GCST90026316 | Cauliflower liking \|\| id:ebi-fl187-GCST90094727 | MR Egger | 4 | 1.57396165656315 | 2.51904710900755 | 0.595869073684618 | -3.363370677 | 6.51129399021794 | 4.82572825250407 | 0.0346183747033233 | 672.696317102962 | 0.999279047927446 |
| ebi-fl187-GCST90094727 | ebi-gm430-GCST90011315 | F_Prevotellaceae abundance \|\| id:ebi-gm430-GCST90011315 | Cauliflower liking \|\| id:ebi-fl187-GCST90094727 | MR Egger | 4 | 1.09169902143024 | 1.09139465555378 | 0.422542405613622 | -1.047434503 | 3.23083254631566 | 2.97933172326358 | 0.350836665788181 | 25.3007122197539 | 0.999279047927446 |
| ebi-fl187-GCST90094727 | ebi-gm430-GCST90011354 | OTU97_1 (Bacteroides) abundance \|\| id:ebi-gm430-GCST90011354 | Cauliflower liking \|\| id:ebi-fl187-GCST90094727 | MR Egger | 4 | -1.222541059 | 0.989212043093177 | 0.341966938596358 | -3.161396664 | 0.716314545357596 | 0.294480921961294 | 0.0423665279536473 | 2.0468756253534 | 0.999279047927446 |
| ebi-fl187-GCST90094727 | ebi-gm430-GCST90011419 | OTU99_1 (Bacteroides) abundance \|\| id:ebi-gm430-GCST90011419 | Cauliflower liking \|\| id:ebi-fl187-GCST90094727 | MR Egger | 4 | -1.215137179 | 0.989394657951996 | 0.344304387591825 | -3.154350709 | 0.724076350357731 | 0.296669314634929 | 0.0426660947202724 | 2.06282489229416 | 0.999279047927446 |
| ebi-fl187-GCST90094727 | ebi-gm430-GCST90011435 | OTU99_155 (Butyrivibrio) abundance \|\| id:ebi-gm430-GCST90011435 | Cauliflower liking \|\| id:ebi-fl187-GCST90094727 | MR Egger | 4 | 1.69821464094075 | 4.50123885602172 | 0.742239408379976 | -7.124213517 | 10.5206427987433 | 5.46418314909467 | 0.000805366168019178 | 37072.9472784846 | 0.999279047927446 |
| ebi-fl187-GCST90094727 | ebi-gm430-GCST90011466 | OTU99_58 (Bacteroides) abundance \|\| id:ebi-gm430-GCST90011466 | Cauliflower liking \|\| id:ebi-fl187-GCST90094727 | MR Egger | 4 | -2.324872705 | 1.83580100838227 | 0.332895431173686 | -5.923042681 | 1.27329727143522 | 0.0977958921877214 | 0.00267704238533185 | 3.57261303787943 | 0.999279047927446 |
| ebi-fl187-GCST90094727 | ebi-gm430-GCST90011472 | OTU99_72 (Parabacteroides) abundance \|\| id:ebi-gm430-GCST90011472 | Cauliflower liking \|\| id:ebi-fl187-GCST90094727 | MR Egger | 4 | 2.03171408948903 | 2.94040587358086 | 0.561010102119201 | -3.731481423 | 7.79490960170752 | 7.62714877605395 | 0.0239573186188588 | 2428.20990852742 | 0.999279047927446 |
| ebi-fl187-GCST90094727 | ebi-gm430-GCST90011505 | TestASV_28 (Holdemanella) abundance \|\| id:ebi-gm430-GCST90011505 | Cauliflower liking \|\| id:ebi-fl187-GCST90094727 | MR Egger | 4 | 2.50355835475176 | 2.31566816291676 | 0.392662896380415 | -2.035151245 | 7.04226795406862 | 12.22592081 | 0.13066071928586 | 1143.97915894528 | 0.999279047927446 |
| ebi-fl187-GCST90094727 | ebi-gm430-GCST90011513 | TestASV_35 (Alphaproteobacteria) abundance \|\| id:ebi-gm430-GCST90011513 | Cauliflower liking \|\| id:ebi-fl187-GCST90094727 | MR Egger | 4 | 4.00658079829448 | 3.65775444094204 | 0.387655116577992 | -3.162617906 | 11.1757795025409 | 54.9586342818749 | 0.0423148197345977 | 71380.4643638662 | 0.999279047927446 |
| ebi-fl187-GCST90094727 | ebi-gm430-GCST90011543 | G_Coprococcus prevalence \|\| id:ebi-gm430-GCST90011543 | Cauliflower liking \|\| id:ebi-fl187-GCST90094727 | MR Egger | 4 | -6.428524983 | 3.01833192645458 | 0.166927319322429 | -12.34445556 | -0.512594407 | 0.00161483098254394 | 4.35382600346525e-06 | 0.59893966826151 | 0.999279047927446 |
| ebi-fl187-GCST90094727 | ebi-gm430-GCST90011547 | G_Parasutterella prevalence \|\| id:ebi-gm430-GCST90011547 | Cauliflower liking \|\| id:ebi-fl187-GCST90094727 | MR Egger | 4 | -1.400911585 | 2.61383279657806 | 0.645614665756385 | -6.524023866 | 3.72220069618726 | 0.246372272250514 | 0.00146775116924127 | 41.3553044997802 | 0.999279047927446 |
| ebi-fl187-GCST90094727 | ebi-gm430-GCST90011569 | OTU97_13 (Subdoligranulum) prevalence \|\| id:ebi-gm430-GCST90011569 | Cauliflower liking \|\| id:ebi-fl187-GCST90094727 | MR Egger | 4 | -1.166193448 | 2.63634425955671 | 0.701472478047318 | -6.333428196 | 4.00104130121377 | 0.311550620756056 | 0.00177593506736563 | 54.6550327639318 | 0.999279047927446 |
| ebi-fl187-GCST90094727 | ebi-gm430-GCST90011590 | OTU97_277 (Prevotella) prevalence \|\| id:ebi-gm430-GCST90011590 | Cauliflower liking \|\| id:ebi-fl187-GCST90094727 | MR Egger | 4 | 0.723869422998447 | 2.34124188209138 | 0.786420071518145 | -3.864964666 | 5.31270351189754 | 2.06239808154746 | 0.020963663156122 | 202.89802479146 | 0.999279047927446 |
| ebi-fl187-GCST90094727 | ebi-gm430-GCST90011616 | OTU97_86 (Phascolarctobacterium) prevalence \|\| id:ebi-gm430-GCST90011616 | Cauliflower liking \|\| id:ebi-fl187-GCST90094727 | MR Egger | 4 | -4.34224206 | 2.86958335888994 | 0.26940183193991 | -9.966625444 | 1.28214132307272 | 0.0130073322621921 | 4.69407005267815e-05 | 3.6043495448589 | 0.999279047927446 |
| ebi-fl187-GCST90094727 | ebi-gm430-GCST90011665 | OTU99_55 (Barnesiella) prevalence \|\| id:ebi-gm430-GCST90011665 | Cauliflower liking \|\| id:ebi-fl187-GCST90094727 | MR Egger | 4 | -1.138315035 | 2.43147702664943 | 0.685734401869395 | -5.904010007 | 3.62737993742295 | 0.320358359990611 | 0.00272848162169133 | 37.6141360088238 | 0.999279047927446 |
| ebi-fl187-GCST90094727 | ebi-gm430-GCST90011687 | TestASV_14 (Lachnospiraceae) prevalence \|\| id:ebi-gm430-GCST90011687 | Cauliflower liking \|\| id:ebi-fl187-GCST90094727 | MR Egger | 4 | -0.077117503 | 2.06947232677547 | 0.973659281226748 | -4.133283263 | 3.97904825746801 | 0.925781065151602 | 0.0160301610841768 | 53.4661240203783 | 0.999279047927446 |
| ebi-fl187-GCST90094727 | ebi-gm430-GCST90011697 | TestASV_23 (Barnesiella) prevalence \|\| id:ebi-gm430-GCST90011697 | Cauliflower liking \|\| id:ebi-fl187-GCST90094727 | MR Egger | 4 | 0.102136465174285 | 4.15041259557969 | 0.982601620006925 | -8.032672222 | 8.23694515251047 | 1.10753460131813 | 0.000324679433079798 | 3777.98150465365 | 0.999279047927446 |
| ebi-fl187-GCST90094727 | ebi-gm430-GCST90011727 | TestASV_6 (Subdoligranulum) prevalence \|\| id:ebi-gm430-GCST90011727 | Cauliflower liking \|\| id:ebi-fl187-GCST90094727 | MR Egger | 4 | 3.49755910414624 | 3.13889468271899 | 0.381115541687188 | -2.654674474 | 9.64979268227546 | 33.0347191595719 | 0.0703217264912695 | 15518.5704959516 | 0.999279047927446 |
| ebi-fl187-GCST90094727 | ebi-hbsm44-GCST90104908 | Sulcal depth of the left posterior inferior temporal sulcus \|\| id:ebi-hbsm44-GCST90104908 | Cauliflower liking \|\| id:ebi-fl187-GCST90094727 | MR Egger | 4 | -1.11264575 | 1.15387886690833 | 0.436651559589552 | -3.374248329 | 1.14895682916542 | 0.328688182900809 | 0.0342438487441286 | 3.15490009274029 | 0.999279047927446 |
| ebi-fl187-GCST90094727 | ebi-hbsm44-GCST90104918 | Sulcal depth of the left inferior frontal sulcus \|\| id:ebi-hbsm44-GCST90104918 | Cauliflower liking \|\| id:ebi-fl187-GCST90094727 | MR Egger | 4 | 0.0584148384227501 | 1.59392141566496 | 0.974094290064331 | -3.065671136 | 3.18250081312607 | 1.06015469740395 | 0.0466225412832212 | 24.1069652467043 | 0.999279047927446 |
| ebi-fl187-GCST90094727 | ebi-hbsm44-GCST90104925 | Sulcal depth of the right anterior inferior temporal sulcus \|\| id:ebi-hbsm44-GCST90104925 | Cauliflower liking \|\| id:ebi-fl187-GCST90094727 | MR Egger | 4 | 0.3993143205637 | 0.51013926262849 | 0.515737490798141 | -0.600558634 | 1.39918727531554 | 1.49080213473877 | 0.548505136769726 | 4.05190554464166 | 0.999279047927446 |
| ebi-fl187-GCST90094727 | ebi-IPs91-GCST90274770 | C-C motif chemokine 4 levels \|\| id:ebi-IPs91-GCST90274770 | Cauliflower liking \|\| id:ebi-fl187-GCST90094727 | MR Egger | 4 | 0.235718903881306 | 0.996328812276991 | 0.835000376357672 | -1.717085568 | 2.18852337594421 | 1.26581844350141 | 0.179588785216024 | 8.92202890052945 | 0.999279047927446 |
| ebi-fl187-GCST90094727 | ebi-IPs91-GCST90274779 | C-X-C motif chemokine 1 levels \|\| id:ebi-IPs91-GCST90274779 | Cauliflower liking \|\| id:ebi-fl187-GCST90094727 | MR Egger | 4 | 0.665537550873854 | 0.682858287008387 | 0.432537253974554 | -0.672864692 | 2.00393979341029 | 1.94553606490703 | 0.510244788144121 | 7.41822487520404 | 0.999279047927446 |
| ebi-fl187-GCST90094727 | ebi-IPs91-GCST90274780 | C-X-C motif chemokine 10 levels \|\| id:ebi-IPs91-GCST90274780 | Cauliflower liking \|\| id:ebi-fl187-GCST90094727 | MR Egger | 4 | -0.191488646 | 0.891911977266496 | 0.849907750407153 | -1.939636121 | 1.55665882979095 | 0.82572900066819 | 0.143756250128697 | 4.74294774616121 | 0.999279047927446 |
| ebi-fl187-GCST90094727 | ebi-IPs91-GCST90274796 | Interleukin-10 receptor subunit alpha levels \|\| id:ebi-IPs91-GCST90274796 | Cauliflower liking \|\| id:ebi-fl187-GCST90094727 | MR Egger | 4 | 0.508054042210367 | 1.46009483793232 | 0.761080970904698 | -2.35373184 | 3.36983992455771 | 1.66205376 | 0.095013923008403 | 29.0738726722536 | 0.999279047927446 |
| ebi-fl187-GCST90094727 | ebi-IPs91-GCST90274807 | Interleukin-20 levels \|\| id:ebi-IPs91-GCST90274807 | Cauliflower liking \|\| id:ebi-fl187-GCST90094727 | MR Egger | 4 | -0.099634611 | 1.27171320688211 | 0.944685292066981 | -2.592192497 | 2.3929232742522 | 0.905168095870405 | 0.0748557390736136 | 10.9454437551665 | 0.999279047927446 |
| ebi-fl187-GCST90094727 | ebi-IPs91-GCST90274808 | Interleukin-20 receptor subunit alpha levels \|\| id:ebi-IPs91-GCST90274808 | Cauliflower liking \|\| id:ebi-fl187-GCST90094727 | MR Egger | 4 | 0.288370707105917 | 1.24401516261191 | 0.838246625761585 | -2.149899012 | 2.72664042582525 | 1.33425182908989 | 0.116495921914663 | 15.2814614809759 | 0.999279047927446 |
| ebi-fl187-GCST90094727 | ebi-IPs91-GCST90274810 | Interleukin-24 levels \|\| id:ebi-IPs91-GCST90274810 | Cauliflower liking \|\| id:ebi-fl187-GCST90094727 | MR Egger | 4 | -0.720020672 | 0.755093846156456 | 0.44094727419126 | -2.200004611 | 0.759963266245293 | 0.486742193813593 | 0.110802647484717 | 2.13819767502528 | 0.999279047927446 |
| ebi-fl187-GCST90094727 | ebi-IPs91-GCST90274823 | Monocyte chemoattractant protein-3 levels \|\| id:ebi-IPs91-GCST90274823 | Cauliflower liking \|\| id:ebi-fl187-GCST90094727 | MR Egger | 4 | -0.434236452 | 0.939201458171419 | 0.689256655002263 | -2.27507131 | 1.40659840584608 | 0.647759073337476 | 0.102789578 | 4.08204629609584 | 0.999279047927446 |
| ebi-fl187-GCST90094727 | ebi-IPs91-GCST90274824 | Monocyte chemoattractant protein-4 levels \|\| id:ebi-IPs91-GCST90274824 | Cauliflower liking \|\| id:ebi-fl187-GCST90094727 | MR Egger | 4 | 0.974221615675912 | 1.18735201543875 | 0.498164966542525 | -1.352988335 | 3.30143156593585 | 2.64910438692415 | 0.258466720383642 | 27.1514802462937 | 0.999279047927446 |
| ebi-fl187-GCST90094727 | ebi-IPs91-GCST90274834 | SIR2-like protein 2 levels \|\| id:ebi-IPs91-GCST90274834 | Cauliflower liking \|\| id:ebi-fl187-GCST90094727 | MR Egger | 4 | 1.54981056789695 | 0.847186816582692 | 0.208843991423452 | -0.110675593 | 3.21029672839903 | 4.71057776341469 | 0.895229120773985 | 24.7864399741517 | 0.999279047927446 |
| ebi-fl187-GCST90094727 | ebi-met1400-GCST90199652 | 1-stearoyl-2-oleoyl-GPS (18:0/18:1) levels \|\| id:ebi-met1400-GCST90199652 | Cauliflower liking \|\| id:ebi-fl187-GCST90094727 | MR Egger | 4 | 0.56535954964694 | 0.863736471883021 | 0.579970246665684 | -1.127563935 | 2.25828303453766 | 1.76008050529167 | 0.323821145638247 | 9.56664945089335 | 0.999279047927446 |
| ebi-fl187-GCST90094727 | ebi-met1400-GCST90199665 | N-acetylaspartate (naa) levels \|\| id:ebi-met1400-GCST90199665 | Cauliflower liking \|\| id:ebi-fl187-GCST90094727 | MR Egger | 4 | 1.54871501373845 | 0.856203968524271 | 0.212203128407667 | -0.129444765 | 3.22687479204602 | 4.70541989623436 | 0.878583115992991 | 25.2007761096729 | 0.999279047927446 |
| ebi-fl187-GCST90094727 | ebi-met1400-GCST90199669 | Acetylcarnitine levels (Biocrates platform) \|\| id:ebi-met1400-GCST90199669 | Cauliflower liking \|\| id:ebi-fl187-GCST90094727 | MR Egger | 4 | 1.10539772440015 | 1.56043859524654 | 0.552137939312106 | -1.953061922 | 4.16385737108337 | 3.02042552687657 | 0.141839105692132 | 64.3191475220477 | 0.999279047927446 |
| ebi-fl187-GCST90094727 | ebi-met1400-GCST90199670 | Iminodiacetate (IDA) levels \|\| id:ebi-met1400-GCST90199670 | Cauliflower liking \|\| id:ebi-fl187-GCST90094727 | MR Egger | 4 | -0.097355039 | 0.852108505700335 | 0.919474046501072 | -1.76748771 | 1.57277763213817 | 0.907233845518297 | 0.170761452641902 | 4.82001785367777 | 0.999279047927446 |
| ebi-fl187-GCST90094727 | ebi-met1400-GCST90199686 | Indolepropionate levels \|\| id:ebi-met1400-GCST90199686 | Cauliflower liking \|\| id:ebi-fl187-GCST90094727 | MR Egger | 4 | -0.957323882 | 0.860038290794217 | 0.381508769906002 | -2.642998932 | 0.728351168311519 | 0.383918924943731 | 0.0711475825664526 | 2.07166196816712 | 0.999279047927446 |
| ebi-fl187-GCST90094727 | ebi-met1400-GCST90199704 | 3-hydroxysebacate levels \|\| id:ebi-met1400-GCST90199704 | Cauliflower liking \|\| id:ebi-fl187-GCST90094727 | MR Egger | 4 | -0.096565929 | 0.878913880541828 | 0.922543848870764 | -1.819237135 | 1.6261052769356 | 0.907950035455401 | 0.162149401901171 | 5.08403519974692 | 0.999279047927446 |
| ebi-fl187-GCST90094727 | ebi-met1400-GCST90199713 | Docosapentaenoate n3 DPA; 22:5n3 levels \|\| id:ebi-met1400-GCST90199713 | Cauliflower liking \|\| id:ebi-fl187-GCST90094727 | MR Egger | 4 | 0.235993878665564 | 0.839396449768346 | 0.805014731020766 | -1.409223163 | 1.88121092021152 | 1.2661665595142 | 0.244333016404668 | 6.56144544041812 | 0.999279047927446 |
| ebi-fl187-GCST90094727 | ebi-met1400-GCST90199715 | Myristoleate (14:1n5) levels \|\| id:ebi-met1400-GCST90199715 | Cauliflower liking \|\| id:ebi-fl187-GCST90094727 | MR Egger | 4 | -0.60634266 | 0.869293095748505 | 0.557660610902049 | -2.310157128 | 1.09747180727884 | 0.545341726132083 | 0.0992456560574993 | 2.9965805061374 | 0.999279047927446 |
| ebi-fl187-GCST90094727 | ebi-met1400-GCST90199725 | Campesterol levels \|\| id:ebi-met1400-GCST90199725 | Cauliflower liking \|\| id:ebi-fl187-GCST90094727 | MR Egger | 4 | -2.027785289 | 1.07238533634043 | 0.199193751491992 | -4.129660548 | 0.0740899706485116 | 0.131626713761978 | 0.0160883391177931 | 1.07690369086119 | 0.999279047927446 |
| ebi-fl187-GCST90094727 | ebi-met1400-GCST90199737 | N-acetylproline levels \|\| id:ebi-met1400-GCST90199737 | Cauliflower liking \|\| id:ebi-fl187-GCST90094727 | MR Egger | 4 | 0.964083059665119 | 0.950334972484324 | 0.417121691534829 | -0.898573486 | 2.82673960573439 | 2.6223819858986 | 0.407150050757293 | 16.8903019100073 | 0.999279047927446 |
| ebi-fl187-GCST90094727 | ebi-met1400-GCST90199775 | Gamma-glutamylisoleucine levels \|\| id:ebi-met1400-GCST90199775 | Cauliflower liking \|\| id:ebi-fl187-GCST90094727 | MR Egger | 4 | -0.779795442 | 0.902665601856319 | 0.478708546354968 | -2.549020022 | 0.989429137386142 | 0.45849979139785 | 0.078158221829969 | 2.68969858563574 | 0.999279047927446 |
| ebi-fl187-GCST90094727 | ebi-met1400-GCST90199777 | 1-palmitoleoyl-GPC (16:1) levels \|\| id:ebi-met1400-GCST90199777 | Cauliflower liking \|\| id:ebi-fl187-GCST90094727 | MR Egger | 4 | -1.517197124 | 0.840116173752838 | 0.212679402599813 | -3.163824825 | 0.129430576148555 | 0.21932576907777 | 0.0422637799808476 | 1.13818009187428 | 0.999279047927446 |
| ebi-fl187-GCST90094727 | ebi-met1400-GCST90199782 | Glutamine degradant levels \|\| id:ebi-met1400-GCST90199782 | Cauliflower liking \|\| id:ebi-fl187-GCST90094727 | MR Egger | 4 | 0.56921045171886 | 1.23040853148294 | 0.689091128177295 | -1.84239027 | 2.98081117342542 | 1.76687147023455 | 0.158438262911503 | 19.7037933574955 | 0.999279047927446 |
| ebi-fl187-GCST90094727 | ebi-met1400-GCST90199783 | 5-methyluridine (ribothymidine) levels \|\| id:ebi-met1400-GCST90199783 | Cauliflower liking \|\| id:ebi-fl187-GCST90094727 | MR Egger | 4 | 0.867869266284366 | 1.29835687616113 | 0.572673008488081 | -1.676910211 | 3.41264874356018 | 2.38183039656504 | 0.186950722856205 | 30.3455153921214 | 0.999279047927446 |
| ebi-fl187-GCST90094727 | ebi-met1400-GCST90199787 | Homostachydrine levels \|\| id:ebi-met1400-GCST90199787 | Cauliflower liking \|\| id:ebi-fl187-GCST90094727 | MR Egger | 4 | -0.249455658 | 0.83216119703155 | 0.792638948960125 | -1.880491604 | 1.38158028797274 | 0.779224832287799 | 0.152515110226456 | 3.98118808262593 | 0.999279047927446 |
| ebi-fl187-GCST90094727 | ebi-met1400-GCST90199802 | Beta-hydroxyisovaleroylcarnitine levels \|\| id:ebi-met1400-GCST90199802 | Cauliflower liking \|\| id:ebi-fl187-GCST90094727 | MR Egger | 4 | 0.537378060120872 | 0.840155728591435 | 0.587910254412759 | -1.109327168 | 2.18408328816008 | 1.71151348861189 | 0.329780773529899 | 8.88250212511208 | 0.999279047927446 |
| ebi-fl187-GCST90094727 | ebi-met1400-GCST90199873 | Pregnenolone sulfate levels \|\| id:ebi-met1400-GCST90199873 | Cauliflower liking \|\| id:ebi-fl187-GCST90094727 | MR Egger | 4 | 0.631381524037599 | 1.24955876530684 | 0.663541180275229 | -1.817753656 | 3.08051670403901 | 1.88020633605019 | 0.162390125615425 | 21.7696479556604 | 0.999279047927446 |
| ebi-fl187-GCST90094727 | ebi-met1400-GCST90199911 | Beta-citrylglutamate levels \|\| id:ebi-met1400-GCST90199911 | Cauliflower liking \|\| id:ebi-fl187-GCST90094727 | MR Egger | 4 | 0.578854936088443 | 1.12481792050066 | 0.658044769696289 | -1.625788188 | 2.78349806026974 | 1.7839944730528 | 0.196756532915139 | 16.1755049894867 | 0.999279047927446 |
| ebi-fl187-GCST90094727 | ebi-met1400-GCST90199934 | 2-hydroxydecanoate levels \|\| id:ebi-met1400-GCST90199934 | Cauliflower liking \|\| id:ebi-fl187-GCST90094727 | MR Egger | 4 | -0.299087033 | 0.848977743068243 | 0.758279824072983 | -1.96308341 | 1.36490934295323 | 0.741494871761933 | 0.140424765587249 | 3.91536808019546 | 0.999279047927446 |
| ebi-fl187-GCST90094727 | ebi-met1400-GCST90199945 | Sphingomyelin (d18:1/14:0, d16:1/16:0) levels \|\| id:ebi-met1400-GCST90199945 | Cauliflower liking \|\| id:ebi-fl187-GCST90094727 | MR Egger | 4 | -1.705475867 | 0.816291578678902 | 0.171875797910081 | -3.305407361 | -0.105544373 | 0.181685907317047 | 0.0366842650544674 | 0.899834544009727 | 0.999279047927446 |
| ebi-fl187-GCST90094727 | ebi-met1400-GCST90199979 | Carnitine C14:1 levels \|\| id:ebi-met1400-GCST90199979 | Cauliflower liking \|\| id:ebi-fl187-GCST90094727 | MR Egger | 4 | 0.903864874806102 | 1.49765243326099 | 0.607493457649362 | -2.031533894 | 3.83926364399764 | 2.46912756280512 | 0.131134220756234 | 46.4912277378527 | 0.999279047927446 |
| ebi-fl187-GCST90094727 | ebi-met1400-GCST90199986 | Eugenol sulfate levels \|\| id:ebi-met1400-GCST90199986 | Cauliflower liking \|\| id:ebi-fl187-GCST90094727 | MR Egger | 4 | 0.147699841653207 | 0.92230990362127 | 0.887482139227668 | -1.660027569 | 1.9554272527509 | 1.15916491111687 | 0.190133738147724 | 7.06693774736927 | 0.999279047927446 |
| ebi-fl187-GCST90094727 | ebi-met1400-GCST90200002 | 6-hydroxyindole sulfate levels \|\| id:ebi-met1400-GCST90200002 | Cauliflower liking \|\| id:ebi-fl187-GCST90094727 | MR Egger | 4 | -0.705743139 | 0.844444874317466 | 0.491236226323329 | -2.360855092 | 0.949368814978911 | 0.493741519464051 | 0.0943395196955719 | 2.58407811306794 | 0.999279047927446 |
| ebi-fl187-GCST90094727 | ebi-met1400-GCST90200023 | Trans 3,4-methyleneheptanoate levels \|\| id:ebi-met1400-GCST90200023 | Cauliflower liking \|\| id:ebi-fl187-GCST90094727 | MR Egger | 4 | -0.701458463 | 0.918617954964484 | 0.524886430972501 | -2.501949655 | 1.0990327283523 | 0.495861580202194 | 0.0819251170945669 | 3.00126158424402 | 0.999279047927446 |
| ebi-fl187-GCST90094727 | ebi-met1400-GCST90200032 | 2-hydroxybutyrate/2-hydroxyisobutyrate levels \|\| id:ebi-met1400-GCST90200032 | Cauliflower liking \|\| id:ebi-fl187-GCST90094727 | MR Egger | 4 | -0.346992456 | 0.859259778175199 | 0.725425867089196 | -2.031141621 | 1.33715670934035 | 0.706810660499926 | 0.131185671297677 | 3.80820027716845 | 0.999279047927446 |
| ebi-fl187-GCST90094727 | ebi-met1400-GCST90200051 | 1-palmitoyl-2-dihomo-linolenoyl-GPC (16:0/20:3n3 or 6) levels \|\| id:ebi-met1400-GCST90200051 | Cauliflower liking \|\| id:ebi-fl187-GCST90094727 | MR Egger | 4 | -0.515217101 | 0.837528908242306 | 0.601116892009427 | -2.156773762 | 1.1263395587881 | 0.597370890502447 | 0.115697788214411 | 3.08434574529954 | 0.999279047927446 |
| ebi-fl187-GCST90094727 | ebi-met1400-GCST90200069 | Phenylacetylglutamate levels \|\| id:ebi-met1400-GCST90200069 | Cauliflower liking \|\| id:ebi-fl187-GCST90094727 | MR Egger | 4 | 0.367996110406041 | 0.933976456061811 | 0.731614477185097 | -1.462597743 | 2.19858996428719 | 1.44483641913594 | 0.231633767380987 | 9.0122968756451 | 0.999279047927446 |
| ebi-fl187-GCST90094727 | ebi-met1400-GCST90200072 | Thioproline levels \|\| id:ebi-met1400-GCST90200072 | Cauliflower liking \|\| id:ebi-fl187-GCST90094727 | MR Egger | 4 | 0.368944067025832 | 0.849916337283929 | 0.706561414634288 | -1.296891954 | 2.03478008810233 | 1.44620671076988 | 0.273380152055902 | 7.65056948921495 | 0.999279047927446 |
| ebi-fl187-GCST90094727 | ebi-met1400-GCST90200081 | 1-oleoyl-2-docosahexaenoyl-GPC (18:1/22:6) levels \|\| id:ebi-met1400-GCST90200081 | Cauliflower liking \|\| id:ebi-fl187-GCST90094727 | MR Egger | 4 | -0.359013419 | 0.818618547098422 | 0.703806415276835 | -1.963505771 | 1.24547893360266 | 0.698364980131385 | 0.140365468145275 | 3.47459850288204 | 0.999279047927446 |
| ebi-fl187-GCST90094727 | ebi-met1400-GCST90200097 | Furaneol sulfate levels \|\| id:ebi-met1400-GCST90200097 | Cauliflower liking \|\| id:ebi-fl187-GCST90094727 | MR Egger | 4 | -0.255659649 | 1.3734521627018 | 0.869502003187431 | -2.947625888 | 2.43630658977275 | 0.774405493533326 | 0.0524641138922046 | 11.4307442540014 | 0.999279047927446 |
| ebi-fl187-GCST90094727 | ebi-met1400-GCST90200124 | Sphingomyelin (d18:2/24:2) levels \|\| id:ebi-met1400-GCST90200124 | Cauliflower liking \|\| id:ebi-fl187-GCST90094727 | MR Egger | 4 | -0.034963713 | 0.777510661385741 | 0.968218327318232 | -1.558884609 | 1.48895718328513 | 0.965640455787182 | 0.210370585773814 | 4.43247085338937 | 0.999279047927446 |
| ebi-fl187-GCST90094727 | ebi-met1400-GCST90200137 | Lignoceroylcarnitine (C24) levels \|\| id:ebi-met1400-GCST90200137 | Cauliflower liking \|\| id:ebi-fl187-GCST90094727 | MR Egger | 4 | -1.710143459 | 0.857004145470363 | 0.18411849778984 | -3.389871584 | -0.030415334 | 0.180839847663093 | 0.0337130059088517 | 0.970042558389135 | 0.999279047927446 |
| ebi-fl187-GCST90094727 | ebi-met1400-GCST90200165 | Octadecenedioate (C18:1-DC) levels \|\| id:ebi-met1400-GCST90200165 | Cauliflower liking \|\| id:ebi-fl187-GCST90094727 | MR Egger | 4 | 0.439096531 | 0.835352241650486 | 0.651602023662108 | -1.198193863 | 2.07638692428795 | 1.5513050286477 | 0.301738701491334 | 7.97560034564134 | 0.999279047927446 |
| ebi-fl187-GCST90094727 | ebi-met1400-GCST90200176 | Glutamine conjugate of C6H10O2 (1) levels \|\| id:ebi-met1400-GCST90200176 | Cauliflower liking \|\| id:ebi-fl187-GCST90094727 | MR Egger | 4 | -0.918540048 | 0.838222145804466 | 0.387496836518271 | -2.561455454 | 0.724375357401376 | 0.399101284526199 | 0.0771923087772573 | 2.0634417836896 | 0.999279047927446 |
| ebi-fl187-GCST90094727 | ebi-met1400-GCST90200182 | Glycine conjugate of C10H12O2 levels \|\| id:ebi-met1400-GCST90200182 | Cauliflower liking \|\| id:ebi-fl187-GCST90094727 | MR Egger | 4 | -1.426999897 | 0.984797991792725 | 0.284348329811907 | -3.357203961 | 0.503204166849942 | 0.240027951685899 | 0.0348325159880859 | 1.65401252123832 | 0.999279047927446 |
| ebi-fl187-GCST90094727 | ebi-met1400-GCST90200208 | Glucuronide of piperine metabolite C17H21NO3 (4) levels \|\| id:ebi-met1400-GCST90200208 | Cauliflower liking \|\| id:ebi-fl187-GCST90094727 | MR Egger | 4 | -0.691302948 | 0.873647873454145 | 0.511714374629253 | -2.40365278 | 1.02104688348237 | 0.500922966857036 | 0.0903871850022072 | 2.77609949594876 | 0.999279047927446 |
| ebi-fl187-GCST90094727 | ebi-met1400-GCST90200227 | 3-ethylcatechol sulfate (2) levels \|\| id:ebi-met1400-GCST90200227 | Cauliflower liking \|\| id:ebi-fl187-GCST90094727 | MR Egger | 4 | -0.683002811 | 1.11499408977391 | 0.602536814225179 | -2.868391227 | 1.50238560507537 | 0.505097999114278 | 0.0567902157032796 | 4.49239337357393 | 0.999279047927446 |
| ebi-fl187-GCST90094727 | ebi-met1400-GCST90200259 | Pentose acid levels \|\| id:ebi-met1400-GCST90200259 | Cauliflower liking \|\| id:ebi-fl187-GCST90094727 | MR Egger | 4 | -0.177383025 | 0.83994294455934 | 0.852307580646646 | -1.823671196 | 1.46890514634151 | 0.837458955372099 | 0.161432013122396 | 4.34447597082978 | 0.999279047927446 |
| ebi-fl187-GCST90094727 | ebi-met1400-GCST90200291 | Sarcosine levels \|\| id:ebi-met1400-GCST90200291 | Cauliflower liking \|\| id:ebi-fl187-GCST90094727 | MR Egger | 4 | -0.925115148 | 0.854668327487849 | 0.39220679950475 | -2.600265069 | 0.750034774322634 | 0.396485762080876 | 0.0742538931683794 | 2.11707363513428 | 0.999279047927446 |
| ebi-fl187-GCST90094727 | ebi-met1400-GCST90200306 | Butyrate/isobutyrate (4:0) levels \|\| id:ebi-met1400-GCST90200306 | Cauliflower liking \|\| id:ebi-fl187-GCST90094727 | MR Egger | 4 | 0.628516960074652 | 0.861859376969811 | 0.541684502074765 | -1.060727419 | 2.31776133893548 | 1.874828072 | 0.346203883504305 | 10.1529198994042 | 0.999279047927446 |
| ebi-fl187-GCST90094727 | ebi-met1400-GCST90200321 | Cholate levels \|\| id:ebi-met1400-GCST90200321 | Cauliflower liking \|\| id:ebi-fl187-GCST90094727 | MR Egger | 4 | 2.2264778551623 | 0.878102196179357 | 0.126658562838548 | 0.505397550650763 | 3.94755815967384 | 9.26716822127947 | 1.65764438709057 | 51.8087036702885 | 0.999279047927446 |
| ebi-fl187-GCST90094727 | ebi-met1400-GCST90200336 | Ceramide (d18:1/16:0) levels \|\| id:ebi-met1400-GCST90200336 | Cauliflower liking \|\| id:ebi-fl187-GCST90094727 | MR Egger | 4 | -1.643399624 | 0.845116994163187 | 0.19125834177795 | -3.299828932 | 0.01302968477282 | 0.193321702812247 | 0.0368894774578351 | 1.01311494100031 | 0.999279047927446 |
| ebi-fl187-GCST90094727 | ebi-met1400-GCST90200358 | Arachidonate (20:4n6) levels \|\| id:ebi-met1400-GCST90200358 | Cauliflower liking \|\| id:ebi-fl187-GCST90094727 | MR Egger | 4 | 0.468506477132282 | 1.09632057414485 | 0.710739823847288 | -1.680281848 | 2.61729480245619 | 1.59760634889549 | 0.186321454273226 | 13.6986159537395 | 0.999279047927446 |
| ebi-fl187-GCST90094727 | ebi-met1400-GCST90200413 | 2'-deoxyuridine levels \|\| id:ebi-met1400-GCST90200413 | Cauliflower liking \|\| id:ebi-fl187-GCST90094727 | MR Egger | 4 | 2.03523611089922 | 0.849096954593043 | 0.138733543374692 | 0.371006079896859 | 3.69946614190159 | 7.6540591189261 | 1.44919188429705 | 40.4257170018815 | 0.999279047927446 |
| ebi-fl187-GCST90094727 | ebi-met1400-GCST90200432 | Threonine levels \|\| id:ebi-met1400-GCST90200432 | Cauliflower liking \|\| id:ebi-fl187-GCST90094727 | MR Egger | 4 | -1.442663795 | 0.852513851359924 | 0.232673245519466 | -3.113590944 | 0.228263353387296 | 0.236297471488006 | 0.0444410830278667 | 1.25641616331926 | 0.999279047927446 |
| ebi-fl187-GCST90094727 | ebi-met1400-GCST90200436 | Caffeine levels \|\| id:ebi-met1400-GCST90200436 | Cauliflower liking \|\| id:ebi-fl187-GCST90094727 | MR Egger | 4 | -0.335880025 | 0.872485703735716 | 0.737343362239034 | -2.045952005 | 1.37419195391868 | 0.714708847578266 | 0.129257078036022 | 3.95188212953645 | 0.999279047927446 |
| ebi-fl187-GCST90094727 | ebi-met1400-GCST90200442 | Valine levels \|\| id:ebi-met1400-GCST90200442 | Cauliflower liking \|\| id:ebi-fl187-GCST90094727 | MR Egger | 4 | -0.579859014 | 1.09401896881343 | 0.649052812542018 | -2.724136192 | 1.56441816531297 | 0.559977310206974 | 0.0656028464848808 | 4.77989301910712 | 0.999279047927446 |
| ebi-fl187-GCST90094727 | ebi-met1400-GCST90200459 | Margarate (17:0) levels \|\| id:ebi-met1400-GCST90200459 | Cauliflower liking \|\| id:ebi-fl187-GCST90094727 | MR Egger | 4 | -0.251847437 | 0.836443920935387 | 0.791762322217016 | -1.891277522 | 1.38758264766703 | 0.777363325618979 | 0.150878934443532 | 4.00515646697891 | 0.999279047927446 |
| ebi-fl187-GCST90094727 | ebi-met1400-GCST90200462 | X-11299 levels \|\| id:ebi-met1400-GCST90200462 | Cauliflower liking \|\| id:ebi-fl187-GCST90094727 | MR Egger | 4 | -0.190468613 | 1.12930583279339 | 0.881578623335938 | -2.403908045 | 2.02297081914042 | 0.826571700816617 | 0.0903641152663584 | 7.56075323237554 | 0.999279047927446 |
| ebi-fl187-GCST90094727 | ebi-met1400-GCST90200497 | X-12740 levels \|\| id:ebi-met1400-GCST90200497 | Cauliflower liking \|\| id:ebi-fl187-GCST90094727 | MR Egger | 4 | 0.534678664801907 | 0.970507416548751 | 0.637007244261955 | -1.367515872 | 2.43687320123746 | 1.70689966717801 | 0.254738978542253 | 11.4372228800044 | 0.999279047927446 |
| ebi-fl187-GCST90094727 | ebi-met1400-GCST90200527 | X-13866 levels \|\| id:ebi-met1400-GCST90200527 | Cauliflower liking \|\| id:ebi-fl187-GCST90094727 | MR Egger | 4 | -0.223566957 | 1.37430320794848 | 0.885723838709055 | -2.917201244 | 2.47006733092724 | 0.79966134742865 | 0.054084845948241 | 11.823242894754 | 0.999279047927446 |
| ebi-fl187-GCST90094727 | ebi-met1400-GCST90200547 | X-17654 levels \|\| id:ebi-met1400-GCST90200547 | Cauliflower liking \|\| id:ebi-fl187-GCST90094727 | MR Egger | 4 | 0.346336132503808 | 0.861404647550853 | 0.726537550899808 | -1.342016977 | 2.03468924170348 | 1.41387778623346 | 0.261318064230592 | 7.64987449409705 | 0.999279047927446 |
| ebi-fl187-GCST90094727 | ebi-met1400-GCST90200553 | X-17653 levels \|\| id:ebi-met1400-GCST90200553 | Cauliflower liking \|\| id:ebi-fl187-GCST90094727 | MR Egger | 4 | 0.344930037240321 | 0.859257219109137 | 0.726935076433905 | -1.339214112 | 2.02907418669423 | 1.41189113641185 | 0.262051530774885 | 7.60704039844292 | 0.999279047927446 |
| ebi-fl187-GCST90094727 | ebi-met1400-GCST90200557 | X-17685 levels \|\| id:ebi-met1400-GCST90200557 | Cauliflower liking \|\| id:ebi-fl187-GCST90094727 | MR Egger | 4 | -0.07224392 | 0.905187248473414 | 0.943654740627558 | -1.846410927 | 1.70192308738631 | 0.930303948746772 | 0.157802515973929 | 5.48448439945547 | 0.999279047927446 |
| ebi-fl187-GCST90094727 | ebi-met1400-GCST90200561 | X-18779 levels \|\| id:ebi-met1400-GCST90200561 | Cauliflower liking \|\| id:ebi-fl187-GCST90094727 | MR Egger | 4 | 0.39970306655771 | 0.849698489692136 | 0.6843754137935 | -1.265705973 | 2.0651121063543 | 1.49138179075869 | 0.282040113067721 | 7.88618193920717 | 0.999279047927446 |
| ebi-fl187-GCST90094727 | ebi-met1400-GCST90200570 | X-18921 levels \|\| id:ebi-met1400-GCST90200570 | Cauliflower liking \|\| id:ebi-fl187-GCST90094727 | MR Egger | 4 | -0.971449491 | 0.853832475247822 | 0.373163662865992 | -2.644961143 | 0.70206216006473 | 0.378533958531022 | 0.0710081128548951 | 2.01790967257465 | 0.999279047927446 |
| ebi-fl187-GCST90094727 | ebi-met1400-GCST90200575 | X-21283 levels \|\| id:ebi-met1400-GCST90200575 | Cauliflower liking \|\| id:ebi-fl187-GCST90094727 | MR Egger | 4 | 0.708054341451858 | 1.20329223221117 | 0.615843445449246 | -1.650398434 | 3.06650711658576 | 2.03003765359747 | 0.191973404710406 | 21.466790575706 | 0.999279047927446 |
| ebi-fl187-GCST90094727 | ebi-met1400-GCST90200578 | X-18922 levels \|\| id:ebi-met1400-GCST90200578 | Cauliflower liking \|\| id:ebi-fl187-GCST90094727 | MR Egger | 4 | -0.230014008 | 0.856546152553113 | 0.813449287264346 | -1.908844467 | 1.44881645113796 | 0.794522472860936 | 0.148251597288804 | 4.25807189551766 | 0.999279047927446 |
| ebi-fl187-GCST90094727 | ebi-met1400-GCST90200623 | X-24241 levels \|\| id:ebi-met1400-GCST90200623 | Cauliflower liking \|\| id:ebi-fl187-GCST90094727 | MR Egger | 4 | 0.631027389943897 | 0.986195063421568 | 0.587780070577109 | -1.301914934 | 2.56396971425017 | 1.87954060876872 | 0.272010411903338 | 12.9872708742711 | 0.999279047927446 |
| ebi-fl187-GCST90094727 | ebi-met1400-GCST90200636 | X-24544 levels \|\| id:ebi-met1400-GCST90200636 | Cauliflower liking \|\| id:ebi-fl187-GCST90094727 | MR Egger | 4 | 0.80713869577903 | 1.17634483863063 | 0.563488571662873 | -1.498497188 | 3.11277457949506 | 2.24148523117651 | 0.223465734934481 | 22.4833397525374 | 0.999279047927446 |
| ebi-fl187-GCST90094727 | ebi-met1400-GCST90200659 | X-25172 levels \|\| id:ebi-met1400-GCST90200659 | Cauliflower liking \|\| id:ebi-fl187-GCST90094727 | MR Egger | 4 | -0.521356476 | 1.3434284744404 | 0.735369522207162 | -3.154476286 | 2.1117633337682 | 0.593714641754967 | 0.0426607371669361 | 8.26279851787064 | 0.999279047927446 |
| ebi-fl187-GCST90094727 | ebi-met1400-GCST90200670 | X-26111 levels \|\| id:ebi-met1400-GCST90200670 | Cauliflower liking \|\| id:ebi-fl187-GCST90094727 | MR Egger | 4 | 0.105996418289954 | 1.15828902579702 | 0.935426857666926 | -2.164250072 | 2.37624290885212 | 1.11181789429008 | 0.114836021030262 | 10.7643840231792 | 0.999279047927446 |
| ebi-fl187-GCST90094727 | ebi-met1400-GCST90200673 | Carnitine C4 levels \|\| id:ebi-met1400-GCST90200673 | Cauliflower liking \|\| id:ebi-fl187-GCST90094727 | MR Egger | 4 | 0.231683567141405 | 0.854772478358019 | 0.811766816066414 | -1.44367049 | 1.90703762472312 | 1.26072073223865 | 0.236059711662541 | 6.73311321742402 | 0.999279047927446 |
| ebi-fl187-GCST90094727 | ebi-met1400-GCST90200710 | X-13431 levels \|\| id:ebi-met1400-GCST90200710 | Cauliflower liking \|\| id:ebi-fl187-GCST90094727 | MR Egger | 4 | -0.853371514 | 0.843154760061793 | 0.418013963003996 | -2.505954844 | 0.799211815717333 | 0.425976323028032 | 0.0815976477741375 | 2.22378748322197 | 0.999279047927446 |
| ebi-fl187-GCST90094727 | ebi-met1400-GCST90200730 | Adenosine 5'-diphosphate (ADP) to fructose ratio \|\| id:ebi-met1400-GCST90200730 | Cauliflower liking \|\| id:ebi-fl187-GCST90094727 | MR Egger | 4 | -1.36641159 | 1.14708597180839 | 0.355773842683535 | -3.614700095 | 0.881876914782121 | 0.255020437503615 | 0.0269249989561967 | 2.41542900894217 | 0.999279047927446 |
| ebi-fl187-GCST90094727 | ebi-met1400-GCST90200731 | Adenosine 5'-diphosphate (ADP) to mannose ratio \|\| id:ebi-met1400-GCST90200731 | Cauliflower liking \|\| id:ebi-fl187-GCST90094727 | MR Egger | 4 | -1.558344371 | 1.4142770297349 | 0.385391436744479 | -4.330327349 | 1.21363860762952 | 0.21048426680945 | 0.0131632378129037 | 3.36570889351262 | 0.999279047927446 |
| ebi-fl187-GCST90094727 | ebi-met1400-GCST90200748 | Aspartate to glutamate ratio \|\| id:ebi-met1400-GCST90200748 | Cauliflower liking \|\| id:ebi-fl187-GCST90094727 | MR Egger | 4 | -0.390128861 | 1.04292833006879 | 0.744286327741813 | -2.434268388 | 1.65401066606943 | 0.676969633984457 | 0.0876618580778164 | 5.22790521882656 | 0.999279047927446 |
| ebi-fl187-GCST90094727 | ebi-met1400-GCST90200774 | Uridine to pseudouridine ratio \|\| id:ebi-met1400-GCST90200774 | Cauliflower liking \|\| id:ebi-fl187-GCST90094727 | MR Egger | 4 | -0.026301955 | 0.813747291585507 | 0.977150848373262 | -1.621246647 | 1.56864273639633 | 0.974040928562304 | 0.197652143055576 | 4.80012873044208 | 0.999279047927446 |
| ebi-fl187-GCST90094727 | ebi-met1400-GCST90200799 | Spermidine to adenosine 5'-diphosphate (ADP) ratio \|\| id:ebi-met1400-GCST90200799 | Cauliflower liking \|\| id:ebi-fl187-GCST90094727 | MR Egger | 4 | 1.98909380515859 | 1.49626680448048 | 0.315087167645846 | -0.943589132 | 4.92177674194033 | 7.30890746616315 | 0.389228333642037 | 137.246247849121 | 0.999279047927446 |
| ebi-fl187-GCST90094727 | ebi-met1400-GCST90200811 | Choline to taurocholate ratio \|\| id:ebi-met1400-GCST90200811 | Cauliflower liking \|\| id:ebi-fl187-GCST90094727 | MR Egger | 4 | 1.39148384726442 | 1.05089046343606 | 0.316533024334183 | -0.668261461 | 3.4512291555991 | 4.02081189940324 | 0.512598976842148 | 31.539134997847 | 0.999279047927446 |
| ebi-fl187-GCST90094727 | ebi-met1400-GCST90200812 | Glutamate to kynurenine ratio \|\| id:ebi-met1400-GCST90200812 | Cauliflower liking \|\| id:ebi-fl187-GCST90094727 | MR Egger | 4 | -0.013468185 | 1.84403022440385 | 0.994835595665346 | -3.627767424 | 3.60083105519718 | 0.986622105561434 | 0.0265754499262182 | 36.6286622384572 | 0.999279047927446 |
| ebi-fl187-GCST90094727 | ebi-met1400-GCST90200876 | Cortisone to 4-cholesten-3-one ratio \|\| id:ebi-met1400-GCST90200876 | Cauliflower liking \|\| id:ebi-fl187-GCST90094727 | MR Egger | 4 | 0.889627542332565 | 1.83731526973578 | 0.676078871931249 | -2.711510386 | 4.4907654710147 | 2.43422283746542 | 0.0664363862863237 | 89.1896918791018 | 0.999279047927446 |
| ebi-fl187-GCST90094727 | ebi-met1400-GCST90200906 | 2'-deoxyuridine to cytidine ratio \|\| id:ebi-met1400-GCST90200906 | Cauliflower liking \|\| id:ebi-fl187-GCST90094727 | MR Egger | 4 | 0.932066171563967 | 1.35543360023561 | 0.562711243167874 | -1.724583685 | 3.58871602802576 | 2.5397513221293 | 0.178247243347617 | 36.1875822431549 | 0.999279047927446 |
| ebi-fl187-GCST90094727 | ebi-met1400-GCST90200907 | Retinol (Vitamin A) to linoleoyl-arachidonoyl-glycerol (18:2 to 20:4) [1] ratio \|\| id:ebi-met1400-GCST90200907 | Cauliflower liking \|\| id:ebi-fl187-GCST90094727 | MR Egger | 4 | -0.936845914 | 1.47338150693798 | 0.589929580801333 | -3.824673667 | 1.95098183993825 | 0.39186185435618 | 0.0218255567628046 | 7.03559201573981 | 0.999279047927446 |
| ebi-fl187-GCST90094727 | ebi-met1400-GCST90200916 | Fructose to sucrose ratio \|\| id:ebi-met1400-GCST90200916 | Cauliflower liking \|\| id:ebi-fl187-GCST90094727 | MR Egger | 4 | -1.651080682 | 0.998954968814304 | 0.240180583703398 | -3.609032421 | 0.306871057168543 | 0.191842475902142 | 0.0270780343437448 | 1.35916570209853 | 0.999279047927446 |
| ebi-fl187-GCST90094727 | ebi-met1400-GCST90200917 | Alpha-ketobutyrate to 3-methyl-2-oxobutyrate ratio \|\| id:ebi-met1400-GCST90200917 | Cauliflower liking \|\| id:ebi-fl187-GCST90094727 | MR Egger | 4 | -2.145508551 | 0.857515503401819 | 0.129442603229311 | -3.826238937 | -0.464778164 | 0.117008517159482 | 0.0217914205965252 | 0.628274463668697 | 0.999279047927446 |
| ebi-fl187-GCST90094727 | ebi-met1400-GCST90200924 | Alpha-tocopherol to glycerol ratio \|\| id:ebi-met1400-GCST90200924 | Cauliflower liking \|\| id:ebi-fl187-GCST90094727 | MR Egger | 4 | -0.576136839 | 0.795995119598346 | 0.544402818941391 | -2.136287273 | 0.984013595401464 | 0.56206552744321 | 0.118092475185832 | 2.67517178078349 | 0.999279047927446 |
| ebi-fl187-GCST90094727 | ebi-met1400-GCST90200938 | Alpha-ketoglutarate to alanine ratio \|\| id:ebi-met1400-GCST90200938 | Cauliflower liking \|\| id:ebi-fl187-GCST90094727 | MR Egger | 4 | 0.909866606998327 | 0.846522168615952 | 0.394907194177431 | -0.749316843 | 2.56905005748559 | 2.48399116424662 | 0.472689363279633 | 13.0534185521858 | 0.999279047927446 |
| ebi-fl187-GCST90094727 | ebi-met1400-GCST90200943 | Phosphate to EDTA ratio \|\| id:ebi-met1400-GCST90200943 | Cauliflower liking \|\| id:ebi-fl187-GCST90094727 | MR Egger | 4 | -0.735957429 | 1.03999400740174 | 0.5525088613589 | -2.774345684 | 1.30243082543054 | 0.479046586210064 | 0.062390286325533 | 3.67822693683649 | 0.999279047927446 |
| ebi-fl187-GCST90094727 | ebi-met1400-GCST90200947 | Adenosine 5'-diphosphate (ADP) to glucose ratio \|\| id:ebi-met1400-GCST90200947 | Cauliflower liking \|\| id:ebi-fl187-GCST90094727 | MR Egger | 4 | -1.093346451 | 1.28681969350866 | 0.485004304667611 | -3.61551305 | 1.42882014851045 | 0.335093242264722 | 0.0269031190295096 | 4.17377185479189 | 0.999279047927446 |
| ebi-fl187-GCST90094727 | ebi-met1400-GCST90200975 | Arachidonate (20:4n6) to caffeine ratio \|\| id:ebi-met1400-GCST90200975 | Cauliflower liking \|\| id:ebi-fl187-GCST90094727 | MR Egger | 4 | 0.307302614160679 | 0.872928166900142 | 0.758444085432695 | -1.403636593 | 2.01824182128496 | 1.35975238614544 | 0.245701819781677 | 7.52508285559754 | 0.999279047927446 |
| ebi-fl187-GCST90094727 | ebi-met1400-GCST90200985 | Cholesterol to oleoyl-linoleoyl-glycerol (18:1 to 18:2) [2] ratio \|\| id:ebi-met1400-GCST90200985 | Cauliflower liking \|\| id:ebi-fl187-GCST90094727 | MR Egger | 4 | -1.435065862 | 1.38861258718617 | 0.409987409401686 | -4.156746533 | 1.28661480923243 | 0.238099681859767 | 0.0156584192948967 | 3.6205096717648 | 0.999279047927446 |
| ebi-fl187-GCST90094727 | ebi-met1400-GCST90201016 | Bilirubin (Z,Z) to etiocholanolone glucuronide ratio \|\| id:ebi-met1400-GCST90201016 | Cauliflower liking \|\| id:ebi-fl187-GCST90094727 | MR Egger | 4 | -0.916099362 | 0.878680441628474 | 0.406604271369297 | -2.638313028 | 0.806114303510532 | 0.400076555242098 | 0.0714817556431141 | 2.2391902467186 | 0.999279047927446 |
| ebi-fl187-GCST90094727 | ebi-pw11-GCST90275189 | Placental weight (fetal genotype, adjusted for sex and gestational age) \|\| id:ebi-pw11-GCST90275189 | Cauliflower liking \|\| id:ebi-fl187-GCST90094727 | MR Egger | 4 | 0.0240755604710548 | 0.3233197571833 | 0.947419104901321 | -0.609631164 | 0.657782284550322 | 1.02436771667365 | 0.543551313611872 | 1.93050626994246 | 0.999279047927446 |
| ebi-fl187-GCST90094727 | ebi-pw11-GCST90275192 | Placental weight (fetal effect, conditional on maternal and paternal genotype) \|\| id:ebi-pw11-GCST90275192 | Cauliflower liking \|\| id:ebi-fl187-GCST90094727 | MR Egger | 4 | 0.0401378933115978 | 0.672327206088685 | 0.957823331726733 | -1.277623431 | 1.35789921724542 | 1.04095430493253 | 0.278698861198456 | 3.88801683759288 | 0.999279047927446 |
| ebi-fl187-GCST90094727 | ebi-pw11-GCST90275193 | Placental weight (maternal effect, conditional on fetal and paternal genotype) \|\| id:ebi-pw11-GCST90275193 | Cauliflower liking \|\| id:ebi-fl187-GCST90094727 | MR Egger | 4 | -0.139055007 | 0.523080317530301 | 0.815259216104434 | -1.164292429 | 0.886182415563646 | 0.870180161319361 | 0.312143447515628 | 2.42585106040349 | 0.999279047927446 |
| ebi-fl187-GCST90094727 | ebi-pw11-GCST90275195 | Placental weight (fetal effect, conditional on maternal genotype) \|\| id:ebi-pw11-GCST90275195 | Cauliflower liking \|\| id:ebi-fl187-GCST90094727 | MR Egger | 4 | 0.109993240124147 | 0.428657994936423 | 0.821471974218149 | -0.73017643 | 0.950162910199536 | 1.1162705245832 | 0.481823974410455 | 2.58613093210627 | 0.999279047927446 |
| ebi-fl187-GCST90094727 | ebi-pw11-GCST90275197 | Placental weight (fetal genotype, adjusted for sex) \|\| id:ebi-pw11-GCST90275197 | Cauliflower liking \|\| id:ebi-fl187-GCST90094727 | MR Egger | 4 | -0.065226816 | 0.328136781252858 | 0.860810011120146 | -0.708374907 | 0.577921275561135 | 0.936854945871999 | 0.492443814579432 | 1.78232960516403 | 0.999279047927446 |
| ebi-fl187-GCST90094727 | prot-a-1030 | Protein FAM171B \|\| id:prot-a-1030 | Cauliflower liking \|\| id:ebi-fl187-GCST90094727 | MR Egger | 4 | 2.17777561931347 | 1.38826086848787 | 0.257265619983574 | -0.543215683 | 4.8987669215497 | 8.82665057489811 | 0.580877328531701 | 134.124291902188 | 0.999279047927446 |
| ebi-fl187-GCST90094727 | prot-a-1036 | BRISC complex subunit Abro1 \|\| id:prot-a-1036 | Cauliflower liking \|\| id:ebi-fl187-GCST90094727 | MR Egger | 4 | 0.0446011562585782 | 2.19285180494938 | 0.985619402657287 | -4.253388381 | 4.34259069395937 | 1.04561074143152 | 0.0142159830358807 | 76.9065227383504 | 0.999279047927446 |
| ebi-fl187-GCST90094727 | prot-a-1055 | Phenylalanine--tRNA ligase, mitochondrial \|\| id:prot-a-1055 | Cauliflower liking \|\| id:ebi-fl187-GCST90094727 | MR Egger | 4 | -0.527647842 | 1.38826086848787 | 0.740454109175083 | -3.248639144 | 2.19334346061655 | 0.589991091340253 | 0.0388270098705233 | 8.96513764571722 | 0.999279047927446 |
| ebi-fl187-GCST90094727 | prot-a-1083 | Fc receptor-like protein 5 \|\| id:prot-a-1083 | Cauliflower liking \|\| id:ebi-fl187-GCST90094727 | MR Egger | 4 | -0.437518231 | 1.38920305880591 | 0.782627442447225 | -3.160356226 | 2.28531976456987 | 0.645636755917229 | 0.0424106306221481 | 9.82882863273515 | 0.999279047927446 |
| ebi-fl187-GCST90094727 | prot-a-1103 | Fibroblast growth factor receptor 3 \|\| id:prot-a-1103 | Cauliflower liking \|\| id:ebi-fl187-GCST90094727 | MR Egger | 4 | -0.770856628 | 2.11424862900052 | 0.750351549558067 | -4.914783941 | 3.37307068495913 | 0.462616608240849 | 0.0073373030135522 | 29.1679552861558 | 0.999279047927446 |
| ebi-fl187-GCST90094727 | prot-a-1105 | Fibroblast growth factor receptor 4 \|\| id:prot-a-1105 | Cauliflower liking \|\| id:ebi-fl187-GCST90094727 | MR Egger | 4 | 0.348052884842014 | 1.72482249499729 | 0.858743259480047 | -3.032599205 | 3.7287049750367 | 1.41630714893931 | 0.0481902189265698 | 41.6251676132272 | 0.999279047927446 |
| ebi-fl187-GCST90094727 | prot-a-1120 | Filamin-A \|\| id:prot-a-1120 | Cauliflower liking \|\| id:ebi-fl187-GCST90094727 | MR Egger | 4 | -0.751496547 | 1.38920305880591 | 0.642732065651246 | -3.474334543 | 1.97134144791832 | 0.471660162537433 | 0.0309824444615756 | 7.18030203203417 | 0.999279047927446 |
| ebi-fl187-GCST90094727 | prot-a-1166 | Galanin peptides \|\| id:prot-a-1166 | Cauliflower liking \|\| id:ebi-fl187-GCST90094727 | MR Egger | 4 | -0.933292793 | 1.64807938771246 | 0.628266566435331 | -4.163528393 | 2.2969428068172 | 0.393256663262638 | 0.0155525853610553 | 9.94373601624591 | 0.999279047927446 |
| ebi-fl187-GCST90094727 | prot-a-1205 | ADP-ribosylation factor-binding protein GGA1 \|\| id:prot-a-1205 | Cauliflower liking \|\| id:ebi-fl187-GCST90094727 | MR Egger | 4 | 0.88820391546987 | 1.38920305880591 | 0.588046665814171 | -1.83463408 | 3.61104191072946 | 2.43075987801014 | 0.159671918261485 | 37.0045882136127 | 0.999279047927446 |
| ebi-fl187-GCST90094727 | prot-a-1227 | Guanine nucleotide-binding protein G(I)/G(S)/G(O) subunit gamma-T2 \|\| id:prot-a-1227 | Cauliflower liking \|\| id:ebi-fl187-GCST90094727 | MR Egger | 4 | -1.23374194 | 1.42153485028679 | 0.476948145299868 | -4.019950247 | 1.55246636648428 | 0.291200880177142 | 0.0179538581820522 | 4.7231047363798 | 0.999279047927446 |
| ebi-fl187-GCST90094727 | prot-a-1240 | Golgi SNAP receptor complex member 1 \|\| id:prot-a-1240 | Cauliflower liking \|\| id:ebi-fl187-GCST90094727 | MR Egger | 4 | -0.348782041 | 1.38920305880591 | 0.825202521937609 | -3.071620037 | 2.37405595397555 | 0.705546893607299 | 0.0463460117738701 | 10.7408685240866 | 0.999279047927446 |
| ebi-fl187-GCST90094727 | prot-a-1283 | Glutathione S-transferase A1 \|\| id:prot-a-1283 | Cauliflower liking \|\| id:ebi-fl187-GCST90094727 | MR Egger | 4 | -0.738755476 | 1.38920305880591 | 0.64803325246431 | -3.461593471 | 1.98408251943437 | 0.477708064963009 | 0.0313797194826446 | 7.27237206364822 | 0.999279047927446 |
| ebi-fl187-GCST90094727 | prot-a-1324 | E3 ubiquitin-protein ligase HECW1 \|\| id:prot-a-1324 | Cauliflower liking \|\| id:ebi-fl187-GCST90094727 | MR Egger | 4 | 0.517603077228151 | 1.46977557086853 | 0.758361280144841 | -2.363157042 | 3.39836319613047 | 1.67800078728949 | 0.0941226046610416 | 29.915094809415 | 0.999279047927446 |
| ebi-fl187-GCST90094727 | prot-a-1339 | Histidine triad nucleotide-binding protein 2, mitochondrial \|\| id:prot-a-1339 | Cauliflower liking \|\| id:ebi-fl187-GCST90094727 | MR Egger | 4 | -1.637119557 | 1.38920305880591 | 0.359832204376062 | -4.359957552 | 1.08571843855264 | 0.194539596297604 | 0.0127789300784747 | 2.96156675834571 | 0.999279047927446 |
| ebi-fl187-GCST90094727 | prot-a-1466 | Interleukin-11 \|\| id:prot-a-1466 | Cauliflower liking \|\| id:ebi-fl187-GCST90094727 | MR Egger | 4 | -1.013051728 | 1.38826086848787 | 0.541451097686284 | -3.73404303 | 1.70793957454047 | 0.363109176693637 | 0.0238960279138024 | 5.51758119277117 | 0.999279047927446 |
| ebi-fl187-GCST90094727 | prot-a-147 | Rho GTPase-activating protein 1 \|\| id:prot-a-147 | Cauliflower liking \|\| id:ebi-fl187-GCST90094727 | MR Egger | 4 | 0.342658363197957 | 1.38826086848787 | 0.828066944114657 | -2.378332939 | 3.06364966543418 | 1.40868742024379 | 0.0927049936398859 | 21.4055378253034 | 0.999279047927446 |
| ebi-fl187-GCST90094727 | prot-a-1473 | Interleukin-12 receptor subunit beta-1 \|\| id:prot-a-1473 | Cauliflower liking \|\| id:ebi-fl187-GCST90094727 | MR Egger | 4 | -1.185760054 | 1.91334437881642 | 0.598630456393537 | -4.935915036 | 2.56439492854292 | 0.305513884200843 | 0.0071838844216113 | 12.992794421733 | 0.999279047927446 |
| ebi-fl187-GCST90094727 | prot-a-1501 | X-linked interleukin-1 receptor accessory protein-like 2 \|\| id:prot-a-1501 | Cauliflower liking \|\| id:ebi-fl187-GCST90094727 | MR Egger | 4 | 0.527958794946757 | 1.38920305880591 | 0.740475726336096 | -2.1948792 | 3.25079679020635 | 1.69546797631423 | 0.111372014396842 | 25.8108975964486 | 0.999279047927446 |
| ebi-fl187-GCST90094727 | prot-a-1503 | Interleukin-1 receptor-like 2 \|\| id:prot-a-1503 | Cauliflower liking \|\| id:ebi-fl187-GCST90094727 | MR Egger | 4 | -0.786460032 | 1.38920305880591 | 0.628361613288411 | -3.509298027 | 1.93637796361258 | 0.455454238503149 | 0.0299179086342556 | 6.93359171279002 | 0.999279047927446 |
| ebi-fl187-GCST90094727 | prot-a-1536 | Interleukin-5 receptor subunit alpha \|\| id:prot-a-1536 | Cauliflower liking \|\| id:ebi-fl187-GCST90094727 | MR Egger | 4 | 0.137775831510649 | 1.73022673417341 | 0.943782998622631 | -3.253468567 | 3.52902023049052 | 1.14771823917086 | 0.038639949851827 | 34.0905503650175 | 0.999279047927446 |
| ebi-fl187-GCST90094727 | prot-a-1559 | Insulin-induced gene 1 protein \|\| id:prot-a-1559 | Cauliflower liking \|\| id:ebi-fl187-GCST90094727 | MR Egger | 4 | 0.214761580204214 | 1.38920305880591 | 0.891333312084923 | -2.508076415 | 2.9375995754638 | 1.23956632458234 | 0.0814247160523416 | 18.8704947039773 | 0.999279047927446 |
| ebi-fl187-GCST90094727 | prot-a-1567 | Interferon regulatory factor 1 \|\| id:prot-a-1567 | Cauliflower liking \|\| id:ebi-fl187-GCST90094727 | MR Egger | 4 | -0.022241869 | 1.38920305880591 | 0.988679575420347 | -2.745079864 | 2.70059612649064 | 0.978003657901289 | 0.0642431700212538 | 14.8886045715344 | 0.999279047927446 |
| ebi-fl187-GCST90094727 | prot-a-1613 | Potassium voltage-gated channel subfamily E regulatory beta subunit 5 \|\| id:prot-a-1613 | Cauliflower liking \|\| id:ebi-fl187-GCST90094727 | MR Egger | 3 | -2.832041401 | 1.72782959654237 | 0.348748704643308 | -6.21858741 | 0.554504608151015 | 0.0588925076457478 | 0.00199205719019407 | 1.74107825512107 | 0.999279047927446 |
| ebi-fl187-GCST90094727 | prot-a-1649 | Kin of IRRE-like protein 2 \|\| id:prot-a-1649 | Cauliflower liking \|\| id:ebi-fl187-GCST90094727 | MR Egger | 4 | -0.294654068 | 1.38920305880591 | 0.851679437309354 | -3.017492063 | 2.42818392727456 | 0.744789189331966 | 0.0489237623332842 | 11.3382722442093 | 0.999279047927446 |
| ebi-fl187-GCST90094727 | prot-a-165 | Armadillo repeat-containing protein 5 \|\| id:prot-a-165 | Cauliflower liking \|\| id:ebi-fl187-GCST90094727 | MR Egger | 4 | 0.259573121041804 | 1.38826086848787 | 0.868927782162195 | -2.461418181 | 2.98056442327803 | 1.29637657265167 | 0.0853138746009188 | 19.69893204 | 0.999279047927446 |
| ebi-fl187-GCST90094727 | prot-a-1656 | Kallikrein-11 \|\| id:prot-a-1656 | Cauliflower liking \|\| id:ebi-fl187-GCST90094727 | MR Egger | 4 | 0.419202539056341 | 1.79734474988002 | 0.837276529766568 | -3.103593171 | 3.94199824882118 | 1.52074833453279 | 0.0448876233834031 | 51.5214511855699 | 0.999279047927446 |
| ebi-fl187-GCST90094727 | prot-a-1697 | Laminin subunit gamma-2 \|\| id:prot-a-1697 | Cauliflower liking \|\| id:ebi-fl187-GCST90094727 | MR Egger | 4 | 0.378595597418716 | 1.5865469352816 | 0.833616047015825 | -2.731036396 | 3.48822759057066 | 1.46023239457343 | 0.0651517316890393 | 32.7278890504208 | 0.999279047927446 |
| ebi-fl187-GCST90094727 | prot-a-1698 | Lysosome-associated membrane glycoprotein 3 \|\| id:prot-a-1698 | Cauliflower liking \|\| id:ebi-fl187-GCST90094727 | MR Egger | 4 | -1.419141126 | 1.38826086848787 | 0.414182818709233 | -4.140132428 | 1.3018501765685 | 0.241921708039429 | 0.0159207430142766 | 3.67609179849411 | 0.999279047927446 |
| ebi-fl187-GCST90094727 | prot-a-1724 | Leptin receptor \|\| id:prot-a-1724 | Cauliflower liking \|\| id:ebi-fl187-GCST90094727 | MR Egger | 4 | 1.57746377648898 | 1.8478473368498 | 0.483214454260154 | -2.044317004 | 5.19924455671459 | 4.84265815955264 | 0.129468586347438 | 181.135352689714 | 0.999279047927446 |
| ebi-fl187-GCST90094727 | prot-a-1743 | Leukocyte immunoglobulin-like receptor subfamily B member 2 \|\| id:prot-a-1743 | Cauliflower liking \|\| id:ebi-fl187-GCST90094727 | MR Egger | 4 | -2.160626043 | 1.38920305880591 | 0.260132251301793 | -4.883464039 | 0.562211951927234 | 0.11525294510997 | 0.00757074320564286 | 1.7545491896517 | 0.999279047927446 |
| ebi-fl187-GCST90094727 | prot-a-1746 | Leukocyte immunoglobulin-like receptor subfamily B member 4 \|\| id:prot-a-1746 | Cauliflower liking \|\| id:ebi-fl187-GCST90094727 | MR Egger | 4 | -1.204457937 | 1.38826086848787 | 0.477076101922427 | -3.92544924 | 1.51653336475892 | 0.299854495352007 | 0.0197332699114246 | 4.55640239992622 | 0.999279047927446 |
| ebi-fl187-GCST90094727 | prot-a-1750 | Leucine-rich repeat and immunoglobulin-like domain-containing nogo receptor-interacting protein 3 \|\| id:prot-a-1750 | Cauliflower liking \|\| id:ebi-fl187-GCST90094727 | MR Egger | 4 | -2.136700298 | 1.38920305880591 | 0.263874275353205 | -4.859538294 | 0.586137696960834 | 0.11804371012735 | 0.00775406316569864 | 1.79703430354688 | 0.999279047927446 |
| ebi-fl187-GCST90094727 | prot-a-1753 | Lipase member N \|\| id:prot-a-1753 | Cauliflower liking \|\| id:ebi-fl187-GCST90094727 | MR Egger | 4 | -0.096514482 | 1.38920305880591 | 0.95093313253876 | -2.819352478 | 2.62632351283089 | 0.907996747506368 | 0.0596445513853944 | 13.8228568131042 | 0.999279047927446 |
| ebi-fl187-GCST90094727 | prot-a-1760 | Ig Kappa chain V-I region HK102- like \|\| id:prot-a-1760 | Cauliflower liking \|\| id:ebi-fl187-GCST90094727 | MR Egger | 4 | -0.488036771 | 1.38826086848787 | 0.75876144353662 | -3.209028073 | 2.23295453101508 | 0.613830301338066 | 0.0403958560030775 | 9.3273834527006 | 0.999279047927446 |
| ebi-fl187-GCST90094727 | prot-a-1792 | Leucine-rich repeat neuronal protein 1 \|\| id:prot-a-1792 | Cauliflower liking \|\| id:ebi-fl187-GCST90094727 | MR Egger | 4 | -1.144054606 | 1.38826086848787 | 0.496523763252685 | -3.865045909 | 1.57693669574106 | 0.318524906869522 | 0.0209619600779668 | 4.8401063602292 | 0.999279047927446 |
| ebi-fl187-GCST90094727 | prot-a-1795 | Leucine-rich repeat transmembrane neuronal protein 1 \|\| id:prot-a-1795 | Cauliflower liking \|\| id:ebi-fl187-GCST90094727 | MR Egger | 4 | -1.227598993 | 1.38920305880591 | 0.470092520572745 | -3.950436988 | 1.49523900209553 | 0.292995217341731 | 0.0192462895317707 | 4.46040247100191 | 0.999279047927446 |
| ebi-fl187-GCST90094727 | prot-a-1820 | LysM and putative peptidoglycan-binding domain-containing protein 4 \|\| id:prot-a-1820 | Cauliflower liking \|\| id:ebi-fl187-GCST90094727 | MR Egger | 4 | -2.237171531 | 1.38920305880591 | 0.248607081566459 | -4.960009526 | 0.485666464421351 | 0.106760045224354 | 0.00701286102706702 | 1.62525782449064 | 0.999279047927446 |
| ebi-fl187-GCST90094727 | prot-a-1844 | Mitogen-activated protein kinase kinase kinase 7:TGF-beta-activated kinase 1 and MAP3K7-binding protein 1 fusion \|\| id:prot-a-1844 | Cauliflower liking \|\| id:ebi-fl187-GCST90094727 | MR Egger | 4 | 1.059123963 | 1.38920305880591 | 0.525467323696017 | -1.663714032 | 3.78196195824559 | 2.88384352868137 | 0.189434107562887 | 43.9020913652339 | 0.999279047927446 |
| ebi-fl187-GCST90094727 | prot-a-1856 | Megakaryocyte-associated tyrosine-protein kinase \|\| id:prot-a-1856 | Cauliflower liking \|\| id:ebi-fl187-GCST90094727 | MR Egger | 4 | -3.688230628 | 1.38826086848787 | 0.117273775284304 | -6.409221931 | -0.967239326 | 0.0250162259068821 | 0.0016463049433565 | 0.380131008626052 | 0.999279047927446 |
| ebi-fl187-GCST90094727 | prot-a-1910 | Stromelysin-2 \|\| id:prot-a-1910 | Cauliflower liking \|\| id:ebi-fl187-GCST90094727 | MR Egger | 4 | -2.909910792 | 1.38920305880591 | 0.171209446116323 | -5.632748788 | -0.187072797 | 0.0544805897402503 | 0.00357872464102208 | 0.829383357529783 | 0.999279047927446 |
| ebi-fl187-GCST90094727 | prot-a-1940 | 39S ribosomal protein L14, mitochondrial \|\| id:prot-a-1940 | Cauliflower liking \|\| id:ebi-fl187-GCST90094727 | MR Egger | 4 | 1.48834968031436 | 1.38826086848787 | 0.395883628533226 | -1.232641622 | 4.20934098255058 | 4.429778932 | 0.291521470135765 | 67.312165298444 | 0.999279047927446 |
| ebi-fl187-GCST90094727 | prot-a-1942 | 39S ribosomal protein L33, mitochondrial \|\| id:prot-a-1942 | Cauliflower liking \|\| id:ebi-fl187-GCST90094727 | MR Egger | 4 | 2.42003918439877 | 2.20646750963794 | 0.387156790074937 | -1.904637134 | 6.74471550328913 | 11.2462999857514 | 0.148876655025044 | 849.557396008357 | 0.999279047927446 |
| ebi-fl187-GCST90094727 | prot-a-1948 | Mesothelin \|\| id:prot-a-1948 | Cauliflower liking \|\| id:ebi-fl187-GCST90094727 | MR Egger | 4 | -0.695089572 | 1.38920305880591 | 0.666458506722422 | -3.417927567 | 2.02774842347137 | 0.499029746996376 | 0.0327802995652878 | 7.59696194634459 | 0.999279047927446 |
| ebi-fl187-GCST90094727 | prot-a-1980 | Unconventional myosin-VI \|\| id:prot-a-1980 | Cauliflower liking \|\| id:ebi-fl187-GCST90094727 | MR Egger | 4 | 1.56595760213348 | 1.40688233005759 | 0.381525373283363 | -1.191531765 | 4.32344696904636 | 4.78725702917862 | 0.303755625364788 | 75.4482483604956 | 0.999279047927446 |
| ebi-fl187-GCST90094727 | prot-a-2039 | Neurofascin \|\| id:prot-a-2039 | Cauliflower liking \|\| id:ebi-fl187-GCST90094727 | MR Egger | 4 | -0.392180768 | 1.38920305880591 | 0.804241537552598 | -3.115018763 | 2.33065722737763 | 0.675581979398448 | 0.044377674475089 | 10.2846986978582 | 0.999279047927446 |
| ebi-fl187-GCST90094727 | prot-a-2041 | NFU1 iron-sulfur cluster scaffold homolog, mitochondrial \|\| id:prot-a-2041 | Cauliflower liking \|\| id:ebi-fl187-GCST90094727 | MR Egger | 4 | -1.351848237 | 1.38920305880591 | 0.433139432254942 | -4.074686233 | 1.37098975780397 | 0.258761565593492 | 0.0169975471145593 | 3.93924766774391 | 0.999279047927446 |
| ebi-fl187-GCST90094727 | prot-a-2088 | Glucocorticoid receptor \|\| id:prot-a-2088 | Cauliflower liking \|\| id:ebi-fl187-GCST90094727 | MR Egger | 4 | 1.1800528810266 | 1.38826086848787 | 0.484838497266875 | -1.540938421 | 3.90104418326282 | 3.25454630208881 | 0.214180016020618 | 49.4540612576119 | 0.999279047927446 |
| ebi-fl187-GCST90094727 | prot-a-2111 | Neuron-specific protein family member 2 \|\| id:prot-a-2111 | Cauliflower liking \|\| id:ebi-fl187-GCST90094727 | MR Egger | 4 | -1.347800912 | 1.38920305880591 | 0.434292888463407 | -4.070638908 | 1.37503708289916 | 0.259810979999695 | 0.0170664811186152 | 3.95522339135127 | 0.999279047927446 |
| ebi-fl187-GCST90094727 | prot-a-2125 | Nuclear migration protein nudC \|\| id:prot-a-2125 | Cauliflower liking \|\| id:ebi-fl187-GCST90094727 | MR Egger | 4 | 0.163735508713069 | 1.38920305880591 | 0.916946279809282 | -2.559102487 | 2.88657350397266 | 1.17790272887998 | 0.0773741536328415 | 17.9317611057395 | 0.999279047927446 |
| ebi-fl187-GCST90094727 | prot-a-2142 | Ornithine decarboxylase \|\| id:prot-a-2142 | Cauliflower liking \|\| id:ebi-fl187-GCST90094727 | MR Egger | 4 | 0.937037254223128 | 1.38920305880591 | 0.569505032286926 | -1.785800741 | 3.65987524948272 | 2.5524080686879 | 0.16766275278787 | 38.856495201088 | 0.999279047927446 |
| ebi-fl187-GCST90094727 | prot-a-2186 | Prostate and testis expressed protein 1 \|\| id:prot-a-2186 | Cauliflower liking \|\| id:ebi-fl187-GCST90094727 | MR Egger | 4 | 0.0545545655061904 | 1.38920305880591 | 0.972242330617763 | -2.66828343 | 2.77739256076578 | 1.05607009984116 | 0.0693712037069963 | 16.0770463273081 | 0.999279047927446 |
| ebi-fl187-GCST90094727 | prot-a-2227 | Platelet-derived growth factor C \|\| id:prot-a-2227 | Cauliflower liking \|\| id:ebi-fl187-GCST90094727 | MR Egger | 4 | 0.15728851175265 | 3.31496646806957 | 0.966468068523972 | -6.340045766 | 6.654622789 | 1.17033322008541 | 0.00176422149421913 | 776.365014553753 | 0.999279047927446 |
| ebi-fl187-GCST90094727 | prot-a-2229 | Platelet-derived growth factor receptor alpha \|\| id:prot-a-2229 | Cauliflower liking \|\| id:ebi-fl187-GCST90094727 | MR Egger | 4 | -3.878207492 | 1.38826086848787 | 0.107810207725953 | -6.599198795 | -1.15721619 | 0.0206878751398358 | 0.00136145840851573 | 0.314360082632294 | 0.999279047927446 |
| ebi-fl187-GCST90094727 | prot-a-2243 | Platelet endothelial aggregation receptor 1 \|\| id:prot-a-2243 | Cauliflower liking \|\| id:ebi-fl187-GCST90094727 | MR Egger | 4 | 1.08537078812844 | 3.08458684823728 | 0.758552281950536 | -4.960419434 | 7.13116101067351 | 2.96053734735384 | 0.00700998698608039 | 1250.3277684368 | 0.999279047927446 |
| ebi-fl187-GCST90094727 | prot-a-2246 | E3 ubiquitin-protein ligase pellino homolog 2 \|\| id:prot-a-2246 | Cauliflower liking \|\| id:ebi-fl187-GCST90094727 | MR Egger | 4 | 1.39690078789749 | 1.38826086848787 | 0.420261726713403 | -1.324090514 | 4.11789209013371 | 4.04265149725785 | 0.266044813033616 | 61.4296175968526 | 0.999279047927446 |
| ebi-fl187-GCST90094727 | prot-a-2251 | Platelet factor 4 variant \|\| id:prot-a-2251 | Cauliflower liking \|\| id:ebi-fl187-GCST90094727 | MR Egger | 4 | 0.164813682517884 | 1.58796317512018 | 0.92680668845668 | -2.947594141 | 3.27722150575344 | 1.17917339762466 | 0.0524657795126392 | 26.5020345562716 | 0.999279047927446 |
| ebi-fl187-GCST90094727 | prot-a-2346 | Protein phosphatase 1L \|\| id:prot-a-2346 | Cauliflower liking \|\| id:ebi-fl187-GCST90094727 | MR Egger | 4 | -0.682527336 | 2.35551402943341 | 0.79928041957915 | -5.299334834 | 3.93428016153528 | 0.505338217552029 | 0.00499491525074338 | 51.1253347252801 | 0.999279047927446 |
| ebi-fl187-GCST90094727 | prot-a-2358 | PR domain zinc finger protein 4 \|\| id:prot-a-2358 | Cauliflower liking \|\| id:ebi-fl187-GCST90094727 | MR Egger | 4 | -1.03306756 | 1.60160250887878 | 0.585026069168201 | -4.172208477 | 2.1060733575157 | 0.355913498325539 | 0.0154181718138449 | 8.21591689467198 | 0.999279047927446 |
| ebi-fl187-GCST90094727 | prot-a-2390 | Trypsin-1 \|\| id:prot-a-2390 | Cauliflower liking \|\| id:ebi-fl187-GCST90094727 | MR Egger | 4 | 0.211687685576092 | 1.77422255753232 | 0.915931680297561 | -3.265788527 | 3.68916389833945 | 1.23576187854475 | 0.0381668276337278 | 40.0113793873441 | 0.999279047927446 |
| ebi-fl187-GCST90094727 | prot-a-242 | Brain-derived neurotrophic factor \|\| id:prot-a-242 | Cauliflower liking \|\| id:ebi-fl187-GCST90094727 | MR Egger | 4 | -1.476959888 | 1.52017639371709 | 0.433748414792736 | -4.45650562 | 1.5025858432489 | 0.228330785364791 | 0.0116028372515996 | 4.49329301228584 | 0.999279047927446 |
| ebi-fl187-GCST90094727 | prot-a-2464 | Dihydropteridine reductase \|\| id:prot-a-2464 | Cauliflower liking \|\| id:ebi-fl187-GCST90094727 | MR Egger | 4 | 0.606021686906375 | 1.38920305880591 | 0.705238763763265 | -2.116816308 | 3.32885968216596 | 1.83312413138638 | 0.120414381164416 | 27.9065012714953 | 0.999279047927446 |
| ebi-fl187-GCST90094727 | prot-a-2474 | Ras-related protein Rab-27A \|\| id:prot-a-2474 | Cauliflower liking \|\| id:ebi-fl187-GCST90094727 | MR Egger | 4 | 2.61807411443199 | 2.90778934943363 | 0.462949719890749 | -3.08119301 | 8.31734123932191 | 13.7092956100556 | 0.0459044594685275 | 4094.25986712134 | 0.999279047927446 |
| ebi-fl187-GCST90094727 | prot-a-2478 | Ras-related protein Rab-7b \|\| id:prot-a-2478 | Cauliflower liking \|\| id:ebi-fl187-GCST90094727 | MR Egger | 4 | -0.757894382 | 1.8956379636905 | 0.727954391922559 | -4.473344791 | 2.95755602682319 | 0.468652191309165 | 0.0114090909360747 | 19.2508656166824 | 0.999279047927446 |
| ebi-fl187-GCST90094727 | prot-a-2528 | E3 ubiquitin-protein ligase rififylin \|\| id:prot-a-2528 | Cauliflower liking \|\| id:ebi-fl187-GCST90094727 | MR Egger | 4 | 2.72580548286461 | 1.38920305880591 | 0.188755044023389 | 0.00296748760502164 | 5.4486434781242 | 15.2687076573826 | 1.00297189495487 | 232.442638422191 | 0.999279047927446 |
| ebi-fl187-GCST90094727 | prot-a-2543 | Protein RIC-3 \|\| id:prot-a-2543 | Cauliflower liking \|\| id:ebi-fl187-GCST90094727 | MR Egger | 4 | -0.370053407 | 1.38920305880591 | 0.814897152580131 | -3.092891402 | 2.3527845880248 | 0.690697441411864 | 0.0453705799598819 | 10.514808406565 | 0.999279047927446 |
| ebi-fl187-GCST90094727 | prot-a-255 | Bone morphogenetic protein 6 \|\| id:prot-a-255 | Cauliflower liking \|\| id:ebi-fl187-GCST90094727 | MR Egger | 4 | 0.480406227383161 | 2.37634062205239 | 0.858488311423501 | -4.177221392 | 5.13803384660584 | 1.6167310292292 | 0.0153410752367436 | 170.380444690872 | 0.999279047927446 |
| ebi-fl187-GCST90094727 | prot-a-2568 | RING finger protein 165 \|\| id:prot-a-2568 | Cauliflower liking \|\| id:ebi-fl187-GCST90094727 | MR Egger | 4 | -0.816125376 | 1.38920305880591 | 0.616374277201937 | -3.538963371 | 1.90671261938468 | 0.44214147193084 | 0.0290434187287746 | 6.7309252752497 | 0.999279047927446 |
| ebi-fl187-GCST90094727 | prot-a-2573 | E3 ubiquitin-protein ligase RNF8 \|\| id:prot-a-2573 | Cauliflower liking \|\| id:ebi-fl187-GCST90094727 | MR Egger | 4 | 2.31003157329557 | 2.94226130112503 | 0.514618362028711 | -3.456800577 | 8.07686372350064 | 10.0747427428224 | 0.0315304801587552 | 3219.12133348433 | 0.999279047927446 |
| ebi-fl187-GCST90094727 | prot-a-260 | Cytoplasmic tyrosine-protein kinase BMX \|\| id:prot-a-260 | Cauliflower liking \|\| id:ebi-fl187-GCST90094727 | MR Egger | 4 | -1.041705078 | 1.65272769016967 | 0.592915183382701 | -4.281051351 | 2.19764119438433 | 0.352852527539612 | 0.0138281162364714 | 9.00375033460545 | 0.999279047927446 |
| ebi-fl187-GCST90094727 | prot-a-2623 | Serum amyloid A-1 protein \|\| id:prot-a-2623 | Cauliflower liking \|\| id:ebi-fl187-GCST90094727 | MR Egger | 4 | -1.660540819 | 1.38920305880591 | 0.35447429894257 | -4.383378814 | 1.06229717662024 | 0.190036177198384 | 0.012483109181967 | 2.89300911477608 | 0.999279047927446 |
| ebi-fl187-GCST90094727 | prot-a-2640 | Lysosome membrane protein 2 \|\| id:prot-a-2640 | Cauliflower liking \|\| id:ebi-fl187-GCST90094727 | MR Egger | 4 | -2.641024324 | 1.38920305880591 | 0.197652770676791 | -5.363862319 | 0.0818136717435514 | 0.0712882099545673 | 0.00468278472746198 | 1.08525357758243 | 0.999279047927446 |
| ebi-fl187-GCST90094727 | prot-a-2641 | Scavenger receptor class F member 2 \|\| id:prot-a-2641 | Cauliflower liking \|\| id:ebi-fl187-GCST90094727 | MR Egger | 4 | -1.375992945 | 1.38826086848787 | 0.426066113542204 | -4.096984247 | 1.34499835747391 | 0.252588664653391 | 0.0166227299354654 | 3.83818023628361 | 0.999279047927446 |
| ebi-fl187-GCST90094727 | prot-a-2669 | Semaphorin-3A \|\| id:prot-a-2669 | Cauliflower liking \|\| id:ebi-fl187-GCST90094727 | MR Egger | 4 | -0.494852819 | 1.5338652688346 | 0.777588390021097 | -3.501228746 | 2.5115231077887 | 0.609660631077183 | 0.0301603012938567 | 12.3236860754152 | 0.999279047927446 |
| ebi-fl187-GCST90094727 | prot-a-2683 | Sentrin-specific protease 7 \|\| id:prot-a-2683 | Cauliflower liking \|\| id:ebi-fl187-GCST90094727 | MR Egger | 4 | 0.612793822783753 | 2.88685359258926 | 0.851564860774476 | -5.045439219 | 6.2710268642587 | 1.84558042734294 | 0.0064386317752902 | 529.020331130494 | 0.999279047927446 |
| ebi-fl187-GCST90094727 | prot-a-270 | Bromodomain testis-specific protein \|\| id:prot-a-270 | Cauliflower liking \|\| id:ebi-fl187-GCST90094727 | MR Egger | 4 | 3.43335726019044 | 1.38826086848787 | 0.131907083516973 | 0.712365957954215 | 6.15434856242666 | 30.9804778764349 | 2.03880929383077 | 470.760071751928 | 0.999279047927446 |
| ebi-fl187-GCST90094727 | prot-a-2708 | 14-3-3 protein sigma \|\| id:prot-a-2708 | Cauliflower liking \|\| id:ebi-fl187-GCST90094727 | MR Egger | 4 | -1.406642981 | 1.38920305880591 | 0.417847976061484 | -4.129480976 | 1.31619501451495 | 0.244964254092676 | 0.0160912283892437 | 3.72920477738626 | 0.999279047927446 |
| ebi-fl187-GCST90094727 | prot-a-2713 | Beta-sarcoglycan \|\| id:prot-a-2713 | Cauliflower liking \|\| id:ebi-fl187-GCST90094727 | MR Egger | 4 | 1.16660535223358 | 1.38826086848787 | 0.489170697835686 | -1.55438595 | 3.8875966544698 | 3.2110736519569 | 0.211319103304233 | 48.7934779064781 | 0.999279047927446 |
| ebi-fl187-GCST90094727 | prot-a-2720 | Protein shisa-3 homolog \|\| id:prot-a-2720 | Cauliflower liking \|\| id:ebi-fl187-GCST90094727 | MR Egger | 4 | -1.618435249 | 1.75014851930001 | 0.452724285784345 | -5.048726346 | 1.81185584931615 | 0.198208603766179 | 0.00641750191805682 | 6.12179803116192 | 0.999279047927446 |
| ebi-fl187-GCST90094727 | prot-a-2730 | Sialic acid-binding Ig-like lectin 8 \|\| id:prot-a-2730 | Cauliflower liking \|\| id:ebi-fl187-GCST90094727 | MR Egger | 4 | -0.435496188 | 1.38826086848787 | 0.783444908534881 | -3.15648749 | 2.28549511471765 | 0.646943582094785 | 0.0425750239560476 | 9.83055227040408 | 0.999279047927446 |
| ebi-fl187-GCST90094727 | prot-a-2761 | Sodium- and chloride-dependent neutral and basic amino acid transporter B(0+) \|\| id:prot-a-2761 | Cauliflower liking \|\| id:ebi-fl187-GCST90094727 | MR Egger | 4 | 1.24211098025525 | 1.825089805 | 0.56636148386099 | -2.335065037 | 4.81928699756525 | 3.46291590149467 | 0.0968041864625494 | 123.876734871006 | 0.999279047927446 |
| ebi-fl187-GCST90094727 | prot-a-2770 | Secreted Ly-6/uPAR-related protein 1 \|\| id:prot-a-2770 | Cauliflower liking \|\| id:ebi-fl187-GCST90094727 | MR Egger | 4 | -0.193077594 | 1.57254475006168 | 0.913506542631199 | -3.275265305 | 2.88911011562223 | 0.824418001355662 | 0.0378068375700025 | 17.9773047587175 | 0.999279047927446 |
| ebi-fl187-GCST90094727 | prot-a-2784 | N-lysine methyltransferase SMYD2 \|\| id:prot-a-2784 | Cauliflower liking \|\| id:ebi-fl187-GCST90094727 | MR Egger | 4 | -0.440631034 | 1.98976995316497 | 0.845297594278043 | -4.340580142 | 3.45931807401617 | 0.64363014028567 | 0.0130289673541983 | 31.7952870877878 | 0.999279047927446 |
| ebi-fl187-GCST90094727 | prot-a-2837 | Serine palmitoyltransferase 2 \|\| id:prot-a-2837 | Cauliflower liking \|\| id:ebi-fl187-GCST90094727 | MR Egger | 4 | 0.542524674276165 | 1.38920305880591 | 0.733816673005785 | -2.180313321 | 3.26536266953575 | 1.72034469406972 | 0.113006117904968 | 26.1896074414546 | 0.999279047927446 |
| ebi-fl187-GCST90094727 | prot-a-2858 | Alpha-2,8-sialyltransferase 8B \|\| id:prot-a-2858 | Cauliflower liking \|\| id:ebi-fl187-GCST90094727 | MR Egger | 4 | -0.176812776 | 1.38826086848787 | 0.910303920026805 | -2.897804078 | 2.54417852630441 | 0.837936651746697 | 0.0551441795067365 | 12.7327641579052 | 0.999279047927446 |
| ebi-fl187-GCST90094727 | prot-a-2911 | Synaptotagmin-11 \|\| id:prot-a-2911 | Cauliflower liking \|\| id:ebi-fl187-GCST90094727 | MR Egger | 4 | -1.472820346 | 1.38826086848787 | 0.399909431566028 | -4.193811648 | 1.24817095619152 | 0.229277929349013 | 0.0150886624503171 | 3.48396480202699 | 0.999279047927446 |
| ebi-fl187-GCST90094727 | prot-a-2962 | Transforming growth factor beta-1 \|\| id:prot-a-2962 | Cauliflower liking \|\| id:ebi-fl187-GCST90094727 | MR Egger | 4 | -2.896908446 | 1.38826086848787 | 0.172197493490571 | -5.617899749 | -0.175917144 | 0.0551935905068155 | 0.00363226176607108 | 0.838687470569936 | 0.999279047927446 |
| ebi-fl187-GCST90094727 | prot-a-2978 | Tyrosine-protein kinase receptor Tie-1, soluble \|\| id:prot-a-2978 | Cauliflower liking \|\| id:ebi-fl187-GCST90094727 | MR Egger | 4 | 1.7267078140715 | 1.38826086848787 | 0.339586355732031 | -0.994283488 | 4.44769911630773 | 5.62211436156616 | 0.369988450696539 | 85.4301528467256 | 0.999279047927446 |
| ebi-fl187-GCST90094727 | prot-a-299 | Uncharacterized protein C1orf226 \|\| id:prot-a-299 | Cauliflower liking \|\| id:ebi-fl187-GCST90094727 | MR Egger | 4 | -0.858584057 | 1.777214976 | 0.6767337309537 | -4.34192541 | 2.62475729546288 | 0.423761680081916 | 0.0130114516963309 | 13.8012241598289 | 0.999279047927446 |
| ebi-fl187-GCST90094727 | prot-a-3015 | Transmembrane protein 70, mitochondrial \|\| id:prot-a-3015 | Cauliflower liking \|\| id:ebi-fl187-GCST90094727 | MR Egger | 4 | -0.262219617 | 1.38920305880591 | 0.867702945499961 | -2.985057613 | 2.4606183780179 | 0.769342044375163 | 0.0505365919257953 | 11.7120517765116 | 0.999279047927446 |
| ebi-fl187-GCST90094727 | prot-a-3016 | Transmembrane protein 87B \|\| id:prot-a-3016 | Cauliflower liking \|\| id:ebi-fl187-GCST90094727 | MR Egger | 4 | -2.35064294 | 3.0497294920828 | 0.521443737961861 | -8.328112744 | 3.62682686494324 | 0.0953078653175666 | 0.000241627628027931 | 37.5933384171672 | 0.999279047927446 |
| ebi-fl187-GCST90094727 | prot-a-3123 | Thioredoxin domain-containing protein 12 \|\| id:prot-a-3123 | Cauliflower liking \|\| id:ebi-fl187-GCST90094727 | MR Egger | 4 | -0.778541907 | 1.38920305880591 | 0.631593227415071 | -3.501379902 | 1.94429608839481 | 0.459074897494127 | 0.0301557427254342 | 6.98871068864408 | 0.999279047927446 |
| ebi-fl187-GCST90094727 | prot-a-3133 | PolyUbiquitin K48-linked \|\| id:prot-a-3133 | Cauliflower liking \|\| id:ebi-fl187-GCST90094727 | MR Egger | 4 | 1.42305980824785 | 2.2233124719833 | 0.587671915256519 | -2.934632637 | 5.78075225333512 | 4.14979862200785 | 0.0531502411609528 | 324.002830976233 | 0.999279047927446 |
| ebi-fl187-GCST90094727 | prot-a-3135 | Ubiquitin-conjugating enzyme E2 B \|\| id:prot-a-3135 | Cauliflower liking \|\| id:ebi-fl187-GCST90094727 | MR Egger | 4 | -1.435278468 | 1.8915948874353 | 0.5272209963503 | -5.142804447 | 2.27224751139422 | 0.238049065741909 | 0.00584128514455569 | 9.70117984283166 | 0.999279047927446 |
| ebi-fl187-GCST90094727 | prot-a-3162 | NKG2D ligand 3 \|\| id:prot-a-3162 | Cauliflower liking \|\| id:ebi-fl187-GCST90094727 | MR Egger | 4 | 0.208779645203921 | 1.79321161645553 | 0.917950711923637 | -3.305915123 | 3.72347441345676 | 1.23217345328317 | 0.0366656429039524 | 41.4080130260614 | 0.999279047927446 |
| ebi-fl187-GCST90094727 | prot-a-3188 | Vesicle-associated membrane protein 8 \|\| id:prot-a-3188 | Cauliflower liking \|\| id:ebi-fl187-GCST90094727 | MR Egger | 4 | 0.1769713839189 | 1.46019738932947 | 0.914613835516781 | -2.685015499 | 3.03895826700466 | 1.19359693657169 | 0.0682201366328429 | 20.8834768927661 | 0.999279047927446 |
| ebi-fl187-GCST90094727 | prot-a-3201 | Vitrin \|\| id:prot-a-3201 | Cauliflower liking \|\| id:ebi-fl187-GCST90094727 | MR Egger | 4 | 2.68927763989882 | 1.91267919627401 | 0.294948896381461 | -1.059573585 | 6.43812886459588 | 14.7210381881333 | 0.346603575856812 | 625.235803758719 | 0.999279047927446 |
| ebi-fl187-GCST90094727 | prot-a-3232 | WNT1-inducible-signaling pathway protein 3 \|\| id:prot-a-3232 | Cauliflower liking \|\| id:ebi-fl187-GCST90094727 | MR Egger | 4 | 1.53548785078438 | 1.45075937070636 | 0.400818177974033 | -1.308000516 | 4.37897621736886 | 4.64359035665815 | 0.270360097045711 | 79.7563384392587 | 0.999279047927446 |
| ebi-fl187-GCST90094727 | prot-a-327 | Carbonic anhydrase-related protein 10 \|\| id:prot-a-327 | Cauliflower liking \|\| id:ebi-fl187-GCST90094727 | MR Egger | 4 | 0.425965806898233 | 1.38920305880591 | 0.788106006831517 | -2.296872188 | 3.14880380215782 | 1.53106842225982 | 0.10057292543924 | 23.3081667198533 | 0.999279047927446 |
| ebi-fl187-GCST90094727 | prot-a-332 | Carbonic anhydrase 5A, mitochondrial \|\| id:prot-a-332 | Cauliflower liking \|\| id:ebi-fl187-GCST90094727 | MR Egger | 4 | 0.134824043422666 | 1.38920305880591 | 0.931535353799977 | -2.588013952 | 2.85766203868226 | 1.14433541329291 | 0.0751691815501759 | 17.4207502477881 | 0.999279047927446 |
| ebi-fl187-GCST90094727 | prot-a-338 | Cell adhesion molecule 3 \|\| id:prot-a-338 | Cauliflower liking \|\| id:ebi-fl187-GCST90094727 | MR Egger | 4 | -1.528354127 | 1.51111068171753 | 0.418282589429925 | -4.490131063 | 1.43342280885995 | 0.216892350905403 | 0.0112191732894126 | 4.19302658651914 | 0.999279047927446 |
| ebi-fl187-GCST90094727 | prot-a-34 | A disintegrin and metalloproteinase with thrombospondin motifs 3 \|\| id:prot-a-34 | Cauliflower liking \|\| id:ebi-fl187-GCST90094727 | MR Egger | 4 | 0.447889539203429 | 2.42587600073886 | 0.870545418147378 | -4.306827422 | 5.2026065006516 | 1.5650058143165 | 0.0134762362524289 | 181.745344394881 | 0.999279047927446 |
| ebi-fl187-GCST90094727 | prot-a-358 | Cysteine--tRNA ligase, cytoplasmic \|\| id:prot-a-358 | Cauliflower liking \|\| id:ebi-fl187-GCST90094727 | MR Egger | 4 | 1.5546205597309 | 2.04144243712301 | 0.525885296825789 | -2.446606617 | 5.55584773649199 | 4.73329018347783 | 0.0865869110860587 | 258.746220184945 | 0.999279047927446 |
| ebi-fl187-GCST90094727 | prot-a-382 | Coiled-coil domain-containing protein 167 \|\| id:prot-a-382 | Cauliflower liking \|\| id:ebi-fl187-GCST90094727 | MR Egger | 4 | -0.786807059 | 1.38826086848787 | 0.62800287425214 | -3.507798362 | 1.9341842428715 | 0.45529621067984 | 0.0299628091433046 | 6.91839802029184 | 0.999279047927446 |
| ebi-fl187-GCST90094727 | prot-a-40 | Pituitary adenylate cyclase-activating polypeptide 27 \|\| id:prot-a-40 | Cauliflower liking \|\| id:ebi-fl187-GCST90094727 | MR Egger | 4 | -1.969838323 | 1.38920305880591 | 0.291957759124627 | -4.692676318 | 0.752999672086724 | 0.139479404979843 | 0.00916213253006849 | 2.12335985641824 | 0.999279047927446 |
| ebi-fl187-GCST90094727 | prot-a-403 | C-C motif chemokine 25 \|\| id:prot-a-403 | Cauliflower liking \|\| id:ebi-fl187-GCST90094727 | MR Egger | 4 | 2.06744274754522 | 1.38826086848787 | 0.274865151691665 | -0.653548555 | 4.78843404978144 | 7.90458323475297 | 0.520196551749504 | 120.113130133214 | 0.999279047927446 |
| ebi-fl187-GCST90094727 | prot-a-415 | Cyclin-C \|\| id:prot-a-415 | Cauliflower liking \|\| id:ebi-fl187-GCST90094727 | MR Egger | 4 | -1.521094336 | 1.74298565186317 | 0.474851339766421 | -4.937346213 | 1.89515754195878 | 0.218472673641535 | 0.00717361036572728 | 6.6535965427001 | 0.999279047927446 |
| ebi-fl187-GCST90094727 | prot-a-44 | Adhesion G protein-coupled receptor F5 \|\| id:prot-a-44 | Cauliflower liking \|\| id:ebi-fl187-GCST90094727 | MR Egger | 4 | -0.047379066 | 1.38920305880591 | 0.975890982622458 | -2.770217061 | 2.67545892969329 | 0.953725804448787 | 0.0626484047517178 | 14.5190115163554 | 0.999279047927446 |
| ebi-fl187-GCST90094727 | prot-a-455 | CD59 glycoprotein \|\| id:prot-a-455 | Cauliflower liking \|\| id:ebi-fl187-GCST90094727 | MR Egger | 4 | -2.040658191 | 1.80717931700752 | 0.376038282080994 | -5.582729653 | 1.50141327002006 | 0.129943155269334 | 0.00376228176724889 | 4.48802738496043 | 0.999279047927446 |
| ebi-fl187-GCST90094727 | prot-a-478 | Cadherin-12 \|\| id:prot-a-478 | Cauliflower liking \|\| id:ebi-fl187-GCST90094727 | MR Egger | 4 | -0.333457059 | 1.57061445101043 | 0.851537881870853 | -3.411861383 | 2.74494726499686 | 0.716442662755585 | 0.0329797552525904 | 15.563793153862 | 0.999279047927446 |
| ebi-fl187-GCST90094727 | prot-a-516 | Centrin-2 \|\| id:prot-a-516 | Cauliflower liking \|\| id:ebi-fl187-GCST90094727 | MR Egger | 4 | -0.325667385 | 1.67517929991108 | 0.863813894286343 | -3.609018813 | 2.95768404259836 | 0.722045310432044 | 0.0270784028114289 | 19.2533301889158 | 0.999279047927446 |
| ebi-fl187-GCST90094727 | prot-a-519 | Cryptic protein \|\| id:prot-a-519 | Cauliflower liking \|\| id:ebi-fl187-GCST90094727 | MR Egger | 4 | -0.100699079 | 1.38920305880591 | 0.948811189946602 | -2.823537074 | 2.62213891611361 | 0.904205086117293 | 0.0593954844771948 | 13.7651345882076 | 0.999279047927446 |
| ebi-fl187-GCST90094727 | prot-a-583 | Chloride intracellular channel protein 4 \|\| id:prot-a-583 | Cauliflower liking \|\| id:ebi-fl187-GCST90094727 | MR Egger | 4 | 0.182918974989999 | 1.38826086848787 | 0.907232497653851 | -2.538072327 | 2.90391027722622 | 1.200717116 | 0.0790185750256442 | 18.2453504412439 | 0.999279047927446 |
| ebi-fl187-GCST90094727 | prot-a-602 | CCR4-NOT transcription complex subunit 1 \|\| id:prot-a-602 | Cauliflower liking \|\| id:ebi-fl187-GCST90094727 | MR Egger | 4 | 0.758323441399103 | 1.84626867925429 | 0.721092828927047 | -2.86036317 | 4.37701005273751 | 2.13469427861501 | 0.0572479657494777 | 79.5996784076687 | 0.999279047927446 |
| ebi-fl187-GCST90094727 | prot-a-606 | Ciliary neurotrophic factor receptor subunit alpha \|\| id:prot-a-606 | Cauliflower liking \|\| id:ebi-fl187-GCST90094727 | MR Egger | 4 | -1.373154258 | 1.38920305880591 | 0.427122049280787 | -4.095992253 | 1.3496837372335 | 0.253306703406832 | 0.0166392277605683 | 3.8562057635207 | 0.999279047927446 |
| ebi-fl187-GCST90094727 | prot-a-626 | Collagen alpha-2(IX) chain \|\| id:prot-a-626 | Cauliflower liking \|\| id:ebi-fl187-GCST90094727 | MR Egger | 4 | -1.153540719 | 2.03136610660972 | 0.627376997140447 | -5.135018288 | 2.82793685026166 | 0.315517630098574 | 0.00588694384586774 | 16.9105358415979 | 0.999279047927446 |
| ebi-fl187-GCST90094727 | prot-a-647 | Probable serine carboxypeptidase CPVL \|\| id:prot-a-647 | Cauliflower liking \|\| id:ebi-fl187-GCST90094727 | MR Egger | 4 | -0.642076611 | 2.87560921922224 | 0.844046420222967 | -6.27827068 | 4.99411745896079 | 0.526198579083615 | 0.00187664310546775 | 147.542675441531 | 0.999279047927446 |
| ebi-fl187-GCST90094727 | prot-a-651 | Cellular retinoic acid-binding protein 2 \|\| id:prot-a-651 | Cauliflower liking \|\| id:ebi-fl187-GCST90094727 | MR Egger | 4 | 0.936100644690204 | 2.4191843564599 | 0.736086456244051 | -3.805500694 | 5.67770198335161 | 2.55001857814334 | 0.0222480549098954 | 292.277000178743 | 0.999279047927446 |
| ebi-fl187-GCST90094727 | prot-a-671 | Cytotoxic and regulatory T-cell molecule \|\| id:prot-a-671 | Cauliflower liking \|\| id:ebi-fl187-GCST90094727 | MR Egger | 4 | -0.914031363 | 2.19228888987531 | 0.717218854201386 | -5.210917587 | 3.38285486145666 | 0.40090476938923 | 0.00545666442622878 | 29.4547403990009 | 0.999279047927446 |
| ebi-fl187-GCST90094727 | prot-a-688 | Chorionic somatomammotropin hormone \|\| id:prot-a-688 | Cauliflower liking \|\| id:ebi-fl187-GCST90094727 | MR Egger | 4 | -0.094906892 | 1.38920305880591 | 0.95174849443934 | -2.817744887 | 2.62793110335581 | 0.909457608392762 | 0.0597405125134984 | 13.8450961778508 | 0.999279047927446 |
| ebi-fl187-GCST90094727 | prot-a-69 | Aldo-keto reductase family 1 member C1 \|\| id:prot-a-69 | Cauliflower liking \|\| id:ebi-fl187-GCST90094727 | MR Egger | 4 | -1.653630733 | 1.38920305880591 | 0.356045264606637 | -4.376468728 | 1.06920726228275 | 0.191353890976021 | 0.0125696672531687 | 2.91306928450572 | 0.999279047927446 |
| ebi-fl187-GCST90094727 | prot-a-741 | Stromal cell-derived factor 1 \|\| id:prot-a-741 | Cauliflower liking \|\| id:ebi-fl187-GCST90094727 | MR Egger | 4 | -2.024422013 | 1.38920305880591 | 0.28237495124163 | -4.747260009 | 0.698415981795925 | 0.132070155903809 | 0.00867543327871503 | 2.01056541155715 | 0.999279047927446 |
| ebi-fl187-GCST90094727 | prot-a-742 | Growth-regulated alpha protein \|\| id:prot-a-742 | Cauliflower liking \|\| id:ebi-fl187-GCST90094727 | MR Egger | 4 | -0.009089431 | 1.38826086848787 | 0.995370373550697 | -2.730080733 | 2.71190187149515 | 0.990951753260358 | 0.0652140245332332 | 15.0578864641202 | 0.999279047927446 |
| ebi-fl187-GCST90094727 | prot-a-788 | Beta-defensin 112 \|\| id:prot-a-788 | Cauliflower liking \|\| id:ebi-fl187-GCST90094727 | MR Egger | 4 | 0.09639551740488 | 1.38920305880591 | 0.950993467664922 | -2.626442478 | 2.81923351266447 | 1.1011945194718 | 0.0723353396169405 | 16.7639963555344 | 0.999279047927446 |
| ebi-fl187-GCST90094727 | prot-a-838 | DnaJ homolog subfamily B member 1 \|\| id:prot-a-838 | Cauliflower liking \|\| id:ebi-fl187-GCST90094727 | MR Egger | 4 | 0.687267043499981 | 1.38920305880591 | 0.669800837013207 | -2.035570952 | 3.41010503875957 | 1.98827423422086 | 0.130605891548538 | 30.2684234500814 | 0.999279047927446 |
| ebi-fl187-GCST90094727 | prot-a-85 | Junctional adhesion molecule-like \|\| id:prot-a-85 | Cauliflower liking \|\| id:ebi-fl187-GCST90094727 | MR Egger | 4 | 0.911064900039171 | 1.38920305880591 | 0.579300774173579 | -1.811773095 | 3.63390289529876 | 2.48696949767398 | 0.163364219536352 | 37.8602933978726 | 0.999279047927446 |
| ebi-fl187-GCST90094727 | prot-a-856 | Dipeptidase 1 \|\| id:prot-a-856 | Cauliflower liking \|\| id:ebi-fl187-GCST90094727 | MR Egger | 4 | -2.056499904 | 3.1169549396296 | 0.577213365499032 | -8.165731586 | 4.05273177778409 | 0.127900852649142 | 0.000284228636507393 | 57.5544685060335 | 0.999279047927446 |
| ebi-fl187-GCST90094727 | prot-a-860 | Dipeptidyl peptidase 2 \|\| id:prot-a-860 | Cauliflower liking \|\| id:ebi-fl187-GCST90094727 | MR Egger | 4 | -0.369551793 | 1.71014448467847 | 0.848951722043268 | -3.721434983 | 2.9823313969003 | 0.691043991942467 | 0.0241992174519334 | 19.7337703067595 | 0.999279047927446 |
| ebi-fl187-GCST90094727 | prot-a-861 | Dermatopontin \|\| id:prot-a-861 | Cauliflower liking \|\| id:ebi-fl187-GCST90094727 | MR Egger | 4 | -1.048953355 | 1.61828538851824 | 0.58334162500342 | -4.220792716 | 2.12288600685042 | 0.350304201570114 | 0.0146869972692365 | 8.35521593611996 | 0.999279047927446 |
| ebi-fl187-GCST90094727 | prot-a-877 | Dual specificity protein phosphatase 6 \|\| id:prot-a-877 | Cauliflower liking \|\| id:ebi-fl187-GCST90094727 | MR Egger | 4 | 0.39955668337622 | 1.38920305880591 | 0.800704757864707 | -2.323281312 | 3.12239467863581 | 1.49116349352526 | 0.0979516477981301 | 22.7006754292183 | 0.999279047927446 |
| ebi-fl187-GCST90094727 | prot-a-879 | Dynactin-associated protein \|\| id:prot-a-879 | Cauliflower liking \|\| id:ebi-fl187-GCST90094727 | MR Egger | 4 | -1.414783471 | 1.38920305880591 | 0.415627266307527 | -4.137621467 | 1.30805452390507 | 0.242978219497928 | 0.0159607696152834 | 3.69897044900957 | 0.999279047927446 |
| ebi-fl187-GCST90094727 | prot-a-937 | EMI domain-containing protein 1 \|\| id:prot-a-937 | Cauliflower liking \|\| id:ebi-fl187-GCST90094727 | MR Egger | 4 | -0.992744069 | 1.38826086848787 | 0.548756297802206 | -3.713735371 | 1.72824723362225 | 0.370558456777778 | 0.0243862611996952 | 5.63077581942919 | 0.999279047927446 |
| ebi-fl187-GCST90094727 | prot-b-1 | SRC proto-oncogene, non-receptor tyrosine kinase \|\| id:prot-b-1 | Cauliflower liking \|\| id:ebi-fl187-GCST90094727 | MR Egger | 4 | 0.957934344612708 | 1.62533497639787 | 0.615317371332294 | -2.227722209 | 4.14359089835253 | 2.60630717666408 | 0.107773636571007 | 63.0287453894646 | 0.999279047927446 |
| ebi-fl187-GCST90094727 | prot-b-10 | coagulation factor III, tissue factor \|\| id:prot-b-10 | Cauliflower liking \|\| id:ebi-fl187-GCST90094727 | MR Egger | 4 | -0.857735024 | 1.62533497639787 | 0.650388240454923 | -4.043391578 | 2.32792152934739 | 0.424121620367175 | 0.0175378903087945 | 10.2566013183853 | 0.999279047927446 |
| ebi-fl187-GCST90094727 | prot-b-29 | myeloperoxidase \|\| id:prot-b-29 | Cauliflower liking \|\| id:ebi-fl187-GCST90094727 | MR Egger | 4 | 4.06688393672979 | 2.51257174159821 | 0.246945373348792 | -0.857756677 | 8.99152455026228 | 58.3747793861571 | 0.424112437211269 | 8034.6968619669 | 0.999279047927446 |
| ebi-fl187-GCST90094727 | prot-b-33 | adrenomedullin \|\| id:prot-b-33 | Cauliflower liking \|\| id:ebi-fl187-GCST90094727 | MR Egger | 4 | 0.347502699060821 | 1.62533497639787 | 0.850516571448763 | -2.838153855 | 3.53315925280064 | 1.41552813120605 | 0.0585336278603586 | 34.2319443280687 | 0.999279047927446 |
| ebi-fl187-GCST90094727 | ukb-rsfMRI191-edge-pheno102 | Net25_Pair11_15 (Postcentral\|Precentral)&(Cerebellum) (Motor)&(Subcortical-cerebellum) \|\| id:ukb-rsfMRI191-edge-pheno102 | Cauliflower liking \|\| id:ebi-fl187-GCST90094727 | MR Egger | 4 | -0.956381651 | 0.422925485555196 | 0.152148522517317 | -1.785315603 | -0.127447699 | 0.384280835532756 | 0.167744112125359 | 0.880339456847195 | 0.999279047927446 |
| ebi-fl187-GCST90094727 | ukb-rsfMRI191-edge-pheno1167 | Net100_Pair11_45 (Angular\|Temporal)&(Frontal) (Default_mode\|Central_executive)&(Salience\|Default_mode) \|\| id:ukb-rsfMRI191-edge-pheno1167 | Cauliflower liking \|\| id:ebi-fl187-GCST90094727 | MR Egger | 4 | 0.0176976401070502 | 0.423437982762582 | 0.970459287071813 | -0.812240806 | 0.847636086321711 | 1.01785517127755 | 0.443862341580704 | 2.33412266065848 | 0.999279047927446 |
| ebi-fl187-GCST90094727 | ukb-rsfMRI191-edge-pheno1317 | Net100_Pair26_48 (Parietal\|Frontal)&(Temporal) (Attention\|Central_executive)&(Default_mode\|Central_executive) \|\| id:ukb-rsfMRI191-edge-pheno1317 | Cauliflower liking \|\| id:ebi-fl187-GCST90094727 | MR Egger | 4 | -0.052273294 | 0.421761171029429 | 0.912695460974194 | -0.878925189 | 0.774378600984265 | 0.949069456187299 | 0.415228964398704 | 2.16924374235794 | 0.999279047927446 |
| ebi-fl187-GCST90094727 | ukb-rsfMRI191-edge-pheno286 | Net100_Pair10_13 (OccipitalPrecuneus)&(Frontal_Sup) (Default_mode\|Central_executive)&(Default_mode) \|\| id:ukb-rsfMRI191-edge-pheno286 | Cauliflower liking \|\| id:ebi-fl187-GCST90094727 | MR Egger | 4 | 0.9142112027952 | 0.417460221778527 | 0.1599396165107 | 0.0959891681092865 | 1.73243323748111 | 2.49480657910905 | 1.10074714075667 | 5.65439567064172 | 0.999279047927446 |
| ebi-fl187-GCST90094727 | ukb-rsfMRI191-edge-pheno599 | Net100_Pair11_29 (Angular\|Temporal)&(Parietal) (Default_mode\|Central_executive)&(Central_executive\|Salience) \|\| id:ukb-rsfMRI191-edge-pheno599 | Cauliflower liking \|\| id:ebi-fl187-GCST90094727 | MR Egger | 4 | -0.53757002 | 0.424710224812489 | 0.333092583597028 | -1.370002061 | 0.294862020346449 | 0.584166040706407 | 0.254106435859604 | 1.34294104736152 | 0.999279047927446 |
| ebi-fl187-GCST90094727 | ukb-rsfMRI191-edge-pheno621 | Net100_Pair5_30 (Precuneus\|Angular\|Cingulate)&(Paracentral) (Default_mode\|Central_executive)&(Motor) \|\| id:ukb-rsfMRI191-edge-pheno621 | Cauliflower liking \|\| id:ebi-fl187-GCST90094727 | MR Egger | 4 | -0.136729183 | 0.426933963485305 | 0.779135582363559 | -0.973519752 | 0.700061385018428 | 0.872206402111659 | 0.377751105292571 | 2.01387632551164 | 0.999279047927446 |
| ebi-fl187-GCST90094727 | ukb-rsfMRI191-edge-pheno624 | Net100_Pair8_30 (Calcarine\|Lingual)&(Paracentral) (Visual)&(Motor) \|\| id:ukb-rsfMRI191-edge-pheno624 | Cauliflower liking \|\| id:ebi-fl187-GCST90094727 | MR Egger | 4 | 0.346392585639276 | 0.425470475610642 | 0.50108344368791 | -0.487529547 | 1.18031471783613 | 1.41395760632069 | 0.614141730181669 | 3.2553985736822 | 0.999279047927446 |
| ebi-fl187-GCST90094727 | ukb-rsfMRI191-edge-pheno882 | Net100_Pair6_38 (Postcentral\|Precentral)&(Subcortical) (Motor)&(Subcortical-cerebellum) \|\| id:ukb-rsfMRI191-edge-pheno882 | Cauliflower liking \|\| id:ebi-fl187-GCST90094727 | MR Egger | 4 | -0.788991954 | 0.433056721221485 | 0.210053431321781 | -1.637783128 | 0.0597992193926398 | 0.454302522287557 | 0.194410548266914 | 1.06162337176002 | 0.999279047927446 |
| ebi-fl187-GCST90094727 | ukb-rsfMRI191-edge-pheno899 | Net100_Pair23_38 (Cerebellum)&(Subcortical) (Subcortical-cerebellum)&(Subcortical-cerebellum) \|\| id:ukb-rsfMRI191-edge-pheno899 | Cauliflower liking \|\| id:ebi-fl187-GCST90094727 | MR Egger | 4 | -0.929151738 | 0.586327862077664 | 0.253900015073376 | -2.078354348 | 0.220050871632154 | 0.394888537265641 | 0.125135973147143 | 1.24614012215686 | 0.999279047927446 |
| ebi-fl187-GCST90094727 | ebi-a-GCST90001392 | IgD+ CD38+ B cell Absolute Count \|\| id:ebi-a-GCST90001392 | Cauliflower liking \|\| id:ebi-fl187-GCST90094727 | Weighted median | 4 | -0.518102841 | 0.444639481104273 | 0.243929929312498 | -1.389596224 | 0.35339054190503 | 0.595649518522851 | 0.249175895562697 | 1.42388712244933 | 0.999489402457283 |
| ebi-fl187-GCST90094727 | ebi-a-GCST90001402 | Switched memory B cell %B cell \|\| id:ebi-a-GCST90001402 | Cauliflower liking \|\| id:ebi-fl187-GCST90094727 | Weighted median | 4 | -0.207713766 | 0.423265610918182 | 0.623609683253883 | -1.037314363 | 0.621886831641728 | 0.812439551452768 | 0.354405208688272 | 1.86243883719369 | 0.999489402457283 |
| ebi-fl187-GCST90094727 | ebi-a-GCST90001407 | Memory B cell Absolute Count \|\| id:ebi-a-GCST90001407 | Cauliflower liking \|\| id:ebi-fl187-GCST90094727 | Weighted median | 4 | 0.237586518489244 | 0.399896830055498 | 0.552432166325681 | -0.546211268 | 1.02138430539802 | 1.26818471346988 | 0.579139864491249 | 2.77703637081089 | 0.999489402457283 |
| ebi-fl187-GCST90094727 | ebi-a-GCST90001411 | IgD+ CD24+ B cell %B cell \|\| id:ebi-a-GCST90001411 | Cauliflower liking \|\| id:ebi-fl187-GCST90094727 | Weighted median | 4 | 0.763842274600653 | 0.422180577118058 | 0.070407735176883 | -0.063631657 | 1.59131620575205 | 2.14650786890779 | 0.938350571168297 | 4.91020752035826 | 0.999489402457283 |
| ebi-fl187-GCST90094727 | ebi-a-GCST90001418 | CD24+ CD27+ B cell Absolute Count \|\| id:ebi-a-GCST90001418 | Cauliflower liking \|\| id:ebi-fl187-GCST90094727 | Weighted median | 4 | 0.120027798597167 | 0.398254915875038 | 0.763121427440233 | -0.660551837 | 0.900607433712242 | 1.12752819484581 | 0.516566195733223 | 2.4610976108642 | 0.999489402457283 |
| ebi-fl187-GCST90094727 | ebi-a-GCST90001436 | Memory B cell %lymphocyte \|\| id:ebi-a-GCST90001436 | Cauliflower liking \|\| id:ebi-fl187-GCST90094727 | Weighted median | 4 | 0.526814894033767 | 0.408261873533974 | 0.196917105663466 | -0.273378378 | 1.32700816616036 | 1.69352963779315 | 0.760804861267439 | 3.76974803934067 | 0.999489402457283 |
| ebi-fl187-GCST90094727 | ebi-a-GCST90001439 | IgD+ CD24+ B cell %lymphocyte \|\| id:ebi-a-GCST90001439 | Cauliflower liking \|\| id:ebi-fl187-GCST90094727 | Weighted median | 4 | 0.416635977107569 | 0.404969950589648 | 0.303570310373923 | -0.377105126 | 1.21037708026328 | 1.51685024420867 | 0.685843970039878 | 3.35474942386987 | 0.999489402457283 |
| ebi-fl187-GCST90094727 | ebi-a-GCST90001457 | CD11c+ HLA DR++ monocyte %monocyte \|\| id:ebi-a-GCST90001457 | Cauliflower liking \|\| id:ebi-fl187-GCST90094727 | Weighted median | 4 | -0.103258259 | 0.442867914982262 | 0.815638609854057 | -0.971279372 | 0.764762854230924 | 0.901894021032652 | 0.378598359797564 | 2.14848481015442 | 0.999489402457283 |
| ebi-fl187-GCST90094727 | ebi-a-GCST90001483 | CD39+ resting CD4 regulatory T cell Absolute Count \|\| id:ebi-a-GCST90001483 | Cauliflower liking \|\| id:ebi-fl187-GCST90094727 | Weighted median | 4 | -0.119812676 | 0.400306609311594 | 0.764709425270974 | -0.90441363 | 0.664788278723753 | 0.887086594182778 | 0.404779165978539 | 1.9440788749007 | 0.999489402457283 |
| ebi-fl187-GCST90094727 | ebi-a-GCST90001525 | CD33dim HLA DR+ CD11b+ Absolute Count \|\| id:ebi-a-GCST90001525 | Cauliflower liking \|\| id:ebi-fl187-GCST90094727 | Weighted median | 4 | -0.239080539 | 0.527641318606806 | 0.650468195374461 | -1.273257524 | 0.795096444984429 | 0.787351466937407 | 0.279918294443805 | 2.21465457883082 | 0.999489402457283 |
| ebi-fl187-GCST90094727 | ebi-a-GCST90001540 | Naive CD4+ T cell Absolute Count \|\| id:ebi-a-GCST90001540 | Cauliflower liking \|\| id:ebi-fl187-GCST90094727 | Weighted median | 4 | -0.710369384 | 0.405042574988784 | 0.0794623643605666 | -1.504252831 | 0.0835140634432461 | 0.491462625726327 | 0.222183240366908 | 1.08710050356161 | 0.999489402457283 |
| ebi-fl187-GCST90094727 | ebi-a-GCST90001624 | HLA DR+ CD4+ T cell Absolute Count \|\| id:ebi-a-GCST90001624 | Cauliflower liking \|\| id:ebi-fl187-GCST90094727 | Weighted median | 4 | -0.323751635 | 0.402819015768019 | 0.421561661449715 | -1.113276906 | 0.465773636041648 | 0.723429894833672 | 0.328480794903684 | 1.5932463049857 | 0.999489402457283 |
| ebi-fl187-GCST90094727 | ebi-a-GCST90001672 | CD39+ CD8+ T cell Absolute Count \|\| id:ebi-a-GCST90001672 | Cauliflower liking \|\| id:ebi-fl187-GCST90094727 | Weighted median | 4 | 0.0665673680127123 | 0.461348243515887 | 0.885272450181084 | -0.837675189 | 0.970809925303851 | 1.0688329668027 | 0.432715336229787 | 2.64008186277365 | 0.999489402457283 |
| ebi-fl187-GCST90094727 | ebi-a-GCST90001722 | CD19 on CD20- CD38- B cell \|\| id:ebi-a-GCST90001722 | Cauliflower liking \|\| id:ebi-fl187-GCST90094727 | Weighted median | 4 | -0.743789838 | 0.408694147775449 | 0.0687714924353421 | -1.544830368 | 0.0572506912334789 | 0.475309152919207 | 0.213348058801581 | 1.05892123939541 | 0.999489402457283 |
| ebi-fl187-GCST90094727 | ebi-a-GCST90001742 | CD19 on transitional B cell \|\| id:ebi-a-GCST90001742 | Cauliflower liking \|\| id:ebi-fl187-GCST90094727 | Weighted median | 4 | -0.408432822 | 0.392233002195794 | 0.29773563021241 | -1.177209507 | 0.360343861840318 | 0.664691123303954 | 0.308137395322482 | 1.43382236659945 | 0.999489402457283 |
| ebi-fl187-GCST90094727 | ebi-a-GCST90001784 | CD25 on IgD+ CD38dim B cell \|\| id:ebi-a-GCST90001784 | Cauliflower liking \|\| id:ebi-fl187-GCST90094727 | Weighted median | 4 | 0.5477120054997 | 0.406694268385635 | 0.17806353598007 | -0.249408761 | 1.34483277153554 | 1.7292918777563 | 0.779261376976067 | 3.83754474022361 | 0.999489402457283 |
| ebi-fl187-GCST90094727 | ebi-a-GCST90001794 | CD25 on IgD+ B cell \|\| id:ebi-a-GCST90001794 | Cauliflower liking \|\| id:ebi-fl187-GCST90094727 | Weighted median | 4 | 0.716407152382898 | 0.405817386108125 | 0.0775060248566432 | -0.078994924 | 1.51180922915482 | 2.04706518919356 | 0.924044614524169 | 4.53492810081028 | 0.999489402457283 |
| ebi-fl187-GCST90094727 | ebi-a-GCST90001802 | CD27 on IgD- CD38- B cell \|\| id:ebi-a-GCST90001802 | Cauliflower liking \|\| id:ebi-fl187-GCST90094727 | Weighted median | 4 | 0.172137292187188 | 0.399781783337977 | 0.66677514688798 | -0.611435003 | 0.955709587529623 | 1.18784090329517 | 0.542571718038774 | 2.60051522154764 | 0.999489402457283 |
| ebi-fl187-GCST90094727 | ebi-a-GCST90001804 | CD27 on IgD- CD38dim B cell \|\| id:ebi-a-GCST90001804 | Cauliflower liking \|\| id:ebi-fl187-GCST90094727 | Weighted median | 4 | 0.946574970757317 | 0.428224051802147 | 0.0270727949461446 | 0.107255829225109 | 1.78589411228953 | 2.57686867707363 | 1.11321901201034 | 5.96491086411798 | 0.999489402457283 |
| ebi-fl187-GCST90094727 | ebi-a-GCST90001805 | CD27 on memory B cell \|\| id:ebi-a-GCST90001805 | Cauliflower liking \|\| id:ebi-fl187-GCST90094727 | Weighted median | 4 | 0.450791471038884 | 0.396086746634058 | 0.255073305116374 | -0.325538552 | 1.22712149444164 | 1.56955395051062 | 0.72213833958943 | 3.41139566826503 | 0.999489402457283 |
| ebi-fl187-GCST90094727 | ebi-a-GCST90001806 | CD27 on unswitched memory B cell \|\| id:ebi-a-GCST90001806 | Cauliflower liking \|\| id:ebi-fl187-GCST90094727 | Weighted median | 4 | 0.602749296756723 | 0.461359313116757 | 0.191394840952343 | -0.301514957 | 1.50701355046557 | 1.82713523837735 | 0.739696762662837 | 4.51323210784679 | 0.999489402457283 |
| ebi-fl187-GCST90094727 | ebi-a-GCST90001839 | CD3 on Effector Memory CD8+ T cell \|\| id:ebi-a-GCST90001839 | Cauliflower liking \|\| id:ebi-fl187-GCST90094727 | Weighted median | 4 | 0.227598614713906 | 0.466533444538796 | 0.625655676808028 | -0.686806937 | 1.14200416600995 | 1.25558125243515 | 0.503180192935166 | 3.13304121187803 | 0.999489402457283 |
| ebi-fl187-GCST90094727 | ebi-a-GCST90001859 | CD3 on CD8+ T cell \|\| id:ebi-a-GCST90001859 | Cauliflower liking \|\| id:ebi-fl187-GCST90094727 | Weighted median | 4 | -0.078464734 | 0.483844195371139 | 0.871172437613901 | -1.026799357 | 0.86986988873573 | 0.924534663818867 | 0.358151442918537 | 2.38660030973848 | 0.999489402457283 |
| ebi-fl187-GCST90094727 | ebi-a-GCST90001905 | CD86 on monocyte \|\| id:ebi-a-GCST90001905 | Cauliflower liking \|\| id:ebi-fl187-GCST90094727 | Weighted median | 4 | 0.248844081623147 | 0.437904963467372 | 0.569858114479564 | -0.609449647 | 1.10713781001919 | 1.28254204560536 | 0.543649986281252 | 3.02568590132291 | 0.999489402457283 |
| ebi-fl187-GCST90094727 | ebi-a-GCST90001943 | CD25 on activated & secreting CD4 regulatory T cell \|\| id:ebi-a-GCST90001943 | Cauliflower liking \|\| id:ebi-fl187-GCST90094727 | Weighted median | 4 | 0.531892918252221 | 0.415686379271507 | 0.200702249151228 | -0.282852385 | 1.34663822162438 | 1.702151294 | 0.75363102680289 | 3.84447949400792 | 0.999489402457283 |
| ebi-fl187-GCST90094727 | ebi-a-GCST90001949 | CD33 on CD33dim HLA DR+ CD11b- \|\| id:ebi-a-GCST90001949 | Cauliflower liking \|\| id:ebi-fl187-GCST90094727 | Weighted median | 4 | -0.510523118 | 0.619643552917748 | 0.409997485817303 | -1.725024482 | 0.703978245820373 | 0.600181530976255 | 0.178168689861843 | 2.02177986718275 | 0.999489402457283 |
| ebi-fl187-GCST90094727 | ebi-a-GCST90001955 | CD33 on Immature Myeloid-Derived Suppressor Cells \|\| id:ebi-a-GCST90001955 | Cauliflower liking \|\| id:ebi-fl187-GCST90094727 | Weighted median | 4 | -0.478375469 | 0.625916104750627 | 0.444700107595547 | -1.705171034 | 0.748420096260371 | 0.619789441535149 | 0.181741299509772 | 2.11365800109621 | 0.999489402457283 |
| ebi-fl187-GCST90094727 | ebi-a-GCST90001956 | CD33 on CD33+ HLA DR+ \|\| id:ebi-a-GCST90001956 | Cauliflower liking \|\| id:ebi-fl187-GCST90094727 | Weighted median | 4 | -0.321960125 | 0.584395649180498 | 0.581682799516172 | -1.467375597 | 0.823455347758317 | 0.724727088510584 | 0.230529694811079 | 2.27835877391613 | 0.999489402457283 |
| ebi-fl187-GCST90094727 | ebi-a-GCST90001957 | CD33 on CD33+ HLA DR+ CD14- \|\| id:ebi-a-GCST90001957 | Cauliflower liking \|\| id:ebi-fl187-GCST90094727 | Weighted median | 4 | -0.369790514 | 0.587565889548253 | 0.529113347481645 | -1.521419658 | 0.781838629163887 | 0.690879044724343 | 0.218401611196496 | 2.18548687358254 | 0.999489402457283 |
| ebi-fl187-GCST90094727 | ebi-a-GCST90002064 | CD4 on resting CD4 regulatory T cell \|\| id:ebi-a-GCST90002064 | Cauliflower liking \|\| id:ebi-fl187-GCST90094727 | Weighted median | 4 | 0.375375187776877 | 0.434724971339545 | 0.387875040219452 | -0.476685756 | 1.22743613160239 | 1.4555374120318 | 0.620837593101436 | 3.41246918898818 | 0.999489402457283 |
| ebi-fl187-GCST90094727 | ebi-a-GCST90002080 | SSC-A on T cell \|\| id:ebi-a-GCST90002080 | Cauliflower liking \|\| id:ebi-fl187-GCST90094727 | Weighted median | 4 | 0.430291452340202 | 0.42970043898769 | 0.316645348747127 | -0.411921408 | 1.27250431275608 | 1.53770562614812 | 0.662376331437046 | 3.56978122626699 | 0.999489402457283 |
| ebi-fl187-GCST90094727 | ebi-a-GCST90002121 | CD8 on CD39+ CD8+ T cell \|\| id:ebi-a-GCST90002121 | Cauliflower liking \|\| id:ebi-fl187-GCST90094727 | Weighted median | 4 | -0.416364717 | 0.456841859299861 | 0.362085753088384 | -1.311774761 | 0.479045327294521 | 0.659439717788729 | 0.269341614913427 | 1.61453231628188 | 0.999489402457283 |
| ebi-fl187-GCST90094727 | ebi-cfmet338-GCST90026053 | 1-palmitoyl-2-docosahexaenoyl-gpc (16:0/22:6) levels \|\| id:ebi-cfmet338-GCST90026053 | Cauliflower liking \|\| id:ebi-fl187-GCST90094727 | Weighted median | 4 | 0.595328114200344 | 0.242952677925662 | 0.0142702696436507 | 0.119140865466046 | 1.07151536293464 | 1.81362592376402 | 1.12652859608768 | 2.91980070703228 | 0.999489402457283 |
| ebi-fl187-GCST90094727 | ebi-cfmet338-GCST90026063 | 1-stearoyl-2-oleoyl-gpc (18:0/18:1) levels \|\| id:ebi-cfmet338-GCST90026063 | Cauliflower liking \|\| id:ebi-fl187-GCST90094727 | Weighted median | 4 | 0.356589554960508 | 0.197945312703362 | 0.0716312085225735 | -0.031383258 | 0.744562367859097 | 1.42844944952473 | 0.969104085059281 | 2.10551979019125 | 0.999489402457283 |
| ebi-fl187-GCST90094727 | ebi-cfmet338-GCST90026113 | Adenosine 3',5'-cyclic monophosphate (camp) levels \|\| id:ebi-cfmet338-GCST90026113 | Cauliflower liking \|\| id:ebi-fl187-GCST90094727 | Weighted median | 4 | 0.434186193803855 | 0.343124454715385 | 0.205731620750325 | -0.238337737 | 1.10671012504601 | 1.54370626990187 | 0.78793653048475 | 3.02439213761071 | 0.999489402457283 |
| ebi-fl187-GCST90094727 | ebi-cfmet338-GCST90026117 | Alpha-ketoglutaramate levels \|\| id:ebi-cfmet338-GCST90026117 | Cauliflower liking \|\| id:ebi-fl187-GCST90094727 | Weighted median | 4 | 0.275319248297414 | 0.146171633308376 | 0.0596280300421533 | -0.011177153 | 0.561815649581831 | 1.31695104214126 | 0.988885079311669 | 1.75385399545536 | 0.999489402457283 |
| ebi-fl187-GCST90094727 | ebi-cfmet338-GCST90026124 | Arginine levels \|\| id:ebi-cfmet338-GCST90026124 | Cauliflower liking \|\| id:ebi-fl187-GCST90094727 | Weighted median | 4 | -0.114023349 | 0.114184357994954 | 0.317993383112646 | -0.337824691 | 0.109777992277521 | 0.892237122444332 | 0.713320328366328 | 1.116030276 | 0.999489402457283 |
| ebi-fl187-GCST90094727 | ebi-cfmet338-GCST90026169 | Glutamate levels \|\| id:ebi-cfmet338-GCST90026169 | Cauliflower liking \|\| id:ebi-fl187-GCST90094727 | Weighted median | 4 | 0.200222987262526 | 0.130777052917656 | 0.125763119946559 | -0.056100036 | 0.456546010981131 | 1.22167514578601 | 0.945444552237143 | 1.57861204900879 | 0.999489402457283 |
| ebi-fl187-GCST90094727 | ebi-cfmet338-GCST90026170 | Glutamine levels \|\| id:ebi-cfmet338-GCST90026170 | Cauliflower liking \|\| id:ebi-fl187-GCST90094727 | Weighted median | 4 | 0.032608220636264 | 0.0775119553217688 | 0.673984138683077 | -0.119315212 | 0.184531653066931 | 1.03314569477868 | 0.887527997372968 | 1.20265504840314 | 0.999489402457283 |
| ebi-fl187-GCST90094727 | ebi-cfmet338-GCST90026191 | Kynurenate levels \|\| id:ebi-cfmet338-GCST90026191 | Cauliflower liking \|\| id:ebi-fl187-GCST90094727 | Weighted median | 4 | -0.332962119 | 0.584445834693813 | 0.568877328077033 | -1.478475955 | 0.812551716659859 | 0.716797346397981 | 0.227984882889998 | 2.253651336 | 0.999489402457283 |
| ebi-fl187-GCST90094727 | ebi-cfmet338-GCST90026202 | Methylsuccinoylcarnitine levels \|\| id:ebi-cfmet338-GCST90026202 | Cauliflower liking \|\| id:ebi-fl187-GCST90094727 | Weighted median | 4 | 0.430189821926973 | 0.389676924867344 | 0.269608169645369 | -0.333576951 | 1.19395659466697 | 1.53754935643092 | 0.716356772282943 | 3.30011261836356 | 0.999489402457283 |
| ebi-fl187-GCST90094727 | ebi-cfmet338-GCST90026222 | N-acetylputrescine levels \|\| id:ebi-cfmet338-GCST90026222 | Cauliflower liking \|\| id:ebi-fl187-GCST90094727 | Weighted median | 4 | -0.070100777 | 0.142586548892815 | 0.62297621302588 | -0.349570412 | 0.209368859258297 | 0.932299861187877 | 0.704990880015663 | 1.23289968113009 | 0.999489402457283 |
| ebi-fl187-GCST90094727 | ebi-cfmet338-GCST90026252 | Propionylcarnitine (c3) levels \|\| id:ebi-cfmet338-GCST90026252 | Cauliflower liking \|\| id:ebi-fl187-GCST90094727 | Weighted median | 4 | -0.017910972 | 0.21778392438404 | 0.934454324806422 | -0.444767464 | 0.408945519821138 | 0.982248476111016 | 0.640973308224234 | 1.5052297130677 | 0.999489402457283 |
| ebi-fl187-GCST90094727 | ebi-cfmet338-GCST90026316 | Ethyl beta-glucopyranoside levels \|\| id:ebi-cfmet338-GCST90026316 | Cauliflower liking \|\| id:ebi-fl187-GCST90094727 | Weighted median | 4 | -0.689069985 | 0.71452334215653 | 0.3348568736092 | -2.089535735 | 0.711395765858963 | 0.502042759431313 | 0.12374457270868 | 2.03683221639769 | 0.999489402457283 |
| ebi-fl187-GCST90094727 | ebi-gm430-GCST90011315 | F_Prevotellaceae abundance \|\| id:ebi-gm430-GCST90011315 | Cauliflower liking \|\| id:ebi-fl187-GCST90094727 | Weighted median | 4 | -0.066105019 | 0.315673953133199 | 0.834128837891831 | -0.684825967 | 0.55261592886148 | 0.936032557664785 | 0.504177965331918 | 1.73779302003346 | 0.999489402457283 |
| ebi-fl187-GCST90094727 | ebi-gm430-GCST90011354 | OTU97_1 (Bacteroides) abundance \|\| id:ebi-gm430-GCST90011354 | Cauliflower liking \|\| id:ebi-fl187-GCST90094727 | Weighted median | 4 | -0.537485238 | 0.294543215840293 | 0.0680297920403908 | -1.114789941 | 0.0398194646673301 | 0.584215569516527 | 0.327984167550337 | 1.04062288803358 | 0.999489402457283 |
| ebi-fl187-GCST90094727 | ebi-gm430-GCST90011419 | OTU99_1 (Bacteroides) abundance \|\| id:ebi-gm430-GCST90011419 | Cauliflower liking \|\| id:ebi-fl187-GCST90094727 | Weighted median | 4 | -0.530464136 | 0.277327285900736 | 0.0557771233125843 | -1.074025616 | 0.0130973448602578 | 0.588331840602754 | 0.341630472487999 | 1.01318349076481 | 0.999489402457283 |
| ebi-fl187-GCST90094727 | ebi-gm430-GCST90011435 | OTU99_155 (Butyrivibrio) abundance \|\| id:ebi-gm430-GCST90011435 | Cauliflower liking \|\| id:ebi-fl187-GCST90094727 | Weighted median | 4 | -0.469675332 | 0.9233524400439 | 0.610988378131249 | -2.279446114 | 1.34009545057956 | 0.625205219521698 | 0.102340876158254 | 3.81940805268012 | 0.999489402457283 |
| ebi-fl187-GCST90094727 | ebi-gm430-GCST90011466 | OTU99_58 (Bacteroides) abundance \|\| id:ebi-gm430-GCST90011466 | Cauliflower liking \|\| id:ebi-fl187-GCST90094727 | Weighted median | 4 | -0.644057951 | 0.5283992307101 | 0.22288780472232 | -1.679720443 | 0.391604541656603 | 0.525157033054485 | 0.186426085523365 | 1.47935257339309 | 0.999489402457283 |
| ebi-fl187-GCST90094727 | ebi-gm430-GCST90011472 | OTU99_72 (Parabacteroides) abundance \|\| id:ebi-gm430-GCST90011472 | Cauliflower liking \|\| id:ebi-fl187-GCST90094727 | Weighted median | 4 | -0.187907788 | 0.828239264576201 | 0.820519998959605 | -1.811256746 | 1.43544117066047 | 0.828691119050556 | 0.163448594227135 | 4.201498178 | 0.999489402457283 |
| ebi-fl187-GCST90094727 | ebi-gm430-GCST90011505 | TestASV_28 (Holdemanella) abundance \|\| id:ebi-gm430-GCST90011505 | Cauliflower liking \|\| id:ebi-fl187-GCST90094727 | Weighted median | 4 | -0.064330415 | 0.695379776903426 | 0.926291896356831 | -1.427274777 | 1.29861394799171 | 0.937695120050822 | 0.239961981772442 | 3.66421435459284 | 0.999489402457283 |
| ebi-fl187-GCST90094727 | ebi-gm430-GCST90011513 | TestASV_35 (Alphaproteobacteria) abundance \|\| id:ebi-gm430-GCST90011513 | Cauliflower liking \|\| id:ebi-fl187-GCST90094727 | Weighted median | 4 | 0.404531605050719 | 0.815348620128152 | 0.6197917106895 | -1.19355169 | 2.0026149005019 | 1.49860039878148 | 0.30314268085414 | 7.40840302955754 | 0.999489402457283 |
| ebi-fl187-GCST90094727 | ebi-gm430-GCST90011543 | G_Coprococcus prevalence \|\| id:ebi-gm430-GCST90011543 | Cauliflower liking \|\| id:ebi-fl187-GCST90094727 | Weighted median | 4 | 0.129430471037275 | 0.888101122364141 | 0.884127875572684 | -1.611247729 | 1.87010867087099 | 1.13817997223872 | 0.19963836407355 | 6.48900152642037 | 0.999489402457283 |
| ebi-fl187-GCST90094727 | ebi-gm430-GCST90011547 | G_Parasutterella prevalence \|\| id:ebi-gm430-GCST90011547 | Cauliflower liking \|\| id:ebi-fl187-GCST90094727 | Weighted median | 4 | 1.14589558294477 | 0.76380287101502 | 0.133549545463739 | -0.351158044 | 2.64294921013421 | 3.14525693432668 | 0.703872502066565 | 14.0545924920853 | 0.999489402457283 |
| ebi-fl187-GCST90094727 | ebi-gm430-GCST90011569 | OTU97_13 (Subdoligranulum) prevalence \|\| id:ebi-gm430-GCST90011569 | Cauliflower liking \|\| id:ebi-fl187-GCST90094727 | Weighted median | 4 | -0.097328788 | 0.77401027569118 | 0.89993289451114 | -1.614388928 | 1.41973135271753 | 0.907257661987028 | 0.199012244103548 | 4.13600916336531 | 0.999489402457283 |
| ebi-fl187-GCST90094727 | ebi-gm430-GCST90011590 | OTU97_277 (Prevotella) prevalence \|\| id:ebi-gm430-GCST90011590 | Cauliflower liking \|\| id:ebi-fl187-GCST90094727 | Weighted median | 4 | 7.70723869823187e-05 | 0.691235518283428 | 0.999911036302558 | -1.354744543 | 1.3548986882225 | 1.00007707535714 | 0.258013197195411 | 3.87636821498474 | 0.999911036302558 |
| ebi-fl187-GCST90094727 | ebi-gm430-GCST90011616 | OTU97_86 (Phascolarctobacterium) prevalence \|\| id:ebi-gm430-GCST90011616 | Cauliflower liking \|\| id:ebi-fl187-GCST90094727 | Weighted median | 4 | 0.164202103154079 | 0.856160937357862 | 0.847907622113265 | -1.513873334 | 1.84227754037549 | 1.17845245998609 | 0.220055974803391 | 6.31089522421759 | 0.999489402457283 |
| ebi-fl187-GCST90094727 | ebi-gm430-GCST90011665 | OTU99_55 (Barnesiella) prevalence \|\| id:ebi-gm430-GCST90011665 | Cauliflower liking \|\| id:ebi-fl187-GCST90094727 | Weighted median | 4 | -0.437119203 | 0.61348890705764 | 0.476146779648064 | -1.639557461 | 0.765319055039836 | 0.645894434400933 | 0.194065905094788 | 2.14968013153231 | 0.999489402457283 |
| ebi-fl187-GCST90094727 | ebi-gm430-GCST90011687 | TestASV_14 (Lachnospiraceae) prevalence \|\| id:ebi-gm430-GCST90011687 | Cauliflower liking \|\| id:ebi-fl187-GCST90094727 | Weighted median | 4 | 0.669102330678006 | 0.591372603867552 | 0.25787014875157 | -0.489987973 | 1.82819263425841 | 1.95248384888196 | 0.612633762346163 | 6.22262992092633 | 0.999489402457283 |
| ebi-fl187-GCST90094727 | ebi-gm430-GCST90011697 | TestASV_23 (Barnesiella) prevalence \|\| id:ebi-gm430-GCST90011697 | Cauliflower liking \|\| id:ebi-fl187-GCST90094727 | Weighted median | 4 | -0.031079839 | 0.866296158355482 | 0.971380681973496 | -1.72902031 | 1.66686063099145 | 0.969398173832012 | 0.177458178881623 | 5.29551709225927 | 0.999489402457283 |
| ebi-fl187-GCST90094727 | ebi-gm430-GCST90011727 | TestASV_6 (Subdoligranulum) prevalence \|\| id:ebi-gm430-GCST90011727 | Cauliflower liking \|\| id:ebi-fl187-GCST90094727 | Weighted median | 4 | -0.391635424 | 0.956101020650542 | 0.682086776807351 | -2.265593425 | 1.48232257642546 | 0.675950504341438 | 0.1037684375303 | 4.40316049074198 | 0.999489402457283 |
| ebi-fl187-GCST90094727 | ebi-hbsm44-GCST90104908 | Sulcal depth of the left posterior inferior temporal sulcus \|\| id:ebi-hbsm44-GCST90104908 | Cauliflower liking \|\| id:ebi-fl187-GCST90094727 | Weighted median | 4 | -0.260581715 | 0.272465293129037 | 0.338877768219669 | -0.79461369 | 0.27345025948756 | 0.770603183929065 | 0.451755719160273 | 1.31449197408154 | 0.999489402457283 |
| ebi-fl187-GCST90094727 | ebi-hbsm44-GCST90104918 | Sulcal depth of the left inferior frontal sulcus \|\| id:ebi-hbsm44-GCST90104918 | Cauliflower liking \|\| id:ebi-fl187-GCST90094727 | Weighted median | 4 | -0.328022531 | 0.45845509146217 | 0.474302952199801 | -1.226594511 | 0.570549447914277 | 0.720346789135691 | 0.293289673787218 | 1.76923888903965 | 0.999489402457283 |
| ebi-fl187-GCST90094727 | ebi-hbsm44-GCST90104925 | Sulcal depth of the right anterior inferior temporal sulcus \|\| id:ebi-hbsm44-GCST90104925 | Cauliflower liking \|\| id:ebi-fl187-GCST90094727 | Weighted median | 4 | -0.091372412 | 0.149002553332675 | 0.539726153280371 | -0.383417416 | 0.200672592990976 | 0.912677755872045 | 0.681528359012947 | 1.22222454142632 | 0.999489402457283 |
| ebi-fl187-GCST90094727 | ebi-IPs91-GCST90274770 | C-C motif chemokine 4 levels \|\| id:ebi-IPs91-GCST90274770 | Cauliflower liking \|\| id:ebi-fl187-GCST90094727 | Weighted median | 4 | -0.027770856 | 0.204506789330475 | 0.891983896853049 | -0.428604163 | 0.373062450679436 | 0.972611208894592 | 0.651417733071658 | 1.45217502631808 | 0.999489402457283 |
| ebi-fl187-GCST90094727 | ebi-IPs91-GCST90274779 | C-X-C motif chemokine 1 levels \|\| id:ebi-IPs91-GCST90274779 | Cauliflower liking \|\| id:ebi-fl187-GCST90094727 | Weighted median | 4 | -0.387511851 | 0.202462193730274 | 0.0556206530769294 | -0.78433775 | 0.00931404893579696 | 0.678743590565811 | 0.456421866879372 | 1.00935755967171 | 0.999489402457283 |
| ebi-fl187-GCST90094727 | ebi-IPs91-GCST90274780 | C-X-C motif chemokine 10 levels \|\| id:ebi-IPs91-GCST90274780 | Cauliflower liking \|\| id:ebi-fl187-GCST90094727 | Weighted median | 4 | 0.0112971594380216 | 0.199426362774834 | 0.954825378380509 | -0.379578512 | 0.402172830476697 | 1.01136121332545 | 0.684149709616469 | 1.49506970395781 | 0.999489402457283 |
| ebi-fl187-GCST90094727 | ebi-IPs91-GCST90274796 | Interleukin-10 receptor subunit alpha levels \|\| id:ebi-IPs91-GCST90274796 | Cauliflower liking \|\| id:ebi-fl187-GCST90094727 | Weighted median | 4 | 0.209490842548012 | 0.255303068467588 | 0.411898375566676 | -0.290903172 | 0.709884856744484 | 1.23305008346219 | 0.74758806223331 | 2.03375707175432 | 0.999489402457283 |
| ebi-fl187-GCST90094727 | ebi-IPs91-GCST90274807 | Interleukin-20 levels \|\| id:ebi-IPs91-GCST90274807 | Cauliflower liking \|\| id:ebi-fl187-GCST90094727 | Weighted median | 4 | -0.071172081 | 0.227265102185336 | 0.75415328306556 | -0.516611682 | 0.374267518830659 | 0.9313016186026 | 0.596538389415282 | 1.45392605103246 | 0.999489402457283 |
| ebi-fl187-GCST90094727 | ebi-IPs91-GCST90274808 | Interleukin-20 receptor subunit alpha levels \|\| id:ebi-IPs91-GCST90274808 | Cauliflower liking \|\| id:ebi-fl187-GCST90094727 | Weighted median | 4 | -0.087953534 | 0.235214972658604 | 0.708457663563492 | -0.54897488 | 0.373067812395097 | 0.915803429442404 | 0.577541556177238 | 1.45218281248854 | 0.999489402457283 |
| ebi-fl187-GCST90094727 | ebi-IPs91-GCST90274810 | Interleukin-24 levels \|\| id:ebi-IPs91-GCST90274810 | Cauliflower liking \|\| id:ebi-fl187-GCST90094727 | Weighted median | 4 | 0.039867339074718 | 0.220304789674531 | 0.856395465878844 | -0.391930049 | 0.4716647268368 | 1.04067270843021 | 0.675751382003563 | 1.60265996476461 | 0.999489402457283 |
| ebi-fl187-GCST90094727 | ebi-IPs91-GCST90274823 | Monocyte chemoattractant protein-3 levels \|\| id:ebi-IPs91-GCST90274823 | Cauliflower liking \|\| id:ebi-fl187-GCST90094727 | Weighted median | 4 | -0.201765937 | 0.228291344504824 | 0.376799346116002 | -0.649216972 | 0.245685098341391 | 0.817286202108058 | 0.522454713243591 | 1.27849690934797 | 0.999489402457283 |
| ebi-fl187-GCST90094727 | ebi-IPs91-GCST90274824 | Monocyte chemoattractant protein-4 levels \|\| id:ebi-IPs91-GCST90274824 | Cauliflower liking \|\| id:ebi-fl187-GCST90094727 | Weighted median | 4 | -0.291611328 | 0.213917278567462 | 0.172820526607116 | -0.710889194 | 0.127666537500621 | 0.747058840039461 | 0.491207224458359 | 1.13617406807586 | 0.999489402457283 |
| ebi-fl187-GCST90094727 | ebi-IPs91-GCST90274834 | SIR2-like protein 2 levels \|\| id:ebi-IPs91-GCST90274834 | Cauliflower liking \|\| id:ebi-fl187-GCST90094727 | Weighted median | 4 | -0.262846461 | 0.211471575890574 | 0.213889978695616 | -0.67733075 | 0.151637827966847 | 0.768859938405208 | 0.507971086417623 | 1.16373868649377 | 0.999489402457283 |
| ebi-fl187-GCST90094727 | ebi-met1400-GCST90199652 | 1-stearoyl-2-oleoyl-GPS (18:0/18:1) levels \|\| id:ebi-met1400-GCST90199652 | Cauliflower liking \|\| id:ebi-fl187-GCST90094727 | Weighted median | 4 | 0.0340902958338786 | 0.244672441958958 | 0.889189111627837 | -0.44546769 | 0.513648282073437 | 1.03467802962569 | 0.640524638740897 | 1.67137774293043 | 0.999489402457283 |
| ebi-fl187-GCST90094727 | ebi-met1400-GCST90199665 | N-acetylaspartate (naa) levels \|\| id:ebi-met1400-GCST90199665 | Cauliflower liking \|\| id:ebi-fl187-GCST90094727 | Weighted median | 4 | -0.189195929 | 0.258952235757148 | 0.465010710354059 | -0.696742311 | 0.318350452910524 | 0.82762433505625 | 0.498205662023641 | 1.37485799979688 | 0.999489402457283 |
| ebi-fl187-GCST90094727 | ebi-met1400-GCST90199669 | Acetylcarnitine levels (Biocrates platform) \|\| id:ebi-met1400-GCST90199669 | Cauliflower liking \|\| id:ebi-fl187-GCST90094727 | Weighted median | 4 | 0.105080026329291 | 0.253360356856539 | 0.678328340253154 | -0.391506273 | 0.601666325768107 | 1.11079950000547 | 0.676037809622175 | 1.82515757499123 | 0.999489402457283 |
| ebi-fl187-GCST90094727 | ebi-met1400-GCST90199670 | Iminodiacetate (IDA) levels \|\| id:ebi-met1400-GCST90199670 | Cauliflower liking \|\| id:ebi-fl187-GCST90094727 | Weighted median | 4 | 0.116283734012911 | 0.237581295604388 | 0.62452442929038 | -0.349375605 | 0.581943073397512 | 1.1233145494662 | 0.705128230573061 | 1.78951220832124 | 0.999489402457283 |
| ebi-fl187-GCST90094727 | ebi-met1400-GCST90199686 | Indolepropionate levels \|\| id:ebi-met1400-GCST90199686 | Cauliflower liking \|\| id:ebi-fl187-GCST90094727 | Weighted median | 4 | 0.198493028895353 | 0.248225284651798 | 0.423914356709148 | -0.288028529 | 0.685014586812877 | 1.21956352567966 | 0.749740202579069 | 1.98380077265676 | 0.999489402457283 |
| ebi-fl187-GCST90094727 | ebi-met1400-GCST90199704 | 3-hydroxysebacate levels \|\| id:ebi-met1400-GCST90199704 | Cauliflower liking \|\| id:ebi-fl187-GCST90094727 | Weighted median | 4 | 0.0277637505552727 | 0.253238788530027 | 0.912699115129023 | -0.468584275 | 0.524111776074126 | 1.0281527552091 | 0.625887726276361 | 1.68895800902364 | 0.999489402457283 |
| ebi-fl187-GCST90094727 | ebi-met1400-GCST90199713 | Docosapentaenoate n3 DPA; 22:5n3 levels \|\| id:ebi-met1400-GCST90199713 | Cauliflower liking \|\| id:ebi-fl187-GCST90094727 | Weighted median | 4 | 0.396868983799607 | 0.231398927647946 | 0.0863295220443442 | -0.056672914 | 0.85041088198958 | 1.48716107509358 | 0.944903083027764 | 2.34060836820076 | 0.999489402457283 |
| ebi-fl187-GCST90094727 | ebi-met1400-GCST90199715 | Myristoleate (14:1n5) levels \|\| id:ebi-met1400-GCST90199715 | Cauliflower liking \|\| id:ebi-fl187-GCST90094727 | Weighted median | 4 | 0.274250567595186 | 0.229025964350305 | 0.23112537664234 | -0.174640323 | 0.723141457721783 | 1.31554439373963 | 0.839759008851325 | 2.06089727369172 | 0.999489402457283 |
| ebi-fl187-GCST90094727 | ebi-met1400-GCST90199725 | Campesterol levels \|\| id:ebi-met1400-GCST90199725 | Cauliflower liking \|\| id:ebi-fl187-GCST90094727 | Weighted median | 4 | -0.294263834 | 0.325457534442489 | 0.365913580392972 | -0.932160602 | 0.343632933038871 | 0.745079887752908 | 0.393702157110556 | 1.41006095371226 | 0.999489402457283 |
| ebi-fl187-GCST90094727 | ebi-met1400-GCST90199737 | N-acetylproline levels \|\| id:ebi-met1400-GCST90199737 | Cauliflower liking \|\| id:ebi-fl187-GCST90094727 | Weighted median | 4 | 0.0570183462080917 | 0.282629065101203 | 0.84011789150132 | -0.496934621 | 0.61097131380645 | 1.05867523289346 | 0.608392758383674 | 1.84221990366823 | 0.999489402457283 |
| ebi-fl187-GCST90094727 | ebi-met1400-GCST90199775 | Gamma-glutamylisoleucine levels \|\| id:ebi-met1400-GCST90199775 | Cauliflower liking \|\| id:ebi-fl187-GCST90094727 | Weighted median | 4 | -0.118630835 | 0.235188087213746 | 0.613974452023468 | -0.579599486 | 0.34233781608626 | 0.888135608963466 | 0.560122658729558 | 1.40823594192384 | 0.999489402457283 |
| ebi-fl187-GCST90094727 | ebi-met1400-GCST90199777 | 1-palmitoleoyl-GPC (16:1) levels \|\| id:ebi-met1400-GCST90199777 | Cauliflower liking \|\| id:ebi-fl187-GCST90094727 | Weighted median | 4 | -0.4573582 | 0.238259668914318 | 0.0549111289068531 | -0.924347151 | 0.00963075095458038 | 0.632953575406549 | 0.396790378661775 | 1.00967727587324 | 0.999489402457283 |
| ebi-fl187-GCST90094727 | ebi-met1400-GCST90199782 | Glutamine degradant levels \|\| id:ebi-met1400-GCST90199782 | Cauliflower liking \|\| id:ebi-fl187-GCST90094727 | Weighted median | 4 | -0.29041799 | 0.245926331238915 | 0.237636658864481 | -0.772433599 | 0.191597619158787 | 0.747950866195549 | 0.461887649997374 | 1.21118306204085 | 0.999489402457283 |
| ebi-fl187-GCST90094727 | ebi-met1400-GCST90199783 | 5-methyluridine (ribothymidine) levels \|\| id:ebi-met1400-GCST90199783 | Cauliflower liking \|\| id:ebi-fl187-GCST90094727 | Weighted median | 4 | 0.0293170664139725 | 0.258005082466109 | 0.909531261839518 | -0.476372895 | 0.535007028047545 | 1.02975104218982 | 0.621031859253057 | 1.70746024232381 | 0.999489402457283 |
| ebi-fl187-GCST90094727 | ebi-met1400-GCST90199787 | Homostachydrine levels \|\| id:ebi-met1400-GCST90199787 | Cauliflower liking \|\| id:ebi-fl187-GCST90094727 | Weighted median | 4 | -0.05190046 | 0.232969773432434 | 0.823708558915012 | -0.508521216 | 0.404720295875349 | 0.949423367691946 | 0.601384238984706 | 1.49888319760645 | 0.999489402457283 |
| ebi-fl187-GCST90094727 | ebi-met1400-GCST90199802 | Beta-hydroxyisovaleroylcarnitine levels \|\| id:ebi-met1400-GCST90199802 | Cauliflower liking \|\| id:ebi-fl187-GCST90094727 | Weighted median | 4 | -0.034798915 | 0.243836117746809 | 0.886515778121265 | -0.512717705 | 0.443119876204914 | 0.96579960495298 | 0.598865824356838 | 1.55755903741726 | 0.999489402457283 |
| ebi-fl187-GCST90094727 | ebi-met1400-GCST90199873 | Pregnenolone sulfate levels \|\| id:ebi-met1400-GCST90199873 | Cauliflower liking \|\| id:ebi-fl187-GCST90094727 | Weighted median | 4 | -0.182929652 | 0.263014062384318 | 0.486733981141497 | -0.698437214 | 0.332577910583966 | 0.832826741630738 | 0.497361967091782 | 1.39455854582318 | 0.999489402457283 |
| ebi-fl187-GCST90094727 | ebi-met1400-GCST90199911 | Beta-citrylglutamate levels \|\| id:ebi-met1400-GCST90199911 | Cauliflower liking \|\| id:ebi-fl187-GCST90094727 | Weighted median | 4 | -0.326759062 | 0.257258391772225 | 0.204027998505932 | -0.83098551 | 0.177467385992153 | 0.721257500518559 | 0.435619767178634 | 1.19418910997434 | 0.999489402457283 |
| ebi-fl187-GCST90094727 | ebi-met1400-GCST90199934 | 2-hydroxydecanoate levels \|\| id:ebi-met1400-GCST90199934 | Cauliflower liking \|\| id:ebi-fl187-GCST90094727 | Weighted median | 4 | -0.21597864 | 0.236111980931789 | 0.360333550811027 | -0.678758123 | 0.246800842168636 | 0.805752512194568 | 0.507246539142568 | 1.27992418046914 | 0.999489402457283 |
| ebi-fl187-GCST90094727 | ebi-met1400-GCST90199945 | Sphingomyelin (d18:1/14:0, d16:1/16:0) levels \|\| id:ebi-met1400-GCST90199945 | Cauliflower liking \|\| id:ebi-fl187-GCST90094727 | Weighted median | 4 | -0.244082218 | 0.243079250513745 | 0.315317840473294 | -0.720517549 | 0.232353113192767 | 0.783423220270168 | 0.486500403082428 | 1.26156512547533 | 0.999489402457283 |
| ebi-fl187-GCST90094727 | ebi-met1400-GCST90199979 | Carnitine C14:1 levels \|\| id:ebi-met1400-GCST90199979 | Cauliflower liking \|\| id:ebi-fl187-GCST90094727 | Weighted median | 4 | 0.0627825881196792 | 0.261870234758115 | 0.810526441129126 | -0.450483072 | 0.576048248245584 | 1.06479531491258 | 0.63732020569736 | 1.77899437758948 | 0.999489402457283 |
| ebi-fl187-GCST90094727 | ebi-met1400-GCST90199986 | Eugenol sulfate levels \|\| id:ebi-met1400-GCST90199986 | Cauliflower liking \|\| id:ebi-fl187-GCST90094727 | Weighted median | 4 | 0.120034641815587 | 0.2544467565292 | 0.63710697063581 | -0.378681001 | 0.61875028461282 | 1.12753591079392 | 0.68476401687825 | 1.85660636189053 | 0.999489402457283 |
| ebi-fl187-GCST90094727 | ebi-met1400-GCST90200002 | 6-hydroxyindole sulfate levels \|\| id:ebi-met1400-GCST90200002 | Cauliflower liking \|\| id:ebi-fl187-GCST90094727 | Weighted median | 4 | -0.042679356 | 0.23641747454968 | 0.856740125352452 | -0.506057606 | 0.420698893882959 | 0.958218587630099 | 0.60286764149808 | 1.52302561702965 | 0.999489402457283 |
| ebi-fl187-GCST90094727 | ebi-met1400-GCST90200023 | Trans 3,4-methyleneheptanoate levels \|\| id:ebi-met1400-GCST90200023 | Cauliflower liking \|\| id:ebi-fl187-GCST90094727 | Weighted median | 4 | -0.359668275 | 0.261250891296388 | 0.168600078624918 | -0.871720022 | 0.15238347216043 | 0.697907801294406 | 0.418231562856336 | 1.16460674507941 | 0.999489402457283 |
| ebi-fl187-GCST90094727 | ebi-met1400-GCST90200032 | 2-hydroxybutyrate/2-hydroxyisobutyrate levels \|\| id:ebi-met1400-GCST90200032 | Cauliflower liking \|\| id:ebi-fl187-GCST90094727 | Weighted median | 4 | 0.207352499740287 | 0.24355262031304 | 0.394565891019164 | -0.270010636 | 0.684715635553844 | 1.23041621674324 | 0.763371375019797 | 1.98320780155725 | 0.999489402457283 |
| ebi-fl187-GCST90094727 | ebi-met1400-GCST90200051 | 1-palmitoyl-2-dihomo-linolenoyl-GPC (16:0/20:3n3 or 6) levels \|\| id:ebi-met1400-GCST90200051 | Cauliflower liking \|\| id:ebi-fl187-GCST90094727 | Weighted median | 4 | 0.137131042628157 | 0.231819919025285 | 0.554157830807199 | -0.317235999 | 0.591498083917715 | 1.14697844174222 | 0.728158890323164 | 1.80669296674734 | 0.999489402457283 |
| ebi-fl187-GCST90094727 | ebi-met1400-GCST90200069 | Phenylacetylglutamate levels \|\| id:ebi-met1400-GCST90200069 | Cauliflower liking \|\| id:ebi-fl187-GCST90094727 | Weighted median | 4 | 0.107622407978073 | 0.259987733996276 | 0.678909357384588 | -0.401953551 | 0.617198366610774 | 1.11362716925386 | 0.669011820129738 | 1.85372729566999 | 0.999489402457283 |
| ebi-fl187-GCST90094727 | ebi-met1400-GCST90200072 | Thioproline levels \|\| id:ebi-met1400-GCST90200072 | Cauliflower liking \|\| id:ebi-fl187-GCST90094727 | Weighted median | 4 | 0.155033082536292 | 0.23777557157866 | 0.514391921197706 | -0.311007038 | 0.621073202830467 | 1.16769659083098 | 0.732708719224623 | 1.86092411958904 | 0.999489402457283 |
| ebi-fl187-GCST90094727 | ebi-met1400-GCST90200081 | 1-oleoyl-2-docosahexaenoyl-GPC (18:1/22:6) levels \|\| id:ebi-met1400-GCST90200081 | Cauliflower liking \|\| id:ebi-fl187-GCST90094727 | Weighted median | 4 | 0.194836291371266 | 0.233918907060211 | 0.404888510284401 | -0.263644766 | 0.653317349209279 | 1.21511204587924 | 0.768246398072003 | 1.92190589861047 | 0.999489402457283 |
| ebi-fl187-GCST90094727 | ebi-met1400-GCST90200097 | Furaneol sulfate levels \|\| id:ebi-met1400-GCST90200097 | Cauliflower liking \|\| id:ebi-fl187-GCST90094727 | Weighted median | 4 | -0.473995346 | 0.322617667811375 | 0.141773954076983 | -1.106325975 | 0.158335282733391 | 0.622510149613057 | 0.330771995903999 | 1.17155893234608 | 0.999489402457283 |
| ebi-fl187-GCST90094727 | ebi-met1400-GCST90200124 | Sphingomyelin (d18:2/24:2) levels \|\| id:ebi-met1400-GCST90200124 | Cauliflower liking \|\| id:ebi-fl187-GCST90094727 | Weighted median | 4 | -0.222310989 | 0.234904755627136 | 0.343950839403041 | -0.68272431 | 0.23810233246424 | 0.800666327540666 | 0.505238689147542 | 1.26883902960616 | 0.999489402457283 |
| ebi-fl187-GCST90094727 | ebi-met1400-GCST90200137 | Lignoceroylcarnitine (C24) levels \|\| id:ebi-met1400-GCST90200137 | Cauliflower liking \|\| id:ebi-fl187-GCST90094727 | Weighted median | 4 | -0.210739606 | 0.260982695636217 | 0.419387125889669 | -0.72226569 | 0.300786477264151 | 0.809984954497317 | 0.485650674813075 | 1.35092085842265 | 0.999489402457283 |
| ebi-fl187-GCST90094727 | ebi-met1400-GCST90200165 | Octadecenedioate (C18:1-DC) levels \|\| id:ebi-met1400-GCST90200165 | Cauliflower liking \|\| id:ebi-fl187-GCST90094727 | Weighted median | 4 | -0.194166582 | 0.232110719025617 | 0.402859223913964 | -0.649103591 | 0.260770427175474 | 0.823520709020519 | 0.522513952889519 | 1.29792966184973 | 0.999489402457283 |
| ebi-fl187-GCST90094727 | ebi-met1400-GCST90200176 | Glutamine conjugate of C6H10O2 (1) levels \|\| id:ebi-met1400-GCST90200176 | Cauliflower liking \|\| id:ebi-fl187-GCST90094727 | Weighted median | 4 | 0.269577287385224 | 0.245638861319569 | 0.272443091780208 | -0.211874881 | 0.751029455571579 | 1.30941082925465 | 0.809065920913733 | 2.11918049623569 | 0.999489402457283 |
| ebi-fl187-GCST90094727 | ebi-met1400-GCST90200182 | Glycine conjugate of C10H12O2 levels \|\| id:ebi-met1400-GCST90200182 | Cauliflower liking \|\| id:ebi-fl187-GCST90094727 | Weighted median | 4 | -0.323825702 | 0.288903163852609 | 0.26233905788104 | -0.890075903 | 0.242424499336708 | 0.723376314571578 | 0.410624583945821 | 1.27433503239105 | 0.999489402457283 |
| ebi-fl187-GCST90094727 | ebi-met1400-GCST90200208 | Glucuronide of piperine metabolite C17H21NO3 (4) levels \|\| id:ebi-met1400-GCST90200208 | Cauliflower liking \|\| id:ebi-fl187-GCST90094727 | Weighted median | 4 | 0.2124456383139 | 0.24779394472777 | 0.39125259858657 | -0.273230493 | 0.698121769980329 | 1.23669888270145 | 0.760917381016533 | 2.00997396646638 | 0.999489402457283 |
| ebi-fl187-GCST90094727 | ebi-met1400-GCST90200227 | 3-ethylcatechol sulfate (2) levels \|\| id:ebi-met1400-GCST90200227 | Cauliflower liking \|\| id:ebi-fl187-GCST90094727 | Weighted median | 4 | 0.0842077431214951 | 0.328922397881203 | 0.797942364383652 | -0.560480157 | 0.728895642968653 | 1.08785486470139 | 0.570934859810773 | 2.07279024273752 | 0.999489402457283 |
| ebi-fl187-GCST90094727 | ebi-met1400-GCST90200259 | Pentose acid levels \|\| id:ebi-met1400-GCST90200259 | Cauliflower liking \|\| id:ebi-fl187-GCST90094727 | Weighted median | 4 | -0.400732421 | 0.247332528712321 | 0.105185624813741 | -0.885504177 | 0.0840393355622041 | 0.66982926949834 | 0.412506144735405 | 1.08767167714428 | 0.999489402457283 |
| ebi-fl187-GCST90094727 | ebi-met1400-GCST90200291 | Sarcosine levels \|\| id:ebi-met1400-GCST90200291 | Cauliflower liking \|\| id:ebi-fl187-GCST90094727 | Weighted median | 4 | 0.0153049389481108 | 0.248741996189849 | 0.950937622816948 | -0.472229374 | 0.502839251480214 | 1.0154226593272 | 0.623610456746247 | 1.65340905676102 | 0.999489402457283 |
| ebi-fl187-GCST90094727 | ebi-met1400-GCST90200306 | Butyrate/isobutyrate (4:0) levels \|\| id:ebi-met1400-GCST90200306 | Cauliflower liking \|\| id:ebi-fl187-GCST90094727 | Weighted median | 4 | 0.00549283433146333 | 0.252983445606022 | 0.982677509286366 | -0.490354719 | 0.501340387719267 | 1.00550794760486 | 0.612409122465469 | 1.65093267818449 | 0.999911036302558 |
| ebi-fl187-GCST90094727 | ebi-met1400-GCST90200321 | Cholate levels \|\| id:ebi-met1400-GCST90200321 | Cauliflower liking \|\| id:ebi-fl187-GCST90094727 | Weighted median | 4 | 0.169288302444253 | 0.266242736738772 | 0.524879438170024 | -0.352547462 | 0.691124066452247 | 1.18446157287007 | 0.702895208513293 | 1.99595786201649 | 0.999489402457283 |
| ebi-fl187-GCST90094727 | ebi-met1400-GCST90200336 | Ceramide (d18:1/16:0) levels \|\| id:ebi-met1400-GCST90200336 | Cauliflower liking \|\| id:ebi-fl187-GCST90094727 | Weighted median | 4 | 0.0818547771831542 | 0.244557896919435 | 0.737847548227445 | -0.397478701 | 0.561188255145247 | 1.08529818832468 | 0.672012255832752 | 1.75275398232317 | 0.999489402457283 |
| ebi-fl187-GCST90094727 | ebi-met1400-GCST90200358 | Arachidonate (20:4n6) levels \|\| id:ebi-met1400-GCST90200358 | Cauliflower liking \|\| id:ebi-fl187-GCST90094727 | Weighted median | 4 | 0.444077707181998 | 0.267948281481816 | 0.0974535430213185 | -0.081100925 | 0.969256338886358 | 1.55905163042373 | 0.922100624183238 | 2.63598345189266 | 0.999489402457283 |
| ebi-fl187-GCST90094727 | ebi-met1400-GCST90200413 | 2'-deoxyuridine levels \|\| id:ebi-met1400-GCST90200413 | Cauliflower liking \|\| id:ebi-fl187-GCST90094727 | Weighted median | 4 | 0.0465686122339283 | 0.261058436074371 | 0.858421521701952 | -0.465105922 | 0.558243146939695 | 1.04766995957891 | 0.628068575090229 | 1.7475995261288 | 0.999489402457283 |
| ebi-fl187-GCST90094727 | ebi-met1400-GCST90200432 | Threonine levels \|\| id:ebi-met1400-GCST90200432 | Cauliflower liking \|\| id:ebi-fl187-GCST90094727 | Weighted median | 4 | 0.0617997747050594 | 0.239902435701553 | 0.796712710727261 | -0.408408999 | 0.532008548680104 | 1.06374933387955 | 0.664706958557794 | 1.70234812613413 | 0.999489402457283 |
| ebi-fl187-GCST90094727 | ebi-met1400-GCST90200436 | Caffeine levels \|\| id:ebi-met1400-GCST90200436 | Cauliflower liking \|\| id:ebi-fl187-GCST90094727 | Weighted median | 4 | -0.191877912 | 0.241901747418251 | 0.427657826067576 | -0.666005337 | 0.282249513171828 | 0.825407634899231 | 0.513756769452967 | 1.32610956051318 | 0.999489402457283 |
| ebi-fl187-GCST90094727 | ebi-met1400-GCST90200442 | Valine levels \|\| id:ebi-met1400-GCST90200442 | Cauliflower liking \|\| id:ebi-fl187-GCST90094727 | Weighted median | 4 | 0.148977737537633 | 0.26516491792721 | 0.574231511756644 | -0.370745502 | 0.668700976674965 | 1.16064715005797 | 0.690219578986827 | 1.95170036891026 | 0.999489402457283 |
| ebi-fl187-GCST90094727 | ebi-met1400-GCST90200459 | Margarate (17:0) levels \|\| id:ebi-met1400-GCST90200459 | Cauliflower liking \|\| id:ebi-fl187-GCST90094727 | Weighted median | 4 | 0.266818171197101 | 0.232509873234851 | 0.25115177636112 | -0.18890118 | 0.72253752273741 | 1.30580299214842 | 0.827868312315158 | 2.05965300149651 | 0.999489402457283 |
| ebi-fl187-GCST90094727 | ebi-met1400-GCST90200462 | X-11299 levels \|\| id:ebi-met1400-GCST90200462 | Cauliflower liking \|\| id:ebi-fl187-GCST90094727 | Weighted median | 4 | 0.288851862628821 | 0.257204687507609 | 0.261419333616178 | -0.215269325 | 0.792973050143734 | 1.33489396619792 | 0.806324247744799 | 2.20995698191578 | 0.999489402457283 |
| ebi-fl187-GCST90094727 | ebi-met1400-GCST90200497 | X-12740 levels \|\| id:ebi-met1400-GCST90200497 | Cauliflower liking \|\| id:ebi-fl187-GCST90094727 | Weighted median | 4 | 0.109589899888649 | 0.268085264149945 | 0.682695627748063 | -0.415857218 | 0.635037017622541 | 1.11582037855404 | 0.659774467779546 | 1.88709199582501 | 0.999489402457283 |
| ebi-fl187-GCST90094727 | ebi-met1400-GCST90200527 | X-13866 levels \|\| id:ebi-met1400-GCST90200527 | Cauliflower liking \|\| id:ebi-fl187-GCST90094727 | Weighted median | 4 | 0.365923987862034 | 0.256670146258384 | 0.153966927450843 | -0.137149499 | 0.868997474528467 | 1.44184564071926 | 0.871839877369567 | 2.38451911368571 | 0.999489402457283 |
| ebi-fl187-GCST90094727 | ebi-met1400-GCST90200547 | X-17654 levels \|\| id:ebi-met1400-GCST90200547 | Cauliflower liking \|\| id:ebi-fl187-GCST90094727 | Weighted median | 4 | 0.245653069359568 | 0.239696480205863 | 0.30543371078332 | -0.224152032 | 0.71545817056306 | 1.27845596104947 | 0.799193622252701 | 2.04512348301264 | 0.999489402457283 |
| ebi-fl187-GCST90094727 | ebi-met1400-GCST90200553 | X-17653 levels \|\| id:ebi-met1400-GCST90200553 | Cauliflower liking \|\| id:ebi-fl187-GCST90094727 | Weighted median | 4 | 0.244686931532256 | 0.243813596616228 | 0.315580144899772 | -0.233187718 | 0.722561580900063 | 1.2772213928625 | 0.792004886126143 | 2.05970255355949 | 0.999489402457283 |
| ebi-fl187-GCST90094727 | ebi-met1400-GCST90200557 | X-17685 levels \|\| id:ebi-met1400-GCST90200557 | Cauliflower liking \|\| id:ebi-fl187-GCST90094727 | Weighted median | 4 | 0.12050776197493 | 0.264297240647941 | 0.648422028772256 | -0.39751483 | 0.638530353644895 | 1.12806949697894 | 0.671987977196928 | 1.8936957701572 | 0.999489402457283 |
| ebi-fl187-GCST90094727 | ebi-met1400-GCST90200561 | X-18779 levels \|\| id:ebi-met1400-GCST90200561 | Cauliflower liking \|\| id:ebi-fl187-GCST90094727 | Weighted median | 4 | 0.217569194706146 | 0.244478629877254 | 0.373502754477792 | -0.26160892 | 0.696747309265564 | 1.24305143911105 | 0.769812023045233 | 2.00721323390561 | 0.999489402457283 |
| ebi-fl187-GCST90094727 | ebi-met1400-GCST90200570 | X-18921 levels \|\| id:ebi-met1400-GCST90200570 | Cauliflower liking \|\| id:ebi-fl187-GCST90094727 | Weighted median | 4 | -0.024048881 | 0.246687645118428 | 0.922339521 | -0.507556665 | 0.459458903509265 | 0.976237989177771 | 0.601964584369434 | 1.58321707997519 | 0.999489402457283 |
| ebi-fl187-GCST90094727 | ebi-met1400-GCST90200575 | X-21283 levels \|\| id:ebi-met1400-GCST90200575 | Cauliflower liking \|\| id:ebi-fl187-GCST90094727 | Weighted median | 4 | 0.305726302246346 | 0.264545614798509 | 0.247817909018198 | -0.212783103 | 0.824235707251424 | 1.35761068069999 | 0.808331443064154 | 2.28013740671029 | 0.999489402457283 |
| ebi-fl187-GCST90094727 | ebi-met1400-GCST90200578 | X-18922 levels \|\| id:ebi-met1400-GCST90200578 | Cauliflower liking \|\| id:ebi-fl187-GCST90094727 | Weighted median | 4 | -0.036904697 | 0.228848676487698 | 0.87188670019141 | -0.485448103 | 0.411638708684601 | 0.963767980729384 | 0.615421362718376 | 1.50928904478775 | 0.999489402457283 |
| ebi-fl187-GCST90094727 | ebi-met1400-GCST90200623 | X-24241 levels \|\| id:ebi-met1400-GCST90200623 | Cauliflower liking \|\| id:ebi-fl187-GCST90094727 | Weighted median | 4 | -0.040905969 | 0.253174919130407 | 0.871642935107513 | -0.537128811 | 0.455316872074503 | 0.959919387479956 | 0.584423837103727 | 1.57667290750215 | 0.999489402457283 |
| ebi-fl187-GCST90094727 | ebi-met1400-GCST90200636 | X-24544 levels \|\| id:ebi-met1400-GCST90200636 | Cauliflower liking \|\| id:ebi-fl187-GCST90094727 | Weighted median | 4 | -0.036403016 | 0.244381001297921 | 0.881585123454797 | -0.515389779 | 0.442583746084848 | 0.96425160589677 | 0.597267746814715 | 1.55672420691241 | 0.999489402457283 |
| ebi-fl187-GCST90094727 | ebi-met1400-GCST90200659 | X-25172 levels \|\| id:ebi-met1400-GCST90200659 | Cauliflower liking \|\| id:ebi-fl187-GCST90094727 | Weighted median | 4 | -0.067958847 | 0.273443437620646 | 0.803724505223019 | -0.603907984 | 0.467990291019767 | 0.934298922254298 | 0.546671074118198 | 1.59678189948789 | 0.999489402457283 |
| ebi-fl187-GCST90094727 | ebi-met1400-GCST90200670 | X-26111 levels \|\| id:ebi-met1400-GCST90200670 | Cauliflower liking \|\| id:ebi-fl187-GCST90094727 | Weighted median | 4 | 0.368898076345275 | 0.257731568985847 | 0.152336604934035 | -0.136255799 | 0.874051951557535 | 1.44614020026846 | 0.872619388886221 | 2.39660212168996 | 0.999489402457283 |
| ebi-fl187-GCST90094727 | ebi-met1400-GCST90200673 | Carnitine C4 levels \|\| id:ebi-met1400-GCST90200673 | Cauliflower liking \|\| id:ebi-fl187-GCST90094727 | Weighted median | 4 | -0.251763045 | 0.247455725354274 | 0.308960125886108 | -0.736776267 | 0.23325017633746 | 0.777428931640342 | 0.478654485229258 | 1.26269733681062 | 0.999489402457283 |
| ebi-fl187-GCST90094727 | ebi-met1400-GCST90200710 | X-13431 levels \|\| id:ebi-met1400-GCST90200710 | Cauliflower liking \|\| id:ebi-fl187-GCST90094727 | Weighted median | 4 | -0.321520155 | 0.237435126483711 | 0.175692048602328 | -0.786893003 | 0.14385269269793 | 0.725046016425347 | 0.455257082495979 | 1.15471399819224 | 0.999489402457283 |
| ebi-fl187-GCST90094727 | ebi-met1400-GCST90200730 | Adenosine 5'-diphosphate (ADP) to fructose ratio \|\| id:ebi-met1400-GCST90200730 | Cauliflower liking \|\| id:ebi-fl187-GCST90094727 | Weighted median | 4 | 0.211832897107428 | 0.329405993447701 | 0.520175163894739 | -0.43380285 | 0.857468644264921 | 1.23594133844901 | 0.648040003946469 | 2.35718625823185 | 0.999489402457283 |
| ebi-fl187-GCST90094727 | ebi-met1400-GCST90200731 | Adenosine 5'-diphosphate (ADP) to mannose ratio \|\| id:ebi-met1400-GCST90200731 | Cauliflower liking \|\| id:ebi-fl187-GCST90094727 | Weighted median | 4 | -0.010136204 | 0.357433387184572 | 0.977376381169696 | -0.710705643 | 0.690433235020033 | 0.989914994321543 | 0.491297394674474 | 1.9945794677619 | 0.999489402457283 |
| ebi-fl187-GCST90094727 | ebi-met1400-GCST90200748 | Aspartate to glutamate ratio \|\| id:ebi-met1400-GCST90200748 | Cauliflower liking \|\| id:ebi-fl187-GCST90094727 | Weighted median | 4 | -0.184700778 | 0.25340029848847 | 0.466069350936028 | -0.681365363 | 0.311963806604915 | 0.831353005388276 | 0.505925748038252 | 1.36610524814772 | 0.999489402457283 |
| ebi-fl187-GCST90094727 | ebi-met1400-GCST90200774 | Uridine to pseudouridine ratio \|\| id:ebi-met1400-GCST90200774 | Cauliflower liking \|\| id:ebi-fl187-GCST90094727 | Weighted median | 4 | 0.257082503564956 | 0.229983236581194 | 0.263638970447102 | -0.19368464 | 0.707849647264096 | 1.29315181199286 | 0.8239176938762 | 2.02962215921494 | 0.999489402457283 |
| ebi-fl187-GCST90094727 | ebi-met1400-GCST90200799 | Spermidine to adenosine 5'-diphosphate (ADP) ratio \|\| id:ebi-met1400-GCST90200799 | Cauliflower liking \|\| id:ebi-fl187-GCST90094727 | Weighted median | 4 | -0.547618857 | 0.36289488864442 | 0.131291409708347 | -1.258892839 | 0.16365512443897 | 0.578325247113068 | 0.283968251078522 | 1.17780804782964 | 0.999489402457283 |
| ebi-fl187-GCST90094727 | ebi-met1400-GCST90200811 | Choline to taurocholate ratio \|\| id:ebi-met1400-GCST90200811 | Cauliflower liking \|\| id:ebi-fl187-GCST90094727 | Weighted median | 4 | 0.239649220486206 | 0.277175120094184 | 0.387251218271067 | -0.303614015 | 0.782912455870806 | 1.27080330036463 | 0.738145724724088 | 2.18783496825277 | 0.999489402457283 |
| ebi-fl187-GCST90094727 | ebi-met1400-GCST90200812 | Glutamate to kynurenine ratio \|\| id:ebi-met1400-GCST90200812 | Cauliflower liking \|\| id:ebi-fl187-GCST90094727 | Weighted median | 4 | 0.393201426453384 | 0.304684747950092 | 0.196870553204773 | -0.20398068 | 0.990383532435564 | 1.48171681623593 | 0.815478126456752 | 2.69226684602286 | 0.999489402457283 |
| ebi-fl187-GCST90094727 | ebi-met1400-GCST90200876 | Cortisone to 4-cholesten-3-one ratio \|\| id:ebi-met1400-GCST90200876 | Cauliflower liking \|\| id:ebi-fl187-GCST90094727 | Weighted median | 4 | -0.160993662 | 0.295147986803347 | 0.585431963876554 | -0.739483716 | 0.417496392509624 | 0.851297466932687 | 0.477360305514705 | 1.51815592715571 | 0.999489402457283 |
| ebi-fl187-GCST90094727 | ebi-met1400-GCST90200906 | 2'-deoxyuridine to cytidine ratio \|\| id:ebi-met1400-GCST90200906 | Cauliflower liking \|\| id:ebi-fl187-GCST90094727 | Weighted median | 4 | 0.394281231763824 | 0.277017963549101 | 0.154647570774645 | -0.148673977 | 0.937236440320061 | 1.4833176460594 | 0.86185005221891 | 2.55291652352577 | 0.999489402457283 |
| ebi-fl187-GCST90094727 | ebi-met1400-GCST90200907 | Retinol (Vitamin A) to linoleoyl-arachidonoyl-glycerol (18:2 to 20:4) [1] ratio \|\| id:ebi-met1400-GCST90200907 | Cauliflower liking \|\| id:ebi-fl187-GCST90094727 | Weighted median | 4 | -0.683456264 | 0.312883480457559 | 0.0289343724973608 | -1.296707886 | -0.070204642 | 0.5048690127143 | 0.273430477290374 | 0.932203032101735 | 0.999489402457283 |
| ebi-fl187-GCST90094727 | ebi-met1400-GCST90200916 | Fructose to sucrose ratio \|\| id:ebi-met1400-GCST90200916 | Cauliflower liking \|\| id:ebi-fl187-GCST90094727 | Weighted median | 4 | -0.18211475 | 0.25888758855064 | 0.481774693153579 | -0.689534423 | 0.32530492390452 | 0.833505690437742 | 0.50180964560992 | 1.38445273435845 | 0.999489402457283 |
| ebi-fl187-GCST90094727 | ebi-met1400-GCST90200917 | Alpha-ketobutyrate to 3-methyl-2-oxobutyrate ratio \|\| id:ebi-met1400-GCST90200917 | Cauliflower liking \|\| id:ebi-fl187-GCST90094727 | Weighted median | 4 | -0.009971668 | 0.261030182631345 | 0.969527259271402 | -0.521590826 | 0.501647490152659 | 0.990077884431706 | 0.593575521252044 | 1.6514397614866 | 0.999489402457283 |
| ebi-fl187-GCST90094727 | ebi-met1400-GCST90200924 | Alpha-tocopherol to glycerol ratio \|\| id:ebi-met1400-GCST90200924 | Cauliflower liking \|\| id:ebi-fl187-GCST90094727 | Weighted median | 4 | -0.177975253 | 0.23962934936615 | 0.457656921784089 | -0.647648778 | 0.291698271261623 | 0.836963135144042 | 0.523274666273648 | 1.33869903272522 | 0.999489402457283 |
| ebi-fl187-GCST90094727 | ebi-met1400-GCST90200938 | Alpha-ketoglutarate to alanine ratio \|\| id:ebi-met1400-GCST90200938 | Cauliflower liking \|\| id:ebi-fl187-GCST90094727 | Weighted median | 4 | 0.0263916673906337 | 0.257363559958354 | 0.918323105876852 | -0.47804091 | 0.530824244909007 | 1.02674301148698 | 0.619996832313554 | 1.70033322219331 | 0.999489402457283 |
| ebi-fl187-GCST90094727 | ebi-met1400-GCST90200943 | Phosphate to EDTA ratio \|\| id:ebi-met1400-GCST90200943 | Cauliflower liking \|\| id:ebi-fl187-GCST90094727 | Weighted median | 4 | -6.48E-05 | 0.245620119887985 | 0.999789564824571 | -0.481480215 | 0.481350654790238 | 0.999935221907978 | 0.617868136796523 | 1.61825863556618 | 0.999911036302558 |
| ebi-fl187-GCST90094727 | ebi-met1400-GCST90200947 | Adenosine 5'-diphosphate (ADP) to glucose ratio \|\| id:ebi-met1400-GCST90200947 | Cauliflower liking \|\| id:ebi-fl187-GCST90094727 | Weighted median | 4 | 0.343966560220073 | 0.347171724946694 | 0.321798984170074 | -0.336490021 | 1.02442314111559 | 1.4105314668572 | 0.714273011502786 | 2.78548816342412 | 0.999489402457283 |
| ebi-fl187-GCST90094727 | ebi-met1400-GCST90200975 | Arachidonate (20:4n6) to caffeine ratio \|\| id:ebi-met1400-GCST90200975 | Cauliflower liking \|\| id:ebi-fl187-GCST90094727 | Weighted median | 4 | 0.430782255559185 | 0.251274588314635 | 0.086457405723876 | -0.061715938 | 0.923280448655869 | 1.53846052225684 | 0.940149910162236 | 2.51753550466688 | 0.999489402457283 |
| ebi-fl187-GCST90094727 | ebi-met1400-GCST90200985 | Cholesterol to oleoyl-linoleoyl-glycerol (18:1 to 18:2) [2] ratio \|\| id:ebi-met1400-GCST90200985 | Cauliflower liking \|\| id:ebi-fl187-GCST90094727 | Weighted median | 4 | -0.341540302 | 0.292473837601111 | 0.242902133326979 | -0.914789024 | 0.23170841934445 | 0.710674825174493 | 0.400601134380031 | 1.26075206431561 | 0.999489402457283 |
| ebi-fl187-GCST90094727 | ebi-met1400-GCST90201016 | Bilirubin (Z,Z) to etiocholanolone glucuronide ratio \|\| id:ebi-met1400-GCST90201016 | Cauliflower liking \|\| id:ebi-fl187-GCST90094727 | Weighted median | 4 | -0.041844328 | 0.257437140142002 | 0.87087911678464 | -0.546421123 | 0.462732466662924 | 0.959019061353317 | 0.579018342266756 | 1.5884083333845 | 0.999489402457283 |
| ebi-fl187-GCST90094727 | ebi-pw11-GCST90275189 | Placental weight (fetal genotype, adjusted for sex and gestational age) \|\| id:ebi-pw11-GCST90275189 | Cauliflower liking \|\| id:ebi-fl187-GCST90094727 | Weighted median | 4 | -0.033582686 | 0.0890940432390212 | 0.706221719764623 | -0.208207011 | 0.141041638714639 | 0.966974952602062 | 0.812038918499602 | 1.15147259282526 | 0.999489402457283 |
| ebi-fl187-GCST90094727 | ebi-pw11-GCST90275192 | Placental weight (fetal effect, conditional on maternal and paternal genotype) \|\| id:ebi-pw11-GCST90275192 | Cauliflower liking \|\| id:ebi-fl187-GCST90094727 | Weighted median | 4 | -0.033348895 | 0.193409984485665 | 0.863102696090529 | -0.412432464 | 0.345734675071429 | 0.967201049568367 | 0.662037906498715 | 1.41302765461507 | 0.999489402457283 |
| ebi-fl187-GCST90094727 | ebi-pw11-GCST90275193 | Placental weight (maternal effect, conditional on fetal and paternal genotype) \|\| id:ebi-pw11-GCST90275193 | Cauliflower liking \|\| id:ebi-fl187-GCST90094727 | Weighted median | 4 | -0.001761811 | 0.151970457572199 | 0.990750240245995 | -0.299623908 | 0.296100285949422 | 0.998239740185687 | 0.741096889084558 | 1.34460499505928 | 0.999911036302558 |
| ebi-fl187-GCST90094727 | ebi-pw11-GCST90275195 | Placental weight (fetal effect, conditional on maternal genotype) \|\| id:ebi-pw11-GCST90275195 | Cauliflower liking \|\| id:ebi-fl187-GCST90094727 | Weighted median | 4 | -0.046743005 | 0.13049472041735 | 0.720194815149243 | -0.302512657 | 0.209026646832858 | 0.954332624614155 | 0.738959135043857 | 1.23247783972369 | 0.999489402457283 |
| ebi-fl187-GCST90094727 | ebi-pw11-GCST90275197 | Placental weight (fetal genotype, adjusted for sex) \|\| id:ebi-pw11-GCST90275197 | Cauliflower liking \|\| id:ebi-fl187-GCST90094727 | Weighted median | 4 | -0.03272955 | 0.0911248799361667 | 0.719466053239426 | -0.211334315 | 0.145875214407406 | 0.96780026552123 | 0.809503392558533 | 1.15705179564796 | 0.999489402457283 |
| ebi-fl187-GCST90094727 | prot-a-1030 | Protein FAM171B \|\| id:prot-a-1030 | Cauliflower liking \|\| id:ebi-fl187-GCST90094727 | Weighted median | 4 | 0.141916350666118 | 0.399657410395005 | 0.722518417587694 | -0.641412174 | 0.925244875040328 | 1.15248024030041 | 0.526548321070325 | 2.52248587856666 | 0.999489402457283 |
| ebi-fl187-GCST90094727 | prot-a-1036 | BRISC complex subunit Abro1 \|\| id:prot-a-1036 | Cauliflower liking \|\| id:ebi-fl187-GCST90094727 | Weighted median | 4 | 0.585396340447432 | 0.416166777199735 | 0.15953451855571 | -0.230290543 | 1.40108322375891 | 1.79570255417267 | 0.794302789966971 | 4.0595950357877 | 0.999489402457283 |
| ebi-fl187-GCST90094727 | prot-a-1055 | Phenylalanine--tRNA ligase, mitochondrial \|\| id:prot-a-1055 | Cauliflower liking \|\| id:ebi-fl187-GCST90094727 | Weighted median | 4 | -0.522554748 | 0.40012664453579 | 0.191561608905375 | -1.306802971 | 0.261693475207121 | 0.59300363622867 | 0.270684059214017 | 1.29912826636899 | 0.999489402457283 |
| ebi-fl187-GCST90094727 | prot-a-1083 | Fc receptor-like protein 5 \|\| id:prot-a-1083 | Cauliflower liking \|\| id:ebi-fl187-GCST90094727 | Weighted median | 4 | -0.032238661 | 0.380603075369008 | 0.932496594968671 | -0.778220688 | 0.713743366953468 | 0.968275465133146 | 0.459222384471699 | 2.04161950305928 | 0.999489402457283 |
| ebi-fl187-GCST90094727 | prot-a-1103 | Fibroblast growth factor receptor 3 \|\| id:prot-a-1103 | Cauliflower liking \|\| id:ebi-fl187-GCST90094727 | Weighted median | 4 | 0.102762553862618 | 0.445503228759346 | 0.817574104283886 | -0.770423775 | 0.975948882230935 | 1.10822823331887 | 0.462816896746262 | 2.65368405034358 | 0.999489402457283 |
| ebi-fl187-GCST90094727 | prot-a-1105 | Fibroblast growth factor receptor 4 \|\| id:prot-a-1105 | Cauliflower liking \|\| id:ebi-fl187-GCST90094727 | Weighted median | 4 | -1.035434861 | 0.421993792586579 | 0.0141405423028849 | -1.862542694 | -0.208327027 | 0.355071940425406 | 0.155277305247689 | 0.811941466116728 | 0.999489402457283 |
| ebi-fl187-GCST90094727 | prot-a-1120 | Filamin-A \|\| id:prot-a-1120 | Cauliflower liking \|\| id:ebi-fl187-GCST90094727 | Weighted median | 4 | -0.093902754 | 0.396001309358705 | 0.812557970572156 | -0.87006532 | 0.682259812720722 | 0.910371288246388 | 0.418924184240688 | 1.9783433700911 | 0.999489402457283 |
| ebi-fl187-GCST90094727 | prot-a-1166 | Galanin peptides \|\| id:prot-a-1166 | Cauliflower liking \|\| id:ebi-fl187-GCST90094727 | Weighted median | 4 | -0.200423388 | 0.423916687399123 | 0.636363316874892 | -1.031300095 | 0.630453319475992 | 0.818384185815477 | 0.356543119143168 | 1.87846192966053 | 0.999489402457283 |
| ebi-fl187-GCST90094727 | prot-a-1205 | ADP-ribosylation factor-binding protein GGA1 \|\| id:prot-a-1205 | Cauliflower liking \|\| id:ebi-fl187-GCST90094727 | Weighted median | 4 | 0.141880394607874 | 0.38237280432493 | 0.710598410247948 | -0.607570302 | 0.891331091084736 | 1.15243880239874 | 0.544672652783022 | 2.43837318890197 | 0.999489402457283 |
| ebi-fl187-GCST90094727 | prot-a-1227 | Guanine nucleotide-binding protein G(I)/G(S)/G(O) subunit gamma-T2 \|\| id:prot-a-1227 | Cauliflower liking \|\| id:ebi-fl187-GCST90094727 | Weighted median | 4 | -0.701293621 | 0.413089251943697 | 0.0895680557749406 | -1.510948555 | 0.108361312787739 | 0.495943325930809 | 0.220700532083599 | 1.11445033780954 | 0.999489402457283 |
| ebi-fl187-GCST90094727 | prot-a-1240 | Golgi SNAP receptor complex member 1 \|\| id:prot-a-1240 | Cauliflower liking \|\| id:ebi-fl187-GCST90094727 | Weighted median | 4 | -0.412151868 | 0.396495875084124 | 0.298578815953664 | -1.189283784 | 0.364980046727712 | 0.662223697536963 | 0.304439230370836 | 1.44048526546773 | 0.999489402457283 |
| ebi-fl187-GCST90094727 | prot-a-1283 | Glutathione S-transferase A1 \|\| id:prot-a-1283 | Cauliflower liking \|\| id:ebi-fl187-GCST90094727 | Weighted median | 4 | -0.444190685 | 0.387004554426142 | 0.251065160258003 | -1.202719612 | 0.314338241837671 | 0.641343114759071 | 0.30037619351621 | 1.36935283064192 | 0.999489402457283 |
| ebi-fl187-GCST90094727 | prot-a-1324 | E3 ubiquitin-protein ligase HECW1 \|\| id:prot-a-1324 | Cauliflower liking \|\| id:ebi-fl187-GCST90094727 | Weighted median | 4 | -0.023678098 | 0.408926955566488 | 0.953825893219415 | -0.825174931 | 0.777818735268728 | 0.976600029017706 | 0.438158338480611 | 2.17671908284267 | 0.999489402457283 |
| ebi-fl187-GCST90094727 | prot-a-1339 | Histidine triad nucleotide-binding protein 2, mitochondrial \|\| id:prot-a-1339 | Cauliflower liking \|\| id:ebi-fl187-GCST90094727 | Weighted median | 4 | 0.217446383603868 | 0.385477890950912 | 0.572689069855505 | -0.538090283 | 0.972983049867656 | 1.24289878796744 | 0.583862200140584 | 2.64582532789239 | 0.999489402457283 |
| ebi-fl187-GCST90094727 | prot-a-1466 | Interleukin-11 \|\| id:prot-a-1466 | Cauliflower liking \|\| id:ebi-fl187-GCST90094727 | Weighted median | 4 | -0.099661365 | 0.384361555541406 | 0.795410978952491 | -0.853010013 | 0.653687284287921 | 0.905143879927668 | 0.426130341548568 | 1.92261701054473 | 0.999489402457283 |
| ebi-fl187-GCST90094727 | prot-a-147 | Rho GTPase-activating protein 1 \|\| id:prot-a-147 | Cauliflower liking \|\| id:ebi-fl187-GCST90094727 | Weighted median | 4 | -0.127520788 | 0.392253718907283 | 0.745107118284489 | -0.896338077 | 0.641296501343547 | 0.880275116736016 | 0.408061215936361 | 1.89894126391432 | 0.999489402457283 |
| ebi-fl187-GCST90094727 | prot-a-1473 | Interleukin-12 receptor subunit beta-1 \|\| id:prot-a-1473 | Cauliflower liking \|\| id:ebi-fl187-GCST90094727 | Weighted median | 4 | 0.25993347392734 | 0.429174427273778 | 0.544741030146524 | -0.581248404 | 1.10111535138395 | 1.29684380987045 | 0.559199823591033 | 3.0075185939781 | 0.999489402457283 |
| ebi-fl187-GCST90094727 | prot-a-1501 | X-linked interleukin-1 receptor accessory protein-like 2 \|\| id:prot-a-1501 | Cauliflower liking \|\| id:ebi-fl187-GCST90094727 | Weighted median | 4 | 0.000714672560621438 | 0.396337451590727 | 0.998561261644981 | -0.776106733 | 0.777536077678447 | 1.0007149279999 | 0.460194187169363 | 2.17610390361863 | 0.999911036302558 |
| ebi-fl187-GCST90094727 | prot-a-1503 | Interleukin-1 receptor-like 2 \|\| id:prot-a-1503 | Cauliflower liking \|\| id:ebi-fl187-GCST90094727 | Weighted median | 4 | 0.0136186727581085 | 0.373089281716739 | 0.970881725394717 | -0.717636319 | 0.744873664922917 | 1.01371182929103 | 0.487904143632099 | 2.10617533434894 | 0.999489402457283 |
| ebi-fl187-GCST90094727 | prot-a-1536 | Interleukin-5 receptor subunit alpha \|\| id:prot-a-1536 | Cauliflower liking \|\| id:ebi-fl187-GCST90094727 | Weighted median | 4 | 0.185665418459424 | 0.430597307649183 | 0.666336699160074 | -0.658305305 | 1.02963614145182 | 1.20401935032914 | 0.517727982721469 | 2.80004682834944 | 0.999489402457283 |
| ebi-fl187-GCST90094727 | prot-a-1559 | Insulin-induced gene 1 protein \|\| id:prot-a-1559 | Cauliflower liking \|\| id:ebi-fl187-GCST90094727 | Weighted median | 4 | 0.41477681600104 | 0.382733207648783 | 0.278487592885544 | -0.335380271 | 1.16493390299266 | 1.51403279508766 | 0.715066115744411 | 3.20571098829734 | 0.999489402457283 |
| ebi-fl187-GCST90094727 | prot-a-1567 | Interferon regulatory factor 1 \|\| id:prot-a-1567 | Cauliflower liking \|\| id:ebi-fl187-GCST90094727 | Weighted median | 4 | -0.302442428 | 0.38963685010968 | 0.437621354050235 | -1.066130654 | 0.461245798707216 | 0.739011033741215 | 0.344338307198102 | 1.58604865208058 | 0.999489402457283 |
| ebi-fl187-GCST90094727 | prot-a-1613 | Potassium voltage-gated channel subfamily E regulatory beta subunit 5 \|\| id:prot-a-1613 | Cauliflower liking \|\| id:ebi-fl187-GCST90094727 | Weighted median | 3 | -0.769106474 | 0.44910538208423 | 0.0867990268480835 | -1.649353023 | 0.111140074658691 | 0.463426967308451 | 0.19217420067578 | 1.11755143652732 | 0.999489402457283 |
| ebi-fl187-GCST90094727 | prot-a-1649 | Kin of IRRE-like protein 2 \|\| id:prot-a-1649 | Cauliflower liking \|\| id:ebi-fl187-GCST90094727 | Weighted median | 4 | -0.342712431 | 0.412579924841018 | 0.4061673855622 | -1.151369084 | 0.465944221706464 | 0.709842310868804 | 0.316203563763705 | 1.59351811314848 | 0.999489402457283 |
| ebi-fl187-GCST90094727 | prot-a-165 | Armadillo repeat-containing protein 5 \|\| id:prot-a-165 | Cauliflower liking \|\| id:ebi-fl187-GCST90094727 | Weighted median | 4 | -0.276549594 | 0.411400854068003 | 0.50144719076859 | -1.082895268 | 0.529796080059596 | 0.758396006372914 | 0.338613727594995 | 1.69858589776467 | 0.999489402457283 |
| ebi-fl187-GCST90094727 | prot-a-1656 | Kallikrein-11 \|\| id:prot-a-1656 | Cauliflower liking \|\| id:ebi-fl187-GCST90094727 | Weighted median | 4 | -0.370002959 | 0.447482880588531 | 0.408319883224914 | -1.247069405 | 0.507063487150612 | 0.690732286893632 | 0.287345657965103 | 1.66040821892374 | 0.999489402457283 |
| ebi-fl187-GCST90094727 | prot-a-1697 | Laminin subunit gamma-2 \|\| id:prot-a-1697 | Cauliflower liking \|\| id:ebi-fl187-GCST90094727 | Weighted median | 4 | -0.15836506 | 0.411331512943114 | 0.700233042267868 | -0.964574826 | 0.647844704908065 | 0.853538132062921 | 0.381145218396107 | 1.91141671920002 | 0.999489402457283 |
| ebi-fl187-GCST90094727 | prot-a-1698 | Lysosome-associated membrane glycoprotein 3 \|\| id:prot-a-1698 | Cauliflower liking \|\| id:ebi-fl187-GCST90094727 | Weighted median | 4 | -0.341762469 | 0.398589919049707 | 0.39120795745155 | -1.122998711 | 0.439473771883583 | 0.710516954146983 | 0.325302841414081 | 1.55189035526345 | 0.999489402457283 |
| ebi-fl187-GCST90094727 | prot-a-1724 | Leptin receptor \|\| id:prot-a-1724 | Cauliflower liking \|\| id:ebi-fl187-GCST90094727 | Weighted median | 4 | -0.483893389 | 0.423524098861853 | 0.253229467608596 | -1.314000623 | 0.346213845029205 | 0.616378911341903 | 0.268742764549483 | 1.41370489726087 | 0.999489402457283 |
| ebi-fl187-GCST90094727 | prot-a-1743 | Leukocyte immunoglobulin-like receptor subfamily B member 2 \|\| id:prot-a-1743 | Cauliflower liking \|\| id:ebi-fl187-GCST90094727 | Weighted median | 4 | -0.323325279 | 0.422582158342161 | 0.444201193388257 | -1.151586309 | 0.504935751416402 | 0.72373839922053 | 0.316134883710049 | 1.65687906490796 | 0.999489402457283 |
| ebi-fl187-GCST90094727 | prot-a-1746 | Leukocyte immunoglobulin-like receptor subfamily B member 4 \|\| id:prot-a-1746 | Cauliflower liking \|\| id:ebi-fl187-GCST90094727 | Weighted median | 4 | 0.0576666082084891 | 0.379550787502838 | 0.879239118513492 | -0.686252935 | 0.801586151714051 | 1.05936175431654 | 0.503459032640315 | 2.22907377512557 | 0.999489402457283 |
| ebi-fl187-GCST90094727 | prot-a-1750 | Leucine-rich repeat and immunoglobulin-like domain-containing nogo receptor-interacting protein 3 \|\| id:prot-a-1750 | Cauliflower liking \|\| id:ebi-fl187-GCST90094727 | Weighted median | 4 | -0.035886218 | 0.400476118150048 | 0.928598024490459 | -0.82081941 | 0.749046973603183 | 0.964750058457579 | 0.440070908423951 | 2.11498342080193 | 0.999489402457283 |
| ebi-fl187-GCST90094727 | prot-a-1753 | Lipase member N \|\| id:prot-a-1753 | Cauliflower liking \|\| id:ebi-fl187-GCST90094727 | Weighted median | 4 | 0.406240406878152 | 0.383754787035133 | 0.289784849011165 | -0.345918976 | 1.15839978946701 | 1.50116339908765 | 0.707569815125384 | 3.18483279329977 | 0.999489402457283 |
| ebi-fl187-GCST90094727 | prot-a-1760 | Ig Kappa chain V-I region HK102- like \|\| id:prot-a-1760 | Cauliflower liking \|\| id:ebi-fl187-GCST90094727 | Weighted median | 4 | -0.472260623 | 0.390991420169137 | 0.227103355246836 | -1.238603806 | 0.294082560713526 | 0.623590969701645 | 0.289788536534543 | 1.34189468687656 | 0.999489402457283 |
| ebi-fl187-GCST90094727 | prot-a-1792 | Leucine-rich repeat neuronal protein 1 \|\| id:prot-a-1792 | Cauliflower liking \|\| id:ebi-fl187-GCST90094727 | Weighted median | 4 | -0.27695987 | 0.396704616904315 | 0.485082537642695 | -1.054500919 | 0.50058117899213 | 0.758084918341497 | 0.348366246807402 | 1.64967975136397 | 0.999489402457283 |
| ebi-fl187-GCST90094727 | prot-a-1795 | Leucine-rich repeat transmembrane neuronal protein 1 \|\| id:prot-a-1795 | Cauliflower liking \|\| id:ebi-fl187-GCST90094727 | Weighted median | 4 | -0.276978374 | 0.40348638231321 | 0.492421341764316 | -1.067811684 | 0.513854934856381 | 0.758070890612342 | 0.343759950396809 | 1.67172317348323 | 0.999489402457283 |
| ebi-fl187-GCST90094727 | prot-a-1820 | LysM and putative peptidoglycan-binding domain-containing protein 4 \|\| id:prot-a-1820 | Cauliflower liking \|\| id:ebi-fl187-GCST90094727 | Weighted median | 4 | -0.072176636 | 0.412117609846738 | 0.860972758566956 | -0.879927152 | 0.735573878801276 | 0.930366544607818 | 0.414813128971567 | 2.08667914988974 | 0.999489402457283 |
| ebi-fl187-GCST90094727 | prot-a-1844 | Mitogen-activated protein kinase kinase kinase 7:TGF-beta-activated kinase 1 and MAP3K7-binding protein 1 fusion \|\| id:prot-a-1844 | Cauliflower liking \|\| id:ebi-fl187-GCST90094727 | Weighted median | 4 | -0.28379129 | 0.378918812262369 | 0.453887270164108 | -1.026472162 | 0.458889582233621 | 0.752923771179077 | 0.358268647555071 | 1.58231597734039 | 0.999489402457283 |
| ebi-fl187-GCST90094727 | prot-a-1856 | Megakaryocyte-associated tyrosine-protein kinase \|\| id:prot-a-1856 | Cauliflower liking \|\| id:ebi-fl187-GCST90094727 | Weighted median | 4 | -0.599699986 | 0.434269813785341 | 0.167297813775432 | -1.450868821 | 0.251468848989291 | 0.548976311953059 | 0.234366576997033 | 1.28591284195527 | 0.999489402457283 |
| ebi-fl187-GCST90094727 | prot-a-1910 | Stromelysin-2 \|\| id:prot-a-1910 | Cauliflower liking \|\| id:ebi-fl187-GCST90094727 | Weighted median | 4 | -0.515565433 | 0.41777947958315 | 0.217180112004917 | -1.334413213 | 0.303282347366466 | 0.597162843790323 | 0.263312638671832 | 1.35429679259788 | 0.999489402457283 |
| ebi-fl187-GCST90094727 | prot-a-1940 | 39S ribosomal protein L14, mitochondrial \|\| id:prot-a-1940 | Cauliflower liking \|\| id:ebi-fl187-GCST90094727 | Weighted median | 4 | -0.60606997 | 0.418820152352811 | 0.147872042420068 | -1.426957468 | 0.214817528925072 | 0.545490456027805 | 0.24003813599171 | 1.23963567867274 | 0.999489402457283 |
| ebi-fl187-GCST90094727 | prot-a-1942 | 39S ribosomal protein L33, mitochondrial \|\| id:prot-a-1942 | Cauliflower liking \|\| id:ebi-fl187-GCST90094727 | Weighted median | 4 | 0.696169775777407 | 0.438013707415465 | 0.111974859223493 | -0.162337091 | 1.55467664231172 | 2.00605433572862 | 0.850154576983356 | 4.73355564605094 | 0.999489402457283 |
| ebi-fl187-GCST90094727 | prot-a-1948 | Mesothelin \|\| id:prot-a-1948 | Cauliflower liking \|\| id:ebi-fl187-GCST90094727 | Weighted median | 4 | 0.561952773480264 | 0.396286600318185 | 0.156177228397472 | -0.214768963 | 1.33867451010391 | 1.75409450724208 | 0.806727802503854 | 3.81398475518901 | 0.999489402457283 |
| ebi-fl187-GCST90094727 | prot-a-1980 | Unconventional myosin-VI \|\| id:prot-a-1980 | Cauliflower liking \|\| id:ebi-fl187-GCST90094727 | Weighted median | 4 | -0.198977009 | 0.416649495571284 | 0.632960201383896 | -1.01561002 | 0.617656002386539 | 0.819568735875892 | 0.362181424426335 | 1.85457582174214 | 0.999489402457283 |
| ebi-fl187-GCST90094727 | prot-a-2039 | Neurofascin \|\| id:prot-a-2039 | Cauliflower liking \|\| id:ebi-fl187-GCST90094727 | Weighted median | 4 | -0.162236713 | 0.388495236091324 | 0.676237521394778 | -0.923687376 | 0.599213949559661 | 0.85023991772343 | 0.397052257522571 | 1.82068708587874 | 0.999489402457283 |
| ebi-fl187-GCST90094727 | prot-a-2041 | NFU1 iron-sulfur cluster scaffold homolog, mitochondrial \|\| id:prot-a-2041 | Cauliflower liking \|\| id:ebi-fl187-GCST90094727 | Weighted median | 4 | 0.190499930357231 | 0.40544664735266 | 0.63846073842984 | -0.604175498 | 0.985175359168445 | 1.20985428937987 | 0.546524851511199 | 2.67828150446124 | 0.999489402457283 |
| ebi-fl187-GCST90094727 | prot-a-2088 | Glucocorticoid receptor \|\| id:prot-a-2088 | Cauliflower liking \|\| id:ebi-fl187-GCST90094727 | Weighted median | 4 | 0.41444690839921 | 0.383204035485412 | 0.27946113573328 | -0.336633001 | 1.16552681795062 | 1.51353338654301 | 0.714170891707814 | 3.20761226588547 | 0.999489402457283 |
| ebi-fl187-GCST90094727 | prot-a-2111 | Neuron-specific protein family member 2 \|\| id:prot-a-2111 | Cauliflower liking \|\| id:ebi-fl187-GCST90094727 | Weighted median | 4 | 0.459232123168403 | 0.413746696175667 | 0.267026845461048 | -0.351711401 | 1.27017564767271 | 1.58285807817495 | 0.703483116970661 | 3.56147807275408 | 0.999489402457283 |
| ebi-fl187-GCST90094727 | prot-a-2125 | Nuclear migration protein nudC \|\| id:prot-a-2125 | Cauliflower liking \|\| id:ebi-fl187-GCST90094727 | Weighted median | 4 | 0.0285964019466553 | 0.413209038236692 | 0.944825922241734 | -0.781293313 | 0.838486116890572 | 1.02900920454366 | 0.457813532065915 | 2.31286292097438 | 0.999489402457283 |
| ebi-fl187-GCST90094727 | prot-a-2142 | Ornithine decarboxylase \|\| id:prot-a-2142 | Cauliflower liking \|\| id:ebi-fl187-GCST90094727 | Weighted median | 4 | -0.337424142 | 0.385448159465229 | 0.381352316160337 | -1.092902534 | 0.418054250747103 | 0.713606105524631 | 0.335242028675743 | 1.51900307921886 | 0.999489402457283 |
| ebi-fl187-GCST90094727 | prot-a-2186 | Prostate and testis expressed protein 1 \|\| id:prot-a-2186 | Cauliflower liking \|\| id:ebi-fl187-GCST90094727 | Weighted median | 4 | 0.629069000760145 | 0.398096047961261 | 0.114062437170246 | -0.151199253 | 1.40933725476422 | 1.87586333870832 | 0.859676388283605 | 4.09324172847826 | 0.999489402457283 |
| ebi-fl187-GCST90094727 | prot-a-2227 | Platelet-derived growth factor C \|\| id:prot-a-2227 | Cauliflower liking \|\| id:ebi-fl187-GCST90094727 | Weighted median | 4 | -0.157799302 | 0.46082386452878 | 0.732028318075934 | -1.061014076 | 0.745415472937721 | 0.854021165503545 | 0.346104655881255 | 2.10731678622164 | 0.999489402457283 |
| ebi-fl187-GCST90094727 | prot-a-2229 | Platelet-derived growth factor receptor alpha \|\| id:prot-a-2229 | Cauliflower liking \|\| id:ebi-fl187-GCST90094727 | Weighted median | 4 | -0.457037428 | 0.435029669048277 | 0.293447350226035 | -1.309695579 | 0.395620723657902 | 0.633156642037169 | 0.26990220777668 | 1.48530586932984 | 0.999489402457283 |
| ebi-fl187-GCST90094727 | prot-a-2243 | Platelet endothelial aggregation receptor 1 \|\| id:prot-a-2243 | Cauliflower liking \|\| id:ebi-fl187-GCST90094727 | Weighted median | 4 | 0.242750948472598 | 0.461481110881901 | 0.598870314440426 | -0.661752029 | 1.14725392580112 | 1.27475110587092 | 0.515946588841123 | 3.14953217457851 | 0.999489402457283 |
| ebi-fl187-GCST90094727 | prot-a-2246 | E3 ubiquitin-protein ligase pellino homolog 2 \|\| id:prot-a-2246 | Cauliflower liking \|\| id:ebi-fl187-GCST90094727 | Weighted median | 4 | 0.666563243631325 | 0.406158936942995 | 0.100768578973922 | -0.129508273 | 1.4626347600396 | 1.94753261090455 | 0.878527320525621 | 4.31731965747795 | 0.999489402457283 |
| ebi-fl187-GCST90094727 | prot-a-2251 | Platelet factor 4 variant \|\| id:prot-a-2251 | Cauliflower liking \|\| id:ebi-fl187-GCST90094727 | Weighted median | 4 | -0.271374628 | 0.427646500075893 | 0.525704340971557 | -1.109561768 | 0.566812512499022 | 0.762330852687732 | 0.329703416074337 | 1.76263969563656 | 0.999489402457283 |
| ebi-fl187-GCST90094727 | prot-a-2346 | Protein phosphatase 1L \|\| id:prot-a-2346 | Cauliflower liking \|\| id:ebi-fl187-GCST90094727 | Weighted median | 4 | 0.856032126876247 | 0.467037533271872 | 0.0668177520055601 | -0.059361438 | 1.77142569208912 | 2.35380255014319 | 0.942366100359624 | 5.87922935995498 | 0.999489402457283 |
| ebi-fl187-GCST90094727 | prot-a-2358 | PR domain zinc finger protein 4 \|\| id:prot-a-2358 | Cauliflower liking \|\| id:ebi-fl187-GCST90094727 | Weighted median | 4 | 0.0820048650017098 | 0.432976671838648 | 0.849780983623383 | -0.766629412 | 0.93063914180546 | 1.08546109058677 | 0.464576327766716 | 2.53612960617625 | 0.999489402457283 |
| ebi-fl187-GCST90094727 | prot-a-2390 | Trypsin-1 \|\| id:prot-a-2390 | Cauliflower liking \|\| id:ebi-fl187-GCST90094727 | Weighted median | 4 | 0.0974159953152729 | 0.413630487156128 | 0.813809392282655 | -0.71329976 | 0.908131750141284 | 1.10231883772737 | 0.490024563515356 | 2.47968553105103 | 0.999489402457283 |
| ebi-fl187-GCST90094727 | prot-a-242 | Brain-derived neurotrophic factor \|\| id:prot-a-242 | Cauliflower liking \|\| id:ebi-fl187-GCST90094727 | Weighted median | 4 | 0.135165704776473 | 0.404943413695688 | 0.738538695754669 | -0.658523386 | 0.928854795620021 | 1.14472645527759 | 0.517615088119311 | 2.5316083079679 | 0.999489402457283 |
| ebi-fl187-GCST90094727 | prot-a-2464 | Dihydropteridine reductase \|\| id:prot-a-2464 | Cauliflower liking \|\| id:ebi-fl187-GCST90094727 | Weighted median | 4 | 0.0590210272127084 | 0.399815766469065 | 0.882642228598511 | -0.724617875 | 0.842659929492075 | 1.06079754612129 | 0.484509676813902 | 2.32253655129611 | 0.999489402457283 |
| ebi-fl187-GCST90094727 | prot-a-2474 | Ras-related protein Rab-27A \|\| id:prot-a-2474 | Cauliflower liking \|\| id:ebi-fl187-GCST90094727 | Weighted median | 4 | -0.219320052 | 0.504500591382201 | 0.663760563670931 | -1.208141211 | 0.76950110676881 | 0.803064654296854 | 0.298752080587986 | 2.15868902975218 | 0.999489402457283 |
| ebi-fl187-GCST90094727 | prot-a-2478 | Ras-related protein Rab-7b \|\| id:prot-a-2478 | Cauliflower liking \|\| id:ebi-fl187-GCST90094727 | Weighted median | 4 | -0.556239174 | 0.44981822940327 | 0.216240614702618 | -1.437882903 | 0.325404555916387 | 0.573361326517169 | 0.237429888989287 | 1.38459067704135 | 0.999489402457283 |
| ebi-fl187-GCST90094727 | prot-a-2528 | E3 ubiquitin-protein ligase rififylin \|\| id:prot-a-2528 | Cauliflower liking \|\| id:ebi-fl187-GCST90094727 | Weighted median | 4 | -0.180233875 | 0.419792018347749 | 0.667675169394921 | -1.003026231 | 0.642558480504971 | 0.835074885051091 | 0.36676783568453 | 1.9013392009732 | 0.999489402457283 |
| ebi-fl187-GCST90094727 | prot-a-2543 | Protein RIC-3 \|\| id:prot-a-2543 | Cauliflower liking \|\| id:ebi-fl187-GCST90094727 | Weighted median | 4 | -0.209906999 | 0.386096008015592 | 0.586671820807824 | -0.966655175 | 0.546841176883227 | 0.810659634761176 | 0.380353127631264 | 1.72778661641037 | 0.999489402457283 |
| ebi-fl187-GCST90094727 | prot-a-255 | Bone morphogenetic protein 6 \|\| id:prot-a-255 | Cauliflower liking \|\| id:ebi-fl187-GCST90094727 | Weighted median | 4 | -0.497788295 | 0.464584420162827 | 0.283958127155532 | -1.408373759 | 0.4127971682929 | 0.607873611026631 | 0.244540642045677 | 1.51103850832916 | 0.999489402457283 |
| ebi-fl187-GCST90094727 | prot-a-2568 | RING finger protein 165 \|\| id:prot-a-2568 | Cauliflower liking \|\| id:ebi-fl187-GCST90094727 | Weighted median | 4 | 0.000874679420970539 | 0.385798696730025 | 0.998191044672571 | -0.755290766 | 0.757040125011819 | 1.00087506206457 | 0.469873971410881 | 2.13195654752874 | 0.999911036302558 |
| ebi-fl187-GCST90094727 | prot-a-2573 | E3 ubiquitin-protein ligase RNF8 \|\| id:prot-a-2573 | Cauliflower liking \|\| id:ebi-fl187-GCST90094727 | Weighted median | 4 | -0.041474907 | 0.485351111489167 | 0.931900934538425 | -0.992763085 | 0.909813271712589 | 0.959373408782326 | 0.370551410041711 | 2.48385868340109 | 0.999489402457283 |
| ebi-fl187-GCST90094727 | prot-a-260 | Cytoplasmic tyrosine-protein kinase BMX \|\| id:prot-a-260 | Cauliflower liking \|\| id:ebi-fl187-GCST90094727 | Weighted median | 4 | 0.179657743348876 | 0.407756713906108 | 0.659502362759338 | -0.619545416 | 0.978860902604848 | 1.19680767762923 | 0.538189034169515 | 2.66142289473168 | 0.999489402457283 |
| ebi-fl187-GCST90094727 | prot-a-2623 | Serum amyloid A-1 protein \|\| id:prot-a-2623 | Cauliflower liking \|\| id:ebi-fl187-GCST90094727 | Weighted median | 4 | -0.327700453 | 0.393175049248791 | 0.404578498842775 | -1.09832355 | 0.442922643267964 | 0.720578834421394 | 0.333429593523298 | 1.55725186576702 | 0.999489402457283 |
| ebi-fl187-GCST90094727 | prot-a-2640 | Lysosome membrane protein 2 \|\| id:prot-a-2640 | Cauliflower liking \|\| id:ebi-fl187-GCST90094727 | Weighted median | 4 | 0.102173193408914 | 0.418011614584349 | 0.806900376474712 | -0.717129571 | 0.921475957994238 | 1.10757527985585 | 0.488151450849391 | 2.51299673167671 | 0.999489402457283 |
| ebi-fl187-GCST90094727 | prot-a-2641 | Scavenger receptor class F member 2 \|\| id:prot-a-2641 | Cauliflower liking \|\| id:ebi-fl187-GCST90094727 | Weighted median | 4 | -0.252413214 | 0.402103495292071 | 0.530179547057204 | -1.040536065 | 0.535709636834051 | 0.776923636056104 | 0.353265258153136 | 1.70866034044304 | 0.999489402457283 |
| ebi-fl187-GCST90094727 | prot-a-2669 | Semaphorin-3A \|\| id:prot-a-2669 | Cauliflower liking \|\| id:ebi-fl187-GCST90094727 | Weighted median | 4 | 0.057377450139697 | 0.405451397310121 | 0.887463119481177 | -0.737307289 | 0.852062188867534 | 1.05905547560112 | 0.478400376863372 | 2.34447662385735 | 0.999489402457283 |
| ebi-fl187-GCST90094727 | prot-a-2683 | Sentrin-specific protease 7 \|\| id:prot-a-2683 | Cauliflower liking \|\| id:ebi-fl187-GCST90094727 | Weighted median | 4 | 0.0803734598632812 | 0.471418295627745 | 0.864622538404542 | -0.8436064 | 1.00435331929366 | 1.0836917074688 | 0.430156406851368 | 2.73014117221423 | 0.999489402457283 |
| ebi-fl187-GCST90094727 | prot-a-270 | Bromodomain testis-specific protein \|\| id:prot-a-270 | Cauliflower liking \|\| id:ebi-fl187-GCST90094727 | Weighted median | 4 | 0.649706250445109 | 0.445549770386172 | 0.144781975747587 | -0.2235713 | 1.52298380040201 | 1.91497822238477 | 0.799657874618912 | 4.58588817618478 | 0.999489402457283 |
| ebi-fl187-GCST90094727 | prot-a-2708 | 14-3-3 protein sigma \|\| id:prot-a-2708 | Cauliflower liking \|\| id:ebi-fl187-GCST90094727 | Weighted median | 4 | 0.267826782869142 | 0.42502469106998 | 0.528600337359507 | -0.565221612 | 1.1008751773663 | 1.30712070470611 | 0.568234205495656 | 3.0067963528895 | 0.999489402457283 |
| ebi-fl187-GCST90094727 | prot-a-2713 | Beta-sarcoglycan \|\| id:prot-a-2713 | Cauliflower liking \|\| id:ebi-fl187-GCST90094727 | Weighted median | 4 | -0.258807589 | 0.403928312206999 | 0.521700369477906 | -1.050507081 | 0.532891903110393 | 0.771971544714084 | 0.349760347400314 | 1.70385256727279 | 0.999489402457283 |
| ebi-fl187-GCST90094727 | prot-a-2720 | Protein shisa-3 homolog \|\| id:prot-a-2720 | Cauliflower liking \|\| id:ebi-fl187-GCST90094727 | Weighted median | 4 | -0.186585814 | 0.410433275433507 | 0.649392043500646 | -0.991035034 | 0.617863405819999 | 0.829787351499556 | 0.37119229552356 | 1.85496050702632 | 0.999489402457283 |
| ebi-fl187-GCST90094727 | prot-a-2730 | Sialic acid-binding Ig-like lectin 8 \|\| id:prot-a-2730 | Cauliflower liking \|\| id:ebi-fl187-GCST90094727 | Weighted median | 4 | -0.807859881 | 0.401610471846908 | 0.044267306247634 | -1.595016406 | -0.020703356 | 0.445811134919327 | 0.20290519965823 | 0.979509486956595 | 0.999489402457283 |
| ebi-fl187-GCST90094727 | prot-a-2761 | Sodium- and chloride-dependent neutral and basic amino acid transporter B(0+) \|\| id:prot-a-2761 | Cauliflower liking \|\| id:ebi-fl187-GCST90094727 | Weighted median | 4 | -0.092979177 | 0.423468886091533 | 0.826209527579967 | -0.922978194 | 0.73701983991782 | 0.911212474438518 | 0.397333939848811 | 2.08969858927306 | 0.999489402457283 |
| ebi-fl187-GCST90094727 | prot-a-2770 | Secreted Ly-6/uPAR-related protein 1 \|\| id:prot-a-2770 | Cauliflower liking \|\| id:ebi-fl187-GCST90094727 | Weighted median | 4 | 0.199089894380087 | 0.396120735508102 | 0.615246216809043 | -0.577306747 | 0.975486535975966 | 1.22029165833158 | 0.561408346866619 | 2.65245741304846 | 0.999489402457283 |
| ebi-fl187-GCST90094727 | prot-a-2784 | N-lysine methyltransferase SMYD2 \|\| id:prot-a-2784 | Cauliflower liking \|\| id:ebi-fl187-GCST90094727 | Weighted median | 4 | 0.225601921331131 | 0.430296760217925 | 0.600074212090709 | -0.617779729 | 1.06898357135826 | 1.2530767428578 | 0.539140147101166 | 2.91241773022049 | 0.999489402457283 |
| ebi-fl187-GCST90094727 | prot-a-2837 | Serine palmitoyltransferase 2 \|\| id:prot-a-2837 | Cauliflower liking \|\| id:ebi-fl187-GCST90094727 | Weighted median | 4 | 0.134226764443627 | 0.38031725641185 | 0.724138169271407 | -0.611195058 | 0.879648587010853 | 1.1436521298813 | 0.542701921046987 | 2.41005263378972 | 0.999489402457283 |
| ebi-fl187-GCST90094727 | prot-a-2858 | Alpha-2,8-sialyltransferase 8B \|\| id:prot-a-2858 | Cauliflower liking \|\| id:ebi-fl187-GCST90094727 | Weighted median | 4 | 0.338182109334859 | 0.381819820166412 | 0.375773145722331 | -0.410184738 | 1.08654895686103 | 1.40239586953135 | 0.663527659913344 | 2.96402741542897 | 0.999489402457283 |
| ebi-fl187-GCST90094727 | prot-a-2911 | Synaptotagmin-11 \|\| id:prot-a-2911 | Cauliflower liking \|\| id:ebi-fl187-GCST90094727 | Weighted median | 4 | -0.249962912 | 0.393679105109682 | 0.525467049371577 | -1.021573958 | 0.521648134198291 | 0.778829667913252 | 0.360027825367408 | 1.68480214273121 | 0.999489402457283 |
| ebi-fl187-GCST90094727 | prot-a-2962 | Transforming growth factor beta-1 \|\| id:prot-a-2962 | Cauliflower liking \|\| id:ebi-fl187-GCST90094727 | Weighted median | 4 | -0.116264102 | 0.411709950454209 | 0.777642006177581 | -0.923215605 | 0.690687400509048 | 0.890240077723218 | 0.397239619315369 | 1.99508648545817 | 0.999489402457283 |
| ebi-fl187-GCST90094727 | prot-a-2978 | Tyrosine-protein kinase receptor Tie-1, soluble \|\| id:prot-a-2978 | Cauliflower liking \|\| id:ebi-fl187-GCST90094727 | Weighted median | 4 | -0.141743093 | 0.40421205137563 | 0.72583954892899 | -0.933998714 | 0.650512527803112 | 0.867844183187284 | 0.39297915328767 | 1.91652284858143 | 0.999489402457283 |
| ebi-fl187-GCST90094727 | prot-a-299 | Uncharacterized protein C1orf226 \|\| id:prot-a-299 | Cauliflower liking \|\| id:ebi-fl187-GCST90094727 | Weighted median | 4 | 0.136848432354429 | 0.420210024440623 | 0.744676504676356 | -0.686763216 | 0.960460080258049 | 1.14665433965029 | 0.503202192973885 | 2.61289833986687 | 0.999489402457283 |
| ebi-fl187-GCST90094727 | prot-a-3015 | Transmembrane protein 70, mitochondrial \|\| id:prot-a-3015 | Cauliflower liking \|\| id:ebi-fl187-GCST90094727 | Weighted median | 4 | -0.305802941 | 0.402260222327928 | 0.447128032610443 | -1.094232977 | 0.482627094931598 | 0.736531745490027 | 0.334796305091568 | 1.62032556472275 | 0.999489402457283 |
| ebi-fl187-GCST90094727 | prot-a-3016 | Transmembrane protein 87B \|\| id:prot-a-3016 | Cauliflower liking \|\| id:ebi-fl187-GCST90094727 | Weighted median | 4 | -0.783984935 | 0.428269910551377 | 0.0671623485414973 | -1.623393959 | 0.0554240900616337 | 0.456582928167242 | 0.197228177458643 | 1.056988778 | 0.999489402457283 |
| ebi-fl187-GCST90094727 | prot-a-3123 | Thioredoxin domain-containing protein 12 \|\| id:prot-a-3123 | Cauliflower liking \|\| id:ebi-fl187-GCST90094727 | Weighted median | 4 | -0.42315884 | 0.376894508537252 | 0.261543088215918 | -1.161872077 | 0.315554396848977 | 0.654974588761103 | 0.312899859723911 | 1.37101918901881 | 0.999489402457283 |
| ebi-fl187-GCST90094727 | prot-a-3133 | PolyUbiquitin K48-linked \|\| id:prot-a-3133 | Cauliflower liking \|\| id:ebi-fl187-GCST90094727 | Weighted median | 4 | -0.095710777 | 0.413354128371627 | 0.816890123873658 | -0.905884868 | 0.714463314836471 | 0.908726802964236 | 0.404184077110218 | 2.04308989193658 | 0.999489402457283 |
| ebi-fl187-GCST90094727 | prot-a-3135 | Ubiquitin-conjugating enzyme E2 B \|\| id:prot-a-3135 | Cauliflower liking \|\| id:ebi-fl187-GCST90094727 | Weighted median | 4 | 0.0195640143211246 | 0.405688844640884 | 0.961537574930576 | -0.775586121 | 0.814714149817258 | 1.01975664380057 | 0.460433831876692 | 2.25852997886111 | 0.999489402457283 |
| ebi-fl187-GCST90094727 | prot-a-3162 | NKG2D ligand 3 \|\| id:prot-a-3162 | Cauliflower liking \|\| id:ebi-fl187-GCST90094727 | Weighted median | 4 | 0.945409350848726 | 0.404602562708479 | 0.0194580626918993 | 0.152388327940108 | 1.73843037375734 | 2.57386677751863 | 1.16461240016691 | 5.68840773759982 | 0.999489402457283 |
| ebi-fl187-GCST90094727 | prot-a-3188 | Vesicle-associated membrane protein 8 \|\| id:prot-a-3188 | Cauliflower liking \|\| id:ebi-fl187-GCST90094727 | Weighted median | 4 | -0.823860566 | 0.414168622811332 | 0.046680106573193 | -1.635631067 | -0.012090065 | 0.43873461695794 | 0.194829382181975 | 0.98798272602149 | 0.999489402457283 |
| ebi-fl187-GCST90094727 | prot-a-3201 | Vitrin \|\| id:prot-a-3201 | Cauliflower liking \|\| id:ebi-fl187-GCST90094727 | Weighted median | 4 | -0.366323239 | 0.42750488131264 | 0.391507513700295 | -1.204232807 | 0.471586328035037 | 0.693278670049529 | 0.299922009423934 | 1.60253432306888 | 0.999489402457283 |
| ebi-fl187-GCST90094727 | prot-a-3232 | WNT1-inducible-signaling pathway protein 3 \|\| id:prot-a-3232 | Cauliflower liking \|\| id:ebi-fl187-GCST90094727 | Weighted median | 4 | -0.510185815 | 0.420898395133112 | 0.225460581334895 | -1.33514667 | 0.314775039052252 | 0.600384007847022 | 0.263119580911162 | 1.36995109079379 | 0.999489402457283 |
| ebi-fl187-GCST90094727 | prot-a-327 | Carbonic anhydrase-related protein 10 \|\| id:prot-a-327 | Cauliflower liking \|\| id:ebi-fl187-GCST90094727 | Weighted median | 4 | 0.0525803508152506 | 0.384945636890344 | 0.89135359522337 | -0.701913097 | 0.807073799120326 | 1.05398724741082 | 0.495636195850725 | 2.24133977099446 | 0.999489402457283 |
| ebi-fl187-GCST90094727 | prot-a-332 | Carbonic anhydrase 5A, mitochondrial \|\| id:prot-a-332 | Cauliflower liking \|\| id:ebi-fl187-GCST90094727 | Weighted median | 4 | -0.10414278 | 0.395739460793628 | 0.792427291043004 | -0.879792124 | 0.671506562686933 | 0.901096629236816 | 0.414869144212594 | 1.95718372057543 | 0.999489402457283 |
| ebi-fl187-GCST90094727 | prot-a-338 | Cell adhesion molecule 3 \|\| id:prot-a-338 | Cauliflower liking \|\| id:ebi-fl187-GCST90094727 | Weighted median | 4 | -0.01806019 | 0.407279820139198 | 0.964630642266415 | -0.816328637 | 0.780208257503266 | 0.982101917894725 | 0.442051610695185 | 2.18192662077546 | 0.999489402457283 |
| ebi-fl187-GCST90094727 | prot-a-34 | A disintegrin and metalloproteinase with thrombospondin motifs 3 \|\| id:prot-a-34 | Cauliflower liking \|\| id:ebi-fl187-GCST90094727 | Weighted median | 4 | -0.3879138 | 0.456587080072974 | 0.395550658356204 | -1.282824477 | 0.506996876790157 | 0.678470824824898 | 0.277253098467873 | 1.66029762221726 | 0.999489402457283 |
| ebi-fl187-GCST90094727 | prot-a-358 | Cysteine--tRNA ligase, cytoplasmic \|\| id:prot-a-358 | Cauliflower liking \|\| id:ebi-fl187-GCST90094727 | Weighted median | 4 | -0.131901092 | 0.416233887430321 | 0.751325729027081 | -0.947719512 | 0.683917327130756 | 0.876427676297266 | 0.387623988286667 | 1.98162522184194 | 0.999489402457283 |
| ebi-fl187-GCST90094727 | prot-a-382 | Coiled-coil domain-containing protein 167 \|\| id:prot-a-382 | Cauliflower liking \|\| id:ebi-fl187-GCST90094727 | Weighted median | 4 | -0.695036464 | 0.39811172348326 | 0.0808400274802207 | -1.475335442 | 0.0852625139440164 | 0.499056250024743 | 0.22870199789603 | 1.08900290762647 | 0.999489402457283 |
| ebi-fl187-GCST90094727 | prot-a-40 | Pituitary adenylate cyclase-activating polypeptide 27 \|\| id:prot-a-40 | Cauliflower liking \|\| id:ebi-fl187-GCST90094727 | Weighted median | 4 | -0.180450616 | 0.408130949414037 | 0.658388613897721 | -0.980387277 | 0.61948604515549 | 0.834893910333543 | 0.375165777806317 | 1.85797288224912 | 0.999489402457283 |
| ebi-fl187-GCST90094727 | prot-a-403 | C-C motif chemokine 25 \|\| id:prot-a-403 | Cauliflower liking \|\| id:ebi-fl187-GCST90094727 | Weighted median | 4 | 0.17978619432354 | 0.404298842679514 | 0.656546403523732 | -0.612639537 | 0.972211925975388 | 1.19696141861579 | 0.541918565314419 | 2.6437858552114 | 0.999489402457283 |
| ebi-fl187-GCST90094727 | prot-a-415 | Cyclin-C \|\| id:prot-a-415 | Cauliflower liking \|\| id:ebi-fl187-GCST90094727 | Weighted median | 4 | -0.172521308 | 0.411480944974607 | 0.675019365864372 | -0.97902396 | 0.633981344336706 | 0.841540357235729 | 0.375677596338942 | 1.88510089437832 | 0.999489402457283 |
| ebi-fl187-GCST90094727 | prot-a-44 | Adhesion G protein-coupled receptor F5 \|\| id:prot-a-44 | Cauliflower liking \|\| id:ebi-fl187-GCST90094727 | Weighted median | 4 | -0.48256204 | 0.391136134171965 | 0.217298139645515 | -1.249188863 | 0.284064782786126 | 0.617200073016126 | 0.286737285730871 | 1.32851899312688 | 0.999489402457283 |
| ebi-fl187-GCST90094727 | prot-a-455 | CD59 glycoprotein \|\| id:prot-a-455 | Cauliflower liking \|\| id:ebi-fl187-GCST90094727 | Weighted median | 4 | 0.024828344860496 | 0.43248759149349 | 0.954220011394348 | -0.822847334 | 0.872504024187737 | 1.02513913501922 | 0.439179381941343 | 2.39289522541455 | 0.999489402457283 |
| ebi-fl187-GCST90094727 | prot-a-478 | Cadherin-12 \|\| id:prot-a-478 | Cauliflower liking \|\| id:ebi-fl187-GCST90094727 | Weighted median | 4 | -0.623486583 | 0.411718491720871 | 0.12993667174064 | -1.430454827 | 0.183481660906779 | 0.536072115635163 | 0.239200102919799 | 1.20139293275268 | 0.999489402457283 |
| ebi-fl187-GCST90094727 | prot-a-516 | Centrin-2 \|\| id:prot-a-516 | Cauliflower liking \|\| id:ebi-fl187-GCST90094727 | Weighted median | 4 | 0.0120739604543426 | 0.422424661833486 | 0.977197555090527 | -0.815878377 | 0.840026297647974 | 1.01214714496055 | 0.442250693980551 | 2.31642789258539 | 0.999489402457283 |
| ebi-fl187-GCST90094727 | prot-a-519 | Cryptic protein \|\| id:prot-a-519 | Cauliflower liking \|\| id:ebi-fl187-GCST90094727 | Weighted median | 4 | -0.027822608 | 0.38382898114499 | 0.942214357392827 | -0.780127411 | 0.724482194891752 | 0.972560875870591 | 0.458347608967426 | 2.06366224840805 | 0.999489402457283 |
| ebi-fl187-GCST90094727 | prot-a-583 | Chloride intracellular channel protein 4 \|\| id:prot-a-583 | Cauliflower liking \|\| id:ebi-fl187-GCST90094727 | Weighted median | 4 | 0.452425879158762 | 0.403510843166061 | 0.262192697636105 | -0.338455373 | 1.24330713176424 | 1.5721213397413 | 0.712870591636488 | 3.46706055189649 | 0.999489402457283 |
| ebi-fl187-GCST90094727 | prot-a-602 | CCR4-NOT transcription complex subunit 1 \|\| id:prot-a-602 | Cauliflower liking \|\| id:ebi-fl187-GCST90094727 | Weighted median | 4 | 0.41215923926937 | 0.43886011523069 | 0.347649335449401 | -0.448006587 | 1.27232506512152 | 1.51007488040482 | 0.638900475847184 | 3.56914140877091 | 0.999489402457283 |
| ebi-fl187-GCST90094727 | prot-a-606 | Ciliary neurotrophic factor receptor subunit alpha \|\| id:prot-a-606 | Cauliflower liking \|\| id:ebi-fl187-GCST90094727 | Weighted median | 4 | -0.217872337 | 0.402259504229622 | 0.588079379374017 | -1.006300965 | 0.570556291651974 | 0.804228105576915 | 0.365568733204768 | 1.76925099728797 | 0.999489402457283 |
| ebi-fl187-GCST90094727 | prot-a-626 | Collagen alpha-2(IX) chain \|\| id:prot-a-626 | Cauliflower liking \|\| id:ebi-fl187-GCST90094727 | Weighted median | 4 | 0.397714607550972 | 0.410011492838371 | 0.332042286396293 | -0.405907918 | 1.20133713351418 | 1.48841918568991 | 0.666371525147961 | 3.32455933173001 | 0.999489402457283 |
| ebi-fl187-GCST90094727 | prot-a-647 | Probable serine carboxypeptidase CPVL \|\| id:prot-a-647 | Cauliflower liking \|\| id:ebi-fl187-GCST90094727 | Weighted median | 4 | 0.0839794945196448 | 0.486599573181315 | 0.86297811627066 | -0.869755669 | 1.03771465795502 | 1.08760659168457 | 0.419053924640159 | 2.82275866833003 | 0.999489402457283 |
| ebi-fl187-GCST90094727 | prot-a-651 | Cellular retinoic acid-binding protein 2 \|\| id:prot-a-651 | Cauliflower liking \|\| id:ebi-fl187-GCST90094727 | Weighted median | 4 | -0.575607224 | 0.411767813483728 | 0.162145288469421 | -1.382672139 | 0.231457690192349 | 0.56236328449267 | 0.250907197657964 | 1.26043599664486 | 0.999489402457283 |
| ebi-fl187-GCST90094727 | prot-a-671 | Cytotoxic and regulatory T-cell molecule \|\| id:prot-a-671 | Cauliflower liking \|\| id:ebi-fl187-GCST90094727 | Weighted median | 4 | 0.131997986097636 | 0.46169748223328 | 0.774956910316722 | -0.772929079 | 1.03692505127486 | 1.14110602118881 | 0.461658850692717 | 2.82053067896244 | 0.999489402457283 |
| ebi-fl187-GCST90094727 | prot-a-688 | Chorionic somatomammotropin hormone \|\| id:prot-a-688 | Cauliflower liking \|\| id:ebi-fl187-GCST90094727 | Weighted median | 4 | -0.006959537 | 0.3971576119123 | 0.986019094366269 | -0.785388457 | 0.771469382166286 | 0.993064624313636 | 0.455942553518479 | 2.1629421085023 | 0.999911036302558 |
| ebi-fl187-GCST90094727 | prot-a-69 | Aldo-keto reductase family 1 member C1 \|\| id:prot-a-69 | Cauliflower liking \|\| id:ebi-fl187-GCST90094727 | Weighted median | 4 | -0.243841815 | 0.389190619055287 | 0.530963631418206 | -1.006655429 | 0.518971798170606 | 0.78361157991792 | 0.365439175359414 | 1.6802990746067 | 0.999489402457283 |
| ebi-fl187-GCST90094727 | prot-a-741 | Stromal cell-derived factor 1 \|\| id:prot-a-741 | Cauliflower liking \|\| id:ebi-fl187-GCST90094727 | Weighted median | 4 | -0.171491516 | 0.40695835560953 | 0.673464337986397 | -0.969129893 | 0.626146861053398 | 0.84240741502368 | 0.379413024473627 | 1.87038980507167 | 0.999489402457283 |
| ebi-fl187-GCST90094727 | prot-a-742 | Growth-regulated alpha protein \|\| id:prot-a-742 | Cauliflower liking \|\| id:ebi-fl187-GCST90094727 | Weighted median | 4 | -0.277175377 | 0.391696495 | 0.479176349 | -1.044900508 | 0.490549753954613 | 0.757921563516863 | 0.351726811724789 | 1.63321383896467 | 0.999489402457283 |
| ebi-fl187-GCST90094727 | prot-a-788 | Beta-defensin 112 \|\| id:prot-a-788 | Cauliflower liking \|\| id:ebi-fl187-GCST90094727 | Weighted median | 4 | -0.194053248 | 0.388996442303015 | 0.617880763462573 | -0.956486275 | 0.568379779205655 | 0.823614047540434 | 0.384240632845109 | 1.76540438808662 | 0.999489402457283 |
| ebi-fl187-GCST90094727 | prot-a-838 | DnaJ homolog subfamily B member 1 \|\| id:prot-a-838 | Cauliflower liking \|\| id:ebi-fl187-GCST90094727 | Weighted median | 4 | 0.334814211376497 | 0.388090612854706 | 0.388290472815654 | -0.42584339 | 1.09547181257172 | 1.39768068792771 | 0.653218634799236 | 2.99059335012154 | 0.999489402457283 |
| ebi-fl187-GCST90094727 | prot-a-85 | Junctional adhesion molecule-like \|\| id:prot-a-85 | Cauliflower liking \|\| id:ebi-fl187-GCST90094727 | Weighted median | 4 | -0.150923684 | 0.394245654393253 | 0.701855934607436 | -0.923645167 | 0.621797798521358 | 0.859913321223281 | 0.397069017141623 | 1.86227302583398 | 0.999489402457283 |
| ebi-fl187-GCST90094727 | prot-a-856 | Dipeptidase 1 \|\| id:prot-a-856 | Cauliflower liking \|\| id:ebi-fl187-GCST90094727 | Weighted median | 4 | -0.262136758 | 0.434126379493888 | 0.545959389237846 | -1.113024461 | 0.588750946273848 | 0.769405794473065 | 0.328563728517289 | 1.80173654359287 | 0.999489402457283 |
| ebi-fl187-GCST90094727 | prot-a-860 | Dipeptidyl peptidase 2 \|\| id:prot-a-860 | Cauliflower liking \|\| id:ebi-fl187-GCST90094727 | Weighted median | 4 | -0.309579449 | 0.424956053185988 | 0.466309060286661 | -1.142493313 | 0.523334415368473 | 0.733755473035076 | 0.319022606122425 | 1.68764558961166 | 0.999489402457283 |
| ebi-fl187-GCST90094727 | prot-a-861 | Dermatopontin \|\| id:prot-a-861 | Cauliflower liking \|\| id:ebi-fl187-GCST90094727 | Weighted median | 4 | -0.136703756 | 0.414409509006535 | 0.74149365074868 | -0.948946393 | 0.675538882152107 | 0.872228580781336 | 0.387148711178206 | 1.96509164350965 | 0.999489402457283 |
| ebi-fl187-GCST90094727 | prot-a-877 | Dual specificity protein phosphatase 6 \|\| id:prot-a-877 | Cauliflower liking \|\| id:ebi-fl187-GCST90094727 | Weighted median | 4 | -0.217012943 | 0.413414363361969 | 0.599632961666397 | -1.027305095 | 0.593279209436883 | 0.804919551362986 | 0.357970357982137 | 1.80991378118163 | 0.999489402457283 |
| ebi-fl187-GCST90094727 | prot-a-879 | Dynactin-associated protein \|\| id:prot-a-879 | Cauliflower liking \|\| id:ebi-fl187-GCST90094727 | Weighted median | 4 | -0.140397384 | 0.40351625133602 | 0.72788907869393 | -0.931289237 | 0.650494468339221 | 0.869012834735852 | 0.394045364929218 | 1.91648823751878 | 0.999489402457283 |
| ebi-fl187-GCST90094727 | prot-a-937 | EMI domain-containing protein 1 \|\| id:prot-a-937 | Cauliflower liking \|\| id:ebi-fl187-GCST90094727 | Weighted median | 4 | -0.264689118 | 0.396656812124543 | 0.504580481955554 | -1.04213647 | 0.512758233433929 | 0.767444497320761 | 0.352700342699658 | 1.66989079726938 | 0.999489402457283 |
| ebi-fl187-GCST90094727 | prot-b-1 | SRC proto-oncogene, non-receptor tyrosine kinase \|\| id:prot-b-1 | Cauliflower liking \|\| id:ebi-fl187-GCST90094727 | Weighted median | 4 | 0.154279505615991 | 0.470115336457787 | 0.742780305928935 | -0.767146554 | 1.07570556507325 | 1.16681697310165 | 0.464336137928681 | 2.93206093066833 | 0.999489402457283 |
| ebi-fl187-GCST90094727 | prot-b-10 | coagulation factor III, tissue factor \|\| id:prot-b-10 | Cauliflower liking \|\| id:ebi-fl187-GCST90094727 | Weighted median | 4 | -0.022987697 | 0.482806918267275 | 0.962024983454867 | -0.969289256 | 0.923313863118851 | 0.977274507415411 | 0.3793525646837 | 2.51761962816927 | 0.999489402457283 |
| ebi-fl187-GCST90094727 | prot-b-29 | myeloperoxidase \|\| id:prot-b-29 | Cauliflower liking \|\| id:ebi-fl187-GCST90094727 | Weighted median | 4 | 0.348055133424434 | 0.556378896794787 | 0.531595621571653 | -0.742447504 | 1.43855777114222 | 1.41631033362624 | 0.475947605021569 | 4.21461299515432 | 0.999489402457283 |
| ebi-fl187-GCST90094727 | prot-b-33 | adrenomedullin \|\| id:prot-b-33 | Cauliflower liking \|\| id:ebi-fl187-GCST90094727 | Weighted median | 4 | 0.0131427921706164 | 0.494207969177552 | 0.978783840600268 | -0.955504827 | 0.981790411758618 | 1.01322953827573 | 0.384617929858441 | 2.6692309888213 | 0.999489402457283 |
| ebi-fl187-GCST90094727 | ukb-rsfMRI191-edge-pheno102 | Net25_Pair11_15 (Postcentral\|Precentral)&(Cerebellum) (Motor)&(Subcortical-cerebellum) \|\| id:ukb-rsfMRI191-edge-pheno102 | Cauliflower liking \|\| id:ebi-fl187-GCST90094727 | Weighted median | 4 | -0.013026579 | 0.122139244335863 | 0.915063863844257 | -0.252419498 | 0.226366339480225 | 0.987057899247063 | 0.776918753589678 | 1.25403498366392 | 0.999489402457283 |
| ebi-fl187-GCST90094727 | ukb-rsfMRI191-edge-pheno1167 | Net100_Pair11_45 (Angular\|Temporal)&(Frontal) (Default_mode\|Central_executive)&(Salience\|Default_mode) \|\| id:ukb-rsfMRI191-edge-pheno1167 | Cauliflower liking \|\| id:ebi-fl187-GCST90094727 | Weighted median | 4 | -0.301647892 | 0.121273015255153 | 0.0128700332289076 | -0.539343002 | -0.063952782 | 0.739598437632889 | 0.583131242713242 | 0.938049291277648 | 0.999489402457283 |
| ebi-fl187-GCST90094727 | ukb-rsfMRI191-edge-pheno1317 | Net100_Pair26_48 (Parietal\|Frontal)&(Temporal) (Attention\|Central_executive)&(Default_mode\|Central_executive) \|\| id:ukb-rsfMRI191-edge-pheno1317 | Cauliflower liking \|\| id:ebi-fl187-GCST90094727 | Weighted median | 4 | -0.151515545 | 0.123215549552899 | 0.218817420093065 | -0.393018022 | 0.0899869317735212 | 0.859404522424854 | 0.675016582007813 | 1.09415998488129 | 0.999489402457283 |
| ebi-fl187-GCST90094727 | ukb-rsfMRI191-edge-pheno286 | Net100_Pair10_13 (OccipitalPrecuneus)&(Frontal_Sup) (Default_mode\|Central_executive)&(Default_mode) \|\| id:ukb-rsfMRI191-edge-pheno286 | Cauliflower liking \|\| id:ebi-fl187-GCST90094727 | Weighted median | 4 | 0.237946094746363 | 0.124909854628603 | 0.0567875417340676 | -0.00687722 | 0.482769409818424 | 1.26864080457734 | 0.993146373636064 | 1.62055617758155 | 0.999489402457283 |
| ebi-fl187-GCST90094727 | ukb-rsfMRI191-edge-pheno599 | Net100_Pair11_29 (Angular\|Temporal)&(Parietal) (Default_mode\|Central_executive)&(Central_executive\|Salience) \|\| id:ukb-rsfMRI191-edge-pheno599 | Cauliflower liking \|\| id:ebi-fl187-GCST90094727 | Weighted median | 4 | -0.00515702 | 0.12184216300176 | 0.966239286764855 | -0.243967659 | 0.233653619609669 | 0.994856254724283 | 0.783512973166292 | 1.26320686633221 | 0.999489402457283 |
| ebi-fl187-GCST90094727 | ukb-rsfMRI191-edge-pheno621 | Net100_Pair5_30 (Precuneus\|Angular\|Cingulate)&(Paracentral) (Default_mode\|Central_executive)&(Motor) \|\| id:ukb-rsfMRI191-edge-pheno621 | Cauliflower liking \|\| id:ebi-fl187-GCST90094727 | Weighted median | 4 | -0.045538943 | 0.114728648949801 | 0.691421007018499 | -0.270407095 | 0.179329208985448 | 0.955482392538497 | 0.763068789687073 | 1.19641454976226 | 0.999489402457283 |
| ebi-fl187-GCST90094727 | ukb-rsfMRI191-edge-pheno624 | Net100_Pair8_30 (Calcarine\|Lingual)&(Paracentral) (Visual)&(Motor) \|\| id:ukb-rsfMRI191-edge-pheno624 | Cauliflower liking \|\| id:ebi-fl187-GCST90094727 | Weighted median | 4 | 0.032406786500188 | 0.121002775244607 | 0.788838999675447 | -0.204758653 | 0.269572225979617 | 1.03293760492712 | 0.814843952842054 | 1.30940420181211 | 0.999489402457283 |
| ebi-fl187-GCST90094727 | ukb-rsfMRI191-edge-pheno882 | Net100_Pair6_38 (Postcentral\|Precentral)&(Subcortical) (Motor)&(Subcortical-cerebellum) \|\| id:ukb-rsfMRI191-edge-pheno882 | Cauliflower liking \|\| id:ebi-fl187-GCST90094727 | Weighted median | 4 | -0.05202929 | 0.129059358484991 | 0.686843525187213 | -0.304985633 | 0.200927052717565 | 0.949301061490164 | 0.73713396505565 | 1.22253558792169 | 0.999489402457283 |
| ebi-fl187-GCST90094727 | ukb-rsfMRI191-edge-pheno899 | Net100_Pair23_38 (Cerebellum)&(Subcortical) (Subcortical-cerebellum)&(Subcortical-cerebellum) \|\| id:ukb-rsfMRI191-edge-pheno899 | Cauliflower liking \|\| id:ebi-fl187-GCST90094727 | Weighted median | 4 | 0.105572563651752 | 0.132776912351847 | 0.426548147593393 | -0.154670185 | 0.365815311861373 | 1.11134674497521 | 0.856697683018807 | 1.44168895521559 | 0.999489402457283 |
| ebi-fl187-GCST90094727 | ebi-a-GCST90001392 | IgD+ CD38+ B cell Absolute Count \|\| id:ebi-a-GCST90001392 | Cauliflower liking \|\| id:ebi-fl187-GCST90094727 | Weighted mode | 4 | -1.008520312 | 0.681122665902784 | 0.235274456887525 | -2.343520737 | 0.326480113576877 | 0.364758309094775 | 0.0959890902921692 | 1.38608068530194 | 0.999228396881742 |
| ebi-fl187-GCST90094727 | ebi-a-GCST90001402 | Switched memory B cell %B cell \|\| id:ebi-a-GCST90001402 | Cauliflower liking \|\| id:ebi-fl187-GCST90094727 | Weighted mode | 4 | -0.237960104 | 0.576242135047774 | 0.707382442148243 | -1.367394689 | 0.891474480636783 | 0.788234137810816 | 0.254769850416659 | 2.43872285120962 | 0.999228396881742 |
| ebi-fl187-GCST90094727 | ebi-a-GCST90001407 | Memory B cell Absolute Count \|\| id:ebi-a-GCST90001407 | Cauliflower liking \|\| id:ebi-fl187-GCST90094727 | Weighted mode | 4 | 0.301338401247268 | 0.498780896899737 | 0.588372917832588 | -0.676272157 | 1.27894895917075 | 1.35166666984038 | 0.508509105698127 | 3.59286149625407 | 0.999228396881742 |
| ebi-fl187-GCST90094727 | ebi-a-GCST90001411 | IgD+ CD24+ B cell %B cell \|\| id:ebi-a-GCST90001411 | Cauliflower liking \|\| id:ebi-fl187-GCST90094727 | Weighted mode | 4 | 0.87288920096654 | 0.559642461187812 | 0.21672660747484 | -0.224010023 | 1.96978842489465 | 2.39381709061863 | 0.799307122904639 | 7.16915951219603 | 0.999228396881742 |
| ebi-fl187-GCST90094727 | ebi-a-GCST90001418 | CD24+ CD27+ B cell Absolute Count \|\| id:ebi-a-GCST90001418 | Cauliflower liking \|\| id:ebi-fl187-GCST90094727 | Weighted mode | 4 | 0.146849640476412 | 0.49929676604974 | 0.787847114800021 | -0.831772021 | 1.1254713019339 | 1.1581798065732 | 0.43527728204329 | 3.08166890322689 | 0.999228396881742 |
| ebi-fl187-GCST90094727 | ebi-a-GCST90001436 | Memory B cell %lymphocyte \|\| id:ebi-a-GCST90001436 | Cauliflower liking \|\| id:ebi-fl187-GCST90094727 | Weighted mode | 4 | 0.788246269075431 | 0.569431213833661 | 0.260275383865748 | -0.32783891 | 1.90433144818941 | 2.19953564817659 | 0.720479072303583 | 6.71491685682313 | 0.999228396881742 |
| ebi-fl187-GCST90094727 | ebi-a-GCST90001439 | IgD+ CD24+ B cell %lymphocyte \|\| id:ebi-a-GCST90001439 | Cauliflower liking \|\| id:ebi-fl187-GCST90094727 | Weighted mode | 4 | 0.746614787629157 | 0.571625856846317 | 0.282631504568549 | -0.373771892 | 1.86700146704794 | 2.1098456383445 | 0.688133862911351 | 6.46887016838288 | 0.999228396881742 |
| ebi-fl187-GCST90094727 | ebi-a-GCST90001457 | CD11c+ HLA DR++ monocyte %monocyte \|\| id:ebi-a-GCST90001457 | Cauliflower liking \|\| id:ebi-fl187-GCST90094727 | Weighted mode | 4 | -0.151784367 | 0.588554886266666 | 0.813168468043272 | -1.305351944 | 1.00178320993743 | 0.859173526808161 | 0.271077114229513 | 2.72313342004585 | 0.999228396881742 |
| ebi-fl187-GCST90094727 | ebi-a-GCST90001483 | CD39+ resting CD4 regulatory T cell Absolute Count \|\| id:ebi-a-GCST90001483 | Cauliflower liking \|\| id:ebi-fl187-GCST90094727 | Weighted mode | 4 | -0.178281199 | 0.568090825376581 | 0.774209774007994 | -1.291739217 | 0.935176818701689 | 0.836707109172383 | 0.274792443614866 | 2.54766389253701 | 0.999228396881742 |
| ebi-fl187-GCST90094727 | ebi-a-GCST90001525 | CD33dim HLA DR+ CD11b+ Absolute Count \|\| id:ebi-a-GCST90001525 | Cauliflower liking \|\| id:ebi-fl187-GCST90094727 | Weighted mode | 4 | -0.643217483 | 0.805172013288574 | 0.482769860721291 | -2.221354629 | 0.934919663315392 | 0.525598596167123 | 0.108462083405742 | 2.54700883127444 | 0.999228396881742 |
| ebi-fl187-GCST90094727 | ebi-a-GCST90001540 | Naive CD4+ T cell Absolute Count \|\| id:ebi-a-GCST90001540 | Cauliflower liking \|\| id:ebi-fl187-GCST90094727 | Weighted mode | 4 | -0.764575834 | 0.524167101356246 | 0.24074745512475 | -1.791943353 | 0.262791684251917 | 0.465531351483937 | 0.166636022178181 | 1.30055576448366 | 0.999228396881742 |
| ebi-fl187-GCST90094727 | ebi-a-GCST90001624 | HLA DR+ CD4+ T cell Absolute Count \|\| id:ebi-a-GCST90001624 | Cauliflower liking \|\| id:ebi-fl187-GCST90094727 | Weighted mode | 4 | -0.638462646 | 0.55327115852641 | 0.332096137783235 | -1.722874117 | 0.445948824282858 | 0.528103682369824 | 0.178552229710793 | 1.56197152947517 | 0.999228396881742 |
| ebi-fl187-GCST90094727 | ebi-a-GCST90001672 | CD39+ CD8+ T cell Absolute Count \|\| id:ebi-a-GCST90001672 | Cauliflower liking \|\| id:ebi-fl187-GCST90094727 | Weighted mode | 4 | -0.126428894 | 0.804207621252802 | 0.885064683080777 | -1.702675832 | 1.44981804352178 | 0.881236808422447 | 0.182195347102693 | 4.26233888443303 | 0.999228396881742 |
| ebi-fl187-GCST90094727 | ebi-a-GCST90001722 | CD19 on CD20- CD38- B cell \|\| id:ebi-a-GCST90001722 | Cauliflower liking \|\| id:ebi-fl187-GCST90094727 | Weighted mode | 4 | -0.866234629 | 0.547750260359874 | 0.211919403195437 | -1.939825139 | 0.207355881799093 | 0.420532031151847 | 0.143729080218243 | 1.23042037809028 | 0.999228396881742 |
| ebi-fl187-GCST90094727 | ebi-a-GCST90001742 | CD19 on transitional B cell \|\| id:ebi-a-GCST90001742 | Cauliflower liking \|\| id:ebi-fl187-GCST90094727 | Weighted mode | 4 | -0.421554467 | 0.489096430380686 | 0.452127246657516 | -1.380183471 | 0.5370745364719 | 0.656026255589665 | 0.251532400020543 | 1.71099408262255 | 0.999228396881742 |
| ebi-fl187-GCST90094727 | ebi-a-GCST90001784 | CD25 on IgD+ CD38dim B cell \|\| id:ebi-a-GCST90001784 | Cauliflower liking \|\| id:ebi-fl187-GCST90094727 | Weighted mode | 4 | 0.553717102343033 | 0.504981630640874 | 0.352988525692644 | -0.436046894 | 1.54348109839915 | 1.73970768561956 | 0.646587404340279 | 4.68085646439685 | 0.999228396881742 |
| ebi-fl187-GCST90094727 | ebi-a-GCST90001794 | CD25 on IgD+ B cell \|\| id:ebi-a-GCST90001794 | Cauliflower liking \|\| id:ebi-fl187-GCST90094727 | Weighted mode | 4 | 0.847819298272096 | 0.522623833109002 | 0.203206311450926 | -0.176523415 | 1.87216201116574 | 2.33455033900027 | 0.838179153277756 | 6.5023393435797 | 0.999228396881742 |
| ebi-fl187-GCST90094727 | ebi-a-GCST90001802 | CD27 on IgD- CD38- B cell \|\| id:ebi-a-GCST90001802 | Cauliflower liking \|\| id:ebi-fl187-GCST90094727 | Weighted mode | 4 | 0.11670689963336 | 0.542379143907111 | 0.843428315127125 | -0.946356222 | 1.1797700216913 | 1.12378999815416 | 0.388152792246977 | 3.25362585346997 | 0.999228396881742 |
| ebi-fl187-GCST90094727 | ebi-a-GCST90001804 | CD27 on IgD- CD38dim B cell \|\| id:ebi-a-GCST90001804 | Cauliflower liking \|\| id:ebi-fl187-GCST90094727 | Weighted mode | 4 | 0.962703318346876 | 0.567191585332021 | 0.188203298464631 | -0.148992189 | 2.07439882559764 | 2.61876627206892 | 0.861575844724408 | 7.95975981652482 | 0.999228396881742 |
| ebi-fl187-GCST90094727 | ebi-a-GCST90001805 | CD27 on memory B cell \|\| id:ebi-a-GCST90001805 | Cauliflower liking \|\| id:ebi-fl187-GCST90094727 | Weighted mode | 4 | 0.707560777150862 | 0.519859481052871 | 0.266711884138025 | -0.311363806 | 1.72648536001449 | 2.0290359467057 | 0.732447358858614 | 5.62086383851456 | 0.999228396881742 |
| ebi-fl187-GCST90094727 | ebi-a-GCST90001806 | CD27 on unswitched memory B cell \|\| id:ebi-a-GCST90001806 | Cauliflower liking \|\| id:ebi-fl187-GCST90094727 | Weighted mode | 4 | 0.43387817619598 | 0.588848694541368 | 0.514620942159176 | -0.720265265 | 1.58802161749706 | 1.54323085441128 | 0.486623154695424 | 4.89405702755265 | 0.999228396881742 |
| ebi-fl187-GCST90094727 | ebi-a-GCST90001839 | CD3 on Effector Memory CD8+ T cell \|\| id:ebi-a-GCST90001839 | Cauliflower liking \|\| id:ebi-fl187-GCST90094727 | Weighted mode | 4 | 0.0479810410105019 | 0.618794254255803 | 0.943076278056511 | -1.164855697 | 1.26081777935188 | 1.04915076429759 | 0.311967676553119 | 3.52830568342166 | 0.999228396881742 |
| ebi-fl187-GCST90094727 | ebi-a-GCST90001859 | CD3 on CD8+ T cell \|\| id:ebi-a-GCST90001859 | Cauliflower liking \|\| id:ebi-fl187-GCST90094727 | Weighted mode | 4 | -0.187646936 | 0.638639274127712 | 0.788048453071195 | -1.439379913 | 1.06408604146058 | 0.828907313048038 | 0.237074720036472 | 2.89818894869438 | 0.999228396881742 |
| ebi-fl187-GCST90094727 | ebi-a-GCST90001905 | CD86 on monocyte \|\| id:ebi-a-GCST90001905 | Cauliflower liking \|\| id:ebi-fl187-GCST90094727 | Weighted mode | 4 | 0.381480299082226 | 0.555446925852341 | 0.54153549926832 | -0.707195676 | 1.47015627375281 | 1.46445081090218 | 0.493024862296975 | 4.34991486547026 | 0.999228396881742 |
| ebi-fl187-GCST90094727 | ebi-a-GCST90001943 | CD25 on activated & secreting CD4 regulatory T cell \|\| id:ebi-a-GCST90001943 | Cauliflower liking \|\| id:ebi-fl187-GCST90094727 | Weighted mode | 4 | 0.673971814113197 | 0.553203735889333 | 0.310178832885676 | -0.410307508 | 1.75825113645629 | 1.96201462278168 | 0.663446203597233 | 5.8022811181027 | 0.999228396881742 |
| ebi-fl187-GCST90094727 | ebi-a-GCST90001949 | CD33 on CD33dim HLA DR+ CD11b- \|\| id:ebi-a-GCST90001949 | Cauliflower liking \|\| id:ebi-fl187-GCST90094727 | Weighted mode | 4 | -0.631595774 | 0.820251426314293 | 0.497375180651798 | -2.23928857 | 0.976097021539685 | 0.531742582599442 | 0.106534269141774 | 2.65407719438376 | 0.999228396881742 |
| ebi-fl187-GCST90094727 | ebi-a-GCST90001955 | CD33 on Immature Myeloid-Derived Suppressor Cells \|\| id:ebi-a-GCST90001955 | Cauliflower liking \|\| id:ebi-fl187-GCST90094727 | Weighted mode | 4 | -0.688994431 | 0.764488680988174 | 0.433882252213489 | -2.187392246 | 0.809403383597102 | 0.502080692016211 | 0.112208980849316 | 2.24656724789256 | 0.999228396881742 |
| ebi-fl187-GCST90094727 | ebi-a-GCST90001956 | CD33 on CD33+ HLA DR+ \|\| id:ebi-a-GCST90001956 | Cauliflower liking \|\| id:ebi-fl187-GCST90094727 | Weighted mode | 4 | -0.643814237 | 0.827545866396196 | 0.493300641472896 | -2.265804135 | 0.978175661475365 | 0.525285036706976 | 0.103746574757929 | 2.65959980300131 | 0.999228396881742 |
| ebi-fl187-GCST90094727 | ebi-a-GCST90001957 | CD33 on CD33+ HLA DR+ CD14- \|\| id:ebi-a-GCST90001957 | Cauliflower liking \|\| id:ebi-fl187-GCST90094727 | Weighted mode | 4 | -0.428595055 | 0.796698884363081 | 0.627950376381932 | -1.990124868 | 1.13293475863623 | 0.651423666719899 | 0.136678357617609 | 3.10475484897198 | 0.999228396881742 |
| ebi-fl187-GCST90094727 | ebi-a-GCST90002064 | CD4 on resting CD4 regulatory T cell \|\| id:ebi-a-GCST90002064 | Cauliflower liking \|\| id:ebi-fl187-GCST90094727 | Weighted mode | 4 | 0.505561949996213 | 0.62304914760509 | 0.47651931932674 | -0.715614379 | 1.72673827930219 | 1.65791692514472 | 0.48889165459114 | 5.6222856431862 | 0.999228396881742 |
| ebi-fl187-GCST90094727 | ebi-a-GCST90002080 | SSC-A on T cell \|\| id:ebi-a-GCST90002080 | Cauliflower liking \|\| id:ebi-fl187-GCST90094727 | Weighted mode | 4 | 0.489508891480996 | 0.544953835091427 | 0.435245840410651 | -0.578600625 | 1.55761840826019 | 1.63151477236903 | 0.560682422641323 | 4.74750116102923 | 0.999228396881742 |
| ebi-fl187-GCST90094727 | ebi-a-GCST90002121 | CD8 on CD39+ CD8+ T cell \|\| id:ebi-a-GCST90002121 | Cauliflower liking \|\| id:ebi-fl187-GCST90094727 | Weighted mode | 4 | -0.330002859 | 0.605831820908032 | 0.62383427836474 | -1.517433228 | 0.857427510376402 | 0.718921678317078 | 0.21927399167974 | 2.35708929998923 | 0.999228396881742 |
| ebi-fl187-GCST90094727 | ebi-cfmet338-GCST90026053 | 1-palmitoyl-2-docosahexaenoyl-gpc (16:0/22:6) levels \|\| id:ebi-cfmet338-GCST90026053 | Cauliflower liking \|\| id:ebi-fl187-GCST90094727 | Weighted mode | 4 | 0.516850400662881 | 0.294308281092666 | 0.177321752206157 | -0.05999383 | 1.09369463160451 | 1.67673827061335 | 0.941770344026834 | 2.985283244 | 0.999228396881742 |
| ebi-fl187-GCST90094727 | ebi-cfmet338-GCST90026063 | 1-stearoyl-2-oleoyl-gpc (18:0/18:1) levels \|\| id:ebi-cfmet338-GCST90026063 | Cauliflower liking \|\| id:ebi-fl187-GCST90094727 | Weighted mode | 4 | 0.275267144051977 | 0.258904154845548 | 0.36568557218606 | -0.232184999 | 0.782719287549251 | 1.31688242518857 | 0.792799442282095 | 2.18741238865992 | 0.999228396881742 |
| ebi-fl187-GCST90094727 | ebi-cfmet338-GCST90026113 | Adenosine 3',5'-cyclic monophosphate (camp) levels \|\| id:ebi-cfmet338-GCST90026113 | Cauliflower liking \|\| id:ebi-fl187-GCST90094727 | Weighted mode | 4 | 0.518727731330184 | 0.435885909082528 | 0.319618045831912 | -0.33560865 | 1.37306411313194 | 1.67988901936307 | 0.714902827962518 | 3.94742754818783 | 0.999228396881742 |
| ebi-fl187-GCST90094727 | ebi-cfmet338-GCST90026117 | Alpha-ketoglutaramate levels \|\| id:ebi-cfmet338-GCST90026117 | Cauliflower liking \|\| id:ebi-fl187-GCST90094727 | Weighted mode | 4 | 0.239331102254578 | 0.181955338875492 | 0.279895933448172 | -0.117301362 | 0.595963566450542 | 1.2703990989613 | 0.889317146434142 | 1.81477876268654 | 0.999228396881742 |
| ebi-fl187-GCST90094727 | ebi-cfmet338-GCST90026124 | Arginine levels \|\| id:ebi-cfmet338-GCST90026124 | Cauliflower liking \|\| id:ebi-fl187-GCST90094727 | Weighted mode | 4 | -0.146280875 | 0.142527903725919 | 0.38025694378794 | -0.425635567 | 0.133073815912362 | 0.863915016636295 | 0.653354402844927 | 1.14233431766868 | 0.999228396881742 |
| ebi-fl187-GCST90094727 | ebi-cfmet338-GCST90026169 | Glutamate levels \|\| id:ebi-cfmet338-GCST90026169 | Cauliflower liking \|\| id:ebi-fl187-GCST90094727 | Weighted mode | 4 | 0.288053977643134 | 0.20764450214509 | 0.259462011426946 | -0.118929247 | 0.69503720184751 | 1.33382929914222 | 0.887870618439068 | 2.00378361700716 | 0.999228396881742 |
| ebi-fl187-GCST90094727 | ebi-cfmet338-GCST90026170 | Glutamine levels \|\| id:ebi-cfmet338-GCST90026170 | Cauliflower liking \|\| id:ebi-fl187-GCST90094727 | Weighted mode | 4 | 0.0276839770108246 | 0.100361097367683 | 0.800578112333224 | -0.169023774 | 0.224391727851483 | 1.02807073909098 | 0.844488826413726 | 1.25156119479226 | 0.999228396881742 |
| ebi-fl187-GCST90094727 | ebi-cfmet338-GCST90026191 | Kynurenate levels \|\| id:ebi-cfmet338-GCST90026191 | Cauliflower liking \|\| id:ebi-fl187-GCST90094727 | Weighted mode | 4 | -1.03233302 | 0.923361685623381 | 0.345021396315397 | -2.842121924 | 0.777455884084383 | 0.356175027119663 | 0.0583018226069613 | 2.17592940102263 | 0.999228396881742 |
| ebi-fl187-GCST90094727 | ebi-cfmet338-GCST90026202 | Methylsuccinoylcarnitine levels \|\| id:ebi-cfmet338-GCST90026202 | Cauliflower liking \|\| id:ebi-fl187-GCST90094727 | Weighted mode | 4 | 0.208720186419959 | 0.518842620608548 | 0.714425190170706 | -0.80821135 | 1.22565172281271 | 1.23210019192605 | 0.44565447364995 | 3.40638537858954 | 0.999228396881742 |
| ebi-fl187-GCST90094727 | ebi-cfmet338-GCST90026222 | N-acetylputrescine levels \|\| id:ebi-cfmet338-GCST90026222 | Cauliflower liking \|\| id:ebi-fl187-GCST90094727 | Weighted mode | 4 | -0.084739807 | 0.196716022807703 | 0.69570863532557 | -0.470303211 | 0.300823598193597 | 0.91875130641344 | 0.624812789314584 | 1.3509710067913 | 0.999228396881742 |
| ebi-fl187-GCST90094727 | ebi-cfmet338-GCST90026252 | Propionylcarnitine (c3) levels \|\| id:ebi-cfmet338-GCST90026252 | Cauliflower liking \|\| id:ebi-fl187-GCST90094727 | Weighted mode | 4 | -0.103724741 | 0.321515170576809 | 0.768165288937025 | -0.733894476 | 0.526444992987619 | 0.901473401631143 | 0.480035857054464 | 1.69290331525427 | 0.999228396881742 |
| ebi-fl187-GCST90094727 | ebi-cfmet338-GCST90026316 | Ethyl beta-glucopyranoside levels \|\| id:ebi-cfmet338-GCST90026316 | Cauliflower liking \|\| id:ebi-fl187-GCST90094727 | Weighted mode | 4 | -0.729009917 | 0.970259635522631 | 0.507008367906279 | -2.630718802 | 1.17269896911901 | 0.482386356504524 | 0.0720266707027663 | 3.23070044292319 | 0.999228396881742 |
| ebi-fl187-GCST90094727 | ebi-gm430-GCST90011315 | F_Prevotellaceae abundance \|\| id:ebi-gm430-GCST90011315 | Cauliflower liking \|\| id:ebi-fl187-GCST90094727 | Weighted mode | 4 | -0.129264484 | 0.394760657275958 | 0.764846702391961 | -0.902995372 | 0.644466404098132 | 0.878741521592459 | 0.405353654297309 | 1.90497027369652 | 0.999228396881742 |
| ebi-fl187-GCST90094727 | ebi-gm430-GCST90011354 | OTU97_1 (Bacteroides) abundance \|\| id:ebi-gm430-GCST90011354 | Cauliflower liking \|\| id:ebi-fl187-GCST90094727 | Weighted mode | 4 | -0.666858973 | 0.344435583063172 | 0.148278196144926 | -1.341952716 | 0.00823476995891648 | 0.513318395241493 | 0.261334857342604 | 1.00826876893757 | 0.999228396881742 |
| ebi-fl187-GCST90094727 | ebi-gm430-GCST90011419 | OTU99_1 (Bacteroides) abundance \|\| id:ebi-gm430-GCST90011419 | Cauliflower liking \|\| id:ebi-fl187-GCST90094727 | Weighted mode | 4 | -0.648608794 | 0.341495281876808 | 0.153734561415897 | -1.317939547 | 0.0207219581822652 | 0.522772555255222 | 0.267686289207131 | 1.02093814867226 | 0.999228396881742 |
| ebi-fl187-GCST90094727 | ebi-gm430-GCST90011435 | OTU99_155 (Butyrivibrio) abundance \|\| id:ebi-gm430-GCST90011435 | Cauliflower liking \|\| id:ebi-fl187-GCST90094727 | Weighted mode | 4 | -0.577866631 | 1.10337969286685 | 0.636697415668706 | -2.740490829 | 1.58475756658718 | 0.561094111170457 | 0.0645386616419233 | 4.87810861862772 | 0.999228396881742 |
| ebi-fl187-GCST90094727 | ebi-gm430-GCST90011466 | OTU99_58 (Bacteroides) abundance \|\| id:ebi-gm430-GCST90011466 | Cauliflower liking \|\| id:ebi-fl187-GCST90094727 | Weighted mode | 4 | -0.789752867 | 0.703125069038067 | 0.343126241560428 | -2.167878002 | 0.588372268701463 | 0.453956969344126 | 0.114420158839555 | 1.8010543955377 | 0.999228396881742 |
| ebi-fl187-GCST90094727 | ebi-gm430-GCST90011472 | OTU99_72 (Parabacteroides) abundance \|\| id:ebi-gm430-GCST90011472 | Cauliflower liking \|\| id:ebi-fl187-GCST90094727 | Weighted mode | 4 | -0.387013512 | 0.972906899598764 | 0.717399607204203 | -2.293911035 | 1.51988401095991 | 0.679081918937494 | 0.100871178617171 | 4.57169489788564 | 0.999228396881742 |
| ebi-fl187-GCST90094727 | ebi-gm430-GCST90011505 | TestASV_28 (Holdemanella) abundance \|\| id:ebi-gm430-GCST90011505 | Cauliflower liking \|\| id:ebi-fl187-GCST90094727 | Weighted mode | 4 | -0.116523055 | 0.754295503474456 | 0.887039382090533 | -1.594942242 | 1.36189613180572 | 0.890009577565579 | 0.202920248469503 | 3.90358800628765 | 0.999228396881742 |
| ebi-fl187-GCST90094727 | ebi-gm430-GCST90011513 | TestASV_35 (Alphaproteobacteria) abundance \|\| id:ebi-gm430-GCST90011513 | Cauliflower liking \|\| id:ebi-fl187-GCST90094727 | Weighted mode | 4 | 0.0716191039683007 | 1.17056970676187 | 0.955061344341536 | -2.222697521 | 2.36593572922157 | 1.07424609005438 | 0.108316528240754 | 10.6540034170235 | 0.999228396881742 |
| ebi-fl187-GCST90094727 | ebi-gm430-GCST90011543 | G_Coprococcus prevalence \|\| id:ebi-gm430-GCST90011543 | Cauliflower liking \|\| id:ebi-fl187-GCST90094727 | Weighted mode | 4 | 0.922680559037223 | 1.24449272290514 | 0.512208172135277 | -1.516525178 | 3.36188629593129 | 2.51602571415216 | 0.219473193796882 | 28.8435470626701 | 0.999228396881742 |
| ebi-fl187-GCST90094727 | ebi-gm430-GCST90011547 | G_Parasutterella prevalence \|\| id:ebi-gm430-GCST90011547 | Cauliflower liking \|\| id:ebi-fl187-GCST90094727 | Weighted mode | 4 | 1.18228740932763 | 0.974527240544976 | 0.311866719892366 | -0.727785982 | 3.09236080079579 | 3.261826809 | 0.482977127201905 | 22.0290227704683 | 0.999228396881742 |
| ebi-fl187-GCST90094727 | ebi-gm430-GCST90011569 | OTU97_13 (Subdoligranulum) prevalence \|\| id:ebi-gm430-GCST90011569 | Cauliflower liking \|\| id:ebi-fl187-GCST90094727 | Weighted mode | 4 | -0.085851181 | 1.12942639616723 | 0.94419401164427 | -2.299526917 | 2.12782455592071 | 0.917730797234898 | 0.100306285692981 | 8.39658063674408 | 0.999228396881742 |
| ebi-fl187-GCST90094727 | ebi-gm430-GCST90011590 | OTU97_277 (Prevotella) prevalence \|\| id:ebi-gm430-GCST90011590 | Cauliflower liking \|\| id:ebi-fl187-GCST90094727 | Weighted mode | 4 | -0.067212292 | 0.879152875710027 | 0.943873192480532 | -1.790351929 | 1.65592734402969 | 0.934996687610811 | 0.166901421920744 | 5.23793503843441 | 0.999228396881742 |
| ebi-fl187-GCST90094727 | ebi-gm430-GCST90011616 | OTU97_86 (Phascolarctobacterium) prevalence \|\| id:ebi-gm430-GCST90011616 | Cauliflower liking \|\| id:ebi-fl187-GCST90094727 | Weighted mode | 4 | 0.0730618290625564 | 1.13354484976413 | 0.952663003206917 | -2.148686076 | 2.29480973460025 | 1.07579705038158 | 0.116637309641557 | 9.92254791512564 | 0.999228396881742 |
| ebi-fl187-GCST90094727 | ebi-gm430-GCST90011665 | OTU99_55 (Barnesiella) prevalence \|\| id:ebi-gm430-GCST90011665 | Cauliflower liking \|\| id:ebi-fl187-GCST90094727 | Weighted mode | 4 | -0.28103455 | 0.851200947292135 | 0.762989287491541 | -1.949388407 | 1.38731930655583 | 0.755002249598601 | 0.142361112051021 | 4.00410188348805 | 0.999228396881742 |
| ebi-fl187-GCST90094727 | ebi-gm430-GCST90011687 | TestASV_14 (Lachnospiraceae) prevalence \|\| id:ebi-gm430-GCST90011687 | Cauliflower liking \|\| id:ebi-fl187-GCST90094727 | Weighted mode | 4 | 0.853415808510726 | 0.702296479997715 | 0.311209682661626 | -0.523085292 | 2.22991690930625 | 2.34765230231388 | 0.592689105031571 | 9.29909337926177 | 0.999228396881742 |
| ebi-fl187-GCST90094727 | ebi-gm430-GCST90011697 | TestASV_23 (Barnesiella) prevalence \|\| id:ebi-gm430-GCST90011697 | Cauliflower liking \|\| id:ebi-fl187-GCST90094727 | Weighted mode | 4 | -0.854016875 | 1.51426650136795 | 0.612178850574656 | -3.821979218 | 2.11394546767951 | 0.425701503211664 | 0.0218844439235811 | 8.28084873755457 | 0.999228396881742 |
| ebi-fl187-GCST90094727 | ebi-gm430-GCST90011727 | TestASV_6 (Subdoligranulum) prevalence \|\| id:ebi-gm430-GCST90011727 | Cauliflower liking \|\| id:ebi-fl187-GCST90094727 | Weighted mode | 4 | -0.09710271 | 1.18878376520774 | 0.940043693418362 | -2.42711889 | 2.23291346973198 | 0.907462795774555 | 0.0882908421304846 | 9.32700046623116 | 0.999228396881742 |
| ebi-fl187-GCST90094727 | ebi-hbsm44-GCST90104908 | Sulcal depth of the left posterior inferior temporal sulcus \|\| id:ebi-hbsm44-GCST90104908 | Cauliflower liking \|\| id:ebi-fl187-GCST90094727 | Weighted mode | 4 | -0.403739799 | 0.374621287767166 | 0.360094034949673 | -1.137997523 | 0.330517924954888 | 0.667817865494247 | 0.320460093673677 | 1.39168873216218 | 0.999228396881742 |
| ebi-fl187-GCST90094727 | ebi-hbsm44-GCST90104918 | Sulcal depth of the left inferior frontal sulcus \|\| id:ebi-hbsm44-GCST90104918 | Cauliflower liking \|\| id:ebi-fl187-GCST90094727 | Weighted mode | 4 | -0.776327061 | 0.643733013786889 | 0.314262761714746 | -2.038043768 | 0.48538964632361 | 0.460092804608506 | 0.130283326202003 | 1.62480798597592 | 0.999228396881742 |
| ebi-fl187-GCST90094727 | ebi-hbsm44-GCST90104925 | Sulcal depth of the right anterior inferior temporal sulcus \|\| id:ebi-hbsm44-GCST90104925 | Cauliflower liking \|\| id:ebi-fl187-GCST90094727 | Weighted mode | 4 | -0.103329383 | 0.201453765270967 | 0.643388558154897 | -0.498178763 | 0.291519996600657 | 0.901829876826541 | 0.607636302145808 | 1.33846039788093 | 0.999228396881742 |
| ebi-fl187-GCST90094727 | ebi-IPs91-GCST90274770 | C-C motif chemokine 4 levels \|\| id:ebi-IPs91-GCST90274770 | Cauliflower liking \|\| id:ebi-fl187-GCST90094727 | Weighted mode | 4 | -0.058996322 | 0.267152352808575 | 0.839397713335264 | -0.582614934 | 0.464622289166916 | 0.94271023612044 | 0.558436181969378 | 1.59141298142997 | 0.999228396881742 |
| ebi-fl187-GCST90094727 | ebi-IPs91-GCST90274779 | C-X-C motif chemokine 1 levels \|\| id:ebi-IPs91-GCST90274779 | Cauliflower liking \|\| id:ebi-fl187-GCST90094727 | Weighted mode | 4 | -0.455584226 | 0.275684912125751 | 0.196991139235708 | -0.995926654 | 0.0847582014998715 | 0.634077415034474 | 0.369380997512517 | 1.08845384836879 | 0.999228396881742 |
| ebi-fl187-GCST90094727 | ebi-IPs91-GCST90274780 | C-X-C motif chemokine 10 levels \|\| id:ebi-IPs91-GCST90274780 | Cauliflower liking \|\| id:ebi-fl187-GCST90094727 | Weighted mode | 4 | -0.030390681 | 0.272538382117636 | 0.918254281377506 | -0.56456591 | 0.503784547918678 | 0.970066472936787 | 0.568606919780597 | 1.65497275741759 | 0.999228396881742 |
| ebi-fl187-GCST90094727 | ebi-IPs91-GCST90274796 | Interleukin-10 receptor subunit alpha levels \|\| id:ebi-IPs91-GCST90274796 | Cauliflower liking \|\| id:ebi-fl187-GCST90094727 | Weighted mode | 4 | 0.250054603588479 | 0.455108953636933 | 0.620960273388439 | -0.641958946 | 1.14206815271687 | 1.28409553099742 | 0.526260497974225 | 3.13324169128177 | 0.999228396881742 |
| ebi-fl187-GCST90094727 | ebi-IPs91-GCST90274807 | Interleukin-20 levels \|\| id:ebi-IPs91-GCST90274807 | Cauliflower liking \|\| id:ebi-fl187-GCST90094727 | Weighted mode | 4 | -0.080564549 | 0.302357361781782 | 0.807153718715006 | -0.673184978 | 0.512055879842106 | 0.922595348823677 | 0.51008138970356 | 1.66871835525259 | 0.999228396881742 |
| ebi-fl187-GCST90094727 | ebi-IPs91-GCST90274808 | Interleukin-20 receptor subunit alpha levels \|\| id:ebi-IPs91-GCST90274808 | Cauliflower liking \|\| id:ebi-fl187-GCST90094727 | Weighted mode | 4 | -0.21889696 | 0.335197497235782 | 0.560313797319374 | -0.875884054 | 0.438090134800245 | 0.803404496863374 | 0.416493653842521 | 1.5497445870432 | 0.999228396881742 |
| ebi-fl187-GCST90094727 | ebi-IPs91-GCST90274810 | Interleukin-24 levels \|\| id:ebi-IPs91-GCST90274810 | Cauliflower liking \|\| id:ebi-fl187-GCST90094727 | Weighted mode | 4 | 0.0411064062987927 | 0.297421446024166 | 0.898830447436085 | -0.541839628 | 0.624052440506158 | 1.04196297107021 | 0.581677197898161 | 1.86647652169364 | 0.999228396881742 |
| ebi-fl187-GCST90094727 | ebi-IPs91-GCST90274823 | Monocyte chemoattractant protein-3 levels \|\| id:ebi-IPs91-GCST90274823 | Cauliflower liking \|\| id:ebi-fl187-GCST90094727 | Weighted mode | 4 | -0.24634388 | 0.332725280758586 | 0.512748566601264 | -0.89848543 | 0.405797670334594 | 0.78165338377531 | 0.407185904407925 | 1.50049892629707 | 0.999228396881742 |
| ebi-fl187-GCST90094727 | ebi-IPs91-GCST90274824 | Monocyte chemoattractant protein-4 levels \|\| id:ebi-IPs91-GCST90274824 | Cauliflower liking \|\| id:ebi-fl187-GCST90094727 | Weighted mode | 4 | -0.3977748 | 0.24527761378126 | 0.203311310544228 | -0.878518923 | 0.0829693234204098 | 0.671813303258467 | 0.415397692433653 | 1.08650847767321 | 0.999228396881742 |
| ebi-fl187-GCST90094727 | ebi-IPs91-GCST90274834 | SIR2-like protein 2 levels \|\| id:ebi-IPs91-GCST90274834 | Cauliflower liking \|\| id:ebi-fl187-GCST90094727 | Weighted mode | 4 | -0.436950985 | 0.285396821430992 | 0.223266331757285 | -0.996328755 | 0.122426785013916 | 0.646003094482237 | 0.369232498915603 | 1.13023636680479 | 0.999228396881742 |
| ebi-fl187-GCST90094727 | ebi-met1400-GCST90199652 | 1-stearoyl-2-oleoyl-GPS (18:0/18:1) levels \|\| id:ebi-met1400-GCST90199652 | Cauliflower liking \|\| id:ebi-fl187-GCST90094727 | Weighted mode | 4 | -0.003496103 | 0.319819742317595 | 0.991964425343444 | -0.630342798 | 0.623350591689182 | 0.996510000999903 | 0.532409260787436 | 1.86516699695292 | 0.999228396881742 |
| ebi-fl187-GCST90094727 | ebi-met1400-GCST90199665 | N-acetylaspartate (naa) levels \|\| id:ebi-met1400-GCST90199665 | Cauliflower liking \|\| id:ebi-fl187-GCST90094727 | Weighted mode | 4 | 0.108518150465661 | 0.476804922408151 | 0.834590547728787 | -0.826019497 | 1.04305579838564 | 1.11462513932006 | 0.437788440673625 | 2.8378757540802 | 0.999228396881742 |
| ebi-fl187-GCST90094727 | ebi-met1400-GCST90199669 | Acetylcarnitine levels (Biocrates platform) \|\| id:ebi-met1400-GCST90199669 | Cauliflower liking \|\| id:ebi-fl187-GCST90094727 | Weighted mode | 4 | 0.483075049318106 | 0.403183316680118 | 0.316882802355291 | -0.307164251 | 1.27331435001114 | 1.62105155926112 | 0.735529779207426 | 3.57267405354344 | 0.999228396881742 |
| ebi-fl187-GCST90094727 | ebi-met1400-GCST90199670 | Iminodiacetate (IDA) levels \|\| id:ebi-met1400-GCST90199670 | Cauliflower liking \|\| id:ebi-fl187-GCST90094727 | Weighted mode | 4 | 0.133397219900468 | 0.311528363171424 | 0.697385182953624 | -0.477198372 | 0.743992811716459 | 1.14270381288766 | 0.620519423456821 | 2.10432091990568 | 0.999228396881742 |
| ebi-fl187-GCST90094727 | ebi-met1400-GCST90199686 | Indolepropionate levels \|\| id:ebi-met1400-GCST90199686 | Cauliflower liking \|\| id:ebi-fl187-GCST90094727 | Weighted mode | 4 | 0.158194004532364 | 0.314314979798868 | 0.649384513540509 | -0.457863356 | 0.774251364938146 | 1.17139342829905 | 0.632633916010382 | 2.1689677539193 | 0.999228396881742 |
| ebi-fl187-GCST90094727 | ebi-met1400-GCST90199704 | 3-hydroxysebacate levels \|\| id:ebi-met1400-GCST90199704 | Cauliflower liking \|\| id:ebi-fl187-GCST90094727 | Weighted mode | 4 | 0.0389403250532112 | 0.346203793554333 | 0.917548383639845 | -0.63961911 | 0.717499760419705 | 1.0397084372532 | 0.527493302543097 | 2.04930305140163 | 0.999228396881742 |
| ebi-fl187-GCST90094727 | ebi-met1400-GCST90199713 | Docosapentaenoate n3 DPA; 22:5n3 levels \|\| id:ebi-met1400-GCST90199713 | Cauliflower liking \|\| id:ebi-fl187-GCST90094727 | Weighted mode | 4 | 0.379588214611271 | 0.33944310740127 | 0.344929219061824 | -0.285720276 | 1.04489670511776 | 1.46168256596687 | 0.751472791603816 | 2.84310483030488 | 0.999228396881742 |
| ebi-fl187-GCST90094727 | ebi-met1400-GCST90199715 | Myristoleate (14:1n5) levels \|\| id:ebi-met1400-GCST90199715 | Cauliflower liking \|\| id:ebi-fl187-GCST90094727 | Weighted mode | 4 | 0.40389798268424 | 0.322352184580752 | 0.298986706854665 | -0.227912299 | 1.03570826446251 | 1.49765115281389 | 0.796194083716067 | 2.81710078157859 | 0.999228396881742 |
| ebi-fl187-GCST90094727 | ebi-met1400-GCST90199725 | Campesterol levels \|\| id:ebi-met1400-GCST90199725 | Cauliflower liking \|\| id:ebi-fl187-GCST90094727 | Weighted mode | 4 | -0.340283598 | 0.423765530270794 | 0.480703076336974 | -1.170864037 | 0.490296841714336 | 0.711568495015922 | 0.310098888581438 | 1.63280083142334 | 0.999228396881742 |
| ebi-fl187-GCST90094727 | ebi-met1400-GCST90199737 | N-acetylproline levels \|\| id:ebi-met1400-GCST90199737 | Cauliflower liking \|\| id:ebi-fl187-GCST90094727 | Weighted mode | 4 | 0.262945090518544 | 0.377028297706176 | 0.535732709819174 | -0.476030373 | 1.00192055402265 | 1.30075529319212 | 0.621244612907246 | 2.7235074519993 | 0.999228396881742 |
| ebi-fl187-GCST90094727 | ebi-met1400-GCST90199775 | Gamma-glutamylisoleucine levels \|\| id:ebi-met1400-GCST90199775 | Cauliflower liking \|\| id:ebi-fl187-GCST90094727 | Weighted mode | 4 | -0.320764575 | 0.343889600501647 | 0.419752833556856 | -0.994788192 | 0.353259041687739 | 0.725594053649445 | 0.3698017631183 | 1.42369989329394 | 0.999228396881742 |
| ebi-fl187-GCST90094727 | ebi-met1400-GCST90199777 | 1-palmitoleoyl-GPC (16:1) levels \|\| id:ebi-met1400-GCST90199777 | Cauliflower liking \|\| id:ebi-fl187-GCST90094727 | Weighted mode | 4 | -0.482868414 | 0.326167399403869 | 0.235333919439598 | -1.122156517 | 0.156419689156869 | 0.617011008243257 | 0.325576925007623 | 1.1693168497259 | 0.999228396881742 |
| ebi-fl187-GCST90094727 | ebi-met1400-GCST90199782 | Glutamine degradant levels \|\| id:ebi-met1400-GCST90199782 | Cauliflower liking \|\| id:ebi-fl187-GCST90094727 | Weighted mode | 4 | -0.280103754 | 0.262973526933618 | 0.364934165760822 | -0.795531866 | 0.235324359259482 | 0.755705330292111 | 0.451341117933587 | 1.26531912015147 | 0.999228396881742 |
| ebi-fl187-GCST90094727 | ebi-met1400-GCST90199783 | 5-methyluridine (ribothymidine) levels \|\| id:ebi-met1400-GCST90199783 | Cauliflower liking \|\| id:ebi-fl187-GCST90094727 | Weighted mode | 4 | 0.337812308940241 | 0.365136225212914 | 0.423116388443386 | -0.377854692 | 1.05347931035755 | 1.40187735886402 | 0.685330077046982 | 2.86761109006577 | 0.999228396881742 |
| ebi-fl187-GCST90094727 | ebi-met1400-GCST90199787 | Homostachydrine levels \|\| id:ebi-met1400-GCST90199787 | Cauliflower liking \|\| id:ebi-fl187-GCST90094727 | Weighted mode | 4 | 0.109870969292604 | 0.313912780963142 | 0.749466101074896 | -0.505398081 | 0.725140019980363 | 1.1161340456018 | 0.603265378897648 | 2.06502022381564 | 0.999228396881742 |
| ebi-fl187-GCST90094727 | ebi-met1400-GCST90199802 | Beta-hydroxyisovaleroylcarnitine levels \|\| id:ebi-met1400-GCST90199802 | Cauliflower liking \|\| id:ebi-fl187-GCST90094727 | Weighted mode | 4 | 0.0211106630631183 | 0.309138507556342 | 0.949852579712476 | -0.584800812 | 0.627022137873548 | 1.02133506945122 | 0.55721684180794 | 1.87202763058348 | 0.999228396881742 |
| ebi-fl187-GCST90094727 | ebi-met1400-GCST90199873 | Pregnenolone sulfate levels \|\| id:ebi-met1400-GCST90199873 | Cauliflower liking \|\| id:ebi-fl187-GCST90094727 | Weighted mode | 4 | -0.109148353 | 0.391277695658684 | 0.798404871700955 | -0.876052637 | 0.657755930095018 | 0.896597394362961 | 0.416423446209597 | 1.93045539317167 | 0.999228396881742 |
| ebi-fl187-GCST90094727 | ebi-met1400-GCST90199911 | Beta-citrylglutamate levels \|\| id:ebi-met1400-GCST90199911 | Cauliflower liking \|\| id:ebi-fl187-GCST90094727 | Weighted mode | 4 | -0.557737182 | 0.370058579750669 | 0.228872431164774 | -1.283051998 | 0.167577634564803 | 0.572503069642871 | 0.277190024751783 | 1.18243708461014 | 0.999228396881742 |
| ebi-fl187-GCST90094727 | ebi-met1400-GCST90199934 | 2-hydroxydecanoate levels \|\| id:ebi-met1400-GCST90199934 | Cauliflower liking \|\| id:ebi-fl187-GCST90094727 | Weighted mode | 4 | -0.146104273 | 0.317414720940895 | 0.676617259699007 | -0.768237126 | 0.476028579668623 | 0.864067599241743 | 0.463830021697893 | 1.60966901910821 | 0.999228396881742 |
| ebi-fl187-GCST90094727 | ebi-met1400-GCST90199945 | Sphingomyelin (d18:1/14:0, d16:1/16:0) levels \|\| id:ebi-met1400-GCST90199945 | Cauliflower liking \|\| id:ebi-fl187-GCST90094727 | Weighted mode | 4 | -0.056247694 | 0.33589552080563 | 0.877662962497534 | -0.714602915 | 0.602107526816656 | 0.945304960558348 | 0.489386401344747 | 1.82596301409431 | 0.999228396881742 |
| ebi-fl187-GCST90094727 | ebi-met1400-GCST90199979 | Carnitine C14:1 levels \|\| id:ebi-met1400-GCST90199979 | Cauliflower liking \|\| id:ebi-fl187-GCST90094727 | Weighted mode | 4 | 0.342925633448044 | 0.445912875205475 | 0.497867835965839 | -0.531063602 | 1.21691486885078 | 1.40906397080103 | 0.58797926109349 | 3.37675391835609 | 0.999228396881742 |
| ebi-fl187-GCST90094727 | ebi-met1400-GCST90199986 | Eugenol sulfate levels \|\| id:ebi-met1400-GCST90199986 | Cauliflower liking \|\| id:ebi-fl187-GCST90094727 | Weighted mode | 4 | 0.0361664772022803 | 0.336683480434635 | 0.921236921392117 | -0.623733144 | 0.696066098854165 | 1.03682844042205 | 0.535939957138747 | 2.00584636496831 | 0.999228396881742 |
| ebi-fl187-GCST90094727 | ebi-met1400-GCST90200002 | 6-hydroxyindole sulfate levels \|\| id:ebi-met1400-GCST90200002 | Cauliflower liking \|\| id:ebi-fl187-GCST90094727 | Weighted mode | 4 | -0.068956579 | 0.332738365787727 | 0.84909255184114 | -0.721123776 | 0.583210617456841 | 0.933367206481183 | 0.486205562485119 | 1.79178193207354 | 0.999228396881742 |
| ebi-fl187-GCST90094727 | ebi-met1400-GCST90200023 | Trans 3,4-methyleneheptanoate levels \|\| id:ebi-met1400-GCST90200023 | Cauliflower liking \|\| id:ebi-fl187-GCST90094727 | Weighted mode | 4 | -0.485654158 | 0.356563574334776 | 0.266436890142519 | -1.184518764 | 0.213210447537292 | 0.615294565126373 | 0.305893351020761 | 1.23764508319881 | 0.999228396881742 |
| ebi-fl187-GCST90094727 | ebi-met1400-GCST90200032 | 2-hydroxybutyrate/2-hydroxyisobutyrate levels \|\| id:ebi-met1400-GCST90200032 | Cauliflower liking \|\| id:ebi-fl187-GCST90094727 | Weighted mode | 4 | 0.0168825899127556 | 0.349978120651299 | 0.964557594661648 | -0.669074527 | 0.702839706389302 | 1.01702590621467 | 0.512182369689457 | 2.0194793009742 | 0.999228396881742 |
| ebi-fl187-GCST90094727 | ebi-met1400-GCST90200051 | 1-palmitoyl-2-dihomo-linolenoyl-GPC (16:0/20:3n3 or 6) levels \|\| id:ebi-met1400-GCST90200051 | Cauliflower liking \|\| id:ebi-fl187-GCST90094727 | Weighted mode | 4 | 0.165140302220654 | 0.328861347431816 | 0.650097277503387 | -0.479427939 | 0.809708543187014 | 1.17955860179352 | 0.619137475078398 | 2.24725291404612 | 0.999228396881742 |
| ebi-fl187-GCST90094727 | ebi-met1400-GCST90200069 | Phenylacetylglutamate levels \|\| id:ebi-met1400-GCST90200069 | Cauliflower liking \|\| id:ebi-fl187-GCST90094727 | Weighted mode | 4 | 0.0828150780713105 | 0.35439787776812 | 0.830272786989266 | -0.611804762 | 0.777434918496825 | 1.08634090171801 | 0.542371134241072 | 2.17588378186247 | 0.999228396881742 |
| ebi-fl187-GCST90094727 | ebi-met1400-GCST90200072 | Thioproline levels \|\| id:ebi-met1400-GCST90200072 | Cauliflower liking \|\| id:ebi-fl187-GCST90094727 | Weighted mode | 4 | 0.252255922955694 | 0.298354138439421 | 0.459937090391866 | -0.332518188 | 0.837030034296959 | 1.28692534888289 | 0.717115625569871 | 2.30949765218298 | 0.999228396881742 |
| ebi-fl187-GCST90094727 | ebi-met1400-GCST90200081 | 1-oleoyl-2-docosahexaenoyl-GPC (18:1/22:6) levels \|\| id:ebi-met1400-GCST90200081 | Cauliflower liking \|\| id:ebi-fl187-GCST90094727 | Weighted mode | 4 | 0.268869530313752 | 0.313908446937948 | 0.454675476305896 | -0.346391026 | 0.88413008631213 | 1.30848441235851 | 0.707235885634514 | 2.42087752072862 | 0.999228396881742 |
| ebi-fl187-GCST90094727 | ebi-met1400-GCST90200097 | Furaneol sulfate levels \|\| id:ebi-met1400-GCST90200097 | Cauliflower liking \|\| id:ebi-fl187-GCST90094727 | Weighted mode | 4 | -0.279323494 | 0.401974715825794 | 0.537114717399286 | -1.067193937 | 0.508546948666676 | 0.756295206410537 | 0.343972372487454 | 1.6628731985166 | 0.999228396881742 |
| ebi-fl187-GCST90094727 | ebi-met1400-GCST90200124 | Sphingomyelin (d18:2/24:2) levels \|\| id:ebi-met1400-GCST90200124 | Cauliflower liking \|\| id:ebi-fl187-GCST90094727 | Weighted mode | 4 | -0.262288273 | 0.343359937623184 | 0.500517596974412 | -0.935273751 | 0.410697204387151 | 0.769289226154307 | 0.392478409429445 | 1.50786871139591 | 0.999228396881742 |
| ebi-fl187-GCST90094727 | ebi-met1400-GCST90200137 | Lignoceroylcarnitine (C24) levels \|\| id:ebi-met1400-GCST90200137 | Cauliflower liking \|\| id:ebi-fl187-GCST90094727 | Weighted mode | 4 | -0.256546276 | 0.346671660325022 | 0.512935434437964 | -0.936022731 | 0.422930177831607 | 0.773719188788124 | 0.392184561185141 | 1.52642771375273 | 0.999228396881742 |
| ebi-fl187-GCST90094727 | ebi-met1400-GCST90200165 | Octadecenedioate (C18:1-DC) levels \|\| id:ebi-met1400-GCST90200165 | Cauliflower liking \|\| id:ebi-fl187-GCST90094727 | Weighted mode | 4 | -0.162689661 | 0.304949394719032 | 0.630685334940537 | -0.760390475 | 0.435011152720909 | 0.849854890671688 | 0.467483850807322 | 1.54498028958923 | 0.999228396881742 |
| ebi-fl187-GCST90094727 | ebi-met1400-GCST90200176 | Glutamine conjugate of C6H10O2 (1) levels \|\| id:ebi-met1400-GCST90200176 | Cauliflower liking \|\| id:ebi-fl187-GCST90094727 | Weighted mode | 4 | 0.29462430515155 | 0.337614885 | 0.447071255203055 | -0.367100869 | 0.956349479739791 | 1.3426218478095 | 0.69273976525019 | 2.60217980350005 | 0.999228396881742 |
| ebi-fl187-GCST90094727 | ebi-met1400-GCST90200182 | Glycine conjugate of C10H12O2 levels \|\| id:ebi-met1400-GCST90200182 | Cauliflower liking \|\| id:ebi-fl187-GCST90094727 | Weighted mode | 4 | -0.326611981 | 0.370339180242954 | 0.442758728297783 | -1.052476774 | 0.399252812678207 | 0.721363591799246 | 0.349072104881594 | 1.49071044147174 | 0.999228396881742 |
| ebi-fl187-GCST90094727 | ebi-met1400-GCST90200208 | Glucuronide of piperine metabolite C17H21NO3 (4) levels \|\| id:ebi-met1400-GCST90200208 | Cauliflower liking \|\| id:ebi-fl187-GCST90094727 | Weighted mode | 4 | 0.347179891559482 | 0.351600194017419 | 0.396233426496454 | -0.341956489 | 1.03631627183362 | 1.41507126185133 | 0.71037911354504 | 2.81881412042749 | 0.999228396881742 |
| ebi-fl187-GCST90094727 | ebi-met1400-GCST90200227 | 3-ethylcatechol sulfate (2) levels \|\| id:ebi-met1400-GCST90200227 | Cauliflower liking \|\| id:ebi-fl187-GCST90094727 | Weighted mode | 4 | 0.131954275602577 | 0.420334918422078 | 0.774139936681805 | -0.691902165 | 0.95581071570985 | 1.14105614396979 | 0.500622895704741 | 2.60077822021772 | 0.999228396881742 |
| ebi-fl187-GCST90094727 | ebi-met1400-GCST90200259 | Pentose acid levels \|\| id:ebi-met1400-GCST90200259 | Cauliflower liking \|\| id:ebi-fl187-GCST90094727 | Weighted mode | 4 | -0.250457845 | 0.323682800612078 | 0.495444654772185 | -0.884876134 | 0.383960444315682 | 0.778444294731637 | 0.412765297664527 | 1.46808736933292 | 0.999228396881742 |
| ebi-fl187-GCST90094727 | ebi-met1400-GCST90200291 | Sarcosine levels \|\| id:ebi-met1400-GCST90200291 | Cauliflower liking \|\| id:ebi-fl187-GCST90094727 | Weighted mode | 4 | 0.0335442621336151 | 0.291034444233148 | 0.915521837794473 | -0.536883249 | 0.603971772830585 | 1.03411321477059 | 0.584567367218587 | 1.82937023332556 | 0.999228396881742 |
| ebi-fl187-GCST90094727 | ebi-met1400-GCST90200306 | Butyrate/isobutyrate (4:0) levels \|\| id:ebi-met1400-GCST90200306 | Cauliflower liking \|\| id:ebi-fl187-GCST90094727 | Weighted mode | 4 | -0.028554381 | 0.299953709964192 | 0.930161500976441 | -0.616463652 | 0.559354890642411 | 0.971849442673978 | 0.539850163775273 | 1.74954348929102 | 0.999228396881742 |
| ebi-fl187-GCST90094727 | ebi-met1400-GCST90200321 | Cholate levels \|\| id:ebi-met1400-GCST90200321 | Cauliflower liking \|\| id:ebi-fl187-GCST90094727 | Weighted mode | 4 | -0.232812286 | 0.448831315464278 | 0.639798033127047 | -1.112521665 | 0.646897092084102 | 0.792302285618498 | 0.328728970848839 | 1.90960629413145 | 0.999228396881742 |
| ebi-fl187-GCST90094727 | ebi-met1400-GCST90200336 | Ceramide (d18:1/16:0) levels \|\| id:ebi-met1400-GCST90200336 | Cauliflower liking \|\| id:ebi-fl187-GCST90094727 | Weighted mode | 4 | 0.349699123073645 | 0.398595923050831 | 0.444895369853135 | -0.431548886 | 1.13094713225327 | 1.41864064813642 | 0.6495023101233 | 3.09858988517354 | 0.999228396881742 |
| ebi-fl187-GCST90094727 | ebi-met1400-GCST90200358 | Arachidonate (20:4n6) levels \|\| id:ebi-met1400-GCST90200358 | Cauliflower liking \|\| id:ebi-fl187-GCST90094727 | Weighted mode | 4 | 0.231845742231276 | 0.415429811979848 | 0.615727863431172 | -0.582396689 | 1.04608817371178 | 1.26092520631656 | 0.558558070947213 | 2.84649431925353 | 0.999228396881742 |
| ebi-fl187-GCST90094727 | ebi-met1400-GCST90200413 | 2'-deoxyuridine levels \|\| id:ebi-met1400-GCST90200413 | Cauliflower liking \|\| id:ebi-fl187-GCST90094727 | Weighted mode | 4 | 0.228670559895858 | 0.48257101633005 | 0.667952198721424 | -0.717168632 | 1.17450975190276 | 1.25692788834023 | 0.488132383569875 | 3.23655583949037 | 0.999228396881742 |
| ebi-fl187-GCST90094727 | ebi-met1400-GCST90200432 | Threonine levels \|\| id:ebi-met1400-GCST90200432 | Cauliflower liking \|\| id:ebi-fl187-GCST90094727 | Weighted mode | 4 | -0.129348425 | 0.363914354359629 | 0.745784902147642 | -0.84262056 | 0.583923709158454 | 0.878667762049619 | 0.430580681185704 | 1.79306009256906 | 0.999228396881742 |
| ebi-fl187-GCST90094727 | ebi-met1400-GCST90200436 | Caffeine levels \|\| id:ebi-met1400-GCST90200436 | Cauliflower liking \|\| id:ebi-fl187-GCST90094727 | Weighted mode | 4 | -0.22068349 | 0.329634890575624 | 0.551108001771689 | -0.866767875 | 0.425400895948363 | 0.80197047213527 | 0.4203078436542 | 1.53020374919773 | 0.999228396881742 |
| ebi-fl187-GCST90094727 | ebi-met1400-GCST90200442 | Valine levels \|\| id:ebi-met1400-GCST90200442 | Cauliflower liking \|\| id:ebi-fl187-GCST90094727 | Weighted mode | 4 | 0.0949590320107516 | 0.321459338466402 | 0.786953622020048 | -0.535101271 | 0.725019335404899 | 1.09961380523676 | 0.585609981608748 | 2.06477102276427 | 0.999228396881742 |
| ebi-fl187-GCST90094727 | ebi-met1400-GCST90200459 | Margarate (17:0) levels \|\| id:ebi-met1400-GCST90200459 | Cauliflower liking \|\| id:ebi-fl187-GCST90094727 | Weighted mode | 4 | 0.203307774247049 | 0.300121385703292 | 0.546703657368448 | -0.384930142 | 0.791545690225502 | 1.22544957203243 | 0.680498172968297 | 2.20680482806298 | 0.999228396881742 |
| ebi-fl187-GCST90094727 | ebi-met1400-GCST90200462 | X-11299 levels \|\| id:ebi-met1400-GCST90200462 | Cauliflower liking \|\| id:ebi-fl187-GCST90094727 | Weighted mode | 4 | 0.319609240936402 | 0.337571229398627 | 0.413594491366739 | -0.342030369 | 0.981248850557712 | 1.37658974430526 | 0.710326632695932 | 2.66778582823827 | 0.999228396881742 |
| ebi-fl187-GCST90094727 | ebi-met1400-GCST90200497 | X-12740 levels \|\| id:ebi-met1400-GCST90200497 | Cauliflower liking \|\| id:ebi-fl187-GCST90094727 | Weighted mode | 4 | 0.17028495788518 | 0.334174951525593 | 0.645472459202363 | -0.484697947 | 0.825267862875342 | 1.18564266141263 | 0.61588319797471 | 2.28249207834267 | 0.999228396881742 |
| ebi-fl187-GCST90094727 | ebi-met1400-GCST90200527 | X-13866 levels \|\| id:ebi-met1400-GCST90200527 | Cauliflower liking \|\| id:ebi-fl187-GCST90094727 | Weighted mode | 4 | 0.481089579951711 | 0.338808222324162 | 0.250703231172171 | -0.182974536 | 1.14515369570707 | 1.61783620409972 | 0.832789361778922 | 3.14292437370328 | 0.999228396881742 |
| ebi-fl187-GCST90094727 | ebi-met1400-GCST90200547 | X-17654 levels \|\| id:ebi-met1400-GCST90200547 | Cauliflower liking \|\| id:ebi-fl187-GCST90094727 | Weighted mode | 4 | 0.248036081025676 | 0.337268435722251 | 0.515358348658742 | -0.413010053 | 0.909082215041288 | 1.28150616941688 | 0.661655631177012 | 2.48204350551981 | 0.999228396881742 |
| ebi-fl187-GCST90094727 | ebi-met1400-GCST90200553 | X-17653 levels \|\| id:ebi-met1400-GCST90200553 | Cauliflower liking \|\| id:ebi-fl187-GCST90094727 | Weighted mode | 4 | 0.234938566971836 | 0.348759802370726 | 0.5487961898044 | -0.448630646 | 0.918507779618459 | 1.26483106394372 | 0.638501888580285 | 2.50554876802976 | 0.999228396881742 |
| ebi-fl187-GCST90094727 | ebi-met1400-GCST90200557 | X-17685 levels \|\| id:ebi-met1400-GCST90200557 | Cauliflower liking \|\| id:ebi-fl187-GCST90094727 | Weighted mode | 4 | 0.219113450444205 | 0.364058349731945 | 0.58970985558913 | -0.494440915 | 0.932667815918817 | 1.24497251136025 | 0.609911804514855 | 2.54127980893161 | 0.999228396881742 |
| ebi-fl187-GCST90094727 | ebi-met1400-GCST90200561 | X-18779 levels \|\| id:ebi-met1400-GCST90200561 | Cauliflower liking \|\| id:ebi-fl187-GCST90094727 | Weighted mode | 4 | 0.348269877191307 | 0.326005454445172 | 0.363715414234208 | -0.290700814 | 0.987240567903845 | 1.41661451010118 | 0.747739358060976 | 2.68381843030597 | 0.999228396881742 |
| ebi-fl187-GCST90094727 | ebi-met1400-GCST90200570 | X-18921 levels \|\| id:ebi-met1400-GCST90200570 | Cauliflower liking \|\| id:ebi-fl187-GCST90094727 | Weighted mode | 4 | 0.0374204197238408 | 0.321550719835273 | 0.914708657835415 | -0.592818991 | 0.667659830600976 | 1.03812937917181 | 0.552766841494022 | 1.94966942117366 | 0.999228396881742 |
| ebi-fl187-GCST90094727 | ebi-met1400-GCST90200575 | X-21283 levels \|\| id:ebi-met1400-GCST90200575 | Cauliflower liking \|\| id:ebi-fl187-GCST90094727 | Weighted mode | 4 | 0.492286214749685 | 0.417813373660264 | 0.323649756724349 | -0.326627998 | 1.3112004271238 | 1.63605231450351 | 0.721352037792047 | 3.71062537507371 | 0.999228396881742 |
| ebi-fl187-GCST90094727 | ebi-met1400-GCST90200578 | X-18922 levels \|\| id:ebi-met1400-GCST90200578 | Cauliflower liking \|\| id:ebi-fl187-GCST90094727 | Weighted mode | 4 | -0.037046716 | 0.304185326437143 | 0.910765369938171 | -0.633249955 | 0.559156524246134 | 0.963631117719973 | 0.530863711042576 | 1.74919647307324 | 0.999228396881742 |
| ebi-fl187-GCST90094727 | ebi-met1400-GCST90200623 | X-24241 levels \|\| id:ebi-met1400-GCST90200623 | Cauliflower liking \|\| id:ebi-fl187-GCST90094727 | Weighted mode | 4 | -0.123065345 | 0.371947390208368 | 0.762506670666881 | -0.85208223 | 0.605951539344706 | 0.884205881829636 | 0.426525881563709 | 1.83299554670833 | 0.999228396881742 |
| ebi-fl187-GCST90094727 | ebi-met1400-GCST90200636 | X-24544 levels \|\| id:ebi-met1400-GCST90200636 | Cauliflower liking \|\| id:ebi-fl187-GCST90094727 | Weighted mode | 4 | -0.285046316 | 0.376900716016009 | 0.504444228448696 | -1.023771719 | 0.453679087845865 | 0.751979425175533 | 0.359237439074284 | 1.57409277091076 | 0.999228396881742 |
| ebi-fl187-GCST90094727 | ebi-met1400-GCST90200659 | X-25172 levels \|\| id:ebi-met1400-GCST90200659 | Cauliflower liking \|\| id:ebi-fl187-GCST90094727 | Weighted mode | 4 | -0.106337828 | 0.454397662127956 | 0.830030970929816 | -0.996957246 | 0.784281589285611 | 0.899120848128555 | 0.369000512425354 | 2.19083245772714 | 0.999228396881742 |
| ebi-fl187-GCST90094727 | ebi-met1400-GCST90200670 | X-26111 levels \|\| id:ebi-met1400-GCST90200670 | Cauliflower liking \|\| id:ebi-fl187-GCST90094727 | Weighted mode | 4 | 0.407524320836449 | 0.362376620187553 | 0.342622495463359 | -0.302733855 | 1.11778249640405 | 1.5030920015443 | 0.738795697186583 | 3.05806540794708 | 0.999228396881742 |
| ebi-fl187-GCST90094727 | ebi-met1400-GCST90200673 | Carnitine C4 levels \|\| id:ebi-met1400-GCST90200673 | Cauliflower liking \|\| id:ebi-fl187-GCST90094727 | Weighted mode | 4 | -0.090656314 | 0.336826942678424 | 0.805265218548715 | -0.750837122 | 0.569524493229581 | 0.91333155584957 | 0.471971289739434 | 1.76742642835569 | 0.999228396881742 |
| ebi-fl187-GCST90094727 | ebi-met1400-GCST90200710 | X-13431 levels \|\| id:ebi-met1400-GCST90200710 | Cauliflower liking \|\| id:ebi-fl187-GCST90094727 | Weighted mode | 4 | -0.267641797 | 0.300427285694343 | 0.438629926216594 | -0.856479277 | 0.321195682742721 | 0.765181822276108 | 0.424654544448996 | 1.37877535704109 | 0.999228396881742 |
| ebi-fl187-GCST90094727 | ebi-met1400-GCST90200730 | Adenosine 5'-diphosphate (ADP) to fructose ratio \|\| id:ebi-met1400-GCST90200730 | Cauliflower liking \|\| id:ebi-fl187-GCST90094727 | Weighted mode | 4 | 0.105094799797896 | 0.449128725589789 | 0.830046750085568 | -0.775197502 | 0.985387101953882 | 1.11081591048822 | 0.460612799900624 | 2.6788486712918 | 0.999228396881742 |
| ebi-fl187-GCST90094727 | ebi-met1400-GCST90200731 | Adenosine 5'-diphosphate (ADP) to mannose ratio \|\| id:ebi-met1400-GCST90200731 | Cauliflower liking \|\| id:ebi-fl187-GCST90094727 | Weighted mode | 4 | 0.356787951307285 | 0.660669718642248 | 0.626681791146354 | -0.938124697 | 1.65170059984609 | 1.42873287679161 | 0.391361068121446 | 5.21584234993907 | 0.999228396881742 |
| ebi-fl187-GCST90094727 | ebi-met1400-GCST90200748 | Aspartate to glutamate ratio \|\| id:ebi-met1400-GCST90200748 | Cauliflower liking \|\| id:ebi-fl187-GCST90094727 | Weighted mode | 4 | -0.22228424 | 0.413775291970127 | 0.628411294515732 | -1.033283812 | 0.588715332542799 | 0.800687744727637 | 0.35583654 | 1.80167237817478 | 0.999228396881742 |
| ebi-fl187-GCST90094727 | ebi-met1400-GCST90200774 | Uridine to pseudouridine ratio \|\| id:ebi-met1400-GCST90200774 | Cauliflower liking \|\| id:ebi-fl187-GCST90094727 | Weighted mode | 4 | 0.276456074724493 | 0.306755681204112 | 0.433892766054579 | -0.32478506 | 0.877697209884552 | 1.31844903820857 | 0.722682670047978 | 2.40535429781054 | 0.999228396881742 |
| ebi-fl187-GCST90094727 | ebi-met1400-GCST90200799 | Spermidine to adenosine 5'-diphosphate (ADP) ratio \|\| id:ebi-met1400-GCST90200799 | Cauliflower liking \|\| id:ebi-fl187-GCST90094727 | Weighted mode | 4 | -0.74458452 | 0.4801484692815 | 0.218751867895635 | -1.68567552 | 0.196506479414619 | 0.474931583348599 | 0.185319202804152 | 1.21714320722814 | 0.999228396881742 |
| ebi-fl187-GCST90094727 | ebi-met1400-GCST90200811 | Choline to taurocholate ratio \|\| id:ebi-met1400-GCST90200811 | Cauliflower liking \|\| id:ebi-fl187-GCST90094727 | Weighted mode | 4 | -0.040932659 | 0.429103904786141 | 0.930018975205023 | -0.881976312 | 0.800110994743419 | 0.95989376832565 | 0.413963980726352 | 2.22578796554645 | 0.999228396881742 |
| ebi-fl187-GCST90094727 | ebi-met1400-GCST90200812 | Glutamate to kynurenine ratio \|\| id:ebi-met1400-GCST90200812 | Cauliflower liking \|\| id:ebi-fl187-GCST90094727 | Weighted mode | 4 | 0.805482151717085 | 0.605472258136902 | 0.275494869545797 | -0.381243474 | 1.99220777766541 | 2.23777518590201 | 0.68301157365607 | 7.3317026764767 | 0.999228396881742 |
| ebi-fl187-GCST90094727 | ebi-met1400-GCST90200876 | Cortisone to 4-cholesten-3-one ratio \|\| id:ebi-met1400-GCST90200876 | Cauliflower liking \|\| id:ebi-fl187-GCST90094727 | Weighted mode | 4 | -0.215583344 | 0.515094058025272 | 0.703715020361417 | -1.225167698 | 0.794001009655916 | 0.806071086210511 | 0.293708441932908 | 2.21222989624866 | 0.999228396881742 |
| ebi-fl187-GCST90094727 | ebi-met1400-GCST90200906 | 2'-deoxyuridine to cytidine ratio \|\| id:ebi-met1400-GCST90200906 | Cauliflower liking \|\| id:ebi-fl187-GCST90094727 | Weighted mode | 4 | 0.576785370400835 | 0.364336769511386 | 0.211554706220017 | -0.137314698 | 1.29088543864315 | 1.78030619718551 | 0.87169586215725 | 3.63600458982721 | 0.999228396881742 |
| ebi-fl187-GCST90094727 | ebi-met1400-GCST90200907 | Retinol (Vitamin A) to linoleoyl-arachidonoyl-glycerol (18:2 to 20:4) [1] ratio \|\| id:ebi-met1400-GCST90200907 | Cauliflower liking \|\| id:ebi-fl187-GCST90094727 | Weighted mode | 4 | -0.730772682 | 0.56216332189605 | 0.284488716446307 | -1.832612793 | 0.371067428756667 | 0.481536771432891 | 0.159994987386115 | 1.449280793 | 0.999228396881742 |
| ebi-fl187-GCST90094727 | ebi-met1400-GCST90200916 | Fructose to sucrose ratio \|\| id:ebi-met1400-GCST90200916 | Cauliflower liking \|\| id:ebi-fl187-GCST90094727 | Weighted mode | 4 | -0.116229941 | 0.372584631200691 | 0.77549846724435 | -0.846495818 | 0.614035936604164 | 0.890270490474678 | 0.428915299031528 | 1.84787427261195 | 0.999228396881742 |
| ebi-fl187-GCST90094727 | ebi-met1400-GCST90200917 | Alpha-ketobutyrate to 3-methyl-2-oxobutyrate ratio \|\| id:ebi-met1400-GCST90200917 | Cauliflower liking \|\| id:ebi-fl187-GCST90094727 | Weighted mode | 4 | -0.05246236 | 0.325371723050272 | 0.882152409112585 | -0.690190937 | 0.585266217469491 | 0.948890036880696 | 0.501480308834918 | 1.79546890721057 | 0.999228396881742 |
| ebi-fl187-GCST90094727 | ebi-met1400-GCST90200924 | Alpha-tocopherol to glycerol ratio \|\| id:ebi-met1400-GCST90200924 | Cauliflower liking \|\| id:ebi-fl187-GCST90094727 | Weighted mode | 4 | -0.057599846 | 0.303145402748533 | 0.861433225076216 | -0.651764835 | 0.536565143490354 | 0.94402762839553 | 0.521125264469587 | 1.71012273619311 | 0.999228396881742 |
| ebi-fl187-GCST90094727 | ebi-met1400-GCST90200938 | Alpha-ketoglutarate to alanine ratio \|\| id:ebi-met1400-GCST90200938 | Cauliflower liking \|\| id:ebi-fl187-GCST90094727 | Weighted mode | 4 | -0.053014921 | 0.353478655291007 | 0.890295944303108 | -0.745833086 | 0.639803243104717 | 0.948365861557763 | 0.474338970308284 | 1.89610777032226 | 0.999228396881742 |
| ebi-fl187-GCST90094727 | ebi-met1400-GCST90200943 | Phosphate to EDTA ratio \|\| id:ebi-met1400-GCST90200943 | Cauliflower liking \|\| id:ebi-fl187-GCST90094727 | Weighted mode | 4 | -0.041710891 | 0.34860530944683 | 0.912322730334238 | -0.724977297 | 0.641555515757556 | 0.959147038764722 | 0.484335564568152 | 1.89943318077706 | 0.999228396881742 |
| ebi-fl187-GCST90094727 | ebi-met1400-GCST90200947 | Adenosine 5'-diphosphate (ADP) to glucose ratio \|\| id:ebi-met1400-GCST90200947 | Cauliflower liking \|\| id:ebi-fl187-GCST90094727 | Weighted mode | 4 | 0.459580823151229 | 0.462382120923262 | 0.393514945089639 | -0.446688134 | 1.36584978016082 | 1.583410117 | 0.639743391470292 | 3.91905196998149 | 0.999228396881742 |
| ebi-fl187-GCST90094727 | ebi-met1400-GCST90200975 | Arachidonate (20:4n6) to caffeine ratio \|\| id:ebi-met1400-GCST90200975 | Cauliflower liking \|\| id:ebi-fl187-GCST90094727 | Weighted mode | 4 | 0.455134056022183 | 0.374073924776446 | 0.310709399147093 | -0.278050837 | 1.18831894858402 | 1.57638469273134 | 0.757258324142986 | 3.28156009680057 | 0.999228396881742 |
| ebi-fl187-GCST90094727 | ebi-met1400-GCST90200985 | Cholesterol to oleoyl-linoleoyl-glycerol (18:1 to 18:2) [2] ratio \|\| id:ebi-met1400-GCST90200985 | Cauliflower liking \|\| id:ebi-fl187-GCST90094727 | Weighted mode | 4 | -0.371459758 | 0.458955206236144 | 0.477544676848389 | -1.271011962 | 0.528092446670344 | 0.689726761563638 | 0.280547574658487 | 1.69569459367512 | 0.999228396881742 |
| ebi-fl187-GCST90094727 | ebi-met1400-GCST90201016 | Bilirubin (Z,Z) to etiocholanolone glucuronide ratio \|\| id:ebi-met1400-GCST90201016 | Cauliflower liking \|\| id:ebi-fl187-GCST90094727 | Weighted mode | 4 | -0.044583717 | 0.355230642228539 | 0.908061028140769 | -0.740835776 | 0.651668341492731 | 0.956395529914256 | 0.476715321731365 | 1.91873927256614 | 0.999228396881742 |
| ebi-fl187-GCST90094727 | ebi-pw11-GCST90275189 | Placental weight (fetal genotype, adjusted for sex and gestational age) \|\| id:ebi-pw11-GCST90275189 | Cauliflower liking \|\| id:ebi-fl187-GCST90094727 | Weighted mode | 4 | -0.012068414 | 0.124242171888535 | 0.928743965946393 | -0.25558307 | 0.231446243330616 | 0.988004117659976 | 0.77446479873155 | 1.26042156869086 | 0.999228396881742 |
| ebi-fl187-GCST90094727 | ebi-pw11-GCST90275192 | Placental weight (fetal effect, conditional on maternal and paternal genotype) \|\| id:ebi-pw11-GCST90275192 | Cauliflower liking \|\| id:ebi-fl187-GCST90094727 | Weighted mode | 4 | -0.032478989 | 0.243195189285597 | 0.902212927351757 | -0.50914156 | 0.444183582171664 | 0.968042789331074 | 0.601011289662087 | 1.55921670373741 | 0.999228396881742 |
| ebi-fl187-GCST90094727 | ebi-pw11-GCST90275193 | Placental weight (maternal effect, conditional on fetal and paternal genotype) \|\| id:ebi-pw11-GCST90275193 | Cauliflower liking \|\| id:ebi-fl187-GCST90094727 | Weighted mode | 4 | 0.0139008644425763 | 0.168667455605374 | 0.939506973188301 | -0.316687349 | 0.34448907742911 | 1.01399793070553 | 0.728558504397988 | 1.4112686864107 | 0.999228396881742 |
| ebi-fl187-GCST90094727 | ebi-pw11-GCST90275195 | Placental weight (fetal effect, conditional on maternal genotype) \|\| id:ebi-pw11-GCST90275195 | Cauliflower liking \|\| id:ebi-fl187-GCST90094727 | Weighted mode | 4 | 0.0116265242988253 | 0.166011517047189 | 0.948573348106152 | -0.313756049 | 0.337009097711316 | 1.01169437503356 | 0.730697260662934 | 1.40075180731613 | 0.999228396881742 |
| ebi-fl187-GCST90094727 | ebi-pw11-GCST90275197 | Placental weight (fetal genotype, adjusted for sex) \|\| id:ebi-pw11-GCST90275197 | Cauliflower liking \|\| id:ebi-fl187-GCST90094727 | Weighted mode | 4 | -0.01016264 | 0.121868128830469 | 0.938793766389065 | -0.249024173 | 0.228698892212849 | 0.989888824845894 | 0.779561128979664 | 1.25696350052408 | 0.999228396881742 |
| ebi-fl187-GCST90094727 | prot-a-1030 | Protein FAM171B \|\| id:prot-a-1030 | Cauliflower liking \|\| id:ebi-fl187-GCST90094727 | Weighted mode | 4 | 0.205447731817003 | 0.520914804133378 | 0.719652030777187 | -0.815545284 | 1.22644074791842 | 1.22807479004645 | 0.442398028886587 | 3.4090741627926 | 0.999228396881742 |
| ebi-fl187-GCST90094727 | prot-a-1036 | BRISC complex subunit Abro1 \|\| id:prot-a-1036 | Cauliflower liking \|\| id:ebi-fl187-GCST90094727 | Weighted mode | 4 | 0.643890382752237 | 0.610389420535884 | 0.368924501138428 | -0.552472881 | 1.84025364700257 | 1.90387328613003 | 0.575524844481127 | 6.29813556164982 | 0.999228396881742 |
| ebi-fl187-GCST90094727 | prot-a-1055 | Phenylalanine--tRNA ligase, mitochondrial \|\| id:prot-a-1055 | Cauliflower liking \|\| id:ebi-fl187-GCST90094727 | Weighted mode | 4 | -0.696042221 | 0.53544762677494 | 0.284489806892745 | -1.74551957 | 0.353435127405597 | 0.498554573037679 | 0.174554272316951 | 1.42395060858471 | 0.999228396881742 |
| ebi-fl187-GCST90094727 | prot-a-1083 | Fc receptor-like protein 5 \|\| id:prot-a-1083 | Cauliflower liking \|\| id:ebi-fl187-GCST90094727 | Weighted mode | 4 | -0.018983721 | 0.467561497320683 | 0.970164519369979 | -0.935404255 | 0.89743681414013 | 0.981195335375881 | 0.392427192666654 | 2.4533067640427 | 0.999228396881742 |
| ebi-fl187-GCST90094727 | prot-a-1103 | Fibroblast growth factor receptor 3 \|\| id:prot-a-1103 | Cauliflower liking \|\| id:ebi-fl187-GCST90094727 | Weighted mode | 4 | 0.146779416588398 | 0.746476682117155 | 0.856684216294053 | -1.31631488 | 1.60987371353802 | 1.15809847753981 | 0.268121543606449 | 5.00217948039509 | 0.999228396881742 |
| ebi-fl187-GCST90094727 | prot-a-1105 | Fibroblast growth factor receptor 4 \|\| id:prot-a-1105 | Cauliflower liking \|\| id:ebi-fl187-GCST90094727 | Weighted mode | 4 | -1.119426065 | 0.57164655433708 | 0.145100006491705 | -2.239853312 | 0.00100118127143345 | 0.326467111690917 | 0.106474121738509 | 1.0010016826207 | 0.999228396881742 |
| ebi-fl187-GCST90094727 | prot-a-1120 | Filamin-A \|\| id:prot-a-1120 | Cauliflower liking \|\| id:ebi-fl187-GCST90094727 | Weighted mode | 4 | -0.245502849 | 0.513025129727754 | 0.664977991167546 | -1.251032103 | 0.760026405660836 | 0.782311055295712 | 0.286209246981695 | 2.13833268383894 | 0.999228396881742 |
| ebi-fl187-GCST90094727 | prot-a-1166 | Galanin peptides \|\| id:prot-a-1166 | Cauliflower liking \|\| id:ebi-fl187-GCST90094727 | Weighted mode | 4 | -0.071546566 | 0.586701670697636 | 0.910650985173241 | -1.22148184 | 1.07838870882683 | 0.930952926073112 | 0.294793006943192 | 2.93993863542056 | 0.999228396881742 |
| ebi-fl187-GCST90094727 | prot-a-1205 | ADP-ribosylation factor-binding protein GGA1 \|\| id:prot-a-1205 | Cauliflower liking \|\| id:ebi-fl187-GCST90094727 | Weighted mode | 4 | 0.175201148974583 | 0.528967596097289 | 0.762270091053338 | -0.861575339 | 1.21197763732527 | 1.19148585866837 | 0.422495983230814 | 3.36012319111481 | 0.999228396881742 |
| ebi-fl187-GCST90094727 | prot-a-1227 | Guanine nucleotide-binding protein G(I)/G(S)/G(O) subunit gamma-T2 \|\| id:prot-a-1227 | Cauliflower liking \|\| id:ebi-fl187-GCST90094727 | Weighted mode | 4 | -0.807282607 | 0.579649865818193 | 0.257976596345065 | -1.943396344 | 0.328831130116675 | 0.446068564423219 | 0.143216709631726 | 1.38934321754947 | 0.999228396881742 |
| ebi-fl187-GCST90094727 | prot-a-1240 | Golgi SNAP receptor complex member 1 \|\| id:prot-a-1240 | Cauliflower liking \|\| id:ebi-fl187-GCST90094727 | Weighted mode | 4 | -0.51574032 | 0.569675221917506 | 0.432030896604751 | -1.632303755 | 0.600823114874601 | 0.597058416624833 | 0.195478719910871 | 1.82361923090702 | 0.999228396881742 |
| ebi-fl187-GCST90094727 | prot-a-1283 | Glutathione S-transferase A1 \|\| id:prot-a-1283 | Cauliflower liking \|\| id:ebi-fl187-GCST90094727 | Weighted mode | 4 | -0.636153382 | 0.525527360041528 | 0.31275663948571 | -1.666187008 | 0.393880243239643 | 0.529324622378825 | 0.188966220365124 | 1.48272297194233 | 0.999228396881742 |
| ebi-fl187-GCST90094727 | prot-a-1324 | E3 ubiquitin-protein ligase HECW1 \|\| id:prot-a-1324 | Cauliflower liking \|\| id:ebi-fl187-GCST90094727 | Weighted mode | 4 | 0.30892810423127 | 0.56836982534257 | 0.624550877649853 | -0.805076753 | 1.42293296190271 | 1.3619644475024 | 0.447053612339819 | 4.14927226860326 | 0.999228396881742 |
| ebi-fl187-GCST90094727 | prot-a-1339 | Histidine triad nucleotide-binding protein 2, mitochondrial \|\| id:prot-a-1339 | Cauliflower liking \|\| id:ebi-fl187-GCST90094727 | Weighted mode | 4 | 0.51039943682368 | 0.604987601437274 | 0.460819414882196 | -0.675376262 | 1.69617513564074 | 1.66595650643741 | 0.508964880434694 | 5.45305027523849 | 0.999228396881742 |
| ebi-fl187-GCST90094727 | prot-a-1466 | Interleukin-11 \|\| id:prot-a-1466 | Cauliflower liking \|\| id:ebi-fl187-GCST90094727 | Weighted mode | 4 | -0.104636348 | 0.492830613188361 | 0.845467256265829 | -1.07058435 | 0.861311653473574 | 0.90065198659918 | 0.342808138865773 | 2.36626237535938 | 0.999228396881742 |
| ebi-fl187-GCST90094727 | prot-a-147 | Rho GTPase-activating protein 1 \|\| id:prot-a-147 | Cauliflower liking \|\| id:ebi-fl187-GCST90094727 | Weighted mode | 4 | -0.185929007 | 0.544203742912647 | 0.755143586101586 | -1.252568343 | 0.880710328746974 | 0.830332540387177 | 0.285769898275841 | 2.41261284615892 | 0.999228396881742 |
| ebi-fl187-GCST90094727 | prot-a-1473 | Interleukin-12 receptor subunit beta-1 \|\| id:prot-a-1473 | Cauliflower liking \|\| id:ebi-fl187-GCST90094727 | Weighted mode | 4 | -0.110719176 | 0.606909488976907 | 0.866875961238258 | -1.300261774 | 1.07882342245624 | 0.895190104555006 | 0.272460460542522 | 2.94121694464411 | 0.999228396881742 |
| ebi-fl187-GCST90094727 | prot-a-1501 | X-linked interleukin-1 receptor accessory protein-like 2 \|\| id:prot-a-1501 | Cauliflower liking \|\| id:ebi-fl187-GCST90094727 | Weighted mode | 4 | 0.0756444510157004 | 0.53285249503313 | 0.896108033746186 | -0.968746439 | 1.12003534128064 | 1.07857901830043 | 0.379558539694044 | 3.06496252107954 | 0.999228396881742 |
| ebi-fl187-GCST90094727 | prot-a-1503 | Interleukin-1 receptor-like 2 \|\| id:prot-a-1503 | Cauliflower liking \|\| id:ebi-fl187-GCST90094727 | Weighted mode | 4 | 0.0558071587784141 | 0.516971338976711 | 0.920850030332595 | -0.957456666 | 1.06907098317277 | 1.0573937549709 | 0.383867950048813 | 2.91267232106584 | 0.999228396881742 |
| ebi-fl187-GCST90094727 | prot-a-1536 | Interleukin-5 receptor subunit alpha \|\| id:prot-a-1536 | Cauliflower liking \|\| id:ebi-fl187-GCST90094727 | Weighted mode | 4 | 0.444691412754048 | 0.592251782325867 | 0.507272375666825 | -0.716122081 | 1.60550490611275 | 1.56000872275285 | 0.488643506662887 | 4.98037358909164 | 0.999228396881742 |
| ebi-fl187-GCST90094727 | prot-a-1559 | Insulin-induced gene 1 protein \|\| id:prot-a-1559 | Cauliflower liking \|\| id:ebi-fl187-GCST90094727 | Weighted mode | 4 | 0.427847577551107 | 0.531591768647793 | 0.479786149728216 | -0.614072289 | 1.46976744410078 | 1.53395225438872 | 0.541142686538352 | 4.34822381837269 | 0.999228396881742 |
| ebi-fl187-GCST90094727 | prot-a-1567 | Interferon regulatory factor 1 \|\| id:prot-a-1567 | Cauliflower liking \|\| id:ebi-fl187-GCST90094727 | Weighted mode | 4 | -0.113475572 | 0.481788922032726 | 0.828960419989389 | -1.057781859 | 0.83083071493744 | 0.89272600343541 | 0.347225150955757 | 2.29522462591227 | 0.999228396881742 |
| ebi-fl187-GCST90094727 | prot-a-1613 | Potassium voltage-gated channel subfamily E regulatory beta subunit 5 \|\| id:prot-a-1613 | Cauliflower liking \|\| id:ebi-fl187-GCST90094727 | Weighted mode | 3 | -0.820623734 | 0.559469156183127 | 0.280108859283792 | -1.91718328 | 0.27593581236426 | 0.440157028072251 | 0.147020495041079 | 1.31776327720339 | 0.999228396881742 |
| ebi-fl187-GCST90094727 | prot-a-1649 | Kin of IRRE-like protein 2 \|\| id:prot-a-1649 | Cauliflower liking \|\| id:ebi-fl187-GCST90094727 | Weighted mode | 4 | -0.383032284 | 0.531724735290081 | 0.523359322061248 | -1.425212766 | 0.659148196833549 | 0.681790887765251 | 0.240457296747477 | 1.93314497387822 | 0.999228396881742 |
| ebi-fl187-GCST90094727 | prot-a-165 | Armadillo repeat-containing protein 5 \|\| id:prot-a-165 | Cauliflower liking \|\| id:ebi-fl187-GCST90094727 | Weighted mode | 4 | -0.344081549 | 0.613918720261371 | 0.614292397311206 | -1.547362241 | 0.859199142347508 | 0.708871117702986 | 0.212808571849755 | 2.36126889601161 | 0.999228396881742 |
| ebi-fl187-GCST90094727 | prot-a-1656 | Kallikrein-11 \|\| id:prot-a-1656 | Cauliflower liking \|\| id:ebi-fl187-GCST90094727 | Weighted mode | 4 | -0.625617372 | 0.639266823433348 | 0.399925673448419 | -1.878580346 | 0.627345602247598 | 0.53493107525655 | 0.152806884809479 | 1.87263326277415 | 0.999228396881742 |
| ebi-fl187-GCST90094727 | prot-a-1697 | Laminin subunit gamma-2 \|\| id:prot-a-1697 | Cauliflower liking \|\| id:ebi-fl187-GCST90094727 | Weighted mode | 4 | -0.345298241 | 0.581397821920703 | 0.59437756737321 | -1.484837972 | 0.794241490173922 | 0.708009164763732 | 0.226539043426525 | 2.21276195841279 | 0.999228396881742 |
| ebi-fl187-GCST90094727 | prot-a-1698 | Lysosome-associated membrane glycoprotein 3 \|\| id:prot-a-1698 | Cauliflower liking \|\| id:ebi-fl187-GCST90094727 | Weighted mode | 4 | -0.021091071 | 0.535297205481108 | 0.971046346814075 | -1.070273594 | 1.02809145181546 | 0.97912979025077 | 0.34291468529594 | 2.79572496386135 | 0.999228396881742 |
| ebi-fl187-GCST90094727 | prot-a-1724 | Leptin receptor \|\| id:prot-a-1724 | Cauliflower liking \|\| id:ebi-fl187-GCST90094727 | Weighted mode | 4 | -0.612476169 | 0.546786202500669 | 0.344244843456242 | -1.684177126 | 0.459224788091851 | 0.542007104979871 | 0.185597092212594 | 1.58284646783236 | 0.999228396881742 |
| ebi-fl187-GCST90094727 | prot-a-1743 | Leukocyte immunoglobulin-like receptor subfamily B member 2 \|\| id:prot-a-1743 | Cauliflower liking \|\| id:ebi-fl187-GCST90094727 | Weighted mode | 4 | -0.504378237 | 0.553717520219508 | 0.42951122123376 | -1.589664577 | 0.580908102298793 | 0.603880929342541 | 0.20399402455547 | 1.78766107300584 | 0.999228396881742 |
| ebi-fl187-GCST90094727 | prot-a-1746 | Leukocyte immunoglobulin-like receptor subfamily B member 4 \|\| id:prot-a-1746 | Cauliflower liking \|\| id:ebi-fl187-GCST90094727 | Weighted mode | 4 | 0.194583785194334 | 0.51563389260527 | 0.731014654900547 | -0.816058644 | 1.20522621470066 | 1.21480526131612 | 0.442170977706765 | 3.33751398740602 | 0.999228396881742 |
| ebi-fl187-GCST90094727 | prot-a-1750 | Leucine-rich repeat and immunoglobulin-like domain-containing nogo receptor-interacting protein 3 \|\| id:prot-a-1750 | Cauliflower liking \|\| id:ebi-fl187-GCST90094727 | Weighted mode | 4 | 0.138431461467308 | 0.523397490222515 | 0.808535065336252 | -0.887427619 | 1.16429054230344 | 1.14847096435789 | 0.411713475506312 | 3.20364922316691 | 0.999228396881742 |
| ebi-fl187-GCST90094727 | prot-a-1753 | Lipase member N \|\| id:prot-a-1753 | Cauliflower liking \|\| id:ebi-fl187-GCST90094727 | Weighted mode | 4 | 0.365723868261702 | 0.522093312897176 | 0.534058061658476 | -0.657579025 | 1.38902676154017 | 1.44155712801539 | 0.518104134529431 | 4.0109445473146 | 0.999228396881742 |
| ebi-fl187-GCST90094727 | prot-a-1760 | Ig Kappa chain V-I region HK102- like \|\| id:prot-a-1760 | Cauliflower liking \|\| id:ebi-fl187-GCST90094727 | Weighted mode | 4 | -0.489269733 | 0.472578178890364 | 0.37665379958786 | -1.415522964 | 0.4369834973698 | 0.613073938260395 | 0.242798605340499 | 1.54803053027017 | 0.999228396881742 |
| ebi-fl187-GCST90094727 | prot-a-1792 | Leucine-rich repeat neuronal protein 1 \|\| id:prot-a-1792 | Cauliflower liking \|\| id:ebi-fl187-GCST90094727 | Weighted mode | 4 | -0.234632059 | 0.525848708081569 | 0.68569470991573 | -1.265295527 | 0.79603140893267 | 0.790861786651641 | 0.282155899203057 | 2.21672616930013 | 0.999228396881742 |
| ebi-fl187-GCST90094727 | prot-a-1795 | Leucine-rich repeat transmembrane neuronal protein 1 \|\| id:prot-a-1795 | Cauliflower liking \|\| id:ebi-fl187-GCST90094727 | Weighted mode | 4 | -0.393250489 | 0.529172640439402 | 0.511299857054561 | -1.430428864 | 0.64392788606422 | 0.67485968135271 | 0.239206313156733 | 1.90394468902277 | 0.999228396881742 |
| ebi-fl187-GCST90094727 | prot-a-1820 | LysM and putative peptidoglycan-binding domain-containing protein 4 \|\| id:prot-a-1820 | Cauliflower liking \|\| id:ebi-fl187-GCST90094727 | Weighted mode | 4 | -0.210911098 | 0.534567293682207 | 0.71955402792353 | -1.258662994 | 0.836840797436783 | 0.809846060469467 | 0.284033527333526 | 2.30906065144832 | 0.999228396881742 |
| ebi-fl187-GCST90094727 | prot-a-1844 | Mitogen-activated protein kinase kinase kinase 7:TGF-beta-activated kinase 1 and MAP3K7-binding protein 1 fusion \|\| id:prot-a-1844 | Cauliflower liking \|\| id:ebi-fl187-GCST90094727 | Weighted mode | 4 | -0.618513392 | 0.556259310456372 | 0.347263041588143 | -1.70878164 | 0.471754856938664 | 0.538744745062629 | 0.181086286548327 | 1.60280441918024 | 0.999228396881742 |
| ebi-fl187-GCST90094727 | prot-a-1856 | Megakaryocyte-associated tyrosine-protein kinase \|\| id:prot-a-1856 | Cauliflower liking \|\| id:ebi-fl187-GCST90094727 | Weighted mode | 4 | -0.909988629 | 0.583379149957874 | 0.216697144440087 | -2.053411763 | 0.2334345048571 | 0.402528801138325 | 0.12829643900373 | 1.26293010939413 | 0.999228396881742 |
| ebi-fl187-GCST90094727 | prot-a-1910 | Stromelysin-2 \|\| id:prot-a-1910 | Cauliflower liking \|\| id:ebi-fl187-GCST90094727 | Weighted mode | 4 | -0.573208333 | 0.477989334115549 | 0.31652823743787 | -1.510067428 | 0.363650762045065 | 0.563713952352628 | 0.220895083012505 | 1.43857172257217 | 0.999228396881742 |
| ebi-fl187-GCST90094727 | prot-a-1940 | 39S ribosomal protein L14, mitochondrial \|\| id:prot-a-1940 | Cauliflower liking \|\| id:ebi-fl187-GCST90094727 | Weighted mode | 4 | -0.634917539 | 0.577309206983382 | 0.351762466273903 | -1.766443585 | 0.496608506208939 | 0.529979188876063 | 0.170939842064906 | 1.64313911402282 | 0.999228396881742 |
| ebi-fl187-GCST90094727 | prot-a-1942 | 39S ribosomal protein L33, mitochondrial \|\| id:prot-a-1942 | Cauliflower liking \|\| id:ebi-fl187-GCST90094727 | Weighted mode | 4 | 0.943887994755074 | 0.557807609125451 | 0.189196599580339 | -0.149414919 | 2.03719090864096 | 2.56995398674114 | 0.861211707543373 | 7.6690358899168 | 0.999228396881742 |
| ebi-fl187-GCST90094727 | prot-a-1948 | Mesothelin \|\| id:prot-a-1948 | Cauliflower liking \|\| id:ebi-fl187-GCST90094727 | Weighted mode | 4 | 0.705217435093429 | 0.544726188015851 | 0.28608829768491 | -0.362445893 | 1.7728807636045 | 2.02428678806057 | 0.695971969324603 | 5.8877902859984 | 0.999228396881742 |
| ebi-fl187-GCST90094727 | prot-a-1980 | Unconventional myosin-VI \|\| id:prot-a-1980 | Cauliflower liking \|\| id:ebi-fl187-GCST90094727 | Weighted mode | 4 | -0.636288736 | 0.689676822560612 | 0.424265025596991 | -1.988055308 | 0.71547783661898 | 0.529252981468064 | 0.136961514617526 | 2.04516370292091 | 0.999228396881742 |
| ebi-fl187-GCST90094727 | prot-a-2039 | Neurofascin \|\| id:prot-a-2039 | Cauliflower liking \|\| id:ebi-fl187-GCST90094727 | Weighted mode | 4 | -0.127278093 | 0.554187876062633 | 0.833119471883647 | -1.21348633 | 0.958930144035391 | 0.880488780739164 | 0.29715947538481 | 2.60890382850356 | 0.999228396881742 |
| ebi-fl187-GCST90094727 | prot-a-2041 | NFU1 iron-sulfur cluster scaffold homolog, mitochondrial \|\| id:prot-a-2041 | Cauliflower liking \|\| id:ebi-fl187-GCST90094727 | Weighted mode | 4 | 0.247849793676882 | 0.536569786404136 | 0.675578335927615 | -0.803826988 | 1.29952657502899 | 1.28126746326477 | 0.447612673914857 | 3.6675599420878 | 0.999228396881742 |
| ebi-fl187-GCST90094727 | prot-a-2088 | Glucocorticoid receptor \|\| id:prot-a-2088 | Cauliflower liking \|\| id:ebi-fl187-GCST90094727 | Weighted mode | 4 | 0.502262128981183 | 0.501270683437033 | 0.390185187045079 | -0.480228411 | 1.48475266851777 | 1.65245511249209 | 0.618642071288109 | 4.41387358786593 | 0.999228396881742 |
| ebi-fl187-GCST90094727 | prot-a-2111 | Neuron-specific protein family member 2 \|\| id:prot-a-2111 | Cauliflower liking \|\| id:ebi-fl187-GCST90094727 | Weighted mode | 4 | 0.612009661358628 | 0.537588227596274 | 0.337621821780416 | -0.441663265 | 1.66568258744732 | 1.8441337616487 | 0.642966108372512 | 5.28928241561723 | 0.999228396881742 |
| ebi-fl187-GCST90094727 | prot-a-2125 | Nuclear migration protein nudC \|\| id:prot-a-2125 | Cauliflower liking \|\| id:ebi-fl187-GCST90094727 | Weighted mode | 4 | -0.279858015 | 0.583557347103331 | 0.664322834521068 | -1.423630416 | 0.863914385028737 | 0.755891058806744 | 0.240838085514952 | 2.37242914285044 | 0.999228396881742 |
| ebi-fl187-GCST90094727 | prot-a-2142 | Ornithine decarboxylase \|\| id:prot-a-2142 | Cauliflower liking \|\| id:ebi-fl187-GCST90094727 | Weighted mode | 4 | -0.499108632 | 0.534340249578186 | 0.41917421915906 | -1.546415521 | 0.548198256975237 | 0.607071542640623 | 0.213010137316236 | 1.73013295295395 | 0.999228396881742 |
| ebi-fl187-GCST90094727 | prot-a-2186 | Prostate and testis expressed protein 1 \|\| id:prot-a-2186 | Cauliflower liking \|\| id:ebi-fl187-GCST90094727 | Weighted mode | 4 | 0.640687646541192 | 0.48040791822199 | 0.27453828450271 | -0.300911873 | 1.58228716625629 | 1.89778543630769 | 0.740142996325812 | 4.86607261048262 | 0.999228396881742 |
| ebi-fl187-GCST90094727 | prot-a-2227 | Platelet-derived growth factor C \|\| id:prot-a-2227 | Cauliflower liking \|\| id:ebi-fl187-GCST90094727 | Weighted mode | 4 | -0.286895538 | 0.59358838587517 | 0.66194641705032 | -1.450328774 | 0.876537698523203 | 0.750590133044361 | 0.234493180132937 | 2.40256687850863 | 0.999228396881742 |
| ebi-fl187-GCST90094727 | prot-a-2229 | Platelet-derived growth factor receptor alpha \|\| id:prot-a-2229 | Cauliflower liking \|\| id:ebi-fl187-GCST90094727 | Weighted mode | 4 | -0.471573446 | 0.604177301958428 | 0.492008431655471 | -1.655760958 | 0.712614065622262 | 0.624019634092272 | 0.190946697979052 | 2.03931520080736 | 0.999228396881742 |
| ebi-fl187-GCST90094727 | prot-a-2243 | Platelet endothelial aggregation receptor 1 \|\| id:prot-a-2243 | Cauliflower liking \|\| id:ebi-fl187-GCST90094727 | Weighted mode | 4 | 0.032811015158885 | 0.51864399678448 | 0.953536294543773 | -0.983731219 | 1.04935324885647 | 1.03335523231247 | 0.373913340414315 | 2.85580352646513 | 0.999228396881742 |
| ebi-fl187-GCST90094727 | prot-a-2246 | E3 ubiquitin-protein ligase pellino homolog 2 \|\| id:prot-a-2246 | Cauliflower liking \|\| id:ebi-fl187-GCST90094727 | Weighted mode | 4 | 0.738272049189569 | 0.491417818701054 | 0.230025174517426 | -0.224906875 | 1.70145097384363 | 2.09231696825879 | 0.798590583673887 | 5.48189571122139 | 0.999228396881742 |
| ebi-fl187-GCST90094727 | prot-a-2251 | Platelet factor 4 variant \|\| id:prot-a-2251 | Cauliflower liking \|\| id:ebi-fl187-GCST90094727 | Weighted mode | 4 | -0.122976259 | 0.65166948610133 | 0.862364816794895 | -1.400248452 | 1.15429593349267 | 0.884284655878477 | 0.246535704037059 | 3.17178948045827 | 0.999228396881742 |
| ebi-fl187-GCST90094727 | prot-a-2346 | Protein phosphatase 1L \|\| id:prot-a-2346 | Cauliflower liking \|\| id:ebi-fl187-GCST90094727 | Weighted mode | 4 | 0.702943905099016 | 0.633664790658083 | 0.34821717614536 | -0.539039085 | 1.94492689478886 | 2.01968973907515 | 0.583308493278315 | 6.99312060278739 | 0.999228396881742 |
| ebi-fl187-GCST90094727 | prot-a-2358 | PR domain zinc finger protein 4 \|\| id:prot-a-2358 | Cauliflower liking \|\| id:ebi-fl187-GCST90094727 | Weighted mode | 4 | -0.322696389 | 0.728505366907186 | 0.687791189974453 | -1.750566909 | 1.10517412977592 | 0.724193693902739 | 0.173675457443696 | 3.01975025146266 | 0.999228396881742 |
| ebi-fl187-GCST90094727 | prot-a-2390 | Trypsin-1 \|\| id:prot-a-2390 | Cauliflower liking \|\| id:ebi-fl187-GCST90094727 | Weighted mode | 4 | 0.0751351964459204 | 0.548076769223843 | 0.899643804375955 | -0.999095271 | 1.14936566412465 | 1.07802988684221 | 0.368212422891084 | 3.15619018989155 | 0.999228396881742 |
| ebi-fl187-GCST90094727 | prot-a-242 | Brain-derived neurotrophic factor \|\| id:prot-a-242 | Cauliflower liking \|\| id:ebi-fl187-GCST90094727 | Weighted mode | 4 | 0.393308778160461 | 0.639940751605076 | 0.582288955043424 | -0.860975095 | 1.64759265130641 | 1.48187588960381 | 0.422749660201279 | 5.19445988707249 | 0.999228396881742 |
| ebi-fl187-GCST90094727 | prot-a-2464 | Dihydropteridine reductase \|\| id:prot-a-2464 | Cauliflower liking \|\| id:ebi-fl187-GCST90094727 | Weighted mode | 4 | -0.026150438 | 0.553425436486887 | 0.96528206019206 | -1.110864293 | 1.05856341782117 | 0.974188523910314 | 0.329274248558955 | 2.88222745711812 | 0.999228396881742 |
| ebi-fl187-GCST90094727 | prot-a-2474 | Ras-related protein Rab-27A \|\| id:prot-a-2474 | Cauliflower liking \|\| id:ebi-fl187-GCST90094727 | Weighted mode | 4 | -0.88782464 | 1.05621707630038 | 0.46230174042304 | -2.958010109 | 1.18236082969006 | 0.411550049264511 | 0.0519221338093731 | 3.26206630242623 | 0.999228396881742 |
| ebi-fl187-GCST90094727 | prot-a-2478 | Ras-related protein Rab-7b \|\| id:prot-a-2478 | Cauliflower liking \|\| id:ebi-fl187-GCST90094727 | Weighted mode | 4 | -0.698603215 | 0.734452649238029 | 0.411681879279439 | -2.138130408 | 0.740923977198927 | 0.497279411188654 | 0.11787501534902 | 2.09787300608136 | 0.999228396881742 |
| ebi-fl187-GCST90094727 | prot-a-2528 | E3 ubiquitin-protein ligase rififylin \|\| id:prot-a-2528 | Cauliflower liking \|\| id:ebi-fl187-GCST90094727 | Weighted mode | 4 | -0.040297002 | 0.658455607555305 | 0.955049500290976 | -1.330869993 | 1.25027598840241 | 0.960504124748874 | 0.264247267937972 | 3.49130638457977 | 0.999228396881742 |
| ebi-fl187-GCST90094727 | prot-a-2543 | Protein RIC-3 \|\| id:prot-a-2543 | Cauliflower liking \|\| id:ebi-fl187-GCST90094727 | Weighted mode | 4 | -0.195512102 | 0.477383021877859 | 0.709623353514959 | -1.131182825 | 0.740158620758053 | 0.822413390556317 | 0.322651390494512 | 2.09626799974334 | 0.999228396881742 |
| ebi-fl187-GCST90094727 | prot-a-255 | Bone morphogenetic protein 6 \|\| id:prot-a-255 | Cauliflower liking \|\| id:ebi-fl187-GCST90094727 | Weighted mode | 4 | -0.287288939 | 0.814621220237008 | 0.747661605087277 | -1.883946531 | 1.30936865271638 | 0.750294908093049 | 0.151989090975011 | 3.70383456798826 | 0.999228396881742 |
| ebi-fl187-GCST90094727 | prot-a-2568 | RING finger protein 165 \|\| id:prot-a-2568 | Cauliflower liking \|\| id:ebi-fl187-GCST90094727 | Weighted mode | 4 | -0.104428309 | 0.532333976054291 | 0.85701307778602 | -1.147802902 | 0.938946284466887 | 0.900839377528516 | 0.317333216298584 | 2.55728534683996 | 0.999228396881742 |
| ebi-fl187-GCST90094727 | prot-a-2573 | E3 ubiquitin-protein ligase RNF8 \|\| id:prot-a-2573 | Cauliflower liking \|\| id:ebi-fl187-GCST90094727 | Weighted mode | 4 | 0.119444205195074 | 0.758577955457989 | 0.884884786032946 | -1.367368588 | 1.60625699789273 | 1.12687036880076 | 0.254776500314468 | 4.98412069603679 | 0.999228396881742 |
| ebi-fl187-GCST90094727 | prot-a-260 | Cytoplasmic tyrosine-protein kinase BMX \|\| id:prot-a-260 | Cauliflower liking \|\| id:ebi-fl187-GCST90094727 | Weighted mode | 4 | -0.165107265 | 0.576446129863351 | 0.793195582681227 | -1.294941679 | 0.964727149976661 | 0.847802760024254 | 0.273913838770594 | 2.62407158079632 | 0.999228396881742 |
| ebi-fl187-GCST90094727 | prot-a-2623 | Serum amyloid A-1 protein \|\| id:prot-a-2623 | Cauliflower liking \|\| id:ebi-fl187-GCST90094727 | Weighted mode | 4 | -0.391898362 | 0.496953885164235 | 0.487919329702201 | -1.365927977 | 0.582131253150762 | 0.675772794820245 | 0.255143798598255 | 1.78984898997379 | 0.999228396881742 |
| ebi-fl187-GCST90094727 | prot-a-2640 | Lysosome membrane protein 2 \|\| id:prot-a-2640 | Cauliflower liking \|\| id:ebi-fl187-GCST90094727 | Weighted mode | 4 | 0.774650124106906 | 0.682940154472694 | 0.339112319754445 | -0.563912579 | 2.11321282687339 | 2.16983282216341 | 0.568978529870734 | 8.27478407174217 | 0.999228396881742 |
| ebi-fl187-GCST90094727 | prot-a-2641 | Scavenger receptor class F member 2 \|\| id:prot-a-2641 | Cauliflower liking \|\| id:ebi-fl187-GCST90094727 | Weighted mode | 4 | -0.342073872 | 0.471049696444035 | 0.520248334214253 | -1.265331277 | 0.581183532664118 | 0.710295731544659 | 0.282145812126734 | 1.78815351696215 | 0.999228396881742 |
| ebi-fl187-GCST90094727 | prot-a-2669 | Semaphorin-3A \|\| id:prot-a-2669 | Cauliflower liking \|\| id:ebi-fl187-GCST90094727 | Weighted mode | 4 | 0.138850788943701 | 0.535874502954825 | 0.812312168921543 | -0.911463237 | 1.18916481473516 | 1.1489526507742 | 0.40193566646149 | 3.28433703170143 | 0.999228396881742 |
| ebi-fl187-GCST90094727 | prot-a-2683 | Sentrin-specific protease 7 \|\| id:prot-a-2683 | Cauliflower liking \|\| id:ebi-fl187-GCST90094727 | Weighted mode | 4 | 0.155576375710599 | 0.779183566711264 | 0.854509529194411 | -1.371623415 | 1.68277616646468 | 1.16833116478268 | 0.253694773157333 | 5.38047234325924 | 0.999228396881742 |
| ebi-fl187-GCST90094727 | prot-a-270 | Bromodomain testis-specific protein \|\| id:prot-a-270 | Cauliflower liking \|\| id:ebi-fl187-GCST90094727 | Weighted mode | 4 | 0.133066544320889 | 0.626496555301849 | 0.84540958916555 | -1.094866704 | 1.36099979271251 | 1.1423260111104 | 0.334584202688541 | 3.90009063540312 | 0.999228396881742 |
| ebi-fl187-GCST90094727 | prot-a-2708 | 14-3-3 protein sigma \|\| id:prot-a-2708 | Cauliflower liking \|\| id:ebi-fl187-GCST90094727 | Weighted mode | 4 | 0.321931517490286 | 0.541151438866848 | 0.593794782917933 | -0.738725303 | 1.38258833766931 | 1.37979028122033 | 0.477722479131112 | 3.98520334151486 | 0.999228396881742 |
| ebi-fl187-GCST90094727 | prot-a-2713 | Beta-sarcoglycan \|\| id:prot-a-2713 | Cauliflower liking \|\| id:ebi-fl187-GCST90094727 | Weighted mode | 4 | -0.26738442 | 0.505872663737382 | 0.633717393851295 | -1.258894841 | 0.724126000549827 | 0.765378787703737 | 0.283967682502651 | 2.06292731448894 | 0.999228396881742 |
| ebi-fl187-GCST90094727 | prot-a-2720 | Protein shisa-3 homolog \|\| id:prot-a-2720 | Cauliflower liking \|\| id:ebi-fl187-GCST90094727 | Weighted mode | 4 | -0.546965869 | 0.578866924196136 | 0.414423229479057 | -1.681545041 | 0.587613301992366 | 0.57870300980933 | 0.186086242968757 | 1.79968797381011 | 0.999228396881742 |
| ebi-fl187-GCST90094727 | prot-a-2730 | Sialic acid-binding Ig-like lectin 8 \|\| id:prot-a-2730 | Cauliflower liking \|\| id:ebi-fl187-GCST90094727 | Weighted mode | 4 | -0.851929307 | 0.567431192207869 | 0.230253105455942 | -1.964094444 | 0.260235829714324 | 0.426591112279085 | 0.140282863139759 | 1.29723597738525 | 0.999228396881742 |
| ebi-fl187-GCST90094727 | prot-a-2761 | Sodium- and chloride-dependent neutral and basic amino acid transporter B(0+) \|\| id:prot-a-2761 | Cauliflower liking \|\| id:ebi-fl187-GCST90094727 | Weighted mode | 4 | 0.374245157245772 | 0.784442255625976 | 0.665901779885514 | -1.163261664 | 1.91175197827268 | 1.45389353930516 | 0.312465360052692 | 6.76493043349449 | 0.999228396881742 |
| ebi-fl187-GCST90094727 | prot-a-2770 | Secreted Ly-6/uPAR-related protein 1 \|\| id:prot-a-2770 | Cauliflower liking \|\| id:ebi-fl187-GCST90094727 | Weighted mode | 4 | 0.325394670052634 | 0.554933739192654 | 0.598829401226059 | -0.762275459 | 1.41306479887024 | 1.38457698923422 | 0.466603481142869 | 4.10852793987173 | 0.999228396881742 |
| ebi-fl187-GCST90094727 | prot-a-2784 | N-lysine methyltransferase SMYD2 \|\| id:prot-a-2784 | Cauliflower liking \|\| id:ebi-fl187-GCST90094727 | Weighted mode | 4 | -0.201560206 | 0.685543835410835 | 0.787915098337477 | -1.545226124 | 1.14210571120411 | 0.817454360256892 | 0.213263641826422 | 3.13335937330981 | 0.999228396881742 |
| ebi-fl187-GCST90094727 | prot-a-2837 | Serine palmitoyltransferase 2 \|\| id:prot-a-2837 | Cauliflower liking \|\| id:ebi-fl187-GCST90094727 | Weighted mode | 4 | 0.125352745823739 | 0.455760859593298 | 0.801139706576209 | -0.767938539 | 1.0186440306266 | 1.13354823695823 | 0.463968536195242 | 2.76943694511731 | 0.999228396881742 |
| ebi-fl187-GCST90094727 | prot-a-2858 | Alpha-2,8-sialyltransferase 8B \|\| id:prot-a-2858 | Cauliflower liking \|\| id:ebi-fl187-GCST90094727 | Weighted mode | 4 | 0.373864948249152 | 0.514049205830271 | 0.519664108841995 | -0.633671495 | 1.38140139167648 | 1.45334086097468 | 0.530639978024424 | 3.98047592652621 | 0.999228396881742 |
| ebi-fl187-GCST90094727 | prot-a-2911 | Synaptotagmin-11 \|\| id:prot-a-2911 | Cauliflower liking \|\| id:ebi-fl187-GCST90094727 | Weighted mode | 4 | -0.310632028 | 0.510367214229676 | 0.585751206294046 | -1.310951768 | 0.689687711528973 | 0.732983543406974 | 0.269563372392654 | 1.99309301607507 | 0.999228396881742 |
| ebi-fl187-GCST90094727 | prot-a-2962 | Transforming growth factor beta-1 \|\| id:prot-a-2962 | Cauliflower liking \|\| id:ebi-fl187-GCST90094727 | Weighted mode | 4 | -0.476416893 | 0.651738091918518 | 0.517701316866542 | -1.753823553 | 0.800989767062907 | 0.621004535770303 | 0.17311077815471 | 2.22774478607355 | 0.999228396881742 |
| ebi-fl187-GCST90094727 | prot-a-2978 | Tyrosine-protein kinase receptor Tie-1, soluble \|\| id:prot-a-2978 | Cauliflower liking \|\| id:ebi-fl187-GCST90094727 | Weighted mode | 4 | -0.357068281 | 0.609153422898001 | 0.598945006336555 | -1.55100899 | 0.836872427477785 | 0.699724717918431 | 0.212033925688102 | 2.30913368828647 | 0.999228396881742 |
| ebi-fl187-GCST90094727 | prot-a-299 | Uncharacterized protein C1orf226 \|\| id:prot-a-299 | Cauliflower liking \|\| id:ebi-fl187-GCST90094727 | Weighted mode | 4 | 0.219991483619683 | 0.518315121071393 | 0.699846807812682 | -0.795906154 | 1.23588912091961 | 1.24606611856923 | 0.451172218268598 | 3.44143701446133 | 0.999228396881742 |
| ebi-fl187-GCST90094727 | prot-a-3015 | Transmembrane protein 70, mitochondrial \|\| id:prot-a-3015 | Cauliflower liking \|\| id:ebi-fl187-GCST90094727 | Weighted mode | 4 | -0.468197741 | 0.521224568005023 | 0.435242475210864 | -1.489797894 | 0.553402412689533 | 0.626129700152711 | 0.22541820933331 | 1.73916030374301 | 0.999228396881742 |
| ebi-fl187-GCST90094727 | prot-a-3016 | Transmembrane protein 87B \|\| id:prot-a-3016 | Cauliflower liking \|\| id:ebi-fl187-GCST90094727 | Weighted mode | 4 | -1.280088734 | 0.648705556038885 | 0.142987001836336 | -2.551551623 | -0.008625844 | 0.278012630282029 | 0.0779606065975975 | 0.991411252035001 | 0.999228396881742 |
| ebi-fl187-GCST90094727 | prot-a-3123 | Thioredoxin domain-containing protein 12 \|\| id:prot-a-3123 | Cauliflower liking \|\| id:ebi-fl187-GCST90094727 | Weighted mode | 4 | -0.475481122 | 0.473031155641982 | 0.388866140487107 | -1.402622187 | 0.451659943284192 | 0.621585925988235 | 0.245951187672891 | 1.57091765663889 | 0.999228396881742 |
| ebi-fl187-GCST90094727 | prot-a-3133 | PolyUbiquitin K48-linked \|\| id:prot-a-3133 | Cauliflower liking \|\| id:ebi-fl187-GCST90094727 | Weighted mode | 4 | -0.418679107 | 0.572333439254669 | 0.517417684212512 | -1.540452648 | 0.703094434172619 | 0.657915281960036 | 0.214284084272228 | 2.01999378398377 | 0.999228396881742 |
| ebi-fl187-GCST90094727 | prot-a-3135 | Ubiquitin-conjugating enzyme E2 B \|\| id:prot-a-3135 | Cauliflower liking \|\| id:ebi-fl187-GCST90094727 | Weighted mode | 4 | 0.117760635211355 | 0.526521873657512 | 0.837388744525758 | -0.914222237 | 1.14974350758008 | 1.12497479978159 | 0.400828254211125 | 3.15738296102507 | 0.999228396881742 |
| ebi-fl187-GCST90094727 | prot-a-3162 | NKG2D ligand 3 \|\| id:prot-a-3162 | Cauliflower liking \|\| id:ebi-fl187-GCST90094727 | Weighted mode | 4 | 1.12450499517052 | 0.530179745501044 | 0.124066133211691 | 0.0853526939884715 | 2.16365729635256 | 3.07869250401213 | 1.08910111838535 | 8.70290864113027 | 0.999228396881742 |
| ebi-fl187-GCST90094727 | prot-a-3188 | Vesicle-associated membrane protein 8 \|\| id:prot-a-3188 | Cauliflower liking \|\| id:ebi-fl187-GCST90094727 | Weighted mode | 4 | -0.936059677 | 0.556537159760202 | 0.19117177338317 | -2.026872511 | 0.154753155690258 | 0.392170071489346 | 0.131746914581684 | 1.16736976695271 | 0.999228396881742 |
| ebi-fl187-GCST90094727 | prot-a-3201 | Vitrin \|\| id:prot-a-3201 | Cauliflower liking \|\| id:ebi-fl187-GCST90094727 | Weighted mode | 4 | -0.889818578 | 0.803816551177805 | 0.349082744512049 | -2.465299018 | 0.685661862705155 | 0.410730261661799 | 0.0849834270051797 | 1.98508525473428 | 0.999228396881742 |
| ebi-fl187-GCST90094727 | prot-a-3232 | WNT1-inducible-signaling pathway protein 3 \|\| id:prot-a-3232 | Cauliflower liking \|\| id:ebi-fl187-GCST90094727 | Weighted mode | 4 | -0.562533296 | 0.535459907518388 | 0.370619332303085 | -1.612034715 | 0.486968122940052 | 0.569763853674063 | 0.199481313335199 | 1.62737473262984 | 0.999228396881742 |
| ebi-fl187-GCST90094727 | prot-a-327 | Carbonic anhydrase-related protein 10 \|\| id:prot-a-327 | Cauliflower liking \|\| id:ebi-fl187-GCST90094727 | Weighted mode | 4 | -0.003899359 | 0.489961769053706 | 0.994149750242185 | -0.964224426 | 0.956425708329687 | 0.996108233612789 | 0.3812787948791 | 2.60237817155769 | 0.999228396881742 |
| ebi-fl187-GCST90094727 | prot-a-332 | Carbonic anhydrase 5A, mitochondrial \|\| id:prot-a-332 | Cauliflower liking \|\| id:ebi-fl187-GCST90094727 | Weighted mode | 4 | -0.517278656 | 0.579834035103408 | 0.438058134019538 | -1.653753365 | 0.619196053071107 | 0.596140646478939 | 0.191330426389133 | 1.85743416293621 | 0.999228396881742 |
| ebi-fl187-GCST90094727 | prot-a-338 | Cell adhesion molecule 3 \|\| id:prot-a-338 | Cauliflower liking \|\| id:ebi-fl187-GCST90094727 | Weighted mode | 4 | 0.0213543366460534 | 0.534741080289044 | 0.970654726126211 | -1.026738181 | 1.06944685401258 | 1.02158397215125 | 0.358173354004204 | 2.91376731543259 | 0.999228396881742 |
| ebi-fl187-GCST90094727 | prot-a-34 | A disintegrin and metalloproteinase with thrombospondin motifs 3 \|\| id:prot-a-34 | Cauliflower liking \|\| id:ebi-fl187-GCST90094727 | Weighted mode | 4 | -0.22155713 | 0.758045125934169 | 0.789124438791512 | -1.707325577 | 1.26421131709655 | 0.801270144489759 | 0.181350151733402 | 3.54029946109158 | 0.999228396881742 |
| ebi-fl187-GCST90094727 | prot-a-358 | Cysteine--tRNA ligase, cytoplasmic \|\| id:prot-a-358 | Cauliflower liking \|\| id:ebi-fl187-GCST90094727 | Weighted mode | 4 | -0.347106132 | 0.536179713296991 | 0.563515652136308 | -1.398018371 | 0.703806105589614 | 0.706730317241316 | 0.247086112246713 | 2.0214318674831 | 0.999228396881742 |
| ebi-fl187-GCST90094727 | prot-a-382 | Coiled-coil domain-containing protein 167 \|\| id:prot-a-382 | Cauliflower liking \|\| id:ebi-fl187-GCST90094727 | Weighted mode | 4 | -0.844388822 | 0.465530077789789 | 0.167349993470405 | -1.756827774 | 0.068050130530484 | 0.429819974489141 | 0.172591495504583 | 1.07041896780447 | 0.999228396881742 |
| ebi-fl187-GCST90094727 | prot-a-40 | Pituitary adenylate cyclase-activating polypeptide 27 \|\| id:prot-a-40 | Cauliflower liking \|\| id:ebi-fl187-GCST90094727 | Weighted mode | 4 | 0.0421051750375328 | 0.524015729301673 | 0.941018161044808 | -0.984965654 | 1.06917600446881 | 1.04300417098505 | 0.37345205315357 | 2.91297822975113 | 0.999228396881742 |
| ebi-fl187-GCST90094727 | prot-a-403 | C-C motif chemokine 25 \|\| id:prot-a-403 | Cauliflower liking \|\| id:ebi-fl187-GCST90094727 | Weighted mode | 4 | -0.007118408 | 0.549347839801365 | 0.990474917565749 | -1.083840174 | 1.0696033580023 | 0.992906867847731 | 0.33829392052501 | 2.91422336732861 | 0.999228396881742 |
| ebi-fl187-GCST90094727 | prot-a-415 | Cyclin-C \|\| id:prot-a-415 | Cauliflower liking \|\| id:ebi-fl187-GCST90094727 | Weighted mode | 4 | -0.555828091 | 0.558489845350387 | 0.392977638191882 | -1.650468188 | 0.53881200626804 | 0.573597074118608 | 0.191960014298451 | 1.71396946723442 | 0.999228396881742 |
| ebi-fl187-GCST90094727 | prot-a-44 | Adhesion G protein-coupled receptor F5 \|\| id:prot-a-44 | Cauliflower liking \|\| id:ebi-fl187-GCST90094727 | Weighted mode | 4 | -0.553464128 | 0.532944525171025 | 0.375386103809338 | -1.598035398 | 0.491107141081678 | 0.574954639999723 | 0.202293554262235 | 1.63412442508517 | 0.999228396881742 |
| ebi-fl187-GCST90094727 | prot-a-455 | CD59 glycoprotein \|\| id:prot-a-455 | Cauliflower liking \|\| id:ebi-fl187-GCST90094727 | Weighted mode | 4 | 0.0964516318943014 | 0.550580140618122 | 0.872093147279147 | -0.982685444 | 1.17558870750582 | 1.10125631417379 | 0.374304574106651 | 3.24004982413622 | 0.999228396881742 |
| ebi-fl187-GCST90094727 | prot-a-478 | Cadherin-12 \|\| id:prot-a-478 | Cauliflower liking \|\| id:ebi-fl187-GCST90094727 | Weighted mode | 4 | -0.79523912 | 0.559261864782502 | 0.250178621572738 | -1.891392375 | 0.3009141346451 | 0.451473265578653 | 0.150861606555064 | 1.35109332444936 | 0.999228396881742 |
| ebi-fl187-GCST90094727 | prot-a-516 | Centrin-2 \|\| id:prot-a-516 | Cauliflower liking \|\| id:ebi-fl187-GCST90094727 | Weighted mode | 4 | -0.062238714 | 0.725145450076433 | 0.937009507214909 | -1.483523796 | 1.35904636829529 | 0.939658550501542 | 0.226836951252775 | 3.89247953939717 | 0.999228396881742 |
| ebi-fl187-GCST90094727 | prot-a-519 | Cryptic protein \|\| id:prot-a-519 | Cauliflower liking \|\| id:ebi-fl187-GCST90094727 | Weighted mode | 4 | 0.153097238750087 | 0.473186821755102 | 0.767524959846011 | -0.774348932 | 1.08054340939009 | 1.16543829919661 | 0.461003828203956 | 2.94628015243589 | 0.999228396881742 |
| ebi-fl187-GCST90094727 | prot-a-583 | Chloride intracellular channel protein 4 \|\| id:prot-a-583 | Cauliflower liking \|\| id:ebi-fl187-GCST90094727 | Weighted mode | 4 | 0.492143093186744 | 0.529202509810269 | 0.420983972094436 | -0.545093826 | 1.52938001241487 | 1.63581817689468 | 0.579787381632875 | 4.61531449739867 | 0.999228396881742 |
| ebi-fl187-GCST90094727 | prot-a-602 | CCR4-NOT transcription complex subunit 1 \|\| id:prot-a-602 | Cauliflower liking \|\| id:ebi-fl187-GCST90094727 | Weighted mode | 4 | 0.554014057370417 | 0.677310523935786 | 0.4733008882448 | -0.77351457 | 1.88154268428456 | 1.74022437727628 | 0.46138863295063 | 6.563622653 | 0.999228396881742 |
| ebi-fl187-GCST90094727 | prot-a-606 | Ciliary neurotrophic factor receptor subunit alpha \|\| id:prot-a-606 | Cauliflower liking \|\| id:ebi-fl187-GCST90094727 | Weighted mode | 4 | -0.232390127 | 0.488822130135442 | 0.666966726555666 | -1.190481502 | 0.725701248456594 | 0.792636834259447 | 0.304074816278944 | 2.06617949724744 | 0.999228396881742 |
| ebi-fl187-GCST90094727 | prot-a-626 | Collagen alpha-2(IX) chain \|\| id:prot-a-626 | Cauliflower liking \|\| id:ebi-fl187-GCST90094727 | Weighted mode | 4 | 0.432790462043446 | 0.506004029270638 | 0.455250927184095 | -0.558977435 | 1.4245583594139 | 1.54155317295465 | 0.571793460799317 | 4.15602196940933 | 0.999228396881742 |
| ebi-fl187-GCST90094727 | prot-a-647 | Probable serine carboxypeptidase CPVL \|\| id:prot-a-647 | Cauliflower liking \|\| id:ebi-fl187-GCST90094727 | Weighted mode | 4 | 0.298345560009058 | 0.684129774395409 | 0.692243461028742 | -1.042548798 | 1.63923991782406 | 1.3476273935619 | 0.352554944552352 | 5.1512526485322 | 0.999228396881742 |
| ebi-fl187-GCST90094727 | prot-a-651 | Cellular retinoic acid-binding protein 2 \|\| id:prot-a-651 | Cauliflower liking \|\| id:ebi-fl187-GCST90094727 | Weighted mode | 4 | -0.719906365 | 0.488850754018643 | 0.237252235031983 | -1.678053843 | 0.238241112445076 | 0.486797834931293 | 0.186737042177296 | 1.26901513128179 | 0.999228396881742 |
| ebi-fl187-GCST90094727 | prot-a-671 | Cytotoxic and regulatory T-cell molecule \|\| id:prot-a-671 | Cauliflower liking \|\| id:ebi-fl187-GCST90094727 | Weighted mode | 4 | 0.392326080845623 | 0.799263870947616 | 0.657189418213558 | -1.174231106 | 1.95888326790295 | 1.48042036943118 | 0.30905651999314 | 7.09140344386009 | 0.999228396881742 |
| ebi-fl187-GCST90094727 | prot-a-688 | Chorionic somatomammotropin hormone \|\| id:prot-a-688 | Cauliflower liking \|\| id:ebi-fl187-GCST90094727 | Weighted mode | 4 | -0.084187244 | 0.499282841376077 | 0.876825768394816 | -1.062781613 | 0.894407125065926 | 0.919259114197111 | 0.345493443386448 | 2.44588525545259 | 0.999228396881742 |
| ebi-fl187-GCST90094727 | prot-a-69 | Aldo-keto reductase family 1 member C1 \|\| id:prot-a-69 | Cauliflower liking \|\| id:ebi-fl187-GCST90094727 | Weighted mode | 4 | 0.0521982120731528 | 0.541582614278399 | 0.929295758569855 | -1.009303712 | 1.11370013605881 | 1.05358455499699 | 0.364472669218527 | 3.04560673070018 | 0.999228396881742 |
| ebi-fl187-GCST90094727 | prot-a-741 | Stromal cell-derived factor 1 \|\| id:prot-a-741 | Cauliflower liking \|\| id:ebi-fl187-GCST90094727 | Weighted mode | 4 | -0.252715206 | 0.531684938840796 | 0.667029210612916 | -1.294817686 | 0.789387273796861 | 0.776689046452235 | 0.273947804173165 | 2.20204676105951 | 0.999228396881742 |
| ebi-fl187-GCST90094727 | prot-a-742 | Growth-regulated alpha protein \|\| id:prot-a-742 | Cauliflower liking \|\| id:ebi-fl187-GCST90094727 | Weighted mode | 4 | -0.379505393 | 0.513774758016473 | 0.513653721199404 | -1.386503919 | 0.627493132758463 | 0.684199735546358 | 0.249947616102357 | 1.87290955369624 | 0.999228396881742 |
| ebi-fl187-GCST90094727 | prot-a-788 | Beta-defensin 112 \|\| id:prot-a-788 | Cauliflower liking \|\| id:ebi-fl187-GCST90094727 | Weighted mode | 4 | -0.101000214 | 0.53712691584106 | 0.862847082164614 | -1.15376897 | 0.951768540573956 | 0.903932839015281 | 0.315445621156113 | 2.59028663785398 | 0.999228396881742 |
| ebi-fl187-GCST90094727 | prot-a-838 | DnaJ homolog subfamily B member 1 \|\| id:prot-a-838 | Cauliflower liking \|\| id:ebi-fl187-GCST90094727 | Weighted mode | 4 | 0.405251517716809 | 0.483024299641698 | 0.463064303229286 | -0.54147611 | 1.35197914501454 | 1.49967964862617 | 0.581888686658066 | 3.86506749498829 | 0.999228396881742 |
| ebi-fl187-GCST90094727 | prot-a-85 | Junctional adhesion molecule-like \|\| id:prot-a-85 | Cauliflower liking \|\| id:ebi-fl187-GCST90094727 | Weighted mode | 4 | -0.091155899 | 0.521845180343591 | 0.872454650048687 | -1.113972453 | 0.931660654179532 | 0.912875383177496 | 0.328252400511494 | 2.53872161761169 | 0.999228396881742 |
| ebi-fl187-GCST90094727 | prot-a-856 | Dipeptidase 1 \|\| id:prot-a-856 | Cauliflower liking \|\| id:ebi-fl187-GCST90094727 | Weighted mode | 4 | -1.017911102 | 0.636586434503316 | 0.208115739301113 | -2.265620513 | 0.229798309833446 | 0.361348973572363 | 0.103765626596075 | 1.25834618828138 | 0.999228396881742 |
| ebi-fl187-GCST90094727 | prot-a-860 | Dipeptidyl peptidase 2 \|\| id:prot-a-860 | Cauliflower liking \|\| id:ebi-fl187-GCST90094727 | Weighted mode | 4 | -0.355852637 | 0.691237552392868 | 0.64221706242544 | -1.71067824 | 0.998972965261181 | 0.700575851287607 | 0.180743163771042 | 2.71549149172297 | 0.999228396881742 |
| ebi-fl187-GCST90094727 | prot-a-861 | Dermatopontin \|\| id:prot-a-861 | Cauliflower liking \|\| id:ebi-fl187-GCST90094727 | Weighted mode | 4 | -0.104734009 | 0.537091595571527 | 0.857850571918598 | -1.157433536 | 0.947965518251469 | 0.900564032596812 | 0.314291765067485 | 2.58045442785588 | 0.999228396881742 |
| ebi-fl187-GCST90094727 | prot-a-877 | Dual specificity protein phosphatase 6 \|\| id:prot-a-877 | Cauliflower liking \|\| id:ebi-fl187-GCST90094727 | Weighted mode | 4 | -0.281927887 | 0.522542467484346 | 0.626992499895159 | -1.306111123 | 0.742255349466487 | 0.754328079581297 | 0.270871396317454 | 2.10066791614255 | 0.999228396881742 |
| ebi-fl187-GCST90094727 | prot-a-879 | Dynactin-associated protein \|\| id:prot-a-879 | Cauliflower liking \|\| id:ebi-fl187-GCST90094727 | Weighted mode | 4 | -0.114236312 | 0.509459082727845 | 0.836981294580315 | -1.112776114 | 0.884303490237912 | 0.892047129613193 | 0.328645336561006 | 2.42129734679324 | 0.999228396881742 |
| ebi-fl187-GCST90094727 | prot-a-937 | EMI domain-containing protein 1 \|\| id:prot-a-937 | Cauliflower liking \|\| id:ebi-fl187-GCST90094727 | Weighted mode | 4 | -0.236758336 | 0.473691551304457 | 0.651563593292776 | -1.165193776 | 0.691677104950862 | 0.789181982160263 | 0.311862224712012 | 1.99706200884551 | 0.999228396881742 |
| ebi-fl187-GCST90094727 | prot-b-1 | SRC proto-oncogene, non-receptor tyrosine kinase \|\| id:prot-b-1 | Cauliflower liking \|\| id:ebi-fl187-GCST90094727 | Weighted mode | 4 | 0.408843977819751 | 0.658441824482311 | 0.578629853023259 | -0.881701998 | 1.69938995380508 | 1.50507687678986 | 0.414077552357198 | 5.47060904932476 | 0.999228396881742 |
| ebi-fl187-GCST90094727 | prot-b-10 | coagulation factor III, tissue factor \|\| id:prot-b-10 | Cauliflower liking \|\| id:ebi-fl187-GCST90094727 | Weighted mode | 4 | -0.008469766 | 0.53660497704404 | 0.988397750799528 | -1.060215522 | 1.04327598851262 | 0.991566000926727 | 0.346381149699899 | 2.83850069510324 | 0.999228396881742 |
| ebi-fl187-GCST90094727 | prot-b-29 | myeloperoxidase \|\| id:prot-b-29 | Cauliflower liking \|\| id:ebi-fl187-GCST90094727 | Weighted mode | 4 | -0.16210779 | 0.757867388126241 | 0.844338120072946 | -1.64752787 | 1.3233122911615 | 0.850349540792233 | 0.192525268238235 | 3.75584123654246 | 0.999228396881742 |
| ebi-fl187-GCST90094727 | prot-b-33 | adrenomedullin \|\| id:prot-b-33 | Cauliflower liking \|\| id:ebi-fl187-GCST90094727 | Weighted mode | 4 | -0.085135032 | 0.586418671774058 | 0.893775679786091 | -1.234515629 | 1.06424556459207 | 0.918388264146062 | 0.290975668484363 | 2.89865131374908 | 0.999228396881742 |
| ebi-fl187-GCST90094727 | ukb-rsfMRI191-edge-pheno102 | Net25_Pair11_15 (Postcentral\|Precentral)&(Cerebellum) (Motor)&(Subcortical-cerebellum) \|\| id:ukb-rsfMRI191-edge-pheno102 | Cauliflower liking \|\| id:ebi-fl187-GCST90094727 | Weighted mode | 4 | 0.0934834749011781 | 0.184967713929451 | 0.648068567772829 | -0.269053244 | 0.456020194202901 | 1.09799245875749 | 0.764102570381763 | 1.57778220649878 | 0.999228396881742 |
| ebi-fl187-GCST90094727 | ukb-rsfMRI191-edge-pheno1167 | Net100_Pair11_45 (Angular\|Temporal)&(Frontal) (Default_mode\|Central_executive)&(Salience\|Default_mode) \|\| id:ukb-rsfMRI191-edge-pheno1167 | Cauliflower liking \|\| id:ebi-fl187-GCST90094727 | Weighted mode | 4 | -0.315903874 | 0.165290297646201 | 0.151945530935301 | -0.639872858 | 0.00806510892435897 | 0.729129534767769 | 0.527359469398235 | 1.00809771952574 | 0.999228396881742 |
| ebi-fl187-GCST90094727 | ukb-rsfMRI191-edge-pheno1317 | Net100_Pair26_48 (Parietal\|Frontal)&(Temporal) (Attention\|Central_executive)&(Default_mode\|Central_executive) \|\| id:ukb-rsfMRI191-edge-pheno1317 | Cauliflower liking \|\| id:ebi-fl187-GCST90094727 | Weighted mode | 4 | -0.182043608 | 0.160960426594646 | 0.340303776213294 | -0.497526044 | 0.133438828550395 | 0.833564989875255 | 0.608033047713852 | 1.14275136023976 | 0.999228396881742 |
| ebi-fl187-GCST90094727 | ukb-rsfMRI191-edge-pheno286 | Net100_Pair10_13 (OccipitalPrecuneus)&(Frontal_Sup) (Default_mode\|Central_executive)&(Default_mode) \|\| id:ukb-rsfMRI191-edge-pheno286 | Cauliflower liking \|\| id:ebi-fl187-GCST90094727 | Weighted mode | 4 | 0.147316479091525 | 0.168424092691572 | 0.446131373338185 | -0.182794743 | 0.477427700767007 | 1.15872061585566 | 0.832939105120615 | 1.61192271722491 | 0.999228396881742 |
| ebi-fl187-GCST90094727 | ukb-rsfMRI191-edge-pheno599 | Net100_Pair11_29 (Angular\|Temporal)&(Parietal) (Default_mode\|Central_executive)&(Central_executive\|Salience) \|\| id:ukb-rsfMRI191-edge-pheno599 | Cauliflower liking \|\| id:ebi-fl187-GCST90094727 | Weighted mode | 4 | -0.013518002 | 0.152294775837382 | 0.934864501655924 | -0.312015762 | 0.28497975888101 | 0.986572956108043 | 0.731969990532486 | 1.32973511252244 | 0.999228396881742 |
| ebi-fl187-GCST90094727 | ukb-rsfMRI191-edge-pheno621 | Net100_Pair5_30 (Precuneus\|Angular\|Cingulate)&(Paracentral) (Default_mode\|Central_executive)&(Motor) \|\| id:ukb-rsfMRI191-edge-pheno621 | Cauliflower liking \|\| id:ebi-fl187-GCST90094727 | Weighted mode | 4 | -0.032579136 | 0.139249372694921 | 0.830071227268908 | -0.305507906 | 0.240349634576586 | 0.96794584752927 | 0.736749079782754 | 1.27169370069037 | 0.999228396881742 |
| ebi-fl187-GCST90094727 | ukb-rsfMRI191-edge-pheno624 | Net100_Pair8_30 (Calcarine\|Lingual)&(Paracentral) (Visual)&(Motor) \|\| id:ukb-rsfMRI191-edge-pheno624 | Cauliflower liking \|\| id:ebi-fl187-GCST90094727 | Weighted mode | 4 | 0.0356192969933301 | 0.155329511519077 | 0.833369053475719 | -0.268826546 | 0.340065139570722 | 1.03626126360749 | 0.764275811166138 | 1.40503911122731 | 0.999228396881742 |
| ebi-fl187-GCST90094727 | ukb-rsfMRI191-edge-pheno882 | Net100_Pair6_38 (Postcentral\|Precentral)&(Subcortical) (Motor)&(Subcortical-cerebellum) \|\| id:ukb-rsfMRI191-edge-pheno882 | Cauliflower liking \|\| id:ebi-fl187-GCST90094727 | Weighted mode | 4 | 0.0135989682119384 | 0.164224686030525 | 0.93922064377327 | -0.308281416 | 0.335479352831767 | 1.01369185475628 | 0.734708529878322 | 1.3986106525392 | 0.999228396881742 |
| ebi-fl187-GCST90094727 | ukb-rsfMRI191-edge-pheno899 | Net100_Pair23_38 (Cerebellum)&(Subcortical) (Subcortical-cerebellum)&(Subcortical-cerebellum) \|\| id:ukb-rsfMRI191-edge-pheno899 | Cauliflower liking \|\| id:ebi-fl187-GCST90094727 | Weighted mode | 4 | 0.253122604403968 | 0.212802968255032 | 0.319816413159464 | -0.163971213 | 0.67021642218383 | 1.28804118667619 | 0.84876645464942 | 1.95466030671473 | 0.999228396881742 |
| ebi-fl187-GCST90094753 | ebi-a-GCST90001392 | IgD+ CD38+ B cell Absolute Count \|\| id:ebi-a-GCST90001392 | F-deep fried food liking (derived food-liking factor) \|\| id:ebi-fl187-GCST90094753 | Inverse variance weighted | 8 | 0.186547229068939 | 0.110940650943504 | 0.0926649189006554 | -0.030896447 | 0.403990904918208 | 1.20508153562062 | 0.969575970591163 | 1.49779032437068 | 0.998074519377826 |
| ebi-fl187-GCST90094753 | ebi-a-GCST90001402 | Switched memory B cell %B cell \|\| id:ebi-a-GCST90001402 | F-deep fried food liking (derived food-liking factor) \|\| id:ebi-fl187-GCST90094753 | Inverse variance weighted | 8 | -0.034001649 | 0.117100589757963 | 0.77153874275341 | -0.263518804 | 0.195515507402332 | 0.966569911223197 | 0.76834317403344 | 1.21593764981031 | 0.998074519377826 |
| ebi-fl187-GCST90094753 | ebi-a-GCST90001407 | Memory B cell Absolute Count \|\| id:ebi-a-GCST90001407 | F-deep fried food liking (derived food-liking factor) \|\| id:ebi-fl187-GCST90094753 | Inverse variance weighted | 8 | 0.202441353021936 | 0.134847638465443 | 0.133288350873138 | -0.061860018 | 0.466742724414205 | 1.22438827634387 | 0.940014462338143 | 1.59479104983072 | 0.998074519377826 |
| ebi-fl187-GCST90094753 | ebi-a-GCST90001411 | IgD+ CD24+ B cell %B cell \|\| id:ebi-a-GCST90001411 | F-deep fried food liking (derived food-liking factor) \|\| id:ebi-fl187-GCST90094753 | Inverse variance weighted | 8 | 0.146745695174297 | 0.133628771696891 | 0.2721348800774 | -0.115166697 | 0.408658087700203 | 1.15805942547993 | 0.891217567915285 | 1.50479712387177 | 0.998074519377826 |
| ebi-fl187-GCST90094753 | ebi-a-GCST90001418 | CD24+ CD27+ B cell Absolute Count \|\| id:ebi-a-GCST90001418 | F-deep fried food liking (derived food-liking factor) \|\| id:ebi-fl187-GCST90094753 | Inverse variance weighted | 8 | 0.227502870655036 | 0.130414313268587 | 0.0810784860012964 | -0.028109183 | 0.483114924661466 | 1.25546104374452 | 0.972282203976215 | 1.62111620053744 | 0.998074519377826 |
| ebi-fl187-GCST90094753 | ebi-a-GCST90001436 | Memory B cell %lymphocyte \|\| id:ebi-a-GCST90001436 | F-deep fried food liking (derived food-liking factor) \|\| id:ebi-fl187-GCST90094753 | Inverse variance weighted | 8 | 0.155943653850537 | 0.118311231877169 | 0.187476910324378 | -0.075946361 | 0.387833668329788 | 1.16876034608935 | 0.926865921731333 | 1.47378462684147 | 0.998074519377826 |
| ebi-fl187-GCST90094753 | ebi-a-GCST90001439 | IgD+ CD24+ B cell %lymphocyte \|\| id:ebi-a-GCST90001439 | F-deep fried food liking (derived food-liking factor) \|\| id:ebi-fl187-GCST90094753 | Inverse variance weighted | 8 | 0.195771195464507 | 0.121203612377636 | 0.106261816586178 | -0.041787885 | 0.433330275724674 | 1.21624859030191 | 0.959073193004598 | 1.54238554909155 | 0.998074519377826 |
| ebi-fl187-GCST90094753 | ebi-a-GCST90001457 | CD11c+ HLA DR++ monocyte %monocyte \|\| id:ebi-a-GCST90001457 | F-deep fried food liking (derived food-liking factor) \|\| id:ebi-fl187-GCST90094753 | Inverse variance weighted | 8 | -0.000839426 | 0.12923202828002 | 0.994817383862166 | -0.254134201 | 0.252455349855414 | 0.99916092664566 | 0.77558771041395 | 1.2871820220083 | 0.999355215041207 |
| ebi-fl187-GCST90094753 | ebi-a-GCST90001483 | CD39+ resting CD4 regulatory T cell Absolute Count \|\| id:ebi-a-GCST90001483 | F-deep fried food liking (derived food-liking factor) \|\| id:ebi-fl187-GCST90094753 | Inverse variance weighted | 8 | -0.138573848 | 0.117959416534177 | 0.24009127633977 | -0.369774305 | 0.0926266081852966 | 0.870598956710367 | 0.690890243772368 | 1.09705202853451 | 0.998074519377826 |
| ebi-fl187-GCST90094753 | ebi-a-GCST90001525 | CD33dim HLA DR+ CD11b+ Absolute Count \|\| id:ebi-a-GCST90001525 | F-deep fried food liking (derived food-liking factor) \|\| id:ebi-fl187-GCST90094753 | Inverse variance weighted | 8 | -0.01109158 | 0.14970903816725 | 0.940940697616161 | -0.304521295 | 0.282338134426665 | 0.988969704405543 | 0.737476323369728 | 1.32622708721409 | 0.998074519377826 |
| ebi-fl187-GCST90094753 | ebi-a-GCST90001540 | Naive CD4+ T cell Absolute Count \|\| id:ebi-a-GCST90001540 | F-deep fried food liking (derived food-liking factor) \|\| id:ebi-fl187-GCST90094753 | Inverse variance weighted | 8 | -0.035601091 | 0.122610968680883 | 0.77154227261677 | -0.27591859 | 0.204716407137946 | 0.965025173479074 | 0.758874708167407 | 1.22717699697389 | 0.998074519377826 |
| ebi-fl187-GCST90094753 | ebi-a-GCST90001624 | HLA DR+ CD4+ T cell Absolute Count \|\| id:ebi-a-GCST90001624 | F-deep fried food liking (derived food-liking factor) \|\| id:ebi-fl187-GCST90094753 | Inverse variance weighted | 8 | 0.0918152644059278 | 0.119690791000861 | 0.443019783896152 | -0.142778686 | 0.326409214767615 | 1.09616230318089 | 0.866945914972171 | 1.38598241731539 | 0.998074519377826 |
| ebi-fl187-GCST90094753 | ebi-a-GCST90001672 | CD39+ CD8+ T cell Absolute Count \|\| id:ebi-a-GCST90001672 | F-deep fried food liking (derived food-liking factor) \|\| id:ebi-fl187-GCST90094753 | Inverse variance weighted | 8 | 0.0366776934799216 | 0.122923996071922 | 0.765415439476583 | -0.204253339 | 0.277608725780888 | 1.03735861950446 | 0.815255809077775 | 1.31996962607051 | 0.998074519377826 |
| ebi-fl187-GCST90094753 | ebi-a-GCST90001722 | CD19 on CD20- CD38- B cell \|\| id:ebi-a-GCST90001722 | F-deep fried food liking (derived food-liking factor) \|\| id:ebi-fl187-GCST90094753 | Inverse variance weighted | 8 | 0.0974597969070675 | 0.120351776939027 | 0.418060253055167 | -0.138429686 | 0.33334927970756 | 1.10236712210459 | 0.870724473330119 | 1.39563468022154 | 0.998074519377826 |
| ebi-fl187-GCST90094753 | ebi-a-GCST90001742 | CD19 on transitional B cell \|\| id:ebi-a-GCST90001742 | F-deep fried food liking (derived food-liking factor) \|\| id:ebi-fl187-GCST90094753 | Inverse variance weighted | 8 | -0.035863322 | 0.112337029726311 | 0.749538797247022 | -0.256043901 | 0.184317256013021 | 0.964772147358006 | 0.774107984307791 | 1.2023972303426 | 0.998074519377826 |
| ebi-fl187-GCST90094753 | ebi-a-GCST90001784 | CD25 on IgD+ CD38dim B cell \|\| id:ebi-a-GCST90001784 | F-deep fried food liking (derived food-liking factor) \|\| id:ebi-fl187-GCST90094753 | Inverse variance weighted (multiplicative random effects) | 8 | -0.030622861 | 0.171240585406941 | 0.858071613054026 | -0.366254409 | 0.305008686119762 | 0.969841268809496 | 0.693326390521839 | 1.35663678686465 | 0.998074519377826 |
| ebi-fl187-GCST90094753 | ebi-a-GCST90001794 | CD25 on IgD+ B cell \|\| id:ebi-a-GCST90001794 | F-deep fried food liking (derived food-liking factor) \|\| id:ebi-fl187-GCST90094753 | Inverse variance weighted (multiplicative random effects) | 8 | -0.00713247 | 0.160540469122565 | 0.964563352496528 | -0.321791789 | 0.307526849590174 | 0.992892905807006 | 0.724849095905995 | 1.36005732499352 | 0.998074519377826 |
| ebi-fl187-GCST90094753 | ebi-a-GCST90001802 | CD27 on IgD- CD38- B cell \|\| id:ebi-a-GCST90001802 | F-deep fried food liking (derived food-liking factor) \|\| id:ebi-fl187-GCST90094753 | Inverse variance weighted | 8 | 0.0396007481831868 | 0.113269931440479 | 0.726628440048733 | -0.182408317 | 0.261609813806525 | 1.04039531154246 | 0.833261035931166 | 1.29901958402499 | 0.998074519377826 |
| ebi-fl187-GCST90094753 | ebi-a-GCST90001804 | CD27 on IgD- CD38dim B cell \|\| id:ebi-a-GCST90001804 | F-deep fried food liking (derived food-liking factor) \|\| id:ebi-fl187-GCST90094753 | Inverse variance weighted | 8 | -0.024229327 | 0.112157900719738 | 0.828965369306883 | -0.244058812 | 0.195600158908727 | 0.976061847240962 | 0.78344155721151 | 1.21604058512081 | 0.998074519377826 |
| ebi-fl187-GCST90094753 | ebi-a-GCST90001805 | CD27 on memory B cell \|\| id:ebi-a-GCST90001805 | F-deep fried food liking (derived food-liking factor) \|\| id:ebi-fl187-GCST90094753 | Inverse variance weighted | 8 | 0.0573260321987858 | 0.113446594177558 | 0.613339054635171 | -0.165029292 | 0.279681356786799 | 1.0590010225492 | 0.847868867619274 | 1.32270827316642 | 0.998074519377826 |
| ebi-fl187-GCST90094753 | ebi-a-GCST90001806 | CD27 on unswitched memory B cell \|\| id:ebi-a-GCST90001806 | F-deep fried food liking (derived food-liking factor) \|\| id:ebi-fl187-GCST90094753 | Inverse variance weighted | 8 | -0.081186586 | 0.113065579578551 | 0.472727295946604 | -0.302795122 | 0.140421950230217 | 0.922021639300552 | 0.7387504347871 | 1.15075925956439 | 0.998074519377826 |
| ebi-fl187-GCST90094753 | ebi-a-GCST90001839 | CD3 on Effector Memory CD8+ T cell \|\| id:ebi-a-GCST90001839 | F-deep fried food liking (derived food-liking factor) \|\| id:ebi-fl187-GCST90094753 | Inverse variance weighted | 8 | 0.0520450985530057 | 0.124562860088099 | 0.676077292619631 | -0.192098107 | 0.296188304325679 | 1.05342324930636 | 0.825225903901112 | 1.34472335021629 | 0.998074519377826 |
| ebi-fl187-GCST90094753 | ebi-a-GCST90001859 | CD3 on CD8+ T cell \|\| id:ebi-a-GCST90001859 | F-deep fried food liking (derived food-liking factor) \|\| id:ebi-fl187-GCST90094753 | Inverse variance weighted | 8 | 0.0317223179083779 | 0.122149791036069 | 0.795095084164184 | -0.207691273 | 0.271135908339073 | 1.03223083348827 | 0.812457826052528 | 1.31145329571235 | 0.998074519377826 |
| ebi-fl187-GCST90094753 | ebi-a-GCST90001905 | CD86 on monocyte \|\| id:ebi-a-GCST90001905 | F-deep fried food liking (derived food-liking factor) \|\| id:ebi-fl187-GCST90094753 | Inverse variance weighted | 8 | -0.00217386 | 0.123353109862968 | 0.985939558125132 | -0.243945955 | 0.239598235736863 | 0.997828501526988 | 0.783529979054309 | 1.27073851042856 | 0.999355215041207 |
| ebi-fl187-GCST90094753 | ebi-a-GCST90001943 | CD25 on activated & secreting CD4 regulatory T cell \|\| id:ebi-a-GCST90001943 | F-deep fried food liking (derived food-liking factor) \|\| id:ebi-fl187-GCST90094753 | Inverse variance weighted | 8 | -0.031403864 | 0.113558922589299 | 0.782131447993281 | -0.253979353 | 0.191171623837569 | 0.969084115422311 | 0.775707818143482 | 1.21066721360559 | 0.998074519377826 |
| ebi-fl187-GCST90094753 | ebi-a-GCST90001949 | CD33 on CD33dim HLA DR+ CD11b- \|\| id:ebi-a-GCST90001949 | F-deep fried food liking (derived food-liking factor) \|\| id:ebi-fl187-GCST90094753 | Inverse variance weighted | 8 | 0.120284921125248 | 0.199298474847557 | 0.54614844744848 | -0.27034009 | 0.51090993182646 | 1.12781814502051 | 0.763119921069907 | 1.66680718602416 | 0.998074519377826 |
| ebi-fl187-GCST90094753 | ebi-a-GCST90001955 | CD33 on Immature Myeloid-Derived Suppressor Cells \|\| id:ebi-a-GCST90001955 | F-deep fried food liking (derived food-liking factor) \|\| id:ebi-fl187-GCST90094753 | Inverse variance weighted | 8 | 0.31813307867059 | 0.166898470501731 | 0.0566310898002435 | -0.008987924 | 0.645254080853983 | 1.37455917356389 | 0.991052347131598 | 1.90647136561169 | 0.998074519377826 |
| ebi-fl187-GCST90094753 | ebi-a-GCST90001956 | CD33 on CD33+ HLA DR+ \|\| id:ebi-a-GCST90001956 | F-deep fried food liking (derived food-liking factor) \|\| id:ebi-fl187-GCST90094753 | Inverse variance weighted | 8 | 0.203794759374675 | 0.226182376398696 | 0.367577886720062 | -0.239522698 | 0.647112217116119 | 1.22604649308271 | 0.787003409447184 | 1.91001714243691 | 0.998074519377826 |
| ebi-fl187-GCST90094753 | ebi-a-GCST90001957 | CD33 on CD33+ HLA DR+ CD14- \|\| id:ebi-a-GCST90001957 | F-deep fried food liking (derived food-liking factor) \|\| id:ebi-fl187-GCST90094753 | Inverse variance weighted | 8 | 0.217462697915744 | 0.216268708007509 | 0.314646108635346 | -0.20642397 | 0.641349365610462 | 1.2429190651713 | 0.813488108786511 | 1.89904165270561 | 0.998074519377826 |
| ebi-fl187-GCST90094753 | ebi-a-GCST90002064 | CD4 on resting CD4 regulatory T cell \|\| id:ebi-a-GCST90002064 | F-deep fried food liking (derived food-liking factor) \|\| id:ebi-fl187-GCST90094753 | Inverse variance weighted | 8 | 0.109667509320159 | 0.119846521864767 | 0.360156842879443 | -0.125231674 | 0.344566692175102 | 1.11590698009979 | 0.882292475088872 | 1.4113782259222 | 0.998074519377826 |
| ebi-fl187-GCST90094753 | ebi-a-GCST90002080 | SSC-A on T cell \|\| id:ebi-a-GCST90002080 | F-deep fried food liking (derived food-liking factor) \|\| id:ebi-fl187-GCST90094753 | Inverse variance weighted (multiplicative random effects) | 8 | 0.0465175272068084 | 0.164440827810701 | 0.777266468252222 | -0.275786495 | 0.368821549715783 | 1.04761644069763 | 0.758974958182928 | 1.44602953626759 | 0.998074519377826 |
| ebi-fl187-GCST90094753 | ebi-a-GCST90002121 | CD8 on CD39+ CD8+ T cell \|\| id:ebi-a-GCST90002121 | F-deep fried food liking (derived food-liking factor) \|\| id:ebi-fl187-GCST90094753 | Inverse variance weighted | 8 | 0.106554222737284 | 0.120609319787548 | 0.376984592169791 | -0.129840044 | 0.342948489520877 | 1.11243824425703 | 0.878235898746678 | 1.40909617683782 | 0.998074519377826 |
| ebi-fl187-GCST90094753 | ebi-cfmet338-GCST90026053 | 1-palmitoyl-2-docosahexaenoyl-gpc (16:0/22:6) levels \|\| id:ebi-cfmet338-GCST90026053 | F-deep fried food liking (derived food-liking factor) \|\| id:ebi-fl187-GCST90094753 | Inverse variance weighted | 8 | -0.038992156 | 0.0549588849809444 | 0.478027495306686 | -0.14671157 | 0.0687272589911544 | 0.961758253560634 | 0.863543013095354 | 1.07114402440294 | 0.998074519377826 |
| ebi-fl187-GCST90094753 | ebi-cfmet338-GCST90026063 | 1-stearoyl-2-oleoyl-gpc (18:0/18:1) levels \|\| id:ebi-cfmet338-GCST90026063 | F-deep fried food liking (derived food-liking factor) \|\| id:ebi-fl187-GCST90094753 | Inverse variance weighted | 8 | -0.027833304 | 0.0567507196604437 | 0.623816585756555 | -0.139064714 | 0.0833981069467232 | 0.972550473964275 | 0.870171714237444 | 1.08697445450409 | 0.998074519377826 |
| ebi-fl187-GCST90094753 | ebi-cfmet338-GCST90026113 | Adenosine 3',5'-cyclic monophosphate (camp) levels \|\| id:ebi-cfmet338-GCST90026113 | F-deep fried food liking (derived food-liking factor) \|\| id:ebi-fl187-GCST90094753 | Inverse variance weighted | 8 | 0.022390905256619 | 0.0975433501462233 | 0.818442824064663 | -0.168794061 | 0.213575871543217 | 1.02264346305247 | 0.844682838589175 | 1.23809743106738 | 0.998074519377826 |
| ebi-fl187-GCST90094753 | ebi-cfmet338-GCST90026117 | Alpha-ketoglutaramate levels \|\| id:ebi-cfmet338-GCST90026117 | F-deep fried food liking (derived food-liking factor) \|\| id:ebi-fl187-GCST90094753 | Inverse variance weighted | 8 | 0.0194953280114163 | 0.0378505174856136 | 0.606510345978077 | -0.054691686 | 0.0936823422832191 | 1.01968660288536 | 0.946777007318752 | 1.09821083535654 | 0.998074519377826 |
| ebi-fl187-GCST90094753 | ebi-cfmet338-GCST90026124 | Arginine levels \|\| id:ebi-cfmet338-GCST90026124 | F-deep fried food liking (derived food-liking factor) \|\| id:ebi-fl187-GCST90094753 | Inverse variance weighted | 8 | -0.078941823 | 0.0317815192707618 | 0.0129955653967278 | -0.141233601 | -0.016650045 | 0.924093683700636 | 0.868286455451609 | 0.983487800476236 | 0.998074519377826 |
| ebi-fl187-GCST90094753 | ebi-cfmet338-GCST90026169 | Glutamate levels \|\| id:ebi-cfmet338-GCST90026169 | F-deep fried food liking (derived food-liking factor) \|\| id:ebi-fl187-GCST90094753 | Inverse variance weighted | 8 | -0.031420562 | 0.0382691024661139 | 0.411622024253291 | -0.106428003 | 0.0435868788421991 | 0.969067934223127 | 0.89903977415514 | 1.04455073972893 | 0.998074519377826 |
| ebi-fl187-GCST90094753 | ebi-cfmet338-GCST90026170 | Glutamine levels \|\| id:ebi-cfmet338-GCST90026170 | F-deep fried food liking (derived food-liking factor) \|\| id:ebi-fl187-GCST90094753 | Inverse variance weighted | 8 | -0.02217643 | 0.0201834418811359 | 0.271879860455122 | -0.061735976 | 0.0173831157789071 | 0.978067659049391 | 0.94013107082078 | 1.01753508140432 | 0.998074519377826 |
| ebi-fl187-GCST90094753 | ebi-cfmet338-GCST90026191 | Kynurenate levels \|\| id:ebi-cfmet338-GCST90026191 | F-deep fried food liking (derived food-liking factor) \|\| id:ebi-fl187-GCST90094753 | Inverse variance weighted (multiplicative random effects) | 8 | 0.0081877775267722 | 0.201496555663773 | 0.967587019753177 | -0.386745472 | 0.403121026627768 | 1.00822138904912 | 0.679263964913217 | 1.49648799559976 | 0.998074519377826 |
| ebi-fl187-GCST90094753 | ebi-cfmet338-GCST90026202 | Methylsuccinoylcarnitine levels \|\| id:ebi-cfmet338-GCST90026202 | F-deep fried food liking (derived food-liking factor) \|\| id:ebi-fl187-GCST90094753 | Inverse variance weighted | 8 | 0.121252792710767 | 0.0916925883184429 | 0.186040382966487 | -0.05846468 | 0.300970265814915 | 1.12891025658333 | 0.943211553672392 | 1.35116916502668 | 0.998074519377826 |
| ebi-fl187-GCST90094753 | ebi-cfmet338-GCST90026222 | N-acetylputrescine levels \|\| id:ebi-cfmet338-GCST90026222 | F-deep fried food liking (derived food-liking factor) \|\| id:ebi-fl187-GCST90094753 | Inverse variance weighted | 8 | -0.002980062 | 0.0359874815529059 | 0.934003982783427 | -0.073515525 | 0.0675554022829116 | 0.997024374415081 | 0.929121720689017 | 1.06988953227852 | 0.998074519377826 |
| ebi-fl187-GCST90094753 | ebi-cfmet338-GCST90026252 | Propionylcarnitine (c3) levels \|\| id:ebi-cfmet338-GCST90026252 | F-deep fried food liking (derived food-liking factor) \|\| id:ebi-fl187-GCST90094753 | Inverse variance weighted | 8 | 0.0121678205391613 | 0.0589330151507181 | 0.83642484472279 | -0.103340889 | 0.127676530234569 | 1.01224214963593 | 0.901819500588764 | 1.13618542161777 | 0.998074519377826 |
| ebi-fl187-GCST90094753 | ebi-cfmet338-GCST90026316 | Ethyl beta-glucopyranoside levels \|\| id:ebi-cfmet338-GCST90026316 | F-deep fried food liking (derived food-liking factor) \|\| id:ebi-fl187-GCST90094753 | Inverse variance weighted | 8 | 0.243341565470421 | 0.186832471874963 | 0.192759651124854 | -0.122850079 | 0.609533210345348 | 1.27550421792054 | 0.88439624183362 | 1.83957250492157 | 0.998074519377826 |
| ebi-fl187-GCST90094753 | ebi-gm430-GCST90011315 | F_Prevotellaceae abundance \|\| id:ebi-gm430-GCST90011315 | F-deep fried food liking (derived food-liking factor) \|\| id:ebi-fl187-GCST90094753 | Inverse variance weighted | 8 | -0.095603396 | 0.0832366416763921 | 0.250731911154079 | -0.258747214 | 0.0675404212098644 | 0.908824387556607 | 0.772018153635772 | 1.06987350430534 | 0.998074519377826 |
| ebi-fl187-GCST90094753 | ebi-gm430-GCST90011354 | OTU97_1 (Bacteroides) abundance \|\| id:ebi-gm430-GCST90011354 | F-deep fried food liking (derived food-liking factor) \|\| id:ebi-fl187-GCST90094753 | Inverse variance weighted | 8 | -0.00581136 | 0.0759234878139054 | 0.938987644566702 | -0.154621396 | 0.142998676057969 | 0.994205493232922 | 0.856739480935094 | 1.15372827419564 | 0.998074519377826 |
| ebi-fl187-GCST90094753 | ebi-gm430-GCST90011419 | OTU99_1 (Bacteroides) abundance \|\| id:ebi-gm430-GCST90011419 | F-deep fried food liking (derived food-liking factor) \|\| id:ebi-fl187-GCST90094753 | Inverse variance weighted | 8 | -0.004814496 | 0.0742771247313453 | 0.948318900712011 | -0.15039766 | 0.140768668967878 | 0.9951970756008 | 0.860365775353576 | 1.15115831853884 | 0.998074519377826 |
| ebi-fl187-GCST90094753 | ebi-gm430-GCST90011435 | OTU99_155 (Butyrivibrio) abundance \|\| id:ebi-gm430-GCST90011435 | F-deep fried food liking (derived food-liking factor) \|\| id:ebi-fl187-GCST90094753 | Inverse variance weighted | 8 | -0.163798261 | 0.230917969313229 | 0.478116442507701 | -0.616397481 | 0.288800958374661 | 0.84891326311258 | 0.539885887427744 | 1.33482601614568 | 0.998074519377826 |
| ebi-fl187-GCST90094753 | ebi-gm430-GCST90011466 | OTU99_58 (Bacteroides) abundance \|\| id:ebi-gm430-GCST90011466 | F-deep fried food liking (derived food-liking factor) \|\| id:ebi-fl187-GCST90094753 | Inverse variance weighted | 8 | 0.0557505926455036 | 0.131336854293124 | 0.671211764639978 | -0.201669642 | 0.313170827060026 | 1.05733394398687 | 0.817364906569551 | 1.36775516066484 | 0.998074519377826 |
| ebi-fl187-GCST90094753 | ebi-gm430-GCST90011472 | OTU99_72 (Parabacteroides) abundance \|\| id:ebi-gm430-GCST90011472 | F-deep fried food liking (derived food-liking factor) \|\| id:ebi-fl187-GCST90094753 | Inverse variance weighted | 7 | -0.161554809 | 0.211490978977324 | 0.444935714819019 | -0.576077127 | 0.252967510232311 | 0.850819897971241 | 0.562099090306702 | 1.28784143448588 | 0.998074519377826 |
| ebi-fl187-GCST90094753 | ebi-gm430-GCST90011505 | TestASV_28 (Holdemanella) abundance \|\| id:ebi-gm430-GCST90011505 | F-deep fried food liking (derived food-liking factor) \|\| id:ebi-fl187-GCST90094753 | Inverse variance weighted | 8 | -0.127405129 | 0.171837797200893 | 0.45843476675665 | -0.464207212 | 0.20939695338488 | 0.880376933999088 | 0.628633280835729 | 1.23293431885635 | 0.998074519377826 |
| ebi-fl187-GCST90094753 | ebi-gm430-GCST90011513 | TestASV_35 (Alphaproteobacteria) abundance \|\| id:ebi-gm430-GCST90011513 | F-deep fried food liking (derived food-liking factor) \|\| id:ebi-fl187-GCST90094753 | Inverse variance weighted | 8 | 0.306621031268404 | 0.206686274747419 | 0.137939121077648 | -0.098484067 | 0.711726129773346 | 1.35882591794961 | 0.906210130930913 | 2.03750522342414 | 0.998074519377826 |
| ebi-fl187-GCST90094753 | ebi-gm430-GCST90011543 | G_Coprococcus prevalence \|\| id:ebi-gm430-GCST90011543 | F-deep fried food liking (derived food-liking factor) \|\| id:ebi-fl187-GCST90094753 | Inverse variance weighted | 8 | -0.161388222 | 0.230960136538095 | 0.484695217287348 | -0.61407009 | 0.291293645185546 | 0.850961644575061 | 0.541143876487959 | 1.3381574697612 | 0.998074519377826 |
| ebi-fl187-GCST90094753 | ebi-gm430-GCST90011547 | G_Parasutterella prevalence \|\| id:ebi-gm430-GCST90011547 | F-deep fried food liking (derived food-liking factor) \|\| id:ebi-fl187-GCST90094753 | Inverse variance weighted | 8 | -0.109798046 | 0.237541517738907 | 0.643918713986178 | -0.575379421 | 0.355783328308525 | 0.896015070441276 | 0.562491407133144 | 1.42729825962986 | 0.998074519377826 |
| ebi-fl187-GCST90094753 | ebi-gm430-GCST90011569 | OTU97_13 (Subdoligranulum) prevalence \|\| id:ebi-gm430-GCST90011569 | F-deep fried food liking (derived food-liking factor) \|\| id:ebi-fl187-GCST90094753 | Inverse variance weighted | 8 | 0.152579413260736 | 0.209156699880254 | 0.46569703515863 | -0.257367719 | 0.562526545026034 | 1.16483496176425 | 0.773083884242224 | 1.7551012455502 | 0.998074519377826 |
| ebi-fl187-GCST90094753 | ebi-gm430-GCST90011590 | OTU97_277 (Prevotella) prevalence \|\| id:ebi-gm430-GCST90011590 | F-deep fried food liking (derived food-liking factor) \|\| id:ebi-fl187-GCST90094753 | Inverse variance weighted | 8 | -0.088107556 | 0.229398905138468 | 0.700919075038075 | -0.53772941 | 0.361514298003731 | 0.915662386381215 | 0.584072937987043 | 1.43550154664406 | 0.998074519377826 |
| ebi-fl187-GCST90094753 | ebi-gm430-GCST90011616 | OTU97_86 (Phascolarctobacterium) prevalence \|\| id:ebi-gm430-GCST90011616 | F-deep fried food liking (derived food-liking factor) \|\| id:ebi-fl187-GCST90094753 | Inverse variance weighted | 8 | -0.33762646 | 0.227335035846645 | 0.137504290050195 | -0.78320313 | 0.107950210426648 | 0.713461744748457 | 0.456940026339667 | 1.11399227880537 | 0.998074519377826 |
| ebi-fl187-GCST90094753 | ebi-gm430-GCST90011665 | OTU99_55 (Barnesiella) prevalence \|\| id:ebi-gm430-GCST90011665 | F-deep fried food liking (derived food-liking factor) \|\| id:ebi-fl187-GCST90094753 | Inverse variance weighted | 8 | -0.172271674 | 0.151180018412384 | 0.254489034252243 | -0.46858451 | 0.124041161797742 | 0.841750460143119 | 0.62588757893288 | 1.1320624677665 | 0.998074519377826 |
| ebi-fl187-GCST90094753 | ebi-gm430-GCST90011687 | TestASV_14 (Lachnospiraceae) prevalence \|\| id:ebi-gm430-GCST90011687 | F-deep fried food liking (derived food-liking factor) \|\| id:ebi-fl187-GCST90094753 | Inverse variance weighted | 8 | 0.091465577709235 | 0.164723526077881 | 0.57871188294168 | -0.231392533 | 0.414323688821882 | 1.09577905681803 | 0.793427957923887 | 1.51334690108841 | 0.998074519377826 |
| ebi-fl187-GCST90094753 | ebi-gm430-GCST90011697 | TestASV_23 (Barnesiella) prevalence \|\| id:ebi-gm430-GCST90011697 | F-deep fried food liking (derived food-liking factor) \|\| id:ebi-fl187-GCST90094753 | Inverse variance weighted | 8 | 0.0587101477877726 | 0.217154190608186 | 0.786882102645365 | -0.366912066 | 0.484332361379817 | 1.0604678172458 | 0.692870569381948 | 1.62309100878281 | 0.998074519377826 |
| ebi-fl187-GCST90094753 | ebi-gm430-GCST90011727 | TestASV_6 (Subdoligranulum) prevalence \|\| id:ebi-gm430-GCST90011727 | F-deep fried food liking (derived food-liking factor) \|\| id:ebi-fl187-GCST90094753 | Inverse variance weighted | 8 | 0.318230761173214 | 0.261919636297945 | 0.22436841679958 | -0.195131726 | 0.831593248317187 | 1.37469345050212 | 0.822726276500401 | 2.29697547876058 | 0.998074519377826 |
| ebi-fl187-GCST90094753 | ebi-hbsm44-GCST90104908 | Sulcal depth of the left posterior inferior temporal sulcus \|\| id:ebi-hbsm44-GCST90104908 | F-deep fried food liking (derived food-liking factor) \|\| id:ebi-fl187-GCST90094753 | Inverse variance weighted (multiplicative random effects) | 7 | 0.0680104381093454 | 0.128360503479253 | 0.596223361031647 | -0.183576149 | 0.319597024928681 | 1.07037648112721 | 0.832288495629486 | 1.37657292797703 | 0.998074519377826 |
| ebi-fl187-GCST90094753 | ebi-hbsm44-GCST90104918 | Sulcal depth of the left inferior frontal sulcus \|\| id:ebi-hbsm44-GCST90104918 | F-deep fried food liking (derived food-liking factor) \|\| id:ebi-fl187-GCST90094753 | Inverse variance weighted | 7 | 0.0591435875192796 | 0.144428604135868 | 0.682172390222892 | -0.223936477 | 0.342223651625581 | 1.06092756576121 | 0.799365911207487 | 1.40807518060382 | 0.998074519377826 |
| ebi-fl187-GCST90094753 | ebi-hbsm44-GCST90104925 | Sulcal depth of the right anterior inferior temporal sulcus \|\| id:ebi-hbsm44-GCST90104925 | F-deep fried food liking (derived food-liking factor) \|\| id:ebi-fl187-GCST90094753 | Inverse variance weighted | 7 | 0.0400714251848893 | 0.044074055706245 | 0.363253021348938 | -0.046313724 | 0.12645657436913 | 1.04088511694931 | 0.954742389600801 | 1.13480017069274 | 0.998074519377826 |
| ebi-fl187-GCST90094753 | ebi-IPs91-GCST90274770 | C-C motif chemokine 4 levels \|\| id:ebi-IPs91-GCST90274770 | F-deep fried food liking (derived food-liking factor) \|\| id:ebi-fl187-GCST90094753 | Inverse variance weighted (multiplicative random effects) | 8 | 0.0219583267528179 | 0.0696115237123873 | 0.752426861781994 | -0.11448026 | 0.158396913229097 | 1.02220118514016 | 0.891829543206171 | 1.17163113832885 | 0.998074519377826 |
| ebi-fl187-GCST90094753 | ebi-IPs91-GCST90274779 | C-X-C motif chemokine 1 levels \|\| id:ebi-IPs91-GCST90274779 | F-deep fried food liking (derived food-liking factor) \|\| id:ebi-fl187-GCST90094753 | Inverse variance weighted | 8 | -0.017134745 | 0.0484264723231225 | 0.723466199537665 | -0.11205063 | 0.0777811412527148 | 0.983011220354717 | 0.893998992958467 | 1.08088607140988 | 0.998074519377826 |
| ebi-fl187-GCST90094753 | ebi-IPs91-GCST90274780 | C-X-C motif chemokine 10 levels \|\| id:ebi-IPs91-GCST90274780 | F-deep fried food liking (derived food-liking factor) \|\| id:ebi-fl187-GCST90094753 | Inverse variance weighted | 8 | 0.0925933436728269 | 0.0662025405206409 | 0.161921769881673 | -0.037163636 | 0.222350323093283 | 1.09701553624058 | 0.963518456385483 | 1.24900885787676 | 0.998074519377826 |
| ebi-fl187-GCST90094753 | ebi-IPs91-GCST90274796 | Interleukin-10 receptor subunit alpha levels \|\| id:ebi-IPs91-GCST90274796 | F-deep fried food liking (derived food-liking factor) \|\| id:ebi-fl187-GCST90094753 | Inverse variance weighted | 8 | -0.07001741 | 0.0657911626364906 | 0.287221211048669 | -0.198968088 | 0.0589332691006225 | 0.932377587381427 | 0.81957604687039 | 1.06070445661608 | 0.998074519377826 |
| ebi-fl187-GCST90094753 | ebi-IPs91-GCST90274807 | Interleukin-20 levels \|\| id:ebi-IPs91-GCST90274807 | F-deep fried food liking (derived food-liking factor) \|\| id:ebi-fl187-GCST90094753 | Inverse variance weighted | 8 | -0.040226315 | 0.0524553911452953 | 0.443160523871073 | -0.143038881 | 0.0625862518522889 | 0.960572022892961 | 0.866720368906688 | 1.0645862774964 | 0.998074519377826 |
| ebi-fl187-GCST90094753 | ebi-IPs91-GCST90274808 | Interleukin-20 receptor subunit alpha levels \|\| id:ebi-IPs91-GCST90274808 | F-deep fried food liking (derived food-liking factor) \|\| id:ebi-fl187-GCST90094753 | Inverse variance weighted | 8 | -0.027774745 | 0.0644892637462945 | 0.666695322175154 | -0.154173702 | 0.098624211732261 | 0.972607426609355 | 0.857123123947763 | 1.1036514823433 | 0.998074519377826 |
| ebi-fl187-GCST90094753 | ebi-IPs91-GCST90274810 | Interleukin-24 levels \|\| id:ebi-IPs91-GCST90274810 | F-deep fried food liking (derived food-liking factor) \|\| id:ebi-fl187-GCST90094753 | Inverse variance weighted | 8 | 0.013974516016628 | 0.0539842484586227 | 0.795741068415076 | -0.091834611 | 0.119783642995529 | 1.01407261599952 | 0.912256014213417 | 1.12725293612538 | 0.998074519377826 |
| ebi-fl187-GCST90094753 | ebi-IPs91-GCST90274823 | Monocyte chemoattractant protein-3 levels \|\| id:ebi-IPs91-GCST90274823 | F-deep fried food liking (derived food-liking factor) \|\| id:ebi-fl187-GCST90094753 | Inverse variance weighted (multiplicative random effects) | 8 | 0.047142048437624 | 0.0841624421064271 | 0.575389636581398 | -0.117816338 | 0.212100434966221 | 1.04827090374836 | 0.888859287217716 | 1.23627204378442 | 0.998074519377826 |
| ebi-fl187-GCST90094753 | ebi-IPs91-GCST90274824 | Monocyte chemoattractant protein-4 levels \|\| id:ebi-IPs91-GCST90274824 | F-deep fried food liking (derived food-liking factor) \|\| id:ebi-fl187-GCST90094753 | Inverse variance weighted | 8 | -0.031036233 | 0.0662021257133534 | 0.639205299385881 | -0.160792399 | 0.0987199337607765 | 0.96944044705548 | 0.851468818507956 | 1.10375713115835 | 0.998074519377826 |
| ebi-fl187-GCST90094753 | ebi-IPs91-GCST90274834 | SIR2-like protein 2 levels \|\| id:ebi-IPs91-GCST90274834 | F-deep fried food liking (derived food-liking factor) \|\| id:ebi-fl187-GCST90094753 | Inverse variance weighted | 8 | -0.059182218 | 0.0483677355386818 | 0.221107402254345 | -0.153982979 | 0.0356185441524515 | 0.942535007132718 | 0.857286612626246 | 1.03626048346795 | 0.998074519377826 |
| ebi-fl187-GCST90094753 | ebi-met1400-GCST90199652 | 1-stearoyl-2-oleoyl-GPS (18:0/18:1) levels \|\| id:ebi-met1400-GCST90199652 | F-deep fried food liking (derived food-liking factor) \|\| id:ebi-fl187-GCST90094753 | Inverse variance weighted | 8 | -0.055163491 | 0.0647157050290132 | 0.393993625607946 | -0.182006273 | 0.0716792904846631 | 0.94633041844669 | 0.833596111059977 | 1.07431074712998 | 0.998074519377826 |
| ebi-fl187-GCST90094753 | ebi-met1400-GCST90199665 | N-acetylaspartate (naa) levels \|\| id:ebi-met1400-GCST90199665 | F-deep fried food liking (derived food-liking factor) \|\| id:ebi-fl187-GCST90094753 | Inverse variance weighted | 8 | -0.053335033 | 0.0842740473843592 | 0.52681453895596 | -0.218512166 | 0.111842100197981 | 0.948062327409867 | 0.803713701766995 | 1.11833626162864 | 0.998074519377826 |
| ebi-fl187-GCST90094753 | ebi-met1400-GCST90199669 | Acetylcarnitine levels (Biocrates platform) \|\| id:ebi-met1400-GCST90199669 | F-deep fried food liking (derived food-liking factor) \|\| id:ebi-fl187-GCST90094753 | Inverse variance weighted | 8 | -0.0002383 | 0.0621419966529331 | 0.996940310781909 | -0.122036613 | 0.121560013725113 | 0.999761728676486 | 0.885115961022031 | 1.12925713481888 | 0.999355215041207 |
| ebi-fl187-GCST90094753 | ebi-met1400-GCST90199670 | Iminodiacetate (IDA) levels \|\| id:ebi-met1400-GCST90199670 | F-deep fried food liking (derived food-liking factor) \|\| id:ebi-fl187-GCST90094753 | Inverse variance weighted (multiplicative random effects) | 8 | 0.030646392326247 | 0.0908764288607587 | 0.735942410438524 | -0.147471408 | 0.208764192893334 | 1.03112082717702 | 0.862887109429093 | 1.23215441350338 | 0.998074519377826 |
| ebi-fl187-GCST90094753 | ebi-met1400-GCST90199686 | Indolepropionate levels \|\| id:ebi-met1400-GCST90199686 | F-deep fried food liking (derived food-liking factor) \|\| id:ebi-fl187-GCST90094753 | Inverse variance weighted | 8 | 0.0149882878315112 | 0.0651812759783565 | 0.818132370265316 | -0.112767013 | 0.14274358874909 | 1.01510117551009 | 0.89335877677528 | 1.15343401028809 | 0.998074519377826 |
| ebi-fl187-GCST90094753 | ebi-met1400-GCST90199704 | 3-hydroxysebacate levels \|\| id:ebi-met1400-GCST90199704 | F-deep fried food liking (derived food-liking factor) \|\| id:ebi-fl187-GCST90094753 | Inverse variance weighted | 8 | -0.04683402 | 0.0658658839281255 | 0.477052471705514 | -0.175931152 | 0.0822631127543938 | 0.954245770403187 | 0.838675722291063 | 1.08574144467288 | 0.998074519377826 |
| ebi-fl187-GCST90094753 | ebi-met1400-GCST90199713 | Docosapentaenoate n3 DPA; 22:5n3 levels \|\| id:ebi-met1400-GCST90199713 | F-deep fried food liking (derived food-liking factor) \|\| id:ebi-fl187-GCST90094753 | Inverse variance weighted | 8 | -0.052162924 | 0.0613378795035594 | 0.395091945047163 | -0.172385168 | 0.0680593201446203 | 0.949174211287005 | 0.841654932594936 | 1.07042880458695 | 0.998074519377826 |
| ebi-fl187-GCST90094753 | ebi-met1400-GCST90199715 | Myristoleate (14:1n5) levels \|\| id:ebi-met1400-GCST90199715 | F-deep fried food liking (derived food-liking factor) \|\| id:ebi-fl187-GCST90094753 | Inverse variance weighted | 8 | -0.013334332 | 0.0587818299893013 | 0.820544696046059 | -0.128546719 | 0.101878054704755 | 0.986754176294907 | 0.879372478184909 | 1.10724843975695 | 0.998074519377826 |
| ebi-fl187-GCST90094753 | ebi-met1400-GCST90199725 | Campesterol levels \|\| id:ebi-met1400-GCST90199725 | F-deep fried food liking (derived food-liking factor) \|\| id:ebi-fl187-GCST90094753 | Inverse variance weighted | 8 | -0.147667919 | 0.0932887342742762 | 0.113441371520625 | -0.330513838 | 0.0351780001396025 | 0.862717559455125 | 0.718554417835975 | 1.03580406565965 | 0.998074519377826 |
| ebi-fl187-GCST90094753 | ebi-met1400-GCST90199737 | N-acetylproline levels \|\| id:ebi-met1400-GCST90199737 | F-deep fried food liking (derived food-liking factor) \|\| id:ebi-fl187-GCST90094753 | Inverse variance weighted | 8 | -0.046673443 | 0.0687773786567036 | 0.497381074465629 | -0.181477106 | 0.0881302187011944 | 0.954399011941196 | 0.83403733984178 | 1.09213032856194 | 0.998074519377826 |
| ebi-fl187-GCST90094753 | ebi-met1400-GCST90199775 | Gamma-glutamylisoleucine levels \|\| id:ebi-met1400-GCST90199775 | F-deep fried food liking (derived food-liking factor) \|\| id:ebi-fl187-GCST90094753 | Inverse variance weighted | 8 | 0.0361130883420355 | 0.0763886951575858 | 0.636388442204465 | -0.113608754 | 0.185834930850904 | 1.036773087 | 0.892607116389101 | 1.204223464 | 0.998074519377826 |
| ebi-fl187-GCST90094753 | ebi-met1400-GCST90199777 | 1-palmitoleoyl-GPC (16:1) levels \|\| id:ebi-met1400-GCST90199777 | F-deep fried food liking (derived food-liking factor) \|\| id:ebi-fl187-GCST90094753 | Inverse variance weighted | 8 | 0.0810249885978386 | 0.0629486324876335 | 0.198038377617191 | -0.042354331 | 0.2044043082736 | 1.08439799381356 | 0.958530083395734 | 1.22679405618759 | 0.998074519377826 |
| ebi-fl187-GCST90094753 | ebi-met1400-GCST90199782 | Glutamine degradant levels \|\| id:ebi-met1400-GCST90199782 | F-deep fried food liking (derived food-liking factor) \|\| id:ebi-fl187-GCST90094753 | Inverse variance weighted | 8 | -0.075917842 | 0.0534162727257863 | 0.15524428636495 | -0.180613737 | 0.0287780523620392 | 0.926892354886036 | 0.834757732688457 | 1.02919614147117 | 0.998074519377826 |
| ebi-fl187-GCST90094753 | ebi-met1400-GCST90199783 | 5-methyluridine (ribothymidine) levels \|\| id:ebi-met1400-GCST90199783 | F-deep fried food liking (derived food-liking factor) \|\| id:ebi-fl187-GCST90094753 | Inverse variance weighted | 8 | -0.079266986 | 0.0637266810531479 | 0.213551575313934 | -0.204171281 | 0.0456373088267162 | 0.923793251617776 | 0.815322710018103 | 1.04669471517063 | 0.998074519377826 |
| ebi-fl187-GCST90094753 | ebi-met1400-GCST90199787 | Homostachydrine levels \|\| id:ebi-met1400-GCST90199787 | F-deep fried food liking (derived food-liking factor) \|\| id:ebi-fl187-GCST90094753 | Inverse variance weighted | 8 | 0.00780309999428983 | 0.0853009746915904 | 0.927113369195425 | -0.15938681 | 0.174993010389807 | 1.00783362352011 | 0.852666474910679 | 1.19123789029476 | 0.998074519377826 |
| ebi-fl187-GCST90094753 | ebi-met1400-GCST90199802 | Beta-hydroxyisovaleroylcarnitine levels \|\| id:ebi-met1400-GCST90199802 | F-deep fried food liking (derived food-liking factor) \|\| id:ebi-fl187-GCST90094753 | Inverse variance weighted | 8 | -0.032316041 | 0.0629998510387408 | 0.60798332427917 | -0.155795749 | 0.0911636667547665 | 0.968200542381316 | 0.855733956767386 | 1.09544827905233 | 0.998074519377826 |
| ebi-fl187-GCST90094753 | ebi-met1400-GCST90199873 | Pregnenolone sulfate levels \|\| id:ebi-met1400-GCST90199873 | F-deep fried food liking (derived food-liking factor) \|\| id:ebi-fl187-GCST90094753 | Inverse variance weighted | 8 | 0.0377409339721025 | 0.0707069620038399 | 0.593503814691203 | -0.100844712 | 0.176326579499629 | 1.03846216775826 | 0.904073414140053 | 1.19282754807137 | 0.998074519377826 |
| ebi-fl187-GCST90094753 | ebi-met1400-GCST90199911 | Beta-citrylglutamate levels \|\| id:ebi-met1400-GCST90199911 | F-deep fried food liking (derived food-liking factor) \|\| id:ebi-fl187-GCST90094753 | Inverse variance weighted | 8 | -0.02020836 | 0.0634154426294159 | 0.749979622563703 | -0.144502628 | 0.10408590731161 | 0.979994460149623 | 0.865452638167398 | 1.10969578180221 | 0.998074519377826 |
| ebi-fl187-GCST90094753 | ebi-met1400-GCST90199934 | 2-hydroxydecanoate levels \|\| id:ebi-met1400-GCST90199934 | F-deep fried food liking (derived food-liking factor) \|\| id:ebi-fl187-GCST90094753 | Inverse variance weighted | 8 | -0.071530231 | 0.0818045247926853 | 0.381898098609404 | -0.2318671 | 0.0888066376672639 | 0.930968133140335 | 0.793051513230066 | 1.09286931613403 | 0.998074519377826 |
| ebi-fl187-GCST90094753 | ebi-met1400-GCST90199945 | Sphingomyelin (d18:1/14:0, d16:1/16:0) levels \|\| id:ebi-met1400-GCST90199945 | F-deep fried food liking (derived food-liking factor) \|\| id:ebi-fl187-GCST90094753 | Inverse variance weighted | 8 | -0.107818223 | 0.0722518274071576 | 0.135632047209417 | -0.249431805 | 0.0337953588874845 | 0.897790779465165 | 0.779243419874114 | 1.03437290984489 | 0.998074519377826 |
| ebi-fl187-GCST90094753 | ebi-met1400-GCST90199979 | Carnitine C14:1 levels \|\| id:ebi-met1400-GCST90199979 | F-deep fried food liking (derived food-liking factor) \|\| id:ebi-fl187-GCST90094753 | Inverse variance weighted | 8 | -0.043009569 | 0.0618238143680185 | 0.486629482165469 | -0.164184245 | 0.0781651075683422 | 0.957902224246686 | 0.848585660019798 | 1.08130117494012 | 0.998074519377826 |
| ebi-fl187-GCST90094753 | ebi-met1400-GCST90199986 | Eugenol sulfate levels \|\| id:ebi-met1400-GCST90199986 | F-deep fried food liking (derived food-liking factor) \|\| id:ebi-fl187-GCST90094753 | Inverse variance weighted | 8 | 0.0549748585915329 | 0.0931584602176541 | 0.555108847973836 | -0.127615723 | 0.237565440618135 | 1.05651405209025 | 0.880191551150451 | 1.26815798311766 | 0.998074519377826 |
| ebi-fl187-GCST90094753 | ebi-met1400-GCST90200002 | 6-hydroxyindole sulfate levels \|\| id:ebi-met1400-GCST90200002 | F-deep fried food liking (derived food-liking factor) \|\| id:ebi-fl187-GCST90094753 | Inverse variance weighted | 8 | -0.006443288 | 0.063294510519798 | 0.918916589962346 | -0.130500529 | 0.11761395229005 | 0.993577425141991 | 0.877656028714602 | 1.12480979729339 | 0.998074519377826 |
| ebi-fl187-GCST90094753 | ebi-met1400-GCST90200023 | Trans 3,4-methyleneheptanoate levels \|\| id:ebi-met1400-GCST90200023 | F-deep fried food liking (derived food-liking factor) \|\| id:ebi-fl187-GCST90094753 | Inverse variance weighted | 8 | -0.018882768 | 0.0687671619345858 | 0.783631324471692 | -0.153666406 | 0.115900869064165 | 0.981294394282911 | 0.8575580497607 | 1.12288455401914 | 0.998074519377826 |
| ebi-fl187-GCST90094753 | ebi-met1400-GCST90200032 | 2-hydroxybutyrate/2-hydroxyisobutyrate levels \|\| id:ebi-met1400-GCST90200032 | F-deep fried food liking (derived food-liking factor) \|\| id:ebi-fl187-GCST90094753 | Inverse variance weighted | 8 | 0.0587153663406429 | 0.064427755103702 | 0.362118088024035 | -0.067563034 | 0.184993766343899 | 1.06047335136761 | 0.934668803161162 | 1.2032109397011 | 0.998074519377826 |
| ebi-fl187-GCST90094753 | ebi-met1400-GCST90200051 | 1-palmitoyl-2-dihomo-linolenoyl-GPC (16:0/20:3n3 or 6) levels \|\| id:ebi-met1400-GCST90200051 | F-deep fried food liking (derived food-liking factor) \|\| id:ebi-fl187-GCST90094753 | Inverse variance weighted | 8 | 0.0151351744877865 | 0.0627499078056037 | 0.809401386423473 | -0.107854645 | 0.13812499378677 | 1.01525029127884 | 0.897758080742245 | 1.14811904905337 | 0.998074519377826 |
| ebi-fl187-GCST90094753 | ebi-met1400-GCST90200069 | Phenylacetylglutamate levels \|\| id:ebi-met1400-GCST90200069 | F-deep fried food liking (derived food-liking factor) \|\| id:ebi-fl187-GCST90094753 | Inverse variance weighted | 8 | -0.07876143 | 0.0704476030717937 | 0.263560873581171 | -0.216838732 | 0.0593158723398201 | 0.924260399243053 | 0.80505978945913 | 1.06111036322265 | 0.998074519377826 |
| ebi-fl187-GCST90094753 | ebi-met1400-GCST90200072 | Thioproline levels \|\| id:ebi-met1400-GCST90200072 | F-deep fried food liking (derived food-liking factor) \|\| id:ebi-fl187-GCST90094753 | Inverse variance weighted | 8 | 0.0220766745669843 | 0.0637261441280369 | 0.729019270607964 | -0.102826568 | 0.146979917057936 | 1.02232216757492 | 0.902283444803553 | 1.15833070010792 | 0.998074519377826 |
| ebi-fl187-GCST90094753 | ebi-met1400-GCST90200081 | 1-oleoyl-2-docosahexaenoyl-GPC (18:1/22:6) levels \|\| id:ebi-met1400-GCST90200081 | F-deep fried food liking (derived food-liking factor) \|\| id:ebi-fl187-GCST90094753 | Inverse variance weighted (multiplicative random effects) | 7 | 0.0331422920568392 | 0.117353795416418 | 0.777626491058602 | -0.196871147 | 0.263155731073019 | 1.03369761573704 | 0.821296453040171 | 1.30102931386723 | 0.998074519377826 |
| ebi-fl187-GCST90094753 | ebi-met1400-GCST90200097 | Furaneol sulfate levels \|\| id:ebi-met1400-GCST90200097 | F-deep fried food liking (derived food-liking factor) \|\| id:ebi-fl187-GCST90094753 | Inverse variance weighted | 8 | 0.0607455416135106 | 0.0759371433071556 | 0.423742637151869 | -0.088091259 | 0.209582342495536 | 1.06262848505243 | 0.915677308868811 | 1.23316291264197 | 0.998074519377826 |
| ebi-fl187-GCST90094753 | ebi-met1400-GCST90200124 | Sphingomyelin (d18:2/24:2) levels \|\| id:ebi-met1400-GCST90200124 | F-deep fried food liking (derived food-liking factor) \|\| id:ebi-fl187-GCST90094753 | Inverse variance weighted | 8 | 0.111078117184546 | 0.0647252586062646 | 0.0861349745462312 | -0.01578339 | 0.237939624052825 | 1.11748219800803 | 0.984340515274638 | 1.26863259561804 | 0.998074519377826 |
| ebi-fl187-GCST90094753 | ebi-met1400-GCST90200137 | Lignoceroylcarnitine (C24) levels \|\| id:ebi-met1400-GCST90200137 | F-deep fried food liking (derived food-liking factor) \|\| id:ebi-fl187-GCST90094753 | Inverse variance weighted | 8 | -0.010692633 | 0.0642158721263196 | 0.867755019515332 | -0.136555743 | 0.115170475972774 | 0.989364329600723 | 0.872357691276766 | 1.12206470634044 | 0.998074519377826 |
| ebi-fl187-GCST90094753 | ebi-met1400-GCST90200165 | Octadecenedioate (C18:1-DC) levels \|\| id:ebi-met1400-GCST90200165 | F-deep fried food liking (derived food-liking factor) \|\| id:ebi-fl187-GCST90094753 | Inverse variance weighted | 8 | -0.02782715 | 0.0626425595513834 | 0.656882698034736 | -0.150606567 | 0.0949522664962551 | 0.972556458439072 | 0.860186057722435 | 1.09960636580878 | 0.998074519377826 |
| ebi-fl187-GCST90094753 | ebi-met1400-GCST90200176 | Glutamine conjugate of C6H10O2 (1) levels \|\| id:ebi-met1400-GCST90200176 | F-deep fried food liking (derived food-liking factor) \|\| id:ebi-fl187-GCST90094753 | Inverse variance weighted | 8 | -0.013489818 | 0.0629267137641772 | 0.830255830325312 | -0.136826177 | 0.10984654112901 | 0.986600761984745 | 0.872121807937791 | 1.11610678082976 | 0.998074519377826 |
| ebi-fl187-GCST90094753 | ebi-met1400-GCST90200182 | Glycine conjugate of C10H12O2 levels \|\| id:ebi-met1400-GCST90200182 | F-deep fried food liking (derived food-liking factor) \|\| id:ebi-fl187-GCST90094753 | Inverse variance weighted | 8 | 0.00570773185160787 | 0.098739161018721 | 0.953903030516394 | -0.187821024 | 0.199236487448301 | 1.0057240519886 | 0.82876302286184 | 1.22047055774228 | 0.998074519377826 |
| ebi-fl187-GCST90094753 | ebi-met1400-GCST90200208 | Glucuronide of piperine metabolite C17H21NO3 (4) levels \|\| id:ebi-met1400-GCST90200208 | F-deep fried food liking (derived food-liking factor) \|\| id:ebi-fl187-GCST90094753 | Inverse variance weighted | 8 | -0.010869785 | 0.0678245700878006 | 0.872673713829563 | -0.143805943 | 0.122066371937116 | 0.989189077214889 | 0.866055796109932 | 1.12982908823699 | 0.998074519377826 |
| ebi-fl187-GCST90094753 | ebi-met1400-GCST90200227 | 3-ethylcatechol sulfate (2) levels \|\| id:ebi-met1400-GCST90200227 | F-deep fried food liking (derived food-liking factor) \|\| id:ebi-fl187-GCST90094753 | Inverse variance weighted | 8 | -0.144616528 | 0.0831117700265361 | 0.0818546902654226 | -0.307515598 | 0.0182825409663784 | 0.865354068301582 | 0.735271399035036 | 1.01845068978457 | 0.998074519377826 |
| ebi-fl187-GCST90094753 | ebi-met1400-GCST90200259 | Pentose acid levels \|\| id:ebi-met1400-GCST90200259 | F-deep fried food liking (derived food-liking factor) \|\| id:ebi-fl187-GCST90094753 | Inverse variance weighted | 8 | -0.101955588 | 0.0678336645262517 | 0.132832999493705 | -0.23490957 | 0.0309983946384793 | 0.903069658058754 | 0.790642343942552 | 1.03148384798071 | 0.998074519377826 |
| ebi-fl187-GCST90094753 | ebi-met1400-GCST90200291 | Sarcosine levels \|\| id:ebi-met1400-GCST90200291 | F-deep fried food liking (derived food-liking factor) \|\| id:ebi-fl187-GCST90094753 | Inverse variance weighted | 8 | 0.00609162544218609 | 0.0640749614190698 | 0.924258992857797 | -0.119495299 | 0.131678549823563 | 1.00611021712447 | 0.887368179381017 | 1.14074156874584 | 0.998074519377826 |
| ebi-fl187-GCST90094753 | ebi-met1400-GCST90200306 | Butyrate/isobutyrate (4:0) levels \|\| id:ebi-met1400-GCST90200306 | F-deep fried food liking (derived food-liking factor) \|\| id:ebi-fl187-GCST90094753 | Inverse variance weighted | 8 | -0.113896182 | 0.0646653551241689 | 0.0781847844274684 | -0.240640278 | 0.012847914446048 | 0.892350593486825 | 0.78612436204235 | 1.0129308035023 | 0.998074519377826 |
| ebi-fl187-GCST90094753 | ebi-met1400-GCST90200321 | Cholate levels \|\| id:ebi-met1400-GCST90200321 | F-deep fried food liking (derived food-liking factor) \|\| id:ebi-fl187-GCST90094753 | Inverse variance weighted | 8 | 0.044853219106504 | 0.0659172266445661 | 0.496221085870561 | -0.084344545 | 0.174050983329853 | 1.04587433427241 | 0.919114525112752 | 1.1901162403625 | 0.998074519377826 |
| ebi-fl187-GCST90094753 | ebi-met1400-GCST90200336 | Ceramide (d18:1/16:0) levels \|\| id:ebi-met1400-GCST90200336 | F-deep fried food liking (derived food-liking factor) \|\| id:ebi-fl187-GCST90094753 | Inverse variance weighted | 8 | -0.087699607 | 0.0736481408199741 | 0.23373530295554 | -0.232049963 | 0.0566507489883866 | 0.916036006184596 | 0.792906506308679 | 1.0582861383407 | 0.998074519377826 |
| ebi-fl187-GCST90094753 | ebi-met1400-GCST90200358 | Arachidonate (20:4n6) levels \|\| id:ebi-met1400-GCST90200358 | F-deep fried food liking (derived food-liking factor) \|\| id:ebi-fl187-GCST90094753 | Inverse variance weighted | 8 | -0.040679759 | 0.0628918803460102 | 0.517748112079507 | -0.163947844 | 0.082588326916716 | 0.960136556231813 | 0.848786289989865 | 1.08609460058985 | 0.998074519377826 |
| ebi-fl187-GCST90094753 | ebi-met1400-GCST90200413 | 2'-deoxyuridine levels \|\| id:ebi-met1400-GCST90200413 | F-deep fried food liking (derived food-liking factor) \|\| id:ebi-fl187-GCST90094753 | Inverse variance weighted | 8 | -0.021319557 | 0.0637140999291141 | 0.737917149266354 | -0.146199193 | 0.103560079212613 | 0.9789060986309 | 0.863985586585867 | 1.10911242596468 | 0.998074519377826 |
| ebi-fl187-GCST90094753 | ebi-met1400-GCST90200432 | Threonine levels \|\| id:ebi-met1400-GCST90200432 | F-deep fried food liking (derived food-liking factor) \|\| id:ebi-fl187-GCST90094753 | Inverse variance weighted | 8 | -0.035616818 | 0.0649663637834702 | 0.5835307298901 | -0.162950891 | 0.0917172553491356 | 0.965009997429288 | 0.849632912282882 | 1.09605487461204 | 0.998074519377826 |
| ebi-fl187-GCST90094753 | ebi-met1400-GCST90200436 | Caffeine levels \|\| id:ebi-met1400-GCST90200436 | F-deep fried food liking (derived food-liking factor) \|\| id:ebi-fl187-GCST90094753 | Inverse variance weighted | 8 | 0.0742045340577487 | 0.0654647520307983 | 0.257002817706461 | -0.05410638 | 0.202515448038113 | 1.07702707168676 | 0.947331324108331 | 1.22447900077408 | 0.998074519377826 |
| ebi-fl187-GCST90094753 | ebi-met1400-GCST90200442 | Valine levels \|\| id:ebi-met1400-GCST90200442 | F-deep fried food liking (derived food-liking factor) \|\| id:ebi-fl187-GCST90094753 | Inverse variance weighted | 8 | 0.0637842329724258 | 0.0627087918564099 | 0.30908218879773 | -0.059124999 | 0.186693465010989 | 1.0658623959879 | 0.942588939055793 | 1.20525777474016 | 0.998074519377826 |
| ebi-fl187-GCST90094753 | ebi-met1400-GCST90200459 | Margarate (17:0) levels \|\| id:ebi-met1400-GCST90200459 | F-deep fried food liking (derived food-liking factor) \|\| id:ebi-fl187-GCST90094753 | Inverse variance weighted | 8 | -0.022692601 | 0.0627006249788869 | 0.717411924988243 | -0.145585826 | 0.100200624093133 | 0.977562939592547 | 0.864515689118351 | 1.10539266423191 | 0.998074519377826 |
| ebi-fl187-GCST90094753 | ebi-met1400-GCST90200462 | X-11299 levels \|\| id:ebi-met1400-GCST90200462 | F-deep fried food liking (derived food-liking factor) \|\| id:ebi-fl187-GCST90094753 | Inverse variance weighted | 8 | 0.00991684930991758 | 0.0635297195731981 | 0.875955934314883 | -0.114601401 | 0.134435099673386 | 1.00996618420742 | 0.891721512332728 | 1.14389041773156 | 0.998074519377826 |
| ebi-fl187-GCST90094753 | ebi-met1400-GCST90200497 | X-12740 levels \|\| id:ebi-met1400-GCST90200497 | F-deep fried food liking (derived food-liking factor) \|\| id:ebi-fl187-GCST90094753 | Inverse variance weighted | 8 | 0.100375268644372 | 0.0927555789409335 | 0.279186749012905 | -0.081425666 | 0.282176203368602 | 1.10558573189632 | 0.921801228406129 | 1.32601234724563 | 0.998074519377826 |
| ebi-fl187-GCST90094753 | ebi-met1400-GCST90200527 | X-13866 levels \|\| id:ebi-met1400-GCST90200527 | F-deep fried food liking (derived food-liking factor) \|\| id:ebi-fl187-GCST90094753 | Inverse variance weighted | 8 | 0.00250336019625084 | 0.0626387842234814 | 0.968121014862398 | -0.120268657 | 0.125275377274274 | 1.00250649621871 | 0.886682191442798 | 1.13346053936795 | 0.998074519377826 |
| ebi-fl187-GCST90094753 | ebi-met1400-GCST90200547 | X-17654 levels \|\| id:ebi-met1400-GCST90200547 | F-deep fried food liking (derived food-liking factor) \|\| id:ebi-fl187-GCST90094753 | Inverse variance weighted | 8 | 0.0123817345761086 | 0.0646690809475549 | 0.848162886210961 | -0.114369664 | 0.139133133233316 | 1.01245870560188 | 0.891928181121741 | 1.14927709679477 | 0.998074519377826 |
| ebi-fl187-GCST90094753 | ebi-met1400-GCST90200553 | X-17653 levels \|\| id:ebi-met1400-GCST90200553 | F-deep fried food liking (derived food-liking factor) \|\| id:ebi-fl187-GCST90094753 | Inverse variance weighted | 8 | 0.0998705231411716 | 0.0643497140557657 | 0.120663090258472 | -0.026254916 | 0.225995962690472 | 1.10502783327999 | 0.974086747262006 | 1.25357060421543 | 0.998074519377826 |
| ebi-fl187-GCST90094753 | ebi-met1400-GCST90200557 | X-17685 levels \|\| id:ebi-met1400-GCST90200557 | F-deep fried food liking (derived food-liking factor) \|\| id:ebi-fl187-GCST90094753 | Inverse variance weighted | 8 | -0.037271305 | 0.0816888683265075 | 0.648203532144766 | -0.197381487 | 0.122838876572829 | 0.963414720323886 | 0.820877419289583 | 1.13070222365237 | 0.998074519377826 |
| ebi-fl187-GCST90094753 | ebi-met1400-GCST90200561 | X-18779 levels \|\| id:ebi-met1400-GCST90200561 | F-deep fried food liking (derived food-liking factor) \|\| id:ebi-fl187-GCST90094753 | Inverse variance weighted | 8 | 0.0888584175123522 | 0.0637459181517502 | 0.163333675588679 | -0.036083582 | 0.213800417089783 | 1.09292590620302 | 0.964559670224712 | 1.23837547154694 | 0.998074519377826 |
| ebi-fl187-GCST90094753 | ebi-met1400-GCST90200570 | X-18921 levels \|\| id:ebi-met1400-GCST90200570 | F-deep fried food liking (derived food-liking factor) \|\| id:ebi-fl187-GCST90094753 | Inverse variance weighted | 8 | 0.0364383183860651 | 0.0800399151179379 | 0.64892807158112 | -0.120439915 | 0.193316552017223 | 1.03711033140573 | 0.886530352704104 | 1.21326679478905 | 0.998074519377826 |
| ebi-fl187-GCST90094753 | ebi-met1400-GCST90200575 | X-21283 levels \|\| id:ebi-met1400-GCST90200575 | F-deep fried food liking (derived food-liking factor) \|\| id:ebi-fl187-GCST90094753 | Inverse variance weighted | 8 | -0.013940264 | 0.0676282665379682 | 0.836688795805696 | -0.146491666 | 0.118611138524308 | 0.98615645165356 | 0.863732930391981 | 1.12593200156974 | 0.998074519377826 |
| ebi-fl187-GCST90094753 | ebi-met1400-GCST90200578 | X-18922 levels \|\| id:ebi-met1400-GCST90200578 | F-deep fried food liking (derived food-liking factor) \|\| id:ebi-fl187-GCST90094753 | Inverse variance weighted | 8 | -0.02454788 | 0.064221486140839 | 0.702284983189884 | -0.150421993 | 0.101326232989816 | 0.975750968993592 | 0.860344840583156 | 1.10663760457562 | 0.998074519377826 |
| ebi-fl187-GCST90094753 | ebi-met1400-GCST90200623 | X-24241 levels \|\| id:ebi-met1400-GCST90200623 | F-deep fried food liking (derived food-liking factor) \|\| id:ebi-fl187-GCST90094753 | Inverse variance weighted | 8 | 0.0021553727407552 | 0.06458361054946 | 0.973376845368138 | -0.124428504 | 0.128739249417697 | 1.00215769722633 | 0.883001390233372 | 1.13739350947628 | 0.998074519377826 |
| ebi-fl187-GCST90094753 | ebi-met1400-GCST90200636 | X-24544 levels \|\| id:ebi-met1400-GCST90200636 | F-deep fried food liking (derived food-liking factor) \|\| id:ebi-fl187-GCST90094753 | Inverse variance weighted | 8 | 0.0716694195898849 | 0.0668077399070375 | 0.283373783752602 | -0.059273751 | 0.202612589807679 | 1.07430014277397 | 0.942448737906829 | 1.22459795460861 | 0.998074519377826 |
| ebi-fl187-GCST90094753 | ebi-met1400-GCST90200659 | X-25172 levels \|\| id:ebi-met1400-GCST90200659 | F-deep fried food liking (derived food-liking factor) \|\| id:ebi-fl187-GCST90094753 | Inverse variance weighted | 8 | 0.0288227729328883 | 0.0648016399057434 | 0.656475408356003 | -0.098188441 | 0.155833987148145 | 1.02924216873931 | 0.906478069768433 | 1.16863217902426 | 0.998074519377826 |
| ebi-fl187-GCST90094753 | ebi-met1400-GCST90200670 | X-26111 levels \|\| id:ebi-met1400-GCST90200670 | F-deep fried food liking (derived food-liking factor) \|\| id:ebi-fl187-GCST90094753 | Inverse variance weighted | 8 | 0.0550225384726596 | 0.0673697598848702 | 0.414085844365981 | -0.077022191 | 0.187067267847005 | 1.05656442775561 | 0.925869307503751 | 1.20570838772945 | 0.998074519377826 |
| ebi-fl187-GCST90094753 | ebi-met1400-GCST90200673 | Carnitine C4 levels \|\| id:ebi-met1400-GCST90200673 | F-deep fried food liking (derived food-liking factor) \|\| id:ebi-fl187-GCST90094753 | Inverse variance weighted | 8 | -0.054715711 | 0.0677292647399287 | 0.419171632403576 | -0.18746507 | 0.0780336482644622 | 0.946754261875189 | 0.829058077074471 | 1.08115903718325 | 0.998074519377826 |
| ebi-fl187-GCST90094753 | ebi-met1400-GCST90200710 | X-13431 levels \|\| id:ebi-met1400-GCST90200710 | F-deep fried food liking (derived food-liking factor) \|\| id:ebi-fl187-GCST90094753 | Inverse variance weighted | 8 | -0.048601773 | 0.0631799308266 | 0.441739175146868 | -0.172434437 | 0.0752308918219977 | 0.952560389826762 | 0.841613465691244 | 1.0781330542539 | 0.998074519377826 |
| ebi-fl187-GCST90094753 | ebi-met1400-GCST90200730 | Adenosine 5'-diphosphate (ADP) to fructose ratio \|\| id:ebi-met1400-GCST90200730 | F-deep fried food liking (derived food-liking factor) \|\| id:ebi-fl187-GCST90094753 | Inverse variance weighted | 8 | -0.142317836 | 0.0906434200704703 | 0.116395437045721 | -0.319978939 | 0.0353432678230862 | 0.867345539455656 | 0.726164330766241 | 1.03597526474457 | 0.998074519377826 |
| ebi-fl187-GCST90094753 | ebi-met1400-GCST90200731 | Adenosine 5'-diphosphate (ADP) to mannose ratio \|\| id:ebi-met1400-GCST90200731 | F-deep fried food liking (derived food-liking factor) \|\| id:ebi-fl187-GCST90094753 | Inverse variance weighted | 8 | -0.090678907 | 0.104814971158238 | 0.386965709196181 | -0.29611625 | 0.114758436826661 | 0.913310921892146 | 0.743700967672763 | 1.12160246699385 | 0.998074519377826 |
| ebi-fl187-GCST90094753 | ebi-met1400-GCST90200748 | Aspartate to glutamate ratio \|\| id:ebi-met1400-GCST90200748 | F-deep fried food liking (derived food-liking factor) \|\| id:ebi-fl187-GCST90094753 | Inverse variance weighted (multiplicative random effects) | 8 | -0.078657241 | 0.0901573301927721 | 0.382966092349908 | -0.255365608 | 0.0980511266551554 | 0.924356702172797 | 0.774633234307015 | 1.10301917734803 | 0.998074519377826 |
| ebi-fl187-GCST90094753 | ebi-met1400-GCST90200774 | Uridine to pseudouridine ratio \|\| id:ebi-met1400-GCST90200774 | F-deep fried food liking (derived food-liking factor) \|\| id:ebi-fl187-GCST90094753 | Inverse variance weighted | 8 | 0.0584726661258284 | 0.0609996358805182 | 0.337773386224858 | -0.06108662 | 0.178031952451644 | 1.06021600548764 | 0.940741749008252 | 1.19486349944304 | 0.998074519377826 |
| ebi-fl187-GCST90094753 | ebi-met1400-GCST90200799 | Spermidine to adenosine 5'-diphosphate (ADP) ratio \|\| id:ebi-met1400-GCST90200799 | F-deep fried food liking (derived food-liking factor) \|\| id:ebi-fl187-GCST90094753 | Inverse variance weighted | 8 | 0.108916771671493 | 0.104780553242095 | 0.298583852087423 | -0.096453113 | 0.314286656025998 | 1.11506954110525 | 0.908052472745755 | 1.36928219328664 | 0.998074519377826 |
| ebi-fl187-GCST90094753 | ebi-met1400-GCST90200811 | Choline to taurocholate ratio \|\| id:ebi-met1400-GCST90200811 | F-deep fried food liking (derived food-liking factor) \|\| id:ebi-fl187-GCST90094753 | Inverse variance weighted | 8 | 0.0535817357061324 | 0.077186447419075 | 0.487565696965689 | -0.097703701 | 0.204867172647519 | 1.05504322294629 | 0.906917582506787 | 1.22736202688689 | 0.998074519377826 |
| ebi-fl187-GCST90094753 | ebi-met1400-GCST90200812 | Glutamate to kynurenine ratio \|\| id:ebi-met1400-GCST90200812 | F-deep fried food liking (derived food-liking factor) \|\| id:ebi-fl187-GCST90094753 | Inverse variance weighted | 8 | 0.00393732626093343 | 0.0830915374884042 | 0.962206060069617 | -0.158922087 | 0.166796739738206 | 1.00394508771309 | 0.853062820878994 | 1.1815140860373 | 0.998074519377826 |
| ebi-fl187-GCST90094753 | ebi-met1400-GCST90200876 | Cortisone to 4-cholesten-3-one ratio \|\| id:ebi-met1400-GCST90200876 | F-deep fried food liking (derived food-liking factor) \|\| id:ebi-fl187-GCST90094753 | Inverse variance weighted | 8 | 0.113694853579614 | 0.0828455263689997 | 0.16994776395364 | -0.048682378 | 0.276072085262853 | 1.12041018355884 | 0.95248361130946 | 1.31794286486102 | 0.998074519377826 |
| ebi-fl187-GCST90094753 | ebi-met1400-GCST90200906 | 2'-deoxyuridine to cytidine ratio \|\| id:ebi-met1400-GCST90200906 | F-deep fried food liking (derived food-liking factor) \|\| id:ebi-fl187-GCST90094753 | Inverse variance weighted | 8 | 0.0508219913456358 | 0.0714770787870662 | 0.477068731792261 | -0.089273083 | 0.190917065768286 | 1.05213558737255 | 0.914595778839137 | 1.21035906771933 | 0.998074519377826 |
| ebi-fl187-GCST90094753 | ebi-met1400-GCST90200907 | Retinol (Vitamin A) to linoleoyl-arachidonoyl-glycerol (18:2 to 20:4) [1] ratio \|\| id:ebi-met1400-GCST90200907 | F-deep fried food liking (derived food-liking factor) \|\| id:ebi-fl187-GCST90094753 | Inverse variance weighted | 7 | -0.020811077 | 0.10850453986162 | 0.847899546551946 | -0.233479975 | 0.191857820962234 | 0.979403978866986 | 0.791773450611029 | 1.21149825506302 | 0.998074519377826 |
| ebi-fl187-GCST90094753 | ebi-met1400-GCST90200916 | Fructose to sucrose ratio \|\| id:ebi-met1400-GCST90200916 | F-deep fried food liking (derived food-liking factor) \|\| id:ebi-fl187-GCST90094753 | Inverse variance weighted | 8 | 0.0854727052616303 | 0.0914556639852582 | 0.350004390843768 | -0.093780396 | 0.264725806672736 | 1.08923183064048 | 0.910482685791608 | 1.30307363269505 | 0.998074519377826 |
| ebi-fl187-GCST90094753 | ebi-met1400-GCST90200917 | Alpha-ketobutyrate to 3-methyl-2-oxobutyrate ratio \|\| id:ebi-met1400-GCST90200917 | F-deep fried food liking (derived food-liking factor) \|\| id:ebi-fl187-GCST90094753 | Inverse variance weighted | 8 | 0.0347361051421573 | 0.0642618353205406 | 0.588824547282906 | -0.091217092 | 0.160689302370417 | 1.03534645014115 | 0.912819523492996 | 1.17432005367062 | 0.998074519377826 |
| ebi-fl187-GCST90094753 | ebi-met1400-GCST90200924 | Alpha-tocopherol to glycerol ratio \|\| id:ebi-met1400-GCST90200924 | F-deep fried food liking (derived food-liking factor) \|\| id:ebi-fl187-GCST90094753 | Inverse variance weighted | 8 | 0.0100685801965618 | 0.0628991323261785 | 0.872821974411721 | -0.113213719 | 0.133350879555872 | 1.01011943889847 | 0.892959797100996 | 1.14265086082612 | 0.998074519377826 |
| ebi-fl187-GCST90094753 | ebi-met1400-GCST90200938 | Alpha-ketoglutarate to alanine ratio \|\| id:ebi-met1400-GCST90200938 | F-deep fried food liking (derived food-liking factor) \|\| id:ebi-fl187-GCST90094753 | Inverse variance weighted | 8 | -0.033339416 | 0.0634986078475408 | 0.599554216320539 | -0.157796687 | 0.0911178556033512 | 0.967210217461651 | 0.854023398242044 | 1.09539809645482 | 0.998074519377826 |
| ebi-fl187-GCST90094753 | ebi-met1400-GCST90200943 | Phosphate to EDTA ratio \|\| id:ebi-met1400-GCST90200943 | F-deep fried food liking (derived food-liking factor) \|\| id:ebi-fl187-GCST90094753 | Inverse variance weighted (multiplicative random effects) | 8 | -0.032283191 | 0.0974802126994758 | 0.7405108998442 | -0.223344407 | 0.158778026379311 | 0.968232349036602 | 0.799839331265447 | 1.17207774746177 | 0.998074519377826 |
| ebi-fl187-GCST90094753 | ebi-met1400-GCST90200947 | Adenosine 5'-diphosphate (ADP) to glucose ratio \|\| id:ebi-met1400-GCST90200947 | F-deep fried food liking (derived food-liking factor) \|\| id:ebi-fl187-GCST90094753 | Inverse variance weighted | 8 | -0.166279288 | 0.100457038268753 | 0.0978782873725879 | -0.363175083 | 0.0306165072452939 | 0.846809697575226 | 0.695464658961406 | 1.03109001250808 | 0.998074519377826 |
| ebi-fl187-GCST90094753 | ebi-met1400-GCST90200975 | Arachidonate (20:4n6) to caffeine ratio \|\| id:ebi-met1400-GCST90200975 | F-deep fried food liking (derived food-liking factor) \|\| id:ebi-fl187-GCST90094753 | Inverse variance weighted | 8 | -0.066177268 | 0.0654573075986432 | 0.312016939985734 | -0.194473591 | 0.0621190546867322 | 0.935964932759782 | 0.823267919569643 | 1.06408902197229 | 0.998074519377826 |
| ebi-fl187-GCST90094753 | ebi-met1400-GCST90200985 | Cholesterol to oleoyl-linoleoyl-glycerol (18:1 to 18:2) [2] ratio \|\| id:ebi-met1400-GCST90200985 | F-deep fried food liking (derived food-liking factor) \|\| id:ebi-fl187-GCST90094753 | Inverse variance weighted (multiplicative random effects) | 8 | 0.0902006147458327 | 0.125795497578227 | 0.473348380041419 | -0.156358561 | 0.336759789999157 | 1.09439381322073 | 0.855252475624713 | 1.40040263261553 | 0.998074519377826 |
| ebi-fl187-GCST90094753 | ebi-met1400-GCST90201016 | Bilirubin (Z,Z) to etiocholanolone glucuronide ratio \|\| id:ebi-met1400-GCST90201016 | F-deep fried food liking (derived food-liking factor) \|\| id:ebi-fl187-GCST90094753 | Inverse variance weighted | 8 | 0.0338927554750046 | 0.0656932260254359 | 0.605907028163853 | -0.094865968 | 0.162651478484859 | 1.03447365914277 | 0.909494828133051 | 1.1766265385554 | 0.998074519377826 |
| ebi-fl187-GCST90094753 | ebi-pw11-GCST90275189 | Placental weight (fetal genotype, adjusted for sex and gestational age) \|\| id:ebi-pw11-GCST90275189 | F-deep fried food liking (derived food-liking factor) \|\| id:ebi-fl187-GCST90094753 | Inverse variance weighted (multiplicative random effects) | 8 | 0.0228526064525726 | 0.0344818327117828 | 0.507494919993003 | -0.044731786 | 0.0904369985676667 | 1.0231157277766 | 0.956253928454114 | 1.09465254079119 | 0.998074519377826 |
| ebi-fl187-GCST90094753 | ebi-pw11-GCST90275192 | Placental weight (fetal effect, conditional on maternal and paternal genotype) \|\| id:ebi-pw11-GCST90275192 | F-deep fried food liking (derived food-liking factor) \|\| id:ebi-fl187-GCST90094753 | Inverse variance weighted | 8 | 0.0477382625972296 | 0.0472726714072218 | 0.312567613136075 | -0.044916173 | 0.140392698555384 | 1.04889608405644 | 0.956077623247912 | 1.15072559842105 | 0.998074519377826 |
| ebi-fl187-GCST90094753 | ebi-pw11-GCST90275193 | Placental weight (maternal effect, conditional on fetal and paternal genotype) \|\| id:ebi-pw11-GCST90275193 | F-deep fried food liking (derived food-liking factor) \|\| id:ebi-fl187-GCST90094753 | Inverse variance weighted (multiplicative random effects) | 8 | -0.022645378 | 0.053960093166939 | 0.674727278985106 | -0.128407161 | 0.0831164046072991 | 0.977609104005842 | 0.879495210429674 | 1.0866682943824 | 0.998074519377826 |
| ebi-fl187-GCST90094753 | ebi-pw11-GCST90275195 | Placental weight (fetal effect, conditional on maternal genotype) \|\| id:ebi-pw11-GCST90275195 | F-deep fried food liking (derived food-liking factor) \|\| id:ebi-fl187-GCST90094753 | Inverse variance weighted | 8 | 0.0275966285392028 | 0.0404865555322184 | 0.49547641317893 | -0.05175702 | 0.106950277382351 | 1.02798094260505 | 0.949559562508457 | 1.11287891785067 | 0.998074519377826 |
| ebi-fl187-GCST90094753 | ebi-pw11-GCST90275197 | Placental weight (fetal genotype, adjusted for sex) \|\| id:ebi-pw11-GCST90275197 | F-deep fried food liking (derived food-liking factor) \|\| id:ebi-fl187-GCST90094753 | Inverse variance weighted (multiplicative random effects) | 8 | 0.0245539927386856 | 0.033070082618391 | 0.45779440760974 | -0.040263369 | 0.0893713546707319 | 1.02485792449949 | 0.960536430131597 | 1.09348665231313 | 0.998074519377826 |
| ebi-fl187-GCST90094753 | prot-a-1030 | Protein FAM171B \|\| id:prot-a-1030 | F-deep fried food liking (derived food-liking factor) \|\| id:ebi-fl187-GCST90094753 | Inverse variance weighted | 8 | 0.102999494650065 | 0.101125659527097 | 0.308426261024249 | -0.095206798 | 0.301205787323174 | 1.10849084890009 | 0.909184897386692 | 1.35148743190421 | 0.998074519377826 |
| ebi-fl187-GCST90094753 | prot-a-1036 | BRISC complex subunit Abro1 \|\| id:prot-a-1036 | F-deep fried food liking (derived food-liking factor) \|\| id:ebi-fl187-GCST90094753 | Inverse variance weighted | 8 | 0.113199822640007 | 0.101125659527097 | 0.262970545679642 | -0.08500647 | 0.311406115313116 | 1.11985568311169 | 0.918506341615797 | 1.3653435955504 | 0.998074519377826 |
| ebi-fl187-GCST90094753 | prot-a-1055 | Phenylalanine--tRNA ligase, mitochondrial \|\| id:prot-a-1055 | F-deep fried food liking (derived food-liking factor) \|\| id:ebi-fl187-GCST90094753 | Inverse variance weighted (multiplicative random effects) | 8 | -0.135172278 | 0.156767559742999 | 0.388551892736984 | -0.442436695 | 0.172092139247929 | 0.873565402755751 | 0.642469011217121 | 1.18778726999787 | 0.998074519377826 |
| ebi-fl187-GCST90094753 | prot-a-1083 | Fc receptor-like protein 5 \|\| id:prot-a-1083 | F-deep fried food liking (derived food-liking factor) \|\| id:ebi-fl187-GCST90094753 | Inverse variance weighted | 8 | -0.104255121 | 0.133836924911593 | 0.435996632116618 | -0.366575494 | 0.158065251915656 | 0.900995405328638 | 0.693103809510146 | 1.17124261803878 | 0.998074519377826 |
| ebi-fl187-GCST90094753 | prot-a-1103 | Fibroblast growth factor receptor 3 \|\| id:prot-a-1103 | F-deep fried food liking (derived food-liking factor) \|\| id:ebi-fl187-GCST90094753 | Inverse variance weighted | 8 | -0.022582902 | 0.119984400280464 | 0.850707864132973 | -0.257752327 | 0.21258652235533 | 0.977670182830054 | 0.772786606981806 | 1.23687312611185 | 0.998074519377826 |
| ebi-fl187-GCST90094753 | prot-a-1105 | Fibroblast growth factor receptor 4 \|\| id:prot-a-1105 | F-deep fried food liking (derived food-liking factor) \|\| id:ebi-fl187-GCST90094753 | Inverse variance weighted | 8 | 0.0479859508915823 | 0.101206557660081 | 0.635400966369529 | -0.150378902 | 0.246350803905342 | 1.04915591551573 | 0.860381914122966 | 1.27934829520873 | 0.998074519377826 |
| ebi-fl187-GCST90094753 | prot-a-1120 | Filamin-A \|\| id:prot-a-1120 | F-deep fried food liking (derived food-liking factor) \|\| id:ebi-fl187-GCST90094753 | Inverse variance weighted | 8 | 0.000863997428481518 | 0.104811475564824 | 0.993422834687839 | -0.204566495 | 0.206294489535537 | 1.00086437078178 | 0.815000546916285 | 1.22911511224213 | 0.999355215041207 |
| ebi-fl187-GCST90094753 | prot-a-1166 | Galanin peptides \|\| id:prot-a-1166 | F-deep fried food liking (derived food-liking factor) \|\| id:ebi-fl187-GCST90094753 | Inverse variance weighted | 8 | 0.0503286765004588 | 0.101206557660081 | 0.618986879429835 | -0.148036177 | 0.248693529514218 | 1.05161668127072 | 0.862399915755687 | 1.28234897072988 | 0.998074519377826 |
| ebi-fl187-GCST90094753 | prot-a-1205 | ADP-ribosylation factor-binding protein GGA1 \|\| id:prot-a-1205 | F-deep fried food liking (derived food-liking factor) \|\| id:ebi-fl187-GCST90094753 | Inverse variance weighted | 8 | 0.074828383944399 | 0.101206557660081 | 0.459686604244405 | -0.123536469 | 0.273193236958159 | 1.07769918453031 | 0.883789409679073 | 1.31415416344381 | 0.998074519377826 |
| ebi-fl187-GCST90094753 | prot-a-1227 | Guanine nucleotide-binding protein G(I)/G(S)/G(O) subunit gamma-T2 \|\| id:prot-a-1227 | F-deep fried food liking (derived food-liking factor) \|\| id:ebi-fl187-GCST90094753 | Inverse variance weighted | 8 | 0.0447002691043679 | 0.101125659527097 | 0.658469688031876 | -0.153506024 | 0.242906561777477 | 1.04571438002356 | 0.857695597794875 | 1.27494948953858 | 0.998074519377826 |
| ebi-fl187-GCST90094753 | prot-a-1240 | Golgi SNAP receptor complex member 1 \|\| id:prot-a-1240 | F-deep fried food liking (derived food-liking factor) \|\| id:ebi-fl187-GCST90094753 | Inverse variance weighted | 8 | -0.072833153 | 0.101206557660081 | 0.471741411152341 | -0.271198006 | 0.125531700472046 | 0.929755944501372 | 0.762465509050743 | 1.1337511088362 | 0.998074519377826 |
| ebi-fl187-GCST90094753 | prot-a-1283 | Glutathione S-transferase A1 \|\| id:prot-a-1283 | F-deep fried food liking (derived food-liking factor) \|\| id:ebi-fl187-GCST90094753 | Inverse variance weighted | 8 | -0.00618821 | 0.101125659527097 | 0.951205289487899 | -0.204394502 | 0.192018082890294 | 0.993830897753213 | 0.815140732726899 | 1.21169242766804 | 0.998074519377826 |
| ebi-fl187-GCST90094753 | prot-a-1324 | E3 ubiquitin-protein ligase HECW1 \|\| id:prot-a-1324 | F-deep fried food liking (derived food-liking factor) \|\| id:ebi-fl187-GCST90094753 | Inverse variance weighted | 8 | 0.112550052245942 | 0.114038370194109 | 0.323667654741943 | -0.110965153 | 0.336065257826396 | 1.1191282703944 | 0.894969935103611 | 1.39943034561375 | 0.998074519377826 |
| ebi-fl187-GCST90094753 | prot-a-1339 | Histidine triad nucleotide-binding protein 2, mitochondrial \|\| id:prot-a-1339 | F-deep fried food liking (derived food-liking factor) \|\| id:ebi-fl187-GCST90094753 | Inverse variance weighted | 8 | 0.0817634620075147 | 0.101125659527097 | 0.418783651150209 | -0.116442831 | 0.279969754680624 | 1.08519908865471 | 0.890080980859408 | 1.32308979445887 | 0.998074519377826 |
| ebi-fl187-GCST90094753 | prot-a-1466 | Interleukin-11 \|\| id:prot-a-1466 | F-deep fried food liking (derived food-liking factor) \|\| id:ebi-fl187-GCST90094753 | Inverse variance weighted | 8 | -0.019387954 | 0.101125659527097 | 0.847960434995161 | -0.217594247 | 0.178818338726941 | 0.980798783665579 | 0.804451784485915 | 1.19580349325053 | 0.998074519377826 |
| ebi-fl187-GCST90094753 | prot-a-147 | Rho GTPase-activating protein 1 \|\| id:prot-a-147 | F-deep fried food liking (derived food-liking factor) \|\| id:ebi-fl187-GCST90094753 | Inverse variance weighted | 8 | -0.018557905 | 0.113950143759544 | 0.870628773376548 | -0.241900187 | 0.204784376946371 | 0.981613232806253 | 0.78513454059591 | 1.22726041079406 | 0.998074519377826 |
| ebi-fl187-GCST90094753 | prot-a-1473 | Interleukin-12 receptor subunit beta-1 \|\| id:prot-a-1473 | F-deep fried food liking (derived food-liking factor) \|\| id:ebi-fl187-GCST90094753 | Inverse variance weighted | 8 | -0.040662483 | 0.142217695537579 | 0.774941549512721 | -0.319409167 | 0.238084199771037 | 0.960153142809791 | 0.726578196847668 | 1.26881602234591 | 0.998074519377826 |
| ebi-fl187-GCST90094753 | prot-a-1501 | X-linked interleukin-1 receptor accessory protein-like 2 \|\| id:prot-a-1501 | F-deep fried food liking (derived food-liking factor) \|\| id:ebi-fl187-GCST90094753 | Inverse variance weighted | 8 | 0.0353373095575448 | 0.10991051997162 | 0.747824085029276 | -0.18008731 | 0.250761928701921 | 1.03596909214724 | 0.835197287497746 | 1.28500412531246 | 0.998074519377826 |
| ebi-fl187-GCST90094753 | prot-a-1503 | Interleukin-1 receptor-like 2 \|\| id:prot-a-1503 | F-deep fried food liking (derived food-liking factor) \|\| id:ebi-fl187-GCST90094753 | Inverse variance weighted | 8 | 0.024259054704093 | 0.101125659527097 | 0.810415378219438 | -0.173947238 | 0.222465347377202 | 1.02455569948852 | 0.840341234599025 | 1.24915253248913 | 0.998074519377826 |
| ebi-fl187-GCST90094753 | prot-a-1536 | Interleukin-5 receptor subunit alpha \|\| id:prot-a-1536 | F-deep fried food liking (derived food-liking factor) \|\| id:ebi-fl187-GCST90094753 | Inverse variance weighted | 8 | -0.205289311 | 0.101125659527097 | 0.0423522952166884 | -0.403495604 | -0.007083019 | 0.814411663838201 | 0.667980963263685 | 0.992942006842616 | 0.998074519377826 |
| ebi-fl187-GCST90094753 | prot-a-1559 | Insulin-induced gene 1 protein \|\| id:prot-a-1559 | F-deep fried food liking (derived food-liking factor) \|\| id:ebi-fl187-GCST90094753 | Inverse variance weighted | 8 | 0.0854676189564758 | 0.105041350222376 | 0.415841533890775 | -0.120413427 | 0.291348665392332 | 1.0892262904891 | 0.886553835223382 | 1.33823109748738 | 0.998074519377826 |
| ebi-fl187-GCST90094753 | prot-a-1567 | Interferon regulatory factor 1 \|\| id:prot-a-1567 | F-deep fried food liking (derived food-liking factor) \|\| id:ebi-fl187-GCST90094753 | Inverse variance weighted | 8 | 0.111252822798862 | 0.122283216149376 | 0.36292992384369 | -0.128422281 | 0.350927926451639 | 1.11767744547684 | 0.879481912345558 | 1.42038495003955 | 0.998074519377826 |
| ebi-fl187-GCST90094753 | prot-a-1613 | Potassium voltage-gated channel subfamily E regulatory beta subunit 5 \|\| id:prot-a-1613 | F-deep fried food liking (derived food-liking factor) \|\| id:ebi-fl187-GCST90094753 | Inverse variance weighted | 8 | 0.0184932138039486 | 0.101125659527097 | 0.854897194650025 | -0.179713079 | 0.216699506477058 | 1.01866527228387 | 0.835509902469457 | 1.24197084186576 | 0.998074519377826 |
| ebi-fl187-GCST90094753 | prot-a-1649 | Kin of IRRE-like protein 2 \|\| id:prot-a-1649 | F-deep fried food liking (derived food-liking factor) \|\| id:ebi-fl187-GCST90094753 | Inverse variance weighted | 8 | 0.0714955993625556 | 0.101125659527097 | 0.47956796446006 | -0.126710693 | 0.269701892035665 | 1.07411342390716 | 0.880988511601797 | 1.3095739980989 | 0.998074519377826 |
| ebi-fl187-GCST90094753 | prot-a-165 | Armadillo repeat-containing protein 5 \|\| id:prot-a-165 | F-deep fried food liking (derived food-liking factor) \|\| id:ebi-fl187-GCST90094753 | Inverse variance weighted | 8 | -0.185797361 | 0.105462656868284 | 0.0781139091481191 | -0.392504169 | 0.0209094461717576 | 0.830441857599786 | 0.675363530923704 | 1.02112958025815 | 0.998074519377826 |
| ebi-fl187-GCST90094753 | prot-a-1656 | Kallikrein-11 \|\| id:prot-a-1656 | F-deep fried food liking (derived food-liking factor) \|\| id:ebi-fl187-GCST90094753 | Inverse variance weighted | 8 | 0.00753387604369033 | 0.107185963865497 | 0.943964515063471 | -0.202550613 | 0.217618365220065 | 1.00756232709182 | 0.816645148582836 | 1.24311256209185 | 0.998074519377826 |
| ebi-fl187-GCST90094753 | prot-a-1697 | Laminin subunit gamma-2 \|\| id:prot-a-1697 | F-deep fried food liking (derived food-liking factor) \|\| id:ebi-fl187-GCST90094753 | Inverse variance weighted | 8 | 0.0174834460565611 | 0.105498220173179 | 0.868375209670269 | -0.189293065 | 0.224259957595993 | 1.01763717610307 | 0.827543946587169 | 1.25139628711906 | 0.998074519377826 |
| ebi-fl187-GCST90094753 | prot-a-1698 | Lysosome-associated membrane glycoprotein 3 \|\| id:prot-a-1698 | F-deep fried food liking (derived food-liking factor) \|\| id:ebi-fl187-GCST90094753 | Inverse variance weighted | 8 | -0.024172494 | 0.102226849755505 | 0.813076247795672 | -0.22453712 | 0.176192131186401 | 0.976117320527742 | 0.798885921619814 | 1.19266718520006 | 0.998074519377826 |
| ebi-fl187-GCST90094753 | prot-a-1724 | Leptin receptor \|\| id:prot-a-1724 | F-deep fried food liking (derived food-liking factor) \|\| id:ebi-fl187-GCST90094753 | Inverse variance weighted | 8 | -0.050997396 | 0.101125659527097 | 0.61405247916485 | -0.249203689 | 0.147208896280926 | 0.950281144688623 | 0.779421197639592 | 1.15859596414041 | 0.998074519377826 |
| ebi-fl187-GCST90094753 | prot-a-1743 | Leukocyte immunoglobulin-like receptor subfamily B member 2 \|\| id:prot-a-1743 | F-deep fried food liking (derived food-liking factor) \|\| id:ebi-fl187-GCST90094753 | Inverse variance weighted | 8 | -0.083671379 | 0.101125659527097 | 0.408010332310141 | -0.281877672 | 0.114534913364032 | 0.919733449880605 | 0.754365958981567 | 1.12135179054381 | 0.998074519377826 |
| ebi-fl187-GCST90094753 | prot-a-1746 | Leukocyte immunoglobulin-like receptor subfamily B member 4 \|\| id:prot-a-1746 | F-deep fried food liking (derived food-liking factor) \|\| id:ebi-fl187-GCST90094753 | Inverse variance weighted (multiplicative random effects) | 8 | 0.00564536682135249 | 0.176358231201579 | 0.974463443791985 | -0.340016766 | 0.351307499976448 | 1.00566133193346 | 0.711758389083873 | 1.42092419289631 | 0.998074519377826 |
| ebi-fl187-GCST90094753 | prot-a-1750 | Leucine-rich repeat and immunoglobulin-like domain-containing nogo receptor-interacting protein 3 \|\| id:prot-a-1750 | F-deep fried food liking (derived food-liking factor) \|\| id:ebi-fl187-GCST90094753 | Inverse variance weighted | 8 | 0.044159613088696 | 0.117131144420024 | 0.70616617131664 | -0.18541743 | 0.273736656151944 | 1.04514916106161 | 0.830757428411033 | 1.31486849411274 | 0.998074519377826 |
| ebi-fl187-GCST90094753 | prot-a-1753 | Lipase member N \|\| id:prot-a-1753 | F-deep fried food liking (derived food-liking factor) \|\| id:ebi-fl187-GCST90094753 | Inverse variance weighted | 8 | -0.008363242 | 0.116065346962178 | 0.942557084116645 | -0.235851322 | 0.219124837783834 | 0.991671632359177 | 0.78989810543017 | 1.24498668836578 | 0.998074519377826 |
| ebi-fl187-GCST90094753 | prot-a-1760 | Ig Kappa chain V-I region HK102- like \|\| id:prot-a-1760 | F-deep fried food liking (derived food-liking factor) \|\| id:ebi-fl187-GCST90094753 | Inverse variance weighted | 8 | 0.00315875666591957 | 0.101125659527097 | 0.975081365040344 | -0.195047536 | 0.201365049339029 | 1.00316375079479 | 0.822795544711451 | 1.22307117166219 | 0.998074519377826 |
| ebi-fl187-GCST90094753 | prot-a-1792 | Leucine-rich repeat neuronal protein 1 \|\| id:prot-a-1792 | F-deep fried food liking (derived food-liking factor) \|\| id:ebi-fl187-GCST90094753 | Inverse variance weighted | 7 | -0.112129434 | 0.146935124700805 | 0.4453906107864 | -0.400122278 | 0.175863410789609 | 0.893928545600788 | 0.670238085626964 | 1.19227519560072 | 0.998074519377826 |
| ebi-fl187-GCST90094753 | prot-a-1795 | Leucine-rich repeat transmembrane neuronal protein 1 \|\| id:prot-a-1795 | F-deep fried food liking (derived food-liking factor) \|\| id:ebi-fl187-GCST90094753 | Inverse variance weighted | 8 | -0.021929121 | 0.101125659527097 | 0.828325080739022 | -0.220135413 | 0.17627717214631 | 0.978309574661083 | 0.802410133681515 | 1.19276861507513 | 0.998074519377826 |
| ebi-fl187-GCST90094753 | prot-a-1820 | LysM and putative peptidoglycan-binding domain-containing protein 4 \|\| id:prot-a-1820 | F-deep fried food liking (derived food-liking factor) \|\| id:ebi-fl187-GCST90094753 | Inverse variance weighted (multiplicative random effects) | 8 | 0.100027167755175 | 0.144013853734617 | 0.487327145571651 | -0.182239986 | 0.382294321075023 | 1.10520094349644 | 0.833401312130253 | 1.46564339139714 | 0.998074519377826 |
| ebi-fl187-GCST90094753 | prot-a-1844 | Mitogen-activated protein kinase kinase kinase 7:TGF-beta-activated kinase 1 and MAP3K7-binding protein 1 fusion \|\| id:prot-a-1844 | F-deep fried food liking (derived food-liking factor) \|\| id:ebi-fl187-GCST90094753 | Inverse variance weighted | 8 | 0.128552570400379 | 0.133586214276525 | 0.335889319055464 | -0.13327641 | 0.390381550382367 | 1.13718120179094 | 0.875223138609621 | 1.47754444399292 | 0.998074519377826 |
| ebi-fl187-GCST90094753 | prot-a-1856 | Megakaryocyte-associated tyrosine-protein kinase \|\| id:prot-a-1856 | F-deep fried food liking (derived food-liking factor) \|\| id:ebi-fl187-GCST90094753 | Inverse variance weighted | 8 | 0.093958753929713 | 0.124760635512897 | 0.451382507073302 | -0.150572092 | 0.33848959953499 | 1.09851443557912 | 0.860215713379874 | 1.40282715882315 | 0.998074519377826 |
| ebi-fl187-GCST90094753 | prot-a-1910 | Stromelysin-2 \|\| id:prot-a-1910 | F-deep fried food liking (derived food-liking factor) \|\| id:ebi-fl187-GCST90094753 | Inverse variance weighted | 8 | 0.0807107885703482 | 0.101206557660081 | 0.425168969695097 | -0.117654064 | 0.279075641584108 | 1.08405732945527 | 0.889003537360104 | 1.32190733125247 | 0.998074519377826 |
| ebi-fl187-GCST90094753 | prot-a-1940 | 39S ribosomal protein L14, mitochondrial \|\| id:prot-a-1940 | F-deep fried food liking (derived food-liking factor) \|\| id:ebi-fl187-GCST90094753 | Inverse variance weighted | 8 | 0.0828540298241147 | 0.101125659527097 | 0.412605249632467 | -0.115352263 | 0.281060322497224 | 1.08638321742441 | 0.891052204027406 | 1.32453350069387 | 0.998074519377826 |
| ebi-fl187-GCST90094753 | prot-a-1942 | 39S ribosomal protein L33, mitochondrial \|\| id:prot-a-1942 | F-deep fried food liking (derived food-liking factor) \|\| id:ebi-fl187-GCST90094753 | Inverse variance weighted | 8 | -0.071525154 | 0.101206557660081 | 0.479737697779863 | -0.269890007 | 0.126839698599513 | 0.930972859223372 | 0.763463465028834 | 1.13523502343076 | 0.998074519377826 |
| ebi-fl187-GCST90094753 | prot-a-1948 | Mesothelin \|\| id:prot-a-1948 | F-deep fried food liking (derived food-liking factor) \|\| id:ebi-fl187-GCST90094753 | Inverse variance weighted | 8 | -0.05526369 | 0.101125659527097 | 0.584732363594969 | -0.253469983 | 0.142942602450717 | 0.946235601977192 | 0.776103040941577 | 1.15366358230329 | 0.998074519377826 |
| ebi-fl187-GCST90094753 | prot-a-1980 | Unconventional myosin-VI \|\| id:prot-a-1980 | F-deep fried food liking (derived food-liking factor) \|\| id:ebi-fl187-GCST90094753 | Inverse variance weighted | 8 | 0.0203816688942171 | 0.105652559736747 | 0.84702772168807 | -0.186697348 | 0.227460685978242 | 1.02059079346049 | 0.829694807025249 | 1.25540808364323 | 0.998074519377826 |
| ebi-fl187-GCST90094753 | prot-a-2039 | Neurofascin \|\| id:prot-a-2039 | F-deep fried food liking (derived food-liking factor) \|\| id:ebi-fl187-GCST90094753 | Inverse variance weighted | 8 | -0.066734765 | 0.101125659527097 | 0.509305675394594 | -0.264941058 | 0.13147152724789 | 0.93544328033645 | 0.767251171886241 | 1.14050543393156 | 0.998074519377826 |
| ebi-fl187-GCST90094753 | prot-a-2041 | NFU1 iron-sulfur cluster scaffold homolog, mitochondrial \|\| id:prot-a-2041 | F-deep fried food liking (derived food-liking factor) \|\| id:ebi-fl187-GCST90094753 | Inverse variance weighted | 8 | 0.0831880569995144 | 0.106876988684172 | 0.436360760759208 | -0.126290841 | 0.292666954820493 | 1.08674615955482 | 0.881358474480736 | 1.33999643675402 | 0.998074519377826 |
| ebi-fl187-GCST90094753 | prot-a-2088 | Glucocorticoid receptor \|\| id:prot-a-2088 | F-deep fried food liking (derived food-liking factor) \|\| id:ebi-fl187-GCST90094753 | Inverse variance weighted (multiplicative random effects) | 8 | -0.023951965 | 0.145695298646411 | 0.869418111231222 | -0.30951475 | 0.261610820225648 | 0.976332606649871 | 0.733802947381622 | 1.2990208913838 | 0.998074519377826 |
| ebi-fl187-GCST90094753 | prot-a-2111 | Neuron-specific protein family member 2 \|\| id:prot-a-2111 | F-deep fried food liking (derived food-liking factor) \|\| id:ebi-fl187-GCST90094753 | Inverse variance weighted | 8 | 0.0850114742363821 | 0.101125659527097 | 0.400542994203972 | -0.113194818 | 0.283217766909491 | 1.08872955896704 | 0.892976674848969 | 1.32739419287637 | 0.998074519377826 |
| ebi-fl187-GCST90094753 | prot-a-2125 | Nuclear migration protein nudC \|\| id:prot-a-2125 | F-deep fried food liking (derived food-liking factor) \|\| id:ebi-fl187-GCST90094753 | Inverse variance weighted | 8 | 0.0846825326495567 | 0.115494612629601 | 0.4634265146955 | -0.141686908 | 0.311051973403575 | 1.0883714894335 | 0.867892944199108 | 1.36486015577049 | 0.998074519377826 |
| ebi-fl187-GCST90094753 | prot-a-2142 | Ornithine decarboxylase \|\| id:prot-a-2142 | F-deep fried food liking (derived food-liking factor) \|\| id:ebi-fl187-GCST90094753 | Inverse variance weighted | 8 | -0.059942893 | 0.101125659527097 | 0.553344113814908 | -0.258149186 | 0.138263399220534 | 0.941818316040899 | 0.772479980214733 | 1.14827796596559 | 0.998074519377826 |
| ebi-fl187-GCST90094753 | prot-a-2186 | Prostate and testis expressed protein 1 \|\| id:prot-a-2186 | F-deep fried food liking (derived food-liking factor) \|\| id:ebi-fl187-GCST90094753 | Inverse variance weighted (multiplicative random effects) | 8 | 0.0162950866803405 | 0.149945476810655 | 0.913461530544756 | -0.277598048 | 0.310188221229225 | 1.01642857569131 | 0.757601279770612 | 1.36368176383582 | 0.998074519377826 |
| ebi-fl187-GCST90094753 | prot-a-2227 | Platelet-derived growth factor C \|\| id:prot-a-2227 | F-deep fried food liking (derived food-liking factor) \|\| id:ebi-fl187-GCST90094753 | Inverse variance weighted | 8 | -0.00808478 | 0.129305401371976 | 0.950145004259736 | -0.261523366 | 0.245353807143094 | 0.991947814386772 | 0.769877886066522 | 1.27807342472709 | 0.998074519377826 |
| ebi-fl187-GCST90094753 | prot-a-2229 | Platelet-derived growth factor receptor alpha \|\| id:prot-a-2229 | F-deep fried food liking (derived food-liking factor) \|\| id:ebi-fl187-GCST90094753 | Inverse variance weighted | 8 | -0.036552758 | 0.101206557660081 | 0.717972470401787 | -0.234917611 | 0.161812094558325 | 0.964107227746317 | 0.790635986282754 | 1.17563931154061 | 0.998074519377826 |
| ebi-fl187-GCST90094753 | prot-a-2243 | Platelet endothelial aggregation receptor 1 \|\| id:prot-a-2243 | F-deep fried food liking (derived food-liking factor) \|\| id:ebi-fl187-GCST90094753 | Inverse variance weighted | 8 | -0.092654033 | 0.101125659527097 | 0.359547984889555 | -0.290860326 | 0.105552259629295 | 0.911508797676027 | 0.747620094026453 | 1.11132418039502 | 0.998074519377826 |
| ebi-fl187-GCST90094753 | prot-a-2246 | E3 ubiquitin-protein ligase pellino homolog 2 \|\| id:prot-a-2246 | F-deep fried food liking (derived food-liking factor) \|\| id:ebi-fl187-GCST90094753 | Inverse variance weighted (multiplicative random effects) | 8 | 0.105368482649069 | 0.179158666930351 | 0.556445428100858 | -0.245782505 | 0.456519469832556 | 1.11111996335887 | 0.78209230795931 | 1.57857015138786 | 0.998074519377826 |
| ebi-fl187-GCST90094753 | prot-a-2251 | Platelet factor 4 variant \|\| id:prot-a-2251 | F-deep fried food liking (derived food-liking factor) \|\| id:ebi-fl187-GCST90094753 | Inverse variance weighted | 8 | 0.0622320957675637 | 0.101125659527097 | 0.538294737972524 | -0.135974197 | 0.260438388440673 | 1.06420931454431 | 0.872865154820181 | 1.29749877046722 | 0.998074519377826 |
| ebi-fl187-GCST90094753 | prot-a-2346 | Protein phosphatase 1L \|\| id:prot-a-2346 | F-deep fried food liking (derived food-liking factor) \|\| id:ebi-fl187-GCST90094753 | Inverse variance weighted | 8 | 0.0922477330705725 | 0.130515382831349 | 0.479693343809768 | -0.163562417 | 0.348057883420017 | 1.09663646155031 | 0.849113497993513 | 1.41631422847876 | 0.998074519377826 |
| ebi-fl187-GCST90094753 | prot-a-2358 | PR domain zinc finger protein 4 \|\| id:prot-a-2358 | F-deep fried food liking (derived food-liking factor) \|\| id:ebi-fl187-GCST90094753 | Inverse variance weighted | 8 | 0.0914577001188104 | 0.101206557660081 | 0.366168471318946 | -0.106907153 | 0.28982255313257 | 1.09577042475343 | 0.898609102370885 | 1.33619036419324 | 0.998074519377826 |
| ebi-fl187-GCST90094753 | prot-a-2390 | Trypsin-1 \|\| id:prot-a-2390 | F-deep fried food liking (derived food-liking factor) \|\| id:ebi-fl187-GCST90094753 | Inverse variance weighted | 8 | 0.123560064096724 | 0.116690558538676 | 0.289659252550843 | -0.105153431 | 0.352273558832529 | 1.1315179661056 | 0.900186395816096 | 1.422297553 | 0.998074519377826 |
| ebi-fl187-GCST90094753 | prot-a-242 | Brain-derived neurotrophic factor \|\| id:prot-a-242 | F-deep fried food liking (derived food-liking factor) \|\| id:ebi-fl187-GCST90094753 | Inverse variance weighted | 8 | 0.0757638743219752 | 0.101125659527097 | 0.453733509538996 | -0.122442418 | 0.273970166995084 | 1.07870783346449 | 0.884756849234966 | 1.31517556601433 | 0.998074519377826 |
| ebi-fl187-GCST90094753 | prot-a-2464 | Dihydropteridine reductase \|\| id:prot-a-2464 | F-deep fried food liking (derived food-liking factor) \|\| id:ebi-fl187-GCST90094753 | Inverse variance weighted | 8 | 0.0727474895957803 | 0.1058404107557 | 0.491873607087511 | -0.134699715 | 0.280194694676952 | 1.07545893805421 | 0.873978314442252 | 1.32338744374773 | 0.998074519377826 |
| ebi-fl187-GCST90094753 | prot-a-2474 | Ras-related protein Rab-27A \|\| id:prot-a-2474 | F-deep fried food liking (derived food-liking factor) \|\| id:ebi-fl187-GCST90094753 | Inverse variance weighted (multiplicative random effects) | 8 | 0.0560401340692432 | 0.191250675834795 | 0.769507419914281 | -0.318811191 | 0.430891458705442 | 1.05764013028705 | 0.727012803223706 | 1.53862853615992 | 0.998074519377826 |
| ebi-fl187-GCST90094753 | prot-a-2478 | Ras-related protein Rab-7b \|\| id:prot-a-2478 | F-deep fried food liking (derived food-liking factor) \|\| id:ebi-fl187-GCST90094753 | Inverse variance weighted | 8 | -0.049038404 | 0.136344680160578 | 0.719097696433709 | -0.316273977 | 0.218197168767542 | 0.952144562505986 | 0.728859731670309 | 1.24383228832265 | 0.998074519377826 |
| ebi-fl187-GCST90094753 | prot-a-2528 | E3 ubiquitin-protein ligase rififylin \|\| id:prot-a-2528 | F-deep fried food liking (derived food-liking factor) \|\| id:ebi-fl187-GCST90094753 | Inverse variance weighted | 8 | 0.0924061385201781 | 0.101125659527097 | 0.360834882779814 | -0.105800154 | 0.290612431193287 | 1.09681018850134 | 0.899604412318564 | 1.33724620858612 | 0.998074519377826 |
| ebi-fl187-GCST90094753 | prot-a-2543 | Protein RIC-3 \|\| id:prot-a-2543 | F-deep fried food liking (derived food-liking factor) \|\| id:ebi-fl187-GCST90094753 | Inverse variance weighted | 8 | 0.127467504055698 | 0.101125659527097 | 0.207494002523083 | -0.070738789 | 0.325673796728807 | 1.13594795393994 | 0.931705232356498 | 1.38496351554952 | 0.998074519377826 |
| ebi-fl187-GCST90094753 | prot-a-255 | Bone morphogenetic protein 6 \|\| id:prot-a-255 | F-deep fried food liking (derived food-liking factor) \|\| id:ebi-fl187-GCST90094753 | Inverse variance weighted (multiplicative random effects) | 8 | -0.10150626 | 0.159911367074435 | 0.525581200384565 | -0.414932539 | 0.211920019667418 | 0.903475523749814 | 0.66038482924871 | 1.23604902151316 | 0.998074519377826 |
| ebi-fl187-GCST90094753 | prot-a-2568 | RING finger protein 165 \|\| id:prot-a-2568 | F-deep fried food liking (derived food-liking factor) \|\| id:ebi-fl187-GCST90094753 | Inverse variance weighted | 8 | -0.185772397 | 0.117653115603474 | 0.114339708066321 | -0.416372503 | 0.0448277097612912 | 0.830462589398209 | 0.659434583100401 | 1.04584765504325 | 0.998074519377826 |
| ebi-fl187-GCST90094753 | prot-a-2573 | E3 ubiquitin-protein ligase RNF8 \|\| id:prot-a-2573 | F-deep fried food liking (derived food-liking factor) \|\| id:ebi-fl187-GCST90094753 | Inverse variance weighted | 8 | -0.092833436 | 0.101125659527097 | 0.358618447387852 | -0.291039729 | 0.105372856395329 | 0.911345284717751 | 0.747485980594358 | 1.11112482312628 | 0.998074519377826 |
| ebi-fl187-GCST90094753 | prot-a-260 | Cytoplasmic tyrosine-protein kinase BMX \|\| id:prot-a-260 | F-deep fried food liking (derived food-liking factor) \|\| id:ebi-fl187-GCST90094753 | Inverse variance weighted | 7 | -0.033056303 | 0.106795492646334 | 0.756919148012933 | -0.242375469 | 0.176262862461486 | 0.967484085677792 | 0.784761468850295 | 1.1927515470543 | 0.998074519377826 |
| ebi-fl187-GCST90094753 | prot-a-2623 | Serum amyloid A-1 protein \|\| id:prot-a-2623 | F-deep fried food liking (derived food-liking factor) \|\| id:ebi-fl187-GCST90094753 | Inverse variance weighted | 8 | -0.043562679 | 0.101125659527097 | 0.666630019223882 | -0.241768971 | 0.154643614141803 | 0.957372545505297 | 0.785237569087523 | 1.1672418984644 | 0.998074519377826 |
| ebi-fl187-GCST90094753 | prot-a-2640 | Lysosome membrane protein 2 \|\| id:prot-a-2640 | F-deep fried food liking (derived food-liking factor) \|\| id:ebi-fl187-GCST90094753 | Inverse variance weighted | 8 | 0.106798494139651 | 0.113863800543189 | 0.34827036593929 | -0.116374555 | 0.329971543204302 | 1.11271001429848 | 0.890141753872202 | 1.39092854653112 | 0.998074519377826 |
| ebi-fl187-GCST90094753 | prot-a-2641 | Scavenger receptor class F member 2 \|\| id:prot-a-2641 | F-deep fried food liking (derived food-liking factor) \|\| id:ebi-fl187-GCST90094753 | Inverse variance weighted | 8 | 0.154345477895133 | 0.11313090164084 | 0.172470416248222 | -0.067391089 | 0.376082045111179 | 1.16689395321596 | 0.934829527990695 | 1.4565666330402 | 0.998074519377826 |
| ebi-fl187-GCST90094753 | prot-a-2669 | Semaphorin-3A \|\| id:prot-a-2669 | F-deep fried food liking (derived food-liking factor) \|\| id:ebi-fl187-GCST90094753 | Inverse variance weighted | 8 | 0.0500312537775739 | 0.101125659527097 | 0.620781253867743 | -0.148175039 | 0.248237546450683 | 1.05130395308249 | 0.862280169163287 | 1.28176437461078 | 0.998074519377826 |
| ebi-fl187-GCST90094753 | prot-a-2683 | Sentrin-specific protease 7 \|\| id:prot-a-2683 | F-deep fried food liking (derived food-liking factor) \|\| id:ebi-fl187-GCST90094753 | Inverse variance weighted | 8 | -0.031518999 | 0.101554348968243 | 0.756282779961581 | -0.230565523 | 0.167527524736408 | 0.968972546535582 | 0.794084402331295 | 1.18237783437526 | 0.998074519377826 |
| ebi-fl187-GCST90094753 | prot-a-270 | Bromodomain testis-specific protein \|\| id:prot-a-270 | F-deep fried food liking (derived food-liking factor) \|\| id:ebi-fl187-GCST90094753 | Inverse variance weighted | 8 | 0.00629242444272569 | 0.101092374827652 | 0.950368281913976 | -0.19184863 | 0.204433479104923 | 1.00631226333516 | 0.825431804466761 | 1.22682984331204 | 0.998074519377826 |
| ebi-fl187-GCST90094753 | prot-a-2708 | 14-3-3 protein sigma \|\| id:prot-a-2708 | F-deep fried food liking (derived food-liking factor) \|\| id:ebi-fl187-GCST90094753 | Inverse variance weighted | 8 | 0.124266761365537 | 0.101206557660081 | 0.219502207867832 | -0.074098092 | 0.322631614379296 | 1.13231788938036 | 0.928580603371861 | 1.38075660632483 | 0.998074519377826 |
| ebi-fl187-GCST90094753 | prot-a-2713 | Beta-sarcoglycan \|\| id:prot-a-2713 | F-deep fried food liking (derived food-liking factor) \|\| id:ebi-fl187-GCST90094753 | Inverse variance weighted | 8 | 0.0586451933611556 | 0.139492437374757 | 0.674179797192157 | -0.214759984 | 0.33205037061568 | 1.06039893740383 | 0.806735046347005 | 1.39382305446975 | 0.998074519377826 |
| ebi-fl187-GCST90094753 | prot-a-2720 | Protein shisa-3 homolog \|\| id:prot-a-2720 | F-deep fried food liking (derived food-liking factor) \|\| id:ebi-fl187-GCST90094753 | Inverse variance weighted | 8 | -0.065607833 | 0.109984915180028 | 0.550830137885519 | -0.281178266 | 0.149962601192273 | 0.936498056331303 | 0.754893751358566 | 1.16179079232533 | 0.998074519377826 |
| ebi-fl187-GCST90094753 | prot-a-2730 | Sialic acid-binding Ig-like lectin 8 \|\| id:prot-a-2730 | F-deep fried food liking (derived food-liking factor) \|\| id:ebi-fl187-GCST90094753 | Inverse variance weighted | 8 | -0.030871275 | 0.101677625102518 | 0.761418054447493 | -0.23015942 | 0.168416870308257 | 0.96960037695574 | 0.79440694797795 | 1.18342984459749 | 0.998074519377826 |
| ebi-fl187-GCST90094753 | prot-a-2761 | Sodium- and chloride-dependent neutral and basic amino acid transporter B(0+) \|\| id:prot-a-2761 | F-deep fried food liking (derived food-liking factor) \|\| id:ebi-fl187-GCST90094753 | Inverse variance weighted | 8 | 0.105553538987684 | 0.112774724964708 | 0.34928964002875 | -0.115484922 | 0.326591999918511 | 1.11132560217784 | 0.890934005689397 | 1.38623577747521 | 0.998074519377826 |
| ebi-fl187-GCST90094753 | prot-a-2770 | Secreted Ly-6/uPAR-related protein 1 \|\| id:prot-a-2770 | F-deep fried food liking (derived food-liking factor) \|\| id:ebi-fl187-GCST90094753 | Inverse variance weighted | 8 | 0.0296981941588072 | 0.140904080824789 | 0.833067629525426 | -0.246473804 | 0.305870192575393 | 1.03014358368175 | 0.781551834599242 | 1.3578060418025 | 0.998074519377826 |
| ebi-fl187-GCST90094753 | prot-a-2784 | N-lysine methyltransferase SMYD2 \|\| id:prot-a-2784 | F-deep fried food liking (derived food-liking factor) \|\| id:ebi-fl187-GCST90094753 | Inverse variance weighted | 8 | -0.051102584 | 0.114547129842413 | 0.655505403034439 | -0.275614959 | 0.173409790064789 | 0.950181191740136 | 0.759105161170286 | 1.18935339043779 | 0.998074519377826 |
| ebi-fl187-GCST90094753 | prot-a-2837 | Serine palmitoyltransferase 2 \|\| id:prot-a-2837 | F-deep fried food liking (derived food-liking factor) \|\| id:ebi-fl187-GCST90094753 | Inverse variance weighted | 8 | -0.002975937 | 0.101206557660081 | 0.976541918341061 | -0.20134079 | 0.19538891647264 | 0.997028487168714 | 0.817633743030146 | 1.21578373287523 | 0.998074519377826 |
| ebi-fl187-GCST90094753 | prot-a-2858 | Alpha-2,8-sialyltransferase 8B \|\| id:prot-a-2858 | F-deep fried food liking (derived food-liking factor) \|\| id:ebi-fl187-GCST90094753 | Inverse variance weighted | 8 | 0.143074302938429 | 0.101125659527097 | 0.157122156351573 | -0.05513199 | 0.341280595611538 | 1.15381553036533 | 0.94636022987406 | 1.40674791277884 | 0.998074519377826 |
| ebi-fl187-GCST90094753 | prot-a-2911 | Synaptotagmin-11 \|\| id:prot-a-2911 | F-deep fried food liking (derived food-liking factor) \|\| id:ebi-fl187-GCST90094753 | Inverse variance weighted | 8 | 0.104811426139117 | 0.101206557660081 | 0.300379960811535 | -0.093553427 | 0.303176279152876 | 1.11050117911484 | 0.910689360840106 | 1.35415315237438 | 0.998074519377826 |
| ebi-fl187-GCST90094753 | prot-a-2962 | Transforming growth factor beta-1 \|\| id:prot-a-2962 | F-deep fried food liking (derived food-liking factor) \|\| id:ebi-fl187-GCST90094753 | Inverse variance weighted | 8 | -0.045046291 | 0.101206557660081 | 0.65625282908489 | -0.243411144 | 0.153318562110809 | 0.95595322887071 | 0.783949131586898 | 1.16569626646374 | 0.998074519377826 |
| ebi-fl187-GCST90094753 | prot-a-2978 | Tyrosine-protein kinase receptor Tie-1, soluble \|\| id:prot-a-2978 | F-deep fried food liking (derived food-liking factor) \|\| id:ebi-fl187-GCST90094753 | Inverse variance weighted | 8 | -0.022639825 | 0.101125659527097 | 0.822852120204142 | -0.220846118 | 0.17556646738815 | 0.977614532405592 | 0.801840059582694 | 1.19192120990812 | 0.998074519377826 |
| ebi-fl187-GCST90094753 | prot-a-299 | Uncharacterized protein C1orf226 \|\| id:prot-a-299 | F-deep fried food liking (derived food-liking factor) \|\| id:ebi-fl187-GCST90094753 | Inverse variance weighted | 8 | -0.047857848 | 0.101206557660081 | 0.636303796109804 | -0.246222701 | 0.150507005499163 | 0.953269287044861 | 0.781748109821336 | 1.16242344843111 | 0.998074519377826 |
| ebi-fl187-GCST90094753 | prot-a-3015 | Transmembrane protein 70, mitochondrial \|\| id:prot-a-3015 | F-deep fried food liking (derived food-liking factor) \|\| id:ebi-fl187-GCST90094753 | Inverse variance weighted | 8 | 0.0255250867897178 | 0.101206557660081 | 0.800880422379818 | -0.172839766 | 0.223889939803477 | 1.02585364132222 | 0.841272404299374 | 1.25093333388309 | 0.998074519377826 |
| ebi-fl187-GCST90094753 | prot-a-3016 | Transmembrane protein 87B \|\| id:prot-a-3016 | F-deep fried food liking (derived food-liking factor) \|\| id:ebi-fl187-GCST90094753 | Inverse variance weighted | 8 | 0.039957772752174 | 0.126437178127641 | 0.751980749900759 | -0.207859096 | 0.28777464188235 | 1.04076682454583 | 0.812321487688313 | 1.33345676495367 | 0.998074519377826 |
| ebi-fl187-GCST90094753 | prot-a-3123 | Thioredoxin domain-containing protein 12 \|\| id:prot-a-3123 | F-deep fried food liking (derived food-liking factor) \|\| id:ebi-fl187-GCST90094753 | Inverse variance weighted (multiplicative random effects) | 8 | 0.169016859428034 | 0.196610055040028 | 0.389979127937523 | -0.216338848 | 0.55437256730649 | 1.18414010268052 | 0.805462325966334 | 1.74084837685487 | 0.998074519377826 |
| ebi-fl187-GCST90094753 | prot-a-3133 | PolyUbiquitin K48-linked \|\| id:prot-a-3133 | F-deep fried food liking (derived food-liking factor) \|\| id:ebi-fl187-GCST90094753 | Inverse variance weighted | 8 | -0.070357645 | 0.121419293887592 | 0.562278715061707 | -0.308339461 | 0.167624171448043 | 0.932060413941601 | 0.734665885559287 | 1.18249211282709 | 0.998074519377826 |
| ebi-fl187-GCST90094753 | prot-a-3135 | Ubiquitin-conjugating enzyme E2 B \|\| id:prot-a-3135 | F-deep fried food liking (derived food-liking factor) \|\| id:ebi-fl187-GCST90094753 | Inverse variance weighted | 8 | 0.00602310115612107 | 0.101206557660081 | 0.952543548393795 | -0.192341752 | 0.204387954169881 | 1.00604127650222 | 0.825024866526346 | 1.22677399323441 | 0.998074519377826 |
| ebi-fl187-GCST90094753 | prot-a-3162 | NKG2D ligand 3 \|\| id:prot-a-3162 | F-deep fried food liking (derived food-liking factor) \|\| id:ebi-fl187-GCST90094753 | Inverse variance weighted | 8 | 0.11333104116501 | 0.101206557660081 | 0.262799260837061 | -0.085033812 | 0.31169589417877 | 1.12000263856408 | 0.918481228328058 | 1.36573930059946 | 0.998074519377826 |
| ebi-fl187-GCST90094753 | prot-a-3188 | Vesicle-associated membrane protein 8 \|\| id:prot-a-3188 | F-deep fried food liking (derived food-liking factor) \|\| id:ebi-fl187-GCST90094753 | Inverse variance weighted | 8 | 0.0561894960880995 | 0.108565221770757 | 0.604762211317069 | -0.156598339 | 0.268977330758784 | 1.05779811335018 | 0.855047429416164 | 1.30862547516366 | 0.998074519377826 |
| ebi-fl187-GCST90094753 | prot-a-3201 | Vitrin \|\| id:prot-a-3201 | F-deep fried food liking (derived food-liking factor) \|\| id:ebi-fl187-GCST90094753 | Inverse variance weighted | 8 | 0.0422815538407142 | 0.101125659527097 | 0.67586738553808 | -0.155924739 | 0.240487846513823 | 1.04318815103706 | 0.85562358317775 | 1.27186947608717 | 0.998074519377826 |
| ebi-fl187-GCST90094753 | prot-a-3232 | WNT1-inducible-signaling pathway protein 3 \|\| id:prot-a-3232 | F-deep fried food liking (derived food-liking factor) \|\| id:ebi-fl187-GCST90094753 | Inverse variance weighted | 8 | 0.167840005838605 | 0.101125659527097 | 0.0969713389094084 | -0.030366287 | 0.366046298511715 | 1.18274736283648 | 0.970090137218438 | 1.44202200458169 | 0.998074519377826 |
| ebi-fl187-GCST90094753 | prot-a-327 | Carbonic anhydrase-related protein 10 \|\| id:prot-a-327 | F-deep fried food liking (derived food-liking factor) \|\| id:ebi-fl187-GCST90094753 | Inverse variance weighted | 8 | -0.22180411 | 0.138781493610469 | 0.109992819175095 | -0.493815838 | 0.0502076170472516 | 0.801072270669227 | 0.610293165609524 | 1.05148938083592 | 0.998074519377826 |
| ebi-fl187-GCST90094753 | prot-a-332 | Carbonic anhydrase 5A, mitochondrial \|\| id:prot-a-332 | F-deep fried food liking (derived food-liking factor) \|\| id:ebi-fl187-GCST90094753 | Inverse variance weighted | 8 | -0.011131773 | 0.12643833187359 | 0.929843991807089 | -0.258950903 | 0.236687357777407 | 0.98892995622375 | 0.771860918039974 | 1.26704492410388 | 0.998074519377826 |
| ebi-fl187-GCST90094753 | prot-a-338 | Cell adhesion molecule 3 \|\| id:prot-a-338 | F-deep fried food liking (derived food-liking factor) \|\| id:ebi-fl187-GCST90094753 | Inverse variance weighted | 8 | 0.0440037518366081 | 0.101125659527097 | 0.663461296261294 | -0.154202541 | 0.242210044509717 | 1.04498627549885 | 0.857098406001871 | 1.27406177439394 | 0.998074519377826 |
| ebi-fl187-GCST90094753 | prot-a-34 | A disintegrin and metalloproteinase with thrombospondin motifs 3 \|\| id:prot-a-34 | F-deep fried food liking (derived food-liking factor) \|\| id:ebi-fl187-GCST90094753 | Inverse variance weighted | 8 | 0.0904487788742686 | 0.101125659527097 | 0.37109739283824 | -0.107757514 | 0.288655071547378 | 1.0946654362097 | 0.897845285128535 | 1.33463129681704 | 0.998074519377826 |
| ebi-fl187-GCST90094753 | prot-a-358 | Cysteine--tRNA ligase, cytoplasmic \|\| id:prot-a-358 | F-deep fried food liking (derived food-liking factor) \|\| id:ebi-fl187-GCST90094753 | Inverse variance weighted | 8 | -0.047110315 | 0.101125659527097 | 0.64131631549824 | -0.245316607 | 0.15109597785209 | 0.953982153414972 | 0.782456767344504 | 1.16310828535984 | 0.998074519377826 |
| ebi-fl187-GCST90094753 | prot-a-382 | Coiled-coil domain-containing protein 167 \|\| id:prot-a-382 | F-deep fried food liking (derived food-liking factor) \|\| id:ebi-fl187-GCST90094753 | Inverse variance weighted | 8 | -0.066775712 | 0.103928951878711 | 0.520540091211419 | -0.270476458 | 0.136925033407903 | 0.935404977665753 | 0.763015862737302 | 1.14674217794488 | 0.998074519377826 |
| ebi-fl187-GCST90094753 | prot-a-40 | Pituitary adenylate cyclase-activating polypeptide 27 \|\| id:prot-a-40 | F-deep fried food liking (derived food-liking factor) \|\| id:ebi-fl187-GCST90094753 | Inverse variance weighted | 8 | 0.0663558671720541 | 0.122139664863362 | 0.586937990354719 | -0.173037876 | 0.305749610304244 | 1.06860693163586 | 0.84110575655319 | 1.35764232433711 | 0.998074519377826 |
| ebi-fl187-GCST90094753 | prot-a-403 | C-C motif chemokine 25 \|\| id:prot-a-403 | F-deep fried food liking (derived food-liking factor) \|\| id:ebi-fl187-GCST90094753 | Inverse variance weighted | 8 | -0.126465706 | 0.101206557660081 | 0.211452955852418 | -0.324830559 | 0.0718991469120593 | 0.881204368958352 | 0.722649789688521 | 1.07454696721905 | 0.998074519377826 |
| ebi-fl187-GCST90094753 | prot-a-415 | Cyclin-C \|\| id:prot-a-415 | F-deep fried food liking (derived food-liking factor) \|\| id:ebi-fl187-GCST90094753 | Inverse variance weighted | 8 | -0.063271045 | 0.101125659527097 | 0.531533013819973 | -0.261477338 | 0.134935247803714 | 0.938689012364713 | 0.769913323354599 | 1.14446267548018 | 0.998074519377826 |
| ebi-fl187-GCST90094753 | prot-a-44 | Adhesion G protein-coupled receptor F5 \|\| id:prot-a-44 | F-deep fried food liking (derived food-liking factor) \|\| id:ebi-fl187-GCST90094753 | Inverse variance weighted | 8 | 0.0369320094224672 | 0.101125659527097 | 0.714955338353659 | -0.161274283 | 0.235138302095576 | 1.0376224698888 | 0.85105860796962 | 1.26508372036413 | 0.998074519377826 |
| ebi-fl187-GCST90094753 | prot-a-455 | CD59 glycoprotein \|\| id:prot-a-455 | F-deep fried food liking (derived food-liking factor) \|\| id:ebi-fl187-GCST90094753 | Inverse variance weighted | 8 | 0.0351242157059745 | 0.101206557660081 | 0.728550193755218 | -0.163240637 | 0.233489068719734 | 1.03574835702277 | 0.849386769674666 | 1.26299902161917 | 0.998074519377826 |
| ebi-fl187-GCST90094753 | prot-a-478 | Cadherin-12 \|\| id:prot-a-478 | F-deep fried food liking (derived food-liking factor) \|\| id:ebi-fl187-GCST90094753 | Inverse variance weighted | 8 | 0.100057883979217 | 0.106364238920159 | 0.346853585577114 | -0.108416024 | 0.308531792262728 | 1.1052348916176 | 0.89725423920234 | 1.36142479163423 | 0.998074519377826 |
| ebi-fl187-GCST90094753 | prot-a-516 | Centrin-2 \|\| id:prot-a-516 | F-deep fried food liking (derived food-liking factor) \|\| id:ebi-fl187-GCST90094753 | Inverse variance weighted | 8 | -0.006215361 | 0.113027862197379 | 0.956146721386815 | -0.227749971 | 0.215319249022021 | 0.993803914515425 | 0.796323339040617 | 1.24025778485416 | 0.998074519377826 |
| ebi-fl187-GCST90094753 | prot-a-519 | Cryptic protein \|\| id:prot-a-519 | F-deep fried food liking (derived food-liking factor) \|\| id:ebi-fl187-GCST90094753 | Inverse variance weighted | 8 | 0.0202952484746418 | 0.101206557660081 | 0.841063799387058 | -0.178069605 | 0.218660101488401 | 1.02050259738693 | 0.836884170524472 | 1.24440823228955 | 0.998074519377826 |
| ebi-fl187-GCST90094753 | prot-a-583 | Chloride intracellular channel protein 4 \|\| id:prot-a-583 | F-deep fried food liking (derived food-liking factor) \|\| id:ebi-fl187-GCST90094753 | Inverse variance weighted | 8 | 0.189427137749383 | 0.101125659527097 | 0.0610427484546713 | -0.008779155 | 0.387633430422492 | 1.20855706259372 | 0.991259269330486 | 1.47348954883587 | 0.998074519377826 |
| ebi-fl187-GCST90094753 | prot-a-602 | CCR4-NOT transcription complex subunit 1 \|\| id:prot-a-602 | F-deep fried food liking (derived food-liking factor) \|\| id:ebi-fl187-GCST90094753 | Inverse variance weighted | 8 | 0.191883683194582 | 0.101125659527097 | 0.0577649200932491 | -0.006322609 | 0.390089975867691 | 1.21152958751754 | 0.993697336158485 | 1.47711369248988 | 0.998074519377826 |
| ebi-fl187-GCST90094753 | prot-a-606 | Ciliary neurotrophic factor receptor subunit alpha \|\| id:prot-a-606 | F-deep fried food liking (derived food-liking factor) \|\| id:ebi-fl187-GCST90094753 | Inverse variance weighted | 8 | -0.079369911 | 0.101125659527097 | 0.43253318851273 | -0.277576203 | 0.118836381869945 | 0.923698175306725 | 0.757617829290908 | 1.12618563882471 | 0.998074519377826 |
| ebi-fl187-GCST90094753 | prot-a-626 | Collagen alpha-2(IX) chain \|\| id:prot-a-626 | F-deep fried food liking (derived food-liking factor) \|\| id:ebi-fl187-GCST90094753 | Inverse variance weighted | 8 | -0.094857201 | 0.101206557660081 | 0.348623091773929 | -0.293222054 | 0.103507652085812 | 0.909502801351638 | 0.74585650193133 | 1.10905427991112 | 0.998074519377826 |
| ebi-fl187-GCST90094753 | prot-a-647 | Probable serine carboxypeptidase CPVL \|\| id:prot-a-647 | F-deep fried food liking (derived food-liking factor) \|\| id:ebi-fl187-GCST90094753 | Inverse variance weighted | 8 | -0.089121322 | 0.12964665396057 | 0.491819950067377 | -0.343228763 | 0.164986120059789 | 0.914734589684063 | 0.709475890830812 | 1.17937674891902 | 0.998074519377826 |
| ebi-fl187-GCST90094753 | prot-a-651 | Cellular retinoic acid-binding protein 2 \|\| id:prot-a-651 | F-deep fried food liking (derived food-liking factor) \|\| id:ebi-fl187-GCST90094753 | Inverse variance weighted | 8 | 0.0457476050294294 | 0.101125659527097 | 0.650992551800076 | -0.152458688 | 0.243953897702539 | 1.046810168 | 0.85859436378001 | 1.27628548943983 | 0.998074519377826 |
| ebi-fl187-GCST90094753 | prot-a-671 | Cytotoxic and regulatory T-cell molecule \|\| id:prot-a-671 | F-deep fried food liking (derived food-liking factor) \|\| id:ebi-fl187-GCST90094753 | Inverse variance weighted | 8 | 0.136794498068117 | 0.101125659527097 | 0.176145743554015 | -0.061411795 | 0.335000790741226 | 1.14659249733456 | 0.940435893600625 | 1.397941491 | 0.998074519377826 |
| ebi-fl187-GCST90094753 | prot-a-688 | Chorionic somatomammotropin hormone \|\| id:prot-a-688 | F-deep fried food liking (derived food-liking factor) \|\| id:ebi-fl187-GCST90094753 | Inverse variance weighted | 8 | 0.0125114923569903 | 0.101125659527097 | 0.901535207938533 | -0.1856948 | 0.210717785030099 | 1.01259008852055 | 0.830527032893274 | 1.23456389348113 | 0.998074519377826 |
| ebi-fl187-GCST90094753 | prot-a-69 | Aldo-keto reductase family 1 member C1 \|\| id:prot-a-69 | F-deep fried food liking (derived food-liking factor) \|\| id:ebi-fl187-GCST90094753 | Inverse variance weighted | 8 | 0.0346837803809699 | 0.101206557660081 | 0.731821984002668 | -0.163681073 | 0.233048633394729 | 1.03529227730271 | 0.849012752108031 | 1.26244287471697 | 0.998074519377826 |
| ebi-fl187-GCST90094753 | prot-a-741 | Stromal cell-derived factor 1 \|\| id:prot-a-741 | F-deep fried food liking (derived food-liking factor) \|\| id:ebi-fl187-GCST90094753 | Inverse variance weighted | 8 | 0.113118262025787 | 0.125753604392654 | 0.368374139747306 | -0.133358803 | 0.35959532663539 | 1.11976435071896 | 0.875151029318322 | 1.43274950166912 | 0.998074519377826 |
| ebi-fl187-GCST90094753 | prot-a-742 | Growth-regulated alpha protein \|\| id:prot-a-742 | F-deep fried food liking (derived food-liking factor) \|\| id:ebi-fl187-GCST90094753 | Inverse variance weighted | 8 | 0.1686208215389 | 0.101125659527097 | 0.0954272368560238 | -0.029585471 | 0.366827114212009 | 1.18367123118539 | 0.970847894624221 | 1.44314839769852 | 0.998074519377826 |
| ebi-fl187-GCST90094753 | prot-a-788 | Beta-defensin 112 \|\| id:prot-a-788 | F-deep fried food liking (derived food-liking factor) \|\| id:ebi-fl187-GCST90094753 | Inverse variance weighted | 8 | 0.0347269363348519 | 0.132200228131466 | 0.792793849057811 | -0.224385511 | 0.293839383472525 | 1.03533695729258 | 0.799007049139115 | 1.34156840830226 | 0.998074519377826 |
| ebi-fl187-GCST90094753 | prot-a-838 | DnaJ homolog subfamily B member 1 \|\| id:prot-a-838 | F-deep fried food liking (derived food-liking factor) \|\| id:ebi-fl187-GCST90094753 | Inverse variance weighted | 8 | 0.0724049328342113 | 0.101125659527097 | 0.473997695436532 | -0.12580136 | 0.270611225507321 | 1.07509059541592 | 0.881789988293104 | 1.31076537916823 | 0.998074519377826 |
| ebi-fl187-GCST90094753 | prot-a-85 | Junctional adhesion molecule-like \|\| id:prot-a-85 | F-deep fried food liking (derived food-liking factor) \|\| id:ebi-fl187-GCST90094753 | Inverse variance weighted | 8 | -0.098933795 | 0.141983211514954 | 0.485928438805826 | -0.377220889 | 0.17935330001379 | 0.905802675107169 | 0.685764579227128 | 1.19644337296628 | 0.998074519377826 |
| ebi-fl187-GCST90094753 | prot-a-856 | Dipeptidase 1 \|\| id:prot-a-856 | F-deep fried food liking (derived food-liking factor) \|\| id:ebi-fl187-GCST90094753 | Inverse variance weighted | 8 | -0.010996969 | 0.110866826700702 | 0.920986782718473 | -0.228295949 | 0.20630201156676 | 0.989063276852449 | 0.795888682438453 | 1.22912435771915 | 0.998074519377826 |
| ebi-fl187-GCST90094753 | prot-a-860 | Dipeptidyl peptidase 2 \|\| id:prot-a-860 | F-deep fried food liking (derived food-liking factor) \|\| id:ebi-fl187-GCST90094753 | Inverse variance weighted | 8 | 0.112347728525776 | 0.101125659527097 | 0.266580604755684 | -0.085858564 | 0.310554021198885 | 1.11890186710354 | 0.917724021120902 | 1.36418068983165 | 0.998074519377826 |
| ebi-fl187-GCST90094753 | prot-a-861 | Dermatopontin \|\| id:prot-a-861 | F-deep fried food liking (derived food-liking factor) \|\| id:ebi-fl187-GCST90094753 | Inverse variance weighted | 8 | 0.0155478695185935 | 0.101125659527097 | 0.877808423563842 | -0.182658423 | 0.213754162191703 | 1.01566936649826 | 0.833052658643784 | 1.23831819194046 | 0.998074519377826 |
| ebi-fl187-GCST90094753 | prot-a-877 | Dual specificity protein phosphatase 6 \|\| id:prot-a-877 | F-deep fried food liking (derived food-liking factor) \|\| id:ebi-fl187-GCST90094753 | Inverse variance weighted | 8 | 0.00784780182930025 | 0.101125659527097 | 0.93814269743057 | -0.190358491 | 0.206054094502409 | 1.00787867653943 | 0.8266627297979 | 1.2288196745863 | 0.998074519377826 |
| ebi-fl187-GCST90094753 | prot-a-879 | Dynactin-associated protein \|\| id:prot-a-879 | F-deep fried food liking (derived food-liking factor) \|\| id:ebi-fl187-GCST90094753 | Inverse variance weighted | 8 | -0.00678165 | 0.101125659527097 | 0.94653265310808 | -0.204987943 | 0.191424642785122 | 0.993241293605403 | 0.814657139030878 | 1.21097357410569 | 0.998074519377826 |
| ebi-fl187-GCST90094753 | prot-a-937 | EMI domain-containing protein 1 \|\| id:prot-a-937 | F-deep fried food liking (derived food-liking factor) \|\| id:ebi-fl187-GCST90094753 | Inverse variance weighted | 8 | -0.142361726 | 0.126925689882558 | 0.262026276222124 | -0.391136078 | 0.106412625884057 | 0.867307471826863 | 0.676288121534375 | 1.11228073765372 | 0.998074519377826 |
| ebi-fl187-GCST90094753 | prot-b-1 | SRC proto-oncogene, non-receptor tyrosine kinase \|\| id:prot-b-1 | F-deep fried food liking (derived food-liking factor) \|\| id:ebi-fl187-GCST90094753 | Inverse variance weighted | 7 | 0.0488821008917092 | 0.154984047514925 | 0.752457318752262 | -0.254886632 | 0.352650834020962 | 1.05009653799602 | 0.775004353490444 | 1.42283425137536 | 0.998074519377826 |
| ebi-fl187-GCST90094753 | prot-b-10 | coagulation factor III, tissue factor \|\| id:prot-b-10 | F-deep fried food liking (derived food-liking factor) \|\| id:ebi-fl187-GCST90094753 | Inverse variance weighted | 7 | -0.107231341 | 0.111746646350569 | 0.337259905440026 | -0.326254768 | 0.111792085508832 | 0.898317830900794 | 0.721621317856956 | 1.11828032978686 | 0.998074519377826 |
| ebi-fl187-GCST90094753 | prot-b-29 | myeloperoxidase \|\| id:prot-b-29 | F-deep fried food liking (derived food-liking factor) \|\| id:ebi-fl187-GCST90094753 | Inverse variance weighted | 7 | 0.105140913954148 | 0.111635064660462 | 0.34628123810532 | -0.113663813 | 0.323945640688654 | 1.11086713600779 | 0.892557972031773 | 1.38257214940681 | 0.998074519377826 |
| ebi-fl187-GCST90094753 | prot-b-33 | adrenomedullin \|\| id:prot-b-33 | F-deep fried food liking (derived food-liking factor) \|\| id:ebi-fl187-GCST90094753 | Inverse variance weighted | 7 | 0.018970893833091 | 0.111863969104317 | 0.865333407874947 | -0.200282486 | 0.238224273277552 | 1.01915198457794 | 0.818499506084163 | 1.26899376230332 | 0.998074519377826 |
| ebi-fl187-GCST90094753 | ukb-rsfMRI191-edge-pheno102 | Net25_Pair11_15 (Postcentral\|Precentral)&(Cerebellum) (Motor)&(Subcortical-cerebellum) \|\| id:ukb-rsfMRI191-edge-pheno102 | F-deep fried food liking (derived food-liking factor) \|\| id:ebi-fl187-GCST90094753 | Inverse variance weighted | 8 | -0.034238651 | 0.0317778319014388 | 0.28128453258572 | -0.096523201 | 0.0280458995696014 | 0.966340858945707 | 0.907988830666997 | 1.02844288842403 | 0.998074519377826 |
| ebi-fl187-GCST90094753 | ukb-rsfMRI191-edge-pheno1167 | Net100_Pair11_45 (Angular\|Temporal)&(Frontal) (Default_mode\|Central_executive)&(Salience\|Default_mode) \|\| id:ukb-rsfMRI191-edge-pheno1167 | F-deep fried food liking (derived food-liking factor) \|\| id:ebi-fl187-GCST90094753 | Inverse variance weighted | 8 | 0.0163879883823098 | 0.0314019427485937 | 0.601755159282297 | -0.045159819 | 0.0779357961695534 | 1.01652300802231 | 0.955844707092944 | 1.08105324868243 | 0.998074519377826 |
| ebi-fl187-GCST90094753 | ukb-rsfMRI191-edge-pheno1317 | Net100_Pair26_48 (Parietal\|Frontal)&(Temporal) (Attention\|Central_executive)&(Default_mode\|Central_executive) \|\| id:ukb-rsfMRI191-edge-pheno1317 | F-deep fried food liking (derived food-liking factor) \|\| id:ebi-fl187-GCST90094753 | Inverse variance weighted | 8 | -0.012097526 | 0.0366381867714003 | 0.74125716467626 | -0.083908372 | 0.0597133201478413 | 0.987975354953827 | 0.91951550560564 | 1.06153218303071 | 0.998074519377826 |
| ebi-fl187-GCST90094753 | ukb-rsfMRI191-edge-pheno286 | Net100_Pair10_13 (OccipitalPrecuneus)&(Frontal_Sup) (Default_mode\|Central_executive)&(Default_mode) \|\| id:ukb-rsfMRI191-edge-pheno286 | F-deep fried food liking (derived food-liking factor) \|\| id:ebi-fl187-GCST90094753 | Inverse variance weighted | 8 | -0.009629724 | 0.0309586829120508 | 0.75576198808553 | -0.070308742 | 0.0510492948162836 | 0.990416493625546 | 0.932105995021828 | 1.05237476862548 | 0.998074519377826 |
| ebi-fl187-GCST90094753 | ukb-rsfMRI191-edge-pheno599 | Net100_Pair11_29 (Angular\|Temporal)&(Parietal) (Default_mode\|Central_executive)&(Central_executive\|Salience) \|\| id:ukb-rsfMRI191-edge-pheno599 | F-deep fried food liking (derived food-liking factor) \|\| id:ebi-fl187-GCST90094753 | Inverse variance weighted | 8 | 0.00460959707819789 | 0.0386833203806633 | 0.905146699270141 | -0.071209711 | 0.0804289050242979 | 1.00462023761406 | 0.931266574926562 | 1.08375179459577 | 0.998074519377826 |
| ebi-fl187-GCST90094753 | ukb-rsfMRI191-edge-pheno621 | Net100_Pair5_30 (Precuneus\|Angular\|Cingulate)&(Paracentral) (Default_mode\|Central_executive)&(Motor) \|\| id:ukb-rsfMRI191-edge-pheno621 | F-deep fried food liking (derived food-liking factor) \|\| id:ebi-fl187-GCST90094753 | Inverse variance weighted | 8 | 0.0209781817722352 | 0.0348608618312011 | 0.547327992953427 | -0.047349107 | 0.0893054709613894 | 1.02119977062527 | 0.953754376736891 | 1.09341461172954 | 0.998074519377826 |
| ebi-fl187-GCST90094753 | ukb-rsfMRI191-edge-pheno624 | Net100_Pair8_30 (Calcarine\|Lingual)&(Paracentral) (Visual)&(Motor) \|\| id:ukb-rsfMRI191-edge-pheno624 | F-deep fried food liking (derived food-liking factor) \|\| id:ebi-fl187-GCST90094753 | Inverse variance weighted | 8 | 0.00265144507593569 | 0.0315534474661374 | 0.933032355672319 | -0.059193312 | 0.0644962021095649 | 1.00265496326517 | 0.942524550279134 | 1.06662152732526 | 0.998074519377826 |
| ebi-fl187-GCST90094753 | ukb-rsfMRI191-edge-pheno882 | Net100_Pair6_38 (Postcentral\|Precentral)&(Subcortical) (Motor)&(Subcortical-cerebellum) \|\| id:ukb-rsfMRI191-edge-pheno882 | F-deep fried food liking (derived food-liking factor) \|\| id:ebi-fl187-GCST90094753 | Inverse variance weighted | 8 | 0.0517171413010188 | 0.0317296802569089 | 0.103116401819853 | -0.010473032 | 0.11390731460456 | 1.05307782815712 | 0.989581619242666 | 1.12064825234408 | 0.998074519377826 |
| ebi-fl187-GCST90094753 | ukb-rsfMRI191-edge-pheno899 | Net100_Pair23_38 (Cerebellum)&(Subcortical) (Subcortical-cerebellum)&(Subcortical-cerebellum) \|\| id:ukb-rsfMRI191-edge-pheno899 | F-deep fried food liking (derived food-liking factor) \|\| id:ebi-fl187-GCST90094753 | Inverse variance weighted | 8 | -0.061938863 | 0.0315345722776122 | 0.0495118440627531 | -0.123746624 | -0.000131101 | 0.93994035039935 | 0.883603696122892 | 0.999868907503955 | 0.998074519377826 |
| ebi-fl187-GCST90094753 | ebi-a-GCST90001392 | IgD+ CD38+ B cell Absolute Count \|\| id:ebi-a-GCST90001392 | F-deep fried food liking (derived food-liking factor) \|\| id:ebi-fl187-GCST90094753 | MR Egger | 8 | 0.551302564306738 | 0.399753157895213 | 0.217064694281419 | -0.232213625 | 1.33481875378136 | 1.73551216240411 | 0.792776748150015 | 3.79930727393464 | 0.999279047927446 |
| ebi-fl187-GCST90094753 | ebi-a-GCST90001402 | Switched memory B cell %B cell \|\| id:ebi-a-GCST90001402 | F-deep fried food liking (derived food-liking factor) \|\| id:ebi-fl187-GCST90094753 | MR Egger | 8 | 0.313273072505813 | 0.421473012220557 | 0.485386176162621 | -0.512814031 | 1.13936017645811 | 1.36789501455056 | 0.59880814073546 | 3.12476842504868 | 0.999279047927446 |
| ebi-fl187-GCST90094753 | ebi-a-GCST90001407 | Memory B cell Absolute Count \|\| id:ebi-a-GCST90001407 | F-deep fried food liking (derived food-liking factor) \|\| id:ebi-fl187-GCST90094753 | MR Egger | 8 | 0.0892425652344735 | 0.521797208149158 | 0.869822777158275 | -0.933479963 | 1.11196509320682 | 1.09334583185191 | 0.393183064443022 | 3.04032705406919 | 0.999279047927446 |
| ebi-fl187-GCST90094753 | ebi-a-GCST90001411 | IgD+ CD24+ B cell %B cell \|\| id:ebi-a-GCST90001411 | F-deep fried food liking (derived food-liking factor) \|\| id:ebi-fl187-GCST90094753 | MR Egger | 8 | 0.0933159771315018 | 0.518171036357296 | 0.863012445912951 | -0.922299254 | 1.1089312083918 | 1.09780856287103 | 0.397603797126329 | 3.03111703012748 | 0.999279047927446 |
| ebi-fl187-GCST90094753 | ebi-a-GCST90001418 | CD24+ CD27+ B cell Absolute Count \|\| id:ebi-a-GCST90001418 | F-deep fried food liking (derived food-liking factor) \|\| id:ebi-fl187-GCST90094753 | MR Egger | 8 | 0.349998048530018 | 0.504051871513193 | 0.513425067955228 | -0.63794362 | 1.33793971669588 | 1.41906477932824 | 0.52837785347541 | 3.81118329370633 | 0.999279047927446 |
| ebi-fl187-GCST90094753 | ebi-a-GCST90001436 | Memory B cell %lymphocyte \|\| id:ebi-a-GCST90001436 | F-deep fried food liking (derived food-liking factor) \|\| id:ebi-fl187-GCST90094753 | MR Egger | 8 | -0.194789059 | 0.435369752205774 | 0.670277652368555 | -1.048113774 | 0.658535654946236 | 0.82300824561904 | 0.350598433801288 | 1.93196120419875 | 0.999279047927446 |
| ebi-fl187-GCST90094753 | ebi-a-GCST90001439 | IgD+ CD24+ B cell %lymphocyte \|\| id:ebi-a-GCST90001439 | F-deep fried food liking (derived food-liking factor) \|\| id:ebi-fl187-GCST90094753 | MR Egger | 8 | 0.108340743015675 | 0.469108999698143 | 0.825027426671209 | -0.811112896 | 1.02779438242404 | 1.11442741405589 | 0.444363260670511 | 2.79489456289722 | 0.999279047927446 |
| ebi-fl187-GCST90094753 | ebi-a-GCST90001457 | CD11c+ HLA DR++ monocyte %monocyte \|\| id:ebi-a-GCST90001457 | F-deep fried food liking (derived food-liking factor) \|\| id:ebi-fl187-GCST90094753 | MR Egger | 8 | 0.113821008723974 | 0.508744252248004 | 0.830391164847864 | -0.883317726 | 1.11095974313006 | 1.1205515379834 | 0.413409056061235 | 3.03727199699025 | 0.999279047927446 |
| ebi-fl187-GCST90094753 | ebi-a-GCST90001483 | CD39+ resting CD4 regulatory T cell Absolute Count \|\| id:ebi-a-GCST90001483 | F-deep fried food liking (derived food-liking factor) \|\| id:ebi-fl187-GCST90094753 | MR Egger | 8 | 0.185316749084468 | 0.427158201876291 | 0.679572325212599 | -0.651913327 | 1.022546825 | 1.20359961883284 | 0.521047887641862 | 2.78026660660847 | 0.999279047927446 |
| ebi-fl187-GCST90094753 | ebi-a-GCST90001525 | CD33dim HLA DR+ CD11b+ Absolute Count \|\| id:ebi-a-GCST90001525 | F-deep fried food liking (derived food-liking factor) \|\| id:ebi-fl187-GCST90094753 | MR Egger | 8 | 0.300013763184631 | 0.545666072048751 | 0.602297582324644 | -0.769491738 | 1.36951926440018 | 1.34987738605985 | 0.463248460060307 | 3.93345928696352 | 0.999279047927446 |
| ebi-fl187-GCST90094753 | ebi-a-GCST90001540 | Naive CD4+ T cell Absolute Count \|\| id:ebi-a-GCST90001540 | F-deep fried food liking (derived food-liking factor) \|\| id:ebi-fl187-GCST90094753 | MR Egger | 8 | 0.33928175166998 | 0.452678322866705 | 0.481898368952676 | -0.547967761 | 1.22653126448872 | 1.40393885160881 | 0.57812350240768 | 3.40938275445982 | 0.999279047927446 |
| ebi-fl187-GCST90094753 | ebi-a-GCST90001624 | HLA DR+ CD4+ T cell Absolute Count \|\| id:ebi-a-GCST90001624 | F-deep fried food liking (derived food-liking factor) \|\| id:ebi-fl187-GCST90094753 | MR Egger | 8 | 0.483716474257118 | 0.439110675325043 | 0.312860002308065 | -0.376940449 | 1.3443733978942 | 1.62209167570131 | 0.68595692183976 | 3.83578227816778 | 0.999279047927446 |
| ebi-fl187-GCST90094753 | ebi-a-GCST90001672 | CD39+ CD8+ T cell Absolute Count \|\| id:ebi-a-GCST90001672 | F-deep fried food liking (derived food-liking factor) \|\| id:ebi-fl187-GCST90094753 | MR Egger | 8 | 0.343797649017033 | 0.443939260424004 | 0.468088601494874 | -0.526323301 | 1.21391859944808 | 1.410293232 | 0.590773076032467 | 3.36665139640686 | 0.999279047927446 |
| ebi-fl187-GCST90094753 | ebi-a-GCST90001722 | CD19 on CD20- CD38- B cell \|\| id:ebi-a-GCST90001722 | F-deep fried food liking (derived food-liking factor) \|\| id:ebi-fl187-GCST90094753 | MR Egger | 8 | -0.005098916 | 0.468338436537721 | 0.991666380321586 | -0.923042251 | 0.912844419806485 | 0.99491406159749 | 0.397308488301957 | 2.49139904912406 | 0.999279047927446 |
| ebi-fl187-GCST90094753 | ebi-a-GCST90001742 | CD19 on transitional B cell \|\| id:ebi-a-GCST90001742 | F-deep fried food liking (derived food-liking factor) \|\| id:ebi-fl187-GCST90094753 | MR Egger | 8 | 0.332135059472902 | 0.407239531166707 | 0.445902739183947 | -0.466054422 | 1.13032454055965 | 1.39394110074993 | 0.627473135017476 | 3.09666132926282 | 0.999279047927446 |
| ebi-fl187-GCST90094753 | ebi-a-GCST90001784 | CD25 on IgD+ CD38dim B cell \|\| id:ebi-a-GCST90001784 | F-deep fried food liking (derived food-liking factor) \|\| id:ebi-fl187-GCST90094753 | MR Egger | 8 | -0.559332407 | 0.631628479945991 | 0.409948405544718 | -1.797324228 | 0.678659413490068 | 0.571590526221406 | 0.165741782653176 | 1.97123335127711 | 0.999279047927446 |
| ebi-fl187-GCST90094753 | ebi-a-GCST90001794 | CD25 on IgD+ B cell \|\| id:ebi-a-GCST90001794 | F-deep fried food liking (derived food-liking factor) \|\| id:ebi-fl187-GCST90094753 | MR Egger | 8 | -0.429580848 | 0.60231804197092 | 0.502497761710638 | -1.61012421 | 0.750962514461473 | 0.650781814191984 | 0.199862787563584 | 2.11903864068879 | 0.999279047927446 |
| ebi-fl187-GCST90094753 | ebi-a-GCST90001802 | CD27 on IgD- CD38- B cell \|\| id:ebi-a-GCST90001802 | F-deep fried food liking (derived food-liking factor) \|\| id:ebi-fl187-GCST90094753 | MR Egger | 8 | 0.549721821196689 | 0.410466523031331 | 0.22898634368532 | -0.254792564 | 1.3542362063381 | 1.73277093067338 | 0.77507726025596 | 3.87380104171182 | 0.999279047927446 |
| ebi-fl187-GCST90094753 | ebi-a-GCST90001804 | CD27 on IgD- CD38dim B cell \|\| id:ebi-a-GCST90001804 | F-deep fried food liking (derived food-liking factor) \|\| id:ebi-fl187-GCST90094753 | MR Egger | 8 | 0.204048015125992 | 0.406342653812912 | 0.633449245759955 | -0.592383586 | 1.0004796165993 | 1.22635703573016 | 0.553007571237035 | 2.71958587424155 | 0.999279047927446 |
| ebi-fl187-GCST90094753 | ebi-a-GCST90001805 | CD27 on memory B cell \|\| id:ebi-a-GCST90001805 | F-deep fried food liking (derived food-liking factor) \|\| id:ebi-fl187-GCST90094753 | MR Egger | 8 | 0.706342845006968 | 0.411035071418626 | 0.136516630621385 | -0.099285895 | 1.51197158498748 | 2.02656622288839 | 0.905483797748341 | 4.53566443261038 | 0.999279047927446 |
| ebi-fl187-GCST90094753 | ebi-a-GCST90001806 | CD27 on unswitched memory B cell \|\| id:ebi-a-GCST90001806 | F-deep fried food liking (derived food-liking factor) \|\| id:ebi-fl187-GCST90094753 | MR Egger | 8 | 0.396657038487023 | 0.409726841075102 | 0.370381344010086 | -0.40640757 | 1.19972164699422 | 1.48684591167454 | 0.666038654710485 | 3.31919288682163 | 0.999279047927446 |
| ebi-fl187-GCST90094753 | ebi-a-GCST90001839 | CD3 on Effector Memory CD8+ T cell \|\| id:ebi-a-GCST90001839 | F-deep fried food liking (derived food-liking factor) \|\| id:ebi-fl187-GCST90094753 | MR Egger | 8 | 0.0865413518338386 | 0.477306733672187 | 0.86209268998759 | -0.848979846 | 1.02206254983133 | 1.09039645667861 | 0.427851183415237 | 2.77892051915481 | 0.999279047927446 |
| ebi-fl187-GCST90094753 | ebi-a-GCST90001859 | CD3 on CD8+ T cell \|\| id:ebi-a-GCST90001859 | F-deep fried food liking (derived food-liking factor) \|\| id:ebi-fl187-GCST90094753 | MR Egger | 8 | -0.289861156 | 0.443112428492117 | 0.53726734939166 | -1.158361516 | 0.578639203974131 | 0.748367466795034 | 0.314000244090794 | 1.78360964966408 | 0.999279047927446 |
| ebi-fl187-GCST90094753 | ebi-a-GCST90001905 | CD86 on monocyte \|\| id:ebi-a-GCST90001905 | F-deep fried food liking (derived food-liking factor) \|\| id:ebi-fl187-GCST90094753 | MR Egger | 8 | -0.395249801 | 0.448909742633112 | 0.412481083863054 | -1.275112896 | 0.484613294946854 | 0.673511774578578 | 0.279399423314618 | 1.62354705358568 | 0.999279047927446 |
| ebi-fl187-GCST90094753 | ebi-a-GCST90001943 | CD25 on activated & secreting CD4 regulatory T cell \|\| id:ebi-a-GCST90001943 | F-deep fried food liking (derived food-liking factor) \|\| id:ebi-fl187-GCST90094753 | MR Egger | 8 | 0.355967968887255 | 0.415755635949369 | 0.424757674567594 | -0.458913078 | 1.17084901534802 | 1.42756182113786 | 0.63197017489321 | 3.22472932130826 | 0.999279047927446 |
| ebi-fl187-GCST90094753 | ebi-a-GCST90001949 | CD33 on CD33dim HLA DR+ CD11b- \|\| id:ebi-a-GCST90001949 | F-deep fried food liking (derived food-liking factor) \|\| id:ebi-fl187-GCST90094753 | MR Egger | 8 | -0.986324754 | 0.622043669331729 | 0.16391969852751 | -2.205530346 | 0.232880837612505 | 0.372944839266715 | 0.110192069863064 | 1.2622310598986 | 0.999279047927446 |
| ebi-fl187-GCST90094753 | ebi-a-GCST90001955 | CD33 on Immature Myeloid-Derived Suppressor Cells \|\| id:ebi-a-GCST90001955 | F-deep fried food liking (derived food-liking factor) \|\| id:ebi-fl187-GCST90094753 | MR Egger | 8 | -0.453718532 | 0.605287281911907 | 0.481846055541106 | -1.640081605 | 0.73264454026827 | 0.63526151369474 | 0.193964213229087 | 2.08057550443648 | 0.999279047927446 |
| ebi-fl187-GCST90094753 | ebi-a-GCST90001956 | CD33 on CD33+ HLA DR+ \|\| id:ebi-a-GCST90001956 | F-deep fried food liking (derived food-liking factor) \|\| id:ebi-fl187-GCST90094753 | MR Egger | 8 | -1.407057298 | 0.604550029002308 | 0.0588460937050253 | -2.591975355 | -0.222139241 | 0.244862782242767 | 0.0748719951683281 | 0.800803851865722 | 0.999279047927446 |
| ebi-fl187-GCST90094753 | ebi-a-GCST90001957 | CD33 on CD33+ HLA DR+ CD14- \|\| id:ebi-a-GCST90001957 | F-deep fried food liking (derived food-liking factor) \|\| id:ebi-fl187-GCST90094753 | MR Egger | 8 | -1.284936613 | 0.605404850269418 | 0.0780197552809667 | -2.471530119 | -0.098343106 | 0.276668120246115 | 0.0844555330267452 | 0.906337880033021 | 0.999279047927446 |
| ebi-fl187-GCST90094753 | ebi-a-GCST90002064 | CD4 on resting CD4 regulatory T cell \|\| id:ebi-a-GCST90002064 | F-deep fried food liking (derived food-liking factor) \|\| id:ebi-fl187-GCST90094753 | MR Egger | 8 | 0.091812289895573 | 0.443241241884358 | 0.842752852968044 | -0.776940544 | 0.960565123988914 | 1.09615904263962 | 0.459810631827858 | 2.61317282287297 | 0.999279047927446 |
| ebi-fl187-GCST90094753 | ebi-a-GCST90002080 | SSC-A on T cell \|\| id:ebi-a-GCST90002080 | F-deep fried food liking (derived food-liking factor) \|\| id:ebi-fl187-GCST90094753 | MR Egger | 8 | -0.253772609 | 0.631774790589878 | 0.701834410818953 | -1.492051199 | 0.984505980475343 | 0.775868207374071 | 0.224910845249821 | 2.67648931978006 | 0.999279047927446 |
| ebi-fl187-GCST90094753 | ebi-a-GCST90002121 | CD8 on CD39+ CD8+ T cell \|\| id:ebi-a-GCST90002121 | F-deep fried food liking (derived food-liking factor) \|\| id:ebi-fl187-GCST90094753 | MR Egger | 8 | -0.244589271 | 0.438459114882306 | 0.597137532548809 | -1.103969137 | 0.614790593676946 | 0.783026083338121 | 0.331552491443681 | 1.84926931032272 | 0.999279047927446 |
| ebi-fl187-GCST90094753 | ebi-cfmet338-GCST90026053 | 1-palmitoyl-2-docosahexaenoyl-gpc (16:0/22:6) levels \|\| id:ebi-cfmet338-GCST90026053 | F-deep fried food liking (derived food-liking factor) \|\| id:ebi-fl187-GCST90094753 | MR Egger | 8 | -0.138357139 | 0.175558614111768 | 0.4606329872366 | -0.482452023 | 0.205737744648257 | 0.870787643967665 | 0.617267979573539 | 1.22843099914341 | 0.999279047927446 |
| ebi-fl187-GCST90094753 | ebi-cfmet338-GCST90026063 | 1-stearoyl-2-oleoyl-gpc (18:0/18:1) levels \|\| id:ebi-cfmet338-GCST90026063 | F-deep fried food liking (derived food-liking factor) \|\| id:ebi-fl187-GCST90094753 | MR Egger | 8 | 0.0476693961916512 | 0.183909372977378 | 0.804144316286242 | -0.312792975 | 0.408131767227312 | 1.04882385284048 | 0.731401315367653 | 1.50400532672573 | 0.999279047927446 |
| ebi-fl187-GCST90094753 | ebi-cfmet338-GCST90026113 | Adenosine 3',5'-cyclic monophosphate (camp) levels \|\| id:ebi-cfmet338-GCST90026113 | F-deep fried food liking (derived food-liking factor) \|\| id:ebi-fl187-GCST90094753 | MR Egger | 8 | 0.151285147455006 | 0.315728494338234 | 0.64878693700907 | -0.467542701 | 0.770112996357944 | 1.16332833090464 | 0.626539973978819 | 2.16001031329426 | 0.999279047927446 |
| ebi-fl187-GCST90094753 | ebi-cfmet338-GCST90026117 | Alpha-ketoglutaramate levels \|\| id:ebi-cfmet338-GCST90026117 | F-deep fried food liking (derived food-liking factor) \|\| id:ebi-fl187-GCST90094753 | MR Egger | 8 | -0.071460898 | 0.118350700712046 | 0.568083200535211 | -0.303428271 | 0.160506475519538 | 0.93103268223843 | 0.738282843322098 | 1.1741053760583 | 0.999279047927446 |
| ebi-fl187-GCST90094753 | ebi-cfmet338-GCST90026124 | Arginine levels \|\| id:ebi-cfmet338-GCST90026124 | F-deep fried food liking (derived food-liking factor) \|\| id:ebi-fl187-GCST90094753 | MR Egger | 8 | -0.264123548 | 0.085479265331596 | 0.0213902073857599 | -0.431662908 | -0.096584188 | 0.76787866406133 | 0.649428256946392 | 0.90793345748934 | 0.999279047927446 |
| ebi-fl187-GCST90094753 | ebi-cfmet338-GCST90026169 | Glutamate levels \|\| id:ebi-cfmet338-GCST90026169 | F-deep fried food liking (derived food-liking factor) \|\| id:ebi-fl187-GCST90094753 | MR Egger | 8 | -0.148751676 | 0.115512073497239 | 0.245265078316774 | -0.37515534 | 0.0776519879875279 | 0.861783089696501 | 0.687182523482703 | 1.08074648045898 | 0.999279047927446 |
| ebi-fl187-GCST90094753 | ebi-cfmet338-GCST90026170 | Glutamine levels \|\| id:ebi-cfmet338-GCST90026170 | F-deep fried food liking (derived food-liking factor) \|\| id:ebi-fl187-GCST90094753 | MR Egger | 8 | -0.109812233 | 0.0546260449786716 | 0.0911224252211573 | -0.216879281 | -0.002745185 | 0.896002358991051 | 0.805027145216428 | 0.997258579524909 | 0.999279047927446 |
| ebi-fl187-GCST90094753 | ebi-cfmet338-GCST90026191 | Kynurenate levels \|\| id:ebi-cfmet338-GCST90026191 | F-deep fried food liking (derived food-liking factor) \|\| id:ebi-fl187-GCST90094753 | MR Egger | 8 | 0.89426048378484 | 0.540956871788788 | 0.149394729555751 | -0.166014985 | 1.95453595249086 | 2.44552661400181 | 0.847033541363441 | 7.06064179012837 | 0.999279047927446 |
| ebi-fl187-GCST90094753 | ebi-cfmet338-GCST90026202 | Methylsuccinoylcarnitine levels \|\| id:ebi-cfmet338-GCST90026202 | F-deep fried food liking (derived food-liking factor) \|\| id:ebi-fl187-GCST90094753 | MR Egger | 8 | -0.211651607 | 0.279272992600043 | 0.477231329685438 | -0.759026673 | 0.335723458464136 | 0.809246584279358 | 0.468121841188647 | 1.39895210295026 | 0.999279047927446 |
| ebi-fl187-GCST90094753 | ebi-cfmet338-GCST90026222 | N-acetylputrescine levels \|\| id:ebi-cfmet338-GCST90026222 | F-deep fried food liking (derived food-liking factor) \|\| id:ebi-fl187-GCST90094753 | MR Egger | 8 | -0.043041848 | 0.117266827870051 | 0.726185141313507 | -0.272884831 | 0.186801134378839 | 0.957871303993863 | 0.761180447069528 | 1.20538755106921 | 0.999279047927446 |
| ebi-fl187-GCST90094753 | ebi-cfmet338-GCST90026252 | Propionylcarnitine (c3) levels \|\| id:ebi-cfmet338-GCST90026252 | F-deep fried food liking (derived food-liking factor) \|\| id:ebi-fl187-GCST90094753 | MR Egger | 8 | -0.153620795 | 0.180006790772586 | 0.426180191496253 | -0.506434105 | 0.199192514803817 | 0.857597164397582 | 0.60264070535436 | 1.22041689160427 | 0.999279047927446 |
| ebi-fl187-GCST90094753 | ebi-cfmet338-GCST90026316 | Ethyl beta-glucopyranoside levels \|\| id:ebi-cfmet338-GCST90026316 | F-deep fried food liking (derived food-liking factor) \|\| id:ebi-fl187-GCST90094753 | MR Egger | 8 | 0.0860364426009663 | 0.610691350815004 | 0.892572750394283 | -1.110918605 | 1.28299149019837 | 1.08984604440596 | 0.329256365571073 | 3.60741514730387 | 0.999279047927446 |
| ebi-fl187-GCST90094753 | ebi-gm430-GCST90011315 | F_Prevotellaceae abundance \|\| id:ebi-gm430-GCST90011315 | F-deep fried food liking (derived food-liking factor) \|\| id:ebi-fl187-GCST90094753 | MR Egger | 8 | 0.186131724476524 | 0.277341934769793 | 0.527116960257942 | -0.357458468 | 0.729721916625318 | 1.20458092271902 | 0.699451748198721 | 2.07450364248194 | 0.999279047927446 |
| ebi-fl187-GCST90094753 | ebi-gm430-GCST90011354 | OTU97_1 (Bacteroides) abundance \|\| id:ebi-gm430-GCST90011354 | F-deep fried food liking (derived food-liking factor) \|\| id:ebi-fl187-GCST90094753 | MR Egger | 8 | -0.312321717 | 0.242267917420179 | 0.244809394059461 | -0.787166835 | 0.162523400891578 | 0.731746075019267 | 0.455132435479286 | 1.17647584871035 | 0.999279047927446 |
| ebi-fl187-GCST90094753 | ebi-gm430-GCST90011419 | OTU99_1 (Bacteroides) abundance \|\| id:ebi-gm430-GCST90011419 | F-deep fried food liking (derived food-liking factor) \|\| id:ebi-fl187-GCST90094753 | MR Egger | 8 | -0.296096749 | 0.242306680817179 | 0.267536331749235 | -0.771017843 | 0.178824345305912 | 0.743715470740038 | 0.462542033231085 | 1.19581067596022 | 0.999279047927446 |
| ebi-fl187-GCST90094753 | ebi-gm430-GCST90011435 | OTU99_155 (Butyrivibrio) abundance \|\| id:ebi-gm430-GCST90011435 | F-deep fried food liking (derived food-liking factor) \|\| id:ebi-fl187-GCST90094753 | MR Egger | 8 | -1.100679891 | 0.783591733759472 | 0.209718923073455 | -2.636519689 | 0.435159907254182 | 0.332644844590285 | 0.0716100616476679 | 1.54521012950557 | 0.999279047927446 |
| ebi-fl187-GCST90094753 | ebi-gm430-GCST90011466 | OTU99_58 (Bacteroides) abundance \|\| id:ebi-gm430-GCST90011466 | F-deep fried food liking (derived food-liking factor) \|\| id:ebi-fl187-GCST90094753 | MR Egger | 8 | -0.429978794 | 0.432620331889766 | 0.358650978266075 | -1.277914644 | 0.417957056547726 | 0.650522889593928 | 0.278617712049909 | 1.51885544810528 | 0.999279047927446 |
| ebi-fl187-GCST90094753 | ebi-gm430-GCST90011472 | OTU99_72 (Parabacteroides) abundance \|\| id:ebi-gm430-GCST90011472 | F-deep fried food liking (derived food-liking factor) \|\| id:ebi-fl187-GCST90094753 | MR Egger | 7 | 0.51054282045145 | 0.678184198910553 | 0.48547741874238 | -0.818698209 | 1.83978385031614 | 1.66619539445091 | 0.441005377640615 | 6.29517741335079 | 0.999279047927446 |
| ebi-fl187-GCST90094753 | ebi-gm430-GCST90011505 | TestASV_28 (Holdemanella) abundance \|\| id:ebi-gm430-GCST90011505 | F-deep fried food liking (derived food-liking factor) \|\| id:ebi-fl187-GCST90094753 | MR Egger | 8 | -0.50847491 | 0.598335642016464 | 0.428029106988149 | -1.681212769 | 0.664262948110342 | 0.601412087170393 | 0.186148084539239 | 1.94305785896177 | 0.999279047927446 |
| ebi-fl187-GCST90094753 | ebi-gm430-GCST90011513 | TestASV_35 (Alphaproteobacteria) abundance \|\| id:ebi-gm430-GCST90011513 | F-deep fried food liking (derived food-liking factor) \|\| id:ebi-fl187-GCST90094753 | MR Egger | 8 | 0.731186949650621 | 0.7090833133828 | 0.342222195605717 | -0.658616345 | 2.12099024388091 | 2.07754508619303 | 0.517566973626985 | 8.33939143164015 | 0.999279047927446 |
| ebi-fl187-GCST90094753 | ebi-gm430-GCST90011543 | G_Coprococcus prevalence \|\| id:ebi-gm430-GCST90011543 | F-deep fried food liking (derived food-liking factor) \|\| id:ebi-fl187-GCST90094753 | MR Egger | 8 | 0.676813901294531 | 0.752485750163597 | 0.40307172204789 | -0.798058169 | 2.15168597161518 | 1.96759877094983 | 0.450202332709864 | 8.59934443284697 | 0.999279047927446 |
| ebi-fl187-GCST90094753 | ebi-gm430-GCST90011547 | G_Parasutterella prevalence \|\| id:ebi-gm430-GCST90011547 | F-deep fried food liking (derived food-liking factor) \|\| id:ebi-fl187-GCST90094753 | MR Egger | 8 | -0.85192061 | 0.786846885573281 | 0.320521756248164 | -2.394140506 | 0.69029928554901 | 0.426594822289217 | 0.0912510749880242 | 1.99431231279031 | 0.999279047927446 |
| ebi-fl187-GCST90094753 | ebi-gm430-GCST90011569 | OTU97_13 (Subdoligranulum) prevalence \|\| id:ebi-gm430-GCST90011569 | F-deep fried food liking (derived food-liking factor) \|\| id:ebi-fl187-GCST90094753 | MR Egger | 8 | -0.545583977 | 0.664132816190821 | 0.442773383074754 | -1.847284297 | 0.756116342285657 | 0.579503267667144 | 0.157664756069785 | 2.12998799229586 | 0.999279047927446 |
| ebi-fl187-GCST90094753 | ebi-gm430-GCST90011590 | OTU97_277 (Prevotella) prevalence \|\| id:ebi-gm430-GCST90011590 | F-deep fried food liking (derived food-liking factor) \|\| id:ebi-fl187-GCST90094753 | MR Egger | 8 | -0.217134136 | 0.812505218951372 | 0.798232525219035 | -1.809644365 | 1.37537609319327 | 0.804822006498753 | 0.16371234827632 | 3.95656448010507 | 0.999279047927446 |
| ebi-fl187-GCST90094753 | ebi-gm430-GCST90011616 | OTU97_86 (Phascolarctobacterium) prevalence \|\| id:ebi-gm430-GCST90011616 | F-deep fried food liking (derived food-liking factor) \|\| id:ebi-fl187-GCST90094753 | MR Egger | 8 | -0.524399537 | 0.74104108622785 | 0.505705854643422 | -1.976840066 | 0.928040992388086 | 0.591910678367094 | 0.138506217090092 | 2.52954891502886 | 0.999279047927446 |
| ebi-fl187-GCST90094753 | ebi-gm430-GCST90011665 | OTU99_55 (Barnesiella) prevalence \|\| id:ebi-gm430-GCST90011665 | F-deep fried food liking (derived food-liking factor) \|\| id:ebi-fl187-GCST90094753 | MR Egger | 8 | 0.0818200077839703 | 0.493433894491877 | 0.873746791151639 | -0.885310425 | 1.04895044098805 | 1.08526045381474 | 0.412586076191749 | 2.85465341798594 | 0.999279047927446 |
| ebi-fl187-GCST90094753 | ebi-gm430-GCST90011687 | TestASV_14 (Lachnospiraceae) prevalence \|\| id:ebi-gm430-GCST90011687 | F-deep fried food liking (derived food-liking factor) \|\| id:ebi-fl187-GCST90094753 | MR Egger | 8 | -0.249356972 | 0.539788491214791 | 0.660390554821838 | -1.307342415 | 0.808628470606508 | 0.779301734691109 | 0.270538079812808 | 2.24482702809447 | 0.999279047927446 |
| ebi-fl187-GCST90094753 | ebi-gm430-GCST90011697 | TestASV_23 (Barnesiella) prevalence \|\| id:ebi-gm430-GCST90011697 | F-deep fried food liking (derived food-liking factor) \|\| id:ebi-fl187-GCST90094753 | MR Egger | 8 | 0.348437728504654 | 0.732022397020501 | 0.650915681182504 | -1.08632617 | 1.78320162666484 | 1.41685231066421 | 0.337453967806723 | 5.94887202921942 | 0.999279047927446 |
| ebi-fl187-GCST90094753 | ebi-gm430-GCST90011727 | TestASV_6 (Subdoligranulum) prevalence \|\| id:ebi-gm430-GCST90011727 | F-deep fried food liking (derived food-liking factor) \|\| id:ebi-fl187-GCST90094753 | MR Egger | 8 | -1.102871095 | 0.791242650541914 | 0.212799330464718 | -2.65370669 | 0.447964499729453 | 0.331916749730746 | 0.0703898156465258 | 1.56512313237263 | 0.999279047927446 |
| ebi-fl187-GCST90094753 | ebi-hbsm44-GCST90104908 | Sulcal depth of the left posterior inferior temporal sulcus \|\| id:ebi-hbsm44-GCST90104908 | F-deep fried food liking (derived food-liking factor) \|\| id:ebi-fl187-GCST90094753 | MR Egger | 7 | 0.308495981322634 | 0.704704701423444 | 0.679820228395239 | -1.072725233 | 1.68971719611259 | 1.36137603860553 | 0.342075011715709 | 5.41794827161932 | 0.999279047927446 |
| ebi-fl187-GCST90094753 | ebi-hbsm44-GCST90104918 | Sulcal depth of the left inferior frontal sulcus \|\| id:ebi-hbsm44-GCST90104918 | F-deep fried food liking (derived food-liking factor) \|\| id:ebi-fl187-GCST90094753 | MR Egger | 7 | 0.119034420678986 | 0.801858876576307 | 0.887790335536195 | -1.452608977 | 1.69067781876855 | 1.12640868937299 | 0.233959097149339 | 5.42315537610871 | 0.999279047927446 |
| ebi-fl187-GCST90094753 | ebi-hbsm44-GCST90104925 | Sulcal depth of the right anterior inferior temporal sulcus \|\| id:ebi-hbsm44-GCST90104925 | F-deep fried food liking (derived food-liking factor) \|\| id:ebi-fl187-GCST90094753 | MR Egger | 7 | 0.160967586714396 | 0.227954874585548 | 0.511636919007518 | -0.285823967 | 0.60775914090207 | 1.17464689403155 | 0.751394874243943 | 1.83631186870462 | 0.999279047927446 |
| ebi-fl187-GCST90094753 | ebi-IPs91-GCST90274770 | C-C motif chemokine 4 levels \|\| id:ebi-IPs91-GCST90274770 | F-deep fried food liking (derived food-liking factor) \|\| id:ebi-fl187-GCST90094753 | MR Egger | 8 | 0.0760996153561991 | 0.236066451674134 | 0.75811268251584 | -0.38659063 | 0.538789860637502 | 1.079070061 | 0.679369151409147 | 1.71393151072013 | 0.999279047927446 |
| ebi-fl187-GCST90094753 | ebi-IPs91-GCST90274779 | C-X-C motif chemokine 1 levels \|\| id:ebi-IPs91-GCST90274779 | F-deep fried food liking (derived food-liking factor) \|\| id:ebi-fl187-GCST90094753 | MR Egger | 8 | 0.0469153371922017 | 0.153025560212253 | 0.769520056206783 | -0.253014761 | 0.346845435208218 | 1.04803327588371 | 0.776456420602725 | 1.41459806141717 | 0.999279047927446 |
| ebi-fl187-GCST90094753 | ebi-IPs91-GCST90274780 | C-X-C motif chemokine 10 levels \|\| id:ebi-IPs91-GCST90274780 | F-deep fried food liking (derived food-liking factor) \|\| id:ebi-fl187-GCST90094753 | MR Egger | 8 | 0.15959359499557 | 0.224235383676672 | 0.50335561821435 | -0.279907757 | 0.599094947001847 | 1.17303404719944 | 0.75585346042279 | 1.82047043234996 | 0.999279047927446 |
| ebi-fl187-GCST90094753 | ebi-IPs91-GCST90274796 | Interleukin-10 receptor subunit alpha levels \|\| id:ebi-IPs91-GCST90274796 | F-deep fried food liking (derived food-liking factor) \|\| id:ebi-fl187-GCST90094753 | MR Egger | 8 | -0.106471654 | 0.223378591813371 | 0.650479481887041 | -0.544293694 | 0.331350385523876 | 0.899000530482244 | 0.580251473513703 | 1.39284774050345 | 0.999279047927446 |
| ebi-fl187-GCST90094753 | ebi-IPs91-GCST90274807 | Interleukin-20 levels \|\| id:ebi-IPs91-GCST90274807 | F-deep fried food liking (derived food-liking factor) \|\| id:ebi-fl187-GCST90094753 | MR Egger | 8 | -0.280646526 | 0.165177997382745 | 0.140222438717335 | -0.604395401 | 0.0431023484755241 | 0.755295265241621 | 0.546404682373401 | 1.04404474577061 | 0.999279047927446 |
| ebi-fl187-GCST90094753 | ebi-IPs91-GCST90274808 | Interleukin-20 receptor subunit alpha levels \|\| id:ebi-IPs91-GCST90274808 | F-deep fried food liking (derived food-liking factor) \|\| id:ebi-fl187-GCST90094753 | MR Egger | 8 | -0.444042055 | 0.168688327658945 | 0.0389383817517645 | -0.774671177 | -0.113412933 | 0.641438444739987 | 0.460855295935081 | 0.892781925301803 | 0.999279047927446 |
| ebi-fl187-GCST90094753 | ebi-IPs91-GCST90274810 | Interleukin-24 levels \|\| id:ebi-IPs91-GCST90274810 | F-deep fried food liking (derived food-liking factor) \|\| id:ebi-fl187-GCST90094753 | MR Egger | 8 | -0.037289625 | 0.170408365574944 | 0.83403990597649 | -0.371290021 | 0.296710771600243 | 0.963397071132968 | 0.689843843029689 | 1.34542610772803 | 0.999279047927446 |
| ebi-fl187-GCST90094753 | ebi-IPs91-GCST90274823 | Monocyte chemoattractant protein-3 levels \|\| id:ebi-IPs91-GCST90274823 | F-deep fried food liking (derived food-liking factor) \|\| id:ebi-fl187-GCST90094753 | MR Egger | 8 | -0.057126821 | 0.283820959395868 | 0.847131603915741 | -0.613415901 | 0.499162259341221 | 0.944474282527417 | 0.541498002437535 | 1.6473406482392 | 0.999279047927446 |
| ebi-fl187-GCST90094753 | ebi-IPs91-GCST90274824 | Monocyte chemoattractant protein-4 levels \|\| id:ebi-IPs91-GCST90274824 | F-deep fried food liking (derived food-liking factor) \|\| id:ebi-fl187-GCST90094753 | MR Egger | 8 | -0.065741446 | 0.225181792740681 | 0.780157617878504 | -0.50709776 | 0.375614867667625 | 0.93637293586657 | 0.602240892610275 | 1.45588631689076 | 0.999279047927446 |
| ebi-fl187-GCST90094753 | ebi-IPs91-GCST90274834 | SIR2-like protein 2 levels \|\| id:ebi-IPs91-GCST90274834 | F-deep fried food liking (derived food-liking factor) \|\| id:ebi-fl187-GCST90094753 | MR Egger | 8 | 0.0301579205741776 | 0.152410008526009 | 0.849677634268746 | -0.268565696 | 0.328881537285156 | 1.03061727677506 | 0.76447519809289 | 1.38941325217223 | 0.999279047927446 |
| ebi-fl187-GCST90094753 | ebi-met1400-GCST90199652 | 1-stearoyl-2-oleoyl-GPS (18:0/18:1) levels \|\| id:ebi-met1400-GCST90199652 | F-deep fried food liking (derived food-liking factor) \|\| id:ebi-fl187-GCST90094753 | MR Egger | 8 | -0.168215841 | 0.208663352291645 | 0.450912387451182 | -0.577196011 | 0.240764329726673 | 0.845171392556259 | 0.56147051840067 | 1.27222117526346 | 0.999279047927446 |
| ebi-fl187-GCST90094753 | ebi-met1400-GCST90199665 | N-acetylaspartate (naa) levels \|\| id:ebi-met1400-GCST90199665 | F-deep fried food liking (derived food-liking factor) \|\| id:ebi-fl187-GCST90094753 | MR Egger | 8 | 0.186756231272631 | 0.274921012206493 | 0.522270420722944 | -0.352088953 | 0.725601415197357 | 1.20533342663917 | 0.703217566126621 | 2.0659732341102 | 0.999279047927446 |
| ebi-fl187-GCST90094753 | ebi-met1400-GCST90199669 | Acetylcarnitine levels (Biocrates platform) \|\| id:ebi-met1400-GCST90199669 | F-deep fried food liking (derived food-liking factor) \|\| id:ebi-fl187-GCST90094753 | MR Egger | 8 | -0.058805241 | 0.200340444846465 | 0.779008032511547 | -0.451472513 | 0.333862031082456 | 0.942890387837764 | 0.636689927 | 1.39635047731997 | 0.999279047927446 |
| ebi-fl187-GCST90094753 | ebi-met1400-GCST90199670 | Iminodiacetate (IDA) levels \|\| id:ebi-met1400-GCST90199670 | F-deep fried food liking (derived food-liking factor) \|\| id:ebi-fl187-GCST90094753 | MR Egger | 8 | 0.229783664387684 | 0.305013646213744 | 0.479743390903367 | -0.368043082 | 0.827610410966623 | 1.25832775937548 | 0.69208736440624 | 2.28784519332084 | 0.999279047927446 |
| ebi-fl187-GCST90094753 | ebi-met1400-GCST90199686 | Indolepropionate levels \|\| id:ebi-met1400-GCST90199686 | F-deep fried food liking (derived food-liking factor) \|\| id:ebi-fl187-GCST90094753 | MR Egger | 8 | -0.17377497 | 0.211708009112338 | 0.443129031321148 | -0.588722668 | 0.241172727610742 | 0.840486010736943 | 0.555035797059365 | 1.27274085381009 | 0.999279047927446 |
| ebi-fl187-GCST90094753 | ebi-met1400-GCST90199704 | 3-hydroxysebacate levels \|\| id:ebi-met1400-GCST90199704 | F-deep fried food liking (derived food-liking factor) \|\| id:ebi-fl187-GCST90094753 | MR Egger | 8 | 0.00805645632067325 | 0.212316826190679 | 0.970962199164081 | -0.408084523 | 0.424197435654404 | 1.00808899689343 | 0.664922675179229 | 1.52836331740932 | 0.999279047927446 |
| ebi-fl187-GCST90094753 | ebi-met1400-GCST90199713 | Docosapentaenoate n3 DPA; 22:5n3 levels \|\| id:ebi-met1400-GCST90199713 | F-deep fried food liking (derived food-liking factor) \|\| id:ebi-fl187-GCST90094753 | MR Egger | 8 | -0.182220221 | 0.197783063427514 | 0.392424814318332 | -0.569875025 | 0.205434583493878 | 0.833417784253807 | 0.565596119577522 | 1.22805864302844 | 0.999279047927446 |
| ebi-fl187-GCST90094753 | ebi-met1400-GCST90199715 | Myristoleate (14:1n5) levels \|\| id:ebi-met1400-GCST90199715 | F-deep fried food liking (derived food-liking factor) \|\| id:ebi-fl187-GCST90094753 | MR Egger | 8 | 0.0922961400172876 | 0.189562510622712 | 0.643610745770959 | -0.279246381 | 0.463838660837803 | 1.09668954765793 | 0.756353529266444 | 1.59016639362895 | 0.999279047927446 |
| ebi-fl187-GCST90094753 | ebi-met1400-GCST90199725 | Campesterol levels \|\| id:ebi-met1400-GCST90199725 | F-deep fried food liking (derived food-liking factor) \|\| id:ebi-fl187-GCST90094753 | MR Egger | 8 | -0.49678828 | 0.287299439549928 | 0.134509951234446 | -1.059895182 | 0.0663186212735828 | 0.608481797792054 | 0.346492127120892 | 1.06856713115178 | 0.999279047927446 |
| ebi-fl187-GCST90094753 | ebi-met1400-GCST90199737 | N-acetylproline levels \|\| id:ebi-met1400-GCST90199737 | F-deep fried food liking (derived food-liking factor) \|\| id:ebi-fl187-GCST90094753 | MR Egger | 8 | -0.115027674 | 0.221725380442049 | 0.622481634313852 | -0.54960942 | 0.319554071770402 | 0.891341476674175 | 0.577175199703345 | 1.37651380109202 | 0.999279047927446 |
| ebi-fl187-GCST90094753 | ebi-met1400-GCST90199775 | Gamma-glutamylisoleucine levels \|\| id:ebi-met1400-GCST90199775 | F-deep fried food liking (derived food-liking factor) \|\| id:ebi-fl187-GCST90094753 | MR Egger | 8 | 0.570452703212884 | 0.198492510177571 | 0.0282814760874783 | 0.181407383264844 | 0.959498023160924 | 1.769067733 | 1.19890349304025 | 2.61038579127633 | 0.999279047927446 |
| ebi-fl187-GCST90094753 | ebi-met1400-GCST90199777 | 1-palmitoleoyl-GPC (16:1) levels \|\| id:ebi-met1400-GCST90199777 | F-deep fried food liking (derived food-liking factor) \|\| id:ebi-fl187-GCST90094753 | MR Egger | 8 | 0.2109917594806 | 0.202945567196358 | 0.33857316589154 | -0.186781552 | 0.608765071185461 | 1.2349021787841 | 0.829624946316451 | 1.83815999981278 | 0.999279047927446 |
| ebi-fl187-GCST90094753 | ebi-met1400-GCST90199782 | Glutamine degradant levels \|\| id:ebi-met1400-GCST90199782 | F-deep fried food liking (derived food-liking factor) \|\| id:ebi-fl187-GCST90094753 | MR Egger | 8 | -0.150866682 | 0.17227490571294 | 0.414854501586673 | -0.488525497 | 0.186792132915241 | 0.859962339233753 | 0.613530379647413 | 1.20537670086588 | 0.999279047927446 |
| ebi-fl187-GCST90094753 | ebi-met1400-GCST90199783 | 5-methyluridine (ribothymidine) levels \|\| id:ebi-met1400-GCST90199783 | F-deep fried food liking (derived food-liking factor) \|\| id:ebi-fl187-GCST90094753 | MR Egger | 8 | -0.110714964 | 0.205498746789937 | 0.609442889187746 | -0.513492507 | 0.292062580165942 | 0.895193875458307 | 0.598402001693988 | 1.33918682155056 | 0.999279047927446 |
| ebi-fl187-GCST90094753 | ebi-met1400-GCST90199787 | Homostachydrine levels \|\| id:ebi-met1400-GCST90199787 | F-deep fried food liking (derived food-liking factor) \|\| id:ebi-fl187-GCST90094753 | MR Egger | 8 | 0.355436371651921 | 0.256806503670581 | 0.215622046182831 | -0.147904376 | 0.85877711884626 | 1.4268031348961 | 0.86251358839279 | 2.36027259529068 | 0.999279047927446 |
| ebi-fl187-GCST90094753 | ebi-met1400-GCST90199802 | Beta-hydroxyisovaleroylcarnitine levels \|\| id:ebi-met1400-GCST90199802 | F-deep fried food liking (derived food-liking factor) \|\| id:ebi-fl187-GCST90094753 | MR Egger | 8 | 0.156564067607174 | 0.203104810601403 | 0.470050099876675 | -0.241521361 | 0.554649496385924 | 1.16948568606846 | 0.785432025861522 | 1.74133053515205 | 0.999279047927446 |
| ebi-fl187-GCST90094753 | ebi-met1400-GCST90199873 | Pregnenolone sulfate levels \|\| id:ebi-met1400-GCST90199873 | F-deep fried food liking (derived food-liking factor) \|\| id:ebi-fl187-GCST90094753 | MR Egger | 8 | 0.169044487811669 | 0.240055175827492 | 0.507710397548638 | -0.301463657 | 0.639552632433554 | 1.18417281900951 | 0.739734710185077 | 1.89563264501957 | 0.999279047927446 |
| ebi-fl187-GCST90094753 | ebi-met1400-GCST90199911 | Beta-citrylglutamate levels \|\| id:ebi-met1400-GCST90199911 | F-deep fried food liking (derived food-liking factor) \|\| id:ebi-fl187-GCST90094753 | MR Egger | 8 | 0.201016507646822 | 0.204466740786891 | 0.363512061004907 | -0.199738304 | 0.601771319589129 | 1.22264495464784 | 0.818945039436903 | 1.82534921531934 | 0.999279047927446 |
| ebi-fl187-GCST90094753 | ebi-met1400-GCST90199934 | 2-hydroxydecanoate levels \|\| id:ebi-met1400-GCST90199934 | F-deep fried food liking (derived food-liking factor) \|\| id:ebi-fl187-GCST90094753 | MR Egger | 8 | -0.084475411 | 0.284876527391056 | 0.776819532826352 | -0.642833405 | 0.473882582601707 | 0.918994252170786 | 0.525800505775216 | 1.60621837797165 | 0.999279047927446 |
| ebi-fl187-GCST90094753 | ebi-met1400-GCST90199945 | Sphingomyelin (d18:1/14:0, d16:1/16:0) levels \|\| id:ebi-met1400-GCST90199945 | F-deep fried food liking (derived food-liking factor) \|\| id:ebi-fl187-GCST90094753 | MR Egger | 8 | 0.15515813040179 | 0.225118651523864 | 0.516433299477726 | -0.286074427 | 0.596390687388563 | 1.16784261792721 | 0.751206704116645 | 1.81555405825466 | 0.999279047927446 |
| ebi-fl187-GCST90094753 | ebi-met1400-GCST90199979 | Carnitine C14:1 levels \|\| id:ebi-met1400-GCST90199979 | F-deep fried food liking (derived food-liking factor) \|\| id:ebi-fl187-GCST90094753 | MR Egger | 8 | -0.420389742 | 0.199466842717361 | 0.0796401881301914 | -0.811344754 | -0.029434731 | 0.656790790781761 | 0.444260243606117 | 0.970994251824587 | 0.999279047927446 |
| ebi-fl187-GCST90094753 | ebi-met1400-GCST90199986 | Eugenol sulfate levels \|\| id:ebi-met1400-GCST90199986 | F-deep fried food liking (derived food-liking factor) \|\| id:ebi-fl187-GCST90094753 | MR Egger | 8 | 0.082171958205481 | 0.324736686066419 | 0.808682510555718 | -0.554311946 | 0.718655862895662 | 1.08564247891192 | 0.574467389552928 | 2.05167362578276 | 0.999279047927446 |
| ebi-fl187-GCST90094753 | ebi-met1400-GCST90200002 | 6-hydroxyindole sulfate levels \|\| id:ebi-met1400-GCST90200002 | F-deep fried food liking (derived food-liking factor) \|\| id:ebi-fl187-GCST90094753 | MR Egger | 8 | 0.248251239770327 | 0.20408302608903 | 0.269500854211988 | -0.151751491 | 0.648253970904827 | 1.28178192634024 | 0.859201773273233 | 1.91219915717054 | 0.999279047927446 |
| ebi-fl187-GCST90094753 | ebi-met1400-GCST90200023 | Trans 3,4-methyleneheptanoate levels \|\| id:ebi-met1400-GCST90200023 | F-deep fried food liking (derived food-liking factor) \|\| id:ebi-fl187-GCST90094753 | MR Egger | 8 | -0.19510388 | 0.221616690691187 | 0.41253097205905 | -0.629472594 | 0.239264833446455 | 0.822749186177582 | 0.532872767169845 | 1.27031491391662 | 0.999279047927446 |
| ebi-fl187-GCST90094753 | ebi-met1400-GCST90200032 | 2-hydroxybutyrate/2-hydroxyisobutyrate levels \|\| id:ebi-met1400-GCST90200032 | F-deep fried food liking (derived food-liking factor) \|\| id:ebi-fl187-GCST90094753 | MR Egger | 8 | 0.263713095099491 | 0.207621163142964 | 0.251057254442079 | -0.143224385 | 0.6706505748597 | 1.30175466292712 | 0.866559604396056 | 1.95550911195951 | 0.999279047927446 |
| ebi-fl187-GCST90094753 | ebi-met1400-GCST90200051 | 1-palmitoyl-2-dihomo-linolenoyl-GPC (16:0/20:3n3 or 6) levels \|\| id:ebi-met1400-GCST90200051 | F-deep fried food liking (derived food-liking factor) \|\| id:ebi-fl187-GCST90094753 | MR Egger | 8 | 0.00757151540580993 | 0.20231685031678 | 0.971360980096835 | -0.388969511 | 0.404112542026699 | 1.00760025180884 | 0.677754933623366 | 1.49797252233566 | 0.999279047927446 |
| ebi-fl187-GCST90094753 | ebi-met1400-GCST90200069 | Phenylacetylglutamate levels \|\| id:ebi-met1400-GCST90200069 | F-deep fried food liking (derived food-liking factor) \|\| id:ebi-fl187-GCST90094753 | MR Egger | 8 | -0.266983925 | 0.227762727975655 | 0.285547530730036 | -0.713398872 | 0.179431021454117 | 0.765685379469369 | 0.489975998264697 | 1.19653636588222 | 0.999279047927446 |
| ebi-fl187-GCST90094753 | ebi-met1400-GCST90200072 | Thioproline levels \|\| id:ebi-met1400-GCST90200072 | F-deep fried food liking (derived food-liking factor) \|\| id:ebi-fl187-GCST90094753 | MR Egger | 8 | 0.259772319214487 | 0.205432712688818 | 0.2529433884975 | -0.142875798 | 0.66242043608457 | 1.29663483421782 | 0.866861728468515 | 1.93948104766068 | 0.999279047927446 |
| ebi-fl187-GCST90094753 | ebi-met1400-GCST90200081 | 1-oleoyl-2-docosahexaenoyl-GPC (18:1/22:6) levels \|\| id:ebi-met1400-GCST90200081 | F-deep fried food liking (derived food-liking factor) \|\| id:ebi-fl187-GCST90094753 | MR Egger | 7 | -0.311352368 | 0.633084036964937 | 0.643687587272874 | -1.55219708 | 0.929492344918234 | 0.732455736771056 | 0.211782159954283 | 2.53322284768764 | 0.999279047927446 |
| ebi-fl187-GCST90094753 | ebi-met1400-GCST90200097 | Furaneol sulfate levels \|\| id:ebi-met1400-GCST90200097 | F-deep fried food liking (derived food-liking factor) \|\| id:ebi-fl187-GCST90094753 | MR Egger | 8 | 0.164770791711349 | 0.245719970038519 | 0.527452095862555 | -0.31684035 | 0.646381932986845 | 1.17912282301119 | 0.728447042730638 | 1.90862279642749 | 0.999279047927446 |
| ebi-fl187-GCST90094753 | ebi-met1400-GCST90200124 | Sphingomyelin (d18:2/24:2) levels \|\| id:ebi-met1400-GCST90200124 | F-deep fried food liking (derived food-liking factor) \|\| id:ebi-fl187-GCST90094753 | MR Egger | 8 | 0.278040068785221 | 0.213624483669471 | 0.240812161505384 | -0.140663919 | 0.696744056777385 | 1.32053910854696 | 0.868781243327864 | 2.00720670547891 | 0.999279047927446 |
| ebi-fl187-GCST90094753 | ebi-met1400-GCST90200137 | Lignoceroylcarnitine (C24) levels \|\| id:ebi-met1400-GCST90200137 | F-deep fried food liking (derived food-liking factor) \|\| id:ebi-fl187-GCST90094753 | MR Egger | 8 | 0.0768949993014831 | 0.207153970695507 | 0.72324477466667 | -0.329126783 | 0.482916781864677 | 1.07992867717375 | 0.71955178384167 | 1.62079501986041 | 0.999279047927446 |
| ebi-fl187-GCST90094753 | ebi-met1400-GCST90200165 | Octadecenedioate (C18:1-DC) levels \|\| id:ebi-met1400-GCST90200165 | F-deep fried food liking (derived food-liking factor) \|\| id:ebi-fl187-GCST90094753 | MR Egger | 8 | -0.147487286 | 0.205516475090581 | 0.499951824656961 | -0.550299577 | 0.255325005348504 | 0.862873408971676 | 0.576776995370343 | 1.29088109596384 | 0.999279047927446 |
| ebi-fl187-GCST90094753 | ebi-met1400-GCST90200176 | Glutamine conjugate of C6H10O2 (1) levels \|\| id:ebi-met1400-GCST90200176 | F-deep fried food liking (derived food-liking factor) \|\| id:ebi-fl187-GCST90094753 | MR Egger | 8 | 0.247020189280612 | 0.202928883944976 | 0.269199526188756 | -0.150720423 | 0.644760801812766 | 1.28020495893452 | 0.860088125690304 | 1.90553117515154 | 0.999279047927446 |
| ebi-fl187-GCST90094753 | ebi-met1400-GCST90200182 | Glycine conjugate of C10H12O2 levels \|\| id:ebi-met1400-GCST90200182 | F-deep fried food liking (derived food-liking factor) \|\| id:ebi-fl187-GCST90094753 | MR Egger | 8 | 0.414837395120195 | 0.295965383706111 | 0.210574652598774 | -0.165254757 | 0.994929547184172 | 1.51412451663894 | 0.847677724791491 | 2.704533793 | 0.999279047927446 |
| ebi-fl187-GCST90094753 | ebi-met1400-GCST90200208 | Glucuronide of piperine metabolite C17H21NO3 (4) levels \|\| id:ebi-met1400-GCST90200208 | F-deep fried food liking (derived food-liking factor) \|\| id:ebi-fl187-GCST90094753 | MR Egger | 8 | 0.337015269683605 | 0.211391927260675 | 0.161985929997507 | -0.077312908 | 0.751343447114527 | 1.40076045274415 | 0.925600180820932 | 2.11984600546615 | 0.999279047927446 |
| ebi-fl187-GCST90094753 | ebi-met1400-GCST90200227 | 3-ethylcatechol sulfate (2) levels \|\| id:ebi-met1400-GCST90200227 | F-deep fried food liking (derived food-liking factor) \|\| id:ebi-fl187-GCST90094753 | MR Egger | 8 | -0.128692419 | 0.268481606118951 | 0.648671462988957 | -0.654916367 | 0.397531528843964 | 0.879244362688113 | 0.519485506827796 | 1.48814671277266 | 0.999279047927446 |
| ebi-fl187-GCST90094753 | ebi-met1400-GCST90200259 | Pentose acid levels \|\| id:ebi-met1400-GCST90200259 | F-deep fried food liking (derived food-liking factor) \|\| id:ebi-fl187-GCST90094753 | MR Egger | 8 | -0.190457813 | 0.233171789277982 | 0.445247923996401 | -0.64747452 | 0.266558894173564 | 0.826580628106459 | 0.523365859255216 | 1.30546447132252 | 0.999279047927446 |
| ebi-fl187-GCST90094753 | ebi-met1400-GCST90200291 | Sarcosine levels \|\| id:ebi-met1400-GCST90200291 | F-deep fried food liking (derived food-liking factor) \|\| id:ebi-fl187-GCST90094753 | MR Egger | 8 | 0.254831955317618 | 0.206620335964375 | 0.263573484754228 | -0.150143903 | 0.659807813807793 | 1.29024478387935 | 0.860584127 | 1.93442052975958 | 0.999279047927446 |
| ebi-fl187-GCST90094753 | ebi-met1400-GCST90200306 | Butyrate/isobutyrate (4:0) levels \|\| id:ebi-met1400-GCST90200306 | F-deep fried food liking (derived food-liking factor) \|\| id:ebi-fl187-GCST90094753 | MR Egger | 8 | 0.0355883082417091 | 0.208638405388375 | 0.870165285288296 | -0.373342966 | 0.444519582802923 | 1.03622915166214 | 0.688429084361479 | 1.55974068955899 | 0.999279047927446 |
| ebi-fl187-GCST90094753 | ebi-met1400-GCST90200321 | Cholate levels \|\| id:ebi-met1400-GCST90200321 | F-deep fried food liking (derived food-liking factor) \|\| id:ebi-fl187-GCST90094753 | MR Egger | 8 | -0.012825311 | 0.21252362932581 | 0.953838603906629 | -0.429371625 | 0.403721002371277 | 0.987256582716736 | 0.650917987100595 | 1.49738612149751 | 0.999279047927446 |
| ebi-fl187-GCST90094753 | ebi-met1400-GCST90200336 | Ceramide (d18:1/16:0) levels \|\| id:ebi-met1400-GCST90200336 | F-deep fried food liking (derived food-liking factor) \|\| id:ebi-fl187-GCST90094753 | MR Egger | 8 | -0.392703572 | 0.22055299219955 | 0.12528284588277 | -0.824987437 | 0.0395802927093277 | 0.675228874666684 | 0.438240498171559 | 1.04037402998103 | 0.999279047927446 |
| ebi-fl187-GCST90094753 | ebi-met1400-GCST90200358 | Arachidonate (20:4n6) levels \|\| id:ebi-met1400-GCST90200358 | F-deep fried food liking (derived food-liking factor) \|\| id:ebi-fl187-GCST90094753 | MR Egger | 8 | -0.097264768 | 0.20288999582936 | 0.64862961387512 | -0.49492916 | 0.300399623813066 | 0.907315746141298 | 0.609614090927666 | 1.35039835109983 | 0.999279047927446 |
| ebi-fl187-GCST90094753 | ebi-met1400-GCST90200413 | 2'-deoxyuridine levels \|\| id:ebi-met1400-GCST90200413 | F-deep fried food liking (derived food-liking factor) \|\| id:ebi-fl187-GCST90094753 | MR Egger | 8 | 0.14209361898394 | 0.205432244826474 | 0.51499648834785 | -0.260553581 | 0.544740818843829 | 1.1526845566428 | 0.770624864514566 | 1.72416145430212 | 0.999279047927446 |
| ebi-fl187-GCST90094753 | ebi-met1400-GCST90200432 | Threonine levels \|\| id:ebi-met1400-GCST90200432 | F-deep fried food liking (derived food-liking factor) \|\| id:ebi-fl187-GCST90094753 | MR Egger | 8 | -0.455643074 | 0.206084001824903 | 0.069052142360117 | -0.859567718 | -0.051718431 | 0.634040101950441 | 0.423345047282531 | 0.949596206361273 | 0.999279047927446 |
| ebi-fl187-GCST90094753 | ebi-met1400-GCST90200436 | Caffeine levels \|\| id:ebi-met1400-GCST90200436 | F-deep fried food liking (derived food-liking factor) \|\| id:ebi-fl187-GCST90094753 | MR Egger | 8 | 0.124090093080736 | 0.211314235875442 | 0.578461271906999 | -0.290085809 | 0.538265995396602 | 1.13211786239073 | 0.748199362408894 | 1.71303387671653 | 0.999279047927446 |
| ebi-fl187-GCST90094753 | ebi-met1400-GCST90200442 | Valine levels \|\| id:ebi-met1400-GCST90200442 | F-deep fried food liking (derived food-liking factor) \|\| id:ebi-fl187-GCST90094753 | MR Egger | 8 | 0.374368605867711 | 0.20227263440165 | 0.113662138601513 | -0.022085758 | 0.770822969294945 | 1.45407303153784 | 0.978156347153087 | 2.1615444066783 | 0.999279047927446 |
| ebi-fl187-GCST90094753 | ebi-met1400-GCST90200459 | Margarate (17:0) levels \|\| id:ebi-met1400-GCST90200459 | F-deep fried food liking (derived food-liking factor) \|\| id:ebi-fl187-GCST90094753 | MR Egger | 8 | 0.162804272730728 | 0.202215344549987 | 0.451476385194317 | -0.233537803 | 0.559146348048703 | 1.17680633405554 | 0.791727665820373 | 1.74917867299515 | 0.999279047927446 |
| ebi-fl187-GCST90094753 | ebi-met1400-GCST90200462 | X-11299 levels \|\| id:ebi-met1400-GCST90200462 | F-deep fried food liking (derived food-liking factor) \|\| id:ebi-fl187-GCST90094753 | MR Egger | 8 | 0.103697424519591 | 0.205027956240074 | 0.63105610220124 | -0.29815737 | 0.505552218750136 | 1.10926476781278 | 0.742184533191646 | 1.65790079162564 | 0.999279047927446 |
| ebi-fl187-GCST90094753 | ebi-met1400-GCST90200497 | X-12740 levels \|\| id:ebi-met1400-GCST90200497 | F-deep fried food liking (derived food-liking factor) \|\| id:ebi-fl187-GCST90094753 | MR Egger | 8 | 0.273245756352469 | 0.313583895456351 | 0.417053566259723 | -0.341378679 | 0.887870191446916 | 1.31422318383695 | 0.71078969628921 | 2.42994881038872 | 0.999279047927446 |
| ebi-fl187-GCST90094753 | ebi-met1400-GCST90200527 | X-13866 levels \|\| id:ebi-met1400-GCST90200527 | F-deep fried food liking (derived food-liking factor) \|\| id:ebi-fl187-GCST90094753 | MR Egger | 8 | -0.091325642 | 0.202107932434165 | 0.66724093615477 | -0.48745719 | 0.304805905573773 | 0.9127204423926 | 0.614186169236064 | 1.35636171520683 | 0.999279047927446 |
| ebi-fl187-GCST90094753 | ebi-met1400-GCST90200547 | X-17654 levels \|\| id:ebi-met1400-GCST90200547 | F-deep fried food liking (derived food-liking factor) \|\| id:ebi-fl187-GCST90094753 | MR Egger | 8 | -0.167695225 | 0.208634532019361 | 0.452186907038893 | -0.576618908 | 0.241228457421187 | 0.845611516380374 | 0.561794638328456 | 1.2728117853931 | 0.999279047927446 |
| ebi-fl187-GCST90094753 | ebi-met1400-GCST90200553 | X-17653 levels \|\| id:ebi-met1400-GCST90200553 | F-deep fried food liking (derived food-liking factor) \|\| id:ebi-fl187-GCST90094753 | MR Egger | 8 | -0.028244949 | 0.207590047384998 | 0.896223196974504 | -0.435121442 | 0.378631543403205 | 0.972150209954201 | 0.647186066511206 | 1.46028488500784 | 0.999279047927446 |
| ebi-fl187-GCST90094753 | ebi-met1400-GCST90200557 | X-17685 levels \|\| id:ebi-met1400-GCST90200557 | F-deep fried food liking (derived food-liking factor) \|\| id:ebi-fl187-GCST90094753 | MR Egger | 8 | -0.156254014 | 0.280681677003331 | 0.5978711428843 | -0.706390101 | 0.393882073286092 | 0.855341894265719 | 0.493422190828451 | 1.48272568539672 | 0.999279047927446 |
| ebi-fl187-GCST90094753 | ebi-met1400-GCST90200561 | X-18779 levels \|\| id:ebi-met1400-GCST90200561 | F-deep fried food liking (derived food-liking factor) \|\| id:ebi-fl187-GCST90094753 | MR Egger | 8 | 0.171182818116008 | 0.205553408706764 | 0.436850301615293 | -0.231701863 | 0.574067499181266 | 1.18670768085404 | 0.793182565169578 | 1.77547412366142 | 0.999279047927446 |
| ebi-fl187-GCST90094753 | ebi-met1400-GCST90200570 | X-18921 levels \|\| id:ebi-met1400-GCST90200570 | F-deep fried food liking (derived food-liking factor) \|\| id:ebi-fl187-GCST90094753 | MR Egger | 8 | -0.386476594 | 0.211727635125891 | 0.117744664219087 | -0.801462759 | 0.028509570710354 | 0.679446628223635 | 0.448672184610743 | 1.02891985828134 | 0.999279047927446 |
| ebi-fl187-GCST90094753 | ebi-met1400-GCST90200575 | X-21283 levels \|\| id:ebi-met1400-GCST90200575 | F-deep fried food liking (derived food-liking factor) \|\| id:ebi-fl187-GCST90094753 | MR Egger | 8 | 0.296856211629383 | 0.217396063427828 | 0.221068693347078 | -0.129240073 | 0.722952495947926 | 1.3456218007708 | 0.878762973229733 | 2.06050787967859 | 0.999279047927446 |
| ebi-fl187-GCST90094753 | ebi-met1400-GCST90200578 | X-18922 levels \|\| id:ebi-met1400-GCST90200578 | F-deep fried food liking (derived food-liking factor) \|\| id:ebi-fl187-GCST90094753 | MR Egger | 8 | 0.0795525147318343 | 0.20717487697705 | 0.714227109051714 | -0.326510244 | 0.485615273606852 | 1.08280242111516 | 0.721436984506991 | 1.62517462834829 | 0.999279047927446 |
| ebi-fl187-GCST90094753 | ebi-met1400-GCST90200623 | X-24241 levels \|\| id:ebi-met1400-GCST90200623 | F-deep fried food liking (derived food-liking factor) \|\| id:ebi-fl187-GCST90094753 | MR Egger | 8 | 0.190195793373657 | 0.208203319768858 | 0.396197864686074 | -0.217882713 | 0.598274300120619 | 1.2094863838954 | 0.804219760358105 | 1.81897708180783 | 0.999279047927446 |
| ebi-fl187-GCST90094753 | ebi-met1400-GCST90200636 | X-24544 levels \|\| id:ebi-met1400-GCST90200636 | F-deep fried food liking (derived food-liking factor) \|\| id:ebi-fl187-GCST90094753 | MR Egger | 8 | 0.258773689264702 | 0.218249540859952 | 0.28057262580047 | -0.168995411 | 0.686542789350208 | 1.29534062216544 | 0.844512778997617 | 1.9868347396987 | 0.999279047927446 |
| ebi-fl187-GCST90094753 | ebi-met1400-GCST90200659 | X-25172 levels \|\| id:ebi-met1400-GCST90200659 | F-deep fried food liking (derived food-liking factor) \|\| id:ebi-fl187-GCST90094753 | MR Egger | 8 | -0.036683472 | 0.221420554657781 | 0.87385547663388 | -0.470667759 | 0.397300814848045 | 0.963981213838183 | 0.624585056450564 | 1.48780341610124 | 0.999279047927446 |
| ebi-fl187-GCST90094753 | ebi-met1400-GCST90200670 | X-26111 levels \|\| id:ebi-met1400-GCST90200670 | F-deep fried food liking (derived food-liking factor) \|\| id:ebi-fl187-GCST90094753 | MR Egger | 8 | 0.233733238801069 | 0.222005369329255 | 0.332961153024888 | -0.201397285 | 0.668863762686409 | 1.26330744584546 | 0.8175875516838 | 1.95201810428959 | 0.999279047927446 |
| ebi-fl187-GCST90094753 | ebi-met1400-GCST90200673 | Carnitine C4 levels \|\| id:ebi-met1400-GCST90200673 | F-deep fried food liking (derived food-liking factor) \|\| id:ebi-fl187-GCST90094753 | MR Egger | 8 | 0.140805115527868 | 0.22057803220592 | 0.546825062089314 | -0.291527828 | 0.573138058651471 | 1.15120027506413 | 0.74712122272634 | 1.77382469269402 | 0.999279047927446 |
| ebi-fl187-GCST90094753 | ebi-met1400-GCST90200710 | X-13431 levels \|\| id:ebi-met1400-GCST90200710 | F-deep fried food liking (derived food-liking factor) \|\| id:ebi-fl187-GCST90094753 | MR Egger | 8 | 0.176603362019708 | 0.203701439386231 | 0.419273698991784 | -0.222651459 | 0.575858183216721 | 1.19315774758076 | 0.800393770587283 | 1.77865628510255 | 0.999279047927446 |
| ebi-fl187-GCST90094753 | ebi-met1400-GCST90200730 | Adenosine 5'-diphosphate (ADP) to fructose ratio \|\| id:ebi-met1400-GCST90200730 | F-deep fried food liking (derived food-liking factor) \|\| id:ebi-fl187-GCST90094753 | MR Egger | 8 | -0.021362575 | 0.30948838287954 | 0.947212292754536 | -0.627959806 | 0.585234655252542 | 0.978863988422662 | 0.533679500980413 | 1.79541223912569 | 0.999279047927446 |
| ebi-fl187-GCST90094753 | ebi-met1400-GCST90200731 | Adenosine 5'-diphosphate (ADP) to mannose ratio \|\| id:ebi-met1400-GCST90200731 | F-deep fried food liking (derived food-liking factor) \|\| id:ebi-fl187-GCST90094753 | MR Egger | 8 | -0.098267117 | 0.36286955123811 | 0.795615547823537 | -0.809491437 | 0.612957203873664 | 0.906406755166328 | 0.445084362122678 | 1.84588198491841 | 0.999279047927446 |
| ebi-fl187-GCST90094753 | ebi-met1400-GCST90200748 | Aspartate to glutamate ratio \|\| id:ebi-met1400-GCST90200748 | F-deep fried food liking (derived food-liking factor) \|\| id:ebi-fl187-GCST90094753 | MR Egger | 8 | -0.334660791 | 0.294083671655206 | 0.298520977868753 | -0.911064787 | 0.241743205659243 | 0.715580776782407 | 0.402095849484804 | 1.27346713167171 | 0.999279047927446 |
| ebi-fl187-GCST90094753 | ebi-met1400-GCST90200774 | Uridine to pseudouridine ratio \|\| id:ebi-met1400-GCST90200774 | F-deep fried food liking (derived food-liking factor) \|\| id:ebi-fl187-GCST90094753 | MR Egger | 8 | -0.12355303 | 0.196737819723267 | 0.553134976670737 | -0.509159156 | 0.262053097115977 | 0.883774773830253 | 0.601000714137292 | 1.29959554537283 | 0.999279047927446 |
| ebi-fl187-GCST90094753 | ebi-met1400-GCST90200799 | Spermidine to adenosine 5'-diphosphate (ADP) ratio \|\| id:ebi-met1400-GCST90200799 | F-deep fried food liking (derived food-liking factor) \|\| id:ebi-fl187-GCST90094753 | MR Egger | 8 | 0.306753885941316 | 0.35291364201854 | 0.418144359404728 | -0.384956852 | 0.998464624297655 | 1.35900645631491 | 0.680479996639647 | 2.71411144695798 | 0.999279047927446 |
| ebi-fl187-GCST90094753 | ebi-met1400-GCST90200811 | Choline to taurocholate ratio \|\| id:ebi-met1400-GCST90200811 | F-deep fried food liking (derived food-liking factor) \|\| id:ebi-fl187-GCST90094753 | MR Egger | 8 | -0.2711943 | 0.230172602079019 | 0.283316093598441 | -0.7223326 | 0.179944000222723 | 0.762468334526946 | 0.485618180869185 | 1.19715032109331 | 0.999279047927446 |
| ebi-fl187-GCST90094753 | ebi-met1400-GCST90200812 | Glutamate to kynurenine ratio \|\| id:ebi-met1400-GCST90200812 | F-deep fried food liking (derived food-liking factor) \|\| id:ebi-fl187-GCST90094753 | MR Egger | 8 | 0.239878725014563 | 0.270868736951856 | 0.409923456480775 | -0.291023999 | 0.770781449440201 | 1.27109498894736 | 0.747497738297321 | 2.16145466153162 | 0.999279047927446 |
| ebi-fl187-GCST90094753 | ebi-met1400-GCST90200876 | Cortisone to 4-cholesten-3-one ratio \|\| id:ebi-met1400-GCST90200876 | F-deep fried food liking (derived food-liking factor) \|\| id:ebi-fl187-GCST90094753 | MR Egger | 8 | 0.203448367155301 | 0.287161198964047 | 0.505226706942847 | -0.359387583 | 0.766284317124832 | 1.22562187366359 | 0.698103725903365 | 2.15175613804214 | 0.999279047927446 |
| ebi-fl187-GCST90094753 | ebi-met1400-GCST90200906 | 2'-deoxyuridine to cytidine ratio \|\| id:ebi-met1400-GCST90200906 | F-deep fried food liking (derived food-liking factor) \|\| id:ebi-fl187-GCST90094753 | MR Egger | 8 | 0.401345075615943 | 0.21226168365004 | 0.107532289003471 | -0.014687824 | 0.817377975570022 | 1.49383266479642 | 0.985419515581188 | 2.26455432953004 | 0.999279047927446 |
| ebi-fl187-GCST90094753 | ebi-met1400-GCST90200907 | Retinol (Vitamin A) to linoleoyl-arachidonoyl-glycerol (18:2 to 20:4) [1] ratio \|\| id:ebi-met1400-GCST90200907 | F-deep fried food liking (derived food-liking factor) \|\| id:ebi-fl187-GCST90094753 | MR Egger | 7 | -0.954234023 | 0.426653267715525 | 0.0755404978189209 | -1.790474428 | -0.117993618 | 0.385107014704969 | 0.166880977913281 | 0.888701724003805 | 0.999279047927446 |
| ebi-fl187-GCST90094753 | ebi-met1400-GCST90200916 | Fructose to sucrose ratio \|\| id:ebi-met1400-GCST90200916 | F-deep fried food liking (derived food-liking factor) \|\| id:ebi-fl187-GCST90094753 | MR Egger | 8 | -0.464798417 | 0.213192463881776 | 0.0720432228484286 | -0.882655647 | -0.046941188 | 0.628261739092021 | 0.413682856195628 | 0.954143510893459 | 0.999279047927446 |
| ebi-fl187-GCST90094753 | ebi-met1400-GCST90200917 | Alpha-ketobutyrate to 3-methyl-2-oxobutyrate ratio \|\| id:ebi-met1400-GCST90200917 | F-deep fried food liking (derived food-liking factor) \|\| id:ebi-fl187-GCST90094753 | MR Egger | 8 | -0.056696017 | 0.20707405543374 | 0.793422946742757 | -0.462561166 | 0.349169131644354 | 0.944881253547397 | 0.629668892222919 | 1.4178889799581 | 0.999279047927446 |
| ebi-fl187-GCST90094753 | ebi-met1400-GCST90200924 | Alpha-tocopherol to glycerol ratio \|\| id:ebi-met1400-GCST90200924 | F-deep fried food liking (derived food-liking factor) \|\| id:ebi-fl187-GCST90094753 | MR Egger | 8 | -0.344864454 | 0.192408385373624 | 0.123250047699009 | -0.721984889 | 0.0322559812365836 | 0.708316356342292 | 0.485787064768834 | 1.03278184424438 | 0.999279047927446 |
| ebi-fl187-GCST90094753 | ebi-met1400-GCST90200938 | Alpha-ketoglutarate to alanine ratio \|\| id:ebi-met1400-GCST90200938 | F-deep fried food liking (derived food-liking factor) \|\| id:ebi-fl187-GCST90094753 | MR Egger | 8 | 0.0562688621360653 | 0.204791600289785 | 0.792715264721915 | -0.345122674 | 0.457660398704045 | 1.05788206993759 | 0.70813347826706 | 1.58037221546723 | 0.999279047927446 |
| ebi-fl187-GCST90094753 | ebi-met1400-GCST90200943 | Phosphate to EDTA ratio \|\| id:ebi-met1400-GCST90200943 | F-deep fried food liking (derived food-liking factor) \|\| id:ebi-fl187-GCST90094753 | MR Egger | 8 | -0.710925726 | 0.19620139891002 | 0.0110514449922699 | -1.095480468 | -0.326370984 | 0.491189280160902 | 0.334378909971457 | 0.721537458703899 | 0.999279047927446 |
| ebi-fl187-GCST90094753 | ebi-met1400-GCST90200947 | Adenosine 5'-diphosphate (ADP) to glucose ratio \|\| id:ebi-met1400-GCST90200947 | F-deep fried food liking (derived food-liking factor) \|\| id:ebi-fl187-GCST90094753 | MR Egger | 8 | -0.097112523 | 0.346474837850841 | 0.788670132876229 | -0.776203206 | 0.581978158704152 | 0.907453890515305 | 0.460149792944568 | 1.78957499500725 | 0.999279047927446 |
| ebi-fl187-GCST90094753 | ebi-met1400-GCST90200975 | Arachidonate (20:4n6) to caffeine ratio \|\| id:ebi-met1400-GCST90200975 | F-deep fried food liking (derived food-liking factor) \|\| id:ebi-fl187-GCST90094753 | MR Egger | 8 | -0.130037229 | 0.211173710387824 | 0.560655232532559 | -0.543937701 | 0.283863243409939 | 0.878062740958006 | 0.580458075791879 | 1.32825127121691 | 0.999279047927446 |
| ebi-fl187-GCST90094753 | ebi-met1400-GCST90200985 | Cholesterol to oleoyl-linoleoyl-glycerol (18:1 to 18:2) [2] ratio \|\| id:ebi-met1400-GCST90200985 | F-deep fried food liking (derived food-liking factor) \|\| id:ebi-fl187-GCST90094753 | MR Egger | 8 | 0.434411941487418 | 0.41236500473122 | 0.332691574741204 | -0.373823468 | 1.24264735076061 | 1.54405479735455 | 0.688098372637119 | 3.46477380566472 | 0.999279047927446 |
| ebi-fl187-GCST90094753 | ebi-met1400-GCST90201016 | Bilirubin (Z,Z) to etiocholanolone glucuronide ratio \|\| id:ebi-met1400-GCST90201016 | F-deep fried food liking (derived food-liking factor) \|\| id:ebi-fl187-GCST90094753 | MR Egger | 8 | -0.079828263 | 0.211991284030417 | 0.719454988019523 | -0.49533118 | 0.335674653817548 | 0.923274893341287 | 0.609369063283375 | 1.39888382925334 | 0.999279047927446 |
| ebi-fl187-GCST90094753 | ebi-pw11-GCST90275189 | Placental weight (fetal genotype, adjusted for sex and gestational age) \|\| id:ebi-pw11-GCST90275189 | F-deep fried food liking (derived food-liking factor) \|\| id:ebi-fl187-GCST90094753 | MR Egger | 8 | 0.12112781663893 | 0.107720823673084 | 0.303780763088465 | -0.090004998 | 0.332260631038174 | 1.12876917862987 | 0.913926617673635 | 1.39411615110629 | 0.999279047927446 |
| ebi-fl187-GCST90094753 | ebi-pw11-GCST90275192 | Placental weight (fetal effect, conditional on maternal and paternal genotype) \|\| id:ebi-pw11-GCST90275192 | F-deep fried food liking (derived food-liking factor) \|\| id:ebi-fl187-GCST90094753 | MR Egger | 8 | -0.008759865 | 0.145359632286993 | 0.953902994627978 | -0.293664744 | 0.276145014132396 | 0.991278390681809 | 0.745526391424126 | 1.31803898444918 | 0.999279047927446 |
| ebi-fl187-GCST90094753 | ebi-pw11-GCST90275193 | Placental weight (maternal effect, conditional on fetal and paternal genotype) \|\| id:ebi-pw11-GCST90275193 | F-deep fried food liking (derived food-liking factor) \|\| id:ebi-fl187-GCST90094753 | MR Egger | 8 | 0.0506490206949015 | 0.173650191059597 | 0.780358439268235 | -0.289705354 | 0.391003395171711 | 1.05195361453377 | 0.748484073092851 | 1.47846353304219 | 0.999279047927446 |
| ebi-fl187-GCST90094753 | ebi-pw11-GCST90275195 | Placental weight (fetal effect, conditional on maternal genotype) \|\| id:ebi-pw11-GCST90275195 | F-deep fried food liking (derived food-liking factor) \|\| id:ebi-fl187-GCST90094753 | MR Egger | 8 | 0.124491833857853 | 0.128583227180249 | 0.370344595016142 | -0.127531291 | 0.376514959131141 | 1.13257277167224 | 0.880265870638208 | 1.45719733766724 | 0.999279047927446 |
| ebi-fl187-GCST90094753 | ebi-pw11-GCST90275197 | Placental weight (fetal genotype, adjusted for sex) \|\| id:ebi-pw11-GCST90275197 | F-deep fried food liking (derived food-liking factor) \|\| id:ebi-fl187-GCST90094753 | MR Egger | 8 | 0.147651203464559 | 0.0977718348175904 | 0.18174566008206 | -0.043981593 | 0.339283999707037 | 1.15910853280633 | 0.956971572499185 | 1.40394200771892 | 0.999279047927446 |
| ebi-fl187-GCST90094753 | prot-a-1030 | Protein FAM171B \|\| id:prot-a-1030 | F-deep fried food liking (derived food-liking factor) \|\| id:ebi-fl187-GCST90094753 | MR Egger | 8 | 0.0625084459854146 | 0.339926759903854 | 0.860158605787378 | -0.603748003 | 0.728764895396968 | 1.0645034496605 | 0.546758538114185 | 2.07251924816299 | 0.999279047927446 |
| ebi-fl187-GCST90094753 | prot-a-1036 | BRISC complex subunit Abro1 \|\| id:prot-a-1036 | F-deep fried food liking (derived food-liking factor) \|\| id:ebi-fl187-GCST90094753 | MR Egger | 8 | 0.194896687784415 | 0.321023817040392 | 0.566028486510728 | -0.434309994 | 0.824103369183584 | 1.21518543650463 | 0.647711437950914 | 2.27983567769703 | 0.999279047927446 |
| ebi-fl187-GCST90094753 | prot-a-1055 | Phenylalanine--tRNA ligase, mitochondrial \|\| id:prot-a-1055 | F-deep fried food liking (derived food-liking factor) \|\| id:ebi-fl187-GCST90094753 | MR Egger | 8 | -0.959926027 | 0.403835693953746 | 0.0549911785828701 | -1.751443987 | -0.168408067 | 0.382921210837079 | 0.173523197225721 | 0.845008944355717 | 0.999279047927446 |
| ebi-fl187-GCST90094753 | prot-a-1083 | Fc receptor-like protein 5 \|\| id:prot-a-1083 | F-deep fried food liking (derived food-liking factor) \|\| id:ebi-fl187-GCST90094753 | MR Egger | 8 | -0.422245784 | 0.438048607567545 | 0.372308969677023 | -1.280821055 | 0.436329486676773 | 0.655572890161037 | 0.277809110231985 | 1.54701843274761 | 0.999279047927446 |
| ebi-fl187-GCST90094753 | prot-a-1103 | Fibroblast growth factor receptor 3 \|\| id:prot-a-1103 | F-deep fried food liking (derived food-liking factor) \|\| id:ebi-fl187-GCST90094753 | MR Egger | 8 | 0.21988602568301 | 0.397969369822739 | 0.6005538004706 | -0.560133939 | 0.999905990535579 | 1.24593471793615 | 0.57113256170459 | 2.71802629625162 | 0.999279047927446 |
| ebi-fl187-GCST90094753 | prot-a-1105 | Fibroblast growth factor receptor 4 \|\| id:prot-a-1105 | F-deep fried food liking (derived food-liking factor) \|\| id:ebi-fl187-GCST90094753 | MR Egger | 8 | -0.162656967 | 0.321650419739721 | 0.631107198171304 | -0.79309179 | 0.467777855671151 | 0.849882676204942 | 0.452443769569514 | 1.59644272259627 | 0.999279047927446 |
| ebi-fl187-GCST90094753 | prot-a-1120 | Filamin-A \|\| id:prot-a-1120 | F-deep fried food liking (derived food-liking factor) \|\| id:ebi-fl187-GCST90094753 | MR Egger | 8 | 0.214063033702954 | 0.347485681133407 | 0.560500273707096 | -0.467008901 | 0.895134968724431 | 1.23870073222668 | 0.626874510377852 | 2.44766612554409 | 0.999279047927446 |
| ebi-fl187-GCST90094753 | prot-a-1166 | Galanin peptides \|\| id:prot-a-1166 | F-deep fried food liking (derived food-liking factor) \|\| id:ebi-fl187-GCST90094753 | MR Egger | 8 | 0.340226361193181 | 0.321650419739721 | 0.330883656751912 | -0.290208461 | 0.970661183883033 | 1.4052656521736 | 0.748107599692577 | 2.63968920244946 | 0.999279047927446 |
| ebi-fl187-GCST90094753 | prot-a-1205 | ADP-ribosylation factor-binding protein GGA1 \|\| id:prot-a-1205 | F-deep fried food liking (derived food-liking factor) \|\| id:ebi-fl187-GCST90094753 | MR Egger | 8 | -0.003929433 | 0.321650419739721 | 0.99064898496534 | -0.634364255 | 0.626505390183492 | 0.996078277611448 | 0.530272499165706 | 1.87106051452867 | 0.999279047927446 |
| ebi-fl187-GCST90094753 | prot-a-1227 | Guanine nucleotide-binding protein G(I)/G(S)/G(O) subunit gamma-T2 \|\| id:prot-a-1227 | F-deep fried food liking (derived food-liking factor) \|\| id:ebi-fl187-GCST90094753 | MR Egger | 8 | 0.582325937808405 | 0.321023817040392 | 0.11961622486683 | -0.046880744 | 1.21153261920757 | 1.79019748003337 | 0.954201185412333 | 3.35862820808901 | 0.999279047927446 |
| ebi-fl187-GCST90094753 | prot-a-1240 | Golgi SNAP receptor complex member 1 \|\| id:prot-a-1240 | F-deep fried food liking (derived food-liking factor) \|\| id:ebi-fl187-GCST90094753 | MR Egger | 8 | 0.125267606814814 | 0.321650419739721 | 0.710388953781222 | -0.505167216 | 0.755702429504667 | 1.13345173189299 | 0.60340466815104 | 2.12910654547612 | 0.999279047927446 |
| ebi-fl187-GCST90094753 | prot-a-1283 | Glutathione S-transferase A1 \|\| id:prot-a-1283 | F-deep fried food liking (derived food-liking factor) \|\| id:ebi-fl187-GCST90094753 | MR Egger | 8 | 0.367569337482879 | 0.321023817040392 | 0.295822678056776 | -0.261637344 | 0.996776018882048 | 1.44421993363287 | 0.76979014217075 | 2.70953225098573 | 0.999279047927446 |
| ebi-fl187-GCST90094753 | prot-a-1324 | E3 ubiquitin-protein ligase HECW1 \|\| id:prot-a-1324 | F-deep fried food liking (derived food-liking factor) \|\| id:ebi-fl187-GCST90094753 | MR Egger | 8 | 0.407502413421328 | 0.36986589288186 | 0.312789673852935 | -0.317434737 | 1.13243956346977 | 1.50305907304454 | 0.728014191885596 | 3.1032177699862 | 0.999279047927446 |
| ebi-fl187-GCST90094753 | prot-a-1339 | Histidine triad nucleotide-binding protein 2, mitochondrial \|\| id:prot-a-1339 | F-deep fried food liking (derived food-liking factor) \|\| id:ebi-fl187-GCST90094753 | MR Egger | 8 | 0.00618141070436431 | 0.325426668429186 | 0.985461163932041 | -0.631654859 | 0.64401768082557 | 1.00620055504954 | 0.531711165314775 | 1.90411566095782 | 0.999279047927446 |
| ebi-fl187-GCST90094753 | prot-a-1466 | Interleukin-11 \|\| id:prot-a-1466 | F-deep fried food liking (derived food-liking factor) \|\| id:ebi-fl187-GCST90094753 | MR Egger | 8 | 0.103542197088756 | 0.321023817040392 | 0.757988559675471 | -0.525664484 | 0.732748878487925 | 1.10909259285624 | 0.591162415677031 | 2.08079259930592 | 0.999279047927446 |
| ebi-fl187-GCST90094753 | prot-a-147 | Rho GTPase-activating protein 1 \|\| id:prot-a-147 | F-deep fried food liking (derived food-liking factor) \|\| id:ebi-fl187-GCST90094753 | MR Egger | 8 | -0.47625943 | 0.337490170940173 | 0.207881186501361 | -1.137740165 | 0.18522130475448 | 0.621102328588151 | 0.320542577179433 | 1.20348474755564 | 0.999279047927446 |
| ebi-fl187-GCST90094753 | prot-a-1473 | Interleukin-12 receptor subunit beta-1 \|\| id:prot-a-1473 | F-deep fried food liking (derived food-liking factor) \|\| id:ebi-fl187-GCST90094753 | MR Egger | 8 | 0.580825709495573 | 0.407836276099177 | 0.204265552696493 | -0.218533392 | 1.38018481064996 | 1.7875137886665 | 0.803696642232522 | 3.97563629953357 | 0.999279047927446 |
| ebi-fl187-GCST90094753 | prot-a-1501 | X-linked interleukin-1 receptor accessory protein-like 2 \|\| id:prot-a-1501 | F-deep fried food liking (derived food-liking factor) \|\| id:ebi-fl187-GCST90094753 | MR Egger | 8 | -0.339148066 | 0.340706538018453 | 0.357963766069564 | -1.00693288 | 0.328636748877366 | 0.712376962729878 | 0.365337797729311 | 1.38907318153884 | 0.999279047927446 |
| ebi-fl187-GCST90094753 | prot-a-1503 | Interleukin-1 receptor-like 2 \|\| id:prot-a-1503 | F-deep fried food liking (derived food-liking factor) \|\| id:ebi-fl187-GCST90094753 | MR Egger | 8 | 0.427001531174242 | 0.321023817040392 | 0.231801819884174 | -0.20220515 | 1.05620821257341 | 1.53265500848409 | 0.816927317927186 | 2.87544720745983 | 0.999279047927446 |
| ebi-fl187-GCST90094753 | prot-a-1536 | Interleukin-5 receptor subunit alpha \|\| id:prot-a-1536 | F-deep fried food liking (derived food-liking factor) \|\| id:ebi-fl187-GCST90094753 | MR Egger | 8 | -0.447849384 | 0.321023817040392 | 0.212449999557269 | -1.077056065 | 0.181357297760586 | 0.639000920777949 | 0.340596745826346 | 1.19884344685798 | 0.999279047927446 |
| ebi-fl187-GCST90094753 | prot-a-1559 | Insulin-induced gene 1 protein \|\| id:prot-a-1559 | F-deep fried food liking (derived food-liking factor) \|\| id:ebi-fl187-GCST90094753 | MR Egger | 8 | 0.590914082329055 | 0.321023817040392 | 0.115263524489902 | -0.038292599 | 1.22012076372822 | 1.80563816321799 | 0.962431293186846 | 3.3875968077404 | 0.999279047927446 |
| ebi-fl187-GCST90094753 | prot-a-1567 | Interferon regulatory factor 1 \|\| id:prot-a-1567 | F-deep fried food liking (derived food-liking factor) \|\| id:ebi-fl187-GCST90094753 | MR Egger | 8 | -0.393207092 | 0.358775355376651 | 0.315121327853169 | -1.096406788 | 0.309992604715721 | 0.674888969126533 | 0.334069311404053 | 1.36341503125315 | 0.999279047927446 |
| ebi-fl187-GCST90094753 | prot-a-1613 | Potassium voltage-gated channel subfamily E regulatory beta subunit 5 \|\| id:prot-a-1613 | F-deep fried food liking (derived food-liking factor) \|\| id:ebi-fl187-GCST90094753 | MR Egger | 8 | 0.138827976825819 | 0.321023817040392 | 0.680522476701681 | -0.490378705 | 0.768034658224988 | 1.14892644102984 | 0.612394433692205 | 2.1555257433332 | 0.999279047927446 |
| ebi-fl187-GCST90094753 | prot-a-1649 | Kin of IRRE-like protein 2 \|\| id:prot-a-1649 | F-deep fried food liking (derived food-liking factor) \|\| id:ebi-fl187-GCST90094753 | MR Egger | 8 | 0.717000372781866 | 0.321023817040392 | 0.0669446446067367 | 0.0877936913826967 | 1.34620705418103 | 2.04827991028485 | 1.09176285870631 | 3.84282223691683 | 0.999279047927446 |
| ebi-fl187-GCST90094753 | prot-a-165 | Armadillo repeat-containing protein 5 \|\| id:prot-a-165 | F-deep fried food liking (derived food-liking factor) \|\| id:ebi-fl187-GCST90094753 | MR Egger | 8 | -0.623411944 | 0.321023817040392 | 0.100170827409489 | -1.252618626 | 0.00579473709928879 | 0.536112128782522 | 0.285755529489783 | 1.00581155906557 | 0.999279047927446 |
| ebi-fl187-GCST90094753 | prot-a-1656 | Kallikrein-11 \|\| id:prot-a-1656 | F-deep fried food liking (derived food-liking factor) \|\| id:ebi-fl187-GCST90094753 | MR Egger | 8 | 0.109975325469683 | 0.365300848432372 | 0.773533438228082 | -0.606014337 | 0.825964988397132 | 1.11625052716159 | 0.54552080372174 | 2.28408381657998 | 0.999279047927446 |
| ebi-fl187-GCST90094753 | prot-a-1697 | Laminin subunit gamma-2 \|\| id:prot-a-1697 | F-deep fried food liking (derived food-liking factor) \|\| id:ebi-fl187-GCST90094753 | MR Egger | 8 | -0.188675262 | 0.350700208166557 | 0.609940225308462 | -0.87604767 | 0.498697145879658 | 0.828055363976095 | 0.41642551448746 | 1.64657462608534 | 0.999279047927446 |
| ebi-fl187-GCST90094753 | prot-a-1698 | Lysosome-associated membrane glycoprotein 3 \|\| id:prot-a-1698 | F-deep fried food liking (derived food-liking factor) \|\| id:ebi-fl187-GCST90094753 | MR Egger | 8 | -0.447838975 | 0.321023817040392 | 0.212459283752495 | -1.077045657 | 0.181367706011793 | 0.639007571694667 | 0.340600290861286 | 1.19885592478667 | 0.999279047927446 |
| ebi-fl187-GCST90094753 | prot-a-1724 | Leptin receptor \|\| id:prot-a-1724 | F-deep fried food liking (derived food-liking factor) \|\| id:ebi-fl187-GCST90094753 | MR Egger | 8 | -0.167952752 | 0.321023817040392 | 0.619600662228087 | -0.797159433 | 0.461253929605273 | 0.845393777080467 | 0.450607127552963 | 1.58606154813291 | 0.999279047927446 |
| ebi-fl187-GCST90094753 | prot-a-1743 | Leukocyte immunoglobulin-like receptor subfamily B member 2 \|\| id:prot-a-1743 | F-deep fried food liking (derived food-liking factor) \|\| id:ebi-fl187-GCST90094753 | MR Egger | 8 | -0.195922621 | 0.321023817040392 | 0.564045026779079 | -0.825129302 | 0.433284060607362 | 0.822075843795011 | 0.438178331383541 | 1.54231426920958 | 0.999279047927446 |
| ebi-fl187-GCST90094753 | prot-a-1746 | Leukocyte immunoglobulin-like receptor subfamily B member 4 \|\| id:prot-a-1746 | F-deep fried food liking (derived food-liking factor) \|\| id:ebi-fl187-GCST90094753 | MR Egger | 8 | 1.15675974342903 | 0.347127850069134 | 0.0157601749565138 | 0.476389157293528 | 1.83713032956453 | 3.1796138017585 | 1.61024953439413 | 6.27849517257404 | 0.999279047927446 |
| ebi-fl187-GCST90094753 | prot-a-1750 | Leucine-rich repeat and immunoglobulin-like domain-containing nogo receptor-interacting protein 3 \|\| id:prot-a-1750 | F-deep fried food liking (derived food-liking factor) \|\| id:ebi-fl187-GCST90094753 | MR Egger | 8 | 0.484514415829263 | 0.354152912548663 | 0.220302412238646 | -0.209625293 | 1.17865412442464 | 1.62338652662216 | 0.810888034663181 | 3.24999716627097 | 0.999279047927446 |
| ebi-fl187-GCST90094753 | prot-a-1753 | Lipase member N \|\| id:prot-a-1753 | F-deep fried food liking (derived food-liking factor) \|\| id:ebi-fl187-GCST90094753 | MR Egger | 8 | 0.478670915694079 | 0.338367218894746 | 0.206909449542767 | -0.184528833 | 1.14187066472778 | 1.61392792980509 | 0.831495964748217 | 3.13262297477739 | 0.999279047927446 |
| ebi-fl187-GCST90094753 | prot-a-1760 | Ig Kappa chain V-I region HK102- like \|\| id:prot-a-1760 | F-deep fried food liking (derived food-liking factor) \|\| id:ebi-fl187-GCST90094753 | MR Egger | 8 | -0.060325169 | 0.321023817040392 | 0.857137461159999 | -0.68953185 | 0.568881512428367 | 0.941458350763399 | 0.501810936689526 | 1.76629037236493 | 0.999279047927446 |
| ebi-fl187-GCST90094753 | prot-a-1792 | Leucine-rich repeat neuronal protein 1 \|\| id:prot-a-1792 | F-deep fried food liking (derived food-liking factor) \|\| id:ebi-fl187-GCST90094753 | MR Egger | 7 | 0.809124590259653 | 0.702732443523012 | 0.301634345705957 | -0.568230999 | 2.18648017956476 | 2.24594100721164 | 0.566526739130008 | 8.90381805388582 | 0.999279047927446 |
| ebi-fl187-GCST90094753 | prot-a-1795 | Leucine-rich repeat transmembrane neuronal protein 1 \|\| id:prot-a-1795 | F-deep fried food liking (derived food-liking factor) \|\| id:ebi-fl187-GCST90094753 | MR Egger | 8 | -0.026361619 | 0.321023817040392 | 0.937224306666305 | -0.6555683 | 0.602845062502075 | 0.973982815326602 | 0.519146947373963 | 1.82731022372398 | 0.999279047927446 |
| ebi-fl187-GCST90094753 | prot-a-1820 | LysM and putative peptidoglycan-binding domain-containing protein 4 \|\| id:prot-a-1820 | F-deep fried food liking (derived food-liking factor) \|\| id:ebi-fl187-GCST90094753 | MR Egger | 8 | 0.834169567053089 | 0.379798687161246 | 0.0704559176674129 | 0.089764140217046 | 1.57857499388913 | 2.30290084926651 | 1.09391624242812 | 4.84804239653741 | 0.999279047927446 |
| ebi-fl187-GCST90094753 | prot-a-1844 | Mitogen-activated protein kinase kinase kinase 7:TGF-beta-activated kinase 1 and MAP3K7-binding protein 1 fusion \|\| id:prot-a-1844 | F-deep fried food liking (derived food-liking factor) \|\| id:ebi-fl187-GCST90094753 | MR Egger | 8 | 0.202031594396925 | 0.456956139620969 | 0.673889598020609 | -0.693602439 | 1.09766562805402 | 1.22388667546194 | 0.499772422457145 | 2.99716136198314 | 0.999279047927446 |
| ebi-fl187-GCST90094753 | prot-a-1856 | Megakaryocyte-associated tyrosine-protein kinase \|\| id:prot-a-1856 | F-deep fried food liking (derived food-liking factor) \|\| id:ebi-fl187-GCST90094753 | MR Egger | 8 | -0.266997974 | 0.39915107110871 | 0.528432669645905 | -1.049334073 | 0.515338125428189 | 0.76567462276279 | 0.350170859603977 | 1.67420449721591 | 0.999279047927446 |
| ebi-fl187-GCST90094753 | prot-a-1910 | Stromelysin-2 \|\| id:prot-a-1910 | F-deep fried food liking (derived food-liking factor) \|\| id:ebi-fl187-GCST90094753 | MR Egger | 8 | 0.211836281627989 | 0.321650419739721 | 0.534598942408954 | -0.418598541 | 0.842271104317841 | 1.23594552152496 | 0.657968289503606 | 2.3216336661605 | 0.999279047927446 |
| ebi-fl187-GCST90094753 | prot-a-1940 | 39S ribosomal protein L14, mitochondrial \|\| id:prot-a-1940 | F-deep fried food liking (derived food-liking factor) \|\| id:ebi-fl187-GCST90094753 | MR Egger | 8 | -0.098249158 | 0.321023817040392 | 0.769908321750376 | -0.727455839 | 0.530957523593637 | 0.906423033242542 | 0.4831366050124 | 1.70055985547095 | 0.999279047927446 |
| ebi-fl187-GCST90094753 | prot-a-1942 | 39S ribosomal protein L33, mitochondrial \|\| id:prot-a-1942 | F-deep fried food liking (derived food-liking factor) \|\| id:ebi-fl187-GCST90094753 | MR Egger | 8 | -0.271640175 | 0.321650419739721 | 0.430758726471987 | -0.902074997 | 0.358794648141168 | 0.762128444969382 | 0.405726903480203 | 1.43160278909084 | 0.999279047927446 |
| ebi-fl187-GCST90094753 | prot-a-1948 | Mesothelin \|\| id:prot-a-1948 | F-deep fried food liking (derived food-liking factor) \|\| id:ebi-fl187-GCST90094753 | MR Egger | 8 | -0.087726287 | 0.321023817040392 | 0.793808133890579 | -0.716932968 | 0.541480394887711 | 0.916011567134672 | 0.488247432453664 | 1.7185491112729 | 0.999279047927446 |
| ebi-fl187-GCST90094753 | prot-a-1980 | Unconventional myosin-VI \|\| id:prot-a-1980 | F-deep fried food liking (derived food-liking factor) \|\| id:ebi-fl187-GCST90094753 | MR Egger | 8 | 0.218793236647063 | 0.352119959511243 | 0.557217010648628 | -0.471361884 | 0.908948357289099 | 1.24457391780603 | 0.624151667038284 | 2.48171128699083 | 0.999279047927446 |
| ebi-fl187-GCST90094753 | prot-a-2039 | Neurofascin \|\| id:prot-a-2039 | F-deep fried food liking (derived food-liking factor) \|\| id:ebi-fl187-GCST90094753 | MR Egger | 8 | 0.239850246073045 | 0.321023817040392 | 0.483218597819765 | -0.389356435 | 0.869056927472214 | 1.27105879002296 | 0.677492744625061 | 2.38466088458075 | 0.999279047927446 |
| ebi-fl187-GCST90094753 | prot-a-2041 | NFU1 iron-sulfur cluster scaffold homolog, mitochondrial \|\| id:prot-a-2041 | F-deep fried food liking (derived food-liking factor) \|\| id:ebi-fl187-GCST90094753 | MR Egger | 8 | 0.276163049977306 | 0.356941056305822 | 0.468489759732096 | -0.42344142 | 0.975767520336717 | 1.31806275661027 | 0.654789531863556 | 2.65320281681758 | 0.999279047927446 |
| ebi-fl187-GCST90094753 | prot-a-2088 | Glucocorticoid receptor \|\| id:prot-a-2088 | F-deep fried food liking (derived food-liking factor) \|\| id:ebi-fl187-GCST90094753 | MR Egger | 8 | 0.365041179895089 | 0.470713206240918 | 0.467495700528942 | -0.557556704 | 1.28763906412729 | 1.44057332958636 | 0.572606402838125 | 3.62421989630146 | 0.999279047927446 |
| ebi-fl187-GCST90094753 | prot-a-2111 | Neuron-specific protein family member 2 \|\| id:prot-a-2111 | F-deep fried food liking (derived food-liking factor) \|\| id:ebi-fl187-GCST90094753 | MR Egger | 8 | 0.440689580800756 | 0.321023817040392 | 0.218924524691215 | -0.188517101 | 1.06989626219992 | 1.55377830485626 | 0.828186340835522 | 2.91507708060772 | 0.999279047927446 |
| ebi-fl187-GCST90094753 | prot-a-2125 | Nuclear migration protein nudC \|\| id:prot-a-2125 | F-deep fried food liking (derived food-liking factor) \|\| id:ebi-fl187-GCST90094753 | MR Egger | 8 | 0.189062436756936 | 0.393460958696484 | 0.647881737406074 | -0.582121042 | 0.960245915802044 | 1.20811638099695 | 0.558712057003961 | 2.61233880983318 | 0.999279047927446 |
| ebi-fl187-GCST90094753 | prot-a-2142 | Ornithine decarboxylase \|\| id:prot-a-2142 | F-deep fried food liking (derived food-liking factor) \|\| id:ebi-fl187-GCST90094753 | MR Egger | 8 | -0.484225259 | 0.321023817040392 | 0.182188115459072 | -1.11343194 | 0.14498142287004 | 0.616174387742069 | 0.328429873107206 | 1.15601809457017 | 0.999279047927446 |
| ebi-fl187-GCST90094753 | prot-a-2186 | Prostate and testis expressed protein 1 \|\| id:prot-a-2186 | F-deep fried food liking (derived food-liking factor) \|\| id:ebi-fl187-GCST90094753 | MR Egger | 8 | 0.534215748937054 | 0.463367520224178 | 0.292804799221402 | -0.373984591 | 1.44241608857644 | 1.70610969910085 | 0.68798751315168 | 4.23090572099416 | 0.999279047927446 |
| ebi-fl187-GCST90094753 | prot-a-2227 | Platelet-derived growth factor C \|\| id:prot-a-2227 | F-deep fried food liking (derived food-liking factor) \|\| id:ebi-fl187-GCST90094753 | MR Egger | 8 | 0.241127343962935 | 0.430215311477967 | 0.595444353686209 | -0.602094667 | 1.08434935445975 | 1.27268309349813 | 0.547663261876903 | 2.95751489870801 | 0.999279047927446 |
| ebi-fl187-GCST90094753 | prot-a-2229 | Platelet-derived growth factor receptor alpha \|\| id:prot-a-2229 | F-deep fried food liking (derived food-liking factor) \|\| id:ebi-fl187-GCST90094753 | MR Egger | 8 | 0.0381429799673951 | 0.321650419739721 | 0.909474537136603 | -0.592291843 | 0.668577802657248 | 1.03887976125435 | 0.553058308483568 | 1.95145998493928 | 0.999279047927446 |
| ebi-fl187-GCST90094753 | prot-a-2243 | Platelet endothelial aggregation receptor 1 \|\| id:prot-a-2243 | F-deep fried food liking (derived food-liking factor) \|\| id:ebi-fl187-GCST90094753 | MR Egger | 8 | -0.126511267 | 0.33641996157791 | 0.719816380305612 | -0.785894392 | 0.532871857450147 | 0.881164221196571 | 0.45571193438197 | 1.70381841276545 | 0.999279047927446 |
| ebi-fl187-GCST90094753 | prot-a-2246 | E3 ubiquitin-protein ligase pellino homolog 2 \|\| id:prot-a-2246 | F-deep fried food liking (derived food-liking factor) \|\| id:ebi-fl187-GCST90094753 | MR Egger | 8 | 0.423903303398919 | 0.598969513750078 | 0.505666217136298 | -0.750076944 | 1.59788355034907 | 1.52791384256533 | 0.472330208579205 | 4.94256066603303 | 0.999279047927446 |
| ebi-fl187-GCST90094753 | prot-a-2251 | Platelet factor 4 variant \|\| id:prot-a-2251 | F-deep fried food liking (derived food-liking factor) \|\| id:ebi-fl187-GCST90094753 | MR Egger | 8 | 0.0472706661770546 | 0.321023817040392 | 0.887758316773905 | -0.581936015 | 0.676477347576224 | 1.04840573865321 | 0.558815443420988 | 1.96693667968822 | 0.999279047927446 |
| ebi-fl187-GCST90094753 | prot-a-2346 | Protein phosphatase 1L \|\| id:prot-a-2346 | F-deep fried food liking (derived food-liking factor) \|\| id:ebi-fl187-GCST90094753 | MR Egger | 8 | -0.19095388 | 0.431159002306381 | 0.673369443993189 | -1.036025524 | 0.654117764696189 | 0.826170690409932 | 0.354862274355376 | 1.92344483766927 | 0.999279047927446 |
| ebi-fl187-GCST90094753 | prot-a-2358 | PR domain zinc finger protein 4 \|\| id:prot-a-2358 | F-deep fried food liking (derived food-liking factor) \|\| id:ebi-fl187-GCST90094753 | MR Egger | 8 | 0.168112014076648 | 0.33396437556053 | 0.632637283797775 | -0.486458162 | 0.822682190175286 | 1.18306912362151 | 0.614800064735804 | 2.27659792434802 | 0.999279047927446 |
| ebi-fl187-GCST90094753 | prot-a-2390 | Trypsin-1 \|\| id:prot-a-2390 | F-deep fried food liking (derived food-liking factor) \|\| id:ebi-fl187-GCST90094753 | MR Egger | 8 | -0.311218371 | 0.354250799176789 | 0.413452780350922 | -1.005549937 | 0.383113195300266 | 0.732553889813155 | 0.365843388484157 | 1.46684406052516 | 0.999279047927446 |
| ebi-fl187-GCST90094753 | prot-a-242 | Brain-derived neurotrophic factor \|\| id:prot-a-242 | F-deep fried food liking (derived food-liking factor) \|\| id:ebi-fl187-GCST90094753 | MR Egger | 8 | 0.431039002214862 | 0.321023817040392 | 0.227934324460285 | -0.198167679 | 1.06024568361403 | 1.53885556756193 | 0.820232305722268 | 2.88708021044248 | 0.999279047927446 |
| ebi-fl187-GCST90094753 | prot-a-2464 | Dihydropteridine reductase \|\| id:prot-a-2464 | F-deep fried food liking (derived food-liking factor) \|\| id:ebi-fl187-GCST90094753 | MR Egger | 8 | -0.061190915 | 0.358309230642075 | 0.870012710551603 | -0.763477007 | 0.641095177312362 | 0.940643639890738 | 0.466043171331818 | 1.89855899988484 | 0.999279047927446 |
| ebi-fl187-GCST90094753 | prot-a-2474 | Ras-related protein Rab-27A \|\| id:prot-a-2474 | F-deep fried food liking (derived food-liking factor) \|\| id:ebi-fl187-GCST90094753 | MR Egger | 8 | 0.751607276791521 | 0.583538395743414 | 0.245180979822453 | -0.392127979 | 1.89534253244861 | 2.12040535753665 | 0.675617643647965 | 6.65482750863867 | 0.999279047927446 |
| ebi-fl187-GCST90094753 | prot-a-2478 | Ras-related protein Rab-7b \|\| id:prot-a-2478 | F-deep fried food liking (derived food-liking factor) \|\| id:ebi-fl187-GCST90094753 | MR Egger | 8 | -0.186456333 | 0.463816548737808 | 0.701609523034481 | -1.095536768 | 0.722624102693391 | 0.829894800315203 | 0.334360084858026 | 2.05983133388261 | 0.999279047927446 |
| ebi-fl187-GCST90094753 | prot-a-2528 | E3 ubiquitin-protein ligase rififylin \|\| id:prot-a-2528 | F-deep fried food liking (derived food-liking factor) \|\| id:ebi-fl187-GCST90094753 | MR Egger | 8 | -0.043482238 | 0.321023817040392 | 0.896687082504036 | -0.672688919 | 0.585724443485293 | 0.957449560241482 | 0.510334482952076 | 1.79629182630149 | 0.999279047927446 |
| ebi-fl187-GCST90094753 | prot-a-2543 | Protein RIC-3 \|\| id:prot-a-2543 | F-deep fried food liking (derived food-liking factor) \|\| id:ebi-fl187-GCST90094753 | MR Egger | 8 | 0.281768913252641 | 0.321023817040392 | 0.413856813224554 | -0.347437768 | 0.91097559465181 | 1.32547238549096 | 0.706495979115783 | 2.48674740781669 | 0.999279047927446 |
| ebi-fl187-GCST90094753 | prot-a-255 | Bone morphogenetic protein 6 \|\| id:prot-a-255 | F-deep fried food liking (derived food-liking factor) \|\| id:ebi-fl187-GCST90094753 | MR Egger | 8 | 0.0625291468139824 | 0.544391729181721 | 0.912303065614782 | -1.004478642 | 1.12953693601016 | 1.06452548599201 | 0.366235524719461 | 3.09422334492147 | 0.999279047927446 |
| ebi-fl187-GCST90094753 | prot-a-2568 | RING finger protein 165 \|\| id:prot-a-2568 | F-deep fried food liking (derived food-liking factor) \|\| id:ebi-fl187-GCST90094753 | MR Egger | 8 | 0.191673083055022 | 0.369398257053542 | 0.622419397453034 | -0.532347501 | 0.915693666879965 | 1.21127446608257 | 0.587224839620839 | 2.49850778303411 | 0.999279047927446 |
| ebi-fl187-GCST90094753 | prot-a-2573 | E3 ubiquitin-protein ligase RNF8 \|\| id:prot-a-2573 | F-deep fried food liking (derived food-liking factor) \|\| id:ebi-fl187-GCST90094753 | MR Egger | 8 | -0.023982454 | 0.321023817040392 | 0.942876994473092 | -0.653189135 | 0.605224227623049 | 0.976302840035829 | 0.520383554146355 | 1.83166287225509 | 0.999279047927446 |
| ebi-fl187-GCST90094753 | prot-a-260 | Cytoplasmic tyrosine-protein kinase BMX \|\| id:prot-a-260 | F-deep fried food liking (derived food-liking factor) \|\| id:ebi-fl187-GCST90094753 | MR Egger | 7 | -0.018101954 | 0.324343465414579 | 0.957653870218306 | -0.653815146 | 0.617611238069741 | 0.982060902076558 | 0.520057890168791 | 1.85449280478064 | 0.999279047927446 |
| ebi-fl187-GCST90094753 | prot-a-2623 | Serum amyloid A-1 protein \|\| id:prot-a-2623 | F-deep fried food liking (derived food-liking factor) \|\| id:ebi-fl187-GCST90094753 | MR Egger | 8 | 0.324953794773431 | 0.321023817040392 | 0.350487663995871 | -0.304252887 | 0.9541604761726 | 1.38396669800855 | 0.73767429489753 | 2.59648985256123 | 0.999279047927446 |
| ebi-fl187-GCST90094753 | prot-a-2640 | Lysosome membrane protein 2 \|\| id:prot-a-2640 | F-deep fried food liking (derived food-liking factor) \|\| id:ebi-fl187-GCST90094753 | MR Egger | 8 | 0.5867447098443 | 0.331374583599021 | 0.127011657160629 | -0.062749474 | 1.23623889369838 | 1.79812545765996 | 0.939178732900682 | 3.4426409459878 | 0.999279047927446 |
| ebi-fl187-GCST90094753 | prot-a-2641 | Scavenger receptor class F member 2 \|\| id:prot-a-2641 | F-deep fried food liking (derived food-liking factor) \|\| id:ebi-fl187-GCST90094753 | MR Egger | 8 | 0.482310561745755 | 0.36134767023754 | 0.23037103204195 | -0.225930872 | 1.19055199541133 | 1.61981275907393 | 0.797773248292633 | 3.2888961620035 | 0.999279047927446 |
| ebi-fl187-GCST90094753 | prot-a-2669 | Semaphorin-3A \|\| id:prot-a-2669 | F-deep fried food liking (derived food-liking factor) \|\| id:ebi-fl187-GCST90094753 | MR Egger | 8 | 0.0310076871417694 | 0.321023817040392 | 0.926197537452071 | -0.598198994 | 0.660214368540938 | 1.0314934330923 | 0.54980093960548 | 1.93520713747055 | 0.999279047927446 |
| ebi-fl187-GCST90094753 | prot-a-2683 | Sentrin-specific protease 7 \|\| id:prot-a-2683 | F-deep fried food liking (derived food-liking factor) \|\| id:ebi-fl187-GCST90094753 | MR Egger | 8 | 0.202731619298535 | 0.333740420142811 | 0.565815245056615 | -0.451399604 | 0.856862842778445 | 1.22474372655506 | 0.636736348825642 | 2.35575870374369 | 0.999279047927446 |
| ebi-fl187-GCST90094753 | prot-a-270 | Bromodomain testis-specific protein \|\| id:prot-a-270 | F-deep fried food liking (derived food-liking factor) \|\| id:ebi-fl187-GCST90094753 | MR Egger | 8 | 0.0833818720211251 | 0.320960389064698 | 0.803711320099401 | -0.545700491 | 0.712464234587933 | 1.08695680769794 | 0.579435751879827 | 2.03900967099099 | 0.999279047927446 |
| ebi-fl187-GCST90094753 | prot-a-2708 | 14-3-3 protein sigma \|\| id:prot-a-2708 | F-deep fried food liking (derived food-liking factor) \|\| id:ebi-fl187-GCST90094753 | MR Egger | 8 | 0.14607810895188 | 0.321650419739721 | 0.665685341934656 | -0.484356714 | 0.776512931641732 | 1.15728657896229 | 0.616093393732903 | 2.17387857014885 | 0.999279047927446 |
| ebi-fl187-GCST90094753 | prot-a-2713 | Beta-sarcoglycan \|\| id:prot-a-2713 | F-deep fried food liking (derived food-liking factor) \|\| id:ebi-fl187-GCST90094753 | MR Egger | 8 | -0.400807886 | 0.436174905558035 | 0.393581327718532 | -1.255710701 | 0.454094928625252 | 0.66977872236837 | 0.284873316466095 | 1.57474747899317 | 0.999279047927446 |
| ebi-fl187-GCST90094753 | prot-a-2720 | Protein shisa-3 homolog \|\| id:prot-a-2720 | F-deep fried food liking (derived food-liking factor) \|\| id:ebi-fl187-GCST90094753 | MR Egger | 8 | 0.150025432800019 | 0.365537984685051 | 0.695748754544383 | -0.566429017 | 0.86647988278272 | 1.16186379180199 | 0.567548530386859 | 2.37852341857065 | 0.999279047927446 |
| ebi-fl187-GCST90094753 | prot-a-2730 | Sialic acid-binding Ig-like lectin 8 \|\| id:prot-a-2730 | F-deep fried food liking (derived food-liking factor) \|\| id:ebi-fl187-GCST90094753 | MR Egger | 8 | 0.315294889967952 | 0.321023817040392 | 0.363953171464936 | -0.313911791 | 0.944501571367121 | 1.37066344626609 | 0.730583469039444 | 2.57153133426372 | 0.999279047927446 |
| ebi-fl187-GCST90094753 | prot-a-2761 | Sodium- and chloride-dependent neutral and basic amino acid transporter B(0+) \|\| id:prot-a-2761 | F-deep fried food liking (derived food-liking factor) \|\| id:ebi-fl187-GCST90094753 | MR Egger | 8 | -0.216747332 | 0.360981494173952 | 0.570182328690005 | -0.92427106 | 0.49077639686894 | 0.805133375278339 | 0.396820571946151 | 1.63358403725868 | 0.999279047927446 |
| ebi-fl187-GCST90094753 | prot-a-2770 | Secreted Ly-6/uPAR-related protein 1 \|\| id:prot-a-2770 | F-deep fried food liking (derived food-liking factor) \|\| id:ebi-fl187-GCST90094753 | MR Egger | 8 | 0.104183367731509 | 0.482698037991743 | 0.836266385476306 | -0.841904787 | 1.05027152219533 | 1.10980393848947 | 0.430888989623592 | 2.85842713911692 | 0.999279047927446 |
| ebi-fl187-GCST90094753 | prot-a-2784 | N-lysine methyltransferase SMYD2 \|\| id:prot-a-2784 | F-deep fried food liking (derived food-liking factor) \|\| id:ebi-fl187-GCST90094753 | MR Egger | 8 | -0.177598325 | 0.389435835558136 | 0.664401833389773 | -0.940892562 | 0.585695913186583 | 0.837278670275558 | 0.390279331271825 | 1.79624057829018 | 0.999279047927446 |
| ebi-fl187-GCST90094753 | prot-a-2837 | Serine palmitoyltransferase 2 \|\| id:prot-a-2837 | F-deep fried food liking (derived food-liking factor) \|\| id:ebi-fl187-GCST90094753 | MR Egger | 8 | -0.172531006 | 0.321650419739721 | 0.610981203048916 | -0.802965829 | 0.457903816192644 | 0.841532195441575 | 0.447998305389486 | 1.58075695252694 | 0.999279047927446 |
| ebi-fl187-GCST90094753 | prot-a-2858 | Alpha-2,8-sialyltransferase 8B \|\| id:prot-a-2858 | F-deep fried food liking (derived food-liking factor) \|\| id:ebi-fl187-GCST90094753 | MR Egger | 8 | 0.47374919812228 | 0.321023817040392 | 0.190466263393826 | -0.155457483 | 1.10295587952145 | 1.60600414765728 | 0.856023471468149 | 3.01305911375159 | 0.999279047927446 |
| ebi-fl187-GCST90094753 | prot-a-2911 | Synaptotagmin-11 \|\| id:prot-a-2911 | F-deep fried food liking (derived food-liking factor) \|\| id:ebi-fl187-GCST90094753 | MR Egger | 8 | 0.346281474342369 | 0.321650419739721 | 0.323040351682568 | -0.284153348 | 0.976716297032222 | 1.41380050838511 | 0.752651218035661 | 2.65572130837275 | 0.999279047927446 |
| ebi-fl187-GCST90094753 | prot-a-2962 | Transforming growth factor beta-1 \|\| id:prot-a-2962 | F-deep fried food liking (derived food-liking factor) \|\| id:ebi-fl187-GCST90094753 | MR Egger | 8 | -0.222962868 | 0.321650419739721 | 0.514117621822316 | -0.853397691 | 0.407471954318538 | 0.800144559413633 | 0.425965172367326 | 1.50301329191097 | 0.999279047927446 |
| ebi-fl187-GCST90094753 | prot-a-2978 | Tyrosine-protein kinase receptor Tie-1, soluble \|\| id:prot-a-2978 | F-deep fried food liking (derived food-liking factor) \|\| id:ebi-fl187-GCST90094753 | MR Egger | 8 | 0.187897995514295 | 0.321023817040392 | 0.579671622715303 | -0.441308686 | 0.817104676913463 | 1.20671041919583 | 0.643194130976325 | 2.26393551443859 | 0.999279047927446 |
| ebi-fl187-GCST90094753 | prot-a-299 | Uncharacterized protein C1orf226 \|\| id:prot-a-299 | F-deep fried food liking (derived food-liking factor) \|\| id:ebi-fl187-GCST90094753 | MR Egger | 8 | -0.092896062 | 0.321650419739721 | 0.782444910665828 | -0.723330885 | 0.53753876022785 | 0.911288212427188 | 0.485133637310895 | 1.7117885511133 | 0.999279047927446 |
| ebi-fl187-GCST90094753 | prot-a-3015 | Transmembrane protein 70, mitochondrial \|\| id:prot-a-3015 | F-deep fried food liking (derived food-liking factor) \|\| id:ebi-fl187-GCST90094753 | MR Egger | 8 | -0.199265838 | 0.321650419739721 | 0.558355757511865 | -0.829700661 | 0.431168984285754 | 0.819332054453456 | 0.436179832375668 | 1.53905560419572 | 0.999279047927446 |
| ebi-fl187-GCST90094753 | prot-a-3016 | Transmembrane protein 87B \|\| id:prot-a-3016 | F-deep fried food liking (derived food-liking factor) \|\| id:ebi-fl187-GCST90094753 | MR Egger | 8 | 0.762260912550614 | 0.321023817040392 | 0.0551830896741889 | 0.133054231151445 | 1.39146759394978 | 2.14311614530209 | 1.14231194554326 | 4.02074654841342 | 0.999279047927446 |
| ebi-fl187-GCST90094753 | prot-a-3123 | Thioredoxin domain-containing protein 12 \|\| id:prot-a-3123 | F-deep fried food liking (derived food-liking factor) \|\| id:ebi-fl187-GCST90094753 | MR Egger | 8 | -0.827376882 | 0.521645621775643 | 0.163813999854239 | -1.849802301 | 0.195048536841719 | 0.437194596609169 | 0.157268255092849 | 1.21536997527827 | 0.999279047927446 |
| ebi-fl187-GCST90094753 | prot-a-3133 | PolyUbiquitin K48-linked \|\| id:prot-a-3133 | F-deep fried food liking (derived food-liking factor) \|\| id:ebi-fl187-GCST90094753 | MR Egger | 8 | -0.568784204 | 0.356880906789073 | 0.16209775020091 | -1.268270781 | 0.130702373621867 | 0.566213420582281 | 0.281317661270255 | 1.13962854731505 | 0.999279047927446 |
| ebi-fl187-GCST90094753 | prot-a-3135 | Ubiquitin-conjugating enzyme E2 B \|\| id:prot-a-3135 | F-deep fried food liking (derived food-liking factor) \|\| id:ebi-fl187-GCST90094753 | MR Egger | 8 | -0.092225155 | 0.321650419739721 | 0.783966748979515 | -0.722659978 | 0.538209667713069 | 0.911899807649062 | 0.485459226307333 | 1.71293738820396 | 0.999279047927446 |
| ebi-fl187-GCST90094753 | prot-a-3162 | NKG2D ligand 3 \|\| id:prot-a-3162 | F-deep fried food liking (derived food-liking factor) \|\| id:ebi-fl187-GCST90094753 | MR Egger | 8 | -0.178053652 | 0.322734764384451 | 0.601079754314723 | -0.81061379 | 0.45450648592889 | 0.836897520836955 | 0.444585100367148 | 1.57539571120273 | 0.999279047927446 |
| ebi-fl187-GCST90094753 | prot-a-3188 | Vesicle-associated membrane protein 8 \|\| id:prot-a-3188 | F-deep fried food liking (derived food-liking factor) \|\| id:ebi-fl187-GCST90094753 | MR Egger | 8 | 0.116456877699645 | 0.371350773148817 | 0.764438471058911 | -0.611390638 | 0.844304393071326 | 1.1235090611274 | 0.5425957900292 | 2.3263590201602 | 0.999279047927446 |
| ebi-fl187-GCST90094753 | prot-a-3201 | Vitrin \|\| id:prot-a-3201 | F-deep fried food liking (derived food-liking factor) \|\| id:ebi-fl187-GCST90094753 | MR Egger | 8 | -0.054168295 | 0.321023817040392 | 0.871548869962154 | -0.683374976 | 0.575038386738778 | 0.947272672153542 | 0.504910054202941 | 1.7771987464687 | 0.999279047927446 |
| ebi-fl187-GCST90094753 | prot-a-3232 | WNT1-inducible-signaling pathway protein 3 \|\| id:prot-a-3232 | F-deep fried food liking (derived food-liking factor) \|\| id:ebi-fl187-GCST90094753 | MR Egger | 8 | -0.190830716 | 0.321023817040392 | 0.573930861993118 | -0.820037398 | 0.438375964920756 | 0.826272450622886 | 0.440415183604891 | 1.55018761403749 | 0.999279047927446 |
| ebi-fl187-GCST90094753 | prot-a-327 | Carbonic anhydrase-related protein 10 \|\| id:prot-a-327 | F-deep fried food liking (derived food-liking factor) \|\| id:ebi-fl187-GCST90094753 | MR Egger | 8 | -0.617453434 | 0.444387893825652 | 0.214064525581685 | -1.488453706 | 0.253546837511744 | 0.539316094166873 | 0.225721417434947 | 1.28858773231494 | 0.999279047927446 |
| ebi-fl187-GCST90094753 | prot-a-332 | Carbonic anhydrase 5A, mitochondrial \|\| id:prot-a-332 | F-deep fried food liking (derived food-liking factor) \|\| id:ebi-fl187-GCST90094753 | MR Egger | 8 | -0.274335823 | 0.419015639887133 | 0.536926453435991 | -1.095606477 | 0.546934831195634 | 0.760076781144401 | 0.334336777829064 | 1.72794843865547 | 0.999279047927446 |
| ebi-fl187-GCST90094753 | prot-a-338 | Cell adhesion molecule 3 \|\| id:prot-a-338 | F-deep fried food liking (derived food-liking factor) \|\| id:ebi-fl187-GCST90094753 | MR Egger | 8 | 0.442892020696249 | 0.321023817040392 | 0.216914041067239 | -0.186314661 | 1.07209870209542 | 1.55720417944022 | 0.830012381607867 | 2.92150443800454 | 0.999279047927446 |
| ebi-fl187-GCST90094753 | prot-a-34 | A disintegrin and metalloproteinase with thrombospondin motifs 3 \|\| id:prot-a-34 | F-deep fried food liking (derived food-liking factor) \|\| id:ebi-fl187-GCST90094753 | MR Egger | 8 | 0.147931453627031 | 0.321023817040392 | 0.661164061993915 | -0.481275228 | 0.7771381350262 | 1.1594334186835 | 0.617994804960762 | 2.17523811133892 | 0.999279047927446 |
| ebi-fl187-GCST90094753 | prot-a-358 | Cysteine--tRNA ligase, cytoplasmic \|\| id:prot-a-358 | F-deep fried food liking (derived food-liking factor) \|\| id:ebi-fl187-GCST90094753 | MR Egger | 8 | -0.213961439 | 0.321023817040392 | 0.529872613111467 | -0.84316812 | 0.415245242790944 | 0.807379518100319 | 0.430344976932154 | 1.51474217474292 | 0.999279047927446 |
| ebi-fl187-GCST90094753 | prot-a-382 | Coiled-coil domain-containing protein 167 \|\| id:prot-a-382 | F-deep fried food liking (derived food-liking factor) \|\| id:ebi-fl187-GCST90094753 | MR Egger | 8 | 0.152603137406998 | 0.343636158030724 | 0.672550027527517 | -0.520923732 | 0.826130007147216 | 1.16486259680706 | 0.593971623685857 | 2.28446076433734 | 0.999279047927446 |
| ebi-fl187-GCST90094753 | prot-a-40 | Pituitary adenylate cyclase-activating polypeptide 27 \|\| id:prot-a-40 | F-deep fried food liking (derived food-liking factor) \|\| id:ebi-fl187-GCST90094753 | MR Egger | 8 | 0.0922050716179893 | 0.418651728327257 | 0.832984583510578 | -0.728352316 | 0.912762459139412 | 1.09658967844383 | 0.482703678387012 | 2.49119486076387 | 0.999279047927446 |
| ebi-fl187-GCST90094753 | prot-a-403 | C-C motif chemokine 25 \|\| id:prot-a-403 | F-deep fried food liking (derived food-liking factor) \|\| id:ebi-fl187-GCST90094753 | MR Egger | 8 | -0.080766642 | 0.321650419739721 | 0.810114583496292 | -0.711201465 | 0.549668180448576 | 0.922408917608924 | 0.491053858905673 | 1.73267798603718 | 0.999279047927446 |
| ebi-fl187-GCST90094753 | prot-a-415 | Cyclin-C \|\| id:prot-a-415 | F-deep fried food liking (derived food-liking factor) \|\| id:ebi-fl187-GCST90094753 | MR Egger | 8 | -0.458935507 | 0.321023817040392 | 0.202769082824758 | -1.088142188 | 0.170271174715669 | 0.631956000523605 | 0.336841701294775 | 1.18562631961147 | 0.999279047927446 |
| ebi-fl187-GCST90094753 | prot-a-44 | Adhesion G protein-coupled receptor F5 \|\| id:prot-a-44 | F-deep fried food liking (derived food-liking factor) \|\| id:ebi-fl187-GCST90094753 | MR Egger | 8 | 0.241890148685215 | 0.321023817040392 | 0.479664956063037 | -0.387316533 | 0.871096830084384 | 1.2736542725347 | 0.678876174395822 | 2.38953032545236 | 0.999279047927446 |
| ebi-fl187-GCST90094753 | prot-a-455 | CD59 glycoprotein \|\| id:prot-a-455 | F-deep fried food liking (derived food-liking factor) \|\| id:ebi-fl187-GCST90094753 | MR Egger | 8 | -0.009465436 | 0.321650419739721 | 0.977477891720876 | -0.639900259 | 0.620969386346089 | 0.990579219890843 | 0.527345019321898 | 1.8607309350173 | 0.999279047927446 |
| ebi-fl187-GCST90094753 | prot-a-478 | Cadherin-12 \|\| id:prot-a-478 | F-deep fried food liking (derived food-liking factor) \|\| id:ebi-fl187-GCST90094753 | MR Egger | 8 | 0.165469968802637 | 0.363620702043642 | 0.665066463036228 | -0.547226607 | 0.878166544808176 | 1.17994752695031 | 0.578552139746189 | 2.40648347954767 | 0.999279047927446 |
| ebi-fl187-GCST90094753 | prot-a-516 | Centrin-2 \|\| id:prot-a-516 | F-deep fried food liking (derived food-liking factor) \|\| id:ebi-fl187-GCST90094753 | MR Egger | 8 | -0.17708849 | 0.380978808694509 | 0.658446757080445 | -0.923806955 | 0.569629974842707 | 0.837705652503563 | 0.397004781121695 | 1.7676128691793 | 0.999279047927446 |
| ebi-fl187-GCST90094753 | prot-a-519 | Cryptic protein \|\| id:prot-a-519 | F-deep fried food liking (derived food-liking factor) \|\| id:ebi-fl187-GCST90094753 | MR Egger | 8 | 0.320100906214838 | 0.321650419739721 | 0.358072788731518 | -0.310333916 | 0.95053572890469 | 1.37726673209724 | 0.733202087087251 | 2.58709526984214 | 0.999279047927446 |
| ebi-fl187-GCST90094753 | prot-a-583 | Chloride intracellular channel protein 4 \|\| id:prot-a-583 | F-deep fried food liking (derived food-liking factor) \|\| id:ebi-fl187-GCST90094753 | MR Egger | 8 | 0.146383322464131 | 0.321023817040392 | 0.664436508718932 | -0.482823359 | 0.7755900038633 | 1.1576398523729 | 0.617038808139864 | 2.17187316279495 | 0.999279047927446 |
| ebi-fl187-GCST90094753 | prot-a-602 | CCR4-NOT transcription complex subunit 1 \|\| id:prot-a-602 | F-deep fried food liking (derived food-liking factor) \|\| id:ebi-fl187-GCST90094753 | MR Egger | 8 | 0.0490675112409091 | 0.321023817040392 | 0.883529632384951 | -0.58013917 | 0.678274192640078 | 1.05029125481252 | 0.559820450843015 | 1.97047413733192 | 0.999279047927446 |
| ebi-fl187-GCST90094753 | prot-a-606 | Ciliary neurotrophic factor receptor subunit alpha \|\| id:prot-a-606 | F-deep fried food liking (derived food-liking factor) \|\| id:ebi-fl187-GCST90094753 | MR Egger | 8 | 0.329111606597671 | 0.321023817040392 | 0.344815387875895 | -0.300095075 | 0.95831828799684 | 1.38973295029891 | 0.740747790884543 | 2.60730804318732 | 0.999279047927446 |
| ebi-fl187-GCST90094753 | prot-a-626 | Collagen alpha-2(IX) chain \|\| id:prot-a-626 | F-deep fried food liking (derived food-liking factor) \|\| id:ebi-fl187-GCST90094753 | MR Egger | 8 | -0.304262495 | 0.321650419739721 | 0.380698288535642 | -0.934697318 | 0.326172327383819 | 0.737667206855121 | 0.39270471219355 | 1.38565413445114 | 0.999279047927446 |
| ebi-fl187-GCST90094753 | prot-a-647 | Probable serine carboxypeptidase CPVL \|\| id:prot-a-647 | F-deep fried food liking (derived food-liking factor) \|\| id:ebi-fl187-GCST90094753 | MR Egger | 8 | 0.256580898094776 | 0.418993610424652 | 0.562763372540866 | -0.564646578 | 1.07780837452709 | 1.29250332262669 | 0.568561053045709 | 2.93823298316343 | 0.999279047927446 |
| ebi-fl187-GCST90094753 | prot-a-651 | Cellular retinoic acid-binding protein 2 \|\| id:prot-a-651 | F-deep fried food liking (derived food-liking factor) \|\| id:ebi-fl187-GCST90094753 | MR Egger | 8 | -0.313500161 | 0.321023817040392 | 0.366499834830465 | -0.942706842 | 0.315706520822044 | 0.730884261640301 | 0.389571897310118 | 1.37122776976966 | 0.999279047927446 |
| ebi-fl187-GCST90094753 | prot-a-671 | Cytotoxic and regulatory T-cell molecule \|\| id:prot-a-671 | F-deep fried food liking (derived food-liking factor) \|\| id:ebi-fl187-GCST90094753 | MR Egger | 8 | -0.017712935 | 0.321023817040392 | 0.95778935962258 | -0.646919617 | 0.611493745985799 | 0.98244301648095 | 0.523656357123637 | 1.84318259007463 | 0.999279047927446 |
| ebi-fl187-GCST90094753 | prot-a-688 | Chorionic somatomammotropin hormone \|\| id:prot-a-688 | F-deep fried food liking (derived food-liking factor) \|\| id:ebi-fl187-GCST90094753 | MR Egger | 8 | -0.427902542 | 0.321023817040392 | 0.23093367070366 | -1.057109223 | 0.20130413934278 | 0.65187494209441 | 0.347458785650556 | 1.22299667667048 | 0.999279047927446 |
| ebi-fl187-GCST90094753 | prot-a-69 | Aldo-keto reductase family 1 member C1 \|\| id:prot-a-69 | F-deep fried food liking (derived food-liking factor) \|\| id:ebi-fl187-GCST90094753 | MR Egger | 8 | -0.09385682 | 0.321650419739721 | 0.78026743459588 | -0.724291643 | 0.536578002367745 | 0.910413105564491 | 0.484667765186636 | 1.71014472659309 | 0.999279047927446 |
| ebi-fl187-GCST90094753 | prot-a-741 | Stromal cell-derived factor 1 \|\| id:prot-a-741 | F-deep fried food liking (derived food-liking factor) \|\| id:ebi-fl187-GCST90094753 | MR Egger | 8 | 0.730990864681594 | 0.33954166158437 | 0.074808791305606 | 0.06548920797623 | 1.39649252138696 | 2.07713775076664 | 1.06768121481027 | 4.04100135490963 | 0.999279047927446 |
| ebi-fl187-GCST90094753 | prot-a-742 | Growth-regulated alpha protein \|\| id:prot-a-742 | F-deep fried food liking (derived food-liking factor) \|\| id:ebi-fl187-GCST90094753 | MR Egger | 8 | 0.102753649729561 | 0.321023817040392 | 0.759759826408433 | -0.526453032 | 0.731960331128731 | 1.10821836555116 | 0.590696439861439 | 2.07915244255233 | 0.999279047927446 |
| ebi-fl187-GCST90094753 | prot-a-788 | Beta-defensin 112 \|\| id:prot-a-788 | F-deep fried food liking (derived food-liking factor) \|\| id:ebi-fl187-GCST90094753 | MR Egger | 8 | -0.200359551 | 0.441872424097359 | 0.666174227514419 | -1.066429503 | 0.665710399844889 | 0.818436430216273 | 0.344235417450834 | 1.9458723778788 | 0.999279047927446 |
| ebi-fl187-GCST90094753 | prot-a-838 | DnaJ homolog subfamily B member 1 \|\| id:prot-a-838 | F-deep fried food liking (derived food-liking factor) \|\| id:ebi-fl187-GCST90094753 | MR Egger | 8 | 0.301978031829898 | 0.321023817040392 | 0.383184400673814 | -0.32722865 | 0.931184713229067 | 1.35253151374075 | 0.720918886387274 | 2.53751362352177 | 0.999279047927446 |
| ebi-fl187-GCST90094753 | prot-a-85 | Junctional adhesion molecule-like \|\| id:prot-a-85 | F-deep fried food liking (derived food-liking factor) \|\| id:ebi-fl187-GCST90094753 | MR Egger | 8 | 0.283032254862188 | 0.458458276692058 | 0.559683864971644 | -0.615545967 | 1.18161047717862 | 1.32714796810161 | 0.540345803538625 | 3.25961952087287 | 0.999279047927446 |
| ebi-fl187-GCST90094753 | prot-a-856 | Dipeptidase 1 \|\| id:prot-a-856 | F-deep fried food liking (derived food-liking factor) \|\| id:ebi-fl187-GCST90094753 | MR Egger | 8 | 0.489136582816236 | 0.320960389064698 | 0.178350744789042 | -0.13994578 | 1.11821894538304 | 1.63090745834374 | 0.869405373497079 | 3.05940038877639 | 0.999279047927446 |
| ebi-fl187-GCST90094753 | prot-a-860 | Dipeptidyl peptidase 2 \|\| id:prot-a-860 | F-deep fried food liking (derived food-liking factor) \|\| id:ebi-fl187-GCST90094753 | MR Egger | 8 | 0.271865765034987 | 0.321023817040392 | 0.429545267030673 | -0.357340916 | 0.901072446434156 | 1.31241081806615 | 0.699533974499476 | 2.46224231869435 | 0.999279047927446 |
| ebi-fl187-GCST90094753 | prot-a-861 | Dermatopontin \|\| id:prot-a-861 | F-deep fried food liking (derived food-liking factor) \|\| id:ebi-fl187-GCST90094753 | MR Egger | 8 | 0.558969379183207 | 0.321023817040392 | 0.132289162467371 | -0.070237302 | 1.18817606058238 | 1.7488691502186 | 0.932172587036899 | 3.28109123473425 | 0.999279047927446 |
| ebi-fl187-GCST90094753 | prot-a-877 | Dual specificity protein phosphatase 6 \|\| id:prot-a-877 | F-deep fried food liking (derived food-liking factor) \|\| id:ebi-fl187-GCST90094753 | MR Egger | 8 | 0.328571537000874 | 0.321023817040392 | 0.345547971033309 | -0.300635144 | 0.957778218400043 | 1.3889826004236 | 0.740347843533216 | 2.60590029555877 | 0.999279047927446 |
| ebi-fl187-GCST90094753 | prot-a-879 | Dynactin-associated protein \|\| id:prot-a-879 | F-deep fried food liking (derived food-liking factor) \|\| id:ebi-fl187-GCST90094753 | MR Egger | 8 | -0.065177842 | 0.321023817040392 | 0.845820871560897 | -0.694384523 | 0.564028839825618 | 0.936900828642923 | 0.499381711389857 | 1.75773990655122 | 0.999279047927446 |
| ebi-fl187-GCST90094753 | prot-a-937 | EMI domain-containing protein 1 \|\| id:prot-a-937 | F-deep fried food liking (derived food-liking factor) \|\| id:ebi-fl187-GCST90094753 | MR Egger | 8 | -0.242286224 | 0.43308223374412 | 0.596106841650108 | -1.091127402 | 0.60655495422373 | 0.78483150785385 | 0.335837656132657 | 1.83410193726709 | 0.999279047927446 |
| ebi-fl187-GCST90094753 | prot-b-1 | SRC proto-oncogene, non-receptor tyrosine kinase \|\| id:prot-b-1 | F-deep fried food liking (derived food-liking factor) \|\| id:ebi-fl187-GCST90094753 | MR Egger | 7 | -0.318690459 | 0.465312873378997 | 0.52386208363829 | -1.230703691 | 0.593322773012956 | 0.727100582055597 | 0.292086966483755 | 1.80999262921576 | 0.999279047927446 |
| ebi-fl187-GCST90094753 | prot-b-10 | coagulation factor III, tissue factor \|\| id:prot-b-10 | F-deep fried food liking (derived food-liking factor) \|\| id:ebi-fl187-GCST90094753 | MR Egger | 7 | -0.538279597 | 0.326889401354473 | 0.160543023954936 | -1.178982824 | 0.102423629778692 | 0.583751677187934 | 0.307591454320081 | 1.10785269172375 | 0.999279047927446 |
| ebi-fl187-GCST90094753 | prot-b-29 | myeloperoxidase \|\| id:prot-b-29 | F-deep fried food liking (derived food-liking factor) \|\| id:ebi-fl187-GCST90094753 | MR Egger | 7 | 0.0637027273448504 | 0.326790326616019 | 0.853117672220024 | -0.576806313 | 0.704211767512247 | 1.06577552574467 | 0.561689365221807 | 2.02225205176845 | 0.999279047927446 |
| ebi-fl187-GCST90094753 | prot-b-33 | adrenomedullin \|\| id:prot-b-33 | F-deep fried food liking (derived food-liking factor) \|\| id:ebi-fl187-GCST90094753 | MR Egger | 7 | 0.0655980985608878 | 0.327532531514709 | 0.849153226972194 | -0.576365663 | 0.707561860329718 | 1.06779748157205 | 0.561936927964327 | 2.02903814451572 | 0.999279047927446 |
| ebi-fl187-GCST90094753 | ukb-rsfMRI191-edge-pheno102 | Net25_Pair11_15 (Postcentral\|Precentral)&(Cerebellum) (Motor)&(Subcortical-cerebellum) \|\| id:ukb-rsfMRI191-edge-pheno102 | F-deep fried food liking (derived food-liking factor) \|\| id:ebi-fl187-GCST90094753 | MR Egger | 8 | 0.00203517761569036 | 0.108558443508359 | 0.985650569071157 | -0.210739372 | 0.214809726892073 | 1.0020372499953 | 0.809985144456746 | 1.23962600703199 | 0.999279047927446 |
| ebi-fl187-GCST90094753 | ukb-rsfMRI191-edge-pheno1167 | Net100_Pair11_45 (Angular\|Temporal)&(Frontal) (Default_mode\|Central_executive)&(Salience\|Default_mode) \|\| id:ukb-rsfMRI191-edge-pheno1167 | F-deep fried food liking (derived food-liking factor) \|\| id:ebi-fl187-GCST90094753 | MR Egger | 8 | 0.00922084860789007 | 0.100335737462138 | 0.929769063138747 | -0.187437197 | 0.205878894033681 | 1.00926349159983 | 0.829081185482054 | 1.22860440366166 | 0.999279047927446 |
| ebi-fl187-GCST90094753 | ukb-rsfMRI191-edge-pheno1317 | Net100_Pair26_48 (Parietal\|Frontal)&(Temporal) (Attention\|Central_executive)&(Default_mode\|Central_executive) \|\| id:ukb-rsfMRI191-edge-pheno1317 | F-deep fried food liking (derived food-liking factor) \|\| id:ebi-fl187-GCST90094753 | MR Egger | 8 | 0.0167149046572308 | 0.125838770533616 | 0.898672219682677 | -0.229929086 | 0.263358894903119 | 1.01685538026346 | 0.794589948383891 | 1.30129366281787 | 0.999279047927446 |
| ebi-fl187-GCST90094753 | ukb-rsfMRI191-edge-pheno286 | Net100_Pair10_13 (OccipitalPrecuneus)&(Frontal_Sup) (Default_mode\|Central_executive)&(Default_mode) \|\| id:ukb-rsfMRI191-edge-pheno286 | F-deep fried food liking (derived food-liking factor) \|\| id:ebi-fl187-GCST90094753 | MR Egger | 8 | -0.048456372 | 0.0989197019202189 | 0.641630357087182 | -0.242338988 | 0.145426243401555 | 0.952698902401945 | 0.784790097931093 | 1.15653242953833 | 0.999279047927446 |
| ebi-fl187-GCST90094753 | ukb-rsfMRI191-edge-pheno599 | Net100_Pair11_29 (Angular\|Temporal)&(Parietal) (Default_mode\|Central_executive)&(Central_executive\|Salience) \|\| id:ukb-rsfMRI191-edge-pheno599 | F-deep fried food liking (derived food-liking factor) \|\| id:ebi-fl187-GCST90094753 | MR Egger | 8 | -0.09383936 | 0.126620348345157 | 0.48660953058884 | -0.342015242 | 0.154336523135011 | 0.910429002153941 | 0.710337377395843 | 1.1668835040073 | 0.999279047927446 |
| ebi-fl187-GCST90094753 | ukb-rsfMRI191-edge-pheno621 | Net100_Pair5_30 (Precuneus\|Angular\|Cingulate)&(Paracentral) (Default_mode\|Central_executive)&(Motor) \|\| id:ukb-rsfMRI191-edge-pheno621 | F-deep fried food liking (derived food-liking factor) \|\| id:ebi-fl187-GCST90094753 | MR Egger | 8 | -0.13942364 | 0.10115601778746 | 0.217299723887678 | -0.337689435 | 0.0588421547763265 | 0.869859443059771 | 0.713416815825796 | 1.06060781564899 | 0.999279047927446 |
| ebi-fl187-GCST90094753 | ukb-rsfMRI191-edge-pheno624 | Net100_Pair8_30 (Calcarine\|Lingual)&(Paracentral) (Visual)&(Motor) \|\| id:ukb-rsfMRI191-edge-pheno624 | F-deep fried food liking (derived food-liking factor) \|\| id:ebi-fl187-GCST90094753 | MR Egger | 8 | -0.06992945 | 0.100827272747707 | 0.513899574608178 | -0.267550905 | 0.127692004633394 | 0.932459602655054 | 0.765251374863922 | 1.13620300354015 | 0.999279047927446 |
| ebi-fl187-GCST90094753 | ukb-rsfMRI191-edge-pheno882 | Net100_Pair6_38 (Postcentral\|Precentral)&(Subcortical) (Motor)&(Subcortical-cerebellum) \|\| id:ukb-rsfMRI191-edge-pheno882 | F-deep fried food liking (derived food-liking factor) \|\| id:ebi-fl187-GCST90094753 | MR Egger | 8 | -0.062250067 | 0.101381322336829 | 0.561745588590224 | -0.260957459 | 0.136457325056659 | 0.939647882741663 | 0.770313689214993 | 1.14620596245754 | 0.999279047927446 |
| ebi-fl187-GCST90094753 | ukb-rsfMRI191-edge-pheno899 | Net100_Pair23_38 (Cerebellum)&(Subcortical) (Subcortical-cerebellum)&(Subcortical-cerebellum) \|\| id:ukb-rsfMRI191-edge-pheno899 | F-deep fried food liking (derived food-liking factor) \|\| id:ebi-fl187-GCST90094753 | MR Egger | 8 | 0.0603758092591751 | 0.100761056985213 | 0.570957138250586 | -0.137115862 | 0.257867480950192 | 1.06223566954364 | 0.871869203393699 | 1.2941673054385 | 0.999279047927446 |
| ebi-fl187-GCST90094753 | ebi-a-GCST90001392 | IgD+ CD38+ B cell Absolute Count \|\| id:ebi-a-GCST90001392 | F-deep fried food liking (derived food-liking factor) \|\| id:ebi-fl187-GCST90094753 | Weighted median | 8 | 0.31128460851497 | 0.146429580545633 | 0.0335172957739836 | 0.0242826306455288 | 0.598286586384411 | 1.36517770710023 | 1.02457985463843 | 1.81899943037738 | 0.999489402457283 |
| ebi-fl187-GCST90094753 | ebi-a-GCST90001402 | Switched memory B cell %B cell \|\| id:ebi-a-GCST90001402 | F-deep fried food liking (derived food-liking factor) \|\| id:ebi-fl187-GCST90094753 | Weighted median | 8 | -0.069761552 | 0.158367560656584 | 0.659572079356486 | -0.380161971 | 0.240638866966731 | 0.932616173930839 | 0.683750652597888 | 1.27206156889609 | 0.999489402457283 |
| ebi-fl187-GCST90094753 | ebi-a-GCST90001407 | Memory B cell Absolute Count \|\| id:ebi-a-GCST90001407 | F-deep fried food liking (derived food-liking factor) \|\| id:ebi-fl187-GCST90094753 | Weighted median | 8 | 0.258040727757939 | 0.15164409380532 | 0.0888264961467734 | -0.039181696 | 0.555263151616366 | 1.29439153521584 | 0.961575978667278 | 1.74239943967876 | 0.999489402457283 |
| ebi-fl187-GCST90094753 | ebi-a-GCST90001411 | IgD+ CD24+ B cell %B cell \|\| id:ebi-a-GCST90001411 | F-deep fried food liking (derived food-liking factor) \|\| id:ebi-fl187-GCST90094753 | Weighted median | 8 | 0.243615592276399 | 0.162564013117719 | 0.133981964116897 | -0.075009873 | 0.562241057987128 | 1.27585378816101 | 0.927734326359387 | 1.7546002584087 | 0.999489402457283 |
| ebi-fl187-GCST90094753 | ebi-a-GCST90001418 | CD24+ CD27+ B cell Absolute Count \|\| id:ebi-a-GCST90001418 | F-deep fried food liking (derived food-liking factor) \|\| id:ebi-fl187-GCST90094753 | Weighted median | 8 | 0.367312304988122 | 0.158144524929885 | 0.0201986680830742 | 0.057349036125547 | 0.677275573850697 | 1.44384876988297 | 1.05902538401137 | 1.96850736702472 | 0.999489402457283 |
| ebi-fl187-GCST90094753 | ebi-a-GCST90001436 | Memory B cell %lymphocyte \|\| id:ebi-a-GCST90001436 | F-deep fried food liking (derived food-liking factor) \|\| id:ebi-fl187-GCST90094753 | Weighted median | 8 | 0.163021911014135 | 0.151238287034093 | 0.281072006953694 | -0.133405132 | 0.459448953600957 | 1.17706248003856 | 0.87511048539521 | 1.58320132718879 | 0.999489402457283 |
| ebi-fl187-GCST90094753 | ebi-a-GCST90001439 | IgD+ CD24+ B cell %lymphocyte \|\| id:ebi-a-GCST90001439 | F-deep fried food liking (derived food-liking factor) \|\| id:ebi-fl187-GCST90094753 | Weighted median | 8 | 0.186526483555274 | 0.152907000181266 | 0.222514603266103 | -0.113171237 | 0.486224203910556 | 1.20505653584448 | 0.892997732948805 | 1.62616454779369 | 0.999489402457283 |
| ebi-fl187-GCST90094753 | ebi-a-GCST90001457 | CD11c+ HLA DR++ monocyte %monocyte \|\| id:ebi-a-GCST90001457 | F-deep fried food liking (derived food-liking factor) \|\| id:ebi-fl187-GCST90094753 | Weighted median | 8 | 0.0315514189424586 | 0.165727981155604 | 0.849010773545672 | -0.293275424 | 0.356378262007443 | 1.03205444137928 | 0.745816696497178 | 1.42814766010635 | 0.999489402457283 |
| ebi-fl187-GCST90094753 | ebi-a-GCST90001483 | CD39+ resting CD4 regulatory T cell Absolute Count \|\| id:ebi-a-GCST90001483 | F-deep fried food liking (derived food-liking factor) \|\| id:ebi-fl187-GCST90094753 | Weighted median | 8 | -0.180922392 | 0.152853152288909 | 0.236557447287498 | -0.48051457 | 0.118669786595474 | 0.834500120159214 | 0.618465066110419 | 1.12599803724631 | 0.999489402457283 |
| ebi-fl187-GCST90094753 | ebi-a-GCST90001525 | CD33dim HLA DR+ CD11b+ Absolute Count \|\| id:ebi-a-GCST90001525 | F-deep fried food liking (derived food-liking factor) \|\| id:ebi-fl187-GCST90094753 | Weighted median | 8 | 0.145698968047125 | 0.19371663191038 | 0.451976368142908 | -0.23398563 | 0.52538356659147 | 1.15684788744988 | 0.791373187453099 | 1.69110737628645 | 0.999489402457283 |
| ebi-fl187-GCST90094753 | ebi-a-GCST90001540 | Naive CD4+ T cell Absolute Count \|\| id:ebi-a-GCST90001540 | F-deep fried food liking (derived food-liking factor) \|\| id:ebi-fl187-GCST90094753 | Weighted median | 8 | 0.0380320447029983 | 0.141017065596642 | 0.787392467581587 | -0.238361404 | 0.314425493272416 | 1.03876451924567 | 0.787917883062149 | 1.36947231385353 | 0.999489402457283 |
| ebi-fl187-GCST90094753 | ebi-a-GCST90001624 | HLA DR+ CD4+ T cell Absolute Count \|\| id:ebi-a-GCST90001624 | F-deep fried food liking (derived food-liking factor) \|\| id:ebi-fl187-GCST90094753 | Weighted median | 8 | 0.0612856197444711 | 0.155420866149937 | 0.693344496101017 | -0.243339278 | 0.365910517398347 | 1.06320254246252 | 0.784005472905405 | 1.44182621852073 | 0.999489402457283 |
| ebi-fl187-GCST90094753 | ebi-a-GCST90001672 | CD39+ CD8+ T cell Absolute Count \|\| id:ebi-a-GCST90001672 | F-deep fried food liking (derived food-liking factor) \|\| id:ebi-fl187-GCST90094753 | Weighted median | 8 | 0.0598053830770328 | 0.164296338588706 | 0.715851119828279 | -0.262215441 | 0.381826206710897 | 1.0616299152916 | 0.769345257681144 | 1.46495746323188 | 0.999489402457283 |
| ebi-fl187-GCST90094753 | ebi-a-GCST90001722 | CD19 on CD20- CD38- B cell \|\| id:ebi-a-GCST90001722 | F-deep fried food liking (derived food-liking factor) \|\| id:ebi-fl187-GCST90094753 | Weighted median | 8 | 0.102497608054076 | 0.15234527290503 | 0.501075755459685 | -0.196099127 | 0.401094342947935 | 1.10793465178686 | 0.821930755241567 | 1.49345815909919 | 0.999489402457283 |
| ebi-fl187-GCST90094753 | ebi-a-GCST90001742 | CD19 on transitional B cell \|\| id:ebi-a-GCST90001742 | F-deep fried food liking (derived food-liking factor) \|\| id:ebi-fl187-GCST90094753 | Weighted median | 8 | -0.02191609 | 0.140864896304228 | 0.876362246158599 | -0.298011287 | 0.254179106584058 | 0.978322322464774 | 0.742292961492898 | 1.28940272410494 | 0.999489402457283 |
| ebi-fl187-GCST90094753 | ebi-a-GCST90001784 | CD25 on IgD+ CD38dim B cell \|\| id:ebi-a-GCST90001784 | F-deep fried food liking (derived food-liking factor) \|\| id:ebi-fl187-GCST90094753 | Weighted median | 8 | 0.0475865493107987 | 0.156964001011897 | 0.761761411776539 | -0.260062893 | 0.355235991294116 | 1.04873696465497 | 0.771003093833596 | 1.4265172602162 | 0.999489402457283 |
| ebi-fl187-GCST90094753 | ebi-a-GCST90001794 | CD25 on IgD+ B cell \|\| id:ebi-a-GCST90001794 | F-deep fried food liking (derived food-liking factor) \|\| id:ebi-fl187-GCST90094753 | Weighted median | 8 | 0.0486407893393815 | 0.149642356755722 | 0.745145550050003 | -0.24465823 | 0.341939808580597 | 1.0498431681421 | 0.782972088966553 | 1.40767556497373 | 0.999489402457283 |
| ebi-fl187-GCST90094753 | ebi-a-GCST90001802 | CD27 on IgD- CD38- B cell \|\| id:ebi-a-GCST90001802 | F-deep fried food liking (derived food-liking factor) \|\| id:ebi-fl187-GCST90094753 | Weighted median | 8 | 0.0499082882525308 | 0.145390898255438 | 0.731395582550863 | -0.235057872 | 0.33487444883319 | 1.05117468688772 | 0.790525098776984 | 1.39776488319344 | 0.999489402457283 |
| ebi-fl187-GCST90094753 | ebi-a-GCST90001804 | CD27 on IgD- CD38dim B cell \|\| id:ebi-a-GCST90001804 | F-deep fried food liking (derived food-liking factor) \|\| id:ebi-fl187-GCST90094753 | Weighted median | 8 | -0.032199981 | 0.149087904702612 | 0.82900346422345 | -0.324412275 | 0.260012311828126 | 0.968312918152901 | 0.722952126128148 | 1.29694605434439 | 0.999489402457283 |
| ebi-fl187-GCST90094753 | ebi-a-GCST90001805 | CD27 on memory B cell \|\| id:ebi-a-GCST90001805 | F-deep fried food liking (derived food-liking factor) \|\| id:ebi-fl187-GCST90094753 | Weighted median | 8 | -0.036391249 | 0.151653257567239 | 0.810358537619535 | -0.333631634 | 0.260849135401428 | 0.964262952339862 | 0.716317600494862 | 1.29803182361126 | 0.999489402457283 |
| ebi-fl187-GCST90094753 | ebi-a-GCST90001806 | CD27 on unswitched memory B cell \|\| id:ebi-a-GCST90001806 | F-deep fried food liking (derived food-liking factor) \|\| id:ebi-fl187-GCST90094753 | Weighted median | 8 | 0.0357058096118912 | 0.148474822003108 | 0.809955115033527 | -0.255304842 | 0.326716460737982 | 1.03635091716095 | 0.774680307244661 | 1.38640832025324 | 0.999489402457283 |
| ebi-fl187-GCST90094753 | ebi-a-GCST90001839 | CD3 on Effector Memory CD8+ T cell \|\| id:ebi-a-GCST90001839 | F-deep fried food liking (derived food-liking factor) \|\| id:ebi-fl187-GCST90094753 | Weighted median | 8 | -0.017811473 | 0.164095539072966 | 0.913564727690892 | -0.339438729 | 0.30381578375428 | 0.982346213854782 | 0.712169930643952 | 1.35501941650676 | 0.999489402457283 |
| ebi-fl187-GCST90094753 | ebi-a-GCST90001859 | CD3 on CD8+ T cell \|\| id:ebi-a-GCST90001859 | F-deep fried food liking (derived food-liking factor) \|\| id:ebi-fl187-GCST90094753 | Weighted median | 8 | 0.152671464465374 | 0.154832293598913 | 0.324111488994078 | -0.150799831 | 0.456142759919245 | 1.16494219116091 | 0.860019830750271 | 1.57797560035664 | 0.999489402457283 |
| ebi-fl187-GCST90094753 | ebi-a-GCST90001905 | CD86 on monocyte \|\| id:ebi-a-GCST90001905 | F-deep fried food liking (derived food-liking factor) \|\| id:ebi-fl187-GCST90094753 | Weighted median | 8 | 0.0239556421636024 | 0.166502002379013 | 0.885598387380617 | -0.302388282 | 0.350299566826467 | 1.02424488359597 | 0.739051048583207 | 1.41949271783545 | 0.999489402457283 |
| ebi-fl187-GCST90094753 | ebi-a-GCST90001943 | CD25 on activated & secreting CD4 regulatory T cell \|\| id:ebi-a-GCST90001943 | F-deep fried food liking (derived food-liking factor) \|\| id:ebi-fl187-GCST90094753 | Weighted median | 8 | -0.110626197 | 0.152010782482614 | 0.466765227821748 | -0.40856733 | 0.18731493687244 | 0.895273342435183 | 0.66460172304568 | 1.2060070413329 | 0.999489402457283 |
| ebi-fl187-GCST90094753 | ebi-a-GCST90001949 | CD33 on CD33dim HLA DR+ CD11b- \|\| id:ebi-a-GCST90001949 | F-deep fried food liking (derived food-liking factor) \|\| id:ebi-fl187-GCST90094753 | Weighted median | 8 | 0.346374757297032 | 0.211948330445877 | 0.102208151939169 | -0.06904397 | 0.761793484970951 | 1.41393239802527 | 0.933285642254551 | 2.14211462779604 | 0.999489402457283 |
| ebi-fl187-GCST90094753 | ebi-a-GCST90001955 | CD33 on Immature Myeloid-Derived Suppressor Cells \|\| id:ebi-a-GCST90001955 | F-deep fried food liking (derived food-liking factor) \|\| id:ebi-fl187-GCST90094753 | Weighted median | 8 | 0.234824512717452 | 0.227143738351553 | 0.301222845472708 | -0.210377214 | 0.680026239886495 | 1.26468681280619 | 0.810278539540441 | 1.97392952723764 | 0.999489402457283 |
| ebi-fl187-GCST90094753 | ebi-a-GCST90001956 | CD33 on CD33+ HLA DR+ \|\| id:ebi-a-GCST90001956 | F-deep fried food liking (derived food-liking factor) \|\| id:ebi-fl187-GCST90094753 | Weighted median | 8 | 0.204464751046327 | 0.247654620824378 | 0.409028523665722 | -0.280938306 | 0.689867807862108 | 1.22686820926289 | 0.755074917809322 | 1.99345199714348 | 0.999489402457283 |
| ebi-fl187-GCST90094753 | ebi-a-GCST90001957 | CD33 on CD33+ HLA DR+ CD14- \|\| id:ebi-a-GCST90001957 | F-deep fried food liking (derived food-liking factor) \|\| id:ebi-fl187-GCST90094753 | Weighted median | 8 | 0.202187672355553 | 0.235435264177385 | 0.390460553315571 | -0.259265445 | 0.663640790143227 | 1.22407771210375 | 0.771618173337242 | 1.94184934601621 | 0.999489402457283 |
| ebi-fl187-GCST90094753 | ebi-a-GCST90002064 | CD4 on resting CD4 regulatory T cell \|\| id:ebi-a-GCST90002064 | F-deep fried food liking (derived food-liking factor) \|\| id:ebi-fl187-GCST90094753 | Weighted median | 8 | 0.0673913597507719 | 0.162491833074918 | 0.678334513673709 | -0.251092633 | 0.385875352577611 | 1.06971403928508 | 0.777950304291389 | 1.47090131533004 | 0.999489402457283 |
| ebi-fl187-GCST90094753 | ebi-a-GCST90002080 | SSC-A on T cell \|\| id:ebi-a-GCST90002080 | F-deep fried food liking (derived food-liking factor) \|\| id:ebi-fl187-GCST90094753 | Weighted median | 8 | 0.00512263071851667 | 0.158908821687603 | 0.974283616681072 | -0.30633866 | 0.316583921226219 | 1.0051357738241 | 0.736137277142287 | 1.37243141353062 | 0.999489402457283 |
| ebi-fl187-GCST90094753 | ebi-a-GCST90002121 | CD8 on CD39+ CD8+ T cell \|\| id:ebi-a-GCST90002121 | F-deep fried food liking (derived food-liking factor) \|\| id:ebi-fl187-GCST90094753 | Weighted median | 8 | 0.179206505008142 | 0.161570238289129 | 0.267363141736811 | -0.137471162 | 0.495884172054836 | 1.1962677539449 | 0.871559483633544 | 1.64194936318319 | 0.999489402457283 |
| ebi-fl187-GCST90094753 | ebi-cfmet338-GCST90026053 | 1-palmitoyl-2-docosahexaenoyl-gpc (16:0/22:6) levels \|\| id:ebi-cfmet338-GCST90026053 | F-deep fried food liking (derived food-liking factor) \|\| id:ebi-fl187-GCST90094753 | Weighted median | 8 | -0.084033018 | 0.0688811270105038 | 0.222475645878675 | -0.219040027 | 0.0509739910739517 | 0.919400898937697 | 0.803289564393586 | 1.05229552385082 | 0.999489402457283 |
| ebi-fl187-GCST90094753 | ebi-cfmet338-GCST90026063 | 1-stearoyl-2-oleoyl-gpc (18:0/18:1) levels \|\| id:ebi-cfmet338-GCST90026063 | F-deep fried food liking (derived food-liking factor) \|\| id:ebi-fl187-GCST90094753 | Weighted median | 8 | 0.000666637227707354 | 0.05906711928396 | 0.990995188754142 | -0.115104917 | 0.116438191024269 | 1.00066685947969 | 0.891272629735099 | 1.12348806667445 | 0.999911036302558 |
| ebi-fl187-GCST90094753 | ebi-cfmet338-GCST90026113 | Adenosine 3',5'-cyclic monophosphate (camp) levels \|\| id:ebi-cfmet338-GCST90026113 | F-deep fried food liking (derived food-liking factor) \|\| id:ebi-fl187-GCST90094753 | Weighted median | 8 | 0.0552521358602225 | 0.112819501278291 | 0.624318436827022 | -0.165874087 | 0.276378358365672 | 1.05680704003852 | 0.847152895337285 | 1.31834657713153 | 0.999489402457283 |
| ebi-fl187-GCST90094753 | ebi-cfmet338-GCST90026117 | Alpha-ketoglutaramate levels \|\| id:ebi-cfmet338-GCST90026117 | F-deep fried food liking (derived food-liking factor) \|\| id:ebi-fl187-GCST90094753 | Weighted median | 8 | -0.018919561 | 0.0466262962987776 | 0.684911465655518 | -0.110307102 | 0.0724679797536203 | 0.981258290511804 | 0.895559065316385 | 1.07515837870279 | 0.999489402457283 |
| ebi-fl187-GCST90094753 | ebi-cfmet338-GCST90026124 | Arginine levels \|\| id:ebi-cfmet338-GCST90026124 | F-deep fried food liking (derived food-liking factor) \|\| id:ebi-fl187-GCST90094753 | Weighted median | 8 | -0.129620962 | 0.037406033509755 | 0.000529754332030829 | -0.202936788 | -0.056305137 | 0.878428325106426 | 0.816329841493478 | 0.945250661102343 | 0.999489402457283 |
| ebi-fl187-GCST90094753 | ebi-cfmet338-GCST90026169 | Glutamate levels \|\| id:ebi-cfmet338-GCST90026169 | F-deep fried food liking (derived food-liking factor) \|\| id:ebi-fl187-GCST90094753 | Weighted median | 8 | -0.052313371 | 0.0399635694333748 | 0.19052537528061 | -0.130641967 | 0.0260152247745409 | 0.949031421015569 | 0.87753190317863 | 1.02635657440194 | 0.999489402457283 |
| ebi-fl187-GCST90094753 | ebi-cfmet338-GCST90026170 | Glutamine levels \|\| id:ebi-cfmet338-GCST90026170 | F-deep fried food liking (derived food-liking factor) \|\| id:ebi-fl187-GCST90094753 | Weighted median | 8 | -0.023791494 | 0.024152428991691 | 0.32459658183176 | -0.071130255 | 0.0235472665820924 | 0.976489292173531 | 0.931340572399401 | 1.02382669239137 | 0.999489402457283 |
| ebi-fl187-GCST90094753 | ebi-cfmet338-GCST90026191 | Kynurenate levels \|\| id:ebi-cfmet338-GCST90026191 | F-deep fried food liking (derived food-liking factor) \|\| id:ebi-fl187-GCST90094753 | Weighted median | 8 | 0.290647243878151 | 0.196031168075064 | 0.138165211647047 | -0.093573846 | 0.674868333305277 | 1.33729276252729 | 0.910670765960471 | 1.96377439526318 | 0.999489402457283 |
| ebi-fl187-GCST90094753 | ebi-cfmet338-GCST90026202 | Methylsuccinoylcarnitine levels \|\| id:ebi-cfmet338-GCST90026202 | F-deep fried food liking (derived food-liking factor) \|\| id:ebi-fl187-GCST90094753 | Weighted median | 8 | 0.108830813255945 | 0.121345232418023 | 0.369788665299176 | -0.129005842 | 0.346667468795271 | 1.11497369561369 | 0.878968830345592 | 1.4143463328747 | 0.999489402457283 |
| ebi-fl187-GCST90094753 | ebi-cfmet338-GCST90026222 | N-acetylputrescine levels \|\| id:ebi-cfmet338-GCST90026222 | F-deep fried food liking (derived food-liking factor) \|\| id:ebi-fl187-GCST90094753 | Weighted median | 8 | 0.0199108855336969 | 0.0424871160953806 | 0.639331634169492 | -0.063363862 | 0.103185633080643 | 1.0201104293796 | 0.938601889974907 | 1.10869720085143 | 0.999489402457283 |
| ebi-fl187-GCST90094753 | ebi-cfmet338-GCST90026252 | Propionylcarnitine (c3) levels \|\| id:ebi-cfmet338-GCST90026252 | F-deep fried food liking (derived food-liking factor) \|\| id:ebi-fl187-GCST90094753 | Weighted median | 8 | -0.022658865 | 0.0743824206648052 | 0.760650551586351 | -0.168448409 | 0.123130679924595 | 0.977595919492804 | 0.844974855323399 | 1.13103221449496 | 0.999489402457283 |
| ebi-fl187-GCST90094753 | ebi-cfmet338-GCST90026316 | Ethyl beta-glucopyranoside levels \|\| id:ebi-cfmet338-GCST90026316 | F-deep fried food liking (derived food-liking factor) \|\| id:ebi-fl187-GCST90094753 | Weighted median | 8 | 0.463786563699398 | 0.239996634269007 | 0.0533013837634502 | -0.006606839 | 0.934179966866652 | 1.59008355266816 | 0.993414937710187 | 2.54512551451427 | 0.999489402457283 |
| ebi-fl187-GCST90094753 | ebi-gm430-GCST90011315 | F_Prevotellaceae abundance \|\| id:ebi-gm430-GCST90011315 | F-deep fried food liking (derived food-liking factor) \|\| id:ebi-fl187-GCST90094753 | Weighted median | 8 | -0.171561791 | 0.105239205218574 | 0.103057550487627 | -0.377830633 | 0.0347070513472955 | 0.842348216973374 | 0.685346565853557 | 1.03531636983476 | 0.999489402457283 |
| ebi-fl187-GCST90094753 | ebi-gm430-GCST90011354 | OTU97_1 (Bacteroides) abundance \|\| id:ebi-gm430-GCST90011354 | F-deep fried food liking (derived food-liking factor) \|\| id:ebi-fl187-GCST90094753 | Weighted median | 8 | -0.090357158 | 0.0930645205240054 | 0.33159371683292 | -0.272763618 | 0.0920493022473718 | 0.913604825739956 | 0.761272717372139 | 1.09641887666298 | 0.999489402457283 |
| ebi-fl187-GCST90094753 | ebi-gm430-GCST90011419 | OTU99_1 (Bacteroides) abundance \|\| id:ebi-gm430-GCST90011419 | F-deep fried food liking (derived food-liking factor) \|\| id:ebi-fl187-GCST90094753 | Weighted median | 8 | -0.086629431 | 0.0894525386189749 | 0.332824587558588 | -0.261956407 | 0.0886975442934928 | 0.917016850328733 | 0.769544569661331 | 1.09275009783632 | 0.999489402457283 |
| ebi-fl187-GCST90094753 | ebi-gm430-GCST90011435 | OTU99_155 (Butyrivibrio) abundance \|\| id:ebi-gm430-GCST90011435 | F-deep fried food liking (derived food-liking factor) \|\| id:ebi-fl187-GCST90094753 | Weighted median | 8 | -0.296125873 | 0.301648404789746 | 0.326251530382443 | -0.887356747 | 0.295105000201801 | 0.743693811018892 | 0.411742655825019 | 1.34326739462925 | 0.999489402457283 |
| ebi-fl187-GCST90094753 | ebi-gm430-GCST90011466 | OTU99_58 (Bacteroides) abundance \|\| id:ebi-gm430-GCST90011466 | F-deep fried food liking (derived food-liking factor) \|\| id:ebi-fl187-GCST90094753 | Weighted median | 8 | -0.063490104 | 0.169862523596363 | 0.708572862346482 | -0.39642065 | 0.269440442124011 | 0.938483406369322 | 0.672723654954038 | 1.30923165484755 | 0.999489402457283 |
| ebi-fl187-GCST90094753 | ebi-gm430-GCST90011472 | OTU99_72 (Parabacteroides) abundance \|\| id:ebi-gm430-GCST90011472 | F-deep fried food liking (derived food-liking factor) \|\| id:ebi-fl187-GCST90094753 | Weighted median | 7 | -0.11213839 | 0.281024598874496 | 0.689868099287895 | -0.662946604 | 0.438669823879913 | 0.89392053935323 | 0.515330620004713 | 1.55064321749455 | 0.999489402457283 |
| ebi-fl187-GCST90094753 | ebi-gm430-GCST90011505 | TestASV_28 (Holdemanella) abundance \|\| id:ebi-gm430-GCST90011505 | F-deep fried food liking (derived food-liking factor) \|\| id:ebi-fl187-GCST90094753 | Weighted median | 8 | -0.2299484 | 0.210962501375586 | 0.275714661151737 | -0.643434903 | 0.183538102750435 | 0.794574601538123 | 0.525484332988777 | 1.20146074349842 | 0.999489402457283 |
| ebi-fl187-GCST90094753 | ebi-gm430-GCST90011513 | TestASV_35 (Alphaproteobacteria) abundance \|\| id:ebi-gm430-GCST90011513 | F-deep fried food liking (derived food-liking factor) \|\| id:ebi-fl187-GCST90094753 | Weighted median | 8 | 0.231032603504548 | 0.26268509420477 | 0.379128039645229 | -0.283830181 | 0.745895388145897 | 1.25990031594529 | 0.75289448953698 | 2.10832836231175 | 0.999489402457283 |
| ebi-fl187-GCST90094753 | ebi-gm430-GCST90011543 | G_Coprococcus prevalence \|\| id:ebi-gm430-GCST90011543 | F-deep fried food liking (derived food-liking factor) \|\| id:ebi-fl187-GCST90094753 | Weighted median | 8 | -0.019581835 | 0.296228256153839 | 0.947295126440132 | -0.600189217 | 0.561025546684261 | 0.980608643426712 | 0.548707801185865 | 1.75246881762021 | 0.999489402457283 |
| ebi-fl187-GCST90094753 | ebi-gm430-GCST90011547 | G_Parasutterella prevalence \|\| id:ebi-gm430-GCST90011547 | F-deep fried food liking (derived food-liking factor) \|\| id:ebi-fl187-GCST90094753 | Weighted median | 8 | -0.309688282 | 0.278602687973701 | 0.266320156413895 | -0.85574955 | 0.236372986801104 | 0.733675620753525 | 0.424964539480248 | 1.26664666455797 | 0.999489402457283 |
| ebi-fl187-GCST90094753 | ebi-gm430-GCST90011569 | OTU97_13 (Subdoligranulum) prevalence \|\| id:ebi-gm430-GCST90011569 | F-deep fried food liking (derived food-liking factor) \|\| id:ebi-fl187-GCST90094753 | Weighted median | 8 | 0.0575460877701688 | 0.271733884431389 | 0.832283594261216 | -0.475052326 | 0.590144501255691 | 1.05923408726697 | 0.621852516735913 | 1.80424911282423 | 0.999489402457283 |
| ebi-fl187-GCST90094753 | ebi-gm430-GCST90011590 | OTU97_277 (Prevotella) prevalence \|\| id:ebi-gm430-GCST90011590 | F-deep fried food liking (derived food-liking factor) \|\| id:ebi-fl187-GCST90094753 | Weighted median | 8 | 0.0268544107545307 | 0.258313226688019 | 0.91720057665805 | -0.479439514 | 0.533148335063047 | 1.0272182399479 | 0.6191303087223 | 1.70428954553885 | 0.999489402457283 |
| ebi-fl187-GCST90094753 | ebi-gm430-GCST90011616 | OTU97_86 (Phascolarctobacterium) prevalence \|\| id:ebi-gm430-GCST90011616 | F-deep fried food liking (derived food-liking factor) \|\| id:ebi-fl187-GCST90094753 | Weighted median | 8 | -0.306085635 | 0.296919232935574 | 0.302600988166294 | -0.888047332 | 0.275876061418376 | 0.736323561588297 | 0.411458410634712 | 1.3176845419534 | 0.999489402457283 |
| ebi-fl187-GCST90094753 | ebi-gm430-GCST90011665 | OTU99_55 (Barnesiella) prevalence \|\| id:ebi-gm430-GCST90011665 | F-deep fried food liking (derived food-liking factor) \|\| id:ebi-fl187-GCST90094753 | Weighted median | 8 | -0.217433477 | 0.20261504525967 | 0.28321014970053 | -0.614558966 | 0.179692011610197 | 0.804581126210414 | 0.540879389017974 | 1.1968486908502 | 0.999489402457283 |
| ebi-fl187-GCST90094753 | ebi-gm430-GCST90011687 | TestASV_14 (Lachnospiraceae) prevalence \|\| id:ebi-gm430-GCST90011687 | F-deep fried food liking (derived food-liking factor) \|\| id:ebi-fl187-GCST90094753 | Weighted median | 8 | 0.179253167725596 | 0.205908354888065 | 0.384000837368711 | -0.224327208 | 0.582833543306203 | 1.1963235763515 | 0.799053634963433 | 1.79110642478954 | 0.999489402457283 |
| ebi-fl187-GCST90094753 | ebi-gm430-GCST90011697 | TestASV_23 (Barnesiella) prevalence \|\| id:ebi-gm430-GCST90011697 | F-deep fried food liking (derived food-liking factor) \|\| id:ebi-fl187-GCST90094753 | Weighted median | 8 | 0.0411809447892335 | 0.280448330449289 | 0.883258559270476 | -0.508497783 | 0.590859672469839 | 1.04204064031181 | 0.601398331439867 | 1.80553992137242 | 0.999489402457283 |
| ebi-fl187-GCST90094753 | ebi-gm430-GCST90011727 | TestASV_6 (Subdoligranulum) prevalence \|\| id:ebi-gm430-GCST90011727 | F-deep fried food liking (derived food-liking factor) \|\| id:ebi-fl187-GCST90094753 | Weighted median | 8 | 0.197331912299376 | 0.332919709169965 | 0.553361030405136 | -0.455190718 | 0.849854542272506 | 1.21814829201479 | 0.63432697904539 | 2.33930655696164 | 0.999489402457283 |
| ebi-fl187-GCST90094753 | ebi-hbsm44-GCST90104908 | Sulcal depth of the left posterior inferior temporal sulcus \|\| id:ebi-hbsm44-GCST90104908 | F-deep fried food liking (derived food-liking factor) \|\| id:ebi-fl187-GCST90094753 | Weighted median | 7 | 0.107261798188381 | 0.10541908163233 | 0.308925182386101 | -0.099359602 | 0.313883198187748 | 1.11322565679357 | 0.905417059860801 | 1.36872985708266 | 0.999489402457283 |
| ebi-fl187-GCST90094753 | ebi-hbsm44-GCST90104918 | Sulcal depth of the left inferior frontal sulcus \|\| id:ebi-hbsm44-GCST90104918 | F-deep fried food liking (derived food-liking factor) \|\| id:ebi-fl187-GCST90094753 | Weighted median | 7 | 0.115323269108217 | 0.178279241087633 | 0.517716895059748 | -0.234104043 | 0.464750581639978 | 1.12223616322347 | 0.791279484186107 | 1.59161716083408 | 0.999489402457283 |
| ebi-fl187-GCST90094753 | ebi-hbsm44-GCST90104925 | Sulcal depth of the right anterior inferior temporal sulcus \|\| id:ebi-hbsm44-GCST90104925 | F-deep fried food liking (derived food-liking factor) \|\| id:ebi-fl187-GCST90094753 | Weighted median | 7 | 0.0878084260210728 | 0.0570164570567853 | 0.1235471772399 | -0.02394383 | 0.199560681852372 | 1.09177894555574 | 0.976340549451663 | 1.22086629161132 | 0.999489402457283 |
| ebi-fl187-GCST90094753 | ebi-IPs91-GCST90274770 | C-C motif chemokine 4 levels \|\| id:ebi-IPs91-GCST90274770 | F-deep fried food liking (derived food-liking factor) \|\| id:ebi-fl187-GCST90094753 | Weighted median | 8 | 0.0217276915970249 | 0.0687899824818628 | 0.752112219838611 | -0.113100674 | 0.156556057261476 | 1.02196545679524 | 0.893060747532246 | 1.16947631812134 | 0.999489402457283 |
| ebi-fl187-GCST90094753 | ebi-IPs91-GCST90274779 | C-X-C motif chemokine 1 levels \|\| id:ebi-IPs91-GCST90274779 | F-deep fried food liking (derived food-liking factor) \|\| id:ebi-fl187-GCST90094753 | Weighted median | 8 | -0.0144521 | 0.0619631632379402 | 0.815577407233284 | -0.1358999 | 0.106995700122044 | 0.985651830497489 | 0.872930008609502 | 1.11292946900815 | 0.999489402457283 |
| ebi-fl187-GCST90094753 | ebi-IPs91-GCST90274780 | C-X-C motif chemokine 10 levels \|\| id:ebi-IPs91-GCST90274780 | F-deep fried food liking (derived food-liking factor) \|\| id:ebi-fl187-GCST90094753 | Weighted median | 8 | 0.0887058609434687 | 0.0675567727512193 | 0.189163903549911 | -0.043705414 | 0.221117135535858 | 1.09275918589419 | 0.957235904574374 | 1.24746954501992 | 0.999489402457283 |
| ebi-fl187-GCST90094753 | ebi-IPs91-GCST90274796 | Interleukin-10 receptor subunit alpha levels \|\| id:ebi-IPs91-GCST90274796 | F-deep fried food liking (derived food-liking factor) \|\| id:ebi-fl187-GCST90094753 | Weighted median | 8 | -0.015941731 | 0.076207061820799 | 0.834300167086916 | -0.165307572 | 0.133424110166905 | 0.98418466583899 | 0.84763295568242 | 1.14273454091079 | 0.999489402457283 |
| ebi-fl187-GCST90094753 | ebi-IPs91-GCST90274807 | Interleukin-20 levels \|\| id:ebi-IPs91-GCST90274807 | F-deep fried food liking (derived food-liking factor) \|\| id:ebi-fl187-GCST90094753 | Weighted median | 8 | -0.036337973 | 0.0712997990754725 | 0.610296393184074 | -0.176085579 | 0.103409633591393 | 0.964314326585456 | 0.838546218500252 | 1.10894557740789 | 0.999489402457283 |
| ebi-fl187-GCST90094753 | ebi-IPs91-GCST90274808 | Interleukin-20 receptor subunit alpha levels \|\| id:ebi-IPs91-GCST90274808 | F-deep fried food liking (derived food-liking factor) \|\| id:ebi-fl187-GCST90094753 | Weighted median | 8 | -0.055242633 | 0.072584409979576 | 0.446608543343651 | -0.197508076 | 0.0870228106217397 | 0.946255527338934 | 0.820773511625168 | 1.09092156403361 | 0.999489402457283 |
| ebi-fl187-GCST90094753 | ebi-IPs91-GCST90274810 | Interleukin-24 levels \|\| id:ebi-IPs91-GCST90274810 | F-deep fried food liking (derived food-liking factor) \|\| id:ebi-fl187-GCST90094753 | Weighted median | 8 | -0.006325809 | 0.0666381297336846 | 0.924372202485738 | -0.136936543 | 0.124284925530844 | 0.993694157058835 | 0.872025560480432 | 1.13233845717648 | 0.999489402457283 |
| ebi-fl187-GCST90094753 | ebi-IPs91-GCST90274823 | Monocyte chemoattractant protein-3 levels \|\| id:ebi-IPs91-GCST90274823 | F-deep fried food liking (derived food-liking factor) \|\| id:ebi-fl187-GCST90094753 | Weighted median | 8 | 0.0846692742438864 | 0.077814490208276 | 0.276554778901497 | -0.067847127 | 0.237185675052107 | 1.08835705945844 | 0.93440330810314 | 1.26767647181988 | 0.999489402457283 |
| ebi-fl187-GCST90094753 | ebi-IPs91-GCST90274824 | Monocyte chemoattractant protein-4 levels \|\| id:ebi-IPs91-GCST90274824 | F-deep fried food liking (derived food-liking factor) \|\| id:ebi-fl187-GCST90094753 | Weighted median | 8 | -0.041216342 | 0.0717584691557757 | 0.565713396564402 | -0.181862941 | 0.0994302577539644 | 0.959621501254622 | 0.833715600531188 | 1.10454143485315 | 0.999489402457283 |
| ebi-fl187-GCST90094753 | ebi-IPs91-GCST90274834 | SIR2-like protein 2 levels \|\| id:ebi-IPs91-GCST90274834 | F-deep fried food liking (derived food-liking factor) \|\| id:ebi-fl187-GCST90094753 | Weighted median | 8 | -0.055036642 | 0.0625397271293461 | 0.378844735714235 | -0.177614508 | 0.0675412228044006 | 0.946450467130726 | 0.837265120674941 | 1.06987436191044 | 0.999489402457283 |
| ebi-fl187-GCST90094753 | ebi-met1400-GCST90199652 | 1-stearoyl-2-oleoyl-GPS (18:0/18:1) levels \|\| id:ebi-met1400-GCST90199652 | F-deep fried food liking (derived food-liking factor) \|\| id:ebi-fl187-GCST90094753 | Weighted median | 8 | -0.017615911 | 0.0896375792212735 | 0.844200260392152 | -0.193305567 | 0.158073743794069 | 0.982538341591421 | 0.824230078346547 | 1.17125256413092 | 0.999489402457283 |
| ebi-fl187-GCST90094753 | ebi-met1400-GCST90199665 | N-acetylaspartate (naa) levels \|\| id:ebi-met1400-GCST90199665 | F-deep fried food liking (derived food-liking factor) \|\| id:ebi-fl187-GCST90094753 | Weighted median | 8 | -0.023382604 | 0.087016582202685 | 0.788149509201754 | -0.193935105 | 0.147169896913734 | 0.976888650558674 | 0.823711357018433 | 1.15855078051205 | 0.999489402457283 |
| ebi-fl187-GCST90094753 | ebi-met1400-GCST90199669 | Acetylcarnitine levels (Biocrates platform) \|\| id:ebi-met1400-GCST90199669 | F-deep fried food liking (derived food-liking factor) \|\| id:ebi-fl187-GCST90094753 | Weighted median | 8 | -0.02530708 | 0.0801039277362392 | 0.752057098616689 | -0.182310778 | 0.131696618348725 | 0.975010459826842 | 0.833342315395591 | 1.14076218044979 | 0.999489402457283 |
| ebi-fl187-GCST90094753 | ebi-met1400-GCST90199670 | Iminodiacetate (IDA) levels \|\| id:ebi-met1400-GCST90199670 | F-deep fried food liking (derived food-liking factor) \|\| id:ebi-fl187-GCST90094753 | Weighted median | 8 | 0.0600849105786543 | 0.0843325869266942 | 0.476170314843282 | -0.10520696 | 0.225376780954975 | 1.06192671152889 | 0.900138210885418 | 1.25279465644427 | 0.999489402457283 |
| ebi-fl187-GCST90094753 | ebi-met1400-GCST90199686 | Indolepropionate levels \|\| id:ebi-met1400-GCST90199686 | F-deep fried food liking (derived food-liking factor) \|\| id:ebi-fl187-GCST90094753 | Weighted median | 8 | -0.036305522 | 0.0847930290473068 | 0.668530009609736 | -0.202499859 | 0.129888814748328 | 0.964345619490518 | 0.816686597655267 | 1.13870176944314 | 0.999489402457283 |
| ebi-fl187-GCST90094753 | ebi-met1400-GCST90199704 | 3-hydroxysebacate levels \|\| id:ebi-met1400-GCST90199704 | F-deep fried food liking (derived food-liking factor) \|\| id:ebi-fl187-GCST90094753 | Weighted median | 8 | -0.115283352 | 0.0916528995700428 | 0.208454881119259 | -0.294923035 | 0.0643563313871871 | 0.891113609511875 | 0.744588892598825 | 1.06647234863484 | 0.999489402457283 |
| ebi-fl187-GCST90094753 | ebi-met1400-GCST90199713 | Docosapentaenoate n3 DPA; 22:5n3 levels \|\| id:ebi-met1400-GCST90199713 | F-deep fried food liking (derived food-liking factor) \|\| id:ebi-fl187-GCST90094753 | Weighted median | 8 | -0.095870945 | 0.0813393216993381 | 0.238535839917682 | -0.255296016 | 0.0635541251260127 | 0.908581265090253 | 0.774687144276933 | 1.06561716090373 | 0.999489402457283 |
| ebi-fl187-GCST90094753 | ebi-met1400-GCST90199715 | Myristoleate (14:1n5) levels \|\| id:ebi-met1400-GCST90199715 | F-deep fried food liking (derived food-liking factor) \|\| id:ebi-fl187-GCST90094753 | Weighted median | 8 | -0.017019697 | 0.0738373181017774 | 0.817701205655862 | -0.161740841 | 0.12770144609583 | 0.983124319467289 | 0.850661632710508 | 1.13621373100877 | 0.999489402457283 |
| ebi-fl187-GCST90094753 | ebi-met1400-GCST90199725 | Campesterol levels \|\| id:ebi-met1400-GCST90199725 | F-deep fried food liking (derived food-liking factor) \|\| id:ebi-fl187-GCST90094753 | Weighted median | 8 | -0.101252356 | 0.115164695394682 | 0.379294937290654 | -0.326975159 | 0.124470447381356 | 0.90370494911034 | 0.721101656003454 | 1.13254855019028 | 0.999489402457283 |
| ebi-fl187-GCST90094753 | ebi-met1400-GCST90199737 | N-acetylproline levels \|\| id:ebi-met1400-GCST90199737 | F-deep fried food liking (derived food-liking factor) \|\| id:ebi-fl187-GCST90094753 | Weighted median | 8 | -0.096552606 | 0.0887341909447022 | 0.276546321954183 | -0.270471621 | 0.0773664079761125 | 0.907962131837317 | 0.76301955378169 | 1.0804378849332 | 0.999489402457283 |
| ebi-fl187-GCST90094753 | ebi-met1400-GCST90199775 | Gamma-glutamylisoleucine levels \|\| id:ebi-met1400-GCST90199775 | F-deep fried food liking (derived food-liking factor) \|\| id:ebi-fl187-GCST90094753 | Weighted median | 8 | 0.108752311316676 | 0.0844896450523121 | 0.198035969691551 | -0.056847393 | 0.274352015619208 | 1.1148861714518 | 0.944738232046866 | 1.31567785988871 | 0.999489402457283 |
| ebi-fl187-GCST90094753 | ebi-met1400-GCST90199777 | 1-palmitoleoyl-GPC (16:1) levels \|\| id:ebi-met1400-GCST90199777 | F-deep fried food liking (derived food-liking factor) \|\| id:ebi-fl187-GCST90094753 | Weighted median | 8 | 0.0988537616094938 | 0.0806112834146251 | 0.22008512584303 | -0.059144354 | 0.256851877102159 | 1.10390485448524 | 0.94257069559589 | 1.2928536113524 | 0.999489402457283 |
| ebi-fl187-GCST90094753 | ebi-met1400-GCST90199782 | Glutamine degradant levels \|\| id:ebi-met1400-GCST90199782 | F-deep fried food liking (derived food-liking factor) \|\| id:ebi-fl187-GCST90094753 | Weighted median | 8 | -0.062673091 | 0.0679210584566278 | 0.356145679502886 | -0.195798366 | 0.0704521833415649 | 0.939250472719603 | 0.822177997161729 | 1.07299326125175 | 0.999489402457283 |
| ebi-fl187-GCST90094753 | ebi-met1400-GCST90199783 | 5-methyluridine (ribothymidine) levels \|\| id:ebi-met1400-GCST90199783 | F-deep fried food liking (derived food-liking factor) \|\| id:ebi-fl187-GCST90094753 | Weighted median | 8 | -0.079278021 | 0.0793363121788063 | 0.317666205981447 | -0.234777193 | 0.0762211506560759 | 0.923783057452044 | 0.790747013905534 | 1.07920121382513 | 0.999489402457283 |
| ebi-fl187-GCST90094753 | ebi-met1400-GCST90199787 | Homostachydrine levels \|\| id:ebi-met1400-GCST90199787 | F-deep fried food liking (derived food-liking factor) \|\| id:ebi-fl187-GCST90094753 | Weighted median | 8 | -0.001300834 | 0.0855320047406245 | 0.987865649427903 | -0.168943563 | 0.166341895320283 | 0.998700011746417 | 0.844556566057889 | 1.18097680314989 | 0.999911036302558 |
| ebi-fl187-GCST90094753 | ebi-met1400-GCST90199802 | Beta-hydroxyisovaleroylcarnitine levels \|\| id:ebi-met1400-GCST90199802 | F-deep fried food liking (derived food-liking factor) \|\| id:ebi-fl187-GCST90094753 | Weighted median | 8 | -0.019209412 | 0.079051471481758 | 0.808006331895262 | -0.174150296 | 0.135731471889607 | 0.980973912812162 | 0.840170613618194 | 1.14537428710321 | 0.999489402457283 |
| ebi-fl187-GCST90094753 | ebi-met1400-GCST90199873 | Pregnenolone sulfate levels \|\| id:ebi-met1400-GCST90199873 | F-deep fried food liking (derived food-liking factor) \|\| id:ebi-fl187-GCST90094753 | Weighted median | 8 | 0.096162405445392 | 0.0812466538124322 | 0.236576968181237 | -0.063081036 | 0.255405846917759 | 1.10093784777737 | 0.938867388523333 | 1.29098545703566 | 0.999489402457283 |
| ebi-fl187-GCST90094753 | ebi-met1400-GCST90199911 | Beta-citrylglutamate levels \|\| id:ebi-met1400-GCST90199911 | F-deep fried food liking (derived food-liking factor) \|\| id:ebi-fl187-GCST90094753 | Weighted median | 8 | -0.092582202 | 0.0855557674415992 | 0.279196117926892 | -0.260271507 | 0.0751071016887422 | 0.911574274203158 | 0.770842268562403 | 1.07799960027976 | 0.999489402457283 |
| ebi-fl187-GCST90094753 | ebi-met1400-GCST90199934 | 2-hydroxydecanoate levels \|\| id:ebi-met1400-GCST90199934 | F-deep fried food liking (derived food-liking factor) \|\| id:ebi-fl187-GCST90094753 | Weighted median | 8 | -0.036212146 | 0.0947424358187746 | 0.702300596072867 | -0.22190732 | 0.149483027971123 | 0.964435670383786 | 0.800989596259283 | 1.16123376215181 | 0.999489402457283 |
| ebi-fl187-GCST90094753 | ebi-met1400-GCST90199945 | Sphingomyelin (d18:1/14:0, d16:1/16:0) levels \|\| id:ebi-met1400-GCST90199945 | F-deep fried food liking (derived food-liking factor) \|\| id:ebi-fl187-GCST90094753 | Weighted median | 8 | -0.15292511 | 0.0861895061589349 | 0.0760153265257887 | -0.321856542 | 0.0160063223773641 | 0.858193989815216 | 0.724802161710314 | 1.0161351097754 | 0.999489402457283 |
| ebi-fl187-GCST90094753 | ebi-met1400-GCST90199979 | Carnitine C14:1 levels \|\| id:ebi-met1400-GCST90199979 | F-deep fried food liking (derived food-liking factor) \|\| id:ebi-fl187-GCST90094753 | Weighted median | 8 | -0.116075167 | 0.0815915847885673 | 0.15484155577304 | -0.275994673 | 0.0438443391437645 | 0.890408291424684 | 0.75881697279592 | 1.04481970469979 | 0.999489402457283 |
| ebi-fl187-GCST90094753 | ebi-met1400-GCST90199986 | Eugenol sulfate levels \|\| id:ebi-met1400-GCST90199986 | F-deep fried food liking (derived food-liking factor) \|\| id:ebi-fl187-GCST90094753 | Weighted median | 8 | -0.03373638 | 0.095642375521111 | 0.724287378998897 | -0.221195436 | 0.153722676036569 | 0.966826345821385 | 0.801560011293987 | 1.16616743575484 | 0.999489402457283 |
| ebi-fl187-GCST90094753 | ebi-met1400-GCST90200002 | 6-hydroxyindole sulfate levels \|\| id:ebi-met1400-GCST90200002 | F-deep fried food liking (derived food-liking factor) \|\| id:ebi-fl187-GCST90094753 | Weighted median | 8 | 0.0202622548974482 | 0.0777980508463165 | 0.794519132 | -0.132221925 | 0.172746434556229 | 1.02046892791114 | 0.876146534891758 | 1.18856468793861 | 0.999489402457283 |
| ebi-fl187-GCST90094753 | ebi-met1400-GCST90200023 | Trans 3,4-methyleneheptanoate levels \|\| id:ebi-met1400-GCST90200023 | F-deep fried food liking (derived food-liking factor) \|\| id:ebi-fl187-GCST90094753 | Weighted median | 8 | -0.038912321 | 0.088551406036488 | 0.660348545374013 | -0.212473077 | 0.134648434549806 | 0.961835037912723 | 0.80858208539234 | 1.14413447548455 | 0.999489402457283 |
| ebi-fl187-GCST90094753 | ebi-met1400-GCST90200032 | 2-hydroxybutyrate/2-hydroxyisobutyrate levels \|\| id:ebi-met1400-GCST90200032 | F-deep fried food liking (derived food-liking factor) \|\| id:ebi-fl187-GCST90094753 | Weighted median | 8 | 0.0232256460138868 | 0.0889181836703768 | 0.793936401091466 | -0.151053994 | 0.197505286007825 | 1.02349746161535 | 0.859801273313073 | 1.2183595052105 | 0.999489402457283 |
| ebi-fl187-GCST90094753 | ebi-met1400-GCST90200051 | 1-palmitoyl-2-dihomo-linolenoyl-GPC (16:0/20:3n3 or 6) levels \|\| id:ebi-met1400-GCST90200051 | F-deep fried food liking (derived food-liking factor) \|\| id:ebi-fl187-GCST90094753 | Weighted median | 8 | 0.0139221897651958 | 0.0791079835298644 | 0.860302039564137 | -0.141129458 | 0.16897383748373 | 1.0140195547691 | 0.868376886126228 | 1.18408915976682 | 0.999489402457283 |
| ebi-fl187-GCST90094753 | ebi-met1400-GCST90200069 | Phenylacetylglutamate levels \|\| id:ebi-met1400-GCST90200069 | F-deep fried food liking (derived food-liking factor) \|\| id:ebi-fl187-GCST90094753 | Weighted median | 8 | -0.106276301 | 0.0837404215180152 | 0.204399725908065 | -0.270407528 | 0.0578549247504659 | 0.899176170093112 | 0.763068459505464 | 1.05956126844413 | 0.999489402457283 |
| ebi-fl187-GCST90094753 | ebi-met1400-GCST90200072 | Thioproline levels \|\| id:ebi-met1400-GCST90200072 | F-deep fried food liking (derived food-liking factor) \|\| id:ebi-fl187-GCST90094753 | Weighted median | 8 | 0.0319940732537851 | 0.0879101002429073 | 0.715902207952393 | -0.140309723 | 0.204297869729883 | 1.03251138585404 | 0.869089016658594 | 1.22666348496383 | 0.999489402457283 |
| ebi-fl187-GCST90094753 | ebi-met1400-GCST90200081 | 1-oleoyl-2-docosahexaenoyl-GPC (18:1/22:6) levels \|\| id:ebi-met1400-GCST90200081 | F-deep fried food liking (derived food-liking factor) \|\| id:ebi-fl187-GCST90094753 | Weighted median | 7 | 0.0164403523542436 | 0.0980905383510173 | 0.866894938353546 | -0.175817103 | 0.208697807522238 | 1.01657623859825 | 0.838771378233885 | 1.23207261919039 | 0.999489402457283 |
| ebi-fl187-GCST90094753 | ebi-met1400-GCST90200097 | Furaneol sulfate levels \|\| id:ebi-met1400-GCST90200097 | F-deep fried food liking (derived food-liking factor) \|\| id:ebi-fl187-GCST90094753 | Weighted median | 8 | 0.0505644112502141 | 0.0982266519393306 | 0.606711776087777 | -0.141959827 | 0.243088649051302 | 1.05186461308784 | 0.867656112524461 | 1.27518166275264 | 0.999489402457283 |
| ebi-fl187-GCST90094753 | ebi-met1400-GCST90200124 | Sphingomyelin (d18:2/24:2) levels \|\| id:ebi-met1400-GCST90200124 | F-deep fried food liking (derived food-liking factor) \|\| id:ebi-fl187-GCST90094753 | Weighted median | 8 | 0.0726985083557835 | 0.0860156492833581 | 0.39801163287751 | -0.095892164 | 0.241289180951165 | 1.07540626203194 | 0.908561986258927 | 1.27288907736441 | 0.999489402457283 |
| ebi-fl187-GCST90094753 | ebi-met1400-GCST90200137 | Lignoceroylcarnitine (C24) levels \|\| id:ebi-met1400-GCST90200137 | F-deep fried food liking (derived food-liking factor) \|\| id:ebi-fl187-GCST90094753 | Weighted median | 8 | -0.05636058 | 0.0831556732860359 | 0.497916319950616 | -0.2193457 | 0.106624539597836 | 0.945198254809464 | 0.803044058086237 | 1.1125164701722 | 0.999489402457283 |
| ebi-fl187-GCST90094753 | ebi-met1400-GCST90200165 | Octadecenedioate (C18:1-DC) levels \|\| id:ebi-met1400-GCST90200165 | F-deep fried food liking (derived food-liking factor) \|\| id:ebi-fl187-GCST90094753 | Weighted median | 8 | -0.028188389 | 0.0856717773338041 | 0.742134738561734 | -0.196105073 | 0.139728294304984 | 0.972205196521576 | 0.821925868052747 | 1.1499613053703 | 0.999489402457283 |
| ebi-fl187-GCST90094753 | ebi-met1400-GCST90200176 | Glutamine conjugate of C6H10O2 (1) levels \|\| id:ebi-met1400-GCST90200176 | F-deep fried food liking (derived food-liking factor) \|\| id:ebi-fl187-GCST90094753 | Weighted median | 8 | 0.0473003181252474 | 0.0795346213452886 | 0.552034953464812 | -0.10858754 | 0.203188175962013 | 1.04843682638676 | 0.897100359472869 | 1.22530301912913 | 0.999489402457283 |
| ebi-fl187-GCST90094753 | ebi-met1400-GCST90200182 | Glycine conjugate of C10H12O2 levels \|\| id:ebi-met1400-GCST90200182 | F-deep fried food liking (derived food-liking factor) \|\| id:ebi-fl187-GCST90094753 | Weighted median | 8 | 0.0909419137787165 | 0.0963694769904936 | 0.345333379045393 | -0.097942261 | 0.279826088680084 | 1.09520538706835 | 0.906701254155086 | 1.32289972509332 | 0.999489402457283 |
| ebi-fl187-GCST90094753 | ebi-met1400-GCST90200208 | Glucuronide of piperine metabolite C17H21NO3 (4) levels \|\| id:ebi-met1400-GCST90200208 | F-deep fried food liking (derived food-liking factor) \|\| id:ebi-fl187-GCST90094753 | Weighted median | 8 | -0.101446286 | 0.091798056387992 | 0.269115061533753 | -0.281370477 | 0.0784779043494262 | 0.903529710079132 | 0.754748666889035 | 1.08163945537078 | 0.999489402457283 |
| ebi-fl187-GCST90094753 | ebi-met1400-GCST90200227 | 3-ethylcatechol sulfate (2) levels \|\| id:ebi-met1400-GCST90200227 | F-deep fried food liking (derived food-liking factor) \|\| id:ebi-fl187-GCST90094753 | Weighted median | 8 | -0.125978134 | 0.105620584334617 | 0.232970330747242 | -0.332994479 | 0.0810382111713352 | 0.881634124274642 | 0.716774151153561 | 1.08441233244053 | 0.999489402457283 |
| ebi-fl187-GCST90094753 | ebi-met1400-GCST90200259 | Pentose acid levels \|\| id:ebi-met1400-GCST90200259 | F-deep fried food liking (derived food-liking factor) \|\| id:ebi-fl187-GCST90094753 | Weighted median | 8 | -0.137409067 | 0.0822954690592763 | 0.0949779591280037 | -0.298708186 | 0.0238900526231676 | 0.8716136050663 | 0.741775838363322 | 1.02417770604786 | 0.999489402457283 |
| ebi-fl187-GCST90094753 | ebi-met1400-GCST90200291 | Sarcosine levels \|\| id:ebi-met1400-GCST90200291 | F-deep fried food liking (derived food-liking factor) \|\| id:ebi-fl187-GCST90094753 | Weighted median | 8 | 0.0604243434586343 | 0.0813813960808842 | 0.45779444768923 | -0.099083193 | 0.219931879777167 | 1.06228722555261 | 0.905667359831595 | 1.24599185045389 | 0.999489402457283 |
| ebi-fl187-GCST90094753 | ebi-met1400-GCST90200306 | Butyrate/isobutyrate (4:0) levels \|\| id:ebi-met1400-GCST90200306 | F-deep fried food liking (derived food-liking factor) \|\| id:ebi-fl187-GCST90094753 | Weighted median | 8 | -0.084959686 | 0.0783335353244814 | 0.278103756432645 | -0.238493415 | 0.068574043322865 | 0.918549314131897 | 0.787813875876969 | 1.07097992092734 | 0.999489402457283 |
| ebi-fl187-GCST90094753 | ebi-met1400-GCST90200321 | Cholate levels \|\| id:ebi-met1400-GCST90200321 | F-deep fried food liking (derived food-liking factor) \|\| id:ebi-fl187-GCST90094753 | Weighted median | 8 | 0.0405908239221799 | 0.0857265864650068 | 0.635862448098163 | -0.127433286 | 0.208614933393593 | 1.04142589179145 | 0.880352146085028 | 1.23197051647655 | 0.999489402457283 |
| ebi-fl187-GCST90094753 | ebi-met1400-GCST90200336 | Ceramide (d18:1/16:0) levels \|\| id:ebi-met1400-GCST90200336 | F-deep fried food liking (derived food-liking factor) \|\| id:ebi-fl187-GCST90094753 | Weighted median | 8 | -0.052135878 | 0.087249031346839 | 0.550138555010046 | -0.22314398 | 0.118872223127635 | 0.949199882402062 | 0.799999657249862 | 1.12622600345776 | 0.999489402457283 |
| ebi-fl187-GCST90094753 | ebi-met1400-GCST90200358 | Arachidonate (20:4n6) levels \|\| id:ebi-met1400-GCST90200358 | F-deep fried food liking (derived food-liking factor) \|\| id:ebi-fl187-GCST90094753 | Weighted median | 8 | -0.082573919 | 0.0819841778890996 | 0.313841866846062 | -0.243262908 | 0.078115069667646 | 0.920743374916347 | 0.784065349887149 | 1.08124707025296 | 0.999489402457283 |
| ebi-fl187-GCST90094753 | ebi-met1400-GCST90200413 | 2'-deoxyuridine levels \|\| id:ebi-met1400-GCST90200413 | F-deep fried food liking (derived food-liking factor) \|\| id:ebi-fl187-GCST90094753 | Weighted median | 8 | 0.000440248605439939 | 0.0799596188669319 | 0.995606960170469 | -0.156280604 | 0.157161101584626 | 1.00044034552908 | 0.855319150399909 | 1.170184117 | 0.999911036302558 |
| ebi-fl187-GCST90094753 | ebi-met1400-GCST90200432 | Threonine levels \|\| id:ebi-met1400-GCST90200432 | F-deep fried food liking (derived food-liking factor) \|\| id:ebi-fl187-GCST90094753 | Weighted median | 8 | 0.0279800466264617 | 0.0877039733218169 | 0.749705077845246 | -0.143919741 | 0.199879834337223 | 1.02837516466291 | 0.865957246059863 | 1.22125599630626 | 0.999489402457283 |
| ebi-fl187-GCST90094753 | ebi-met1400-GCST90200436 | Caffeine levels \|\| id:ebi-met1400-GCST90200436 | F-deep fried food liking (derived food-liking factor) \|\| id:ebi-fl187-GCST90094753 | Weighted median | 8 | 0.0893378264473859 | 0.0848862302307772 | 0.292596848026402 | -0.077039185 | 0.255714837699709 | 1.09344999026304 | 0.925853573504007 | 1.29138442127648 | 0.999489402457283 |
| ebi-fl187-GCST90094753 | ebi-met1400-GCST90200442 | Valine levels \|\| id:ebi-met1400-GCST90200442 | F-deep fried food liking (derived food-liking factor) \|\| id:ebi-fl187-GCST90094753 | Weighted median | 8 | 0.161335846558046 | 0.0805711889566289 | 0.0452415980546671 | 0.00341631620305355 | 0.319255376913039 | 1.17507954897292 | 1.00342215846236 | 1.37610270489776 | 0.999489402457283 |
| ebi-fl187-GCST90094753 | ebi-met1400-GCST90200459 | Margarate (17:0) levels \|\| id:ebi-met1400-GCST90200459 | F-deep fried food liking (derived food-liking factor) \|\| id:ebi-fl187-GCST90094753 | Weighted median | 8 | 0.0229892562766241 | 0.0806009047789181 | 0.775473068405865 | -0.134988517 | 0.180967029643304 | 1.02325554591363 | 0.873725944546769 | 1.19837566776863 | 0.999489402457283 |
| ebi-fl187-GCST90094753 | ebi-met1400-GCST90200462 | X-11299 levels \|\| id:ebi-met1400-GCST90200462 | F-deep fried food liking (derived food-liking factor) \|\| id:ebi-fl187-GCST90094753 | Weighted median | 8 | -0.021512321 | 0.0801157858158459 | 0.788302630768931 | -0.178539262 | 0.135514618785407 | 0.978717418212697 | 0.836491214238582 | 1.1451259360624 | 0.999489402457283 |
| ebi-fl187-GCST90094753 | ebi-met1400-GCST90200497 | X-12740 levels \|\| id:ebi-met1400-GCST90200497 | F-deep fried food liking (derived food-liking factor) \|\| id:ebi-fl187-GCST90094753 | Weighted median | 8 | 0.0998930177454919 | 0.0976881527314656 | 0.306510989831065 | -0.091575762 | 0.291361797099165 | 1.10505269072344 | 0.912492181658039 | 1.33824867086121 | 0.999489402457283 |
| ebi-fl187-GCST90094753 | ebi-met1400-GCST90200527 | X-13866 levels \|\| id:ebi-met1400-GCST90200527 | F-deep fried food liking (derived food-liking factor) \|\| id:ebi-fl187-GCST90094753 | Weighted median | 8 | -0.02135104 | 0.0730468290096051 | 0.77006300014363 | -0.164522824 | 0.12182074530205 | 0.978875280305066 | 0.848298394808258 | 1.12955160619973 | 0.999489402457283 |
| ebi-fl187-GCST90094753 | ebi-met1400-GCST90200547 | X-17654 levels \|\| id:ebi-met1400-GCST90200547 | F-deep fried food liking (derived food-liking factor) \|\| id:ebi-fl187-GCST90094753 | Weighted median | 8 | -0.006348225 | 0.08363230218661 | 0.939493585663433 | -0.170267537 | 0.157571087465875 | 0.99367188258802 | 0.843439135143652 | 1.17066397455917 | 0.999489402457283 |
| ebi-fl187-GCST90094753 | ebi-met1400-GCST90200553 | X-17653 levels \|\| id:ebi-met1400-GCST90200553 | F-deep fried food liking (derived food-liking factor) \|\| id:ebi-fl187-GCST90094753 | Weighted median | 8 | 0.0290409547861793 | 0.085012788168825 | 0.73264674595793 | -0.13758411 | 0.195666019597076 | 1.02946675520262 | 0.871461048304184 | 1.21612067702823 | 0.999489402457283 |
| ebi-fl187-GCST90094753 | ebi-met1400-GCST90200557 | X-17685 levels \|\| id:ebi-met1400-GCST90200557 | F-deep fried food liking (derived food-liking factor) \|\| id:ebi-fl187-GCST90094753 | Weighted median | 8 | 0.0282481931313823 | 0.0943576852498675 | 0.764655058039273 | -0.15669287 | 0.213189256221123 | 1.02865095684376 | 0.854966604426619 | 1.23761885614844 | 0.999489402457283 |
| ebi-fl187-GCST90094753 | ebi-met1400-GCST90200561 | X-18779 levels \|\| id:ebi-met1400-GCST90200561 | F-deep fried food liking (derived food-liking factor) \|\| id:ebi-fl187-GCST90094753 | Weighted median | 8 | 0.0502560413905786 | 0.0809386871565588 | 0.534655580035254 | -0.108383785 | 0.208895868217434 | 1.05154029975155 | 0.897283166129619 | 1.23231666851738 | 0.999489402457283 |
| ebi-fl187-GCST90094753 | ebi-met1400-GCST90200570 | X-18921 levels \|\| id:ebi-met1400-GCST90200570 | F-deep fried food liking (derived food-liking factor) \|\| id:ebi-fl187-GCST90094753 | Weighted median | 8 | -0.087822447 | 0.0877949072346461 | 0.317158725577829 | -0.259900466 | 0.084255570832621 | 0.91592348693175 | 0.771128335836241 | 1.08790689555386 | 0.999489402457283 |
| ebi-fl187-GCST90094753 | ebi-met1400-GCST90200575 | X-21283 levels \|\| id:ebi-met1400-GCST90200575 | F-deep fried food liking (derived food-liking factor) \|\| id:ebi-fl187-GCST90094753 | Weighted median | 8 | -0.057036141 | 0.0938747421867023 | 0.543468012888466 | -0.241030635 | 0.12695835391514 | 0.944559931625653 | 0.785817552139966 | 1.13536973309263 | 0.999489402457283 |
| ebi-fl187-GCST90094753 | ebi-met1400-GCST90200578 | X-18922 levels \|\| id:ebi-met1400-GCST90200578 | F-deep fried food liking (derived food-liking factor) \|\| id:ebi-fl187-GCST90094753 | Weighted median | 8 | -0.047070324 | 0.0785190704045797 | 0.548855289781074 | -0.200967702 | 0.106827053832697 | 0.954020304554461 | 0.817938848790832 | 1.11274179340873 | 0.999489402457283 |
| ebi-fl187-GCST90094753 | ebi-met1400-GCST90200623 | X-24241 levels \|\| id:ebi-met1400-GCST90200623 | F-deep fried food liking (derived food-liking factor) \|\| id:ebi-fl187-GCST90094753 | Weighted median | 8 | -0.010041186 | 0.0826230994437408 | 0.903271243184791 | -0.171982461 | 0.151900088481174 | 0.990009057972131 | 0.841993940485884 | 1.16404392922504 | 0.999489402457283 |
| ebi-fl187-GCST90094753 | ebi-met1400-GCST90200636 | X-24544 levels \|\| id:ebi-met1400-GCST90200636 | F-deep fried food liking (derived food-liking factor) \|\| id:ebi-fl187-GCST90094753 | Weighted median | 8 | 0.103805712618709 | 0.0813513056323163 | 0.20194923309016 | -0.055642846 | 0.263254271658049 | 1.10938489448993 | 0.945876898890255 | 1.30115752437383 | 0.999489402457283 |
| ebi-fl187-GCST90094753 | ebi-met1400-GCST90200659 | X-25172 levels \|\| id:ebi-met1400-GCST90200659 | F-deep fried food liking (derived food-liking factor) \|\| id:ebi-fl187-GCST90094753 | Weighted median | 8 | 0.0602025777806969 | 0.0843492433496572 | 0.475394208208292 | -0.105121939 | 0.225527094746025 | 1.06205167282559 | 0.900214744441343 | 1.25298298291217 | 0.999489402457283 |
| ebi-fl187-GCST90094753 | ebi-met1400-GCST90200670 | X-26111 levels \|\| id:ebi-met1400-GCST90200670 | F-deep fried food liking (derived food-liking factor) \|\| id:ebi-fl187-GCST90094753 | Weighted median | 8 | 0.0466924314076091 | 0.0869507159546965 | 0.591268432780309 | -0.123730972 | 0.217115834678814 | 1.04779968923894 | 0.883617526885758 | 1.24248801700282 | 0.999489402457283 |
| ebi-fl187-GCST90094753 | ebi-met1400-GCST90200673 | Carnitine C4 levels \|\| id:ebi-met1400-GCST90200673 | F-deep fried food liking (derived food-liking factor) \|\| id:ebi-fl187-GCST90094753 | Weighted median | 8 | 0.0107625243859053 | 0.0877048973419727 | 0.902334429492288 | -0.161139074 | 0.182664123176172 | 1.01082064868573 | 0.851173686401756 | 1.20041115007774 | 0.999489402457283 |
| ebi-fl187-GCST90094753 | ebi-met1400-GCST90200710 | X-13431 levels \|\| id:ebi-met1400-GCST90200710 | F-deep fried food liking (derived food-liking factor) \|\| id:ebi-fl187-GCST90094753 | Weighted median | 8 | -0.075036334 | 0.0803956224396191 | 0.350645195305748 | -0.232611754 | 0.0825390860281916 | 0.927709778352272 | 0.792461183742384 | 1.08604112164338 | 0.999489402457283 |
| ebi-fl187-GCST90094753 | ebi-met1400-GCST90200730 | Adenosine 5'-diphosphate (ADP) to fructose ratio \|\| id:ebi-met1400-GCST90200730 | F-deep fried food liking (derived food-liking factor) \|\| id:ebi-fl187-GCST90094753 | Weighted median | 8 | -0.129630152 | 0.114099369026947 | 0.255907899093856 | -0.353264915 | 0.0940046116919338 | 0.878420253173752 | 0.702391094865964 | 1.09856481214797 | 0.999489402457283 |
| ebi-fl187-GCST90094753 | ebi-met1400-GCST90200731 | Adenosine 5'-diphosphate (ADP) to mannose ratio \|\| id:ebi-met1400-GCST90200731 | F-deep fried food liking (derived food-liking factor) \|\| id:ebi-fl187-GCST90094753 | Weighted median | 8 | -0.123194219 | 0.115209017019122 | 0.284929753480322 | -0.349003892 | 0.102615454763664 | 0.88409193879223 | 0.70539038491959 | 1.10806522593373 | 0.999489402457283 |
| ebi-fl187-GCST90094753 | ebi-met1400-GCST90200748 | Aspartate to glutamate ratio \|\| id:ebi-met1400-GCST90200748 | F-deep fried food liking (derived food-liking factor) \|\| id:ebi-fl187-GCST90094753 | Weighted median | 8 | -0.12432117 | 0.0864130369678761 | 0.150239694538805 | -0.293690722 | 0.0450483829176815 | 0.883096171741561 | 0.745507024716295 | 1.0460784710129 | 0.999489402457283 |
| ebi-fl187-GCST90094753 | ebi-met1400-GCST90200774 | Uridine to pseudouridine ratio \|\| id:ebi-met1400-GCST90200774 | F-deep fried food liking (derived food-liking factor) \|\| id:ebi-fl187-GCST90094753 | Weighted median | 8 | 0.0575000344287768 | 0.0797241462047588 | 0.470763476290564 | -0.098759292 | 0.213759360990104 | 1.05918530712119 | 0.905960753660852 | 1.23832462972384 | 0.999489402457283 |
| ebi-fl187-GCST90094753 | ebi-met1400-GCST90200799 | Spermidine to adenosine 5'-diphosphate (ADP) ratio \|\| id:ebi-met1400-GCST90200799 | F-deep fried food liking (derived food-liking factor) \|\| id:ebi-fl187-GCST90094753 | Weighted median | 8 | 0.221601650961157 | 0.112791473542287 | 0.0494487205691014 | 0.000530362818274149 | 0.44267293910404 | 1.24807410970571 | 1.0005305034855 | 1.55686306203683 | 0.999489402457283 |
| ebi-fl187-GCST90094753 | ebi-met1400-GCST90200811 | Choline to taurocholate ratio \|\| id:ebi-met1400-GCST90200811 | F-deep fried food liking (derived food-liking factor) \|\| id:ebi-fl187-GCST90094753 | Weighted median | 8 | 0.00377705097231989 | 0.0977233042112706 | 0.969169068516912 | -0.187760625 | 0.19531472722641 | 1.00378419301847 | 0.828813080386557 | 1.21569353814229 | 0.999489402457283 |
| ebi-fl187-GCST90094753 | ebi-met1400-GCST90200812 | Glutamate to kynurenine ratio \|\| id:ebi-met1400-GCST90200812 | F-deep fried food liking (derived food-liking factor) \|\| id:ebi-fl187-GCST90094753 | Weighted median | 8 | 0.0417772727484463 | 0.0837367748651111 | 0.617841456202721 | -0.122346806 | 0.205901351484064 | 1.04266222359559 | 0.884841446973084 | 1.22863199529391 | 0.999489402457283 |
| ebi-fl187-GCST90094753 | ebi-met1400-GCST90200876 | Cortisone to 4-cholesten-3-one ratio \|\| id:ebi-met1400-GCST90200876 | F-deep fried food liking (derived food-liking factor) \|\| id:ebi-fl187-GCST90094753 | Weighted median | 8 | 0.0411732515104576 | 0.0953212228291726 | 0.665783477541718 | -0.145656345 | 0.228002848255636 | 1.0420326236335 | 0.864454726131047 | 1.25608890308955 | 0.999489402457283 |
| ebi-fl187-GCST90094753 | ebi-met1400-GCST90200906 | 2'-deoxyuridine to cytidine ratio \|\| id:ebi-met1400-GCST90200906 | F-deep fried food liking (derived food-liking factor) \|\| id:ebi-fl187-GCST90094753 | Weighted median | 8 | 0.0554496831993945 | 0.0851235656809686 | 0.514786851109474 | -0.111392506 | 0.222291871934093 | 1.05701583007958 | 0.894587549444698 | 1.24893585399478 | 0.999489402457283 |
| ebi-fl187-GCST90094753 | ebi-met1400-GCST90200907 | Retinol (Vitamin A) to linoleoyl-arachidonoyl-glycerol (18:2 to 20:4) [1] ratio \|\| id:ebi-met1400-GCST90200907 | F-deep fried food liking (derived food-liking factor) \|\| id:ebi-fl187-GCST90094753 | Weighted median | 7 | -0.115697615 | 0.102498611964533 | 0.258993815111751 | -0.316594894 | 0.0851996646963818 | 0.890744530582057 | 0.728625865907087 | 1.0889344667638 | 0.999489402457283 |
| ebi-fl187-GCST90094753 | ebi-met1400-GCST90200916 | Fructose to sucrose ratio \|\| id:ebi-met1400-GCST90200916 | F-deep fried food liking (derived food-liking factor) \|\| id:ebi-fl187-GCST90094753 | Weighted median | 8 | -0.013425535 | 0.0878317333776772 | 0.878512489391535 | -0.185575732 | 0.158724662869452 | 0.986664185974046 | 0.830625928260159 | 1.17201520294815 | 0.999489402457283 |
| ebi-fl187-GCST90094753 | ebi-met1400-GCST90200917 | Alpha-ketobutyrate to 3-methyl-2-oxobutyrate ratio \|\| id:ebi-met1400-GCST90200917 | F-deep fried food liking (derived food-liking factor) \|\| id:ebi-fl187-GCST90094753 | Weighted median | 8 | 0.0221721523145969 | 0.0774323518277605 | 0.774616016498986 | -0.129595257 | 0.173939561897007 | 1.02241978125271 | 0.878450905597436 | 1.18998364329296 | 0.999489402457283 |
| ebi-fl187-GCST90094753 | ebi-met1400-GCST90200924 | Alpha-tocopherol to glycerol ratio \|\| id:ebi-met1400-GCST90200924 | F-deep fried food liking (derived food-liking factor) \|\| id:ebi-fl187-GCST90094753 | Weighted median | 8 | 0.0281943160985297 | 0.0824569724007636 | 0.732405365361532 | -0.13342135 | 0.189809982004026 | 1.02859553767529 | 0.875096292763428 | 1.20901984030178 | 0.999489402457283 |
| ebi-fl187-GCST90094753 | ebi-met1400-GCST90200938 | Alpha-ketoglutarate to alanine ratio \|\| id:ebi-met1400-GCST90200938 | F-deep fried food liking (derived food-liking factor) \|\| id:ebi-fl187-GCST90094753 | Weighted median | 8 | -0.017475363 | 0.0793365259060288 | 0.825661796687556 | -0.172974954 | 0.138024227304666 | 0.982676445103444 | 0.841158682033413 | 1.1480033629645 | 0.999489402457283 |
| ebi-fl187-GCST90094753 | ebi-met1400-GCST90200943 | Phosphate to EDTA ratio \|\| id:ebi-met1400-GCST90200943 | F-deep fried food liking (derived food-liking factor) \|\| id:ebi-fl187-GCST90094753 | Weighted median | 8 | 0.0167527669959118 | 0.0922460775134187 | 0.855889167545807 | -0.164049545 | 0.197555078922212 | 1.01689388151512 | 0.848699972057501 | 1.21842017239142 | 0.999489402457283 |
| ebi-fl187-GCST90094753 | ebi-met1400-GCST90200947 | Adenosine 5'-diphosphate (ADP) to glucose ratio \|\| id:ebi-met1400-GCST90200947 | F-deep fried food liking (derived food-liking factor) \|\| id:ebi-fl187-GCST90094753 | Weighted median | 8 | -0.173245784 | 0.110116696703738 | 0.115651082996278 | -0.389074509 | 0.0425829419388047 | 0.840930902417415 | 0.677683774497678 | 1.04350260291349 | 0.999489402457283 |
| ebi-fl187-GCST90094753 | ebi-met1400-GCST90200975 | Arachidonate (20:4n6) to caffeine ratio \|\| id:ebi-met1400-GCST90200975 | F-deep fried food liking (derived food-liking factor) \|\| id:ebi-fl187-GCST90094753 | Weighted median | 8 | -0.06704985 | 0.0846559342493711 | 0.428344488149892 | -0.232975481 | 0.0988757808876044 | 0.935148582792438 | 0.79217299628285 | 1.10392916194086 | 0.999489402457283 |
| ebi-fl187-GCST90094753 | ebi-met1400-GCST90200985 | Cholesterol to oleoyl-linoleoyl-glycerol (18:1 to 18:2) [2] ratio \|\| id:ebi-met1400-GCST90200985 | F-deep fried food liking (derived food-liking factor) \|\| id:ebi-fl187-GCST90094753 | Weighted median | 8 | 0.137041386665262 | 0.108807681317356 | 0.207856053585214 | -0.076221669 | 0.35030444204728 | 1.14687561289528 | 0.926610783169016 | 1.41949963819276 | 0.999489402457283 |
| ebi-fl187-GCST90094753 | ebi-met1400-GCST90201016 | Bilirubin (Z,Z) to etiocholanolone glucuronide ratio \|\| id:ebi-met1400-GCST90201016 | F-deep fried food liking (derived food-liking factor) \|\| id:ebi-fl187-GCST90094753 | Weighted median | 8 | -0.005816114 | 0.083647124235375 | 0.944566521106991 | -0.169764478 | 0.158132249 | 0.994200766748442 | 0.843863541958594 | 1.17132109097763 | 0.999489402457283 |
| ebi-fl187-GCST90094753 | ebi-pw11-GCST90275189 | Placental weight (fetal genotype, adjusted for sex and gestational age) \|\| id:ebi-pw11-GCST90275189 | F-deep fried food liking (derived food-liking factor) \|\| id:ebi-fl187-GCST90094753 | Weighted median | 8 | 0.034500103544291 | 0.0302052382697735 | 0.253375293249649 | -0.024702163 | 0.0937023705530471 | 1.03510213555503 | 0.975600438216113 | 1.09823283083974 | 0.999489402457283 |
| ebi-fl187-GCST90094753 | ebi-pw11-GCST90275192 | Placental weight (fetal effect, conditional on maternal and paternal genotype) \|\| id:ebi-pw11-GCST90275192 | F-deep fried food liking (derived food-liking factor) \|\| id:ebi-fl187-GCST90094753 | Weighted median | 8 | 0.0472867372158007 | 0.0645661705532525 | 0.463938881311931 | -0.079262957 | 0.173836431500176 | 1.04842258775785 | 0.923796973559536 | 1.18986092613565 | 0.999489402457283 |
| ebi-fl187-GCST90094753 | ebi-pw11-GCST90275193 | Placental weight (maternal effect, conditional on fetal and paternal genotype) \|\| id:ebi-pw11-GCST90275193 | F-deep fried food liking (derived food-liking factor) \|\| id:ebi-fl187-GCST90094753 | Weighted median | 8 | -0.048054804 | 0.0537025913316335 | 0.370876829396233 | -0.153311883 | 0.0572022745633249 | 0.953081552539051 | 0.857862127078831 | 1.05886997119617 | 0.999489402457283 |
| ebi-fl187-GCST90094753 | ebi-pw11-GCST90275195 | Placental weight (fetal effect, conditional on maternal genotype) \|\| id:ebi-pw11-GCST90275195 | F-deep fried food liking (derived food-liking factor) \|\| id:ebi-fl187-GCST90094753 | Weighted median | 8 | -0.008308509 | 0.0432276091814387 | 0.84758259668078 | -0.093034623 | 0.0764176047499798 | 0.991725911024293 | 0.911161952369894 | 1.07941324814855 | 0.999489402457283 |
| ebi-fl187-GCST90094753 | ebi-pw11-GCST90275197 | Placental weight (fetal genotype, adjusted for sex) \|\| id:ebi-pw11-GCST90275197 | F-deep fried food liking (derived food-liking factor) \|\| id:ebi-fl187-GCST90094753 | Weighted median | 8 | 0.0397244911146827 | 0.0329508915880705 | 0.227984681761705 | -0.024859256 | 0.104308238627301 | 1.04052406107398 | 0.975447190318775 | 1.10994252935423 | 0.999489402457283 |
| ebi-fl187-GCST90094753 | prot-a-1030 | Protein FAM171B \|\| id:prot-a-1030 | F-deep fried food liking (derived food-liking factor) \|\| id:ebi-fl187-GCST90094753 | Weighted median | 8 | 0.249154546518064 | 0.135452042605202 | 0.0658519695484942 | -0.016331457 | 0.51464055002426 | 1.28294029170429 | 0.98380117823182 | 1.67303702058634 | 0.999489402457283 |
| ebi-fl187-GCST90094753 | prot-a-1036 | BRISC complex subunit Abro1 \|\| id:prot-a-1036 | F-deep fried food liking (derived food-liking factor) \|\| id:ebi-fl187-GCST90094753 | Weighted median | 8 | 0.163855936874791 | 0.13786309791356 | 0.234620357842131 | -0.106355735 | 0.434067608785368 | 1.17804459008218 | 0.899104748119738 | 1.54352322 | 0.999489402457283 |
| ebi-fl187-GCST90094753 | prot-a-1055 | Phenylalanine--tRNA ligase, mitochondrial \|\| id:prot-a-1055 | F-deep fried food liking (derived food-liking factor) \|\| id:ebi-fl187-GCST90094753 | Weighted median | 8 | -0.28409062 | 0.148812323881186 | 0.0562552494898788 | -0.575762774 | 0.00758153518062593 | 0.752698432364747 | 0.562275815575575 | 1.00761034778704 | 0.999489402457283 |
| ebi-fl187-GCST90094753 | prot-a-1083 | Fc receptor-like protein 5 \|\| id:prot-a-1083 | F-deep fried food liking (derived food-liking factor) \|\| id:ebi-fl187-GCST90094753 | Weighted median | 8 | -0.185405176 | 0.135780916979485 | 0.172104433640795 | -0.451535773 | 0.0807254212514388 | 0.83076760853024 | 0.636649650895966 | 1.08407319223652 | 0.999489402457283 |
| ebi-fl187-GCST90094753 | prot-a-1103 | Fibroblast growth factor receptor 3 \|\| id:prot-a-1103 | F-deep fried food liking (derived food-liking factor) \|\| id:ebi-fl187-GCST90094753 | Weighted median | 8 | 0.0351185072574462 | 0.146507548400939 | 0.810559406355296 | -0.252036288 | 0.322273302123286 | 1.03574244452346 | 0.777216534227993 | 1.38026195293569 | 0.999489402457283 |
| ebi-fl187-GCST90094753 | prot-a-1105 | Fibroblast growth factor receptor 4 \|\| id:prot-a-1105 | F-deep fried food liking (derived food-liking factor) \|\| id:ebi-fl187-GCST90094753 | Weighted median | 8 | -0.013275993 | 0.126492894552836 | 0.916411947412977 | -0.261202066 | 0.234650080311124 | 0.986811744287035 | 0.770125287496502 | 1.26446622968116 | 0.999489402457283 |
| ebi-fl187-GCST90094753 | prot-a-1120 | Filamin-A \|\| id:prot-a-1120 | F-deep fried food liking (derived food-liking factor) \|\| id:ebi-fl187-GCST90094753 | Weighted median | 8 | 0.072527453119084 | 0.140610255642254 | 0.605990708373827 | -0.203068648 | 0.348123554177901 | 1.07522232389147 | 0.816222207529415 | 1.41640724196164 | 0.999489402457283 |
| ebi-fl187-GCST90094753 | prot-a-1166 | Galanin peptides \|\| id:prot-a-1166 | F-deep fried food liking (derived food-liking factor) \|\| id:ebi-fl187-GCST90094753 | Weighted median | 8 | 0.0308187553043745 | 0.130200019125802 | 0.812887087198449 | -0.224373282 | 0.286010792790946 | 1.03129856955127 | 0.799016819952936 | 1.33110682153242 | 0.999489402457283 |
| ebi-fl187-GCST90094753 | prot-a-1205 | ADP-ribosylation factor-binding protein GGA1 \|\| id:prot-a-1205 | F-deep fried food liking (derived food-liking factor) \|\| id:ebi-fl187-GCST90094753 | Weighted median | 8 | 0.10900079070572 | 0.129900040712137 | 0.401405997402167 | -0.145603289 | 0.363604870501509 | 1.11516323210705 | 0.864500591982763 | 1.43850570581012 | 0.999489402457283 |
| ebi-fl187-GCST90094753 | prot-a-1227 | Guanine nucleotide-binding protein G(I)/G(S)/G(O) subunit gamma-T2 \|\| id:prot-a-1227 | F-deep fried food liking (derived food-liking factor) \|\| id:ebi-fl187-GCST90094753 | Weighted median | 8 | 0.00526486802709161 | 0.139726264798431 | 0.969942922963947 | -0.268598611 | 0.279128347032016 | 1.00527875179948 | 0.764450035927378 | 1.32197700480653 | 0.999489402457283 |
| ebi-fl187-GCST90094753 | prot-a-1240 | Golgi SNAP receptor complex member 1 \|\| id:prot-a-1240 | F-deep fried food liking (derived food-liking factor) \|\| id:ebi-fl187-GCST90094753 | Weighted median | 8 | -0.121408681 | 0.131322720104787 | 0.355222777632619 | -0.378801212 | 0.135983850524691 | 0.885671928436592 | 0.684681705450038 | 1.14566339158281 | 0.999489402457283 |
| ebi-fl187-GCST90094753 | prot-a-1283 | Glutathione S-transferase A1 \|\| id:prot-a-1283 | F-deep fried food liking (derived food-liking factor) \|\| id:ebi-fl187-GCST90094753 | Weighted median | 8 | 0.0380100538978176 | 0.130716042467633 | 0.771216989897767 | -0.218193389 | 0.294213497134378 | 1.03874167622867 | 0.803969947415079 | 1.34207040126748 | 0.999489402457283 |
| ebi-fl187-GCST90094753 | prot-a-1324 | E3 ubiquitin-protein ligase HECW1 \|\| id:prot-a-1324 | F-deep fried food liking (derived food-liking factor) \|\| id:ebi-fl187-GCST90094753 | Weighted median | 8 | 0.137340979762589 | 0.136587786189599 | 0.314649240638917 | -0.130371081 | 0.405053040694204 | 1.14721926038704 | 0.877769646691474 | 1.49938202621123 | 0.999489402457283 |
| ebi-fl187-GCST90094753 | prot-a-1339 | Histidine triad nucleotide-binding protein 2, mitochondrial \|\| id:prot-a-1339 | F-deep fried food liking (derived food-liking factor) \|\| id:ebi-fl187-GCST90094753 | Weighted median | 8 | 0.11943756175823 | 0.13146481567018 | 0.363606976689269 | -0.138233477 | 0.377108600471783 | 1.1268628825335 | 0.870895334016059 | 1.45806264706466 | 0.999489402457283 |
| ebi-fl187-GCST90094753 | prot-a-1466 | Interleukin-11 \|\| id:prot-a-1466 | F-deep fried food liking (derived food-liking factor) \|\| id:ebi-fl187-GCST90094753 | Weighted median | 8 | -0.075995233 | 0.134329240594887 | 0.571571510939525 | -0.339280544 | 0.187290078968165 | 0.926820625075525 | 0.71228259433165 | 1.20597706289791 | 0.999489402457283 |
| ebi-fl187-GCST90094753 | prot-a-147 | Rho GTPase-activating protein 1 \|\| id:prot-a-147 | F-deep fried food liking (derived food-liking factor) \|\| id:ebi-fl187-GCST90094753 | Weighted median | 8 | 0.0338178958517211 | 0.136558656600733 | 0.804410068368036 | -0.233837071 | 0.301472862789157 | 1.03439622173285 | 0.791490762121304 | 1.35184842924448 | 0.999489402457283 |
| ebi-fl187-GCST90094753 | prot-a-1473 | Interleukin-12 receptor subunit beta-1 \|\| id:prot-a-1473 | F-deep fried food liking (derived food-liking factor) \|\| id:ebi-fl187-GCST90094753 | Weighted median | 8 | -0.00059077 | 0.137928498133579 | 0.996582541538953 | -0.270930626 | 0.269749086226238 | 0.999409404354729 | 0.762669403648233 | 1.30963580384216 | 0.999911036302558 |
| ebi-fl187-GCST90094753 | prot-a-1501 | X-linked interleukin-1 receptor accessory protein-like 2 \|\| id:prot-a-1501 | F-deep fried food liking (derived food-liking factor) \|\| id:ebi-fl187-GCST90094753 | Weighted median | 8 | -0.043238559 | 0.135072160462448 | 0.748881524268552 | -0.307979993 | 0.221502875634722 | 0.957682899062014 | 0.734930021329067 | 1.24795083686636 | 0.999489402457283 |
| ebi-fl187-GCST90094753 | prot-a-1503 | Interleukin-1 receptor-like 2 \|\| id:prot-a-1503 | F-deep fried food liking (derived food-liking factor) \|\| id:ebi-fl187-GCST90094753 | Weighted median | 8 | 0.0366950513032874 | 0.143211401113337 | 0.797773277379057 | -0.243999295 | 0.317389397485428 | 1.03737662594842 | 0.783488186716757 | 1.37353731978243 | 0.999489402457283 |
| ebi-fl187-GCST90094753 | prot-a-1536 | Interleukin-5 receptor subunit alpha \|\| id:prot-a-1536 | F-deep fried food liking (derived food-liking factor) \|\| id:ebi-fl187-GCST90094753 | Weighted median | 8 | -0.269706036 | 0.131441958305146 | 0.0401791735382312 | -0.527332274 | -0.012079798 | 0.763603933446888 | 0.590177302665934 | 0.987992870179243 | 0.999489402457283 |
| ebi-fl187-GCST90094753 | prot-a-1559 | Insulin-induced gene 1 protein \|\| id:prot-a-1559 | F-deep fried food liking (derived food-liking factor) \|\| id:ebi-fl187-GCST90094753 | Weighted median | 8 | 0.178887081111751 | 0.134468301704125 | 0.18340989683586 | -0.08467079 | 0.442444952451837 | 1.19588569845988 | 0.918814717400096 | 1.55650815849755 | 0.999489402457283 |
| ebi-fl187-GCST90094753 | prot-a-1567 | Interferon regulatory factor 1 \|\| id:prot-a-1567 | F-deep fried food liking (derived food-liking factor) \|\| id:ebi-fl187-GCST90094753 | Weighted median | 8 | 0.138103072480493 | 0.152250266199926 | 0.364364837311765 | -0.160307449 | 0.436513594232347 | 1.14809388105991 | 0.851881838249541 | 1.54730327674986 | 0.999489402457283 |
| ebi-fl187-GCST90094753 | prot-a-1613 | Potassium voltage-gated channel subfamily E regulatory beta subunit 5 \|\| id:prot-a-1613 | F-deep fried food liking (derived food-liking factor) \|\| id:ebi-fl187-GCST90094753 | Weighted median | 8 | 0.105130603042364 | 0.131397827780514 | 0.42365638384098 | -0.152409139 | 0.362670345492171 | 1.1108556820138 | 0.858636906670331 | 1.43716201420646 | 0.999489402457283 |
| ebi-fl187-GCST90094753 | prot-a-1649 | Kin of IRRE-like protein 2 \|\| id:prot-a-1649 | F-deep fried food liking (derived food-liking factor) \|\| id:ebi-fl187-GCST90094753 | Weighted median | 8 | 0.182745978847914 | 0.135385595375091 | 0.177074214897403 | -0.082609788 | 0.448101745783093 | 1.20050941456049 | 0.920710349279572 | 1.56533795408739 | 0.999489402457283 |
| ebi-fl187-GCST90094753 | prot-a-165 | Armadillo repeat-containing protein 5 \|\| id:prot-a-165 | F-deep fried food liking (derived food-liking factor) \|\| id:ebi-fl187-GCST90094753 | Weighted median | 8 | -0.037449251 | 0.145711176448963 | 0.797170940396495 | -0.323043157 | 0.248144655045193 | 0.963243300312365 | 0.723942610766909 | 1.2816453152464 | 0.999489402457283 |
| ebi-fl187-GCST90094753 | prot-a-1656 | Kallikrein-11 \|\| id:prot-a-1656 | F-deep fried food liking (derived food-liking factor) \|\| id:ebi-fl187-GCST90094753 | Weighted median | 8 | 0.041571340431401 | 0.136045172202596 | 0.759931969020166 | -0.225077197 | 0.30821987794849 | 1.04244752785514 | 0.798454578013688 | 1.36100020997395 | 0.999489402457283 |
| ebi-fl187-GCST90094753 | prot-a-1697 | Laminin subunit gamma-2 \|\| id:prot-a-1697 | F-deep fried food liking (derived food-liking factor) \|\| id:ebi-fl187-GCST90094753 | Weighted median | 8 | 0.0079213874987382 | 0.137205840437956 | 0.953960827133055 | -0.26100206 | 0.276844834757131 | 1.00795284469538 | 0.770279333023152 | 1.31896169814408 | 0.999489402457283 |
| ebi-fl187-GCST90094753 | prot-a-1698 | Lysosome-associated membrane glycoprotein 3 \|\| id:prot-a-1698 | F-deep fried food liking (derived food-liking factor) \|\| id:ebi-fl187-GCST90094753 | Weighted median | 8 | -0.153625372 | 0.129879220832514 | 0.236875512859907 | -0.408188644 | 0.100937901307912 | 0.857593239687439 | 0.664853446141812 | 1.10620794556388 | 0.999489402457283 |
| ebi-fl187-GCST90094753 | prot-a-1724 | Leptin receptor \|\| id:prot-a-1724 | F-deep fried food liking (derived food-liking factor) \|\| id:ebi-fl187-GCST90094753 | Weighted median | 8 | -0.044321054 | 0.137463284770327 | 0.747133753575941 | -0.313749092 | 0.22510698411235 | 0.956646772855377 | 0.730702344087632 | 1.25245670199308 | 0.999489402457283 |
| ebi-fl187-GCST90094753 | prot-a-1743 | Leukocyte immunoglobulin-like receptor subfamily B member 2 \|\| id:prot-a-1743 | F-deep fried food liking (derived food-liking factor) \|\| id:ebi-fl187-GCST90094753 | Weighted median | 8 | -0.109194724 | 0.124369528758099 | 0.379951233091526 | -0.352959001 | 0.13456955222033 | 0.896555819433685 | 0.702605999273368 | 1.14404422705144 | 0.999489402457283 |
| ebi-fl187-GCST90094753 | prot-a-1746 | Leukocyte immunoglobulin-like receptor subfamily B member 4 \|\| id:prot-a-1746 | F-deep fried food liking (derived food-liking factor) \|\| id:ebi-fl187-GCST90094753 | Weighted median | 8 | 0.0339503483647419 | 0.161092385344401 | 0.833081735570317 | -0.281790727 | 0.349691423639767 | 1.03453323918583 | 0.754431550235489 | 1.4186297254486 | 0.999489402457283 |
| ebi-fl187-GCST90094753 | prot-a-1750 | Leucine-rich repeat and immunoglobulin-like domain-containing nogo receptor-interacting protein 3 \|\| id:prot-a-1750 | F-deep fried food liking (derived food-liking factor) \|\| id:ebi-fl187-GCST90094753 | Weighted median | 8 | 0.195315312825428 | 0.132924480419066 | 0.141732318978409 | -0.065216669 | 0.455847294446797 | 1.21569425005144 | 0.936864452092297 | 1.5775094319221 | 0.999489402457283 |
| ebi-fl187-GCST90094753 | prot-a-1753 | Lipase member N \|\| id:prot-a-1753 | F-deep fried food liking (derived food-liking factor) \|\| id:ebi-fl187-GCST90094753 | Weighted median | 8 | 0.0822839097046067 | 0.12972573298205 | 0.525890703579031 | -0.171978527 | 0.336546346349425 | 1.08576402501845 | 0.841997253231742 | 1.40010375746417 | 0.999489402457283 |
| ebi-fl187-GCST90094753 | prot-a-1760 | Ig Kappa chain V-I region HK102- like \|\| id:prot-a-1760 | F-deep fried food liking (derived food-liking factor) \|\| id:ebi-fl187-GCST90094753 | Weighted median | 8 | 0.005966274449582 | 0.130355843674097 | 0.963494258238924 | -0.249531179 | 0.261463728050811 | 1.00598410811419 | 0.779165986716074 | 1.29882982962791 | 0.999489402457283 |
| ebi-fl187-GCST90094753 | prot-a-1792 | Leucine-rich repeat neuronal protein 1 \|\| id:prot-a-1792 | F-deep fried food liking (derived food-liking factor) \|\| id:ebi-fl187-GCST90094753 | Weighted median | 7 | -0.261848551 | 0.174964480221123 | 0.13450226653414 | -0.604778932 | 0.0810818305347998 | 0.769627574440075 | 0.546195159602798 | 1.08445963484785 | 0.999489402457283 |
| ebi-fl187-GCST90094753 | prot-a-1795 | Leucine-rich repeat transmembrane neuronal protein 1 \|\| id:prot-a-1795 | F-deep fried food liking (derived food-liking factor) \|\| id:ebi-fl187-GCST90094753 | Weighted median | 8 | -0.00566939 | 0.128970367574683 | 0.964937199760503 | -0.25845131 | 0.247112530542824 | 0.994346650759477 | 0.772246630552031 | 1.28032318013458 | 0.999489402457283 |
| ebi-fl187-GCST90094753 | prot-a-1820 | LysM and putative peptidoglycan-binding domain-containing protein 4 \|\| id:prot-a-1820 | F-deep fried food liking (derived food-liking factor) \|\| id:ebi-fl187-GCST90094753 | Weighted median | 8 | 0.150529773083565 | 0.136964557260744 | 0.271749835319786 | -0.117920759 | 0.418980305314624 | 1.16244991430636 | 0.888766476437631 | 1.52041041049065 | 0.999489402457283 |
| ebi-fl187-GCST90094753 | prot-a-1844 | Mitogen-activated protein kinase kinase kinase 7:TGF-beta-activated kinase 1 and MAP3K7-binding protein 1 fusion \|\| id:prot-a-1844 | F-deep fried food liking (derived food-liking factor) \|\| id:ebi-fl187-GCST90094753 | Weighted median | 8 | 0.119449210442252 | 0.132373986387471 | 0.366864779414792 | -0.140003803 | 0.378902223761695 | 1.12687600907961 | 0.869354929342488 | 1.46068020894482 | 0.999489402457283 |
| ebi-fl187-GCST90094753 | prot-a-1856 | Megakaryocyte-associated tyrosine-protein kinase \|\| id:prot-a-1856 | F-deep fried food liking (derived food-liking factor) \|\| id:ebi-fl187-GCST90094753 | Weighted median | 8 | 0.024779846453064 | 0.137345804853599 | 0.856823332378492 | -0.244417931 | 0.293977623966119 | 1.02508941860937 | 0.783160258860325 | 1.34175388020079 | 0.999489402457283 |
| ebi-fl187-GCST90094753 | prot-a-1910 | Stromelysin-2 \|\| id:prot-a-1910 | F-deep fried food liking (derived food-liking factor) \|\| id:ebi-fl187-GCST90094753 | Weighted median | 8 | 0.13671088642233 | 0.13373216198318 | 0.306651326401759 | -0.125404151 | 0.398825923909363 | 1.14649663285656 | 0.882140312584787 | 1.49007420973643 | 0.999489402457283 |
| ebi-fl187-GCST90094753 | prot-a-1940 | 39S ribosomal protein L14, mitochondrial \|\| id:prot-a-1940 | F-deep fried food liking (derived food-liking factor) \|\| id:ebi-fl187-GCST90094753 | Weighted median | 8 | 0.0845839233713454 | 0.133780247154842 | 0.527216877880024 | -0.177625361 | 0.346793207794836 | 1.08826417119788 | 0.837256033459306 | 1.41452418254872 | 0.999489402457283 |
| ebi-fl187-GCST90094753 | prot-a-1942 | 39S ribosomal protein L33, mitochondrial \|\| id:prot-a-1942 | F-deep fried food liking (derived food-liking factor) \|\| id:ebi-fl187-GCST90094753 | Weighted median | 8 | -0.084792554 | 0.128495759945485 | 0.509326985760789 | -0.336644243 | 0.167059135788802 | 0.918702846137346 | 0.714162863011426 | 1.18182415134537 | 0.999489402457283 |
| ebi-fl187-GCST90094753 | prot-a-1948 | Mesothelin \|\| id:prot-a-1948 | F-deep fried food liking (derived food-liking factor) \|\| id:ebi-fl187-GCST90094753 | Weighted median | 8 | -0.045575081 | 0.130639023463583 | 0.727192398124755 | -0.301627567 | 0.210477405115996 | 0.955447864019512 | 0.73961347016748 | 1.2342671647837 | 0.999489402457283 |
| ebi-fl187-GCST90094753 | prot-a-1980 | Unconventional myosin-VI \|\| id:prot-a-1980 | F-deep fried food liking (derived food-liking factor) \|\| id:ebi-fl187-GCST90094753 | Weighted median | 8 | 0.141492033848528 | 0.131817599017567 | 0.283095008374578 | -0.11687046 | 0.399854527922959 | 1.15199132728685 | 0.889700437292562 | 1.49160769458825 | 0.999489402457283 |
| ebi-fl187-GCST90094753 | prot-a-2039 | Neurofascin \|\| id:prot-a-2039 | F-deep fried food liking (derived food-liking factor) \|\| id:ebi-fl187-GCST90094753 | Weighted median | 8 | -0.054190456 | 0.124307309480779 | 0.662880685131576 | -0.297832783 | 0.189451870490395 | 0.947251679467684 | 0.742425475771261 | 1.20858695389219 | 0.999489402457283 |
| ebi-fl187-GCST90094753 | prot-a-2041 | NFU1 iron-sulfur cluster scaffold homolog, mitochondrial \|\| id:prot-a-2041 | F-deep fried food liking (derived food-liking factor) \|\| id:ebi-fl187-GCST90094753 | Weighted median | 8 | 0.104133469845921 | 0.133786895667894 | 0.436360770002053 | -0.158088846 | 0.366355785354994 | 1.1097485630011 | 0.853773924488224 | 1.4424683604869 | 0.999489402457283 |
| ebi-fl187-GCST90094753 | prot-a-2088 | Glucocorticoid receptor \|\| id:prot-a-2088 | F-deep fried food liking (derived food-liking factor) \|\| id:ebi-fl187-GCST90094753 | Weighted median | 8 | 0.0310016017796014 | 0.163367249249638 | 0.849492248644138 | -0.289198207 | 0.351201410308892 | 1.03148715610029 | 0.748863760839802 | 1.42077345551704 | 0.999489402457283 |
| ebi-fl187-GCST90094753 | prot-a-2111 | Neuron-specific protein family member 2 \|\| id:prot-a-2111 | F-deep fried food liking (derived food-liking factor) \|\| id:ebi-fl187-GCST90094753 | Weighted median | 8 | 0.185560629470157 | 0.133988874071013 | 0.166084330286738 | -0.077057564 | 0.448178822649343 | 1.20389318896864 | 0.925836557486346 | 1.56545861008135 | 0.999489402457283 |
| ebi-fl187-GCST90094753 | prot-a-2125 | Nuclear migration protein nudC \|\| id:prot-a-2125 | F-deep fried food liking (derived food-liking factor) \|\| id:ebi-fl187-GCST90094753 | Weighted median | 8 | 0.088038923148209 | 0.135026977961169 | 0.514394801411378 | -0.176613954 | 0.3526917999521 | 1.09203062647091 | 0.838103268782093 | 1.42289254029924 | 0.999489402457283 |
| ebi-fl187-GCST90094753 | prot-a-2142 | Ornithine decarboxylase \|\| id:prot-a-2142 | F-deep fried food liking (derived food-liking factor) \|\| id:ebi-fl187-GCST90094753 | Weighted median | 8 | -0.080426602 | 0.130118977514445 | 0.536509109733897 | -0.335459798 | 0.174606594223188 | 0.922722627365813 | 0.715009251197955 | 1.19077766564051 | 0.999489402457283 |
| ebi-fl187-GCST90094753 | prot-a-2186 | Prostate and testis expressed protein 1 \|\| id:prot-a-2186 | F-deep fried food liking (derived food-liking factor) \|\| id:ebi-fl187-GCST90094753 | Weighted median | 8 | -0.239610335 | 0.156863448374071 | 0.126634481162453 | -0.547062694 | 0.0678420234553265 | 0.786934441858507 | 0.578646979754055 | 1.07019622922143 | 0.999489402457283 |
| ebi-fl187-GCST90094753 | prot-a-2227 | Platelet-derived growth factor C \|\| id:prot-a-2227 | F-deep fried food liking (derived food-liking factor) \|\| id:ebi-fl187-GCST90094753 | Weighted median | 8 | -0.072777451 | 0.138880773972967 | 0.600258882106225 | -0.344983768 | 0.199428866217269 | 0.929807734997375 | 0.708231849566146 | 1.22070537295175 | 0.999489402457283 |
| ebi-fl187-GCST90094753 | prot-a-2229 | Platelet-derived growth factor receptor alpha \|\| id:prot-a-2229 | F-deep fried food liking (derived food-liking factor) \|\| id:ebi-fl187-GCST90094753 | Weighted median | 8 | 0.0577127292595422 | 0.133293451660299 | 0.665032948281946 | -0.203542436 | 0.318967894513728 | 1.05941061432082 | 0.815835582793658 | 1.37570715644979 | 0.999489402457283 |
| ebi-fl187-GCST90094753 | prot-a-2243 | Platelet endothelial aggregation receptor 1 \|\| id:prot-a-2243 | F-deep fried food liking (derived food-liking factor) \|\| id:ebi-fl187-GCST90094753 | Weighted median | 8 | -0.072153622 | 0.132649807948751 | 0.586482320144355 | -0.332147245 | 0.187840001858665 | 0.930387957033185 | 0.717381683995633 | 1.20664043967654 | 0.999489402457283 |
| ebi-fl187-GCST90094753 | prot-a-2246 | E3 ubiquitin-protein ligase pellino homolog 2 \|\| id:prot-a-2246 | F-deep fried food liking (derived food-liking factor) \|\| id:ebi-fl187-GCST90094753 | Weighted median | 8 | 0.152850996667025 | 0.141260387200278 | 0.279229698882538 | -0.124019362 | 0.42972135557957 | 1.1651513545725 | 0.883362736830971 | 1.53682923498946 | 0.999489402457283 |
| ebi-fl187-GCST90094753 | prot-a-2251 | Platelet factor 4 variant \|\| id:prot-a-2251 | F-deep fried food liking (derived food-liking factor) \|\| id:ebi-fl187-GCST90094753 | Weighted median | 8 | 0.0321781237679329 | 0.130663627741444 | 0.805475647453163 | -0.223922587 | 0.288278834141162 | 1.03270143759449 | 0.799377014462498 | 1.33412925304942 | 0.999489402457283 |
| ebi-fl187-GCST90094753 | prot-a-2346 | Protein phosphatase 1L \|\| id:prot-a-2346 | F-deep fried food liking (derived food-liking factor) \|\| id:ebi-fl187-GCST90094753 | Weighted median | 8 | 0.0807593609916183 | 0.145438259855143 | 0.578701256578484 | -0.204299628 | 0.365818350307699 | 1.08410998602338 | 0.815218072164633 | 1.44169333571675 | 0.999489402457283 |
| ebi-fl187-GCST90094753 | prot-a-2358 | PR domain zinc finger protein 4 \|\| id:prot-a-2358 | F-deep fried food liking (derived food-liking factor) \|\| id:ebi-fl187-GCST90094753 | Weighted median | 8 | 0.00953913146095128 | 0.132187257204406 | 0.942471573500469 | -0.249547893 | 0.268626155581586 | 1.00958477399005 | 0.77915296422792 | 1.30816599906355 | 0.999489402457283 |
| ebi-fl187-GCST90094753 | prot-a-2390 | Trypsin-1 \|\| id:prot-a-2390 | F-deep fried food liking (derived food-liking factor) \|\| id:ebi-fl187-GCST90094753 | Weighted median | 8 | 0.184136664034096 | 0.146187187935999 | 0.207815467955405 | -0.102390224 | 0.470663552388654 | 1.2021801066533 | 0.902677236321198 | 1.60105622550417 | 0.999489402457283 |
| ebi-fl187-GCST90094753 | prot-a-242 | Brain-derived neurotrophic factor \|\| id:prot-a-242 | F-deep fried food liking (derived food-liking factor) \|\| id:ebi-fl187-GCST90094753 | Weighted median | 8 | 0.0464367639156021 | 0.131435001390473 | 0.7238591088144 | -0.211175839 | 0.304049366640929 | 1.04753183516252 | 0.809631689691295 | 1.35533596282199 | 0.999489402457283 |
| ebi-fl187-GCST90094753 | prot-a-2464 | Dihydropteridine reductase \|\| id:prot-a-2464 | F-deep fried food liking (derived food-liking factor) \|\| id:ebi-fl187-GCST90094753 | Weighted median | 8 | 0.0692381171090756 | 0.130924917799267 | 0.59691759379914 | -0.187374722 | 0.325850955995639 | 1.07169136681853 | 0.829132983980662 | 1.38520889640563 | 0.999489402457283 |
| ebi-fl187-GCST90094753 | prot-a-2474 | Ras-related protein Rab-27A \|\| id:prot-a-2474 | F-deep fried food liking (derived food-liking factor) \|\| id:ebi-fl187-GCST90094753 | Weighted median | 8 | 0.10981856106726 | 0.169051868244576 | 0.515941202566702 | -0.221523101 | 0.44116022282663 | 1.116075553 | 0.801297411409341 | 1.55450975033641 | 0.999489402457283 |
| ebi-fl187-GCST90094753 | prot-a-2478 | Ras-related protein Rab-7b \|\| id:prot-a-2478 | F-deep fried food liking (derived food-liking factor) \|\| id:ebi-fl187-GCST90094753 | Weighted median | 8 | -0.018641313 | 0.139756158420985 | 0.893889259529545 | -0.292563384 | 0.255280757495999 | 0.981531361640778 | 0.7463479373815 | 1.29082397851118 | 0.999489402457283 |
| ebi-fl187-GCST90094753 | prot-a-2528 | E3 ubiquitin-protein ligase rififylin \|\| id:prot-a-2528 | F-deep fried food liking (derived food-liking factor) \|\| id:ebi-fl187-GCST90094753 | Weighted median | 8 | 0.10509237821916 | 0.130061408662488 | 0.419078366045014 | -0.149827983 | 0.360012739197637 | 1.11081322056329 | 0.860856045771072 | 1.43334767414334 | 0.999489402457283 |
| ebi-fl187-GCST90094753 | prot-a-2543 | Protein RIC-3 \|\| id:prot-a-2543 | F-deep fried food liking (derived food-liking factor) \|\| id:ebi-fl187-GCST90094753 | Weighted median | 8 | 0.163308460860648 | 0.12526553540771 | 0.192336482084966 | -0.082211989 | 0.40882891025976 | 1.17739981544094 | 0.92107668029942 | 1.50505419912458 | 0.999489402457283 |
| ebi-fl187-GCST90094753 | prot-a-255 | Bone morphogenetic protein 6 \|\| id:prot-a-255 | F-deep fried food liking (derived food-liking factor) \|\| id:ebi-fl187-GCST90094753 | Weighted median | 8 | -0.083996217 | 0.149722935485889 | 0.574790024901583 | -0.37745317 | 0.209460736826828 | 0.919434734562518 | 0.685605307538584 | 1.23301296215895 | 0.999489402457283 |
| ebi-fl187-GCST90094753 | prot-a-2568 | RING finger protein 165 \|\| id:prot-a-2568 | F-deep fried food liking (derived food-liking factor) \|\| id:ebi-fl187-GCST90094753 | Weighted median | 8 | -0.100229292 | 0.134445178203272 | 0.455967660332457 | -0.363741841 | 0.163283257159867 | 0.904629969731478 | 0.695070610040816 | 1.17737014098225 | 0.999489402457283 |
| ebi-fl187-GCST90094753 | prot-a-2573 | E3 ubiquitin-protein ligase RNF8 \|\| id:prot-a-2573 | F-deep fried food liking (derived food-liking factor) \|\| id:ebi-fl187-GCST90094753 | Weighted median | 8 | -0.104725781 | 0.134590718991308 | 0.436506811954921 | -0.36852359 | 0.159072028557397 | 0.900571442831229 | 0.691754890984872 | 1.17242239153298 | 0.999489402457283 |
| ebi-fl187-GCST90094753 | prot-a-260 | Cytoplasmic tyrosine-protein kinase BMX \|\| id:prot-a-260 | F-deep fried food liking (derived food-liking factor) \|\| id:ebi-fl187-GCST90094753 | Weighted median | 7 | -0.074598462 | 0.132514668283618 | 0.573472376639308 | -0.334327212 | 0.185130287726338 | 0.928116085292224 | 0.715819519205004 | 1.20337521493524 | 0.999489402457283 |
| ebi-fl187-GCST90094753 | prot-a-2623 | Serum amyloid A-1 protein \|\| id:prot-a-2623 | F-deep fried food liking (derived food-liking factor) \|\| id:ebi-fl187-GCST90094753 | Weighted median | 8 | -0.108728403 | 0.134365057803419 | 0.418399268531786 | -0.372083916 | 0.154627110772166 | 0.896974000294333 | 0.689296397226697 | 1.16722263519885 | 0.999489402457283 |
| ebi-fl187-GCST90094753 | prot-a-2640 | Lysosome membrane protein 2 \|\| id:prot-a-2640 | F-deep fried food liking (derived food-liking factor) \|\| id:ebi-fl187-GCST90094753 | Weighted median | 8 | 0.168821076938818 | 0.139428580849453 | 0.225969449828743 | -0.104458942 | 0.442101095403745 | 1.18390829147667 | 0.900811782604657 | 1.55597303420524 | 0.999489402457283 |
| ebi-fl187-GCST90094753 | prot-a-2641 | Scavenger receptor class F member 2 \|\| id:prot-a-2641 | F-deep fried food liking (derived food-liking factor) \|\| id:ebi-fl187-GCST90094753 | Weighted median | 8 | 0.268696360569648 | 0.131582097526298 | 0.0411472213883706 | 0.010795449418103 | 0.526597271721193 | 1.30825784206578 | 1.01085393053603 | 1.69316112805638 | 0.999489402457283 |
| ebi-fl187-GCST90094753 | prot-a-2669 | Semaphorin-3A \|\| id:prot-a-2669 | F-deep fried food liking (derived food-liking factor) \|\| id:ebi-fl187-GCST90094753 | Weighted median | 8 | 0.0309724210245025 | 0.129423873651156 | 0.810865248801212 | -0.222698371 | 0.284643213380768 | 1.03145705696536 | 0.800356223271809 | 1.32928767145017 | 0.999489402457283 |
| ebi-fl187-GCST90094753 | prot-a-2683 | Sentrin-specific protease 7 \|\| id:prot-a-2683 | F-deep fried food liking (derived food-liking factor) \|\| id:ebi-fl187-GCST90094753 | Weighted median | 8 | -0.007893562 | 0.134459564583807 | 0.953186408461795 | -0.271434308 | 0.255647185025302 | 0.992137510787173 | 0.762285357763832 | 1.29129705862189 | 0.999489402457283 |
| ebi-fl187-GCST90094753 | prot-a-270 | Bromodomain testis-specific protein \|\| id:prot-a-270 | F-deep fried food liking (derived food-liking factor) \|\| id:ebi-fl187-GCST90094753 | Weighted median | 8 | 0.0469067607694797 | 0.1333597292914 | 0.725039954398534 | -0.214478309 | 0.308291830180624 | 1.04802428754585 | 0.80696231565081 | 1.36109814050011 | 0.999489402457283 |
| ebi-fl187-GCST90094753 | prot-a-2708 | 14-3-3 protein sigma \|\| id:prot-a-2708 | F-deep fried food liking (derived food-liking factor) \|\| id:ebi-fl187-GCST90094753 | Weighted median | 8 | 0.112598087553697 | 0.123766632555916 | 0.362948429338683 | -0.129984512 | 0.355180687363293 | 1.11918202935644 | 0.878109030743208 | 1.4264383703858 | 0.999489402457283 |
| ebi-fl187-GCST90094753 | prot-a-2713 | Beta-sarcoglycan \|\| id:prot-a-2713 | F-deep fried food liking (derived food-liking factor) \|\| id:ebi-fl187-GCST90094753 | Weighted median | 8 | -0.007084385 | 0.143545536008038 | 0.960638079410146 | -0.288433636 | 0.274264865093874 | 0.992940649622619 | 0.749436539060501 | 1.31556320286833 | 0.999489402457283 |
| ebi-fl187-GCST90094753 | prot-a-2720 | Protein shisa-3 homolog \|\| id:prot-a-2720 | F-deep fried food liking (derived food-liking factor) \|\| id:ebi-fl187-GCST90094753 | Weighted median | 8 | -0.093339692 | 0.137355070986038 | 0.496789191610781 | -0.362555631 | 0.175876247187281 | 0.91088402776907 | 0.695895599179438 | 1.19229050021749 | 0.999489402457283 |
| ebi-fl187-GCST90094753 | prot-a-2730 | Sialic acid-binding Ig-like lectin 8 \|\| id:prot-a-2730 | F-deep fried food liking (derived food-liking factor) \|\| id:ebi-fl187-GCST90094753 | Weighted median | 8 | -0.038211037 | 0.145171426420385 | 0.792386249210589 | -0.322747033 | 0.246324958563733 | 0.962509794064807 | 0.724157019025239 | 1.27931523044229 | 0.999489402457283 |
| ebi-fl187-GCST90094753 | prot-a-2761 | Sodium- and chloride-dependent neutral and basic amino acid transporter B(0+) \|\| id:prot-a-2761 | F-deep fried food liking (derived food-liking factor) \|\| id:ebi-fl187-GCST90094753 | Weighted median | 8 | 0.0912151144322898 | 0.137544489667575 | 0.507222915574694 | -0.178372085 | 0.360802314180736 | 1.09550463877191 | 0.83663106743162 | 1.43447985652155 | 0.999489402457283 |
| ebi-fl187-GCST90094753 | prot-a-2770 | Secreted Ly-6/uPAR-related protein 1 \|\| id:prot-a-2770 | F-deep fried food liking (derived food-liking factor) \|\| id:ebi-fl187-GCST90094753 | Weighted median | 8 | -0.048939194 | 0.138044811709662 | 0.722951672996889 | -0.319507025 | 0.221628636554727 | 0.952239029407309 | 0.726507098393488 | 1.24810779018082 | 0.999489402457283 |
| ebi-fl187-GCST90094753 | prot-a-2784 | N-lysine methyltransferase SMYD2 \|\| id:prot-a-2784 | F-deep fried food liking (derived food-liking factor) \|\| id:ebi-fl187-GCST90094753 | Weighted median | 8 | -0.091734008 | 0.135007222173634 | 0.496837058300742 | -0.356348163 | 0.172880147794302 | 0.912347794792109 | 0.700228783947737 | 1.18872362539748 | 0.999489402457283 |
| ebi-fl187-GCST90094753 | prot-a-2837 | Serine palmitoyltransferase 2 \|\| id:prot-a-2837 | F-deep fried food liking (derived food-liking factor) \|\| id:ebi-fl187-GCST90094753 | Weighted median | 8 | -0.053256366 | 0.12731135572947 | 0.675716153780333 | -0.302786624 | 0.196273890740374 | 0.948136910790789 | 0.738756712713904 | 1.21686014642284 | 0.999489402457283 |
| ebi-fl187-GCST90094753 | prot-a-2858 | Alpha-2,8-sialyltransferase 8B \|\| id:prot-a-2858 | F-deep fried food liking (derived food-liking factor) \|\| id:ebi-fl187-GCST90094753 | Weighted median | 8 | 0.179441013624236 | 0.127880426807759 | 0.160559075542948 | -0.071204623 | 0.430086650167444 | 1.19654832193687 | 0.93127131317539 | 1.53739073294133 | 0.999489402457283 |
| ebi-fl187-GCST90094753 | prot-a-2911 | Synaptotagmin-11 \|\| id:prot-a-2911 | F-deep fried food liking (derived food-liking factor) \|\| id:ebi-fl187-GCST90094753 | Weighted median | 8 | 0.126183982209144 | 0.126285514590445 | 0.317699748705169 | -0.121335626 | 0.373703590806417 | 1.13449087522073 | 0.885736633113355 | 1.45310637252867 | 0.999489402457283 |
| ebi-fl187-GCST90094753 | prot-a-2962 | Transforming growth factor beta-1 \|\| id:prot-a-2962 | F-deep fried food liking (derived food-liking factor) \|\| id:ebi-fl187-GCST90094753 | Weighted median | 8 | -0.077982267 | 0.124494531891575 | 0.53105844349322 | -0.32199155 | 0.166027015306848 | 0.92498082889756 | 0.724704314267006 | 1.18060499569869 | 0.999489402457283 |
| ebi-fl187-GCST90094753 | prot-a-2978 | Tyrosine-protein kinase receptor Tie-1, soluble \|\| id:prot-a-2978 | F-deep fried food liking (derived food-liking factor) \|\| id:ebi-fl187-GCST90094753 | Weighted median | 8 | -0.037516543 | 0.130195399991075 | 0.773227577495389 | -0.292699527 | 0.217666440773589 | 0.963178483526142 | 0.746246333745365 | 1.24317232685296 | 0.999489402457283 |
| ebi-fl187-GCST90094753 | prot-a-299 | Uncharacterized protein C1orf226 \|\| id:prot-a-299 | F-deep fried food liking (derived food-liking factor) \|\| id:ebi-fl187-GCST90094753 | Weighted median | 8 | -0.042177774 | 0.130927815118226 | 0.747342658152926 | -0.298796292 | 0.214440743685178 | 0.958699333658328 | 0.741710486719261 | 1.23916869022887 | 0.999489402457283 |
| ebi-fl187-GCST90094753 | prot-a-3015 | Transmembrane protein 70, mitochondrial \|\| id:prot-a-3015 | F-deep fried food liking (derived food-liking factor) \|\| id:ebi-fl187-GCST90094753 | Weighted median | 8 | -0.022907407 | 0.128513734304042 | 0.858527659911814 | -0.274794326 | 0.228979512332173 | 0.977352975721899 | 0.759728363422461 | 1.25731627926768 | 0.999489402457283 |
| ebi-fl187-GCST90094753 | prot-a-3016 | Transmembrane protein 87B \|\| id:prot-a-3016 | F-deep fried food liking (derived food-liking factor) \|\| id:ebi-fl187-GCST90094753 | Weighted median | 8 | 0.0327323127985167 | 0.150415665806841 | 0.827731128616881 | -0.262082392 | 0.327547017779925 | 1.03327390801684 | 0.769447624626403 | 1.38756028976861 | 0.999489402457283 |
| ebi-fl187-GCST90094753 | prot-a-3123 | Thioredoxin domain-containing protein 12 \|\| id:prot-a-3123 | F-deep fried food liking (derived food-liking factor) \|\| id:ebi-fl187-GCST90094753 | Weighted median | 8 | -0.009670911 | 0.159069405404421 | 0.951521039336123 | -0.321446945 | 0.302105123865458 | 0.990375702145941 | 0.725099098907709 | 1.35270342064776 | 0.999489402457283 |
| ebi-fl187-GCST90094753 | prot-a-3133 | PolyUbiquitin K48-linked \|\| id:prot-a-3133 | F-deep fried food liking (derived food-liking factor) \|\| id:ebi-fl187-GCST90094753 | Weighted median | 8 | 0.0143820629797391 | 0.149322229175338 | 0.923269906343901 | -0.278289506 | 0.307053632163403 | 1.01448598244194 | 0.757077611119211 | 1.35941387442395 | 0.999489402457283 |
| ebi-fl187-GCST90094753 | prot-a-3135 | Ubiquitin-conjugating enzyme E2 B \|\| id:prot-a-3135 | F-deep fried food liking (derived food-liking factor) \|\| id:ebi-fl187-GCST90094753 | Weighted median | 8 | -0.053397997 | 0.124108070719121 | 0.667010863906523 | -0.296649816 | 0.189853821189715 | 0.948002634787032 | 0.74330426002926 | 1.20907284390887 | 0.999489402457283 |
| ebi-fl187-GCST90094753 | prot-a-3162 | NKG2D ligand 3 \|\| id:prot-a-3162 | F-deep fried food liking (derived food-liking factor) \|\| id:ebi-fl187-GCST90094753 | Weighted median | 8 | 0.0732477704573976 | 0.137132299811545 | 0.593245061301207 | -0.195531537 | 0.342027078088026 | 1.07599710418425 | 0.822397407065712 | 1.40779841748746 | 0.999489402457283 |
| ebi-fl187-GCST90094753 | prot-a-3188 | Vesicle-associated membrane protein 8 \|\| id:prot-a-3188 | F-deep fried food liking (derived food-liking factor) \|\| id:ebi-fl187-GCST90094753 | Weighted median | 8 | 0.141496467963203 | 0.137598892940266 | 0.303796679781507 | -0.128197362 | 0.411190298126124 | 1.15199643535982 | 0.879679746481133 | 1.50861241535951 | 0.999489402457283 |
| ebi-fl187-GCST90094753 | prot-a-3201 | Vitrin \|\| id:prot-a-3201 | F-deep fried food liking (derived food-liking factor) \|\| id:ebi-fl187-GCST90094753 | Weighted median | 8 | 0.0300210060573229 | 0.124476359107289 | 0.809416870260615 | -0.213952658 | 0.27399466990761 | 1.03047617996787 | 0.807386607581839 | 1.315207792 | 0.999489402457283 |
| ebi-fl187-GCST90094753 | prot-a-3232 | WNT1-inducible-signaling pathway protein 3 \|\| id:prot-a-3232 | F-deep fried food liking (derived food-liking factor) \|\| id:ebi-fl187-GCST90094753 | Weighted median | 8 | 0.204439277746054 | 0.136935122715732 | 0.135446478340815 | -0.063953563 | 0.472832118268889 | 1.22683695727865 | 0.938048558889654 | 1.60453198875581 | 0.999489402457283 |
| ebi-fl187-GCST90094753 | prot-a-327 | Carbonic anhydrase-related protein 10 \|\| id:prot-a-327 | F-deep fried food liking (derived food-liking factor) \|\| id:ebi-fl187-GCST90094753 | Weighted median | 8 | -0.26340874 | 0.152811531700427 | 0.084753146786216 | -0.562919343 | 0.0361018617275113 | 0.768427745644076 | 0.569543940645629 | 1.03676144742454 | 0.999489402457283 |
| ebi-fl187-GCST90094753 | prot-a-332 | Carbonic anhydrase 5A, mitochondrial \|\| id:prot-a-332 | F-deep fried food liking (derived food-liking factor) \|\| id:ebi-fl187-GCST90094753 | Weighted median | 8 | -0.074032692 | 0.150428021159557 | 0.622615463450456 | -0.368871613 | 0.220806229567811 | 0.928641334290828 | 0.69151418592203 | 1.24708175957895 | 0.999489402457283 |
| ebi-fl187-GCST90094753 | prot-a-338 | Cell adhesion molecule 3 \|\| id:prot-a-338 | F-deep fried food liking (derived food-liking factor) \|\| id:ebi-fl187-GCST90094753 | Weighted median | 8 | -0.00800725 | 0.133230537330947 | 0.952075423889264 | -0.269139103 | 0.253124603181066 | 0.992024722644061 | 0.764036968302138 | 1.28804376118599 | 0.999489402457283 |
| ebi-fl187-GCST90094753 | prot-a-34 | A disintegrin and metalloproteinase with thrombospondin motifs 3 \|\| id:prot-a-34 | F-deep fried food liking (derived food-liking factor) \|\| id:ebi-fl187-GCST90094753 | Weighted median | 8 | 0.066746099913025 | 0.122406734917913 | 0.585560083736101 | -0.173171101 | 0.306663300352135 | 1.06902401842298 | 0.840993708067828 | 1.35888335549002 | 0.999489402457283 |
| ebi-fl187-GCST90094753 | prot-a-358 | Cysteine--tRNA ligase, cytoplasmic \|\| id:prot-a-358 | F-deep fried food liking (derived food-liking factor) \|\| id:ebi-fl187-GCST90094753 | Weighted median | 8 | -0.030946516 | 0.133610040203995 | 0.816834573999049 | -0.292822195 | 0.230929162418382 | 0.969527425524388 | 0.746154798821989 | 1.25976999722842 | 0.999489402457283 |
| ebi-fl187-GCST90094753 | prot-a-382 | Coiled-coil domain-containing protein 167 \|\| id:prot-a-382 | F-deep fried food liking (derived food-liking factor) \|\| id:ebi-fl187-GCST90094753 | Weighted median | 8 | -0.117178199 | 0.137482468732509 | 0.394039973952916 | -0.386643838 | 0.152287439612124 | 0.889426684002934 | 0.679333004568868 | 1.16449491029588 | 0.999489402457283 |
| ebi-fl187-GCST90094753 | prot-a-40 | Pituitary adenylate cyclase-activating polypeptide 27 \|\| id:prot-a-40 | F-deep fried food liking (derived food-liking factor) \|\| id:ebi-fl187-GCST90094753 | Weighted median | 8 | 0.0389484089553522 | 0.129314117679203 | 0.763267905727292 | -0.214507262 | 0.29240407960659 | 1.03971684218843 | 0.806938951965357 | 1.33964423119916 | 0.999489402457283 |
| ebi-fl187-GCST90094753 | prot-a-403 | C-C motif chemokine 25 \|\| id:prot-a-403 | F-deep fried food liking (derived food-liking factor) \|\| id:ebi-fl187-GCST90094753 | Weighted median | 8 | -0.139922313 | 0.131530281329109 | 0.287417934886168 | -0.397721664 | 0.117877038562346 | 0.869425775992267 | 0.671849001237238 | 1.12510575823992 | 0.999489402457283 |
| ebi-fl187-GCST90094753 | prot-a-415 | Cyclin-C \|\| id:prot-a-415 | F-deep fried food liking (derived food-liking factor) \|\| id:ebi-fl187-GCST90094753 | Weighted median | 8 | -0.001972816 | 0.135507693988057 | 0.988384247519019 | -0.267567896 | 0.263622264205271 | 0.998029128711116 | 0.765238372060033 | 1.30163642875677 | 0.999911036302558 |
| ebi-fl187-GCST90094753 | prot-a-44 | Adhesion G protein-coupled receptor F5 \|\| id:prot-a-44 | F-deep fried food liking (derived food-liking factor) \|\| id:ebi-fl187-GCST90094753 | Weighted median | 8 | 0.0795961834018837 | 0.130915888999742 | 0.543190904226628 | -0.176998959 | 0.336191325841379 | 1.08284970668926 | 0.837780656620694 | 1.39960678014067 | 0.999489402457283 |
| ebi-fl187-GCST90094753 | prot-a-455 | CD59 glycoprotein \|\| id:prot-a-455 | F-deep fried food liking (derived food-liking factor) \|\| id:ebi-fl187-GCST90094753 | Weighted median | 8 | 0.0704215260082235 | 0.132993927376402 | 0.596452170636412 | -0.190246572 | 0.331089623665971 | 1.0729603666439 | 0.826755254402107 | 1.3924845862893 | 0.999489402457283 |
| ebi-fl187-GCST90094753 | prot-a-478 | Cadherin-12 \|\| id:prot-a-478 | F-deep fried food liking (derived food-liking factor) \|\| id:ebi-fl187-GCST90094753 | Weighted median | 8 | 0.139929354215305 | 0.13016535834594 | 0.282369245718056 | -0.115194748 | 0.395053456573347 | 1.15019253973243 | 0.891192568908059 | 1.48446354313422 | 0.999489402457283 |
| ebi-fl187-GCST90094753 | prot-a-516 | Centrin-2 \|\| id:prot-a-516 | F-deep fried food liking (derived food-liking factor) \|\| id:ebi-fl187-GCST90094753 | Weighted median | 8 | -0.006342632 | 0.135105221481472 | 0.962556371830767 | -0.271148866 | 0.258463602351777 | 0.993677440277944 | 0.762502977297608 | 1.29493901626032 | 0.999489402457283 |
| ebi-fl187-GCST90094753 | prot-a-519 | Cryptic protein \|\| id:prot-a-519 | F-deep fried food liking (derived food-liking factor) \|\| id:ebi-fl187-GCST90094753 | Weighted median | 8 | 0.0876880088720786 | 0.127644759837645 | 0.492102185447317 | -0.16249572 | 0.337871738153864 | 1.09164748456303 | 0.850019727953791 | 1.4019606738087 | 0.999489402457283 |
| ebi-fl187-GCST90094753 | prot-a-583 | Chloride intracellular channel protein 4 \|\| id:prot-a-583 | F-deep fried food liking (derived food-liking factor) \|\| id:ebi-fl187-GCST90094753 | Weighted median | 8 | 0.219591988523783 | 0.130832210459648 | 0.0932642564267219 | -0.036839144 | 0.476023121024693 | 1.24556842068624 | 0.963831160927589 | 1.60966023252218 | 0.999489402457283 |
| ebi-fl187-GCST90094753 | prot-a-602 | CCR4-NOT transcription complex subunit 1 \|\| id:prot-a-602 | F-deep fried food liking (derived food-liking factor) \|\| id:ebi-fl187-GCST90094753 | Weighted median | 8 | 0.207835125572692 | 0.124919712462195 | 0.0961624734055143 | -0.037007511 | 0.452677761998594 | 1.23101019071611 | 0.96366889734624 | 1.57251738000469 | 0.999489402457283 |
| ebi-fl187-GCST90094753 | prot-a-606 | Ciliary neurotrophic factor receptor subunit alpha \|\| id:prot-a-606 | F-deep fried food liking (derived food-liking factor) \|\| id:ebi-fl187-GCST90094753 | Weighted median | 8 | -0.027677093 | 0.13624041121321 | 0.839018630923335 | -0.294708299 | 0.239354112659892 | 0.972702408202691 | 0.74474879953312 | 1.2704283316958 | 0.999489402457283 |
| ebi-fl187-GCST90094753 | prot-a-626 | Collagen alpha-2(IX) chain \|\| id:prot-a-626 | F-deep fried food liking (derived food-liking factor) \|\| id:ebi-fl187-GCST90094753 | Weighted median | 8 | -0.101110658 | 0.133232931558893 | 0.447911542423448 | -0.362247204 | 0.160025887794268 | 0.903833010943243 | 0.696110265386463 | 1.17354125099304 | 0.999489402457283 |
| ebi-fl187-GCST90094753 | prot-a-647 | Probable serine carboxypeptidase CPVL \|\| id:prot-a-647 | F-deep fried food liking (derived food-liking factor) \|\| id:ebi-fl187-GCST90094753 | Weighted median | 8 | -0.139226281 | 0.137630230922028 | 0.311730947418888 | -0.408981533 | 0.130528971811984 | 0.870031134845437 | 0.664326500059119 | 1.13943095079464 | 0.999489402457283 |
| ebi-fl187-GCST90094753 | prot-a-651 | Cellular retinoic acid-binding protein 2 \|\| id:prot-a-651 | F-deep fried food liking (derived food-liking factor) \|\| id:ebi-fl187-GCST90094753 | Weighted median | 8 | 0.0203880088497704 | 0.138946152773466 | 0.883342639537383 | -0.251946451 | 0.292722468285763 | 1.02059726398127 | 0.777286360183442 | 1.34007082666447 | 0.999489402457283 |
| ebi-fl187-GCST90094753 | prot-a-671 | Cytotoxic and regulatory T-cell molecule \|\| id:prot-a-671 | F-deep fried food liking (derived food-liking factor) \|\| id:ebi-fl187-GCST90094753 | Weighted median | 8 | 0.114595651505321 | 0.127478801306209 | 0.368685214424083 | -0.135262799 | 0.364454102065491 | 1.12141990143574 | 0.873486330140464 | 1.43972784912835 | 0.999489402457283 |
| ebi-fl187-GCST90094753 | prot-a-688 | Chorionic somatomammotropin hormone \|\| id:prot-a-688 | F-deep fried food liking (derived food-liking factor) \|\| id:ebi-fl187-GCST90094753 | Weighted median | 8 | -0.053415229 | 0.128645546774854 | 0.677986404900381 | -0.305560501 | 0.198730042421101 | 0.947986299100096 | 0.736710331816283 | 1.2198526129882 | 0.999489402457283 |
| ebi-fl187-GCST90094753 | prot-a-69 | Aldo-keto reductase family 1 member C1 \|\| id:prot-a-69 | F-deep fried food liking (derived food-liking factor) \|\| id:ebi-fl187-GCST90094753 | Weighted median | 8 | -0.040207037 | 0.13082675244711 | 0.758591906037472 | -0.296627472 | 0.216213397886848 | 0.960590540866526 | 0.743320868845597 | 1.24136725588679 | 0.999489402457283 |
| ebi-fl187-GCST90094753 | prot-a-741 | Stromal cell-derived factor 1 \|\| id:prot-a-741 | F-deep fried food liking (derived food-liking factor) \|\| id:ebi-fl187-GCST90094753 | Weighted median | 8 | 0.00434654854263005 | 0.149468524730328 | 0.976800766231453 | -0.28861176 | 0.297304857014073 | 1.00435600848582 | 0.749303058411358 | 1.34622564322669 | 0.999489402457283 |
| ebi-fl187-GCST90094753 | prot-a-742 | Growth-regulated alpha protein \|\| id:prot-a-742 | F-deep fried food liking (derived food-liking factor) \|\| id:ebi-fl187-GCST90094753 | Weighted median | 8 | 0.164354123789245 | 0.130193321744902 | 0.206810208596703 | -0.090824787 | 0.419533034409253 | 1.17863162269544 | 0.913177697641445 | 1.52125101785298 | 0.999489402457283 |
| ebi-fl187-GCST90094753 | prot-a-788 | Beta-defensin 112 \|\| id:prot-a-788 | F-deep fried food liking (derived food-liking factor) \|\| id:ebi-fl187-GCST90094753 | Weighted median | 8 | 0.103245325535116 | 0.148580348365127 | 0.487131295990055 | -0.187972157 | 0.394462808330765 | 1.1087633836839 | 0.828637778457273 | 1.48358700623926 | 0.999489402457283 |
| ebi-fl187-GCST90094753 | prot-a-838 | DnaJ homolog subfamily B member 1 \|\| id:prot-a-838 | F-deep fried food liking (derived food-liking factor) \|\| id:ebi-fl187-GCST90094753 | Weighted median | 8 | 0.10468087847585 | 0.129937094556899 | 0.420457744877793 | -0.149995827 | 0.359357583807371 | 1.1103562152434 | 0.860711568291163 | 1.43240891623823 | 0.999489402457283 |
| ebi-fl187-GCST90094753 | prot-a-85 | Junctional adhesion molecule-like \|\| id:prot-a-85 | F-deep fried food liking (derived food-liking factor) \|\| id:ebi-fl187-GCST90094753 | Weighted median | 8 | -0.011050487 | 0.139210643449169 | 0.936730686746478 | -0.283903348 | 0.261802374650051 | 0.989010345833878 | 0.752839404871997 | 1.29926974841701 | 0.999489402457283 |
| ebi-fl187-GCST90094753 | prot-a-856 | Dipeptidase 1 \|\| id:prot-a-856 | F-deep fried food liking (derived food-liking factor) \|\| id:ebi-fl187-GCST90094753 | Weighted median | 8 | 0.0826854132142655 | 0.133331518851336 | 0.535159669319628 | -0.178644364 | 0.344015190162884 | 1.0862000506122 | 0.836403301857198 | 1.41060006258965 | 0.999489402457283 |
| ebi-fl187-GCST90094753 | prot-a-860 | Dipeptidyl peptidase 2 \|\| id:prot-a-860 | F-deep fried food liking (derived food-liking factor) \|\| id:ebi-fl187-GCST90094753 | Weighted median | 8 | 0.139692281042755 | 0.130319759874653 | 0.283756228051654 | -0.115734448 | 0.395119010397075 | 1.14991989225797 | 0.89071172189644 | 1.48456085858532 | 0.999489402457283 |
| ebi-fl187-GCST90094753 | prot-a-861 | Dermatopontin \|\| id:prot-a-861 | F-deep fried food liking (derived food-liking factor) \|\| id:ebi-fl187-GCST90094753 | Weighted median | 8 | 0.0640852322591086 | 0.134722468574049 | 0.634299999970573 | -0.199970806 | 0.328141270664245 | 1.06618326809751 | 0.818754655332927 | 1.3883850965206 | 0.999489402457283 |
| ebi-fl187-GCST90094753 | prot-a-877 | Dual specificity protein phosphatase 6 \|\| id:prot-a-877 | F-deep fried food liking (derived food-liking factor) \|\| id:ebi-fl187-GCST90094753 | Weighted median | 8 | 0.0780535505925097 | 0.127830948113768 | 0.541464559071564 | -0.172495108 | 0.328602208895496 | 1.08118055497921 | 0.841562405968645 | 1.38902520380491 | 0.999489402457283 |
| ebi-fl187-GCST90094753 | prot-a-879 | Dynactin-associated protein \|\| id:prot-a-879 | F-deep fried food liking (derived food-liking factor) \|\| id:ebi-fl187-GCST90094753 | Weighted median | 8 | -0.025158939 | 0.121931214401231 | 0.836527563557558 | -0.264144119 | 0.213826241297964 | 0.975154909634293 | 0.767862867934365 | 1.23840745202586 | 0.999489402457283 |
| ebi-fl187-GCST90094753 | prot-a-937 | EMI domain-containing protein 1 \|\| id:prot-a-937 | F-deep fried food liking (derived food-liking factor) \|\| id:ebi-fl187-GCST90094753 | Weighted median | 8 | -0.249101084 | 0.136544916478265 | 0.0681043038244313 | -0.51672912 | 0.0185269522473226 | 0.779501174266366 | 0.596468336888568 | 1.01869964104425 | 0.999489402457283 |
| ebi-fl187-GCST90094753 | prot-b-1 | SRC proto-oncogene, non-receptor tyrosine kinase \|\| id:prot-b-1 | F-deep fried food liking (derived food-liking factor) \|\| id:ebi-fl187-GCST90094753 | Weighted median | 7 | -0.03626757 | 0.137875923986601 | 0.792515908599115 | -0.306504381 | 0.233969241014658 | 0.96438221920869 | 0.736015293679861 | 1.26360562438314 | 0.999489402457283 |
| ebi-fl187-GCST90094753 | prot-b-10 | coagulation factor III, tissue factor \|\| id:prot-b-10 | F-deep fried food liking (derived food-liking factor) \|\| id:ebi-fl187-GCST90094753 | Weighted median | 7 | -0.07981006 | 0.148767389755178 | 0.591629963147492 | -0.371394144 | 0.211774023940738 | 0.923291699777261 | 0.689772018540715 | 1.23586857681045 | 0.999489402457283 |
| ebi-fl187-GCST90094753 | prot-b-29 | myeloperoxidase \|\| id:prot-b-29 | F-deep fried food liking (derived food-liking factor) \|\| id:ebi-fl187-GCST90094753 | Weighted median | 7 | 0.149223194303233 | 0.13557367620813 | 0.271036080988153 | -0.116501211 | 0.414947599671169 | 1.16093207372021 | 0.890029019093296 | 1.5142913892463 | 0.999489402457283 |
| ebi-fl187-GCST90094753 | prot-b-33 | adrenomedullin \|\| id:prot-b-33 | F-deep fried food liking (derived food-liking factor) \|\| id:ebi-fl187-GCST90094753 | Weighted median | 7 | 0.0478841328504327 | 0.13297264137196 | 0.718768502308483 | -0.212742244 | 0.308510509939474 | 1.04904909795362 | 0.808364470965418 | 1.36139581766005 | 0.999489402457283 |
| ebi-fl187-GCST90094753 | ukb-rsfMRI191-edge-pheno102 | Net25_Pair11_15 (Postcentral\|Precentral)&(Cerebellum) (Motor)&(Subcortical-cerebellum) \|\| id:ukb-rsfMRI191-edge-pheno102 | F-deep fried food liking (derived food-liking factor) \|\| id:ebi-fl187-GCST90094753 | Weighted median | 8 | -0.013061439 | 0.0411361422061268 | 0.750851066460576 | -0.093688278 | 0.0675653993082403 | 0.987023491010703 | 0.910566561507661 | 1.06990022804473 | 0.999489402457283 |
| ebi-fl187-GCST90094753 | ukb-rsfMRI191-edge-pheno1167 | Net100_Pair11_45 (Angular\|Temporal)&(Frontal) (Default_mode\|Central_executive)&(Salience\|Default_mode) \|\| id:ukb-rsfMRI191-edge-pheno1167 | F-deep fried food liking (derived food-liking factor) \|\| id:ebi-fl187-GCST90094753 | Weighted median | 8 | 0.0206702016331427 | 0.0376595727713667 | 0.583094814561231 | -0.053142561 | 0.0944829642650213 | 1.02088530980421 | 0.948244820117346 | 1.09909043916006 | 0.999489402457283 |
| ebi-fl187-GCST90094753 | ukb-rsfMRI191-edge-pheno1317 | Net100_Pair26_48 (Parietal\|Frontal)&(Temporal) (Attention\|Central_executive)&(Default_mode\|Central_executive) \|\| id:ukb-rsfMRI191-edge-pheno1317 | F-deep fried food liking (derived food-liking factor) \|\| id:ebi-fl187-GCST90094753 | Weighted median | 8 | 0.0150928945094635 | 0.0450208518536592 | 0.737442305731389 | -0.073147975 | 0.103333764142636 | 1.01520736742595 | 0.929463282405034 | 1.10886144550979 | 0.999489402457283 |
| ebi-fl187-GCST90094753 | ukb-rsfMRI191-edge-pheno286 | Net100_Pair10_13 (OccipitalPrecuneus)&(Frontal_Sup) (Default_mode\|Central_executive)&(Default_mode) \|\| id:ukb-rsfMRI191-edge-pheno286 | F-deep fried food liking (derived food-liking factor) \|\| id:ebi-fl187-GCST90094753 | Weighted median | 8 | -0.026522959 | 0.0409154729367585 | 0.516831113398378 | -0.106717286 | 0.0536713680369478 | 0.973825685593744 | 0.898779734801344 | 1.05513779316768 | 0.999489402457283 |
| ebi-fl187-GCST90094753 | ukb-rsfMRI191-edge-pheno599 | Net100_Pair11_29 (Angular\|Temporal)&(Parietal) (Default_mode\|Central_executive)&(Central_executive\|Salience) \|\| id:ukb-rsfMRI191-edge-pheno599 | F-deep fried food liking (derived food-liking factor) \|\| id:ebi-fl187-GCST90094753 | Weighted median | 8 | 0.0117356228028818 | 0.0430820911943138 | 0.785313381236613 | -0.072705276 | 0.0961765215437369 | 1.0118047553975 | 0.929874846136068 | 1.10095338883399 | 0.999489402457283 |
| ebi-fl187-GCST90094753 | ukb-rsfMRI191-edge-pheno621 | Net100_Pair5_30 (Precuneus\|Angular\|Cingulate)&(Paracentral) (Default_mode\|Central_executive)&(Motor) \|\| id:ukb-rsfMRI191-edge-pheno621 | F-deep fried food liking (derived food-liking factor) \|\| id:ebi-fl187-GCST90094753 | Weighted median | 8 | 0.0413362259542284 | 0.0464019160803035 | 0.373020296658143 | -0.04961153 | 0.132283981471623 | 1.04220246216002 | 0.951599020794748 | 1.14143241890399 | 0.999489402457283 |
| ebi-fl187-GCST90094753 | ukb-rsfMRI191-edge-pheno624 | Net100_Pair8_30 (Calcarine\|Lingual)&(Paracentral) (Visual)&(Motor) \|\| id:ukb-rsfMRI191-edge-pheno624 | F-deep fried food liking (derived food-liking factor) \|\| id:ebi-fl187-GCST90094753 | Weighted median | 8 | -0.007855547 | 0.0407742633561848 | 0.847225678061798 | -0.087773104 | 0.0720620087879805 | 0.992175226786865 | 0.915968683172957 | 1.07472198420531 | 0.999489402457283 |
| ebi-fl187-GCST90094753 | ukb-rsfMRI191-edge-pheno882 | Net100_Pair6_38 (Postcentral\|Precentral)&(Subcortical) (Motor)&(Subcortical-cerebellum) \|\| id:ukb-rsfMRI191-edge-pheno882 | F-deep fried food liking (derived food-liking factor) \|\| id:ebi-fl187-GCST90094753 | Weighted median | 8 | 0.0658614094347141 | 0.0405545055766536 | 0.104371112385859 | -0.013625421 | 0.145348240364955 | 1.06807868127983 | 0.986466984394103 | 1.15644222001525 | 0.999489402457283 |
| ebi-fl187-GCST90094753 | ukb-rsfMRI191-edge-pheno899 | Net100_Pair23_38 (Cerebellum)&(Subcortical) (Subcortical-cerebellum)&(Subcortical-cerebellum) \|\| id:ukb-rsfMRI191-edge-pheno899 | F-deep fried food liking (derived food-liking factor) \|\| id:ebi-fl187-GCST90094753 | Weighted median | 8 | -0.037193243 | 0.044991028909421 | 0.408417712404682 | -0.12537566 | 0.0509891735413172 | 0.963489929556956 | 0.882165446250436 | 1.05231150041455 | 0.999489402457283 |
| ebi-fl187-GCST90094753 | ebi-a-GCST90001392 | IgD+ CD38+ B cell Absolute Count \|\| id:ebi-a-GCST90001392 | F-deep fried food liking (derived food-liking factor) \|\| id:ebi-fl187-GCST90094753 | Weighted mode | 8 | 0.310702570376483 | 0.20564134855158 | 0.174566487772136 | -0.092354473 | 0.71375961353758 | 1.36438335280356 | 0.911781890389542 | 2.04165267267171 | 0.999228396881742 |
| ebi-fl187-GCST90094753 | ebi-a-GCST90001402 | Switched memory B cell %B cell \|\| id:ebi-a-GCST90001402 | F-deep fried food liking (derived food-liking factor) \|\| id:ebi-fl187-GCST90094753 | Weighted mode | 8 | -0.162006481 | 0.239708871890158 | 0.520833095428954 | -0.63183587 | 0.307822907677064 | 0.850435692655048 | 0.531614928606494 | 1.36046004057388 | 0.999228396881742 |
| ebi-fl187-GCST90094753 | ebi-a-GCST90001407 | Memory B cell Absolute Count \|\| id:ebi-a-GCST90001407 | F-deep fried food liking (derived food-liking factor) \|\| id:ebi-fl187-GCST90094753 | Weighted mode | 8 | 0.314871211366044 | 0.197303411365194 | 0.154547639559561 | -0.071843475 | 0.701585897641824 | 1.3700828484956 | 0.930676558643313 | 2.01694884684554 | 0.999228396881742 |
| ebi-fl187-GCST90094753 | ebi-a-GCST90001411 | IgD+ CD24+ B cell %B cell \|\| id:ebi-a-GCST90001411 | F-deep fried food liking (derived food-liking factor) \|\| id:ebi-fl187-GCST90094753 | Weighted mode | 8 | 0.312920619227931 | 0.218723910594776 | 0.195612199473565 | -0.115778246 | 0.741619483993691 | 1.36741298042112 | 0.890672712047887 | 2.09933259853104 | 0.999228396881742 |
| ebi-fl187-GCST90094753 | ebi-a-GCST90001418 | CD24+ CD27+ B cell Absolute Count \|\| id:ebi-a-GCST90001418 | F-deep fried food liking (derived food-liking factor) \|\| id:ebi-fl187-GCST90094753 | Weighted mode | 8 | 0.426511478717567 | 0.222204992064911 | 0.0964076903598665 | -0.009010306 | 0.862033263164791 | 1.53190411113652 | 0.991030165431288 | 2.36797050945034 | 0.999228396881742 |
| ebi-fl187-GCST90094753 | ebi-a-GCST90001436 | Memory B cell %lymphocyte \|\| id:ebi-a-GCST90001436 | F-deep fried food liking (derived food-liking factor) \|\| id:ebi-fl187-GCST90094753 | Weighted mode | 8 | 0.163904694287002 | 0.207891684928953 | 0.456320091000445 | -0.243563008 | 0.57137239674775 | 1.17810202988815 | 0.78383008677403 | 1.77069548138781 | 0.999228396881742 |
| ebi-fl187-GCST90094753 | ebi-a-GCST90001439 | IgD+ CD24+ B cell %lymphocyte \|\| id:ebi-a-GCST90001439 | F-deep fried food liking (derived food-liking factor) \|\| id:ebi-fl187-GCST90094753 | Weighted mode | 8 | 0.18214239542092 | 0.225544128168412 | 0.445908718954452 | -0.259924096 | 0.624208886631007 | 1.19978502561049 | 0.771110114086942 | 1.86676854755514 | 0.999228396881742 |
| ebi-fl187-GCST90094753 | ebi-a-GCST90001457 | CD11c+ HLA DR++ monocyte %monocyte \|\| id:ebi-a-GCST90001457 | F-deep fried food liking (derived food-liking factor) \|\| id:ebi-fl187-GCST90094753 | Weighted mode | 8 | 0.00531446546028169 | 0.24812603634634 | 0.983509630566841 | -0.481012566 | 0.491641496699108 | 1.00532861228167 | 0.618157150028269 | 1.63499786199347 | 0.999228396881742 |
| ebi-fl187-GCST90094753 | ebi-a-GCST90001483 | CD39+ resting CD4 regulatory T cell Absolute Count \|\| id:ebi-a-GCST90001483 | F-deep fried food liking (derived food-liking factor) \|\| id:ebi-fl187-GCST90094753 | Weighted mode | 8 | -0.223718443 | 0.242624321226207 | 0.387164771088009 | -0.699262113 | 0.251825226343921 | 0.799540218618782 | 0.496951862922597 | 1.28637119384044 | 0.999228396881742 |
| ebi-fl187-GCST90094753 | ebi-a-GCST90001525 | CD33dim HLA DR+ CD11b+ Absolute Count \|\| id:ebi-a-GCST90001525 | F-deep fried food liking (derived food-liking factor) \|\| id:ebi-fl187-GCST90094753 | Weighted mode | 8 | 0.242956878190302 | 0.319190691498524 | 0.471417130653486 | -0.382656877 | 0.86857063352741 | 1.27501364203737 | 0.682046885013969 | 2.38350152035092 | 0.999228396881742 |
| ebi-fl187-GCST90094753 | ebi-a-GCST90001540 | Naive CD4+ T cell Absolute Count \|\| id:ebi-a-GCST90001540 | F-deep fried food liking (derived food-liking factor) \|\| id:ebi-fl187-GCST90094753 | Weighted mode | 8 | 0.0610095816886023 | 0.210352823007252 | 0.78019321331679 | -0.351281951 | 0.473301114782815 | 1.0629090986024 | 0.70378529262622 | 1.60528468515722 | 0.999228396881742 |
| ebi-fl187-GCST90094753 | ebi-a-GCST90001624 | HLA DR+ CD4+ T cell Absolute Count \|\| id:ebi-a-GCST90001624 | F-deep fried food liking (derived food-liking factor) \|\| id:ebi-fl187-GCST90094753 | Weighted mode | 8 | 0.0250392086221101 | 0.263136930313562 | 0.926857189069692 | -0.490709175 | 0.540787592036691 | 1.02535532250566 | 0.612192089005803 | 1.71735890788475 | 0.999228396881742 |
| ebi-fl187-GCST90094753 | ebi-a-GCST90001672 | CD39+ CD8+ T cell Absolute Count \|\| id:ebi-a-GCST90001672 | F-deep fried food liking (derived food-liking factor) \|\| id:ebi-fl187-GCST90094753 | Weighted mode | 8 | 0.199923647183421 | 0.230449281994738 | 0.414395007238634 | -0.251756946 | 0.651604239893108 | 1.22130950417955 | 0.777433673839633 | 1.91861628225148 | 0.999228396881742 |
| ebi-fl187-GCST90094753 | ebi-a-GCST90001722 | CD19 on CD20- CD38- B cell \|\| id:ebi-a-GCST90001722 | F-deep fried food liking (derived food-liking factor) \|\| id:ebi-fl187-GCST90094753 | Weighted mode | 8 | 0.0401241490330579 | 0.225898475049989 | 0.864051768310692 | -0.402636862 | 0.482885160131036 | 1.04093999786493 | 0.66855483286951 | 1.62074376832234 | 0.999228396881742 |
| ebi-fl187-GCST90094753 | ebi-a-GCST90001742 | CD19 on transitional B cell \|\| id:ebi-a-GCST90001742 | F-deep fried food liking (derived food-liking factor) \|\| id:ebi-fl187-GCST90094753 | Weighted mode | 8 | -0.017137583 | 0.210957969949741 | 0.937527451477028 | -0.430615204 | 0.396340037953228 | 0.983008429936178 | 0.650109021839624 | 1.48637465542505 | 0.999228396881742 |
| ebi-fl187-GCST90094753 | ebi-a-GCST90001784 | CD25 on IgD+ CD38dim B cell \|\| id:ebi-a-GCST90001784 | F-deep fried food liking (derived food-liking factor) \|\| id:ebi-fl187-GCST90094753 | Weighted mode | 8 | -0.006756882 | 0.21593772524838 | 0.975911048510543 | -0.429994823 | 0.416481059654423 | 0.993265894565632 | 0.650512462209957 | 1.51661527583286 | 0.999228396881742 |
| ebi-fl187-GCST90094753 | ebi-a-GCST90001794 | CD25 on IgD+ B cell \|\| id:ebi-a-GCST90001794 | F-deep fried food liking (derived food-liking factor) \|\| id:ebi-fl187-GCST90094753 | Weighted mode | 8 | -0.025430659 | 0.187454072505901 | 0.895906245869403 | -0.392840641 | 0.341979322878611 | 0.974889976240828 | 0.675136327831453 | 1.40773118938448 | 0.999228396881742 |
| ebi-fl187-GCST90094753 | ebi-a-GCST90001802 | CD27 on IgD- CD38- B cell \|\| id:ebi-a-GCST90001802 | F-deep fried food liking (derived food-liking factor) \|\| id:ebi-fl187-GCST90094753 | Weighted mode | 8 | -0.069564639 | 0.249044890341764 | 0.788067732814742 | -0.557692624 | 0.418563345718199 | 0.932799835859163 | 0.572528579416523 | 1.51977659292683 | 0.999228396881742 |
| ebi-fl187-GCST90094753 | ebi-a-GCST90001804 | CD27 on IgD- CD38dim B cell \|\| id:ebi-a-GCST90001804 | F-deep fried food liking (derived food-liking factor) \|\| id:ebi-fl187-GCST90094753 | Weighted mode | 8 | -0.13048658 | 0.216821282476518 | 0.566269855820751 | -0.555456293 | 0.294483133863888 | 0.87766827136214 | 0.573810375540132 | 1.34243232153255 | 0.999228396881742 |
| ebi-fl187-GCST90094753 | ebi-a-GCST90001805 | CD27 on memory B cell \|\| id:ebi-a-GCST90001805 | F-deep fried food liking (derived food-liking factor) \|\| id:ebi-fl187-GCST90094753 | Weighted mode | 8 | -0.078271177 | 0.256406671315913 | 0.769043791631981 | -0.580828253 | 0.4242858989054 | 0.924713631588415 | 0.559434821250713 | 1.52849852737755 | 0.999228396881742 |
| ebi-fl187-GCST90094753 | ebi-a-GCST90001806 | CD27 on unswitched memory B cell \|\| id:ebi-a-GCST90001806 | F-deep fried food liking (derived food-liking factor) \|\| id:ebi-fl187-GCST90094753 | Weighted mode | 8 | 0.0749212892186404 | 0.20734439457725 | 0.728507079699463 | -0.331473724 | 0.481316302590051 | 1.07779931311977 | 0.71786501848048 | 1.6182030457764 | 0.999228396881742 |
| ebi-fl187-GCST90094753 | ebi-a-GCST90001839 | CD3 on Effector Memory CD8+ T cell \|\| id:ebi-a-GCST90001839 | F-deep fried food liking (derived food-liking factor) \|\| id:ebi-fl187-GCST90094753 | Weighted mode | 8 | -0.133009714 | 0.264947600590274 | 0.631054397948777 | -0.652307011 | 0.386287582992809 | 0.875456587738486 | 0.52084279941864 | 1.47150779058516 | 0.999228396881742 |
| ebi-fl187-GCST90094753 | ebi-a-GCST90001859 | CD3 on CD8+ T cell \|\| id:ebi-a-GCST90001859 | F-deep fried food liking (derived food-liking factor) \|\| id:ebi-fl187-GCST90094753 | Weighted mode | 8 | 0.224356005269629 | 0.253778702231022 | 0.406001507297754 | -0.273050251 | 0.721762261642431 | 1.25151648659358 | 0.761054542837668 | 2.05805685145176 | 0.999228396881742 |
| ebi-fl187-GCST90094753 | ebi-a-GCST90001905 | CD86 on monocyte \|\| id:ebi-a-GCST90001905 | F-deep fried food liking (derived food-liking factor) \|\| id:ebi-fl187-GCST90094753 | Weighted mode | 8 | 0.0706706842549695 | 0.252137716466103 | 0.787360460688045 | -0.42351924 | 0.564860608528531 | 1.07322773687505 | 0.654738578362828 | 1.75920254779863 | 0.999228396881742 |
| ebi-fl187-GCST90094753 | ebi-a-GCST90001943 | CD25 on activated & secreting CD4 regulatory T cell \|\| id:ebi-a-GCST90001943 | F-deep fried food liking (derived food-liking factor) \|\| id:ebi-fl187-GCST90094753 | Weighted mode | 8 | -0.186119953 | 0.251920305599335 | 0.484055590688474 | -0.679883752 | 0.307643845806516 | 0.830174007006823 | 0.506675888928567 | 1.36021645586323 | 0.999228396881742 |
| ebi-fl187-GCST90094753 | ebi-a-GCST90001949 | CD33 on CD33dim HLA DR+ CD11b- \|\| id:ebi-a-GCST90001949 | F-deep fried food liking (derived food-liking factor) \|\| id:ebi-fl187-GCST90094753 | Weighted mode | 8 | 0.38512878381053 | 0.351654209189814 | 0.309677841241129 | -0.304113466 | 1.07437103382257 | 1.46980359616097 | 0.73777714893048 | 2.92815061352798 | 0.999228396881742 |
| ebi-fl187-GCST90094753 | ebi-a-GCST90001955 | CD33 on Immature Myeloid-Derived Suppressor Cells \|\| id:ebi-a-GCST90001955 | F-deep fried food liking (derived food-liking factor) \|\| id:ebi-fl187-GCST90094753 | Weighted mode | 8 | 0.183365947638815 | 0.286620891102097 | 0.542697401148893 | -0.378410999 | 0.745142894198925 | 1.20125392369307 | 0.684948929535938 | 2.10674245474864 | 0.999228396881742 |
| ebi-fl187-GCST90094753 | ebi-a-GCST90001956 | CD33 on CD33+ HLA DR+ \|\| id:ebi-a-GCST90001956 | F-deep fried food liking (derived food-liking factor) \|\| id:ebi-fl187-GCST90094753 | Weighted mode | 8 | 0.177626239079293 | 0.359143525754139 | 0.636037387054789 | -0.526295071 | 0.881547549557405 | 1.19437882566915 | 0.590789753800786 | 2.4146335816241 | 0.999228396881742 |
| ebi-fl187-GCST90094753 | ebi-a-GCST90001957 | CD33 on CD33+ HLA DR+ CD14- \|\| id:ebi-a-GCST90001957 | F-deep fried food liking (derived food-liking factor) \|\| id:ebi-fl187-GCST90094753 | Weighted mode | 8 | 0.156705307389176 | 0.350036473892179 | 0.66790830123827 | -0.529366181 | 0.842776796217846 | 1.1696508756372 | 0.588978156686877 | 2.32280799439937 | 0.999228396881742 |
| ebi-fl187-GCST90094753 | ebi-a-GCST90002064 | CD4 on resting CD4 regulatory T cell \|\| id:ebi-a-GCST90002064 | F-deep fried food liking (derived food-liking factor) \|\| id:ebi-fl187-GCST90094753 | Weighted mode | 8 | -0.058747071 | 0.244294410081082 | 0.81685178270048 | -0.537564115 | 0.420069972533673 | 0.942945236981589 | 0.5841694903934 | 1.5220680548508 | 0.999228396881742 |
| ebi-fl187-GCST90094753 | ebi-a-GCST90002080 | SSC-A on T cell \|\| id:ebi-a-GCST90002080 | F-deep fried food liking (derived food-liking factor) \|\| id:ebi-fl187-GCST90094753 | Weighted mode | 8 | -0.07417805 | 0.217428949886393 | 0.742994456517327 | -0.500338792 | 0.351982691774922 | 0.928506358563372 | 0.606325206915788 | 1.42188391321879 | 0.999228396881742 |
| ebi-fl187-GCST90094753 | ebi-a-GCST90002121 | CD8 on CD39+ CD8+ T cell \|\| id:ebi-a-GCST90002121 | F-deep fried food liking (derived food-liking factor) \|\| id:ebi-fl187-GCST90094753 | Weighted mode | 8 | 0.383478428027296 | 0.30223624450566 | 0.24507148452857 | -0.208904611 | 0.97586146725839 | 1.46737989782822 | 0.811472637349753 | 2.65345208876378 | 0.999228396881742 |
| ebi-fl187-GCST90094753 | ebi-cfmet338-GCST90026053 | 1-palmitoyl-2-docosahexaenoyl-gpc (16:0/22:6) levels \|\| id:ebi-cfmet338-GCST90026053 | F-deep fried food liking (derived food-liking factor) \|\| id:ebi-fl187-GCST90094753 | Weighted mode | 8 | -0.106987039 | 0.0889603721176866 | 0.268212602050128 | -0.281349369 | 0.0673752899407993 | 0.898537318488744 | 0.754764598239933 | 1.06969684932187 | 0.999228396881742 |
| ebi-fl187-GCST90094753 | ebi-cfmet338-GCST90026063 | 1-stearoyl-2-oleoyl-gpc (18:0/18:1) levels \|\| id:ebi-cfmet338-GCST90026063 | F-deep fried food liking (derived food-liking factor) \|\| id:ebi-fl187-GCST90094753 | Weighted mode | 8 | 0.00880098739238966 | 0.0645518156135468 | 0.895390839789457 | -0.117720571 | 0.135322545994941 | 1.00883982994926 | 0.888944414575258 | 1.14490600965004 | 0.999228396881742 |
| ebi-fl187-GCST90094753 | ebi-cfmet338-GCST90026113 | Adenosine 3',5'-cyclic monophosphate (camp) levels \|\| id:ebi-cfmet338-GCST90026113 | F-deep fried food liking (derived food-liking factor) \|\| id:ebi-fl187-GCST90094753 | Weighted mode | 8 | 0.0697416881654814 | 0.129687600693209 | 0.607403123229982 | -0.184446009 | 0.323929385524172 | 1.07223117547686 | 0.831564835543787 | 1.38254967565177 | 0.999228396881742 |
| ebi-fl187-GCST90094753 | ebi-cfmet338-GCST90026117 | Alpha-ketoglutaramate levels \|\| id:ebi-cfmet338-GCST90026117 | F-deep fried food liking (derived food-liking factor) \|\| id:ebi-fl187-GCST90094753 | Weighted mode | 8 | -0.037652819 | 0.062148045809298 | 0.563730558088635 | -0.159462989 | 0.0841573505090005 | 0.963047234292599 | 0.852601522393329 | 1.08780004623398 | 0.999228396881742 |
| ebi-fl187-GCST90094753 | ebi-cfmet338-GCST90026124 | Arginine levels \|\| id:ebi-cfmet338-GCST90026124 | F-deep fried food liking (derived food-liking factor) \|\| id:ebi-fl187-GCST90094753 | Weighted mode | 8 | -0.121823257 | 0.0401282509510531 | 0.0189575801553835 | -0.200474629 | -0.043171885 | 0.885304826207047 | 0.818342252127622 | 0.95774675359578 | 0.999228396881742 |
| ebi-fl187-GCST90094753 | ebi-cfmet338-GCST90026169 | Glutamate levels \|\| id:ebi-cfmet338-GCST90026169 | F-deep fried food liking (derived food-liking factor) \|\| id:ebi-fl187-GCST90094753 | Weighted mode | 8 | -0.065773689 | 0.0445002639529388 | 0.182920476352109 | -0.152994206 | 0.0214468286040953 | 0.936342745218226 | 0.858134693750951 | 1.02167846482297 | 0.999228396881742 |
| ebi-fl187-GCST90094753 | ebi-cfmet338-GCST90026170 | Glutamine levels \|\| id:ebi-cfmet338-GCST90026170 | F-deep fried food liking (derived food-liking factor) \|\| id:ebi-fl187-GCST90094753 | Weighted mode | 8 | -0.042747907 | 0.032434961230889 | 0.229008483308996 | -0.106320431 | 0.0208246168202662 | 0.958152903079471 | 0.899136490522099 | 1.021042962 | 0.999228396881742 |
| ebi-fl187-GCST90094753 | ebi-cfmet338-GCST90026191 | Kynurenate levels \|\| id:ebi-cfmet338-GCST90026191 | F-deep fried food liking (derived food-liking factor) \|\| id:ebi-fl187-GCST90094753 | Weighted mode | 8 | 0.376203395564932 | 0.208088004089656 | 0.113562621592686 | -0.031649092 | 0.784055883580658 | 1.45674339878713 | 0.968846497986346 | 2.1903380301425 | 0.999228396881742 |
| ebi-fl187-GCST90094753 | ebi-cfmet338-GCST90026202 | Methylsuccinoylcarnitine levels \|\| id:ebi-cfmet338-GCST90026202 | F-deep fried food liking (derived food-liking factor) \|\| id:ebi-fl187-GCST90094753 | Weighted mode | 8 | 0.286062122435764 | 0.175566039769337 | 0.14725762501941 | -0.058047316 | 0.630171560383664 | 1.33117514852637 | 0.943605299212544 | 1.8779327304891 | 0.999228396881742 |
| ebi-fl187-GCST90094753 | ebi-cfmet338-GCST90026222 | N-acetylputrescine levels \|\| id:ebi-cfmet338-GCST90026222 | F-deep fried food liking (derived food-liking factor) \|\| id:ebi-fl187-GCST90094753 | Weighted mode | 8 | 0.0189436440902757 | 0.0535371189278096 | 0.73387578462288 | -0.085989109 | 0.123876397188783 | 1.01912421332685 | 0.91760422478579 | 1.13187595930209 | 0.999228396881742 |
| ebi-fl187-GCST90094753 | ebi-cfmet338-GCST90026252 | Propionylcarnitine (c3) levels \|\| id:ebi-cfmet338-GCST90026252 | F-deep fried food liking (derived food-liking factor) \|\| id:ebi-fl187-GCST90094753 | Weighted mode | 8 | -0.092531468 | 0.105211089964317 | 0.408314359812345 | -0.298745204 | 0.113682268558193 | 0.911620523846429 | 0.741748379804125 | 1.12039608326141 | 0.999228396881742 |
| ebi-fl187-GCST90094753 | ebi-cfmet338-GCST90026316 | Ethyl beta-glucopyranoside levels \|\| id:ebi-cfmet338-GCST90026316 | F-deep fried food liking (derived food-liking factor) \|\| id:ebi-fl187-GCST90094753 | Weighted mode | 8 | 0.445459362280155 | 0.334546810927319 | 0.224737390910808 | -0.210252387 | 1.1011711116977 | 1.5612071908348 | 0.810379690747372 | 3.00768629883412 | 0.999228396881742 |
| ebi-fl187-GCST90094753 | ebi-gm430-GCST90011315 | F_Prevotellaceae abundance \|\| id:ebi-gm430-GCST90011315 | F-deep fried food liking (derived food-liking factor) \|\| id:ebi-fl187-GCST90094753 | Weighted mode | 8 | -0.18699132 | 0.162390696381632 | 0.287337924706262 | -0.505277085 | 0.131294444430853 | 0.829450935592514 | 0.603338376017563 | 1.14030348789759 | 0.999228396881742 |
| ebi-fl187-GCST90094753 | ebi-gm430-GCST90011354 | OTU97_1 (Bacteroides) abundance \|\| id:ebi-gm430-GCST90011354 | F-deep fried food liking (derived food-liking factor) \|\| id:ebi-fl187-GCST90094753 | Weighted mode | 8 | -0.107931103 | 0.110794777784713 | 0.362433618121194 | -0.325088868 | 0.10922666127356 | 0.89768944224382 | 0.722463147192483 | 1.11541514310809 | 0.999228396881742 |
| ebi-fl187-GCST90094753 | ebi-gm430-GCST90011419 | OTU99_1 (Bacteroides) abundance \|\| id:ebi-gm430-GCST90011419 | F-deep fried food liking (derived food-liking factor) \|\| id:ebi-fl187-GCST90094753 | Weighted mode | 8 | -0.11141319 | 0.113542713071925 | 0.359161350557674 | -0.333956907 | 0.111130527924387 | 0.894569045842911 | 0.716084639570316 | 1.11754076761161 | 0.999228396881742 |
| ebi-fl187-GCST90094753 | ebi-gm430-GCST90011435 | OTU99_155 (Butyrivibrio) abundance \|\| id:ebi-gm430-GCST90011435 | F-deep fried food liking (derived food-liking factor) \|\| id:ebi-fl187-GCST90094753 | Weighted mode | 8 | -0.34300758 | 0.348700754062671 | 0.358046006134613 | -1.026461058 | 0.34044589784407 | 0.709632832438672 | 0.358272625703822 | 1.40557419335529 | 0.999228396881742 |
| ebi-fl187-GCST90094753 | ebi-gm430-GCST90011466 | OTU99_58 (Bacteroides) abundance \|\| id:ebi-gm430-GCST90011466 | F-deep fried food liking (derived food-liking factor) \|\| id:ebi-fl187-GCST90094753 | Weighted mode | 8 | -0.08222606 | 0.221966893187047 | 0.722007689252305 | -0.517281171 | 0.352829050175306 | 0.921063719061426 | 0.596139146956782 | 1.42308784602057 | 0.999228396881742 |
| ebi-fl187-GCST90094753 | ebi-gm430-GCST90011472 | OTU99_72 (Parabacteroides) abundance \|\| id:ebi-gm430-GCST90011472 | F-deep fried food liking (derived food-liking factor) \|\| id:ebi-fl187-GCST90094753 | Weighted mode | 7 | -0.323698155 | 0.446490090043428 | 0.495751111341934 | -1.198818731 | 0.551422421533425 | 0.723468584835328 | 0.301550213391429 | 1.73572018854525 | 0.999228396881742 |
| ebi-fl187-GCST90094753 | ebi-gm430-GCST90011505 | TestASV_28 (Holdemanella) abundance \|\| id:ebi-gm430-GCST90011505 | F-deep fried food liking (derived food-liking factor) \|\| id:ebi-fl187-GCST90094753 | Weighted mode | 8 | -0.274756649 | 0.290536578565955 | 0.375799271377984 | -0.844208343 | 0.294695045086509 | 0.759756988427249 | 0.429897554988477 | 1.3427168281511 | 0.999228396881742 |
| ebi-fl187-GCST90094753 | ebi-gm430-GCST90011513 | TestASV_35 (Alphaproteobacteria) abundance \|\| id:ebi-gm430-GCST90011513 | F-deep fried food liking (derived food-liking factor) \|\| id:ebi-fl187-GCST90094753 | Weighted mode | 8 | 0.331688158323364 | 0.362749163266645 | 0.390928720077254 | -0.379300202 | 1.04267651832599 | 1.39331828604829 | 0.684340141766724 | 2.8367996084881 | 0.999228396881742 |
| ebi-fl187-GCST90094753 | ebi-gm430-GCST90011543 | G_Coprococcus prevalence \|\| id:ebi-gm430-GCST90011543 | F-deep fried food liking (derived food-liking factor) \|\| id:ebi-fl187-GCST90094753 | Weighted mode | 8 | 0.0278182157117208 | 0.409478760610254 | 0.947736631865375 | -0.774760155 | 0.830396586507818 | 1.02820875523477 | 0.460814291710633 | 2.29422841990611 | 0.999228396881742 |
| ebi-fl187-GCST90094753 | ebi-gm430-GCST90011547 | G_Parasutterella prevalence \|\| id:ebi-gm430-GCST90011547 | F-deep fried food liking (derived food-liking factor) \|\| id:ebi-fl187-GCST90094753 | Weighted mode | 8 | -0.329754347 | 0.368146042217692 | 0.400156380175591 | -1.051320589 | 0.391811896086986 | 0.719100361142177 | 0.3494759300306 | 1.47965935550849 | 0.999228396881742 |
| ebi-fl187-GCST90094753 | ebi-gm430-GCST90011569 | OTU97_13 (Subdoligranulum) prevalence \|\| id:ebi-gm430-GCST90011569 | F-deep fried food liking (derived food-liking factor) \|\| id:ebi-fl187-GCST90094753 | Weighted mode | 8 | -0.063862789 | 0.382091064830792 | 0.87198560586502 | -0.812761276 | 0.685035698460543 | 0.938133713332894 | 0.443631384847474 | 1.98384265440181 | 0.999228396881742 |
| ebi-fl187-GCST90094753 | ebi-gm430-GCST90011590 | OTU97_277 (Prevotella) prevalence \|\| id:ebi-gm430-GCST90011590 | F-deep fried food liking (derived food-liking factor) \|\| id:ebi-fl187-GCST90094753 | Weighted mode | 8 | 0.0217802107613077 | 0.326579149416337 | 0.948691778086601 | -0.618314922 | 0.661875343617328 | 1.02201913097641 | 0.538851680053173 | 1.93842413923382 | 0.999228396881742 |
| ebi-fl187-GCST90094753 | ebi-gm430-GCST90011616 | OTU97_86 (Phascolarctobacterium) prevalence \|\| id:ebi-gm430-GCST90011616 | F-deep fried food liking (derived food-liking factor) \|\| id:ebi-fl187-GCST90094753 | Weighted mode | 8 | -0.288652938 | 0.400980414304566 | 0.494933767818643 | -1.07457455 | 0.497268674037131 | 0.749272204192077 | 0.341442991311575 | 1.64422421973967 | 0.999228396881742 |
| ebi-fl187-GCST90094753 | ebi-gm430-GCST90011665 | OTU99_55 (Barnesiella) prevalence \|\| id:ebi-gm430-GCST90011665 | F-deep fried food liking (derived food-liking factor) \|\| id:ebi-fl187-GCST90094753 | Weighted mode | 8 | -0.31199216 | 0.261666607417528 | 0.271979714688388 | -0.824858711 | 0.200874390507276 | 0.731987266963241 | 0.438296914811873 | 1.22247120819067 | 0.999228396881742 |
| ebi-fl187-GCST90094753 | ebi-gm430-GCST90011687 | TestASV_14 (Lachnospiraceae) prevalence \|\| id:ebi-gm430-GCST90011687 | F-deep fried food liking (derived food-liking factor) \|\| id:ebi-fl187-GCST90094753 | Weighted mode | 8 | 0.280985542083788 | 0.315424918985571 | 0.402606406663513 | -0.337247299 | 0.899218383295508 | 1.32443445523445 | 0.713732312697601 | 2.45768139539925 | 0.999228396881742 |
| ebi-fl187-GCST90094753 | ebi-gm430-GCST90011697 | TestASV_23 (Barnesiella) prevalence \|\| id:ebi-gm430-GCST90011697 | F-deep fried food liking (derived food-liking factor) \|\| id:ebi-fl187-GCST90094753 | Weighted mode | 8 | 0.0109407922763571 | 0.370347162281107 | 0.977256953178654 | -0.714939646 | 0.736821230347326 | 1.01100086161301 | 0.489221637488512 | 2.08928359634593 | 0.999228396881742 |
| ebi-fl187-GCST90094753 | ebi-gm430-GCST90011727 | TestASV_6 (Subdoligranulum) prevalence \|\| id:ebi-gm430-GCST90011727 | F-deep fried food liking (derived food-liking factor) \|\| id:ebi-fl187-GCST90094753 | Weighted mode | 8 | 0.00257647425762642 | 0.470375499693596 | 0.995782455104461 | -0.919359505 | 0.924512453657074 | 1.0025797962198 | 0.398774372241633 | 2.52063903238762 | 0.999228396881742 |
| ebi-fl187-GCST90094753 | ebi-hbsm44-GCST90104908 | Sulcal depth of the left posterior inferior temporal sulcus \|\| id:ebi-hbsm44-GCST90104908 | F-deep fried food liking (derived food-liking factor) \|\| id:ebi-fl187-GCST90094753 | Weighted mode | 7 | 0.116097835551569 | 0.124755819547261 | 0.387970584104691 | -0.128423571 | 0.3606192418642 | 1.12310574642853 | 0.879480777896284 | 1.43421726700836 | 0.999228396881742 |
| ebi-fl187-GCST90094753 | ebi-hbsm44-GCST90104918 | Sulcal depth of the left inferior frontal sulcus \|\| id:ebi-hbsm44-GCST90104918 | F-deep fried food liking (derived food-liking factor) \|\| id:ebi-fl187-GCST90094753 | Weighted mode | 7 | 0.287193497152007 | 0.237558817234941 | 0.272161490458299 | -0.178421785 | 0.752808778932492 | 1.3326820587116 | 0.836589488476134 | 2.12295456024304 | 0.999228396881742 |
| ebi-fl187-GCST90094753 | ebi-hbsm44-GCST90104925 | Sulcal depth of the right anterior inferior temporal sulcus \|\| id:ebi-hbsm44-GCST90104925 | F-deep fried food liking (derived food-liking factor) \|\| id:ebi-fl187-GCST90094753 | Weighted mode | 7 | 0.0923974336837569 | 0.0768268130484531 | 0.274405369064815 | -0.05818312 | 0.242977987258725 | 1.09680064098962 | 0.943477162181774 | 1.27504055667165 | 0.999228396881742 |
| ebi-fl187-GCST90094753 | ebi-IPs91-GCST90274770 | C-C motif chemokine 4 levels \|\| id:ebi-IPs91-GCST90274770 | F-deep fried food liking (derived food-liking factor) \|\| id:ebi-fl187-GCST90094753 | Weighted mode | 8 | 0.000960272684486507 | 0.096036149016964 | 0.992301029614597 | -0.187270579 | 0.189191124757736 | 1.00096073389392 | 0.829219336366579 | 1.20827186108275 | 0.999228396881742 |
| ebi-fl187-GCST90094753 | ebi-IPs91-GCST90274779 | C-X-C motif chemokine 1 levels \|\| id:ebi-IPs91-GCST90274779 | F-deep fried food liking (derived food-liking factor) \|\| id:ebi-fl187-GCST90094753 | Weighted mode | 8 | -0.006524251 | 0.0771026372700991 | 0.934934514174932 | -0.15764542 | 0.144596918472128 | 0.993496986135886 | 0.854152594025381 | 1.15557368597274 | 0.999228396881742 |
| ebi-fl187-GCST90094753 | ebi-IPs91-GCST90274780 | C-X-C motif chemokine 10 levels \|\| id:ebi-IPs91-GCST90274780 | F-deep fried food liking (derived food-liking factor) \|\| id:ebi-fl187-GCST90094753 | Weighted mode | 8 | 0.0733682283685234 | 0.0899840581752837 | 0.44172863793111 | -0.103000526 | 0.249736982392079 | 1.07612672435453 | 0.902126499274057 | 1.28368773980357 | 0.999228396881742 |
| ebi-fl187-GCST90094753 | ebi-IPs91-GCST90274796 | Interleukin-10 receptor subunit alpha levels \|\| id:ebi-IPs91-GCST90274796 | F-deep fried food liking (derived food-liking factor) \|\| id:ebi-fl187-GCST90094753 | Weighted mode | 8 | 0.0522003588805738 | 0.115571179329096 | 0.665166829039076 | -0.174319153 | 0.278719870365601 | 1.05358681684256 | 0.840028757506119 | 1.32143711831955 | 0.999228396881742 |
| ebi-fl187-GCST90094753 | ebi-IPs91-GCST90274807 | Interleukin-20 levels \|\| id:ebi-IPs91-GCST90274807 | F-deep fried food liking (derived food-liking factor) \|\| id:ebi-fl187-GCST90094753 | Weighted mode | 8 | -0.036935334 | 0.103854625019694 | 0.732582813570206 | -0.240490399 | 0.166619731048849 | 0.96373845445484 | 0.786242194100893 | 1.18130496628602 | 0.999228396881742 |
| ebi-fl187-GCST90094753 | ebi-IPs91-GCST90274808 | Interleukin-20 receptor subunit alpha levels \|\| id:ebi-IPs91-GCST90274808 | F-deep fried food liking (derived food-liking factor) \|\| id:ebi-fl187-GCST90094753 | Weighted mode | 8 | -0.146145853 | 0.0895956746052488 | 0.146875042435538 | -0.321753375 | 0.0294616696364049 | 0.864031672736723 | 0.724876941195641 | 1.02989995827544 | 0.999228396881742 |
| ebi-fl187-GCST90094753 | ebi-IPs91-GCST90274810 | Interleukin-24 levels \|\| id:ebi-IPs91-GCST90274810 | F-deep fried food liking (derived food-liking factor) \|\| id:ebi-fl187-GCST90094753 | Weighted mode | 8 | -0.012102329 | 0.0848353211180823 | 0.890580706938386 | -0.178379558 | 0.154174900556362 | 0.987970609807546 | 0.836624815386006 | 1.16669492452617 | 0.999228396881742 |
| ebi-fl187-GCST90094753 | ebi-IPs91-GCST90274823 | Monocyte chemoattractant protein-3 levels \|\| id:ebi-IPs91-GCST90274823 | F-deep fried food liking (derived food-liking factor) \|\| id:ebi-fl187-GCST90094753 | Weighted mode | 8 | 0.0988913789473903 | 0.111329685718019 | 0.403881154615633 | -0.119314805 | 0.317097562954708 | 1.10394638122822 | 0.887528358361275 | 1.37313653264789 | 0.999228396881742 |
| ebi-fl187-GCST90094753 | ebi-IPs91-GCST90274824 | Monocyte chemoattractant protein-4 levels \|\| id:ebi-IPs91-GCST90274824 | F-deep fried food liking (derived food-liking factor) \|\| id:ebi-fl187-GCST90094753 | Weighted mode | 8 | -0.099204839 | 0.0919197630996563 | 0.316251885558595 | -0.279367574 | 0.0809578968884144 | 0.905557195786682 | 0.756261869569126 | 1.08432524213901 | 0.999228396881742 |
| ebi-fl187-GCST90094753 | ebi-IPs91-GCST90274834 | SIR2-like protein 2 levels \|\| id:ebi-IPs91-GCST90274834 | F-deep fried food liking (derived food-liking factor) \|\| id:ebi-fl187-GCST90094753 | Weighted mode | 8 | -0.058360271 | 0.0722209764676013 | 0.445633554007246 | -0.199913384 | 0.0831928433054305 | 0.943310039364462 | 0.818801670965706 | 1.08675136106684 | 0.999228396881742 |
| ebi-fl187-GCST90094753 | ebi-met1400-GCST90199652 | 1-stearoyl-2-oleoyl-GPS (18:0/18:1) levels \|\| id:ebi-met1400-GCST90199652 | F-deep fried food liking (derived food-liking factor) \|\| id:ebi-fl187-GCST90094753 | Weighted mode | 8 | -0.0117011 | 0.132458650694628 | 0.932082373377207 | -0.271320055 | 0.247917855673043 | 0.988367091947012 | 0.762372456199489 | 1.2813546718537 | 0.999228396881742 |
| ebi-fl187-GCST90094753 | ebi-met1400-GCST90199665 | N-acetylaspartate (naa) levels \|\| id:ebi-met1400-GCST90199665 | F-deep fried food liking (derived food-liking factor) \|\| id:ebi-fl187-GCST90094753 | Weighted mode | 8 | -0.015610716 | 0.112848599184226 | 0.893872399910216 | -0.236793971 | 0.205572538229634 | 0.984510499484157 | 0.789153860187709 | 1.22822807122048 | 0.999228396881742 |
| ebi-fl187-GCST90094753 | ebi-met1400-GCST90199669 | Acetylcarnitine levels (Biocrates platform) \|\| id:ebi-met1400-GCST90199669 | F-deep fried food liking (derived food-liking factor) \|\| id:ebi-fl187-GCST90094753 | Weighted mode | 8 | -0.026627254 | 0.0999773595810533 | 0.797657492903642 | -0.222582879 | 0.169328370478948 | 0.973724125369238 | 0.800448663553189 | 1.18450903286827 | 0.999228396881742 |
| ebi-fl187-GCST90094753 | ebi-met1400-GCST90199670 | Iminodiacetate (IDA) levels \|\| id:ebi-met1400-GCST90199670 | F-deep fried food liking (derived food-liking factor) \|\| id:ebi-fl187-GCST90094753 | Weighted mode | 8 | 0.100813016309792 | 0.0939171540305985 | 0.318684552158407 | -0.083264606 | 0.284890638209765 | 1.10606980541267 | 0.920107649378506 | 1.32961661091718 | 0.999228396881742 |
| ebi-fl187-GCST90094753 | ebi-met1400-GCST90199686 | Indolepropionate levels \|\| id:ebi-met1400-GCST90199686 | F-deep fried food liking (derived food-liking factor) \|\| id:ebi-fl187-GCST90094753 | Weighted mode | 8 | -0.078391227 | 0.10634938356435 | 0.485022520989891 | -0.286836018 | 0.130053565102553 | 0.924602626556038 | 0.75063480899003 | 1.13888938641757 | 0.999228396881742 |
| ebi-fl187-GCST90094753 | ebi-met1400-GCST90199704 | 3-hydroxysebacate levels \|\| id:ebi-met1400-GCST90199704 | F-deep fried food liking (derived food-liking factor) \|\| id:ebi-fl187-GCST90094753 | Weighted mode | 8 | -0.149812845 | 0.136827055621951 | 0.309794486455494 | -0.417993874 | 0.118368183962779 | 0.860869077252912 | 0.658366261514636 | 1.12565848448145 | 0.999228396881742 |
| ebi-fl187-GCST90094753 | ebi-met1400-GCST90199713 | Docosapentaenoate n3 DPA; 22:5n3 levels \|\| id:ebi-met1400-GCST90199713 | F-deep fried food liking (derived food-liking factor) \|\| id:ebi-fl187-GCST90094753 | Weighted mode | 8 | -0.107143108 | 0.101863462006231 | 0.327826311159241 | -0.306795494 | 0.092509277469961 | 0.898397095922788 | 0.735801061551934 | 1.09692331818623 | 0.999228396881742 |
| ebi-fl187-GCST90094753 | ebi-met1400-GCST90199715 | Myristoleate (14:1n5) levels \|\| id:ebi-met1400-GCST90199715 | F-deep fried food liking (derived food-liking factor) \|\| id:ebi-fl187-GCST90094753 | Weighted mode | 8 | -0.039095506 | 0.0994392820047066 | 0.705900753971496 | -0.233996498 | 0.155805487042607 | 0.961658860870621 | 0.791364586920433 | 1.16859887335843 | 0.999228396881742 |
| ebi-fl187-GCST90094753 | ebi-met1400-GCST90199725 | Campesterol levels \|\| id:ebi-met1400-GCST90199725 | F-deep fried food liking (derived food-liking factor) \|\| id:ebi-fl187-GCST90094753 | Weighted mode | 8 | 0.0682840633765631 | 0.26088981931599 | 0.8010599716667 | -0.443059982 | 0.579628109235903 | 1.07066940325151 | 0.642068693058401 | 1.78537434304505 | 0.999228396881742 |
| ebi-fl187-GCST90094753 | ebi-met1400-GCST90199737 | N-acetylproline levels \|\| id:ebi-met1400-GCST90199737 | F-deep fried food liking (derived food-liking factor) \|\| id:ebi-fl187-GCST90094753 | Weighted mode | 8 | -0.112988102 | 0.117940190473049 | 0.369967146246562 | -0.344150875 | 0.118174671304608 | 0.893161286865631 | 0.708821976217948 | 1.12544067639091 | 0.999228396881742 |
| ebi-fl187-GCST90094753 | ebi-met1400-GCST90199775 | Gamma-glutamylisoleucine levels \|\| id:ebi-met1400-GCST90199775 | F-deep fried food liking (derived food-liking factor) \|\| id:ebi-fl187-GCST90094753 | Weighted mode | 8 | 0.166356370448849 | 0.110414677920508 | 0.175626199037198 | -0.050056398 | 0.382769139173044 | 1.18099389806467 | 0.951175778314503 | 1.46633947064706 | 0.999228396881742 |
| ebi-fl187-GCST90094753 | ebi-met1400-GCST90199777 | 1-palmitoleoyl-GPC (16:1) levels \|\| id:ebi-met1400-GCST90199777 | F-deep fried food liking (derived food-liking factor) \|\| id:ebi-fl187-GCST90094753 | Weighted mode | 8 | -0.012362898 | 0.114325110502133 | 0.916920529641975 | -0.236440114 | 0.21171431904162 | 0.987713209119458 | 0.789433156780392 | 1.23579479160444 | 0.999228396881742 |
| ebi-fl187-GCST90094753 | ebi-met1400-GCST90199782 | Glutamine degradant levels \|\| id:ebi-met1400-GCST90199782 | F-deep fried food liking (derived food-liking factor) \|\| id:ebi-fl187-GCST90094753 | Weighted mode | 8 | -0.07539985 | 0.0883056990022613 | 0.421443229666651 | -0.24847902 | 0.0976793204830628 | 0.927372602656259 | 0.77998622508194 | 1.1026091442411 | 0.999228396881742 |
| ebi-fl187-GCST90094753 | ebi-met1400-GCST90199783 | 5-methyluridine (ribothymidine) levels \|\| id:ebi-met1400-GCST90199783 | F-deep fried food liking (derived food-liking factor) \|\| id:ebi-fl187-GCST90094753 | Weighted mode | 8 | -0.067537824 | 0.101099256475521 | 0.52551755498069 | -0.265692367 | 0.130616718701712 | 0.93469236615241 | 0.766674945983557 | 1.1395309367033 | 0.999228396881742 |
| ebi-fl187-GCST90094753 | ebi-met1400-GCST90199787 | Homostachydrine levels \|\| id:ebi-met1400-GCST90199787 | F-deep fried food liking (derived food-liking factor) \|\| id:ebi-fl187-GCST90094753 | Weighted mode | 8 | 0.01063597518118 | 0.121324044940616 | 0.932597446919528 | -0.227159153 | 0.248431103264787 | 1.01069273823017 | 0.796793960126959 | 1.28201249285128 | 0.999228396881742 |
| ebi-fl187-GCST90094753 | ebi-met1400-GCST90199802 | Beta-hydroxyisovaleroylcarnitine levels \|\| id:ebi-met1400-GCST90199802 | F-deep fried food liking (derived food-liking factor) \|\| id:ebi-fl187-GCST90094753 | Weighted mode | 8 | 0.0161416152894196 | 0.100788771797317 | 0.87728415954322 | -0.181404377 | 0.21368760801216 | 1.01627259495362 | 0.83409800008187 | 1.23823577943167 | 0.999228396881742 |
| ebi-fl187-GCST90094753 | ebi-met1400-GCST90199873 | Pregnenolone sulfate levels \|\| id:ebi-met1400-GCST90199873 | F-deep fried food liking (derived food-liking factor) \|\| id:ebi-fl187-GCST90094753 | Weighted mode | 8 | 0.0946462586053515 | 0.101918677002983 | 0.383977704994124 | -0.105114348 | 0.294406865531198 | 1.09926992906273 | 0.900221577875097 | 1.34232994036191 | 0.999228396881742 |
| ebi-fl187-GCST90094753 | ebi-met1400-GCST90199911 | Beta-citrylglutamate levels \|\| id:ebi-met1400-GCST90199911 | F-deep fried food liking (derived food-liking factor) \|\| id:ebi-fl187-GCST90094753 | Weighted mode | 8 | -0.097630039 | 0.13031517637664 | 0.478161300987975 | -0.353047784 | 0.157787707159562 | 0.906984390962125 | 0.702543622064312 | 1.17091759089891 | 0.999228396881742 |
| ebi-fl187-GCST90094753 | ebi-met1400-GCST90199934 | 2-hydroxydecanoate levels \|\| id:ebi-met1400-GCST90199934 | F-deep fried food liking (derived food-liking factor) \|\| id:ebi-fl187-GCST90094753 | Weighted mode | 8 | 0.118176561113538 | 0.215268925525022 | 0.600089700064231 | -0.303750533 | 0.540103655142581 | 1.12544280326076 | 0.73804496141157 | 1.71618474433987 | 0.999228396881742 |
| ebi-fl187-GCST90094753 | ebi-met1400-GCST90199945 | Sphingomyelin (d18:1/14:0, d16:1/16:0) levels \|\| id:ebi-met1400-GCST90199945 | F-deep fried food liking (derived food-liking factor) \|\| id:ebi-fl187-GCST90094753 | Weighted mode | 8 | -0.149549435 | 0.129426327795214 | 0.285806713051071 | -0.403225038 | 0.104126167426802 | 0.861095868648613 | 0.668161720940191 | 1.10974045918156 | 0.999228396881742 |
| ebi-fl187-GCST90094753 | ebi-met1400-GCST90199979 | Carnitine C14:1 levels \|\| id:ebi-met1400-GCST90199979 | F-deep fried food liking (derived food-liking factor) \|\| id:ebi-fl187-GCST90094753 | Weighted mode | 8 | -0.156703186 | 0.110249941112515 | 0.198199641734301 | -0.37279307 | 0.0593866990735619 | 0.85495778502254 | 0.688807753025667 | 1.06118552086537 | 0.999228396881742 |
| ebi-fl187-GCST90094753 | ebi-met1400-GCST90199986 | Eugenol sulfate levels \|\| id:ebi-met1400-GCST90199986 | F-deep fried food liking (derived food-liking factor) \|\| id:ebi-fl187-GCST90094753 | Weighted mode | 8 | -0.082179834 | 0.145696780717903 | 0.590327752589404 | -0.367745525 | 0.203385855781882 | 0.921106297179465 | 0.692293330874898 | 1.22554526075159 | 0.999228396881742 |
| ebi-fl187-GCST90094753 | ebi-met1400-GCST90200002 | 6-hydroxyindole sulfate levels \|\| id:ebi-met1400-GCST90200002 | F-deep fried food liking (derived food-liking factor) \|\| id:ebi-fl187-GCST90094753 | Weighted mode | 8 | 0.0326989471156919 | 0.0976838157671264 | 0.747625859926176 | -0.158761332 | 0.22415922601926 | 1.0332394327025 | 0.853199966381417 | 1.25127023834659 | 0.999228396881742 |
| ebi-fl187-GCST90094753 | ebi-met1400-GCST90200023 | Trans 3,4-methyleneheptanoate levels \|\| id:ebi-met1400-GCST90200023 | F-deep fried food liking (derived food-liking factor) \|\| id:ebi-fl187-GCST90094753 | Weighted mode | 8 | -0.08498995 | 0.116760569890063 | 0.490300666316148 | -0.313840667 | 0.143860767306091 | 0.918521515791669 | 0.730635433467334 | 1.15472332209296 | 0.999228396881742 |
| ebi-fl187-GCST90094753 | ebi-met1400-GCST90200032 | 2-hydroxybutyrate/2-hydroxyisobutyrate levels \|\| id:ebi-met1400-GCST90200032 | F-deep fried food liking (derived food-liking factor) \|\| id:ebi-fl187-GCST90094753 | Weighted mode | 8 | -0.065068687 | 0.190873017137359 | 0.743181563357884 | -0.4391798 | 0.309042426864426 | 0.937003101492858 | 0.644564876243225 | 1.36212015976485 | 0.999228396881742 |
| ebi-fl187-GCST90094753 | ebi-met1400-GCST90200051 | 1-palmitoyl-2-dihomo-linolenoyl-GPC (16:0/20:3n3 or 6) levels \|\| id:ebi-met1400-GCST90200051 | F-deep fried food liking (derived food-liking factor) \|\| id:ebi-fl187-GCST90094753 | Weighted mode | 8 | -0.027351554 | 0.104391427596373 | 0.800856827439646 | -0.231958752 | 0.177255644396571 | 0.973019112927728 | 0.792978831596442 | 1.19393627723531 | 0.999228396881742 |
| ebi-fl187-GCST90094753 | ebi-met1400-GCST90200069 | Phenylacetylglutamate levels \|\| id:ebi-met1400-GCST90200069 | F-deep fried food liking (derived food-liking factor) \|\| id:ebi-fl187-GCST90094753 | Weighted mode | 8 | -0.125058564 | 0.109781118765696 | 0.292112973415238 | -0.340229557 | 0.0901124286361427 | 0.88244522142171 | 0.71160694970816 | 1.09429730714318 | 0.999228396881742 |
| ebi-fl187-GCST90094753 | ebi-met1400-GCST90200072 | Thioproline levels \|\| id:ebi-met1400-GCST90200072 | F-deep fried food liking (derived food-liking factor) \|\| id:ebi-fl187-GCST90094753 | Weighted mode | 8 | 0.0542606612721979 | 0.127004411710277 | 0.682042737401101 | -0.194667986 | 0.303189308224341 | 1.05575976197446 | 0.823107896302525 | 1.35417079584752 | 0.999228396881742 |
| ebi-fl187-GCST90094753 | ebi-met1400-GCST90200081 | 1-oleoyl-2-docosahexaenoyl-GPC (18:1/22:6) levels \|\| id:ebi-met1400-GCST90200081 | F-deep fried food liking (derived food-liking factor) \|\| id:ebi-fl187-GCST90094753 | Weighted mode | 7 | 0.0109048800385495 | 0.128567503311905 | 0.935165171145121 | -0.241087426 | 0.262897186529883 | 1.01096455496157 | 0.785772926045658 | 1.30069298331776 | 0.999228396881742 |
| ebi-fl187-GCST90094753 | ebi-met1400-GCST90200097 | Furaneol sulfate levels \|\| id:ebi-met1400-GCST90200097 | F-deep fried food liking (derived food-liking factor) \|\| id:ebi-fl187-GCST90094753 | Weighted mode | 8 | 0.0473923627748484 | 0.144323133998222 | 0.752231346399677 | -0.23548098 | 0.330265705411363 | 1.0485333338285 | 0.790190692402158 | 1.39133776532763 | 0.999228396881742 |
| ebi-fl187-GCST90094753 | ebi-met1400-GCST90200124 | Sphingomyelin (d18:2/24:2) levels \|\| id:ebi-met1400-GCST90200124 | F-deep fried food liking (derived food-liking factor) \|\| id:ebi-fl187-GCST90094753 | Weighted mode | 8 | 0.050232860861626 | 0.198621624423059 | 0.807606094921792 | -0.339065523 | 0.439531244730822 | 1.0515159247737 | 0.712435766625691 | 1.55197954938386 | 0.999228396881742 |
| ebi-fl187-GCST90094753 | ebi-met1400-GCST90200137 | Lignoceroylcarnitine (C24) levels \|\| id:ebi-met1400-GCST90200137 | F-deep fried food liking (derived food-liking factor) \|\| id:ebi-fl187-GCST90094753 | Weighted mode | 8 | -0.071118386 | 0.134119882418585 | 0.612330088248509 | -0.333993355 | 0.19175658372163 | 0.931351626775871 | 0.716058540163177 | 1.21137561253078 | 0.999228396881742 |
| ebi-fl187-GCST90094753 | ebi-met1400-GCST90200165 | Octadecenedioate (C18:1-DC) levels \|\| id:ebi-met1400-GCST90200165 | F-deep fried food liking (derived food-liking factor) \|\| id:ebi-fl187-GCST90094753 | Weighted mode | 8 | -0.178422647 | 0.192202226113274 | 0.384141262583019 | -0.55513901 | 0.198293716548846 | 0.83658876733238 | 0.573992465063855 | 1.21932047583386 | 0.999228396881742 |
| ebi-fl187-GCST90094753 | ebi-met1400-GCST90200176 | Glutamine conjugate of C6H10O2 (1) levels \|\| id:ebi-met1400-GCST90200176 | F-deep fried food liking (derived food-liking factor) \|\| id:ebi-fl187-GCST90094753 | Weighted mode | 8 | 0.0532207089766524 | 0.0967736225756927 | 0.599453159230527 | -0.136455591 | 0.24289700922501 | 1.0546623928911 | 0.872445063575125 | 1.27493731057485 | 0.999228396881742 |
| ebi-fl187-GCST90094753 | ebi-met1400-GCST90200182 | Glycine conjugate of C10H12O2 levels \|\| id:ebi-met1400-GCST90200182 | F-deep fried food liking (derived food-liking factor) \|\| id:ebi-fl187-GCST90094753 | Weighted mode | 8 | 0.0986798839353118 | 0.112089535879624 | 0.407867663804881 | -0.121015606 | 0.318375374259374 | 1.10371292676308 | 0.886020131910356 | 1.37489226353966 | 0.999228396881742 |
| ebi-fl187-GCST90094753 | ebi-met1400-GCST90200208 | Glucuronide of piperine metabolite C17H21NO3 (4) levels \|\| id:ebi-met1400-GCST90200208 | F-deep fried food liking (derived food-liking factor) \|\| id:ebi-fl187-GCST90094753 | Weighted mode | 8 | -0.144117904 | 0.163212660717793 | 0.406533550239797 | -0.464014719 | 0.175778911252086 | 0.865785662660728 | 0.628754299914306 | 1.1921744531545 | 0.999228396881742 |
| ebi-fl187-GCST90094753 | ebi-met1400-GCST90200227 | 3-ethylcatechol sulfate (2) levels \|\| id:ebi-met1400-GCST90200227 | F-deep fried food liking (derived food-liking factor) \|\| id:ebi-fl187-GCST90094753 | Weighted mode | 8 | -0.119827958 | 0.14409946781317 | 0.433102047125289 | -0.402262915 | 0.162606998542337 | 0.887073037079892 | 0.668804883546176 | 1.17657420343853 | 0.999228396881742 |
| ebi-fl187-GCST90094753 | ebi-met1400-GCST90200259 | Pentose acid levels \|\| id:ebi-met1400-GCST90200259 | F-deep fried food liking (derived food-liking factor) \|\| id:ebi-fl187-GCST90094753 | Weighted mode | 8 | -0.177358973 | 0.10844253169278 | 0.145956529535715 | -0.389906335 | 0.035188389373113 | 0.837479098386552 | 0.677120294093408 | 1.03581482692586 | 0.999228396881742 |
| ebi-fl187-GCST90094753 | ebi-met1400-GCST90200291 | Sarcosine levels \|\| id:ebi-met1400-GCST90200291 | F-deep fried food liking (derived food-liking factor) \|\| id:ebi-fl187-GCST90094753 | Weighted mode | 8 | 0.0839993627404763 | 0.092055487191921 | 0.391854821184768 | -0.096429392 | 0.264428117636641 | 1.08762820070718 | 0.908074012484713 | 1.30268577969402 | 0.999228396881742 |
| ebi-fl187-GCST90094753 | ebi-met1400-GCST90200306 | Butyrate/isobutyrate (4:0) levels \|\| id:ebi-met1400-GCST90200306 | F-deep fried food liking (derived food-liking factor) \|\| id:ebi-fl187-GCST90094753 | Weighted mode | 8 | -0.056653205 | 0.101813356115756 | 0.595241303734211 | -0.256207383 | 0.142900972945851 | 0.944921706636246 | 0.773981441532617 | 1.15361555685922 | 0.999228396881742 |
| ebi-fl187-GCST90094753 | ebi-met1400-GCST90200321 | Cholate levels \|\| id:ebi-met1400-GCST90200321 | F-deep fried food liking (derived food-liking factor) \|\| id:ebi-fl187-GCST90094753 | Weighted mode | 8 | 0.0354106180935888 | 0.09778736574336 | 0.727948577767643 | -0.156252619 | 0.227073854950574 | 1.03604504030856 | 0.855343087363321 | 1.25492254676051 | 0.999228396881742 |
| ebi-fl187-GCST90094753 | ebi-met1400-GCST90200336 | Ceramide (d18:1/16:0) levels \|\| id:ebi-met1400-GCST90200336 | F-deep fried food liking (derived food-liking factor) \|\| id:ebi-fl187-GCST90094753 | Weighted mode | 8 | -0.044642616 | 0.143037174578884 | 0.764052100398202 | -0.324995478 | 0.235710246529284 | 0.956339201435195 | 0.72253062104825 | 1.26580748491299 | 0.999228396881742 |
| ebi-fl187-GCST90094753 | ebi-met1400-GCST90200358 | Arachidonate (20:4n6) levels \|\| id:ebi-met1400-GCST90200358 | F-deep fried food liking (derived food-liking factor) \|\| id:ebi-fl187-GCST90094753 | Weighted mode | 8 | -0.105517274 | 0.0986843786061027 | 0.320440142684613 | -0.298938656 | 0.0879041076476638 | 0.899858928171188 | 0.741604900688632 | 1.0918834137389 | 0.999228396881742 |
| ebi-fl187-GCST90094753 | ebi-met1400-GCST90200413 | 2'-deoxyuridine levels \|\| id:ebi-met1400-GCST90200413 | F-deep fried food liking (derived food-liking factor) \|\| id:ebi-fl187-GCST90094753 | Weighted mode | 8 | 0.0252075099137629 | 0.100525447350578 | 0.809202757850891 | -0.171822367 | 0.222237386720896 | 1.02552790565342 | 0.842128749829237 | 1.24886780731229 | 0.999228396881742 |
| ebi-fl187-GCST90094753 | ebi-met1400-GCST90200432 | Threonine levels \|\| id:ebi-met1400-GCST90200432 | F-deep fried food liking (derived food-liking factor) \|\| id:ebi-fl187-GCST90094753 | Weighted mode | 8 | 0.050679513267149 | 0.157678437725613 | 0.757283500831929 | -0.258370225 | 0.359729251209351 | 1.05198569179442 | 0.772309251230029 | 1.43294139488505 | 0.999228396881742 |
| ebi-fl187-GCST90094753 | ebi-met1400-GCST90200436 | Caffeine levels \|\| id:ebi-met1400-GCST90200436 | F-deep fried food liking (derived food-liking factor) \|\| id:ebi-fl187-GCST90094753 | Weighted mode | 8 | 0.0584640978604344 | 0.111590263671605 | 0.616509469717681 | -0.160252819 | 0.277181014656781 | 1.06020692131445 | 0.85192837811136 | 1.31940518109626 | 0.999228396881742 |
| ebi-fl187-GCST90094753 | ebi-met1400-GCST90200442 | Valine levels \|\| id:ebi-met1400-GCST90200442 | F-deep fried food liking (derived food-liking factor) \|\| id:ebi-fl187-GCST90094753 | Weighted mode | 8 | 0.162359124741233 | 0.0907516329047765 | 0.116739992172279 | -0.015514076 | 0.340232325234595 | 1.17628259766066 | 0.984605647589154 | 1.40527403326113 | 0.999228396881742 |
| ebi-fl187-GCST90094753 | ebi-met1400-GCST90200459 | Margarate (17:0) levels \|\| id:ebi-met1400-GCST90200459 | F-deep fried food liking (derived food-liking factor) \|\| id:ebi-fl187-GCST90094753 | Weighted mode | 8 | 0.0377349481789342 | 0.0993379678168981 | 0.715308621194958 | -0.156967469 | 0.232437365100055 | 1.03845595175712 | 0.854731863868041 | 1.26167141922098 | 0.999228396881742 |
| ebi-fl187-GCST90094753 | ebi-met1400-GCST90200462 | X-11299 levels \|\| id:ebi-met1400-GCST90200462 | F-deep fried food liking (derived food-liking factor) \|\| id:ebi-fl187-GCST90094753 | Weighted mode | 8 | -0.037117959 | 0.108270304075137 | 0.741793342993214 | -0.249327755 | 0.175091837136524 | 0.96356246792381 | 0.779324504144733 | 1.19135562227746 | 0.999228396881742 |
| ebi-fl187-GCST90094753 | ebi-met1400-GCST90200497 | X-12740 levels \|\| id:ebi-met1400-GCST90200497 | F-deep fried food liking (derived food-liking factor) \|\| id:ebi-fl187-GCST90094753 | Weighted mode | 8 | 0.106754282386942 | 0.118378585638663 | 0.397127855100379 | -0.125267745 | 0.338776310238722 | 1.11266082052597 | 0.88226064967043 | 1.4032294220489 | 0.999228396881742 |
| ebi-fl187-GCST90094753 | ebi-met1400-GCST90200527 | X-13866 levels \|\| id:ebi-met1400-GCST90200527 | F-deep fried food liking (derived food-liking factor) \|\| id:ebi-fl187-GCST90094753 | Weighted mode | 8 | -0.044713055 | 0.104387026742628 | 0.68127554257244 | -0.249311627 | 0.15988551788229 | 0.956271840337809 | 0.779337073105343 | 1.17337653267196 | 0.999228396881742 |
| ebi-fl187-GCST90094753 | ebi-met1400-GCST90200547 | X-17654 levels \|\| id:ebi-met1400-GCST90200547 | F-deep fried food liking (derived food-liking factor) \|\| id:ebi-fl187-GCST90094753 | Weighted mode | 8 | -0.0414599 | 0.105056349797176 | 0.704853455752637 | -0.247370345 | 0.164450546007951 | 0.959387806410169 | 0.780851455389645 | 1.17874527445073 | 0.999228396881742 |
| ebi-fl187-GCST90094753 | ebi-met1400-GCST90200553 | X-17653 levels \|\| id:ebi-met1400-GCST90200553 | F-deep fried food liking (derived food-liking factor) \|\| id:ebi-fl187-GCST90094753 | Weighted mode | 8 | 0.00277139422994702 | 0.110006386134015 | 0.980604142141704 | -0.212841123 | 0.218383911052617 | 1.00277523809307 | 0.808284545168536 | 1.24406458609566 | 0.999228396881742 |
| ebi-fl187-GCST90094753 | ebi-met1400-GCST90200557 | X-17685 levels \|\| id:ebi-met1400-GCST90200557 | F-deep fried food liking (derived food-liking factor) \|\| id:ebi-fl187-GCST90094753 | Weighted mode | 8 | 0.0341117392184837 | 0.144287436321482 | 0.819879383541285 | -0.248691636 | 0.316915114408588 | 1.0347002168625 | 0.779820404874043 | 1.37288602873662 | 0.999228396881742 |
| ebi-fl187-GCST90094753 | ebi-met1400-GCST90200561 | X-18779 levels \|\| id:ebi-met1400-GCST90200561 | F-deep fried food liking (derived food-liking factor) \|\| id:ebi-fl187-GCST90094753 | Weighted mode | 8 | -0.005330394 | 0.123748691319485 | 0.966845209506927 | -0.247877828 | 0.237217041478442 | 0.994683787831164 | 0.78045528685159 | 1.26771623492416 | 0.999228396881742 |
| ebi-fl187-GCST90094753 | ebi-met1400-GCST90200570 | X-18921 levels \|\| id:ebi-met1400-GCST90200570 | F-deep fried food liking (derived food-liking factor) \|\| id:ebi-fl187-GCST90094753 | Weighted mode | 8 | -0.08883862 | 0.0949143170996068 | 0.380437717474956 | -0.274870681 | 0.0971934419193909 | 0.914993223636454 | 0.759670356599587 | 1.1020735402236 | 0.999228396881742 |
| ebi-fl187-GCST90094753 | ebi-met1400-GCST90200575 | X-21283 levels \|\| id:ebi-met1400-GCST90200575 | F-deep fried food liking (derived food-liking factor) \|\| id:ebi-fl187-GCST90094753 | Weighted mode | 8 | -0.075296572 | 0.14502378730641 | 0.619627123953843 | -0.359543195 | 0.208950051471211 | 0.927468384708324 | 0.697995101069187 | 1.23238344125313 | 0.999228396881742 |
| ebi-fl187-GCST90094753 | ebi-met1400-GCST90200578 | X-18922 levels \|\| id:ebi-met1400-GCST90200578 | F-deep fried food liking (derived food-liking factor) \|\| id:ebi-fl187-GCST90094753 | Weighted mode | 8 | -0.102184523 | 0.119671924806544 | 0.42143192568064 | -0.336741496 | 0.132372449715275 | 0.902862937404751 | 0.714093412386972 | 1.14153340389225 | 0.999228396881742 |
| ebi-fl187-GCST90094753 | ebi-met1400-GCST90200623 | X-24241 levels \|\| id:ebi-met1400-GCST90200623 | F-deep fried food liking (derived food-liking factor) \|\| id:ebi-fl187-GCST90094753 | Weighted mode | 8 | -0.018069149 | 0.106148109941948 | 0.869647995207327 | -0.226119445 | 0.189981146293525 | 0.98209311906392 | 0.797622824173466 | 1.2092267990352 | 0.999228396881742 |
| ebi-fl187-GCST90094753 | ebi-met1400-GCST90200636 | X-24544 levels \|\| id:ebi-met1400-GCST90200636 | F-deep fried food liking (derived food-liking factor) \|\| id:ebi-fl187-GCST90094753 | Weighted mode | 8 | 0.157368940800348 | 0.0984559349579728 | 0.153993032966798 | -0.035604692 | 0.350342573317974 | 1.17042735265723 | 0.965021699162429 | 1.4195537665497 | 0.999228396881742 |
| ebi-fl187-GCST90094753 | ebi-met1400-GCST90200659 | X-25172 levels \|\| id:ebi-met1400-GCST90200659 | F-deep fried food liking (derived food-liking factor) \|\| id:ebi-fl187-GCST90094753 | Weighted mode | 8 | 0.0316814423633253 | 0.10620253388898 | 0.774125715333124 | -0.176475524 | 0.239838408785727 | 1.03218864135265 | 0.838219295110058 | 1.27104374422392 | 0.999228396881742 |
| ebi-fl187-GCST90094753 | ebi-met1400-GCST90200670 | X-26111 levels \|\| id:ebi-met1400-GCST90200670 | F-deep fried food liking (derived food-liking factor) \|\| id:ebi-fl187-GCST90094753 | Weighted mode | 8 | 0.0678618749547557 | 0.117765429183004 | 0.582494284079126 | -0.162958366 | 0.298682116153444 | 1.07021747443214 | 0.849626560823224 | 1.34808102217301 | 0.999228396881742 |
| ebi-fl187-GCST90094753 | ebi-met1400-GCST90200673 | Carnitine C4 levels \|\| id:ebi-met1400-GCST90200673 | F-deep fried food liking (derived food-liking factor) \|\| id:ebi-fl187-GCST90094753 | Weighted mode | 8 | 0.0592734555512842 | 0.139179267168307 | 0.682984963401185 | -0.213517908 | 0.332064819201166 | 1.06106535528331 | 0.807737694974759 | 1.39384319338679 | 0.999228396881742 |
| ebi-fl187-GCST90094753 | ebi-met1400-GCST90200710 | X-13431 levels \|\| id:ebi-met1400-GCST90200710 | F-deep fried food liking (derived food-liking factor) \|\| id:ebi-fl187-GCST90094753 | Weighted mode | 8 | -0.082942686 | 0.110118755956633 | 0.475887184915332 | -0.298775447 | 0.132890075926528 | 0.920403897968941 | 0.74172594720859 | 1.14212444445898 | 0.999228396881742 |
| ebi-fl187-GCST90094753 | ebi-met1400-GCST90200730 | Adenosine 5'-diphosphate (ADP) to fructose ratio \|\| id:ebi-met1400-GCST90200730 | F-deep fried food liking (derived food-liking factor) \|\| id:ebi-fl187-GCST90094753 | Weighted mode | 8 | -0.042903515 | 0.172935845593626 | 0.811185860224808 | -0.381857773 | 0.29605074209026 | 0.958003818344642 | 0.682592129580531 | 1.34453837978898 | 0.999228396881742 |
| ebi-fl187-GCST90094753 | ebi-met1400-GCST90200731 | Adenosine 5'-diphosphate (ADP) to mannose ratio \|\| id:ebi-met1400-GCST90200731 | F-deep fried food liking (derived food-liking factor) \|\| id:ebi-fl187-GCST90094753 | Weighted mode | 8 | -0.134466731 | 0.130467957207106 | 0.336994453480817 | -0.390183927 | 0.121250465012853 | 0.874181961453626 | 0.676932356748046 | 1.12890762882434 | 0.999228396881742 |
| ebi-fl187-GCST90094753 | ebi-met1400-GCST90200748 | Aspartate to glutamate ratio \|\| id:ebi-met1400-GCST90200748 | F-deep fried food liking (derived food-liking factor) \|\| id:ebi-fl187-GCST90094753 | Weighted mode | 8 | -0.174622713 | 0.102228309858738 | 0.131360156494155 | -0.3749902 | 0.0257447742050692 | 0.839773796645033 | 0.687296013955685 | 1.02607903321418 | 0.999228396881742 |
| ebi-fl187-GCST90094753 | ebi-met1400-GCST90200774 | Uridine to pseudouridine ratio \|\| id:ebi-met1400-GCST90200774 | F-deep fried food liking (derived food-liking factor) \|\| id:ebi-fl187-GCST90094753 | Weighted mode | 8 | 0.0303020564436973 | 0.104414176155174 | 0.780064281244271 | -0.174349729 | 0.234953841707839 | 1.03076583639852 | 0.840003072998061 | 1.26485038405187 | 0.999228396881742 |
| ebi-fl187-GCST90094753 | ebi-met1400-GCST90200799 | Spermidine to adenosine 5'-diphosphate (ADP) ratio \|\| id:ebi-met1400-GCST90200799 | F-deep fried food liking (derived food-liking factor) \|\| id:ebi-fl187-GCST90094753 | Weighted mode | 8 | 0.22013309920884 | 0.128877318941106 | 0.131375710703066 | -0.032466446 | 0.472732644333408 | 1.24624259345224 | 0.968054931483107 | 1.6043723875825 | 0.999228396881742 |
| ebi-fl187-GCST90094753 | ebi-met1400-GCST90200811 | Choline to taurocholate ratio \|\| id:ebi-met1400-GCST90200811 | F-deep fried food liking (derived food-liking factor) \|\| id:ebi-fl187-GCST90094753 | Weighted mode | 8 | -0.087065407 | 0.1730757646363 | 0.630369335637182 | -0.426293905 | 0.252163092139988 | 0.916617140910203 | 0.65292441601454 | 1.2868058880979 | 0.999228396881742 |
| ebi-fl187-GCST90094753 | ebi-met1400-GCST90200812 | Glutamate to kynurenine ratio \|\| id:ebi-met1400-GCST90200812 | F-deep fried food liking (derived food-liking factor) \|\| id:ebi-fl187-GCST90094753 | Weighted mode | 8 | 0.0705555407941226 | 0.0938593465921288 | 0.47673085694423 | -0.113408779 | 0.254519860114695 | 1.07310416883331 | 0.892785633917794 | 1.28984216750229 | 0.999228396881742 |
| ebi-fl187-GCST90094753 | ebi-met1400-GCST90200876 | Cortisone to 4-cholesten-3-one ratio \|\| id:ebi-met1400-GCST90200876 | F-deep fried food liking (derived food-liking factor) \|\| id:ebi-fl187-GCST90094753 | Weighted mode | 8 | -0.011993488 | 0.172374082624993 | 0.946475281634996 | -0.34984669 | 0.325859713564955 | 0.988078146830374 | 0.704796133502579 | 1.38522102752168 | 0.999228396881742 |
| ebi-fl187-GCST90094753 | ebi-met1400-GCST90200906 | 2'-deoxyuridine to cytidine ratio \|\| id:ebi-met1400-GCST90200906 | F-deep fried food liking (derived food-liking factor) \|\| id:ebi-fl187-GCST90094753 | Weighted mode | 8 | 0.0880064509331409 | 0.146578705220623 | 0.567158954823379 | -0.199287811 | 0.375300713165561 | 1.09199516639328 | 0.819314051553893 | 1.45542901548523 | 0.999228396881742 |
| ebi-fl187-GCST90094753 | ebi-met1400-GCST90200907 | Retinol (Vitamin A) to linoleoyl-arachidonoyl-glycerol (18:2 to 20:4) [1] ratio \|\| id:ebi-met1400-GCST90200907 | F-deep fried food liking (derived food-liking factor) \|\| id:ebi-fl187-GCST90094753 | Weighted mode | 7 | -0.153702583 | 0.134440647916115 | 0.296481122646556 | -0.417206252 | 0.10980108734509 | 0.857527026572002 | 0.658885009257443 | 1.11605605070626 | 0.999228396881742 |
| ebi-fl187-GCST90094753 | ebi-met1400-GCST90200916 | Fructose to sucrose ratio \|\| id:ebi-met1400-GCST90200916 | F-deep fried food liking (derived food-liking factor) \|\| id:ebi-fl187-GCST90094753 | Weighted mode | 8 | -0.053278058 | 0.102902099003945 | 0.620585191276561 | -0.254966172 | 0.148410055666642 | 0.948116344130665 | 0.774942711947447 | 1.15998845869352 | 0.999228396881742 |
| ebi-fl187-GCST90094753 | ebi-met1400-GCST90200917 | Alpha-ketobutyrate to 3-methyl-2-oxobutyrate ratio \|\| id:ebi-met1400-GCST90200917 | F-deep fried food liking (derived food-liking factor) \|\| id:ebi-fl187-GCST90094753 | Weighted mode | 8 | 0.00925419171916814 | 0.106054318114111 | 0.932909361290832 | -0.198612272 | 0.217120655222825 | 1.00929714414578 | 0.819867717561319 | 1.24249400648542 | 0.999228396881742 |
| ebi-fl187-GCST90094753 | ebi-met1400-GCST90200924 | Alpha-tocopherol to glycerol ratio \|\| id:ebi-met1400-GCST90200924 | F-deep fried food liking (derived food-liking factor) \|\| id:ebi-fl187-GCST90094753 | Weighted mode | 8 | 0.11645708647956 | 0.142442936655598 | 0.440538962997237 | -0.162731069 | 0.395645242324531 | 1.12350929569355 | 0.849819700237569 | 1.48534228749575 | 0.999228396881742 |
| ebi-fl187-GCST90094753 | ebi-met1400-GCST90200938 | Alpha-ketoglutarate to alanine ratio \|\| id:ebi-met1400-GCST90200938 | F-deep fried food liking (derived food-liking factor) \|\| id:ebi-fl187-GCST90094753 | Weighted mode | 8 | 0.0500934356086086 | 0.1220512111522 | 0.69376234919615 | -0.189126938 | 0.289313809466921 | 1.05136932711978 | 0.827681435593357 | 1.33551075869651 | 0.999228396881742 |
| ebi-fl187-GCST90094753 | ebi-met1400-GCST90200943 | Phosphate to EDTA ratio \|\| id:ebi-met1400-GCST90200943 | F-deep fried food liking (derived food-liking factor) \|\| id:ebi-fl187-GCST90094753 | Weighted mode | 8 | 0.0739604912929024 | 0.167652352336598 | 0.672405923263492 | -0.254638119 | 0.402559101872634 | 1.07676426309194 | 0.775196976042754 | 1.49564731817013 | 0.999228396881742 |
| ebi-fl187-GCST90094753 | ebi-met1400-GCST90200947 | Adenosine 5'-diphosphate (ADP) to glucose ratio \|\| id:ebi-met1400-GCST90200947 | F-deep fried food liking (derived food-liking factor) \|\| id:ebi-fl187-GCST90094753 | Weighted mode | 8 | -0.177653225 | 0.142667467562769 | 0.253114440924152 | -0.457281461 | 0.101975011844448 | 0.837232704878696 | 0.633002149568063 | 1.10735580060321 | 0.999228396881742 |
| ebi-fl187-GCST90094753 | ebi-met1400-GCST90200975 | Arachidonate (20:4n6) to caffeine ratio \|\| id:ebi-met1400-GCST90200975 | F-deep fried food liking (derived food-liking factor) \|\| id:ebi-fl187-GCST90094753 | Weighted mode | 8 | -0.065888196 | 0.110418719005779 | 0.569487134679461 | -0.282308885 | 0.150532493020354 | 0.936235533101624 | 0.754040736321268 | 1.16245307610095 | 0.999228396881742 |
| ebi-fl187-GCST90094753 | ebi-met1400-GCST90200985 | Cholesterol to oleoyl-linoleoyl-glycerol (18:1 to 18:2) [2] ratio \|\| id:ebi-met1400-GCST90200985 | F-deep fried food liking (derived food-liking factor) \|\| id:ebi-fl187-GCST90094753 | Weighted mode | 8 | 0.356856583898227 | 0.233197926032245 | 0.169802334349232 | -0.100211351 | 0.813924518921427 | 1.42883093779576 | 0.904646199837543 | 2.25674727774121 | 0.999228396881742 |
| ebi-fl187-GCST90094753 | ebi-met1400-GCST90201016 | Bilirubin (Z,Z) to etiocholanolone glucuronide ratio \|\| id:ebi-met1400-GCST90201016 | F-deep fried food liking (derived food-liking factor) \|\| id:ebi-fl187-GCST90094753 | Weighted mode | 8 | -0.010646107 | 0.106761892718212 | 0.923363788182066 | -0.219899416 | 0.198607203085599 | 0.989410362581105 | 0.80259952227619 | 1.21970277630692 | 0.999228396881742 |
| ebi-fl187-GCST90094753 | ebi-pw11-GCST90275189 | Placental weight (fetal genotype, adjusted for sex and gestational age) \|\| id:ebi-pw11-GCST90275189 | F-deep fried food liking (derived food-liking factor) \|\| id:ebi-fl187-GCST90094753 | Weighted mode | 8 | 0.0342436321899663 | 0.0377833838756785 | 0.394894928883392 | -0.0398118 | 0.108299064586296 | 1.03483669554879 | 0.960970276542746 | 1.11438096743953 | 0.999228396881742 |
| ebi-fl187-GCST90094753 | ebi-pw11-GCST90275192 | Placental weight (fetal effect, conditional on maternal and paternal genotype) \|\| id:ebi-pw11-GCST90275192 | F-deep fried food liking (derived food-liking factor) \|\| id:ebi-fl187-GCST90094753 | Weighted mode | 8 | 0.0410162531393398 | 0.0801062911098378 | 0.624388895384741 | -0.115992077 | 0.198024583714622 | 1.04186903905053 | 0.890482278172418 | 1.21899236081362 | 0.999228396881742 |
| ebi-fl187-GCST90094753 | ebi-pw11-GCST90275193 | Placental weight (maternal effect, conditional on fetal and paternal genotype) \|\| id:ebi-pw11-GCST90275193 | F-deep fried food liking (derived food-liking factor) \|\| id:ebi-fl187-GCST90094753 | Weighted mode | 8 | -0.050115324 | 0.0696502306145783 | 0.495130214278871 | -0.186629776 | 0.0863991277356575 | 0.951119730988046 | 0.82975087298754 | 1.09024138705348 | 0.999228396881742 |
| ebi-fl187-GCST90094753 | ebi-pw11-GCST90275195 | Placental weight (fetal effect, conditional on maternal genotype) \|\| id:ebi-pw11-GCST90275195 | F-deep fried food liking (derived food-liking factor) \|\| id:ebi-fl187-GCST90094753 | Weighted mode | 8 | -0.037269141 | 0.0681326853125281 | 0.601367744564854 | -0.170809204 | 0.096270922448105 | 0.963416805716956 | 0.842982395817577 | 1.10105732473531 | 0.999228396881742 |
| ebi-fl187-GCST90094753 | ebi-pw11-GCST90275197 | Placental weight (fetal genotype, adjusted for sex) \|\| id:ebi-pw11-GCST90275197 | F-deep fried food liking (derived food-liking factor) \|\| id:ebi-fl187-GCST90094753 | Weighted mode | 8 | 0.0450063509923596 | 0.0398283610208499 | 0.295701297038465 | -0.033057237 | 0.123069938593225 | 1.04603450324476 | 0.967483182548094 | 1.13096351617886 | 0.999228396881742 |
| ebi-fl187-GCST90094753 | prot-a-1030 | Protein FAM171B \|\| id:prot-a-1030 | F-deep fried food liking (derived food-liking factor) \|\| id:ebi-fl187-GCST90094753 | Weighted mode | 8 | 0.27875090245647 | 0.207275249733589 | 0.220618789908731 | -0.127508587 | 0.685010391934305 | 1.32147812591256 | 0.880285856768307 | 1.98379245087087 | 0.999228396881742 |
| ebi-fl187-GCST90094753 | prot-a-1036 | BRISC complex subunit Abro1 \|\| id:prot-a-1036 | F-deep fried food liking (derived food-liking factor) \|\| id:ebi-fl187-GCST90094753 | Weighted mode | 8 | 0.234906956033554 | 0.198965506199726 | 0.276302858523049 | -0.155065436 | 0.624879348185018 | 1.26479108207896 | 0.856359138832576 | 1.86802056376398 | 0.999228396881742 |
| ebi-fl187-GCST90094753 | prot-a-1055 | Phenylalanine--tRNA ligase, mitochondrial \|\| id:prot-a-1055 | F-deep fried food liking (derived food-liking factor) \|\| id:ebi-fl187-GCST90094753 | Weighted mode | 8 | -0.396258784 | 0.18247485252352 | 0.066461785702985 | -0.753909495 | -0.038608073 | 0.672832554834275 | 0.470523443061501 | 0.962127718651515 | 0.999228396881742 |
| ebi-fl187-GCST90094753 | prot-a-1083 | Fc receptor-like protein 5 \|\| id:prot-a-1083 | F-deep fried food liking (derived food-liking factor) \|\| id:ebi-fl187-GCST90094753 | Weighted mode | 8 | -0.199798169 | 0.150432700711805 | 0.225792630699206 | -0.494646263 | 0.0950499241212527 | 0.818896014777252 | 0.60978657342443 | 1.09971375599855 | 0.999228396881742 |
| ebi-fl187-GCST90094753 | prot-a-1103 | Fibroblast growth factor receptor 3 \|\| id:prot-a-1103 | F-deep fried food liking (derived food-liking factor) \|\| id:ebi-fl187-GCST90094753 | Weighted mode | 8 | 0.205048225015201 | 0.294410449995759 | 0.508593508475089 | -0.371996257 | 0.782092707006889 | 1.22758426380552 | 0.689356822798191 | 2.18604222792192 | 0.999228396881742 |
| ebi-fl187-GCST90094753 | prot-a-1105 | Fibroblast growth factor receptor 4 \|\| id:prot-a-1105 | F-deep fried food liking (derived food-liking factor) \|\| id:ebi-fl187-GCST90094753 | Weighted mode | 8 | -0.01000693 | 0.156489036665206 | 0.950800586295775 | -0.316725442 | 0.296711581789353 | 0.990042972653884 | 0.728530751660212 | 1.34542719777805 | 0.999228396881742 |
| ebi-fl187-GCST90094753 | prot-a-1120 | Filamin-A \|\| id:prot-a-1120 | F-deep fried food liking (derived food-liking factor) \|\| id:ebi-fl187-GCST90094753 | Weighted mode | 8 | 0.214120006049079 | 0.228656483419067 | 0.380226844340382 | -0.234046701 | 0.66228671355045 | 1.23877130592389 | 0.791324859012469 | 1.93922171267996 | 0.999228396881742 |
| ebi-fl187-GCST90094753 | prot-a-1166 | Galanin peptides \|\| id:prot-a-1166 | F-deep fried food liking (derived food-liking factor) \|\| id:ebi-fl187-GCST90094753 | Weighted mode | 8 | 0.0164410941625053 | 0.179446273749727 | 0.929565792229021 | -0.335273602 | 0.36815579071197 | 1.01657699270318 | 0.715142394916694 | 1.44506714947842 | 0.999228396881742 |
| ebi-fl187-GCST90094753 | prot-a-1205 | ADP-ribosylation factor-binding protein GGA1 \|\| id:prot-a-1205 | F-deep fried food liking (derived food-liking factor) \|\| id:ebi-fl187-GCST90094753 | Weighted mode | 8 | 0.125427560740281 | 0.170285227799109 | 0.48532917611458 | -0.208331486 | 0.459186607226534 | 1.13363304644744 | 0.811937846275352 | 1.58278603453826 | 0.999228396881742 |
| ebi-fl187-GCST90094753 | prot-a-1227 | Guanine nucleotide-binding protein G(I)/G(S)/G(O) subunit gamma-T2 \|\| id:prot-a-1227 | F-deep fried food liking (derived food-liking factor) \|\| id:ebi-fl187-GCST90094753 | Weighted mode | 8 | -0.089457455 | 0.241404338884472 | 0.721916405768094 | -0.56260996 | 0.383695048921708 | 0.914427168333732 | 0.569720175137841 | 1.46769779740473 | 0.999228396881742 |
| ebi-fl187-GCST90094753 | prot-a-1240 | Golgi SNAP receptor complex member 1 \|\| id:prot-a-1240 | F-deep fried food liking (derived food-liking factor) \|\| id:ebi-fl187-GCST90094753 | Weighted mode | 8 | -0.138057005 | 0.184493457363255 | 0.478659727886835 | -0.499664182 | 0.223550171069121 | 0.871049035864129 | 0.606734377954386 | 1.25050837804491 | 0.999228396881742 |
| ebi-fl187-GCST90094753 | prot-a-1283 | Glutathione S-transferase A1 \|\| id:prot-a-1283 | F-deep fried food liking (derived food-liking factor) \|\| id:ebi-fl187-GCST90094753 | Weighted mode | 8 | 0.116838974314862 | 0.177949224435412 | 0.532430360603526 | -0.231941506 | 0.465619454208269 | 1.12393843216221 | 0.792992507588009 | 1.59300067428575 | 0.999228396881742 |
| ebi-fl187-GCST90094753 | prot-a-1324 | E3 ubiquitin-protein ligase HECW1 \|\| id:prot-a-1324 | F-deep fried food liking (derived food-liking factor) \|\| id:ebi-fl187-GCST90094753 | Weighted mode | 8 | 0.149948828689677 | 0.168817102939302 | 0.403902538151678 | -0.180932693 | 0.480830350450708 | 1.16177479166881 | 0.834491523867067 | 1.61741686758237 | 0.999228396881742 |
| ebi-fl187-GCST90094753 | prot-a-1339 | Histidine triad nucleotide-binding protein 2, mitochondrial \|\| id:prot-a-1339 | F-deep fried food liking (derived food-liking factor) \|\| id:ebi-fl187-GCST90094753 | Weighted mode | 8 | 0.128080216599003 | 0.175000945257805 | 0.488013300070231 | -0.214921636 | 0.4710820693043 | 1.13664417677031 | 0.806604646381523 | 1.60172643485463 | 0.999228396881742 |
| ebi-fl187-GCST90094753 | prot-a-1466 | Interleukin-11 \|\| id:prot-a-1466 | F-deep fried food liking (derived food-liking factor) \|\| id:ebi-fl187-GCST90094753 | Weighted mode | 8 | -0.133572643 | 0.1763895574997 | 0.473608654762909 | -0.479296176 | 0.212150889208181 | 0.874963906236084 | 0.619219059588822 | 1.23633442052682 | 0.999228396881742 |
| ebi-fl187-GCST90094753 | prot-a-147 | Rho GTPase-activating protein 1 \|\| id:prot-a-147 | F-deep fried food liking (derived food-liking factor) \|\| id:ebi-fl187-GCST90094753 | Weighted mode | 8 | 0.070659283667557 | 0.199374862635717 | 0.733472099666168 | -0.320115447 | 0.461434014433563 | 1.07321550151817 | 0.726065210113202 | 1.58634719947443 | 0.999228396881742 |
| ebi-fl187-GCST90094753 | prot-a-1473 | Interleukin-12 receptor subunit beta-1 \|\| id:prot-a-1473 | F-deep fried food liking (derived food-liking factor) \|\| id:ebi-fl187-GCST90094753 | Weighted mode | 8 | 0.034366969801956 | 0.183337252732879 | 0.85662383166877 | -0.324974046 | 0.3937079851584 | 1.034964338 | 0.722546106682265 | 1.48246758292521 | 0.999228396881742 |
| ebi-fl187-GCST90094753 | prot-a-1501 | X-linked interleukin-1 receptor accessory protein-like 2 \|\| id:prot-a-1501 | F-deep fried food liking (derived food-liking factor) \|\| id:ebi-fl187-GCST90094753 | Weighted mode | 8 | -0.051146338 | 0.15418025774393 | 0.749803498760374 | -0.353339643 | 0.251046967178335 | 0.950139618827081 | 0.702338608345766 | 1.28537045313681 | 0.999228396881742 |
| ebi-fl187-GCST90094753 | prot-a-1503 | Interleukin-1 receptor-like 2 \|\| id:prot-a-1503 | F-deep fried food liking (derived food-liking factor) \|\| id:ebi-fl187-GCST90094753 | Weighted mode | 8 | 0.228105372793645 | 0.228608016594071 | 0.351610143527004 | -0.21996634 | 0.676177085318024 | 1.25621768962581 | 0.802545811415946 | 1.96634617149714 | 0.999228396881742 |
| ebi-fl187-GCST90094753 | prot-a-1536 | Interleukin-5 receptor subunit alpha \|\| id:prot-a-1536 | F-deep fried food liking (derived food-liking factor) \|\| id:ebi-fl187-GCST90094753 | Weighted mode | 8 | -0.323463852 | 0.160625439399729 | 0.0838969946973017 | -0.638289713 | -0.008637991 | 0.723638115578165 | 0.528195016971885 | 0.991399209556327 | 0.999228396881742 |
| ebi-fl187-GCST90094753 | prot-a-1559 | Insulin-induced gene 1 protein \|\| id:prot-a-1559 | F-deep fried food liking (derived food-liking factor) \|\| id:ebi-fl187-GCST90094753 | Weighted mode | 8 | 0.206197009383081 | 0.168695334599965 | 0.26114832667967 | -0.124445846 | 0.536839865199012 | 1.22899530375317 | 0.882986076917494 | 1.71059260857233 | 0.999228396881742 |
| ebi-fl187-GCST90094753 | prot-a-1567 | Interferon regulatory factor 1 \|\| id:prot-a-1567 | F-deep fried food liking (derived food-liking factor) \|\| id:ebi-fl187-GCST90094753 | Weighted mode | 8 | 0.380309411825687 | 0.3573239437202 | 0.322511053123896 | -0.320045518 | 1.08066434151728 | 1.46273710758234 | 0.726115985071428 | 2.94663647390695 | 0.999228396881742 |
| ebi-fl187-GCST90094753 | prot-a-1613 | Potassium voltage-gated channel subfamily E regulatory beta subunit 5 \|\| id:prot-a-1613 | F-deep fried food liking (derived food-liking factor) \|\| id:ebi-fl187-GCST90094753 | Weighted mode | 8 | 0.137315168098544 | 0.17585511470708 | 0.460481271541755 | -0.207360857 | 0.481991192924421 | 1.14718964913106 | 0.812726319305776 | 1.61929552398106 | 0.999228396881742 |
| ebi-fl187-GCST90094753 | prot-a-1649 | Kin of IRRE-like protein 2 \|\| id:prot-a-1649 | F-deep fried food liking (derived food-liking factor) \|\| id:ebi-fl187-GCST90094753 | Weighted mode | 8 | 0.249477644257278 | 0.16117365637851 | 0.165577078349104 | -0.066422722 | 0.565378010759158 | 1.28335487378375 | 0.935735224580169 | 1.76011299863532 | 0.999228396881742 |
| ebi-fl187-GCST90094753 | prot-a-165 | Armadillo repeat-containing protein 5 \|\| id:prot-a-165 | F-deep fried food liking (derived food-liking factor) \|\| id:ebi-fl187-GCST90094753 | Weighted mode | 8 | 0.00784150528684591 | 0.278252842503522 | 0.978304222384164 | -0.537534066 | 0.553217076593749 | 1.00787233040853 | 0.58418704434519 | 1.73883800443014 | 0.999228396881742 |
| ebi-fl187-GCST90094753 | prot-a-1656 | Kallikrein-11 \|\| id:prot-a-1656 | F-deep fried food liking (derived food-liking factor) \|\| id:ebi-fl187-GCST90094753 | Weighted mode | 8 | 0.0894000400086154 | 0.155618017693746 | 0.583622223560112 | -0.215611275 | 0.394411354688357 | 1.09351801979711 | 0.80604857247785 | 1.48351067224781 | 0.999228396881742 |
| ebi-fl187-GCST90094753 | prot-a-1697 | Laminin subunit gamma-2 \|\| id:prot-a-1697 | F-deep fried food liking (derived food-liking factor) \|\| id:ebi-fl187-GCST90094753 | Weighted mode | 8 | -0.014741226 | 0.172649804252628 | 0.934348286916356 | -0.353134842 | 0.323652390205977 | 0.985366893819192 | 0.702482462524122 | 1.38216676889858 | 0.999228396881742 |
| ebi-fl187-GCST90094753 | prot-a-1698 | Lysosome-associated membrane glycoprotein 3 \|\| id:prot-a-1698 | F-deep fried food liking (derived food-liking factor) \|\| id:ebi-fl187-GCST90094753 | Weighted mode | 8 | -0.172098377 | 0.163846991375246 | 0.328456268152963 | -0.49323848 | 0.149041726040239 | 0.84189634581109 | 0.610645624839294 | 1.16072142050735 | 0.999228396881742 |
| ebi-fl187-GCST90094753 | prot-a-1724 | Leptin receptor \|\| id:prot-a-1724 | F-deep fried food liking (derived food-liking factor) \|\| id:ebi-fl187-GCST90094753 | Weighted mode | 8 | -0.047115309 | 0.155006551925548 | 0.769997039925845 | -0.350928151 | 0.256697532611681 | 0.953977388914331 | 0.704034336246204 | 1.29265408191902 | 0.999228396881742 |
| ebi-fl187-GCST90094753 | prot-a-1743 | Leukocyte immunoglobulin-like receptor subfamily B member 2 \|\| id:prot-a-1743 | F-deep fried food liking (derived food-liking factor) \|\| id:ebi-fl187-GCST90094753 | Weighted mode | 8 | -0.111187789 | 0.146305136751269 | 0.472086004409876 | -0.397945857 | 0.175570279330523 | 0.894770705321872 | 0.671698394622037 | 1.19192575345175 | 0.999228396881742 |
| ebi-fl187-GCST90094753 | prot-a-1746 | Leukocyte immunoglobulin-like receptor subfamily B member 4 \|\| id:prot-a-1746 | F-deep fried food liking (derived food-liking factor) \|\| id:ebi-fl187-GCST90094753 | Weighted mode | 8 | 0.373635086792836 | 0.185396302157926 | 0.0837047980077646 | 0.0102583345632999 | 0.737011839022371 | 1.45300683231948 | 1.01031113165963 | 2.08968186988002 | 0.999228396881742 |
| ebi-fl187-GCST90094753 | prot-a-1750 | Leucine-rich repeat and immunoglobulin-like domain-containing nogo receptor-interacting protein 3 \|\| id:prot-a-1750 | F-deep fried food liking (derived food-liking factor) \|\| id:ebi-fl187-GCST90094753 | Weighted mode | 8 | 0.199313451961976 | 0.149779050242028 | 0.224991639049075 | -0.094253487 | 0.492880390436351 | 1.22056449428006 | 0.910052047080896 | 1.63702470586795 | 0.999228396881742 |
| ebi-fl187-GCST90094753 | prot-a-1753 | Lipase member N \|\| id:prot-a-1753 | F-deep fried food liking (derived food-liking factor) \|\| id:ebi-fl187-GCST90094753 | Weighted mode | 8 | 0.110973744517495 | 0.157405579844827 | 0.503575515287053 | -0.197541192 | 0.419488681013356 | 1.11736556949717 | 0.820746331766262 | 1.52118354670062 | 0.999228396881742 |
| ebi-fl187-GCST90094753 | prot-a-1760 | Ig Kappa chain V-I region HK102- like \|\| id:prot-a-1760 | F-deep fried food liking (derived food-liking factor) \|\| id:ebi-fl187-GCST90094753 | Weighted mode | 8 | 0.00106859226263456 | 0.172275528770932 | 0.99522397785053 | -0.336591444 | 0.338728628653662 | 1.00106916341077 | 0.714200571141252 | 1.40316251544097 | 0.999228396881742 |
| ebi-fl187-GCST90094753 | prot-a-1792 | Leucine-rich repeat neuronal protein 1 \|\| id:prot-a-1792 | F-deep fried food liking (derived food-liking factor) \|\| id:ebi-fl187-GCST90094753 | Weighted mode | 7 | -0.303694187 | 0.325034796078158 | 0.386186387505184 | -0.940762388 | 0.333374013014463 | 0.738086548181735 | 0.39033013903039 | 1.39566919930929 | 0.999228396881742 |
| ebi-fl187-GCST90094753 | prot-a-1795 | Leucine-rich repeat transmembrane neuronal protein 1 \|\| id:prot-a-1795 | F-deep fried food liking (derived food-liking factor) \|\| id:ebi-fl187-GCST90094753 | Weighted mode | 8 | 0.0320839872203233 | 0.155511584464081 | 0.842419187926703 | -0.272718718 | 0.336886692769922 | 1.03260422722204 | 0.761306899191232 | 1.40058035886654 | 0.999228396881742 |
| ebi-fl187-GCST90094753 | prot-a-1820 | LysM and putative peptidoglycan-binding domain-containing protein 4 \|\| id:prot-a-1820 | F-deep fried food liking (derived food-liking factor) \|\| id:ebi-fl187-GCST90094753 | Weighted mode | 8 | 0.150075363887586 | 0.167057934570957 | 0.398847398855657 | -0.177358188 | 0.477508915646661 | 1.16192180637307 | 0.837479755701749 | 1.61205363465055 | 0.999228396881742 |
| ebi-fl187-GCST90094753 | prot-a-1844 | Mitogen-activated protein kinase kinase kinase 7:TGF-beta-activated kinase 1 and MAP3K7-binding protein 1 fusion \|\| id:prot-a-1844 | F-deep fried food liking (derived food-liking factor) \|\| id:ebi-fl187-GCST90094753 | Weighted mode | 8 | 0.131299390311329 | 0.16248783842173 | 0.445645680431459 | -0.187176773 | 0.449775553617921 | 1.1403091277163 | 0.829297126090474 | 1.567960223 | 0.999228396881742 |
| ebi-fl187-GCST90094753 | prot-a-1856 | Megakaryocyte-associated tyrosine-protein kinase \|\| id:prot-a-1856 | F-deep fried food liking (derived food-liking factor) \|\| id:ebi-fl187-GCST90094753 | Weighted mode | 8 | -0.05034869 | 0.174467003013322 | 0.781256954959118 | -0.392304016 | 0.291606636101702 | 0.950897798319431 | 0.675498720517318 | 1.33857636644563 | 0.999228396881742 |
| ebi-fl187-GCST90094753 | prot-a-1910 | Stromelysin-2 \|\| id:prot-a-1910 | F-deep fried food liking (derived food-liking factor) \|\| id:ebi-fl187-GCST90094753 | Weighted mode | 8 | 0.153347026965516 | 0.157590157592705 | 0.362933255897192 | -0.155529682 | 0.462223735847218 | 1.16572944831085 | 0.855961669969321 | 1.58760046662817 | 0.999228396881742 |
| ebi-fl187-GCST90094753 | prot-a-1940 | 39S ribosomal protein L14, mitochondrial \|\| id:prot-a-1940 | F-deep fried food liking (derived food-liking factor) \|\| id:ebi-fl187-GCST90094753 | Weighted mode | 8 | 0.0768969085930058 | 0.157633874485152 | 0.640584783300018 | -0.232065485 | 0.385859302583904 | 1.07993073907439 | 0.792894198614465 | 1.47087770756264 | 0.999228396881742 |
| ebi-fl187-GCST90094753 | prot-a-1942 | 39S ribosomal protein L33, mitochondrial \|\| id:prot-a-1942 | F-deep fried food liking (derived food-liking factor) \|\| id:ebi-fl187-GCST90094753 | Weighted mode | 8 | -0.104659364 | 0.160914892430658 | 0.536187693524504 | -0.420052553 | 0.210733825385562 | 0.900631257969364 | 0.657012290978543 | 1.23458369648365 | 0.999228396881742 |
| ebi-fl187-GCST90094753 | prot-a-1948 | Mesothelin \|\| id:prot-a-1948 | F-deep fried food liking (derived food-liking factor) \|\| id:ebi-fl187-GCST90094753 | Weighted mode | 8 | -0.075910636 | 0.170266827412425 | 0.669180851593263 | -0.409633618 | 0.257812345742643 | 0.926899034276963 | 0.663893444380338 | 1.29409595322254 | 0.999228396881742 |
| ebi-fl187-GCST90094753 | prot-a-1980 | Unconventional myosin-VI \|\| id:prot-a-1980 | F-deep fried food liking (derived food-liking factor) \|\| id:ebi-fl187-GCST90094753 | Weighted mode | 8 | 0.140183230201893 | 0.159493135627293 | 0.408595135929288 | -0.172423316 | 0.452789776031388 | 1.15048458306805 | 0.841622825655439 | 1.57269353388374 | 0.999228396881742 |
| ebi-fl187-GCST90094753 | prot-a-2039 | Neurofascin \|\| id:prot-a-2039 | F-deep fried food liking (derived food-liking factor) \|\| id:ebi-fl187-GCST90094753 | Weighted mode | 8 | -0.027956037 | 0.167120579232421 | 0.871879204394011 | -0.355512372 | 0.299600298139766 | 0.972431116699235 | 0.700814273274919 | 1.34931937431298 | 0.999228396881742 |
| ebi-fl187-GCST90094753 | prot-a-2041 | NFU1 iron-sulfur cluster scaffold homolog, mitochondrial \|\| id:prot-a-2041 | F-deep fried food liking (derived food-liking factor) \|\| id:ebi-fl187-GCST90094753 | Weighted mode | 8 | 0.147752119045187 | 0.167102361229125 | 0.405931092128043 | -0.179768509 | 0.475272747054271 | 1.15922551081928 | 0.835463591359937 | 1.60845283843767 | 0.999228396881742 |
| ebi-fl187-GCST90094753 | prot-a-2088 | Glucocorticoid receptor \|\| id:prot-a-2088 | F-deep fried food liking (derived food-liking factor) \|\| id:ebi-fl187-GCST90094753 | Weighted mode | 8 | 0.211532500459204 | 0.295631431362846 | 0.497449363383462 | -0.367905105 | 0.790970105930381 | 1.23557012157248 | 0.692182863256776 | 2.20553499134565 | 0.999228396881742 |
| ebi-fl187-GCST90094753 | prot-a-2111 | Neuron-specific protein family member 2 \|\| id:prot-a-2111 | F-deep fried food liking (derived food-liking factor) \|\| id:ebi-fl187-GCST90094753 | Weighted mode | 8 | 0.202625482038317 | 0.163510128243571 | 0.255197881304964 | -0.117854369 | 0.523105333395716 | 1.22461374250965 | 0.88882548345021 | 1.68725902470989 | 0.999228396881742 |
| ebi-fl187-GCST90094753 | prot-a-2125 | Nuclear migration protein nudC \|\| id:prot-a-2125 | F-deep fried food liking (derived food-liking factor) \|\| id:ebi-fl187-GCST90094753 | Weighted mode | 8 | 0.194637589967622 | 0.208733337595737 | 0.382130465289976 | -0.214479752 | 0.603754931655267 | 1.21487062539623 | 0.806961151141796 | 1.82897359353954 | 0.999228396881742 |
| ebi-fl187-GCST90094753 | prot-a-2142 | Ornithine decarboxylase \|\| id:prot-a-2142 | F-deep fried food liking (derived food-liking factor) \|\| id:ebi-fl187-GCST90094753 | Weighted mode | 8 | -0.266031729 | 0.178520128373968 | 0.179787542587973 | -0.615931181 | 0.0838677226147916 | 0.766414809541128 | 0.540137695311404 | 1.08748503461754 | 0.999228396881742 |
| ebi-fl187-GCST90094753 | prot-a-2186 | Prostate and testis expressed protein 1 \|\| id:prot-a-2186 | F-deep fried food liking (derived food-liking factor) \|\| id:ebi-fl187-GCST90094753 | Weighted mode | 8 | -0.277276847 | 0.308376653651232 | 0.398446083489888 | -0.881695088 | 0.327141394060007 | 0.757844660971668 | 0.414080413606845 | 1.3869975765349 | 0.999228396881742 |
| ebi-fl187-GCST90094753 | prot-a-2227 | Platelet-derived growth factor C \|\| id:prot-a-2227 | F-deep fried food liking (derived food-liking factor) \|\| id:ebi-fl187-GCST90094753 | Weighted mode | 8 | -0.100635161 | 0.211234652665575 | 0.648291190244279 | -0.51465508 | 0.313384758254351 | 0.904262883104067 | 0.59770671995331 | 1.36804779746086 | 0.999228396881742 |
| ebi-fl187-GCST90094753 | prot-a-2229 | Platelet-derived growth factor receptor alpha \|\| id:prot-a-2229 | F-deep fried food liking (derived food-liking factor) \|\| id:ebi-fl187-GCST90094753 | Weighted mode | 8 | 0.107147319240498 | 0.195847830545347 | 0.601311410571057 | -0.276714429 | 0.491009067109379 | 1.113098223 | 0.758271006686005 | 1.63396416787023 | 0.999228396881742 |
| ebi-fl187-GCST90094753 | prot-a-2243 | Platelet endothelial aggregation receptor 1 \|\| id:prot-a-2243 | F-deep fried food liking (derived food-liking factor) \|\| id:ebi-fl187-GCST90094753 | Weighted mode | 8 | -0.016452539 | 0.164987412757891 | 0.923362457107414 | -0.339827868 | 0.306922789905848 | 0.983682064719282 | 0.711892851682278 | 1.35923601727953 | 0.999228396881742 |
| ebi-fl187-GCST90094753 | prot-a-2246 | E3 ubiquitin-protein ligase pellino homolog 2 \|\| id:prot-a-2246 | F-deep fried food liking (derived food-liking factor) \|\| id:ebi-fl187-GCST90094753 | Weighted mode | 8 | 0.173134728345306 | 0.15874520633949 | 0.311541866000125 | -0.138005876 | 0.484275332770708 | 1.18902628983763 | 0.871093573115154 | 1.62299844879943 | 0.999228396881742 |
| ebi-fl187-GCST90094753 | prot-a-2251 | Platelet factor 4 variant \|\| id:prot-a-2251 | F-deep fried food liking (derived food-liking factor) \|\| id:ebi-fl187-GCST90094753 | Weighted mode | 8 | 0.0158742879922794 | 0.163458265608413 | 0.925356991411704 | -0.304503913 | 0.336252488584768 | 1.01600095385781 | 0.737489142728785 | 1.39969238654894 | 0.999228396881742 |
| ebi-fl187-GCST90094753 | prot-a-2346 | Protein phosphatase 1L \|\| id:prot-a-2346 | F-deep fried food liking (derived food-liking factor) \|\| id:ebi-fl187-GCST90094753 | Weighted mode | 8 | 0.108289005842484 | 0.194864935446779 | 0.595713257131819 | -0.273646268 | 0.490224279318171 | 1.11436975822324 | 0.760601076899904 | 1.63268235578104 | 0.999228396881742 |
| ebi-fl187-GCST90094753 | prot-a-2358 | PR domain zinc finger protein 4 \|\| id:prot-a-2358 | F-deep fried food liking (derived food-liking factor) \|\| id:ebi-fl187-GCST90094753 | Weighted mode | 8 | -0.013150703 | 0.205037874934709 | 0.950653558116889 | -0.415024938 | 0.388723531497004 | 0.986935389318227 | 0.660323813181259 | 1.47509667718336 | 0.999228396881742 |
| ebi-fl187-GCST90094753 | prot-a-2390 | Trypsin-1 \|\| id:prot-a-2390 | F-deep fried food liking (derived food-liking factor) \|\| id:ebi-fl187-GCST90094753 | Weighted mode | 8 | 0.294419923795205 | 0.223426368809934 | 0.229074636940449 | -0.143495759 | 0.732335606662675 | 1.3423474689751 | 0.866324474198945 | 2.07993284401899 | 0.999228396881742 |
| ebi-fl187-GCST90094753 | prot-a-242 | Brain-derived neurotrophic factor \|\| id:prot-a-242 | F-deep fried food liking (derived food-liking factor) \|\| id:ebi-fl187-GCST90094753 | Weighted mode | 8 | 0.159453167220499 | 0.182323223955043 | 0.410811342603676 | -0.197900352 | 0.516806686172383 | 1.17286933220366 | 0.8204516056465 | 1.67666497445622 | 0.999228396881742 |
| ebi-fl187-GCST90094753 | prot-a-2464 | Dihydropteridine reductase \|\| id:prot-a-2464 | F-deep fried food liking (derived food-liking factor) \|\| id:ebi-fl187-GCST90094753 | Weighted mode | 8 | 0.0666167613527868 | 0.15348301509827 | 0.677327521112263 | -0.234209948 | 0.367443470945396 | 1.06888576133675 | 0.791195688314981 | 1.44403816610993 | 0.999228396881742 |
| ebi-fl187-GCST90094753 | prot-a-2474 | Ras-related protein Rab-27A \|\| id:prot-a-2474 | F-deep fried food liking (derived food-liking factor) \|\| id:ebi-fl187-GCST90094753 | Weighted mode | 8 | 0.390570127385117 | 0.393084888915472 | 0.353513415816475 | -0.379876255 | 1.16101650965944 | 1.47782310116894 | 0.683946038954355 | 3.19317752272899 | 0.999228396881742 |
| ebi-fl187-GCST90094753 | prot-a-2478 | Ras-related protein Rab-7b \|\| id:prot-a-2478 | F-deep fried food liking (derived food-liking factor) \|\| id:ebi-fl187-GCST90094753 | Weighted mode | 8 | -0.031729185 | 0.17868201331628 | 0.864087165533334 | -0.381945931 | 0.318487561257684 | 0.968768903860784 | 0.682531956067558 | 1.37504651722817 | 0.999228396881742 |
| ebi-fl187-GCST90094753 | prot-a-2528 | E3 ubiquitin-protein ligase rififylin \|\| id:prot-a-2528 | F-deep fried food liking (derived food-liking factor) \|\| id:ebi-fl187-GCST90094753 | Weighted mode | 8 | 0.138309632914819 | 0.182450268646175 | 0.473154622237944 | -0.219292894 | 0.495912159461322 | 1.1483310563253 | 0.803086464791974 | 1.64199531773052 | 0.999228396881742 |
| ebi-fl187-GCST90094753 | prot-a-2543 | Protein RIC-3 \|\| id:prot-a-2543 | F-deep fried food liking (derived food-liking factor) \|\| id:ebi-fl187-GCST90094753 | Weighted mode | 8 | 0.161677048816833 | 0.148486485559566 | 0.312287304902672 | -0.129356463 | 0.452710560513581 | 1.17548055717785 | 0.878660699791577 | 1.57256895708537 | 0.999228396881742 |
| ebi-fl187-GCST90094753 | prot-a-255 | Bone morphogenetic protein 6 \|\| id:prot-a-255 | F-deep fried food liking (derived food-liking factor) \|\| id:ebi-fl187-GCST90094753 | Weighted mode | 8 | -0.089548253 | 0.165427924768015 | 0.605083399028049 | -0.413786985 | 0.234690480018968 | 0.914344144644926 | 0.661141769333828 | 1.26451731477932 | 0.999228396881742 |
| ebi-fl187-GCST90094753 | prot-a-2568 | RING finger protein 165 \|\| id:prot-a-2568 | F-deep fried food liking (derived food-liking factor) \|\| id:ebi-fl187-GCST90094753 | Weighted mode | 8 | 0.0208340485179774 | 0.210139467737841 | 0.923803512746097 | -0.391039308 | 0.432707405284145 | 1.02105259238598 | 0.676353569242789 | 1.54142514186081 | 0.999228396881742 |
| ebi-fl187-GCST90094753 | prot-a-2573 | E3 ubiquitin-protein ligase RNF8 \|\| id:prot-a-2573 | F-deep fried food liking (derived food-liking factor) \|\| id:ebi-fl187-GCST90094753 | Weighted mode | 8 | -0.078088008 | 0.157436331149766 | 0.635088423923912 | -0.386663218 | 0.230487200554865 | 0.924883025395079 | 0.679319839404253 | 1.25921334995034 | 0.999228396881742 |
| ebi-fl187-GCST90094753 | prot-a-260 | Cytoplasmic tyrosine-protein kinase BMX \|\| id:prot-a-260 | F-deep fried food liking (derived food-liking factor) \|\| id:ebi-fl187-GCST90094753 | Weighted mode | 7 | -0.112029454 | 0.175592856403481 | 0.547031359060987 | -0.456191452 | 0.232132544806052 | 0.894017924936781 | 0.633692503599617 | 1.26128689477644 | 0.999228396881742 |
| ebi-fl187-GCST90094753 | prot-a-2623 | Serum amyloid A-1 protein \|\| id:prot-a-2623 | F-deep fried food liking (derived food-liking factor) \|\| id:ebi-fl187-GCST90094753 | Weighted mode | 8 | -0.211902292 | 0.212484837814473 | 0.351855774738858 | -0.628372574 | 0.204567990176375 | 0.809043743799379 | 0.533459260392725 | 1.2269948766079 | 0.999228396881742 |
| ebi-fl187-GCST90094753 | prot-a-2640 | Lysosome membrane protein 2 \|\| id:prot-a-2640 | F-deep fried food liking (derived food-liking factor) \|\| id:ebi-fl187-GCST90094753 | Weighted mode | 8 | 0.245851761154275 | 0.180216767683468 | 0.21474194504997 | -0.107373104 | 0.599076625813872 | 1.27871000499622 | 0.898190492444498 | 1.8204370794745 | 0.999228396881742 |
| ebi-fl187-GCST90094753 | prot-a-2641 | Scavenger receptor class F member 2 \|\| id:prot-a-2641 | F-deep fried food liking (derived food-liking factor) \|\| id:ebi-fl187-GCST90094753 | Weighted mode | 8 | 0.356194907731086 | 0.227637458005716 | 0.161620819710289 | -0.08997451 | 0.802364325422288 | 1.42788582713043 | 0.913954481710503 | 2.23080905681883 | 0.999228396881742 |
| ebi-fl187-GCST90094753 | prot-a-2669 | Semaphorin-3A \|\| id:prot-a-2669 | F-deep fried food liking (derived food-liking factor) \|\| id:ebi-fl187-GCST90094753 | Weighted mode | 8 | 0.0038961232441358 | 0.167917482376086 | 0.982136221647196 | -0.325222142 | 0.333014388701264 | 1.00390372299896 | 0.722366867642804 | 1.39516737297192 | 0.999228396881742 |
| ebi-fl187-GCST90094753 | prot-a-2683 | Sentrin-specific protease 7 \|\| id:prot-a-2683 | F-deep fried food liking (derived food-liking factor) \|\| id:ebi-fl187-GCST90094753 | Weighted mode | 8 | -0.001787571 | 0.161384807657439 | 0.99147152242362 | -0.318101794 | 0.314526652304547 | 0.998214026048899 | 0.727528726795021 | 1.36961085535457 | 0.999228396881742 |
| ebi-fl187-GCST90094753 | prot-a-270 | Bromodomain testis-specific protein \|\| id:prot-a-270 | F-deep fried food liking (derived food-liking factor) \|\| id:ebi-fl187-GCST90094753 | Weighted mode | 8 | 0.0653955653163332 | 0.163808259001947 | 0.701630300352965 | -0.255668622 | 0.386459752960149 | 1.06758123898247 | 0.774398544665475 | 1.47176116184424 | 0.999228396881742 |
| ebi-fl187-GCST90094753 | prot-a-2708 | 14-3-3 protein sigma \|\| id:prot-a-2708 | F-deep fried food liking (derived food-liking factor) \|\| id:ebi-fl187-GCST90094753 | Weighted mode | 8 | 0.118456486286409 | 0.169267183140643 | 0.506623550451191 | -0.213307193 | 0.450220165242069 | 1.12575788712992 | 0.807907915703392 | 1.56865751133511 | 0.999228396881742 |
| ebi-fl187-GCST90094753 | prot-a-2713 | Beta-sarcoglycan \|\| id:prot-a-2713 | F-deep fried food liking (derived food-liking factor) \|\| id:ebi-fl187-GCST90094753 | Weighted mode | 8 | -0.083338748 | 0.167174590808364 | 0.633402400508775 | -0.411000946 | 0.244323450273974 | 0.920039433175412 | 0.662986304617722 | 1.27675723118566 | 0.999228396881742 |
| ebi-fl187-GCST90094753 | prot-a-2720 | Protein shisa-3 homolog \|\| id:prot-a-2720 | F-deep fried food liking (derived food-liking factor) \|\| id:ebi-fl187-GCST90094753 | Weighted mode | 8 | 0.00969277090013421 | 0.201910647408065 | 0.963052996209717 | -0.386052098 | 0.405437639819942 | 1.00973989794481 | 0.679735111904602 | 1.49995879813354 | 0.999228396881742 |
| ebi-fl187-GCST90094753 | prot-a-2730 | Sialic acid-binding Ig-like lectin 8 \|\| id:prot-a-2730 | F-deep fried food liking (derived food-liking factor) \|\| id:ebi-fl187-GCST90094753 | Weighted mode | 8 | -0.170231124 | 0.229761538535414 | 0.482859907551571 | -0.62056374 | 0.280101491265287 | 0.843469847718368 | 0.53764126237249 | 1.3232641052709 | 0.999228396881742 |
| ebi-fl187-GCST90094753 | prot-a-2761 | Sodium- and chloride-dependent neutral and basic amino acid transporter B(0+) \|\| id:prot-a-2761 | F-deep fried food liking (derived food-liking factor) \|\| id:ebi-fl187-GCST90094753 | Weighted mode | 8 | -0.046481932 | 0.169082241025214 | 0.79132458723511 | -0.377883124 | 0.284919260592593 | 0.954581807973115 | 0.685310592191123 | 1.32965466825747 | 0.999228396881742 |
| ebi-fl187-GCST90094753 | prot-a-2770 | Secreted Ly-6/uPAR-related protein 1 \|\| id:prot-a-2770 | F-deep fried food liking (derived food-liking factor) \|\| id:ebi-fl187-GCST90094753 | Weighted mode | 8 | -0.031428983 | 0.149663476500634 | 0.839650859830318 | -0.324769397 | 0.261911430740775 | 0.969059773533799 | 0.722693989727656 | 1.2994114494231 | 0.999228396881742 |
| ebi-fl187-GCST90094753 | prot-a-2784 | N-lysine methyltransferase SMYD2 \|\| id:prot-a-2784 | F-deep fried food liking (derived food-liking factor) \|\| id:ebi-fl187-GCST90094753 | Weighted mode | 8 | -0.11195382 | 0.1765015781444 | 0.546051354578339 | -0.457896913 | 0.233989272987607 | 0.894085545260662 | 0.632612686776075 | 1.26363093715035 | 0.999228396881742 |
| ebi-fl187-GCST90094753 | prot-a-2837 | Serine palmitoyltransferase 2 \|\| id:prot-a-2837 | F-deep fried food liking (derived food-liking factor) \|\| id:ebi-fl187-GCST90094753 | Weighted mode | 8 | -0.055491767 | 0.154226516140079 | 0.729601910756114 | -0.357775739 | 0.246792204327001 | 0.946019811928535 | 0.699229867454433 | 1.27991312473451 | 0.999228396881742 |
| ebi-fl187-GCST90094753 | prot-a-2858 | Alpha-2,8-sialyltransferase 8B \|\| id:prot-a-2858 | F-deep fried food liking (derived food-liking factor) \|\| id:ebi-fl187-GCST90094753 | Weighted mode | 8 | 0.187767337107932 | 0.150792075762171 | 0.253121000508093 | -0.107785131 | 0.483319805601788 | 1.20655276263532 | 0.897820489150591 | 1.62144837037545 | 0.999228396881742 |
| ebi-fl187-GCST90094753 | prot-a-2911 | Synaptotagmin-11 \|\| id:prot-a-2911 | F-deep fried food liking (derived food-liking factor) \|\| id:ebi-fl187-GCST90094753 | Weighted mode | 8 | 0.160996406533933 | 0.161277157852972 | 0.351403056722897 | -0.155106823 | 0.477099635925758 | 1.17468074763088 | 0.856323697652969 | 1.61139398878793 | 0.999228396881742 |
| ebi-fl187-GCST90094753 | prot-a-2962 | Transforming growth factor beta-1 \|\| id:prot-a-2962 | F-deep fried food liking (derived food-liking factor) \|\| id:ebi-fl187-GCST90094753 | Weighted mode | 8 | -0.127455802 | 0.154249018148084 | 0.435889678805826 | -0.429783878 | 0.174872273333996 | 0.880332323694459 | 0.650649699369109 | 1.19109407242136 | 0.999228396881742 |
| ebi-fl187-GCST90094753 | prot-a-2978 | Tyrosine-protein kinase receptor Tie-1, soluble \|\| id:prot-a-2978 | F-deep fried food liking (derived food-liking factor) \|\| id:ebi-fl187-GCST90094753 | Weighted mode | 8 | -0.202923503 | 0.21264256895992 | 0.371719196051936 | -0.619702938 | 0.213855932333617 | 0.816340686791202 | 0.538104264188996 | 1.23844422217154 | 0.999228396881742 |
| ebi-fl187-GCST90094753 | prot-a-299 | Uncharacterized protein C1orf226 \|\| id:prot-a-299 | F-deep fried food liking (derived food-liking factor) \|\| id:ebi-fl187-GCST90094753 | Weighted mode | 8 | -0.023843648 | 0.174819368711118 | 0.895352511988134 | -0.366489611 | 0.318802314400027 | 0.976438365647625 | 0.693163337755417 | 1.37547938556009 | 0.999228396881742 |
| ebi-fl187-GCST90094753 | prot-a-3015 | Transmembrane protein 70, mitochondrial \|\| id:prot-a-3015 | F-deep fried food liking (derived food-liking factor) \|\| id:ebi-fl187-GCST90094753 | Weighted mode | 8 | -0.084293748 | 0.170236783237711 | 0.635652867619558 | -0.417957844 | 0.249370346772923 | 0.919161214323677 | 0.658389983244682 | 1.28321718042147 | 0.999228396881742 |
| ebi-fl187-GCST90094753 | prot-a-3016 | Transmembrane protein 87B \|\| id:prot-a-3016 | F-deep fried food liking (derived food-liking factor) \|\| id:ebi-fl187-GCST90094753 | Weighted mode | 8 | 0.0736090673840057 | 0.22002209240008 | 0.747762908344332 | -0.357634234 | 0.504852368488163 | 1.07638592886739 | 0.699328819132951 | 1.65674091523954 | 0.999228396881742 |
| ebi-fl187-GCST90094753 | prot-a-3123 | Thioredoxin domain-containing protein 12 \|\| id:prot-a-3123 | F-deep fried food liking (derived food-liking factor) \|\| id:ebi-fl187-GCST90094753 | Weighted mode | 8 | -0.231983492 | 0.177484587957898 | 0.232487854520865 | -0.579853284 | 0.115886300487351 | 0.792959213440596 | 0.55998051846826 | 1.12286819530842 | 0.999228396881742 |
| ebi-fl187-GCST90094753 | prot-a-3133 | PolyUbiquitin K48-linked \|\| id:prot-a-3133 | F-deep fried food liking (derived food-liking factor) \|\| id:ebi-fl187-GCST90094753 | Weighted mode | 8 | 0.0969953935724919 | 0.244564158831702 | 0.703471548235017 | -0.382350358 | 0.576341144882628 | 1.10185529799277 | 0.682255977666024 | 1.77951551537602 | 0.999228396881742 |
| ebi-fl187-GCST90094753 | prot-a-3135 | Ubiquitin-conjugating enzyme E2 B \|\| id:prot-a-3135 | F-deep fried food liking (derived food-liking factor) \|\| id:ebi-fl187-GCST90094753 | Weighted mode | 8 | -0.055119848 | 0.150903033342119 | 0.725699450104318 | -0.350889794 | 0.240650096859262 | 0.946371719933697 | 0.704061341475775 | 1.27207585409102 | 0.999228396881742 |
| ebi-fl187-GCST90094753 | prot-a-3162 | NKG2D ligand 3 \|\| id:prot-a-3162 | F-deep fried food liking (derived food-liking factor) \|\| id:ebi-fl187-GCST90094753 | Weighted mode | 8 | -0.014704073 | 0.178594723630091 | 0.936687448397689 | -0.364749731 | 0.335341585345069 | 0.985403503992421 | 0.694370408323949 | 1.39841798273685 | 0.999228396881742 |
| ebi-fl187-GCST90094753 | prot-a-3188 | Vesicle-associated membrane protein 8 \|\| id:prot-a-3188 | F-deep fried food liking (derived food-liking factor) \|\| id:ebi-fl187-GCST90094753 | Weighted mode | 8 | 0.18684448134935 | 0.177699733963931 | 0.327984243566881 | -0.161446997 | 0.535135959918654 | 1.20543980210029 | 0.850911630952178 | 1.70768040256023 | 0.999228396881742 |
| ebi-fl187-GCST90094753 | prot-a-3201 | Vitrin \|\| id:prot-a-3201 | F-deep fried food liking (derived food-liking factor) \|\| id:ebi-fl187-GCST90094753 | Weighted mode | 8 | 0.0133176469209914 | 0.148208242938825 | 0.93091736027951 | -0.277170509 | 0.303805803081089 | 1.01340672176395 | 0.75792525283399 | 1.35500589256828 | 0.999228396881742 |
| ebi-fl187-GCST90094753 | prot-a-3232 | WNT1-inducible-signaling pathway protein 3 \|\| id:prot-a-3232 | F-deep fried food liking (derived food-liking factor) \|\| id:ebi-fl187-GCST90094753 | Weighted mode | 8 | 0.325196610765697 | 0.238079035145067 | 0.214227174313454 | -0.141438298 | 0.791831519650028 | 1.38430278805796 | 0.868108737874784 | 2.2074356879719 | 0.999228396881742 |
| ebi-fl187-GCST90094753 | prot-a-327 | Carbonic anhydrase-related protein 10 \|\| id:prot-a-327 | F-deep fried food liking (derived food-liking factor) \|\| id:ebi-fl187-GCST90094753 | Weighted mode | 8 | -0.382616711 | 0.241363851957949 | 0.156933728732021 | -0.855689861 | 0.090456438994702 | 0.682074280866636 | 0.424989906105205 | 1.09467382151089 | 0.999228396881742 |
| ebi-fl187-GCST90094753 | prot-a-332 | Carbonic anhydrase 5A, mitochondrial \|\| id:prot-a-332 | F-deep fried food liking (derived food-liking factor) \|\| id:ebi-fl187-GCST90094753 | Weighted mode | 8 | -0.156759911 | 0.186394546886051 | 0.428132155011639 | -0.522093223 | 0.208573401268448 | 0.854909288814016 | 0.593277385729204 | 1.23191935118539 | 0.999228396881742 |
| ebi-fl187-GCST90094753 | prot-a-338 | Cell adhesion molecule 3 \|\| id:prot-a-338 | F-deep fried food liking (derived food-liking factor) \|\| id:ebi-fl187-GCST90094753 | Weighted mode | 8 | -0.074420575 | 0.197954987978354 | 0.718090820255758 | -0.462412352 | 0.313571201103327 | 0.928281199555132 | 0.629762602668992 | 1.36830288396854 | 0.999228396881742 |
| ebi-fl187-GCST90094753 | prot-a-34 | A disintegrin and metalloproteinase with thrombospondin motifs 3 \|\| id:prot-a-34 | F-deep fried food liking (derived food-liking factor) \|\| id:ebi-fl187-GCST90094753 | Weighted mode | 8 | 0.0779687900607325 | 0.162624708762432 | 0.646242108252258 | -0.240775639 | 0.396713219235099 | 1.0810889174241 | 0.786017958292437 | 1.48692944613662 | 0.999228396881742 |
| ebi-fl187-GCST90094753 | prot-a-358 | Cysteine--tRNA ligase, cytoplasmic \|\| id:prot-a-358 | F-deep fried food liking (derived food-liking factor) \|\| id:ebi-fl187-GCST90094753 | Weighted mode | 8 | -0.039549808 | 0.18650767404013 | 0.838106746106033 | -0.405104849 | 0.326005232833309 | 0.961222075974806 | 0.666906882403416 | 1.38542261853944 | 0.999228396881742 |
| ebi-fl187-GCST90094753 | prot-a-382 | Coiled-coil domain-containing protein 167 \|\| id:prot-a-382 | F-deep fried food liking (derived food-liking factor) \|\| id:ebi-fl187-GCST90094753 | Weighted mode | 8 | -0.148843181 | 0.200939940953103 | 0.482956015138352 | -0.542685466 | 0.244999102793718 | 0.86170423549172 | 0.581185401336469 | 1.27762016691553 | 0.999228396881742 |
| ebi-fl187-GCST90094753 | prot-a-40 | Pituitary adenylate cyclase-activating polypeptide 27 \|\| id:prot-a-40 | F-deep fried food liking (derived food-liking factor) \|\| id:ebi-fl187-GCST90094753 | Weighted mode | 8 | 0.0843869784603932 | 0.163701737782249 | 0.622085145914156 | -0.236468428 | 0.405242384513602 | 1.08804986421162 | 0.789410805507911 | 1.49966595180974 | 0.999228396881742 |
| ebi-fl187-GCST90094753 | prot-a-403 | C-C motif chemokine 25 \|\| id:prot-a-403 | F-deep fried food liking (derived food-liking factor) \|\| id:ebi-fl187-GCST90094753 | Weighted mode | 8 | -0.138012527 | 0.159229250976007 | 0.414796887447815 | -0.450101859 | 0.174076804413126 | 0.871087779125425 | 0.637563206500343 | 1.19014697084979 | 0.999228396881742 |
| ebi-fl187-GCST90094753 | prot-a-415 | Cyclin-C \|\| id:prot-a-415 | F-deep fried food liking (derived food-liking factor) \|\| id:ebi-fl187-GCST90094753 | Weighted mode | 8 | 0.0720613600335667 | 0.222027833837675 | 0.754997786974279 | -0.363113194 | 0.507235914355409 | 1.07472128697491 | 0.695507701543909 | 1.66069454315609 | 0.999228396881742 |
| ebi-fl187-GCST90094753 | prot-a-44 | Adhesion G protein-coupled receptor F5 \|\| id:prot-a-44 | F-deep fried food liking (derived food-liking factor) \|\| id:ebi-fl187-GCST90094753 | Weighted mode | 8 | 0.106875588433624 | 0.17022173288749 | 0.550020897708888 | -0.226759008 | 0.440510184893105 | 1.11279580119823 | 0.797112856945996 | 1.55349958838802 | 0.999228396881742 |
| ebi-fl187-GCST90094753 | prot-a-455 | CD59 glycoprotein \|\| id:prot-a-455 | F-deep fried food liking (derived food-liking factor) \|\| id:ebi-fl187-GCST90094753 | Weighted mode | 8 | 0.0962950933591894 | 0.161236947912341 | 0.569162015879271 | -0.219729325 | 0.412319511267378 | 1.10108393861565 | 0.802736049501056 | 1.51031692251881 | 0.999228396881742 |
| ebi-fl187-GCST90094753 | prot-a-478 | Cadherin-12 \|\| id:prot-a-478 | F-deep fried food liking (derived food-liking factor) \|\| id:ebi-fl187-GCST90094753 | Weighted mode | 8 | 0.171571706011656 | 0.171055267490946 | 0.349256034381739 | -0.163696618 | 0.50684003029391 | 1.18716926685358 | 0.84899955376588 | 1.66003723077376 | 0.999228396881742 |
| ebi-fl187-GCST90094753 | prot-a-516 | Centrin-2 \|\| id:prot-a-516 | F-deep fried food liking (derived food-liking factor) \|\| id:ebi-fl187-GCST90094753 | Weighted mode | 8 | 0.0109570220095463 | 0.174313897289104 | 0.951636813448698 | -0.330698217 | 0.35261226069619 | 1.0110172700204 | 0.718421944090911 | 1.42277936898619 | 0.999228396881742 |
| ebi-fl187-GCST90094753 | prot-a-519 | Cryptic protein \|\| id:prot-a-519 | F-deep fried food liking (derived food-liking factor) \|\| id:ebi-fl187-GCST90094753 | Weighted mode | 8 | 0.118814048129523 | 0.146478676973247 | 0.443988526191838 | -0.168284159 | 0.405912254997087 | 1.12616048716786 | 0.845113654132114 | 1.50067087031099 | 0.999228396881742 |
| ebi-fl187-GCST90094753 | prot-a-583 | Chloride intracellular channel protein 4 \|\| id:prot-a-583 | F-deep fried food liking (derived food-liking factor) \|\| id:ebi-fl187-GCST90094753 | Weighted mode | 8 | 0.227659357880726 | 0.162037684660748 | 0.202817618459792 | -0.089934504 | 0.545253219815793 | 1.25565752273305 | 0.913991046018772 | 1.72504514268919 | 0.999228396881742 |
| ebi-fl187-GCST90094753 | prot-a-602 | CCR4-NOT transcription complex subunit 1 \|\| id:prot-a-602 | F-deep fried food liking (derived food-liking factor) \|\| id:ebi-fl187-GCST90094753 | Weighted mode | 8 | 0.208296270353185 | 0.168716435283304 | 0.256814379144023 | -0.122387943 | 0.538980483508461 | 1.23157799555035 | 0.884805048162918 | 1.71425825640694 | 0.999228396881742 |
| ebi-fl187-GCST90094753 | prot-a-606 | Ciliary neurotrophic factor receptor subunit alpha \|\| id:prot-a-606 | F-deep fried food liking (derived food-liking factor) \|\| id:ebi-fl187-GCST90094753 | Weighted mode | 8 | -0.01634944 | 0.164889941071104 | 0.923796051486199 | -0.339533725 | 0.306834844184717 | 0.983783486373086 | 0.712102280988164 | 1.35911648354412 | 0.999228396881742 |
| ebi-fl187-GCST90094753 | prot-a-626 | Collagen alpha-2(IX) chain \|\| id:prot-a-626 | F-deep fried food liking (derived food-liking factor) \|\| id:ebi-fl187-GCST90094753 | Weighted mode | 8 | -0.094863442 | 0.174377635438182 | 0.603321206195072 | -0.436643608 | 0.246916723257435 | 0.909497124913662 | 0.646201691709552 | 1.28007250807077 | 0.999228396881742 |
| ebi-fl187-GCST90094753 | prot-a-647 | Probable serine carboxypeptidase CPVL \|\| id:prot-a-647 | F-deep fried food liking (derived food-liking factor) \|\| id:ebi-fl187-GCST90094753 | Weighted mode | 8 | -0.212305693 | 0.193860977245142 | 0.309697351882595 | -0.592273208 | 0.167661822512561 | 0.80871744060615 | 0.553068614508172 | 1.18253663575209 | 0.999228396881742 |
| ebi-fl187-GCST90094753 | prot-a-651 | Cellular retinoic acid-binding protein 2 \|\| id:prot-a-651 | F-deep fried food liking (derived food-liking factor) \|\| id:ebi-fl187-GCST90094753 | Weighted mode | 8 | 0.170059762763983 | 0.225559485673386 | 0.475472847777317 | -0.272036829 | 0.612156354683819 | 1.1853756905312 | 0.761826203157197 | 1.84440430360515 | 0.999228396881742 |
| ebi-fl187-GCST90094753 | prot-a-671 | Cytotoxic and regulatory T-cell molecule \|\| id:prot-a-671 | F-deep fried food liking (derived food-liking factor) \|\| id:ebi-fl187-GCST90094753 | Weighted mode | 8 | 0.0240550432416033 | 0.182847889795266 | 0.89903554706857 | -0.334326821 | 0.382436907240325 | 1.02434669970177 | 0.715819799225297 | 1.46585238676759 | 0.999228396881742 |
| ebi-fl187-GCST90094753 | prot-a-688 | Chorionic somatomammotropin hormone \|\| id:prot-a-688 | F-deep fried food liking (derived food-liking factor) \|\| id:ebi-fl187-GCST90094753 | Weighted mode | 8 | -0.108312849 | 0.177411700022023 | 0.560810284469116 | -0.456039781 | 0.239414082765617 | 0.897346818208307 | 0.633788623648539 | 1.27050452170171 | 0.999228396881742 |
| ebi-fl187-GCST90094753 | prot-a-69 | Aldo-keto reductase family 1 member C1 \|\| id:prot-a-69 | F-deep fried food liking (derived food-liking factor) \|\| id:ebi-fl187-GCST90094753 | Weighted mode | 8 | -0.075261602 | 0.162734580681906 | 0.657768531562967 | -0.39422138 | 0.243698176298815 | 0.927500818670133 | 0.674204784280356 | 1.27595915764971 | 0.999228396881742 |
| ebi-fl187-GCST90094753 | prot-a-741 | Stromal cell-derived factor 1 \|\| id:prot-a-741 | F-deep fried food liking (derived food-liking factor) \|\| id:ebi-fl187-GCST90094753 | Weighted mode | 8 | -0.036441873 | 0.27458827398436 | 0.898153566022263 | -0.57463489 | 0.501751143648013 | 0.964214138794301 | 0.562910355283286 | 1.65161094786213 | 0.999228396881742 |
| ebi-fl187-GCST90094753 | prot-a-742 | Growth-regulated alpha protein \|\| id:prot-a-742 | F-deep fried food liking (derived food-liking factor) \|\| id:ebi-fl187-GCST90094753 | Weighted mode | 8 | 0.156718512113754 | 0.168301384815106 | 0.382753778210672 | -0.173152202 | 0.486589226351362 | 1.16966632065684 | 0.841009601655377 | 1.62675824269547 | 0.999228396881742 |
| ebi-fl187-GCST90094753 | prot-a-788 | Beta-defensin 112 \|\| id:prot-a-788 | F-deep fried food liking (derived food-liking factor) \|\| id:ebi-fl187-GCST90094753 | Weighted mode | 8 | 0.0333208467550203 | 0.186051461545569 | 0.862936825078071 | -0.331340018 | 0.397981711384336 | 1.03388220378187 | 0.717961007957604 | 1.48881680126002 | 0.999228396881742 |
| ebi-fl187-GCST90094753 | prot-a-838 | DnaJ homolog subfamily B member 1 \|\| id:prot-a-838 | F-deep fried food liking (derived food-liking factor) \|\| id:ebi-fl187-GCST90094753 | Weighted mode | 8 | 0.21759150879839 | 0.197718135658764 | 0.307507298821396 | -0.169936037 | 0.605119054689569 | 1.24307917698499 | 0.843718781576626 | 1.83147024102764 | 0.999228396881742 |
| ebi-fl187-GCST90094753 | prot-a-85 | Junctional adhesion molecule-like \|\| id:prot-a-85 | F-deep fried food liking (derived food-liking factor) \|\| id:ebi-fl187-GCST90094753 | Weighted mode | 8 | 0.0162056271112452 | 0.147257164339792 | 0.915458474651008 | -0.272418415 | 0.304829669217237 | 1.01633765049604 | 0.761535556523221 | 1.35639394768602 | 0.999228396881742 |
| ebi-fl187-GCST90094753 | prot-a-856 | Dipeptidase 1 \|\| id:prot-a-856 | F-deep fried food liking (derived food-liking factor) \|\| id:ebi-fl187-GCST90094753 | Weighted mode | 8 | 0.0830011369187127 | 0.147246103892287 | 0.590557983704033 | -0.205601227 | 0.371603500547596 | 1.08654304385865 | 0.814157675892648 | 1.45005792012369 | 0.999228396881742 |
| ebi-fl187-GCST90094753 | prot-a-860 | Dipeptidyl peptidase 2 \|\| id:prot-a-860 | F-deep fried food liking (derived food-liking factor) \|\| id:ebi-fl187-GCST90094753 | Weighted mode | 8 | 0.171404317190585 | 0.173570910723922 | 0.356285821851857 | -0.168794668 | 0.511603302209473 | 1.18697056462032 | 0.8446823260372 | 1.66796330152294 | 0.999228396881742 |
| ebi-fl187-GCST90094753 | prot-a-861 | Dermatopontin \|\| id:prot-a-861 | F-deep fried food liking (derived food-liking factor) \|\| id:ebi-fl187-GCST90094753 | Weighted mode | 8 | 0.162515664145418 | 0.163817502127722 | 0.354217747028185 | -0.15856664 | 0.483597968315754 | 1.17646674665056 | 0.853366093558299 | 1.6218994595899 | 0.999228396881742 |
| ebi-fl187-GCST90094753 | prot-a-877 | Dual specificity protein phosphatase 6 \|\| id:prot-a-877 | F-deep fried food liking (derived food-liking factor) \|\| id:ebi-fl187-GCST90094753 | Weighted mode | 8 | 0.115760993263602 | 0.164382351577582 | 0.504043802689996 | -0.206428416 | 0.437950402355663 | 1.12272750062744 | 0.813484491986164 | 1.54952805257237 | 0.999228396881742 |
| ebi-fl187-GCST90094753 | prot-a-879 | Dynactin-associated protein \|\| id:prot-a-879 | F-deep fried food liking (derived food-liking factor) \|\| id:ebi-fl187-GCST90094753 | Weighted mode | 8 | -0.03316027 | 0.15222801667322 | 0.833773100232996 | -0.331527183 | 0.265206642304842 | 0.967383504247302 | 0.717826643230992 | 1.30370034759025 | 0.999228396881742 |
| ebi-fl187-GCST90094753 | prot-a-937 | EMI domain-containing protein 1 \|\| id:prot-a-937 | F-deep fried food liking (derived food-liking factor) \|\| id:ebi-fl187-GCST90094753 | Weighted mode | 8 | -0.292353327 | 0.158356711179843 | 0.107368855837972 | -0.602732481 | 0.0180258265835933 | 0.746504728849114 | 0.547314065566825 | 1.01818927240058 | 0.999228396881742 |
| ebi-fl187-GCST90094753 | prot-b-1 | SRC proto-oncogene, non-receptor tyrosine kinase \|\| id:prot-b-1 | F-deep fried food liking (derived food-liking factor) \|\| id:ebi-fl187-GCST90094753 | Weighted mode | 7 | -0.029801789 | 0.159996129312168 | 0.858374460079132 | -0.343394202 | 0.283790624602434 | 0.970637905738819 | 0.709358525674178 | 1.32815481869571 | 0.999228396881742 |
| ebi-fl187-GCST90094753 | prot-b-10 | coagulation factor III, tissue factor \|\| id:prot-b-10 | F-deep fried food liking (derived food-liking factor) \|\| id:ebi-fl187-GCST90094753 | Weighted mode | 7 | -0.247048884 | 0.181968256961244 | 0.22341348910308 | -0.603706667 | 0.109608899864277 | 0.781102509355053 | 0.546781139393516 | 1.11584157931545 | 0.999228396881742 |
| ebi-fl187-GCST90094753 | prot-b-29 | myeloperoxidase \|\| id:prot-b-29 | F-deep fried food liking (derived food-liking factor) \|\| id:ebi-fl187-GCST90094753 | Weighted mode | 7 | 0.183670341760821 | 0.159487352694718 | 0.293287353356805 | -0.12892487 | 0.496265553042468 | 1.20161963398368 | 0.879040005761581 | 1.64257569088011 | 0.999228396881742 |
| ebi-fl187-GCST90094753 | prot-b-33 | adrenomedullin \|\| id:prot-b-33 | F-deep fried food liking (derived food-liking factor) \|\| id:ebi-fl187-GCST90094753 | Weighted mode | 7 | 0.0320014503040179 | 0.161048444652576 | 0.849054145079654 | -0.283653501 | 0.347656401823067 | 1.03251900277049 | 0.753027522628284 | 1.41574571851131 | 0.999228396881742 |
| ebi-fl187-GCST90094753 | ukb-rsfMRI191-edge-pheno102 | Net25_Pair11_15 (Postcentral\|Precentral)&(Cerebellum) (Motor)&(Subcortical-cerebellum) \|\| id:ukb-rsfMRI191-edge-pheno102 | F-deep fried food liking (derived food-liking factor) \|\| id:ebi-fl187-GCST90094753 | Weighted mode | 8 | -0.017777305 | 0.051604583557754 | 0.740594998133929 | -0.118922289 | 0.0833676785764137 | 0.98237977887208 | 0.887876795901412 | 1.08694138014608 | 0.999228396881742 |
| ebi-fl187-GCST90094753 | ukb-rsfMRI191-edge-pheno1167 | Net100_Pair11_45 (Angular\|Temporal)&(Frontal) (Default_mode\|Central_executive)&(Salience\|Default_mode) \|\| id:ukb-rsfMRI191-edge-pheno1167 | F-deep fried food liking (derived food-liking factor) \|\| id:ebi-fl187-GCST90094753 | Weighted mode | 8 | 0.0201273941338787 | 0.0499631507970949 | 0.699081841821873 | -0.077800381 | 0.118055169696185 | 1.02033131597176 | 0.925149085051142 | 1.12530619245558 | 0.999228396881742 |
| ebi-fl187-GCST90094753 | ukb-rsfMRI191-edge-pheno1317 | Net100_Pair26_48 (Parietal\|Frontal)&(Temporal) (Attention\|Central_executive)&(Default_mode\|Central_executive) \|\| id:ukb-rsfMRI191-edge-pheno1317 | F-deep fried food liking (derived food-liking factor) \|\| id:ebi-fl187-GCST90094753 | Weighted mode | 8 | 0.0452577966635671 | 0.0666653304024183 | 0.519021051039119 | -0.085406251 | 0.175921844252307 | 1.04629755716303 | 0.918139213721422 | 1.19234486640442 | 0.999228396881742 |
| ebi-fl187-GCST90094753 | ukb-rsfMRI191-edge-pheno286 | Net100_Pair10_13 (OccipitalPrecuneus)&(Frontal_Sup) (Default_mode\|Central_executive)&(Default_mode) \|\| id:ukb-rsfMRI191-edge-pheno286 | F-deep fried food liking (derived food-liking factor) \|\| id:ebi-fl187-GCST90094753 | Weighted mode | 8 | -0.019946083 | 0.050514323470468 | 0.704701470729428 | -0.118954157 | 0.0790619905340378 | 0.980251523644554 | 0.887848501050411 | 1.08227141057358 | 0.999228396881742 |
| ebi-fl187-GCST90094753 | ukb-rsfMRI191-edge-pheno599 | Net100_Pair11_29 (Angular\|Temporal)&(Parietal) (Default_mode\|Central_executive)&(Central_executive\|Salience) \|\| id:ukb-rsfMRI191-edge-pheno599 | F-deep fried food liking (derived food-liking factor) \|\| id:ebi-fl187-GCST90094753 | Weighted mode | 8 | 0.006010131854497 | 0.0589463115295526 | 0.921648097510825 | -0.109524639 | 0.12154490245242 | 1.00602822893406 | 0.896260081367874 | 1.1292400704353 | 0.999228396881742 |
| ebi-fl187-GCST90094753 | ukb-rsfMRI191-edge-pheno621 | Net100_Pair5_30 (Precuneus\|Angular\|Cingulate)&(Paracentral) (Default_mode\|Central_executive)&(Motor) \|\| id:ukb-rsfMRI191-edge-pheno621 | F-deep fried food liking (derived food-liking factor) \|\| id:ebi-fl187-GCST90094753 | Weighted mode | 8 | 0.093745316749716 | 0.103502627292149 | 0.39518436370642 | -0.109119833 | 0.296610466242328 | 1.09827999677567 | 0.896622966270884 | 1.3452911610488 | 0.999228396881742 |
| ebi-fl187-GCST90094753 | ukb-rsfMRI191-edge-pheno624 | Net100_Pair8_30 (Calcarine\|Lingual)&(Paracentral) (Visual)&(Motor) \|\| id:ukb-rsfMRI191-edge-pheno624 | F-deep fried food liking (derived food-liking factor) \|\| id:ebi-fl187-GCST90094753 | Weighted mode | 8 | -0.014917538 | 0.0496991774595186 | 0.772775448212753 | -0.112327926 | 0.0824928494624431 | 0.98519317690038 | 0.893751125048784 | 1.08599090799252 | 0.999228396881742 |
| ebi-fl187-GCST90094753 | ukb-rsfMRI191-edge-pheno882 | Net100_Pair6_38 (Postcentral\|Precentral)&(Subcortical) (Motor)&(Subcortical-cerebellum) \|\| id:ukb-rsfMRI191-edge-pheno882 | F-deep fried food liking (derived food-liking factor) \|\| id:ebi-fl187-GCST90094753 | Weighted mode | 8 | 0.0762298651303672 | 0.0558246216617102 | 0.214345293506644 | -0.033186393 | 0.185646123587319 | 1.07921061853734 | 0.967358233664608 | 1.20399611915389 | 0.999228396881742 |
| ebi-fl187-GCST90094753 | ukb-rsfMRI191-edge-pheno899 | Net100_Pair23_38 (Cerebellum)&(Subcortical) (Subcortical-cerebellum)&(Subcortical-cerebellum) \|\| id:ukb-rsfMRI191-edge-pheno899 | F-deep fried food liking (derived food-liking factor) \|\| id:ebi-fl187-GCST90094753 | Weighted mode | 8 | 0.00240983322719174 | 0.0663488126481396 | 0.972040731535106 | -0.12763384 | 0.132453506017545 | 1.00241273920912 | 0.880175606 | 1.141625936 | 0.999228396881742 |
| ebi-fl187-GCST90094756 | ebi-a-GCST90001392 | IgD+ CD38+ B cell Absolute Count \|\| id:ebi-a-GCST90001392 | Dried fruit liking \|\| id:ebi-fl187-GCST90094756 | Inverse variance weighted | 5 | 0.178145803859987 | 0.277906766993153 | 0.521504994327623 | -0.366551459 | 0.722843067166567 | 1.19499954407952 | 0.693120467969118 | 2.06028241314883 | 0.998074519377826 |
| ebi-fl187-GCST90094756 | ebi-a-GCST90001402 | Switched memory B cell %B cell \|\| id:ebi-a-GCST90001402 | Dried fruit liking \|\| id:ebi-fl187-GCST90094756 | Inverse variance weighted | 5 | 0.122157755846229 | 0.292830018767151 | 0.676559419507206 | -0.451789081 | 0.696104592629846 | 1.12993234115346 | 0.636488403105747 | 2.00592357905445 | 0.998074519377826 |
| ebi-fl187-GCST90094756 | ebi-a-GCST90001407 | Memory B cell Absolute Count \|\| id:ebi-a-GCST90001407 | Dried fruit liking \|\| id:ebi-fl187-GCST90094756 | Inverse variance weighted | 5 | 0.245321840324428 | 0.315032132745653 | 0.436144638572954 | -0.37214114 | 0.862784820505907 | 1.27803256943869 | 0.689256954030778 | 2.36975084399821 | 0.998074519377826 |
| ebi-fl187-GCST90094756 | ebi-a-GCST90001411 | IgD+ CD24+ B cell %B cell \|\| id:ebi-a-GCST90001411 | Dried fruit liking \|\| id:ebi-fl187-GCST90094756 | Inverse variance weighted | 5 | 0.328403175150044 | 0.294600300012309 | 0.264961219208887 | -0.249013413 | 0.905819763174171 | 1.38874876842697 | 0.779569517046805 | 2.4739591526276 | 0.998074519377826 |
| ebi-fl187-GCST90094756 | ebi-a-GCST90001418 | CD24+ CD27+ B cell Absolute Count \|\| id:ebi-a-GCST90001418 | Dried fruit liking \|\| id:ebi-fl187-GCST90094756 | Inverse variance weighted | 5 | 0.1470417867572 | 0.289370192471857 | 0.611352215483077 | -0.42012379 | 0.71420736400204 | 1.15840236789699 | 0.656965488702943 | 2.04256702829047 | 0.998074519377826 |
| ebi-fl187-GCST90094756 | ebi-a-GCST90001436 | Memory B cell %lymphocyte \|\| id:ebi-a-GCST90001436 | Dried fruit liking \|\| id:ebi-fl187-GCST90094756 | Inverse variance weighted | 5 | 0.279984571154867 | 0.286321045606222 | 0.328138952155273 | -0.281204678 | 0.841173820543062 | 1.32310939812996 | 0.75487381342858 | 2.31908757235681 | 0.998074519377826 |
| ebi-fl187-GCST90094756 | ebi-a-GCST90001439 | IgD+ CD24+ B cell %lymphocyte \|\| id:ebi-a-GCST90001439 | Dried fruit liking \|\| id:ebi-fl187-GCST90094756 | Inverse variance weighted | 5 | 0.433612643997486 | 0.289307128504428 | 0.13392631414348 | -0.133429328 | 1.00065461586617 | 1.54282113132947 | 0.87508931121685 | 2.72006184142146 | 0.998074519377826 |
| ebi-fl187-GCST90094756 | ebi-a-GCST90001457 | CD11c+ HLA DR++ monocyte %monocyte \|\| id:ebi-a-GCST90001457 | Dried fruit liking \|\| id:ebi-fl187-GCST90094756 | Inverse variance weighted | 5 | -0.076183804 | 0.30900172390916 | 0.805257934700269 | -0.681827183 | 0.529459574905563 | 0.926645869728541 | 0.505692155681813 | 1.69801441101501 | 0.998074519377826 |
| ebi-fl187-GCST90094756 | ebi-a-GCST90001483 | CD39+ resting CD4 regulatory T cell Absolute Count \|\| id:ebi-a-GCST90001483 | Dried fruit liking \|\| id:ebi-fl187-GCST90094756 | Inverse variance weighted | 5 | 0.396894974476372 | 0.420736389017466 | 0.345510049290168 | -0.427748348 | 1.2215382969506 | 1.48719972791868 | 0.651975465087209 | 3.39240224388743 | 0.998074519377826 |
| ebi-fl187-GCST90094756 | ebi-a-GCST90001525 | CD33dim HLA DR+ CD11b+ Absolute Count \|\| id:ebi-a-GCST90001525 | Dried fruit liking \|\| id:ebi-fl187-GCST90094756 | Inverse variance weighted | 5 | 0.112191484321409 | 0.373006604402103 | 0.763585208716226 | -0.61890146 | 0.843284428949532 | 1.1187270588283 | 0.538535715623885 | 2.32398742710074 | 0.998074519377826 |
| ebi-fl187-GCST90094756 | ebi-a-GCST90001540 | Naive CD4+ T cell Absolute Count \|\| id:ebi-a-GCST90001540 | Dried fruit liking \|\| id:ebi-fl187-GCST90094756 | Inverse variance weighted | 5 | -0.360240555 | 0.275743463509073 | 0.191405110215848 | -0.900697743 | 0.180216633911389 | 0.697508517029355 | 0.406286077533803 | 1.19747674909685 | 0.998074519377826 |
| ebi-fl187-GCST90094756 | ebi-a-GCST90001624 | HLA DR+ CD4+ T cell Absolute Count \|\| id:ebi-a-GCST90001624 | Dried fruit liking \|\| id:ebi-fl187-GCST90094756 | Inverse variance weighted | 5 | -0.062669615 | 0.296962837722201 | 0.832859795158335 | -0.644716777 | 0.519377546638936 | 0.939253737500604 | 0.524811159528911 | 1.68098099171661 | 0.998074519377826 |
| ebi-fl187-GCST90094756 | ebi-a-GCST90001672 | CD39+ CD8+ T cell Absolute Count \|\| id:ebi-a-GCST90001672 | Dried fruit liking \|\| id:ebi-fl187-GCST90094756 | Inverse variance weighted | 5 | 0.165129165411758 | 0.408348783107575 | 0.685931352348039 | -0.635234449 | 0.965492780302605 | 1.17954546534794 | 0.529811259781609 | 2.62608141887434 | 0.998074519377826 |
| ebi-fl187-GCST90094756 | ebi-a-GCST90001722 | CD19 on CD20- CD38- B cell \|\| id:ebi-a-GCST90001722 | Dried fruit liking \|\| id:ebi-fl187-GCST90094756 | Inverse variance weighted | 5 | -0.143075294 | 0.388772819221651 | 0.712860006970118 | -0.90507002 | 0.618919431636954 | 0.866688809938974 | 0.404513560188428 | 1.85692042789255 | 0.998074519377826 |
| ebi-fl187-GCST90094756 | ebi-a-GCST90001742 | CD19 on transitional B cell \|\| id:ebi-a-GCST90001742 | Dried fruit liking \|\| id:ebi-fl187-GCST90094756 | Inverse variance weighted | 5 | -0.253184297 | 0.3772758171977 | 0.502166119359496 | -0.992644899 | 0.486276304327027 | 0.776324794012863 | 0.370595206706505 | 1.62624927385099 | 0.998074519377826 |
| ebi-fl187-GCST90094756 | ebi-a-GCST90001784 | CD25 on IgD+ CD38dim B cell \|\| id:ebi-a-GCST90001784 | Dried fruit liking \|\| id:ebi-fl187-GCST90094756 | Inverse variance weighted | 5 | 0.145812662561841 | 0.286059801305771 | 0.610242101979758 | -0.414864548 | 0.706489873121152 | 1.15697942218629 | 0.660429731176341 | 2.02686420700389 | 0.998074519377826 |
| ebi-fl187-GCST90094756 | ebi-a-GCST90001794 | CD25 on IgD+ B cell \|\| id:ebi-a-GCST90001794 | Dried fruit liking \|\| id:ebi-fl187-GCST90094756 | Inverse variance weighted | 5 | 0.29834375024861 | 0.285321962486739 | 0.295727770445364 | -0.260887296 | 0.857574796722619 | 1.34762495468135 | 0.770367738074515 | 2.35743649262768 | 0.998074519377826 |
| ebi-fl187-GCST90094756 | ebi-a-GCST90001802 | CD27 on IgD- CD38- B cell \|\| id:ebi-a-GCST90001802 | Dried fruit liking \|\| id:ebi-fl187-GCST90094756 | Inverse variance weighted | 5 | 0.313583408893798 | 0.324432852617152 | 0.33376466963658 | -0.322304982 | 0.949471800023417 | 1.36831958802541 | 0.724477203955693 | 2.58434424817116 | 0.998074519377826 |
| ebi-fl187-GCST90094756 | ebi-a-GCST90001804 | CD27 on IgD- CD38dim B cell \|\| id:ebi-a-GCST90001804 | Dried fruit liking \|\| id:ebi-fl187-GCST90094756 | Inverse variance weighted | 5 | 0.250924882583624 | 0.428510101285538 | 0.558161027041095 | -0.588954916 | 1.09080468110328 | 1.28521353878459 | 0.554906906169814 | 2.97666837790216 | 0.998074519377826 |
| ebi-fl187-GCST90094756 | ebi-a-GCST90001805 | CD27 on memory B cell \|\| id:ebi-a-GCST90001805 | Dried fruit liking \|\| id:ebi-fl187-GCST90094756 | Inverse variance weighted | 5 | 0.300122492813968 | 0.306480612261961 | 0.327454287785744 | -0.300579507 | 0.900824492847411 | 1.35002416570719 | 0.740389035544468 | 2.46163187256428 | 0.998074519377826 |
| ebi-fl187-GCST90094756 | ebi-a-GCST90001806 | CD27 on unswitched memory B cell \|\| id:ebi-a-GCST90001806 | Dried fruit liking \|\| id:ebi-fl187-GCST90094756 | Inverse variance weighted (multiplicative random effects) | 5 | 0.541477699826763 | 0.455956326757777 | 0.235004899022253 | -0.352196701 | 1.43515210027201 | 1.71854447968454 | 0.703141799945844 | 4.20028382451688 | 0.998074519377826 |
| ebi-fl187-GCST90094756 | ebi-a-GCST90001839 | CD3 on Effector Memory CD8+ T cell \|\| id:ebi-a-GCST90001839 | Dried fruit liking \|\| id:ebi-fl187-GCST90094756 | Inverse variance weighted | 5 | -0.44970277 | 0.314249665254726 | 0.152419727965373 | -1.065632114 | 0.166226573656092 | 0.637817701850816 | 0.344510016271581 | 1.18084061879223 | 0.998074519377826 |
| ebi-fl187-GCST90094756 | ebi-a-GCST90001859 | CD3 on CD8+ T cell \|\| id:ebi-a-GCST90001859 | Dried fruit liking \|\| id:ebi-fl187-GCST90094756 | Inverse variance weighted | 5 | -0.24742466 | 0.308091006441576 | 0.421922992279309 | -0.851283032 | 0.356433712902861 | 0.780809044965006 | 0.426866896413808 | 1.42822685436857 | 0.998074519377826 |
| ebi-fl187-GCST90094756 | ebi-a-GCST90001905 | CD86 on monocyte \|\| id:ebi-a-GCST90001905 | Dried fruit liking \|\| id:ebi-fl187-GCST90094756 | Inverse variance weighted | 5 | 0.341864905798595 | 0.313832438411908 | 0.276011456094266 | -0.273246673 | 0.956976485085935 | 1.40757013010649 | 0.760905069369262 | 2.60381189576029 | 0.998074519377826 |
| ebi-fl187-GCST90094756 | ebi-a-GCST90001943 | CD25 on activated & secreting CD4 regulatory T cell \|\| id:ebi-a-GCST90001943 | Dried fruit liking \|\| id:ebi-fl187-GCST90094756 | Inverse variance weighted | 5 | 0.394583685974697 | 0.286899965957768 | 0.169027479392674 | -0.167740247 | 0.956907619251922 | 1.48376634958023 | 0.845573446144637 | 2.60363258825662 | 0.998074519377826 |
| ebi-fl187-GCST90094756 | ebi-a-GCST90001949 | CD33 on CD33dim HLA DR+ CD11b- \|\| id:ebi-a-GCST90001949 | Dried fruit liking \|\| id:ebi-fl187-GCST90094756 | Inverse variance weighted | 5 | 0.0530619694935644 | 0.406857067503704 | 0.896234784240875 | -0.744377883 | 0.850501821800824 | 1.05449498961513 | 0.475029732194046 | 2.34082123236273 | 0.998074519377826 |
| ebi-fl187-GCST90094756 | ebi-a-GCST90001955 | CD33 on Immature Myeloid-Derived Suppressor Cells \|\| id:ebi-a-GCST90001955 | Dried fruit liking \|\| id:ebi-fl187-GCST90094756 | Inverse variance weighted | 5 | -0.044970653 | 0.421408866029031 | 0.915015072055107 | -0.87093203 | 0.780990724845256 | 0.956025538312396 | 0.418561255750867 | 2.18363457522099 | 0.998074519377826 |
| ebi-fl187-GCST90094756 | ebi-a-GCST90001956 | CD33 on CD33+ HLA DR+ \|\| id:ebi-a-GCST90001956 | Dried fruit liking \|\| id:ebi-fl187-GCST90094756 | Inverse variance weighted | 5 | -0.309277917 | 0.419087063702751 | 0.46052653580022 | -1.130688562 | 0.512132727420768 | 0.733976756739377 | 0.32281090446254 | 1.66884659714514 | 0.998074519377826 |
| ebi-fl187-GCST90094756 | ebi-a-GCST90001957 | CD33 on CD33+ HLA DR+ CD14- \|\| id:ebi-a-GCST90001957 | Dried fruit liking \|\| id:ebi-fl187-GCST90094756 | Inverse variance weighted | 5 | -0.284470242 | 0.419379883614793 | 0.497574141999517 | -1.106454814 | 0.537514329858072 | 0.752412745409072 | 0.330729382373994 | 1.71174673199684 | 0.998074519377826 |
| ebi-fl187-GCST90094756 | ebi-a-GCST90002064 | CD4 on resting CD4 regulatory T cell \|\| id:ebi-a-GCST90002064 | Dried fruit liking \|\| id:ebi-fl187-GCST90094756 | Inverse variance weighted | 5 | -0.03730095 | 0.303773006442369 | 0.902271667076056 | -0.632696042 | 0.558094143026403 | 0.963386161036989 | 0.531157844892959 | 1.74733914635983 | 0.998074519377826 |
| ebi-fl187-GCST90094756 | ebi-a-GCST90002080 | SSC-A on T cell \|\| id:ebi-a-GCST90002080 | Dried fruit liking \|\| id:ebi-fl187-GCST90094756 | Inverse variance weighted | 5 | 0.137612187592242 | 0.291994519147227 | 0.637438324747191 | -0.43469707 | 0.709921445120806 | 1.1475304374277 | 0.647460772706494 | 2.03383148498473 | 0.998074519377826 |
| ebi-fl187-GCST90094756 | ebi-a-GCST90002121 | CD8 on CD39+ CD8+ T cell \|\| id:ebi-a-GCST90002121 | Dried fruit liking \|\| id:ebi-fl187-GCST90094756 | Inverse variance weighted | 5 | -0.521479489 | 0.308314024255824 | 0.0907621974769904 | -1.125774977 | 0.0828159983210435 | 0.593641611576967 | 0.324400966706658 | 1.0863419014234 | 0.998074519377826 |
| ebi-fl187-GCST90094756 | ebi-cfmet338-GCST90026053 | 1-palmitoyl-2-docosahexaenoyl-gpc (16:0/22:6) levels \|\| id:ebi-cfmet338-GCST90026053 | Dried fruit liking \|\| id:ebi-fl187-GCST90094756 | Inverse variance weighted | 5 | -0.093585023 | 0.161205600657054 | 0.561555860197329 | -0.409548 | 0.222377954734021 | 0.910660587445805 | 0.663950287957947 | 1.24904337051762 | 0.998074519377826 |
| ebi-fl187-GCST90094756 | ebi-cfmet338-GCST90026063 | 1-stearoyl-2-oleoyl-gpc (18:0/18:1) levels \|\| id:ebi-cfmet338-GCST90026063 | Dried fruit liking \|\| id:ebi-fl187-GCST90094756 | Inverse variance weighted | 4 | -0.065201742 | 0.147764832047312 | 0.659029519783038 | -0.354820813 | 0.224417328446962 | 0.936878436238488 | 0.701299095506462 | 1.25159323591426 | 0.998074519377826 |
| ebi-fl187-GCST90094756 | ebi-cfmet338-GCST90026113 | Adenosine 3',5'-cyclic monophosphate (camp) levels \|\| id:ebi-cfmet338-GCST90026113 | Dried fruit liking \|\| id:ebi-fl187-GCST90094756 | Inverse variance weighted | 5 | -0.438453166 | 0.25497128431742 | 0.0855019335999845 | -0.938196883 | 0.061290551492927 | 0.645033409554301 | 0.391332818429343 | 1.06320778592294 | 0.998074519377826 |
| ebi-fl187-GCST90094756 | ebi-cfmet338-GCST90026117 | Alpha-ketoglutaramate levels \|\| id:ebi-cfmet338-GCST90026117 | Dried fruit liking \|\| id:ebi-fl187-GCST90094756 | Inverse variance weighted | 5 | 0.0788645890433472 | 0.102641290271313 | 0.442278387768505 | -0.12231234 | 0.280041517975121 | 1.08205778966904 | 0.884871944531332 | 1.32318474714845 | 0.998074519377826 |
| ebi-fl187-GCST90094756 | ebi-cfmet338-GCST90026124 | Arginine levels \|\| id:ebi-cfmet338-GCST90026124 | Dried fruit liking \|\| id:ebi-fl187-GCST90094756 | Inverse variance weighted | 5 | -0.111236362 | 0.0834356768877111 | 0.182466503184487 | -0.274770289 | 0.0522975646271377 | 0.894727244348142 | 0.759746625511425 | 1.05368923651347 | 0.998074519377826 |
| ebi-fl187-GCST90094756 | ebi-cfmet338-GCST90026169 | Glutamate levels \|\| id:ebi-cfmet338-GCST90026169 | Dried fruit liking \|\| id:ebi-fl187-GCST90094756 | Inverse variance weighted | 5 | 0.0279857393199766 | 0.114900122984194 | 0.807567125423615 | -0.197218502 | 0.253189980368996 | 1.0283810189042 | 0.821011221341032 | 1.28812797261775 | 0.998074519377826 |
| ebi-fl187-GCST90094756 | ebi-cfmet338-GCST90026170 | Glutamine levels \|\| id:ebi-cfmet338-GCST90026170 | Dried fruit liking \|\| id:ebi-fl187-GCST90094756 | Inverse variance weighted | 5 | -0.046433634 | 0.0526500287293671 | 0.377814720893257 | -0.14962769 | 0.0567604221438358 | 0.954627913145603 | 0.861028485863337 | 1.05840221028573 | 0.998074519377826 |
| ebi-fl187-GCST90094756 | ebi-cfmet338-GCST90026191 | Kynurenate levels \|\| id:ebi-cfmet338-GCST90026191 | Dried fruit liking \|\| id:ebi-fl187-GCST90094756 | Inverse variance weighted | 5 | 0.04002318516821 | 0.427430155345474 | 0.925397688494524 | -0.797739919 | 0.877786289645339 | 1.04083490584501 | 0.450345632276245 | 2.40556857573976 | 0.998074519377826 |
| ebi-fl187-GCST90094756 | ebi-cfmet338-GCST90026202 | Methylsuccinoylcarnitine levels \|\| id:ebi-cfmet338-GCST90026202 | Dried fruit liking \|\| id:ebi-fl187-GCST90094756 | Inverse variance weighted | 5 | 0.0307722467726921 | 0.308177704773487 | 0.920461601184314 | -0.573256055 | 0.634800548128727 | 1.03125060648443 | 0.563687051571478 | 1.88664580889285 | 0.998074519377826 |
| ebi-fl187-GCST90094756 | ebi-cfmet338-GCST90026222 | N-acetylputrescine levels \|\| id:ebi-cfmet338-GCST90026222 | Dried fruit liking \|\| id:ebi-fl187-GCST90094756 | Inverse variance weighted | 5 | -0.154683406 | 0.0963625493312398 | 0.108444680500337 | -0.343554002 | 0.034187191169759 | 0.856686356726507 | 0.709245179304047 | 1.0347782899582 | 0.998074519377826 |
| ebi-fl187-GCST90094756 | ebi-cfmet338-GCST90026252 | Propionylcarnitine (c3) levels \|\| id:ebi-cfmet338-GCST90026252 | Dried fruit liking \|\| id:ebi-fl187-GCST90094756 | Inverse variance weighted (multiplicative random effects) | 5 | -0.155464888 | 0.247810874272848 | 0.530427889815457 | -0.641174201 | 0.330244426034943 | 0.856017133268739 | 0.526673640050529 | 1.3913081588426 | 0.998074519377826 |
| ebi-fl187-GCST90094756 | ebi-cfmet338-GCST90026316 | Ethyl beta-glucopyranoside levels \|\| id:ebi-cfmet338-GCST90026316 | Dried fruit liking \|\| id:ebi-fl187-GCST90094756 | Inverse variance weighted | 5 | -0.074563308 | 0.568742424433825 | 0.895694565889002 | -1.189298459 | 1.04017184434207 | 0.928148713379573 | 0.304434762503261 | 2.82970324106443 | 0.998074519377826 |
| ebi-fl187-GCST90094756 | ebi-gm430-GCST90011315 | F_Prevotellaceae abundance \|\| id:ebi-gm430-GCST90011315 | Dried fruit liking \|\| id:ebi-fl187-GCST90094756 | Inverse variance weighted | 3 | -0.243528449 | 0.284077862163885 | 0.391301479628707 | -0.800321059 | 0.313264160583508 | 0.783857175560266 | 0.449184726120583 | 1.36788282403079 | 0.998074519377826 |
| ebi-fl187-GCST90094756 | ebi-gm430-GCST90011354 | OTU97_1 (Bacteroides) abundance \|\| id:ebi-gm430-GCST90011354 | Dried fruit liking \|\| id:ebi-fl187-GCST90094756 | Inverse variance weighted | 3 | -0.11599337 | 0.25766046119342 | 0.652581262722844 | -0.621007874 | 0.389021134296946 | 0.890481127486428 | 0.537402530740512 | 1.47553573541391 | 0.998074519377826 |
| ebi-fl187-GCST90094756 | ebi-gm430-GCST90011419 | OTU99_1 (Bacteroides) abundance \|\| id:ebi-gm430-GCST90011419 | Dried fruit liking \|\| id:ebi-fl187-GCST90094756 | Inverse variance weighted | 3 | -0.097156848 | 0.257751659667152 | 0.706218375675305 | -0.602350101 | 0.408036405194496 | 0.907413669175786 | 0.547523387832951 | 1.50386190855884 | 0.998074519377826 |
| ebi-fl187-GCST90094756 | ebi-gm430-GCST90011435 | OTU99_155 (Butyrivibrio) abundance \|\| id:ebi-gm430-GCST90011435 | Dried fruit liking \|\| id:ebi-fl187-GCST90094756 | Inverse variance weighted | 3 | 0.553739744166687 | 0.812888621920185 | 0.495744936426316 | -1.039521955 | 2.14700144313025 | 1.73974707622013 | 0.353623689667456 | 8.55915476721248 | 0.998074519377826 |
| ebi-fl187-GCST90094756 | ebi-gm430-GCST90011466 | OTU99_58 (Bacteroides) abundance \|\| id:ebi-gm430-GCST90011466 | Dried fruit liking \|\| id:ebi-fl187-GCST90094756 | Inverse variance weighted | 3 | 0.548131689433649 | 0.488363511814508 | 0.261698945454691 | -0.409060794 | 1.50532417259008 | 1.73001778608988 | 0.66427384741452 | 4.50561399012848 | 0.998074519377826 |
| ebi-fl187-GCST90094756 | ebi-gm430-GCST90011472 | OTU99_72 (Parabacteroides) abundance \|\| id:ebi-gm430-GCST90011472 | Dried fruit liking \|\| id:ebi-fl187-GCST90094756 | Inverse variance weighted | 3 | -0.105650653 | 0.712585898267835 | 0.882134627445911 | -1.502319014 | 1.29101770765019 | 0.899738914309931 | 0.222613317795298 | 3.6364855523514 | 0.998074519377826 |
| ebi-fl187-GCST90094756 | ebi-gm430-GCST90011505 | TestASV_28 (Holdemanella) abundance \|\| id:ebi-gm430-GCST90011505 | Dried fruit liking \|\| id:ebi-fl187-GCST90094756 | Inverse variance weighted | 4 | 0.280360352202454 | 0.543612189798154 | 0.606038838462073 | -0.78511954 | 1.34584024420684 | 1.32360669099642 | 0.456065180585624 | 3.84141290988467 | 0.998074519377826 |
| ebi-fl187-GCST90094756 | ebi-gm430-GCST90011513 | TestASV_35 (Alphaproteobacteria) abundance \|\| id:ebi-gm430-GCST90011513 | Dried fruit liking \|\| id:ebi-fl187-GCST90094756 | Inverse variance weighted | 3 | 1.57046988485277 | 0.714092292314727 | 0.0278599108958964 | 0.170848991915904 | 2.97009077778963 | 4.80890729567133 | 1.18631159285426 | 19.4936891097228 | 0.998074519377826 |
| ebi-fl187-GCST90094756 | ebi-gm430-GCST90011543 | G_Coprococcus prevalence \|\| id:ebi-gm430-GCST90011543 | Dried fruit liking \|\| id:ebi-fl187-GCST90094756 | Inverse variance weighted | 3 | 0.512444856287628 | 0.762106536782362 | 0.501325390796114 | -0.981283956 | 2.00617366838106 | 1.66936757364414 | 0.37482952521284 | 7.43481478507356 | 0.998074519377826 |
| ebi-fl187-GCST90094756 | ebi-gm430-GCST90011547 | G_Parasutterella prevalence \|\| id:ebi-gm430-GCST90011547 | Dried fruit liking \|\| id:ebi-fl187-GCST90094756 | Inverse variance weighted | 3 | -0.065953339 | 0.853445628395256 | 0.938401676156631 | -1.738706771 | 1.60680009238224 | 0.93617454585792 | 0.175747535539038 | 4.9868282796922 | 0.998074519377826 |
| ebi-fl187-GCST90094756 | ebi-gm430-GCST90011569 | OTU97_13 (Subdoligranulum) prevalence \|\| id:ebi-gm430-GCST90011569 | Dried fruit liking \|\| id:ebi-fl187-GCST90094756 | Inverse variance weighted | 3 | 1.03164909497612 | 0.699413242925782 | 0.140206946110904 | -0.339200861 | 2.40249905111066 | 2.80568886915862 | 0.712339353410785 | 11.0507583117918 | 0.998074519377826 |
| ebi-fl187-GCST90094756 | ebi-gm430-GCST90011590 | OTU97_277 (Prevotella) prevalence \|\| id:ebi-gm430-GCST90011590 | Dried fruit liking \|\| id:ebi-fl187-GCST90094756 | Inverse variance weighted | 3 | 0.201989149365554 | 0.893439794723704 | 0.821138955352876 | -1.549152848 | 1.95313114702401 | 1.22383472865605 | 0.212427856243188 | 7.05072992569287 | 0.998074519377826 |
| ebi-fl187-GCST90094756 | ebi-gm430-GCST90011616 | OTU97_86 (Phascolarctobacterium) prevalence \|\| id:ebi-gm430-GCST90011616 | Dried fruit liking \|\| id:ebi-fl187-GCST90094756 | Inverse variance weighted | 3 | 0.589796692386675 | 0.748720776856824 | 0.430849281732635 | -0.87769603 | 2.05728941502605 | 1.80362168809915 | 0.415739660698965 | 7.8247314396524 | 0.998074519377826 |
| ebi-fl187-GCST90094756 | ebi-gm430-GCST90011665 | OTU99_55 (Barnesiella) prevalence \|\| id:ebi-gm430-GCST90011665 | Dried fruit liking \|\| id:ebi-fl187-GCST90094756 | Inverse variance weighted | 3 | 0.0396955875394332 | 0.582816676138138 | 0.945698146854713 | -1.102625098 | 1.18201627277018 | 1.04049398664312 | 0.331998410512083 | 3.26094252852179 | 0.998074519377826 |
| ebi-fl187-GCST90094756 | ebi-gm430-GCST90011687 | TestASV_14 (Lachnospiraceae) prevalence \|\| id:ebi-gm430-GCST90011687 | Dried fruit liking \|\| id:ebi-fl187-GCST90094756 | Inverse variance weighted | 3 | 0.251164047059112 | 0.541860955940561 | 0.64299081391339 | -0.810883427 | 1.31321152070261 | 1.28552095296629 | 0.444465240322931 | 3.718095299 | 0.998074519377826 |
| ebi-fl187-GCST90094756 | ebi-gm430-GCST90011697 | TestASV_23 (Barnesiella) prevalence \|\| id:ebi-gm430-GCST90011697 | Dried fruit liking \|\| id:ebi-fl187-GCST90094756 | Inverse variance weighted | 3 | 0.220600943459108 | 0.715873969452064 | 0.757963541388232 | -1.182512037 | 1.62371392358515 | 1.24682577729299 | 0.306507811846333 | 5.07189200026541 | 0.998074519377826 |
| ebi-fl187-GCST90094756 | ebi-gm430-GCST90011727 | TestASV_6 (Subdoligranulum) prevalence \|\| id:ebi-gm430-GCST90011727 | Dried fruit liking \|\| id:ebi-fl187-GCST90094756 | Inverse variance weighted | 3 | 1.79198845091716 | 0.813115051557413 | 0.0275344757378347 | 0.198282949864626 | 3.38569395196969 | 6.00137404744446 | 1.21930734786601 | 29.5384838944625 | 0.998074519377826 |
| ebi-fl187-GCST90094756 | ebi-hbsm44-GCST90104908 | Sulcal depth of the left posterior inferior temporal sulcus \|\| id:ebi-hbsm44-GCST90104908 | Dried fruit liking \|\| id:ebi-fl187-GCST90094756 | Inverse variance weighted | 4 | -0.069290767 | 0.218660555789464 | 0.751329069961993 | -0.497865457 | 0.359283922095304 | 0.93305533869468 | 0.607826708473655 | 1.43230340643114 | 0.998074519377826 |
| ebi-fl187-GCST90094756 | ebi-hbsm44-GCST90104918 | Sulcal depth of the left inferior frontal sulcus \|\| id:ebi-hbsm44-GCST90104918 | Dried fruit liking \|\| id:ebi-fl187-GCST90094756 | Inverse variance weighted | 4 | -0.242483913 | 0.467014765022462 | 0.603606576880251 | -1.157832853 | 0.672865026297301 | 0.784676370450831 | 0.314166288327706 | 1.95984429017297 | 0.998074519377826 |
| ebi-fl187-GCST90094756 | ebi-hbsm44-GCST90104925 | Sulcal depth of the right anterior inferior temporal sulcus \|\| id:ebi-hbsm44-GCST90104925 | Dried fruit liking \|\| id:ebi-fl187-GCST90094756 | Inverse variance weighted | 4 | -0.061462062 | 0.12321352923706 | 0.617902252443424 | -0.30296058 | 0.180036454883529 | 0.940388621130207 | 0.738628212723307 | 1.19726100833688 | 0.998074519377826 |
| ebi-fl187-GCST90094756 | ebi-IPs91-GCST90274770 | C-C motif chemokine 4 levels \|\| id:ebi-IPs91-GCST90274770 | Dried fruit liking \|\| id:ebi-fl187-GCST90094756 | Inverse variance weighted | 5 | 0.239194755135083 | 0.144123411546869 | 0.0969844153467014 | -0.043287131 | 0.521676641766947 | 1.27022589551171 | 0.957636383019299 | 1.68485017302858 | 0.998074519377826 |
| ebi-fl187-GCST90094756 | ebi-IPs91-GCST90274779 | C-X-C motif chemokine 1 levels \|\| id:ebi-IPs91-GCST90274779 | Dried fruit liking \|\| id:ebi-fl187-GCST90094756 | Inverse variance weighted | 5 | 0.112801925741495 | 0.135950007007267 | 0.406690929877087 | -0.153660088 | 0.379263939475737 | 1.11941018464571 | 0.857563467595172 | 1.46120865549753 | 0.998074519377826 |
| ebi-fl187-GCST90094756 | ebi-IPs91-GCST90274780 | C-X-C motif chemokine 10 levels \|\| id:ebi-IPs91-GCST90274780 | Dried fruit liking \|\| id:ebi-fl187-GCST90094756 | Inverse variance weighted | 5 | -0.089846162 | 0.13545226604399 | 0.507135250867636 | -0.355332603 | 0.175640279486389 | 0.914071793468787 | 0.700940269312495 | 1.19200919136057 | 0.998074519377826 |
| ebi-fl187-GCST90094756 | ebi-IPs91-GCST90274796 | Interleukin-10 receptor subunit alpha levels \|\| id:ebi-IPs91-GCST90274796 | Dried fruit liking \|\| id:ebi-fl187-GCST90094756 | Inverse variance weighted (multiplicative random effects) | 5 | -0.073223851 | 0.271407680223584 | 0.787319624075059 | -0.605182905 | 0.458735201775797 | 0.929392760809685 | 0.545974556193836 | 1.58207171753034 | 0.998074519377826 |
| ebi-fl187-GCST90094756 | ebi-IPs91-GCST90274807 | Interleukin-20 levels \|\| id:ebi-IPs91-GCST90274807 | Dried fruit liking \|\| id:ebi-fl187-GCST90094756 | Inverse variance weighted | 5 | 0.0377674896270844 | 0.150995682289886 | 0.802492247226232 | -0.258184048 | 0.333719026915261 | 1.03848974516747 | 0.772453050845953 | 1.39615080765998 | 0.998074519377826 |
| ebi-fl187-GCST90094756 | ebi-IPs91-GCST90274808 | Interleukin-20 receptor subunit alpha levels \|\| id:ebi-IPs91-GCST90274808 | Dried fruit liking \|\| id:ebi-fl187-GCST90094756 | Inverse variance weighted | 5 | 0.0428653580205674 | 0.152698209780138 | 0.77892558664621 | -0.256423133 | 0.342153849189638 | 1.04379734644812 | 0.773814472955663 | 1.40797689695648 | 0.998074519377826 |
| ebi-fl187-GCST90094756 | ebi-IPs91-GCST90274810 | Interleukin-24 levels \|\| id:ebi-IPs91-GCST90274810 | Dried fruit liking \|\| id:ebi-fl187-GCST90094756 | Inverse variance weighted | 5 | 0.2662522675417 | 0.189433562525659 | 0.159867323196237 | -0.105037515 | 0.637542050091991 | 1.3050642425122 | 0.900290747537606 | 1.89182514842274 | 0.998074519377826 |
| ebi-fl187-GCST90094756 | ebi-IPs91-GCST90274823 | Monocyte chemoattractant protein-3 levels \|\| id:ebi-IPs91-GCST90274823 | Dried fruit liking \|\| id:ebi-fl187-GCST90094756 | Inverse variance weighted | 5 | 0.0830777489297371 | 0.172525323124335 | 0.630133149848289 | -0.255071884 | 0.421227382253433 | 1.08662628929507 | 0.774860795560347 | 1.52383073108415 | 0.998074519377826 |
| ebi-fl187-GCST90094756 | ebi-IPs91-GCST90274824 | Monocyte chemoattractant protein-4 levels \|\| id:ebi-IPs91-GCST90274824 | Dried fruit liking \|\| id:ebi-fl187-GCST90094756 | Inverse variance weighted | 5 | 0.186678717670161 | 0.135220372158573 | 0.167416836093102 | -0.078353212 | 0.451710647100964 | 1.20524000052404 | 0.924637775921726 | 1.57099731017927 | 0.998074519377826 |
| ebi-fl187-GCST90094756 | ebi-IPs91-GCST90274834 | SIR2-like protein 2 levels \|\| id:ebi-IPs91-GCST90274834 | Dried fruit liking \|\| id:ebi-fl187-GCST90094756 | Inverse variance weighted | 5 | 0.20973117919168 | 0.182479625539703 | 0.250415705567731 | -0.147928887 | 0.567391245249497 | 1.23334646619511 | 0.862492447302113 | 1.76366008819453 | 0.998074519377826 |
| ebi-fl187-GCST90094756 | ebi-met1400-GCST90199652 | 1-stearoyl-2-oleoyl-GPS (18:0/18:1) levels \|\| id:ebi-met1400-GCST90199652 | Dried fruit liking \|\| id:ebi-fl187-GCST90094756 | Inverse variance weighted | 5 | -0.217752996 | 0.18980363861728 | 0.251276633279685 | -0.589768127 | 0.154262136020028 | 0.804324088664967 | 0.554455832968436 | 1.16679670613828 | 0.998074519377826 |
| ebi-fl187-GCST90094756 | ebi-met1400-GCST90199665 | N-acetylaspartate (naa) levels \|\| id:ebi-met1400-GCST90199665 | Dried fruit liking \|\| id:ebi-fl187-GCST90094756 | Inverse variance weighted | 5 | -0.047731743 | 0.194382901430303 | 0.806026528477609 | -0.42872223 | 0.333258743467008 | 0.953389505864806 | 0.651340826906348 | 1.39550833042411 | 0.998074519377826 |
| ebi-fl187-GCST90094756 | ebi-met1400-GCST90199669 | Acetylcarnitine levels (Biocrates platform) \|\| id:ebi-met1400-GCST90199669 | Dried fruit liking \|\| id:ebi-fl187-GCST90094756 | Inverse variance weighted | 5 | -0.211497561 | 0.171481959155404 | 0.217444587277047 | -0.547602201 | 0.124607078872135 | 0.809371255048166 | 0.578334879944614 | 1.13270330255886 | 0.998074519377826 |
| ebi-fl187-GCST90094756 | ebi-met1400-GCST90199670 | Iminodiacetate (IDA) levels \|\| id:ebi-met1400-GCST90199670 | Dried fruit liking \|\| id:ebi-fl187-GCST90094756 | Inverse variance weighted | 5 | 0.214495398337635 | 0.189398856304874 | 0.257421636453715 | -0.15672636 | 0.585717156695189 | 1.23923641841378 | 0.854937972021858 | 1.79627873714767 | 0.998074519377826 |
| ebi-fl187-GCST90094756 | ebi-met1400-GCST90199686 | Indolepropionate levels \|\| id:ebi-met1400-GCST90199686 | Dried fruit liking \|\| id:ebi-fl187-GCST90094756 | Inverse variance weighted | 5 | 0.123943419424995 | 0.177831966795758 | 0.485822013628315 | -0.224607235 | 0.472494074344681 | 1.13195182270224 | 0.79882990918622 | 1.60398967813339 | 0.998074519377826 |
| ebi-fl187-GCST90094756 | ebi-met1400-GCST90199704 | 3-hydroxysebacate levels \|\| id:ebi-met1400-GCST90199704 | Dried fruit liking \|\| id:ebi-fl187-GCST90094756 | Inverse variance weighted | 5 | 0.0422609809960132 | 0.18151690635038 | 0.815900262689458 | -0.313512155 | 0.398034117442758 | 1.04316668990999 | 0.73087549482854 | 1.48889482632476 | 0.998074519377826 |
| ebi-fl187-GCST90094756 | ebi-met1400-GCST90199713 | Docosapentaenoate n3 DPA; 22:5n3 levels \|\| id:ebi-met1400-GCST90199713 | Dried fruit liking \|\| id:ebi-fl187-GCST90094756 | Inverse variance weighted | 5 | -0.08822617 | 0.196337274833195 | 0.653171789986046 | -0.473047229 | 0.296594888717206 | 0.915553782546386 | 0.623100642293708 | 1.34527020490516 | 0.998074519377826 |
| ebi-fl187-GCST90094756 | ebi-met1400-GCST90199715 | Myristoleate (14:1n5) levels \|\| id:ebi-met1400-GCST90199715 | Dried fruit liking \|\| id:ebi-fl187-GCST90094756 | Inverse variance weighted | 5 | -0.07822186 | 0.162176658343267 | 0.629575073991077 | -0.396088111 | 0.239644389866091 | 0.924759236248327 | 0.672947399364783 | 1.27079716161147 | 0.998074519377826 |
| ebi-fl187-GCST90094756 | ebi-met1400-GCST90199725 | Campesterol levels \|\| id:ebi-met1400-GCST90199725 | Dried fruit liking \|\| id:ebi-fl187-GCST90094756 | Inverse variance weighted | 5 | -0.312015067 | 0.220761125725853 | 0.157549737237827 | -0.744706874 | 0.120676739145976 | 0.731970499343179 | 0.474873477446335 | 1.12826013107723 | 0.998074519377826 |
| ebi-fl187-GCST90094756 | ebi-met1400-GCST90199737 | N-acetylproline levels \|\| id:ebi-met1400-GCST90199737 | Dried fruit liking \|\| id:ebi-fl187-GCST90094756 | Inverse variance weighted | 5 | -0.183056227 | 0.190582591444565 | 0.336799280712484 | -0.556598106 | 0.190485652012447 | 0.832721332816079 | 0.573155565296824 | 1.20983701478651 | 0.998074519377826 |
| ebi-fl187-GCST90094756 | ebi-met1400-GCST90199775 | Gamma-glutamylisoleucine levels \|\| id:ebi-met1400-GCST90199775 | Dried fruit liking \|\| id:ebi-fl187-GCST90094756 | Inverse variance weighted | 5 | -0.424353798 | 0.169900208501683 | 0.0125014284519161 | -0.757358207 | -0.091349389 | 0.654192389079875 | 0.468903538493585 | 0.912698768076985 | 0.998074519377826 |
| ebi-fl187-GCST90094756 | ebi-met1400-GCST90199777 | 1-palmitoleoyl-GPC (16:1) levels \|\| id:ebi-met1400-GCST90199777 | Dried fruit liking \|\| id:ebi-fl187-GCST90094756 | Inverse variance weighted | 5 | 0.0733819429371939 | 0.216421147655841 | 0.734556912750326 | -0.350803506 | 0.497567392342643 | 1.0761414830696 | 0.704122095700901 | 1.644715453 | 0.998074519377826 |
| ebi-fl187-GCST90094756 | ebi-met1400-GCST90199782 | Glutamine degradant levels \|\| id:ebi-met1400-GCST90199782 | Dried fruit liking \|\| id:ebi-fl187-GCST90094756 | Inverse variance weighted | 5 | 0.254182579279612 | 0.147345916468101 | 0.0845141744093653 | -0.034615417 | 0.54298057555709 | 1.28940720181583 | 0.965976843105076 | 1.72112918022992 | 0.998074519377826 |
| ebi-fl187-GCST90094756 | ebi-met1400-GCST90199783 | 5-methyluridine (ribothymidine) levels \|\| id:ebi-met1400-GCST90199783 | Dried fruit liking \|\| id:ebi-fl187-GCST90094756 | Inverse variance weighted | 5 | 0.0269037399025723 | 0.198543531463742 | 0.892212218385135 | -0.362241582 | 0.416049061571506 | 1.02726891299835 | 0.696114179033954 | 1.51596024243795 | 0.998074519377826 |
| ebi-fl187-GCST90094756 | ebi-met1400-GCST90199787 | Homostachydrine levels \|\| id:ebi-met1400-GCST90199787 | Dried fruit liking \|\| id:ebi-fl187-GCST90094756 | Inverse variance weighted | 5 | -0.144568442 | 0.210908012053126 | 0.493055379688783 | -0.557948146 | 0.268811261437288 | 0.865395680803284 | 0.572382304807352 | 1.30840817066321 | 0.998074519377826 |
| ebi-fl187-GCST90094756 | ebi-met1400-GCST90199802 | Beta-hydroxyisovaleroylcarnitine levels \|\| id:ebi-met1400-GCST90199802 | Dried fruit liking \|\| id:ebi-fl187-GCST90094756 | Inverse variance weighted | 5 | -0.171857159 | 0.174608787009999 | 0.324996941406127 | -0.514090382 | 0.170376063528306 | 0.842099450896157 | 0.598044339445128 | 1.18575068507052 | 0.998074519377826 |
| ebi-fl187-GCST90094756 | ebi-met1400-GCST90199873 | Pregnenolone sulfate levels \|\| id:ebi-met1400-GCST90199873 | Dried fruit liking \|\| id:ebi-fl187-GCST90094756 | Inverse variance weighted (multiplicative random effects) | 5 | -0.05770489 | 0.270043440812061 | 0.830790810927028 | -0.586990034 | 0.471580253951241 | 0.943928469029892 | 0.555998304591517 | 1.60252458917068 | 0.998074519377826 |
| ebi-fl187-GCST90094756 | ebi-met1400-GCST90199911 | Beta-citrylglutamate levels \|\| id:ebi-met1400-GCST90199911 | Dried fruit liking \|\| id:ebi-fl187-GCST90094756 | Inverse variance weighted | 5 | -0.26381791 | 0.180894539012049 | 0.144728319039242 | -0.618371207 | 0.0907353863596713 | 0.768113392611233 | 0.538821351923926 | 1.09497922048204 | 0.998074519377826 |
| ebi-fl187-GCST90094756 | ebi-met1400-GCST90199934 | 2-hydroxydecanoate levels \|\| id:ebi-met1400-GCST90199934 | Dried fruit liking \|\| id:ebi-fl187-GCST90094756 | Inverse variance weighted | 5 | 0.0770483997028584 | 0.175630408242035 | 0.660881651990601 | -0.2671872 | 0.421283999857247 | 1.08009435137321 | 0.765529750535961 | 1.52391700917117 | 0.998074519377826 |
| ebi-fl187-GCST90094756 | ebi-met1400-GCST90199945 | Sphingomyelin (d18:1/14:0, d16:1/16:0) levels \|\| id:ebi-met1400-GCST90199945 | Dried fruit liking \|\| id:ebi-fl187-GCST90094756 | Inverse variance weighted | 5 | -0.095239218 | 0.168736108200814 | 0.572463556716734 | -0.42596199 | 0.235483553922332 | 0.909155421973544 | 0.653141167397778 | 1.26552056823651 | 0.998074519377826 |
| ebi-fl187-GCST90094756 | ebi-met1400-GCST90199979 | Carnitine C14:1 levels \|\| id:ebi-met1400-GCST90199979 | Dried fruit liking \|\| id:ebi-fl187-GCST90094756 | Inverse variance weighted (multiplicative random effects) | 5 | 0.0157879284520141 | 0.266579950927197 | 0.95277370160836 | -0.506708775 | 0.53828463226932 | 1.01591321627107 | 0.602475200557249 | 1.71306580260836 | 0.998074519377826 |
| ebi-fl187-GCST90094756 | ebi-met1400-GCST90199986 | Eugenol sulfate levels \|\| id:ebi-met1400-GCST90199986 | Dried fruit liking \|\| id:ebi-fl187-GCST90094756 | Inverse variance weighted | 5 | -0.176739835 | 0.190268001173048 | 0.352941216363855 | -0.549665117 | 0.196185447377865 | 0.837997773921946 | 0.577143053291685 | 1.21675252797893 | 0.998074519377826 |
| ebi-fl187-GCST90094756 | ebi-met1400-GCST90200002 | 6-hydroxyindole sulfate levels \|\| id:ebi-met1400-GCST90200002 | Dried fruit liking \|\| id:ebi-fl187-GCST90094756 | Inverse variance weighted | 5 | -0.065409348 | 0.174629481811926 | 0.707987173669791 | -0.407683133 | 0.276864435944142 | 0.936683954803467 | 0.665189622232396 | 1.31898755161237 | 0.998074519377826 |
| ebi-fl187-GCST90094756 | ebi-met1400-GCST90200023 | Trans 3,4-methyleneheptanoate levels \|\| id:ebi-met1400-GCST90200023 | Dried fruit liking \|\| id:ebi-fl187-GCST90094756 | Inverse variance weighted | 5 | -0.175760406 | 0.190093549669233 | 0.35517426004367 | -0.548343763 | 0.196822951556104 | 0.838818935418108 | 0.577906167677085 | 1.21752846010311 | 0.998074519377826 |
| ebi-fl187-GCST90094756 | ebi-met1400-GCST90200032 | 2-hydroxybutyrate/2-hydroxyisobutyrate levels \|\| id:ebi-met1400-GCST90200032 | Dried fruit liking \|\| id:ebi-fl187-GCST90094756 | Inverse variance weighted | 5 | -0.299299668 | 0.245692244903962 | 0.223152038768398 | -0.780856468 | 0.182257132255913 | 0.741337221283928 | 0.458013569412962 | 1.19992269304461 | 0.998074519377826 |
| ebi-fl187-GCST90094756 | ebi-met1400-GCST90200051 | 1-palmitoyl-2-dihomo-linolenoyl-GPC (16:0/20:3n3 or 6) levels \|\| id:ebi-met1400-GCST90200051 | Dried fruit liking \|\| id:ebi-fl187-GCST90094756 | Inverse variance weighted (multiplicative random effects) | 5 | -0.059274375 | 0.2691245795497 | 0.82567736519409 | -0.586758551 | 0.468209800716284 | 0.942448149278771 | 0.556127023596514 | 1.59713244707094 | 0.998074519377826 |
| ebi-fl187-GCST90094756 | ebi-met1400-GCST90200069 | Phenylacetylglutamate levels \|\| id:ebi-met1400-GCST90200069 | Dried fruit liking \|\| id:ebi-fl187-GCST90094756 | Inverse variance weighted | 5 | -0.069636803 | 0.194277325257985 | 0.720013914206073 | -0.45042036 | 0.311146754712267 | 0.932732524241315 | 0.637360174388725 | 1.36498952513304 | 0.998074519377826 |
| ebi-fl187-GCST90094756 | ebi-met1400-GCST90200072 | Thioproline levels \|\| id:ebi-met1400-GCST90200072 | Dried fruit liking \|\| id:ebi-fl187-GCST90094756 | Inverse variance weighted | 5 | 0.0157824002848049 | 0.175851436186499 | 0.928487084550182 | -0.328886415 | 0.360451215210342 | 1.01590760014847 | 0.719724762300138 | 1.43397630052504 | 0.998074519377826 |
| ebi-fl187-GCST90094756 | ebi-met1400-GCST90200081 | 1-oleoyl-2-docosahexaenoyl-GPC (18:1/22:6) levels \|\| id:ebi-met1400-GCST90200081 | Dried fruit liking \|\| id:ebi-fl187-GCST90094756 | Inverse variance weighted | 5 | -0.169626261 | 0.169321586231364 | 0.316440494810389 | -0.50149657 | 0.162244048228846 | 0.843980186152291 | 0.605623623136751 | 1.17614724294996 | 0.998074519377826 |
| ebi-fl187-GCST90094756 | ebi-met1400-GCST90200097 | Furaneol sulfate levels \|\| id:ebi-met1400-GCST90200097 | Dried fruit liking \|\| id:ebi-fl187-GCST90094756 | Inverse variance weighted | 5 | 0.00557747024405386 | 0.208491068643801 | 0.978657854802241 | -0.403065024 | 0.414219964785905 | 1.00559305328907 | 0.668268644211427 | 1.51318993878052 | 0.998074519377826 |
| ebi-fl187-GCST90094756 | ebi-met1400-GCST90200124 | Sphingomyelin (d18:2/24:2) levels \|\| id:ebi-met1400-GCST90200124 | Dried fruit liking \|\| id:ebi-fl187-GCST90094756 | Inverse variance weighted | 4 | 0.228703788663577 | 0.179724182758158 | 0.203186107948693 | -0.12355561 | 0.580963186869566 | 1.256969655 | 0.883772493693584 | 1.78775954826094 | 0.998074519377826 |
| ebi-fl187-GCST90094756 | ebi-met1400-GCST90200137 | Lignoceroylcarnitine (C24) levels \|\| id:ebi-met1400-GCST90200137 | Dried fruit liking \|\| id:ebi-fl187-GCST90094756 | Inverse variance weighted | 5 | -0.042225928 | 0.177099816589132 | 0.811547520511325 | -0.389341568 | 0.304889712725722 | 0.958653169713171 | 0.677502816999776 | 1.35647539278263 | 0.998074519377826 |
| ebi-fl187-GCST90094756 | ebi-met1400-GCST90200165 | Octadecenedioate (C18:1-DC) levels \|\| id:ebi-met1400-GCST90200165 | Dried fruit liking \|\| id:ebi-fl187-GCST90094756 | Inverse variance weighted | 5 | 0.153434126052988 | 0.244216821651182 | 0.529827017744966 | -0.325230844 | 0.632099096489305 | 1.16583098670392 | 0.722360581510628 | 1.881556005 | 0.998074519377826 |
| ebi-fl187-GCST90094756 | ebi-met1400-GCST90200176 | Glutamine conjugate of C6H10O2 (1) levels \|\| id:ebi-met1400-GCST90200176 | Dried fruit liking \|\| id:ebi-fl187-GCST90094756 | Inverse variance weighted | 5 | 0.112186712487845 | 0.173547773974023 | 0.518000077754056 | -0.227966925 | 0.452340349476931 | 1.1187217204617 | 0.796150592477902 | 1.57198688245344 | 0.998074519377826 |
| ebi-fl187-GCST90094756 | ebi-met1400-GCST90200182 | Glycine conjugate of C10H12O2 levels \|\| id:ebi-met1400-GCST90200182 | Dried fruit liking \|\| id:ebi-fl187-GCST90094756 | Inverse variance weighted | 5 | -0.124859849 | 0.216972033678344 | 0.56497673754403 | -0.550125035 | 0.300405336978105 | 0.882620594047772 | 0.576877675947049 | 1.35040606617051 | 0.998074519377826 |
| ebi-fl187-GCST90094756 | ebi-met1400-GCST90200208 | Glucuronide of piperine metabolite C17H21NO3 (4) levels \|\| id:ebi-met1400-GCST90200208 | Dried fruit liking \|\| id:ebi-fl187-GCST90094756 | Inverse variance weighted | 5 | -0.20862494 | 0.245795235360631 | 0.396006302806748 | -0.690383601 | 0.273133721669746 | 0.811699614911686 | 0.501383700911172 | 1.31407595350712 | 0.998074519377826 |
| ebi-fl187-GCST90094756 | ebi-met1400-GCST90200227 | 3-ethylcatechol sulfate (2) levels \|\| id:ebi-met1400-GCST90200227 | Dried fruit liking \|\| id:ebi-fl187-GCST90094756 | Inverse variance weighted | 5 | -0.141968163 | 0.258850404164012 | 0.583378552523063 | -0.649314955 | 0.365378628922122 | 0.867648879175905 | 0.522403523923309 | 1.4410595316844 | 0.998074519377826 |
| ebi-fl187-GCST90094756 | ebi-met1400-GCST90200259 | Pentose acid levels \|\| id:ebi-met1400-GCST90200259 | Dried fruit liking \|\| id:ebi-fl187-GCST90094756 | Inverse variance weighted | 5 | -0.0699652 | 0.173775938234435 | 0.687229776907942 | -0.410566039 | 0.270635638951897 | 0.932426267787037 | 0.663274704557506 | 1.31079737985679 | 0.998074519377826 |
| ebi-fl187-GCST90094756 | ebi-met1400-GCST90200291 | Sarcosine levels \|\| id:ebi-met1400-GCST90200291 | Dried fruit liking \|\| id:ebi-fl187-GCST90094756 | Inverse variance weighted | 5 | 0.0985534173531373 | 0.262643572529905 | 0.707484693315818 | -0.416227985 | 0.613334819511752 | 1.10357335278744 | 0.659529890549067 | 1.84657915 | 0.998074519377826 |
| ebi-fl187-GCST90094756 | ebi-met1400-GCST90200306 | Butyrate/isobutyrate (4:0) levels \|\| id:ebi-met1400-GCST90200306 | Dried fruit liking \|\| id:ebi-fl187-GCST90094756 | Inverse variance weighted | 5 | -0.041062196 | 0.178619048824321 | 0.818179585530849 | -0.391155531 | 0.309031140076734 | 0.959769434637402 | 0.676274965924838 | 1.36210478589056 | 0.998074519377826 |
| ebi-fl187-GCST90094756 | ebi-met1400-GCST90200321 | Cholate levels \|\| id:ebi-met1400-GCST90200321 | Dried fruit liking \|\| id:ebi-fl187-GCST90094756 | Inverse variance weighted | 5 | 0.363515607460156 | 0.185587706840967 | 0.0501447949607321 | -0.000236298 | 0.727267512868451 | 1.43837730614657 | 0.999763729968022 | 2.06941821634563 | 0.998074519377826 |
| ebi-fl187-GCST90094756 | ebi-met1400-GCST90200336 | Ceramide (d18:1/16:0) levels \|\| id:ebi-met1400-GCST90200336 | Dried fruit liking \|\| id:ebi-fl187-GCST90094756 | Inverse variance weighted | 5 | -0.165942501 | 0.181931389359628 | 0.361707745153717 | -0.522528024 | 0.190643022641517 | 0.847094940321778 | 0.593019484127446 | 1.21002742258051 | 0.998074519377826 |
| ebi-fl187-GCST90094756 | ebi-met1400-GCST90200358 | Arachidonate (20:4n6) levels \|\| id:ebi-met1400-GCST90200358 | Dried fruit liking \|\| id:ebi-fl187-GCST90094756 | Inverse variance weighted | 5 | -0.232846096 | 0.192082915258375 | 0.225429484406113 | -0.60932861 | 0.143636418289665 | 0.792275498813664 | 0.543715792162812 | 1.15446429010927 | 0.998074519377826 |
| ebi-fl187-GCST90094756 | ebi-met1400-GCST90200413 | 2'-deoxyuridine levels \|\| id:ebi-met1400-GCST90200413 | Dried fruit liking \|\| id:ebi-fl187-GCST90094756 | Inverse variance weighted | 5 | -0.041649128 | 0.218317626386182 | 0.848703374044098 | -0.469551675 | 0.386253419953233 | 0.9592062803875 | 0.625282534944767 | 1.47145752026497 | 0.998074519377826 |
| ebi-fl187-GCST90094756 | ebi-met1400-GCST90200432 | Threonine levels \|\| id:ebi-met1400-GCST90200432 | Dried fruit liking \|\| id:ebi-fl187-GCST90094756 | Inverse variance weighted | 5 | -0.055032056 | 0.176423918476232 | 0.755093106755522 | -0.400822936 | 0.2907588243845 | 0.946454808073799 | 0.669768642425933 | 1.33744198665597 | 0.998074519377826 |
| ebi-fl187-GCST90094756 | ebi-met1400-GCST90200436 | Caffeine levels \|\| id:ebi-met1400-GCST90200436 | Dried fruit liking \|\| id:ebi-fl187-GCST90094756 | Inverse variance weighted | 5 | 0.00691086257417854 | 0.187284606039426 | 0.970564481703322 | -0.360166965 | 0.373988690411454 | 1.0069347976906 | 0.697559848083852 | 1.4535207116427 | 0.998074519377826 |
| ebi-fl187-GCST90094756 | ebi-met1400-GCST90200442 | Valine levels \|\| id:ebi-met1400-GCST90200442 | Dried fruit liking \|\| id:ebi-fl187-GCST90094756 | Inverse variance weighted | 5 | -0.580844723 | 0.176583544027349 | 0.00100419789319875 | -0.926948469 | -0.234740976 | 0.559425607506716 | 0.395759542202569 | 0.790775652792907 | 0.598008988154769 |
| ebi-fl187-GCST90094756 | ebi-met1400-GCST90200459 | Margarate (17:0) levels \|\| id:ebi-met1400-GCST90200459 | Dried fruit liking \|\| id:ebi-fl187-GCST90094756 | Inverse variance weighted (multiplicative random effects) | 5 | -0.238275039 | 0.274376409812547 | 0.385162301139378 | -0.776052802 | 0.299502724034572 | 0.787985934267413 | 0.460219006169374 | 1.34918772210544 | 0.998074519377826 |
| ebi-fl187-GCST90094756 | ebi-met1400-GCST90200462 | X-11299 levels \|\| id:ebi-met1400-GCST90200462 | Dried fruit liking \|\| id:ebi-fl187-GCST90094756 | Inverse variance weighted | 5 | -0.166207822 | 0.175183648522092 | 0.3427409948276 | -0.509567773 | 0.17715212891572 | 0.84687021747875 | 0.600755185140488 | 1.19381269274403 | 0.998074519377826 |
| ebi-fl187-GCST90094756 | ebi-met1400-GCST90200497 | X-12740 levels \|\| id:ebi-met1400-GCST90200497 | Dried fruit liking \|\| id:ebi-fl187-GCST90094756 | Inverse variance weighted | 5 | 0.655363279301189 | 0.229665664294905 | 0.00432332363017139 | 0.205218577283175 | 1.1055079813192 | 1.92584200885246 | 1.22779340338218 | 3.02075856804909 | 0.877504772527479 |
| ebi-fl187-GCST90094756 | ebi-met1400-GCST90200527 | X-13866 levels \|\| id:ebi-met1400-GCST90200527 | Dried fruit liking \|\| id:ebi-fl187-GCST90094756 | Inverse variance weighted | 5 | -0.187689221 | 0.235576930148507 | 0.425612755224404 | -0.649420004 | 0.274041562025425 | 0.828872263247802 | 0.522348648965359 | 1.31526946636574 | 0.998074519377826 |
| ebi-fl187-GCST90094756 | ebi-met1400-GCST90200547 | X-17654 levels \|\| id:ebi-met1400-GCST90200547 | Dried fruit liking \|\| id:ebi-fl187-GCST90094756 | Inverse variance weighted | 5 | -0.055949968 | 0.238353567469895 | 0.814414278351098 | -0.523122961 | 0.41122302392116 | 0.94558644398547 | 0.592666779875239 | 1.50866178670805 | 0.998074519377826 |
| ebi-fl187-GCST90094756 | ebi-met1400-GCST90200553 | X-17653 levels \|\| id:ebi-met1400-GCST90200553 | Dried fruit liking \|\| id:ebi-fl187-GCST90094756 | Inverse variance weighted | 5 | 0.0683981418420215 | 0.178117913816044 | 0.700974856697573 | -0.280712969 | 0.417509252921469 | 1.0707915505411 | 0.755245082944213 | 1.51817545139172 | 0.998074519377826 |
| ebi-fl187-GCST90094756 | ebi-met1400-GCST90200557 | X-17685 levels \|\| id:ebi-met1400-GCST90200557 | Dried fruit liking \|\| id:ebi-fl187-GCST90094756 | Inverse variance weighted | 5 | -0.229138208 | 0.187257039423143 | 0.22108200759082 | -0.596162006 | 0.137885588958874 | 0.795218620087846 | 0.550922019320063 | 1.14784421670943 | 0.998074519377826 |
| ebi-fl187-GCST90094756 | ebi-met1400-GCST90200561 | X-18779 levels \|\| id:ebi-met1400-GCST90200561 | Dried fruit liking \|\| id:ebi-fl187-GCST90094756 | Inverse variance weighted | 5 | -0.050557216 | 0.208236299151846 | 0.808169850407393 | -0.458700362 | 0.357585930419183 | 0.950699531969285 | 0.63210461892823 | 1.42987343079238 | 0.998074519377826 |
| ebi-fl187-GCST90094756 | ebi-met1400-GCST90200570 | X-18921 levels \|\| id:ebi-met1400-GCST90200570 | Dried fruit liking \|\| id:ebi-fl187-GCST90094756 | Inverse variance weighted | 5 | 0.031724485763595 | 0.190556369186355 | 0.867776489879049 | -0.341765998 | 0.405214969368851 | 1.03223307121769 | 0.710514447172041 | 1.49962483881416 | 0.998074519377826 |
| ebi-fl187-GCST90094756 | ebi-met1400-GCST90200575 | X-21283 levels \|\| id:ebi-met1400-GCST90200575 | Dried fruit liking \|\| id:ebi-fl187-GCST90094756 | Inverse variance weighted | 5 | -0.052425246 | 0.235682927896305 | 0.823971589341949 | -0.514363785 | 0.409513292733828 | 0.948925254417114 | 0.597880854637089 | 1.50608458438958 | 0.998074519377826 |
| ebi-fl187-GCST90094756 | ebi-met1400-GCST90200578 | X-18922 levels \|\| id:ebi-met1400-GCST90200578 | Dried fruit liking \|\| id:ebi-fl187-GCST90094756 | Inverse variance weighted | 5 | -0.253395454 | 0.196077755296348 | 0.19624590883569 | -0.637707854 | 0.130916946581791 | 0.77616088535549 | 0.528502441407135 | 1.13987310702267 | 0.998074519377826 |
| ebi-fl187-GCST90094756 | ebi-met1400-GCST90200623 | X-24241 levels \|\| id:ebi-met1400-GCST90200623 | Dried fruit liking \|\| id:ebi-fl187-GCST90094756 | Inverse variance weighted (multiplicative random effects) | 5 | -0.166602567 | 0.296708706023233 | 0.574455286986413 | -0.748151631 | 0.414946497000127 | 0.846535985990783 | 0.473240468027838 | 1.51428971948196 | 0.998074519377826 |
| ebi-fl187-GCST90094756 | ebi-met1400-GCST90200636 | X-24544 levels \|\| id:ebi-met1400-GCST90200636 | Dried fruit liking \|\| id:ebi-fl187-GCST90094756 | Inverse variance weighted (multiplicative random effects) | 5 | -0.06132366 | 0.265090859681666 | 0.817057882463008 | -0.580901745 | 0.458254425449465 | 0.940518782644492 | 0.559393708861652 | 1.58131127771737 | 0.998074519377826 |
| ebi-fl187-GCST90094756 | ebi-met1400-GCST90200659 | X-25172 levels \|\| id:ebi-met1400-GCST90200659 | Dried fruit liking \|\| id:ebi-fl187-GCST90094756 | Inverse variance weighted | 5 | -0.173516729 | 0.178909998703289 | 0.332118849605694 | -0.524180327 | 0.177146868075985 | 0.840703086600763 | 0.592040445197812 | 1.1938064123033 | 0.998074519377826 |
| ebi-fl187-GCST90094756 | ebi-met1400-GCST90200670 | X-26111 levels \|\| id:ebi-met1400-GCST90200670 | Dried fruit liking \|\| id:ebi-fl187-GCST90094756 | Inverse variance weighted | 5 | -0.347604586 | 0.177706459298384 | 0.0504580274853221 | -0.695909246 | 0.000700074467088696 | 0.706378132973892 | 0.498620872785262 | 1.00070031957641 | 0.998074519377826 |
| ebi-fl187-GCST90094756 | ebi-met1400-GCST90200673 | Carnitine C4 levels \|\| id:ebi-met1400-GCST90200673 | Dried fruit liking \|\| id:ebi-fl187-GCST90094756 | Inverse variance weighted | 5 | -0.376025631 | 0.206671732668959 | 0.0688452039709027 | -0.781102227 | 0.0290509652061419 | 0.686584735083572 | 0.457901022245908 | 1.02947706064876 | 0.998074519377826 |
| ebi-fl187-GCST90094756 | ebi-met1400-GCST90200710 | X-13431 levels \|\| id:ebi-met1400-GCST90200710 | Dried fruit liking \|\| id:ebi-fl187-GCST90094756 | Inverse variance weighted | 5 | -0.338521427 | 0.174296251982907 | 0.0521106391214326 | -0.680142081 | 0.00309922678058261 | 0.7128235054805 | 0.50654501683384 | 1.0031040343492 | 0.998074519377826 |
| ebi-fl187-GCST90094756 | ebi-met1400-GCST90200730 | Adenosine 5'-diphosphate (ADP) to fructose ratio \|\| id:ebi-met1400-GCST90200730 | Dried fruit liking \|\| id:ebi-fl187-GCST90094756 | Inverse variance weighted | 5 | 0.212577633428922 | 0.30467534172576 | 0.485353233652634 | -0.384586036 | 0.809741303211411 | 1.23686213168651 | 0.680732376342232 | 2.24732653531232 | 0.998074519377826 |
| ebi-fl187-GCST90094756 | ebi-met1400-GCST90200731 | Adenosine 5'-diphosphate (ADP) to mannose ratio \|\| id:ebi-met1400-GCST90200731 | Dried fruit liking \|\| id:ebi-fl187-GCST90094756 | Inverse variance weighted | 5 | 0.382087629352313 | 0.234285325391656 | 0.102918905514864 | -0.077111608 | 0.841286867119959 | 1.46534048634476 | 0.925786522273592 | 2.31934975208738 | 0.998074519377826 |
| ebi-fl187-GCST90094756 | ebi-met1400-GCST90200748 | Aspartate to glutamate ratio \|\| id:ebi-met1400-GCST90200748 | Dried fruit liking \|\| id:ebi-fl187-GCST90094756 | Inverse variance weighted | 5 | 0.502452454682473 | 0.23101216492911 | 0.0296297933100151 | 0.0496686114214172 | 0.955236297943529 | 1.65276964710129 | 1.05092277485976 | 2.59928471599051 | 0.998074519377826 |
| ebi-fl187-GCST90094756 | ebi-met1400-GCST90200774 | Uridine to pseudouridine ratio \|\| id:ebi-met1400-GCST90200774 | Dried fruit liking \|\| id:ebi-fl187-GCST90094756 | Inverse variance weighted | 5 | 0.087356278071446 | 0.168245315581564 | 0.603607560258537 | -0.24240454 | 0.417117096611311 | 1.09128541152767 | 0.784738654787762 | 1.51758020603077 | 0.998074519377826 |
| ebi-fl187-GCST90094756 | ebi-met1400-GCST90200799 | Spermidine to adenosine 5'-diphosphate (ADP) ratio \|\| id:ebi-met1400-GCST90200799 | Dried fruit liking \|\| id:ebi-fl187-GCST90094756 | Inverse variance weighted | 5 | -0.430348933 | 0.245576830637573 | 0.0797049846718561 | -0.911679522 | 0.0509816545986937 | 0.650282149936637 | 0.401848743345813 | 1.0523035881745 | 0.998074519377826 |
| ebi-fl187-GCST90094756 | ebi-met1400-GCST90200811 | Choline to taurocholate ratio \|\| id:ebi-met1400-GCST90200811 | Dried fruit liking \|\| id:ebi-fl187-GCST90094756 | Inverse variance weighted | 5 | 0.0805346856367959 | 0.184661983292133 | 0.662750054125639 | -0.281402802 | 0.442472172889376 | 1.08386644058796 | 0.754724270089841 | 1.55655052790732 | 0.998074519377826 |
| ebi-fl187-GCST90094756 | ebi-met1400-GCST90200812 | Glutamate to kynurenine ratio \|\| id:ebi-met1400-GCST90200812 | Dried fruit liking \|\| id:ebi-fl187-GCST90094756 | Inverse variance weighted | 5 | -0.362244638 | 0.215025080412069 | 0.0920537519975619 | -0.783693795 | 0.0592045199188724 | 0.696112051766277 | 0.456715876764015 | 1.06099221259311 | 0.998074519377826 |
| ebi-fl187-GCST90094756 | ebi-met1400-GCST90200876 | Cortisone to 4-cholesten-3-one ratio \|\| id:ebi-met1400-GCST90200876 | Dried fruit liking \|\| id:ebi-fl187-GCST90094756 | Inverse variance weighted | 5 | -0.097631798 | 0.186470418307848 | 0.600572127827168 | -0.463113817 | 0.267850222286045 | 0.906982795524759 | 0.629321000700902 | 1.30715134321232 | 0.998074519377826 |
| ebi-fl187-GCST90094756 | ebi-met1400-GCST90200906 | 2'-deoxyuridine to cytidine ratio \|\| id:ebi-met1400-GCST90200906 | Dried fruit liking \|\| id:ebi-fl187-GCST90094756 | Inverse variance weighted | 5 | 0.126860580079616 | 0.274432937406348 | 0.643891718089108 | -0.411027977 | 0.664749137396057 | 1.13525872906582 | 0.662968383317796 | 1.94400278256157 | 0.998074519377826 |
| ebi-fl187-GCST90094756 | ebi-met1400-GCST90200907 | Retinol (Vitamin A) to linoleoyl-arachidonoyl-glycerol (18:2 to 20:4) [1] ratio \|\| id:ebi-met1400-GCST90200907 | Dried fruit liking \|\| id:ebi-fl187-GCST90094756 | Inverse variance weighted | 5 | 0.138203133971381 | 0.183966985896926 | 0.452508965611758 | -0.222372158 | 0.498778426329356 | 1.14820876679305 | 0.800617352422126 | 1.64670846585061 | 0.998074519377826 |
| ebi-fl187-GCST90094756 | ebi-met1400-GCST90200916 | Fructose to sucrose ratio \|\| id:ebi-met1400-GCST90200916 | Dried fruit liking \|\| id:ebi-fl187-GCST90094756 | Inverse variance weighted | 5 | 0.15587469768117 | 0.190513207358836 | 0.413252610431253 | -0.217531189 | 0.529280584104488 | 1.16867975563161 | 0.8045025131071 | 1.69771050925397 | 0.998074519377826 |
| ebi-fl187-GCST90094756 | ebi-met1400-GCST90200917 | Alpha-ketobutyrate to 3-methyl-2-oxobutyrate ratio \|\| id:ebi-met1400-GCST90200917 | Dried fruit liking \|\| id:ebi-fl187-GCST90094756 | Inverse variance weighted | 5 | -0.216103326 | 0.238405236485012 | 0.364695721759184 | -0.68337759 | 0.251170937271847 | 0.80565205257628 | 0.504908734526387 | 1.28552981050965 | 0.998074519377826 |
| ebi-fl187-GCST90094756 | ebi-met1400-GCST90200924 | Alpha-tocopherol to glycerol ratio \|\| id:ebi-met1400-GCST90200924 | Dried fruit liking \|\| id:ebi-fl187-GCST90094756 | Inverse variance weighted | 5 | 0.131752788113044 | 0.164566991865414 | 0.423361651637595 | -0.190798516 | 0.454304092169256 | 1.14082625859222 | 0.826299057466429 | 1.57507689320631 | 0.998074519377826 |
| ebi-fl187-GCST90094756 | ebi-met1400-GCST90200938 | Alpha-ketoglutarate to alanine ratio \|\| id:ebi-met1400-GCST90200938 | Dried fruit liking \|\| id:ebi-fl187-GCST90094756 | Inverse variance weighted | 5 | -0.15542537 | 0.175120381658604 | 0.374791232026539 | -0.498661318 | 0.187810577989112 | 0.856050961575446 | 0.607343155035984 | 1.20660493616797 | 0.998074519377826 |
| ebi-fl187-GCST90094756 | ebi-met1400-GCST90200943 | Phosphate to EDTA ratio \|\| id:ebi-met1400-GCST90200943 | Dried fruit liking \|\| id:ebi-fl187-GCST90094756 | Inverse variance weighted | 5 | -0.089721388 | 0.183823430142592 | 0.625489907632988 | -0.450015311 | 0.270572534840993 | 0.914185852723683 | 0.637618388769139 | 1.31071466576338 | 0.998074519377826 |
| ebi-fl187-GCST90094756 | ebi-met1400-GCST90200947 | Adenosine 5'-diphosphate (ADP) to glucose ratio \|\| id:ebi-met1400-GCST90200947 | Dried fruit liking \|\| id:ebi-fl187-GCST90094756 | Inverse variance weighted | 5 | 0.519899136087603 | 0.234944472142237 | 0.0269073251464725 | 0.0594079706888182 | 0.980390301486389 | 1.68185800236517 | 1.06120809423557 | 2.66549638613277 | 0.998074519377826 |
| ebi-fl187-GCST90094756 | ebi-met1400-GCST90200975 | Arachidonate (20:4n6) to caffeine ratio \|\| id:ebi-met1400-GCST90200975 | Dried fruit liking \|\| id:ebi-fl187-GCST90094756 | Inverse variance weighted | 5 | -0.022278764 | 0.208513612742456 | 0.914911463775755 | -0.430965445 | 0.386407917031279 | 0.977967574950839 | 0.649881367089941 | 1.47168487371459 | 0.998074519377826 |
| ebi-fl187-GCST90094756 | ebi-met1400-GCST90200985 | Cholesterol to oleoyl-linoleoyl-glycerol (18:1 to 18:2) [2] ratio \|\| id:ebi-met1400-GCST90200985 | Dried fruit liking \|\| id:ebi-fl187-GCST90094756 | Inverse variance weighted | 5 | 0.203562784937274 | 0.176491555562827 | 0.248752227826284 | -0.142360664 | 0.549486233840415 | 1.22576211462279 | 0.86730839318533 | 1.73236275983263 | 0.998074519377826 |
| ebi-fl187-GCST90094756 | ebi-met1400-GCST90201016 | Bilirubin (Z,Z) to etiocholanolone glucuronide ratio \|\| id:ebi-met1400-GCST90201016 | Dried fruit liking \|\| id:ebi-fl187-GCST90094756 | Inverse variance weighted | 5 | -0.096417212 | 0.220514105437627 | 0.661938539720032 | -0.528624858 | 0.33579043509277 | 0.908085073429957 | 0.589414941748046 | 1.39904580318357 | 0.998074519377826 |
| ebi-fl187-GCST90094756 | ebi-pw11-GCST90275189 | Placental weight (fetal genotype, adjusted for sex and gestational age) \|\| id:ebi-pw11-GCST90275189 | Dried fruit liking \|\| id:ebi-fl187-GCST90094756 | Inverse variance weighted (multiplicative random effects) | 5 | 0.00802085697409827 | 0.102676791728542 | 0.937734565856872 | -0.193225655 | 0.209267368762041 | 1.00805311022263 | 0.824295946802487 | 1.23277455987904 | 0.998074519377826 |
| ebi-fl187-GCST90094756 | ebi-pw11-GCST90275192 | Placental weight (fetal effect, conditional on maternal and paternal genotype) \|\| id:ebi-pw11-GCST90275192 | Dried fruit liking \|\| id:ebi-fl187-GCST90094756 | Inverse variance weighted | 5 | 0.0734820486381839 | 0.165331280567931 | 0.656714818043549 | -0.250567261 | 0.397531358551328 | 1.07624921635939 | 0.778359124825913 | 1.48814645935226 | 0.998074519377826 |
| ebi-fl187-GCST90094756 | ebi-pw11-GCST90275193 | Placental weight (maternal effect, conditional on fetal and paternal genotype) \|\| id:ebi-pw11-GCST90275193 | Dried fruit liking \|\| id:ebi-fl187-GCST90094756 | Inverse variance weighted | 5 | -0.090418575 | 0.122850677350185 | 0.461728339447745 | -0.331205902 | 0.150368753004734 | 0.913548716940786 | 0.718057304233842 | 1.1622627515984 | 0.998074519377826 |
| ebi-fl187-GCST90094756 | ebi-pw11-GCST90275195 | Placental weight (fetal effect, conditional on maternal genotype) \|\| id:ebi-pw11-GCST90275195 | Dried fruit liking \|\| id:ebi-fl187-GCST90094756 | Inverse variance weighted | 5 | 0.0507757685338497 | 0.126705050212733 | 0.688611634902365 | -0.19756613 | 0.299117666950807 | 1.05208695583127 | 0.82072586432763 | 1.34866830782421 | 0.998074519377826 |
| ebi-fl187-GCST90094756 | ebi-pw11-GCST90275197 | Placental weight (fetal genotype, adjusted for sex) \|\| id:ebi-pw11-GCST90275197 | Dried fruit liking \|\| id:ebi-fl187-GCST90094756 | Inverse variance weighted (multiplicative random effects) | 5 | 0.0154536979095173 | 0.10699848626401 | 0.885161618291261 | -0.194263335 | 0.225170730986977 | 1.01557372378321 | 0.82344103473197 | 1.25253654473827 | 0.998074519377826 |
| ebi-fl187-GCST90094756 | prot-a-1030 | Protein FAM171B \|\| id:prot-a-1030 | Dried fruit liking \|\| id:ebi-fl187-GCST90094756 | Inverse variance weighted | 5 | 0.0192244812489294 | 0.289790668691478 | 0.947107774527977 | -0.548765229 | 0.587214191884227 | 1.01941046146791 | 0.577662651058676 | 1.79896984346434 | 0.998074519377826 |
| ebi-fl187-GCST90094756 | prot-a-1036 | BRISC complex subunit Abro1 \|\| id:prot-a-1036 | Dried fruit liking \|\| id:ebi-fl187-GCST90094756 | Inverse variance weighted | 5 | -0.114179886 | 0.286720114655262 | 0.690462363920353 | -0.676151311 | 0.447791538629852 | 0.892097465518897 | 0.508570560630234 | 1.56485245036405 | 0.998074519377826 |
| ebi-fl187-GCST90094756 | prot-a-1055 | Phenylalanine--tRNA ligase, mitochondrial \|\| id:prot-a-1055 | Dried fruit liking \|\| id:ebi-fl187-GCST90094756 | Inverse variance weighted (multiplicative random effects) | 5 | -0.083976998 | 0.451680128205727 | 0.852506406191226 | -0.96927005 | 0.801316053053922 | 0.919452404885277 | 0.379359850969406 | 2.22847178658739 | 0.998074519377826 |
| ebi-fl187-GCST90094756 | prot-a-1083 | Fc receptor-like protein 5 \|\| id:prot-a-1083 | Dried fruit liking \|\| id:ebi-fl187-GCST90094756 | Inverse variance weighted | 5 | 0.0719879042289026 | 0.286917093262408 | 0.801890331623375 | -0.490369599 | 0.634345407023222 | 1.07464234535738 | 0.612400010186167 | 1.88578731421663 | 0.998074519377826 |
| ebi-fl187-GCST90094756 | prot-a-1103 | Fibroblast growth factor receptor 3 \|\| id:prot-a-1103 | Dried fruit liking \|\| id:ebi-fl187-GCST90094756 | Inverse variance weighted | 5 | 0.262687948379183 | 0.328798019363091 | 0.424328627178629 | -0.38175617 | 0.907132066330841 | 1.30042085719388 | 0.68266148655593 | 2.4772078682167 | 0.998074519377826 |
| ebi-fl187-GCST90094756 | prot-a-1105 | Fibroblast growth factor receptor 4 \|\| id:prot-a-1105 | Dried fruit liking \|\| id:ebi-fl187-GCST90094756 | Inverse variance weighted | 5 | -0.031980153 | 0.286917093262408 | 0.911250550423348 | -0.594337656 | 0.530377349852941 | 0.968525804276859 | 0.55192801118168 | 1.69957352144854 | 0.998074519377826 |
| ebi-fl187-GCST90094756 | prot-a-1120 | Filamin-A \|\| id:prot-a-1120 | Dried fruit liking \|\| id:ebi-fl187-GCST90094756 | Inverse variance weighted | 5 | -0.454918018 | 0.286917093262408 | 0.112844100162989 | -1.01727552 | 0.107439485283275 | 0.634499983703915 | 0.361578713291984 | 1.1134234802013 | 0.998074519377826 |
| ebi-fl187-GCST90094756 | prot-a-1166 | Galanin peptides \|\| id:prot-a-1166 | Dried fruit liking \|\| id:ebi-fl187-GCST90094756 | Inverse variance weighted | 5 | -0.50253903 | 0.422000724764807 | 0.233712972573571 | -1.32966045 | 0.324582390998275 | 0.604992613848469 | 0.264567079780867 | 1.38345278299312 | 0.998074519377826 |
| ebi-fl187-GCST90094756 | prot-a-1205 | ADP-ribosylation factor-binding protein GGA1 \|\| id:prot-a-1205 | Dried fruit liking \|\| id:ebi-fl187-GCST90094756 | Inverse variance weighted | 5 | 0.171713818657492 | 0.286720114655262 | 0.549246218981815 | -0.390257606 | 0.733685243381806 | 1.18733799060776 | 0.676882483002829 | 2.08274189292999 | 0.998074519377826 |
| ebi-fl187-GCST90094756 | prot-a-1227 | Guanine nucleotide-binding protein G(I)/G(S)/G(O) subunit gamma-T2 \|\| id:prot-a-1227 | Dried fruit liking \|\| id:ebi-fl187-GCST90094756 | Inverse variance weighted | 5 | -0.52417531 | 0.35573607913052 | 0.140617628846053 | -1.221418026 | 0.173067404643236 | 0.59204341511004 | 0.294811819688293 | 1.1889462428805 | 0.998074519377826 |
| ebi-fl187-GCST90094756 | prot-a-1240 | Golgi SNAP receptor complex member 1 \|\| id:prot-a-1240 | Dried fruit liking \|\| id:ebi-fl187-GCST90094756 | Inverse variance weighted | 5 | -0.184019078 | 0.286917093262408 | 0.521284442472748 | -0.746376581 | 0.378338424471101 | 0.831919932037914 | 0.474081239265393 | 1.45985691058855 | 0.998074519377826 |
| ebi-fl187-GCST90094756 | prot-a-1283 | Glutathione S-transferase A1 \|\| id:prot-a-1283 | Dried fruit liking \|\| id:ebi-fl187-GCST90094756 | Inverse variance weighted | 5 | -0.305964682 | 0.315412203641758 | 0.332023032739498 | -0.924172602 | 0.312243236651419 | 0.736412627259791 | 0.396859644294843 | 1.36648703233925 | 0.998074519377826 |
| ebi-fl187-GCST90094756 | prot-a-1324 | E3 ubiquitin-protein ligase HECW1 \|\| id:prot-a-1324 | Dried fruit liking \|\| id:ebi-fl187-GCST90094756 | Inverse variance weighted | 5 | -0.262983128 | 0.41652150572377 | 0.527792435898375 | -1.079365279 | 0.553399023414951 | 0.768754867784824 | 0.339811142470046 | 1.73915440926119 | 0.998074519377826 |
| ebi-fl187-GCST90094756 | prot-a-1339 | Histidine triad nucleotide-binding protein 2, mitochondrial \|\| id:prot-a-1339 | Dried fruit liking \|\| id:ebi-fl187-GCST90094756 | Inverse variance weighted | 5 | 0.00783475742376369 | 0.286917093262408 | 0.978215117390827 | -0.554522745 | 0.570192260218083 | 1.00786552944699 | 0.574346305229918 | 1.76860705152587 | 0.998074519377826 |
| ebi-fl187-GCST90094756 | prot-a-1466 | Interleukin-11 \|\| id:prot-a-1466 | Dried fruit liking \|\| id:ebi-fl187-GCST90094756 | Inverse variance weighted | 5 | -0.364055213 | 0.286917093262408 | 0.204493996812876 | -0.926412716 | 0.198302289461312 | 0.694852828543145 | 0.395971628250138 | 1.21933092900638 | 0.998074519377826 |
| ebi-fl187-GCST90094756 | prot-a-147 | Rho GTPase-activating protein 1 \|\| id:prot-a-147 | Dried fruit liking \|\| id:ebi-fl187-GCST90094756 | Inverse variance weighted | 5 | 0.0601221005488102 | 0.286917093262408 | 0.834022651652778 | -0.502235402 | 0.622479603343129 | 1.06196620528598 | 0.605176334009267 | 1.86354316550692 | 0.998074519377826 |
| ebi-fl187-GCST90094756 | prot-a-1473 | Interleukin-12 receptor subunit beta-1 \|\| id:prot-a-1473 | Dried fruit liking \|\| id:ebi-fl187-GCST90094756 | Inverse variance weighted | 5 | -0.173420857 | 0.315137369775008 | 0.582111964501792 | -0.791090102 | 0.444248387462864 | 0.84078369042341 | 0.453350327697323 | 1.55931775250415 | 0.998074519377826 |
| ebi-fl187-GCST90094756 | prot-a-1501 | X-linked interleukin-1 receptor accessory protein-like 2 \|\| id:prot-a-1501 | Dried fruit liking \|\| id:ebi-fl187-GCST90094756 | Inverse variance weighted | 5 | 0.415644413173397 | 0.371175409430725 | 0.262796484796938 | -0.311859389 | 1.14314821565762 | 1.51534693564942 | 0.732084459891938 | 3.1366276176946 | 0.998074519377826 |
| ebi-fl187-GCST90094756 | prot-a-1503 | Interleukin-1 receptor-like 2 \|\| id:prot-a-1503 | Dried fruit liking \|\| id:ebi-fl187-GCST90094756 | Inverse variance weighted | 5 | -0.498023205 | 0.305237772245652 | 0.102765673387618 | -1.096289239 | 0.100242828564304 | 0.607730832322309 | 0.334108583466935 | 1.10543931772923 | 0.998074519377826 |
| ebi-fl187-GCST90094756 | prot-a-1536 | Interleukin-5 receptor subunit alpha \|\| id:prot-a-1536 | Dried fruit liking \|\| id:ebi-fl187-GCST90094756 | Inverse variance weighted | 5 | 0.0450625783539253 | 0.312510210513081 | 0.885346063227638 | -0.567457434 | 0.657582590959563 | 1.04609332065854 | 0.566965153819332 | 1.93012079870278 | 0.998074519377826 |
| ebi-fl187-GCST90094756 | prot-a-1559 | Insulin-induced gene 1 protein \|\| id:prot-a-1559 | Dried fruit liking \|\| id:ebi-fl187-GCST90094756 | Inverse variance weighted | 5 | 0.172559456455899 | 0.323421559054484 | 0.593657437624352 | -0.461346799 | 0.806465712202688 | 1.18834247314644 | 0.630434005416571 | 2.23997725590752 | 0.998074519377826 |
| ebi-fl187-GCST90094756 | prot-a-1567 | Interferon regulatory factor 1 \|\| id:prot-a-1567 | Dried fruit liking \|\| id:ebi-fl187-GCST90094756 | Inverse variance weighted | 5 | -0.378696756 | 0.286917093262408 | 0.186874361335991 | -0.941054259 | 0.183660747048538 | 0.684753228667659 | 0.390216229634642 | 1.20160810484433 | 0.998074519377826 |
| ebi-fl187-GCST90094756 | prot-a-1613 | Potassium voltage-gated channel subfamily E regulatory beta subunit 5 \|\| id:prot-a-1613 | Dried fruit liking \|\| id:ebi-fl187-GCST90094756 | Inverse variance weighted | 5 | 0.18186197014054 | 0.286917093262408 | 0.526179695725944 | -0.380495533 | 0.744219472934859 | 1.19944862272843 | 0.683522617501542 | 2.10479794190844 | 0.998074519377826 |
| ebi-fl187-GCST90094756 | prot-a-1649 | Kin of IRRE-like protein 2 \|\| id:prot-a-1649 | Dried fruit liking \|\| id:ebi-fl187-GCST90094756 | Inverse variance weighted | 5 | -0.227982954 | 0.368027980666574 | 0.535605596447456 | -0.949317796 | 0.493351887949004 | 0.796137830559874 | 0.387004949641059 | 1.63779674093692 | 0.998074519377826 |
| ebi-fl187-GCST90094756 | prot-a-165 | Armadillo repeat-containing protein 5 \|\| id:prot-a-165 | Dried fruit liking \|\| id:ebi-fl187-GCST90094756 | Inverse variance weighted | 5 | -0.037094263 | 0.333973954801087 | 0.911561369415247 | -0.691683215 | 0.617494688043479 | 0.963585300273452 | 0.500732518952095 | 1.85427667619068 | 0.998074519377826 |
| ebi-fl187-GCST90094756 | prot-a-1656 | Kallikrein-11 \|\| id:prot-a-1656 | Dried fruit liking \|\| id:ebi-fl187-GCST90094756 | Inverse variance weighted (multiplicative random effects) | 5 | -0.226610936 | 0.459945251983829 | 0.622230861877775 | -1.128103629 | 0.674881758301826 | 0.797230896130142 | 0.323646428385257 | 1.96380075910463 | 0.998074519377826 |
| ebi-fl187-GCST90094756 | prot-a-1697 | Laminin subunit gamma-2 \|\| id:prot-a-1697 | Dried fruit liking \|\| id:ebi-fl187-GCST90094756 | Inverse variance weighted | 5 | -0.003135628 | 0.286720114655262 | 0.991274350425591 | -0.565107053 | 0.558835796791155 | 0.9968692830138 | 0.568299305550093 | 1.74863554769706 | 0.999355215041207 |
| ebi-fl187-GCST90094756 | prot-a-1698 | Lysosome-associated membrane glycoprotein 3 \|\| id:prot-a-1698 | Dried fruit liking \|\| id:ebi-fl187-GCST90094756 | Inverse variance weighted | 5 | 0.052386023664375 | 0.372871131713131 | 0.888269974827189 | -0.678441394 | 0.783213441822112 | 1.05378244897156 | 0.507407224069417 | 2.1884935749527 | 0.998074519377826 |
| ebi-fl187-GCST90094756 | prot-a-1724 | Leptin receptor \|\| id:prot-a-1724 | Dried fruit liking \|\| id:ebi-fl187-GCST90094756 | Inverse variance weighted | 5 | 0.317607053779728 | 0.286720114655262 | 0.267980576852893 | -0.244364371 | 0.879578478504041 | 1.37383631136306 | 0.78320220613751 | 2.40988367452104 | 0.998074519377826 |
| ebi-fl187-GCST90094756 | prot-a-1743 | Leukocyte immunoglobulin-like receptor subfamily B member 2 \|\| id:prot-a-1743 | Dried fruit liking \|\| id:ebi-fl187-GCST90094756 | Inverse variance weighted | 5 | -0.159308478 | 0.390374885609685 | 0.683206866847297 | -0.924443253 | 0.605826298136168 | 0.85273326923008 | 0.396752248040202 | 1.83276599450582 | 0.998074519377826 |
| ebi-fl187-GCST90094756 | prot-a-1746 | Leukocyte immunoglobulin-like receptor subfamily B member 4 \|\| id:prot-a-1746 | Dried fruit liking \|\| id:ebi-fl187-GCST90094756 | Inverse variance weighted | 5 | -0.241011657 | 0.344372196172677 | 0.484015952076367 | -0.915981161 | 0.433957847686945 | 0.785832466034027 | 0.400123847394447 | 1.54335381081235 | 0.998074519377826 |
| ebi-fl187-GCST90094756 | prot-a-1750 | Leucine-rich repeat and immunoglobulin-like domain-containing nogo receptor-interacting protein 3 \|\| id:prot-a-1750 | Dried fruit liking \|\| id:ebi-fl187-GCST90094756 | Inverse variance weighted | 5 | 0.0705794890508358 | 0.428646946072038 | 0.869214388410692 | -0.769568525 | 0.91072750335203 | 1.07312986811515 | 0.463212889864836 | 2.48613054394251 | 0.998074519377826 |
| ebi-fl187-GCST90094756 | prot-a-1753 | Lipase member N \|\| id:prot-a-1753 | Dried fruit liking \|\| id:ebi-fl187-GCST90094756 | Inverse variance weighted | 5 | 0.446834091039238 | 0.286720114655262 | 0.119130636833022 | -0.115137334 | 1.00880551576355 | 1.56335490318166 | 0.891243737714976 | 2.74232339580685 | 0.998074519377826 |
| ebi-fl187-GCST90094756 | prot-a-1760 | Ig Kappa chain V-I region HK102- like \|\| id:prot-a-1760 | Dried fruit liking \|\| id:ebi-fl187-GCST90094756 | Inverse variance weighted | 5 | 0.175306052102022 | 0.286917093262408 | 0.541200253795445 | -0.387051451 | 0.737663554896342 | 1.19161085581743 | 0.679056156118491 | 2.09104419260162 | 0.998074519377826 |
| ebi-fl187-GCST90094756 | prot-a-1792 | Leucine-rich repeat neuronal protein 1 \|\| id:prot-a-1792 | Dried fruit liking \|\| id:ebi-fl187-GCST90094756 | Inverse variance weighted | 5 | -0.145489725 | 0.286917093262408 | 0.612099162404296 | -0.707847228 | 0.416867777369527 | 0.86459877341344 | 0.492703735277858 | 1.51720189124699 | 0.998074519377826 |
| ebi-fl187-GCST90094756 | prot-a-1795 | Leucine-rich repeat transmembrane neuronal protein 1 \|\| id:prot-a-1795 | Dried fruit liking \|\| id:ebi-fl187-GCST90094756 | Inverse variance weighted | 5 | -0.166851063 | 0.316323392307937 | 0.597867324092935 | -0.786844912 | 0.453142785595632 | 0.846325650876934 | 0.455278976730115 | 1.57324880774555 | 0.998074519377826 |
| ebi-fl187-GCST90094756 | prot-a-1820 | LysM and putative peptidoglycan-binding domain-containing protein 4 \|\| id:prot-a-1820 | Dried fruit liking \|\| id:ebi-fl187-GCST90094756 | Inverse variance weighted | 5 | -0.049329161 | 0.286917093262408 | 0.863493918196635 | -0.611686663 | 0.513028342199519 | 0.951867760768677 | 0.542435191493191 | 1.67034191033335 | 0.998074519377826 |
| ebi-fl187-GCST90094756 | prot-a-1844 | Mitogen-activated protein kinase kinase kinase 7:TGF-beta-activated kinase 1 and MAP3K7-binding protein 1 fusion \|\| id:prot-a-1844 | Dried fruit liking \|\| id:ebi-fl187-GCST90094756 | Inverse variance weighted | 5 | -0.373749606 | 0.319308965651872 | 0.241801751132279 | -0.999595179 | 0.252095966190407 | 0.688149198353429 | 0.368028396582114 | 1.28671951292978 | 0.998074519377826 |
| ebi-fl187-GCST90094756 | prot-a-1856 | Megakaryocyte-associated tyrosine-protein kinase \|\| id:prot-a-1856 | Dried fruit liking \|\| id:ebi-fl187-GCST90094756 | Inverse variance weighted | 5 | -0.108946872 | 0.286720114655262 | 0.703962946041528 | -0.670918297 | 0.45302455251953 | 0.896778060073782 | 0.511238893058996 | 1.57306280869537 | 0.998074519377826 |
| ebi-fl187-GCST90094756 | prot-a-1910 | Stromelysin-2 \|\| id:prot-a-1910 | Dried fruit liking \|\| id:ebi-fl187-GCST90094756 | Inverse variance weighted | 5 | 0.135553245398027 | 0.286917093262408 | 0.63660745151878 | -0.426804257 | 0.697910748192346 | 1.14517016925261 | 0.652591279642879 | 2.00954986291529 | 0.998074519377826 |
| ebi-fl187-GCST90094756 | prot-a-1940 | 39S ribosomal protein L14, mitochondrial \|\| id:prot-a-1940 | Dried fruit liking \|\| id:ebi-fl187-GCST90094756 | Inverse variance weighted | 5 | -0.01763165 | 0.286720114655262 | 0.950965561328624 | -0.579603075 | 0.544339774703287 | 0.982522877992742 | 0.560120648478868 | 1.72347012808895 | 0.998074519377826 |
| ebi-fl187-GCST90094756 | prot-a-1942 | 39S ribosomal protein L33, mitochondrial \|\| id:prot-a-1942 | Dried fruit liking \|\| id:ebi-fl187-GCST90094756 | Inverse variance weighted | 5 | -0.049013583 | 0.286917093262408 | 0.864358710126653 | -0.611371086 | 0.513343919488009 | 0.952168196018399 | 0.542606398733242 | 1.67086911548688 | 0.998074519377826 |
| ebi-fl187-GCST90094756 | prot-a-1948 | Mesothelin \|\| id:prot-a-1948 | Dried fruit liking \|\| id:ebi-fl187-GCST90094756 | Inverse variance weighted | 5 | -0.110507235 | 0.286720114655262 | 0.699927380989158 | -0.67247866 | 0.451464189262005 | 0.895379851678879 | 0.510441796716098 | 1.57061017328562 | 0.998074519377826 |
| ebi-fl187-GCST90094756 | prot-a-1980 | Unconventional myosin-VI \|\| id:prot-a-1980 | Dried fruit liking \|\| id:ebi-fl187-GCST90094756 | Inverse variance weighted | 5 | -0.323763139 | 0.286720114655262 | 0.258815259944558 | -0.885734564 | 0.238208285569504 | 0.723421572333414 | 0.412411119674699 | 1.26897347416371 | 0.998074519377826 |
| ebi-fl187-GCST90094756 | prot-a-2039 | Neurofascin \|\| id:prot-a-2039 | Dried fruit liking \|\| id:ebi-fl187-GCST90094756 | Inverse variance weighted | 5 | -0.303936551 | 0.339293247220988 | 0.37036327448655 | -0.968951315 | 0.361078214006106 | 0.737907684804218 | 0.379480785280518 | 1.43487568386529 | 0.998074519377826 |
| ebi-fl187-GCST90094756 | prot-a-2041 | NFU1 iron-sulfur cluster scaffold homolog, mitochondrial \|\| id:prot-a-2041 | Dried fruit liking \|\| id:ebi-fl187-GCST90094756 | Inverse variance weighted | 5 | 0.154872404818841 | 0.390656392203197 | 0.691779358503356 | -0.610814124 | 0.920558933537108 | 1.16750898308069 | 0.542908694163217 | 2.51069330852218 | 0.998074519377826 |
| ebi-fl187-GCST90094756 | prot-a-2088 | Glucocorticoid receptor \|\| id:prot-a-2088 | Dried fruit liking \|\| id:ebi-fl187-GCST90094756 | Inverse variance weighted | 5 | -0.085115988 | 0.286917093262408 | 0.766728325091856 | -0.647473491 | 0.477241514678089 | 0.918405754070101 | 0.523366397739077 | 1.61162262757568 | 0.998074519377826 |
| ebi-fl187-GCST90094756 | prot-a-2111 | Neuron-specific protein family member 2 \|\| id:prot-a-2111 | Dried fruit liking \|\| id:ebi-fl187-GCST90094756 | Inverse variance weighted | 5 | -0.103267347 | 0.42961350031493 | 0.8100413582486 | -0.945309808 | 0.738775113644115 | 0.901885824802383 | 0.388559173671401 | 2.09336980335292 | 0.998074519377826 |
| ebi-fl187-GCST90094756 | prot-a-2125 | Nuclear migration protein nudC \|\| id:prot-a-2125 | Dried fruit liking \|\| id:ebi-fl187-GCST90094756 | Inverse variance weighted | 5 | -0.013746014 | 0.286720114655262 | 0.961762247946983 | -0.575717439 | 0.548225410289378 | 0.986348030613462 | 0.562301307081776 | 1.73017993243535 | 0.998074519377826 |
| ebi-fl187-GCST90094756 | prot-a-2142 | Ornithine decarboxylase \|\| id:prot-a-2142 | Dried fruit liking \|\| id:ebi-fl187-GCST90094756 | Inverse variance weighted | 5 | -0.237869093 | 0.345823599779871 | 0.491557571077894 | -0.915683349 | 0.439945162077641 | 0.788305878710338 | 0.400243026923597 | 1.55262207360805 | 0.998074519377826 |
| ebi-fl187-GCST90094756 | prot-a-2186 | Prostate and testis expressed protein 1 \|\| id:prot-a-2186 | Dried fruit liking \|\| id:ebi-fl187-GCST90094756 | Inverse variance weighted (multiplicative random effects) | 5 | 0.258017479834244 | 0.492406483111912 | 0.600283508132518 | -0.707099227 | 1.22313418673359 | 1.29436144364998 | 0.493072416110083 | 3.39782046626113 | 0.998074519377826 |
| ebi-fl187-GCST90094756 | prot-a-2227 | Platelet-derived growth factor C \|\| id:prot-a-2227 | Dried fruit liking \|\| id:ebi-fl187-GCST90094756 | Inverse variance weighted | 5 | -0.027948032 | 0.286917093262408 | 0.922402359700611 | -0.590305535 | 0.534409470953819 | 0.972438901348071 | 0.554157944420978 | 1.70644024212828 | 0.998074519377826 |
| ebi-fl187-GCST90094756 | prot-a-2229 | Platelet-derived growth factor receptor alpha \|\| id:prot-a-2229 | Dried fruit liking \|\| id:ebi-fl187-GCST90094756 | Inverse variance weighted | 5 | -0.483054857 | 0.304469722014507 | 0.112615775981386 | -1.079815512 | 0.113705798332796 | 0.616895981496202 | 0.339658182736039 | 1.12042244623887 | 0.998074519377826 |
| ebi-fl187-GCST90094756 | prot-a-2243 | Platelet endothelial aggregation receptor 1 \|\| id:prot-a-2243 | Dried fruit liking \|\| id:ebi-fl187-GCST90094756 | Inverse variance weighted | 5 | -0.005584402 | 0.286917093262408 | 0.984471379623935 | -0.567941905 | 0.556773101056237 | 0.994431162048349 | 0.566690542577985 | 1.74503236202631 | 0.999355215041207 |
| ebi-fl187-GCST90094756 | prot-a-2246 | E3 ubiquitin-protein ligase pellino homolog 2 \|\| id:prot-a-2246 | Dried fruit liking \|\| id:ebi-fl187-GCST90094756 | Inverse variance weighted | 5 | -0.054622686 | 0.393049359959998 | 0.889472615792376 | -0.824999432 | 0.715754059340184 | 0.946842337260888 | 0.438235241512648 | 2.04572870163338 | 0.998074519377826 |
| ebi-fl187-GCST90094756 | prot-a-2251 | Platelet factor 4 variant \|\| id:prot-a-2251 | Dried fruit liking \|\| id:ebi-fl187-GCST90094756 | Inverse variance weighted | 5 | 0.0976647717767997 | 0.286917093262408 | 0.733560060296572 | -0.464692731 | 0.660022274571119 | 1.10259310282123 | 0.628328141279756 | 1.93483543155147 | 0.998074519377826 |
| ebi-fl187-GCST90094756 | prot-a-2346 | Protein phosphatase 1L \|\| id:prot-a-2346 | Dried fruit liking \|\| id:ebi-fl187-GCST90094756 | Inverse variance weighted | 5 | -0.176457087 | 0.416106392740398 | 0.671516780745301 | -0.992025617 | 0.639111442786378 | 0.838234749563927 | 0.370824780848601 | 1.89479649598545 | 0.998074519377826 |
| ebi-fl187-GCST90094756 | prot-a-2358 | PR domain zinc finger protein 4 \|\| id:prot-a-2358 | Dried fruit liking \|\| id:ebi-fl187-GCST90094756 | Inverse variance weighted | 5 | 0.0169081976792335 | 0.286917093262408 | 0.953007381726638 | -0.545449305 | 0.579265700473553 | 1.01705195031004 | 0.579581315979692 | 1.78472742497053 | 0.998074519377826 |
| ebi-fl187-GCST90094756 | prot-a-2390 | Trypsin-1 \|\| id:prot-a-2390 | Dried fruit liking \|\| id:ebi-fl187-GCST90094756 | Inverse variance weighted | 5 | -0.399113459 | 0.339572021167287 | 0.239857682073621 | -1.06467462 | 0.266447702893663 | 0.670914576010355 | 0.344840040538811 | 1.30531932312685 | 0.998074519377826 |
| ebi-fl187-GCST90094756 | prot-a-242 | Brain-derived neurotrophic factor \|\| id:prot-a-242 | Dried fruit liking \|\| id:ebi-fl187-GCST90094756 | Inverse variance weighted | 5 | -0.150821591 | 0.348256272675111 | 0.664959230090386 | -0.833403885 | 0.53176070380517 | 0.860001117223747 | 0.434567547925412 | 1.70192626015654 | 0.998074519377826 |
| ebi-fl187-GCST90094756 | prot-a-2464 | Dihydropteridine reductase \|\| id:prot-a-2464 | Dried fruit liking \|\| id:ebi-fl187-GCST90094756 | Inverse variance weighted | 5 | -0.02525803 | 0.297403904655233 | 0.932318334672774 | -0.608169683 | 0.55765362330079 | 0.975058285448883 | 0.54434628412021 | 1.74656957851587 | 0.998074519377826 |
| ebi-fl187-GCST90094756 | prot-a-2474 | Ras-related protein Rab-27A \|\| id:prot-a-2474 | Dried fruit liking \|\| id:ebi-fl187-GCST90094756 | Inverse variance weighted | 5 | 0.0816353949291817 | 0.286720114655262 | 0.775857480612108 | -0.48033603 | 0.643606819653496 | 1.08506011927691 | 0.618575497081128 | 1.90333349445752 | 0.998074519377826 |
| ebi-fl187-GCST90094756 | prot-a-2478 | Ras-related protein Rab-7b \|\| id:prot-a-2478 | Dried fruit liking \|\| id:ebi-fl187-GCST90094756 | Inverse variance weighted | 5 | 0.124145918563442 | 0.297026534667343 | 0.675974619864827 | -0.458026089 | 0.706317926511434 | 1.13218106518102 | 0.632530973648282 | 2.02651572453619 | 0.998074519377826 |
| ebi-fl187-GCST90094756 | prot-a-2528 | E3 ubiquitin-protein ligase rififylin \|\| id:prot-a-2528 | Dried fruit liking \|\| id:ebi-fl187-GCST90094756 | Inverse variance weighted | 5 | -0.02354591 | 0.286720114655262 | 0.934550030494211 | -0.585517334 | 0.538425515087168 | 0.976729132359448 | 0.556817726344503 | 1.71330716114702 | 0.998074519377826 |
| ebi-fl187-GCST90094756 | prot-a-2543 | Protein RIC-3 \|\| id:prot-a-2543 | Dried fruit liking \|\| id:ebi-fl187-GCST90094756 | Inverse variance weighted (multiplicative random effects) | 5 | 0.258185364741174 | 0.545584994112647 | 0.6360514790599 | -0.811161224 | 1.32753195320196 | 1.29457876564252 | 0.444341786300842 | 3.77172310172469 | 0.998074519377826 |
| ebi-fl187-GCST90094756 | prot-a-255 | Bone morphogenetic protein 6 \|\| id:prot-a-255 | Dried fruit liking \|\| id:ebi-fl187-GCST90094756 | Inverse variance weighted | 5 | 0.0215590198207204 | 0.343468834032748 | 0.949950858298102 | -0.651639895 | 0.694757934524906 | 1.02179309460305 | 0.521190378136384 | 2.00322410385187 | 0.998074519377826 |
| ebi-fl187-GCST90094756 | prot-a-2568 | RING finger protein 165 \|\| id:prot-a-2568 | Dried fruit liking \|\| id:ebi-fl187-GCST90094756 | Inverse variance weighted | 5 | -0.416628404 | 0.286917093262408 | 0.146477588328802 | -0.978985906 | 0.145729099277047 | 0.659265855305757 | 0.375691892515527 | 1.15688274522472 | 0.998074519377826 |
| ebi-fl187-GCST90094756 | prot-a-2573 | E3 ubiquitin-protein ligase RNF8 \|\| id:prot-a-2573 | Dried fruit liking \|\| id:ebi-fl187-GCST90094756 | Inverse variance weighted | 5 | -0.027575028 | 0.406516938800126 | 0.945919035246657 | -0.824348228 | 0.769198172055863 | 0.972801692457365 | 0.438520714847456 | 2.15803518695145 | 0.998074519377826 |
| ebi-fl187-GCST90094756 | prot-a-260 | Cytoplasmic tyrosine-protein kinase BMX \|\| id:prot-a-260 | Dried fruit liking \|\| id:ebi-fl187-GCST90094756 | Inverse variance weighted | 5 | -0.682897561 | 0.286720114655262 | 0.0172302759869217 | -1.244868986 | -0.120926137 | 0.505151163272143 | 0.287978634889291 | 0.886099407524791 | 0.998074519377826 |
| ebi-fl187-GCST90094756 | prot-a-2623 | Serum amyloid A-1 protein \|\| id:prot-a-2623 | Dried fruit liking \|\| id:ebi-fl187-GCST90094756 | Inverse variance weighted | 5 | 0.0823605826575317 | 0.286917093262408 | 0.774071284458969 | -0.47999692 | 0.644718085451851 | 1.08584727694398 | 0.618785297577281 | 1.9054497795326 | 0.998074519377826 |
| ebi-fl187-GCST90094756 | prot-a-2640 | Lysosome membrane protein 2 \|\| id:prot-a-2640 | Dried fruit liking \|\| id:ebi-fl187-GCST90094756 | Inverse variance weighted | 5 | 0.100875593283997 | 0.391083282348356 | 0.796454059313743 | -0.66564764 | 0.867398826686775 | 1.10613902208002 | 0.513940571367642 | 2.38071015275594 | 0.998074519377826 |
| ebi-fl187-GCST90094756 | prot-a-2641 | Scavenger receptor class F member 2 \|\| id:prot-a-2641 | Dried fruit liking \|\| id:ebi-fl187-GCST90094756 | Inverse variance weighted | 5 | -0.308095931 | 0.425995701468273 | 0.469534178082974 | -1.143047506 | 0.526855643644362 | 0.73484482005766 | 0.318845855011884 | 1.69359864987251 | 0.998074519377826 |
| ebi-fl187-GCST90094756 | prot-a-2669 | Semaphorin-3A \|\| id:prot-a-2669 | Dried fruit liking \|\| id:ebi-fl187-GCST90094756 | Inverse variance weighted | 5 | -0.141987694 | 0.286917093262408 | 0.620689348514627 | -0.704345197 | 0.420369808531875 | 0.867631933271087 | 0.494432223956667 | 1.52252449407039 | 0.998074519377826 |
| ebi-fl187-GCST90094756 | prot-a-2683 | Sentrin-specific protease 7 \|\| id:prot-a-2683 | Dried fruit liking \|\| id:ebi-fl187-GCST90094756 | Inverse variance weighted | 5 | -0.2745711 | 0.286917093262408 | 0.338582309888394 | -0.836928603 | 0.287786402331725 | 0.759897973230709 | 0.433038516076877 | 1.33347244709666 | 0.998074519377826 |
| ebi-fl187-GCST90094756 | prot-a-270 | Bromodomain testis-specific protein \|\| id:prot-a-270 | Dried fruit liking \|\| id:ebi-fl187-GCST90094756 | Inverse variance weighted | 5 | -0.167843056 | 0.286720114655262 | 0.558285586833805 | -0.72981448 | 0.394128369064584 | 0.845486518596261 | 0.481998401954541 | 1.48309091944966 | 0.998074519377826 |
| ebi-fl187-GCST90094756 | prot-a-2708 | 14-3-3 protein sigma \|\| id:prot-a-2708 | Dried fruit liking \|\| id:ebi-fl187-GCST90094756 | Inverse variance weighted | 5 | 0.0307320995190628 | 0.286917093262408 | 0.914700581254437 | -0.531625403 | 0.593089602313382 | 1.03120920543585 | 0.587649026339986 | 1.80957064116772 | 0.998074519377826 |
| ebi-fl187-GCST90094756 | prot-a-2713 | Beta-sarcoglycan \|\| id:prot-a-2713 | Dried fruit liking \|\| id:ebi-fl187-GCST90094756 | Inverse variance weighted | 5 | -0.494123968 | 0.286917093262408 | 0.0850362299486718 | -1.05648147 | 0.0682335351152883 | 0.610105145083642 | 0.347676972415974 | 1.0706153055549 | 0.998074519377826 |
| ebi-fl187-GCST90094756 | prot-a-2720 | Protein shisa-3 homolog \|\| id:prot-a-2720 | Dried fruit liking \|\| id:ebi-fl187-GCST90094756 | Inverse variance weighted | 5 | -0.249282121 | 0.29090184278979 | 0.39148432312382 | -0.819449733 | 0.320885491151797 | 0.779360068745567 | 0.440674076386684 | 1.37834773884477 | 0.998074519377826 |
| ebi-fl187-GCST90094756 | prot-a-2730 | Sialic acid-binding Ig-like lectin 8 \|\| id:prot-a-2730 | Dried fruit liking \|\| id:ebi-fl187-GCST90094756 | Inverse variance weighted | 5 | -0.158402473 | 0.286917093262408 | 0.580890477349998 | -0.720759976 | 0.403955029690254 | 0.853506199542309 | 0.486382476506492 | 1.49773659176521 | 0.998074519377826 |
| ebi-fl187-GCST90094756 | prot-a-2761 | Sodium- and chloride-dependent neutral and basic amino acid transporter B(0+) \|\| id:prot-a-2761 | Dried fruit liking \|\| id:ebi-fl187-GCST90094756 | Inverse variance weighted | 5 | -0.291121432 | 0.286720114655262 | 0.309938745865768 | -0.853092857 | 0.270849992483437 | 0.74742491102535 | 0.42609504086873 | 1.31107838402033 | 0.998074519377826 |
| ebi-fl187-GCST90094756 | prot-a-2770 | Secreted Ly-6/uPAR-related protein 1 \|\| id:prot-a-2770 | Dried fruit liking \|\| id:ebi-fl187-GCST90094756 | Inverse variance weighted | 5 | -0.220554675 | 0.338494663286846 | 0.514675351480641 | -0.884004215 | 0.442894865016488 | 0.802073784257974 | 0.413125352523117 | 1.55720860863389 | 0.998074519377826 |
| ebi-fl187-GCST90094756 | prot-a-2784 | N-lysine methyltransferase SMYD2 \|\| id:prot-a-2784 | Dried fruit liking \|\| id:ebi-fl187-GCST90094756 | Inverse variance weighted | 5 | 0.18558188826877 | 0.384086627488851 | 0.628969946708643 | -0.567227902 | 0.938391678146919 | 1.20391878256354 | 0.567095305765688 | 2.5558674534473 | 0.998074519377826 |
| ebi-fl187-GCST90094756 | prot-a-2837 | Serine palmitoyltransferase 2 \|\| id:prot-a-2837 | Dried fruit liking \|\| id:ebi-fl187-GCST90094756 | Inverse variance weighted | 5 | -0.036607176 | 0.286672518138208 | 0.898388886361911 | -0.598485312 | 0.525270959213417 | 0.96405476450041 | 0.549643544436534 | 1.69091695584038 | 0.998074519377826 |
| ebi-fl187-GCST90094756 | prot-a-2858 | Alpha-2,8-sialyltransferase 8B \|\| id:prot-a-2858 | Dried fruit liking \|\| id:ebi-fl187-GCST90094756 | Inverse variance weighted | 5 | 0.506252222000641 | 0.286668130341841 | 0.0773980120333963 | -0.055617313 | 1.06812175747065 | 1.65906173385306 | 0.945901050227341 | 2.90990884942349 | 0.998074519377826 |
| ebi-fl187-GCST90094756 | prot-a-2911 | Synaptotagmin-11 \|\| id:prot-a-2911 | Dried fruit liking \|\| id:ebi-fl187-GCST90094756 | Inverse variance weighted | 5 | -0.181174897 | 0.386525311771187 | 0.639264697994592 | -0.938764508 | 0.576414714146948 | 0.834289431279294 | 0.391110751183497 | 1.77964643783921 | 0.998074519377826 |
| ebi-fl187-GCST90094756 | prot-a-2962 | Transforming growth factor beta-1 \|\| id:prot-a-2962 | Dried fruit liking \|\| id:ebi-fl187-GCST90094756 | Inverse variance weighted | 5 | -0.102918031 | 0.371563621714622 | 0.781790629848837 | -0.831182729 | 0.625346667744401 | 0.902200923123919 | 0.435533862884123 | 1.86889373031877 | 0.998074519377826 |
| ebi-fl187-GCST90094756 | prot-a-2978 | Tyrosine-protein kinase receptor Tie-1, soluble \|\| id:prot-a-2978 | Dried fruit liking \|\| id:ebi-fl187-GCST90094756 | Inverse variance weighted | 5 | -0.03427555 | 0.374753363151078 | 0.927125800844855 | -0.768792142 | 0.70024104192495 | 0.966305202694681 | 0.463572660408435 | 2.01423816480489 | 0.998074519377826 |
| ebi-fl187-GCST90094756 | prot-a-299 | Uncharacterized protein C1orf226 \|\| id:prot-a-299 | Dried fruit liking \|\| id:ebi-fl187-GCST90094756 | Inverse variance weighted | 5 | 0.432988895789385 | 0.343344081743137 | 0.207275260801572 | -0.239965504 | 1.10594329600593 | 1.54185909947739 | 0.786654996713258 | 3.02207383487554 | 0.998074519377826 |
| ebi-fl187-GCST90094756 | prot-a-3015 | Transmembrane protein 70, mitochondrial \|\| id:prot-a-3015 | Dried fruit liking \|\| id:ebi-fl187-GCST90094756 | Inverse variance weighted | 5 | 0.126997084176122 | 0.296769007957818 | 0.668700311984312 | -0.454670171 | 0.708664339773446 | 1.13541370711026 | 0.634657261533424 | 2.03127635092216 | 0.998074519377826 |
| ebi-fl187-GCST90094756 | prot-a-3016 | Transmembrane protein 87B \|\| id:prot-a-3016 | Dried fruit liking \|\| id:ebi-fl187-GCST90094756 | Inverse variance weighted | 5 | 0.137119944465908 | 0.322352149180666 | 0.670564686525096 | -0.494690268 | 0.768930156860013 | 1.14696571246001 | 0.60975974019863 | 2.15745687822939 | 0.998074519377826 |
| ebi-fl187-GCST90094756 | prot-a-3123 | Thioredoxin domain-containing protein 12 \|\| id:prot-a-3123 | Dried fruit liking \|\| id:ebi-fl187-GCST90094756 | Inverse variance weighted | 5 | -0.449866146 | 0.286668130341841 | 0.116579799829572 | -1.011735681 | 0.112003389447115 | 0.637713506398193 | 0.363587359733317 | 1.11851665179166 | 0.998074519377826 |
| ebi-fl187-GCST90094756 | prot-a-3133 | PolyUbiquitin K48-linked \|\| id:prot-a-3133 | Dried fruit liking \|\| id:ebi-fl187-GCST90094756 | Inverse variance weighted | 5 | 0.329876240321985 | 0.286917093262408 | 0.250256457301459 | -0.232481262 | 0.892233743116304 | 1.39079599334799 | 0.792564599908764 | 2.44057518508331 | 0.998074519377826 |
| ebi-fl187-GCST90094756 | prot-a-3135 | Ubiquitin-conjugating enzyme E2 B \|\| id:prot-a-3135 | Dried fruit liking \|\| id:ebi-fl187-GCST90094756 | Inverse variance weighted | 5 | 0.176028778018193 | 0.319479793887756 | 0.581643519999287 | -0.450151618 | 0.802209174038194 | 1.19247237514859 | 0.637531483044061 | 2.23046297055459 | 0.998074519377826 |
| ebi-fl187-GCST90094756 | prot-a-3162 | NKG2D ligand 3 \|\| id:prot-a-3162 | Dried fruit liking \|\| id:ebi-fl187-GCST90094756 | Inverse variance weighted | 5 | -0.309632238 | 0.286720114655262 | 0.280181764481589 | -0.871603663 | 0.252339186845667 | 0.733716739837867 | 0.418280230743719 | 1.28703250775471 | 0.998074519377826 |
| ebi-fl187-GCST90094756 | prot-a-3188 | Vesicle-associated membrane protein 8 \|\| id:prot-a-3188 | Dried fruit liking \|\| id:ebi-fl187-GCST90094756 | Inverse variance weighted | 5 | 0.297912885083653 | 0.430982465860818 | 0.489413906273763 | -0.546812748 | 1.14263851817086 | 1.34704443510476 | 0.578791628425407 | 3.13502929384639 | 0.998074519377826 |
| ebi-fl187-GCST90094756 | prot-a-3201 | Vitrin \|\| id:prot-a-3201 | Dried fruit liking \|\| id:ebi-fl187-GCST90094756 | Inverse variance weighted | 5 | -0.052255466 | 0.286917093262408 | 0.855482740609056 | -0.614612969 | 0.510102036524865 | 0.949086376314186 | 0.540850180558511 | 1.66546112414165 | 0.998074519377826 |
| ebi-fl187-GCST90094756 | prot-a-3232 | WNT1-inducible-signaling pathway protein 3 \|\| id:prot-a-3232 | Dried fruit liking \|\| id:ebi-fl187-GCST90094756 | Inverse variance weighted | 5 | -0.177677266 | 0.320765406 | 0.579635960065432 | -0.806377463 | 0.451022930128731 | 0.837212576559565 | 0.446472503513812 | 1.56991728008581 | 0.998074519377826 |
| ebi-fl187-GCST90094756 | prot-a-327 | Carbonic anhydrase-related protein 10 \|\| id:prot-a-327 | Dried fruit liking \|\| id:ebi-fl187-GCST90094756 | Inverse variance weighted | 5 | -0.339923667 | 0.360826475090449 | 0.346156987476875 | -1.047143558 | 0.367296224077129 | 0.711824656329065 | 0.35093875487471 | 1.4438255516661 | 0.998074519377826 |
| ebi-fl187-GCST90094756 | prot-a-332 | Carbonic anhydrase 5A, mitochondrial \|\| id:prot-a-332 | Dried fruit liking \|\| id:ebi-fl187-GCST90094756 | Inverse variance weighted | 5 | 0.232085922776881 | 0.356101315106737 | 0.5145680953464 | -0.465872655 | 0.930044500386087 | 1.26122809239279 | 0.627587199155939 | 2.53462196676436 | 0.998074519377826 |
| ebi-fl187-GCST90094756 | prot-a-338 | Cell adhesion molecule 3 \|\| id:prot-a-338 | Dried fruit liking \|\| id:ebi-fl187-GCST90094756 | Inverse variance weighted | 5 | 0.109743689440457 | 0.286720114655262 | 0.701901088704129 | -0.452227735 | 0.671715114164771 | 1.1159919932659 | 0.636209265928091 | 1.95759193669832 | 0.998074519377826 |
| ebi-fl187-GCST90094756 | prot-a-34 | A disintegrin and metalloproteinase with thrombospondin motifs 3 \|\| id:prot-a-34 | Dried fruit liking \|\| id:ebi-fl187-GCST90094756 | Inverse variance weighted (multiplicative random effects) | 5 | -0.076334026 | 0.456604916700755 | 0.867230460640278 | -0.971279663 | 0.818611610621924 | 0.926506677444059 | 0.378598249873252 | 2.26734968699885 | 0.998074519377826 |
| ebi-fl187-GCST90094756 | prot-a-358 | Cysteine--tRNA ligase, cytoplasmic \|\| id:prot-a-358 | Dried fruit liking \|\| id:ebi-fl187-GCST90094756 | Inverse variance weighted | 5 | -0.310550741 | 0.286476042485175 | 0.278348222415599 | -0.872043784 | 0.25094230275813 | 0.733043128483853 | 0.41809617726055 | 1.28523592762372 | 0.998074519377826 |
| ebi-fl187-GCST90094756 | prot-a-382 | Coiled-coil domain-containing protein 167 \|\| id:prot-a-382 | Dried fruit liking \|\| id:ebi-fl187-GCST90094756 | Inverse variance weighted (multiplicative random effects) | 5 | -0.331603973 | 0.465132390955157 | 0.475892882097279 | -1.24326346 | 0.580055512997688 | 0.717771523281327 | 0.288441363570744 | 1.78613758184941 | 0.998074519377826 |
| ebi-fl187-GCST90094756 | prot-a-40 | Pituitary adenylate cyclase-activating polypeptide 27 \|\| id:prot-a-40 | Dried fruit liking \|\| id:ebi-fl187-GCST90094756 | Inverse variance weighted | 5 | -0.331824437 | 0.301910281801292 | 0.271731894891902 | -0.923568589 | 0.259919715250148 | 0.717613298081596 | 0.397099424774863 | 1.29682596713785 | 0.998074519377826 |
| ebi-fl187-GCST90094756 | prot-a-403 | C-C motif chemokine 25 \|\| id:prot-a-403 | Dried fruit liking \|\| id:ebi-fl187-GCST90094756 | Inverse variance weighted | 5 | -0.265894603 | 0.286917093262408 | 0.354066872997652 | -0.828252106 | 0.296462899943699 | 0.766519912257398 | 0.43681212089053 | 1.34509265605827 | 0.998074519377826 |
| ebi-fl187-GCST90094756 | prot-a-415 | Cyclin-C \|\| id:prot-a-415 | Dried fruit liking \|\| id:ebi-fl187-GCST90094756 | Inverse variance weighted | 5 | -0.000385469 | 0.325978149322956 | 0.999056501777916 | -0.639302642 | 0.638531703532331 | 0.999614605143021 | 0.527660263974738 | 1.89369832643505 | 0.999355215041207 |
| ebi-fl187-GCST90094756 | prot-a-44 | Adhesion G protein-coupled receptor F5 \|\| id:prot-a-44 | Dried fruit liking \|\| id:ebi-fl187-GCST90094756 | Inverse variance weighted | 5 | -0.063980623 | 0.30324620744479 | 0.832898405307162 | -0.65834319 | 0.530381943668773 | 0.938023175501916 | 0.517708368977787 | 1.69958132899422 | 0.998074519377826 |
| ebi-fl187-GCST90094756 | prot-a-455 | CD59 glycoprotein \|\| id:prot-a-455 | Dried fruit liking \|\| id:ebi-fl187-GCST90094756 | Inverse variance weighted | 5 | 0.268526685170461 | 0.399420966257597 | 0.501399152438101 | -0.514338409 | 1.05139177903535 | 1.30803588172533 | 0.597896026609471 | 2.86163110596904 | 0.998074519377826 |
| ebi-fl187-GCST90094756 | prot-a-478 | Cadherin-12 \|\| id:prot-a-478 | Dried fruit liking \|\| id:ebi-fl187-GCST90094756 | Inverse variance weighted | 5 | -0.093801936 | 0.286720114655262 | 0.74355061020651 | -0.655773361 | 0.468169488823021 | 0.910463074431735 | 0.519040501844207 | 1.5970680649359 | 0.998074519377826 |
| ebi-fl187-GCST90094756 | prot-a-516 | Centrin-2 \|\| id:prot-a-516 | Dried fruit liking \|\| id:ebi-fl187-GCST90094756 | Inverse variance weighted | 5 | 0.45701930055045 | 0.286917093262408 | 0.111191179594477 | -0.105338202 | 1.01937680334477 | 1.57935936646041 | 0.900020082296613 | 2.77146705666971 | 0.998074519377826 |
| ebi-fl187-GCST90094756 | prot-a-519 | Cryptic protein \|\| id:prot-a-519 | Dried fruit liking \|\| id:ebi-fl187-GCST90094756 | Inverse variance weighted | 5 | 0.265093562280484 | 0.286720114655262 | 0.355188270375579 | -0.296877862 | 0.827064987004797 | 1.30355293345815 | 0.743134771484172 | 2.28659768797204 | 0.998074519377826 |
| ebi-fl187-GCST90094756 | prot-a-583 | Chloride intracellular channel protein 4 \|\| id:prot-a-583 | Dried fruit liking \|\| id:ebi-fl187-GCST90094756 | Inverse variance weighted | 5 | 0.182476554868402 | 0.286720114655262 | 0.524497846275113 | -0.37949487 | 0.744447979592716 | 1.20018601210472 | 0.684206935485049 | 2.10527895720711 | 0.998074519377826 |
| ebi-fl187-GCST90094756 | prot-a-602 | CCR4-NOT transcription complex subunit 1 \|\| id:prot-a-602 | Dried fruit liking \|\| id:ebi-fl187-GCST90094756 | Inverse variance weighted | 5 | 0.337722506192375 | 0.353810941042397 | 0.339816362571977 | -0.355746938 | 1.03119195063547 | 1.40175147207759 | 0.700649905492962 | 2.80440655749356 | 0.998074519377826 |
| ebi-fl187-GCST90094756 | prot-a-606 | Ciliary neurotrophic factor receptor subunit alpha \|\| id:prot-a-606 | Dried fruit liking \|\| id:ebi-fl187-GCST90094756 | Inverse variance weighted | 5 | 0.0708827590061729 | 0.286917093262408 | 0.804869822358617 | -0.491474744 | 0.633240261800492 | 1.07345536551662 | 0.611723593078922 | 1.88370439655049 | 0.998074519377826 |
| ebi-fl187-GCST90094756 | prot-a-626 | Collagen alpha-2(IX) chain \|\| id:prot-a-626 | Dried fruit liking \|\| id:ebi-fl187-GCST90094756 | Inverse variance weighted | 5 | -0.183760482 | 0.308064404604299 | 0.550841114047296 | -0.787566715 | 0.420045751181978 | 0.832135091423152 | 0.454950473745397 | 1.52203118875162 | 0.998074519377826 |
| ebi-fl187-GCST90094756 | prot-a-647 | Probable serine carboxypeptidase CPVL \|\| id:prot-a-647 | Dried fruit liking \|\| id:ebi-fl187-GCST90094756 | Inverse variance weighted | 5 | -0.523612107 | 0.286720114655262 | 0.0678181279419795 | -1.085583532 | 0.0383593174055291 | 0.59237694973183 | 0.337704666893443 | 1.03910453415294 | 0.998074519377826 |
| ebi-fl187-GCST90094756 | prot-a-651 | Cellular retinoic acid-binding protein 2 \|\| id:prot-a-651 | Dried fruit liking \|\| id:ebi-fl187-GCST90094756 | Inverse variance weighted | 5 | -0.02841693 | 0.286917093262408 | 0.921104682157499 | -0.590774433 | 0.533940573068182 | 0.97198303368936 | 0.553898161843041 | 1.7056402834709 | 0.998074519377826 |
| ebi-fl187-GCST90094756 | prot-a-671 | Cytotoxic and regulatory T-cell molecule \|\| id:prot-a-671 | Dried fruit liking \|\| id:ebi-fl187-GCST90094756 | Inverse variance weighted | 5 | 0.146185752357376 | 0.362948930894746 | 0.687115787786693 | -0.565194152 | 0.857565656911078 | 1.15741115993582 | 0.568249809098239 | 2.35741494620088 | 0.998074519377826 |
| ebi-fl187-GCST90094756 | prot-a-688 | Chorionic somatomammotropin hormone \|\| id:prot-a-688 | Dried fruit liking \|\| id:ebi-fl187-GCST90094756 | Inverse variance weighted | 5 | -0.591464965 | 0.395256719740989 | 0.134547598205473 | -1.366168136 | 0.183238205500193 | 0.553515809094226 | 0.255082530827186 | 1.20110048274847 | 0.998074519377826 |
| ebi-fl187-GCST90094756 | prot-a-69 | Aldo-keto reductase family 1 member C1 \|\| id:prot-a-69 | Dried fruit liking \|\| id:ebi-fl187-GCST90094756 | Inverse variance weighted | 5 | -0.134877848 | 0.286917093262408 | 0.638288250445366 | -0.69723535 | 0.427479655284415 | 0.873822644781008 | 0.497960087722797 | 1.53338798300866 | 0.998074519377826 |
| ebi-fl187-GCST90094756 | prot-a-741 | Stromal cell-derived factor 1 \|\| id:prot-a-741 | Dried fruit liking \|\| id:ebi-fl187-GCST90094756 | Inverse variance weighted | 5 | 0.0544518186493162 | 0.308886406888357 | 0.860070609319536 | -0.550965539 | 0.659869176150497 | 1.05596159753199 | 0.57639301177159 | 1.934539234 | 0.998074519377826 |
| ebi-fl187-GCST90094756 | prot-a-742 | Growth-regulated alpha protein \|\| id:prot-a-742 | Dried fruit liking \|\| id:ebi-fl187-GCST90094756 | Inverse variance weighted | 5 | -0.65981024 | 0.294063546609228 | 0.0248473621611981 | -1.236174791 | -0.083445688 | 0.516949421737083 | 0.290493292949779 | 0.919941048967708 | 0.998074519377826 |
| ebi-fl187-GCST90094756 | prot-a-788 | Beta-defensin 112 \|\| id:prot-a-788 | Dried fruit liking \|\| id:ebi-fl187-GCST90094756 | Inverse variance weighted | 5 | -0.06745866 | 0.376939613885619 | 0.857965888901725 | -0.806260303 | 0.67134298364526 | 0.934766363460163 | 0.44652481531963 | 1.95686359252188 | 0.998074519377826 |
| ebi-fl187-GCST90094756 | prot-a-838 | DnaJ homolog subfamily B member 1 \|\| id:prot-a-838 | Dried fruit liking \|\| id:ebi-fl187-GCST90094756 | Inverse variance weighted | 5 | -0.193055379 | 0.332563952075827 | 0.56157342188903 | -0.844880725 | 0.458769966695067 | 0.824436316108142 | 0.429608596530353 | 1.58212671908194 | 0.998074519377826 |
| ebi-fl187-GCST90094756 | prot-a-85 | Junctional adhesion molecule-like \|\| id:prot-a-85 | Dried fruit liking \|\| id:ebi-fl187-GCST90094756 | Inverse variance weighted | 5 | -0.09771415 | 0.286668130341841 | 0.733207792841968 | -0.659583686 | 0.464155384991145 | 0.906908105953545 | 0.51706655176047 | 1.59067011750019 | 0.998074519377826 |
| ebi-fl187-GCST90094756 | prot-a-856 | Dipeptidase 1 \|\| id:prot-a-856 | Dried fruit liking \|\| id:ebi-fl187-GCST90094756 | Inverse variance weighted | 5 | -0.093360919 | 0.332883741479833 | 0.779123813993211 | -0.745813053 | 0.559091213813151 | 0.910864692145366 | 0.474348472764069 | 1.74908223602469 | 0.998074519377826 |
| ebi-fl187-GCST90094756 | prot-a-860 | Dipeptidyl peptidase 2 \|\| id:prot-a-860 | Dried fruit liking \|\| id:ebi-fl187-GCST90094756 | Inverse variance weighted | 5 | -0.149382934 | 0.318199926084422 | 0.638739080252314 | -0.773054789 | 0.474288920977271 | 0.861239253825399 | 0.46160081911666 | 1.60687117875818 | 0.998074519377826 |
| ebi-fl187-GCST90094756 | prot-a-861 | Dermatopontin \|\| id:prot-a-861 | Dried fruit liking \|\| id:ebi-fl187-GCST90094756 | Inverse variance weighted | 5 | 0.485691112012805 | 0.286831156999317 | 0.0903984352694085 | -0.076497956 | 1.04788017973147 | 1.62529788367519 | 0.926354808028594 | 2.85159982739303 | 0.998074519377826 |
| ebi-fl187-GCST90094756 | prot-a-877 | Dual specificity protein phosphatase 6 \|\| id:prot-a-877 | Dried fruit liking \|\| id:ebi-fl187-GCST90094756 | Inverse variance weighted | 5 | -0.271610599 | 0.286917093262408 | 0.343816210750519 | -0.833968102 | 0.290746904062744 | 0.762150985874215 | 0.434322426925722 | 1.33742604401218 | 0.998074519377826 |
| ebi-fl187-GCST90094756 | prot-a-879 | Dynactin-associated protein \|\| id:prot-a-879 | Dried fruit liking \|\| id:ebi-fl187-GCST90094756 | Inverse variance weighted (multiplicative random effects) | 5 | -0.103129542 | 0.459560275586202 | 0.822438932092299 | -1.003867682 | 0.79760859782596 | 0.9020101174268 | 0.366459348309428 | 2.22022512372453 | 0.998074519377826 |
| ebi-fl187-GCST90094756 | prot-a-937 | EMI domain-containing protein 1 \|\| id:prot-a-937 | Dried fruit liking \|\| id:ebi-fl187-GCST90094756 | Inverse variance weighted | 5 | -0.522304565 | 0.286720114655262 | 0.0685076491503287 | -1.08427599 | 0.0396668592554716 | 0.593152013988596 | 0.338146518685231 | 1.04046409546577 | 0.998074519377826 |
| ebi-fl187-GCST90094756 | prot-b-1 | SRC proto-oncogene, non-receptor tyrosine kinase \|\| id:prot-b-1 | Dried fruit liking \|\| id:ebi-fl187-GCST90094756 | Inverse variance weighted | 5 | -0.281973674 | 0.310871818714763 | 0.364384795256037 | -0.891282439 | 0.327335090682695 | 0.754293541804815 | 0.410129449479159 | 1.38726625930178 | 0.998074519377826 |
| ebi-fl187-GCST90094756 | prot-b-10 | coagulation factor III, tissue factor \|\| id:prot-b-10 | Dried fruit liking \|\| id:ebi-fl187-GCST90094756 | Inverse variance weighted | 5 | -0.519428364 | 0.310586689486824 | 0.094443451422559 | -1.128178276 | 0.0893215470353233 | 0.594860494258259 | 0.323622270285579 | 1.09343218968497 | 0.998074519377826 |
| ebi-fl187-GCST90094756 | prot-b-29 | myeloperoxidase \|\| id:prot-b-29 | Dried fruit liking \|\| id:ebi-fl187-GCST90094756 | Inverse variance weighted | 5 | 0.101958183474425 | 0.310586689486824 | 0.742702922141381 | -0.506791728 | 0.7107080948686 | 1.10733716576685 | 0.602425225773162 | 2.03543202746008 | 0.998074519377826 |
| ebi-fl187-GCST90094756 | prot-b-33 | adrenomedullin \|\| id:prot-b-33 | Dried fruit liking \|\| id:ebi-fl187-GCST90094756 | Inverse variance weighted | 5 | -0.400156331 | 0.313048125961497 | 0.201158404214796 | -1.013730658 | 0.213417995653332 | 0.670215262268244 | 0.362862734495825 | 1.23790198076253 | 0.998074519377826 |
| ebi-fl187-GCST90094756 | ukb-rsfMRI191-edge-pheno102 | Net25_Pair11_15 (Postcentral\|Precentral)&(Cerebellum) (Motor)&(Subcortical-cerebellum) \|\| id:ukb-rsfMRI191-edge-pheno102 | Dried fruit liking \|\| id:ebi-fl187-GCST90094756 | Inverse variance weighted | 5 | 0.0368046059464137 | 0.0881439177443069 | 0.676275221905803 | -0.135957473 | 0.209566684725255 | 1.0374902816001 | 0.872879752802921 | 1.23314360421153 | 0.998074519377826 |
| ebi-fl187-GCST90094756 | ukb-rsfMRI191-edge-pheno1167 | Net100_Pair11_45 (Angular\|Temporal)&(Frontal) (Default_mode\|Central_executive)&(Salience\|Default_mode) \|\| id:ukb-rsfMRI191-edge-pheno1167 | Dried fruit liking \|\| id:ebi-fl187-GCST90094756 | Inverse variance weighted | 5 | 0.068277942741263 | 0.123004136153118 | 0.578835455554656 | -0.172810164 | 0.309366049601374 | 1.07066285009462 | 0.841297308102183 | 1.36256104415527 | 0.998074519377826 |
| ebi-fl187-GCST90094756 | ukb-rsfMRI191-edge-pheno1317 | Net100_Pair26_48 (Parietal\|Frontal)&(Temporal) (Attention\|Central_executive)&(Default_mode\|Central_executive) \|\| id:ukb-rsfMRI191-edge-pheno1317 | Dried fruit liking \|\| id:ebi-fl187-GCST90094756 | Inverse variance weighted | 5 | 0.218456677590321 | 0.0879085876240108 | 0.0129535549793852 | 0.0461558458472595 | 0.390757509333382 | 1.24415511566199 | 1.04723760587195 | 1.4781000444871 | 0.998074519377826 |
| ebi-fl187-GCST90094756 | ukb-rsfMRI191-edge-pheno286 | Net100_Pair10_13 (OccipitalPrecuneus)&(Frontal_Sup) (Default_mode\|Central_executive)&(Default_mode) \|\| id:ukb-rsfMRI191-edge-pheno286 | Dried fruit liking \|\| id:ebi-fl187-GCST90094756 | Inverse variance weighted (multiplicative random effects) | 5 | -0.099245353 | 0.160203215292373 | 0.53558923866619 | -0.413243655 | 0.21475294934268 | 0.905520508927376 | 0.66150108540577 | 1.2395556261032 | 0.998074519377826 |
| ebi-fl187-GCST90094756 | ukb-rsfMRI191-edge-pheno599 | Net100_Pair11_29 (Angular\|Temporal)&(Parietal) (Default_mode\|Central_executive)&(Central_executive\|Salience) \|\| id:ukb-rsfMRI191-edge-pheno599 | Dried fruit liking \|\| id:ebi-fl187-GCST90094756 | Inverse variance weighted | 5 | 0.104632657285552 | 0.0934856134106377 | 0.263038589280594 | -0.078599145 | 0.287864459570402 | 1.11030267383597 | 0.924410404719124 | 1.33357653833622 | 0.998074519377826 |
| ebi-fl187-GCST90094756 | ukb-rsfMRI191-edge-pheno621 | Net100_Pair5_30 (Precuneus\|Angular\|Cingulate)&(Paracentral) (Default_mode\|Central_executive)&(Motor) \|\| id:ukb-rsfMRI191-edge-pheno621 | Dried fruit liking \|\| id:ebi-fl187-GCST90094756 | Inverse variance weighted | 5 | 0.14337618404135 | 0.0889829245397038 | 0.107119252932255 | -0.031030348 | 0.31778271613917 | 1.15416389805037 | 0.96944615182303 | 1.37407766388865 | 0.998074519377826 |
| ebi-fl187-GCST90094756 | ukb-rsfMRI191-edge-pheno624 | Net100_Pair8_30 (Calcarine\|Lingual)&(Paracentral) (Visual)&(Motor) \|\| id:ukb-rsfMRI191-edge-pheno624 | Dried fruit liking \|\| id:ebi-fl187-GCST90094756 | Inverse variance weighted | 5 | -0.043720695 | 0.0886736401692175 | 0.621976007122471 | -0.21752103 | 0.130079639377299 | 0.95722127648901 | 0.804510685812986 | 1.13891908251949 | 0.998074519377826 |
| ebi-fl187-GCST90094756 | ukb-rsfMRI191-edge-pheno882 | Net100_Pair6_38 (Postcentral\|Precentral)&(Subcortical) (Motor)&(Subcortical-cerebellum) \|\| id:ukb-rsfMRI191-edge-pheno882 | Dried fruit liking \|\| id:ebi-fl187-GCST90094756 | Inverse variance weighted | 5 | -0.193470417 | 0.103430881015644 | 0.0614104552578641 | -0.396194943 | 0.00925411021948505 | 0.824094215367063 | 0.672875510536817 | 1.00929706188838 | 0.998074519377826 |
| ebi-fl187-GCST90094756 | ukb-rsfMRI191-edge-pheno899 | Net100_Pair23_38 (Cerebellum)&(Subcortical) (Subcortical-cerebellum)&(Subcortical-cerebellum) \|\| id:ukb-rsfMRI191-edge-pheno899 | Dried fruit liking \|\| id:ebi-fl187-GCST90094756 | Inverse variance weighted | 5 | 0.0536306159060581 | 0.0978588742511069 | 0.583664167621878 | -0.138172778 | 0.245434009438228 | 1.05509479493037 | 0.870948198383047 | 1.27817593325976 | 0.998074519377826 |
| ebi-fl187-GCST90094756 | ebi-a-GCST90001392 | IgD+ CD38+ B cell Absolute Count \|\| id:ebi-a-GCST90001392 | Dried fruit liking \|\| id:ebi-fl187-GCST90094756 | MR Egger | 5 | 0.628059824317611 | 0.970653872135586 | 0.563697246014718 | -1.274421765 | 2.53054141370336 | 1.87397121652064 | 0.279592591691942 | 12.560304617145 | 0.999279047927446 |
| ebi-fl187-GCST90094756 | ebi-a-GCST90001402 | Switched memory B cell %B cell \|\| id:ebi-a-GCST90001402 | Dried fruit liking \|\| id:ebi-fl187-GCST90094756 | MR Egger | 5 | -0.908258679 | 1.02040109041913 | 0.438984391228007 | -2.908244816 | 1.09172745795308 | 0.403225758430841 | 0.0545714287319213 | 2.97941644630279 | 0.999279047927446 |
| ebi-fl187-GCST90094756 | ebi-a-GCST90001407 | Memory B cell Absolute Count \|\| id:ebi-a-GCST90001407 | Dried fruit liking \|\| id:ebi-fl187-GCST90094756 | MR Egger | 5 | 1.22315901876981 | 1.1185978296438 | 0.354127038493055 | -0.969292727 | 3.41561076487166 | 3.39790484210963 | 0.379351248012726 | 30.4355327062079 | 0.999279047927446 |
| ebi-fl187-GCST90094756 | ebi-a-GCST90001411 | IgD+ CD24+ B cell %B cell \|\| id:ebi-a-GCST90001411 | Dried fruit liking \|\| id:ebi-fl187-GCST90094756 | MR Egger | 5 | 1.6530304789788 | 1.02153940725931 | 0.204055279545554 | -0.349186759 | 3.65524771720705 | 5.22278340419838 | 0.705261403879321 | 38.6771009119869 | 0.999279047927446 |
| ebi-fl187-GCST90094756 | ebi-a-GCST90001418 | CD24+ CD27+ B cell Absolute Count \|\| id:ebi-a-GCST90001418 | Dried fruit liking \|\| id:ebi-fl187-GCST90094756 | MR Egger | 5 | 1.03880577632148 | 1.01668019739335 | 0.382101023832569 | -0.953887411 | 3.03149896321245 | 2.82584031307099 | 0.385240520736963 | 20.7282802434727 | 0.999279047927446 |
| ebi-fl187-GCST90094756 | ebi-a-GCST90001436 | Memory B cell %lymphocyte \|\| id:ebi-a-GCST90001436 | Dried fruit liking \|\| id:ebi-fl187-GCST90094756 | MR Egger | 5 | 1.12510274588457 | 0.997616411320215 | 0.341459283016268 | -0.83022542 | 3.08043091207219 | 3.08053334478188 | 0.435951003037207 | 21.7677803748582 | 0.999279047927446 |
| ebi-fl187-GCST90094756 | ebi-a-GCST90001439 | IgD+ CD24+ B cell %lymphocyte \|\| id:ebi-a-GCST90001439 | Dried fruit liking \|\| id:ebi-fl187-GCST90094756 | MR Egger | 5 | 1.79302370709679 | 1.00311759233575 | 0.171827633410621 | -0.173086774 | 3.75913418807487 | 6.00759022412586 | 0.841064629235759 | 42.9112568124606 | 0.999279047927446 |
| ebi-fl187-GCST90094756 | ebi-a-GCST90001457 | CD11c+ HLA DR++ monocyte %monocyte \|\| id:ebi-a-GCST90001457 | Dried fruit liking \|\| id:ebi-fl187-GCST90094756 | MR Egger | 5 | 1.02184236692553 | 1.08555694699335 | 0.415990440103841 | -1.105849249 | 3.14953398303251 | 2.77830871571674 | 0.330929721076195 | 23.3251921124676 | 0.999279047927446 |
| ebi-fl187-GCST90094756 | ebi-a-GCST90001483 | CD39+ resting CD4 regulatory T cell Absolute Count \|\| id:ebi-a-GCST90001483 | Dried fruit liking \|\| id:ebi-fl187-GCST90094756 | MR Egger | 5 | 0.884907257452024 | 1.6573874355838 | 0.630427988401283 | -2.363572116 | 4.13338663119627 | 2.42275968815932 | 0.094083544863788 | 62.3888535988726 | 0.999279047927446 |
| ebi-fl187-GCST90094756 | ebi-a-GCST90001525 | CD33dim HLA DR+ CD11b+ Absolute Count \|\| id:ebi-a-GCST90001525 | Dried fruit liking \|\| id:ebi-fl187-GCST90094756 | MR Egger | 5 | 1.39184550752007 | 1.30100404717146 | 0.363125850130297 | -1.158122425 | 3.94181343997612 | 4.02226633025175 | 0.314075327629288 | 51.5119304454661 | 0.999279047927446 |
| ebi-fl187-GCST90094756 | ebi-a-GCST90001540 | Naive CD4+ T cell Absolute Count \|\| id:ebi-a-GCST90001540 | Dried fruit liking \|\| id:ebi-fl187-GCST90094756 | MR Egger | 5 | 0.528496311579777 | 0.956048852770726 | 0.618927733750632 | -1.34535944 | 2.4023520630104 | 1.69637956352695 | 0.260446076334702 | 11.0491341011938 | 0.999279047927446 |
| ebi-fl187-GCST90094756 | ebi-a-GCST90001624 | HLA DR+ CD4+ T cell Absolute Count \|\| id:ebi-a-GCST90001624 | Dried fruit liking \|\| id:ebi-fl187-GCST90094756 | MR Egger | 5 | -0.131951538 | 1.20102459223291 | 0.919452925286548 | -2.485959738 | 2.22205666324659 | 0.876383465757751 | 0.0832456221487267 | 9.22628673110728 | 0.999279047927446 |
| ebi-fl187-GCST90094756 | ebi-a-GCST90001672 | CD39+ CD8+ T cell Absolute Count \|\| id:ebi-a-GCST90001672 | Dried fruit liking \|\| id:ebi-fl187-GCST90094756 | MR Egger | 5 | 0.268046284916007 | 1.62332054846613 | 0.879347288492664 | -2.91366199 | 3.44975455990963 | 1.30740765186789 | 0.0542766051059164 | 31.4926617983404 | 0.999279047927446 |
| ebi-fl187-GCST90094756 | ebi-a-GCST90001722 | CD19 on CD20- CD38- B cell \|\| id:ebi-a-GCST90001722 | Dried fruit liking \|\| id:ebi-fl187-GCST90094756 | MR Egger | 5 | 0.865074936580194 | 1.48334223798923 | 0.600709336062931 | -2.04227585 | 3.77242572303909 | 2.37518406747971 | 0.12973312153861 | 43.4854205888394 | 0.999279047927446 |
| ebi-fl187-GCST90094756 | ebi-a-GCST90001742 | CD19 on transitional B cell \|\| id:ebi-a-GCST90001742 | Dried fruit liking \|\| id:ebi-fl187-GCST90094756 | MR Egger | 5 | 0.426665782639624 | 1.49483790443977 | 0.793889830401403 | -2.50321651 | 3.35654807534158 | 1.53214050818711 | 0.0818213955680674 | 28.6899840870474 | 0.999279047927446 |
| ebi-fl187-GCST90094756 | ebi-a-GCST90001784 | CD25 on IgD+ CD38dim B cell \|\| id:ebi-a-GCST90001784 | Dried fruit liking \|\| id:ebi-fl187-GCST90094756 | MR Egger | 5 | 0.173186709151478 | 1.01700547741519 | 0.875618068358285 | -1.820144027 | 2.16651744488525 | 1.18908809798914 | 0.162002416599152 | 8.72783588332493 | 0.999279047927446 |
| ebi-fl187-GCST90094756 | ebi-a-GCST90001794 | CD25 on IgD+ B cell \|\| id:ebi-a-GCST90001794 | Dried fruit liking \|\| id:ebi-fl187-GCST90094756 | MR Egger | 5 | 0.0821625994687973 | 1.0144252211264 | 0.9405473392281 | -1.906110834 | 2.07043603287654 | 1.08563231871737 | 0.148657417165844 | 7.92827935473135 | 0.999279047927446 |
| ebi-fl187-GCST90094756 | ebi-a-GCST90001802 | CD27 on IgD- CD38- B cell \|\| id:ebi-a-GCST90001802 | Dried fruit liking \|\| id:ebi-fl187-GCST90094756 | MR Egger | 5 | -1.094920273 | 1.02725758380972 | 0.36465297727511 | -3.108345137 | 0.918504591227619 | 0.334566279837921 | 0.0446748249024961 | 2.50554077937375 | 0.999279047927446 |
| ebi-fl187-GCST90094756 | ebi-a-GCST90001804 | CD27 on IgD- CD38dim B cell \|\| id:ebi-a-GCST90001804 | Dried fruit liking \|\| id:ebi-fl187-GCST90094756 | MR Egger | 5 | -0.11638711 | 1.74401047396412 | 0.950991001065191 | -3.534647639 | 3.30187341885623 | 0.890130578044906 | 0.0291690332061078 | 27.1634798579697 | 0.999279047927446 |
| ebi-fl187-GCST90094756 | ebi-a-GCST90001805 | CD27 on memory B cell \|\| id:ebi-a-GCST90001805 | Dried fruit liking \|\| id:ebi-fl187-GCST90094756 | MR Egger | 5 | -0.839464028 | 1.05441324529828 | 0.48412799570481 | -2.906113989 | 1.2271859327655 | 0.431941970192438 | 0.054687835019963 | 3.41161549996665 | 0.999279047927446 |
| ebi-fl187-GCST90094756 | ebi-a-GCST90001806 | CD27 on unswitched memory B cell \|\| id:ebi-a-GCST90001806 | Dried fruit liking \|\| id:ebi-fl187-GCST90094756 | MR Egger | 5 | -0.643146359 | 1.7315489236147 | 0.73499987720506 | -4.03698225 | 2.75068953095311 | 0.525635979854948 | 0.0176506574039013 | 15.6534216825829 | 0.999279047927446 |
| ebi-fl187-GCST90094756 | ebi-a-GCST90001839 | CD3 on Effector Memory CD8+ T cell \|\| id:ebi-a-GCST90001839 | Dried fruit liking \|\| id:ebi-fl187-GCST90094756 | MR Egger | 5 | 0.393333497607401 | 1.11506783096965 | 0.747608512235237 | -1.792199451 | 2.57886644630792 | 1.48191252120899 | 0.166593352485461 | 13.1821869705613 | 0.999279047927446 |
| ebi-fl187-GCST90094756 | ebi-a-GCST90001859 | CD3 on CD8+ T cell \|\| id:ebi-a-GCST90001859 | Dried fruit liking \|\| id:ebi-fl187-GCST90094756 | MR Egger | 5 | 0.800318359816553 | 1.09338838379712 | 0.517189238498045 | -1.342722872 | 2.94335959205891 | 2.22624956408876 | 0.261133666015508 | 18.9795027092028 | 0.999279047927446 |
| ebi-fl187-GCST90094756 | ebi-a-GCST90001905 | CD86 on monocyte \|\| id:ebi-a-GCST90001905 | Dried fruit liking \|\| id:ebi-fl187-GCST90094756 | MR Egger | 5 | -0.119223802 | 1.10874746912563 | 0.921156421272858 | -2.292368841 | 2.05392123757247 | 0.887609129909379 | 0.101026861566196 | 7.79842069014747 | 0.999279047927446 |
| ebi-fl187-GCST90094756 | ebi-a-GCST90001943 | CD25 on activated & secreting CD4 regulatory T cell \|\| id:ebi-a-GCST90001943 | Dried fruit liking \|\| id:ebi-fl187-GCST90094756 | MR Egger | 5 | 1.17989005336335 | 1.02397250948726 | 0.332699812690577 | -0.827096065 | 3.18687617195838 | 3.2540164150608 | 0.437317385352079 | 24.2126729559594 | 0.999279047927446 |
| ebi-fl187-GCST90094756 | ebi-a-GCST90001949 | CD33 on CD33dim HLA DR+ CD11b- \|\| id:ebi-a-GCST90001949 | Dried fruit liking \|\| id:ebi-fl187-GCST90094756 | MR Egger | 5 | -0.054513671 | 1.42210155288656 | 0.971830279945052 | -2.841832714 | 2.73280537302513 | 0.946945563424516 | 0.0583186864729395 | 15.3759618798248 | 0.999279047927446 |
| ebi-fl187-GCST90094756 | ebi-a-GCST90001955 | CD33 on Immature Myeloid-Derived Suppressor Cells \|\| id:ebi-a-GCST90001955 | Dried fruit liking \|\| id:ebi-fl187-GCST90094756 | MR Egger | 5 | 0.375550982879461 | 1.47319675131685 | 0.815259670463074 | -2.51191465 | 3.26301661546049 | 1.45579331087269 | 0.0811127878958068 | 26.1282372232605 | 0.999279047927446 |
| ebi-fl187-GCST90094756 | ebi-a-GCST90001956 | CD33 on CD33+ HLA DR+ \|\| id:ebi-a-GCST90001956 | Dried fruit liking \|\| id:ebi-fl187-GCST90094756 | MR Egger | 5 | -0.149282682 | 1.46012742219295 | 0.925017254228899 | -3.011132429 | 2.71256706571006 | 0.861325599421283 | 0.0492358910136633 | 15.0679062152558 | 0.999279047927446 |
| ebi-fl187-GCST90094756 | ebi-a-GCST90001957 | CD33 on CD33+ HLA DR+ CD14- \|\| id:ebi-a-GCST90001957 | Dried fruit liking \|\| id:ebi-fl187-GCST90094756 | MR Egger | 5 | 0.0952289496380198 | 1.46114957536564 | 0.952135426034757 | -2.768624218 | 2.95908211735468 | 1.09991065044605 | 0.062748273325205 | 19.2802666090049 | 0.999279047927446 |
| ebi-fl187-GCST90094756 | ebi-a-GCST90002064 | CD4 on resting CD4 regulatory T cell \|\| id:ebi-a-GCST90002064 | Dried fruit liking \|\| id:ebi-fl187-GCST90094756 | MR Egger | 5 | 0.411135830708621 | 1.07803901908312 | 0.728333479148754 | -1.701820647 | 2.52409230811154 | 1.50853024737499 | 0.182351224490406 | 12.4795625233927 | 0.999279047927446 |
| ebi-fl187-GCST90094756 | ebi-a-GCST90002080 | SSC-A on T cell \|\| id:ebi-a-GCST90002080 | Dried fruit liking \|\| id:ebi-fl187-GCST90094756 | MR Egger | 5 | -0.573138705 | 1.03353024037557 | 0.617867243028888 | -2.598857976 | 1.45258056603464 | 0.563753203836303 | 0.074358448858495 | 4.27412997063059 | 0.999279047927446 |
| ebi-fl187-GCST90094756 | ebi-a-GCST90002121 | CD8 on CD39+ CD8+ T cell \|\| id:ebi-a-GCST90002121 | Dried fruit liking \|\| id:ebi-fl187-GCST90094756 | MR Egger | 5 | -1.690788267 | 1.09484696345133 | 0.220212026431062 | -3.836688315 | 0.455111781619932 | 0.18437413070036 | 0.0215648993730507 | 1.57634958009565 | 0.999279047927446 |
| ebi-fl187-GCST90094756 | ebi-cfmet338-GCST90026053 | 1-palmitoyl-2-docosahexaenoyl-gpc (16:0/22:6) levels \|\| id:ebi-cfmet338-GCST90026053 | Dried fruit liking \|\| id:ebi-fl187-GCST90094756 | MR Egger | 5 | -0.470746265 | 0.633564359573309 | 0.511367810367308 | -1.71253241 | 0.771039879734536 | 0.624536024938931 | 0.180408345778607 | 2.1620133190799 | 0.999279047927446 |
| ebi-fl187-GCST90094756 | ebi-cfmet338-GCST90026063 | 1-stearoyl-2-oleoyl-gpc (18:0/18:1) levels \|\| id:ebi-cfmet338-GCST90026063 | Dried fruit liking \|\| id:ebi-fl187-GCST90094756 | MR Egger | 4 | -0.140762589 | 0.530935625782558 | 0.815740523414737 | -1.181396415 | 0.899871237715836 | 0.868695525249465 | 0.306849949306923 | 2.45928642743112 | 0.999279047927446 |
| ebi-fl187-GCST90094756 | ebi-cfmet338-GCST90026113 | Adenosine 3',5'-cyclic monophosphate (camp) levels \|\| id:ebi-cfmet338-GCST90026113 | Dried fruit liking \|\| id:ebi-fl187-GCST90094756 | MR Egger | 5 | -1.078728969 | 1.00693809451693 | 0.36255928606113 | -3.052327635 | 0.894869695924859 | 0.340027436401761 | 0.0472488183140533 | 2.44701691241165 | 0.999279047927446 |
| ebi-fl187-GCST90094756 | ebi-cfmet338-GCST90026117 | Alpha-ketoglutaramate levels \|\| id:ebi-cfmet338-GCST90026117 | Dried fruit liking \|\| id:ebi-fl187-GCST90094756 | MR Egger | 5 | 0.118565526959913 | 0.40733924727132 | 0.789959388194947 | -0.679819398 | 0.916950451611699 | 1.12588064722093 | 0.506708496826457 | 2.50164984350118 | 0.999279047927446 |
| ebi-fl187-GCST90094756 | ebi-cfmet338-GCST90026124 | Arginine levels \|\| id:ebi-cfmet338-GCST90026124 | Dried fruit liking \|\| id:ebi-fl187-GCST90094756 | MR Egger | 5 | -0.146691339 | 0.34981243925259 | 0.703183036343972 | -0.83232372 | 0.538941042002813 | 0.863560483785113 | 0.435037206282426 | 1.7141906448136 | 0.999279047927446 |
| ebi-fl187-GCST90094756 | ebi-cfmet338-GCST90026169 | Glutamate levels \|\| id:ebi-cfmet338-GCST90026169 | Dried fruit liking \|\| id:ebi-fl187-GCST90094756 | MR Egger | 5 | 0.635027430197162 | 0.378543456748202 | 0.192026963192752 | -0.106917745 | 1.37697260542364 | 1.88707390355805 | 0.89859958423294 | 3.96288622871952 | 0.999279047927446 |
| ebi-fl187-GCST90094756 | ebi-cfmet338-GCST90026170 | Glutamine levels \|\| id:ebi-cfmet338-GCST90026170 | Dried fruit liking \|\| id:ebi-fl187-GCST90094756 | MR Egger | 5 | -0.14956994 | 0.207201711517391 | 0.522559206172085 | -0.555685294 | 0.256545414763255 | 0.86107821226636 | 0.573678987468831 | 1.29245746111647 | 0.999279047927446 |
| ebi-fl187-GCST90094756 | ebi-cfmet338-GCST90026191 | Kynurenate levels \|\| id:ebi-cfmet338-GCST90026191 | Dried fruit liking \|\| id:ebi-fl187-GCST90094756 | MR Egger | 5 | 0.115084853999876 | 1.95061652214323 | 0.956662880400436 | -3.708123529 | 3.93829323740062 | 1.12196863705944 | 0.0245234977457959 | 51.3309168045094 | 0.999279047927446 |
| ebi-fl187-GCST90094756 | ebi-cfmet338-GCST90026202 | Methylsuccinoylcarnitine levels \|\| id:ebi-cfmet338-GCST90026202 | Dried fruit liking \|\| id:ebi-fl187-GCST90094756 | MR Egger | 5 | -0.226504493 | 1.3970433933958 | 0.881507184089347 | -2.964709544 | 2.51170055847441 | 0.797315760299023 | 0.0515754474866371 | 12.32587312 | 0.999279047927446 |
| ebi-fl187-GCST90094756 | ebi-cfmet338-GCST90026222 | N-acetylputrescine levels \|\| id:ebi-cfmet338-GCST90026222 | Dried fruit liking \|\| id:ebi-fl187-GCST90094756 | MR Egger | 5 | 0.0402351850059955 | 0.382284227352683 | 0.922820446275028 | -0.709041901 | 0.789512270617254 | 1.04105558606747 | 0.492115467194552 | 2.20232202710633 | 0.999279047927446 |
| ebi-fl187-GCST90094756 | ebi-cfmet338-GCST90026252 | Propionylcarnitine (c3) levels \|\| id:ebi-cfmet338-GCST90026252 | Dried fruit liking \|\| id:ebi-fl187-GCST90094756 | MR Egger | 5 | -1.709165989 | 0.618918788043202 | 0.0700622975211371 | -2.922246814 | -0.496085165 | 0.181016699576804 | 0.053812644385186 | 0.608909781335693 | 0.999279047927446 |
| ebi-fl187-GCST90094756 | ebi-cfmet338-GCST90026316 | Ethyl beta-glucopyranoside levels \|\| id:ebi-cfmet338-GCST90026316 | Dried fruit liking \|\| id:ebi-fl187-GCST90094756 | MR Egger | 5 | -1.367174357 | 2.48999519726754 | 0.621185996405138 | -6.247564944 | 3.51321622915191 | 0.254825990362772 | 0.0019351606278501 | 33.5560182601017 | 0.999279047927446 |
| ebi-fl187-GCST90094756 | ebi-gm430-GCST90011315 | F_Prevotellaceae abundance \|\| id:ebi-gm430-GCST90011315 | Dried fruit liking \|\| id:ebi-fl187-GCST90094756 | MR Egger | 3 | -13.48847618 | 9.45799737953003 | 0.389308785422761 | -32.02615105 | 5.04919868145394 | 1.38684915004845e-06 | 1.23372772342274e-14 | 155.897490870526 | 0.999279047927446 |
| ebi-fl187-GCST90094756 | ebi-gm430-GCST90011354 | OTU97_1 (Bacteroides) abundance \|\| id:ebi-gm430-GCST90011354 | Dried fruit liking \|\| id:ebi-fl187-GCST90094756 | MR Egger | 3 | 11.7400757548188 | 8.58968779425315 | 0.402125149341819 | -5.095712322 | 28.575863831555 | 125501.84703033 | 0.00612294357417584 | 2572408747069.74 | 0.999279047927446 |
| ebi-fl187-GCST90094756 | ebi-gm430-GCST90011419 | OTU99_1 (Bacteroides) abundance \|\| id:ebi-gm430-GCST90011419 | Dried fruit liking \|\| id:ebi-fl187-GCST90094756 | MR Egger | 3 | 11.2302960547528 | 8.59323599551824 | 0.415806848646831 | -5.612446496 | 28.0730386059686 | 75379.9086343483 | 0.00365212351194891 | 1555842951951.67 | 0.999279047927446 |
| ebi-fl187-GCST90094756 | ebi-gm430-GCST90011435 | OTU99_155 (Butyrivibrio) abundance \|\| id:ebi-gm430-GCST90011435 | Dried fruit liking \|\| id:ebi-fl187-GCST90094756 | MR Egger | 3 | -28.49114745 | 26.8997811801567 | 0.481715117289755 | -81.21471856 | 24.2324236661415 | 4.23108627189685e-13 | 5.35668218298018e-36 | 33420110487.6344 | 0.999279047927446 |
| ebi-fl187-GCST90094756 | ebi-gm430-GCST90011466 | OTU99_58 (Bacteroides) abundance \|\| id:ebi-gm430-GCST90011466 | Dried fruit liking \|\| id:ebi-fl187-GCST90094756 | MR Egger | 3 | 12.2066666373526 | 19.7772477767301 | 0.647964467820863 | -26.55673901 | 50.9700722797435 | 200118.833654098 | 2.92789024536565e-12 | 1.36779538257847e+22 | 0.999279047927446 |
| ebi-fl187-GCST90094756 | ebi-gm430-GCST90011472 | OTU99_72 (Parabacteroides) abundance \|\| id:ebi-gm430-GCST90011472 | Dried fruit liking \|\| id:ebi-fl187-GCST90094756 | MR Egger | 3 | -27.83582612 | 23.6758480193128 | 0.448699213222235 | -74.24048824 | 18.5688359987617 | 8.14806185550503e-13 | 5.72486994332478e-33 | 115969293.029178 | 0.999279047927446 |
| ebi-fl187-GCST90094756 | ebi-gm430-GCST90011505 | TestASV_28 (Holdemanella) abundance \|\| id:ebi-gm430-GCST90011505 | Dried fruit liking \|\| id:ebi-fl187-GCST90094756 | MR Egger | 4 | -0.669218452 | 2.12832657753926 | 0.782961393444093 | -4.840738544 | 3.50230163998077 | 0.512108658924993 | 0.0079012165003879 | 33.1917595895619 | 0.999279047927446 |
| ebi-fl187-GCST90094756 | ebi-gm430-GCST90011513 | TestASV_35 (Alphaproteobacteria) abundance \|\| id:ebi-gm430-GCST90011513 | Dried fruit liking \|\| id:ebi-fl187-GCST90094756 | MR Egger | 3 | 14.5532238394674 | 23.8452317165127 | 0.651149387089887 | -32.18343032 | 61.2898780038322 | 2091148.17571922 | 1.05417764232093e-14 | 4.14816300143325e+26 | 0.999279047927446 |
| ebi-fl187-GCST90094756 | ebi-gm430-GCST90011543 | G_Coprococcus prevalence \|\| id:ebi-gm430-GCST90011543 | Dried fruit liking \|\| id:ebi-fl187-GCST90094756 | MR Egger | 3 | -5.792531603 | 25.5293523832909 | 0.857957886835807 | -55.83006227 | 44.244999067844 | 0.0030502503693934 | 5.66646264091115e-25 | 16419462909383100000 | 0.999279047927446 |
| ebi-fl187-GCST90094756 | ebi-gm430-GCST90011547 | G_Parasutterella prevalence \|\| id:ebi-gm430-GCST90011547 | Dried fruit liking \|\| id:ebi-fl187-GCST90094756 | MR Egger | 3 | 39.5043438662591 | 22.7555733126737 | 0.33270121281601 | -5.096579827 | 84.1052675590996 | 143389896848348000 | 0.00611763419535566 | 3.36088459388253e+36 | 0.999279047927446 |
| ebi-fl187-GCST90094756 | ebi-gm430-GCST90011569 | OTU97_13 (Subdoligranulum) prevalence \|\| id:ebi-gm430-GCST90011569 | Dried fruit liking \|\| id:ebi-fl187-GCST90094756 | MR Egger | 3 | 1.67756112745713 | 23.4241919705665 | 0.954485160708138 | -44.23385513 | 47.5889773897676 | 5.35248600845175 | 6.15858303948422e-20 | 4.6518990305067e+20 | 0.999279047927446 |
| ebi-fl187-GCST90094756 | ebi-gm430-GCST90011590 | OTU97_277 (Prevotella) prevalence \|\| id:ebi-gm430-GCST90011590 | Dried fruit liking \|\| id:ebi-fl187-GCST90094756 | MR Egger | 3 | -35.06962226 | 23.0608703504202 | 0.370309757839144 | -80.26892814 | 10.1296836296936 | 5.8810730148146e-16 | 1.37926412037545e-35 | 25076.4297386101 | 0.999279047927446 |
| ebi-fl187-GCST90094756 | ebi-gm430-GCST90011616 | OTU97_86 (Phascolarctobacterium) prevalence \|\| id:ebi-gm430-GCST90011616 | Dried fruit liking \|\| id:ebi-fl187-GCST90094756 | MR Egger | 3 | 17.815611848347 | 24.9832834792881 | 0.605636479050242 | -31.15162377 | 66.7828474677517 | 54603680.580748 | 2.95816022905455e-14 | 1.00791089802369e+29 | 0.999279047927446 |
| ebi-fl187-GCST90094756 | ebi-gm430-GCST90011665 | OTU99_55 (Barnesiella) prevalence \|\| id:ebi-gm430-GCST90011665 | Dried fruit liking \|\| id:ebi-fl187-GCST90094756 | MR Egger | 3 | -8.169168837 | 26.254633126801 | 0.807959900509177 | -59.62824977 | 43.2899120919667 | 0.000283253348457646 | 1.26993110726598e-26 | 6317859209323570000 | 0.999279047927446 |
| ebi-fl187-GCST90094756 | ebi-gm430-GCST90011687 | TestASV_14 (Lachnospiraceae) prevalence \|\| id:ebi-gm430-GCST90011687 | Dried fruit liking \|\| id:ebi-fl187-GCST90094756 | MR Egger | 3 | 11.5687393883888 | 18.1000449548778 | 0.637945612190887 | -23.90734872 | 47.0448274999493 | 105740.090788333 | 4.14162117670584e-11 | 2.69965946253394e+20 | 0.999279047927446 |
| ebi-fl187-GCST90094756 | ebi-gm430-GCST90011697 | TestASV_23 (Barnesiella) prevalence \|\| id:ebi-gm430-GCST90011697 | Dried fruit liking \|\| id:ebi-fl187-GCST90094756 | MR Egger | 3 | 5.23324190486695 | 25.0325735564144 | 0.868799586526693 | -43.83060227 | 54.2970860754392 | 187.399350671862 | 9.21746076164175e-20 | 3.80999903773719e+23 | 0.999279047927446 |
| ebi-fl187-GCST90094756 | ebi-gm430-GCST90011727 | TestASV_6 (Subdoligranulum) prevalence \|\| id:ebi-gm430-GCST90011727 | Dried fruit liking \|\| id:ebi-fl187-GCST90094756 | MR Egger | 3 | -10.91756188 | 28.7626567884396 | 0.769050346429592 | -67.29236918 | 45.4572454291681 | 1.81369026255284e-05 | 5.96067427642363e-30 | 55186245983779500000 | 0.999279047927446 |
| ebi-fl187-GCST90094756 | ebi-hbsm44-GCST90104908 | Sulcal depth of the left posterior inferior temporal sulcus \|\| id:ebi-hbsm44-GCST90104908 | Dried fruit liking \|\| id:ebi-fl187-GCST90094756 | MR Egger | 4 | 0.32989191068746 | 0.809199805273986 | 0.723008404883294 | -1.256139708 | 1.91592352902447 | 1.39081778780027 | 0.2847511301765 | 6.79320962716679 | 0.999279047927446 |
| ebi-fl187-GCST90094756 | ebi-hbsm44-GCST90104918 | Sulcal depth of the left inferior frontal sulcus \|\| id:ebi-hbsm44-GCST90104918 | Dried fruit liking \|\| id:ebi-fl187-GCST90094756 | MR Egger | 4 | 0.814068150984694 | 1.96906358356584 | 0.719405758996829 | -3.045296473 | 4.67343277477374 | 2.25707144228864 | 0.0475822030669977 | 107.064641131093 | 0.999279047927446 |
| ebi-fl187-GCST90094756 | ebi-hbsm44-GCST90104925 | Sulcal depth of the right anterior inferior temporal sulcus \|\| id:ebi-hbsm44-GCST90104925 | Dried fruit liking \|\| id:ebi-fl187-GCST90094756 | MR Egger | 4 | 0.0249446238463947 | 0.529387933185107 | 0.966699788286332 | -1.012655725 | 1.0625449728892 | 1.02525834408887 | 0.363252997309924 | 2.89372608046784 | 0.999279047927446 |
| ebi-fl187-GCST90094756 | ebi-IPs91-GCST90274770 | C-C motif chemokine 4 levels \|\| id:ebi-IPs91-GCST90274770 | Dried fruit liking \|\| id:ebi-fl187-GCST90094756 | MR Egger | 5 | 0.616378772850304 | 0.661959965685897 | 0.420465659859771 | -0.68106276 | 1.91382030559466 | 1.85220861478055 | 0.50607886614459 | 6.77893700403431 | 0.999279047927446 |
| ebi-fl187-GCST90094756 | ebi-IPs91-GCST90274779 | C-X-C motif chemokine 1 levels \|\| id:ebi-IPs91-GCST90274779 | Dried fruit liking \|\| id:ebi-fl187-GCST90094756 | MR Egger | 5 | -0.283155671 | 0.569721805385547 | 0.653324450537747 | -1.399810409 | 0.833499067847289 | 0.753402496030249 | 0.246643720873682 | 2.30135727361699 | 0.999279047927446 |
| ebi-fl187-GCST90094756 | ebi-IPs91-GCST90274780 | C-X-C motif chemokine 10 levels \|\| id:ebi-IPs91-GCST90274780 | Dried fruit liking \|\| id:ebi-fl187-GCST90094756 | MR Egger | 5 | 0.264919678072798 | 0.569320815525176 | 0.673395272924485 | -0.85094912 | 1.38078847650214 | 1.30332628589492 | 0.427009456189209 | 3.97803697993979 | 0.999279047927446 |
| ebi-fl187-GCST90094756 | ebi-IPs91-GCST90274796 | Interleukin-10 receptor subunit alpha levels \|\| id:ebi-IPs91-GCST90274796 | Dried fruit liking \|\| id:ebi-fl187-GCST90094756 | MR Egger | 5 | -1.677466344 | 0.890733164177406 | 0.156193643225566 | -3.423303346 | 0.068370657591714 | 0.186846782256696 | 0.0326045527326746 | 1.07076212104252 | 0.999279047927446 |
| ebi-fl187-GCST90094756 | ebi-IPs91-GCST90274807 | Interleukin-20 levels \|\| id:ebi-IPs91-GCST90274807 | Dried fruit liking \|\| id:ebi-fl187-GCST90094756 | MR Egger | 5 | -0.44650042 | 0.62789919651262 | 0.528323489147797 | -1.677182845 | 0.784182005212457 | 0.639863491473065 | 0.186899760656687 | 2.19061429657027 | 0.999279047927446 |
| ebi-fl187-GCST90094756 | ebi-IPs91-GCST90274808 | Interleukin-20 receptor subunit alpha levels \|\| id:ebi-IPs91-GCST90274808 | Dried fruit liking \|\| id:ebi-fl187-GCST90094756 | MR Egger | 5 | 0.139220156828778 | 0.637118431603355 | 0.841048624730083 | -1.109531969 | 1.38797228277136 | 1.14937711537184 | 0.329713240948853 | 4.00671732059869 | 0.999279047927446 |
| ebi-fl187-GCST90094756 | ebi-IPs91-GCST90274810 | Interleukin-24 levels \|\| id:ebi-IPs91-GCST90274810 | Dried fruit liking \|\| id:ebi-fl187-GCST90094756 | MR Egger | 5 | -0.4421842 | 0.811485376869892 | 0.623714469765519 | -2.032695539 | 1.14832713861893 | 0.642631251846543 | 0.130981977875959 | 3.15291410732053 | 0.999279047927446 |
| ebi-fl187-GCST90094756 | ebi-IPs91-GCST90274823 | Monocyte chemoattractant protein-3 levels \|\| id:ebi-IPs91-GCST90274823 | Dried fruit liking \|\| id:ebi-fl187-GCST90094756 | MR Egger | 5 | -0.270223392 | 0.811953415367298 | 0.761180510552451 | -1.861652086 | 1.32120530222732 | 0.763208980593254 | 0.155415658121414 | 3.74793605161163 | 0.999279047927446 |
| ebi-fl187-GCST90094756 | ebi-IPs91-GCST90274824 | Monocyte chemoattractant protein-4 levels \|\| id:ebi-IPs91-GCST90274824 | Dried fruit liking \|\| id:ebi-fl187-GCST90094756 | MR Egger | 5 | -0.193764033 | 0.567575710331312 | 0.755323692861565 | -1.306212426 | 0.918684358894288 | 0.823852282993352 | 0.270843957748899 | 2.50599123508088 | 0.999279047927446 |
| ebi-fl187-GCST90094756 | ebi-IPs91-GCST90274834 | SIR2-like protein 2 levels \|\| id:ebi-IPs91-GCST90274834 | Dried fruit liking \|\| id:ebi-fl187-GCST90094756 | MR Egger | 5 | -0.334649932 | 0.821562365715522 | 0.711085365395362 | -1.944912168 | 1.27561230527361 | 0.71558854749955 | 0.142999782696077 | 3.58089333884395 | 0.999279047927446 |
| ebi-fl187-GCST90094756 | ebi-met1400-GCST90199652 | 1-stearoyl-2-oleoyl-GPS (18:0/18:1) levels \|\| id:ebi-met1400-GCST90199652 | Dried fruit liking \|\| id:ebi-fl187-GCST90094756 | MR Egger | 5 | 1.06911956550891 | 0.708564634406381 | 0.228469661705451 | -0.319667118 | 2.45790624894542 | 2.91281382892898 | 0.726390799306951 | 11.6803302163174 | 0.999279047927446 |
| ebi-fl187-GCST90094756 | ebi-met1400-GCST90199665 | N-acetylaspartate (naa) levels \|\| id:ebi-met1400-GCST90199665 | Dried fruit liking \|\| id:ebi-fl187-GCST90094756 | MR Egger | 5 | 1.06189271065075 | 0.702520490366569 | 0.227830413066838 | -0.31504745 | 2.43883287176923 | 2.89183922764545 | 0.729754246267198 | 11.4596580442329 | 0.999279047927446 |
| ebi-fl187-GCST90094756 | ebi-met1400-GCST90199669 | Acetylcarnitine levels (Biocrates platform) \|\| id:ebi-met1400-GCST90199669 | Dried fruit liking \|\| id:ebi-fl187-GCST90094756 | MR Egger | 5 | 0.54997905013418 | 0.680639414893508 | 0.478202022180425 | -0.784074203 | 1.88403230332546 | 1.7332167068296 | 0.456542171541581 | 6.57998393158217 | 0.999279047927446 |
| ebi-fl187-GCST90094756 | ebi-met1400-GCST90199670 | Iminodiacetate (IDA) levels \|\| id:ebi-met1400-GCST90199670 | Dried fruit liking \|\| id:ebi-fl187-GCST90094756 | MR Egger | 5 | -0.230312769 | 0.82652948900141 | 0.798616699431446 | -1.850310567 | 1.38968502986472 | 0.794285136216521 | 0.15718834121745 | 4.01358569425796 | 0.999279047927446 |
| ebi-fl187-GCST90094756 | ebi-met1400-GCST90199686 | Indolepropionate levels \|\| id:ebi-met1400-GCST90199686 | Dried fruit liking \|\| id:ebi-fl187-GCST90094756 | MR Egger | 5 | 0.507628568857113 | 0.70429452196654 | 0.523143744883987 | -0.872788694 | 1.88804583191153 | 1.66134675038208 | 0.417784849034458 | 6.60644595270485 | 0.999279047927446 |
| ebi-fl187-GCST90094756 | ebi-met1400-GCST90199704 | 3-hydroxysebacate levels \|\| id:ebi-met1400-GCST90199704 | Dried fruit liking \|\| id:ebi-fl187-GCST90094756 | MR Egger | 5 | 0.421058190816456 | 0.768644264306483 | 0.621959467318933 | -1.085484567 | 1.92760094885716 | 1.52357293378221 | 0.337738089428427 | 6.87300176442093 | 0.999279047927446 |
| ebi-fl187-GCST90094756 | ebi-met1400-GCST90199713 | Docosapentaenoate n3 DPA; 22:5n3 levels \|\| id:ebi-met1400-GCST90199713 | Dried fruit liking \|\| id:ebi-fl187-GCST90094756 | MR Egger | 5 | 0.439255745158071 | 0.84271051423968 | 0.638230668829301 | -1.212456863 | 2.09096835306784 | 1.55155203857331 | 0.297465548890222 | 8.09274800857499 | 0.999279047927446 |
| ebi-fl187-GCST90094756 | ebi-met1400-GCST90199715 | Myristoleate (14:1n5) levels \|\| id:ebi-met1400-GCST90199715 | Dried fruit liking \|\| id:ebi-fl187-GCST90094756 | MR Egger | 5 | 0.179152952344417 | 0.716821143402898 | 0.818780723232758 | -1.225816489 | 1.5841223934141 | 1.19620369233549 | 0.293517948363965 | 4.87501115871367 | 0.999279047927446 |
| ebi-fl187-GCST90094756 | ebi-met1400-GCST90199725 | Campesterol levels \|\| id:ebi-met1400-GCST90199725 | Dried fruit liking \|\| id:ebi-fl187-GCST90094756 | MR Egger | 5 | -0.490902702 | 0.875231319560111 | 0.614041981040773 | -2.206356088 | 1.22455068430041 | 0.612073624620859 | 0.110101117176966 | 3.40263688109871 | 0.999279047927446 |
| ebi-fl187-GCST90094756 | ebi-met1400-GCST90199737 | N-acetylproline levels \|\| id:ebi-met1400-GCST90199737 | Dried fruit liking \|\| id:ebi-fl187-GCST90094756 | MR Egger | 5 | 0.254723910262688 | 0.76443210745995 | 0.760897984752451 | -1.24356302 | 1.75301084088419 | 1.29010538684151 | 0.288354970782169 | 5.77195497841723 | 0.999279047927446 |
| ebi-fl187-GCST90094756 | ebi-met1400-GCST90199775 | Gamma-glutamylisoleucine levels \|\| id:ebi-met1400-GCST90199775 | Dried fruit liking \|\| id:ebi-fl187-GCST90094756 | MR Egger | 5 | -1.457091395 | 0.674491527938871 | 0.11954401160556 | -2.77909479 | -0.135088 | 0.232912741587319 | 0.0620946906830833 | 0.873639027700323 | 0.999279047927446 |
| ebi-fl187-GCST90094756 | ebi-met1400-GCST90199777 | 1-palmitoleoyl-GPC (16:1) levels \|\| id:ebi-met1400-GCST90199777 | Dried fruit liking \|\| id:ebi-fl187-GCST90094756 | MR Egger | 5 | -0.695421217 | 0.879043743955302 | 0.486654833965946 | -2.418346955 | 1.02750452082373 | 0.49886427344704 | 0.0890687304103728 | 2.79408454768837 | 0.999279047927446 |
| ebi-fl187-GCST90094756 | ebi-met1400-GCST90199782 | Glutamine degradant levels \|\| id:ebi-met1400-GCST90199782 | Dried fruit liking \|\| id:ebi-fl187-GCST90094756 | MR Egger | 5 | 0.281618247522846 | 0.584710840685022 | 0.663014536233349 | -0.864415 | 1.42765149526549 | 1.32527269727014 | 0.421297939754243 | 4.16889701182541 | 0.999279047927446 |
| ebi-fl187-GCST90094756 | ebi-met1400-GCST90199783 | 5-methyluridine (ribothymidine) levels \|\| id:ebi-met1400-GCST90199783 | Dried fruit liking \|\| id:ebi-fl187-GCST90094756 | MR Egger | 5 | 0.752731680894598 | 0.800313090539067 | 0.416323740926593 | -0.815881977 | 2.32134533835117 | 2.12279089092128 | 0.442249101959335 | 10.1893732437532 | 0.999279047927446 |
| ebi-fl187-GCST90094756 | ebi-met1400-GCST90199787 | Homostachydrine levels \|\| id:ebi-met1400-GCST90199787 | Dried fruit liking \|\| id:ebi-fl187-GCST90094756 | MR Egger | 5 | -0.359993841 | 0.957931752618067 | 0.732063501057974 | -2.237540076 | 1.51755239438436 | 0.697680623249234 | 0.106720706588668 | 4.5610478754938 | 0.999279047927446 |
| ebi-fl187-GCST90094756 | ebi-met1400-GCST90199802 | Beta-hydroxyisovaleroylcarnitine levels \|\| id:ebi-met1400-GCST90199802 | Dried fruit liking \|\| id:ebi-fl187-GCST90094756 | MR Egger | 5 | -1.089693882 | 0.690208104466549 | 0.21249977468652 | -2.442501766 | 0.263114003083938 | 0.336319431492906 | 0.0869430679056089 | 1.3009750256629 | 0.999279047927446 |
| ebi-fl187-GCST90094756 | ebi-met1400-GCST90199873 | Pregnenolone sulfate levels \|\| id:ebi-met1400-GCST90199873 | Dried fruit liking \|\| id:ebi-fl187-GCST90094756 | MR Egger | 5 | 1.38735373042331 | 0.882587233733321 | 0.214013106299567 | -0.342517248 | 3.11722470854062 | 4.00423972253312 | 0.709980873747092 | 22.5836164724963 | 0.999279047927446 |
| ebi-fl187-GCST90094756 | ebi-met1400-GCST90199911 | Beta-citrylglutamate levels \|\| id:ebi-met1400-GCST90199911 | Dried fruit liking \|\| id:ebi-fl187-GCST90094756 | MR Egger | 5 | -0.65500527 | 0.796269552928123 | 0.471029814577714 | -2.215693594 | 0.905683053492207 | 0.519439325006334 | 0.109077832276741 | 2.47362096157617 | 0.999279047927446 |
| ebi-fl187-GCST90094756 | ebi-met1400-GCST90199934 | 2-hydroxydecanoate levels \|\| id:ebi-met1400-GCST90199934 | Dried fruit liking \|\| id:ebi-fl187-GCST90094756 | MR Egger | 5 | -0.430292084 | 0.696781724915621 | 0.580585756885488 | -1.795984265 | 0.935400096762588 | 0.650319119123812 | 0.165964019369324 | 2.54823279349994 | 0.999279047927446 |
| ebi-fl187-GCST90094756 | ebi-met1400-GCST90199945 | Sphingomyelin (d18:1/14:0, d16:1/16:0) levels \|\| id:ebi-met1400-GCST90199945 | Dried fruit liking \|\| id:ebi-fl187-GCST90094756 | MR Egger | 5 | -0.83219021 | 0.669786495122223 | 0.302332296247597 | -2.144971741 | 0.480591319998278 | 0.435095291727544 | 0.11707134533048 | 1.61703030189907 | 0.999279047927446 |
| ebi-fl187-GCST90094756 | ebi-met1400-GCST90199979 | Carnitine C14:1 levels \|\| id:ebi-met1400-GCST90199979 | Dried fruit liking \|\| id:ebi-fl187-GCST90094756 | MR Egger | 5 | 1.59251317850123 | 0.779397960554471 | 0.133626839891068 | 0.064893175814472 | 3.120133181 | 4.91608842389148 | 1.06704503207926 | 22.649395915771 | 0.999279047927446 |
| ebi-fl187-GCST90094756 | ebi-met1400-GCST90199986 | Eugenol sulfate levels \|\| id:ebi-met1400-GCST90199986 | Dried fruit liking \|\| id:ebi-fl187-GCST90094756 | MR Egger | 5 | 0.0965376319403317 | 0.751412266840279 | 0.905902122883546 | -1.376230411 | 1.56930567494728 | 1.10135102634007 | 0.252528690477891 | 4.80331197585849 | 0.999279047927446 |
| ebi-fl187-GCST90094756 | ebi-met1400-GCST90200002 | 6-hydroxyindole sulfate levels \|\| id:ebi-met1400-GCST90200002 | Dried fruit liking \|\| id:ebi-fl187-GCST90094756 | MR Egger | 5 | -0.274788748 | 0.745756756418085 | 0.736989169240026 | -1.73647199 | 1.18689449473094 | 0.759732601420301 | 0.176140731894375 | 3.27688899355193 | 0.999279047927446 |
| ebi-fl187-GCST90094756 | ebi-met1400-GCST90200023 | Trans 3,4-methyleneheptanoate levels \|\| id:ebi-met1400-GCST90200023 | Dried fruit liking \|\| id:ebi-fl187-GCST90094756 | MR Egger | 5 | 0.781497020153389 | 0.753157685667258 | 0.375734263407979 | -0.694692044 | 2.25768608406121 | 2.18474041907925 | 0.499228164746197 | 9.56094033914368 | 0.999279047927446 |
| ebi-fl187-GCST90094756 | ebi-met1400-GCST90200032 | 2-hydroxybutyrate/2-hydroxyisobutyrate levels \|\| id:ebi-met1400-GCST90200032 | Dried fruit liking \|\| id:ebi-fl187-GCST90094756 | MR Egger | 5 | 0.556746990716446 | 1.00799669281462 | 0.619207497926813 | -1.418926527 | 2.53242050863311 | 1.74498679923322 | 0.24197362963818 | 12.5839288109672 | 0.999279047927446 |
| ebi-fl187-GCST90094756 | ebi-met1400-GCST90200051 | 1-palmitoyl-2-dihomo-linolenoyl-GPC (16:0/20:3n3 or 6) levels \|\| id:ebi-met1400-GCST90200051 | Dried fruit liking \|\| id:ebi-fl187-GCST90094756 | MR Egger | 5 | -1.338912971 | 0.968551617606087 | 0.260791297749438 | -3.237274142 | 0.5594481994113 | 0.262130457148955 | 0.039270795963358 | 1.74970674465659 | 0.999279047927446 |
| ebi-fl187-GCST90094756 | ebi-met1400-GCST90200069 | Phenylacetylglutamate levels \|\| id:ebi-met1400-GCST90200069 | Dried fruit liking \|\| id:ebi-fl187-GCST90094756 | MR Egger | 5 | -0.434756087 | 0.77796701212401 | 0.615275655493565 | -1.959571431 | 1.09005925633319 | 0.647422562322067 | 0.140918801384131 | 2.9744503223604 | 0.999279047927446 |
| ebi-fl187-GCST90094756 | ebi-met1400-GCST90200072 | Thioproline levels \|\| id:ebi-met1400-GCST90200072 | Dried fruit liking \|\| id:ebi-fl187-GCST90094756 | MR Egger | 5 | -0.561661982 | 0.696796043126343 | 0.479179232638193 | -1.927382227 | 0.804058262149965 | 0.570260512906548 | 0.14552866129775 | 2.23459110858644 | 0.999279047927446 |
| ebi-fl187-GCST90094756 | ebi-met1400-GCST90200081 | 1-oleoyl-2-docosahexaenoyl-GPC (18:1/22:6) levels \|\| id:ebi-met1400-GCST90200081 | Dried fruit liking \|\| id:ebi-fl187-GCST90094756 | MR Egger | 5 | 0.120503266934184 | 0.673358958122383 | 0.869374071972658 | -1.199280291 | 1.44028682485406 | 1.12806442627198 | 0.301411062126741 | 4.22190659109002 | 0.999279047927446 |
| ebi-fl187-GCST90094756 | ebi-met1400-GCST90200097 | Furaneol sulfate levels \|\| id:ebi-met1400-GCST90200097 | Dried fruit liking \|\| id:ebi-fl187-GCST90094756 | MR Egger | 5 | -0.582415102 | 0.879841860568363 | 0.555306907491497 | -2.306905149 | 1.14207494440897 | 0.558547786280974 | 0.099568926199411 | 3.13326297136688 | 0.999279047927446 |
| ebi-fl187-GCST90094756 | ebi-met1400-GCST90200124 | Sphingomyelin (d18:2/24:2) levels \|\| id:ebi-met1400-GCST90200124 | Dried fruit liking \|\| id:ebi-fl187-GCST90094756 | MR Egger | 4 | 0.766140338095893 | 0.649569263441169 | 0.359511920219749 | -0.507015418 | 2.03929609444058 | 2.15144635258471 | 0.602290484146943 | 7.68519764114484 | 0.999279047927446 |
| ebi-fl187-GCST90094756 | ebi-met1400-GCST90200137 | Lignoceroylcarnitine (C24) levels \|\| id:ebi-met1400-GCST90200137 | Dried fruit liking \|\| id:ebi-fl187-GCST90094756 | MR Egger | 5 | 0.429549045863032 | 0.703039837264156 | 0.58438721149783 | -0.948409035 | 1.80750712690078 | 1.53656444719302 | 0.387356804532443 | 6.09523383286753 | 0.999279047927446 |
| ebi-fl187-GCST90094756 | ebi-met1400-GCST90200165 | Octadecenedioate (C18:1-DC) levels \|\| id:ebi-met1400-GCST90200165 | Dried fruit liking \|\| id:ebi-fl187-GCST90094756 | MR Egger | 5 | -0.304912238 | 1.0845678815849 | 0.79688058690278 | -2.430665286 | 1.82084080974802 | 0.737188068535268 | 0.0879782823829447 | 6.17704999087492 | 0.999279047927446 |
| ebi-fl187-GCST90094756 | ebi-met1400-GCST90200176 | Glutamine conjugate of C6H10O2 (1) levels \|\| id:ebi-met1400-GCST90200176 | Dried fruit liking \|\| id:ebi-fl187-GCST90094756 | MR Egger | 5 | 0.818169338110013 | 0.68792084080462 | 0.319861517996646 | -0.53015551 | 2.16649418608707 | 2.26634712227715 | 0.588513442914622 | 8.72763288671226 | 0.999279047927446 |
| ebi-fl187-GCST90094756 | ebi-met1400-GCST90200182 | Glycine conjugate of C10H12O2 levels \|\| id:ebi-met1400-GCST90200182 | Dried fruit liking \|\| id:ebi-fl187-GCST90094756 | MR Egger | 5 | 0.893446703348648 | 0.799350787270551 | 0.345131464340956 | -0.67328084 | 2.46017424639893 | 2.44353730182859 | 0.510032494951862 | 11.7068512389418 | 0.999279047927446 |
| ebi-fl187-GCST90094756 | ebi-met1400-GCST90200208 | Glucuronide of piperine metabolite C17H21NO3 (4) levels \|\| id:ebi-met1400-GCST90200208 | Dried fruit liking \|\| id:ebi-fl187-GCST90094756 | MR Egger | 5 | 0.445694980177204 | 1.06028747885919 | 0.702520617065508 | -1.632468478 | 2.52385843874122 | 1.5615750825292 | 0.195446522654185 | 12.476644277221 | 0.999279047927446 |
| ebi-fl187-GCST90094756 | ebi-met1400-GCST90200227 | 3-ethylcatechol sulfate (2) levels \|\| id:ebi-met1400-GCST90200227 | Dried fruit liking \|\| id:ebi-fl187-GCST90094756 | MR Egger | 5 | -0.251609518 | 1.1789128229607 | 0.844676834369971 | -2.562278651 | 2.05905961526228 | 0.777548297613753 | 0.0771287904793893 | 7.83859504815655 | 0.999279047927446 |
| ebi-fl187-GCST90094756 | ebi-met1400-GCST90200259 | Pentose acid levels \|\| id:ebi-met1400-GCST90200259 | Dried fruit liking \|\| id:ebi-fl187-GCST90094756 | MR Egger | 5 | -0.055994962 | 0.689749887028602 | 0.940410250693573 | -1.407904741 | 1.29591481615803 | 0.945543899133298 | 0.244655362848653 | 3.65433749245576 | 0.999279047927446 |
| ebi-fl187-GCST90094756 | ebi-met1400-GCST90200291 | Sarcosine levels \|\| id:ebi-met1400-GCST90200291 | Dried fruit liking \|\| id:ebi-fl187-GCST90094756 | MR Egger | 5 | -0.74998053 | 1.09416133123454 | 0.542282499850207 | -2.894536739 | 1.39457567940822 | 0.472375749896387 | 0.0553246489094311 | 4.03326281302683 | 0.999279047927446 |
| ebi-fl187-GCST90094756 | ebi-met1400-GCST90200306 | Butyrate/isobutyrate (4:0) levels \|\| id:ebi-met1400-GCST90200306 | Dried fruit liking \|\| id:ebi-fl187-GCST90094756 | MR Egger | 5 | -1.038984742 | 0.708961657560981 | 0.239029777810706 | -2.428549591 | 0.35058010660879 | 0.353813711800875 | 0.0881646146397379 | 1.41989099787761 | 0.999279047927446 |
| ebi-fl187-GCST90094756 | ebi-met1400-GCST90200321 | Cholate levels \|\| id:ebi-met1400-GCST90200321 | Dried fruit liking \|\| id:ebi-fl187-GCST90094756 | MR Egger | 5 | -0.227541788 | 0.779135633621668 | 0.78928460402257 | -1.75464763 | 1.29956405429391 | 0.796489137428954 | 0.172968180438385 | 3.66769740211441 | 0.999279047927446 |
| ebi-fl187-GCST90094756 | ebi-met1400-GCST90200336 | Ceramide (d18:1/16:0) levels \|\| id:ebi-met1400-GCST90200336 | Dried fruit liking \|\| id:ebi-fl187-GCST90094756 | MR Egger | 5 | 0.0509330919372196 | 0.823761798172519 | 0.954587103784097 | -1.563640032 | 1.66550621635536 | 1.0522524867524 | 0.209372559522869 | 5.28834962136325 | 0.999279047927446 |
| ebi-fl187-GCST90094756 | ebi-met1400-GCST90200358 | Arachidonate (20:4n6) levels \|\| id:ebi-met1400-GCST90200358 | Dried fruit liking \|\| id:ebi-fl187-GCST90094756 | MR Egger | 5 | 0.769675868284889 | 0.688848774884889 | 0.345270336565106 | -0.58046773 | 2.11981946705927 | 2.15906631848658 | 0.559636546263784 | 8.32963357869407 | 0.999279047927446 |
| ebi-fl187-GCST90094756 | ebi-met1400-GCST90200413 | 2'-deoxyuridine levels \|\| id:ebi-met1400-GCST90200413 | Dried fruit liking \|\| id:ebi-fl187-GCST90094756 | MR Egger | 5 | -1.088658923 | 0.780308612107591 | 0.257311246616449 | -2.618063802 | 0.440745956974764 | 0.336667688471356 | 0.0729439601013979 | 1.55386590340156 | 0.999279047927446 |
| ebi-fl187-GCST90094756 | ebi-met1400-GCST90200432 | Threonine levels \|\| id:ebi-met1400-GCST90200432 | Dried fruit liking \|\| id:ebi-fl187-GCST90094756 | MR Egger | 5 | 0.738441768754742 | 0.700185323395955 | 0.369021111671979 | -0.633921465 | 2.11080500261081 | 2.09267210552087 | 0.530507350567026 | 8.2548838136633 | 0.999279047927446 |
| ebi-fl187-GCST90094756 | ebi-met1400-GCST90200436 | Caffeine levels \|\| id:ebi-met1400-GCST90200436 | Dried fruit liking \|\| id:ebi-fl187-GCST90094756 | MR Egger | 5 | -0.710665965 | 0.743563939525612 | 0.409704408027157 | -2.168051286 | 0.746719356599871 | 0.491316888696922 | 0.114400333327298 | 2.1100662742669 | 0.999279047927446 |
| ebi-fl187-GCST90094756 | ebi-met1400-GCST90200442 | Valine levels \|\| id:ebi-met1400-GCST90200442 | Dried fruit liking \|\| id:ebi-fl187-GCST90094756 | MR Egger | 5 | -1.85144363 | 0.685988036027623 | 0.073852025751162 | -3.19598018 | -0.506907079 | 0.157010337831518 | 0.0409263904656311 | 0.60235573930395 | 0.999279047927446 |
| ebi-fl187-GCST90094756 | ebi-met1400-GCST90200459 | Margarate (17:0) levels \|\| id:ebi-met1400-GCST90200459 | Dried fruit liking \|\| id:ebi-fl187-GCST90094756 | MR Egger | 5 | 0.513382971493473 | 1.17593705001641 | 0.691932418845318 | -1.791453647 | 2.81821958952563 | 1.67093436755081 | 0.16671764490965 | 16.7470075658489 | 0.999279047927446 |
| ebi-fl187-GCST90094756 | ebi-met1400-GCST90200462 | X-11299 levels \|\| id:ebi-met1400-GCST90200462 | Dried fruit liking \|\| id:ebi-fl187-GCST90094756 | MR Egger | 5 | 0.741903913390041 | 0.696561365167464 | 0.364951838505265 | -0.623356362 | 2.10716418911827 | 2.09992979536092 | 0.536141927774462 | 8.22488396635819 | 0.999279047927446 |
| ebi-fl187-GCST90094756 | ebi-met1400-GCST90200497 | X-12740 levels \|\| id:ebi-met1400-GCST90200497 | Dried fruit liking \|\| id:ebi-fl187-GCST90094756 | MR Egger | 5 | 0.60246806144892 | 1.06003480299432 | 0.60955859070927 | -1.475200152 | 2.68013627531779 | 1.82662145568649 | 0.228732941011616 | 14.5870810195405 | 0.999279047927446 |
| ebi-fl187-GCST90094756 | ebi-met1400-GCST90200527 | X-13866 levels \|\| id:ebi-met1400-GCST90200527 | Dried fruit liking \|\| id:ebi-fl187-GCST90094756 | MR Egger | 5 | 1.44281188185665 | 0.684893646813706 | 0.125770320884718 | 0.10042033410179 | 2.78520342961152 | 4.23258061648207 | 1.10563555674572 | 16.2031137346462 | 0.999279047927446 |
| ebi-fl187-GCST90094756 | ebi-met1400-GCST90200547 | X-17654 levels \|\| id:ebi-met1400-GCST90200547 | Dried fruit liking \|\| id:ebi-fl187-GCST90094756 | MR Egger | 5 | -1.021449146 | 0.931488343710915 | 0.352963017082292 | -2.8471663 | 0.804268007202632 | 0.360072763734605 | 0.0580084667788362 | 2.23505985217281 | 0.999279047927446 |
| ebi-fl187-GCST90094756 | ebi-met1400-GCST90200553 | X-17653 levels \|\| id:ebi-met1400-GCST90200553 | Dried fruit liking \|\| id:ebi-fl187-GCST90094756 | MR Egger | 5 | -0.405178871 | 0.767621917707929 | 0.634163028697584 | -1.909717829 | 1.09936008802814 | 0.666857518932405 | 0.148122176424584 | 3.00224423709505 | 0.999279047927446 |
| ebi-fl187-GCST90094756 | ebi-met1400-GCST90200557 | X-17685 levels \|\| id:ebi-met1400-GCST90200557 | Dried fruit liking \|\| id:ebi-fl187-GCST90094756 | MR Egger | 5 | 0.125148420366786 | 0.743726983732487 | 0.877074178109933 | -1.332556468 | 1.58285330848246 | 1.13331664785731 | 0.26380199722418 | 4.86882827963975 | 0.999279047927446 |
| ebi-fl187-GCST90094756 | ebi-met1400-GCST90200561 | X-18779 levels \|\| id:ebi-met1400-GCST90200561 | Dried fruit liking \|\| id:ebi-fl187-GCST90094756 | MR Egger | 5 | -0.312126921 | 0.941029427998082 | 0.761946322243136 | -2.1565446 | 1.53229075815483 | 0.731888630500139 | 0.115724304780349 | 4.6287680748835 | 0.999279047927446 |
| ebi-fl187-GCST90094756 | ebi-met1400-GCST90200570 | X-18921 levels \|\| id:ebi-met1400-GCST90200570 | Dried fruit liking \|\| id:ebi-fl187-GCST90094756 | MR Egger | 5 | 1.3883678658977 | 0.701571236585966 | 0.142203893230856 | 0.013288242189202 | 2.76344748960619 | 4.00830262390168 | 1.01337692324922 | 15.8544067426192 | 0.999279047927446 |
| ebi-fl187-GCST90094756 | ebi-met1400-GCST90200575 | X-21283 levels \|\| id:ebi-met1400-GCST90200575 | Dried fruit liking \|\| id:ebi-fl187-GCST90094756 | MR Egger | 5 | -0.33208919 | 1.06846707075742 | 0.776289528133501 | -2.426284648 | 1.76210626912356 | 0.717423333328751 | 0.088364528759094 | 5.82469285393622 | 0.999279047927446 |
| ebi-fl187-GCST90094756 | ebi-met1400-GCST90200578 | X-18922 levels \|\| id:ebi-met1400-GCST90200578 | Dried fruit liking \|\| id:ebi-fl187-GCST90094756 | MR Egger | 5 | 1.08760858104104 | 0.702331639807306 | 0.219243877880337 | -0.288961433 | 2.46417859506336 | 2.96716983514653 | 0.749041093127474 | 11.7538235370288 | 0.999279047927446 |
| ebi-fl187-GCST90094756 | ebi-met1400-GCST90200623 | X-24241 levels \|\| id:ebi-met1400-GCST90200623 | Dried fruit liking \|\| id:ebi-fl187-GCST90094756 | MR Egger | 5 | -1.111287737 | 1.23760612246271 | 0.435393888664454 | -3.536995737 | 1.31442026255844 | 0.329134848784195 | 0.029100621795344 | 3.72259223345967 | 0.999279047927446 |
| ebi-fl187-GCST90094756 | ebi-met1400-GCST90200636 | X-24544 levels \|\| id:ebi-met1400-GCST90200636 | Dried fruit liking \|\| id:ebi-fl187-GCST90094756 | MR Egger | 5 | 1.45464323125414 | 0.813738252778254 | 0.171800975622866 | -0.140283744 | 3.04957020669952 | 4.28295516844997 | 0.869111595042443 | 21.1062711389283 | 0.999279047927446 |
| ebi-fl187-GCST90094756 | ebi-met1400-GCST90200659 | X-25172 levels \|\| id:ebi-met1400-GCST90200659 | Dried fruit liking \|\| id:ebi-fl187-GCST90094756 | MR Egger | 5 | -1.050844317 | 0.709682818094523 | 0.235262098117543 | -2.44182264 | 0.340134006755741 | 0.349642415517376 | 0.0870021332794446 | 1.40513587564765 | 0.999279047927446 |
| ebi-fl187-GCST90094756 | ebi-met1400-GCST90200670 | X-26111 levels \|\| id:ebi-met1400-GCST90200670 | Dried fruit liking \|\| id:ebi-fl187-GCST90094756 | MR Egger | 5 | 0.47793902497127 | 0.707614057473286 | 0.547807833417943 | -0.908984528 | 1.86486257761891 | 1.61274714308212 | 0.402933183851278 | 6.45504875686671 | 0.999279047927446 |
| ebi-fl187-GCST90094756 | ebi-met1400-GCST90200673 | Carnitine C4 levels \|\| id:ebi-met1400-GCST90200673 | Dried fruit liking \|\| id:ebi-fl187-GCST90094756 | MR Egger | 5 | -1.783938313 | 0.700797697302057 | 0.0842673309213568 | -3.1575018 | -0.410374826 | 0.167975303544054 | 0.0425318615748322 | 0.663401543124868 | 0.999279047927446 |
| ebi-fl187-GCST90094756 | ebi-met1400-GCST90200710 | X-13431 levels \|\| id:ebi-met1400-GCST90200710 | Dried fruit liking \|\| id:ebi-fl187-GCST90094756 | MR Egger | 5 | 0.19739556686655 | 0.691517834060114 | 0.793871377142321 | -1.157979388 | 1.55277052162437 | 1.21822583518503 | 0.314120255249049 | 4.72454151145277 | 0.999279047927446 |
| ebi-fl187-GCST90094756 | ebi-met1400-GCST90200730 | Adenosine 5'-diphosphate (ADP) to fructose ratio \|\| id:ebi-met1400-GCST90200730 | Dried fruit liking \|\| id:ebi-fl187-GCST90094756 | MR Egger | 5 | 0.229078754136758 | 1.38285860592402 | 0.878961996397862 | -2.481324113 | 2.93948162174783 | 1.25744106379599 | 0.0836324134389296 | 18.9060432899617 | 0.999279047927446 |
| ebi-fl187-GCST90094756 | ebi-met1400-GCST90200731 | Adenosine 5'-diphosphate (ADP) to mannose ratio \|\| id:ebi-met1400-GCST90200731 | Dried fruit liking \|\| id:ebi-fl187-GCST90094756 | MR Egger | 5 | 0.425218813879451 | 0.921998519637719 | 0.676041539887895 | -1.381898285 | 2.23233591236938 | 1.52992515189777 | 0.251101438357178 | 9.32161514375691 | 0.999279047927446 |
| ebi-fl187-GCST90094756 | ebi-met1400-GCST90200748 | Aspartate to glutamate ratio \|\| id:ebi-met1400-GCST90200748 | Dried fruit liking \|\| id:ebi-fl187-GCST90094756 | MR Egger | 5 | 2.21236496752813 | 0.679482341650387 | 0.0472759066690252 | 0.880579577893373 | 3.54415035716289 | 9.13730027884002 | 2.41229741559173 | 34.6102664812622 | 0.999279047927446 |
| ebi-fl187-GCST90094756 | ebi-met1400-GCST90200774 | Uridine to pseudouridine ratio \|\| id:ebi-met1400-GCST90200774 | Dried fruit liking \|\| id:ebi-fl187-GCST90094756 | MR Egger | 5 | -0.011940709 | 0.667693211137714 | 0.986854661176117 | -1.320619403 | 1.2967379847394 | 0.988130298269192 | 0.266969888813644 | 3.6573468667065 | 0.999279047927446 |
| ebi-fl187-GCST90094756 | ebi-met1400-GCST90200799 | Spermidine to adenosine 5'-diphosphate (ADP) ratio \|\| id:ebi-met1400-GCST90200799 | Dried fruit liking \|\| id:ebi-fl187-GCST90094756 | MR Egger | 5 | -1.024223869 | 0.964098469014272 | 0.366010136555774 | -2.913856869 | 0.865409130006569 | 0.35907504646533 | 0.0542660287957273 | 2.37597797103278 | 0.999279047927446 |
| ebi-fl187-GCST90094756 | ebi-met1400-GCST90200811 | Choline to taurocholate ratio \|\| id:ebi-met1400-GCST90200811 | Dried fruit liking \|\| id:ebi-fl187-GCST90094756 | MR Egger | 5 | -0.056770976 | 0.732305839445364 | 0.943087980051172 | -1.492090422 | 1.37854846887905 | 0.944810428443558 | 0.224902023720048 | 3.96913611949916 | 0.999279047927446 |
| ebi-fl187-GCST90094756 | ebi-met1400-GCST90200812 | Glutamate to kynurenine ratio \|\| id:ebi-met1400-GCST90200812 | Dried fruit liking \|\| id:ebi-fl187-GCST90094756 | MR Egger | 5 | -1.875456364 | 0.679446107784248 | 0.0701363323998845 | -3.207170735 | -0.543741993 | 0.153284997158429 | 0.0404709544982113 | 0.580571687650658 | 0.999279047927446 |
| ebi-fl187-GCST90094756 | ebi-met1400-GCST90200876 | Cortisone to 4-cholesten-3-one ratio \|\| id:ebi-met1400-GCST90200876 | Dried fruit liking \|\| id:ebi-fl187-GCST90094756 | MR Egger | 5 | 0.663514309478094 | 0.740507993121674 | 0.436265714133644 | -0.787881357 | 2.11490997599657 | 1.94160375515092 | 0.454807349657116 | 8.28883953801154 | 0.999279047927446 |
| ebi-fl187-GCST90094756 | ebi-met1400-GCST90200906 | 2'-deoxyuridine to cytidine ratio \|\| id:ebi-met1400-GCST90200906 | Dried fruit liking \|\| id:ebi-fl187-GCST90094756 | MR Egger | 5 | -0.895630457 | 1.0901750626872 | 0.471541508676604 | -3.03237358 | 1.24111266615578 | 0.408350070426792 | 0.0482010931087467 | 3.45946054877555 | 0.999279047927446 |
| ebi-fl187-GCST90094756 | ebi-met1400-GCST90200907 | Retinol (Vitamin A) to linoleoyl-arachidonoyl-glycerol (18:2 to 20:4) [1] ratio \|\| id:ebi-met1400-GCST90200907 | Dried fruit liking \|\| id:ebi-fl187-GCST90094756 | MR Egger | 5 | 0.981125604038709 | 0.731883186606877 | 0.272541969956342 | -0.453365442 | 2.41561664978819 | 2.6674570531821 | 0.635485858148253 | 11.196672655005 | 0.999279047927446 |
| ebi-fl187-GCST90094756 | ebi-met1400-GCST90200916 | Fructose to sucrose ratio \|\| id:ebi-met1400-GCST90200916 | Dried fruit liking \|\| id:ebi-fl187-GCST90094756 | MR Egger | 5 | 0.104682555609674 | 0.871219328484238 | 0.911954588823664 | -1.602907328 | 1.81227243943878 | 1.11035807746093 | 0.201310390995123 | 6.12434884303907 | 0.999279047927446 |
| ebi-fl187-GCST90094756 | ebi-met1400-GCST90200917 | Alpha-ketobutyrate to 3-methyl-2-oxobutyrate ratio \|\| id:ebi-met1400-GCST90200917 | Dried fruit liking \|\| id:ebi-fl187-GCST90094756 | MR Egger | 5 | 0.883124890575262 | 0.873452765686296 | 0.386448693794006 | -0.82884253 | 2.5950923113204 | 2.41844528760529 | 0.436554292423182 | 13.3978240751565 | 0.999279047927446 |
| ebi-fl187-GCST90094756 | ebi-met1400-GCST90200924 | Alpha-tocopherol to glycerol ratio \|\| id:ebi-met1400-GCST90200924 | Dried fruit liking \|\| id:ebi-fl187-GCST90094756 | MR Egger | 5 | 0.769210870029471 | 0.654654564128797 | 0.324770655906796 | -0.513912076 | 2.05233381572191 | 2.15806258979928 | 0.598150983779461 | 7.78605112719886 | 0.999279047927446 |
| ebi-fl187-GCST90094756 | ebi-met1400-GCST90200938 | Alpha-ketoglutarate to alanine ratio \|\| id:ebi-met1400-GCST90200938 | Dried fruit liking \|\| id:ebi-fl187-GCST90094756 | MR Egger | 5 | 0.242641838687494 | 0.695111137207802 | 0.750101117152827 | -1.11977599 | 1.60505966761479 | 1.27461202563935 | 0.326352892668624 | 4.97815662861025 | 0.999279047927446 |
| ebi-fl187-GCST90094756 | ebi-met1400-GCST90200943 | Phosphate to EDTA ratio \|\| id:ebi-met1400-GCST90200943 | Dried fruit liking \|\| id:ebi-fl187-GCST90094756 | MR Egger | 5 | 0.205148411644295 | 0.824469088405923 | 0.819558328050977 | -1.410811002 | 1.8211078249199 | 1.22770725749589 | 0.24394536282023 | 6.17869957716243 | 0.999279047927446 |
| ebi-fl187-GCST90094756 | ebi-met1400-GCST90200947 | Adenosine 5'-diphosphate (ADP) to glucose ratio \|\| id:ebi-met1400-GCST90200947 | Dried fruit liking \|\| id:ebi-fl187-GCST90094756 | MR Egger | 5 | 0.785018622992954 | 0.924410223423575 | 0.458157360938392 | -1.026825415 | 2.59686266090316 | 2.19244777029241 | 0.358142110402135 | 13.4215639151254 | 0.999279047927446 |
| ebi-fl187-GCST90094756 | ebi-met1400-GCST90200975 | Arachidonate (20:4n6) to caffeine ratio \|\| id:ebi-met1400-GCST90200975 | Dried fruit liking \|\| id:ebi-fl187-GCST90094756 | MR Egger | 5 | 1.10734049107068 | 0.715547320291681 | 0.219476750476274 | -0.295132257 | 2.50981323884237 | 3.02629921267405 | 0.744433124685673 | 12.3026321921108 | 0.999279047927446 |
| ebi-fl187-GCST90094756 | ebi-met1400-GCST90200985 | Cholesterol to oleoyl-linoleoyl-glycerol (18:1 to 18:2) [2] ratio \|\| id:ebi-met1400-GCST90200985 | Dried fruit liking \|\| id:ebi-fl187-GCST90094756 | MR Egger | 5 | 0.851966071404517 | 0.699896157468726 | 0.310518471077751 | -0.519830397 | 2.22376254004322 | 2.34425128954162 | 0.594621388850669 | 9.24203907151692 | 0.999279047927446 |
| ebi-fl187-GCST90094756 | ebi-met1400-GCST90201016 | Bilirubin (Z,Z) to etiocholanolone glucuronide ratio \|\| id:ebi-met1400-GCST90201016 | Dried fruit liking \|\| id:ebi-fl187-GCST90094756 | MR Egger | 5 | -1.630386222 | 0.717834586418194 | 0.107800138494122 | -3.037342012 | -0.223430433 | 0.195853916397553 | 0.047962203203362 | 0.799770527755297 | 0.999279047927446 |
| ebi-fl187-GCST90094756 | ebi-pw11-GCST90275189 | Placental weight (fetal genotype, adjusted for sex and gestational age) \|\| id:ebi-pw11-GCST90275189 | Dried fruit liking \|\| id:ebi-fl187-GCST90094756 | MR Egger | 5 | 0.370896510999269 | 0.482561529418142 | 0.498094781956116 | -0.574924087 | 1.31671710865883 | 1.44903310663859 | 0.562747587234247 | 3.73115228170792 | 0.999279047927446 |
| ebi-fl187-GCST90094756 | ebi-pw11-GCST90275192 | Placental weight (fetal effect, conditional on maternal and paternal genotype) \|\| id:ebi-pw11-GCST90275192 | Dried fruit liking \|\| id:ebi-fl187-GCST90094756 | MR Egger | 5 | 0.216379504926924 | 0.837907167067721 | 0.812925941498266 | -1.425918543 | 1.85867755237966 | 1.24157347285388 | 0.240287647386955 | 6.41524733067965 | 0.999279047927446 |
| ebi-fl187-GCST90094756 | ebi-pw11-GCST90275193 | Placental weight (maternal effect, conditional on fetal and paternal genotype) \|\| id:ebi-pw11-GCST90275193 | Dried fruit liking \|\| id:ebi-fl187-GCST90094756 | MR Egger | 5 | 0.19253642235865 | 0.588741865984542 | 0.765134519328948 | -0.961397635 | 1.34647047968835 | 1.21232065848115 | 0.382358115281668 | 3.84383466765831 | 0.999279047927446 |
| ebi-fl187-GCST90094756 | ebi-pw11-GCST90275195 | Placental weight (fetal effect, conditional on maternal genotype) \|\| id:ebi-pw11-GCST90275195 | Dried fruit liking \|\| id:ebi-fl187-GCST90094756 | MR Egger | 5 | 0.299133613359732 | 0.631643874685395 | 0.66812995527168 | -0.938888381 | 1.53715560774311 | 1.34868981441202 | 0.391062306111227 | 4.6513411982524 | 0.999279047927446 |
| ebi-fl187-GCST90094756 | ebi-pw11-GCST90275197 | Placental weight (fetal genotype, adjusted for sex) \|\| id:ebi-pw11-GCST90275197 | Dried fruit liking \|\| id:ebi-fl187-GCST90094756 | MR Egger | 5 | 0.419981304983275 | 0.49142246956098 | 0.455577242346829 | -0.543206735 | 1.3831693453228 | 1.52193310278786 | 0.580882525993481 | 3.98751944792961 | 0.999279047927446 |
| ebi-fl187-GCST90094756 | prot-a-1030 | Protein FAM171B \|\| id:prot-a-1030 | Dried fruit liking \|\| id:ebi-fl187-GCST90094756 | MR Egger | 5 | 1.42910116776491 | 1.16705538857374 | 0.308136305568379 | -0.858327394 | 3.71652972936944 | 4.17494492986742 | 0.423870458075149 | 41.1214436754529 | 0.999279047927446 |
| ebi-fl187-GCST90094756 | prot-a-1036 | BRISC complex subunit Abro1 \|\| id:prot-a-1036 | Dried fruit liking \|\| id:ebi-fl187-GCST90094756 | MR Egger | 5 | 0.838646675495435 | 1.16695035192794 | 0.524264476328139 | -1.448576014 | 3.1258693652742 | 2.31323430083153 | 0.234904550769753 | 22.7796903593771 | 0.999279047927446 |
| ebi-fl187-GCST90094756 | prot-a-1055 | Phenylalanine--tRNA ligase, mitochondrial \|\| id:prot-a-1055 | Dried fruit liking \|\| id:ebi-fl187-GCST90094756 | MR Egger | 5 | -0.540226002 | 2.10526318435817 | 0.814073419389228 | -4.666541843 | 3.5860898394943 | 0.582616565073541 | 0.00940473641012933 | 36.0926715109737 | 0.999279047927446 |
| ebi-fl187-GCST90094756 | prot-a-1083 | Fc receptor-like protein 5 \|\| id:prot-a-1083 | Dried fruit liking \|\| id:ebi-fl187-GCST90094756 | MR Egger | 5 | 0.662507417745874 | 1.16705538857374 | 0.609961949573118 | -1.624921144 | 2.94993597935041 | 1.93964975428135 | 0.196927203511328 | 19.1047305918164 | 0.999279047927446 |
| ebi-fl187-GCST90094756 | prot-a-1103 | Fibroblast growth factor receptor 3 \|\| id:prot-a-1103 | Dried fruit liking \|\| id:ebi-fl187-GCST90094756 | MR Egger | 5 | -0.532191504 | 1.46994022404664 | 0.741315151370497 | -3.413274343 | 2.34889133506441 | 0.587316451905107 | 0.0329331890757032 | 10.473951182969 | 0.999279047927446 |
| ebi-fl187-GCST90094756 | prot-a-1105 | Fibroblast growth factor receptor 4 \|\| id:prot-a-1105 | Dried fruit liking \|\| id:ebi-fl187-GCST90094756 | MR Egger | 5 | -0.856761035 | 1.16705538857374 | 0.516046989334132 | -3.144189597 | 1.43066752657578 | 0.424534911552251 | 0.0431018397730722 | 4.18148951589014 | 0.999279047927446 |
| ebi-fl187-GCST90094756 | prot-a-1120 | Filamin-A \|\| id:prot-a-1120 | Dried fruit liking \|\| id:ebi-fl187-GCST90094756 | MR Egger | 5 | -0.517956684 | 1.16705538857374 | 0.687235520597377 | -2.805385245 | 1.76947187781237 | 0.595736583391098 | 0.0604834657069662 | 5.86775365204679 | 0.999279047927446 |
| ebi-fl187-GCST90094756 | prot-a-1166 | Galanin peptides \|\| id:prot-a-1166 | Dried fruit liking \|\| id:ebi-fl187-GCST90094756 | MR Egger | 5 | -3.578633583 | 1.1653753266492 | 0.0545281316651547 | -5.862769223 | -1.294497943 | 0.0279138141162013 | 0.00284335887464546 | 0.27403541123911 | 0.999279047927446 |
| ebi-fl187-GCST90094756 | prot-a-1205 | ADP-ribosylation factor-binding protein GGA1 \|\| id:prot-a-1205 | Dried fruit liking \|\| id:ebi-fl187-GCST90094756 | MR Egger | 5 | 0.581957911949767 | 1.28465823165935 | 0.681301299016254 | -1.935972222 | 3.0998880460021 | 1.78953876228867 | 0.144283924591182 | 22.1954662711565 | 0.999279047927446 |
| ebi-fl187-GCST90094756 | prot-a-1227 | Guanine nucleotide-binding protein G(I)/G(S)/G(O) subunit gamma-T2 \|\| id:prot-a-1227 | Dried fruit liking \|\| id:ebi-fl187-GCST90094756 | MR Egger | 5 | 1.43754783429288 | 1.19430635764558 | 0.315033703503719 | -0.903292627 | 3.77838829527821 | 4.21035865085596 | 0.405233179099954 | 43.74548009 | 0.999279047927446 |
| ebi-fl187-GCST90094756 | prot-a-1240 | Golgi SNAP receptor complex member 1 \|\| id:prot-a-1240 | Dried fruit liking \|\| id:ebi-fl187-GCST90094756 | MR Egger | 5 | 0.557894504554746 | 1.16705538857374 | 0.665297593019825 | -1.729534057 | 2.84532306615928 | 1.74699034506142 | 0.177367033638348 | 17.2071168081933 | 0.999279047927446 |
| ebi-fl187-GCST90094756 | prot-a-1283 | Glutathione S-transferase A1 \|\| id:prot-a-1283 | Dried fruit liking \|\| id:ebi-fl187-GCST90094756 | MR Egger | 5 | -1.385028885 | 1.33574366791282 | 0.376025035303201 | -4.003086474 | 1.23302870400366 | 0.250316569266766 | 0.0182591952922412 | 3.43160713528864 | 0.999279047927446 |
| ebi-fl187-GCST90094756 | prot-a-1324 | E3 ubiquitin-protein ligase HECW1 \|\| id:prot-a-1324 | Dried fruit liking \|\| id:ebi-fl187-GCST90094756 | MR Egger | 5 | -1.469079946 | 1.8196538255015 | 0.478545508191638 | -5.035601444 | 2.0974415516027 | 0.230137126307306 | 0.00650228617711531 | 8.14530389194306 | 0.999279047927446 |
| ebi-fl187-GCST90094756 | prot-a-1339 | Histidine triad nucleotide-binding protein 2, mitochondrial \|\| id:prot-a-1339 | Dried fruit liking \|\| id:ebi-fl187-GCST90094756 | MR Egger | 5 | -1.267664166 | 1.16705538857374 | 0.356868844594717 | -3.555092728 | 1.01976439530462 | 0.281488364466824 | 0.0285787247481529 | 2.77254146322008 | 0.999279047927446 |
| ebi-fl187-GCST90094756 | prot-a-1466 | Interleukin-11 \|\| id:prot-a-1466 | Dried fruit liking \|\| id:ebi-fl187-GCST90094756 | MR Egger | 5 | -0.08900534 | 1.16705538857374 | 0.944009625768073 | -2.376433902 | 2.19842322132823 | 0.914840688059352 | 0.0928812111363147 | 9.01079426387545 | 0.999279047927446 |
| ebi-fl187-GCST90094756 | prot-a-147 | Rho GTPase-activating protein 1 \|\| id:prot-a-147 | Dried fruit liking \|\| id:ebi-fl187-GCST90094756 | MR Egger | 5 | -0.864352403 | 1.16705538857374 | 0.512620088956067 | -3.151780965 | 1.4230761585461 | 0.421324312634783 | 0.0427758766629705 | 4.14986647300767 | 0.999279047927446 |
| ebi-fl187-GCST90094756 | prot-a-1473 | Interleukin-12 receptor subunit beta-1 \|\| id:prot-a-1473 | Dried fruit liking \|\| id:ebi-fl187-GCST90094756 | MR Egger | 5 | -1.113852235 | 1.37234718585576 | 0.476416327231108 | -3.80365272 | 1.57594824894513 | 0.328291864545261 | 0.0222892067570358 | 4.83532453628409 | 0.999279047927446 |
| ebi-fl187-GCST90094756 | prot-a-1501 | X-linked interleukin-1 receptor accessory protein-like 2 \|\| id:prot-a-1501 | Dried fruit liking \|\| id:ebi-fl187-GCST90094756 | MR Egger | 5 | -0.076866375 | 1.71848729763378 | 0.9671340228559 | -3.445101478 | 3.29136872885319 | 0.926013584359396 | 0.0319015245795427 | 26.8796294133234 | 0.999279047927446 |
| ebi-fl187-GCST90094756 | prot-a-1503 | Interleukin-1 receptor-like 2 \|\| id:prot-a-1503 | Dried fruit liking \|\| id:ebi-fl187-GCST90094756 | MR Egger | 5 | -1.528680322 | 1.29556378065302 | 0.323069603981045 | -4.067985332 | 1.0106246882788 | 0.216821613352318 | 0.0171118284585524 | 2.74731669561624 | 0.999279047927446 |
| ebi-fl187-GCST90094756 | prot-a-1536 | Interleukin-5 receptor subunit alpha \|\| id:prot-a-1536 | Dried fruit liking \|\| id:ebi-fl187-GCST90094756 | MR Egger | 5 | 0.262709253240851 | 1.46294995726001 | 0.868930382893665 | -2.604672663 | 3.13009116947047 | 1.30044856277548 | 0.0739273323893822 | 22.8760650461088 | 0.999279047927446 |
| ebi-fl187-GCST90094756 | prot-a-1559 | Insulin-induced gene 1 protein \|\| id:prot-a-1559 | Dried fruit liking \|\| id:ebi-fl187-GCST90094756 | MR Egger | 5 | 0.0635856797580247 | 1.5186861463196 | 0.969233956315241 | -2.913039167 | 3.04021052654444 | 1.06565078659163 | 0.0543104203559754 | 20.9096448070194 | 0.999279047927446 |
| ebi-fl187-GCST90094756 | prot-a-1567 | Interferon regulatory factor 1 \|\| id:prot-a-1567 | Dried fruit liking \|\| id:ebi-fl187-GCST90094756 | MR Egger | 5 | -1.009465627 | 1.16705538857374 | 0.450682547942122 | -3.296894188 | 1.27796293499469 | 0.36441366051392 | 0.0369978976503008 | 3.58932059395204 | 0.999279047927446 |
| ebi-fl187-GCST90094756 | prot-a-1613 | Potassium voltage-gated channel subfamily E regulatory beta subunit 5 \|\| id:prot-a-1613 | Dried fruit liking \|\| id:ebi-fl187-GCST90094756 | MR Egger | 5 | 1.21883829935451 | 1.16705538857374 | 0.373059065187793 | -1.068590262 | 3.50626686095905 | 3.38325512014688 | 0.343492410475291 | 33.3236335328681 | 0.999279047927446 |
| ebi-fl187-GCST90094756 | prot-a-1649 | Kin of IRRE-like protein 2 \|\| id:prot-a-1649 | Dried fruit liking \|\| id:ebi-fl187-GCST90094756 | MR Egger | 5 | -2.640330505 | 1.16695035192794 | 0.108665646855314 | -4.927553195 | -0.353107815 | 0.071337688212227 | 0.00724420677854469 | 0.702501449094096 | 0.999279047927446 |
| ebi-fl187-GCST90094756 | prot-a-165 | Armadillo repeat-containing protein 5 \|\| id:prot-a-165 | Dried fruit liking \|\| id:ebi-fl187-GCST90094756 | MR Egger | 5 | 1.49308605329623 | 1.2766631581202 | 0.326660371314541 | -1.009173737 | 3.99534584321183 | 4.45080978284612 | 0.364520044739309 | 54.344632096284 | 0.999279047927446 |
| ebi-fl187-GCST90094756 | prot-a-1656 | Kallikrein-11 \|\| id:prot-a-1656 | Dried fruit liking \|\| id:ebi-fl187-GCST90094756 | MR Egger | 5 | -0.783582367 | 2.13596458021058 | 0.738078158015826 | -4.970072944 | 3.40290821015114 | 0.456766770643446 | 0.00694264159032258 | 30.0513687837295 | 0.999279047927446 |
| ebi-fl187-GCST90094756 | prot-a-1697 | Laminin subunit gamma-2 \|\| id:prot-a-1697 | Dried fruit liking \|\| id:ebi-fl187-GCST90094756 | MR Egger | 5 | 0.365995114892647 | 1.31539217667007 | 0.798903163739909 | -2.212173551 | 2.94416378116598 | 1.44194819856556 | 0.109462467462617 | 18.9947719573976 | 0.999279047927446 |
| ebi-fl187-GCST90094756 | prot-a-1698 | Lysosome-associated membrane glycoprotein 3 \|\| id:prot-a-1698 | Dried fruit liking \|\| id:ebi-fl187-GCST90094756 | MR Egger | 5 | 1.92744331246924 | 1.35193370909664 | 0.24919708371109 | -0.722346757 | 4.57723338229866 | 6.87191841463855 | 0.485611305810923 | 97.2449820100258 | 0.999279047927446 |
| ebi-fl187-GCST90094756 | prot-a-1724 | Leptin receptor \|\| id:prot-a-1724 | Dried fruit liking \|\| id:ebi-fl187-GCST90094756 | MR Egger | 5 | 1.0646320385945 | 1.16695035192794 | 0.428868675018077 | -1.222590651 | 3.35185472837327 | 2.89977178362625 | 0.294466318402192 | 28.5556475278444 | 0.999279047927446 |
| ebi-fl187-GCST90094756 | prot-a-1743 | Leukocyte immunoglobulin-like receptor subfamily B member 2 \|\| id:prot-a-1743 | Dried fruit liking \|\| id:ebi-fl187-GCST90094756 | MR Egger | 5 | 1.17287233542699 | 1.6541599104696 | 0.529432284786149 | -2.069281089 | 4.41502575994739 | 3.23126058608474 | 0.126276530656059 | 82.6839708134134 | 0.999279047927446 |
| ebi-fl187-GCST90094756 | prot-a-1746 | Leukocyte immunoglobulin-like receptor subfamily B member 4 \|\| id:prot-a-1746 | Dried fruit liking \|\| id:ebi-fl187-GCST90094756 | MR Egger | 5 | -2.20472421 | 1.16695035192794 | 0.155261123206199 | -4.4919469 | 0.0824984798047375 | 0.110280935492943 | 0.0111988195926049 | 1.08599702251027 | 0.999279047927446 |
| ebi-fl187-GCST90094756 | prot-a-1750 | Leucine-rich repeat and immunoglobulin-like domain-containing nogo receptor-interacting protein 3 \|\| id:prot-a-1750 | Dried fruit liking \|\| id:ebi-fl187-GCST90094756 | MR Egger | 5 | 0.456877398017597 | 2.00008847399229 | 0.83399807145634 | -3.463296011 | 4.37705080704248 | 1.57913526726652 | 0.0313263397109595 | 79.6029225033432 | 0.999279047927446 |
| ebi-fl187-GCST90094756 | prot-a-1753 | Lipase member N \|\| id:prot-a-1753 | Dried fruit liking \|\| id:ebi-fl187-GCST90094756 | MR Egger | 5 | 1.28305567687592 | 1.16695035192794 | 0.351871876202652 | -1.004167013 | 3.57027836665469 | 3.60764670272803 | 0.366349672290298 | 35.5264811630326 | 0.999279047927446 |
| ebi-fl187-GCST90094756 | prot-a-1760 | Ig Kappa chain V-I region HK102- like \|\| id:prot-a-1760 | Dried fruit liking \|\| id:ebi-fl187-GCST90094756 | MR Egger | 5 | -0.892772378 | 1.16705538857374 | 0.499955601532968 | -3.18020094 | 1.39465618355914 | 0.409518836468274 | 0.0415772997536812 | 4.03358752049501 | 0.999279047927446 |
| ebi-fl187-GCST90094756 | prot-a-1792 | Leucine-rich repeat neuronal protein 1 \|\| id:prot-a-1792 | Dried fruit liking \|\| id:ebi-fl187-GCST90094756 | MR Egger | 5 | 0.704264968766882 | 1.16705538857374 | 0.588779225065937 | -1.583163593 | 2.99169353037142 | 2.02235964097671 | 0.205324506505698 | 19.9193880314426 | 0.999279047927446 |
| ebi-fl187-GCST90094756 | prot-a-1795 | Leucine-rich repeat transmembrane neuronal protein 1 \|\| id:prot-a-1795 | Dried fruit liking \|\| id:ebi-fl187-GCST90094756 | MR Egger | 5 | 0.059400296153806 | 1.48048231177093 | 0.970516464220478 | -2.842345035 | 2.96114562722483 | 1.06119994998815 | 0.0582888162591348 | 19.320092706092 | 0.999279047927446 |
| ebi-fl187-GCST90094756 | prot-a-1820 | LysM and putative peptidoglycan-binding domain-containing protein 4 \|\| id:prot-a-1820 | Dried fruit liking \|\| id:ebi-fl187-GCST90094756 | MR Egger | 5 | -0.100977806 | 1.16705538857374 | 0.936501645761598 | -2.388406368 | 2.18645075540773 | 0.903953094820323 | 0.0917758243082039 | 8.90355607039965 | 0.999279047927446 |
| ebi-fl187-GCST90094756 | prot-a-1844 | Mitogen-activated protein kinase kinase kinase 7:TGF-beta-activated kinase 1 and MAP3K7-binding protein 1 fusion \|\| id:prot-a-1844 | Dried fruit liking \|\| id:ebi-fl187-GCST90094756 | MR Egger | 5 | -2.548597533 | 1.16535942990239 | 0.116585571798964 | -4.832702016 | -0.264493051 | 0.0781912497717428 | 0.0079649706874026 | 0.767594983185158 | 0.999279047927446 |
| ebi-fl187-GCST90094756 | prot-a-1856 | Megakaryocyte-associated tyrosine-protein kinase \|\| id:prot-a-1856 | Dried fruit liking \|\| id:ebi-fl187-GCST90094756 | MR Egger | 5 | -1.361171676 | 1.16695035192794 | 0.327732818898318 | -3.648394365 | 0.926051014100827 | 0.256360229869955 | 0.0260328945542547 | 2.52452017281479 | 0.999279047927446 |
| ebi-fl187-GCST90094756 | prot-a-1910 | Stromelysin-2 \|\| id:prot-a-1910 | Dried fruit liking \|\| id:ebi-fl187-GCST90094756 | MR Egger | 5 | 0.799584357623315 | 1.16705538857374 | 0.542452300982476 | -1.487844204 | 3.08701291922785 | 2.22461609158556 | 0.225859037094332 | 21.9115286180665 | 0.999279047927446 |
| ebi-fl187-GCST90094756 | prot-a-1940 | 39S ribosomal protein L14, mitochondrial \|\| id:prot-a-1940 | Dried fruit liking \|\| id:ebi-fl187-GCST90094756 | MR Egger | 5 | 0.631633167144535 | 1.16695035192794 | 0.625931997153773 | -1.655589523 | 2.9188558569233 | 1.8806795365506 | 0.190979435812654 | 18.5200857052999 | 0.999279047927446 |
| ebi-fl187-GCST90094756 | prot-a-1942 | 39S ribosomal protein L33, mitochondrial \|\| id:prot-a-1942 | Dried fruit liking \|\| id:ebi-fl187-GCST90094756 | MR Egger | 5 | 1.50654828187113 | 1.16705538857374 | 0.287219351660766 | -0.78088028 | 3.79397684347567 | 4.51113273111253 | 0.458002663339367 | 44.4327514808266 | 0.999279047927446 |
| ebi-fl187-GCST90094756 | prot-a-1948 | Mesothelin \|\| id:prot-a-1948 | Dried fruit liking \|\| id:ebi-fl187-GCST90094756 | MR Egger | 5 | -1.604782186 | 1.16695035192794 | 0.262772637922688 | -3.892004876 | 0.682440503502282 | 0.200933316177932 | 0.0204043967160987 | 1.97870087079841 | 0.999279047927446 |
| ebi-fl187-GCST90094756 | prot-a-1980 | Unconventional myosin-VI \|\| id:prot-a-1980 | Dried fruit liking \|\| id:ebi-fl187-GCST90094756 | MR Egger | 5 | 0.764773255012318 | 1.16695035192794 | 0.559006594634012 | -1.522449435 | 3.05199594479109 | 2.14850715619147 | 0.218176822023318 | 21.1575315718578 | 0.999279047927446 |
| ebi-fl187-GCST90094756 | prot-a-2039 | Neurofascin \|\| id:prot-a-2039 | Dried fruit liking \|\| id:ebi-fl187-GCST90094756 | MR Egger | 5 | -0.033792832 | 1.58641378226728 | 0.984342810164662 | -3.143163845 | 3.07557818136206 | 0.966771768180619 | 0.0431460742331046 | 21.662403089131 | 0.999279047927446 |
| ebi-fl187-GCST90094756 | prot-a-2041 | NFU1 iron-sulfur cluster scaffold homolog, mitochondrial \|\| id:prot-a-2041 | Dried fruit liking \|\| id:ebi-fl187-GCST90094756 | MR Egger | 5 | -0.695992561 | 1.76460991647629 | 0.719639219893318 | -4.154627997 | 2.76264287529083 | 0.498579331907722 | 0.0156916273718135 | 15.8416551907211 | 0.999279047927446 |
| ebi-fl187-GCST90094756 | prot-a-2088 | Glucocorticoid receptor \|\| id:prot-a-2088 | Dried fruit liking \|\| id:ebi-fl187-GCST90094756 | MR Egger | 5 | -0.834733796 | 1.16705538857374 | 0.526095463597632 | -3.122162358 | 1.45269476519622 | 0.433989995752767 | 0.0440617879732366 | 4.2746181005612 | 0.999279047927446 |
| ebi-fl187-GCST90094756 | prot-a-2111 | Neuron-specific protein family member 2 \|\| id:prot-a-2111 | Dried fruit liking \|\| id:ebi-fl187-GCST90094756 | MR Egger | 5 | -2.499129521 | 1.42657802569923 | 0.178095437705996 | -5.295222452 | 0.296963408964227 | 0.0821564829663494 | 0.00501549854482307 | 1.34576605557328 | 0.999279047927446 |
| ebi-fl187-GCST90094756 | prot-a-2125 | Nuclear migration protein nudC \|\| id:prot-a-2125 | Dried fruit liking \|\| id:ebi-fl187-GCST90094756 | MR Egger | 5 | 0.309871828214765 | 1.20479697340133 | 0.813657452996046 | -2.05153024 | 2.67127389608138 | 1.36325037269996 | 0.128538058981879 | 14.4583759346218 | 0.999279047927446 |
| ebi-fl187-GCST90094756 | prot-a-2142 | Ornithine decarboxylase \|\| id:prot-a-2142 | Dried fruit liking \|\| id:ebi-fl187-GCST90094756 | MR Egger | 5 | 1.65017696940593 | 1.1733854287122 | 0.254308874787551 | -0.649658471 | 3.95001240968185 | 5.20790138484791 | 0.522224101050717 | 51.9360113402096 | 0.999279047927446 |
| ebi-fl187-GCST90094756 | prot-a-2186 | Prostate and testis expressed protein 1 \|\| id:prot-a-2186 | Dried fruit liking \|\| id:ebi-fl187-GCST90094756 | MR Egger | 5 | -1.337076109 | 2.11011641789805 | 0.571319846217432 | -5.472904288 | 2.79875206985466 | 0.262612397083987 | 0.00419901926914767 | 16.4241378002051 | 0.999279047927446 |
| ebi-fl187-GCST90094756 | prot-a-2227 | Platelet-derived growth factor C \|\| id:prot-a-2227 | Dried fruit liking \|\| id:ebi-fl187-GCST90094756 | MR Egger | 5 | 0.115462483555248 | 1.16705538857374 | 0.927430185839933 | -2.171966078 | 2.40289104515979 | 1.12239240558572 | 0.113953355334629 | 11.0550909924254 | 0.999279047927446 |
| ebi-fl187-GCST90094756 | prot-a-2229 | Platelet-derived growth factor receptor alpha \|\| id:prot-a-2229 | Dried fruit liking \|\| id:ebi-fl187-GCST90094756 | MR Egger | 5 | -1.294555354 | 1.3469028797249 | 0.407387066389213 | -3.934484998 | 1.34537429012131 | 0.274019678963203 | 0.0195557679863611 | 3.83962340479111 | 0.999279047927446 |
| ebi-fl187-GCST90094756 | prot-a-2243 | Platelet endothelial aggregation receptor 1 \|\| id:prot-a-2243 | Dried fruit liking \|\| id:ebi-fl187-GCST90094756 | MR Egger | 5 | 0.256597984414122 | 1.16705538857374 | 0.840085475502717 | -2.030830577 | 2.54402654601866 | 1.29252540693989 | 0.131226482149257 | 12.7308291758134 | 0.999279047927446 |
| ebi-fl187-GCST90094756 | prot-a-2246 | E3 ubiquitin-protein ligase pellino homolog 2 \|\| id:prot-a-2246 | Dried fruit liking \|\| id:ebi-fl187-GCST90094756 | MR Egger | 5 | -0.357200814 | 1.83961536048874 | 0.858446074780671 | -3.96284692 | 3.24844529273287 | 0.699631987851307 | 0.0190089203025611 | 25.75027464 | 0.999279047927446 |
| ebi-fl187-GCST90094756 | prot-a-2251 | Platelet factor 4 variant \|\| id:prot-a-2251 | Dried fruit liking \|\| id:ebi-fl187-GCST90094756 | MR Egger | 5 | -0.644953635 | 1.16705538857374 | 0.619023944033121 | -2.932382197 | 1.64247492661534 | 0.524686868654973 | 0.0532699872929489 | 5.16794397988153 | 0.999279047927446 |
| ebi-fl187-GCST90094756 | prot-a-2346 | Protein phosphatase 1L \|\| id:prot-a-2346 | Dried fruit liking \|\| id:ebi-fl187-GCST90094756 | MR Egger | 5 | -1.999405041 | 1.62643122758947 | 0.306576850388119 | -5.187210247 | 1.18840016486446 | 0.135415826110319 | 0.00558757303891504 | 3.2818266237287 | 0.999279047927446 |
| ebi-fl187-GCST90094756 | prot-a-2358 | PR domain zinc finger protein 4 \|\| id:prot-a-2358 | Dried fruit liking \|\| id:ebi-fl187-GCST90094756 | MR Egger | 5 | -0.692238637 | 1.16705538857374 | 0.594825395923176 | -2.979667199 | 1.5951899243888 | 0.500454478097369 | 0.050809740593064 | 4.92926517089715 | 0.999279047927446 |
| ebi-fl187-GCST90094756 | prot-a-2390 | Trypsin-1 \|\| id:prot-a-2390 | Dried fruit liking \|\| id:ebi-fl187-GCST90094756 | MR Egger | 5 | -0.564255078 | 1.59282851435184 | 0.746590099162008 | -3.686198966 | 2.55768880999174 | 0.568783688389545 | 0.0250671020891509 | 12.9059547061897 | 0.999279047927446 |
| ebi-fl187-GCST90094756 | prot-a-242 | Brain-derived neurotrophic factor \|\| id:prot-a-242 | Dried fruit liking \|\| id:ebi-fl187-GCST90094756 | MR Egger | 5 | -2.002774217 | 1.20777670089399 | 0.195851088361455 | -4.370016551 | 0.364468116780671 | 0.134960354104851 | 0.01265103118255 | 1.43974802664549 | 0.999279047927446 |
| ebi-fl187-GCST90094756 | prot-a-2464 | Dihydropteridine reductase \|\| id:prot-a-2464 | Dried fruit liking \|\| id:ebi-fl187-GCST90094756 | MR Egger | 5 | -0.508871629 | 1.36683058580437 | 0.734414050128005 | -3.187859578 | 2.17011631884923 | 0.601173542837891 | 0.0412600905177366 | 8.75930285351414 | 0.999279047927446 |
| ebi-fl187-GCST90094756 | prot-a-2474 | Ras-related protein Rab-27A \|\| id:prot-a-2474 | Dried fruit liking \|\| id:ebi-fl187-GCST90094756 | MR Egger | 5 | 0.181060699264501 | 1.16695035192794 | 0.886548966713201 | -2.106161991 | 2.46828338904327 | 1.19848792442091 | 0.121704172978875 | 11.802169718799 | 0.999279047927446 |
| ebi-fl187-GCST90094756 | prot-a-2478 | Ras-related protein Rab-7b \|\| id:prot-a-2478 | Dried fruit liking \|\| id:ebi-fl187-GCST90094756 | MR Egger | 5 | 0.121211748329186 | 1.3959134572627 | 0.936275114942331 | -2.614778628 | 2.85720212456408 | 1.12886392211088 | 0.073183987795549 | 17.4127400409446 | 0.999279047927446 |
| ebi-fl187-GCST90094756 | prot-a-2528 | E3 ubiquitin-protein ligase rififylin \|\| id:prot-a-2528 | Dried fruit liking \|\| id:ebi-fl187-GCST90094756 | MR Egger | 5 | 1.06790093540727 | 1.16695035192794 | 0.427608484683777 | -1.219321754 | 3.35512362518603 | 2.90926634828892 | 0.295430473414953 | 28.6491457277608 | 0.999279047927446 |
| ebi-fl187-GCST90094756 | prot-a-2543 | Protein RIC-3 \|\| id:prot-a-2543 | Dried fruit liking \|\| id:ebi-fl187-GCST90094756 | MR Egger | 5 | 0.560675532974315 | 2.55942837682511 | 0.840658736584213 | -4.455804086 | 5.57715515155153 | 1.75185553684239 | 0.0116109798982922 | 264.31858885715 | 0.999279047927446 |
| ebi-fl187-GCST90094756 | prot-a-255 | Bone morphogenetic protein 6 \|\| id:prot-a-255 | Dried fruit liking \|\| id:ebi-fl187-GCST90094756 | MR Egger | 5 | -1.119053278 | 1.46425316646412 | 0.50033150671616 | -3.988989484 | 1.7508829284801 | 0.326588837217085 | 0.0185184178244411 | 5.75968582229712 | 0.999279047927446 |
| ebi-fl187-GCST90094756 | prot-a-2568 | RING finger protein 165 \|\| id:prot-a-2568 | Dried fruit liking \|\| id:ebi-fl187-GCST90094756 | MR Egger | 5 | 0.390617463097709 | 1.16705538857374 | 0.759884128696908 | -1.896811099 | 2.67804602470225 | 1.4778930566342 | 0.150046340113404 | 14.5566222088242 | 0.999279047927446 |
| ebi-fl187-GCST90094756 | prot-a-2573 | E3 ubiquitin-protein ligase RNF8 \|\| id:prot-a-2573 | Dried fruit liking \|\| id:ebi-fl187-GCST90094756 | MR Egger | 5 | -1.173561371 | 1.78438866171436 | 0.557701591359847 | -4.670963148 | 2.32384040625643 | 0.309263575447013 | 0.00936324699321985 | 10.2148281645809 | 0.999279047927446 |
| ebi-fl187-GCST90094756 | prot-a-260 | Cytoplasmic tyrosine-protein kinase BMX \|\| id:prot-a-260 | Dried fruit liking \|\| id:ebi-fl187-GCST90094756 | MR Egger | 5 | -1.82122854 | 1.16695035192794 | 0.216514445820483 | -4.108451229 | 0.465994150252238 | 0.161826818117978 | 0.0164332060953952 | 1.59359767717677 | 0.999279047927446 |
| ebi-fl187-GCST90094756 | prot-a-2623 | Serum amyloid A-1 protein \|\| id:prot-a-2623 | Dried fruit liking \|\| id:ebi-fl187-GCST90094756 | MR Egger | 5 | 0.500654929357113 | 1.16705538857374 | 0.696871245334568 | -1.786773632 | 2.78808349096165 | 1.64980142033425 | 0.1674997144914 | 16.2498469612416 | 0.999279047927446 |
| ebi-fl187-GCST90094756 | prot-a-2640 | Lysosome membrane protein 2 \|\| id:prot-a-2640 | Dried fruit liking \|\| id:ebi-fl187-GCST90094756 | MR Egger | 5 | -1.194950962 | 1.66804201891914 | 0.525488607804707 | -4.464313319 | 2.07441139506122 | 0.302718798536469 | 0.0115125985278376 | 7.95985986706472 | 0.999279047927446 |
| ebi-fl187-GCST90094756 | prot-a-2641 | Scavenger receptor class F member 2 \|\| id:prot-a-2641 | Dried fruit liking \|\| id:ebi-fl187-GCST90094756 | MR Egger | 5 | -1.993296767 | 1.73083232989498 | 0.332921272232958 | -5.385728134 | 1.39913459917858 | 0.136245514454499 | 0.00458150315917107 | 4.05169211153171 | 0.999279047927446 |
| ebi-fl187-GCST90094756 | prot-a-2669 | Semaphorin-3A \|\| id:prot-a-2669 | Dried fruit liking \|\| id:ebi-fl187-GCST90094756 | MR Egger | 5 | -1.22436245 | 1.16705538857374 | 0.371192175127462 | -3.511791012 | 1.06306611132682 | 0.293945045178277 | 0.0298434166298316 | 2.89523450537126 | 0.999279047927446 |
| ebi-fl187-GCST90094756 | prot-a-2683 | Sentrin-specific protease 7 \|\| id:prot-a-2683 | Dried fruit liking \|\| id:ebi-fl187-GCST90094756 | MR Egger | 5 | -0.978001999 | 1.16705538857374 | 0.463536943814978 | -3.265430561 | 1.30942656261459 | 0.376061720427616 | 0.038180492528615 | 3.7040490628817 | 0.999279047927446 |
| ebi-fl187-GCST90094756 | prot-a-270 | Bromodomain testis-specific protein \|\| id:prot-a-270 | Dried fruit liking \|\| id:ebi-fl187-GCST90094756 | MR Egger | 5 | -0.050606737 | 1.16695035192794 | 0.968134259915211 | -2.337829427 | 2.23661595277135 | 0.950652453458863 | 0.0965369515044991 | 9.3615975352737 | 0.999279047927446 |
| ebi-fl187-GCST90094756 | prot-a-2708 | 14-3-3 protein sigma \|\| id:prot-a-2708 | Dried fruit liking \|\| id:ebi-fl187-GCST90094756 | MR Egger | 5 | -0.844385536 | 1.16705538857374 | 0.521673299316829 | -3.131814098 | 1.44304302522414 | 0.42982138668953 | 0.0436385607779436 | 4.23355906249519 | 0.999279047927446 |
| ebi-fl187-GCST90094756 | prot-a-2713 | Beta-sarcoglycan \|\| id:prot-a-2713 | Dried fruit liking \|\| id:ebi-fl187-GCST90094756 | MR Egger | 5 | -0.355694905 | 1.16705538857374 | 0.780454423775774 | -2.643123466 | 1.93173365706329 | 0.700686363855115 | 0.0711387227863889 | 6.90146464910162 | 0.999279047927446 |
| ebi-fl187-GCST90094756 | prot-a-2720 | Protein shisa-3 homolog \|\| id:prot-a-2720 | Dried fruit liking \|\| id:ebi-fl187-GCST90094756 | MR Egger | 5 | 0.280600759351418 | 1.33030663167886 | 0.846457558293682 | -2.326800239 | 2.88800175744198 | 1.32392493375974 | 0.0976075688634839 | 17.9573905040315 | 0.999279047927446 |
| ebi-fl187-GCST90094756 | prot-a-2730 | Sialic acid-binding Ig-like lectin 8 \|\| id:prot-a-2730 | Dried fruit liking \|\| id:ebi-fl187-GCST90094756 | MR Egger | 5 | 0.0661906110336904 | 1.16705538857374 | 0.958337612017433 | -2.221237951 | 2.35361917263823 | 1.06843035237171 | 0.108474739305261 | 10.5235875668408 | 0.999279047927446 |
| ebi-fl187-GCST90094756 | prot-a-2761 | Sodium- and chloride-dependent neutral and basic amino acid transporter B(0+) \|\| id:prot-a-2761 | Dried fruit liking \|\| id:ebi-fl187-GCST90094756 | MR Egger | 5 | -1.402692358 | 1.16695035192794 | 0.315586458239777 | -3.689915048 | 0.88453033203903 | 0.245933929660398 | 0.0249741235659306 | 2.42184666054574 | 0.999279047927446 |
| ebi-fl187-GCST90094756 | prot-a-2770 | Secreted Ly-6/uPAR-related protein 1 \|\| id:prot-a-2770 | Dried fruit liking \|\| id:ebi-fl187-GCST90094756 | MR Egger | 5 | -0.409524717 | 1.58681274267353 | 0.813037085718669 | -3.519677693 | 2.70062825864006 | 0.663965746787278 | 0.0296089768212834 | 14.8890829820873 | 0.999279047927446 |
| ebi-fl187-GCST90094756 | prot-a-2784 | N-lysine methyltransferase SMYD2 \|\| id:prot-a-2784 | Dried fruit liking \|\| id:ebi-fl187-GCST90094756 | MR Egger | 5 | -1.083832752 | 1.63788911584564 | 0.555435054189391 | -4.294095419 | 2.12642991509221 | 0.33829643137001 | 0.0136489126614938 | 8.38487858454496 | 0.999279047927446 |
| ebi-fl187-GCST90094756 | prot-a-2837 | Serine palmitoyltransferase 2 \|\| id:prot-a-2837 | Dried fruit liking \|\| id:ebi-fl187-GCST90094756 | MR Egger | 5 | -0.874591964 | 1.16693446916665 | 0.507986641736526 | -3.161783524 | 1.4125995953852 | 0.417032149021007 | 0.0423501412008949 | 4.10661708285885 | 0.999279047927446 |
| ebi-fl187-GCST90094756 | prot-a-2858 | Alpha-2,8-sialyltransferase 8B \|\| id:prot-a-2858 | Dried fruit liking \|\| id:ebi-fl187-GCST90094756 | MR Egger | 5 | 2.27787854351058 | 1.16682405332298 | 0.145959067068789 | -0.009096601 | 4.56485368802361 | 9.75596159223433 | 0.99094464790274 | 96.048539936706 | 0.999279047927446 |
| ebi-fl187-GCST90094756 | prot-a-2911 | Synaptotagmin-11 \|\| id:prot-a-2911 | Dried fruit liking \|\| id:ebi-fl187-GCST90094756 | MR Egger | 5 | -2.309214454 | 1.29969469780925 | 0.173686276701094 | -4.856616062 | 0.238187153342232 | 0.0993392564368265 | 0.0077767554731833 | 1.2689466582112 | 0.999279047927446 |
| ebi-fl187-GCST90094756 | prot-a-2962 | Transforming growth factor beta-1 \|\| id:prot-a-2962 | Dried fruit liking \|\| id:ebi-fl187-GCST90094756 | MR Egger | 5 | -2.181131055 | 1.23018230882723 | 0.174337222100434 | -4.59228838 | 0.230026270705018 | 0.112913746776526 | 0.0101296513397456 | 1.25863307467339 | 0.999279047927446 |
| ebi-fl187-GCST90094756 | prot-a-2978 | Tyrosine-protein kinase receptor Tie-1, soluble \|\| id:prot-a-2978 | Dried fruit liking \|\| id:ebi-fl187-GCST90094756 | MR Egger | 5 | 1.9412946684433 | 1.30900095074316 | 0.234695676299503 | -0.624347195 | 4.50693653189989 | 6.96776608045358 | 0.535610963925535 | 90.6437086277966 | 0.999279047927446 |
| ebi-fl187-GCST90094756 | prot-a-299 | Uncharacterized protein C1orf226 \|\| id:prot-a-299 | Dried fruit liking \|\| id:ebi-fl187-GCST90094756 | MR Egger | 5 | 3.07532960776295 | 1.16548034615352 | 0.0777438838184548 | 0.790988129302046 | 5.35967108622386 | 21.6570190568217 | 2.20557474288075 | 212.654989789615 | 0.999279047927446 |
| ebi-fl187-GCST90094756 | prot-a-3015 | Transmembrane protein 70, mitochondrial \|\| id:prot-a-3015 | Dried fruit liking \|\| id:ebi-fl187-GCST90094756 | MR Egger | 5 | -0.196561146 | 1.38048478087991 | 0.895800318542209 | -2.902311316 | 2.50918902486714 | 0.821551095477523 | 0.0548961908563726 | 12.2949551134834 | 0.999279047927446 |
| ebi-fl187-GCST90094756 | prot-a-3016 | Transmembrane protein 87B \|\| id:prot-a-3016 | Dried fruit liking \|\| id:ebi-fl187-GCST90094756 | MR Egger | 5 | -1.181370851 | 1.29442755348582 | 0.428715985494146 | -3.718448856 | 1.35570715336737 | 0.306857793684605 | 0.0242715873814227 | 3.87950339074461 | 0.999279047927446 |
| ebi-fl187-GCST90094756 | prot-a-3123 | Thioredoxin domain-containing protein 12 \|\| id:prot-a-3123 | Dried fruit liking \|\| id:ebi-fl187-GCST90094756 | MR Egger | 5 | -0.822674159 | 1.18591930213219 | 0.537756762902026 | -3.147075991 | 1.50172767298638 | 0.439255443536967 | 0.04297761022354 | 4.48943865592545 | 0.999279047927446 |
| ebi-fl187-GCST90094756 | prot-a-3133 | PolyUbiquitin K48-linked \|\| id:prot-a-3133 | Dried fruit liking \|\| id:ebi-fl187-GCST90094756 | MR Egger | 5 | 0.681482887209091 | 1.17147354704047 | 0.601576617236784 | -1.614605265 | 2.97757103940841 | 1.97680694157865 | 0.198969195048747 | 19.6400537445817 | 0.999279047927446 |
| ebi-fl187-GCST90094756 | prot-a-3135 | Ubiquitin-conjugating enzyme E2 B \|\| id:prot-a-3135 | Dried fruit liking \|\| id:ebi-fl187-GCST90094756 | MR Egger | 5 | 0.482942489409508 | 1.49026700226991 | 0.767168507068648 | -2.437980835 | 3.40386581385854 | 1.62083668705663 | 0.0873370213974049 | 30.080159868914 | 0.999279047927446 |
| ebi-fl187-GCST90094756 | prot-a-3162 | NKG2D ligand 3 \|\| id:prot-a-3162 | Dried fruit liking \|\| id:ebi-fl187-GCST90094756 | MR Egger | 5 | 0.263913646309562 | 1.27225202874248 | 0.848950698845838 | -2.22970033 | 2.75752762264483 | 1.30201575758054 | 0.107560658006311 | 15.7608280240212 | 0.999279047927446 |
| ebi-fl187-GCST90094756 | prot-a-3188 | Vesicle-associated membrane protein 8 \|\| id:prot-a-3188 | Dried fruit liking \|\| id:ebi-fl187-GCST90094756 | MR Egger | 5 | 1.21510684391871 | 1.94913618851586 | 0.577198885401872 | -2.605200086 | 5.03541377340979 | 3.37065417898433 | 0.073888351725279 | 153.763202575509 | 0.999279047927446 |
| ebi-fl187-GCST90094756 | prot-a-3201 | Vitrin \|\| id:prot-a-3201 | Dried fruit liking \|\| id:ebi-fl187-GCST90094756 | MR Egger | 5 | 1.22896637677367 | 1.16705538857374 | 0.369643168802905 | -1.058462185 | 3.51639493837821 | 3.41769510093028 | 0.346989005203122 | 33.6628527929443 | 0.999279047927446 |
| ebi-fl187-GCST90094756 | prot-a-3232 | WNT1-inducible-signaling pathway protein 3 \|\| id:prot-a-3232 | Dried fruit liking \|\| id:ebi-fl187-GCST90094756 | MR Egger | 5 | 0.0179049050129108 | 1.50297081174668 | 0.991242961282577 | -2.927917886 | 2.96372769603641 | 1.01806615879821 | 0.053508332689069 | 19.3700429746687 | 0.999279047927446 |
| ebi-fl187-GCST90094756 | prot-a-327 | Carbonic anhydrase-related protein 10 \|\| id:prot-a-327 | Dried fruit liking \|\| id:ebi-fl187-GCST90094756 | MR Egger | 5 | -0.010561286 | 1.68436548871485 | 0.995390793361943 | -3.311917644 | 3.2907950715726 | 0.989494288257379 | 0.0364462158239623 | 26.8642141401755 | 0.999279047927446 |
| ebi-fl187-GCST90094756 | prot-a-332 | Carbonic anhydrase 5A, mitochondrial \|\| id:prot-a-332 | Dried fruit liking \|\| id:ebi-fl187-GCST90094756 | MR Egger | 5 | -0.330980123 | 1.63857548859354 | 0.852844226769214 | -3.542588081 | 2.88062783438346 | 0.718219444759949 | 0.0289383353286586 | 17.8254611045452 | 0.999279047927446 |
| ebi-fl187-GCST90094756 | prot-a-338 | Cell adhesion molecule 3 \|\| id:prot-a-338 | Dried fruit liking \|\| id:ebi-fl187-GCST90094756 | MR Egger | 5 | -0.724231437 | 1.1695559643399 | 0.57960609286634 | -3.016561127 | 1.56809825313942 | 0.484696945994427 | 0.0489693284395708 | 4.79751585211617 | 0.999279047927446 |
| ebi-fl187-GCST90094756 | prot-a-34 | A disintegrin and metalloproteinase with thrombospondin motifs 3 \|\| id:prot-a-34 | Dried fruit liking \|\| id:ebi-fl187-GCST90094756 | MR Egger | 5 | -0.254245426 | 2.14469231321126 | 0.913126972449328 | -4.45784236 | 3.94935150765708 | 0.775501450286043 | 0.0115873376366135 | 51.9016980652618 | 0.999279047927446 |
| ebi-fl187-GCST90094756 | prot-a-358 | Cysteine--tRNA ligase, cytoplasmic \|\| id:prot-a-358 | Dried fruit liking \|\| id:ebi-fl187-GCST90094756 | MR Egger | 5 | -0.144249821 | 1.16682989780501 | 0.909429558532605 | -2.431236421 | 2.14273677860354 | 0.865671458052495 | 0.087928049276927 | 8.52273056719998 | 0.999279047927446 |
| ebi-fl187-GCST90094756 | prot-a-382 | Coiled-coil domain-containing protein 167 \|\| id:prot-a-382 | Dried fruit liking \|\| id:ebi-fl187-GCST90094756 | MR Egger | 5 | 2.59683004896795 | 1.31820402086341 | 0.143450215519683 | 0.0131501680756703 | 5.18050992986023 | 13.4211262190897 | 1.01323701178816 | 177.77343986956 | 0.999279047927446 |
| ebi-fl187-GCST90094756 | prot-a-40 | Pituitary adenylate cyclase-activating polypeptide 27 \|\| id:prot-a-40 | Dried fruit liking \|\| id:ebi-fl187-GCST90094756 | MR Egger | 5 | -2.336212492 | 1.16705538857374 | 0.139083066344295 | -4.623641054 | -0.048783931 | 0.0966931717003759 | 0.00981698673153011 | 0.952386889089858 | 0.999279047927446 |
| ebi-fl187-GCST90094756 | prot-a-403 | C-C motif chemokine 25 \|\| id:prot-a-403 | Dried fruit liking \|\| id:ebi-fl187-GCST90094756 | MR Egger | 5 | -0.822190338 | 1.16705538857374 | 0.531887201832792 | -3.1096189 | 1.46523822354959 | 0.439468016024809 | 0.0446179560188183 | 4.32857428581726 | 0.999279047927446 |
| ebi-fl187-GCST90094756 | prot-a-415 | Cyclin-C \|\| id:prot-a-415 | Dried fruit liking \|\| id:ebi-fl187-GCST90094756 | MR Egger | 5 | 1.10371920570756 | 1.38438582081496 | 0.483567407130189 | -1.609677003 | 3.81711541450487 | 3.01535993870166 | 0.199952187584847 | 45.4728486332196 | 0.999279047927446 |
| ebi-fl187-GCST90094756 | prot-a-44 | Adhesion G protein-coupled receptor F5 \|\| id:prot-a-44 | Dried fruit liking \|\| id:ebi-fl187-GCST90094756 | MR Egger | 5 | 1.12112135981386 | 1.23707045052663 | 0.431601280606102 | -1.303536723 | 3.54577944284606 | 3.06829293529951 | 0.271569625976071 | 34.6666955222689 | 0.999279047927446 |
| ebi-fl187-GCST90094756 | prot-a-455 | CD59 glycoprotein \|\| id:prot-a-455 | Dried fruit liking \|\| id:ebi-fl187-GCST90094756 | MR Egger | 5 | 1.39363970550683 | 1.7534392081581 | 0.484799632546046 | -2.043101142 | 4.83038055349671 | 4.02948955040952 | 0.12962609792184 | 125.258619191405 | 0.999279047927446 |
| ebi-fl187-GCST90094756 | prot-a-478 | Cadherin-12 \|\| id:prot-a-478 | Dried fruit liking \|\| id:ebi-fl187-GCST90094756 | MR Egger | 5 | -1.0736848 | 1.16695035192794 | 0.425386987544527 | -3.36090749 | 1.21353788971858 | 0.341746925397719 | 0.0347037513487666 | 3.36536992341449 | 0.999279047927446 |
| ebi-fl187-GCST90094756 | prot-a-516 | Centrin-2 \|\| id:prot-a-516 | Dried fruit liking \|\| id:ebi-fl187-GCST90094756 | MR Egger | 5 | -1.059070767 | 1.16705538857374 | 0.431057296893248 | -3.346499328 | 1.22835779492945 | 0.346777898238858 | 0.0352073881323053 | 3.41561578652344 | 0.999279047927446 |
| ebi-fl187-GCST90094756 | prot-a-519 | Cryptic protein \|\| id:prot-a-519 | Dried fruit liking \|\| id:ebi-fl187-GCST90094756 | MR Egger | 5 | 0.394823107694396 | 1.16695035192794 | 0.757403508030848 | -1.892399582 | 2.68204579747316 | 1.48412163800146 | 0.150709734218052 | 14.6149619851184 | 0.999279047927446 |
| ebi-fl187-GCST90094756 | prot-a-583 | Chloride intracellular channel protein 4 \|\| id:prot-a-583 | Dried fruit liking \|\| id:ebi-fl187-GCST90094756 | MR Egger | 5 | -1.636349515 | 1.16695035192794 | 0.255404539778432 | -3.923572205 | 0.650873174711415 | 0.194689457579613 | 0.0197703447315761 | 1.91721416127386 | 0.999279047927446 |
| ebi-fl187-GCST90094756 | prot-a-602 | CCR4-NOT transcription complex subunit 1 \|\| id:prot-a-602 | Dried fruit liking \|\| id:ebi-fl187-GCST90094756 | MR Egger | 5 | -1.543167606 | 1.22874950901112 | 0.298065218190639 | -3.951516643 | 0.865181432089038 | 0.213703101041209 | 0.0192255213959118 | 2.37543702738489 | 0.999279047927446 |
| ebi-fl187-GCST90094756 | prot-a-606 | Ciliary neurotrophic factor receptor subunit alpha \|\| id:prot-a-606 | Dried fruit liking \|\| id:ebi-fl187-GCST90094756 | MR Egger | 5 | 0.878199560350099 | 1.16705538857374 | 0.50641700551532 | -1.409229001 | 3.16562812195464 | 2.40656293221546 | 0.244331589901291 | 23.7036281270597 | 0.999279047927446 |
| ebi-fl187-GCST90094756 | prot-a-626 | Collagen alpha-2(IX) chain \|\| id:prot-a-626 | Dried fruit liking \|\| id:ebi-fl187-GCST90094756 | MR Egger | 5 | 1.21329834971006 | 1.18370107193793 | 0.380791853168515 | -1.106755751 | 3.53335245070841 | 3.36456387921814 | 0.330629868515844 | 34.238558506994 | 0.999279047927446 |
| ebi-fl187-GCST90094756 | prot-a-647 | Probable serine carboxypeptidase CPVL \|\| id:prot-a-647 | Dried fruit liking \|\| id:ebi-fl187-GCST90094756 | MR Egger | 5 | -0.52709174 | 1.26774874130608 | 0.705528931652608 | -3.011879273 | 1.95769579268129 | 0.59031927742126 | 0.0491991332141067 | 7.08298757579006 | 0.999279047927446 |
| ebi-fl187-GCST90094756 | prot-a-651 | Cellular retinoic acid-binding protein 2 \|\| id:prot-a-651 | Dried fruit liking \|\| id:ebi-fl187-GCST90094756 | MR Egger | 5 | -1.61341032 | 1.16705538857374 | 0.260770530354866 | -3.900838882 | 0.674018241279501 | 0.199207094336841 | 0.0202249379924295 | 1.96210571567544 | 0.999279047927446 |
| ebi-fl187-GCST90094756 | prot-a-671 | Cytotoxic and regulatory T-cell molecule \|\| id:prot-a-671 | Dried fruit liking \|\| id:ebi-fl187-GCST90094756 | MR Egger | 5 | -1.978970326 | 1.16682989780501 | 0.188450820816572 | -4.265956926 | 0.30801627331559 | 0.138211476779525 | 0.0140384269665617 | 1.36072313223394 | 0.999279047927446 |
| ebi-fl187-GCST90094756 | prot-a-688 | Chorionic somatomammotropin hormone \|\| id:prot-a-688 | Dried fruit liking \|\| id:ebi-fl187-GCST90094756 | MR Egger | 5 | 0.249707286381582 | 1.79017086691415 | 0.89790245291468 | -3.259027613 | 3.75844218553331 | 1.28364961996497 | 0.0384257445602669 | 42.8815723857188 | 0.999279047927446 |
| ebi-fl187-GCST90094756 | prot-a-69 | Aldo-keto reductase family 1 member C1 \|\| id:prot-a-69 | Dried fruit liking \|\| id:ebi-fl187-GCST90094756 | MR Egger | 5 | -0.84454181 | 1.27937482094332 | 0.556333843265164 | -3.35211646 | 1.66303283855327 | 0.42975422198081 | 0.0350101780077835 | 5.27528569746978 | 0.999279047927446 |
| ebi-fl187-GCST90094756 | prot-a-741 | Stromal cell-derived factor 1 \|\| id:prot-a-741 | Dried fruit liking \|\| id:ebi-fl187-GCST90094756 | MR Egger | 5 | -1.251696975 | 1.22559437812401 | 0.382289047731678 | -3.653861956 | 1.15046800588596 | 0.286019017608863 | 0.0258909457494206 | 3.1596713084832 | 0.999279047927446 |
| ebi-fl187-GCST90094756 | prot-a-742 | Growth-regulated alpha protein \|\| id:prot-a-742 | Dried fruit liking \|\| id:ebi-fl187-GCST90094756 | MR Egger | 5 | -1.243601974 | 1.3366767820593 | 0.420808032460346 | -3.863488467 | 1.37628451909847 | 0.288343738600489 | 0.0209946325544361 | 3.96016035882179 | 0.999279047927446 |
| ebi-fl187-GCST90094756 | prot-a-788 | Beta-defensin 112 \|\| id:prot-a-788 | Dried fruit liking \|\| id:ebi-fl187-GCST90094756 | MR Egger | 5 | 1.94314905408367 | 1.30538887672967 | 0.233347995016522 | -0.615413144 | 4.50171125247382 | 6.98069899341661 | 0.540417578735734 | 90.17130522 | 0.999279047927446 |
| ebi-fl187-GCST90094756 | prot-a-838 | DnaJ homolog subfamily B member 1 \|\| id:prot-a-838 | Dried fruit liking \|\| id:ebi-fl187-GCST90094756 | MR Egger | 5 | -1.96876652 | 1.16705538857374 | 0.190197775551274 | -4.256195082 | 0.31866204120917 | 0.139628979536398 | 0.0141761389697033 | 1.37528645620945 | 0.999279047927446 |
| ebi-fl187-GCST90094756 | prot-a-85 | Junctional adhesion molecule-like \|\| id:prot-a-85 | Dried fruit liking \|\| id:ebi-fl187-GCST90094756 | MR Egger | 5 | 0.00916519165095868 | 1.16682405332298 | 0.994225960980191 | -2.277809953 | 2.29614033616399 | 1.00920732062831 | 0.102508459422077 | 9.93575966073324 | 0.999279047927446 |
| ebi-fl187-GCST90094756 | prot-a-856 | Dipeptidase 1 \|\| id:prot-a-856 | Dried fruit liking \|\| id:ebi-fl187-GCST90094756 | MR Egger | 5 | -1.470456395 | 1.32909879882013 | 0.349318544092288 | -4.075490041 | 1.13457725056525 | 0.229820572259509 | 0.0169838898382528 | 3.10985857401921 | 0.999279047927446 |
| ebi-fl187-GCST90094756 | prot-a-860 | Dipeptidyl peptidase 2 \|\| id:prot-a-860 | Dried fruit liking \|\| id:ebi-fl187-GCST90094756 | MR Egger | 5 | -0.748428241 | 1.45131149338529 | 0.641667449831871 | -3.592998768 | 2.09614228582159 | 0.47310958279974 | 0.0275156933928359 | 8.13472784935242 | 0.999279047927446 |
| ebi-fl187-GCST90094756 | prot-a-861 | Dermatopontin \|\| id:prot-a-861 | Dried fruit liking \|\| id:ebi-fl187-GCST90094756 | MR Egger | 5 | 2.1661920447421 | 1.16548034615352 | 0.160047985489952 | -0.118149434 | 4.450533523 | 8.72499630630349 | 0.888563261380577 | 85.6726401525221 | 0.999279047927446 |
| ebi-fl187-GCST90094756 | prot-a-877 | Dual specificity protein phosphatase 6 \|\| id:prot-a-877 | Dried fruit liking \|\| id:ebi-fl187-GCST90094756 | MR Egger | 5 | 1.57032459377352 | 1.16705538857374 | 0.271109980840539 | -0.717103968 | 3.85775315537806 | 4.80820865509469 | 0.488163949319591 | 47.3588238196427 | 0.999279047927446 |
| ebi-fl187-GCST90094756 | prot-a-879 | Dynactin-associated protein \|\| id:prot-a-879 | Dried fruit liking \|\| id:ebi-fl187-GCST90094756 | MR Egger | 5 | -3.379230585 | 1.1653753266492 | 0.0625178496984183 | -5.663366225 | -1.095094944 | 0.0340736614472273 | 0.00347081367183737 | 0.334507845765644 | 0.999279047927446 |
| ebi-fl187-GCST90094756 | prot-a-937 | EMI domain-containing protein 1 \|\| id:prot-a-937 | Dried fruit liking \|\| id:ebi-fl187-GCST90094756 | MR Egger | 5 | -0.861927408 | 1.29985587410688 | 0.554668994129512 | -3.409644921 | 1.68579010519064 | 0.422347261808147 | 0.0330529346852657 | 5.39671321942727 | 0.999279047927446 |
| ebi-fl187-GCST90094756 | prot-b-1 | SRC proto-oncogene, non-receptor tyrosine kinase \|\| id:prot-b-1 | Dried fruit liking \|\| id:ebi-fl187-GCST90094756 | MR Egger | 5 | -0.706199021 | 1.32279036795783 | 0.630456347012088 | -3.298868142 | 1.88647010013335 | 0.493516482703762 | 0.0369249375341538 | 6.5960441632437 | 0.999279047927446 |
| ebi-fl187-GCST90094756 | prot-b-10 | coagulation factor III, tissue factor \|\| id:prot-b-10 | Dried fruit liking \|\| id:ebi-fl187-GCST90094756 | MR Egger | 5 | -0.228155205 | 1.44463286757649 | 0.884541363410321 | -3.059635625 | 2.60332521572498 | 0.796000707176915 | 0.0469047830287301 | 13.5085823856822 | 0.999279047927446 |
| ebi-fl187-GCST90094756 | prot-b-29 | myeloperoxidase \|\| id:prot-b-29 | Dried fruit liking \|\| id:ebi-fl187-GCST90094756 | MR Egger | 5 | 0.565194134465172 | 1.32084629389869 | 0.697580701202694 | -2.023664602 | 3.15405287050661 | 1.75978938533346 | 0.132170225301952 | 23.4308345442956 | 0.999279047927446 |
| ebi-fl187-GCST90094756 | prot-b-33 | adrenomedullin \|\| id:prot-b-33 | Dried fruit liking \|\| id:ebi-fl187-GCST90094756 | MR Egger | 5 | -1.973601758 | 1.32132173617157 | 0.232111879542982 | -4.56339236 | 0.616188845348235 | 0.138955469908535 | 0.0104266279997454 | 1.85185686282978 | 0.999279047927446 |
| ebi-fl187-GCST90094756 | ukb-rsfMRI191-edge-pheno102 | Net25_Pair11_15 (Postcentral\|Precentral)&(Cerebellum) (Motor)&(Subcortical-cerebellum) \|\| id:ukb-rsfMRI191-edge-pheno102 | Dried fruit liking \|\| id:ebi-fl187-GCST90094756 | MR Egger | 5 | -0.074686758 | 0.362955180540494 | 0.850139924853666 | -0.786078912 | 0.636705395427915 | 0.928034139673399 | 0.455627854011314 | 1.89024300603439 | 0.999279047927446 |
| ebi-fl187-GCST90094756 | ukb-rsfMRI191-edge-pheno1167 | Net100_Pair11_45 (Angular\|Temporal)&(Frontal) (Default_mode\|Central_executive)&(Salience\|Default_mode) \|\| id:ukb-rsfMRI191-edge-pheno1167 | Dried fruit liking \|\| id:ebi-fl187-GCST90094756 | MR Egger | 5 | 0.236461797305466 | 0.576291008523298 | 0.709117827558355 | -0.893068579 | 1.36599217401113 | 1.26675916108246 | 0.409397554396939 | 3.9196100586143 | 0.999279047927446 |
| ebi-fl187-GCST90094756 | ukb-rsfMRI191-edge-pheno1317 | Net100_Pair26_48 (Parietal\|Frontal)&(Temporal) (Attention\|Central_executive)&(Default_mode\|Central_executive) \|\| id:ukb-rsfMRI191-edge-pheno1317 | Dried fruit liking \|\| id:ebi-fl187-GCST90094756 | MR Egger | 5 | 0.035823738654072 | 0.362024096464342 | 0.927416208589703 | -0.67374349 | 0.745390967724183 | 1.03647314023866 | 0.509796582630411 | 2.10726514660652 | 0.999279047927446 |
| ebi-fl187-GCST90094756 | ukb-rsfMRI191-edge-pheno286 | Net100_Pair10_13 (OccipitalPrecuneus)&(Frontal_Sup) (Default_mode\|Central_executive)&(Default_mode) \|\| id:ukb-rsfMRI191-edge-pheno286 | Dried fruit liking \|\| id:ebi-fl187-GCST90094756 | MR Egger | 5 | 0.693512929326107 | 0.598115133405545 | 0.330155657838302 | -0.478792732 | 1.86581859080097 | 2.00073163132079 | 0.619530880220527 | 6.46122281934146 | 0.999279047927446 |
| ebi-fl187-GCST90094756 | ukb-rsfMRI191-edge-pheno599 | Net100_Pair11_29 (Angular\|Temporal)&(Parietal) (Default_mode\|Central_executive)&(Central_executive\|Salience) \|\| id:ukb-rsfMRI191-edge-pheno599 | Dried fruit liking \|\| id:ebi-fl187-GCST90094756 | MR Egger | 5 | 0.394638385296216 | 0.409674868829393 | 0.406459151056944 | -0.408324358 | 1.19760112820183 | 1.48384751281261 | 0.66476322283969 | 3.31216193319879 | 0.999279047927446 |
| ebi-fl187-GCST90094756 | ukb-rsfMRI191-edge-pheno621 | Net100_Pair5_30 (Precuneus\|Angular\|Cingulate)&(Paracentral) (Default_mode\|Central_executive)&(Motor) \|\| id:ukb-rsfMRI191-edge-pheno621 | Dried fruit liking \|\| id:ebi-fl187-GCST90094756 | MR Egger | 5 | -0.004415563 | 0.366442359567954 | 0.991142403069951 | -0.722642588 | 0.713811461545857 | 0.995594171059192 | 0.48546766837401 | 2.04175853104063 | 0.999279047927446 |
| ebi-fl187-GCST90094756 | ukb-rsfMRI191-edge-pheno624 | Net100_Pair8_30 (Calcarine\|Lingual)&(Paracentral) (Visual)&(Motor) \|\| id:ukb-rsfMRI191-edge-pheno624 | Dried fruit liking \|\| id:ebi-fl187-GCST90094756 | MR Egger | 5 | -0.396228494 | 0.36515534973435 | 0.357290061022475 | -1.111932979 | 0.319475991702964 | 0.672852935663216 | 0.328922545726896 | 1.37640632699745 | 0.999279047927446 |
| ebi-fl187-GCST90094756 | ukb-rsfMRI191-edge-pheno882 | Net100_Pair6_38 (Postcentral\|Precentral)&(Subcortical) (Motor)&(Subcortical-cerebellum) \|\| id:ukb-rsfMRI191-edge-pheno882 | Dried fruit liking \|\| id:ebi-fl187-GCST90094756 | MR Egger | 5 | -0.383936021 | 0.47854968064277 | 0.481056219153558 | -1.321893395 | 0.55402135349426 | 0.681175006977121 | 0.266629987951234 | 1.74023707421517 | 0.999279047927446 |
| ebi-fl187-GCST90094756 | ukb-rsfMRI191-edge-pheno899 | Net100_Pair23_38 (Cerebellum)&(Subcortical) (Subcortical-cerebellum)&(Subcortical-cerebellum) \|\| id:ukb-rsfMRI191-edge-pheno899 | Dried fruit liking \|\| id:ebi-fl187-GCST90094756 | MR Egger | 5 | 0.553388795837197 | 0.365033187322131 | 0.226779212330959 | -0.162076251 | 1.26885384298857 | 1.7391366220152 | 0.850376359753035 | 3.55677360423428 | 0.999279047927446 |
| ebi-fl187-GCST90094756 | ebi-a-GCST90001392 | IgD+ CD38+ B cell Absolute Count \|\| id:ebi-a-GCST90001392 | Dried fruit liking \|\| id:ebi-fl187-GCST90094756 | Weighted median | 5 | 0.407049740093914 | 0.357600624687016 | 0.255003464634561 | -0.293847484 | 1.10794696448047 | 1.50237883226769 | 0.745390166482955 | 3.02813513934068 | 0.999489402457283 |
| ebi-fl187-GCST90094756 | ebi-a-GCST90001402 | Switched memory B cell %B cell \|\| id:ebi-a-GCST90001402 | Dried fruit liking \|\| id:ebi-fl187-GCST90094756 | Weighted median | 5 | 0.0225069815347569 | 0.370895242987231 | 0.951611787779024 | -0.704447695 | 0.749461657789729 | 1.02276217458919 | 0.494381548406083 | 2.11586065285591 | 0.999489402457283 |
| ebi-fl187-GCST90094756 | ebi-a-GCST90001407 | Memory B cell Absolute Count \|\| id:ebi-a-GCST90001407 | Dried fruit liking \|\| id:ebi-fl187-GCST90094756 | Weighted median | 5 | 0.545786823432372 | 0.403076603739137 | 0.175719676020888 | -0.24424332 | 1.33581696676108 | 1.72596587864826 | 0.783297019324056 | 3.8031016852697 | 0.999489402457283 |
| ebi-fl187-GCST90094756 | ebi-a-GCST90001411 | IgD+ CD24+ B cell %B cell \|\| id:ebi-a-GCST90001411 | Dried fruit liking \|\| id:ebi-fl187-GCST90094756 | Weighted median | 5 | 0.454572259829537 | 0.375799299077469 | 0.226426873455115 | -0.281994366 | 1.19113888602138 | 1.57549933453154 | 0.754277933849722 | 3.29082695080387 | 0.999489402457283 |
| ebi-fl187-GCST90094756 | ebi-a-GCST90001418 | CD24+ CD27+ B cell Absolute Count \|\| id:ebi-a-GCST90001418 | Dried fruit liking \|\| id:ebi-fl187-GCST90094756 | Weighted median | 5 | 0.410046627783406 | 0.375472169738131 | 0.274796791513516 | -0.325878825 | 1.14597208047014 | 1.50688804632422 | 0.721892657544572 | 3.14549754790188 | 0.999489402457283 |
| ebi-fl187-GCST90094756 | ebi-a-GCST90001436 | Memory B cell %lymphocyte \|\| id:ebi-a-GCST90001436 | Dried fruit liking \|\| id:ebi-fl187-GCST90094756 | Weighted median | 5 | 0.50327245627618 | 0.372494928394111 | 0.176668080392918 | -0.226817603 | 1.23336251592864 | 1.65412547666117 | 0.797066151207201 | 3.4327528378862 | 0.999489402457283 |
| ebi-fl187-GCST90094756 | ebi-a-GCST90001439 | IgD+ CD24+ B cell %lymphocyte \|\| id:ebi-a-GCST90001439 | Dried fruit liking \|\| id:ebi-fl187-GCST90094756 | Weighted median | 5 | 0.559712939958997 | 0.38554487776052 | 0.146572455958162 | -0.19595502 | 1.31538090036962 | 1.75017002430022 | 0.822049209248806 | 3.72617001451546 | 0.999489402457283 |
| ebi-fl187-GCST90094756 | ebi-a-GCST90001457 | CD11c+ HLA DR++ monocyte %monocyte \|\| id:ebi-a-GCST90001457 | Dried fruit liking \|\| id:ebi-fl187-GCST90094756 | Weighted median | 5 | -0.114178937 | 0.383151137337643 | 0.765703365792667 | -0.865155167 | 0.636797291746455 | 0.89209831181571 | 0.420986224550773 | 1.89041672038953 | 0.999489402457283 |
| ebi-fl187-GCST90094756 | ebi-a-GCST90001483 | CD39+ resting CD4 regulatory T cell Absolute Count \|\| id:ebi-a-GCST90001483 | Dried fruit liking \|\| id:ebi-fl187-GCST90094756 | Weighted median | 5 | 0.439377482395094 | 0.431333856580402 | 0.30836998429505 | -0.406036877 | 1.28479184129268 | 1.55174093172902 | 0.66628559668938 | 3.61391561091452 | 0.999489402457283 |
| ebi-fl187-GCST90094756 | ebi-a-GCST90001525 | CD33dim HLA DR+ CD11b+ Absolute Count \|\| id:ebi-a-GCST90001525 | Dried fruit liking \|\| id:ebi-fl187-GCST90094756 | Weighted median | 5 | 0.220362093551978 | 0.451834018818797 | 0.625758208535656 | -0.665232583 | 1.10595677043682 | 1.2465280086343 | 0.51415393016429 | 3.02211455587491 | 0.999489402457283 |
| ebi-fl187-GCST90094756 | ebi-a-GCST90001540 | Naive CD4+ T cell Absolute Count \|\| id:ebi-a-GCST90001540 | Dried fruit liking \|\| id:ebi-fl187-GCST90094756 | Weighted median | 5 | -0.488720268 | 0.359618024662225 | 0.174147141743984 | -1.193571596 | 0.216131060448695 | 0.613410893720495 | 0.303136646608507 | 1.24126504909491 | 0.999489402457283 |
| ebi-fl187-GCST90094756 | ebi-a-GCST90001624 | HLA DR+ CD4+ T cell Absolute Count \|\| id:ebi-a-GCST90001624 | Dried fruit liking \|\| id:ebi-fl187-GCST90094756 | Weighted median | 5 | -0.188062965 | 0.374073839615688 | 0.615145047508124 | -0.921247691 | 0.545121760741659 | 0.828562535228805 | 0.398022122706873 | 1.72481838475691 | 0.999489402457283 |
| ebi-fl187-GCST90094756 | ebi-a-GCST90001672 | CD39+ CD8+ T cell Absolute Count \|\| id:ebi-a-GCST90001672 | Dried fruit liking \|\| id:ebi-fl187-GCST90094756 | Weighted median | 5 | 0.315300600222906 | 0.427926354093299 | 0.461237640615287 | -0.523435054 | 1.15403625424577 | 1.37067127312617 | 0.592481841440699 | 3.17096593948753 | 0.999489402457283 |
| ebi-fl187-GCST90094756 | ebi-a-GCST90001722 | CD19 on CD20- CD38- B cell \|\| id:ebi-a-GCST90001722 | Dried fruit liking \|\| id:ebi-fl187-GCST90094756 | Weighted median | 5 | -0.255115776 | 0.379540309188009 | 0.501475198240921 | -0.999014782 | 0.488783230046043 | 0.774826786451026 | 0.368242061229978 | 1.63033127447949 | 0.999489402457283 |
| ebi-fl187-GCST90094756 | ebi-a-GCST90001742 | CD19 on transitional B cell \|\| id:ebi-a-GCST90001742 | Dried fruit liking \|\| id:ebi-fl187-GCST90094756 | Weighted median | 5 | -0.21183245 | 0.39472480281517 | 0.591503474802812 | -0.985493064 | 0.561828163018217 | 0.809100250553087 | 0.373255142877572 | 1.75387594233308 | 0.999489402457283 |
| ebi-fl187-GCST90094756 | ebi-a-GCST90001784 | CD25 on IgD+ CD38dim B cell \|\| id:ebi-a-GCST90001784 | Dried fruit liking \|\| id:ebi-fl187-GCST90094756 | Weighted median | 5 | 0.176543224952726 | 0.339480528807335 | 0.603036166641065 | -0.488838612 | 0.841925061415102 | 1.19308599673084 | 0.613338304749855 | 2.32083042029434 | 0.999489402457283 |
| ebi-fl187-GCST90094756 | ebi-a-GCST90001794 | CD25 on IgD+ B cell \|\| id:ebi-a-GCST90001794 | Dried fruit liking \|\| id:ebi-fl187-GCST90094756 | Weighted median | 5 | 0.119006992778403 | 0.366031709329701 | 0.745084538998599 | -0.598415158 | 0.836429143064617 | 1.12637779477113 | 0.549682105691521 | 2.30811031215464 | 0.999489402457283 |
| ebi-fl187-GCST90094756 | ebi-a-GCST90001802 | CD27 on IgD- CD38- B cell \|\| id:ebi-a-GCST90001802 | Dried fruit liking \|\| id:ebi-fl187-GCST90094756 | Weighted median | 5 | -0.069267422 | 0.375514518745497 | 0.853652575143242 | -0.805275879 | 0.666741034776299 | 0.933077121393769 | 0.446964601532844 | 1.94787889573959 | 0.999489402457283 |
| ebi-fl187-GCST90094756 | ebi-a-GCST90001804 | CD27 on IgD- CD38dim B cell \|\| id:ebi-a-GCST90001804 | Dried fruit liking \|\| id:ebi-fl187-GCST90094756 | Weighted median | 5 | 0.250230414620781 | 0.37986989595824 | 0.51007133229399 | -0.494314581 | 0.994775410698931 | 1.28432130900485 | 0.609988861719711 | 2.70411695733858 | 0.999489402457283 |
| ebi-fl187-GCST90094756 | ebi-a-GCST90001805 | CD27 on memory B cell \|\| id:ebi-a-GCST90001805 | Dried fruit liking \|\| id:ebi-fl187-GCST90094756 | Weighted median | 5 | 0.190363828554096 | 0.374768377164685 | 0.611487975548463 | -0.544182191 | 0.92490984779688 | 1.20968963723454 | 0.580316177304811 | 2.52164091862633 | 0.999489402457283 |
| ebi-fl187-GCST90094756 | ebi-a-GCST90001806 | CD27 on unswitched memory B cell \|\| id:ebi-a-GCST90001806 | Dried fruit liking \|\| id:ebi-fl187-GCST90094756 | Weighted median | 5 | 0.535445069212461 | 0.408785876904836 | 0.190249160191493 | -0.26577525 | 1.33666538794594 | 1.70820834403511 | 0.766611404420971 | 3.80632968646632 | 0.999489402457283 |
| ebi-fl187-GCST90094756 | ebi-a-GCST90001839 | CD3 on Effector Memory CD8+ T cell \|\| id:ebi-a-GCST90001839 | Dried fruit liking \|\| id:ebi-fl187-GCST90094756 | Weighted median | 5 | -0.48943321 | 0.408908313894704 | 0.231335191753663 | -1.290893505 | 0.312027085228377 | 0.612973723117148 | 0.275024937051674 | 1.36619169614246 | 0.999489402457283 |
| ebi-fl187-GCST90094756 | ebi-a-GCST90001859 | CD3 on CD8+ T cell \|\| id:ebi-a-GCST90001859 | Dried fruit liking \|\| id:ebi-fl187-GCST90094756 | Weighted median | 5 | -0.052846239 | 0.392610594120386 | 0.892926423950818 | -0.822363003 | 0.7166705256183 | 0.948525847687999 | 0.439392141707977 | 2.04760440237956 | 0.999489402457283 |
| ebi-fl187-GCST90094756 | ebi-a-GCST90001905 | CD86 on monocyte \|\| id:ebi-a-GCST90001905 | Dried fruit liking \|\| id:ebi-fl187-GCST90094756 | Weighted median | 5 | 0.255135407030932 | 0.391438950139542 | 0.514537619905769 | -0.512084935 | 1.02235574930443 | 1.29063637028039 | 0.599244888674024 | 2.77973541664476 | 0.999489402457283 |
| ebi-fl187-GCST90094756 | ebi-a-GCST90001943 | CD25 on activated & secreting CD4 regulatory T cell \|\| id:ebi-a-GCST90001943 | Dried fruit liking \|\| id:ebi-fl187-GCST90094756 | Weighted median | 5 | 0.456824354948647 | 0.373334564201215 | 0.221090844276541 | -0.274911391 | 1.18856010078303 | 1.57905150730712 | 0.759639431220036 | 3.28235154766032 | 0.999489402457283 |
| ebi-fl187-GCST90094756 | ebi-a-GCST90001949 | CD33 on CD33dim HLA DR+ CD11b- \|\| id:ebi-a-GCST90001949 | Dried fruit liking \|\| id:ebi-fl187-GCST90094756 | Weighted median | 5 | -0.029962894 | 0.506666557169201 | 0.952842746542044 | -1.023029346 | 0.963103557971321 | 0.970481543490623 | 0.359504226195068 | 2.61981461587854 | 0.999489402457283 |
| ebi-fl187-GCST90094756 | ebi-a-GCST90001955 | CD33 on Immature Myeloid-Derived Suppressor Cells \|\| id:ebi-a-GCST90001955 | Dried fruit liking \|\| id:ebi-fl187-GCST90094756 | Weighted median | 5 | -0.221750537 | 0.53401498668237 | 0.677957784822477 | -1.26841991 | 0.824918837358079 | 0.801115188376483 | 0.281275711651456 | 2.28169556937344 | 0.999489402457283 |
| ebi-fl187-GCST90094756 | ebi-a-GCST90001956 | CD33 on CD33+ HLA DR+ \|\| id:ebi-a-GCST90001956 | Dried fruit liking \|\| id:ebi-fl187-GCST90094756 | Weighted median | 5 | -0.210561783 | 0.504856337538949 | 0.676625250590648 | -1.200080205 | 0.778956638179383 | 0.810129001085447 | 0.301170055607219 | 2.17919738759039 | 0.999489402457283 |
| ebi-fl187-GCST90094756 | ebi-a-GCST90001957 | CD33 on CD33+ HLA DR+ CD14- \|\| id:ebi-a-GCST90001957 | Dried fruit liking \|\| id:ebi-fl187-GCST90094756 | Weighted median | 5 | -0.170659834 | 0.513382618912789 | 0.739570397009484 | -1.176889767 | 0.835570098658284 | 0.843108321136844 | 0.308235934706283 | 2.30612839430139 | 0.999489402457283 |
| ebi-fl187-GCST90094756 | ebi-a-GCST90002064 | CD4 on resting CD4 regulatory T cell \|\| id:ebi-a-GCST90002064 | Dried fruit liking \|\| id:ebi-fl187-GCST90094756 | Weighted median | 5 | 0.10048666481888 | 0.357388745700716 | 0.778580719016733 | -0.599995277 | 0.800968606392283 | 1.10570889677736 | 0.548814228272227 | 2.22769764599866 | 0.999489402457283 |
| ebi-fl187-GCST90094756 | ebi-a-GCST90002080 | SSC-A on T cell \|\| id:ebi-a-GCST90002080 | Dried fruit liking \|\| id:ebi-fl187-GCST90094756 | Weighted median | 5 | -0.076064082 | 0.366639967621126 | 0.835648595712162 | -0.794678419 | 0.642550254462614 | 0.926756816156865 | 0.451726478395371 | 1.90132356054073 | 0.999489402457283 |
| ebi-fl187-GCST90094756 | ebi-a-GCST90002121 | CD8 on CD39+ CD8+ T cell \|\| id:ebi-a-GCST90002121 | Dried fruit liking \|\| id:ebi-fl187-GCST90094756 | Weighted median | 5 | -0.306969026 | 0.405069909927646 | 0.448560304254272 | -1.100906049 | 0.486967997458665 | 0.735673387302209 | 0.332569622623153 | 1.62737452842461 | 0.999489402457283 |
| ebi-fl187-GCST90094756 | ebi-cfmet338-GCST90026053 | 1-palmitoyl-2-docosahexaenoyl-gpc (16:0/22:6) levels \|\| id:ebi-cfmet338-GCST90026053 | Dried fruit liking \|\| id:ebi-fl187-GCST90094756 | Weighted median | 5 | -0.137232027 | 0.188535939666452 | 0.466685465567073 | -0.506762469 | 0.2322984149245 | 0.871767929121957 | 0.602442852602624 | 1.26149612193486 | 0.999489402457283 |
| ebi-fl187-GCST90094756 | ebi-cfmet338-GCST90026063 | 1-stearoyl-2-oleoyl-gpc (18:0/18:1) levels \|\| id:ebi-cfmet338-GCST90026063 | Dried fruit liking \|\| id:ebi-fl187-GCST90094756 | Weighted median | 4 | -0.052905372 | 0.164773159610978 | 0.748149740370634 | -0.375860765 | 0.2700500211264 | 0.948469760306365 | 0.686697939083773 | 1.31002997826948 | 0.999489402457283 |
| ebi-fl187-GCST90094756 | ebi-cfmet338-GCST90026113 | Adenosine 3',5'-cyclic monophosphate (camp) levels \|\| id:ebi-cfmet338-GCST90026113 | Dried fruit liking \|\| id:ebi-fl187-GCST90094756 | Weighted median | 5 | -0.328603787 | 0.325907785825323 | 0.313323766132539 | -0.967383047 | 0.310175473541543 | 0.71992820539281 | 0.380076379834962 | 1.3636643801574 | 0.999489402457283 |
| ebi-fl187-GCST90094756 | ebi-cfmet338-GCST90026117 | Alpha-ketoglutaramate levels \|\| id:ebi-cfmet338-GCST90026117 | Dried fruit liking \|\| id:ebi-fl187-GCST90094756 | Weighted median | 5 | 0.0876394479329555 | 0.124115338686895 | 0.480117899029634 | -0.155626616 | 0.33090551175927 | 1.09159447442311 | 0.855878702221605 | 1.39222823689627 | 0.999489402457283 |
| ebi-fl187-GCST90094756 | ebi-cfmet338-GCST90026124 | Arginine levels \|\| id:ebi-cfmet338-GCST90026124 | Dried fruit liking \|\| id:ebi-fl187-GCST90094756 | Weighted median | 5 | -0.096707983 | 0.106683179170521 | 0.364672847192229 | -0.305807014 | 0.112391048325253 | 0.907821066751825 | 0.736528745460888 | 1.11895033875794 | 0.999489402457283 |
| ebi-fl187-GCST90094756 | ebi-cfmet338-GCST90026169 | Glutamate levels \|\| id:ebi-cfmet338-GCST90026169 | Dried fruit liking \|\| id:ebi-fl187-GCST90094756 | Weighted median | 5 | 0.136748085705753 | 0.119970554729375 | 0.254350018272003 | -0.098394202 | 0.371890372975328 | 1.14653928250301 | 0.906291571773263 | 1.45047396143213 | 0.999489402457283 |
| ebi-fl187-GCST90094756 | ebi-cfmet338-GCST90026170 | Glutamine levels \|\| id:ebi-cfmet338-GCST90026170 | Dried fruit liking \|\| id:ebi-fl187-GCST90094756 | Weighted median | 5 | -0.041894267 | 0.0678743981870485 | 0.537081552950817 | -0.174928087 | 0.0911395535725223 | 0.958971170231751 | 0.839517390543372 | 1.09542186462681 | 0.999489402457283 |
| ebi-fl187-GCST90094756 | ebi-cfmet338-GCST90026191 | Kynurenate levels \|\| id:ebi-cfmet338-GCST90026191 | Dried fruit liking \|\| id:ebi-fl187-GCST90094756 | Weighted median | 5 | 0.433630962939124 | 0.553978653256604 | 0.433769510946716 | -0.652167197 | 1.51942912332207 | 1.54284939443861 | 0.520915625560795 | 4.56961576331476 | 0.999489402457283 |
| ebi-fl187-GCST90094756 | ebi-cfmet338-GCST90026202 | Methylsuccinoylcarnitine levels \|\| id:ebi-cfmet338-GCST90026202 | Dried fruit liking \|\| id:ebi-fl187-GCST90094756 | Weighted median | 5 | -0.001076832 | 0.337384981894595 | 0.997453396419873 | -0.662351396 | 0.66019773297194 | 0.998923748033565 | 0.515637440035446 | 1.93517494446647 | 0.999911036302558 |
| ebi-fl187-GCST90094756 | ebi-cfmet338-GCST90026222 | N-acetylputrescine levels \|\| id:ebi-cfmet338-GCST90026222 | Dried fruit liking \|\| id:ebi-fl187-GCST90094756 | Weighted median | 5 | -0.11306419 | 0.117262133323381 | 0.334945487987852 | -0.342897972 | 0.116769591097076 | 0.893093330421575 | 0.709710618554504 | 1.12386045240247 | 0.999489402457283 |
| ebi-fl187-GCST90094756 | ebi-cfmet338-GCST90026252 | Propionylcarnitine (c3) levels \|\| id:ebi-cfmet338-GCST90026252 | Dried fruit liking \|\| id:ebi-fl187-GCST90094756 | Weighted median | 5 | 0.0982641030953808 | 0.208140804565206 | 0.63685256883082 | -0.309691874 | 0.506220080043185 | 1.1032541194636 | 0.733672985230298 | 1.65900840921837 | 0.999489402457283 |
| ebi-fl187-GCST90094756 | ebi-cfmet338-GCST90026316 | Ethyl beta-glucopyranoside levels \|\| id:ebi-cfmet338-GCST90026316 | Dried fruit liking \|\| id:ebi-fl187-GCST90094756 | Weighted median | 5 | -0.278840364 | 0.689990775974185 | 0.686123102471581 | -1.631222284 | 1.07354155733102 | 0.756660684178271 | 0.195690238745515 | 2.92572278848146 | 0.999489402457283 |
| ebi-fl187-GCST90094756 | ebi-gm430-GCST90011315 | F_Prevotellaceae abundance \|\| id:ebi-gm430-GCST90011315 | Dried fruit liking \|\| id:ebi-fl187-GCST90094756 | Weighted median | 3 | -0.48670032 | 0.382544371935324 | 0.203276654538034 | -1.236487289 | 0.263086649292605 | 0.614651204203887 | 0.290402528621857 | 1.30093943955023 | 0.999489402457283 |
| ebi-fl187-GCST90094756 | ebi-gm430-GCST90011354 | OTU97_1 (Bacteroides) abundance \|\| id:ebi-gm430-GCST90011354 | Dried fruit liking \|\| id:ebi-fl187-GCST90094756 | Weighted median | 3 | 0.0892151369549336 | 0.330466038788491 | 0.787185148234911 | -0.558498299 | 0.736928572980375 | 1.09331584366807 | 0.572067493421854 | 2.0895078775856 | 0.999489402457283 |
| ebi-fl187-GCST90094756 | ebi-gm430-GCST90011419 | OTU99_1 (Bacteroides) abundance \|\| id:ebi-gm430-GCST90011419 | Dried fruit liking \|\| id:ebi-fl187-GCST90094756 | Weighted median | 3 | 0.0869462451942818 | 0.339502020090702 | 0.797874127230473 | -0.578477714 | 0.752370204572057 | 1.09083804035529 | 0.560751340978215 | 2.12202369094717 | 0.999489402457283 |
| ebi-fl187-GCST90094756 | ebi-gm430-GCST90011435 | OTU99_155 (Butyrivibrio) abundance \|\| id:ebi-gm430-GCST90011435 | Dried fruit liking \|\| id:ebi-fl187-GCST90094756 | Weighted median | 3 | 0.351479200988634 | 1.03202876246959 | 0.733426466981253 | -1.671297173 | 2.37425557542903 | 1.42116818796497 | 0.188003034852175 | 10.7430128458922 | 0.999489402457283 |
| ebi-fl187-GCST90094756 | ebi-gm430-GCST90011466 | OTU99_58 (Bacteroides) abundance \|\| id:ebi-gm430-GCST90011466 | Dried fruit liking \|\| id:ebi-fl187-GCST90094756 | Weighted median | 3 | 0.123680540696068 | 0.627253675981955 | 0.843688351676431 | -1.105736664 | 1.3530977456207 | 1.13165429575435 | 0.330966980880657 | 3.86939338084925 | 0.999489402457283 |
| ebi-fl187-GCST90094756 | ebi-gm430-GCST90011472 | OTU99_72 (Parabacteroides) abundance \|\| id:ebi-gm430-GCST90011472 | Dried fruit liking \|\| id:ebi-fl187-GCST90094756 | Weighted median | 3 | -0.303775531 | 0.918929249009638 | 0.740965125710005 | -2.104876859 | 1.49732579665309 | 0.738026511632376 | 0.121860679334482 | 4.46972013324505 | 0.999489402457283 |
| ebi-fl187-GCST90094756 | ebi-gm430-GCST90011505 | TestASV_28 (Holdemanella) abundance \|\| id:ebi-gm430-GCST90011505 | Dried fruit liking \|\| id:ebi-fl187-GCST90094756 | Weighted median | 4 | 0.127432957603977 | 0.627546085845776 | 0.839083839521177 | -1.102557371 | 1.3574232858617 | 1.13590871164664 | 0.332020896542353 | 3.88616684862825 | 0.999489402457283 |
| ebi-fl187-GCST90094756 | ebi-gm430-GCST90011513 | TestASV_35 (Alphaproteobacteria) abundance \|\| id:ebi-gm430-GCST90011513 | Dried fruit liking \|\| id:ebi-fl187-GCST90094756 | Weighted median | 3 | 1.30933872670482 | 0.866600336568877 | 0.130816201448732 | -0.389197933 | 3.00787538637982 | 3.70372372865064 | 0.677600137331993 | 20.2443427951208 | 0.999489402457283 |
| ebi-fl187-GCST90094756 | ebi-gm430-GCST90011543 | G_Coprococcus prevalence \|\| id:ebi-gm430-GCST90011543 | Dried fruit liking \|\| id:ebi-fl187-GCST90094756 | Weighted median | 3 | 0.848144435674256 | 0.982329886858678 | 0.387917125479152 | -1.077222143 | 2.77351101391726 | 2.33530951204379 | 0.340540185056599 | 16.0147634739078 | 0.999489402457283 |
| ebi-fl187-GCST90094756 | ebi-gm430-GCST90011547 | G_Parasutterella prevalence \|\| id:ebi-gm430-GCST90011547 | Dried fruit liking \|\| id:ebi-fl187-GCST90094756 | Weighted median | 3 | 0.427338587356979 | 0.922181244985605 | 0.643077835304155 | -1.380136653 | 2.23481382752877 | 1.53317168640056 | 0.251544176491217 | 9.34474195653822 | 0.999489402457283 |
| ebi-fl187-GCST90094756 | ebi-gm430-GCST90011569 | OTU97_13 (Subdoligranulum) prevalence \|\| id:ebi-gm430-GCST90011569 | Dried fruit liking \|\| id:ebi-fl187-GCST90094756 | Weighted median | 3 | 1.02669654789204 | 0.823400023156971 | 0.212434585835188 | -0.587167497 | 2.64056059327971 | 2.7918279147769 | 0.555899643961226 | 14.0210615178433 | 0.999489402457283 |
| ebi-fl187-GCST90094756 | ebi-gm430-GCST90011590 | OTU97_277 (Prevotella) prevalence \|\| id:ebi-gm430-GCST90011590 | Dried fruit liking \|\| id:ebi-fl187-GCST90094756 | Weighted median | 3 | 0.173765971298106 | 0.87442319897602 | 0.842481247600602 | -1.540103499 | 1.88763544129111 | 1.1897770912479 | 0.214358914410954 | 6.60373528550576 | 0.999489402457283 |
| ebi-fl187-GCST90094756 | ebi-gm430-GCST90011616 | OTU97_86 (Phascolarctobacterium) prevalence \|\| id:ebi-gm430-GCST90011616 | Dried fruit liking \|\| id:ebi-fl187-GCST90094756 | Weighted median | 3 | 0.551566451724661 | 0.939399337785061 | 0.55710430591445 | -1.28965625 | 2.39278915378338 | 1.7359702026603 | 0.275365423594872 | 10.9439758455591 | 0.999489402457283 |
| ebi-fl187-GCST90094756 | ebi-gm430-GCST90011665 | OTU99_55 (Barnesiella) prevalence \|\| id:ebi-gm430-GCST90011665 | Dried fruit liking \|\| id:ebi-fl187-GCST90094756 | Weighted median | 3 | 0.462065952296823 | 0.687674908854286 | 0.501631454006305 | -0.885776869 | 1.80990877365122 | 1.58734998915107 | 0.412393672917731 | 6.10989002384752 | 0.999489402457283 |
| ebi-fl187-GCST90094756 | ebi-gm430-GCST90011687 | TestASV_14 (Lachnospiraceae) prevalence \|\| id:ebi-gm430-GCST90011687 | Dried fruit liking \|\| id:ebi-fl187-GCST90094756 | Weighted median | 3 | 0.0805341720102541 | 0.671999991664523 | 0.904607899068406 | -1.236585812 | 1.39765415567272 | 1.08386588388553 | 0.290373918715015 | 4.04569824814024 | 0.999489402457283 |
| ebi-fl187-GCST90094756 | ebi-gm430-GCST90011697 | TestASV_23 (Barnesiella) prevalence \|\| id:ebi-gm430-GCST90011697 | Dried fruit liking \|\| id:ebi-fl187-GCST90094756 | Weighted median | 3 | 0.0752797121627695 | 0.929648754290741 | 0.935460633008723 | -1.746831846 | 1.89739127057262 | 1.07818569036185 | 0.174325359045017 | 6.66847548325354 | 0.999489402457283 |
| ebi-fl187-GCST90094756 | ebi-gm430-GCST90011727 | TestASV_6 (Subdoligranulum) prevalence \|\| id:ebi-gm430-GCST90011727 | Dried fruit liking \|\| id:ebi-fl187-GCST90094756 | Weighted median | 3 | 2.39751001441511 | 1.04623439231183 | 0.0219309834744733 | 0.346890605483924 | 4.4481294233463 | 10.9957629740115 | 1.41466196064478 | 85.4669219532373 | 0.999489402457283 |
| ebi-fl187-GCST90094756 | ebi-hbsm44-GCST90104908 | Sulcal depth of the left posterior inferior temporal sulcus \|\| id:ebi-hbsm44-GCST90104908 | Dried fruit liking \|\| id:ebi-fl187-GCST90094756 | Weighted median | 4 | -0.102507502 | 0.243430999360602 | 0.673685936457542 | -0.579632261 | 0.374617256728208 | 0.902571378620283 | 0.560104301024973 | 1.45443463300275 | 0.999489402457283 |
| ebi-fl187-GCST90094756 | ebi-hbsm44-GCST90104918 | Sulcal depth of the left inferior frontal sulcus \|\| id:ebi-hbsm44-GCST90104918 | Dried fruit liking \|\| id:ebi-fl187-GCST90094756 | Weighted median | 4 | 0.18515305179292 | 0.446987200420172 | 0.678709697093807 | -0.690941861 | 1.06124796461646 | 1.20340260896056 | 0.5011038765175 | 2.88997532670754 | 0.999489402457283 |
| ebi-fl187-GCST90094756 | ebi-hbsm44-GCST90104925 | Sulcal depth of the right anterior inferior temporal sulcus \|\| id:ebi-hbsm44-GCST90104925 | Dried fruit liking \|\| id:ebi-fl187-GCST90094756 | Weighted median | 4 | -0.098556056 | 0.145133866344732 | 0.497093503985532 | -0.383018434 | 0.185906322069422 | 0.906144896362838 | 0.681800330861536 | 1.20430943787731 | 0.999489402457283 |
| ebi-fl187-GCST90094756 | ebi-IPs91-GCST90274770 | C-C motif chemokine 4 levels \|\| id:ebi-IPs91-GCST90274770 | Dried fruit liking \|\| id:ebi-fl187-GCST90094756 | Weighted median | 5 | 0.108694439729383 | 0.187860554849324 | 0.562865130640686 | -0.259512248 | 0.476901127234058 | 1.11482165308632 | 0.771427759662267 | 1.61107414482236 | 0.999489402457283 |
| ebi-fl187-GCST90094756 | ebi-IPs91-GCST90274779 | C-X-C motif chemokine 1 levels \|\| id:ebi-IPs91-GCST90274779 | Dried fruit liking \|\| id:ebi-fl187-GCST90094756 | Weighted median | 5 | 0.0411371256020904 | 0.167775525316297 | 0.806308190397515 | -0.287702904 | 0.369977155222032 | 1.04199497993839 | 0.749984376488179 | 1.44770154186527 | 0.999489402457283 |
| ebi-fl187-GCST90094756 | ebi-IPs91-GCST90274780 | C-X-C motif chemokine 10 levels \|\| id:ebi-IPs91-GCST90274780 | Dried fruit liking \|\| id:ebi-fl187-GCST90094756 | Weighted median | 5 | 0.0036108523606749 | 0.17261990985854 | 0.983311118276303 | -0.334724171 | 0.341945875683413 | 1.00361737934168 | 0.715535424583274 | 1.40768410551202 | 0.999911036302558 |
| ebi-fl187-GCST90094756 | ebi-IPs91-GCST90274796 | Interleukin-10 receptor subunit alpha levels \|\| id:ebi-IPs91-GCST90274796 | Dried fruit liking \|\| id:ebi-fl187-GCST90094756 | Weighted median | 5 | 0.0458117583323262 | 0.215396524224624 | 0.83157212929843 | -0.376365429 | 0.467988945812589 | 1.04687732647398 | 0.686351474375092 | 1.59677975148687 | 0.999489402457283 |
| ebi-fl187-GCST90094756 | ebi-IPs91-GCST90274807 | Interleukin-20 levels \|\| id:ebi-IPs91-GCST90274807 | Dried fruit liking \|\| id:ebi-fl187-GCST90094756 | Weighted median | 5 | 0.15846316269065 | 0.18950788458332 | 0.403052283410325 | -0.212972291 | 0.529898616473958 | 1.1717087608321 | 0.808178530650261 | 1.69876007360164 | 0.999489402457283 |
| ebi-fl187-GCST90094756 | ebi-IPs91-GCST90274808 | Interleukin-20 receptor subunit alpha levels \|\| id:ebi-IPs91-GCST90274808 | Dried fruit liking \|\| id:ebi-fl187-GCST90094756 | Weighted median | 5 | 0.0865784669565728 | 0.184397390090426 | 0.638697200570728 | -0.274840418 | 0.447997351533808 | 1.09043692762797 | 0.759693347224021 | 1.56517455033615 | 0.999489402457283 |
| ebi-fl187-GCST90094756 | ebi-IPs91-GCST90274810 | Interleukin-24 levels \|\| id:ebi-IPs91-GCST90274810 | Dried fruit liking \|\| id:ebi-fl187-GCST90094756 | Weighted median | 5 | 0.337675329573873 | 0.21540224329006 | 0.116962833326962 | -0.084513067 | 0.759863726422391 | 1.40168534374303 | 0.918959647000279 | 2.13798484979985 | 0.999489402457283 |
| ebi-fl187-GCST90094756 | ebi-IPs91-GCST90274823 | Monocyte chemoattractant protein-3 levels \|\| id:ebi-IPs91-GCST90274823 | Dried fruit liking \|\| id:ebi-fl187-GCST90094756 | Weighted median | 5 | 0.0119293193991019 | 0.208351133304786 | 0.95434139624268 | -0.396438902 | 0.420297540676482 | 1.01200075751652 | 0.672711376847239 | 1.52241446846613 | 0.999489402457283 |
| ebi-fl187-GCST90094756 | ebi-IPs91-GCST90274824 | Monocyte chemoattractant protein-4 levels \|\| id:ebi-IPs91-GCST90274824 | Dried fruit liking \|\| id:ebi-fl187-GCST90094756 | Weighted median | 5 | 0.114382063860233 | 0.177504401021453 | 0.519322556926973 | -0.233526562 | 0.462290689862281 | 1.12118040557743 | 0.791736565242014 | 1.58770676641228 | 0.999489402457283 |
| ebi-fl187-GCST90094756 | ebi-IPs91-GCST90274834 | SIR2-like protein 2 levels \|\| id:ebi-IPs91-GCST90274834 | Dried fruit liking \|\| id:ebi-fl187-GCST90094756 | Weighted median | 5 | 0.0404162978932718 | 0.166786325273789 | 0.808529265307975 | -0.2864849 | 0.367317495429899 | 1.0412441517258 | 0.750898417279385 | 1.44385626411539 | 0.999489402457283 |
| ebi-fl187-GCST90094756 | ebi-met1400-GCST90199652 | 1-stearoyl-2-oleoyl-GPS (18:0/18:1) levels \|\| id:ebi-met1400-GCST90199652 | Dried fruit liking \|\| id:ebi-fl187-GCST90094756 | Weighted median | 5 | -0.141109906 | 0.23774384533725 | 0.552821443022927 | -0.607087843 | 0.32486803104238 | 0.868393864914069 | 0.544935498510614 | 1.38384800895056 | 0.999489402457283 |
| ebi-fl187-GCST90094756 | ebi-met1400-GCST90199665 | N-acetylaspartate (naa) levels \|\| id:ebi-met1400-GCST90199665 | Dried fruit liking \|\| id:ebi-fl187-GCST90094756 | Weighted median | 5 | -0.162076069 | 0.246299416997021 | 0.510509295141716 | -0.644822927 | 0.320670787886531 | 0.850376514425071 | 0.524755454038249 | 1.37805183485148 | 0.999489402457283 |
| ebi-fl187-GCST90094756 | ebi-met1400-GCST90199669 | Acetylcarnitine levels (Biocrates platform) \|\| id:ebi-met1400-GCST90199669 | Dried fruit liking \|\| id:ebi-fl187-GCST90094756 | Weighted median | 5 | -0.315253487 | 0.226625852918662 | 0.164202780896219 | -0.759440158 | 0.128933185073733 | 0.7296039059789 | 0.467928319448213 | 1.13761411202337 | 0.999489402457283 |
| ebi-fl187-GCST90094756 | ebi-met1400-GCST90199670 | Iminodiacetate (IDA) levels \|\| id:ebi-met1400-GCST90199670 | Dried fruit liking \|\| id:ebi-fl187-GCST90094756 | Weighted median | 5 | 0.113316205904798 | 0.251468903021986 | 0.652265488161689 | -0.379562844 | 0.606195255827891 | 1.11998602315674 | 0.684160428672361 | 1.83344233237898 | 0.999489402457283 |
| ebi-fl187-GCST90094756 | ebi-met1400-GCST90199686 | Indolepropionate levels \|\| id:ebi-met1400-GCST90199686 | Dried fruit liking \|\| id:ebi-fl187-GCST90094756 | Weighted median | 5 | 0.185285740020629 | 0.219569527147399 | 0.398748154568258 | -0.245070533 | 0.615642013229532 | 1.20356229691408 | 0.782649333542214 | 1.85084448484357 | 0.999489402457283 |
| ebi-fl187-GCST90094756 | ebi-met1400-GCST90199704 | 3-hydroxysebacate levels \|\| id:ebi-met1400-GCST90199704 | Dried fruit liking \|\| id:ebi-fl187-GCST90094756 | Weighted median | 5 | -0.155509153 | 0.238629968459977 | 0.514610939246436 | -0.623223891 | 0.312205585201057 | 0.855979242131763 | 0.536212955830691 | 1.36643558308915 | 0.999489402457283 |
| ebi-fl187-GCST90094756 | ebi-met1400-GCST90199713 | Docosapentaenoate n3 DPA; 22:5n3 levels \|\| id:ebi-met1400-GCST90199713 | Dried fruit liking \|\| id:ebi-fl187-GCST90094756 | Weighted median | 5 | -0.056094942 | 0.232865418206567 | 0.809640399962299 | -0.512511162 | 0.400321277253044 | 0.945449368366888 | 0.598989528822768 | 1.49230406398278 | 0.999489402457283 |
| ebi-fl187-GCST90094756 | ebi-met1400-GCST90199715 | Myristoleate (14:1n5) levels \|\| id:ebi-met1400-GCST90199715 | Dried fruit liking \|\| id:ebi-fl187-GCST90094756 | Weighted median | 5 | -0.021921412 | 0.216524196152297 | 0.9193581031966 | -0.446308836 | 0.402466012616577 | 0.97831711617037 | 0.639986090601066 | 1.4955080959541 | 0.999489402457283 |
| ebi-fl187-GCST90094756 | ebi-met1400-GCST90199725 | Campesterol levels \|\| id:ebi-met1400-GCST90199725 | Dried fruit liking \|\| id:ebi-fl187-GCST90094756 | Weighted median | 5 | -0.280973711 | 0.280903556909606 | 0.317189661988042 | -0.831544682 | 0.269597260949181 | 0.755048184987841 | 0.435376248726731 | 1.30943698311679 | 0.999489402457283 |
| ebi-fl187-GCST90094756 | ebi-met1400-GCST90199737 | N-acetylproline levels \|\| id:ebi-met1400-GCST90199737 | Dried fruit liking \|\| id:ebi-fl187-GCST90094756 | Weighted median | 5 | -0.238624374 | 0.242184058401609 | 0.324475878142209 | -0.713305129 | 0.236056380248795 | 0.787710711260356 | 0.490021932494894 | 1.26624569940196 | 0.999489402457283 |
| ebi-fl187-GCST90094756 | ebi-met1400-GCST90199775 | Gamma-glutamylisoleucine levels \|\| id:ebi-met1400-GCST90199775 | Dried fruit liking \|\| id:ebi-fl187-GCST90094756 | Weighted median | 5 | -0.221622054 | 0.219386430653584 | 0.312404104094184 | -0.651619458 | 0.208375350007583 | 0.801218124243961 | 0.521201029671739 | 1.2316753921636 | 0.999489402457283 |
| ebi-fl187-GCST90094756 | ebi-met1400-GCST90199777 | 1-palmitoleoyl-GPC (16:1) levels \|\| id:ebi-met1400-GCST90199777 | Dried fruit liking \|\| id:ebi-fl187-GCST90094756 | Weighted median | 5 | -0.070107004 | 0.224989249760872 | 0.75534333745265 | -0.511085934 | 0.370871925046167 | 0.932294054923046 | 0.599843834176389 | 1.44899748121653 | 0.999489402457283 |
| ebi-fl187-GCST90094756 | ebi-met1400-GCST90199782 | Glutamine degradant levels \|\| id:ebi-met1400-GCST90199782 | Dried fruit liking \|\| id:ebi-fl187-GCST90094756 | Weighted median | 5 | 0.289153571433069 | 0.174769793120557 | 0.098029507782823 | -0.053395223 | 0.631702365949361 | 1.33529677622287 | 0.94800526486903 | 1.88080968182969 | 0.999489402457283 |
| ebi-fl187-GCST90094756 | ebi-met1400-GCST90199783 | 5-methyluridine (ribothymidine) levels \|\| id:ebi-met1400-GCST90199783 | Dried fruit liking \|\| id:ebi-fl187-GCST90094756 | Weighted median | 5 | -0.049184956 | 0.233541042736337 | 0.833195360237954 | -0.5069254 | 0.408555487523567 | 0.952005034142775 | 0.602344703720202 | 1.50464273934113 | 0.999489402457283 |
| ebi-fl187-GCST90094756 | ebi-met1400-GCST90199787 | Homostachydrine levels \|\| id:ebi-met1400-GCST90199787 | Dried fruit liking \|\| id:ebi-fl187-GCST90094756 | Weighted median | 5 | -0.198816606 | 0.242066129462747 | 0.411457308774359 | -0.673266219 | 0.275633008086124 | 0.819700207927007 | 0.51003995183129 | 1.31736431325253 | 0.999489402457283 |
| ebi-fl187-GCST90094756 | ebi-met1400-GCST90199802 | Beta-hydroxyisovaleroylcarnitine levels \|\| id:ebi-met1400-GCST90199802 | Dried fruit liking \|\| id:ebi-fl187-GCST90094756 | Weighted median | 5 | -0.287542576 | 0.229750373886931 | 0.210736270365786 | -0.737853309 | 0.162768156443507 | 0.7501046293552 | 0.478139231701915 | 1.17676383294747 | 0.999489402457283 |
| ebi-fl187-GCST90094756 | ebi-met1400-GCST90199873 | Pregnenolone sulfate levels \|\| id:ebi-met1400-GCST90199873 | Dried fruit liking \|\| id:ebi-fl187-GCST90094756 | Weighted median | 5 | 0.210978296168002 | 0.221643566475998 | 0.341157344542035 | -0.223443094 | 0.645399686460959 | 1.23488555302196 | 0.799760401638179 | 1.90674897874259 | 0.999489402457283 |
| ebi-fl187-GCST90094756 | ebi-met1400-GCST90199911 | Beta-citrylglutamate levels \|\| id:ebi-met1400-GCST90199911 | Dried fruit liking \|\| id:ebi-fl187-GCST90094756 | Weighted median | 5 | -0.331872044 | 0.24616504464081 | 0.177604082490273 | -0.814355532 | 0.15061144348458 | 0.717579135528007 | 0.442924686404003 | 1.16254485593393 | 0.999489402457283 |
| ebi-fl187-GCST90094756 | ebi-met1400-GCST90199934 | 2-hydroxydecanoate levels \|\| id:ebi-met1400-GCST90199934 | Dried fruit liking \|\| id:ebi-fl187-GCST90094756 | Weighted median | 5 | -0.004727298 | 0.223423792427548 | 0.983119269460886 | -0.442637931 | 0.433183334960393 | 0.995283857890216 | 0.642339736067014 | 1.54215892643062 | 0.999911036302558 |
| ebi-fl187-GCST90094756 | ebi-met1400-GCST90199945 | Sphingomyelin (d18:1/14:0, d16:1/16:0) levels \|\| id:ebi-met1400-GCST90199945 | Dried fruit liking \|\| id:ebi-fl187-GCST90094756 | Weighted median | 5 | -0.127051321 | 0.211015515719639 | 0.547111110219463 | -0.540641732 | 0.286539089698529 | 0.88068847352558 | 0.582374404185675 | 1.33181022693699 | 0.999489402457283 |
| ebi-fl187-GCST90094756 | ebi-met1400-GCST90199979 | Carnitine C14:1 levels \|\| id:ebi-met1400-GCST90199979 | Dried fruit liking \|\| id:ebi-fl187-GCST90094756 | Weighted median | 5 | -0.048784876 | 0.256954233241959 | 0.849420223388175 | -0.552415173 | 0.454845420812607 | 0.952385988420743 | 0.575558057828461 | 1.5759297582634 | 0.999489402457283 |
| ebi-fl187-GCST90094756 | ebi-met1400-GCST90199986 | Eugenol sulfate levels \|\| id:ebi-met1400-GCST90199986 | Dried fruit liking \|\| id:ebi-fl187-GCST90094756 | Weighted median | 5 | -0.19660518 | 0.241766453939631 | 0.416101739845192 | -0.67046743 | 0.277257069448817 | 0.82151491958753 | 0.511469445741923 | 1.31950553199895 | 0.999489402457283 |
| ebi-fl187-GCST90094756 | ebi-met1400-GCST90200002 | 6-hydroxyindole sulfate levels \|\| id:ebi-met1400-GCST90200002 | Dried fruit liking \|\| id:ebi-fl187-GCST90094756 | Weighted median | 5 | -0.038673457 | 0.22316813463384 | 0.8624211919442 | -0.476083001 | 0.398736087151893 | 0.962064813649003 | 0.621211919137663 | 1.48994035231378 | 0.999489402457283 |
| ebi-fl187-GCST90094756 | ebi-met1400-GCST90200023 | Trans 3,4-methyleneheptanoate levels \|\| id:ebi-met1400-GCST90200023 | Dried fruit liking \|\| id:ebi-fl187-GCST90094756 | Weighted median | 5 | -0.124157205 | 0.249422916017689 | 0.618640623949347 | -0.61302612 | 0.364711710498894 | 0.883240980162011 | 0.541709109318869 | 1.44009878293988 | 0.999489402457283 |
| ebi-fl187-GCST90094756 | ebi-met1400-GCST90200032 | 2-hydroxybutyrate/2-hydroxyisobutyrate levels \|\| id:ebi-met1400-GCST90200032 | Dried fruit liking \|\| id:ebi-fl187-GCST90094756 | Weighted median | 5 | -0.160590089 | 0.279116761665657 | 0.565053981164064 | -0.707658942 | 0.386478763495683 | 0.851641096306862 | 0.492796513220292 | 1.47178914107805 | 0.999489402457283 |
| ebi-fl187-GCST90094756 | ebi-met1400-GCST90200051 | 1-palmitoyl-2-dihomo-linolenoyl-GPC (16:0/20:3n3 or 6) levels \|\| id:ebi-met1400-GCST90200051 | Dried fruit liking \|\| id:ebi-fl187-GCST90094756 | Weighted median | 5 | -0.208075049 | 0.257020655284728 | 0.41818995720691 | -0.711835533 | 0.29568543528855 | 0.812146083616844 | 0.490742595815166 | 1.34404730047645 | 0.999489402457283 |
| ebi-fl187-GCST90094756 | ebi-met1400-GCST90200069 | Phenylacetylglutamate levels \|\| id:ebi-met1400-GCST90200069 | Dried fruit liking \|\| id:ebi-fl187-GCST90094756 | Weighted median | 5 | -0.166705783 | 0.250938523246914 | 0.506479648746886 | -0.658545289 | 0.325133722143099 | 0.84644861392066 | 0.51760375096278 | 1.38421573389975 | 0.999489402457283 |
| ebi-fl187-GCST90094756 | ebi-met1400-GCST90200072 | Thioproline levels \|\| id:ebi-met1400-GCST90200072 | Dried fruit liking \|\| id:ebi-fl187-GCST90094756 | Weighted median | 5 | 0.007468314758289 | 0.217587665189578 | 0.972619388896236 | -0.419003509 | 0.433940138529861 | 1.00749627217588 | 0.657701887379109 | 1.54332647955929 | 0.999489402457283 |
| ebi-fl187-GCST90094756 | ebi-met1400-GCST90200081 | 1-oleoyl-2-docosahexaenoyl-GPC (18:1/22:6) levels \|\| id:ebi-met1400-GCST90200081 | Dried fruit liking \|\| id:ebi-fl187-GCST90094756 | Weighted median | 5 | -0.190058731 | 0.209242200884009 | 0.363709545026712 | -0.600173445 | 0.220055982914157 | 0.82691056738275 | 0.54871645596061 | 1.24614649154671 | 0.999489402457283 |
| ebi-fl187-GCST90094756 | ebi-met1400-GCST90200097 | Furaneol sulfate levels \|\| id:ebi-met1400-GCST90200097 | Dried fruit liking \|\| id:ebi-fl187-GCST90094756 | Weighted median | 5 | 0.0933055697943013 | 0.271384749369419 | 0.730987020104845 | -0.438608539 | 0.625219678558362 | 1.09779713766659 | 0.644933196434419 | 1.86865641609365 | 0.999489402457283 |
| ebi-fl187-GCST90094756 | ebi-met1400-GCST90200124 | Sphingomyelin (d18:2/24:2) levels \|\| id:ebi-met1400-GCST90200124 | Dried fruit liking \|\| id:ebi-fl187-GCST90094756 | Weighted median | 4 | 0.313274274575177 | 0.212568516761384 | 0.140547112030118 | -0.103360018 | 0.729908567427489 | 1.36789665885623 | 0.901802249739515 | 2.07489088638948 | 0.999489402457283 |
| ebi-fl187-GCST90094756 | ebi-met1400-GCST90200137 | Lignoceroylcarnitine (C24) levels \|\| id:ebi-met1400-GCST90200137 | Dried fruit liking \|\| id:ebi-fl187-GCST90094756 | Weighted median | 5 | -0.102254436 | 0.226956053143023 | 0.652315859687952 | -0.5470883 | 0.342579428241263 | 0.902799817742478 | 0.578632163162217 | 1.40857622995173 | 0.999489402457283 |
| ebi-fl187-GCST90094756 | ebi-met1400-GCST90200165 | Octadecenedioate (C18:1-DC) levels \|\| id:ebi-met1400-GCST90200165 | Dried fruit liking \|\| id:ebi-fl187-GCST90094756 | Weighted median | 5 | 0.0847739715515504 | 0.248126652847228 | 0.732609752498958 | -0.401554268 | 0.571102211132118 | 1.08847101347757 | 0.669278998262088 | 1.77021712956387 | 0.999489402457283 |
| ebi-fl187-GCST90094756 | ebi-met1400-GCST90200176 | Glutamine conjugate of C6H10O2 (1) levels \|\| id:ebi-met1400-GCST90200176 | Dried fruit liking \|\| id:ebi-fl187-GCST90094756 | Weighted median | 5 | 0.0197946888078582 | 0.225998170697709 | 0.930204277180847 | -0.423161726 | 0.462751103375368 | 1.01999190277405 | 0.654972698588636 | 1.58843793636971 | 0.999489402457283 |
| ebi-fl187-GCST90094756 | ebi-met1400-GCST90200182 | Glycine conjugate of C10H12O2 levels \|\| id:ebi-met1400-GCST90200182 | Dried fruit liking \|\| id:ebi-fl187-GCST90094756 | Weighted median | 5 | -0.135017752 | 0.27967258025673 | 0.629258494652136 | -0.683176009 | 0.413140505366606 | 0.873700401676243 | 0.505010524545503 | 1.51155739293993 | 0.999489402457283 |
| ebi-fl187-GCST90094756 | ebi-met1400-GCST90200208 | Glucuronide of piperine metabolite C17H21NO3 (4) levels \|\| id:ebi-met1400-GCST90200208 | Dried fruit liking \|\| id:ebi-fl187-GCST90094756 | Weighted median | 5 | -0.079230119 | 0.261121097758137 | 0.761567599912849 | -0.591027471 | 0.432567232186262 | 0.923827309378272 | 0.553758022011281 | 1.54120909066616 | 0.999489402457283 |
| ebi-fl187-GCST90094756 | ebi-met1400-GCST90200227 | 3-ethylcatechol sulfate (2) levels \|\| id:ebi-met1400-GCST90200227 | Dried fruit liking \|\| id:ebi-fl187-GCST90094756 | Weighted median | 5 | -0.232689991 | 0.312865751199686 | 0.457035298312353 | -0.845906864 | 0.380526880908271 | 0.792399185979489 | 0.429167984775938 | 1.46305524227016 | 0.999489402457283 |
| ebi-fl187-GCST90094756 | ebi-met1400-GCST90200259 | Pentose acid levels \|\| id:ebi-met1400-GCST90200259 | Dried fruit liking \|\| id:ebi-fl187-GCST90094756 | Weighted median | 5 | -0.055637216 | 0.197934212824759 | 0.778641803759764 | -0.443588273 | 0.332313840952128 | 0.945882224415634 | 0.641729583632483 | 1.39419033388035 | 0.999489402457283 |
| ebi-fl187-GCST90094756 | ebi-met1400-GCST90200291 | Sarcosine levels \|\| id:ebi-met1400-GCST90200291 | Dried fruit liking \|\| id:ebi-fl187-GCST90094756 | Weighted median | 5 | 0.289929885874953 | 0.2485055269925 | 0.243334001574388 | -0.197140947 | 0.777000718780254 | 1.33633378886632 | 0.821074897088074 | 2.17493921882044 | 0.999489402457283 |
| ebi-fl187-GCST90094756 | ebi-met1400-GCST90200306 | Butyrate/isobutyrate (4:0) levels \|\| id:ebi-met1400-GCST90200306 | Dried fruit liking \|\| id:ebi-fl187-GCST90094756 | Weighted median | 5 | 0.0248466664312927 | 0.231636978676071 | 0.914578319029778 | -0.429161812 | 0.478855144636391 | 1.02515791735052 | 0.651054572362078 | 1.61422528943085 | 0.999489402457283 |
| ebi-fl187-GCST90094756 | ebi-met1400-GCST90200321 | Cholate levels \|\| id:ebi-met1400-GCST90200321 | Dried fruit liking \|\| id:ebi-fl187-GCST90094756 | Weighted median | 5 | 0.23757955879798 | 0.234120013951043 | 0.310212234715515 | -0.221295669 | 0.696454786142023 | 1.26817588732652 | 0.801479672924517 | 2.00662616349084 | 0.999489402457283 |
| ebi-fl187-GCST90094756 | ebi-met1400-GCST90200336 | Ceramide (d18:1/16:0) levels \|\| id:ebi-met1400-GCST90200336 | Dried fruit liking \|\| id:ebi-fl187-GCST90094756 | Weighted median | 5 | -0.265690678 | 0.226698715437812 | 0.241197438836944 | -0.710020161 | 0.178638803847737 | 0.766676240340458 | 0.491634285685201 | 1.19558882408569 | 0.999489402457283 |
| ebi-fl187-GCST90094756 | ebi-met1400-GCST90200358 | Arachidonate (20:4n6) levels \|\| id:ebi-met1400-GCST90200358 | Dried fruit liking \|\| id:ebi-fl187-GCST90094756 | Weighted median | 5 | -0.327385943 | 0.236863253883208 | 0.166918655251086 | -0.79163792 | 0.136866035087348 | 0.720805499843387 | 0.45310204220524 | 1.14667452407803 | 0.999489402457283 |
| ebi-fl187-GCST90094756 | ebi-met1400-GCST90200413 | 2'-deoxyuridine levels \|\| id:ebi-met1400-GCST90200413 | Dried fruit liking \|\| id:ebi-fl187-GCST90094756 | Weighted median | 5 | 0.0368322313318566 | 0.252175871525705 | 0.88387582766093 | -0.457432477 | 0.531096939522238 | 1.03751894306492 | 0.632906563423708 | 1.70079695712983 | 0.999489402457283 |
| ebi-fl187-GCST90094756 | ebi-met1400-GCST90200432 | Threonine levels \|\| id:ebi-met1400-GCST90200432 | Dried fruit liking \|\| id:ebi-fl187-GCST90094756 | Weighted median | 5 | 0.0463077131276751 | 0.228951492885373 | 0.839713523073901 | -0.402437213 | 0.495052639183007 | 1.04739665907629 | 0.668688322590216 | 1.64058459581693 | 0.999489402457283 |
| ebi-fl187-GCST90094756 | ebi-met1400-GCST90200436 | Caffeine levels \|\| id:ebi-met1400-GCST90200436 | Dried fruit liking \|\| id:ebi-fl187-GCST90094756 | Weighted median | 5 | 0.0606889370301272 | 0.242649541884635 | 0.802502728644524 | -0.414904165 | 0.536282039124012 | 1.06256833711208 | 0.660403567406173 | 1.70963866150458 | 0.999489402457283 |
| ebi-fl187-GCST90094756 | ebi-met1400-GCST90200442 | Valine levels \|\| id:ebi-met1400-GCST90200442 | Dried fruit liking \|\| id:ebi-fl187-GCST90094756 | Weighted median | 5 | -0.417768447 | 0.248438023720433 | 0.0926502841593126 | -0.904706973 | 0.0691700795341464 | 0.658514691852626 | 0.404660443985563 | 1.07161845401729 | 0.999489402457283 |
| ebi-fl187-GCST90094756 | ebi-met1400-GCST90200459 | Margarate (17:0) levels \|\| id:ebi-met1400-GCST90200459 | Dried fruit liking \|\| id:ebi-fl187-GCST90094756 | Weighted median | 5 | -0.337836696 | 0.274933345202388 | 0.219148709386408 | -0.876706053 | 0.201032660666308 | 0.713311765101577 | 0.416151437494022 | 1.22266470421513 | 0.999489402457283 |
| ebi-fl187-GCST90094756 | ebi-met1400-GCST90200462 | X-11299 levels \|\| id:ebi-met1400-GCST90200462 | Dried fruit liking \|\| id:ebi-fl187-GCST90094756 | Weighted median | 5 | -0.160404084 | 0.220043438323052 | 0.466022741517883 | -0.591689223 | 0.270881055113606 | 0.851799520857054 | 0.553391692707413 | 1.31111911019583 | 0.999489402457283 |
| ebi-fl187-GCST90094756 | ebi-met1400-GCST90200497 | X-12740 levels \|\| id:ebi-met1400-GCST90200497 | Dried fruit liking \|\| id:ebi-fl187-GCST90094756 | Weighted median | 5 | 0.420820906 | 0.266022170740281 | 0.113672028266188 | -0.100582549 | 0.94222436077095 | 1.52321145612934 | 0.9043104598316 | 2.56568207838239 | 0.999489402457283 |
| ebi-fl187-GCST90094756 | ebi-met1400-GCST90200527 | X-13866 levels \|\| id:ebi-met1400-GCST90200527 | Dried fruit liking \|\| id:ebi-fl187-GCST90094756 | Weighted median | 5 | -0.125748958 | 0.249284521719191 | 0.613952569976444 | -0.614346621 | 0.362848704289053 | 0.881836196673302 | 0.540994254223958 | 1.437418368 | 0.999489402457283 |
| ebi-fl187-GCST90094756 | ebi-met1400-GCST90200547 | X-17654 levels \|\| id:ebi-met1400-GCST90200547 | Dried fruit liking \|\| id:ebi-fl187-GCST90094756 | Weighted median | 5 | -0.101395283 | 0.245394122103266 | 0.679464769473702 | -0.582367762 | 0.379577196150344 | 0.903575793979213 | 0.558574228453246 | 1.46166646056336 | 0.999489402457283 |
| ebi-fl187-GCST90094756 | ebi-met1400-GCST90200553 | X-17653 levels \|\| id:ebi-met1400-GCST90200553 | Dried fruit liking \|\| id:ebi-fl187-GCST90094756 | Weighted median | 5 | 0.0518390426118738 | 0.232538675643434 | 0.823592679382717 | -0.403936762 | 0.507614846873003 | 1.05320620754945 | 0.667686343317054 | 1.66132395356478 | 0.999489402457283 |
| ebi-fl187-GCST90094756 | ebi-met1400-GCST90200557 | X-17685 levels \|\| id:ebi-met1400-GCST90200557 | Dried fruit liking \|\| id:ebi-fl187-GCST90094756 | Weighted median | 5 | -0.129241402 | 0.238970193825562 | 0.58862720801568 | -0.597622982 | 0.33914017763388 | 0.878761804849176 | 0.550117722823301 | 1.4037401043882 | 0.999489402457283 |
| ebi-fl187-GCST90094756 | ebi-met1400-GCST90200561 | X-18779 levels \|\| id:ebi-met1400-GCST90200561 | Dried fruit liking \|\| id:ebi-fl187-GCST90094756 | Weighted median | 5 | -0.188566463 | 0.227752090975628 | 0.407701007473009 | -0.634960561 | 0.257827635528663 | 0.828145460756996 | 0.529956388804669 | 1.29411573982402 | 0.999489402457283 |
| ebi-fl187-GCST90094756 | ebi-met1400-GCST90200570 | X-18921 levels \|\| id:ebi-met1400-GCST90200570 | Dried fruit liking \|\| id:ebi-fl187-GCST90094756 | Weighted median | 5 | -0.011484254 | 0.230375784658673 | 0.960241849185977 | -0.463020792 | 0.440052284031615 | 0.988581438428156 | 0.629379546419346 | 1.55278840242693 | 0.999489402457283 |
| ebi-fl187-GCST90094756 | ebi-met1400-GCST90200575 | X-21283 levels \|\| id:ebi-met1400-GCST90200575 | Dried fruit liking \|\| id:ebi-fl187-GCST90094756 | Weighted median | 5 | -0.289816489 | 0.266967619475798 | 0.277661900662064 | -0.813073023 | 0.233440045611479 | 0.748400895102739 | 0.44349310562383 | 1.26293710699904 | 0.999489402457283 |
| ebi-fl187-GCST90094756 | ebi-met1400-GCST90200578 | X-18922 levels \|\| id:ebi-met1400-GCST90200578 | Dried fruit liking \|\| id:ebi-fl187-GCST90094756 | Weighted median | 5 | -0.343879193 | 0.233657647677369 | 0.14109585777142 | -0.801848182 | 0.114089796845639 | 0.709014577082087 | 0.448499289322551 | 1.12085276940842 | 0.999489402457283 |
| ebi-fl187-GCST90094756 | ebi-met1400-GCST90200623 | X-24241 levels \|\| id:ebi-met1400-GCST90200623 | Dried fruit liking \|\| id:ebi-fl187-GCST90094756 | Weighted median | 5 | -0.01758883 | 0.279173317042787 | 0.949763906801945 | -0.564768531 | 0.529590871773302 | 0.982564950906803 | 0.568491719719726 | 1.69823736962512 | 0.999489402457283 |
| ebi-fl187-GCST90094756 | ebi-met1400-GCST90200636 | X-24544 levels \|\| id:ebi-met1400-GCST90200636 | Dried fruit liking \|\| id:ebi-fl187-GCST90094756 | Weighted median | 5 | 0.0413371336150137 | 0.232446649988591 | 0.858852614776337 | -0.4142583 | 0.496932567592652 | 1.04220340812676 | 0.660830236529293 | 1.64367167824483 | 0.999489402457283 |
| ebi-fl187-GCST90094756 | ebi-met1400-GCST90200659 | X-25172 levels \|\| id:ebi-met1400-GCST90200659 | Dried fruit liking \|\| id:ebi-fl187-GCST90094756 | Weighted median | 5 | -0.076322172 | 0.2272106375188 | 0.736939243031477 | -0.521655022 | 0.369010677088403 | 0.926517660007175 | 0.593537417168548 | 1.44630304589778 | 0.999489402457283 |
| ebi-fl187-GCST90094756 | ebi-met1400-GCST90200670 | X-26111 levels \|\| id:ebi-met1400-GCST90200670 | Dried fruit liking \|\| id:ebi-fl187-GCST90094756 | Weighted median | 5 | -0.335256431 | 0.225822775459536 | 0.13764988811982 | -0.77786907 | 0.107356209343146 | 0.715154675325345 | 0.45938388373543 | 1.11333076267485 | 0.999489402457283 |
| ebi-fl187-GCST90094756 | ebi-met1400-GCST90200673 | Carnitine C4 levels \|\| id:ebi-met1400-GCST90200673 | Dried fruit liking \|\| id:ebi-fl187-GCST90094756 | Weighted median | 5 | -0.137968223 | 0.259371618695204 | 0.594772669464484 | -0.646336595 | 0.37040015011675 | 0.871126373501783 | 0.52396174913033 | 1.44831404175958 | 0.999489402457283 |
| ebi-fl187-GCST90094756 | ebi-met1400-GCST90200710 | X-13431 levels \|\| id:ebi-met1400-GCST90200710 | Dried fruit liking \|\| id:ebi-fl187-GCST90094756 | Weighted median | 5 | -0.252192826 | 0.220045287688994 | 0.251755805484182 | -0.68348159 | 0.179095938210527 | 0.777094879788149 | 0.504856226859047 | 1.19613549376218 | 0.999489402457283 |
| ebi-fl187-GCST90094756 | ebi-met1400-GCST90200730 | Adenosine 5'-diphosphate (ADP) to fructose ratio \|\| id:ebi-met1400-GCST90200730 | Dried fruit liking \|\| id:ebi-fl187-GCST90094756 | Weighted median | 5 | 0.172237396082488 | 0.331455982207238 | 0.603315334505947 | -0.477416329 | 0.821891121208675 | 1.18795981674834 | 0.620384191563219 | 2.27479769052905 | 0.999489402457283 |
| ebi-fl187-GCST90094756 | ebi-met1400-GCST90200731 | Adenosine 5'-diphosphate (ADP) to mannose ratio \|\| id:ebi-met1400-GCST90200731 | Dried fruit liking \|\| id:ebi-fl187-GCST90094756 | Weighted median | 5 | 0.341441889625633 | 0.29392176316938 | 0.245367054504556 | -0.234644766 | 0.917528545437617 | 1.4069748310963 | 0.790851737014004 | 2.50309644992715 | 0.999489402457283 |
| ebi-fl187-GCST90094756 | ebi-met1400-GCST90200748 | Aspartate to glutamate ratio \|\| id:ebi-met1400-GCST90200748 | Dried fruit liking \|\| id:ebi-fl187-GCST90094756 | Weighted median | 5 | 0.434044641625545 | 0.228580109283784 | 0.057581150924623 | -0.013972373 | 0.882061655821762 | 1.5434877703816 | 0.986124787979412 | 2.41587527902939 | 0.999489402457283 |
| ebi-fl187-GCST90094756 | ebi-met1400-GCST90200774 | Uridine to pseudouridine ratio \|\| id:ebi-met1400-GCST90200774 | Dried fruit liking \|\| id:ebi-fl187-GCST90094756 | Weighted median | 5 | 0.0221863243182146 | 0.226795428449011 | 0.922071040027937 | -0.422332715 | 0.466705364078276 | 1.02243427109222 | 0.655515902843503 | 1.59473146901435 | 0.999489402457283 |
| ebi-fl187-GCST90094756 | ebi-met1400-GCST90200799 | Spermidine to adenosine 5'-diphosphate (ADP) ratio \|\| id:ebi-met1400-GCST90200799 | Dried fruit liking \|\| id:ebi-fl187-GCST90094756 | Weighted median | 5 | -0.357868079 | 0.290534025912773 | 0.218038894851279 | -0.927314769 | 0.211578612139235 | 0.699165303753805 | 0.395614601784273 | 1.23562709710019 | 0.999489402457283 |
| ebi-fl187-GCST90094756 | ebi-met1400-GCST90200811 | Choline to taurocholate ratio \|\| id:ebi-met1400-GCST90200811 | Dried fruit liking \|\| id:ebi-fl187-GCST90094756 | Weighted median | 5 | 0.153119643853276 | 0.233777194435125 | 0.512479923035427 | -0.305083657 | 0.611322944946121 | 1.16546441125448 | 0.737061711264223 | 1.84286779945597 | 0.999489402457283 |
| ebi-fl187-GCST90094756 | ebi-met1400-GCST90200812 | Glutamate to kynurenine ratio \|\| id:ebi-met1400-GCST90200812 | Dried fruit liking \|\| id:ebi-fl187-GCST90094756 | Weighted median | 5 | -0.421540251 | 0.239982893677492 | 0.0789957505178199 | -0.891906723 | 0.0488262201223078 | 0.656035581455356 | 0.409873491959467 | 1.05003785943304 | 0.999489402457283 |
| ebi-fl187-GCST90094756 | ebi-met1400-GCST90200876 | Cortisone to 4-cholesten-3-one ratio \|\| id:ebi-met1400-GCST90200876 | Dried fruit liking \|\| id:ebi-fl187-GCST90094756 | Weighted median | 5 | -0.194489583 | 0.241089283381959 | 0.419832512303198 | -0.667024579 | 0.278045412352528 | 0.82325475399397 | 0.513233393848486 | 1.32054616495542 | 0.999489402457283 |
| ebi-fl187-GCST90094756 | ebi-met1400-GCST90200906 | 2'-deoxyuridine to cytidine ratio \|\| id:ebi-met1400-GCST90200906 | Dried fruit liking \|\| id:ebi-fl187-GCST90094756 | Weighted median | 5 | 0.29982527808714 | 0.278186227381577 | 0.281129177541195 | -0.245419728 | 0.845070283755031 | 1.34962297826593 | 0.782376084494959 | 2.32814143934269 | 0.999489402457283 |
| ebi-fl187-GCST90094756 | ebi-met1400-GCST90200907 | Retinol (Vitamin A) to linoleoyl-arachidonoyl-glycerol (18:2 to 20:4) [1] ratio \|\| id:ebi-met1400-GCST90200907 | Dried fruit liking \|\| id:ebi-fl187-GCST90094756 | Weighted median | 5 | 0.0583042918933192 | 0.239819851693109 | 0.807914733040961 | -0.411742617 | 0.528351201211813 | 1.06003750745908 | 0.662494768719642 | 1.69613341912377 | 0.999489402457283 |
| ebi-fl187-GCST90094756 | ebi-met1400-GCST90200916 | Fructose to sucrose ratio \|\| id:ebi-met1400-GCST90200916 | Dried fruit liking \|\| id:ebi-fl187-GCST90094756 | Weighted median | 5 | 0.331180066968451 | 0.236813029335021 | 0.161966716647451 | -0.132973471 | 0.795333604465093 | 1.39261053288936 | 0.875488318043336 | 2.21517986744671 | 0.999489402457283 |
| ebi-fl187-GCST90094756 | ebi-met1400-GCST90200917 | Alpha-ketobutyrate to 3-methyl-2-oxobutyrate ratio \|\| id:ebi-met1400-GCST90200917 | Dried fruit liking \|\| id:ebi-fl187-GCST90094756 | Weighted median | 5 | -0.269092593 | 0.254761059572153 | 0.290851818175017 | -0.76842427 | 0.230239083498563 | 0.764072504406463 | 0.463743226997505 | 1.25890095639738 | 0.999489402457283 |
| ebi-fl187-GCST90094756 | ebi-met1400-GCST90200924 | Alpha-tocopherol to glycerol ratio \|\| id:ebi-met1400-GCST90200924 | Dried fruit liking \|\| id:ebi-fl187-GCST90094756 | Weighted median | 5 | 0.281104835238006 | 0.209991955774272 | 0.180686030929004 | -0.130479398 | 0.692689068555579 | 1.32459246062246 | 0.877674574544231 | 1.99908398582583 | 0.999489402457283 |
| ebi-fl187-GCST90094756 | ebi-met1400-GCST90200938 | Alpha-ketoglutarate to alanine ratio \|\| id:ebi-met1400-GCST90200938 | Dried fruit liking \|\| id:ebi-fl187-GCST90094756 | Weighted median | 5 | -0.100051259 | 0.224479091883073 | 0.65581097750303 | -0.540030279 | 0.339927760686103 | 0.904791037797264 | 0.582730607318022 | 1.40484610177937 | 0.999489402457283 |
| ebi-fl187-GCST90094756 | ebi-met1400-GCST90200943 | Phosphate to EDTA ratio \|\| id:ebi-met1400-GCST90200943 | Dried fruit liking \|\| id:ebi-fl187-GCST90094756 | Weighted median | 5 | 0.00143282884170986 | 0.222602485918647 | 0.994864279650163 | -0.434868044 | 0.437733701242257 | 1.0014338558314 | 0.647350083455489 | 1.54919230449793 | 0.999911036302558 |
| ebi-fl187-GCST90094756 | ebi-met1400-GCST90200947 | Adenosine 5'-diphosphate (ADP) to glucose ratio \|\| id:ebi-met1400-GCST90200947 | Dried fruit liking \|\| id:ebi-fl187-GCST90094756 | Weighted median | 5 | 0.45176564491906 | 0.306635962930889 | 0.140671144510612 | -0.149240842 | 1.0527721322636 | 1.57108371397956 | 0.86136163748929 | 2.86558389519929 | 0.999489402457283 |
| ebi-fl187-GCST90094756 | ebi-met1400-GCST90200975 | Arachidonate (20:4n6) to caffeine ratio \|\| id:ebi-met1400-GCST90200975 | Dried fruit liking \|\| id:ebi-fl187-GCST90094756 | Weighted median | 5 | 0.114833102766382 | 0.246622934223635 | 0.641485979870165 | -0.368547848 | 0.598214053844706 | 1.12168621562259 | 0.691738110305366 | 1.8188674985137 | 0.999489402457283 |
| ebi-fl187-GCST90094756 | ebi-met1400-GCST90200985 | Cholesterol to oleoyl-linoleoyl-glycerol (18:1 to 18:2) [2] ratio \|\| id:ebi-met1400-GCST90200985 | Dried fruit liking \|\| id:ebi-fl187-GCST90094756 | Weighted median | 5 | 0.268739787791381 | 0.22930650246862 | 0.241210070788687 | -0.180700957 | 0.718180532629875 | 1.30831465730283 | 0.834684928023561 | 2.05069863495259 | 0.999489402457283 |
| ebi-fl187-GCST90094756 | ebi-met1400-GCST90201016 | Bilirubin (Z,Z) to etiocholanolone glucuronide ratio \|\| id:ebi-met1400-GCST90201016 | Dried fruit liking \|\| id:ebi-fl187-GCST90094756 | Weighted median | 5 | 0.0977762998412616 | 0.234017862848101 | 0.676081987769118 | -0.360898711 | 0.556451311023539 | 1.10271607975344 | 0.697049598110379 | 1.7444709183438 | 0.999489402457283 |
| ebi-fl187-GCST90094756 | ebi-pw11-GCST90275189 | Placental weight (fetal genotype, adjusted for sex and gestational age) \|\| id:ebi-pw11-GCST90275189 | Dried fruit liking \|\| id:ebi-fl187-GCST90094756 | Weighted median | 5 | -0.020308505 | 0.0962670769942619 | 0.832918465776021 | -0.208991976 | 0.168374966064973 | 0.979896323908698 | 0.811401746505698 | 1.18338025490439 | 0.999489402457283 |
| ebi-fl187-GCST90094756 | ebi-pw11-GCST90275192 | Placental weight (fetal effect, conditional on maternal and paternal genotype) \|\| id:ebi-pw11-GCST90275192 | Dried fruit liking \|\| id:ebi-fl187-GCST90094756 | Weighted median | 5 | -0.102475974 | 0.178967491427344 | 0.566918631031499 | -0.453252257 | 0.248300309228706 | 0.902599835384141 | 0.635557789396184 | 1.2818448242283 | 0.999489402457283 |
| ebi-fl187-GCST90094756 | ebi-pw11-GCST90275193 | Placental weight (maternal effect, conditional on fetal and paternal genotype) \|\| id:ebi-pw11-GCST90275193 | Dried fruit liking \|\| id:ebi-fl187-GCST90094756 | Weighted median | 5 | -0.055038655 | 0.140061964230478 | 0.694349525026005 | -0.329560105 | 0.219482795227019 | 0.946448562594531 | 0.719240054275467 | 1.24543242038934 | 0.999489402457283 |
| ebi-fl187-GCST90094756 | ebi-pw11-GCST90275195 | Placental weight (fetal effect, conditional on maternal genotype) \|\| id:ebi-pw11-GCST90275195 | Dried fruit liking \|\| id:ebi-fl187-GCST90094756 | Weighted median | 5 | -0.082025178 | 0.120741235463013 | 0.496918119928799 | -0.318677999 | 0.154627643889177 | 0.921248763554595 | 0.727109641555556 | 1.16722325746526 | 0.999489402457283 |
| ebi-fl187-GCST90094756 | ebi-pw11-GCST90275197 | Placental weight (fetal genotype, adjusted for sex) \|\| id:ebi-pw11-GCST90275197 | Dried fruit liking \|\| id:ebi-fl187-GCST90094756 | Weighted median | 5 | -0.044309931 | 0.0963920519300849 | 0.645742704327736 | -0.233238353 | 0.144618490611111 | 0.956657413568069 | 0.79196478387957 | 1.15559861443778 | 0.999489402457283 |
| ebi-fl187-GCST90094756 | prot-a-1030 | Protein FAM171B \|\| id:prot-a-1030 | Dried fruit liking \|\| id:ebi-fl187-GCST90094756 | Weighted median | 5 | -0.077293645 | 0.372326360552566 | 0.835544047154408 | -0.807053311 | 0.65246602201228 | 0.925618010899818 | 0.44617085767979 | 1.92027042410965 | 0.999489402457283 |
| ebi-fl187-GCST90094756 | prot-a-1036 | BRISC complex subunit Abro1 \|\| id:prot-a-1036 | Dried fruit liking \|\| id:ebi-fl187-GCST90094756 | Weighted median | 5 | -0.208766501 | 0.373175196145025 | 0.575866278137054 | -0.940189886 | 0.52265688305859 | 0.811584717427623 | 0.390553667709734 | 1.68650254246648 | 0.999489402457283 |
| ebi-fl187-GCST90094756 | prot-a-1055 | Phenylalanine--tRNA ligase, mitochondrial \|\| id:prot-a-1055 | Dried fruit liking \|\| id:ebi-fl187-GCST90094756 | Weighted median | 5 | 0.370742376572492 | 0.385406214010311 | 0.336073587975788 | -0.384653803 | 1.1261385560327 | 1.44880977796308 | 0.680686247031432 | 3.08372584560901 | 0.999489402457283 |
| ebi-fl187-GCST90094756 | prot-a-1083 | Fc receptor-like protein 5 \|\| id:prot-a-1083 | Dried fruit liking \|\| id:ebi-fl187-GCST90094756 | Weighted median | 5 | 0.154131266458415 | 0.351838967950834 | 0.661333130752055 | -0.535473111 | 0.84373564364205 | 1.16664401795619 | 0.585392269258137 | 2.32503628098442 | 0.999489402457283 |
| ebi-fl187-GCST90094756 | prot-a-1103 | Fibroblast growth factor receptor 3 \|\| id:prot-a-1103 | Dried fruit liking \|\| id:ebi-fl187-GCST90094756 | Weighted median | 5 | 0.097866769869761 | 0.386523529788163 | 0.800115641408232 | -0.659719349 | 0.855452888254561 | 1.10281584702151 | 0.516996409943097 | 2.35243953159293 | 0.999489402457283 |
| ebi-fl187-GCST90094756 | prot-a-1105 | Fibroblast growth factor receptor 4 \|\| id:prot-a-1105 | Dried fruit liking \|\| id:ebi-fl187-GCST90094756 | Weighted median | 5 | -0.138023471 | 0.373626587945736 | 0.711818010657443 | -0.870331583 | 0.594284641627423 | 0.871078246649404 | 0.418812655014633 | 1.81173444188139 | 0.999489402457283 |
| ebi-fl187-GCST90094756 | prot-a-1120 | Filamin-A \|\| id:prot-a-1120 | Dried fruit liking \|\| id:ebi-fl187-GCST90094756 | Weighted median | 5 | -0.319210691 | 0.345166898021774 | 0.355069433200225 | -0.995737811 | 0.357316429547562 | 0.726722419610876 | 0.369450759238887 | 1.42948813057818 | 0.999489402457283 |
| ebi-fl187-GCST90094756 | prot-a-1166 | Galanin peptides \|\| id:prot-a-1166 | Dried fruit liking \|\| id:ebi-fl187-GCST90094756 | Weighted median | 5 | -0.331623254 | 0.393897820813996 | 0.399842965812941 | -1.103662983 | 0.440416474760745 | 0.717757684234073 | 0.331654013049126 | 1.55335401655685 | 0.999489402457283 |
| ebi-fl187-GCST90094756 | prot-a-1205 | ADP-ribosylation factor-binding protein GGA1 \|\| id:prot-a-1205 | Dried fruit liking \|\| id:ebi-fl187-GCST90094756 | Weighted median | 5 | 0.225941544150502 | 0.375485608259703 | 0.547352832633603 | -0.510010248 | 0.96189333633952 | 1.25350238858951 | 0.600489424941979 | 2.61664597732329 | 0.999489402457283 |
| ebi-fl187-GCST90094756 | prot-a-1227 | Guanine nucleotide-binding protein G(I)/G(S)/G(O) subunit gamma-T2 \|\| id:prot-a-1227 | Dried fruit liking \|\| id:ebi-fl187-GCST90094756 | Weighted median | 5 | -0.654901588 | 0.419354465933036 | 0.118361084970815 | -1.476836341 | 0.167033164970467 | 0.519493184300596 | 0.22835899667949 | 1.18179345880359 | 0.999489402457283 |
| ebi-fl187-GCST90094756 | prot-a-1240 | Golgi SNAP receptor complex member 1 \|\| id:prot-a-1240 | Dried fruit liking \|\| id:ebi-fl187-GCST90094756 | Weighted median | 5 | -0.115158525 | 0.369123182480541 | 0.755056804702624 | -0.838639963 | 0.608322912583115 | 0.891224851218192 | 0.432298065275053 | 1.83734742121393 | 0.999489402457283 |
| ebi-fl187-GCST90094756 | prot-a-1283 | Glutathione S-transferase A1 \|\| id:prot-a-1283 | Dried fruit liking \|\| id:ebi-fl187-GCST90094756 | Weighted median | 5 | -0.174783662 | 0.388988431266547 | 0.65319456481105 | -0.937200987 | 0.587633663273875 | 0.839638646860586 | 0.391722739244295 | 1.79972461813663 | 0.999489402457283 |
| ebi-fl187-GCST90094756 | prot-a-1324 | E3 ubiquitin-protein ligase HECW1 \|\| id:prot-a-1324 | Dried fruit liking \|\| id:ebi-fl187-GCST90094756 | Weighted median | 5 | -0.074332899 | 0.425670567606788 | 0.861373978803566 | -0.908647211 | 0.759981413887855 | 0.928362591767309 | 0.403069122806909 | 2.13823647862451 | 0.999489402457283 |
| ebi-fl187-GCST90094756 | prot-a-1339 | Histidine triad nucleotide-binding protein 2, mitochondrial \|\| id:prot-a-1339 | Dried fruit liking \|\| id:ebi-fl187-GCST90094756 | Weighted median | 5 | 0.12471241879484 | 0.360902234366323 | 0.729675405146602 | -0.582655961 | 0.832080798152833 | 1.13282262772194 | 0.558413271634219 | 2.2980956418232 | 0.999489402457283 |
| ebi-fl187-GCST90094756 | prot-a-1466 | Interleukin-11 \|\| id:prot-a-1466 | Dried fruit liking \|\| id:ebi-fl187-GCST90094756 | Weighted median | 5 | -0.526996522 | 0.361933066714104 | 0.145375835018056 | -1.236385332 | 0.182392289229531 | 0.590375489560255 | 0.290432138528853 | 1.20008488192461 | 0.999489402457283 |
| ebi-fl187-GCST90094756 | prot-a-147 | Rho GTPase-activating protein 1 \|\| id:prot-a-147 | Dried fruit liking \|\| id:ebi-fl187-GCST90094756 | Weighted median | 5 | -0.179342556 | 0.367266445373659 | 0.62532511523588 | -0.899184789 | 0.540499676948832 | 0.83581953536898 | 0.406901234967211 | 1.71686452551738 | 0.999489402457283 |
| ebi-fl187-GCST90094756 | prot-a-1473 | Interleukin-12 receptor subunit beta-1 \|\| id:prot-a-1473 | Dried fruit liking \|\| id:ebi-fl187-GCST90094756 | Weighted median | 5 | -0.238464164 | 0.386650006547518 | 0.537403436878462 | -0.996298177 | 0.51936984895046 | 0.787836920767591 | 0.369243789582876 | 1.68096805209841 | 0.999489402457283 |
| ebi-fl187-GCST90094756 | prot-a-1501 | X-linked interleukin-1 receptor accessory protein-like 2 \|\| id:prot-a-1501 | Dried fruit liking \|\| id:ebi-fl187-GCST90094756 | Weighted median | 5 | 0.130318630276023 | 0.417585303919412 | 0.754982257853068 | -0.688148565 | 0.948785825958071 | 1.1391913063428 | 0.502505564536358 | 2.58257206294703 | 0.999489402457283 |
| ebi-fl187-GCST90094756 | prot-a-1503 | Interleukin-1 receptor-like 2 \|\| id:prot-a-1503 | Dried fruit liking \|\| id:ebi-fl187-GCST90094756 | Weighted median | 5 | -0.403715735 | 0.398147408726818 | 0.31058962836067 | -1.184084656 | 0.376653185996987 | 0.667833936030797 | 0.306026170489235 | 1.45739877540988 | 0.999489402457283 |
| ebi-fl187-GCST90094756 | prot-a-1536 | Interleukin-5 receptor subunit alpha \|\| id:prot-a-1536 | Dried fruit liking \|\| id:ebi-fl187-GCST90094756 | Weighted median | 5 | -0.08182468 | 0.381016456596321 | 0.829959381214743 | -0.828616935 | 0.66496757507178 | 0.921433490387347 | 0.436652788166127 | 1.94442747239344 | 0.999489402457283 |
| ebi-fl187-GCST90094756 | prot-a-1559 | Insulin-induced gene 1 protein \|\| id:prot-a-1559 | Dried fruit liking \|\| id:ebi-fl187-GCST90094756 | Weighted median | 5 | 0.0845942111103692 | 0.383917743815384 | 0.825602768710541 | -0.667884567 | 0.837072988988521 | 1.08827536703325 | 0.512792208887715 | 2.30959685807295 | 0.999489402457283 |
| ebi-fl187-GCST90094756 | prot-a-1567 | Interferon regulatory factor 1 \|\| id:prot-a-1567 | Dried fruit liking \|\| id:ebi-fl187-GCST90094756 | Weighted median | 5 | -0.404553032 | 0.361700455910219 | 0.263364042315627 | -1.113485926 | 0.304379861165241 | 0.667274994504596 | 0.328412142946198 | 1.35578396796385 | 0.999489402457283 |
| ebi-fl187-GCST90094756 | prot-a-1613 | Potassium voltage-gated channel subfamily E regulatory beta subunit 5 \|\| id:prot-a-1613 | Dried fruit liking \|\| id:ebi-fl187-GCST90094756 | Weighted median | 5 | 0.310261532104258 | 0.368376692436798 | 0.399653709168781 | -0.411756785 | 1.03227984928038 | 1.36378174020383 | 0.662485382794312 | 2.80745912772967 | 0.999489402457283 |
| ebi-fl187-GCST90094756 | prot-a-1649 | Kin of IRRE-like protein 2 \|\| id:prot-a-1649 | Dried fruit liking \|\| id:ebi-fl187-GCST90094756 | Weighted median | 5 | -0.022276303 | 0.409366375753716 | 0.956603298430448 | -0.8246344 | 0.780081793208768 | 0.977969981414572 | 0.438395240581185 | 2.181650702 | 0.999489402457283 |
| ebi-fl187-GCST90094756 | prot-a-165 | Armadillo repeat-containing protein 5 \|\| id:prot-a-165 | Dried fruit liking \|\| id:ebi-fl187-GCST90094756 | Weighted median | 5 | -0.157450251 | 0.397480126464492 | 0.692015718819214 | -0.936511299 | 0.621610796737919 | 0.854319313969664 | 0.391992999016522 | 1.8619248100164 | 0.999489402457283 |
| ebi-fl187-GCST90094756 | prot-a-1656 | Kallikrein-11 \|\| id:prot-a-1656 | Dried fruit liking \|\| id:ebi-fl187-GCST90094756 | Weighted median | 5 | -0.42737523 | 0.394808277728593 | 0.279035736201541 | -1.201199455 | 0.346448993856549 | 0.652218773935528 | 0.300833159633323 | 1.41403736739813 | 0.999489402457283 |
| ebi-fl187-GCST90094756 | prot-a-1697 | Laminin subunit gamma-2 \|\| id:prot-a-1697 | Dried fruit liking \|\| id:ebi-fl187-GCST90094756 | Weighted median | 5 | 0.124876844700913 | 0.346274533911915 | 0.718376362933605 | -0.553821242 | 0.803574931168266 | 1.13300890842319 | 0.574749352586105 | 2.2335113224403 | 0.999489402457283 |
| ebi-fl187-GCST90094756 | prot-a-1698 | Lysosome-associated membrane glycoprotein 3 \|\| id:prot-a-1698 | Dried fruit liking \|\| id:ebi-fl187-GCST90094756 | Weighted median | 5 | -0.259950966 | 0.388339614178228 | 0.503246216834288 | -1.02109661 | 0.501194677609016 | 0.771089394334937 | 0.360199724904791 | 1.65069213812692 | 0.999489402457283 |
| ebi-fl187-GCST90094756 | prot-a-1724 | Leptin receptor \|\| id:prot-a-1724 | Dried fruit liking \|\| id:ebi-fl187-GCST90094756 | Weighted median | 5 | 0.278571438796616 | 0.333004865237963 | 0.402851945574644 | -0.374118097 | 0.931260974663023 | 1.32124098989095 | 0.687895668568998 | 2.53770714532841 | 0.999489402457283 |
| ebi-fl187-GCST90094756 | prot-a-1743 | Leukocyte immunoglobulin-like receptor subfamily B member 2 \|\| id:prot-a-1743 | Dried fruit liking \|\| id:ebi-fl187-GCST90094756 | Weighted median | 5 | -0.421210646 | 0.412141971632279 | 0.30677911890789 | -1.229008911 | 0.386587618073637 | 0.656251849807736 | 0.292582409320581 | 1.47194936078401 | 0.999489402457283 |
| ebi-fl187-GCST90094756 | prot-a-1746 | Leukocyte immunoglobulin-like receptor subfamily B member 4 \|\| id:prot-a-1746 | Dried fruit liking \|\| id:ebi-fl187-GCST90094756 | Weighted median | 5 | -0.569196031 | 0.413193087411986 | 0.168340851461574 | -1.379054483 | 0.24066242007499 | 0.565980286295292 | 0.25181653745564 | 1.27209153025278 | 0.999489402457283 |
| ebi-fl187-GCST90094756 | prot-a-1750 | Leucine-rich repeat and immunoglobulin-like domain-containing nogo receptor-interacting protein 3 \|\| id:prot-a-1750 | Dried fruit liking \|\| id:ebi-fl187-GCST90094756 | Weighted median | 5 | 0.0264030902590938 | 0.386307327502744 | 0.945509115603786 | -0.730759272 | 0.783565452164472 | 1.02675473990433 | 0.481543229131471 | 2.1892640829307 | 0.999489402457283 |
| ebi-fl187-GCST90094756 | prot-a-1753 | Lipase member N \|\| id:prot-a-1753 | Dried fruit liking \|\| id:ebi-fl187-GCST90094756 | Weighted median | 5 | 0.362751020183495 | 0.363243963660877 | 0.317967690560221 | -0.349207149 | 1.07470918895881 | 1.43727796148528 | 0.705247024209691 | 2.92914095013193 | 0.999489402457283 |
| ebi-fl187-GCST90094756 | prot-a-1760 | Ig Kappa chain V-I region HK102- like \|\| id:prot-a-1760 | Dried fruit liking \|\| id:ebi-fl187-GCST90094756 | Weighted median | 5 | 0.161195803686232 | 0.350699152250175 | 0.645773731255355 | -0.526174535 | 0.848566142096576 | 1.17491499898057 | 0.590860969925153 | 2.33629453474373 | 0.999489402457283 |
| ebi-fl187-GCST90094756 | prot-a-1792 | Leucine-rich repeat neuronal protein 1 \|\| id:prot-a-1792 | Dried fruit liking \|\| id:ebi-fl187-GCST90094756 | Weighted median | 5 | -0.22908415 | 0.34977996080079 | 0.512507743668762 | -0.914652873 | 0.456484573595071 | 0.795261609763272 | 0.400655680461591 | 1.57851506619011 | 0.999489402457283 |
| ebi-fl187-GCST90094756 | prot-a-1795 | Leucine-rich repeat transmembrane neuronal protein 1 \|\| id:prot-a-1795 | Dried fruit liking \|\| id:ebi-fl187-GCST90094756 | Weighted median | 5 | -0.416697504 | 0.376411066901881 | 0.268281922454173 | -1.154463195 | 0.321068187433703 | 0.659220301492562 | 0.315226706822277 | 1.37859958085643 | 0.999489402457283 |
| ebi-fl187-GCST90094756 | prot-a-1820 | LysM and putative peptidoglycan-binding domain-containing protein 4 \|\| id:prot-a-1820 | Dried fruit liking \|\| id:ebi-fl187-GCST90094756 | Weighted median | 5 | -0.035964217 | 0.353818486371416 | 0.919037702560823 | -0.729448451 | 0.657520015812254 | 0.964674811365411 | 0.482174859939071 | 1.93000002488824 | 0.999489402457283 |
| ebi-fl187-GCST90094756 | prot-a-1844 | Mitogen-activated protein kinase kinase kinase 7:TGF-beta-activated kinase 1 and MAP3K7-binding protein 1 fusion \|\| id:prot-a-1844 | Dried fruit liking \|\| id:ebi-fl187-GCST90094756 | Weighted median | 5 | -0.319803761 | 0.370261339816064 | 0.38773929572491 | -1.045515987 | 0.405908465246643 | 0.726291549967856 | 0.351510397839818 | 1.50066518315367 | 0.999489402457283 |
| ebi-fl187-GCST90094756 | prot-a-1856 | Megakaryocyte-associated tyrosine-protein kinase \|\| id:prot-a-1856 | Dried fruit liking \|\| id:ebi-fl187-GCST90094756 | Weighted median | 5 | -0.073860727 | 0.386568294214636 | 0.848472529291667 | -0.831534583 | 0.683813130056259 | 0.928801042108639 | 0.435380645557663 | 1.98141875304799 | 0.999489402457283 |
| ebi-fl187-GCST90094756 | prot-a-1910 | Stromelysin-2 \|\| id:prot-a-1910 | Dried fruit liking \|\| id:ebi-fl187-GCST90094756 | Weighted median | 5 | 0.157168131502821 | 0.340683898805675 | 0.644560962399631 | -0.51057231 | 0.824908573161943 | 1.17019234355954 | 0.600152007417774 | 2.28167214972279 | 0.999489402457283 |
| ebi-fl187-GCST90094756 | prot-a-1940 | 39S ribosomal protein L14, mitochondrial \|\| id:prot-a-1940 | Dried fruit liking \|\| id:ebi-fl187-GCST90094756 | Weighted median | 5 | -0.079028691 | 0.333573736827376 | 0.812722572789413 | -0.732833215 | 0.574775833473467 | 0.92401341346557 | 0.480545570706004 | 1.77673219838425 | 0.999489402457283 |
| ebi-fl187-GCST90094756 | prot-a-1942 | 39S ribosomal protein L33, mitochondrial \|\| id:prot-a-1942 | Dried fruit liking \|\| id:ebi-fl187-GCST90094756 | Weighted median | 5 | -0.037059616 | 0.363812376858877 | 0.918864119782953 | -0.750131874 | 0.676012643070169 | 0.963618686956263 | 0.472304263879148 | 1.96602284769741 | 0.999489402457283 |
| ebi-fl187-GCST90094756 | prot-a-1948 | Mesothelin \|\| id:prot-a-1948 | Dried fruit liking \|\| id:ebi-fl187-GCST90094756 | Weighted median | 5 | -0.178999405 | 0.377638865182556 | 0.635502583219313 | -0.919171581 | 0.561172770789577 | 0.836106396906754 | 0.39884931872448 | 1.75272684226723 | 0.999489402457283 |
| ebi-fl187-GCST90094756 | prot-a-1980 | Unconventional myosin-VI \|\| id:prot-a-1980 | Dried fruit liking \|\| id:ebi-fl187-GCST90094756 | Weighted median | 5 | -0.362173097 | 0.367179890784811 | 0.323954429751331 | -1.081845682 | 0.357499489431969 | 0.69616185422707 | 0.338969318212827 | 1.42974983646332 | 0.999489402457283 |
| ebi-fl187-GCST90094756 | prot-a-2039 | Neurofascin \|\| id:prot-a-2039 | Dried fruit liking \|\| id:ebi-fl187-GCST90094756 | Weighted median | 5 | -0.386223591 | 0.377834657874825 | 0.306684993991476 | -1.12677952 | 0.354332338636944 | 0.679618552236787 | 0.324075255456646 | 1.42522876636663 | 0.999489402457283 |
| ebi-fl187-GCST90094756 | prot-a-2041 | NFU1 iron-sulfur cluster scaffold homolog, mitochondrial \|\| id:prot-a-2041 | Dried fruit liking \|\| id:ebi-fl187-GCST90094756 | Weighted median | 5 | -0.004437279 | 0.383850548413263 | 0.990776728297585 | -0.756784354 | 0.74790979545652 | 0.995572550745785 | 0.469172697051406 | 2.11257967487795 | 0.999911036302558 |
| ebi-fl187-GCST90094756 | prot-a-2088 | Glucocorticoid receptor \|\| id:prot-a-2088 | Dried fruit liking \|\| id:ebi-fl187-GCST90094756 | Weighted median | 5 | -0.007694613 | 0.353084885860535 | 0.982613452820173 | -0.699740989 | 0.684351763252721 | 0.992334914717533 | 0.496713941346868 | 1.98248629844636 | 0.999911036302558 |
| ebi-fl187-GCST90094756 | prot-a-2111 | Neuron-specific protein family member 2 \|\| id:prot-a-2111 | Dried fruit liking \|\| id:ebi-fl187-GCST90094756 | Weighted median | 5 | -0.226325796 | 0.444489011376825 | 0.610624670926309 | -1.097524258 | 0.644872666198511 | 0.797458250550738 | 0.333696207463043 | 1.90574434814898 | 0.999489402457283 |
| ebi-fl187-GCST90094756 | prot-a-2125 | Nuclear migration protein nudC \|\| id:prot-a-2125 | Dried fruit liking \|\| id:ebi-fl187-GCST90094756 | Weighted median | 5 | 0.103336361393228 | 0.355149574324563 | 0.771077516131837 | -0.592756804 | 0.799429527069372 | 1.10886432550457 | 0.552801217402797 | 2.22427167970722 | 0.999489402457283 |
| ebi-fl187-GCST90094756 | prot-a-2142 | Ornithine decarboxylase \|\| id:prot-a-2142 | Dried fruit liking \|\| id:ebi-fl187-GCST90094756 | Weighted median | 5 | -0.272164344 | 0.394242152782015 | 0.489975270317966 | -1.044878963 | 0.500550275763683 | 0.761729065437879 | 0.351734389627167 | 1.64962877172147 | 0.999489402457283 |
| ebi-fl187-GCST90094756 | prot-a-2186 | Prostate and testis expressed protein 1 \|\| id:prot-a-2186 | Dried fruit liking \|\| id:ebi-fl187-GCST90094756 | Weighted median | 5 | -0.407123236 | 0.429340141767991 | 0.343000483685959 | -1.248629914 | 0.434383441949549 | 0.665562164084582 | 0.286897602161172 | 1.54401079313344 | 0.999489402457283 |
| ebi-fl187-GCST90094756 | prot-a-2227 | Platelet-derived growth factor C \|\| id:prot-a-2227 | Dried fruit liking \|\| id:ebi-fl187-GCST90094756 | Weighted median | 5 | -0.057110157 | 0.342138850237689 | 0.867432213873399 | -0.727702304 | 0.613481989410917 | 0.94449002139663 | 0.483017543752231 | 1.84685093130986 | 0.999489402457283 |
| ebi-fl187-GCST90094756 | prot-a-2229 | Platelet-derived growth factor receptor alpha \|\| id:prot-a-2229 | Dried fruit liking \|\| id:ebi-fl187-GCST90094756 | Weighted median | 5 | -0.629370354 | 0.378898413662941 | 0.0967027989232325 | -1.372011245 | 0.113270536916364 | 0.532927250973304 | 0.253596401892097 | 1.11993487569594 | 0.999489402457283 |
| ebi-fl187-GCST90094756 | prot-a-2243 | Platelet endothelial aggregation receptor 1 \|\| id:prot-a-2243 | Dried fruit liking \|\| id:ebi-fl187-GCST90094756 | Weighted median | 5 | -0.039786301 | 0.347818550855255 | 0.908930151777734 | -0.721510661 | 0.641938058639171 | 0.960994780798875 | 0.486017493577786 | 1.90015993441782 | 0.999489402457283 |
| ebi-fl187-GCST90094756 | prot-a-2246 | E3 ubiquitin-protein ligase pellino homolog 2 \|\| id:prot-a-2246 | Dried fruit liking \|\| id:ebi-fl187-GCST90094756 | Weighted median | 5 | 0.267139389115865 | 0.422144117640998 | 0.52685405898558 | -0.560263081 | 1.09454185969222 | 1.30622250684209 | 0.571058809099538 | 2.98781353197446 | 0.999489402457283 |
| ebi-fl187-GCST90094756 | prot-a-2251 | Platelet factor 4 variant \|\| id:prot-a-2251 | Dried fruit liking \|\| id:ebi-fl187-GCST90094756 | Weighted median | 5 | 0.150473677418398 | 0.359784115881529 | 0.675776593068086 | -0.55470319 | 0.855650544546194 | 1.16238470773411 | 0.574242677040445 | 2.35290455202263 | 0.999489402457283 |
| ebi-fl187-GCST90094756 | prot-a-2346 | Protein phosphatase 1L \|\| id:prot-a-2346 | Dried fruit liking \|\| id:ebi-fl187-GCST90094756 | Weighted median | 5 | -0.29174887 | 0.409064235101696 | 0.475715567181875 | -1.093514771 | 0.510017031047756 | 0.746956095691291 | 0.335036844189033 | 1.66531955684127 | 0.999489402457283 |
| ebi-fl187-GCST90094756 | prot-a-2358 | PR domain zinc finger protein 4 \|\| id:prot-a-2358 | Dried fruit liking \|\| id:ebi-fl187-GCST90094756 | Weighted median | 5 | 0.0030373040370246 | 0.352015182765556 | 0.993115672144925 | -0.686912454 | 0.692987062257515 | 1.00304192131844 | 0.503127101369266 | 1.99967978903164 | 0.999911036302558 |
| ebi-fl187-GCST90094756 | prot-a-2390 | Trypsin-1 \|\| id:prot-a-2390 | Dried fruit liking \|\| id:ebi-fl187-GCST90094756 | Weighted median | 5 | -0.360351984 | 0.376863210914399 | 0.338977390555238 | -1.099003877 | 0.378299909774976 | 0.697430798647464 | 0.333202829439783 | 1.459800686 | 0.999489402457283 |
| ebi-fl187-GCST90094756 | prot-a-242 | Brain-derived neurotrophic factor \|\| id:prot-a-242 | Dried fruit liking \|\| id:ebi-fl187-GCST90094756 | Weighted median | 5 | -0.457851655 | 0.392320881093923 | 0.24319679634928 | -1.226800581 | 0.311097272425751 | 0.632641318727781 | 0.293229241563185 | 1.36492198400132 | 0.999489402457283 |
| ebi-fl187-GCST90094756 | prot-a-2464 | Dihydropteridine reductase \|\| id:prot-a-2464 | Dried fruit liking \|\| id:ebi-fl187-GCST90094756 | Weighted median | 5 | -0.077423447 | 0.377937493042889 | 0.837683219159389 | -0.818180933 | 0.663334039383009 | 0.925497871340909 | 0.441233558179458 | 1.94125377360391 | 0.999489402457283 |
| ebi-fl187-GCST90094756 | prot-a-2474 | Ras-related protein Rab-27A \|\| id:prot-a-2474 | Dried fruit liking \|\| id:ebi-fl187-GCST90094756 | Weighted median | 5 | 0.158649675334361 | 0.382612074086513 | 0.678398945867446 | -0.59126999 | 0.908569340543926 | 1.17192731971211 | 0.553623741536275 | 2.48077085508736 | 0.999489402457283 |
| ebi-fl187-GCST90094756 | prot-a-2478 | Ras-related protein Rab-7b \|\| id:prot-a-2478 | Dried fruit liking \|\| id:ebi-fl187-GCST90094756 | Weighted median | 5 | 0.313404301138313 | 0.381266367662506 | 0.411071900790761 | -0.433877779 | 1.06068638175683 | 1.36807453332146 | 0.647991448497408 | 2.88835282172744 | 0.999489402457283 |
| ebi-fl187-GCST90094756 | prot-a-2528 | E3 ubiquitin-protein ligase rififylin \|\| id:prot-a-2528 | Dried fruit liking \|\| id:ebi-fl187-GCST90094756 | Weighted median | 5 | -0.08008565 | 0.372993555646858 | 0.829993000764322 | -0.811153019 | 0.650981719533456 | 0.923037285287206 | 0.444345432192316 | 1.91742227623851 | 0.999489402457283 |
| ebi-fl187-GCST90094756 | prot-a-2543 | Protein RIC-3 \|\| id:prot-a-2543 | Dried fruit liking \|\| id:ebi-fl187-GCST90094756 | Weighted median | 5 | 0.479020430579101 | 0.40581695073543 | 0.237846939760018 | -0.316380793 | 1.27442165402054 | 1.61449212023065 | 0.728781882384047 | 3.57663228092332 | 0.999489402457283 |
| ebi-fl187-GCST90094756 | prot-a-255 | Bone morphogenetic protein 6 \|\| id:prot-a-255 | Dried fruit liking \|\| id:ebi-fl187-GCST90094756 | Weighted median | 5 | -0.043275841 | 0.398438521050528 | 0.913508976342188 | -0.824215342 | 0.737663660620032 | 0.957647195616544 | 0.438578992045729 | 2.09104441367454 | 0.999489402457283 |
| ebi-fl187-GCST90094756 | prot-a-2568 | RING finger protein 165 \|\| id:prot-a-2568 | Dried fruit liking \|\| id:ebi-fl187-GCST90094756 | Weighted median | 5 | -0.380160601 | 0.381456261515682 | 0.318957062286859 | -1.127814874 | 0.367493671138113 | 0.683751588909206 | 0.323739896556334 | 1.44411065892372 | 0.999489402457283 |
| ebi-fl187-GCST90094756 | prot-a-2573 | E3 ubiquitin-protein ligase RNF8 \|\| id:prot-a-2573 | Dried fruit liking \|\| id:ebi-fl187-GCST90094756 | Weighted median | 5 | -0.063495157 | 0.418695939602743 | 0.879463150625884 | -0.884139198 | 0.757148884916485 | 0.938478664618782 | 0.41306959128044 | 2.13218843152921 | 0.999489402457283 |
| ebi-fl187-GCST90094756 | prot-a-260 | Cytoplasmic tyrosine-protein kinase BMX \|\| id:prot-a-260 | Dried fruit liking \|\| id:ebi-fl187-GCST90094756 | Weighted median | 5 | -0.456949232 | 0.362625114210899 | 0.207627993244189 | -1.167694456 | 0.253795992031223 | 0.633212486290878 | 0.311083331522845 | 1.28890882977197 | 0.999489402457283 |
| ebi-fl187-GCST90094756 | prot-a-2623 | Serum amyloid A-1 protein \|\| id:prot-a-2623 | Dried fruit liking \|\| id:ebi-fl187-GCST90094756 | Weighted median | 5 | 0.0917498443835141 | 0.340390907087905 | 0.787512177674481 | -0.575416334 | 0.758916022275808 | 1.09609059456406 | 0.562470644675578 | 2.13595963249736 | 0.999489402457283 |
| ebi-fl187-GCST90094756 | prot-a-2640 | Lysosome membrane protein 2 \|\| id:prot-a-2640 | Dried fruit liking \|\| id:ebi-fl187-GCST90094756 | Weighted median | 5 | -0.226326796 | 0.418593055902138 | 0.588725032150843 | -1.046769186 | 0.594115593182534 | 0.79745745286514 | 0.351070161227725 | 1.81142819687729 | 0.999489402457283 |
| ebi-fl187-GCST90094756 | prot-a-2641 | Scavenger receptor class F member 2 \|\| id:prot-a-2641 | Dried fruit liking \|\| id:ebi-fl187-GCST90094756 | Weighted median | 5 | -0.701200924 | 0.405072573026696 | 0.0834428265208895 | -1.495143167 | 0.0927413189987979 | 0.495989300464752 | 0.224216501969012 | 1.09717787948325 | 0.999489402457283 |
| ebi-fl187-GCST90094756 | prot-a-2669 | Semaphorin-3A \|\| id:prot-a-2669 | Dried fruit liking \|\| id:ebi-fl187-GCST90094756 | Weighted median | 5 | -0.078678024 | 0.364972527434285 | 0.829321028083491 | -0.794024178 | 0.636668129743066 | 0.924337490999873 | 0.452022112991353 | 1.89017256614675 | 0.999489402457283 |
| ebi-fl187-GCST90094756 | prot-a-2683 | Sentrin-specific protease 7 \|\| id:prot-a-2683 | Dried fruit liking \|\| id:ebi-fl187-GCST90094756 | Weighted median | 5 | -0.250888351 | 0.337625206387527 | 0.457422824514023 | -0.912633756 | 0.410857053304867 | 0.778109241661069 | 0.401465468414279 | 1.5081097618428 | 0.999489402457283 |
| ebi-fl187-GCST90094756 | prot-a-270 | Bromodomain testis-specific protein \|\| id:prot-a-270 | Dried fruit liking \|\| id:ebi-fl187-GCST90094756 | Weighted median | 5 | -0.062289006 | 0.3483082255888 | 0.858068830879796 | -0.744973128 | 0.620395116438273 | 0.939611294512398 | 0.474747057233227 | 1.85966267999749 | 0.999489402457283 |
| ebi-fl187-GCST90094756 | prot-a-2708 | 14-3-3 protein sigma \|\| id:prot-a-2708 | Dried fruit liking \|\| id:ebi-fl187-GCST90094756 | Weighted median | 5 | -0.141748162 | 0.366019254199433 | 0.698556564280885 | -0.8591459 | 0.575649576205507 | 0.867839783981489 | 0.42352365933325 | 1.77828528362904 | 0.999489402457283 |
| ebi-fl187-GCST90094756 | prot-a-2713 | Beta-sarcoglycan \|\| id:prot-a-2713 | Dried fruit liking \|\| id:ebi-fl187-GCST90094756 | Weighted median | 5 | -0.541416175 | 0.350795766530648 | 0.122735143868091 | -1.228975877 | 0.146143527487863 | 0.581923563008869 | 0.29259207447567 | 1.1573622894324 | 0.999489402457283 |
| ebi-fl187-GCST90094756 | prot-a-2720 | Protein shisa-3 homolog \|\| id:prot-a-2720 | Dried fruit liking \|\| id:ebi-fl187-GCST90094756 | Weighted median | 5 | -0.083567901 | 0.385357429039446 | 0.828318889365698 | -0.838868462 | 0.671732660006588 | 0.919828627349228 | 0.432199296846314 | 1.95762628459811 | 0.999489402457283 |
| ebi-fl187-GCST90094756 | prot-a-2730 | Sialic acid-binding Ig-like lectin 8 \|\| id:prot-a-2730 | Dried fruit liking \|\| id:ebi-fl187-GCST90094756 | Weighted median | 5 | -0.096984431 | 0.336016550437872 | 0.77286459451023 | -0.75557687 | 0.561608007844955 | 0.907570135970679 | 0.469739557957374 | 1.75348985997169 | 0.999489402457283 |
| ebi-fl187-GCST90094756 | prot-a-2761 | Sodium- and chloride-dependent neutral and basic amino acid transporter B(0+) \|\| id:prot-a-2761 | Dried fruit liking \|\| id:ebi-fl187-GCST90094756 | Weighted median | 5 | -0.384552911 | 0.382306502520185 | 0.314475253730015 | -1.133873656 | 0.364767834247875 | 0.680754926426253 | 0.321784357293668 | 1.44017960895059 | 0.999489402457283 |
| ebi-fl187-GCST90094756 | prot-a-2770 | Secreted Ly-6/uPAR-related protein 1 \|\| id:prot-a-2770 | Dried fruit liking \|\| id:ebi-fl187-GCST90094756 | Weighted median | 5 | -0.054188809 | 0.409236740455834 | 0.894656579821601 | -0.856292821 | 0.747915201999308 | 0.947253239400954 | 0.424733731470179 | 2.11259109666123 | 0.999489402457283 |
| ebi-fl187-GCST90094756 | prot-a-2784 | N-lysine methyltransferase SMYD2 \|\| id:prot-a-2784 | Dried fruit liking \|\| id:ebi-fl187-GCST90094756 | Weighted median | 5 | 0.0267904955266107 | 0.408721724381266 | 0.947738461519584 | -0.774304084 | 0.827885075313892 | 1.0271525871581 | 0.46102450359634 | 2.28847366913352 | 0.999489402457283 |
| ebi-fl187-GCST90094756 | prot-a-2837 | Serine palmitoyltransferase 2 \|\| id:prot-a-2837 | Dried fruit liking \|\| id:ebi-fl187-GCST90094756 | Weighted median | 5 | -0.028724132 | 0.336183851331996 | 0.931910213551022 | -0.687644481 | 0.63019621657958 | 0.971684484120901 | 0.502758933789781 | 1.87797903373724 | 0.999489402457283 |
| ebi-fl187-GCST90094756 | prot-a-2858 | Alpha-2,8-sialyltransferase 8B \|\| id:prot-a-2858 | Dried fruit liking \|\| id:ebi-fl187-GCST90094756 | Weighted median | 5 | 0.359848827246112 | 0.369178679978428 | 0.329695158551283 | -0.363741386 | 1.08343904000383 | 1.43311275058277 | 0.695070926913398 | 2.95482385517587 | 0.999489402457283 |
| ebi-fl187-GCST90094756 | prot-a-2911 | Synaptotagmin-11 \|\| id:prot-a-2911 | Dried fruit liking \|\| id:ebi-fl187-GCST90094756 | Weighted median | 5 | -0.280546694 | 0.402187324955015 | 0.485457220366589 | -1.068833851 | 0.50774046287282 | 0.755370671911108 | 0.343408749795094 | 1.66153265554211 | 0.999489402457283 |
| ebi-fl187-GCST90094756 | prot-a-2962 | Transforming growth factor beta-1 \|\| id:prot-a-2962 | Dried fruit liking \|\| id:ebi-fl187-GCST90094756 | Weighted median | 5 | -0.040907467 | 0.40438771337598 | 0.919424223446955 | -0.833507385 | 0.751692451575683 | 0.959917950270389 | 0.434522572608664 | 2.12058597029703 | 0.999489402457283 |
| ebi-fl187-GCST90094756 | prot-a-2978 | Tyrosine-protein kinase receptor Tie-1, soluble \|\| id:prot-a-2978 | Dried fruit liking \|\| id:ebi-fl187-GCST90094756 | Weighted median | 5 | -0.128130043 | 0.402718050781175 | 0.750361184203784 | -0.917457423 | 0.661197336291521 | 0.879738967600248 | 0.399533595769077 | 1.93711031890713 | 0.999489402457283 |
| ebi-fl187-GCST90094756 | prot-a-299 | Uncharacterized protein C1orf226 \|\| id:prot-a-299 | Dried fruit liking \|\| id:ebi-fl187-GCST90094756 | Weighted median | 5 | 0.147648490992821 | 0.378602006211286 | 0.696548834888416 | -0.594411441 | 0.889708423166941 | 1.15910538876145 | 0.551887288429827 | 2.43441972740177 | 0.999489402457283 |
| ebi-fl187-GCST90094756 | prot-a-3015 | Transmembrane protein 70, mitochondrial \|\| id:prot-a-3015 | Dried fruit liking \|\| id:ebi-fl187-GCST90094756 | Weighted median | 5 | 0.421032422872131 | 0.382957274302098 | 0.271583161952546 | -0.329563835 | 1.17162868050424 | 1.52353367494549 | 0.719237371368685 | 3.22724451077373 | 0.999489402457283 |
| ebi-fl187-GCST90094756 | prot-a-3016 | Transmembrane protein 87B \|\| id:prot-a-3016 | Dried fruit liking \|\| id:ebi-fl187-GCST90094756 | Weighted median | 5 | 0.226187147693279 | 0.408756710057496 | 0.580021052548332 | -0.574976004 | 1.02735029940597 | 1.25381029102644 | 0.56271837162407 | 2.79365367323396 | 0.999489402457283 |
| ebi-fl187-GCST90094756 | prot-a-3123 | Thioredoxin domain-containing protein 12 \|\| id:prot-a-3123 | Dried fruit liking \|\| id:ebi-fl187-GCST90094756 | Weighted median | 5 | -0.314155164 | 0.376065013632882 | 0.403506820304736 | -1.051242591 | 0.422932262292327 | 0.730405686385722 | 0.349503189607371 | 1.52643089553466 | 0.999489402457283 |
| ebi-fl187-GCST90094756 | prot-a-3133 | PolyUbiquitin K48-linked \|\| id:prot-a-3133 | Dried fruit liking \|\| id:ebi-fl187-GCST90094756 | Weighted median | 5 | 0.374953377941269 | 0.361337044732488 | 0.299417572641374 | -0.33326723 | 1.08317398561695 | 1.45492358150429 | 0.716578677437704 | 2.95404076993526 | 0.999489402457283 |
| ebi-fl187-GCST90094756 | prot-a-3135 | Ubiquitin-conjugating enzyme E2 B \|\| id:prot-a-3135 | Dried fruit liking \|\| id:ebi-fl187-GCST90094756 | Weighted median | 5 | 0.210938773415695 | 0.386246714247059 | 0.584980395798878 | -0.546104787 | 0.96798233333993 | 1.23483674791059 | 0.579201535694127 | 2.63262733266585 | 0.999489402457283 |
| ebi-fl187-GCST90094756 | prot-a-3162 | NKG2D ligand 3 \|\| id:prot-a-3162 | Dried fruit liking \|\| id:ebi-fl187-GCST90094756 | Weighted median | 5 | -0.55484452 | 0.371136999033574 | 0.134918164492652 | -1.282273038 | 0.172583997970197 | 0.574161524812926 | 0.277406028760873 | 1.1883716372281 | 0.999489402457283 |
| ebi-fl187-GCST90094756 | prot-a-3188 | Vesicle-associated membrane protein 8 \|\| id:prot-a-3188 | Dried fruit liking \|\| id:ebi-fl187-GCST90094756 | Weighted median | 5 | 0.592826169749189 | 0.409272194772625 | 0.147480410069943 | -0.209347332 | 1.39499967150353 | 1.80909400411724 | 0.811113461046742 | 4.03497324715753 | 0.999489402457283 |
| ebi-fl187-GCST90094756 | prot-a-3201 | Vitrin \|\| id:prot-a-3201 | Dried fruit liking \|\| id:ebi-fl187-GCST90094756 | Weighted median | 5 | 0.00400767687015774 | 0.383880236532467 | 0.991670304669254 | -0.748397587 | 0.756412940473794 | 1.00401571834606 | 0.473124085950326 | 2.13061983657242 | 0.999911036302558 |
| ebi-fl187-GCST90094756 | prot-a-3232 | WNT1-inducible-signaling pathway protein 3 \|\| id:prot-a-3232 | Dried fruit liking \|\| id:ebi-fl187-GCST90094756 | Weighted median | 5 | -0.311794924 | 0.383612699962765 | 0.416340924519004 | -1.063675816 | 0.440085967582658 | 0.732131655213298 | 0.345184640150444 | 1.55284070673524 | 0.999489402457283 |
| ebi-fl187-GCST90094756 | prot-a-327 | Carbonic anhydrase-related protein 10 \|\| id:prot-a-327 | Dried fruit liking \|\| id:ebi-fl187-GCST90094756 | Weighted median | 5 | -0.531964904 | 0.391448136964761 | 0.174157205029734 | -1.299203252 | 0.235273444569292 | 0.587449553001778 | 0.272749018629914 | 1.2652546984605 | 0.999489402457283 |
| ebi-fl187-GCST90094756 | prot-a-332 | Carbonic anhydrase 5A, mitochondrial \|\| id:prot-a-332 | Dried fruit liking \|\| id:ebi-fl187-GCST90094756 | Weighted median | 5 | 0.252459555998064 | 0.377228600961577 | 0.503337174483999 | -0.486908502 | 0.991827613882754 | 1.28718743609089 | 0.614523258091084 | 2.69615750716575 | 0.999489402457283 |
| ebi-fl187-GCST90094756 | prot-a-338 | Cell adhesion molecule 3 \|\| id:prot-a-338 | Dried fruit liking \|\| id:ebi-fl187-GCST90094756 | Weighted median | 5 | -0.031826418 | 0.380266370174539 | 0.933298883056765 | -0.777148503 | 0.713495667985699 | 0.968674712410174 | 0.459715020056325 | 2.04111385864218 | 0.999489402457283 |
| ebi-fl187-GCST90094756 | prot-a-34 | A disintegrin and metalloproteinase with thrombospondin motifs 3 \|\| id:prot-a-34 | Dried fruit liking \|\| id:ebi-fl187-GCST90094756 | Weighted median | 5 | -0.104771977 | 0.401986063817304 | 0.794373345539505 | -0.892664662 | 0.683120708209948 | 0.900529840807912 | 0.409562950612599 | 1.98004725030072 | 0.999489402457283 |
| ebi-fl187-GCST90094756 | prot-a-358 | Cysteine--tRNA ligase, cytoplasmic \|\| id:prot-a-358 | Dried fruit liking \|\| id:ebi-fl187-GCST90094756 | Weighted median | 5 | -0.157784026 | 0.367847067738454 | 0.667967473055045 | -0.878764279 | 0.563196226423283 | 0.854034210942678 | 0.415295784408265 | 1.75627699785042 | 0.999489402457283 |
| ebi-fl187-GCST90094756 | prot-a-382 | Coiled-coil domain-containing protein 167 \|\| id:prot-a-382 | Dried fruit liking \|\| id:ebi-fl187-GCST90094756 | Weighted median | 5 | -0.602782442 | 0.409249769778523 | 0.14077901951927 | -1.404911991 | 0.199347106841463 | 0.54728672206532 | 0.245388651988466 | 1.22060557292226 | 0.999489402457283 |
| ebi-fl187-GCST90094756 | prot-a-40 | Pituitary adenylate cyclase-activating polypeptide 27 \|\| id:prot-a-40 | Dried fruit liking \|\| id:ebi-fl187-GCST90094756 | Weighted median | 5 | -0.426597229 | 0.394973726570419 | 0.280113314079435 | -1.200745733 | 0.347551275136971 | 0.652726398593659 | 0.300969685172133 | 1.41559689367843 | 0.999489402457283 |
| ebi-fl187-GCST90094756 | prot-a-403 | C-C motif chemokine 25 \|\| id:prot-a-403 | Dried fruit liking \|\| id:ebi-fl187-GCST90094756 | Weighted median | 5 | -0.262197641 | 0.361015531740191 | 0.467668358448486 | -0.969788083 | 0.445392800957653 | 0.769358951612914 | 0.379163380580379 | 1.5611032782778 | 0.999489402457283 |
| ebi-fl187-GCST90094756 | prot-a-415 | Cyclin-C \|\| id:prot-a-415 | Dried fruit liking \|\| id:ebi-fl187-GCST90094756 | Weighted median | 5 | 0.135653595970052 | 0.374092092868415 | 0.716888126354532 | -0.597566906 | 0.868874097992146 | 1.14528509350043 | 0.550148572150341 | 2.38422493812423 | 0.999489402457283 |
| ebi-fl187-GCST90094756 | prot-a-44 | Adhesion G protein-coupled receptor F5 \|\| id:prot-a-44 | Dried fruit liking \|\| id:ebi-fl187-GCST90094756 | Weighted median | 5 | 0.286521736398433 | 0.374403060631292 | 0.444107117144975 | -0.447308262 | 1.02035173523577 | 1.33178711583497 | 0.639346791293294 | 2.77417036584514 | 0.999489402457283 |
| ebi-fl187-GCST90094756 | prot-a-455 | CD59 glycoprotein \|\| id:prot-a-455 | Dried fruit liking \|\| id:ebi-fl187-GCST90094756 | Weighted median | 5 | 0.683355647904881 | 0.38268864479407 | 0.0741526989508044 | -0.066714096 | 1.43342539170126 | 1.98051249664793 | 0.935462615712705 | 4.19303741645541 | 0.999489402457283 |
| ebi-fl187-GCST90094756 | prot-a-478 | Cadherin-12 \|\| id:prot-a-478 | Dried fruit liking \|\| id:ebi-fl187-GCST90094756 | Weighted median | 5 | -0.117545539 | 0.346076474043345 | 0.734118673409497 | -0.795855428 | 0.560764350187028 | 0.889100022153808 | 0.451195104838369 | 1.7520111386782 | 0.999489402457283 |
| ebi-fl187-GCST90094756 | prot-a-516 | Centrin-2 \|\| id:prot-a-516 | Dried fruit liking \|\| id:ebi-fl187-GCST90094756 | Weighted median | 5 | 0.482494657927985 | 0.375482111655023 | 0.198792149594965 | -0.253450281 | 1.21843959677183 | 1.62011098786935 | 0.77611833185852 | 3.38190647646432 | 0.999489402457283 |
| ebi-fl187-GCST90094756 | prot-a-519 | Cryptic protein \|\| id:prot-a-519 | Dried fruit liking \|\| id:ebi-fl187-GCST90094756 | Weighted median | 5 | 0.278622725771182 | 0.372967941969815 | 0.455038195121716 | -0.45239444 | 1.00963989203202 | 1.32130875408169 | 0.636103215371319 | 2.74461248021483 | 0.999489402457283 |
| ebi-fl187-GCST90094756 | prot-a-583 | Chloride intracellular channel protein 4 \|\| id:prot-a-583 | Dried fruit liking \|\| id:ebi-fl187-GCST90094756 | Weighted median | 5 | 0.405861494032758 | 0.371614015043183 | 0.274763334900742 | -0.322501975 | 1.1342249635174 | 1.50059469674381 | 0.724334500917551 | 3.10876320407658 | 0.999489402457283 |
| ebi-fl187-GCST90094756 | prot-a-602 | CCR4-NOT transcription complex subunit 1 \|\| id:prot-a-602 | Dried fruit liking \|\| id:ebi-fl187-GCST90094756 | Weighted median | 5 | 0.208649577826952 | 0.393643207275661 | 0.596079033140025 | -0.562891108 | 0.980190264087246 | 1.23201319813634 | 0.569560021435961 | 2.66496324049458 | 0.999489402457283 |
| ebi-fl187-GCST90094756 | prot-a-606 | Ciliary neurotrophic factor receptor subunit alpha \|\| id:prot-a-606 | Dried fruit liking \|\| id:ebi-fl187-GCST90094756 | Weighted median | 5 | 0.135295932809343 | 0.344935940088863 | 0.694884619149654 | -0.54077851 | 0.811370375383514 | 1.14487554045936 | 0.58229475371855 | 2.25099057611575 | 0.999489402457283 |
| ebi-fl187-GCST90094756 | prot-a-626 | Collagen alpha-2(IX) chain \|\| id:prot-a-626 | Dried fruit liking \|\| id:ebi-fl187-GCST90094756 | Weighted median | 5 | -0.221039811 | 0.385221981396971 | 0.566104135999954 | -0.976074895 | 0.533995272553325 | 0.80168476379499 | 0.376787129393906 | 1.70573358366796 | 0.999489402457283 |
| ebi-fl187-GCST90094756 | prot-a-647 | Probable serine carboxypeptidase CPVL \|\| id:prot-a-647 | Dried fruit liking \|\| id:ebi-fl187-GCST90094756 | Weighted median | 5 | -0.722456254 | 0.385168206632016 | 0.0606978239660636 | -1.477385939 | 0.032473430889474 | 0.48555813586263 | 0.228233525600643 | 1.0330064467169 | 0.999489402457283 |
| ebi-fl187-GCST90094756 | prot-a-651 | Cellular retinoic acid-binding protein 2 \|\| id:prot-a-651 | Dried fruit liking \|\| id:ebi-fl187-GCST90094756 | Weighted median | 5 | 0.0864110626044447 | 0.390419327391736 | 0.824836384201754 | -0.678810819 | 0.851632944292247 | 1.09025439901903 | 0.507219809983522 | 2.34347048593974 | 0.999489402457283 |
| ebi-fl187-GCST90094756 | prot-a-671 | Cytotoxic and regulatory T-cell molecule \|\| id:prot-a-671 | Dried fruit liking \|\| id:ebi-fl187-GCST90094756 | Weighted median | 5 | 0.0465364700511834 | 0.391720876314937 | 0.905433748119806 | -0.721236448 | 0.814309387628459 | 1.04763628572079 | 0.486150784257997 | 2.25761599630867 | 0.999489402457283 |
| ebi-fl187-GCST90094756 | prot-a-688 | Chorionic somatomammotropin hormone \|\| id:prot-a-688 | Dried fruit liking \|\| id:ebi-fl187-GCST90094756 | Weighted median | 5 | -0.278509587 | 0.411596825325861 | 0.498624045980702 | -1.085239365 | 0.528220190680401 | 0.75691101124083 | 0.337820913849254 | 1.69591122233856 | 0.999489402457283 |
| ebi-fl187-GCST90094756 | prot-a-69 | Aldo-keto reductase family 1 member C1 \|\| id:prot-a-69 | Dried fruit liking \|\| id:ebi-fl187-GCST90094756 | Weighted median | 5 | -0.302019305 | 0.379065658141734 | 0.425598167394947 | -1.044987995 | 0.440949385258269 | 0.739323792328103 | 0.351696041584219 | 1.55418203582913 | 0.999489402457283 |
| ebi-fl187-GCST90094756 | prot-a-741 | Stromal cell-derived factor 1 \|\| id:prot-a-741 | Dried fruit liking \|\| id:ebi-fl187-GCST90094756 | Weighted median | 5 | 0.0704591042552246 | 0.385762230182391 | 0.85507339657175 | -0.685634867 | 0.826553075412711 | 1.07300068737117 | 0.503770300939111 | 2.28542745166345 | 0.999489402457283 |
| ebi-fl187-GCST90094756 | prot-a-742 | Growth-regulated alpha protein \|\| id:prot-a-742 | Dried fruit liking \|\| id:ebi-fl187-GCST90094756 | Weighted median | 5 | -0.822277361 | 0.379389775859704 | 0.0302067848548064 | -1.565881322 | -0.078673401 | 0.439429773760838 | 0.208903820491238 | 0.924341764614112 | 0.999489402457283 |
| ebi-fl187-GCST90094756 | prot-a-788 | Beta-defensin 112 \|\| id:prot-a-788 | Dried fruit liking \|\| id:ebi-fl187-GCST90094756 | Weighted median | 5 | -0.114263898 | 0.413379681357665 | 0.782230166259332 | -0.924488074 | 0.695960277191287 | 0.892022521618407 | 0.396734465893063 | 2.00563411420105 | 0.999489402457283 |
| ebi-fl187-GCST90094756 | prot-a-838 | DnaJ homolog subfamily B member 1 \|\| id:prot-a-838 | Dried fruit liking \|\| id:ebi-fl187-GCST90094756 | Weighted median | 5 | -0.221293854 | 0.412707368844077 | 0.59182006054194 | -1.030200297 | 0.587612588733101 | 0.801481127086291 | 0.35693546025842 | 1.79968669016645 | 0.999489402457283 |
| ebi-fl187-GCST90094756 | prot-a-85 | Junctional adhesion molecule-like \|\| id:prot-a-85 | Dried fruit liking \|\| id:ebi-fl187-GCST90094756 | Weighted median | 5 | -0.06780167 | 0.338992324822983 | 0.841473186317881 | -0.732226627 | 0.596623286432119 | 0.934445783626178 | 0.48083715231629 | 1.81597640350878 | 0.999489402457283 |
| ebi-fl187-GCST90094756 | prot-a-856 | Dipeptidase 1 \|\| id:prot-a-856 | Dried fruit liking \|\| id:ebi-fl187-GCST90094756 | Weighted median | 5 | 0.107658752910989 | 0.396286667494761 | 0.785876636056763 | -0.669063115 | 0.884380621200721 | 1.11366764469415 | 0.512188214330603 | 2.42148411099141 | 0.999489402457283 |
| ebi-fl187-GCST90094756 | prot-a-860 | Dipeptidyl peptidase 2 \|\| id:prot-a-860 | Dried fruit liking \|\| id:ebi-fl187-GCST90094756 | Weighted median | 5 | -0.035854343 | 0.372651902335125 | 0.923350584960867 | -0.766252071 | 0.694543385754449 | 0.964780810499083 | 0.464751664264059 | 2.00279436068552 | 0.999489402457283 |
| ebi-fl187-GCST90094756 | prot-a-861 | Dermatopontin \|\| id:prot-a-861 | Dried fruit liking \|\| id:ebi-fl187-GCST90094756 | Weighted median | 5 | 0.323263687767247 | 0.372455466204028 | 0.385434984391912 | -0.406749026 | 1.05327640152714 | 1.38162962170675 | 0.665811270656969 | 2.86702928548205 | 0.999489402457283 |
| ebi-fl187-GCST90094756 | prot-a-877 | Dual specificity protein phosphatase 6 \|\| id:prot-a-877 | Dried fruit liking \|\| id:ebi-fl187-GCST90094756 | Weighted median | 5 | -0.435637481 | 0.358747004552699 | 0.224621488441699 | -1.13878161 | 0.267506648005698 | 0.646852179694527 | 0.320208923643224 | 1.30670231677166 | 0.999489402457283 |
| ebi-fl187-GCST90094756 | prot-a-879 | Dynactin-associated protein \|\| id:prot-a-879 | Dried fruit liking \|\| id:ebi-fl187-GCST90094756 | Weighted median | 5 | -0.255346355 | 0.407629625845796 | 0.531041096335381 | -1.054300421 | 0.543607712064128 | 0.77464814854704 | 0.348436100553142 | 1.72220890170316 | 0.999489402457283 |
| ebi-fl187-GCST90094756 | prot-a-937 | EMI domain-containing protein 1 \|\| id:prot-a-937 | Dried fruit liking \|\| id:ebi-fl187-GCST90094756 | Weighted median | 5 | -0.600533119 | 0.386850685718541 | 0.120575127002099 | -1.358760463 | 0.157694225287779 | 0.548519132313312 | 0.256979114807677 | 1.17080813644689 | 0.999489402457283 |
| ebi-fl187-GCST90094756 | prot-b-1 | SRC proto-oncogene, non-receptor tyrosine kinase \|\| id:prot-b-1 | Dried fruit liking \|\| id:ebi-fl187-GCST90094756 | Weighted median | 5 | -0.275855179 | 0.380522484457576 | 0.46849011582905 | -1.021679248 | 0.469968890871083 | 0.758922831020066 | 0.359989919899697 | 1.59994441956595 | 0.999489402457283 |
| ebi-fl187-GCST90094756 | prot-b-10 | coagulation factor III, tissue factor \|\| id:prot-b-10 | Dried fruit liking \|\| id:ebi-fl187-GCST90094756 | Weighted median | 5 | -0.643626846 | 0.395368049245709 | 0.103542230305187 | -1.418548222 | 0.131294530771612 | 0.525383479572006 | 0.242065186772041 | 1.14030358635226 | 0.999489402457283 |
| ebi-fl187-GCST90094756 | prot-b-29 | myeloperoxidase \|\| id:prot-b-29 | Dried fruit liking \|\| id:ebi-fl187-GCST90094756 | Weighted median | 5 | 0.125633135588436 | 0.377064256578133 | 0.738992675877895 | -0.613412807 | 0.864679078481577 | 1.13386611684471 | 0.541499677935599 | 2.37424401770633 | 0.999489402457283 |
| ebi-fl187-GCST90094756 | prot-b-33 | adrenomedullin \|\| id:prot-b-33 | Dried fruit liking \|\| id:ebi-fl187-GCST90094756 | Weighted median | 5 | -0.33219744 | 0.398424861359514 | 0.404406723574903 | -1.113110168 | 0.4487152887298 | 0.717345676474917 | 0.328535569690833 | 1.56629865083255 | 0.999489402457283 |
| ebi-fl187-GCST90094756 | ukb-rsfMRI191-edge-pheno102 | Net25_Pair11_15 (Postcentral\|Precentral)&(Cerebellum) (Motor)&(Subcortical-cerebellum) \|\| id:ukb-rsfMRI191-edge-pheno102 | Dried fruit liking \|\| id:ebi-fl187-GCST90094756 | Weighted median | 5 | 0.0182386729805669 | 0.109857207973756 | 0.868139926354465 | -0.197081455 | 0.233558800609128 | 1.0184060133841 | 0.821123746242854 | 1.26308709599801 | 0.999489402457283 |
| ebi-fl187-GCST90094756 | ukb-rsfMRI191-edge-pheno1167 | Net100_Pair11_45 (Angular\|Temporal)&(Frontal) (Default_mode\|Central_executive)&(Salience\|Default_mode) \|\| id:ukb-rsfMRI191-edge-pheno1167 | Dried fruit liking \|\| id:ebi-fl187-GCST90094756 | Weighted median | 5 | 0.028803389284493 | 0.12451767495542 | 0.817066455328735 | -0.215251254 | 0.272858032197115 | 1.02922221846435 | 0.806338819169943 | 1.31371372653388 | 0.999489402457283 |
| ebi-fl187-GCST90094756 | ukb-rsfMRI191-edge-pheno1317 | Net100_Pair26_48 (Parietal\|Frontal)&(Temporal) (Attention\|Central_executive)&(Default_mode\|Central_executive) \|\| id:ukb-rsfMRI191-edge-pheno1317 | Dried fruit liking \|\| id:ebi-fl187-GCST90094756 | Weighted median | 5 | 0.184257070946219 | 0.114238252156588 | 0.106762061999726 | -0.039649903 | 0.408164045173132 | 1.20232486616261 | 0.961125867270668 | 1.50405387371167 | 0.999489402457283 |
| ebi-fl187-GCST90094756 | ukb-rsfMRI191-edge-pheno286 | Net100_Pair10_13 (OccipitalPrecuneus)&(Frontal_Sup) (Default_mode\|Central_executive)&(Default_mode) \|\| id:ukb-rsfMRI191-edge-pheno286 | Dried fruit liking \|\| id:ebi-fl187-GCST90094756 | Weighted median | 5 | 0.0913440447398046 | 0.120669046762805 | 0.449061895711715 | -0.145167287 | 0.327855376394903 | 1.09564589162756 | 0.864877598302778 | 1.38798822191266 | 0.999489402457283 |
| ebi-fl187-GCST90094756 | ukb-rsfMRI191-edge-pheno599 | Net100_Pair11_29 (Angular\|Temporal)&(Parietal) (Default_mode\|Central_executive)&(Central_executive\|Salience) \|\| id:ukb-rsfMRI191-edge-pheno599 | Dried fruit liking \|\| id:ebi-fl187-GCST90094756 | Weighted median | 5 | 0.0557801718989245 | 0.11883850887412 | 0.638799529253133 | -0.177143305 | 0.2887036492922 | 1.0573652195981 | 0.837659734678922 | 1.33469613177037 | 0.999489402457283 |
| ebi-fl187-GCST90094756 | ukb-rsfMRI191-edge-pheno621 | Net100_Pair5_30 (Precuneus\|Angular\|Cingulate)&(Paracentral) (Default_mode\|Central_executive)&(Motor) \|\| id:ukb-rsfMRI191-edge-pheno621 | Dried fruit liking \|\| id:ebi-fl187-GCST90094756 | Weighted median | 5 | 0.154995795876731 | 0.115491681470993 | 0.179579265566442 | -0.0713679 | 0.381359491559877 | 1.16765305213744 | 0.931119270506874 | 1.46427390491411 | 0.999489402457283 |
| ebi-fl187-GCST90094756 | ukb-rsfMRI191-edge-pheno624 | Net100_Pair8_30 (Calcarine\|Lingual)&(Paracentral) (Visual)&(Motor) \|\| id:ukb-rsfMRI191-edge-pheno624 | Dried fruit liking \|\| id:ebi-fl187-GCST90094756 | Weighted median | 5 | -0.068499583 | 0.113336012443506 | 0.545581859734435 | -0.290638167 | 0.153639001646998 | 0.93379384973614 | 0.747786202699493 | 1.16606986148881 | 0.999489402457283 |
| ebi-fl187-GCST90094756 | ukb-rsfMRI191-edge-pheno882 | Net100_Pair6_38 (Postcentral\|Precentral)&(Subcortical) (Motor)&(Subcortical-cerebellum) \|\| id:ukb-rsfMRI191-edge-pheno882 | Dried fruit liking \|\| id:ebi-fl187-GCST90094756 | Weighted median | 5 | -0.123521081 | 0.128115949599283 | 0.334978208254703 | -0.374628342 | 0.127586180181516 | 0.883803009567212 | 0.687544762652864 | 1.13608277184192 | 0.999489402457283 |
| ebi-fl187-GCST90094756 | ukb-rsfMRI191-edge-pheno899 | Net100_Pair23_38 (Cerebellum)&(Subcortical) (Subcortical-cerebellum)&(Subcortical-cerebellum) \|\| id:ukb-rsfMRI191-edge-pheno899 | Dried fruit liking \|\| id:ebi-fl187-GCST90094756 | Weighted median | 5 | 0.0776836457516522 | 0.117968855146569 | 0.510210609834859 | -0.15353531 | 0.308902601838927 | 1.08078069501771 | 0.857670479031673 | 1.36192971459359 | 0.999489402457283 |
| ebi-fl187-GCST90094756 | ebi-a-GCST90001392 | IgD+ CD38+ B cell Absolute Count \|\| id:ebi-a-GCST90001392 | Dried fruit liking \|\| id:ebi-fl187-GCST90094756 | Weighted mode | 5 | 0.497526550801837 | 0.475395709606822 | 0.354377005229855 | -0.43424904 | 1.42930214163121 | 1.64464828163742 | 0.6477509194898 | 4.17578406901126 | 0.999228396881742 |
| ebi-fl187-GCST90094756 | ebi-a-GCST90001402 | Switched memory B cell %B cell \|\| id:ebi-a-GCST90001402 | Dried fruit liking \|\| id:ebi-fl187-GCST90094756 | Weighted mode | 5 | 0.00409681668099593 | 0.481561059811252 | 0.993619570935806 | -0.939762861 | 0.94795649391105 | 1.0041052201063 | 0.39072047961329 | 2.58043114106176 | 0.999228396881742 |
| ebi-fl187-GCST90094756 | ebi-a-GCST90001407 | Memory B cell Absolute Count \|\| id:ebi-a-GCST90001407 | Dried fruit liking \|\| id:ebi-fl187-GCST90094756 | Weighted mode | 5 | 0.580260021470445 | 0.496045582603764 | 0.307038499815506 | -0.39198932 | 1.55250936337382 | 1.78650289947241 | 0.675711330226562 | 4.72330781955841 | 0.999228396881742 |
| ebi-fl187-GCST90094756 | ebi-a-GCST90001411 | IgD+ CD24+ B cell %B cell \|\| id:ebi-a-GCST90001411 | Dried fruit liking \|\| id:ebi-fl187-GCST90094756 | Weighted mode | 5 | 0.590824949909986 | 0.453088849390704 | 0.262213364312892 | -0.297229195 | 1.47887909471577 | 1.80547722949285 | 0.742873729981628 | 4.38802436357224 | 0.999228396881742 |
| ebi-fl187-GCST90094756 | ebi-a-GCST90001418 | CD24+ CD27+ B cell Absolute Count \|\| id:ebi-a-GCST90001418 | Dried fruit liking \|\| id:ebi-fl187-GCST90094756 | Weighted mode | 5 | 0.389792214311277 | 0.455972336577164 | 0.440808446478024 | -0.503913565 | 1.28349799400252 | 1.47667393029319 | 0.60416160107753 | 3.6092427796114 | 0.999228396881742 |
| ebi-fl187-GCST90094756 | ebi-a-GCST90001436 | Memory B cell %lymphocyte \|\| id:ebi-a-GCST90001436 | Dried fruit liking \|\| id:ebi-fl187-GCST90094756 | Weighted mode | 5 | 0.542657347680274 | 0.493473813155612 | 0.333212286834554 | -0.424551326 | 1.50986602146527 | 1.72057295319815 | 0.654063180380427 | 4.52612435018149 | 0.999228396881742 |
| ebi-fl187-GCST90094756 | ebi-a-GCST90001439 | IgD+ CD24+ B cell %lymphocyte \|\| id:ebi-a-GCST90001439 | Dried fruit liking \|\| id:ebi-fl187-GCST90094756 | Weighted mode | 5 | 0.700950830818999 | 0.53421187283707 | 0.2597106950169 | -0.34610444 | 1.74800610157966 | 2.01566835619118 | 0.707438598402159 | 5.74314001430947 | 0.999228396881742 |
| ebi-fl187-GCST90094756 | ebi-a-GCST90001457 | CD11c+ HLA DR++ monocyte %monocyte \|\| id:ebi-a-GCST90001457 | Dried fruit liking \|\| id:ebi-fl187-GCST90094756 | Weighted mode | 5 | -0.144806282 | 0.492485201692185 | 0.783360032161993 | -1.110077278 | 0.820464712872279 | 0.865189879346574 | 0.329533494480545 | 2.27155521323776 | 0.999228396881742 |
| ebi-fl187-GCST90094756 | ebi-a-GCST90001483 | CD39+ resting CD4 regulatory T cell Absolute Count \|\| id:ebi-a-GCST90001483 | Dried fruit liking \|\| id:ebi-fl187-GCST90094756 | Weighted mode | 5 | 0.16086536709274 | 0.683189631498141 | 0.82541384405823 | -1.178186311 | 1.4999170448291 | 1.17452682820711 | 0.30783655247641 | 4.48131730647538 | 0.999228396881742 |
| ebi-fl187-GCST90094756 | ebi-a-GCST90001525 | CD33dim HLA DR+ CD11b+ Absolute Count \|\| id:ebi-a-GCST90001525 | Dried fruit liking \|\| id:ebi-fl187-GCST90094756 | Weighted mode | 5 | 0.477825994407538 | 0.605136119873602 | 0.473924259341392 | -0.708240801 | 1.6638927893598 | 1.61256486366515 | 0.492509858877623 | 5.2798241347967 | 0.999228396881742 |
| ebi-fl187-GCST90094756 | ebi-a-GCST90001540 | Naive CD4+ T cell Absolute Count \|\| id:ebi-a-GCST90001540 | Dried fruit liking \|\| id:ebi-fl187-GCST90094756 | Weighted mode | 5 | -0.746131421 | 0.510250919596746 | 0.217481686854791 | -1.746223223 | 0.253960381417448 | 0.474197479329575 | 0.17443148973455 | 1.28912073 | 0.999228396881742 |
| ebi-fl187-GCST90094756 | ebi-a-GCST90001624 | HLA DR+ CD4+ T cell Absolute Count \|\| id:ebi-a-GCST90001624 | Dried fruit liking \|\| id:ebi-fl187-GCST90094756 | Weighted mode | 5 | -0.211228027 | 0.450115575467171 | 0.66330963839716 | -1.093454554 | 0.670998501351118 | 0.809589437933706 | 0.335057019398759 | 1.95618960375746 | 0.999228396881742 |
| ebi-fl187-GCST90094756 | ebi-a-GCST90001672 | CD39+ CD8+ T cell Absolute Count \|\| id:ebi-a-GCST90001672 | Dried fruit liking \|\| id:ebi-fl187-GCST90094756 | Weighted mode | 5 | 0.588934230503774 | 0.637644021131885 | 0.407961559373315 | -0.660848051 | 1.83871651192227 | 1.80206680375261 | 0.516413204049429 | 6.28846191329437 | 0.999228396881742 |
| ebi-fl187-GCST90094756 | ebi-a-GCST90001722 | CD19 on CD20- CD38- B cell \|\| id:ebi-a-GCST90001722 | Dried fruit liking \|\| id:ebi-fl187-GCST90094756 | Weighted mode | 5 | 0.00500151257993498 | 0.550736188905577 | 0.993188989465669 | -1.074441418 | 1.08444444283487 | 1.00501404102232 | 0.341488451449459 | 2.95779613736517 | 0.999228396881742 |
| ebi-fl187-GCST90094756 | ebi-a-GCST90001742 | CD19 on transitional B cell \|\| id:ebi-a-GCST90001742 | Dried fruit liking \|\| id:ebi-fl187-GCST90094756 | Weighted mode | 5 | -0.284367397 | 0.489998608752472 | 0.592806345550094 | -1.24476467 | 0.676029876601957 | 0.752490131633887 | 0.288008677322595 | 1.9660567295066 | 0.999228396881742 |
| ebi-fl187-GCST90094756 | ebi-a-GCST90001784 | CD25 on IgD+ CD38dim B cell \|\| id:ebi-a-GCST90001784 | Dried fruit liking \|\| id:ebi-fl187-GCST90094756 | Weighted mode | 5 | 0.125021010378004 | 0.483915220056127 | 0.808883170160827 | -0.823452821 | 1.07349484168801 | 1.13317226119427 | 0.438913545258136 | 2.92558611465256 | 0.999228396881742 |
| ebi-fl187-GCST90094756 | ebi-a-GCST90001794 | CD25 on IgD+ B cell \|\| id:ebi-a-GCST90001794 | Dried fruit liking \|\| id:ebi-fl187-GCST90094756 | Weighted mode | 5 | 0.063273306697041 | 0.50274558629462 | 0.905918555859425 | -0.922108042 | 1.0486546558345 | 1.06531795797954 | 0.397679830888726 | 2.85380917874923 | 0.999228396881742 |
| ebi-fl187-GCST90094756 | ebi-a-GCST90001802 | CD27 on IgD- CD38- B cell \|\| id:ebi-a-GCST90001802 | Dried fruit liking \|\| id:ebi-fl187-GCST90094756 | Weighted mode | 5 | -0.151448374 | 0.481736037279125 | 0.768946950817913 | -1.095651007 | 0.792754259442729 | 0.859462252048668 | 0.334321890301118 | 2.20947351676929 | 0.999228396881742 |
| ebi-fl187-GCST90094756 | ebi-a-GCST90001804 | CD27 on IgD- CD38dim B cell \|\| id:ebi-a-GCST90001804 | Dried fruit liking \|\| id:ebi-fl187-GCST90094756 | Weighted mode | 5 | 0.222645499998678 | 0.493072421719786 | 0.674995133368635 | -0.743776447 | 1.18906744656946 | 1.2493775908643 | 0.475315518223246 | 3.28401725739729 | 0.999228396881742 |
| ebi-fl187-GCST90094756 | ebi-a-GCST90001805 | CD27 on memory B cell \|\| id:ebi-a-GCST90001805 | Dried fruit liking \|\| id:ebi-fl187-GCST90094756 | Weighted mode | 5 | 0.0818499254882896 | 0.50093781038228 | 0.878131807453882 | -0.899988183 | 1.06368803383756 | 1.08529292280181 | 0.406574464259177 | 2.89703567692053 | 0.999228396881742 |
| ebi-fl187-GCST90094756 | ebi-a-GCST90001806 | CD27 on unswitched memory B cell \|\| id:ebi-a-GCST90001806 | Dried fruit liking \|\| id:ebi-fl187-GCST90094756 | Weighted mode | 5 | 0.480960604228825 | 0.477485852708693 | 0.370788046551903 | -0.454911667 | 1.41683287553786 | 1.61762755596134 | 0.634504013064968 | 4.12403839207482 | 0.999228396881742 |
| ebi-fl187-GCST90094756 | ebi-a-GCST90001839 | CD3 on Effector Memory CD8+ T cell \|\| id:ebi-a-GCST90001839 | Dried fruit liking \|\| id:ebi-fl187-GCST90094756 | Weighted mode | 5 | -0.530778979 | 0.543088656136589 | 0.383742679790047 | -1.595232745 | 0.533674787401203 | 0.588146637526169 | 0.202861308122597 | 1.70518700897013 | 0.999228396881742 |
| ebi-fl187-GCST90094756 | ebi-a-GCST90001859 | CD3 on CD8+ T cell \|\| id:ebi-a-GCST90001859 | Dried fruit liking \|\| id:ebi-fl187-GCST90094756 | Weighted mode | 5 | 0.0015890119193247 | 0.514301313932382 | 0.99768276584734 | -1.006441563 | 1.00961958722679 | 1.00159027506773 | 0.365517338416953 | 2.74455675195877 | 0.999264242604493 |
| ebi-fl187-GCST90094756 | ebi-a-GCST90001905 | CD86 on monocyte \|\| id:ebi-a-GCST90001905 | Dried fruit liking \|\| id:ebi-fl187-GCST90094756 | Weighted mode | 5 | 0.321519335422861 | 0.450502090985176 | 0.514849367126339 | -0.561464763 | 1.20450343375381 | 1.37922167360258 | 0.570372990473448 | 3.33510256745523 | 0.999228396881742 |
| ebi-fl187-GCST90094756 | ebi-a-GCST90001943 | CD25 on activated & secreting CD4 regulatory T cell \|\| id:ebi-a-GCST90001943 | Dried fruit liking \|\| id:ebi-fl187-GCST90094756 | Weighted mode | 5 | 0.592635426662111 | 0.489309307388874 | 0.292492293632848 | -0.366410816 | 1.5516816691443 | 1.80874896485006 | 0.693217957800844 | 4.71939998240215 | 0.999228396881742 |
| ebi-fl187-GCST90094756 | ebi-a-GCST90001949 | CD33 on CD33dim HLA DR+ CD11b- \|\| id:ebi-a-GCST90001949 | Dried fruit liking \|\| id:ebi-fl187-GCST90094756 | Weighted mode | 5 | -0.224470287 | 0.658343925385659 | 0.750288699870307 | -1.51482438 | 1.0658838070445 | 0.798939315461704 | 0.219846790848357 | 2.90340389926682 | 0.999228396881742 |
| ebi-fl187-GCST90094756 | ebi-a-GCST90001955 | CD33 on Immature Myeloid-Derived Suppressor Cells \|\| id:ebi-a-GCST90001955 | Dried fruit liking \|\| id:ebi-fl187-GCST90094756 | Weighted mode | 5 | -0.220550088 | 0.689173481332518 | 0.764971159273048 | -1.571330112 | 1.13022993497132 | 0.802077463046247 | 0.207768642949127 | 3.09636838165336 | 0.999228396881742 |
| ebi-fl187-GCST90094756 | ebi-a-GCST90001956 | CD33 on CD33+ HLA DR+ \|\| id:ebi-a-GCST90001956 | Dried fruit liking \|\| id:ebi-fl187-GCST90094756 | Weighted mode | 5 | -0.212481969 | 0.644684245683619 | 0.758246211122977 | -1.47606309 | 1.05109915301781 | 0.808574895990386 | 0.228535643886647 | 2.8607938407636 | 0.999228396881742 |
| ebi-fl187-GCST90094756 | ebi-a-GCST90001957 | CD33 on CD33+ HLA DR+ CD14- \|\| id:ebi-a-GCST90001957 | Dried fruit liking \|\| id:ebi-fl187-GCST90094756 | Weighted mode | 5 | -0.144856777 | 0.604417646022458 | 0.822371448251982 | -1.329515363 | 1.03980180909446 | 0.865146192976291 | 0.264605467747639 | 2.82865634483114 | 0.999228396881742 |
| ebi-fl187-GCST90094756 | ebi-a-GCST90002064 | CD4 on resting CD4 regulatory T cell \|\| id:ebi-a-GCST90002064 | Dried fruit liking \|\| id:ebi-fl187-GCST90094756 | Weighted mode | 5 | 0.169020099702335 | 0.431733077571742 | 0.715393973472194 | -0.677176732 | 1.01521693174295 | 1.18414393962548 | 0.508049328720058 | 2.75996205581917 | 0.999228396881742 |
| ebi-fl187-GCST90094756 | ebi-a-GCST90002080 | SSC-A on T cell \|\| id:ebi-a-GCST90002080 | Dried fruit liking \|\| id:ebi-fl187-GCST90094756 | Weighted mode | 5 | -0.106017667 | 0.479460176158003 | 0.835828749124967 | -1.045759612 | 0.833724278355749 | 0.899408758158437 | 0.351424771426466 | 2.30187562182527 | 0.999228396881742 |
| ebi-fl187-GCST90094756 | ebi-a-GCST90002121 | CD8 on CD39+ CD8+ T cell \|\| id:ebi-a-GCST90002121 | Dried fruit liking \|\| id:ebi-fl187-GCST90094756 | Weighted mode | 5 | -0.147445972 | 0.537097718554967 | 0.797277303282985 | -1.200157501 | 0.905265556087348 | 0.862909058070625 | 0.301146777364199 | 2.47258844679523 | 0.999228396881742 |
| ebi-fl187-GCST90094756 | ebi-cfmet338-GCST90026053 | 1-palmitoyl-2-docosahexaenoyl-gpc (16:0/22:6) levels \|\| id:ebi-cfmet338-GCST90026053 | Dried fruit liking \|\| id:ebi-fl187-GCST90094756 | Weighted mode | 5 | -0.192077121 | 0.247805999828458 | 0.481545554654085 | -0.677776881 | 0.29362263833993 | 0.825243222187678 | 0.507744515077522 | 1.34127766138985 | 0.999228396881742 |
| ebi-fl187-GCST90094756 | ebi-cfmet338-GCST90026063 | 1-stearoyl-2-oleoyl-gpc (18:0/18:1) levels \|\| id:ebi-cfmet338-GCST90026063 | Dried fruit liking \|\| id:ebi-fl187-GCST90094756 | Weighted mode | 4 | -0.038663844 | 0.228049921446129 | 0.876158712910753 | -0.48564169 | 0.408314001786348 | 0.962074061524509 | 0.615302236590777 | 1.50427943344833 | 0.999228396881742 |
| ebi-fl187-GCST90094756 | ebi-cfmet338-GCST90026113 | Adenosine 3',5'-cyclic monophosphate (camp) levels \|\| id:ebi-cfmet338-GCST90026113 | Dried fruit liking \|\| id:ebi-fl187-GCST90094756 | Weighted mode | 5 | -0.208352324 | 0.419447616674918 | 0.645439412027735 | -1.030469653 | 0.61376500481486 | 0.81192092719173 | 0.356839330706433 | 1.84737369254346 | 0.999228396881742 |
| ebi-fl187-GCST90094756 | ebi-cfmet338-GCST90026117 | Alpha-ketoglutaramate levels \|\| id:ebi-cfmet338-GCST90026117 | Dried fruit liking \|\| id:ebi-fl187-GCST90094756 | Weighted mode | 5 | 0.0857659186176214 | 0.162312934987176 | 0.625180968359861 | -0.232367434 | 0.403899271192485 | 1.08955125478842 | 0.792654821495083 | 1.49765308255099 | 0.999228396881742 |
| ebi-fl187-GCST90094756 | ebi-cfmet338-GCST90026124 | Arginine levels \|\| id:ebi-cfmet338-GCST90026124 | Dried fruit liking \|\| id:ebi-fl187-GCST90094756 | Weighted mode | 5 | -0.090529744 | 0.133998985494916 | 0.536338379812232 | -0.353167756 | 0.172108267610044 | 0.913447163960985 | 0.702459342053164 | 1.18780642721557 | 0.999228396881742 |
| ebi-fl187-GCST90094756 | ebi-cfmet338-GCST90026169 | Glutamate levels \|\| id:ebi-cfmet338-GCST90026169 | Dried fruit liking \|\| id:ebi-fl187-GCST90094756 | Weighted mode | 5 | 0.203860438528799 | 0.170741036264885 | 0.29845348626695 | -0.130791993 | 0.538512869607973 | 1.22612702142378 | 0.877400261201711 | 1.71345683281023 | 0.999228396881742 |
| ebi-fl187-GCST90094756 | ebi-cfmet338-GCST90026170 | Glutamine levels \|\| id:ebi-cfmet338-GCST90026170 | Dried fruit liking \|\| id:ebi-fl187-GCST90094756 | Weighted mode | 5 | 0.0331126204998201 | 0.0975088471349681 | 0.751249804639647 | -0.15800472 | 0.224229960884358 | 1.03366694477433 | 0.853845751905456 | 1.25135874990849 | 0.999228396881742 |
| ebi-fl187-GCST90094756 | ebi-cfmet338-GCST90026191 | Kynurenate levels \|\| id:ebi-cfmet338-GCST90026191 | Dried fruit liking \|\| id:ebi-fl187-GCST90094756 | Weighted mode | 5 | 0.715984699099557 | 0.783627326837962 | 0.412576383880521 | -0.819924862 | 2.25189425970196 | 2.04620058242401 | 0.440464749121941 | 9.50572510481023 | 0.999228396881742 |
| ebi-fl187-GCST90094756 | ebi-cfmet338-GCST90026202 | Methylsuccinoylcarnitine levels \|\| id:ebi-cfmet338-GCST90026202 | Dried fruit liking \|\| id:ebi-fl187-GCST90094756 | Weighted mode | 5 | 0.196559371450115 | 0.505389619482309 | 0.71714667058597 | -0.794004283 | 1.18712302563544 | 1.21720758611241 | 0.452031106089735 | 3.2776379495341 | 0.999228396881742 |
| ebi-fl187-GCST90094756 | ebi-cfmet338-GCST90026222 | N-acetylputrescine levels \|\| id:ebi-cfmet338-GCST90026222 | Dried fruit liking \|\| id:ebi-fl187-GCST90094756 | Weighted mode | 5 | -0.082191419 | 0.159166250713341 | 0.632819235886505 | -0.39415727 | 0.229774432749012 | 0.921095626939659 | 0.674248008885387 | 1.25831614300189 | 0.999228396881742 |
| ebi-fl187-GCST90094756 | ebi-cfmet338-GCST90026252 | Propionylcarnitine (c3) levels \|\| id:ebi-cfmet338-GCST90026252 | Dried fruit liking \|\| id:ebi-fl187-GCST90094756 | Weighted mode | 5 | 0.141226990243747 | 0.242939700872601 | 0.592204810455762 | -0.334934823 | 0.617388803954045 | 1.15168603981182 | 0.715384711128677 | 1.85408034818745 | 0.999228396881742 |
| ebi-fl187-GCST90094756 | ebi-cfmet338-GCST90026316 | Ethyl beta-glucopyranoside levels \|\| id:ebi-cfmet338-GCST90026316 | Dried fruit liking \|\| id:ebi-fl187-GCST90094756 | Weighted mode | 5 | -0.547005259 | 0.948316633065036 | 0.594969573477295 | -2.405705859 | 1.31169534214489 | 0.578680215592002 | 0.0902018033364312 | 3.71246227382636 | 0.999228396881742 |
| ebi-fl187-GCST90094756 | ebi-gm430-GCST90011315 | F_Prevotellaceae abundance \|\| id:ebi-gm430-GCST90011315 | Dried fruit liking \|\| id:ebi-fl187-GCST90094756 | Weighted mode | 3 | -0.517178809 | 0.444940456132359 | 0.365038905450713 | -1.389262103 | 0.354904485431648 | 0.596200172391487 | 0.249259164476074 | 1.42604443975723 | 0.999228396881742 |
| ebi-fl187-GCST90094756 | ebi-gm430-GCST90011354 | OTU97_1 (Bacteroides) abundance \|\| id:ebi-gm430-GCST90011354 | Dried fruit liking \|\| id:ebi-fl187-GCST90094756 | Weighted mode | 3 | 0.12828701616845 | 0.411805413954074 | 0.784877127303921 | -0.678851595 | 0.935425627518435 | 1.13687925860326 | 0.507199127960422 | 2.54829785263973 | 0.999228396881742 |
| ebi-fl187-GCST90094756 | ebi-gm430-GCST90011419 | OTU99_1 (Bacteroides) abundance \|\| id:ebi-gm430-GCST90011419 | Dried fruit liking \|\| id:ebi-fl187-GCST90094756 | Weighted mode | 3 | 0.136304404681745 | 0.397832584893853 | 0.7645441727278 | -0.643447462 | 0.916056271073697 | 1.14603069761316 | 0.525477733436567 | 2.49941391670832 | 0.999228396881742 |
| ebi-fl187-GCST90094756 | ebi-gm430-GCST90011435 | OTU99_155 (Butyrivibrio) abundance \|\| id:ebi-gm430-GCST90011435 | Dried fruit liking \|\| id:ebi-fl187-GCST90094756 | Weighted mode | 3 | 0.116872039101823 | 1.1646706047233 | 0.929221402570835 | -2.165882346 | 2.39962642435948 | 1.12397559556142 | 0.114648730088875 | 11.0190591595593 | 0.999228396881742 |
| ebi-fl187-GCST90094756 | ebi-gm430-GCST90011466 | OTU99_58 (Bacteroides) abundance \|\| id:ebi-gm430-GCST90011466 | Dried fruit liking \|\| id:ebi-fl187-GCST90094756 | Weighted mode | 3 | 0.0707895314011937 | 0.734388766744523 | 0.931998023113892 | -1.368612451 | 1.51019151422046 | 1.07335529450861 | 0.254459790032293 | 4.52759781065388 | 0.999228396881742 |
| ebi-fl187-GCST90094756 | ebi-gm430-GCST90011472 | OTU99_72 (Parabacteroides) abundance \|\| id:ebi-gm430-GCST90011472 | Dried fruit liking \|\| id:ebi-fl187-GCST90094756 | Weighted mode | 3 | -0.52487406 | 1.08707136140827 | 0.676897540394563 | -2.655533929 | 1.6057858081551 | 0.591629869419552 | 0.0702613141257606 | 4.98177278271348 | 0.999228396881742 |
| ebi-fl187-GCST90094756 | ebi-gm430-GCST90011505 | TestASV_28 (Holdemanella) abundance \|\| id:ebi-gm430-GCST90011505 | Dried fruit liking \|\| id:ebi-fl187-GCST90094756 | Weighted mode | 4 | 0.0384701578278976 | 0.789343523396051 | 0.964192147684339 | -1.508643148 | 1.58558346368416 | 1.03921971532164 | 0.221209923543388 | 4.88213909852633 | 0.999228396881742 |
| ebi-fl187-GCST90094756 | ebi-gm430-GCST90011513 | TestASV_35 (Alphaproteobacteria) abundance \|\| id:ebi-gm430-GCST90011513 | Dried fruit liking \|\| id:ebi-fl187-GCST90094756 | Weighted mode | 3 | 1.16365161385195 | 1.06254881438384 | 0.387730267929345 | -0.918944062 | 3.24624729004427 | 3.20160297430257 | 0.398940074601529 | 25.6937376253847 | 0.999228396881742 |
| ebi-fl187-GCST90094756 | ebi-gm430-GCST90011543 | G_Coprococcus prevalence \|\| id:ebi-gm430-GCST90011543 | Dried fruit liking \|\| id:ebi-fl187-GCST90094756 | Weighted mode | 3 | 1.0300365883728 | 1.02579341545395 | 0.421060889179069 | -0.980518506 | 3.04059168266255 | 2.80116832301501 | 0.375116548268121 | 20.9176161651345 | 0.999228396881742 |
| ebi-fl187-GCST90094756 | ebi-gm430-GCST90011547 | G_Parasutterella prevalence \|\| id:ebi-gm430-GCST90011547 | Dried fruit liking \|\| id:ebi-fl187-GCST90094756 | Weighted mode | 3 | 0.724618635253936 | 1.07012050901868 | 0.568142592669115 | -1.372817562 | 2.82205483293054 | 2.06394383444206 | 0.25339200501972 | 16.8113597404143 | 0.999228396881742 |
| ebi-fl187-GCST90094756 | ebi-gm430-GCST90011569 | OTU97_13 (Subdoligranulum) prevalence \|\| id:ebi-gm430-GCST90011569 | Dried fruit liking \|\| id:ebi-fl187-GCST90094756 | Weighted mode | 3 | 1.02068339231941 | 0.963304746056541 | 0.400396941772938 | -0.86739391 | 2.90876069459023 | 2.7750905916895 | 0.420044798645412 | 18.3340629783268 | 0.999228396881742 |
| ebi-fl187-GCST90094756 | ebi-gm430-GCST90011590 | OTU97_277 (Prevotella) prevalence \|\| id:ebi-gm430-GCST90011590 | Dried fruit liking \|\| id:ebi-fl187-GCST90094756 | Weighted mode | 3 | 0.144221460577328 | 1.10453589772122 | 0.908062683132816 | -2.020668899 | 2.30911182011091 | 1.15513989814895 | 0.132566761648112 | 10.0654807261377 | 0.999228396881742 |
| ebi-fl187-GCST90094756 | ebi-gm430-GCST90011616 | OTU97_86 (Phascolarctobacterium) prevalence \|\| id:ebi-gm430-GCST90011616 | Dried fruit liking \|\| id:ebi-fl187-GCST90094756 | Weighted mode | 3 | 0.454716037972978 | 1.13247326038913 | 0.726874270861328 | -1.764931552 | 2.67436362833567 | 1.57572587318611 | 0.171198504212409 | 14.503117529272 | 0.999228396881742 |
| ebi-fl187-GCST90094756 | ebi-gm430-GCST90011665 | OTU99_55 (Barnesiella) prevalence \|\| id:ebi-gm430-GCST90011665 | Dried fruit liking \|\| id:ebi-fl187-GCST90094756 | Weighted mode | 3 | 0.606787130907634 | 0.871066091397282 | 0.558124698243313 | -1.100502408 | 2.31407667004631 | 1.83452782241122 | 0.332703888529387 | 10.1155785887473 | 0.999228396881742 |
| ebi-fl187-GCST90094756 | ebi-gm430-GCST90011687 | TestASV_14 (Lachnospiraceae) prevalence \|\| id:ebi-gm430-GCST90011687 | Dried fruit liking \|\| id:ebi-fl187-GCST90094756 | Weighted mode | 3 | -0.086237851 | 0.788507420441412 | 0.922895040925512 | -1.631712395 | 1.45923669325362 | 0.917376006641056 | 0.195594352425853 | 4.30267401447454 | 0.999228396881742 |
| ebi-fl187-GCST90094756 | ebi-gm430-GCST90011697 | TestASV_23 (Barnesiella) prevalence \|\| id:ebi-gm430-GCST90011697 | Dried fruit liking \|\| id:ebi-fl187-GCST90094756 | Weighted mode | 3 | -0.158236327 | 1.06513299022663 | 0.895526955509133 | -2.245896988 | 1.92942433400706 | 0.853648018192147 | 0.105832567275932 | 6.88554532617016 | 0.999228396881742 |
| ebi-fl187-GCST90094756 | ebi-gm430-GCST90011727 | TestASV_6 (Subdoligranulum) prevalence \|\| id:ebi-gm430-GCST90011727 | Dried fruit liking \|\| id:ebi-fl187-GCST90094756 | Weighted mode | 3 | 2.43521702318028 | 1.30280581331876 | 0.202527234052731 | -0.118282371 | 4.98871641728505 | 11.4182964795063 | 0.888445146114676 | 146.747939435618 | 0.999228396881742 |
| ebi-fl187-GCST90094756 | ebi-hbsm44-GCST90104908 | Sulcal depth of the left posterior inferior temporal sulcus \|\| id:ebi-hbsm44-GCST90104908 | Dried fruit liking \|\| id:ebi-fl187-GCST90094756 | Weighted mode | 4 | -0.228381538 | 0.34771713247019 | 0.558195852733426 | -0.909907118 | 0.453144041458047 | 0.795820565970582 | 0.402561613095376 | 1.57325078353083 | 0.999228396881742 |
| ebi-fl187-GCST90094756 | ebi-hbsm44-GCST90104918 | Sulcal depth of the left inferior frontal sulcus \|\| id:ebi-hbsm44-GCST90104918 | Dried fruit liking \|\| id:ebi-fl187-GCST90094756 | Weighted mode | 4 | 0.296351686323416 | 0.579199779175386 | 0.644172692478732 | -0.838879881 | 1.43158325350717 | 1.34494307175243 | 0.432194361576762 | 4.18532037219462 | 0.999228396881742 |
| ebi-fl187-GCST90094756 | ebi-hbsm44-GCST90104925 | Sulcal depth of the right anterior inferior temporal sulcus \|\| id:ebi-hbsm44-GCST90104925 | Dried fruit liking \|\| id:ebi-fl187-GCST90094756 | Weighted mode | 4 | -0.143778002 | 0.206606400190452 | 0.536556080053036 | -0.548726547 | 0.261170542204953 | 0.86607999460011 | 0.577684997125527 | 1.2984490869226 | 0.999228396881742 |
| ebi-fl187-GCST90094756 | ebi-IPs91-GCST90274770 | C-C motif chemokine 4 levels \|\| id:ebi-IPs91-GCST90274770 | Dried fruit liking \|\| id:ebi-fl187-GCST90094756 | Weighted mode | 5 | 0.0363915311075168 | 0.248159363377655 | 0.890505628737372 | -0.450000821 | 0.522783883327721 | 1.0370618089709 | 0.637627628057423 | 1.68671674234469 | 0.999228396881742 |
| ebi-fl187-GCST90094756 | ebi-IPs91-GCST90274779 | C-X-C motif chemokine 1 levels \|\| id:ebi-IPs91-GCST90274779 | Dried fruit liking \|\| id:ebi-fl187-GCST90094756 | Weighted mode | 5 | -0.033170901 | 0.236341988599118 | 0.89516630163973 | -0.496401199 | 0.430059396449178 | 0.967373220211975 | 0.60871737535994 | 1.53734883389838 | 0.999228396881742 |
| ebi-fl187-GCST90094756 | ebi-IPs91-GCST90274780 | C-X-C motif chemokine 10 levels \|\| id:ebi-IPs91-GCST90274780 | Dried fruit liking \|\| id:ebi-fl187-GCST90094756 | Weighted mode | 5 | 0.040105710682128 | 0.226174751226032 | 0.867872707372808 | -0.403196802 | 0.483408223085151 | 1.0409208048249 | 0.66818058729364 | 1.6215917410979 | 0.999228396881742 |
| ebi-fl187-GCST90094756 | ebi-IPs91-GCST90274796 | Interleukin-10 receptor subunit alpha levels \|\| id:ebi-IPs91-GCST90274796 | Dried fruit liking \|\| id:ebi-fl187-GCST90094756 | Weighted mode | 5 | 0.00128635394628551 | 0.26234523088562 | 0.99632255320909 | -0.512910299 | 0.515483006482101 | 1.00128718165439 | 0.598750497961112 | 1.67444707529999 | 0.999228396881742 |
| ebi-fl187-GCST90094756 | ebi-IPs91-GCST90274807 | Interleukin-20 levels \|\| id:ebi-IPs91-GCST90274807 | Dried fruit liking \|\| id:ebi-fl187-GCST90094756 | Weighted mode | 5 | 0.221832118695187 | 0.279112860861192 | 0.471236836667964 | -0.325229089 | 0.768893325983123 | 1.24836178366618 | 0.722361849825646 | 2.15737741866401 | 0.999228396881742 |
| ebi-fl187-GCST90094756 | ebi-IPs91-GCST90274808 | Interleukin-20 receptor subunit alpha levels \|\| id:ebi-IPs91-GCST90274808 | Dried fruit liking \|\| id:ebi-fl187-GCST90094756 | Weighted mode | 5 | 0.0856564659655613 | 0.241482831063566 | 0.740717767495672 | -0.387649883 | 0.55896281485015 | 1.08943200704014 | 0.678649908598838 | 1.7488576700967 | 0.999228396881742 |
| ebi-fl187-GCST90094756 | ebi-IPs91-GCST90274810 | Interleukin-24 levels \|\| id:ebi-IPs91-GCST90274810 | Dried fruit liking \|\| id:ebi-fl187-GCST90094756 | Weighted mode | 5 | 0.342337310773562 | 0.312161518838011 | 0.334376906986897 | -0.269499266 | 0.954173887696064 | 1.40823523032452 | 0.763761840009595 | 2.59652467567932 | 0.999228396881742 |
| ebi-fl187-GCST90094756 | ebi-IPs91-GCST90274823 | Monocyte chemoattractant protein-3 levels \|\| id:ebi-IPs91-GCST90274823 | Dried fruit liking \|\| id:ebi-fl187-GCST90094756 | Weighted mode | 5 | -0.133518589 | 0.301617776676545 | 0.680885664635977 | -0.724689432 | 0.457652252816775 | 0.875011202832577 | 0.484475008146226 | 1.58035934198575 | 0.999228396881742 |
| ebi-fl187-GCST90094756 | ebi-IPs91-GCST90274824 | Monocyte chemoattractant protein-4 levels \|\| id:ebi-IPs91-GCST90274824 | Dried fruit liking \|\| id:ebi-fl187-GCST90094756 | Weighted mode | 5 | 0.0866501774546878 | 0.234654465194173 | 0.730645568322611 | -0.373272574 | 0.546572929235267 | 1.09051512620701 | 0.688477545962823 | 1.72732320387178 | 0.999228396881742 |
| ebi-fl187-GCST90094756 | ebi-IPs91-GCST90274834 | SIR2-like protein 2 levels \|\| id:ebi-IPs91-GCST90274834 | Dried fruit liking \|\| id:ebi-fl187-GCST90094756 | Weighted mode | 5 | 0.0168752128079763 | 0.195620593037761 | 0.935401350789413 | -0.36654115 | 0.400291575161988 | 1.01701840353567 | 0.693127614009055 | 1.49225974008985 | 0.999228396881742 |
| ebi-fl187-GCST90094756 | ebi-met1400-GCST90199652 | 1-stearoyl-2-oleoyl-GPS (18:0/18:1) levels \|\| id:ebi-met1400-GCST90199652 | Dried fruit liking \|\| id:ebi-fl187-GCST90094756 | Weighted mode | 5 | -0.203756047 | 0.332905209384244 | 0.573583019067039 | -0.856250258 | 0.448738163153924 | 0.815661329751336 | 0.424751809777715 | 1.56633447942197 | 0.999228396881742 |
| ebi-fl187-GCST90094756 | ebi-met1400-GCST90199665 | N-acetylaspartate (naa) levels \|\| id:ebi-met1400-GCST90199665 | Dried fruit liking \|\| id:ebi-fl187-GCST90094756 | Weighted mode | 5 | -0.182084806 | 0.314579359676861 | 0.593740254574263 | -0.798660351 | 0.434490739153591 | 0.833530649173854 | 0.449931310690133 | 1.54417647006273 | 0.999228396881742 |
| ebi-fl187-GCST90094756 | ebi-met1400-GCST90199669 | Acetylcarnitine levels (Biocrates platform) \|\| id:ebi-met1400-GCST90199669 | Dried fruit liking \|\| id:ebi-fl187-GCST90094756 | Weighted mode | 5 | -0.351034669 | 0.296460230471978 | 0.301933242218903 | -0.932096721 | 0.230027382275119 | 0.703959347549175 | 0.393727307922889 | 1.25863447373306 | 0.999228396881742 |
| ebi-fl187-GCST90094756 | ebi-met1400-GCST90199670 | Iminodiacetate (IDA) levels \|\| id:ebi-met1400-GCST90199670 | Dried fruit liking \|\| id:ebi-fl187-GCST90094756 | Weighted mode | 5 | -0.060952253 | 0.375004903949879 | 0.878763399663247 | -0.795961865 | 0.674057358568351 | 0.940868162172532 | 0.451147083607301 | 1.96218246943266 | 0.999228396881742 |
| ebi-fl187-GCST90094756 | ebi-met1400-GCST90199686 | Indolepropionate levels \|\| id:ebi-met1400-GCST90199686 | Dried fruit liking \|\| id:ebi-fl187-GCST90094756 | Weighted mode | 5 | 0.276644160681967 | 0.289867635403435 | 0.393935666584558 | -0.291496405 | 0.8447847260727 | 1.3186970432807 | 0.747144699800932 | 2.32747671558213 | 0.999228396881742 |
| ebi-fl187-GCST90094756 | ebi-met1400-GCST90199704 | 3-hydroxysebacate levels \|\| id:ebi-met1400-GCST90199704 | Dried fruit liking \|\| id:ebi-fl187-GCST90094756 | Weighted mode | 5 | -0.223293089 | 0.325712774857768 | 0.530662861729193 | -0.861690128 | 0.415103949887469 | 0.799880378928505 | 0.422447488469682 | 1.51452816754224 | 0.999228396881742 |
| ebi-fl187-GCST90094756 | ebi-met1400-GCST90199713 | Docosapentaenoate n3 DPA; 22:5n3 levels \|\| id:ebi-met1400-GCST90199713 | Dried fruit liking \|\| id:ebi-fl187-GCST90094756 | Weighted mode | 5 | 0.116778459509505 | 0.3126901895072 | 0.727753811256521 | -0.496094312 | 0.729651230943617 | 1.12387041930467 | 0.608904211436708 | 2.07435700996025 | 0.999228396881742 |
| ebi-fl187-GCST90094756 | ebi-met1400-GCST90199715 | Myristoleate (14:1n5) levels \|\| id:ebi-met1400-GCST90199715 | Dried fruit liking \|\| id:ebi-fl187-GCST90094756 | Weighted mode | 5 | 0.175329884561638 | 0.335337490921422 | 0.628704949117589 | -0.481931598 | 0.832591366767625 | 1.19163925517344 | 0.617589304883178 | 2.29926927691683 | 0.999228396881742 |
| ebi-fl187-GCST90094756 | ebi-met1400-GCST90199725 | Campesterol levels \|\| id:ebi-met1400-GCST90199725 | Dried fruit liking \|\| id:ebi-fl187-GCST90094756 | Weighted mode | 5 | -0.292411036 | 0.34606476632583 | 0.445710794571288 | -0.970697978 | 0.385875906246305 | 0.746461650481138 | 0.37881853889506 | 1.47090212972228 | 0.999228396881742 |
| ebi-fl187-GCST90094756 | ebi-met1400-GCST90199737 | N-acetylproline levels \|\| id:ebi-met1400-GCST90199737 | Dried fruit liking \|\| id:ebi-fl187-GCST90094756 | Weighted mode | 5 | -0.23575595 | 0.347300986793283 | 0.534495647492843 | -0.916465884 | 0.444953983821875 | 0.789973443196586 | 0.399929945121868 | 1.56041838968999 | 0.999228396881742 |
| ebi-fl187-GCST90094756 | ebi-met1400-GCST90199775 | Gamma-glutamylisoleucine levels \|\| id:ebi-met1400-GCST90199775 | Dried fruit liking \|\| id:ebi-fl187-GCST90094756 | Weighted mode | 5 | -0.177445248 | 0.320534561030506 | 0.609344246919189 | -0.805692987 | 0.450802492013371 | 0.837406848109912 | 0.446778207665271 | 1.56957124861998 | 0.999228396881742 |
| ebi-fl187-GCST90094756 | ebi-met1400-GCST90199777 | 1-palmitoleoyl-GPC (16:1) levels \|\| id:ebi-met1400-GCST90199777 | Dried fruit liking \|\| id:ebi-fl187-GCST90094756 | Weighted mode | 5 | -0.157097841 | 0.287589183518132 | 0.613928393394161 | -0.72077264 | 0.406576958911489 | 0.85462043799327 | 0.486376316715127 | 1.50166870370786 | 0.999228396881742 |
| ebi-fl187-GCST90094756 | ebi-met1400-GCST90199782 | Glutamine degradant levels \|\| id:ebi-met1400-GCST90199782 | Dried fruit liking \|\| id:ebi-fl187-GCST90094756 | Weighted mode | 5 | 0.374695525553775 | 0.237325754485611 | 0.189520211544515 | -0.090462953 | 0.839854004345572 | 1.45454847434839 | 0.913508175794037 | 2.31602882195359 | 0.999228396881742 |
| ebi-fl187-GCST90094756 | ebi-met1400-GCST90199783 | 5-methyluridine (ribothymidine) levels \|\| id:ebi-met1400-GCST90199783 | Dried fruit liking \|\| id:ebi-fl187-GCST90094756 | Weighted mode | 5 | -0.059196142 | 0.353899236386544 | 0.875274647630593 | -0.752838645 | 0.634446361193002 | 0.942521882781096 | 0.471027572922599 | 1.88597770191937 | 0.999228396881742 |
| ebi-fl187-GCST90094756 | ebi-met1400-GCST90199787 | Homostachydrine levels \|\| id:ebi-met1400-GCST90199787 | Dried fruit liking \|\| id:ebi-fl187-GCST90094756 | Weighted mode | 5 | -0.130471515 | 0.336151096794015 | 0.717688726007054 | -0.789327665 | 0.528384634232717 | 0.877681492925602 | 0.454150033531755 | 1.69619012693577 | 0.999228396881742 |
| ebi-fl187-GCST90094756 | ebi-met1400-GCST90199802 | Beta-hydroxyisovaleroylcarnitine levels \|\| id:ebi-met1400-GCST90199802 | Dried fruit liking \|\| id:ebi-fl187-GCST90094756 | Weighted mode | 5 | -0.400834466 | 0.354318024739977 | 0.321164154269301 | -1.095297795 | 0.293628862396566 | 0.669760920003538 | 0.33443999769776 | 1.34128600960396 | 0.999228396881742 |
| ebi-fl187-GCST90094756 | ebi-met1400-GCST90199873 | Pregnenolone sulfate levels \|\| id:ebi-met1400-GCST90199873 | Dried fruit liking \|\| id:ebi-fl187-GCST90094756 | Weighted mode | 5 | 0.314057799898183 | 0.274164773904924 | 0.315875469877205 | -0.223305157 | 0.851420756751834 | 1.36896886052143 | 0.799870725932983 | 2.34297328345325 | 0.999228396881742 |
| ebi-fl187-GCST90094756 | ebi-met1400-GCST90199911 | Beta-citrylglutamate levels \|\| id:ebi-met1400-GCST90199911 | Dried fruit liking \|\| id:ebi-fl187-GCST90094756 | Weighted mode | 5 | -0.469577529 | 0.32253724039176 | 0.219132638489941 | -1.10175052 | 0.162595461974542 | 0.62526636927872 | 0.332288895802976 | 1.17656062988877 | 0.999228396881742 |
| ebi-fl187-GCST90094756 | ebi-met1400-GCST90199934 | 2-hydroxydecanoate levels \|\| id:ebi-met1400-GCST90199934 | Dried fruit liking \|\| id:ebi-fl187-GCST90094756 | Weighted mode | 5 | -0.044706709 | 0.275608091753941 | 0.879003955361127 | -0.584898569 | 0.495485150496665 | 0.956277908085683 | 0.557162372383151 | 1.64129432068659 | 0.999228396881742 |
| ebi-fl187-GCST90094756 | ebi-met1400-GCST90199945 | Sphingomyelin (d18:1/14:0, d16:1/16:0) levels \|\| id:ebi-met1400-GCST90199945 | Dried fruit liking \|\| id:ebi-fl187-GCST90094756 | Weighted mode | 5 | -0.082661414 | 0.280621897678349 | 0.782980882673869 | -0.632680333 | 0.467357505561845 | 0.920662818097732 | 0.531166188858866 | 1.59577179874466 | 0.999228396881742 |
| ebi-fl187-GCST90094756 | ebi-met1400-GCST90199979 | Carnitine C14:1 levels \|\| id:ebi-met1400-GCST90199979 | Dried fruit liking \|\| id:ebi-fl187-GCST90094756 | Weighted mode | 5 | -0.23963885 | 0.392462210136748 | 0.57445325337349 | -1.008864781 | 0.52958708228226 | 0.786912003350389 | 0.364632682489197 | 1.69823093418201 | 0.999228396881742 |
| ebi-fl187-GCST90094756 | ebi-met1400-GCST90199986 | Eugenol sulfate levels \|\| id:ebi-met1400-GCST90199986 | Dried fruit liking \|\| id:ebi-fl187-GCST90094756 | Weighted mode | 5 | -0.187474393 | 0.350324039953927 | 0.620915029206623 | -0.874109511 | 0.499160725425848 | 0.829050347497132 | 0.417233395969542 | 1.64733812135999 | 0.999228396881742 |
| ebi-fl187-GCST90094756 | ebi-met1400-GCST90200002 | 6-hydroxyindole sulfate levels \|\| id:ebi-met1400-GCST90200002 | Dried fruit liking \|\| id:ebi-fl187-GCST90094756 | Weighted mode | 5 | -0.014104373 | 0.306413736884667 | 0.965492366668633 | -0.614675297 | 0.586466551326294 | 0.985994627706718 | 0.540816471391989 | 1.79762536330344 | 0.999228396881742 |
| ebi-fl187-GCST90094756 | ebi-met1400-GCST90200023 | Trans 3,4-methyleneheptanoate levels \|\| id:ebi-met1400-GCST90200023 | Dried fruit liking \|\| id:ebi-fl187-GCST90094756 | Weighted mode | 5 | -0.135440557 | 0.32334374342183 | 0.696822181886741 | -0.769194294 | 0.498313179972233 | 0.873331074687135 | 0.463386270932262 | 1.64594251892646 | 0.999228396881742 |
| ebi-fl187-GCST90094756 | ebi-met1400-GCST90200032 | 2-hydroxybutyrate/2-hydroxyisobutyrate levels \|\| id:ebi-met1400-GCST90200032 | Dried fruit liking \|\| id:ebi-fl187-GCST90094756 | Weighted mode | 5 | 0.00644128047641779 | 0.432883747625002 | 0.988840566188306 | -0.842010865 | 0.854893425821422 | 1.00646207013688 | 0.430843284146849 | 2.35112379813481 | 0.999228396881742 |
| ebi-fl187-GCST90094756 | ebi-met1400-GCST90200051 | 1-palmitoyl-2-dihomo-linolenoyl-GPC (16:0/20:3n3 or 6) levels \|\| id:ebi-met1400-GCST90200051 | Dried fruit liking \|\| id:ebi-fl187-GCST90094756 | Weighted mode | 5 | -0.38368314 | 0.37748792672607 | 0.366912707452139 | -1.123559477 | 0.356193196178916 | 0.681347284540943 | 0.325120473844797 | 1.42788338323143 | 0.999228396881742 |
| ebi-fl187-GCST90094756 | ebi-met1400-GCST90200069 | Phenylacetylglutamate levels \|\| id:ebi-met1400-GCST90200069 | Dried fruit liking \|\| id:ebi-fl187-GCST90094756 | Weighted mode | 5 | -0.253160708 | 0.356997412127679 | 0.517384145291097 | -0.952875636 | 0.446554219397127 | 0.776343106960118 | 0.385630494493212 | 1.56291742569933 | 0.999228396881742 |
| ebi-fl187-GCST90094756 | ebi-met1400-GCST90200072 | Thioproline levels \|\| id:ebi-met1400-GCST90200072 | Dried fruit liking \|\| id:ebi-fl187-GCST90094756 | Weighted mode | 5 | 0.0157164701645707 | 0.301074606520686 | 0.960871274275312 | -0.574389759 | 0.605822698945116 | 1.01584062344616 | 0.563048359400323 | 1.83275939804272 | 0.999228396881742 |
| ebi-fl187-GCST90094756 | ebi-met1400-GCST90200081 | 1-oleoyl-2-docosahexaenoyl-GPC (18:1/22:6) levels \|\| id:ebi-met1400-GCST90200081 | Dried fruit liking \|\| id:ebi-fl187-GCST90094756 | Weighted mode | 5 | -0.259988143 | 0.285885204165239 | 0.414572198922365 | -0.820323143 | 0.300346857191193 | 0.771060728237489 | 0.440289355032516 | 1.35032709702059 | 0.999228396881742 |
| ebi-fl187-GCST90094756 | ebi-met1400-GCST90200097 | Furaneol sulfate levels \|\| id:ebi-met1400-GCST90200097 | Dried fruit liking \|\| id:ebi-fl187-GCST90094756 | Weighted mode | 5 | 0.228948208179113 | 0.424013582217418 | 0.617885285918932 | -0.602118413 | 1.06001482932525 | 1.25727692066247 | 0.547650256982279 | 2.88641379251953 | 0.999228396881742 |
| ebi-fl187-GCST90094756 | ebi-met1400-GCST90200124 | Sphingomyelin (d18:2/24:2) levels \|\| id:ebi-met1400-GCST90200124 | Dried fruit liking \|\| id:ebi-fl187-GCST90094756 | Weighted mode | 4 | 0.348650067512387 | 0.296474673161395 | 0.324426556086207 | -0.232440292 | 0.92974042690872 | 1.41715319562164 | 0.792597072411961 | 2.53385137261368 | 0.999228396881742 |
| ebi-fl187-GCST90094756 | ebi-met1400-GCST90200137 | Lignoceroylcarnitine (C24) levels \|\| id:ebi-met1400-GCST90200137 | Dried fruit liking \|\| id:ebi-fl187-GCST90094756 | Weighted mode | 5 | -0.219406188 | 0.290080680804727 | 0.49153409363816 | -0.787964323 | 0.349151945891518 | 0.802995484382049 | 0.454769617756466 | 1.41786461267793 | 0.999228396881742 |
| ebi-fl187-GCST90094756 | ebi-met1400-GCST90200165 | Octadecenedioate (C18:1-DC) levels \|\| id:ebi-met1400-GCST90200165 | Dried fruit liking \|\| id:ebi-fl187-GCST90094756 | Weighted mode | 5 | -0.208566436 | 0.417457970592472 | 0.643580955533627 | -1.026784059 | 0.609651186177463 | 0.811747103531318 | 0.358156922166841 | 1.83978954282093 | 0.999228396881742 |
| ebi-fl187-GCST90094756 | ebi-met1400-GCST90200176 | Glutamine conjugate of C6H10O2 (1) levels \|\| id:ebi-met1400-GCST90200176 | Dried fruit liking \|\| id:ebi-fl187-GCST90094756 | Weighted mode | 5 | -0.012451669 | 0.326013372398716 | 0.97136340049573 | -0.651437878 | 0.6265345413425 | 0.987625532705569 | 0.521295677788047 | 1.87111505890626 | 0.999228396881742 |
| ebi-fl187-GCST90094756 | ebi-met1400-GCST90200182 | Glycine conjugate of C10H12O2 levels \|\| id:ebi-met1400-GCST90200182 | Dried fruit liking \|\| id:ebi-fl187-GCST90094756 | Weighted mode | 5 | -0.111642314 | 0.374140316218444 | 0.78025902532152 | -0.844957334 | 0.621672705690538 | 0.894364101725852 | 0.429575686145045 | 1.86204008339944 | 0.999228396881742 |
| ebi-fl187-GCST90094756 | ebi-met1400-GCST90200208 | Glucuronide of piperine metabolite C17H21NO3 (4) levels \|\| id:ebi-met1400-GCST90200208 | Dried fruit liking \|\| id:ebi-fl187-GCST90094756 | Weighted mode | 5 | -0.096838592 | 0.3705819486682 | 0.806752858888315 | -0.823179211 | 0.629502027417435 | 0.907702504781017 | 0.439033652635076 | 1.87667581343833 | 0.999228396881742 |
| ebi-fl187-GCST90094756 | ebi-met1400-GCST90200227 | 3-ethylcatechol sulfate (2) levels \|\| id:ebi-met1400-GCST90200227 | Dried fruit liking \|\| id:ebi-fl187-GCST90094756 | Weighted mode | 5 | -0.082568928 | 0.441503355560648 | 0.86074949269138 | -0.947915505 | 0.782777648892727 | 0.920747970347729 | 0.387548024023076 | 2.18754005271094 | 0.999228396881742 |
| ebi-fl187-GCST90094756 | ebi-met1400-GCST90200259 | Pentose acid levels \|\| id:ebi-met1400-GCST90200259 | Dried fruit liking \|\| id:ebi-fl187-GCST90094756 | Weighted mode | 5 | -0.122371059 | 0.271595080788852 | 0.675646330137754 | -0.654697417 | 0.409955299286969 | 0.884819987109989 | 0.519599260495603 | 1.50675043078886 | 0.999228396881742 |
| ebi-fl187-GCST90094756 | ebi-met1400-GCST90200291 | Sarcosine levels \|\| id:ebi-met1400-GCST90200291 | Dried fruit liking \|\| id:ebi-fl187-GCST90094756 | Weighted mode | 5 | 0.467074678431756 | 0.417450277039601 | 0.32584477278944 | -0.351127865 | 1.28527722142937 | 1.59532053500437 | 0.703893745033153 | 3.61567015954482 | 0.999228396881742 |
| ebi-fl187-GCST90094756 | ebi-met1400-GCST90200306 | Butyrate/isobutyrate (4:0) levels \|\| id:ebi-met1400-GCST90200306 | Dried fruit liking \|\| id:ebi-fl187-GCST90094756 | Weighted mode | 5 | 0.00277547010973556 | 0.301463607611704 | 0.993095133966814 | -0.588093201 | 0.593644141028676 | 1.00277932529272 | 0.555385283927914 | 1.81057439643116 | 0.999228396881742 |
| ebi-fl187-GCST90094756 | ebi-met1400-GCST90200321 | Cholate levels \|\| id:ebi-met1400-GCST90200321 | Dried fruit liking \|\| id:ebi-fl187-GCST90094756 | Weighted mode | 5 | 0.192261459701874 | 0.289217774719308 | 0.542567285722048 | -0.374605379 | 0.759128298151719 | 1.21198736139626 | 0.687560551268097 | 2.13641309332695 | 0.999228396881742 |
| ebi-fl187-GCST90094756 | ebi-met1400-GCST90200336 | Ceramide (d18:1/16:0) levels \|\| id:ebi-met1400-GCST90200336 | Dried fruit liking \|\| id:ebi-fl187-GCST90094756 | Weighted mode | 5 | -0.239540779 | 0.32949832889434 | 0.507498693944048 | -0.885357504 | 0.406275945700419 | 0.786989180108956 | 0.412566652861723 | 1.50121674961488 | 0.999228396881742 |
| ebi-fl187-GCST90094756 | ebi-met1400-GCST90200358 | Arachidonate (20:4n6) levels \|\| id:ebi-met1400-GCST90200358 | Dried fruit liking \|\| id:ebi-fl187-GCST90094756 | Weighted mode | 5 | -0.366525118 | 0.359874986801912 | 0.366040584807284 | -1.071880092 | 0.338829856299323 | 0.69313872612153 | 0.342364235705681 | 1.40330456146826 | 0.999228396881742 |
| ebi-fl187-GCST90094756 | ebi-met1400-GCST90200413 | 2'-deoxyuridine levels \|\| id:ebi-met1400-GCST90200413 | Dried fruit liking \|\| id:ebi-fl187-GCST90094756 | Weighted mode | 5 | 0.162000510939513 | 0.343458860333245 | 0.661737309598536 | -0.511178855 | 0.835179877192673 | 1.17586084211461 | 0.599788098498758 | 2.30522866905694 | 0.999228396881742 |
| ebi-fl187-GCST90094756 | ebi-met1400-GCST90200432 | Threonine levels \|\| id:ebi-met1400-GCST90200432 | Dried fruit liking \|\| id:ebi-fl187-GCST90094756 | Weighted mode | 5 | 0.0334545353037491 | 0.303188562924581 | 0.917452493253853 | -0.560795048 | 0.627704118635928 | 1.03402043123276 | 0.570755105691962 | 1.8733047528511 | 0.999228396881742 |
| ebi-fl187-GCST90094756 | ebi-met1400-GCST90200436 | Caffeine levels \|\| id:ebi-met1400-GCST90200436 | Dried fruit liking \|\| id:ebi-fl187-GCST90094756 | Weighted mode | 5 | 0.0689624207613084 | 0.349312184257017 | 0.853122539069668 | -0.61568946 | 0.753614301905062 | 1.07139594614783 | 0.540268273299688 | 2.12466533785386 | 0.999228396881742 |
| ebi-fl187-GCST90094756 | ebi-met1400-GCST90200442 | Valine levels \|\| id:ebi-met1400-GCST90200442 | Dried fruit liking \|\| id:ebi-fl187-GCST90094756 | Weighted mode | 5 | -0.348327339 | 0.325661710131852 | 0.345050002250576 | -0.98662429 | 0.289969613319555 | 0.705867780665246 | 0.372833145545715 | 1.33638687904745 | 0.999228396881742 |
| ebi-fl187-GCST90094756 | ebi-met1400-GCST90200459 | Margarate (17:0) levels \|\| id:ebi-met1400-GCST90200459 | Dried fruit liking \|\| id:ebi-fl187-GCST90094756 | Weighted mode | 5 | -0.613844412 | 0.498536907657519 | 0.285657351231107 | -1.590976751 | 0.363287926676639 | 0.54126601438108 | 0.203726524364777 | 1.43804985255338 | 0.999228396881742 |
| ebi-fl187-GCST90094756 | ebi-met1400-GCST90200462 | X-11299 levels \|\| id:ebi-met1400-GCST90200462 | Dried fruit liking \|\| id:ebi-fl187-GCST90094756 | Weighted mode | 5 | -0.197688771 | 0.293565389237749 | 0.537595368675257 | -0.773076934 | 0.377699391924965 | 0.820625215778491 | 0.461590597258177 | 1.45892431252198 | 0.999228396881742 |
| ebi-fl187-GCST90094756 | ebi-met1400-GCST90200497 | X-12740 levels \|\| id:ebi-met1400-GCST90200497 | Dried fruit liking \|\| id:ebi-fl187-GCST90094756 | Weighted mode | 5 | 0.369693155698985 | 0.319994009459844 | 0.31227640368486 | -0.257495103 | 0.996881414240279 | 1.44729045369505 | 0.772985411735669 | 2.70981783815748 | 0.999228396881742 |
| ebi-fl187-GCST90094756 | ebi-met1400-GCST90200527 | X-13866 levels \|\| id:ebi-met1400-GCST90200527 | Dried fruit liking \|\| id:ebi-fl187-GCST90094756 | Weighted mode | 5 | -0.221713747 | 0.328070453054032 | 0.536217506897091 | -0.864731835 | 0.421304341075307 | 0.801144661649018 | 0.421164479101304 | 1.52394800781463 | 0.999228396881742 |
| ebi-fl187-GCST90094756 | ebi-met1400-GCST90200547 | X-17654 levels \|\| id:ebi-met1400-GCST90200547 | Dried fruit liking \|\| id:ebi-fl187-GCST90094756 | Weighted mode | 5 | -0.056331697 | 0.424684923604523 | 0.900880345483768 | -0.888714148 | 0.776050752897429 | 0.945225555058057 | 0.411184135062351 | 2.17287408182549 | 0.999228396881742 |
| ebi-fl187-GCST90094756 | ebi-met1400-GCST90200553 | X-17653 levels \|\| id:ebi-met1400-GCST90200553 | Dried fruit liking \|\| id:ebi-fl187-GCST90094756 | Weighted mode | 5 | -0.228272008 | 0.374589626863662 | 0.575180654039749 | -0.962467677 | 0.505923660601507 | 0.795907737076268 | 0.381949194961288 | 1.65851671974873 | 0.999228396881742 |
| ebi-fl187-GCST90094756 | ebi-met1400-GCST90200557 | X-17685 levels \|\| id:ebi-met1400-GCST90200557 | Dried fruit liking \|\| id:ebi-fl187-GCST90094756 | Weighted mode | 5 | -0.075832414 | 0.324921040875155 | 0.826917932037063 | -0.712677654 | 0.561012826338565 | 0.926971541202707 | 0.49032950539281 | 1.7524465257528 | 0.999228396881742 |
| ebi-fl187-GCST90094756 | ebi-met1400-GCST90200561 | X-18779 levels \|\| id:ebi-met1400-GCST90200561 | Dried fruit liking \|\| id:ebi-fl187-GCST90094756 | Weighted mode | 5 | -0.278663925 | 0.307483380489813 | 0.41604711951628 | -0.88133135 | 0.324003501238859 | 0.756794200454411 | 0.414231057772416 | 1.38265214810643 | 0.999228396881742 |
| ebi-fl187-GCST90094756 | ebi-met1400-GCST90200570 | X-18921 levels \|\| id:ebi-met1400-GCST90200570 | Dried fruit liking \|\| id:ebi-fl187-GCST90094756 | Weighted mode | 5 | -0.209472122 | 0.360010446837163 | 0.591883480872985 | -0.915092597 | 0.496148354270327 | 0.811012248898168 | 0.400479541037097 | 1.64238319430639 | 0.999228396881742 |
| ebi-fl187-GCST90094756 | ebi-met1400-GCST90200575 | X-21283 levels \|\| id:ebi-met1400-GCST90200575 | Dried fruit liking \|\| id:ebi-fl187-GCST90094756 | Weighted mode | 5 | -0.448685665 | 0.440598680468576 | 0.366093041053264 | -1.312259079 | 0.414887748676905 | 0.638466759577124 | 0.269211199612949 | 1.5142007601132 | 0.999228396881742 |
| ebi-fl187-GCST90094756 | ebi-met1400-GCST90200578 | X-18922 levels \|\| id:ebi-met1400-GCST90200578 | Dried fruit liking \|\| id:ebi-fl187-GCST90094756 | Weighted mode | 5 | -0.346574004 | 0.298759420405222 | 0.310553466622997 | -0.932142468 | 0.238994460291402 | 0.7071064888525 | 0.3937092966799 | 1.2699715012923 | 0.999228396881742 |
| ebi-fl187-GCST90094756 | ebi-met1400-GCST90200623 | X-24241 levels \|\| id:ebi-met1400-GCST90200623 | Dried fruit liking \|\| id:ebi-fl187-GCST90094756 | Weighted mode | 5 | 0.178468541199114 | 0.418052875614093 | 0.691423907928426 | -0.640915095 | 0.997852177402735 | 1.19538527729454 | 0.526810122089603 | 2.71244970674558 | 0.999228396881742 |
| ebi-fl187-GCST90094756 | ebi-met1400-GCST90200636 | X-24544 levels \|\| id:ebi-met1400-GCST90200636 | Dried fruit liking \|\| id:ebi-fl187-GCST90094756 | Weighted mode | 5 | 0.000979403464746342 | 0.296029765306031 | 0.997518658562153 | -0.579238937 | 0.581197743464567 | 1.00097988323694 | 0.560324646948904 | 1.78817892823551 | 0.999264242604493 |
| ebi-fl187-GCST90094756 | ebi-met1400-GCST90200659 | X-25172 levels \|\| id:ebi-met1400-GCST90200659 | Dried fruit liking \|\| id:ebi-fl187-GCST90094756 | Weighted mode | 5 | -0.063806358 | 0.28346358451904 | 0.832937091579939 | -0.619394984 | 0.491782267377438 | 0.938186654019696 | 0.538270001095957 | 1.6352280379522 | 0.999228396881742 |
| ebi-fl187-GCST90094756 | ebi-met1400-GCST90200670 | X-26111 levels \|\| id:ebi-met1400-GCST90200670 | Dried fruit liking \|\| id:ebi-fl187-GCST90094756 | Weighted mode | 5 | -0.329317131 | 0.278493892543365 | 0.302497654389276 | -0.87516516 | 0.216530898807466 | 0.719414832125632 | 0.416793176448189 | 1.24176145370913 | 0.999228396881742 |
| ebi-fl187-GCST90094756 | ebi-met1400-GCST90200673 | Carnitine C4 levels \|\| id:ebi-met1400-GCST90200673 | Dried fruit liking \|\| id:ebi-fl187-GCST90094756 | Weighted mode | 5 | -0.048736138 | 0.404446725434797 | 0.909896785683875 | -0.84145172 | 0.743979443766609 | 0.95243240718407 | 0.431084255347779 | 2.10429278963717 | 0.999228396881742 |
| ebi-fl187-GCST90094756 | ebi-met1400-GCST90200710 | X-13431 levels \|\| id:ebi-met1400-GCST90200710 | Dried fruit liking \|\| id:ebi-fl187-GCST90094756 | Weighted mode | 5 | -0.174210945 | 0.302468928848485 | 0.595494249268416 | -0.767050045 | 0.418628155664783 | 0.840119660026005 | 0.464380952438151 | 1.51987509275849 | 0.999228396881742 |
| ebi-fl187-GCST90094756 | ebi-met1400-GCST90200730 | Adenosine 5'-diphosphate (ADP) to fructose ratio \|\| id:ebi-met1400-GCST90200730 | Dried fruit liking \|\| id:ebi-fl187-GCST90094756 | Weighted mode | 5 | 0.111548977575882 | 0.421625229820286 | 0.804414629918451 | -0.714836473 | 0.937934428023644 | 1.11800850001076 | 0.489272114518603 | 2.55469904988583 | 0.999228396881742 |
| ebi-fl187-GCST90094756 | ebi-met1400-GCST90200731 | Adenosine 5'-diphosphate (ADP) to mannose ratio \|\| id:ebi-met1400-GCST90200731 | Dried fruit liking \|\| id:ebi-fl187-GCST90094756 | Weighted mode | 5 | 0.242730260611377 | 0.379365324454205 | 0.557089914339492 | -0.500825775 | 0.98628629654162 | 1.27472473426974 | 0.606030008404904 | 2.68125856083584 | 0.999228396881742 |
| ebi-fl187-GCST90094756 | ebi-met1400-GCST90200748 | Aspartate to glutamate ratio \|\| id:ebi-met1400-GCST90200748 | Dried fruit liking \|\| id:ebi-fl187-GCST90094756 | Weighted mode | 5 | 0.29442146597394 | 0.256277517322782 | 0.31464854412208 | -0.207882468 | 0.796725399926593 | 1.34234953911642 | 0.812302502656709 | 2.21826509123483 | 0.999228396881742 |
| ebi-fl187-GCST90094756 | ebi-met1400-GCST90200774 | Uridine to pseudouridine ratio \|\| id:ebi-met1400-GCST90200774 | Dried fruit liking \|\| id:ebi-fl187-GCST90094756 | Weighted mode | 5 | -0.057874723 | 0.296101965344258 | 0.854563592260504 | -0.638234575 | 0.522485129437755 | 0.943768172819145 | 0.528224141619657 | 1.68621290442216 | 0.999228396881742 |
| ebi-fl187-GCST90094756 | ebi-met1400-GCST90200799 | Spermidine to adenosine 5'-diphosphate (ADP) ratio \|\| id:ebi-met1400-GCST90200799 | Dried fruit liking \|\| id:ebi-fl187-GCST90094756 | Weighted mode | 5 | -0.276946931 | 0.39770987484838 | 0.52455136451588 | -1.056458286 | 0.50256442338508 | 0.758094727131216 | 0.347685033209767 | 1.65295471593515 | 0.999228396881742 |
| ebi-fl187-GCST90094756 | ebi-met1400-GCST90200811 | Choline to taurocholate ratio \|\| id:ebi-met1400-GCST90200811 | Dried fruit liking \|\| id:ebi-fl187-GCST90094756 | Weighted mode | 5 | 0.169802346646896 | 0.352899638901977 | 0.655537435587512 | -0.521880946 | 0.861485638894772 | 1.18507059499359 | 0.59340333819559 | 2.36667410633197 | 0.999228396881742 |
| ebi-fl187-GCST90094756 | ebi-met1400-GCST90200812 | Glutamate to kynurenine ratio \|\| id:ebi-met1400-GCST90200812 | Dried fruit liking \|\| id:ebi-fl187-GCST90094756 | Weighted mode | 5 | -0.254258306 | 0.32141142380858 | 0.47316728056501 | -0.884224696 | 0.375708085071075 | 0.775491462390528 | 0.413034276194198 | 1.45602203715859 | 0.999228396881742 |
| ebi-fl187-GCST90094756 | ebi-met1400-GCST90200876 | Cortisone to 4-cholesten-3-one ratio \|\| id:ebi-met1400-GCST90200876 | Dried fruit liking \|\| id:ebi-fl187-GCST90094756 | Weighted mode | 5 | -0.354344395 | 0.340161540026737 | 0.356371498192419 | -1.021061014 | 0.312372223174249 | 0.701633286550034 | 0.36021254688852 | 1.36666330211801 | 0.999228396881742 |
| ebi-fl187-GCST90094756 | ebi-met1400-GCST90200906 | 2'-deoxyuridine to cytidine ratio \|\| id:ebi-met1400-GCST90200906 | Dried fruit liking \|\| id:ebi-fl187-GCST90094756 | Weighted mode | 5 | 0.398122000881901 | 0.45945909513441 | 0.435098621162712 | -0.502417826 | 1.29866182734534 | 1.48902568127253 | 0.605065945792529 | 3.66438979900775 | 0.999228396881742 |
| ebi-fl187-GCST90094756 | ebi-met1400-GCST90200907 | Retinol (Vitamin A) to linoleoyl-arachidonoyl-glycerol (18:2 to 20:4) [1] ratio \|\| id:ebi-met1400-GCST90200907 | Dried fruit liking \|\| id:ebi-fl187-GCST90094756 | Weighted mode | 5 | 0.0146139410989288 | 0.290775738713751 | 0.962325976873428 | -0.555306507 | 0.584534388977881 | 1.01472124681877 | 0.573896331119414 | 1.79415541259349 | 0.999228396881742 |
| ebi-fl187-GCST90094756 | ebi-met1400-GCST90200916 | Fructose to sucrose ratio \|\| id:ebi-met1400-GCST90200916 | Dried fruit liking \|\| id:ebi-fl187-GCST90094756 | Weighted mode | 5 | 0.361081843588327 | 0.318176013891688 | 0.319830980316908 | -0.262543144 | 0.984706830816035 | 1.43488089187402 | 0.769093182173872 | 2.67702694756135 | 0.999228396881742 |
| ebi-fl187-GCST90094756 | ebi-met1400-GCST90200917 | Alpha-ketobutyrate to 3-methyl-2-oxobutyrate ratio \|\| id:ebi-met1400-GCST90200917 | Dried fruit liking \|\| id:ebi-fl187-GCST90094756 | Weighted mode | 5 | -0.285808336 | 0.372752525057093 | 0.485980195563548 | -1.016403285 | 0.44478661307903 | 0.751406619719939 | 0.361894232542525 | 1.56015724315971 | 0.999228396881742 |
| ebi-fl187-GCST90094756 | ebi-met1400-GCST90200924 | Alpha-tocopherol to glycerol ratio \|\| id:ebi-met1400-GCST90200924 | Dried fruit liking \|\| id:ebi-fl187-GCST90094756 | Weighted mode | 5 | 0.333963471631391 | 0.292536478753883 | 0.317315527071466 | -0.239408027 | 0.907334969989002 | 1.39649213106349 | 0.787093661593844 | 2.47771055375183 | 0.999228396881742 |
| ebi-fl187-GCST90094756 | ebi-met1400-GCST90200938 | Alpha-ketoglutarate to alanine ratio \|\| id:ebi-met1400-GCST90200938 | Dried fruit liking \|\| id:ebi-fl187-GCST90094756 | Weighted mode | 5 | 0.00912047407136085 | 0.346534269143238 | 0.980263514464796 | -0.670086693 | 0.688327641592108 | 1.00916219232864 | 0.511664217927736 | 1.99038411274909 | 0.999228396881742 |
| ebi-fl187-GCST90094756 | ebi-met1400-GCST90200943 | Phosphate to EDTA ratio \|\| id:ebi-met1400-GCST90200943 | Dried fruit liking \|\| id:ebi-fl187-GCST90094756 | Weighted mode | 5 | 0.0106319274871713 | 0.281692463335068 | 0.971701124301993 | -0.541485301 | 0.562749155623905 | 1.01068864726351 | 0.581883338503783 | 1.75549199317847 | 0.999228396881742 |
| ebi-fl187-GCST90094756 | ebi-met1400-GCST90200947 | Adenosine 5'-diphosphate (ADP) to glucose ratio \|\| id:ebi-met1400-GCST90200947 | Dried fruit liking \|\| id:ebi-fl187-GCST90094756 | Weighted mode | 5 | 0.349199305542409 | 0.400936703411006 | 0.432928594181356 | -0.436636633 | 1.13503524422798 | 1.41793176384111 | 0.646206199 | 3.11128319574707 | 0.999228396881742 |
| ebi-fl187-GCST90094756 | ebi-met1400-GCST90200975 | Arachidonate (20:4n6) to caffeine ratio \|\| id:ebi-met1400-GCST90200975 | Dried fruit liking \|\| id:ebi-fl187-GCST90094756 | Weighted mode | 5 | 0.231542531041132 | 0.339095509466563 | 0.532214451275598 | -0.433084668 | 0.896169729595595 | 1.26054293764107 | 0.648505582125456 | 2.45020018552342 | 0.999228396881742 |
| ebi-fl187-GCST90094756 | ebi-met1400-GCST90200985 | Cholesterol to oleoyl-linoleoyl-glycerol (18:1 to 18:2) [2] ratio \|\| id:ebi-met1400-GCST90200985 | Dried fruit liking \|\| id:ebi-fl187-GCST90094756 | Weighted mode | 5 | 0.376292588277398 | 0.302356197797304 | 0.281242802217531 | -0.216325559 | 0.968910735960115 | 1.45687333547685 | 0.805473029862634 | 2.63507260570277 | 0.999228396881742 |
| ebi-fl187-GCST90094756 | ebi-met1400-GCST90201016 | Bilirubin (Z,Z) to etiocholanolone glucuronide ratio \|\| id:ebi-met1400-GCST90201016 | Dried fruit liking \|\| id:ebi-fl187-GCST90094756 | Weighted mode | 5 | 0.0904171000949567 | 0.265688704613204 | 0.750742472286335 | -0.430332761 | 0.611166961136837 | 1.09463075909419 | 0.650292666712366 | 1.8425803643348 | 0.999228396881742 |
| ebi-fl187-GCST90094756 | ebi-pw11-GCST90275189 | Placental weight (fetal genotype, adjusted for sex and gestational age) \|\| id:ebi-pw11-GCST90275189 | Dried fruit liking \|\| id:ebi-fl187-GCST90094756 | Weighted mode | 5 | -0.05193568 | 0.159961716736924 | 0.761697048052305 | -0.365460645 | 0.261589284445163 | 0.949389929298335 | 0.693876946189042 | 1.29899291625627 | 0.999228396881742 |
| ebi-fl187-GCST90094756 | ebi-pw11-GCST90275192 | Placental weight (fetal effect, conditional on maternal and paternal genotype) \|\| id:ebi-pw11-GCST90275192 | Dried fruit liking \|\| id:ebi-fl187-GCST90094756 | Weighted mode | 5 | -0.153404447 | 0.266744933584201 | 0.596026125858351 | -0.676224517 | 0.369415622760188 | 0.857782723940016 | 0.508533331540325 | 1.44688883865542 | 0.999228396881742 |
| ebi-fl187-GCST90094756 | ebi-pw11-GCST90275193 | Placental weight (maternal effect, conditional on fetal and paternal genotype) \|\| id:ebi-pw11-GCST90275193 | Dried fruit liking \|\| id:ebi-fl187-GCST90094756 | Weighted mode | 5 | 0.0970700616098631 | 0.239149533016871 | 0.705593338348184 | -0.371663023 | 0.565803146322931 | 1.10193757443701 | 0.689586578121327 | 1.76086144435148 | 0.999228396881742 |
| ebi-fl187-GCST90094756 | ebi-pw11-GCST90275195 | Placental weight (fetal effect, conditional on maternal genotype) \|\| id:ebi-pw11-GCST90275195 | Dried fruit liking \|\| id:ebi-fl187-GCST90094756 | Weighted mode | 5 | -0.104603018 | 0.143542605390502 | 0.50654343417651 | -0.385946524 | 0.176740488762939 | 0.900682006346404 | 0.679806877810881 | 1.19332137263528 | 0.999228396881742 |
| ebi-fl187-GCST90094756 | ebi-pw11-GCST90275197 | Placental weight (fetal genotype, adjusted for sex) \|\| id:ebi-pw11-GCST90275197 | Dried fruit liking \|\| id:ebi-fl187-GCST90094756 | Weighted mode | 5 | -0.059109698 | 0.154781161283341 | 0.721962774395736 | -0.362480774 | 0.244261378526041 | 0.942603362168952 | 0.695947694045923 | 1.27667798309219 | 0.999228396881742 |
| ebi-fl187-GCST90094756 | prot-a-1030 | Protein FAM171B \|\| id:prot-a-1030 | Dried fruit liking \|\| id:ebi-fl187-GCST90094756 | Weighted mode | 5 | -0.000319759 | 0.506274770948563 | 0.999526306287621 | -0.99261831 | 0.99197879213163 | 0.999680292189884 | 0.370605060630971 | 2.69656513834808 | 0.999854019830666 |
| ebi-fl187-GCST90094756 | prot-a-1036 | BRISC complex subunit Abro1 \|\| id:prot-a-1036 | Dried fruit liking \|\| id:ebi-fl187-GCST90094756 | Weighted mode | 5 | -0.362539372 | 0.481602598375684 | 0.493462204299434 | -1.306480465 | 0.581401720333631 | 0.695906913556319 | 0.270771370545742 | 1.78854371257714 | 0.999228396881742 |
| ebi-fl187-GCST90094756 | prot-a-1055 | Phenylalanine--tRNA ligase, mitochondrial \|\| id:prot-a-1055 | Dried fruit liking \|\| id:ebi-fl187-GCST90094756 | Weighted mode | 5 | 0.54246113551724 | 0.488005588868763 | 0.328622310767811 | -0.414029819 | 1.49895208970002 | 1.72023538897553 | 0.660981241393491 | 4.47699512204785 | 0.999228396881742 |
| ebi-fl187-GCST90094756 | prot-a-1083 | Fc receptor-like protein 5 \|\| id:prot-a-1083 | Dried fruit liking \|\| id:ebi-fl187-GCST90094756 | Weighted mode | 5 | 0.322648787427303 | 0.501744741453349 | 0.555198572127635 | -0.660770906 | 1.30606848067587 | 1.38078031832792 | 0.51645304433104 | 3.69163142401706 | 0.999228396881742 |
| ebi-fl187-GCST90094756 | prot-a-1103 | Fibroblast growth factor receptor 3 \|\| id:prot-a-1103 | Dried fruit liking \|\| id:ebi-fl187-GCST90094756 | Weighted mode | 5 | -0.144525865 | 0.523280071751428 | 0.796083222444274 | -1.170154805 | 0.881103075961846 | 0.865432527986057 | 0.310318898535598 | 2.41356057923239 | 0.999228396881742 |
| ebi-fl187-GCST90094756 | prot-a-1105 | Fibroblast growth factor receptor 4 \|\| id:prot-a-1105 | Dried fruit liking \|\| id:ebi-fl187-GCST90094756 | Weighted mode | 5 | -0.302198819 | 0.53735451000927 | 0.603877690564949 | -1.355413659 | 0.751016020674184 | 0.739191085087891 | 0.257840614410969 | 2.11915202545441 | 0.999228396881742 |
| ebi-fl187-GCST90094756 | prot-a-1120 | Filamin-A \|\| id:prot-a-1120 | Dried fruit liking \|\| id:ebi-fl187-GCST90094756 | Weighted mode | 5 | -0.227281981 | 0.485986300509506 | 0.66436193588131 | -1.17981513 | 0.725251168129136 | 0.796696097554917 | 0.307335550513309 | 2.06524975974671 | 0.999228396881742 |
| ebi-fl187-GCST90094756 | prot-a-1166 | Galanin peptides \|\| id:prot-a-1166 | Dried fruit liking \|\| id:ebi-fl187-GCST90094756 | Weighted mode | 5 | -0.284553727 | 0.412958785486422 | 0.528672149606619 | -1.093952947 | 0.524845492310409 | 0.752349932690462 | 0.334890071161125 | 1.69019767966489 | 0.999228396881742 |
| ebi-fl187-GCST90094756 | prot-a-1205 | ADP-ribosylation factor-binding protein GGA1 \|\| id:prot-a-1205 | Dried fruit liking \|\| id:ebi-fl187-GCST90094756 | Weighted mode | 5 | 0.256644272328528 | 0.502731644783417 | 0.636583744858422 | -0.728709751 | 1.24199829610403 | 1.29258523662998 | 0.482531173766838 | 3.46252570774028 | 0.999228396881742 |
| ebi-fl187-GCST90094756 | prot-a-1227 | Guanine nucleotide-binding protein G(I)/G(S)/G(O) subunit gamma-T2 \|\| id:prot-a-1227 | Dried fruit liking \|\| id:ebi-fl187-GCST90094756 | Weighted mode | 5 | -0.969960932 | 0.608751456032223 | 0.186300594798866 | -2.163113786 | 0.223191922042404 | 0.37909784849195 | 0.114966581833202 | 1.2500604648726 | 0.999228396881742 |
| ebi-fl187-GCST90094756 | prot-a-1240 | Golgi SNAP receptor complex member 1 \|\| id:prot-a-1240 | Dried fruit liking \|\| id:ebi-fl187-GCST90094756 | Weighted mode | 5 | -0.064696705 | 0.534029335734985 | 0.909415591980214 | -1.111394203 | 0.982000793351163 | 0.937351714648616 | 0.329099809221729 | 2.66979260496237 | 0.999228396881742 |
| ebi-fl187-GCST90094756 | prot-a-1283 | Glutathione S-transferase A1 \|\| id:prot-a-1283 | Dried fruit liking \|\| id:ebi-fl187-GCST90094756 | Weighted mode | 5 | 0.171467614801151 | 0.598607663212863 | 0.788761865972539 | -1.001803405 | 1.34473863469836 | 1.18704569939878 | 0.367216603374672 | 3.83718350290235 | 0.999228396881742 |
| ebi-fl187-GCST90094756 | prot-a-1324 | E3 ubiquitin-protein ligase HECW1 \|\| id:prot-a-1324 | Dried fruit liking \|\| id:ebi-fl187-GCST90094756 | Weighted mode | 5 | 0.153589446686094 | 0.629136447580137 | 0.819142808666327 | -1.079517991 | 1.38669688394316 | 1.16601207837415 | 0.339759253346333 | 4.00161041538582 | 0.999228396881742 |
| ebi-fl187-GCST90094756 | prot-a-1339 | Histidine triad nucleotide-binding protein 2, mitochondrial \|\| id:prot-a-1339 | Dried fruit liking \|\| id:ebi-fl187-GCST90094756 | Weighted mode | 5 | 0.277243272812961 | 0.512340250785671 | 0.617145646916816 | -0.726943619 | 1.28143016435288 | 1.3194873273872 | 0.483384140866091 | 3.60178719147869 | 0.999228396881742 |
| ebi-fl187-GCST90094756 | prot-a-1466 | Interleukin-11 \|\| id:prot-a-1466 | Dried fruit liking \|\| id:ebi-fl187-GCST90094756 | Weighted mode | 5 | -0.540273827 | 0.517200961557577 | 0.355172887549543 | -1.553987712 | 0.473440057271972 | 0.582588701791986 | 0.211403275353867 | 1.60550774290292 | 0.999228396881742 |
| ebi-fl187-GCST90094756 | prot-a-147 | Rho GTPase-activating protein 1 \|\| id:prot-a-147 | Dried fruit liking \|\| id:ebi-fl187-GCST90094756 | Weighted mode | 5 | -0.231855231 | 0.486159530130674 | 0.658310955106211 | -1.18472791 | 0.72101744851461 | 0.793060925997327 | 0.305829381418423 | 2.05652455439931 | 0.999228396881742 |
| ebi-fl187-GCST90094756 | prot-a-1473 | Interleukin-12 receptor subunit beta-1 \|\| id:prot-a-1473 | Dried fruit liking \|\| id:ebi-fl187-GCST90094756 | Weighted mode | 5 | -0.293635783 | 0.576480369111219 | 0.637314176337977 | -1.423537306 | 0.836265740853295 | 0.745547983543856 | 0.240860510885585 | 2.30773319263758 | 0.999228396881742 |
| ebi-fl187-GCST90094756 | prot-a-1501 | X-linked interleukin-1 receptor accessory protein-like 2 \|\| id:prot-a-1501 | Dried fruit liking \|\| id:ebi-fl187-GCST90094756 | Weighted mode | 5 | -0.111788327 | 0.528425031729364 | 0.842799621620939 | -1.147501389 | 0.923924735579353 | 0.894233522909491 | 0.317428910771996 | 2.51915804250577 | 0.999228396881742 |
| ebi-fl187-GCST90094756 | prot-a-1503 | Interleukin-1 receptor-like 2 \|\| id:prot-a-1503 | Dried fruit liking \|\| id:ebi-fl187-GCST90094756 | Weighted mode | 5 | -0.408678188 | 0.559763668261209 | 0.505793629994706 | -1.505814977 | 0.688458602228466 | 0.664528051306878 | 0.221836428475548 | 1.99064479178807 | 0.999228396881742 |
| ebi-fl187-GCST90094756 | prot-a-1536 | Interleukin-5 receptor subunit alpha \|\| id:prot-a-1536 | Dried fruit liking \|\| id:ebi-fl187-GCST90094756 | Weighted mode | 5 | -0.316259137 | 0.567309866368792 | 0.606927697770054 | -1.428186475 | 0.795668201101354 | 0.728870548379166 | 0.239743308707865 | 2.21592118319304 | 0.999228396881742 |
| ebi-fl187-GCST90094756 | prot-a-1559 | Insulin-induced gene 1 protein \|\| id:prot-a-1559 | Dried fruit liking \|\| id:ebi-fl187-GCST90094756 | Weighted mode | 5 | -0.227082254 | 0.606924619579372 | 0.727279885036273 | -1.416654509 | 0.96249000014726 | 0.796855234881981 | 0.242524023240026 | 2.61820770114134 | 0.999228396881742 |
| ebi-fl187-GCST90094756 | prot-a-1567 | Interferon regulatory factor 1 \|\| id:prot-a-1567 | Dried fruit liking \|\| id:ebi-fl187-GCST90094756 | Weighted mode | 5 | -0.410170819 | 0.48419085326808 | 0.444634423887687 | -1.359184891 | 0.538843253377061 | 0.663536895727231 | 0.25687006863759 | 1.71402302466197 | 0.999228396881742 |
| ebi-fl187-GCST90094756 | prot-a-1613 | Potassium voltage-gated channel subfamily E regulatory beta subunit 5 \|\| id:prot-a-1613 | Dried fruit liking \|\| id:ebi-fl187-GCST90094756 | Weighted mode | 5 | 0.507205873939656 | 0.509474302840122 | 0.375816789640859 | -0.49136376 | 1.50577550750629 | 1.66064465595089 | 0.611791488476351 | 4.50764799001424 | 0.999228396881742 |
| ebi-fl187-GCST90094756 | prot-a-1649 | Kin of IRRE-like protein 2 \|\| id:prot-a-1649 | Dried fruit liking \|\| id:ebi-fl187-GCST90094756 | Weighted mode | 5 | -0.002767026 | 0.615031217231235 | 0.996625763717472 | -1.208228212 | 1.20269415990765 | 0.997236798822017 | 0.298726090230998 | 3.32907390899728 | 0.999228396881742 |
| ebi-fl187-GCST90094756 | prot-a-165 | Armadillo repeat-containing protein 5 \|\| id:prot-a-165 | Dried fruit liking \|\| id:ebi-fl187-GCST90094756 | Weighted mode | 5 | -0.086329794 | 0.556439816514736 | 0.884219830740628 | -1.176951834 | 1.00429224654425 | 0.917291664204283 | 0.308216804108474 | 2.72997444 | 0.999228396881742 |
| ebi-fl187-GCST90094756 | prot-a-1656 | Kallikrein-11 \|\| id:prot-a-1656 | Dried fruit liking \|\| id:ebi-fl187-GCST90094756 | Weighted mode | 5 | -0.629179118 | 0.44423814674083 | 0.229629652107183 | -1.499885886 | 0.241527649316166 | 0.533029175363862 | 0.223155623896937 | 1.27319265733719 | 0.999228396881742 |
| ebi-fl187-GCST90094756 | prot-a-1697 | Laminin subunit gamma-2 \|\| id:prot-a-1697 | Dried fruit liking \|\| id:ebi-fl187-GCST90094756 | Weighted mode | 5 | 0.23637462395625 | 0.46827837648235 | 0.640257507235182 | -0.681450994 | 1.15420024186166 | 1.26664873825677 | 0.505882427228791 | 3.17148598127103 | 0.999228396881742 |
| ebi-fl187-GCST90094756 | prot-a-1698 | Lysosome-associated membrane glycoprotein 3 \|\| id:prot-a-1698 | Dried fruit liking \|\| id:ebi-fl187-GCST90094756 | Weighted mode | 5 | -0.457508462 | 0.637513856983367 | 0.512655346430839 | -1.707035621 | 0.792018698064515 | 0.63285847399468 | 0.18140274278707 | 2.20784891095606 | 0.999228396881742 |
| ebi-fl187-GCST90094756 | prot-a-1724 | Leptin receptor \|\| id:prot-a-1724 | Dried fruit liking \|\| id:ebi-fl187-GCST90094756 | Weighted mode | 5 | 0.281021475228231 | 0.445901539352243 | 0.562753102513492 | -0.592945542 | 1.15498849235863 | 1.32448204718409 | 0.552696892862399 | 3.17398689221456 | 0.999228396881742 |
| ebi-fl187-GCST90094756 | prot-a-1743 | Leukocyte immunoglobulin-like receptor subfamily B member 2 \|\| id:prot-a-1743 | Dried fruit liking \|\| id:ebi-fl187-GCST90094756 | Weighted mode | 5 | -0.712904521 | 0.576670730971213 | 0.283999490070488 | -1.843179154 | 0.417370111649876 | 0.490218278347003 | 0.158313322825494 | 1.51796422522439 | 0.999228396881742 |
| ebi-fl187-GCST90094756 | prot-a-1746 | Leukocyte immunoglobulin-like receptor subfamily B member 4 \|\| id:prot-a-1746 | Dried fruit liking \|\| id:ebi-fl187-GCST90094756 | Weighted mode | 5 | -0.764261338 | 0.589039688665265 | 0.264238959242278 | -1.918779128 | 0.390256451923844 | 0.465677782510968 | 0.146786059823078 | 1.4773596170216 | 0.999228396881742 |
| ebi-fl187-GCST90094756 | prot-a-1750 | Leucine-rich repeat and immunoglobulin-like domain-containing nogo receptor-interacting protein 3 \|\| id:prot-a-1750 | Dried fruit liking \|\| id:ebi-fl187-GCST90094756 | Weighted mode | 5 | 0.276257219414981 | 0.538727268459347 | 0.635113793650325 | -0.779648227 | 1.3321626655953 | 1.31818688368327 | 0.458567294636525 | 3.78922936859661 | 0.999228396881742 |
| ebi-fl187-GCST90094756 | prot-a-1753 | Lipase member N \|\| id:prot-a-1753 | Dried fruit liking \|\| id:ebi-fl187-GCST90094756 | Weighted mode | 5 | 0.203566038184446 | 0.499811461760005 | 0.704652084526635 | -0.776064427 | 1.18319650323406 | 1.22576610233641 | 0.460213656414752 | 3.26479346428373 | 0.999228396881742 |
| ebi-fl187-GCST90094756 | prot-a-1760 | Ig Kappa chain V-I region HK102- like \|\| id:prot-a-1760 | Dried fruit liking \|\| id:ebi-fl187-GCST90094756 | Weighted mode | 5 | -0.097448687 | 0.474521321526715 | 0.847316882081517 | -1.027510477 | 0.832613102969175 | 0.907148888690022 | 0.357896845 | 2.29931925484041 | 0.999228396881742 |
| ebi-fl187-GCST90094756 | prot-a-1792 | Leucine-rich repeat neuronal protein 1 \|\| id:prot-a-1792 | Dried fruit liking \|\| id:ebi-fl187-GCST90094756 | Weighted mode | 5 | -0.397873379 | 0.46886478593678 | 0.44390883783121 | -1.31684836 | 0.521101601106404 | 0.671747079342715 | 0.267978544431968 | 1.68388159418496 | 0.999228396881742 |
| ebi-fl187-GCST90094756 | prot-a-1795 | Leucine-rich repeat transmembrane neuronal protein 1 \|\| id:prot-a-1795 | Dried fruit liking \|\| id:ebi-fl187-GCST90094756 | Weighted mode | 5 | -0.474258365 | 0.602973829962092 | 0.475537239795949 | -1.656087072 | 0.707570341827147 | 0.622346439319673 | 0.190884437822288 | 2.02905535387051 | 0.999228396881742 |
| ebi-fl187-GCST90094756 | prot-a-1820 | LysM and putative peptidoglycan-binding domain-containing protein 4 \|\| id:prot-a-1820 | Dried fruit liking \|\| id:ebi-fl187-GCST90094756 | Weighted mode | 5 | -0.036579708 | 0.435750947475761 | 0.937132489249526 | -0.890651565 | 0.817492149519355 | 0.964081246295813 | 0.410388271157198 | 2.2648128974019 | 0.999228396881742 |
| ebi-fl187-GCST90094756 | prot-a-1844 | Mitogen-activated protein kinase kinase kinase 7:TGF-beta-activated kinase 1 and MAP3K7-binding protein 1 fusion \|\| id:prot-a-1844 | Dried fruit liking \|\| id:ebi-fl187-GCST90094756 | Weighted mode | 5 | -0.282404867 | 0.481819469006715 | 0.589272403625727 | -1.226771026 | 0.661961292037318 | 0.753968365657421 | 0.29323790807956 | 1.93859075088576 | 0.999228396881742 |
| ebi-fl187-GCST90094756 | prot-a-1856 | Megakaryocyte-associated tyrosine-protein kinase \|\| id:prot-a-1856 | Dried fruit liking \|\| id:ebi-fl187-GCST90094756 | Weighted mode | 5 | -0.080553057 | 0.552155504810992 | 0.891066197051745 | -1.162777846 | 1.00167173254288 | 0.922605951685738 | 0.312616572827814 | 2.72282986914702 | 0.999228396881742 |
| ebi-fl187-GCST90094756 | prot-a-1910 | Stromelysin-2 \|\| id:prot-a-1910 | Dried fruit liking \|\| id:ebi-fl187-GCST90094756 | Weighted mode | 5 | 0.256404436029636 | 0.473638987018505 | 0.617008137634811 | -0.671927979 | 1.1847368505859 | 1.29227526494354 | 0.510722965062062 | 3.26982625537889 | 0.999228396881742 |
| ebi-fl187-GCST90094756 | prot-a-1940 | 39S ribosomal protein L14, mitochondrial \|\| id:prot-a-1940 | Dried fruit liking \|\| id:ebi-fl187-GCST90094756 | Weighted mode | 5 | -0.101314339 | 0.427142161548627 | 0.8241613493331 | -0.938512976 | 0.735884297176746 | 0.903648935719543 | 0.391209140388219 | 2.08732699398773 | 0.999228396881742 |
| ebi-fl187-GCST90094756 | prot-a-1942 | 39S ribosomal protein L33, mitochondrial \|\| id:prot-a-1942 | Dried fruit liking \|\| id:ebi-fl187-GCST90094756 | Weighted mode | 5 | -0.187068376 | 0.489739442338094 | 0.721905688406018 | -1.146957683 | 0.772820930758819 | 0.829387024093727 | 0.317601545573242 | 2.16586740626052 | 0.999228396881742 |
| ebi-fl187-GCST90094756 | prot-a-1948 | Mesothelin \|\| id:prot-a-1948 | Dried fruit liking \|\| id:ebi-fl187-GCST90094756 | Weighted mode | 5 | -0.179684752 | 0.515768477460106 | 0.7451159760071 | -1.190590968 | 0.831221463788744 | 0.835533570156735 | 0.304041532191983 | 2.29612165754395 | 0.999228396881742 |
| ebi-fl187-GCST90094756 | prot-a-1980 | Unconventional myosin-VI \|\| id:prot-a-1980 | Dried fruit liking \|\| id:ebi-fl187-GCST90094756 | Weighted mode | 5 | -0.553470443 | 0.544551032423158 | 0.366926349193611 | -1.620790466 | 0.513849580814607 | 0.57495100947089 | 0.197742328645229 | 1.67171422303148 | 0.999228396881742 |
| ebi-fl187-GCST90094756 | prot-a-2039 | Neurofascin \|\| id:prot-a-2039 | Dried fruit liking \|\| id:ebi-fl187-GCST90094756 | Weighted mode | 5 | -0.892895557 | 0.659468271502395 | 0.247191002454463 | -2.185453369 | 0.399662255374425 | 0.409468395566874 | 0.112426751304005 | 1.49132092694505 | 0.999228396881742 |
| ebi-fl187-GCST90094756 | prot-a-2041 | NFU1 iron-sulfur cluster scaffold homolog, mitochondrial \|\| id:prot-a-2041 | Dried fruit liking \|\| id:ebi-fl187-GCST90094756 | Weighted mode | 5 | 0.0127307953984241 | 0.515670313664658 | 0.981486457335861 | -0.997983019 | 1.02344461018115 | 1.01281217695813 | 0.368622195682116 | 2.78276381023802 | 0.999228396881742 |
| ebi-fl187-GCST90094756 | prot-a-2088 | Glucocorticoid receptor \|\| id:prot-a-2088 | Dried fruit liking \|\| id:ebi-fl187-GCST90094756 | Weighted mode | 5 | 0.186733047249691 | 0.503454697919528 | 0.729517437023378 | -0.800038161 | 1.17350425517197 | 1.20530548248528 | 0.4493118177489 | 3.2333031287438 | 0.999228396881742 |
| ebi-fl187-GCST90094756 | prot-a-2111 | Neuron-specific protein family member 2 \|\| id:prot-a-2111 | Dried fruit liking \|\| id:ebi-fl187-GCST90094756 | Weighted mode | 5 | -0.752286116 | 0.749929348180525 | 0.372553865476255 | -2.222147639 | 0.717575405949572 | 0.471287901209488 | 0.108376105968546 | 2.04945807788027 | 0.999228396881742 |
| ebi-fl187-GCST90094756 | prot-a-2125 | Nuclear migration protein nudC \|\| id:prot-a-2125 | Dried fruit liking \|\| id:ebi-fl187-GCST90094756 | Weighted mode | 5 | 0.452754807013084 | 0.575619619497578 | 0.475526861928407 | -0.675459647 | 1.58096926122834 | 1.57263853929632 | 0.508922442061262 | 4.85966381294373 | 0.999228396881742 |
| ebi-fl187-GCST90094756 | prot-a-2142 | Ornithine decarboxylase \|\| id:prot-a-2142 | Dried fruit liking \|\| id:ebi-fl187-GCST90094756 | Weighted mode | 5 | -0.667086062 | 0.644902368312555 | 0.35938561052425 | -1.931094704 | 0.596922579620236 | 0.513201839295819 | 0.144989391085624 | 1.81651999421858 | 0.999228396881742 |
| ebi-fl187-GCST90094756 | prot-a-2186 | Prostate and testis expressed protein 1 \|\| id:prot-a-2186 | Dried fruit liking \|\| id:ebi-fl187-GCST90094756 | Weighted mode | 5 | -0.476893841 | 0.498578415905562 | 0.392981823040924 | -1.454107536 | 0.500319854157953 | 0.620708419570547 | 0.233608758257121 | 1.64924870540046 | 0.999228396881742 |
| ebi-fl187-GCST90094756 | prot-a-2227 | Platelet-derived growth factor C \|\| id:prot-a-2227 | Dried fruit liking \|\| id:ebi-fl187-GCST90094756 | Weighted mode | 5 | -0.042441657 | 0.477926679020614 | 0.933506423256335 | -0.979177948 | 0.894294633544939 | 0.95844638220552 | 0.3756197508564 | 2.44561013 | 0.999228396881742 |
| ebi-fl187-GCST90094756 | prot-a-2229 | Platelet-derived growth factor receptor alpha \|\| id:prot-a-2229 | Dried fruit liking \|\| id:ebi-fl187-GCST90094756 | Weighted mode | 5 | -0.6359432 | 0.546033679266983 | 0.308881094213039 | -1.706169211 | 0.434282811832227 | 0.529435889061425 | 0.181559980119095 | 1.54385542696357 | 0.999228396881742 |
| ebi-fl187-GCST90094756 | prot-a-2243 | Platelet endothelial aggregation receptor 1 \|\| id:prot-a-2243 | Dried fruit liking \|\| id:ebi-fl187-GCST90094756 | Weighted mode | 5 | -0.055523192 | 0.445618992616049 | 0.906852586211081 | -0.928936418 | 0.817890033424508 | 0.945990083916607 | 0.394973573981998 | 2.26571420929881 | 0.999228396881742 |
| ebi-fl187-GCST90094756 | prot-a-2246 | E3 ubiquitin-protein ligase pellino homolog 2 \|\| id:prot-a-2246 | Dried fruit liking \|\| id:ebi-fl187-GCST90094756 | Weighted mode | 5 | 0.323656704626856 | 0.589390983014039 | 0.612125813793579 | -0.831549622 | 1.47886303133437 | 1.38217273216061 | 0.435374097997676 | 4.38795387762945 | 0.999228396881742 |
| ebi-fl187-GCST90094756 | prot-a-2251 | Platelet factor 4 variant \|\| id:prot-a-2251 | Dried fruit liking \|\| id:ebi-fl187-GCST90094756 | Weighted mode | 5 | 0.455363054349656 | 0.537807629067654 | 0.444844895120551 | -0.598739899 | 1.50946600732226 | 1.57674572352558 | 0.549503630292267 | 4.52431419849564 | 0.999228396881742 |
| ebi-fl187-GCST90094756 | prot-a-2346 | Protein phosphatase 1L \|\| id:prot-a-2346 | Dried fruit liking \|\| id:ebi-fl187-GCST90094756 | Weighted mode | 5 | -0.507167548 | 0.477560594660644 | 0.348102250244097 | -1.443186313 | 0.428851217677163 | 0.602198864900322 | 0.236174034030727 | 1.53549256325128 | 0.999228396881742 |
| ebi-fl187-GCST90094756 | prot-a-2358 | PR domain zinc finger protein 4 \|\| id:prot-a-2358 | Dried fruit liking \|\| id:ebi-fl187-GCST90094756 | Weighted mode | 5 | -0.128085285 | 0.457038451233581 | 0.79318184743431 | -1.023880649 | 0.76771007990117 | 0.879778344474175 | 0.359198309472147 | 2.15482621993169 | 0.999228396881742 |
| ebi-fl187-GCST90094756 | prot-a-2390 | Trypsin-1 \|\| id:prot-a-2390 | Dried fruit liking \|\| id:ebi-fl187-GCST90094756 | Weighted mode | 5 | -0.479049995 | 0.581753823422466 | 0.456510092925116 | -1.619287489 | 0.661187498976027 | 0.619371518481775 | 0.198039754361015 | 1.9370912630355 | 0.999228396881742 |
| ebi-fl187-GCST90094756 | prot-a-242 | Brain-derived neurotrophic factor \|\| id:prot-a-242 | Dried fruit liking \|\| id:ebi-fl187-GCST90094756 | Weighted mode | 5 | -0.622916716 | 0.584866684915505 | 0.346870263325805 | -1.769255418 | 0.523421986646097 | 0.536377692546049 | 0.170459862893163 | 1.68779338536335 | 0.999228396881742 |
| ebi-fl187-GCST90094756 | prot-a-2464 | Dihydropteridine reductase \|\| id:prot-a-2464 | Dried fruit liking \|\| id:ebi-fl187-GCST90094756 | Weighted mode | 5 | -0.260030901 | 0.508669038345599 | 0.636136075484872 | -1.257022216 | 0.736960414291193 | 0.771027760009815 | 0.284499945772085 | 2.08957441131465 | 0.999228396881742 |
| ebi-fl187-GCST90094756 | prot-a-2474 | Ras-related protein Rab-27A \|\| id:prot-a-2474 | Dried fruit liking \|\| id:ebi-fl187-GCST90094756 | Weighted mode | 5 | 0.0910310720926848 | 0.503371680600539 | 0.865284250830087 | -0.895577422 | 1.07763956606974 | 1.09530303808724 | 0.408371727776326 | 2.93773702644823 | 0.999228396881742 |
| ebi-fl187-GCST90094756 | prot-a-2478 | Ras-related protein Rab-7b \|\| id:prot-a-2478 | Dried fruit liking \|\| id:ebi-fl187-GCST90094756 | Weighted mode | 5 | 0.473604306584509 | 0.589601350466479 | 0.466841447865443 | -0.68201434 | 1.62922295349881 | 1.60577146810371 | 0.50559752045252 | 5.09991031100814 | 0.999228396881742 |
| ebi-fl187-GCST90094756 | prot-a-2528 | E3 ubiquitin-protein ligase rififylin \|\| id:prot-a-2528 | Dried fruit liking \|\| id:ebi-fl187-GCST90094756 | Weighted mode | 5 | -0.302258204 | 0.523217450467752 | 0.594432759778796 | -1.327764407 | 0.723247998525355 | 0.739147189197952 | 0.265069186137215 | 2.06111685504032 | 0.999228396881742 |
| ebi-fl187-GCST90094756 | prot-a-2543 | Protein RIC-3 \|\| id:prot-a-2543 | Dried fruit liking \|\| id:ebi-fl187-GCST90094756 | Weighted mode | 5 | 0.535995519383447 | 0.543778406790416 | 0.380089784078402 | -0.529810158 | 1.60180119669266 | 1.70914888644728 | 0.588716722274039 | 4.96196184942785 | 0.999228396881742 |
| ebi-fl187-GCST90094756 | prot-a-255 | Bone morphogenetic protein 6 \|\| id:prot-a-255 | Dried fruit liking \|\| id:ebi-fl187-GCST90094756 | Weighted mode | 5 | -0.097937901 | 0.540228796317568 | 0.864955762044724 | -1.156786342 | 0.960910539655036 | 0.906705207376869 | 0.314495238799095 | 2.61407560961364 | 0.999228396881742 |
| ebi-fl187-GCST90094756 | prot-a-2568 | RING finger protein 165 \|\| id:prot-a-2568 | Dried fruit liking \|\| id:ebi-fl187-GCST90094756 | Weighted mode | 5 | -0.373518859 | 0.465558872753043 | 0.467336967766442 | -1.286014249 | 0.53897653205228 | 0.688308005687352 | 0.276370133262856 | 1.71425148260394 | 0.999228396881742 |
| ebi-fl187-GCST90094756 | prot-a-2573 | E3 ubiquitin-protein ligase RNF8 \|\| id:prot-a-2573 | Dried fruit liking \|\| id:ebi-fl187-GCST90094756 | Weighted mode | 5 | -0.169987214 | 0.631758387118825 | 0.801184569906478 | -1.408233653 | 1.06825922494791 | 0.843675603928219 | 0.24457490610297 | 2.91030889474806 | 0.999228396881742 |
| ebi-fl187-GCST90094756 | prot-a-260 | Cytoplasmic tyrosine-protein kinase BMX \|\| id:prot-a-260 | Dried fruit liking \|\| id:ebi-fl187-GCST90094756 | Weighted mode | 5 | -0.365120116 | 0.484359465433931 | 0.492900478603673 | -1.314464668 | 0.584224436030238 | 0.694113271607855 | 0.268618084485785 | 1.79359939500892 | 0.999228396881742 |
| ebi-fl187-GCST90094756 | prot-a-2623 | Serum amyloid A-1 protein \|\| id:prot-a-2623 | Dried fruit liking \|\| id:ebi-fl187-GCST90094756 | Weighted mode | 5 | 0.0548250688330868 | 0.453620314093369 | 0.909628944988596 | -0.834270747 | 0.943920884456089 | 1.05635580895749 | 0.434191001188846 | 2.5700385131494 | 0.999228396881742 |
| ebi-fl187-GCST90094756 | prot-a-2640 | Lysosome membrane protein 2 \|\| id:prot-a-2640 | Dried fruit liking \|\| id:ebi-fl187-GCST90094756 | Weighted mode | 5 | -0.488248702 | 0.583379284226485 | 0.449718964458633 | -1.631672099 | 0.655174695438589 | 0.613700225805902 | 0.195602234283529 | 1.92547886037072 | 0.999228396881742 |
| ebi-fl187-GCST90094756 | prot-a-2641 | Scavenger receptor class F member 2 \|\| id:prot-a-2641 | Dried fruit liking \|\| id:ebi-fl187-GCST90094756 | Weighted mode | 5 | -0.942421423 | 0.54694327776899 | 0.15997431234708 | -2.014430248 | 0.129587401002406 | 0.389683104226249 | 0.133396384595313 | 1.13835860079779 | 0.999228396881742 |
| ebi-fl187-GCST90094756 | prot-a-2669 | Semaphorin-3A \|\| id:prot-a-2669 | Dried fruit liking \|\| id:ebi-fl187-GCST90094756 | Weighted mode | 5 | -0.094930744 | 0.524592579905957 | 0.865197358736417 | -1.123132201 | 0.93327071270852 | 0.909435916265522 | 0.325259419723343 | 2.54281240032094 | 0.999228396881742 |
| ebi-fl187-GCST90094756 | prot-a-2683 | Sentrin-specific protease 7 \|\| id:prot-a-2683 | Dried fruit liking \|\| id:ebi-fl187-GCST90094756 | Weighted mode | 5 | -0.162392783 | 0.4513028170213 | 0.737167297107319 | -1.046946305 | 0.722160737879031 | 0.850107230876031 | 0.351007985576642 | 2.05887710161488 | 0.999228396881742 |
| ebi-fl187-GCST90094756 | prot-a-270 | Bromodomain testis-specific protein \|\| id:prot-a-270 | Dried fruit liking \|\| id:ebi-fl187-GCST90094756 | Weighted mode | 5 | 0.0105024845467716 | 0.476662680286715 | 0.983476644646772 | -0.923756369 | 0.944761337908732 | 1.0105578292201 | 0.397024864682087 | 2.57219941883432 | 0.999228396881742 |
| ebi-fl187-GCST90094756 | prot-a-2708 | 14-3-3 protein sigma \|\| id:prot-a-2708 | Dried fruit liking \|\| id:ebi-fl187-GCST90094756 | Weighted mode | 5 | -0.264838467 | 0.513915163318514 | 0.633491039651254 | -1.272112187 | 0.742435253224568 | 0.767329889156726 | 0.280239078883152 | 2.10104586819162 | 0.999228396881742 |
| ebi-fl187-GCST90094756 | prot-a-2713 | Beta-sarcoglycan \|\| id:prot-a-2713 | Dried fruit liking \|\| id:ebi-fl187-GCST90094756 | Weighted mode | 5 | -0.654522169 | 0.489186308690825 | 0.251901714925657 | -1.613327334 | 0.30428299616152 | 0.51969032748311 | 0.199223626506108 | 1.35565264630509 | 0.999228396881742 |
| ebi-fl187-GCST90094756 | prot-a-2720 | Protein shisa-3 homolog \|\| id:prot-a-2720 | Dried fruit liking \|\| id:ebi-fl187-GCST90094756 | Weighted mode | 5 | -0.069244329 | 0.554376885073513 | 0.906624644061541 | -1.155823024 | 1.01733436583811 | 0.933098669247496 | 0.314798343775081 | 2.76581228512921 | 0.999228396881742 |
| ebi-fl187-GCST90094756 | prot-a-2730 | Sialic acid-binding Ig-like lectin 8 \|\| id:prot-a-2730 | Dried fruit liking \|\| id:ebi-fl187-GCST90094756 | Weighted mode | 5 | -0.084495543 | 0.444460888643445 | 0.858482506211255 | -0.955638884 | 0.786647798990988 | 0.91897575147222 | 0.38456637256007 | 2.1960225647707 | 0.999228396881742 |
| ebi-fl187-GCST90094756 | prot-a-2761 | Sodium- and chloride-dependent neutral and basic amino acid transporter B(0+) \|\| id:prot-a-2761 | Dried fruit liking \|\| id:ebi-fl187-GCST90094756 | Weighted mode | 5 | -0.617177118 | 0.524842859018108 | 0.304833349177217 | -1.645869122 | 0.411514885557031 | 0.539465136567797 | 0.192844884246026 | 1.50910217146768 | 0.999228396881742 |
| ebi-fl187-GCST90094756 | prot-a-2770 | Secreted Ly-6/uPAR-related protein 1 \|\| id:prot-a-2770 | Dried fruit liking \|\| id:ebi-fl187-GCST90094756 | Weighted mode | 5 | -0.240454965 | 0.588929301294391 | 0.703970579995967 | -1.394756395 | 0.913846465919287 | 0.786270054623263 | 0.247893417049921 | 2.49389679707699 | 0.999228396881742 |
| ebi-fl187-GCST90094756 | prot-a-2784 | N-lysine methyltransferase SMYD2 \|\| id:prot-a-2784 | Dried fruit liking \|\| id:ebi-fl187-GCST90094756 | Weighted mode | 5 | -0.365325079 | 0.752217472817364 | 0.652608478891751 | -1.839671326 | 1.10902116739243 | 0.693971018572282 | 0.158869633836618 | 3.03138971865149 | 0.999228396881742 |
| ebi-fl187-GCST90094756 | prot-a-2837 | Serine palmitoyltransferase 2 \|\| id:prot-a-2837 | Dried fruit liking \|\| id:ebi-fl187-GCST90094756 | Weighted mode | 5 | 0.0925747210054042 | 0.475010172386463 | 0.854977716722383 | -0.838445217 | 1.02359465888287 | 1.09699510707532 | 0.432382261735434 | 2.78318139166292 | 0.999228396881742 |
| ebi-fl187-GCST90094756 | prot-a-2858 | Alpha-2,8-sialyltransferase 8B \|\| id:prot-a-2858 | Dried fruit liking \|\| id:ebi-fl187-GCST90094756 | Weighted mode | 5 | 0.397944545732139 | 0.454183266137676 | 0.430399635430692 | -0.492254656 | 1.28814374736198 | 1.48876146944083 | 0.611246688437002 | 3.6260494409534 | 0.999228396881742 |
| ebi-fl187-GCST90094756 | prot-a-2911 | Synaptotagmin-11 \|\| id:prot-a-2911 | Dried fruit liking \|\| id:ebi-fl187-GCST90094756 | Weighted mode | 5 | -0.312104277 | 0.560316414016176 | 0.607212174923791 | -1.410324449 | 0.786115894073172 | 0.731905203078338 | 0.244064083989776 | 2.19485480016627 | 0.999228396881742 |
| ebi-fl187-GCST90094756 | prot-a-2962 | Transforming growth factor beta-1 \|\| id:prot-a-2962 | Dried fruit liking \|\| id:ebi-fl187-GCST90094756 | Weighted mode | 5 | 0.2160423215264 | 0.608245872519627 | 0.740385489438206 | -0.976119589 | 1.40820423166487 | 1.24115490545926 | 0.376770289612632 | 4.0886066173885 | 0.999228396881742 |
| ebi-fl187-GCST90094756 | prot-a-2978 | Tyrosine-protein kinase receptor Tie-1, soluble \|\| id:prot-a-2978 | Dried fruit liking \|\| id:ebi-fl187-GCST90094756 | Weighted mode | 5 | -0.105071266 | 0.586132116673168 | 0.866445996916829 | -1.253890214 | 1.04374768311255 | 0.900260362735024 | 0.285392396957693 | 2.83983991637991 | 0.999228396881742 |
| ebi-fl187-GCST90094756 | prot-a-299 | Uncharacterized protein C1orf226 \|\| id:prot-a-299 | Dried fruit liking \|\| id:ebi-fl187-GCST90094756 | Weighted mode | 5 | 0.0879697840966605 | 0.44563845056726 | 0.853138475721607 | -0.785481579 | 0.96142114720849 | 1.09195512711914 | 0.455900096991629 | 2.6154107171942 | 0.999228396881742 |
| ebi-fl187-GCST90094756 | prot-a-3015 | Transmembrane protein 70, mitochondrial \|\| id:prot-a-3015 | Dried fruit liking \|\| id:ebi-fl187-GCST90094756 | Weighted mode | 5 | 0.534365762250584 | 0.52178663976746 | 0.363675236055526 | -0.488336052 | 1.55706757619481 | 1.70636565746816 | 0.613646621402734 | 4.74488680526086 | 0.999228396881742 |
| ebi-fl187-GCST90094756 | prot-a-3016 | Transmembrane protein 87B \|\| id:prot-a-3016 | Dried fruit liking \|\| id:ebi-fl187-GCST90094756 | Weighted mode | 5 | 0.556173048995468 | 0.556933940819438 | 0.374487917622377 | -0.535417475 | 1.64776357300157 | 1.74398556585887 | 0.585424838881361 | 5.1953478088422 | 0.999228396881742 |
| ebi-fl187-GCST90094756 | prot-a-3123 | Thioredoxin domain-containing protein 12 \|\| id:prot-a-3123 | Dried fruit liking \|\| id:ebi-fl187-GCST90094756 | Weighted mode | 5 | -0.262306271 | 0.562335948067107 | 0.665158768175805 | -1.364484729 | 0.839872187433662 | 0.769275381054838 | 0.255512300155229 | 2.31607093489255 | 0.999228396881742 |
| ebi-fl187-GCST90094756 | prot-a-3133 | PolyUbiquitin K48-linked \|\| id:prot-a-3133 | Dried fruit liking \|\| id:ebi-fl187-GCST90094756 | Weighted mode | 5 | 0.399927809465534 | 0.510736620089576 | 0.477367574610657 | -0.601115966 | 1.4009715848411 | 1.49171700590623 | 0.548199522629521 | 4.05914185228808 | 0.999228396881742 |
| ebi-fl187-GCST90094756 | prot-a-3135 | Ubiquitin-conjugating enzyme E2 B \|\| id:prot-a-3135 | Dried fruit liking \|\| id:ebi-fl187-GCST90094756 | Weighted mode | 5 | 0.451148717628981 | 0.639513877554025 | 0.51944157197634 | -0.802298482 | 1.70459591763487 | 1.5701147684766 | 0.448297375410155 | 5.49916310335926 | 0.999228396881742 |
| ebi-fl187-GCST90094756 | prot-a-3162 | NKG2D ligand 3 \|\| id:prot-a-3162 | Dried fruit liking \|\| id:ebi-fl187-GCST90094756 | Weighted mode | 5 | -0.609490686 | 0.511801703097422 | 0.299541025632629 | -1.612622024 | 0.393640652440138 | 0.543627675964511 | 0.199364190527704 | 1.48236776771357 | 0.999228396881742 |
| ebi-fl187-GCST90094756 | prot-a-3188 | Vesicle-associated membrane protein 8 \|\| id:prot-a-3188 | Dried fruit liking \|\| id:ebi-fl187-GCST90094756 | Weighted mode | 5 | 0.796842685173729 | 0.472018882447633 | 0.166652385773409 | -0.128314324 | 1.72199969477109 | 2.21852527626191 | 0.879576863198499 | 5.59570698973949 | 0.999228396881742 |
| ebi-fl187-GCST90094756 | prot-a-3201 | Vitrin \|\| id:prot-a-3201 | Dried fruit liking \|\| id:ebi-fl187-GCST90094756 | Weighted mode | 5 | 0.278815746136118 | 0.524561026263249 | 0.623203885841835 | -0.749323865 | 1.30695535761209 | 1.32156381819509 | 0.47268604413705 | 3.69490689904097 | 0.999228396881742 |
| ebi-fl187-GCST90094756 | prot-a-3232 | WNT1-inducible-signaling pathway protein 3 \|\| id:prot-a-3232 | Dried fruit liking \|\| id:ebi-fl187-GCST90094756 | Weighted mode | 5 | -0.667134274 | 0.641857306987619 | 0.357324820187815 | -1.925174596 | 0.590906047765459 | 0.513177097580733 | 0.145850289810422 | 1.80562365576161 | 0.999228396881742 |
| ebi-fl187-GCST90094756 | prot-a-327 | Carbonic anhydrase-related protein 10 \|\| id:prot-a-327 | Dried fruit liking \|\| id:ebi-fl187-GCST90094756 | Weighted mode | 5 | -0.777573266 | 0.665124402101435 | 0.307292769017214 | -2.081217094 | 0.526070562324855 | 0.459519791731051 | 0.124778252890927 | 1.69226955900023 | 0.999228396881742 |
| ebi-fl187-GCST90094756 | prot-a-332 | Carbonic anhydrase 5A, mitochondrial \|\| id:prot-a-332 | Dried fruit liking \|\| id:ebi-fl187-GCST90094756 | Weighted mode | 5 | -0.258701113 | 0.622031803867337 | 0.698829827019554 | -1.477883449 | 0.960481222304476 | 0.772053745177125 | 0.228120005437996 | 2.61295358246885 | 0.999228396881742 |
| ebi-fl187-GCST90094756 | prot-a-338 | Cell adhesion molecule 3 \|\| id:prot-a-338 | Dried fruit liking \|\| id:ebi-fl187-GCST90094756 | Weighted mode | 5 | -0.066866857 | 0.582878324958624 | 0.914196298117874 | -1.209308374 | 1.07557465974846 | 0.935319724161551 | 0.298403591732072 | 2.93167713340099 | 0.999228396881742 |
| ebi-fl187-GCST90094756 | prot-a-34 | A disintegrin and metalloproteinase with thrombospondin motifs 3 \|\| id:prot-a-34 | Dried fruit liking \|\| id:ebi-fl187-GCST90094756 | Weighted mode | 5 | -0.307539683 | 0.689897707526468 | 0.678823732379442 | -1.65973919 | 1.04465982334952 | 0.735253689600862 | 0.190188576686952 | 2.84243143036661 | 0.999228396881742 |
| ebi-fl187-GCST90094756 | prot-a-358 | Cysteine--tRNA ligase, cytoplasmic \|\| id:prot-a-358 | Dried fruit liking \|\| id:ebi-fl187-GCST90094756 | Weighted mode | 5 | 0.112084115600017 | 0.569028569002132 | 0.853451216869361 | -1.00321188 | 1.2273801108442 | 1.11860694898254 | 0.36669975220648 | 3.41227802523153 | 0.999228396881742 |
| ebi-fl187-GCST90094756 | prot-a-382 | Coiled-coil domain-containing protein 167 \|\| id:prot-a-382 | Dried fruit liking \|\| id:ebi-fl187-GCST90094756 | Weighted mode | 5 | -0.617966571 | 0.49524936911538 | 0.28016779449905 | -1.588655335 | 0.352722192428558 | 0.539039422303761 | 0.204200007912309 | 1.42293578618445 | 0.999228396881742 |
| ebi-fl187-GCST90094756 | prot-a-40 | Pituitary adenylate cyclase-activating polypeptide 27 \|\| id:prot-a-40 | Dried fruit liking \|\| id:ebi-fl187-GCST90094756 | Weighted mode | 5 | -0.240835691 | 0.572628383873671 | 0.695674360848396 | -1.363187323 | 0.881515941454126 | 0.785970757897095 | 0.255844018399359 | 2.41455726084265 | 0.999228396881742 |
| ebi-fl187-GCST90094756 | prot-a-403 | C-C motif chemokine 25 \|\| id:prot-a-403 | Dried fruit liking \|\| id:ebi-fl187-GCST90094756 | Weighted mode | 5 | -0.414016008 | 0.499044392144896 | 0.453395538498526 | -1.392143017 | 0.564111000669314 | 0.660990370090551 | 0.248542103667769 | 1.75788432987783 | 0.999228396881742 |
| ebi-fl187-GCST90094756 | prot-a-415 | Cyclin-C \|\| id:prot-a-415 | Dried fruit liking \|\| id:ebi-fl187-GCST90094756 | Weighted mode | 5 | 0.198768522553616 | 0.50109166532537 | 0.711862640381438 | -0.783371141 | 1.18090818659134 | 1.21989955398154 | 0.456863261658674 | 3.25733112441894 | 0.999228396881742 |
| ebi-fl187-GCST90094756 | prot-a-44 | Adhesion G protein-coupled receptor F5 \|\| id:prot-a-44 | Dried fruit liking \|\| id:ebi-fl187-GCST90094756 | Weighted mode | 5 | 0.399025239367879 | 0.507096101336193 | 0.475353710604318 | -0.594883119 | 1.39293359798682 | 1.49037123416056 | 0.551627036681241 | 4.026645302 | 0.999228396881742 |
| ebi-fl187-GCST90094756 | prot-a-455 | CD59 glycoprotein \|\| id:prot-a-455 | Dried fruit liking \|\| id:ebi-fl187-GCST90094756 | Weighted mode | 5 | 0.785678625770208 | 0.441935407477538 | 0.150066262312588 | -0.080514773 | 1.65187202442618 | 2.19389527 | 0.922641273408943 | 5.2167365501655 | 0.999228396881742 |
| ebi-fl187-GCST90094756 | prot-a-478 | Cadherin-12 \|\| id:prot-a-478 | Dried fruit liking \|\| id:ebi-fl187-GCST90094756 | Weighted mode | 5 | -0.120902692 | 0.474953032408485 | 0.81161640707633 | -1.051810636 | 0.810005251317961 | 0.886120181800822 | 0.349304712593895 | 2.24791979118705 | 0.999228396881742 |
| ebi-fl187-GCST90094756 | prot-a-516 | Centrin-2 \|\| id:prot-a-516 | Dried fruit liking \|\| id:ebi-fl187-GCST90094756 | Weighted mode | 5 | 0.805566829855096 | 0.539851381613677 | 0.209925647984874 | -0.252541878 | 1.8636755378179 | 2.23796468456112 | 0.776823680252362 | 6.44739090306269 | 0.999228396881742 |
| ebi-fl187-GCST90094756 | prot-a-519 | Cryptic protein \|\| id:prot-a-519 | Dried fruit liking \|\| id:ebi-fl187-GCST90094756 | Weighted mode | 5 | 0.297324971252953 | 0.507562544808938 | 0.58947502717249 | -0.697497617 | 1.29214755907847 | 1.3462527218032 | 0.497829506713052 | 3.6405965627248 | 0.999228396881742 |
| ebi-fl187-GCST90094756 | prot-a-583 | Chloride intracellular channel protein 4 \|\| id:prot-a-583 | Dried fruit liking \|\| id:ebi-fl187-GCST90094756 | Weighted mode | 5 | 0.611804418417188 | 0.548104416041675 | 0.326854745032183 | -0.462480237 | 1.68608907385887 | 1.84375530505015 | 0.629719852526655 | 5.39832690880057 | 0.999228396881742 |
| ebi-fl187-GCST90094756 | prot-a-602 | CCR4-NOT transcription complex subunit 1 \|\| id:prot-a-602 | Dried fruit liking \|\| id:ebi-fl187-GCST90094756 | Weighted mode | 5 | -0.029360094 | 0.540697107522016 | 0.959299663621672 | -1.089126424 | 1.03040623720116 | 0.971066726644105 | 0.336510332597141 | 2.8022039630034 | 0.999228396881742 |
| ebi-fl187-GCST90094756 | prot-a-606 | Ciliary neurotrophic factor receptor subunit alpha \|\| id:prot-a-606 | Dried fruit liking \|\| id:ebi-fl187-GCST90094756 | Weighted mode | 5 | 0.27676699265228 | 0.479523605415064 | 0.594752400632605 | -0.663099274 | 1.2166332592658 | 1.31885903138523 | 0.515251950353921 | 3.375803126 | 0.999228396881742 |
| ebi-fl187-GCST90094756 | prot-a-626 | Collagen alpha-2(IX) chain \|\| id:prot-a-626 | Dried fruit liking \|\| id:ebi-fl187-GCST90094756 | Weighted mode | 5 | -0.249019487 | 0.576241099603276 | 0.68791261367248 | -1.378452043 | 0.880413067771943 | 0.779564781506601 | 0.25196828749262 | 2.4118957770955 | 0.999228396881742 |
| ebi-fl187-GCST90094756 | prot-a-647 | Probable serine carboxypeptidase CPVL \|\| id:prot-a-647 | Dried fruit liking \|\| id:ebi-fl187-GCST90094756 | Weighted mode | 5 | -0.744315643 | 0.51212134055853 | 0.219778447913502 | -1.74807347 | 0.259442184934035 | 0.475059299084882 | 0.174109046845811 | 1.29620684126127 | 0.999228396881742 |
| ebi-fl187-GCST90094756 | prot-a-651 | Cellular retinoic acid-binding protein 2 \|\| id:prot-a-651 | Dried fruit liking \|\| id:ebi-fl187-GCST90094756 | Weighted mode | 5 | 0.305526229734533 | 0.541570088902892 | 0.602783019452197 | -0.755951145 | 1.3670036039842 | 1.35733908729113 | 0.469563779248527 | 3.92357647524854 | 0.999228396881742 |
| ebi-fl187-GCST90094756 | prot-a-671 | Cytotoxic and regulatory T-cell molecule \|\| id:prot-a-671 | Dried fruit liking \|\| id:ebi-fl187-GCST90094756 | Weighted mode | 5 | 0.0235044998176095 | 0.565160566543833 | 0.968819435549578 | -1.084210211 | 1.13121921024352 | 1.02378290757262 | 0.338168762554437 | 3.09943305798129 | 0.999228396881742 |
| ebi-fl187-GCST90094756 | prot-a-688 | Chorionic somatomammotropin hormone \|\| id:prot-a-688 | Dried fruit liking \|\| id:ebi-fl187-GCST90094756 | Weighted mode | 5 | 0.023938915161815 | 0.602666214843506 | 0.970218527872869 | -1.157286866 | 1.20516469625509 | 1.02422775119326 | 0.314337865765166 | 3.33730867504874 | 0.999228396881742 |
| ebi-fl187-GCST90094756 | prot-a-69 | Aldo-keto reductase family 1 member C1 \|\| id:prot-a-69 | Dried fruit liking \|\| id:ebi-fl187-GCST90094756 | Weighted mode | 5 | -0.497154925 | 0.557332165427811 | 0.422791065637711 | -1.589525969 | 0.595216119704807 | 0.608258742309182 | 0.204022301757556 | 1.81342281901711 | 0.999228396881742 |
| ebi-fl187-GCST90094756 | prot-a-741 | Stromal cell-derived factor 1 \|\| id:prot-a-741 | Dried fruit liking \|\| id:ebi-fl187-GCST90094756 | Weighted mode | 5 | -0.119576583 | 0.538998709421454 | 0.835297199277004 | -1.176014054 | 0.93686088718954 | 0.887296053178002 | 0.308505979372286 | 2.55195794774273 | 0.999228396881742 |
| ebi-fl187-GCST90094756 | prot-a-742 | Growth-regulated alpha protein \|\| id:prot-a-742 | Dried fruit liking \|\| id:ebi-fl187-GCST90094756 | Weighted mode | 5 | -0.867217533 | 0.577026433462734 | 0.20728662230524 | -1.998189343 | 0.263754276446298 | 0.420118891341292 | 0.135580551032365 | 1.3018082720412 | 0.999228396881742 |
| ebi-fl187-GCST90094756 | prot-a-788 | Beta-defensin 112 \|\| id:prot-a-788 | Dried fruit liking \|\| id:ebi-fl187-GCST90094756 | Weighted mode | 5 | 0.465721203684746 | 0.719813722718465 | 0.552886067110698 | -0.945113693 | 1.87655610021294 | 1.5931627695168 | 0.388635383328101 | 6.53097406736026 | 0.999228396881742 |
| ebi-fl187-GCST90094756 | prot-a-838 | DnaJ homolog subfamily B member 1 \|\| id:prot-a-838 | Dried fruit liking \|\| id:ebi-fl187-GCST90094756 | Weighted mode | 5 | -0.717985173 | 0.680490568560615 | 0.350892072317101 | -2.051746687 | 0.615776341334096 | 0.48773396618183 | 0.128510240216202 | 1.85109312197416 | 0.999228396881742 |
| ebi-fl187-GCST90094756 | prot-a-85 | Junctional adhesion molecule-like \|\| id:prot-a-85 | Dried fruit liking \|\| id:ebi-fl187-GCST90094756 | Weighted mode | 5 | -0.112692462 | 0.453089585839713 | 0.815825662330173 | -1.00074805 | 0.775363126006981 | 0.893425379911581 | 0.367604351680406 | 2.17138046876037 | 0.999228396881742 |
| ebi-fl187-GCST90094756 | prot-a-856 | Dipeptidase 1 \|\| id:prot-a-856 | Dried fruit liking \|\| id:ebi-fl187-GCST90094756 | Weighted mode | 5 | 0.33231003726831 | 0.553739345229207 | 0.580768624555226 | -0.753019079 | 1.41763915391755 | 1.39418503083122 | 0.470942591229409 | 4.12736485591509 | 0.999228396881742 |
| ebi-fl187-GCST90094756 | prot-a-860 | Dipeptidyl peptidase 2 \|\| id:prot-a-860 | Dried fruit liking \|\| id:ebi-fl187-GCST90094756 | Weighted mode | 5 | -0.015741802 | 0.568742829156361 | 0.979244630670789 | -1.130477747 | 1.09899414327999 | 0.984381452699348 | 0.322878965107957 | 3.0011457825829 | 0.999228396881742 |
| ebi-fl187-GCST90094756 | prot-a-861 | Dermatopontin \|\| id:prot-a-861 | Dried fruit liking \|\| id:ebi-fl187-GCST90094756 | Weighted mode | 5 | 0.230966502255214 | 0.485807242091618 | 0.659280346740693 | -0.721215692 | 1.18314869675479 | 1.2598170377128 | 0.486160874559203 | 3.26463738973337 | 0.999228396881742 |
| ebi-fl187-GCST90094756 | prot-a-877 | Dual specificity protein phosphatase 6 \|\| id:prot-a-877 | Dried fruit liking \|\| id:ebi-fl187-GCST90094756 | Weighted mode | 5 | -0.495480798 | 0.508730911932844 | 0.385229031629309 | -1.492593385 | 0.50163178930127 | 0.609277897215115 | 0.224788934601852 | 1.65141383267988 | 0.999228396881742 |
| ebi-fl187-GCST90094756 | prot-a-879 | Dynactin-associated protein \|\| id:prot-a-879 | Dried fruit liking \|\| id:ebi-fl187-GCST90094756 | Weighted mode | 5 | -0.121202834 | 0.609342404107636 | 0.852036293867023 | -1.315513946 | 1.0731082778933 | 0.885854259866256 | 0.268336377330118 | 2.92445540754162 | 0.999228396881742 |
| ebi-fl187-GCST90094756 | prot-a-937 | EMI domain-containing protein 1 \|\| id:prot-a-937 | Dried fruit liking \|\| id:ebi-fl187-GCST90094756 | Weighted mode | 5 | -0.855327057 | 0.581322357702333 | 0.215163929800855 | -1.994718879 | 0.284064763677243 | 0.425144121802375 | 0.136051895902201 | 1.32851896774037 | 0.999228396881742 |
| ebi-fl187-GCST90094756 | prot-b-1 | SRC proto-oncogene, non-receptor tyrosine kinase \|\| id:prot-b-1 | Dried fruit liking \|\| id:ebi-fl187-GCST90094756 | Weighted mode | 5 | 0.133353561207115 | 0.498463582106457 | 0.802289758676634 | -0.84363506 | 1.11034218213577 | 1.14265392502133 | 0.430144078678978 | 3.03539687533645 | 0.999228396881742 |
| ebi-fl187-GCST90094756 | prot-b-10 | coagulation factor III, tissue factor \|\| id:prot-b-10 | Dried fruit liking \|\| id:ebi-fl187-GCST90094756 | Weighted mode | 5 | -0.870145622 | 0.611795976484226 | 0.228014449654699 | -2.069265735 | 0.328974492405578 | 0.418890545334991 | 0.126278469480491 | 1.3895424112513 | 0.999228396881742 |
| ebi-fl187-GCST90094756 | prot-b-29 | myeloperoxidase \|\| id:prot-b-29 | Dried fruit liking \|\| id:ebi-fl187-GCST90094756 | Weighted mode | 5 | 0.19347391801024 | 0.470867813913006 | 0.702213477653963 | -0.729426997 | 1.11637483327973 | 1.21345773674649 | 0.482185204390533 | 3.05376371042137 | 0.999228396881742 |
| ebi-fl187-GCST90094756 | prot-b-33 | adrenomedullin \|\| id:prot-b-33 | Dried fruit liking \|\| id:ebi-fl187-GCST90094756 | Weighted mode | 5 | -0.16758626 | 0.503489431774372 | 0.755961935684826 | -1.154425546 | 0.819253026256673 | 0.845703663726528 | 0.315238574865544 | 2.26880446704699 | 0.999228396881742 |
| ebi-fl187-GCST90094756 | ukb-rsfMRI191-edge-pheno102 | Net25_Pair11_15 (Postcentral\|Precentral)&(Cerebellum) (Motor)&(Subcortical-cerebellum) \|\| id:ukb-rsfMRI191-edge-pheno102 | Dried fruit liking \|\| id:ebi-fl187-GCST90094756 | Weighted mode | 5 | 0.0210345772296406 | 0.135933186990463 | 0.884518817113066 | -0.245394469 | 0.287463623730948 | 1.02125736327741 | 0.782395846241495 | 1.33304210018314 | 0.999228396881742 |
| ebi-fl187-GCST90094756 | ukb-rsfMRI191-edge-pheno1167 | Net100_Pair11_45 (Angular\|Temporal)&(Frontal) (Default_mode\|Central_executive)&(Salience\|Default_mode) \|\| id:ukb-rsfMRI191-edge-pheno1167 | Dried fruit liking \|\| id:ebi-fl187-GCST90094756 | Weighted mode | 5 | -0.082938291 | 0.189677918825801 | 0.684495187948101 | -0.454707012 | 0.288830429426866 | 0.920407942487293 | 0.634633880588264 | 1.33486535545256 | 0.999228396881742 |
| ebi-fl187-GCST90094756 | ukb-rsfMRI191-edge-pheno1317 | Net100_Pair26_48 (Parietal\|Frontal)&(Temporal) (Attention\|Central_executive)&(Default_mode\|Central_executive) \|\| id:ukb-rsfMRI191-edge-pheno1317 | Dried fruit liking \|\| id:ebi-fl187-GCST90094756 | Weighted mode | 5 | 0.162072143138403 | 0.142693367065413 | 0.319473669718711 | -0.117606856 | 0.441751142586613 | 1.17594507462917 | 0.889045506548506 | 1.55542861232508 | 0.999228396881742 |
| ebi-fl187-GCST90094756 | ukb-rsfMRI191-edge-pheno286 | Net100_Pair10_13 (OccipitalPrecuneus)&(Frontal_Sup) (Default_mode\|Central_executive)&(Default_mode) \|\| id:ukb-rsfMRI191-edge-pheno286 | Dried fruit liking \|\| id:ebi-fl187-GCST90094756 | Weighted mode | 5 | 0.129107619886895 | 0.138188435643944 | 0.403047825938112 | -0.141741714 | 0.399956953749025 | 1.13781256883684 | 0.867845379873978 | 1.49176048156307 | 0.999228396881742 |
| ebi-fl187-GCST90094756 | ukb-rsfMRI191-edge-pheno599 | Net100_Pair11_29 (Angular\|Temporal)&(Parietal) (Default_mode\|Central_executive)&(Central_executive\|Salience) \|\| id:ukb-rsfMRI191-edge-pheno599 | Dried fruit liking \|\| id:ebi-fl187-GCST90094756 | Weighted mode | 5 | 0.0320606921995644 | 0.169757978661477 | 0.859396916030416 | -0.300664946 | 0.364786330376059 | 1.0325801729653 | 0.7403257803275 | 1.44020624694359 | 0.999228396881742 |
| ebi-fl187-GCST90094756 | ukb-rsfMRI191-edge-pheno621 | Net100_Pair5_30 (Precuneus\|Angular\|Cingulate)&(Paracentral) (Default_mode\|Central_executive)&(Motor) \|\| id:ukb-rsfMRI191-edge-pheno621 | Dried fruit liking \|\| id:ebi-fl187-GCST90094756 | Weighted mode | 5 | 0.198431749514932 | 0.146449203310912 | 0.246902088569002 | -0.088608689 | 0.485472188004319 | 1.2194887938722 | 0.915203632785753 | 1.62494210589308 | 0.999228396881742 |
| ebi-fl187-GCST90094756 | ukb-rsfMRI191-edge-pheno624 | Net100_Pair8_30 (Calcarine\|Lingual)&(Paracentral) (Visual)&(Motor) \|\| id:ukb-rsfMRI191-edge-pheno624 | Dried fruit liking \|\| id:ebi-fl187-GCST90094756 | Weighted mode | 5 | -0.098650858 | 0.152259691477807 | 0.552354410437327 | -0.397079853 | 0.199778137264633 | 0.906058996026743 | 0.672280339666463 | 1.22113180446164 | 0.999228396881742 |
| ebi-fl187-GCST90094756 | ukb-rsfMRI191-edge-pheno882 | Net100_Pair6_38 (Postcentral\|Precentral)&(Subcortical) (Motor)&(Subcortical-cerebellum) \|\| id:ukb-rsfMRI191-edge-pheno882 | Dried fruit liking \|\| id:ebi-fl187-GCST90094756 | Weighted mode | 5 | -0.051395822 | 0.176347177047652 | 0.785198665441921 | -0.397036289 | 0.294244644647609 | 0.949902603413697 | 0.67230962749096 | 1.34211220407408 | 0.999228396881742 |
| ebi-fl187-GCST90094756 | ukb-rsfMRI191-edge-pheno899 | Net100_Pair23_38 (Cerebellum)&(Subcortical) (Subcortical-cerebellum)&(Subcortical-cerebellum) \|\| id:ukb-rsfMRI191-edge-pheno899 | Dried fruit liking \|\| id:ebi-fl187-GCST90094756 | Weighted mode | 5 | 0.161893709974388 | 0.192320083510966 | 0.447288637288382 | -0.215053654 | 0.53884107365588 | 1.17573526574778 | 0.806498167400011 | 1.71401928857375 | 0.999228396881742 |
| ebi-fl187-GCST90094765 | ebi-a-GCST90001392 | IgD+ CD38+ B cell Absolute Count \|\| id:ebi-a-GCST90001392 | Fried fish liking \|\| id:ebi-fl187-GCST90094765 | Inverse variance weighted | 4 | 0.0719962770381923 | 0.365635153515218 | 0.843899982119578 | -0.644648624 | 0.788641177928019 | 1.07465134317046 | 0.524846928402406 | 2.20040443581031 | 0.998074519377826 |
| ebi-fl187-GCST90094765 | ebi-a-GCST90001402 | Switched memory B cell %B cell \|\| id:ebi-a-GCST90001402 | Fried fish liking \|\| id:ebi-fl187-GCST90094765 | Inverse variance weighted | 4 | 0.306188809136151 | 0.347937502854676 | 0.378853382785923 | -0.375768696 | 0.988146314731315 | 1.3582387302209 | 0.686761164961668 | 2.68625039153905 | 0.998074519377826 |
| ebi-fl187-GCST90094765 | ebi-a-GCST90001407 | Memory B cell Absolute Count \|\| id:ebi-a-GCST90001407 | Fried fish liking \|\| id:ebi-fl187-GCST90094765 | Inverse variance weighted | 4 | -0.068471057 | 0.303832764470778 | 0.821701005931032 | -0.663983275 | 0.527041161536146 | 0.933820487440705 | 0.514796668261661 | 1.69391287186957 | 0.998074519377826 |
| ebi-fl187-GCST90094765 | ebi-a-GCST90001411 | IgD+ CD24+ B cell %B cell \|\| id:ebi-a-GCST90001411 | Fried fish liking \|\| id:ebi-fl187-GCST90094765 | Inverse variance weighted | 4 | -0.113962619 | 0.262675428352201 | 0.664394926464243 | -0.628806458 | 0.40088122103938 | 0.892291310419002 | 0.533227851137064 | 1.4931399043607 | 0.998074519377826 |
| ebi-fl187-GCST90094765 | ebi-a-GCST90001418 | CD24+ CD27+ B cell Absolute Count \|\| id:ebi-a-GCST90001418 | Fried fish liking \|\| id:ebi-fl187-GCST90094765 | Inverse variance weighted | 4 | -0.109900036 | 0.352067689670552 | 0.754921980971959 | -0.799952708 | 0.580152635396194 | 0.895923690615306 | 0.449350214234577 | 1.7863110642398 | 0.998074519377826 |
| ebi-fl187-GCST90094765 | ebi-a-GCST90001436 | Memory B cell %lymphocyte \|\| id:ebi-a-GCST90001436 | Fried fish liking \|\| id:ebi-fl187-GCST90094765 | Inverse variance weighted | 4 | -0.2209131 | 0.32189936950281 | 0.492536557920718 | -0.851835864 | 0.410009664683075 | 0.801786352863798 | 0.42663097619945 | 1.50683234809957 | 0.998074519377826 |
| ebi-fl187-GCST90094765 | ebi-a-GCST90001439 | IgD+ CD24+ B cell %lymphocyte \|\| id:ebi-a-GCST90001439 | Fried fish liking \|\| id:ebi-fl187-GCST90094765 | Inverse variance weighted | 4 | -0.253515921 | 0.285746996850801 | 0.37496899950448 | -0.813580035 | 0.306548192887934 | 0.776067389104723 | 0.443268306275282 | 1.3587269468749 | 0.998074519377826 |
| ebi-fl187-GCST90094765 | ebi-a-GCST90001457 | CD11c+ HLA DR++ monocyte %monocyte \|\| id:ebi-a-GCST90001457 | Fried fish liking \|\| id:ebi-fl187-GCST90094765 | Inverse variance weighted | 4 | -0.147388812 | 0.384712705787592 | 0.701635261160622 | -0.901425716 | 0.606648090948468 | 0.862958383262998 | 0.40599041999148 | 1.83427276746953 | 0.998074519377826 |
| ebi-fl187-GCST90094765 | ebi-a-GCST90001483 | CD39+ resting CD4 regulatory T cell Absolute Count \|\| id:ebi-a-GCST90001483 | Fried fish liking \|\| id:ebi-fl187-GCST90094765 | Inverse variance weighted | 3 | -0.042511014 | 0.269267738162972 | 0.874554216670552 | -0.57027578 | 0.485253753104048 | 0.958379910158435 | 0.565369499317738 | 1.6245872005895 | 0.998074519377826 |
| ebi-fl187-GCST90094765 | ebi-a-GCST90001525 | CD33dim HLA DR+ CD11b+ Absolute Count \|\| id:ebi-a-GCST90001525 | Fried fish liking \|\| id:ebi-fl187-GCST90094765 | Inverse variance weighted | 4 | -0.367266269 | 0.308472673512196 | 0.233812302429993 | -0.971872709 | 0.237340171528873 | 0.692625196178851 | 0.378373790337558 | 1.26787233849842 | 0.998074519377826 |
| ebi-fl187-GCST90094765 | ebi-a-GCST90001540 | Naive CD4+ T cell Absolute Count \|\| id:ebi-a-GCST90001540 | Fried fish liking \|\| id:ebi-fl187-GCST90094765 | Inverse variance weighted (multiplicative random effects) | 4 | 0.12600980342151 | 0.406623725518047 | 0.756642406098211 | -0.670972699 | 0.922992305436883 | 1.13429328818374 | 0.511211081568623 | 2.51681019838371 | 0.998074519377826 |
| ebi-fl187-GCST90094765 | ebi-a-GCST90001624 | HLA DR+ CD4+ T cell Absolute Count \|\| id:ebi-a-GCST90001624 | Fried fish liking \|\| id:ebi-fl187-GCST90094765 | Inverse variance weighted | 4 | 0.135792381814926 | 0.228435967363655 | 0.552215211362791 | -0.311942114 | 0.58352687784769 | 1.14544405389022 | 0.732023900777975 | 1.79234869134472 | 0.998074519377826 |
| ebi-fl187-GCST90094765 | ebi-a-GCST90001672 | CD39+ CD8+ T cell Absolute Count \|\| id:ebi-a-GCST90001672 | Fried fish liking \|\| id:ebi-fl187-GCST90094765 | Inverse variance weighted | 4 | 0.47402617885887 | 0.248241277504744 | 0.0561927108818745 | -0.012526725 | 0.960579082768169 | 1.60644904147973 | 0.987551407780035 | 2.61320929983014 | 0.998074519377826 |
| ebi-fl187-GCST90094765 | ebi-a-GCST90001722 | CD19 on CD20- CD38- B cell \|\| id:ebi-a-GCST90001722 | Fried fish liking \|\| id:ebi-fl187-GCST90094765 | Inverse variance weighted | 4 | 0.47633964221747 | 0.228681763430751 | 0.0372530057130319 | 0.0281233858931993 | 0.924555898541742 | 1.61016980473989 | 1.02852258177002 | 2.5207485436385 | 0.998074519377826 |
| ebi-fl187-GCST90094765 | ebi-a-GCST90001742 | CD19 on transitional B cell \|\| id:ebi-a-GCST90001742 | Fried fish liking \|\| id:ebi-fl187-GCST90094765 | Inverse variance weighted | 4 | 0.153949719008351 | 0.323677227533876 | 0.63433993754772 | -0.480457647 | 0.788357084974748 | 1.16643223593443 | 0.618500272258533 | 2.19977940520335 | 0.998074519377826 |
| ebi-fl187-GCST90094765 | ebi-a-GCST90001784 | CD25 on IgD+ CD38dim B cell \|\| id:ebi-a-GCST90001784 | Fried fish liking \|\| id:ebi-fl187-GCST90094765 | Inverse variance weighted (multiplicative random effects) | 4 | -0.204385408 | 0.533199981664804 | 0.701483636772942 | -1.249457372 | 0.840686556151952 | 0.815148146094571 | 0.286660304580135 | 2.31795783882475 | 0.998074519377826 |
| ebi-fl187-GCST90094765 | ebi-a-GCST90001794 | CD25 on IgD+ B cell \|\| id:ebi-a-GCST90001794 | Fried fish liking \|\| id:ebi-fl187-GCST90094765 | Inverse variance weighted (multiplicative random effects) | 4 | -0.268570211 | 0.508507528673228 | 0.597392555411028 | -1.265244967 | 0.728104545493345 | 0.764471746824358 | 0.282170165321205 | 2.0711511120512 | 0.998074519377826 |
| ebi-fl187-GCST90094765 | ebi-a-GCST90001802 | CD27 on IgD- CD38- B cell \|\| id:ebi-a-GCST90001802 | Fried fish liking \|\| id:ebi-fl187-GCST90094765 | Inverse variance weighted | 4 | -0.007750534 | 0.243165322713155 | 0.974572917542834 | -0.484354567 | 0.46885349824626 | 0.992279423672568 | 0.616094716455211 | 1.5981608482363 | 0.998074519377826 |
| ebi-fl187-GCST90094765 | ebi-a-GCST90001804 | CD27 on IgD- CD38dim B cell \|\| id:ebi-a-GCST90001804 | Fried fish liking \|\| id:ebi-fl187-GCST90094765 | Inverse variance weighted | 4 | 0.246104966863018 | 0.293556698316997 | 0.40183089374633 | -0.329266162 | 0.821476095564332 | 1.27903382266382 | 0.71945150072708 | 2.27385378703742 | 0.998074519377826 |
| ebi-fl187-GCST90094765 | ebi-a-GCST90001805 | CD27 on memory B cell \|\| id:ebi-a-GCST90001805 | Fried fish liking \|\| id:ebi-fl187-GCST90094765 | Inverse variance weighted | 4 | 0.218517596243392 | 0.235999802590047 | 0.354486105387332 | -0.244042017 | 0.681077209319885 | 1.24423091022448 | 0.783454715285278 | 1.97600515735529 | 0.998074519377826 |
| ebi-fl187-GCST90094765 | ebi-a-GCST90001806 | CD27 on unswitched memory B cell \|\| id:ebi-a-GCST90001806 | Fried fish liking \|\| id:ebi-fl187-GCST90094765 | Inverse variance weighted | 4 | 0.103362412913668 | 0.245708230082966 | 0.673995084505951 | -0.378225718 | 0.584950543876282 | 1.1088932134825 | 0.685075849228649 | 1.7949022145388 | 0.998074519377826 |
| ebi-fl187-GCST90094765 | ebi-a-GCST90001839 | CD3 on Effector Memory CD8+ T cell \|\| id:ebi-a-GCST90001839 | Fried fish liking \|\| id:ebi-fl187-GCST90094765 | Inverse variance weighted | 4 | -0.102910074 | 0.25894728754463 | 0.691059924300734 | -0.610446758 | 0.404626609370523 | 0.902208101603717 | 0.543108177049515 | 1.49874277905629 | 0.998074519377826 |
| ebi-fl187-GCST90094765 | ebi-a-GCST90001859 | CD3 on CD8+ T cell \|\| id:ebi-a-GCST90001859 | Fried fish liking \|\| id:ebi-fl187-GCST90094765 | Inverse variance weighted | 4 | 0.00997045952043511 | 0.253653005575482 | 0.96864524595085 | -0.487189431 | 0.507130350448379 | 1.01002033015855 | 0.614350644613863 | 1.66051924300457 | 0.998074519377826 |
| ebi-fl187-GCST90094765 | ebi-a-GCST90001905 | CD86 on monocyte \|\| id:ebi-a-GCST90001905 | Fried fish liking \|\| id:ebi-fl187-GCST90094765 | Inverse variance weighted (multiplicative random effects) | 4 | 0.0593155142012881 | 0.425845104157458 | 0.889221827359197 | -0.77534089 | 0.893971918349905 | 1.06110998319821 | 0.460546758476547 | 2.44482102136051 | 0.998074519377826 |
| ebi-fl187-GCST90094765 | ebi-a-GCST90001943 | CD25 on activated & secreting CD4 regulatory T cell \|\| id:ebi-a-GCST90001943 | Fried fish liking \|\| id:ebi-fl187-GCST90094765 | Inverse variance weighted (multiplicative random effects) | 4 | 0.0556368207511369 | 0.384581717653582 | 0.884972691862669 | -0.698143346 | 0.809416987352158 | 1.05721365594392 | 0.497508147392182 | 2.24659781 | 0.998074519377826 |
| ebi-fl187-GCST90094765 | ebi-a-GCST90001949 | CD33 on CD33dim HLA DR+ CD11b- \|\| id:ebi-a-GCST90001949 | Fried fish liking \|\| id:ebi-fl187-GCST90094765 | Inverse variance weighted | 4 | 0.0353683561269785 | 0.38059733181068 | 0.925960328544956 | -0.710602414 | 0.781339126475911 | 1.03600125593288 | 0.491348113195914 | 2.18439548961196 | 0.998074519377826 |
| ebi-fl187-GCST90094765 | ebi-a-GCST90001955 | CD33 on Immature Myeloid-Derived Suppressor Cells \|\| id:ebi-a-GCST90001955 | Fried fish liking \|\| id:ebi-fl187-GCST90094765 | Inverse variance weighted | 4 | 0.175258415660688 | 0.348314034296027 | 0.614850319507551 | -0.507437092 | 0.857953922880902 | 1.1915540930688 | 0.602036567863095 | 2.35833042791494 | 0.998074519377826 |
| ebi-fl187-GCST90094765 | ebi-a-GCST90001956 | CD33 on CD33+ HLA DR+ \|\| id:ebi-a-GCST90001956 | Fried fish liking \|\| id:ebi-fl187-GCST90094765 | Inverse variance weighted | 4 | 0.0223135054343245 | 0.347470118589678 | 0.94879740272673 | -0.658727927 | 0.703354937870093 | 1.02256431369328 | 0.517509225472535 | 2.02052006837991 | 0.998074519377826 |
| ebi-fl187-GCST90094765 | ebi-a-GCST90001957 | CD33 on CD33+ HLA DR+ CD14- \|\| id:ebi-a-GCST90001957 | Fried fish liking \|\| id:ebi-fl187-GCST90094765 | Inverse variance weighted | 4 | 0.0303741006073785 | 0.347867404836268 | 0.930420998404071 | -0.651446013 | 0.712194214086464 | 1.03084009973624 | 0.521291437371873 | 2.03845917090363 | 0.998074519377826 |
| ebi-fl187-GCST90094765 | ebi-a-GCST90002064 | CD4 on resting CD4 regulatory T cell \|\| id:ebi-a-GCST90002064 | Fried fish liking \|\| id:ebi-fl187-GCST90094765 | Inverse variance weighted | 4 | -0.455193155 | 0.249931853749934 | 0.0685659653227622 | -0.945059589 | 0.0346732779315416 | 0.634325432720123 | 0.38865641065495 | 1.03528140425502 | 0.998074519377826 |
| ebi-fl187-GCST90094765 | ebi-a-GCST90002080 | SSC-A on T cell \|\| id:ebi-a-GCST90002080 | Fried fish liking \|\| id:ebi-fl187-GCST90094765 | Inverse variance weighted (multiplicative random effects) | 4 | -0.073779026 | 0.550080336366536 | 0.893304448046508 | -1.151936486 | 1.00437843290098 | 0.928876928464537 | 0.316024200124253 | 2.7302097367685 | 0.998074519377826 |
| ebi-fl187-GCST90094765 | ebi-a-GCST90002121 | CD8 on CD39+ CD8+ T cell \|\| id:ebi-a-GCST90002121 | Fried fish liking \|\| id:ebi-fl187-GCST90094765 | Inverse variance weighted | 4 | 0.428294698849333 | 0.253058601751297 | 0.0905559383604534 | -0.067700161 | 0.924289558281875 | 1.53463827046689 | 0.934540643693665 | 2.52007725621574 | 0.998074519377826 |
| ebi-fl187-GCST90094765 | ebi-cfmet338-GCST90026053 | 1-palmitoyl-2-docosahexaenoyl-gpc (16:0/22:6) levels \|\| id:ebi-cfmet338-GCST90026053 | Fried fish liking \|\| id:ebi-fl187-GCST90094765 | Inverse variance weighted | 4 | -0.029573146 | 0.130478913674789 | 0.820695313308492 | -0.285311817 | 0.226165524988455 | 0.970859860709011 | 0.751779800334101 | 1.25378318054972 | 0.998074519377826 |
| ebi-fl187-GCST90094765 | ebi-cfmet338-GCST90026063 | 1-stearoyl-2-oleoyl-gpc (18:0/18:1) levels \|\| id:ebi-cfmet338-GCST90026063 | Fried fish liking \|\| id:ebi-fl187-GCST90094765 | Inverse variance weighted | 3 | -0.234846722 | 0.121230582483101 | 0.0527220722028608 | -0.472458663 | 0.00276521998340262 | 0.790692036285066 | 0.623467485641852 | 1.00276904673063 | 0.998074519377826 |
| ebi-fl187-GCST90094765 | ebi-cfmet338-GCST90026113 | Adenosine 3',5'-cyclic monophosphate (camp) levels \|\| id:ebi-cfmet338-GCST90026113 | Fried fish liking \|\| id:ebi-fl187-GCST90094765 | Inverse variance weighted | 4 | -0.56576057 | 0.207322621580339 | 0.00635479520666461 | -0.972112909 | -0.159408232 | 0.567928033298421 | 0.378282915910596 | 0.852648209686642 | 0.877504772527479 |
| ebi-fl187-GCST90094765 | ebi-cfmet338-GCST90026117 | Alpha-ketoglutaramate levels \|\| id:ebi-cfmet338-GCST90026117 | Fried fish liking \|\| id:ebi-fl187-GCST90094765 | Inverse variance weighted (multiplicative random effects) | 4 | -0.128402109 | 0.139491886823871 | 0.357312180848539 | -0.401806207 | 0.145001989358396 | 0.879499653466631 | 0.66911040204474 | 1.15604187004733 | 0.998074519377826 |
| ebi-fl187-GCST90094765 | ebi-cfmet338-GCST90026124 | Arginine levels \|\| id:ebi-cfmet338-GCST90026124 | Fried fish liking \|\| id:ebi-fl187-GCST90094765 | Inverse variance weighted | 4 | -0.061134582 | 0.0672617105071233 | 0.36339966791116 | -0.192967535 | 0.0706983703447644 | 0.940696630188196 | 0.824508741510213 | 1.07325745076587 | 0.998074519377826 |
| ebi-fl187-GCST90094765 | ebi-cfmet338-GCST90026169 | Glutamate levels \|\| id:ebi-cfmet338-GCST90026169 | Fried fish liking \|\| id:ebi-fl187-GCST90094765 | Inverse variance weighted (multiplicative random effects) | 4 | -0.021776812 | 0.120947555667487 | 0.857111985374333 | -0.258834021 | 0.215280396830158 | 0.978458590626961 | 0.771951139791045 | 1.24020959905681 | 0.998074519377826 |
| ebi-fl187-GCST90094765 | ebi-cfmet338-GCST90026170 | Glutamine levels \|\| id:ebi-cfmet338-GCST90026170 | Fried fish liking \|\| id:ebi-fl187-GCST90094765 | Inverse variance weighted | 4 | -0.014600651 | 0.0423403642801663 | 0.730214661388685 | -0.097587765 | 0.0683864628615159 | 0.985505421508369 | 0.907022733106377 | 1.07077904486048 | 0.998074519377826 |
| ebi-fl187-GCST90094765 | ebi-cfmet338-GCST90026191 | Kynurenate levels \|\| id:ebi-cfmet338-GCST90026191 | Fried fish liking \|\| id:ebi-fl187-GCST90094765 | Inverse variance weighted | 4 | -0.726727892 | 0.320772823703669 | 0.0234787542639343 | -1.355442626 | -0.098013157 | 0.483488431016173 | 0.257833145430955 | 0.906636974605265 | 0.998074519377826 |
| ebi-fl187-GCST90094765 | ebi-cfmet338-GCST90026202 | Methylsuccinoylcarnitine levels \|\| id:ebi-cfmet338-GCST90026202 | Fried fish liking \|\| id:ebi-fl187-GCST90094765 | Inverse variance weighted | 4 | -0.175158921 | 0.215900494728774 | 0.417196470433605 | -0.59832389 | 0.248006049134304 | 0.839323624411109 | 0.549732275985509 | 1.2814676839407 | 0.998074519377826 |
| ebi-fl187-GCST90094765 | ebi-cfmet338-GCST90026222 | N-acetylputrescine levels \|\| id:ebi-cfmet338-GCST90026222 | Fried fish liking \|\| id:ebi-fl187-GCST90094765 | Inverse variance weighted | 4 | -0.018390344 | 0.0893531609898017 | 0.836934347166206 | -0.19352254 | 0.156741851567834 | 0.981777726535038 | 0.824051262272318 | 1.1696936203488 | 0.998074519377826 |
| ebi-fl187-GCST90094765 | ebi-cfmet338-GCST90026252 | Propionylcarnitine (c3) levels \|\| id:ebi-cfmet338-GCST90026252 | Fried fish liking \|\| id:ebi-fl187-GCST90094765 | Inverse variance weighted | 4 | 0.0369387615399227 | 0.128262821673291 | 0.773352295481848 | -0.214456369 | 0.288333892019573 | 1.03762947606124 | 0.806980020357707 | 1.33420270939777 | 0.998074519377826 |
| ebi-fl187-GCST90094765 | ebi-cfmet338-GCST90026316 | Ethyl beta-glucopyranoside levels \|\| id:ebi-cfmet338-GCST90026316 | Fried fish liking \|\| id:ebi-fl187-GCST90094765 | Inverse variance weighted | 4 | 0.309384553764035 | 0.501338525151237 | 0.537157520698228 | -0.673238956 | 1.29200806306046 | 1.36258625743974 | 0.510053857686576 | 3.64008874942091 | 0.998074519377826 |
| ebi-fl187-GCST90094765 | ebi-gm430-GCST90011315 | F_Prevotellaceae abundance \|\| id:ebi-gm430-GCST90011315 | Fried fish liking \|\| id:ebi-fl187-GCST90094765 | Inverse variance weighted | 3 | 0.0592489559142212 | 0.206339875803817 | 0.774003181264319 | -0.345177201 | 0.463675112489702 | 1.06103935988565 | 0.708094867471277 | 1.58990634580784 | 0.998074519377826 |
| ebi-fl187-GCST90094765 | ebi-gm430-GCST90011354 | OTU97_1 (Bacteroides) abundance \|\| id:ebi-gm430-GCST90011354 | Fried fish liking \|\| id:ebi-fl187-GCST90094765 | Inverse variance weighted | 3 | 0.043855569291478 | 0.188527919552639 | 0.81605565257093 | -0.325659153 | 0.413370291614651 | 1.0448314382453 | 0.722051254474779 | 1.51190476795143 | 0.998074519377826 |
| ebi-fl187-GCST90094765 | ebi-gm430-GCST90011419 | OTU99_1 (Bacteroides) abundance \|\| id:ebi-gm430-GCST90011419 | Fried fish liking \|\| id:ebi-fl187-GCST90094765 | Inverse variance weighted | 3 | 0.0534083814698293 | 0.18859337181195 | 0.777028655321068 | -0.316234627 | 0.423051390221251 | 1.05486034258611 | 0.728888412996489 | 1.52661274691746 | 0.998074519377826 |
| ebi-fl187-GCST90094765 | ebi-gm430-GCST90011435 | OTU99_155 (Butyrivibrio) abundance \|\| id:ebi-gm430-GCST90011435 | Fried fish liking \|\| id:ebi-fl187-GCST90094765 | Inverse variance weighted | 3 | -0.125921394 | 0.571571966263654 | 0.82563194767523 | -1.246202448 | 0.99435965975095 | 0.881684149612914 | 0.287594882241691 | 2.70299295181949 | 0.998074519377826 |
| ebi-fl187-GCST90094765 | ebi-gm430-GCST90011466 | OTU99_58 (Bacteroides) abundance \|\| id:ebi-gm430-GCST90011466 | Fried fish liking \|\| id:ebi-fl187-GCST90094765 | Inverse variance weighted | 3 | 0.615257689176127 | 0.349216600052833 | 0.0780992794685083 | -0.069206847 | 1.29972222527968 | 1.85013329746089 | 0.933133644287263 | 3.66827757130977 | 0.998074519377826 |
| ebi-fl187-GCST90094765 | ebi-gm430-GCST90011472 | OTU99_72 (Parabacteroides) abundance \|\| id:ebi-gm430-GCST90011472 | Fried fish liking \|\| id:ebi-fl187-GCST90094765 | Inverse variance weighted | 2 | 1.06080722451928 | 0.633293573205886 | 0.0939216881441885 | -0.180448179 | 2.30206262800281 | 2.88870187935147 | 0.834895944748532 | 9.99477671469836 | 0.998074519377826 |
| ebi-fl187-GCST90094765 | ebi-gm430-GCST90011505 | TestASV_28 (Holdemanella) abundance \|\| id:ebi-gm430-GCST90011505 | Fried fish liking \|\| id:ebi-fl187-GCST90094765 | Inverse variance weighted | 3 | 0.170681169432643 | 0.44720223597988 | 0.702710315354807 | -0.705835213 | 1.04719755195321 | 1.18611251980176 | 0.493696060600451 | 2.84965391038244 | 0.998074519377826 |
| ebi-fl187-GCST90094765 | ebi-gm430-GCST90011513 | TestASV_35 (Alphaproteobacteria) abundance \|\| id:ebi-gm430-GCST90011513 | Fried fish liking \|\| id:ebi-fl187-GCST90094765 | Inverse variance weighted | 3 | -0.102151706 | 0.515581542650188 | 0.842944059038335 | -1.11269153 | 0.908388117516921 | 0.902892566988741 | 0.328673135999847 | 2.4803213230177 | 0.998074519377826 |
| ebi-fl187-GCST90094765 | ebi-gm430-GCST90011543 | G_Coprococcus prevalence \|\| id:ebi-gm430-GCST90011543 | Fried fish liking \|\| id:ebi-fl187-GCST90094765 | Inverse variance weighted | 3 | -0.671187972 | 0.554807226309285 | 0.226367967871683 | -1.758610135 | 0.416234191925226 | 0.511101043445923 | 0.172284149170686 | 1.5162409186739 | 0.998074519377826 |
| ebi-fl187-GCST90094765 | ebi-gm430-GCST90011547 | G_Parasutterella prevalence \|\| id:ebi-gm430-GCST90011547 | Fried fish liking \|\| id:ebi-fl187-GCST90094765 | Inverse variance weighted | 3 | 0.0795828214986077 | 0.51757717089085 | 0.877798736862888 | -0.934868433 | 1.09403407644467 | 1.08283523785289 | 0.392637520098355 | 2.98629675544557 | 0.998074519377826 |
| ebi-fl187-GCST90094765 | ebi-gm430-GCST90011569 | OTU97_13 (Subdoligranulum) prevalence \|\| id:ebi-gm430-GCST90011569 | Fried fish liking \|\| id:ebi-fl187-GCST90094765 | Inverse variance weighted | 3 | -0.174127496 | 0.595744211037587 | 0.770068214754744 | -1.341786149 | 0.99353115806587 | 0.840189770357617 | 0.261378390586821 | 2.70075444503551 | 0.998074519377826 |
| ebi-fl187-GCST90094765 | ebi-gm430-GCST90011590 | OTU97_277 (Prevotella) prevalence \|\| id:ebi-gm430-GCST90011590 | Fried fish liking \|\| id:ebi-fl187-GCST90094765 | Inverse variance weighted | 3 | 0.332880579414391 | 0.642263615507995 | 0.604254026303186 | -0.925956107 | 1.59171726581006 | 1.3949806991103 | 0.396152473801897 | 4.91217720342544 | 0.998074519377826 |
| ebi-fl187-GCST90094765 | ebi-gm430-GCST90011616 | OTU97_86 (Phascolarctobacterium) prevalence \|\| id:ebi-gm430-GCST90011616 | Fried fish liking \|\| id:ebi-fl187-GCST90094765 | Inverse variance weighted | 3 | 0.582862889408392 | 0.716001950369007 | 0.415615106373456 | -0.820500933 | 1.98622671213165 | 1.79115898755297 | 0.440211082867791 | 7.28798216026586 | 0.998074519377826 |
| ebi-fl187-GCST90094765 | ebi-gm430-GCST90011665 | OTU99_55 (Barnesiella) prevalence \|\| id:ebi-gm430-GCST90011665 | Fried fish liking \|\| id:ebi-fl187-GCST90094765 | Inverse variance weighted | 3 | 0.779129987770893 | 0.365254469048026 | 0.0329152484448152 | 0.0632312284367618 | 1.49502874710502 | 2.17957518331177 | 1.06527313219632 | 4.45946474770666 | 0.998074519377826 |
| ebi-fl187-GCST90094765 | ebi-gm430-GCST90011687 | TestASV_14 (Lachnospiraceae) prevalence \|\| id:ebi-gm430-GCST90011687 | Fried fish liking \|\| id:ebi-fl187-GCST90094765 | Inverse variance weighted | 3 | 0.478274151794207 | 0.389893809127064 | 0.219943645630311 | -0.285917714 | 1.24246601768325 | 1.6132877084823 | 0.751324436814712 | 3.46414558452855 | 0.998074519377826 |
| ebi-fl187-GCST90094765 | ebi-gm430-GCST90011697 | TestASV_23 (Barnesiella) prevalence \|\| id:ebi-gm430-GCST90011697 | Fried fish liking \|\| id:ebi-fl187-GCST90094765 | Inverse variance weighted | 3 | 1.18428225948859 | 0.526852600578511 | 0.0245861630019571 | 0.151651162354713 | 2.21691335662248 | 3.26834015928206 | 1.16375420434026 | 9.17895493475933 | 0.998074519377826 |
| ebi-fl187-GCST90094765 | ebi-gm430-GCST90011727 | TestASV_6 (Subdoligranulum) prevalence \|\| id:ebi-gm430-GCST90011727 | Fried fish liking \|\| id:ebi-fl187-GCST90094765 | Inverse variance weighted | 3 | 0.584455595618675 | 0.631912235983187 | 0.355017952515382 | -0.654092387 | 1.82300357814572 | 1.79401405063086 | 0.51991372901633 | 6.19042397658987 | 0.998074519377826 |
| ebi-fl187-GCST90094765 | ebi-hbsm44-GCST90104908 | Sulcal depth of the left posterior inferior temporal sulcus \|\| id:ebi-hbsm44-GCST90104908 | Fried fish liking \|\| id:ebi-fl187-GCST90094765 | Inverse variance weighted | 3 | 0.0805200555554481 | 0.186157987604769 | 0.665351798681872 | -0.2843496 | 0.445389711260796 | 1.08385058364975 | 0.752503523370676 | 1.56109845494936 | 0.998074519377826 |
| ebi-fl187-GCST90094765 | ebi-hbsm44-GCST90104918 | Sulcal depth of the left inferior frontal sulcus \|\| id:ebi-hbsm44-GCST90104918 | Fried fish liking \|\| id:ebi-fl187-GCST90094765 | Inverse variance weighted | 3 | 0.268503321416772 | 0.3077273436822 | 0.382915796921512 | -0.334642272 | 0.871648915033884 | 1.30800532145417 | 0.715594028448276 | 2.39084991341023 | 0.998074519377826 |
| ebi-fl187-GCST90094765 | ebi-hbsm44-GCST90104925 | Sulcal depth of the right anterior inferior temporal sulcus \|\| id:ebi-hbsm44-GCST90104925 | Fried fish liking \|\| id:ebi-fl187-GCST90094765 | Inverse variance weighted | 3 | 0.120927012322825 | 0.116531333668841 | 0.29939996439174 | -0.107474402 | 0.349328426313753 | 1.12854253966272 | 0.898099512005939 | 1.41811486010468 | 0.998074519377826 |
| ebi-fl187-GCST90094765 | ebi-IPs91-GCST90274770 | C-C motif chemokine 4 levels \|\| id:ebi-IPs91-GCST90274770 | Fried fish liking \|\| id:ebi-fl187-GCST90094765 | Inverse variance weighted | 5 | -0.025291298 | 0.105714046612489 | 0.810917508472265 | -0.23249083 | 0.181908233024161 | 0.975025847249371 | 0.792557017301607 | 1.19950411396405 | 0.998074519377826 |
| ebi-fl187-GCST90094765 | ebi-IPs91-GCST90274779 | C-X-C motif chemokine 1 levels \|\| id:ebi-IPs91-GCST90274779 | Fried fish liking \|\| id:ebi-fl187-GCST90094765 | Inverse variance weighted | 5 | -0.126728063 | 0.105067980198134 | 0.227758488772194 | -0.332661304 | 0.0792051779825639 | 0.880973209056559 | 0.717013002187571 | 1.08242638935072 | 0.998074519377826 |
| ebi-fl187-GCST90094765 | ebi-IPs91-GCST90274780 | C-X-C motif chemokine 10 levels \|\| id:ebi-IPs91-GCST90274780 | Fried fish liking \|\| id:ebi-fl187-GCST90094765 | Inverse variance weighted | 5 | 0.00227539310690074 | 0.142483227697682 | 0.987258683739735 | -0.276991733 | 0.281542519394358 | 1.00227798377836 | 0.758060763836067 | 1.32517234064899 | 0.999355215041207 |
| ebi-fl187-GCST90094765 | ebi-IPs91-GCST90274796 | Interleukin-10 receptor subunit alpha levels \|\| id:ebi-IPs91-GCST90274796 | Fried fish liking \|\| id:ebi-fl187-GCST90094765 | Inverse variance weighted (multiplicative random effects) | 5 | -0.056224513 | 0.182985971742062 | 0.758644467811993 | -0.414877017 | 0.302427991740109 | 0.945326874009857 | 0.660421496004895 | 1.35314023563919 | 0.998074519377826 |
| ebi-fl187-GCST90094765 | ebi-IPs91-GCST90274807 | Interleukin-20 levels \|\| id:ebi-IPs91-GCST90274807 | Fried fish liking \|\| id:ebi-fl187-GCST90094765 | Inverse variance weighted | 5 | -0.054976168 | 0.116854461972805 | 0.638021407764787 | -0.284010913 | 0.174058577637465 | 0.94650770501795 | 0.752758429585864 | 1.19012527850564 | 0.998074519377826 |
| ebi-fl187-GCST90094765 | ebi-IPs91-GCST90274808 | Interleukin-20 receptor subunit alpha levels \|\| id:ebi-IPs91-GCST90274808 | Fried fish liking \|\| id:ebi-fl187-GCST90094765 | Inverse variance weighted | 5 | 0.0107621893029996 | 0.127619375374615 | 0.932793765641628 | -0.239371786 | 0.260896165037246 | 1.01082030997707 | 0.787122186617187 | 1.29809287101071 | 0.998074519377826 |
| ebi-fl187-GCST90094765 | ebi-IPs91-GCST90274810 | Interleukin-24 levels \|\| id:ebi-IPs91-GCST90274810 | Fried fish liking \|\| id:ebi-fl187-GCST90094765 | Inverse variance weighted | 5 | -0.017228701 | 0.119623360715892 | 0.885481084029278 | -0.251690488 | 0.217233086364823 | 0.982918864755872 | 0.777485342154025 | 1.24263370935883 | 0.998074519377826 |
| ebi-fl187-GCST90094765 | ebi-IPs91-GCST90274823 | Monocyte chemoattractant protein-3 levels \|\| id:ebi-IPs91-GCST90274823 | Fried fish liking \|\| id:ebi-fl187-GCST90094765 | Inverse variance weighted | 5 | -0.032217651 | 0.119022813629209 | 0.786633570665275 | -0.265502365 | 0.201067064040318 | 0.968295808908163 | 0.766820629056565 | 1.22270676872981 | 0.998074519377826 |
| ebi-fl187-GCST90094765 | ebi-IPs91-GCST90274824 | Monocyte chemoattractant protein-4 levels \|\| id:ebi-IPs91-GCST90274824 | Fried fish liking \|\| id:ebi-fl187-GCST90094765 | Inverse variance weighted | 5 | -0.108332354 | 0.104830796229103 | 0.301415811932737 | -0.313800715 | 0.0971360063528228 | 0.897329315648451 | 0.730664624249467 | 1.10201024382317 | 0.998074519377826 |
| ebi-fl187-GCST90094765 | ebi-IPs91-GCST90274834 | SIR2-like protein 2 levels \|\| id:ebi-IPs91-GCST90274834 | Fried fish liking \|\| id:ebi-fl187-GCST90094765 | Inverse variance weighted | 5 | -0.026515639 | 0.105067980198134 | 0.800757790087868 | -0.23244888 | 0.179417602584678 | 0.973832814331033 | 0.792590265690404 | 1.19652030982467 | 0.998074519377826 |
| ebi-fl187-GCST90094765 | ebi-met1400-GCST90199652 | 1-stearoyl-2-oleoyl-GPS (18:0/18:1) levels \|\| id:ebi-met1400-GCST90199652 | Fried fish liking \|\| id:ebi-fl187-GCST90094765 | Inverse variance weighted | 4 | -0.119747856 | 0.175349592934478 | 0.494664198642499 | -0.463433058 | 0.223937345948599 | 0.887144096399744 | 0.629120127779844 | 1.25099263721591 | 0.998074519377826 |
| ebi-fl187-GCST90094765 | ebi-met1400-GCST90199665 | N-acetylaspartate (naa) levels \|\| id:ebi-met1400-GCST90199665 | Fried fish liking \|\| id:ebi-fl187-GCST90094765 | Inverse variance weighted | 4 | 0.0459063104234265 | 0.155333398869696 | 0.767585935522281 | -0.258547151 | 0.350359772208031 | 1.04697631559406 | 0.772172621200651 | 1.41957818150882 | 0.998074519377826 |
| ebi-fl187-GCST90094765 | ebi-met1400-GCST90199669 | Acetylcarnitine levels (Biocrates platform) \|\| id:ebi-met1400-GCST90199669 | Fried fish liking \|\| id:ebi-fl187-GCST90094765 | Inverse variance weighted | 4 | -0.038740402 | 0.145100670346771 | 0.789476997326903 | -0.323137716 | 0.245656911679508 | 0.962000409923935 | 0.723874158391815 | 1.27846087329574 | 0.998074519377826 |
| ebi-fl187-GCST90094765 | ebi-met1400-GCST90199670 | Iminodiacetate (IDA) levels \|\| id:ebi-met1400-GCST90199670 | Fried fish liking \|\| id:ebi-fl187-GCST90094765 | Inverse variance weighted | 4 | 0.281543151010723 | 0.153136883516326 | 0.0659878094792552 | -0.018605141 | 0.581691442702722 | 1.3251731776498 | 0.981566866557136 | 1.78906196877035 | 0.998074519377826 |
| ebi-fl187-GCST90094765 | ebi-met1400-GCST90199686 | Indolepropionate levels \|\| id:ebi-met1400-GCST90199686 | Fried fish liking \|\| id:ebi-fl187-GCST90094765 | Inverse variance weighted | 4 | -0.052188675 | 0.150658252278984 | 0.729038849226279 | -0.34747885 | 0.243101499070185 | 0.949149768738646 | 0.706466955644146 | 1.27519804896637 | 0.998074519377826 |
| ebi-fl187-GCST90094765 | ebi-met1400-GCST90199704 | 3-hydroxysebacate levels \|\| id:ebi-met1400-GCST90199704 | Fried fish liking \|\| id:ebi-fl187-GCST90094765 | Inverse variance weighted | 4 | -0.060752007 | 0.183305011414682 | 0.740322891366124 | -0.42002983 | 0.298525815204177 | 0.941056586128003 | 0.657027220702076 | 1.3478703322955 | 0.998074519377826 |
| ebi-fl187-GCST90094765 | ebi-met1400-GCST90199713 | Docosapentaenoate n3 DPA; 22:5n3 levels \|\| id:ebi-met1400-GCST90199713 | Fried fish liking \|\| id:ebi-fl187-GCST90094765 | Inverse variance weighted | 4 | -0.126052454 | 0.159504404494418 | 0.429366779501862 | -0.438681086 | 0.186576179129887 | 0.8815686040539 | 0.644886409828584 | 1.20511642330952 | 0.998074519377826 |
| ebi-fl187-GCST90094765 | ebi-met1400-GCST90199715 | Myristoleate (14:1n5) levels \|\| id:ebi-met1400-GCST90199715 | Fried fish liking \|\| id:ebi-fl187-GCST90094765 | Inverse variance weighted | 4 | 0.0379417216965092 | 0.137219877376268 | 0.782161677738038 | -0.231009238 | 0.306892681353994 | 1.03867069914838 | 0.793732133534899 | 1.35919509326751 | 0.998074519377826 |
| ebi-fl187-GCST90094765 | ebi-met1400-GCST90199725 | Campesterol levels \|\| id:ebi-met1400-GCST90199725 | Fried fish liking \|\| id:ebi-fl187-GCST90094765 | Inverse variance weighted | 4 | 0.397988393396131 | 0.187934682424011 | 0.0342008812956974 | 0.0296364158450707 | 0.766340370947192 | 1.48882674958468 | 1.03007994511401 | 2.15187675557897 | 0.998074519377826 |
| ebi-fl187-GCST90094765 | ebi-met1400-GCST90199737 | N-acetylproline levels \|\| id:ebi-met1400-GCST90199737 | Fried fish liking \|\| id:ebi-fl187-GCST90094765 | Inverse variance weighted | 4 | -0.060161626 | 0.200061088250862 | 0.763630853232453 | -0.452281359 | 0.33195810668627 | 0.941612331981166 | 0.636175150774134 | 1.39369446081021 | 0.998074519377826 |
| ebi-fl187-GCST90094765 | ebi-met1400-GCST90199775 | Gamma-glutamylisoleucine levels \|\| id:ebi-met1400-GCST90199775 | Fried fish liking \|\| id:ebi-fl187-GCST90094765 | Inverse variance weighted | 4 | 0.175935752752247 | 0.143823210391897 | 0.221224756707999 | -0.10595774 | 0.457829245120364 | 1.19236145024825 | 0.899462658910218 | 1.58063907818104 | 0.998074519377826 |
| ebi-fl187-GCST90094765 | ebi-met1400-GCST90199777 | 1-palmitoleoyl-GPC (16:1) levels \|\| id:ebi-met1400-GCST90199777 | Fried fish liking \|\| id:ebi-fl187-GCST90094765 | Inverse variance weighted | 4 | -0.079415718 | 0.176714689270715 | 0.653142571589719 | -0.425776509 | 0.266945072472679 | 0.923655863791766 | 0.653262323751241 | 1.3059687107285 | 0.998074519377826 |
| ebi-fl187-GCST90094765 | ebi-met1400-GCST90199782 | Glutamine degradant levels \|\| id:ebi-met1400-GCST90199782 | Fried fish liking \|\| id:ebi-fl187-GCST90094765 | Inverse variance weighted | 4 | -0.200550851 | 0.155689817623846 | 0.197695961306839 | -0.505702893 | 0.104601191782468 | 0.818279878813809 | 0.6030815244485 | 1.1102677381534 | 0.998074519377826 |
| ebi-fl187-GCST90094765 | ebi-met1400-GCST90199783 | 5-methyluridine (ribothymidine) levels \|\| id:ebi-met1400-GCST90199783 | Fried fish liking \|\| id:ebi-fl187-GCST90094765 | Inverse variance weighted | 4 | -0.143644652 | 0.164055829252209 | 0.381256250863838 | -0.465194077 | 0.177904773436185 | 0.866195494302181 | 0.628013210295701 | 1.19471154754233 | 0.998074519377826 |
| ebi-fl187-GCST90094765 | ebi-met1400-GCST90199787 | Homostachydrine levels \|\| id:ebi-met1400-GCST90199787 | Fried fish liking \|\| id:ebi-fl187-GCST90094765 | Inverse variance weighted | 4 | -0.339670421 | 0.199728441810036 | 0.0890066158872082 | -0.731138167 | 0.0517973248453932 | 0.712004945902312 | 0.481360809176513 | 1.0531622710553 | 0.998074519377826 |
| ebi-fl187-GCST90094765 | ebi-met1400-GCST90199802 | Beta-hydroxyisovaleroylcarnitine levels \|\| id:ebi-met1400-GCST90199802 | Fried fish liking \|\| id:ebi-fl187-GCST90094765 | Inverse variance weighted | 4 | 0.331569404853985 | 0.224272430541579 | 0.139294729786389 | -0.108004559 | 0.77114336871548 | 1.39315283449204 | 0.89762350414881 | 2.16223707521303 | 0.998074519377826 |
| ebi-fl187-GCST90094765 | ebi-met1400-GCST90199873 | Pregnenolone sulfate levels \|\| id:ebi-met1400-GCST90199873 | Fried fish liking \|\| id:ebi-fl187-GCST90094765 | Inverse variance weighted | 4 | -0.282355645 | 0.220031859331423 | 0.199404883201813 | -0.713618089 | 0.148906799170811 | 0.754005478474877 | 0.489868598871638 | 1.16056481856495 | 0.998074519377826 |
| ebi-fl187-GCST90094765 | ebi-met1400-GCST90199911 | Beta-citrylglutamate levels \|\| id:ebi-met1400-GCST90199911 | Fried fish liking \|\| id:ebi-fl187-GCST90094765 | Inverse variance weighted | 4 | 0.215886890024855 | 0.148117745892323 | 0.144968587354221 | -0.074423892 | 0.506197671973808 | 1.24096200588038 | 0.928278120832234 | 1.65897123445935 | 0.998074519377826 |
| ebi-fl187-GCST90094765 | ebi-met1400-GCST90199934 | 2-hydroxydecanoate levels \|\| id:ebi-met1400-GCST90199934 | Fried fish liking \|\| id:ebi-fl187-GCST90094765 | Inverse variance weighted | 4 | -0.065695783 | 0.148610698516708 | 0.658441223367015 | -0.356972752 | 0.225581186121696 | 0.936415694564774 | 0.69979156535076 | 1.25305076031848 | 0.998074519377826 |
| ebi-fl187-GCST90094765 | ebi-met1400-GCST90199945 | Sphingomyelin (d18:1/14:0, d16:1/16:0) levels \|\| id:ebi-met1400-GCST90199945 | Fried fish liking \|\| id:ebi-fl187-GCST90094765 | Inverse variance weighted | 4 | -0.006444404 | 0.223064161648714 | 0.976952034402866 | -0.443650161 | 0.43076135300548 | 0.993576316809728 | 0.641689869926665 | 1.53842836483924 | 0.998074519377826 |
| ebi-fl187-GCST90094765 | ebi-met1400-GCST90199979 | Carnitine C14:1 levels \|\| id:ebi-met1400-GCST90199979 | Fried fish liking \|\| id:ebi-fl187-GCST90094765 | Inverse variance weighted | 4 | 0.032516271988047 | 0.222602885393904 | 0.883863758128797 | -0.403785383 | 0.468817927360098 | 1.0330507027959 | 0.667787424167967 | 1.59810400124976 | 0.998074519377826 |
| ebi-fl187-GCST90094765 | ebi-met1400-GCST90199986 | Eugenol sulfate levels \|\| id:ebi-met1400-GCST90199986 | Fried fish liking \|\| id:ebi-fl187-GCST90094765 | Inverse variance weighted | 4 | -0.016931804 | 0.17947363342127 | 0.924837919382448 | -0.368700125 | 0.334836517561239 | 0.983210733442447 | 0.691632782425265 | 1.39771186519906 | 0.998074519377826 |
| ebi-fl187-GCST90094765 | ebi-met1400-GCST90200002 | 6-hydroxyindole sulfate levels \|\| id:ebi-met1400-GCST90200002 | Fried fish liking \|\| id:ebi-fl187-GCST90094765 | Inverse variance weighted (multiplicative random effects) | 4 | 0.195094472186591 | 0.306286187663805 | 0.524145747651138 | -0.405226456 | 0.795415400007648 | 1.2154258049995 | 0.66682578730231 | 2.21536106669637 | 0.998074519377826 |
| ebi-fl187-GCST90094765 | ebi-met1400-GCST90200023 | Trans 3,4-methyleneheptanoate levels \|\| id:ebi-met1400-GCST90200023 | Fried fish liking \|\| id:ebi-fl187-GCST90094765 | Inverse variance weighted | 4 | -0.153918723 | 0.160471052262159 | 0.337474040821765 | -0.468441985 | 0.160604539481087 | 0.857341700381648 | 0.625976789912448 | 1.17422051912835 | 0.998074519377826 |
| ebi-fl187-GCST90094765 | ebi-met1400-GCST90200032 | 2-hydroxybutyrate/2-hydroxyisobutyrate levels \|\| id:ebi-met1400-GCST90200032 | Fried fish liking \|\| id:ebi-fl187-GCST90094765 | Inverse variance weighted | 4 | 0.0633040690024165 | 0.150559819266499 | 0.674150961001589 | -0.231793177 | 0.358401314764754 | 1.06535073011995 | 0.793110139953757 | 1.43103980266006 | 0.998074519377826 |
| ebi-fl187-GCST90094765 | ebi-met1400-GCST90200051 | 1-palmitoyl-2-dihomo-linolenoyl-GPC (16:0/20:3n3 or 6) levels \|\| id:ebi-met1400-GCST90200051 | Fried fish liking \|\| id:ebi-fl187-GCST90094765 | Inverse variance weighted | 4 | 0.186703126671844 | 0.146651560952205 | 0.202980080502578 | -0.100733933 | 0.474140186138166 | 1.20526941958828 | 0.904173571820244 | 1.60663219880471 | 0.998074519377826 |
| ebi-fl187-GCST90094765 | ebi-met1400-GCST90200069 | Phenylacetylglutamate levels \|\| id:ebi-met1400-GCST90200069 | Fried fish liking \|\| id:ebi-fl187-GCST90094765 | Inverse variance weighted | 4 | -0.074464829 | 0.164239186950507 | 0.650266077842126 | -0.396373635 | 0.247443977570515 | 0.928240120755092 | 0.672755283866291 | 1.28074760978121 | 0.998074519377826 |
| ebi-fl187-GCST90094765 | ebi-met1400-GCST90200072 | Thioproline levels \|\| id:ebi-met1400-GCST90200072 | Fried fish liking \|\| id:ebi-fl187-GCST90094765 | Inverse variance weighted | 4 | 0.0508144358444087 | 0.148802968406221 | 0.732736197860834 | -0.240839382 | 0.342468253920601 | 1.05212763799085 | 0.785967856653688 | 1.40841964115075 | 0.998074519377826 |
| ebi-fl187-GCST90094765 | ebi-met1400-GCST90200081 | 1-oleoyl-2-docosahexaenoyl-GPC (18:1/22:6) levels \|\| id:ebi-met1400-GCST90200081 | Fried fish liking \|\| id:ebi-fl187-GCST90094765 | Inverse variance weighted | 4 | 0.0344389156847705 | 0.184848742316855 | 0.852202741264019 | -0.327864619 | 0.396742450625806 | 1.03503880180861 | 0.720460549588354 | 1.48697291178749 | 0.998074519377826 |
| ebi-fl187-GCST90094765 | ebi-met1400-GCST90200097 | Furaneol sulfate levels \|\| id:ebi-met1400-GCST90200097 | Fried fish liking \|\| id:ebi-fl187-GCST90094765 | Inverse variance weighted | 4 | -0.049501852 | 0.228076808044841 | 0.828176961112179 | -0.496532396 | 0.397528691528935 | 0.951703395352728 | 0.608637518615276 | 1.48814249043762 | 0.998074519377826 |
| ebi-fl187-GCST90094765 | ebi-met1400-GCST90200124 | Sphingomyelin (d18:2/24:2) levels \|\| id:ebi-met1400-GCST90200124 | Fried fish liking \|\| id:ebi-fl187-GCST90094765 | Inverse variance weighted | 4 | -0.186720174 | 0.136084682907588 | 0.170036013207061 | -0.453446153 | 0.0800058043464304 | 0.829675868658729 | 0.635434569556742 | 1.08329335546663 | 0.998074519377826 |
| ebi-fl187-GCST90094765 | ebi-met1400-GCST90200137 | Lignoceroylcarnitine (C24) levels \|\| id:ebi-met1400-GCST90200137 | Fried fish liking \|\| id:ebi-fl187-GCST90094765 | Inverse variance weighted | 4 | -0.081486175 | 0.149993670265056 | 0.586947983822418 | -0.375473769 | 0.212501418653717 | 0.921745452836043 | 0.686963739705426 | 1.23676786810937 | 0.998074519377826 |
| ebi-fl187-GCST90094765 | ebi-met1400-GCST90200165 | Octadecenedioate (C18:1-DC) levels \|\| id:ebi-met1400-GCST90200165 | Fried fish liking \|\| id:ebi-fl187-GCST90094765 | Inverse variance weighted | 4 | -0.186415362 | 0.146300061176651 | 0.202592970612525 | -0.473163482 | 0.100332757605242 | 0.829928802242977 | 0.623028208824799 | 1.10553873329699 | 0.998074519377826 |
| ebi-fl187-GCST90094765 | ebi-met1400-GCST90200176 | Glutamine conjugate of C6H10O2 (1) levels \|\| id:ebi-met1400-GCST90200176 | Fried fish liking \|\| id:ebi-fl187-GCST90094765 | Inverse variance weighted (multiplicative random effects) | 4 | -0.173209574 | 0.269398160161665 | 0.520256479531767 | -0.701229968 | 0.354810820020663 | 0.840961352828043 | 0.495974895319645 | 1.425910875 | 0.998074519377826 |
| ebi-fl187-GCST90094765 | ebi-met1400-GCST90200182 | Glycine conjugate of C10H12O2 levels \|\| id:ebi-met1400-GCST90200182 | Fried fish liking \|\| id:ebi-fl187-GCST90094765 | Inverse variance weighted | 4 | -0.11827378 | 0.171822108166554 | 0.491232285742307 | -0.455045112 | 0.218497552056589 | 0.888452778757167 | 0.634419347404791 | 1.24420597087763 | 0.998074519377826 |
| ebi-fl187-GCST90094765 | ebi-met1400-GCST90200208 | Glucuronide of piperine metabolite C17H21NO3 (4) levels \|\| id:ebi-met1400-GCST90200208 | Fried fish liking \|\| id:ebi-fl187-GCST90094765 | Inverse variance weighted | 4 | 0.168318761674918 | 0.152881131995662 | 0.270906235837682 | -0.131328257 | 0.467965780386416 | 1.18331374560803 | 0.876929868740099 | 1.59674276183186 | 0.998074519377826 |
| ebi-fl187-GCST90094765 | ebi-met1400-GCST90200227 | 3-ethylcatechol sulfate (2) levels \|\| id:ebi-met1400-GCST90200227 | Fried fish liking \|\| id:ebi-fl187-GCST90094765 | Inverse variance weighted | 4 | -0.002052975 | 0.194873033617648 | 0.991594494334173 | -0.38400412 | 0.379898171353051 | 0.997949131373313 | 0.681128620632237 | 1.46213569455404 | 0.999355215041207 |
| ebi-fl187-GCST90094765 | ebi-met1400-GCST90200259 | Pentose acid levels \|\| id:ebi-met1400-GCST90200259 | Fried fish liking \|\| id:ebi-fl187-GCST90094765 | Inverse variance weighted | 4 | -0.099637922 | 0.147123140040154 | 0.498252628487439 | -0.387999277 | 0.188723432352223 | 0.905165098963591 | 0.678412834024256 | 1.20770689363532 | 0.998074519377826 |
| ebi-fl187-GCST90094765 | ebi-met1400-GCST90200291 | Sarcosine levels \|\| id:ebi-met1400-GCST90200291 | Fried fish liking \|\| id:ebi-fl187-GCST90094765 | Inverse variance weighted | 4 | 0.325450510731962 | 0.149506024111252 | 0.0294925986732023 | 0.0324187034739071 | 0.618482317990016 | 1.3846543071126 | 1.03294991449076 | 1.85610892000575 | 0.998074519377826 |
| ebi-fl187-GCST90094765 | ebi-met1400-GCST90200306 | Butyrate/isobutyrate (4:0) levels \|\| id:ebi-met1400-GCST90200306 | Fried fish liking \|\| id:ebi-fl187-GCST90094765 | Inverse variance weighted | 4 | 0.127391223014647 | 0.151090875057455 | 0.399148437848815 | -0.168746892 | 0.423529338127258 | 1.13586130595228 | 0.844722682316249 | 1.5273425626762 | 0.998074519377826 |
| ebi-fl187-GCST90094765 | ebi-met1400-GCST90200321 | Cholate levels \|\| id:ebi-met1400-GCST90200321 | Fried fish liking \|\| id:ebi-fl187-GCST90094765 | Inverse variance weighted | 4 | 0.0661731788470999 | 0.154014585383604 | 0.667446423011183 | -0.235695409 | 0.368041766198965 | 1.06841172745679 | 0.790021271049241 | 1.44490238579417 | 0.998074519377826 |
| ebi-fl187-GCST90094765 | ebi-met1400-GCST90200336 | Ceramide (d18:1/16:0) levels \|\| id:ebi-met1400-GCST90200336 | Fried fish liking \|\| id:ebi-fl187-GCST90094765 | Inverse variance weighted | 4 | -0.117161912 | 0.180273949135906 | 0.515750109463814 | -0.470498852 | 0.236175028570595 | 0.889441170540453 | 0.624690562378843 | 1.26639594624227 | 0.998074519377826 |
| ebi-fl187-GCST90094765 | ebi-met1400-GCST90200358 | Arachidonate (20:4n6) levels \|\| id:ebi-met1400-GCST90200358 | Fried fish liking \|\| id:ebi-fl187-GCST90094765 | Inverse variance weighted | 4 | -0.173633459 | 0.146865097415949 | 0.237100583343552 | -0.46148905 | 0.114222132024523 | 0.84060495745344 | 0.630344332207524 | 1.12100110747512 | 0.998074519377826 |
| ebi-fl187-GCST90094765 | ebi-met1400-GCST90200413 | 2'-deoxyuridine levels \|\| id:ebi-met1400-GCST90200413 | Fried fish liking \|\| id:ebi-fl187-GCST90094765 | Inverse variance weighted | 4 | -0.058245198 | 0.149036646422594 | 0.695936759177929 | -0.350357025 | 0.233866628997574 | 0.943418594730522 | 0.704436543375121 | 1.26347596991339 | 0.998074519377826 |
| ebi-fl187-GCST90094765 | ebi-met1400-GCST90200432 | Threonine levels \|\| id:ebi-met1400-GCST90200432 | Fried fish liking \|\| id:ebi-fl187-GCST90094765 | Inverse variance weighted | 4 | 0.0668027133590113 | 0.149323079103273 | 0.654607703382065 | -0.225870522 | 0.359475948401427 | 1.06908454126969 | 0.797821395549611 | 1.43257847277267 | 0.998074519377826 |
| ebi-fl187-GCST90094765 | ebi-met1400-GCST90200436 | Caffeine levels \|\| id:ebi-met1400-GCST90200436 | Fried fish liking \|\| id:ebi-fl187-GCST90094765 | Inverse variance weighted | 4 | 0.202469435004248 | 0.152716679977284 | 0.184911028256533 | -0.096855258 | 0.501794127759725 | 1.22442266007657 | 0.907687377337739 | 1.65168194241742 | 0.998074519377826 |
| ebi-fl187-GCST90094765 | ebi-met1400-GCST90200442 | Valine levels \|\| id:ebi-met1400-GCST90200442 | Fried fish liking \|\| id:ebi-fl187-GCST90094765 | Inverse variance weighted | 4 | 0.311906590128186 | 0.146438718332323 | 0.03317605615432 | 0.0248867021968333 | 0.598926478059539 | 1.36602708665467 | 1.02519896115414 | 1.82016376545437 | 0.998074519377826 |
| ebi-fl187-GCST90094765 | ebi-met1400-GCST90200459 | Margarate (17:0) levels \|\| id:ebi-met1400-GCST90200459 | Fried fish liking \|\| id:ebi-fl187-GCST90094765 | Inverse variance weighted | 4 | 0.00483982245550176 | 0.146414555449812 | 0.97363024140931 | -0.282132706 | 0.291812351137133 | 1.00485155331366 | 0.754173594360387 | 1.33885176005561 | 0.998074519377826 |
| ebi-fl187-GCST90094765 | ebi-met1400-GCST90200462 | X-11299 levels \|\| id:ebi-met1400-GCST90200462 | Fried fish liking \|\| id:ebi-fl187-GCST90094765 | Inverse variance weighted | 4 | -0.179193269 | 0.179634476558264 | 0.318500595521394 | -0.531276843 | 0.172890304915847 | 0.835944321544799 | 0.587853893035313 | 1.18873569946915 | 0.998074519377826 |
| ebi-fl187-GCST90094765 | ebi-met1400-GCST90200497 | X-12740 levels \|\| id:ebi-met1400-GCST90200497 | Fried fish liking \|\| id:ebi-fl187-GCST90094765 | Inverse variance weighted | 4 | 0.12321898643405 | 0.183954750212552 | 0.502964170357628 | -0.237332324 | 0.483770296850652 | 1.13113209641195 | 0.788729130853547 | 1.62217898323178 | 0.998074519377826 |
| ebi-fl187-GCST90094765 | ebi-met1400-GCST90200527 | X-13866 levels \|\| id:ebi-met1400-GCST90200527 | Fried fish liking \|\| id:ebi-fl187-GCST90094765 | Inverse variance weighted | 4 | -0.133251369 | 0.146250419613605 | 0.362233279758168 | -0.419902191 | 0.153399453858409 | 0.875245055344326 | 0.65711108803272 | 1.16579056548586 | 0.998074519377826 |
| ebi-fl187-GCST90094765 | ebi-met1400-GCST90200547 | X-17654 levels \|\| id:ebi-met1400-GCST90200547 | Fried fish liking \|\| id:ebi-fl187-GCST90094765 | Inverse variance weighted (multiplicative random effects) | 4 | -0.216878296 | 0.331990233560204 | 0.513584101077769 | -0.867579154 | 0.43382256195592 | 0.805027938606715 | 0.419966995220823 | 1.5431450311866 | 0.998074519377826 |
| ebi-fl187-GCST90094765 | ebi-met1400-GCST90200553 | X-17653 levels \|\| id:ebi-met1400-GCST90200553 | Fried fish liking \|\| id:ebi-fl187-GCST90094765 | Inverse variance weighted | 3 | 0.326461342724838 | 0.189323223246103 | 0.0846429220300624 | -0.044612175 | 0.697534860287199 | 1.38605466763067 | 0.956368313616119 | 2.00879463937562 | 0.998074519377826 |
| ebi-fl187-GCST90094765 | ebi-met1400-GCST90200557 | X-17685 levels \|\| id:ebi-met1400-GCST90200557 | Fried fish liking \|\| id:ebi-fl187-GCST90094765 | Inverse variance weighted | 4 | 0.0554050195737242 | 0.158570678482292 | 0.726787023777524 | -0.25539351 | 0.366203549399016 | 1.05696862097449 | 0.774611620365188 | 1.4422487816514 | 0.998074519377826 |
| ebi-fl187-GCST90094765 | ebi-met1400-GCST90200561 | X-18779 levels \|\| id:ebi-met1400-GCST90200561 | Fried fish liking \|\| id:ebi-fl187-GCST90094765 | Inverse variance weighted | 4 | 0.0316031640077287 | 0.148829274959944 | 0.831837829993783 | -0.260102215 | 0.32330854292922 | 1.03210784648542 | 0.77097277686001 | 1.38169159631715 | 0.998074519377826 |
| ebi-fl187-GCST90094765 | ebi-met1400-GCST90200570 | X-18921 levels \|\| id:ebi-met1400-GCST90200570 | Fried fish liking \|\| id:ebi-fl187-GCST90094765 | Inverse variance weighted | 4 | -0.106918871 | 0.207666103652314 | 0.606651186354024 | -0.513944435 | 0.300106691721123 | 0.898598572043662 | 0.598131628565027 | 1.35000283401853 | 0.998074519377826 |
| ebi-fl187-GCST90094765 | ebi-met1400-GCST90200575 | X-21283 levels \|\| id:ebi-met1400-GCST90200575 | Fried fish liking \|\| id:ebi-fl187-GCST90094765 | Inverse variance weighted | 4 | -0.047348115 | 0.15761392251709 | 0.763867746354139 | -0.356271403 | 0.261575173602636 | 0.953755323706785 | 0.700282535895677 | 1.29897458650111 | 0.998074519377826 |
| ebi-fl187-GCST90094765 | ebi-met1400-GCST90200578 | X-18922 levels \|\| id:ebi-met1400-GCST90200578 | Fried fish liking \|\| id:ebi-fl187-GCST90094765 | Inverse variance weighted | 4 | -0.089135411 | 0.209797150197754 | 0.67093528302748 | -0.500337825 | 0.322067003611853 | 0.914721702012609 | 0.606325792999957 | 1.37997723631873 | 0.998074519377826 |
| ebi-fl187-GCST90094765 | ebi-met1400-GCST90200623 | X-24241 levels \|\| id:ebi-met1400-GCST90200623 | Fried fish liking \|\| id:ebi-fl187-GCST90094765 | Inverse variance weighted | 4 | 0.0457684635831333 | 0.221665594138973 | 0.836419679580202 | -0.388696101 | 0.480233028095521 | 1.04683200316383 | 0.677940264127966 | 1.61645103681458 | 0.998074519377826 |
| ebi-fl187-GCST90094765 | ebi-met1400-GCST90200636 | X-24544 levels \|\| id:ebi-met1400-GCST90200636 | Fried fish liking \|\| id:ebi-fl187-GCST90094765 | Inverse variance weighted | 4 | -0.192062292 | 0.14017072705431 | 0.170623673804793 | -0.466796917 | 0.0826723334615659 | 0.825255460436497 | 0.627007412286009 | 1.08618584347707 | 0.998074519377826 |
| ebi-fl187-GCST90094765 | ebi-met1400-GCST90200659 | X-25172 levels \|\| id:ebi-met1400-GCST90200659 | Fried fish liking \|\| id:ebi-fl187-GCST90094765 | Inverse variance weighted | 4 | 0.0353149170792526 | 0.151549931806603 | 0.815742022498039 | -0.261722949 | 0.332352783420194 | 1.03594589449156 | 0.769724246840282 | 1.39424462815007 | 0.998074519377826 |
| ebi-fl187-GCST90094765 | ebi-met1400-GCST90200670 | X-26111 levels \|\| id:ebi-met1400-GCST90200670 | Fried fish liking \|\| id:ebi-fl187-GCST90094765 | Inverse variance weighted | 4 | -0.167474901 | 0.150499817825699 | 0.265798035951252 | -0.462454544 | 0.127504742116035 | 0.845797845852835 | 0.629736032292794 | 1.13599025522599 | 0.998074519377826 |
| ebi-fl187-GCST90094765 | ebi-met1400-GCST90200673 | Carnitine C4 levels \|\| id:ebi-met1400-GCST90200673 | Fried fish liking \|\| id:ebi-fl187-GCST90094765 | Inverse variance weighted | 4 | 0.120157875733672 | 0.149345569782108 | 0.421072070457248 | -0.172559441 | 0.412875192506603 | 1.12767487002406 | 0.841508267199172 | 1.5111564105202 | 0.998074519377826 |
| ebi-fl187-GCST90094765 | ebi-met1400-GCST90200710 | X-13431 levels \|\| id:ebi-met1400-GCST90200710 | Fried fish liking \|\| id:ebi-fl187-GCST90094765 | Inverse variance weighted | 4 | -0.001201263 | 0.147441689475375 | 0.99349940562305 | -0.290186974 | 0.287784448346559 | 0.998799458202429 | 0.748123674527905 | 1.33346984151382 | 0.999355215041207 |
| ebi-fl187-GCST90094765 | ebi-met1400-GCST90200730 | Adenosine 5'-diphosphate (ADP) to fructose ratio \|\| id:ebi-met1400-GCST90200730 | Fried fish liking \|\| id:ebi-fl187-GCST90094765 | Inverse variance weighted | 4 | -0.332970099 | 0.200973286181244 | 0.0975623743295238 | -0.72687774 | 0.0609375422184836 | 0.716791626839063 | 0.483415986834458 | 1.06283252995217 | 0.998074519377826 |
| ebi-fl187-GCST90094765 | ebi-met1400-GCST90200731 | Adenosine 5'-diphosphate (ADP) to mannose ratio \|\| id:ebi-met1400-GCST90200731 | Fried fish liking \|\| id:ebi-fl187-GCST90094765 | Inverse variance weighted | 4 | -0.190451779 | 0.215202872682703 | 0.376163556645798 | -0.61224941 | 0.231345851371957 | 0.826585615481822 | 0.542130024048733 | 1.26029503885223 | 0.998074519377826 |
| ebi-fl187-GCST90094765 | ebi-met1400-GCST90200748 | Aspartate to glutamate ratio \|\| id:ebi-met1400-GCST90200748 | Fried fish liking \|\| id:ebi-fl187-GCST90094765 | Inverse variance weighted | 4 | -0.008371044 | 0.206935120963114 | 0.967732370910662 | -0.413963881 | 0.39722179290885 | 0.991663895449771 | 0.66102482621424 | 1.48768585163531 | 0.998074519377826 |
| ebi-fl187-GCST90094765 | ebi-met1400-GCST90200774 | Uridine to pseudouridine ratio \|\| id:ebi-met1400-GCST90200774 | Fried fish liking \|\| id:ebi-fl187-GCST90094765 | Inverse variance weighted | 4 | -0.148045665 | 0.142360787471312 | 0.298371082153755 | -0.427072808 | 0.130981478765578 | 0.862391733201901 | 0.652416049311332 | 1.139946668 | 0.998074519377826 |
| ebi-fl187-GCST90094765 | ebi-met1400-GCST90200799 | Spermidine to adenosine 5'-diphosphate (ADP) ratio \|\| id:ebi-met1400-GCST90200799 | Fried fish liking \|\| id:ebi-fl187-GCST90094765 | Inverse variance weighted | 4 | 0.0482808904584812 | 0.212423836707693 | 0.820201628377437 | -0.368069829 | 0.464631610405559 | 1.04946539874424 | 0.692068853187281 | 1.59142781543928 | 0.998074519377826 |
| ebi-fl187-GCST90094765 | ebi-met1400-GCST90200811 | Choline to taurocholate ratio \|\| id:ebi-met1400-GCST90200811 | Fried fish liking \|\| id:ebi-fl187-GCST90094765 | Inverse variance weighted | 4 | -0.208787855 | 0.191445531374707 | 0.275455002572451 | -0.584021097 | 0.166445386061611 | 0.811567386994334 | 0.557651481462961 | 1.18109902963928 | 0.998074519377826 |
| ebi-fl187-GCST90094765 | ebi-met1400-GCST90200812 | Glutamate to kynurenine ratio \|\| id:ebi-met1400-GCST90200812 | Fried fish liking \|\| id:ebi-fl187-GCST90094765 | Inverse variance weighted | 4 | 0.135953483871958 | 0.226368263780857 | 0.548116136348015 | -0.307728313 | 0.579635280882437 | 1.14562860214867 | 0.735115011971156 | 1.78538714716468 | 0.998074519377826 |
| ebi-fl187-GCST90094765 | ebi-met1400-GCST90200876 | Cortisone to 4-cholesten-3-one ratio \|\| id:ebi-met1400-GCST90200876 | Fried fish liking \|\| id:ebi-fl187-GCST90094765 | Inverse variance weighted | 4 | -0.097758819 | 0.157698350515256 | 0.535316897820365 | -0.406847586 | 0.211329948079572 | 0.90686759667758 | 0.665745651566791 | 1.2353198792486 | 0.998074519377826 |
| ebi-fl187-GCST90094765 | ebi-met1400-GCST90200906 | 2'-deoxyuridine to cytidine ratio \|\| id:ebi-met1400-GCST90200906 | Fried fish liking \|\| id:ebi-fl187-GCST90094765 | Inverse variance weighted | 4 | 0.194825408067316 | 0.153833999290898 | 0.205346577648353 | -0.106689231 | 0.496340046677476 | 1.21509882151747 | 0.898804950719189 | 1.64269805687178 | 0.998074519377826 |
| ebi-fl187-GCST90094765 | ebi-met1400-GCST90200907 | Retinol (Vitamin A) to linoleoyl-arachidonoyl-glycerol (18:2 to 20:4) [1] ratio \|\| id:ebi-met1400-GCST90200907 | Fried fish liking \|\| id:ebi-fl187-GCST90094765 | Inverse variance weighted | 4 | 0.034455924159877 | 0.155298969603246 | 0.824416379469049 | -0.269930056 | 0.338841904582239 | 1.03505640639002 | 0.763432889819148 | 1.40332146898049 | 0.998074519377826 |
| ebi-fl187-GCST90094765 | ebi-met1400-GCST90200916 | Fructose to sucrose ratio \|\| id:ebi-met1400-GCST90200916 | Fried fish liking \|\| id:ebi-fl187-GCST90094765 | Inverse variance weighted | 4 | 0.153760845106193 | 0.165062951506848 | 0.351580244541961 | -0.16976254 | 0.477284230059615 | 1.16621194813039 | 0.843865177160504 | 1.6116914701214 | 0.998074519377826 |
| ebi-fl187-GCST90094765 | ebi-met1400-GCST90200917 | Alpha-ketobutyrate to 3-methyl-2-oxobutyrate ratio \|\| id:ebi-met1400-GCST90200917 | Fried fish liking \|\| id:ebi-fl187-GCST90094765 | Inverse variance weighted | 4 | -0.027819294 | 0.150131230522996 | 0.852993855659221 | -0.322076506 | 0.266437917325836 | 0.97256409860538 | 0.724642748455982 | 1.30530654989857 | 0.998074519377826 |
| ebi-fl187-GCST90094765 | ebi-met1400-GCST90200924 | Alpha-tocopherol to glycerol ratio \|\| id:ebi-met1400-GCST90200924 | Fried fish liking \|\| id:ebi-fl187-GCST90094765 | Inverse variance weighted | 4 | 0.0101103439356641 | 0.139226287687151 | 0.942110049015208 | -0.26277318 | 0.282993867802479 | 1.01016162614412 | 0.768916283177595 | 1.32709702377113 | 0.998074519377826 |
| ebi-fl187-GCST90094765 | ebi-met1400-GCST90200938 | Alpha-ketoglutarate to alanine ratio \|\| id:ebi-met1400-GCST90200938 | Fried fish liking \|\| id:ebi-fl187-GCST90094765 | Inverse variance weighted | 4 | -0.013576465 | 0.148260661890554 | 0.927038424680449 | -0.304167362 | 0.27701443279871 | 0.986515280029356 | 0.737737387051942 | 1.31918541043505 | 0.998074519377826 |
| ebi-fl187-GCST90094765 | ebi-met1400-GCST90200943 | Phosphate to EDTA ratio \|\| id:ebi-met1400-GCST90200943 | Fried fish liking \|\| id:ebi-fl187-GCST90094765 | Inverse variance weighted | 4 | 0.0260449680534343 | 0.142261456017654 | 0.854736678441287 | -0.252787486 | 0.304877421848036 | 1.02638710206577 | 0.776632909854935 | 1.35645872061195 | 0.998074519377826 |
| ebi-fl187-GCST90094765 | ebi-met1400-GCST90200947 | Adenosine 5'-diphosphate (ADP) to glucose ratio \|\| id:ebi-met1400-GCST90200947 | Fried fish liking \|\| id:ebi-fl187-GCST90094765 | Inverse variance weighted | 4 | -0.231529401 | 0.199450832249558 | 0.245709223101441 | -0.622453032 | 0.159394230558644 | 0.793319371054693 | 0.536626459932041 | 1.17280020923738 | 0.998074519377826 |
| ebi-fl187-GCST90094765 | ebi-met1400-GCST90200975 | Arachidonate (20:4n6) to caffeine ratio \|\| id:ebi-met1400-GCST90200975 | Fried fish liking \|\| id:ebi-fl187-GCST90094765 | Inverse variance weighted | 4 | -0.222913777 | 0.15270125811945 | 0.144344183959484 | -0.522208243 | 0.0763806890586154 | 0.800183840687102 | 0.593209150743368 | 1.07937340159772 | 0.998074519377826 |
| ebi-fl187-GCST90094765 | ebi-met1400-GCST90200985 | Cholesterol to oleoyl-linoleoyl-glycerol (18:1 to 18:2) [2] ratio \|\| id:ebi-met1400-GCST90200985 | Fried fish liking \|\| id:ebi-fl187-GCST90094765 | Inverse variance weighted | 4 | -0.034875072 | 0.149150004058767 | 0.815120463117756 | -0.32720908 | 0.257458935792128 | 0.96572605478896 | 0.720932994512163 | 1.29363868764161 | 0.998074519377826 |
| ebi-fl187-GCST90094765 | ebi-met1400-GCST90201016 | Bilirubin (Z,Z) to etiocholanolone glucuronide ratio \|\| id:ebi-met1400-GCST90201016 | Fried fish liking \|\| id:ebi-fl187-GCST90094765 | Inverse variance weighted | 4 | 0.317417263567389 | 0.15360731030603 | 0.0387888111934545 | 0.0163469353675705 | 0.618487591767207 | 1.37357559541925 | 1.01648127754487 | 1.85611870873645 | 0.998074519377826 |
| ebi-fl187-GCST90094765 | ebi-pw11-GCST90275189 | Placental weight (fetal genotype, adjusted for sex and gestational age) \|\| id:ebi-pw11-GCST90275189 | Fried fish liking \|\| id:ebi-fl187-GCST90094765 | Inverse variance weighted (multiplicative random effects) | 4 | 0.101013427991076 | 0.0895860359425447 | 0.259506743675098 | -0.074575202 | 0.276602058438463 | 1.10629149693603 | 0.928137673201598 | 1.31864152434542 | 0.998074519377826 |
| ebi-fl187-GCST90094765 | ebi-pw11-GCST90275192 | Placental weight (fetal effect, conditional on maternal and paternal genotype) \|\| id:ebi-pw11-GCST90275192 | Fried fish liking \|\| id:ebi-fl187-GCST90094765 | Inverse variance weighted | 4 | 0.0384599844507538 | 0.113155788916621 | 0.733942939262439 | -0.183325362 | 0.260245330727332 | 1.03920914300132 | 0.832497248843223 | 1.2972483024997 | 0.998074519377826 |
| ebi-fl187-GCST90094765 | ebi-pw11-GCST90275193 | Placental weight (maternal effect, conditional on fetal and paternal genotype) \|\| id:ebi-pw11-GCST90275193 | Fried fish liking \|\| id:ebi-fl187-GCST90094765 | Inverse variance weighted | 4 | 0.0160596199183149 | 0.0878598216088927 | 0.854965267987756 | -0.15614563 | 0.188264870271745 | 1.01618926872128 | 0.855434603985863 | 1.20715321200797 | 0.998074519377826 |
| ebi-fl187-GCST90094765 | ebi-pw11-GCST90275195 | Placental weight (fetal effect, conditional on maternal genotype) \|\| id:ebi-pw11-GCST90275195 | Fried fish liking \|\| id:ebi-fl187-GCST90094765 | Inverse variance weighted | 4 | 0.111811606513253 | 0.0791224335694316 | 0.157612592174128 | -0.043268363 | 0.266891576309339 | 1.1183021599552 | 0.957654356312483 | 1.30589884828174 | 0.998074519377826 |
| ebi-fl187-GCST90094765 | ebi-pw11-GCST90275197 | Placental weight (fetal genotype, adjusted for sex) \|\| id:ebi-pw11-GCST90275197 | Fried fish liking \|\| id:ebi-fl187-GCST90094765 | Inverse variance weighted | 4 | 0.047372660698737 | 0.0773048128224274 | 0.540006269205513 | -0.104144772 | 0.198890093830695 | 1.04851267574846 | 0.901094834285978 | 1.22004786774339 | 0.998074519377826 |
| ebi-fl187-GCST90094765 | prot-a-1030 | Protein FAM171B \|\| id:prot-a-1030 | Fried fish liking \|\| id:ebi-fl187-GCST90094765 | Inverse variance weighted | 5 | 0.0173884218729836 | 0.260852680793688 | 0.946852433236145 | -0.493882832 | 0.528659676228612 | 1.01754048055552 | 0.610252280646686 | 1.69665671461637 | 0.998074519377826 |
| ebi-fl187-GCST90094765 | prot-a-1036 | BRISC complex subunit Abro1 \|\| id:prot-a-1036 | Fried fish liking \|\| id:ebi-fl187-GCST90094765 | Inverse variance weighted | 5 | -0.343126705 | 0.324380042178604 | 0.290150151640904 | -0.978911588 | 0.29265817718929 | 0.709548302205561 | 0.375719814283522 | 1.33998467481111 | 0.998074519377826 |
| ebi-fl187-GCST90094765 | prot-a-1055 | Phenylalanine--tRNA ligase, mitochondrial \|\| id:prot-a-1055 | Fried fish liking \|\| id:ebi-fl187-GCST90094765 | Inverse variance weighted | 5 | -0.19079079 | 0.22019766146778 | 0.386242125799737 | -0.622378206 | 0.240796626975989 | 0.826305441843091 | 0.536666614982466 | 1.27226226517149 | 0.998074519377826 |
| ebi-fl187-GCST90094765 | prot-a-1083 | Fc receptor-like protein 5 \|\| id:prot-a-1083 | Fried fish liking \|\| id:ebi-fl187-GCST90094765 | Inverse variance weighted | 5 | -0.198235937 | 0.222740857492875 | 0.373474145369857 | -0.634808018 | 0.238336143634361 | 0.820176320325077 | 0.530037236298074 | 1.2691357330293 | 0.998074519377826 |
| ebi-fl187-GCST90094765 | prot-a-1103 | Fibroblast growth factor receptor 3 \|\| id:prot-a-1103 | Fried fish liking \|\| id:ebi-fl187-GCST90094765 | Inverse variance weighted | 5 | -0.120015031 | 0.216945738935729 | 0.580124422022031 | -0.545228679 | 0.305198617575273 | 0.886907105747963 | 0.579709200830066 | 1.35689447933536 | 0.998074519377826 |
| ebi-fl187-GCST90094765 | prot-a-1105 | Fibroblast growth factor receptor 4 \|\| id:prot-a-1105 | Fried fish liking \|\| id:ebi-fl187-GCST90094765 | Inverse variance weighted | 5 | 0.165093757069547 | 0.233853783165603 | 0.480206789324324 | -0.293259658 | 0.623447172074128 | 1.17950370033787 | 0.745828455275745 | 1.86534714419868 | 0.998074519377826 |
| ebi-fl187-GCST90094765 | prot-a-1120 | Filamin-A \|\| id:prot-a-1120 | Fried fish liking \|\| id:ebi-fl187-GCST90094765 | Inverse variance weighted | 5 | -0.258438094 | 0.284695010507202 | 0.363998814558553 | -0.816440315 | 0.299564126105156 | 0.772256836523577 | 0.442002246170749 | 1.3492705675686 | 0.998074519377826 |
| ebi-fl187-GCST90094765 | prot-a-1166 | Galanin peptides \|\| id:prot-a-1166 | Fried fish liking \|\| id:ebi-fl187-GCST90094765 | Inverse variance weighted | 5 | 0.0283926467282738 | 0.260286561151707 | 0.913137244198225 | -0.481769013 | 0.538554306585619 | 1.02879955990729 | 0.617689723503989 | 1.71352783475375 | 0.998074519377826 |
| ebi-fl187-GCST90094765 | prot-a-1205 | ADP-ribosylation factor-binding protein GGA1 \|\| id:prot-a-1205 | Fried fish liking \|\| id:ebi-fl187-GCST90094765 | Inverse variance weighted | 5 | -0.165926722 | 0.318633186391593 | 0.602544240113896 | -0.790447767 | 0.458594323558864 | 0.847108306513554 | 0.453641624006357 | 1.58184885378641 | 0.998074519377826 |
| ebi-fl187-GCST90094765 | prot-a-1227 | Guanine nucleotide-binding protein G(I)/G(S)/G(O) subunit gamma-T2 \|\| id:prot-a-1227 | Fried fish liking \|\| id:ebi-fl187-GCST90094765 | Inverse variance weighted | 5 | 0.0285578616914709 | 0.216945738935729 | 0.895272237110028 | -0.396655787 | 0.4537715100055 | 1.02896954703054 | 0.672565491832985 | 1.57423825868716 | 0.998074519377826 |
| ebi-fl187-GCST90094765 | prot-a-1240 | Golgi SNAP receptor complex member 1 \|\| id:prot-a-1240 | Fried fish liking \|\| id:ebi-fl187-GCST90094765 | Inverse variance weighted | 5 | 0.197090099084268 | 0.216945738935729 | 0.363626540001799 | -0.228123549 | 0.622303747398297 | 1.21785376327173 | 0.796025905372291 | 1.86321547917646 | 0.998074519377826 |
| ebi-fl187-GCST90094765 | prot-a-1283 | Glutathione S-transferase A1 \|\| id:prot-a-1283 | Fried fish liking \|\| id:ebi-fl187-GCST90094765 | Inverse variance weighted | 5 | 0.135153898187378 | 0.245719383867098 | 0.582296381306512 | -0.346456094 | 0.61676389056689 | 1.14471294004249 | 0.707189868348133 | 1.85292207050632 | 0.998074519377826 |
| ebi-fl187-GCST90094765 | prot-a-1324 | E3 ubiquitin-protein ligase HECW1 \|\| id:prot-a-1324 | Fried fish liking \|\| id:ebi-fl187-GCST90094765 | Inverse variance weighted | 5 | 0.141578269251032 | 0.217047069588232 | 0.514212219221969 | -0.283833987 | 0.566990525643966 | 1.15209067400618 | 0.752891624022164 | 1.76295349660171 | 0.998074519377826 |
| ebi-fl187-GCST90094765 | prot-a-1339 | Histidine triad nucleotide-binding protein 2, mitochondrial \|\| id:prot-a-1339 | Fried fish liking \|\| id:ebi-fl187-GCST90094765 | Inverse variance weighted | 5 | -0.420037095 | 0.216945738935729 | 0.0528506855603075 | -0.845250743 | 0.00517655375840881 | 0.657022447407299 | 0.429449663268496 | 1.00518997526187 | 0.998074519377826 |
| ebi-fl187-GCST90094765 | prot-a-1466 | Interleukin-11 \|\| id:prot-a-1466 | Fried fish liking \|\| id:ebi-fl187-GCST90094765 | Inverse variance weighted | 5 | -0.227770338 | 0.24723123544234 | 0.356901842738089 | -0.712343559 | 0.256802883830921 | 0.796307120612269 | 0.490493349293624 | 1.29279027177637 | 0.998074519377826 |
| ebi-fl187-GCST90094765 | prot-a-147 | Rho GTPase-activating protein 1 \|\| id:prot-a-147 | Fried fish liking \|\| id:ebi-fl187-GCST90094765 | Inverse variance weighted | 5 | -0.234747151 | 0.321414592773641 | 0.465172464806639 | -0.864719753 | 0.39522545116773 | 0.790770770213311 | 0.421169567806192 | 1.48471888479726 | 0.998074519377826 |
| ebi-fl187-GCST90094765 | prot-a-1473 | Interleukin-12 receptor subunit beta-1 \|\| id:prot-a-1473 | Fried fish liking \|\| id:ebi-fl187-GCST90094765 | Inverse variance weighted | 5 | -0.351325058 | 0.216945738935729 | 0.105358126535196 | -0.776538706 | 0.0738885905777516 | 0.70375495567854 | 0.459995438409015 | 1.07668684575459 | 0.998074519377826 |
| ebi-fl187-GCST90094765 | prot-a-1501 | X-linked interleukin-1 receptor accessory protein-like 2 \|\| id:prot-a-1501 | Fried fish liking \|\| id:ebi-fl187-GCST90094765 | Inverse variance weighted | 5 | -0.000864276 | 0.216945738935729 | 0.996821366582998 | -0.426077925 | 0.42434937190938 | 0.999136096974627 | 0.653065450196347 | 1.52859554884975 | 0.999355215041207 |
| ebi-fl187-GCST90094765 | prot-a-1503 | Interleukin-1 receptor-like 2 \|\| id:prot-a-1503 | Fried fish liking \|\| id:ebi-fl187-GCST90094765 | Inverse variance weighted | 5 | -0.406406751 | 0.216945738935729 | 0.0610255079882171 | -0.831620399 | 0.0188068975268247 | 0.666039200351535 | 0.435343284606637 | 1.01898486112113 | 0.998074519377826 |
| ebi-fl187-GCST90094765 | prot-a-1536 | Interleukin-5 receptor subunit alpha \|\| id:prot-a-1536 | Fried fish liking \|\| id:ebi-fl187-GCST90094765 | Inverse variance weighted (multiplicative random effects) | 5 | -0.277804984 | 0.337378679702015 | 0.410268129588876 | -0.939067196 | 0.383457228353284 | 0.757444521016709 | 0.390992384535164 | 1.46734879018247 | 0.998074519377826 |
| ebi-fl187-GCST90094765 | prot-a-1559 | Insulin-induced gene 1 protein \|\| id:prot-a-1559 | Fried fish liking \|\| id:ebi-fl187-GCST90094765 | Inverse variance weighted | 5 | -0.131238831 | 0.242881055787333 | 0.588961212094636 | -0.6072857 | 0.344808038615027 | 0.877008292847553 | 0.544827689659985 | 1.41171889814078 | 0.998074519377826 |
| ebi-fl187-GCST90094765 | prot-a-1567 | Interferon regulatory factor 1 \|\| id:prot-a-1567 | Fried fish liking \|\| id:ebi-fl187-GCST90094765 | Inverse variance weighted | 5 | 0.191537729429515 | 0.216945738935729 | 0.37729942827999 | -0.233675919 | 0.616751377743544 | 1.21111052678724 | 0.791618322877921 | 1.85289888536483 | 0.998074519377826 |
| ebi-fl187-GCST90094765 | prot-a-1613 | Potassium voltage-gated channel subfamily E regulatory beta subunit 5 \|\| id:prot-a-1613 | Fried fish liking \|\| id:ebi-fl187-GCST90094765 | Inverse variance weighted | 5 | -0.257489703 | 0.272754481237396 | 0.345151900369503 | -0.792088487 | 0.277109079800351 | 0.77298958541779 | 0.452897935582214 | 1.31931027328761 | 0.998074519377826 |
| ebi-fl187-GCST90094765 | prot-a-1649 | Kin of IRRE-like protein 2 \|\| id:prot-a-1649 | Fried fish liking \|\| id:ebi-fl187-GCST90094765 | Inverse variance weighted | 5 | 0.237482783812337 | 0.252089413828416 | 0.346163078399748 | -0.256612467 | 0.731578034916033 | 1.26805316556153 | 0.773667977324447 | 2.07835774236304 | 0.998074519377826 |
| ebi-fl187-GCST90094765 | prot-a-165 | Armadillo repeat-containing protein 5 \|\| id:prot-a-165 | Fried fish liking \|\| id:ebi-fl187-GCST90094765 | Inverse variance weighted | 5 | 0.188459329136796 | 0.305651573947078 | 0.53751031826996 | -0.410617756 | 0.787536414073069 | 1.20738797647679 | 0.663240402951245 | 2.1979748508293 | 0.998074519377826 |
| ebi-fl187-GCST90094765 | prot-a-1656 | Kallikrein-11 \|\| id:prot-a-1656 | Fried fish liking \|\| id:ebi-fl187-GCST90094765 | Inverse variance weighted | 5 | -0.204142106 | 0.217047069588232 | 0.346939095512155 | -0.629554362 | 0.221270150854839 | 0.815346497701566 | 0.532829197081083 | 1.24766044157495 | 0.998074519377826 |
| ebi-fl187-GCST90094765 | prot-a-1697 | Laminin subunit gamma-2 \|\| id:prot-a-1697 | Fried fish liking \|\| id:ebi-fl187-GCST90094765 | Inverse variance weighted (multiplicative random effects) | 5 | -0.336340729 | 0.369331562465972 | 0.362467055771435 | -1.060230592 | 0.387549132953001 | 0.714379654134537 | 0.346375929632048 | 1.47336534263079 | 0.998074519377826 |
| ebi-fl187-GCST90094765 | prot-a-1698 | Lysosome-associated membrane glycoprotein 3 \|\| id:prot-a-1698 | Fried fish liking \|\| id:ebi-fl187-GCST90094765 | Inverse variance weighted (multiplicative random effects) | 5 | -0.076730502 | 0.339385513921941 | 0.821134101716523 | -0.741926109 | 0.58846510575006 | 0.926139413125444 | 0.476195826651691 | 1.80122160787378 | 0.998074519377826 |
| ebi-fl187-GCST90094765 | prot-a-1724 | Leptin receptor \|\| id:prot-a-1724 | Fried fish liking \|\| id:ebi-fl187-GCST90094765 | Inverse variance weighted | 5 | -0.081318313 | 0.281823842558468 | 0.772931077976082 | -0.633693044 | 0.471056418868443 | 0.921900192337339 | 0.530628543502114 | 1.60168535040037 | 0.998074519377826 |
| ebi-fl187-GCST90094765 | prot-a-1743 | Leukocyte immunoglobulin-like receptor subfamily B member 2 \|\| id:prot-a-1743 | Fried fish liking \|\| id:ebi-fl187-GCST90094765 | Inverse variance weighted | 5 | -0.342163869 | 0.216945738935729 | 0.114752640885408 | -0.767377518 | 0.0830497788741394 | 0.710231809883783 | 0.464228905421378 | 1.08659589672239 | 0.998074519377826 |
| ebi-fl187-GCST90094765 | prot-a-1746 | Leukocyte immunoglobulin-like receptor subfamily B member 4 \|\| id:prot-a-1746 | Fried fish liking \|\| id:ebi-fl187-GCST90094765 | Inverse variance weighted | 5 | -0.069472471 | 0.217047069588232 | 0.748907529873163 | -0.494884727 | 0.355939785528396 | 0.932885814571024 | 0.609641178256644 | 1.42752158821771 | 0.998074519377826 |
| ebi-fl187-GCST90094765 | prot-a-1750 | Leucine-rich repeat and immunoglobulin-like domain-containing nogo receptor-interacting protein 3 \|\| id:prot-a-1750 | Fried fish liking \|\| id:ebi-fl187-GCST90094765 | Inverse variance weighted | 5 | -0.199275193 | 0.216945738935729 | 0.358331754787657 | -0.624488842 | 0.225938454874623 | 0.81932438960901 | 0.535535101751451 | 1.2534985161808 | 0.998074519377826 |
| ebi-fl187-GCST90094765 | prot-a-1753 | Lipase member N \|\| id:prot-a-1753 | Fried fish liking \|\| id:ebi-fl187-GCST90094765 | Inverse variance weighted | 5 | 0.102353964481962 | 0.250748507672847 | 0.68313146317489 | -0.389113111 | 0.593821039520742 | 1.10777551552553 | 0.677657615448618 | 1.81089471264255 | 0.998074519377826 |
| ebi-fl187-GCST90094765 | prot-a-1760 | Ig Kappa chain V-I region HK102- like \|\| id:prot-a-1760 | Fried fish liking \|\| id:ebi-fl187-GCST90094765 | Inverse variance weighted | 5 | -0.043958923 | 0.229710170975362 | 0.848238151613511 | -0.494190858 | 0.406273012038511 | 0.956993267008062 | 0.610064336206727 | 1.50121234555895 | 0.998074519377826 |
| ebi-fl187-GCST90094765 | prot-a-1792 | Leucine-rich repeat neuronal protein 1 \|\| id:prot-a-1792 | Fried fish liking \|\| id:ebi-fl187-GCST90094765 | Inverse variance weighted | 5 | 0.127543945724845 | 0.268941822089476 | 0.635326184604977 | -0.399582026 | 0.654669917020218 | 1.13603479101655 | 0.670600281235939 | 1.92450716546292 | 0.998074519377826 |
| ebi-fl187-GCST90094765 | prot-a-1795 | Leucine-rich repeat transmembrane neuronal protein 1 \|\| id:prot-a-1795 | Fried fish liking \|\| id:ebi-fl187-GCST90094765 | Inverse variance weighted | 5 | -0.179502932 | 0.216945738935729 | 0.408005085609638 | -0.60471658 | 0.245710716544468 | 0.835685500902251 | 0.546229216942352 | 1.27852966256097 | 0.998074519377826 |
| ebi-fl187-GCST90094765 | prot-a-1820 | LysM and putative peptidoglycan-binding domain-containing protein 4 \|\| id:prot-a-1820 | Fried fish liking \|\| id:ebi-fl187-GCST90094765 | Inverse variance weighted | 5 | -0.187537681 | 0.218582019138217 | 0.390906840205204 | -0.615958439 | 0.240883076070567 | 0.828997879757702 | 0.540122972255078 | 1.2723722558466 | 0.998074519377826 |
| ebi-fl187-GCST90094765 | prot-a-1844 | Mitogen-activated protein kinase kinase kinase 7:TGF-beta-activated kinase 1 and MAP3K7-binding protein 1 fusion \|\| id:prot-a-1844 | Fried fish liking \|\| id:ebi-fl187-GCST90094765 | Inverse variance weighted | 5 | 0.0405393456007445 | 0.217047069588232 | 0.851835679283571 | -0.384872911 | 0.465951601993679 | 1.04137228231451 | 0.680537119632336 | 1.59352987381323 | 0.998074519377826 |
| ebi-fl187-GCST90094765 | prot-a-1856 | Megakaryocyte-associated tyrosine-protein kinase \|\| id:prot-a-1856 | Fried fish liking \|\| id:ebi-fl187-GCST90094765 | Inverse variance weighted (multiplicative random effects) | 5 | 0.0756382453188292 | 0.411477063294406 | 0.854153652320598 | -0.730856799 | 0.882133289375866 | 1.07857232498676 | 0.481496267910732 | 2.41604834296042 | 0.998074519377826 |
| ebi-fl187-GCST90094765 | prot-a-1910 | Stromelysin-2 \|\| id:prot-a-1910 | Fried fish liking \|\| id:ebi-fl187-GCST90094765 | Inverse variance weighted (multiplicative random effects) | 5 | -0.532122961 | 0.423934167868184 | 0.209405486630483 | -1.36303393 | 0.298788008467908 | 0.587356710017828 | 0.255883266284223 | 1.34822378115094 | 0.998074519377826 |
| ebi-fl187-GCST90094765 | prot-a-1940 | 39S ribosomal protein L14, mitochondrial \|\| id:prot-a-1940 | Fried fish liking \|\| id:ebi-fl187-GCST90094765 | Inverse variance weighted | 5 | -0.031585678 | 0.216945738935729 | 0.884243068140995 | -0.456799327 | 0.39362797007847 | 0.968907938574782 | 0.633307415296219 | 1.48234896790863 | 0.998074519377826 |
| ebi-fl187-GCST90094765 | prot-a-1942 | 39S ribosomal protein L33, mitochondrial \|\| id:prot-a-1942 | Fried fish liking \|\| id:ebi-fl187-GCST90094765 | Inverse variance weighted | 5 | 0.026962950049304 | 0.216945738935729 | 0.901090174973348 | -0.398250698 | 0.452176598363333 | 1.02732973954218 | 0.671493664262281 | 1.57172948892569 | 0.998074519377826 |
| ebi-fl187-GCST90094765 | prot-a-1948 | Mesothelin \|\| id:prot-a-1948 | Fried fish liking \|\| id:ebi-fl187-GCST90094765 | Inverse variance weighted | 5 | -0.455515038 | 0.216945738935729 | 0.0357576458731779 | -0.880728686 | -0.030301389 | 0.634121287545324 | 0.414480775325636 | 0.97015309576716 | 0.998074519377826 |
| ebi-fl187-GCST90094765 | prot-a-1980 | Unconventional myosin-VI \|\| id:prot-a-1980 | Fried fish liking \|\| id:ebi-fl187-GCST90094765 | Inverse variance weighted | 5 | 0.196801057466685 | 0.216945738935729 | 0.364330574632866 | -0.228412591 | 0.622014705780714 | 1.21750180371794 | 0.795795854005775 | 1.86267701018417 | 0.998074519377826 |
| ebi-fl187-GCST90094765 | prot-a-2039 | Neurofascin \|\| id:prot-a-2039 | Fried fish liking \|\| id:ebi-fl187-GCST90094765 | Inverse variance weighted | 5 | -0.146048302 | 0.217047069588232 | 0.501018742247488 | -0.571460558 | 0.279363954424511 | 0.86411596367461 | 0.564700058696044 | 1.32228850905648 | 0.998074519377826 |
| ebi-fl187-GCST90094765 | prot-a-2041 | NFU1 iron-sulfur cluster scaffold homolog, mitochondrial \|\| id:prot-a-2041 | Fried fish liking \|\| id:ebi-fl187-GCST90094765 | Inverse variance weighted | 5 | -0.124304561 | 0.29461463895838 | 0.67308154606929 | -0.701749254 | 0.453140131059047 | 0.883110838536499 | 0.495717409437334 | 1.57324463150457 | 0.998074519377826 |
| ebi-fl187-GCST90094765 | prot-a-2088 | Glucocorticoid receptor \|\| id:prot-a-2088 | Fried fish liking \|\| id:ebi-fl187-GCST90094765 | Inverse variance weighted | 5 | 0.0614980675008634 | 0.217047069588232 | 0.776916391198947 | -0.363914189 | 0.486910323893798 | 1.06342844145227 | 0.694950826684727 | 1.62728067464065 | 0.998074519377826 |
| ebi-fl187-GCST90094765 | prot-a-2111 | Neuron-specific protein family member 2 \|\| id:prot-a-2111 | Fried fish liking \|\| id:ebi-fl187-GCST90094765 | Inverse variance weighted | 5 | -0.036498458 | 0.216945738935729 | 0.866396263448833 | -0.461712106 | 0.388715190696304 | 0.964159580997825 | 0.630203745748018 | 1.47508437374727 | 0.998074519377826 |
| ebi-fl187-GCST90094765 | prot-a-2125 | Nuclear migration protein nudC \|\| id:prot-a-2125 | Fried fish liking \|\| id:ebi-fl187-GCST90094765 | Inverse variance weighted | 5 | -0.330203096 | 0.223237879937591 | 0.139099288884176 | -0.767749341 | 0.107343148812557 | 0.718777737820383 | 0.464056326621439 | 1.11331622207932 | 0.998074519377826 |
| ebi-fl187-GCST90094765 | prot-a-2142 | Ornithine decarboxylase \|\| id:prot-a-2142 | Fried fish liking \|\| id:ebi-fl187-GCST90094765 | Inverse variance weighted | 5 | 0.0134384754620922 | 0.216945738935729 | 0.950607469632265 | -0.411775173 | 0.438652123776122 | 1.01352917761762 | 0.662473201270789 | 1.55061577119156 | 0.998074519377826 |
| ebi-fl187-GCST90094765 | prot-a-2186 | Prostate and testis expressed protein 1 \|\| id:prot-a-2186 | Fried fish liking \|\| id:ebi-fl187-GCST90094765 | Inverse variance weighted | 5 | -0.203124043 | 0.267685769904953 | 0.447963084683946 | -0.727788152 | 0.321540065893988 | 0.816176994005637 | 0.482976079146101 | 1.37925026581403 | 0.998074519377826 |
| ebi-fl187-GCST90094765 | prot-a-2227 | Platelet-derived growth factor C \|\| id:prot-a-2227 | Fried fish liking \|\| id:ebi-fl187-GCST90094765 | Inverse variance weighted (multiplicative random effects) | 5 | -0.123674785 | 0.357140292094008 | 0.729123366311801 | -0.823669758 | 0.576320187397133 | 0.883667175883351 | 0.438818339138317 | 1.77947822159621 | 0.998074519377826 |
| ebi-fl187-GCST90094765 | prot-a-2229 | Platelet-derived growth factor receptor alpha \|\| id:prot-a-2229 | Fried fish liking \|\| id:ebi-fl187-GCST90094765 | Inverse variance weighted | 5 | -0.128101077 | 0.216945738935729 | 0.554872111389243 | -0.553314726 | 0.297112570969284 | 0.879764450395731 | 0.575040546131945 | 1.34596680770839 | 0.998074519377826 |
| ebi-fl187-GCST90094765 | prot-a-2243 | Platelet endothelial aggregation receptor 1 \|\| id:prot-a-2243 | Fried fish liking \|\| id:ebi-fl187-GCST90094765 | Inverse variance weighted | 5 | -0.265876026 | 0.217047069588232 | 0.220586737983548 | -0.691288282 | 0.159536230823026 | 0.766534152245249 | 0.500930313710085 | 1.17296675900194 | 0.998074519377826 |
| ebi-fl187-GCST90094765 | prot-a-2246 | E3 ubiquitin-protein ligase pellino homolog 2 \|\| id:prot-a-2246 | Fried fish liking \|\| id:ebi-fl187-GCST90094765 | Inverse variance weighted | 5 | 0.137644796607978 | 0.239409582896968 | 0.565335687178538 | -0.331597986 | 0.606887579086036 | 1.14756785787591 | 0.717775820882526 | 1.83471210664456 | 0.998074519377826 |
| ebi-fl187-GCST90094765 | prot-a-2251 | Platelet factor 4 variant \|\| id:prot-a-2251 | Fried fish liking \|\| id:ebi-fl187-GCST90094765 | Inverse variance weighted | 5 | 0.0460544715352287 | 0.216945738935729 | 0.831884174379403 | -0.379159177 | 0.471268119849258 | 1.04713144826104 | 0.684436657572502 | 1.60202446465415 | 0.998074519377826 |
| ebi-fl187-GCST90094765 | prot-a-2346 | Protein phosphatase 1L \|\| id:prot-a-2346 | Fried fish liking \|\| id:ebi-fl187-GCST90094765 | Inverse variance weighted (multiplicative random effects) | 5 | -0.120965339 | 0.377104429309272 | 0.748381578617452 | -0.86009002 | 0.618159342747466 | 0.886064671215155 | 0.423123990920314 | 1.85550953957484 | 0.998074519377826 |
| ebi-fl187-GCST90094765 | prot-a-2358 | PR domain zinc finger protein 4 \|\| id:prot-a-2358 | Fried fish liking \|\| id:ebi-fl187-GCST90094765 | Inverse variance weighted | 5 | 0.186540153495743 | 0.216945738935729 | 0.389873360021618 | -0.238673495 | 0.611753801809772 | 1.20507300900818 | 0.787672019387941 | 1.84366198277355 | 0.998074519377826 |
| ebi-fl187-GCST90094765 | prot-a-2390 | Trypsin-1 \|\| id:prot-a-2390 | Fried fish liking \|\| id:ebi-fl187-GCST90094765 | Inverse variance weighted (multiplicative random effects) | 5 | -0.33288176 | 0.38189386205957 | 0.383393664387917 | -1.08139373 | 0.415630209438163 | 0.716854949931764 | 0.339122550904962 | 1.51532541221562 | 0.998074519377826 |
| ebi-fl187-GCST90094765 | prot-a-242 | Brain-derived neurotrophic factor \|\| id:prot-a-242 | Fried fish liking \|\| id:ebi-fl187-GCST90094765 | Inverse variance weighted | 5 | 0.00215070719261763 | 0.217047069588232 | 0.992093936187785 | -0.423261549 | 0.427562963585552 | 1.00215302162225 | 0.654907320223523 | 1.53351573227772 | 0.999355215041207 |
| ebi-fl187-GCST90094765 | prot-a-2464 | Dihydropteridine reductase \|\| id:prot-a-2464 | Fried fish liking \|\| id:ebi-fl187-GCST90094765 | Inverse variance weighted | 5 | 0.182316961068706 | 0.289611176111727 | 0.52900656988225 | -0.385320944 | 0.74995486624769 | 1.19999448514237 | 0.680232284621764 | 2.11690447061448 | 0.998074519377826 |
| ebi-fl187-GCST90094765 | prot-a-2474 | Ras-related protein Rab-27A \|\| id:prot-a-2474 | Fried fish liking \|\| id:ebi-fl187-GCST90094765 | Inverse variance weighted (multiplicative random effects) | 5 | -0.175448233 | 0.353192476882332 | 0.619365618293226 | -0.867705487 | 0.516809021952218 | 0.839080832967209 | 0.419913942534581 | 1.67666889078103 | 0.998074519377826 |
| ebi-fl187-GCST90094765 | prot-a-2478 | Ras-related protein Rab-7b \|\| id:prot-a-2478 | Fried fish liking \|\| id:ebi-fl187-GCST90094765 | Inverse variance weighted | 5 | -0.183999268 | 0.217047069588232 | 0.396583334502613 | -0.609411525 | 0.241412987928854 | 0.831936412417818 | 0.543670711655344 | 1.27304667966994 | 0.998074519377826 |
| ebi-fl187-GCST90094765 | prot-a-2528 | E3 ubiquitin-protein ligase rififylin \|\| id:prot-a-2528 | Fried fish liking \|\| id:ebi-fl187-GCST90094765 | Inverse variance weighted | 5 | -0.004733587 | 0.217047069588232 | 0.982600287525908 | -0.430145843 | 0.420678669394844 | 0.995277598768284 | 0.650414229188974 | 1.52299481492764 | 0.999355215041207 |
| ebi-fl187-GCST90094765 | prot-a-2543 | Protein RIC-3 \|\| id:prot-a-2543 | Fried fish liking \|\| id:ebi-fl187-GCST90094765 | Inverse variance weighted | 5 | -0.206508638 | 0.300804350345703 | 0.492384344751868 | -0.796085164 | 0.383067888858148 | 0.813419235258141 | 0.451091460789753 | 1.46677760454509 | 0.998074519377826 |
| ebi-fl187-GCST90094765 | prot-a-255 | Bone morphogenetic protein 6 \|\| id:prot-a-255 | Fried fish liking \|\| id:ebi-fl187-GCST90094765 | Inverse variance weighted | 5 | -0.239264807 | 0.233331869997294 | 0.30516173871322 | -0.696595272 | 0.218065658293151 | 0.787206397082847 | 0.498278923152314 | 1.24366872210392 | 0.998074519377826 |
| ebi-fl187-GCST90094765 | prot-a-2568 | RING finger protein 165 \|\| id:prot-a-2568 | Fried fish liking \|\| id:ebi-fl187-GCST90094765 | Inverse variance weighted | 5 | -0.410286814 | 0.216945738935729 | 0.0585982465861506 | -0.835500462 | 0.0149268345544156 | 0.663459933407042 | 0.433657398035847 | 1.015038796 | 0.998074519377826 |
| ebi-fl187-GCST90094765 | prot-a-2573 | E3 ubiquitin-protein ligase RNF8 \|\| id:prot-a-2573 | Fried fish liking \|\| id:ebi-fl187-GCST90094765 | Inverse variance weighted | 5 | 0.141782338463642 | 0.225880851372602 | 0.530209396188027 | -0.30094413 | 0.584508807153941 | 1.15232580423348 | 0.7401191218792 | 1.79410951541267 | 0.998074519377826 |
| ebi-fl187-GCST90094765 | prot-a-260 | Cytoplasmic tyrosine-protein kinase BMX \|\| id:prot-a-260 | Fried fish liking \|\| id:ebi-fl187-GCST90094765 | Inverse variance weighted | 4 | -0.010788086 | 0.287435222777188 | 0.970060638490441 | -0.574161123 | 0.552584950335522 | 0.989269896400524 | 0.563177107053948 | 1.73773918660113 | 0.998074519377826 |
| ebi-fl187-GCST90094765 | prot-a-2623 | Serum amyloid A-1 protein \|\| id:prot-a-2623 | Fried fish liking \|\| id:ebi-fl187-GCST90094765 | Inverse variance weighted | 5 | -0.408400921 | 0.216945738935729 | 0.0597678049657864 | -0.833614569 | 0.0168127271409798 | 0.664712328146726 | 0.434476000963888 | 1.01695485644779 | 0.998074519377826 |
| ebi-fl187-GCST90094765 | prot-a-2640 | Lysosome membrane protein 2 \|\| id:prot-a-2640 | Fried fish liking \|\| id:ebi-fl187-GCST90094765 | Inverse variance weighted | 5 | -0.09944068 | 0.216876468282798 | 0.646583926608794 | -0.524518557 | 0.325637198231345 | 0.905343653620637 | 0.591840232865867 | 1.38491282889334 | 0.998074519377826 |
| ebi-fl187-GCST90094765 | prot-a-2641 | Scavenger receptor class F member 2 \|\| id:prot-a-2641 | Fried fish liking \|\| id:ebi-fl187-GCST90094765 | Inverse variance weighted | 5 | -0.111159533 | 0.216945738935729 | 0.608382263836552 | -0.536373181 | 0.314054115748228 | 0.894795988441956 | 0.584865612197545 | 1.36896381704415 | 0.998074519377826 |
| ebi-fl187-GCST90094765 | prot-a-2669 | Semaphorin-3A \|\| id:prot-a-2669 | Fried fish liking \|\| id:ebi-fl187-GCST90094765 | Inverse variance weighted | 5 | 0.232171965951011 | 0.244793054749219 | 0.342904587246682 | -0.247622421 | 0.711966353259481 | 1.261336617 | 0.780654646159366 | 2.03799473882613 | 0.998074519377826 |
| ebi-fl187-GCST90094765 | prot-a-2683 | Sentrin-specific protease 7 \|\| id:prot-a-2683 | Fried fish liking \|\| id:ebi-fl187-GCST90094765 | Inverse variance weighted | 5 | 0.266849177942621 | 0.234330008174656 | 0.254796970862646 | -0.192437638 | 0.726135993964946 | 1.30584348147721 | 0.824945761801372 | 2.06707795478936 | 0.998074519377826 |
| ebi-fl187-GCST90094765 | prot-a-270 | Bromodomain testis-specific protein \|\| id:prot-a-270 | Fried fish liking \|\| id:ebi-fl187-GCST90094765 | Inverse variance weighted | 5 | 0.103166225758582 | 0.217047069588232 | 0.634560834442736 | -0.322246031 | 0.528578482151516 | 1.10867568421658 | 0.724519914306003 | 1.69651896173271 | 0.998074519377826 |
| ebi-fl187-GCST90094765 | prot-a-2708 | 14-3-3 protein sigma \|\| id:prot-a-2708 | Fried fish liking \|\| id:ebi-fl187-GCST90094765 | Inverse variance weighted | 5 | 0.171027348823789 | 0.267580438034638 | 0.522717304542657 | -0.35343031 | 0.695485007371681 | 1.18652319859186 | 0.702274932616536 | 2.00468112331924 | 0.998074519377826 |
| ebi-fl187-GCST90094765 | prot-a-2713 | Beta-sarcoglycan \|\| id:prot-a-2713 | Fried fish liking \|\| id:ebi-fl187-GCST90094765 | Inverse variance weighted | 5 | -0.506702673 | 0.216945738935729 | 0.0195110875121891 | -0.931916321 | -0.081489024 | 0.602478877265124 | 0.393798342794665 | 0.921742826479873 | 0.998074519377826 |
| ebi-fl187-GCST90094765 | prot-a-2720 | Protein shisa-3 homolog \|\| id:prot-a-2720 | Fried fish liking \|\| id:ebi-fl187-GCST90094765 | Inverse variance weighted | 5 | -0.246024155 | 0.282558841614731 | 0.383917647025639 | -0.799839484 | 0.307791174681714 | 0.781903337913603 | 0.449401094192757 | 1.36041686978671 | 0.998074519377826 |
| ebi-fl187-GCST90094765 | prot-a-2730 | Sialic acid-binding Ig-like lectin 8 \|\| id:prot-a-2730 | Fried fish liking \|\| id:ebi-fl187-GCST90094765 | Inverse variance weighted | 5 | -0.269474143 | 0.289363117501797 | 0.351715788488684 | -0.836625853 | 0.297677567450891 | 0.763781028465648 | 0.433169638378895 | 1.34672748909002 | 0.998074519377826 |
| ebi-fl187-GCST90094765 | prot-a-2761 | Sodium- and chloride-dependent neutral and basic amino acid transporter B(0+) \|\| id:prot-a-2761 | Fried fish liking \|\| id:ebi-fl187-GCST90094765 | Inverse variance weighted | 5 | 0.354326414937002 | 0.216945738935729 | 0.102416634548573 | -0.070887233 | 0.779540063251031 | 1.42522032376408 | 0.931566935862197 | 2.18046915693737 | 0.998074519377826 |
| ebi-fl187-GCST90094765 | prot-a-2770 | Secreted Ly-6/uPAR-related protein 1 \|\| id:prot-a-2770 | Fried fish liking \|\| id:ebi-fl187-GCST90094765 | Inverse variance weighted | 5 | 0.129819415943435 | 0.307950476054651 | 0.673346650580052 | -0.473763517 | 0.733402349010551 | 1.13862274764325 | 0.62265448228127 | 2.08215278030396 | 0.998074519377826 |
| ebi-fl187-GCST90094765 | prot-a-2784 | N-lysine methyltransferase SMYD2 \|\| id:prot-a-2784 | Fried fish liking \|\| id:ebi-fl187-GCST90094765 | Inverse variance weighted | 5 | -0.211933659 | 0.216945738935729 | 0.328620108214153 | -0.637147307 | 0.2132799897218 | 0.809018367203613 | 0.528798775056526 | 1.23773115473431 | 0.998074519377826 |
| ebi-fl187-GCST90094765 | prot-a-2837 | Serine palmitoyltransferase 2 \|\| id:prot-a-2837 | Fried fish liking \|\| id:ebi-fl187-GCST90094765 | Inverse variance weighted | 5 | 0.0937740551951159 | 0.278961035104995 | 0.736754232571252 | -0.452989574 | 0.640537684000905 | 1.09831156008893 | 0.635724761905732 | 1.8975008609211 | 0.998074519377826 |
| ebi-fl187-GCST90094765 | prot-a-2858 | Alpha-2,8-sialyltransferase 8B \|\| id:prot-a-2858 | Fried fish liking \|\| id:ebi-fl187-GCST90094765 | Inverse variance weighted | 5 | 0.0225334762938848 | 0.245607494802623 | 0.92689994234017 | -0.458857214 | 0.503924166107026 | 1.02278927278563 | 0.632005480295487 | 1.65520383784689 | 0.998074519377826 |
| ebi-fl187-GCST90094765 | prot-a-2911 | Synaptotagmin-11 \|\| id:prot-a-2911 | Fried fish liking \|\| id:ebi-fl187-GCST90094765 | Inverse variance weighted | 5 | -0.176004966 | 0.264645724094952 | 0.506012889571344 | -0.694710585 | 0.342700653420639 | 0.838613818933647 | 0.499218908504252 | 1.40874699520819 | 0.998074519377826 |
| ebi-fl187-GCST90094765 | prot-a-2962 | Transforming growth factor beta-1 \|\| id:prot-a-2962 | Fried fish liking \|\| id:ebi-fl187-GCST90094765 | Inverse variance weighted | 5 | -0.025795632 | 0.217047069588232 | 0.905395701439787 | -0.451207888 | 0.399616624841823 | 0.974534233308362 | 0.636858433225016 | 1.49125287872943 | 0.998074519377826 |
| ebi-fl187-GCST90094765 | prot-a-2978 | Tyrosine-protein kinase receptor Tie-1, soluble \|\| id:prot-a-2978 | Fried fish liking \|\| id:ebi-fl187-GCST90094765 | Inverse variance weighted | 5 | -0.034387954 | 0.216945738935729 | 0.874055358142237 | -0.459601603 | 0.390825693975091 | 0.96619659175763 | 0.631535197341986 | 1.47820083164508 | 0.998074519377826 |
| ebi-fl187-GCST90094765 | prot-a-299 | Uncharacterized protein C1orf226 \|\| id:prot-a-299 | Fried fish liking \|\| id:ebi-fl187-GCST90094765 | Inverse variance weighted | 5 | -0.04407788 | 0.216945738935729 | 0.838998505022582 | -0.469291529 | 0.381135767924082 | 0.956879432427734 | 0.625445221340964 | 1.46394634887468 | 0.998074519377826 |
| ebi-fl187-GCST90094765 | prot-a-3015 | Transmembrane protein 70, mitochondrial \|\| id:prot-a-3015 | Fried fish liking \|\| id:ebi-fl187-GCST90094765 | Inverse variance weighted | 5 | -0.161750593 | 0.216945738935729 | 0.455920642366013 | -0.586964241 | 0.263463055492243 | 0.85065333713389 | 0.556012645583033 | 1.30142921339181 | 0.998074519377826 |
| ebi-fl187-GCST90094765 | prot-a-3016 | Transmembrane protein 87B \|\| id:prot-a-3016 | Fried fish liking \|\| id:ebi-fl187-GCST90094765 | Inverse variance weighted | 5 | 0.426939713609944 | 0.281206212021599 | 0.128952836893433 | -0.124224462 | 0.978103889172277 | 1.53256026641295 | 0.883181577971037 | 2.65940892424815 | 0.998074519377826 |
| ebi-fl187-GCST90094765 | prot-a-3123 | Thioredoxin domain-containing protein 12 \|\| id:prot-a-3123 | Fried fish liking \|\| id:ebi-fl187-GCST90094765 | Inverse variance weighted | 5 | -0.114866855 | 0.256950612946165 | 0.654847153721142 | -0.618490057 | 0.388756346190422 | 0.891484832589213 | 0.538757316816471 | 1.47514508282279 | 0.998074519377826 |
| ebi-fl187-GCST90094765 | prot-a-3133 | PolyUbiquitin K48-linked \|\| id:prot-a-3133 | Fried fish liking \|\| id:ebi-fl187-GCST90094765 | Inverse variance weighted | 5 | 0.0497126836978581 | 0.216945738935729 | 0.818753881799906 | -0.375500965 | 0.474926332011887 | 1.05096909243947 | 0.686945057409789 | 1.60789574267821 | 0.998074519377826 |
| ebi-fl187-GCST90094765 | prot-a-3135 | Ubiquitin-conjugating enzyme E2 B \|\| id:prot-a-3135 | Fried fish liking \|\| id:ebi-fl187-GCST90094765 | Inverse variance weighted | 5 | 0.334632542457361 | 0.227121482589808 | 0.140652999334469 | -0.110525563 | 0.779790648333386 | 1.39742679585068 | 0.895363441346422 | 2.18101561844533 | 0.998074519377826 |
| ebi-fl187-GCST90094765 | prot-a-3162 | NKG2D ligand 3 \|\| id:prot-a-3162 | Fried fish liking \|\| id:ebi-fl187-GCST90094765 | Inverse variance weighted | 5 | -0.098449259 | 0.296933175363159 | 0.740226370913277 | -0.680438283 | 0.483539764279747 | 0.90624167466496 | 0.506394999328911 | 1.62180506124256 | 0.998074519377826 |
| ebi-fl187-GCST90094765 | prot-a-3188 | Vesicle-associated membrane protein 8 \|\| id:prot-a-3188 | Fried fish liking \|\| id:ebi-fl187-GCST90094765 | Inverse variance weighted | 5 | 0.047777774782602 | 0.313180948584807 | 0.878748068083124 | -0.566056884 | 0.661612434008824 | 1.04893752905173 | 0.567759773078745 | 1.93791457588974 | 0.998074519377826 |
| ebi-fl187-GCST90094765 | prot-a-3201 | Vitrin \|\| id:prot-a-3201 | Fried fish liking \|\| id:ebi-fl187-GCST90094765 | Inverse variance weighted | 5 | -0.007871536 | 0.216945738935729 | 0.971056361490026 | -0.433085184 | 0.417342112592589 | 0.992159363687446 | 0.648505247158052 | 1.51792172425209 | 0.998074519377826 |
| ebi-fl187-GCST90094765 | prot-a-3232 | WNT1-inducible-signaling pathway protein 3 \|\| id:prot-a-3232 | Fried fish liking \|\| id:ebi-fl187-GCST90094765 | Inverse variance weighted | 5 | 0.277428604761184 | 0.217047069588232 | 0.201180474616486 | -0.147983652 | 0.702840861154118 | 1.31973189320647 | 0.862445214398754 | 2.01948163299919 | 0.998074519377826 |
| ebi-fl187-GCST90094765 | prot-a-327 | Carbonic anhydrase-related protein 10 \|\| id:prot-a-327 | Fried fish liking \|\| id:ebi-fl187-GCST90094765 | Inverse variance weighted | 5 | -0.18300167 | 0.216945738935729 | 0.398928408773473 | -0.608215318 | 0.24221197866599 | 0.832766765308534 | 0.544321443436533 | 1.2740642386309 | 0.998074519377826 |
| ebi-fl187-GCST90094765 | prot-a-332 | Carbonic anhydrase 5A, mitochondrial \|\| id:prot-a-332 | Fried fish liking \|\| id:ebi-fl187-GCST90094765 | Inverse variance weighted | 5 | -0.204462078 | 0.216945738935729 | 0.345958603253429 | -0.629675727 | 0.220751570126012 | 0.815085650856245 | 0.532764534418111 | 1.24701359664898 | 0.998074519377826 |
| ebi-fl187-GCST90094765 | prot-a-338 | Cell adhesion molecule 3 \|\| id:prot-a-338 | Fried fish liking \|\| id:ebi-fl187-GCST90094765 | Inverse variance weighted | 5 | -0.091868977 | 0.216945738935729 | 0.671955863437499 | -0.517082625 | 0.333344671611171 | 0.912224664398568 | 0.596257519810928 | 1.39562824901723 | 0.998074519377826 |
| ebi-fl187-GCST90094765 | prot-a-34 | A disintegrin and metalloproteinase with thrombospondin motifs 3 \|\| id:prot-a-34 | Fried fish liking \|\| id:ebi-fl187-GCST90094765 | Inverse variance weighted | 5 | -0.001785038 | 0.216945738935729 | 0.993435047692572 | -0.426998687 | 0.423428610014368 | 0.998216553933664 | 0.65246440916516 | 1.52718872409019 | 0.999355215041207 |
| ebi-fl187-GCST90094765 | prot-a-358 | Cysteine--tRNA ligase, cytoplasmic \|\| id:prot-a-358 | Fried fish liking \|\| id:ebi-fl187-GCST90094765 | Inverse variance weighted (multiplicative random effects) | 5 | -0.400566842 | 0.352263345172845 | 0.255486762722602 | -1.091002999 | 0.289869314090375 | 0.669940187849575 | 0.335879437965613 | 1.33625284719535 | 0.998074519377826 |
| ebi-fl187-GCST90094765 | prot-a-382 | Coiled-coil domain-containing protein 167 \|\| id:prot-a-382 | Fried fish liking \|\| id:ebi-fl187-GCST90094765 | Inverse variance weighted | 5 | 0.247135414842351 | 0.217047069588232 | 0.254859221240197 | -0.178276842 | 0.672547671235285 | 1.28035247976897 | 0.836710755119695 | 1.95922242234839 | 0.998074519377826 |
| ebi-fl187-GCST90094765 | prot-a-40 | Pituitary adenylate cyclase-activating polypeptide 27 \|\| id:prot-a-40 | Fried fish liking \|\| id:ebi-fl187-GCST90094765 | Inverse variance weighted | 5 | -0.098547856 | 0.216945738935729 | 0.649648056271489 | -0.523761504 | 0.32666579231576 | 0.90615232675244 | 0.592288457006951 | 1.38633807491073 | 0.998074519377826 |
| ebi-fl187-GCST90094765 | prot-a-403 | C-C motif chemokine 25 \|\| id:prot-a-403 | Fried fish liking \|\| id:ebi-fl187-GCST90094765 | Inverse variance weighted | 4 | -0.085508556 | 0.259264731528935 | 0.74154220427168 | -0.59366743 | 0.42265031753026 | 0.918045287980454 | 0.55229805149557 | 1.52600058700347 | 0.998074519377826 |
| ebi-fl187-GCST90094765 | prot-a-415 | Cyclin-C \|\| id:prot-a-415 | Fried fish liking \|\| id:ebi-fl187-GCST90094765 | Inverse variance weighted | 5 | 0.296914263962602 | 0.217047069588232 | 0.171320912692086 | -0.128497992 | 0.722326520355536 | 1.34569991952343 | 0.879415327904182 | 2.05921845565408 | 0.998074519377826 |
| ebi-fl187-GCST90094765 | prot-a-44 | Adhesion G protein-coupled receptor F5 \|\| id:prot-a-44 | Fried fish liking \|\| id:ebi-fl187-GCST90094765 | Inverse variance weighted (multiplicative random effects) | 5 | 0.170016212782109 | 0.370910186899541 | 0.646682553970717 | -0.556967754 | 0.89700017910521 | 1.18532406856543 | 0.572943739162437 | 2.45223579818573 | 0.998074519377826 |
| ebi-fl187-GCST90094765 | prot-a-455 | CD59 glycoprotein \|\| id:prot-a-455 | Fried fish liking \|\| id:ebi-fl187-GCST90094765 | Inverse variance weighted | 5 | 0.149398213752318 | 0.250776744429683 | 0.55134766365977 | -0.342124205 | 0.640920632834497 | 1.16113527719394 | 0.710259981155126 | 1.89822764581436 | 0.998074519377826 |
| ebi-fl187-GCST90094765 | prot-a-478 | Cadherin-12 \|\| id:prot-a-478 | Fried fish liking \|\| id:ebi-fl187-GCST90094765 | Inverse variance weighted | 5 | 0.121145714669374 | 0.216945738935729 | 0.576561158645666 | -0.304067934 | 0.546359362983403 | 1.12878938155579 | 0.737810742575252 | 1.72695434531864 | 0.998074519377826 |
| ebi-fl187-GCST90094765 | prot-a-516 | Centrin-2 \|\| id:prot-a-516 | Fried fish liking \|\| id:ebi-fl187-GCST90094765 | Inverse variance weighted | 5 | 0.0427826070440826 | 0.216945738935729 | 0.843667757903508 | -0.382431041 | 0.467996255358112 | 1.04371097477216 | 0.682200933064438 | 1.59679142326381 | 0.998074519377826 |
| ebi-fl187-GCST90094765 | prot-a-519 | Cryptic protein \|\| id:prot-a-519 | Fried fish liking \|\| id:ebi-fl187-GCST90094765 | Inverse variance weighted | 5 | -0.135091626 | 0.216945738935729 | 0.533483154537816 | -0.560305274 | 0.290122022486034 | 0.873635860411632 | 0.571034715105367 | 1.33659057217973 | 0.998074519377826 |
| ebi-fl187-GCST90094765 | prot-a-583 | Chloride intracellular channel protein 4 \|\| id:prot-a-583 | Fried fish liking \|\| id:ebi-fl187-GCST90094765 | Inverse variance weighted | 5 | -0.15824538 | 0.216945738935729 | 0.465742479122436 | -0.583459028 | 0.266968268499508 | 0.853640290170933 | 0.55796500806474 | 1.3059990043651 | 0.998074519377826 |
| ebi-fl187-GCST90094765 | prot-a-602 | CCR4-NOT transcription complex subunit 1 \|\| id:prot-a-602 | Fried fish liking \|\| id:ebi-fl187-GCST90094765 | Inverse variance weighted | 5 | -0.344882684 | 0.221048398912301 | 0.118709448880894 | -0.778137546 | 0.0883721773859159 | 0.708303443579072 | 0.459260566791995 | 1.09239461095121 | 0.998074519377826 |
| ebi-fl187-GCST90094765 | prot-a-606 | Ciliary neurotrophic factor receptor subunit alpha \|\| id:prot-a-606 | Fried fish liking \|\| id:ebi-fl187-GCST90094765 | Inverse variance weighted | 5 | -0.316473457 | 0.273248001397592 | 0.246785978893791 | -0.85203954 | 0.219092625445331 | 0.728714353353967 | 0.426544090444144 | 1.24494658507906 | 0.998074519377826 |
| ebi-fl187-GCST90094765 | prot-a-626 | Collagen alpha-2(IX) chain \|\| id:prot-a-626 | Fried fish liking \|\| id:ebi-fl187-GCST90094765 | Inverse variance weighted | 5 | -0.384064368 | 0.222785648507135 | 0.0847225023889104 | -0.82072424 | 0.0525955025913715 | 0.681087585194156 | 0.4401127919602 | 1.05400321731061 | 0.998074519377826 |
| ebi-fl187-GCST90094765 | prot-a-647 | Probable serine carboxypeptidase CPVL \|\| id:prot-a-647 | Fried fish liking \|\| id:ebi-fl187-GCST90094765 | Inverse variance weighted | 5 | -0.229731117 | 0.239090429438521 | 0.336625326622173 | -0.698348359 | 0.238886124512733 | 0.794747267657739 | 0.497406162190763 | 1.26983392539316 | 0.998074519377826 |
| ebi-fl187-GCST90094765 | prot-a-651 | Cellular retinoic acid-binding protein 2 \|\| id:prot-a-651 | Fried fish liking \|\| id:ebi-fl187-GCST90094765 | Inverse variance weighted | 5 | 0.136093491146871 | 0.224852049745933 | 0.545007615230512 | -0.304616526 | 0.5768035086489 | 1.14578900971617 | 0.737406095983379 | 1.78033848911381 | 0.998074519377826 |
| ebi-fl187-GCST90094765 | prot-a-671 | Cytotoxic and regulatory T-cell molecule \|\| id:prot-a-671 | Fried fish liking \|\| id:ebi-fl187-GCST90094765 | Inverse variance weighted | 5 | -0.01278669 | 0.274481892189245 | 0.962844130688131 | -0.550771198 | 0.525197819118341 | 0.98729471281745 | 0.576505039214014 | 1.69079328653611 | 0.998074519377826 |
| ebi-fl187-GCST90094765 | prot-a-688 | Chorionic somatomammotropin hormone \|\| id:prot-a-688 | Fried fish liking \|\| id:ebi-fl187-GCST90094765 | Inverse variance weighted | 5 | -0.170194626 | 0.229138406696045 | 0.457627570828779 | -0.619305903 | 0.278916651436511 | 0.843500633728854 | 0.538317952929093 | 1.32169717771738 | 0.998074519377826 |
| ebi-fl187-GCST90094765 | prot-a-69 | Aldo-keto reductase family 1 member C1 \|\| id:prot-a-69 | Fried fish liking \|\| id:ebi-fl187-GCST90094765 | Inverse variance weighted | 5 | 0.206361198930299 | 0.216945738935729 | 0.341497215540176 | -0.218852449 | 0.631574847244328 | 1.22919710850229 | 0.803440257513266 | 1.88056985870594 | 0.998074519377826 |
| ebi-fl187-GCST90094765 | prot-a-741 | Stromal cell-derived factor 1 \|\| id:prot-a-741 | Fried fish liking \|\| id:ebi-fl187-GCST90094765 | Inverse variance weighted | 5 | -0.214552652 | 0.217047069588232 | 0.322904172877494 | -0.639964908 | 0.210859604559449 | 0.806902325726534 | 0.52731092799407 | 1.2347389911673 | 0.998074519377826 |
| ebi-fl187-GCST90094765 | prot-a-742 | Growth-regulated alpha protein \|\| id:prot-a-742 | Fried fish liking \|\| id:ebi-fl187-GCST90094765 | Inverse variance weighted | 5 | 0.213041952515467 | 0.216945738935729 | 0.326097039448072 | -0.212171696 | 0.638255600829496 | 1.23743656373124 | 0.808825813649976 | 1.89317554338299 | 0.998074519377826 |
| ebi-fl187-GCST90094765 | prot-a-788 | Beta-defensin 112 \|\| id:prot-a-788 | Fried fish liking \|\| id:ebi-fl187-GCST90094765 | Inverse variance weighted | 5 | -0.325788718 | 0.267063708738487 | 0.222506050765003 | -0.849233588 | 0.197656150662131 | 0.721957707651254 | 0.427742633616893 | 1.21854332646179 | 0.998074519377826 |
| ebi-fl187-GCST90094765 | prot-a-838 | DnaJ homolog subfamily B member 1 \|\| id:prot-a-838 | Fried fish liking \|\| id:ebi-fl187-GCST90094765 | Inverse variance weighted | 5 | -0.152424501 | 0.276824597684574 | 0.581895827458527 | -0.695000713 | 0.390151710279152 | 0.85862371658453 | 0.499074092322663 | 1.47720488404915 | 0.998074519377826 |
| ebi-fl187-GCST90094765 | prot-a-85 | Junctional adhesion molecule-like \|\| id:prot-a-85 | Fried fish liking \|\| id:ebi-fl187-GCST90094765 | Inverse variance weighted | 5 | 0.193035285537961 | 0.264853181591996 | 0.466100250228837 | -0.32607695 | 0.712147521458274 | 1.21292559149604 | 0.72174964638354 | 2.03836399210958 | 0.998074519377826 |
| ebi-fl187-GCST90094765 | prot-a-856 | Dipeptidase 1 \|\| id:prot-a-856 | Fried fish liking \|\| id:ebi-fl187-GCST90094765 | Inverse variance weighted | 5 | 0.507691124860017 | 0.297172297031794 | 0.0875609164367528 | -0.074766577 | 1.09014882704233 | 1.66145068084493 | 0.927960067973902 | 2.97471675791733 | 0.998074519377826 |
| ebi-fl187-GCST90094765 | prot-a-860 | Dipeptidyl peptidase 2 \|\| id:prot-a-860 | Fried fish liking \|\| id:ebi-fl187-GCST90094765 | Inverse variance weighted | 5 | -0.081721167 | 0.216945738935729 | 0.706404417136936 | -0.506934816 | 0.343492481068237 | 0.921528875310685 | 0.602339032336041 | 1.40986292177991 | 0.998074519377826 |
| ebi-fl187-GCST90094765 | prot-a-861 | Dermatopontin \|\| id:prot-a-861 | Fried fish liking \|\| id:ebi-fl187-GCST90094765 | Inverse variance weighted | 5 | -0.024434917 | 0.230755558184631 | 0.915668904470765 | -0.476715811 | 0.427845977469746 | 0.975861199243621 | 0.620818934377796 | 1.53394979994228 | 0.998074519377826 |
| ebi-fl187-GCST90094765 | prot-a-877 | Dual specificity protein phosphatase 6 \|\| id:prot-a-877 | Fried fish liking \|\| id:ebi-fl187-GCST90094765 | Inverse variance weighted | 5 | -0.047437648 | 0.227521412457554 | 0.834840525241484 | -0.493379617 | 0.398504320036526 | 0.953669934143934 | 0.610559446445298 | 1.48959507314998 | 0.998074519377826 |
| ebi-fl187-GCST90094765 | prot-a-879 | Dynactin-associated protein \|\| id:prot-a-879 | Fried fish liking \|\| id:ebi-fl187-GCST90094765 | Inverse variance weighted | 5 | 0.0876868244465233 | 0.216945738935729 | 0.686074612509219 | -0.337526824 | 0.512900472760553 | 1.09164619158861 | 0.713532834739579 | 1.67012833830536 | 0.998074519377826 |
| ebi-fl187-GCST90094765 | prot-a-937 | EMI domain-containing protein 1 \|\| id:prot-a-937 | Fried fish liking \|\| id:ebi-fl187-GCST90094765 | Inverse variance weighted | 5 | 0.0377931813523238 | 0.276360922673847 | 0.891226046249179 | -0.503874227 | 0.579460589793063 | 1.0385164261034 | 0.604185368230213 | 1.78507528317971 | 0.998074519377826 |
| ebi-fl187-GCST90094765 | prot-b-1 | SRC proto-oncogene, non-receptor tyrosine kinase \|\| id:prot-b-1 | Fried fish liking \|\| id:ebi-fl187-GCST90094765 | Inverse variance weighted | 2 | -0.29473408 | 0.351601063434517 | 0.401883142570051 | -0.983872164 | 0.394404004301546 | 0.744729599609757 | 0.373860642604651 | 1.48349976791061 | 0.998074519377826 |
| ebi-fl187-GCST90094765 | prot-b-29 | myeloperoxidase \|\| id:prot-b-29 | Fried fish liking \|\| id:ebi-fl187-GCST90094765 | Inverse variance weighted | 2 | -0.495860132 | 0.351601063434517 | 0.158453509484384 | -1.184998216 | 0.193277952777058 | 0.609046821547837 | 0.305746724985162 | 1.21321996451646 | 0.998074519377826 |
| ebi-fl187-GCST90094765 | prot-b-33 | adrenomedullin \|\| id:prot-b-33 | Fried fish liking \|\| id:ebi-fl187-GCST90094765 | Inverse variance weighted | 2 | 0.119176018537887 | 0.351601063434517 | 0.734645601294612 | -0.569962066 | 0.80831410286954 | 1.12656819772441 | 0.565546891865061 | 2.24412143781499 | 0.998074519377826 |
| ebi-fl187-GCST90094765 | ukb-rsfMRI191-edge-pheno102 | Net25_Pair11_15 (Postcentral\|Precentral)&(Cerebellum) (Motor)&(Subcortical-cerebellum) \|\| id:ukb-rsfMRI191-edge-pheno102 | Fried fish liking \|\| id:ebi-fl187-GCST90094765 | Inverse variance weighted | 5 | -0.00532447 | 0.0658606562616731 | 0.935565639672455 | -0.134411356 | 0.123762416243727 | 0.994689679836742 | 0.874230370454865 | 1.13174695436276 | 0.998074519377826 |
| ebi-fl187-GCST90094765 | ukb-rsfMRI191-edge-pheno1167 | Net100_Pair11_45 (Angular\|Temporal)&(Frontal) (Default_mode\|Central_executive)&(Salience\|Default_mode) \|\| id:ukb-rsfMRI191-edge-pheno1167 | Fried fish liking \|\| id:ebi-fl187-GCST90094765 | Inverse variance weighted | 5 | -0.052082119 | 0.0659427276585223 | 0.429639680747798 | -0.181329866 | 0.0771656269020044 | 0.949250911813466 | 0.834160152635776 | 1.08022097523057 | 0.998074519377826 |
| ebi-fl187-GCST90094765 | ukb-rsfMRI191-edge-pheno1317 | Net100_Pair26_48 (Parietal\|Frontal)&(Temporal) (Attention\|Central_executive)&(Default_mode\|Central_executive) \|\| id:ukb-rsfMRI191-edge-pheno1317 | Fried fish liking \|\| id:ebi-fl187-GCST90094765 | Inverse variance weighted | 5 | -0.133405088 | 0.0746343390116177 | 0.0738649182261918 | -0.279688393 | 0.0128782160661022 | 0.875110523178998 | 0.756019285763021 | 1.01296149741168 | 0.998074519377826 |
| ebi-fl187-GCST90094765 | ukb-rsfMRI191-edge-pheno286 | Net100_Pair10_13 (OccipitalPrecuneus)&(Frontal_Sup) (Default_mode\|Central_executive)&(Default_mode) \|\| id:ukb-rsfMRI191-edge-pheno286 | Fried fish liking \|\| id:ebi-fl187-GCST90094765 | Inverse variance weighted | 5 | 0.0253720147576614 | 0.0650117840527277 | 0.696338430798714 | -0.102051082 | 0.152795111501008 | 1.02569662383855 | 0.902983424304394 | 1.16508624171506 | 0.998074519377826 |
| ebi-fl187-GCST90094765 | ukb-rsfMRI191-edge-pheno599 | Net100_Pair11_29 (Angular\|Temporal)&(Parietal) (Default_mode\|Central_executive)&(Central_executive\|Salience) \|\| id:ukb-rsfMRI191-edge-pheno599 | Fried fish liking \|\| id:ebi-fl187-GCST90094765 | Inverse variance weighted | 5 | 0.0791725034307777 | 0.078803382136542 | 0.315048996682717 | -0.075282126 | 0.2336271324184 | 1.08239102213141 | 0.927481783099184 | 1.26317340797344 | 0.998074519377826 |
| ebi-fl187-GCST90094765 | ukb-rsfMRI191-edge-pheno621 | Net100_Pair5_30 (Precuneus\|Angular\|Cingulate)&(Paracentral) (Default_mode\|Central_executive)&(Motor) \|\| id:ukb-rsfMRI191-edge-pheno621 | Fried fish liking \|\| id:ebi-fl187-GCST90094765 | Inverse variance weighted | 5 | -0.001537983 | 0.0915410130116244 | 0.986595347274098 | -0.180958369 | 0.17788240209156 | 0.998463198679173 | 0.834470097869072 | 1.19468482053753 | 0.999355215041207 |
| ebi-fl187-GCST90094765 | ukb-rsfMRI191-edge-pheno624 | Net100_Pair8_30 (Calcarine\|Lingual)&(Paracentral) (Visual)&(Motor) \|\| id:ukb-rsfMRI191-edge-pheno624 | Fried fish liking \|\| id:ebi-fl187-GCST90094765 | Inverse variance weighted | 5 | 0.0811190774348698 | 0.0871015823003041 | 0.351690245352688 | -0.089600024 | 0.251838178743466 | 1.08450002835976 | 0.914296809041909 | 1.28638785554201 | 0.998074519377826 |
| ebi-fl187-GCST90094765 | ukb-rsfMRI191-edge-pheno882 | Net100_Pair6_38 (Postcentral\|Precentral)&(Subcortical) (Motor)&(Subcortical-cerebellum) \|\| id:ukb-rsfMRI191-edge-pheno882 | Fried fish liking \|\| id:ebi-fl187-GCST90094765 | Inverse variance weighted | 5 | 0.0140975387961063 | 0.0839912437151618 | 0.866704952866148 | -0.150525299 | 0.178720376477823 | 1.01419737770544 | 0.860255966214829 | 1.19568635538847 | 0.998074519377826 |
| ebi-fl187-GCST90094765 | ukb-rsfMRI191-edge-pheno899 | Net100_Pair23_38 (Cerebellum)&(Subcortical) (Subcortical-cerebellum)&(Subcortical-cerebellum) \|\| id:ukb-rsfMRI191-edge-pheno899 | Fried fish liking \|\| id:ebi-fl187-GCST90094765 | Inverse variance weighted | 5 | -0.008510006 | 0.083337902004663 | 0.918665809401812 | -0.171852294 | 0.154832281578489 | 0.991526101255479 | 0.842103547493848 | 1.1674621397769 | 0.998074519377826 |
| ebi-fl187-GCST90094765 | ebi-a-GCST90001392 | IgD+ CD38+ B cell Absolute Count \|\| id:ebi-a-GCST90001392 | Fried fish liking \|\| id:ebi-fl187-GCST90094765 | MR Egger | 4 | 0.510353735476052 | 3.0118915520674 | 0.881034299815586 | -5.392953707 | 6.41366117752816 | 1.66588037171972 | 0.00454851848571513 | 610.123366893319 | 0.999279047927446 |
| ebi-fl187-GCST90094765 | ebi-a-GCST90001402 | Switched memory B cell %B cell \|\| id:ebi-a-GCST90001402 | Fried fish liking \|\| id:ebi-fl187-GCST90094765 | MR Egger | 4 | -0.593229742 | 2.80566153960765 | 0.85213271508577 | -6.09232636 | 4.90586687565949 | 0.552539838685592 | 0.00226014488441969 | 135.079956793605 | 0.999279047927446 |
| ebi-fl187-GCST90094765 | ebi-a-GCST90001407 | Memory B cell Absolute Count \|\| id:ebi-a-GCST90001407 | Fried fish liking \|\| id:ebi-fl187-GCST90094765 | MR Egger | 4 | -1.177819937 | 2.38321594525867 | 0.670101696291949 | -5.84892319 | 3.49328331523765 | 0.307949356194165 | 0.00288300193204178 | 32.893771220347 | 0.999279047927446 |
| ebi-fl187-GCST90094765 | ebi-a-GCST90001411 | IgD+ CD24+ B cell %B cell \|\| id:ebi-a-GCST90001411 | Fried fish liking \|\| id:ebi-fl187-GCST90094765 | MR Egger | 4 | -0.543320357 | 2.14662231604505 | 0.823827155989518 | -4.750700096 | 3.66405938255581 | 0.580816528977915 | 0.0086456403027215 | 39.019416552382 | 0.999279047927446 |
| ebi-fl187-GCST90094765 | ebi-a-GCST90001418 | CD24+ CD27+ B cell Absolute Count \|\| id:ebi-a-GCST90001418 | Fried fish liking \|\| id:ebi-fl187-GCST90094765 | MR Egger | 4 | -1.378604138 | 2.76538947948885 | 0.667543669643623 | -6.798767518 | 4.04155924146699 | 0.251929967124331 | 0.00111514870178887 | 56.9150179105734 | 0.999279047927446 |
| ebi-fl187-GCST90094765 | ebi-a-GCST90001436 | Memory B cell %lymphocyte \|\| id:ebi-a-GCST90001436 | Fried fish liking \|\| id:ebi-fl187-GCST90094765 | MR Egger | 4 | -1.13855717 | 2.58164040879097 | 0.702291412152547 | -6.198572371 | 3.92145803144908 | 0.320280799418752 | 0.00203232997940293 | 50.4739838096822 | 0.999279047927446 |
| ebi-fl187-GCST90094765 | ebi-a-GCST90001439 | IgD+ CD24+ B cell %lymphocyte \|\| id:ebi-a-GCST90001439 | Fried fish liking \|\| id:ebi-fl187-GCST90094765 | MR Egger | 4 | -0.992264649 | 2.29982994194679 | 0.708195628897292 | -5.499931335 | 3.51540203766247 | 0.370736152527679 | 0.00408705206720369 | 33.6294455101143 | 0.999279047927446 |
| ebi-fl187-GCST90094765 | ebi-a-GCST90001457 | CD11c+ HLA DR++ monocyte %monocyte \|\| id:ebi-a-GCST90001457 | Fried fish liking \|\| id:ebi-fl187-GCST90094765 | MR Egger | 4 | -3.240632173 | 2.28460811399552 | 0.2918332397293 | -7.718464076 | 1.23719973040484 | 0.0391391445650319 | 0.00044454286366925 | 3.44595035142036 | 0.999279047927446 |
| ebi-fl187-GCST90094765 | ebi-a-GCST90001483 | CD39+ resting CD4 regulatory T cell Absolute Count \|\| id:ebi-a-GCST90001483 | Fried fish liking \|\| id:ebi-fl187-GCST90094765 | MR Egger | 3 | 2.19908836241464 | 2.29548101624243 | 0.513651155069988 | -2.300054429 | 6.69823115424981 | 9.01678970704835 | 0.100253386840547 | 810.970074761116 | 0.999279047927446 |
| ebi-fl187-GCST90094765 | ebi-a-GCST90001525 | CD33dim HLA DR+ CD11b+ Absolute Count \|\| id:ebi-a-GCST90001525 | Fried fish liking \|\| id:ebi-fl187-GCST90094765 | MR Egger | 4 | 1.11401064388536 | 2.09303421120449 | 0.64776476315402 | -2.98833641 | 5.21635769784616 | 3.04655256226308 | 0.0503711640212916 | 184.26182310793 | 0.999279047927446 |
| ebi-fl187-GCST90094765 | ebi-a-GCST90001540 | Naive CD4+ T cell Absolute Count \|\| id:ebi-a-GCST90001540 | Fried fish liking \|\| id:ebi-fl187-GCST90094765 | MR Egger | 4 | 1.93928997470915 | 3.08746598173207 | 0.594089450636223 | -4.112143349 | 7.990723299 | 6.95381183511848 | 0.0163726445925118 | 2953.43240152844 | 0.999279047927446 |
| ebi-fl187-GCST90094765 | ebi-a-GCST90001624 | HLA DR+ CD4+ T cell Absolute Count \|\| id:ebi-a-GCST90001624 | Fried fish liking \|\| id:ebi-fl187-GCST90094765 | MR Egger | 4 | 1.08883171048251 | 1.54036508318489 | 0.552908241822817 | -1.930283853 | 4.10794727352489 | 2.97080128834564 | 0.145107003642831 | 60.8217389462454 | 0.999279047927446 |
| ebi-fl187-GCST90094765 | ebi-a-GCST90001672 | CD39+ CD8+ T cell Absolute Count \|\| id:ebi-a-GCST90001672 | Fried fish liking \|\| id:ebi-fl187-GCST90094765 | MR Egger | 4 | -0.393022877 | 1.66337963353355 | 0.83520916622515 | -3.653246959 | 2.86720120471959 | 0.675013305125953 | 0.0259068735169171 | 17.5877248097702 | 0.999279047927446 |
| ebi-fl187-GCST90094765 | ebi-a-GCST90001722 | CD19 on CD20- CD38- B cell \|\| id:ebi-a-GCST90001722 | Fried fish liking \|\| id:ebi-fl187-GCST90094765 | MR Egger | 4 | -0.903651647 | 1.55435888215435 | 0.619785447047249 | -3.950195056 | 2.14289176214561 | 0.405087718322486 | 0.01925094639848 | 8.52405155253427 | 0.999279047927446 |
| ebi-fl187-GCST90094765 | ebi-a-GCST90001742 | CD19 on transitional B cell \|\| id:ebi-a-GCST90001742 | Fried fish liking \|\| id:ebi-fl187-GCST90094765 | MR Egger | 4 | 0.155921579077894 | 2.70002042587688 | 0.959199789850503 | -5.136118456 | 5.44796161379657 | 1.16873454625519 | 0.00588047078005864 | 232.28419810237 | 0.999279047927446 |
| ebi-fl187-GCST90094765 | ebi-a-GCST90001784 | CD25 on IgD+ CD38dim B cell \|\| id:ebi-a-GCST90001784 | Fried fish liking \|\| id:ebi-fl187-GCST90094765 | MR Egger | 4 | -3.946799019 | 3.5498866494082 | 0.381957789620598 | -10.90457685 | 3.010978814 | 0.0193164344538633 | 1.83739464209037e-05 | 20.3072672284436 | 0.999279047927446 |
| ebi-fl187-GCST90094765 | ebi-a-GCST90001794 | CD25 on IgD+ B cell \|\| id:ebi-a-GCST90001794 | Fried fish liking \|\| id:ebi-fl187-GCST90094765 | MR Egger | 4 | -3.443172156 | 3.58068232506372 | 0.437717394356271 | -10.46130951 | 3.5749652006389 | 0.0319631322845564 | 2.86227267637299e-05 | 35.6933786872694 | 0.999279047927446 |
| ebi-fl187-GCST90094765 | ebi-a-GCST90001802 | CD27 on IgD- CD38- B cell \|\| id:ebi-a-GCST90001802 | Fried fish liking \|\| id:ebi-fl187-GCST90094765 | MR Egger | 4 | -0.041339907 | 2.02796520584057 | 0.985587182466178 | -4.016151711 | 3.9334718961583 | 0.959502932471722 | 0.018022186248106 | 51.0840285827468 | 0.999279047927446 |
| ebi-fl187-GCST90094765 | ebi-a-GCST90001804 | CD27 on IgD- CD38dim B cell \|\| id:ebi-a-GCST90001804 | Fried fish liking \|\| id:ebi-fl187-GCST90094765 | MR Egger | 4 | 1.06591213667699 | 2.37674039839215 | 0.697714928409765 | -3.592499044 | 5.7243233175256 | 2.90348615279734 | 0.027529447083562 | 306.225977365148 | 0.999279047927446 |
| ebi-fl187-GCST90094765 | ebi-a-GCST90001805 | CD27 on memory B cell \|\| id:ebi-a-GCST90001805 | Fried fish liking \|\| id:ebi-fl187-GCST90094765 | MR Egger | 4 | 1.19959384602849 | 1.68337230317314 | 0.550006704878272 | -2.099815868 | 4.49900356024784 | 3.3187687178704 | 0.122478978452697 | 89.9274793263321 | 0.999279047927446 |
| ebi-fl187-GCST90094765 | ebi-a-GCST90001806 | CD27 on unswitched memory B cell \|\| id:ebi-a-GCST90001806 | Fried fish liking \|\| id:ebi-fl187-GCST90094765 | MR Egger | 4 | 1.66070811527981 | 1.72008900120068 | 0.436168337747559 | -1.710666327 | 5.03208255763314 | 5.26303636170929 | 0.180745316985372 | 153.25183637768 | 0.999279047927446 |
| ebi-fl187-GCST90094765 | ebi-a-GCST90001839 | CD3 on Effector Memory CD8+ T cell \|\| id:ebi-a-GCST90001839 | Fried fish liking \|\| id:ebi-fl187-GCST90094765 | MR Egger | 4 | 0.226065720284647 | 1.75046319985876 | 0.909058237506969 | -3.204842151 | 3.65697359200781 | 1.25365805333499 | 0.0405653043074637 | 38.7439103816239 | 0.999279047927446 |
| ebi-fl187-GCST90094765 | ebi-a-GCST90001859 | CD3 on CD8+ T cell \|\| id:ebi-a-GCST90001859 | Fried fish liking \|\| id:ebi-fl187-GCST90094765 | MR Egger | 4 | -0.631136969 | 1.72261236798601 | 0.749207386998586 | -4.007457211 | 2.74518327198562 | 0.531986604607365 | 0.0181795633164318 | 15.5674667512982 | 0.999279047927446 |
| ebi-fl187-GCST90094765 | ebi-a-GCST90001905 | CD86 on monocyte \|\| id:ebi-a-GCST90001905 | Fried fish liking \|\| id:ebi-fl187-GCST90094765 | MR Egger | 4 | 4.01848083696237 | 2.09992338256931 | 0.19578163950437 | -0.097368993 | 8.13433066679822 | 55.6165510082076 | 0.907221186211601 | 3409.53319109007 | 0.999279047927446 |
| ebi-fl187-GCST90094765 | ebi-a-GCST90001943 | CD25 on activated & secreting CD4 regulatory T cell \|\| id:ebi-a-GCST90001943 | Fried fish liking \|\| id:ebi-fl187-GCST90094765 | MR Egger | 4 | 4.19101723563478 | 1.60501130030061 | 0.120681183853838 | 1.04519508704558 | 7.33683938422398 | 66.089985866815 | 2.84395328798107 | 1535.85020201811 | 0.999279047927446 |
| ebi-fl187-GCST90094765 | ebi-a-GCST90001949 | CD33 on CD33dim HLA DR+ CD11b- \|\| id:ebi-a-GCST90001949 | Fried fish liking \|\| id:ebi-fl187-GCST90094765 | MR Egger | 4 | -2.637072386 | 2.53855614279276 | 0.407999834409208 | -7.612642426 | 2.33849765399797 | 0.071570493931738 | 0.000494164339152507 | 10.3656520630723 | 0.999279047927446 |
| ebi-fl187-GCST90094765 | ebi-a-GCST90001955 | CD33 on Immature Myeloid-Derived Suppressor Cells \|\| id:ebi-a-GCST90001955 | Fried fish liking \|\| id:ebi-fl187-GCST90094765 | MR Egger | 4 | -1.435411578 | 2.41081007045656 | 0.611971791887506 | -6.160599316 | 3.28977616057639 | 0.238017381249192 | 0.00211098774463063 | 26.8368558371883 | 0.999279047927446 |
| ebi-fl187-GCST90094765 | ebi-a-GCST90001956 | CD33 on CD33+ HLA DR+ \|\| id:ebi-a-GCST90001956 | Fried fish liking \|\| id:ebi-fl187-GCST90094765 | MR Egger | 4 | -0.332916428 | 2.37134500440924 | 0.901213863156845 | -4.980752636 | 4.31491978083803 | 0.716830098717945 | 0.00686889083394016 | 74.8076221984772 | 0.999279047927446 |
| ebi-fl187-GCST90094765 | ebi-a-GCST90001957 | CD33 on CD33+ HLA DR+ CD14- \|\| id:ebi-a-GCST90001957 | Fried fish liking \|\| id:ebi-fl187-GCST90094765 | MR Egger | 4 | -0.499245915 | 2.3733166951358 | 0.852873204866051 | -5.150946637 | 4.15245480783024 | 0.606988208099575 | 0.00579391739282443 | 63.5899098644773 | 0.999279047927446 |
| ebi-fl187-GCST90094765 | ebi-a-GCST90002064 | CD4 on resting CD4 regulatory T cell \|\| id:ebi-a-GCST90002064 | Fried fish liking \|\| id:ebi-fl187-GCST90094765 | MR Egger | 4 | -0.534962561 | 1.68978619919038 | 0.781546731589978 | -3.846943511 | 2.77701838943731 | 0.585691217441964 | 0.0213448772107482 | 16.0710318828124 | 0.999279047927446 |
| ebi-fl187-GCST90094765 | ebi-a-GCST90002080 | SSC-A on T cell \|\| id:ebi-a-GCST90002080 | Fried fish liking \|\| id:ebi-fl187-GCST90094765 | MR Egger | 4 | -2.491862648 | 4.22744707542251 | 0.615277327632018 | -10.77765892 | 5.7939336194652 | 0.0827556781987651 | 2.08603794927563e-05 | 328.301902490109 | 0.999279047927446 |
| ebi-fl187-GCST90094765 | ebi-a-GCST90002121 | CD8 on CD39+ CD8+ T cell \|\| id:ebi-a-GCST90002121 | Fried fish liking \|\| id:ebi-fl187-GCST90094765 | MR Egger | 4 | 0.849506694110043 | 1.70928964681765 | 0.668449784904679 | -2.500701014 | 4.19971440187264 | 2.33849297498406 | 0.0820274760835919 | 66.667288269079 | 0.999279047927446 |
| ebi-fl187-GCST90094765 | ebi-cfmet338-GCST90026053 | 1-palmitoyl-2-docosahexaenoyl-gpc (16:0/22:6) levels \|\| id:ebi-cfmet338-GCST90026053 | Fried fish liking \|\| id:ebi-fl187-GCST90094765 | MR Egger | 4 | 1.17565181811865 | 0.854140563045894 | 0.3025348491812 | -0.498463685 | 2.8497673216886 | 3.24025431211884 | 0.607463197741834 | 17.2837598166182 | 0.999279047927446 |
| ebi-fl187-GCST90094765 | ebi-cfmet338-GCST90026063 | 1-stearoyl-2-oleoyl-gpc (18:0/18:1) levels \|\| id:ebi-cfmet338-GCST90026063 | Fried fish liking \|\| id:ebi-fl187-GCST90094765 | MR Egger | 3 | 0.340368656605906 | 1.01070393873426 | 0.793203660503545 | -1.640611063 | 2.32134837652505 | 1.40546562925713 | 0.193861544412087 | 10.1894042008879 | 0.999279047927446 |
| ebi-fl187-GCST90094765 | ebi-cfmet338-GCST90026113 | Adenosine 3',5'-cyclic monophosphate (camp) levels \|\| id:ebi-cfmet338-GCST90026113 | Fried fish liking \|\| id:ebi-fl187-GCST90094765 | MR Egger | 4 | -1.370557439 | 1.35624756259019 | 0.418609992383094 | -4.028802662 | 1.2876877837256 | 0.253965349908815 | 0.0177956245868474 | 3.62439647114029 | 0.999279047927446 |
| ebi-fl187-GCST90094765 | ebi-cfmet338-GCST90026117 | Alpha-ketoglutaramate levels \|\| id:ebi-cfmet338-GCST90026117 | Fried fish liking \|\| id:ebi-fl187-GCST90094765 | MR Egger | 4 | -0.161377851 | 1.1208080508913 | 0.898711903938773 | -2.358161631 | 2.03540592876233 | 0.850970470322303 | 0.0945939620821296 | 7.6553590252603 | 0.999279047927446 |
| ebi-fl187-GCST90094765 | ebi-cfmet338-GCST90026124 | Arginine levels \|\| id:ebi-cfmet338-GCST90026124 | Fried fish liking \|\| id:ebi-fl187-GCST90094765 | MR Egger | 4 | -0.125183355 | 0.440268443826358 | 0.802889813932572 | -0.988109505 | 0.737742794788149 | 0.88233510710008 | 0.372279819315231 | 2.09120989328217 | 0.999279047927446 |
| ebi-fl187-GCST90094765 | ebi-cfmet338-GCST90026169 | Glutamate levels \|\| id:ebi-cfmet338-GCST90026169 | Fried fish liking \|\| id:ebi-fl187-GCST90094765 | MR Egger | 4 | 0.975645499020562 | 0.658514686719505 | 0.27663794758082 | -0.315043287 | 2.26633428499079 | 2.65287908926908 | 0.729757284618503 | 9.64398384314901 | 0.999279047927446 |
| ebi-fl187-GCST90094765 | ebi-cfmet338-GCST90026170 | Glutamine levels \|\| id:ebi-cfmet338-GCST90026170 | Fried fish liking \|\| id:ebi-fl187-GCST90094765 | MR Egger | 4 | -0.337715268 | 0.278425637068121 | 0.348972588824426 | -0.883429516 | 0.207998980874188 | 0.713398386489362 | 0.413362843335623 | 1.23121191478839 | 0.999279047927446 |
| ebi-fl187-GCST90094765 | ebi-cfmet338-GCST90026191 | Kynurenate levels \|\| id:ebi-cfmet338-GCST90026191 | Fried fish liking \|\| id:ebi-fl187-GCST90094765 | MR Egger | 4 | -2.629615522 | 2.09140524074829 | 0.335557897748217 | -6.728769794 | 1.46953875004538 | 0.0721061801627797 | 0.00119600338440464 | 4.34722951913335 | 0.999279047927446 |
| ebi-fl187-GCST90094765 | ebi-cfmet338-GCST90026202 | Methylsuccinoylcarnitine levels \|\| id:ebi-cfmet338-GCST90026202 | Fried fish liking \|\| id:ebi-fl187-GCST90094765 | MR Egger | 4 | 1.17090498415991 | 1.41535566318997 | 0.495068830605035 | -1.603192116 | 3.94500208401225 | 3.22490981062788 | 0.201253068480337 | 51.676445806341 | 0.999279047927446 |
| ebi-fl187-GCST90094765 | ebi-cfmet338-GCST90026222 | N-acetylputrescine levels \|\| id:ebi-cfmet338-GCST90026222 | Fried fish liking \|\| id:ebi-fl187-GCST90094765 | MR Egger | 4 | -0.377300462 | 0.668943643767946 | 0.629550058958682 | -1.688430003 | 0.933829080147757 | 0.685710013387515 | 0.184809446543277 | 2.5442326204348 | 0.999279047927446 |
| ebi-fl187-GCST90094765 | ebi-cfmet338-GCST90026252 | Propionylcarnitine (c3) levels \|\| id:ebi-cfmet338-GCST90026252 | Fried fish liking \|\| id:ebi-fl187-GCST90094765 | MR Egger | 4 | -0.244471271 | 0.84091992282488 | 0.798641289362079 | -1.89267432 | 1.40373177752767 | 0.783118486089438 | 0.1506683342353 | 4.07036134279721 | 0.999279047927446 |
| ebi-fl187-GCST90094765 | ebi-cfmet338-GCST90026316 | Ethyl beta-glucopyranoside levels \|\| id:ebi-cfmet338-GCST90026316 | Fried fish liking \|\| id:ebi-fl187-GCST90094765 | MR Egger | 4 | 3.41371084282348 | 3.37507868609111 | 0.418269198558063 | -3.201443382 | 10.028865067562 | 30.3777624634278 | 0.0407034109908992 | 22671.5262878281 | 0.999279047927446 |
| ebi-fl187-GCST90094765 | ebi-gm430-GCST90011315 | F_Prevotellaceae abundance \|\| id:ebi-gm430-GCST90011315 | Fried fish liking \|\| id:ebi-fl187-GCST90094765 | MR Egger | 3 | 0.949875772332577 | 1.83171608160737 | 0.695444797985312 | -2.640287748 | 4.54003929228301 | 2.58538846258741 | 0.0713407384750903 | 93.6944815172329 | 0.999279047927446 |
| ebi-fl187-GCST90094765 | ebi-gm430-GCST90011354 | OTU97_1 (Bacteroides) abundance \|\| id:ebi-gm430-GCST90011354 | Fried fish liking \|\| id:ebi-fl187-GCST90094765 | MR Egger | 3 | -0.232214263 | 1.68638703293926 | 0.912885759266846 | -3.537532847 | 3.07310432175929 | 0.792776242648931 | 0.0290849957610588 | 21.6088795773535 | 0.999279047927446 |
| ebi-fl187-GCST90094765 | ebi-gm430-GCST90011419 | OTU99_1 (Bacteroides) abundance \|\| id:ebi-gm430-GCST90011419 | Fried fish liking \|\| id:ebi-fl187-GCST90094765 | MR Egger | 3 | -0.263579925 | 1.68690000358813 | 0.901325346457041 | -3.569903932 | 3.04274408182931 | 0.768296213754034 | 0.0281585586801316 | 20.9626877133196 | 0.999279047927446 |
| ebi-fl187-GCST90094765 | ebi-gm430-GCST90011435 | OTU99_155 (Butyrivibrio) abundance \|\| id:ebi-gm430-GCST90011435 | Fried fish liking \|\| id:ebi-fl187-GCST90094765 | MR Egger | 3 | -6.361433776 | 5.1281920361588 | 0.431928878365018 | -16.41269017 | 3.68982261523833 | 0.00172688896469654 | 7.44833544665604e-08 | 40.0377442416283 | 0.999279047927446 |
| ebi-fl187-GCST90094765 | ebi-gm430-GCST90011466 | OTU99_58 (Bacteroides) abundance \|\| id:ebi-gm430-GCST90011466 | Fried fish liking \|\| id:ebi-fl187-GCST90094765 | MR Egger | 3 | 0.289647609908688 | 3.16753899831572 | 0.941947359072177 | -5.918728827 | 6.4980240466075 | 1.33595662718915 | 0.00268861570188388 | 663.828641810001 | 0.999279047927446 |
| ebi-fl187-GCST90094765 | ebi-gm430-GCST90011505 | TestASV_28 (Holdemanella) abundance \|\| id:ebi-gm430-GCST90011505 | Fried fish liking \|\| id:ebi-fl187-GCST90094765 | MR Egger | 3 | -0.117812903 | 3.96212880564958 | 0.981075847271128 | -7.883585362 | 7.64795955620094 | 0.888862340649038 | 0.000376879382530517 | 2096.36371010588 | 0.999279047927446 |
| ebi-fl187-GCST90094765 | ebi-gm430-GCST90011513 | TestASV_35 (Alphaproteobacteria) abundance \|\| id:ebi-gm430-GCST90011513 | Fried fish liking \|\| id:ebi-fl187-GCST90094765 | MR Egger | 3 | -1.404793139 | 4.65897441400018 | 0.813563048741667 | -10.53638299 | 7.72679671288695 | 0.245417818687366 | 2.65525972555324e-05 | 2268.32445615901 | 0.999279047927446 |
| ebi-fl187-GCST90094765 | ebi-gm430-GCST90011543 | G_Coprococcus prevalence \|\| id:ebi-gm430-GCST90011543 | Fried fish liking \|\| id:ebi-fl187-GCST90094765 | MR Egger | 3 | 0.167795747653427 | 5.45576562086791 | 0.980426500002823 | -10.52550487 | 10.8610963645545 | 1.18269501774304 | 2.6843016357777e-05 | 52109.1775361882 | 0.999279047927446 |
| ebi-fl187-GCST90094765 | ebi-gm430-GCST90011547 | G_Parasutterella prevalence \|\| id:ebi-gm430-GCST90011547 | Fried fish liking \|\| id:ebi-fl187-GCST90094765 | MR Egger | 3 | 6.23309414102509 | 4.38109326884471 | 0.390027436972817 | -2.353848666 | 14.8200369479607 | 509.328983628296 | 0.0950028235817101 | 2730613.72055658 | 0.999279047927446 |
| ebi-fl187-GCST90094765 | ebi-gm430-GCST90011569 | OTU97_13 (Subdoligranulum) prevalence \|\| id:ebi-gm430-GCST90011569 | Fried fish liking \|\| id:ebi-fl187-GCST90094765 | MR Egger | 3 | 5.84153506802035 | 4.53037257652586 | 0.419946482156897 | -3.037995182 | 14.721065318011 | 344.30747059085 | 0.0479308859386514 | 2473303.63257634 | 0.999279047927446 |
| ebi-fl187-GCST90094765 | ebi-gm430-GCST90011590 | OTU97_277 (Prevotella) prevalence \|\| id:ebi-gm430-GCST90011590 | Fried fish liking \|\| id:ebi-fl187-GCST90094765 | MR Egger | 3 | 2.46746840378396 | 7.80898284854459 | 0.805162212056748 | -12.83813798 | 17.7730747869314 | 11.792555042892 | 2.65746478136085e-06 | 52329707.4019645 | 0.999279047927446 |
| ebi-fl187-GCST90094765 | ebi-gm430-GCST90011616 | OTU97_86 (Phascolarctobacterium) prevalence \|\| id:ebi-gm430-GCST90011616 | Fried fish liking \|\| id:ebi-fl187-GCST90094765 | MR Egger | 3 | 2.55551614692959 | 8.6991877331544 | 0.818100693412047 | -14.49489181 | 19.6059241039122 | 12.8779448541216 | 5.06930557570315e-07 | 327148287.253908 | 0.999279047927446 |
| ebi-fl187-GCST90094765 | ebi-gm430-GCST90011665 | OTU99_55 (Barnesiella) prevalence \|\| id:ebi-gm430-GCST90011665 | Fried fish liking \|\| id:ebi-fl187-GCST90094765 | MR Egger | 3 | 1.74641423048132 | 3.27676070801937 | 0.688263727364496 | -4.676036757 | 8.16886521819929 | 5.73400494855963 | 0.00931586184216757 | 3529.33666333294 | 0.999279047927446 |
| ebi-fl187-GCST90094765 | ebi-gm430-GCST90011687 | TestASV_14 (Lachnospiraceae) prevalence \|\| id:ebi-gm430-GCST90011687 | Fried fish liking \|\| id:ebi-fl187-GCST90094765 | MR Egger | 3 | 4.13820662812133 | 3.44995730471049 | 0.442415908519112 | -2.623709689 | 10.9001229453539 | 62.690293567642 | 0.072533287170033 | 54183.0249383878 | 0.999279047927446 |
| ebi-fl187-GCST90094765 | ebi-gm430-GCST90011697 | TestASV_23 (Barnesiella) prevalence \|\| id:ebi-gm430-GCST90011697 | Fried fish liking \|\| id:ebi-fl187-GCST90094765 | MR Egger | 3 | 2.09740603553941 | 4.74602127128285 | 0.73508842665686 | -7.204795656 | 11.3996077272538 | 8.1450146079517 | 0.000743014010936261 | 89286.6917545081 | 0.999279047927446 |
| ebi-fl187-GCST90094765 | ebi-gm430-GCST90011727 | TestASV_6 (Subdoligranulum) prevalence \|\| id:ebi-gm430-GCST90011727 | Fried fish liking \|\| id:ebi-fl187-GCST90094765 | MR Egger | 3 | 6.03030549261321 | 5.97775634203207 | 0.49721407041461 | -5.686096938 | 17.7467079229961 | 415.842046653093 | 0.00339280950675509 | 50967968.4109407 | 0.999279047927446 |
| ebi-fl187-GCST90094765 | ebi-hbsm44-GCST90104908 | Sulcal depth of the left posterior inferior temporal sulcus \|\| id:ebi-hbsm44-GCST90104908 | Fried fish liking \|\| id:ebi-fl187-GCST90094765 | MR Egger | 3 | -1.335829392 | 1.28189208989547 | 0.486884511997578 | -3.848337888 | 1.17667910446694 | 0.262940004729108 | 0.0213151351554687 | 3.2435846914724 | 0.999279047927446 |
| ebi-fl187-GCST90094765 | ebi-hbsm44-GCST90104918 | Sulcal depth of the left inferior frontal sulcus \|\| id:ebi-hbsm44-GCST90104918 | Fried fish liking \|\| id:ebi-fl187-GCST90094765 | MR Egger | 3 | -0.319131394 | 2.11937388716532 | 0.90485378501041 | -4.473104213 | 3.83484142507912 | 0.726780048665531 | 0.0114118360452243 | 46.2860872733375 | 0.999279047927446 |
| ebi-fl187-GCST90094765 | ebi-hbsm44-GCST90104925 | Sulcal depth of the right anterior inferior temporal sulcus \|\| id:ebi-hbsm44-GCST90104925 | Fried fish liking \|\| id:ebi-fl187-GCST90094765 | MR Egger | 3 | -0.718616539 | 0.753331745461297 | 0.515011594296906 | -2.19514676 | 0.757913681955109 | 0.487426124678227 | 0.111342219693426 | 2.13381974666041 | 0.999279047927446 |
| ebi-fl187-GCST90094765 | ebi-IPs91-GCST90274770 | C-C motif chemokine 4 levels \|\| id:ebi-IPs91-GCST90274770 | Fried fish liking \|\| id:ebi-fl187-GCST90094765 | MR Egger | 5 | 0.53068771825235 | 0.694029126129421 | 0.500125978523479 | -0.829609369 | 1.89098480546601 | 1.70010109722926 | 0.436219653980451 | 6.62589068242593 | 0.999279047927446 |
| ebi-fl187-GCST90094765 | ebi-IPs91-GCST90274779 | C-X-C motif chemokine 1 levels \|\| id:ebi-IPs91-GCST90274779 | Fried fish liking \|\| id:ebi-fl187-GCST90094765 | MR Egger | 5 | 0.377466978107585 | 0.689956269327865 | 0.622387884042476 | -0.97484731 | 1.7297812659902 | 1.45858527775295 | 0.377249951545692 | 5.63942024050853 | 0.999279047927446 |
| ebi-fl187-GCST90094765 | ebi-IPs91-GCST90274780 | C-X-C motif chemokine 10 levels \|\| id:ebi-IPs91-GCST90274780 | Fried fish liking \|\| id:ebi-fl187-GCST90094765 | MR Egger | 5 | 0.450549155386709 | 1.04934123068541 | 0.69662723257549 | -1.606159657 | 2.50725796753011 | 1.5691736690974 | 0.200656727006851 | 12.2712357592903 | 0.999279047927446 |
| ebi-fl187-GCST90094765 | ebi-IPs91-GCST90274796 | Interleukin-10 receptor subunit alpha levels \|\| id:ebi-IPs91-GCST90274796 | Fried fish liking \|\| id:ebi-fl187-GCST90094765 | MR Egger | 5 | 0.978757582568262 | 1.2370702295239 | 0.486615108841281 | -1.445900067 | 3.40341523243511 | 2.66114793062572 | 0.235533984684384 | 30.0666093606961 | 0.999279047927446 |
| ebi-fl187-GCST90094765 | ebi-IPs91-GCST90274807 | Interleukin-20 levels \|\| id:ebi-IPs91-GCST90274807 | Fried fish liking \|\| id:ebi-fl187-GCST90094765 | MR Egger | 5 | -0.151103323 | 0.863416429195485 | 0.872219569726873 | -1.843399524 | 1.54119287814886 | 0.859758861141122 | 0.158278439076845 | 4.67015787887447 | 0.999279047927446 |
| ebi-fl187-GCST90094765 | ebi-IPs91-GCST90274808 | Interleukin-20 receptor subunit alpha levels \|\| id:ebi-IPs91-GCST90274808 | Fried fish liking \|\| id:ebi-fl187-GCST90094765 | MR Egger | 5 | -0.640257178 | 0.88171471128669 | 0.520272168169149 | -2.368418012 | 1.08790365590237 | 0.527156833352489 | 0.0936287286714553 | 2.96804550156138 | 0.999279047927446 |
| ebi-fl187-GCST90094765 | ebi-IPs91-GCST90274810 | Interleukin-24 levels \|\| id:ebi-IPs91-GCST90274810 | Fried fish liking \|\| id:ebi-fl187-GCST90094765 | MR Egger | 5 | -0.161124313 | 0.780158254574078 | 0.849601262196869 | -1.690234492 | 1.36798586550453 | 0.851186250621345 | 0.184476260634853 | 3.92743234686936 | 0.999279047927446 |
| ebi-fl187-GCST90094765 | ebi-IPs91-GCST90274823 | Monocyte chemoattractant protein-3 levels \|\| id:ebi-IPs91-GCST90274823 | Fried fish liking \|\| id:ebi-fl187-GCST90094765 | MR Egger | 5 | 0.445310511360032 | 0.775062492765134 | 0.605851564160966 | -1.073811974 | 1.96443299717969 | 1.56097482100261 | 0.341703466701196 | 7.13086822128981 | 0.999279047927446 |
| ebi-fl187-GCST90094765 | ebi-IPs91-GCST90274824 | Monocyte chemoattractant protein-4 levels \|\| id:ebi-IPs91-GCST90274824 | Fried fish liking \|\| id:ebi-fl187-GCST90094765 | MR Egger | 5 | 0.330703788018955 | 0.688422597987911 | 0.663811447170089 | -1.018604504 | 1.68001208007526 | 1.39194741973365 | 0.361098500232537 | 5.36562078783342 | 0.999279047927446 |
| ebi-fl187-GCST90094765 | ebi-IPs91-GCST90274834 | SIR2-like protein 2 levels \|\| id:ebi-IPs91-GCST90274834 | Fried fish liking \|\| id:ebi-fl187-GCST90094765 | MR Egger | 5 | 0.0503427001141613 | 0.706376462338469 | 0.947668877821657 | -1.334155166 | 1.43484056629756 | 1.05163142884023 | 0.263380594352098 | 4.19897549720801 | 0.999279047927446 |
| ebi-fl187-GCST90094765 | ebi-met1400-GCST90199652 | 1-stearoyl-2-oleoyl-GPS (18:0/18:1) levels \|\| id:ebi-met1400-GCST90199652 | Fried fish liking \|\| id:ebi-fl187-GCST90094765 | MR Egger | 4 | -0.776300453 | 1.39934234071759 | 0.634816982037096 | -3.519011441 | 1.96641053479162 | 0.460105046775256 | 0.0296287104289908 | 7.14498373378146 | 0.999279047927446 |
| ebi-fl187-GCST90094765 | ebi-met1400-GCST90199665 | N-acetylaspartate (naa) levels \|\| id:ebi-met1400-GCST90199665 | Fried fish liking \|\| id:ebi-fl187-GCST90094765 | MR Egger | 4 | -1.22262082 | 1.03236857544363 | 0.357969521617234 | -3.246063227 | 0.800821588298407 | 0.29445743496239 | 0.0389271539285928 | 2.22737015821091 | 0.999279047927446 |
| ebi-fl187-GCST90094765 | ebi-met1400-GCST90199669 | Acetylcarnitine levels (Biocrates platform) \|\| id:ebi-met1400-GCST90199669 | Fried fish liking \|\| id:ebi-fl187-GCST90094765 | MR Egger | 4 | -0.227475912 | 1.07813532147432 | 0.852440628937691 | -2.340621142 | 1.88566931778786 | 0.796541608120267 | 0.0962678236295335 | 6.59076428182774 | 0.999279047927446 |
| ebi-fl187-GCST90094765 | ebi-met1400-GCST90199670 | Iminodiacetate (IDA) levels \|\| id:ebi-met1400-GCST90199670 | Fried fish liking \|\| id:ebi-fl187-GCST90094765 | MR Egger | 4 | -0.791976483 | 1.03885830862552 | 0.525488569337385 | -2.828138767 | 1.2441858023464 | 0.452948664844522 | 0.059122792591855 | 3.47010829479491 | 0.999279047927446 |
| ebi-fl187-GCST90094765 | ebi-met1400-GCST90199686 | Indolepropionate levels \|\| id:ebi-met1400-GCST90199686 | Fried fish liking \|\| id:ebi-fl187-GCST90094765 | MR Egger | 4 | -0.475003871 | 1.03639152549323 | 0.691701713903223 | -2.506331261 | 1.5563235185836 | 0.621882648914362 | 0.0815669387614287 | 4.74135764922961 | 0.999279047927446 |
| ebi-fl187-GCST90094765 | ebi-met1400-GCST90199704 | 3-hydroxysebacate levels \|\| id:ebi-met1400-GCST90199704 | Fried fish liking \|\| id:ebi-fl187-GCST90094765 | MR Egger | 4 | 1.78682718260855 | 1.0604131116931 | 0.234024073486497 | -0.291582516 | 3.86523688152703 | 5.97047914279472 | 0.747080364744568 | 47.7145737951962 | 0.999279047927446 |
| ebi-fl187-GCST90094765 | ebi-met1400-GCST90199713 | Docosapentaenoate n3 DPA; 22:5n3 levels \|\| id:ebi-met1400-GCST90199713 | Fried fish liking \|\| id:ebi-fl187-GCST90094765 | MR Egger | 4 | 0.63331399554082 | 1.2300444990848 | 0.657898211233127 | -1.777573223 | 3.04420121374702 | 1.88384329424198 | 0.169047891480031 | 20.9932553798205 | 0.999279047927446 |
| ebi-fl187-GCST90094765 | ebi-met1400-GCST90199715 | Myristoleate (14:1n5) levels \|\| id:ebi-met1400-GCST90199715 | Fried fish liking \|\| id:ebi-fl187-GCST90094765 | MR Egger | 4 | 0.413392416905812 | 0.944185647007358 | 0.704256588866092 | -1.437211451 | 2.26399628504023 | 1.5119382196547 | 0.237589365324977 | 9.62146254705405 | 0.999279047927446 |
| ebi-fl187-GCST90094765 | ebi-met1400-GCST90199725 | Campesterol levels \|\| id:ebi-met1400-GCST90199725 | Fried fish liking \|\| id:ebi-fl187-GCST90094765 | MR Egger | 4 | 0.505964915223954 | 1.29357994577311 | 0.733432969144102 | -2.029451778 | 3.04138160893925 | 1.6585851426412 | 0.131407541845881 | 20.9341460676319 | 0.999279047927446 |
| ebi-fl187-GCST90094765 | ebi-met1400-GCST90199737 | N-acetylproline levels \|\| id:ebi-met1400-GCST90199737 | Fried fish liking \|\| id:ebi-fl187-GCST90094765 | MR Egger | 4 | -0.97796487 | 1.55235130062957 | 0.593079568183783 | -4.02057342 | 2.06464367883034 | 0.376075683326873 | 0.0179426733078474 | 7.88248869960297 | 0.999279047927446 |
| ebi-fl187-GCST90094765 | ebi-met1400-GCST90199775 | Gamma-glutamylisoleucine levels \|\| id:ebi-met1400-GCST90199775 | Fried fish liking \|\| id:ebi-fl187-GCST90094765 | MR Egger | 4 | -0.26326311 | 0.989737659073512 | 0.81515579095562 | -2.203148922 | 1.67662270138945 | 0.768539659933732 | 0.110454796609374 | 5.34746545213345 | 0.999279047927446 |
| ebi-fl187-GCST90094765 | ebi-met1400-GCST90199777 | 1-palmitoleoyl-GPC (16:1) levels \|\| id:ebi-met1400-GCST90199777 | Fried fish liking \|\| id:ebi-fl187-GCST90094765 | MR Egger | 4 | -1.075455095 | 1.30784299855382 | 0.497336191705674 | -3.638827372 | 1.48791718235219 | 0.341142467800712 | 0.0262831462561293 | 4.42786347582036 | 0.999279047927446 |
| ebi-fl187-GCST90094765 | ebi-met1400-GCST90199782 | Glutamine degradant levels \|\| id:ebi-met1400-GCST90199782 | Fried fish liking \|\| id:ebi-fl187-GCST90094765 | MR Egger | 4 | 0.91388516017832 | 1.04283850917702 | 0.473263759851953 | -1.130078318 | 2.95784863816527 | 2.49399329843267 | 0.323007958156159 | 19.2564994625302 | 0.999279047927446 |
| ebi-fl187-GCST90094765 | ebi-met1400-GCST90199783 | 5-methyluridine (ribothymidine) levels \|\| id:ebi-met1400-GCST90199783 | Fried fish liking \|\| id:ebi-fl187-GCST90094765 | MR Egger | 4 | 0.425402642802918 | 1.32176814763659 | 0.778096044846925 | -2.165262927 | 3.01606821217064 | 1.53020642224345 | 0.114719767757169 | 20.4108824525473 | 0.999279047927446 |
| ebi-fl187-GCST90094765 | ebi-met1400-GCST90199787 | Homostachydrine levels \|\| id:ebi-met1400-GCST90199787 | Fried fish liking \|\| id:ebi-fl187-GCST90094765 | MR Egger | 4 | -1.461717336 | 1.48141104937218 | 0.427800662131574 | -4.365282992 | 1.44184832118018 | 0.23183778937452 | 0.0127110575340675 | 4.2285042324771 | 0.999279047927446 |
| ebi-fl187-GCST90094765 | ebi-met1400-GCST90199802 | Beta-hydroxyisovaleroylcarnitine levels \|\| id:ebi-met1400-GCST90199802 | Fried fish liking \|\| id:ebi-fl187-GCST90094765 | MR Egger | 4 | -2.045465159 | 1.01312047361692 | 0.180945534200077 | -4.031181287 | -0.05974903 | 0.129320021647451 | 0.0177533457650176 | 0.942000917474991 | 0.999279047927446 |
| ebi-fl187-GCST90094765 | ebi-met1400-GCST90199873 | Pregnenolone sulfate levels \|\| id:ebi-met1400-GCST90199873 | Fried fish liking \|\| id:ebi-fl187-GCST90094765 | MR Egger | 4 | -0.607791351 | 1.83963725040022 | 0.772506995511178 | -4.213480362 | 2.9978976595187 | 0.54455226652785 | 0.0147947874133532 | 20.0433546421206 | 0.999279047927446 |
| ebi-fl187-GCST90094765 | ebi-met1400-GCST90199911 | Beta-citrylglutamate levels \|\| id:ebi-met1400-GCST90199911 | Fried fish liking \|\| id:ebi-fl187-GCST90094765 | MR Egger | 4 | -1.045996298 | 1.01859289634698 | 0.412433136896326 | -3.042438375 | 0.950445778724719 | 0.351341603969677 | 0.0477183921874383 | 2.58686257062284 | 0.999279047927446 |
| ebi-fl187-GCST90094765 | ebi-met1400-GCST90199934 | 2-hydroxydecanoate levels \|\| id:ebi-met1400-GCST90199934 | Fried fish liking \|\| id:ebi-fl187-GCST90094765 | MR Egger | 4 | 0.0276695624046972 | 1.02273886461752 | 0.980873166446932 | -1.976898612 | 2.03223773705504 | 1.02805591996301 | 0.138498108256529 | 7.63114375983666 | 0.999279047927446 |
| ebi-fl187-GCST90094765 | ebi-met1400-GCST90199945 | Sphingomyelin (d18:1/14:0, d16:1/16:0) levels \|\| id:ebi-met1400-GCST90199945 | Fried fish liking \|\| id:ebi-fl187-GCST90094765 | MR Egger | 4 | -0.737514309 | 1.80606752984777 | 0.722583818028688 | -4.277406667 | 2.80237804994836 | 0.478301348684797 | 0.0138786073069331 | 16.4837994976205 | 0.999279047927446 |
| ebi-fl187-GCST90094765 | ebi-met1400-GCST90199979 | Carnitine C14:1 levels \|\| id:ebi-met1400-GCST90199979 | Fried fish liking \|\| id:ebi-fl187-GCST90094765 | MR Egger | 4 | 1.19348976106481 | 1.68153936773589 | 0.551445367387899 | -2.1023274 | 4.48930692182716 | 3.2985723744492 | 0.122171754602091 | 89.0596991499147 | 0.999279047927446 |
| ebi-fl187-GCST90094765 | ebi-met1400-GCST90199986 | Eugenol sulfate levels \|\| id:ebi-met1400-GCST90199986 | Fried fish liking \|\| id:ebi-fl187-GCST90094765 | MR Egger | 4 | 0.236888538650936 | 1.50606563022211 | 0.889461003887266 | -2.715000097 | 3.18877717388627 | 1.26729985495126 | 0.066204946612708 | 24.2587450716438 | 0.999279047927446 |
| ebi-fl187-GCST90094765 | ebi-met1400-GCST90200002 | 6-hydroxyindole sulfate levels \|\| id:ebi-met1400-GCST90200002 | Fried fish liking \|\| id:ebi-fl187-GCST90094765 | MR Egger | 4 | -0.109362297 | 2.57317152986995 | 0.969960834875554 | -5.152778496 | 4.93405390108106 | 0.896405593187111 | 0.00578331346905307 | 138.941627805055 | 0.999279047927446 |
| ebi-fl187-GCST90094765 | ebi-met1400-GCST90200023 | Trans 3,4-methyleneheptanoate levels \|\| id:ebi-met1400-GCST90200023 | Fried fish liking \|\| id:ebi-fl187-GCST90094765 | MR Egger | 4 | -1.05195568 | 1.10267578985108 | 0.440765354541169 | -3.213200228 | 1.10928886835784 | 0.349254051705858 | 0.0402276693489828 | 3.03220133323589 | 0.999279047927446 |
| ebi-fl187-GCST90094765 | ebi-met1400-GCST90200032 | 2-hydroxybutyrate/2-hydroxyisobutyrate levels \|\| id:ebi-met1400-GCST90200032 | Fried fish liking \|\| id:ebi-fl187-GCST90094765 | MR Egger | 4 | -0.227243818 | 1.03626398554508 | 0.846768769835037 | -2.25832123 | 1.80383359366338 | 0.796726502340311 | 0.104525812378373 | 6.07288386560067 | 0.999279047927446 |
| ebi-fl187-GCST90094765 | ebi-met1400-GCST90200051 | 1-palmitoyl-2-dihomo-linolenoyl-GPC (16:0/20:3n3 or 6) levels \|\| id:ebi-met1400-GCST90200051 | Fried fish liking \|\| id:ebi-fl187-GCST90094765 | MR Egger | 4 | -0.737041826 | 1.00974770654073 | 0.541352099979886 | -2.716147331 | 1.24206367871105 | 0.478527391071553 | 0.066129037575251 | 3.46275210409907 | 0.999279047927446 |
| ebi-fl187-GCST90094765 | ebi-met1400-GCST90200069 | Phenylacetylglutamate levels \|\| id:ebi-met1400-GCST90200069 | Fried fish liking \|\| id:ebi-fl187-GCST90094765 | MR Egger | 4 | 0.417990273097575 | 1.12840015497444 | 0.746616233470605 | -1.793674031 | 2.62965457684748 | 1.5189059000809 | 0.166347878363226 | 13.868978408387 | 0.999279047927446 |
| ebi-fl187-GCST90094765 | ebi-met1400-GCST90200072 | Thioproline levels \|\| id:ebi-met1400-GCST90200072 | Fried fish liking \|\| id:ebi-fl187-GCST90094765 | MR Egger | 4 | 0.280624736040748 | 1.16320105735452 | 0.831838295326233 | -1.999249336 | 2.56049880845561 | 1.32395667747713 | 0.135436912651018 | 12.9422714201474 | 0.999279047927446 |
| ebi-fl187-GCST90094765 | ebi-met1400-GCST90200081 | 1-oleoyl-2-docosahexaenoyl-GPC (18:1/22:6) levels \|\| id:ebi-met1400-GCST90200081 | Fried fish liking \|\| id:ebi-fl187-GCST90094765 | MR Egger | 4 | 0.54415069067978 | 1.51469872594566 | 0.753793706321691 | -2.424658812 | 3.51296019353327 | 1.72314427823021 | 0.0885083118496135 | 33.5474278239832 | 0.999279047927446 |
| ebi-fl187-GCST90094765 | ebi-met1400-GCST90200097 | Furaneol sulfate levels \|\| id:ebi-met1400-GCST90200097 | Fried fish liking \|\| id:ebi-fl187-GCST90094765 | MR Egger | 4 | -2.649925425 | 1.21986101672249 | 0.16194568737697 | -5.040853018 | -0.258997833 | 0.070656482036717 | 0.00646822844735498 | 0.771824695809316 | 0.999279047927446 |
| ebi-fl187-GCST90094765 | ebi-met1400-GCST90200124 | Sphingomyelin (d18:2/24:2) levels \|\| id:ebi-met1400-GCST90200124 | Fried fish liking \|\| id:ebi-fl187-GCST90094765 | MR Egger | 4 | 0.541183595328574 | 0.936304314218459 | 0.621671209034308 | -1.293972861 | 2.37634005119675 | 1.71803912234031 | 0.274179340168741 | 10.7654297514732 | 0.999279047927446 |
| ebi-fl187-GCST90094765 | ebi-met1400-GCST90200137 | Lignoceroylcarnitine (C24) levels \|\| id:ebi-met1400-GCST90200137 | Fried fish liking \|\| id:ebi-fl187-GCST90094765 | MR Egger | 4 | 0.801305070405916 | 1.03182479519364 | 0.518665337182224 | -1.221071528 | 2.82368166898545 | 2.22844731220057 | 0.294913988909529 | 16.8387313250757 | 0.999279047927446 |
| ebi-fl187-GCST90094765 | ebi-met1400-GCST90200165 | Octadecenedioate (C18:1-DC) levels \|\| id:ebi-met1400-GCST90200165 | Fried fish liking \|\| id:ebi-fl187-GCST90094765 | MR Egger | 4 | 0.020869170658664 | 1.00717126228787 | 0.98534991120634 | -1.953186503 | 1.99492484474288 | 1.02108845456855 | 0.141821436314955 | 7.3516504919451 | 0.999279047927446 |
| ebi-fl187-GCST90094765 | ebi-met1400-GCST90200176 | Glutamine conjugate of C6H10O2 (1) levels \|\| id:ebi-met1400-GCST90200176 | Fried fish liking \|\| id:ebi-fl187-GCST90094765 | MR Egger | 4 | 2.08384052802978 | 1.60009310381109 | 0.322591950015135 | -1.052341955 | 5.22002301149951 | 8.03526940874826 | 0.349119169408714 | 184.938439732534 | 0.999279047927446 |
| ebi-fl187-GCST90094765 | ebi-met1400-GCST90200182 | Glycine conjugate of C10H12O2 levels \|\| id:ebi-met1400-GCST90200182 | Fried fish liking \|\| id:ebi-fl187-GCST90094765 | MR Egger | 4 | -1.184332553 | 1.18006822602025 | 0.421261355945615 | -3.497266276 | 1.12860116985636 | 0.305950316943029 | 0.0302800476689296 | 3.0913292297617 | 0.999279047927446 |
| ebi-fl187-GCST90094765 | ebi-met1400-GCST90200208 | Glucuronide of piperine metabolite C17H21NO3 (4) levels \|\| id:ebi-met1400-GCST90200208 | Fried fish liking \|\| id:ebi-fl187-GCST90094765 | MR Egger | 4 | 0.00597653882389823 | 1.05295572784073 | 0.995986519678026 | -2.057816688 | 2.06976976539172 | 1.00599443396463 | 0.12773254570771 | 7.92299875932655 | 0.999279047927446 |
| ebi-fl187-GCST90094765 | ebi-met1400-GCST90200227 | 3-ethylcatechol sulfate (2) levels \|\| id:ebi-met1400-GCST90200227 | Fried fish liking \|\| id:ebi-fl187-GCST90094765 | MR Egger | 4 | 0.215619485051058 | 1.34414167078302 | 0.887292741420728 | -2.41889819 | 2.85013715978578 | 1.24063021083152 | 0.0890196462094749 | 17.2901531916449 | 0.999279047927446 |
| ebi-fl187-GCST90094765 | ebi-met1400-GCST90200259 | Pentose acid levels \|\| id:ebi-met1400-GCST90200259 | Fried fish liking \|\| id:ebi-fl187-GCST90094765 | MR Egger | 4 | -1.465312037 | 1.01184753718079 | 0.28455878169372 | -3.44853321 | 0.517909135933758 | 0.231005897856354 | 0.031792234732576 | 1.67851443263726 | 0.999279047927446 |
| ebi-fl187-GCST90094765 | ebi-met1400-GCST90200291 | Sarcosine levels \|\| id:ebi-met1400-GCST90200291 | Fried fish liking \|\| id:ebi-fl187-GCST90094765 | MR Egger | 4 | -0.486648814 | 1.02874977532092 | 0.682780058492299 | -2.502998374 | 1.52970074572543 | 0.614682863119373 | 0.0818392457501738 | 4.61679501991045 | 0.999279047927446 |
| ebi-fl187-GCST90094765 | ebi-met1400-GCST90200306 | Butyrate/isobutyrate (4:0) levels \|\| id:ebi-met1400-GCST90200306 | Fried fish liking \|\| id:ebi-fl187-GCST90094765 | MR Egger | 4 | -0.176120561 | 1.04143756966945 | 0.88126501655027 | -2.217338197 | 1.86509707562914 | 0.838516884873356 | 0.108898589924897 | 6.45656263044934 | 0.999279047927446 |
| ebi-fl187-GCST90094765 | ebi-met1400-GCST90200321 | Cholate levels \|\| id:ebi-met1400-GCST90200321 | Fried fish liking \|\| id:ebi-fl187-GCST90094765 | MR Egger | 4 | 0.549460574974061 | 1.06001995623265 | 0.655859882912285 | -1.528178539 | 2.62709968919005 | 1.73231830993836 | 0.216930437957218 | 13.8335899526444 | 0.999279047927446 |
| ebi-fl187-GCST90094765 | ebi-met1400-GCST90200336 | Ceramide (d18:1/16:0) levels \|\| id:ebi-met1400-GCST90200336 | Fried fish liking \|\| id:ebi-fl187-GCST90094765 | MR Egger | 4 | 0.401169041315481 | 1.47346456919266 | 0.810952883009313 | -2.486821514 | 3.2891595969331 | 1.49356972215241 | 0.0831739139724392 | 26.8203143075557 | 0.999279047927446 |
| ebi-fl187-GCST90094765 | ebi-met1400-GCST90200358 | Arachidonate (20:4n6) levels \|\| id:ebi-met1400-GCST90200358 | Fried fish liking \|\| id:ebi-fl187-GCST90094765 | MR Egger | 4 | 0.147050051928976 | 1.01109617511837 | 0.897700559537141 | -1.834698451 | 2.12879855516097 | 1.15841194233112 | 0.159661640269283 | 8.40476288400956 | 0.999279047927446 |
| ebi-fl187-GCST90094765 | ebi-met1400-GCST90200413 | 2'-deoxyuridine levels \|\| id:ebi-met1400-GCST90200413 | Fried fish liking \|\| id:ebi-fl187-GCST90094765 | MR Egger | 4 | 0.708853541359262 | 1.02546738347417 | 0.560863736010726 | -1.30106253 | 2.71876961296864 | 2.03166070798833 | 0.272242373545506 | 15.1616560590032 | 0.999279047927446 |
| ebi-fl187-GCST90094765 | ebi-met1400-GCST90200432 | Threonine levels \|\| id:ebi-met1400-GCST90200432 | Fried fish liking \|\| id:ebi-fl187-GCST90094765 | MR Egger | 4 | -0.117171955 | 1.02779017952834 | 0.919647820294441 | -2.131640706 | 1.89729679726297 | 0.889432238037208 | 0.118642476596024 | 6.66784552006212 | 0.999279047927446 |
| ebi-fl187-GCST90094765 | ebi-met1400-GCST90200436 | Caffeine levels \|\| id:ebi-met1400-GCST90200436 | Fried fish liking \|\| id:ebi-fl187-GCST90094765 | MR Egger | 4 | -0.458697873 | 1.05121962944204 | 0.705170071593191 | -2.519088347 | 1.60169260080464 | 0.632106192462539 | 0.0805329914685639 | 4.96142303003181 | 0.999279047927446 |
| ebi-fl187-GCST90094765 | ebi-met1400-GCST90200442 | Valine levels \|\| id:ebi-met1400-GCST90200442 | Fried fish liking \|\| id:ebi-fl187-GCST90094765 | MR Egger | 4 | -0.483522627 | 1.00819904537128 | 0.67884298862886 | -2.459592756 | 1.49254750197305 | 0.616607483457818 | 0.0854697509341019 | 4.44841343868342 | 0.999279047927446 |
| ebi-fl187-GCST90094765 | ebi-met1400-GCST90200459 | Margarate (17:0) levels \|\| id:ebi-met1400-GCST90200459 | Fried fish liking \|\| id:ebi-fl187-GCST90094765 | MR Egger | 4 | 0.221607484737709 | 1.0075095270669 | 0.846315543890296 | -1.753111188 | 2.19632615778884 | 1.24808139071242 | 0.173234140138701 | 8.99191785519567 | 0.999279047927446 |
| ebi-fl187-GCST90094765 | ebi-met1400-GCST90200462 | X-11299 levels \|\| id:ebi-met1400-GCST90200462 | Fried fish liking \|\| id:ebi-fl187-GCST90094765 | MR Egger | 4 | -1.458378098 | 1.20603847408335 | 0.350123941681304 | -3.822213507 | 0.905457311695582 | 0.232613244944356 | 0.0218793172390658 | 2.47306262495846 | 0.999279047927446 |
| ebi-fl187-GCST90094765 | ebi-met1400-GCST90200497 | X-12740 levels \|\| id:ebi-met1400-GCST90200497 | Fried fish liking \|\| id:ebi-fl187-GCST90094765 | MR Egger | 4 | -0.765606373 | 1.40768778274989 | 0.641051487544937 | -3.524674427 | 1.99346168158512 | 0.465051850758053 | 0.0294613976495265 | 7.34090169333722 | 0.999279047927446 |
| ebi-fl187-GCST90094765 | ebi-met1400-GCST90200527 | X-13866 levels \|\| id:ebi-met1400-GCST90200527 | Fried fish liking \|\| id:ebi-fl187-GCST90094765 | MR Egger | 4 | -0.245507076 | 1.00666985706329 | 0.830058907692239 | -2.218579995 | 1.72756584432405 | 0.78230774854081 | 0.108763443815264 | 5.62694037590876 | 0.999279047927446 |
| ebi-fl187-GCST90094765 | ebi-met1400-GCST90200547 | X-17654 levels \|\| id:ebi-met1400-GCST90200547 | Fried fish liking \|\| id:ebi-fl187-GCST90094765 | MR Egger | 4 | 2.379544899 | 2.08983526851674 | 0.372870768524184 | -1.716532227 | 6.47562202534382 | 10.7999866611532 | 0.179688186542086 | 649.12287293727 | 0.999279047927446 |
| ebi-fl187-GCST90094765 | ebi-met1400-GCST90200553 | X-17653 levels \|\| id:ebi-met1400-GCST90200553 | Fried fish liking \|\| id:ebi-fl187-GCST90094765 | MR Egger | 3 | 1.04878223066451 | 1.10076431320073 | 0.515392256995396 | -1.108715823 | 3.20628028453795 | 2.85417327619444 | 0.329982444900145 | 24.6870862872952 | 0.999279047927446 |
| ebi-fl187-GCST90094765 | ebi-met1400-GCST90200557 | X-17685 levels \|\| id:ebi-met1400-GCST90200557 | Fried fish liking \|\| id:ebi-fl187-GCST90094765 | MR Egger | 4 | -0.015870958 | 1.08907131544394 | 0.989695930704071 | -2.150450736 | 2.11870882018724 | 0.984254321925468 | 0.116431665959914 | 8.32038743276673 | 0.999279047927446 |
| ebi-fl187-GCST90094765 | ebi-met1400-GCST90200561 | X-18779 levels \|\| id:ebi-met1400-GCST90200561 | Fried fish liking \|\| id:ebi-fl187-GCST90094765 | MR Egger | 4 | -1.313083526 | 1.02421997598101 | 0.328365041542021 | -3.320554679 | 0.694387626592781 | 0.268989340561731 | 0.0361327840888689 | 2.00248243140846 | 0.999279047927446 |
| ebi-fl187-GCST90094765 | ebi-met1400-GCST90200570 | X-18921 levels \|\| id:ebi-met1400-GCST90200570 | Fried fish liking \|\| id:ebi-fl187-GCST90094765 | MR Egger | 4 | 2.17351827878478 | 1.02918806006671 | 0.169094718134823 | 0.156309681054033 | 4.19072687651553 | 8.78915239556999 | 1.1691882224728 | 66.0707988224289 | 0.999279047927446 |
| ebi-fl187-GCST90094765 | ebi-met1400-GCST90200575 | X-21283 levels \|\| id:ebi-met1400-GCST90200575 | Fried fish liking \|\| id:ebi-fl187-GCST90094765 | MR Egger | 4 | 0.320161768446737 | 1.08485237060918 | 0.795719163985667 | -1.806148878 | 2.44647241484072 | 1.37735055817537 | 0.164285604006157 | 11.547539856474 | 0.999279047927446 |
| ebi-fl187-GCST90094765 | ebi-met1400-GCST90200578 | X-18922 levels \|\| id:ebi-met1400-GCST90200578 | Fried fish liking \|\| id:ebi-fl187-GCST90094765 | MR Egger | 4 | 2.08529496936203 | 1.03294352852936 | 0.180970414136496 | 0.0607256534444884 | 4.10986428527958 | 8.04696473971686 | 1.06260735152767 | 60.9384467641344 | 0.999279047927446 |
| ebi-fl187-GCST90094765 | ebi-met1400-GCST90200623 | X-24241 levels \|\| id:ebi-met1400-GCST90200623 | Fried fish liking \|\| id:ebi-fl187-GCST90094765 | MR Egger | 4 | -1.187230107 | 1.64880684755003 | 0.54627151179053 | -4.418891529 | 2.04443131383408 | 0.305065092423228 | 0.0120475792835059 | 7.72476432195847 | 0.999279047927446 |
| ebi-fl187-GCST90094765 | ebi-met1400-GCST90200636 | X-24544 levels \|\| id:ebi-met1400-GCST90200636 | Fried fish liking \|\| id:ebi-fl187-GCST90094765 | MR Egger | 4 | -0.994167321 | 0.966685956666399 | 0.411862162409451 | -2.888871796 | 0.900537153709412 | 0.370031433570394 | 0.055638949276565 | 2.46092465099503 | 0.999279047927446 |
| ebi-fl187-GCST90094765 | ebi-met1400-GCST90200659 | X-25172 levels \|\| id:ebi-met1400-GCST90200659 | Fried fish liking \|\| id:ebi-fl187-GCST90094765 | MR Egger | 4 | -0.701486214 | 1.04236198145918 | 0.570304541549728 | -2.744515697 | 1.34154326985484 | 0.495847820022507 | 0.0642794240954127 | 3.82494187029623 | 0.999279047927446 |
| ebi-fl187-GCST90094765 | ebi-met1400-GCST90200670 | X-26111 levels \|\| id:ebi-met1400-GCST90200670 | Fried fish liking \|\| id:ebi-fl187-GCST90094765 | MR Egger | 4 | -0.665631083 | 1.12221571398366 | 0.613227435743912 | -2.865173883 | 1.53391171594695 | 0.513949080576233 | 0.0569732236031086 | 4.63627719690313 | 0.999279047927446 |
| ebi-fl187-GCST90094765 | ebi-met1400-GCST90200673 | Carnitine C4 levels \|\| id:ebi-met1400-GCST90200673 | Fried fish liking \|\| id:ebi-fl187-GCST90094765 | MR Egger | 4 | 0.338286566568701 | 1.02693166613775 | 0.773141557857039 | -1.674499499 | 2.35107263219869 | 1.40254236757589 | 0.187401950865991 | 10.4968229186261 | 0.999279047927446 |
| ebi-fl187-GCST90094765 | ebi-met1400-GCST90200710 | X-13431 levels \|\| id:ebi-met1400-GCST90200710 | Fried fish liking \|\| id:ebi-fl187-GCST90094765 | MR Egger | 4 | -0.622260419 | 1.01442278631981 | 0.602071767679184 | -2.61052908 | 1.36600824216034 | 0.536729831029801 | 0.0734956483812515 | 3.91967303999957 | 0.999279047927446 |
| ebi-fl187-GCST90094765 | ebi-met1400-GCST90200730 | Adenosine 5'-diphosphate (ADP) to fructose ratio \|\| id:ebi-met1400-GCST90200730 | Fried fish liking \|\| id:ebi-fl187-GCST90094765 | MR Egger | 4 | -2.078743056 | 1.38876764902398 | 0.273118946297197 | -4.800727648 | 0.643241535697908 | 0.12508734116105 | 0.00822376086430434 | 1.90263836423755 | 0.999279047927446 |
| ebi-fl187-GCST90094765 | ebi-met1400-GCST90200731 | Adenosine 5'-diphosphate (ADP) to mannose ratio \|\| id:ebi-met1400-GCST90200731 | Fried fish liking \|\| id:ebi-fl187-GCST90094765 | MR Egger | 4 | -2.609674756 | 1.37416479477854 | 0.197955074587429 | -5.303037754 | 0.0836882416294902 | 0.0735584643119707 | 0.00497645368082366 | 1.08728986924679 | 0.999279047927446 |
| ebi-fl187-GCST90094765 | ebi-met1400-GCST90200748 | Aspartate to glutamate ratio \|\| id:ebi-met1400-GCST90200748 | Fried fish liking \|\| id:ebi-fl187-GCST90094765 | MR Egger | 4 | -2.175426323 | 0.995197117298568 | 0.160393311872382 | -4.126012673 | -0.224839973 | 0.113559730255211 | 0.0161471345445805 | 0.798644013278785 | 0.999279047927446 |
| ebi-fl187-GCST90094765 | ebi-met1400-GCST90200774 | Uridine to pseudouridine ratio \|\| id:ebi-met1400-GCST90200774 | Fried fish liking \|\| id:ebi-fl187-GCST90094765 | MR Egger | 4 | -0.475860566 | 0.979747371654314 | 0.675182627660124 | -2.396165414 | 1.44444428253084 | 0.621350113594294 | 0.0910664868647752 | 4.23949552635017 | 0.999279047927446 |
| ebi-fl187-GCST90094765 | ebi-met1400-GCST90200799 | Spermidine to adenosine 5'-diphosphate (ADP) ratio \|\| id:ebi-met1400-GCST90200799 | Fried fish liking \|\| id:ebi-fl187-GCST90094765 | MR Egger | 4 | 2.49575861036357 | 1.43651443900535 | 0.224454241094648 | -0.31980969 | 5.31132691081406 | 12.1309326801096 | 0.726287243584436 | 202.618907311487 | 0.999279047927446 |
| ebi-fl187-GCST90094765 | ebi-met1400-GCST90200811 | Choline to taurocholate ratio \|\| id:ebi-met1400-GCST90200811 | Fried fish liking \|\| id:ebi-fl187-GCST90094765 | MR Egger | 4 | 1.14128024338083 | 1.29398339511194 | 0.470818304849937 | -1.394927211 | 3.67748769780023 | 3.13077395320575 | 0.247851076533108 | 39.5469153621457 | 0.999279047927446 |
| ebi-fl187-GCST90094765 | ebi-met1400-GCST90200812 | Glutamate to kynurenine ratio \|\| id:ebi-met1400-GCST90200812 | Fried fish liking \|\| id:ebi-fl187-GCST90094765 | MR Egger | 4 | 0.759578434400152 | 1.85546777622058 | 0.721944804424131 | -2.877138407 | 4.39629527579248 | 2.1373749867772 | 0.0562956277341191 | 81.1496739263124 | 0.999279047927446 |
| ebi-fl187-GCST90094765 | ebi-met1400-GCST90200876 | Cortisone to 4-cholesten-3-one ratio \|\| id:ebi-met1400-GCST90200876 | Fried fish liking \|\| id:ebi-fl187-GCST90094765 | MR Egger | 4 | 0.358511943932046 | 1.08356170627673 | 0.772195038750648 | -1.765269 | 2.48229288823444 | 1.43119812615922 | 0.171140743369063 | 11.9686758161642 | 0.999279047927446 |
| ebi-fl187-GCST90094765 | ebi-met1400-GCST90200906 | 2'-deoxyuridine to cytidine ratio \|\| id:ebi-met1400-GCST90200906 | Fried fish liking \|\| id:ebi-fl187-GCST90094765 | MR Egger | 4 | -0.886248669 | 1.05677436568366 | 0.489935123245582 | -2.957526426 | 1.18502908740641 | 0.412199151360305 | 0.051947253754717 | 3.27078195864641 | 0.999279047927446 |
| ebi-fl187-GCST90094765 | ebi-met1400-GCST90200907 | Retinol (Vitamin A) to linoleoyl-arachidonoyl-glycerol (18:2 to 20:4) [1] ratio \|\| id:ebi-met1400-GCST90200907 | Fried fish liking \|\| id:ebi-fl187-GCST90094765 | MR Egger | 4 | -0.633651682 | 1.11775379544413 | 0.627923698299204 | -2.824449121 | 1.55714575678003 | 0.530650491638874 | 0.0593413377023666 | 4.74525777778934 | 0.999279047927446 |
| ebi-fl187-GCST90094765 | ebi-met1400-GCST90200916 | Fructose to sucrose ratio \|\| id:ebi-met1400-GCST90200916 | Fried fish liking \|\| id:ebi-fl187-GCST90094765 | MR Egger | 4 | 1.91016297196883 | 1.05586371050635 | 0.212156138510781 | -0.159329901 | 3.97965584456128 | 6.75418945239387 | 0.852715001350938 | 53.498619218092 | 0.999279047927446 |
| ebi-fl187-GCST90094765 | ebi-met1400-GCST90200917 | Alpha-ketobutyrate to 3-methyl-2-oxobutyrate ratio \|\| id:ebi-met1400-GCST90200917 | Fried fish liking \|\| id:ebi-fl187-GCST90094765 | MR Egger | 4 | -0.719147128 | 1.03340661489443 | 0.55848386042521 | -2.744624094 | 1.30632983670967 | 0.487167570174369 | 0.0642724568269919 | 3.69259638025738 | 0.999279047927446 |
| ebi-fl187-GCST90094765 | ebi-met1400-GCST90200924 | Alpha-tocopherol to glycerol ratio \|\| id:ebi-met1400-GCST90200924 | Fried fish liking \|\| id:ebi-fl187-GCST90094765 | MR Egger | 4 | -1.052659243 | 0.958356141069966 | 0.386596423645284 | -2.931037279 | 0.825718793965326 | 0.34900841597399 | 0.0533416792336042 | 2.28352155707797 | 0.999279047927446 |
| ebi-fl187-GCST90094765 | ebi-met1400-GCST90200938 | Alpha-ketoglutarate to alanine ratio \|\| id:ebi-met1400-GCST90200938 | Fried fish liking \|\| id:ebi-fl187-GCST90094765 | MR Egger | 4 | 0.431335104107662 | 1.02007272572442 | 0.713532740913178 | -1.568007438 | 2.43067764652753 | 1.5393112930755 | 0.208460138492272 | 11.3665819956155 | 0.999279047927446 |
| ebi-fl187-GCST90094765 | ebi-met1400-GCST90200943 | Phosphate to EDTA ratio \|\| id:ebi-met1400-GCST90200943 | Fried fish liking \|\| id:ebi-fl187-GCST90094765 | MR Egger | 4 | 0.30014948906703 | 0.980044825008673 | 0.788346961454686 | -1.620738368 | 2.22103734608403 | 1.35006061179316 | 0.197752630959518 | 9.21688701015782 | 0.999279047927446 |
| ebi-fl187-GCST90094765 | ebi-met1400-GCST90200947 | Adenosine 5'-diphosphate (ADP) to glucose ratio \|\| id:ebi-met1400-GCST90200947 | Fried fish liking \|\| id:ebi-fl187-GCST90094765 | MR Egger | 4 | -2.240703546 | 1.37806359254597 | 0.245467573356425 | -4.941708187 | 0.46030109546323 | 0.106383632273188 | 0.00714238741039144 | 1.58455101429686 | 0.999279047927446 |
| ebi-fl187-GCST90094765 | ebi-met1400-GCST90200975 | Arachidonate (20:4n6) to caffeine ratio \|\| id:ebi-met1400-GCST90200975 | Fried fish liking \|\| id:ebi-fl187-GCST90094765 | MR Egger | 4 | 0.948822082043451 | 1.05091375782062 | 0.461894733136121 | -1.110968883 | 3.00861304737186 | 2.58266569859767 | 0.329239811540685 | 20.2592817663812 | 0.999279047927446 |
| ebi-fl187-GCST90094765 | ebi-met1400-GCST90200985 | Cholesterol to oleoyl-linoleoyl-glycerol (18:1 to 18:2) [2] ratio \|\| id:ebi-met1400-GCST90200985 | Fried fish liking \|\| id:ebi-fl187-GCST90094765 | MR Egger | 4 | -0.766487694 | 1.02605236846695 | 0.532930680730001 | -2.777550336 | 1.24457494856134 | 0.464642171338365 | 0.062190667180215 | 3.4714589370849 | 0.999279047927446 |
| ebi-fl187-GCST90094765 | ebi-met1400-GCST90201016 | Bilirubin (Z,Z) to etiocholanolone glucuronide ratio \|\| id:ebi-met1400-GCST90201016 | Fried fish liking \|\| id:ebi-fl187-GCST90094765 | MR Egger | 4 | -0.034980919 | 1.0573052145679 | 0.976611787409771 | -2.10729914 | 2.03733730125044 | 0.965623840858062 | 0.121565855817261 | 7.6701586622737 | 0.999279047927446 |
| ebi-fl187-GCST90094765 | ebi-pw11-GCST90275189 | Placental weight (fetal genotype, adjusted for sex and gestational age) \|\| id:ebi-pw11-GCST90275189 | Fried fish liking \|\| id:ebi-fl187-GCST90094765 | MR Egger | 4 | -0.307349989 | 0.724036030564315 | 0.712508362684865 | -1.726460609 | 1.11176063125942 | 0.735393176599462 | 0.177913000652297 | 3.03970548642459 | 0.999279047927446 |
| ebi-fl187-GCST90094765 | ebi-pw11-GCST90275192 | Placental weight (fetal effect, conditional on maternal and paternal genotype) \|\| id:ebi-pw11-GCST90275192 | Fried fish liking \|\| id:ebi-fl187-GCST90094765 | MR Egger | 4 | -0.660103633 | 0.795879283265135 | 0.494108026557248 | -2.220027029 | 0.899819761833968 | 0.516797774223705 | 0.108606173316233 | 2.45915983675171 | 0.999279047927446 |
| ebi-fl187-GCST90094765 | ebi-pw11-GCST90275193 | Placental weight (maternal effect, conditional on fetal and paternal genotype) \|\| id:ebi-pw11-GCST90275193 | Fried fish liking \|\| id:ebi-fl187-GCST90094765 | MR Egger | 4 | 0.115704363201949 | 0.729485379504134 | 0.888543923867839 | -1.314086981 | 1.54549570703005 | 1.1226639223001 | 0.268719557432484 | 4.69029606358638 | 0.999279047927446 |
| ebi-fl187-GCST90094765 | ebi-pw11-GCST90275195 | Placental weight (fetal effect, conditional on maternal genotype) \|\| id:ebi-pw11-GCST90275195 | Fried fish liking \|\| id:ebi-fl187-GCST90094765 | MR Egger | 4 | -0.23862743 | 0.638985560786029 | 0.744684641560562 | -1.491039129 | 1.01378426959635 | 0.787708304551087 | 0.225138585983536 | 2.75601079374339 | 0.999279047927446 |
| ebi-fl187-GCST90094765 | ebi-pw11-GCST90275197 | Placental weight (fetal genotype, adjusted for sex) \|\| id:ebi-pw11-GCST90275197 | Fried fish liking \|\| id:ebi-fl187-GCST90094765 | MR Egger | 4 | -0.389161622 | 0.596408809745889 | 0.581050746229771 | -1.55812289 | 0.779799644647665 | 0.677624741789253 | 0.210530890258189 | 2.18103523963554 | 0.999279047927446 |
| ebi-fl187-GCST90094765 | prot-a-1030 | Protein FAM171B \|\| id:prot-a-1030 | Fried fish liking \|\| id:ebi-fl187-GCST90094765 | MR Egger | 5 | 0.241768936668706 | 1.94893549487352 | 0.909119302417599 | -3.578144633 | 4.0616825066208 | 1.2734998996881 | 0.027927465907131 | 58.0719353448928 | 0.999279047927446 |
| ebi-fl187-GCST90094765 | prot-a-1036 | BRISC complex subunit Abro1 \|\| id:prot-a-1036 | Fried fish liking \|\| id:ebi-fl187-GCST90094765 | MR Egger | 5 | 2.73577908481736 | 1.63243301701787 | 0.192353004076612 | -0.463789629 | 5.93534779817239 | 15.4217536109447 | 0.628895842289821 | 378.171500658586 | 0.999279047927446 |
| ebi-fl187-GCST90094765 | prot-a-1055 | Phenylalanine--tRNA ligase, mitochondrial \|\| id:prot-a-1055 | Fried fish liking \|\| id:ebi-fl187-GCST90094765 | MR Egger | 5 | 1.6599688934146 | 1.40706821024374 | 0.323137695139848 | -1.097884799 | 4.41782258549234 | 5.25914724778947 | 0.333575918230004 | 82.9155471434862 | 0.999279047927446 |
| ebi-fl187-GCST90094765 | prot-a-1083 | Fc receptor-like protein 5 \|\| id:prot-a-1083 | Fried fish liking \|\| id:ebi-fl187-GCST90094765 | MR Egger | 5 | 2.06651099764712 | 1.40706821024374 | 0.238241645602982 | -0.691342694 | 4.82436468972486 | 7.89722157027714 | 0.500903057597075 | 124.507342457106 | 0.999279047927446 |
| ebi-fl187-GCST90094765 | prot-a-1103 | Fibroblast growth factor receptor 3 \|\| id:prot-a-1103 | Fried fish liking \|\| id:ebi-fl187-GCST90094765 | MR Egger | 5 | 0.240431137062096 | 1.40706821024374 | 0.875197696468532 | -2.517422555 | 2.99828482913983 | 1.27179735111161 | 0.0806672544446935 | 20.05111632 | 0.999279047927446 |
| ebi-fl187-GCST90094765 | prot-a-1105 | Fibroblast growth factor receptor 4 \|\| id:prot-a-1105 | Fried fish liking \|\| id:ebi-fl187-GCST90094765 | MR Egger | 5 | 2.5204739442771 | 1.40706821024374 | 0.171166499412117 | -0.237379748 | 5.27832763635484 | 12.4344885219347 | 0.788691727193647 | 196.04174796189 | 0.999279047927446 |
| ebi-fl187-GCST90094765 | prot-a-1120 | Filamin-A \|\| id:prot-a-1120 | Fried fish liking \|\| id:ebi-fl187-GCST90094765 | MR Egger | 5 | 3.01558705513986 | 1.40706821024374 | 0.121488602092993 | 0.257733363062127 | 5.7734407472176 | 20.401063975255 | 1.29399374609166 | 321.642521518775 | 0.999279047927446 |
| ebi-fl187-GCST90094765 | prot-a-1166 | Galanin peptides \|\| id:prot-a-1166 | Fried fish liking \|\| id:ebi-fl187-GCST90094765 | MR Egger | 5 | -1.071909124 | 1.84024503287459 | 0.601131284345934 | -4.678789389 | 2.53497113996134 | 0.342354296157318 | 0.00929025396651756 | 12.6160667426089 | 0.999279047927446 |
| ebi-fl187-GCST90094765 | prot-a-1205 | ADP-ribosylation factor-binding protein GGA1 \|\| id:prot-a-1205 | Fried fish liking \|\| id:ebi-fl187-GCST90094765 | MR Egger | 5 | -1.041055482 | 2.33086057106542 | 0.68540831855614 | -5.609542201 | 3.52743123749074 | 0.353081813788138 | 0.0036627457750099 | 34.0364237339363 | 0.999279047927446 |
| ebi-fl187-GCST90094765 | prot-a-1227 | Guanine nucleotide-binding protein G(I)/G(S)/G(O) subunit gamma-T2 \|\| id:prot-a-1227 | Fried fish liking \|\| id:ebi-fl187-GCST90094765 | MR Egger | 5 | 0.858327173034675 | 1.40706821024374 | 0.584955528629481 | -1.899526519 | 3.61618086511241 | 2.35921083940645 | 0.149639453883701 | 37.1952425668344 | 0.999279047927446 |
| ebi-fl187-GCST90094765 | prot-a-1240 | Golgi SNAP receptor complex member 1 \|\| id:prot-a-1240 | Fried fish liking \|\| id:ebi-fl187-GCST90094765 | MR Egger | 5 | -1.288954365 | 1.40706821024374 | 0.427188319850048 | -4.046808057 | 1.46889932704415 | 0.275558766382243 | 0.0174780747127625 | 4.34445068910578 | 0.999279047927446 |
| ebi-fl187-GCST90094765 | prot-a-1283 | Glutathione S-transferase A1 \|\| id:prot-a-1283 | Fried fish liking \|\| id:ebi-fl187-GCST90094765 | MR Egger | 5 | 2.73769586305798 | 1.40706821024374 | 0.146893833845765 | -0.020157829 | 5.49554955513572 | 15.4513420408687 | 0.980043981718716 | 243.60536396053 | 0.999279047927446 |
| ebi-fl187-GCST90094765 | prot-a-1324 | E3 ubiquitin-protein ligase HECW1 \|\| id:prot-a-1324 | Fried fish liking \|\| id:ebi-fl187-GCST90094765 | MR Egger | 5 | -0.456555521 | 1.40756120963424 | 0.766966179474082 | -3.215375492 | 2.3022644493885 | 0.63346183766914 | 0.0401402586323358 | 9.9967940779512 | 0.999279047927446 |
| ebi-fl187-GCST90094765 | prot-a-1339 | Histidine triad nucleotide-binding protein 2, mitochondrial \|\| id:prot-a-1339 | Fried fish liking \|\| id:ebi-fl187-GCST90094765 | MR Egger | 5 | -0.215535033 | 1.40706821024374 | 0.887979458994593 | -2.973388725 | 2.54231865902953 | 0.806110029271919 | 0.0511297517524004 | 12.7091049148751 | 0.999279047927446 |
| ebi-fl187-GCST90094765 | prot-a-1466 | Interleukin-11 \|\| id:prot-a-1466 | Fried fish liking \|\| id:ebi-fl187-GCST90094765 | MR Egger | 5 | 2.92866689771937 | 1.40706821024374 | 0.128830574081351 | 0.170813205641636 | 5.68652058979711 | 18.7026812833686 | 1.18626913994185 | 294.865874370137 | 0.999279047927446 |
| ebi-fl187-GCST90094765 | prot-a-147 | Rho GTPase-activating protein 1 \|\| id:prot-a-147 | Fried fish liking \|\| id:ebi-fl187-GCST90094765 | MR Egger | 5 | 2.60281223453541 | 1.74498964645632 | 0.232611849482113 | -0.817367473 | 6.02299194158979 | 13.5016545141064 | 0.44159263042002 | 412.81185885032 | 0.999279047927446 |
| ebi-fl187-GCST90094765 | prot-a-1473 | Interleukin-12 receptor subunit beta-1 \|\| id:prot-a-1473 | Fried fish liking \|\| id:ebi-fl187-GCST90094765 | MR Egger | 5 | 0.32174091988443 | 1.5579508355747 | 0.849610037160831 | -2.731842718 | 3.37532455761083 | 1.37952732155668 | 0.0650992195810371 | 29.2337702843324 | 0.999279047927446 |
| ebi-fl187-GCST90094765 | prot-a-1501 | X-linked interleukin-1 receptor accessory protein-like 2 \|\| id:prot-a-1501 | Fried fish liking \|\| id:ebi-fl187-GCST90094765 | MR Egger | 5 | 1.02063320477811 | 1.40706821024374 | 0.520691578086795 | -1.737220487 | 3.77848689685585 | 2.7749513202107 | 0.176008940436478 | 43.7497936777715 | 0.999279047927446 |
| ebi-fl187-GCST90094765 | prot-a-1503 | Interleukin-1 receptor-like 2 \|\| id:prot-a-1503 | Fried fish liking \|\| id:ebi-fl187-GCST90094765 | MR Egger | 5 | 0.362464219592063 | 1.40706821024374 | 0.813373115272358 | -2.395389472 | 3.1203179116698 | 1.43686580842157 | 0.0911371765867525 | 22.6535803360742 | 0.999279047927446 |
| ebi-fl187-GCST90094765 | prot-a-1536 | Interleukin-5 receptor subunit alpha \|\| id:prot-a-1536 | Fried fish liking \|\| id:ebi-fl187-GCST90094765 | MR Egger | 5 | 3.49504404523434 | 1.40706821024374 | 0.0889609246621253 | 0.7371903531566 | 6.25289773731208 | 32.951739288121 | 2.09005494092807 | 519.51606670691 | 0.999279047927446 |
| ebi-fl187-GCST90094765 | prot-a-1559 | Insulin-induced gene 1 protein \|\| id:prot-a-1559 | Fried fish liking \|\| id:ebi-fl187-GCST90094765 | MR Egger | 5 | 2.28522409054626 | 1.40706821024374 | 0.202817251144847 | -0.472629602 | 5.04307778262399 | 9.82788831413521 | 0.62336092035216 | 154.946172532839 | 0.999279047927446 |
| ebi-fl187-GCST90094765 | prot-a-1567 | Interferon regulatory factor 1 \|\| id:prot-a-1567 | Fried fish liking \|\| id:ebi-fl187-GCST90094765 | MR Egger | 5 | 0.916087706697812 | 1.40706821024374 | 0.561428936903654 | -1.841765985 | 3.67394139877555 | 2.49949248857968 | 0.158537204360919 | 39.4069185567543 | 0.999279047927446 |
| ebi-fl187-GCST90094765 | prot-a-1613 | Potassium voltage-gated channel subfamily E regulatory beta subunit 5 \|\| id:prot-a-1613 | Fried fish liking \|\| id:ebi-fl187-GCST90094765 | MR Egger | 5 | 0.708186591500269 | 1.96321770641572 | 0.742207751340626 | -3.139720113 | 4.55609329607507 | 2.03030614392898 | 0.04329491388667 | 95.2107919388866 | 0.999279047927446 |
| ebi-fl187-GCST90094765 | prot-a-1649 | Kin of IRRE-like protein 2 \|\| id:prot-a-1649 | Fried fish liking \|\| id:ebi-fl187-GCST90094765 | MR Egger | 5 | -1.994849532 | 1.40706821024374 | 0.251285504155556 | -4.752703224 | 0.763004159734575 | 0.136034121362476 | 0.00862833931169602 | 2.1447096024349 | 0.999279047927446 |
| ebi-fl187-GCST90094765 | prot-a-165 | Armadillo repeat-containing protein 5 \|\| id:prot-a-165 | Fried fish liking \|\| id:ebi-fl187-GCST90094765 | MR Egger | 5 | -1.112160194 | 2.15894362748069 | 0.642008692714571 | -5.343689704 | 3.1193693157954 | 0.328847818142746 | 0.00477820797246902 | 22.6321014322375 | 0.999279047927446 |
| ebi-fl187-GCST90094765 | prot-a-1656 | Kallikrein-11 \|\| id:prot-a-1656 | Fried fish liking \|\| id:ebi-fl187-GCST90094765 | MR Egger | 5 | 0.17402964404053 | 1.40756120963424 | 0.909419572406356 | -2.584790327 | 2.93284961492363 | 1.19009084439869 | 0.0754118897926238 | 18.7810731413352 | 0.999279047927446 |
| ebi-fl187-GCST90094765 | prot-a-1697 | Laminin subunit gamma-2 \|\| id:prot-a-1697 | Fried fish liking \|\| id:ebi-fl187-GCST90094765 | MR Egger | 5 | -0.249685107 | 2.7688838733084 | 0.933831115739919 | -5.676697499 | 5.17732728477368 | 0.779046060671957 | 0.00342485036047302 | 177.208549504239 | 0.999279047927446 |
| ebi-fl187-GCST90094765 | prot-a-1698 | Lysosome-associated membrane glycoprotein 3 \|\| id:prot-a-1698 | Fried fish liking \|\| id:ebi-fl187-GCST90094765 | MR Egger | 5 | 3.6503579066963 | 1.40706821024374 | 0.0807728691426636 | 0.892504214618562 | 6.40821159877404 | 38.4884388541915 | 2.44123538039797 | 606.807494814919 | 0.999279047927446 |
| ebi-fl187-GCST90094765 | prot-a-1724 | Leptin receptor \|\| id:prot-a-1724 | Fried fish liking \|\| id:ebi-fl187-GCST90094765 | MR Egger | 5 | 1.21550726854263 | 1.96989908936256 | 0.580876158870085 | -2.645494947 | 5.07650948369325 | 3.37200414217752 | 0.0709702185763859 | 160.213849737891 | 0.999279047927446 |
| ebi-fl187-GCST90094765 | prot-a-1743 | Leukocyte immunoglobulin-like receptor subfamily B member 2 \|\| id:prot-a-1743 | Fried fish liking \|\| id:ebi-fl187-GCST90094765 | MR Egger | 5 | -0.701326693 | 1.40706821024374 | 0.652431076734805 | -3.459180385 | 2.05652699870977 | 0.495926924192732 | 0.0314555328683952 | 7.81876801032909 | 0.999279047927446 |
| ebi-fl187-GCST90094765 | prot-a-1746 | Leukocyte immunoglobulin-like receptor subfamily B member 4 \|\| id:prot-a-1746 | Fried fish liking \|\| id:ebi-fl187-GCST90094765 | MR Egger | 5 | -1.222463968 | 1.40756120963424 | 0.449022950752256 | -3.981283939 | 1.53635600278754 | 0.294503624667913 | 0.018661663512088 | 4.64762344934355 | 0.999279047927446 |
| ebi-fl187-GCST90094765 | prot-a-1750 | Leucine-rich repeat and immunoglobulin-like domain-containing nogo receptor-interacting protein 3 \|\| id:prot-a-1750 | Fried fish liking \|\| id:ebi-fl187-GCST90094765 | MR Egger | 5 | -0.368534597 | 1.40706821024374 | 0.810339712449864 | -3.126388289 | 2.38931909513094 | 0.691747276840385 | 0.0438759787819452 | 10.9060654212231 | 0.999279047927446 |
| ebi-fl187-GCST90094765 | prot-a-1753 | Lipase member N \|\| id:prot-a-1753 | Fried fish liking \|\| id:ebi-fl187-GCST90094765 | MR Egger | 5 | 0.753694561171036 | 1.83892316239759 | 0.709421148151249 | -2.850594837 | 4.3579839594703 | 2.12483586877757 | 0.0578099231566589 | 78.099523796438 | 0.999279047927446 |
| ebi-fl187-GCST90094765 | prot-a-1760 | Ig Kappa chain V-I region HK102- like \|\| id:prot-a-1760 | Fried fish liking \|\| id:ebi-fl187-GCST90094765 | MR Egger | 5 | 1.27543664757046 | 1.53790836983514 | 0.467741220606019 | -1.738863757 | 4.28973705244735 | 3.58026438258695 | 0.175719947735282 | 72.9472846675964 | 0.999279047927446 |
| ebi-fl187-GCST90094765 | prot-a-1792 | Leucine-rich repeat neuronal protein 1 \|\| id:prot-a-1792 | Fried fish liking \|\| id:ebi-fl187-GCST90094765 | MR Egger | 5 | -1.5733578 | 1.75157301487952 | 0.435247369773116 | -5.006440909 | 1.85972530906039 | 0.207347779746713 | 0.00669468795714955 | 6.4219724714693 | 0.999279047927446 |
| ebi-fl187-GCST90094765 | prot-a-1795 | Leucine-rich repeat transmembrane neuronal protein 1 \|\| id:prot-a-1795 | Fried fish liking \|\| id:ebi-fl187-GCST90094765 | MR Egger | 5 | 1.64850060487169 | 1.40706821024374 | 0.325945690664763 | -1.109353087 | 4.40635429694943 | 5.19917835719842 | 0.329772225957924 | 81.9700795342584 | 0.999279047927446 |
| ebi-fl187-GCST90094765 | prot-a-1820 | LysM and putative peptidoglycan-binding domain-containing protein 4 \|\| id:prot-a-1820 | Fried fish liking \|\| id:ebi-fl187-GCST90094765 | MR Egger | 5 | 0.394598241219581 | 1.60018893759023 | 0.821132617821779 | -2.741772076 | 3.53096855889644 | 1.48378794631997 | 0.0644560246254174 | 34.1570346982933 | 0.999279047927446 |
| ebi-fl187-GCST90094765 | prot-a-1844 | Mitogen-activated protein kinase kinase kinase 7:TGF-beta-activated kinase 1 and MAP3K7-binding protein 1 fusion \|\| id:prot-a-1844 | Fried fish liking \|\| id:ebi-fl187-GCST90094765 | MR Egger | 5 | -1.092236846 | 1.40756120963424 | 0.494320496377362 | -3.851056817 | 1.66658312468284 | 0.33546526961992 | 0.0212572595284627 | 5.29404775674318 | 0.999279047927446 |
| ebi-fl187-GCST90094765 | prot-a-1856 | Megakaryocyte-associated tyrosine-protein kinase \|\| id:prot-a-1856 | Fried fish liking \|\| id:ebi-fl187-GCST90094765 | MR Egger | 5 | 2.48517579948117 | 2.74116074099147 | 0.431445827078923 | -2.887499253 | 7.85785085182446 | 12.0032302310064 | 0.0557153685905437 | 2585.95679474693 | 0.999279047927446 |
| ebi-fl187-GCST90094765 | prot-a-1910 | Stromelysin-2 \|\| id:prot-a-1910 | Fried fish liking \|\| id:ebi-fl187-GCST90094765 | MR Egger | 5 | 0.389125588063138 | 3.13078641496317 | 0.908945945550567 | -5.747215785 | 6.52546696139095 | 1.47568986872839 | 0.00319165468938186 | 682.298306239819 | 0.999279047927446 |
| ebi-fl187-GCST90094765 | prot-a-1940 | 39S ribosomal protein L14, mitochondrial \|\| id:prot-a-1940 | Fried fish liking \|\| id:ebi-fl187-GCST90094765 | MR Egger | 5 | 1.26944247781757 | 1.42473407910557 | 0.438568960686493 | -1.523036317 | 4.06192127286449 | 3.55886786137246 | 0.218048815438745 | 58.0858026182117 | 0.999279047927446 |
| ebi-fl187-GCST90094765 | prot-a-1942 | 39S ribosomal protein L33, mitochondrial \|\| id:prot-a-1942 | Fried fish liking \|\| id:ebi-fl187-GCST90094765 | MR Egger | 5 | 1.63093026524857 | 1.40706821024374 | 0.330295603030444 | -1.126923427 | 4.38878395732631 | 5.10862488493525 | 0.324028622245022 | 80.5424163895157 | 0.999279047927446 |
| ebi-fl187-GCST90094765 | prot-a-1948 | Mesothelin \|\| id:prot-a-1948 | Fried fish liking \|\| id:ebi-fl187-GCST90094765 | MR Egger | 5 | -1.26501131 | 1.476311586 | 0.454509050882394 | -4.158582018 | 1.62855939892357 | 0.282236104105428 | 0.0156297048481261 | 5.09652736469685 | 0.999279047927446 |
| ebi-fl187-GCST90094765 | prot-a-1980 | Unconventional myosin-VI \|\| id:prot-a-1980 | Fried fish liking \|\| id:ebi-fl187-GCST90094765 | MR Egger | 5 | 0.562148857907814 | 1.51160128684513 | 0.734689975523839 | -2.400589664 | 3.52488738012427 | 1.75443849158328 | 0.0906644759186026 | 33.9499499617961 | 0.999279047927446 |
| ebi-fl187-GCST90094765 | prot-a-2039 | Neurofascin \|\| id:prot-a-2039 | Fried fish liking \|\| id:ebi-fl187-GCST90094765 | MR Egger | 5 | -1.067665346 | 1.40756120963424 | 0.503288353400851 | -3.826485317 | 1.69115462452062 | 0.343810259031921 | 0.021786052288125 | 5.42574178434461 | 0.999279047927446 |
| ebi-fl187-GCST90094765 | prot-a-2041 | NFU1 iron-sulfur cluster scaffold homolog, mitochondrial \|\| id:prot-a-2041 | Fried fish liking \|\| id:ebi-fl187-GCST90094765 | MR Egger | 5 | 0.363684576045539 | 2.18791406989205 | 0.878551733585013 | -3.924627001 | 4.65199615303395 | 1.43862036725951 | 0.0197495020434472 | 104.793961718168 | 0.999279047927446 |
| ebi-fl187-GCST90094765 | prot-a-2088 | Glucocorticoid receptor \|\| id:prot-a-2088 | Fried fish liking \|\| id:ebi-fl187-GCST90094765 | MR Egger | 5 | 1.82488235512873 | 1.40756120963424 | 0.285526729020006 | -0.933937616 | 4.58370232601184 | 6.20206533500599 | 0.393003164196631 | 97.8760934363304 | 0.999279047927446 |
| ebi-fl187-GCST90094765 | prot-a-2111 | Neuron-specific protein family member 2 \|\| id:prot-a-2111 | Fried fish liking \|\| id:ebi-fl187-GCST90094765 | MR Egger | 5 | -1.472872614 | 1.40706821024374 | 0.372112753908626 | -4.230726306 | 1.28498107767529 | 0.229265945681367 | 0.0145418248902761 | 3.61459955994377 | 0.999279047927446 |
| ebi-fl187-GCST90094765 | prot-a-2125 | Nuclear migration protein nudC \|\| id:prot-a-2125 | Fried fish liking \|\| id:ebi-fl187-GCST90094765 | MR Egger | 5 | -0.813311735 | 1.64785955860192 | 0.655491933936528 | -4.043116469 | 2.41649300035668 | 0.443387251234704 | 0.0175427157999524 | 11.2064891661758 | 0.999279047927446 |
| ebi-fl187-GCST90094765 | prot-a-2142 | Ornithine decarboxylase \|\| id:prot-a-2142 | Fried fish liking \|\| id:ebi-fl187-GCST90094765 | MR Egger | 5 | -0.193545807 | 1.40706821024374 | 0.899307165050176 | -2.951399499 | 2.56430788551748 | 0.824032089255034 | 0.0522665078335224 | 12.991663538816 | 0.999279047927446 |
| ebi-fl187-GCST90094765 | prot-a-2186 | Prostate and testis expressed protein 1 \|\| id:prot-a-2186 | Fried fish liking \|\| id:ebi-fl187-GCST90094765 | MR Egger | 5 | 0.128255995214464 | 1.99407620752136 | 0.952762546502324 | -3.780133372 | 4.03664536195634 | 1.13684399207109 | 0.0228196477308537 | 56.6360303871258 | 0.999279047927446 |
| ebi-fl187-GCST90094765 | prot-a-2227 | Platelet-derived growth factor C \|\| id:prot-a-2227 | Fried fish liking \|\| id:ebi-fl187-GCST90094765 | MR Egger | 5 | 3.67796739344278 | 1.48967124994688 | 0.0901459972789442 | 0.758211743546907 | 6.59772304333866 | 39.5658903958779 | 2.13445585116516 | 733.423313470769 | 0.999279047927446 |
| ebi-fl187-GCST90094765 | prot-a-2229 | Platelet-derived growth factor receptor alpha \|\| id:prot-a-2229 | Fried fish liking \|\| id:ebi-fl187-GCST90094765 | MR Egger | 5 | 0.0602354159491519 | 1.40706821024374 | 0.968543568863244 | -2.697618276 | 2.81808910802689 | 1.06208654922996 | 0.0673657684804386 | 16.7448225337586 | 0.999279047927446 |
| ebi-fl187-GCST90094765 | prot-a-2243 | Platelet endothelial aggregation receptor 1 \|\| id:prot-a-2243 | Fried fish liking \|\| id:ebi-fl187-GCST90094765 | MR Egger | 5 | -0.841334794 | 1.40756120963424 | 0.592135376635377 | -3.600154765 | 1.9174851764906 | 0.43113466305632 | 0.0273194940110734 | 6.80382651352713 | 0.999279047927446 |
| ebi-fl187-GCST90094765 | prot-a-2246 | E3 ubiquitin-protein ligase pellino homolog 2 \|\| id:prot-a-2246 | Fried fish liking \|\| id:ebi-fl187-GCST90094765 | MR Egger | 5 | 1.49517314978654 | 1.60795408725633 | 0.421032962690569 | -1.656416861 | 4.64676316080894 | 4.46010875287125 | 0.190821496496944 | 104.247008081489 | 0.999279047927446 |
| ebi-fl187-GCST90094765 | prot-a-2251 | Platelet factor 4 variant \|\| id:prot-a-2251 | Fried fish liking \|\| id:ebi-fl187-GCST90094765 | MR Egger | 5 | 0.808195962056455 | 1.40706821024374 | 0.605949976431458 | -1.94965773 | 3.56604965413419 | 2.24385633114393 | 0.142322776064532 | 35.3765670825009 | 0.999279047927446 |
| ebi-fl187-GCST90094765 | prot-a-2346 | Protein phosphatase 1L \|\| id:prot-a-2346 | Fried fish liking \|\| id:ebi-fl187-GCST90094765 | MR Egger | 5 | 1.23999545817523 | 2.7099171347731 | 0.678361523241109 | -4.071442126 | 6.55143304233051 | 3.45559777000749 | 0.0170527785114693 | 700.246938646943 | 0.999279047927446 |
| ebi-fl187-GCST90094765 | prot-a-2358 | PR domain zinc finger protein 4 \|\| id:prot-a-2358 | Fried fish liking \|\| id:ebi-fl187-GCST90094765 | MR Egger | 5 | 0.272573202747751 | 1.40706821024374 | 0.858771846746865 | -2.485280489 | 3.03042689482549 | 1.31333959546083 | 0.083302185860609 | 20.7060700170757 | 0.999279047927446 |
| ebi-fl187-GCST90094765 | prot-a-2390 | Trypsin-1 \|\| id:prot-a-2390 | Fried fish liking \|\| id:ebi-fl187-GCST90094765 | MR Egger | 5 | -1.32641862 | 2.80373745588447 | 0.668441886894371 | -6.821744034 | 4.1689067933127 | 0.265426152971981 | 0.00108981858349436 | 64.6447433990462 | 0.999279047927446 |
| ebi-fl187-GCST90094765 | prot-a-242 | Brain-derived neurotrophic factor \|\| id:prot-a-242 | Fried fish liking \|\| id:ebi-fl187-GCST90094765 | MR Egger | 5 | -0.167512101 | 1.40756120963424 | 0.912790227377767 | -2.926332072 | 2.5913078698835 | 0.845766382608261 | 0.053593254276136 | 13.3472166154459 | 0.999279047927446 |
| ebi-fl187-GCST90094765 | prot-a-2464 | Dihydropteridine reductase \|\| id:prot-a-2464 | Fried fish liking \|\| id:ebi-fl187-GCST90094765 | MR Egger | 5 | 0.557353408040495 | 2.15784623218392 | 0.812888311858674 | -3.672025207 | 4.78673202312098 | 1.74604531037582 | 0.0254249270305459 | 119.908868262342 | 0.999279047927446 |
| ebi-fl187-GCST90094765 | prot-a-2474 | Ras-related protein Rab-27A \|\| id:prot-a-2474 | Fried fish liking \|\| id:ebi-fl187-GCST90094765 | MR Egger | 5 | 1.76989650605776 | 2.38840303307285 | 0.512404234255151 | -2.911373439 | 6.45116645088053 | 5.87024579506415 | 0.0544009621462323 | 633.440739556019 | 0.999279047927446 |
| ebi-fl187-GCST90094765 | prot-a-2478 | Ras-related protein Rab-7b \|\| id:prot-a-2478 | Fried fish liking \|\| id:ebi-fl187-GCST90094765 | MR Egger | 5 | 1.62763907881986 | 1.40756120963424 | 0.331259346555046 | -1.131180892 | 4.38645904970296 | 5.09183908579357 | 0.322652014160864 | 80.355380216808 | 0.999279047927446 |
| ebi-fl187-GCST90094765 | prot-a-2528 | E3 ubiquitin-protein ligase rififylin \|\| id:prot-a-2528 | Fried fish liking \|\| id:ebi-fl187-GCST90094765 | MR Egger | 5 | 1.5768474023258 | 1.40756120963424 | 0.34419702531461 | -1.181972569 | 4.33566737320891 | 4.83967418989739 | 0.306673207645047 | 76.3759131233557 | 0.999279047927446 |
| ebi-fl187-GCST90094765 | prot-a-2543 | Protein RIC-3 \|\| id:prot-a-2543 | Fried fish liking \|\| id:ebi-fl187-GCST90094765 | MR Egger | 5 | -2.835217285 | 1.64552431068027 | 0.183364541329487 | -6.060444933 | 0.390010364380979 | 0.0587057685912978 | 0.00233336246543678 | 1.47699610195362 | 0.999279047927446 |
| ebi-fl187-GCST90094765 | prot-a-255 | Bone morphogenetic protein 6 \|\| id:prot-a-255 | Fried fish liking \|\| id:ebi-fl187-GCST90094765 | MR Egger | 5 | 2.07824213384637 | 1.40706821024374 | 0.23617738044531 | -0.679611558 | 4.83609582592411 | 7.99041048934897 | 0.506813821792033 | 125.976555971863 | 0.999279047927446 |
| ebi-fl187-GCST90094765 | prot-a-2568 | RING finger protein 165 \|\| id:prot-a-2568 | Fried fish liking \|\| id:ebi-fl187-GCST90094765 | MR Egger | 5 | 1.61225133015696 | 1.40706821024374 | 0.334983992654462 | -1.145602362 | 4.37010502223469 | 5.01408689548313 | 0.318032289540635 | 79.0519334743316 | 0.999279047927446 |
| ebi-fl187-GCST90094765 | prot-a-2573 | E3 ubiquitin-protein ligase RNF8 \|\| id:prot-a-2573 | Fried fish liking \|\| id:ebi-fl187-GCST90094765 | MR Egger | 5 | -0.354822136 | 1.66638374668244 | 0.845030370957491 | -3.62093428 | 2.91129000728763 | 0.701298167666323 | 0.0267576656668509 | 18.3804942514638 | 0.999279047927446 |
| ebi-fl187-GCST90094765 | prot-a-260 | Cytoplasmic tyrosine-protein kinase BMX \|\| id:prot-a-260 | Fried fish liking \|\| id:ebi-fl187-GCST90094765 | MR Egger | 4 | 3.21204959302672 | 1.66638936432955 | 0.19372997916047 | -0.054073561 | 6.47817274711263 | 24.8299253488822 | 0.947362414955857 | 650.780718232095 | 0.999279047927446 |
| ebi-fl187-GCST90094765 | prot-a-2623 | Serum amyloid A-1 protein \|\| id:prot-a-2623 | Fried fish liking \|\| id:ebi-fl187-GCST90094765 | MR Egger | 5 | -0.381157761 | 1.40706821024374 | 0.804046212442028 | -3.139011453 | 2.37669593130429 | 0.68307011944881 | 0.0433256061439215 | 10.7692616355761 | 0.999279047927446 |
| ebi-fl187-GCST90094765 | prot-a-2640 | Lysosome membrane protein 2 \|\| id:prot-a-2640 | Fried fish liking \|\| id:ebi-fl187-GCST90094765 | MR Egger | 5 | 0.518785973196219 | 1.40571613193338 | 0.736595683307364 | -2.236417645 | 3.27398959178565 | 1.67998686208354 | 0.106840560414361 | 26.41652052 | 0.999279047927446 |
| ebi-fl187-GCST90094765 | prot-a-2641 | Scavenger receptor class F member 2 \|\| id:prot-a-2641 | Fried fish liking \|\| id:ebi-fl187-GCST90094765 | MR Egger | 5 | -1.043705119 | 1.40706821024374 | 0.512025607416923 | -3.801558811 | 1.71414857308459 | 0.352147513406344 | 0.0223359272144943 | 5.55194642279276 | 0.999279047927446 |
| ebi-fl187-GCST90094765 | prot-a-2669 | Semaphorin-3A \|\| id:prot-a-2669 | Fried fish liking \|\| id:ebi-fl187-GCST90094765 | MR Egger | 5 | 0.526001982808092 | 1.82523782676353 | 0.791970161185243 | -3.051464158 | 4.10346812326462 | 1.69215350795105 | 0.0472896341980337 | 60.5499184552818 | 0.999279047927446 |
| ebi-fl187-GCST90094765 | prot-a-2683 | Sentrin-specific protease 7 \|\| id:prot-a-2683 | Fried fish liking \|\| id:ebi-fl187-GCST90094765 | MR Egger | 5 | 2.47210174889592 | 1.40706821024374 | 0.177185318393946 | -0.285751943 | 5.22995544097366 | 11.8473207965006 | 0.751448994876345 | 186.784480400152 | 0.999279047927446 |
| ebi-fl187-GCST90094765 | prot-a-270 | Bromodomain testis-specific protein \|\| id:prot-a-270 | Fried fish liking \|\| id:ebi-fl187-GCST90094765 | MR Egger | 5 | -1.180502003 | 1.40756120963424 | 0.463209847801651 | -3.939321974 | 1.57831796785835 | 0.307124522456634 | 0.0194614056137994 | 4.84679648356604 | 0.999279047927446 |
| ebi-fl187-GCST90094765 | prot-a-2708 | 14-3-3 protein sigma \|\| id:prot-a-2708 | Fried fish liking \|\| id:ebi-fl187-GCST90094765 | MR Egger | 5 | 0.584460554825294 | 1.98933875085169 | 0.788066962191736 | -3.314643397 | 4.48356450649461 | 1.79402294753927 | 0.036347007724231 | 88.5497469479957 | 0.999279047927446 |
| ebi-fl187-GCST90094765 | prot-a-2713 | Beta-sarcoglycan \|\| id:prot-a-2713 | Fried fish liking \|\| id:ebi-fl187-GCST90094765 | MR Egger | 5 | 1.27727881140707 | 1.40706821024374 | 0.430927701919431 | -1.480574881 | 4.0351325034848 | 3.58686589482469 | 0.227506861485375 | 56.5504128686844 | 0.999279047927446 |
| ebi-fl187-GCST90094765 | prot-a-2720 | Protein shisa-3 homolog \|\| id:prot-a-2720 | Fried fish liking \|\| id:ebi-fl187-GCST90094765 | MR Egger | 5 | 2.27170364447243 | 1.52104142181279 | 0.232145400571889 | -0.709537542 | 5.2529448312255 | 9.69590512651468 | 0.491871614696635 | 191.128281066504 | 0.999279047927446 |
| ebi-fl187-GCST90094765 | prot-a-2730 | Sialic acid-binding Ig-like lectin 8 \|\| id:prot-a-2730 | Fried fish liking \|\| id:ebi-fl187-GCST90094765 | MR Egger | 5 | 0.170301949953412 | 2.15154302590737 | 0.941894782467973 | -4.046722381 | 4.38732628073185 | 1.1856628081048 | 0.0174795722334457 | 80.4250971218322 | 0.999279047927446 |
| ebi-fl187-GCST90094765 | prot-a-2761 | Sodium- and chloride-dependent neutral and basic amino acid transporter B(0+) \|\| id:prot-a-2761 | Fried fish liking \|\| id:ebi-fl187-GCST90094765 | MR Egger | 5 | 0.122302714488811 | 1.40706821024374 | 0.936211508720346 | -2.635550978 | 2.88015640656655 | 1.13009614648405 | 0.0716794647478365 | 17.8170596668225 | 0.999279047927446 |
| ebi-fl187-GCST90094765 | prot-a-2770 | Secreted Ly-6/uPAR-related protein 1 \|\| id:prot-a-2770 | Fried fish liking \|\| id:ebi-fl187-GCST90094765 | MR Egger | 5 | 2.43744722867666 | 1.87101818105272 | 0.283645107599145 | -1.229748406 | 6.10464286353998 | 11.4437900444518 | 0.292366125936843 | 447.932639808564 | 0.999279047927446 |
| ebi-fl187-GCST90094765 | prot-a-2784 | N-lysine methyltransferase SMYD2 \|\| id:prot-a-2784 | Fried fish liking \|\| id:ebi-fl187-GCST90094765 | MR Egger | 5 | 1.06484362662592 | 1.40706821024374 | 0.504189363088731 | -1.693010065 | 3.82269731870365 | 2.90038540554475 | 0.183964943229561 | 45.7273834515295 | 0.999279047927446 |
| ebi-fl187-GCST90094765 | prot-a-2837 | Serine palmitoyltransferase 2 \|\| id:prot-a-2837 | Fried fish liking \|\| id:ebi-fl187-GCST90094765 | MR Egger | 5 | -0.629239399 | 2.04602104848523 | 0.77854441889416 | -4.639440654 | 3.38096185564391 | 0.532997044751926 | 0.00966310112910751 | 29.3990351460313 | 0.999279047927446 |
| ebi-fl187-GCST90094765 | prot-a-2858 | Alpha-2,8-sialyltransferase 8B \|\| id:prot-a-2858 | Fried fish liking \|\| id:ebi-fl187-GCST90094765 | MR Egger | 5 | 1.50954586761141 | 1.62122082373399 | 0.420476861380035 | -1.668046947 | 4.68713868213004 | 4.52467552596343 | 0.188615081412694 | 108.542161432244 | 0.999279047927446 |
| ebi-fl187-GCST90094765 | prot-a-2911 | Synaptotagmin-11 \|\| id:prot-a-2911 | Fried fish liking \|\| id:ebi-fl187-GCST90094765 | MR Egger | 5 | -2.185297446 | 1.59648981985582 | 0.264543156610278 | -5.314417493 | 0.943822600515576 | 0.112444282532333 | 0.00492014393748622 | 2.56978593204959 | 0.999279047927446 |
| ebi-fl187-GCST90094765 | prot-a-2962 | Transforming growth factor beta-1 \|\| id:prot-a-2962 | Fried fish liking \|\| id:ebi-fl187-GCST90094765 | MR Egger | 5 | -0.828363046 | 1.40756120963424 | 0.597562496782994 | -3.587183017 | 1.93045692516067 | 0.436763663607967 | 0.0276761840664934 | 6.89265895146299 | 0.999279047927446 |
| ebi-fl187-GCST90094765 | prot-a-2978 | Tyrosine-protein kinase receptor Tie-1, soluble \|\| id:prot-a-2978 | Fried fish liking \|\| id:ebi-fl187-GCST90094765 | MR Egger | 5 | -0.608773831 | 1.40706821024374 | 0.69448238309831 | -3.366627523 | 2.14907986067764 | 0.544017517477034 | 0.0345058113750066 | 8.5769627644879 | 0.999279047927446 |
| ebi-fl187-GCST90094765 | prot-a-299 | Uncharacterized protein C1orf226 \|\| id:prot-a-299 | Fried fish liking \|\| id:ebi-fl187-GCST90094765 | MR Egger | 5 | 1.42193042475335 | 1.40706821024374 | 0.386657669166021 | -1.335923267 | 4.17978411683108 | 4.14511455349133 | 0.262915322237955 | 65.351743349575 | 0.999279047927446 |
| ebi-fl187-GCST90094765 | prot-a-3015 | Transmembrane protein 70, mitochondrial \|\| id:prot-a-3015 | Fried fish liking \|\| id:ebi-fl187-GCST90094765 | MR Egger | 5 | -1.273544042 | 1.40706821024374 | 0.432130093484498 | -4.031397735 | 1.48430964958265 | 0.279838104151078 | 0.0177495035126037 | 4.41191859137148 | 0.999279047927446 |
| ebi-fl187-GCST90094765 | prot-a-3016 | Transmembrane protein 87B \|\| id:prot-a-3016 | Fried fish liking \|\| id:ebi-fl187-GCST90094765 | MR Egger | 5 | 1.45000474384027 | 2.01795661921659 | 0.524325610950423 | -2.50519023 | 5.40519971750478 | 4.2631347387511 | 0.0816600623283632 | 222.560665306201 | 0.999279047927446 |
| ebi-fl187-GCST90094765 | prot-a-3123 | Thioredoxin domain-containing protein 12 \|\| id:prot-a-3123 | Fried fish liking \|\| id:ebi-fl187-GCST90094765 | MR Egger | 5 | 0.635463180614128 | 1.87373177098836 | 0.756854168803935 | -3.037051091 | 4.30797745175131 | 1.8878963759817 | 0.0479761584454573 | 74.2900816141175 | 0.999279047927446 |
| ebi-fl187-GCST90094765 | prot-a-3133 | PolyUbiquitin K48-linked \|\| id:prot-a-3133 | Fried fish liking \|\| id:ebi-fl187-GCST90094765 | MR Egger | 5 | -0.984734948 | 1.40706821024374 | 0.534409151062772 | -3.74258864 | 1.77311874401644 | 0.373538220846432 | 0.02369269182665 | 5.88919163149586 | 0.999279047927446 |
| ebi-fl187-GCST90094765 | prot-a-3135 | Ubiquitin-conjugating enzyme E2 B \|\| id:prot-a-3135 | Fried fish liking \|\| id:ebi-fl187-GCST90094765 | MR Egger | 5 | 0.857627488164134 | 1.6732713299098 | 0.643620485764446 | -2.421984318 | 4.13723929478734 | 2.35756071262739 | 0.0887453436020261 | 62.6296804782135 | 0.999279047927446 |
| ebi-fl187-GCST90094765 | prot-a-3162 | NKG2D ligand 3 \|\| id:prot-a-3162 | Fried fish liking \|\| id:ebi-fl187-GCST90094765 | MR Egger | 5 | 1.94326773912484 | 1.87665427189434 | 0.376583709757634 | -1.734974634 | 5.62151011203774 | 6.98152754713152 | 0.176404674947958 | 276.306321846265 | 0.999279047927446 |
| ebi-fl187-GCST90094765 | prot-a-3188 | Vesicle-associated membrane protein 8 \|\| id:prot-a-3188 | Fried fish liking \|\| id:ebi-fl187-GCST90094765 | MR Egger | 5 | 0.756276964387228 | 2.30835823884201 | 0.764726648811038 | -3.768105184 | 5.28065911251756 | 2.13033014292124 | 0.0230957841198884 | 196.499347858503 | 0.999279047927446 |
| ebi-fl187-GCST90094765 | prot-a-3201 | Vitrin \|\| id:prot-a-3201 | Fried fish liking \|\| id:ebi-fl187-GCST90094765 | MR Egger | 5 | 1.69620415893779 | 1.40706821024374 | 0.314425705238221 | -1.061649533 | 4.45405785101553 | 5.4532085430332 | 0.34588479107646 | 85.975111311664 | 0.999279047927446 |
| ebi-fl187-GCST90094765 | prot-a-3232 | WNT1-inducible-signaling pathway protein 3 \|\| id:prot-a-3232 | Fried fish liking \|\| id:ebi-fl187-GCST90094765 | MR Egger | 5 | 0.468045864940298 | 1.40756120963424 | 0.761374659980002 | -2.290774106 | 3.2268658358234 | 1.59687064138413 | 0.101188101217586 | 25.2005504069226 | 0.999279047927446 |
| ebi-fl187-GCST90094765 | prot-a-327 | Carbonic anhydrase-related protein 10 \|\| id:prot-a-327 | Fried fish liking \|\| id:ebi-fl187-GCST90094765 | MR Egger | 5 | -2.215056115 | 1.40706821024374 | 0.213501366699127 | -4.972909807 | 0.542797577069316 | 0.109147389269946 | 0.00692297417864338 | 1.72081424500988 | 0.999279047927446 |
| ebi-fl187-GCST90094765 | prot-a-332 | Carbonic anhydrase 5A, mitochondrial \|\| id:prot-a-332 | Fried fish liking \|\| id:ebi-fl187-GCST90094765 | MR Egger | 5 | 0.56095736911228 | 1.55161112856511 | 0.741664471909774 | -2.480200443 | 3.60211518109989 | 1.75234934262405 | 0.083726441541447 | 36.6757282653037 | 0.999279047927446 |
| ebi-fl187-GCST90094765 | prot-a-338 | Cell adhesion molecule 3 \|\| id:prot-a-338 | Fried fish liking \|\| id:ebi-fl187-GCST90094765 | MR Egger | 5 | 1.46712443931173 | 1.40706821024374 | 0.373727308391365 | -1.290729253 | 4.22497813138947 | 4.33674661582863 | 0.275070114287815 | 68.3730083095189 | 0.999279047927446 |
| ebi-fl187-GCST90094765 | prot-a-34 | A disintegrin and metalloproteinase with thrombospondin motifs 3 \|\| id:prot-a-34 | Fried fish liking \|\| id:ebi-fl187-GCST90094765 | MR Egger | 5 | 0.360633622231685 | 1.40706821024374 | 0.814288741996988 | -2.39722007 | 3.11848731430942 | 1.434237892 | 0.090970493723013 | 22.612148685613 | 0.999279047927446 |
| ebi-fl187-GCST90094765 | prot-a-358 | Cysteine--tRNA ligase, cytoplasmic \|\| id:prot-a-358 | Fried fish liking \|\| id:ebi-fl187-GCST90094765 | MR Egger | 5 | 0.932793030645751 | 2.52048250420839 | 0.735902675642969 | -4.007352678 | 5.87293873889419 | 2.54159803451179 | 0.018181463778541 | 355.291556703944 | 0.999279047927446 |
| ebi-fl187-GCST90094765 | prot-a-382 | Coiled-coil domain-containing protein 167 \|\| id:prot-a-382 | Fried fish liking \|\| id:ebi-fl187-GCST90094765 | MR Egger | 5 | -0.734034288 | 1.40756120963424 | 0.638074846222939 | -3.492854258 | 2.02478568329974 | 0.479968747015974 | 0.0304139389225877 | 7.57448743151761 | 0.999279047927446 |
| ebi-fl187-GCST90094765 | prot-a-40 | Pituitary adenylate cyclase-activating polypeptide 27 \|\| id:prot-a-40 | Fried fish liking \|\| id:ebi-fl187-GCST90094765 | MR Egger | 5 | -0.792088327 | 1.40706821024374 | 0.612807214476098 | -3.549942019 | 1.96576536518166 | 0.452898007934551 | 0.0287263051866075 | 7.14037549412069 | 0.999279047927446 |
| ebi-fl187-GCST90094765 | prot-a-403 | C-C motif chemokine 25 \|\| id:prot-a-403 | Fried fish liking \|\| id:ebi-fl187-GCST90094765 | MR Egger | 4 | 0.761872388110558 | 1.40960410874211 | 0.643001955609665 | -2.000951665 | 3.52469644124509 | 2.14228365403384 | 0.135206550645914 | 33.9434682152307 | 0.999279047927446 |
| ebi-fl187-GCST90094765 | prot-a-415 | Cyclin-C \|\| id:prot-a-415 | Fried fish liking \|\| id:ebi-fl187-GCST90094765 | MR Egger | 5 | 1.75659345505189 | 1.40756120963424 | 0.300575528237053 | -1.002226516 | 4.51541342593499 | 5.7926707522532 | 0.367061262953075 | 91.4153516883074 | 0.999279047927446 |
| ebi-fl187-GCST90094765 | prot-a-44 | Adhesion G protein-coupled receptor F5 \|\| id:prot-a-44 | Fried fish liking \|\| id:ebi-fl187-GCST90094765 | MR Egger | 5 | 1.35500172118692 | 2.69012144527799 | 0.649135960432911 | -3.917636312 | 6.62763975393178 | 3.87676762926915 | 0.019888048380718 | 755.6964345 | 0.999279047927446 |
| ebi-fl187-GCST90094765 | prot-a-455 | CD59 glycoprotein \|\| id:prot-a-455 | Fried fish liking \|\| id:ebi-fl187-GCST90094765 | MR Egger | 5 | 2.20253342676877 | 1.44468760672254 | 0.224767339768874 | -0.629054282 | 5.03412113594494 | 9.04790669709748 | 0.53309572068808 | 153.564570906172 | 0.999279047927446 |
| ebi-fl187-GCST90094765 | prot-a-478 | Cadherin-12 \|\| id:prot-a-478 | Fried fish liking \|\| id:ebi-fl187-GCST90094765 | MR Egger | 5 | 1.5999077748599 | 1.40706821024374 | 0.338118722194633 | -1.157945917 | 4.35776146693764 | 4.95257565134916 | 0.314130769241487 | 78.0821491685228 | 0.999279047927446 |
| ebi-fl187-GCST90094765 | prot-a-516 | Centrin-2 \|\| id:prot-a-516 | Fried fish liking \|\| id:ebi-fl187-GCST90094765 | MR Egger | 5 | -0.85377901 | 1.40706821024374 | 0.586838378915782 | -3.611632702 | 1.90407468198996 | 0.425802774707057 | 0.027007715294051 | 6.71319291447675 | 0.999279047927446 |
| ebi-fl187-GCST90094765 | prot-a-519 | Cryptic protein \|\| id:prot-a-519 | Fried fish liking \|\| id:ebi-fl187-GCST90094765 | MR Egger | 5 | -0.926569891 | 1.49889233525623 | 0.580222688048564 | -3.864398868 | 2.01125908648506 | 0.395909396502231 | 0.0209755277147649 | 7.4727202276025 | 0.999279047927446 |
| ebi-fl187-GCST90094765 | prot-a-583 | Chloride intracellular channel protein 4 \|\| id:prot-a-583 | Fried fish liking \|\| id:ebi-fl187-GCST90094765 | MR Egger | 5 | 1.28575443369883 | 1.40706821024374 | 0.428210238389502 | -1.472099258 | 4.04360812577656 | 3.61739601347383 | 0.229443318458763 | 57.0317497419223 | 0.999279047927446 |
| ebi-fl187-GCST90094765 | prot-a-602 | CCR4-NOT transcription complex subunit 1 \|\| id:prot-a-602 | Fried fish liking \|\| id:ebi-fl187-GCST90094765 | MR Egger | 5 | -1.242158268 | 1.57024555867819 | 0.486680582331378 | -4.319839563 | 1.83552302672665 | 0.288760322668855 | 0.0133020175031563 | 6.26841183512474 | 0.999279047927446 |
| ebi-fl187-GCST90094765 | prot-a-606 | Ciliary neurotrophic factor receptor subunit alpha \|\| id:prot-a-606 | Fried fish liking \|\| id:ebi-fl187-GCST90094765 | MR Egger | 5 | -1.435632928 | 1.9390919372274 | 0.512758391563914 | -5.236253125 | 2.36498726855233 | 0.237964701719394 | 0.00532015348554138 | 10.6439033043494 | 0.999279047927446 |
| ebi-fl187-GCST90094765 | prot-a-626 | Collagen alpha-2(IX) chain \|\| id:prot-a-626 | Fried fish liking \|\| id:ebi-fl187-GCST90094765 | MR Egger | 5 | 1.77502352412992 | 1.40706821024374 | 0.296295896279834 | -0.982830168 | 4.53287721620766 | 5.90041993693162 | 0.37425040708489 | 93.0258318309411 | 0.999279047927446 |
| ebi-fl187-GCST90094765 | prot-a-647 | Probable serine carboxypeptidase CPVL \|\| id:prot-a-647 | Fried fish liking \|\| id:ebi-fl187-GCST90094765 | MR Egger | 5 | 0.744783465470175 | 1.69762657712145 | 0.690537952632309 | -2.582564626 | 4.07213155662821 | 2.10598536705401 | 0.0755799210471276 | 58.6819131959673 | 0.999279047927446 |
| ebi-fl187-GCST90094765 | prot-a-651 | Cellular retinoic acid-binding protein 2 \|\| id:prot-a-651 | Fried fish liking \|\| id:ebi-fl187-GCST90094765 | MR Egger | 5 | 0.00760228290200221 | 1.68227941175714 | 0.99667804750718 | -3.289665364 | 3.30486993 | 1.00763125362267 | 0.0372663179561697 | 27.2449976000136 | 0.999279047927446 |
| ebi-fl187-GCST90094765 | prot-a-671 | Cytotoxic and regulatory T-cell molecule \|\| id:prot-a-671 | Fried fish liking \|\| id:ebi-fl187-GCST90094765 | MR Egger | 5 | -0.221722353 | 2.05068958499311 | 0.920725535678462 | -4.24107394 | 3.79762923365872 | 0.801137767013958 | 0.0143921272664227 | 44.5953339527162 | 0.999279047927446 |
| ebi-fl187-GCST90094765 | prot-a-688 | Chorionic somatomammotropin hormone \|\| id:prot-a-688 | Fried fish liking \|\| id:ebi-fl187-GCST90094765 | MR Egger | 5 | 0.85613206750378 | 1.60784958055703 | 0.631315325171547 | -2.29525311 | 4.00751724539555 | 2.35403780240257 | 0.100735892740319 | 55.0101242406756 | 0.999279047927446 |
| ebi-fl187-GCST90094765 | prot-a-69 | Aldo-keto reductase family 1 member C1 \|\| id:prot-a-69 | Fried fish liking \|\| id:ebi-fl187-GCST90094765 | MR Egger | 5 | 0.206155175745777 | 1.40706821024374 | 0.892807296224978 | -2.551698516 | 2.96400886782351 | 1.22894389148477 | 0.0779491555836349 | 19.3754900500143 | 0.999279047927446 |
| ebi-fl187-GCST90094765 | prot-a-741 | Stromal cell-derived factor 1 \|\| id:prot-a-741 | Fried fish liking \|\| id:ebi-fl187-GCST90094765 | MR Egger | 5 | -0.442891624 | 1.40756120963424 | 0.773640245268869 | -3.201711595 | 2.31592834646885 | 0.642176799603471 | 0.0406924952553061 | 10.1343267194995 | 0.999279047927446 |
| ebi-fl187-GCST90094765 | prot-a-742 | Growth-regulated alpha protein \|\| id:prot-a-742 | Fried fish liking \|\| id:ebi-fl187-GCST90094765 | MR Egger | 5 | 0.77422343561542 | 1.40706821024374 | 0.620475089754573 | -1.983630256 | 3.53207712769316 | 2.16890717696486 | 0.137568919260779 | 34.1949211171192 | 0.999279047927446 |
| ebi-fl187-GCST90094765 | prot-a-788 | Beta-defensin 112 \|\| id:prot-a-788 | Fried fish liking \|\| id:ebi-fl187-GCST90094765 | MR Egger | 5 | 2.47169394909408 | 1.40706821024374 | 0.177237082016068 | -0.286159743 | 5.22954764117182 | 11.8424904464023 | 0.751142616599888 | 186.70832525514 | 0.999279047927446 |
| ebi-fl187-GCST90094765 | prot-a-838 | DnaJ homolog subfamily B member 1 \|\| id:prot-a-838 | Fried fish liking \|\| id:ebi-fl187-GCST90094765 | MR Egger | 5 | 1.70312943097027 | 1.76704700605927 | 0.406231306250377 | -1.760282701 | 5.16654156284644 | 5.49110456431596 | 0.171996233458441 | 175.307497902489 | 0.999279047927446 |
| ebi-fl187-GCST90094765 | prot-a-85 | Junctional adhesion molecule-like \|\| id:prot-a-85 | Fried fish liking \|\| id:ebi-fl187-GCST90094765 | MR Egger | 5 | 0.425730371484527 | 1.97886316094323 | 0.843454692327435 | -3.452841424 | 4.30430216693325 | 1.53070799696252 | 0.0316555615970282 | 74.0175455356628 | 0.999279047927446 |
| ebi-fl187-GCST90094765 | prot-a-856 | Dipeptidase 1 \|\| id:prot-a-856 | Fried fish liking \|\| id:ebi-fl187-GCST90094765 | MR Egger | 5 | 3.46123932842571 | 1.40706821024374 | 0.0908760640069546 | 0.703385636347968 | 6.21909302050344 | 31.8564326425826 | 2.0205820962226 | 502.247497198255 | 0.999279047927446 |
| ebi-fl187-GCST90094765 | prot-a-860 | Dipeptidyl peptidase 2 \|\| id:prot-a-860 | Fried fish liking \|\| id:ebi-fl187-GCST90094765 | MR Egger | 5 | -0.82883938 | 1.40706821024374 | 0.597240725455733 | -3.586693072 | 1.92901431192552 | 0.436555667579077 | 0.0276897471795283 | 6.88272267925632 | 0.999279047927446 |
| ebi-fl187-GCST90094765 | prot-a-861 | Dermatopontin \|\| id:prot-a-861 | Fried fish liking \|\| id:ebi-fl187-GCST90094765 | MR Egger | 5 | -0.619342918 | 1.69263617128972 | 0.738716235045205 | -3.936909814 | 2.69822397747404 | 0.538298027 | 0.0195084063003251 | 14.8533284395042 | 0.999279047927446 |
| ebi-fl187-GCST90094765 | prot-a-877 | Dual specificity protein phosphatase 6 \|\| id:prot-a-877 | Fried fish liking \|\| id:ebi-fl187-GCST90094765 | MR Egger | 5 | -1.124331508 | 1.58349287925377 | 0.528899491047531 | -4.227977551 | 1.97931453581073 | 0.324869567646099 | 0.014581851800006 | 7.23778004536571 | 0.999279047927446 |
| ebi-fl187-GCST90094765 | prot-a-879 | Dynactin-associated protein \|\| id:prot-a-879 | Fried fish liking \|\| id:ebi-fl187-GCST90094765 | MR Egger | 5 | 1.07297602447817 | 1.40706821024374 | 0.501201002249776 | -1.684877668 | 3.83082971655591 | 2.92406866390122 | 0.185467119206143 | 46.1007729445871 | 0.999279047927446 |
| ebi-fl187-GCST90094765 | prot-a-937 | EMI domain-containing protein 1 \|\| id:prot-a-937 | Fried fish liking \|\| id:ebi-fl187-GCST90094765 | MR Egger | 5 | -1.048217504 | 1.96977033302232 | 0.63151054736549 | -4.908967356 | 2.81253234911556 | 0.350562068144211 | 0.00738010541695191 | 16.6520336334577 | 0.999279047927446 |
| ebi-fl187-GCST90094765 | ukb-rsfMRI191-edge-pheno102 | Net25_Pair11_15 (Postcentral\|Precentral)&(Cerebellum) (Motor)&(Subcortical-cerebellum) \|\| id:ukb-rsfMRI191-edge-pheno102 | Fried fish liking \|\| id:ebi-fl187-GCST90094765 | MR Egger | 5 | 0.24969156866047 | 0.430761063589418 | 0.602811787239363 | -0.594600116 | 1.09398325329573 | 1.2836294440768 | 0.551783171032125 | 2.98614498629751 | 0.999279047927446 |
| ebi-fl187-GCST90094765 | ukb-rsfMRI191-edge-pheno1167 | Net100_Pair11_45 (Angular\|Temporal)&(Frontal) (Default_mode\|Central_executive)&(Salience\|Default_mode) \|\| id:ukb-rsfMRI191-edge-pheno1167 | Fried fish liking \|\| id:ebi-fl187-GCST90094765 | MR Egger | 5 | -0.25769886 | 0.431306438889654 | 0.592276739070648 | -1.10305948 | 0.587661760101496 | 0.772827926375698 | 0.331854227478764 | 1.79977518539938 | 0.999279047927446 |
| ebi-fl187-GCST90094765 | ukb-rsfMRI191-edge-pheno1317 | Net100_Pair26_48 (Parietal\|Frontal)&(Temporal) (Attention\|Central_executive)&(Default_mode\|Central_executive) \|\| id:ukb-rsfMRI191-edge-pheno1317 | Fried fish liking \|\| id:ebi-fl187-GCST90094765 | MR Egger | 5 | 0.528190393607133 | 0.42959849532908 | 0.3065191844987 | -0.313822657 | 1.37020344445213 | 1.69586068990047 | 0.73064859190993 | 3.93615140218356 | 0.999279047927446 |
| ebi-fl187-GCST90094765 | ukb-rsfMRI191-edge-pheno286 | Net100_Pair10_13 (OccipitalPrecuneus)&(Frontal_Sup) (Default_mode\|Central_executive)&(Default_mode) \|\| id:ukb-rsfMRI191-edge-pheno286 | Fried fish liking \|\| id:ebi-fl187-GCST90094765 | MR Egger | 5 | 0.592763662812168 | 0.425217584015794 | 0.257619754725803 | -0.240662802 | 1.42619012748312 | 1.80898092672636 | 0.786106655405185 | 4.16280916941614 | 0.999279047927446 |
| ebi-fl187-GCST90094765 | ukb-rsfMRI191-edge-pheno599 | Net100_Pair11_29 (Angular\|Temporal)&(Parietal) (Default_mode\|Central_executive)&(Central_executive\|Salience) \|\| id:ukb-rsfMRI191-edge-pheno599 | Fried fish liking \|\| id:ebi-fl187-GCST90094765 | MR Egger | 5 | 0.11197319258056 | 0.594851563305014 | 0.862703968973703 | -1.053935871 | 1.27788225665839 | 1.11848287660354 | 0.348563146003714 | 3.58903102521918 | 0.999279047927446 |
| ebi-fl187-GCST90094765 | ukb-rsfMRI191-edge-pheno621 | Net100_Pair5_30 (Precuneus\|Angular\|Cingulate)&(Paracentral) (Default_mode\|Central_executive)&(Motor) \|\| id:ukb-rsfMRI191-edge-pheno621 | Fried fish liking \|\| id:ebi-fl187-GCST90094765 | MR Egger | 5 | -0.004617503 | 0.691357764792752 | 0.995090361161897 | -1.359678722 | 1.35044371580065 | 0.995393141085096 | 0.256743249614284 | 3.85913751114308 | 0.999279047927446 |
| ebi-fl187-GCST90094765 | ukb-rsfMRI191-edge-pheno624 | Net100_Pair8_30 (Calcarine\|Lingual)&(Paracentral) (Visual)&(Motor) \|\| id:ukb-rsfMRI191-edge-pheno624 | Fried fish liking \|\| id:ebi-fl187-GCST90094765 | MR Egger | 5 | 0.413048330517356 | 0.628526670839575 | 0.557989962036618 | -0.818863944 | 1.64496060536292 | 1.51141807178616 | 0.440932293708294 | 5.18080580696378 | 0.999279047927446 |
| ebi-fl187-GCST90094765 | ukb-rsfMRI191-edge-pheno882 | Net100_Pair6_38 (Postcentral\|Precentral)&(Subcortical) (Motor)&(Subcortical-cerebellum) \|\| id:ukb-rsfMRI191-edge-pheno882 | Fried fish liking \|\| id:ebi-fl187-GCST90094765 | MR Egger | 5 | -0.582979069 | 0.529791632320966 | 0.351536482616289 | -1.621370668 | 0.45541253074216 | 0.558232872960102 | 0.197627631492511 | 1.57682373714578 | 0.999279047927446 |
| ebi-fl187-GCST90094765 | ukb-rsfMRI191-edge-pheno899 | Net100_Pair23_38 (Cerebellum)&(Subcortical) (Subcortical-cerebellum)&(Subcortical-cerebellum) \|\| id:ukb-rsfMRI191-edge-pheno899 | Fried fish liking \|\| id:ebi-fl187-GCST90094765 | MR Egger | 5 | 0.390444568058139 | 0.584642124932592 | 0.552023009394408 | -0.755453997 | 1.53634313292602 | 1.47763755834352 | 0.46979727984125 | 4.64756363545827 | 0.999279047927446 |
| ebi-fl187-GCST90094765 | ebi-a-GCST90001392 | IgD+ CD38+ B cell Absolute Count \|\| id:ebi-a-GCST90001392 | Fried fish liking \|\| id:ebi-fl187-GCST90094765 | Weighted median | 4 | -0.12759696 | 0.341671017478301 | 0.708813730526375 | -0.797272154 | 0.542078234719476 | 0.880208067129074 | 0.450556337708684 | 1.71957683556111 | 0.999489402457283 |
| ebi-fl187-GCST90094765 | ebi-a-GCST90001402 | Switched memory B cell %B cell \|\| id:ebi-a-GCST90001402 | Fried fish liking \|\| id:ebi-fl187-GCST90094765 | Weighted median | 4 | 0.278671253044809 | 0.331125575600649 | 0.400018706395687 | -0.370334875 | 0.927677381222082 | 1.32137287514894 | 0.690503059612505 | 2.52862930999786 | 0.999489402457283 |
| ebi-fl187-GCST90094765 | ebi-a-GCST90001407 | Memory B cell Absolute Count \|\| id:ebi-a-GCST90001407 | Fried fish liking \|\| id:ebi-fl187-GCST90094765 | Weighted median | 4 | 0.105716736003558 | 0.283111405926593 | 0.708843039557778 | -0.44918162 | 0.66061509161968 | 1.1115069819997 | 0.638150187578396 | 1.93598277503024 | 0.999489402457283 |
| ebi-fl187-GCST90094765 | ebi-a-GCST90001411 | IgD+ CD24+ B cell %B cell \|\| id:ebi-a-GCST90001411 | Fried fish liking \|\| id:ebi-fl187-GCST90094765 | Weighted median | 4 | 0.000110178266497563 | 0.285768850729452 | 0.99969237537481 | -0.559996769 | 0.560217125696224 | 1.00011018433635 | 0.571210909335044 | 1.75105265755105 | 0.999911036302558 |
| ebi-fl187-GCST90094765 | ebi-a-GCST90001418 | CD24+ CD27+ B cell Absolute Count \|\| id:ebi-a-GCST90001418 | Fried fish liking \|\| id:ebi-fl187-GCST90094765 | Weighted median | 4 | -0.079020288 | 0.304474665471931 | 0.795226394903199 | -0.675790632 | 0.517750056812781 | 0.924021178164002 | 0.508754024425886 | 1.67824743727402 | 0.999489402457283 |
| ebi-fl187-GCST90094765 | ebi-a-GCST90001436 | Memory B cell %lymphocyte \|\| id:ebi-a-GCST90001436 | Fried fish liking \|\| id:ebi-fl187-GCST90094765 | Weighted median | 4 | -0.076005121 | 0.310725503435269 | 0.806762020811466 | -0.685027107 | 0.533016866061832 | 0.926811460650388 | 0.504076565182311 | 1.70406549902243 | 0.999489402457283 |
| ebi-fl187-GCST90094765 | ebi-a-GCST90001439 | IgD+ CD24+ B cell %lymphocyte \|\| id:ebi-a-GCST90001439 | Fried fish liking \|\| id:ebi-fl187-GCST90094765 | Weighted median | 4 | -0.280160905 | 0.291535074240327 | 0.3365595871699 | -0.85156965 | 0.291247840989616 | 0.755662142217702 | 0.426744566344251 | 1.33809617793799 | 0.999489402457283 |
| ebi-fl187-GCST90094765 | ebi-a-GCST90001457 | CD11c+ HLA DR++ monocyte %monocyte \|\| id:ebi-a-GCST90001457 | Fried fish liking \|\| id:ebi-fl187-GCST90094765 | Weighted median | 4 | 0.0305243376530757 | 0.349047743217235 | 0.93031357111839 | -0.653609239 | 0.714657914358856 | 1.03099498174163 | 0.520164984911621 | 2.04348751494111 | 0.999489402457283 |
| ebi-fl187-GCST90094765 | ebi-a-GCST90001483 | CD39+ resting CD4 regulatory T cell Absolute Count \|\| id:ebi-a-GCST90001483 | Fried fish liking \|\| id:ebi-fl187-GCST90094765 | Weighted median | 3 | -0.10892308 | 0.320193981904494 | 0.733722175542984 | -0.736503284 | 0.518657124676915 | 0.89679939678409 | 0.478785167441276 | 1.67977041220883 | 0.999489402457283 |
| ebi-fl187-GCST90094765 | ebi-a-GCST90001525 | CD33dim HLA DR+ CD11b+ Absolute Count \|\| id:ebi-a-GCST90001525 | Fried fish liking \|\| id:ebi-fl187-GCST90094765 | Weighted median | 4 | -0.252939891 | 0.345121258419845 | 0.463618502529112 | -0.929377558 | 0.423497775327534 | 0.776514555798228 | 0.394799373746846 | 1.52729435622955 | 0.999489402457283 |
| ebi-fl187-GCST90094765 | ebi-a-GCST90001540 | Naive CD4+ T cell Absolute Count \|\| id:ebi-a-GCST90001540 | Fried fish liking \|\| id:ebi-fl187-GCST90094765 | Weighted median | 4 | -0.202002124 | 0.321518654073219 | 0.529823822740335 | -0.832178686 | 0.428174437851594 | 0.817093192326666 | 0.435100305936356 | 1.53445372443439 | 0.999489402457283 |
| ebi-fl187-GCST90094765 | ebi-a-GCST90001624 | HLA DR+ CD4+ T cell Absolute Count \|\| id:ebi-a-GCST90001624 | Fried fish liking \|\| id:ebi-fl187-GCST90094765 | Weighted median | 4 | 0.220232347492163 | 0.262540982386771 | 0.401553821264869 | -0.294347978 | 0.734812672970234 | 1.24636628702833 | 0.745017196747892 | 2.08509136194671 | 0.999489402457283 |
| ebi-fl187-GCST90094765 | ebi-a-GCST90001672 | CD39+ CD8+ T cell Absolute Count \|\| id:ebi-a-GCST90001672 | Fried fish liking \|\| id:ebi-fl187-GCST90094765 | Weighted median | 4 | 0.38264520726662 | 0.293451670939092 | 0.192251907831292 | -0.192520068 | 0.957810482307241 | 1.46615775566137 | 0.824877764576948 | 2.60598437344041 | 0.999489402457283 |
| ebi-fl187-GCST90094765 | ebi-a-GCST90001722 | CD19 on CD20- CD38- B cell \|\| id:ebi-a-GCST90001722 | Fried fish liking \|\| id:ebi-fl187-GCST90094765 | Weighted median | 4 | 0.425585652558091 | 0.272492576968803 | 0.118329278115777 | -0.108499798 | 0.959671103416945 | 1.53048649057298 | 0.897179075777288 | 2.61083763661902 | 0.999489402457283 |
| ebi-fl187-GCST90094765 | ebi-a-GCST90001742 | CD19 on transitional B cell \|\| id:ebi-a-GCST90001742 | Fried fish liking \|\| id:ebi-fl187-GCST90094765 | Weighted median | 4 | 0.125997744389219 | 0.329839876813518 | 0.702463812569963 | -0.520488414 | 0.772483902943714 | 1.13427960978682 | 0.594230246612437 | 2.16513757169496 | 0.999489402457283 |
| ebi-fl187-GCST90094765 | ebi-a-GCST90001784 | CD25 on IgD+ CD38dim B cell \|\| id:ebi-a-GCST90001784 | Fried fish liking \|\| id:ebi-fl187-GCST90094765 | Weighted median | 4 | 0.0356013214302429 | 0.30170436185779 | 0.906067114957425 | -0.555739228 | 0.626941870671511 | 1.03624263639519 | 0.573648047829899 | 1.87187737419385 | 0.999489402457283 |
| ebi-fl187-GCST90094765 | ebi-a-GCST90001794 | CD25 on IgD+ B cell \|\| id:ebi-a-GCST90001794 | Fried fish liking \|\| id:ebi-fl187-GCST90094765 | Weighted median | 4 | -0.237081229 | 0.316173898172683 | 0.453348420687475 | -0.856782069 | 0.382619611608659 | 0.788927201797201 | 0.424525981894141 | 1.4661202288692 | 0.999489402457283 |
| ebi-fl187-GCST90094765 | ebi-a-GCST90001802 | CD27 on IgD- CD38- B cell \|\| id:ebi-a-GCST90001802 | Fried fish liking \|\| id:ebi-fl187-GCST90094765 | Weighted median | 4 | -0.12015401 | 0.306633485131805 | 0.69516982281557 | -0.721155641 | 0.480847620963857 | 0.8867838527122 | 0.486190070121457 | 1.61744480144286 | 0.999489402457283 |
| ebi-fl187-GCST90094765 | ebi-a-GCST90001804 | CD27 on IgD- CD38dim B cell \|\| id:ebi-a-GCST90001804 | Fried fish liking \|\| id:ebi-fl187-GCST90094765 | Weighted median | 4 | 0.394038170984956 | 0.313127573136833 | 0.208248940900297 | -0.219691872 | 1.00776821433315 | 1.48295715352961 | 0.802766114283693 | 2.73948025467786 | 0.999489402457283 |
| ebi-fl187-GCST90094765 | ebi-a-GCST90001805 | CD27 on memory B cell \|\| id:ebi-a-GCST90001805 | Fried fish liking \|\| id:ebi-fl187-GCST90094765 | Weighted median | 4 | 0.237472470988004 | 0.313584453344285 | 0.448879769216272 | -0.377153058 | 0.852097999542803 | 1.26804008841942 | 0.685811097284661 | 2.3445605826517 | 0.999489402457283 |
| ebi-fl187-GCST90094765 | ebi-a-GCST90001806 | CD27 on unswitched memory B cell \|\| id:ebi-a-GCST90001806 | Fried fish liking \|\| id:ebi-fl187-GCST90094765 | Weighted median | 4 | 0.241429860072342 | 0.28196426754065 | 0.391863520249017 | -0.311220104 | 0.794079824452016 | 1.27306815887739 | 0.732552620136403 | 2.21240425956797 | 0.999489402457283 |
| ebi-fl187-GCST90094765 | ebi-a-GCST90001839 | CD3 on Effector Memory CD8+ T cell \|\| id:ebi-a-GCST90001839 | Fried fish liking \|\| id:ebi-fl187-GCST90094765 | Weighted median | 4 | -0.094340682 | 0.290618916462176 | 0.745468769417033 | -0.663953758 | 0.475272394664216 | 0.909972698470882 | 0.51481186390484 | 1.60845227163498 | 0.999489402457283 |
| ebi-fl187-GCST90094765 | ebi-a-GCST90001859 | CD3 on CD8+ T cell \|\| id:ebi-a-GCST90001859 | Fried fish liking \|\| id:ebi-fl187-GCST90094765 | Weighted median | 4 | 0.20577908956936 | 0.313460478886105 | 0.511518298172046 | -0.408603449 | 0.820161628186127 | 1.22848178957611 | 0.66457771900336 | 2.27086684396124 | 0.999489402457283 |
| ebi-fl187-GCST90094765 | ebi-a-GCST90001905 | CD86 on monocyte \|\| id:ebi-a-GCST90001905 | Fried fish liking \|\| id:ebi-fl187-GCST90094765 | Weighted median | 4 | -0.036354645 | 0.32045839382606 | 0.909677165667338 | -0.664453097 | 0.591743807097057 | 0.96429824947287 | 0.51455486251993 | 1.80713696763565 | 0.999489402457283 |
| ebi-fl187-GCST90094765 | ebi-a-GCST90001943 | CD25 on activated & secreting CD4 regulatory T cell \|\| id:ebi-a-GCST90001943 | Fried fish liking \|\| id:ebi-fl187-GCST90094765 | Weighted median | 4 | -0.128102915 | 0.299409974363339 | 0.66875946175257 | -0.714946465 | 0.458740634506392 | 0.879762833477247 | 0.489218301398089 | 1.58208031252311 | 0.999489402457283 |
| ebi-fl187-GCST90094765 | ebi-a-GCST90001949 | CD33 on CD33dim HLA DR+ CD11b- \|\| id:ebi-a-GCST90001949 | Fried fish liking \|\| id:ebi-fl187-GCST90094765 | Weighted median | 4 | -0.038415001 | 0.434526753616628 | 0.92955357455535 | -0.890087438 | 0.813257436317577 | 0.962313497168923 | 0.410619847462054 | 2.25524234290468 | 0.999489402457283 |
| ebi-fl187-GCST90094765 | ebi-a-GCST90001955 | CD33 on Immature Myeloid-Derived Suppressor Cells \|\| id:ebi-a-GCST90001955 | Fried fish liking \|\| id:ebi-fl187-GCST90094765 | Weighted median | 4 | -0.156070296 | 0.450885462595143 | 0.729236166263208 | -1.039805803 | 0.727665210256276 | 0.855499049727969 | 0.353523328421659 | 2.0702413822392 | 0.999489402457283 |
| ebi-fl187-GCST90094765 | ebi-a-GCST90001956 | CD33 on CD33+ HLA DR+ \|\| id:ebi-a-GCST90001956 | Fried fish liking \|\| id:ebi-fl187-GCST90094765 | Weighted median | 4 | -0.021495849 | 0.414935378113956 | 0.958683838034965 | -0.83476919 | 0.791777492275664 | 0.978733540352287 | 0.433974635589819 | 2.20731642923925 | 0.999489402457283 |
| ebi-fl187-GCST90094765 | ebi-a-GCST90001957 | CD33 on CD33+ HLA DR+ CD14- \|\| id:ebi-a-GCST90001957 | Fried fish liking \|\| id:ebi-fl187-GCST90094765 | Weighted median | 4 | -0.048910928 | 0.394212633410538 | 0.901257910586795 | -0.821567689 | 0.723745833881864 | 0.952265946531668 | 0.439741735537982 | 2.06214320734069 | 0.999489402457283 |
| ebi-fl187-GCST90094765 | ebi-a-GCST90002064 | CD4 on resting CD4 regulatory T cell \|\| id:ebi-a-GCST90002064 | Fried fish liking \|\| id:ebi-fl187-GCST90094765 | Weighted median | 4 | -0.505785685 | 0.312063898976064 | 0.105065682611159 | -1.117430927 | 0.105859557056869 | 0.603031596410973 | 0.327119108925171 | 1.11166573993435 | 0.999489402457283 |
| ebi-fl187-GCST90094765 | ebi-a-GCST90002080 | SSC-A on T cell \|\| id:ebi-a-GCST90002080 | Fried fish liking \|\| id:ebi-fl187-GCST90094765 | Weighted median | 4 | 0.0884706421468255 | 0.367318075137697 | 0.80966697874581 | -0.631472785 | 0.808414069416711 | 1.09250217862112 | 0.531807985063622 | 2.24434578610003 | 0.999489402457283 |
| ebi-fl187-GCST90094765 | ebi-a-GCST90002121 | CD8 on CD39+ CD8+ T cell \|\| id:ebi-a-GCST90002121 | Fried fish liking \|\| id:ebi-fl187-GCST90094765 | Weighted median | 4 | 0.341269398398798 | 0.314018069043913 | 0.277132864293171 | -0.274206017 | 0.956744813724868 | 1.40673216121138 | 0.7601754501173 | 2.60320873698444 | 0.999489402457283 |
| ebi-fl187-GCST90094765 | ebi-cfmet338-GCST90026053 | 1-palmitoyl-2-docosahexaenoyl-gpc (16:0/22:6) levels \|\| id:ebi-cfmet338-GCST90026053 | Fried fish liking \|\| id:ebi-fl187-GCST90094765 | Weighted median | 4 | -0.122303962 | 0.151820226225667 | 0.4204826894417 | -0.419871606 | 0.175263681226635 | 0.884879357765361 | 0.65713118637771 | 1.19156036729198 | 0.999489402457283 |
| ebi-fl187-GCST90094765 | ebi-cfmet338-GCST90026063 | 1-stearoyl-2-oleoyl-gpc (18:0/18:1) levels \|\| id:ebi-cfmet338-GCST90026063 | Fried fish liking \|\| id:ebi-fl187-GCST90094765 | Weighted median | 3 | -0.188879773 | 0.131530800345827 | 0.150999000695751 | -0.446680142 | 0.0689205952298835 | 0.827886034595106 | 0.639748504148842 | 1.07135113538014 | 0.999489402457283 |
| ebi-fl187-GCST90094765 | ebi-cfmet338-GCST90026113 | Adenosine 3',5'-cyclic monophosphate (camp) levels \|\| id:ebi-cfmet338-GCST90026113 | Fried fish liking \|\| id:ebi-fl187-GCST90094765 | Weighted median | 4 | -0.587169004 | 0.243009964522819 | 0.0156819681411764 | -1.063468534 | -0.110869473 | 0.555898806667142 | 0.34525619816827 | 0.895055570018591 | 0.999489402457283 |
| ebi-fl187-GCST90094765 | ebi-cfmet338-GCST90026117 | Alpha-ketoglutaramate levels \|\| id:ebi-cfmet338-GCST90026117 | Fried fish liking \|\| id:ebi-fl187-GCST90094765 | Weighted median | 4 | -0.050182769 | 0.11320353835662 | 0.657551055616606 | -0.272061704 | 0.171696166611944 | 0.951055585548512 | 0.761807253278311 | 1.18731703184845 | 0.999489402457283 |
| ebi-fl187-GCST90094765 | ebi-cfmet338-GCST90026124 | Arginine levels \|\| id:ebi-cfmet338-GCST90026124 | Fried fish liking \|\| id:ebi-fl187-GCST90094765 | Weighted median | 4 | -0.048073306 | 0.0746471812854917 | 0.519570903494272 | -0.194381782 | 0.0982351690418769 | 0.953063918948354 | 0.823343506857689 | 1.10322219831169 | 0.999489402457283 |
| ebi-fl187-GCST90094765 | ebi-cfmet338-GCST90026169 | Glutamate levels \|\| id:ebi-cfmet338-GCST90026169 | Fried fish liking \|\| id:ebi-fl187-GCST90094765 | Weighted median | 4 | -0.096285599 | 0.0916345112159669 | 0.29337023340199 | -0.275889241 | 0.0833180426476221 | 0.908204596396041 | 0.758896980535138 | 1.08688743014007 | 0.999489402457283 |
| ebi-fl187-GCST90094765 | ebi-cfmet338-GCST90026170 | Glutamine levels \|\| id:ebi-cfmet338-GCST90026170 | Fried fish liking \|\| id:ebi-fl187-GCST90094765 | Weighted median | 4 | -0.013863629 | 0.049968943388177 | 0.781438503355898 | -0.111802758 | 0.0840755001704724 | 0.986232028668601 | 0.894220618049493 | 1.08771101307569 | 0.999489402457283 |
| ebi-fl187-GCST90094765 | ebi-cfmet338-GCST90026191 | Kynurenate levels \|\| id:ebi-cfmet338-GCST90026191 | Fried fish liking \|\| id:ebi-fl187-GCST90094765 | Weighted median | 4 | -0.799653784 | 0.369117921650057 | 0.0302813128092322 | -1.52312491 | -0.076182657 | 0.449484556123282 | 0.218029498746526 | 0.926646932432865 | 0.999489402457283 |
| ebi-fl187-GCST90094765 | ebi-cfmet338-GCST90026202 | Methylsuccinoylcarnitine levels \|\| id:ebi-cfmet338-GCST90026202 | Fried fish liking \|\| id:ebi-fl187-GCST90094765 | Weighted median | 4 | -0.251054494 | 0.264847130056974 | 0.343169024850194 | -0.770154869 | 0.268045881215501 | 0.777979975399402 | 0.46294136767416 | 1.30740712406686 | 0.999489402457283 |
| ebi-fl187-GCST90094765 | ebi-cfmet338-GCST90026222 | N-acetylputrescine levels \|\| id:ebi-cfmet338-GCST90026222 | Fried fish liking \|\| id:ebi-fl187-GCST90094765 | Weighted median | 4 | 0.00603698638838161 | 0.0963944156888757 | 0.950062757824663 | -0.182896068 | 0.194970041138578 | 1.00605524571599 | 0.832854711193592 | 1.21527457770169 | 0.999489402457283 |
| ebi-fl187-GCST90094765 | ebi-cfmet338-GCST90026252 | Propionylcarnitine (c3) levels \|\| id:ebi-cfmet338-GCST90026252 | Fried fish liking \|\| id:ebi-fl187-GCST90094765 | Weighted median | 4 | 0.120300422409538 | 0.149067163890212 | 0.419654033753916 | -0.171871219 | 0.412472063634353 | 1.12783562778571 | 0.842087611226173 | 1.51054734251533 | 0.999489402457283 |
| ebi-fl187-GCST90094765 | ebi-cfmet338-GCST90026316 | Ethyl beta-glucopyranoside levels \|\| id:ebi-cfmet338-GCST90026316 | Fried fish liking \|\| id:ebi-fl187-GCST90094765 | Weighted median | 4 | 0.425350297665543 | 0.518360523754549 | 0.411892118110578 | -0.590636329 | 1.44133692422446 | 1.53012632547442 | 0.553974662470611 | 4.22634234112478 | 0.999489402457283 |
| ebi-fl187-GCST90094765 | ebi-gm430-GCST90011315 | F_Prevotellaceae abundance \|\| id:ebi-gm430-GCST90011315 | Fried fish liking \|\| id:ebi-fl187-GCST90094765 | Weighted median | 3 | 0.131382261040506 | 0.234649678864085 | 0.575542108462551 | -0.32853111 | 0.591295631614114 | 1.14040362988087 | 0.719980529619299 | 1.80632723461723 | 0.999489402457283 |
| ebi-fl187-GCST90094765 | ebi-gm430-GCST90011354 | OTU97_1 (Bacteroides) abundance \|\| id:ebi-gm430-GCST90011354 | Fried fish liking \|\| id:ebi-fl187-GCST90094765 | Weighted median | 3 | 0.000801730633876438 | 0.219816813083661 | 0.997089908331064 | -0.430039223 | 0.431642684277852 | 1.00080205210579 | 0.650483580298904 | 1.53978482752617 | 0.999911036302558 |
| ebi-fl187-GCST90094765 | ebi-gm430-GCST90011419 | OTU99_1 (Bacteroides) abundance \|\| id:ebi-gm430-GCST90011419 | Fried fish liking \|\| id:ebi-fl187-GCST90094765 | Weighted median | 3 | 0.00126618553003732 | 0.209816114440269 | 0.995185003991505 | -0.409973399 | 0.412505769832964 | 1.00126698748137 | 0.663667904282155 | 1.51059825818215 | 0.999911036302558 |
| ebi-fl187-GCST90094765 | ebi-gm430-GCST90011435 | OTU99_155 (Butyrivibrio) abundance \|\| id:ebi-gm430-GCST90011435 | Fried fish liking \|\| id:ebi-fl187-GCST90094765 | Weighted median | 3 | -0.414015274 | 0.678294046152639 | 0.541611916269156 | -1.743471604 | 0.915441056849487 | 0.66099085547248 | 0.174912119746139 | 2.49787671461735 | 0.999489402457283 |
| ebi-fl187-GCST90094765 | ebi-gm430-GCST90011466 | OTU99_58 (Bacteroides) abundance \|\| id:ebi-gm430-GCST90011466 | Fried fish liking \|\| id:ebi-fl187-GCST90094765 | Weighted median | 3 | 0.524507745382953 | 0.402404267491527 | 0.192426345700478 | -0.264204619 | 1.31322010966635 | 1.68962691698388 | 0.76781641383146 | 3.71812723350183 | 0.999489402457283 |
| ebi-fl187-GCST90094765 | ebi-gm430-GCST90011505 | TestASV_28 (Holdemanella) abundance \|\| id:ebi-gm430-GCST90011505 | Fried fish liking \|\| id:ebi-fl187-GCST90094765 | Weighted median | 3 | 0.162968256605884 | 0.507825512054073 | 0.748275653358343 | -0.832369747 | 1.15830626023187 | 1.17699932714195 | 0.435017183219282 | 3.18453493225412 | 0.999489402457283 |
| ebi-fl187-GCST90094765 | ebi-gm430-GCST90011513 | TestASV_35 (Alphaproteobacteria) abundance \|\| id:ebi-gm430-GCST90011513 | Fried fish liking \|\| id:ebi-fl187-GCST90094765 | Weighted median | 3 | -0.140993656 | 0.565285356322961 | 0.80303566693964 | -1.248952954 | 0.966965642683844 | 0.868494821663931 | 0.286804937635616 | 2.62995212521541 | 0.999489402457283 |
| ebi-fl187-GCST90094765 | ebi-gm430-GCST90011543 | G_Coprococcus prevalence \|\| id:ebi-gm430-GCST90011543 | Fried fish liking \|\| id:ebi-fl187-GCST90094765 | Weighted median | 3 | -0.629356037 | 0.662537070818155 | 0.342153806311465 | -1.927928696 | 0.669216621436433 | 0.532934880678696 | 0.145449156082668 | 1.95270701249439 | 0.999489402457283 |
| ebi-fl187-GCST90094765 | ebi-gm430-GCST90011547 | G_Parasutterella prevalence \|\| id:ebi-gm430-GCST90011547 | Fried fish liking \|\| id:ebi-fl187-GCST90094765 | Weighted median | 3 | -0.15533935 | 0.59579685375946 | 0.794304307296412 | -1.323101183 | 1.01242248377636 | 0.856124602648412 | 0.266308149757271 | 2.75226025162187 | 0.999489402457283 |
| ebi-fl187-GCST90094765 | ebi-gm430-GCST90011569 | OTU97_13 (Subdoligranulum) prevalence \|\| id:ebi-gm430-GCST90011569 | Fried fish liking \|\| id:ebi-fl187-GCST90094765 | Weighted median | 3 | 0.190856722176694 | 0.62050795774956 | 0.758400855750213 | -1.025338875 | 1.40705231936583 | 1.21028603250969 | 0.35867489884837 | 4.08389961268879 | 0.999489402457283 |
| ebi-fl187-GCST90094765 | ebi-gm430-GCST90011590 | OTU97_277 (Prevotella) prevalence \|\| id:ebi-gm430-GCST90011590 | Fried fish liking \|\| id:ebi-fl187-GCST90094765 | Weighted median | 3 | 0.429270735823021 | 0.554088560242503 | 0.438497400773728 | -0.656742842 | 1.51528431389833 | 1.53613686538316 | 0.51853754545705 | 4.55071477439362 | 0.999489402457283 |
| ebi-fl187-GCST90094765 | ebi-gm430-GCST90011616 | OTU97_86 (Phascolarctobacterium) prevalence \|\| id:ebi-gm430-GCST90011616 | Fried fish liking \|\| id:ebi-fl187-GCST90094765 | Weighted median | 3 | 0.642032680479755 | 0.661272298737982 | 0.3315955133147 | -0.654061025 | 1.9381263860062 | 1.90033973955483 | 0.519930034734473 | 6.9457251639167 | 0.999489402457283 |
| ebi-fl187-GCST90094765 | ebi-gm430-GCST90011665 | OTU99_55 (Barnesiella) prevalence \|\| id:ebi-gm430-GCST90011665 | Fried fish liking \|\| id:ebi-fl187-GCST90094765 | Weighted median | 3 | 0.81494751847678 | 0.42400903044108 | 0.0546051161700359 | -0.016110181 | 1.6460052181413 | 2.25905711048038 | 0.984018893711658 | 5.18622057058525 | 0.999489402457283 |
| ebi-fl187-GCST90094765 | ebi-gm430-GCST90011687 | TestASV_14 (Lachnospiraceae) prevalence \|\| id:ebi-gm430-GCST90011687 | Fried fish liking \|\| id:ebi-fl187-GCST90094765 | Weighted median | 3 | 0.619799737717134 | 0.441227990454145 | 0.160105934890504 | -0.245007124 | 1.48460659900726 | 1.85855580594665 | 0.782698962608818 | 4.41322890259708 | 0.999489402457283 |
| ebi-fl187-GCST90094765 | ebi-gm430-GCST90011697 | TestASV_23 (Barnesiella) prevalence \|\| id:ebi-gm430-GCST90011697 | Fried fish liking \|\| id:ebi-fl187-GCST90094765 | Weighted median | 3 | 1.35305653380128 | 0.564692777374182 | 0.0165708754276582 | 0.246258690147887 | 2.45985437745468 | 3.86923391939386 | 1.2792304550576 | 11.7031071796316 | 0.999489402457283 |
| ebi-fl187-GCST90094765 | ebi-gm430-GCST90011727 | TestASV_6 (Subdoligranulum) prevalence \|\| id:ebi-gm430-GCST90011727 | Fried fish liking \|\| id:ebi-fl187-GCST90094765 | Weighted median | 3 | 1.02895915105081 | 0.709775680425597 | 0.147143238485678 | -0.362201183 | 2.42011948468498 | 2.79815186503263 | 0.696142302046131 | 11.2472031031189 | 0.999489402457283 |
| ebi-fl187-GCST90094765 | ebi-hbsm44-GCST90104908 | Sulcal depth of the left posterior inferior temporal sulcus \|\| id:ebi-hbsm44-GCST90104908 | Fried fish liking \|\| id:ebi-fl187-GCST90094765 | Weighted median | 3 | 0.0904756685456197 | 0.225862291456644 | 0.688730182076732 | -0.352214423 | 0.533165759800641 | 1.09469487179927 | 0.703129338913353 | 1.7043192425957 | 0.999489402457283 |
| ebi-fl187-GCST90094765 | ebi-hbsm44-GCST90104918 | Sulcal depth of the left inferior frontal sulcus \|\| id:ebi-hbsm44-GCST90104918 | Fried fish liking \|\| id:ebi-fl187-GCST90094765 | Weighted median | 3 | 0.265712862253257 | 0.344653987221647 | 0.440733351287002 | -0.409808953 | 0.941234677207685 | 1.30436047378339 | 0.663777050836054 | 2.56314412109531 | 0.999489402457283 |
| ebi-fl187-GCST90094765 | ebi-hbsm44-GCST90104925 | Sulcal depth of the right anterior inferior temporal sulcus \|\| id:ebi-hbsm44-GCST90104925 | Fried fish liking \|\| id:ebi-fl187-GCST90094765 | Weighted median | 3 | 0.0820077922064761 | 0.131082362266171 | 0.531563993501668 | -0.174913638 | 0.33892922224817 | 1.08546426795829 | 0.839529521225366 | 1.40344400908559 | 0.999489402457283 |
| ebi-fl187-GCST90094765 | ebi-IPs91-GCST90274770 | C-C motif chemokine 4 levels \|\| id:ebi-IPs91-GCST90274770 | Fried fish liking \|\| id:ebi-fl187-GCST90094765 | Weighted median | 5 | -0.06939945 | 0.123212087949824 | 0.573263325288125 | -0.310895142 | 0.172096242297046 | 0.932953937107938 | 0.732790710467562 | 1.18779214355738 | 0.999489402457283 |
| ebi-fl187-GCST90094765 | ebi-IPs91-GCST90274779 | C-X-C motif chemokine 1 levels \|\| id:ebi-IPs91-GCST90274779 | Fried fish liking \|\| id:ebi-fl187-GCST90094765 | Weighted median | 5 | -0.119792879 | 0.130785143036663 | 0.359692209108623 | -0.376131759 | 0.136546001398733 | 0.887104155631874 | 0.686511872755721 | 1.14630760831628 | 0.999489402457283 |
| ebi-fl187-GCST90094765 | ebi-IPs91-GCST90274780 | C-X-C motif chemokine 10 levels \|\| id:ebi-IPs91-GCST90274780 | Fried fish liking \|\| id:ebi-fl187-GCST90094765 | Weighted median | 5 | -0.097583789 | 0.13874729119376 | 0.481856151389595 | -0.36952848 | 0.174360901776622 | 0.907026339575241 | 0.691060102692173 | 1.19048513650009 | 0.999489402457283 |
| ebi-fl187-GCST90094765 | ebi-IPs91-GCST90274796 | Interleukin-10 receptor subunit alpha levels \|\| id:ebi-IPs91-GCST90274796 | Fried fish liking \|\| id:ebi-fl187-GCST90094765 | Weighted median | 5 | -0.100092047 | 0.162225530685405 | 0.53723922039457 | -0.418054087 | 0.217869993167579 | 0.904754134321091 | 0.658326620471471 | 1.24342540331252 | 0.999489402457283 |
| ebi-fl187-GCST90094765 | ebi-IPs91-GCST90274807 | Interleukin-20 levels \|\| id:ebi-IPs91-GCST90274807 | Fried fish liking \|\| id:ebi-fl187-GCST90094765 | Weighted median | 5 | -0.16731628 | 0.147155372511378 | 0.255536521401079 | -0.45574081 | 0.121108250534358 | 0.845932017992184 | 0.633978136782159 | 1.12874709323015 | 0.999489402457283 |
| ebi-fl187-GCST90094765 | ebi-IPs91-GCST90274808 | Interleukin-20 receptor subunit alpha levels \|\| id:ebi-IPs91-GCST90274808 | Fried fish liking \|\| id:ebi-fl187-GCST90094765 | Weighted median | 5 | 0.0224451598791629 | 0.156479432977306 | 0.885943955780409 | -0.284254529 | 0.329144848514682 | 1.02269894769269 | 0.752575068330175 | 1.38977914845416 | 0.999489402457283 |
| ebi-fl187-GCST90094765 | ebi-IPs91-GCST90274810 | Interleukin-24 levels \|\| id:ebi-IPs91-GCST90274810 | Fried fish liking \|\| id:ebi-fl187-GCST90094765 | Weighted median | 5 | -0.012349426 | 0.143471561966714 | 0.931406178130372 | -0.293553688 | 0.268854835070295 | 0.987726514849872 | 0.74560919164311 | 1.30846518400279 | 0.999489402457283 |
| ebi-fl187-GCST90094765 | ebi-IPs91-GCST90274823 | Monocyte chemoattractant protein-3 levels \|\| id:ebi-IPs91-GCST90274823 | Fried fish liking \|\| id:ebi-fl187-GCST90094765 | Weighted median | 5 | -0.076785315 | 0.143280091730889 | 0.59202035975716 | -0.357614295 | 0.204043664332103 | 0.926088649181806 | 0.69934276281666 | 1.22635170011507 | 0.999489402457283 |
| ebi-fl187-GCST90094765 | ebi-IPs91-GCST90274824 | Monocyte chemoattractant protein-4 levels \|\| id:ebi-IPs91-GCST90274824 | Fried fish liking \|\| id:ebi-fl187-GCST90094765 | Weighted median | 5 | -0.086006321 | 0.129100489811314 | 0.505285379292208 | -0.339043281 | 0.167030639211854 | 0.917588431292053 | 0.712451612911569 | 1.18179047388235 | 0.999489402457283 |
| ebi-fl187-GCST90094765 | ebi-IPs91-GCST90274834 | SIR2-like protein 2 levels \|\| id:ebi-IPs91-GCST90274834 | Fried fish liking \|\| id:ebi-fl187-GCST90094765 | Weighted median | 5 | -0.124931544 | 0.132393334968754 | 0.345354039642094 | -0.384422481 | 0.134559392298538 | 0.882557316648388 | 0.680843723022624 | 1.14403260371062 | 0.999489402457283 |
| ebi-fl187-GCST90094765 | ebi-met1400-GCST90199652 | 1-stearoyl-2-oleoyl-GPS (18:0/18:1) levels \|\| id:ebi-met1400-GCST90199652 | Fried fish liking \|\| id:ebi-fl187-GCST90094765 | Weighted median | 4 | -0.058384296 | 0.179067632775497 | 0.744389508099144 | -0.409356856 | 0.292588264430998 | 0.943287376388588 | 0.664077210063939 | 1.33989099606113 | 0.999489402457283 |
| ebi-fl187-GCST90094765 | ebi-met1400-GCST90199665 | N-acetylaspartate (naa) levels \|\| id:ebi-met1400-GCST90199665 | Fried fish liking \|\| id:ebi-fl187-GCST90094765 | Weighted median | 4 | 0.0795627960742661 | 0.18211835119874 | 0.662202635037146 | -0.277389172 | 0.436514764423797 | 1.08281355383487 | 0.757759540715241 | 1.54730508739198 | 0.999489402457283 |
| ebi-fl187-GCST90094765 | ebi-met1400-GCST90199669 | Acetylcarnitine levels (Biocrates platform) \|\| id:ebi-met1400-GCST90199669 | Fried fish liking \|\| id:ebi-fl187-GCST90094765 | Weighted median | 4 | -0.127003147 | 0.17321261485742 | 0.463423578418197 | -0.466499872 | 0.212493578359474 | 0.880730901143085 | 0.627193689185696 | 1.23675817152339 | 0.999489402457283 |
| ebi-fl187-GCST90094765 | ebi-met1400-GCST90199670 | Iminodiacetate (IDA) levels \|\| id:ebi-met1400-GCST90199670 | Fried fish liking \|\| id:ebi-fl187-GCST90094765 | Weighted median | 4 | 0.36617472649588 | 0.185484620384233 | 0.0483645281244575 | 0.00262487054278293 | 0.729724582448978 | 1.44220721245354 | 1.00262831853165 | 2.0745091727502 | 0.999489402457283 |
| ebi-fl187-GCST90094765 | ebi-met1400-GCST90199686 | Indolepropionate levels \|\| id:ebi-met1400-GCST90199686 | Fried fish liking \|\| id:ebi-fl187-GCST90094765 | Weighted median | 4 | -0.014835903 | 0.175768970590124 | 0.932733876413434 | -0.359343085 | 0.329671279554059 | 0.98527360697571 | 0.698134790573263 | 1.39051096394408 | 0.999489402457283 |
| ebi-fl187-GCST90094765 | ebi-met1400-GCST90199704 | 3-hydroxysebacate levels \|\| id:ebi-met1400-GCST90199704 | Fried fish liking \|\| id:ebi-fl187-GCST90094765 | Weighted median | 4 | -0.00141238 | 0.182043599711022 | 0.993809696589665 | -0.358217836 | 0.355393075150838 | 0.998588616656859 | 0.698920808507581 | 1.42674136064994 | 0.999911036302558 |
| ebi-fl187-GCST90094765 | ebi-met1400-GCST90199713 | Docosapentaenoate n3 DPA; 22:5n3 levels \|\| id:ebi-met1400-GCST90199713 | Fried fish liking \|\| id:ebi-fl187-GCST90094765 | Weighted median | 4 | -0.158794133 | 0.180355439441954 | 0.378615022165928 | -0.512290794 | 0.194702528775503 | 0.853171981247688 | 0.599121541660005 | 1.21494952020801 | 0.999489402457283 |
| ebi-fl187-GCST90094765 | ebi-met1400-GCST90199715 | Myristoleate (14:1n5) levels \|\| id:ebi-met1400-GCST90199715 | Fried fish liking \|\| id:ebi-fl187-GCST90094765 | Weighted median | 4 | -0.057964379 | 0.159933724426403 | 0.717032598871138 | -0.371434478 | 0.255505721364923 | 0.943683562251884 | 0.689744197501264 | 1.29111439993342 | 0.999489402457283 |
| ebi-fl187-GCST90094765 | ebi-met1400-GCST90199725 | Campesterol levels \|\| id:ebi-met1400-GCST90199725 | Fried fish liking \|\| id:ebi-fl187-GCST90094765 | Weighted median | 4 | 0.35105646863001 | 0.225614785429506 | 0.11970823728714 | -0.091148511 | 0.793261448071842 | 1.42056754115024 | 0.912882127965827 | 2.21059442084419 | 0.999489402457283 |
| ebi-fl187-GCST90094765 | ebi-met1400-GCST90199737 | N-acetylproline levels \|\| id:ebi-met1400-GCST90199737 | Fried fish liking \|\| id:ebi-fl187-GCST90094765 | Weighted median | 4 | -0.254645934 | 0.192793903457834 | 0.186561578835939 | -0.632521985 | 0.123230116728904 | 0.775190918086847 | 0.531250304893868 | 1.13114468631577 | 0.999489402457283 |
| ebi-fl187-GCST90094765 | ebi-met1400-GCST90199775 | Gamma-glutamylisoleucine levels \|\| id:ebi-met1400-GCST90199775 | Fried fish liking \|\| id:ebi-fl187-GCST90094765 | Weighted median | 4 | 0.0907170639283842 | 0.16544946682458 | 0.5834812747348 | -0.233563891 | 0.414998018904561 | 1.09495915798431 | 0.791707011133827 | 1.51436774058205 | 0.999489402457283 |
| ebi-fl187-GCST90094765 | ebi-met1400-GCST90199777 | 1-palmitoleoyl-GPC (16:1) levels \|\| id:ebi-met1400-GCST90199777 | Fried fish liking \|\| id:ebi-fl187-GCST90094765 | Weighted median | 4 | -0.232921409 | 0.17865843466424 | 0.192327473882006 | -0.583091941 | 0.117249122646465 | 0.792215831878205 | 0.558169867303216 | 1.1243995081833 | 0.999489402457283 |
| ebi-fl187-GCST90094765 | ebi-met1400-GCST90199782 | Glutamine degradant levels \|\| id:ebi-met1400-GCST90199782 | Fried fish liking \|\| id:ebi-fl187-GCST90094765 | Weighted median | 4 | -0.305632683 | 0.160607593569527 | 0.0570434438391773 | -0.620423566 | 0.00915820050630256 | 0.736657156544432 | 0.5377166307161 | 1.00920026513861 | 0.999489402457283 |
| ebi-fl187-GCST90094765 | ebi-met1400-GCST90199783 | 5-methyluridine (ribothymidine) levels \|\| id:ebi-met1400-GCST90199783 | Fried fish liking \|\| id:ebi-fl187-GCST90094765 | Weighted median | 4 | -0.213482646 | 0.191341470286038 | 0.264543929655476 | -0.588511928 | 0.16154663577716 | 0.807766178016531 | 0.555152777831963 | 1.1753272691809 | 0.999489402457283 |
| ebi-fl187-GCST90094765 | ebi-met1400-GCST90199787 | Homostachydrine levels \|\| id:ebi-met1400-GCST90199787 | Fried fish liking \|\| id:ebi-fl187-GCST90094765 | Weighted median | 4 | -0.431551315 | 0.186906245577114 | 0.0209480962900341 | -0.797887556 | -0.065215073 | 0.649500732680382 | 0.450279149584256 | 0.936865946695176 | 0.999489402457283 |
| ebi-fl187-GCST90094765 | ebi-met1400-GCST90199802 | Beta-hydroxyisovaleroylcarnitine levels \|\| id:ebi-met1400-GCST90199802 | Fried fish liking \|\| id:ebi-fl187-GCST90094765 | Weighted median | 4 | 0.324371977742595 | 0.184018184278712 | 0.0779482357567184 | -0.036303663 | 0.685047618928872 | 1.38316171681213 | 0.964347411960647 | 1.98386630287629 | 0.999489402457283 |
| ebi-fl187-GCST90094765 | ebi-met1400-GCST90199873 | Pregnenolone sulfate levels \|\| id:ebi-met1400-GCST90199873 | Fried fish liking \|\| id:ebi-fl187-GCST90094765 | Weighted median | 4 | -0.200042479 | 0.200344251492605 | 0.318040004409552 | -0.592717211 | 0.192632254408948 | 0.818695975348788 | 0.552823104806709 | 1.21243684322249 | 0.999489402457283 |
| ebi-fl187-GCST90094765 | ebi-met1400-GCST90199911 | Beta-citrylglutamate levels \|\| id:ebi-met1400-GCST90199911 | Fried fish liking \|\| id:ebi-fl187-GCST90094765 | Weighted median | 4 | 0.307344665697081 | 0.180388774548173 | 0.0884202486357671 | -0.046217332 | 0.660906663811499 | 1.35980956702467 | 0.954834423165709 | 1.93654733607243 | 0.999489402457283 |
| ebi-fl187-GCST90094765 | ebi-met1400-GCST90199934 | 2-hydroxydecanoate levels \|\| id:ebi-met1400-GCST90199934 | Fried fish liking \|\| id:ebi-fl187-GCST90094765 | Weighted median | 4 | -0.086097676 | 0.178352805939711 | 0.629281780259589 | -0.435669176 | 0.263473823345332 | 0.917504608391038 | 0.646831678026176 | 1.30144322706583 | 0.999489402457283 |
| ebi-fl187-GCST90094765 | ebi-met1400-GCST90199945 | Sphingomyelin (d18:1/14:0, d16:1/16:0) levels \|\| id:ebi-met1400-GCST90199945 | Fried fish liking \|\| id:ebi-fl187-GCST90094765 | Weighted median | 4 | -0.24860498 | 0.19459382199324 | 0.201405161484246 | -0.630008871 | 0.13279891097825 | 0.779887983796722 | 0.532587076280695 | 1.14202032748905 | 0.999489402457283 |
| ebi-fl187-GCST90094765 | ebi-met1400-GCST90199979 | Carnitine C14:1 levels \|\| id:ebi-met1400-GCST90199979 | Fried fish liking \|\| id:ebi-fl187-GCST90094765 | Weighted median | 4 | 0.0987262371070995 | 0.207879002979032 | 0.63484282625685 | -0.308716609 | 0.506169082946003 | 1.10376408854373 | 0.734388859929805 | 1.65892380676256 | 0.999489402457283 |
| ebi-fl187-GCST90094765 | ebi-met1400-GCST90199986 | Eugenol sulfate levels \|\| id:ebi-met1400-GCST90199986 | Fried fish liking \|\| id:ebi-fl187-GCST90094765 | Weighted median | 4 | -0.005041616 | 0.190721801125094 | 0.978910858675556 | -0.378856346 | 0.368773114346291 | 0.99497107175536 | 0.684643957401491 | 1.44595949898886 | 0.999489402457283 |
| ebi-fl187-GCST90094765 | ebi-met1400-GCST90200002 | 6-hydroxyindole sulfate levels \|\| id:ebi-met1400-GCST90200002 | Fried fish liking \|\| id:ebi-fl187-GCST90094765 | Weighted median | 4 | 0.0471387352412754 | 0.217379922993939 | 0.828325606887357 | -0.378925914 | 0.473203384309396 | 1.04826743062678 | 0.684596329909771 | 1.60512780759095 | 0.999489402457283 |
| ebi-fl187-GCST90094765 | ebi-met1400-GCST90200023 | Trans 3,4-methyleneheptanoate levels \|\| id:ebi-met1400-GCST90200023 | Fried fish liking \|\| id:ebi-fl187-GCST90094765 | Weighted median | 4 | -0.138680815 | 0.190597558676915 | 0.466851955199072 | -0.51225203 | 0.234890400148758 | 0.870505836648856 | 0.599144766440934 | 1.26477014251685 | 0.999489402457283 |
| ebi-fl187-GCST90094765 | ebi-met1400-GCST90200032 | 2-hydroxybutyrate/2-hydroxyisobutyrate levels \|\| id:ebi-met1400-GCST90200032 | Fried fish liking \|\| id:ebi-fl187-GCST90094765 | Weighted median | 4 | 0.0416676599366971 | 0.175400292768048 | 0.812224295367029 | -0.302116914 | 0.385452233762071 | 1.0425479407211 | 0.739251631054075 | 1.47027908095654 | 0.999489402457283 |
| ebi-fl187-GCST90094765 | ebi-met1400-GCST90200051 | 1-palmitoyl-2-dihomo-linolenoyl-GPC (16:0/20:3n3 or 6) levels \|\| id:ebi-met1400-GCST90200051 | Fried fish liking \|\| id:ebi-fl187-GCST90094765 | Weighted median | 4 | 0.116784848051636 | 0.176219006077676 | 0.507506305497432 | -0.228604404 | 0.46217409996388 | 1.12387759922113 | 0.795643224643693 | 1.58752166663229 | 0.999489402457283 |
| ebi-fl187-GCST90094765 | ebi-met1400-GCST90200069 | Phenylacetylglutamate levels \|\| id:ebi-met1400-GCST90200069 | Fried fish liking \|\| id:ebi-fl187-GCST90094765 | Weighted median | 4 | -0.104772576 | 0.183737188381065 | 0.568521272939498 | -0.464897465 | 0.255352313573806 | 0.900529301587813 | 0.628199514400095 | 1.29091634811699 | 0.999489402457283 |
| ebi-fl187-GCST90094765 | ebi-met1400-GCST90200072 | Thioproline levels \|\| id:ebi-met1400-GCST90200072 | Fried fish liking \|\| id:ebi-fl187-GCST90094765 | Weighted median | 4 | -0.006418464 | 0.189520429835933 | 0.972983312365198 | -0.377878506 | 0.365041578821061 | 0.993602090681222 | 0.685313757024731 | 1.44057390426859 | 0.999489402457283 |
| ebi-fl187-GCST90094765 | ebi-met1400-GCST90200081 | 1-oleoyl-2-docosahexaenoyl-GPC (18:1/22:6) levels \|\| id:ebi-met1400-GCST90200081 | Fried fish liking \|\| id:ebi-fl187-GCST90094765 | Weighted median | 4 | -0.084893438 | 0.193029431985886 | 0.660085367025367 | -0.463231125 | 0.293444248412814 | 0.918610167865986 | 0.629247180963076 | 1.34103841230604 | 0.999489402457283 |
| ebi-fl187-GCST90094765 | ebi-met1400-GCST90200097 | Furaneol sulfate levels \|\| id:ebi-met1400-GCST90200097 | Fried fish liking \|\| id:ebi-fl187-GCST90094765 | Weighted median | 4 | 0.0221712884200885 | 0.220423874070205 | 0.919880073678918 | -0.409859505 | 0.454202081597691 | 1.02241889799026 | 0.663743496389312 | 1.57491622690714 | 0.999489402457283 |
| ebi-fl187-GCST90094765 | ebi-met1400-GCST90200124 | Sphingomyelin (d18:2/24:2) levels \|\| id:ebi-met1400-GCST90200124 | Fried fish liking \|\| id:ebi-fl187-GCST90094765 | Weighted median | 4 | -0.223926898 | 0.165663107062832 | 0.17647115884889 | -0.548626587 | 0.100772792329552 | 0.799373568428917 | 0.577742744933113 | 1.10602531577745 | 0.999489402457283 |
| ebi-fl187-GCST90094765 | ebi-met1400-GCST90200137 | Lignoceroylcarnitine (C24) levels \|\| id:ebi-met1400-GCST90200137 | Fried fish liking \|\| id:ebi-fl187-GCST90094765 | Weighted median | 4 | -0.10095367 | 0.177410445878684 | 0.569328879642721 | -0.448678144 | 0.246770803680296 | 0.903974912854732 | 0.638471561425378 | 1.27988573405901 | 0.999489402457283 |
| ebi-fl187-GCST90094765 | ebi-met1400-GCST90200165 | Octadecenedioate (C18:1-DC) levels \|\| id:ebi-met1400-GCST90200165 | Fried fish liking \|\| id:ebi-fl187-GCST90094765 | Weighted median | 4 | -0.261329097 | 0.171127993326533 | 0.126736816984909 | -0.596739964 | 0.07408176949409 | 0.770027463855309 | 0.550603701105836 | 1.07689485904394 | 0.999489402457283 |
| ebi-fl187-GCST90094765 | ebi-met1400-GCST90200176 | Glutamine conjugate of C6H10O2 (1) levels \|\| id:ebi-met1400-GCST90200176 | Fried fish liking \|\| id:ebi-fl187-GCST90094765 | Weighted median | 4 | -0.003045804 | 0.179233054153196 | 0.986441768770187 | -0.35434259 | 0.348250981932161 | 0.996958829547835 | 0.701634552949995 | 1.41658774305579 | 0.999911036302558 |
| ebi-fl187-GCST90094765 | ebi-met1400-GCST90200182 | Glycine conjugate of C10H12O2 levels \|\| id:ebi-met1400-GCST90200182 | Fried fish liking \|\| id:ebi-fl187-GCST90094765 | Weighted median | 4 | -0.015521405 | 0.197672729985852 | 0.937413849307041 | -0.402959956 | 0.371917145783416 | 0.984598431207728 | 0.66833886190875 | 1.45051279521297 | 0.999489402457283 |
| ebi-fl187-GCST90094765 | ebi-met1400-GCST90200208 | Glucuronide of piperine metabolite C17H21NO3 (4) levels \|\| id:ebi-met1400-GCST90200208 | Fried fish liking \|\| id:ebi-fl187-GCST90094765 | Weighted median | 4 | 0.137845035712312 | 0.185623951464941 | 0.457721639470059 | -0.225977909 | 0.501667980583596 | 1.14779766884374 | 0.79773572412409 | 1.65147360054567 | 0.999489402457283 |
| ebi-fl187-GCST90094765 | ebi-met1400-GCST90200227 | 3-ethylcatechol sulfate (2) levels \|\| id:ebi-met1400-GCST90200227 | Fried fish liking \|\| id:ebi-fl187-GCST90094765 | Weighted median | 4 | 0.00697788021698239 | 0.238151425495576 | 0.976625181394503 | -0.459798914 | 0.473754674188311 | 1.00700228234851 | 0.63141060072919 | 1.60601294226612 | 0.999489402457283 |
| ebi-fl187-GCST90094765 | ebi-met1400-GCST90200259 | Pentose acid levels \|\| id:ebi-met1400-GCST90200259 | Fried fish liking \|\| id:ebi-fl187-GCST90094765 | Weighted median | 4 | -0.181672245 | 0.176423128125683 | 0.303125970594495 | -0.527461576 | 0.164117086296675 | 0.833874602344115 | 0.590100996657915 | 1.17835227592007 | 0.999489402457283 |
| ebi-fl187-GCST90094765 | ebi-met1400-GCST90200291 | Sarcosine levels \|\| id:ebi-met1400-GCST90200291 | Fried fish liking \|\| id:ebi-fl187-GCST90094765 | Weighted median | 4 | 0.294815525121592 | 0.175742191576391 | 0.0934358571225537 | -0.04963917 | 0.639270220611318 | 1.34287860846712 | 0.951572718195321 | 1.89509737153733 | 0.999489402457283 |
| ebi-fl187-GCST90094765 | ebi-met1400-GCST90200306 | Butyrate/isobutyrate (4:0) levels \|\| id:ebi-met1400-GCST90200306 | Fried fish liking \|\| id:ebi-fl187-GCST90094765 | Weighted median | 4 | 0.181238692069642 | 0.176446396332816 | 0.304345098778069 | -0.164596245 | 0.527073628881961 | 1.19870126563455 | 0.848236114748993 | 1.69396786961737 | 0.999489402457283 |
| ebi-fl187-GCST90094765 | ebi-met1400-GCST90200321 | Cholate levels \|\| id:ebi-met1400-GCST90200321 | Fried fish liking \|\| id:ebi-fl187-GCST90094765 | Weighted median | 4 | 0.122593496384874 | 0.184877894385941 | 0.507263169256316 | -0.239767177 | 0.484954169381319 | 1.13042480576603 | 0.786811027752516 | 1.62410057360445 | 0.999489402457283 |
| ebi-fl187-GCST90094765 | ebi-met1400-GCST90200336 | Ceramide (d18:1/16:0) levels \|\| id:ebi-met1400-GCST90200336 | Fried fish liking \|\| id:ebi-fl187-GCST90094765 | Weighted median | 4 | -0.021787708 | 0.194385756285697 | 0.910756089872103 | -0.40278379 | 0.35920837422641 | 0.978447929580823 | 0.668456610427973 | 1.43219520304849 | 0.999489402457283 |
| ebi-fl187-GCST90094765 | ebi-met1400-GCST90200358 | Arachidonate (20:4n6) levels \|\| id:ebi-met1400-GCST90200358 | Fried fish liking \|\| id:ebi-fl187-GCST90094765 | Weighted median | 4 | -0.17441896 | 0.175069612537457 | 0.319112436160215 | -0.517555401 | 0.168717480637542 | 0.839944920661351 | 0.595975690494647 | 1.18378564930937 | 0.999489402457283 |
| ebi-fl187-GCST90094765 | ebi-met1400-GCST90200413 | 2'-deoxyuridine levels \|\| id:ebi-met1400-GCST90200413 | Fried fish liking \|\| id:ebi-fl187-GCST90094765 | Weighted median | 4 | -0.125272464 | 0.178147505775972 | 0.481934135668466 | -0.474441575 | 0.223896647659095 | 0.882256487000723 | 0.622232429620328 | 1.25094172499142 | 0.999489402457283 |
| ebi-fl187-GCST90094765 | ebi-met1400-GCST90200432 | Threonine levels \|\| id:ebi-met1400-GCST90200432 | Fried fish liking \|\| id:ebi-fl187-GCST90094765 | Weighted median | 4 | 0.0560985436318135 | 0.171145565262427 | 0.743076021049791 | -0.279346764 | 0.39154385154617 | 1.05770190838862 | 0.756277607678284 | 1.47926279404642 | 0.999489402457283 |
| ebi-fl187-GCST90094765 | ebi-met1400-GCST90200436 | Caffeine levels \|\| id:ebi-met1400-GCST90200436 | Fried fish liking \|\| id:ebi-fl187-GCST90094765 | Weighted median | 4 | 0.175169759186333 | 0.176164678879655 | 0.320051366596458 | -0.170113011 | 0.520452529790458 | 1.19144845876656 | 0.843569478226583 | 1.68278898957024 | 0.999489402457283 |
| ebi-fl187-GCST90094765 | ebi-met1400-GCST90200442 | Valine levels \|\| id:ebi-met1400-GCST90200442 | Fried fish liking \|\| id:ebi-fl187-GCST90094765 | Weighted median | 4 | 0.35307942321317 | 0.182050010686017 | 0.0524448765228371 | -0.003738598 | 0.709897444157763 | 1.42344419345576 | 0.996268382124076 | 2.03378267165621 | 0.999489402457283 |
| ebi-fl187-GCST90094765 | ebi-met1400-GCST90200459 | Margarate (17:0) levels \|\| id:ebi-met1400-GCST90200459 | Fried fish liking \|\| id:ebi-fl187-GCST90094765 | Weighted median | 4 | 0.0212609917767475 | 0.16692665687672 | 0.898649723775136 | -0.305915256 | 0.348437239255118 | 1.02148861697941 | 0.736449026667791 | 1.41685161747005 | 0.999489402457283 |
| ebi-fl187-GCST90094765 | ebi-met1400-GCST90200462 | X-11299 levels \|\| id:ebi-met1400-GCST90200462 | Fried fish liking \|\| id:ebi-fl187-GCST90094765 | Weighted median | 4 | -0.055870378 | 0.185187272398162 | 0.762883328537542 | -0.418837432 | 0.307096675716679 | 0.945661706334285 | 0.657811125559591 | 1.35947238968682 | 0.999489402457283 |
| ebi-fl187-GCST90094765 | ebi-met1400-GCST90200497 | X-12740 levels \|\| id:ebi-met1400-GCST90200497 | Fried fish liking \|\| id:ebi-fl187-GCST90094765 | Weighted median | 4 | 0.321679212752154 | 0.206254250772117 | 0.118848608897061 | -0.082579119 | 0.725937544265504 | 1.37944219750818 | 0.920738587278509 | 2.06666778449094 | 0.999489402457283 |
| ebi-fl187-GCST90094765 | ebi-met1400-GCST90200527 | X-13866 levels \|\| id:ebi-met1400-GCST90200527 | Fried fish liking \|\| id:ebi-fl187-GCST90094765 | Weighted median | 4 | -0.146304318 | 0.170111412095607 | 0.389761487739611 | -0.479722686 | 0.187114049730711 | 0.863894764471396 | 0.61895501309457 | 1.20576479435842 | 0.999489402457283 |
| ebi-fl187-GCST90094765 | ebi-met1400-GCST90200547 | X-17654 levels \|\| id:ebi-met1400-GCST90200547 | Fried fish liking \|\| id:ebi-fl187-GCST90094765 | Weighted median | 4 | 0.0239811686123686 | 0.223640130518174 | 0.914605673894808 | -0.414353487 | 0.46231582442799 | 1.02427102926421 | 0.660767337180529 | 1.58774667323385 | 0.999489402457283 |
| ebi-fl187-GCST90094765 | ebi-met1400-GCST90200553 | X-17653 levels \|\| id:ebi-met1400-GCST90200553 | Fried fish liking \|\| id:ebi-fl187-GCST90094765 | Weighted median | 3 | 0.331779930182555 | 0.231444071042764 | 0.15170900932358 | -0.121850449 | 0.785410309426372 | 1.39344615932534 | 0.885280753170938 | 2.19330669054273 | 0.999489402457283 |
| ebi-fl187-GCST90094765 | ebi-met1400-GCST90200557 | X-17685 levels \|\| id:ebi-met1400-GCST90200557 | Fried fish liking \|\| id:ebi-fl187-GCST90094765 | Weighted median | 4 | 0.0687460196909813 | 0.181832399155111 | 0.705375960532142 | -0.287645483 | 0.425137522034998 | 1.07116412000296 | 0.75002744285112 | 1.52980078651531 | 0.999489402457283 |
| ebi-fl187-GCST90094765 | ebi-met1400-GCST90200561 | X-18779 levels \|\| id:ebi-met1400-GCST90200561 | Fried fish liking \|\| id:ebi-fl187-GCST90094765 | Weighted median | 4 | -0.023296801 | 0.17373040967013 | 0.893325534765138 | -0.363808404 | 0.31721480214376 | 0.976972474516429 | 0.69502434603608 | 1.37329752749871 | 0.999489402457283 |
| ebi-fl187-GCST90094765 | ebi-met1400-GCST90200570 | X-18921 levels \|\| id:ebi-met1400-GCST90200570 | Fried fish liking \|\| id:ebi-fl187-GCST90094765 | Weighted median | 4 | 0.0331254794759793 | 0.190231525727202 | 0.861761380302042 | -0.339728311 | 0.405979269901295 | 1.03368023675839 | 0.711963729237913 | 1.50077144099544 | 0.999489402457283 |
| ebi-fl187-GCST90094765 | ebi-met1400-GCST90200575 | X-21283 levels \|\| id:ebi-met1400-GCST90200575 | Fried fish liking \|\| id:ebi-fl187-GCST90094765 | Weighted median | 4 | -0.061138471 | 0.181827732618939 | 0.736686928953344 | -0.417520827 | 0.295243884748357 | 0.940692971886727 | 0.658677773345544 | 1.34345396666795 | 0.999489402457283 |
| ebi-fl187-GCST90094765 | ebi-met1400-GCST90200578 | X-18922 levels \|\| id:ebi-met1400-GCST90200578 | Fried fish liking \|\| id:ebi-fl187-GCST90094765 | Weighted median | 4 | -0.171493153 | 0.200588725398273 | 0.39257931832234 | -0.564647055 | 0.221660748439163 | 0.842406035666764 | 0.56856078196466 | 1.24814786991745 | 0.999489402457283 |
| ebi-fl187-GCST90094765 | ebi-met1400-GCST90200623 | X-24241 levels \|\| id:ebi-met1400-GCST90200623 | Fried fish liking \|\| id:ebi-fl187-GCST90094765 | Weighted median | 4 | 0.118941700638279 | 0.204769678123303 | 0.561337494323099 | -0.282406868 | 0.520290269759954 | 1.12630425355518 | 0.753966856766505 | 1.68251596232877 | 0.999489402457283 |
| ebi-fl187-GCST90094765 | ebi-met1400-GCST90200636 | X-24544 levels \|\| id:ebi-met1400-GCST90200636 | Fried fish liking \|\| id:ebi-fl187-GCST90094765 | Weighted median | 4 | -0.266968055 | 0.176306610701163 | 0.129968528426389 | -0.612529012 | 0.0785929023162897 | 0.765697531544201 | 0.541978464551189 | 1.08176384886139 | 0.999489402457283 |
| ebi-fl187-GCST90094765 | ebi-met1400-GCST90200659 | X-25172 levels \|\| id:ebi-met1400-GCST90200659 | Fried fish liking \|\| id:ebi-fl187-GCST90094765 | Weighted median | 4 | 0.0862543226026383 | 0.170238151429395 | 0.612387379401889 | -0.247412454 | 0.419921099404253 | 1.09008352593423 | 0.780818575206433 | 1.5218414766824 | 0.999489402457283 |
| ebi-fl187-GCST90094765 | ebi-met1400-GCST90200670 | X-26111 levels \|\| id:ebi-met1400-GCST90200670 | Fried fish liking \|\| id:ebi-fl187-GCST90094765 | Weighted median | 4 | -0.141048468 | 0.186761005877365 | 0.45010792821668 | -0.50710004 | 0.225003103495062 | 0.868447218756457 | 0.602239519702331 | 1.25232660277526 | 0.999489402457283 |
| ebi-fl187-GCST90094765 | ebi-met1400-GCST90200673 | Carnitine C4 levels \|\| id:ebi-met1400-GCST90200673 | Fried fish liking \|\| id:ebi-fl187-GCST90094765 | Weighted median | 4 | 0.0399492855448472 | 0.186640262832851 | 0.830512506478932 | -0.32586563 | 0.405764200697235 | 1.04075799137949 | 0.721902183194563 | 1.50044870598258 | 0.999489402457283 |
| ebi-fl187-GCST90094765 | ebi-met1400-GCST90200710 | X-13431 levels \|\| id:ebi-met1400-GCST90200710 | Fried fish liking \|\| id:ebi-fl187-GCST90094765 | Weighted median | 4 | -0.04989742 | 0.178231503868687 | 0.779509363566135 | -0.399231168 | 0.299436327560261 | 0.951327006598704 | 0.670835607967017 | 1.34909814377139 | 0.999489402457283 |
| ebi-fl187-GCST90094765 | ebi-met1400-GCST90200730 | Adenosine 5'-diphosphate (ADP) to fructose ratio \|\| id:ebi-met1400-GCST90200730 | Fried fish liking \|\| id:ebi-fl187-GCST90094765 | Weighted median | 4 | -0.364801443 | 0.234253360545969 | 0.119400607965932 | -0.82393803 | 0.0943351436560552 | 0.694334502157853 | 0.438700632222356 | 1.09892798294952 | 0.999489402457283 |
| ebi-fl187-GCST90094765 | ebi-met1400-GCST90200731 | Adenosine 5'-diphosphate (ADP) to mannose ratio \|\| id:ebi-met1400-GCST90200731 | Fried fish liking \|\| id:ebi-fl187-GCST90094765 | Weighted median | 4 | -0.24613947 | 0.241371709387351 | 0.307845725992499 | -0.719228021 | 0.226949080234925 | 0.781813177708905 | 0.487128163770129 | 1.25476597392494 | 0.999489402457283 |
| ebi-fl187-GCST90094765 | ebi-met1400-GCST90200748 | Aspartate to glutamate ratio \|\| id:ebi-met1400-GCST90200748 | Fried fish liking \|\| id:ebi-fl187-GCST90094765 | Weighted median | 4 | 0.0824342844634904 | 0.173594301692804 | 0.63488144877301 | -0.257810547 | 0.422679115781386 | 1.08592730879849 | 0.772741616569983 | 1.52604453378439 | 0.999489402457283 |
| ebi-fl187-GCST90094765 | ebi-met1400-GCST90200774 | Uridine to pseudouridine ratio \|\| id:ebi-met1400-GCST90200774 | Fried fish liking \|\| id:ebi-fl187-GCST90094765 | Weighted median | 4 | -0.163610445 | 0.164664796270897 | 0.320419113252443 | -0.486353446 | 0.159132555796556 | 0.84907271807618 | 0.614864447778746 | 1.17249335717113 | 0.999489402457283 |
| ebi-fl187-GCST90094765 | ebi-met1400-GCST90200799 | Spermidine to adenosine 5'-diphosphate (ADP) ratio \|\| id:ebi-met1400-GCST90200799 | Fried fish liking \|\| id:ebi-fl187-GCST90094765 | Weighted median | 4 | 0.102350729228446 | 0.240074995074846 | 0.669868746301169 | -0.368196261 | 0.572897719575144 | 1.1077719315967 | 0.691981359325451 | 1.77339842453231 | 0.999489402457283 |
| ebi-fl187-GCST90094765 | ebi-met1400-GCST90200811 | Choline to taurocholate ratio \|\| id:ebi-met1400-GCST90200811 | Fried fish liking \|\| id:ebi-fl187-GCST90094765 | Weighted median | 4 | -0.177967262 | 0.208032090885086 | 0.392285553620678 | -0.585710161 | 0.229775635752421 | 0.836969823438328 | 0.556710367674044 | 1.25831765676141 | 0.999489402457283 |
| ebi-fl187-GCST90094765 | ebi-met1400-GCST90200812 | Glutamate to kynurenine ratio \|\| id:ebi-met1400-GCST90200812 | Fried fish liking \|\| id:ebi-fl187-GCST90094765 | Weighted median | 4 | 0.0970395718960081 | 0.19603393547406 | 0.620590132438133 | -0.287186942 | 0.481266085425165 | 1.10190397718786 | 0.750371440062097 | 1.61812178624756 | 0.999489402457283 |
| ebi-fl187-GCST90094765 | ebi-met1400-GCST90200876 | Cortisone to 4-cholesten-3-one ratio \|\| id:ebi-met1400-GCST90200876 | Fried fish liking \|\| id:ebi-fl187-GCST90094765 | Weighted median | 4 | -0.113649948 | 0.193610521025465 | 0.55720172278073 | -0.493126569 | 0.265826673443197 | 0.892570347446025 | 0.610713966732169 | 1.30450893304902 | 0.999489402457283 |
| ebi-fl187-GCST90094765 | ebi-met1400-GCST90200906 | 2'-deoxyuridine to cytidine ratio \|\| id:ebi-met1400-GCST90200906 | Fried fish liking \|\| id:ebi-fl187-GCST90094765 | Weighted median | 4 | 0.146122361598572 | 0.18277550122335 | 0.424021640504602 | -0.212117621 | 0.504362343996339 | 1.15733779308956 | 0.808869552087905 | 1.65592927049361 | 0.999489402457283 |
| ebi-fl187-GCST90094765 | ebi-met1400-GCST90200907 | Retinol (Vitamin A) to linoleoyl-arachidonoyl-glycerol (18:2 to 20:4) [1] ratio \|\| id:ebi-met1400-GCST90200907 | Fried fish liking \|\| id:ebi-fl187-GCST90094765 | Weighted median | 4 | -0.106965828 | 0.192926284609286 | 0.579278542889341 | -0.485101346 | 0.271169690060614 | 0.898556378137669 | 0.615634801719451 | 1.31149759961073 | 0.999489402457283 |
| ebi-fl187-GCST90094765 | ebi-met1400-GCST90200916 | Fructose to sucrose ratio \|\| id:ebi-met1400-GCST90200916 | Fried fish liking \|\| id:ebi-fl187-GCST90094765 | Weighted median | 4 | 0.0443418943931006 | 0.187223417881619 | 0.812781332665704 | -0.322616005 | 0.411299793441074 | 1.04533968957835 | 0.724251910340669 | 1.50877761039494 | 0.999489402457283 |
| ebi-fl187-GCST90094765 | ebi-met1400-GCST90200917 | Alpha-ketobutyrate to 3-methyl-2-oxobutyrate ratio \|\| id:ebi-met1400-GCST90200917 | Fried fish liking \|\| id:ebi-fl187-GCST90094765 | Weighted median | 4 | -0.008249051 | 0.172323608372509 | 0.961820219572908 | -0.346003323 | 0.32950522151148 | 0.991784879160699 | 0.707510135827967 | 1.39028007758602 | 0.999489402457283 |
| ebi-fl187-GCST90094765 | ebi-met1400-GCST90200924 | Alpha-tocopherol to glycerol ratio \|\| id:ebi-met1400-GCST90200924 | Fried fish liking \|\| id:ebi-fl187-GCST90094765 | Weighted median | 4 | -0.016778267 | 0.165026062863135 | 0.919018291315795 | -0.34022935 | 0.3066728160534 | 0.983361704047998 | 0.71160709669436 | 1.35889628627961 | 0.999489402457283 |
| ebi-fl187-GCST90094765 | ebi-met1400-GCST90200938 | Alpha-ketoglutarate to alanine ratio \|\| id:ebi-met1400-GCST90200938 | Fried fish liking \|\| id:ebi-fl187-GCST90094765 | Weighted median | 4 | -0.007343753 | 0.175585141450325 | 0.966638639894643 | -0.35149063 | 0.336803124417037 | 0.992683146639231 | 0.703638442975307 | 1.40046331956332 | 0.999489402457283 |
| ebi-fl187-GCST90094765 | ebi-met1400-GCST90200943 | Phosphate to EDTA ratio \|\| id:ebi-met1400-GCST90200943 | Fried fish liking \|\| id:ebi-fl187-GCST90094765 | Weighted median | 4 | -0.038704736 | 0.171393326858266 | 0.82133826919179 | -0.374635656 | 0.29722618502837 | 0.962034721806498 | 0.687539733963112 | 1.34611973714811 | 0.999489402457283 |
| ebi-fl187-GCST90094765 | ebi-met1400-GCST90200947 | Adenosine 5'-diphosphate (ADP) to glucose ratio \|\| id:ebi-met1400-GCST90200947 | Fried fish liking \|\| id:ebi-fl187-GCST90094765 | Weighted median | 4 | -0.267697637 | 0.240323418584018 | 0.265319623045028 | -0.738731538 | 0.203336262971568 | 0.765139095536345 | 0.477719500450429 | 1.225484484 | 0.999489402457283 |
| ebi-fl187-GCST90094765 | ebi-met1400-GCST90200975 | Arachidonate (20:4n6) to caffeine ratio \|\| id:ebi-met1400-GCST90200975 | Fried fish liking \|\| id:ebi-fl187-GCST90094765 | Weighted median | 4 | -0.205418714 | 0.179977959094846 | 0.253722292188415 | -0.558175513 | 0.147338086273898 | 0.814306283942373 | 0.572252178429172 | 1.15874565281381 | 0.999489402457283 |
| ebi-fl187-GCST90094765 | ebi-met1400-GCST90200985 | Cholesterol to oleoyl-linoleoyl-glycerol (18:1 to 18:2) [2] ratio \|\| id:ebi-met1400-GCST90200985 | Fried fish liking \|\| id:ebi-fl187-GCST90094765 | Weighted median | 4 | 0.000565945524152886 | 0.166909862848152 | 0.9972945976768 | -0.326577386 | 0.327709276706531 | 1.00056610570154 | 0.72138854776092 | 1.38778545207864 | 0.999911036302558 |
| ebi-fl187-GCST90094765 | ebi-met1400-GCST90201016 | Bilirubin (Z,Z) to etiocholanolone glucuronide ratio \|\| id:ebi-met1400-GCST90201016 | Fried fish liking \|\| id:ebi-fl187-GCST90094765 | Weighted median | 4 | 0.235007686999975 | 0.184095500998149 | 0.201760060067288 | -0.125819495 | 0.595834868956346 | 1.26491849212394 | 0.881773997073059 | 1.81454522023575 | 0.999489402457283 |
| ebi-fl187-GCST90094765 | ebi-pw11-GCST90275189 | Placental weight (fetal genotype, adjusted for sex and gestational age) \|\| id:ebi-pw11-GCST90275189 | Fried fish liking \|\| id:ebi-fl187-GCST90094765 | Weighted median | 4 | 0.0613172960783525 | 0.084336589232766 | 0.46719257476711 | -0.103982419 | 0.226617010974574 | 1.06323622135464 | 0.901241142166584 | 1.25434937388993 | 0.999489402457283 |
| ebi-fl187-GCST90094765 | ebi-pw11-GCST90275192 | Placental weight (fetal effect, conditional on maternal and paternal genotype) \|\| id:ebi-pw11-GCST90275192 | Fried fish liking \|\| id:ebi-fl187-GCST90094765 | Weighted median | 4 | 0.0917707818139739 | 0.135193036271054 | 0.497256396558668 | -0.173207569 | 0.35674913290524 | 1.09611354412492 | 0.840963038636762 | 1.42867741674083 | 0.999489402457283 |
| ebi-fl187-GCST90094765 | ebi-pw11-GCST90275193 | Placental weight (maternal effect, conditional on fetal and paternal genotype) \|\| id:ebi-pw11-GCST90275193 | Fried fish liking \|\| id:ebi-fl187-GCST90094765 | Weighted median | 4 | -0.004469971 | 0.10660379193069 | 0.96655394552674 | -0.213413403 | 0.204473460923577 | 0.995540004192096 | 0.807822111733885 | 1.22687889518094 | 0.999489402457283 |
| ebi-fl187-GCST90094765 | ebi-pw11-GCST90275195 | Placental weight (fetal effect, conditional on maternal genotype) \|\| id:ebi-pw11-GCST90275195 | Fried fish liking \|\| id:ebi-fl187-GCST90094765 | Weighted median | 4 | 0.154557125812504 | 0.0898768293999152 | 0.0854951990194148 | -0.02160146 | 0.330715711436338 | 1.16714095002821 | 0.978630180798417 | 1.39196401660266 | 0.999489402457283 |
| ebi-fl187-GCST90094765 | ebi-pw11-GCST90275197 | Placental weight (fetal genotype, adjusted for sex) \|\| id:ebi-pw11-GCST90275197 | Fried fish liking \|\| id:ebi-fl187-GCST90094765 | Weighted median | 4 | 0.0254892199722702 | 0.0779134398590212 | 0.743556007395286 | -0.127221122 | 0.178199562095952 | 1.02581684787677 | 0.880538944402804 | 1.19506378687376 | 0.999489402457283 |
| ebi-fl187-GCST90094765 | prot-a-1030 | Protein FAM171B \|\| id:prot-a-1030 | Fried fish liking \|\| id:ebi-fl187-GCST90094765 | Weighted median | 5 | -0.044651183 | 0.270217809477144 | 0.868753940394126 | -0.57427809 | 0.48497572361528 | 0.956331008211495 | 0.563111238003824 | 1.62413558022546 | 0.999489402457283 |
| ebi-fl187-GCST90094765 | prot-a-1036 | BRISC complex subunit Abro1 \|\| id:prot-a-1036 | Fried fish liking \|\| id:ebi-fl187-GCST90094765 | Weighted median | 5 | -0.065695775 | 0.299700315288581 | 0.826490445400065 | -0.653108393 | 0.521716842917136 | 0.936415701983592 | 0.520425572735477 | 1.68491790730492 | 0.999489402457283 |
| ebi-fl187-GCST90094765 | prot-a-1055 | Phenylalanine--tRNA ligase, mitochondrial \|\| id:prot-a-1055 | Fried fish liking \|\| id:ebi-fl187-GCST90094765 | Weighted median | 5 | -0.301363997 | 0.269324422900383 | 0.26315603136072 | -0.829239865 | 0.226511872349442 | 0.73980843602336 | 0.43638086847019 | 1.25421750025395 | 0.999489402457283 |
| ebi-fl187-GCST90094765 | prot-a-1083 | Fc receptor-like protein 5 \|\| id:prot-a-1083 | Fried fish liking \|\| id:ebi-fl187-GCST90094765 | Weighted median | 5 | -0.04931265 | 0.275437812309294 | 0.857911243209888 | -0.589170762 | 0.490545462453566 | 0.951883477112918 | 0.554787144735329 | 1.63320683004081 | 0.999489402457283 |
| ebi-fl187-GCST90094765 | prot-a-1103 | Fibroblast growth factor receptor 3 \|\| id:prot-a-1103 | Fried fish liking \|\| id:ebi-fl187-GCST90094765 | Weighted median | 5 | -0.265066063 | 0.254503330199526 | 0.297642014463896 | -0.76389259 | 0.233760464553784 | 0.767155268001569 | 0.465849531940869 | 1.26334184080979 | 0.999489402457283 |
| ebi-fl187-GCST90094765 | prot-a-1105 | Fibroblast growth factor receptor 4 \|\| id:prot-a-1105 | Fried fish liking \|\| id:ebi-fl187-GCST90094765 | Weighted median | 5 | 0.315045324163271 | 0.268197446962255 | 0.240124036458589 | -0.210621672 | 0.84071232020929 | 1.3703214182212 | 0.810080485139065 | 2.31801755959274 | 0.999489402457283 |
| ebi-fl187-GCST90094765 | prot-a-1120 | Filamin-A \|\| id:prot-a-1120 | Fried fish liking \|\| id:ebi-fl187-GCST90094765 | Weighted median | 5 | -0.367422954 | 0.294933957952872 | 0.21284501511647 | -0.945493511 | 0.210647603812034 | 0.692516680548839 | 0.388487800441203 | 1.2344772533236 | 0.999489402457283 |
| ebi-fl187-GCST90094765 | prot-a-1166 | Galanin peptides \|\| id:prot-a-1166 | Fried fish liking \|\| id:ebi-fl187-GCST90094765 | Weighted median | 5 | 0.0144017979670975 | 0.289845333755072 | 0.960371120924121 | -0.553695056 | 0.582498652127039 | 1.01450600350754 | 0.574821882238837 | 1.7905066994739 | 0.999489402457283 |
| ebi-fl187-GCST90094765 | prot-a-1205 | ADP-ribosylation factor-binding protein GGA1 \|\| id:prot-a-1205 | Fried fish liking \|\| id:ebi-fl187-GCST90094765 | Weighted median | 5 | -0.199106472 | 0.285459539518366 | 0.485493352026732 | -0.758607169 | 0.36039422581498 | 0.819462639156017 | 0.468318261103694 | 1.43389458141129 | 0.999489402457283 |
| ebi-fl187-GCST90094765 | prot-a-1227 | Guanine nucleotide-binding protein G(I)/G(S)/G(O) subunit gamma-T2 \|\| id:prot-a-1227 | Fried fish liking \|\| id:ebi-fl187-GCST90094765 | Weighted median | 5 | 0.13325044218706 | 0.254393617445213 | 0.600420209020334 | -0.365361048 | 0.631861932379678 | 1.14253610174333 | 0.693946057802502 | 1.88110981986206 | 0.999489402457283 |
| ebi-fl187-GCST90094765 | prot-a-1240 | Golgi SNAP receptor complex member 1 \|\| id:prot-a-1240 | Fried fish liking \|\| id:ebi-fl187-GCST90094765 | Weighted median | 5 | 0.126244785575559 | 0.266680143560046 | 0.635932094478798 | -0.396448296 | 0.648937866953249 | 1.13455985818229 | 0.672705057477471 | 1.91350734990085 | 0.999489402457283 |
| ebi-fl187-GCST90094765 | prot-a-1283 | Glutathione S-transferase A1 \|\| id:prot-a-1283 | Fried fish liking \|\| id:ebi-fl187-GCST90094765 | Weighted median | 5 | 0.2171558096675 | 0.288613779985257 | 0.451804710800554 | -0.348527199 | 0.782838818438604 | 1.242537686 | 0.705726719628718 | 2.18767386763521 | 0.999489402457283 |
| ebi-fl187-GCST90094765 | prot-a-1324 | E3 ubiquitin-protein ligase HECW1 \|\| id:prot-a-1324 | Fried fish liking \|\| id:ebi-fl187-GCST90094765 | Weighted median | 5 | 0.129198250939168 | 0.255778313701156 | 0.613475902744172 | -0.372127244 | 0.630523745793434 | 1.13791569466037 | 0.68926653197195 | 1.87859422747526 | 0.999489402457283 |
| ebi-fl187-GCST90094765 | prot-a-1339 | Histidine triad nucleotide-binding protein 2, mitochondrial \|\| id:prot-a-1339 | Fried fish liking \|\| id:ebi-fl187-GCST90094765 | Weighted median | 5 | -0.462758538 | 0.253261758336908 | 0.0676705029997381 | -0.959151585 | 0.0336345080488197 | 0.629544625077967 | 0.383217876027708 | 1.03420654347529 | 0.999489402457283 |
| ebi-fl187-GCST90094765 | prot-a-1466 | Interleukin-11 \|\| id:prot-a-1466 | Fried fish liking \|\| id:ebi-fl187-GCST90094765 | Weighted median | 5 | -0.267621893 | 0.293023418340774 | 0.361078313835345 | -0.841947793 | 0.306704007050042 | 0.765197052851774 | 0.430870459161269 | 1.35893867227014 | 0.999489402457283 |
| ebi-fl187-GCST90094765 | prot-a-147 | Rho GTPase-activating protein 1 \|\| id:prot-a-147 | Fried fish liking \|\| id:ebi-fl187-GCST90094765 | Weighted median | 5 | -0.043348959 | 0.29874933892849 | 0.884630781371814 | -0.628897664 | 0.542199745023974 | 0.957577176318893 | 0.53317922005565 | 1.71978579456108 | 0.999489402457283 |
| ebi-fl187-GCST90094765 | prot-a-1473 | Interleukin-12 receptor subunit beta-1 \|\| id:prot-a-1473 | Fried fish liking \|\| id:ebi-fl187-GCST90094765 | Weighted median | 5 | -0.583647617 | 0.266196898617984 | 0.0283404652919007 | -1.105393538 | -0.061901696 | 0.557859792049779 | 0.3310805637541 | 0.939975285945696 | 0.999489402457283 |
| ebi-fl187-GCST90094765 | prot-a-1501 | X-linked interleukin-1 receptor accessory protein-like 2 \|\| id:prot-a-1501 | Fried fish liking \|\| id:ebi-fl187-GCST90094765 | Weighted median | 5 | 0.0257700922876353 | 0.272432961882074 | 0.924638573323351 | -0.508198513 | 0.559738697576501 | 1.02610501189673 | 0.601578338786495 | 1.75021510509086 | 0.999489402457283 |
| ebi-fl187-GCST90094765 | prot-a-1503 | Interleukin-1 receptor-like 2 \|\| id:prot-a-1503 | Fried fish liking \|\| id:ebi-fl187-GCST90094765 | Weighted median | 5 | -0.445513776 | 0.256353103867087 | 0.0822300050953991 | -0.94796586 | 0.0569383072173992 | 0.640495120230514 | 0.387528509519485 | 1.05859050098732 | 0.999489402457283 |
| ebi-fl187-GCST90094765 | prot-a-1536 | Interleukin-5 receptor subunit alpha \|\| id:prot-a-1536 | Fried fish liking \|\| id:ebi-fl187-GCST90094765 | Weighted median | 5 | -0.032342541 | 0.294542117563903 | 0.912563100560362 | -0.609645091 | 0.544960009659448 | 0.968174885905861 | 0.543543743308641 | 1.72453941607891 | 0.999489402457283 |
| ebi-fl187-GCST90094765 | prot-a-1559 | Insulin-induced gene 1 protein \|\| id:prot-a-1559 | Fried fish liking \|\| id:ebi-fl187-GCST90094765 | Weighted median | 5 | -0.319792297 | 0.293703526816395 | 0.276230232098152 | -0.89545121 | 0.25586661545542 | 0.726299875995399 | 0.4084232725312 | 1.29158043958094 | 0.999489402457283 |
| ebi-fl187-GCST90094765 | prot-a-1567 | Interferon regulatory factor 1 \|\| id:prot-a-1567 | Fried fish liking \|\| id:ebi-fl187-GCST90094765 | Weighted median | 5 | 0.305516511922866 | 0.261387226053542 | 0.242473240093726 | -0.206802451 | 0.817835474987808 | 1.3573258969896 | 0.81318027695621 | 2.26559059884557 | 0.999489402457283 |
| ebi-fl187-GCST90094765 | prot-a-1613 | Potassium voltage-gated channel subfamily E regulatory beta subunit 5 \|\| id:prot-a-1613 | Fried fish liking \|\| id:ebi-fl187-GCST90094765 | Weighted median | 5 | -0.246204073 | 0.2928828824612 | 0.400559197413207 | -0.820254522 | 0.327846376888728 | 0.781762672199039 | 0.440319569066969 | 1.38797573076029 | 0.999489402457283 |
| ebi-fl187-GCST90094765 | prot-a-1649 | Kin of IRRE-like protein 2 \|\| id:prot-a-1649 | Fried fish liking \|\| id:ebi-fl187-GCST90094765 | Weighted median | 5 | 0.251068396180179 | 0.271487330200001 | 0.35507586601731 | -0.281046771 | 0.78318356337218 | 1.28539799763774 | 0.754993022866812 | 2.18842818713384 | 0.999489402457283 |
| ebi-fl187-GCST90094765 | prot-a-165 | Armadillo repeat-containing protein 5 \|\| id:prot-a-165 | Fried fish liking \|\| id:ebi-fl187-GCST90094765 | Weighted median | 5 | 0.283936257062121 | 0.302158419035202 | 0.347374877109991 | -0.308294244 | 0.876166758371116 | 1.32834825523378 | 0.73469910521605 | 2.40167583525739 | 0.999489402457283 |
| ebi-fl187-GCST90094765 | prot-a-1656 | Kallikrein-11 \|\| id:prot-a-1656 | Fried fish liking \|\| id:ebi-fl187-GCST90094765 | Weighted median | 5 | -0.187169105 | 0.268256541004096 | 0.485349632720388 | -0.712951926 | 0.338613714952237 | 0.829303484816471 | 0.490195040232622 | 1.40300128210676 | 0.999489402457283 |
| ebi-fl187-GCST90094765 | prot-a-1697 | Laminin subunit gamma-2 \|\| id:prot-a-1697 | Fried fish liking \|\| id:ebi-fl187-GCST90094765 | Weighted median | 5 | -0.047542041 | 0.299270813289607 | 0.873779492842541 | -0.634112835 | 0.539028753078168 | 0.953570383266561 | 0.530405837133654 | 1.71434100491245 | 0.999489402457283 |
| ebi-fl187-GCST90094765 | prot-a-1698 | Lysosome-associated membrane glycoprotein 3 \|\| id:prot-a-1698 | Fried fish liking \|\| id:ebi-fl187-GCST90094765 | Weighted median | 5 | 0.108107229541986 | 0.296780534867943 | 0.715658955595362 | -0.473582619 | 0.689797077883154 | 1.11416721062094 | 0.622767129622408 | 1.99331100531191 | 0.999489402457283 |
| ebi-fl187-GCST90094765 | prot-a-1724 | Leptin receptor \|\| id:prot-a-1724 | Fried fish liking \|\| id:ebi-fl187-GCST90094765 | Weighted median | 5 | -0.272770562 | 0.284392537260246 | 0.337491226277087 | -0.830179935 | 0.28463881113389 | 0.761267431349145 | 0.435970832883817 | 1.32928181961054 | 0.999489402457283 |
| ebi-fl187-GCST90094765 | prot-a-1743 | Leukocyte immunoglobulin-like receptor subfamily B member 2 \|\| id:prot-a-1743 | Fried fish liking \|\| id:ebi-fl187-GCST90094765 | Weighted median | 5 | -0.409684067 | 0.260412553862566 | 0.115669815880149 | -0.920092672 | 0.100724538926703 | 0.663859952511348 | 0.39848211115365 | 1.10597194757994 | 0.999489402457283 |
| ebi-fl187-GCST90094765 | prot-a-1746 | Leukocyte immunoglobulin-like receptor subfamily B member 4 \|\| id:prot-a-1746 | Fried fish liking \|\| id:ebi-fl187-GCST90094765 | Weighted median | 5 | -0.078357589 | 0.268559504004579 | 0.77046253375243 | -0.604734217 | 0.448019038769785 | 0.924633728496497 | 0.546219583267506 | 1.56520849501405 | 0.999489402457283 |
| ebi-fl187-GCST90094765 | prot-a-1750 | Leucine-rich repeat and immunoglobulin-like domain-containing nogo receptor-interacting protein 3 \|\| id:prot-a-1750 | Fried fish liking \|\| id:ebi-fl187-GCST90094765 | Weighted median | 5 | -0.349388502 | 0.261352364381465 | 0.181272111937563 | -0.861639136 | 0.162862132088257 | 0.7051191367849 | 0.422469030151953 | 1.1768744252838 | 0.999489402457283 |
| ebi-fl187-GCST90094765 | prot-a-1753 | Lipase member N \|\| id:prot-a-1753 | Fried fish liking \|\| id:ebi-fl187-GCST90094765 | Weighted median | 5 | 0.197950703017601 | 0.279604694666306 | 0.478966181289142 | -0.350074499 | 0.74597590456356 | 1.2189023041351 | 0.70463559344855 | 2.10849812419296 | 0.999489402457283 |
| ebi-fl187-GCST90094765 | prot-a-1760 | Ig Kappa chain V-I region HK102- like \|\| id:prot-a-1760 | Fried fish liking \|\| id:ebi-fl187-GCST90094765 | Weighted median | 5 | -0.226666181 | 0.283403527448365 | 0.423826602520881 | -0.782137094 | 0.328804733157522 | 0.79718685428216 | 0.457427400431555 | 1.38930654359735 | 0.999489402457283 |
| ebi-fl187-GCST90094765 | prot-a-1792 | Leucine-rich repeat neuronal protein 1 \|\| id:prot-a-1792 | Fried fish liking \|\| id:ebi-fl187-GCST90094765 | Weighted median | 5 | 0.278393083596405 | 0.279154331439394 | 0.318632005151804 | -0.268749406 | 0.825535573217616 | 1.32100536070314 | 0.764334769339336 | 2.28310320687726 | 0.999489402457283 |
| ebi-fl187-GCST90094765 | prot-a-1795 | Leucine-rich repeat transmembrane neuronal protein 1 \|\| id:prot-a-1795 | Fried fish liking \|\| id:ebi-fl187-GCST90094765 | Weighted median | 5 | -0.041164749 | 0.275328424701393 | 0.881150084724126 | -0.580808461 | 0.498478963762095 | 0.959671012417063 | 0.559445893462442 | 1.64621541213515 | 0.999489402457283 |
| ebi-fl187-GCST90094765 | prot-a-1820 | LysM and putative peptidoglycan-binding domain-containing protein 4 \|\| id:prot-a-1820 | Fried fish liking \|\| id:ebi-fl187-GCST90094765 | Weighted median | 5 | -0.337645339 | 0.278977307888146 | 0.226165031228305 | -0.884440862 | 0.209150184872165 | 0.713448275605527 | 0.412945001962618 | 1.23263010702471 | 0.999489402457283 |
| ebi-fl187-GCST90094765 | prot-a-1844 | Mitogen-activated protein kinase kinase kinase 7:TGF-beta-activated kinase 1 and MAP3K7-binding protein 1 fusion \|\| id:prot-a-1844 | Fried fish liking \|\| id:ebi-fl187-GCST90094765 | Weighted median | 5 | 0.120797962314318 | 0.281777665582019 | 0.668141878150233 | -0.431486262 | 0.673082186855075 | 1.12839691063529 | 0.649542985751353 | 1.96026993726738 | 0.999489402457283 |
| ebi-fl187-GCST90094765 | prot-a-1856 | Megakaryocyte-associated tyrosine-protein kinase \|\| id:prot-a-1856 | Fried fish liking \|\| id:ebi-fl187-GCST90094765 | Weighted median | 5 | 0.502334347681517 | 0.302133952384172 | 0.0963882627025391 | -0.089848199 | 1.09451689435449 | 1.652574455 | 0.914069931477529 | 2.98773894113167 | 0.999489402457283 |
| ebi-fl187-GCST90094765 | prot-a-1910 | Stromelysin-2 \|\| id:prot-a-1910 | Fried fish liking \|\| id:ebi-fl187-GCST90094765 | Weighted median | 5 | -0.059421094 | 0.38471163292879 | 0.877250050104207 | -0.813455895 | 0.694613706572777 | 0.942309884592056 | 0.443323337133387 | 2.00293520377595 | 0.999489402457283 |
| ebi-fl187-GCST90094765 | prot-a-1940 | 39S ribosomal protein L14, mitochondrial \|\| id:prot-a-1940 | Fried fish liking \|\| id:ebi-fl187-GCST90094765 | Weighted median | 5 | -0.026339612 | 0.272473788928292 | 0.922989716453671 | -0.560388238 | 0.507709014576354 | 0.974004250171747 | 0.57098734181465 | 1.66148040399218 | 0.999489402457283 |
| ebi-fl187-GCST90094765 | prot-a-1942 | 39S ribosomal protein L33, mitochondrial \|\| id:prot-a-1942 | Fried fish liking \|\| id:ebi-fl187-GCST90094765 | Weighted median | 5 | 0.0768055101778896 | 0.261003054611863 | 0.768551181171533 | -0.434760477 | 0.588371497217141 | 1.07983203962696 | 0.647419720511324 | 1.80105300605301 | 0.999489402457283 |
| ebi-fl187-GCST90094765 | prot-a-1948 | Mesothelin \|\| id:prot-a-1948 | Fried fish liking \|\| id:ebi-fl187-GCST90094765 | Weighted median | 5 | -0.499040265 | 0.280756664390793 | 0.0754887662656925 | -1.049323327 | 0.0512427972420153 | 0.607113047861658 | 0.350174622612101 | 1.05257842539939 | 0.999489402457283 |
| ebi-fl187-GCST90094765 | prot-a-1980 | Unconventional myosin-VI \|\| id:prot-a-1980 | Fried fish liking \|\| id:ebi-fl187-GCST90094765 | Weighted median | 5 | 0.297733148449342 | 0.25731956058316 | 0.247249470665899 | -0.20661319 | 0.802079487192335 | 1.34680234362873 | 0.813334194710192 | 2.23017372760304 | 0.999489402457283 |
| ebi-fl187-GCST90094765 | prot-a-2039 | Neurofascin \|\| id:prot-a-2039 | Fried fish liking \|\| id:ebi-fl187-GCST90094765 | Weighted median | 5 | -0.094357845 | 0.258891165916691 | 0.71550770033453 | -0.601784531 | 0.413068839828394 | 0.909957080045844 | 0.547833138294258 | 1.51144907024738 | 0.999489402457283 |
| ebi-fl187-GCST90094765 | prot-a-2041 | NFU1 iron-sulfur cluster scaffold homolog, mitochondrial \|\| id:prot-a-2041 | Fried fish liking \|\| id:ebi-fl187-GCST90094765 | Weighted median | 5 | 0.0511574884543639 | 0.289911460525342 | 0.859933350934806 | -0.517068974 | 0.619383951084035 | 1.05248863504002 | 0.596265659283427 | 1.8577832039156 | 0.999489402457283 |
| ebi-fl187-GCST90094765 | prot-a-2088 | Glucocorticoid receptor \|\| id:prot-a-2088 | Fried fish liking \|\| id:ebi-fl187-GCST90094765 | Weighted median | 5 | 0.242266277206161 | 0.273169779935323 | 0.375148511620997 | -0.293146491 | 0.777679045879395 | 1.27413342033736 | 0.745912862823696 | 2.17641503951955 | 0.999489402457283 |
| ebi-fl187-GCST90094765 | prot-a-2111 | Neuron-specific protein family member 2 \|\| id:prot-a-2111 | Fried fish liking \|\| id:ebi-fl187-GCST90094765 | Weighted median | 5 | -0.198902532 | 0.260850637388589 | 0.445752674165125 | -0.710169782 | 0.312364716809221 | 0.819629776727718 | 0.491560732332381 | 1.36665304348289 | 0.999489402457283 |
| ebi-fl187-GCST90094765 | prot-a-2125 | Nuclear migration protein nudC \|\| id:prot-a-2125 | Fried fish liking \|\| id:ebi-fl187-GCST90094765 | Weighted median | 5 | -0.260759469 | 0.279570057901009 | 0.35096661716622 | -0.808716782 | 0.287197844967388 | 0.770466218710015 | 0.445429282518063 | 1.33268785297975 | 0.999489402457283 |
| ebi-fl187-GCST90094765 | prot-a-2142 | Ornithine decarboxylase \|\| id:prot-a-2142 | Fried fish liking \|\| id:ebi-fl187-GCST90094765 | Weighted median | 5 | 0.0883099356847706 | 0.265081048789516 | 0.73902622866897 | -0.43124892 | 0.607868791312222 | 1.09232662056824 | 0.649697168063257 | 1.83651323209378 | 0.999489402457283 |
| ebi-fl187-GCST90094765 | prot-a-2186 | Prostate and testis expressed protein 1 \|\| id:prot-a-2186 | Fried fish liking \|\| id:ebi-fl187-GCST90094765 | Weighted median | 5 | -0.365442099 | 0.294388362972962 | 0.214472587155862 | -0.94244329 | 0.211559092391735 | 0.69388981503925 | 0.389674583104377 | 1.23560297820665 | 0.999489402457283 |
| ebi-fl187-GCST90094765 | prot-a-2227 | Platelet-derived growth factor C \|\| id:prot-a-2227 | Fried fish liking \|\| id:ebi-fl187-GCST90094765 | Weighted median | 5 | -0.244212203 | 0.311723345498012 | 0.433376937503419 | -0.85518996 | 0.366765553995975 | 0.783321393334331 | 0.425202411808469 | 1.44305955990586 | 0.999489402457283 |
| ebi-fl187-GCST90094765 | prot-a-2229 | Platelet-derived growth factor receptor alpha \|\| id:prot-a-2229 | Fried fish liking \|\| id:ebi-fl187-GCST90094765 | Weighted median | 5 | -0.086259987 | 0.271555985383325 | 0.750749804102077 | -0.618509719 | 0.445989744120078 | 0.917355699445528 | 0.538746723861312 | 1.56203544640362 | 0.999489402457283 |
| ebi-fl187-GCST90094765 | prot-a-2243 | Platelet endothelial aggregation receptor 1 \|\| id:prot-a-2243 | Fried fish liking \|\| id:ebi-fl187-GCST90094765 | Weighted median | 5 | -0.257266776 | 0.255266219211706 | 0.313532652901205 | -0.757588566 | 0.24305501337338 | 0.773161924986801 | 0.468795534599456 | 1.27513877187428 | 0.999489402457283 |
| ebi-fl187-GCST90094765 | prot-a-2246 | E3 ubiquitin-protein ligase pellino homolog 2 \|\| id:prot-a-2246 | Fried fish liking \|\| id:ebi-fl187-GCST90094765 | Weighted median | 5 | 0.274603801354112 | 0.263934509369946 | 0.29814295029086 | -0.242707837 | 0.791915439719206 | 1.31600917051345 | 0.784500682356881 | 2.20762094390078 | 0.999489402457283 |
| ebi-fl187-GCST90094765 | prot-a-2251 | Platelet factor 4 variant \|\| id:prot-a-2251 | Fried fish liking \|\| id:ebi-fl187-GCST90094765 | Weighted median | 5 | -0.015875796 | 0.253925746594399 | 0.950147612085893 | -0.513570259 | 0.481818667456174 | 0.984249560325232 | 0.59835547658424 | 1.61901617836037 | 0.999489402457283 |
| ebi-fl187-GCST90094765 | prot-a-2346 | Protein phosphatase 1L \|\| id:prot-a-2346 | Fried fish liking \|\| id:ebi-fl187-GCST90094765 | Weighted median | 5 | 0.11742195383161 | 0.280546720196783 | 0.675547319878971 | -0.432449618 | 0.667293525417305 | 1.1245938562771 | 0.648917546234265 | 1.94895537794509 | 0.999489402457283 |
| ebi-fl187-GCST90094765 | prot-a-2358 | PR domain zinc finger protein 4 \|\| id:prot-a-2358 | Fried fish liking \|\| id:ebi-fl187-GCST90094765 | Weighted median | 5 | 0.200631999144606 | 0.248568189360496 | 0.419579639375507 | -0.286561652 | 0.687825650291177 | 1.22217492763807 | 0.750840786266468 | 1.98938520798071 | 0.999489402457283 |
| ebi-fl187-GCST90094765 | prot-a-2390 | Trypsin-1 \|\| id:prot-a-2390 | Fried fish liking \|\| id:ebi-fl187-GCST90094765 | Weighted median | 5 | -0.135786374 | 0.312204174281612 | 0.663614604535764 | -0.747706555 | 0.476133807774366 | 0.873029114446144 | 0.473451142503967 | 1.60983841044218 | 0.999489402457283 |
| ebi-fl187-GCST90094765 | prot-a-242 | Brain-derived neurotrophic factor \|\| id:prot-a-242 | Fried fish liking \|\| id:ebi-fl187-GCST90094765 | Weighted median | 5 | 0.0186815984496433 | 0.268464560124953 | 0.944522517462491 | -0.507508939 | 0.54487213629455 | 1.018857191 | 0.601993314392512 | 1.72438788165554 | 0.999489402457283 |
| ebi-fl187-GCST90094765 | prot-a-2464 | Dihydropteridine reductase \|\| id:prot-a-2464 | Fried fish liking \|\| id:ebi-fl187-GCST90094765 | Weighted median | 5 | 0.461609957954007 | 0.270946576474491 | 0.0884383377525501 | -0.069445332 | 0.992665247844009 | 1.58662633154037 | 0.932911132436034 | 2.69841684637617 | 0.999489402457283 |
| ebi-fl187-GCST90094765 | prot-a-2474 | Ras-related protein Rab-27A \|\| id:prot-a-2474 | Fried fish liking \|\| id:ebi-fl187-GCST90094765 | Weighted median | 5 | -0.15189167 | 0.292068837701345 | 0.603025693573646 | -0.724346592 | 0.4205632519633 | 0.859081340041027 | 0.484641133999431 | 1.5228190449215 | 0.999489402457283 |
| ebi-fl187-GCST90094765 | prot-a-2478 | Ras-related protein Rab-7b \|\| id:prot-a-2478 | Fried fish liking \|\| id:ebi-fl187-GCST90094765 | Weighted median | 5 | -0.14776262 | 0.273689998196367 | 0.58927257446724 | -0.684195017 | 0.38866977628008 | 0.862635862981297 | 0.504496177185305 | 1.47501738517269 | 0.999489402457283 |
| ebi-fl187-GCST90094765 | prot-a-2528 | E3 ubiquitin-protein ligase rififylin \|\| id:prot-a-2528 | Fried fish liking \|\| id:ebi-fl187-GCST90094765 | Weighted median | 5 | 0.0114013661577144 | 0.282842415151103 | 0.967846014101066 | -0.542969768 | 0.565772499853877 | 1.01146660945131 | 0.581020192768717 | 1.76080748099261 | 0.999489402457283 |
| ebi-fl187-GCST90094765 | prot-a-2543 | Protein RIC-3 \|\| id:prot-a-2543 | Fried fish liking \|\| id:ebi-fl187-GCST90094765 | Weighted median | 5 | -0.095838423 | 0.28286481358849 | 0.734750215490106 | -0.650253458 | 0.458576611420646 | 0.908610814625015 | 0.521913476929726 | 1.581820836 | 0.999489402457283 |
| ebi-fl187-GCST90094765 | prot-a-255 | Bone morphogenetic protein 6 \|\| id:prot-a-255 | Fried fish liking \|\| id:ebi-fl187-GCST90094765 | Weighted median | 5 | -0.170344549 | 0.275548814791878 | 0.536442861358714 | -0.710420226 | 0.369731128484085 | 0.843374183214101 | 0.491437639435742 | 1.44734541238788 | 0.999489402457283 |
| ebi-fl187-GCST90094765 | prot-a-2568 | RING finger protein 165 \|\| id:prot-a-2568 | Fried fish liking \|\| id:ebi-fl187-GCST90094765 | Weighted median | 5 | -0.295554462 | 0.266499907338024 | 0.267420365283566 | -0.81789428 | 0.22678535666847 | 0.744118887629456 | 0.441360057342103 | 1.25456055598096 | 0.999489402457283 |
| ebi-fl187-GCST90094765 | prot-a-2573 | E3 ubiquitin-protein ligase RNF8 \|\| id:prot-a-2573 | Fried fish liking \|\| id:ebi-fl187-GCST90094765 | Weighted median | 5 | 0.155329137708635 | 0.299254906561465 | 0.603723827346332 | -0.431210479 | 0.741868754569106 | 1.168042345 | 0.64972214341624 | 2.09985596560316 | 0.999489402457283 |
| ebi-fl187-GCST90094765 | prot-a-260 | Cytoplasmic tyrosine-protein kinase BMX \|\| id:prot-a-260 | Fried fish liking \|\| id:ebi-fl187-GCST90094765 | Weighted median | 4 | -0.082525049 | 0.305479381993519 | 0.787045375099536 | -0.681264638 | 0.516214539337432 | 0.920788372399411 | 0.505976710174461 | 1.67567243649143 | 0.999489402457283 |
| ebi-fl187-GCST90094765 | prot-a-2623 | Serum amyloid A-1 protein \|\| id:prot-a-2623 | Fried fish liking \|\| id:ebi-fl187-GCST90094765 | Weighted median | 5 | -0.553314746 | 0.268341886616001 | 0.0392099443399045 | -1.079264844 | -0.027364648 | 0.57504053444703 | 0.339845273209841 | 0.973006371793639 | 0.999489402457283 |
| ebi-fl187-GCST90094765 | prot-a-2640 | Lysosome membrane protein 2 \|\| id:prot-a-2640 | Fried fish liking \|\| id:ebi-fl187-GCST90094765 | Weighted median | 5 | -0.148234979 | 0.277129116353026 | 0.592722464340664 | -0.691408047 | 0.394938088706273 | 0.862228485250809 | 0.500870323165754 | 1.48429229362004 | 0.999489402457283 |
| ebi-fl187-GCST90094765 | prot-a-2641 | Scavenger receptor class F member 2 \|\| id:prot-a-2641 | Fried fish liking \|\| id:ebi-fl187-GCST90094765 | Weighted median | 5 | -0.189547731 | 0.268904296096598 | 0.480879198248065 | -0.716600152 | 0.337504689024263 | 0.82733322624383 | 0.488409956170245 | 1.40144617979171 | 0.999489402457283 |
| ebi-fl187-GCST90094765 | prot-a-2669 | Semaphorin-3A \|\| id:prot-a-2669 | Fried fish liking \|\| id:ebi-fl187-GCST90094765 | Weighted median | 5 | 0.0616856199365514 | 0.275709216632166 | 0.822964022836221 | -0.478704445 | 0.602075684535597 | 1.06362790875135 | 0.6195855794592 | 1.8259048721925 | 0.999489402457283 |
| ebi-fl187-GCST90094765 | prot-a-2683 | Sentrin-specific protease 7 \|\| id:prot-a-2683 | Fried fish liking \|\| id:ebi-fl187-GCST90094765 | Weighted median | 5 | 0.354770584676624 | 0.276061191035057 | 0.19875183842347 | -0.18630935 | 0.895850519105336 | 1.42585350411355 | 0.830016789774521 | 2.44941818073967 | 0.999489402457283 |
| ebi-fl187-GCST90094765 | prot-a-270 | Bromodomain testis-specific protein \|\| id:prot-a-270 | Fried fish liking \|\| id:ebi-fl187-GCST90094765 | Weighted median | 5 | 0.0680625716351471 | 0.280763133707451 | 0.808454813886517 | -0.48223317 | 0.61835831370175 | 1.07043228508173 | 0.617403084836078 | 1.85587876881034 | 0.999489402457283 |
| ebi-fl187-GCST90094765 | prot-a-2708 | 14-3-3 protein sigma \|\| id:prot-a-2708 | Fried fish liking \|\| id:ebi-fl187-GCST90094765 | Weighted median | 5 | 0.375205460406685 | 0.283251828183161 | 0.185292036469268 | -0.179968123 | 0.93037904364568 | 1.45529038845856 | 0.835296837884254 | 2.5354700493112 | 0.999489402457283 |
| ebi-fl187-GCST90094765 | prot-a-2713 | Beta-sarcoglycan \|\| id:prot-a-2713 | Fried fish liking \|\| id:ebi-fl187-GCST90094765 | Weighted median | 5 | -0.464697308 | 0.272900381332676 | 0.0886037615791394 | -0.999582055 | 0.0701874396610456 | 0.628325265595896 | 0.368033226619181 | 1.07270923066591 | 0.999489402457283 |
| ebi-fl187-GCST90094765 | prot-a-2720 | Protein shisa-3 homolog \|\| id:prot-a-2720 | Fried fish liking \|\| id:ebi-fl187-GCST90094765 | Weighted median | 5 | -0.148170589 | 0.290288712092202 | 0.609753811102664 | -0.717136465 | 0.42079528682342 | 0.862284006334305 | 0.488148085836996 | 1.52317243302317 | 0.999489402457283 |
| ebi-fl187-GCST90094765 | prot-a-2730 | Sialic acid-binding Ig-like lectin 8 \|\| id:prot-a-2730 | Fried fish liking \|\| id:ebi-fl187-GCST90094765 | Weighted median | 5 | 0.00773555590673591 | 0.2952074993036 | 0.9790947913487 | -0.570871143 | 0.586342254541791 | 1.00776555261652 | 0.565032999849338 | 1.79740193813685 | 0.999489402457283 |
| ebi-fl187-GCST90094765 | prot-a-2761 | Sodium- and chloride-dependent neutral and basic amino acid transporter B(0+) \|\| id:prot-a-2761 | Fried fish liking \|\| id:ebi-fl187-GCST90094765 | Weighted median | 5 | 0.337080669363302 | 0.259377830077848 | 0.193747035187632 | -0.171299878 | 0.845461216315884 | 1.40085206502477 | 0.842568868062212 | 2.32905176356369 | 0.999489402457283 |
| ebi-fl187-GCST90094765 | prot-a-2770 | Secreted Ly-6/uPAR-related protein 1 \|\| id:prot-a-2770 | Fried fish liking \|\| id:ebi-fl187-GCST90094765 | Weighted median | 5 | 0.200703416956602 | 0.280351528370855 | 0.474053631952789 | -0.348785579 | 0.750192412563479 | 1.22226221581421 | 0.705544397833952 | 2.11740739320369 | 0.999489402457283 |
| ebi-fl187-GCST90094765 | prot-a-2784 | N-lysine methyltransferase SMYD2 \|\| id:prot-a-2784 | Fried fish liking \|\| id:ebi-fl187-GCST90094765 | Weighted median | 5 | -0.251437045 | 0.25613085466617 | 0.326260393459959 | -0.75345352 | 0.250579430432004 | 0.777682415287791 | 0.470738039140824 | 1.28476963568038 | 0.999489402457283 |
| ebi-fl187-GCST90094765 | prot-a-2837 | Serine palmitoyltransferase 2 \|\| id:prot-a-2837 | Fried fish liking \|\| id:ebi-fl187-GCST90094765 | Weighted median | 5 | 0.314700794881595 | 0.292567777752706 | 0.282083411986035 | -0.25873205 | 0.888133639276898 | 1.36984938368684 | 0.772029861108001 | 2.43058905946219 | 0.999489402457283 |
| ebi-fl187-GCST90094765 | prot-a-2858 | Alpha-2,8-sialyltransferase 8B \|\| id:prot-a-2858 | Fried fish liking \|\| id:ebi-fl187-GCST90094765 | Weighted median | 5 | 0.0259642518654387 | 0.28073189058149 | 0.926310549193267 | -0.524270254 | 0.57619875740516 | 1.0263042593549 | 0.591987207269142 | 1.77926215268894 | 0.999489402457283 |
| ebi-fl187-GCST90094765 | prot-a-2911 | Synaptotagmin-11 \|\| id:prot-a-2911 | Fried fish liking \|\| id:ebi-fl187-GCST90094765 | Weighted median | 5 | -0.354807261 | 0.27679190291225 | 0.199893293087569 | -0.89731939 | 0.18770486896797 | 0.701308599883701 | 0.40766097629734 | 1.20647739388257 | 0.999489402457283 |
| ebi-fl187-GCST90094765 | prot-a-2962 | Transforming growth factor beta-1 \|\| id:prot-a-2962 | Fried fish liking \|\| id:ebi-fl187-GCST90094765 | Weighted median | 5 | -0.033285681 | 0.255795359066867 | 0.896466573882614 | -0.534644584 | 0.468073223174402 | 0.967262192074337 | 0.585877483161194 | 1.59691432954258 | 0.999489402457283 |
| ebi-fl187-GCST90094765 | prot-a-2978 | Tyrosine-protein kinase receptor Tie-1, soluble \|\| id:prot-a-2978 | Fried fish liking \|\| id:ebi-fl187-GCST90094765 | Weighted median | 5 | 0.00188168589986762 | 0.27654134072606 | 0.994570950807756 | -0.540139342 | 0.543902713722946 | 1.00188345738173 | 0.582667056768863 | 1.72271703113182 | 0.999911036302558 |
| ebi-fl187-GCST90094765 | prot-a-299 | Uncharacterized protein C1orf226 \|\| id:prot-a-299 | Fried fish liking \|\| id:ebi-fl187-GCST90094765 | Weighted median | 5 | 0.00438168902083569 | 0.275538572743162 | 0.987312358334252 | -0.535673914 | 0.544437291597433 | 1.00439130265637 | 0.585274732634732 | 1.72363820373797 | 0.999911036302558 |
| ebi-fl187-GCST90094765 | prot-a-3015 | Transmembrane protein 70, mitochondrial \|\| id:prot-a-3015 | Fried fish liking \|\| id:ebi-fl187-GCST90094765 | Weighted median | 5 | -0.018655735 | 0.259728705191523 | 0.94273896984971 | -0.527723997 | 0.490412527569345 | 0.981517206493198 | 0.589946162184072 | 1.63298973431007 | 0.999489402457283 |
| ebi-fl187-GCST90094765 | prot-a-3016 | Transmembrane protein 87B \|\| id:prot-a-3016 | Fried fish liking \|\| id:ebi-fl187-GCST90094765 | Weighted median | 5 | 0.159132352383067 | 0.285918247774904 | 0.577824044411916 | -0.401267413 | 0.719532118021878 | 1.17249311867019 | 0.669471011676056 | 2.05347220320593 | 0.999489402457283 |
| ebi-fl187-GCST90094765 | prot-a-3123 | Thioredoxin domain-containing protein 12 \|\| id:prot-a-3123 | Fried fish liking \|\| id:ebi-fl187-GCST90094765 | Weighted median | 5 | -0.286740593 | 0.276029431838855 | 0.298895707467917 | -0.827758279 | 0.254277093841256 | 0.750706442415143 | 0.437027883639366 | 1.28952907533161 | 0.999489402457283 |
| ebi-fl187-GCST90094765 | prot-a-3133 | PolyUbiquitin K48-linked \|\| id:prot-a-3133 | Fried fish liking \|\| id:ebi-fl187-GCST90094765 | Weighted median | 5 | -0.038257767 | 0.270394174608488 | 0.887483750069026 | -0.568230349 | 0.491714815336361 | 0.962464817344813 | 0.566527107325224 | 1.6351177422033 | 0.999489402457283 |
| ebi-fl187-GCST90094765 | prot-a-3135 | Ubiquitin-conjugating enzyme E2 B \|\| id:prot-a-3135 | Fried fish liking \|\| id:ebi-fl187-GCST90094765 | Weighted median | 5 | 0.320891756272197 | 0.289047182856144 | 0.266925412022603 | -0.245640722 | 0.887424234670239 | 1.37835637438636 | 0.782203202751738 | 2.42886539984484 | 0.999489402457283 |
| ebi-fl187-GCST90094765 | prot-a-3162 | NKG2D ligand 3 \|\| id:prot-a-3162 | Fried fish liking \|\| id:ebi-fl187-GCST90094765 | Weighted median | 5 | -0.124333274 | 0.275408526463285 | 0.651665006353515 | -0.664133986 | 0.415467438029062 | 0.883085482545597 | 0.514719088835436 | 1.51507878063585 | 0.999489402457283 |
| ebi-fl187-GCST90094765 | prot-a-3188 | Vesicle-associated membrane protein 8 \|\| id:prot-a-3188 | Fried fish liking \|\| id:ebi-fl187-GCST90094765 | Weighted median | 5 | 0.0501107959998426 | 0.30378304785508 | 0.868978719233192 | -0.545303978 | 0.645525569795799 | 1.05138757946106 | 0.579665551099153 | 1.90698902177112 | 0.999489402457283 |
| ebi-fl187-GCST90094765 | prot-a-3201 | Vitrin \|\| id:prot-a-3201 | Fried fish liking \|\| id:ebi-fl187-GCST90094765 | Weighted median | 5 | -0.122556024 | 0.297527372011238 | 0.680401636594458 | -0.705709673 | 0.46059762542133 | 0.884656341815334 | 0.493758043205651 | 1.58502095081442 | 0.999489402457283 |
| ebi-fl187-GCST90094765 | prot-a-3232 | WNT1-inducible-signaling pathway protein 3 \|\| id:prot-a-3232 | Fried fish liking \|\| id:ebi-fl187-GCST90094765 | Weighted median | 5 | 0.269108367162289 | 0.265556731670136 | 0.310881410045351 | -0.251382827 | 0.789599561235756 | 1.30879696397485 | 0.777724580662448 | 2.20251427754892 | 0.999489402457283 |
| ebi-fl187-GCST90094765 | prot-a-327 | Carbonic anhydrase-related protein 10 \|\| id:prot-a-327 | Fried fish liking \|\| id:ebi-fl187-GCST90094765 | Weighted median | 5 | -0.193564004 | 0.277673409969644 | 0.485745014699737 | -0.737803888 | 0.350675879559599 | 0.824017094132922 | 0.478162862726063 | 1.42002699154046 | 0.999489402457283 |
| ebi-fl187-GCST90094765 | prot-a-332 | Carbonic anhydrase 5A, mitochondrial \|\| id:prot-a-332 | Fried fish liking \|\| id:ebi-fl187-GCST90094765 | Weighted median | 5 | -0.290924391 | 0.277254379881981 | 0.294037792819215 | -0.834342975 | 0.252494193936581 | 0.747572199342744 | 0.434159641395364 | 1.28723202238234 | 0.999489402457283 |
| ebi-fl187-GCST90094765 | prot-a-338 | Cell adhesion molecule 3 \|\| id:prot-a-338 | Fried fish liking \|\| id:ebi-fl187-GCST90094765 | Weighted median | 5 | -0.226738891 | 0.260814697001237 | 0.384656488882723 | -0.737935697 | 0.28445791488573 | 0.797128892458569 | 0.478099840310306 | 1.32904137926466 | 0.999489402457283 |
| ebi-fl187-GCST90094765 | prot-a-34 | A disintegrin and metalloproteinase with thrombospondin motifs 3 \|\| id:prot-a-34 | Fried fish liking \|\| id:ebi-fl187-GCST90094765 | Weighted median | 5 | -0.058031367 | 0.260871558195442 | 0.823962133800082 | -0.569339621 | 0.453276887124856 | 0.943620348491422 | 0.565899023162589 | 1.57345979696319 | 0.999489402457283 |
| ebi-fl187-GCST90094765 | prot-a-358 | Cysteine--tRNA ligase, cytoplasmic \|\| id:prot-a-358 | Fried fish liking \|\| id:ebi-fl187-GCST90094765 | Weighted median | 5 | -0.367299547 | 0.297181304677068 | 0.216479259465307 | -0.949774904 | 0.215175810229161 | 0.692602147115913 | 0.386828087069794 | 1.24007989653301 | 0.999489402457283 |
| ebi-fl187-GCST90094765 | prot-a-382 | Coiled-coil domain-containing protein 167 \|\| id:prot-a-382 | Fried fish liking \|\| id:ebi-fl187-GCST90094765 | Weighted median | 5 | 0.207292693540063 | 0.278094206944817 | 0.456027180288054 | -0.337771952 | 0.752357339151904 | 1.23034263242504 | 0.713357949152681 | 2.12199639039643 | 0.999489402457283 |
| ebi-fl187-GCST90094765 | prot-a-40 | Pituitary adenylate cyclase-activating polypeptide 27 \|\| id:prot-a-40 | Fried fish liking \|\| id:ebi-fl187-GCST90094765 | Weighted median | 5 | -0.073509138 | 0.261624851797563 | 0.778732225986899 | -0.586293848 | 0.439275571097791 | 0.929127654988932 | 0.556385517644088 | 1.55158279985544 | 0.999489402457283 |
| ebi-fl187-GCST90094765 | prot-a-403 | C-C motif chemokine 25 \|\| id:prot-a-403 | Fried fish liking \|\| id:ebi-fl187-GCST90094765 | Weighted median | 4 | -0.237136175 | 0.31135143037943 | 0.446277824940324 | -0.847384979 | 0.373112628414313 | 0.788883854341955 | 0.428534093789502 | 1.45224789499973 | 0.999489402457283 |
| ebi-fl187-GCST90094765 | prot-a-415 | Cyclin-C \|\| id:prot-a-415 | Fried fish liking \|\| id:ebi-fl187-GCST90094765 | Weighted median | 5 | 0.264934435277258 | 0.26999000612707 | 0.326457162237142 | -0.264245977 | 0.794114847286315 | 1.30334551948932 | 0.767784659266356 | 2.21248174559264 | 0.999489402457283 |
| ebi-fl187-GCST90094765 | prot-a-44 | Adhesion G protein-coupled receptor F5 \|\| id:prot-a-44 | Fried fish liking \|\| id:ebi-fl187-GCST90094765 | Weighted median | 5 | 0.280874136302191 | 0.308952226567764 | 0.363287661079948 | -0.324672228 | 0.886420500375008 | 1.32428691379743 | 0.722764216860031 | 2.42642868745499 | 0.999489402457283 |
| ebi-fl187-GCST90094765 | prot-a-455 | CD59 glycoprotein \|\| id:prot-a-455 | Fried fish liking \|\| id:ebi-fl187-GCST90094765 | Weighted median | 5 | -0.06111547 | 0.267636065278887 | 0.819371906647557 | -0.585682158 | 0.4634512177353 | 0.94071460898963 | 0.556725957095987 | 1.58955041396415 | 0.999489402457283 |
| ebi-fl187-GCST90094765 | prot-a-478 | Cadherin-12 \|\| id:prot-a-478 | Fried fish liking \|\| id:ebi-fl187-GCST90094765 | Weighted median | 5 | 0.209121996191081 | 0.254432799305356 | 0.411125584100265 | -0.28956629 | 0.707810282829578 | 1.23259536129745 | 0.748588167021525 | 2.02954226585885 | 0.999489402457283 |
| ebi-fl187-GCST90094765 | prot-a-516 | Centrin-2 \|\| id:prot-a-516 | Fried fish liking \|\| id:ebi-fl187-GCST90094765 | Weighted median | 5 | 0.0148277378831287 | 0.268896202881045 | 0.956024555287241 | -0.51220882 | 0.541864295529977 | 1.01493821415083 | 0.599170656106168 | 1.71920899003632 | 0.999489402457283 |
| ebi-fl187-GCST90094765 | prot-a-519 | Cryptic protein \|\| id:prot-a-519 | Fried fish liking \|\| id:ebi-fl187-GCST90094765 | Weighted median | 5 | -0.060965566 | 0.263252670446058 | 0.816859778732836 | -0.5769408 | 0.455009667704372 | 0.940855636293198 | 0.561613830034372 | 1.57618862108596 | 0.999489402457283 |
| ebi-fl187-GCST90094765 | prot-a-583 | Chloride intracellular channel protein 4 \|\| id:prot-a-583 | Fried fish liking \|\| id:ebi-fl187-GCST90094765 | Weighted median | 5 | -0.120417893 | 0.264851390885747 | 0.649352340910409 | -0.639526619 | 0.398690832847226 | 0.886549876051722 | 0.527542093123572 | 1.48987292762478 | 0.999489402457283 |
| ebi-fl187-GCST90094765 | prot-a-602 | CCR4-NOT transcription complex subunit 1 \|\| id:prot-a-602 | Fried fish liking \|\| id:ebi-fl187-GCST90094765 | Weighted median | 5 | -0.499513527 | 0.279599772528608 | 0.0740133999425284 | -1.047529082 | 0.0485020267798013 | 0.606825792055119 | 0.350803486 | 1.04969749932386 | 0.999489402457283 |
| ebi-fl187-GCST90094765 | prot-a-606 | Ciliary neurotrophic factor receptor subunit alpha \|\| id:prot-a-606 | Fried fish liking \|\| id:ebi-fl187-GCST90094765 | Weighted median | 5 | -0.444066045 | 0.275574587527721 | 0.107088493160389 | -0.984192236 | 0.0960601467659472 | 0.641423056778322 | 0.373740999436404 | 1.10082527308288 | 0.999489402457283 |
| ebi-fl187-GCST90094765 | prot-a-626 | Collagen alpha-2(IX) chain \|\| id:prot-a-626 | Fried fish liking \|\| id:ebi-fl187-GCST90094765 | Weighted median | 5 | -0.441953503 | 0.255619391390197 | 0.0838177948886892 | -0.94296751 | 0.059060504345573 | 0.642779522222063 | 0.389470361645076 | 1.06083942419357 | 0.999489402457283 |
| ebi-fl187-GCST90094765 | prot-a-647 | Probable serine carboxypeptidase CPVL \|\| id:prot-a-647 | Fried fish liking \|\| id:ebi-fl187-GCST90094765 | Weighted median | 5 | -0.216944095 | 0.276383997480986 | 0.432490360690039 | -0.75865673 | 0.324768540199389 | 0.804974970282816 | 0.468295051437559 | 1.38371033559431 | 0.999489402457283 |
| ebi-fl187-GCST90094765 | prot-a-651 | Cellular retinoic acid-binding protein 2 \|\| id:prot-a-651 | Fried fish liking \|\| id:ebi-fl187-GCST90094765 | Weighted median | 5 | 0.335332951342474 | 0.276687388143122 | 0.225529208496379 | -0.206974329 | 0.877640232102993 | 1.39840590884448 | 0.81304052094301 | 2.40521724996316 | 0.999489402457283 |
| ebi-fl187-GCST90094765 | prot-a-671 | Cytotoxic and regulatory T-cell molecule \|\| id:prot-a-671 | Fried fish liking \|\| id:ebi-fl187-GCST90094765 | Weighted median | 5 | -0.196966164 | 0.289364357026827 | 0.496070420987189 | -0.764120304 | 0.3701879755611 | 0.82121841941534 | 0.465743463485121 | 1.44800677896916 | 0.999489402457283 |
| ebi-fl187-GCST90094765 | prot-a-688 | Chorionic somatomammotropin hormone \|\| id:prot-a-688 | Fried fish liking \|\| id:ebi-fl187-GCST90094765 | Weighted median | 5 | -0.060030127 | 0.263106839146757 | 0.819522828981165 | -0.575719532 | 0.455659277381648 | 0.941736161145695 | 0.562300130234511 | 1.57721286110952 | 0.999489402457283 |
| ebi-fl187-GCST90094765 | prot-a-69 | Aldo-keto reductase family 1 member C1 \|\| id:prot-a-69 | Fried fish liking \|\| id:ebi-fl187-GCST90094765 | Weighted median | 5 | 0.23540875999561 | 0.256956253730153 | 0.359591719808809 | -0.268225497 | 0.739043017306709 | 1.26542591852352 | 0.76473531589758 | 2.09393069992017 | 0.999489402457283 |
| ebi-fl187-GCST90094765 | prot-a-741 | Stromal cell-derived factor 1 \|\| id:prot-a-741 | Fried fish liking \|\| id:ebi-fl187-GCST90094765 | Weighted median | 5 | -0.203192103 | 0.24587018570158 | 0.408565734378921 | -0.685097667 | 0.278713461106414 | 0.816121447094591 | 0.504040999077256 | 1.3214286489137 | 0.999489402457283 |
| ebi-fl187-GCST90094765 | prot-a-742 | Growth-regulated alpha protein \|\| id:prot-a-742 | Fried fish liking \|\| id:ebi-fl187-GCST90094765 | Weighted median | 5 | 0.235159175747792 | 0.253388168679153 | 0.353376954868651 | -0.261481635 | 0.731799986358932 | 1.26511012755734 | 0.769910014797137 | 2.07881908805909 | 0.999489402457283 |
| ebi-fl187-GCST90094765 | prot-a-788 | Beta-defensin 112 \|\| id:prot-a-788 | Fried fish liking \|\| id:ebi-fl187-GCST90094765 | Weighted median | 5 | -0.254608755 | 0.298014965955566 | 0.392911706758789 | -0.838718088 | 0.329500578408574 | 0.775219739588493 | 0.432264293136485 | 1.39027362238754 | 0.999489402457283 |
| ebi-fl187-GCST90094765 | prot-a-838 | DnaJ homolog subfamily B member 1 \|\| id:prot-a-838 | Fried fish liking \|\| id:ebi-fl187-GCST90094765 | Weighted median | 5 | 0.00617100296156337 | 0.275528188292168 | 0.982131278824532 | -0.533864246 | 0.546206252014214 | 1.00619008282745 | 0.586334844211679 | 1.72668994990652 | 0.999911036302558 |
| ebi-fl187-GCST90094765 | prot-a-85 | Junctional adhesion molecule-like \|\| id:prot-a-85 | Fried fish liking \|\| id:ebi-fl187-GCST90094765 | Weighted median | 5 | -0.03004484 | 0.287412608694436 | 0.916744345319348 | -0.593373553 | 0.533283873325124 | 0.970402020021994 | 0.552460383210745 | 1.70452055763707 | 0.999489402457283 |
| ebi-fl187-GCST90094765 | prot-a-856 | Dipeptidase 1 \|\| id:prot-a-856 | Fried fish liking \|\| id:ebi-fl187-GCST90094765 | Weighted median | 5 | 0.773851395324909 | 0.294570898737223 | 0.00861296089728245 | 0.196492433799952 | 1.35121035684987 | 2.16810040619357 | 1.21712611182371 | 3.86209721874539 | 0.999489402457283 |
| ebi-fl187-GCST90094765 | prot-a-860 | Dipeptidyl peptidase 2 \|\| id:prot-a-860 | Fried fish liking \|\| id:ebi-fl187-GCST90094765 | Weighted median | 5 | -0.067891236 | 0.247903629048294 | 0.78419113387319 | -0.553782349 | 0.417999877261131 | 0.934362093314576 | 0.574771706838346 | 1.51892048797165 | 0.999489402457283 |
| ebi-fl187-GCST90094765 | prot-a-861 | Dermatopontin \|\| id:prot-a-861 | Fried fish liking \|\| id:ebi-fl187-GCST90094765 | Weighted median | 5 | -0.117301443 | 0.261777135016281 | 0.654083484585011 | -0.630384628 | 0.395781741509295 | 0.889317074238285 | 0.532386990808766 | 1.48554504934519 | 0.999489402457283 |
| ebi-fl187-GCST90094765 | prot-a-877 | Dual specificity protein phosphatase 6 \|\| id:prot-a-877 | Fried fish liking \|\| id:ebi-fl187-GCST90094765 | Weighted median | 5 | 0.0259515172094232 | 0.277988542491593 | 0.925621833977215 | -0.518906026 | 0.570809060492945 | 1.02629118980641 | 0.595171293832689 | 1.76969826533727 | 0.999489402457283 |
| ebi-fl187-GCST90094765 | prot-a-879 | Dynactin-associated protein \|\| id:prot-a-879 | Fried fish liking \|\| id:ebi-fl187-GCST90094765 | Weighted median | 5 | 0.239144553820177 | 0.250858281150748 | 0.34043535675216 | -0.252537677 | 0.730826784875643 | 1.2701621301021 | 0.776826943596393 | 2.07679696236657 | 0.999489402457283 |
| ebi-fl187-GCST90094765 | prot-a-937 | EMI domain-containing protein 1 \|\| id:prot-a-937 | Fried fish liking \|\| id:ebi-fl187-GCST90094765 | Weighted median | 5 | -0.131987693 | 0.286711362052174 | 0.645264724450103 | -0.693941962 | 0.429966577016601 | 0.876351780619947 | 0.499602767043659 | 1.53720614467421 | 0.999489402457283 |
| ebi-fl187-GCST90094765 | ukb-rsfMRI191-edge-pheno102 | Net25_Pair11_15 (Postcentral\|Precentral)&(Cerebellum) (Motor)&(Subcortical-cerebellum) \|\| id:ukb-rsfMRI191-edge-pheno102 | Fried fish liking \|\| id:ebi-fl187-GCST90094765 | Weighted median | 5 | -0.002488578 | 0.078581318220017 | 0.974736158559514 | -0.156507962 | 0.151530805903458 | 0.997514516134939 | 0.855124709584178 | 1.16361414744263 | 0.999489402457283 |
| ebi-fl187-GCST90094765 | ukb-rsfMRI191-edge-pheno1167 | Net100_Pair11_45 (Angular\|Temporal)&(Frontal) (Default_mode\|Central_executive)&(Salience\|Default_mode) \|\| id:ukb-rsfMRI191-edge-pheno1167 | Fried fish liking \|\| id:ebi-fl187-GCST90094765 | Weighted median | 5 | -0.050282848 | 0.079486408547395 | 0.526996978446393 | -0.206076208 | 0.105510513199615 | 0.950960409632279 | 0.813771057805862 | 1.11127778754665 | 0.999489402457283 |
| ebi-fl187-GCST90094765 | ukb-rsfMRI191-edge-pheno1317 | Net100_Pair26_48 (Parietal\|Frontal)&(Temporal) (Attention\|Central_executive)&(Default_mode\|Central_executive) \|\| id:ukb-rsfMRI191-edge-pheno1317 | Fried fish liking \|\| id:ebi-fl187-GCST90094765 | Weighted median | 5 | -0.111486902 | 0.087869821461884 | 0.20452185372743 | -0.283711752 | 0.0607379477980962 | 0.894503107289233 | 0.752983659211173 | 1.0626204156785 | 0.999489402457283 |
| ebi-fl187-GCST90094765 | ukb-rsfMRI191-edge-pheno286 | Net100_Pair10_13 (OccipitalPrecuneus)&(Frontal_Sup) (Default_mode\|Central_executive)&(Default_mode) \|\| id:ukb-rsfMRI191-edge-pheno286 | Fried fish liking \|\| id:ebi-fl187-GCST90094765 | Weighted median | 5 | 0.0240226455754669 | 0.0832545178944999 | 0.772929858746987 | -0.139156209 | 0.187201500648687 | 1.02431351379696 | 0.870092101191837 | 1.20587024420733 | 0.999489402457283 |
| ebi-fl187-GCST90094765 | ukb-rsfMRI191-edge-pheno599 | Net100_Pair11_29 (Angular\|Temporal)&(Parietal) (Default_mode\|Central_executive)&(Central_executive\|Salience) \|\| id:ukb-rsfMRI191-edge-pheno599 | Fried fish liking \|\| id:ebi-fl187-GCST90094765 | Weighted median | 5 | 0.0855749393205811 | 0.0811223574911929 | 0.291476901407743 | -0.073424881 | 0.244574760003319 | 1.08934319292408 | 0.929205943854697 | 1.27707813302106 | 0.999489402457283 |
| ebi-fl187-GCST90094765 | ukb-rsfMRI191-edge-pheno621 | Net100_Pair5_30 (Precuneus\|Angular\|Cingulate)&(Paracentral) (Default_mode\|Central_executive)&(Motor) \|\| id:ukb-rsfMRI191-edge-pheno621 | Fried fish liking \|\| id:ebi-fl187-GCST90094765 | Weighted median | 5 | -0.058304793 | 0.0913694333950475 | 0.523394640399731 | -0.237388883 | 0.120779296354637 | 0.943362373271866 | 0.788684522722184 | 1.12837584822063 | 0.999489402457283 |
| ebi-fl187-GCST90094765 | ukb-rsfMRI191-edge-pheno624 | Net100_Pair8_30 (Calcarine\|Lingual)&(Paracentral) (Visual)&(Motor) \|\| id:ukb-rsfMRI191-edge-pheno624 | Fried fish liking \|\| id:ebi-fl187-GCST90094765 | Weighted median | 5 | 0.0614508753735808 | 0.0848196336706634 | 0.468765695198042 | -0.104795607 | 0.227697357368081 | 1.06337825718606 | 0.90050856176526 | 1.25570523798178 | 0.999489402457283 |
| ebi-fl187-GCST90094765 | ukb-rsfMRI191-edge-pheno882 | Net100_Pair6_38 (Postcentral\|Precentral)&(Subcortical) (Motor)&(Subcortical-cerebellum) \|\| id:ukb-rsfMRI191-edge-pheno882 | Fried fish liking \|\| id:ebi-fl187-GCST90094765 | Weighted median | 5 | 0.0751362828120996 | 0.0895272440341881 | 0.401325630269132 | -0.100337115 | 0.250609681119108 | 1.07803105797806 | 0.904532434732153 | 1.28480850143249 | 0.999489402457283 |
| ebi-fl187-GCST90094765 | ukb-rsfMRI191-edge-pheno899 | Net100_Pair23_38 (Cerebellum)&(Subcortical) (Subcortical-cerebellum)&(Subcortical-cerebellum) \|\| id:ukb-rsfMRI191-edge-pheno899 | Fried fish liking \|\| id:ebi-fl187-GCST90094765 | Weighted median | 5 | 0.07708027695408 | 0.090197091175359 | 0.392785872358131 | -0.099706022 | 0.253866575657784 | 1.08012878236097 | 0.905103459660299 | 1.28899980884226 | 0.999489402457283 |
| ebi-fl187-GCST90094765 | ebi-a-GCST90001392 | IgD+ CD38+ B cell Absolute Count \|\| id:ebi-a-GCST90001392 | Fried fish liking \|\| id:ebi-fl187-GCST90094765 | Weighted mode | 4 | -0.288688088 | 0.384681540012277 | 0.507474859016059 | -1.042663906 | 0.465287730670082 | 0.749245867921159 | 0.352514364862186 | 1.59247232610335 | 0.999228396881742 |
| ebi-fl187-GCST90094765 | ebi-a-GCST90001402 | Switched memory B cell %B cell \|\| id:ebi-a-GCST90001402 | Fried fish liking \|\| id:ebi-fl187-GCST90094765 | Weighted mode | 4 | 0.361789439660056 | 0.455260834160992 | 0.484858568394866 | -0.530521795 | 1.2541006746156 | 1.43589656725808 | 0.588297918490176 | 3.50468510436856 | 0.999228396881742 |
| ebi-fl187-GCST90094765 | ebi-a-GCST90001407 | Memory B cell Absolute Count \|\| id:ebi-a-GCST90001407 | Fried fish liking \|\| id:ebi-fl187-GCST90094765 | Weighted mode | 4 | 0.300678389796847 | 0.490217868311176 | 0.583011360263694 | -0.660148632 | 1.26150541168675 | 1.35077484869959 | 0.516774519504781 | 3.53073269484705 | 0.999228396881742 |
| ebi-fl187-GCST90094765 | ebi-a-GCST90001411 | IgD+ CD24+ B cell %B cell \|\| id:ebi-a-GCST90001411 | Fried fish liking \|\| id:ebi-fl187-GCST90094765 | Weighted mode | 4 | -0.483160566 | 0.480322641055184 | 0.388566336785461 | -1.424592943 | 0.458271810307733 | 0.616830773272815 | 0.240606383879491 | 1.58133876882878 | 0.999228396881742 |
| ebi-fl187-GCST90094765 | ebi-a-GCST90001418 | CD24+ CD27+ B cell Absolute Count \|\| id:ebi-a-GCST90001418 | Fried fish liking \|\| id:ebi-fl187-GCST90094765 | Weighted mode | 4 | 0.297906416044081 | 0.56002793820871 | 0.631635111900886 | -0.799748343 | 1.39556117493315 | 1.34703572104919 | 0.449442055195487 | 4.03723953467879 | 0.999228396881742 |
| ebi-fl187-GCST90094765 | ebi-a-GCST90001436 | Memory B cell %lymphocyte \|\| id:ebi-a-GCST90001436 | Fried fish liking \|\| id:ebi-fl187-GCST90094765 | Weighted mode | 4 | 0.111293420221458 | 0.480224509913309 | 0.831638381122896 | -0.829946619 | 1.05253345965154 | 1.11772282122148 | 0.436072563598791 | 2.8649000404179 | 0.999228396881742 |
| ebi-fl187-GCST90094765 | ebi-a-GCST90001439 | IgD+ CD24+ B cell %lymphocyte \|\| id:ebi-a-GCST90001439 | Fried fish liking \|\| id:ebi-fl187-GCST90094765 | Weighted mode | 4 | -0.631645618 | 0.483062150012156 | 0.282195185608477 | -1.578447432 | 0.315156195623426 | 0.531716078889099 | 0.20629513684029 | 1.37047335618036 | 0.999228396881742 |
| ebi-fl187-GCST90094765 | ebi-a-GCST90001457 | CD11c+ HLA DR++ monocyte %monocyte \|\| id:ebi-a-GCST90001457 | Fried fish liking \|\| id:ebi-fl187-GCST90094765 | Weighted mode | 4 | 0.3229051373347 | 0.565972269390641 | 0.608250877088426 | -0.786400511 | 1.43221078534036 | 1.38113432660771 | 0.455481348390766 | 4.18794761821428 | 0.999228396881742 |
| ebi-fl187-GCST90094765 | ebi-a-GCST90001483 | CD39+ resting CD4 regulatory T cell Absolute Count \|\| id:ebi-a-GCST90001483 | Fried fish liking \|\| id:ebi-fl187-GCST90094765 | Weighted mode | 3 | -0.282243461 | 0.392624939461755 | 0.546867946591592 | -1.051788342 | 0.487301420576717 | 0.7540900708346 | 0.349312499943711 | 1.62791722318258 | 0.999228396881742 |
| ebi-fl187-GCST90094765 | ebi-a-GCST90001525 | CD33dim HLA DR+ CD11b+ Absolute Count \|\| id:ebi-a-GCST90001525 | Fried fish liking \|\| id:ebi-fl187-GCST90094765 | Weighted mode | 4 | -0.210094089 | 0.425292664403898 | 0.655213778927398 | -1.043667711 | 0.62347953310937 | 0.8105079823978 | 0.352160686659872 | 1.86540750974009 | 0.999228396881742 |
| ebi-fl187-GCST90094765 | ebi-a-GCST90001540 | Naive CD4+ T cell Absolute Count \|\| id:ebi-a-GCST90001540 | Fried fish liking \|\| id:ebi-fl187-GCST90094765 | Weighted mode | 4 | -0.37825204 | 0.38173774471557 | 0.394795183831622 | -1.126458019 | 0.36995393994441 | 0.685057817139815 | 0.32417946269073 | 1.44766793346218 | 0.999228396881742 |
| ebi-fl187-GCST90094765 | ebi-a-GCST90001624 | HLA DR+ CD4+ T cell Absolute Count \|\| id:ebi-a-GCST90001624 | Fried fish liking \|\| id:ebi-fl187-GCST90094765 | Weighted mode | 4 | 0.290536677828136 | 0.344230480713079 | 0.460644093988012 | -0.384155064 | 0.96522842002577 | 1.33714491152263 | 0.681025816152711 | 2.625387279 | 0.999228396881742 |
| ebi-fl187-GCST90094765 | ebi-a-GCST90001672 | CD39+ CD8+ T cell Absolute Count \|\| id:ebi-a-GCST90001672 | Fried fish liking \|\| id:ebi-fl187-GCST90094765 | Weighted mode | 4 | 0.359330922038845 | 0.359220417105094 | 0.390875036890725 | -0.344741095 | 1.06340293956483 | 1.43237072619238 | 0.708403738652014 | 2.89620986636367 | 0.999228396881742 |
| ebi-fl187-GCST90094765 | ebi-a-GCST90001722 | CD19 on CD20- CD38- B cell \|\| id:ebi-a-GCST90001722 | Fried fish liking \|\| id:ebi-fl187-GCST90094765 | Weighted mode | 4 | 0.38963597020097 | 0.32102878946319 | 0.311694848391451 | -0.239580457 | 1.01885239754882 | 1.47644322671225 | 0.786957954403036 | 2.77001406429403 | 0.999228396881742 |
| ebi-fl187-GCST90094765 | ebi-a-GCST90001742 | CD19 on transitional B cell \|\| id:ebi-a-GCST90001742 | Fried fish liking \|\| id:ebi-fl187-GCST90094765 | Weighted mode | 4 | 0.109692970878411 | 0.476650662050722 | 0.832788049548284 | -0.824542327 | 1.04392826849783 | 1.11593539319209 | 0.438435606806565 | 2.84035279627325 | 0.999228396881742 |
| ebi-fl187-GCST90094765 | ebi-a-GCST90001784 | CD25 on IgD+ CD38dim B cell \|\| id:ebi-a-GCST90001784 | Fried fish liking \|\| id:ebi-fl187-GCST90094765 | Weighted mode | 4 | 0.169965022392911 | 0.374339807645388 | 0.680636007536546 | -0.563741001 | 0.903671045377871 | 1.18526339291806 | 0.56907616248292 | 2.46864901960076 | 0.999228396881742 |
| ebi-fl187-GCST90094765 | ebi-a-GCST90001794 | CD25 on IgD+ B cell \|\| id:ebi-a-GCST90001794 | Fried fish liking \|\| id:ebi-fl187-GCST90094765 | Weighted mode | 4 | -0.248189012 | 0.509197669697774 | 0.659363059133927 | -1.246216445 | 0.749838420301852 | 0.780212459584368 | 0.287590856829936 | 2.11665798002284 | 0.999228396881742 |
| ebi-fl187-GCST90094765 | ebi-a-GCST90001802 | CD27 on IgD- CD38- B cell \|\| id:ebi-a-GCST90001802 | Fried fish liking \|\| id:ebi-fl187-GCST90094765 | Weighted mode | 4 | -0.165212896 | 0.432056602691989 | 0.727656016346041 | -1.012043837 | 0.681618045672428 | 0.847713210459598 | 0.363475335590981 | 1.97707414182397 | 0.999228396881742 |
| ebi-fl187-GCST90094765 | ebi-a-GCST90001804 | CD27 on IgD- CD38dim B cell \|\| id:ebi-a-GCST90001804 | Fried fish liking \|\| id:ebi-fl187-GCST90094765 | Weighted mode | 4 | 0.502075250277728 | 0.36088373277774 | 0.258375258233285 | -0.205256866 | 1.2094073665221 | 1.65214633267624 | 0.814438088113977 | 3.3514978540561 | 0.999228396881742 |
| ebi-fl187-GCST90094765 | ebi-a-GCST90001805 | CD27 on memory B cell \|\| id:ebi-a-GCST90001805 | Fried fish liking \|\| id:ebi-fl187-GCST90094765 | Weighted mode | 4 | 0.302661541240868 | 0.369086113303158 | 0.472285537470617 | -0.420747241 | 1.02607032331506 | 1.35345629777232 | 0.656556030993447 | 2.79008015082544 | 0.999228396881742 |
| ebi-fl187-GCST90094765 | ebi-a-GCST90001806 | CD27 on unswitched memory B cell \|\| id:ebi-a-GCST90001806 | Fried fish liking \|\| id:ebi-fl187-GCST90094765 | Weighted mode | 4 | 0.255357666733689 | 0.293816637884152 | 0.44873781497889 | -0.320522944 | 0.831238276986626 | 1.29092325861709 | 0.7257694014134 | 2.29616026301628 | 0.999228396881742 |
| ebi-fl187-GCST90094765 | ebi-a-GCST90001839 | CD3 on Effector Memory CD8+ T cell \|\| id:ebi-a-GCST90001839 | Fried fish liking \|\| id:ebi-fl187-GCST90094765 | Weighted mode | 4 | -0.062088084 | 0.360640832651469 | 0.874270184356175 | -0.768944116 | 0.644767947735783 | 0.939800101547581 | 0.4635022144777 | 1.90554479197928 | 0.999228396881742 |
| ebi-fl187-GCST90094765 | ebi-a-GCST90001859 | CD3 on CD8+ T cell \|\| id:ebi-a-GCST90001859 | Fried fish liking \|\| id:ebi-fl187-GCST90094765 | Weighted mode | 4 | 0.246038438302459 | 0.377535955126686 | 0.561071193683805 | -0.493932034 | 0.986008910350763 | 1.27894873321516 | 0.610222256202234 | 2.68051491987958 | 0.999228396881742 |
| ebi-fl187-GCST90094765 | ebi-a-GCST90001905 | CD86 on monocyte \|\| id:ebi-a-GCST90001905 | Fried fish liking \|\| id:ebi-fl187-GCST90094765 | Weighted mode | 4 | -0.04207102 | 0.366151316044115 | 0.915782767456035 | -0.7597276 | 0.675585559066426 | 0.958801683694422 | 0.467793836778198 | 1.96518337006466 | 0.999228396881742 |
| ebi-fl187-GCST90094765 | ebi-a-GCST90001943 | CD25 on activated & secreting CD4 regulatory T cell \|\| id:ebi-a-GCST90001943 | Fried fish liking \|\| id:ebi-fl187-GCST90094765 | Weighted mode | 4 | -0.32450687 | 0.392272887008734 | 0.468756103014222 | -1.093361728 | 0.444347988803464 | 0.722883741614042 | 0.335088122915397 | 1.55947307037753 | 0.999228396881742 |
| ebi-fl187-GCST90094765 | ebi-a-GCST90001949 | CD33 on CD33dim HLA DR+ CD11b- \|\| id:ebi-a-GCST90001949 | Fried fish liking \|\| id:ebi-fl187-GCST90094765 | Weighted mode | 4 | -0.364991776 | 0.778455666040744 | 0.671133461478549 | -1.890764882 | 1.1607813293719 | 0.694202359927535 | 0.150956300983981 | 3.19242663862105 | 0.999228396881742 |
| ebi-fl187-GCST90094765 | ebi-a-GCST90001955 | CD33 on Immature Myeloid-Derived Suppressor Cells \|\| id:ebi-a-GCST90001955 | Fried fish liking \|\| id:ebi-fl187-GCST90094765 | Weighted mode | 4 | -0.190140335 | 0.607208004920431 | 0.774682885937579 | -1.380268024 | 0.999987354788308 | 0.826843090895232 | 0.25151113287945 | 2.71824745542721 | 0.999228396881742 |
| ebi-fl187-GCST90094765 | ebi-a-GCST90001956 | CD33 on CD33+ HLA DR+ \|\| id:ebi-a-GCST90001956 | Fried fish liking \|\| id:ebi-fl187-GCST90094765 | Weighted mode | 4 | -0.18465224 | 0.53964422411733 | 0.754788972812345 | -1.242354919 | 0.873050439234179 | 0.831393358909587 | 0.288703543239006 | 2.394203097 | 0.999228396881742 |
| ebi-fl187-GCST90094765 | ebi-a-GCST90001957 | CD33 on CD33+ HLA DR+ CD14- \|\| id:ebi-a-GCST90001957 | Fried fish liking \|\| id:ebi-fl187-GCST90094765 | Weighted mode | 4 | -0.213924643 | 0.527000866219162 | 0.712013183988926 | -1.246846341 | 0.818997054346609 | 0.807409226309685 | 0.287409761449462 | 2.26822379115553 | 0.999228396881742 |
| ebi-fl187-GCST90094765 | ebi-a-GCST90002064 | CD4 on resting CD4 regulatory T cell \|\| id:ebi-a-GCST90002064 | Fried fish liking \|\| id:ebi-fl187-GCST90094765 | Weighted mode | 4 | -0.598404894 | 0.353187563323289 | 0.188781674714026 | -1.290652518 | 0.0938427302453305 | 0.549687747459443 | 0.275091222543491 | 1.0983869892805 | 0.999228396881742 |
| ebi-fl187-GCST90094765 | ebi-a-GCST90002080 | SSC-A on T cell \|\| id:ebi-a-GCST90002080 | Fried fish liking \|\| id:ebi-fl187-GCST90094765 | Weighted mode | 4 | 0.267509466142333 | 0.529632854536972 | 0.648268077401217 | -0.770570929 | 1.3055898610348 | 1.30670599924252 | 0.46274879628627 | 3.68986495947597 | 0.999228396881742 |
| ebi-fl187-GCST90094765 | ebi-a-GCST90002121 | CD8 on CD39+ CD8+ T cell \|\| id:ebi-a-GCST90002121 | Fried fish liking \|\| id:ebi-fl187-GCST90094765 | Weighted mode | 4 | 0.273219977900393 | 0.380184111861178 | 0.524271702571806 | -0.471940881 | 1.0183808371483 | 1.31418930563425 | 0.623790389474953 | 2.76870814328694 | 0.999228396881742 |
| ebi-fl187-GCST90094765 | ebi-cfmet338-GCST90026053 | 1-palmitoyl-2-docosahexaenoyl-gpc (16:0/22:6) levels \|\| id:ebi-cfmet338-GCST90026053 | Fried fish liking \|\| id:ebi-fl187-GCST90094765 | Weighted mode | 4 | -0.179360264 | 0.199665389173664 | 0.435224524531982 | -0.570704427 | 0.211983898350744 | 0.835804734434842 | 0.565127207471484 | 1.23612798121907 | 0.999228396881742 |
| ebi-fl187-GCST90094765 | ebi-cfmet338-GCST90026063 | 1-stearoyl-2-oleoyl-gpc (18:0/18:1) levels \|\| id:ebi-cfmet338-GCST90026063 | Fried fish liking \|\| id:ebi-fl187-GCST90094765 | Weighted mode | 3 | -0.17390316 | 0.155766714967539 | 0.380373151972218 | -0.479205922 | 0.131399600935428 | 0.840378275613326 | 0.619274949388644 | 1.14042340453143 | 0.999228396881742 |
| ebi-fl187-GCST90094765 | ebi-cfmet338-GCST90026113 | Adenosine 3',5'-cyclic monophosphate (camp) levels \|\| id:ebi-cfmet338-GCST90026113 | Fried fish liking \|\| id:ebi-fl187-GCST90094765 | Weighted mode | 4 | -0.599880382 | 0.30863889878119 | 0.147187590909024 | -1.204812624 | 0.00505185961987587 | 0.548877287775595 | 0.299748159981917 | 1.00506464177818 | 0.999228396881742 |
| ebi-fl187-GCST90094765 | ebi-cfmet338-GCST90026117 | Alpha-ketoglutaramate levels \|\| id:ebi-cfmet338-GCST90026117 | Fried fish liking \|\| id:ebi-fl187-GCST90094765 | Weighted mode | 4 | 0.0246990554204031 | 0.174165732818241 | 0.896215249670103 | -0.316665781 | 0.366063891744156 | 1.02500660392206 | 0.728574217855423 | 1.44204737463317 | 0.999228396881742 |
| ebi-fl187-GCST90094765 | ebi-cfmet338-GCST90026124 | Arginine levels \|\| id:ebi-cfmet338-GCST90026124 | Fried fish liking \|\| id:ebi-fl187-GCST90094765 | Weighted mode | 4 | -0.133836598 | 0.111530844045969 | 0.316263362439402 | -0.352437053 | 0.0847638561353771 | 0.874732985875247 | 0.702972818782085 | 1.08846000319597 | 0.999228396881742 |
| ebi-fl187-GCST90094765 | ebi-cfmet338-GCST90026169 | Glutamate levels \|\| id:ebi-cfmet338-GCST90026169 | Fried fish liking \|\| id:ebi-fl187-GCST90094765 | Weighted mode | 4 | -0.107888121 | 0.106932189932723 | 0.387322222585522 | -0.317475213 | 0.101698971503463 | 0.897728027937526 | 0.727984725084173 | 1.10705016791584 | 0.999228396881742 |
| ebi-fl187-GCST90094765 | ebi-cfmet338-GCST90026170 | Glutamine levels \|\| id:ebi-cfmet338-GCST90026170 | Fried fish liking \|\| id:ebi-fl187-GCST90094765 | Weighted mode | 4 | -0.057889205 | 0.0746364935113724 | 0.494505583439192 | -0.204176732 | 0.0883983224349603 | 0.943754505068925 | 0.815318265520234 | 1.09242317203525 | 0.999228396881742 |
| ebi-fl187-GCST90094765 | ebi-cfmet338-GCST90026191 | Kynurenate levels \|\| id:ebi-cfmet338-GCST90026191 | Fried fish liking \|\| id:ebi-fl187-GCST90094765 | Weighted mode | 4 | -0.940942515 | 0.53816302769326 | 0.178707578289228 | -1.995742049 | 0.113857019422817 | 0.390259836270085 | 0.135912762790268 | 1.12059189055393 | 0.999228396881742 |
| ebi-fl187-GCST90094765 | ebi-cfmet338-GCST90026202 | Methylsuccinoylcarnitine levels \|\| id:ebi-cfmet338-GCST90026202 | Fried fish liking \|\| id:ebi-fl187-GCST90094765 | Weighted mode | 4 | -0.385006767 | 0.363050087606468 | 0.366742870181401 | -1.096584939 | 0.326571404668614 | 0.680446031583452 | 0.334009802127569 | 1.38620722789695 | 0.999228396881742 |
| ebi-fl187-GCST90094765 | ebi-cfmet338-GCST90026222 | N-acetylputrescine levels \|\| id:ebi-cfmet338-GCST90026222 | Fried fish liking \|\| id:ebi-fl187-GCST90094765 | Weighted mode | 4 | 0.0351151746876748 | 0.128592155972945 | 0.802515615133805 | -0.216925451 | 0.287155800394646 | 1.03573899284525 | 0.804989978250526 | 1.33263182186629 | 0.999228396881742 |
| ebi-fl187-GCST90094765 | ebi-cfmet338-GCST90026252 | Propionylcarnitine (c3) levels \|\| id:ebi-cfmet338-GCST90026252 | Fried fish liking \|\| id:ebi-fl187-GCST90094765 | Weighted mode | 4 | 0.161459152795793 | 0.227075579994544 | 0.528358488431272 | -0.283608984 | 0.606527289585099 | 1.17522445254473 | 0.753061046067498 | 1.83405119820163 | 0.999228396881742 |
| ebi-fl187-GCST90094765 | ebi-cfmet338-GCST90026316 | Ethyl beta-glucopyranoside levels \|\| id:ebi-cfmet338-GCST90026316 | Fried fish liking \|\| id:ebi-fl187-GCST90094765 | Weighted mode | 4 | 0.699138751051267 | 0.890748692823433 | 0.489794544239452 | -1.046728687 | 2.4450061889852 | 2.01201911170408 | 0.351084379531087 | 11.5306209614604 | 0.999228396881742 |
| ebi-fl187-GCST90094765 | ebi-gm430-GCST90011315 | F_Prevotellaceae abundance \|\| id:ebi-gm430-GCST90011315 | Fried fish liking \|\| id:ebi-fl187-GCST90094765 | Weighted mode | 3 | 0.142713599865023 | 0.267688680867168 | 0.647251326948843 | -0.381956215 | 0.667383414364673 | 1.15339942060793 | 0.682524937154895 | 1.94913057536652 | 0.999228396881742 |
| ebi-fl187-GCST90094765 | ebi-gm430-GCST90011354 | OTU97_1 (Bacteroides) abundance \|\| id:ebi-gm430-GCST90011354 | Fried fish liking \|\| id:ebi-fl187-GCST90094765 | Weighted mode | 3 | -0.047838573 | 0.269157874708054 | 0.875303818640631 | -0.575388008 | 0.479710860984094 | 0.953287660601756 | 0.562486577240702 | 1.61560719957712 | 0.999228396881742 |
| ebi-fl187-GCST90094765 | ebi-gm430-GCST90011419 | OTU99_1 (Bacteroides) abundance \|\| id:ebi-gm430-GCST90011419 | Fried fish liking \|\| id:ebi-fl187-GCST90094765 | Weighted mode | 3 | -0.054966627 | 0.250474800157439 | 0.84666076251973 | -0.545897235 | 0.435963981558287 | 0.946516735765762 | 0.57932176228868 | 1.54645309291565 | 0.999228396881742 |
| ebi-fl187-GCST90094765 | ebi-gm430-GCST90011435 | OTU99_155 (Butyrivibrio) abundance \|\| id:ebi-gm430-GCST90011435 | Fried fish liking \|\| id:ebi-fl187-GCST90094765 | Weighted mode | 3 | -0.527283414 | 0.746839561286063 | 0.553336702172229 | -1.991088954 | 0.936522125999457 | 0.590206139500126 | 0.136546651400901 | 2.55109358984503 | 0.999228396881742 |
| ebi-fl187-GCST90094765 | ebi-gm430-GCST90011466 | OTU99_58 (Bacteroides) abundance \|\| id:ebi-gm430-GCST90011466 | Fried fish liking \|\| id:ebi-fl187-GCST90094765 | Weighted mode | 3 | 0.294425489611627 | 0.560529066386812 | 0.651822655882019 | -0.804211481 | 1.39306245972978 | 1.34235494025548 | 0.447440603132649 | 4.02716421579224 | 0.999228396881742 |
| ebi-fl187-GCST90094765 | ebi-gm430-GCST90011505 | TestASV_28 (Holdemanella) abundance \|\| id:ebi-gm430-GCST90011505 | Fried fish liking \|\| id:ebi-fl187-GCST90094765 | Weighted mode | 3 | 0.109839687740478 | 0.601652458330902 | 0.871970595206962 | -1.069399131 | 1.28907850606905 | 1.11609913174256 | 0.343214682677844 | 3.62944050690786 | 0.999228396881742 |
| ebi-fl187-GCST90094765 | ebi-gm430-GCST90011513 | TestASV_35 (Alphaproteobacteria) abundance \|\| id:ebi-gm430-GCST90011513 | Fried fish liking \|\| id:ebi-fl187-GCST90094765 | Weighted mode | 3 | -0.165948382 | 0.660684884942446 | 0.825128267534976 | -1.460890756 | 1.12899399282683 | 0.847089958438082 | 0.232029500946539 | 3.09254381343501 | 0.999228396881742 |
| ebi-fl187-GCST90094765 | ebi-gm430-GCST90011543 | G_Coprococcus prevalence \|\| id:ebi-gm430-GCST90011543 | Fried fish liking \|\| id:ebi-fl187-GCST90094765 | Weighted mode | 3 | -0.410446826 | 0.848527752785323 | 0.676368299573399 | -2.073561222 | 1.25266756900352 | 0.663353779887525 | 0.125737205347934 | 3.49966611770488 | 0.999228396881742 |
| ebi-fl187-GCST90094765 | ebi-gm430-GCST90011547 | G_Parasutterella prevalence \|\| id:ebi-gm430-GCST90011547 | Fried fish liking \|\| id:ebi-fl187-GCST90094765 | Weighted mode | 3 | -0.421360599 | 0.77606900580773 | 0.641588060993866 | -1.942455851 | 1.0997346520072 | 0.656153450218925 | 0.143351467322866 | 3.0033689802736 | 0.999228396881742 |
| ebi-fl187-GCST90094765 | ebi-gm430-GCST90011569 | OTU97_13 (Subdoligranulum) prevalence \|\| id:ebi-gm430-GCST90011569 | Fried fish liking \|\| id:ebi-fl187-GCST90094765 | Weighted mode | 3 | 0.252267415270503 | 0.681774161946946 | 0.746879506149455 | -1.084009942 | 1.58854477268652 | 1.28694013871912 | 0.338236493874675 | 4.89661804873145 | 0.999228396881742 |
| ebi-fl187-GCST90094765 | ebi-gm430-GCST90011590 | OTU97_277 (Prevotella) prevalence \|\| id:ebi-gm430-GCST90011590 | Fried fish liking \|\| id:ebi-fl187-GCST90094765 | Weighted mode | 3 | 0.682395184571506 | 0.728541279710261 | 0.447812131378728 | -0.745545724 | 2.11033609280362 | 1.97861120022252 | 0.474475296878411 | 8.25101392507108 | 0.999228396881742 |
| ebi-fl187-GCST90094765 | ebi-gm430-GCST90011616 | OTU97_86 (Phascolarctobacterium) prevalence \|\| id:ebi-gm430-GCST90011616 | Fried fish liking \|\| id:ebi-fl187-GCST90094765 | Weighted mode | 3 | 0.915327474251391 | 0.888067370916599 | 0.411014923062625 | -0.825284573 | 2.65593952124793 | 2.49759301540233 | 0.438110300472938 | 14.2383570161502 | 0.999228396881742 |
| ebi-fl187-GCST90094765 | ebi-gm430-GCST90011665 | OTU99_55 (Barnesiella) prevalence \|\| id:ebi-gm430-GCST90011665 | Fried fish liking \|\| id:ebi-fl187-GCST90094765 | Weighted mode | 3 | 0.836615922624668 | 0.473618841745027 | 0.21936130789936 | -0.091677007 | 1.76490885244492 | 2.30854146024686 | 0.912399800527776 | 5.84103993731253 | 0.999228396881742 |
| ebi-fl187-GCST90094765 | ebi-gm430-GCST90011687 | TestASV_14 (Lachnospiraceae) prevalence \|\| id:ebi-gm430-GCST90011687 | Fried fish liking \|\| id:ebi-fl187-GCST90094765 | Weighted mode | 3 | 0.695904819713409 | 0.535274702634986 | 0.323221885816803 | -0.353233597 | 1.74504323687798 | 2.00552288987567 | 0.702413092303166 | 5.72614905087688 | 0.999228396881742 |
| ebi-fl187-GCST90094765 | ebi-gm430-GCST90011697 | TestASV_23 (Barnesiella) prevalence \|\| id:ebi-gm430-GCST90011697 | Fried fish liking \|\| id:ebi-fl187-GCST90094765 | Weighted mode | 3 | 0.774766521718133 | 0.728738100011704 | 0.399095460877634 | -0.653560154 | 2.20309319774107 | 2.17008540022013 | 0.520190517705402 | 9.05297287044271 | 0.999228396881742 |
| ebi-fl187-GCST90094765 | ebi-gm430-GCST90011727 | TestASV_6 (Subdoligranulum) prevalence \|\| id:ebi-gm430-GCST90011727 | Fried fish liking \|\| id:ebi-fl187-GCST90094765 | Weighted mode | 3 | 1.07521337338814 | 0.806930916175578 | 0.314240200263927 | -0.506371222 | 2.65679796909227 | 2.93061814974952 | 0.60267860222582 | 14.2505851508947 | 0.999228396881742 |
| ebi-fl187-GCST90094765 | ebi-hbsm44-GCST90104908 | Sulcal depth of the left posterior inferior temporal sulcus \|\| id:ebi-hbsm44-GCST90104908 | Fried fish liking \|\| id:ebi-fl187-GCST90094765 | Weighted mode | 3 | 0.129571620957853 | 0.27904144922713 | 0.688043628322817 | -0.41734962 | 0.676492861443027 | 1.13834063759009 | 0.658790553634221 | 1.96696719471858 | 0.999228396881742 |
| ebi-fl187-GCST90094765 | ebi-hbsm44-GCST90104918 | Sulcal depth of the left inferior frontal sulcus \|\| id:ebi-hbsm44-GCST90104918 | Fried fish liking \|\| id:ebi-fl187-GCST90094765 | Weighted mode | 3 | 0.253989040138216 | 0.442160969209971 | 0.623677847047955 | -0.61264646 | 1.12062453978976 | 1.28915767520044 | 0.541914814066829 | 3.06676892454033 | 0.999228396881742 |
| ebi-fl187-GCST90094765 | ebi-hbsm44-GCST90104925 | Sulcal depth of the right anterior inferior temporal sulcus \|\| id:ebi-hbsm44-GCST90104925 | Fried fish liking \|\| id:ebi-fl187-GCST90094765 | Weighted mode | 3 | 0.049549115660278 | 0.153233957146469 | 0.777104998690803 | -0.25078944 | 0.349887671667358 | 1.05079720154577 | 0.778186208927858 | 1.41890815605393 | 0.999228396881742 |
| ebi-fl187-GCST90094765 | ebi-IPs91-GCST90274770 | C-C motif chemokine 4 levels \|\| id:ebi-IPs91-GCST90274770 | Fried fish liking \|\| id:ebi-fl187-GCST90094765 | Weighted mode | 5 | -0.095138527 | 0.158696498406942 | 0.581143619045804 | -0.406183664 | 0.215906609850622 | 0.90924697046411 | 0.666187801535311 | 1.24098647767621 | 0.999228396881742 |
| ebi-fl187-GCST90094765 | ebi-IPs91-GCST90274779 | C-X-C motif chemokine 1 levels \|\| id:ebi-IPs91-GCST90274779 | Fried fish liking \|\| id:ebi-fl187-GCST90094765 | Weighted mode | 5 | -0.11530241 | 0.171776764707595 | 0.538841965557805 | -0.451984869 | 0.22138004905735 | 0.891096626831033 | 0.636363798729698 | 1.24779756474948 | 0.999228396881742 |
| ebi-fl187-GCST90094765 | ebi-IPs91-GCST90274780 | C-X-C motif chemokine 10 levels \|\| id:ebi-IPs91-GCST90274780 | Fried fish liking \|\| id:ebi-fl187-GCST90094765 | Weighted mode | 5 | -0.146288015 | 0.220362775312112 | 0.543094278779436 | -0.578199055 | 0.285623024220282 | 0.863908848304217 | 0.560907621260698 | 1.33059076020548 | 0.999228396881742 |
| ebi-fl187-GCST90094765 | ebi-IPs91-GCST90274796 | Interleukin-10 receptor subunit alpha levels \|\| id:ebi-IPs91-GCST90274796 | Fried fish liking \|\| id:ebi-fl187-GCST90094765 | Weighted mode | 5 | -0.122972261 | 0.203633245887514 | 0.578491735258016 | -0.522093423 | 0.276148900907842 | 0.884288191462739 | 0.593277266808934 | 1.3180441073807 | 0.999228396881742 |
| ebi-fl187-GCST90094765 | ebi-IPs91-GCST90274807 | Interleukin-20 levels \|\| id:ebi-IPs91-GCST90274807 | Fried fish liking \|\| id:ebi-fl187-GCST90094765 | Weighted mode | 5 | -0.246418764 | 0.27543601439344 | 0.421542445311578 | -0.786273352 | 0.293435824269297 | 0.781594852642989 | 0.45553927040655 | 1.34102711525358 | 0.999228396881742 |
| ebi-fl187-GCST90094765 | ebi-IPs91-GCST90274808 | Interleukin-20 receptor subunit alpha levels \|\| id:ebi-IPs91-GCST90274808 | Fried fish liking \|\| id:ebi-fl187-GCST90094765 | Weighted mode | 5 | 0.0169940313948152 | 0.241878763507818 | 0.947360268399385 | -0.457088345 | 0.491076407870138 | 1.01713925140451 | 0.633124404165508 | 1.63407420396526 | 0.999228396881742 |
| ebi-fl187-GCST90094765 | ebi-IPs91-GCST90274810 | Interleukin-24 levels \|\| id:ebi-IPs91-GCST90274810 | Fried fish liking \|\| id:ebi-fl187-GCST90094765 | Weighted mode | 5 | 0.0411343410003686 | 0.182762307637553 | 0.832955462425895 | -0.317079782 | 0.399348463969973 | 1.04199207840141 | 0.728272649781713 | 1.49085303667071 | 0.999228396881742 |
| ebi-fl187-GCST90094765 | ebi-IPs91-GCST90274823 | Monocyte chemoattractant protein-3 levels \|\| id:ebi-IPs91-GCST90274823 | Fried fish liking \|\| id:ebi-fl187-GCST90094765 | Weighted mode | 5 | -0.12408879 | 0.202622218874859 | 0.573367023949289 | -0.521228339 | 0.27305075938872 | 0.883301409416728 | 0.593790723759586 | 1.31396693929068 | 0.999228396881742 |
| ebi-fl187-GCST90094765 | ebi-IPs91-GCST90274824 | Monocyte chemoattractant protein-4 levels \|\| id:ebi-IPs91-GCST90274824 | Fried fish liking \|\| id:ebi-fl187-GCST90094765 | Weighted mode | 5 | -0.029193875 | 0.180644329333277 | 0.879447751135013 | -0.38325676 | 0.324869010816801 | 0.971228149669211 | 0.681637859362906 | 1.38384936481011 | 0.999228396881742 |
| ebi-fl187-GCST90094765 | ebi-IPs91-GCST90274834 | SIR2-like protein 2 levels \|\| id:ebi-IPs91-GCST90274834 | Fried fish liking \|\| id:ebi-fl187-GCST90094765 | Weighted mode | 5 | -0.139864433 | 0.162863355714204 | 0.438876916010658 | -0.45907661 | 0.179347743962798 | 0.869476099469675 | 0.63186683545087 | 1.19643672548435 | 0.999228396881742 |
| ebi-fl187-GCST90094765 | ebi-met1400-GCST90199652 | 1-stearoyl-2-oleoyl-GPS (18:0/18:1) levels \|\| id:ebi-met1400-GCST90199652 | Fried fish liking \|\| id:ebi-fl187-GCST90094765 | Weighted mode | 4 | -0.011530167 | 0.248579883429378 | 0.965919060099899 | -0.498746739 | 0.475686404247906 | 0.988536050360524 | 0.607291277584818 | 1.6091183241569 | 0.999228396881742 |
| ebi-fl187-GCST90094765 | ebi-met1400-GCST90199665 | N-acetylaspartate (naa) levels \|\| id:ebi-met1400-GCST90199665 | Fried fish liking \|\| id:ebi-fl187-GCST90094765 | Weighted mode | 4 | 0.11744627888249 | 0.220295970694456 | 0.630910876412674 | -0.314333824 | 0.549226381443625 | 1.12462121241259 | 0.730275204309477 | 1.7319126596997 | 0.999228396881742 |
| ebi-fl187-GCST90094765 | ebi-met1400-GCST90199669 | Acetylcarnitine levels (Biocrates platform) \|\| id:ebi-met1400-GCST90199669 | Fried fish liking \|\| id:ebi-fl187-GCST90094765 | Weighted mode | 4 | -0.212882225 | 0.243537854219925 | 0.446389084697223 | -0.69021642 | 0.264451968782944 | 0.808251323016201 | 0.501467529839427 | 1.30271685061368 | 0.999228396881742 |
| ebi-fl187-GCST90094765 | ebi-met1400-GCST90199670 | Iminodiacetate (IDA) levels \|\| id:ebi-met1400-GCST90199670 | Fried fish liking \|\| id:ebi-fl187-GCST90094765 | Weighted mode | 4 | 0.416903191460482 | 0.232403674765836 | 0.17072428692564 | -0.038608011 | 0.87241439400152 | 1.51725562252418 | 0.962127778666329 | 2.39268075938136 | 0.999228396881742 |
| ebi-fl187-GCST90094765 | ebi-met1400-GCST90199686 | Indolepropionate levels \|\| id:ebi-met1400-GCST90199686 | Fried fish liking \|\| id:ebi-fl187-GCST90094765 | Weighted mode | 4 | 0.0301988663224763 | 0.216923603549761 | 0.89810104858366 | -0.394971397 | 0.455369129280009 | 1.03065947703463 | 0.673699309040475 | 1.57675530217513 | 0.999228396881742 |
| ebi-fl187-GCST90094765 | ebi-met1400-GCST90199704 | 3-hydroxysebacate levels \|\| id:ebi-met1400-GCST90199704 | Fried fish liking \|\| id:ebi-fl187-GCST90094765 | Weighted mode | 4 | 0.0163957164856503 | 0.204527683462973 | 0.941155165166345 | -0.384478543 | 0.417269976073078 | 1.01653086384752 | 0.680805554411968 | 1.5178122306113 | 0.999228396881742 |
| ebi-fl187-GCST90094765 | ebi-met1400-GCST90199713 | Docosapentaenoate n3 DPA; 22:5n3 levels \|\| id:ebi-met1400-GCST90199713 | Fried fish liking \|\| id:ebi-fl187-GCST90094765 | Weighted mode | 4 | -0.305487875 | 0.280854604041109 | 0.356300930004797 | -0.855962899 | 0.244987149183063 | 0.73676383823027 | 0.424873883560732 | 1.27760489483277 | 0.999228396881742 |
| ebi-fl187-GCST90094765 | ebi-met1400-GCST90199715 | Myristoleate (14:1n5) levels \|\| id:ebi-met1400-GCST90199715 | Fried fish liking \|\| id:ebi-fl187-GCST90094765 | Weighted mode | 4 | -0.071296081 | 0.231079510778293 | 0.777859313272431 | -0.524211922 | 0.381619759824054 | 0.931186144502238 | 0.59202173962855 | 1.46465505854057 | 0.999228396881742 |
| ebi-fl187-GCST90094765 | ebi-met1400-GCST90199725 | Campesterol levels \|\| id:ebi-met1400-GCST90199725 | Fried fish liking \|\| id:ebi-fl187-GCST90094765 | Weighted mode | 4 | 0.389152429900323 | 0.284687240038466 | 0.265063641391801 | -0.168834561 | 0.947139420375716 | 1.47572947948719 | 0.844648630011186 | 2.57832360019183 | 0.999228396881742 |
| ebi-fl187-GCST90094765 | ebi-met1400-GCST90199737 | N-acetylproline levels \|\| id:ebi-met1400-GCST90199737 | Fried fish liking \|\| id:ebi-fl187-GCST90094765 | Weighted mode | 4 | -0.295564582 | 0.253704126475128 | 0.328233476653866 | -0.79282467 | 0.201695506092984 | 0.744111357121756 | 0.452564642500926 | 1.22347541057949 | 0.999228396881742 |
| ebi-fl187-GCST90094765 | ebi-met1400-GCST90199775 | Gamma-glutamylisoleucine levels \|\| id:ebi-met1400-GCST90199775 | Fried fish liking \|\| id:ebi-fl187-GCST90094765 | Weighted mode | 4 | 0.070534058285827 | 0.212046404168196 | 0.76129777390198 | -0.345076894 | 0.486145010455492 | 1.07308111611172 | 0.708165897747892 | 1.62603577130384 | 0.999228396881742 |
| ebi-fl187-GCST90094765 | ebi-met1400-GCST90199777 | 1-palmitoleoyl-GPC (16:1) levels \|\| id:ebi-met1400-GCST90199777 | Fried fish liking \|\| id:ebi-fl187-GCST90094765 | Weighted mode | 4 | -0.241015503 | 0.198940864647898 | 0.312428852691963 | -0.630939598 | 0.148908591296568 | 0.785829443255253 | 0.532091613774534 | 1.16056689844492 | 0.999228396881742 |
| ebi-fl187-GCST90094765 | ebi-met1400-GCST90199782 | Glutamine degradant levels \|\| id:ebi-met1400-GCST90199782 | Fried fish liking \|\| id:ebi-fl187-GCST90094765 | Weighted mode | 4 | -0.319923399 | 0.224463749848849 | 0.249305507137352 | -0.759872349 | 0.120025550727469 | 0.726204662963781 | 0.46772612905707 | 1.12752566031219 | 0.999228396881742 |
| ebi-fl187-GCST90094765 | ebi-met1400-GCST90199783 | 5-methyluridine (ribothymidine) levels \|\| id:ebi-met1400-GCST90199783 | Fried fish liking \|\| id:ebi-fl187-GCST90094765 | Weighted mode | 4 | -0.275824055 | 0.27042187899101 | 0.382824088830988 | -0.805850938 | 0.254202827599732 | 0.758946451679206 | 0.446707644254462 | 1.28943331040994 | 0.999228396881742 |
| ebi-fl187-GCST90094765 | ebi-met1400-GCST90199787 | Homostachydrine levels \|\| id:ebi-met1400-GCST90199787 | Fried fish liking \|\| id:ebi-fl187-GCST90094765 | Weighted mode | 4 | -0.467996853 | 0.21370930999548 | 0.11626790038042 | -0.8868671 | -0.049126605 | 0.626255494565113 | 0.411944313384441 | 0.952060586176809 | 0.999228396881742 |
| ebi-fl187-GCST90094765 | ebi-met1400-GCST90199802 | Beta-hydroxyisovaleroylcarnitine levels \|\| id:ebi-met1400-GCST90199802 | Fried fish liking \|\| id:ebi-fl187-GCST90094765 | Weighted mode | 4 | 0.315862457131845 | 0.216016387259986 | 0.239851921460331 | -0.107529662 | 0.739254576161418 | 1.37144161064032 | 0.898049884191915 | 2.09437373636337 | 0.999228396881742 |
| ebi-fl187-GCST90094765 | ebi-met1400-GCST90199873 | Pregnenolone sulfate levels \|\| id:ebi-met1400-GCST90199873 | Fried fish liking \|\| id:ebi-fl187-GCST90094765 | Weighted mode | 4 | 0.0429091085227791 | 0.311421291542489 | 0.899138594273517 | -0.567476623 | 0.653294839946058 | 1.04384301410521 | 0.5669542746285 | 1.92186263841159 | 0.999228396881742 |
| ebi-fl187-GCST90094765 | ebi-met1400-GCST90199911 | Beta-citrylglutamate levels \|\| id:ebi-met1400-GCST90199911 | Fried fish liking \|\| id:ebi-fl187-GCST90094765 | Weighted mode | 4 | 0.335479660957017 | 0.217800610462182 | 0.221128672530602 | -0.091409536 | 0.762368857462894 | 1.39861108348652 | 0.912643874244838 | 2.14334749627276 | 0.999228396881742 |
| ebi-fl187-GCST90094765 | ebi-met1400-GCST90199934 | 2-hydroxydecanoate levels \|\| id:ebi-met1400-GCST90199934 | Fried fish liking \|\| id:ebi-fl187-GCST90094765 | Weighted mode | 4 | -0.186562388 | 0.224464434826703 | 0.466860197321869 | -0.62651268 | 0.253387904394778 | 0.829806790461922 | 0.534452361278153 | 1.28838294932399 | 0.999228396881742 |
| ebi-fl187-GCST90094765 | ebi-met1400-GCST90199945 | Sphingomyelin (d18:1/14:0, d16:1/16:0) levels \|\| id:ebi-met1400-GCST90199945 | Fried fish liking \|\| id:ebi-fl187-GCST90094765 | Weighted mode | 4 | -0.240738884 | 0.2005918320287 | 0.316214124876824 | -0.633898875 | 0.152421106348795 | 0.786046848666748 | 0.530519334809122 | 1.16465057493377 | 0.999228396881742 |
| ebi-fl187-GCST90094765 | ebi-met1400-GCST90199979 | Carnitine C14:1 levels \|\| id:ebi-met1400-GCST90199979 | Fried fish liking \|\| id:ebi-fl187-GCST90094765 | Weighted mode | 4 | 0.244582365854639 | 0.429777849119385 | 0.60911368040792 | -0.597782218 | 1.08694695012863 | 1.2770878463244 | 0.550030131110264 | 2.96520731316542 | 0.999228396881742 |
| ebi-fl187-GCST90094765 | ebi-met1400-GCST90199986 | Eugenol sulfate levels \|\| id:ebi-met1400-GCST90199986 | Fried fish liking \|\| id:ebi-fl187-GCST90094765 | Weighted mode | 4 | 0.241508309825104 | 0.285479835527877 | 0.459706231532434 | -0.318032168 | 0.801048787459743 | 1.27316803467727 | 0.727579383403127 | 2.22787627233502 | 0.999228396881742 |
| ebi-fl187-GCST90094765 | ebi-met1400-GCST90200002 | 6-hydroxyindole sulfate levels \|\| id:ebi-met1400-GCST90200002 | Fried fish liking \|\| id:ebi-fl187-GCST90094765 | Weighted mode | 4 | -0.126305536 | 0.405819229051276 | 0.775994977018232 | -0.921711225 | 0.66910015281369 | 0.881345522743998 | 0.397837668468831 | 1.95247959664168 | 0.999228396881742 |
| ebi-fl187-GCST90094765 | ebi-met1400-GCST90200023 | Trans 3,4-methyleneheptanoate levels \|\| id:ebi-met1400-GCST90200023 | Fried fish liking \|\| id:ebi-fl187-GCST90094765 | Weighted mode | 4 | -0.099051823 | 0.22426052097215 | 0.68861709160874 | -0.538602444 | 0.340498799 | 0.905695771348033 | 0.583563245261237 | 1.40564855120459 | 0.999228396881742 |
| ebi-fl187-GCST90094765 | ebi-met1400-GCST90200032 | 2-hydroxybutyrate/2-hydroxyisobutyrate levels \|\| id:ebi-met1400-GCST90200032 | Fried fish liking \|\| id:ebi-fl187-GCST90094765 | Weighted mode | 4 | 0.0269693739560318 | 0.218279258072282 | 0.909481191388429 | -0.400857972 | 0.454796719777704 | 1.0273363390338 | 0.669745176941185 | 1.57585301072209 | 0.999228396881742 |
| ebi-fl187-GCST90094765 | ebi-met1400-GCST90200051 | 1-palmitoyl-2-dihomo-linolenoyl-GPC (16:0/20:3n3 or 6) levels \|\| id:ebi-met1400-GCST90200051 | Fried fish liking \|\| id:ebi-fl187-GCST90094765 | Weighted mode | 4 | 0.0979039456486571 | 0.203741700573888 | 0.663715934880066 | -0.301429787 | 0.497237678773477 | 1.10285684582168 | 0.739759764931378 | 1.64417325736628 | 0.999228396881742 |
| ebi-fl187-GCST90094765 | ebi-met1400-GCST90200069 | Phenylacetylglutamate levels \|\| id:ebi-met1400-GCST90200069 | Fried fish liking \|\| id:ebi-fl187-GCST90094765 | Weighted mode | 4 | -0.120516468 | 0.237139756507765 | 0.646319425098084 | -0.58531039 | 0.344277455220904 | 0.886462489373719 | 0.556932968396031 | 1.41097006221378 | 0.999228396881742 |
| ebi-fl187-GCST90094765 | ebi-met1400-GCST90200072 | Thioproline levels \|\| id:ebi-met1400-GCST90200072 | Fried fish liking \|\| id:ebi-fl187-GCST90094765 | Weighted mode | 4 | -0.09500769 | 0.219211295379706 | 0.693992183342288 | -0.524661829 | 0.334646448463686 | 0.909365940980269 | 0.591755444813422 | 1.39744622861166 | 0.999228396881742 |
| ebi-fl187-GCST90094765 | ebi-met1400-GCST90200081 | 1-oleoyl-2-docosahexaenoyl-GPC (18:1/22:6) levels \|\| id:ebi-met1400-GCST90200081 | Fried fish liking \|\| id:ebi-fl187-GCST90094765 | Weighted mode | 4 | -0.164381642 | 0.227668933048849 | 0.52247178159124 | -0.610612751 | 0.281849466919764 | 0.848418168201718 | 0.54301803246956 | 1.32557916145282 | 0.999228396881742 |
| ebi-fl187-GCST90094765 | ebi-met1400-GCST90200097 | Furaneol sulfate levels \|\| id:ebi-met1400-GCST90200097 | Fried fish liking \|\| id:ebi-fl187-GCST90094765 | Weighted mode | 4 | 0.212063425793424 | 0.311859862406957 | 0.545279815960967 | -0.399181905 | 0.823308756111059 | 1.23622629122536 | 0.670868656209784 | 2.27802481002917 | 0.999228396881742 |
| ebi-fl187-GCST90094765 | ebi-met1400-GCST90200124 | Sphingomyelin (d18:2/24:2) levels \|\| id:ebi-met1400-GCST90200124 | Fried fish liking \|\| id:ebi-fl187-GCST90094765 | Weighted mode | 4 | -0.302365675 | 0.232777476302477 | 0.284784321598562 | -0.758609529 | 0.153878178320565 | 0.739067756696431 | 0.468317156019956 | 1.16634879155488 | 0.999228396881742 |
| ebi-fl187-GCST90094765 | ebi-met1400-GCST90200137 | Lignoceroylcarnitine (C24) levels \|\| id:ebi-met1400-GCST90200137 | Fried fish liking \|\| id:ebi-fl187-GCST90094765 | Weighted mode | 4 | -0.217252015 | 0.265822576019833 | 0.473635007579685 | -0.738264264 | 0.303760234238727 | 0.804727140630837 | 0.477942779 | 1.35494414792519 | 0.999228396881742 |
| ebi-fl187-GCST90094765 | ebi-met1400-GCST90200165 | Octadecenedioate (C18:1-DC) levels \|\| id:ebi-met1400-GCST90200165 | Fried fish liking \|\| id:ebi-fl187-GCST90094765 | Weighted mode | 4 | -0.306466456 | 0.233561831292821 | 0.280839740813103 | -0.764247645 | 0.151314733297267 | 0.736043207571637 | 0.465684158842762 | 1.16336274946228 | 0.999228396881742 |
| ebi-fl187-GCST90094765 | ebi-met1400-GCST90200176 | Glutamine conjugate of C6H10O2 (1) levels \|\| id:ebi-met1400-GCST90200176 | Fried fish liking \|\| id:ebi-fl187-GCST90094765 | Weighted mode | 4 | 0.00626029023358821 | 0.190912630750743 | 0.975900638102466 | -0.367928466 | 0.380449046505045 | 1.00627992680599 | 0.692166693343858 | 1.46294137067011 | 0.999228396881742 |
| ebi-fl187-GCST90094765 | ebi-met1400-GCST90200182 | Glycine conjugate of C10H12O2 levels \|\| id:ebi-met1400-GCST90200182 | Fried fish liking \|\| id:ebi-fl187-GCST90094765 | Weighted mode | 4 | 0.0427308036205729 | 0.264536242053496 | 0.881940879159641 | -0.475760231 | 0.561221838045424 | 1.04365690837094 | 0.62141245995235 | 1.75281284587359 | 0.999228396881742 |
| ebi-fl187-GCST90094765 | ebi-met1400-GCST90200208 | Glucuronide of piperine metabolite C17H21NO3 (4) levels \|\| id:ebi-met1400-GCST90200208 | Fried fish liking \|\| id:ebi-fl187-GCST90094765 | Weighted mode | 4 | 0.0815644763056522 | 0.216134792758559 | 0.731008148223999 | -0.342059718 | 0.505188670112429 | 1.08498317103537 | 0.710305785756053 | 1.65729817359851 | 0.999228396881742 |
| ebi-fl187-GCST90094765 | ebi-met1400-GCST90200227 | 3-ethylcatechol sulfate (2) levels \|\| id:ebi-met1400-GCST90200227 | Fried fish liking \|\| id:ebi-fl187-GCST90094765 | Weighted mode | 4 | -0.00757794 | 0.286033115542485 | 0.980527727780939 | -0.568202847 | 0.553046966014657 | 0.992450699751869 | 0.566542688290938 | 1.73854223484774 | 0.999228396881742 |
| ebi-fl187-GCST90094765 | ebi-met1400-GCST90200259 | Pentose acid levels \|\| id:ebi-met1400-GCST90200259 | Fried fish liking \|\| id:ebi-fl187-GCST90094765 | Weighted mode | 4 | -0.229320843 | 0.20708557915157 | 0.348941875485967 | -0.635208578 | 0.176566892349316 | 0.795073399012437 | 0.52982496699967 | 1.19311423430445 | 0.999228396881742 |
| ebi-fl187-GCST90094765 | ebi-met1400-GCST90200291 | Sarcosine levels \|\| id:ebi-met1400-GCST90200291 | Fried fish liking \|\| id:ebi-fl187-GCST90094765 | Weighted mode | 4 | 0.285582333282117 | 0.207889899034364 | 0.263180622279075 | -0.121881869 | 0.69304653538947 | 1.33053661832068 | 0.885252938295594 | 1.99979871978816 | 0.999228396881742 |
| ebi-fl187-GCST90094765 | ebi-met1400-GCST90200306 | Butyrate/isobutyrate (4:0) levels \|\| id:ebi-met1400-GCST90200306 | Fried fish liking \|\| id:ebi-fl187-GCST90094765 | Weighted mode | 4 | 0.198452677775955 | 0.209649591132986 | 0.413680903064101 | -0.212460521 | 0.609365876396608 | 1.21951431591906 | 0.808592238229873 | 1.8392647077435 | 0.999228396881742 |
| ebi-fl187-GCST90094765 | ebi-met1400-GCST90200321 | Cholate levels \|\| id:ebi-met1400-GCST90200321 | Fried fish liking \|\| id:ebi-fl187-GCST90094765 | Weighted mode | 4 | 0.146040478818252 | 0.230445611610558 | 0.571273881076324 | -0.30563292 | 0.597713877574945 | 1.15724303093304 | 0.736656981921001 | 1.81795797163395 | 0.999228396881742 |
| ebi-fl187-GCST90094765 | ebi-met1400-GCST90200336 | Ceramide (d18:1/16:0) levels \|\| id:ebi-met1400-GCST90200336 | Fried fish liking \|\| id:ebi-fl187-GCST90094765 | Weighted mode | 4 | 0.164990114431092 | 0.324785746859059 | 0.646451445224083 | -0.471589949 | 0.801570178274848 | 1.17938145979707 | 0.624009335858666 | 2.22903816943552 | 0.999228396881742 |
| ebi-fl187-GCST90094765 | ebi-met1400-GCST90200358 | Arachidonate (20:4n6) levels \|\| id:ebi-met1400-GCST90200358 | Fried fish liking \|\| id:ebi-fl187-GCST90094765 | Weighted mode | 4 | -0.28835654 | 0.229485436604865 | 0.297859835561527 | -0.738147996 | 0.161434915734199 | 0.749494319881921 | 0.477998351253604 | 1.17519596890247 | 0.999228396881742 |
| ebi-fl187-GCST90094765 | ebi-met1400-GCST90200413 | 2'-deoxyuridine levels \|\| id:ebi-met1400-GCST90200413 | Fried fish liking \|\| id:ebi-fl187-GCST90094765 | Weighted mode | 4 | -0.147114489 | 0.240827780807829 | 0.584455883537041 | -0.619136939 | 0.324907961475843 | 0.86319514548968 | 0.538408916710476 | 1.38390326770465 | 0.999228396881742 |
| ebi-fl187-GCST90094765 | ebi-met1400-GCST90200432 | Threonine levels \|\| id:ebi-met1400-GCST90200432 | Fried fish liking \|\| id:ebi-fl187-GCST90094765 | Weighted mode | 4 | 0.0239246709009415 | 0.217227554458741 | 0.919255573006013 | -0.401841336 | 0.449690677640074 | 1.02421316192988 | 0.66908689738064 | 1.56782714648443 | 0.999228396881742 |
| ebi-fl187-GCST90094765 | ebi-met1400-GCST90200436 | Caffeine levels \|\| id:ebi-met1400-GCST90200436 | Fried fish liking \|\| id:ebi-fl187-GCST90094765 | Weighted mode | 4 | 0.130420256288302 | 0.228645604005314 | 0.608327583089809 | -0.317725128 | 0.578565640138718 | 1.13930708369539 | 0.727802813856341 | 1.78347844532338 | 0.999228396881742 |
| ebi-fl187-GCST90094765 | ebi-met1400-GCST90200442 | Valine levels \|\| id:ebi-met1400-GCST90200442 | Fried fish liking \|\| id:ebi-fl187-GCST90094765 | Weighted mode | 4 | 0.420086977510545 | 0.220106183789362 | 0.152340964884874 | -0.011321143 | 0.851495097737695 | 1.52209393780294 | 0.988742700267299 | 2.34314746887147 | 0.999228396881742 |
| ebi-fl187-GCST90094765 | ebi-met1400-GCST90200459 | Margarate (17:0) levels \|\| id:ebi-met1400-GCST90200459 | Fried fish liking \|\| id:ebi-fl187-GCST90094765 | Weighted mode | 4 | 0.0980884610940009 | 0.204555825088586 | 0.664356542978237 | -0.302840956 | 0.49901787826763 | 1.10306035871879 | 0.738716575408268 | 1.64710282059715 | 0.999228396881742 |
| ebi-fl187-GCST90094765 | ebi-met1400-GCST90200462 | X-11299 levels \|\| id:ebi-met1400-GCST90200462 | Fried fish liking \|\| id:ebi-fl187-GCST90094765 | Weighted mode | 4 | -0.003298196 | 0.215734332807034 | 0.988762127718473 | -0.426137488 | 0.41954109658817 | 0.996707237359112 | 0.653026552623601 | 1.5212632825003 | 0.999228396881742 |
| ebi-fl187-GCST90094765 | ebi-met1400-GCST90200497 | X-12740 levels \|\| id:ebi-met1400-GCST90200497 | Fried fish liking \|\| id:ebi-fl187-GCST90094765 | Weighted mode | 4 | 0.313987862264326 | 0.250280812681939 | 0.29848941123427 | -0.176562531 | 0.804538255120925 | 1.36887312142642 | 0.838146367727756 | 2.23566395407008 | 0.999228396881742 |
| ebi-fl187-GCST90094765 | ebi-met1400-GCST90200527 | X-13866 levels \|\| id:ebi-met1400-GCST90200527 | Fried fish liking \|\| id:ebi-fl187-GCST90094765 | Weighted mode | 4 | -0.152494732 | 0.223680751495756 | 0.544311504422687 | -0.590909005 | 0.285919541057987 | 0.858563416965002 | 0.553823627517027 | 1.33098536127001 | 0.999228396881742 |
| ebi-fl187-GCST90094765 | ebi-met1400-GCST90200547 | X-17654 levels \|\| id:ebi-met1400-GCST90200547 | Fried fish liking \|\| id:ebi-fl187-GCST90094765 | Weighted mode | 4 | 0.243727599808241 | 0.329207119185651 | 0.512766917777249 | -0.401518354 | 0.888973553412116 | 1.27599670139834 | 0.669303035335864 | 2.43263140314674 | 0.999228396881742 |
| ebi-fl187-GCST90094765 | ebi-met1400-GCST90200553 | X-17653 levels \|\| id:ebi-met1400-GCST90200553 | Fried fish liking \|\| id:ebi-fl187-GCST90094765 | Weighted mode | 3 | 0.412743172796693 | 0.28806562915674 | 0.288289779708928 | -0.15186546 | 0.977351805943902 | 1.5109569212578 | 0.859103856497911 | 2.65740957932996 | 0.999228396881742 |
| ebi-fl187-GCST90094765 | ebi-met1400-GCST90200557 | X-17685 levels \|\| id:ebi-met1400-GCST90200557 | Fried fish liking \|\| id:ebi-fl187-GCST90094765 | Weighted mode | 4 | 0.0453674818876148 | 0.240914220057148 | 0.86264893853018 | -0.426824389 | 0.517559353199625 | 1.04641232683916 | 0.652578141789143 | 1.67792741993917 | 0.999228396881742 |
| ebi-fl187-GCST90094765 | ebi-met1400-GCST90200561 | X-18779 levels \|\| id:ebi-met1400-GCST90200561 | Fried fish liking \|\| id:ebi-fl187-GCST90094765 | Weighted mode | 4 | -0.034389937 | 0.202246565324545 | 0.875799193421258 | -0.430793205 | 0.362013330935614 | 0.966194676022071 | 0.649993312155709 | 1.43621808796359 | 0.999228396881742 |
| ebi-fl187-GCST90094765 | ebi-met1400-GCST90200570 | X-18921 levels \|\| id:ebi-met1400-GCST90200570 | Fried fish liking \|\| id:ebi-fl187-GCST90094765 | Weighted mode | 4 | 0.132297645574099 | 0.243034525145116 | 0.624049144595136 | -0.344050024 | 0.608645314858527 | 1.14144801566009 | 0.708893465681157 | 1.83793988170342 | 0.999228396881742 |
| ebi-fl187-GCST90094765 | ebi-met1400-GCST90200575 | X-21283 levels \|\| id:ebi-met1400-GCST90200575 | Fried fish liking \|\| id:ebi-fl187-GCST90094765 | Weighted mode | 4 | -0.08169655 | 0.234774279426425 | 0.750841230853633 | -0.541854138 | 0.378461037434342 | 0.921551560870234 | 0.58166875781821 | 1.46003591894441 | 0.999228396881742 |
| ebi-fl187-GCST90094765 | ebi-met1400-GCST90200578 | X-18922 levels \|\| id:ebi-met1400-GCST90200578 | Fried fish liking \|\| id:ebi-fl187-GCST90094765 | Weighted mode | 4 | -0.385046453 | 0.347191982408983 | 0.348328266641343 | -1.065542739 | 0.295449832370677 | 0.680419027862643 | 0.344540808392179 | 1.34373067631095 | 0.999228396881742 |
| ebi-fl187-GCST90094765 | ebi-met1400-GCST90200623 | X-24241 levels \|\| id:ebi-met1400-GCST90200623 | Fried fish liking \|\| id:ebi-fl187-GCST90094765 | Weighted mode | 4 | 0.191863873188613 | 0.277479639420673 | 0.538985940976769 | -0.35199622 | 0.735723966453132 | 1.2115055873469 | 0.703282780326843 | 2.08699235816728 | 0.999228396881742 |
| ebi-fl187-GCST90094765 | ebi-met1400-GCST90200636 | X-24544 levels \|\| id:ebi-met1400-GCST90200636 | Fried fish liking \|\| id:ebi-fl187-GCST90094765 | Weighted mode | 4 | -0.327443461 | 0.231284647521307 | 0.251806713370503 | -0.78076137 | 0.125874448625333 | 0.720764041750222 | 0.458057127708629 | 1.13413976653709 | 0.999228396881742 |
| ebi-fl187-GCST90094765 | ebi-met1400-GCST90200659 | X-25172 levels \|\| id:ebi-met1400-GCST90200659 | Fried fish liking \|\| id:ebi-fl187-GCST90094765 | Weighted mode | 4 | 0.0898531335758652 | 0.218062814162688 | 0.707974905908417 | -0.337549982 | 0.517256249334734 | 1.09401359804074 | 0.713516310712409 | 1.67741891072263 | 0.999228396881742 |
| ebi-fl187-GCST90094765 | ebi-met1400-GCST90200670 | X-26111 levels \|\| id:ebi-met1400-GCST90200670 | Fried fish liking \|\| id:ebi-fl187-GCST90094765 | Weighted mode | 4 | -0.119025291 | 0.227001343484975 | 0.636319081965903 | -0.563947924 | 0.325897342311999 | 0.887785347571104 | 0.568958419401474 | 1.38527315263401 | 0.999228396881742 |
| ebi-fl187-GCST90094765 | ebi-met1400-GCST90200673 | Carnitine C4 levels \|\| id:ebi-met1400-GCST90200673 | Fried fish liking \|\| id:ebi-fl187-GCST90094765 | Weighted mode | 4 | -0.051111632 | 0.275976944573774 | 0.864883800531175 | -0.592026443 | 0.489803179636327 | 0.950172595202894 | 0.553205109433379 | 1.63199497849781 | 0.999228396881742 |
| ebi-fl187-GCST90094765 | ebi-met1400-GCST90200710 | X-13431 levels \|\| id:ebi-met1400-GCST90200710 | Fried fish liking \|\| id:ebi-fl187-GCST90094765 | Weighted mode | 4 | -0.123822092 | 0.220897881617927 | 0.614248802212925 | -0.55678194 | 0.309137755594542 | 0.883537014871617 | 0.573050209559665 | 1.36225001513933 | 0.999228396881742 |
| ebi-fl187-GCST90094765 | ebi-met1400-GCST90200730 | Adenosine 5'-diphosphate (ADP) to fructose ratio \|\| id:ebi-met1400-GCST90200730 | Fried fish liking \|\| id:ebi-fl187-GCST90094765 | Weighted mode | 4 | -0.377643902 | 0.30279841955621 | 0.300827359480853 | -0.971128805 | 0.215840999994879 | 0.685474553097615 | 0.37865536882434 | 1.24090505940338 | 0.999228396881742 |
| ebi-fl187-GCST90094765 | ebi-met1400-GCST90200731 | Adenosine 5'-diphosphate (ADP) to mannose ratio \|\| id:ebi-met1400-GCST90200731 | Fried fish liking \|\| id:ebi-fl187-GCST90094765 | Weighted mode | 4 | -0.296096621 | 0.292133935099547 | 0.385431248919393 | -0.868679133 | 0.276485892294769 | 0.743715566378446 | 0.419505294030873 | 1.31848835174153 | 0.999228396881742 |
| ebi-fl187-GCST90094765 | ebi-met1400-GCST90200748 | Aspartate to glutamate ratio \|\| id:ebi-met1400-GCST90200748 | Fried fish liking \|\| id:ebi-fl187-GCST90094765 | Weighted mode | 4 | 0.114164601940658 | 0.203220700643938 | 0.613504353587087 | -0.284147971 | 0.512477175202776 | 1.12093661804245 | 0.752655265071727 | 1.66942152666495 | 0.999228396881742 |
| ebi-fl187-GCST90094765 | ebi-met1400-GCST90200774 | Uridine to pseudouridine ratio \|\| id:ebi-met1400-GCST90200774 | Fried fish liking \|\| id:ebi-fl187-GCST90094765 | Weighted mode | 4 | -0.184777711 | 0.204395601540883 | 0.432622332086674 | -0.58539309 | 0.215837667880821 | 0.831289049611422 | 0.556886912016453 | 1.24090092457308 | 0.999228396881742 |
| ebi-fl187-GCST90094765 | ebi-met1400-GCST90200799 | Spermidine to adenosine 5'-diphosphate (ADP) ratio \|\| id:ebi-met1400-GCST90200799 | Fried fish liking \|\| id:ebi-fl187-GCST90094765 | Weighted mode | 4 | 0.179645563880255 | 0.341492910989573 | 0.63525740810514 | -0.489680542 | 0.848971669419819 | 1.19679310123644 | 0.61282213405947 | 2.33724215814331 | 0.999228396881742 |
| ebi-fl187-GCST90094765 | ebi-met1400-GCST90200811 | Choline to taurocholate ratio \|\| id:ebi-met1400-GCST90200811 | Fried fish liking \|\| id:ebi-fl187-GCST90094765 | Weighted mode | 4 | -0.249533852 | 0.264280669654534 | 0.41472472514883 | -0.767523965 | 0.268456260139233 | 0.779163903827403 | 0.464160925397875 | 1.30794376650116 | 0.999228396881742 |
| ebi-fl187-GCST90094765 | ebi-met1400-GCST90200812 | Glutamate to kynurenine ratio \|\| id:ebi-met1400-GCST90200812 | Fried fish liking \|\| id:ebi-fl187-GCST90094765 | Weighted mode | 4 | 0.0446546154668965 | 0.226343276094427 | 0.85621312915409 | -0.398978206 | 0.488287436611973 | 1.04566664044811 | 0.671005325300089 | 1.62952316728191 | 0.999228396881742 |
| ebi-fl187-GCST90094765 | ebi-met1400-GCST90200876 | Cortisone to 4-cholesten-3-one ratio \|\| id:ebi-met1400-GCST90200876 | Fried fish liking \|\| id:ebi-fl187-GCST90094765 | Weighted mode | 4 | -0.151587338 | 0.239777704386039 | 0.572149941714125 | -0.621551639 | 0.318376962645606 | 0.859342825753699 | 0.537110389506718 | 1.37489444740133 | 0.999228396881742 |
| ebi-fl187-GCST90094765 | ebi-met1400-GCST90200906 | 2'-deoxyuridine to cytidine ratio \|\| id:ebi-met1400-GCST90200906 | Fried fish liking \|\| id:ebi-fl187-GCST90094765 | Weighted mode | 4 | 0.135849221208261 | 0.229909762704151 | 0.596162737980807 | -0.314773914 | 0.586472356108397 | 1.14550916208568 | 0.729953888194343 | 1.79763579815726 | 0.999228396881742 |
| ebi-fl187-GCST90094765 | ebi-met1400-GCST90200907 | Retinol (Vitamin A) to linoleoyl-arachidonoyl-glycerol (18:2 to 20:4) [1] ratio \|\| id:ebi-met1400-GCST90200907 | Fried fish liking \|\| id:ebi-fl187-GCST90094765 | Weighted mode | 4 | -0.107564095 | 0.214484986973724 | 0.650509224482934 | -0.527954669 | 0.312826479803085 | 0.898018962381247 | 0.589810093609234 | 1.36728425900859 | 0.999228396881742 |
| ebi-fl187-GCST90094765 | ebi-met1400-GCST90200916 | Fructose to sucrose ratio \|\| id:ebi-met1400-GCST90200916 | Fried fish liking \|\| id:ebi-fl187-GCST90094765 | Weighted mode | 4 | -0.043378725 | 0.260456147434246 | 0.878317381265669 | -0.553872774 | 0.467115324044033 | 0.957548673834845 | 0.57471973528986 | 1.5953853791021 | 0.999228396881742 |
| ebi-fl187-GCST90094765 | ebi-met1400-GCST90200917 | Alpha-ketobutyrate to 3-methyl-2-oxobutyrate ratio \|\| id:ebi-met1400-GCST90200917 | Fried fish liking \|\| id:ebi-fl187-GCST90094765 | Weighted mode | 4 | 0.0736086864398343 | 0.248775423771578 | 0.786617515760875 | -0.413991144 | 0.561208517032127 | 1.07638551882452 | 0.661006805015482 | 1.75278949678588 | 0.999228396881742 |
| ebi-fl187-GCST90094765 | ebi-met1400-GCST90200924 | Alpha-tocopherol to glycerol ratio \|\| id:ebi-met1400-GCST90200924 | Fried fish liking \|\| id:ebi-fl187-GCST90094765 | Weighted mode | 4 | -0.036913253 | 0.214053514417134 | 0.874062405089962 | -0.456458142 | 0.382631634956883 | 0.963759734698922 | 0.633523527146166 | 1.46613785664922 | 0.999228396881742 |
| ebi-fl187-GCST90094765 | ebi-met1400-GCST90200938 | Alpha-ketoglutarate to alanine ratio \|\| id:ebi-met1400-GCST90200938 | Fried fish liking \|\| id:ebi-fl187-GCST90094765 | Weighted mode | 4 | 0.0273484497028896 | 0.222744415189952 | 0.910045138366531 | -0.409230604 | 0.463927503475196 | 1.02772585114642 | 0.664161056419044 | 1.59030767448104 | 0.999228396881742 |
| ebi-fl187-GCST90094765 | ebi-met1400-GCST90200943 | Phosphate to EDTA ratio \|\| id:ebi-met1400-GCST90200943 | Fried fish liking \|\| id:ebi-fl187-GCST90094765 | Weighted mode | 4 | -0.093905314 | 0.227436643308425 | 0.707425875731682 | -0.539681135 | 0.351870507073316 | 0.910368957526943 | 0.582934100201445 | 1.42172440854343 | 0.999228396881742 |
| ebi-fl187-GCST90094765 | ebi-met1400-GCST90200947 | Adenosine 5'-diphosphate (ADP) to glucose ratio \|\| id:ebi-met1400-GCST90200947 | Fried fish liking \|\| id:ebi-fl187-GCST90094765 | Weighted mode | 4 | -0.291581043 | 0.30675986059096 | 0.411972704028347 | -0.89283037 | 0.309668283732738 | 0.747081465407216 | 0.40949508844763 | 1.36297291884704 | 0.999228396881742 |
| ebi-fl187-GCST90094765 | ebi-met1400-GCST90200975 | Arachidonate (20:4n6) to caffeine ratio \|\| id:ebi-met1400-GCST90200975 | Fried fish liking \|\| id:ebi-fl187-GCST90094765 | Weighted mode | 4 | -0.192360879 | 0.214982450191632 | 0.436838384103153 | -0.613726481 | 0.229004723733942 | 0.825009086604824 | 0.541329850357891 | 1.25734797837313 | 0.999228396881742 |
| ebi-fl187-GCST90094765 | ebi-met1400-GCST90200985 | Cholesterol to oleoyl-linoleoyl-glycerol (18:1 to 18:2) [2] ratio \|\| id:ebi-met1400-GCST90200985 | Fried fish liking \|\| id:ebi-fl187-GCST90094765 | Weighted mode | 4 | 0.0339433138118258 | 0.214754422636294 | 0.884452133693114 | -0.386975355 | 0.454861982178961 | 1.03452596173262 | 0.679107831634898 | 1.57595585802959 | 0.999228396881742 |
| ebi-fl187-GCST90094765 | ebi-met1400-GCST90201016 | Bilirubin (Z,Z) to etiocholanolone glucuronide ratio \|\| id:ebi-met1400-GCST90201016 | Fried fish liking \|\| id:ebi-fl187-GCST90094765 | Weighted mode | 4 | 0.226071923149828 | 0.223345478313524 | 0.385985287815158 | -0.211685214 | 0.663829060644335 | 1.25366582963099 | 0.80921938813332 | 1.94221497338301 | 0.999228396881742 |
| ebi-fl187-GCST90094765 | ebi-pw11-GCST90275189 | Placental weight (fetal genotype, adjusted for sex and gestational age) \|\| id:ebi-pw11-GCST90275189 | Fried fish liking \|\| id:ebi-fl187-GCST90094765 | Weighted mode | 4 | 0.0042121941235656 | 0.125451858747777 | 0.975324157442753 | -0.241673449 | 0.250097837269208 | 1.00422107788223 | 0.785312580276336 | 1.28415104837373 | 0.999228396881742 |
| ebi-fl187-GCST90094765 | ebi-pw11-GCST90275192 | Placental weight (fetal effect, conditional on maternal and paternal genotype) \|\| id:ebi-pw11-GCST90275192 | Fried fish liking \|\| id:ebi-fl187-GCST90094765 | Weighted mode | 4 | 0.10525372020182 | 0.158307688350184 | 0.5536780054738 | -0.205029349 | 0.415536789368181 | 1.11099245582936 | 0.814623407707061 | 1.51518385702171 | 0.999228396881742 |
| ebi-fl187-GCST90094765 | ebi-pw11-GCST90275193 | Placental weight (maternal effect, conditional on fetal and paternal genotype) \|\| id:ebi-pw11-GCST90275193 | Fried fish liking \|\| id:ebi-fl187-GCST90094765 | Weighted mode | 4 | 0.141457012523088 | 0.158599431272594 | 0.438150564968068 | -0.169397873 | 0.452311897817372 | 1.15195098373012 | 0.844172963123518 | 1.57194215745408 | 0.999228396881742 |
| ebi-fl187-GCST90094765 | ebi-pw11-GCST90275195 | Placental weight (fetal effect, conditional on maternal genotype) \|\| id:ebi-pw11-GCST90275195 | Fried fish liking \|\| id:ebi-fl187-GCST90094765 | Weighted mode | 4 | 0.175025698435243 | 0.101272353402388 | 0.182386950889361 | -0.023468114 | 0.373519511103923 | 1.19127683016947 | 0.976805120352333 | 1.45283890975791 | 0.999228396881742 |
| ebi-fl187-GCST90094765 | ebi-pw11-GCST90275197 | Placental weight (fetal genotype, adjusted for sex) \|\| id:ebi-pw11-GCST90275197 | Fried fish liking \|\| id:ebi-fl187-GCST90094765 | Weighted mode | 4 | 0.00318572586448007 | 0.117011411555056 | 0.979989490476884 | -0.226156641 | 0.232528092512389 | 1.00319080568199 | 0.797593156263302 | 1.26178589259692 | 0.999228396881742 |
| ebi-fl187-GCST90094765 | prot-a-1030 | Protein FAM171B \|\| id:prot-a-1030 | Fried fish liking \|\| id:ebi-fl187-GCST90094765 | Weighted mode | 5 | -0.440241215 | 0.524368549655385 | 0.448401482294793 | -1.468003573 | 0.587521142030851 | 0.643881088383739 | 0.230384973235757 | 1.79952212227826 | 0.999228396881742 |
| ebi-fl187-GCST90094765 | prot-a-1036 | BRISC complex subunit Abro1 \|\| id:prot-a-1036 | Fried fish liking \|\| id:ebi-fl187-GCST90094765 | Weighted mode | 5 | 0.0697538018859891 | 0.373132091202871 | 0.860805528869133 | -0.661585097 | 0.801092700643616 | 1.07224416426431 | 0.516032724018144 | 2.22797410762353 | 0.999228396881742 |
| ebi-fl187-GCST90094765 | prot-a-1055 | Phenylalanine--tRNA ligase, mitochondrial \|\| id:prot-a-1055 | Fried fish liking \|\| id:ebi-fl187-GCST90094765 | Weighted mode | 5 | -0.337505121 | 0.372199852160774 | 0.415806642530613 | -1.067016831 | 0.392006589544992 | 0.713548320837307 | 0.344033297606546 | 1.47994746355055 | 0.999228396881742 |
| ebi-fl187-GCST90094765 | prot-a-1083 | Fc receptor-like protein 5 \|\| id:prot-a-1083 | Fried fish liking \|\| id:ebi-fl187-GCST90094765 | Weighted mode | 5 | 0.0647512181118151 | 0.374155767621966 | 0.871008896270723 | -0.668594086 | 0.798096522650868 | 1.06689356756891 | 0.512428501778224 | 2.22130869100747 | 0.999228396881742 |
| ebi-fl187-GCST90094765 | prot-a-1103 | Fibroblast growth factor receptor 3 \|\| id:prot-a-1103 | Fried fish liking \|\| id:ebi-fl187-GCST90094765 | Weighted mode | 5 | -0.322868847 | 0.356335482531683 | 0.416137419013898 | -1.021286393 | 0.375548698451129 | 0.724068811712523 | 0.360131371569503 | 1.455789985 | 0.999228396881742 |
| ebi-fl187-GCST90094765 | prot-a-1105 | Fibroblast growth factor receptor 4 \|\| id:prot-a-1105 | Fried fish liking \|\| id:ebi-fl187-GCST90094765 | Weighted mode | 5 | 0.361142107111237 | 0.34394835990525 | 0.352971965812588 | -0.312996678 | 1.03528089252553 | 1.43496736545709 | 0.731252341563519 | 2.81589708899141 | 0.999228396881742 |
| ebi-fl187-GCST90094765 | prot-a-1120 | Filamin-A \|\| id:prot-a-1120 | Fried fish liking \|\| id:ebi-fl187-GCST90094765 | Weighted mode | 5 | -0.552724407 | 0.44103804002652 | 0.278377578327572 | -1.417158965 | 0.311710151624814 | 0.575380103608992 | 0.242401711230739 | 1.36575877269266 | 0.999228396881742 |
| ebi-fl187-GCST90094765 | prot-a-1166 | Galanin peptides \|\| id:prot-a-1166 | Fried fish liking \|\| id:ebi-fl187-GCST90094765 | Weighted mode | 5 | 0.0832109382461039 | 0.426627593506397 | 0.854865265595113 | -0.752979145 | 0.919401021518641 | 1.08677102594616 | 0.47096139839332 | 2.50778782903499 | 0.999228396881742 |
| ebi-fl187-GCST90094765 | prot-a-1205 | ADP-ribosylation factor-binding protein GGA1 \|\| id:prot-a-1205 | Fried fish liking \|\| id:ebi-fl187-GCST90094765 | Weighted mode | 5 | -0.171971905 | 0.371822223497827 | 0.667754825826787 | -0.900743463 | 0.556799653114592 | 0.842002828955225 | 0.406267502578176 | 1.74507869684255 | 0.999228396881742 |
| ebi-fl187-GCST90094765 | prot-a-1227 | Guanine nucleotide-binding protein G(I)/G(S)/G(O) subunit gamma-T2 \|\| id:prot-a-1227 | Fried fish liking \|\| id:ebi-fl187-GCST90094765 | Weighted mode | 5 | 0.174475553037052 | 0.336224321543032 | 0.631200083490446 | -0.484524117 | 0.833475223261395 | 1.19062163494623 | 0.615990266205882 | 2.30130239936004 | 0.999228396881742 |
| ebi-fl187-GCST90094765 | prot-a-1240 | Golgi SNAP receptor complex member 1 \|\| id:prot-a-1240 | Fried fish liking \|\| id:ebi-fl187-GCST90094765 | Weighted mode | 5 | 0.501338822459878 | 0.408017927117432 | 0.286523110501139 | -0.298376315 | 1.30105395961005 | 1.65093009404864 | 0.742022053402079 | 3.67316599680435 | 0.999228396881742 |
| ebi-fl187-GCST90094765 | prot-a-1283 | Glutathione S-transferase A1 \|\| id:prot-a-1283 | Fried fish liking \|\| id:ebi-fl187-GCST90094765 | Weighted mode | 5 | 0.193894405468106 | 0.326844965073334 | 0.584943761765671 | -0.446721726 | 0.834510537011842 | 1.21396808779603 | 0.639721901432243 | 2.30368620315753 | 0.999228396881742 |
| ebi-fl187-GCST90094765 | prot-a-1324 | E3 ubiquitin-protein ligase HECW1 \|\| id:prot-a-1324 | Fried fish liking \|\| id:ebi-fl187-GCST90094765 | Weighted mode | 5 | 0.0771398729417346 | 0.308799285861923 | 0.815042080766242 | -0.528106727 | 0.682386473231103 | 1.08019315562072 | 0.589720414958325 | 1.97859396394191 | 0.999228396881742 |
| ebi-fl187-GCST90094765 | prot-a-1339 | Histidine triad nucleotide-binding protein 2, mitochondrial \|\| id:prot-a-1339 | Fried fish liking \|\| id:ebi-fl187-GCST90094765 | Weighted mode | 5 | -0.468693246 | 0.362131889646956 | 0.265240870537054 | -1.17847175 | 0.241085257840392 | 0.625819526440955 | 0.30774869647898 | 1.2726295323286 | 0.999228396881742 |
| ebi-fl187-GCST90094765 | prot-a-1466 | Interleukin-11 \|\| id:prot-a-1466 | Fried fish liking \|\| id:ebi-fl187-GCST90094765 | Weighted mode | 5 | -0.473019182 | 0.399187501345031 | 0.301626247259806 | -1.255426685 | 0.30938832017802 | 0.623118118125734 | 0.284954236556002 | 1.36259138951334 | 0.999228396881742 |
| ebi-fl187-GCST90094765 | prot-a-147 | Rho GTPase-activating protein 1 \|\| id:prot-a-147 | Fried fish liking \|\| id:ebi-fl187-GCST90094765 | Weighted mode | 5 | -0.137605248 | 0.375690114435934 | 0.732711824094328 | -0.873957873 | 0.598747375890572 | 0.871442627224834 | 0.4172966694111 | 1.81983779936759 | 0.999228396881742 |
| ebi-fl187-GCST90094765 | prot-a-1473 | Interleukin-12 receptor subunit beta-1 \|\| id:prot-a-1473 | Fried fish liking \|\| id:ebi-fl187-GCST90094765 | Weighted mode | 5 | -0.632933054 | 0.36855799209549 | 0.161053928211754 | -1.355306719 | 0.0894406102679589 | 0.531031969021343 | 0.257868189313179 | 1.09356238500672 | 0.999228396881742 |
| ebi-fl187-GCST90094765 | prot-a-1501 | X-linked interleukin-1 receptor accessory protein-like 2 \|\| id:prot-a-1501 | Fried fish liking \|\| id:ebi-fl187-GCST90094765 | Weighted mode | 5 | 0.216670848350538 | 0.357529994222289 | 0.577207911844818 | -0.48408794 | 0.917429637026225 | 1.24193524981825 | 0.61625900551198 | 2.50284888487706 | 0.999228396881742 |
| ebi-fl187-GCST90094765 | prot-a-1503 | Interleukin-1 receptor-like 2 \|\| id:prot-a-1503 | Fried fish liking \|\| id:ebi-fl187-GCST90094765 | Weighted mode | 5 | -0.517026495 | 0.356005489112249 | 0.220063880768782 | -1.214797253 | 0.180744263958762 | 0.596290988873024 | 0.296770177350848 | 1.19810874052488 | 0.999228396881742 |
| ebi-fl187-GCST90094765 | prot-a-1536 | Interleukin-5 receptor subunit alpha \|\| id:prot-a-1536 | Fried fish liking \|\| id:ebi-fl187-GCST90094765 | Weighted mode | 5 | 0.103296760256814 | 0.360504953396746 | 0.788698085509228 | -0.603292948 | 0.809886468914436 | 1.10882041408663 | 0.547007399953217 | 2.24765279372894 | 0.999228396881742 |
| ebi-fl187-GCST90094765 | prot-a-1559 | Insulin-induced gene 1 protein \|\| id:prot-a-1559 | Fried fish liking \|\| id:ebi-fl187-GCST90094765 | Weighted mode | 5 | -0.352932289 | 0.398066110237425 | 0.425375025370802 | -1.133141865 | 0.427277287281615 | 0.70262476734415 | 0.322019922301817 | 1.53307770574117 | 0.999228396881742 |
| ebi-fl187-GCST90094765 | prot-a-1567 | Interferon regulatory factor 1 \|\| id:prot-a-1567 | Fried fish liking \|\| id:ebi-fl187-GCST90094765 | Weighted mode | 5 | 0.313972177555393 | 0.314830961231745 | 0.375073660327064 | -0.303096506 | 0.931040861569613 | 1.36885165121832 | 0.738527820226559 | 2.53714862422964 | 0.999228396881742 |
| ebi-fl187-GCST90094765 | prot-a-1613 | Potassium voltage-gated channel subfamily E regulatory beta subunit 5 \|\| id:prot-a-1613 | Fried fish liking \|\| id:ebi-fl187-GCST90094765 | Weighted mode | 5 | -0.232988596 | 0.392049969965436 | 0.584304716193757 | -1.001406537 | 0.535429345422261 | 0.79216260752491 | 0.367362368909768 | 1.70818148473666 | 0.999228396881742 |
| ebi-fl187-GCST90094765 | prot-a-1649 | Kin of IRRE-like protein 2 \|\| id:prot-a-1649 | Fried fish liking \|\| id:ebi-fl187-GCST90094765 | Weighted mode | 5 | 0.173924529903831 | 0.410598798646306 | 0.693651113708955 | -0.630849115 | 0.978698175250591 | 1.1899657556014 | 0.532139761028108 | 2.66098984366104 | 0.999228396881742 |
| ebi-fl187-GCST90094765 | prot-a-165 | Armadillo repeat-containing protein 5 \|\| id:prot-a-165 | Fried fish liking \|\| id:ebi-fl187-GCST90094765 | Weighted mode | 5 | 0.413668678832263 | 0.495265171721871 | 0.450563822957426 | -0.557051058 | 1.38438841540713 | 1.5123559683213 | 0.572896012529619 | 3.99238347779355 | 0.999228396881742 |
| ebi-fl187-GCST90094765 | prot-a-1656 | Kallikrein-11 \|\| id:prot-a-1656 | Fried fish liking \|\| id:ebi-fl187-GCST90094765 | Weighted mode | 5 | -0.376703956 | 0.361636472620273 | 0.356383741390311 | -1.085511443 | 0.332103529836817 | 0.686119164952395 | 0.33772901263309 | 1.39389715098716 | 0.999228396881742 |
| ebi-fl187-GCST90094765 | prot-a-1697 | Laminin subunit gamma-2 \|\| id:prot-a-1697 | Fried fish liking \|\| id:ebi-fl187-GCST90094765 | Weighted mode | 5 | 0.315888023206487 | 0.5440678384538 | 0.592646529976963 | -0.75048494 | 1.38226098657593 | 1.37147667346711 | 0.472137538761478 | 3.98389899434508 | 0.999228396881742 |
| ebi-fl187-GCST90094765 | prot-a-1698 | Lysosome-associated membrane glycoprotein 3 \|\| id:prot-a-1698 | Fried fish liking \|\| id:ebi-fl187-GCST90094765 | Weighted mode | 5 | 0.0651837066481789 | 0.347683333704307 | 0.860410152921486 | -0.616275627 | 0.746643040708621 | 1.06735508660005 | 0.539951678648361 | 2.10990524882306 | 0.999228396881742 |
| ebi-fl187-GCST90094765 | prot-a-1724 | Leptin receptor \|\| id:prot-a-1724 | Fried fish liking \|\| id:ebi-fl187-GCST90094765 | Weighted mode | 5 | -0.351348258 | 0.354806053278736 | 0.378105407127607 | -1.046768123 | 0.344071606108296 | 0.703738628343562 | 0.351070534489053 | 1.41067964517062 | 0.999228396881742 |
| ebi-fl187-GCST90094765 | prot-a-1743 | Leukocyte immunoglobulin-like receptor subfamily B member 2 \|\| id:prot-a-1743 | Fried fish liking \|\| id:ebi-fl187-GCST90094765 | Weighted mode | 5 | -0.486241055 | 0.321331317567158 | 0.204779660566858 | -1.116050437 | 0.143568327691169 | 0.614933556797068 | 0.327571005351859 | 1.15438568462099 | 0.999228396881742 |
| ebi-fl187-GCST90094765 | prot-a-1746 | Leukocyte immunoglobulin-like receptor subfamily B member 4 \|\| id:prot-a-1746 | Fried fish liking \|\| id:ebi-fl187-GCST90094765 | Weighted mode | 5 | -0.133716285 | 0.348043419016984 | 0.720384832355446 | -0.815881386 | 0.548448816623924 | 0.874838234433306 | 0.442249363169122 | 1.73056650877235 | 0.999228396881742 |
| ebi-fl187-GCST90094765 | prot-a-1750 | Leucine-rich repeat and immunoglobulin-like domain-containing nogo receptor-interacting protein 3 \|\| id:prot-a-1750 | Fried fish liking \|\| id:ebi-fl187-GCST90094765 | Weighted mode | 5 | -0.416695466 | 0.357761680954147 | 0.308855839626099 | -1.117908361 | 0.284517428501276 | 0.659221644671862 | 0.326962968446413 | 1.32912047767605 | 0.999228396881742 |
| ebi-fl187-GCST90094765 | prot-a-1753 | Lipase member N \|\| id:prot-a-1753 | Fried fish liking \|\| id:ebi-fl187-GCST90094765 | Weighted mode | 5 | 0.211203681388129 | 0.354640139710457 | 0.58353976355624 | -0.483890992 | 0.906298355220625 | 1.23516390934168 | 0.616380388369784 | 2.47514345317711 | 0.999228396881742 |
| ebi-fl187-GCST90094765 | prot-a-1760 | Ig Kappa chain V-I region HK102- like \|\| id:prot-a-1760 | Fried fish liking \|\| id:ebi-fl187-GCST90094765 | Weighted mode | 5 | -0.270429384 | 0.353602501084925 | 0.487028311998202 | -0.963490286 | 0.422631518364216 | 0.763051781939786 | 0.381558809846176 | 1.5259718997347 | 0.999228396881742 |
| ebi-fl187-GCST90094765 | prot-a-1792 | Leucine-rich repeat neuronal protein 1 \|\| id:prot-a-1792 | Fried fish liking \|\| id:ebi-fl187-GCST90094765 | Weighted mode | 5 | 0.498401492619616 | 0.466174245063528 | 0.345236942664491 | -0.415300028 | 1.41210301294413 | 1.64608788288639 | 0.660142190043941 | 4.10457831517333 | 0.999228396881742 |
| ebi-fl187-GCST90094765 | prot-a-1795 | Leucine-rich repeat transmembrane neuronal protein 1 \|\| id:prot-a-1795 | Fried fish liking \|\| id:ebi-fl187-GCST90094765 | Weighted mode | 5 | -0.019357768 | 0.37167176590262 | 0.960959834587198 | -0.747834429 | 0.709118892958192 | 0.980828390244818 | 0.473390604297239 | 2.03219988393811 | 0.999228396881742 |
| ebi-fl187-GCST90094765 | prot-a-1820 | LysM and putative peptidoglycan-binding domain-containing protein 4 \|\| id:prot-a-1820 | Fried fish liking \|\| id:ebi-fl187-GCST90094765 | Weighted mode | 5 | -0.512319185 | 0.385839074206188 | 0.254946872230005 | -1.268563771 | 0.243925400032012 | 0.599104531897194 | 0.281235250120078 | 1.2762491187947 | 0.999228396881742 |
| ebi-fl187-GCST90094765 | prot-a-1844 | Mitogen-activated protein kinase kinase kinase 7:TGF-beta-activated kinase 1 and MAP3K7-binding protein 1 fusion \|\| id:prot-a-1844 | Fried fish liking \|\| id:ebi-fl187-GCST90094765 | Weighted mode | 5 | 0.276500641832667 | 0.395966702949124 | 0.523460018169971 | -0.499594096 | 1.05259537961295 | 1.31850779897886 | 0.606776902937487 | 2.86507744041008 | 0.999228396881742 |
| ebi-fl187-GCST90094765 | prot-a-1856 | Megakaryocyte-associated tyrosine-protein kinase \|\| id:prot-a-1856 | Fried fish liking \|\| id:ebi-fl187-GCST90094765 | Weighted mode | 5 | 0.658889167217341 | 0.356098426962103 | 0.137941410882253 | -0.03906375 | 1.35684208406306 | 1.93264429692544 | 0.961689399850307 | 3.88390885770379 | 0.999228396881742 |
| ebi-fl187-GCST90094765 | prot-a-1910 | Stromelysin-2 \|\| id:prot-a-1910 | Fried fish liking \|\| id:ebi-fl187-GCST90094765 | Weighted mode | 5 | 0.078743917107956 | 0.585021688748427 | 0.899429225989551 | -1.067898593 | 1.22538642705487 | 1.08192722353935 | 0.343730075852009 | 3.40548179886226 | 0.999228396881742 |
| ebi-fl187-GCST90094765 | prot-a-1940 | 39S ribosomal protein L14, mitochondrial \|\| id:prot-a-1940 | Fried fish liking \|\| id:ebi-fl187-GCST90094765 | Weighted mode | 5 | -0.036393215 | 0.347657398748357 | 0.921667776556397 | -0.717801717 | 0.645015286512955 | 0.964261056983126 | 0.487823452338847 | 1.90601616539003 | 0.999228396881742 |
| ebi-fl187-GCST90094765 | prot-a-1942 | 39S ribosomal protein L33, mitochondrial \|\| id:prot-a-1942 | Fried fish liking \|\| id:ebi-fl187-GCST90094765 | Weighted mode | 5 | 0.224906753735457 | 0.365568235410591 | 0.57168388133411 | -0.491606988 | 0.941420495140215 | 1.2522059472208 | 0.61164270172559 | 2.56362044348992 | 0.999228396881742 |
| ebi-fl187-GCST90094765 | prot-a-1948 | Mesothelin \|\| id:prot-a-1948 | Fried fish liking \|\| id:ebi-fl187-GCST90094765 | Weighted mode | 5 | -0.503659997 | 0.346168821514409 | 0.219374064951625 | -1.182150888 | 0.174830892785485 | 0.604314816549154 | 0.306618526862721 | 1.19104478531509 | 0.999228396881742 |
| ebi-fl187-GCST90094765 | prot-a-1980 | Unconventional myosin-VI \|\| id:prot-a-1980 | Fried fish liking \|\| id:ebi-fl187-GCST90094765 | Weighted mode | 5 | 0.482458150611793 | 0.361657233638222 | 0.253082761508754 | -0.226390027 | 1.19130632854271 | 1.62005184304487 | 0.79740703048014 | 3.29137802130081 | 0.999228396881742 |
| ebi-fl187-GCST90094765 | prot-a-2039 | Neurofascin \|\| id:prot-a-2039 | Fried fish liking \|\| id:ebi-fl187-GCST90094765 | Weighted mode | 5 | 0.0146466987103864 | 0.335593853428863 | 0.967279892482532 | -0.643117254 | 0.672410651430959 | 1.01475448720755 | 0.525651278881782 | 1.95895398846625 | 0.999228396881742 |
| ebi-fl187-GCST90094765 | prot-a-2041 | NFU1 iron-sulfur cluster scaffold homolog, mitochondrial \|\| id:prot-a-2041 | Fried fish liking \|\| id:ebi-fl187-GCST90094765 | Weighted mode | 5 | 0.128769328820217 | 0.375241212000185 | 0.748751978955032 | -0.606703447 | 0.864242104340579 | 1.13742772210802 | 0.545145009790392 | 2.37320676110983 | 0.999228396881742 |
| ebi-fl187-GCST90094765 | prot-a-2088 | Glucocorticoid receptor \|\| id:prot-a-2088 | Fried fish liking \|\| id:ebi-fl187-GCST90094765 | Weighted mode | 5 | 0.289067009716699 | 0.35029286629135 | 0.455619930599266 | -0.397507008 | 0.975641027647745 | 1.33518119564455 | 0.671993233158469 | 2.65286722728415 | 0.999228396881742 |
| ebi-fl187-GCST90094765 | prot-a-2111 | Neuron-specific protein family member 2 \|\| id:prot-a-2111 | Fried fish liking \|\| id:ebi-fl187-GCST90094765 | Weighted mode | 5 | -0.199222912 | 0.358860237616633 | 0.608370056549854 | -0.902588977 | 0.504143153991592 | 0.819367226402696 | 0.405518421456632 | 1.65556634712499 | 0.999228396881742 |
| ebi-fl187-GCST90094765 | prot-a-2125 | Nuclear migration protein nudC \|\| id:prot-a-2125 | Fried fish liking \|\| id:ebi-fl187-GCST90094765 | Weighted mode | 5 | -0.071932711 | 0.382977784929039 | 0.860157259771311 | -0.82256917 | 0.678703747024935 | 0.930593512005614 | 0.439301563077652 | 1.97132074495685 | 0.999228396881742 |
| ebi-fl187-GCST90094765 | prot-a-2142 | Ornithine decarboxylase \|\| id:prot-a-2142 | Fried fish liking \|\| id:ebi-fl187-GCST90094765 | Weighted mode | 5 | 0.0561520246382072 | 0.354665973389127 | 0.881873275885523 | -0.638993283 | 0.751297332480897 | 1.0577584768638 | 0.527823525471991 | 2.11974825179821 | 0.999228396881742 |
| ebi-fl187-GCST90094765 | prot-a-2186 | Prostate and testis expressed protein 1 \|\| id:prot-a-2186 | Fried fish liking \|\| id:ebi-fl187-GCST90094765 | Weighted mode | 5 | -0.497868328 | 0.392151038451652 | 0.273069672915402 | -1.266484363 | 0.270747707865109 | 0.607824963466017 | 0.28182066138999 | 1.31094428772634 | 0.999228396881742 |
| ebi-fl187-GCST90094765 | prot-a-2227 | Platelet-derived growth factor C \|\| id:prot-a-2227 | Fried fish liking \|\| id:ebi-fl187-GCST90094765 | Weighted mode | 5 | 0.00523567921549972 | 0.364384194201943 | 0.989224037192094 | -0.708957341 | 0.719428699851308 | 1.00524940933563 | 0.492157081836786 | 2.05325984784825 | 0.999228396881742 |
| ebi-fl187-GCST90094765 | prot-a-2229 | Platelet-derived growth factor receptor alpha \|\| id:prot-a-2229 | Fried fish liking \|\| id:ebi-fl187-GCST90094765 | Weighted mode | 5 | 0.195026603757991 | 0.36359075706113 | 0.620129969818672 | -0.51761128 | 0.907664487597806 | 1.21534331875918 | 0.595942388558279 | 2.47852713754079 | 0.999228396881742 |
| ebi-fl187-GCST90094765 | prot-a-2243 | Platelet endothelial aggregation receptor 1 \|\| id:prot-a-2243 | Fried fish liking \|\| id:ebi-fl187-GCST90094765 | Weighted mode | 5 | -0.321724404 | 0.366650599614011 | 0.429775020084676 | -1.040359579 | 0.396910771011565 | 0.72489794160835 | 0.353327609757326 | 1.48722322070707 | 0.999228396881742 |
| ebi-fl187-GCST90094765 | prot-a-2246 | E3 ubiquitin-protein ligase pellino homolog 2 \|\| id:prot-a-2246 | Fried fish liking \|\| id:ebi-fl187-GCST90094765 | Weighted mode | 5 | 0.346922989671714 | 0.331154189315865 | 0.353940990809348 | -0.302139221 | 0.99598520073081 | 1.41470777406518 | 0.739235140383206 | 2.70739035073871 | 0.999228396881742 |
| ebi-fl187-GCST90094765 | prot-a-2251 | Platelet factor 4 variant \|\| id:prot-a-2251 | Fried fish liking \|\| id:ebi-fl187-GCST90094765 | Weighted mode | 5 | -0.013608998 | 0.343121975735089 | 0.97026303939194 | -0.68612807 | 0.658910074659386 | 0.986483185978907 | 0.50352190101509 | 1.93268470399648 | 0.999228396881742 |
| ebi-fl187-GCST90094765 | prot-a-2346 | Protein phosphatase 1L \|\| id:prot-a-2346 | Fried fish liking \|\| id:ebi-fl187-GCST90094765 | Weighted mode | 5 | 0.43752177358223 | 0.375867095325216 | 0.309107463550909 | -0.299177733 | 1.17422128041965 | 1.54886402258514 | 0.74142762137916 | 3.23562232008054 | 0.999228396881742 |
| ebi-fl187-GCST90094765 | prot-a-2358 | PR domain zinc finger protein 4 \|\| id:prot-a-2358 | Fried fish liking \|\| id:ebi-fl187-GCST90094765 | Weighted mode | 5 | 0.209368569273039 | 0.328340422280077 | 0.558369783157564 | -0.434178658 | 0.85291579694199 | 1.23289932360741 | 0.647796510860777 | 2.34647874242456 | 0.999228396881742 |
| ebi-fl187-GCST90094765 | prot-a-2390 | Trypsin-1 \|\| id:prot-a-2390 | Fried fish liking \|\| id:ebi-fl187-GCST90094765 | Weighted mode | 5 | -0.003706187 | 0.637347357216336 | 0.995638766899506 | -1.252907007 | 1.2454946335143 | 0.996300672803217 | 0.285673134884184 | 3.47465305419974 | 0.999228396881742 |
| ebi-fl187-GCST90094765 | prot-a-242 | Brain-derived neurotrophic factor \|\| id:prot-a-242 | Fried fish liking \|\| id:ebi-fl187-GCST90094765 | Weighted mode | 5 | -0.168121228 | 0.36594923552322 | 0.669797471545182 | -0.88538173 | 0.549139273217859 | 0.845251359996925 | 0.412556657950076 | 1.73176180243129 | 0.999228396881742 |
| ebi-fl187-GCST90094765 | prot-a-2464 | Dihydropteridine reductase \|\| id:prot-a-2464 | Fried fish liking \|\| id:ebi-fl187-GCST90094765 | Weighted mode | 5 | 0.515064157638331 | 0.311336027170625 | 0.173395639985916 | -0.095154456 | 1.12528277089276 | 1.67374588193582 | 0.909232487558065 | 3.081087968 | 0.999228396881742 |
| ebi-fl187-GCST90094765 | prot-a-2474 | Ras-related protein Rab-27A \|\| id:prot-a-2474 | Fried fish liking \|\| id:ebi-fl187-GCST90094765 | Weighted mode | 5 | -0.188352061 | 0.379569941194353 | 0.645765793346889 | -0.932309146 | 0.555605023365649 | 0.828323035345489 | 0.393643679305379 | 1.74299521865735 | 0.999228396881742 |
| ebi-fl187-GCST90094765 | prot-a-2478 | Ras-related protein Rab-7b \|\| id:prot-a-2478 | Fried fish liking \|\| id:ebi-fl187-GCST90094765 | Weighted mode | 5 | -0.116347992 | 0.349524613705405 | 0.755944176214756 | -0.801416235 | 0.568720250597935 | 0.8901653987193 | 0.448693059056377 | 1.7660055601117 | 0.999228396881742 |
| ebi-fl187-GCST90094765 | prot-a-2528 | E3 ubiquitin-protein ligase rififylin \|\| id:prot-a-2528 | Fried fish liking \|\| id:ebi-fl187-GCST90094765 | Weighted mode | 5 | 0.0625392048811611 | 0.40117230400841 | 0.883669847645365 | -0.723758511 | 0.848836920737644 | 1.0645361931147 | 0.484926225990128 | 2.33692723906052 | 0.999228396881742 |
| ebi-fl187-GCST90094765 | prot-a-2543 | Protein RIC-3 \|\| id:prot-a-2543 | Fried fish liking \|\| id:ebi-fl187-GCST90094765 | Weighted mode | 5 | -0.14328503 | 0.340672266947158 | 0.695663891530098 | -0.811002673 | 0.524432613435839 | 0.866507053378468 | 0.444412242597439 | 1.68949997679241 | 0.999228396881742 |
| ebi-fl187-GCST90094765 | prot-a-255 | Bone morphogenetic protein 6 \|\| id:prot-a-255 | Fried fish liking \|\| id:ebi-fl187-GCST90094765 | Weighted mode | 5 | -0.069774902 | 0.384060224595818 | 0.864671175625372 | -0.822532942 | 0.682983138633795 | 0.932603723910858 | 0.439317478433526 | 1.97977487477545 | 0.999228396881742 |
| ebi-fl187-GCST90094765 | prot-a-2568 | RING finger protein 165 \|\| id:prot-a-2568 | Fried fish liking \|\| id:ebi-fl187-GCST90094765 | Weighted mode | 5 | -0.280531823 | 0.36596986853636 | 0.48609061248073 | -0.997832766 | 0.436769119009814 | 0.755381904898542 | 0.368677586703813 | 1.54769870159359 | 0.999228396881742 |
| ebi-fl187-GCST90094765 | prot-a-2573 | E3 ubiquitin-protein ligase RNF8 \|\| id:prot-a-2573 | Fried fish liking \|\| id:ebi-fl187-GCST90094765 | Weighted mode | 5 | -0.065185563 | 0.503955833181284 | 0.903325835253167 | -1.052938996 | 0.922567870474831 | 0.936893594871792 | 0.348910793455997 | 2.51574220280603 | 0.999228396881742 |
| ebi-fl187-GCST90094765 | prot-a-260 | Cytoplasmic tyrosine-protein kinase BMX \|\| id:prot-a-260 | Fried fish liking \|\| id:ebi-fl187-GCST90094765 | Weighted mode | 4 | -0.1588903 | 0.382850253572104 | 0.706022203808582 | -0.909276797 | 0.591496197265772 | 0.853089938028014 | 0.40281543615603 | 1.80668955814976 | 0.999228396881742 |
| ebi-fl187-GCST90094765 | prot-a-2623 | Serum amyloid A-1 protein \|\| id:prot-a-2623 | Fried fish liking \|\| id:ebi-fl187-GCST90094765 | Weighted mode | 5 | -0.612223421 | 0.362595426712175 | 0.166595870884403 | -1.322910457 | 0.0984636155213577 | 0.542144113491649 | 0.266358946428248 | 1.10347425432854 | 0.999228396881742 |
| ebi-fl187-GCST90094765 | prot-a-2640 | Lysosome membrane protein 2 \|\| id:prot-a-2640 | Fried fish liking \|\| id:ebi-fl187-GCST90094765 | Weighted mode | 5 | -0.045770553 | 0.373770931026437 | 0.908443674853927 | -0.778361578 | 0.686820471381723 | 0.955261118434419 | 0.459157689302827 | 1.98738652461234 | 0.999228396881742 |
| ebi-fl187-GCST90094765 | prot-a-2641 | Scavenger receptor class F member 2 \|\| id:prot-a-2641 | Fried fish liking \|\| id:ebi-fl187-GCST90094765 | Weighted mode | 5 | -0.233659697 | 0.338412224767507 | 0.527880495652921 | -0.896947657 | 0.429628263893004 | 0.791631164799127 | 0.407812545607878 | 1.53668617562293 | 0.999228396881742 |
| ebi-fl187-GCST90094765 | prot-a-2669 | Semaphorin-3A \|\| id:prot-a-2669 | Fried fish liking \|\| id:ebi-fl187-GCST90094765 | Weighted mode | 5 | 0.0507610785551758 | 0.471177542201427 | 0.919395491191386 | -0.872746904 | 0.974269061269972 | 1.05207150080984 | 0.41780230864386 | 2.64923007823724 | 0.999228396881742 |
| ebi-fl187-GCST90094765 | prot-a-2683 | Sentrin-specific protease 7 \|\| id:prot-a-2683 | Fried fish liking \|\| id:ebi-fl187-GCST90094765 | Weighted mode | 5 | 0.527326424699365 | 0.418276866136609 | 0.27593801987254 | -0.292496233 | 1.34714908232712 | 1.69439615174131 | 0.746398056765516 | 3.8464439892529 | 0.999228396881742 |
| ebi-fl187-GCST90094765 | prot-a-270 | Bromodomain testis-specific protein \|\| id:prot-a-270 | Fried fish liking \|\| id:ebi-fl187-GCST90094765 | Weighted mode | 5 | -0.210486112 | 0.409619430873142 | 0.634434923960198 | -1.013340197 | 0.592367972097923 | 0.810190306663237 | 0.363004445975901 | 1.80826527136983 | 0.999228396881742 |
| ebi-fl187-GCST90094765 | prot-a-2708 | 14-3-3 protein sigma \|\| id:prot-a-2708 | Fried fish liking \|\| id:ebi-fl187-GCST90094765 | Weighted mode | 5 | 0.460140735555019 | 0.309211832575598 | 0.210943407052633 | -0.145914456 | 1.06619592740319 | 1.58429693621417 | 0.864231629599799 | 2.90431025217152 | 0.999228396881742 |
| ebi-fl187-GCST90094765 | prot-a-2713 | Beta-sarcoglycan \|\| id:prot-a-2713 | Fried fish liking \|\| id:ebi-fl187-GCST90094765 | Weighted mode | 5 | -0.441119166 | 0.367689459270366 | 0.296453715463266 | -1.161790506 | 0.279552173879007 | 0.643316040418617 | 0.312925384055349 | 1.32253741290188 | 0.999228396881742 |
| ebi-fl187-GCST90094765 | prot-a-2720 | Protein shisa-3 homolog \|\| id:prot-a-2720 | Fried fish liking \|\| id:ebi-fl187-GCST90094765 | Weighted mode | 5 | -0.171550069 | 0.361109295036572 | 0.659518424758464 | -0.879324287 | 0.536224149256657 | 0.842358090924241 | 0.415063280481976 | 1.70953969361388 | 0.999228396881742 |
| ebi-fl187-GCST90094765 | prot-a-2730 | Sialic acid-binding Ig-like lectin 8 \|\| id:prot-a-2730 | Fried fish liking \|\| id:ebi-fl187-GCST90094765 | Weighted mode | 5 | 0.122228032924735 | 0.354810520544722 | 0.747828683311724 | -0.573200587 | 0.817656653192389 | 1.13001175228766 | 0.563718318603831 | 2.2651854980885 | 0.999228396881742 |
| ebi-fl187-GCST90094765 | prot-a-2761 | Sodium- and chloride-dependent neutral and basic amino acid transporter B(0+) \|\| id:prot-a-2761 | Fried fish liking \|\| id:ebi-fl187-GCST90094765 | Weighted mode | 5 | 0.191536924633491 | 0.381370097889433 | 0.64189122146135 | -0.555948467 | 0.939022316496781 | 1.2111095520907 | 0.573528030602396 | 2.55747978982775 | 0.999228396881742 |
| ebi-fl187-GCST90094765 | prot-a-2770 | Secreted Ly-6/uPAR-related protein 1 \|\| id:prot-a-2770 | Fried fish liking \|\| id:ebi-fl187-GCST90094765 | Weighted mode | 5 | 0.201837196081512 | 0.377024255108983 | 0.620790701060193 | -0.537130344 | 0.940804736095119 | 1.22364877707824 | 0.584422941173669 | 2.56204235692405 | 0.999228396881742 |
| ebi-fl187-GCST90094765 | prot-a-2784 | N-lysine methyltransferase SMYD2 \|\| id:prot-a-2784 | Fried fish liking \|\| id:ebi-fl187-GCST90094765 | Weighted mode | 5 | -0.323441721 | 0.344935243936767 | 0.401492902649649 | -0.999514799 | 0.352631357185043 | 0.723654130582473 | 0.368057979936945 | 1.42280653933598 | 0.999228396881742 |
| ebi-fl187-GCST90094765 | prot-a-2837 | Serine palmitoyltransferase 2 \|\| id:prot-a-2837 | Fried fish liking \|\| id:ebi-fl187-GCST90094765 | Weighted mode | 5 | 0.379441037271744 | 0.3851116598837 | 0.380269246156941 | -0.375377816 | 1.1342598906438 | 1.46146745524568 | 0.687029658883254 | 3.10887178613818 | 0.999228396881742 |
| ebi-fl187-GCST90094765 | prot-a-2858 | Alpha-2,8-sialyltransferase 8B \|\| id:prot-a-2858 | Fried fish liking \|\| id:ebi-fl187-GCST90094765 | Weighted mode | 5 | 0.00889119149499318 | 0.353761951444827 | 0.981152534415384 | -0.684482233 | 0.702264616326854 | 1.00893083554527 | 0.504351298271412 | 2.01831825238264 | 0.999228396881742 |
| ebi-fl187-GCST90094765 | prot-a-2911 | Synaptotagmin-11 \|\| id:prot-a-2911 | Fried fish liking \|\| id:ebi-fl187-GCST90094765 | Weighted mode | 5 | -0.352478569 | 0.37528502803551 | 0.400790265302222 | -1.088037224 | 0.383080085911651 | 0.702943634407646 | 0.336877059434785 | 1.46679549501912 | 0.999228396881742 |
| ebi-fl187-GCST90094765 | prot-a-2962 | Transforming growth factor beta-1 \|\| id:prot-a-2962 | Fried fish liking \|\| id:ebi-fl187-GCST90094765 | Weighted mode | 5 | -0.022944928 | 0.3453172105581 | 0.950211326918849 | -0.699766661 | 0.653876804659834 | 0.977316305021524 | 0.496701190164619 | 1.92298142016605 | 0.999228396881742 |
| ebi-fl187-GCST90094765 | prot-a-2978 | Tyrosine-protein kinase receptor Tie-1, soluble \|\| id:prot-a-2978 | Fried fish liking \|\| id:ebi-fl187-GCST90094765 | Weighted mode | 5 | -0.034734464 | 0.36192220922762 | 0.928158649963081 | -0.744101994 | 0.674633065819365 | 0.965861853044841 | 0.475160805495665 | 1.96331243734228 | 0.999228396881742 |
| ebi-fl187-GCST90094765 | prot-a-299 | Uncharacterized protein C1orf226 \|\| id:prot-a-299 | Fried fish liking \|\| id:ebi-fl187-GCST90094765 | Weighted mode | 5 | 0.21049104800795 | 0.364784844874289 | 0.594840089308029 | -0.504487248 | 0.925469343961557 | 1.23428400387214 | 0.603815103499444 | 2.52305216180475 | 0.999228396881742 |
| ebi-fl187-GCST90094765 | prot-a-3015 | Transmembrane protein 70, mitochondrial \|\| id:prot-a-3015 | Fried fish liking \|\| id:ebi-fl187-GCST90094765 | Weighted mode | 5 | -0.007284634 | 0.309735354190733 | 0.982362859435147 | -0.614365928 | 0.599796660235244 | 0.992741834657069 | 0.540983809163566 | 1.82174832884935 | 0.999228396881742 |
| ebi-fl187-GCST90094765 | prot-a-3016 | Transmembrane protein 87B \|\| id:prot-a-3016 | Fried fish liking \|\| id:ebi-fl187-GCST90094765 | Weighted mode | 5 | 0.0717807301239464 | 0.341991362590692 | 0.844010398146282 | -0.598522341 | 0.742083800801703 | 1.07441973035216 | 0.549623192246419 | 2.10030758027483 | 0.999228396881742 |
| ebi-fl187-GCST90094765 | prot-a-3123 | Thioredoxin domain-containing protein 12 \|\| id:prot-a-3123 | Fried fish liking \|\| id:ebi-fl187-GCST90094765 | Weighted mode | 5 | -0.398308722 | 0.361606255403337 | 0.332503674623801 | -1.107056982 | 0.310439538793412 | 0.671454702958241 | 0.330530287516263 | 1.36402452408406 | 0.999228396881742 |
| ebi-fl187-GCST90094765 | prot-a-3133 | PolyUbiquitin K48-linked \|\| id:prot-a-3133 | Fried fish liking \|\| id:ebi-fl187-GCST90094765 | Weighted mode | 5 | -0.301856584 | 0.395240746885962 | 0.487591173027743 | -1.076528448 | 0.472815279665586 | 0.739444105230481 | 0.340776497844993 | 1.60450497090564 | 0.999228396881742 |
| ebi-fl187-GCST90094765 | prot-a-3135 | Ubiquitin-conjugating enzyme E2 B \|\| id:prot-a-3135 | Fried fish liking \|\| id:ebi-fl187-GCST90094765 | Weighted mode | 5 | 0.25974623597227 | 0.355706369786575 | 0.505719048841497 | -0.437438249 | 0.956930720753957 | 1.29660101421844 | 0.645688397224119 | 2.60369273677492 | 0.999228396881742 |
| ebi-fl187-GCST90094765 | prot-a-3162 | NKG2D ligand 3 \|\| id:prot-a-3162 | Fried fish liking \|\| id:ebi-fl187-GCST90094765 | Weighted mode | 5 | -0.103209787 | 0.381311265930472 | 0.800036978905515 | -0.850579869 | 0.644160293839104 | 0.901937738473407 | 0.427167159291688 | 1.90438723199467 | 0.999228396881742 |
| ebi-fl187-GCST90094765 | prot-a-3188 | Vesicle-associated membrane protein 8 \|\| id:prot-a-3188 | Fried fish liking \|\| id:ebi-fl187-GCST90094765 | Weighted mode | 5 | 0.0060776392269164 | 0.470704043927388 | 0.990316481941419 | -0.916502287 | 0.928657565324596 | 1.00609614554879 | 0.399915386951829 | 2.5311090473497 | 0.999228396881742 |
| ebi-fl187-GCST90094765 | prot-a-3201 | Vitrin \|\| id:prot-a-3201 | Fried fish liking \|\| id:ebi-fl187-GCST90094765 | Weighted mode | 5 | -0.24899369 | 0.414591288277146 | 0.58049279151053 | -1.061592615 | 0.563605234899438 | 0.779584892453365 | 0.345904478704852 | 1.75699547695102 | 0.999228396881742 |
| ebi-fl187-GCST90094765 | prot-a-3232 | WNT1-inducible-signaling pathway protein 3 \|\| id:prot-a-3232 | Fried fish liking \|\| id:ebi-fl187-GCST90094765 | Weighted mode | 5 | 0.230777265 | 0.365922942565494 | 0.562492911847828 | -0.486431702 | 0.947986232358367 | 1.2595786558624 | 0.614816332267854 | 2.58050788021835 | 0.999228396881742 |
| ebi-fl187-GCST90094765 | prot-a-327 | Carbonic anhydrase-related protein 10 \|\| id:prot-a-327 | Fried fish liking \|\| id:ebi-fl187-GCST90094765 | Weighted mode | 5 | -0.292475486 | 0.352959759581607 | 0.453890300775437 | -0.984276615 | 0.399325642899401 | 0.746413542482349 | 0.373709465130012 | 1.49081901419656 | 0.999228396881742 |
| ebi-fl187-GCST90094765 | prot-a-332 | Carbonic anhydrase 5A, mitochondrial \|\| id:prot-a-332 | Fried fish liking \|\| id:ebi-fl187-GCST90094765 | Weighted mode | 5 | -0.215403717 | 0.374375367744802 | 0.59585986527214 | -0.949179437 | 0.51837200409188 | 0.806215891657525 | 0.387058498884442 | 1.6792915433571 | 0.999228396881742 |
| ebi-fl187-GCST90094765 | prot-a-338 | Cell adhesion molecule 3 \|\| id:prot-a-338 | Fried fish liking \|\| id:ebi-fl187-GCST90094765 | Weighted mode | 5 | -0.259886263 | 0.33906775205046 | 0.486128170397515 | -0.924459057 | 0.404686531199602 | 0.771139288024517 | 0.396745978061456 | 1.49883258915569 | 0.999228396881742 |
| ebi-fl187-GCST90094765 | prot-a-34 | A disintegrin and metalloproteinase with thrombospondin motifs 3 \|\| id:prot-a-34 | Fried fish liking \|\| id:ebi-fl187-GCST90094765 | Weighted mode | 5 | -0.089938753 | 0.341007153384385 | 0.805006876336468 | -0.758312774 | 0.578435267488504 | 0.913987162396307 | 0.468456152103675 | 1.78324594366812 | 0.999228396881742 |
| ebi-fl187-GCST90094765 | prot-a-358 | Cysteine--tRNA ligase, cytoplasmic \|\| id:prot-a-358 | Fried fish liking \|\| id:ebi-fl187-GCST90094765 | Weighted mode | 5 | -0.408424851 | 0.370512832105647 | 0.332184874858067 | -1.134630002 | 0.317780299987333 | 0.664696421926118 | 0.321541068923089 | 1.37407434391239 | 0.999228396881742 |
| ebi-fl187-GCST90094765 | prot-a-382 | Coiled-coil domain-containing protein 167 \|\| id:prot-a-382 | Fried fish liking \|\| id:ebi-fl187-GCST90094765 | Weighted mode | 5 | 0.174119684407146 | 0.404389629574682 | 0.688963964675958 | -0.61848399 | 0.966723358373522 | 1.19019800543896 | 0.538760585466658 | 2.62931500626366 | 0.999228396881742 |
| ebi-fl187-GCST90094765 | prot-a-40 | Pituitary adenylate cyclase-activating polypeptide 27 \|\| id:prot-a-40 | Fried fish liking \|\| id:ebi-fl187-GCST90094765 | Weighted mode | 5 | -0.073588225 | 0.332855339175591 | 0.835855752225232 | -0.72598469 | 0.578808239666382 | 0.929054176261566 | 0.483847894169993 | 1.78391116883895 | 0.999228396881742 |
| ebi-fl187-GCST90094765 | prot-a-403 | C-C motif chemokine 25 \|\| id:prot-a-403 | Fried fish liking \|\| id:ebi-fl187-GCST90094765 | Weighted mode | 4 | -0.303211629 | 0.449596197204281 | 0.548369947004013 | -1.184420175 | 0.577996917682968 | 0.738442804040961 | 0.305923510073189 | 1.7824644294563 | 0.999228396881742 |
| ebi-fl187-GCST90094765 | prot-a-415 | Cyclin-C \|\| id:prot-a-415 | Fried fish liking \|\| id:ebi-fl187-GCST90094765 | Weighted mode | 5 | 0.247011297271498 | 0.336972316417776 | 0.504182261071927 | -0.413454443 | 0.907477037450338 | 1.28019357539097 | 0.661361663408716 | 2.47806258080533 | 0.999228396881742 |
| ebi-fl187-GCST90094765 | prot-a-44 | Adhesion G protein-coupled receptor F5 \|\| id:prot-a-44 | Fried fish liking \|\| id:ebi-fl187-GCST90094765 | Weighted mode | 5 | 0.549595184272545 | 0.593345961780218 | 0.406735201658711 | -0.613362901 | 1.71255326936177 | 1.73255151178603 | 0.541526702957146 | 5.54309644307531 | 0.999228396881742 |
| ebi-fl187-GCST90094765 | prot-a-455 | CD59 glycoprotein \|\| id:prot-a-455 | Fried fish liking \|\| id:ebi-fl187-GCST90094765 | Weighted mode | 5 | -0.077063891 | 0.310013332603087 | 0.81592493650964 | -0.684690023 | 0.530562241032007 | 0.925830699587905 | 0.504246510287284 | 1.69988778665237 | 0.999228396881742 |
| ebi-fl187-GCST90094765 | prot-a-478 | Cadherin-12 \|\| id:prot-a-478 | Fried fish liking \|\| id:ebi-fl187-GCST90094765 | Weighted mode | 5 | 0.302853845689363 | 0.33938124681486 | 0.422627007337692 | -0.362333398 | 0.968041089446488 | 1.35371659846691 | 0.696050267338796 | 2.6327820201423 | 0.999228396881742 |
| ebi-fl187-GCST90094765 | prot-a-516 | Centrin-2 \|\| id:prot-a-516 | Fried fish liking \|\| id:ebi-fl187-GCST90094765 | Weighted mode | 5 | -0.04831732 | 0.342819994779359 | 0.894729593165912 | -0.720244509 | 0.623609870222623 | 0.952831387079194 | 0.486633255049535 | 1.86565065741519 | 0.999228396881742 |
| ebi-fl187-GCST90094765 | prot-a-519 | Cryptic protein \|\| id:prot-a-519 | Fried fish liking \|\| id:ebi-fl187-GCST90094765 | Weighted mode | 5 | -0.022086362 | 0.381321639791117 | 0.95658991710729 | -0.769476776 | 0.725304052431966 | 0.978155756347712 | 0.463255391458772 | 2.06535898192847 | 0.999228396881742 |
| ebi-fl187-GCST90094765 | prot-a-583 | Chloride intracellular channel protein 4 \|\| id:prot-a-583 | Fried fish liking \|\| id:ebi-fl187-GCST90094765 | Weighted mode | 5 | -0.05894511 | 0.35062852232293 | 0.874652376319639 | -0.746177014 | 0.628286793972913 | 0.94275851595921 | 0.474175859954497 | 1.87439659939437 | 0.999228396881742 |
| ebi-fl187-GCST90094765 | prot-a-602 | CCR4-NOT transcription complex subunit 1 \|\| id:prot-a-602 | Fried fish liking \|\| id:ebi-fl187-GCST90094765 | Weighted mode | 5 | -0.525436813 | 0.375401349707561 | 0.234192854794742 | -1.261223459 | 0.210349832286801 | 0.591297021638468 | 0.283307199758367 | 1.23410971587282 | 0.999228396881742 |
| ebi-fl187-GCST90094765 | prot-a-606 | Ciliary neurotrophic factor receptor subunit alpha \|\| id:prot-a-606 | Fried fish liking \|\| id:ebi-fl187-GCST90094765 | Weighted mode | 5 | -0.588442967 | 0.344595260173517 | 0.162891300134729 | -1.263849677 | 0.0869637427234067 | 0.555191062780383 | 0.282564149257287 | 1.09085712749266 | 0.999228396881742 |
| ebi-fl187-GCST90094765 | prot-a-626 | Collagen alpha-2(IX) chain \|\| id:prot-a-626 | Fried fish liking \|\| id:ebi-fl187-GCST90094765 | Weighted mode | 5 | -0.550528793 | 0.359068281476069 | 0.199997993716374 | -1.254302624 | 0.153245038888318 | 0.576644804121705 | 0.285274722474209 | 1.16561056386838 | 0.999228396881742 |
| ebi-fl187-GCST90094765 | prot-a-647 | Probable serine carboxypeptidase CPVL \|\| id:prot-a-647 | Fried fish liking \|\| id:ebi-fl187-GCST90094765 | Weighted mode | 5 | 0.122357542972191 | 0.458827259705807 | 0.802903046184081 | -0.776943886 | 1.02165897199557 | 1.1301581096405 | 0.459809095210692 | 2.77779923470396 | 0.999228396881742 |
| ebi-fl187-GCST90094765 | prot-a-651 | Cellular retinoic acid-binding protein 2 \|\| id:prot-a-651 | Fried fish liking \|\| id:ebi-fl187-GCST90094765 | Weighted mode | 5 | 0.38357759722477 | 0.366748212818455 | 0.354649172666639 | -0.3352489 | 1.10240409434894 | 1.46752542393081 | 0.715160060931002 | 3.01139701101273 | 0.999228396881742 |
| ebi-fl187-GCST90094765 | prot-a-671 | Cytotoxic and regulatory T-cell molecule \|\| id:prot-a-671 | Fried fish liking \|\| id:ebi-fl187-GCST90094765 | Weighted mode | 5 | -0.425419099 | 0.454407589972928 | 0.402169861564123 | -1.316057975 | 0.465219777810268 | 0.653495848576793 | 0.268190434348493 | 1.59236411673123 | 0.999228396881742 |
| ebi-fl187-GCST90094765 | prot-a-688 | Chorionic somatomammotropin hormone \|\| id:prot-a-688 | Fried fish liking \|\| id:ebi-fl187-GCST90094765 | Weighted mode | 5 | 0.0445966578527695 | 0.332216590435184 | 0.899696415132481 | -0.606547859 | 0.695741175105731 | 1.04560603786066 | 0.545229834029164 | 2.00519472372121 | 0.999228396881742 |
| ebi-fl187-GCST90094765 | prot-a-69 | Aldo-keto reductase family 1 member C1 \|\| id:prot-a-69 | Fried fish liking \|\| id:ebi-fl187-GCST90094765 | Weighted mode | 5 | 0.392478786160543 | 0.34994265192227 | 0.32482990567413 | -0.293408812 | 1.07836638392819 | 1.48064645475169 | 0.745717220518648 | 2.93987300232115 | 0.999228396881742 |
| ebi-fl187-GCST90094765 | prot-a-741 | Stromal cell-derived factor 1 \|\| id:prot-a-741 | Fried fish liking \|\| id:ebi-fl187-GCST90094765 | Weighted mode | 5 | -0.299348229 | 0.317983922459978 | 0.399804573180495 | -0.922596717 | 0.323900258602683 | 0.741301221589713 | 0.397485542173245 | 1.38250940682237 | 0.999228396881742 |
| ebi-fl187-GCST90094765 | prot-a-742 | Growth-regulated alpha protein \|\| id:prot-a-742 | Fried fish liking \|\| id:ebi-fl187-GCST90094765 | Weighted mode | 5 | 0.254802694729128 | 0.358625702543914 | 0.516626596491762 | -0.448103682 | 0.9577090717152 | 1.29020703111001 | 0.638838444386302 | 2.60572011242191 | 0.999228396881742 |
| ebi-fl187-GCST90094765 | prot-a-788 | Beta-defensin 112 \|\| id:prot-a-788 | Fried fish liking \|\| id:ebi-fl187-GCST90094765 | Weighted mode | 5 | -0.217556224 | 0.381876709416967 | 0.599349523712516 | -0.966034574 | 0.530922126789486 | 0.80448237269863 | 0.380589248199991 | 1.70049966215213 | 0.999228396881742 |
| ebi-fl187-GCST90094765 | prot-a-838 | DnaJ homolog subfamily B member 1 \|\| id:prot-a-838 | Fried fish liking \|\| id:ebi-fl187-GCST90094765 | Weighted mode | 5 | 0.0915414771309804 | 0.305850540003814 | 0.77961713304199 | -0.507925581 | 0.691008535538456 | 1.09586222897113 | 0.601742551008406 | 1.99572728050071 | 0.999228396881742 |
| ebi-fl187-GCST90094765 | prot-a-85 | Junctional adhesion molecule-like \|\| id:prot-a-85 | Fried fish liking \|\| id:ebi-fl187-GCST90094765 | Weighted mode | 5 | -0.145130815 | 0.482749444387631 | 0.778671827958215 | -1.091319726 | 0.80105809564599 | 0.864909142314544 | 0.335773072601116 | 2.22789700991881 | 0.999228396881742 |
| ebi-fl187-GCST90094765 | prot-a-856 | Dipeptidase 1 \|\| id:prot-a-856 | Fried fish liking \|\| id:ebi-fl187-GCST90094765 | Weighted mode | 5 | 0.875509720984612 | 0.389086806530524 | 0.087629109741509 | 0.112899580184786 | 1.63811986178444 | 2.40009836272933 | 1.11951950536184 | 5.14548618687454 | 0.999228396881742 |
| ebi-fl187-GCST90094765 | prot-a-860 | Dipeptidyl peptidase 2 \|\| id:prot-a-860 | Fried fish liking \|\| id:ebi-fl187-GCST90094765 | Weighted mode | 5 | -0.026299822 | 0.308179826646346 | 0.936092520160946 | -0.630332282 | 0.577732638322133 | 0.974043006395022 | 0.532414859666866 | 1.78199342313754 | 0.999228396881742 |
| ebi-fl187-GCST90094765 | prot-a-861 | Dermatopontin \|\| id:prot-a-861 | Fried fish liking \|\| id:ebi-fl187-GCST90094765 | Weighted mode | 5 | -0.304947924 | 0.410787146596131 | 0.499101490854735 | -1.110090732 | 0.500194883122645 | 0.737161761676309 | 0.329529061041504 | 1.64904260996052 | 0.999228396881742 |
| ebi-fl187-GCST90094765 | prot-a-877 | Dual specificity protein phosphatase 6 \|\| id:prot-a-877 | Fried fish liking \|\| id:ebi-fl187-GCST90094765 | Weighted mode | 5 | 0.238100089622997 | 0.393224255942861 | 0.577517659432921 | -0.532619452 | 1.008819631 | 1.26883618380485 | 0.587065164801552 | 2.74236210536638 | 0.999228396881742 |
| ebi-fl187-GCST90094765 | prot-a-879 | Dynactin-associated protein \|\| id:prot-a-879 | Fried fish liking \|\| id:ebi-fl187-GCST90094765 | Weighted mode | 5 | 0.241142025417512 | 0.298782684793239 | 0.464873513706785 | -0.344472037 | 0.82675608761226 | 1.27270177847432 | 0.708594366491917 | 2.28589146841626 | 0.999228396881742 |
| ebi-fl187-GCST90094765 | prot-a-937 | EMI domain-containing protein 1 \|\| id:prot-a-937 | Fried fish liking \|\| id:ebi-fl187-GCST90094765 | Weighted mode | 5 | -0.408631862 | 0.613542135417911 | 0.541842023083582 | -1.611174447 | 0.793910723768783 | 0.66455883688877 | 0.199652994453711 | 2.21203017212621 | 0.999228396881742 |
| ebi-fl187-GCST90094765 | ukb-rsfMRI191-edge-pheno102 | Net25_Pair11_15 (Postcentral\|Precentral)&(Cerebellum) (Motor)&(Subcortical-cerebellum) \|\| id:ukb-rsfMRI191-edge-pheno102 | Fried fish liking \|\| id:ebi-fl187-GCST90094765 | Weighted mode | 5 | 0.065458204752926 | 0.105997289317359 | 0.570296472502037 | -0.142296482 | 0.273212891814949 | 1.06764811376428 | 0.867364060261319 | 1.31417999320954 | 0.999228396881742 |
| ebi-fl187-GCST90094765 | ukb-rsfMRI191-edge-pheno1167 | Net100_Pair11_45 (Angular\|Temporal)&(Frontal) (Default_mode\|Central_executive)&(Salience\|Default_mode) \|\| id:ukb-rsfMRI191-edge-pheno1167 | Fried fish liking \|\| id:ebi-fl187-GCST90094765 | Weighted mode | 5 | -0.075835172 | 0.103710899930462 | 0.505175993912565 | -0.279108535 | 0.127438192230707 | 0.926968984751963 | 0.756457796236716 | 1.13591465772031 | 0.999228396881742 |
| ebi-fl187-GCST90094765 | ukb-rsfMRI191-edge-pheno1317 | Net100_Pair26_48 (Parietal\|Frontal)&(Temporal) (Attention\|Central_executive)&(Default_mode\|Central_executive) \|\| id:ukb-rsfMRI191-edge-pheno1317 | Fried fish liking \|\| id:ebi-fl187-GCST90094765 | Weighted mode | 5 | -0.111397799 | 0.123502784500497 | 0.418066977674478 | -0.353463257 | 0.130667658479029 | 0.894582813862639 | 0.702251795118229 | 1.13958898563394 | 0.999228396881742 |
| ebi-fl187-GCST90094765 | ukb-rsfMRI191-edge-pheno286 | Net100_Pair10_13 (OccipitalPrecuneus)&(Frontal_Sup) (Default_mode\|Central_executive)&(Default_mode) \|\| id:ukb-rsfMRI191-edge-pheno286 | Fried fish liking \|\| id:ebi-fl187-GCST90094765 | Weighted mode | 5 | -0.0068697 | 0.0984794070266267 | 0.947734671053611 | -0.199889338 | 0.186149937491924 | 0.993153842169999 | 0.818821360430935 | 1.20460286196971 | 0.999228396881742 |
| ebi-fl187-GCST90094765 | ukb-rsfMRI191-edge-pheno599 | Net100_Pair11_29 (Angular\|Temporal)&(Parietal) (Default_mode\|Central_executive)&(Central_executive\|Salience) \|\| id:ukb-rsfMRI191-edge-pheno599 | Fried fish liking \|\| id:ebi-fl187-GCST90094765 | Weighted mode | 5 | 0.205451523002269 | 0.130073166465731 | 0.189367522766063 | -0.049491883 | 0.460394929275103 | 1.22807944591433 | 0.951712882901084 | 1.58469970573467 | 0.999228396881742 |
| ebi-fl187-GCST90094765 | ukb-rsfMRI191-edge-pheno621 | Net100_Pair5_30 (Precuneus\|Angular\|Cingulate)&(Paracentral) (Default_mode\|Central_executive)&(Motor) \|\| id:ukb-rsfMRI191-edge-pheno621 | Fried fish liking \|\| id:ebi-fl187-GCST90094765 | Weighted mode | 5 | -0.118292671 | 0.164375335220818 | 0.511545306304556 | -0.440468328 | 0.203882985614704 | 0.888435994738249 | 0.643734871121455 | 1.22615466732673 | 0.999228396881742 |
| ebi-fl187-GCST90094765 | ukb-rsfMRI191-edge-pheno624 | Net100_Pair8_30 (Calcarine\|Lingual)&(Paracentral) (Visual)&(Motor) \|\| id:ukb-rsfMRI191-edge-pheno624 | Fried fish liking \|\| id:ebi-fl187-GCST90094765 | Weighted mode | 5 | 0.0294065548015775 | 0.111614704029246 | 0.805207735153686 | -0.189358265 | 0.2481713746989 | 1.02984319707356 | 0.827489992801117 | 1.28167956082291 | 0.999228396881742 |
| ebi-fl187-GCST90094765 | ukb-rsfMRI191-edge-pheno882 | Net100_Pair6_38 (Postcentral\|Precentral)&(Subcortical) (Motor)&(Subcortical-cerebellum) \|\| id:ukb-rsfMRI191-edge-pheno882 | Fried fish liking \|\| id:ebi-fl187-GCST90094765 | Weighted mode | 5 | 0.0880061761146667 | 0.125777841372565 | 0.522671319660371 | -0.158518393 | 0.334530745204893 | 1.09199486629288 | 0.853407266947575 | 1.39728454888264 | 0.999228396881742 |
| ebi-fl187-GCST90094765 | ukb-rsfMRI191-edge-pheno899 | Net100_Pair23_38 (Cerebellum)&(Subcortical) (Subcortical-cerebellum)&(Subcortical-cerebellum) \|\| id:ukb-rsfMRI191-edge-pheno899 | Fried fish liking \|\| id:ebi-fl187-GCST90094765 | Weighted mode | 5 | 0.117287009125172 | 0.123241278482713 | 0.395147474581367 | -0.124265897 | 0.35883991495129 | 1.12444210852831 | 0.883144984322555 | 1.43166759464922 | 0.999228396881742 |
| ebi-fl187-GCST90094778 | ebi-a-GCST90001392 | IgD+ CD38+ B cell Absolute Count \|\| id:ebi-a-GCST90001392 | Hard cheese liking \|\| id:ebi-fl187-GCST90094778 | Inverse variance weighted | 3 | 0.248752946610436 | 0.413118665585326 | 0.54708469914141 | -0.560959638 | 1.05846553115767 | 1.2824251664457 | 0.57066117289182 | 2.88194533929691 | 0.998074519377826 |
| ebi-fl187-GCST90094778 | ebi-a-GCST90001402 | Switched memory B cell %B cell \|\| id:ebi-a-GCST90001402 | Hard cheese liking \|\| id:ebi-fl187-GCST90094778 | Inverse variance weighted | 3 | 0.297513887293921 | 0.434694114732374 | 0.493709204262918 | -0.554486578 | 1.14951435216937 | 1.34650707456245 | 0.57436707844157 | 3.15665951253012 | 0.998074519377826 |
| ebi-fl187-GCST90094778 | ebi-a-GCST90001407 | Memory B cell Absolute Count \|\| id:ebi-a-GCST90001407 | Hard cheese liking \|\| id:ebi-fl187-GCST90094778 | Inverse variance weighted | 3 | 1.17523940734989 | 0.458066307991682 | 0.0102981656706787 | 0.277429443686191 | 2.07304937101358 | 3.23891827186461 | 1.31973300036302 | 7.94902572636495 | 0.998074519377826 |
| ebi-fl187-GCST90094778 | ebi-a-GCST90001411 | IgD+ CD24+ B cell %B cell \|\| id:ebi-a-GCST90001411 | Hard cheese liking \|\| id:ebi-fl187-GCST90094778 | Inverse variance weighted | 3 | 1.21515966409542 | 0.435694481587766 | 0.00528681593886586 | 0.361198480183395 | 2.06912084800744 | 3.37083222223578 | 1.43504826125608 | 7.91785905549791 | 0.877504772527479 |
| ebi-fl187-GCST90094778 | ebi-a-GCST90001418 | CD24+ CD27+ B cell Absolute Count \|\| id:ebi-a-GCST90001418 | Hard cheese liking \|\| id:ebi-fl187-GCST90094778 | Inverse variance weighted | 3 | 1.27540041948266 | 0.569647071256614 | 0.025160413765468 | 0.1588921598197 | 2.39190867914563 | 3.58013467880403 | 1.17221152836179 | 10.934344193226 | 0.998074519377826 |
| ebi-fl187-GCST90094778 | ebi-a-GCST90001436 | Memory B cell %lymphocyte \|\| id:ebi-a-GCST90001436 | Hard cheese liking \|\| id:ebi-fl187-GCST90094778 | Inverse variance weighted | 3 | 1.36669801837404 | 0.42464035014366 | 0.00128870323591772 | 0.534402932092466 | 2.19899310465561 | 3.92237766991549 | 1.70642908398861 | 9.01593082877529 | 0.598008988154769 |
| ebi-fl187-GCST90094778 | ebi-a-GCST90001439 | IgD+ CD24+ B cell %lymphocyte \|\| id:ebi-a-GCST90001439 | Hard cheese liking \|\| id:ebi-fl187-GCST90094778 | Inverse variance weighted | 3 | 1.3218715259208 | 0.427595535070821 | 0.00199210851868293 | 0.48378427718199 | 2.15995877465961 | 3.75043384736384 | 1.62220166198998 | 8.67078019523023 | 0.598008988154769 |
| ebi-fl187-GCST90094778 | ebi-a-GCST90001457 | CD11c+ HLA DR++ monocyte %monocyte \|\| id:ebi-a-GCST90001457 | Hard cheese liking \|\| id:ebi-fl187-GCST90094778 | Inverse variance weighted | 3 | -0.478446328 | 0.461466296642037 | 0.299831023826675 | -1.382920269 | 0.426027613781377 | 0.619745525687534 | 0.250844947680144 | 1.53116305575135 | 0.998074519377826 |
| ebi-fl187-GCST90094778 | ebi-a-GCST90001483 | CD39+ resting CD4 regulatory T cell Absolute Count \|\| id:ebi-a-GCST90001483 | Hard cheese liking \|\| id:ebi-fl187-GCST90094778 | Inverse variance weighted | 3 | -0.637476434 | 0.433662372066781 | 0.141566283549444 | -1.487454684 | 0.212501814957604 | 0.528624761535307 | 0.22594703094176 | 1.23676835824538 | 0.998074519377826 |
| ebi-fl187-GCST90094778 | ebi-a-GCST90001525 | CD33dim HLA DR+ CD11b+ Absolute Count \|\| id:ebi-a-GCST90001525 | Hard cheese liking \|\| id:ebi-fl187-GCST90094778 | Inverse variance weighted | 3 | 0.163254694926729 | 0.558319784854855 | 0.769978083593986 | -0.931052083 | 1.25756147324224 | 1.17733651314204 | 0.394138825252075 | 3.51683512602685 | 0.998074519377826 |
| ebi-fl187-GCST90094778 | ebi-a-GCST90001540 | Naive CD4+ T cell Absolute Count \|\| id:ebi-a-GCST90001540 | Hard cheese liking \|\| id:ebi-fl187-GCST90094778 | Inverse variance weighted | 3 | -0.996052384 | 0.410300297569964 | 0.0151983422194668 | -1.800240968 | -0.191863801 | 0.369334558029729 | 0.165259061340478 | 0.825419281996147 | 0.998074519377826 |
| ebi-fl187-GCST90094778 | ebi-a-GCST90001624 | HLA DR+ CD4+ T cell Absolute Count \|\| id:ebi-a-GCST90001624 | Hard cheese liking \|\| id:ebi-fl187-GCST90094778 | Inverse variance weighted | 3 | 0.325145576246079 | 0.418603520964999 | 0.43731291902896 | -0.495317325 | 1.14560847733748 | 1.38423214263286 | 0.609377505989583 | 3.14435404304324 | 0.998074519377826 |
| ebi-fl187-GCST90094778 | ebi-a-GCST90001672 | CD39+ CD8+ T cell Absolute Count \|\| id:ebi-a-GCST90001672 | Hard cheese liking \|\| id:ebi-fl187-GCST90094778 | Inverse variance weighted | 3 | 0.211698123650158 | 0.448964711051977 | 0.637265810256245 | -0.66827271 | 1.09166895731203 | 1.23577477758609 | 0.512593210678697 | 2.97924215362889 | 0.998074519377826 |
| ebi-fl187-GCST90094778 | ebi-a-GCST90001722 | CD19 on CD20- CD38- B cell \|\| id:ebi-a-GCST90001722 | Hard cheese liking \|\| id:ebi-fl187-GCST90094778 | Inverse variance weighted | 3 | 0.820993871905746 | 0.567800497596855 | 0.148199752048216 | -0.291895103 | 1.93388284719558 | 2.27275754528324 | 0.746846873574251 | 6.91631315924408 | 0.998074519377826 |
| ebi-fl187-GCST90094778 | ebi-a-GCST90001742 | CD19 on transitional B cell \|\| id:ebi-a-GCST90001742 | Hard cheese liking \|\| id:ebi-fl187-GCST90094778 | Inverse variance weighted | 3 | 0.103161878504489 | 0.428612082209071 | 0.80979677083733 | -0.736917803 | 0.943241559634268 | 1.10867086453215 | 0.478586743385999 | 2.56829321507365 | 0.998074519377826 |
| ebi-fl187-GCST90094778 | ebi-a-GCST90001784 | CD25 on IgD+ CD38dim B cell \|\| id:ebi-a-GCST90001784 | Hard cheese liking \|\| id:ebi-fl187-GCST90094778 | Inverse variance weighted | 3 | 0.740263971608782 | 0.43053641504739 | 0.0855417061240456 | -0.103587402 | 1.58411534510167 | 2.09648885499391 | 0.901597218002509 | 4.87497679823301 | 0.998074519377826 |
| ebi-fl187-GCST90094778 | ebi-a-GCST90001794 | CD25 on IgD+ B cell \|\| id:ebi-a-GCST90001794 | Hard cheese liking \|\| id:ebi-fl187-GCST90094778 | Inverse variance weighted | 3 | 1.02157749537811 | 0.429342668528843 | 0.0173408966943921 | 0.180065865061576 | 1.86308912569464 | 2.77757291823819 | 1.1972962205141 | 6.4436111832188 | 0.998074519377826 |
| ebi-fl187-GCST90094778 | ebi-a-GCST90001802 | CD27 on IgD- CD38- B cell \|\| id:ebi-a-GCST90001802 | Hard cheese liking \|\| id:ebi-fl187-GCST90094778 | Inverse variance weighted | 3 | -0.104013371 | 0.488747137820884 | 0.831470227917584 | -1.061957761 | 0.8539310191566 | 0.901213247243249 | 0.345778196141068 | 2.34886214940913 | 0.998074519377826 |
| ebi-fl187-GCST90094778 | ebi-a-GCST90001804 | CD27 on IgD- CD38dim B cell \|\| id:ebi-a-GCST90001804 | Hard cheese liking \|\| id:ebi-fl187-GCST90094778 | Inverse variance weighted | 3 | 0.598634979696207 | 0.42391915361121 | 0.157907502621426 | -0.232246561 | 1.42951652077418 | 1.81963326801907 | 0.792750637515471 | 4.17667936598427 | 0.998074519377826 |
| ebi-fl187-GCST90094778 | ebi-a-GCST90001805 | CD27 on memory B cell \|\| id:ebi-a-GCST90001805 | Hard cheese liking \|\| id:ebi-fl187-GCST90094778 | Inverse variance weighted | 3 | 0.80506892404451 | 0.42990836077419 | 0.0611164002574682 | -0.037551463 | 1.64768931116192 | 2.23685066630189 | 0.963144850051747 | 5.19496200708167 | 0.998074519377826 |
| ebi-fl187-GCST90094778 | ebi-a-GCST90001806 | CD27 on unswitched memory B cell \|\| id:ebi-a-GCST90001806 | Hard cheese liking \|\| id:ebi-fl187-GCST90094778 | Inverse variance weighted | 3 | 0.296745910850784 | 0.429741390145992 | 0.489865940847926 | -0.545547214 | 1.13903903553693 | 1.34547338582377 | 0.579524572692626 | 3.12376509515196 | 0.998074519377826 |
| ebi-fl187-GCST90094778 | ebi-a-GCST90001839 | CD3 on Effector Memory CD8+ T cell \|\| id:ebi-a-GCST90001839 | Hard cheese liking \|\| id:ebi-fl187-GCST90094778 | Inverse variance weighted | 3 | -0.418142935 | 0.478622181374411 | 0.382314958221019 | -1.356242411 | 0.51995654004259 | 0.658268131847646 | 0.2576270169093 | 1.68195455043733 | 0.998074519377826 |
| ebi-fl187-GCST90094778 | ebi-a-GCST90001859 | CD3 on CD8+ T cell \|\| id:ebi-a-GCST90001859 | Hard cheese liking \|\| id:ebi-fl187-GCST90094778 | Inverse variance weighted | 3 | 0.296237363241977 | 0.469273032922509 | 0.527864868654634 | -0.623537781 | 1.21601250777009 | 1.34478932250482 | 0.536044670292414 | 3.37370824139797 | 0.998074519377826 |
| ebi-fl187-GCST90094778 | ebi-a-GCST90001905 | CD86 on monocyte \|\| id:ebi-a-GCST90001905 | Hard cheese liking \|\| id:ebi-fl187-GCST90094778 | Inverse variance weighted (multiplicative random effects) | 3 | -0.658049 | 1.14708989895663 | 0.566192349606411 | -2.906345202 | 1.59024720161908 | 0.517860695583083 | 0.0546751919166075 | 4.90496129284433 | 0.998074519377826 |
| ebi-fl187-GCST90094778 | ebi-a-GCST90001943 | CD25 on activated & secreting CD4 regulatory T cell \|\| id:ebi-a-GCST90001943 | Hard cheese liking \|\| id:ebi-fl187-GCST90094778 | Inverse variance weighted | 3 | -0.209946141 | 0.435999265100103 | 0.630140817338256 | -1.0645047 | 0.644612418878658 | 0.810627904631747 | 0.344898640657137 | 1.90524844782121 | 0.998074519377826 |
| ebi-fl187-GCST90094778 | ebi-a-GCST90001949 | CD33 on CD33dim HLA DR+ CD11b- \|\| id:ebi-a-GCST90001949 | Hard cheese liking \|\| id:ebi-fl187-GCST90094778 | Inverse variance weighted | 3 | -0.550125009 | 0.777326879455995 | 0.479122966386359 | -2.073685693 | 0.973435674662214 | 0.576877690928255 | 0.125721555700019 | 2.64702316510282 | 0.998074519377826 |
| ebi-fl187-GCST90094778 | ebi-a-GCST90001955 | CD33 on Immature Myeloid-Derived Suppressor Cells \|\| id:ebi-a-GCST90001955 | Hard cheese liking \|\| id:ebi-fl187-GCST90094778 | Inverse variance weighted | 3 | 0.0525506097981984 | 0.722664403895218 | 0.942030635015744 | -1.363871622 | 1.46897284143283 | 1.05395590122426 | 0.255669004607527 | 4.34477008048213 | 0.998074519377826 |
| ebi-fl187-GCST90094778 | ebi-a-GCST90001956 | CD33 on CD33+ HLA DR+ \|\| id:ebi-a-GCST90001956 | Hard cheese liking \|\| id:ebi-fl187-GCST90094778 | Inverse variance weighted | 3 | -0.218281969 | 0.716842244489832 | 0.760743267539529 | -1.623292768 | 1.18672883010755 | 0.803898735109127 | 0.197248136186369 | 3.27634617393517 | 0.998074519377826 |
| ebi-fl187-GCST90094778 | ebi-a-GCST90001957 | CD33 on CD33+ HLA DR+ CD14- \|\| id:ebi-a-GCST90001957 | Hard cheese liking \|\| id:ebi-fl187-GCST90094778 | Inverse variance weighted | 3 | -0.28747243 | 0.785579047346207 | 0.71441209606734 | -1.827207362 | 1.2522625031798 | 0.750157248607206 | 0.160862170834645 | 3.49824880963707 | 0.998074519377826 |
| ebi-fl187-GCST90094778 | ebi-a-GCST90002064 | CD4 on resting CD4 regulatory T cell \|\| id:ebi-a-GCST90002064 | Hard cheese liking \|\| id:ebi-fl187-GCST90094778 | Inverse variance weighted | 3 | 0.194304381767924 | 0.514589175990535 | 0.705734249096931 | -0.814290403 | 1.20289916670937 | 1.21446588797693 | 0.442953534290256 | 3.32975646175365 | 0.998074519377826 |
| ebi-fl187-GCST90094778 | ebi-a-GCST90002080 | SSC-A on T cell \|\| id:ebi-a-GCST90002080 | Hard cheese liking \|\| id:ebi-fl187-GCST90094778 | Inverse variance weighted | 3 | 0.148538212665658 | 0.444999163792473 | 0.738534736957158 | -0.723660148 | 1.02073657369891 | 1.16013712885964 | 0.484973926944231 | 2.77523817875979 | 0.998074519377826 |
| ebi-fl187-GCST90094778 | ebi-a-GCST90002121 | CD8 on CD39+ CD8+ T cell \|\| id:ebi-a-GCST90002121 | Hard cheese liking \|\| id:ebi-fl187-GCST90094778 | Inverse variance weighted | 3 | -0.554857794 | 0.46983882470463 | 0.237621204925543 | -1.475741891 | 0.366026302196381 | 0.574153903392279 | 0.228609061192213 | 1.44199316974326 | 0.998074519377826 |
| ebi-fl187-GCST90094778 | ebi-cfmet338-GCST90026053 | 1-palmitoyl-2-docosahexaenoyl-gpc (16:0/22:6) levels \|\| id:ebi-cfmet338-GCST90026053 | Hard cheese liking \|\| id:ebi-fl187-GCST90094778 | Inverse variance weighted | 3 | 0.36005498950535 | 0.214626635202703 | 0.0934276282121177 | -0.060613215 | 0.780723194502648 | 1.43340823480297 | 0.941187206013684 | 2.18305046485205 | 0.998074519377826 |
| ebi-fl187-GCST90094778 | ebi-cfmet338-GCST90026063 | 1-stearoyl-2-oleoyl-gpc (18:0/18:1) levels \|\| id:ebi-cfmet338-GCST90026063 | Hard cheese liking \|\| id:ebi-fl187-GCST90094778 | Inverse variance weighted | 3 | 0.138101315836559 | 0.175654374220166 | 0.431744167468622 | -0.206181258 | 0.482383889308084 | 1.14809186426953 | 0.813685576192419 | 1.6199315403499 | 0.998074519377826 |
| ebi-fl187-GCST90094778 | ebi-cfmet338-GCST90026113 | Adenosine 3',5'-cyclic monophosphate (camp) levels \|\| id:ebi-cfmet338-GCST90026113 | Hard cheese liking \|\| id:ebi-fl187-GCST90094778 | Inverse variance weighted | 2 | 0.370032674683045 | 0.39920209271843 | 0.353962517098631 | -0.412403427 | 1.15246877641117 | 1.44778191970583 | 0.662057130417062 | 3.16599941413918 | 0.998074519377826 |
| ebi-fl187-GCST90094778 | ebi-cfmet338-GCST90026117 | Alpha-ketoglutaramate levels \|\| id:ebi-cfmet338-GCST90026117 | Hard cheese liking \|\| id:ebi-fl187-GCST90094778 | Inverse variance weighted (multiplicative random effects) | 3 | 0.0209043341927782 | 0.264021054546504 | 0.936892021785264 | -0.496576933 | 0.538385601103926 | 1.02112436027854 | 0.608610412505298 | 1.71323877759844 | 0.998074519377826 |
| ebi-fl187-GCST90094778 | ebi-cfmet338-GCST90026124 | Arginine levels \|\| id:ebi-cfmet338-GCST90026124 | Hard cheese liking \|\| id:ebi-fl187-GCST90094778 | Inverse variance weighted | 3 | -0.014422478 | 0.110652118203932 | 0.896296853325581 | -0.23130063 | 0.202455673299467 | 0.985681027360491 | 0.793500879956856 | 1.22440581004933 | 0.998074519377826 |
| ebi-fl187-GCST90094778 | ebi-cfmet338-GCST90026169 | Glutamate levels \|\| id:ebi-cfmet338-GCST90026169 | Hard cheese liking \|\| id:ebi-fl187-GCST90094778 | Inverse variance weighted | 3 | 0.0447832808068482 | 0.11578029258121 | 0.69890770708594 | -0.182146093 | 0.27171265426602 | 1.04580119015763 | 0.833479566280303 | 1.31220988921917 | 0.998074519377826 |
| ebi-fl187-GCST90094778 | ebi-cfmet338-GCST90026170 | Glutamine levels \|\| id:ebi-cfmet338-GCST90026170 | Hard cheese liking \|\| id:ebi-fl187-GCST90094778 | Inverse variance weighted | 3 | -0.091222274 | 0.069594262475759 | 0.18993449816804 | -0.227627028 | 0.0451824806460441 | 0.912814793529757 | 0.796421247067232 | 1.04621875716537 | 0.998074519377826 |
| ebi-fl187-GCST90094778 | ebi-cfmet338-GCST90026191 | Kynurenate levels \|\| id:ebi-cfmet338-GCST90026191 | Hard cheese liking \|\| id:ebi-fl187-GCST90094778 | Inverse variance weighted | 3 | 0.00379394010429364 | 0.529304532469246 | 0.994280985467724 | -1.033642944 | 1.04123082374402 | 1.00380114620534 | 0.355708770422434 | 2.83270142573805 | 0.999355215041207 |
| ebi-fl187-GCST90094778 | ebi-cfmet338-GCST90026202 | Methylsuccinoylcarnitine levels \|\| id:ebi-cfmet338-GCST90026202 | Hard cheese liking \|\| id:ebi-fl187-GCST90094778 | Inverse variance weighted | 3 | 0.1168937300396 | 0.470659998011581 | 0.803854872004109 | -0.805599866 | 1.0393873261423 | 1.12399997591054 | 0.446819814108812 | 2.82748415794122 | 0.998074519377826 |
| ebi-fl187-GCST90094778 | ebi-cfmet338-GCST90026222 | N-acetylputrescine levels \|\| id:ebi-cfmet338-GCST90026222 | Hard cheese liking \|\| id:ebi-fl187-GCST90094778 | Inverse variance weighted | 3 | 0.0229783064239681 | 0.128037783593513 | 0.857572562802489 | -0.227975749 | 0.273932362267254 | 1.02324434147752 | 0.796143566545148 | 1.31512584709982 | 0.998074519377826 |
| ebi-fl187-GCST90094778 | ebi-cfmet338-GCST90026252 | Propionylcarnitine (c3) levels \|\| id:ebi-cfmet338-GCST90026252 | Hard cheese liking \|\| id:ebi-fl187-GCST90094778 | Inverse variance weighted | 3 | -0.230728436 | 0.330321531411399 | 0.48486674145175 | -0.878158638 | 0.416701765628925 | 0.79395504641989 | 0.415547380995794 | 1.51695003882599 | 0.998074519377826 |
| ebi-fl187-GCST90094778 | ebi-cfmet338-GCST90026316 | Ethyl beta-glucopyranoside levels \|\| id:ebi-cfmet338-GCST90026316 | Hard cheese liking \|\| id:ebi-fl187-GCST90094778 | Inverse variance weighted | 3 | -0.074774641 | 0.6902129328098 | 0.913729474473987 | -1.427591989 | 1.27804270775235 | 0.927952585646233 | 0.23988587516967 | 3.58960693537491 | 0.998074519377826 |
| ebi-fl187-GCST90094778 | ebi-gm430-GCST90011315 | F_Prevotellaceae abundance \|\| id:ebi-gm430-GCST90011315 | Hard cheese liking \|\| id:ebi-fl187-GCST90094778 | Inverse variance weighted | 3 | -0.411072635 | 0.314378990767262 | 0.19101909239692 | -1.027255457 | 0.205110187260826 | 0.66293877753048 | 0.357988127497256 | 1.22766033003979 | 0.998074519377826 |
| ebi-fl187-GCST90094778 | ebi-gm430-GCST90011354 | OTU97_1 (Bacteroides) abundance \|\| id:ebi-gm430-GCST90011354 | Hard cheese liking \|\| id:ebi-fl187-GCST90094778 | Inverse variance weighted | 3 | 0.224227705262905 | 0.312202240970302 | 0.472625969090923 | -0.387688687 | 0.836144097564697 | 1.25135592732004 | 0.678623574697377 | 2.30745248945599 | 0.998074519377826 |
| ebi-fl187-GCST90094778 | ebi-gm430-GCST90011419 | OTU99_1 (Bacteroides) abundance \|\| id:ebi-gm430-GCST90011419 | Hard cheese liking \|\| id:ebi-fl187-GCST90094778 | Inverse variance weighted | 3 | 0.24042165324474 | 0.317211065074042 | 0.448496908279062 | -0.381312034 | 0.862155340789863 | 1.27178528967502 | 0.682964747940563 | 2.36825960331196 | 0.998074519377826 |
| ebi-fl187-GCST90094778 | ebi-gm430-GCST90011435 | OTU99_155 (Butyrivibrio) abundance \|\| id:ebi-gm430-GCST90011435 | Hard cheese liking \|\| id:ebi-fl187-GCST90094778 | Inverse variance weighted | 3 | -1.329673124 | 0.909405487397049 | 0.143704360348587 | -3.112107879 | 0.452761631120201 | 0.264563726652955 | 0.0445070409168084 | 1.57264927118679 | 0.998074519377826 |
| ebi-fl187-GCST90094778 | ebi-gm430-GCST90011466 | OTU99_58 (Bacteroides) abundance \|\| id:ebi-gm430-GCST90011466 | Hard cheese liking \|\| id:ebi-fl187-GCST90094778 | Inverse variance weighted | 3 | 0.0379189365917588 | 0.510961503433267 | 0.940842536139349 | -0.96356561 | 1.03940348332096 | 1.03864703319732 | 0.381530070297875 | 2.82752984247699 | 0.998074519377826 |
| ebi-fl187-GCST90094778 | ebi-gm430-GCST90011472 | OTU99_72 (Parabacteroides) abundance \|\| id:ebi-gm430-GCST90011472 | Hard cheese liking \|\| id:ebi-fl187-GCST90094778 | Inverse variance weighted | 3 | -0.645006897 | 0.785596731585081 | 0.411623081085818 | -2.184776491 | 0.894762697162028 | 0.524658923655449 | 0.112502876288963 | 2.44675509863652 | 0.998074519377826 |
| ebi-fl187-GCST90094778 | ebi-gm430-GCST90011505 | TestASV_28 (Holdemanella) abundance \|\| id:ebi-gm430-GCST90011505 | Hard cheese liking \|\| id:ebi-fl187-GCST90094778 | Inverse variance weighted | 3 | 0.0929298572870613 | 0.707195104051779 | 0.895453944030606 | -1.293172547 | 1.47903226122855 | 1.09738475902432 | 0.274398857531387 | 4.38869651343613 | 0.998074519377826 |
| ebi-fl187-GCST90094778 | ebi-gm430-GCST90011513 | TestASV_35 (Alphaproteobacteria) abundance \|\| id:ebi-gm430-GCST90011513 | Hard cheese liking \|\| id:ebi-fl187-GCST90094778 | Inverse variance weighted | 2 | -1.008556391 | 0.944930626380987 | 0.285821132817832 | -2.860620419 | 0.843507636635716 | 0.364745149042632 | 0.0572332406708711 | 2.32450621685384 | 0.998074519377826 |
| ebi-fl187-GCST90094778 | ebi-gm430-GCST90011543 | G_Coprococcus prevalence \|\| id:ebi-gm430-GCST90011543 | Hard cheese liking \|\| id:ebi-fl187-GCST90094778 | Inverse variance weighted | 3 | 0.0324376512531437 | 0.884473136041333 | 0.970744503848431 | -1.701129695 | 1.76600499789416 | 1.03296948678312 | 0.182477263845711 | 5.84744607704765 | 0.998074519377826 |
| ebi-fl187-GCST90094778 | ebi-gm430-GCST90011547 | G_Parasutterella prevalence \|\| id:ebi-gm430-GCST90011547 | Hard cheese liking \|\| id:ebi-fl187-GCST90094778 | Inverse variance weighted | 3 | 0.241631683956656 | 0.75832464198208 | 0.750000397677585 | -1.244684614 | 1.72794798224153 | 1.27332512036782 | 0.28803173488949 | 5.62909105408746 | 0.998074519377826 |
| ebi-fl187-GCST90094778 | ebi-gm430-GCST90011569 | OTU97_13 (Subdoligranulum) prevalence \|\| id:ebi-gm430-GCST90011569 | Hard cheese liking \|\| id:ebi-fl187-GCST90094778 | Inverse variance weighted | 3 | -0.867921062 | 0.757941297913678 | 0.252166379026641 | -2.353486006 | 0.617643881431003 | 0.419823429320591 | 0.0950372835083347 | 1.8545533426473 | 0.998074519377826 |
| ebi-fl187-GCST90094778 | ebi-gm430-GCST90011590 | OTU97_277 (Prevotella) prevalence \|\| id:ebi-gm430-GCST90011590 | Hard cheese liking \|\| id:ebi-fl187-GCST90094778 | Inverse variance weighted | 3 | 0.242844745021855 | 1.08570011476013 | 0.823009646990385 | -1.88512748 | 2.37081696995171 | 1.27487067873349 | 0.151809705508547 | 10.7061353030744 | 0.998074519377826 |
| ebi-fl187-GCST90094778 | ebi-gm430-GCST90011616 | OTU97_86 (Phascolarctobacterium) prevalence \|\| id:ebi-gm430-GCST90011616 | Hard cheese liking \|\| id:ebi-fl187-GCST90094778 | Inverse variance weighted | 3 | 1.44686576278669 | 0.814898845954374 | 0.0758127618182772 | -0.150335975 | 3.04406750085726 | 4.24977382038275 | 0.860418848390994 | 20.9904484986404 | 0.998074519377826 |
| ebi-fl187-GCST90094778 | ebi-gm430-GCST90011665 | OTU99_55 (Barnesiella) prevalence \|\| id:ebi-gm430-GCST90011665 | Hard cheese liking \|\| id:ebi-fl187-GCST90094778 | Inverse variance weighted | 3 | 0.108801221984009 | 0.565515859919683 | 0.847434473305505 | -0.999609863 | 1.21721230742659 | 1.11494070261202 | 0.36802299238475 | 3.37775844461746 | 0.998074519377826 |
| ebi-fl187-GCST90094778 | ebi-gm430-GCST90011687 | TestASV_14 (Lachnospiraceae) prevalence \|\| id:ebi-gm430-GCST90011687 | Hard cheese liking \|\| id:ebi-fl187-GCST90094778 | Inverse variance weighted | 3 | 0.664353979113983 | 0.615472193471989 | 0.280399826714859 | -0.54197152 | 1.87067947831908 | 1.94323474551976 | 0.581600484282069 | 6.49270655415043 | 0.998074519377826 |
| ebi-fl187-GCST90094778 | ebi-gm430-GCST90011697 | TestASV_23 (Barnesiella) prevalence \|\| id:ebi-gm430-GCST90011697 | Hard cheese liking \|\| id:ebi-fl187-GCST90094778 | Inverse variance weighted | 3 | 0.0789684904884249 | 0.803638706562947 | 0.921722923862893 | -1.496163374 | 1.6541003553518 | 1.08217022287793 | 0.22398787134485 | 5.22837412692163 | 0.998074519377826 |
| ebi-fl187-GCST90094778 | ebi-gm430-GCST90011727 | TestASV_6 (Subdoligranulum) prevalence \|\| id:ebi-gm430-GCST90011727 | Hard cheese liking \|\| id:ebi-fl187-GCST90094778 | Inverse variance weighted | 2 | -1.834167134 | 1.44190230751889 | 0.203356511732361 | -4.660295657 | 0.991961388383175 | 0.159746493744939 | 0.00946366398868144 | 2.6965182084151 | 0.998074519377826 |
| ebi-fl187-GCST90094778 | ebi-hbsm44-GCST90104908 | Sulcal depth of the left posterior inferior temporal sulcus \|\| id:ebi-hbsm44-GCST90104908 | Hard cheese liking \|\| id:ebi-fl187-GCST90094778 | Inverse variance weighted | 3 | 0.454495322708006 | 0.269479181744282 | 0.0916861937018454 | -0.073683874 | 0.982674518926799 | 1.57537812481059 | 0.928965317972231 | 2.67159191857572 | 0.998074519377826 |
| ebi-fl187-GCST90094778 | ebi-hbsm44-GCST90104918 | Sulcal depth of the left inferior frontal sulcus \|\| id:ebi-hbsm44-GCST90104918 | Hard cheese liking \|\| id:ebi-fl187-GCST90094778 | Inverse variance weighted | 3 | -0.555957707 | 0.604150769225868 | 0.357452541969937 | -1.740093214 | 0.628177801056613 | 0.573522731574118 | 0.175504040366692 | 1.8741923145757 | 0.998074519377826 |
| ebi-fl187-GCST90094778 | ebi-hbsm44-GCST90104925 | Sulcal depth of the right anterior inferior temporal sulcus \|\| id:ebi-hbsm44-GCST90104925 | Hard cheese liking \|\| id:ebi-fl187-GCST90094778 | Inverse variance weighted | 3 | -0.143785 | 0.15164854190622 | 0.343054993206052 | -0.441016142 | 0.153446142025233 | 0.866073933843205 | 0.643382320852698 | 1.16584499538087 | 0.998074519377826 |
| ebi-fl187-GCST90094778 | ebi-IPs91-GCST90274770 | C-C motif chemokine 4 levels \|\| id:ebi-IPs91-GCST90274770 | Hard cheese liking \|\| id:ebi-fl187-GCST90094778 | Inverse variance weighted | 4 | 0.138532460390279 | 0.169576260031524 | 0.413966658709271 | -0.193837009 | 0.470901930052067 | 1.1485869645462 | 0.823792163811654 | 1.60143792703895 | 0.998074519377826 |
| ebi-fl187-GCST90094778 | ebi-IPs91-GCST90274779 | C-X-C motif chemokine 1 levels \|\| id:ebi-IPs91-GCST90274779 | Hard cheese liking \|\| id:ebi-fl187-GCST90094778 | Inverse variance weighted | 4 | 0.175960863903879 | 0.239418450930201 | 0.462369250223101 | -0.2932993 | 0.645221027727073 | 1.19239139219337 | 0.745798889741867 | 1.9064083518131 | 0.998074519377826 |
| ebi-fl187-GCST90094778 | ebi-IPs91-GCST90274780 | C-X-C motif chemokine 10 levels \|\| id:ebi-IPs91-GCST90274780 | Hard cheese liking \|\| id:ebi-fl187-GCST90094778 | Inverse variance weighted | 4 | 0.0900241178020452 | 0.168485414411066 | 0.593124299178238 | -0.240207294 | 0.420255530047735 | 1.09420067310221 | 0.786464814381658 | 1.52235051222052 | 0.998074519377826 |
| ebi-fl187-GCST90094778 | ebi-IPs91-GCST90274796 | Interleukin-10 receptor subunit alpha levels \|\| id:ebi-IPs91-GCST90274796 | Hard cheese liking \|\| id:ebi-fl187-GCST90094778 | Inverse variance weighted | 4 | 0.31755317614054 | 0.190911844974595 | 0.096242438681465 | -0.05663404 | 0.691740392290746 | 1.37376229429992 | 0.944939816263926 | 1.99718840158712 | 0.998074519377826 |
| ebi-fl187-GCST90094778 | ebi-IPs91-GCST90274807 | Interleukin-20 levels \|\| id:ebi-IPs91-GCST90274807 | Hard cheese liking \|\| id:ebi-fl187-GCST90094778 | Inverse variance weighted | 4 | -0.204232352 | 0.188892441101781 | 0.279603815318731 | -0.574461536 | 0.165996832728394 | 0.815272919022788 | 0.563007946492477 | 1.18056936253353 | 0.998074519377826 |
| ebi-fl187-GCST90094778 | ebi-IPs91-GCST90274808 | Interleukin-20 receptor subunit alpha levels \|\| id:ebi-IPs91-GCST90274808 | Hard cheese liking \|\| id:ebi-fl187-GCST90094778 | Inverse variance weighted | 4 | 0.0531148053567685 | 0.191710958385905 | 0.781736569683972 | -0.322638673 | 0.428868283793142 | 1.05455070624005 | 0.724235492876833 | 1.53551876836906 | 0.998074519377826 |
| ebi-fl187-GCST90094778 | ebi-IPs91-GCST90274810 | Interleukin-24 levels \|\| id:ebi-IPs91-GCST90274810 | Hard cheese liking \|\| id:ebi-fl187-GCST90094778 | Inverse variance weighted | 4 | 0.318185947887294 | 0.192501964694771 | 0.0983517982574219 | -0.059117903 | 0.695489798689045 | 1.3746318473518 | 0.942595627833609 | 2.00469072840573 | 0.998074519377826 |
| ebi-fl187-GCST90094778 | ebi-IPs91-GCST90274823 | Monocyte chemoattractant protein-3 levels \|\| id:ebi-IPs91-GCST90274823 | Hard cheese liking \|\| id:ebi-fl187-GCST90094778 | Inverse variance weighted | 4 | 0.0355869468760576 | 0.191718333978723 | 0.852741992305897 | -0.340180988 | 0.411354881474355 | 1.03622774097632 | 0.711641512730004 | 1.50886072827553 | 0.998074519377826 |
| ebi-fl187-GCST90094778 | ebi-IPs91-GCST90274824 | Monocyte chemoattractant protein-4 levels \|\| id:ebi-IPs91-GCST90274824 | Hard cheese liking \|\| id:ebi-fl187-GCST90094778 | Inverse variance weighted | 4 | -0.035596291 | 0.170718255302044 | 0.834831564186858 | -0.370204071 | 0.299011489468824 | 0.965029806145074 | 0.690593385955928 | 1.3485251172212 | 0.998074519377826 |
| ebi-fl187-GCST90094778 | ebi-IPs91-GCST90274834 | SIR2-like protein 2 levels \|\| id:ebi-IPs91-GCST90274834 | Hard cheese liking \|\| id:ebi-fl187-GCST90094778 | Inverse variance weighted | 4 | 0.0150275588956263 | 0.232139156495704 | 0.948384890330723 | -0.439965188 | 0.470020305627206 | 1.0151410403962 | 0.644058841774938 | 1.60002668243284 | 0.998074519377826 |
| ebi-fl187-GCST90094778 | ebi-met1400-GCST90199652 | 1-stearoyl-2-oleoyl-GPS (18:0/18:1) levels \|\| id:ebi-met1400-GCST90199652 | Hard cheese liking \|\| id:ebi-fl187-GCST90094778 | Inverse variance weighted | 3 | -0.175265532 | 0.255659169 | 0.493000896344212 | -0.676357503 | 0.325826439136825 | 0.839234147804193 | 0.508465708273829 | 1.38517493585099 | 0.998074519377826 |
| ebi-fl187-GCST90094778 | ebi-met1400-GCST90199665 | N-acetylaspartate (naa) levels \|\| id:ebi-met1400-GCST90199665 | Hard cheese liking \|\| id:ebi-fl187-GCST90094778 | Inverse variance weighted | 3 | -0.157380504 | 0.334195189257685 | 0.63769507504657 | -0.812403075 | 0.497642066742062 | 0.854378902196637 | 0.443790322307849 | 1.64483827570347 | 0.998074519377826 |
| ebi-fl187-GCST90094778 | ebi-met1400-GCST90199669 | Acetylcarnitine levels (Biocrates platform) \|\| id:ebi-met1400-GCST90199669 | Hard cheese liking \|\| id:ebi-fl187-GCST90094778 | Inverse variance weighted | 3 | -0.513238477 | 0.245690954687274 | 0.0367113606101745 | -0.994792748 | -0.031684206 | 0.598554033310188 | 0.369800078398671 | 0.968812479281459 | 0.998074519377826 |
| ebi-fl187-GCST90094778 | ebi-met1400-GCST90199670 | Iminodiacetate (IDA) levels \|\| id:ebi-met1400-GCST90199670 | Hard cheese liking \|\| id:ebi-fl187-GCST90094778 | Inverse variance weighted | 3 | 0.338309686915948 | 0.253082160588759 | 0.181301534330166 | -0.157731348 | 0.834350721669916 | 1.40257479521732 | 0.854079201374043 | 2.30331806817688 | 0.998074519377826 |
| ebi-fl187-GCST90094778 | ebi-met1400-GCST90199686 | Indolepropionate levels \|\| id:ebi-met1400-GCST90199686 | Hard cheese liking \|\| id:ebi-fl187-GCST90094778 | Inverse variance weighted (multiplicative random effects) | 3 | 0.448627466284945 | 0.478157884076201 | 0.348120427537282 | -0.488561987 | 1.3858169190743 | 1.56616110069575 | 0.613507992930535 | 3.9980906876469 | 0.998074519377826 |
| ebi-fl187-GCST90094778 | ebi-met1400-GCST90199704 | 3-hydroxysebacate levels \|\| id:ebi-met1400-GCST90199704 | Hard cheese liking \|\| id:ebi-fl187-GCST90094778 | Inverse variance weighted | 3 | -0.348286787 | 0.333867836259699 | 0.296861415166325 | -1.002667746 | 0.306094172130718 | 0.705896405313935 | 0.366899340172622 | 1.35811019665692 | 0.998074519377826 |
| ebi-fl187-GCST90094778 | ebi-met1400-GCST90199713 | Docosapentaenoate n3 DPA; 22:5n3 levels \|\| id:ebi-met1400-GCST90199713 | Hard cheese liking \|\| id:ebi-fl187-GCST90094778 | Inverse variance weighted | 3 | -0.00161966 | 0.272957566822893 | 0.995265587454998 | -0.53661649 | 0.53337717146877 | 0.998381651436499 | 0.584723326091549 | 1.70467959365967 | 0.999355215041207 |
| ebi-fl187-GCST90094778 | ebi-met1400-GCST90199715 | Myristoleate (14:1n5) levels \|\| id:ebi-met1400-GCST90199715 | Hard cheese liking \|\| id:ebi-fl187-GCST90094778 | Inverse variance weighted | 3 | 0.0281867656488698 | 0.282526721976433 | 0.92052952617427 | -0.525565609 | 0.581939140722679 | 1.02858777134578 | 0.591220869682946 | 1.78950517076546 | 0.998074519377826 |
| ebi-fl187-GCST90094778 | ebi-met1400-GCST90199725 | Campesterol levels \|\| id:ebi-met1400-GCST90199725 | Hard cheese liking \|\| id:ebi-fl187-GCST90094778 | Inverse variance weighted | 3 | 0.0664831203141305 | 0.317356043809956 | 0.834065242498224 | -0.555534726 | 0.688500966181644 | 1.06874292387809 | 0.573765372146907 | 1.99072912515716 | 0.998074519377826 |
| ebi-fl187-GCST90094778 | ebi-met1400-GCST90199737 | N-acetylproline levels \|\| id:ebi-met1400-GCST90199737 | Hard cheese liking \|\| id:ebi-fl187-GCST90094778 | Inverse variance weighted | 3 | -0.3453518 | 0.272653167343498 | 0.20528615239282 | -0.879752008 | 0.189048407881451 | 0.707971245288979 | 0.414885787237462 | 1.20809943260156 | 0.998074519377826 |
| ebi-fl187-GCST90094778 | ebi-met1400-GCST90199775 | Gamma-glutamylisoleucine levels \|\| id:ebi-met1400-GCST90199775 | Hard cheese liking \|\| id:ebi-fl187-GCST90094778 | Inverse variance weighted | 3 | 0.209340875259371 | 0.243571392291379 | 0.390084500554165 | -0.268059054 | 0.686740804150475 | 1.23286518014948 | 0.764862611854167 | 1.98722820133718 | 0.998074519377826 |
| ebi-fl187-GCST90094778 | ebi-met1400-GCST90199777 | 1-palmitoleoyl-GPC (16:1) levels \|\| id:ebi-met1400-GCST90199777 | Hard cheese liking \|\| id:ebi-fl187-GCST90094778 | Inverse variance weighted | 3 | 0.125082940586989 | 0.249001201789889 | 0.615429289388772 | -0.362959415 | 0.613125296095171 | 1.13324244096232 | 0.69561466450219 | 1.84619228940105 | 0.998074519377826 |
| ebi-fl187-GCST90094778 | ebi-met1400-GCST90199782 | Glutamine degradant levels \|\| id:ebi-met1400-GCST90199782 | Hard cheese liking \|\| id:ebi-fl187-GCST90094778 | Inverse variance weighted | 2 | 0.109250592876918 | 0.37810434721121 | 0.772625020516765 | -0.631833928 | 0.850335113410889 | 1.11544183710028 | 0.531615961256368 | 2.34043103034983 | 0.998074519377826 |
| ebi-fl187-GCST90094778 | ebi-met1400-GCST90199783 | 5-methyluridine (ribothymidine) levels \|\| id:ebi-met1400-GCST90199783 | Hard cheese liking \|\| id:ebi-fl187-GCST90094778 | Inverse variance weighted | 3 | -0.02700272 | 0.31190207246539 | 0.931009854187849 | -0.638330782 | 0.584325342500609 | 0.973358594441325 | 0.528173325282329 | 1.79378038992476 | 0.998074519377826 |
| ebi-fl187-GCST90094778 | ebi-met1400-GCST90199787 | Homostachydrine levels \|\| id:ebi-met1400-GCST90199787 | Hard cheese liking \|\| id:ebi-fl187-GCST90094778 | Inverse variance weighted | 3 | -0.000199447 | 0.246804181208379 | 0.999355215041207 | -0.483935642 | 0.483536748219642 | 0.99980057293944 | 0.616352867801498 | 1.62180016978839 | 0.999355215041207 |
| ebi-fl187-GCST90094778 | ebi-met1400-GCST90199802 | Beta-hydroxyisovaleroylcarnitine levels \|\| id:ebi-met1400-GCST90199802 | Hard cheese liking \|\| id:ebi-fl187-GCST90094778 | Inverse variance weighted | 3 | -0.426207137 | 0.248982532827258 | 0.0869343931391938 | -0.914212901 | 0.0617986275855716 | 0.652981071730593 | 0.400831996385262 | 1.06374811363266 | 0.998074519377826 |
| ebi-fl187-GCST90094778 | ebi-met1400-GCST90199873 | Pregnenolone sulfate levels \|\| id:ebi-met1400-GCST90199873 | Hard cheese liking \|\| id:ebi-fl187-GCST90094778 | Inverse variance weighted (multiplicative random effects) | 3 | -0.111277395 | 0.445413945395443 | 0.802719463057501 | -0.984288728 | 0.761733938173049 | 0.894690532000587 | 0.373704938371106 | 2.14198707552692 | 0.998074519377826 |
| ebi-fl187-GCST90094778 | ebi-met1400-GCST90199911 | Beta-citrylglutamate levels \|\| id:ebi-met1400-GCST90199911 | Hard cheese liking \|\| id:ebi-fl187-GCST90094778 | Inverse variance weighted | 3 | -0.110102612 | 0.288898531342897 | 0.703120363023353 | -0.676343733 | 0.456138509839083 | 0.895742217044866 | 0.50847270977554 | 1.57796889384809 | 0.998074519377826 |
| ebi-fl187-GCST90094778 | ebi-met1400-GCST90199934 | 2-hydroxydecanoate levels \|\| id:ebi-met1400-GCST90199934 | Hard cheese liking \|\| id:ebi-fl187-GCST90094778 | Inverse variance weighted | 3 | 0.0689680669514476 | 0.251622136676213 | 0.784012540337271 | -0.424211321 | 0.562147454836825 | 1.07140199547014 | 0.654285603054071 | 1.75443602998326 | 0.998074519377826 |
| ebi-fl187-GCST90094778 | ebi-met1400-GCST90199945 | Sphingomyelin (d18:1/14:0, d16:1/16:0) levels \|\| id:ebi-met1400-GCST90199945 | Hard cheese liking \|\| id:ebi-fl187-GCST90094778 | Inverse variance weighted | 3 | 0.145445061162904 | 0.241882904377772 | 0.547637792285868 | -0.328645431 | 0.619535553743337 | 1.15655419309435 | 0.719898224793112 | 1.85806487013985 | 0.998074519377826 |
| ebi-fl187-GCST90094778 | ebi-met1400-GCST90199979 | Carnitine C14:1 levels \|\| id:ebi-met1400-GCST90199979 | Hard cheese liking \|\| id:ebi-fl187-GCST90094778 | Inverse variance weighted | 3 | -0.228659038 | 0.344461268287472 | 0.5068078294596 | -0.903803124 | 0.446485047650506 | 0.795599756394782 | 0.405026361432777 | 1.56280931971015 | 0.998074519377826 |
| ebi-fl187-GCST90094778 | ebi-met1400-GCST90199986 | Eugenol sulfate levels \|\| id:ebi-met1400-GCST90199986 | Hard cheese liking \|\| id:ebi-fl187-GCST90094778 | Inverse variance weighted | 3 | 0.515439930546153 | 0.341237653598363 | 0.130915549566703 | -0.153385871 | 1.18426573159894 | 1.67437494847851 | 0.857798658738722 | 3.26828614096298 | 0.998074519377826 |
| ebi-fl187-GCST90094778 | ebi-met1400-GCST90200002 | 6-hydroxyindole sulfate levels \|\| id:ebi-met1400-GCST90200002 | Hard cheese liking \|\| id:ebi-fl187-GCST90094778 | Inverse variance weighted | 3 | 0.112277643607445 | 0.359149564848377 | 0.7545687326734 | -0.591655503 | 0.816210790710264 | 1.11882345170546 | 0.553410353178165 | 2.26191271792694 | 0.998074519377826 |
| ebi-fl187-GCST90094778 | ebi-met1400-GCST90200023 | Trans 3,4-methyleneheptanoate levels \|\| id:ebi-met1400-GCST90200023 | Hard cheese liking \|\| id:ebi-fl187-GCST90094778 | Inverse variance weighted | 3 | -0.058502918 | 0.271342367408208 | 0.829295236821341 | -0.590333958 | 0.473328122382814 | 0.943175488457375 | 0.554142193690246 | 1.60532804062934 | 0.998074519377826 |
| ebi-fl187-GCST90094778 | ebi-met1400-GCST90200032 | 2-hydroxybutyrate/2-hydroxyisobutyrate levels \|\| id:ebi-met1400-GCST90200032 | Hard cheese liking \|\| id:ebi-fl187-GCST90094778 | Inverse variance weighted | 3 | -0.061397906 | 0.279639347161854 | 0.826213036714367 | -0.609491026 | 0.486695214609097 | 0.940448955195601 | 0.543627490786167 | 1.62693066910479 | 0.998074519377826 |
| ebi-fl187-GCST90094778 | ebi-met1400-GCST90200051 | 1-palmitoyl-2-dihomo-linolenoyl-GPC (16:0/20:3n3 or 6) levels \|\| id:ebi-met1400-GCST90200051 | Hard cheese liking \|\| id:ebi-fl187-GCST90094778 | Inverse variance weighted | 3 | 0.240277699737202 | 0.247966149951622 | 0.332548206500223 | -0.245735954 | 0.726291353642381 | 1.27160222489846 | 0.782128715490222 | 2.06739912030109 | 0.998074519377826 |
| ebi-fl187-GCST90094778 | ebi-met1400-GCST90200069 | Phenylacetylglutamate levels \|\| id:ebi-met1400-GCST90200069 | Hard cheese liking \|\| id:ebi-fl187-GCST90094778 | Inverse variance weighted | 3 | -0.633425458 | 0.348704210704679 | 0.0692918358732159 | -1.316885711 | 0.0500347947447005 | 0.530770551124011 | 0.267968535231194 | 1.05130767572181 | 0.998074519377826 |
| ebi-fl187-GCST90094778 | ebi-met1400-GCST90200072 | Thioproline levels \|\| id:ebi-met1400-GCST90200072 | Hard cheese liking \|\| id:ebi-fl187-GCST90094778 | Inverse variance weighted | 3 | -0.182993305 | 0.252151498731155 | 0.468005771385734 | -0.677210242 | 0.31122363259792 | 0.832773731209224 | 0.508032304226613 | 1.36509446667546 | 0.998074519377826 |
| ebi-fl187-GCST90094778 | ebi-met1400-GCST90200081 | 1-oleoyl-2-docosahexaenoyl-GPC (18:1/22:6) levels \|\| id:ebi-met1400-GCST90200081 | Hard cheese liking \|\| id:ebi-fl187-GCST90094778 | Inverse variance weighted | 3 | -0.103909839 | 0.303778034525549 | 0.732306917903614 | -0.699314786 | 0.491495109037651 | 0.901306556789685 | 0.496925687447997 | 1.63475853599758 | 0.998074519377826 |
| ebi-fl187-GCST90094778 | ebi-met1400-GCST90200097 | Furaneol sulfate levels \|\| id:ebi-met1400-GCST90200097 | Hard cheese liking \|\| id:ebi-fl187-GCST90094778 | Inverse variance weighted | 3 | -0.275575979 | 0.327056857330454 | 0.399455832539256 | -0.91660742 | 0.365455461134147 | 0.759134751426242 | 0.399873344965276 | 1.44117025572941 | 0.998074519377826 |
| ebi-fl187-GCST90094778 | ebi-met1400-GCST90200124 | Sphingomyelin (d18:2/24:2) levels \|\| id:ebi-met1400-GCST90200124 | Hard cheese liking \|\| id:ebi-fl187-GCST90094778 | Inverse variance weighted | 3 | 0.209093667506565 | 0.235448945844713 | 0.374506474829315 | -0.252386266 | 0.670573601362201 | 1.23256044398691 | 0.776944572557287 | 1.95535859537674 | 0.998074519377826 |
| ebi-fl187-GCST90094778 | ebi-met1400-GCST90200137 | Lignoceroylcarnitine (C24) levels \|\| id:ebi-met1400-GCST90200137 | Hard cheese liking \|\| id:ebi-fl187-GCST90094778 | Inverse variance weighted | 3 | 0.196519021969726 | 0.253861083667953 | 0.438859645222553 | -0.301048702 | 0.694086745958913 | 1.21715847340962 | 0.740041730342293 | 2.00188001385762 | 0.998074519377826 |
| ebi-fl187-GCST90094778 | ebi-met1400-GCST90200165 | Octadecenedioate (C18:1-DC) levels \|\| id:ebi-met1400-GCST90200165 | Hard cheese liking \|\| id:ebi-fl187-GCST90094778 | Inverse variance weighted | 3 | -0.233376369 | 0.397184721805866 | 0.556816656072849 | -1.011858423 | 0.545105686050423 | 0.791855487820882 | 0.363542735055713 | 1.72479065905678 | 0.998074519377826 |
| ebi-fl187-GCST90094778 | ebi-met1400-GCST90200176 | Glutamine conjugate of C6H10O2 (1) levels \|\| id:ebi-met1400-GCST90200176 | Hard cheese liking \|\| id:ebi-fl187-GCST90094778 | Inverse variance weighted | 3 | 0.23267403257907 | 0.249483900152012 | 0.351015462930383 | -0.256314412 | 0.721662476877014 | 1.26197005115195 | 0.773898607744975 | 2.05785149897731 | 0.998074519377826 |
| ebi-fl187-GCST90094778 | ebi-met1400-GCST90200182 | Glycine conjugate of C10H12O2 levels \|\| id:ebi-met1400-GCST90200182 | Hard cheese liking \|\| id:ebi-fl187-GCST90094778 | Inverse variance weighted | 3 | 0.133482040448848 | 0.291333659411995 | 0.646826124955471 | -0.437531932 | 0.704496012896359 | 1.14280074176243 | 0.645627909909174 | 2.02282694928179 | 0.998074519377826 |
| ebi-fl187-GCST90094778 | ebi-met1400-GCST90200208 | Glucuronide of piperine metabolite C17H21NO3 (4) levels \|\| id:ebi-met1400-GCST90200208 | Hard cheese liking \|\| id:ebi-fl187-GCST90094778 | Inverse variance weighted | 3 | 0.279587347882186 | 0.258399136857476 | 0.279253419044092 | -0.22687496 | 0.786049656122839 | 1.32258393265518 | 0.79702043520924 | 2.19470942229784 | 0.998074519377826 |
| ebi-fl187-GCST90094778 | ebi-met1400-GCST90200227 | 3-ethylcatechol sulfate (2) levels \|\| id:ebi-met1400-GCST90200227 | Hard cheese liking \|\| id:ebi-fl187-GCST90094778 | Inverse variance weighted | 3 | -0.229642629 | 0.330705471942004 | 0.487429973846874 | -0.877825354 | 0.418540096325554 | 0.794817596767711 | 0.415685899294606 | 1.51974125945483 | 0.998074519377826 |
| ebi-fl187-GCST90094778 | ebi-met1400-GCST90200259 | Pentose acid levels \|\| id:ebi-met1400-GCST90200259 | Hard cheese liking \|\| id:ebi-fl187-GCST90094778 | Inverse variance weighted | 3 | -0.280963812 | 0.361657521745586 | 0.437230687618495 | -0.989812554 | 0.427884930932856 | 0.755055659175206 | 0.371646347999587 | 1.53400955376302 | 0.998074519377826 |
| ebi-fl187-GCST90094778 | ebi-met1400-GCST90200291 | Sarcosine levels \|\| id:ebi-met1400-GCST90200291 | Hard cheese liking \|\| id:ebi-fl187-GCST90094778 | Inverse variance weighted | 3 | 0.0705644381281957 | 0.253137154109798 | 0.780429240664362 | -0.425584384 | 0.566713260183401 | 1.07311371664207 | 0.653387844186397 | 1.76246475824678 | 0.998074519377826 |
| ebi-fl187-GCST90094778 | ebi-met1400-GCST90200306 | Butyrate/isobutyrate (4:0) levels \|\| id:ebi-met1400-GCST90200306 | Hard cheese liking \|\| id:ebi-fl187-GCST90094778 | Inverse variance weighted | 3 | -0.314009367 | 0.255493314272954 | 0.219059942925691 | -0.814776263 | 0.186757528590756 | 0.730512185139049 | 0.442738373077196 | 1.20533499034108 | 0.998074519377826 |
| ebi-fl187-GCST90094778 | ebi-met1400-GCST90200321 | Cholate levels \|\| id:ebi-met1400-GCST90200321 | Hard cheese liking \|\| id:ebi-fl187-GCST90094778 | Inverse variance weighted | 3 | -0.172636515 | 0.259787998219017 | 0.506352148783199 | -0.681820991 | 0.336547961738597 | 0.841443411516368 | 0.505695286703903 | 1.40010601917844 | 0.998074519377826 |
| ebi-fl187-GCST90094778 | ebi-met1400-GCST90200336 | Ceramide (d18:1/16:0) levels \|\| id:ebi-met1400-GCST90200336 | Hard cheese liking \|\| id:ebi-fl187-GCST90094778 | Inverse variance weighted | 3 | 0.303670700637142 | 0.378034894885959 | 0.421808588772014 | -0.437277693 | 1.04461909461362 | 1.35482284032632 | 0.645792074351002 | 2.84231566408511 | 0.998074519377826 |
| ebi-fl187-GCST90094778 | ebi-met1400-GCST90200358 | Arachidonate (20:4n6) levels \|\| id:ebi-met1400-GCST90200358 | Hard cheese liking \|\| id:ebi-fl187-GCST90094778 | Inverse variance weighted | 3 | 0.068269990354052 | 0.310153224733929 | 0.825780053010685 | -0.53963033 | 0.676170310832552 | 1.07065433580291 | 0.582963716670799 | 1.96633285055869 | 0.998074519377826 |
| ebi-fl187-GCST90094778 | ebi-met1400-GCST90200413 | 2'-deoxyuridine levels \|\| id:ebi-met1400-GCST90200413 | Hard cheese liking \|\| id:ebi-fl187-GCST90094778 | Inverse variance weighted | 3 | -0.02173932 | 0.324926252489293 | 0.946657016646818 | -0.658594775 | 0.615116135027129 | 0.978495276101201 | 0.517578137588736 | 1.84987142195166 | 0.998074519377826 |
| ebi-fl187-GCST90094778 | ebi-met1400-GCST90200432 | Threonine levels \|\| id:ebi-met1400-GCST90200432 | Hard cheese liking \|\| id:ebi-fl187-GCST90094778 | Inverse variance weighted | 3 | 0.478724427522372 | 0.25282405427895 | 0.0582903147944887 | -0.016810719 | 0.974259573909114 | 1.61401429635014 | 0.983329792800851 | 2.64920494415472 | 0.998074519377826 |
| ebi-fl187-GCST90094778 | ebi-met1400-GCST90200436 | Caffeine levels \|\| id:ebi-met1400-GCST90200436 | Hard cheese liking \|\| id:ebi-fl187-GCST90094778 | Inverse variance weighted | 3 | -0.34291189 | 0.258076569265158 | 0.183939863791339 | -0.848741966 | 0.162918185473722 | 0.709700740334737 | 0.42795297281157 | 1.1769403949285 | 0.998074519377826 |
| ebi-fl187-GCST90094778 | ebi-met1400-GCST90200442 | Valine levels \|\| id:ebi-met1400-GCST90200442 | Hard cheese liking \|\| id:ebi-fl187-GCST90094778 | Inverse variance weighted | 3 | 0.787968001844743 | 0.247899300493747 | 0.00147997869536489 | 0.302085372876999 | 1.27385063081249 | 2.19892367463301 | 1.35267670368195 | 3.57459052388503 | 0.598008988154769 |
| ebi-fl187-GCST90094778 | ebi-met1400-GCST90200459 | Margarate (17:0) levels \|\| id:ebi-met1400-GCST90200459 | Hard cheese liking \|\| id:ebi-fl187-GCST90094778 | Inverse variance weighted | 3 | -0.189382315 | 0.386787739970301 | 0.624396780114261 | -0.947486285 | 0.568721655611279 | 0.827470092208319 | 0.387714403025145 | 1.76600804137482 | 0.998074519377826 |
| ebi-fl187-GCST90094778 | ebi-met1400-GCST90200462 | X-11299 levels \|\| id:ebi-met1400-GCST90200462 | Hard cheese liking \|\| id:ebi-fl187-GCST90094778 | Inverse variance weighted | 3 | 0.0975228099548282 | 0.307675106978245 | 0.751268754967494 | -0.5055204 | 0.700566019632189 | 1.1024365878053 | 0.603191592998312 | 2.01489285367941 | 0.998074519377826 |
| ebi-fl187-GCST90094778 | ebi-met1400-GCST90200497 | X-12740 levels \|\| id:ebi-met1400-GCST90200497 | Hard cheese liking \|\| id:ebi-fl187-GCST90094778 | Inverse variance weighted | 3 | -0.426026761 | 0.288120650695135 | 0.139236516959829 | -0.990743236 | 0.138689714253849 | 0.653098864237171 | 0.37130062427762 | 1.14876759848635 | 0.998074519377826 |
| ebi-fl187-GCST90094778 | ebi-met1400-GCST90200527 | X-13866 levels \|\| id:ebi-met1400-GCST90200527 | Hard cheese liking \|\| id:ebi-fl187-GCST90094778 | Inverse variance weighted | 3 | 0.169211597649911 | 0.247564529402342 | 0.494287781662177 | -0.31601488 | 0.654438075278501 | 1.18437072247309 | 0.729048601859262 | 1.92406103608743 | 0.998074519377826 |
| ebi-fl187-GCST90094778 | ebi-met1400-GCST90200547 | X-17654 levels \|\| id:ebi-met1400-GCST90200547 | Hard cheese liking \|\| id:ebi-fl187-GCST90094778 | Inverse variance weighted | 3 | 0.205947194894377 | 0.255340298702604 | 0.419920199406794 | -0.294519791 | 0.706414180351481 | 1.22868832126567 | 0.744889204419204 | 2.02671079384453 | 0.998074519377826 |
| ebi-fl187-GCST90094778 | ebi-met1400-GCST90200553 | X-17653 levels \|\| id:ebi-met1400-GCST90200553 | Hard cheese liking \|\| id:ebi-fl187-GCST90094778 | Inverse variance weighted | 3 | 0.162531818399696 | 0.254692108680308 | 0.523375915521738 | -0.336664715 | 0.6617283514131 | 1.17648575174704 | 0.714148243235862 | 1.93813922693732 | 0.998074519377826 |
| ebi-fl187-GCST90094778 | ebi-met1400-GCST90200557 | X-17685 levels \|\| id:ebi-met1400-GCST90200557 | Hard cheese liking \|\| id:ebi-fl187-GCST90094778 | Inverse variance weighted | 3 | -0.208223102 | 0.267728854438312 | 0.436723048748244 | -0.732971657 | 0.316525452714427 | 0.812025851922185 | 0.480479047720027 | 1.37235117185416 | 0.998074519377826 |
| ebi-fl187-GCST90094778 | ebi-met1400-GCST90200561 | X-18779 levels \|\| id:ebi-met1400-GCST90200561 | Hard cheese liking \|\| id:ebi-fl187-GCST90094778 | Inverse variance weighted | 3 | 0.198507920600886 | 0.273514422618216 | 0.467981219883986 | -0.337580348 | 0.73459618893259 | 1.21958168719579 | 0.713494644727712 | 2.08464002180555 | 0.998074519377826 |
| ebi-fl187-GCST90094778 | ebi-met1400-GCST90200570 | X-18921 levels \|\| id:ebi-met1400-GCST90200570 | Hard cheese liking \|\| id:ebi-fl187-GCST90094778 | Inverse variance weighted | 3 | 0.240506938165286 | 0.435679269610933 | 0.580929512023831 | -0.61342443 | 1.09443830660272 | 1.27189375840771 | 0.541493384139051 | 2.98750415067133 | 0.998074519377826 |
| ebi-fl187-GCST90094778 | ebi-met1400-GCST90200575 | X-21283 levels \|\| id:ebi-met1400-GCST90200575 | Hard cheese liking \|\| id:ebi-fl187-GCST90094778 | Inverse variance weighted | 3 | -0.067693611 | 0.406740693879477 | 0.867819036186989 | -0.864905371 | 0.729518149052468 | 0.934546764610923 | 0.421091398218853 | 2.07408096897488 | 0.998074519377826 |
| ebi-fl187-GCST90094778 | ebi-met1400-GCST90200578 | X-18922 levels \|\| id:ebi-met1400-GCST90200578 | Hard cheese liking \|\| id:ebi-fl187-GCST90094778 | Inverse variance weighted | 3 | -0.018475391 | 0.253898314381788 | 0.941991619166109 | -0.516116087 | 0.479165305516288 | 0.981694233129891 | 0.596834104055416 | 1.61472603661905 | 0.998074519377826 |
| ebi-fl187-GCST90094778 | ebi-met1400-GCST90200623 | X-24241 levels \|\| id:ebi-met1400-GCST90200623 | Hard cheese liking \|\| id:ebi-fl187-GCST90094778 | Inverse variance weighted | 3 | -0.290031715 | 0.25567142331575 | 0.256629993016425 | -0.791147705 | 0.211084274713821 | 0.74823983678702 | 0.45332421427881 | 1.23501643133215 | 0.998074519377826 |
| ebi-fl187-GCST90094778 | ebi-met1400-GCST90200636 | X-24544 levels \|\| id:ebi-met1400-GCST90200636 | Hard cheese liking \|\| id:ebi-fl187-GCST90094778 | Inverse variance weighted (multiplicative random effects) | 3 | -0.101429921 | 0.434181908401638 | 0.815286537906127 | -0.952426462 | 0.749566619376862 | 0.903544496536733 | 0.385803748818661 | 2.11608274860374 | 0.998074519377826 |
| ebi-fl187-GCST90094778 | ebi-met1400-GCST90200659 | X-25172 levels \|\| id:ebi-met1400-GCST90200659 | Hard cheese liking \|\| id:ebi-fl187-GCST90094778 | Inverse variance weighted | 3 | -0.152920122 | 0.270573497230561 | 0.571957892177639 | -0.683244177 | 0.377403932615825 | 0.858198270272727 | 0.504976100520842 | 1.45849332342552 | 0.998074519377826 |
| ebi-fl187-GCST90094778 | ebi-met1400-GCST90200670 | X-26111 levels \|\| id:ebi-met1400-GCST90200670 | Hard cheese liking \|\| id:ebi-fl187-GCST90094778 | Inverse variance weighted | 3 | 0.204691662672311 | 0.254301974384248 | 0.420868302999484 | -0.293740207 | 0.703123532465437 | 1.22714663151055 | 0.745470134121194 | 2.02005256320957 | 0.998074519377826 |
| ebi-fl187-GCST90094778 | ebi-met1400-GCST90200673 | Carnitine C4 levels \|\| id:ebi-met1400-GCST90200673 | Hard cheese liking \|\| id:ebi-fl187-GCST90094778 | Inverse variance weighted | 3 | -0.2218239 | 0.252628071697772 | 0.379908375987924 | -0.716974921 | 0.273327120414295 | 0.801056417858935 | 0.488226949570448 | 1.3143301187236 | 0.998074519377826 |
| ebi-fl187-GCST90094778 | ebi-met1400-GCST90200710 | X-13431 levels \|\| id:ebi-met1400-GCST90200710 | Hard cheese liking \|\| id:ebi-fl187-GCST90094778 | Inverse variance weighted | 3 | -0.021929068 | 0.25017361705939 | 0.93015057119396 | -0.512269357 | 0.468411221730101 | 0.978309626335882 | 0.599134384982971 | 1.59745417550798 | 0.998074519377826 |
| ebi-fl187-GCST90094778 | ebi-met1400-GCST90200730 | Adenosine 5'-diphosphate (ADP) to fructose ratio \|\| id:ebi-met1400-GCST90200730 | Hard cheese liking \|\| id:ebi-fl187-GCST90094778 | Inverse variance weighted | 3 | 0.287717290034987 | 0.403533207858317 | 0.475848651020471 | -0.503207797 | 1.07864237743729 | 1.3333802909378 | 0.604588149514458 | 2.94068450016628 | 0.998074519377826 |
| ebi-fl187-GCST90094778 | ebi-met1400-GCST90200731 | Adenosine 5'-diphosphate (ADP) to mannose ratio \|\| id:ebi-met1400-GCST90200731 | Hard cheese liking \|\| id:ebi-fl187-GCST90094778 | Inverse variance weighted | 3 | 0.476907057941917 | 0.382935413676359 | 0.212985430909557 | -0.273646353 | 1.22746046874758 | 1.61108369966078 | 0.760601012073464 | 3.41255223975691 | 0.998074519377826 |
| ebi-fl187-GCST90094778 | ebi-met1400-GCST90200748 | Aspartate to glutamate ratio \|\| id:ebi-met1400-GCST90200748 | Hard cheese liking \|\| id:ebi-fl187-GCST90094778 | Inverse variance weighted | 3 | -0.008421346 | 0.24472561823577 | 0.972549108 | -0.488083558 | 0.47124086527679 | 0.991614013743017 | 0.613801582740013 | 1.60198080275728 | 0.998074519377826 |
| ebi-fl187-GCST90094778 | ebi-met1400-GCST90200774 | Uridine to pseudouridine ratio \|\| id:ebi-met1400-GCST90200774 | Hard cheese liking \|\| id:ebi-fl187-GCST90094778 | Inverse variance weighted | 3 | -0.071871 | 0.35023345294555 | 0.837408964140358 | -0.758328568 | 0.61458656763366 | 0.930650941909644 | 0.468448753302577 | 1.84889204970917 | 0.998074519377826 |
| ebi-fl187-GCST90094778 | ebi-met1400-GCST90200799 | Spermidine to adenosine 5'-diphosphate (ADP) ratio \|\| id:ebi-met1400-GCST90200799 | Hard cheese liking \|\| id:ebi-fl187-GCST90094778 | Inverse variance weighted | 3 | 0.118759698069582 | 0.537280118866284 | 0.825062301264196 | -0.934309335 | 1.1718287310475 | 1.12609928194114 | 0.392857104541198 | 3.22789018737315 | 0.998074519377826 |
| ebi-fl187-GCST90094778 | ebi-met1400-GCST90200811 | Choline to taurocholate ratio \|\| id:ebi-met1400-GCST90200811 | Hard cheese liking \|\| id:ebi-fl187-GCST90094778 | Inverse variance weighted | 3 | -0.21269315 | 0.26356020394691 | 0.419666339190776 | -0.72927115 | 0.303884849294055 | 0.808404157620607 | 0.482260357403377 | 1.35511300488599 | 0.998074519377826 |
| ebi-fl187-GCST90094778 | ebi-met1400-GCST90200812 | Glutamate to kynurenine ratio \|\| id:ebi-met1400-GCST90200812 | Hard cheese liking \|\| id:ebi-fl187-GCST90094778 | Inverse variance weighted | 3 | 0.427431982752838 | 0.245684787332518 | 0.0819015856170923 | -0.0541102 | 0.908974165924573 | 1.53331488426403 | 0.947327704839444 | 2.48177533739931 | 0.998074519377826 |
| ebi-fl187-GCST90094778 | ebi-met1400-GCST90200876 | Cortisone to 4-cholesten-3-one ratio \|\| id:ebi-met1400-GCST90200876 | Hard cheese liking \|\| id:ebi-fl187-GCST90094778 | Inverse variance weighted | 3 | -0.2669597 | 0.442829945525985 | 0.546608980894173 | -1.134906393 | 0.60098699338307 | 0.765703928828417 | 0.321452210077813 | 1.82391810739565 | 0.998074519377826 |
| ebi-fl187-GCST90094778 | ebi-met1400-GCST90200906 | 2'-deoxyuridine to cytidine ratio \|\| id:ebi-met1400-GCST90200906 | Hard cheese liking \|\| id:ebi-fl187-GCST90094778 | Inverse variance weighted | 3 | 0.409202623369996 | 0.440548694778147 | 0.352968042039757 | -0.454272818 | 1.27267806513516 | 1.50561676272256 | 0.634909494626259 | 3.57040153813665 | 0.998074519377826 |
| ebi-fl187-GCST90094778 | ebi-met1400-GCST90200907 | Retinol (Vitamin A) to linoleoyl-arachidonoyl-glycerol (18:2 to 20:4) [1] ratio \|\| id:ebi-met1400-GCST90200907 | Hard cheese liking \|\| id:ebi-fl187-GCST90094778 | Inverse variance weighted | 3 | -0.087571398 | 0.396474734733176 | 0.825189683108452 | -0.864661878 | 0.68951908195726 | 0.916153457681394 | 0.421193943408898 | 1.99275694998952 | 0.998074519377826 |
| ebi-fl187-GCST90094778 | ebi-met1400-GCST90200916 | Fructose to sucrose ratio \|\| id:ebi-met1400-GCST90200916 | Hard cheese liking \|\| id:ebi-fl187-GCST90094778 | Inverse variance weighted | 3 | 0.206784522223139 | 0.259836068841732 | 0.426132678693029 | -0.302494173 | 0.716063217152935 | 1.22971756642302 | 0.738972794457644 | 2.04636125241823 | 0.998074519377826 |
| ebi-fl187-GCST90094778 | ebi-met1400-GCST90200917 | Alpha-ketobutyrate to 3-methyl-2-oxobutyrate ratio \|\| id:ebi-met1400-GCST90200917 | Hard cheese liking \|\| id:ebi-fl187-GCST90094778 | Inverse variance weighted (multiplicative random effects) | 3 | 0.122018638924604 | 0.509737314155972 | 0.810814403983008 | -0.877066497 | 1.12110377467031 | 1.12977515937809 | 0.416001465112885 | 3.06823897940221 | 0.998074519377826 |
| ebi-fl187-GCST90094778 | ebi-met1400-GCST90200924 | Alpha-tocopherol to glycerol ratio \|\| id:ebi-met1400-GCST90200924 | Hard cheese liking \|\| id:ebi-fl187-GCST90094778 | Inverse variance weighted | 3 | -0.011806897 | 0.235862782949198 | 0.960075906730796 | -0.474097951 | 0.450484157795621 | 0.988262531109741 | 0.622446280118646 | 1.56907167990348 | 0.998074519377826 |
| ebi-fl187-GCST90094778 | ebi-met1400-GCST90200938 | Alpha-ketoglutarate to alanine ratio \|\| id:ebi-met1400-GCST90200938 | Hard cheese liking \|\| id:ebi-fl187-GCST90094778 | Inverse variance weighted | 3 | -0.455419915 | 0.300444822171021 | 0.129565172665195 | -1.044291766 | 0.133451936580336 | 0.63418160973696 | 0.351940987590234 | 1.14276633955698 | 0.998074519377826 |
| ebi-fl187-GCST90094778 | ebi-met1400-GCST90200943 | Phosphate to EDTA ratio \|\| id:ebi-met1400-GCST90200943 | Hard cheese liking \|\| id:ebi-fl187-GCST90094778 | Inverse variance weighted | 3 | 0.306388069004016 | 0.240511204232347 | 0.202697611758052 | -0.165013891 | 0.777790029299416 | 1.3585093996567 | 0.847881925831216 | 2.17665659890831 | 0.998074519377826 |
| ebi-fl187-GCST90094778 | ebi-met1400-GCST90200947 | Adenosine 5'-diphosphate (ADP) to glucose ratio \|\| id:ebi-met1400-GCST90200947 | Hard cheese liking \|\| id:ebi-fl187-GCST90094778 | Inverse variance weighted | 3 | 0.472189319472676 | 0.338919473810219 | 0.163553335905751 | -0.192092849 | 1.13647148814071 | 1.60350092894246 | 0.825230242970388 | 3.11575496780673 | 0.998074519377826 |
| ebi-fl187-GCST90094778 | ebi-met1400-GCST90200975 | Arachidonate (20:4n6) to caffeine ratio \|\| id:ebi-met1400-GCST90200975 | Hard cheese liking \|\| id:ebi-fl187-GCST90094778 | Inverse variance weighted | 3 | 0.373673270056805 | 0.258291785909824 | 0.147978205413792 | -0.13257863 | 0.87992517044006 | 1.45306231392213 | 0.875834064280221 | 2.41071930660282 | 0.998074519377826 |
| ebi-fl187-GCST90094778 | ebi-met1400-GCST90200985 | Cholesterol to oleoyl-linoleoyl-glycerol (18:1 to 18:2) [2] ratio \|\| id:ebi-met1400-GCST90200985 | Hard cheese liking \|\| id:ebi-fl187-GCST90094778 | Inverse variance weighted | 3 | 0.155017305263143 | 0.335745995758246 | 0.644289252855394 | -0.503044846 | 0.813079456949306 | 1.16767816790824 | 0.604686675751584 | 2.25484099201434 | 0.998074519377826 |
| ebi-fl187-GCST90094778 | ebi-met1400-GCST90201016 | Bilirubin (Z,Z) to etiocholanolone glucuronide ratio \|\| id:ebi-met1400-GCST90201016 | Hard cheese liking \|\| id:ebi-fl187-GCST90094778 | Inverse variance weighted (multiplicative random effects) | 3 | -0.293594508 | 0.50111164755911 | 0.557951845732761 | -1.275773337 | 0.688584321506974 | 0.745578756594273 | 0.279214957471076 | 1.99089506994712 | 0.998074519377826 |
| ebi-fl187-GCST90094778 | ebi-pw11-GCST90275189 | Placental weight (fetal genotype, adjusted for sex and gestational age) \|\| id:ebi-pw11-GCST90275189 | Hard cheese liking \|\| id:ebi-fl187-GCST90094778 | Inverse variance weighted | 3 | 0.0677901496146642 | 0.0908970269872893 | 0.455793910408071 | -0.110368023 | 0.245948322509751 | 1.07014071547263 | 0.895504508138296 | 1.27883348492916 | 0.998074519377826 |
| ebi-fl187-GCST90094778 | ebi-pw11-GCST90275192 | Placental weight (fetal effect, conditional on maternal and paternal genotype) \|\| id:ebi-pw11-GCST90275192 | Hard cheese liking \|\| id:ebi-fl187-GCST90094778 | Inverse variance weighted | 3 | -0.080324576 | 0.188622739856367 | 0.670218764881937 | -0.450025146 | 0.289375994207266 | 0.922816773677024 | 0.637612118006931 | 1.33559380966849 | 0.998074519377826 |
| ebi-fl187-GCST90094778 | ebi-pw11-GCST90275193 | Placental weight (maternal effect, conditional on fetal and paternal genotype) \|\| id:ebi-pw11-GCST90275193 | Hard cheese liking \|\| id:ebi-fl187-GCST90094778 | Inverse variance weighted | 3 | 0.136092709100112 | 0.146755764172988 | 0.353749258616108 | -0.151548589 | 0.423734006879168 | 1.14578811365594 | 0.859376125307833 | 1.52765519396408 | 0.998074519377826 |
| ebi-fl187-GCST90094778 | ebi-pw11-GCST90275195 | Placental weight (fetal effect, conditional on maternal genotype) \|\| id:ebi-pw11-GCST90275195 | Hard cheese liking \|\| id:ebi-fl187-GCST90094778 | Inverse variance weighted | 3 | 0.0261953451419068 | 0.120492212453124 | 0.827894444276973 | -0.209969391 | 0.26236008155003 | 1.02654145877539 | 0.810609057307301 | 1.29999456221867 | 0.998074519377826 |
| ebi-fl187-GCST90094778 | ebi-pw11-GCST90275197 | Placental weight (fetal genotype, adjusted for sex) \|\| id:ebi-pw11-GCST90275197 | Hard cheese liking \|\| id:ebi-fl187-GCST90094778 | Inverse variance weighted | 3 | 0.0718826950356368 | 0.0919061360304962 | 0.434137225798774 | -0.108253332 | 0.252018721655409 | 1.07452928905056 | 0.89740022781051 | 1.28662012471798 | 0.998074519377826 |
| ebi-fl187-GCST90094778 | prot-a-1030 | Protein FAM171B \|\| id:prot-a-1030 | Hard cheese liking \|\| id:ebi-fl187-GCST90094778 | Inverse variance weighted | 4 | -0.014792577 | 0.445054526141871 | 0.973485056519164 | -0.887099448 | 0.857514294721372 | 0.985316296146461 | 0.411848610354597 | 2.35729386731666 | 0.998074519377826 |
| ebi-fl187-GCST90094778 | prot-a-1036 | BRISC complex subunit Abro1 \|\| id:prot-a-1036 | Hard cheese liking \|\| id:ebi-fl187-GCST90094778 | Inverse variance weighted | 4 | 0.379154943137083 | 0.338916404945775 | 0.263256610069455 | -0.285121211 | 1.0434310968308 | 1.46104939778348 | 0.751923107777092 | 2.83894100431916 | 0.998074519377826 |
| ebi-fl187-GCST90094778 | prot-a-1055 | Phenylalanine--tRNA ligase, mitochondrial \|\| id:prot-a-1055 | Hard cheese liking \|\| id:ebi-fl187-GCST90094778 | Inverse variance weighted (multiplicative random effects) | 4 | -0.507017369 | 0.876373063076811 | 0.562899217373451 | -2.224708572 | 1.2106738350654 | 0.602289309491106 | 0.108098917074896 | 3.35574510960126 | 0.998074519377826 |
| ebi-fl187-GCST90094778 | prot-a-1083 | Fc receptor-like protein 5 \|\| id:prot-a-1083 | Hard cheese liking \|\| id:ebi-fl187-GCST90094778 | Inverse variance weighted | 4 | -0.648990887 | 0.338916404945775 | 0.0555053091549462 | -1.313267041 | 0.0152852662373708 | 0.522572845593865 | 0.268939981560492 | 1.01540268340744 | 0.998074519377826 |
| ebi-fl187-GCST90094778 | prot-a-1103 | Fibroblast growth factor receptor 3 \|\| id:prot-a-1103 | Hard cheese liking \|\| id:ebi-fl187-GCST90094778 | Inverse variance weighted | 4 | 0.541495910348933 | 0.339040591624451 | 0.110234096149276 | -0.123023649 | 1.20601546993286 | 1.71857577556184 | 0.884242750648948 | 3.34014917756511 | 0.998074519377826 |
| ebi-fl187-GCST90094778 | prot-a-1105 | Fibroblast growth factor receptor 4 \|\| id:prot-a-1105 | Hard cheese liking \|\| id:ebi-fl187-GCST90094778 | Inverse variance weighted | 4 | 0.0513765083427374 | 0.360217740150114 | 0.886585358603565 | -0.654650262 | 0.75740327903696 | 1.05271917622901 | 0.519623762804408 | 2.13273091672953 | 0.998074519377826 |
| ebi-fl187-GCST90094778 | prot-a-1120 | Filamin-A \|\| id:prot-a-1120 | Hard cheese liking \|\| id:ebi-fl187-GCST90094778 | Inverse variance weighted | 4 | 0.323067952179739 | 0.452811555787381 | 0.475554299250206 | -0.564442697 | 1.21057860152301 | 1.38135921408609 | 0.568676983758345 | 3.35542554532398 | 0.998074519377826 |
| ebi-fl187-GCST90094778 | prot-a-1166 | Galanin peptides \|\| id:prot-a-1166 | Hard cheese liking \|\| id:ebi-fl187-GCST90094778 | Inverse variance weighted | 4 | -0.452193783 | 0.461431177958157 | 0.327095502311658 | -1.356598892 | 0.452211326086067 | 0.636230867235667 | 0.257535194252271 | 1.57178407245937 | 0.998074519377826 |
| ebi-fl187-GCST90094778 | prot-a-1205 | ADP-ribosylation factor-binding protein GGA1 \|\| id:prot-a-1205 | Hard cheese liking \|\| id:ebi-fl187-GCST90094778 | Inverse variance weighted (multiplicative random effects) | 4 | -0.283884872 | 0.570638496162121 | 0.61884611483379 | -1.402336324 | 0.834566580554924 | 0.752853314271511 | 0.246021505927695 | 2.30381531351237 | 0.998074519377826 |
| ebi-fl187-GCST90094778 | prot-a-1227 | Guanine nucleotide-binding protein G(I)/G(S)/G(O) subunit gamma-T2 \|\| id:prot-a-1227 | Hard cheese liking \|\| id:ebi-fl187-GCST90094778 | Inverse variance weighted | 4 | 0.0078564430547627 | 0.426493311189546 | 0.98530298143354 | -0.828070447 | 0.843783332986274 | 1.00788738588394 | 0.436891478850159 | 2.32514716308389 | 0.999355215041207 |
| ebi-fl187-GCST90094778 | prot-a-1240 | Golgi SNAP receptor complex member 1 \|\| id:prot-a-1240 | Hard cheese liking \|\| id:ebi-fl187-GCST90094778 | Inverse variance weighted | 4 | -0.099301705 | 0.492915635152488 | 0.840340614632744 | -1.06541635 | 0.866812939778084 | 0.905469482029331 | 0.34458435719279 | 2.37931573436968 | 0.998074519377826 |
| ebi-fl187-GCST90094778 | prot-a-1283 | Glutathione S-transferase A1 \|\| id:prot-a-1283 | Hard cheese liking \|\| id:ebi-fl187-GCST90094778 | Inverse variance weighted | 4 | 0.124975322523411 | 0.339040591624451 | 0.712414988568526 | -0.539544237 | 0.789494882107334 | 1.13312049016743 | 0.583013907963861 | 2.20228373234086 | 0.998074519377826 |
| ebi-fl187-GCST90094778 | prot-a-1324 | E3 ubiquitin-protein ligase HECW1 \|\| id:prot-a-1324 | Hard cheese liking \|\| id:ebi-fl187-GCST90094778 | Inverse variance weighted | 4 | 0.0416459731881346 | 0.374365305014984 | 0.911422693433685 | -0.692110025 | 0.775401971017504 | 1.04252533149121 | 0.500518846975451 | 2.17146481769579 | 0.998074519377826 |
| ebi-fl187-GCST90094778 | prot-a-1339 | Histidine triad nucleotide-binding protein 2, mitochondrial \|\| id:prot-a-1339 | Hard cheese liking \|\| id:ebi-fl187-GCST90094778 | Inverse variance weighted | 4 | 0.169533827064715 | 0.422613046783502 | 0.688305155346283 | -0.658787745 | 0.997855398760379 | 1.18475242305218 | 0.517478270223264 | 2.71245844453025 | 0.998074519377826 |
| ebi-fl187-GCST90094778 | prot-a-1466 | Interleukin-11 \|\| id:prot-a-1466 | Hard cheese liking \|\| id:ebi-fl187-GCST90094778 | Inverse variance weighted | 4 | 0.0696612006827091 | 0.338916404945775 | 0.837149505594019 | -0.594614953 | 0.733937354376428 | 1.07214487776158 | 0.551774984265965 | 2.08326704125521 | 0.998074519377826 |
| ebi-fl187-GCST90094778 | prot-a-147 | Rho GTPase-activating protein 1 \|\| id:prot-a-147 | Hard cheese liking \|\| id:ebi-fl187-GCST90094778 | Inverse variance weighted | 4 | -0.066055003 | 0.338916404945775 | 0.845470781741488 | -0.730331157 | 0.598221150497712 | 0.936079375518239 | 0.481749429029244 | 1.81888040643101 | 0.998074519377826 |
| ebi-fl187-GCST90094778 | prot-a-1473 | Interleukin-12 receptor subunit beta-1 \|\| id:prot-a-1473 | Hard cheese liking \|\| id:ebi-fl187-GCST90094778 | Inverse variance weighted | 4 | 0.61407520537378 | 0.339040591624451 | 0.0701076683439649 | -0.050444354 | 1.2785947649577 | 1.84794683778581 | 0.950806835597657 | 3.59158915084579 | 0.998074519377826 |
| ebi-fl187-GCST90094778 | prot-a-1501 | X-linked interleukin-1 receptor accessory protein-like 2 \|\| id:prot-a-1501 | Hard cheese liking \|\| id:ebi-fl187-GCST90094778 | Inverse variance weighted | 4 | -0.081613618 | 0.338916404945775 | 0.809704303445221 | -0.745889772 | 0.58266253580372 | 0.921627990477361 | 0.47431208271631 | 1.79080015834084 | 0.998074519377826 |
| ebi-fl187-GCST90094778 | prot-a-1503 | Interleukin-1 receptor-like 2 \|\| id:prot-a-1503 | Hard cheese liking \|\| id:ebi-fl187-GCST90094778 | Inverse variance weighted | 4 | 0.398325295100363 | 0.338916404945775 | 0.239878438305585 | -0.265950859 | 1.06260144879408 | 1.48932842235639 | 0.766476792323296 | 2.8938895108816 | 0.998074519377826 |
| ebi-fl187-GCST90094778 | prot-a-1536 | Interleukin-5 receptor subunit alpha \|\| id:prot-a-1536 | Hard cheese liking \|\| id:ebi-fl187-GCST90094778 | Inverse variance weighted | 4 | -0.624696682 | 0.338916404945775 | 0.0652972463988749 | -1.288972836 | 0.0395794716035407 | 0.535423807521682 | 0.27555367665208 | 1.04037317572424 | 0.998074519377826 |
| ebi-fl187-GCST90094778 | prot-a-1559 | Insulin-induced gene 1 protein \|\| id:prot-a-1559 | Hard cheese liking \|\| id:ebi-fl187-GCST90094778 | Inverse variance weighted | 4 | 0.400737763054783 | 0.339040591624451 | 0.23721531667896 | -0.263781797 | 1.06525732263871 | 1.49292571688312 | 0.768141132432586 | 2.90158553164915 | 0.998074519377826 |
| ebi-fl187-GCST90094778 | prot-a-1567 | Interferon regulatory factor 1 \|\| id:prot-a-1567 | Hard cheese liking \|\| id:ebi-fl187-GCST90094778 | Inverse variance weighted | 4 | -0.438138761 | 0.338916404945775 | 0.196092864779167 | -1.102414915 | 0.226137392737052 | 0.645236243046762 | 0.332068198281435 | 1.25374790930221 | 0.998074519377826 |
| ebi-fl187-GCST90094778 | prot-a-1613 | Potassium voltage-gated channel subfamily E regulatory beta subunit 5 \|\| id:prot-a-1613 | Hard cheese liking \|\| id:ebi-fl187-GCST90094778 | Inverse variance weighted | 4 | 0.262369492171121 | 0.338916404945775 | 0.438846702963543 | -0.401906662 | 0.92664564586484 | 1.30000679603248 | 0.669043190248756 | 2.52602177910559 | 0.998074519377826 |
| ebi-fl187-GCST90094778 | prot-a-1649 | Kin of IRRE-like protein 2 \|\| id:prot-a-1649 | Hard cheese liking \|\| id:ebi-fl187-GCST90094778 | Inverse variance weighted | 4 | 0.460472652115233 | 0.457349654952059 | 0.314017212018466 | -0.435932672 | 1.35687797582127 | 1.58482287788314 | 0.646661263143915 | 3.88404826052309 | 0.998074519377826 |
| ebi-fl187-GCST90094778 | prot-a-165 | Armadillo repeat-containing protein 5 \|\| id:prot-a-165 | Hard cheese liking \|\| id:ebi-fl187-GCST90094778 | Inverse variance weighted (multiplicative random effects) | 4 | -0.473074029 | 0.557963773316313 | 0.396517076982757 | -1.566683025 | 0.620534966432768 | 0.623083943022552 | 0.208736408789248 | 1.85992277199956 | 0.998074519377826 |
| ebi-fl187-GCST90094778 | prot-a-1656 | Kallikrein-11 \|\| id:prot-a-1656 | Hard cheese liking \|\| id:ebi-fl187-GCST90094778 | Inverse variance weighted | 4 | 0.139140707156767 | 0.411264049696295 | 0.735118657015211 | -0.66693683 | 0.945218244561506 | 1.14928580136449 | 0.513278431160061 | 2.57337494239287 | 0.998074519377826 |
| ebi-fl187-GCST90094778 | prot-a-1697 | Laminin subunit gamma-2 \|\| id:prot-a-1697 | Hard cheese liking \|\| id:ebi-fl187-GCST90094778 | Inverse variance weighted | 4 | -0.092798924 | 0.338916404945775 | 0.784230131219457 | -0.757075078 | 0.571477229227136 | 0.911376737436895 | 0.469036317190181 | 1.77088111751553 | 0.998074519377826 |
| ebi-fl187-GCST90094778 | prot-a-1698 | Lysosome-associated membrane glycoprotein 3 \|\| id:prot-a-1698 | Hard cheese liking \|\| id:ebi-fl187-GCST90094778 | Inverse variance weighted | 4 | -0.450106108 | 0.339040591624451 | 0.184314360625637 | -1.114625668 | 0.21441345110787 | 0.63756049745972 | 0.328038051039436 | 1.23913487058311 | 0.998074519377826 |
| ebi-fl187-GCST90094778 | prot-a-1724 | Leptin receptor \|\| id:prot-a-1724 | Hard cheese liking \|\| id:ebi-fl187-GCST90094778 | Inverse variance weighted | 4 | 0.135216373097779 | 0.338916404945775 | 0.689917711797949 | -0.529059781 | 0.799492526791498 | 1.14478445811487 | 0.589158647740814 | 2.22441181261909 | 0.998074519377826 |
| ebi-fl187-GCST90094778 | prot-a-1743 | Leukocyte immunoglobulin-like receptor subfamily B member 2 \|\| id:prot-a-1743 | Hard cheese liking \|\| id:ebi-fl187-GCST90094778 | Inverse variance weighted | 4 | -0.319687823 | 0.338916404945775 | 0.345545614014657 | -0.983963977 | 0.344588330409958 | 0.726375759282441 | 0.37382631905683 | 1.41140876598614 | 0.998074519377826 |
| ebi-fl187-GCST90094778 | prot-a-1746 | Leukocyte immunoglobulin-like receptor subfamily B member 4 \|\| id:prot-a-1746 | Hard cheese liking \|\| id:ebi-fl187-GCST90094778 | Inverse variance weighted | 4 | 0.46378447034489 | 0.365216948522815 | 0.204124674548921 | -0.252040749 | 1.17960968944961 | 1.59008022406307 | 0.777213066955071 | 3.25310423416058 | 0.998074519377826 |
| ebi-fl187-GCST90094778 | prot-a-1750 | Leucine-rich repeat and immunoglobulin-like domain-containing nogo receptor-interacting protein 3 \|\| id:prot-a-1750 | Hard cheese liking \|\| id:ebi-fl187-GCST90094778 | Inverse variance weighted | 4 | -0.015506912 | 0.485981460707979 | 0.974545064284471 | -0.968030575 | 0.937016750491043 | 0.984612700596267 | 0.379830349176979 | 2.5523557353332 | 0.998074519377826 |
| ebi-fl187-GCST90094778 | prot-a-1753 | Lipase member N \|\| id:prot-a-1753 | Hard cheese liking \|\| id:ebi-fl187-GCST90094778 | Inverse variance weighted | 4 | -0.130297408 | 0.338916404945775 | 0.700642769242225 | -0.794573562 | 0.533978745426655 | 0.877834316910792 | 0.451773847404686 | 1.70570539302591 | 0.998074519377826 |
| ebi-fl187-GCST90094778 | prot-a-1760 | Ig Kappa chain V-I region HK102- like \|\| id:prot-a-1760 | Hard cheese liking \|\| id:ebi-fl187-GCST90094778 | Inverse variance weighted | 4 | 0.0122047715297702 | 0.42492921704801 | 0.977086395433125 | -0.820656494 | 0.84506603694387 | 1.01227955367715 | 0.440142608707132 | 2.32813155218663 | 0.998074519377826 |
| ebi-fl187-GCST90094778 | prot-a-1792 | Leucine-rich repeat neuronal protein 1 \|\| id:prot-a-1792 | Hard cheese liking \|\| id:ebi-fl187-GCST90094778 | Inverse variance weighted | 3 | -0.659092517 | 0.411824264423766 | 0.109505049260361 | -1.466268076 | 0.148083040919148 | 0.517320580993966 | 0.230785152819576 | 1.15960918737764 | 0.998074519377826 |
| ebi-fl187-GCST90094778 | prot-a-1795 | Leucine-rich repeat transmembrane neuronal protein 1 \|\| id:prot-a-1795 | Hard cheese liking \|\| id:ebi-fl187-GCST90094778 | Inverse variance weighted | 4 | 0.0230499053606947 | 0.338916404945775 | 0.94577720256106 | -0.641226248 | 0.687326059054413 | 1.02331760730723 | 0.526646228865902 | 1.98839157678964 | 0.998074519377826 |
| ebi-fl187-GCST90094778 | prot-a-1820 | LysM and putative peptidoglycan-binding domain-containing protein 4 \|\| id:prot-a-1820 | Hard cheese liking \|\| id:ebi-fl187-GCST90094778 | Inverse variance weighted | 4 | 0.692524144463908 | 0.339040591624451 | 0.0410921002058631 | 0.0280045848799854 | 1.35704370404783 | 1.9987543159013 | 1.02840039950302 | 3.88469201029548 | 0.998074519377826 |
| ebi-fl187-GCST90094778 | prot-a-1844 | Mitogen-activated protein kinase kinase kinase 7:TGF-beta-activated kinase 1 and MAP3K7-binding protein 1 fusion \|\| id:prot-a-1844 | Hard cheese liking \|\| id:ebi-fl187-GCST90094778 | Inverse variance weighted | 4 | 0.0615874578936544 | 0.338916404945775 | 0.855803468115693 | -0.602688696 | 0.725863611587373 | 1.06352350598722 | 0.547338030479383 | 2.06651499585493 | 0.998074519377826 |
| ebi-fl187-GCST90094778 | prot-a-1856 | Megakaryocyte-associated tyrosine-protein kinase \|\| id:prot-a-1856 | Hard cheese liking \|\| id:ebi-fl187-GCST90094778 | Inverse variance weighted | 4 | 0.0775083371436617 | 0.339040591624451 | 0.819171438240043 | -0.587011222 | 0.742027896727585 | 1.08059124146542 | 0.555986523997274 | 2.10019016780614 | 0.998074519377826 |
| ebi-fl187-GCST90094778 | prot-a-1910 | Stromelysin-2 \|\| id:prot-a-1910 | Hard cheese liking \|\| id:ebi-fl187-GCST90094778 | Inverse variance weighted | 4 | 0.136124416848839 | 0.4870345615991 | 0.779863884291321 | -0.818463324 | 1.09071215758307 | 1.14582444459352 | 0.441108975587874 | 2.976392979 | 0.998074519377826 |
| ebi-fl187-GCST90094778 | prot-a-1940 | 39S ribosomal protein L14, mitochondrial \|\| id:prot-a-1940 | Hard cheese liking \|\| id:ebi-fl187-GCST90094778 | Inverse variance weighted | 4 | 0.164998081188178 | 0.502446635526913 | 0.742617358890196 | -0.819797324 | 1.14979348682093 | 1.1793908556801 | 0.440520928282706 | 3.15754076857205 | 0.998074519377826 |
| ebi-fl187-GCST90094778 | prot-a-1942 | 39S ribosomal protein L33, mitochondrial \|\| id:prot-a-1942 | Hard cheese liking \|\| id:ebi-fl187-GCST90094778 | Inverse variance weighted | 4 | -0.738526465 | 0.389867871391357 | 0.0581852839623681 | -1.502667493 | 0.0256145628823802 | 0.477817477787717 | 0.222535755152484 | 1.02594543480429 | 0.998074519377826 |
| ebi-fl187-GCST90094778 | prot-a-1948 | Mesothelin \|\| id:prot-a-1948 | Hard cheese liking \|\| id:ebi-fl187-GCST90094778 | Inverse variance weighted | 4 | 0.353536377070087 | 0.353826510065122 | 0.317707496059429 | -0.339963583 | 1.04703633679773 | 1.42409479040513 | 0.711796244018187 | 2.84919454001393 | 0.998074519377826 |
| ebi-fl187-GCST90094778 | prot-a-1980 | Unconventional myosin-VI \|\| id:prot-a-1980 | Hard cheese liking \|\| id:ebi-fl187-GCST90094778 | Inverse variance weighted | 4 | -0.578308908 | 0.338916404945775 | 0.0879435373991657 | -1.242585062 | 0.0859672452134698 | 0.560846006992531 | 0.288637107822614 | 1.08977063251609 | 0.998074519377826 |
| ebi-fl187-GCST90094778 | prot-a-2039 | Neurofascin \|\| id:prot-a-2039 | Hard cheese liking \|\| id:ebi-fl187-GCST90094778 | Inverse variance weighted | 4 | -0.48405373 | 0.338916404945775 | 0.153222991856723 | -1.148329883 | 0.180222424049216 | 0.616280088512642 | 0.317166031565101 | 1.19748368267235 | 0.998074519377826 |
| ebi-fl187-GCST90094778 | prot-a-2041 | NFU1 iron-sulfur cluster scaffold homolog, mitochondrial \|\| id:prot-a-2041 | Hard cheese liking \|\| id:ebi-fl187-GCST90094778 | Inverse variance weighted | 4 | 0.475208748465058 | 0.53410975524466 | 0.37361563168245 | -0.571646372 | 1.52206386874459 | 1.60834990301909 | 0.564595139576156 | 4.5816714123388 | 0.998074519377826 |
| ebi-fl187-GCST90094778 | prot-a-2088 | Glucocorticoid receptor \|\| id:prot-a-2088 | Hard cheese liking \|\| id:ebi-fl187-GCST90094778 | Inverse variance weighted | 4 | 0.637834952377863 | 0.413487398617779 | 0.122934145829575 | -0.172600349 | 1.44827025366871 | 1.8923793494926 | 0.841473843589343 | 4.25574678246767 | 0.998074519377826 |
| ebi-fl187-GCST90094778 | prot-a-2111 | Neuron-specific protein family member 2 \|\| id:prot-a-2111 | Hard cheese liking \|\| id:ebi-fl187-GCST90094778 | Inverse variance weighted | 4 | 0.590433477763928 | 0.338916404945775 | 0.0814872264831485 | -0.073842676 | 1.25470963145765 | 1.8047705737742 | 0.928817807745366 | 3.50681995629245 | 0.998074519377826 |
| ebi-fl187-GCST90094778 | prot-a-2125 | Nuclear migration protein nudC \|\| id:prot-a-2125 | Hard cheese liking \|\| id:ebi-fl187-GCST90094778 | Inverse variance weighted (multiplicative random effects) | 4 | 0.0249287817906763 | 0.600451042895135 | 0.966883931890405 | -1.151955262 | 1.20181282586514 | 1.02524210201771 | 0.316018266311134 | 3.32614117538007 | 0.998074519377826 |
| ebi-fl187-GCST90094778 | prot-a-2142 | Ornithine decarboxylase \|\| id:prot-a-2142 | Hard cheese liking \|\| id:ebi-fl187-GCST90094778 | Inverse variance weighted | 4 | -0.617863816 | 0.509820977203697 | 0.225541821073666 | -1.617112931 | 0.381385299321528 | 0.539094814166938 | 0.19847087177365 | 1.46431169503368 | 0.998074519377826 |
| ebi-fl187-GCST90094778 | prot-a-2186 | Prostate and testis expressed protein 1 \|\| id:prot-a-2186 | Hard cheese liking \|\| id:ebi-fl187-GCST90094778 | Inverse variance weighted (multiplicative random effects) | 4 | 0.796918155377535 | 0.712645934804928 | 0.263459133339215 | -0.599867877 | 2.19370418759519 | 2.2186927151349 | 0.548884151611948 | 8.96837219609297 | 0.998074519377826 |
| ebi-fl187-GCST90094778 | prot-a-2227 | Platelet-derived growth factor C \|\| id:prot-a-2227 | Hard cheese liking \|\| id:ebi-fl187-GCST90094778 | Inverse variance weighted | 4 | 0.421124613800037 | 0.338916404945775 | 0.214029315186925 | -0.24315154 | 1.08540076749376 | 1.52367413740326 | 0.784152674354426 | 2.96062610371492 | 0.998074519377826 |
| ebi-fl187-GCST90094778 | prot-a-2229 | Platelet-derived growth factor receptor alpha \|\| id:prot-a-2229 | Hard cheese liking \|\| id:ebi-fl187-GCST90094778 | Inverse variance weighted | 4 | 0.0842832668558181 | 0.338916404945775 | 0.803604816292538 | -0.579992887 | 0.748559420549537 | 1.08793702666572 | 0.559902349227407 | 2.11395250551008 | 0.998074519377826 |
| ebi-fl187-GCST90094778 | prot-a-2243 | Platelet endothelial aggregation receptor 1 \|\| id:prot-a-2243 | Hard cheese liking \|\| id:ebi-fl187-GCST90094778 | Inverse variance weighted | 4 | -0.361791846 | 0.516489054231671 | 0.483625665227293 | -1.374110392 | 0.650526700339776 | 0.696427316918932 | 0.253064623947642 | 1.91655001076425 | 0.998074519377826 |
| ebi-fl187-GCST90094778 | prot-a-2246 | E3 ubiquitin-protein ligase pellino homolog 2 \|\| id:prot-a-2246 | Hard cheese liking \|\| id:ebi-fl187-GCST90094778 | Inverse variance weighted | 4 | 0.663792189310222 | 0.339040591624451 | 0.0502470738988649 | -0.00072737 | 1.32831174889414 | 1.942143363 | 0.99927289419593 | 3.77466542220684 | 0.998074519377826 |
| ebi-fl187-GCST90094778 | prot-a-2251 | Platelet factor 4 variant \|\| id:prot-a-2251 | Hard cheese liking \|\| id:ebi-fl187-GCST90094778 | Inverse variance weighted | 4 | 0.033892958460227 | 0.338916404945775 | 0.920341238137611 | -0.630383195 | 0.698169112153946 | 1.03447386912566 | 0.532387753464874 | 2.01006912525536 | 0.998074519377826 |
| ebi-fl187-GCST90094778 | prot-a-2346 | Protein phosphatase 1L \|\| id:prot-a-2346 | Hard cheese liking \|\| id:ebi-fl187-GCST90094778 | Inverse variance weighted | 4 | 0.427851835728819 | 0.350219887756159 | 0.221833843913118 | -0.258579144 | 1.11428281573089 | 1.53395878624393 | 0.772147917545048 | 3.04738186094723 | 0.998074519377826 |
| ebi-fl187-GCST90094778 | prot-a-2358 | PR domain zinc finger protein 4 \|\| id:prot-a-2358 | Hard cheese liking \|\| id:ebi-fl187-GCST90094778 | Inverse variance weighted | 4 | 0.16322152481348 | 0.339040591624451 | 0.630216760816732 | -0.501298035 | 0.827741084397403 | 1.17729746140425 | 0.605743872575988 | 2.2881441734353 | 0.998074519377826 |
| ebi-fl187-GCST90094778 | prot-a-2390 | Trypsin-1 \|\| id:prot-a-2390 | Hard cheese liking \|\| id:ebi-fl187-GCST90094778 | Inverse variance weighted | 4 | -0.313254619 | 0.338916404945775 | 0.355339072302938 | -0.977530773 | 0.351021534843636 | 0.731063746258752 | 0.376238972415296 | 1.42051791621402 | 0.998074519377826 |
| ebi-fl187-GCST90094778 | prot-a-242 | Brain-derived neurotrophic factor \|\| id:prot-a-242 | Hard cheese liking \|\| id:ebi-fl187-GCST90094778 | Inverse variance weighted | 4 | 0.494843827286611 | 0.338916404945775 | 0.144269091759317 | -0.169432326 | 1.15911998098033 | 1.64024205800051 | 0.844143878796633 | 3.18712730899503 | 0.998074519377826 |
| ebi-fl187-GCST90094778 | prot-a-2464 | Dihydropteridine reductase \|\| id:prot-a-2464 | Hard cheese liking \|\| id:ebi-fl187-GCST90094778 | Inverse variance weighted | 4 | 0.152712107220374 | 0.338916404945775 | 0.652285576545902 | -0.511564046 | 0.816988260914093 | 1.16498953858313 | 0.59955710991576 | 2.26367197146377 | 0.998074519377826 |
| ebi-fl187-GCST90094778 | prot-a-2474 | Ras-related protein Rab-27A \|\| id:prot-a-2474 | Hard cheese liking \|\| id:ebi-fl187-GCST90094778 | Inverse variance weighted (multiplicative random effects) | 4 | 0.509605327245088 | 0.595506971600059 | 0.392135857607647 | -0.657588337 | 1.6767989915812 | 1.66463407956355 | 0.518099309927717 | 5.34840824094317 | 0.998074519377826 |
| ebi-fl187-GCST90094778 | prot-a-2478 | Ras-related protein Rab-7b \|\| id:prot-a-2478 | Hard cheese liking \|\| id:ebi-fl187-GCST90094778 | Inverse variance weighted | 4 | -0.120137836 | 0.339040591624451 | 0.72307924830848 | -0.784657395 | 0.544381723814586 | 0.886798195781202 | 0.456275997287194 | 1.72354242764562 | 0.998074519377826 |
| ebi-fl187-GCST90094778 | prot-a-2528 | E3 ubiquitin-protein ligase rififylin \|\| id:prot-a-2528 | Hard cheese liking \|\| id:ebi-fl187-GCST90094778 | Inverse variance weighted | 4 | -0.331239358 | 0.46765577031284 | 0.478760602923886 | -1.247844667 | 0.585365952309194 | 0.718033281816037 | 0.287122975964162 | 1.79564798694432 | 0.998074519377826 |
| ebi-fl187-GCST90094778 | prot-a-2543 | Protein RIC-3 \|\| id:prot-a-2543 | Hard cheese liking \|\| id:ebi-fl187-GCST90094778 | Inverse variance weighted | 4 | -0.109175265 | 0.338916404945775 | 0.747353934944918 | -0.773451419 | 0.555100888267745 | 0.896573265431693 | 0.461417770758466 | 1.74211673504796 | 0.998074519377826 |
| ebi-fl187-GCST90094778 | prot-a-255 | Bone morphogenetic protein 6 \|\| id:prot-a-255 | Hard cheese liking \|\| id:ebi-fl187-GCST90094778 | Inverse variance weighted | 4 | -0.484964205 | 0.338916404945775 | 0.152451501136171 | -1.149240359 | 0.179311948563582 | 0.615719235959761 | 0.316877391088463 | 1.19639389932062 | 0.998074519377826 |
| ebi-fl187-GCST90094778 | prot-a-2568 | RING finger protein 165 \|\| id:prot-a-2568 | Hard cheese liking \|\| id:ebi-fl187-GCST90094778 | Inverse variance weighted | 4 | 0.439136899371996 | 0.420809264689016 | 0.29669210597677 | -0.385649259 | 1.26392305816247 | 1.55136765410853 | 0.68000899060702 | 3.5392790852159 | 0.998074519377826 |
| ebi-fl187-GCST90094778 | prot-a-2573 | E3 ubiquitin-protein ligase RNF8 \|\| id:prot-a-2573 | Hard cheese liking \|\| id:ebi-fl187-GCST90094778 | Inverse variance weighted (multiplicative random effects) | 4 | -0.423137661 | 0.607722351461295 | 0.486260973785458 | -1.61427347 | 0.767998148139061 | 0.65498846071893 | 0.199035223065897 | 2.15544704633972 | 0.998074519377826 |
| ebi-fl187-GCST90094778 | prot-a-260 | Cytoplasmic tyrosine-protein kinase BMX \|\| id:prot-a-260 | Hard cheese liking \|\| id:ebi-fl187-GCST90094778 | Inverse variance weighted | 4 | 0.289792183268726 | 0.338916404945775 | 0.392520673014444 | -0.37448397 | 0.954068336962444 | 1.336149785 | 0.687644031909092 | 2.59625062505832 | 0.998074519377826 |
| ebi-fl187-GCST90094778 | prot-a-2623 | Serum amyloid A-1 protein \|\| id:prot-a-2623 | Hard cheese liking \|\| id:ebi-fl187-GCST90094778 | Inverse variance weighted | 4 | 0.624633598314712 | 0.408784320354385 | 0.126505761775118 | -0.17658367 | 1.42585086620931 | 1.86756155435576 | 0.838128650349339 | 4.16139712901352 | 0.998074519377826 |
| ebi-fl187-GCST90094778 | prot-a-2640 | Lysosome membrane protein 2 \|\| id:prot-a-2640 | Hard cheese liking \|\| id:ebi-fl187-GCST90094778 | Inverse variance weighted | 4 | 0.684094947948568 | 0.338916404945775 | 0.0435416134317753 | 0.0198187942548499 | 1.34837110164229 | 1.9819772309956 | 1.02001649043114 | 3.85114729128014 | 0.998074519377826 |
| ebi-fl187-GCST90094778 | prot-a-2641 | Scavenger receptor class F member 2 \|\| id:prot-a-2641 | Hard cheese liking \|\| id:ebi-fl187-GCST90094778 | Inverse variance weighted | 4 | 0.359532629437846 | 0.338916404945775 | 0.288767246872191 | -0.304743524 | 1.02380878313156 | 1.43265967510655 | 0.737312452903578 | 2.78377740209525 | 0.998074519377826 |
| ebi-fl187-GCST90094778 | prot-a-2669 | Semaphorin-3A \|\| id:prot-a-2669 | Hard cheese liking \|\| id:ebi-fl187-GCST90094778 | Inverse variance weighted | 4 | 0.318637489359495 | 0.338916404945775 | 0.347132708504731 | -0.345638664 | 0.982913643053213 | 1.37525269079762 | 0.707768182795279 | 2.67223083704675 | 0.998074519377826 |
| ebi-fl187-GCST90094778 | prot-a-2683 | Sentrin-specific protease 7 \|\| id:prot-a-2683 | Hard cheese liking \|\| id:ebi-fl187-GCST90094778 | Inverse variance weighted | 4 | -0.238582344 | 0.338916404945775 | 0.481459977525393 | -0.902858498 | 0.425693809288738 | 0.787743819290303 | 0.405409140602335 | 1.53065203194015 | 0.998074519377826 |
| ebi-fl187-GCST90094778 | prot-a-270 | Bromodomain testis-specific protein \|\| id:prot-a-270 | Hard cheese liking \|\| id:ebi-fl187-GCST90094778 | Inverse variance weighted | 4 | 0.103227101102621 | 0.338916404945775 | 0.760686183645227 | -0.561049053 | 0.767503254796339 | 1.10874317728459 | 0.570610149701471 | 2.15438059385778 | 0.998074519377826 |
| ebi-fl187-GCST90094778 | prot-a-2708 | 14-3-3 protein sigma \|\| id:prot-a-2708 | Hard cheese liking \|\| id:ebi-fl187-GCST90094778 | Inverse variance weighted | 4 | 0.470967912401561 | 0.338916404945775 | 0.164641701555421 | -0.193308241 | 1.13524406609528 | 1.60154359716208 | 0.824227873914142 | 3.11193296755462 | 0.998074519377826 |
| ebi-fl187-GCST90094778 | prot-a-2713 | Beta-sarcoglycan \|\| id:prot-a-2713 | Hard cheese liking \|\| id:ebi-fl187-GCST90094778 | Inverse variance weighted | 4 | 0.0593760869932911 | 0.338916404945775 | 0.860927276753741 | -0.604900067 | 0.72365224068701 | 1.06117425953919 | 0.546129000385798 | 2.06195021380125 | 0.998074519377826 |
| ebi-fl187-GCST90094778 | prot-a-2720 | Protein shisa-3 homolog \|\| id:prot-a-2720 | Hard cheese liking \|\| id:ebi-fl187-GCST90094778 | Inverse variance weighted | 4 | 0.464977346621689 | 0.338916404945775 | 0.170078098898567 | -0.199298807 | 1.12925350031541 | 1.59197812479589 | 0.819305042612302 | 3.0933464558547 | 0.998074519377826 |
| ebi-fl187-GCST90094778 | prot-a-2730 | Sialic acid-binding Ig-like lectin 8 \|\| id:prot-a-2730 | Hard cheese liking \|\| id:ebi-fl187-GCST90094778 | Inverse variance weighted | 4 | 0.26822316891983 | 0.515683907258605 | 0.602972296744256 | -0.742517289 | 1.2789636271467 | 1.30763893182224 | 0.47591439217033 | 3.59291419664656 | 0.998074519377826 |
| ebi-fl187-GCST90094778 | prot-a-2761 | Sodium- and chloride-dependent neutral and basic amino acid transporter B(0+) \|\| id:prot-a-2761 | Hard cheese liking \|\| id:ebi-fl187-GCST90094778 | Inverse variance weighted (multiplicative random effects) | 4 | -0.561832984 | 0.566733219487505 | 0.321512977499205 | -1.672630094 | 0.548964126172924 | 0.57016300575799 | 0.187752608639115 | 1.73145851602969 | 0.998074519377826 |
| ebi-fl187-GCST90094778 | prot-a-2770 | Secreted Ly-6/uPAR-related protein 1 \|\| id:prot-a-2770 | Hard cheese liking \|\| id:ebi-fl187-GCST90094778 | Inverse variance weighted | 4 | 0.172222734717856 | 0.338916404945775 | 0.611343262278689 | -0.492053419 | 0.836498888411575 | 1.18794239976398 | 0.611369706216516 | 2.30827129772311 | 0.998074519377826 |
| ebi-fl187-GCST90094778 | prot-a-2784 | N-lysine methyltransferase SMYD2 \|\| id:prot-a-2784 | Hard cheese liking \|\| id:ebi-fl187-GCST90094778 | Inverse variance weighted | 4 | 0.635455567876679 | 0.347659307410489 | 0.0675778951892231 | -0.045956675 | 1.31686781040124 | 1.88788200397697 | 0.955083340616377 | 3.73171461522923 | 0.998074519377826 |
| ebi-fl187-GCST90094778 | prot-a-2837 | Serine palmitoyltransferase 2 \|\| id:prot-a-2837 | Hard cheese liking \|\| id:ebi-fl187-GCST90094778 | Inverse variance weighted | 4 | -0.176806434 | 0.416998442928297 | 0.671567398125368 | -0.994123382 | 0.640510514521935 | 0.837941966221149 | 0.37004769296069 | 1.89744930751171 | 0.998074519377826 |
| ebi-fl187-GCST90094778 | prot-a-2858 | Alpha-2,8-sialyltransferase 8B \|\| id:prot-a-2858 | Hard cheese liking \|\| id:ebi-fl187-GCST90094778 | Inverse variance weighted | 4 | 0.258160887413076 | 0.338916404945775 | 0.446224523088813 | -0.406115266 | 0.922437041106794 | 1.29454707820113 | 0.666233368756366 | 2.51541309137269 | 0.998074519377826 |
| ebi-fl187-GCST90094778 | prot-a-2911 | Synaptotagmin-11 \|\| id:prot-a-2911 | Hard cheese liking \|\| id:ebi-fl187-GCST90094778 | Inverse variance weighted | 4 | 0.165609178588857 | 0.338916404945775 | 0.625094360738663 | -0.498666975 | 0.829885332282576 | 1.18011179862714 | 0.607339719310183 | 2.2930557857154 | 0.998074519377826 |
| ebi-fl187-GCST90094778 | prot-a-2962 | Transforming growth factor beta-1 \|\| id:prot-a-2962 | Hard cheese liking \|\| id:ebi-fl187-GCST90094778 | Inverse variance weighted | 4 | 0.211018789815861 | 0.338916404945775 | 0.53352911069539 | -0.453257364 | 0.87529494350958 | 1.23493555905515 | 0.63555454379428 | 2.39958293101669 | 0.998074519377826 |
| ebi-fl187-GCST90094778 | prot-a-2978 | Tyrosine-protein kinase receptor Tie-1, soluble \|\| id:prot-a-2978 | Hard cheese liking \|\| id:ebi-fl187-GCST90094778 | Inverse variance weighted (multiplicative random effects) | 4 | -0.401611573 | 0.676463130397042 | 0.552717319569274 | -1.727479309 | 0.924256162441617 | 0.669240646255994 | 0.177731852932965 | 2.51999309752353 | 0.998074519377826 |
| ebi-fl187-GCST90094778 | prot-a-299 | Uncharacterized protein C1orf226 \|\| id:prot-a-299 | Hard cheese liking \|\| id:ebi-fl187-GCST90094778 | Inverse variance weighted | 4 | -0.063342549 | 0.339040591624451 | 0.851794860856777 | -0.727862108 | 0.601177010759323 | 0.938621894787237 | 0.482940361355121 | 1.82426471645875 | 0.998074519377826 |
| ebi-fl187-GCST90094778 | prot-a-3015 | Transmembrane protein 70, mitochondrial \|\| id:prot-a-3015 | Hard cheese liking \|\| id:ebi-fl187-GCST90094778 | Inverse variance weighted | 4 | 0.349648878770935 | 0.339040591624451 | 0.3024052514545 | -0.314870681 | 1.01416843835486 | 1.4185693713169 | 0.729883256075526 | 2.7570697703883 | 0.998074519377826 |
| ebi-fl187-GCST90094778 | prot-a-3016 | Transmembrane protein 87B \|\| id:prot-a-3016 | Hard cheese liking \|\| id:ebi-fl187-GCST90094778 | Inverse variance weighted | 4 | 0.59986204132138 | 0.476906682509561 | 0.208457408639267 | -0.334875056 | 1.53459913904012 | 1.82186744062755 | 0.715427468853944 | 4.63946537660325 | 0.998074519377826 |
| ebi-fl187-GCST90094778 | prot-a-3123 | Thioredoxin domain-containing protein 12 \|\| id:prot-a-3123 | Hard cheese liking \|\| id:ebi-fl187-GCST90094778 | Inverse variance weighted | 4 | 0.123872148030142 | 0.351390726805867 | 0.724448558902262 | -0.564853677 | 0.812597972569642 | 1.1318711497918 | 0.568443317282908 | 2.25375558262286 | 0.998074519377826 |
| ebi-fl187-GCST90094778 | prot-a-3133 | PolyUbiquitin K48-linked \|\| id:prot-a-3133 | Hard cheese liking \|\| id:ebi-fl187-GCST90094778 | Inverse variance weighted | 4 | -0.475565574 | 0.373810301995274 | 0.203298164247569 | -1.208233766 | 0.25710261792904 | 0.621533433901137 | 0.298724431035131 | 1.29317782318082 | 0.998074519377826 |
| ebi-fl187-GCST90094778 | prot-a-3135 | Ubiquitin-conjugating enzyme E2 B \|\| id:prot-a-3135 | Hard cheese liking \|\| id:ebi-fl187-GCST90094778 | Inverse variance weighted | 4 | -0.253802879 | 0.338916404945775 | 0.453938141500988 | -0.918079032 | 0.410473274902211 | 0.775844722423343 | 0.399285318978289 | 1.50753109293478 | 0.998074519377826 |
| ebi-fl187-GCST90094778 | prot-a-3162 | NKG2D ligand 3 \|\| id:prot-a-3162 | Hard cheese liking \|\| id:ebi-fl187-GCST90094778 | Inverse variance weighted | 4 | 0.340547618302721 | 0.338916404945775 | 0.314986890528478 | -0.323728535 | 1.00482377199644 | 1.40571717627893 | 0.723446605875765 | 2.73142587668031 | 0.998074519377826 |
| ebi-fl187-GCST90094778 | prot-a-3188 | Vesicle-associated membrane protein 8 \|\| id:prot-a-3188 | Hard cheese liking \|\| id:ebi-fl187-GCST90094778 | Inverse variance weighted | 4 | -0.114901344 | 0.338916404945775 | 0.734590430627477 | -0.779177498 | 0.549374809691918 | 0.891454086861499 | 0.458783206406566 | 1.73216974354076 | 0.998074519377826 |
| ebi-fl187-GCST90094778 | prot-a-3201 | Vitrin \|\| id:prot-a-3201 | Hard cheese liking \|\| id:ebi-fl187-GCST90094778 | Inverse variance weighted | 4 | -0.771200299 | 0.338916404945775 | 0.0228766564748448 | -1.435476452 | -0.106924145 | 0.46245764770538 | 0.238001940389865 | 0.898593833188346 | 0.998074519377826 |
| ebi-fl187-GCST90094778 | prot-a-3232 | WNT1-inducible-signaling pathway protein 3 \|\| id:prot-a-3232 | Hard cheese liking \|\| id:ebi-fl187-GCST90094778 | Inverse variance weighted | 4 | -0.479154937 | 0.338916404945775 | 0.157425033059513 | -1.14343109 | 0.185121217174322 | 0.619306524061811 | 0.318723574264937 | 1.20336429970726 | 0.998074519377826 |
| ebi-fl187-GCST90094778 | prot-a-327 | Carbonic anhydrase-related protein 10 \|\| id:prot-a-327 | Hard cheese liking \|\| id:ebi-fl187-GCST90094778 | Inverse variance weighted (multiplicative random effects) | 4 | -0.704910035 | 0.757269396505793 | 0.351927187222944 | -2.189158052 | 0.779337982053706 | 0.494153028685415 | 0.112011016350811 | 2.18002856963811 | 0.998074519377826 |
| ebi-fl187-GCST90094778 | prot-a-332 | Carbonic anhydrase 5A, mitochondrial \|\| id:prot-a-332 | Hard cheese liking \|\| id:ebi-fl187-GCST90094778 | Inverse variance weighted | 4 | 0.0679189538456324 | 0.339040591624451 | 0.84122491850158 | -0.596600606 | 0.732438513429556 | 1.07027856300199 | 0.550680437817815 | 2.08014689419672 | 0.998074519377826 |
| ebi-fl187-GCST90094778 | prot-a-338 | Cell adhesion molecule 3 \|\| id:prot-a-338 | Hard cheese liking \|\| id:ebi-fl187-GCST90094778 | Inverse variance weighted | 4 | 0.613971412381488 | 0.338916404945775 | 0.0700523807014784 | -0.050304741 | 1.27824756607521 | 1.84775504380753 | 0.950939589762212 | 3.59034237155895 | 0.998074519377826 |
| ebi-fl187-GCST90094778 | prot-a-34 | A disintegrin and metalloproteinase with thrombospondin motifs 3 \|\| id:prot-a-34 | Hard cheese liking \|\| id:ebi-fl187-GCST90094778 | Inverse variance weighted | 4 | -0.346731336 | 0.338916404945775 | 0.306280149629292 | -1.011007489 | 0.317544817965558 | 0.70699524710766 | 0.36385221758779 | 1.37375081220227 | 0.998074519377826 |
| ebi-fl187-GCST90094778 | prot-a-358 | Cysteine--tRNA ligase, cytoplasmic \|\| id:prot-a-358 | Hard cheese liking \|\| id:ebi-fl187-GCST90094778 | Inverse variance weighted | 4 | -0.2723324 | 0.338916404945775 | 0.421663518105189 | -0.936608554 | 0.391943753357405 | 0.761601062561199 | 0.391954877580544 | 1.47985447221576 | 0.998074519377826 |
| ebi-fl187-GCST90094778 | prot-a-382 | Coiled-coil domain-containing protein 167 \|\| id:prot-a-382 | Hard cheese liking \|\| id:ebi-fl187-GCST90094778 | Inverse variance weighted | 4 | -0.080230211 | 0.340205867723898 | 0.813565816211245 | -0.747033712 | 0.586573289414314 | 0.922903859009282 | 0.473769808148954 | 1.79781724863825 | 0.998074519377826 |
| ebi-fl187-GCST90094778 | prot-a-40 | Pituitary adenylate cyclase-activating polypeptide 27 \|\| id:prot-a-40 | Hard cheese liking \|\| id:ebi-fl187-GCST90094778 | Inverse variance weighted | 4 | 0.426047461801149 | 0.380599972875015 | 0.262965215029173 | -0.319928485 | 1.17202340863618 | 1.53119344660756 | 0.726200969454424 | 3.22851864642281 | 0.998074519377826 |
| ebi-fl187-GCST90094778 | prot-a-403 | C-C motif chemokine 25 \|\| id:prot-a-403 | Hard cheese liking \|\| id:ebi-fl187-GCST90094778 | Inverse variance weighted | 4 | 0.177863338582303 | 0.338916404945775 | 0.599722212532226 | -0.486412815 | 0.842139492276021 | 1.19466204586948 | 0.614827944651489 | 2.32132813131981 | 0.998074519377826 |
| ebi-fl187-GCST90094778 | prot-a-415 | Cyclin-C \|\| id:prot-a-415 | Hard cheese liking \|\| id:ebi-fl187-GCST90094778 | Inverse variance weighted | 4 | -0.094827588 | 0.338916404945775 | 0.779633719043299 | -0.759103741 | 0.569448565995315 | 0.90952973506566 | 0.468085764960286 | 1.76729223764965 | 0.998074519377826 |
| ebi-fl187-GCST90094778 | prot-a-44 | Adhesion G protein-coupled receptor F5 \|\| id:prot-a-44 | Hard cheese liking \|\| id:ebi-fl187-GCST90094778 | Inverse variance weighted | 4 | -0.696929561 | 0.416673421138172 | 0.0944053119538724 | -1.513609466 | 0.119750344369816 | 0.498112381844942 | 0.220114048101433 | 1.12721540077672 | 0.998074519377826 |
| ebi-fl187-GCST90094778 | prot-a-455 | CD59 glycoprotein \|\| id:prot-a-455 | Hard cheese liking \|\| id:ebi-fl187-GCST90094778 | Inverse variance weighted | 4 | 0.132379182406407 | 0.338916404945775 | 0.696096340191958 | -0.531896971 | 0.796655336100126 | 1.14154108950995 | 0.587489461329474 | 2.21810967653896 | 0.998074519377826 |
| ebi-fl187-GCST90094778 | prot-a-478 | Cadherin-12 \|\| id:prot-a-478 | Hard cheese liking \|\| id:ebi-fl187-GCST90094778 | Inverse variance weighted | 4 | -0.023923476 | 0.338916404945775 | 0.943725556870274 | -0.688199629 | 0.64035267807433 | 0.976360422275777 | 0.502479905294038 | 1.89714984448721 | 0.998074519377826 |
| ebi-fl187-GCST90094778 | prot-a-516 | Centrin-2 \|\| id:prot-a-516 | Hard cheese liking \|\| id:ebi-fl187-GCST90094778 | Inverse variance weighted | 4 | -0.074676949 | 0.339040591624451 | 0.82566897305724 | -0.739196509 | 0.589842610574955 | 0.928043243197005 | 0.477497426505625 | 1.80370450904089 | 0.998074519377826 |
| ebi-fl187-GCST90094778 | prot-a-519 | Cryptic protein \|\| id:prot-a-519 | Hard cheese liking \|\| id:ebi-fl187-GCST90094778 | Inverse variance weighted | 4 | 0.0490228064609775 | 0.339040591624451 | 0.885032433484871 | -0.615496753 | 0.713542366044901 | 1.05024430282261 | 0.5403723969504 | 2.04120917692357 | 0.998074519377826 |
| ebi-fl187-GCST90094778 | prot-a-583 | Chloride intracellular channel protein 4 \|\| id:prot-a-583 | Hard cheese liking \|\| id:ebi-fl187-GCST90094778 | Inverse variance weighted | 4 | 0.423074869928314 | 0.542158517555342 | 0.435183293444173 | -0.639555824 | 1.48570556433678 | 1.52664859175837 | 0.52752668645257 | 4.41808155411179 | 0.998074519377826 |
| ebi-fl187-GCST90094778 | prot-a-602 | CCR4-NOT transcription complex subunit 1 \|\| id:prot-a-602 | Hard cheese liking \|\| id:ebi-fl187-GCST90094778 | Inverse variance weighted | 4 | 0.309702929007947 | 0.338916404945775 | 0.360820212528083 | -0.354573225 | 0.973979082701666 | 1.36302014023691 | 0.701472750589084 | 2.64846196966493 | 0.998074519377826 |
| ebi-fl187-GCST90094778 | prot-a-606 | Ciliary neurotrophic factor receptor subunit alpha \|\| id:prot-a-606 | Hard cheese liking \|\| id:ebi-fl187-GCST90094778 | Inverse variance weighted | 4 | 0.153021543876362 | 0.338916404945775 | 0.651627551721658 | -0.51125461 | 0.817297697570081 | 1.16535008483044 | 0.599742663569997 | 2.26437254293453 | 0.998074519377826 |
| ebi-fl187-GCST90094778 | prot-a-626 | Collagen alpha-2(IX) chain \|\| id:prot-a-626 | Hard cheese liking \|\| id:ebi-fl187-GCST90094778 | Inverse variance weighted | 4 | 0.0768393125289961 | 0.339040591624451 | 0.820705629124475 | -0.587680247 | 0.741358872112919 | 1.07986854110561 | 0.555614679727651 | 2.09878555879979 | 0.998074519377826 |
| ebi-fl187-GCST90094778 | prot-a-647 | Probable serine carboxypeptidase CPVL \|\| id:prot-a-647 | Hard cheese liking \|\| id:ebi-fl187-GCST90094778 | Inverse variance weighted | 4 | 0.377158156652148 | 0.338916404945775 | 0.265779149474823 | -0.287117997 | 1.04143431034587 | 1.45813490487057 | 0.750423175897996 | 2.83327790117575 | 0.998074519377826 |
| ebi-fl187-GCST90094778 | prot-a-651 | Cellular retinoic acid-binding protein 2 \|\| id:prot-a-651 | Hard cheese liking \|\| id:ebi-fl187-GCST90094778 | Inverse variance weighted | 4 | -0.10863099 | 0.338916404945775 | 0.748570808519925 | -0.772907144 | 0.555645163743781 | 0.89706138109518 | 0.461668977491929 | 1.74306518454873 | 0.998074519377826 |
| ebi-fl187-GCST90094778 | prot-a-671 | Cytotoxic and regulatory T-cell molecule \|\| id:prot-a-671 | Hard cheese liking \|\| id:ebi-fl187-GCST90094778 | Inverse variance weighted | 4 | 0.308564247723085 | 0.381139718640743 | 0.418179865454665 | -0.438469601 | 1.05559809625894 | 1.36146897801963 | 0.645022808489235 | 2.87369338527932 | 0.998074519377826 |
| ebi-fl187-GCST90094778 | prot-a-688 | Chorionic somatomammotropin hormone \|\| id:prot-a-688 | Hard cheese liking \|\| id:ebi-fl187-GCST90094778 | Inverse variance weighted | 4 | -0.56508755 | 0.338916404945775 | 0.0954475960831498 | -1.229363704 | 0.0991886034636774 | 0.568310388874135 | 0.292478621484346 | 1.10427454992486 | 0.998074519377826 |
| ebi-fl187-GCST90094778 | prot-a-69 | Aldo-keto reductase family 1 member C1 \|\| id:prot-a-69 | Hard cheese liking \|\| id:ebi-fl187-GCST90094778 | Inverse variance weighted | 4 | -0.361000932 | 0.339040591624451 | 0.286979114929116 | -1.025520491 | 0.303518627822934 | 0.696978349049334 | 0.35860976354369 | 1.35461682426935 | 0.998074519377826 |
| ebi-fl187-GCST90094778 | prot-a-741 | Stromal cell-derived factor 1 \|\| id:prot-a-741 | Hard cheese liking \|\| id:ebi-fl187-GCST90094778 | Inverse variance weighted | 4 | 0.529096337285986 | 0.339040591624451 | 0.118625408037947 | -0.135423222 | 1.19361589686991 | 1.69739774030813 | 0.873346213869844 | 3.29898846877296 | 0.998074519377826 |
| ebi-fl187-GCST90094778 | prot-a-742 | Growth-regulated alpha protein \|\| id:prot-a-742 | Hard cheese liking \|\| id:ebi-fl187-GCST90094778 | Inverse variance weighted | 4 | -0.036234474 | 0.40714496314798 | 0.929084697875362 | -0.834238601 | 0.761769654231511 | 0.96441413737495 | 0.434204958691909 | 2.14206358022875 | 0.998074519377826 |
| ebi-fl187-GCST90094778 | prot-a-788 | Beta-defensin 112 \|\| id:prot-a-788 | Hard cheese liking \|\| id:ebi-fl187-GCST90094778 | Inverse variance weighted | 4 | 0.228931839772929 | 0.5155780514713 | 0.657021290377005 | -0.781601141 | 1.23946482065668 | 1.25725634121158 | 0.457672625878534 | 3.45376458660265 | 0.998074519377826 |
| ebi-fl187-GCST90094778 | prot-a-838 | DnaJ homolog subfamily B member 1 \|\| id:prot-a-838 | Hard cheese liking \|\| id:ebi-fl187-GCST90094778 | Inverse variance weighted | 4 | 0.423300028361063 | 0.388022305507027 | 0.275309520676379 | -0.33722369 | 1.18382374715484 | 1.52699236826329 | 0.713749163185149 | 3.266841929 | 0.998074519377826 |
| ebi-fl187-GCST90094778 | prot-a-85 | Junctional adhesion molecule-like \|\| id:prot-a-85 | Hard cheese liking \|\| id:ebi-fl187-GCST90094778 | Inverse variance weighted | 4 | -0.303947669 | 0.338916404945775 | 0.369813936948657 | -0.968223823 | 0.36032848482737 | 0.737899480556561 | 0.379756955164506 | 1.43380031886387 | 0.998074519377826 |
| ebi-fl187-GCST90094778 | prot-a-856 | Dipeptidase 1 \|\| id:prot-a-856 | Hard cheese liking \|\| id:ebi-fl187-GCST90094778 | Inverse variance weighted | 4 | 0.298968059603167 | 0.508786324079317 | 0.556793928798385 | -0.698253136 | 1.29618925479863 | 1.34846655222826 | 0.497453529099151 | 3.65534052149814 | 0.998074519377826 |
| ebi-fl187-GCST90094778 | prot-a-860 | Dipeptidyl peptidase 2 \|\| id:prot-a-860 | Hard cheese liking \|\| id:ebi-fl187-GCST90094778 | Inverse variance weighted | 4 | 0.0916661976582111 | 0.338916404945775 | 0.786799879303295 | -0.572609956 | 0.75594235135193 | 1.09599891400963 | 0.564051366635975 | 2.12961742593478 | 0.998074519377826 |
| ebi-fl187-GCST90094778 | prot-a-861 | Dermatopontin \|\| id:prot-a-861 | Hard cheese liking \|\| id:ebi-fl187-GCST90094778 | Inverse variance weighted | 4 | 0.321040011073765 | 0.423311193359442 | 0.448210423989427 | -0.508649928 | 1.15072995005827 | 1.37856073748567 | 0.601306838639345 | 3.16049907437835 | 0.998074519377826 |
| ebi-fl187-GCST90094778 | prot-a-877 | Dual specificity protein phosphatase 6 \|\| id:prot-a-877 | Hard cheese liking \|\| id:ebi-fl187-GCST90094778 | Inverse variance weighted | 4 | 0.03707727270298 | 0.482410843319293 | 0.938736278767488 | -0.90844798 | 0.982602525608794 | 1.03777320928089 | 0.403149434642297 | 2.67139958873246 | 0.998074519377826 |
| ebi-fl187-GCST90094778 | prot-a-879 | Dynactin-associated protein \|\| id:prot-a-879 | Hard cheese liking \|\| id:ebi-fl187-GCST90094778 | Inverse variance weighted | 4 | -0.27634711 | 0.338916404945775 | 0.414852377006101 | -0.940623264 | 0.387929043262424 | 0.75854958458532 | 0.390384446898038 | 1.47392519565424 | 0.998074519377826 |
| ebi-fl187-GCST90094778 | prot-a-937 | EMI domain-containing protein 1 \|\| id:prot-a-937 | Hard cheese liking \|\| id:ebi-fl187-GCST90094778 | Inverse variance weighted | 4 | -0.202253773 | 0.338916404945775 | 0.550663648960675 | -0.866529927 | 0.462022380626675 | 0.816887597564853 | 0.4204078671107 | 1.58728082716769 | 0.998074519377826 |
| ebi-fl187-GCST90094778 | prot-b-1 | SRC proto-oncogene, non-receptor tyrosine kinase \|\| id:prot-b-1 | Hard cheese liking \|\| id:ebi-fl187-GCST90094778 | Inverse variance weighted | 3 | -0.059910303 | 0.433199076031912 | 0.890005385581088 | -0.908980492 | 0.789159886038411 | 0.94184901084118 | 0.402934809959795 | 2.20154609950682 | 0.998074519377826 |
| ebi-fl187-GCST90094778 | prot-b-10 | coagulation factor III, tissue factor \|\| id:prot-b-10 | Hard cheese liking \|\| id:ebi-fl187-GCST90094778 | Inverse variance weighted | 3 | -0.192113015 | 0.706630660074687 | 0.785720524075659 | -1.577109109 | 1.1928830789614 | 0.825213601883747 | 0.206571411382077 | 3.29657179654159 | 0.998074519377826 |
| ebi-fl187-GCST90094778 | prot-b-29 | myeloperoxidase \|\| id:prot-b-29 | Hard cheese liking \|\| id:ebi-fl187-GCST90094778 | Inverse variance weighted | 3 | -0.129949684 | 0.433199076031912 | 0.764194832084524 | -0.979019873 | 0.71912050507665 | 0.87813961432933 | 0.375679131734653 | 2.05262714139555 | 0.998074519377826 |
| ebi-fl187-GCST90094778 | prot-b-33 | adrenomedullin \|\| id:prot-b-33 | Hard cheese liking \|\| id:ebi-fl187-GCST90094778 | Inverse variance weighted | 3 | -0.05532897 | 0.433199076031912 | 0.898369130591404 | -0.904399159 | 0.793741219502323 | 0.946173834397606 | 0.404785023675327 | 2.21165525535039 | 0.998074519377826 |
| ebi-fl187-GCST90094778 | ukb-rsfMRI191-edge-pheno102 | Net25_Pair11_15 (Postcentral\|Precentral)&(Cerebellum) (Motor)&(Subcortical-cerebellum) \|\| id:ukb-rsfMRI191-edge-pheno102 | Hard cheese liking \|\| id:ebi-fl187-GCST90094778 | Inverse variance weighted | 4 | -0.074878108 | 0.121358671616002 | 0.537235770864945 | -0.312741104 | 0.162984888277036 | 0.927856577646106 | 0.731439254420383 | 1.17701890277049 | 0.998074519377826 |
| ebi-fl187-GCST90094778 | ukb-rsfMRI191-edge-pheno1167 | Net100_Pair11_45 (Angular\|Temporal)&(Frontal) (Default_mode\|Central_executive)&(Salience\|Default_mode) \|\| id:ukb-rsfMRI191-edge-pheno1167 | Hard cheese liking \|\| id:ebi-fl187-GCST90094778 | Inverse variance weighted | 4 | -0.126833383 | 0.142549311885787 | 0.373599641338996 | -0.406230034 | 0.152563268769213 | 0.880880430442005 | 0.666156911177459 | 1.16481615624789 | 0.998074519377826 |
| ebi-fl187-GCST90094778 | ukb-rsfMRI191-edge-pheno1317 | Net100_Pair26_48 (Parietal\|Frontal)&(Temporal) (Attention\|Central_executive)&(Default_mode\|Central_executive) \|\| id:ukb-rsfMRI191-edge-pheno1317 | Hard cheese liking \|\| id:ebi-fl187-GCST90094778 | Inverse variance weighted | 4 | -0.046150193 | 0.104685703768453 | 0.659325046581361 | -0.251334173 | 0.159033786216268 | 0.954898532182398 | 0.77776242127092 | 1.17237755621325 | 0.998074519377826 |
| ebi-fl187-GCST90094778 | ukb-rsfMRI191-edge-pheno286 | Net100_Pair10_13 (OccipitalPrecuneus)&(Frontal_Sup) (Default_mode\|Central_executive)&(Default_mode) \|\| id:ukb-rsfMRI191-edge-pheno286 | Hard cheese liking \|\| id:ebi-fl187-GCST90094778 | Inverse variance weighted | 4 | 0.00949581483132128 | 0.103617854222337 | 0.926981963569509 | -0.193595179 | 0.212586809107101 | 1.00954104312746 | 0.823991405418465 | 1.23687348078746 | 0.998074519377826 |
| ebi-fl187-GCST90094778 | ukb-rsfMRI191-edge-pheno599 | Net100_Pair11_29 (Angular\|Temporal)&(Parietal) (Default_mode\|Central_executive)&(Central_executive\|Salience) \|\| id:ukb-rsfMRI191-edge-pheno599 | Hard cheese liking \|\| id:ebi-fl187-GCST90094778 | Inverse variance weighted | 4 | 0.0295164036247248 | 0.138251149539491 | 0.830938212145908 | -0.241455849 | 0.300488656722128 | 1.02995633035045 | 0.785483482533394 | 1.35051858634579 | 0.998074519377826 |
| ebi-fl187-GCST90094778 | ukb-rsfMRI191-edge-pheno621 | Net100_Pair5_30 (Precuneus\|Angular\|Cingulate)&(Paracentral) (Default_mode\|Central_executive)&(Motor) \|\| id:ukb-rsfMRI191-edge-pheno621 | Hard cheese liking \|\| id:ebi-fl187-GCST90094778 | Inverse variance weighted | 4 | -0.03170514 | 0.105964740496228 | 0.764783922728032 | -0.239396032 | 0.175985751222886 | 0.968792197891231 | 0.787103102999422 | 1.1924210679876 | 0.998074519377826 |
| ebi-fl187-GCST90094778 | ukb-rsfMRI191-edge-pheno624 | Net100_Pair8_30 (Calcarine\|Lingual)&(Paracentral) (Visual)&(Motor) \|\| id:ukb-rsfMRI191-edge-pheno624 | Hard cheese liking \|\| id:ebi-fl187-GCST90094778 | Inverse variance weighted | 4 | -0.137043341 | 0.105609112512148 | 0.194408686015463 | -0.344037202 | 0.0699505193490125 | 0.871932434737088 | 0.708902555179755 | 1.07245511416413 | 0.998074519377826 |
| ebi-fl187-GCST90094778 | ukb-rsfMRI191-edge-pheno882 | Net100_Pair6_38 (Postcentral\|Precentral)&(Subcortical) (Motor)&(Subcortical-cerebellum) \|\| id:ukb-rsfMRI191-edge-pheno882 | Hard cheese liking \|\| id:ebi-fl187-GCST90094778 | Inverse variance weighted | 4 | -0.086020045 | 0.106198556165082 | 0.417944429115729 | -0.294169215 | 0.122129125514612 | 0.917575838623653 | 0.745150390410139 | 1.12989999127892 | 0.998074519377826 |
| ebi-fl187-GCST90094778 | ukb-rsfMRI191-edge-pheno899 | Net100_Pair23_38 (Cerebellum)&(Subcortical) (Subcortical-cerebellum)&(Subcortical-cerebellum) \|\| id:ukb-rsfMRI191-edge-pheno899 | Hard cheese liking \|\| id:ebi-fl187-GCST90094778 | Inverse variance weighted | 4 | 0.0947091907520632 | 0.105546465818524 | 0.369546994310918 | -0.112161882 | 0.30158026375637 | 1.09933911065603 | 0.893899539316316 | 1.35199362687035 | 0.998074519377826 |
| ebi-fl187-GCST90094778 | ebi-a-GCST90001392 | IgD+ CD38+ B cell Absolute Count \|\| id:ebi-a-GCST90001392 | Hard cheese liking \|\| id:ebi-fl187-GCST90094778 | MR Egger | 3 | -1.026845748 | 1.40857535774348 | 0.598978912445352 | -3.787653449 | 1.73396195360175 | 0.35813482849504 | 0.0226486858466291 | 5.66304624690889 | 0.999279047927446 |
| ebi-fl187-GCST90094778 | ebi-a-GCST90001402 | Switched memory B cell %B cell \|\| id:ebi-a-GCST90001402 | Hard cheese liking \|\| id:ebi-fl187-GCST90094778 | MR Egger | 3 | 1.18364420202317 | 1.48068900562326 | 0.570684429067184 | -1.718506249 | 4.08579465304477 | 3.26625543560137 | 0.179333828023229 | 59.4891922410403 | 0.999279047927446 |
| ebi-fl187-GCST90094778 | ebi-a-GCST90001407 | Memory B cell Absolute Count \|\| id:ebi-a-GCST90001407 | Hard cheese liking \|\| id:ebi-fl187-GCST90094778 | MR Egger | 3 | 1.8555857386306 | 2.08796151679035 | 0.537469856484721 | -2.236818834 | 5.94799031153969 | 6.3954432118786 | 0.106797705766005 | 382.982888845759 | 0.999279047927446 |
[truncated: 2,615,975 more chars]
