# Supplementary material for: Mendelian Randomization Mediation Analysis Reveals the Impact of Dietary Preferences on Preeclampsia and Fetal Growth Restriction via Immune Modulation
Source: Food Sci Nutr. 2025 Nov 26;13(12):e71280. doi: 10.1002/fsn3.71280 (PMC12648301; doi:10.1002/fsn3.71280)
Supplement: Supplementary file 1 — Data S1: fsn371280‐sup‐0001‐Supinfo1.zip. [file FSN3-13-e71280-s001.zip › fsn371280-sup-0015-Supinfo15@Supplementary Material 5_PE and FGR_Reverse MR.docx]

| **id.exposure** | **id.outcome** | **outcome** | **exposure** | **method** | **nsnp** | **b** | **se** | **pval** | **lo_ci** | **up_ci** | **or** | **or_lci95** | **or_uci95** | **p_fdr** |
| --- | --- | --- | --- | --- | --- | --- | --- | --- | --- | --- | --- | --- | --- | --- |
| finngen_R11_O15_PREECLAMPS | ebi-fl187-GCST90094707 | Black olive liking \|\| id:ebi-fl187-GCST90094707 | Pre-eclampsia \|\| id:finngen_R11_O15_PREECLAMPS | Inverse variance weighted (multiplicative random effects) | 42 | -0.012540065 | 0.0228521486309719 | 0.58317858627564 | -0.057330276 | 0.0322501466452926 | 0.987538234306419 | 0.944282144140435 | 1.03277581840201 | 0.952193991303069 |
| finngen_R11_O15_PREECLAMPS | ebi-fl187-GCST90094716 | F-burn/spicy liking (derived food-liking factor) \|\| id:ebi-fl187-GCST90094716 | Pre-eclampsia \|\| id:finngen_R11_O15_PREECLAMPS | Inverse variance weighted (multiplicative random effects) | 41 | -0.004015648 | 0.0214125831143624 | 0.851239766091264 | -0.04598431 | 0.0379530153914152 | 0.995992404418227 | 0.955056946518507 | 1.03868242964461 | 0.952193991303069 |
| finngen_R11_O15_PREECLAMPS | ebi-fl187-GCST90094726 | F-capsicum liking (derived food-liking factor) \|\| id:ebi-fl187-GCST90094726 | Pre-eclampsia \|\| id:finngen_R11_O15_PREECLAMPS | Inverse variance weighted (multiplicative random effects) | 40 | 0.0162502949827285 | 0.0281656426832253 | 0.563970176018143 | -0.038954365 | 0.0714549546418501 | 1.01638304914952 | 0.961794599952686 | 1.07406976775424 | 0.952193991303069 |
| finngen_R11_O15_PREECLAMPS | ebi-fl187-GCST90094729 | F-cheese liking (derived food-liking factor) \|\| id:ebi-fl187-GCST90094729 | Pre-eclampsia \|\| id:finngen_R11_O15_PREECLAMPS | Inverse variance weighted | 40 | -0.003589291 | 0.020574452458878 | 0.861508849274206 | -0.043915218 | 0.0367366357473876 | 0.996417142733285 | 0.957035093486773 | 1.0374197655757 | 0.952193991303069 |
| finngen_R11_O15_PREECLAMPS | ebi-fl187-GCST90094733 | Chilli pepper liking \|\| id:ebi-fl187-GCST90094733 | Pre-eclampsia \|\| id:finngen_R11_O15_PREECLAMPS | Inverse variance weighted (multiplicative random effects) | 41 | -0.017465139 | 0.023461486276066 | 0.456623975153339 | -0.063449653 | 0.0285193736967617 | 0.982686492104444 | 0.938521370310777 | 1.02892994481817 | 0.952193991303069 |
| finngen_R11_O15_PREECLAMPS | ebi-fl187-GCST90094734 | Chips liking \|\| id:ebi-fl187-GCST90094734 | Pre-eclampsia \|\| id:finngen_R11_O15_PREECLAMPS | Inverse variance weighted | 40 | -0.002178594 | 0.0115091894254116 | 0.849864178279081 | -0.024736606 | 0.0203794169203464 | 0.997823777060789 | 0.975566837005643 | 1.02058849511927 | 0.952193991303069 |
| finngen_R11_O15_PREECLAMPS | ebi-fl187-GCST90094750 | Curry liking \|\| id:ebi-fl187-GCST90094750 | Pre-eclampsia \|\| id:finngen_R11_O15_PREECLAMPS | Inverse variance weighted (multiplicative random effects) | 42 | 0.0201008773762775 | 0.0191599737861296 | 0.294128520293417 | -0.017452671 | 0.0576544259970916 | 1.0203042604523 | 0.982698744473034 | 1.05934884902631 | 0.952193991303069 |
| finngen_R11_O15_PREECLAMPS | ebi-fl187-GCST90094764 | Fried chicken liking \|\| id:ebi-fl187-GCST90094764 | Pre-eclampsia \|\| id:finngen_R11_O15_PREECLAMPS | Inverse variance weighted (multiplicative random effects) | 42 | -0.003825898 | 0.0186035623523945 | 0.837061144830508 | -0.04028888 | 0.0326370840495403 | 0.996181411262542 | 0.960511926027852 | 1.03317551532021 | 0.952193991303069 |
| finngen_R11_O15_PREECLAMPS | ebi-fl187-GCST90094766 | Fruit liking \|\| id:ebi-fl187-GCST90094766 | Pre-eclampsia \|\| id:finngen_R11_O15_PREECLAMPS | Inverse variance weighted | 42 | 0.00641070625475284 | 0.00821221610209577 | 0.435019855469806 | -0.009685237 | 0.0225066498148605 | 1.00643129881286 | 0.990361513552686 | 1.02276183531868 | 0.952193991303069 |
| finngen_R11_O15_PREECLAMPS | ebi-fl187-GCST90094770 | Gherkins liking \|\| id:ebi-fl187-GCST90094770 | Pre-eclampsia \|\| id:finngen_R11_O15_PREECLAMPS | Inverse variance weighted | 42 | -0.013049068 | 0.0182283275733445 | 0.474073127994707 | -0.048776591 | 0.0226784535733187 | 0.987035701499879 | 0.952393879759393 | 1.02293756474105 | 0.952193991303069 |
| finngen_R11_O15_PREECLAMPS | ebi-fl187-GCST90094775 | Green olives liking \|\| id:ebi-fl187-GCST90094775 | Pre-eclampsia \|\| id:finngen_R11_O15_PREECLAMPS | Inverse variance weighted (multiplicative random effects) | 42 | -0.006258155 | 0.0238363533035688 | 0.792900007564338 | -0.052977408 | 0.0404610972335195 | 0.993761386226275 | 0.948401438794468 | 1.04129079982173 | 0.952193991303069 |
| finngen_R11_O15_PREECLAMPS | ebi-fl187-GCST90094780 | F-healthy breakfast food liking (derived food-liking factor) \|\| id:ebi-fl187-GCST90094780 | Pre-eclampsia \|\| id:finngen_R11_O15_PREECLAMPS | Inverse variance weighted (multiplicative random effects) | 38 | 0.000352608209417802 | 0.0292369619387 | 0.990377460326582 | -0.056951837 | 0.0576570536092698 | 1.00035267 | 0.944639564766388 | 1.0593516325879 | 0.993723220497197 |
| finngen_R11_O15_PREECLAMPS | ebi-fl187-GCST90094784 | Ice cream liking \|\| id:ebi-fl187-GCST90094784 | Pre-eclampsia \|\| id:finngen_R11_O15_PREECLAMPS | Inverse variance weighted (multiplicative random effects) | 40 | -0.000120225 | 0.0152824777288806 | 0.993723220497197 | -0.030073881 | 0.029833431272076 | 0.999879782150215 | 0.970373838297979 | 1.03028290674687 | 0.993723220497197 |
| finngen_R11_O15_PREECLAMPS | ebi-fl187-GCST90094798 | Mayonnaise liking \|\| id:ebi-fl187-GCST90094798 | Pre-eclampsia \|\| id:finngen_R11_O15_PREECLAMPS | Inverse variance weighted | 41 | 0.00772337030632672 | 0.0160615442751437 | 0.63061528972677 | -0.023757256 | 0.0392039970856084 | 1.00775327246301 | 0.976522725560362 | 1.03998261543492 | 0.952193991303069 |
| finngen_R11_O15_PREECLAMPS | ebi-fl187-GCST90094807 | Oranges liking \|\| id:ebi-fl187-GCST90094807 | Pre-eclampsia \|\| id:finngen_R11_O15_PREECLAMPS | Inverse variance weighted (multiplicative random effects) | 42 | 0.00335721054884503 | 0.0133418954328452 | 0.801327682025353 | -0.022792904 | 0.0295073255972217 | 1.00336285229192 | 0.977464891394573 | 1.02994698042099 | 0.952193991303069 |
| finngen_R11_O15_PREECLAMPS | ebi-fl187-GCST90094812 | Plum liking \|\| id:ebi-fl187-GCST90094812 | Pre-eclampsia \|\| id:finngen_R11_O15_PREECLAMPS | Inverse variance weighted | 42 | -0.003702076 | 0.0121719717075659 | 0.761015455510553 | -0.02755914 | 0.0201549887499203 | 0.996304768437131 | 0.972817148111634 | 1.02035947201114 | 0.952193991303069 |
| finngen_R11_O15_PREECLAMPS | ebi-fl187-GCST90094817 | Prawns liking \|\| id:ebi-fl187-GCST90094817 | Pre-eclampsia \|\| id:finngen_R11_O15_PREECLAMPS | Inverse variance weighted (multiplicative random effects) | 41 | 0.018243112191132 | 0.0228392487287136 | 0.42442872826647 | -0.026521815 | 0.0630080396994106 | 1.01841053431287 | 0.973826799263336 | 1.06503540176138 | 0.952193991303069 |
| finngen_R11_O15_PREECLAMPS | ebi-fl187-GCST90094840 | F-small fish liking (derived food-liking factor) \|\| id:ebi-fl187-GCST90094840 | Pre-eclampsia \|\| id:finngen_R11_O15_PREECLAMPS | Inverse variance weighted (multiplicative random effects) | 40 | 0.0323669133376774 | 0.0209841745982485 | 0.122965746340835 | -0.008762069 | 0.0734958955502445 | 1.03289641925924 | 0.991276206179462 | 1.07626411919083 | 0.952193991303069 |
| finngen_R11_O15_PREECLAMPS | ebi-fl187-GCST90094846 | Spicy food liking \|\| id:ebi-fl187-GCST90094846 | Pre-eclampsia \|\| id:finngen_R11_O15_PREECLAMPS | Inverse variance weighted (multiplicative random effects) | 41 | 0.0210799264586948 | 0.021243493017729 | 0.321051011933179 | -0.02055732 | 0.0627171727734437 | 1.02130367756165 | 0.979652541322479 | 1.06472566323655 | 0.952193991303069 |
| finngen_R11_O15_PREECLAMPS | ebi-fl187-GCST90094852 | F-strong vegetable liking (derived food-liking factor) \|\| id:ebi-fl187-GCST90094852 | Pre-eclampsia \|\| id:finngen_R11_O15_PREECLAMPS | Inverse variance weighted (multiplicative random effects) | 39 | 0.00719518942286036 | 0.0212770011434784 | 0.735236996354449 | -0.034507733 | 0.048898111664078 | 1.00722113699351 | 0.966080869129728 | 1.05011335098725 | 0.952193991303069 |
| finngen_R11_O15_PREECLAMPS | ebi-fl187-GCST90094853 | F-sweetened coffee drink liking (derived food-liking factor) \|\| id:ebi-fl187-GCST90094853 | Pre-eclampsia \|\| id:finngen_R11_O15_PREECLAMPS | Inverse variance weighted | 42 | 0.00678600190274227 | 0.0180160235219197 | 0.706422724656995 | -0.028525404 | 0.0420974080057049 | 1.0068090789845 | 0.971877604059279 | 1.04299606996992 | 0.952193991303069 |
| finngen_R11_O15_PREECLAMPS | ebi-fl187-GCST90094707 | Black olive liking \|\| id:ebi-fl187-GCST90094707 | Pre-eclampsia \|\| id:finngen_R11_O15_PREECLAMPS | MR Egger | 42 | 0.0694314562455601 | 0.0439942582750817 | 0.122397822384265 | -0.01679729 | 0.15566020246472 | 1.07189858673318 | 0.983342997917895 | 1.16842910629697 | 0.353458059892231 |
| finngen_R11_O15_PREECLAMPS | ebi-fl187-GCST90094716 | F-burn/spicy liking (derived food-liking factor) \|\| id:ebi-fl187-GCST90094716 | Pre-eclampsia \|\| id:finngen_R11_O15_PREECLAMPS | MR Egger | 41 | 0.0606745731600377 | 0.0414728650304307 | 0.151482025668099 | -0.020612242 | 0.141961388619682 | 1.06255307462814 | 0.979598737888599 | 1.15253214682082 | 0.353458059892231 |
| finngen_R11_O15_PREECLAMPS | ebi-fl187-GCST90094726 | F-capsicum liking (derived food-liking factor) \|\| id:ebi-fl187-GCST90094726 | Pre-eclampsia \|\| id:finngen_R11_O15_PREECLAMPS | MR Egger | 40 | 0.109693383770992 | 0.0538557795137074 | 0.0486809477337747 | 0.00413605592412573 | 0.215250711617859 | 1.11593585395363 | 1.00414462120819 | 1.240172784 | 0.255574975602317 |
| finngen_R11_O15_PREECLAMPS | ebi-fl187-GCST90094729 | F-cheese liking (derived food-liking factor) \|\| id:ebi-fl187-GCST90094729 | Pre-eclampsia \|\| id:finngen_R11_O15_PREECLAMPS | MR Egger | 40 | 0.0434507643447208 | 0.0405964796455185 | 0.291231829089128 | -0.036118336 | 0.123019864449937 | 1.04440857090574 | 0.964526148794221 | 1.13090688556758 | 0.599574926922347 |
| finngen_R11_O15_PREECLAMPS | ebi-fl187-GCST90094733 | Chilli pepper liking \|\| id:ebi-fl187-GCST90094733 | Pre-eclampsia \|\| id:finngen_R11_O15_PREECLAMPS | MR Egger | 41 | 0.0661379611912563 | 0.0447080271598115 | 0.147084695540313 | -0.021489772 | 0.153765694424487 | 1.06837410116283 | 0.978739487924355 | 1.16621760347703 | 0.353458059892231 |
| finngen_R11_O15_PREECLAMPS | ebi-fl187-GCST90094734 | Chips liking \|\| id:ebi-fl187-GCST90094734 | Pre-eclampsia \|\| id:finngen_R11_O15_PREECLAMPS | MR Egger | 40 | 0.0170793497348227 | 0.0227599159692503 | 0.457627260363697 | -0.027530086 | 0.0616887850345533 | 1.0172260357391 | 0.972845413509335 | 1.06363127524324 | 0.640678164509176 |
| finngen_R11_O15_PREECLAMPS | ebi-fl187-GCST90094750 | Curry liking \|\| id:ebi-fl187-GCST90094750 | Pre-eclampsia \|\| id:finngen_R11_O15_PREECLAMPS | MR Egger | 42 | 0.0957233663810401 | 0.0364392621680186 | 0.0121547444235339 | 0.0243024125317238 | 0.167144320230356 | 1.10045459914505 | 1.02460012296098 | 1.18192482866373 | 0.217709411479153 |
| finngen_R11_O15_PREECLAMPS | ebi-fl187-GCST90094764 | Fried chicken liking \|\| id:ebi-fl187-GCST90094764 | Pre-eclampsia \|\| id:finngen_R11_O15_PREECLAMPS | MR Egger | 42 | -0.032930437 | 0.0374349809096616 | 0.384292438546888 | -0.106302999 | 0.0404421259698551 | 0.967605867189256 | 0.899152164413928 | 1.04127104540679 | 0.620780093037281 |
| finngen_R11_O15_PREECLAMPS | ebi-fl187-GCST90094766 | Fruit liking \|\| id:ebi-fl187-GCST90094766 | Pre-eclampsia \|\| id:finngen_R11_O15_PREECLAMPS | MR Egger | 42 | 0.0333262173664601 | 0.0159552420723924 | 0.0431405662228114 | 0.00205394290457091 | 0.0645984918283492 | 1.03388775637637 | 1.0020560536902 | 1.06673063732166 | 0.255574975602317 |
| finngen_R11_O15_PREECLAMPS | ebi-fl187-GCST90094770 | Gherkins liking \|\| id:ebi-fl187-GCST90094770 | Pre-eclampsia \|\| id:finngen_R11_O15_PREECLAMPS | MR Egger | 42 | 0.0350727101727457 | 0.036017183832665 | 0.3360195340979 | -0.03552097 | 0.105666390484769 | 1.03569501162516 | 0.965102495684047 | 1.11145102401269 | 0.599574926922347 |
| finngen_R11_O15_PREECLAMPS | ebi-fl187-GCST90094775 | Green olives liking \|\| id:ebi-fl187-GCST90094775 | Pre-eclampsia \|\| id:finngen_R11_O15_PREECLAMPS | MR Egger | 42 | 0.0832698055618795 | 0.045631965867711 | 0.0755040913794296 | -0.006168848 | 0.172708458662593 | 1.08683500312238 | 0.993850140735824 | 1.18851955198949 | 0.279568058634993 |
| finngen_R11_O15_PREECLAMPS | ebi-fl187-GCST90094780 | F-healthy breakfast food liking (derived food-liking factor) \|\| id:ebi-fl187-GCST90094780 | Pre-eclampsia \|\| id:finngen_R11_O15_PREECLAMPS | MR Egger | 38 | 0.00624023285271386 | 0.0579929177964606 | 0.914907798825265 | -0.107425886 | 0.119906351733777 | 1.00625974366864 | 0.898143084935303 | 1.12739126839797 | 0.932307942385575 |
| finngen_R11_O15_PREECLAMPS | ebi-fl187-GCST90094784 | Ice cream liking \|\| id:ebi-fl187-GCST90094784 | Pre-eclampsia \|\| id:finngen_R11_O15_PREECLAMPS | MR Egger | 40 | -0.003147573 | 0.0306797398048582 | 0.918824304537079 | -0.063279863 | 0.056984717157924 | 0.996857375554662 | 0.938680735034246 | 1.05863963124957 | 0.932307942385575 |
| finngen_R11_O15_PREECLAMPS | ebi-fl187-GCST90094798 | Mayonnaise liking \|\| id:ebi-fl187-GCST90094798 | Pre-eclampsia \|\| id:finngen_R11_O15_PREECLAMPS | MR Egger | 41 | 0.0562224633657685 | 0.0312645826154522 | 0.0798765881814265 | -0.005056119 | 0.117501045292055 | 1.05783298664913 | 0.994956642091416 | 1.12468280556512 | 0.279568058634993 |
| finngen_R11_O15_PREECLAMPS | ebi-fl187-GCST90094807 | Oranges liking \|\| id:ebi-fl187-GCST90094807 | Pre-eclampsia \|\| id:finngen_R11_O15_PREECLAMPS | MR Egger | 42 | 0.00241186026150335 | 0.0271224784689256 | 0.929585444435711 | -0.050748198 | 0.0555719180605975 | 1.0024147711362 | 0.950517983170133 | 1.0571450421598 | 0.932307942385575 |
| finngen_R11_O15_PREECLAMPS | ebi-fl187-GCST90094812 | Plum liking \|\| id:ebi-fl187-GCST90094812 | Pre-eclampsia \|\| id:finngen_R11_O15_PREECLAMPS | MR Egger | 42 | 0.00211313617693743 | 0.024721453007278 | 0.932307942385575 | -0.046340912 | 0.0505671840712022 | 1.00211537042267 | 0.954716432686828 | 1.05186752972416 | 0.932307942385575 |
| finngen_R11_O15_PREECLAMPS | ebi-fl187-GCST90094817 | Prawns liking \|\| id:ebi-fl187-GCST90094817 | Pre-eclampsia \|\| id:finngen_R11_O15_PREECLAMPS | MR Egger | 41 | 0.0442013826411173 | 0.0460088368723488 | 0.342614243955627 | -0.045975938 | 0.134378702910921 | 1.04519281738603 | 0.955064943041515 | 1.14382590783448 | 0.599574926922347 |
| finngen_R11_O15_PREECLAMPS | ebi-fl187-GCST90094840 | F-small fish liking (derived food-liking factor) \|\| id:ebi-fl187-GCST90094840 | Pre-eclampsia \|\| id:finngen_R11_O15_PREECLAMPS | MR Egger | 40 | 0.0225725621325894 | 0.0423313593375966 | 0.596976103439238 | -0.060396902 | 0.105542026434279 | 1.02282925014344 | 0.941390819366985 | 1.11131280805614 | 0.783531136 |
| finngen_R11_O15_PREECLAMPS | ebi-fl187-GCST90094846 | Spicy food liking \|\| id:ebi-fl187-GCST90094846 | Pre-eclampsia \|\| id:finngen_R11_O15_PREECLAMPS | MR Egger | 41 | 0.0974483520304566 | 0.0404232990622102 | 0.0207342296646812 | 0.0182186858685246 | 0.176678018192389 | 1.10235450572109 | 1.01838565859242 | 1.19324682749674 | 0.217709411479153 |
| finngen_R11_O15_PREECLAMPS | ebi-fl187-GCST90094852 | F-strong vegetable liking (derived food-liking factor) \|\| id:ebi-fl187-GCST90094852 | Pre-eclampsia \|\| id:finngen_R11_O15_PREECLAMPS | MR Egger | 39 | 0.0326426628156245 | 0.0423395468187404 | 0.44561838806796 | -0.050342849 | 0.115628174580356 | 1.03318127918081 | 0.950903352392099 | 1.12257839134164 | 0.640678164509176 |
| finngen_R11_O15_PREECLAMPS | ebi-fl187-GCST90094853 | F-sweetened coffee drink liking (derived food-liking factor) \|\| id:ebi-fl187-GCST90094853 | Pre-eclampsia \|\| id:finngen_R11_O15_PREECLAMPS | MR Egger | 42 | -0.007198189 | 0.0365322964015557 | 0.844796907899423 | -0.07880149 | 0.0644051118270623 | 0.992827655793944 | 0.924223373756197 | 1.06652437289399 | 0.932307942385575 |
| finngen_R11_O15_PREECLAMPS | ebi-fl187-GCST90094707 | Black olive liking \|\| id:ebi-fl187-GCST90094707 | Pre-eclampsia \|\| id:finngen_R11_O15_PREECLAMPS | Weighted median | 42 | 0.00159357126994385 | 0.0311171175684073 | 0.959156552771973 | -0.059395979 | 0.0625831217040222 | 1.00159484167938 | 0.942333550816845 | 1.06458294518872 | 0.996813049002282 |
| finngen_R11_O15_PREECLAMPS | ebi-fl187-GCST90094716 | F-burn/spicy liking (derived food-liking factor) \|\| id:ebi-fl187-GCST90094716 | Pre-eclampsia \|\| id:finngen_R11_O15_PREECLAMPS | Weighted median | 41 | 0.0133423838435531 | 0.0260313176418299 | 0.608265276582041 | -0.037678999 | 0.0643637664215397 | 1.01343179063761 | 0.963022022568754 | 1.06648027792286 | 0.912397914873061 |
| finngen_R11_O15_PREECLAMPS | ebi-fl187-GCST90094726 | F-capsicum liking (derived food-liking factor) \|\| id:ebi-fl187-GCST90094726 | Pre-eclampsia \|\| id:finngen_R11_O15_PREECLAMPS | Weighted median | 40 | 0.00483632612783404 | 0.0306585729503195 | 0.874655438119255 | -0.055254477 | 0.0649271291104603 | 1.00484804002951 | 0.94624432003379 | 1.06708126239013 | 0.996813049002282 |
| finngen_R11_O15_PREECLAMPS | ebi-fl187-GCST90094729 | F-cheese liking (derived food-liking factor) \|\| id:ebi-fl187-GCST90094729 | Pre-eclampsia \|\| id:finngen_R11_O15_PREECLAMPS | Weighted median | 40 | 0.00247696225300404 | 0.0329074009345006 | 0.939999353907167 | -0.062021544 | 0.0669754680846252 | 1.00248003245841 | 0.939862638568337 | 1.06926924663018 | 0.996813049002282 |
| finngen_R11_O15_PREECLAMPS | ebi-fl187-GCST90094733 | Chilli pepper liking \|\| id:ebi-fl187-GCST90094733 | Pre-eclampsia \|\| id:finngen_R11_O15_PREECLAMPS | Weighted median | 41 | -0.018420673 | 0.0271080846346171 | 0.496804058819755 | -0.071552519 | 0.0347111727187893 | 0.981747950460546 | 0.930947383839762 | 1.03532063676692 | 0.823323008440611 |
| finngen_R11_O15_PREECLAMPS | ebi-fl187-GCST90094734 | Chips liking \|\| id:ebi-fl187-GCST90094734 | Pre-eclampsia \|\| id:finngen_R11_O15_PREECLAMPS | Weighted median | 40 | 0.0140449888482016 | 0.0167833255200849 | 0.402681481032852 | -0.018850329 | 0.0469403068675679 | 1.0141440830864 | 0.981326227161612 | 1.0480594452611 | 0.823323008440611 |
| finngen_R11_O15_PREECLAMPS | ebi-fl187-GCST90094750 | Curry liking \|\| id:ebi-fl187-GCST90094750 | Pre-eclampsia \|\| id:finngen_R11_O15_PREECLAMPS | Weighted median | 42 | 0.0201841444123007 | 0.0246076713204218 | 0.412080505554479 | -0.028046891 | 0.0684151802003274 | 1.02038922170109 | 0.972342771242015 | 1.07080979522664 | 0.823323008440611 |
| finngen_R11_O15_PREECLAMPS | ebi-fl187-GCST90094764 | Fried chicken liking \|\| id:ebi-fl187-GCST90094764 | Pre-eclampsia \|\| id:finngen_R11_O15_PREECLAMPS | Weighted median | 42 | -0.000346234 | 0.0246559522297596 | 0.988795995121283 | -0.0486719 | 0.0479794327148733 | 0.9996538262765 | 0.952493591559169 | 1.04914907695436 | 0.996813049002282 |
| finngen_R11_O15_PREECLAMPS | ebi-fl187-GCST90094766 | Fruit liking \|\| id:ebi-fl187-GCST90094766 | Pre-eclampsia \|\| id:finngen_R11_O15_PREECLAMPS | Weighted median | 42 | 0.0116179326748295 | 0.0110500827377392 | 0.293080080700851 | -0.010040229 | 0.0332760948407983 | 1.01168568297323 | 0.990010005349297 | 1.03383593660945 | 0.823323008440611 |
| finngen_R11_O15_PREECLAMPS | ebi-fl187-GCST90094770 | Gherkins liking \|\| id:ebi-fl187-GCST90094770 | Pre-eclampsia \|\| id:finngen_R11_O15_PREECLAMPS | Weighted median | 42 | -0.018837617 | 0.0285703260896964 | 0.509676148082283 | -0.074835456 | 0.0371602218695726 | 0.981338701766607 | 0.927896153139609 | 1.03785929527404 | 0.823323008440611 |
| finngen_R11_O15_PREECLAMPS | ebi-fl187-GCST90094775 | Green olives liking \|\| id:ebi-fl187-GCST90094775 | Pre-eclampsia \|\| id:finngen_R11_O15_PREECLAMPS | Weighted median | 42 | 0.0256480023397627 | 0.0305462454872363 | 0.401107459428273 | -0.034222639 | 0.0855186434947457 | 1.02597974243654 | 0.966356332256639 | 1.08928186917557 | 0.823323008440611 |
| finngen_R11_O15_PREECLAMPS | ebi-fl187-GCST90094780 | F-healthy breakfast food liking (derived food-liking factor) \|\| id:ebi-fl187-GCST90094780 | Pre-eclampsia \|\| id:finngen_R11_O15_PREECLAMPS | Weighted median | 38 | 0.0274873892441439 | 0.0405807311804702 | 0.498183429846379 | -0.052050844 | 0.107025622357866 | 1.02786865282489 | 0.949280600516831 | 1.1129627708444 | 0.823323008440611 |
| finngen_R11_O15_PREECLAMPS | ebi-fl187-GCST90094784 | Ice cream liking \|\| id:ebi-fl187-GCST90094784 | Pre-eclampsia \|\| id:finngen_R11_O15_PREECLAMPS | Weighted median | 40 | 7.36006586055105e-05 | 0.0184266005527395 | 0.996813049002282 | -0.036042536 | 0.0361897377419748 | 1.0000736033672 | 0.964599262006528 | 1.03685255789164 | 0.996813049002282 |
| finngen_R11_O15_PREECLAMPS | ebi-fl187-GCST90094798 | Mayonnaise liking \|\| id:ebi-fl187-GCST90094798 | Pre-eclampsia \|\| id:finngen_R11_O15_PREECLAMPS | Weighted median | 41 | 0.0177710857257383 | 0.021992497635293 | 0.419060542617362 | -0.02533421 | 0.0608763810909126 | 1.0179299310257 | 0.974984008517376 | 1.06276752790404 | 0.823323008440611 |
| finngen_R11_O15_PREECLAMPS | ebi-fl187-GCST90094807 | Oranges liking \|\| id:ebi-fl187-GCST90094807 | Pre-eclampsia \|\| id:finngen_R11_O15_PREECLAMPS | Weighted median | 42 | -0.015332327 | 0.0173579622940091 | 0.377072993107387 | -0.049353933 | 0.0186892790957482 | 0.984784614699472 | 0.951844180914768 | 1.01886501676857 | 0.823323008440611 |
| finngen_R11_O15_PREECLAMPS | ebi-fl187-GCST90094812 | Plum liking \|\| id:ebi-fl187-GCST90094812 | Pre-eclampsia \|\| id:finngen_R11_O15_PREECLAMPS | Weighted median | 42 | -0.013943607 | 0.0168516975268611 | 0.407993150242111 | -0.046972934 | 0.0190857201588421 | 0.986153154835793 | 0.954113221129612 | 1.01926901677477 | 0.823323008440611 |
| finngen_R11_O15_PREECLAMPS | ebi-fl187-GCST90094817 | Prawns liking \|\| id:ebi-fl187-GCST90094817 | Pre-eclampsia \|\| id:finngen_R11_O15_PREECLAMPS | Weighted median | 41 | 0.0185280461243673 | 0.0272186809768593 | 0.496054593721781 | -0.034820569 | 0.0718766608390115 | 1.01870075537701 | 0.965778691743709 | 1.07452280514911 | 0.823323008440611 |
| finngen_R11_O15_PREECLAMPS | ebi-fl187-GCST90094840 | F-small fish liking (derived food-liking factor) \|\| id:ebi-fl187-GCST90094840 | Pre-eclampsia \|\| id:finngen_R11_O15_PREECLAMPS | Weighted median | 40 | 0.0309668045210795 | 0.0269777708425872 | 0.251024825454045 | -0.021909626 | 0.0838432353725503 | 1.03145126379953 | 0.978328646205971 | 1.08745840543411 | 0.823323008440611 |
| finngen_R11_O15_PREECLAMPS | ebi-fl187-GCST90094846 | Spicy food liking \|\| id:ebi-fl187-GCST90094846 | Pre-eclampsia \|\| id:finngen_R11_O15_PREECLAMPS | Weighted median | 41 | 0.0074750508191223 | 0.0252684321481198 | 0.76736321964637 | -0.042051076 | 0.057001177829437 | 1.00750305875492 | 0.958820806406961 | 1.05865705731221 | 0.996813049002282 |
| finngen_R11_O15_PREECLAMPS | ebi-fl187-GCST90094852 | F-strong vegetable liking (derived food-liking factor) \|\| id:ebi-fl187-GCST90094852 | Pre-eclampsia \|\| id:finngen_R11_O15_PREECLAMPS | Weighted median | 39 | 0.00844320088299415 | 0.0285715330767754 | 0.767603588746647 | -0.047557004 | 0.064443405713474 | 1.00847894523165 | 0.953556115120619 | 1.06656521503918 | 0.996813049002282 |
| finngen_R11_O15_PREECLAMPS | ebi-fl187-GCST90094853 | F-sweetened coffee drink liking (derived food-liking factor) \|\| id:ebi-fl187-GCST90094853 | Pre-eclampsia \|\| id:finngen_R11_O15_PREECLAMPS | Weighted median | 42 | 0.0212353050010976 | 0.0246474040695045 | 0.388927735065958 | -0.027073607 | 0.0695442169773264 | 1.02146237856747 | 0.973289597984403 | 1.07201946161704 | 0.823323008440611 |
| finngen_R11_O15_PREECLAMPS | ebi-fl187-GCST90094707 | Black olive liking \|\| id:ebi-fl187-GCST90094707 | Pre-eclampsia \|\| id:finngen_R11_O15_PREECLAMPS | Weighted mode | 42 | 0.0101340498818234 | 0.0408495983734748 | 0.805309303214141 | -0.069931163 | 0.090199262693834 | 1.01018557326509 | 0.932458005373567 | 1.09439233354438 | 0.955522814956416 |
| finngen_R11_O15_PREECLAMPS | ebi-fl187-GCST90094716 | F-burn/spicy liking (derived food-liking factor) \|\| id:ebi-fl187-GCST90094716 | Pre-eclampsia \|\| id:finngen_R11_O15_PREECLAMPS | Weighted mode | 41 | 0.0293411802819339 | 0.0299523367327903 | 0.333173664043794 | -0.0293654 | 0.088047760278203 | 1.02977587376988 | 0.971061574010372 | 1.09204027693016 | 0.955522814956416 |
| finngen_R11_O15_PREECLAMPS | ebi-fl187-GCST90094726 | F-capsicum liking (derived food-liking factor) \|\| id:ebi-fl187-GCST90094726 | Pre-eclampsia \|\| id:finngen_R11_O15_PREECLAMPS | Weighted mode | 40 | 0.0192249128746614 | 0.0373011092748374 | 0.609184376027697 | -0.053885261 | 0.0923350870533427 | 1.01941090147179 | 0.947540819862924 | 1.09673226129706 | 0.955522814956416 |
| finngen_R11_O15_PREECLAMPS | ebi-fl187-GCST90094729 | F-cheese liking (derived food-liking factor) \|\| id:ebi-fl187-GCST90094729 | Pre-eclampsia \|\| id:finngen_R11_O15_PREECLAMPS | Weighted mode | 40 | 0.00252523980827912 | 0.0449870900862414 | 0.955522814956416 | -0.085649457 | 0.0906999363773122 | 1.00252843091186 | 0.917915944058112 | 1.09494040417601 | 0.955522814956416 |
| finngen_R11_O15_PREECLAMPS | ebi-fl187-GCST90094733 | Chilli pepper liking \|\| id:ebi-fl187-GCST90094733 | Pre-eclampsia \|\| id:finngen_R11_O15_PREECLAMPS | Weighted mode | 41 | -0.01011178 | 0.0371719790088843 | 0.78700053992362 | -0.082968858 | 0.0627452992820229 | 0.98993917258408 | 0.920379808843501 | 1.06475561067321 | 0.955522814956416 |
| finngen_R11_O15_PREECLAMPS | ebi-fl187-GCST90094734 | Chips liking \|\| id:ebi-fl187-GCST90094734 | Pre-eclampsia \|\| id:finngen_R11_O15_PREECLAMPS | Weighted mode | 40 | 0.0194138698272268 | 0.0199763628983292 | 0.337118366894671 | -0.019739801 | 0.0585675411079521 | 1.01960354444919 | 0.980453752761045 | 1.060316598 | 0.955522814956416 |
| finngen_R11_O15_PREECLAMPS | ebi-fl187-GCST90094750 | Curry liking \|\| id:ebi-fl187-GCST90094750 | Pre-eclampsia \|\| id:finngen_R11_O15_PREECLAMPS | Weighted mode | 42 | 0.0251520371494124 | 0.0318587198889235 | 0.434369706470516 | -0.037291054 | 0.0875951281317024 | 1.02547101836343 | 0.963395694529875 | 1.09154609624501 | 0.955522814956416 |
| finngen_R11_O15_PREECLAMPS | ebi-fl187-GCST90094764 | Fried chicken liking \|\| id:ebi-fl187-GCST90094764 | Pre-eclampsia \|\| id:finngen_R11_O15_PREECLAMPS | Weighted mode | 42 | 0.00336956348082629 | 0.031914117722667 | 0.916428708803813 | -0.059182107 | 0.0659212342172537 | 1.00337524684154 | 0.9425351110451 | 1.06814258076604 | 0.955522814956416 |
| finngen_R11_O15_PREECLAMPS | ebi-fl187-GCST90094766 | Fruit liking \|\| id:ebi-fl187-GCST90094766 | Pre-eclampsia \|\| id:finngen_R11_O15_PREECLAMPS | Weighted mode | 42 | 0.0136897533764012 | 0.0137086718928741 | 0.323836352975184 | -0.013179244 | 0.0405587502864343 | 1.01378388711556 | 0.986907222427627 | 1.0413924900124 | 0.955522814956416 |
| finngen_R11_O15_PREECLAMPS | ebi-fl187-GCST90094770 | Gherkins liking \|\| id:ebi-fl187-GCST90094770 | Pre-eclampsia \|\| id:finngen_R11_O15_PREECLAMPS | Weighted mode | 42 | -0.007516715 | 0.033984987567227 | 0.826052067938102 | -0.074127291 | 0.0590938605419294 | 0.99251146476219 | 0.928553490074529 | 1.06087481035188 | 0.955522814956416 |
| finngen_R11_O15_PREECLAMPS | ebi-fl187-GCST90094775 | Green olives liking \|\| id:ebi-fl187-GCST90094775 | Pre-eclampsia \|\| id:finngen_R11_O15_PREECLAMPS | Weighted mode | 42 | 0.018660424336899 | 0.0401689698350251 | 0.644712568442465 | -0.060070757 | 0.0973916052135481 | 1.01883561808836 | 0.941697899942004 | 1.10229195238665 | 0.955522814956416 |
| finngen_R11_O15_PREECLAMPS | ebi-fl187-GCST90094780 | F-healthy breakfast food liking (derived food-liking factor) \|\| id:ebi-fl187-GCST90094780 | Pre-eclampsia \|\| id:finngen_R11_O15_PREECLAMPS | Weighted mode | 38 | 0.0227033807466205 | 0.0535418304281665 | 0.673998643604961 | -0.082238607 | 0.127645368385827 | 1.02296306400082 | 0.921052163080475 | 1.13615001653116 | 0.955522814956416 |
| finngen_R11_O15_PREECLAMPS | ebi-fl187-GCST90094784 | Ice cream liking \|\| id:ebi-fl187-GCST90094784 | Pre-eclampsia \|\| id:finngen_R11_O15_PREECLAMPS | Weighted mode | 40 | 0.00286548577017259 | 0.0229291054592182 | 0.901188042827851 | -0.042075561 | 0.0478065324702402 | 1.00286959519875 | 0.958797330217459 | 1.04896769450329 | 0.955522814956416 |
| finngen_R11_O15_PREECLAMPS | ebi-fl187-GCST90094798 | Mayonnaise liking \|\| id:ebi-fl187-GCST90094798 | Pre-eclampsia \|\| id:finngen_R11_O15_PREECLAMPS | Weighted mode | 41 | 0.0204497545460285 | 0.0293405244638778 | 0.48984652636416 | -0.037057673 | 0.0779571824952291 | 1.0206602834155 | 0.963620558469442 | 1.08107636868651 | 0.955522814956416 |
| finngen_R11_O15_PREECLAMPS | ebi-fl187-GCST90094807 | Oranges liking \|\| id:ebi-fl187-GCST90094807 | Pre-eclampsia \|\| id:finngen_R11_O15_PREECLAMPS | Weighted mode | 42 | -0.019445863 | 0.0218538458690391 | 0.378757181186154 | -0.062279401 | 0.0233876748049179 | 0.980741988084016 | 0.939620319253549 | 1.02366331110751 | 0.955522814956416 |
| finngen_R11_O15_PREECLAMPS | ebi-fl187-GCST90094812 | Plum liking \|\| id:ebi-fl187-GCST90094812 | Pre-eclampsia \|\| id:finngen_R11_O15_PREECLAMPS | Weighted mode | 42 | -0.010573135 | 0.0206895141794443 | 0.612065262294685 | -0.051124583 | 0.0299783124646871 | 0.989482563790687 | 0.950160289226294 | 1.03043218617676 | 0.955522814956416 |
| finngen_R11_O15_PREECLAMPS | ebi-fl187-GCST90094817 | Prawns liking \|\| id:ebi-fl187-GCST90094817 | Pre-eclampsia \|\| id:finngen_R11_O15_PREECLAMPS | Weighted mode | 41 | 0.0270651111788206 | 0.0351308203550316 | 0.445583746491556 | -0.041791297 | 0.0959215190746826 | 1.027434698 | 0.959069920727847 | 1.10067267879392 | 0.955522814956416 |
| finngen_R11_O15_PREECLAMPS | ebi-fl187-GCST90094840 | F-small fish liking (derived food-liking factor) \|\| id:ebi-fl187-GCST90094840 | Pre-eclampsia \|\| id:finngen_R11_O15_PREECLAMPS | Weighted mode | 40 | 0.0240576294085563 | 0.0348899947291453 | 0.494573561640995 | -0.04432676 | 0.0924420190776811 | 1.02434934883677 | 0.956641314031059 | 1.09684954336841 | 0.955522814956416 |
| finngen_R11_O15_PREECLAMPS | ebi-fl187-GCST90094846 | Spicy food liking \|\| id:ebi-fl187-GCST90094846 | Pre-eclampsia \|\| id:finngen_R11_O15_PREECLAMPS | Weighted mode | 41 | 0.0354965071193119 | 0.033974617014654 | 0.30239089074984 | -0.031093742 | 0.102086756468034 | 1.0361340290292 | 0.969384696533999 | 1.10747954857428 | 0.955522814956416 |
| finngen_R11_O15_PREECLAMPS | ebi-fl187-GCST90094852 | F-strong vegetable liking (derived food-liking factor) \|\| id:ebi-fl187-GCST90094852 | Pre-eclampsia \|\| id:finngen_R11_O15_PREECLAMPS | Weighted mode | 39 | 0.0132754933308507 | 0.0328574466000374 | 0.688453389176119 | -0.051125102 | 0.077676088666924 | 1.01336400393272 | 0.950159796201087 | 1.08077252749728 | 0.955522814956416 |
| finngen_R11_O15_PREECLAMPS | ebi-fl187-GCST90094853 | F-sweetened coffee drink liking (derived food-liking factor) \|\| id:ebi-fl187-GCST90094853 | Pre-eclampsia \|\| id:finngen_R11_O15_PREECLAMPS | Weighted mode | 42 | 0.0240394016306348 | 0.0324410924397133 | 0.462906339306256 | -0.03954514 | 0.0876239428124729 | 1.0243306773945 | 0.961226563675607 | 1.09157754925048 | 0.955522814956416 |

| **id.exposure** | **id.outcome** | **outcome** | **exposure** | **method** | **nsnp** | **b** | **se** | **pval** | **lo_ci** | **up_ci** | **or** | **or_lci95** | **or_uci95** | **p_fdr** |
| --- | --- | --- | --- | --- | --- | --- | --- | --- | --- | --- | --- | --- | --- | --- |
| finngen_R11_O15_POOR_FETGRO | ebi-fl187-GCST90094697 | Banana liking \|\| id:ebi-fl187-GCST90094697 | Poor fetal growth \|\| id:finngen_R11_O15_POOR_FETGRO | Inverse variance weighted | 26 | -0.023404484 | 0.0122413739239917 | 0.055886915952167 | -0.047397577 | 0.000588609151429276 | 0.976867276922036 | 0.953708150132481 | 1.00058878241579 | 0.492374452442311 |
| finngen_R11_O15_POOR_FETGRO | ebi-fl187-GCST90094727 | Cauliflower liking \|\| id:ebi-fl187-GCST90094727 | Poor fetal growth \|\| id:finngen_R11_O15_POOR_FETGRO | Inverse variance weighted | 26 | -0.016703251 | 0.011579217198991 | 0.149155758256139 | -0.039398517 | 0.00599201432791196 | 0.983435474456484 | 0.961367511410896 | 1.00601000235599 | 0.492374452442311 |
| finngen_R11_O15_POOR_FETGRO | ebi-fl187-GCST90094753 | F-deep fried food liking (derived food-liking factor) \|\| id:ebi-fl187-GCST90094753 | Poor fetal growth \|\| id:finngen_R11_O15_POOR_FETGRO | Inverse variance weighted | 24 | 0.0135775660602517 | 0.0228843799105405 | 0.55297269549347 | -0.031275819 | 0.0584309506849111 | 1.01367015980161 | 0.969208210588695 | 1.06017177903197 | 0.749840104856726 |
| finngen_R11_O15_POOR_FETGRO | ebi-fl187-GCST90094756 | Dried fruit liking \|\| id:ebi-fl187-GCST90094756 | Poor fetal growth \|\| id:finngen_R11_O15_POOR_FETGRO | Inverse variance weighted | 26 | -0.017554142 | 0.0135694406475319 | 0.19578545884853 | -0.044150245 | 0.00904196204374998 | 0.982599034716025 | 0.956810190444242 | 1.00908296406896 | 0.492374452442311 |
| finngen_R11_O15_POOR_FETGRO | ebi-fl187-GCST90094765 | Fried fish liking \|\| id:ebi-fl187-GCST90094765 | Poor fetal growth \|\| id:finngen_R11_O15_POOR_FETGRO | Inverse variance weighted | 26 | 0.0188378360644516 | 0.0150888509807742 | 0.21186242569789 | -0.010736312 | 0.0484119839867691 | 1.01901638751029 | 0.989321116631151 | 1.04960298588437 | 0.492374452442311 |
| finngen_R11_O15_POOR_FETGRO | ebi-fl187-GCST90094778 | Hard cheese liking \|\| id:ebi-fl187-GCST90094778 | Poor fetal growth \|\| id:finngen_R11_O15_POOR_FETGRO | Inverse variance weighted | 26 | -0.004505513 | 0.0118372485847129 | 0.703484033564979 | -0.02770652 | 0.018695494449326 | 0.995504621819748 | 0.972673785216712 | 1.01887134939457 | 0.749840104856726 |
| finngen_R11_O15_POOR_FETGRO | ebi-fl187-GCST90094781 | Herring liking \|\| id:ebi-fl187-GCST90094781 | Poor fetal growth \|\| id:finngen_R11_O15_POOR_FETGRO | Inverse variance weighted | 26 | -0.02168793 | 0.0178286024355535 | 0.223806569291959 | -0.056631991 | 0.0132561305919685 | 0.978545561942776 | 0.944941752498869 | 1.01334438262008 | 0.492374452442311 |
| finngen_R11_O15_POOR_FETGRO | ebi-fl187-GCST90094785 | F-highly palatable foods liking (derived food-liking factor) \|\| id:ebi-fl187-GCST90094785 | Poor fetal growth \|\| id:finngen_R11_O15_POOR_FETGRO | Inverse variance weighted | 24 | -0.002905622 | 0.00694708998586738 | 0.675764146826077 | -0.016521919 | 0.0107106741278676 | 0.997098594990329 | 0.983613819700067 | 1.01076823873301 | 0.749840104856726 |
| finngen_R11_O15_POOR_FETGRO | ebi-fl187-GCST90094803 | Mushroom liking \|\| id:ebi-fl187-GCST90094803 | Poor fetal growth \|\| id:finngen_R11_O15_POOR_FETGRO | Inverse variance weighted | 26 | -0.008789376 | 0.0118989544935628 | 0.460108687099519 | -0.032111326 | 0.0145325751726996 | 0.991249138007785 | 0.968398767688704 | 1.01463868644351 | 0.723027936870673 |
| finngen_R11_O15_POOR_FETGRO | ebi-fl187-GCST90094838 | Shellfish liking \|\| id:ebi-fl187-GCST90094838 | Poor fetal growth \|\| id:finngen_R11_O15_POOR_FETGRO | Inverse variance weighted | 26 | -0.00637202 | 0.0199843674667609 | 0.749840104856726 | -0.04554138 | 0.0327973405533497 | 0.993648238584381 | 0.955480064068782 | 1.0333411016839 | 0.749840104856726 |
| finngen_R11_O15_POOR_FETGRO | ebi-fl187-GCST90094852 | F-strong vegetable liking (derived food-liking factor) \|\| id:ebi-fl187-GCST90094852 | Poor fetal growth \|\| id:finngen_R11_O15_POOR_FETGRO | Inverse variance weighted | 24 | -0.020024225 | 0.0192479988949602 | 0.298187717206776 | -0.057750303 | 0.0177018529914105 | 0.980174928435677 | 0.943885603722827 | 1.01785945939272 | 0.546677481545756 |
| finngen_R11_O15_POOR_FETGRO | ebi-fl187-GCST90094697 | Banana liking \|\| id:ebi-fl187-GCST90094697 | Poor fetal growth \|\| id:finngen_R11_O15_POOR_FETGRO | MR Egger | 26 | -0.021062938 | 0.0253980947447268 | 0.415100606563841 | -0.070843204 | 0.0287173272463267 | 0.979157335981775 | 0.931607952934479 | 1.02913364531395 | 0.91715959663518 |
| finngen_R11_O15_POOR_FETGRO | ebi-fl187-GCST90094727 | Cauliflower liking \|\| id:ebi-fl187-GCST90094727 | Poor fetal growth \|\| id:finngen_R11_O15_POOR_FETGRO | MR Egger | 26 | -0.053519383 | 0.0240251152627765 | 0.0355374950700408 | -0.100608609 | -0.006430157 | 0.94788756800926 | 0.904286893544944 | 0.993590472227556 | 0.390912445770449 |
| finngen_R11_O15_POOR_FETGRO | ebi-fl187-GCST90094753 | F-deep fried food liking (derived food-liking factor) \|\| id:ebi-fl187-GCST90094753 | Poor fetal growth \|\| id:finngen_R11_O15_POOR_FETGRO | MR Egger | 24 | 0.0510518666990988 | 0.0479756384175788 | 0.298814899016457 | -0.042980385 | 0.145084117997553 | 1.05237747521354 | 0.957930180067015 | 1.15613681809185 | 0.91715959663518 |
| finngen_R11_O15_POOR_FETGRO | ebi-fl187-GCST90094756 | Dried fruit liking \|\| id:ebi-fl187-GCST90094756 | Poor fetal growth \|\| id:finngen_R11_O15_POOR_FETGRO | MR Egger | 26 | 0.00297830286800106 | 0.0283342561785694 | 0.91715959663518 | -0.052556839 | 0.058513444977997 | 1.00298274241834 | 0.948800390428131 | 1.06025924076093 | 0.91715959663518 |
| finngen_R11_O15_POOR_FETGRO | ebi-fl187-GCST90094765 | Fried fish liking \|\| id:ebi-fl187-GCST90094765 | Poor fetal growth \|\| id:finngen_R11_O15_POOR_FETGRO | MR Egger | 26 | 0.0101274456559788 | 0.0318886459871857 | 0.753544255109684 | -0.0523743 | 0.0726291917908628 | 1.01017890179345 | 0.948973599085985 | 1.07533172114745 | 0.91715959663518 |
| finngen_R11_O15_POOR_FETGRO | ebi-fl187-GCST90094778 | Hard cheese liking \|\| id:ebi-fl187-GCST90094778 | Poor fetal growth \|\| id:finngen_R11_O15_POOR_FETGRO | MR Egger | 26 | 0.00596504192164866 | 0.0245598595765266 | 0.810163904181587 | -0.042172283 | 0.0541023666916408 | 1.00598286821144 | 0.958704597984969 | 1.05559265415225 | 0.91715959663518 |
| finngen_R11_O15_POOR_FETGRO | ebi-fl187-GCST90094781 | Herring liking \|\| id:ebi-fl187-GCST90094781 | Poor fetal growth \|\| id:finngen_R11_O15_POOR_FETGRO | MR Egger | 26 | 0.00456195779334585 | 0.0370014117641638 | 0.902903014325076 | -0.067960809 | 0.077084724851107 | 1.00457237936436 | 0.934297088649882 | 1.08013358667325 | 0.91715959663518 |
| finngen_R11_O15_POOR_FETGRO | ebi-fl187-GCST90094785 | F-highly palatable foods liking (derived food-liking factor) \|\| id:ebi-fl187-GCST90094785 | Poor fetal growth \|\| id:finngen_R11_O15_POOR_FETGRO | MR Egger | 24 | 0.00257638238129489 | 0.0148319601040213 | 0.863685893677497 | -0.026494259 | 0.0316470241851767 | 1.00257970410645 | 0.973853634301665 | 1.03215311591147 | 0.91715959663518 |
| finngen_R11_O15_POOR_FETGRO | ebi-fl187-GCST90094803 | Mushroom liking \|\| id:ebi-fl187-GCST90094803 | Poor fetal growth \|\| id:finngen_R11_O15_POOR_FETGRO | MR Egger | 26 | -0.016007448 | 0.0246870727191966 | 0.522873237002937 | -0.064394111 | 0.0323792143006162 | 0.984119990076929 | 0.937635394506204 | 1.03290912495796 | 0.91715959663518 |
| finngen_R11_O15_POOR_FETGRO | ebi-fl187-GCST90094838 | Shellfish liking \|\| id:ebi-fl187-GCST90094838 | Poor fetal growth \|\| id:finngen_R11_O15_POOR_FETGRO | MR Egger | 26 | -0.012210901 | 0.0414691669995325 | 0.770940326509433 | -0.093490468 | 0.0690686665109154 | 0.98786334971191 | 0.910746698506461 | 1.07150978346051 | 0.91715959663518 |
| finngen_R11_O15_POOR_FETGRO | ebi-fl187-GCST90094852 | F-strong vegetable liking (derived food-liking factor) \|\| id:ebi-fl187-GCST90094852 | Poor fetal growth \|\| id:finngen_R11_O15_POOR_FETGRO | MR Egger | 24 | -0.006930612 | 0.0411366664493967 | 0.867746789360989 | -0.087558478 | 0.0736972540059948 | 0.99309334907069 | 0.916165294134492 | 1.076480856 | 0.91715959663518 |
| finngen_R11_O15_POOR_FETGRO | ebi-fl187-GCST90094697 | Banana liking \|\| id:ebi-fl187-GCST90094697 | Poor fetal growth \|\| id:finngen_R11_O15_POOR_FETGRO | Weighted median | 26 | -0.011116905 | 0.0167227085344239 | 0.506191856851144 | -0.043893414 | 0.0216596032862448 | 0.988944659005501 | 0.957055960642073 | 1.02189587526248 | 0.797627494489227 |
| finngen_R11_O15_POOR_FETGRO | ebi-fl187-GCST90094727 | Cauliflower liking \|\| id:ebi-fl187-GCST90094727 | Poor fetal growth \|\| id:finngen_R11_O15_POOR_FETGRO | Weighted median | 26 | -0.011544022 | 0.0167752749284684 | 0.49135393423887 | -0.044423561 | 0.0213355163962566 | 0.988522354100739 | 0.956548714617103 | 1.02156474586662 | 0.797627494489227 |
| finngen_R11_O15_POOR_FETGRO | ebi-fl187-GCST90094753 | F-deep fried food liking (derived food-liking factor) \|\| id:ebi-fl187-GCST90094753 | Poor fetal growth \|\| id:finngen_R11_O15_POOR_FETGRO | Weighted median | 24 | 0.00461044728431561 | 0.0327121045069133 | 0.887917262997999 | -0.059505278 | 0.0687261721178656 | 1.00462109174869 | 0.942230560909933 | 1.07114286020575 | 0.986364968167403 |
| finngen_R11_O15_POOR_FETGRO | ebi-fl187-GCST90094756 | Dried fruit liking \|\| id:ebi-fl187-GCST90094756 | Poor fetal growth \|\| id:finngen_R11_O15_POOR_FETGRO | Weighted median | 26 | -0.011842913 | 0.017873159601539 | 0.507581132856781 | -0.046874306 | 0.0231884799875295 | 0.988226938441246 | 0.954207328522337 | 1.02345942298859 | 0.797627494489227 |
| finngen_R11_O15_POOR_FETGRO | ebi-fl187-GCST90094765 | Fried fish liking \|\| id:ebi-fl187-GCST90094765 | Poor fetal growth \|\| id:finngen_R11_O15_POOR_FETGRO | Weighted median | 26 | 0.0170732690496223 | 0.0203344963224612 | 0.401120950939331 | -0.022782344 | 0.0569288818416464 | 1.0172198503266 | 0.977475214218403 | 1.0585805234211 | 0.797627494489227 |
| finngen_R11_O15_POOR_FETGRO | ebi-fl187-GCST90094778 | Hard cheese liking \|\| id:ebi-fl187-GCST90094778 | Poor fetal growth \|\| id:finngen_R11_O15_POOR_FETGRO | Weighted median | 26 | -0.008683216 | 0.0164464243066082 | 0.597520287096072 | -0.040918207 | 0.0235517760542203 | 0.991354374649672 | 0.959907640244031 | 1.02383130931971 | 0.821590394757099 |
| finngen_R11_O15_POOR_FETGRO | ebi-fl187-GCST90094781 | Herring liking \|\| id:ebi-fl187-GCST90094781 | Poor fetal growth \|\| id:finngen_R11_O15_POOR_FETGRO | Weighted median | 26 | -0.047894639 | 0.0250057747604684 | 0.0554487578085685 | -0.096905958 | 0.0011166794724621 | 0.953234215441631 | 0.907641358901921 | 1.00111730319113 | 0.609936335894254 |
| finngen_R11_O15_POOR_FETGRO | ebi-fl187-GCST90094785 | F-highly palatable foods liking (derived food-liking factor) \|\| id:ebi-fl187-GCST90094785 | Poor fetal growth \|\| id:finngen_R11_O15_POOR_FETGRO | Weighted median | 24 | 0.0120260224492545 | 0.00971289330646153 | 0.215660309677219 | -0.007011248 | 0.0310632933299191 | 1.01209862580853 | 0.993013273028722 | 1.03155079210496 | 0.797627494489227 |
| finngen_R11_O15_POOR_FETGRO | ebi-fl187-GCST90094803 | Mushroom liking \|\| id:ebi-fl187-GCST90094803 | Poor fetal growth \|\| id:finngen_R11_O15_POOR_FETGRO | Weighted median | 26 | -0.001137956 | 0.0170856598439587 | 0.946897768194411 | -0.034625849 | 0.0323499373233059 | 0.998862691255514 | 0.965966765829156 | 1.03287888494362 | 0.986364968167403 |
| finngen_R11_O15_POOR_FETGRO | ebi-fl187-GCST90094838 | Shellfish liking \|\| id:ebi-fl187-GCST90094838 | Poor fetal growth \|\| id:finngen_R11_O15_POOR_FETGRO | Weighted median | 26 | 0.0194630524753092 | 0.0277553864606039 | 0.483156279649325 | -0.034937505 | 0.0738636099380928 | 1.0196536924847 | 0.965665763665837 | 1.07665994976441 | 0.797627494489227 |
| finngen_R11_O15_POOR_FETGRO | ebi-fl187-GCST90094852 | F-strong vegetable liking (derived food-liking factor) \|\| id:ebi-fl187-GCST90094852 | Poor fetal growth \|\| id:finngen_R11_O15_POOR_FETGRO | Weighted median | 24 | -0.000468846 | 0.027434225514643 | 0.986364968167403 | -0.054239928 | 0.0533022363071124 | 0.999531264189383 | 0.947204818553261 | 1.05474838020564 | 0.986364968167403 |
| finngen_R11_O15_POOR_FETGRO | ebi-fl187-GCST90094697 | Banana liking \|\| id:ebi-fl187-GCST90094697 | Poor fetal growth \|\| id:finngen_R11_O15_POOR_FETGRO | Weighted mode | 26 | -0.013698864 | 0.0274616835169229 | 0.622256280015487 | -0.067523763 | 0.0401260361259851 | 0.986394538875455 | 0.934705508701758 | 1.04094196221729 | 0.867921561904123 |
| finngen_R11_O15_POOR_FETGRO | ebi-fl187-GCST90094727 | Cauliflower liking \|\| id:ebi-fl187-GCST90094727 | Poor fetal growth \|\| id:finngen_R11_O15_POOR_FETGRO | Weighted mode | 26 | 0.00853686478775037 | 0.0328781123782198 | 0.797258638020271 | -0.055904235 | 0.0729779650490612 | 1.00857340773132 | 0.94562968933381 | 1.07570683350625 | 0.867921561904123 |
| finngen_R11_O15_POOR_FETGRO | ebi-fl187-GCST90094753 | F-deep fried food liking (derived food-liking factor) \|\| id:ebi-fl187-GCST90094753 | Poor fetal growth \|\| id:finngen_R11_O15_POOR_FETGRO | Weighted mode | 24 | -0.047587893 | 0.0709062800344712 | 0.508817389631573 | -0.186564202 | 0.0913884159244522 | 0.953526661184854 | 0.829805285239314 | 1.0956945078123 | 0.867921561904123 |
| finngen_R11_O15_POOR_FETGRO | ebi-fl187-GCST90094756 | Dried fruit liking \|\| id:ebi-fl187-GCST90094756 | Poor fetal growth \|\| id:finngen_R11_O15_POOR_FETGRO | Weighted mode | 26 | -0.006375778 | 0.0297240978787919 | 0.831898266240652 | -0.06463501 | 0.0518834539508672 | 0.993644504252588 | 0.937409546304796 | 1.05325298288604 | 0.867921561904123 |
| finngen_R11_O15_POOR_FETGRO | ebi-fl187-GCST90094765 | Fried fish liking \|\| id:ebi-fl187-GCST90094765 | Poor fetal growth \|\| id:finngen_R11_O15_POOR_FETGRO | Weighted mode | 26 | 0.00927061793750919 | 0.036154427076994 | 0.79972702851202 | -0.061592059 | 0.0801332950084174 | 1.0093137232172 | 0.940266381646724 | 1.08343147405787 | 0.867921561904123 |
| finngen_R11_O15_POOR_FETGRO | ebi-fl187-GCST90094778 | Hard cheese liking \|\| id:ebi-fl187-GCST90094778 | Poor fetal growth \|\| id:finngen_R11_O15_POOR_FETGRO | Weighted mode | 26 | -0.02167567 | 0.0312363181127688 | 0.494127144424016 | -0.082898854 | 0.0395475132054844 | 0.978557558873522 | 0.920444241952499 | 1.04033992759544 | 0.867921561904123 |
| finngen_R11_O15_POOR_FETGRO | ebi-fl187-GCST90094781 | Herring liking \|\| id:ebi-fl187-GCST90094781 | Poor fetal growth \|\| id:finngen_R11_O15_POOR_FETGRO | Weighted mode | 26 | -0.051111009 | 0.0404723891311802 | 0.218291351676078 | -0.130436892 | 0.0282148738063162 | 0.950173187006176 | 0.877711882204115 | 1.02861668345914 | 0.867921561904123 |
| finngen_R11_O15_POOR_FETGRO | ebi-fl187-GCST90094785 | F-highly palatable foods liking (derived food-liking factor) \|\| id:ebi-fl187-GCST90094785 | Poor fetal growth \|\| id:finngen_R11_O15_POOR_FETGRO | Weighted mode | 24 | 0.0173322867252139 | 0.0187639131688699 | 0.365230719635707 | -0.019444983 | 0.054109556536199 | 1.01748336237346 | 0.980742851149729 | 1.05560024372663 | 0.867921561904123 |
| finngen_R11_O15_POOR_FETGRO | ebi-fl187-GCST90094803 | Mushroom liking \|\| id:ebi-fl187-GCST90094803 | Poor fetal growth \|\| id:finngen_R11_O15_POOR_FETGRO | Weighted mode | 26 | 0.00483143103836151 | 0.0287555666255251 | 0.867921561904123 | -0.05152948 | 0.0611923416243907 | 1.00484312122049 | 0.949775650592932 | 1.06310337355331 | 0.867921561904123 |
| finngen_R11_O15_POOR_FETGRO | ebi-fl187-GCST90094838 | Shellfish liking \|\| id:ebi-fl187-GCST90094838 | Poor fetal growth \|\| id:finngen_R11_O15_POOR_FETGRO | Weighted mode | 26 | 0.0301542111327623 | 0.0444545544160241 | 0.503802544041595 | -0.056976716 | 0.11728513778817 | 1.03061345376775 | 0.944616064001807 | 1.12444000432016 | 0.867921561904123 |
| finngen_R11_O15_POOR_FETGRO | ebi-fl187-GCST90094852 | F-strong vegetable liking (derived food-liking factor) \|\| id:ebi-fl187-GCST90094852 | Poor fetal growth \|\| id:finngen_R11_O15_POOR_FETGRO | Weighted mode | 24 | 0.010504447167236 | 0.042141149419165 | 0.805365969081452 | -0.072092206 | 0.0931011000287995 | 1.01055981256352 | 0.930445099519378 | 1.09757269429001 | 0.867921561904123 |
