# Supplementary material for: Mendelian Randomization Mediation Analysis Reveals the Impact of Dietary Preferences on Preeclampsia and Fetal Growth Restriction via Immune Modulation
Source: Food Sci Nutr. 2025 Nov 26;13(12):e71280. doi: 10.1002/fsn3.71280 (PMC12648301; doi:10.1002/fsn3.71280)
Supplement: Supplementary file 1 — Data S1: fsn371280‐sup‐0001‐Supinfo1.zip. [file FSN3-13-e71280-s001.zip › fsn371280-sup-0016-Supinfo16@Supplementary Material 6_Dietary→Immune Cell→PE_FGR_Overall MR Mediation Analysis.docx]

| **id.exposure** | **id.outcome** | **outcome** | **exposure** | **method** | **nsnp** | **b** | **se** | **pval** | **lo_ci** | **up_ci** | **or** | **or_lci95** | **or_uci95** |
| --- | --- | --- | --- | --- | --- | --- | --- | --- | --- | --- | --- | --- | --- |
| ebi-a-GCST90002115 | finngen_R11_O15_PREECLAMPS | Pre-eclampsia \|\| id:finngen_R11_O15_PREECLAMPS | \|\| id:ebi-a-GCST90002115 | Inverse variance weighted | 20 | 0.0657027564987089 | 0.0270419967845183 | 0.0151131490479552 | 0.0127004428010531 | 0.118705070196365 | 1.06790924090263 | 1.01278143594445 | 1.12603776721256 |
| ebi-a-GCST90002115 | finngen_R11_O15_PREECLAMPS | Pre-eclampsia \|\| id:finngen_R11_O15_PREECLAMPS | \|\| id:ebi-a-GCST90002115 | Inverse variance weighted | 20 | 0.0657027564987089 | 0.0270419967845183 | 0.0151131490479552 | 0.0127004428010531 | 0.118705070196365 | 1.06790924090263 | 1.01278143594445 | 1.12603776721256 |
| ebi-fl187-GCST90094733 | ebi-a-GCST90002115 | HLA DR on HLA DR+ CD8+ T cell \|\| id:ebi-a-GCST90002115 | Chilli pepper liking \|\| id:ebi-fl187-GCST90094733 | Inverse variance weighted | 12 | -0.449906713 | 0.120522309218552 | 0.000189231721755638 | -0.686130439 | -0.213682987 | 0.637687636843246 | 0.503520708273885 | 0.80760436562131 |

| **id.exposure** | **id.outcome** | **outcome** | **exposure** | **method** | **nsnp** | **b** | **se** | **pval** | **lo_ci** | **up_ci** | **or** | **or_lci95** | **or_uci95** |
| --- | --- | --- | --- | --- | --- | --- | --- | --- | --- | --- | --- | --- | --- |
| ebi-fl187-GCST90094778 | finngen_R11_O15_POOR_FETGRO | Poor fetal growth \|\| id:finngen_R11_O15_POOR_FETGRO | Hard cheese liking \|\| id:ebi-fl187-GCST90094778 | Inverse variance weighted | 4 | -1.087435885 | 0.331916036989648 | 0.00105199922519077 | -1.737991318 | -0.436880453 | 0.337079697586799 | 0.175873319665549 | 0.646048660145151 |
| ebi-a-GCST90001411 | finngen_R11_O15_POOR_FETGRO | Poor fetal growth \|\| id:finngen_R11_O15_POOR_FETGRO | \|\| id:ebi-a-GCST90001411 | Inverse variance weighted | 20 | -0.124195673 | 0.0422883294547234 | 0.00331534278737066 | -0.207080799 | -0.041310548 | 0.883207003969508 | 0.812953961464326 | 0.959531103650358 |
| ebi-fl187-GCST90094778 | ebi-a-GCST90001411 | IgD+ CD24+ B cell %B cell \|\| id:ebi-a-GCST90001411 | Hard cheese liking \|\| id:ebi-fl187-GCST90094778 | Inverse variance weighted | 3 | 1.21515966409542 | 0.435694481587766 | 0.00528681593886586 | 0.361198480183395 | 2.06912084800744 | 3.37083222223578 | 1.43504826125608 | 7.91785905549791 |
