# Supplementary material for: Mendelian Randomization Mediation Analysis Reveals the Impact of Dietary Preferences on Preeclampsia and Fetal Growth Restriction via Immune Modulation
Source: Food Sci Nutr. 2025 Nov 26;13(12):e71280. doi: 10.1002/fsn3.71280 (PMC12648301; doi:10.1002/fsn3.71280)
Supplement: Supplementary file 1 — Data S1: fsn371280‐sup‐0001‐Supinfo1.zip. [file FSN3-13-e71280-s001.zip › fsn371280-sup-0017-Supinfo17@Supplementary Material 7_PE and FGR_Exposure_Outcome_Mediator_Heterogeneity.docx]

| **id.exposure** | **id.outcome** | **outcome** | **exposure** | **method** | **Q** | **Q_df** | **Q_pval** |
| --- | --- | --- | --- | --- | --- | --- | --- |
| ebi-fl187-GCST90094733 | finngen_R11_O15_PREECLAMPS | Pre-eclampsia \|\| id:finngen_R11_O15_PREECLAMPS | Chilli pepper liking \|\| id:ebi-fl187-GCST90094733 | MR Egger | 14.8950716687142 | 11 | 0.187351177440087 |
| ebi-fl187-GCST90094733 | finngen_R11_O15_PREECLAMPS | Pre-eclampsia \|\| id:finngen_R11_O15_PREECLAMPS | Chilli pepper liking \|\| id:ebi-fl187-GCST90094733 | Inverse variance weighted | 14.8951317062106 | 12 | 0.247221588293041 |
|  |  |  |  |  |  |  |  |
| **id.exposure** | **id.outcome** | **outcome** | **exposure** | **method** | **Q** | **Q_df** | **Q_pval** |
| ebi-fl187-GCST90094733 | ebi-a-GCST90002115 | HLA DR on HLA DR+ CD8+ T cell \|\| id:ebi-a-GCST90002115 | Chilli pepper liking \|\| id:ebi-fl187-GCST90094733 | MR Egger | 6.63977135819125 | 10 | 0.7589586005602 |
| ebi-fl187-GCST90094733 | ebi-a-GCST90002115 | HLA DR on HLA DR+ CD8+ T cell \|\| id:ebi-a-GCST90002115 | Chilli pepper liking \|\| id:ebi-fl187-GCST90094733 | Inverse variance weighted | 7.48409984866367 | 11 | 0.758635453092072 |
|  |  |  |  |  |  |  |  |
| **id.exposure** | **id.outcome** | **outcome** | **exposure** | **method** | **Q** | **Q_df** | **Q_pval** |
| ebi-a-GCST90002115 | finngen_R11_O15_PREECLAMPS | Pre-eclampsia \|\| id:finngen_R11_O15_PREECLAMPS | \|\| id:ebi-a-GCST90002115 | MR Egger | 19.2945985750778 | 18 | 0.373897065816095 |
| ebi-a-GCST90002115 | finngen_R11_O15_PREECLAMPS | Pre-eclampsia \|\| id:finngen_R11_O15_PREECLAMPS | \|\| id:ebi-a-GCST90002115 | Inverse variance weighted | 19.9049885893994 | 19 | 0.400315676630896 |

| **id.exposure** | **id.outcome** | **outcome** | **exposure** | **method** | **Q** | **Q_df** | **Q_pval** |
| --- | --- | --- | --- | --- | --- | --- | --- |
| ebi-fl187-GCST90094778 | finngen_R11_O15_POOR_FETGRO | Poor fetal growth \|\| id:finngen_R11_O15_POOR_FETGRO | Hard cheese liking \|\| id:ebi-fl187-GCST90094778 | MR Egger | 2.88915609597235 | 2 | 0.235845571077098 |
| ebi-fl187-GCST90094778 | finngen_R11_O15_POOR_FETGRO | Poor fetal growth \|\| id:finngen_R11_O15_POOR_FETGRO | Hard cheese liking \|\| id:ebi-fl187-GCST90094778 | Inverse variance weighted | 2.92684871804869 | 3 | 0.403041719354059 |
|  |  |  |  |  |  |  |  |
| **id.exposure** | **id.outcome** | **outcome** | **exposure** | **method** | **Q** | **Q_df** | **Q_pval** |
| ebi-fl187-GCST90094778 | ebi-a-GCST90001411 | IgD+ CD24+ B cell %B cell \|\| id:ebi-a-GCST90001411 | Hard cheese liking \|\| id:ebi-fl187-GCST90094778 | MR Egger | 0.364164520793434 | 1 | 0.546202415097142 |
| ebi-fl187-GCST90094778 | ebi-a-GCST90001411 | IgD+ CD24+ B cell %B cell \|\| id:ebi-a-GCST90001411 | Hard cheese liking \|\| id:ebi-fl187-GCST90094778 | Inverse variance weighted | 1.15011623498537 | 2 | 0.562672166761148 |
|  |  |  |  |  |  |  |  |
| **id.exposure** | **id.outcome** | **outcome** | **exposure** | **method** | **Q** | **Q_df** | **Q_pval** |
| ebi-a-GCST90001411 | finngen_R11_O15_POOR_FETGRO | Poor fetal growth \|\| id:finngen_R11_O15_POOR_FETGRO | \|\| id:ebi-a-GCST90001411 | MR Egger | 16.3532957933718 | 18 | 0.567905520132518 |
| ebi-a-GCST90001411 | finngen_R11_O15_POOR_FETGRO | Poor fetal growth \|\| id:finngen_R11_O15_POOR_FETGRO | \|\| id:ebi-a-GCST90001411 | Inverse variance weighted | 16.3559536200604 | 19 | 0.633412240891264 |
