# Supplementary material for: Mendelian Randomization Mediation Analysis Reveals the Impact of Dietary Preferences on Preeclampsia and Fetal Growth Restriction via Immune Modulation
Source: Food Sci Nutr. 2025 Nov 26;13(12):e71280. doi: 10.1002/fsn3.71280 (PMC12648301; doi:10.1002/fsn3.71280)
Supplement: Supplementary file 1 — Data S1: fsn371280‐sup‐0001‐Supinfo1.zip. [file FSN3-13-e71280-s001.zip › fsn371280-sup-0018-Supinfo18@Supplementary Material 8_PE and FGR_Exposure_Outcome_Mediator_Horizontal Pleiotropy.docx]

| **id.exposure** | **id.outcome** | **outcome** | **exposure** | **egger_intercept** | **se** | **pval** |
| --- | --- | --- | --- | --- | --- | --- |
| ebi-fl187-GCST90094733 | finngen_R11_O15_PREECLAMPS | Pre-eclampsia \|\| id:finngen_R11_O15_PREECLAMPS | Chilli pepper liking \|\| id:ebi-fl187-GCST90094733 | 0.000273009716186956 | 0.0410007472988435 | 0.994806429560085 |
|  |  |  |  |  |  |  |
| **id.exposure** | **id.outcome** | **outcome** | **exposure** | **egger_intercept** | **se** | **pval** |
| ebi-a-GCST90002115 | finngen_R11_O15_PREECLAMPS | Pre-eclampsia \|\| id:finngen_R11_O15_PREECLAMPS | \|\| id:ebi-a-GCST90002115 | -0.007926718 | 0.0105044014136037 | 0.460243141618904 |
|  |  |  |  |  |  |  |
| **id.exposure** | **id.outcome** | **outcome** | **exposure** | **egger_intercept** | **se** | **pval** |
| ebi-fl187-GCST90094733 | ebi-a-GCST90002115 | HLA DR on HLA DR+ CD8+ T cell \|\| id:ebi-a-GCST90002115 | Chilli pepper liking \|\| id:ebi-fl187-GCST90094733 | -0.054128923 | 0.058907917070541 | 0.379791988717061 |

| **id.exposure** | **id.outcome** | **outcome** | **exposure** | **egger_intercept** | **se** | **pval** |
| --- | --- | --- | --- | --- | --- | --- |
| ebi-fl187-GCST90094778 | finngen_R11_O15_POOR_FETGRO | Poor fetal growth \|\| id:finngen_R11_O15_POOR_FETGRO | Hard cheese liking \|\| id:ebi-fl187-GCST90094778 | -0.009019964 | 0.05584022215509 | 0.886517724015441 |
|  |  |  |  |  |  |  |
| **id.exposure** | **id.outcome** | **outcome** | **exposure** | **egger_intercept** | **se** | **pval** |
| ebi-fl187-GCST90094778 | ebi-a-GCST90001411 | IgD+ CD24+ B cell %B cell \|\| id:ebi-a-GCST90001411 | Hard cheese liking \|\| id:ebi-fl187-GCST90094778 | -0.05487999 | 0.061903625788415 | 0.538241719373457 |
|  |  |  |  |  |  |  |
| **id.exposure** | **id.outcome** | **outcome** | **exposure** | **egger_intercept** | **se** | **pval** |
| ebi-a-GCST90001411 | finngen_R11_O15_POOR_FETGRO | Poor fetal growth \|\| id:finngen_R11_O15_POOR_FETGRO | \|\| id:ebi-a-GCST90001411 | -0.00083176 | 0.0161337202773295 | 0.959451798740482 |
