# Supplementary material for: Mendelian Randomization Mediation Analysis Reveals the Impact of Dietary Preferences on Preeclampsia and Fetal Growth Restriction via Immune Modulation
Source: Food Sci Nutr. 2025 Nov 26;13(12):e71280. doi: 10.1002/fsn3.71280 (PMC12648301; doi:10.1002/fsn3.71280)
Supplement: Supplementary file 1 — Data S1: fsn371280‐sup‐0001‐Supinfo1.zip. [file FSN3-13-e71280-s001.zip › fsn371280-sup-0019-Supinfo19@Supplementary Material 9_DietaryPreferences_to_PE_MR-PRESSO_Results.docx]

| **Exposure** | **MR Analysis** | **Causal Estimate** | **Sd** | **T-stat** | **P-value** | **lo_ci** | **up_ci** | **or** | **or_lci95** | **or_uci95** | **id.exposure** | **id.outcome** |
| --- | --- | --- | --- | --- | --- | --- | --- | --- | --- | --- | --- | --- |
| beta.exposure | Raw | -0.123789699 | 0.0409910408383669 | -3.019920858 | 0.00651796961039605 | -0.204132139 | -0.043447259 | 0.883565635887621 | 0.815354623708987 | 0.957483051203172 | ebi-fl187-GCST90094707 | finngen_R11_O15_PREECLAMPS |
| beta.exposure | Outlier-corrected |  |  |  |  |  |  |  |  |  | ebi-fl187-GCST90094707 | finngen_R11_O15_PREECLAMPS |
| beta.exposure | Raw | -0.19298044 | 0.0715011917992961 | -2.698982151 | 0.0193457610812598 | -0.333122776 | -0.052838104 | 0.824498100819386 | 0.716682197133225 | 0.948533563375798 | ebi-fl187-GCST90094716 | finngen_R11_O15_PREECLAMPS |
| beta.exposure | Outlier-corrected |  |  |  |  |  |  |  |  |  | ebi-fl187-GCST90094716 | finngen_R11_O15_PREECLAMPS |
| beta.exposure | Raw | -0.191129381 | 0.0404183397006856 | -4.728778618 | 0.00016755141149204 | -0.270349326 | -0.111909435 | 0.826025709566626 | 0.76311287231668 | 0.894125230509669 | ebi-fl187-GCST90094726 | finngen_R11_O15_PREECLAMPS |
| beta.exposure | Outlier-corrected |  |  |  |  |  |  |  |  |  | ebi-fl187-GCST90094726 | finngen_R11_O15_PREECLAMPS |
| beta.exposure | Raw | -0.176648738 | 0.0372987520083228 | -4.736049548 | 0.000318727789618351 | -0.249754292 | -0.103543184 | 0.838074116760764 | 0.778992164534841 | 0.901637085918239 | ebi-fl187-GCST90094729 | finngen_R11_O15_PREECLAMPS |
| beta.exposure | Outlier-corrected |  |  |  |  |  |  |  |  |  | ebi-fl187-GCST90094729 | finngen_R11_O15_PREECLAMPS |
| beta.exposure | Raw | -0.271826343 | 0.0820457591189633 | -3.313106565 | 0.00618867835240784 | -0.432636031 | -0.111016655 | 0.761986573754748 | 0.648796590638026 | 0.894923843560144 | ebi-fl187-GCST90094733 | finngen_R11_O15_PREECLAMPS |
| beta.exposure | Outlier-corrected |  |  |  |  |  |  |  |  |  | ebi-fl187-GCST90094733 | finngen_R11_O15_PREECLAMPS |
| beta.exposure | Raw | -0.302738497 | 0.147265318374315 | -2.055735185 | 0.0855600376249229 | -0.591378521 | -0.014098473 | 0.738792267854419 | 0.553563659642625 | 0.986000445538365 | ebi-fl187-GCST90094750 | finngen_R11_O15_PREECLAMPS |
| beta.exposure | Outlier-corrected |  |  |  |  |  |  |  |  |  | ebi-fl187-GCST90094750 | finngen_R11_O15_PREECLAMPS |
| beta.exposure | Raw | -0.549083646 | 0.240618154283303 | -2.281970984 | 0.0519113761177921 | -1.020695229 | -0.077472064 | 0.57747874283488 | 0.360344331381918 | 0.925452877660801 | ebi-fl187-GCST90094766 | finngen_R11_O15_PREECLAMPS |
| beta.exposure | Outlier-corrected |  |  |  |  |  |  |  |  |  | ebi-fl187-GCST90094766 | finngen_R11_O15_PREECLAMPS |
| beta.exposure | Raw | -0.132434398 | 0.0501540381490966 | -2.640553033 | 0.0166171911397005 | -0.230736312 | -0.034132483 | 0.875960397376027 | 0.793948792954975 | 0.966443459048979 | ebi-fl187-GCST90094770 | finngen_R11_O15_PREECLAMPS |
| beta.exposure | Outlier-corrected |  |  |  |  |  |  |  |  |  | ebi-fl187-GCST90094770 | finngen_R11_O15_PREECLAMPS |
| beta.exposure | Raw | -0.12023846 | 0.0537360547205326 | -2.23757514 | 0.0347961506584862 | -0.225561127 | -0.014915793 | 0.88670896675148 | 0.798068275116481 | 0.985194896517746 | ebi-fl187-GCST90094775 | finngen_R11_O15_PREECLAMPS |
| beta.exposure | Outlier-corrected |  |  |  |  |  |  |  |  |  | ebi-fl187-GCST90094775 | finngen_R11_O15_PREECLAMPS |
| beta.exposure | Raw | -0.155472817 | 0.0756145291567995 | -2.056123586 | 0.085514050228558 | -0.303677294 | -0.00726834 | 0.856010345702854 | 0.738099017033645 | 0.992758010835988 | ebi-fl187-GCST90094780 | finngen_R11_O15_PREECLAMPS |
| beta.exposure | Outlier-corrected |  |  |  |  |  |  |  |  |  | ebi-fl187-GCST90094780 | finngen_R11_O15_PREECLAMPS |
| beta.exposure | Raw | 0.317349789148296 | 0.14141881762013 | 2.24404216135324 | 0.0597206873372531 | 0.0401689066128416 | 0.594530671683751 | 1.37348291733061 | 1.04098658886258 | 1.81218023784553 | ebi-fl187-GCST90094784 | finngen_R11_O15_PREECLAMPS |
| beta.exposure | Outlier-corrected |  |  |  |  |  |  |  |  |  | ebi-fl187-GCST90094784 | finngen_R11_O15_PREECLAMPS |
| beta.exposure | Raw | 0.233394141277673 | 0.0347649993391066 | 6.71348038874064 | 0.000150599929328013 | 0.165254742573024 | 0.301533539982322 | 1.26287913404313 | 1.17969359861995 | 1.35193045810137 | ebi-fl187-GCST90094798 | finngen_R11_O15_PREECLAMPS |
| beta.exposure | Outlier-corrected |  |  |  |  |  |  |  |  |  | ebi-fl187-GCST90094798 | finngen_R11_O15_PREECLAMPS |
| beta.exposure | Raw | -0.347356477 | 0.15172450643563 | -2.289389403 | 0.0619959453994081 | -0.64473651 | -0.049976445 | 0.706553413134109 | 0.524800803720089 | 0.951251831309553 | ebi-fl187-GCST90094807 | finngen_R11_O15_PREECLAMPS |
| beta.exposure | Outlier-corrected |  |  |  |  |  |  |  |  |  | ebi-fl187-GCST90094807 | finngen_R11_O15_PREECLAMPS |
| beta.exposure | Raw | -0.451555006 | 0.224699693437687 | -2.009593334 | 0.114852210815684 | -0.891966405 | -0.011143607 | 0.636637406435575 | 0.409849030542523 | 0.988918252988189 | ebi-fl187-GCST90094812 | finngen_R11_O15_PREECLAMPS |
| beta.exposure | Outlier-corrected |  |  |  |  |  |  |  |  |  | ebi-fl187-GCST90094812 | finngen_R11_O15_PREECLAMPS |
| beta.exposure | Raw | -0.185321471 | 0.0589860665563963 | -3.141783852 | 0.0056390126227607 | -0.300934162 | -0.069708781 | 0.830837150536712 | 0.740126499703624 | 0.932665390292581 | ebi-fl187-GCST90094817 | finngen_R11_O15_PREECLAMPS |
| beta.exposure | Outlier-corrected |  |  |  |  |  |  |  |  |  | ebi-fl187-GCST90094817 | finngen_R11_O15_PREECLAMPS |
| beta.exposure | Raw | -0.125404539 | 0.0480827737757341 | -2.608097029 | 0.0148891500324293 | -0.219646776 | -0.031162303 | 0.882139969979326 | 0.80280231689615 | 0.969318237201586 | ebi-fl187-GCST90094840 | finngen_R11_O15_PREECLAMPS |
| beta.exposure | Outlier-corrected |  |  |  |  |  |  |  |  |  | ebi-fl187-GCST90094840 | finngen_R11_O15_PREECLAMPS |
| beta.exposure | Raw | -0.2545956 | 0.0561614742172533 | -4.533278429 | 0.000396154890510236 | -0.364672089 | -0.14451911 | 0.775229937881226 | 0.694424322880837 | 0.865438373607281 | ebi-fl187-GCST90094846 | finngen_R11_O15_PREECLAMPS |
| beta.exposure | Outlier-corrected |  |  |  |  |  |  |  |  |  | ebi-fl187-GCST90094846 | finngen_R11_O15_PREECLAMPS |
| beta.exposure | Raw | -0.219692983 | 0.0595541116497558 | -3.688964153 | 0.0012138065229233 | -0.336419042 | -0.102966924 | 0.802765222693217 | 0.714323711564882 | 0.902156812566003 | ebi-fl187-GCST90094852 | finngen_R11_O15_PREECLAMPS |
| beta.exposure | Outlier-corrected |  |  |  |  |  |  |  |  |  | ebi-fl187-GCST90094852 | finngen_R11_O15_PREECLAMPS |
| beta.exposure | Raw | 0.491244761325726 | 0.0563897822583599 | 8.71159173970562 | 0.00318384119784468 | 0.380720788099341 | 0.601768734552111 | 1.63434932916268 | 1.46333896670979 | 1.82534449673015 | ebi-fl187-GCST90094853 | finngen_R11_O15_PREECLAMPS |
| beta.exposure | Outlier-corrected |  |  |  |  |  |  |  |  |  | ebi-fl187-GCST90094853 | finngen_R11_O15_PREECLAMPS |
