# Supplementary material for: Mendelian Randomization Mediation Analysis Reveals the Impact of Dietary Preferences on Preeclampsia and Fetal Growth Restriction via Immune Modulation
Source: Food Sci Nutr. 2025 Nov 26;13(12):e71280. doi: 10.1002/fsn3.71280 (PMC12648301; doi:10.1002/fsn3.71280)
Supplement: Supplementary file 1 — Data S1: fsn371280‐sup‐0001‐Supinfo1.zip. [file FSN3-13-e71280-s001.zip › fsn371280-sup-0020-FigureS1-S1@Supplementary Material 1_Fig_S1_FDR.docx]

Forest plot showing the associations between dietary preferences and the risks of PE. ORs and 95% CIs are provided, with IVW as the primary analytical method. Estimates are per 1-SD increase in inverse-normalized dietary preference scores.

Forest plot showing the associations between dietary preferences and the risks of FGR. ORs and 95% CIs are provided, with IVW as the primary analytical method. Estimates are per 1-SD increase in inverse-normalized dietary preference scores.

Forest plot showing immune cell traits significantly associated with PE. ORs and 95% CIs are provided, with IVW as the primary analytical method. Estimates are per 1-SD increase in inverse-normalized immune traits. Forest plot showing immune cell traits significantly associated with FGR. ORs and 95% CIs are provided, with IVW as the primary analytical method. Estimates are per 1-SD increase in inverse-normalized immune traits.

PE

Forest plot showing dietary preferences significantly associated immune cell traits. ORs and 95% CIs are provided, with IVW as the primary analytical method. Estimates are per 1-SD increase in inverse-normalized traits.

FGR

Forest plot showing dietary preferences significantly associated immune cell traits. ORs and 95% CIs are provided, with IVW as the primary analytical method. Estimates are per 1-SD increase in inverse-normalized traits.
