# Supplementary material for: Mendelian Randomization Mediation Analysis Reveals the Impact of Dietary Preferences on Preeclampsia and Fetal Growth Restriction via Immune Modulation
Source: Food Sci Nutr. 2025 Nov 26;13(12):e71280. doi: 10.1002/fsn3.71280 (PMC12648301; doi:10.1002/fsn3.71280)
Supplement: Supplementary file 1 — Data S1: fsn371280‐sup‐0001‐Supinfo1.zip. [file FSN3-13-e71280-s001.zip › fsn371280-sup-0001-Supinfo1@Supplementary Material 10_DietaryPreferences_to_FGR_MR-PRESSO_Results.docx]

| **Exposure** | **MR Analysis** | **Causal Estimate** | **Sd** | **T-stat** | **P-value** | **lo_ci** | **up_ci** | **or** | **or_lci95** | **or_uci95** | **id.exposure** | **id.outcome** |
| --- | --- | --- | --- | --- | --- | --- | --- | --- | --- | --- | --- | --- |
| beta.exposure | Raw | -0.948101799 | 0.448875047738298 | -2.112173096 | 0.125108520790423 | -1.827896893 | -0.068306706 | 0.387475832648931 | 0.160751289671252 | 0.933973974292987 | ebi-fl187-GCST90094727 | finngen_R11_O15_POOR_FETGRO |
| beta.exposure | Outlier-corrected |  |  |  |  |  |  |  |  |  | ebi-fl187-GCST90094727 | finngen_R11_O15_POOR_FETGRO |
| beta.exposure | Raw | -0.177548415 | 0.0555702698224774 | -3.195025249 | 0.0151703076056721 | -0.286466144 | -0.068630686 | 0.837320459328262 | 0.750912500965485 | 0.933671434032921 | ebi-fl187-GCST90094753 | finngen_R11_O15_POOR_FETGRO |
| beta.exposure | Outlier-corrected |  |  |  |  |  |  |  |  |  | ebi-fl187-GCST90094753 | finngen_R11_O15_POOR_FETGRO |
| beta.exposure | Raw | -0.550951233 | 0.24000271595062 | -2.295604161 | 0.0833411944539858 | -1.021356557 | -0.08054591 | 0.576401257408119 | 0.360106104355391 | 0.922612545367386 | ebi-fl187-GCST90094756 | finngen_R11_O15_POOR_FETGRO |
| beta.exposure | Outlier-corrected |  |  |  |  |  |  |  |  |  | ebi-fl187-GCST90094756 | finngen_R11_O15_POOR_FETGRO |
| beta.exposure | Raw | -0.464490138 | 0.0371185311260071 | -12.51369932 | 0.000234607360888589 | -0.537242459 | -0.391737817 | 0.628455449157817 | 0.584357422307074 | 0.675881295418214 | ebi-fl187-GCST90094765 | finngen_R11_O15_POOR_FETGRO |
| beta.exposure | Outlier-corrected |  |  |  |  |  |  |  |  |  | ebi-fl187-GCST90094765 | finngen_R11_O15_POOR_FETGRO |
| beta.exposure | Raw | -1.087498321 | 0.327871937467545 | -3.316838669 | 0.045160617120955 | -1.730127318 | -0.444869323 | 0.337058652499644 | 0.177261839877528 | 0.640908022298365 | ebi-fl187-GCST90094778 | finngen_R11_O15_POOR_FETGRO |
| beta.exposure | Outlier-corrected |  |  |  |  |  |  |  |  |  | ebi-fl187-GCST90094778 | finngen_R11_O15_POOR_FETGRO |
| beta.exposure | Raw | -0.191532604 | 0.0767776520069404 | -2.494640033 | 0.0232008572425166 | -0.342016802 | -0.041048406 | 0.825692703526906 | 0.710336269366017 | 0.959782669222364 | ebi-fl187-GCST90094781 | finngen_R11_O15_POOR_FETGRO |
| beta.exposure | Outlier-corrected |  |  |  |  |  |  |  |  |  | ebi-fl187-GCST90094781 | finngen_R11_O15_POOR_FETGRO |
| beta.exposure | Raw | -0.503858465 | 0.195821213306937 | -2.573053533 | 0.020427516503365 | -0.887668043 | -0.120048887 | 0.604194891687657 | 0.411614501830686 | 0.886877079203639 | ebi-fl187-GCST90094785 | finngen_R11_O15_POOR_FETGRO |
| beta.exposure | Outlier-corrected |  |  |  |  |  |  |  |  |  | ebi-fl187-GCST90094785 | finngen_R11_O15_POOR_FETGRO |
| beta.exposure | Raw | -0.622948512 | 0.232542295730866 | -2.678861106 | 0.0365916590372557 | -1.078731411 | -0.167165612 | 0.536360638272075 | 0.340026606100412 | 0.846059482188501 | ebi-fl187-GCST90094803 | finngen_R11_O15_POOR_FETGRO |
| beta.exposure | Outlier-corrected |  |  |  |  |  |  |  |  |  | ebi-fl187-GCST90094803 | finngen_R11_O15_POOR_FETGRO |
| beta.exposure | Raw | 0.131305881673821 | 0.0475112238128043 | 2.76368131856948 | 0.00967300369424362 | 0.0381838830007244 | 0.224427880346917 | 1.14031652990022 | 1.03892225545691 | 1.25160644267055 | ebi-fl187-GCST90094838 | finngen_R11_O15_POOR_FETGRO |
| beta.exposure | Outlier-corrected |  |  |  |  |  |  |  |  |  | ebi-fl187-GCST90094838 | finngen_R11_O15_POOR_FETGRO |
| beta.exposure | Raw | -0.197706539 | 0.0715653835027592 | -2.762600142 | 0.0110804349514682 | -0.33797469 | -0.057438387 | 0.820610635327354 | 0.71321333888886 | 0.944180062394066 | ebi-fl187-GCST90094852 | finngen_R11_O15_POOR_FETGRO |
| beta.exposure | Outlier-corrected |  |  |  |  |  |  |  |  |  | ebi-fl187-GCST90094852 | finngen_R11_O15_POOR_FETGRO |
