# Supplementary material for: Mendelian Randomization Mediation Analysis Reveals the Impact of Dietary Preferences on Preeclampsia and Fetal Growth Restriction via Immune Modulation
Source: Food Sci Nutr. 2025 Nov 26;13(12):e71280. doi: 10.1002/fsn3.71280 (PMC12648301; doi:10.1002/fsn3.71280)
Supplement: Supplementary file 1 — Data S1: fsn371280‐sup‐0001‐Supinfo1.zip. [file FSN3-13-e71280-s001.zip › fsn371280-sup-0002-Supinfo2@Supplementary Material 11_ImmuneCells_to_PE_MR-PRESSO_Results.docx]

| Exposure | MR Analysis | Causal Estimate | Sd | T-stat | P-value | lo_ci | up_ci | or | or_lci95 | or_uci95 | id.exposure | id.outcome |
| --- | --- | --- | --- | --- | --- | --- | --- | --- | --- | --- | --- | --- |
| beta.exposure | Raw | 0.0515282201235502 | 0.0192078825349933 | 2.68266010215729 | 0.0157339552510813 | 0.0138807703549634 | 0.089175669892137 | 1.05287889824549 | 1.01397755554698 | 1.0932726945545 | ebi-a-GCST90001414 | finngen_R11_O15_PREECLAMPS |
| beta.exposure | Outlier-corrected |  |  |  |  |  |  |  |  |  | ebi-a-GCST90001414 | finngen_R11_O15_PREECLAMPS |
| beta.exposure | Raw | -0.047358223 | 0.0147209442098479 | -3.21706424 | 0.00396915847155571 | -0.076211274 | -0.018505173 | 0.953745682563191 | 0.926620415218048 | 0.981664996873478 | ebi-a-GCST90001422 | finngen_R11_O15_PREECLAMPS |
| beta.exposure | Outlier-corrected |  |  |  |  |  |  |  |  |  | ebi-a-GCST90001422 | finngen_R11_O15_PREECLAMPS |
| beta.exposure | Raw | -0.047361605 | 0.0170386574160752 | -2.77965592 | 0.0109283722968224 | -0.080757373 | -0.013965836 | 0.953742457229561 | 0.922417467226747 | 0.986131233460997 | ebi-a-GCST90001444 | finngen_R11_O15_PREECLAMPS |
| beta.exposure | Outlier-corrected |  |  |  |  |  |  |  |  |  | ebi-a-GCST90001444 | finngen_R11_O15_PREECLAMPS |
| beta.exposure | Raw | -0.044938318 | 0.0206845622153128 | -2.172553505 | 0.0394995092121895 | -0.08548006 | -0.004396576 | 0.956056451349825 | 0.918071449136473 | 0.995613074589521 | ebi-a-GCST90001459 | finngen_R11_O15_PREECLAMPS |
| beta.exposure | Outlier-corrected |  |  |  |  |  |  |  |  |  | ebi-a-GCST90001459 | finngen_R11_O15_PREECLAMPS |
| beta.exposure | Raw | 0.0722073972609298 | 0.0341403163043888 | 2.11501840279222 | 0.0722510324106227 | 0.00529237730432768 | 0.139122417217532 | 1.07487824775262 | 1.00530640667174 | 1.14926478118925 | ebi-a-GCST90001476 | finngen_R11_O15_PREECLAMPS |
| beta.exposure | Outlier-corrected |  |  |  |  |  |  |  |  |  | ebi-a-GCST90001476 | finngen_R11_O15_PREECLAMPS |
| beta.exposure | Raw | -0.040663735 | 0.0186804018507516 | -2.176812652 | 0.0471007559969965 | -0.077277323 | -0.004050147 | 0.960151941080571 | 0.925633118921656 | 0.995958043327981 | ebi-a-GCST90001478 | finngen_R11_O15_PREECLAMPS |
| beta.exposure | Outlier-corrected |  |  |  |  |  |  |  |  |  | ebi-a-GCST90001478 | finngen_R11_O15_PREECLAMPS |
| beta.exposure | Raw | 0.019385350253623 | 0.00788839832348856 | 2.45745073444136 | 0.0194164194021083 | 0.00392408953958538 | 0.0348466109676605 | 1.01957446620551 | 1.00393179885967 | 1.03546086827713 | ebi-a-GCST90001485 | finngen_R11_O15_PREECLAMPS |
| beta.exposure | Outlier-corrected |  |  |  |  |  |  |  |  |  | ebi-a-GCST90001485 | finngen_R11_O15_PREECLAMPS |
| beta.exposure | Raw | -0.048040833 | 0.0172070897110384 | -2.791920895 | 0.0109256988223745 | -0.081766729 | -0.014314937 | 0.953094868261729 | 0.921486889659814 | 0.985787034085958 | ebi-a-GCST90001515 | finngen_R11_O15_PREECLAMPS |
| beta.exposure | Outlier-corrected |  |  |  |  |  |  |  |  |  | ebi-a-GCST90001515 | finngen_R11_O15_PREECLAMPS |
| beta.exposure | Raw | -0.024976537 | 0.00736448072154248 | -3.391486465 | 0.00202491396014728 | -0.039410919 | -0.010542154 | 0.975332796299967 | 0.961355588789892 | 0.989513219282088 | ebi-a-GCST90001517 | finngen_R11_O15_PREECLAMPS |
| beta.exposure | Outlier-corrected |  |  |  |  |  |  |  |  |  | ebi-a-GCST90001517 | finngen_R11_O15_PREECLAMPS |
| beta.exposure | Raw | -0.024400205 | 0.00569026120934037 | -4.288064112 | 0.000193253439156395 | -0.035553117 | -0.013247293 | 0.975895073623264 | 0.96507147130991 | 0.986840066290101 | ebi-a-GCST90001518 | finngen_R11_O15_PREECLAMPS |
| beta.exposure | Outlier-corrected |  |  |  |  |  |  |  |  |  | ebi-a-GCST90001518 | finngen_R11_O15_PREECLAMPS |
| beta.exposure | Raw | -0.025768854 | 0.00969993868507756 | -2.656599661 | 0.0130892288198937 | -0.044780734 | -0.006756974 | 0.974560329470152 | 0.956207122898053 | 0.993265803018109 | ebi-a-GCST90001520 | finngen_R11_O15_PREECLAMPS |
| beta.exposure | Outlier-corrected |  |  |  |  |  |  |  |  |  | ebi-a-GCST90001520 | finngen_R11_O15_PREECLAMPS |
| beta.exposure | Raw | -0.021438339 | 0.00699183236506445 | -3.066197552 | 0.00546785175362594 | -0.035142331 | -0.007734348 | 0.978789828495329 | 0.965467990729197 | 0.992295485262373 | ebi-a-GCST90001531 | finngen_R11_O15_PREECLAMPS |
| beta.exposure | Outlier-corrected |  |  |  |  |  |  |  |  |  | ebi-a-GCST90001531 | finngen_R11_O15_PREECLAMPS |
| beta.exposure | Raw | -0.019297029 | 0.00787322946179982 | -2.450967389 | 0.0226601798877303 | -0.034728558 | -0.003865499 | 0.980887967131993 | 0.965867557310008 | 0.996141962510831 | ebi-a-GCST90001532 | finngen_R11_O15_PREECLAMPS |
| beta.exposure | Outlier-corrected |  |  |  |  |  |  |  |  |  | ebi-a-GCST90001532 | finngen_R11_O15_PREECLAMPS |
| beta.exposure | Raw | 0.0458883655807117 | 0.0232699952285445 | 1.97199720627454 | 0.0589344554198297 | 0.000279174932764505 | 0.0914975562286589 | 1.04695752793732 | 1.00027921390571 | 1.09581409877018 | ebi-a-GCST90001588 | finngen_R11_O15_PREECLAMPS |
| beta.exposure | Outlier-corrected |  |  |  |  |  |  |  |  |  | ebi-a-GCST90001588 | finngen_R11_O15_PREECLAMPS |
| beta.exposure | Raw | 0.0291407899166667 | 0.0144818245727481 | 2.01223193736954 | 0.0560502212039365 | 0.000756413754080417 | 0.057525166079253 | 1.02956953728101 | 1.00075669990711 | 1.05921192653061 | ebi-a-GCST90001624 | finngen_R11_O15_PREECLAMPS |
| beta.exposure | Outlier-corrected |  |  |  |  |  |  |  |  |  | ebi-a-GCST90001624 | finngen_R11_O15_PREECLAMPS |
| beta.exposure | Raw | 0.0578228121750278 | 0.0231628618976036 | 2.49635871554413 | 0.0192151119742308 | 0.0124236028557247 | 0.103222021494331 | 1.05952724374928 | 1.01250109639348 | 1.10873754531786 | ebi-a-GCST90001631 | finngen_R11_O15_PREECLAMPS |
| beta.exposure | Outlier-corrected |  |  |  |  |  |  |  |  |  | ebi-a-GCST90001631 | finngen_R11_O15_PREECLAMPS |
| beta.exposure | Raw | 0.059436663399615 | 0.023334817101725 | 2.54712360249102 | 0.0171225152903427 | 0.0137004218802339 | 0.105172904918996 | 1.06123854360935 | 1.01379470273053 | 1.11090267428774 | ebi-a-GCST90001632 | finngen_R11_O15_PREECLAMPS |
| beta.exposure | Outlier-corrected |  |  |  |  |  |  |  |  |  | ebi-a-GCST90001632 | finngen_R11_O15_PREECLAMPS |
| beta.exposure | Raw | -0.024198966 | 0.011905031683301 | -2.032667108 | 0.0524163767605428 | -0.047532828 | -0.000865104 | 0.976091481107334 | 0.953579168123258 | 0.999135269875314 | ebi-a-GCST90001646 | finngen_R11_O15_PREECLAMPS |
| beta.exposure | Outlier-corrected |  |  |  |  |  |  |  |  |  | ebi-a-GCST90001646 | finngen_R11_O15_PREECLAMPS |
| beta.exposure | Raw | 0.06410746385165 | 0.0266555276379986 | 2.40503451000017 | 0.0332122851303097 | 0.0118626296811727 | 0.116352298022127 | 1.06620697131298 | 1.01193326972185 | 1.12339157105575 | ebi-a-GCST90001684 | finngen_R11_O15_PREECLAMPS |
| beta.exposure | Outlier-corrected |  |  |  |  |  |  |  |  |  | ebi-a-GCST90001684 | finngen_R11_O15_PREECLAMPS |
| beta.exposure | Raw | 0.0745292306439606 | 0.0376148498954863 | 1.98137785611379 | 0.0691037161878823 | 0.000804124848807417 | 0.148254336439114 | 1.07737683548068 | 1.00080444824387 | 1.15980784025004 | ebi-a-GCST90001687 | finngen_R11_O15_PREECLAMPS |
| beta.exposure | Outlier-corrected |  |  |  |  |  |  |  |  |  | ebi-a-GCST90001687 | finngen_R11_O15_PREECLAMPS |
| beta.exposure | Raw | 0.0340286197183812 | 0.0145978301958798 | 2.33107381451703 | 0.0331527459110934 | 0.00541687253445692 | 0.0626403669023055 | 1.03461421667192 | 1.00543157031517 | 1.06464388919487 | ebi-a-GCST90001701 | finngen_R11_O15_PREECLAMPS |
| beta.exposure | Outlier-corrected |  |  |  |  |  |  |  |  |  | ebi-a-GCST90001701 | finngen_R11_O15_PREECLAMPS |
| beta.exposure | Raw | -0.048627664 | 0.0243510021623105 | -1.996947121 | 0.0568271709047879 | -0.096355628 | -0.000899699 | 0.952535727333674 | 0.908140998348804 | 0.99910070517332 | ebi-a-GCST90001732 | finngen_R11_O15_PREECLAMPS |
| beta.exposure | Outlier-corrected |  |  |  |  |  |  |  |  |  | ebi-a-GCST90001732 | finngen_R11_O15_PREECLAMPS |
| beta.exposure | Raw | -0.038018509 | 0.0172073662920885 | -2.209432196 | 0.0358123534214595 | -0.071744947 | -0.004292071 | 0.962695122111434 | 0.930768260751811 | 0.995717126611685 | ebi-a-GCST90001798 | finngen_R11_O15_PREECLAMPS |
| beta.exposure | Outlier-corrected |  |  |  |  |  |  |  |  |  | ebi-a-GCST90001798 | finngen_R11_O15_PREECLAMPS |
| beta.exposure | Raw | 0.0512037835486687 | 0.0160100999615301 | 3.19821760461858 | 0.00385729344824864 | 0.0198239876240697 | 0.0825835794732676 | 1.05253736122852 | 1.02002178776714 | 1.08608944442939 | ebi-a-GCST90001836 | finngen_R11_O15_PREECLAMPS |
| beta.exposure | Outlier-corrected |  |  |  |  |  |  |  |  |  | ebi-a-GCST90001836 | finngen_R11_O15_PREECLAMPS |
| beta.exposure | Raw | -0.025514564 | 0.0115197843221085 | -2.214847411 | 0.0385431026197244 | -0.048093342 | -0.002935787 | 0.974808181288743 | 0.95304482405325 | 0.997068518001178 | ebi-a-GCST90001943 | finngen_R11_O15_PREECLAMPS |
| beta.exposure | Outlier-corrected |  |  |  |  |  |  |  |  |  | ebi-a-GCST90001943 | finngen_R11_O15_PREECLAMPS |
| beta.exposure | Raw | -0.038366839 | 0.00919484670130662 | -4.172645819 | 0.000816832059780531 | -0.056388738 | -0.020344939 | 0.962359845351019 | 0.945171640164483 | 0.979860622757225 | ebi-a-GCST90001960 | finngen_R11_O15_PREECLAMPS |
| beta.exposure | Outlier-corrected |  |  |  |  |  |  |  |  |  | ebi-a-GCST90001960 | finngen_R11_O15_PREECLAMPS |
| beta.exposure | Raw | 0.0427338261467995 | 0.0206617702889203 | 2.06825579557019 | 0.0511532957592431 | 0.00223675638051558 | 0.0832308959130833 | 1.04366006285608 | 1.00223925978622 | 1.08679271557682 | ebi-a-GCST90001988 | finngen_R11_O15_PREECLAMPS |
| beta.exposure | Outlier-corrected |  |  |  |  |  |  |  |  |  | ebi-a-GCST90001988 | finngen_R11_O15_PREECLAMPS |
| beta.exposure | Raw | 0.0435824833875853 | 0.0218401351965208 | 1.99552260072676 | 0.0597877369105052 | 0.000775818402404503 | 0.0863891483727662 | 1.04454614846365 | 1.00077611942734 | 1.09023050719333 | ebi-a-GCST90001991 | finngen_R11_O15_PREECLAMPS |
| beta.exposure | Outlier-corrected |  |  |  |  |  |  |  |  |  | ebi-a-GCST90001991 | finngen_R11_O15_PREECLAMPS |
| beta.exposure | Raw | -0.03106028 | 0.0114609981852251 | -2.710085077 | 0.0124896476581367 | -0.053523837 | -0.008596724 | 0.969417134706955 | 0.947883346427421 | 0.991440122463847 | ebi-a-GCST90002019 | finngen_R11_O15_PREECLAMPS |
| beta.exposure | Outlier-corrected |  |  |  |  |  |  |  |  |  | ebi-a-GCST90002019 | finngen_R11_O15_PREECLAMPS |
| beta.exposure | Raw | -0.046764176 | 0.0174901199687052 | -2.67374817 | 0.0145914368679337 | -0.081044811 | -0.012483541 | 0.954312420574331 | 0.9221523675753 | 0.987594055044351 | ebi-a-GCST90002037 | finngen_R11_O15_PREECLAMPS |
| beta.exposure | Outlier-corrected |  |  |  |  |  |  |  |  |  | ebi-a-GCST90002037 | finngen_R11_O15_PREECLAMPS |
| beta.exposure | Raw | -0.052018771 | 0.0214404151049712 | -2.426201661 | 0.0266723601036317 | -0.094041984 | -0.009995557 | 0.949311047410233 | 0.910244545422024 | 0.990054232423098 | ebi-a-GCST90002042 | finngen_R11_O15_PREECLAMPS |
| beta.exposure | Outlier-corrected |  |  |  |  |  |  |  |  |  | ebi-a-GCST90002042 | finngen_R11_O15_PREECLAMPS |
| beta.exposure | Raw | -0.044374257 | 0.0190814341635516 | -2.325519987 | 0.0301437482988913 | -0.081773867 | -0.006974646 | 0.95659587821007 | 0.921480311786884 | 0.993049620815046 | ebi-a-GCST90002056 | finngen_R11_O15_PREECLAMPS |
| beta.exposure | Outlier-corrected |  |  |  |  |  |  |  |  |  | ebi-a-GCST90002056 | finngen_R11_O15_PREECLAMPS |
| beta.exposure | Raw | -0.040956187 | 0.0183504278407269 | -2.231892766 | 0.0348208498260151 | -0.076923026 | -0.004989349 | 0.959871183713234 | 0.925961126051081 | 0.99502307753708 | ebi-a-GCST90002058 | finngen_R11_O15_PREECLAMPS |
| beta.exposure | Outlier-corrected |  |  |  |  |  |  |  |  |  | ebi-a-GCST90002058 | finngen_R11_O15_PREECLAMPS |
| beta.exposure | Raw | 0.0404053990441739 | 0.016808821420683 | 2.40382106710085 | 0.0230896292292004 | 0.00746010905963519 | 0.0733506890287126 | 1.04123280342476 | 1.007488005 | 1.07610784996776 | ebi-a-GCST90002063 | finngen_R11_O15_PREECLAMPS |
| beta.exposure | Outlier-corrected |  |  |  |  |  |  |  |  |  | ebi-a-GCST90002063 | finngen_R11_O15_PREECLAMPS |
| beta.exposure | Raw | 0.0293143985819453 | 0.0143705133717636 | 2.03989918965211 | 0.0530067070921187 | 0.00114819237328871 | 0.0574806047906019 | 1.02974829499068 | 1.00114885179851 | 1.05916472773383 | ebi-a-GCST90002066 | finngen_R11_O15_PREECLAMPS |
| beta.exposure | Outlier-corrected |  |  |  |  |  |  |  |  |  | ebi-a-GCST90002066 | finngen_R11_O15_PREECLAMPS |
| beta.exposure | Raw | 0.0419398658222304 | 0.0117519835127894 | 3.56874784385021 | 0.00119120848159672 | 0.0189059781371632 | 0.0649737535072977 | 1.04283176703436 | 1.01908582776493 | 1.06713101557022 | ebi-a-GCST90002070 | finngen_R11_O15_PREECLAMPS |
| beta.exposure | Outlier-corrected |  |  |  |  |  |  |  |  |  | ebi-a-GCST90002070 | finngen_R11_O15_PREECLAMPS |
| beta.exposure | Raw | -0.057094496 | 0.0277318780694914 | -2.058803813 | 0.0489248656780427 | -0.111448977 | -0.002740015 | 0.944504812940192 | 0.894537031919939 | 0.997263735132912 | ebi-a-GCST90002081 | finngen_R11_O15_PREECLAMPS |
| beta.exposure | Outlier-corrected | -0.035318098 | 0.0259170607617886 | -1.362735452 | 0.184224805029426 | -0.086115537 | 0.0154793415829828 | 0.965298308426602 | 0.917488221623646 | 1.01559976715808 | ebi-a-GCST90002081 | finngen_R11_O15_PREECLAMPS |
| beta.exposure | Raw | 0.0377065043476999 | 0.0162179268505321 | 2.32498917372185 | 0.0345138433485383 | 0.0059193677206571 | 0.0694936409747427 | 1.03842641451136 | 1.00593692179702 | 1.07196524452903 | ebi-a-GCST90002094 | finngen_R11_O15_PREECLAMPS |
| beta.exposure | Outlier-corrected |  |  |  |  |  |  |  |  |  | ebi-a-GCST90002094 | finngen_R11_O15_PREECLAMPS |
| beta.exposure | Raw | 0.0334626941873389 | 0.0160198639409653 | 2.08882511803172 | 0.0554672316786538 | 0.0020637608630469 | 0.0648616275116308 | 1.0340288677195 | 1.00206589188322 | 1.06701136915045 | ebi-a-GCST90002104 | finngen_R11_O15_PREECLAMPS |
| beta.exposure | Outlier-corrected |  |  |  |  |  |  |  |  |  | ebi-a-GCST90002104 | finngen_R11_O15_PREECLAMPS |
| beta.exposure | Raw | 0.0260437137551376 | 0.0117690165915641 | 2.21290483809884 | 0.0366640019197355 | 0.00297644123567202 | 0.0491109862746033 | 1.02638581467098 | 1.00298087523497 | 1.05033691725279 | ebi-a-GCST90002105 | finngen_R11_O15_PREECLAMPS |
| beta.exposure | Outlier-corrected |  |  |  |  |  |  |  |  |  | ebi-a-GCST90002105 | finngen_R11_O15_PREECLAMPS |
| beta.exposure | Raw | 0.0473439962677557 | 0.0165737652283567 | 2.85656250196866 | 0.0104826453028257 | 0.0148594164201766 | 0.0798285761153348 | 1.04848262115998 | 1.01497036641792 | 1.08310138231352 | ebi-a-GCST90002106 | finngen_R11_O15_PREECLAMPS |
| beta.exposure | Outlier-corrected |  |  |  |  |  |  |  |  |  | ebi-a-GCST90002106 | finngen_R11_O15_PREECLAMPS |
| beta.exposure | Raw | 0.0430096338018653 | 0.0196766385506647 | 2.18582222218095 | 0.0440395266544613 | 0.00444342224256247 | 0.0815758453611682 | 1.0439479519899 | 1.00445330888125 | 1.08499550633939 | ebi-a-GCST90002111 | finngen_R11_O15_PREECLAMPS |
| beta.exposure | Outlier-corrected |  |  |  |  |  |  |  |  |  | ebi-a-GCST90002111 | finngen_R11_O15_PREECLAMPS |
| beta.exposure | Raw | 0.0396347258299822 | 0.0144288610009535 | 2.74690606745488 | 0.0189954472445069 | 0.0113541582681134 | 0.067915293391851 | 1.04043066232745 | 1.01141886137433 | 1.07027464530395 | ebi-a-GCST90002112 | finngen_R11_O15_PREECLAMPS |
| beta.exposure | Outlier-corrected |  |  |  |  |  |  |  |  |  | ebi-a-GCST90002112 | finngen_R11_O15_PREECLAMPS |
| beta.exposure | Raw | 0.0657027564987089 | 0.0270419967845183 | 2.42965624995282 | 0.0251984045016157 | 0.0127004428010531 | 0.118705070196365 | 1.06790924090263 | 1.01278143594445 | 1.12603776721256 | ebi-a-GCST90002115 | finngen_R11_O15_PREECLAMPS |
| beta.exposure | Outlier-corrected |  |  |  |  |  |  |  |  |  | ebi-a-GCST90002115 | finngen_R11_O15_PREECLAMPS |
| beta.exposure | Raw | 0.0372638785455445 | 0.012289064951127 | 3.03227940398566 | 0.00633606237856978 | 0.0131773112413356 | 0.0613504458497535 | 1.03796688189466 | 1.01326451462149 | 1.06327146797653 | ebi-a-GCST90002116 | finngen_R11_O15_PREECLAMPS |
| beta.exposure | Outlier-corrected |  |  |  |  |  |  |  |  |  | ebi-a-GCST90002116 | finngen_R11_O15_PREECLAMPS |
