# Supplementary material for: Mendelian Randomization Mediation Analysis Reveals the Impact of Dietary Preferences on Preeclampsia and Fetal Growth Restriction via Immune Modulation
Source: Food Sci Nutr. 2025 Nov 26;13(12):e71280. doi: 10.1002/fsn3.71280 (PMC12648301; doi:10.1002/fsn3.71280)
Supplement: Supplementary file 1 — Data S1: fsn371280‐sup‐0001‐Supinfo1.zip. [file FSN3-13-e71280-s001.zip › fsn371280-sup-0003-Supinfo3@Supplementary Material 12_ImmuneCells_to_FGR_MR-PRESSO_Results.docx]

| **Exposure** | **MR Analysis** | **Causal Estimate** | **Sd** | **T-stat** | **P-value** | **lo_ci** | **up_ci** | **or** | **or_lci95** | **or_uci95** | **id.exposure** | **id.outcome** |
| --- | --- | --- | --- | --- | --- | --- | --- | --- | --- | --- | --- | --- |
| beta.exposure | Raw | 0.0269962539689498 | 0.0108012161407096 | 2.49937170196988 | 0.0185776981555866 | 0.00582587033315907 | 0.0481666376047406 | 1.02736395421901 | 1.00584287371953 | 1.04934550117698 | ebi-a-GCST90001392 | finngen_R11_O15_POOR_FETGRO |
| beta.exposure | Outlier-corrected |  |  |  |  |  |  |  |  |  | ebi-a-GCST90001392 | finngen_R11_O15_POOR_FETGRO |
| beta.exposure | Raw | -0.108668194 | 0.0216848705496458 | -5.011244771 | 0.000303544360208757 | -0.15117054 | -0.066165848 | 0.897028007267014 | 0.859701072370478 | 0.93597562185507 | ebi-a-GCST90001402 | finngen_R11_O15_POOR_FETGRO |
| beta.exposure | Outlier-corrected |  |  |  |  |  |  |  |  |  | ebi-a-GCST90001402 | finngen_R11_O15_POOR_FETGRO |
| beta.exposure | Raw | -0.031398076 | 0.00864716593139492 | -3.631025019 | 0.00224700434734351 | -0.048346521 | -0.014449631 | 0.96908972507898 | 0.952803563362596 | 0.985654264284337 | ebi-a-GCST90001407 | finngen_R11_O15_POOR_FETGRO |
| beta.exposure | Outlier-corrected |  |  |  |  |  |  |  |  |  | ebi-a-GCST90001407 | finngen_R11_O15_POOR_FETGRO |
| beta.exposure | Raw | -0.124195676 | 0.0392357229357574 | -3.165372437 | 0.00509434013164857 | -0.201097693 | -0.047293659 | 0.883207001639361 | 0.817832531248815 | 0.953807262415522 | ebi-a-GCST90001411 | finngen_R11_O15_POOR_FETGRO |
| beta.exposure | Outlier-corrected |  |  |  |  |  |  |  |  |  | ebi-a-GCST90001411 | finngen_R11_O15_POOR_FETGRO |
| beta.exposure | Raw | -0.039016348 | 0.0148550085364547 | -2.62647768 | 0.0145194564761083 | -0.068132165 | -0.009900532 | 0.961734986234459 | 0.934137005121784 | 0.990148317298286 | ebi-a-GCST90001418 | finngen_R11_O15_POOR_FETGRO |
| beta.exposure | Outlier-corrected |  |  |  |  |  |  |  |  |  | ebi-a-GCST90001418 | finngen_R11_O15_POOR_FETGRO |
| beta.exposure | Raw | -0.037402933 | 0.0135956034367657 | -2.751105013 | 0.0104761971327854 | -0.064050316 | -0.01075555 | 0.96328791687724 | 0.937957804526261 | 0.989302084085076 | ebi-a-GCST90001436 | finngen_R11_O15_POOR_FETGRO |
| beta.exposure | Outlier-corrected |  |  |  |  |  |  |  |  |  | ebi-a-GCST90001436 | finngen_R11_O15_POOR_FETGRO |
| beta.exposure | Raw | -0.107196217 | 0.0405176666155033 | -2.645666112 | 0.0191908127843315 | -0.186610844 | -0.027781591 | 0.89834938384072 | 0.829766582166005 | 0.972600768447848 | ebi-a-GCST90001439 | finngen_R11_O15_POOR_FETGRO |
| beta.exposure | Outlier-corrected |  |  |  |  |  |  |  |  |  | ebi-a-GCST90001439 | finngen_R11_O15_POOR_FETGRO |
| beta.exposure | Raw | -0.045178537 | 0.0196386209999339 | -2.300494347 | 0.0352112654760287 | -0.083670234 | -0.006686839 | 0.955826816544395 | 0.919734503496388 | 0.993335467738033 | ebi-a-GCST90001457 | finngen_R11_O15_POOR_FETGRO |
| beta.exposure | Outlier-corrected |  |  |  |  |  |  |  |  |  | ebi-a-GCST90001457 | finngen_R11_O15_POOR_FETGRO |
| beta.exposure | Raw | -0.033688777 | 0.0150819346576158 | -2.233717239 | 0.0339818380134303 | -0.063249369 | -0.004128186 | 0.966872370309306 | 0.938709359137449 | 0.99588032373144 | ebi-a-GCST90001483 | finngen_R11_O15_POOR_FETGRO |
| beta.exposure | Outlier-corrected |  |  |  |  |  |  |  |  |  | ebi-a-GCST90001483 | finngen_R11_O15_POOR_FETGRO |
| beta.exposure | Raw | 0.043519680674393 | 0.0213095310905615 | 2.04226364669605 | 0.0513897804813422 | 0.0017529997368925 | 0.0852863616118936 | 1.04448055019137 | 1.00175453713916 | 1.08902887811579 | ebi-a-GCST90001525 | finngen_R11_O15_POOR_FETGRO |
| beta.exposure | Outlier-corrected |  |  |  |  |  |  |  |  |  | ebi-a-GCST90001525 | finngen_R11_O15_POOR_FETGRO |
| beta.exposure | Raw | 0.0719932073414823 | 0.0315056944320235 | 2.28508555800332 | 0.0314405025419844 | 0.0102420462547163 | 0.133744368428248 | 1.07464804432183 | 1.01029467553417 | 1.14310056969678 | ebi-a-GCST90001540 | finngen_R11_O15_POOR_FETGRO |
| beta.exposure | Outlier-corrected |  |  |  |  |  |  |  |  |  | ebi-a-GCST90001540 | finngen_R11_O15_POOR_FETGRO |
| beta.exposure | Raw | 0.0372295092468859 | 0.0139259399065341 | 2.67339292692321 | 0.013572843737995 | 0.00993466703007904 | 0.0645243514636928 | 1.03793120831394 | 1.00998417966258 | 1.06665155245495 | ebi-a-GCST90001624 | finngen_R11_O15_POOR_FETGRO |
| beta.exposure | Outlier-corrected |  |  |  |  |  |  |  |  |  | ebi-a-GCST90001624 | finngen_R11_O15_POOR_FETGRO |
| beta.exposure | Raw | -0.060765381 | 0.0243643471184787 | -2.494028714 | 0.0202590783130994 | -0.108519502 | -0.013011261 | 0.941044000397299 | 0.897161398516382 | 0.987073019579522 | ebi-a-GCST90001672 | finngen_R11_O15_POOR_FETGRO |
| beta.exposure | Outlier-corrected |  |  |  |  |  |  |  |  |  | ebi-a-GCST90001672 | finngen_R11_O15_POOR_FETGRO |
| beta.exposure | Raw | -0.075671089 | 0.0284680109544012 | -2.658109441 | 0.0160119907172385 | -0.13146839 | -0.019873787 | 0.92712109704306 | 0.876806990440025 | 0.980322394727899 | ebi-a-GCST90001722 | finngen_R11_O15_POOR_FETGRO |
| beta.exposure | Outlier-corrected |  |  |  |  |  |  |  |  |  | ebi-a-GCST90001722 | finngen_R11_O15_POOR_FETGRO |
| beta.exposure | Raw | 0.0457840688016201 | 0.0191269631989476 | 2.39369252324066 | 0.0248505063341421 | 0.00829522093168287 | 0.0832729166715574 | 1.04684833933342 | 1.00832972160776 | 1.08683838439054 | ebi-a-GCST90001742 | finngen_R11_O15_POOR_FETGRO |
| beta.exposure | Outlier-corrected |  |  |  |  |  |  |  |  |  | ebi-a-GCST90001742 | finngen_R11_O15_POOR_FETGRO |
| beta.exposure | Raw | -0.062125752 | 0.0160583129380494 | -3.868759551 | 0.000830187526646901 | -0.093600045 | -0.030651458 | 0.939764702485622 | 0.910646907275626 | 0.969813534732166 | ebi-a-GCST90001784 | finngen_R11_O15_POOR_FETGRO |
| beta.exposure | Outlier-corrected |  |  |  |  |  |  |  |  |  | ebi-a-GCST90001784 | finngen_R11_O15_POOR_FETGRO |
| beta.exposure | Raw | -0.033425655 | 0.0145310256083338 | -2.300295633 | 0.0297082623613982 | -0.061906465 | -0.004944845 | 0.967126809846641 | 0.939970802906034 | 0.995067361063176 | ebi-a-GCST90001794 | finngen_R11_O15_POOR_FETGRO |
| beta.exposure | Outlier-corrected |  |  |  |  |  |  |  |  |  | ebi-a-GCST90001794 | finngen_R11_O15_POOR_FETGRO |
| beta.exposure | Raw | -0.04488499 | 0.0195873151652138 | -2.291533551 | 0.0293843207303844 | -0.083276128 | -0.006493852 | 0.956107437551899 | 0.920097047957096 | 0.993527187345878 | ebi-a-GCST90001802 | finngen_R11_O15_POOR_FETGRO |
| beta.exposure | Outlier-corrected |  |  |  |  |  |  |  |  |  | ebi-a-GCST90001802 | finngen_R11_O15_POOR_FETGRO |
| beta.exposure | Raw | -0.033918019 | 0.0149778075913397 | -2.264551686 | 0.030927147578426 | -0.063274522 | -0.004561517 | 0.966650747958286 | 0.938685748126036 | 0.995448871354178 | ebi-a-GCST90001804 | finngen_R11_O15_POOR_FETGRO |
| beta.exposure | Outlier-corrected |  |  |  |  |  |  |  |  |  | ebi-a-GCST90001804 | finngen_R11_O15_POOR_FETGRO |
| beta.exposure | Raw | -0.031659635 | 0.0132155121848865 | -2.395641894 | 0.0247447741136749 | -0.057562039 | -0.005757231 | 0.968836284271488 | 0.94406332027076 | 0.994259310330771 | ebi-a-GCST90001805 | finngen_R11_O15_POOR_FETGRO |
| beta.exposure | Outlier-corrected |  |  |  |  |  |  |  |  |  | ebi-a-GCST90001805 | finngen_R11_O15_POOR_FETGRO |
| beta.exposure | Raw | -0.058677373 | 0.0255378424972521 | -2.297663691 | 0.028987272018794 | -0.108731545 | -0.008623202 | 0.943010960457739 | 0.896971181807813 | 0.991413871013266 | ebi-a-GCST90001806 | finngen_R11_O15_POOR_FETGRO |
| beta.exposure | Outlier-corrected |  |  |  |  |  |  |  |  |  | ebi-a-GCST90001806 | finngen_R11_O15_POOR_FETGRO |
| beta.exposure | Raw | 0.0566870497778053 | 0.0287291998844285 | 1.97315101032557 | 0.0632050791097762 | 0.000377818004325403 | 0.112996281551285 | 1.05832455566023 | 1.00037788938654 | 1.1196277696624 | ebi-a-GCST90001839 | finngen_R11_O15_POOR_FETGRO |
| beta.exposure | Outlier-corrected |  |  |  |  |  |  |  |  |  | ebi-a-GCST90001839 | finngen_R11_O15_POOR_FETGRO |
| beta.exposure | Raw | 0.0466791582129131 | 0.0190287493719183 | 2.45308597536104 | 0.0225563364702842 | 0.00938280944395316 | 0.0839755069818731 | 1.04778578168196 | 1.00942696599661 | 1.08760225482085 | ebi-a-GCST90001859 | finngen_R11_O15_POOR_FETGRO |
| beta.exposure | Outlier-corrected |  |  |  |  |  |  |  |  |  | ebi-a-GCST90001859 | finngen_R11_O15_POOR_FETGRO |
| beta.exposure | Raw | 0.0596578194551982 | 0.0284651693449404 | 2.09581818159119 | 0.0534742140872751 | 0.00386608753911507 | 0.115449551371281 | 1.06147326889419 | 1.00387357049569 | 1.1223778906945 | ebi-a-GCST90001905 | finngen_R11_O15_POOR_FETGRO |
| beta.exposure | Outlier-corrected |  |  |  |  |  |  |  |  |  | ebi-a-GCST90001905 | finngen_R11_O15_POOR_FETGRO |
| beta.exposure | Raw | -0.033052109 | 0.0121949609381862 | -2.710308761 | 0.0134704822420259 | -0.056954233 | -0.009149986 | 0.967488142980403 | 0.944637301678031 | 0.990891747705624 | ebi-a-GCST90001943 | finngen_R11_O15_POOR_FETGRO |
| beta.exposure | Outlier-corrected |  |  |  |  |  |  |  |  |  | ebi-a-GCST90001943 | finngen_R11_O15_POOR_FETGRO |
| beta.exposure | Raw | -0.032777851 | 0.016500744068353 | -1.986446849 | 0.0601888011930299 | -0.065119309 | -0.000436393 | 0.967753521126253 | 0.936955669054018 | 0.999563702515221 | ebi-a-GCST90001949 | finngen_R11_O15_POOR_FETGRO |
| beta.exposure | Outlier-corrected |  |  |  |  |  |  |  |  |  | ebi-a-GCST90001949 | finngen_R11_O15_POOR_FETGRO |
| beta.exposure | Raw | -0.034373427 | 0.0137875621236887 | -2.493075052 | 0.0210913083613771 | -0.061397049 | -0.007349805 | 0.966210627965505 | 0.940449761067299 | 0.992677138366236 | ebi-a-GCST90001955 | finngen_R11_O15_POOR_FETGRO |
| beta.exposure | Outlier-corrected |  |  |  |  |  |  |  |  |  | ebi-a-GCST90001955 | finngen_R11_O15_POOR_FETGRO |
| beta.exposure | Raw | -0.031621818 | 0.0113389342455914 | -2.788782243 | 0.0107046266518189 | -0.05384613 | -0.009397507 | 0.968872922644437 | 0.947577899480539 | 0.990646511224223 | ebi-a-GCST90001956 | finngen_R11_O15_POOR_FETGRO |
| beta.exposure | Outlier-corrected |  |  |  |  |  |  |  |  |  | ebi-a-GCST90001956 | finngen_R11_O15_POOR_FETGRO |
| beta.exposure | Raw | -0.030294614 | 0.0118865356469268 | -2.54864956 | 0.0191320743000085 | -0.053592224 | -0.006997004 | 0.970159668963583 | 0.947818525622653 | 0.993027418054756 | ebi-a-GCST90001957 | finngen_R11_O15_POOR_FETGRO |
| beta.exposure | Outlier-corrected |  |  |  |  |  |  |  |  |  | ebi-a-GCST90001957 | finngen_R11_O15_POOR_FETGRO |
| beta.exposure | Raw | -0.096152829 | 0.0456159119976843 | -2.107879134 | 0.0567450886697218 | -0.185560017 | -0.006745642 | 0.908325186960514 | 0.830638981963688 | 0.993277059206115 | ebi-a-GCST90002064 | finngen_R11_O15_POOR_FETGRO |
| beta.exposure | Outlier-corrected |  |  |  |  |  |  |  |  |  | ebi-a-GCST90002064 | finngen_R11_O15_POOR_FETGRO |
| beta.exposure | Raw | 0.0576330017883725 | 0.027946612515633 | 2.06225358283167 | 0.0517723554190612 | 0.00285764125773178 | 0.112408362319013 | 1.05932615355858 | 1.00286172820659 | 1.11896971242484 | ebi-a-GCST90002080 | finngen_R11_O15_POOR_FETGRO |
| beta.exposure | Outlier-corrected |  |  |  |  |  |  |  |  |  | ebi-a-GCST90002080 | finngen_R11_O15_POOR_FETGRO |
| beta.exposure | Raw | 0.0673151922035765 | 0.0300902806897219 | 2.23710748655694 | 0.0348307398747363 | 0.00833824205172164 | 0.126292142355431 | 1.0696325648934 | 1.00837310201489 | 1.13461358855598 | ebi-a-GCST90002121 | finngen_R11_O15_POOR_FETGRO |
| beta.exposure | Outlier-corrected |  |  |  |  |  |  |  |  |  | ebi-a-GCST90002121 | finngen_R11_O15_POOR_FETGRO |
