# Supplementary material for: Mendelian Randomization Mediation Analysis Reveals the Impact of Dietary Preferences on Preeclampsia and Fetal Growth Restriction via Immune Modulation
Source: Food Sci Nutr. 2025 Nov 26;13(12):e71280. doi: 10.1002/fsn3.71280 (PMC12648301; doi:10.1002/fsn3.71280)
Supplement: Supplementary file 1 — Data S1: fsn371280‐sup‐0001‐Supinfo1.zip. [file FSN3-13-e71280-s001.zip › fsn371280-sup-0004-Supinfo4@Supplementary Material 13_PE(DietaryPreferences_to_ImmuneCells_MR-PRESSO_Results).docx]

| **Exposure** | **MR Analysis** | **Causal Estimate** | **Sd** | **T-stat** | **P-value** | **lo_ci** | **up_ci** | **or** | **or_lci95** | **or_uci95** | **id.exposure** | **id.outcome** |
| --- | --- | --- | --- | --- | --- | --- | --- | --- | --- | --- | --- | --- |
| beta.exposure | Raw | 0.171885387575809 | 0.059798383732664 | 2.87441527423624 | 0.0097089013138162 | 0.0546805554597875 | 0.28909021969183 | 1.18754171837866 | 1.05620316244613 | 1.33521218552654 | ebi-fl187-GCST90094707 | ebi-a-GCST90001588 |
| beta.exposure | Outlier-corrected |  |  |  |  |  |  |  |  |  | ebi-fl187-GCST90094707 | ebi-a-GCST90001588 |
| beta.exposure | Raw | -0.209399223 | 0.084788715236133 | -2.469659107 | 0.0231734677952624 | -0.375585105 | -0.043213341 | 0.81107137283119 | 0.686887260264578 | 0.957707050168442 | ebi-fl187-GCST90094707 | ebi-a-GCST90001732 |
| beta.exposure | Outlier-corrected |  |  |  |  |  |  |  |  |  | ebi-fl187-GCST90094707 | ebi-a-GCST90001732 |
| beta.exposure | Raw | 0.328165262492814 | 0.130097580848195 | 2.52245476321144 | 0.024384573319728 | 0.0731740040303527 | 0.583156520955275 | 1.38841840681741 | 1.07591773464981 | 1.79168500556116 | ebi-fl187-GCST90094716 | ebi-a-GCST90001517 |
| beta.exposure | Outlier-corrected |  |  |  |  |  |  |  |  |  | ebi-fl187-GCST90094716 | ebi-a-GCST90001517 |
| beta.exposure | Raw | 0.422935256393357 | 0.131261880183859 | 3.22207221015689 | 0.00614477361397043 | 0.165661971232992 | 0.680208541553721 | 1.52643546582982 | 1.18017410149392 | 1.97428941068412 | ebi-fl187-GCST90094716 | ebi-a-GCST90001520 |
| beta.exposure | Outlier-corrected |  |  |  |  |  |  |  |  |  | ebi-fl187-GCST90094716 | ebi-a-GCST90001520 |
| beta.exposure | Raw | 0.290704636204046 | 0.0952108323081444 | 3.05327271232329 | 0.00654121066178151 | 0.104091404880083 | 0.477317867528009 | 1.33736951507182 | 1.10970188244752 | 1.6117456842541 | ebi-fl187-GCST90094726 | ebi-a-GCST90001517 |
| beta.exposure | Outlier-corrected |  |  |  |  |  |  |  |  |  | ebi-fl187-GCST90094726 | ebi-a-GCST90001517 |
| beta.exposure | Raw | 0.334590605689931 | 0.0886682379126919 | 3.7735113899454 | 0.00128515919466876 | 0.160800859381055 | 0.508380351998807 | 1.39736819351695 | 1.1744510646128 | 1.66259619245743 | ebi-fl187-GCST90094726 | ebi-a-GCST90001520 |
| beta.exposure | Outlier-corrected |  |  |  |  |  |  |  |  |  | ebi-fl187-GCST90094726 | ebi-a-GCST90001520 |
| beta.exposure | Raw | -0.25855732 | 0.0889319281553113 | -2.907362126 | 0.0090313565952091 | -0.432863899 | -0.084250741 | 0.77216476950864 | 0.648648767602485 | 0.919200746305475 | ebi-fl187-GCST90094726 | ebi-a-GCST90002115 |
| beta.exposure | Outlier-corrected |  |  |  |  |  |  |  |  |  | ebi-fl187-GCST90094726 | ebi-a-GCST90002115 |
| beta.exposure | Raw | -0.261301718 | 0.107156345638741 | -2.438509046 | 0.0298503507843009 | -0.471328156 | -0.051275281 | 0.770048546944047 | 0.62417271903069 | 0.950017113166207 | ebi-fl187-GCST90094729 | ebi-a-GCST90002063 |
| beta.exposure | Outlier-corrected |  |  |  |  |  |  |  |  |  | ebi-fl187-GCST90094729 | ebi-a-GCST90002063 |
| beta.exposure | Raw | -0.288008662 | 0.105746393554024 | -2.723579049 | 0.017393689457434 | -0.495271593 | -0.080745731 | 0.749755097854173 | 0.609405374379504 | 0.9224282068905 | ebi-fl187-GCST90094729 | ebi-a-GCST90002066 |
| beta.exposure | Outlier-corrected |  |  |  |  |  |  |  |  |  | ebi-fl187-GCST90094729 | ebi-a-GCST90002066 |
| beta.exposure | Raw | -0.25899566 | 0.108821208332953 | -2.380010885 | 0.0333072419008042 | -0.472285229 | -0.045706092 | 0.771826372516828 | 0.6235756259149 | 0.955322697930119 | ebi-fl187-GCST90094729 | ebi-a-GCST90002070 |
| beta.exposure | Outlier-corrected |  |  |  |  |  |  |  |  |  | ebi-fl187-GCST90094729 | ebi-a-GCST90002070 |
| beta.exposure | Raw | 0.317758720824653 | 0.122813367181939 | 2.58733009375043 | 0.0252581462163581 | 0.0770445211480521 | 0.558472920501253 | 1.37404469285852 | 1.0800901621762 | 1.74800112443267 | ebi-fl187-GCST90094733 | ebi-a-GCST90001520 |
| beta.exposure | Outlier-corrected |  |  |  |  |  |  |  |  |  | ebi-fl187-GCST90094733 | ebi-a-GCST90001520 |
| beta.exposure | Raw | -0.449906713 | 0.0994124754277615 | -4.525656473 | 0.000863974127717652 | -0.644755165 | -0.255058261 | 0.637687636843246 | 0.524791013695713 | 0.774871351776817 | ebi-fl187-GCST90094733 | ebi-a-GCST90002115 |
| beta.exposure | Outlier-corrected |  |  |  |  |  |  |  |  |  | ebi-fl187-GCST90094733 | ebi-a-GCST90002115 |
| beta.exposure | Raw | -0.646465685 | 0.260295743584591 | -2.483581469 | 0.0889875192932375 | -1.156645343 | -0.136286028 | 0.523894115299111 | 0.314539585554159 | 0.872593011024297 | ebi-fl187-GCST90094734 | ebi-a-GCST90001422 |
| beta.exposure | Outlier-corrected |  |  |  |  |  |  |  |  |  | ebi-fl187-GCST90094734 | ebi-a-GCST90001422 |
| beta.exposure | Raw | -0.678791634 | 0.28968425151512 | -2.343212067 | 0.100926414469503 | -1.246572767 | -0.111010501 | 0.507229541190593 | 0.28748840015302 | 0.894929351304181 | ebi-fl187-GCST90094734 | ebi-a-GCST90001444 |
| beta.exposure | Outlier-corrected |  |  |  |  |  |  |  |  |  | ebi-fl187-GCST90094734 | ebi-a-GCST90001444 |
| beta.exposure | Raw | -0.457626575 | 0.216339719100726 | -2.115314642 | 0.0880267525542242 | -0.881652425 | -0.033600726 | 0.632783729052728 | 0.414098080034692 | 0.966957508521484 | ebi-fl187-GCST90094750 | ebi-a-GCST90002115 |
| beta.exposure | Outlier-corrected |  |  |  |  |  |  |  |  |  | ebi-fl187-GCST90094750 | ebi-a-GCST90002115 |
| beta.exposure | Raw | 0.722147368063046 | 0.274495483425944 | 2.63081694113876 | 0.0301399294253656 | 0.184136220548197 | 1.2601585155779 | 2.0588495749909 | 1.2021795735035 | 3.52598036588408 | ebi-fl187-GCST90094766 | ebi-a-GCST90002037 |
| beta.exposure | Outlier-corrected |  |  |  |  |  |  |  |  |  | ebi-fl187-GCST90094766 | ebi-a-GCST90002037 |
| beta.exposure | Raw | -0.421243141 | 0.12892447074413 | -3.267363738 | 0.00427851288545624 | -0.673935103 | -0.168551178 | 0.656230525715251 | 0.509698908407494 | 0.844888022668134 | ebi-fl187-GCST90094770 | ebi-a-GCST90002111 |
| beta.exposure | Outlier-corrected |  |  |  |  |  |  |  |  |  | ebi-fl187-GCST90094770 | ebi-a-GCST90002111 |
| beta.exposure | Raw | -0.190476662 | 0.0757645249693716 | -2.514061331 | 0.0401577020423848 | -0.338975131 | -0.041978194 | 0.826565047502823 | 0.712500167754012 | 0.958890690379768 | ebi-fl187-GCST90094780 | ebi-a-GCST90001701 |
| beta.exposure | Outlier-corrected |  |  |  |  |  |  |  |  |  | ebi-fl187-GCST90094780 | ebi-a-GCST90001701 |
| beta.exposure | Raw | -0.215514679 | 0.0457953081862379 | -4.706042773 | 0.00219298106175504 | -0.305273483 | -0.125755875 | 0.80612643694046 | 0.736921811117771 | 0.88183009721023 | ebi-fl187-GCST90094780 | ebi-a-GCST90002115 |
| beta.exposure | Outlier-corrected |  |  |  |  |  |  |  |  |  | ebi-fl187-GCST90094780 | ebi-a-GCST90002115 |
| beta.exposure | Raw | 0.430413020038393 | 0.21566128145016 | 1.99578253984298 | 0.086153506566793 | 0.0077169083960793 | 0.853109131680706 | 1.53789257284469 | 1.00774676047285 | 2.34693244213568 | ebi-fl187-GCST90094784 | ebi-a-GCST90001414 |
| beta.exposure | Outlier-corrected |  |  |  |  |  |  |  |  |  | ebi-fl187-GCST90094784 | ebi-a-GCST90001414 |
| beta.exposure | Raw | -0.397705234 | 0.185105393471091 | -2.148534015 | 0.068762315040166 | -0.760511805 | -0.034898663 | 0.671860039846901 | 0.467427134051973 | 0.965703272786233 | ebi-fl187-GCST90094784 | ebi-a-GCST90001646 |
| beta.exposure | Outlier-corrected |  |  |  |  |  |  |  |  |  | ebi-fl187-GCST90094784 | ebi-a-GCST90001646 |
| beta.exposure | Raw | 0.435260524351919 | 0.150869757795434 | 2.88500843848436 | 0.02035446217354 | 0.139555799072868 | 0.73096524963097 | 1.54536561188616 | 1.14976295963535 | 2.07708454545943 | ebi-fl187-GCST90094798 | ebi-a-GCST90001646 |
| beta.exposure | Outlier-corrected |  |  |  |  |  |  |  |  |  | ebi-fl187-GCST90094798 | ebi-a-GCST90001646 |
| beta.exposure | Raw | 0.441138741058341 | 0.104998186853123 | 4.2013938933577 | 0.00299081257913597 | 0.23534229482622 | 0.646935187290462 | 1.55447635707682 | 1.26534181457052 | 1.90967904236296 | ebi-fl187-GCST90094798 | ebi-a-GCST90001798 |
| beta.exposure | Outlier-corrected |  |  |  |  |  |  |  |  |  | ebi-fl187-GCST90094798 | ebi-a-GCST90001798 |
| beta.exposure | Raw | -0.651620536 | 0.164400920329686 | -3.963606374 | 0.00415597958834731 | -0.97384634 | -0.329394732 | 0.521200468063199 | 0.377627756576789 | 0.719359006794987 | ebi-fl187-GCST90094798 | ebi-a-GCST90002042 |
| beta.exposure | Outlier-corrected |  |  |  |  |  |  |  |  |  | ebi-fl187-GCST90094798 | ebi-a-GCST90002042 |
| beta.exposure | Raw | -0.364889829 | 0.113272987799706 | -3.221331369 | 0.0122147701547689 | -0.586904885 | -0.142874773 | 0.694273135535063 | 0.556045649359899 | 0.866862616910262 | ebi-fl187-GCST90094798 | ebi-a-GCST90002104 |
| beta.exposure | Outlier-corrected |  |  |  |  |  |  |  |  |  | ebi-fl187-GCST90094798 | ebi-a-GCST90002104 |
| beta.exposure | Raw | -0.46324833 | 0.127861274879546 | -3.623054211 | 0.00675263796652302 | -0.713856429 | -0.212640232 | 0.629236354571333 | 0.489751857608332 | 0.808446938512407 | ebi-fl187-GCST90094798 | ebi-a-GCST90002106 |
| beta.exposure | Outlier-corrected |  |  |  |  |  |  |  |  |  | ebi-fl187-GCST90094798 | ebi-a-GCST90002106 |
| beta.exposure | Raw | 0.562878295221526 | 0.193304790535411 | 2.91186935234496 | 0.0435965121066805 | 0.18400090577212 | 0.941755684670932 | 1.75571871134693 | 1.20201691184922 | 2.56447988625337 | ebi-fl187-GCST90094812 | ebi-a-GCST90001485 |
| beta.exposure | Outlier-corrected |  |  |  |  |  |  |  |  |  | ebi-fl187-GCST90094812 | ebi-a-GCST90001485 |
| beta.exposure | Raw | 0.280974700060837 | 0.0952307247288187 | 2.95046268797121 | 0.00992408723258827 | 0.0943224795923525 | 0.467626920529322 | 1.32442009576353 | 1.09891406614366 | 1.59620178147121 | ebi-fl187-GCST90094817 | ebi-a-GCST90001624 |
| beta.exposure | Outlier-corrected |  |  |  |  |  |  |  |  |  | ebi-fl187-GCST90094817 | ebi-a-GCST90001624 |
| beta.exposure | Raw | -0.256048719 | 0.122221417396687 | -2.094957862 | 0.0469071327336279 | -0.495602697 | -0.016494741 | 0.774104254037615 | 0.609203631171592 | 0.983640552119998 | ebi-fl187-GCST90094840 | ebi-a-GCST90001836 |
| beta.exposure | Outlier-corrected |  |  |  |  |  |  |  |  |  | ebi-fl187-GCST90094840 | ebi-a-GCST90001836 |
| beta.exposure | Raw | 0.409237940202467 | 0.149638496045653 | 2.73484398077359 | 0.0161173740255287 | 0.115946487952987 | 0.702529392451948 | 1.50566993727651 | 1.12293577993319 | 2.01885272562347 | ebi-fl187-GCST90094846 | ebi-a-GCST90001517 |
| beta.exposure | Outlier-corrected |  |  |  |  |  |  |  |  |  | ebi-fl187-GCST90094846 | ebi-a-GCST90001517 |
| beta.exposure | Raw | 0.434761280465544 | 0.152250609150743 | 2.85556348766453 | 0.0127096740368759 | 0.136350086530088 | 0.733172474 | 1.54459429010707 | 1.14608305160949 | 2.08167420125525 | ebi-fl187-GCST90094846 | ebi-a-GCST90001520 |
| beta.exposure | Outlier-corrected |  |  |  |  |  |  |  |  |  | ebi-fl187-GCST90094846 | ebi-a-GCST90001520 |
| beta.exposure | Raw | -0.225619632 | 0.182856660031605 | -1.233860622 | 0.237567905029446 | -0.584018686 | 0.132779421323938 | 0.798021585549003 | 0.557652825921755 | 1.14199807012455 | ebi-fl187-GCST90094846 | ebi-a-GCST90001836 |
| beta.exposure | Outlier-corrected |  |  |  |  |  |  |  |  |  | ebi-fl187-GCST90094846 | ebi-a-GCST90001836 |
| beta.exposure | Raw | 0.260368073227286 | 0.102247552889909 | 2.54644796739173 | 0.0232771699697067 | 0.0599628695630651 | 0.460773276891508 | 1.2974075397718 | 1.06179712082238 | 1.5852993865278 | ebi-fl187-GCST90094846 | ebi-a-GCST90001960 |
| beta.exposure | Outlier-corrected |  |  |  |  |  |  |  |  |  | ebi-fl187-GCST90094846 | ebi-a-GCST90001960 |
| beta.exposure | Raw | -0.259944528 | 0.127238564196823 | -2.042969678 | 0.0603533605809975 | -0.509332114 | -0.010556943 | 0.771094358387051 | 0.600896775175638 | 0.989498586279723 | ebi-fl187-GCST90094846 | ebi-a-GCST90002063 |
| beta.exposure | Outlier-corrected |  |  |  |  |  |  |  |  |  | ebi-fl187-GCST90094846 | ebi-a-GCST90002063 |
| beta.exposure | Raw | -0.272339699 | 0.112968041890346 | -2.410767635 | 0.0302385313883136 | -0.493757061 | -0.050922337 | 0.76159550381629 | 0.610329037667236 | 0.950352474871811 | ebi-fl187-GCST90094846 | ebi-a-GCST90002066 |
| beta.exposure | Outlier-corrected |  |  |  |  |  |  |  |  |  | ebi-fl187-GCST90094846 | ebi-a-GCST90002066 |
| beta.exposure | Raw | -0.305391324 | 0.140574317985013 | -2.1724546 | 0.0474858617605203 | -0.580916987 | -0.02986566 | 0.736834976948712 | 0.559385182375316 | 0.970575911484793 | ebi-fl187-GCST90094846 | ebi-a-GCST90002115 |
| beta.exposure | Outlier-corrected |  |  |  |  |  |  |  |  |  | ebi-fl187-GCST90094846 | ebi-a-GCST90002115 |
| beta.exposure | Raw | -1.07308565 | 0.308351355298561 | -3.480074375 | 0.0400531048211245 | -1.677454307 | -0.468716994 | 0.341951744379892 | 0.186849031462961 | 0.625804664701355 | ebi-fl187-GCST90094853 | ebi-a-GCST90002042 |
| beta.exposure | Outlier-corrected |  |  |  |  |  |  |  |  |  | ebi-fl187-GCST90094853 | ebi-a-GCST90002042 |
