# Supplementary material for: Mendelian Randomization Mediation Analysis Reveals the Impact of Dietary Preferences on Preeclampsia and Fetal Growth Restriction via Immune Modulation
Source: Food Sci Nutr. 2025 Nov 26;13(12):e71280. doi: 10.1002/fsn3.71280 (PMC12648301; doi:10.1002/fsn3.71280)
Supplement: Supplementary file 1 — Data S1: fsn371280‐sup‐0001‐Supinfo1.zip. [file FSN3-13-e71280-s001.zip › fsn371280-sup-0005-Supinfo5@Supplementary Material 14_FGR(DietaryPreferences_to_ImmuneCells_MR-PRESSO_Results).docx]

| **Exposure** | **MR Analysis** | **Causal Estimate** | **Sd** | **T-stat** | **P-value** | **lo_ci** | **up_ci** | **or** | **or_lci95** | **or_uci95** | **id.exposure** | **id.outcome** |
| --- | --- | --- | --- | --- | --- | --- | --- | --- | --- | --- | --- | --- |
| beta.exposure | Raw | -0.71846377 | 0.27778314997125 | -2.586419551 | 0.0813258910265516 | -1.262918744 | -0.174008796 | 0.48750059405256 | 0.282827320093791 | 0.840289506412559 | ebi-fl187-GCST90094727 | ebi-a-GCST90001722 |
| beta.exposure | Outlier-corrected |  |  |  |  |  |  |  |  |  | ebi-fl187-GCST90094727 | ebi-a-GCST90001722 |
| beta.exposure | Raw | 0.686572846209857 | 0.209430922423576 | 3.27827828987572 | 0.04648607339994 | 0.276088238259647 | 1.09705745416007 | 1.9868944586091 | 1.31796415375983 | 2.99533912086271 | ebi-fl187-GCST90094727 | ebi-a-GCST90001794 |
| beta.exposure | Outlier-corrected |  |  |  |  |  |  |  |  |  | ebi-fl187-GCST90094727 | ebi-a-GCST90001794 |
| beta.exposure | Raw | 0.47633964221747 | 0.186637642115192 | 2.55221635260199 | 0.0837806995946795 | 0.110529863671695 | 0.842149420763246 | 1.61016980473989 | 1.11686970238421 | 2.32135117871092 | ebi-fl187-GCST90094765 | ebi-a-GCST90001722 |
| beta.exposure | Outlier-corrected |  |  |  |  |  |  |  |  |  | ebi-fl187-GCST90094765 | ebi-a-GCST90001722 |
| beta.exposure | Raw | -0.268570211 | 0.508507528673228 | -0.528153853 | 0.633967728322401 | -1.265244967 | 0.728104545493345 | 0.764471746824358 | 0.282170165321205 | 2.0711511120512 | ebi-fl187-GCST90094765 | ebi-a-GCST90001794 |
| beta.exposure | Outlier-corrected | -0.193601365 | 0.468898325715885 | -0.412885597 | 0.750721940457322 | -1.112642083 | 0.725439353327373 | 0.823986308527197 | 0.328689388036934 | 2.06563844575349 | ebi-fl187-GCST90094765 | ebi-a-GCST90001794 |
| beta.exposure | Raw | 0.224137513392215 | 0.0948551639710987 | 2.36294476767239 | 0.0320605679529892 | 0.0382213920088613 | 0.410053634775568 | 1.25124307027752 | 1.0389612251311 | 1.50689860511394 | ebi-fl187-GCST90094781 | ebi-a-GCST90001457 |
| beta.exposure | Outlier-corrected |  |  |  |  |  |  |  |  |  | ebi-fl187-GCST90094781 | ebi-a-GCST90001457 |
| beta.exposure | Raw | -0.853731468 | 0.229147299849641 | -3.725688534 | 0.00254248355235409 | -1.302860175 | -0.40460276 | 0.425823018891041 | 0.271753417991766 | 0.667241813396342 | ebi-fl187-GCST90094785 | ebi-a-GCST90001943 |
| beta.exposure | Outlier-corrected |  |  |  |  |  |  |  |  |  | ebi-fl187-GCST90094785 | ebi-a-GCST90001943 |
| beta.exposure | Raw | 0.770819788393465 | 0.305867828339927 | 2.5201074352181 | 0.053167689618494 | 0.171318844847208 | 1.37032073193972 | 2.16153753102943 | 1.18686911580024 | 3.93661309056691 | ebi-fl187-GCST90094803 | ebi-a-GCST90001802 |
| beta.exposure | Outlier-corrected |  |  |  |  |  |  |  |  |  | ebi-fl187-GCST90094803 | ebi-a-GCST90001802 |
| beta.exposure | Raw | 0.990742408225223 | 0.140123437316039 | 7.07049746425126 | 0.000875400322710503 | 0.716100471085787 | 1.26538434536466 | 2.69323320880493 | 2.04643748884297 | 3.54445476910743 | ebi-fl187-GCST90094803 | ebi-a-GCST90001804 |
| beta.exposure | Outlier-corrected |  |  |  |  |  |  |  |  |  | ebi-fl187-GCST90094803 | ebi-a-GCST90001804 |
| beta.exposure | Raw | 0.653198824229861 | 0.19009226993848 | 3.4362198128375 | 0.0185107571514976 | 0.280617975150439 | 1.02577967330928 | 1.92167811825249 | 1.32394772638151 | 2.78926933185146 | ebi-fl187-GCST90094803 | ebi-a-GCST90001805 |
| beta.exposure | Outlier-corrected |  |  |  |  |  |  |  |  |  | ebi-fl187-GCST90094803 | ebi-a-GCST90001805 |
| beta.exposure | Raw | 0.217905250323849 | 0.0686053202689224 | 3.1762150438143 | 0.00352603701039219 | 0.0834388225967615 | 0.352371678050937 | 1.24346924372912 | 1.08701871227657 | 1.42243711413393 | ebi-fl187-GCST90094838 | ebi-a-GCST90001672 |
| beta.exposure | Outlier-corrected |  |  |  |  |  |  |  |  |  | ebi-fl187-GCST90094838 | ebi-a-GCST90001672 |
| beta.exposure | Raw | 0.16135391972078 | 0.0821607759376533 | 1.96388018344936 | 0.0591944850989684 | 0.000318798882979948 | 0.322389040558581 | 1.17510078656875 | 1.00031884970474 | 1.38042171153935 | ebi-fl187-GCST90094838 | ebi-a-GCST90001839 |
| beta.exposure | Outlier-corrected |  |  |  |  |  |  |  |  |  | ebi-fl187-GCST90094838 | ebi-a-GCST90001839 |
| beta.exposure | Raw | -0.176816504 | 0.0782119761796542 | -2.260734387 | 0.0340089903910128 | -0.330111977 | -0.023521031 | 0.837933527875763 | 0.718843234798778 | 0.976753432665839 | ebi-fl187-GCST90094852 | ebi-a-GCST90001392 |
| beta.exposure | Outlier-corrected |  |  |  |  |  |  |  |  |  | ebi-fl187-GCST90094852 | ebi-a-GCST90001392 |
| beta.exposure | Raw | -0.266643887 | 0.0762669154182592 | -3.496193414 | 0.00204266656266353 | -0.416127042 | -0.117160733 | 0.765945785860065 | 0.659596468966864 | 0.889442218809491 | ebi-fl187-GCST90094852 | ebi-a-GCST90001525 |
| beta.exposure | Outlier-corrected |  |  |  |  |  |  |  |  |  | ebi-fl187-GCST90094852 | ebi-a-GCST90001525 |
