# Supplementary material for: Mendelian Randomization Mediation Analysis Reveals the Impact of Dietary Preferences on Preeclampsia and Fetal Growth Restriction via Immune Modulation
Source: Food Sci Nutr. 2025 Nov 26;13(12):e71280. doi: 10.1002/fsn3.71280 (PMC12648301; doi:10.1002/fsn3.71280)
Supplement: Supplementary file 1 — Data S1: fsn371280‐sup‐0001‐Supinfo1.zip. [file FSN3-13-e71280-s001.zip › fsn371280-sup-0006-Supinfo6@Supplementary Material 1_Dietary_Preferences_to_PE and FGR_MR_Analysis.docx]

| id.exposure | id.outcome | outcome | exposure | method | nsnp | b | se | pval | lo_ci | up_ci | or | or_lci95 | or_uci95 | p_fdr |
| --- | --- | --- | --- | --- | --- | --- | --- | --- | --- | --- | --- | --- | --- | --- |
| ebi-fl187-GCST90094687 | finngen_R11_O15_PREECLAMPS | Pre-eclampsia \|\| id:finngen_R11_O15_PREECLAMPS | Adding salt to food liking \|\| id:ebi-fl187-GCST90094687 | Inverse variance weighted | 13 | -0.037387507 | 0.0836559313994595 | 0.654932840518727 | -0.201353132 | 0.126578118722336 | 0.963302776617951 | 0.817623651195578 | 1.13493810762806 | 0.941775027862301 |
| ebi-fl187-GCST90094687 | finngen_R11_O15_PREECLAMPS | Pre-eclampsia \|\| id:finngen_R11_O15_PREECLAMPS | Adding salt to food liking \|\| id:ebi-fl187-GCST90094687 | MR Egger | 13 | -0.072785148 | 0.323409111401942 | 0.826062026516907 | -0.706667007 | 0.561096710069991 | 0.929800577822379 | 0.493285578149648 | 1.75259353367221 | 0.954596128018958 |
| ebi-fl187-GCST90094687 | finngen_R11_O15_PREECLAMPS | Pre-eclampsia \|\| id:finngen_R11_O15_PREECLAMPS | Adding salt to food liking \|\| id:ebi-fl187-GCST90094687 | Weighted median | 13 | 0.0862560310386137 | 0.107908798792066 | 0.424092153098392 | -0.125245215 | 0.297757276671064 | 1.09008538827373 | 0.882280527995254 | 1.34683483996633 | 0.831443826469217 |
| ebi-fl187-GCST90094687 | finngen_R11_O15_PREECLAMPS | Pre-eclampsia \|\| id:finngen_R11_O15_PREECLAMPS | Adding salt to food liking \|\| id:ebi-fl187-GCST90094687 | Weighted mode | 13 | 0.137929418692605 | 0.169091859093793 | 0.430561633050382 | -0.193490625 | 0.469349462516439 | 1.14789452751836 | 0.824077561777897 | 1.59895367550577 | 0.905055003276494 |
| ebi-fl187-GCST90094688 | finngen_R11_O15_PREECLAMPS | Pre-eclampsia \|\| id:finngen_R11_O15_PREECLAMPS | Alcohol liking \|\| id:ebi-fl187-GCST90094688 | Inverse variance weighted | 17 | -0.098467781 | 0.0623408463608043 | 0.114219893516219 | -0.22065584 | 0.0237202777764405 | 0.906224889721414 | 0.801992646622194 | 1.02400384119415 | 0.512568357226114 |
| ebi-fl187-GCST90094688 | finngen_R11_O15_PREECLAMPS | Pre-eclampsia \|\| id:finngen_R11_O15_PREECLAMPS | Alcohol liking \|\| id:ebi-fl187-GCST90094688 | MR Egger | 17 | 0.270817687160499 | 0.254800791310172 | 0.304657871242997 | -0.228591864 | 0.770227238128436 | 1.31103602989388 | 0.795653202114593 | 2.16025709079261 | 0.954596128018958 |
| ebi-fl187-GCST90094688 | finngen_R11_O15_PREECLAMPS | Pre-eclampsia \|\| id:finngen_R11_O15_PREECLAMPS | Alcohol liking \|\| id:ebi-fl187-GCST90094688 | Weighted median | 17 | -0.101191865 | 0.0798716241001173 | 0.205179837277675 | -0.257740248 | 0.0553565185601831 | 0.903759616704093 | 0.772795941397633 | 1.05691735816825 | 0.649149713120124 |
| ebi-fl187-GCST90094688 | finngen_R11_O15_PREECLAMPS | Pre-eclampsia \|\| id:finngen_R11_O15_PREECLAMPS | Alcohol liking \|\| id:ebi-fl187-GCST90094688 | Weighted mode | 17 | -0.05148406 | 0.144204760246293 | 0.725741622299698 | -0.33412539 | 0.231157270439357 | 0.949818790291771 | 0.715964002068445 | 1.26005739364684 | 0.936018697441052 |
| ebi-fl187-GCST90094689 | finngen_R11_O15_PREECLAMPS | Pre-eclampsia \|\| id:finngen_R11_O15_PREECLAMPS | Aniseed liking \|\| id:ebi-fl187-GCST90094689 | Inverse variance weighted (multiplicative random effects) | 8 | 0.0271643326865217 | 0.16541182462873 | 0.869556039969639 | -0.297042844 | 0.351371508958832 | 1.02753664674748 | 0.743012178373998 | 1.42101514771887 | 0.953358127900067 |
| ebi-fl187-GCST90094689 | finngen_R11_O15_PREECLAMPS | Pre-eclampsia \|\| id:finngen_R11_O15_PREECLAMPS | Aniseed liking \|\| id:ebi-fl187-GCST90094689 | MR Egger | 8 | 0.059828228942786 | 0.664868866556658 | 0.931227831661328 | -1.24331475 | 1.36297120739384 | 1.06165416942318 | 0.288426569803633 | 3.90778691512704 | 0.970712810886627 |
| ebi-fl187-GCST90094689 | finngen_R11_O15_PREECLAMPS | Pre-eclampsia \|\| id:finngen_R11_O15_PREECLAMPS | Aniseed liking \|\| id:ebi-fl187-GCST90094689 | Weighted median | 8 | -0.072476408 | 0.158244041962661 | 0.646949548915057 | -0.382634731 | 0.237681913955737 | 0.930087688759476 | 0.682061990206853 | 1.26830569831283 | 0.900892362507883 |
| ebi-fl187-GCST90094689 | finngen_R11_O15_PREECLAMPS | Pre-eclampsia \|\| id:finngen_R11_O15_PREECLAMPS | Aniseed liking \|\| id:ebi-fl187-GCST90094689 | Weighted mode | 8 | -0.152845727 | 0.231450461062045 | 0.530132076668895 | -0.606488631 | 0.300797176195321 | 0.858262117852943 | 0.545262127984778 | 1.35093531190925 | 0.905055003276494 |
| ebi-fl187-GCST90094691 | finngen_R11_O15_PREECLAMPS | Pre-eclampsia \|\| id:finngen_R11_O15_PREECLAMPS | Apples liking \|\| id:ebi-fl187-GCST90094691 | Inverse variance weighted | 3 | 0.121374188041299 | 0.328749091016009 | 0.711978602283564 | -0.52297403 | 0.765722406432676 | 1.12904730933568 | 0.592755052436732 | 2.15054738289929 | 0.941775027862301 |
| ebi-fl187-GCST90094691 | finngen_R11_O15_PREECLAMPS | Pre-eclampsia \|\| id:finngen_R11_O15_PREECLAMPS | Apples liking \|\| id:ebi-fl187-GCST90094691 | MR Egger | 3 | 1.55484716705491 | 1.00083414481194 | 0.364098191812223 | -0.406787757 | 3.51648209088632 | 4.73436290323858 | 0.665785483763938 | 33.6657867228431 | 0.954596128018958 |
| ebi-fl187-GCST90094691 | finngen_R11_O15_PREECLAMPS | Pre-eclampsia \|\| id:finngen_R11_O15_PREECLAMPS | Apples liking \|\| id:ebi-fl187-GCST90094691 | Weighted median | 3 | -0.065268233 | 0.384264154400254 | 0.865126125885809 | -0.818425976 | 0.687889509560363 | 0.93681614460791 | 0.441125450520342 | 1.98951225272267 | 0.988450091947753 |
| ebi-fl187-GCST90094691 | finngen_R11_O15_PREECLAMPS | Pre-eclampsia \|\| id:finngen_R11_O15_PREECLAMPS | Apples liking \|\| id:ebi-fl187-GCST90094691 | Weighted mode | 3 | -0.171450062 | 0.466763203185099 | 0.748608811950888 | -1.08630594 | 0.743405816408645 | 0.842442336994737 | 0.337460794427333 | 2.10308605586471 | 0.94527722864985 |
| ebi-fl187-GCST90094692 | finngen_R11_O15_PREECLAMPS | Pre-eclampsia \|\| id:finngen_R11_O15_PREECLAMPS | Asparagus liking \|\| id:ebi-fl187-GCST90094692 | Inverse variance weighted | 8 | -0.204245558 | 0.153408903154883 | 0.183063979920586 | -0.504927008 | 0.0964358921459145 | 0.815262152431309 | 0.603549627980207 | 1.10123898081291 | 0.644285579970435 |
| ebi-fl187-GCST90094692 | finngen_R11_O15_PREECLAMPS | Pre-eclampsia \|\| id:finngen_R11_O15_PREECLAMPS | Asparagus liking \|\| id:ebi-fl187-GCST90094692 | MR Egger | 8 | -0.822132945 | 0.805599247636146 | 0.346852173077391 | -2.401107471 | 0.756841579979867 | 0.439493238990537 | 0.0906175414211641 | 2.13153330016611 | 0.954596128018958 |
| ebi-fl187-GCST90094692 | finngen_R11_O15_PREECLAMPS | Pre-eclampsia \|\| id:finngen_R11_O15_PREECLAMPS | Asparagus liking \|\| id:ebi-fl187-GCST90094692 | Weighted median | 8 | -0.106675569 | 0.16680947437957 | 0.522494166108113 | -0.433622139 | 0.220271000976144 | 0.898817230038147 | 0.648157122782654 | 1.24641446435874 | 0.881409801621768 |
| ebi-fl187-GCST90094692 | finngen_R11_O15_PREECLAMPS | Pre-eclampsia \|\| id:finngen_R11_O15_PREECLAMPS | Asparagus liking \|\| id:ebi-fl187-GCST90094692 | Weighted mode | 8 | -0.070598767 | 0.238179021669917 | 0.775517794843284 | -0.537429649 | 0.396232115673939 | 0.931835700551249 | 0.584248046441303 | 1.48621425114012 | 0.947148782226634 |
| ebi-fl187-GCST90094693 | finngen_R11_O15_PREECLAMPS | Pre-eclampsia \|\| id:finngen_R11_O15_PREECLAMPS | Aubergine liking \|\| id:ebi-fl187-GCST90094693 | Inverse variance weighted (multiplicative random effects) | 10 | -0.061792264 | 0.14685443415073 | 0.673921429281348 | -0.349626955 | 0.226042426739979 | 0.940078154400092 | 0.704951019034078 | 1.25362885153523 | 0.941775027862301 |
| ebi-fl187-GCST90094693 | finngen_R11_O15_PREECLAMPS | Pre-eclampsia \|\| id:finngen_R11_O15_PREECLAMPS | Aubergine liking \|\| id:ebi-fl187-GCST90094693 | MR Egger | 10 | 0.110310945959028 | 0.846018789179759 | 0.899478280817253 | -1.547885881 | 1.76850777275136 | 1.11662522658463 | 0.212697165991881 | 5.86209924721223 | 0.970712810886627 |
| ebi-fl187-GCST90094693 | finngen_R11_O15_PREECLAMPS | Pre-eclampsia \|\| id:finngen_R11_O15_PREECLAMPS | Aubergine liking \|\| id:ebi-fl187-GCST90094693 | Weighted median | 10 | -0.17081746 | 0.126388436991644 | 0.176526487762165 | -0.418538796 | 0.0769038767450144 | 0.84297543636775 | 0.658007600861541 | 1.07993826422216 | 0.612323741171272 |
| ebi-fl187-GCST90094693 | finngen_R11_O15_PREECLAMPS | Pre-eclampsia \|\| id:finngen_R11_O15_PREECLAMPS | Aubergine liking \|\| id:ebi-fl187-GCST90094693 | Weighted mode | 10 | -0.201183746 | 0.157561596779953 | 0.233610631470087 | -0.510004475 | 0.107636984085037 | 0.81776215754689 | 0.600492891424991 | 1.1136434017209 | 0.905055003276494 |
| ebi-fl187-GCST90094694 | finngen_R11_O15_PREECLAMPS | Pre-eclampsia \|\| id:finngen_R11_O15_PREECLAMPS | Avocado liking \|\| id:ebi-fl187-GCST90094694 | Inverse variance weighted | 11 | -0.168036128 | 0.0890834691723557 | 0.059257592615311 | -0.342639728 | 0.00656747124853041 | 0.84532329401465 | 0.709893920463511 | 1.00658908437646 | 0.366852117259508 |
| ebi-fl187-GCST90094694 | finngen_R11_O15_PREECLAMPS | Pre-eclampsia \|\| id:finngen_R11_O15_PREECLAMPS | Avocado liking \|\| id:ebi-fl187-GCST90094694 | MR Egger | 11 | 0.716877574445627 | 0.744765991738452 | 0.360918414242493 | -0.742863769 | 2.17661891825299 | 2.04802840036253 | 0.475749525888786 | 8.81644668138256 | 0.954596128018958 |
| ebi-fl187-GCST90094694 | finngen_R11_O15_PREECLAMPS | Pre-eclampsia \|\| id:finngen_R11_O15_PREECLAMPS | Avocado liking \|\| id:ebi-fl187-GCST90094694 | Weighted median | 11 | -0.198058658 | 0.121467225686564 | 0.102984831647176 | -0.43613442 | 0.0400171041981277 | 0.820321733173278 | 0.646530813103369 | 1.04082857657833 | 0.529128962601009 |
| ebi-fl187-GCST90094694 | finngen_R11_O15_PREECLAMPS | Pre-eclampsia \|\| id:finngen_R11_O15_PREECLAMPS | Avocado liking \|\| id:ebi-fl187-GCST90094694 | Weighted mode | 11 | -0.240736648 | 0.190089209643273 | 0.234059290127181 | -0.613311498 | 0.131838203372344 | 0.786048606976113 | 0.541554539437942 | 1.14092370672463 | 0.905055003276494 |
| ebi-fl187-GCST90094695 | finngen_R11_O15_PREECLAMPS | Pre-eclampsia \|\| id:finngen_R11_O15_PREECLAMPS | Bacon liking \|\| id:ebi-fl187-GCST90094695 | Inverse variance weighted | 4 | -0.056664531 | 0.181778250793259 | 0.75525075659065 | -0.412949903 | 0.299620840462648 | 0.944911004465308 | 0.661695431187161 | 1.34934709275194 | 0.941775027862301 |
| ebi-fl187-GCST90094695 | finngen_R11_O15_PREECLAMPS | Pre-eclampsia \|\| id:finngen_R11_O15_PREECLAMPS | Bacon liking \|\| id:ebi-fl187-GCST90094695 | MR Egger | 4 | -0.858602424 | 0.503992506895316 | 0.230568546922998 | -1.846427738 | 0.129222889478109 | 0.423753896929181 | 0.157799863190432 | 1.13794373158592 | 0.954596128018958 |
| ebi-fl187-GCST90094695 | finngen_R11_O15_PREECLAMPS | Pre-eclampsia \|\| id:finngen_R11_O15_PREECLAMPS | Bacon liking \|\| id:ebi-fl187-GCST90094695 | Weighted median | 4 | -0.129502376 | 0.197819714340582 | 0.512694101168637 | -0.517229016 | 0.258224264233291 | 0.878532501131021 | 0.596170239485954 | 1.29462912507848 | 0.881409801621768 |
| ebi-fl187-GCST90094695 | finngen_R11_O15_PREECLAMPS | Pre-eclampsia \|\| id:finngen_R11_O15_PREECLAMPS | Bacon liking \|\| id:ebi-fl187-GCST90094695 | Weighted mode | 4 | -0.29443652 | 0.225372260809987 | 0.282535830864522 | -0.736166151 | 0.14729311100808 | 0.744951234211309 | 0.47894660894359 | 1.15869353909198 | 0.905055003276494 |
| ebi-fl187-GCST90094696 | finngen_R11_O15_PREECLAMPS | Pre-eclampsia \|\| id:finngen_R11_O15_PREECLAMPS | Baked/steamed fish liking \|\| id:ebi-fl187-GCST90094696 | Inverse variance weighted | 7 | -0.12270389 | 0.145013306323251 | 0.397465683836692 | -0.40692997 | 0.161522190716758 | 0.884525540930254 | 0.665690806949577 | 1.17529853858598 | 0.861823321954233 |
| ebi-fl187-GCST90094696 | finngen_R11_O15_PREECLAMPS | Pre-eclampsia \|\| id:finngen_R11_O15_PREECLAMPS | Baked/steamed fish liking \|\| id:ebi-fl187-GCST90094696 | MR Egger | 7 | -0.059087671 | 1.64979320531334 | 0.972815595852832 | -3.292682353 | 3.17450701142279 | 0.942624124742909 | 0.0371540553077619 | 23.9150271265734 | 0.986189238231469 |
| ebi-fl187-GCST90094696 | finngen_R11_O15_PREECLAMPS | Pre-eclampsia \|\| id:finngen_R11_O15_PREECLAMPS | Baked/steamed fish liking \|\| id:ebi-fl187-GCST90094696 | Weighted median | 7 | -0.20586572 | 0.180002908735646 | 0.252756385402965 | -0.558671421 | 0.146939980849848 | 0.813942364904486 | 0.571968464340584 | 1.15828444169575 | 0.672512525447174 |
| ebi-fl187-GCST90094696 | finngen_R11_O15_PREECLAMPS | Pre-eclampsia \|\| id:finngen_R11_O15_PREECLAMPS | Baked/steamed fish liking \|\| id:ebi-fl187-GCST90094696 | Weighted mode | 7 | -0.33501258 | 0.276390603002978 | 0.27103499430449 | -0.876738162 | 0.206713001656526 | 0.715329087292004 | 0.416138075257308 | 1.22962961947094 | 0.905055003276494 |
| ebi-fl187-GCST90094697 | finngen_R11_O15_PREECLAMPS | Pre-eclampsia \|\| id:finngen_R11_O15_PREECLAMPS | Banana liking \|\| id:ebi-fl187-GCST90094697 | Inverse variance weighted | 2 | 0.0329464047628593 | 0.227671908782121 | 0.884939833554121 | -0.413290536 | 0.479183345975817 | 1.03349514733944 | 0.661470073740257 | 1.61475516728153 | 0.953358127900067 |
| ebi-fl187-GCST90094698 | finngen_R11_O15_PREECLAMPS | Pre-eclampsia \|\| id:finngen_R11_O15_PREECLAMPS | BBQ/grilled meat liking \|\| id:ebi-fl187-GCST90094698 | Inverse variance weighted (multiplicative random effects) | 4 | -0.066730177 | 0.216946901016791 | 0.758396154458476 | -0.491946103 | 0.358485748705956 | 0.935447572289405 | 0.611435319302644 | 1.43116063609176 | 0.941775027862301 |
| ebi-fl187-GCST90094698 | finngen_R11_O15_PREECLAMPS | Pre-eclampsia \|\| id:finngen_R11_O15_PREECLAMPS | BBQ/grilled meat liking \|\| id:ebi-fl187-GCST90094698 | MR Egger | 4 | -0.841671431 | 0.661779720073027 | 0.33131475896639 | -2.138759682 | 0.455416820316417 | 0.430989551760716 | 0.117800862934668 | 1.57683050106279 | 0.954596128018958 |
| ebi-fl187-GCST90094698 | finngen_R11_O15_PREECLAMPS | Pre-eclampsia \|\| id:finngen_R11_O15_PREECLAMPS | BBQ/grilled meat liking \|\| id:ebi-fl187-GCST90094698 | Weighted median | 4 | -0.238519526 | 0.17277147502241 | 0.167417605009607 | -0.577151617 | 0.100112565435518 | 0.787793305963371 | 0.561495445215435 | 1.10529532912342 | 0.612323741171272 |
| ebi-fl187-GCST90094698 | finngen_R11_O15_PREECLAMPS | Pre-eclampsia \|\| id:finngen_R11_O15_PREECLAMPS | BBQ/grilled meat liking \|\| id:ebi-fl187-GCST90094698 | Weighted mode | 4 | -0.271416452 | 0.165620291355243 | 0.199786394112415 | -0.596032223 | 0.0531993193177415 | 0.762298969561195 | 0.550993524154017 | 1.05463983426351 | 0.905055003276494 |
| ebi-fl187-GCST90094699 | finngen_R11_O15_PREECLAMPS | Pre-eclampsia \|\| id:finngen_R11_O15_PREECLAMPS | Beans liking \|\| id:ebi-fl187-GCST90094699 | Inverse variance weighted | 9 | -0.042229068 | 0.088942021782574 | 0.63493437020617 | -0.216555431 | 0.132097294301269 | 0.958650158968303 | 0.805287895702542 | 1.14121934800497 | 0.941775027862301 |
| ebi-fl187-GCST90094699 | finngen_R11_O15_PREECLAMPS | Pre-eclampsia \|\| id:finngen_R11_O15_PREECLAMPS | Beans liking \|\| id:ebi-fl187-GCST90094699 | MR Egger | 9 | 0.652239461651435 | 0.338475964494904 | 0.0953439751948118 | -0.011173429 | 1.31565235206145 | 1.91983541622907 | 0.988888762152451 | 3.72718162696569 | 0.954596128018958 |
| ebi-fl187-GCST90094699 | finngen_R11_O15_PREECLAMPS | Pre-eclampsia \|\| id:finngen_R11_O15_PREECLAMPS | Beans liking \|\| id:ebi-fl187-GCST90094699 | Weighted median | 9 | 0.0785067900337053 | 0.0910543523791497 | 0.388579116807784 | -0.099959741 | 0.256973320696839 | 1.08167069971785 | 0.904873846954177 | 1.2930106296766 | 0.819869554194876 |
| ebi-fl187-GCST90094699 | finngen_R11_O15_PREECLAMPS | Pre-eclampsia \|\| id:finngen_R11_O15_PREECLAMPS | Beans liking \|\| id:ebi-fl187-GCST90094699 | Weighted mode | 9 | 0.107640846102517 | 0.106628490072027 | 0.342292088947576 | -0.101350994 | 0.31663268664369 | 1.11364770263949 | 0.903615813092853 | 1.37249834235334 | 0.905055003276494 |
| ebi-fl187-GCST90094700 | finngen_R11_O15_PREECLAMPS | Pre-eclampsia \|\| id:finngen_R11_O15_PREECLAMPS | Beef/steak liking \|\| id:ebi-fl187-GCST90094700 | Inverse variance weighted (multiplicative random effects) | 5 | -0.024851842 | 0.200502832218453 | 0.901356775469155 | -0.417837393 | 0.368133709167113 | 0.975454422717701 | 0.658469291350978 | 1.44503524051564 | 0.953358127900067 |
| ebi-fl187-GCST90094700 | finngen_R11_O15_PREECLAMPS | Pre-eclampsia \|\| id:finngen_R11_O15_PREECLAMPS | Beef/steak liking \|\| id:ebi-fl187-GCST90094700 | MR Egger | 5 | -0.668598066 | 0.783958062528303 | 0.45642155585056 | -2.205155869 | 0.867959736571838 | 0.512426462544159 | 0.110233342055369 | 2.38204589119351 | 0.954596128018958 |
| ebi-fl187-GCST90094700 | finngen_R11_O15_PREECLAMPS | Pre-eclampsia \|\| id:finngen_R11_O15_PREECLAMPS | Beef/steak liking \|\| id:ebi-fl187-GCST90094700 | Weighted median | 5 | -0.085244696 | 0.17247188153359 | 0.621127712552252 | -0.423289584 | 0.252800191541084 | 0.918287555372634 | 0.654888960239121 | 1.28762597256846 | 0.881409801621768 |
| ebi-fl187-GCST90094700 | finngen_R11_O15_PREECLAMPS | Pre-eclampsia \|\| id:finngen_R11_O15_PREECLAMPS | Beef/steak liking \|\| id:ebi-fl187-GCST90094700 | Weighted mode | 5 | -0.275261212 | 0.242781492353022 | 0.320229717590127 | -0.751112937 | 0.200590512602324 | 0.759373739471674 | 0.471841130762919 | 1.22212422487801 | 0.905055003276494 |
| ebi-fl187-GCST90094701 | finngen_R11_O15_PREECLAMPS | Pre-eclampsia \|\| id:finngen_R11_O15_PREECLAMPS | Beer liking \|\| id:ebi-fl187-GCST90094701 | Inverse variance weighted | 9 | -0.097325086 | 0.070524876881738 | 0.167583344214327 | -0.235553845 | 0.0409036725672825 | 0.907261020222228 | 0.790133117296498 | 1.04175175144039 | 0.628437540803727 |
| ebi-fl187-GCST90094701 | finngen_R11_O15_PREECLAMPS | Pre-eclampsia \|\| id:finngen_R11_O15_PREECLAMPS | Beer liking \|\| id:ebi-fl187-GCST90094701 | MR Egger | 9 | 0.149013181991052 | 0.183858180598801 | 0.444341542458257 | -0.211348852 | 0.509375215964703 | 1.16068828929089 | 0.809491624860682 | 1.66425107255293 | 0.954596128018958 |
| ebi-fl187-GCST90094701 | finngen_R11_O15_PREECLAMPS | Pre-eclampsia \|\| id:finngen_R11_O15_PREECLAMPS | Beer liking \|\| id:ebi-fl187-GCST90094701 | Weighted median | 9 | -0.080871619 | 0.0817623387948489 | 0.322611285975063 | -0.241125803 | 0.0793825647693798 | 0.922312090945223 | 0.785742771131707 | 1.08261841452075 | 0.763001295401339 |
| ebi-fl187-GCST90094701 | finngen_R11_O15_PREECLAMPS | Pre-eclampsia \|\| id:finngen_R11_O15_PREECLAMPS | Beer liking \|\| id:ebi-fl187-GCST90094701 | Weighted mode | 9 | -0.05841668 | 0.0992177571851683 | 0.572258844945029 | -0.252883484 | 0.13605012427442 | 0.943256829465248 | 0.77655835811062 | 1.14573932150775 | 0.905055003276494 |
| ebi-fl187-GCST90094702 | finngen_R11_O15_PREECLAMPS | Pre-eclampsia \|\| id:finngen_R11_O15_PREECLAMPS | Beetroot liking \|\| id:ebi-fl187-GCST90094702 | Inverse variance weighted | 6 | 0.0255017215157698 | 0.195521599643552 | 0.896226883082262 | -0.357720614 | 0.408724056817133 | 1.02582967225088 | 0.69926841367274 | 1.50489639728364 | 0.953358127900067 |
| ebi-fl187-GCST90094702 | finngen_R11_O15_PREECLAMPS | Pre-eclampsia \|\| id:finngen_R11_O15_PREECLAMPS | Beetroot liking \|\| id:ebi-fl187-GCST90094702 | MR Egger | 6 | 0.46471468831627 | 0.927418753130019 | 0.642631529364103 | -1.353026068 | 2.28245544445111 | 1.59156003342936 | 0.258456967782261 | 9.8007160021457 | 0.954596128018958 |
| ebi-fl187-GCST90094702 | finngen_R11_O15_PREECLAMPS | Pre-eclampsia \|\| id:finngen_R11_O15_PREECLAMPS | Beetroot liking \|\| id:ebi-fl187-GCST90094702 | Weighted median | 6 | -0.061812762 | 0.207285577817085 | 0.765549775906121 | -0.468092494 | 0.344466970629814 | 0.940058885161149 | 0.626195601384182 | 1.41123748812194 | 0.927373305772456 |
| ebi-fl187-GCST90094702 | finngen_R11_O15_PREECLAMPS | Pre-eclampsia \|\| id:finngen_R11_O15_PREECLAMPS | Beetroot liking \|\| id:ebi-fl187-GCST90094702 | Weighted mode | 6 | -0.34568323 | 0.373244773845747 | 0.396867825111371 | -1.077242987 | 0.38587652689543 | 0.707736641449493 | 0.340533086907164 | 1.47090304263668 | 0.905055003276494 |
| ebi-fl187-GCST90094704 | finngen_R11_O15_PREECLAMPS | Pre-eclampsia \|\| id:finngen_R11_O15_PREECLAMPS | Biscuits liking \|\| id:ebi-fl187-GCST90094704 | Inverse variance weighted | 9 | 0.0764799648563835 | 0.156542186895342 | 0.625154531299372 | -0.230342721 | 0.383302651171254 | 1.07948056257241 | 0.7942613454451 | 1.46712198907102 | 0.941775027862301 |
| ebi-fl187-GCST90094704 | finngen_R11_O15_PREECLAMPS | Pre-eclampsia \|\| id:finngen_R11_O15_PREECLAMPS | Biscuits liking \|\| id:ebi-fl187-GCST90094704 | MR Egger | 9 | -0.478195384 | 0.698660552463379 | 0.515707242469758 | -1.847570067 | 0.891179298732902 | 0.619901066339843 | 0.15761970669043 | 2.43800309059074 | 0.954596128018958 |
| ebi-fl187-GCST90094704 | finngen_R11_O15_PREECLAMPS | Pre-eclampsia \|\| id:finngen_R11_O15_PREECLAMPS | Biscuits liking \|\| id:ebi-fl187-GCST90094704 | Weighted median | 9 | 0.0555278776246071 | 0.173007860999613 | 0.748243881485498 | -0.28356753 | 0.394623285183848 | 1.05709848605643 | 0.753092264151495 | 1.4838251067176 | 0.927373305772456 |
| ebi-fl187-GCST90094704 | finngen_R11_O15_PREECLAMPS | Pre-eclampsia \|\| id:finngen_R11_O15_PREECLAMPS | Biscuits liking \|\| id:ebi-fl187-GCST90094704 | Weighted mode | 9 | -0.048513419 | 0.215788794567682 | 0.827756217882398 | -0.471459456 | 0.374432618568388 | 0.952644555888286 | 0.624090770194195 | 1.45416611365875 | 0.963239370826664 |
| ebi-fl187-GCST90094705 | finngen_R11_O15_PREECLAMPS | Pre-eclampsia \|\| id:finngen_R11_O15_PREECLAMPS | Bitter ale liking \|\| id:ebi-fl187-GCST90094705 | Inverse variance weighted | 4 | 0.0526353380023399 | 0.143112960763882 | 0.713030752688785 | -0.227866065 | 0.333136741099549 | 1.05404520479822 | 0.796230895803647 | 1.39533808548935 | 0.941775027862301 |
| ebi-fl187-GCST90094705 | finngen_R11_O15_PREECLAMPS | Pre-eclampsia \|\| id:finngen_R11_O15_PREECLAMPS | Bitter ale liking \|\| id:ebi-fl187-GCST90094705 | MR Egger | 4 | 0.296434284546213 | 0.392749827983757 | 0.529159747883854 | -0.473355378 | 1.06622394739438 | 1.34505416624796 | 0.622908663615121 | 2.90439163205931 | 0.954596128018958 |
| ebi-fl187-GCST90094705 | finngen_R11_O15_PREECLAMPS | Pre-eclampsia \|\| id:finngen_R11_O15_PREECLAMPS | Bitter ale liking \|\| id:ebi-fl187-GCST90094705 | Weighted median | 4 | -0.022274684 | 0.161903906815933 | 0.890572633543276 | -0.339606341 | 0.295056973302758 | 0.977971564956629 | 0.712050572417938 | 1.34320288321086 | 0.988450091947753 |
| ebi-fl187-GCST90094705 | finngen_R11_O15_PREECLAMPS | Pre-eclampsia \|\| id:finngen_R11_O15_PREECLAMPS | Bitter ale liking \|\| id:ebi-fl187-GCST90094705 | Weighted mode | 4 | -0.014588418 | 0.180361394752713 | 0.940627753573966 | -0.368096751 | 0.338919915973343 | 0.98551747764998 | 0.692050221582081 | 1.40343094831071 | 0.981296058194618 |
| ebi-fl187-GCST90094706 | finngen_R11_O15_PREECLAMPS | Pre-eclampsia \|\| id:finngen_R11_O15_PREECLAMPS | F-bitter food liking (derived food-liking factor) \|\| id:ebi-fl187-GCST90094706 | Inverse variance weighted | 6 | -0.278908836 | 0.146760153382859 | 0.0573754288590357 | -0.566558736 | 0.00874106496991855 | 0.756608875819514 | 0.567474913272509 | 1.00877937963395 | 0.366852117259508 |
| ebi-fl187-GCST90094706 | finngen_R11_O15_PREECLAMPS | Pre-eclampsia \|\| id:finngen_R11_O15_PREECLAMPS | F-bitter food liking (derived food-liking factor) \|\| id:ebi-fl187-GCST90094706 | MR Egger | 6 | -1.969518067 | 2.0948832789553 | 0.400367786712564 | -6.075489294 | 2.13645315944339 | 0.139524081230711 | 0.00229852125813553 | 8.46934487743868 | 0.954596128018958 |
| ebi-fl187-GCST90094706 | finngen_R11_O15_PREECLAMPS | Pre-eclampsia \|\| id:finngen_R11_O15_PREECLAMPS | F-bitter food liking (derived food-liking factor) \|\| id:ebi-fl187-GCST90094706 | Weighted median | 6 | -0.263624294 | 0.189205562495698 | 0.163522406150517 | -0.634467197 | 0.107218608048582 | 0.768262125791508 | 0.5302179148024 | 1.11317757746011 | 0.612323741171272 |
| ebi-fl187-GCST90094706 | finngen_R11_O15_PREECLAMPS | Pre-eclampsia \|\| id:finngen_R11_O15_PREECLAMPS | F-bitter food liking (derived food-liking factor) \|\| id:ebi-fl187-GCST90094706 | Weighted mode | 6 | -0.429230856 | 0.277883566229777 | 0.183089606218386 | -0.973882646 | 0.115420933899196 | 0.651009622412536 | 0.377614046599603 | 1.12234577153613 | 0.905055003276494 |
| ebi-fl187-GCST90094707 | finngen_R11_O15_PREECLAMPS | Pre-eclampsia \|\| id:finngen_R11_O15_PREECLAMPS | Black olive liking \|\| id:ebi-fl187-GCST90094707 | Inverse variance weighted | 22 | -0.123789699 | 0.0521191198011884 | 0.0175427574954136 | -0.225943174 | -0.021636224 | 0.883565635887621 | 0.797763434057947 | 0.978596159704141 | 0.241212915561936 |
| ebi-fl187-GCST90094707 | finngen_R11_O15_PREECLAMPS | Pre-eclampsia \|\| id:finngen_R11_O15_PREECLAMPS | Black olive liking \|\| id:ebi-fl187-GCST90094707 | MR Egger | 22 | -0.411638297 | 0.244579655930278 | 0.107912992167614 | -0.891014422 | 0.0677378291154417 | 0.662563884386645 | 0.410239385690116 | 1.07008472664085 | 0.954596128018958 |
| ebi-fl187-GCST90094707 | finngen_R11_O15_PREECLAMPS | Pre-eclampsia \|\| id:finngen_R11_O15_PREECLAMPS | Black olive liking \|\| id:ebi-fl187-GCST90094707 | Weighted median | 22 | -0.076809716 | 0.0686269064252097 | 0.263039395451113 | -0.211318453 | 0.057699020190733 | 0.926066052021866 | 0.809516232959694 | 1.05939609088737 | 0.687594209161682 |
| ebi-fl187-GCST90094707 | finngen_R11_O15_PREECLAMPS | Pre-eclampsia \|\| id:finngen_R11_O15_PREECLAMPS | Black olive liking \|\| id:ebi-fl187-GCST90094707 | Weighted mode | 22 | -0.05349098 | 0.126697677585184 | 0.677174599082293 | -0.301818428 | 0.19483646828747 | 0.947914491362977 | 0.739472320282327 | 1.21511226085227 | 0.936018697441052 |
| ebi-fl187-GCST90094708 | finngen_R11_O15_PREECLAMPS | Pre-eclampsia \|\| id:finngen_R11_O15_PREECLAMPS | Black pepper liking \|\| id:ebi-fl187-GCST90094708 | Inverse variance weighted | 8 | -0.192943508 | 0.124657860145494 | 0.121674181732317 | -0.437272914 | 0.0513858981104965 | 0.82452855227621 | 0.645795161037517 | 1.052729061 | 0.528322104890326 |
| ebi-fl187-GCST90094708 | finngen_R11_O15_PREECLAMPS | Pre-eclampsia \|\| id:finngen_R11_O15_PREECLAMPS | Black pepper liking \|\| id:ebi-fl187-GCST90094708 | MR Egger | 8 | -0.594842447 | 0.772684337061436 | 0.470608434882187 | -2.109303748 | 0.919618853576218 | 0.55164947301542 | 0.121322408038719 | 2.50833416512035 | 0.954596128018958 |
| ebi-fl187-GCST90094708 | finngen_R11_O15_PREECLAMPS | Pre-eclampsia \|\| id:finngen_R11_O15_PREECLAMPS | Black pepper liking \|\| id:ebi-fl187-GCST90094708 | Weighted median | 8 | -0.153267863 | 0.166318699386298 | 0.35677318481632 | -0.479252513 | 0.172716788172964 | 0.857899891715043 | 0.61924609699804 | 1.18852945181665 | 0.783962747510833 |
| ebi-fl187-GCST90094708 | finngen_R11_O15_PREECLAMPS | Pre-eclampsia \|\| id:finngen_R11_O15_PREECLAMPS | Black pepper liking \|\| id:ebi-fl187-GCST90094708 | Weighted mode | 8 | -0.134667324 | 0.233083585939849 | 0.581523740393794 | -0.591511153 | 0.32217650409602 | 0.874006624054074 | 0.553490244120028 | 1.38012835276776 | 0.905055003276494 |
| ebi-fl187-GCST90094709 | finngen_R11_O15_PREECLAMPS | Pre-eclampsia \|\| id:finngen_R11_O15_PREECLAMPS | Blue cheese liking \|\| id:ebi-fl187-GCST90094709 | Inverse variance weighted | 15 | -0.046267333 | 0.0714066810009298 | 0.517022961434049 | -0.186224428 | 0.0936897619281407 | 0.954786682240554 | 0.830087279583341 | 1.09821898372121 | 0.894908414040245 |
| ebi-fl187-GCST90094709 | finngen_R11_O15_PREECLAMPS | Pre-eclampsia \|\| id:finngen_R11_O15_PREECLAMPS | Blue cheese liking \|\| id:ebi-fl187-GCST90094709 | MR Egger | 15 | 0.140156840763737 | 0.378177797173388 | 0.716896044176642 | -0.601071642 | 0.881385323223578 | 1.15045422282689 | 0.548223821680953 | 2.41424189624231 | 0.954596128018958 |
| ebi-fl187-GCST90094709 | finngen_R11_O15_PREECLAMPS | Pre-eclampsia \|\| id:finngen_R11_O15_PREECLAMPS | Blue cheese liking \|\| id:ebi-fl187-GCST90094709 | Weighted median | 15 | -0.115616839 | 0.0855817656964634 | 0.176710878324595 | -0.2833571 | 0.0521234216970539 | 0.890816483988649 | 0.753250754108061 | 1.05350575995846 | 0.612323741171272 |
| ebi-fl187-GCST90094709 | finngen_R11_O15_PREECLAMPS | Pre-eclampsia \|\| id:finngen_R11_O15_PREECLAMPS | Blue cheese liking \|\| id:ebi-fl187-GCST90094709 | Weighted mode | 15 | -0.12399917 | 0.127737873274723 | 0.348155420940507 | -0.374365401 | 0.126367061992532 | 0.883380574418774 | 0.687725570132372 | 1.13469859657863 | 0.905055003276494 |
| ebi-fl187-GCST90094711 | finngen_R11_O15_PREECLAMPS | Pre-eclampsia \|\| id:finngen_R11_O15_PREECLAMPS | Broad bean liking \|\| id:ebi-fl187-GCST90094711 | Inverse variance weighted | 11 | -0.003994787 | 0.0884992459490325 | 0.963996339943155 | -0.177453309 | 0.169463735095725 | 0.996013181582653 | 0.837400097450431 | 1.18466938433229 | 0.989654366060349 |
| ebi-fl187-GCST90094711 | finngen_R11_O15_PREECLAMPS | Pre-eclampsia \|\| id:finngen_R11_O15_PREECLAMPS | Broad bean liking \|\| id:ebi-fl187-GCST90094711 | MR Egger | 11 | 0.184010777878238 | 0.246032021516748 | 0.473601013602553 | -0.298211984 | 0.666233540051065 | 1.2020287783463 | 0.742144000199241 | 1.94689060827116 | 0.954596128018958 |
| ebi-fl187-GCST90094711 | finngen_R11_O15_PREECLAMPS | Pre-eclampsia \|\| id:finngen_R11_O15_PREECLAMPS | Broad bean liking \|\| id:ebi-fl187-GCST90094711 | Weighted median | 11 | 0.107561229309572 | 0.114971762221707 | 0.349507629140705 | -0.117783425 | 0.332905883264118 | 1.11355904111045 | 0.88888854312132 | 1.39501599793888 | 0.783962747510833 |
| ebi-fl187-GCST90094711 | finngen_R11_O15_PREECLAMPS | Pre-eclampsia \|\| id:finngen_R11_O15_PREECLAMPS | Broad bean liking \|\| id:ebi-fl187-GCST90094711 | Weighted mode | 11 | 0.121899079119274 | 0.138896435224487 | 0.400740579320689 | -0.150337934 | 0.394136092159268 | 1.12964009175446 | 0.860417163144593 | 1.48310237354548 | 0.905055003276494 |
| ebi-fl187-GCST90094712 | finngen_R11_O15_PREECLAMPS | Pre-eclampsia \|\| id:finngen_R11_O15_PREECLAMPS | Broccoli liking \|\| id:ebi-fl187-GCST90094712 | Inverse variance weighted | 5 | 0.355292598477378 | 0.284582753796954 | 0.211859543899942 | -0.202489599 | 0.913074795919409 | 1.42659801362577 | 0.81669497702726 | 2.49197307407102 | 0.673947184049608 |
| ebi-fl187-GCST90094712 | finngen_R11_O15_PREECLAMPS | Pre-eclampsia \|\| id:finngen_R11_O15_PREECLAMPS | Broccoli liking \|\| id:ebi-fl187-GCST90094712 | MR Egger | 5 | 0.372182589774771 | 3.66916813347906 | 0.925604467229141 | -6.819386952 | 7.56375213139373 | 1.45089787622531 | 0.00109239040496918 | 1927.06255717662 | 0.970712810886627 |
| ebi-fl187-GCST90094712 | finngen_R11_O15_PREECLAMPS | Pre-eclampsia \|\| id:finngen_R11_O15_PREECLAMPS | Broccoli liking \|\| id:ebi-fl187-GCST90094712 | Weighted median | 5 | 0.491139828530671 | 0.259424928848978 | 0.0583330436404358 | -0.017333032 | 0.999612689074668 | 1.63417784131698 | 0.982816320828567 | 2.71722921206682 | 0.482867972356941 |
| ebi-fl187-GCST90094712 | finngen_R11_O15_PREECLAMPS | Pre-eclampsia \|\| id:finngen_R11_O15_PREECLAMPS | Broccoli liking \|\| id:ebi-fl187-GCST90094712 | Weighted mode | 5 | 0.594415780679599 | 0.36438811026233 | 0.178168349654049 | -0.119784915 | 1.30861647679377 | 1.81197204659818 | 0.88711122013041 | 3.70104968029889 | 0.905055003276494 |
| ebi-fl187-GCST90094714 | finngen_R11_O15_PREECLAMPS | Pre-eclampsia \|\| id:finngen_R11_O15_PREECLAMPS | Brussel sprout liking \|\| id:ebi-fl187-GCST90094714 | Inverse variance weighted | 5 | -0.075257395 | 0.146132610916485 | 0.606557320200181 | -0.361677313 | 0.211162522038951 | 0.927504720192317 | 0.696507085536536 | 1.23511307184527 | 0.941775027862301 |
| ebi-fl187-GCST90094714 | finngen_R11_O15_PREECLAMPS | Pre-eclampsia \|\| id:finngen_R11_O15_PREECLAMPS | Brussel sprout liking \|\| id:ebi-fl187-GCST90094714 | MR Egger | 5 | -0.77119186 | 0.545365285226923 | 0.252250011303245 | -1.840107819 | 0.29772409932951 | 0.462461550418736 | 0.158800303532051 | 1.34679015630808 | 0.954596128018958 |
| ebi-fl187-GCST90094714 | finngen_R11_O15_PREECLAMPS | Pre-eclampsia \|\| id:finngen_R11_O15_PREECLAMPS | Brussel sprout liking \|\| id:ebi-fl187-GCST90094714 | Weighted median | 5 | -0.04655886 | 0.181537717997968 | 0.797588448442747 | -0.402372787 | 0.309255067714162 | 0.954508376971661 | 0.668731404951925 | 1.36240983295015 | 0.950725430543754 |
| ebi-fl187-GCST90094714 | finngen_R11_O15_PREECLAMPS | Pre-eclampsia \|\| id:finngen_R11_O15_PREECLAMPS | Brussel sprout liking \|\| id:ebi-fl187-GCST90094714 | Weighted mode | 5 | -0.073700671 | 0.203048709550969 | 0.734994547655054 | -0.471676142 | 0.324274799908501 | 0.928949713993579 | 0.623955553489802 | 1.38302731068305 | 0.936018697441052 |
| ebi-fl187-GCST90094716 | finngen_R11_O15_PREECLAMPS | Pre-eclampsia \|\| id:finngen_R11_O15_PREECLAMPS | F-burn/spicy liking (derived food-liking factor) \|\| id:ebi-fl187-GCST90094716 | Inverse variance weighted | 13 | -0.19298044 | 0.0759915857827134 | 0.0111011845799446 | -0.341923949 | -0.044036932 | 0.824498100819386 | 0.710402229772774 | 0.956918615630206 | 0.166517768699169 |
| ebi-fl187-GCST90094716 | finngen_R11_O15_PREECLAMPS | Pre-eclampsia \|\| id:finngen_R11_O15_PREECLAMPS | F-burn/spicy liking (derived food-liking factor) \|\| id:ebi-fl187-GCST90094716 | MR Egger | 13 | -0.74707274 | 0.446398222681894 | 0.122384132572522 | -1.622013256 | 0.127867776499983 | 0.47375131827231 | 0.197500679051182 | 1.13640273361586 | 0.954596128018958 |
| ebi-fl187-GCST90094716 | finngen_R11_O15_PREECLAMPS | Pre-eclampsia \|\| id:finngen_R11_O15_PREECLAMPS | F-burn/spicy liking (derived food-liking factor) \|\| id:ebi-fl187-GCST90094716 | Weighted median | 13 | -0.214305384 | 0.0984997566341208 | 0.0295781076907929 | -0.407364907 | -0.021245861 | 0.80710187131343 | 0.665401336327432 | 0.978978242323657 | 0.367261503827345 |
| ebi-fl187-GCST90094716 | finngen_R11_O15_PREECLAMPS | Pre-eclampsia \|\| id:finngen_R11_O15_PREECLAMPS | F-burn/spicy liking (derived food-liking factor) \|\| id:ebi-fl187-GCST90094716 | Weighted mode | 13 | -0.216728666 | 0.138905243811051 | 0.144670179567962 | -0.488982943 | 0.0555256123455871 | 0.805148404189498 | 0.61324978686488 | 1.05709609143612 | 0.905055003276494 |
| ebi-fl187-GCST90094717 | finngen_R11_O15_PREECLAMPS | Pre-eclampsia \|\| id:finngen_R11_O15_PREECLAMPS | Adding butter to bread liking \|\| id:ebi-fl187-GCST90094717 | Inverse variance weighted | 3 | -0.280693081 | 0.14416500847788 | 0.0515315666737004 | -0.563256498 | 0.00187033550978127 | 0.755260103506394 | 0.569351948320181 | 1.0018720856782 | 0.366852117259508 |
| ebi-fl187-GCST90094717 | finngen_R11_O15_PREECLAMPS | Pre-eclampsia \|\| id:finngen_R11_O15_PREECLAMPS | Adding butter to bread liking \|\| id:ebi-fl187-GCST90094717 | MR Egger | 3 | -0.252082756 | 0.437870302838234 | 0.667454276551517 | -1.11030855 | 0.606143037250406 | 0.777180418821937 | 0.329457291384681 | 1.83334659512813 | 0.954596128018958 |
| ebi-fl187-GCST90094717 | finngen_R11_O15_PREECLAMPS | Pre-eclampsia \|\| id:finngen_R11_O15_PREECLAMPS | Adding butter to bread liking \|\| id:ebi-fl187-GCST90094717 | Weighted median | 3 | -0.265626139 | 0.161491083177212 | 0.100003901873457 | -0.582148662 | 0.0508963838967261 | 0.766725722669583 | 0.558696625662813 | 1.05221386133445 | 0.529128962601009 |
| ebi-fl187-GCST90094717 | finngen_R11_O15_PREECLAMPS | Pre-eclampsia \|\| id:finngen_R11_O15_PREECLAMPS | Adding butter to bread liking \|\| id:ebi-fl187-GCST90094717 | Weighted mode | 3 | -0.266298276 | 0.170029380993181 | 0.257800849178954 | -0.599555862 | 0.066959311238283 | 0.766210551571638 | 0.549055438193368 | 1.06925197075082 | 0.905055003276494 |
| ebi-fl187-GCST90094718 | finngen_R11_O15_PREECLAMPS | Pre-eclampsia \|\| id:finngen_R11_O15_PREECLAMPS | Butternut squash liking \|\| id:ebi-fl187-GCST90094718 | Inverse variance weighted | 2 | -0.056285616 | 0.202057955804299 | 0.78058117870117 | -0.452319209 | 0.339747977838668 | 0.945269113784717 | 0.636151072218524 | 1.40459355724926 | 0.945152512750355 |
| ebi-fl187-GCST90094719 | finngen_R11_O15_PREECLAMPS | Pre-eclampsia \|\| id:finngen_R11_O15_PREECLAMPS | Cabbage liking \|\| id:ebi-fl187-GCST90094719 | Inverse variance weighted | 5 | 0.185035038727098 | 0.191665884948996 | 0.334342428933287 | -0.190630096 | 0.56070017322713 | 1.20326060010887 | 0.826438234614142 | 1.75189870353753 | 0.802417492460712 |
| ebi-fl187-GCST90094719 | finngen_R11_O15_PREECLAMPS | Pre-eclampsia \|\| id:finngen_R11_O15_PREECLAMPS | Cabbage liking \|\| id:ebi-fl187-GCST90094719 | MR Egger | 5 | -0.380094079 | 1.45523388048255 | 0.810849581898109 | -3.232352484 | 2.47216432712757 | 0.683797075502175 | 0.039464549764448 | 11.8480622040835 | 0.954596128018958 |
| ebi-fl187-GCST90094719 | finngen_R11_O15_PREECLAMPS | Pre-eclampsia \|\| id:finngen_R11_O15_PREECLAMPS | Cabbage liking \|\| id:ebi-fl187-GCST90094719 | Weighted median | 5 | 0.237867667803068 | 0.223716120123124 | 0.287665547134863 | -0.200615928 | 0.676351263244392 | 1.26854131285836 | 0.818226629446648 | 1.966688695 | 0.715820742437076 |
| ebi-fl187-GCST90094719 | finngen_R11_O15_PREECLAMPS | Pre-eclampsia \|\| id:finngen_R11_O15_PREECLAMPS | Cabbage liking \|\| id:ebi-fl187-GCST90094719 | Weighted mode | 5 | 0.243421657392142 | 0.273706168674134 | 0.424066056796755 | -0.293042433 | 0.779885747993445 | 1.27560637959563 | 0.745990485255312 | 2.18122304215203 | 0.905055003276494 |
| ebi-fl187-GCST90094720 | finngen_R11_O15_PREECLAMPS | Pre-eclampsia \|\| id:finngen_R11_O15_PREECLAMPS | F-caffeinated/sweet liking (derived food-liking factor) \|\| id:ebi-fl187-GCST90094720 | Inverse variance weighted | 7 | 0.054334381765103 | 0.084565104222404 | 0.520538593484431 | -0.111413223 | 0.220081986041015 | 1.05583759597345 | 0.894569016488323 | 1.24617889567332 | 0.894908414040245 |
| ebi-fl187-GCST90094720 | finngen_R11_O15_PREECLAMPS | Pre-eclampsia \|\| id:finngen_R11_O15_PREECLAMPS | F-caffeinated/sweet liking (derived food-liking factor) \|\| id:ebi-fl187-GCST90094720 | MR Egger | 7 | -0.789318082 | 0.574535967546262 | 0.227885739538019 | -1.915408578 | 0.336772414369542 | 0.454154385753532 | 0.147281644190888 | 1.40042031192865 | 0.954596128018958 |
| ebi-fl187-GCST90094720 | finngen_R11_O15_PREECLAMPS | Pre-eclampsia \|\| id:finngen_R11_O15_PREECLAMPS | F-caffeinated/sweet liking (derived food-liking factor) \|\| id:ebi-fl187-GCST90094720 | Weighted median | 7 | 0.0629173620145585 | 0.107856159190193 | 0.559661007340774 | -0.14848071 | 0.274315434027336 | 1.06493883119532 | 0.86201663531255 | 1.31562973117852 | 0.881409801621768 |
| ebi-fl187-GCST90094720 | finngen_R11_O15_PREECLAMPS | Pre-eclampsia \|\| id:finngen_R11_O15_PREECLAMPS | F-caffeinated/sweet liking (derived food-liking factor) \|\| id:ebi-fl187-GCST90094720 | Weighted mode | 7 | 0.0781724458865363 | 0.139206778067936 | 0.594755857150487 | -0.194672839 | 0.351017730899691 | 1.08130910990132 | 0.823103901401939 | 1.42051251265377 | 0.905055003276494 |
| ebi-fl187-GCST90094721 | finngen_R11_O15_PREECLAMPS | Pre-eclampsia \|\| id:finngen_R11_O15_PREECLAMPS | Cake liking \|\| id:ebi-fl187-GCST90094721 | Inverse variance weighted (multiplicative random effects) | 13 | -0.132026196 | 0.172920740310205 | 0.445161086700811 | -0.470950847 | 0.206898454590038 | 0.87631803838508 | 0.624408268930812 | 1.2298576790375 | 0.887682351940796 |
| ebi-fl187-GCST90094721 | finngen_R11_O15_PREECLAMPS | Pre-eclampsia \|\| id:finngen_R11_O15_PREECLAMPS | Cake liking \|\| id:ebi-fl187-GCST90094721 | MR Egger | 13 | -0.307130179 | 0.744087979228711 | 0.687712913114213 | -1.765542618 | 1.1512822602185 | 0.735554840829463 | 0.171093922589045 | 3.1622451322669 | 0.954596128018958 |
| ebi-fl187-GCST90094721 | finngen_R11_O15_PREECLAMPS | Pre-eclampsia \|\| id:finngen_R11_O15_PREECLAMPS | Cake liking \|\| id:ebi-fl187-GCST90094721 | Weighted median | 13 | -0.140094454 | 0.154020747543324 | 0.363043151531862 | -0.441975119 | 0.161786211316624 | 0.869276125028424 | 0.642765627873966 | 1.17560888257796 | 0.783962747510833 |
| ebi-fl187-GCST90094721 | finngen_R11_O15_PREECLAMPS | Pre-eclampsia \|\| id:finngen_R11_O15_PREECLAMPS | Cake liking \|\| id:ebi-fl187-GCST90094721 | Weighted mode | 13 | -0.064611925 | 0.170684733966126 | 0.711637545700149 | -0.399154003 | 0.269930153851307 | 0.93743118666492 | 0.670887374530455 | 1.30987295795668 | 0.936018697441052 |
| ebi-fl187-GCST90094722 | finngen_R11_O15_PREECLAMPS | Pre-eclampsia \|\| id:finngen_R11_O15_PREECLAMPS | F-cake/biscuits liking (derived food-liking factor) \|\| id:ebi-fl187-GCST90094722 | Inverse variance weighted (multiplicative random effects) | 19 | 0.0300143743695274 | 0.0786015366080855 | 0.702569065509363 | -0.124044637 | 0.184073386121375 | 1.03046934619423 | 0.883340409999112 | 1.20210403761221 | 0.941775027862301 |
| ebi-fl187-GCST90094722 | finngen_R11_O15_PREECLAMPS | Pre-eclampsia \|\| id:finngen_R11_O15_PREECLAMPS | F-cake/biscuits liking (derived food-liking factor) \|\| id:ebi-fl187-GCST90094722 | MR Egger | 19 | -0.113849662 | 0.323850656923923 | 0.729495423760148 | -0.74859695 | 0.520897625080835 | 0.892392105805355 | 0.473029771759954 | 1.68353815773744 | 0.954596128018958 |
| ebi-fl187-GCST90094722 | finngen_R11_O15_PREECLAMPS | Pre-eclampsia \|\| id:finngen_R11_O15_PREECLAMPS | F-cake/biscuits liking (derived food-liking factor) \|\| id:ebi-fl187-GCST90094722 | Weighted median | 19 | -0.047073021 | 0.0781543945845193 | 0.546969105147701 | -0.200255634 | 0.106109592595052 | 0.954017731917844 | 0.818521484265607 | 1.11194373062825 | 0.881409801621768 |
| ebi-fl187-GCST90094722 | finngen_R11_O15_PREECLAMPS | Pre-eclampsia \|\| id:finngen_R11_O15_PREECLAMPS | F-cake/biscuits liking (derived food-liking factor) \|\| id:ebi-fl187-GCST90094722 | Weighted mode | 19 | -0.06047636 | 0.0984455797042709 | 0.546695824219993 | -0.253429696 | 0.13247697669608 | 0.94131602191392 | 0.776134308551692 | 1.1416527311688 | 0.905055003276494 |
| ebi-fl187-GCST90094723 | finngen_R11_O15_PREECLAMPS | Pre-eclampsia \|\| id:finngen_R11_O15_PREECLAMPS | Cake icing liking \|\| id:ebi-fl187-GCST90094723 | Inverse variance weighted | 9 | 0.014335468589089 | 0.109807001215746 | 0.896130112572324 | -0.200886254 | 0.22955719097195 | 1.01443871418699 | 0.818005471281333 | 1.25804281385721 | 0.953358127900067 |
| ebi-fl187-GCST90094723 | finngen_R11_O15_PREECLAMPS | Pre-eclampsia \|\| id:finngen_R11_O15_PREECLAMPS | Cake icing liking \|\| id:ebi-fl187-GCST90094723 | MR Egger | 9 | -0.656174959 | 0.792368322397481 | 0.434924843980426 | -2.209216871 | 0.896866952443683 | 0.518832097636306 | 0.109786591883765 | 2.45190911675888 | 0.954596128018958 |
| ebi-fl187-GCST90094723 | finngen_R11_O15_PREECLAMPS | Pre-eclampsia \|\| id:finngen_R11_O15_PREECLAMPS | Cake icing liking \|\| id:ebi-fl187-GCST90094723 | Weighted median | 9 | -0.038477936 | 0.141754680649644 | 0.786052394694744 | -0.31631711 | 0.239361238182772 | 0.962252935759708 | 0.728828294804522 | 1.2704373841942 | 0.944530700076749 |
| ebi-fl187-GCST90094723 | finngen_R11_O15_PREECLAMPS | Pre-eclampsia \|\| id:finngen_R11_O15_PREECLAMPS | Cake icing liking \|\| id:ebi-fl187-GCST90094723 | Weighted mode | 9 | -0.058134972 | 0.215821553924135 | 0.794465306975019 | -0.481145218 | 0.364875273398966 | 0.943522589435322 | 0.618075155557715 | 1.44033434893761 | 0.962401062920957 |
| ebi-fl187-GCST90094724 | finngen_R11_O15_PREECLAMPS | Pre-eclampsia \|\| id:finngen_R11_O15_PREECLAMPS | Capers liking \|\| id:ebi-fl187-GCST90094724 | Inverse variance weighted | 12 | -0.130900641 | 0.075303360581975 | 0.0821560731277661 | -0.278495228 | 0.0166939455017394 | 0.877304937992456 | 0.756921879787937 | 1.01683406805677 | 0.482089972461316 |
| ebi-fl187-GCST90094724 | finngen_R11_O15_PREECLAMPS | Pre-eclampsia \|\| id:finngen_R11_O15_PREECLAMPS | Capers liking \|\| id:ebi-fl187-GCST90094724 | MR Egger | 12 | -0.434235445 | 0.357309410362576 | 0.252167734788825 | -1.134561889 | 0.26609099920995 | 0.647759725676017 | 0.321562970620704 | 1.30485379394879 | 0.954596128018958 |
| ebi-fl187-GCST90094724 | finngen_R11_O15_PREECLAMPS | Pre-eclampsia \|\| id:finngen_R11_O15_PREECLAMPS | Capers liking \|\| id:ebi-fl187-GCST90094724 | Weighted median | 12 | -0.118886231 | 0.102250066575453 | 0.244950928467919 | -0.319296362 | 0.0815238991874649 | 0.887908811246725 | 0.726660163086367 | 1.08493914643821 | 0.672512525447174 |
| ebi-fl187-GCST90094724 | finngen_R11_O15_PREECLAMPS | Pre-eclampsia \|\| id:finngen_R11_O15_PREECLAMPS | Capers liking \|\| id:ebi-fl187-GCST90094724 | Weighted mode | 12 | -0.151788014 | 0.137592718951178 | 0.293503218784925 | -0.421469743 | 0.117893714990902 | 0.859170393401001 | 0.656081838966124 | 1.12512452114216 | 0.905055003276494 |
| ebi-fl187-GCST90094725 | finngen_R11_O15_PREECLAMPS | Pre-eclampsia \|\| id:finngen_R11_O15_PREECLAMPS | F-carbohydrate liking (derived food-liking factor) \|\| id:ebi-fl187-GCST90094725 | Inverse variance weighted | 3 | -0.191981734 | 0.0982706653683377 | 0.0507483449735411 | -0.384592238 | 0.000628769794462886 | 0.825321943414348 | 0.680728154387876 | 1.00062896751163 | 0.366852117259508 |
| ebi-fl187-GCST90094725 | finngen_R11_O15_PREECLAMPS | Pre-eclampsia \|\| id:finngen_R11_O15_PREECLAMPS | F-carbohydrate liking (derived food-liking factor) \|\| id:ebi-fl187-GCST90094725 | MR Egger | 3 | -0.397597337 | 0.339372606984018 | 0.449807750342295 | -1.062767647 | 0.267572972490289 | 0.67193253543386 | 0.345498268664918 | 1.30678898600345 | 0.954596128018958 |
| ebi-fl187-GCST90094725 | finngen_R11_O15_PREECLAMPS | Pre-eclampsia \|\| id:finngen_R11_O15_PREECLAMPS | F-carbohydrate liking (derived food-liking factor) \|\| id:ebi-fl187-GCST90094725 | Weighted median | 3 | -0.231520125 | 0.117861163680956 | 0.0494899611208775 | -0.462528006 | -0.000512244 | 0.793326729492712 | 0.62968977217108 | 0.9994878868012 | 0.447765620977764 |
| ebi-fl187-GCST90094725 | finngen_R11_O15_PREECLAMPS | Pre-eclampsia \|\| id:finngen_R11_O15_PREECLAMPS | F-carbohydrate liking (derived food-liking factor) \|\| id:ebi-fl187-GCST90094725 | Weighted mode | 3 | -0.240184742 | 0.129680055729138 | 0.205202642760632 | -0.494357651 | 0.0139881670872743 | 0.786482551173515 | 0.609962590117916 | 1.01408645927093 | 0.905055003276494 |
| ebi-fl187-GCST90094726 | finngen_R11_O15_PREECLAMPS | Pre-eclampsia \|\| id:finngen_R11_O15_PREECLAMPS | F-capsicum liking (derived food-liking factor) \|\| id:ebi-fl187-GCST90094726 | Inverse variance weighted | 19 | -0.191129381 | 0.0511653188602207 | 0.000187323197654419 | -0.291413406 | -0.090845356 | 0.826025709498053 | 0.747206714710271 | 0.913158914820955 | 0.0185764371202363 |
| ebi-fl187-GCST90094726 | finngen_R11_O15_PREECLAMPS | Pre-eclampsia \|\| id:finngen_R11_O15_PREECLAMPS | F-capsicum liking (derived food-liking factor) \|\| id:ebi-fl187-GCST90094726 | MR Egger | 19 | -0.486588549 | 0.230995104240914 | 0.0503234766109786 | -0.939338953 | -0.033838144 | 0.614719908305325 | 0.39088614413216 | 0.966727962450222 | 0.954596128018958 |
| ebi-fl187-GCST90094726 | finngen_R11_O15_PREECLAMPS | Pre-eclampsia \|\| id:finngen_R11_O15_PREECLAMPS | F-capsicum liking (derived food-liking factor) \|\| id:ebi-fl187-GCST90094726 | Weighted median | 19 | -0.218415095 | 0.0681032813306956 | 0.00134073112573102 | -0.351897527 | -0.084932664 | 0.803791722244004 | 0.703352193099382 | 0.918574135527992 | 0.199768937733922 |
| ebi-fl187-GCST90094726 | finngen_R11_O15_PREECLAMPS | Pre-eclampsia \|\| id:finngen_R11_O15_PREECLAMPS | F-capsicum liking (derived food-liking factor) \|\| id:ebi-fl187-GCST90094726 | Weighted mode | 19 | -0.216780735 | 0.0980827459542872 | 0.0402822012590307 | -0.409022917 | -0.024538553 | 0.805106481915093 | 0.664299008599574 | 0.975760070134949 | 0.905055003276494 |
| ebi-fl187-GCST90094727 | finngen_R11_O15_PREECLAMPS | Pre-eclampsia \|\| id:finngen_R11_O15_PREECLAMPS | Cauliflower liking \|\| id:ebi-fl187-GCST90094727 | Inverse variance weighted | 4 | 0.0106369352916674 | 0.247090975692782 | 0.965662745064947 | -0.473661377 | 0.49493524764952 | 1.01069370860734 | 0.622718083493951 | 1.64039201637923 | 0.989654366060349 |
| ebi-fl187-GCST90094727 | finngen_R11_O15_PREECLAMPS | Pre-eclampsia \|\| id:finngen_R11_O15_PREECLAMPS | Cauliflower liking \|\| id:ebi-fl187-GCST90094727 | MR Egger | 4 | 0.00782820555223798 | 1.08693020016866 | 0.994907395663276 | -2.122554987 | 2.1382113978828 | 1.00785892606316 | 0.119725340734858 | 8.48424910391121 | 0.994907395663276 |
| ebi-fl187-GCST90094727 | finngen_R11_O15_PREECLAMPS | Pre-eclampsia \|\| id:finngen_R11_O15_PREECLAMPS | Cauliflower liking \|\| id:ebi-fl187-GCST90094727 | Weighted median | 4 | 0.0224728029536029 | 0.290431165193669 | 0.938323334090417 | -0.546772281 | 0.591717886733194 | 1.02272721862658 | 0.578815050962924 | 1.80709012659496 | 0.991561537443065 |
| ebi-fl187-GCST90094727 | finngen_R11_O15_PREECLAMPS | Pre-eclampsia \|\| id:finngen_R11_O15_PREECLAMPS | Cauliflower liking \|\| id:ebi-fl187-GCST90094727 | Weighted mode | 4 | 0.0277472571856747 | 0.328693359897334 | 0.938042798220652 | -0.616491728 | 0.671986242584449 | 1.02813579764555 | 0.539835007265037 | 1.95812276746489 | 0.981296058194618 |
| ebi-fl187-GCST90094729 | finngen_R11_O15_PREECLAMPS | Pre-eclampsia \|\| id:finngen_R11_O15_PREECLAMPS | F-cheese liking (derived food-liking factor) \|\| id:ebi-fl187-GCST90094729 | Inverse variance weighted | 15 | -0.176648737 | 0.0576012442499462 | 0.00216398234881876 | -0.289547176 | -0.063750299 | 0.838074116896163 | 0.748602475889539 | 0.938239249845765 | 0.054263705841589 |
| ebi-fl187-GCST90094729 | finngen_R11_O15_PREECLAMPS | Pre-eclampsia \|\| id:finngen_R11_O15_PREECLAMPS | F-cheese liking (derived food-liking factor) \|\| id:ebi-fl187-GCST90094729 | MR Egger | 15 | 0.0799094363962143 | 0.306835390078466 | 0.798610908029199 | -0.521487928 | 0.681306800950007 | 1.08318896573648 | 0.593636601893822 | 1.97645888368439 | 0.954596128018958 |
| ebi-fl187-GCST90094729 | finngen_R11_O15_PREECLAMPS | Pre-eclampsia \|\| id:finngen_R11_O15_PREECLAMPS | F-cheese liking (derived food-liking factor) \|\| id:ebi-fl187-GCST90094729 | Weighted median | 15 | -0.190179344 | 0.0703198137833216 | 0.00684090045311993 | -0.328006179 | -0.052352509 | 0.826810837167533 | 0.720358568658646 | 0.94899427896112 | 0.313340475930495 |
| ebi-fl187-GCST90094729 | finngen_R11_O15_PREECLAMPS | Pre-eclampsia \|\| id:finngen_R11_O15_PREECLAMPS | F-cheese liking (derived food-liking factor) \|\| id:ebi-fl187-GCST90094729 | Weighted mode | 15 | -0.183159858 | 0.108075357816632 | 0.112242229910367 | -0.39498756 | 0.0286678429896812 | 0.832635041449671 | 0.673688420115452 | 1.02908272066052 | 0.905055003276494 |
| ebi-fl187-GCST90094730 | finngen_R11_O15_PREECLAMPS | Pre-eclampsia \|\| id:finngen_R11_O15_PREECLAMPS | Cheesecake liking \|\| id:ebi-fl187-GCST90094730 | Inverse variance weighted (multiplicative random effects) | 7 | 0.0660131614266523 | 0.203077045052813 | 0.74513216915658 | -0.332017847 | 0.464044169730167 | 1.0682407766461 | 0.717474518060805 | 1.59049322054495 | 0.941775027862301 |
| ebi-fl187-GCST90094730 | finngen_R11_O15_PREECLAMPS | Pre-eclampsia \|\| id:finngen_R11_O15_PREECLAMPS | Cheesecake liking \|\| id:ebi-fl187-GCST90094730 | MR Egger | 7 | 1.02868178510333 | 0.955935981498597 | 0.331043004194726 | -0.844952739 | 2.90231630884058 | 2.79737586061296 | 0.429577660158042 | 18.2162910954475 | 0.954596128018958 |
| ebi-fl187-GCST90094730 | finngen_R11_O15_PREECLAMPS | Pre-eclampsia \|\| id:finngen_R11_O15_PREECLAMPS | Cheesecake liking \|\| id:ebi-fl187-GCST90094730 | Weighted median | 7 | 0.0904554887852402 | 0.163864257593771 | 0.580938240938698 | -0.230718456 | 0.411629433669032 | 1.09467278134196 | 0.793962970002857 | 1.50927504617329 | 0.881409801621768 |
| ebi-fl187-GCST90094730 | finngen_R11_O15_PREECLAMPS | Pre-eclampsia \|\| id:finngen_R11_O15_PREECLAMPS | Cheesecake liking \|\| id:ebi-fl187-GCST90094730 | Weighted mode | 7 | 0.10726408630003 | 0.24480946287872 | 0.676609551377777 | -0.372562461 | 0.587090633542321 | 1.11322820398108 | 0.68896661670983 | 1.79874757946491 | 0.936018697441052 |
| ebi-fl187-GCST90094731 | finngen_R11_O15_PREECLAMPS | Pre-eclampsia \|\| id:finngen_R11_O15_PREECLAMPS | Cherry liking \|\| id:ebi-fl187-GCST90094731 | Inverse variance weighted (multiplicative random effects) | 6 | -0.241668303 | 0.227041771777288 | 0.287137479709357 | -0.686670176 | 0.203333569653742 | 0.785316621499252 | 0.503249013001407 | 1.22548118341024 | 0.75876791114856 |
| ebi-fl187-GCST90094731 | finngen_R11_O15_PREECLAMPS | Pre-eclampsia \|\| id:finngen_R11_O15_PREECLAMPS | Cherry liking \|\| id:ebi-fl187-GCST90094731 | MR Egger | 6 | -0.065563495 | 0.887440095998504 | 0.944653406567523 | -1.804946083 | 1.67381909325221 | 0.936539579380221 | 0.164483324775316 | 5.33249425097535 | 0.970712810886627 |
| ebi-fl187-GCST90094731 | finngen_R11_O15_PREECLAMPS | Pre-eclampsia \|\| id:finngen_R11_O15_PREECLAMPS | Cherry liking \|\| id:ebi-fl187-GCST90094731 | Weighted median | 6 | -0.045436892 | 0.186793812397338 | 0.80781464828096 | -0.411552765 | 0.320678979850017 | 0.955579904976978 | 0.662620557065646 | 1.37806312384804 | 0.955272877729072 |
| ebi-fl187-GCST90094731 | finngen_R11_O15_PREECLAMPS | Pre-eclampsia \|\| id:finngen_R11_O15_PREECLAMPS | Cherry liking \|\| id:ebi-fl187-GCST90094731 | Weighted mode | 6 | -0.009629487 | 0.19471520208663 | 0.962472056714683 | -0.391271283 | 0.372012309560233 | 0.990416728514506 | 0.676196690745476 | 1.45065083805712 | 0.981296058194618 |
| ebi-fl187-GCST90094732 | finngen_R11_O15_PREECLAMPS | Pre-eclampsia \|\| id:finngen_R11_O15_PREECLAMPS | Chicken liking \|\| id:ebi-fl187-GCST90094732 | Inverse variance weighted | 2 | -0.157108021 | 0.373819054980084 | 0.674282192485488 | -0.889793368 | 0.575577327258648 | 0.854611738242267 | 0.410740616031152 | 1.77815680903122 | 0.941775027862301 |
| ebi-fl187-GCST90094733 | finngen_R11_O15_PREECLAMPS | Pre-eclampsia \|\| id:finngen_R11_O15_PREECLAMPS | Chilli pepper liking \|\| id:ebi-fl187-GCST90094733 | Inverse variance weighted | 13 | -0.271826343 | 0.0820457591189633 | 0.000922658175447595 | -0.432636031 | -0.111016655 | 0.761986573754748 | 0.648796590638026 | 0.894923843560144 | 0.0380596497372133 |
| ebi-fl187-GCST90094733 | finngen_R11_O15_PREECLAMPS | Pre-eclampsia \|\| id:finngen_R11_O15_PREECLAMPS | Chilli pepper liking \|\| id:ebi-fl187-GCST90094733 | MR Egger | 13 | -0.275935156 | 0.62298558018479 | 0.666404921390612 | -1.496986893 | 0.945116581451621 | 0.758862137041904 | 0.22380348912102 | 2.57311333839129 | 0.954596128018958 |
| ebi-fl187-GCST90094733 | finngen_R11_O15_PREECLAMPS | Pre-eclampsia \|\| id:finngen_R11_O15_PREECLAMPS | Chilli pepper liking \|\| id:ebi-fl187-GCST90094733 | Weighted median | 13 | -0.267370208 | 0.104505825032822 | 0.0105147810714931 | -0.472201625 | -0.06253879 | 0.765389665970773 | 0.623627761529605 | 0.939376623224688 | 0.313340475930495 |
| ebi-fl187-GCST90094733 | finngen_R11_O15_PREECLAMPS | Pre-eclampsia \|\| id:finngen_R11_O15_PREECLAMPS | Chilli pepper liking \|\| id:ebi-fl187-GCST90094733 | Weighted mode | 13 | -0.249063352 | 0.161457037252023 | 0.148877160927413 | -0.565519145 | 0.0673924410766055 | 0.779530587047436 | 0.568065162033357 | 1.06971519599513 | 0.905055003276494 |
| ebi-fl187-GCST90094734 | finngen_R11_O15_PREECLAMPS | Pre-eclampsia \|\| id:finngen_R11_O15_PREECLAMPS | Chips liking \|\| id:ebi-fl187-GCST90094734 | Inverse variance weighted | 3 | -0.511020665 | 0.189148220685051 | 0.0068987163889247 | -0.881751178 | -0.140290153 | 0.59988298659045 | 0.414057188726111 | 0.869106025444996 | 0.126476467130286 |
| ebi-fl187-GCST90094734 | finngen_R11_O15_PREECLAMPS | Pre-eclampsia \|\| id:finngen_R11_O15_PREECLAMPS | Chips liking \|\| id:ebi-fl187-GCST90094734 | MR Egger | 3 | -0.18633924 | 0.620073014670514 | 0.814153981454997 | -1.401682349 | 1.02900386877591 | 0.82999198075569 | 0.246182450627619 | 2.79827699481624 | 0.954596128018958 |
| ebi-fl187-GCST90094734 | finngen_R11_O15_PREECLAMPS | Pre-eclampsia \|\| id:finngen_R11_O15_PREECLAMPS | Chips liking \|\| id:ebi-fl187-GCST90094734 | Weighted median | 3 | -0.457628714 | 0.214524172799094 | 0.0329061260590618 | -0.878096092 | -0.037161335 | 0.632782376082327 | 0.415573372306544 | 0.963520673276039 | 0.377154829446169 |
| ebi-fl187-GCST90094734 | finngen_R11_O15_PREECLAMPS | Pre-eclampsia \|\| id:finngen_R11_O15_PREECLAMPS | Chips liking \|\| id:ebi-fl187-GCST90094734 | Weighted mode | 3 | -0.455008252 | 0.2394453239258 | 0.197780316940403 | -0.924321087 | 0.0143045829222586 | 0.634442732522766 | 0.396800720869017 | 1.01440738305469 | 0.905055003276494 |
| ebi-fl187-GCST90094735 | finngen_R11_O15_PREECLAMPS | Pre-eclampsia \|\| id:finngen_R11_O15_PREECLAMPS | F-chocolate/coffee liking (derived food-liking factor) \|\| id:ebi-fl187-GCST90094735 | Inverse variance weighted (multiplicative random effects) | 12 | -0.024057975 | 0.0609017307988309 | 0.692821186025958 | -0.143425367 | 0.0953094178007932 | 0.976229111659294 | 0.866385458780432 | 1.09999916179643 | 0.941775027862301 |
| ebi-fl187-GCST90094735 | finngen_R11_O15_PREECLAMPS | Pre-eclampsia \|\| id:finngen_R11_O15_PREECLAMPS | F-chocolate/coffee liking (derived food-liking factor) \|\| id:ebi-fl187-GCST90094735 | MR Egger | 12 | -0.069921687 | 0.18462364625026 | 0.71280835243678 | -0.431784033 | 0.29194065995475 | 0.932466841606087 | 0.649349599402972 | 1.33902355756324 | 0.954596128018958 |
| ebi-fl187-GCST90094735 | finngen_R11_O15_PREECLAMPS | Pre-eclampsia \|\| id:finngen_R11_O15_PREECLAMPS | F-chocolate/coffee liking (derived food-liking factor) \|\| id:ebi-fl187-GCST90094735 | Weighted median | 12 | -0.075287599 | 0.062281854163704 | 0.226731711464804 | -0.197360033 | 0.0467848354485995 | 0.927476706861131 | 0.820895030906052 | 1.04789651463783 | 0.649149713120124 |
| ebi-fl187-GCST90094735 | finngen_R11_O15_PREECLAMPS | Pre-eclampsia \|\| id:finngen_R11_O15_PREECLAMPS | F-chocolate/coffee liking (derived food-liking factor) \|\| id:ebi-fl187-GCST90094735 | Weighted mode | 12 | -0.124520375 | 0.0869224280628626 | 0.179788113476769 | -0.294888334 | 0.0458475839425251 | 0.882920271629003 | 0.744614730924642 | 1.04691483216483 | 0.905055003276494 |
| ebi-fl187-GCST90094737 | finngen_R11_O15_PREECLAMPS | Pre-eclampsia \|\| id:finngen_R11_O15_PREECLAMPS | F-coffee/alcohol liking (derived food-liking factor) \|\| id:ebi-fl187-GCST90094737 | Inverse variance weighted | 13 | -0.093653797 | 0.0592082777011246 | 0.11370239636977 | -0.209702021 | 0.0223944273525643 | 0.910597959475002 | 0.810825818852053 | 1.02264706490721 | 0.512568357226114 |
| ebi-fl187-GCST90094737 | finngen_R11_O15_PREECLAMPS | Pre-eclampsia \|\| id:finngen_R11_O15_PREECLAMPS | F-coffee/alcohol liking (derived food-liking factor) \|\| id:ebi-fl187-GCST90094737 | MR Egger | 13 | 0.393501153483356 | 0.343445576478876 | 0.276215274694477 | -0.279652176 | 1.06665448338195 | 1.48216099337914 | 0.756046666589112 | 2.90564234639837 | 0.954596128018958 |
| ebi-fl187-GCST90094737 | finngen_R11_O15_PREECLAMPS | Pre-eclampsia \|\| id:finngen_R11_O15_PREECLAMPS | F-coffee/alcohol liking (derived food-liking factor) \|\| id:ebi-fl187-GCST90094737 | Weighted median | 13 | -0.130791291 | 0.0756947463731882 | 0.0840100909363854 | -0.279152993 | 0.0175704123667499 | 0.877400877159438 | 0.75642416644451 | 1.01772568010177 | 0.529128962601009 |
| ebi-fl187-GCST90094737 | finngen_R11_O15_PREECLAMPS | Pre-eclampsia \|\| id:finngen_R11_O15_PREECLAMPS | F-coffee/alcohol liking (derived food-liking factor) \|\| id:ebi-fl187-GCST90094737 | Weighted mode | 13 | -0.128449746 | 0.117056800912785 | 0.294029486743692 | -0.357881076 | 0.10098158405701 | 0.879457757813716 | 0.699156216851381 | 1.10625626882345 | 0.905055003276494 |
| ebi-fl187-GCST90094738 | finngen_R11_O15_PREECLAMPS | Pre-eclampsia \|\| id:finngen_R11_O15_PREECLAMPS | Coffee difference liking \|\| id:ebi-fl187-GCST90094738 | Inverse variance weighted | 4 | 0.0435986546312813 | 0.114014725365369 | 0.702168439338521 | -0.179870207 | 0.267067516347405 | 1.04456304021055 | 0.835378630602825 | 1.30612862838799 | 0.941775027862301 |
| ebi-fl187-GCST90094738 | finngen_R11_O15_PREECLAMPS | Pre-eclampsia \|\| id:finngen_R11_O15_PREECLAMPS | Coffee difference liking \|\| id:ebi-fl187-GCST90094738 | MR Egger | 4 | -1.838543046 | 0.935325248779322 | 0.188256071051445 | -3.671780534 | -0.005305558 | 0.159048984426784 | 0.0254311485944772 | 0.99470849117231 | 0.954596128018958 |
| ebi-fl187-GCST90094738 | finngen_R11_O15_PREECLAMPS | Pre-eclampsia \|\| id:finngen_R11_O15_PREECLAMPS | Coffee difference liking \|\| id:ebi-fl187-GCST90094738 | Weighted median | 4 | -0.016253422 | 0.106195274293866 | 0.878357095369574 | -0.22439616 | 0.191889315214621 | 0.98387795174569 | 0.798998540386958 | 1.21153641089567 | 0.988450091947753 |
| ebi-fl187-GCST90094738 | finngen_R11_O15_PREECLAMPS | Pre-eclampsia \|\| id:finngen_R11_O15_PREECLAMPS | Coffee difference liking \|\| id:ebi-fl187-GCST90094738 | Weighted mode | 4 | -0.055700301 | 0.136530457939521 | 0.710665487431021 | -0.323299999 | 0.211899396297989 | 0.94582255524285 | 0.723756695637633 | 1.23602353028041 | 0.936018697441052 |
| ebi-fl187-GCST90094739 | finngen_R11_O15_PREECLAMPS | Pre-eclampsia \|\| id:finngen_R11_O15_PREECLAMPS | Coffee max liking \|\| id:ebi-fl187-GCST90094739 | Inverse variance weighted (multiplicative random effects) | 5 | 0.118174274350089 | 0.195851112082131 | 0.546250387557328 | -0.265693905 | 0.502042454031065 | 1.12544022964224 | 0.766673766341163 | 1.6520921493661 | 0.910417312595546 |
| ebi-fl187-GCST90094739 | finngen_R11_O15_PREECLAMPS | Pre-eclampsia \|\| id:finngen_R11_O15_PREECLAMPS | Coffee max liking \|\| id:ebi-fl187-GCST90094739 | MR Egger | 5 | -0.274462809 | 0.553984235295234 | 0.654311736592462 | -1.36027191 | 0.811346292315811 | 0.759980268253724 | 0.256590997798298 | 2.25093636601009 | 0.954596128018958 |
| ebi-fl187-GCST90094739 | finngen_R11_O15_PREECLAMPS | Pre-eclampsia \|\| id:finngen_R11_O15_PREECLAMPS | Coffee max liking \|\| id:ebi-fl187-GCST90094739 | Weighted median | 5 | 0.0566091252013806 | 0.177513070858557 | 0.749801460434044 | -0.291316494 | 0.404534744084152 | 1.05824208938063 | 0.747279131463977 | 1.49860510294562 | 0.927373305772456 |
| ebi-fl187-GCST90094739 | finngen_R11_O15_PREECLAMPS | Pre-eclampsia \|\| id:finngen_R11_O15_PREECLAMPS | Coffee max liking \|\| id:ebi-fl187-GCST90094739 | Weighted mode | 5 | -0.154140416 | 0.196749448512078 | 0.477160799534432 | -0.539769335 | 0.231488503336136 | 0.857151654970626 | 0.582882687602151 | 1.26047483523882 | 0.905055003276494 |
| ebi-fl187-GCST90094740 | finngen_R11_O15_PREECLAMPS | Pre-eclampsia \|\| id:finngen_R11_O15_PREECLAMPS | Coffee with sugar liking \|\| id:ebi-fl187-GCST90094740 | Inverse variance weighted | 3 | 0.0588078755537184 | 0.196591747311782 | 0.76483547717302 | -0.326511949 | 0.444127700284811 | 1.06057145946071 | 0.721435754433315 | 1.55912957420049 | 0.941775027862301 |
| ebi-fl187-GCST90094740 | finngen_R11_O15_PREECLAMPS | Pre-eclampsia \|\| id:finngen_R11_O15_PREECLAMPS | Coffee with sugar liking \|\| id:ebi-fl187-GCST90094740 | MR Egger | 3 | -3.735932213 | 3.62356264227002 | 0.49028042909552 | -10.83811499 | 3.36625056599545 | 0.0238509265617348 | 1.963660774489e-05 | 28.9697031811058 | 0.954596128018958 |
| ebi-fl187-GCST90094740 | finngen_R11_O15_PREECLAMPS | Pre-eclampsia \|\| id:finngen_R11_O15_PREECLAMPS | Coffee with sugar liking \|\| id:ebi-fl187-GCST90094740 | Weighted median | 3 | 0.00124299962104751 | 0.225423300895176 | 0.995600431823091 | -0.44058667 | 0.443072669375592 | 1.00124377246526 | 0.643658694961159 | 1.55748551172909 | 0.999849666244992 |
| ebi-fl187-GCST90094740 | finngen_R11_O15_PREECLAMPS | Pre-eclampsia \|\| id:finngen_R11_O15_PREECLAMPS | Coffee with sugar liking \|\| id:ebi-fl187-GCST90094740 | Weighted mode | 3 | -0.074663584 | 0.2984386899638 | 0.825800373884323 | -0.659603417 | 0.510276248026458 | 0.928055646305339 | 0.517056349785016 | 1.66575129189948 | 0.963239370826664 |
| ebi-fl187-GCST90094741 | finngen_R11_O15_PREECLAMPS | Pre-eclampsia \|\| id:finngen_R11_O15_PREECLAMPS | Coffee without sugar liking \|\| id:ebi-fl187-GCST90094741 | Inverse variance weighted | 5 | 0.0366613874382427 | 0.146497351346013 | 0.802391775859963 | -0.250473421 | 0.323796196076427 | 1.03734170442948 | 0.778432169531784 | 1.38236554688627 | 0.945152512750355 |
| ebi-fl187-GCST90094741 | finngen_R11_O15_PREECLAMPS | Pre-eclampsia \|\| id:finngen_R11_O15_PREECLAMPS | Coffee without sugar liking \|\| id:ebi-fl187-GCST90094741 | MR Egger | 5 | -0.125928007 | 0.480810289586627 | 0.810345871588802 | -1.068316175 | 0.816460160394238 | 0.881678318993425 | 0.343586570342647 | 2.26247684072122 | 0.954596128018958 |
| ebi-fl187-GCST90094741 | finngen_R11_O15_PREECLAMPS | Pre-eclampsia \|\| id:finngen_R11_O15_PREECLAMPS | Coffee without sugar liking \|\| id:ebi-fl187-GCST90094741 | Weighted median | 5 | 0.00738008860544506 | 0.143967485034117 | 0.9591165962932 | -0.274796182 | 0.289556359272315 | 1.00740738857677 | 0.759726953427374 | 1.33583472585863 | 0.996099865434941 |
| ebi-fl187-GCST90094741 | finngen_R11_O15_PREECLAMPS | Pre-eclampsia \|\| id:finngen_R11_O15_PREECLAMPS | Coffee without sugar liking \|\| id:ebi-fl187-GCST90094741 | Weighted mode | 5 | -0.210191133 | 0.218999475852441 | 0.39151782476873 | -0.639430106 | 0.21904783952338 | 0.810429331257158 | 0.527593010570745 | 1.24489083024699 | 0.905055003276494 |
| ebi-fl187-GCST90094742 | finngen_R11_O15_PREECLAMPS | Pre-eclampsia \|\| id:finngen_R11_O15_PREECLAMPS | F-cooking flavour liking (derived food-liking factor) \|\| id:ebi-fl187-GCST90094742 | Inverse variance weighted | 13 | -0.046620832 | 0.067079985463123 | 0.487053038439327 | -0.178097604 | 0.0848559393692986 | 0.954449225461089 | 0.836860738843048 | 1.0885602367279 | 0.894908414040245 |
| ebi-fl187-GCST90094742 | finngen_R11_O15_PREECLAMPS | Pre-eclampsia \|\| id:finngen_R11_O15_PREECLAMPS | F-cooking flavour liking (derived food-liking factor) \|\| id:ebi-fl187-GCST90094742 | MR Egger | 13 | 0.17350996623435 | 0.38495166728876 | 0.660937907472953 | -0.580995302 | 0.928015234120321 | 1.18947254127264 | 0.559341376029201 | 2.52948375906973 | 0.954596128018958 |
| ebi-fl187-GCST90094742 | finngen_R11_O15_PREECLAMPS | Pre-eclampsia \|\| id:finngen_R11_O15_PREECLAMPS | F-cooking flavour liking (derived food-liking factor) \|\| id:ebi-fl187-GCST90094742 | Weighted median | 13 | -1.60E-05 | 0.0846804185768338 | 0.999849666244992 | -0.165989576 | 0.165957665313828 | 0.999984045030516 | 0.847055064262658 | 1.18052312358942 | 0.999849666244992 |
| ebi-fl187-GCST90094742 | finngen_R11_O15_PREECLAMPS | Pre-eclampsia \|\| id:finngen_R11_O15_PREECLAMPS | F-cooking flavour liking (derived food-liking factor) \|\| id:ebi-fl187-GCST90094742 | Weighted mode | 13 | 0.0783619187817195 | 0.143469730805258 | 0.594943711477223 | -0.202838754 | 0.359562591160025 | 1.08151400807968 | 0.816409873968577 | 1.43270260070077 | 0.905055003276494 |
| ebi-fl187-GCST90094743 | finngen_R11_O15_PREECLAMPS | Pre-eclampsia \|\| id:finngen_R11_O15_PREECLAMPS | F-cooking vegetables liking (derived food-liking factor) \|\| id:ebi-fl187-GCST90094743 | Inverse variance weighted | 12 | 0.0111496625552963 | 0.0672281840868545 | 0.868276539235587 | -0.120617578 | 0.142916903365531 | 1.01121205169988 | 0.886372863043781 | 1.15363393458558 | 0.953358127900067 |
| ebi-fl187-GCST90094743 | finngen_R11_O15_PREECLAMPS | Pre-eclampsia \|\| id:finngen_R11_O15_PREECLAMPS | F-cooking vegetables liking (derived food-liking factor) \|\| id:ebi-fl187-GCST90094743 | MR Egger | 12 | -0.022012192 | 0.300976993819644 | 0.943140250414137 | -0.6119271 | 0.567902715396023 | 0.978228307939169 | 0.542304785687456 | 1.76456237840584 | 0.970712810886627 |
| ebi-fl187-GCST90094743 | finngen_R11_O15_PREECLAMPS | Pre-eclampsia \|\| id:finngen_R11_O15_PREECLAMPS | F-cooking vegetables liking (derived food-liking factor) \|\| id:ebi-fl187-GCST90094743 | Weighted median | 12 | 0.0205211484891748 | 0.087607788791313 | 0.814799548883639 | -0.151190118 | 0.192232414520148 | 1.02073315497901 | 0.85968424206689 | 1.21195215951432 | 0.95594592743041 |
| ebi-fl187-GCST90094743 | finngen_R11_O15_PREECLAMPS | Pre-eclampsia \|\| id:finngen_R11_O15_PREECLAMPS | F-cooking vegetables liking (derived food-liking factor) \|\| id:ebi-fl187-GCST90094743 | Weighted mode | 12 | 0.0193728895112921 | 0.118710888782332 | 0.873325360735145 | -0.213300453 | 0.252046231524663 | 1.01956176162995 | 0.807913361156156 | 1.28665551995625 | 0.981296058194618 |
| ebi-fl187-GCST90094744 | finngen_R11_O15_PREECLAMPS | Pre-eclampsia \|\| id:finngen_R11_O15_PREECLAMPS | Coriander liking \|\| id:ebi-fl187-GCST90094744 | Inverse variance weighted | 2 | -0.093550264 | 0.286517577484522 | 0.744040082340811 | -0.655124716 | 0.468024187646662 | 0.910692241037862 | 0.519377283842196 | 1.59683602592552 | 0.941775027862301 |
| ebi-fl187-GCST90094745 | finngen_R11_O15_PREECLAMPS | Pre-eclampsia \|\| id:finngen_R11_O15_PREECLAMPS | Corn flake liking \|\| id:ebi-fl187-GCST90094745 | Inverse variance weighted | 6 | -0.178370052 | 0.126001466440463 | 0.156887126013519 | -0.425332927 | 0.0685928217935927 | 0.836632768209302 | 0.653552163971388 | 1.07100003248127 | 0.62762387754309 |
| ebi-fl187-GCST90094745 | finngen_R11_O15_PREECLAMPS | Pre-eclampsia \|\| id:finngen_R11_O15_PREECLAMPS | Corn flake liking \|\| id:ebi-fl187-GCST90094745 | MR Egger | 6 | -0.388531649 | 0.756412516984943 | 0.634567417159034 | -1.871100183 | 1.09403688399413 | 0.678051761679136 | 0.153954190887758 | 2.98630513963319 | 0.954596128018958 |
| ebi-fl187-GCST90094745 | finngen_R11_O15_PREECLAMPS | Pre-eclampsia \|\| id:finngen_R11_O15_PREECLAMPS | Corn flake liking \|\| id:ebi-fl187-GCST90094745 | Weighted median | 6 | -0.191372943 | 0.156167327787257 | 0.220411284117441 | -0.497460906 | 0.114715018964808 | 0.825824544818729 | 0.608072654901996 | 1.12155377047004 | 0.649149713120124 |
| ebi-fl187-GCST90094745 | finngen_R11_O15_PREECLAMPS | Pre-eclampsia \|\| id:finngen_R11_O15_PREECLAMPS | Corn flake liking \|\| id:ebi-fl187-GCST90094745 | Weighted mode | 6 | -0.251289385 | 0.232158237730063 | 0.328488445282015 | -0.706319531 | 0.203740760571956 | 0.77779725583429 | 0.493457012484669 | 1.22598028982747 | 0.905055003276494 |
| ebi-fl187-GCST90094746 | finngen_R11_O15_PREECLAMPS | Pre-eclampsia \|\| id:finngen_R11_O15_PREECLAMPS | Cream liking \|\| id:ebi-fl187-GCST90094746 | Inverse variance weighted | 5 | -0.074622795 | 0.175069294544943 | 0.669927734775384 | -0.417758612 | 0.268513022480298 | 0.928093501979788 | 0.658521168259268 | 1.30801801055844 | 0.941775027862301 |
| ebi-fl187-GCST90094746 | finngen_R11_O15_PREECLAMPS | Pre-eclampsia \|\| id:finngen_R11_O15_PREECLAMPS | Cream liking \|\| id:ebi-fl187-GCST90094746 | MR Egger | 5 | 0.404067518062952 | 1.00967229262382 | 0.715804838128416 | -1.574890175 | 2.38302521160564 | 1.49790507919341 | 0.207030288434766 | 10.8376394740927 | 0.954596128018958 |
| ebi-fl187-GCST90094746 | finngen_R11_O15_PREECLAMPS | Pre-eclampsia \|\| id:finngen_R11_O15_PREECLAMPS | Cream liking \|\| id:ebi-fl187-GCST90094746 | Weighted median | 5 | -0.15711462 | 0.200447044846036 | 0.43314509996802 | -0.549990828 | 0.235761587731639 | 0.85460609811032 | 0.576955102151015 | 1.26587247465953 | 0.838163894743311 |
| ebi-fl187-GCST90094746 | finngen_R11_O15_PREECLAMPS | Pre-eclampsia \|\| id:finngen_R11_O15_PREECLAMPS | Cream liking \|\| id:ebi-fl187-GCST90094746 | Weighted mode | 5 | -0.178372001 | 0.252708109688061 | 0.519225497225336 | -0.673679896 | 0.316935893959117 | 0.836631137948473 | 0.509829003868057 | 1.37291455698763 | 0.905055003276494 |
| ebi-fl187-GCST90094747 | finngen_R11_O15_PREECLAMPS | Pre-eclampsia \|\| id:finngen_R11_O15_PREECLAMPS | Crisps liking \|\| id:ebi-fl187-GCST90094747 | Inverse variance weighted | 9 | -0.132080174 | 0.112355747877904 | 0.239773412001723 | -0.35229744 | 0.0881370916065054 | 0.876270737927652 | 0.703070969390923 | 1.09213783469607 | 0.71932023600517 |
| ebi-fl187-GCST90094747 | finngen_R11_O15_PREECLAMPS | Pre-eclampsia \|\| id:finngen_R11_O15_PREECLAMPS | Crisps liking \|\| id:ebi-fl187-GCST90094747 | MR Egger | 9 | 0.25618088450868 | 0.517048723997673 | 0.635443767392151 | -0.757234615 | 1.26959638354412 | 1.29198640713107 | 0.468961494809004 | 3.55941563366793 | 0.954596128018958 |
| ebi-fl187-GCST90094747 | finngen_R11_O15_PREECLAMPS | Pre-eclampsia \|\| id:finngen_R11_O15_PREECLAMPS | Crisps liking \|\| id:ebi-fl187-GCST90094747 | Weighted median | 9 | -0.18652241 | 0.154354017486988 | 0.226890911896512 | -0.489056284 | 0.11601146463465 | 0.829839965328222 | 0.613204812455491 | 1.12300874694444 | 0.649149713120124 |
| ebi-fl187-GCST90094747 | finngen_R11_O15_PREECLAMPS | Pre-eclampsia \|\| id:finngen_R11_O15_PREECLAMPS | Crisps liking \|\| id:ebi-fl187-GCST90094747 | Weighted mode | 9 | -0.336647687 | 0.31525867872234 | 0.316750710364019 | -0.954554698 | 0.281259322999065 | 0.714160403367901 | 0.384983540486059 | 1.32479710975351 | 0.905055003276494 |
| ebi-fl187-GCST90094749 | finngen_R11_O15_PREECLAMPS | Pre-eclampsia \|\| id:finngen_R11_O15_PREECLAMPS | Cucumber liking \|\| id:ebi-fl187-GCST90094749 | Inverse variance weighted | 3 | -0.050307161 | 0.283089027799162 | 0.858952368437033 | -0.605161656 | 0.504547333421789 | 0.950937288726701 | 0.54598615781219 | 1.65623562823354 | 0.953358127900067 |
| ebi-fl187-GCST90094749 | finngen_R11_O15_PREECLAMPS | Pre-eclampsia \|\| id:finngen_R11_O15_PREECLAMPS | Cucumber liking \|\| id:ebi-fl187-GCST90094749 | MR Egger | 3 | 0.850357005164491 | 0.561345452239248 | 0.371444062074103 | -0.249880081 | 1.95059409155342 | 2.3404822670501 | 0.778894181507722 | 7.03286450512746 | 0.954596128018958 |
| ebi-fl187-GCST90094749 | finngen_R11_O15_PREECLAMPS | Pre-eclampsia \|\| id:finngen_R11_O15_PREECLAMPS | Cucumber liking \|\| id:ebi-fl187-GCST90094749 | Weighted median | 3 | -0.110188529 | 0.297353908414763 | 0.710962611365119 | -0.69300219 | 0.472625131156302 | 0.895665260200723 | 0.500072500621021 | 1.60419990568205 | 0.925493743359756 |
| ebi-fl187-GCST90094749 | finngen_R11_O15_PREECLAMPS | Pre-eclampsia \|\| id:finngen_R11_O15_PREECLAMPS | Cucumber liking \|\| id:ebi-fl187-GCST90094749 | Weighted mode | 3 | -0.203265027 | 0.385439237602581 | 0.650602667657441 | -0.958725933 | 0.552195878339362 | 0.81606193402183 | 0.383381028038439 | 1.73706321245682 | 0.934683055540254 |
| ebi-fl187-GCST90094750 | finngen_R11_O15_PREECLAMPS | Pre-eclampsia \|\| id:finngen_R11_O15_PREECLAMPS | Curry liking \|\| id:ebi-fl187-GCST90094750 | Inverse variance weighted | 7 | -0.302738497 | 0.147265318374315 | 0.0398080484919415 | -0.591378521 | -0.014098473 | 0.738792267854419 | 0.553563659642625 | 0.986000445538365 | 0.328416400058518 |
| ebi-fl187-GCST90094750 | finngen_R11_O15_PREECLAMPS | Pre-eclampsia \|\| id:finngen_R11_O15_PREECLAMPS | Curry liking \|\| id:ebi-fl187-GCST90094750 | MR Egger | 7 | 0.625301245791954 | 0.524100892007938 | 0.286354626993717 | -0.401936503 | 1.65253899412751 | 1.8688088434445 | 0.669023225614745 | 5.22021711597121 | 0.954596128018958 |
| ebi-fl187-GCST90094750 | finngen_R11_O15_PREECLAMPS | Pre-eclampsia \|\| id:finngen_R11_O15_PREECLAMPS | Curry liking \|\| id:ebi-fl187-GCST90094750 | Weighted median | 7 | -0.353753483 | 0.180482868858029 | 0.0499912182847319 | -0.707499906 | -7.06E-06 | 0.702048012730192 | 0.49287489192734 | 0.999992939894109 | 0.447765620977764 |
| ebi-fl187-GCST90094750 | finngen_R11_O15_PREECLAMPS | Pre-eclampsia \|\| id:finngen_R11_O15_PREECLAMPS | Curry liking \|\| id:ebi-fl187-GCST90094750 | Weighted mode | 7 | -0.402546185 | 0.247997189754331 | 0.155675384337301 | -0.888620677 | 0.0835283070257977 | 0.668615458279863 | 0.411222570577359 | 1.08711598787765 | 0.905055003276494 |
| ebi-fl187-GCST90094751 | finngen_R11_O15_PREECLAMPS | Pre-eclampsia \|\| id:finngen_R11_O15_PREECLAMPS | F-dairy liking (derived food-liking factor) \|\| id:ebi-fl187-GCST90094751 | Inverse variance weighted | 2 | -0.353665325 | 0.428117983095862 | 0.408751264411004 | -1.192776572 | 0.485445922147923 | 0.702109906868674 | 0.303377743537958 | 1.62489942595759 | 0.864666136254048 |
| ebi-fl187-GCST90094752 | finngen_R11_O15_PREECLAMPS | Pre-eclampsia \|\| id:finngen_R11_O15_PREECLAMPS | Dark chocolate liking \|\| id:ebi-fl187-GCST90094752 | Inverse variance weighted | 17 | -0.004883078 | 0.0663717448034409 | 0.941351240338754 | -0.134971698 | 0.125205541897824 | 0.995128825 | 0.87374064018015 | 1.13338138648835 | 0.977922867344962 |
| ebi-fl187-GCST90094752 | finngen_R11_O15_PREECLAMPS | Pre-eclampsia \|\| id:finngen_R11_O15_PREECLAMPS | Dark chocolate liking \|\| id:ebi-fl187-GCST90094752 | MR Egger | 17 | 0.0766611851388613 | 0.164974939433533 | 0.648831417441903 | -0.246689696 | 0.400012066428587 | 1.07967620407145 | 0.781383122106611 | 1.49184269874605 | 0.954596128018958 |
| ebi-fl187-GCST90094752 | finngen_R11_O15_PREECLAMPS | Pre-eclampsia \|\| id:finngen_R11_O15_PREECLAMPS | Dark chocolate liking \|\| id:ebi-fl187-GCST90094752 | Weighted median | 17 | -0.029124304 | 0.0789177697720312 | 0.712093279924698 | -0.183803133 | 0.125554524758132 | 0.971295721023817 | 0.832099600864627 | 1.13377698619117 | 0.925493743359756 |
| ebi-fl187-GCST90094752 | finngen_R11_O15_PREECLAMPS | Pre-eclampsia \|\| id:finngen_R11_O15_PREECLAMPS | Dark chocolate liking \|\| id:ebi-fl187-GCST90094752 | Weighted mode | 17 | -0.027224017 | 0.0907237206687031 | 0.767982701291372 | -0.205042509 | 0.150594475660793 | 0.973143216626766 | 0.814612687010984 | 1.16252513024502 | 0.945697706548879 |
| ebi-fl187-GCST90094753 | finngen_R11_O15_PREECLAMPS | Pre-eclampsia \|\| id:finngen_R11_O15_PREECLAMPS | F-deep fried food liking (derived food-liking factor) \|\| id:ebi-fl187-GCST90094753 | Inverse variance weighted | 7 | -0.07147778 | 0.0880834379561752 | 0.417091173131301 | -0.244121318 | 0.101165758849794 | 0.931016964986237 | 0.783392588923771 | 1.10646003210598 | 0.871139791983097 |
| ebi-fl187-GCST90094753 | finngen_R11_O15_PREECLAMPS | Pre-eclampsia \|\| id:finngen_R11_O15_PREECLAMPS | F-deep fried food liking (derived food-liking factor) \|\| id:ebi-fl187-GCST90094753 | MR Egger | 7 | -0.397600609 | 0.26190291014863 | 0.189441096982404 | -0.910930313 | 0.115729094763764 | 0.671930336921797 | 0.402149924642403 | 1.12269168787564 | 0.954596128018958 |
| ebi-fl187-GCST90094753 | finngen_R11_O15_PREECLAMPS | Pre-eclampsia \|\| id:finngen_R11_O15_PREECLAMPS | F-deep fried food liking (derived food-liking factor) \|\| id:ebi-fl187-GCST90094753 | Weighted median | 7 | -0.199776883 | 0.0876897210204444 | 0.0227131971104213 | -0.371648736 | -0.02790503 | 0.818913446322508 | 0.689596430345086 | 0.972480719240699 | 0.367261503827345 |
| ebi-fl187-GCST90094753 | finngen_R11_O15_PREECLAMPS | Pre-eclampsia \|\| id:finngen_R11_O15_PREECLAMPS | F-deep fried food liking (derived food-liking factor) \|\| id:ebi-fl187-GCST90094753 | Weighted mode | 7 | -0.191690167 | 0.105712680340294 | 0.119724309949108 | -0.398887021 | 0.0155066861821176 | 0.825562615176871 | 0.671066513660938 | 1.0156275387063 | 0.905055003276494 |
| ebi-fl187-GCST90094754 | finngen_R11_O15_PREECLAMPS | Pre-eclampsia \|\| id:finngen_R11_O15_PREECLAMPS | F-dessert liking (derived food-liking factor) \|\| id:ebi-fl187-GCST90094754 | Inverse variance weighted | 14 | 0.023044015643239 | 0.0762511994701719 | 0.762490575483158 | -0.126408335 | 0.172496366604776 | 1.0233115802734 | 0.881254925793566 | 1.18826750316167 | 0.941775027862301 |
| ebi-fl187-GCST90094754 | finngen_R11_O15_PREECLAMPS | Pre-eclampsia \|\| id:finngen_R11_O15_PREECLAMPS | F-dessert liking (derived food-liking factor) \|\| id:ebi-fl187-GCST90094754 | MR Egger | 14 | -0.040333085 | 0.506233849639582 | 0.937810730162308 | -1.03255143 | 0.951885260375784 | 0.960469467972709 | 0.356097243257901 | 2.59058899324211 | 0.970712810886627 |
| ebi-fl187-GCST90094754 | finngen_R11_O15_PREECLAMPS | Pre-eclampsia \|\| id:finngen_R11_O15_PREECLAMPS | F-dessert liking (derived food-liking factor) \|\| id:ebi-fl187-GCST90094754 | Weighted median | 14 | 0.0573810384240248 | 0.0816721703322808 | 0.482319018439022 | -0.102696415 | 0.217458492275295 | 1.05905927580011 | 0.90240088688917 | 1.2429138379116 | 0.876408948139198 |
| ebi-fl187-GCST90094754 | finngen_R11_O15_PREECLAMPS | Pre-eclampsia \|\| id:finngen_R11_O15_PREECLAMPS | F-dessert liking (derived food-liking factor) \|\| id:ebi-fl187-GCST90094754 | Weighted mode | 14 | 0.0677067955758812 | 0.14977053667204 | 0.658669267327024 | -0.225843456 | 0.36125704745308 | 1.07005151863944 | 0.797842989182736 | 1.43513231057585 | 0.934683055540254 |
| ebi-fl187-GCST90094755 | finngen_R11_O15_PREECLAMPS | Pre-eclampsia \|\| id:finngen_R11_O15_PREECLAMPS | Diet fizzy drinks liking \|\| id:ebi-fl187-GCST90094755 | Inverse variance weighted (multiplicative random effects) | 4 | 0.327840135753643 | 0.32519041611688 | 0.313383316613907 | -0.30953308 | 0.965213351342728 | 1.38796706824329 | 0.733789497361243 | 2.6253477181883 | 0.796595336096731 |
| ebi-fl187-GCST90094755 | finngen_R11_O15_PREECLAMPS | Pre-eclampsia \|\| id:finngen_R11_O15_PREECLAMPS | Diet fizzy drinks liking \|\| id:ebi-fl187-GCST90094755 | MR Egger | 4 | -1.589220883 | 1.90883867047128 | 0.492677176911698 | -5.330544677 | 2.15210291075278 | 0.204084555479225 | 0.00484143227475411 | 8.60293058365009 | 0.954596128018958 |
| ebi-fl187-GCST90094755 | finngen_R11_O15_PREECLAMPS | Pre-eclampsia \|\| id:finngen_R11_O15_PREECLAMPS | Diet fizzy drinks liking \|\| id:ebi-fl187-GCST90094755 | Weighted median | 4 | 0.0939621295146574 | 0.250177649586132 | 0.707227949414591 | -0.396386064 | 0.584310322703475 | 1.09851814371417 | 0.67274692264736 | 1.79375344790953 | 0.925493743359756 |
| ebi-fl187-GCST90094755 | finngen_R11_O15_PREECLAMPS | Pre-eclampsia \|\| id:finngen_R11_O15_PREECLAMPS | Diet fizzy drinks liking \|\| id:ebi-fl187-GCST90094755 | Weighted mode | 4 | 0.0181861028174217 | 0.320450260380506 | 0.958311347412613 | -0.609896408 | 0.646268613163214 | 1.01835247702105 | 0.543407159049548 | 1.90840652388304 | 0.981296058194618 |
| ebi-fl187-GCST90094756 | finngen_R11_O15_PREECLAMPS | Pre-eclampsia \|\| id:finngen_R11_O15_PREECLAMPS | Dried fruit liking \|\| id:ebi-fl187-GCST90094756 | Inverse variance weighted | 5 | -0.261203846 | 0.236680775970674 | 0.269761296816085 | -0.725098167 | 0.202690474568584 | 0.770123916676294 | 0.484277026484474 | 1.22469333584184 | 0.754417186011084 |
| ebi-fl187-GCST90094756 | finngen_R11_O15_PREECLAMPS | Pre-eclampsia \|\| id:finngen_R11_O15_PREECLAMPS | Dried fruit liking \|\| id:ebi-fl187-GCST90094756 | MR Egger | 5 | -0.232745762 | 1.63302097649899 | 0.895699458493191 | -3.433466876 | 2.96797535199145 | 0.792354994710248 | 0.0322748536772573 | 19.4524952435239 | 0.970712810886627 |
| ebi-fl187-GCST90094756 | finngen_R11_O15_PREECLAMPS | Pre-eclampsia \|\| id:finngen_R11_O15_PREECLAMPS | Dried fruit liking \|\| id:ebi-fl187-GCST90094756 | Weighted median | 5 | 0.012071576000624 | 0.249143322896849 | 0.961355750130855 | -0.476249337 | 0.500392488878447 | 1.0121447315454 | 0.621108597660902 | 1.64936850246986 | 0.996099865434941 |
| ebi-fl187-GCST90094756 | finngen_R11_O15_PREECLAMPS | Pre-eclampsia \|\| id:finngen_R11_O15_PREECLAMPS | Dried fruit liking \|\| id:ebi-fl187-GCST90094756 | Weighted mode | 5 | 0.0378819347145125 | 0.298147717167829 | 0.905025939905491 | -0.546487591 | 0.622251460363458 | 1.03860860201831 | 0.578979857215237 | 1.86311805971069 | 0.981296058194618 |
| ebi-fl187-GCST90094757 | finngen_R11_O15_PREECLAMPS | Pre-eclampsia \|\| id:finngen_R11_O15_PREECLAMPS | Eggs liking \|\| id:ebi-fl187-GCST90094757 | Inverse variance weighted | 4 | 0.172965345574509 | 0.247914326953211 | 0.485376132584946 | -0.312946735 | 0.658877426402803 | 1.18882490632604 | 0.731288863447239 | 1.93262160624039 | 0.894908414040245 |
| ebi-fl187-GCST90094757 | finngen_R11_O15_PREECLAMPS | Pre-eclampsia \|\| id:finngen_R11_O15_PREECLAMPS | Eggs liking \|\| id:ebi-fl187-GCST90094757 | MR Egger | 4 | -2.281344511 | 1.28623342370249 | 0.218117934250375 | -4.802362022 | 0.239672999365957 | 0.102146776873858 | 0.00821033114857186 | 1.27083351900278 | 0.954596128018958 |
| ebi-fl187-GCST90094757 | finngen_R11_O15_PREECLAMPS | Pre-eclampsia \|\| id:finngen_R11_O15_PREECLAMPS | Eggs liking \|\| id:ebi-fl187-GCST90094757 | Weighted median | 4 | 0.176707419323407 | 0.280897034435613 | 0.529295021845252 | -0.373850768 | 0.727265606817209 | 1.1932819108188 | 0.68807958754331 | 2.06941427193222 | 0.881409801621768 |
| ebi-fl187-GCST90094757 | finngen_R11_O15_PREECLAMPS | Pre-eclampsia \|\| id:finngen_R11_O15_PREECLAMPS | Eggs liking \|\| id:ebi-fl187-GCST90094757 | Weighted mode | 4 | 0.0818386871528416 | 0.358152063994142 | 0.833945495547917 | -0.620139358 | 0.78381673258136 | 1.08528072598442 | 0.537869475808835 | 2.18981427124502 | 0.963239370826664 |
| ebi-fl187-GCST90094758 | finngen_R11_O15_PREECLAMPS | Pre-eclampsia \|\| id:finngen_R11_O15_PREECLAMPS | Extra virgin olive oil liking \|\| id:ebi-fl187-GCST90094758 | Inverse variance weighted | 2 | -0.586503114 | 0.371916744057144 | 0.114801458939559 | -1.315459933 | 0.142453703978643 | 0.556269097017941 | 0.268350871503982 | 1.15309969579348 | 0.512568357226114 |
| ebi-fl187-GCST90094759 | finngen_R11_O15_PREECLAMPS | Pre-eclampsia \|\| id:finngen_R11_O15_PREECLAMPS | F-fatty/salty food liking (derived food-liking factor) \|\| id:ebi-fl187-GCST90094759 | Inverse variance weighted (multiplicative random effects) | 15 | -0.285520674 | 0.303835272716388 | 0.347360205283854 | -0.881037808 | 0.309996460919317 | 0.751622802264942 | 0.414352669896925 | 1.36342028886923 | 0.802417492460712 |
| ebi-fl187-GCST90094759 | finngen_R11_O15_PREECLAMPS | Pre-eclampsia \|\| id:finngen_R11_O15_PREECLAMPS | F-fatty/salty food liking (derived food-liking factor) \|\| id:ebi-fl187-GCST90094759 | MR Egger | 15 | 1.56258357840091 | 2.44888507628918 | 0.534500826447625 | -3.237231171 | 6.3623983279277 | 4.77113192888119 | 0.0392724834845152 | 579.634845139475 | 0.954596128018958 |
| ebi-fl187-GCST90094759 | finngen_R11_O15_PREECLAMPS | Pre-eclampsia \|\| id:finngen_R11_O15_PREECLAMPS | F-fatty/salty food liking (derived food-liking factor) \|\| id:ebi-fl187-GCST90094759 | Weighted median | 15 | -0.637339631 | 0.285384738846357 | 0.0255315013597591 | -1.196693719 | -0.077985543 | 0.528697084119586 | 0.302191692654231 | 0.924977799030304 | 0.367261503827345 |
| ebi-fl187-GCST90094759 | finngen_R11_O15_PREECLAMPS | Pre-eclampsia \|\| id:finngen_R11_O15_PREECLAMPS | F-fatty/salty food liking (derived food-liking factor) \|\| id:ebi-fl187-GCST90094759 | Weighted mode | 15 | -0.733879353 | 0.390012951421964 | 0.0808364521303073 | -1.498304738 | 0.0305460313782101 | 0.480043116338615 | 0.223508744995822 | 1.03101734810598 | 0.905055003276494 |
| ebi-fl187-GCST90094760 | finngen_R11_O15_PREECLAMPS | Pre-eclampsia \|\| id:finngen_R11_O15_PREECLAMPS | F-fatty/dairy food liking (derived food-liking factor) \|\| id:ebi-fl187-GCST90094760 | Inverse variance weighted | 5 | -0.098871757 | 0.104894012901993 | 0.345892072473507 | -0.304464023 | 0.106720507880355 | 0.905858870264831 | 0.7375185616872 | 1.11262324159037 | 0.802417492460712 |
| ebi-fl187-GCST90094760 | finngen_R11_O15_PREECLAMPS | Pre-eclampsia \|\| id:finngen_R11_O15_PREECLAMPS | F-fatty/dairy food liking (derived food-liking factor) \|\| id:ebi-fl187-GCST90094760 | MR Egger | 5 | 0.46401936693729 | 0.53811988090341 | 0.451941786971307 | -0.5906956 | 1.51873433350797 | 1.59045377236024 | 0.553941828955457 | 4.56644194352458 | 0.954596128018958 |
| ebi-fl187-GCST90094760 | finngen_R11_O15_PREECLAMPS | Pre-eclampsia \|\| id:finngen_R11_O15_PREECLAMPS | F-fatty/dairy food liking (derived food-liking factor) \|\| id:ebi-fl187-GCST90094760 | Weighted median | 5 | -0.108606381 | 0.131496592653563 | 0.408846388021344 | -0.366339703 | 0.149126940376301 | 0.897083456903872 | 0.693267256358468 | 1.16082033482695 | 0.819869554194876 |
| ebi-fl187-GCST90094760 | finngen_R11_O15_PREECLAMPS | Pre-eclampsia \|\| id:finngen_R11_O15_PREECLAMPS | F-fatty/dairy food liking (derived food-liking factor) \|\| id:ebi-fl187-GCST90094760 | Weighted mode | 5 | -0.15435835 | 0.186577033154804 | 0.454556890108606 | -0.520049335 | 0.21133263479006 | 0.85696487245363 | 0.594491217917375 | 1.23532319819994 | 0.905055003276494 |
| ebi-fl187-GCST90094761 | finngen_R11_O15_PREECLAMPS | Pre-eclampsia \|\| id:finngen_R11_O15_PREECLAMPS | F-fatty food liking (derived food-liking factor) \|\| id:ebi-fl187-GCST90094761 | Inverse variance weighted | 6 | -0.220231103 | 0.117108656231945 | 0.0600303464606468 | -0.449764069 | 0.00930186365510996 | 0.802333355243189 | 0.63777860576096 | 1.00934526044147 | 0.366852117259508 |
| ebi-fl187-GCST90094761 | finngen_R11_O15_PREECLAMPS | Pre-eclampsia \|\| id:finngen_R11_O15_PREECLAMPS | F-fatty food liking (derived food-liking factor) \|\| id:ebi-fl187-GCST90094761 | MR Egger | 6 | -0.298915851 | 0.34690340774899 | 0.437461458325143 | -0.97884653 | 0.381014828422538 | 0.741621813717263 | 0.37574425873249 | 1.46376931063864 | 0.954596128018958 |
| ebi-fl187-GCST90094761 | finngen_R11_O15_PREECLAMPS | Pre-eclampsia \|\| id:finngen_R11_O15_PREECLAMPS | F-fatty food liking (derived food-liking factor) \|\| id:ebi-fl187-GCST90094761 | Weighted median | 6 | -0.20476154 | 0.150571414551884 | 0.173862678227722 | -0.499881512 | 0.0903584326331275 | 0.814841600464843 | 0.60660253032641 | 1.09456654176967 | 0.612323741171272 |
| ebi-fl187-GCST90094761 | finngen_R11_O15_PREECLAMPS | Pre-eclampsia \|\| id:finngen_R11_O15_PREECLAMPS | F-fatty food liking (derived food-liking factor) \|\| id:ebi-fl187-GCST90094761 | Weighted mode | 6 | -0.187689058 | 0.218771345301717 | 0.430130291261123 | -0.616480895 | 0.241102778892226 | 0.828872398492007 | 0.539840855611464 | 1.27265183033194 | 0.905055003276494 |
| ebi-fl187-GCST90094762 | finngen_R11_O15_PREECLAMPS | Pre-eclampsia \|\| id:finngen_R11_O15_PREECLAMPS | F-fish liking (derived food-liking factor) \|\| id:ebi-fl187-GCST90094762 | Inverse variance weighted | 26 | -0.044976588 | 0.0481390077446456 | 0.350145814891947 | -0.139329043 | 0.0493758675766066 | 0.956019864287785 | 0.869941733310701 | 1.05061516871315 | 0.802417492460712 |
| ebi-fl187-GCST90094762 | finngen_R11_O15_PREECLAMPS | Pre-eclampsia \|\| id:finngen_R11_O15_PREECLAMPS | F-fish liking (derived food-liking factor) \|\| id:ebi-fl187-GCST90094762 | MR Egger | 26 | 0.0543962290745565 | 0.217870861049878 | 0.804965701541881 | -0.372630659 | 0.481423116732317 | 1.05590289870736 | 0.68891963241401 | 1.61837590197832 | 0.954596128018958 |
| ebi-fl187-GCST90094762 | finngen_R11_O15_PREECLAMPS | Pre-eclampsia \|\| id:finngen_R11_O15_PREECLAMPS | F-fish liking (derived food-liking factor) \|\| id:ebi-fl187-GCST90094762 | Weighted median | 26 | -0.082038266 | 0.0668039329614199 | 0.219429904506001 | -0.212973975 | 0.0488974422685247 | 0.921236705668667 | 0.80817716980194 | 1.05011264804627 | 0.649149713120124 |
| ebi-fl187-GCST90094762 | finngen_R11_O15_PREECLAMPS | Pre-eclampsia \|\| id:finngen_R11_O15_PREECLAMPS | F-fish liking (derived food-liking factor) \|\| id:ebi-fl187-GCST90094762 | Weighted mode | 26 | -0.082961511 | 0.109702847764133 | 0.456573790383326 | -0.297979092 | 0.132056070992591 | 0.920386571642188 | 0.742316859766191 | 1.14117230413718 | 0.905055003276494 |
| ebi-fl187-GCST90094764 | finngen_R11_O15_PREECLAMPS | Pre-eclampsia \|\| id:finngen_R11_O15_PREECLAMPS | Fried chicken liking \|\| id:ebi-fl187-GCST90094764 | Inverse variance weighted | 2 | -0.500166681 | 0.183164597960069 | 0.00632009010580443 | -0.859169293 | -0.141164069 | 0.60642957101741 | 0.423513752079597 | 0.868346831238769 | 0.126476467130286 |
| ebi-fl187-GCST90094765 | finngen_R11_O15_PREECLAMPS | Pre-eclampsia \|\| id:finngen_R11_O15_PREECLAMPS | Fried fish liking \|\| id:ebi-fl187-GCST90094765 | Inverse variance weighted | 5 | 0.0447243159928053 | 0.195323382842433 | 0.818887772440363 | -0.338109514 | 0.427558146363974 | 1.04573952650294 | 0.713117187036338 | 1.53350834501044 | 0.945152512750355 |
| ebi-fl187-GCST90094765 | finngen_R11_O15_PREECLAMPS | Pre-eclampsia \|\| id:finngen_R11_O15_PREECLAMPS | Fried fish liking \|\| id:ebi-fl187-GCST90094765 | MR Egger | 5 | 1.54654916324261 | 1.11969251107801 | 0.261109748281804 | -0.648048158 | 3.74114648495551 | 4.69523968860234 | 0.523065722450874 | 42.1462825553381 | 0.954596128018958 |
| ebi-fl187-GCST90094765 | finngen_R11_O15_PREECLAMPS | Pre-eclampsia \|\| id:finngen_R11_O15_PREECLAMPS | Fried fish liking \|\| id:ebi-fl187-GCST90094765 | Weighted median | 5 | -0.077279644 | 0.18247036469557 | 0.671916727199487 | -0.434921559 | 0.280362270454402 | 0.925630969940581 | 0.647315441058602 | 1.32360923000997 | 0.912898347777426 |
| ebi-fl187-GCST90094765 | finngen_R11_O15_PREECLAMPS | Pre-eclampsia \|\| id:finngen_R11_O15_PREECLAMPS | Fried fish liking \|\| id:ebi-fl187-GCST90094765 | Weighted mode | 5 | -0.089286872 | 0.234864768262687 | 0.723149058063452 | -0.549621818 | 0.371048073875929 | 0.914583167709465 | 0.577168043842209 | 1.44925274290854 | 0.936018697441052 |
| ebi-fl187-GCST90094766 | finngen_R11_O15_PREECLAMPS | Pre-eclampsia \|\| id:finngen_R11_O15_PREECLAMPS | Fruit liking \|\| id:ebi-fl187-GCST90094766 | Inverse variance weighted | 9 | -0.549083646 | 0.240618154283303 | 0.0224910534165932 | -1.020695229 | -0.077472064 | 0.57747874283488 | 0.360344331381918 | 0.925452877660801 | 0.258296218889823 |
| ebi-fl187-GCST90094766 | finngen_R11_O15_PREECLAMPS | Pre-eclampsia \|\| id:finngen_R11_O15_PREECLAMPS | Fruit liking \|\| id:ebi-fl187-GCST90094766 | MR Egger | 9 | 0.548737972826512 | 1.12213005618265 | 0.639780151082692 | -1.650636937 | 2.74811288294451 | 1.7310669851668 | 0.191927623820136 | 15.6131402426089 | 0.954596128018958 |
| ebi-fl187-GCST90094766 | finngen_R11_O15_PREECLAMPS | Pre-eclampsia \|\| id:finngen_R11_O15_PREECLAMPS | Fruit liking \|\| id:ebi-fl187-GCST90094766 | Weighted median | 9 | -0.621721924 | 0.282862921763369 | 0.0279518213758233 | -1.176133251 | -0.067310597 | 0.537018935215867 | 0.308469208607761 | 0.934904777309846 | 0.367261503827345 |
| ebi-fl187-GCST90094766 | finngen_R11_O15_PREECLAMPS | Pre-eclampsia \|\| id:finngen_R11_O15_PREECLAMPS | Fruit liking \|\| id:ebi-fl187-GCST90094766 | Weighted mode | 9 | -0.665550294 | 0.403876940392264 | 0.137985682216587 | -1.457149097 | 0.126048509627055 | 0.513990604158265 | 0.232899302554331 | 1.13433719322259 | 0.905055003276494 |
| ebi-fl187-GCST90094767 | finngen_R11_O15_PREECLAMPS | Pre-eclampsia \|\| id:finngen_R11_O15_PREECLAMPS | F-fruit liking (derived food-liking factor) \|\| id:ebi-fl187-GCST90094767 | Inverse variance weighted | 14 | -0.090658142 | 0.0727006744788262 | 0.212395476185331 | -0.233151464 | 0.051835180115225 | 0.913329886789605 | 0.792033599986849 | 1.05320213955186 | 0.673947184049608 |
| ebi-fl187-GCST90094767 | finngen_R11_O15_PREECLAMPS | Pre-eclampsia \|\| id:finngen_R11_O15_PREECLAMPS | F-fruit liking (derived food-liking factor) \|\| id:ebi-fl187-GCST90094767 | MR Egger | 14 | -0.431593876 | 0.284575057646996 | 0.155253883076062 | -0.989360989 | 0.126173236688325 | 0.649473089545817 | 0.371814208387965 | 1.13447868459091 | 0.954596128018958 |
| ebi-fl187-GCST90094767 | finngen_R11_O15_PREECLAMPS | Pre-eclampsia \|\| id:finngen_R11_O15_PREECLAMPS | F-fruit liking (derived food-liking factor) \|\| id:ebi-fl187-GCST90094767 | Weighted median | 14 | -0.045976929 | 0.0839531822914043 | 0.583932503978196 | -0.210525167 | 0.118571308018632 | 0.955063995957722 | 0.810158665987133 | 1.12588715602188 | 0.881409801621768 |
| ebi-fl187-GCST90094767 | finngen_R11_O15_PREECLAMPS | Pre-eclampsia \|\| id:finngen_R11_O15_PREECLAMPS | F-fruit liking (derived food-liking factor) \|\| id:ebi-fl187-GCST90094767 | Weighted mode | 14 | -0.02610822 | 0.109425148719632 | 0.815140020592068 | -0.240581512 | 0.188365071254127 | 0.974229652540381 | 0.786170560716336 | 1.20727417600595 | 0.963239370826664 |
| ebi-fl187-GCST90094769 | finngen_R11_O15_PREECLAMPS | Pre-eclampsia \|\| id:finngen_R11_O15_PREECLAMPS | Garlic liking \|\| id:ebi-fl187-GCST90094769 | Inverse variance weighted | 8 | 0.0952874663213927 | 0.10520044360383 | 0.365057298423861 | -0.110905403 | 0.301480335784899 | 1.09997501545252 | 0.895023411327016 | 1.35185853163978 | 0.825129510136124 |
| ebi-fl187-GCST90094769 | finngen_R11_O15_PREECLAMPS | Pre-eclampsia \|\| id:finngen_R11_O15_PREECLAMPS | Garlic liking \|\| id:ebi-fl187-GCST90094769 | MR Egger | 8 | 0.511120921983368 | 0.211549133162668 | 0.0521400962172712 | 0.0964846209845382 | 0.925757222982198 | 1.66715890303698 | 1.10129264421695 | 2.52377860014828 | 0.954596128018958 |
| ebi-fl187-GCST90094769 | finngen_R11_O15_PREECLAMPS | Pre-eclampsia \|\| id:finngen_R11_O15_PREECLAMPS | Garlic liking \|\| id:ebi-fl187-GCST90094769 | Weighted median | 8 | 0.228673320490626 | 0.111827199066864 | 0.0408665863807403 | 0.00949201031957325 | 0.447854630661679 | 1.25693135821357 | 1.00953720232401 | 1.56495118319925 | 0.43493724076645 |
| ebi-fl187-GCST90094769 | finngen_R11_O15_PREECLAMPS | Pre-eclampsia \|\| id:finngen_R11_O15_PREECLAMPS | Garlic liking \|\| id:ebi-fl187-GCST90094769 | Weighted mode | 8 | 0.261094772073646 | 0.115147324064962 | 0.0576910888317269 | 0.0354060169063213 | 0.486783527240971 | 1.29835070699197 | 1.03604027328228 | 1.62707435397856 | 0.905055003276494 |
| ebi-fl187-GCST90094770 | finngen_R11_O15_PREECLAMPS | Pre-eclampsia \|\| id:finngen_R11_O15_PREECLAMPS | Gherkins liking \|\| id:ebi-fl187-GCST90094770 | Inverse variance weighted | 19 | -0.132434398 | 0.0597316174821663 | 0.0266123377037999 | -0.249508368 | -0.015360427 | 0.875960397376027 | 0.779183760742207 | 0.984756942367837 | 0.258296218889823 |
| ebi-fl187-GCST90094770 | finngen_R11_O15_PREECLAMPS | Pre-eclampsia \|\| id:finngen_R11_O15_PREECLAMPS | Gherkins liking \|\| id:ebi-fl187-GCST90094770 | MR Egger | 19 | -0.122923126 | 0.261531578408564 | 0.644319097809773 | -0.63552502 | 0.389678767267711 | 0.884331641693217 | 0.529657334562297 | 1.4765064155037 | 0.954596128018958 |
| ebi-fl187-GCST90094770 | finngen_R11_O15_PREECLAMPS | Pre-eclampsia \|\| id:finngen_R11_O15_PREECLAMPS | Gherkins liking \|\| id:ebi-fl187-GCST90094770 | Weighted median | 19 | -0.13430231 | 0.0840864755766957 | 0.110222746896659 | -0.299111802 | 0.0305071824340915 | 0.874325707507446 | 0.741476506372979 | 1.03097729494866 | 0.529780299600071 |
| ebi-fl187-GCST90094770 | finngen_R11_O15_PREECLAMPS | Pre-eclampsia \|\| id:finngen_R11_O15_PREECLAMPS | Gherkins liking \|\| id:ebi-fl187-GCST90094770 | Weighted mode | 19 | -0.133124287 | 0.123388518884828 | 0.294876881144687 | -0.374965784 | 0.108717209638976 | 0.875356289611846 | 0.687312795215514 | 1.11484703776357 | 0.905055003276494 |
| ebi-fl187-GCST90094771 | finngen_R11_O15_PREECLAMPS | Pre-eclampsia \|\| id:finngen_R11_O15_PREECLAMPS | Globe artichoke liking \|\| id:ebi-fl187-GCST90094771 | Inverse variance weighted | 9 | -0.159703387 | 0.0827907803860544 | 0.053730905245751 | -0.321973316 | 0.00256654288028205 | 0.852396583656938 | 0.724717528265525 | 1.00256983927097 | 0.366852117259508 |
| ebi-fl187-GCST90094771 | finngen_R11_O15_PREECLAMPS | Pre-eclampsia \|\| id:finngen_R11_O15_PREECLAMPS | Globe artichoke liking \|\| id:ebi-fl187-GCST90094771 | MR Egger | 9 | -0.306058435 | 0.339230322597553 | 0.396924374610769 | -0.970949868 | 0.358832996960158 | 0.736343589717455 | 0.378723130358778 | 1.43165769041976 | 0.954596128018958 |
| ebi-fl187-GCST90094771 | finngen_R11_O15_PREECLAMPS | Pre-eclampsia \|\| id:finngen_R11_O15_PREECLAMPS | Globe artichoke liking \|\| id:ebi-fl187-GCST90094771 | Weighted median | 9 | -0.166585734 | 0.106061375227829 | 0.116263014942116 | -0.37446603 | 0.0412945609889966 | 0.8465502352989 | 0.687656368711882 | 1.04215903973528 | 0.539545353082283 |
| ebi-fl187-GCST90094771 | finngen_R11_O15_PREECLAMPS | Pre-eclampsia \|\| id:finngen_R11_O15_PREECLAMPS | Globe artichoke liking \|\| id:ebi-fl187-GCST90094771 | Weighted mode | 9 | -0.176945351 | 0.135527091454091 | 0.22797870891295 | -0.44257845 | 0.0886877482527298 | 0.837825569603753 | 0.642377944282736 | 1.09273939326425 | 0.905055003276494 |
| ebi-fl187-GCST90094772 | finngen_R11_O15_PREECLAMPS | Pre-eclampsia \|\| id:finngen_R11_O15_PREECLAMPS | F-glutamate liking (derived food-liking factor) \|\| id:ebi-fl187-GCST90094772 | Inverse variance weighted | 9 | -0.003844817 | 0.0531776115674819 | 0.942362035805146 | -0.108072935 | 0.100383301920281 | 0.996162565092301 | 0.897562129968313 | 1.10559461340721 | 0.977922867344962 |
| ebi-fl187-GCST90094772 | finngen_R11_O15_PREECLAMPS | Pre-eclampsia \|\| id:finngen_R11_O15_PREECLAMPS | F-glutamate liking (derived food-liking factor) \|\| id:ebi-fl187-GCST90094772 | MR Egger | 9 | 0.28980538716932 | 0.26014155015296 | 0.302050277355067 | -0.220072051 | 0.799682825469122 | 1.33616742739542 | 0.802460977658886 | 2.22483515552504 | 0.954596128018958 |
| ebi-fl187-GCST90094772 | finngen_R11_O15_PREECLAMPS | Pre-eclampsia \|\| id:finngen_R11_O15_PREECLAMPS | F-glutamate liking (derived food-liking factor) \|\| id:ebi-fl187-GCST90094772 | Weighted median | 9 | 0.0347993812118411 | 0.0686607437146705 | 0.612273527599102 | -0.099775676 | 0.169374438892595 | 1.03541196486802 | 0.905040417128518 | 1.18456360257733 | 0.881409801621768 |
| ebi-fl187-GCST90094772 | finngen_R11_O15_PREECLAMPS | Pre-eclampsia \|\| id:finngen_R11_O15_PREECLAMPS | F-glutamate liking (derived food-liking factor) \|\| id:ebi-fl187-GCST90094772 | Weighted mode | 9 | 0.0480038650618014 | 0.0922587849750353 | 0.61693097188195 | -0.132823353 | 0.228831083612871 | 1.04917471044173 | 0.875619753622366 | 1.25712967127192 | 0.92526935022822 |
| ebi-fl187-GCST90094773 | finngen_R11_O15_PREECLAMPS | Pre-eclampsia \|\| id:finngen_R11_O15_PREECLAMPS | Goat cheese liking \|\| id:ebi-fl187-GCST90094773 | Inverse variance weighted | 7 | -0.183160177 | 0.109843434787969 | 0.0954218077009669 | -0.39845331 | 0.0321329548534313 | 0.832634775839077 | 0.67135762587306 | 1.03265479264501 | 0.507890266795469 |
| ebi-fl187-GCST90094773 | finngen_R11_O15_PREECLAMPS | Pre-eclampsia \|\| id:finngen_R11_O15_PREECLAMPS | Goat cheese liking \|\| id:ebi-fl187-GCST90094773 | MR Egger | 7 | -1.072531687 | 0.551315869071712 | 0.109311710363033 | -2.15311079 | 0.00804741647889284 | 0.342141225566948 | 0.116122363013033 | 1.00807988396959 | 0.954596128018958 |
| ebi-fl187-GCST90094773 | finngen_R11_O15_PREECLAMPS | Pre-eclampsia \|\| id:finngen_R11_O15_PREECLAMPS | Goat cheese liking \|\| id:ebi-fl187-GCST90094773 | Weighted median | 7 | -0.299502672 | 0.135522187801898 | 0.0271058601634197 | -0.56512616 | -0.033879184 | 0.741186741723431 | 0.568288446733787 | 0.966688288780119 | 0.367261503827345 |
| ebi-fl187-GCST90094773 | finngen_R11_O15_PREECLAMPS | Pre-eclampsia \|\| id:finngen_R11_O15_PREECLAMPS | Goat cheese liking \|\| id:ebi-fl187-GCST90094773 | Weighted mode | 7 | -0.324632842 | 0.174105647091971 | 0.111514482258577 | -0.66587991 | 0.0166142263511904 | 0.722792684083101 | 0.513821212186356 | 1.01675301013959 | 0.905055003276494 |
| ebi-fl187-GCST90094774 | finngen_R11_O15_PREECLAMPS | Pre-eclampsia \|\| id:finngen_R11_O15_PREECLAMPS | Grapefruit liking \|\| id:ebi-fl187-GCST90094774 | Inverse variance weighted | 8 | -0.101211748 | 0.0779394426732138 | 0.194083052346184 | -0.253973056 | 0.0515495592240697 | 0.903741646762066 | 0.775712702525352 | 1.05290136597385 | 0.654280117715626 |
| ebi-fl187-GCST90094774 | finngen_R11_O15_PREECLAMPS | Pre-eclampsia \|\| id:finngen_R11_O15_PREECLAMPS | Grapefruit liking \|\| id:ebi-fl187-GCST90094774 | MR Egger | 8 | -0.121244043 | 0.171755624286048 | 0.506713902661739 | -0.457885066 | 0.215396980702207 | 0.885817755679841 | 0.632620181281332 | 1.24035419592269 | 0.954596128018958 |
| ebi-fl187-GCST90094774 | finngen_R11_O15_PREECLAMPS | Pre-eclampsia \|\| id:finngen_R11_O15_PREECLAMPS | Grapefruit liking \|\| id:ebi-fl187-GCST90094774 | Weighted median | 8 | -0.100521944 | 0.0957537084031271 | 0.293811513265443 | -0.288199212 | 0.0871553248135124 | 0.904365267114154 | 0.749612245514032 | 1.09106613620167 | 0.71718574364164 |
| ebi-fl187-GCST90094774 | finngen_R11_O15_PREECLAMPS | Pre-eclampsia \|\| id:finngen_R11_O15_PREECLAMPS | Grapefruit liking \|\| id:ebi-fl187-GCST90094774 | Weighted mode | 8 | -0.093064306 | 0.0981476974019334 | 0.374601290608563 | -0.285433793 | 0.0993051812955686 | 0.911134907324201 | 0.751688106906186 | 1.10440329136176 | 0.905055003276494 |
| ebi-fl187-GCST90094775 | finngen_R11_O15_PREECLAMPS | Pre-eclampsia \|\| id:finngen_R11_O15_PREECLAMPS | Green olives liking \|\| id:ebi-fl187-GCST90094775 | Inverse variance weighted | 25 | -0.12023846 | 0.0537360547205326 | 0.0252487742459328 | -0.225561127 | -0.014915793 | 0.88670896675148 | 0.798068275116481 | 0.985194896517746 | 0.258296218889823 |
| ebi-fl187-GCST90094775 | finngen_R11_O15_PREECLAMPS | Pre-eclampsia \|\| id:finngen_R11_O15_PREECLAMPS | Green olives liking \|\| id:ebi-fl187-GCST90094775 | MR Egger | 25 | -0.318611497 | 0.239195343032403 | 0.195905262567401 | -0.787434369 | 0.150211375685491 | 0.727157997748978 | 0.455010688544037 | 1.16207985219479 | 0.954596128018958 |
| ebi-fl187-GCST90094775 | finngen_R11_O15_PREECLAMPS | Pre-eclampsia \|\| id:finngen_R11_O15_PREECLAMPS | Green olives liking \|\| id:ebi-fl187-GCST90094775 | Weighted median | 25 | -0.080763232 | 0.0702484889435295 | 0.2502759933336 | -0.21845027 | 0.0569238064700632 | 0.922412063381077 | 0.803763449455571 | 1.05857515074523 | 0.672512525447174 |
| ebi-fl187-GCST90094775 | finngen_R11_O15_PREECLAMPS | Pre-eclampsia \|\| id:finngen_R11_O15_PREECLAMPS | Green olives liking \|\| id:ebi-fl187-GCST90094775 | Weighted mode | 25 | -0.036693951 | 0.122226859984701 | 0.766600586879648 | -0.276258596 | 0.202870694683806 | 0.963971112712738 | 0.758616729795943 | 1.22491407010571 | 0.945697706548879 |
| ebi-fl187-GCST90094776 | finngen_R11_O15_PREECLAMPS | Pre-eclampsia \|\| id:finngen_R11_O15_PREECLAMPS | Haddock liking \|\| id:ebi-fl187-GCST90094776 | Inverse variance weighted | 5 | -0.351009245 | 0.215273967342397 | 0.102990919544691 | -0.772946221 | 0.0709277305451684 | 0.703977245241977 | 0.461650936839517 | 1.07350364154197 | 0.512568357226114 |
| ebi-fl187-GCST90094776 | finngen_R11_O15_PREECLAMPS | Pre-eclampsia \|\| id:finngen_R11_O15_PREECLAMPS | Haddock liking \|\| id:ebi-fl187-GCST90094776 | MR Egger | 5 | 0.91217913839666 | 1.83548464624857 | 0.653348178311767 | -2.685370768 | 4.50972904504386 | 2.48974211887578 | 0.0681959044321441 | 90.8971861304658 | 0.954596128018958 |
| ebi-fl187-GCST90094776 | finngen_R11_O15_PREECLAMPS | Pre-eclampsia \|\| id:finngen_R11_O15_PREECLAMPS | Haddock liking \|\| id:ebi-fl187-GCST90094776 | Weighted median | 5 | -0.317336675 | 0.226905929111439 | 0.161951423666185 | -0.762072296 | 0.127398946286427 | 0.728085585808263 | 0.466698287305595 | 1.13587007855172 | 0.612323741171272 |
| ebi-fl187-GCST90094776 | finngen_R11_O15_PREECLAMPS | Pre-eclampsia \|\| id:finngen_R11_O15_PREECLAMPS | Haddock liking \|\| id:ebi-fl187-GCST90094776 | Weighted mode | 5 | -0.328381919 | 0.319927231985712 | 0.362704607146553 | -0.955439294 | 0.298675455802622 | 0.720087951990992 | 0.38464313617271 | 1.34807204351036 | 0.905055003276494 |
| ebi-fl187-GCST90094777 | finngen_R11_O15_PREECLAMPS | Pre-eclampsia \|\| id:finngen_R11_O15_PREECLAMPS | Ham liking \|\| id:ebi-fl187-GCST90094777 | Inverse variance weighted | 10 | -0.065303854 | 0.10437162504178 | 0.531520981956372 | -0.269872239 | 0.139264530704502 | 0.936782774580908 | 0.763477030344321 | 1.14942811882072 | 0.894908414040245 |
| ebi-fl187-GCST90094777 | finngen_R11_O15_PREECLAMPS | Pre-eclampsia \|\| id:finngen_R11_O15_PREECLAMPS | Ham liking \|\| id:ebi-fl187-GCST90094777 | MR Egger | 10 | -0.397779899 | 0.369319491781232 | 0.312856533861972 | -1.121646103 | 0.326086304864867 | 0.671809877398602 | 0.325743146311522 | 1.38553494211878 | 0.954596128018958 |
| ebi-fl187-GCST90094777 | finngen_R11_O15_PREECLAMPS | Pre-eclampsia \|\| id:finngen_R11_O15_PREECLAMPS | Ham liking \|\| id:ebi-fl187-GCST90094777 | Weighted median | 10 | -0.079817231 | 0.146453806529569 | 0.585753957551182 | -0.366866692 | 0.207232229920383 | 0.923285078970246 | 0.692902008493621 | 1.23026824370497 | 0.881409801621768 |
| ebi-fl187-GCST90094777 | finngen_R11_O15_PREECLAMPS | Pre-eclampsia \|\| id:finngen_R11_O15_PREECLAMPS | Ham liking \|\| id:ebi-fl187-GCST90094777 | Weighted mode | 10 | -0.219391561 | 0.242206974279323 | 0.388646623591567 | -0.694117231 | 0.255334108337827 | 0.803007230072489 | 0.499515210034748 | 1.29089284689416 | 0.905055003276494 |
| ebi-fl187-GCST90094778 | finngen_R11_O15_PREECLAMPS | Pre-eclampsia \|\| id:finngen_R11_O15_PREECLAMPS | Hard cheese liking \|\| id:ebi-fl187-GCST90094778 | Inverse variance weighted | 4 | -0.495850256 | 0.382023002010241 | 0.194301368291307 | -1.24461534 | 0.252914828204402 | 0.609052836413664 | 0.288051688878973 | 1.28777359017459 | 0.654280117715626 |
| ebi-fl187-GCST90094778 | finngen_R11_O15_PREECLAMPS | Pre-eclampsia \|\| id:finngen_R11_O15_PREECLAMPS | Hard cheese liking \|\| id:ebi-fl187-GCST90094778 | MR Egger | 4 | -1.20518721 | 1.41310703282129 | 0.483575359041602 | -3.974876994 | 1.56450257455885 | 0.299635899494083 | 0.0187816116016301 | 4.7802965033009 | 0.954596128018958 |
| ebi-fl187-GCST90094778 | finngen_R11_O15_PREECLAMPS | Pre-eclampsia \|\| id:finngen_R11_O15_PREECLAMPS | Hard cheese liking \|\| id:ebi-fl187-GCST90094778 | Weighted median | 4 | -0.425420163 | 0.32824658256846 | 0.194962269166384 | -1.068783465 | 0.217943139081161 | 0.653495153116197 | 0.343426053349166 | 1.24351635812606 | 0.649149713120124 |
| ebi-fl187-GCST90094778 | finngen_R11_O15_PREECLAMPS | Pre-eclampsia \|\| id:finngen_R11_O15_PREECLAMPS | Hard cheese liking \|\| id:ebi-fl187-GCST90094778 | Weighted mode | 4 | -0.974458702 | 0.51460375160586 | 0.154602725607032 | -1.983082055 | 0.0341646508490929 | 0.37739658218646 | 0.137644355357332 | 1.03475496598659 | 0.905055003276494 |
| ebi-fl187-GCST90094779 | finngen_R11_O15_PREECLAMPS | Pre-eclampsia \|\| id:finngen_R11_O15_PREECLAMPS | F-healthy food liking (derived food-liking factor) \|\| id:ebi-fl187-GCST90094779 | Inverse variance weighted | 6 | -0.168684571 | 0.1070079436401 | 0.114939571014341 | -0.37842014 | 0.0410509988738543 | 0.844775328288334 | 0.684942668258685 | 1.04190524018448 | 0.512568357226114 |
| ebi-fl187-GCST90094779 | finngen_R11_O15_PREECLAMPS | Pre-eclampsia \|\| id:finngen_R11_O15_PREECLAMPS | F-healthy food liking (derived food-liking factor) \|\| id:ebi-fl187-GCST90094779 | MR Egger | 6 | 2.15669685485347 | 1.04260766334961 | 0.107405198509079 | 0.113185834688224 | 4.20020787501871 | 8.64254288453558 | 1.11984001873395 | 66.7001949041635 | 0.954596128018958 |
| ebi-fl187-GCST90094779 | finngen_R11_O15_PREECLAMPS | Pre-eclampsia \|\| id:finngen_R11_O15_PREECLAMPS | F-healthy food liking (derived food-liking factor) \|\| id:ebi-fl187-GCST90094779 | Weighted median | 6 | -0.034901527 | 0.109113686603058 | 0.749071523385692 | -0.248764352 | 0.178961299061146 | 0.965700507309786 | 0.779763701163283 | 1.19597445793786 | 0.927373305772456 |
| ebi-fl187-GCST90094779 | finngen_R11_O15_PREECLAMPS | Pre-eclampsia \|\| id:finngen_R11_O15_PREECLAMPS | F-healthy food liking (derived food-liking factor) \|\| id:ebi-fl187-GCST90094779 | Weighted mode | 6 | -0.005621268 | 0.136119835073668 | 0.968657858778169 | -0.272416145 | 0.261173608398989 | 0.994394501420973 | 0.761537285138518 | 1.29845306822555 | 0.981296058194618 |
| ebi-fl187-GCST90094780 | finngen_R11_O15_PREECLAMPS | Pre-eclampsia \|\| id:finngen_R11_O15_PREECLAMPS | F-healthy breakfast food liking (derived food-liking factor) \|\| id:ebi-fl187-GCST90094780 | Inverse variance weighted | 7 | -0.155472817 | 0.0756145291693872 | 0.0397706055915754 | -0.303677294 | -0.007268339 | 0.85601034582354 | 0.738099017119497 | 0.992758011000447 | 0.328416400058518 |
| ebi-fl187-GCST90094780 | finngen_R11_O15_PREECLAMPS | Pre-eclampsia \|\| id:finngen_R11_O15_PREECLAMPS | F-healthy breakfast food liking (derived food-liking factor) \|\| id:ebi-fl187-GCST90094780 | MR Egger | 7 | 0.602652666812512 | 0.674902561368787 | 0.412794932395587 | -0.720156353 | 1.92546168709533 | 1.82695869093122 | 0.486676156504933 | 6.85831433029179 | 0.954596128018958 |
| ebi-fl187-GCST90094780 | finngen_R11_O15_PREECLAMPS | Pre-eclampsia \|\| id:finngen_R11_O15_PREECLAMPS | F-healthy breakfast food liking (derived food-liking factor) \|\| id:ebi-fl187-GCST90094780 | Weighted median | 7 | -0.114559144 | 0.0953724222651113 | 0.229682615603765 | -0.301489091 | 0.0723708039010971 | 0.89175919488577 | 0.739715895591617 | 1.07505390434701 | 0.649149713120124 |
| ebi-fl187-GCST90094780 | finngen_R11_O15_PREECLAMPS | Pre-eclampsia \|\| id:finngen_R11_O15_PREECLAMPS | F-healthy breakfast food liking (derived food-liking factor) \|\| id:ebi-fl187-GCST90094780 | Weighted mode | 7 | -0.067051933 | 0.13352320008436 | 0.633438050274407 | -0.328757405 | 0.194653538972679 | 0.935146634925319 | 0.719817619465066 | 1.21489000152849 | 0.932459680062628 |
| ebi-fl187-GCST90094781 | finngen_R11_O15_PREECLAMPS | Pre-eclampsia \|\| id:finngen_R11_O15_PREECLAMPS | Herring liking \|\| id:ebi-fl187-GCST90094781 | Inverse variance weighted (multiplicative random effects) | 18 | -0.049036973 | 0.0825167616313184 | 0.552333673239876 | -0.210769826 | 0.112695880031024 | 0.952145925578865 | 0.809960477623286 | 1.11929148229142 | 0.911350560845795 |
| ebi-fl187-GCST90094781 | finngen_R11_O15_PREECLAMPS | Pre-eclampsia \|\| id:finngen_R11_O15_PREECLAMPS | Herring liking \|\| id:ebi-fl187-GCST90094781 | MR Egger | 18 | 0.0517271358848435 | 0.363455537814102 | 0.888603684356461 | -0.660645718 | 0.764099990000483 | 1.05308835328434 | 0.516517701890326 | 2.14706112833013 | 0.970712810886627 |
| ebi-fl187-GCST90094781 | finngen_R11_O15_PREECLAMPS | Pre-eclampsia \|\| id:finngen_R11_O15_PREECLAMPS | Herring liking \|\| id:ebi-fl187-GCST90094781 | Weighted median | 18 | -0.118221774 | 0.0820042700660082 | 0.1494000417002 | -0.278950143 | 0.0425065955852992 | 0.8884989850167 | 0.756577622909718 | 1.04342293833598 | 0.612323741171272 |
| ebi-fl187-GCST90094781 | finngen_R11_O15_PREECLAMPS | Pre-eclampsia \|\| id:finngen_R11_O15_PREECLAMPS | Herring liking \|\| id:ebi-fl187-GCST90094781 | Weighted mode | 18 | -0.048385621 | 0.125373943795382 | 0.704335275339255 | -0.294118551 | 0.197347308441592 | 0.952766309152894 | 0.745188143230838 | 1.21816704694351 | 0.936018697441052 |
| ebi-fl187-GCST90094783 | finngen_R11_O15_PREECLAMPS | Pre-eclampsia \|\| id:finngen_R11_O15_PREECLAMPS | Horseradish liking \|\| id:ebi-fl187-GCST90094783 | Inverse variance weighted (multiplicative random effects) | 23 | -0.03998112 | 0.0634192178320367 | 0.5284159460264 | -0.164282787 | 0.0843205471715316 | 0.960807579240263 | 0.848502042832226 | 1.08797758605758 | 0.894908414040245 |
| ebi-fl187-GCST90094783 | finngen_R11_O15_PREECLAMPS | Pre-eclampsia \|\| id:finngen_R11_O15_PREECLAMPS | Horseradish liking \|\| id:ebi-fl187-GCST90094783 | MR Egger | 23 | -0.09435628 | 0.214149847 | 0.663998588678732 | -0.514089979 | 0.325377419275809 | 0.909958504577826 | 0.598044580171885 | 1.38455310441159 | 0.954596128018958 |
| ebi-fl187-GCST90094783 | finngen_R11_O15_PREECLAMPS | Pre-eclampsia \|\| id:finngen_R11_O15_PREECLAMPS | Horseradish liking \|\| id:ebi-fl187-GCST90094783 | Weighted median | 23 | -0.023686499 | 0.0750459440112616 | 0.752285914462042 | -0.170776549 | 0.123403551078496 | 0.976591824106022 | 0.843009923462102 | 1.13134088267183 | 0.927373305772456 |
| ebi-fl187-GCST90094783 | finngen_R11_O15_PREECLAMPS | Pre-eclampsia \|\| id:finngen_R11_O15_PREECLAMPS | Horseradish liking \|\| id:ebi-fl187-GCST90094783 | Weighted mode | 23 | 0.0815966279953213 | 0.145685578273078 | 0.581078940274696 | -0.203947105 | 0.367140361410554 | 1.08501805563838 | 0.81550550586778 | 1.44360053070219 | 0.905055003276494 |
| ebi-fl187-GCST90094784 | finngen_R11_O15_PREECLAMPS | Pre-eclampsia \|\| id:finngen_R11_O15_PREECLAMPS | Ice cream liking \|\| id:ebi-fl187-GCST90094784 | Inverse variance weighted | 8 | 0.317349789148296 | 0.14141881762013 | 0.0248296887714432 | 0.0401689066128416 | 0.594530671683751 | 1.37348291733061 | 1.04098658886258 | 1.81218023784553 | 0.258296218889823 |
| ebi-fl187-GCST90094784 | finngen_R11_O15_PREECLAMPS | Pre-eclampsia \|\| id:finngen_R11_O15_PREECLAMPS | Ice cream liking \|\| id:ebi-fl187-GCST90094784 | MR Egger | 8 | -0.168735487 | 0.691373254726171 | 0.815316765854736 | -1.523827066 | 1.18635609236542 | 0.8447323166024 | 0.217876461724091 | 3.27512518362857 | 0.954596128018958 |
| ebi-fl187-GCST90094784 | finngen_R11_O15_PREECLAMPS | Pre-eclampsia \|\| id:finngen_R11_O15_PREECLAMPS | Ice cream liking \|\| id:ebi-fl187-GCST90094784 | Weighted median | 8 | 0.292841839100374 | 0.158092581027983 | 0.063976445583403 | -0.01701962 | 0.60270329791522 | 1.34023080155867 | 0.983124395825752 | 1.82705119420609 | 0.501710020627739 |
| ebi-fl187-GCST90094784 | finngen_R11_O15_PREECLAMPS | Pre-eclampsia \|\| id:finngen_R11_O15_PREECLAMPS | Ice cream liking \|\| id:ebi-fl187-GCST90094784 | Weighted mode | 8 | 0.132387971466083 | 0.214432986872538 | 0.556522040215488 | -0.287900683 | 0.552676625736257 | 1.1415511226268 | 0.749836060155894 | 1.73789850183996 | 0.905055003276494 |
| ebi-fl187-GCST90094785 | finngen_R11_O15_PREECLAMPS | Pre-eclampsia \|\| id:finngen_R11_O15_PREECLAMPS | F-highly palatable foods liking (derived food-liking factor) \|\| id:ebi-fl187-GCST90094785 | Inverse variance weighted | 17 | 0.126384002748276 | 0.19920934387049 | 0.525801485077935 | -0.264066311 | 0.516834316734436 | 1.13471781939322 | 0.76792261606905 | 1.67671130229187 | 0.894908414040245 |
| ebi-fl187-GCST90094785 | finngen_R11_O15_PREECLAMPS | Pre-eclampsia \|\| id:finngen_R11_O15_PREECLAMPS | F-highly palatable foods liking (derived food-liking factor) \|\| id:ebi-fl187-GCST90094785 | MR Egger | 17 | 0.0506578816817468 | 1.46934584390211 | 0.97295179879212 | -2.829259972 | 2.93057573572988 | 1.05196293592221 | 0.0590565409748145 | 18.7384157671207 | 0.986189238231469 |
| ebi-fl187-GCST90094785 | finngen_R11_O15_PREECLAMPS | Pre-eclampsia \|\| id:finngen_R11_O15_PREECLAMPS | F-highly palatable foods liking (derived food-liking factor) \|\| id:ebi-fl187-GCST90094785 | Weighted median | 17 | 0.0313413166560328 | 0.242841646473005 | 0.897309606433418 | -0.44462831 | 0.507310943743123 | 1.03183762715881 | 0.641062508002671 | 1.66081914872532 | 0.988450091947753 |
| ebi-fl187-GCST90094785 | finngen_R11_O15_PREECLAMPS | Pre-eclampsia \|\| id:finngen_R11_O15_PREECLAMPS | F-highly palatable foods liking (derived food-liking factor) \|\| id:ebi-fl187-GCST90094785 | Weighted mode | 17 | -0.020620942 | 0.420466684646669 | 0.961492033625638 | -0.844735644 | 0.803493759791924 | 0.979590215596955 | 0.429670929276773 | 2.23333003261006 | 0.981296058194618 |
| ebi-fl187-GCST90094786 | finngen_R11_O15_PREECLAMPS | Pre-eclampsia \|\| id:finngen_R11_O15_PREECLAMPS | Jam liking \|\| id:ebi-fl187-GCST90094786 | Inverse variance weighted | 5 | -0.186505703 | 0.174883181111128 | 0.286216305429883 | -0.529276738 | 0.156265332124686 | 0.829853829403346 | 0.589030839374601 | 1.16913637137671 | 0.75876791114856 |
| ebi-fl187-GCST90094786 | finngen_R11_O15_PREECLAMPS | Pre-eclampsia \|\| id:finngen_R11_O15_PREECLAMPS | Jam liking \|\| id:ebi-fl187-GCST90094786 | MR Egger | 5 | 0.822372120163859 | 1.74609056189789 | 0.669786139389148 | -2.599965381 | 4.24470962148372 | 2.27589212903224 | 0.0742761495242587 | 69.7355075103779 | 0.954596128018958 |
| ebi-fl187-GCST90094786 | finngen_R11_O15_PREECLAMPS | Pre-eclampsia \|\| id:finngen_R11_O15_PREECLAMPS | Jam liking \|\| id:ebi-fl187-GCST90094786 | Weighted median | 5 | -0.138551596 | 0.222410895155434 | 0.533315224896413 | -0.574476951 | 0.297373758183691 | 0.870618329407467 | 0.562999268109954 | 1.34631840294367 | 0.881409801621768 |
| ebi-fl187-GCST90094786 | finngen_R11_O15_PREECLAMPS | Pre-eclampsia \|\| id:finngen_R11_O15_PREECLAMPS | Jam liking \|\| id:ebi-fl187-GCST90094786 | Weighted mode | 5 | -0.048557714 | 0.279705097970786 | 0.870608658044322 | -0.596779706 | 0.499664278099089 | 0.952602359299459 | 0.550581819668332 | 1.64816785176368 | 0.981296058194618 |
| ebi-fl187-GCST90094787 | finngen_R11_O15_PREECLAMPS | Pre-eclampsia \|\| id:finngen_R11_O15_PREECLAMPS | Ketchup liking \|\| id:ebi-fl187-GCST90094787 | Inverse variance weighted | 10 | 0.0678529900057083 | 0.117565400565618 | 0.563837446106042 | -0.162575195 | 0.29828117511432 | 1.07020796564665 | 0.849952175581088 | 1.34754062950719 | 0.921120580272246 |
| ebi-fl187-GCST90094787 | finngen_R11_O15_PREECLAMPS | Pre-eclampsia \|\| id:finngen_R11_O15_PREECLAMPS | Ketchup liking \|\| id:ebi-fl187-GCST90094787 | MR Egger | 10 | -1.673621515 | 0.676913220484437 | 0.0385639884459713 | -3.000371427 | -0.346871603 | 0.187566559042207 | 0.0497685795326054 | 0.706896085870503 | 0.954596128018958 |
| ebi-fl187-GCST90094787 | finngen_R11_O15_PREECLAMPS | Pre-eclampsia \|\| id:finngen_R11_O15_PREECLAMPS | Ketchup liking \|\| id:ebi-fl187-GCST90094787 | Weighted median | 10 | 0.00552332582371825 | 0.140694333656999 | 0.968684988448455 | -0.270237568 | 0.281284219791436 | 1.00553860751009 | 0.763198161227554 | 1.32483009336267 | 0.996099865434941 |
| ebi-fl187-GCST90094787 | finngen_R11_O15_PREECLAMPS | Pre-eclampsia \|\| id:finngen_R11_O15_PREECLAMPS | Ketchup liking \|\| id:ebi-fl187-GCST90094787 | Weighted mode | 10 | -0.183142493 | 0.213020606076417 | 0.41224661458994 | -0.600662881 | 0.23437789443101 | 0.832649500159598 | 0.548447959625143 | 1.2641221066624 | 0.905055003276494 |
| ebi-fl187-GCST90094788 | finngen_R11_O15_PREECLAMPS | Pre-eclampsia \|\| id:finngen_R11_O15_PREECLAMPS | Kiwi liking \|\| id:ebi-fl187-GCST90094788 | Inverse variance weighted | 3 | -0.380956497 | 0.273442041232015 | 0.163562586268805 | -0.916902898 | 0.154989903489322 | 0.683207610331768 | 0.399755208427808 | 1.16764617189357 | 0.62762387754309 |
| ebi-fl187-GCST90094788 | finngen_R11_O15_PREECLAMPS | Pre-eclampsia \|\| id:finngen_R11_O15_PREECLAMPS | Kiwi liking \|\| id:ebi-fl187-GCST90094788 | MR Egger | 3 | -1.04789893 | 0.604406200932625 | 0.333060552364167 | -2.232535084 | 0.13673722399993 | 0.350673765818477 | 0.107256181801961 | 1.14652682919824 | 0.954596128018958 |
| ebi-fl187-GCST90094788 | finngen_R11_O15_PREECLAMPS | Pre-eclampsia \|\| id:finngen_R11_O15_PREECLAMPS | Kiwi liking \|\| id:ebi-fl187-GCST90094788 | Weighted median | 3 | -0.287458492 | 0.238631976535556 | 0.228353851885172 | -0.755177166 | 0.180260182458543 | 0.75016770442253 | 0.469927352412205 | 1.19752889860503 | 0.649149713120124 |
| ebi-fl187-GCST90094788 | finngen_R11_O15_PREECLAMPS | Pre-eclampsia \|\| id:finngen_R11_O15_PREECLAMPS | Kiwi liking \|\| id:ebi-fl187-GCST90094788 | Weighted mode | 3 | -0.171923171 | 0.240680565724577 | 0.549147462281964 | -0.64365708 | 0.299810737876674 | 0.842043864119011 | 0.525367595360816 | 1.34960335460644 | 0.905055003276494 |
| ebi-fl187-GCST90094789 | finngen_R11_O15_PREECLAMPS | Pre-eclampsia \|\| id:finngen_R11_O15_PREECLAMPS | Lager liking \|\| id:ebi-fl187-GCST90094789 | Inverse variance weighted | 10 | -0.09735425 | 0.102817832686416 | 0.343709346216828 | -0.298877202 | 0.104168702311793 | 0.907234561580925 | 0.741650477172984 | 1.10978766286826 | 0.802417492460712 |
| ebi-fl187-GCST90094789 | finngen_R11_O15_PREECLAMPS | Pre-eclampsia \|\| id:finngen_R11_O15_PREECLAMPS | Lager liking \|\| id:ebi-fl187-GCST90094789 | MR Egger | 10 | 0.358748208930416 | 0.388252906978603 | 0.382507496400593 | -0.402227489 | 1.11972390660848 | 1.43153630813088 | 0.668828577407253 | 3.06400813410393 | 0.954596128018958 |
| ebi-fl187-GCST90094789 | finngen_R11_O15_PREECLAMPS | Pre-eclampsia \|\| id:finngen_R11_O15_PREECLAMPS | Lager liking \|\| id:ebi-fl187-GCST90094789 | Weighted median | 10 | -0.019871698 | 0.129126880393564 | 0.877694256761757 | -0.272960383 | 0.233216987658264 | 0.980324442910174 | 0.761122940070409 | 1.26265543024921 | 0.988450091947753 |
| ebi-fl187-GCST90094789 | finngen_R11_O15_PREECLAMPS | Pre-eclampsia \|\| id:finngen_R11_O15_PREECLAMPS | Lager liking \|\| id:ebi-fl187-GCST90094789 | Weighted mode | 10 | 0.0234507993787663 | 0.17960933213038 | 0.89899077958448 | -0.328583492 | 0.375485090354311 | 1.02372793145734 | 0.719942816541103 | 1.45569738813567 | 0.981296058194618 |
| ebi-fl187-GCST90094790 | finngen_R11_O15_PREECLAMPS | Pre-eclampsia \|\| id:finngen_R11_O15_PREECLAMPS | Lamb liking \|\| id:ebi-fl187-GCST90094790 | Inverse variance weighted | 7 | 0.0221498198289758 | 0.125745345163105 | 0.860177492148952 | -0.224311057 | 0.268610696348663 | 1.0223969483326 | 0.799066540714197 | 1.30814577597699 | 0.953358127900067 |
| ebi-fl187-GCST90094790 | finngen_R11_O15_PREECLAMPS | Pre-eclampsia \|\| id:finngen_R11_O15_PREECLAMPS | Lamb liking \|\| id:ebi-fl187-GCST90094790 | MR Egger | 7 | -1.389831242 | 0.689336077539204 | 0.0998546802425452 | -2.740929954 | -0.03873253 | 0.249117341575402 | 0.064510327340474 | 0.962007982784784 | 0.954596128018958 |
| ebi-fl187-GCST90094790 | finngen_R11_O15_PREECLAMPS | Pre-eclampsia \|\| id:finngen_R11_O15_PREECLAMPS | Lamb liking \|\| id:ebi-fl187-GCST90094790 | Weighted median | 7 | -0.015982028 | 0.14578808403349 | 0.912706733794366 | -0.301726672 | 0.269762616945402 | 0.984145007186376 | 0.739540173959419 | 1.30965352427631 | 0.988450091947753 |
| ebi-fl187-GCST90094790 | finngen_R11_O15_PREECLAMPS | Pre-eclampsia \|\| id:finngen_R11_O15_PREECLAMPS | Lamb liking \|\| id:ebi-fl187-GCST90094790 | Weighted mode | 7 | -0.100032446 | 0.248389906912917 | 0.701108132516306 | -0.586876664 | 0.386811771598134 | 0.904808060201539 | 0.556061341988066 | 1.47227934040278 | 0.936018697441052 |
| ebi-fl187-GCST90094791 | finngen_R11_O15_PREECLAMPS | Pre-eclampsia \|\| id:finngen_R11_O15_PREECLAMPS | F-acquired taste liking (derived food-liking factor) \|\| id:ebi-fl187-GCST90094791 | Inverse variance weighted (multiplicative random effects) | 34 | -0.060762949 | 0.0573909451316547 | 0.289711384256723 | -0.173249201 | 0.0517233038302149 | 0.941046289652065 | 0.840928028553287 | 1.05308431779998 | 0.75876791114856 |
| ebi-fl187-GCST90094791 | finngen_R11_O15_PREECLAMPS | Pre-eclampsia \|\| id:finngen_R11_O15_PREECLAMPS | F-acquired taste liking (derived food-liking factor) \|\| id:ebi-fl187-GCST90094791 | MR Egger | 34 | -0.218234139 | 0.320917842967271 | 0.501375199353585 | -0.847233111 | 0.41076483359695 | 0.803937186885942 | 0.428599179278036 | 1.5079706908137 | 0.954596128018958 |
| ebi-fl187-GCST90094791 | finngen_R11_O15_PREECLAMPS | Pre-eclampsia \|\| id:finngen_R11_O15_PREECLAMPS | F-acquired taste liking (derived food-liking factor) \|\| id:ebi-fl187-GCST90094791 | Weighted median | 34 | -0.139450011 | 0.0714855041592506 | 0.0510873527290066 | -0.279561599 | 0.000661577511840955 | 0.869836504687505 | 0.756115150600425 | 1.00066179640251 | 0.447765620977764 |
| ebi-fl187-GCST90094791 | finngen_R11_O15_PREECLAMPS | Pre-eclampsia \|\| id:finngen_R11_O15_PREECLAMPS | F-acquired taste liking (derived food-liking factor) \|\| id:ebi-fl187-GCST90094791 | Weighted mode | 34 | -0.176856204 | 0.118099354384427 | 0.143762817595504 | -0.408330939 | 0.0546185302791058 | 0.8379002623034 | 0.664758847848915 | 1.05613765328577 | 0.905055003276494 |
| ebi-fl187-GCST90094792 | finngen_R11_O15_PREECLAMPS | Pre-eclampsia \|\| id:finngen_R11_O15_PREECLAMPS | Lemon liking \|\| id:ebi-fl187-GCST90094792 | Inverse variance weighted | 5 | -0.217283756 | 0.194261357695978 | 0.263348173728449 | -0.598036017 | 0.163468505530973 | 0.804701598358614 | 0.549890552156388 | 1.17758826708619 | 0.754417186011084 |
| ebi-fl187-GCST90094792 | finngen_R11_O15_PREECLAMPS | Pre-eclampsia \|\| id:finngen_R11_O15_PREECLAMPS | Lemon liking \|\| id:ebi-fl187-GCST90094792 | MR Egger | 5 | -1.506429969 | 2.15392856011567 | 0.534659963051066 | -5.728129946 | 2.71527000921467 | 0.221700042953899 | 0.00325315512317324 | 15.1086890064489 | 0.954596128018958 |
| ebi-fl187-GCST90094792 | finngen_R11_O15_PREECLAMPS | Pre-eclampsia \|\| id:finngen_R11_O15_PREECLAMPS | Lemon liking \|\| id:ebi-fl187-GCST90094792 | Weighted median | 5 | -0.306006154 | 0.248274106213762 | 0.217749859102439 | -0.792623402 | 0.180611094044285 | 0.736382087647607 | 0.452655738165913 | 1.19794919910966 | 0.649149713120124 |
| ebi-fl187-GCST90094792 | finngen_R11_O15_PREECLAMPS | Pre-eclampsia \|\| id:finngen_R11_O15_PREECLAMPS | Lemon liking \|\| id:ebi-fl187-GCST90094792 | Weighted mode | 5 | -0.325694364 | 0.319888624463587 | 0.366179571260213 | -0.952676068 | 0.301287339474111 | 0.722025830455927 | 0.385707461571665 | 1.35159765310558 | 0.905055003276494 |
| ebi-fl187-GCST90094793 | finngen_R11_O15_PREECLAMPS | Pre-eclampsia \|\| id:finngen_R11_O15_PREECLAMPS | F-lentils/beans liking (derived food-liking factor) \|\| id:ebi-fl187-GCST90094793 | Inverse variance weighted | 2 | -0.049135872 | 0.277081176183527 | 0.85924616405413 | -0.592214977 | 0.493943233719724 | 0.952051764113687 | 0.553100821388293 | 1.63876553152985 | 0.953358127900067 |
| ebi-fl187-GCST90094794 | finngen_R11_O15_PREECLAMPS | Pre-eclampsia \|\| id:finngen_R11_O15_PREECLAMPS | Liver liking \|\| id:ebi-fl187-GCST90094794 | Inverse variance weighted | 11 | -0.025703457 | 0.0907181496348993 | 0.77692148496834 | -0.20351103 | 0.152104116202252 | 0.974624064623814 | 0.815861205024893 | 1.16428145068482 | 0.945152512750355 |
| ebi-fl187-GCST90094794 | finngen_R11_O15_PREECLAMPS | Pre-eclampsia \|\| id:finngen_R11_O15_PREECLAMPS | Liver liking \|\| id:ebi-fl187-GCST90094794 | MR Egger | 11 | 0.630143124506712 | 0.398096634574865 | 0.147906238967131 | -0.150126279 | 1.41041252827345 | 1.87787933058429 | 0.860599293721029 | 4.09764545005404 | 0.954596128018958 |
| ebi-fl187-GCST90094794 | finngen_R11_O15_PREECLAMPS | Pre-eclampsia \|\| id:finngen_R11_O15_PREECLAMPS | Liver liking \|\| id:ebi-fl187-GCST90094794 | Weighted median | 11 | -0.048563495 | 0.108231297844717 | 0.653647338631858 | -0.260696839 | 0.163569848519875 | 0.952596852004762 | 0.770514474125268 | 1.17770761344822 | 0.901791235705063 |
| ebi-fl187-GCST90094794 | finngen_R11_O15_PREECLAMPS | Pre-eclampsia \|\| id:finngen_R11_O15_PREECLAMPS | Liver liking \|\| id:ebi-fl187-GCST90094794 | Weighted mode | 11 | 0.00803976806672879 | 0.14339501862729 | 0.956392636471005 | -0.273014468 | 0.289094004576218 | 1.00807217378863 | 0.761081775881163 | 1.33521723915986 | 0.981296058194618 |
| ebi-fl187-GCST90094795 | finngen_R11_O15_PREECLAMPS | Pre-eclampsia \|\| id:finngen_R11_O15_PREECLAMPS | F-low caloric food liking (derived food-liking factor) \|\| id:ebi-fl187-GCST90094795 | Inverse variance weighted | 7 | -0.037945348 | 0.0720074653779327 | 0.598218527907655 | -0.17907998 | 0.103189283883444 | 0.962765556268883 | 0.836039029988545 | 1.10870124849366 | 0.941775027862301 |
| ebi-fl187-GCST90094795 | finngen_R11_O15_PREECLAMPS | Pre-eclampsia \|\| id:finngen_R11_O15_PREECLAMPS | F-low caloric food liking (derived food-liking factor) \|\| id:ebi-fl187-GCST90094795 | MR Egger | 7 | 0.239562965560514 | 0.485763577291911 | 0.642788388948889 | -0.712533646 | 1.19165957705266 | 1.27069369204759 | 0.490400121829354 | 3.2925409010632 | 0.954596128018958 |
| ebi-fl187-GCST90094795 | finngen_R11_O15_PREECLAMPS | Pre-eclampsia \|\| id:finngen_R11_O15_PREECLAMPS | F-low caloric food liking (derived food-liking factor) \|\| id:ebi-fl187-GCST90094795 | Weighted median | 7 | -0.062535168 | 0.0980147210402515 | 0.523461884472174 | -0.254644021 | 0.129573685133701 | 0.939380026016988 | 0.775192400799319 | 1.13834298732776 | 0.881409801621768 |
| ebi-fl187-GCST90094795 | finngen_R11_O15_PREECLAMPS | Pre-eclampsia \|\| id:finngen_R11_O15_PREECLAMPS | F-low caloric food liking (derived food-liking factor) \|\| id:ebi-fl187-GCST90094795 | Weighted mode | 7 | -0.082508579 | 0.124997340565801 | 0.533705462357479 | -0.327503367 | 0.16248620819714 | 0.920803537962231 | 0.720720864733458 | 1.1764320932173 | 0.905055003276494 |
| ebi-fl187-GCST90094796 | finngen_R11_O15_PREECLAMPS | Pre-eclampsia \|\| id:finngen_R11_O15_PREECLAMPS | Mackerel liking \|\| id:ebi-fl187-GCST90094796 | Inverse variance weighted | 13 | -0.104162745 | 0.0746210184340706 | 0.162747574033218 | -0.250419942 | 0.0420944507124806 | 0.901078639067507 | 0.77847380092564 | 1.04299298552927 | 0.62762387754309 |
| ebi-fl187-GCST90094796 | finngen_R11_O15_PREECLAMPS | Pre-eclampsia \|\| id:finngen_R11_O15_PREECLAMPS | Mackerel liking \|\| id:ebi-fl187-GCST90094796 | MR Egger | 13 | 0.0656171392340707 | 0.479189305470126 | 0.893557386701203 | -0.873593899 | 1.00482817795552 | 1.06781781334848 | 0.417448581864125 | 2.73143791125746 | 0.970712810886627 |
| ebi-fl187-GCST90094796 | finngen_R11_O15_PREECLAMPS | Pre-eclampsia \|\| id:finngen_R11_O15_PREECLAMPS | Mackerel liking \|\| id:ebi-fl187-GCST90094796 | Weighted median | 13 | -0.036859841 | 0.103027863414343 | 0.720518618991488 | -0.238794454 | 0.165074770799536 | 0.96381121222369 | 0.787576749156623 | 1.17948130617473 | 0.925493743359756 |
| ebi-fl187-GCST90094796 | finngen_R11_O15_PREECLAMPS | Pre-eclampsia \|\| id:finngen_R11_O15_PREECLAMPS | Mackerel liking \|\| id:ebi-fl187-GCST90094796 | Weighted mode | 13 | 0.00572875435401388 | 0.146154400488898 | 0.969378215545474 | -0.280733871 | 0.292191379312253 | 1.00574519504714 | 0.75522929745468 | 1.33935931877843 | 0.981296058194618 |
| ebi-fl187-GCST90094797 | finngen_R11_O15_PREECLAMPS | Pre-eclampsia \|\| id:finngen_R11_O15_PREECLAMPS | Marzipan liking \|\| id:ebi-fl187-GCST90094797 | Inverse variance weighted | 7 | 0.106452661671793 | 0.141414331320211 | 0.451587306436081 | -0.170719428 | 0.383624751059407 | 1.11232526958067 | 0.843058079022544 | 1.46759462501351 | 0.887682351940796 |
| ebi-fl187-GCST90094797 | finngen_R11_O15_PREECLAMPS | Pre-eclampsia \|\| id:finngen_R11_O15_PREECLAMPS | Marzipan liking \|\| id:ebi-fl187-GCST90094797 | MR Egger | 7 | 0.623333741258929 | 1.47198575285216 | 0.689554582574261 | -2.261758334 | 3.50842581684916 | 1.86513556835132 | 0.104167162932691 | 33.3956555059202 | 0.954596128018958 |
| ebi-fl187-GCST90094797 | finngen_R11_O15_PREECLAMPS | Pre-eclampsia \|\| id:finngen_R11_O15_PREECLAMPS | Marzipan liking \|\| id:ebi-fl187-GCST90094797 | Weighted median | 7 | 0.116752872814876 | 0.15854303142538 | 0.461480456943408 | -0.193991469 | 0.427497214408622 | 1.12384166354333 | 0.823664931106268 | 1.5334149081951 | 0.848896149192195 |
| ebi-fl187-GCST90094797 | finngen_R11_O15_PREECLAMPS | Pre-eclampsia \|\| id:finngen_R11_O15_PREECLAMPS | Marzipan liking \|\| id:ebi-fl187-GCST90094797 | Weighted mode | 7 | 0.308472746814946 | 0.24506729984921 | 0.25488726625688 | -0.171859161 | 0.788804654519398 | 1.36134440807095 | 0.8420977651173 | 2.20076417983119 | 0.905055003276494 |
| ebi-fl187-GCST90094798 | finngen_R11_O15_PREECLAMPS | Pre-eclampsia \|\| id:finngen_R11_O15_PREECLAMPS | Mayonnaise liking \|\| id:ebi-fl187-GCST90094798 | Inverse variance weighted | 9 | 0.233394141277673 | 0.112855444507258 | 0.0386324563797533 | 0.012197470043448 | 0.454590812511899 | 1.26287913404313 | 1.01227216255882 | 1.5755285645414 | 0.328416400058518 |
| ebi-fl187-GCST90094798 | finngen_R11_O15_PREECLAMPS | Pre-eclampsia \|\| id:finngen_R11_O15_PREECLAMPS | Mayonnaise liking \|\| id:ebi-fl187-GCST90094798 | MR Egger | 9 | 0.241664908516881 | 0.557464205790273 | 0.677691729075885 | -0.850964935 | 1.33429475186582 | 1.27336742673777 | 0.42700270331202 | 3.7973169511574 | 0.954596128018958 |
| ebi-fl187-GCST90094798 | finngen_R11_O15_PREECLAMPS | Pre-eclampsia \|\| id:finngen_R11_O15_PREECLAMPS | Mayonnaise liking \|\| id:ebi-fl187-GCST90094798 | Weighted median | 9 | 0.223548871110218 | 0.134420286096934 | 0.0963006191449666 | -0.03991489 | 0.487012631860208 | 1.25050675243647 | 0.960871215767562 | 1.62744716693385 | 0.529128962601009 |
| ebi-fl187-GCST90094798 | finngen_R11_O15_PREECLAMPS | Pre-eclampsia \|\| id:finngen_R11_O15_PREECLAMPS | Mayonnaise liking \|\| id:ebi-fl187-GCST90094798 | Weighted mode | 9 | 0.186931702157572 | 0.192824007919849 | 0.360716934201697 | -0.191003353 | 0.564866757680476 | 1.2055449461194 | 0.826129817831458 | 1.75921336543566 | 0.905055003276494 |
| ebi-fl187-GCST90094799 | finngen_R11_O15_PREECLAMPS | Pre-eclampsia \|\| id:finngen_R11_O15_PREECLAMPS | F-meat liking (derived food-liking factor) \|\| id:ebi-fl187-GCST90094799 | Inverse variance weighted | 10 | 0.0285321986192805 | 0.0736389000371681 | 0.698414984391121 | -0.115800045 | 0.17286444269213 | 1.0289431408496 | 0.890653295669452 | 1.18870495651809 | 0.941775027862301 |
| ebi-fl187-GCST90094799 | finngen_R11_O15_PREECLAMPS | Pre-eclampsia \|\| id:finngen_R11_O15_PREECLAMPS | F-meat liking (derived food-liking factor) \|\| id:ebi-fl187-GCST90094799 | MR Egger | 10 | -0.147128836 | 0.238493877044705 | 0.554441928380449 | -0.614576835 | 0.320319162813153 | 0.863182761070061 | 0.540869723917213 | 1.37756736245525 | 0.954596128018958 |
| ebi-fl187-GCST90094799 | finngen_R11_O15_PREECLAMPS | Pre-eclampsia \|\| id:finngen_R11_O15_PREECLAMPS | F-meat liking (derived food-liking factor) \|\| id:ebi-fl187-GCST90094799 | Weighted median | 10 | 0.0095988935108165 | 0.0904425898820191 | 0.915477266367717 | -0.167668583 | 0.186866369679574 | 1.00964511064856 | 0.845634046036535 | 1.20546618745351 | 0.988450091947753 |
| ebi-fl187-GCST90094799 | finngen_R11_O15_PREECLAMPS | Pre-eclampsia \|\| id:finngen_R11_O15_PREECLAMPS | F-meat liking (derived food-liking factor) \|\| id:ebi-fl187-GCST90094799 | Weighted mode | 10 | -0.103792911 | 0.138780383229797 | 0.473612892490387 | -0.375802462 | 0.168216639979627 | 0.901411950457154 | 0.686737976297797 | 1.18319290977236 | 0.905055003276494 |
| ebi-fl187-GCST90094800 | finngen_R11_O15_PREECLAMPS | Pre-eclampsia \|\| id:finngen_R11_O15_PREECLAMPS | Melon liking \|\| id:ebi-fl187-GCST90094800 | Inverse variance weighted | 2 | -0.47388329 | 0.645269248016328 | 0.462707295961854 | -1.738611016 | 0.790844435889917 | 0.622579909490689 | 0.175764364978522 | 2.20525783908927 | 0.887682351940796 |
| ebi-fl187-GCST90094801 | finngen_R11_O15_PREECLAMPS | Pre-eclampsia \|\| id:finngen_R11_O15_PREECLAMPS | Milk chocolate liking \|\| id:ebi-fl187-GCST90094801 | Inverse variance weighted | 5 | 0.293016320785589 | 0.169975689543408 | 0.0847309648568373 | -0.040136031 | 0.626168672290668 | 1.34046466768958 | 0.960658751162629 | 1.8704306010324 | 0.482089972461316 |
| ebi-fl187-GCST90094801 | finngen_R11_O15_PREECLAMPS | Pre-eclampsia \|\| id:finngen_R11_O15_PREECLAMPS | Milk chocolate liking \|\| id:ebi-fl187-GCST90094801 | MR Egger | 5 | 0.287122937377966 | 1.24825937186956 | 0.832869104982983 | -2.159465431 | 2.7337113062423 | 1.33258802828409 | 0.115386786697392 | 15.3898977859847 | 0.954596128018958 |
| ebi-fl187-GCST90094801 | finngen_R11_O15_PREECLAMPS | Pre-eclampsia \|\| id:finngen_R11_O15_PREECLAMPS | Milk chocolate liking \|\| id:ebi-fl187-GCST90094801 | Weighted median | 5 | 0.501029948642499 | 0.181810639148423 | 0.00585525369204206 | 0.144681095911589 | 0.857378801373409 | 1.65042024371203 | 1.15567096330096 | 2.3569744913156 | 0.313340475930495 |
| ebi-fl187-GCST90094801 | finngen_R11_O15_PREECLAMPS | Pre-eclampsia \|\| id:finngen_R11_O15_PREECLAMPS | Milk chocolate liking \|\| id:ebi-fl187-GCST90094801 | Weighted mode | 5 | 0.59313042280808 | 0.335412626344929 | 0.151729466288152 | -0.064278325 | 1.25053917044414 | 1.80964451024416 | 0.937743965778361 | 3.49222535464528 | 0.905055003276494 |
| ebi-fl187-GCST90094802 | finngen_R11_O15_PREECLAMPS | Pre-eclampsia \|\| id:finngen_R11_O15_PREECLAMPS | Milky sweets liking \|\| id:ebi-fl187-GCST90094802 | Inverse variance weighted (multiplicative random effects) | 10 | 0.0942766585945535 | 0.0854115712023213 | 0.269683124017024 | -0.073130021 | 0.261683338151103 | 1.09886371395826 | 0.92947997028892 | 1.29911509709972 | 0.754417186011084 |
| ebi-fl187-GCST90094802 | finngen_R11_O15_PREECLAMPS | Pre-eclampsia \|\| id:finngen_R11_O15_PREECLAMPS | Milky sweets liking \|\| id:ebi-fl187-GCST90094802 | MR Egger | 10 | 0.230970720907158 | 0.544195333437436 | 0.682438555395642 | -0.835652133 | 1.29759357444453 | 1.2598223524536 | 0.43359162996459 | 3.66047739406627 | 0.954596128018958 |
| ebi-fl187-GCST90094802 | finngen_R11_O15_PREECLAMPS | Pre-eclampsia \|\| id:finngen_R11_O15_PREECLAMPS | Milky sweets liking \|\| id:ebi-fl187-GCST90094802 | Weighted median | 10 | 0.0701806428107834 | 0.0909236132168899 | 0.440195117883175 | -0.108029639 | 0.248390924715888 | 1.07270193964667 | 0.897600991955708 | 1.28196098448442 | 0.840885545699912 |
| ebi-fl187-GCST90094802 | finngen_R11_O15_PREECLAMPS | Pre-eclampsia \|\| id:finngen_R11_O15_PREECLAMPS | Milky sweets liking \|\| id:ebi-fl187-GCST90094802 | Weighted mode | 10 | -0.05975122 | 0.16565066634734 | 0.726640222369239 | -0.384426526 | 0.264924086364603 | 0.941998855215969 | 0.680840969057551 | 1.30333203135017 | 0.936018697441052 |
| ebi-fl187-GCST90094803 | finngen_R11_O15_PREECLAMPS | Pre-eclampsia \|\| id:finngen_R11_O15_PREECLAMPS | Mushroom liking \|\| id:ebi-fl187-GCST90094803 | Inverse variance weighted | 7 | 0.178631738551961 | 0.167710928000534 | 0.286823046393529 | -0.15008168 | 0.507345157433007 | 1.19558037692687 | 0.860637676385402 | 1.6608759724487 | 0.75876791114856 |
| ebi-fl187-GCST90094803 | finngen_R11_O15_PREECLAMPS | Pre-eclampsia \|\| id:finngen_R11_O15_PREECLAMPS | Mushroom liking \|\| id:ebi-fl187-GCST90094803 | MR Egger | 7 | 0.971699360346498 | 0.697673216137848 | 0.222447450633988 | -0.395740143 | 2.33913886397668 | 2.64243108868491 | 0.673181603971981 | 10.3723007539867 | 0.954596128018958 |
| ebi-fl187-GCST90094803 | finngen_R11_O15_PREECLAMPS | Pre-eclampsia \|\| id:finngen_R11_O15_PREECLAMPS | Mushroom liking \|\| id:ebi-fl187-GCST90094803 | Weighted median | 7 | 0.315070975170173 | 0.17643957823455 | 0.0741452558972856 | -0.030750598 | 0.66089254850989 | 1.37035656879618 | 0.969717392212348 | 1.93652000131562 | 0.529128962601009 |
| ebi-fl187-GCST90094803 | finngen_R11_O15_PREECLAMPS | Pre-eclampsia \|\| id:finngen_R11_O15_PREECLAMPS | Mushroom liking \|\| id:ebi-fl187-GCST90094803 | Weighted mode | 7 | 0.327956057307241 | 0.220482924452652 | 0.187458921395535 | -0.104190475 | 0.760102589234439 | 1.38812797286816 | 0.901053653222634 | 2.13849559586988 | 0.905055003276494 |
| ebi-fl187-GCST90094804 | finngen_R11_O15_PREECLAMPS | Pre-eclampsia \|\| id:finngen_R11_O15_PREECLAMPS | F-oily fish liking (derived food-liking factor) \|\| id:ebi-fl187-GCST90094804 | Inverse variance weighted | 11 | -0.049032901 | 0.0709918424085473 | 0.489764399801186 | -0.188176912 | 0.0901111100624192 | 0.952149802446965 | 0.828468128165395 | 1.09429586423246 | 0.894908414040245 |
| ebi-fl187-GCST90094804 | finngen_R11_O15_PREECLAMPS | Pre-eclampsia \|\| id:finngen_R11_O15_PREECLAMPS | F-oily fish liking (derived food-liking factor) \|\| id:ebi-fl187-GCST90094804 | MR Egger | 11 | -0.245905496 | 0.578154565591134 | 0.680586536292088 | -1.379088444 | 0.887277452644002 | 0.781996123261974 | 0.251807985434499 | 2.42850891222362 | 0.954596128018958 |
| ebi-fl187-GCST90094804 | finngen_R11_O15_PREECLAMPS | Pre-eclampsia \|\| id:finngen_R11_O15_PREECLAMPS | F-oily fish liking (derived food-liking factor) \|\| id:ebi-fl187-GCST90094804 | Weighted median | 11 | 0.0406667406801838 | 0.0886534408741755 | 0.646438212699311 | -0.133094003 | 0.214427484793568 | 1.04150495646998 | 0.875382799252432 | 1.23915226033444 | 0.900892362507883 |
| ebi-fl187-GCST90094804 | finngen_R11_O15_PREECLAMPS | Pre-eclampsia \|\| id:finngen_R11_O15_PREECLAMPS | F-oily fish liking (derived food-liking factor) \|\| id:ebi-fl187-GCST90094804 | Weighted mode | 11 | 0.121760608279621 | 0.16652640354702 | 0.481451388750371 | -0.204631143 | 0.448152359231779 | 1.1294836803719 | 0.814947860468902 | 1.56541718324462 | 0.905055003276494 |
| ebi-fl187-GCST90094805 | finngen_R11_O15_PREECLAMPS | Pre-eclampsia \|\| id:finngen_R11_O15_PREECLAMPS | Onion liking \|\| id:ebi-fl187-GCST90094805 | Inverse variance weighted | 5 | 0.0529671717010007 | 0.140741176041932 | 0.706660778593341 | -0.222885533 | 0.328819876743188 | 1.05439503055586 | 0.800206441010019 | 1.38932758283932 | 0.941775027862301 |
| ebi-fl187-GCST90094805 | finngen_R11_O15_PREECLAMPS | Pre-eclampsia \|\| id:finngen_R11_O15_PREECLAMPS | Onion liking \|\| id:ebi-fl187-GCST90094805 | MR Egger | 5 | 0.178190521068693 | 0.337312941129167 | 0.633899518484588 | -0.482942844 | 0.83932388568186 | 1.19505298231833 | 0.616965085903291 | 2.3148013772238 | 0.954596128018958 |
| ebi-fl187-GCST90094805 | finngen_R11_O15_PREECLAMPS | Pre-eclampsia \|\| id:finngen_R11_O15_PREECLAMPS | Onion liking \|\| id:ebi-fl187-GCST90094805 | Weighted median | 5 | 0.104932475947451 | 0.159973693381614 | 0.511865418903853 | -0.208615963 | 0.418480914975414 | 1.11063561320617 | 0.811706901211908 | 1.51965132177658 | 0.881409801621768 |
| ebi-fl187-GCST90094805 | finngen_R11_O15_PREECLAMPS | Pre-eclampsia \|\| id:finngen_R11_O15_PREECLAMPS | Onion liking \|\| id:ebi-fl187-GCST90094805 | Weighted mode | 5 | 0.112217404207736 | 0.181211633441592 | 0.569272396109249 | -0.242957397 | 0.467392205753257 | 1.1187560564823 | 0.784304926537675 | 1.59582717329227 | 0.905055003276494 |
| ebi-fl187-GCST90094806 | finngen_R11_O15_PREECLAMPS | Pre-eclampsia \|\| id:finngen_R11_O15_PREECLAMPS | Orange juice liking \|\| id:ebi-fl187-GCST90094806 | Inverse variance weighted | 2 | 0.042077725035824 | 0.283545364898923 | 0.882028233131391 | -0.51367119 | 0.597826640237713 | 1.04297554091172 | 0.598295087031909 | 1.81816298097411 | 0.953358127900067 |
| ebi-fl187-GCST90094807 | finngen_R11_O15_PREECLAMPS | Pre-eclampsia \|\| id:finngen_R11_O15_PREECLAMPS | Oranges liking \|\| id:ebi-fl187-GCST90094807 | Inverse variance weighted | 7 | -0.347356477 | 0.15523668912756 | 0.0252476301864331 | -0.651620388 | -0.043092567 | 0.706553413134109 | 0.521200545061165 | 0.957822723598407 | 0.258296218889823 |
| ebi-fl187-GCST90094807 | finngen_R11_O15_PREECLAMPS | Pre-eclampsia \|\| id:finngen_R11_O15_PREECLAMPS | Oranges liking \|\| id:ebi-fl187-GCST90094807 | MR Egger | 7 | 1.62535185491099 | 1.1091365944971 | 0.202702030159822 | -0.54855587 | 3.79925958012531 | 5.08020621825417 | 0.577783602642326 | 44.6680990979333 | 0.954596128018958 |
| ebi-fl187-GCST90094807 | finngen_R11_O15_PREECLAMPS | Pre-eclampsia \|\| id:finngen_R11_O15_PREECLAMPS | Oranges liking \|\| id:ebi-fl187-GCST90094807 | Weighted median | 7 | -0.318311797 | 0.204453629952854 | 0.119496621823593 | -0.719040911 | 0.0824173180508529 | 0.727375959661793 | 0.487219318458412 | 1.08590888466397 | 0.539545353082283 |
| ebi-fl187-GCST90094807 | finngen_R11_O15_PREECLAMPS | Pre-eclampsia \|\| id:finngen_R11_O15_PREECLAMPS | Oranges liking \|\| id:ebi-fl187-GCST90094807 | Weighted mode | 7 | -0.304598988 | 0.22073056592873 | 0.216816361515782 | -0.737230897 | 0.128032921001367 | 0.737419028825345 | 0.478436923813703 | 1.13659041977592 | 0.905055003276494 |
| ebi-fl187-GCST90094809 | finngen_R11_O15_PREECLAMPS | Pre-eclampsia \|\| id:finngen_R11_O15_PREECLAMPS | Pears liking \|\| id:ebi-fl187-GCST90094809 | Inverse variance weighted | 4 | 0.0551377741849018 | 0.317850651059255 | 0.862281458580491 | -0.567849502 | 0.678125050261041 | 1.05668618872544 | 0.566742908700188 | 1.97018027804523 | 0.953358127900067 |
| ebi-fl187-GCST90094809 | finngen_R11_O15_PREECLAMPS | Pre-eclampsia \|\| id:finngen_R11_O15_PREECLAMPS | Pears liking \|\| id:ebi-fl187-GCST90094809 | MR Egger | 4 | 5.78725685251038 | 7.68926734134246 | 0.530192606868337 | -9.283707137 | 20.8582208415416 | 326.117208658139 | 9.29259947916217e-05 | 1144485286.61396 | 0.954596128018958 |
| ebi-fl187-GCST90094809 | finngen_R11_O15_PREECLAMPS | Pre-eclampsia \|\| id:finngen_R11_O15_PREECLAMPS | Pears liking \|\| id:ebi-fl187-GCST90094809 | Weighted median | 4 | 0.137700872692756 | 0.267260438815629 | 0.606391583985808 | -0.386129587 | 0.66153133277139 | 1.14763221079271 | 0.679682441702311 | 1.93775741499265 | 0.881409801621768 |
| ebi-fl187-GCST90094809 | finngen_R11_O15_PREECLAMPS | Pre-eclampsia \|\| id:finngen_R11_O15_PREECLAMPS | Pears liking \|\| id:ebi-fl187-GCST90094809 | Weighted mode | 4 | 0.493570200935303 | 0.546448422427286 | 0.432979829461128 | -0.577468707 | 1.56460910889278 | 1.63815433226638 | 0.561317428642206 | 4.78080579613297 | 0.905055003276494 |
| ebi-fl187-GCST90094810 | finngen_R11_O15_PREECLAMPS | Pre-eclampsia \|\| id:finngen_R11_O15_PREECLAMPS | Pizza liking \|\| id:ebi-fl187-GCST90094810 | Inverse variance weighted | 3 | -0.016826203 | 0.199905078259161 | 0.932920505782903 | -0.408640156 | 0.374987750787373 | 0.983314567299604 | 0.664553324835872 | 1.45497359222815 | 0.977922867344962 |
| ebi-fl187-GCST90094810 | finngen_R11_O15_PREECLAMPS | Pre-eclampsia \|\| id:finngen_R11_O15_PREECLAMPS | Pizza liking \|\| id:ebi-fl187-GCST90094810 | MR Egger | 3 | -1.935492764 | 1.96309832292908 | 0.504507769808447 | -5.783165477 | 1.91217994935674 | 0.14435311933456 | 0.00307895361064065 | 6.76782624772382 | 0.954596128018958 |
| ebi-fl187-GCST90094810 | finngen_R11_O15_PREECLAMPS | Pre-eclampsia \|\| id:finngen_R11_O15_PREECLAMPS | Pizza liking \|\| id:ebi-fl187-GCST90094810 | Weighted median | 3 | -0.020660111 | 0.231442127090524 | 0.928869835632904 | -0.47428668 | 0.432966458552482 | 0.979551847338174 | 0.62232881798915 | 1.54182450480762 | 0.991561537443065 |
| ebi-fl187-GCST90094810 | finngen_R11_O15_PREECLAMPS | Pre-eclampsia \|\| id:finngen_R11_O15_PREECLAMPS | Pizza liking \|\| id:ebi-fl187-GCST90094810 | Weighted mode | 3 | -0.020023075 | 0.288352599006477 | 0.950957905425172 | -0.585194169 | 0.545148019043216 | 0.980176055474031 | 0.556997699590672 | 1.72486367615282 | 0.981296058194618 |
| ebi-fl187-GCST90094811 | finngen_R11_O15_PREECLAMPS | Pre-eclampsia \|\| id:finngen_R11_O15_PREECLAMPS | Plain yoghurt liking \|\| id:ebi-fl187-GCST90094811 | Inverse variance weighted (multiplicative random effects) | 7 | 0.0934219795058258 | 0.222217165849704 | 0.674187078971657 | -0.342123666 | 0.528967624571245 | 1.09792493935324 | 0.71026036453245 | 1.69717927769674 | 0.941775027862301 |
| ebi-fl187-GCST90094811 | finngen_R11_O15_PREECLAMPS | Pre-eclampsia \|\| id:finngen_R11_O15_PREECLAMPS | Plain yoghurt liking \|\| id:ebi-fl187-GCST90094811 | MR Egger | 7 | 2.55535284647621 | 1.81186938867475 | 0.217511341250632 | -0.995911155 | 6.10661684827872 | 12.8758420515873 | 0.369386722484649 | 448.81772528874 | 0.954596128018958 |
| ebi-fl187-GCST90094811 | finngen_R11_O15_PREECLAMPS | Pre-eclampsia \|\| id:finngen_R11_O15_PREECLAMPS | Plain yoghurt liking \|\| id:ebi-fl187-GCST90094811 | Weighted median | 7 | 0.00230509495266849 | 0.17919523041019 | 0.98973661883597 | -0.348917557 | 0.353527746556641 | 1.00230775372656 | 0.705451287639073 | 1.42408249978893 | 0.999849666244992 |
| ebi-fl187-GCST90094811 | finngen_R11_O15_PREECLAMPS | Pre-eclampsia \|\| id:finngen_R11_O15_PREECLAMPS | Plain yoghurt liking \|\| id:ebi-fl187-GCST90094811 | Weighted mode | 7 | -0.00207897 | 0.274391664258312 | 0.994200400119083 | -0.539886632 | 0.535728692307986 | 0.99792318992226 | 0.582814321364938 | 1.70869290008584 | 0.994200400119083 |
| ebi-fl187-GCST90094812 | finngen_R11_O15_PREECLAMPS | Pre-eclampsia \|\| id:finngen_R11_O15_PREECLAMPS | Plum liking \|\| id:ebi-fl187-GCST90094812 | Inverse variance weighted | 5 | -0.451555006 | 0.224699693437687 | 0.0444742473264661 | -0.891966405 | -0.011143607 | 0.636637406435575 | 0.409849030542523 | 0.988918252988189 | 0.349440514707948 |
| ebi-fl187-GCST90094812 | finngen_R11_O15_PREECLAMPS | Pre-eclampsia \|\| id:finngen_R11_O15_PREECLAMPS | Plum liking \|\| id:ebi-fl187-GCST90094812 | MR Egger | 5 | -0.282053303 | 0.98156745589446 | 0.792549921512255 | -2.205925516 | 1.64181891093853 | 0.754233480845058 | 0.110148533865455 | 5.164554839 | 0.954596128018958 |
| ebi-fl187-GCST90094812 | finngen_R11_O15_PREECLAMPS | Pre-eclampsia \|\| id:finngen_R11_O15_PREECLAMPS | Plum liking \|\| id:ebi-fl187-GCST90094812 | Weighted median | 5 | -0.319406307 | 0.224893053585224 | 0.155532391857828 | -0.760196692 | 0.121384078262613 | 0.726580274843827 | 0.46757444990844 | 1.12905847591867 | 0.612323741171272 |
| ebi-fl187-GCST90094812 | finngen_R11_O15_PREECLAMPS | Pre-eclampsia \|\| id:finngen_R11_O15_PREECLAMPS | Plum liking \|\| id:ebi-fl187-GCST90094812 | Weighted mode | 5 | -0.115131225 | 0.238738368116817 | 0.654830524547171 | -0.583058427 | 0.352795976200189 | 0.89124918178373 | 0.558188574355845 | 1.4230407796269 | 0.934683055540254 |
| ebi-fl187-GCST90094813 | finngen_R11_O15_PREECLAMPS | Pre-eclampsia \|\| id:finngen_R11_O15_PREECLAMPS | Pollock liking \|\| id:ebi-fl187-GCST90094813 | Inverse variance weighted | 3 | -0.258601989 | 0.189578200464199 | 0.172538807135468 | -0.630175262 | 0.112971283654378 | 0.772130278041104 | 0.532498465993929 | 1.11959978167267 | 0.632642292830049 |
| ebi-fl187-GCST90094813 | finngen_R11_O15_PREECLAMPS | Pre-eclampsia \|\| id:finngen_R11_O15_PREECLAMPS | Pollock liking \|\| id:ebi-fl187-GCST90094813 | MR Egger | 3 | -0.496614436 | 2.30736311556691 | 0.865038985290191 | -5.019046142 | 4.02581727065501 | 0.608587588133145 | 0.00661082949302824 | 56.0260785458646 | 0.970712810886627 |
| ebi-fl187-GCST90094813 | finngen_R11_O15_PREECLAMPS | Pre-eclampsia \|\| id:finngen_R11_O15_PREECLAMPS | Pollock liking \|\| id:ebi-fl187-GCST90094813 | Weighted median | 3 | -0.233212358 | 0.224282745326227 | 0.298426282589139 | -0.672806539 | 0.206381822542072 | 0.791985371000448 | 0.51027446103012 | 1.22922245924766 | 0.71718574364164 |
| ebi-fl187-GCST90094813 | finngen_R11_O15_PREECLAMPS | Pre-eclampsia \|\| id:finngen_R11_O15_PREECLAMPS | Pollock liking \|\| id:ebi-fl187-GCST90094813 | Weighted mode | 3 | -0.23302292 | 0.248537018414492 | 0.447435833464408 | -0.720155476 | 0.254109636502223 | 0.792135417897108 | 0.486676583703476 | 1.2893131523035 | 0.905055003276494 |
| ebi-fl187-GCST90094814 | finngen_R11_O15_PREECLAMPS | Pre-eclampsia \|\| id:finngen_R11_O15_PREECLAMPS | Pork chop liking \|\| id:ebi-fl187-GCST90094814 | Inverse variance weighted | 9 | -0.023318356 | 0.0983933249971753 | 0.812663585537759 | -0.216169273 | 0.169532560815666 | 0.976951415741103 | 0.805598924045003 | 1.1847509228615 | 0.945152512750355 |
| ebi-fl187-GCST90094814 | finngen_R11_O15_PREECLAMPS | Pre-eclampsia \|\| id:finngen_R11_O15_PREECLAMPS | Pork chop liking \|\| id:ebi-fl187-GCST90094814 | MR Egger | 9 | -0.974744834 | 0.479818143994586 | 0.0817331110358008 | -1.915188397 | -0.034301272 | 0.37728861237608 | 0.147314076503716 | 0.966280347452585 | 0.954596128018958 |
| ebi-fl187-GCST90094814 | finngen_R11_O15_PREECLAMPS | Pre-eclampsia \|\| id:finngen_R11_O15_PREECLAMPS | Pork chop liking \|\| id:ebi-fl187-GCST90094814 | Weighted median | 9 | -0.020757153 | 0.129589884436542 | 0.87274265728807 | -0.274753326 | 0.233239020816727 | 0.979456794148749 | 0.759759512897448 | 1.26268325084287 | 0.988450091947753 |
| ebi-fl187-GCST90094814 | finngen_R11_O15_PREECLAMPS | Pre-eclampsia \|\| id:finngen_R11_O15_PREECLAMPS | Pork chop liking \|\| id:ebi-fl187-GCST90094814 | Weighted mode | 9 | -0.210858056 | 0.212875738784836 | 0.350928598105468 | -0.628094504 | 0.20637839232491 | 0.809889017857853 | 0.533607620219429 | 1.22921824275491 | 0.905055003276494 |
| ebi-fl187-GCST90094815 | finngen_R11_O15_PREECLAMPS | Pre-eclampsia \|\| id:finngen_R11_O15_PREECLAMPS | Porridge liking \|\| id:ebi-fl187-GCST90094815 | Inverse variance weighted | 8 | -0.054124875 | 0.147754038730561 | 0.714128250641113 | -0.343722791 | 0.23547304126949 | 0.947313803642959 | 0.709125477086056 | 1.26550726432804 | 0.941775027862301 |
| ebi-fl187-GCST90094815 | finngen_R11_O15_PREECLAMPS | Pre-eclampsia \|\| id:finngen_R11_O15_PREECLAMPS | Porridge liking \|\| id:ebi-fl187-GCST90094815 | MR Egger | 8 | 0.0926271484336604 | 1.13566718891316 | 0.937647829616534 | -2.133280542 | 2.31853483870345 | 1.09705262121524 | 0.118448081900337 | 10.1607762186295 | 0.970712810886627 |
| ebi-fl187-GCST90094815 | finngen_R11_O15_PREECLAMPS | Pre-eclampsia \|\| id:finngen_R11_O15_PREECLAMPS | Porridge liking \|\| id:ebi-fl187-GCST90094815 | Weighted median | 8 | 0.0662411991401814 | 0.157443257619716 | 0.673951800372597 | -0.242347586 | 0.374829984074824 | 1.06848440360732 | 0.784783350594792 | 1.4547440639341 | 0.912898347777426 |
| ebi-fl187-GCST90094815 | finngen_R11_O15_PREECLAMPS | Pre-eclampsia \|\| id:finngen_R11_O15_PREECLAMPS | Porridge liking \|\| id:ebi-fl187-GCST90094815 | Weighted mode | 8 | 0.156801548641546 | 0.27729693045299 | 0.589414538277701 | -0.386700435 | 0.700303532329407 | 1.16976344971936 | 0.679294557292612 | 2.01436403929542 | 0.905055003276494 |
| ebi-fl187-GCST90094816 | finngen_R11_O15_PREECLAMPS | Pre-eclampsia \|\| id:finngen_R11_O15_PREECLAMPS | Potatoes liking \|\| id:ebi-fl187-GCST90094816 | Inverse variance weighted | 2 | -0.395966393 | 0.535883118686729 | 0.45996505169202 | -1.446297306 | 0.65436451952356 | 0.67302931398454 | 0.235440440115114 | 1.92391951553026 | 0.887682351940796 |
| ebi-fl187-GCST90094817 | finngen_R11_O15_PREECLAMPS | Pre-eclampsia \|\| id:finngen_R11_O15_PREECLAMPS | Prawns liking \|\| id:ebi-fl187-GCST90094817 | Inverse variance weighted | 19 | -0.185321471 | 0.0607968363868908 | 0.00230209661146135 | -0.304483271 | -0.066159672 | 0.830837150536712 | 0.737504366051557 | 0.93598140226292 | 0.054263705841589 |
| ebi-fl187-GCST90094817 | finngen_R11_O15_PREECLAMPS | Pre-eclampsia \|\| id:finngen_R11_O15_PREECLAMPS | Prawns liking \|\| id:ebi-fl187-GCST90094817 | MR Egger | 19 | -0.49047467 | 0.194012696551531 | 0.0216653710589 | -0.870739555 | -0.110209785 | 0.612335667910635 | 0.418641826039243 | 0.895646222793838 | 0.954596128018958 |
| ebi-fl187-GCST90094817 | finngen_R11_O15_PREECLAMPS | Pre-eclampsia \|\| id:finngen_R11_O15_PREECLAMPS | Prawns liking \|\| id:ebi-fl187-GCST90094817 | Weighted median | 19 | -0.217713336 | 0.0879765935162462 | 0.013335806651062 | -0.390147459 | -0.045279212 | 0.804355988755527 | 0.676957043725331 | 0.955730592721908 | 0.331172531834706 |
| ebi-fl187-GCST90094817 | finngen_R11_O15_PREECLAMPS | Pre-eclampsia \|\| id:finngen_R11_O15_PREECLAMPS | Prawns liking \|\| id:ebi-fl187-GCST90094817 | Weighted mode | 19 | -0.302665886 | 0.120107419222647 | 0.0214003564034823 | -0.538076428 | -0.067255344 | 0.738845913988767 | 0.583870289668518 | 0.934956435149008 | 0.905055003276494 |
| ebi-fl187-GCST90094818 | finngen_R11_O15_PREECLAMPS | Pre-eclampsia \|\| id:finngen_R11_O15_PREECLAMPS | Raw carrot liking \|\| id:ebi-fl187-GCST90094818 | Inverse variance weighted | 11 | -0.071262487 | 0.14809682372253 | 0.630382541039795 | -0.361532261 | 0.219007287621895 | 0.931217427692789 | 0.69660812218031 | 1.24484034858026 | 0.941775027862301 |
| ebi-fl187-GCST90094818 | finngen_R11_O15_PREECLAMPS | Pre-eclampsia \|\| id:finngen_R11_O15_PREECLAMPS | Raw carrot liking \|\| id:ebi-fl187-GCST90094818 | MR Egger | 11 | -0.084109105 | 1.15266759435693 | 0.943426669665339 | -2.34333759 | 2.17511937983487 | 0.919330946923869 | 0.0960066719889607 | 8.80323598831881 | 0.970712810886627 |
| ebi-fl187-GCST90094818 | finngen_R11_O15_PREECLAMPS | Pre-eclampsia \|\| id:finngen_R11_O15_PREECLAMPS | Raw carrot liking \|\| id:ebi-fl187-GCST90094818 | Weighted median | 11 | -0.099991321 | 0.184717834281693 | 0.588287548178196 | -0.462038276 | 0.262055634011926 | 0.904845270990942 | 0.629998225433774 | 1.29959884231569 | 0.881409801621768 |
| ebi-fl187-GCST90094818 | finngen_R11_O15_PREECLAMPS | Pre-eclampsia \|\| id:finngen_R11_O15_PREECLAMPS | Raw carrot liking \|\| id:ebi-fl187-GCST90094818 | Weighted mode | 11 | -0.259161069 | 0.292596433276392 | 0.396562928696156 | -0.832650078 | 0.314327940152787 | 0.771698716239253 | 0.434895251391041 | 1.36933872407322 | 0.905055003276494 |
| ebi-fl187-GCST90094819 | finngen_R11_O15_PREECLAMPS | Pre-eclampsia \|\| id:finngen_R11_O15_PREECLAMPS | Red meat liking \|\| id:ebi-fl187-GCST90094819 | Inverse variance weighted | 6 | -0.190889609 | 0.132538206995192 | 0.149793574162284 | -0.450664495 | 0.0688852767213498 | 0.826223790796516 | 0.637204591836776 | 1.07131329752412 | 0.62762387754309 |
| ebi-fl187-GCST90094819 | finngen_R11_O15_PREECLAMPS | Pre-eclampsia \|\| id:finngen_R11_O15_PREECLAMPS | Red meat liking \|\| id:ebi-fl187-GCST90094819 | MR Egger | 6 | -0.456047323 | 0.551565215444274 | 0.454806094246637 | -1.537115145 | 0.625020499184382 | 0.633783843781271 | 0.215000452682396 | 1.86828425534311 | 0.954596128018958 |
| ebi-fl187-GCST90094819 | finngen_R11_O15_PREECLAMPS | Pre-eclampsia \|\| id:finngen_R11_O15_PREECLAMPS | Red meat liking \|\| id:ebi-fl187-GCST90094819 | Weighted median | 6 | -0.246221176 | 0.160893922194322 | 0.125934482353245 | -0.561573263 | 0.0691309119122779 | 0.781749301941022 | 0.570311108257703 | 1.07157648209287 | 0.551889349136278 |
| ebi-fl187-GCST90094819 | finngen_R11_O15_PREECLAMPS | Pre-eclampsia \|\| id:finngen_R11_O15_PREECLAMPS | Red meat liking \|\| id:ebi-fl187-GCST90094819 | Weighted mode | 6 | -0.344868861 | 0.217512775611647 | 0.173707039088069 | -0.771193901 | 0.0814561789697168 | 0.708313234704505 | 0.462460606206089 | 1.08486567661069 | 0.905055003276494 |
| ebi-fl187-GCST90094820 | finngen_R11_O15_PREECLAMPS | Pre-eclampsia \|\| id:finngen_R11_O15_PREECLAMPS | Red wine liking \|\| id:ebi-fl187-GCST90094820 | Inverse variance weighted | 6 | 0.026298748569779 | 0.115011804898642 | 0.819132177716975 | -0.199124389 | 0.251721886171117 | 1.02664761216929 | 0.819447956655413 | 1.28623826688744 | 0.945152512750355 |
| ebi-fl187-GCST90094820 | finngen_R11_O15_PREECLAMPS | Pre-eclampsia \|\| id:finngen_R11_O15_PREECLAMPS | Red wine liking \|\| id:ebi-fl187-GCST90094820 | MR Egger | 6 | 0.46199564900754 | 0.38693941469668 | 0.298453607606728 | -0.296405604 | 1.22039690181303 | 1.58723839714827 | 0.743485806188121 | 3.38853238140281 | 0.954596128018958 |
| ebi-fl187-GCST90094820 | finngen_R11_O15_PREECLAMPS | Pre-eclampsia \|\| id:finngen_R11_O15_PREECLAMPS | Red wine liking \|\| id:ebi-fl187-GCST90094820 | Weighted median | 6 | 0.0914993638806311 | 0.147113440253373 | 0.533965092556762 | -0.196842979 | 0.379841706777243 | 1.09581607962249 | 0.821319587597968 | 1.46205313801305 | 0.881409801621768 |
| ebi-fl187-GCST90094820 | finngen_R11_O15_PREECLAMPS | Pre-eclampsia \|\| id:finngen_R11_O15_PREECLAMPS | Red wine liking \|\| id:ebi-fl187-GCST90094820 | Weighted mode | 6 | 0.166408928617551 | 0.188894398234592 | 0.418664667783831 | -0.203824092 | 0.53664194915735 | 1.1810559705724 | 0.815605830222918 | 1.71025408835474 | 0.905055003276494 |
| ebi-fl187-GCST90094821 | finngen_R11_O15_PREECLAMPS | Pre-eclampsia \|\| id:finngen_R11_O15_PREECLAMPS | Roast chicken liking \|\| id:ebi-fl187-GCST90094821 | Inverse variance weighted | 2 | -0.094140678 | 0.430076631185409 | 0.826733555299709 | -0.937090875 | 0.748809519141534 | 0.910154714505806 | 0.391765875066157 | 2.1144812681739 | 0.947298865447583 |
| ebi-fl187-GCST90094822 | finngen_R11_O15_PREECLAMPS | Pre-eclampsia \|\| id:finngen_R11_O15_PREECLAMPS | Salad dressing liking \|\| id:ebi-fl187-GCST90094822 | Inverse variance weighted | 6 | 0.00259121892364333 | 0.169135798458042 | 0.987776610767233 | -0.328914946 | 0.334097383901406 | 1.00259457903303 | 0.71970422782836 | 1.39667915101667 | 0.992699677017842 |
| ebi-fl187-GCST90094822 | finngen_R11_O15_PREECLAMPS | Pre-eclampsia \|\| id:finngen_R11_O15_PREECLAMPS | Salad dressing liking \|\| id:ebi-fl187-GCST90094822 | MR Egger | 6 | -1.201476106 | 0.872975881568054 | 0.240751876703029 | -2.912508834 | 0.50955662219417 | 0.300749945398281 | 0.0543392306288253 | 1.66455300545032 | 0.954596128018958 |
| ebi-fl187-GCST90094822 | finngen_R11_O15_PREECLAMPS | Pre-eclampsia \|\| id:finngen_R11_O15_PREECLAMPS | Salad dressing liking \|\| id:ebi-fl187-GCST90094822 | Weighted median | 6 | -0.18956935 | 0.226554039929057 | 0.402732469633615 | -0.633615268 | 0.254476568234727 | 0.827315340567389 | 0.530669815099445 | 1.28978632901868 | 0.819869554194876 |
| ebi-fl187-GCST90094822 | finngen_R11_O15_PREECLAMPS | Pre-eclampsia \|\| id:finngen_R11_O15_PREECLAMPS | Salad dressing liking \|\| id:ebi-fl187-GCST90094822 | Weighted mode | 6 | -0.20318011 | 0.302994742767238 | 0.532215382679625 | -0.797049805 | 0.390689586186893 | 0.816131235086969 | 0.450656529298404 | 1.47799965069083 | 0.905055003276494 |
| ebi-fl187-GCST90094823 | finngen_R11_O15_PREECLAMPS | Pre-eclampsia \|\| id:finngen_R11_O15_PREECLAMPS | Salad leaves liking \|\| id:ebi-fl187-GCST90094823 | Inverse variance weighted | 4 | -0.229268912 | 0.241154310035459 | 0.341749298123575 | -0.70193136 | 0.243393535406592 | 0.795114688663433 | 0.495627144405808 | 1.27557050751585 | 0.802417492460712 |
| ebi-fl187-GCST90094823 | finngen_R11_O15_PREECLAMPS | Pre-eclampsia \|\| id:finngen_R11_O15_PREECLAMPS | Salad leaves liking \|\| id:ebi-fl187-GCST90094823 | MR Egger | 4 | 0.621395109165295 | 1.1251498299433 | 0.636234918774114 | -1.583898558 | 2.82668877585417 | 1.86152325928003 | 0.205173655686064 | 16.8894433998035 | 0.954596128018958 |
| ebi-fl187-GCST90094823 | finngen_R11_O15_PREECLAMPS | Pre-eclampsia \|\| id:finngen_R11_O15_PREECLAMPS | Salad leaves liking \|\| id:ebi-fl187-GCST90094823 | Weighted median | 4 | -0.23216913 | 0.28158414273144 | 0.409649456076469 | -0.78407405 | 0.319735789801219 | 0.79281202370704 | 0.456542241552816 | 1.37676396119796 | 0.819869554194876 |
| ebi-fl187-GCST90094823 | finngen_R11_O15_PREECLAMPS | Pre-eclampsia \|\| id:finngen_R11_O15_PREECLAMPS | Salad leaves liking \|\| id:ebi-fl187-GCST90094823 | Weighted mode | 4 | -0.247965199 | 0.379138546964619 | 0.559759575333071 | -0.991076751 | 0.4951463533187 | 0.780387101266156 | 0.371176810853466 | 1.64073834898867 | 0.905055003276494 |
| ebi-fl187-GCST90094824 | finngen_R11_O15_PREECLAMPS | Pre-eclampsia \|\| id:finngen_R11_O15_PREECLAMPS | F-salad vegetables liking (derived food-liking factor) \|\| id:ebi-fl187-GCST90094824 | Inverse variance weighted | 10 | 0.0490597992535537 | 0.0784097010861806 | 0.531521361066327 | -0.104623215 | 0.202742813382468 | 1.05028315501087 | 0.900663815389951 | 1.22475743651587 | 0.894908414040245 |
| ebi-fl187-GCST90094824 | finngen_R11_O15_PREECLAMPS | Pre-eclampsia \|\| id:finngen_R11_O15_PREECLAMPS | F-salad vegetables liking (derived food-liking factor) \|\| id:ebi-fl187-GCST90094824 | MR Egger | 10 | -0.13591126 | 0.502707703295474 | 0.793728385649393 | -1.121218358 | 0.849395838725363 | 0.872920092213154 | 0.325882511028089 | 2.33823375481399 | 0.954596128018958 |
| ebi-fl187-GCST90094824 | finngen_R11_O15_PREECLAMPS | Pre-eclampsia \|\| id:finngen_R11_O15_PREECLAMPS | F-salad vegetables liking (derived food-liking factor) \|\| id:ebi-fl187-GCST90094824 | Weighted median | 10 | -0.003886997 | 0.101191374770163 | 0.96935892945011 | -0.202222091 | 0.194448097958324 | 0.996120548001621 | 0.816913478547516 | 1.21464043893032 | 0.996099865434941 |
| ebi-fl187-GCST90094824 | finngen_R11_O15_PREECLAMPS | Pre-eclampsia \|\| id:finngen_R11_O15_PREECLAMPS | F-salad vegetables liking (derived food-liking factor) \|\| id:ebi-fl187-GCST90094824 | Weighted mode | 10 | -0.034485523 | 0.137215378374357 | 0.80720795411561 | -0.303427664 | 0.234456618923486 | 0.966102326147871 | 0.738283291436066 | 1.264221628 | 0.963239370826664 |
| ebi-fl187-GCST90094825 | finngen_R11_O15_PREECLAMPS | Pre-eclampsia \|\| id:finngen_R11_O15_PREECLAMPS | Salami liking \|\| id:ebi-fl187-GCST90094825 | Inverse variance weighted | 10 | -0.074226007 | 0.111934131861946 | 0.50725229906707 | -0.293616905 | 0.145164891629162 | 0.928461831420766 | 0.745562057635761 | 1.15623020723293 | 0.894908414040245 |
| ebi-fl187-GCST90094825 | finngen_R11_O15_PREECLAMPS | Pre-eclampsia \|\| id:finngen_R11_O15_PREECLAMPS | Salami liking \|\| id:ebi-fl187-GCST90094825 | MR Egger | 10 | -1.297224954 | 0.728733654271488 | 0.112931774973104 | -2.725542917 | 0.131093008044026 | 0.273289131545194 | 0.0655106262483372 | 1.14007381241638 | 0.954596128018958 |
| ebi-fl187-GCST90094825 | finngen_R11_O15_PREECLAMPS | Pre-eclampsia \|\| id:finngen_R11_O15_PREECLAMPS | Salami liking \|\| id:ebi-fl187-GCST90094825 | Weighted median | 10 | -0.101504649 | 0.123910675178814 | 0.412686017212186 | -0.344369572 | 0.141360274585602 | 0.903476979280413 | 0.70866697609399 | 1.1518395517578 | 0.819869554194876 |
| ebi-fl187-GCST90094825 | finngen_R11_O15_PREECLAMPS | Pre-eclampsia \|\| id:finngen_R11_O15_PREECLAMPS | Salami liking \|\| id:ebi-fl187-GCST90094825 | Weighted mode | 10 | -0.145183891 | 0.173767531220358 | 0.425056761631147 | -0.485768252 | 0.19540047049598 | 0.864863238184082 | 0.61522436787964 | 1.21579778 | 0.905055003276494 |
| ebi-fl187-GCST90094826 | finngen_R11_O15_PREECLAMPS | Pre-eclampsia \|\| id:finngen_R11_O15_PREECLAMPS | Salmon liking \|\| id:ebi-fl187-GCST90094826 | Inverse variance weighted | 12 | -0.132388898 | 0.133190234804556 | 0.32023089364382 | -0.393441758 | 0.128663962595263 | 0.876000254411529 | 0.674730614201836 | 1.13730788195645 | 0.800577234109551 |
| ebi-fl187-GCST90094826 | finngen_R11_O15_PREECLAMPS | Pre-eclampsia \|\| id:finngen_R11_O15_PREECLAMPS | Salmon liking \|\| id:ebi-fl187-GCST90094826 | MR Egger | 12 | -1.047336405 | 1.13783303324304 | 0.378999133254723 | -3.27748915 | 1.18281634008436 | 0.350871083986014 | 0.0377228544176848 | 3.26355254600789 | 0.954596128018958 |
| ebi-fl187-GCST90094826 | finngen_R11_O15_PREECLAMPS | Pre-eclampsia \|\| id:finngen_R11_O15_PREECLAMPS | Salmon liking \|\| id:ebi-fl187-GCST90094826 | Weighted median | 12 | -0.152775122 | 0.160873184149523 | 0.342284113508382 | -0.468086562 | 0.162536319421497 | 0.858322718425694 | 0.626199315967798 | 1.17649104714697 | 0.783962747510833 |
| ebi-fl187-GCST90094826 | finngen_R11_O15_PREECLAMPS | Pre-eclampsia \|\| id:finngen_R11_O15_PREECLAMPS | Salmon liking \|\| id:ebi-fl187-GCST90094826 | Weighted mode | 12 | -0.219852111 | 0.270999162299902 | 0.434416594760612 | -0.751010469 | 0.311306247431263 | 0.802637490701047 | 0.471889482157756 | 1.36520724838598 | 0.905055003276494 |
| ebi-fl187-GCST90094827 | finngen_R11_O15_PREECLAMPS | Pre-eclampsia \|\| id:finngen_R11_O15_PREECLAMPS | F-salty food liking (derived food-liking factor) \|\| id:ebi-fl187-GCST90094827 | Inverse variance weighted | 8 | -0.092819155 | 0.0804323756120287 | 0.248499183523434 | -0.250466611 | 0.0648283014201569 | 0.91135830018688 | 0.778437470844902 | 1.06697581042448 | 0.732185094310117 |
| ebi-fl187-GCST90094827 | finngen_R11_O15_PREECLAMPS | Pre-eclampsia \|\| id:finngen_R11_O15_PREECLAMPS | F-salty food liking (derived food-liking factor) \|\| id:ebi-fl187-GCST90094827 | MR Egger | 8 | -0.183799937 | 0.256758473248622 | 0.500982232401784 | -0.687046544 | 0.319446670897182 | 0.832102260324199 | 0.503059641552045 | 1.37636597024651 | 0.954596128018958 |
| ebi-fl187-GCST90094827 | finngen_R11_O15_PREECLAMPS | Pre-eclampsia \|\| id:finngen_R11_O15_PREECLAMPS | F-salty food liking (derived food-liking factor) \|\| id:ebi-fl187-GCST90094827 | Weighted median | 8 | -0.091398712 | 0.0949692960591506 | 0.335847332150381 | -0.277538532 | 0.0947411084553432 | 0.912653752507599 | 0.757646370337445 | 1.09937419959555 | 0.781894570162605 |
| ebi-fl187-GCST90094827 | finngen_R11_O15_PREECLAMPS | Pre-eclampsia \|\| id:finngen_R11_O15_PREECLAMPS | F-salty food liking (derived food-liking factor) \|\| id:ebi-fl187-GCST90094827 | Weighted mode | 8 | -0.082883282 | 0.148881836945827 | 0.595070777755082 | -0.374691683 | 0.208925118134461 | 0.920458574777446 | 0.687501214640482 | 1.23235271420485 | 0.905055003276494 |
| ebi-fl187-GCST90094828 | finngen_R11_O15_PREECLAMPS | Pre-eclampsia \|\| id:finngen_R11_O15_PREECLAMPS | Salty food liking \|\| id:ebi-fl187-GCST90094828 | Inverse variance weighted | 13 | -0.13335246 | 0.0840922174072209 | 0.112787326659308 | -0.298173206 | 0.0314682864450886 | 0.8751565803408 | 0.742172779990871 | 1.03196864768231 | 0.512568357226114 |
| ebi-fl187-GCST90094828 | finngen_R11_O15_PREECLAMPS | Pre-eclampsia \|\| id:finngen_R11_O15_PREECLAMPS | Salty food liking \|\| id:ebi-fl187-GCST90094828 | MR Egger | 13 | -0.076683462 | 0.261927396443689 | 0.775149694113407 | -0.590061159 | 0.43669423547143 | 0.92618297972843 | 0.554293383897252 | 1.54758280877775 | 0.954596128018958 |
| ebi-fl187-GCST90094828 | finngen_R11_O15_PREECLAMPS | Pre-eclampsia \|\| id:finngen_R11_O15_PREECLAMPS | Salty food liking \|\| id:ebi-fl187-GCST90094828 | Weighted median | 13 | -0.190377535 | 0.11288332687334 | 0.0916998689161863 | -0.411628856 | 0.0308737857121016 | 0.826646986887022 | 0.662570139599986 | 1.03135532389363 | 0.529128962601009 |
| ebi-fl187-GCST90094828 | finngen_R11_O15_PREECLAMPS | Pre-eclampsia \|\| id:finngen_R11_O15_PREECLAMPS | Salty food liking \|\| id:ebi-fl187-GCST90094828 | Weighted mode | 13 | -0.224099123 | 0.184854002364372 | 0.248724871958515 | -0.586412968 | 0.138214721173018 | 0.799235907413386 | 0.556319244866914 | 1.14822207139663 | 0.905055003276494 |
| ebi-fl187-GCST90094829 | finngen_R11_O15_PREECLAMPS | Pre-eclampsia \|\| id:finngen_R11_O15_PREECLAMPS | Salty pretzels liking \|\| id:ebi-fl187-GCST90094829 | Inverse variance weighted | 5 | -0.099416184 | 0.137002120141147 | 0.468050694659692 | -0.36794034 | 0.169107971293644 | 0.905365830665235 | 0.69215847486712 | 1.18424799680957 | 0.887682351940796 |
| ebi-fl187-GCST90094829 | finngen_R11_O15_PREECLAMPS | Pre-eclampsia \|\| id:finngen_R11_O15_PREECLAMPS | Salty pretzels liking \|\| id:ebi-fl187-GCST90094829 | MR Egger | 5 | -0.229348639 | 0.389877431062804 | 0.597710875108395 | -0.993508404 | 0.534811125982425 | 0.795051299369611 | 0.37027533413088 | 1.70712578009822 | 0.954596128018958 |
| ebi-fl187-GCST90094829 | finngen_R11_O15_PREECLAMPS | Pre-eclampsia \|\| id:finngen_R11_O15_PREECLAMPS | Salty pretzels liking \|\| id:ebi-fl187-GCST90094829 | Weighted median | 5 | -0.089907867 | 0.169800688999081 | 0.596465172278923 | -0.422717217 | 0.242901483881714 | 0.914015392777567 | 0.655263904410948 | 1.2749430154944 | 0.881409801621768 |
| ebi-fl187-GCST90094829 | finngen_R11_O15_PREECLAMPS | Pre-eclampsia \|\| id:finngen_R11_O15_PREECLAMPS | Salty pretzels liking \|\| id:ebi-fl187-GCST90094829 | Weighted mode | 5 | -0.125767875 | 0.218223381848556 | 0.595271075980513 | -0.553485703 | 0.301949953528753 | 0.881819515476261 | 0.57494223545035 | 1.35249353748676 | 0.905055003276494 |
| ebi-fl187-GCST90094830 | finngen_R11_O15_PREECLAMPS | Pre-eclampsia \|\| id:finngen_R11_O15_PREECLAMPS | Sardines liking \|\| id:ebi-fl187-GCST90094830 | Inverse variance weighted | 15 | -0.062363707 | 0.0744439771323061 | 0.402184216911975 | -0.208273902 | 0.0835464882212072 | 0.939541107002963 | 0.811984601932617 | 1.08713575312553 | 0.861823321954233 |
| ebi-fl187-GCST90094830 | finngen_R11_O15_PREECLAMPS | Pre-eclampsia \|\| id:finngen_R11_O15_PREECLAMPS | Sardines liking \|\| id:ebi-fl187-GCST90094830 | MR Egger | 15 | -0.176657899 | 0.355797548851047 | 0.627822526002377 | -0.874021095 | 0.520705296548763 | 0.838066438687612 | 0.4172702878111 | 1.68321439645014 | 0.954596128018958 |
| ebi-fl187-GCST90094830 | finngen_R11_O15_PREECLAMPS | Pre-eclampsia \|\| id:finngen_R11_O15_PREECLAMPS | Sardines liking \|\| id:ebi-fl187-GCST90094830 | Weighted median | 15 | -0.054060514 | 0.0936620749465687 | 0.563813268270678 | -0.237638181 | 0.12951715290798 | 0.947374775342029 | 0.78848792949506 | 1.13827863608403 | 0.881409801621768 |
| ebi-fl187-GCST90094830 | finngen_R11_O15_PREECLAMPS | Pre-eclampsia \|\| id:finngen_R11_O15_PREECLAMPS | Sardines liking \|\| id:ebi-fl187-GCST90094830 | Weighted mode | 15 | -0.006321419 | 0.138655794197089 | 0.964280385342498 | -0.278086776 | 0.265443937396884 | 0.993698518906566 | 0.757231109285785 | 1.30400974599214 | 0.981296058194618 |
| ebi-fl187-GCST90094831 | finngen_R11_O15_PREECLAMPS | Pre-eclampsia \|\| id:finngen_R11_O15_PREECLAMPS | F-sauces liking (derived food-liking factor) \|\| id:ebi-fl187-GCST90094831 | Inverse variance weighted | 8 | 0.0278645451760083 | 0.0692404772701317 | 0.687366533485427 | -0.10784679 | 0.163575880625466 | 1.02825639269908 | 0.897765132244671 | 1.17771471752633 | 0.941775027862301 |
| ebi-fl187-GCST90094831 | finngen_R11_O15_PREECLAMPS | Pre-eclampsia \|\| id:finngen_R11_O15_PREECLAMPS | F-sauces liking (derived food-liking factor) \|\| id:ebi-fl187-GCST90094831 | MR Egger | 8 | 0.562536815546346 | 0.455123954274677 | 0.262646877511797 | -0.329506135 | 1.45457976592471 | 1.75511927144576 | 0.719278872510494 | 4.28268335791465 | 0.954596128018958 |
| ebi-fl187-GCST90094831 | finngen_R11_O15_PREECLAMPS | Pre-eclampsia \|\| id:finngen_R11_O15_PREECLAMPS | F-sauces liking (derived food-liking factor) \|\| id:ebi-fl187-GCST90094831 | Weighted median | 8 | 0.0662315192676658 | 0.0884347202826395 | 0.453898687128777 | -0.107100532 | 0.239563571021639 | 1.06847406086456 | 0.898435346510838 | 1.27069446140345 | 0.848896149192195 |
| ebi-fl187-GCST90094831 | finngen_R11_O15_PREECLAMPS | Pre-eclampsia \|\| id:finngen_R11_O15_PREECLAMPS | F-sauces liking (derived food-liking factor) \|\| id:ebi-fl187-GCST90094831 | Weighted mode | 8 | 0.106180426351954 | 0.1144800571864 | 0.384531157710571 | -0.118200486 | 0.330561338437297 | 1.11202249656976 | 0.888517899593915 | 1.39174915152797 | 0.905055003276494 |
| ebi-fl187-GCST90094832 | finngen_R11_O15_PREECLAMPS | Pre-eclampsia \|\| id:finngen_R11_O15_PREECLAMPS | Sausages liking \|\| id:ebi-fl187-GCST90094832 | Inverse variance weighted (multiplicative random effects) | 3 | -0.079603149 | 0.317331371169761 | 0.801928683410358 | -0.701572636 | 0.542366338705644 | 0.923482758929181 | 0.495804969478494 | 1.72007232387461 | 0.945152512750355 |
| ebi-fl187-GCST90094832 | finngen_R11_O15_PREECLAMPS | Pre-eclampsia \|\| id:finngen_R11_O15_PREECLAMPS | Sausages liking \|\| id:ebi-fl187-GCST90094832 | MR Egger | 3 | -0.574333583 | 1.61395183144492 | 0.782351338795817 | -3.737679173 | 2.58901200662335 | 0.563079989872261 | 0.0238092963259112 | 13.3166083807985 | 0.954596128018958 |
| ebi-fl187-GCST90094832 | finngen_R11_O15_PREECLAMPS | Pre-eclampsia \|\| id:finngen_R11_O15_PREECLAMPS | Sausages liking \|\| id:ebi-fl187-GCST90094832 | Weighted median | 3 | -0.097750951 | 0.189719107472269 | 0.606385031355357 | -0.469600402 | 0.274098499179684 | 0.906874731454149 | 0.625252067775697 | 1.31534435619823 | 0.881409801621768 |
| ebi-fl187-GCST90094832 | finngen_R11_O15_PREECLAMPS | Pre-eclampsia \|\| id:finngen_R11_O15_PREECLAMPS | Sausages liking \|\| id:ebi-fl187-GCST90094832 | Weighted mode | 3 | -0.323246529 | 0.209786018161978 | 0.263269366062844 | -0.734427125 | 0.0879340666340164 | 0.723795395842527 | 0.479780234573818 | 1.0919161259492 | 0.905055003276494 |
| ebi-fl187-GCST90094833 | finngen_R11_O15_PREECLAMPS | Pre-eclampsia \|\| id:finngen_R11_O15_PREECLAMPS | F-savoury food liking (derived food-liking factor) \|\| id:ebi-fl187-GCST90094833 | Inverse variance weighted | 20 | -0.034559568 | 0.0466094224720384 | 0.458407479071505 | -0.125914036 | 0.056794899894504 | 0.966030793304675 | 0.88169063700697 | 1.05843870224232 | 0.887682351940796 |
| ebi-fl187-GCST90094833 | finngen_R11_O15_PREECLAMPS | Pre-eclampsia \|\| id:finngen_R11_O15_PREECLAMPS | F-savoury food liking (derived food-liking factor) \|\| id:ebi-fl187-GCST90094833 | MR Egger | 20 | -0.117542485 | 0.203047200477583 | 0.569835652671063 | -0.515514998 | 0.280430028257535 | 0.889102737700055 | 0.597192962459006 | 1.3236989179021 | 0.954596128018958 |
| ebi-fl187-GCST90094833 | finngen_R11_O15_PREECLAMPS | Pre-eclampsia \|\| id:finngen_R11_O15_PREECLAMPS | F-savoury food liking (derived food-liking factor) \|\| id:ebi-fl187-GCST90094833 | Weighted median | 20 | -0.005341915 | 0.0629153329400382 | 0.932335801463772 | -0.128655967 | 0.117972137960801 | 0.994672328051833 | 0.879276413474616 | 1.12521276020856 | 0.991561537443065 |
| ebi-fl187-GCST90094833 | finngen_R11_O15_PREECLAMPS | Pre-eclampsia \|\| id:finngen_R11_O15_PREECLAMPS | F-savoury food liking (derived food-liking factor) \|\| id:ebi-fl187-GCST90094833 | Weighted mode | 20 | 0.0384178431054255 | 0.0926930538504439 | 0.683176889381769 | -0.143260542 | 0.220096228652296 | 1.03916535025271 | 0.86652827209035 | 1.24619664464131 | 0.936018697441052 |
| ebi-fl187-GCST90094834 | finngen_R11_O15_PREECLAMPS | Pre-eclampsia \|\| id:finngen_R11_O15_PREECLAMPS | Savoury biscuit liking \|\| id:ebi-fl187-GCST90094834 | Inverse variance weighted | 2 | -0.731409761 | 0.430995325907316 | 0.0896919701917842 | -1.5761606 | 0.113341077984753 | 0.481230092344047 | 0.206767439168898 | 1.12001387988508 | 0.493305836054813 |
| ebi-fl187-GCST90094835 | finngen_R11_O15_PREECLAMPS | Pre-eclampsia \|\| id:finngen_R11_O15_PREECLAMPS | F-savour/caloric food liking (derived food-liking factor) \|\| id:ebi-fl187-GCST90094835 | Inverse variance weighted (multiplicative random effects) | 12 | 0.0276306107401908 | 0.0808900457022486 | 0.732665232574445 | -0.130913879 | 0.186175100316598 | 1.02801587625361 | 0.877293324659858 | 1.20463317356168 | 0.941775027862301 |
| ebi-fl187-GCST90094835 | finngen_R11_O15_PREECLAMPS | Pre-eclampsia \|\| id:finngen_R11_O15_PREECLAMPS | F-savour/caloric food liking (derived food-liking factor) \|\| id:ebi-fl187-GCST90094835 | MR Egger | 12 | -0.632620325 | 0.236136703171973 | 0.023130699224187 | -1.095448263 | -0.169792387 | 0.531198064248988 | 0.334389678775673 | 0.843839990800578 | 0.954596128018958 |
| ebi-fl187-GCST90094835 | finngen_R11_O15_PREECLAMPS | Pre-eclampsia \|\| id:finngen_R11_O15_PREECLAMPS | F-savour/caloric food liking (derived food-liking factor) \|\| id:ebi-fl187-GCST90094835 | Weighted median | 12 | 0.00926813843537103 | 0.0862597167580368 | 0.914436294993834 | -0.159800906 | 0.178337183281123 | 1.00931122062477 | 0.852313462221896 | 1.195228264 | 0.988450091947753 |
| ebi-fl187-GCST90094835 | finngen_R11_O15_PREECLAMPS | Pre-eclampsia \|\| id:finngen_R11_O15_PREECLAMPS | F-savour/caloric food liking (derived food-liking factor) \|\| id:ebi-fl187-GCST90094835 | Weighted mode | 12 | 0.0219220885036297 | 0.122563703885746 | 0.861297228651383 | -0.218302771 | 0.262146948119692 | 1.02216414303007 | 0.803882012565561 | 1.29971751944279 | 0.981296058194618 |
| ebi-fl187-GCST90094836 | finngen_R11_O15_PREECLAMPS | Pre-eclampsia \|\| id:finngen_R11_O15_PREECLAMPS | F-seafood liking (derived food-liking factor) \|\| id:ebi-fl187-GCST90094836 | Inverse variance weighted | 28 | -0.057254784 | 0.0485112258397987 | 0.23790563457317 | -0.152336787 | 0.0378272184400079 | 0.944353432373124 | 0.858699033492215 | 1.03855177477964 | 0.71932023600517 |
| ebi-fl187-GCST90094836 | finngen_R11_O15_PREECLAMPS | Pre-eclampsia \|\| id:finngen_R11_O15_PREECLAMPS | F-seafood liking (derived food-liking factor) \|\| id:ebi-fl187-GCST90094836 | MR Egger | 28 | -0.208141581 | 0.241535543967435 | 0.396707306389134 | -0.681551247 | 0.265268085234323 | 0.812092051814732 | 0.505831713454538 | 1.30378045321971 | 0.954596128018958 |
| ebi-fl187-GCST90094836 | finngen_R11_O15_PREECLAMPS | Pre-eclampsia \|\| id:finngen_R11_O15_PREECLAMPS | F-seafood liking (derived food-liking factor) \|\| id:ebi-fl187-GCST90094836 | Weighted median | 28 | -0.107462832 | 0.0667433430113959 | 0.107378069654321 | -0.238279784 | 0.0233541202815203 | 0.89810990276063 | 0.787982195184424 | 1.02362896314926 | 0.529780299600071 |
| ebi-fl187-GCST90094836 | finngen_R11_O15_PREECLAMPS | Pre-eclampsia \|\| id:finngen_R11_O15_PREECLAMPS | F-seafood liking (derived food-liking factor) \|\| id:ebi-fl187-GCST90094836 | Weighted mode | 28 | -0.153240144 | 0.120251242218911 | 0.213404714450294 | -0.388932579 | 0.0824522907166827 | 0.857923671821518 | 0.677779965224705 | 1.08594686245661 | 0.905055003276494 |
| ebi-fl187-GCST90094837 | finngen_R11_O15_PREECLAMPS | Pre-eclampsia \|\| id:finngen_R11_O15_PREECLAMPS | F-sharp flavour liking (derived food-liking factor) \|\| id:ebi-fl187-GCST90094837 | Inverse variance weighted (multiplicative random effects) | 43 | -0.073504329 | 0.0552671062357733 | 0.183523771264306 | -0.181827857 | 0.0348191994859959 | 0.92913212381503 | 0.833744851437951 | 1.03543248514954 | 0.644285579970435 |
| ebi-fl187-GCST90094837 | finngen_R11_O15_PREECLAMPS | Pre-eclampsia \|\| id:finngen_R11_O15_PREECLAMPS | F-sharp flavour liking (derived food-liking factor) \|\| id:ebi-fl187-GCST90094837 | MR Egger | 43 | -0.210476951 | 0.217298328777247 | 0.338420129368119 | -0.636381675 | 0.215427773554555 | 0.810197729308061 | 0.529203795165632 | 1.24039239055436 | 0.954596128018958 |
| ebi-fl187-GCST90094837 | finngen_R11_O15_PREECLAMPS | Pre-eclampsia \|\| id:finngen_R11_O15_PREECLAMPS | F-sharp flavour liking (derived food-liking factor) \|\| id:ebi-fl187-GCST90094837 | Weighted median | 43 | -0.119763041 | 0.0680134269008266 | 0.0782598166539264 | -0.253069358 | 0.0135432758835203 | 0.887130625539066 | 0.776414029767587 | 1.01363540146862 | 0.529128962601009 |
| ebi-fl187-GCST90094837 | finngen_R11_O15_PREECLAMPS | Pre-eclampsia \|\| id:finngen_R11_O15_PREECLAMPS | F-sharp flavour liking (derived food-liking factor) \|\| id:ebi-fl187-GCST90094837 | Weighted mode | 43 | -0.081972111 | 0.0963197352305877 | 0.399572368076933 | -0.270758792 | 0.106814569947344 | 0.921297652311914 | 0.762800467672299 | 1.11272790215447 | 0.905055003276494 |
| ebi-fl187-GCST90094838 | finngen_R11_O15_PREECLAMPS | Pre-eclampsia \|\| id:finngen_R11_O15_PREECLAMPS | Shellfish liking \|\| id:ebi-fl187-GCST90094838 | Inverse variance weighted | 30 | -0.016868395 | 0.0426350099439284 | 0.692365779438456 | -0.100433015 | 0.0666962242051705 | 0.9832730794937 | 0.904445694882028 | 1.06897070142297 | 0.941775027862301 |
| ebi-fl187-GCST90094838 | finngen_R11_O15_PREECLAMPS | Pre-eclampsia \|\| id:finngen_R11_O15_PREECLAMPS | Shellfish liking \|\| id:ebi-fl187-GCST90094838 | MR Egger | 30 | -0.036679129 | 0.119965517343865 | 0.762058743389052 | -0.271811543 | 0.198453284644307 | 0.963985400351689 | 0.761997851126336 | 1.21951505600393 | 0.954596128018958 |
| ebi-fl187-GCST90094838 | finngen_R11_O15_PREECLAMPS | Pre-eclampsia \|\| id:finngen_R11_O15_PREECLAMPS | Shellfish liking \|\| id:ebi-fl187-GCST90094838 | Weighted median | 30 | 0.0305167889135702 | 0.0606578033852502 | 0.614895728078493 | -0.088372506 | 0.149406083548661 | 1.03098719905846 | 0.915419814085038 | 1.16114441512805 | 0.881409801621768 |
| ebi-fl187-GCST90094838 | finngen_R11_O15_PREECLAMPS | Pre-eclampsia \|\| id:finngen_R11_O15_PREECLAMPS | Shellfish liking \|\| id:ebi-fl187-GCST90094838 | Weighted mode | 30 | 0.0568094115320259 | 0.0844358745628323 | 0.506393669303537 | -0.108684903 | 0.222303725675177 | 1.05845406203255 | 0.897013019432541 | 1.24895065864477 | 0.905055003276494 |
| ebi-fl187-GCST90094840 | finngen_R11_O15_PREECLAMPS | Pre-eclampsia \|\| id:finngen_R11_O15_PREECLAMPS | F-small fish liking (derived food-liking factor) \|\| id:ebi-fl187-GCST90094840 | Inverse variance weighted | 27 | -0.12540454 | 0.0480827737702834 | 0.00910471404395545 | -0.219646776 | -0.031162303 | 0.882139969717199 | 0.802802316666175 | 0.969318236903199 | 0.150227781725265 |
| ebi-fl187-GCST90094840 | finngen_R11_O15_PREECLAMPS | Pre-eclampsia \|\| id:finngen_R11_O15_PREECLAMPS | F-small fish liking (derived food-liking factor) \|\| id:ebi-fl187-GCST90094840 | MR Egger | 27 | 0.0378853982043076 | 0.172373621275274 | 0.827822183776619 | -0.299966899 | 0.375737695903844 | 1.03861219923483 | 0.740842742544607 | 1.45606515182197 | 0.954596128018958 |
| ebi-fl187-GCST90094840 | finngen_R11_O15_PREECLAMPS | Pre-eclampsia \|\| id:finngen_R11_O15_PREECLAMPS | F-small fish liking (derived food-liking factor) \|\| id:ebi-fl187-GCST90094840 | Weighted median | 27 | -0.11019013 | 0.0663088021090755 | 0.0965584404900165 | -0.240155382 | 0.019775122288064 | 0.895663826681479 | 0.786505642767868 | 1.01997194527755 | 0.529128962601009 |
| ebi-fl187-GCST90094840 | finngen_R11_O15_PREECLAMPS | Pre-eclampsia \|\| id:finngen_R11_O15_PREECLAMPS | F-small fish liking (derived food-liking factor) \|\| id:ebi-fl187-GCST90094840 | Weighted mode | 27 | -0.068284499 | 0.0990727594228212 | 0.496780341494594 | -0.262467107 | 0.125898109465829 | 0.933994715209819 | 0.769151663295401 | 1.13416660155468 | 0.905055003276494 |
| ebi-fl187-GCST90094841 | finngen_R11_O15_PREECLAMPS | Pre-eclampsia \|\| id:finngen_R11_O15_PREECLAMPS | Smoked fish liking \|\| id:ebi-fl187-GCST90094841 | Inverse variance weighted | 6 | 0.00250355365402303 | 0.109664197835666 | 0.981786459495792 | -0.212438274 | 0.217445381411929 | 1.0025066901614 | 0.808610226971916 | 1.24289754234492 | 0.992699677017842 |
| ebi-fl187-GCST90094841 | finngen_R11_O15_PREECLAMPS | Pre-eclampsia \|\| id:finngen_R11_O15_PREECLAMPS | Smoked fish liking \|\| id:ebi-fl187-GCST90094841 | MR Egger | 6 | -0.190698103 | 0.660775790759158 | 0.787227462321849 | -1.485818653 | 1.10442244715532 | 0.826382032973501 | 0.22631698982104 | 3.0174812105862 | 0.954596128018958 |
| ebi-fl187-GCST90094841 | finngen_R11_O15_PREECLAMPS | Pre-eclampsia \|\| id:finngen_R11_O15_PREECLAMPS | Smoked fish liking \|\| id:ebi-fl187-GCST90094841 | Weighted median | 6 | 0.0305700153712187 | 0.136531113804757 | 0.822831131966636 | -0.237030968 | 0.298170998428543 | 1.03104207631539 | 0.788966855161379 | 1.34739217012521 | 0.957826864554912 |
| ebi-fl187-GCST90094841 | finngen_R11_O15_PREECLAMPS | Pre-eclampsia \|\| id:finngen_R11_O15_PREECLAMPS | Smoked fish liking \|\| id:ebi-fl187-GCST90094841 | Weighted mode | 6 | 0.060242867087881 | 0.189597920280661 | 0.763523103339752 | -0.311369057 | 0.431854790837976 | 1.06209446301367 | 0.732443512824656 | 1.54011146062855 | 0.945697706548879 |
| ebi-fl187-GCST90094843 | finngen_R11_O15_PREECLAMPS | Pre-eclampsia \|\| id:finngen_R11_O15_PREECLAMPS | Soft cheese liking \|\| id:ebi-fl187-GCST90094843 | Inverse variance weighted | 2 | -0.090573401 | 0.261458344585855 | 0.729029965774425 | -0.603031757 | 0.421884953953844 | 0.913407286035262 | 0.547150292339574 | 1.52483308857397 | 0.941775027862301 |
| ebi-fl187-GCST90094845 | finngen_R11_O15_PREECLAMPS | Pre-eclampsia \|\| id:finngen_R11_O15_PREECLAMPS | Soya milk liking \|\| id:ebi-fl187-GCST90094845 | Inverse variance weighted | 3 | -0.273503606 | 0.232649662894582 | 0.239753618117897 | -0.729496945 | 0.182489733670611 | 0.760709593534301 | 0.48215147786414 | 1.20020182922302 | 0.71932023600517 |
| ebi-fl187-GCST90094845 | finngen_R11_O15_PREECLAMPS | Pre-eclampsia \|\| id:finngen_R11_O15_PREECLAMPS | Soya milk liking \|\| id:ebi-fl187-GCST90094845 | MR Egger | 3 | -1.451976015 | 0.611304080335881 | 0.253687067433485 | -2.650132012 | -0.253820017 | 0.234107231424808 | 0.0706418868612463 | 0.775831425808853 | 0.954596128018958 |
| ebi-fl187-GCST90094845 | finngen_R11_O15_PREECLAMPS | Pre-eclampsia \|\| id:finngen_R11_O15_PREECLAMPS | Soya milk liking \|\| id:ebi-fl187-GCST90094845 | Weighted median | 3 | -0.189821652 | 0.227869378653428 | 0.404828741190034 | -0.636445634 | 0.256802329873803 | 0.827106633366417 | 0.529169948799105 | 1.29278955562619 | 0.819869554194876 |
| ebi-fl187-GCST90094845 | finngen_R11_O15_PREECLAMPS | Pre-eclampsia \|\| id:finngen_R11_O15_PREECLAMPS | Soya milk liking \|\| id:ebi-fl187-GCST90094845 | Weighted mode | 3 | -0.0879122 | 0.324749329233063 | 0.811994039670767 | -0.724420885 | 0.54859648538661 | 0.915841284140381 | 0.48460512970846 | 1.7308220782568 | 0.963239370826664 |
| ebi-fl187-GCST90094846 | finngen_R11_O15_PREECLAMPS | Pre-eclampsia \|\| id:finngen_R11_O15_PREECLAMPS | Spicy food liking \|\| id:ebi-fl187-GCST90094846 | Inverse variance weighted | 16 | -0.2545956 | 0.0732131742740865 | 0.000506195932485055 | -0.398093421 | -0.111097778 | 0.775229937881226 | 0.671599283139962 | 0.894851247871394 | 0.027840776286678 |
| ebi-fl187-GCST90094846 | finngen_R11_O15_PREECLAMPS | Pre-eclampsia \|\| id:finngen_R11_O15_PREECLAMPS | Spicy food liking \|\| id:ebi-fl187-GCST90094846 | MR Egger | 16 | -0.219987369 | 0.453563527351511 | 0.635163572698574 | -1.108971883 | 0.668997144471204 | 0.802528934530879 | 0.329897960564795 | 1.95227848531295 | 0.954596128018958 |
| ebi-fl187-GCST90094846 | finngen_R11_O15_PREECLAMPS | Pre-eclampsia \|\| id:finngen_R11_O15_PREECLAMPS | Spicy food liking \|\| id:ebi-fl187-GCST90094846 | Weighted median | 16 | -0.231923263 | 0.0970123896478571 | 0.0168182972869883 | -0.422067546 | -0.041778979 | 0.793006974281798 | 0.655689748488398 | 0.959081734477809 | 0.357989470823037 |
| ebi-fl187-GCST90094846 | finngen_R11_O15_PREECLAMPS | Pre-eclampsia \|\| id:finngen_R11_O15_PREECLAMPS | Spicy food liking \|\| id:ebi-fl187-GCST90094846 | Weighted mode | 16 | -0.229141109 | 0.143623119630889 | 0.13146428766888 | -0.510642423 | 0.0523602057616814 | 0.795216313635626 | 0.600109930414103 | 1.05375524287004 | 0.905055003276494 |
| ebi-fl187-GCST90094847 | finngen_R11_O15_PREECLAMPS | Pre-eclampsia \|\| id:finngen_R11_O15_PREECLAMPS | Spinach liking \|\| id:ebi-fl187-GCST90094847 | Inverse variance weighted | 5 | 0.0778488812618706 | 0.169877520256581 | 0.646762237782048 | -0.255111058 | 0.410808820964769 | 1.08095929312225 | 0.774830441721615 | 1.50803702393403 | 0.941775027862301 |
| ebi-fl187-GCST90094847 | finngen_R11_O15_PREECLAMPS | Pre-eclampsia \|\| id:finngen_R11_O15_PREECLAMPS | Spinach liking \|\| id:ebi-fl187-GCST90094847 | MR Egger | 5 | 0.364719408077443 | 0.572824537583562 | 0.569576034957744 | -0.758016686 | 1.48745550174122 | 1.44010986825605 | 0.468594876975142 | 4.42581968893028 | 0.954596128018958 |
| ebi-fl187-GCST90094847 | finngen_R11_O15_PREECLAMPS | Pre-eclampsia \|\| id:finngen_R11_O15_PREECLAMPS | Spinach liking \|\| id:ebi-fl187-GCST90094847 | Weighted median | 5 | -0.002623774 | 0.197470946630638 | 0.989398911699434 | -0.389666829 | 0.384419281681756 | 0.997379665371506 | 0.677282487721181 | 1.46876113723776 | 0.999849666244992 |
| ebi-fl187-GCST90094847 | finngen_R11_O15_PREECLAMPS | Pre-eclampsia \|\| id:finngen_R11_O15_PREECLAMPS | Spinach liking \|\| id:ebi-fl187-GCST90094847 | Weighted mode | 5 | -0.013803237 | 0.247144049622464 | 0.958138965649949 | -0.498205574 | 0.470599100349015 | 0.986291590951698 | 0.607620011082286 | 1.60095303749023 | 0.981296058194618 |
| ebi-fl187-GCST90094848 | finngen_R11_O15_PREECLAMPS | Pre-eclampsia \|\| id:finngen_R11_O15_PREECLAMPS | Spirits liking \|\| id:ebi-fl187-GCST90094848 | Inverse variance weighted | 2 | 0.302708329276109 | 0.213637125310109 | 0.156504109764757 | -0.116020436 | 0.721437094883922 | 1.35351962481474 | 0.890457025436384 | 2.05738774856744 | 0.62762387754309 |
| ebi-fl187-GCST90094849 | finngen_R11_O15_PREECLAMPS | Pre-eclampsia \|\| id:finngen_R11_O15_PREECLAMPS | Strawberries liking \|\| id:ebi-fl187-GCST90094849 | Inverse variance weighted | 5 | -0.053409546 | 0.232264168237359 | 0.818129281672419 | -0.508647316 | 0.401828223398092 | 0.947991686436678 | 0.601308409145653 | 1.49455458111741 | 0.945152512750355 |
| ebi-fl187-GCST90094849 | finngen_R11_O15_PREECLAMPS | Pre-eclampsia \|\| id:finngen_R11_O15_PREECLAMPS | Strawberries liking \|\| id:ebi-fl187-GCST90094849 | MR Egger | 5 | 3.67414831326976 | 2.35792927979226 | 0.217066911624513 | -0.947393075 | 8.29568970166259 | 39.4150732630102 | 0.387750543549267 | 4006.56562878826 | 0.954596128018958 |
| ebi-fl187-GCST90094849 | finngen_R11_O15_PREECLAMPS | Pre-eclampsia \|\| id:finngen_R11_O15_PREECLAMPS | Strawberries liking \|\| id:ebi-fl187-GCST90094849 | Weighted median | 5 | -0.090808961 | 0.300033775730503 | 0.762147107427453 | -0.678875162 | 0.497257239064631 | 0.913192149216218 | 0.507187175133469 | 1.64420541818843 | 0.927373305772456 |
| ebi-fl187-GCST90094849 | finngen_R11_O15_PREECLAMPS | Pre-eclampsia \|\| id:finngen_R11_O15_PREECLAMPS | Strawberries liking \|\| id:ebi-fl187-GCST90094849 | Weighted mode | 5 | -0.206402628 | 0.406481701443771 | 0.638328103130121 | -1.003106763 | 0.590301506359115 | 0.813505469872292 | 0.366738300369652 | 1.80453241138187 | 0.932459680062628 |
| ebi-fl187-GCST90094850 | finngen_R11_O15_PREECLAMPS | Pre-eclampsia \|\| id:finngen_R11_O15_PREECLAMPS | F-strong alcohol liking (derived food-liking factor) \|\| id:ebi-fl187-GCST90094850 | Inverse variance weighted (multiplicative random effects) | 5 | 0.00296485050351302 | 0.170328631403055 | 0.986112205752174 | -0.330879267 | 0.336808968053501 | 1.00296925001966 | 0.718291885306516 | 1.40047150338575 | 0.992699677017842 |
| ebi-fl187-GCST90094850 | finngen_R11_O15_PREECLAMPS | Pre-eclampsia \|\| id:finngen_R11_O15_PREECLAMPS | F-strong alcohol liking (derived food-liking factor) \|\| id:ebi-fl187-GCST90094850 | MR Egger | 5 | 0.404213305410003 | 2.05377344710954 | 0.856551422406817 | -3.621182651 | 4.42960926174471 | 1.49812347072001 | 0.0267510206581088 | 83.8986281011993 | 0.970712810886627 |
| ebi-fl187-GCST90094850 | finngen_R11_O15_PREECLAMPS | Pre-eclampsia \|\| id:finngen_R11_O15_PREECLAMPS | F-strong alcohol liking (derived food-liking factor) \|\| id:ebi-fl187-GCST90094850 | Weighted median | 5 | 0.167758563764475 | 0.122766451547031 | 0.171786660028469 | -0.072863681 | 0.408380808796656 | 1.18265104136045 | 0.929727560670165 | 1.50437993321696 | 0.612323741171272 |
| ebi-fl187-GCST90094850 | finngen_R11_O15_PREECLAMPS | Pre-eclampsia \|\| id:finngen_R11_O15_PREECLAMPS | F-strong alcohol liking (derived food-liking factor) \|\| id:ebi-fl187-GCST90094850 | Weighted mode | 5 | 0.172673062185787 | 0.132606880498636 | 0.262786582041974 | -0.087236424 | 0.432582547963114 | 1.18847748332928 | 0.916460397159246 | 1.54123269566144 | 0.905055003276494 |
| ebi-fl187-GCST90094851 | finngen_R11_O15_PREECLAMPS | Pre-eclampsia \|\| id:finngen_R11_O15_PREECLAMPS | F-strong flavour liking (derived food-liking factor) \|\| id:ebi-fl187-GCST90094851 | Inverse variance weighted | 41 | -0.06434859 | 0.0511947726383874 | 0.208776545747996 | -0.164690345 | 0.0359931640815905 | 0.937678077080557 | 0.848156299555332 | 1.03664876002036 | 0.673947184049608 |
| ebi-fl187-GCST90094851 | finngen_R11_O15_PREECLAMPS | Pre-eclampsia \|\| id:finngen_R11_O15_PREECLAMPS | F-strong flavour liking (derived food-liking factor) \|\| id:ebi-fl187-GCST90094851 | MR Egger | 41 | -0.334342506 | 0.296533451542726 | 0.26641885469921 | -0.915548071 | 0.246863059083044 | 0.715808571548389 | 0.400297174700243 | 1.28000381587963 | 0.954596128018958 |
| ebi-fl187-GCST90094851 | finngen_R11_O15_PREECLAMPS | Pre-eclampsia \|\| id:finngen_R11_O15_PREECLAMPS | F-strong flavour liking (derived food-liking factor) \|\| id:ebi-fl187-GCST90094851 | Weighted median | 41 | -0.11364354 | 0.0671992709143441 | 0.0908095997589182 | -0.24535411 | 0.0180670314877349 | 0.89257606728929 | 0.782427423416753 | 1.01823122765632 | 0.529128962601009 |
| ebi-fl187-GCST90094851 | finngen_R11_O15_PREECLAMPS | Pre-eclampsia \|\| id:finngen_R11_O15_PREECLAMPS | F-strong flavour liking (derived food-liking factor) \|\| id:ebi-fl187-GCST90094851 | Weighted mode | 41 | -0.259703139 | 0.141770788860351 | 0.074427167400967 | -0.537573885 | 0.0181676074529656 | 0.771280515147871 | 0.584163783146437 | 1.01833364239502 | 0.905055003276494 |
| ebi-fl187-GCST90094852 | finngen_R11_O15_PREECLAMPS | Pre-eclampsia \|\| id:finngen_R11_O15_PREECLAMPS | F-strong vegetable liking (derived food-liking factor) \|\| id:ebi-fl187-GCST90094852 | Inverse variance weighted | 24 | -0.219692983 | 0.0595541116470868 | 0.000225168934790744 | -0.336419042 | -0.102966924 | 0.802765222469877 | 0.714323711369884 | 0.902156812310291 | 0.0185764371202363 |
| ebi-fl187-GCST90094852 | finngen_R11_O15_PREECLAMPS | Pre-eclampsia \|\| id:finngen_R11_O15_PREECLAMPS | F-strong vegetable liking (derived food-liking factor) \|\| id:ebi-fl187-GCST90094852 | MR Egger | 24 | -0.474644533 | 0.273886442408024 | 0.0970926003493009 | -1.01146196 | 0.062172894364885 | 0.622106155527256 | 0.363686895075694 | 1.06414631372503 | 0.954596128018958 |
| ebi-fl187-GCST90094852 | finngen_R11_O15_PREECLAMPS | Pre-eclampsia \|\| id:finngen_R11_O15_PREECLAMPS | F-strong vegetable liking (derived food-liking factor) \|\| id:ebi-fl187-GCST90094852 | Weighted median | 24 | -0.130746278 | 0.0793942956327453 | 0.0995998024983593 | -0.286359098 | 0.0248665409526782 | 0.877440371649214 | 0.75099288753071 | 1.02517829207594 | 0.529128962601009 |
| ebi-fl187-GCST90094852 | finngen_R11_O15_PREECLAMPS | Pre-eclampsia \|\| id:finngen_R11_O15_PREECLAMPS | F-strong vegetable liking (derived food-liking factor) \|\| id:ebi-fl187-GCST90094852 | Weighted mode | 24 | -0.132158715 | 0.108568596872987 | 0.235839295599725 | -0.344953165 | 0.0806357349378333 | 0.876201917713979 | 0.708253523883436 | 1.08397597006804 | 0.905055003276494 |
| ebi-fl187-GCST90094853 | finngen_R11_O15_PREECLAMPS | Pre-eclampsia \|\| id:finngen_R11_O15_PREECLAMPS | F-sweetened coffee drink liking (derived food-liking factor) \|\| id:ebi-fl187-GCST90094853 | Inverse variance weighted | 4 | 0.491244761325726 | 0.157021715182946 | 0.00175692157146806 | 0.183482199567151 | 0.799007323084301 | 1.63434932916268 | 1.20139357989561 | 2.22333278155738 | 0.054263705841589 |
| ebi-fl187-GCST90094853 | finngen_R11_O15_PREECLAMPS | Pre-eclampsia \|\| id:finngen_R11_O15_PREECLAMPS | F-sweetened coffee drink liking (derived food-liking factor) \|\| id:ebi-fl187-GCST90094853 | MR Egger | 4 | 0.239365910762272 | 0.994181385778513 | 0.83216702480201 | -1.709229605 | 2.18796142688816 | 1.27044332042775 | 0.181005184340084 | 8.91701658327612 | 0.954596128018958 |
| ebi-fl187-GCST90094853 | finngen_R11_O15_PREECLAMPS | Pre-eclampsia \|\| id:finngen_R11_O15_PREECLAMPS | F-sweetened coffee drink liking (derived food-liking factor) \|\| id:ebi-fl187-GCST90094853 | Weighted median | 4 | 0.460446414474097 | 0.178071470096765 | 0.00971703697252239 | 0.111426333084438 | 0.809466495863757 | 1.58478129641471 | 1.11787139083486 | 2.24670903831808 | 0.313340475930495 |
| ebi-fl187-GCST90094853 | finngen_R11_O15_PREECLAMPS | Pre-eclampsia \|\| id:finngen_R11_O15_PREECLAMPS | F-sweetened coffee drink liking (derived food-liking factor) \|\| id:ebi-fl187-GCST90094853 | Weighted mode | 4 | 0.436705529130205 | 0.241782210310577 | 0.168631282466638 | -0.037187603 | 0.910598661338935 | 1.54760028674862 | 0.96349536369655 | 2.48581024651273 | 0.905055003276494 |
| ebi-fl187-GCST90094854 | finngen_R11_O15_PREECLAMPS | Pre-eclampsia \|\| id:finngen_R11_O15_PREECLAMPS | F-sweet food liking (derived food-liking factor) \|\| id:ebi-fl187-GCST90094854 | Inverse variance weighted (multiplicative random effects) | 13 | -0.122307834 | 0.1214263884889 | 0.313810283916894 | -0.360303555 | 0.115687887917464 | 0.884875932098617 | 0.697464575103111 | 1.12264542624497 | 0.796595336096731 |
| ebi-fl187-GCST90094854 | finngen_R11_O15_PREECLAMPS | Pre-eclampsia \|\| id:finngen_R11_O15_PREECLAMPS | F-sweet food liking (derived food-liking factor) \|\| id:ebi-fl187-GCST90094854 | MR Egger | 13 | -0.878620874 | 0.445196126071293 | 0.0740801319231271 | -1.751205281 | -0.006036467 | 0.415355344140806 | 0.173564623139166 | 0.993981715778563 | 0.954596128018958 |
| ebi-fl187-GCST90094854 | finngen_R11_O15_PREECLAMPS | Pre-eclampsia \|\| id:finngen_R11_O15_PREECLAMPS | F-sweet food liking (derived food-liking factor) \|\| id:ebi-fl187-GCST90094854 | Weighted median | 13 | -0.110246756 | 0.120746991407532 | 0.361221838612256 | -0.346910859 | 0.126417346836997 | 0.895613109831227 | 0.706868336060108 | 1.13475565615568 | 0.783962747510833 |
| ebi-fl187-GCST90094854 | finngen_R11_O15_PREECLAMPS | Pre-eclampsia \|\| id:finngen_R11_O15_PREECLAMPS | F-sweet food liking (derived food-liking factor) \|\| id:ebi-fl187-GCST90094854 | Weighted mode | 13 | -0.115927568 | 0.154334389455223 | 0.467040832475444 | -0.418422971 | 0.186567835768647 | 0.890539724923435 | 0.658083819246931 | 1.20510636862981 | 0.905055003276494 |
| ebi-fl187-GCST90094857 | finngen_R11_O15_PREECLAMPS | Pre-eclampsia \|\| id:finngen_R11_O15_PREECLAMPS | Tea with sugar liking \|\| id:ebi-fl187-GCST90094857 | Inverse variance weighted | 5 | 0.0421787280251184 | 0.161061472992934 | 0.793414201192021 | -0.273501759 | 0.357859215091269 | 1.04308088987931 | 0.760710998232828 | 1.4302642466836 | 0.945152512750355 |
| ebi-fl187-GCST90094857 | finngen_R11_O15_PREECLAMPS | Pre-eclampsia \|\| id:finngen_R11_O15_PREECLAMPS | Tea with sugar liking \|\| id:ebi-fl187-GCST90094857 | MR Egger | 5 | -0.67150818 | 0.83651959232111 | 0.480832166990951 | -2.311086581 | 0.968070221222661 | 0.510937410958802 | 0.0991534547773727 | 2.63285871887601 | 0.954596128018958 |
| ebi-fl187-GCST90094857 | finngen_R11_O15_PREECLAMPS | Pre-eclampsia \|\| id:finngen_R11_O15_PREECLAMPS | Tea with sugar liking \|\| id:ebi-fl187-GCST90094857 | Weighted median | 5 | 0.0733584845414638 | 0.204402068901452 | 0.719675089504053 | -0.327269571 | 0.47398653958831 | 1.07611623881292 | 0.720889386315172 | 1.6063853642736 | 0.925493743359756 |
| ebi-fl187-GCST90094857 | finngen_R11_O15_PREECLAMPS | Pre-eclampsia \|\| id:finngen_R11_O15_PREECLAMPS | Tea with sugar liking \|\| id:ebi-fl187-GCST90094857 | Weighted mode | 5 | 0.212788416253593 | 0.311936168287587 | 0.532596117707394 | -0.398606474 | 0.824183306097263 | 1.23712286845881 | 0.671254805877886 | 2.28001792800897 | 0.905055003276494 |
| ebi-fl187-GCST90094859 | finngen_R11_O15_PREECLAMPS | Pre-eclampsia \|\| id:finngen_R11_O15_PREECLAMPS | Tinned tuna liking \|\| id:ebi-fl187-GCST90094859 | Inverse variance weighted | 2 | 0.0339371803155742 | 0.268654714753938 | 0.899476513586697 | -0.492626061 | 0.560500421233292 | 1.03451961649097 | 0.611019710694255 | 1.7515487932273 | 0.953358127900067 |
| ebi-fl187-GCST90094860 | finngen_R11_O15_PREECLAMPS | Pre-eclampsia \|\| id:finngen_R11_O15_PREECLAMPS | Tomatoes liking \|\| id:ebi-fl187-GCST90094860 | Inverse variance weighted | 4 | 0.174353627762968 | 0.226572486744409 | 0.441580570196044 | -0.269728446 | 0.618435701782009 | 1.19047647692645 | 0.763586821045475 | 1.85602239726295 | 0.887682351940796 |
| ebi-fl187-GCST90094860 | finngen_R11_O15_PREECLAMPS | Pre-eclampsia \|\| id:finngen_R11_O15_PREECLAMPS | Tomatoes liking \|\| id:ebi-fl187-GCST90094860 | MR Egger | 4 | -0.067736706 | 2.49639409525373 | 0.980817022847846 | -4.960669133 | 4.82519572027587 | 0.934506490746558 | 0.00700823681994426 | 124.610854867543 | 0.987444164894115 |
| ebi-fl187-GCST90094860 | finngen_R11_O15_PREECLAMPS | Pre-eclampsia \|\| id:finngen_R11_O15_PREECLAMPS | Tomatoes liking \|\| id:ebi-fl187-GCST90094860 | Weighted median | 4 | 0.201230701350268 | 0.273035636575247 | 0.461114693837799 | -0.333919146 | 0.736380549037753 | 1.22290686554751 | 0.716111680138849 | 2.08836309095428 | 0.848896149192195 |
| ebi-fl187-GCST90094860 | finngen_R11_O15_PREECLAMPS | Pre-eclampsia \|\| id:finngen_R11_O15_PREECLAMPS | Tomatoes liking \|\| id:ebi-fl187-GCST90094860 | Weighted mode | 4 | 0.227526068418038 | 0.414138286838632 | 0.620986141092765 | -0.584184974 | 1.03923711062176 | 1.25549016797008 | 0.557560102777705 | 2.82705945783568 | 0.92526935022822 |
| ebi-fl187-GCST90094861 | finngen_R11_O15_PREECLAMPS | Pre-eclampsia \|\| id:finngen_R11_O15_PREECLAMPS | Turnip liking \|\| id:ebi-fl187-GCST90094861 | Inverse variance weighted | 3 | -0.17232902 | 0.205427267164861 | 0.401536120728805 | -0.574966464 | 0.230308423291452 | 0.841702190453815 | 0.562723739996792 | 1.25898825135543 | 0.861823321954233 |
| ebi-fl187-GCST90094861 | finngen_R11_O15_PREECLAMPS | Pre-eclampsia \|\| id:finngen_R11_O15_PREECLAMPS | Turnip liking \|\| id:ebi-fl187-GCST90094861 | MR Egger | 3 | 0.904408769542466 | 2.42957836217696 | 0.773136156303161 | -3.85756482 | 5.66638235940931 | 2.47047087356665 | 0.021119366405696 | 288.987189288742 | 0.954596128018958 |
| ebi-fl187-GCST90094861 | finngen_R11_O15_PREECLAMPS | Pre-eclampsia \|\| id:finngen_R11_O15_PREECLAMPS | Turnip liking \|\| id:ebi-fl187-GCST90094861 | Weighted median | 3 | -0.117894532 | 0.232086007823512 | 0.611468936155931 | -0.572783107 | 0.33699404362965 | 0.88878978681547 | 0.563953709031094 | 1.40073072044276 | 0.881409801621768 |
| ebi-fl187-GCST90094861 | finngen_R11_O15_PREECLAMPS | Pre-eclampsia \|\| id:finngen_R11_O15_PREECLAMPS | Turnip liking \|\| id:ebi-fl187-GCST90094861 | Weighted mode | 3 | -0.113581787 | 0.286490973205063 | 0.730067843148954 | -0.675104094 | 0.447940520977149 | 0.892631188240745 | 0.509103423244276 | 1.56508560312267 | 0.936018697441052 |
| ebi-fl187-GCST90094862 | finngen_R11_O15_PREECLAMPS | Pre-eclampsia \|\| id:finngen_R11_O15_PREECLAMPS | Vegetables liking \|\| id:ebi-fl187-GCST90094862 | Inverse variance weighted | 3 | 0.353255055752965 | 0.414303288321813 | 0.393854287121051 | -0.458779389 | 1.16528950086372 | 1.42369421853034 | 0.632054667505986 | 3.20685113500488 | 0.861823321954233 |
| ebi-fl187-GCST90094862 | finngen_R11_O15_PREECLAMPS | Pre-eclampsia \|\| id:finngen_R11_O15_PREECLAMPS | Vegetables liking \|\| id:ebi-fl187-GCST90094862 | MR Egger | 3 | -7.190924624 | 5.34591163622764 | 0.406978082338609 | -17.66891143 | 3.28706218313194 | 0.000753392193936536 | 2.12074896797169e-08 | 26.7641199621739 | 0.954596128018958 |
| ebi-fl187-GCST90094862 | finngen_R11_O15_PREECLAMPS | Pre-eclampsia \|\| id:finngen_R11_O15_PREECLAMPS | Vegetables liking \|\| id:ebi-fl187-GCST90094862 | Weighted median | 3 | 0.429427591893517 | 0.404369406512453 | 0.28824996339748 | -0.363136445 | 1.22199162865793 | 1.53637783667402 | 0.695491530772625 | 3.39394047602692 | 0.715820742437076 |
| ebi-fl187-GCST90094862 | finngen_R11_O15_PREECLAMPS | Pre-eclampsia \|\| id:finngen_R11_O15_PREECLAMPS | Vegetables liking \|\| id:ebi-fl187-GCST90094862 | Weighted mode | 3 | 0.45154729874821 | 0.451134313211252 | 0.422297539768274 | -0.432675955 | 1.33577055264226 | 1.57074071131455 | 0.648770688549702 | 3.8029251717526 | 0.905055003276494 |
| ebi-fl187-GCST90094863 | finngen_R11_O15_PREECLAMPS | Pre-eclampsia \|\| id:finngen_R11_O15_PREECLAMPS | F-vegetarian liking (derived food-liking factor) \|\| id:ebi-fl187-GCST90094863 | Inverse variance weighted | 13 | -0.025206096 | 0.067448582900364 | 0.70862133802577 | -0.157405319 | 0.106993126188053 | 0.975108924979422 | 0.854357701407454 | 1.11292660440484 | 0.941775027862301 |
| ebi-fl187-GCST90094863 | finngen_R11_O15_PREECLAMPS | Pre-eclampsia \|\| id:finngen_R11_O15_PREECLAMPS | F-vegetarian liking (derived food-liking factor) \|\| id:ebi-fl187-GCST90094863 | MR Egger | 13 | 0.342776388908357 | 0.421354266298287 | 0.433181478295846 | -0.483077973 | 1.168630751 | 1.40885369138923 | 0.616881721357398 | 3.21758394684402 | 0.954596128018958 |
| ebi-fl187-GCST90094863 | finngen_R11_O15_PREECLAMPS | Pre-eclampsia \|\| id:finngen_R11_O15_PREECLAMPS | F-vegetarian liking (derived food-liking factor) \|\| id:ebi-fl187-GCST90094863 | Weighted median | 13 | -0.033362107 | 0.0932506849759752 | 0.720516970968213 | -0.21613345 | 0.149409235362446 | 0.967188270344507 | 0.805627783878499 | 1.1611480748448 | 0.925493743359756 |
| ebi-fl187-GCST90094863 | finngen_R11_O15_PREECLAMPS | Pre-eclampsia \|\| id:finngen_R11_O15_PREECLAMPS | F-vegetarian liking (derived food-liking factor) \|\| id:ebi-fl187-GCST90094863 | Weighted mode | 13 | -0.017540844 | 0.140949840076638 | 0.9030213912021 | -0.29380253 | 0.258720842825752 | 0.982612101307545 | 0.745423675519071 | 1.29527216983508 | 0.981296058194618 |
| ebi-fl187-GCST90094864 | finngen_R11_O15_PREECLAMPS | Pre-eclampsia \|\| id:finngen_R11_O15_PREECLAMPS | Vinegar liking \|\| id:ebi-fl187-GCST90094864 | Inverse variance weighted (multiplicative random effects) | 11 | -0.132735294 | 0.166031888051925 | 0.424025685472664 | -0.458157794 | 0.192687207027038 | 0.875696864035666 | 0.632447671799038 | 1.21250347163198 | 0.87455297628737 |
| ebi-fl187-GCST90094864 | finngen_R11_O15_PREECLAMPS | Pre-eclampsia \|\| id:finngen_R11_O15_PREECLAMPS | Vinegar liking \|\| id:ebi-fl187-GCST90094864 | MR Egger | 11 | 0.330882185044859 | 0.959082004399913 | 0.738020825441433 | -1.548918544 | 2.21068291366869 | 1.39219576116457 | 0.212477634922739 | 9.1219437665022 | 0.954596128018958 |
| ebi-fl187-GCST90094864 | finngen_R11_O15_PREECLAMPS | Pre-eclampsia \|\| id:finngen_R11_O15_PREECLAMPS | Vinegar liking \|\| id:ebi-fl187-GCST90094864 | Weighted median | 11 | -0.083589398 | 0.167212201562828 | 0.617145413989398 | -0.411325313 | 0.244146516911468 | 0.919808853784135 | 0.66277128826834 | 1.27653135021917 | 0.881409801621768 |
| ebi-fl187-GCST90094864 | finngen_R11_O15_PREECLAMPS | Pre-eclampsia \|\| id:finngen_R11_O15_PREECLAMPS | Vinegar liking \|\| id:ebi-fl187-GCST90094864 | Weighted mode | 11 | -0.031179617 | 0.259755212727231 | 0.906833111187136 | -0.540299834 | 0.477940600102871 | 0.969301454572483 | 0.582573550950106 | 1.61274968337311 | 0.981296058194618 |
| ebi-fl187-GCST90094865 | finngen_R11_O15_PREECLAMPS | Pre-eclampsia \|\| id:finngen_R11_O15_PREECLAMPS | White bread liking \|\| id:ebi-fl187-GCST90094865 | Inverse variance weighted (multiplicative random effects) | 4 | 0.203588546211061 | 0.394022589614437 | 0.605370780453194 | -0.568695729 | 0.975872821855357 | 1.22579369222296 | 0.566263518106983 | 2.65348221781387 | 0.941775027862301 |
| ebi-fl187-GCST90094865 | finngen_R11_O15_PREECLAMPS | Pre-eclampsia \|\| id:finngen_R11_O15_PREECLAMPS | White bread liking \|\| id:ebi-fl187-GCST90094865 | MR Egger | 4 | -0.484615399 | 3.70650067865711 | 0.907940213800702 | -7.749356729 | 6.78012593091674 | 0.615934039909307 | 0.000431019713719081 | 880.179558947643 | 0.970712810886627 |
| ebi-fl187-GCST90094865 | finngen_R11_O15_PREECLAMPS | Pre-eclampsia \|\| id:finngen_R11_O15_PREECLAMPS | White bread liking \|\| id:ebi-fl187-GCST90094865 | Weighted median | 4 | -0.086072346 | 0.278071384235543 | 0.756915903669101 | -0.63109226 | 0.458947566620738 | 0.917527848907897 | 0.532010390092264 | 1.58240772962264 | 0.927373305772456 |
| ebi-fl187-GCST90094865 | finngen_R11_O15_PREECLAMPS | Pre-eclampsia \|\| id:finngen_R11_O15_PREECLAMPS | White bread liking \|\| id:ebi-fl187-GCST90094865 | Weighted mode | 4 | -0.230801046 | 0.306663307376854 | 0.506350114974528 | -0.831861128 | 0.37025903650847 | 0.793897399426748 | 0.435238497332371 | 1.44810967935873 | 0.905055003276494 |
| ebi-fl187-GCST90094867 | finngen_R11_O15_PREECLAMPS | Pre-eclampsia \|\| id:finngen_R11_O15_PREECLAMPS | White wine liking \|\| id:ebi-fl187-GCST90094867 | Inverse variance weighted | 5 | 0.0986406824915728 | 0.190132348271699 | 0.603900143803976 | -0.27401872 | 0.471300085104104 | 1.10366966047094 | 0.760317841885691 | 1.6020756745929 | 0.941775027862301 |
| ebi-fl187-GCST90094867 | finngen_R11_O15_PREECLAMPS | Pre-eclampsia \|\| id:finngen_R11_O15_PREECLAMPS | White wine liking \|\| id:ebi-fl187-GCST90094867 | MR Egger | 5 | 0.283371267901692 | 0.559727007964558 | 0.647530192056066 | -0.813693668 | 1.38043620351223 | 1.32759796484122 | 0.443217939255437 | 3.97663587175963 | 0.954596128018958 |
| ebi-fl187-GCST90094867 | finngen_R11_O15_PREECLAMPS | Pre-eclampsia \|\| id:finngen_R11_O15_PREECLAMPS | White wine liking \|\| id:ebi-fl187-GCST90094867 | Weighted median | 5 | 0.217425646026728 | 0.199874093198839 | 0.276677649261391 | -0.174327577 | 0.609178868696454 | 1.2428730135252 | 0.840021681101295 | 1.8389207832398 | 0.710775340343918 |
| ebi-fl187-GCST90094867 | finngen_R11_O15_PREECLAMPS | Pre-eclampsia \|\| id:finngen_R11_O15_PREECLAMPS | White wine liking \|\| id:ebi-fl187-GCST90094867 | Weighted mode | 5 | 0.285576552674729 | 0.270394649455138 | 0.350466936795403 | -0.24439696 | 0.8155500656068 | 1.33052892703311 | 0.783176682531764 | 2.2604187090313 | 0.905055003276494 |
| ebi-fl187-GCST90094870 | finngen_R11_O15_PREECLAMPS | Pre-eclampsia \|\| id:finngen_R11_O15_PREECLAMPS | Whole milk liking \|\| id:ebi-fl187-GCST90094870 | Inverse variance weighted | 5 | -0.043724524 | 0.124969146433934 | 0.726426842320023 | -0.288664051 | 0.201215003202096 | 0.957217611818353 | 0.749263877711874 | 1.22288766832501 | 0.941775027862301 |
| ebi-fl187-GCST90094870 | finngen_R11_O15_PREECLAMPS | Pre-eclampsia \|\| id:finngen_R11_O15_PREECLAMPS | Whole milk liking \|\| id:ebi-fl187-GCST90094870 | MR Egger | 5 | -0.121841266 | 0.687771360365276 | 0.870673115771479 | -1.469873132 | 1.22619060060862 | 0.885288883054758 | 0.229954657218288 | 3.4082214987121 | 0.970712810886627 |
| ebi-fl187-GCST90094870 | finngen_R11_O15_PREECLAMPS | Pre-eclampsia \|\| id:finngen_R11_O15_PREECLAMPS | Whole milk liking \|\| id:ebi-fl187-GCST90094870 | Weighted median | 5 | -0.081282636 | 0.161036564994283 | 0.613736924117307 | -0.396914304 | 0.234349031148208 | 0.921933082917007 | 0.672391644687333 | 1.26408562047509 | 0.881409801621768 |
| ebi-fl187-GCST90094870 | finngen_R11_O15_PREECLAMPS | Pre-eclampsia \|\| id:finngen_R11_O15_PREECLAMPS | Whole milk liking \|\| id:ebi-fl187-GCST90094870 | Weighted mode | 5 | -0.12856645 | 0.19901617990546 | 0.553466594668175 | -0.518638163 | 0.261505262651799 | 0.879355127361342 | 0.595330739850398 | 1.29888377712697 | 0.905055003276494 |
| ebi-fl187-GCST90094872 | finngen_R11_O15_PREECLAMPS | Pre-eclampsia \|\| id:finngen_R11_O15_PREECLAMPS | F-wine liking (derived food-liking factor) \|\| id:ebi-fl187-GCST90094872 | Inverse variance weighted | 9 | 0.174380614485784 | 0.259187191798054 | 0.501075834327731 | -0.333626281 | 0.682387510409969 | 1.19050860441866 | 0.716321434826987 | 1.97859601609882 | 0.894908414040245 |
| ebi-fl187-GCST90094872 | finngen_R11_O15_PREECLAMPS | Pre-eclampsia \|\| id:finngen_R11_O15_PREECLAMPS | F-wine liking (derived food-liking factor) \|\| id:ebi-fl187-GCST90094872 | MR Egger | 9 | 0.886010734922084 | 0.877199685437004 | 0.346105819936662 | -0.833300649 | 2.60532211837861 | 2.42543462448513 | 0.434612413494263 | 13.5355846611798 | 0.954596128018958 |
| ebi-fl187-GCST90094872 | finngen_R11_O15_PREECLAMPS | Pre-eclampsia \|\| id:finngen_R11_O15_PREECLAMPS | F-wine liking (derived food-liking factor) \|\| id:ebi-fl187-GCST90094872 | Weighted median | 9 | 0.0455099253112884 | 0.347771076720964 | 0.895884688021173 | -0.636121385 | 0.727141235684378 | 1.046561392 | 0.529341559650857 | 2.0691569125393 | 0.988450091947753 |
| ebi-fl187-GCST90094872 | finngen_R11_O15_PREECLAMPS | Pre-eclampsia \|\| id:finngen_R11_O15_PREECLAMPS | F-wine liking (derived food-liking factor) \|\| id:ebi-fl187-GCST90094872 | Weighted mode | 9 | -0.018065931 | 0.552370616140378 | 0.974710178609419 | -1.100712339 | 1.06458047623846 | 0.982096279244322 | 0.332634051066416 | 2.89962226841581 | 0.981296058194618 |
| ebi-fl187-GCST90094873 | finngen_R11_O15_PREECLAMPS | Pre-eclampsia \|\| id:finngen_R11_O15_PREECLAMPS | Whiskey liking \|\| id:ebi-fl187-GCST90094873 | Inverse variance weighted (multiplicative random effects) | 5 | -0.002403121 | 0.262644103244835 | 0.992699677017842 | -0.517185564 | 0.512379320867858 | 0.997599763692821 | 0.596196144915458 | 1.66925817452425 | 0.992699677017842 |
| ebi-fl187-GCST90094873 | finngen_R11_O15_PREECLAMPS | Pre-eclampsia \|\| id:finngen_R11_O15_PREECLAMPS | Whiskey liking \|\| id:ebi-fl187-GCST90094873 | MR Egger | 5 | -1.750637673 | 6.76614754555187 | 0.812576525783726 | -15.01228686 | 11.5110115162868 | 0.173663167822552 | 3.02166737114723e-07 | 99808.7881748331 | 0.954596128018958 |
| ebi-fl187-GCST90094873 | finngen_R11_O15_PREECLAMPS | Pre-eclampsia \|\| id:finngen_R11_O15_PREECLAMPS | Whiskey liking \|\| id:ebi-fl187-GCST90094873 | Weighted median | 5 | 0.225585070297544 | 0.188296785790479 | 0.230905602653467 | -0.14347663 | 0.594646770446882 | 1.25305562739743 | 0.866341046469318 | 1.81239064194331 | 0.649149713120124 |
| ebi-fl187-GCST90094873 | finngen_R11_O15_PREECLAMPS | Pre-eclampsia \|\| id:finngen_R11_O15_PREECLAMPS | Whiskey liking \|\| id:ebi-fl187-GCST90094873 | Weighted mode | 5 | 0.224832530996894 | 0.21732478600573 | 0.359324383145058 | -0.20112405 | 0.650789111568126 | 1.25211300851527 | 0.81781097615776 | 1.91705300099902 | 0.905055003276494 |

| id.exposure | id.outcome | outcome | exposure | method | nsnp | b | se | pval | lo_ci | up_ci | or | or_lci95 | or_uci95 | p_fdr |
| --- | --- | --- | --- | --- | --- | --- | --- | --- | --- | --- | --- | --- | --- | --- |
| ebi-fl187-GCST90094687 | finngen_R11_O15_POOR_FETGRO | Poor fetal growth \|\| id:finngen_R11_O15_POOR_FETGRO | Adding salt to food liking \|\| id:ebi-fl187-GCST90094687 | Inverse variance weighted | 13 | 0.116129443707253 | 0.114858137082595 | 0.311983648346781 | -0.108992505 | 0.341251392389139 | 1.12314124629086 | 0.896737138540649 | 1.40670683180654 | 0.888224550184161 |
| ebi-fl187-GCST90094687 | finngen_R11_O15_POOR_FETGRO | Poor fetal growth \|\| id:finngen_R11_O15_POOR_FETGRO | Adding salt to food liking \|\| id:ebi-fl187-GCST90094687 | MR Egger | 13 | 0.235681453778003 | 0.445060102973393 | 0.606946203548912 | -0.636636348 | 1.10799925560585 | 1.26577103935759 | 0.529069038514774 | 3.02829348807502 | 0.970850396659599 |
| ebi-fl187-GCST90094687 | finngen_R11_O15_POOR_FETGRO | Poor fetal growth \|\| id:finngen_R11_O15_POOR_FETGRO | Adding salt to food liking \|\| id:ebi-fl187-GCST90094687 | Weighted median | 13 | 0.096533732222479 | 0.144966485126541 | 0.505472596308777 | -0.187600579 | 0.380668043070499 | 1.10134673139018 | 0.828945739764274 | 1.46326178488928 | 0.918328271889597 |
| ebi-fl187-GCST90094687 | finngen_R11_O15_POOR_FETGRO | Poor fetal growth \|\| id:finngen_R11_O15_POOR_FETGRO | Adding salt to food liking \|\| id:ebi-fl187-GCST90094687 | Weighted mode | 13 | 0.0816232379949284 | 0.194206092872322 | 0.681700245281553 | -0.299020704 | 0.46226718002468 | 1.08504692835256 | 0.741544056322111 | 1.58766944012281 | 0.972577670507659 |
| ebi-fl187-GCST90094688 | finngen_R11_O15_POOR_FETGRO | Poor fetal growth \|\| id:finngen_R11_O15_POOR_FETGRO | Alcohol liking \|\| id:ebi-fl187-GCST90094688 | Inverse variance weighted | 17 | 0.0811016558343784 | 0.0811428542924465 | 0.317556280619045 | -0.077938339 | 0.240141650247574 | 1.08448113479811 | 0.925021462923078 | 1.27142923583255 | 0.888224550184161 |
| ebi-fl187-GCST90094688 | finngen_R11_O15_POOR_FETGRO | Poor fetal growth \|\| id:finngen_R11_O15_POOR_FETGRO | Alcohol liking \|\| id:ebi-fl187-GCST90094688 | MR Egger | 17 | 0.464121116211527 | 0.337943582666849 | 0.189818525878675 | -0.198248306 | 1.12649053823855 | 1.59061560811048 | 0.820166175820615 | 3.08481145328047 | 0.970850396659599 |
| ebi-fl187-GCST90094688 | finngen_R11_O15_POOR_FETGRO | Poor fetal growth \|\| id:finngen_R11_O15_POOR_FETGRO | Alcohol liking \|\| id:ebi-fl187-GCST90094688 | Weighted median | 17 | -0.052058924 | 0.106021599685823 | 0.623410977537746 | -0.25986126 | 0.155743410956381 | 0.94927292983063 | 0.771158569066771 | 1.16852633356552 | 0.918328271889597 |
| ebi-fl187-GCST90094688 | finngen_R11_O15_POOR_FETGRO | Poor fetal growth \|\| id:finngen_R11_O15_POOR_FETGRO | Alcohol liking \|\| id:ebi-fl187-GCST90094688 | Weighted mode | 17 | -0.058838084 | 0.169747228472278 | 0.733392134076437 | -0.391542652 | 0.273866483956077 | 0.942859420966193 | 0.676013216797133 | 1.31503921168376 | 0.972577670507659 |
| ebi-fl187-GCST90094689 | finngen_R11_O15_POOR_FETGRO | Poor fetal growth \|\| id:finngen_R11_O15_POOR_FETGRO | Aniseed liking \|\| id:ebi-fl187-GCST90094689 | Inverse variance weighted | 9 | -0.205464083 | 0.138188999025679 | 0.137058696414442 | -0.476314521 | 0.0653863553685344 | 0.814269340380367 | 0.621068112677989 | 1.06757140666027 | 0.822352178486651 |
| ebi-fl187-GCST90094689 | finngen_R11_O15_POOR_FETGRO | Poor fetal growth \|\| id:finngen_R11_O15_POOR_FETGRO | Aniseed liking \|\| id:ebi-fl187-GCST90094689 | MR Egger | 9 | -0.386303214 | 0.529497077406502 | 0.489342692938546 | -1.424117486 | 0.651511057876002 | 0.679564441093835 | 0.240720809086041 | 1.91843751004556 | 0.970850396659599 |
| ebi-fl187-GCST90094689 | finngen_R11_O15_POOR_FETGRO | Poor fetal growth \|\| id:finngen_R11_O15_POOR_FETGRO | Aniseed liking \|\| id:ebi-fl187-GCST90094689 | Weighted median | 9 | -0.230074362 | 0.178793967240979 | 0.198159550921158 | -0.580510538 | 0.120361813522169 | 0.794474521377671 | 0.559612590094246 | 1.12790486899514 | 0.918328271889597 |
| ebi-fl187-GCST90094689 | finngen_R11_O15_POOR_FETGRO | Poor fetal growth \|\| id:finngen_R11_O15_POOR_FETGRO | Aniseed liking \|\| id:ebi-fl187-GCST90094689 | Weighted mode | 9 | -0.325145748 | 0.263697803038886 | 0.252567425495679 | -0.841993441 | 0.191701946432715 | 0.722422054743331 | 0.430850790962327 | 1.21130942805954 | 0.972577670507659 |
| ebi-fl187-GCST90094691 | finngen_R11_O15_POOR_FETGRO | Poor fetal growth \|\| id:finngen_R11_O15_POOR_FETGRO | Apples liking \|\| id:ebi-fl187-GCST90094691 | Inverse variance weighted | 3 | 0.449169104204327 | 0.518028867153863 | 0.3859017379227 | -0.566167475 | 1.4645056838259 | 1.56700962271085 | 0.567696987444481 | 4.32540459431017 | 0.888224550184161 |
| ebi-fl187-GCST90094691 | finngen_R11_O15_POOR_FETGRO | Poor fetal growth \|\| id:finngen_R11_O15_POOR_FETGRO | Apples liking \|\| id:ebi-fl187-GCST90094691 | MR Egger | 3 | 2.72316097151693 | 1.32948294816945 | 0.289135976711491 | 0.11737439310481 | 5.32894754992904 | 15.2283827300829 | 1.12454037104785 | 206.22082278629 | 0.970850396659599 |
| ebi-fl187-GCST90094691 | finngen_R11_O15_POOR_FETGRO | Poor fetal growth \|\| id:finngen_R11_O15_POOR_FETGRO | Apples liking \|\| id:ebi-fl187-GCST90094691 | Weighted median | 3 | -0.023247473 | 0.513572822176502 | 0.963895154093095 | -1.029850205 | 0.983355258000661 | 0.977020667162757 | 0.35706044245692 | 2.67341119474004 | 0.987966417243977 |
| ebi-fl187-GCST90094691 | finngen_R11_O15_POOR_FETGRO | Poor fetal growth \|\| id:finngen_R11_O15_POOR_FETGRO | Apples liking \|\| id:ebi-fl187-GCST90094691 | Weighted mode | 3 | -0.031487598 | 0.580254778790973 | 0.961656978831553 | -1.168786965 | 1.1058117682234 | 0.969002973753611 | 0.3107436557785 | 3.02167637434452 | 0.988620775239461 |
| ebi-fl187-GCST90094692 | finngen_R11_O15_POOR_FETGRO | Poor fetal growth \|\| id:finngen_R11_O15_POOR_FETGRO | Asparagus liking \|\| id:ebi-fl187-GCST90094692 | Inverse variance weighted | 8 | -0.092304829 | 0.178763223418016 | 0.605608266726867 | -0.442680747 | 0.258071088752552 | 0.911827155683069 | 0.64231223443645 | 1.29443083482685 | 0.96571624475698 |
| ebi-fl187-GCST90094692 | finngen_R11_O15_POOR_FETGRO | Poor fetal growth \|\| id:finngen_R11_O15_POOR_FETGRO | Asparagus liking \|\| id:ebi-fl187-GCST90094692 | MR Egger | 8 | 0.712110483655111 | 0.924976531658332 | 0.47059201918561 | -1.100843518 | 2.52506448570544 | 2.03828849698336 | 0.332590419205217 | 12.4917007737711 | 0.970850396659599 |
| ebi-fl187-GCST90094692 | finngen_R11_O15_POOR_FETGRO | Poor fetal growth \|\| id:finngen_R11_O15_POOR_FETGRO | Asparagus liking \|\| id:ebi-fl187-GCST90094692 | Weighted median | 8 | -0.074988571 | 0.205043025694584 | 0.714573161547401 | -0.476872902 | 0.326895758957431 | 0.92775408919468 | 0.620721416876396 | 1.38665692308285 | 0.918328271889597 |
| ebi-fl187-GCST90094692 | finngen_R11_O15_POOR_FETGRO | Poor fetal growth \|\| id:finngen_R11_O15_POOR_FETGRO | Asparagus liking \|\| id:ebi-fl187-GCST90094692 | Weighted mode | 8 | -0.163606272 | 0.309397333053808 | 0.613297175544974 | -0.770025045 | 0.442812500552706 | 0.849076260976735 | 0.463001472285707 | 1.55708035526367 | 0.972577670507659 |
| ebi-fl187-GCST90094693 | finngen_R11_O15_POOR_FETGRO | Poor fetal growth \|\| id:finngen_R11_O15_POOR_FETGRO | Aubergine liking \|\| id:ebi-fl187-GCST90094693 | Inverse variance weighted | 10 | -0.12039278 | 0.126233740083109 | 0.340220831844198 | -0.36781091 | 0.127025350642356 | 0.886572140584841 | 0.692248066166075 | 1.13544580169707 | 0.888224550184161 |
| ebi-fl187-GCST90094693 | finngen_R11_O15_POOR_FETGRO | Poor fetal growth \|\| id:finngen_R11_O15_POOR_FETGRO | Aubergine liking \|\| id:ebi-fl187-GCST90094693 | MR Egger | 10 | 0.255178628538814 | 0.717220018701772 | 0.731198450689608 | -1.150572608 | 1.66092986519429 | 1.29069215473597 | 0.316455512494409 | 5.26420356898167 | 0.970850396659599 |
| ebi-fl187-GCST90094693 | finngen_R11_O15_POOR_FETGRO | Poor fetal growth \|\| id:finngen_R11_O15_POOR_FETGRO | Aubergine liking \|\| id:ebi-fl187-GCST90094693 | Weighted median | 10 | -0.199629958 | 0.164559061472823 | 0.225083784018665 | -0.522165718 | 0.122905802763729 | 0.81903377413194 | 0.593234377237795 | 1.13077789977757 | 0.918328271889597 |
| ebi-fl187-GCST90094693 | finngen_R11_O15_POOR_FETGRO | Poor fetal growth \|\| id:finngen_R11_O15_POOR_FETGRO | Aubergine liking \|\| id:ebi-fl187-GCST90094693 | Weighted mode | 10 | -0.28249725 | 0.241695631624104 | 0.272503989933594 | -0.756220688 | 0.191226187884104 | 0.753898715103196 | 0.469437228408667 | 1.21073327431003 | 0.972577670507659 |
| ebi-fl187-GCST90094694 | finngen_R11_O15_POOR_FETGRO | Poor fetal growth \|\| id:finngen_R11_O15_POOR_FETGRO | Avocado liking \|\| id:ebi-fl187-GCST90094694 | Inverse variance weighted (multiplicative random effects) | 11 | -0.193689086 | 0.194165333112354 | 0.318498970911655 | -0.574253139 | 0.186874966801107 | 0.823914030775177 | 0.563125288106206 | 1.20547655103738 | 0.888224550184161 |
| ebi-fl187-GCST90094694 | finngen_R11_O15_POOR_FETGRO | Poor fetal growth \|\| id:finngen_R11_O15_POOR_FETGRO | Avocado liking \|\| id:ebi-fl187-GCST90094694 | MR Egger | 11 | 1.23034820478725 | 1.63879361766595 | 0.471966366189707 | -1.981687286 | 4.44238369541251 | 3.42242103222275 | 0.137836471468591 | 84.9772603506444 | 0.970850396659599 |
| ebi-fl187-GCST90094694 | finngen_R11_O15_POOR_FETGRO | Poor fetal growth \|\| id:finngen_R11_O15_POOR_FETGRO | Avocado liking \|\| id:ebi-fl187-GCST90094694 | Weighted median | 11 | -0.084472752 | 0.174252690146551 | 0.627838384843112 | -0.426008025 | 0.257062520825686 | 0.918996695984879 | 0.653111101177748 | 1.29312597153554 | 0.918328271889597 |
| ebi-fl187-GCST90094694 | finngen_R11_O15_POOR_FETGRO | Poor fetal growth \|\| id:finngen_R11_O15_POOR_FETGRO | Avocado liking \|\| id:ebi-fl187-GCST90094694 | Weighted mode | 11 | -0.017397723 | 0.281096103542553 | 0.951868069519328 | -0.568346086 | 0.533550640328202 | 0.982752743905676 | 0.566461543294645 | 1.7049753281341 | 0.988620775239461 |
| ebi-fl187-GCST90094695 | finngen_R11_O15_POOR_FETGRO | Poor fetal growth \|\| id:finngen_R11_O15_POOR_FETGRO | Bacon liking \|\| id:ebi-fl187-GCST90094695 | Inverse variance weighted | 4 | -0.218104696 | 0.207288988734402 | 0.292718373005054 | -0.624391114 | 0.188181722110165 | 0.804041257509636 | 0.535587441096596 | 1.20705284361043 | 0.888224550184161 |
| ebi-fl187-GCST90094695 | finngen_R11_O15_POOR_FETGRO | Poor fetal growth \|\| id:finngen_R11_O15_POOR_FETGRO | Bacon liking \|\| id:ebi-fl187-GCST90094695 | MR Egger | 4 | 0.343820022860668 | 0.769830520899949 | 0.698853882787714 | -1.165047798 | 1.85268784382457 | 1.41032478644426 | 0.311907753077359 | 6.37693671809986 | 0.970850396659599 |
| ebi-fl187-GCST90094695 | finngen_R11_O15_POOR_FETGRO | Poor fetal growth \|\| id:finngen_R11_O15_POOR_FETGRO | Bacon liking \|\| id:ebi-fl187-GCST90094695 | Weighted median | 4 | -0.154693216 | 0.230597601194626 | 0.502324889669495 | -0.606664515 | 0.297278082039002 | 0.856677952003796 | 0.545166233819703 | 1.34618959855121 | 0.918328271889597 |
| ebi-fl187-GCST90094695 | finngen_R11_O15_POOR_FETGRO | Poor fetal growth \|\| id:finngen_R11_O15_POOR_FETGRO | Bacon liking \|\| id:ebi-fl187-GCST90094695 | Weighted mode | 4 | -0.080489741 | 0.280234641806652 | 0.792638505363625 | -0.629749639 | 0.468770157152712 | 0.92266436934427 | 0.532725158059951 | 1.5980276613136 | 0.972577670507659 |
| ebi-fl187-GCST90094696 | finngen_R11_O15_POOR_FETGRO | Poor fetal growth \|\| id:finngen_R11_O15_POOR_FETGRO | Baked/steamed fish liking \|\| id:ebi-fl187-GCST90094696 | Inverse variance weighted | 7 | -0.007902346 | 0.220735032471594 | 0.971441717565997 | -0.44054301 | 0.424738317507589 | 0.992128795316327 | 0.64368679794012 | 1.52919020499686 | 0.994304114950957 |
| ebi-fl187-GCST90094696 | finngen_R11_O15_POOR_FETGRO | Poor fetal growth \|\| id:finngen_R11_O15_POOR_FETGRO | Baked/steamed fish liking \|\| id:ebi-fl187-GCST90094696 | MR Egger | 7 | -0.686328029 | 2.49480206875475 | 0.7942429746791 | -5.576140083 | 4.20348402620645 | 0.50342122768186 | 0.00378715544723365 | 66.9190731702954 | 0.970850396659599 |
| ebi-fl187-GCST90094696 | finngen_R11_O15_POOR_FETGRO | Poor fetal growth \|\| id:finngen_R11_O15_POOR_FETGRO | Baked/steamed fish liking \|\| id:ebi-fl187-GCST90094696 | Weighted median | 7 | 0.0754385998285262 | 0.2194468479578 | 0.731021467301952 | -0.354677222 | 0.505554421825815 | 1.07835701437972 | 0.701399802981886 | 1.65790444411058 | 0.918328271889597 |
| ebi-fl187-GCST90094696 | finngen_R11_O15_POOR_FETGRO | Poor fetal growth \|\| id:finngen_R11_O15_POOR_FETGRO | Baked/steamed fish liking \|\| id:ebi-fl187-GCST90094696 | Weighted mode | 7 | 0.105451152671945 | 0.25337501433653 | 0.691750598784878 | -0.391163875 | 0.602066180771543 | 1.11121182346864 | 0.676269323033613 | 1.82588751930587 | 0.972577670507659 |
| ebi-fl187-GCST90094697 | finngen_R11_O15_POOR_FETGRO | Poor fetal growth \|\| id:finngen_R11_O15_POOR_FETGRO | Banana liking \|\| id:ebi-fl187-GCST90094697 | Inverse variance weighted | 2 | -0.722219998 | 0.302343828499978 | 0.0169063836455658 | -1.314813902 | -0.129626094 | 0.485672865583678 | 0.268524290512001 | 0.878423817504582 | 0.405753207493579 |
| ebi-fl187-GCST90094698 | finngen_R11_O15_POOR_FETGRO | Poor fetal growth \|\| id:finngen_R11_O15_POOR_FETGRO | BBQ/grilled meat liking \|\| id:ebi-fl187-GCST90094698 | Inverse variance weighted | 4 | 0.0128404269550009 | 0.176384022651377 | 0.941966759306226 | -0.332872257 | 0.3585531113517 | 1.01292321922037 | 0.71686176206245 | 1.43125704610587 | 0.994304114950957 |
| ebi-fl187-GCST90094698 | finngen_R11_O15_POOR_FETGRO | Poor fetal growth \|\| id:finngen_R11_O15_POOR_FETGRO | BBQ/grilled meat liking \|\| id:ebi-fl187-GCST90094698 | MR Egger | 4 | -0.363866807 | 0.580223145245754 | 0.594630362386875 | -1.501104172 | 0.773370557726264 | 0.694983755580887 | 0.222883922123753 | 2.16705815259808 | 0.970850396659599 |
| ebi-fl187-GCST90094698 | finngen_R11_O15_POOR_FETGRO | Poor fetal growth \|\| id:finngen_R11_O15_POOR_FETGRO | BBQ/grilled meat liking \|\| id:ebi-fl187-GCST90094698 | Weighted median | 4 | -0.083794442 | 0.207623181792265 | 0.686514940856102 | -0.490735879 | 0.323146994093294 | 0.919620271769564 | 0.612175741405752 | 1.3814684036769 | 0.918328271889597 |
| ebi-fl187-GCST90094698 | finngen_R11_O15_POOR_FETGRO | Poor fetal growth \|\| id:finngen_R11_O15_POOR_FETGRO | BBQ/grilled meat liking \|\| id:ebi-fl187-GCST90094698 | Weighted mode | 4 | -0.091410449 | 0.234593893766004 | 0.72280773054019 | -0.55121448 | 0.368393583272272 | 0.912643041037653 | 0.576249541524628 | 1.44541081655482 | 0.972577670507659 |
| ebi-fl187-GCST90094699 | finngen_R11_O15_POOR_FETGRO | Poor fetal growth \|\| id:finngen_R11_O15_POOR_FETGRO | Beans liking \|\| id:ebi-fl187-GCST90094699 | Inverse variance weighted | 9 | -0.166284442 | 0.089934927353996 | 0.0644662244185315 | -0.342556899 | 0.00998801573775554 | 0.846805333032236 | 0.709952722288517 | 1.01003806245062 | 0.706473758635168 |
| ebi-fl187-GCST90094699 | finngen_R11_O15_POOR_FETGRO | Poor fetal growth \|\| id:finngen_R11_O15_POOR_FETGRO | Beans liking \|\| id:ebi-fl187-GCST90094699 | MR Egger | 9 | -0.035121861 | 0.411248508066666 | 0.934332355428775 | -0.841168937 | 0.770925214591926 | 0.965487753574125 | 0.431206175844973 | 2.16176542572698 | 0.993285374120483 |
| ebi-fl187-GCST90094699 | finngen_R11_O15_POOR_FETGRO | Poor fetal growth \|\| id:finngen_R11_O15_POOR_FETGRO | Beans liking \|\| id:ebi-fl187-GCST90094699 | Weighted median | 9 | -0.289272563 | 0.121772268380572 | 0.0175242019542172 | -0.527946209 | -0.050598917 | 0.748808079900861 | 0.589815083244968 | 0.950659887231018 | 0.652429624709108 |
| ebi-fl187-GCST90094699 | finngen_R11_O15_POOR_FETGRO | Poor fetal growth \|\| id:finngen_R11_O15_POOR_FETGRO | Beans liking \|\| id:ebi-fl187-GCST90094699 | Weighted mode | 9 | -0.342233322 | 0.19136779545907 | 0.111519495338924 | -0.717314201 | 0.0328475574558732 | 0.710182484432126 | 0.488061331977818 | 1.03339299417621 | 0.972577670507659 |
| ebi-fl187-GCST90094700 | finngen_R11_O15_POOR_FETGRO | Poor fetal growth \|\| id:finngen_R11_O15_POOR_FETGRO | Beef/steak liking \|\| id:ebi-fl187-GCST90094700 | Inverse variance weighted | 5 | 0.0743044175030472 | 0.166770954083174 | 0.655923985751581 | -0.252566652 | 0.401175487506068 | 1.07713465423413 | 0.776804435156183 | 1.49357935001852 | 0.96571624475698 |
| ebi-fl187-GCST90094700 | finngen_R11_O15_POOR_FETGRO | Poor fetal growth \|\| id:finngen_R11_O15_POOR_FETGRO | Beef/steak liking \|\| id:ebi-fl187-GCST90094700 | MR Egger | 5 | -0.258927401 | 0.626687939215141 | 0.707240018047348 | -1.487235762 | 0.969380959729808 | 0.771879058555608 | 0.225996501030972 | 2.63631197084348 | 0.970850396659599 |
| ebi-fl187-GCST90094700 | finngen_R11_O15_POOR_FETGRO | Poor fetal growth \|\| id:finngen_R11_O15_POOR_FETGRO | Beef/steak liking \|\| id:ebi-fl187-GCST90094700 | Weighted median | 5 | 0.059048943819328 | 0.195310558150123 | 0.762397768298287 | -0.32375975 | 0.441857637793569 | 1.06082716040245 | 0.723424024013203 | 1.55559426683761 | 0.92576871864792 |
| ebi-fl187-GCST90094700 | finngen_R11_O15_POOR_FETGRO | Poor fetal growth \|\| id:finngen_R11_O15_POOR_FETGRO | Beef/steak liking \|\| id:ebi-fl187-GCST90094700 | Weighted mode | 5 | -0.023441043 | 0.254206186443456 | 0.930962705220344 | -0.521685169 | 0.474803082267466 | 0.976831563871737 | 0.593519524299836 | 1.60769758215074 | 0.988620775239461 |
| ebi-fl187-GCST90094701 | finngen_R11_O15_POOR_FETGRO | Poor fetal growth \|\| id:finngen_R11_O15_POOR_FETGRO | Beer liking \|\| id:ebi-fl187-GCST90094701 | Inverse variance weighted | 9 | 0.0356485970745046 | 0.0887883845089878 | 0.688051534966327 | -0.138376637 | 0.209673830712121 | 1.03629162659145 | 0.870770665905549 | 1.23327573767861 | 0.96571624475698 |
| ebi-fl187-GCST90094701 | finngen_R11_O15_POOR_FETGRO | Poor fetal growth \|\| id:finngen_R11_O15_POOR_FETGRO | Beer liking \|\| id:ebi-fl187-GCST90094701 | MR Egger | 9 | 0.169850565767131 | 0.258281088225818 | 0.53180430346917 | -0.336380367 | 0.676081498689734 | 1.18512773943281 | 0.714351338347049 | 1.96615822407933 | 0.970850396659599 |
| ebi-fl187-GCST90094701 | finngen_R11_O15_POOR_FETGRO | Poor fetal growth \|\| id:finngen_R11_O15_POOR_FETGRO | Beer liking \|\| id:ebi-fl187-GCST90094701 | Weighted median | 9 | -0.015696145 | 0.105705693513238 | 0.881956771541717 | -0.222879305 | 0.191487013848709 | 0.984426397067479 | 0.800211425205784 | 1.21104910617096 | 0.969669326094948 |
| ebi-fl187-GCST90094701 | finngen_R11_O15_POOR_FETGRO | Poor fetal growth \|\| id:finngen_R11_O15_POOR_FETGRO | Beer liking \|\| id:ebi-fl187-GCST90094701 | Weighted mode | 9 | 0.00540159441109067 | 0.119124093380528 | 0.964944348647055 | -0.228081629 | 0.238884817436925 | 1.00541620932494 | 0.796059275967267 | 1.26983226562504 | 0.988620775239461 |
| ebi-fl187-GCST90094702 | finngen_R11_O15_POOR_FETGRO | Poor fetal growth \|\| id:finngen_R11_O15_POOR_FETGRO | Beetroot liking \|\| id:ebi-fl187-GCST90094702 | Inverse variance weighted | 6 | -0.131151487 | 0.196502757273585 | 0.504498639894572 | -0.516296891 | 0.253993917097514 | 0.877084897227675 | 0.596726203485801 | 1.28916396238528 | 0.914435393876931 |
| ebi-fl187-GCST90094702 | finngen_R11_O15_POOR_FETGRO | Poor fetal growth \|\| id:finngen_R11_O15_POOR_FETGRO | Beetroot liking \|\| id:ebi-fl187-GCST90094702 | MR Egger | 6 | -0.40237849 | 0.905650645199638 | 0.679806133208074 | -2.177453755 | 1.37269677464574 | 0.668727591115587 | 0.113329728881015 | 3.94597777242341 | 0.970850396659599 |
| ebi-fl187-GCST90094702 | finngen_R11_O15_POOR_FETGRO | Poor fetal growth \|\| id:finngen_R11_O15_POOR_FETGRO | Beetroot liking \|\| id:ebi-fl187-GCST90094702 | Weighted median | 6 | -0.401902028 | 0.242304895945071 | 0.0971838339179276 | -0.876819624 | 0.0730155684170276 | 0.669046290526672 | 0.416104177376025 | 1.07574728446668 | 0.918328271889597 |
| ebi-fl187-GCST90094702 | finngen_R11_O15_POOR_FETGRO | Poor fetal growth \|\| id:finngen_R11_O15_POOR_FETGRO | Beetroot liking \|\| id:ebi-fl187-GCST90094702 | Weighted mode | 6 | -0.437439369 | 0.38988064496846 | 0.312840925887793 | -1.201605433 | 0.32672669524873 | 0.645687674001842 | 0.300711052720304 | 1.38642250953671 | 0.972577670507659 |
| ebi-fl187-GCST90094704 | finngen_R11_O15_POOR_FETGRO | Poor fetal growth \|\| id:finngen_R11_O15_POOR_FETGRO | Biscuits liking \|\| id:ebi-fl187-GCST90094704 | Inverse variance weighted (multiplicative random effects) | 9 | -0.050704248 | 0.227014064274179 | 0.823260865263697 | -0.495651814 | 0.394243318291436 | 0.95055975921249 | 0.609173710113495 | 1.48326140940285 | 0.994304114950957 |
| ebi-fl187-GCST90094704 | finngen_R11_O15_POOR_FETGRO | Poor fetal growth \|\| id:finngen_R11_O15_POOR_FETGRO | Biscuits liking \|\| id:ebi-fl187-GCST90094704 | MR Egger | 9 | 1.39217095702984 | 0.899942546080858 | 0.165796775911032 | -0.371716433 | 3.15605834734832 | 4.02357558789416 | 0.689549748157867 | 23.4778716905447 | 0.970850396659599 |
| ebi-fl187-GCST90094704 | finngen_R11_O15_POOR_FETGRO | Poor fetal growth \|\| id:finngen_R11_O15_POOR_FETGRO | Biscuits liking \|\| id:ebi-fl187-GCST90094704 | Weighted median | 9 | -0.131716198 | 0.241199461306981 | 0.585005257856009 | -0.604467142 | 0.341034745979738 | 0.876589737542126 | 0.546365484118111 | 1.40640210683232 | 0.918328271889597 |
| ebi-fl187-GCST90094704 | finngen_R11_O15_POOR_FETGRO | Poor fetal growth \|\| id:finngen_R11_O15_POOR_FETGRO | Biscuits liking \|\| id:ebi-fl187-GCST90094704 | Weighted mode | 9 | -0.26893648 | 0.395547176225779 | 0.51574995330606 | -1.044208946 | 0.506335984979724 | 0.764191795246307 | 0.35197013672752 | 1.65920070762671 | 0.972577670507659 |
| ebi-fl187-GCST90094705 | finngen_R11_O15_POOR_FETGRO | Poor fetal growth \|\| id:finngen_R11_O15_POOR_FETGRO | Bitter ale liking \|\| id:ebi-fl187-GCST90094705 | Inverse variance weighted | 4 | 0.174017310029273 | 0.272283908946352 | 0.522756294027294 | -0.359659152 | 0.707693771564122 | 1.19007616589534 | 0.697914168528187 | 2.02930581509601 | 0.922579066800346 |
| ebi-fl187-GCST90094705 | finngen_R11_O15_POOR_FETGRO | Poor fetal growth \|\| id:finngen_R11_O15_POOR_FETGRO | Bitter ale liking \|\| id:ebi-fl187-GCST90094705 | MR Egger | 4 | 0.397022789847935 | 0.902350699376178 | 0.702927486536615 | -1.371584581 | 2.16563016062524 | 1.48738982705303 | 0.253704625360401 | 8.72009524650216 | 0.970850396659599 |
| ebi-fl187-GCST90094705 | finngen_R11_O15_POOR_FETGRO | Poor fetal growth \|\| id:finngen_R11_O15_POOR_FETGRO | Bitter ale liking \|\| id:ebi-fl187-GCST90094705 | Weighted median | 4 | 0.0780125727659272 | 0.218946156994596 | 0.721608874137565 | -0.351121895 | 0.507147040475335 | 1.08113625145766 | 0.703897947025549 | 1.66054695734678 | 0.918328271889597 |
| ebi-fl187-GCST90094705 | finngen_R11_O15_POOR_FETGRO | Poor fetal growth \|\| id:finngen_R11_O15_POOR_FETGRO | Bitter ale liking \|\| id:ebi-fl187-GCST90094705 | Weighted mode | 4 | 0.080678723440437 | 0.20485056074962 | 0.72002156786968 | -0.320828376 | 0.482185822509691 | 1.08402256957347 | 0.725547761983688 | 1.61961071746932 | 0.972577670507659 |
| ebi-fl187-GCST90094706 | finngen_R11_O15_POOR_FETGRO | Poor fetal growth \|\| id:finngen_R11_O15_POOR_FETGRO | F-bitter food liking (derived food-liking factor) \|\| id:ebi-fl187-GCST90094706 | Inverse variance weighted | 6 | -0.081357627 | 0.19466153357831 | 0.67598803428078 | -0.462894233 | 0.300178978598007 | 0.92186394884858 | 0.629459204981014 | 1.35010042503442 | 0.96571624475698 |
| ebi-fl187-GCST90094706 | finngen_R11_O15_POOR_FETGRO | Poor fetal growth \|\| id:finngen_R11_O15_POOR_FETGRO | F-bitter food liking (derived food-liking factor) \|\| id:ebi-fl187-GCST90094706 | MR Egger | 6 | 3.36348456119595 | 2.77625433932079 | 0.292371563965029 | -2.077973944 | 8.8049430662647 | 28.8896835614105 | 0.125183584406966 | 6667.11869796876 | 0.970850396659599 |
| ebi-fl187-GCST90094706 | finngen_R11_O15_POOR_FETGRO | Poor fetal growth \|\| id:finngen_R11_O15_POOR_FETGRO | F-bitter food liking (derived food-liking factor) \|\| id:ebi-fl187-GCST90094706 | Weighted median | 6 | 0.0459309745742629 | 0.234463911772788 | 0.844690125607629 | -0.413618293 | 0.505480241648927 | 1.04700213869428 | 0.661253308446503 | 1.65778146502701 | 0.964459621029606 |
| ebi-fl187-GCST90094706 | finngen_R11_O15_POOR_FETGRO | Poor fetal growth \|\| id:finngen_R11_O15_POOR_FETGRO | F-bitter food liking (derived food-liking factor) \|\| id:ebi-fl187-GCST90094706 | Weighted mode | 6 | 0.0874102441648117 | 0.328757589802019 | 0.800946316888661 | -0.556954632 | 0.731775120176769 | 1.0913443055272 | 0.572951257204099 | 2.07876739640766 | 0.972577670507659 |
| ebi-fl187-GCST90094707 | finngen_R11_O15_POOR_FETGRO | Poor fetal growth \|\| id:finngen_R11_O15_POOR_FETGRO | Black olive liking \|\| id:ebi-fl187-GCST90094707 | Inverse variance weighted | 22 | -0.008705661 | 0.0691832012888533 | 0.899862614777376 | -0.144304736 | 0.126893413278953 | 0.9913321232957 | 0.865623921287434 | 1.13529600385389 | 0.994304114950957 |
| ebi-fl187-GCST90094707 | finngen_R11_O15_POOR_FETGRO | Poor fetal growth \|\| id:finngen_R11_O15_POOR_FETGRO | Black olive liking \|\| id:ebi-fl187-GCST90094707 | MR Egger | 22 | -0.25779208 | 0.324598401087961 | 0.43640883572527 | -0.894004946 | 0.378420786165301 | 0.772755886834093 | 0.409014387580543 | 1.45997715182856 | 0.970850396659599 |
| ebi-fl187-GCST90094707 | finngen_R11_O15_POOR_FETGRO | Poor fetal growth \|\| id:finngen_R11_O15_POOR_FETGRO | Black olive liking \|\| id:ebi-fl187-GCST90094707 | Weighted median | 22 | -0.054086874 | 0.0993176068281924 | 0.586039007877874 | -0.248749383 | 0.140575635422986 | 0.94734980289769 | 0.779775373595333 | 1.1509361278137 | 0.918328271889597 |
| ebi-fl187-GCST90094707 | finngen_R11_O15_POOR_FETGRO | Poor fetal growth \|\| id:finngen_R11_O15_POOR_FETGRO | Black olive liking \|\| id:ebi-fl187-GCST90094707 | Weighted mode | 22 | -0.062352729 | 0.160441163704018 | 0.701459890349285 | -0.37681741 | 0.252111951632356 | 0.93955142108873 | 0.686041326686608 | 1.28674008187426 | 0.972577670507659 |
| ebi-fl187-GCST90094708 | finngen_R11_O15_POOR_FETGRO | Poor fetal growth \|\| id:finngen_R11_O15_POOR_FETGRO | Black pepper liking \|\| id:ebi-fl187-GCST90094708 | Inverse variance weighted | 8 | -0.130493337 | 0.230836697012969 | 0.571865744852879 | -0.582933263 | 0.321946589059743 | 0.877662340718239 | 0.558258443612285 | 1.3798110769821 | 0.941896520934154 |
| ebi-fl187-GCST90094708 | finngen_R11_O15_POOR_FETGRO | Poor fetal growth \|\| id:finngen_R11_O15_POOR_FETGRO | Black pepper liking \|\| id:ebi-fl187-GCST90094708 | MR Egger | 8 | 1.07293843872225 | 1.46248325286691 | 0.490828309001891 | -1.793528737 | 3.93940561434139 | 2.92395876263551 | 0.166372049427093 | 51.3880479024786 | 0.970850396659599 |
| ebi-fl187-GCST90094708 | finngen_R11_O15_POOR_FETGRO | Poor fetal growth \|\| id:finngen_R11_O15_POOR_FETGRO | Black pepper liking \|\| id:ebi-fl187-GCST90094708 | Weighted median | 8 | -0.002704648 | 0.219135090256847 | 0.99015245853578 | -0.432209425 | 0.426800129161377 | 0.99729900652242 | 0.649073430477344 | 1.53234635976264 | 0.99015245853578 |
| ebi-fl187-GCST90094708 | finngen_R11_O15_POOR_FETGRO | Poor fetal growth \|\| id:finngen_R11_O15_POOR_FETGRO | Black pepper liking \|\| id:ebi-fl187-GCST90094708 | Weighted mode | 8 | -0.030805056 | 0.250184701826772 | 0.905465475083303 | -0.521167071 | 0.459556959772798 | 0.9696645851314 | 0.593827104776556 | 1.58337233193801 | 0.988620775239461 |
| ebi-fl187-GCST90094709 | finngen_R11_O15_POOR_FETGRO | Poor fetal growth \|\| id:finngen_R11_O15_POOR_FETGRO | Blue cheese liking \|\| id:ebi-fl187-GCST90094709 | Inverse variance weighted | 16 | 0.011602484925586 | 0.0773872256610202 | 0.880821696298056 | -0.140076477 | 0.163281447221186 | 1.0116700548272 | 0.869291751709644 | 1.17736801001642 | 0.994304114950957 |
| ebi-fl187-GCST90094709 | finngen_R11_O15_POOR_FETGRO | Poor fetal growth \|\| id:finngen_R11_O15_POOR_FETGRO | Blue cheese liking \|\| id:ebi-fl187-GCST90094709 | MR Egger | 16 | 0.256369101029553 | 0.403236265163127 | 0.535176011463699 | -0.533973979 | 1.04671218074928 | 1.29222960320368 | 0.586270507695259 | 2.84827110604703 | 0.970850396659599 |
| ebi-fl187-GCST90094709 | finngen_R11_O15_POOR_FETGRO | Poor fetal growth \|\| id:finngen_R11_O15_POOR_FETGRO | Blue cheese liking \|\| id:ebi-fl187-GCST90094709 | Weighted median | 16 | 0.0481105889233108 | 0.104279090144496 | 0.644537676191598 | -0.156276428 | 0.252497605606523 | 1.04928668839348 | 0.855322722745175 | 1.28723641400063 | 0.918328271889597 |
| ebi-fl187-GCST90094709 | finngen_R11_O15_POOR_FETGRO | Poor fetal growth \|\| id:finngen_R11_O15_POOR_FETGRO | Blue cheese liking \|\| id:ebi-fl187-GCST90094709 | Weighted mode | 16 | 0.0545531136804902 | 0.174400675185339 | 0.758736906837968 | -0.28727221 | 0.396378437043754 | 1.05606856661256 | 0.750307460080671 | 1.48643173195584 | 0.972577670507659 |
| ebi-fl187-GCST90094711 | finngen_R11_O15_POOR_FETGRO | Poor fetal growth \|\| id:finngen_R11_O15_POOR_FETGRO | Broad bean liking \|\| id:ebi-fl187-GCST90094711 | Inverse variance weighted | 11 | -0.045522712 | 0.117651115021094 | 0.698808242159245 | -0.276118898 | 0.185073473079546 | 0.955497900711487 | 0.758722714934116 | 1.2033068475396 | 0.96571624475698 |
| ebi-fl187-GCST90094711 | finngen_R11_O15_POOR_FETGRO | Poor fetal growth \|\| id:finngen_R11_O15_POOR_FETGRO | Broad bean liking \|\| id:ebi-fl187-GCST90094711 | MR Egger | 11 | 0.0783443811940208 | 0.328387449196555 | 0.816780217743988 | -0.565295019 | 0.721983781619269 | 1.08149504109923 | 0.568192494315547 | 2.05851280265711 | 0.970850396659599 |
| ebi-fl187-GCST90094711 | finngen_R11_O15_POOR_FETGRO | Poor fetal growth \|\| id:finngen_R11_O15_POOR_FETGRO | Broad bean liking \|\| id:ebi-fl187-GCST90094711 | Weighted median | 11 | 0.0561529164115885 | 0.153330146081204 | 0.714199214758117 | -0.24437417 | 0.356680002730748 | 1.05775942014507 | 0.783194531605684 | 1.42857865543545 | 0.918328271889597 |
| ebi-fl187-GCST90094711 | finngen_R11_O15_POOR_FETGRO | Poor fetal growth \|\| id:finngen_R11_O15_POOR_FETGRO | Broad bean liking \|\| id:ebi-fl187-GCST90094711 | Weighted mode | 11 | 0.0522233887876487 | 0.178176437666554 | 0.775438355947581 | -0.297002429 | 0.401449206614094 | 1.05361108112845 | 0.743042207481398 | 1.49398822718217 | 0.972577670507659 |
| ebi-fl187-GCST90094712 | finngen_R11_O15_POOR_FETGRO | Poor fetal growth \|\| id:finngen_R11_O15_POOR_FETGRO | Broccoli liking \|\| id:ebi-fl187-GCST90094712 | Inverse variance weighted | 5 | -0.128041191 | 0.250429937589374 | 0.60915116904229 | -0.618883869 | 0.362801486206798 | 0.879817137438432 | 0.538545189176855 | 1.43735049701865 | 0.96571624475698 |
| ebi-fl187-GCST90094712 | finngen_R11_O15_POOR_FETGRO | Poor fetal growth \|\| id:finngen_R11_O15_POOR_FETGRO | Broccoli liking \|\| id:ebi-fl187-GCST90094712 | MR Egger | 5 | 0.218407568788492 | 2.7970166470207 | 0.942676283270808 | -5.263745059 | 5.70056019694907 | 1.24409401819519 | 0.00517588438193301 | 299.034872477393 | 0.993285374120483 |
| ebi-fl187-GCST90094712 | finngen_R11_O15_POOR_FETGRO | Poor fetal growth \|\| id:finngen_R11_O15_POOR_FETGRO | Broccoli liking \|\| id:ebi-fl187-GCST90094712 | Weighted median | 5 | -0.097551827 | 0.304270787733692 | 0.748507029332328 | -0.693922571 | 0.49881891661005 | 0.907055330065337 | 0.499612454895822 | 1.64677514288849 | 0.918328271889597 |
| ebi-fl187-GCST90094712 | finngen_R11_O15_POOR_FETGRO | Poor fetal growth \|\| id:finngen_R11_O15_POOR_FETGRO | Broccoli liking \|\| id:ebi-fl187-GCST90094712 | Weighted mode | 5 | 0.0059274595990948 | 0.390657675124094 | 0.988620775239461 | -0.759761584 | 0.77161650284232 | 1.00594506174924 | 0.467777939627888 | 2.16326034541657 | 0.988620775239461 |
| ebi-fl187-GCST90094714 | finngen_R11_O15_POOR_FETGRO | Poor fetal growth \|\| id:finngen_R11_O15_POOR_FETGRO | Brussel sprout liking \|\| id:ebi-fl187-GCST90094714 | Inverse variance weighted | 5 | -0.1891651 | 0.212546984492414 | 0.373470220964731 | -0.60575719 | 0.227426989314001 | 0.82764985018281 | 0.545661101103958 | 1.25536578129127 | 0.888224550184161 |
| ebi-fl187-GCST90094714 | finngen_R11_O15_POOR_FETGRO | Poor fetal growth \|\| id:finngen_R11_O15_POOR_FETGRO | Brussel sprout liking \|\| id:ebi-fl187-GCST90094714 | MR Egger | 5 | -1.05777562 | 0.802656663380457 | 0.27915347089357 | -2.63098268 | 0.515431440369157 | 0.347227317499647 | 0.0720076669598182 | 1.67436073279918 | 0.970850396659599 |
| ebi-fl187-GCST90094714 | finngen_R11_O15_POOR_FETGRO | Poor fetal growth \|\| id:finngen_R11_O15_POOR_FETGRO | Brussel sprout liking \|\| id:ebi-fl187-GCST90094714 | Weighted median | 5 | -0.097986228 | 0.23429752975022 | 0.675791530277659 | -0.557209387 | 0.361236929877235 | 0.906661389815842 | 0.572805313656585 | 1.43510343948316 | 0.918328271889597 |
| ebi-fl187-GCST90094714 | finngen_R11_O15_POOR_FETGRO | Poor fetal growth \|\| id:finngen_R11_O15_POOR_FETGRO | Brussel sprout liking \|\| id:ebi-fl187-GCST90094714 | Weighted mode | 5 | -0.08709317 | 0.248167837151536 | 0.743333763737261 | -0.573502131 | 0.399315790882778 | 0.916591692866987 | 0.563548358687139 | 1.49080432669521 | 0.972577670507659 |
| ebi-fl187-GCST90094716 | finngen_R11_O15_POOR_FETGRO | Poor fetal growth \|\| id:finngen_R11_O15_POOR_FETGRO | F-burn/spicy liking (derived food-liking factor) \|\| id:ebi-fl187-GCST90094716 | Inverse variance weighted | 13 | 0.0142548049544707 | 0.100835868418718 | 0.887580486826203 | -0.183383497 | 0.211893107055159 | 1.0143568891734 | 0.83244885275571 | 1.23601575665273 | 0.994304114950957 |
| ebi-fl187-GCST90094716 | finngen_R11_O15_POOR_FETGRO | Poor fetal growth \|\| id:finngen_R11_O15_POOR_FETGRO | F-burn/spicy liking (derived food-liking factor) \|\| id:ebi-fl187-GCST90094716 | MR Egger | 13 | -0.024631438 | 0.596391761630762 | 0.967796233785209 | -1.193559291 | 1.14429641449047 | 0.975669440151931 | 0.303140376765819 | 3.14023115825896 | 0.993285374120483 |
| ebi-fl187-GCST90094716 | finngen_R11_O15_POOR_FETGRO | Poor fetal growth \|\| id:finngen_R11_O15_POOR_FETGRO | F-burn/spicy liking (derived food-liking factor) \|\| id:ebi-fl187-GCST90094716 | Weighted median | 13 | 0.015397142860426 | 0.132053548317381 | 0.907178843325578 | -0.243427812 | 0.274222097562493 | 1.01551628958552 | 0.783936064890521 | 1.31550694068087 | 0.969669326094948 |
| ebi-fl187-GCST90094716 | finngen_R11_O15_POOR_FETGRO | Poor fetal growth \|\| id:finngen_R11_O15_POOR_FETGRO | F-burn/spicy liking (derived food-liking factor) \|\| id:ebi-fl187-GCST90094716 | Weighted mode | 13 | 0.00273218647989681 | 0.167476501108654 | 0.987252112084786 | -0.325521756 | 0.330986128652858 | 1.00273592230292 | 0.722150469211263 | 1.39234047853612 | 0.988620775239461 |
| ebi-fl187-GCST90094717 | finngen_R11_O15_POOR_FETGRO | Poor fetal growth \|\| id:finngen_R11_O15_POOR_FETGRO | Adding butter to bread liking \|\| id:ebi-fl187-GCST90094717 | Inverse variance weighted | 3 | 0.0102305603413787 | 0.19534439032954 | 0.958232349796388 | -0.372644445 | 0.393105565387278 | 1.01028307144372 | 0.688910134949847 | 1.48157478409005 | 0.994304114950957 |
| ebi-fl187-GCST90094717 | finngen_R11_O15_POOR_FETGRO | Poor fetal growth \|\| id:finngen_R11_O15_POOR_FETGRO | Adding butter to bread liking \|\| id:ebi-fl187-GCST90094717 | MR Egger | 3 | -0.198964027 | 0.545821605121577 | 0.777467613261001 | -1.268774373 | 0.870846319423927 | 0.819579375847576 | 0.28117602770758 | 2.38893179760429 | 0.970850396659599 |
| ebi-fl187-GCST90094717 | finngen_R11_O15_POOR_FETGRO | Poor fetal growth \|\| id:finngen_R11_O15_POOR_FETGRO | Adding butter to bread liking \|\| id:ebi-fl187-GCST90094717 | Weighted median | 3 | -0.053247631 | 0.202041162633023 | 0.792127902400085 | -0.44924831 | 0.34275304799789 | 0.948145193491753 | 0.638107630818254 | 1.40882080784513 | 0.932273608209331 |
| ebi-fl187-GCST90094717 | finngen_R11_O15_POOR_FETGRO | Poor fetal growth \|\| id:finngen_R11_O15_POOR_FETGRO | Adding butter to bread liking \|\| id:ebi-fl187-GCST90094717 | Weighted mode | 3 | -0.098512979 | 0.211458523949092 | 0.687117226397673 | -0.512971686 | 0.315945727595092 | 0.906183930863953 | 0.59871374317583 | 1.37155581597345 | 0.972577670507659 |
| ebi-fl187-GCST90094718 | finngen_R11_O15_POOR_FETGRO | Poor fetal growth \|\| id:finngen_R11_O15_POOR_FETGRO | Butternut squash liking \|\| id:ebi-fl187-GCST90094718 | Inverse variance weighted | 3 | -0.024310272 | 0.217508977802539 | 0.911008349808077 | -0.450627869 | 0.402007324360109 | 0.97598284249652 | 0.637227930566685 | 1.49482228125263 | 0.994304114950957 |
| ebi-fl187-GCST90094718 | finngen_R11_O15_POOR_FETGRO | Poor fetal growth \|\| id:finngen_R11_O15_POOR_FETGRO | Butternut squash liking \|\| id:ebi-fl187-GCST90094718 | MR Egger | 3 | -1.065535321 | 1.66646646460454 | 0.637835923224383 | -4.331809592 | 2.20073894928084 | 0.344543363974011 | 0.0131437411482699 | 9.03168499131262 | 0.970850396659599 |
| ebi-fl187-GCST90094718 | finngen_R11_O15_POOR_FETGRO | Poor fetal growth \|\| id:finngen_R11_O15_POOR_FETGRO | Butternut squash liking \|\| id:ebi-fl187-GCST90094718 | Weighted median | 3 | -0.140568568 | 0.267156480798527 | 0.598773106224924 | -0.66419527 | 0.383058134408088 | 0.868864086654866 | 0.514687545440753 | 1.46676329700598 | 0.918328271889597 |
| ebi-fl187-GCST90094718 | finngen_R11_O15_POOR_FETGRO | Poor fetal growth \|\| id:finngen_R11_O15_POOR_FETGRO | Butternut squash liking \|\| id:ebi-fl187-GCST90094718 | Weighted mode | 3 | -0.240198269 | 0.355264607611259 | 0.568675406642034 | -0.9365169 | 0.456120361922232 | 0.786471912610827 | 0.391990803504766 | 1.57794025725966 | 0.972577670507659 |
| ebi-fl187-GCST90094719 | finngen_R11_O15_POOR_FETGRO | Poor fetal growth \|\| id:finngen_R11_O15_POOR_FETGRO | Cabbage liking \|\| id:ebi-fl187-GCST90094719 | Inverse variance weighted | 5 | -0.711218706 | 0.365728564143587 | 0.0518155844873387 | -1.428046692 | 0.00560927924884569 | 0.491045392453195 | 0.239776823058002 | 1.00562504071206 | 0.706473758635168 |
| ebi-fl187-GCST90094719 | finngen_R11_O15_POOR_FETGRO | Poor fetal growth \|\| id:finngen_R11_O15_POOR_FETGRO | Cabbage liking \|\| id:ebi-fl187-GCST90094719 | MR Egger | 5 | -4.31165788 | 2.42584955067753 | 0.173573829531737 | -9.066323 | 0.443007238969051 | 0.013411296827532 | 0.000115490417168005 | 1.55738360815271 | 0.970850396659599 |
| ebi-fl187-GCST90094719 | finngen_R11_O15_POOR_FETGRO | Poor fetal growth \|\| id:finngen_R11_O15_POOR_FETGRO | Cabbage liking \|\| id:ebi-fl187-GCST90094719 | Weighted median | 5 | -0.40830504 | 0.348255737892687 | 0.241024961330291 | -1.090886287 | 0.274276205880479 | 0.664776064341281 | 0.335918641524579 | 1.31557812247448 | 0.918328271889597 |
| ebi-fl187-GCST90094719 | finngen_R11_O15_POOR_FETGRO | Poor fetal growth \|\| id:finngen_R11_O15_POOR_FETGRO | Cabbage liking \|\| id:ebi-fl187-GCST90094719 | Weighted mode | 5 | -0.255462384 | 0.429333625514248 | 0.583856994768381 | -1.09695629 | 0.586031521899299 | 0.774558271712356 | 0.333885790158119 | 1.79684351344787 | 0.972577670507659 |
| ebi-fl187-GCST90094720 | finngen_R11_O15_POOR_FETGRO | Poor fetal growth \|\| id:finngen_R11_O15_POOR_FETGRO | F-caffeinated/sweet liking (derived food-liking factor) \|\| id:ebi-fl187-GCST90094720 | Inverse variance weighted | 8 | -0.094097994 | 0.0926865845319908 | 0.309997270179593 | -0.275763699 | 0.0875677119726179 | 0.910193564626143 | 0.758992259904501 | 1.09151617065382 | 0.888224550184161 |
| ebi-fl187-GCST90094720 | finngen_R11_O15_POOR_FETGRO | Poor fetal growth \|\| id:finngen_R11_O15_POOR_FETGRO | F-caffeinated/sweet liking (derived food-liking factor) \|\| id:ebi-fl187-GCST90094720 | MR Egger | 8 | -0.704502924 | 0.45249181890825 | 0.170490861028026 | -1.591386889 | 0.182381041403819 | 0.494354244993096 | 0.203642985635065 | 1.20007138365493 | 0.970850396659599 |
| ebi-fl187-GCST90094720 | finngen_R11_O15_POOR_FETGRO | Poor fetal growth \|\| id:finngen_R11_O15_POOR_FETGRO | F-caffeinated/sweet liking (derived food-liking factor) \|\| id:ebi-fl187-GCST90094720 | Weighted median | 8 | -0.099702064 | 0.119760572050627 | 0.405120240064544 | -0.334432785 | 0.135028657197195 | 0.905107041820321 | 0.715743951768172 | 1.14456958403761 | 0.918328271889597 |
| ebi-fl187-GCST90094720 | finngen_R11_O15_POOR_FETGRO | Poor fetal growth \|\| id:finngen_R11_O15_POOR_FETGRO | F-caffeinated/sweet liking (derived food-liking factor) \|\| id:ebi-fl187-GCST90094720 | Weighted mode | 8 | -0.112147249 | 0.145767836402334 | 0.466844262792141 | -0.397852208 | 0.17355771052734 | 0.893912620229263 | 0.671761301158072 | 1.18952933315388 | 0.972577670507659 |
| ebi-fl187-GCST90094721 | finngen_R11_O15_POOR_FETGRO | Poor fetal growth \|\| id:finngen_R11_O15_POOR_FETGRO | Cake liking \|\| id:ebi-fl187-GCST90094721 | Inverse variance weighted (multiplicative random effects) | 13 | 0.206567837139765 | 0.18440473440868 | 0.26263396493132 | -0.154865442 | 0.568001116580778 | 1.22945113383664 | 0.85653042249245 | 1.76473602197768 | 0.888224550184161 |
| ebi-fl187-GCST90094721 | finngen_R11_O15_POOR_FETGRO | Poor fetal growth \|\| id:finngen_R11_O15_POOR_FETGRO | Cake liking \|\| id:ebi-fl187-GCST90094721 | MR Egger | 13 | 0.541718065306621 | 0.788056631769678 | 0.506059532464527 | -1.002872933 | 2.08630906357519 | 1.71895760810197 | 0.366824064937363 | 8.05512925918917 | 0.970850396659599 |
| ebi-fl187-GCST90094721 | finngen_R11_O15_POOR_FETGRO | Poor fetal growth \|\| id:finngen_R11_O15_POOR_FETGRO | Cake liking \|\| id:ebi-fl187-GCST90094721 | Weighted median | 13 | 0.0616239403987425 | 0.192538929661188 | 0.748923120674846 | -0.315752362 | 0.43900024253467 | 1.06356230669671 | 0.729240015539877 | 1.5511556635967 | 0.918328271889597 |
| ebi-fl187-GCST90094721 | finngen_R11_O15_POOR_FETGRO | Poor fetal growth \|\| id:finngen_R11_O15_POOR_FETGRO | Cake liking \|\| id:ebi-fl187-GCST90094721 | Weighted mode | 13 | -0.210013361 | 0.293724517077182 | 0.488286174852457 | -0.785713414 | 0.365686692882856 | 0.810573416160043 | 0.455794415623137 | 1.44150353857914 | 0.972577670507659 |
| ebi-fl187-GCST90094722 | finngen_R11_O15_POOR_FETGRO | Poor fetal growth \|\| id:finngen_R11_O15_POOR_FETGRO | F-cake/biscuits liking (derived food-liking factor) \|\| id:ebi-fl187-GCST90094722 | Inverse variance weighted (multiplicative random effects) | 18 | -0.019554288 | 0.0955839540528571 | 0.837902548244309 | -0.206898838 | 0.167790261780782 | 0.98063565683537 | 0.813101900755135 | 1.18268852964659 | 0.994304114950957 |
| ebi-fl187-GCST90094722 | finngen_R11_O15_POOR_FETGRO | Poor fetal growth \|\| id:finngen_R11_O15_POOR_FETGRO | F-cake/biscuits liking (derived food-liking factor) \|\| id:ebi-fl187-GCST90094722 | MR Egger | 18 | 0.327937386192768 | 0.385555830089703 | 0.407564187217884 | -0.427752041 | 1.08362681316859 | 1.38810205521383 | 0.651973057486314 | 2.95537874389743 | 0.970850396659599 |
| ebi-fl187-GCST90094722 | finngen_R11_O15_POOR_FETGRO | Poor fetal growth \|\| id:finngen_R11_O15_POOR_FETGRO | F-cake/biscuits liking (derived food-liking factor) \|\| id:ebi-fl187-GCST90094722 | Weighted median | 18 | -0.066463043 | 0.111488935425388 | 0.551081531544574 | -0.284981356 | 0.152055270638632 | 0.935697495981391 | 0.752028274831709 | 1.16422458209005 | 0.918328271889597 |
| ebi-fl187-GCST90094722 | finngen_R11_O15_POOR_FETGRO | Poor fetal growth \|\| id:finngen_R11_O15_POOR_FETGRO | F-cake/biscuits liking (derived food-liking factor) \|\| id:ebi-fl187-GCST90094722 | Weighted mode | 18 | -0.162471833 | 0.194128623179778 | 0.414243462699567 | -0.542963934 | 0.218020268365442 | 0.850040032908921 | 0.581023581892139 | 1.24361227335165 | 0.972577670507659 |
| ebi-fl187-GCST90094723 | finngen_R11_O15_POOR_FETGRO | Poor fetal growth \|\| id:finngen_R11_O15_POOR_FETGRO | Cake icing liking \|\| id:ebi-fl187-GCST90094723 | Inverse variance weighted | 9 | -0.112375559 | 0.165294394244431 | 0.496598811988878 | -0.436352572 | 0.211601453919412 | 0.893708554354237 | 0.646389787126478 | 1.23565532134507 | 0.914435393876931 |
| ebi-fl187-GCST90094723 | finngen_R11_O15_POOR_FETGRO | Poor fetal growth \|\| id:finngen_R11_O15_POOR_FETGRO | Cake icing liking \|\| id:ebi-fl187-GCST90094723 | MR Egger | 9 | -2.153816495 | 0.985210014155041 | 0.0650474668063055 | -4.084828123 | -0.222804867 | 0.116040443813158 | 0.0168260310781561 | 0.800270993070708 | 0.970850396659599 |
| ebi-fl187-GCST90094723 | finngen_R11_O15_POOR_FETGRO | Poor fetal growth \|\| id:finngen_R11_O15_POOR_FETGRO | Cake icing liking \|\| id:ebi-fl187-GCST90094723 | Weighted median | 9 | -0.180885788 | 0.184753051345375 | 0.327546415781766 | -0.543001768 | 0.181230192929301 | 0.834530666913519 | 0.581001599951669 | 1.19869107774756 | 0.918328271889597 |
| ebi-fl187-GCST90094723 | finngen_R11_O15_POOR_FETGRO | Poor fetal growth \|\| id:finngen_R11_O15_POOR_FETGRO | Cake icing liking \|\| id:ebi-fl187-GCST90094723 | Weighted mode | 9 | -0.342231428 | 0.268761038329296 | 0.238639341486147 | -0.869003063 | 0.184540206962264 | 0.710183829150261 | 0.419369425690966 | 1.20266533583255 | 0.972577670507659 |
| ebi-fl187-GCST90094724 | finngen_R11_O15_POOR_FETGRO | Poor fetal growth \|\| id:finngen_R11_O15_POOR_FETGRO | Capers liking \|\| id:ebi-fl187-GCST90094724 | Inverse variance weighted | 12 | -0.02981915 | 0.0999283240228983 | 0.765393781989594 | -0.225678665 | 0.166040365401471 | 0.970621054801543 | 0.797974477785846 | 1.1806207569923 | 0.989124272109629 |
| ebi-fl187-GCST90094724 | finngen_R11_O15_POOR_FETGRO | Poor fetal growth \|\| id:finngen_R11_O15_POOR_FETGRO | Capers liking \|\| id:ebi-fl187-GCST90094724 | MR Egger | 12 | -0.179120319 | 0.47421578914076 | 0.713532772989019 | -1.108583266 | 0.750342627311091 | 0.83600530568468 | 0.330026189311461 | 2.11772548291113 | 0.970850396659599 |
| ebi-fl187-GCST90094724 | finngen_R11_O15_POOR_FETGRO | Poor fetal growth \|\| id:finngen_R11_O15_POOR_FETGRO | Capers liking \|\| id:ebi-fl187-GCST90094724 | Weighted median | 12 | -0.022630721 | 0.12845076172037 | 0.860150909096758 | -0.274394214 | 0.229132772300597 | 0.97762343324871 | 0.760032401055124 | 1.25750899 | 0.969669326094948 |
| ebi-fl187-GCST90094724 | finngen_R11_O15_POOR_FETGRO | Poor fetal growth \|\| id:finngen_R11_O15_POOR_FETGRO | Capers liking \|\| id:ebi-fl187-GCST90094724 | Weighted mode | 12 | 0.00446010556195076 | 0.182616843306258 | 0.980952393766153 | -0.353468907 | 0.362389118442216 | 1.0044700666364 | 0.702247827016769 | 1.43675790219918 | 0.988620775239461 |
| ebi-fl187-GCST90094725 | finngen_R11_O15_POOR_FETGRO | Poor fetal growth \|\| id:finngen_R11_O15_POOR_FETGRO | F-carbohydrate liking (derived food-liking factor) \|\| id:ebi-fl187-GCST90094725 | Inverse variance weighted | 3 | -0.158253916 | 0.129848393543778 | 0.222935625116285 | -0.412756767 | 0.0962489352636909 | 0.85363300330009 | 0.661823240220996 | 1.101033116 | 0.888224550184161 |
| ebi-fl187-GCST90094725 | finngen_R11_O15_POOR_FETGRO | Poor fetal growth \|\| id:finngen_R11_O15_POOR_FETGRO | F-carbohydrate liking (derived food-liking factor) \|\| id:ebi-fl187-GCST90094725 | MR Egger | 3 | 0.189399187147678 | 0.447382560840451 | 0.745051710974151 | -0.687470632 | 1.06626900639496 | 1.2085232831687 | 0.50284634529548 | 2.90452250399201 | 0.970850396659599 |
| ebi-fl187-GCST90094725 | finngen_R11_O15_POOR_FETGRO | Poor fetal growth \|\| id:finngen_R11_O15_POOR_FETGRO | F-carbohydrate liking (derived food-liking factor) \|\| id:ebi-fl187-GCST90094725 | Weighted median | 3 | -0.163126767 | 0.143259089505997 | 0.254835171978597 | -0.443914582 | 0.117661048773019 | 0.84948349540443 | 0.641520215802793 | 1.12486277312632 | 0.918328271889597 |
| ebi-fl187-GCST90094725 | finngen_R11_O15_POOR_FETGRO | Poor fetal growth \|\| id:finngen_R11_O15_POOR_FETGRO | F-carbohydrate liking (derived food-liking factor) \|\| id:ebi-fl187-GCST90094725 | Weighted mode | 3 | -0.071320995 | 0.16336896126262 | 0.705037038043905 | -0.391524159 | 0.248882169397105 | 0.93116294580046 | 0.676025718359495 | 1.28259089570717 | 0.972577670507659 |
| ebi-fl187-GCST90094726 | finngen_R11_O15_POOR_FETGRO | Poor fetal growth \|\| id:finngen_R11_O15_POOR_FETGRO | F-capsicum liking (derived food-liking factor) \|\| id:ebi-fl187-GCST90094726 | Inverse variance weighted | 19 | 0.0151088594754044 | 0.0679352517629767 | 0.824001830267594 | -0.118044234 | 0.148261952930839 | 1.01522357530637 | 0.888656742920592 | 1.1598166739505 | 0.994304114950957 |
| ebi-fl187-GCST90094726 | finngen_R11_O15_POOR_FETGRO | Poor fetal growth \|\| id:finngen_R11_O15_POOR_FETGRO | F-capsicum liking (derived food-liking factor) \|\| id:ebi-fl187-GCST90094726 | MR Egger | 19 | -0.491757114 | 0.306827651177191 | 0.12741426901318 | -1.09313931 | 0.109625082686606 | 0.61155088517528 | 0.33516266094939 | 1.11585963692758 | 0.970850396659599 |
| ebi-fl187-GCST90094726 | finngen_R11_O15_POOR_FETGRO | Poor fetal growth \|\| id:finngen_R11_O15_POOR_FETGRO | F-capsicum liking (derived food-liking factor) \|\| id:ebi-fl187-GCST90094726 | Weighted median | 19 | 0.00935712089033632 | 0.091542911460297 | 0.918585482569336 | -0.170066986 | 0.188781227352519 | 1.00940103561093 | 0.843608305118946 | 1.20777669507266 | 0.969669326094948 |
| ebi-fl187-GCST90094726 | finngen_R11_O15_POOR_FETGRO | Poor fetal growth \|\| id:finngen_R11_O15_POOR_FETGRO | F-capsicum liking (derived food-liking factor) \|\| id:ebi-fl187-GCST90094726 | Weighted mode | 19 | -0.001987319 | 0.135080599927084 | 0.988423709985043 | -0.266745295 | 0.262770656645031 | 0.99801465419929 | 0.765868117010582 | 1.30052841719584 | 0.988620775239461 |
| ebi-fl187-GCST90094727 | finngen_R11_O15_POOR_FETGRO | Poor fetal growth \|\| id:finngen_R11_O15_POOR_FETGRO | Cauliflower liking \|\| id:ebi-fl187-GCST90094727 | Inverse variance weighted | 4 | -0.948116893 | 0.448877811203064 | 0.0346698370460099 | -1.827917403 | -0.068316383 | 0.387469984171072 | 0.160747992649124 | 0.933964935793856 | 0.568056449122264 |
| ebi-fl187-GCST90094727 | finngen_R11_O15_POOR_FETGRO | Poor fetal growth \|\| id:finngen_R11_O15_POOR_FETGRO | Cauliflower liking \|\| id:ebi-fl187-GCST90094727 | MR Egger | 4 | 1.06400264290207 | 1.84709363561307 | 0.622770259008035 | -2.556300883 | 4.68430616870368 | 2.89794725399239 | 0.077591229281821 | 108.235149315899 | 0.970850396659599 |
| ebi-fl187-GCST90094727 | finngen_R11_O15_POOR_FETGRO | Poor fetal growth \|\| id:finngen_R11_O15_POOR_FETGRO | Cauliflower liking \|\| id:ebi-fl187-GCST90094727 | Weighted median | 4 | -0.48816562 | 0.455579991137079 | 0.283933169604818 | -1.381102403 | 0.404771162645459 | 0.613751215158808 | 0.25130136502025 | 1.49895944289264 | 0.918328271889597 |
| ebi-fl187-GCST90094727 | finngen_R11_O15_POOR_FETGRO | Poor fetal growth \|\| id:finngen_R11_O15_POOR_FETGRO | Cauliflower liking \|\| id:ebi-fl187-GCST90094727 | Weighted mode | 4 | -0.456407506 | 0.460753711787632 | 0.394921082808565 | -1.359484781 | 0.446669769472216 | 0.633555607009263 | 0.256793047601602 | 1.56309803135952 | 0.972577670507659 |
| ebi-fl187-GCST90094729 | finngen_R11_O15_POOR_FETGRO | Poor fetal growth \|\| id:finngen_R11_O15_POOR_FETGRO | F-cheese liking (derived food-liking factor) \|\| id:ebi-fl187-GCST90094729 | Inverse variance weighted | 16 | -0.009452862 | 0.085792466088355 | 0.912264331799428 | -0.177606095 | 0.158700371717188 | 0.990591676035073 | 0.837272163940748 | 1.17198673369418 | 0.994304114950957 |
| ebi-fl187-GCST90094729 | finngen_R11_O15_POOR_FETGRO | Poor fetal growth \|\| id:finngen_R11_O15_POOR_FETGRO | F-cheese liking (derived food-liking factor) \|\| id:ebi-fl187-GCST90094729 | MR Egger | 16 | 0.751887505856506 | 0.417283922931181 | 0.0931347000432854 | -0.065988983 | 1.56976399480162 | 2.1209996400111 | 0.936141177619212 | 4.80551393366557 | 0.970850396659599 |
| ebi-fl187-GCST90094729 | finngen_R11_O15_POOR_FETGRO | Poor fetal growth \|\| id:finngen_R11_O15_POOR_FETGRO | F-cheese liking (derived food-liking factor) \|\| id:ebi-fl187-GCST90094729 | Weighted median | 16 | 0.0577489816026424 | 0.103257567910475 | 0.575976662728156 | -0.144635852 | 0.260133814707173 | 1.05944902113406 | 0.865337347039337 | 1.29710364659772 | 0.918328271889597 |
| ebi-fl187-GCST90094729 | finngen_R11_O15_POOR_FETGRO | Poor fetal growth \|\| id:finngen_R11_O15_POOR_FETGRO | F-cheese liking (derived food-liking factor) \|\| id:ebi-fl187-GCST90094729 | Weighted mode | 16 | 0.0844334847698023 | 0.167067712575854 | 0.620633247074482 | -0.243019232 | 0.411886201418477 | 1.08810046657191 | 0.784256430901837 | 1.50966262908745 | 0.972577670507659 |
| ebi-fl187-GCST90094730 | finngen_R11_O15_POOR_FETGRO | Poor fetal growth \|\| id:finngen_R11_O15_POOR_FETGRO | Cheesecake liking \|\| id:ebi-fl187-GCST90094730 | Inverse variance weighted | 7 | -0.128469963 | 0.159602458023975 | 0.420855892280453 | -0.441290781 | 0.184350854314265 | 0.879439977397338 | 0.643205647306423 | 1.20243762952562 | 0.889015330589552 |
| ebi-fl187-GCST90094730 | finngen_R11_O15_POOR_FETGRO | Poor fetal growth \|\| id:finngen_R11_O15_POOR_FETGRO | Cheesecake liking \|\| id:ebi-fl187-GCST90094730 | MR Egger | 7 | -0.266867126 | 0.755678768394854 | 0.73837779384255 | -1.747997512 | 1.21426326040678 | 0.765774816538754 | 0.174122272384775 | 3.36781194969194 | 0.970850396659599 |
| ebi-fl187-GCST90094730 | finngen_R11_O15_POOR_FETGRO | Poor fetal growth \|\| id:finngen_R11_O15_POOR_FETGRO | Cheesecake liking \|\| id:ebi-fl187-GCST90094730 | Weighted median | 7 | -0.159047646 | 0.199500666355377 | 0.42531825324836 | -0.550068952 | 0.231973660346971 | 0.852955718320533 | 0.576910030043584 | 1.26108651180971 | 0.918328271889597 |
| ebi-fl187-GCST90094730 | finngen_R11_O15_POOR_FETGRO | Poor fetal growth \|\| id:finngen_R11_O15_POOR_FETGRO | Cheesecake liking \|\| id:ebi-fl187-GCST90094730 | Weighted mode | 7 | -0.275713289 | 0.276848038657854 | 0.357750673579044 | -0.818335445 | 0.266908866983389 | 0.759030522129267 | 0.441165387915036 | 1.30592142834828 | 0.972577670507659 |
| ebi-fl187-GCST90094731 | finngen_R11_O15_POOR_FETGRO | Poor fetal growth \|\| id:finngen_R11_O15_POOR_FETGRO | Cherry liking \|\| id:ebi-fl187-GCST90094731 | Inverse variance weighted | 6 | -0.335960636 | 0.187758024955302 | 0.0735620545938193 | -0.703966365 | 0.0320450928563244 | 0.714651236753597 | 0.494619566231987 | 1.0325640655184 | 0.706473758635168 |
| ebi-fl187-GCST90094731 | finngen_R11_O15_POOR_FETGRO | Poor fetal growth \|\| id:finngen_R11_O15_POOR_FETGRO | Cherry liking \|\| id:ebi-fl187-GCST90094731 | MR Egger | 6 | -0.022296478 | 0.659688903352195 | 0.974657175191064 | -1.315286729 | 1.27069377215098 | 0.97795025092175 | 0.268397354902849 | 3.56332383984967 | 0.993285374120483 |
| ebi-fl187-GCST90094731 | finngen_R11_O15_POOR_FETGRO | Poor fetal growth \|\| id:finngen_R11_O15_POOR_FETGRO | Cherry liking \|\| id:ebi-fl187-GCST90094731 | Weighted median | 6 | -0.387786117 | 0.231435731374959 | 0.0938230005934939 | -0.841400151 | 0.0658279161609953 | 0.678557459423143 | 0.431106486551928 | 1.06804290842729 | 0.918328271889597 |
| ebi-fl187-GCST90094731 | finngen_R11_O15_POOR_FETGRO | Poor fetal growth \|\| id:finngen_R11_O15_POOR_FETGRO | Cherry liking \|\| id:ebi-fl187-GCST90094731 | Weighted mode | 6 | -0.392196829 | 0.293085205216542 | 0.2384700487435 | -0.966643831 | 0.182250173042481 | 0.675571128760755 | 0.380357442051289 | 1.19991434255554 | 0.972577670507659 |
| ebi-fl187-GCST90094732 | finngen_R11_O15_POOR_FETGRO | Poor fetal growth \|\| id:finngen_R11_O15_POOR_FETGRO | Chicken liking \|\| id:ebi-fl187-GCST90094732 | Inverse variance weighted | 2 | -0.404687403 | 0.309045734104463 | 0.190373784885779 | -1.010417042 | 0.201042235552809 | 0.667185338204685 | 0.364067116578895 | 1.22267641114694 | 0.888224550184161 |
| ebi-fl187-GCST90094733 | finngen_R11_O15_POOR_FETGRO | Poor fetal growth \|\| id:finngen_R11_O15_POOR_FETGRO | Chilli pepper liking \|\| id:ebi-fl187-GCST90094733 | Inverse variance weighted | 13 | 0.0841333431270158 | 0.10047379564631 | 0.40238685557602 | -0.112795296 | 0.281061982593783 | 1.08777393131622 | 0.893333510039681 | 1.3245356995492 | 0.888224550184161 |
| ebi-fl187-GCST90094733 | finngen_R11_O15_POOR_FETGRO | Poor fetal growth \|\| id:finngen_R11_O15_POOR_FETGRO | Chilli pepper liking \|\| id:ebi-fl187-GCST90094733 | MR Egger | 13 | 0.119302064003244 | 0.762928061803172 | 0.878571280054918 | -1.376036937 | 1.61464106513746 | 1.12671020548667 | 0.252577552924153 | 5.02608356305138 | 0.993285374120483 |
| ebi-fl187-GCST90094733 | finngen_R11_O15_POOR_FETGRO | Poor fetal growth \|\| id:finngen_R11_O15_POOR_FETGRO | Chilli pepper liking \|\| id:ebi-fl187-GCST90094733 | Weighted median | 13 | 0.107253188027037 | 0.134575370817628 | 0.425465629026693 | -0.156514539 | 0.371020914829588 | 1.11321607178232 | 0.855119085228116 | 1.44921338312063 | 0.918328271889597 |
| ebi-fl187-GCST90094733 | finngen_R11_O15_POOR_FETGRO | Poor fetal growth \|\| id:finngen_R11_O15_POOR_FETGRO | Chilli pepper liking \|\| id:ebi-fl187-GCST90094733 | Weighted mode | 13 | 0.14920466285348 | 0.191280532292771 | 0.450484938055912 | -0.22570518 | 0.524114506147311 | 1.16091056016516 | 0.797953319236799 | 1.68896262000891 | 0.972577670507659 |
| ebi-fl187-GCST90094734 | finngen_R11_O15_POOR_FETGRO | Poor fetal growth \|\| id:finngen_R11_O15_POOR_FETGRO | Chips liking \|\| id:ebi-fl187-GCST90094734 | Inverse variance weighted | 4 | -0.265981522 | 0.223427915130901 | 0.23386647622102 | -0.703900235 | 0.17193719190921 | 0.766453290087702 | 0.494652276290051 | 1.18760323977929 | 0.888224550184161 |
| ebi-fl187-GCST90094734 | finngen_R11_O15_POOR_FETGRO | Poor fetal growth \|\| id:finngen_R11_O15_POOR_FETGRO | Chips liking \|\| id:ebi-fl187-GCST90094734 | MR Egger | 4 | -0.211652475 | 0.798040107362755 | 0.815677698811445 | -1.775811086 | 1.35250613508264 | 0.809245881597574 | 0.169346039616879 | 3.86710488396544 | 0.970850396659599 |
| ebi-fl187-GCST90094734 | finngen_R11_O15_POOR_FETGRO | Poor fetal growth \|\| id:finngen_R11_O15_POOR_FETGRO | Chips liking \|\| id:ebi-fl187-GCST90094734 | Weighted median | 4 | -0.269230462 | 0.267752622194145 | 0.314646802279083 | -0.794025601 | 0.255564677667037 | 0.763967170083736 | 0.452021469522658 | 1.29119052150795 | 0.918328271889597 |
| ebi-fl187-GCST90094734 | finngen_R11_O15_POOR_FETGRO | Poor fetal growth \|\| id:finngen_R11_O15_POOR_FETGRO | Chips liking \|\| id:ebi-fl187-GCST90094734 | Weighted mode | 4 | -0.194437739 | 0.349159496551381 | 0.616459840069896 | -0.878790353 | 0.489914873948944 | 0.823297435742281 | 0.415284956367595 | 1.63217727323558 | 0.972577670507659 |
| ebi-fl187-GCST90094735 | finngen_R11_O15_POOR_FETGRO | Poor fetal growth \|\| id:finngen_R11_O15_POOR_FETGRO | F-chocolate/coffee liking (derived food-liking factor) \|\| id:ebi-fl187-GCST90094735 | Inverse variance weighted (multiplicative random effects) | 13 | 0.0309399724014748 | 0.0700624162166066 | 0.658774454821852 | -0.106382363 | 0.168262308186024 | 1.03142358814716 | 0.899080806765018 | 1.18324694530421 | 0.96571624475698 |
| ebi-fl187-GCST90094735 | finngen_R11_O15_POOR_FETGRO | Poor fetal growth \|\| id:finngen_R11_O15_POOR_FETGRO | F-chocolate/coffee liking (derived food-liking factor) \|\| id:ebi-fl187-GCST90094735 | MR Egger | 13 | 0.182495902756291 | 0.216003694215427 | 0.416179149118286 | -0.240871338 | 0.605863143418527 | 1.20020923339377 | 0.785942740922256 | 1.83283352453046 | 0.970850396659599 |
| ebi-fl187-GCST90094735 | finngen_R11_O15_POOR_FETGRO | Poor fetal growth \|\| id:finngen_R11_O15_POOR_FETGRO | F-chocolate/coffee liking (derived food-liking factor) \|\| id:ebi-fl187-GCST90094735 | Weighted median | 13 | 0.0901481413744151 | 0.0789417969750797 | 0.253471874135679 | -0.064577781 | 0.244874063445571 | 1.09433638819434 | 0.937463194885759 | 1.27746042410996 | 0.918328271889597 |
| ebi-fl187-GCST90094735 | finngen_R11_O15_POOR_FETGRO | Poor fetal growth \|\| id:finngen_R11_O15_POOR_FETGRO | F-chocolate/coffee liking (derived food-liking factor) \|\| id:ebi-fl187-GCST90094735 | Weighted mode | 13 | 0.0906330959712816 | 0.118220164403656 | 0.458109064995479 | -0.141078426 | 0.322344618202448 | 1.09486722036066 | 0.868421202000031 | 1.38036039131647 | 0.972577670507659 |
| ebi-fl187-GCST90094737 | finngen_R11_O15_POOR_FETGRO | Poor fetal growth \|\| id:finngen_R11_O15_POOR_FETGRO | F-coffee/alcohol liking (derived food-liking factor) \|\| id:ebi-fl187-GCST90094737 | Inverse variance weighted | 13 | -0.130565904 | 0.0803886306948866 | 0.104336607254292 | -0.28812762 | 0.0269958120151563 | 0.877598653652314 | 0.749665913538517 | 1.02736350017171 | 0.761090127545161 |
| ebi-fl187-GCST90094737 | finngen_R11_O15_POOR_FETGRO | Poor fetal growth \|\| id:finngen_R11_O15_POOR_FETGRO | F-coffee/alcohol liking (derived food-liking factor) \|\| id:ebi-fl187-GCST90094737 | MR Egger | 13 | 0.786247724736309 | 0.444593635825469 | 0.104668738116873 | -0.085155801 | 1.65765125095423 | 2.19514416840345 | 0.918369189974121 | 5.2469725385838 | 0.970850396659599 |
| ebi-fl187-GCST90094737 | finngen_R11_O15_POOR_FETGRO | Poor fetal growth \|\| id:finngen_R11_O15_POOR_FETGRO | F-coffee/alcohol liking (derived food-liking factor) \|\| id:ebi-fl187-GCST90094737 | Weighted median | 13 | -0.106229801 | 0.0992632941252439 | 0.284537347060765 | -0.300785858 | 0.0883262553062235 | 0.899217982977997 | 0.740236271699305 | 1.09234444707065 | 0.918328271889597 |
| ebi-fl187-GCST90094737 | finngen_R11_O15_POOR_FETGRO | Poor fetal growth \|\| id:finngen_R11_O15_POOR_FETGRO | F-coffee/alcohol liking (derived food-liking factor) \|\| id:ebi-fl187-GCST90094737 | Weighted mode | 13 | -0.066052566 | 0.134994514066861 | 0.633452138181734 | -0.330641814 | 0.198536681142881 | 0.936081656529136 | 0.718462466155174 | 1.21961676353052 | 0.972577670507659 |
| ebi-fl187-GCST90094738 | finngen_R11_O15_POOR_FETGRO | Poor fetal growth \|\| id:finngen_R11_O15_POOR_FETGRO | Coffee difference liking \|\| id:ebi-fl187-GCST90094738 | Inverse variance weighted | 5 | -0.107249025 | 0.0819470978184817 | 0.19061590207633 | -0.267865337 | 0.0533672864458523 | 0.898301945242553 | 0.765010792813182 | 1.05481699396575 | 0.888224550184161 |
| ebi-fl187-GCST90094738 | finngen_R11_O15_POOR_FETGRO | Poor fetal growth \|\| id:finngen_R11_O15_POOR_FETGRO | Coffee difference liking \|\| id:ebi-fl187-GCST90094738 | MR Egger | 5 | -0.154207714 | 0.32203941089991 | 0.664782749723934 | -0.785404959 | 0.476989531341606 | 0.857093972084108 | 0.45593502922621 | 1.61121657669001 | 0.970850396659599 |
| ebi-fl187-GCST90094738 | finngen_R11_O15_POOR_FETGRO | Poor fetal growth \|\| id:finngen_R11_O15_POOR_FETGRO | Coffee difference liking \|\| id:ebi-fl187-GCST90094738 | Weighted median | 5 | -0.105933364 | 0.101567855914893 | 0.296956999544792 | -0.305006362 | 0.0931396332575981 | 0.89948458383184 | 0.737118684880132 | 1.09761498812462 | 0.918328271889597 |
| ebi-fl187-GCST90094738 | finngen_R11_O15_POOR_FETGRO | Poor fetal growth \|\| id:finngen_R11_O15_POOR_FETGRO | Coffee difference liking \|\| id:ebi-fl187-GCST90094738 | Weighted mode | 5 | -0.109105799 | 0.11861814435243 | 0.409723763363983 | -0.341597362 | 0.12338576413171 | 0.896635549515538 | 0.710634275669245 | 1.13132075975072 | 0.972577670507659 |
| ebi-fl187-GCST90094739 | finngen_R11_O15_POOR_FETGRO | Poor fetal growth \|\| id:finngen_R11_O15_POOR_FETGRO | Coffee max liking \|\| id:ebi-fl187-GCST90094739 | Inverse variance weighted | 6 | -0.113807241 | 0.172082020436789 | 0.508384913900586 | -0.451088001 | 0.223473518905658 | 0.892429963076909 | 0.636934788681729 | 1.25041252754593 | 0.914435393876931 |
| ebi-fl187-GCST90094739 | finngen_R11_O15_POOR_FETGRO | Poor fetal growth \|\| id:finngen_R11_O15_POOR_FETGRO | Coffee max liking \|\| id:ebi-fl187-GCST90094739 | MR Egger | 6 | -0.08409407 | 0.534705392714172 | 0.882650060027959 | -1.132116639 | 0.963928500142772 | 0.919344769653715 | 0.322350234647911 | 2.62197670311241 | 0.993285374120483 |
| ebi-fl187-GCST90094739 | finngen_R11_O15_POOR_FETGRO | Poor fetal growth \|\| id:finngen_R11_O15_POOR_FETGRO | Coffee max liking \|\| id:ebi-fl187-GCST90094739 | Weighted median | 6 | -0.214067877 | 0.188028197172803 | 0.254916698280762 | -0.582603143 | 0.154467389423531 | 0.807293586467766 | 0.558442766165584 | 1.16703621971306 | 0.918328271889597 |
| ebi-fl187-GCST90094739 | finngen_R11_O15_POOR_FETGRO | Poor fetal growth \|\| id:finngen_R11_O15_POOR_FETGRO | Coffee max liking \|\| id:ebi-fl187-GCST90094739 | Weighted mode | 6 | -0.270522038 | 0.215884473958451 | 0.265581454487414 | -0.693655607 | 0.152611530626817 | 0.762981084980666 | 0.499745851248302 | 1.16487237379592 | 0.972577670507659 |
| ebi-fl187-GCST90094740 | finngen_R11_O15_POOR_FETGRO | Poor fetal growth \|\| id:finngen_R11_O15_POOR_FETGRO | Coffee with sugar liking \|\| id:ebi-fl187-GCST90094740 | Inverse variance weighted | 4 | -0.306575988 | 0.177482959971692 | 0.084104018885139 | -0.654442589 | 0.0412906139771667 | 0.735962592047512 | 0.519731685960601 | 1.04215492632934 | 0.706473758635168 |
| ebi-fl187-GCST90094740 | finngen_R11_O15_POOR_FETGRO | Poor fetal growth \|\| id:finngen_R11_O15_POOR_FETGRO | Coffee with sugar liking \|\| id:ebi-fl187-GCST90094740 | MR Egger | 4 | -1.755958584 | 1.7809879654063 | 0.428097111057671 | -5.246694996 | 1.7347778281777 | 0.172741575587962 | 0.00526489015840491 | 5.66766846768403 | 0.970850396659599 |
| ebi-fl187-GCST90094740 | finngen_R11_O15_POOR_FETGRO | Poor fetal growth \|\| id:finngen_R11_O15_POOR_FETGRO | Coffee with sugar liking \|\| id:ebi-fl187-GCST90094740 | Weighted median | 4 | -0.447600106 | 0.206706177369675 | 0.0303576031325104 | -0.852744213 | -0.042455998 | 0.639160229546049 | 0.426243622139682 | 0.958432637613713 | 0.652429624709108 |
| ebi-fl187-GCST90094740 | finngen_R11_O15_POOR_FETGRO | Poor fetal growth \|\| id:finngen_R11_O15_POOR_FETGRO | Coffee with sugar liking \|\| id:ebi-fl187-GCST90094740 | Weighted mode | 4 | -0.45392593 | 0.28024887116255 | 0.203731041720887 | -1.003213718 | 0.0953618572284087 | 0.635129775407267 | 0.366699078181923 | 1.10005684663532 | 0.972577670507659 |
| ebi-fl187-GCST90094741 | finngen_R11_O15_POOR_FETGRO | Poor fetal growth \|\| id:finngen_R11_O15_POOR_FETGRO | Coffee without sugar liking \|\| id:ebi-fl187-GCST90094741 | Inverse variance weighted | 6 | 0.00861244141062194 | 0.119001268464691 | 0.942305321364563 | -0.224630045 | 0.241854927601416 | 1.00864963518387 | 0.798811688654569 | 1.27360941384083 | 0.994304114950957 |
| ebi-fl187-GCST90094741 | finngen_R11_O15_POOR_FETGRO | Poor fetal growth \|\| id:finngen_R11_O15_POOR_FETGRO | Coffee without sugar liking \|\| id:ebi-fl187-GCST90094741 | MR Egger | 6 | 0.2782512757044 | 0.393647261677592 | 0.518659571304415 | -0.493297357 | 1.04979990859248 | 1.32081804499935 | 0.610609672895099 | 2.85707938383021 | 0.970850396659599 |
| ebi-fl187-GCST90094741 | finngen_R11_O15_POOR_FETGRO | Poor fetal growth \|\| id:finngen_R11_O15_POOR_FETGRO | Coffee without sugar liking \|\| id:ebi-fl187-GCST90094741 | Weighted median | 6 | 0.0705198641387196 | 0.153691993892446 | 0.646349863923165 | -0.230716444 | 0.371756172167914 | 1.0730658847486 | 0.793964567623165 | 1.4502793197162 | 0.918328271889597 |
| ebi-fl187-GCST90094741 | finngen_R11_O15_POOR_FETGRO | Poor fetal growth \|\| id:finngen_R11_O15_POOR_FETGRO | Coffee without sugar liking \|\| id:ebi-fl187-GCST90094741 | Weighted mode | 6 | 0.114522815814911 | 0.194752082440405 | 0.582057976238578 | -0.267191266 | 0.496236897398104 | 1.1213382250175 | 0.765526638421368 | 1.64252862248966 | 0.972577670507659 |
| ebi-fl187-GCST90094742 | finngen_R11_O15_POOR_FETGRO | Poor fetal growth \|\| id:finngen_R11_O15_POOR_FETGRO | F-cooking flavour liking (derived food-liking factor) \|\| id:ebi-fl187-GCST90094742 | Inverse variance weighted | 12 | 0.0381760337147539 | 0.0948921118617038 | 0.687455987621541 | -0.147812506 | 0.224164572963693 | 1.03891410069103 | 0.862592831163218 | 1.25127692883692 | 0.96571624475698 |
| ebi-fl187-GCST90094742 | finngen_R11_O15_POOR_FETGRO | Poor fetal growth \|\| id:finngen_R11_O15_POOR_FETGRO | F-cooking flavour liking (derived food-liking factor) \|\| id:ebi-fl187-GCST90094742 | MR Egger | 12 | -0.567296776 | 0.59476880211143 | 0.362666377960311 | -1.733043629 | 0.598450075701512 | 0.567056248519366 | 0.176745642393848 | 1.81929684166318 | 0.970850396659599 |
| ebi-fl187-GCST90094742 | finngen_R11_O15_POOR_FETGRO | Poor fetal growth \|\| id:finngen_R11_O15_POOR_FETGRO | F-cooking flavour liking (derived food-liking factor) \|\| id:ebi-fl187-GCST90094742 | Weighted median | 12 | -0.073444708 | 0.120099845608983 | 0.540848489206308 | -0.308840406 | 0.161950988968242 | 0.929187520612355 | 0.734297950355383 | 1.1758026126096 | 0.918328271889597 |
| ebi-fl187-GCST90094742 | finngen_R11_O15_POOR_FETGRO | Poor fetal growth \|\| id:finngen_R11_O15_POOR_FETGRO | F-cooking flavour liking (derived food-liking factor) \|\| id:ebi-fl187-GCST90094742 | Weighted mode | 12 | -0.15596065 | 0.188035983035312 | 0.424500729778308 | -0.524511177 | 0.21258987681769 | 0.855592857346141 | 0.591844601110628 | 1.23687727516315 | 0.972577670507659 |
| ebi-fl187-GCST90094743 | finngen_R11_O15_POOR_FETGRO | Poor fetal growth \|\| id:finngen_R11_O15_POOR_FETGRO | F-cooking vegetables liking (derived food-liking factor) \|\| id:ebi-fl187-GCST90094743 | Inverse variance weighted (multiplicative random effects) | 12 | -0.015004952 | 0.129385343772499 | 0.907675500670954 | -0.268600226 | 0.238590322065308 | 0.985107061607987 | 0.764448801689349 | 1.26945836095938 | 0.994304114950957 |
| ebi-fl187-GCST90094743 | finngen_R11_O15_POOR_FETGRO | Poor fetal growth \|\| id:finngen_R11_O15_POOR_FETGRO | F-cooking vegetables liking (derived food-liking factor) \|\| id:ebi-fl187-GCST90094743 | MR Egger | 12 | -0.574846976 | 0.580281888066629 | 0.345228485827353 | -1.712199476 | 0.562505524914772 | 0.562790982916571 | 0.180468419757912 | 1.75506435351449 | 0.970850396659599 |
| ebi-fl187-GCST90094743 | finngen_R11_O15_POOR_FETGRO | Poor fetal growth \|\| id:finngen_R11_O15_POOR_FETGRO | F-cooking vegetables liking (derived food-liking factor) \|\| id:ebi-fl187-GCST90094743 | Weighted median | 12 | -0.121244544 | 0.12891996731099 | 0.346979783067335 | -0.37392768 | 0.131438592007781 | 0.885817311864607 | 0.688026668220719 | 1.14046787172982 | 0.918328271889597 |
| ebi-fl187-GCST90094743 | finngen_R11_O15_POOR_FETGRO | Poor fetal growth \|\| id:finngen_R11_O15_POOR_FETGRO | F-cooking vegetables liking (derived food-liking factor) \|\| id:ebi-fl187-GCST90094743 | Weighted mode | 12 | -0.204698447 | 0.191681784696936 | 0.308438219368942 | -0.580394745 | 0.170997851475476 | 0.814893013179577 | 0.55967739336105 | 1.18648819981998 | 0.972577670507659 |
| ebi-fl187-GCST90094744 | finngen_R11_O15_POOR_FETGRO | Poor fetal growth \|\| id:finngen_R11_O15_POOR_FETGRO | Coriander liking \|\| id:ebi-fl187-GCST90094744 | Inverse variance weighted | 2 | -0.004391669 | 0.199840008522462 | 0.982467161201541 | -0.396078085 | 0.387294747974135 | 0.995617960545872 | 0.672954145969226 | 1.4729905882872 | 0.994304114950957 |
| ebi-fl187-GCST90094745 | finngen_R11_O15_POOR_FETGRO | Poor fetal growth \|\| id:finngen_R11_O15_POOR_FETGRO | Corn flake liking \|\| id:ebi-fl187-GCST90094745 | Inverse variance weighted | 6 | 0.153600434985114 | 0.167140894207098 | 0.35810197749769 | -0.173995718 | 0.481196587631026 | 1.16602489093392 | 0.840300496102482 | 1.61800933426039 | 0.888224550184161 |
| ebi-fl187-GCST90094745 | finngen_R11_O15_POOR_FETGRO | Poor fetal growth \|\| id:finngen_R11_O15_POOR_FETGRO | Corn flake liking \|\| id:ebi-fl187-GCST90094745 | MR Egger | 6 | -0.509508205 | 1.00343117137506 | 0.638336076623884 | -2.476233301 | 1.45721689085525 | 0.600790972142033 | 0.0840592559436973 | 4.29399223387288 | 0.970850396659599 |
| ebi-fl187-GCST90094745 | finngen_R11_O15_POOR_FETGRO | Poor fetal growth \|\| id:finngen_R11_O15_POOR_FETGRO | Corn flake liking \|\| id:ebi-fl187-GCST90094745 | Weighted median | 6 | 0.129426272443545 | 0.203864826378032 | 0.52551765136027 | -0.270148787 | 0.529001332144488 | 1.13817519349346 | 0.763265921644831 | 1.69723648645573 | 0.918328271889597 |
| ebi-fl187-GCST90094745 | finngen_R11_O15_POOR_FETGRO | Poor fetal growth \|\| id:finngen_R11_O15_POOR_FETGRO | Corn flake liking \|\| id:ebi-fl187-GCST90094745 | Weighted mode | 6 | 0.1579246139393 | 0.29656959536292 | 0.617181846941459 | -0.423351793 | 0.739201020850623 | 1.17107790842965 | 0.65484822158351 | 2.09426157453046 | 0.972577670507659 |
| ebi-fl187-GCST90094746 | finngen_R11_O15_POOR_FETGRO | Poor fetal growth \|\| id:finngen_R11_O15_POOR_FETGRO | Cream liking \|\| id:ebi-fl187-GCST90094746 | Inverse variance weighted | 5 | -0.207953991 | 0.232839386771649 | 0.371791814185795 | -0.664319189 | 0.248411206877791 | 0.81224440624706 | 0.514623769854734 | 1.28198698568834 | 0.888224550184161 |
| ebi-fl187-GCST90094746 | finngen_R11_O15_POOR_FETGRO | Poor fetal growth \|\| id:finngen_R11_O15_POOR_FETGRO | Cream liking \|\| id:ebi-fl187-GCST90094746 | MR Egger | 5 | 0.397284636319433 | 1.34582097375294 | 0.787093217281588 | -2.240524472 | 3.03509374487519 | 1.48777934582582 | 0.106402684488657 | 20.8029279759561 | 0.970850396659599 |
| ebi-fl187-GCST90094746 | finngen_R11_O15_POOR_FETGRO | Poor fetal growth \|\| id:finngen_R11_O15_POOR_FETGRO | Cream liking \|\| id:ebi-fl187-GCST90094746 | Weighted median | 5 | -0.136492981 | 0.277119749063377 | 0.622336581457407 | -0.679647689 | 0.406661727206699 | 0.872412443738134 | 0.506795510488204 | 1.50179600299924 | 0.918328271889597 |
| ebi-fl187-GCST90094746 | finngen_R11_O15_POOR_FETGRO | Poor fetal growth \|\| id:finngen_R11_O15_POOR_FETGRO | Cream liking \|\| id:ebi-fl187-GCST90094746 | Weighted mode | 5 | -0.140971829 | 0.305622776020416 | 0.668578268796654 | -0.73999247 | 0.458048812492776 | 0.868513778682659 | 0.477117508437442 | 1.58098617305413 | 0.972577670507659 |
| ebi-fl187-GCST90094747 | finngen_R11_O15_POOR_FETGRO | Poor fetal growth \|\| id:finngen_R11_O15_POOR_FETGRO | Crisps liking \|\| id:ebi-fl187-GCST90094747 | Inverse variance weighted | 10 | 0.042692246403726 | 0.14220520265217 | 0.764012566170695 | -0.236029951 | 0.321414443601978 | 1.04361666864098 | 0.78975701972835 | 1.37907701211687 | 0.989124272109629 |
| ebi-fl187-GCST90094747 | finngen_R11_O15_POOR_FETGRO | Poor fetal growth \|\| id:finngen_R11_O15_POOR_FETGRO | Crisps liking \|\| id:ebi-fl187-GCST90094747 | MR Egger | 10 | 0.596273685315194 | 0.67765429798567 | 0.404564925383614 | -0.731928739 | 1.92447610936711 | 1.81534164709205 | 0.48098040933614 | 6.85155825829861 | 0.970850396659599 |
| ebi-fl187-GCST90094747 | finngen_R11_O15_POOR_FETGRO | Poor fetal growth \|\| id:finngen_R11_O15_POOR_FETGRO | Crisps liking \|\| id:ebi-fl187-GCST90094747 | Weighted median | 10 | 0.0927458442794435 | 0.190976038845432 | 0.627221536877986 | -0.281567192 | 0.467058880416491 | 1.09718284453231 | 0.754600210981906 | 1.59529533230528 | 0.918328271889597 |
| ebi-fl187-GCST90094747 | finngen_R11_O15_POOR_FETGRO | Poor fetal growth \|\| id:finngen_R11_O15_POOR_FETGRO | Crisps liking \|\| id:ebi-fl187-GCST90094747 | Weighted mode | 10 | 0.271119417932782 | 0.270567350691381 | 0.342502310498744 | -0.259192589 | 0.801431425287889 | 1.31143166949299 | 0.771674392406362 | 2.22872890518763 | 0.972577670507659 |
| ebi-fl187-GCST90094748 | finngen_R11_O15_POOR_FETGRO | Poor fetal growth \|\| id:finngen_R11_O15_POOR_FETGRO | Croissant liking \|\| id:ebi-fl187-GCST90094748 | Inverse variance weighted | 2 | 0.0236620042302133 | 0.728241628740025 | 0.974079720673285 | -1.403691588 | 1.45101559656066 | 1.0239441705976 | 0.245688307748078 | 4.2674463189183 | 0.994304114950957 |
| ebi-fl187-GCST90094749 | finngen_R11_O15_POOR_FETGRO | Poor fetal growth \|\| id:finngen_R11_O15_POOR_FETGRO | Cucumber liking \|\| id:ebi-fl187-GCST90094749 | Inverse variance weighted | 3 | -0.060437087 | 0.282996434892136 | 0.830889179533898 | -0.6151101 | 0.494235925245379 | 0.941352990361367 | 0.540581374276552 | 1.63924525451547 | 0.994304114950957 |
| ebi-fl187-GCST90094749 | finngen_R11_O15_POOR_FETGRO | Poor fetal growth \|\| id:finngen_R11_O15_POOR_FETGRO | Cucumber liking \|\| id:ebi-fl187-GCST90094749 | MR Egger | 3 | -0.59074919 | 0.740634880270389 | 0.571369423768492 | -2.042393555 | 0.86089517582024 | 0.553912144076761 | 0.129717852205257 | 2.36527708514803 | 0.970850396659599 |
| ebi-fl187-GCST90094749 | finngen_R11_O15_POOR_FETGRO | Poor fetal growth \|\| id:finngen_R11_O15_POOR_FETGRO | Cucumber liking \|\| id:ebi-fl187-GCST90094749 | Weighted median | 3 | -0.11254337 | 0.346458310336592 | 0.745302491195015 | -0.791601658 | 0.566514918127546 | 0.893558592513863 | 0.453118472772916 | 1.76211522202827 | 0.918328271889597 |
| ebi-fl187-GCST90094749 | finngen_R11_O15_POOR_FETGRO | Poor fetal growth \|\| id:finngen_R11_O15_POOR_FETGRO | Cucumber liking \|\| id:ebi-fl187-GCST90094749 | Weighted mode | 3 | -0.183852758 | 0.429157163906124 | 0.710082667182433 | -1.0250008 | 0.657295282964232 | 0.83205830849424 | 0.358796178531151 | 1.9295663392195 | 0.972577670507659 |
| ebi-fl187-GCST90094750 | finngen_R11_O15_POOR_FETGRO | Poor fetal growth \|\| id:finngen_R11_O15_POOR_FETGRO | Curry liking \|\| id:ebi-fl187-GCST90094750 | Inverse variance weighted | 7 | 0.103798471920124 | 0.176937610265278 | 0.557446301042808 | -0.242999244 | 0.450596188040069 | 1.10937686179738 | 0.784272106524332 | 1.56924747323423 | 0.927237411635561 |
| ebi-fl187-GCST90094750 | finngen_R11_O15_POOR_FETGRO | Poor fetal growth \|\| id:finngen_R11_O15_POOR_FETGRO | Curry liking \|\| id:ebi-fl187-GCST90094750 | MR Egger | 7 | 0.745441825968393 | 0.697839427464823 | 0.334260781213176 | -0.622323452 | 2.11320710379945 | 2.1073723211373 | 0.536696000492526 | 8.2747367146766 | 0.970850396659599 |
| ebi-fl187-GCST90094750 | finngen_R11_O15_POOR_FETGRO | Poor fetal growth \|\| id:finngen_R11_O15_POOR_FETGRO | Curry liking \|\| id:ebi-fl187-GCST90094750 | Weighted median | 7 | 0.0402665801550426 | 0.235523477294554 | 0.864250255171205 | -0.421359435 | 0.501892595652369 | 1.04108827067583 | 0.656154214004074 | 1.65184458806517 | 0.969669326094948 |
| ebi-fl187-GCST90094750 | finngen_R11_O15_POOR_FETGRO | Poor fetal growth \|\| id:finngen_R11_O15_POOR_FETGRO | Curry liking \|\| id:ebi-fl187-GCST90094750 | Weighted mode | 7 | -0.098221088 | 0.366498700258187 | 0.797675783915343 | -0.816558541 | 0.620116364052431 | 0.906448476306732 | 0.441949993156634 | 1.85914436683245 | 0.972577670507659 |
| ebi-fl187-GCST90094751 | finngen_R11_O15_POOR_FETGRO | Poor fetal growth \|\| id:finngen_R11_O15_POOR_FETGRO | F-dairy liking (derived food-liking factor) \|\| id:ebi-fl187-GCST90094751 | Inverse variance weighted | 2 | -0.004838876 | 0.421795247153366 | 0.990846790746342 | -0.831557561 | 0.821879807927879 | 0.995172812009465 | 0.435370641649289 | 2.2747719552496 | 0.996780005062188 |
| ebi-fl187-GCST90094752 | finngen_R11_O15_POOR_FETGRO | Poor fetal growth \|\| id:finngen_R11_O15_POOR_FETGRO | Dark chocolate liking \|\| id:ebi-fl187-GCST90094752 | Inverse variance weighted (multiplicative random effects) | 17 | -0.033629297 | 0.0958945041793495 | 0.725820507368399 | -0.221582526 | 0.154323930692358 | 0.966929881533012 | 0.801249795726599 | 1.16686881018621 | 0.96571624475698 |
| ebi-fl187-GCST90094752 | finngen_R11_O15_POOR_FETGRO | Poor fetal growth \|\| id:finngen_R11_O15_POOR_FETGRO | Dark chocolate liking \|\| id:ebi-fl187-GCST90094752 | MR Egger | 17 | 0.33924514810569 | 0.216271342060153 | 0.137589596261986 | -0.084646682 | 0.76313697854359 | 1.40388746338329 | 0.918836868356865 | 2.14499447912805 | 0.970850396659599 |
| ebi-fl187-GCST90094752 | finngen_R11_O15_POOR_FETGRO | Poor fetal growth \|\| id:finngen_R11_O15_POOR_FETGRO | Dark chocolate liking \|\| id:ebi-fl187-GCST90094752 | Weighted median | 17 | 0.0293289708451484 | 0.108033090451717 | 0.786020830408961 | -0.182415886 | 0.241073828130513 | 1.0297633008632 | 0.8332547290022 | 1.27261498662539 | 0.932273608209331 |
| ebi-fl187-GCST90094752 | finngen_R11_O15_POOR_FETGRO | Poor fetal growth \|\| id:finngen_R11_O15_POOR_FETGRO | Dark chocolate liking \|\| id:ebi-fl187-GCST90094752 | Weighted mode | 17 | 0.0220113777947399 | 0.117719759662012 | 0.854025810428795 | -0.208719351 | 0.252742106732284 | 1.02225541541656 | 0.811622984746297 | 1.28755118323212 | 0.982450744327861 |
| ebi-fl187-GCST90094753 | finngen_R11_O15_POOR_FETGRO | Poor fetal growth \|\| id:finngen_R11_O15_POOR_FETGRO | F-deep fried food liking (derived food-liking factor) \|\| id:ebi-fl187-GCST90094753 | Inverse variance weighted | 8 | -0.177540668 | 0.0850379415072681 | 0.0368175201745895 | -0.344215034 | -0.010866303 | 0.837326945942756 | 0.708776500829167 | 0.989192522017333 | 0.568056449122264 |
| ebi-fl187-GCST90094753 | finngen_R11_O15_POOR_FETGRO | Poor fetal growth \|\| id:finngen_R11_O15_POOR_FETGRO | F-deep fried food liking (derived food-liking factor) \|\| id:ebi-fl187-GCST90094753 | MR Egger | 8 | 0.0724626207410425 | 0.265826896374643 | 0.794304505382158 | -0.448558096 | 0.593483337635343 | 1.07515261693095 | 0.638548213267193 | 1.81028327333175 | 0.970850396659599 |
| ebi-fl187-GCST90094753 | finngen_R11_O15_POOR_FETGRO | Poor fetal growth \|\| id:finngen_R11_O15_POOR_FETGRO | F-deep fried food liking (derived food-liking factor) \|\| id:ebi-fl187-GCST90094753 | Weighted median | 8 | -0.149800326 | 0.105704250494037 | 0.156434798054369 | -0.356980657 | 0.0573800051343883 | 0.860879854731743 | 0.699786033703339 | 1.0590581814857 | 0.918328271889597 |
| ebi-fl187-GCST90094753 | finngen_R11_O15_POOR_FETGRO | Poor fetal growth \|\| id:finngen_R11_O15_POOR_FETGRO | F-deep fried food liking (derived food-liking factor) \|\| id:ebi-fl187-GCST90094753 | Weighted mode | 8 | -0.097829656 | 0.139837961839578 | 0.50675668169792 | -0.371912061 | 0.176252749468502 | 0.906803359348105 | 0.689414866352056 | 1.19273948482726 | 0.972577670507659 |
| ebi-fl187-GCST90094754 | finngen_R11_O15_POOR_FETGRO | Poor fetal growth \|\| id:finngen_R11_O15_POOR_FETGRO | F-dessert liking (derived food-liking factor) \|\| id:ebi-fl187-GCST90094754 | Inverse variance weighted | 14 | -0.142749656 | 0.094837267811481 | 0.132271039147005 | -0.3286307 | 0.0431313894093564 | 0.866971083171528 | 0.719908829696205 | 1.04407506624526 | 0.822352178486651 |
| ebi-fl187-GCST90094754 | finngen_R11_O15_POOR_FETGRO | Poor fetal growth \|\| id:finngen_R11_O15_POOR_FETGRO | F-dessert liking (derived food-liking factor) \|\| id:ebi-fl187-GCST90094754 | MR Egger | 14 | -0.602995909 | 0.614481485080564 | 0.345811729045569 | -1.80737962 | 0.601387801558693 | 0.547169906728812 | 0.164083535184491 | 1.824649295 | 0.970850396659599 |
| ebi-fl187-GCST90094754 | finngen_R11_O15_POOR_FETGRO | Poor fetal growth \|\| id:finngen_R11_O15_POOR_FETGRO | F-dessert liking (derived food-liking factor) \|\| id:ebi-fl187-GCST90094754 | Weighted median | 14 | -0.142172298 | 0.11197658884217 | 0.204205440568004 | -0.361646412 | 0.077301815797624 | 0.867471779667394 | 0.696528608139982 | 1.08036809935032 | 0.918328271889597 |
| ebi-fl187-GCST90094754 | finngen_R11_O15_POOR_FETGRO | Poor fetal growth \|\| id:finngen_R11_O15_POOR_FETGRO | F-dessert liking (derived food-liking factor) \|\| id:ebi-fl187-GCST90094754 | Weighted mode | 14 | -0.228307287 | 0.177547602534175 | 0.220913068904246 | -0.576300588 | 0.119686013661373 | 0.79587965854008 | 0.561973497163441 | 1.12714288854382 | 0.972577670507659 |
| ebi-fl187-GCST90094755 | finngen_R11_O15_POOR_FETGRO | Poor fetal growth \|\| id:finngen_R11_O15_POOR_FETGRO | Diet fizzy drinks liking \|\| id:ebi-fl187-GCST90094755 | Inverse variance weighted | 4 | -0.263171961 | 0.34268553060461 | 0.44250543441323 | -0.934835601 | 0.408491678899761 | 0.768609714985636 | 0.392650411533124 | 1.50454673322166 | 0.895673650378586 |
| ebi-fl187-GCST90094755 | finngen_R11_O15_POOR_FETGRO | Poor fetal growth \|\| id:finngen_R11_O15_POOR_FETGRO | Diet fizzy drinks liking \|\| id:ebi-fl187-GCST90094755 | MR Egger | 4 | -2.710473013 | 1.7538196047326 | 0.262259794624178 | -6.147959438 | 0.727013412532557 | 0.0665053413998743 | 0.00213783971664225 | 2.06889244328413 | 0.970850396659599 |
| ebi-fl187-GCST90094755 | finngen_R11_O15_POOR_FETGRO | Poor fetal growth \|\| id:finngen_R11_O15_POOR_FETGRO | Diet fizzy drinks liking \|\| id:ebi-fl187-GCST90094755 | Weighted median | 4 | -0.109415216 | 0.300513379730825 | 0.71578757468501 | -0.69842144 | 0.479591008232561 | 0.896358157934884 | 0.497369812367393 | 1.61541357621223 | 0.918328271889597 |
| ebi-fl187-GCST90094755 | finngen_R11_O15_POOR_FETGRO | Poor fetal growth \|\| id:finngen_R11_O15_POOR_FETGRO | Diet fizzy drinks liking \|\| id:ebi-fl187-GCST90094755 | Weighted mode | 4 | -0.023948279 | 0.351071476620441 | 0.949906708233145 | -0.712048374 | 0.66415181471235 | 0.976336205084197 | 0.490638157171705 | 1.94284193233796 | 0.988620775239461 |
| ebi-fl187-GCST90094756 | finngen_R11_O15_POOR_FETGRO | Poor fetal growth \|\| id:finngen_R11_O15_POOR_FETGRO | Dried fruit liking \|\| id:ebi-fl187-GCST90094756 | Inverse variance weighted | 5 | -0.550937717 | 0.25977790721515 | 0.0339383997262147 | -1.060102415 | -0.041773019 | 0.576409048390099 | 0.34642032987079 | 0.959087450756437 | 0.568056449122264 |
| ebi-fl187-GCST90094756 | finngen_R11_O15_POOR_FETGRO | Poor fetal growth \|\| id:finngen_R11_O15_POOR_FETGRO | Dried fruit liking \|\| id:ebi-fl187-GCST90094756 | MR Egger | 5 | 0.118474075744328 | 1.60892196229494 | 0.945935140518254 | -3.03501297 | 3.27196112184241 | 1.12577768877505 | 0.0480740393342551 | 26.3629897153374 | 0.993285374120483 |
| ebi-fl187-GCST90094756 | finngen_R11_O15_POOR_FETGRO | Poor fetal growth \|\| id:finngen_R11_O15_POOR_FETGRO | Dried fruit liking \|\| id:ebi-fl187-GCST90094756 | Weighted median | 5 | -0.443509799 | 0.333909459289257 | 0.184100912476836 | -1.097972339 | 0.210952741468263 | 0.641779945069493 | 0.333546718177998 | 1.23485399629566 | 0.918328271889597 |
| ebi-fl187-GCST90094756 | finngen_R11_O15_POOR_FETGRO | Poor fetal growth \|\| id:finngen_R11_O15_POOR_FETGRO | Dried fruit liking \|\| id:ebi-fl187-GCST90094756 | Weighted mode | 5 | -0.348639259 | 0.412061474311392 | 0.445151392574074 | -1.156279748 | 0.459001231098367 | 0.705647640712677 | 0.314654600470524 | 1.58249265098545 | 0.972577670507659 |
| ebi-fl187-GCST90094757 | finngen_R11_O15_POOR_FETGRO | Poor fetal growth \|\| id:finngen_R11_O15_POOR_FETGRO | Eggs liking \|\| id:ebi-fl187-GCST90094757 | Inverse variance weighted | 5 | 0.192098570580218 | 0.291802820724403 | 0.510334868049465 | -0.379834958 | 0.764032099200048 | 1.21178995791738 | 0.683974284354277 | 2.14691536757951 | 0.914435393876931 |
| ebi-fl187-GCST90094757 | finngen_R11_O15_POOR_FETGRO | Poor fetal growth \|\| id:finngen_R11_O15_POOR_FETGRO | Eggs liking \|\| id:ebi-fl187-GCST90094757 | MR Egger | 5 | -1.108592243 | 1.85327702755195 | 0.591868779367218 | -4.741015217 | 2.52383073052594 | 0.330023226562439 | 0.00872977906092595 | 12.4762985764648 | 0.970850396659599 |
| ebi-fl187-GCST90094757 | finngen_R11_O15_POOR_FETGRO | Poor fetal growth \|\| id:finngen_R11_O15_POOR_FETGRO | Eggs liking \|\| id:ebi-fl187-GCST90094757 | Weighted median | 5 | 0.312010020955735 | 0.339969201827932 | 0.358744703006372 | -0.354329615 | 0.978349656538482 | 1.36616838327378 | 0.701643657223516 | 2.66006260049797 | 0.918328271889597 |
| ebi-fl187-GCST90094757 | finngen_R11_O15_POOR_FETGRO | Poor fetal growth \|\| id:finngen_R11_O15_POOR_FETGRO | Eggs liking \|\| id:ebi-fl187-GCST90094757 | Weighted mode | 5 | 0.407611385897556 | 0.476101965109464 | 0.440175905309933 | -0.525548466 | 1.3407712375121 | 1.50322287403839 | 0.59123100548775 | 3.82199003106756 | 0.972577670507659 |
| ebi-fl187-GCST90094758 | finngen_R11_O15_POOR_FETGRO | Poor fetal growth \|\| id:finngen_R11_O15_POOR_FETGRO | Extra virgin olive oil liking \|\| id:ebi-fl187-GCST90094758 | Inverse variance weighted | 2 | 0.563003263708893 | 0.623328823871965 | 0.36640897207254 | -0.658721231 | 1.78472775849794 | 1.75593813456873 | 0.517512690685174 | 5.95795772341443 | 0.888224550184161 |
| ebi-fl187-GCST90094759 | finngen_R11_O15_POOR_FETGRO | Poor fetal growth \|\| id:finngen_R11_O15_POOR_FETGRO | F-fatty/salty food liking (derived food-liking factor) \|\| id:ebi-fl187-GCST90094759 | Inverse variance weighted (multiplicative random effects) | 16 | 0.0495691039246361 | 0.324053633566771 | 0.87842529264114 | -0.585576018 | 0.684714225715508 | 1.05081820536794 | 0.556785051287553 | 1.98320500555684 | 0.994304114950957 |
| ebi-fl187-GCST90094759 | finngen_R11_O15_POOR_FETGRO | Poor fetal growth \|\| id:finngen_R11_O15_POOR_FETGRO | F-fatty/salty food liking (derived food-liking factor) \|\| id:ebi-fl187-GCST90094759 | MR Egger | 16 | -1.701765528 | 2.73603086856602 | 0.543949872212628 | -7.06438603 | 3.66085497441305 | 0.18236127573312 | 0.000855019715383113 | 38.8945825326494 | 0.970850396659599 |
| ebi-fl187-GCST90094759 | finngen_R11_O15_POOR_FETGRO | Poor fetal growth \|\| id:finngen_R11_O15_POOR_FETGRO | F-fatty/salty food liking (derived food-liking factor) \|\| id:ebi-fl187-GCST90094759 | Weighted median | 16 | -0.105303488 | 0.364916234310523 | 0.772911273751028 | -0.820539307 | 0.609932331525394 | 0.900051326604647 | 0.440194190682802 | 1.84030686380986 | 0.931145077826041 |
| ebi-fl187-GCST90094759 | finngen_R11_O15_POOR_FETGRO | Poor fetal growth \|\| id:finngen_R11_O15_POOR_FETGRO | F-fatty/salty food liking (derived food-liking factor) \|\| id:ebi-fl187-GCST90094759 | Weighted mode | 16 | -0.294958679 | 0.607944845958487 | 0.634562886552406 | -1.486530578 | 0.896613218613743 | 0.744562352545119 | 0.226155926454815 | 2.45128706338935 | 0.972577670507659 |
| ebi-fl187-GCST90094760 | finngen_R11_O15_POOR_FETGRO | Poor fetal growth \|\| id:finngen_R11_O15_POOR_FETGRO | F-fatty/dairy food liking (derived food-liking factor) \|\| id:ebi-fl187-GCST90094760 | Inverse variance weighted | 5 | -0.263591424 | 0.160568746287952 | 0.100670641703898 | -0.578306167 | 0.0511233188121933 | 0.768287379390419 | 0.560847544746714 | 1.05245267249439 | 0.761090127545161 |
| ebi-fl187-GCST90094760 | finngen_R11_O15_POOR_FETGRO | Poor fetal growth \|\| id:finngen_R11_O15_POOR_FETGRO | F-fatty/dairy food liking (derived food-liking factor) \|\| id:ebi-fl187-GCST90094760 | MR Egger | 5 | -0.010128303 | 0.937896241672117 | 0.992061835256798 | -1.848404936 | 1.82814833111574 | 0.989922815967904 | 0.157488169749528 | 6.22235424497186 | 0.993285374120483 |
| ebi-fl187-GCST90094760 | finngen_R11_O15_POOR_FETGRO | Poor fetal growth \|\| id:finngen_R11_O15_POOR_FETGRO | F-fatty/dairy food liking (derived food-liking factor) \|\| id:ebi-fl187-GCST90094760 | Weighted median | 5 | -0.174679597 | 0.198819737348615 | 0.379627536435666 | -0.564366283 | 0.215007087833275 | 0.839726028099451 | 0.568720440637382 | 1.23987068493162 | 0.918328271889597 |
| ebi-fl187-GCST90094760 | finngen_R11_O15_POOR_FETGRO | Poor fetal growth \|\| id:finngen_R11_O15_POOR_FETGRO | F-fatty/dairy food liking (derived food-liking factor) \|\| id:ebi-fl187-GCST90094760 | Weighted mode | 5 | -0.103115812 | 0.245889179558954 | 0.696496128558689 | -0.585058604 | 0.378826979868101 | 0.902022502341242 | 0.557073214135229 | 1.46057030581342 | 0.972577670507659 |
| ebi-fl187-GCST90094761 | finngen_R11_O15_POOR_FETGRO | Poor fetal growth \|\| id:finngen_R11_O15_POOR_FETGRO | F-fatty food liking (derived food-liking factor) \|\| id:ebi-fl187-GCST90094761 | Inverse variance weighted | 6 | -0.337136502 | 0.183472124868989 | 0.0661309131100996 | -0.696741867 | 0.0224688622991426 | 0.713811396252026 | 0.49820588326198 | 1.02272318841995 | 0.706473758635168 |
| ebi-fl187-GCST90094761 | finngen_R11_O15_POOR_FETGRO | Poor fetal growth \|\| id:finngen_R11_O15_POOR_FETGRO | F-fatty food liking (derived food-liking factor) \|\| id:ebi-fl187-GCST90094761 | MR Egger | 6 | -0.274561131 | 0.606801418350784 | 0.674382267419431 | -1.463891911 | 0.914769648689005 | 0.759905548831234 | 0.231334188319173 | 2.49620018268887 | 0.970850396659599 |
| ebi-fl187-GCST90094761 | finngen_R11_O15_POOR_FETGRO | Poor fetal growth \|\| id:finngen_R11_O15_POOR_FETGRO | F-fatty food liking (derived food-liking factor) \|\| id:ebi-fl187-GCST90094761 | Weighted median | 6 | -0.306173325 | 0.206582077080012 | 0.138316227474853 | -0.711074196 | 0.0987275459050866 | 0.736258996179301 | 0.491116358660412 | 1.10376553314889 | 0.918328271889597 |
| ebi-fl187-GCST90094761 | finngen_R11_O15_POOR_FETGRO | Poor fetal growth \|\| id:finngen_R11_O15_POOR_FETGRO | F-fatty food liking (derived food-liking factor) \|\| id:ebi-fl187-GCST90094761 | Weighted mode | 6 | -0.349856741 | 0.31707797478437 | 0.320114748401057 | -0.971329572 | 0.271616089511813 | 0.70478904981511 | 0.378579354961237 | 1.31208318211157 | 0.972577670507659 |
| ebi-fl187-GCST90094762 | finngen_R11_O15_POOR_FETGRO | Poor fetal growth \|\| id:finngen_R11_O15_POOR_FETGRO | F-fish liking (derived food-liking factor) \|\| id:ebi-fl187-GCST90094762 | Inverse variance weighted | 27 | -0.001472565 | 0.0586943135705779 | 0.979984193745725 | -0.11651342 | 0.113568289124209 | 0.998528518218413 | 0.890018152788434 | 1.12026838842743 | 0.994304114950957 |
| ebi-fl187-GCST90094762 | finngen_R11_O15_POOR_FETGRO | Poor fetal growth \|\| id:finngen_R11_O15_POOR_FETGRO | F-fish liking (derived food-liking factor) \|\| id:ebi-fl187-GCST90094762 | MR Egger | 27 | -0.061476058 | 0.265181685310812 | 0.818560138360054 | -0.581232161 | 0.458280045262915 | 0.940375459989692 | 0.559208906397359 | 1.58135179113628 | 0.970850396659599 |
| ebi-fl187-GCST90094762 | finngen_R11_O15_POOR_FETGRO | Poor fetal growth \|\| id:finngen_R11_O15_POOR_FETGRO | F-fish liking (derived food-liking factor) \|\| id:ebi-fl187-GCST90094762 | Weighted median | 27 | -0.030480579 | 0.0795670092951027 | 0.701659986850726 | -0.186431918 | 0.125470758778175 | 0.969979269424675 | 0.829915062588577 | 1.13368201822847 | 0.918328271889597 |
| ebi-fl187-GCST90094762 | finngen_R11_O15_POOR_FETGRO | Poor fetal growth \|\| id:finngen_R11_O15_POOR_FETGRO | F-fish liking (derived food-liking factor) \|\| id:ebi-fl187-GCST90094762 | Weighted mode | 27 | -0.041584192 | 0.128380352355771 | 0.748594467362176 | -0.293209683 | 0.210041298488974 | 0.959268569079101 | 0.745865729125423 | 1.23372901004857 | 0.972577670507659 |
| ebi-fl187-GCST90094764 | finngen_R11_O15_POOR_FETGRO | Poor fetal growth \|\| id:finngen_R11_O15_POOR_FETGRO | Fried chicken liking \|\| id:ebi-fl187-GCST90094764 | Inverse variance weighted | 2 | -0.192983995 | 0.241708579579378 | 0.424629127240921 | -0.666732811 | 0.280764821156881 | 0.824495170228186 | 0.513383160628239 | 1.32414215709321 | 0.889015330589552 |
| ebi-fl187-GCST90094765 | finngen_R11_O15_POOR_FETGRO | Poor fetal growth \|\| id:finngen_R11_O15_POOR_FETGRO | Fried fish liking \|\| id:ebi-fl187-GCST90094765 | Inverse variance weighted | 5 | -0.464498352 | 0.182688507673922 | 0.0110040500171779 | -0.822567827 | -0.106428877 | 0.628450286975578 | 0.439302153009476 | 0.899038988300078 | 0.392515819825973 |
| ebi-fl187-GCST90094765 | finngen_R11_O15_POOR_FETGRO | Poor fetal growth \|\| id:finngen_R11_O15_POOR_FETGRO | Fried fish liking \|\| id:ebi-fl187-GCST90094765 | MR Egger | 5 | -0.061101213 | 1.15174918783079 | 0.961026452662196 | -2.318529621 | 2.19632719516242 | 0.940728021065447 | 0.098418191294215 | 8.99192718317848 | 0.993285374120483 |
| ebi-fl187-GCST90094765 | finngen_R11_O15_POOR_FETGRO | Poor fetal growth \|\| id:finngen_R11_O15_POOR_FETGRO | Fried fish liking \|\| id:ebi-fl187-GCST90094765 | Weighted median | 5 | -0.49483203 | 0.213108600626369 | 0.0202344871808703 | -0.912524887 | -0.077139173 | 0.609673305410357 | 0.401509177666449 | 0.925761003746666 | 0.652429624709108 |
| ebi-fl187-GCST90094765 | finngen_R11_O15_POOR_FETGRO | Poor fetal growth \|\| id:finngen_R11_O15_POOR_FETGRO | Fried fish liking \|\| id:ebi-fl187-GCST90094765 | Weighted mode | 5 | -0.523933484 | 0.256658574664606 | 0.110771786084776 | -1.026984291 | -0.020882678 | 0.592186603945955 | 0.358085214799247 | 0.979333855182064 | 0.972577670507659 |
| ebi-fl187-GCST90094766 | finngen_R11_O15_POOR_FETGRO | Poor fetal growth \|\| id:finngen_R11_O15_POOR_FETGRO | Fruit liking \|\| id:ebi-fl187-GCST90094766 | Inverse variance weighted | 9 | -0.292567748 | 0.27146394377683 | 0.281149477956658 | -0.824637078 | 0.239501581772584 | 0.746344679941289 | 0.438394066522255 | 1.27061569444939 | 0.888224550184161 |
| ebi-fl187-GCST90094766 | finngen_R11_O15_POOR_FETGRO | Poor fetal growth \|\| id:finngen_R11_O15_POOR_FETGRO | Fruit liking \|\| id:ebi-fl187-GCST90094766 | MR Egger | 9 | -1.408432575 | 1.26650760816259 | 0.302840518026382 | -3.890787487 | 1.07392233679818 | 0.244526259454951 | 0.0204292519274348 | 2.92683705577751 | 0.970850396659599 |
| ebi-fl187-GCST90094766 | finngen_R11_O15_POOR_FETGRO | Poor fetal growth \|\| id:finngen_R11_O15_POOR_FETGRO | Fruit liking \|\| id:ebi-fl187-GCST90094766 | Weighted median | 9 | -0.591973174 | 0.343490433707739 | 0.0848152887117378 | -1.265214424 | 0.0812680761212236 | 0.553234578982532 | 0.282178783745873 | 1.08466162947821 | 0.918328271889597 |
| ebi-fl187-GCST90094766 | finngen_R11_O15_POOR_FETGRO | Poor fetal growth \|\| id:finngen_R11_O15_POOR_FETGRO | Fruit liking \|\| id:ebi-fl187-GCST90094766 | Weighted mode | 9 | -0.688438422 | 0.458918980832727 | 0.171970173421778 | -1.587919624 | 0.21104278054356 | 0.502359931148365 | 0.20435029521467 | 1.23496518641326 | 0.972577670507659 |
| ebi-fl187-GCST90094767 | finngen_R11_O15_POOR_FETGRO | Poor fetal growth \|\| id:finngen_R11_O15_POOR_FETGRO | F-fruit liking (derived food-liking factor) \|\| id:ebi-fl187-GCST90094767 | Inverse variance weighted | 14 | -0.023818806 | 0.0745809771964714 | 0.749447187418561 | -0.169997521 | 0.122359909634196 | 0.976462623219484 | 0.843666908064328 | 1.13016078434592 | 0.983649433486861 |
| ebi-fl187-GCST90094767 | finngen_R11_O15_POOR_FETGRO | Poor fetal growth \|\| id:finngen_R11_O15_POOR_FETGRO | F-fruit liking (derived food-liking factor) \|\| id:ebi-fl187-GCST90094767 | MR Egger | 14 | -0.736442136 | 0.297935976605807 | 0.029395690657091 | -1.32039665 | -0.152487622 | 0.478814445192743 | 0.267029363673043 | 0.858569521237934 | 0.970850396659599 |
| ebi-fl187-GCST90094767 | finngen_R11_O15_POOR_FETGRO | Poor fetal growth \|\| id:finngen_R11_O15_POOR_FETGRO | F-fruit liking (derived food-liking factor) \|\| id:ebi-fl187-GCST90094767 | Weighted median | 14 | -0.066010899 | 0.104457598367093 | 0.527426306222468 | -0.270747792 | 0.138725993543768 | 0.936120661217531 | 0.762808858600883 | 1.14880927571511 | 0.918328271889597 |
| ebi-fl187-GCST90094767 | finngen_R11_O15_POOR_FETGRO | Poor fetal growth \|\| id:finngen_R11_O15_POOR_FETGRO | F-fruit liking (derived food-liking factor) \|\| id:ebi-fl187-GCST90094767 | Weighted mode | 14 | -0.194759296 | 0.144303478993973 | 0.20016265510607 | -0.477594115 | 0.0880755228835787 | 0.823032741533922 | 0.620273905911301 | 1.09207059523427 | 0.972577670507659 |
| ebi-fl187-GCST90094768 | finngen_R11_O15_POOR_FETGRO | Poor fetal growth \|\| id:finngen_R11_O15_POOR_FETGRO | Fruit juice liking \|\| id:ebi-fl187-GCST90094768 | Inverse variance weighted | 2 | -0.067256751 | 0.194902860970552 | 0.730035494548432 | -0.449266359 | 0.314752856423122 | 0.934955119781526 | 0.638096113680627 | 1.36992070201387 | 0.96571624475698 |
| ebi-fl187-GCST90094769 | finngen_R11_O15_POOR_FETGRO | Poor fetal growth \|\| id:finngen_R11_O15_POOR_FETGRO | Garlic liking \|\| id:ebi-fl187-GCST90094769 | Inverse variance weighted | 8 | 0.197390922877917 | 0.117059579427163 | 0.0917489612241346 | -0.032045853 | 0.426827698555157 | 1.21822017777124 | 0.968462174359363 | 1.53238860620519 | 0.733991689793077 |
| ebi-fl187-GCST90094769 | finngen_R11_O15_POOR_FETGRO | Poor fetal growth \|\| id:finngen_R11_O15_POOR_FETGRO | Garlic liking \|\| id:ebi-fl187-GCST90094769 | MR Egger | 8 | 0.469614378819453 | 0.289081389610569 | 0.155393626898156 | -0.096985145 | 1.03621390245617 | 1.59937732051494 | 0.907569488143728 | 2.81852557495018 | 0.970850396659599 |
| ebi-fl187-GCST90094769 | finngen_R11_O15_POOR_FETGRO | Poor fetal growth \|\| id:finngen_R11_O15_POOR_FETGRO | Garlic liking \|\| id:ebi-fl187-GCST90094769 | Weighted median | 8 | 0.355473469673051 | 0.141928997642217 | 0.0122594579604342 | 0.077292634294306 | 0.633654305051795 | 1.42685606745078 | 1.08035817999257 | 1.88448449312905 | 0.652429624709108 |
| ebi-fl187-GCST90094769 | finngen_R11_O15_POOR_FETGRO | Poor fetal growth \|\| id:finngen_R11_O15_POOR_FETGRO | Garlic liking \|\| id:ebi-fl187-GCST90094769 | Weighted mode | 8 | 0.358857737096146 | 0.153710608695766 | 0.0522562803127055 | 0.0575849440524445 | 0.660130530139847 | 1.43169311026385 | 1.05927524596529 | 1.93504489959934 | 0.972577670507659 |
| ebi-fl187-GCST90094770 | finngen_R11_O15_POOR_FETGRO | Poor fetal growth \|\| id:finngen_R11_O15_POOR_FETGRO | Gherkins liking \|\| id:ebi-fl187-GCST90094770 | Inverse variance weighted | 19 | 0.0683561839191903 | 0.0792574224962396 | 0.38843563400234 | -0.086988364 | 0.22370073201182 | 1.07074662329439 | 0.916687761990905 | 1.25069666993951 | 0.888224550184161 |
| ebi-fl187-GCST90094770 | finngen_R11_O15_POOR_FETGRO | Poor fetal growth \|\| id:finngen_R11_O15_POOR_FETGRO | Gherkins liking \|\| id:ebi-fl187-GCST90094770 | MR Egger | 19 | -0.368880826 | 0.346800461269676 | 0.302355053699901 | -1.04860973 | 0.310848077857013 | 0.691507815132214 | 0.350424595298922 | 1.36458189523205 | 0.970850396659599 |
| ebi-fl187-GCST90094770 | finngen_R11_O15_POOR_FETGRO | Poor fetal growth \|\| id:finngen_R11_O15_POOR_FETGRO | Gherkins liking \|\| id:ebi-fl187-GCST90094770 | Weighted median | 19 | 0.0516598130556102 | 0.111929222629106 | 0.644411232560264 | -0.167721463 | 0.271041089408658 | 1.0530174587834 | 0.845589329549729 | 1.31132895100877 | 0.918328271889597 |
| ebi-fl187-GCST90094770 | finngen_R11_O15_POOR_FETGRO | Poor fetal growth \|\| id:finngen_R11_O15_POOR_FETGRO | Gherkins liking \|\| id:ebi-fl187-GCST90094770 | Weighted mode | 19 | 0.101075331846205 | 0.144087262690019 | 0.491970008371618 | -0.181335703 | 0.383486366718642 | 1.10635998276434 | 0.8341552832344 | 1.46739154695055 | 0.972577670507659 |
| ebi-fl187-GCST90094771 | finngen_R11_O15_POOR_FETGRO | Poor fetal growth \|\| id:finngen_R11_O15_POOR_FETGRO | Globe artichoke liking \|\| id:ebi-fl187-GCST90094771 | Inverse variance weighted | 9 | 0.0136204979425126 | 0.115591549485553 | 0.906199989061493 | -0.212938939 | 0.240179934934196 | 1.01371367950374 | 0.808205485505257 | 1.2714779130342 | 0.994304114950957 |
| ebi-fl187-GCST90094771 | finngen_R11_O15_POOR_FETGRO | Poor fetal growth \|\| id:finngen_R11_O15_POOR_FETGRO | Globe artichoke liking \|\| id:ebi-fl187-GCST90094771 | MR Egger | 9 | 0.0852957177420411 | 0.505259140136307 | 0.870716052687463 | -0.905012197 | 1.0756036324092 | 1.08903906725938 | 0.404536950966029 | 2.93176207311843 | 0.993285374120483 |
| ebi-fl187-GCST90094771 | finngen_R11_O15_POOR_FETGRO | Poor fetal growth \|\| id:finngen_R11_O15_POOR_FETGRO | Globe artichoke liking \|\| id:ebi-fl187-GCST90094771 | Weighted median | 9 | 0.0633068068096795 | 0.148742784101938 | 0.670390139149327 | -0.22822905 | 0.354842663649477 | 1.06535364684891 | 0.795941928432064 | 1.42595628187358 | 0.918328271889597 |
| ebi-fl187-GCST90094771 | finngen_R11_O15_POOR_FETGRO | Poor fetal growth \|\| id:finngen_R11_O15_POOR_FETGRO | Globe artichoke liking \|\| id:ebi-fl187-GCST90094771 | Weighted mode | 9 | 0.130928086007611 | 0.191307777564806 | 0.513066708557359 | -0.244035158 | 0.505891330034631 | 1.13988580462531 | 0.783460088873633 | 1.6584630998298 | 0.972577670507659 |
| ebi-fl187-GCST90094772 | finngen_R11_O15_POOR_FETGRO | Poor fetal growth \|\| id:finngen_R11_O15_POOR_FETGRO | F-glutamate liking (derived food-liking factor) \|\| id:ebi-fl187-GCST90094772 | Inverse variance weighted | 9 | -0.08221686 | 0.0878265128625421 | 0.349207275318033 | -0.254356825 | 0.0899231051793823 | 0.921072193291991 | 0.775415065007979 | 1.09409015060468 | 0.888224550184161 |
| ebi-fl187-GCST90094772 | finngen_R11_O15_POOR_FETGRO | Poor fetal growth \|\| id:finngen_R11_O15_POOR_FETGRO | F-glutamate liking (derived food-liking factor) \|\| id:ebi-fl187-GCST90094772 | MR Egger | 9 | -0.396024323 | 0.443173438780907 | 0.401207943959253 | -1.264644263 | 0.472595617028587 | 0.672990326606718 | 0.282339716963755 | 1.60415255981984 | 0.970850396659599 |
| ebi-fl187-GCST90094772 | finngen_R11_O15_POOR_FETGRO | Poor fetal growth \|\| id:finngen_R11_O15_POOR_FETGRO | F-glutamate liking (derived food-liking factor) \|\| id:ebi-fl187-GCST90094772 | Weighted median | 9 | -0.073198067 | 0.097348838994613 | 0.452102084604664 | -0.264001792 | 0.117605657208998 | 0.929416724806474 | 0.767972163718235 | 1.12480046694365 | 0.918328271889597 |
| ebi-fl187-GCST90094772 | finngen_R11_O15_POOR_FETGRO | Poor fetal growth \|\| id:finngen_R11_O15_POOR_FETGRO | F-glutamate liking (derived food-liking factor) \|\| id:ebi-fl187-GCST90094772 | Weighted mode | 9 | 0.0763825921858383 | 0.171388288121648 | 0.667649882973532 | -0.259538453 | 0.412303636904269 | 1.07937545578457 | 0.771407544849914 | 1.51029294738987 | 0.972577670507659 |
| ebi-fl187-GCST90094773 | finngen_R11_O15_POOR_FETGRO | Poor fetal growth \|\| id:finngen_R11_O15_POOR_FETGRO | Goat cheese liking \|\| id:ebi-fl187-GCST90094773 | Inverse variance weighted | 8 | 0.0159600452479408 | 0.125092455220056 | 0.898476615664477 | -0.229221167 | 0.26114125747925 | 1.01608808704746 | 0.7951526525428 | 1.29841106275402 | 0.994304114950957 |
| ebi-fl187-GCST90094773 | finngen_R11_O15_POOR_FETGRO | Poor fetal growth \|\| id:finngen_R11_O15_POOR_FETGRO | Goat cheese liking \|\| id:ebi-fl187-GCST90094773 | MR Egger | 8 | -1.067399661 | 0.72960345780807 | 0.193796910595024 | -2.497422438 | 0.362623116564636 | 0.343901616610548 | 0.0822968507072353 | 1.43709414018866 | 0.970850396659599 |
| ebi-fl187-GCST90094773 | finngen_R11_O15_POOR_FETGRO | Poor fetal growth \|\| id:finngen_R11_O15_POOR_FETGRO | Goat cheese liking \|\| id:ebi-fl187-GCST90094773 | Weighted median | 8 | 0.0851704594739551 | 0.163637692562705 | 0.602727730150181 | -0.235559418 | 0.405900336896857 | 1.08890266465489 | 0.790128713786433 | 1.50065298527173 | 0.918328271889597 |
| ebi-fl187-GCST90094773 | finngen_R11_O15_POOR_FETGRO | Poor fetal growth \|\| id:finngen_R11_O15_POOR_FETGRO | Goat cheese liking \|\| id:ebi-fl187-GCST90094773 | Weighted mode | 8 | 0.114851617031418 | 0.237447173364887 | 0.643367409248937 | -0.350544843 | 0.580248076826597 | 1.12170698301071 | 0.70430425008794 | 1.78648156045899 | 0.972577670507659 |
| ebi-fl187-GCST90094774 | finngen_R11_O15_POOR_FETGRO | Poor fetal growth \|\| id:finngen_R11_O15_POOR_FETGRO | Grapefruit liking \|\| id:ebi-fl187-GCST90094774 | Inverse variance weighted | 8 | -0.011037753 | 0.103569109248353 | 0.915127110941213 | -0.214033207 | 0.191957701399526 | 0.989022939757263 | 0.807321575967842 | 1.21161926608173 | 0.994304114950957 |
| ebi-fl187-GCST90094774 | finngen_R11_O15_POOR_FETGRO | Poor fetal growth \|\| id:finngen_R11_O15_POOR_FETGRO | Grapefruit liking \|\| id:ebi-fl187-GCST90094774 | MR Egger | 8 | -0.134010745 | 0.228426958866079 | 0.578815323473849 | -0.581727584 | 0.313706094470008 | 0.87458066726447 | 0.558931929986801 | 1.36848747140073 | 0.970850396659599 |
| ebi-fl187-GCST90094774 | finngen_R11_O15_POOR_FETGRO | Poor fetal growth \|\| id:finngen_R11_O15_POOR_FETGRO | Grapefruit liking \|\| id:ebi-fl187-GCST90094774 | Weighted median | 8 | -0.027571008 | 0.124393428482574 | 0.824591337034235 | -0.271382127 | 0.216240112263516 | 0.972805603546388 | 0.762325135427165 | 1.24140041868221 | 0.948590034332616 |
| ebi-fl187-GCST90094774 | finngen_R11_O15_POOR_FETGRO | Poor fetal growth \|\| id:finngen_R11_O15_POOR_FETGRO | Grapefruit liking \|\| id:ebi-fl187-GCST90094774 | Weighted mode | 8 | -0.080468359 | 0.140207370935571 | 0.583980895220419 | -0.355274806 | 0.194338087963325 | 0.922684097704474 | 0.700980782939659 | 1.21450682369135 | 0.972577670507659 |
| ebi-fl187-GCST90094775 | finngen_R11_O15_POOR_FETGRO | Poor fetal growth \|\| id:finngen_R11_O15_POOR_FETGRO | Green olives liking \|\| id:ebi-fl187-GCST90094775 | Inverse variance weighted | 25 | -0.071463915 | 0.0643425699129021 | 0.26670671924352 | -0.197575352 | 0.0546475219238791 | 0.931029873103545 | 0.820718295422125 | 1.0561682728973 | 0.888224550184161 |
| ebi-fl187-GCST90094775 | finngen_R11_O15_POOR_FETGRO | Poor fetal growth \|\| id:finngen_R11_O15_POOR_FETGRO | Green olives liking \|\| id:ebi-fl187-GCST90094775 | MR Egger | 25 | 0.136345154081995 | 0.284669731099432 | 0.63648993357819 | -0.421607519 | 0.694297827036882 | 1.14607739862827 | 0.65599145314002 | 2.00230261744915 | 0.970850396659599 |
| ebi-fl187-GCST90094775 | finngen_R11_O15_POOR_FETGRO | Poor fetal growth \|\| id:finngen_R11_O15_POOR_FETGRO | Green olives liking \|\| id:ebi-fl187-GCST90094775 | Weighted median | 25 | -0.053995744 | 0.090949909426689 | 0.552721739567252 | -0.232257566 | 0.124266078946095 | 0.947436139226515 | 0.792741913640176 | 1.13231711666488 | 0.918328271889597 |
| ebi-fl187-GCST90094775 | finngen_R11_O15_POOR_FETGRO | Poor fetal growth \|\| id:finngen_R11_O15_POOR_FETGRO | Green olives liking \|\| id:ebi-fl187-GCST90094775 | Weighted mode | 25 | -0.062123545 | 0.14644986577822 | 0.675200624077613 | -0.349165282 | 0.224918192172379 | 0.93976677636462 | 0.705276551344008 | 1.25222027058146 | 0.972577670507659 |
| ebi-fl187-GCST90094776 | finngen_R11_O15_POOR_FETGRO | Poor fetal growth \|\| id:finngen_R11_O15_POOR_FETGRO | Haddock liking \|\| id:ebi-fl187-GCST90094776 | Inverse variance weighted | 5 | 0.0277295377091622 | 0.220580265025976 | 0.899960076900923 | -0.404607782 | 0.460066857160074 | 1.02811757977883 | 0.667238462650473 | 1.58417989522286 | 0.994304114950957 |
| ebi-fl187-GCST90094776 | finngen_R11_O15_POOR_FETGRO | Poor fetal growth \|\| id:finngen_R11_O15_POOR_FETGRO | Haddock liking \|\| id:ebi-fl187-GCST90094776 | MR Egger | 5 | -1.182666657 | 1.75245510784187 | 0.548118371649503 | -4.617478669 | 2.25214535392956 | 0.306460423035103 | 0.00987766956719579 | 9.5081122371982 | 0.970850396659599 |
| ebi-fl187-GCST90094776 | finngen_R11_O15_POOR_FETGRO | Poor fetal growth \|\| id:finngen_R11_O15_POOR_FETGRO | Haddock liking \|\| id:ebi-fl187-GCST90094776 | Weighted median | 5 | -0.064414374 | 0.270465721704271 | 0.81175626155054 | -0.594527188 | 0.465698440759311 | 0.937616395371668 | 0.5518234127513 | 1.59312650488422 | 0.940899303160853 |
| ebi-fl187-GCST90094776 | finngen_R11_O15_POOR_FETGRO | Poor fetal growth \|\| id:finngen_R11_O15_POOR_FETGRO | Haddock liking \|\| id:ebi-fl187-GCST90094776 | Weighted mode | 5 | -0.072585415 | 0.369188920180503 | 0.853719705885888 | -0.796195699 | 0.651024868490634 | 0.929986308428451 | 0.451041602547695 | 1.91750501279518 | 0.982450744327861 |
| ebi-fl187-GCST90094777 | finngen_R11_O15_POOR_FETGRO | Poor fetal growth \|\| id:finngen_R11_O15_POOR_FETGRO | Ham liking \|\| id:ebi-fl187-GCST90094777 | Inverse variance weighted | 10 | -0.05239326 | 0.1363344394589 | 0.700756460754334 | -0.319608762 | 0.214822241150733 | 0.948955606992495 | 0.726433190095458 | 1.23964152012955 | 0.96571624475698 |
| ebi-fl187-GCST90094777 | finngen_R11_O15_POOR_FETGRO | Poor fetal growth \|\| id:finngen_R11_O15_POOR_FETGRO | Ham liking \|\| id:ebi-fl187-GCST90094777 | MR Egger | 10 | -0.042195897 | 0.47789426214067 | 0.931812118968397 | -0.978868651 | 0.894476856781518 | 0.958681959242888 | 0.375735947039629 | 2.44605581717431 | 0.993285374120483 |
| ebi-fl187-GCST90094777 | finngen_R11_O15_POOR_FETGRO | Poor fetal growth \|\| id:finngen_R11_O15_POOR_FETGRO | Ham liking \|\| id:ebi-fl187-GCST90094777 | Weighted median | 10 | -0.08815618 | 0.181238537906048 | 0.626676723961382 | -0.443383715 | 0.267071353851676 | 0.915617863951029 | 0.64186086835268 | 1.30613364067179 | 0.918328271889597 |
| ebi-fl187-GCST90094777 | finngen_R11_O15_POOR_FETGRO | Poor fetal growth \|\| id:finngen_R11_O15_POOR_FETGRO | Ham liking \|\| id:ebi-fl187-GCST90094777 | Weighted mode | 10 | -0.032588204 | 0.24154988069574 | 0.895649774644438 | -0.50602597 | 0.440849562430738 | 0.967937070403135 | 0.602886714394924 | 1.55402690072696 | 0.988620775239461 |
| ebi-fl187-GCST90094778 | finngen_R11_O15_POOR_FETGRO | Poor fetal growth \|\| id:finngen_R11_O15_POOR_FETGRO | Hard cheese liking \|\| id:ebi-fl187-GCST90094778 | Inverse variance weighted | 4 | -1.087435885 | 0.331916036989648 | 0.00105199922519077 | -1.737991318 | -0.436880453 | 0.337079697586799 | 0.175873319665549 | 0.646048660145151 | 0.176735869832049 |
| ebi-fl187-GCST90094778 | finngen_R11_O15_POOR_FETGRO | Poor fetal growth \|\| id:finngen_R11_O15_POOR_FETGRO | Hard cheese liking \|\| id:ebi-fl187-GCST90094778 | MR Egger | 4 | -0.889120753 | 1.29090475592068 | 0.562142590211193 | -3.419294074 | 1.64105256888941 | 0.411016979488514 | 0.0327355356396395 | 5.1605985399945 | 0.970850396659599 |
| ebi-fl187-GCST90094778 | finngen_R11_O15_POOR_FETGRO | Poor fetal growth \|\| id:finngen_R11_O15_POOR_FETGRO | Hard cheese liking \|\| id:ebi-fl187-GCST90094778 | Weighted median | 4 | -0.987623401 | 0.466141097242207 | 0.0341139673050514 | -1.901259952 | -0.073986851 | 0.372460829835177 | 0.14938028859675 | 0.928683905116826 | 0.652429624709108 |
| ebi-fl187-GCST90094778 | finngen_R11_O15_POOR_FETGRO | Poor fetal growth \|\| id:finngen_R11_O15_POOR_FETGRO | Hard cheese liking \|\| id:ebi-fl187-GCST90094778 | Weighted mode | 4 | -0.727565123 | 0.540987005857353 | 0.271298610202227 | -1.787899654 | 0.332769408578524 | 0.483083808942886 | 0.167311212254061 | 1.3948256265599 | 0.972577670507659 |
| ebi-fl187-GCST90094779 | finngen_R11_O15_POOR_FETGRO | Poor fetal growth \|\| id:finngen_R11_O15_POOR_FETGRO | F-healthy food liking (derived food-liking factor) \|\| id:ebi-fl187-GCST90094779 | Inverse variance weighted | 7 | 0.0868332309168619 | 0.110969797295299 | 0.433924149454424 | -0.130667572 | 0.304334033615649 | 1.09071476704833 | 0.877509434808039 | 1.35572183713049 | 0.889015330589552 |
| ebi-fl187-GCST90094779 | finngen_R11_O15_POOR_FETGRO | Poor fetal growth \|\| id:finngen_R11_O15_POOR_FETGRO | F-healthy food liking (derived food-liking factor) \|\| id:ebi-fl187-GCST90094779 | MR Egger | 7 | -1.535973039 | 1.58981123677816 | 0.378349004732821 | -4.652003063 | 1.58005698491992 | 0.215246146308066 | 0.00954246860612772 | 4.85523247838842 | 0.970850396659599 |
| ebi-fl187-GCST90094779 | finngen_R11_O15_POOR_FETGRO | Poor fetal growth \|\| id:finngen_R11_O15_POOR_FETGRO | F-healthy food liking (derived food-liking factor) \|\| id:ebi-fl187-GCST90094779 | Weighted median | 7 | 0.00295478100045482 | 0.139981496804238 | 0.983159209383976 | -0.271408953 | 0.27731851473676 | 1.00295915066858 | 0.762304686064647 | 1.31958661188727 | 0.989627362077292 |
| ebi-fl187-GCST90094779 | finngen_R11_O15_POOR_FETGRO | Poor fetal growth \|\| id:finngen_R11_O15_POOR_FETGRO | F-healthy food liking (derived food-liking factor) \|\| id:ebi-fl187-GCST90094779 | Weighted mode | 7 | -0.117030445 | 0.219842279242923 | 0.613618831931117 | -0.547921312 | 0.313860422280989 | 0.88955811012322 | 0.578150356172828 | 1.36869868337406 | 0.972577670507659 |
| ebi-fl187-GCST90094780 | finngen_R11_O15_POOR_FETGRO | Poor fetal growth \|\| id:finngen_R11_O15_POOR_FETGRO | F-healthy breakfast food liking (derived food-liking factor) \|\| id:ebi-fl187-GCST90094780 | Inverse variance weighted (multiplicative random effects) | 9 | 0.0328562540202732 | 0.117914302548719 | 0.78051712393264 | -0.198255779 | 0.263968287015763 | 1.03340198118401 | 0.82016004661069 | 1.30208690258471 | 0.994304114950957 |
| ebi-fl187-GCST90094780 | finngen_R11_O15_POOR_FETGRO | Poor fetal growth \|\| id:finngen_R11_O15_POOR_FETGRO | F-healthy breakfast food liking (derived food-liking factor) \|\| id:ebi-fl187-GCST90094780 | MR Egger | 9 | 0.423990212958381 | 0.953357068893621 | 0.669937938582868 | -1.444589642 | 2.29257006798988 | 1.52804663865483 | 0.235842836678894 | 9.90034958358042 | 0.970850396659599 |
| ebi-fl187-GCST90094780 | finngen_R11_O15_POOR_FETGRO | Poor fetal growth \|\| id:finngen_R11_O15_POOR_FETGRO | F-healthy breakfast food liking (derived food-liking factor) \|\| id:ebi-fl187-GCST90094780 | Weighted median | 9 | 0.167091469919443 | 0.118358212236938 | 0.158025417549984 | -0.064890626 | 0.399073565903841 | 1.18186236521968 | 0.937169959738389 | 1.49044326037998 | 0.918328271889597 |
| ebi-fl187-GCST90094780 | finngen_R11_O15_POOR_FETGRO | Poor fetal growth \|\| id:finngen_R11_O15_POOR_FETGRO | F-healthy breakfast food liking (derived food-liking factor) \|\| id:ebi-fl187-GCST90094780 | Weighted mode | 9 | 0.273541813522128 | 0.172752185300465 | 0.151980805480994 | -0.06505247 | 0.612136096711039 | 1.31461232663447 | 0.937018297049729 | 1.84436694009143 | 0.972577670507659 |
| ebi-fl187-GCST90094781 | finngen_R11_O15_POOR_FETGRO | Poor fetal growth \|\| id:finngen_R11_O15_POOR_FETGRO | Herring liking \|\| id:ebi-fl187-GCST90094781 | Inverse variance weighted | 18 | -0.191540384 | 0.0778165497759011 | 0.0138382445628417 | -0.344060822 | -0.039019947 | 0.825686279791513 | 0.708885811087882 | 0.961731525687753 | 0.392515819825973 |
| ebi-fl187-GCST90094781 | finngen_R11_O15_POOR_FETGRO | Poor fetal growth \|\| id:finngen_R11_O15_POOR_FETGRO | Herring liking \|\| id:ebi-fl187-GCST90094781 | MR Egger | 18 | -0.137233815 | 0.33870313045557 | 0.690715244538584 | -0.80109195 | 0.526624320926722 | 0.87176637045072 | 0.448838586931083 | 1.69320692733914 | 0.970850396659599 |
| ebi-fl187-GCST90094781 | finngen_R11_O15_POOR_FETGRO | Poor fetal growth \|\| id:finngen_R11_O15_POOR_FETGRO | Herring liking \|\| id:ebi-fl187-GCST90094781 | Weighted median | 18 | -0.115208087 | 0.107810991366068 | 0.285244673200461 | -0.32651763 | 0.0961014560529297 | 0.891180681474981 | 0.72143165602277 | 1.10087074832927 | 0.918328271889597 |
| ebi-fl187-GCST90094781 | finngen_R11_O15_POOR_FETGRO | Poor fetal growth \|\| id:finngen_R11_O15_POOR_FETGRO | Herring liking \|\| id:ebi-fl187-GCST90094781 | Weighted mode | 18 | -0.106621686 | 0.150188107226272 | 0.487377744234623 | -0.400990376 | 0.187747004013067 | 0.898865662003818 | 0.669656505570917 | 1.20652822993295 | 0.972577670507659 |
| ebi-fl187-GCST90094783 | finngen_R11_O15_POOR_FETGRO | Poor fetal growth \|\| id:finngen_R11_O15_POOR_FETGRO | Horseradish liking \|\| id:ebi-fl187-GCST90094783 | Inverse variance weighted | 23 | 0.0990416142513723 | 0.0636211874953531 | 0.119532351479809 | -0.025655913 | 0.223739141742264 | 1.1041122454074 | 0.974670403098513 | 1.25074470978406 | 0.803257401944318 |
| ebi-fl187-GCST90094783 | finngen_R11_O15_POOR_FETGRO | Poor fetal growth \|\| id:finngen_R11_O15_POOR_FETGRO | Horseradish liking \|\| id:ebi-fl187-GCST90094783 | MR Egger | 23 | 0.140737453195321 | 0.214959274844574 | 0.519757417873287 | -0.280582726 | 0.562057631890686 | 1.15112238480344 | 0.755343455292507 | 1.75427844844756 | 0.970850396659599 |
| ebi-fl187-GCST90094783 | finngen_R11_O15_POOR_FETGRO | Poor fetal growth \|\| id:finngen_R11_O15_POOR_FETGRO | Horseradish liking \|\| id:ebi-fl187-GCST90094783 | Weighted median | 23 | 0.1657447414241 | 0.0897412415316812 | 0.0647589817828611 | -0.010148092 | 0.341637574826195 | 1.18027178877258 | 0.989903226166945 | 1.4072501821885 | 0.900738564797977 |
| ebi-fl187-GCST90094783 | finngen_R11_O15_POOR_FETGRO | Poor fetal growth \|\| id:finngen_R11_O15_POOR_FETGRO | Horseradish liking \|\| id:ebi-fl187-GCST90094783 | Weighted mode | 23 | 0.146517506072547 | 0.124076018137253 | 0.250264425308587 | -0.096671489 | 0.389706501621562 | 1.1577951990878 | 0.907854196808688 | 1.47654736602295 | 0.972577670507659 |
| ebi-fl187-GCST90094784 | finngen_R11_O15_POOR_FETGRO | Poor fetal growth \|\| id:finngen_R11_O15_POOR_FETGRO | Ice cream liking \|\| id:ebi-fl187-GCST90094784 | Inverse variance weighted | 8 | 0.0192743712169491 | 0.18835848205652 | 0.918496228076473 | -0.349908254 | 0.388456996047728 | 1.01946132109192 | 0.704752745270233 | 1.47470356401941 | 0.994304114950957 |
| ebi-fl187-GCST90094784 | finngen_R11_O15_POOR_FETGRO | Poor fetal growth \|\| id:finngen_R11_O15_POOR_FETGRO | Ice cream liking \|\| id:ebi-fl187-GCST90094784 | MR Egger | 8 | 1.22919920288667 | 0.815406310157315 | 0.182414241345786 | -0.368997165 | 2.82739557079501 | 3.41849092223643 | 0.691427370629143 | 16.9013849925835 | 0.970850396659599 |
| ebi-fl187-GCST90094784 | finngen_R11_O15_POOR_FETGRO | Poor fetal growth \|\| id:finngen_R11_O15_POOR_FETGRO | Ice cream liking \|\| id:ebi-fl187-GCST90094784 | Weighted median | 8 | -0.051310104 | 0.214876729990525 | 0.811269543077398 | -0.472468495 | 0.36984828629794 | 0.949984030543082 | 0.623461355792932 | 1.44751499014578 | 0.940899303160853 |
| ebi-fl187-GCST90094784 | finngen_R11_O15_POOR_FETGRO | Poor fetal growth \|\| id:finngen_R11_O15_POOR_FETGRO | Ice cream liking \|\| id:ebi-fl187-GCST90094784 | Weighted mode | 8 | -0.194294164 | 0.363811115836897 | 0.609838311197225 | -0.907363951 | 0.518775622780066 | 0.823415649183608 | 0.403586697241209 | 1.67996947361037 | 0.972577670507659 |
| ebi-fl187-GCST90094785 | finngen_R11_O15_POOR_FETGRO | Poor fetal growth \|\| id:finngen_R11_O15_POOR_FETGRO | F-highly palatable foods liking (derived food-liking factor) \|\| id:ebi-fl187-GCST90094785 | Inverse variance weighted | 17 | -0.50389848 | 0.241837261699895 | 0.0371941722639578 | -0.977899513 | -0.029897447 | 0.604170715016039 | 0.376100263400368 | 0.970545060465474 | 0.568056449122264 |
| ebi-fl187-GCST90094785 | finngen_R11_O15_POOR_FETGRO | Poor fetal growth \|\| id:finngen_R11_O15_POOR_FETGRO | F-highly palatable foods liking (derived food-liking factor) \|\| id:ebi-fl187-GCST90094785 | MR Egger | 17 | -0.910863703 | 1.72530651054309 | 0.60526158770231 | -4.292464464 | 2.47073705726508 | 0.402176712588014 | 0.0136711915863048 | 11.8311638840707 | 0.970850396659599 |
| ebi-fl187-GCST90094785 | finngen_R11_O15_POOR_FETGRO | Poor fetal growth \|\| id:finngen_R11_O15_POOR_FETGRO | F-highly palatable foods liking (derived food-liking factor) \|\| id:ebi-fl187-GCST90094785 | Weighted median | 17 | -0.807821147 | 0.321043433091743 | 0.0118615619048277 | -1.437066276 | -0.178576018 | 0.445828403321726 | 0.237623859973638 | 0.836460468365633 | 0.652429624709108 |
| ebi-fl187-GCST90094785 | finngen_R11_O15_POOR_FETGRO | Poor fetal growth \|\| id:finngen_R11_O15_POOR_FETGRO | F-highly palatable foods liking (derived food-liking factor) \|\| id:ebi-fl187-GCST90094785 | Weighted mode | 17 | -1.042392149 | 0.560289775850327 | 0.0813029548498426 | -2.14056011 | 0.0557758113057298 | 0.352610176062057 | 0.117588961816497 | 1.05736060886858 | 0.972577670507659 |
| ebi-fl187-GCST90094786 | finngen_R11_O15_POOR_FETGRO | Poor fetal growth \|\| id:finngen_R11_O15_POOR_FETGRO | Jam liking \|\| id:ebi-fl187-GCST90094786 | Inverse variance weighted | 6 | -0.14120619 | 0.294411688651672 | 0.63149652187713 | -0.718253099 | 0.435840720068403 | 0.868310256616988 | 0.48760330687973 | 1.54626248655083 | 0.96571624475698 |
| ebi-fl187-GCST90094786 | finngen_R11_O15_POOR_FETGRO | Poor fetal growth \|\| id:finngen_R11_O15_POOR_FETGRO | Jam liking \|\| id:ebi-fl187-GCST90094786 | MR Egger | 6 | 3.40700296395001 | 2.95968730606738 | 0.313804790341483 | -2.393984156 | 9.20799008384208 | 30.1746740202224 | 0.09126534320499 | 9976.5247163055 | 0.970850396659599 |
| ebi-fl187-GCST90094786 | finngen_R11_O15_POOR_FETGRO | Poor fetal growth \|\| id:finngen_R11_O15_POOR_FETGRO | Jam liking \|\| id:ebi-fl187-GCST90094786 | Weighted median | 6 | 0.108419967204423 | 0.276108158442503 | 0.694561688347966 | -0.432752023 | 0.649591957751728 | 1.11451570716111 | 0.648721339610099 | 1.914759367 | 0.918328271889597 |
| ebi-fl187-GCST90094786 | finngen_R11_O15_POOR_FETGRO | Poor fetal growth \|\| id:finngen_R11_O15_POOR_FETGRO | Jam liking \|\| id:ebi-fl187-GCST90094786 | Weighted mode | 6 | 0.28557375855537 | 0.470728047497987 | 0.570562592663781 | -0.637053215 | 1.20820073165142 | 1.33052520938167 | 0.528848533325083 | 3.3474562587317 | 0.972577670507659 |
| ebi-fl187-GCST90094787 | finngen_R11_O15_POOR_FETGRO | Poor fetal growth \|\| id:finngen_R11_O15_POOR_FETGRO | Ketchup liking \|\| id:ebi-fl187-GCST90094787 | Inverse variance weighted | 10 | -0.076593065 | 0.12603162951996 | 0.543367285336332 | -0.323615059 | 0.170428928985644 | 0.926266707383537 | 0.723528704837198 | 1.18581337197975 | 0.927237411635561 |
| ebi-fl187-GCST90094787 | finngen_R11_O15_POOR_FETGRO | Poor fetal growth \|\| id:finngen_R11_O15_POOR_FETGRO | Ketchup liking \|\| id:ebi-fl187-GCST90094787 | MR Egger | 10 | -0.879501576 | 0.897792692388529 | 0.355962412062055 | -2.639175253 | 0.880172101014484 | 0.414989700941826 | 0.0714201488156725 | 2.41131466040848 | 0.970850396659599 |
| ebi-fl187-GCST90094787 | finngen_R11_O15_POOR_FETGRO | Poor fetal growth \|\| id:finngen_R11_O15_POOR_FETGRO | Ketchup liking \|\| id:ebi-fl187-GCST90094787 | Weighted median | 10 | -0.150682908 | 0.174734155383508 | 0.388492084099486 | -0.493161852 | 0.191796036616368 | 0.860120392773579 | 0.610692418979739 | 1.21142340574809 | 0.918328271889597 |
| ebi-fl187-GCST90094787 | finngen_R11_O15_POOR_FETGRO | Poor fetal growth \|\| id:finngen_R11_O15_POOR_FETGRO | Ketchup liking \|\| id:ebi-fl187-GCST90094787 | Weighted mode | 10 | -0.31874836 | 0.288936856138807 | 0.298572991940749 | -0.885064598 | 0.247567877785512 | 0.727058483106097 | 0.412687513514994 | 1.28090630451637 | 0.972577670507659 |
| ebi-fl187-GCST90094788 | finngen_R11_O15_POOR_FETGRO | Poor fetal growth \|\| id:finngen_R11_O15_POOR_FETGRO | Kiwi liking \|\| id:ebi-fl187-GCST90094788 | Inverse variance weighted | 3 | -0.459173446 | 0.263267056680592 | 0.0811354474398785 | -0.975176877 | 0.0568299848787215 | 0.631805651096506 | 0.377125642694602 | 1.05847583819893 | 0.706473758635168 |
| ebi-fl187-GCST90094788 | finngen_R11_O15_POOR_FETGRO | Poor fetal growth \|\| id:finngen_R11_O15_POOR_FETGRO | Kiwi liking \|\| id:ebi-fl187-GCST90094788 | MR Egger | 3 | -0.799033898 | 0.672858592070273 | 0.445560017791005 | -2.117836739 | 0.519768942003929 | 0.449763271282837 | 0.120291569321349 | 1.68163904865723 | 0.970850396659599 |
| ebi-fl187-GCST90094788 | finngen_R11_O15_POOR_FETGRO | Poor fetal growth \|\| id:finngen_R11_O15_POOR_FETGRO | Kiwi liking \|\| id:ebi-fl187-GCST90094788 | Weighted median | 3 | -0.510542811 | 0.30463339320893 | 0.0937528251395815 | -1.107624262 | 0.0865386394148735 | 0.600169711491951 | 0.330342837608032 | 1.09039349907059 | 0.918328271889597 |
| ebi-fl187-GCST90094788 | finngen_R11_O15_POOR_FETGRO | Poor fetal growth \|\| id:finngen_R11_O15_POOR_FETGRO | Kiwi liking \|\| id:ebi-fl187-GCST90094788 | Weighted mode | 3 | -0.554877388 | 0.370115697635548 | 0.27257585772435 | -1.280304156 | 0.170549378934241 | 0.574142653412214 | 0.277952746655865 | 1.1859562117418 | 0.972577670507659 |
| ebi-fl187-GCST90094789 | finngen_R11_O15_POOR_FETGRO | Poor fetal growth \|\| id:finngen_R11_O15_POOR_FETGRO | Lager liking \|\| id:ebi-fl187-GCST90094789 | Inverse variance weighted | 10 | 0.0979297329358132 | 0.166254202892123 | 0.555837034382249 | -0.227928505 | 0.423787970604375 | 1.10288528587454 | 0.796181180986948 | 1.52773763415354 | 0.927237411635561 |
| ebi-fl187-GCST90094789 | finngen_R11_O15_POOR_FETGRO | Poor fetal growth \|\| id:finngen_R11_O15_POOR_FETGRO | Lager liking \|\| id:ebi-fl187-GCST90094789 | MR Egger | 10 | -0.184679035 | 0.672944044573725 | 0.790704269387646 | -1.503649362 | 1.13429129239425 | 0.831371082077476 | 0.222317361346963 | 3.108969412 | 0.970850396659599 |
| ebi-fl187-GCST90094789 | finngen_R11_O15_POOR_FETGRO | Poor fetal growth \|\| id:finngen_R11_O15_POOR_FETGRO | Lager liking \|\| id:ebi-fl187-GCST90094789 | Weighted median | 10 | 0.0727306054770852 | 0.187773882885419 | 0.698511337573174 | -0.295306205 | 0.440767415932506 | 1.07544078003114 | 0.744303643087932 | 1.55389924810209 | 0.918328271889597 |
| ebi-fl187-GCST90094789 | finngen_R11_O15_POOR_FETGRO | Poor fetal growth \|\| id:finngen_R11_O15_POOR_FETGRO | Lager liking \|\| id:ebi-fl187-GCST90094789 | Weighted mode | 10 | 0.0695541487691429 | 0.25751285215252 | 0.793174902403112 | -0.435171041 | 0.574279338988082 | 1.07203010874403 | 0.647153967458357 | 1.77585027959777 | 0.972577670507659 |
| ebi-fl187-GCST90094790 | finngen_R11_O15_POOR_FETGRO | Poor fetal growth \|\| id:finngen_R11_O15_POOR_FETGRO | Lamb liking \|\| id:ebi-fl187-GCST90094790 | Inverse variance weighted | 7 | 0.0263674739277474 | 0.139875317277043 | 0.850479242722937 | -0.247788148 | 0.300523095790751 | 1.02671817131852 | 0.780525281656281 | 1.35056509774896 | 0.994304114950957 |
| ebi-fl187-GCST90094790 | finngen_R11_O15_POOR_FETGRO | Poor fetal growth \|\| id:finngen_R11_O15_POOR_FETGRO | Lamb liking \|\| id:ebi-fl187-GCST90094790 | MR Egger | 7 | -0.488460885 | 0.910219687152415 | 0.614523837720174 | -2.272491472 | 1.29556970140205 | 0.61357002239152 | 0.103055101040475 | 3.65307654426221 | 0.970850396659599 |
| ebi-fl187-GCST90094790 | finngen_R11_O15_POOR_FETGRO | Poor fetal growth \|\| id:finngen_R11_O15_POOR_FETGRO | Lamb liking \|\| id:ebi-fl187-GCST90094790 | Weighted median | 7 | -0.043517914 | 0.16107376579247 | 0.787026533124229 | -0.359222495 | 0.272186666453948 | 0.957415402319786 | 0.698218983519189 | 1.31283204014169 | 0.932273608209331 |
| ebi-fl187-GCST90094790 | finngen_R11_O15_POOR_FETGRO | Poor fetal growth \|\| id:finngen_R11_O15_POOR_FETGRO | Lamb liking \|\| id:ebi-fl187-GCST90094790 | Weighted mode | 7 | -0.100891543 | 0.239595571231016 | 0.688355942574733 | -0.570498863 | 0.368715776364251 | 0.904031075842762 | 0.565243389418836 | 1.44587659296593 | 0.972577670507659 |
| ebi-fl187-GCST90094791 | finngen_R11_O15_POOR_FETGRO | Poor fetal growth \|\| id:finngen_R11_O15_POOR_FETGRO | F-acquired taste liking (derived food-liking factor) \|\| id:ebi-fl187-GCST90094791 | Inverse variance weighted | 35 | -0.083466523 | 0.0662760783463462 | 0.207894439686122 | -0.213367637 | 0.0464345901381007 | 0.91992188199357 | 0.807859083742443 | 1.04752955806386 | 0.888224550184161 |
| ebi-fl187-GCST90094791 | finngen_R11_O15_POOR_FETGRO | Poor fetal growth \|\| id:finngen_R11_O15_POOR_FETGRO | F-acquired taste liking (derived food-liking factor) \|\| id:ebi-fl187-GCST90094791 | MR Egger | 35 | -0.146630684 | 0.377057664780903 | 0.699861904696866 | -0.885663707 | 0.592402338799055 | 0.863612864428201 | 0.412440342816273 | 1.80832741654984 | 0.970850396659599 |
| ebi-fl187-GCST90094791 | finngen_R11_O15_POOR_FETGRO | Poor fetal growth \|\| id:finngen_R11_O15_POOR_FETGRO | F-acquired taste liking (derived food-liking factor) \|\| id:ebi-fl187-GCST90094791 | Weighted median | 35 | -0.098276179 | 0.0903921112130214 | 0.276939264593356 | -0.275444717 | 0.0788923594327467 | 0.906398541353013 | 0.759234404052108 | 1.08208783925245 | 0.918328271889597 |
| ebi-fl187-GCST90094791 | finngen_R11_O15_POOR_FETGRO | Poor fetal growth \|\| id:finngen_R11_O15_POOR_FETGRO | F-acquired taste liking (derived food-liking factor) \|\| id:ebi-fl187-GCST90094791 | Weighted mode | 35 | -0.139399931 | 0.163891858632201 | 0.400964033063839 | -0.460627974 | 0.181828111520941 | 0.869880066531189 | 0.630887340038476 | 1.1994080117413 | 0.972577670507659 |
| ebi-fl187-GCST90094792 | finngen_R11_O15_POOR_FETGRO | Poor fetal growth \|\| id:finngen_R11_O15_POOR_FETGRO | Lemon liking \|\| id:ebi-fl187-GCST90094792 | Inverse variance weighted | 5 | -0.221407745 | 0.257875180159701 | 0.390569551708022 | -0.726843098 | 0.284027607928818 | 0.801389850810819 | 0.483432733289887 | 1.32846960654086 | 0.888224550184161 |
| ebi-fl187-GCST90094792 | finngen_R11_O15_POOR_FETGRO | Poor fetal growth \|\| id:finngen_R11_O15_POOR_FETGRO | Lemon liking \|\| id:ebi-fl187-GCST90094792 | MR Egger | 5 | -1.590614456 | 2.86045325000774 | 0.616944790778977 | -7.197102826 | 4.015873914 | 0.203800346965788 | 0.000748751933878687 | 55.4717517832885 | 0.970850396659599 |
| ebi-fl187-GCST90094792 | finngen_R11_O15_POOR_FETGRO | Poor fetal growth \|\| id:finngen_R11_O15_POOR_FETGRO | Lemon liking \|\| id:ebi-fl187-GCST90094792 | Weighted median | 5 | -0.406351203 | 0.324049319794861 | 0.209849457530635 | -1.04148787 | 0.228785463497701 | 0.666076198182903 | 0.352929178343944 | 1.25707232217968 | 0.918328271889597 |
| ebi-fl187-GCST90094792 | finngen_R11_O15_POOR_FETGRO | Poor fetal growth \|\| id:finngen_R11_O15_POOR_FETGRO | Lemon liking \|\| id:ebi-fl187-GCST90094792 | Weighted mode | 5 | -0.465503201 | 0.446647834767826 | 0.356157196529239 | -1.340932957 | 0.409926554904649 | 0.627819106337815 | 0.261601491651079 | 1.50670712080088 | 0.972577670507659 |
| ebi-fl187-GCST90094793 | finngen_R11_O15_POOR_FETGRO | Poor fetal growth \|\| id:finngen_R11_O15_POOR_FETGRO | F-lentils/beans liking (derived food-liking factor) \|\| id:ebi-fl187-GCST90094793 | Inverse variance weighted | 2 | -0.285963699 | 0.41663901972866 | 0.49248711787555 | -1.102576178 | 0.53064877938924 | 0.751289887816546 | 0.332014652186669 | 1.70003489851422 | 0.914435393876931 |
| ebi-fl187-GCST90094794 | finngen_R11_O15_POOR_FETGRO | Poor fetal growth \|\| id:finngen_R11_O15_POOR_FETGRO | Liver liking \|\| id:ebi-fl187-GCST90094794 | Inverse variance weighted | 12 | -0.121584446 | 0.0974445802536353 | 0.212130250615475 | -0.312575824 | 0.0694069308619929 | 0.885516271499002 | 0.73156015722214 | 1.07187229833156 | 0.888224550184161 |
| ebi-fl187-GCST90094794 | finngen_R11_O15_POOR_FETGRO | Poor fetal growth \|\| id:finngen_R11_O15_POOR_FETGRO | Liver liking \|\| id:ebi-fl187-GCST90094794 | MR Egger | 12 | 0.265015972888545 | 0.505438236500099 | 0.611473722503487 | -0.725642971 | 1.25567491642874 | 1.30345179550235 | 0.484013262562813 | 3.51020667120212 | 0.970850396659599 |
| ebi-fl187-GCST90094794 | finngen_R11_O15_POOR_FETGRO | Poor fetal growth \|\| id:finngen_R11_O15_POOR_FETGRO | Liver liking \|\| id:ebi-fl187-GCST90094794 | Weighted median | 12 | -0.103154868 | 0.14476250599932 | 0.47610568575851 | -0.38688938 | 0.180579643926696 | 0.90198727385074 | 0.679166220416682 | 1.19791152405893 | 0.918328271889597 |
| ebi-fl187-GCST90094794 | finngen_R11_O15_POOR_FETGRO | Poor fetal growth \|\| id:finngen_R11_O15_POOR_FETGRO | Liver liking \|\| id:ebi-fl187-GCST90094794 | Weighted mode | 12 | -0.118711856 | 0.206730749551439 | 0.577363085737014 | -0.523904125 | 0.286480413003334 | 0.88806365400809 | 0.59220399030225 | 1.33173208300688 | 0.972577670507659 |
| ebi-fl187-GCST90094795 | finngen_R11_O15_POOR_FETGRO | Poor fetal growth \|\| id:finngen_R11_O15_POOR_FETGRO | F-low caloric food liking (derived food-liking factor) \|\| id:ebi-fl187-GCST90094795 | Inverse variance weighted | 7 | -0.08998732 | 0.095654376509758 | 0.346830498290387 | -0.277469898 | 0.0974952583920632 | 0.91394277438781 | 0.757698372855106 | 1.10240621437288 | 0.888224550184161 |
| ebi-fl187-GCST90094795 | finngen_R11_O15_POOR_FETGRO | Poor fetal growth \|\| id:finngen_R11_O15_POOR_FETGRO | F-low caloric food liking (derived food-liking factor) \|\| id:ebi-fl187-GCST90094795 | MR Egger | 7 | -0.769023922 | 0.612466834259792 | 0.264736061106081 | -1.969458917 | 0.431411073532923 | 0.463465225993362 | 0.139532334400039 | 1.5394282381118 | 0.970850396659599 |
| ebi-fl187-GCST90094795 | finngen_R11_O15_POOR_FETGRO | Poor fetal growth \|\| id:finngen_R11_O15_POOR_FETGRO | F-low caloric food liking (derived food-liking factor) \|\| id:ebi-fl187-GCST90094795 | Weighted median | 7 | -0.119864456 | 0.125460616891305 | 0.339377942691867 | -0.365767265 | 0.126038353381818 | 0.887040661852341 | 0.693664222484112 | 1.13432567267438 | 0.918328271889597 |
| ebi-fl187-GCST90094795 | finngen_R11_O15_POOR_FETGRO | Poor fetal growth \|\| id:finngen_R11_O15_POOR_FETGRO | F-low caloric food liking (derived food-liking factor) \|\| id:ebi-fl187-GCST90094795 | Weighted mode | 7 | -0.137840651 | 0.163636608355664 | 0.431876589430935 | -0.458568403 | 0.182887101514668 | 0.871237511631184 | 0.632188036335798 | 1.20067884560554 | 0.972577670507659 |
| ebi-fl187-GCST90094796 | finngen_R11_O15_POOR_FETGRO | Poor fetal growth \|\| id:finngen_R11_O15_POOR_FETGRO | Mackerel liking \|\| id:ebi-fl187-GCST90094796 | Inverse variance weighted | 14 | -0.165319373 | 0.0935382291121794 | 0.0771614293809612 | -0.348654302 | 0.0180155559470273 | 0.847622952873911 | 0.705637025297031 | 1.01817881500231 | 0.706473758635168 |
| ebi-fl187-GCST90094796 | finngen_R11_O15_POOR_FETGRO | Poor fetal growth \|\| id:finngen_R11_O15_POOR_FETGRO | Mackerel liking \|\| id:ebi-fl187-GCST90094796 | MR Egger | 14 | 0.0788684850280515 | 0.625845955402331 | 0.901803652475241 | -1.147789588 | 1.30552655761662 | 1.08206200535785 | 0.317337441334604 | 3.6896313858045 | 0.993285374120483 |
| ebi-fl187-GCST90094796 | finngen_R11_O15_POOR_FETGRO | Poor fetal growth \|\| id:finngen_R11_O15_POOR_FETGRO | Mackerel liking \|\| id:ebi-fl187-GCST90094796 | Weighted median | 14 | -0.10094082 | 0.128409967566273 | 0.431819195676173 | -0.352624356 | 0.150742716802628 | 0.903986529562637 | 0.702841161820279 | 1.16269747707187 | 0.918328271889597 |
| ebi-fl187-GCST90094796 | finngen_R11_O15_POOR_FETGRO | Poor fetal growth \|\| id:finngen_R11_O15_POOR_FETGRO | Mackerel liking \|\| id:ebi-fl187-GCST90094796 | Weighted mode | 14 | -0.078040674 | 0.182678438558916 | 0.676222533467108 | -0.436090414 | 0.280009065444012 | 0.924926805183968 | 0.646559265472719 | 1.32314180715104 | 0.972577670507659 |
| ebi-fl187-GCST90094797 | finngen_R11_O15_POOR_FETGRO | Poor fetal growth \|\| id:finngen_R11_O15_POOR_FETGRO | Marzipan liking \|\| id:ebi-fl187-GCST90094797 | Inverse variance weighted | 7 | 0.15352996394039 | 0.187301317731817 | 0.412389969728361 | -0.213580619 | 0.520640546694752 | 1.16594272283695 | 0.807687042754308 | 1.6831054120921 | 0.888224550184161 |
| ebi-fl187-GCST90094797 | finngen_R11_O15_POOR_FETGRO | Poor fetal growth \|\| id:finngen_R11_O15_POOR_FETGRO | Marzipan liking \|\| id:ebi-fl187-GCST90094797 | MR Egger | 7 | -0.18486648 | 1.96845513982065 | 0.928824363749796 | -4.043038554 | 3.67330559408777 | 0.831215260337387 | 0.0175440827001084 | 39.3818714165935 | 0.993285374120483 |
| ebi-fl187-GCST90094797 | finngen_R11_O15_POOR_FETGRO | Poor fetal growth \|\| id:finngen_R11_O15_POOR_FETGRO | Marzipan liking \|\| id:ebi-fl187-GCST90094797 | Weighted median | 7 | 0.14552607518284 | 0.205250147486155 | 0.478313227871327 | -0.256764214 | 0.547816364255703 | 1.1566478939943 | 0.77355058474758 | 1.7294723539224 | 0.918328271889597 |
| ebi-fl187-GCST90094797 | finngen_R11_O15_POOR_FETGRO | Poor fetal growth \|\| id:finngen_R11_O15_POOR_FETGRO | Marzipan liking \|\| id:ebi-fl187-GCST90094797 | Weighted mode | 7 | 0.0838740621460119 | 0.300980026490714 | 0.789853485097307 | -0.50604679 | 0.673794914067812 | 1.08749192878473 | 0.602874162497009 | 1.96166757300333 | 0.972577670507659 |
| ebi-fl187-GCST90094798 | finngen_R11_O15_POOR_FETGRO | Poor fetal growth \|\| id:finngen_R11_O15_POOR_FETGRO | Mayonnaise liking \|\| id:ebi-fl187-GCST90094798 | Inverse variance weighted | 10 | -0.207250168 | 0.150047246383736 | 0.167207413530628 | -0.501342771 | 0.0868424348133425 | 0.812816283846384 | 0.605716774478585 | 1.09072480592033 | 0.888224550184161 |
| ebi-fl187-GCST90094798 | finngen_R11_O15_POOR_FETGRO | Poor fetal growth \|\| id:finngen_R11_O15_POOR_FETGRO | Mayonnaise liking \|\| id:ebi-fl187-GCST90094798 | MR Egger | 10 | -0.173376675 | 0.829169313841464 | 0.839598770914738 | -1.79854853 | 1.45179517979033 | 0.840820838713081 | 0.165538988728612 | 4.27077444560935 | 0.988143168845807 |
| ebi-fl187-GCST90094798 | finngen_R11_O15_POOR_FETGRO | Poor fetal growth \|\| id:finngen_R11_O15_POOR_FETGRO | Mayonnaise liking \|\| id:ebi-fl187-GCST90094798 | Weighted median | 10 | 0.0141758301974474 | 0.182946326722615 | 0.938236705865968 | -0.34439897 | 0.372750630573773 | 1.01427678374774 | 0.708646142963434 | 1.45172227953938 | 0.977210005210024 |
| ebi-fl187-GCST90094798 | finngen_R11_O15_POOR_FETGRO | Poor fetal growth \|\| id:finngen_R11_O15_POOR_FETGRO | Mayonnaise liking \|\| id:ebi-fl187-GCST90094798 | Weighted mode | 10 | 0.0989496493217399 | 0.23280319798893 | 0.680792180442132 | -0.357344619 | 0.555243917380042 | 1.10401071047134 | 0.699531384569004 | 1.74236592627847 | 0.972577670507659 |
| ebi-fl187-GCST90094799 | finngen_R11_O15_POOR_FETGRO | Poor fetal growth \|\| id:finngen_R11_O15_POOR_FETGRO | F-meat liking (derived food-liking factor) \|\| id:ebi-fl187-GCST90094799 | Inverse variance weighted | 10 | 0.0576731394924296 | 0.080798231093764 | 0.475355742015162 | -0.100691393 | 0.216037672436207 | 1.05936867333155 | 0.904212035588172 | 1.24114913523158 | 0.914435393876931 |
| ebi-fl187-GCST90094799 | finngen_R11_O15_POOR_FETGRO | Poor fetal growth \|\| id:finngen_R11_O15_POOR_FETGRO | F-meat liking (derived food-liking factor) \|\| id:ebi-fl187-GCST90094799 | MR Egger | 10 | 0.00719023243142444 | 0.255270499679505 | 0.978218851825416 | -0.493139947 | 0.507520411803254 | 1.00721614421933 | 0.610705796677492 | 1.66116707372895 | 0.993285374120483 |
| ebi-fl187-GCST90094799 | finngen_R11_O15_POOR_FETGRO | Poor fetal growth \|\| id:finngen_R11_O15_POOR_FETGRO | F-meat liking (derived food-liking factor) \|\| id:ebi-fl187-GCST90094799 | Weighted median | 10 | 0.00812196130073717 | 0.105937447352948 | 0.938888044221395 | -0.199515436 | 0.215759358112516 | 1.00815503390593 | 0.819127577062582 | 1.24080375371395 | 0.977210005210024 |
| ebi-fl187-GCST90094799 | finngen_R11_O15_POOR_FETGRO | Poor fetal growth \|\| id:finngen_R11_O15_POOR_FETGRO | F-meat liking (derived food-liking factor) \|\| id:ebi-fl187-GCST90094799 | Weighted mode | 10 | -0.03654736 | 0.146485907786706 | 0.808579704324775 | -0.32365974 | 0.250565018797007 | 0.964112432002017 | 0.723496377577488 | 1.28475112018276 | 0.97297033017346 |
| ebi-fl187-GCST90094800 | finngen_R11_O15_POOR_FETGRO | Poor fetal growth \|\| id:finngen_R11_O15_POOR_FETGRO | Melon liking \|\| id:ebi-fl187-GCST90094800 | Inverse variance weighted | 2 | -0.448441007 | 0.456867987431763 | 0.326319180706547 | -1.343902262 | 0.4470202486666 | 0.638622984905983 | 0.260825869216982 | 1.5636459607116 | 0.888224550184161 |
| ebi-fl187-GCST90094801 | finngen_R11_O15_POOR_FETGRO | Poor fetal growth \|\| id:finngen_R11_O15_POOR_FETGRO | Milk chocolate liking \|\| id:ebi-fl187-GCST90094801 | Inverse variance weighted | 5 | 0.00542663283735854 | 0.196515349860835 | 0.977969780275595 | -0.379743453 | 0.390596718564594 | 1.00544138367973 | 0.684036874387193 | 1.47786239875074 | 0.994304114950957 |
| ebi-fl187-GCST90094801 | finngen_R11_O15_POOR_FETGRO | Poor fetal growth \|\| id:finngen_R11_O15_POOR_FETGRO | Milk chocolate liking \|\| id:ebi-fl187-GCST90094801 | MR Egger | 5 | -0.067446786 | 1.44334846794561 | 0.96566564440076 | -2.896409783 | 2.76151621097472 | 0.934777462354699 | 0.0552211203759119 | 15.8238170138152 | 0.993285374120483 |
| ebi-fl187-GCST90094801 | finngen_R11_O15_POOR_FETGRO | Poor fetal growth \|\| id:finngen_R11_O15_POOR_FETGRO | Milk chocolate liking \|\| id:ebi-fl187-GCST90094801 | Weighted median | 5 | 0.098729792718768 | 0.240355475529928 | 0.681244323242313 | -0.372366939 | 0.569826524757427 | 1.10376801310718 | 0.689101337750497 | 1.76796032748333 | 0.918328271889597 |
| ebi-fl187-GCST90094801 | finngen_R11_O15_POOR_FETGRO | Poor fetal growth \|\| id:finngen_R11_O15_POOR_FETGRO | Milk chocolate liking \|\| id:ebi-fl187-GCST90094801 | Weighted mode | 5 | 0.331903337170904 | 0.401369259421621 | 0.454753667655717 | -0.454780411 | 1.11858708563728 | 1.39361813093031 | 0.634587300853005 | 3.06052688455482 | 0.972577670507659 |
| ebi-fl187-GCST90094802 | finngen_R11_O15_POOR_FETGRO | Poor fetal growth \|\| id:finngen_R11_O15_POOR_FETGRO | Milky sweets liking \|\| id:ebi-fl187-GCST90094802 | Inverse variance weighted | 10 | -0.046142591 | 0.07681610061559 | 0.548047107099449 | -0.196702148 | 0.104416965950598 | 0.954905791266455 | 0.82143526263516 | 1.11006321699536 | 0.927237411635561 |
| ebi-fl187-GCST90094802 | finngen_R11_O15_POOR_FETGRO | Poor fetal growth \|\| id:finngen_R11_O15_POOR_FETGRO | Milky sweets liking \|\| id:ebi-fl187-GCST90094802 | MR Egger | 10 | 0.501462697057045 | 0.463226910779454 | 0.31055867219707 | -0.406462048 | 1.40938744218478 | 1.65113461501621 | 0.666002371211349 | 4.09344716287741 | 0.970850396659599 |
| ebi-fl187-GCST90094802 | finngen_R11_O15_POOR_FETGRO | Poor fetal growth \|\| id:finngen_R11_O15_POOR_FETGRO | Milky sweets liking \|\| id:ebi-fl187-GCST90094802 | Weighted median | 10 | -0.08389428 | 0.109546259190178 | 0.443774918137034 | -0.298604948 | 0.130816388192604 | 0.919528463671194 | 0.741852421960552 | 1.13975848898215 | 0.918328271889597 |
| ebi-fl187-GCST90094802 | finngen_R11_O15_POOR_FETGRO | Poor fetal growth \|\| id:finngen_R11_O15_POOR_FETGRO | Milky sweets liking \|\| id:ebi-fl187-GCST90094802 | Weighted mode | 10 | -0.214396343 | 0.191657627264066 | 0.292259985814598 | -0.590045293 | 0.161252606106386 | 0.807028461278346 | 0.554302178285562 | 1.17498173889146 | 0.972577670507659 |
| ebi-fl187-GCST90094803 | finngen_R11_O15_POOR_FETGRO | Poor fetal growth \|\| id:finngen_R11_O15_POOR_FETGRO | Mushroom liking \|\| id:ebi-fl187-GCST90094803 | Inverse variance weighted | 7 | -0.622949593 | 0.232535515984984 | 0.00738547752910677 | -1.078719204 | -0.167179981 | 0.536360058356539 | 0.340030756863456 | 0.846047324818185 | 0.392515819825973 |
| ebi-fl187-GCST90094803 | finngen_R11_O15_POOR_FETGRO | Poor fetal growth \|\| id:finngen_R11_O15_POOR_FETGRO | Mushroom liking \|\| id:ebi-fl187-GCST90094803 | MR Egger | 7 | 1.07291826815674 | 0.802694054065313 | 0.238931118126677 | -0.500362078 | 2.64619861412475 | 2.92389978532854 | 0.606311088172285 | 14.1003358200419 | 0.970850396659599 |
| ebi-fl187-GCST90094803 | finngen_R11_O15_POOR_FETGRO | Poor fetal growth \|\| id:finngen_R11_O15_POOR_FETGRO | Mushroom liking \|\| id:ebi-fl187-GCST90094803 | Weighted median | 7 | -0.439425527 | 0.251507477224746 | 0.0806083999413952 | -0.932380182 | 0.0535291282977953 | 0.644406508870216 | 0.3936157173053 | 1.05498772131656 | 0.918328271889597 |
| ebi-fl187-GCST90094803 | finngen_R11_O15_POOR_FETGRO | Poor fetal growth \|\| id:finngen_R11_O15_POOR_FETGRO | Mushroom liking \|\| id:ebi-fl187-GCST90094803 | Weighted mode | 7 | -0.342010317 | 0.287820233283147 | 0.279621579953556 | -0.906137974 | 0.222117340149949 | 0.710340876024161 | 0.404081788665029 | 1.24871789401293 | 0.972577670507659 |
| ebi-fl187-GCST90094804 | finngen_R11_O15_POOR_FETGRO | Poor fetal growth \|\| id:finngen_R11_O15_POOR_FETGRO | F-oily fish liking (derived food-liking factor) \|\| id:ebi-fl187-GCST90094804 | Inverse variance weighted | 12 | 0.0739543004903543 | 0.0790531220875371 | 0.349530000602317 | -0.080989819 | 0.228898419781927 | 1.07675759707763 | 0.922203080529692 | 1.25721432441807 | 0.888224550184161 |
| ebi-fl187-GCST90094804 | finngen_R11_O15_POOR_FETGRO | Poor fetal growth \|\| id:finngen_R11_O15_POOR_FETGRO | F-oily fish liking (derived food-liking factor) \|\| id:ebi-fl187-GCST90094804 | MR Egger | 12 | -0.445013341 | 0.57329524824758 | 0.455575850660843 | -1.568672028 | 0.678645345368708 | 0.640815726726728 | 0.208321644109618 | 1.97120561992217 | 0.970850396659599 |
| ebi-fl187-GCST90094804 | finngen_R11_O15_POOR_FETGRO | Poor fetal growth \|\| id:finngen_R11_O15_POOR_FETGRO | F-oily fish liking (derived food-liking factor) \|\| id:ebi-fl187-GCST90094804 | Weighted median | 12 | 0.0916536619879516 | 0.100219000211381 | 0.360436431667068 | -0.104775578 | 0.288082902402259 | 1.09598517501476 | 0.900526597506553 | 1.33386788039138 | 0.918328271889597 |
| ebi-fl187-GCST90094804 | finngen_R11_O15_POOR_FETGRO | Poor fetal growth \|\| id:finngen_R11_O15_POOR_FETGRO | F-oily fish liking (derived food-liking factor) \|\| id:ebi-fl187-GCST90094804 | Weighted mode | 12 | 0.106074069 | 0.140700423432153 | 0.466740851831338 | -0.16969876 | 0.38184689939802 | 1.11190423161473 | 0.843919000084144 | 1.46498777745196 | 0.972577670507659 |
| ebi-fl187-GCST90094805 | finngen_R11_O15_POOR_FETGRO | Poor fetal growth \|\| id:finngen_R11_O15_POOR_FETGRO | Onion liking \|\| id:ebi-fl187-GCST90094805 | Inverse variance weighted (multiplicative random effects) | 5 | -0.047435451 | 0.422189249596262 | 0.910541224336441 | -0.87492638 | 0.78005547811384 | 0.953672029631302 | 0.41689271006357 | 2.18159329282204 | 0.994304114950957 |
| ebi-fl187-GCST90094805 | finngen_R11_O15_POOR_FETGRO | Poor fetal growth \|\| id:finngen_R11_O15_POOR_FETGRO | Onion liking \|\| id:ebi-fl187-GCST90094805 | MR Egger | 5 | 0.35476264338844 | 1.14021674411862 | 0.776063831743944 | -1.880062175 | 2.58958746186095 | 1.42584218104493 | 0.152580618749162 | 13.3242736981503 | 0.970850396659599 |
| ebi-fl187-GCST90094805 | finngen_R11_O15_POOR_FETGRO | Poor fetal growth \|\| id:finngen_R11_O15_POOR_FETGRO | Onion liking \|\| id:ebi-fl187-GCST90094805 | Weighted median | 5 | 0.0229839518318904 | 0.225921604889521 | 0.918967661985409 | -0.419822394 | 0.465790297415351 | 1.02325011812554 | 0.657163525799316 | 1.59327285087893 | 0.969669326094948 |
| ebi-fl187-GCST90094805 | finngen_R11_O15_POOR_FETGRO | Poor fetal growth \|\| id:finngen_R11_O15_POOR_FETGRO | Onion liking \|\| id:ebi-fl187-GCST90094805 | Weighted mode | 5 | 0.0770629803545564 | 0.239668772022733 | 0.763902448266092 | -0.392687813 | 0.546813773519114 | 1.08011009996756 | 0.675239515811869 | 1.72773926989334 | 0.972577670507659 |
| ebi-fl187-GCST90094806 | finngen_R11_O15_POOR_FETGRO | Poor fetal growth \|\| id:finngen_R11_O15_POOR_FETGRO | Orange juice liking \|\| id:ebi-fl187-GCST90094806 | Inverse variance weighted | 3 | -0.072987516 | 0.270116607316405 | 0.787000672 | -0.602416066 | 0.456441034368605 | 0.929612435261442 | 0.547487271309394 | 1.57844634036131 | 0.994304114950957 |
| ebi-fl187-GCST90094806 | finngen_R11_O15_POOR_FETGRO | Poor fetal growth \|\| id:finngen_R11_O15_POOR_FETGRO | Orange juice liking \|\| id:ebi-fl187-GCST90094806 | MR Egger | 3 | -1.769561136 | 1.11336938177156 | 0.357524556594394 | -3.951765124 | 0.412642852457199 | 0.170407758279335 | 0.0192207448154293 | 1.51080534914951 | 0.970850396659599 |
| ebi-fl187-GCST90094806 | finngen_R11_O15_POOR_FETGRO | Poor fetal growth \|\| id:finngen_R11_O15_POOR_FETGRO | Orange juice liking \|\| id:ebi-fl187-GCST90094806 | Weighted median | 3 | -0.133411742 | 0.266118743049086 | 0.61614304085488 | -0.655004478 | 0.388180994743277 | 0.8751047008813 | 0.519439736525953 | 1.47429660007593 | 0.918328271889597 |
| ebi-fl187-GCST90094806 | finngen_R11_O15_POOR_FETGRO | Poor fetal growth \|\| id:finngen_R11_O15_POOR_FETGRO | Orange juice liking \|\| id:ebi-fl187-GCST90094806 | Weighted mode | 3 | -0.284172833 | 0.300729258153643 | 0.444430396112541 | -0.873602179 | 0.305256513385257 | 0.752636553335235 | 0.417445125784198 | 1.35697304011445 | 0.972577670507659 |
| ebi-fl187-GCST90094807 | finngen_R11_O15_POOR_FETGRO | Poor fetal growth \|\| id:finngen_R11_O15_POOR_FETGRO | Oranges liking \|\| id:ebi-fl187-GCST90094807 | Inverse variance weighted | 7 | -0.228454137 | 0.206031672622385 | 0.267503448216561 | -0.632276215 | 0.175367941646535 | 0.795762792680567 | 0.531380886216932 | 1.1916846063527 | 0.888224550184161 |
| ebi-fl187-GCST90094807 | finngen_R11_O15_POOR_FETGRO | Poor fetal growth \|\| id:finngen_R11_O15_POOR_FETGRO | Oranges liking \|\| id:ebi-fl187-GCST90094807 | MR Egger | 7 | 2.28611583693788 | 1.47606907034415 | 0.182116150887356 | -0.606979541 | 5.17921121481241 | 9.83665620686396 | 0.544994519170882 | 177.542712684962 | 0.970850396659599 |
| ebi-fl187-GCST90094807 | finngen_R11_O15_POOR_FETGRO | Poor fetal growth \|\| id:finngen_R11_O15_POOR_FETGRO | Oranges liking \|\| id:ebi-fl187-GCST90094807 | Weighted median | 7 | -0.102289898 | 0.257276237890483 | 0.690934040640405 | -0.606551325 | 0.401971527860645 | 0.902767802784575 | 0.54522794466393 | 1.49476877280536 | 0.918328271889597 |
| ebi-fl187-GCST90094807 | finngen_R11_O15_POOR_FETGRO | Poor fetal growth \|\| id:finngen_R11_O15_POOR_FETGRO | Oranges liking \|\| id:ebi-fl187-GCST90094807 | Weighted mode | 7 | -0.041791619 | 0.312917955018425 | 0.898121737631427 | -0.65511081 | 0.571527573227824 | 0.959069612011684 | 0.519384506170238 | 1.77097027299994 | 0.988620775239461 |
| ebi-fl187-GCST90094809 | finngen_R11_O15_POOR_FETGRO | Poor fetal growth \|\| id:finngen_R11_O15_POOR_FETGRO | Pears liking \|\| id:ebi-fl187-GCST90094809 | Inverse variance weighted | 4 | -0.125142278 | 0.286795139998181 | 0.66258478658462 | -0.687260752 | 0.4369761964833 | 0.882371351698719 | 0.502951893656698 | 1.54801922831622 | 0.96571624475698 |
| ebi-fl187-GCST90094809 | finngen_R11_O15_POOR_FETGRO | Poor fetal growth \|\| id:finngen_R11_O15_POOR_FETGRO | Pears liking \|\| id:ebi-fl187-GCST90094809 | MR Egger | 4 | 3.08810287315769 | 6.41094765205887 | 0.677581629186219 | -9.477354525 | 15.6535602711931 | 21.9354241949658 | 7.65662235046489e-05 | 6284270.17278545 | 0.970850396659599 |
| ebi-fl187-GCST90094809 | finngen_R11_O15_POOR_FETGRO | Poor fetal growth \|\| id:finngen_R11_O15_POOR_FETGRO | Pears liking \|\| id:ebi-fl187-GCST90094809 | Weighted median | 4 | -0.213576501 | 0.329724277665776 | 0.517151367211496 | -0.859836085 | 0.432683083020853 | 0.807690368501321 | 0.423231450434007 | 1.5413876513686 | 0.918328271889597 |
| ebi-fl187-GCST90094809 | finngen_R11_O15_POOR_FETGRO | Poor fetal growth \|\| id:finngen_R11_O15_POOR_FETGRO | Pears liking \|\| id:ebi-fl187-GCST90094809 | Weighted mode | 4 | -0.238635047 | 0.431060325787966 | 0.618438668502058 | -1.083513285 | 0.606243191952398 | 0.787702304562171 | 0.338404523123084 | 1.83353022260544 | 0.972577670507659 |
| ebi-fl187-GCST90094810 | finngen_R11_O15_POOR_FETGRO | Poor fetal growth \|\| id:finngen_R11_O15_POOR_FETGRO | Pizza liking \|\| id:ebi-fl187-GCST90094810 | Inverse variance weighted | 3 | -0.219975817 | 0.26537465497332 | 0.407146567826172 | -0.740110141 | 0.300158506530152 | 0.80253820533464 | 0.477061368627676 | 1.35007278596983 | 0.888224550184161 |
| ebi-fl187-GCST90094810 | finngen_R11_O15_POOR_FETGRO | Poor fetal growth \|\| id:finngen_R11_O15_POOR_FETGRO | Pizza liking \|\| id:ebi-fl187-GCST90094810 | MR Egger | 3 | 1.33865404693844 | 3.08394236019458 | 0.73928521354019 | -4.705872979 | 7.38318107291982 | 3.8139067097864 | 0.00904201727988237 | 1608.69902597034 | 0.970850396659599 |
| ebi-fl187-GCST90094810 | finngen_R11_O15_POOR_FETGRO | Poor fetal growth \|\| id:finngen_R11_O15_POOR_FETGRO | Pizza liking \|\| id:ebi-fl187-GCST90094810 | Weighted median | 3 | -0.264576298 | 0.310707254766469 | 0.394475453332093 | -0.873562518 | 0.344409921173046 | 0.767531085416813 | 0.417461682431006 | 1.41115698008637 | 0.918328271889597 |
| ebi-fl187-GCST90094810 | finngen_R11_O15_POOR_FETGRO | Poor fetal growth \|\| id:finngen_R11_O15_POOR_FETGRO | Pizza liking \|\| id:ebi-fl187-GCST90094810 | Weighted mode | 3 | -0.514544895 | 0.424047070542233 | 0.348827725660386 | -1.345677154 | 0.316587362951273 | 0.59777258182686 | 0.260363342185619 | 1.37243613707033 | 0.972577670507659 |
| ebi-fl187-GCST90094811 | finngen_R11_O15_POOR_FETGRO | Poor fetal growth \|\| id:finngen_R11_O15_POOR_FETGRO | Plain yoghurt liking \|\| id:ebi-fl187-GCST90094811 | Inverse variance weighted | 7 | -0.101858477 | 0.212581393657103 | 0.631831581140662 | -0.518518008 | 0.314801055062109 | 0.903157360610154 | 0.595402275817682 | 1.36998673191849 | 0.96571624475698 |
| ebi-fl187-GCST90094811 | finngen_R11_O15_POOR_FETGRO | Poor fetal growth \|\| id:finngen_R11_O15_POOR_FETGRO | Plain yoghurt liking \|\| id:ebi-fl187-GCST90094811 | MR Egger | 7 | 1.16968513420851 | 1.94986707359562 | 0.574734600088051 | -2.65205433 | 4.99142459845593 | 3.22097830296436 | 0.0705062211327826 | 147.145898070878 | 0.970850396659599 |
| ebi-fl187-GCST90094811 | finngen_R11_O15_POOR_FETGRO | Poor fetal growth \|\| id:finngen_R11_O15_POOR_FETGRO | Plain yoghurt liking \|\| id:ebi-fl187-GCST90094811 | Weighted median | 7 | -0.109426627 | 0.221604166060549 | 0.621452246594654 | -0.543770792 | 0.324917538499895 | 0.896347929705044 | 0.580554967469266 | 1.383916521 | 0.918328271889597 |
| ebi-fl187-GCST90094811 | finngen_R11_O15_POOR_FETGRO | Poor fetal growth \|\| id:finngen_R11_O15_POOR_FETGRO | Plain yoghurt liking \|\| id:ebi-fl187-GCST90094811 | Weighted mode | 7 | -0.322508303 | 0.388400498708554 | 0.438125358634265 | -1.083773281 | 0.438756674267347 | 0.724329917524747 | 0.338316550895157 | 1.55077789730717 | 0.972577670507659 |
| ebi-fl187-GCST90094812 | finngen_R11_O15_POOR_FETGRO | Poor fetal growth \|\| id:finngen_R11_O15_POOR_FETGRO | Plum liking \|\| id:ebi-fl187-GCST90094812 | Inverse variance weighted | 5 | -0.192740165 | 0.221356371804103 | 0.383905216897134 | -0.626598654 | 0.241118323493102 | 0.824696231047049 | 0.534406414324511 | 1.27267161335046 | 0.888224550184161 |
| ebi-fl187-GCST90094812 | finngen_R11_O15_POOR_FETGRO | Poor fetal growth \|\| id:finngen_R11_O15_POOR_FETGRO | Plum liking \|\| id:ebi-fl187-GCST90094812 | MR Egger | 5 | -0.580124719 | 0.944597276010981 | 0.582550840634878 | -2.43153538 | 1.27128594202089 | 0.55982854097744 | 0.0879017663110099 | 3.56543455775443 | 0.970850396659599 |
| ebi-fl187-GCST90094812 | finngen_R11_O15_POOR_FETGRO | Poor fetal growth \|\| id:finngen_R11_O15_POOR_FETGRO | Plum liking \|\| id:ebi-fl187-GCST90094812 | Weighted median | 5 | -0.279313974 | 0.265228919571817 | 0.292292746784508 | -0.799162656 | 0.240534708814251 | 0.756302406984276 | 0.4497053646374 | 1.27192908021324 | 0.918328271889597 |
| ebi-fl187-GCST90094812 | finngen_R11_O15_POOR_FETGRO | Poor fetal growth \|\| id:finngen_R11_O15_POOR_FETGRO | Plum liking \|\| id:ebi-fl187-GCST90094812 | Weighted mode | 5 | -0.445462445 | 0.300495499395279 | 0.212364711502074 | -1.034433624 | 0.143508734069768 | 0.640527998724635 | 0.355427629763992 | 1.15431689264735 | 0.972577670507659 |
| ebi-fl187-GCST90094813 | finngen_R11_O15_POOR_FETGRO | Poor fetal growth \|\| id:finngen_R11_O15_POOR_FETGRO | Pollock liking \|\| id:ebi-fl187-GCST90094813 | Inverse variance weighted | 4 | 0.181489799239263 | 0.276546119261734 | 0.511648375145426 | -0.360540595 | 0.723520192992261 | 1.19900230591169 | 0.697299268003738 | 2.06167795600473 | 0.914435393876931 |
| ebi-fl187-GCST90094813 | finngen_R11_O15_POOR_FETGRO | Poor fetal growth \|\| id:finngen_R11_O15_POOR_FETGRO | Pollock liking \|\| id:ebi-fl187-GCST90094813 | MR Egger | 4 | -4.001878854 | 3.03988786247293 | 0.318643997322701 | -9.960059064 | 1.95630135662962 | 0.0182812587883967 | 4.72499451787047e-05 | 7.07311768562549 | 0.970850396659599 |
| ebi-fl187-GCST90094813 | finngen_R11_O15_POOR_FETGRO | Poor fetal growth \|\| id:finngen_R11_O15_POOR_FETGRO | Pollock liking \|\| id:ebi-fl187-GCST90094813 | Weighted median | 4 | 0.100967259555024 | 0.264800581986744 | 0.70298402251928 | -0.418041881 | 0.619976400249041 | 1.10624042236683 | 0.658334656042216 | 1.85888417212516 | 0.918328271889597 |
| ebi-fl187-GCST90094813 | finngen_R11_O15_POOR_FETGRO | Poor fetal growth \|\| id:finngen_R11_O15_POOR_FETGRO | Pollock liking \|\| id:ebi-fl187-GCST90094813 | Weighted mode | 4 | -0.150986386 | 0.422577112498613 | 0.744524674846215 | -0.979237527 | 0.677264754132827 | 0.859859404392079 | 0.375597372606922 | 1.96848606844561 | 0.972577670507659 |
| ebi-fl187-GCST90094814 | finngen_R11_O15_POOR_FETGRO | Poor fetal growth \|\| id:finngen_R11_O15_POOR_FETGRO | Pork chop liking \|\| id:ebi-fl187-GCST90094814 | Inverse variance weighted | 9 | -0.088853482 | 0.130289598505019 | 0.495258482812842 | -0.344221095 | 0.166514131381916 | 0.914979625023961 | 0.708772204905867 | 1.18118022746134 | 0.914435393876931 |
| ebi-fl187-GCST90094814 | finngen_R11_O15_POOR_FETGRO | Poor fetal growth \|\| id:finngen_R11_O15_POOR_FETGRO | Pork chop liking \|\| id:ebi-fl187-GCST90094814 | MR Egger | 9 | -0.066897659 | 0.640353275983732 | 0.91972677578609 | -1.32199008 | 1.18819476149127 | 0.935290914637933 | 0.266604209879865 | 3.28115259469624 | 0.993285374120483 |
| ebi-fl187-GCST90094814 | finngen_R11_O15_POOR_FETGRO | Poor fetal growth \|\| id:finngen_R11_O15_POOR_FETGRO | Pork chop liking \|\| id:ebi-fl187-GCST90094814 | Weighted median | 9 | -0.112464059 | 0.177580169236837 | 0.526528422953293 | -0.460521191 | 0.235593072797392 | 0.893629464551207 | 0.630954712123944 | 1.26565917421535 | 0.918328271889597 |
| ebi-fl187-GCST90094814 | finngen_R11_O15_POOR_FETGRO | Poor fetal growth \|\| id:finngen_R11_O15_POOR_FETGRO | Pork chop liking \|\| id:ebi-fl187-GCST90094814 | Weighted mode | 9 | -0.13301791 | 0.24599603618851 | 0.603417758416005 | -0.615170141 | 0.349134321257081 | 0.87544941295618 | 0.540548918166734 | 1.41783962355266 | 0.972577670507659 |
| ebi-fl187-GCST90094815 | finngen_R11_O15_POOR_FETGRO | Poor fetal growth \|\| id:finngen_R11_O15_POOR_FETGRO | Porridge liking \|\| id:ebi-fl187-GCST90094815 | Inverse variance weighted | 9 | -0.006368261 | 0.12496984012801 | 0.959358679924578 | -0.251309148 | 0.238572625193312 | 0.993651972943971 | 0.777781884589392 | 1.26943589571604 | 0.994304114950957 |
| ebi-fl187-GCST90094815 | finngen_R11_O15_POOR_FETGRO | Poor fetal growth \|\| id:finngen_R11_O15_POOR_FETGRO | Porridge liking \|\| id:ebi-fl187-GCST90094815 | MR Egger | 9 | 0.663036688489398 | 0.438546225760187 | 0.174317299633162 | -0.196513914 | 1.52258729097937 | 1.94067662587155 | 0.821589899613393 | 4.58407018876014 | 0.970850396659599 |
| ebi-fl187-GCST90094815 | finngen_R11_O15_POOR_FETGRO | Poor fetal growth \|\| id:finngen_R11_O15_POOR_FETGRO | Porridge liking \|\| id:ebi-fl187-GCST90094815 | Weighted median | 9 | 0.182600900946796 | 0.167798349132067 | 0.276499624469822 | -0.146283863 | 0.511485665245647 | 1.20033525980766 | 0.863912435295242 | 1.66776709892495 | 0.918328271889597 |
| ebi-fl187-GCST90094815 | finngen_R11_O15_POOR_FETGRO | Poor fetal growth \|\| id:finngen_R11_O15_POOR_FETGRO | Porridge liking \|\| id:ebi-fl187-GCST90094815 | Weighted mode | 9 | 0.154692561707452 | 0.182553732049538 | 0.421408705040627 | -0.203112753 | 0.512497876524545 | 1.16729903351215 | 0.816186208704141 | 1.66945608625486 | 0.972577670507659 |
| ebi-fl187-GCST90094816 | finngen_R11_O15_POOR_FETGRO | Poor fetal growth \|\| id:finngen_R11_O15_POOR_FETGRO | Potatoes liking \|\| id:ebi-fl187-GCST90094816 | Inverse variance weighted | 2 | 0.179022818599168 | 0.498610262755351 | 0.719562480508813 | -0.798253296 | 1.15629893359966 | 1.19604803599722 | 0.450114494480473 | 3.17814894200184 | 0.96571624475698 |
| ebi-fl187-GCST90094817 | finngen_R11_O15_POOR_FETGRO | Poor fetal growth \|\| id:finngen_R11_O15_POOR_FETGRO | Prawns liking \|\| id:ebi-fl187-GCST90094817 | Inverse variance weighted | 20 | 0.0276923827285034 | 0.0787674338879325 | 0.725159662526093 | -0.126691788 | 0.182076553148851 | 1.02807938079969 | 0.881005167392128 | 1.19970603163901 | 0.96571624475698 |
| ebi-fl187-GCST90094817 | finngen_R11_O15_POOR_FETGRO | Poor fetal growth \|\| id:finngen_R11_O15_POOR_FETGRO | Prawns liking \|\| id:ebi-fl187-GCST90094817 | MR Egger | 20 | -0.187351692 | 0.259932333869876 | 0.480307478975781 | -0.696819067 | 0.322115682029739 | 0.829152078654137 | 0.498167423475106 | 1.38004441306233 | 0.970850396659599 |
| ebi-fl187-GCST90094817 | finngen_R11_O15_POOR_FETGRO | Poor fetal growth \|\| id:finngen_R11_O15_POOR_FETGRO | Prawns liking \|\| id:ebi-fl187-GCST90094817 | Weighted median | 20 | -0.015201876 | 0.102897201980234 | 0.88254916051216 | -0.216880392 | 0.186476639444601 | 0.984913088787309 | 0.805026250870763 | 1.20499647237005 | 0.969669326094948 |
| ebi-fl187-GCST90094817 | finngen_R11_O15_POOR_FETGRO | Poor fetal growth \|\| id:finngen_R11_O15_POOR_FETGRO | Prawns liking \|\| id:ebi-fl187-GCST90094817 | Weighted mode | 20 | -0.065120521 | 0.137708830647884 | 0.641681857756963 | -0.335029829 | 0.204788786653596 | 0.936954533484961 | 0.7153167485033 | 1.22726582266509 | 0.972577670507659 |
| ebi-fl187-GCST90094818 | finngen_R11_O15_POOR_FETGRO | Poor fetal growth \|\| id:finngen_R11_O15_POOR_FETGRO | Raw carrot liking \|\| id:ebi-fl187-GCST90094818 | Inverse variance weighted (multiplicative random effects) | 12 | 0.294324131749121 | 0.213277896554035 | 0.167585728835545 | -0.123700545 | 0.712348808995029 | 1.34221888892307 | 0.883644412565764 | 2.03877433067319 | 0.888224550184161 |
| ebi-fl187-GCST90094818 | finngen_R11_O15_POOR_FETGRO | Poor fetal growth \|\| id:finngen_R11_O15_POOR_FETGRO | Raw carrot liking \|\| id:ebi-fl187-GCST90094818 | MR Egger | 12 | -2.105303857 | 1.57198086821612 | 0.210127227154558 | -5.186386358 | 0.975778645081997 | 0.121808656278507 | 0.00559217847558474 | 2.65323233318728 | 0.970850396659599 |
| ebi-fl187-GCST90094818 | finngen_R11_O15_POOR_FETGRO | Poor fetal growth \|\| id:finngen_R11_O15_POOR_FETGRO | Raw carrot liking \|\| id:ebi-fl187-GCST90094818 | Weighted median | 12 | 0.188000418762795 | 0.232577391196956 | 0.418897825499141 | -0.267851268 | 0.643852105508829 | 1.20683402072669 | 0.765021555840548 | 1.90380041250355 | 0.918328271889597 |
| ebi-fl187-GCST90094818 | finngen_R11_O15_POOR_FETGRO | Poor fetal growth \|\| id:finngen_R11_O15_POOR_FETGRO | Raw carrot liking \|\| id:ebi-fl187-GCST90094818 | Weighted mode | 12 | 0.209266906086558 | 0.384581792725358 | 0.597200899344741 | -0.544513408 | 0.963047219828259 | 1.2327739895046 | 0.580123998569164 | 2.61966702454546 | 0.972577670507659 |
| ebi-fl187-GCST90094819 | finngen_R11_O15_POOR_FETGRO | Poor fetal growth \|\| id:finngen_R11_O15_POOR_FETGRO | Red meat liking \|\| id:ebi-fl187-GCST90094819 | Inverse variance weighted | 6 | 0.180101798111958 | 0.162507202263581 | 0.267745384180677 | -0.138412318 | 0.498615914548577 | 1.19733924379248 | 0.870739595828603 | 1.64644087806908 | 0.888224550184161 |
| ebi-fl187-GCST90094819 | finngen_R11_O15_POOR_FETGRO | Poor fetal growth \|\| id:finngen_R11_O15_POOR_FETGRO | Red meat liking \|\| id:ebi-fl187-GCST90094819 | MR Egger | 6 | -0.321508992 | 0.620740787465981 | 0.631826063085035 | -1.538160936 | 0.895142950963839 | 0.725054109971167 | 0.214775724771318 | 2.44768566347907 | 0.970850396659599 |
| ebi-fl187-GCST90094819 | finngen_R11_O15_POOR_FETGRO | Poor fetal growth \|\| id:finngen_R11_O15_POOR_FETGRO | Red meat liking \|\| id:ebi-fl187-GCST90094819 | Weighted median | 6 | 0.146688467795759 | 0.224737496473248 | 0.513943092390497 | -0.293797025 | 0.587173960883324 | 1.15799315467109 | 0.745427779074925 | 1.79889747056277 | 0.918328271889597 |
| ebi-fl187-GCST90094819 | finngen_R11_O15_POOR_FETGRO | Poor fetal growth \|\| id:finngen_R11_O15_POOR_FETGRO | Red meat liking \|\| id:ebi-fl187-GCST90094819 | Weighted mode | 6 | -0.022627923 | 0.274842640751929 | 0.937578208548976 | -0.561319499 | 0.516063653195617 | 0.977626168636219 | 0.570455851356888 | 1.67541961981629 | 0.988620775239461 |
| ebi-fl187-GCST90094820 | finngen_R11_O15_POOR_FETGRO | Poor fetal growth \|\| id:finngen_R11_O15_POOR_FETGRO | Red wine liking \|\| id:ebi-fl187-GCST90094820 | Inverse variance weighted | 6 | 0.0790689434026205 | 0.191076799992524 | 0.679015357304986 | -0.295441585 | 0.453579471387967 | 1.08227893549063 | 0.74420288637554 | 1.57393597317447 | 0.96571624475698 |
| ebi-fl187-GCST90094820 | finngen_R11_O15_POOR_FETGRO | Poor fetal growth \|\| id:finngen_R11_O15_POOR_FETGRO | Red wine liking \|\| id:ebi-fl187-GCST90094820 | MR Egger | 6 | 1.40316745624161 | 0.499528580277523 | 0.0483669543596627 | 0.424091438897666 | 2.38224347358556 | 4.06806499924823 | 1.52820132444007 | 10.8291705899235 | 0.970850396659599 |
| ebi-fl187-GCST90094820 | finngen_R11_O15_POOR_FETGRO | Poor fetal growth \|\| id:finngen_R11_O15_POOR_FETGRO | Red wine liking \|\| id:ebi-fl187-GCST90094820 | Weighted median | 6 | 0.0820426336353798 | 0.191521152478593 | 0.668379035365965 | -0.293338825 | 0.457424092493423 | 1.08550208774326 | 0.745769412397067 | 1.57999880781863 | 0.918328271889597 |
| ebi-fl187-GCST90094820 | finngen_R11_O15_POOR_FETGRO | Poor fetal growth \|\| id:finngen_R11_O15_POOR_FETGRO | Red wine liking \|\| id:ebi-fl187-GCST90094820 | Weighted mode | 6 | 0.0963951957062735 | 0.234685509299146 | 0.698271672011137 | -0.363588403 | 0.5563787939326 | 1.10119416521912 | 0.695177269077203 | 1.74434441897432 | 0.972577670507659 |
| ebi-fl187-GCST90094821 | finngen_R11_O15_POOR_FETGRO | Poor fetal growth \|\| id:finngen_R11_O15_POOR_FETGRO | Roast chicken liking \|\| id:ebi-fl187-GCST90094821 | Inverse variance weighted | 2 | -0.330233267 | 0.304519204240839 | 0.278169084951971 | -0.927090908 | 0.266624373048454 | 0.718756051617998 | 0.395703174719821 | 1.30554995446595 | 0.888224550184161 |
| ebi-fl187-GCST90094822 | finngen_R11_O15_POOR_FETGRO | Poor fetal growth \|\| id:finngen_R11_O15_POOR_FETGRO | Salad dressing liking \|\| id:ebi-fl187-GCST90094822 | Inverse variance weighted | 6 | -0.308769651 | 0.290102913900875 | 0.287172337485588 | -0.877371363 | 0.259832059991424 | 0.734349907125273 | 0.415874659874062 | 1.29671229850408 | 0.888224550184161 |
| ebi-fl187-GCST90094822 | finngen_R11_O15_POOR_FETGRO | Poor fetal growth \|\| id:finngen_R11_O15_POOR_FETGRO | Salad dressing liking \|\| id:ebi-fl187-GCST90094822 | MR Egger | 6 | 1.15732369209603 | 1.4941569294022 | 0.481833444341412 | -1.77122389 | 4.08587127372433 | 3.18140744643778 | 0.170124647582724 | 59.4937505180033 | 0.970850396659599 |
| ebi-fl187-GCST90094822 | finngen_R11_O15_POOR_FETGRO | Poor fetal growth \|\| id:finngen_R11_O15_POOR_FETGRO | Salad dressing liking \|\| id:ebi-fl187-GCST90094822 | Weighted median | 6 | -0.207551195 | 0.298265469755613 | 0.486516121344368 | -0.792151515 | 0.377049126055115 | 0.812571641374558 | 0.452869390897082 | 1.45797593221794 | 0.918328271889597 |
| ebi-fl187-GCST90094822 | finngen_R11_O15_POOR_FETGRO | Poor fetal growth \|\| id:finngen_R11_O15_POOR_FETGRO | Salad dressing liking \|\| id:ebi-fl187-GCST90094822 | Weighted mode | 6 | -0.328671342 | 0.360025631068951 | 0.403161199299707 | -1.034321578 | 0.376978895340297 | 0.719879572372898 | 0.355467455922894 | 1.45787354112157 | 0.972577670507659 |
| ebi-fl187-GCST90094823 | finngen_R11_O15_POOR_FETGRO | Poor fetal growth \|\| id:finngen_R11_O15_POOR_FETGRO | Salad leaves liking \|\| id:ebi-fl187-GCST90094823 | Inverse variance weighted | 4 | -0.274218041 | 0.320499625980732 | 0.392221554551911 | -0.902397308 | 0.353961226079838 | 0.760166309887159 | 0.40559615450051 | 1.42469994420702 | 0.888224550184161 |
| ebi-fl187-GCST90094823 | finngen_R11_O15_POOR_FETGRO | Poor fetal growth \|\| id:finngen_R11_O15_POOR_FETGRO | Salad leaves liking \|\| id:ebi-fl187-GCST90094823 | MR Egger | 4 | -1.011175853 | 1.495978015 | 0.56876953868541 | -3.943292761 | 1.92094105584388 | 0.363790963418838 | 0.0193842817362733 | 6.82738039333976 | 0.970850396659599 |
| ebi-fl187-GCST90094823 | finngen_R11_O15_POOR_FETGRO | Poor fetal growth \|\| id:finngen_R11_O15_POOR_FETGRO | Salad leaves liking \|\| id:ebi-fl187-GCST90094823 | Weighted median | 4 | -0.348289115 | 0.381352443559391 | 0.361084881115966 | -1.095739904 | 0.399161674296415 | 0.705894761888993 | 0.334292171153297 | 1.49057458672527 | 0.918328271889597 |
| ebi-fl187-GCST90094823 | finngen_R11_O15_POOR_FETGRO | Poor fetal growth \|\| id:finngen_R11_O15_POOR_FETGRO | Salad leaves liking \|\| id:ebi-fl187-GCST90094823 | Weighted mode | 4 | -0.494394626 | 0.511011807736074 | 0.404668161786931 | -1.49597777 | 0.507188516684798 | 0.609940037102857 | 0.224029448412537 | 1.66061583196852 | 0.972577670507659 |
| ebi-fl187-GCST90094824 | finngen_R11_O15_POOR_FETGRO | Poor fetal growth \|\| id:finngen_R11_O15_POOR_FETGRO | F-salad vegetables liking (derived food-liking factor) \|\| id:ebi-fl187-GCST90094824 | Inverse variance weighted (multiplicative random effects) | 10 | 0.0719719988769702 | 0.162612145548282 | 0.658055693125518 | -0.246747806 | 0.390691804151602 | 1.0746252529286 | 0.781337717059804 | 1.47800292884553 | 0.96571624475698 |
| ebi-fl187-GCST90094824 | finngen_R11_O15_POOR_FETGRO | Poor fetal growth \|\| id:finngen_R11_O15_POOR_FETGRO | F-salad vegetables liking (derived food-liking factor) \|\| id:ebi-fl187-GCST90094824 | MR Egger | 10 | 0.738461722811324 | 1.06936971980009 | 0.509379047095915 | -1.357502928 | 2.83442637361949 | 2.09271386323509 | 0.257302478247726 | 17.0206340148806 | 0.970850396659599 |
| ebi-fl187-GCST90094824 | finngen_R11_O15_POOR_FETGRO | Poor fetal growth \|\| id:finngen_R11_O15_POOR_FETGRO | F-salad vegetables liking (derived food-liking factor) \|\| id:ebi-fl187-GCST90094824 | Weighted median | 10 | -0.080944913 | 0.154389749345741 | 0.600077250701466 | -0.383548822 | 0.221658995740746 | 0.922244493749047 | 0.681438808239265 | 1.24814568229258 | 0.918328271889597 |
| ebi-fl187-GCST90094824 | finngen_R11_O15_POOR_FETGRO | Poor fetal growth \|\| id:finngen_R11_O15_POOR_FETGRO | F-salad vegetables liking (derived food-liking factor) \|\| id:ebi-fl187-GCST90094824 | Weighted mode | 10 | -0.197584065 | 0.217383722773099 | 0.387085349279985 | -0.623656162 | 0.22848803113864 | 0.820711144237725 | 0.5359812166269 | 1.25669848379196 | 0.972577670507659 |
| ebi-fl187-GCST90094825 | finngen_R11_O15_POOR_FETGRO | Poor fetal growth \|\| id:finngen_R11_O15_POOR_FETGRO | Salami liking \|\| id:ebi-fl187-GCST90094825 | Inverse variance weighted | 11 | 0.0586690637878494 | 0.141593317922311 | 0.678618460346287 | -0.218853839 | 0.33619196691558 | 1.06042424988104 | 0.803439140767314 | 1.39960767739275 | 0.96571624475698 |
| ebi-fl187-GCST90094825 | finngen_R11_O15_POOR_FETGRO | Poor fetal growth \|\| id:finngen_R11_O15_POOR_FETGRO | Salami liking \|\| id:ebi-fl187-GCST90094825 | MR Egger | 11 | -0.883395316 | 1.01950113666313 | 0.408723259935209 | -2.881617544 | 1.11482691202935 | 0.413376980835656 | 0.0560440357999681 | 3.04904038129423 | 0.970850396659599 |
| ebi-fl187-GCST90094825 | finngen_R11_O15_POOR_FETGRO | Poor fetal growth \|\| id:finngen_R11_O15_POOR_FETGRO | Salami liking \|\| id:ebi-fl187-GCST90094825 | Weighted median | 11 | 0.0195264852895136 | 0.17389746719559 | 0.910595573766255 | -0.32131255 | 0.36036552099287 | 1.01971837403937 | 0.725196555081628 | 1.43385342231314 | 0.969669326094948 |
| ebi-fl187-GCST90094825 | finngen_R11_O15_POOR_FETGRO | Poor fetal growth \|\| id:finngen_R11_O15_POOR_FETGRO | Salami liking \|\| id:ebi-fl187-GCST90094825 | Weighted mode | 11 | -0.067255093 | 0.299256544591178 | 0.826706816487254 | -0.65379792 | 0.519287734731187 | 0.934956670323262 | 0.520066848913557 | 1.6808300263862 | 0.97297033017346 |
| ebi-fl187-GCST90094826 | finngen_R11_O15_POOR_FETGRO | Poor fetal growth \|\| id:finngen_R11_O15_POOR_FETGRO | Salmon liking \|\| id:ebi-fl187-GCST90094826 | Inverse variance weighted | 12 | 0.153481902808573 | 0.148405458917175 | 0.301039596525587 | -0.137392797 | 0.444356602286237 | 1.16588668765662 | 0.871627786390737 | 1.5594865029298 | 0.888224550184161 |
| ebi-fl187-GCST90094826 | finngen_R11_O15_POOR_FETGRO | Poor fetal growth \|\| id:finngen_R11_O15_POOR_FETGRO | Salmon liking \|\| id:ebi-fl187-GCST90094826 | MR Egger | 12 | -1.968600318 | 1.17754849025405 | 0.125517926044827 | -4.276595359 | 0.339394722507002 | 0.139652188081273 | 0.0138898716976485 | 1.40409746471516 | 0.970850396659599 |
| ebi-fl187-GCST90094826 | finngen_R11_O15_POOR_FETGRO | Poor fetal growth \|\| id:finngen_R11_O15_POOR_FETGRO | Salmon liking \|\| id:ebi-fl187-GCST90094826 | Weighted median | 12 | 0.118412457191248 | 0.180592525474557 | 0.512024516033109 | -0.235548893 | 0.47237380712138 | 1.12570832211994 | 0.790137030100888 | 1.60379678234835 | 0.918328271889597 |
| ebi-fl187-GCST90094826 | finngen_R11_O15_POOR_FETGRO | Poor fetal growth \|\| id:finngen_R11_O15_POOR_FETGRO | Salmon liking \|\| id:ebi-fl187-GCST90094826 | Weighted mode | 12 | 0.156581510569827 | 0.246526114310066 | 0.538317032266037 | -0.326609673 | 0.639772694617556 | 1.16950608554151 | 0.721365256073593 | 1.89604984798311 | 0.972577670507659 |
| ebi-fl187-GCST90094827 | finngen_R11_O15_POOR_FETGRO | Poor fetal growth \|\| id:finngen_R11_O15_POOR_FETGRO | F-salty food liking (derived food-liking factor) \|\| id:ebi-fl187-GCST90094827 | Inverse variance weighted | 8 | -0.036908649 | 0.106899499277799 | 0.729895205855803 | -0.246431667 | 0.172614369969872 | 0.963764172520179 | 0.781584767588601 | 1.1884077309992 | 0.96571624475698 |
| ebi-fl187-GCST90094827 | finngen_R11_O15_POOR_FETGRO | Poor fetal growth \|\| id:finngen_R11_O15_POOR_FETGRO | F-salty food liking (derived food-liking factor) \|\| id:ebi-fl187-GCST90094827 | MR Egger | 8 | 0.110551979169975 | 0.342928953722516 | 0.758105096529318 | -0.56158877 | 0.782692728466105 | 1.11689440278732 | 0.570302264490992 | 2.18735429376389 | 0.970850396659599 |
| ebi-fl187-GCST90094827 | finngen_R11_O15_POOR_FETGRO | Poor fetal growth \|\| id:finngen_R11_O15_POOR_FETGRO | F-salty food liking (derived food-liking factor) \|\| id:ebi-fl187-GCST90094827 | Weighted median | 8 | 0.0150199075565231 | 0.135772276311648 | 0.911913140930887 | -0.251093754 | 0.281133569127353 | 1.01513327323758 | 0.777949432257633 | 1.32463052186248 | 0.969669326094948 |
| ebi-fl187-GCST90094827 | finngen_R11_O15_POOR_FETGRO | Poor fetal growth \|\| id:finngen_R11_O15_POOR_FETGRO | F-salty food liking (derived food-liking factor) \|\| id:ebi-fl187-GCST90094827 | Weighted mode | 8 | 0.0410097388173343 | 0.177645660621931 | 0.824032374388693 | -0.307175756 | 0.38919523363632 | 1.04186225200223 | 0.735521317260547 | 1.47579264757414 | 0.97297033017346 |
| ebi-fl187-GCST90094828 | finngen_R11_O15_POOR_FETGRO | Poor fetal growth \|\| id:finngen_R11_O15_POOR_FETGRO | Salty food liking \|\| id:ebi-fl187-GCST90094828 | Inverse variance weighted | 13 | 0.133900879701821 | 0.111734156417102 | 0.230765975939887 | -0.085098067 | 0.352899826279342 | 1.14327949182406 | 0.918422213188008 | 1.4231885701984 | 0.888224550184161 |
| ebi-fl187-GCST90094828 | finngen_R11_O15_POOR_FETGRO | Poor fetal growth \|\| id:finngen_R11_O15_POOR_FETGRO | Salty food liking \|\| id:ebi-fl187-GCST90094828 | MR Egger | 13 | -0.030890402 | 0.353108646350361 | 0.931861187156429 | -0.722983349 | 0.661202545171744 | 0.969581831797781 | 0.485302268321424 | 1.93712040910904 | 0.993285374120483 |
| ebi-fl187-GCST90094828 | finngen_R11_O15_POOR_FETGRO | Poor fetal growth \|\| id:finngen_R11_O15_POOR_FETGRO | Salty food liking \|\| id:ebi-fl187-GCST90094828 | Weighted median | 13 | 0.0756376317166764 | 0.149003687245833 | 0.611718073080862 | -0.216409595 | 0.36768485871851 | 1.07857166317267 | 0.805405344071941 | 1.44438678134117 | 0.918328271889597 |
| ebi-fl187-GCST90094828 | finngen_R11_O15_POOR_FETGRO | Poor fetal growth \|\| id:finngen_R11_O15_POOR_FETGRO | Salty food liking \|\| id:ebi-fl187-GCST90094828 | Weighted mode | 13 | -0.02080205 | 0.249594607339062 | 0.934952700289843 | -0.51000748 | 0.46840338061881 | 0.979412820379146 | 0.600491087031875 | 1.59744164974111 | 0.988620775239461 |
| ebi-fl187-GCST90094829 | finngen_R11_O15_POOR_FETGRO | Poor fetal growth \|\| id:finngen_R11_O15_POOR_FETGRO | Salty pretzels liking \|\| id:ebi-fl187-GCST90094829 | Inverse variance weighted | 5 | 0.195949772025105 | 0.278307194258562 | 0.481384648979762 | -0.349532329 | 0.741431872771885 | 1.21646580318599 | 0.705017729173896 | 2.09893877712107 | 0.914435393876931 |
| ebi-fl187-GCST90094829 | finngen_R11_O15_POOR_FETGRO | Poor fetal growth \|\| id:finngen_R11_O15_POOR_FETGRO | Salty pretzels liking \|\| id:ebi-fl187-GCST90094829 | MR Egger | 5 | -0.00830253 | 0.908929284371078 | 0.993285374120483 | -1.789803928 | 1.77319886691682 | 0.991731840368079 | 0.16699290912139 | 5.88966350951413 | 0.993285374120483 |
| ebi-fl187-GCST90094829 | finngen_R11_O15_POOR_FETGRO | Poor fetal growth \|\| id:finngen_R11_O15_POOR_FETGRO | Salty pretzels liking \|\| id:ebi-fl187-GCST90094829 | Weighted median | 5 | 0.0864737398864527 | 0.260377631159484 | 0.739806997430928 | -0.423866417 | 0.596813896959042 | 1.090322735 | 0.654511307531614 | 1.8163225807194 | 0.918328271889597 |
| ebi-fl187-GCST90094829 | finngen_R11_O15_POOR_FETGRO | Poor fetal growth \|\| id:finngen_R11_O15_POOR_FETGRO | Salty pretzels liking \|\| id:ebi-fl187-GCST90094829 | Weighted mode | 5 | 0.236953098630406 | 0.383434235887562 | 0.570039778700497 | -0.514578004 | 0.988484200970028 | 1.26738167444498 | 0.597752790862104 | 2.68715819193808 | 0.972577670507659 |
| ebi-fl187-GCST90094830 | finngen_R11_O15_POOR_FETGRO | Poor fetal growth \|\| id:finngen_R11_O15_POOR_FETGRO | Sardines liking \|\| id:ebi-fl187-GCST90094830 | Inverse variance weighted | 16 | -0.065169642 | 0.0892380847870877 | 0.465213205327904 | -0.240076288 | 0.109737004064986 | 0.936908510751394 | 0.786567852852979 | 1.11598453246534 | 0.914435393876931 |
| ebi-fl187-GCST90094830 | finngen_R11_O15_POOR_FETGRO | Poor fetal growth \|\| id:finngen_R11_O15_POOR_FETGRO | Sardines liking \|\| id:ebi-fl187-GCST90094830 | MR Egger | 16 | 0.00379529104123313 | 0.421759400867024 | 0.992947137915885 | -0.822853135 | 0.830443716740599 | 1.00380250227831 | 0.439176834624262 | 2.29433654997367 | 0.993285374120483 |
| ebi-fl187-GCST90094830 | finngen_R11_O15_POOR_FETGRO | Poor fetal growth \|\| id:finngen_R11_O15_POOR_FETGRO | Sardines liking \|\| id:ebi-fl187-GCST90094830 | Weighted median | 16 | -0.057385518 | 0.119495383674647 | 0.631062370464495 | -0.29159647 | 0.176825434041853 | 0.944229981573144 | 0.747069940317248 | 1.19342274395756 | 0.918328271889597 |
| ebi-fl187-GCST90094830 | finngen_R11_O15_POOR_FETGRO | Poor fetal growth \|\| id:finngen_R11_O15_POOR_FETGRO | Sardines liking \|\| id:ebi-fl187-GCST90094830 | Weighted mode | 16 | -0.087282337 | 0.164915317595094 | 0.604373126752913 | -0.410516359 | 0.235951685558559 | 0.916418320370789 | 0.663307656540246 | 1.2661131371401 | 0.972577670507659 |
| ebi-fl187-GCST90094831 | finngen_R11_O15_POOR_FETGRO | Poor fetal growth \|\| id:finngen_R11_O15_POOR_FETGRO | F-sauces liking (derived food-liking factor) \|\| id:ebi-fl187-GCST90094831 | Inverse variance weighted | 8 | 0.0341831283826823 | 0.0919089446201003 | 0.709949548391246 | -0.145958403 | 0.214324659838079 | 1.03477408588287 | 0.864193650237424 | 1.23902485110896 | 0.96571624475698 |
| ebi-fl187-GCST90094831 | finngen_R11_O15_POOR_FETGRO | Poor fetal growth \|\| id:finngen_R11_O15_POOR_FETGRO | F-sauces liking (derived food-liking factor) \|\| id:ebi-fl187-GCST90094831 | MR Egger | 8 | -0.083573147 | 0.609669138152016 | 0.895452131828809 | -1.278524658 | 1.11137836378272 | 0.919823801863176 | 0.278447803364863 | 3.03854372794378 | 0.993285374120483 |
| ebi-fl187-GCST90094831 | finngen_R11_O15_POOR_FETGRO | Poor fetal growth \|\| id:finngen_R11_O15_POOR_FETGRO | F-sauces liking (derived food-liking factor) \|\| id:ebi-fl187-GCST90094831 | Weighted median | 8 | 0.145368587681361 | 0.125831739756749 | 0.247983281150967 | -0.101261622 | 0.391998797604589 | 1.1564657507504 | 0.903696574831994 | 1.47993593193305 | 0.918328271889597 |
| ebi-fl187-GCST90094831 | finngen_R11_O15_POOR_FETGRO | Poor fetal growth \|\| id:finngen_R11_O15_POOR_FETGRO | F-sauces liking (derived food-liking factor) \|\| id:ebi-fl187-GCST90094831 | Weighted mode | 8 | 0.174012936202721 | 0.170718888220764 | 0.341991675712419 | -0.160596085 | 0.508621957115419 | 1.19007096071999 | 0.851635990443411 | 1.66299793273368 | 0.972577670507659 |
| ebi-fl187-GCST90094832 | finngen_R11_O15_POOR_FETGRO | Poor fetal growth \|\| id:finngen_R11_O15_POOR_FETGRO | Sausages liking \|\| id:ebi-fl187-GCST90094832 | Inverse variance weighted (multiplicative random effects) | 3 | 0.234228731053932 | 0.441912219046066 | 0.596087996834328 | -0.631919218 | 1.10037668038422 | 1.26393356000241 | 0.531570621335362 | 3.00529784751309 | 0.96571624475698 |
| ebi-fl187-GCST90094832 | finngen_R11_O15_POOR_FETGRO | Poor fetal growth \|\| id:finngen_R11_O15_POOR_FETGRO | Sausages liking \|\| id:ebi-fl187-GCST90094832 | MR Egger | 3 | 0.910990917621895 | 2.33455309961935 | 0.763147133496644 | -3.664733158 | 5.48671499287582 | 2.48678551246477 | 0.0256110044730595 | 241.462695909088 | 0.970850396659599 |
| ebi-fl187-GCST90094832 | finngen_R11_O15_POOR_FETGRO | Poor fetal growth \|\| id:finngen_R11_O15_POOR_FETGRO | Sausages liking \|\| id:ebi-fl187-GCST90094832 | Weighted median | 3 | 0.097818855803878 | 0.251293907988622 | 0.697083265978075 | -0.394717204 | 0.590354915461576 | 1.10276300789623 | 0.673870580308594 | 1.80462879241212 | 0.918328271889597 |
| ebi-fl187-GCST90094832 | finngen_R11_O15_POOR_FETGRO | Poor fetal growth \|\| id:finngen_R11_O15_POOR_FETGRO | Sausages liking \|\| id:ebi-fl187-GCST90094832 | Weighted mode | 3 | -0.013379832 | 0.261393736407662 | 0.963829359494701 | -0.525711555 | 0.498951891468325 | 0.986709280182428 | 0.591134589761749 | 1.64699413713977 | 0.988620775239461 |
| ebi-fl187-GCST90094833 | finngen_R11_O15_POOR_FETGRO | Poor fetal growth \|\| id:finngen_R11_O15_POOR_FETGRO | F-savoury food liking (derived food-liking factor) \|\| id:ebi-fl187-GCST90094833 | Inverse variance weighted | 21 | -0.044092188 | 0.0600291473857934 | 0.462636149395413 | -0.161749317 | 0.0735649406649309 | 0.956865741665774 | 0.850654422342313 | 1.07633843253582 | 0.914435393876931 |
| ebi-fl187-GCST90094833 | finngen_R11_O15_POOR_FETGRO | Poor fetal growth \|\| id:finngen_R11_O15_POOR_FETGRO | F-savoury food liking (derived food-liking factor) \|\| id:ebi-fl187-GCST90094833 | MR Egger | 21 | -0.431090633 | 0.255351957128081 | 0.107715082537432 | -0.931580469 | 0.069399203361196 | 0.649800015034614 | 0.393930623143482 | 1.07186401546952 | 0.970850396659599 |
| ebi-fl187-GCST90094833 | finngen_R11_O15_POOR_FETGRO | Poor fetal growth \|\| id:finngen_R11_O15_POOR_FETGRO | F-savoury food liking (derived food-liking factor) \|\| id:ebi-fl187-GCST90094833 | Weighted median | 21 | -0.042124989 | 0.079509841250423 | 0.59624471706352 | -0.197964278 | 0.113714299787667 | 0.958749939826464 | 0.820399158983714 | 1.12043197150022 | 0.918328271889597 |
| ebi-fl187-GCST90094833 | finngen_R11_O15_POOR_FETGRO | Poor fetal growth \|\| id:finngen_R11_O15_POOR_FETGRO | F-savoury food liking (derived food-liking factor) \|\| id:ebi-fl187-GCST90094833 | Weighted mode | 21 | -0.040148506 | 0.123671491806976 | 0.748825893476361 | -0.28254463 | 0.202247618227883 | 0.960646767024936 | 0.753862996562733 | 1.22415109270941 | 0.972577670507659 |
| ebi-fl187-GCST90094834 | finngen_R11_O15_POOR_FETGRO | Poor fetal growth \|\| id:finngen_R11_O15_POOR_FETGRO | Savoury biscuit liking \|\| id:ebi-fl187-GCST90094834 | Inverse variance weighted | 2 | -0.133342306 | 0.616598428011156 | 0.828789220731049 | -1.341875225 | 1.07519061322482 | 0.875165466722351 | 0.261355109244938 | 2.93055144916087 | 0.994304114950957 |
| ebi-fl187-GCST90094835 | finngen_R11_O15_POOR_FETGRO | Poor fetal growth \|\| id:finngen_R11_O15_POOR_FETGRO | F-savour/caloric food liking (derived food-liking factor) \|\| id:ebi-fl187-GCST90094835 | Inverse variance weighted | 12 | -0.118641107 | 0.0797672883670453 | 0.136924941451804 | -0.274984992 | 0.0377027782486557 | 0.888126485994247 | 0.759583522854877 | 1.0384225452389 | 0.822352178486651 |
| ebi-fl187-GCST90094835 | finngen_R11_O15_POOR_FETGRO | Poor fetal growth \|\| id:finngen_R11_O15_POOR_FETGRO | F-savour/caloric food liking (derived food-liking factor) \|\| id:ebi-fl187-GCST90094835 | MR Egger | 12 | -0.047972817 | 0.29962740338743 | 0.875983743988627 | -0.635242527 | 0.539296894082419 | 0.953159696887688 | 0.529806980133358 | 1.71480075166686 | 0.993285374120483 |
| ebi-fl187-GCST90094835 | finngen_R11_O15_POOR_FETGRO | Poor fetal growth \|\| id:finngen_R11_O15_POOR_FETGRO | F-savour/caloric food liking (derived food-liking factor) \|\| id:ebi-fl187-GCST90094835 | Weighted median | 12 | -0.074003696 | 0.103607483719235 | 0.47506036558306 | -0.277074364 | 0.12906697187948 | 0.928668261281825 | 0.757998127014517 | 1.13776631996307 | 0.918328271889597 |
| ebi-fl187-GCST90094835 | finngen_R11_O15_POOR_FETGRO | Poor fetal growth \|\| id:finngen_R11_O15_POOR_FETGRO | F-savour/caloric food liking (derived food-liking factor) \|\| id:ebi-fl187-GCST90094835 | Weighted mode | 12 | -0.044163756 | 0.145523900043365 | 0.767186178041213 | -0.3293906 | 0.241063087908566 | 0.956797263182132 | 0.719361978886907 | 1.27260131853138 | 0.972577670507659 |
| ebi-fl187-GCST90094836 | finngen_R11_O15_POOR_FETGRO | Poor fetal growth \|\| id:finngen_R11_O15_POOR_FETGRO | F-seafood liking (derived food-liking factor) \|\| id:ebi-fl187-GCST90094836 | Inverse variance weighted | 29 | 0.0735326595001887 | 0.0612831330431798 | 0.230184339653177 | -0.046582281 | 0.193647600264821 | 1.07630368763837 | 0.954486021022144 | 1.2136685111254 | 0.888224550184161 |
| ebi-fl187-GCST90094836 | finngen_R11_O15_POOR_FETGRO | Poor fetal growth \|\| id:finngen_R11_O15_POOR_FETGRO | F-seafood liking (derived food-liking factor) \|\| id:ebi-fl187-GCST90094836 | MR Egger | 29 | -0.386310501 | 0.308554692071351 | 0.221307493087482 | -0.991077697 | 0.218456695470482 | 0.679559489024796 | 0.37117645947294 | 1.24415513790768 | 0.970850396659599 |
| ebi-fl187-GCST90094836 | finngen_R11_O15_POOR_FETGRO | Poor fetal growth \|\| id:finngen_R11_O15_POOR_FETGRO | F-seafood liking (derived food-liking factor) \|\| id:ebi-fl187-GCST90094836 | Weighted median | 29 | 0.0565884581177859 | 0.0842675437115427 | 0.501880880317803 | -0.108575928 | 0.22175284379241 | 1.05822021882891 | 0.897110776801491 | 1.24826282382972 | 0.918328271889597 |
| ebi-fl187-GCST90094836 | finngen_R11_O15_POOR_FETGRO | Poor fetal growth \|\| id:finngen_R11_O15_POOR_FETGRO | F-seafood liking (derived food-liking factor) \|\| id:ebi-fl187-GCST90094836 | Weighted mode | 29 | 0.0431861418850192 | 0.135991949023538 | 0.7531718495095 | -0.223358078 | 0.309730361971153 | 1.04413223350492 | 0.799828396897874 | 1.36305753243115 | 0.972577670507659 |
| ebi-fl187-GCST90094837 | finngen_R11_O15_POOR_FETGRO | Poor fetal growth \|\| id:finngen_R11_O15_POOR_FETGRO | F-sharp flavour liking (derived food-liking factor) \|\| id:ebi-fl187-GCST90094837 | Inverse variance weighted | 43 | 0.00019167822388106 | 0.0560538155771326 | 0.997271607724694 | -0.1096738 | 0.110057156755061 | 1.00019169659533 | 0.896126403782527 | 1.11634187511454 | 0.997271607724694 |
| ebi-fl187-GCST90094837 | finngen_R11_O15_POOR_FETGRO | Poor fetal growth \|\| id:finngen_R11_O15_POOR_FETGRO | F-sharp flavour liking (derived food-liking factor) \|\| id:ebi-fl187-GCST90094837 | MR Egger | 43 | -0.22274168 | 0.218825290949334 | 0.314695944241195 | -0.651639251 | 0.206155889865127 | 0.800321561343663 | 0.521190713901512 | 1.2289447690977 | 0.970850396659599 |
| ebi-fl187-GCST90094837 | finngen_R11_O15_POOR_FETGRO | Poor fetal growth \|\| id:finngen_R11_O15_POOR_FETGRO | F-sharp flavour liking (derived food-liking factor) \|\| id:ebi-fl187-GCST90094837 | Weighted median | 43 | 0.0371492804977156 | 0.0809375474463835 | 0.646243515667469 | -0.121488312 | 0.195786873492627 | 1.03784793973168 | 0.885601403757272 | 1.21626765883099 | 0.918328271889597 |
| ebi-fl187-GCST90094837 | finngen_R11_O15_POOR_FETGRO | Poor fetal growth \|\| id:finngen_R11_O15_POOR_FETGRO | F-sharp flavour liking (derived food-liking factor) \|\| id:ebi-fl187-GCST90094837 | Weighted mode | 43 | 0.0131446345470487 | 0.136419596620936 | 0.923697381097092 | -0.254237775 | 0.280527043924083 | 1.01323140502767 | 0.775507383985979 | 1.32382734366448 | 0.988620775239461 |
| ebi-fl187-GCST90094838 | finngen_R11_O15_POOR_FETGRO | Poor fetal growth \|\| id:finngen_R11_O15_POOR_FETGRO | Shellfish liking \|\| id:ebi-fl187-GCST90094838 | Inverse variance weighted | 31 | 0.131304685009158 | 0.0534456067990975 | 0.0140184221366419 | 0.0265512956829265 | 0.236058074335389 | 1.14031516532454 | 1.02690692180254 | 1.26624784453364 | 0.392515819825973 |
| ebi-fl187-GCST90094838 | finngen_R11_O15_POOR_FETGRO | Poor fetal growth \|\| id:finngen_R11_O15_POOR_FETGRO | Shellfish liking \|\| id:ebi-fl187-GCST90094838 | MR Egger | 31 | 0.173145034505136 | 0.151465153969896 | 0.262332294076517 | -0.123726667 | 0.470016736286133 | 1.18903854419576 | 0.883621330503114 | 1.60002097140207 | 0.970850396659599 |
| ebi-fl187-GCST90094838 | finngen_R11_O15_POOR_FETGRO | Poor fetal growth \|\| id:finngen_R11_O15_POOR_FETGRO | Shellfish liking \|\| id:ebi-fl187-GCST90094838 | Weighted median | 31 | 0.149613386680631 | 0.0760309212149775 | 0.0490914479502368 | 0.000592781099274659 | 0.298633992261987 | 1.16138514895356 | 1.00059295682871 | 1.34801614882921 | 0.81367942751543 |
| ebi-fl187-GCST90094838 | finngen_R11_O15_POOR_FETGRO | Poor fetal growth \|\| id:finngen_R11_O15_POOR_FETGRO | Shellfish liking \|\| id:ebi-fl187-GCST90094838 | Weighted mode | 31 | 0.18249416189033 | 0.108335342172232 | 0.102456900627996 | -0.029843109 | 0.394831432547905 | 1.20020714399219 | 0.970597799888902 | 1.48413399314811 | 0.972577670507659 |
| ebi-fl187-GCST90094840 | finngen_R11_O15_POOR_FETGRO | Poor fetal growth \|\| id:finngen_R11_O15_POOR_FETGRO | F-small fish liking (derived food-liking factor) \|\| id:ebi-fl187-GCST90094840 | Inverse variance weighted | 28 | -0.108417579 | 0.0602093828436083 | 0.0717539934539116 | -0.226427969 | 0.00959281151645071 | 0.8972528443744 | 0.797376775907224 | 1.00963897001136 | 0.706473758635168 |
| ebi-fl187-GCST90094840 | finngen_R11_O15_POOR_FETGRO | Poor fetal growth \|\| id:finngen_R11_O15_POOR_FETGRO | F-small fish liking (derived food-liking factor) \|\| id:ebi-fl187-GCST90094840 | MR Egger | 28 | 0.0781010452944917 | 0.219604151234156 | 0.724975991372651 | -0.352323091 | 0.508525181713437 | 1.08123190654691 | 0.703052935113931 | 1.66283700322738 | 0.970850396659599 |
| ebi-fl187-GCST90094840 | finngen_R11_O15_POOR_FETGRO | Poor fetal growth \|\| id:finngen_R11_O15_POOR_FETGRO | F-small fish liking (derived food-liking factor) \|\| id:ebi-fl187-GCST90094840 | Weighted median | 28 | -0.083684528 | 0.0818488329069709 | 0.30657843646029 | -0.24410824 | 0.0767391848010753 | 0.9197213569483 | 0.783402833998618 | 1.0797604217351 | 0.918328271889597 |
| ebi-fl187-GCST90094840 | finngen_R11_O15_POOR_FETGRO | Poor fetal growth \|\| id:finngen_R11_O15_POOR_FETGRO | F-small fish liking (derived food-liking factor) \|\| id:ebi-fl187-GCST90094840 | Weighted mode | 28 | -0.088566761 | 0.123735667770805 | 0.480275949757393 | -0.33108867 | 0.153955147444031 | 0.915242005870777 | 0.718141488455332 | 1.16643856785398 | 0.972577670507659 |
| ebi-fl187-GCST90094841 | finngen_R11_O15_POOR_FETGRO | Poor fetal growth \|\| id:finngen_R11_O15_POOR_FETGRO | Smoked fish liking \|\| id:ebi-fl187-GCST90094841 | Inverse variance weighted | 7 | -0.126730876 | 0.134397529689774 | 0.345703718424561 | -0.390150034 | 0.136688282430704 | 0.880970731274021 | 0.676955300598694 | 1.14647071774916 | 0.888224550184161 |
| ebi-fl187-GCST90094841 | finngen_R11_O15_POOR_FETGRO | Poor fetal growth \|\| id:finngen_R11_O15_POOR_FETGRO | Smoked fish liking \|\| id:ebi-fl187-GCST90094841 | MR Egger | 7 | 0.474745982860714 | 0.866029224083357 | 0.607139462597292 | -1.222671296 | 2.17216326206409 | 1.60760578619334 | 0.294442572076828 | 8.77725101222101 | 0.970850396659599 |
| ebi-fl187-GCST90094841 | finngen_R11_O15_POOR_FETGRO | Poor fetal growth \|\| id:finngen_R11_O15_POOR_FETGRO | Smoked fish liking \|\| id:ebi-fl187-GCST90094841 | Weighted median | 7 | -0.122438972 | 0.174360121525864 | 0.482542662555329 | -0.46418481 | 0.219306865970316 | 0.884759898228009 | 0.628647363153143 | 1.24521333166197 | 0.918328271889597 |
| ebi-fl187-GCST90094841 | finngen_R11_O15_POOR_FETGRO | Poor fetal growth \|\| id:finngen_R11_O15_POOR_FETGRO | Smoked fish liking \|\| id:ebi-fl187-GCST90094841 | Weighted mode | 7 | -0.102771565 | 0.243759711713625 | 0.687997130529499 | -0.5805406 | 0.374997469648323 | 0.902333074116071 | 0.559595767157806 | 1.45498773298289 | 0.972577670507659 |
| ebi-fl187-GCST90094843 | finngen_R11_O15_POOR_FETGRO | Poor fetal growth \|\| id:finngen_R11_O15_POOR_FETGRO | Soft cheese liking \|\| id:ebi-fl187-GCST90094843 | Inverse variance weighted | 3 | 0.121138585212607 | 0.250392253472012 | 0.628531179812641 | -0.369630232 | 0.611907402017751 | 1.12878133392938 | 0.69098978959819 | 1.84394519138744 | 0.96571624475698 |
| ebi-fl187-GCST90094843 | finngen_R11_O15_POOR_FETGRO | Poor fetal growth \|\| id:finngen_R11_O15_POOR_FETGRO | Soft cheese liking \|\| id:ebi-fl187-GCST90094843 | MR Egger | 3 | 1.12759029977892 | 2.27510893205946 | 0.707067204123345 | -3.331623207 | 5.58680380661546 | 3.08820587646198 | 0.0357350525752492 | 266.881250988275 | 0.970850396659599 |
| ebi-fl187-GCST90094843 | finngen_R11_O15_POOR_FETGRO | Poor fetal growth \|\| id:finngen_R11_O15_POOR_FETGRO | Soft cheese liking \|\| id:ebi-fl187-GCST90094843 | Weighted median | 3 | 0.12119080747398 | 0.292254927299586 | 0.678379847318629 | -0.45162885 | 0.694010464981169 | 1.12884028298245 | 0.636590396389095 | 2.00172731431694 | 0.918328271889597 |
| ebi-fl187-GCST90094843 | finngen_R11_O15_POOR_FETGRO | Poor fetal growth \|\| id:finngen_R11_O15_POOR_FETGRO | Soft cheese liking \|\| id:ebi-fl187-GCST90094843 | Weighted mode | 3 | 0.121019079726287 | 0.350314333410612 | 0.762701246559554 | -0.565597014 | 0.807635173211087 | 1.12864644642718 | 0.568020929198986 | 2.24259835430547 | 0.972577670507659 |
| ebi-fl187-GCST90094845 | finngen_R11_O15_POOR_FETGRO | Poor fetal growth \|\| id:finngen_R11_O15_POOR_FETGRO | Soya milk liking \|\| id:ebi-fl187-GCST90094845 | Inverse variance weighted | 3 | 0.0151247678419287 | 0.265263290538985 | 0.954530899957638 | -0.504791282 | 0.53504081729834 | 1.01523972598357 | 0.603631551282676 | 1.70751793710088 | 0.994304114950957 |
| ebi-fl187-GCST90094845 | finngen_R11_O15_POOR_FETGRO | Poor fetal growth \|\| id:finngen_R11_O15_POOR_FETGRO | Soya milk liking \|\| id:ebi-fl187-GCST90094845 | MR Egger | 3 | 0.0953348444222242 | 1.48529280076803 | 0.959193968087676 | -2.815839045 | 3.00650873392756 | 1.10002713141428 | 0.0598544770608625 | 20.2166947113596 | 0.993285374120483 |
| ebi-fl187-GCST90094845 | finngen_R11_O15_POOR_FETGRO | Poor fetal growth \|\| id:finngen_R11_O15_POOR_FETGRO | Soya milk liking \|\| id:ebi-fl187-GCST90094845 | Weighted median | 3 | 0.0406026316442206 | 0.291258857637299 | 0.889130960910639 | -0.530264729 | 0.611469992613328 | 1.0414381887315 | 0.588449169305481 | 1.84313880879201 | 0.969669326094948 |
| ebi-fl187-GCST90094845 | finngen_R11_O15_POOR_FETGRO | Poor fetal growth \|\| id:finngen_R11_O15_POOR_FETGRO | Soya milk liking \|\| id:ebi-fl187-GCST90094845 | Weighted mode | 3 | 0.0856616009049091 | 0.362295046032378 | 0.835099371350237 | -0.624436689 | 0.795759891128371 | 1.08943760122178 | 0.535563031939281 | 2.21612437038117 | 0.975345067302185 |
| ebi-fl187-GCST90094846 | finngen_R11_O15_POOR_FETGRO | Poor fetal growth \|\| id:finngen_R11_O15_POOR_FETGRO | Spicy food liking \|\| id:ebi-fl187-GCST90094846 | Inverse variance weighted | 16 | 0.11014793719621 | 0.0972144785762595 | 0.257197264221029 | -0.080392441 | 0.300688315205679 | 1.11644322172251 | 0.922754148932108 | 1.35078825575874 | 0.888224550184161 |
| ebi-fl187-GCST90094846 | finngen_R11_O15_POOR_FETGRO | Poor fetal growth \|\| id:finngen_R11_O15_POOR_FETGRO | Spicy food liking \|\| id:ebi-fl187-GCST90094846 | MR Egger | 16 | -0.295803861 | 0.602138620437792 | 0.63085743701036 | -1.475995557 | 0.884387834928727 | 0.743933327954176 | 0.228551078076771 | 2.42150157898216 | 0.970850396659599 |
| ebi-fl187-GCST90094846 | finngen_R11_O15_POOR_FETGRO | Poor fetal growth \|\| id:finngen_R11_O15_POOR_FETGRO | Spicy food liking \|\| id:ebi-fl187-GCST90094846 | Weighted median | 16 | 0.0524180299242073 | 0.124994221322297 | 0.674950402891297 | -0.192570644 | 0.29740670371591 | 1.05381617714618 | 0.824836046536983 | 1.34636275885065 | 0.918328271889597 |
| ebi-fl187-GCST90094846 | finngen_R11_O15_POOR_FETGRO | Poor fetal growth \|\| id:finngen_R11_O15_POOR_FETGRO | Spicy food liking \|\| id:ebi-fl187-GCST90094846 | Weighted mode | 16 | 0.0101947955402488 | 0.183686792449387 | 0.956471872796347 | -0.349831318 | 0.370220908741048 | 1.01024693951672 | 0.704806968180336 | 1.44805446722223 | 0.988620775239461 |
| ebi-fl187-GCST90094847 | finngen_R11_O15_POOR_FETGRO | Poor fetal growth \|\| id:finngen_R11_O15_POOR_FETGRO | Spinach liking \|\| id:ebi-fl187-GCST90094847 | Inverse variance weighted | 5 | -0.21983615 | 0.347674594512958 | 0.527188038171626 | -0.901278355 | 0.461606055543992 | 0.80265030168032 | 0.40605025146965 | 1.58662013988597 | 0.922579066800346 |
| ebi-fl187-GCST90094847 | finngen_R11_O15_POOR_FETGRO | Poor fetal growth \|\| id:finngen_R11_O15_POOR_FETGRO | Spinach liking \|\| id:ebi-fl187-GCST90094847 | MR Egger | 5 | -0.615027439 | 1.34668953159132 | 0.678927613493551 | -3.254538921 | 2.02448404281023 | 0.540626060808514 | 0.0385986135701664 | 7.57220300397621 | 0.970850396659599 |
| ebi-fl187-GCST90094847 | finngen_R11_O15_POOR_FETGRO | Poor fetal growth \|\| id:finngen_R11_O15_POOR_FETGRO | Spinach liking \|\| id:ebi-fl187-GCST90094847 | Weighted median | 5 | -0.178130584 | 0.300879848875456 | 0.553828971029824 | -0.767855088 | 0.411593920064025 | 0.836833139559257 | 0.464007256658592 | 1.5092214473272 | 0.918328271889597 |
| ebi-fl187-GCST90094847 | finngen_R11_O15_POOR_FETGRO | Poor fetal growth \|\| id:finngen_R11_O15_POOR_FETGRO | Spinach liking \|\| id:ebi-fl187-GCST90094847 | Weighted mode | 5 | -0.102363899 | 0.431223459492422 | 0.824024449067569 | -0.94756188 | 0.74283408135717 | 0.90270099967766 | 0.387685094947609 | 2.10188399151422 | 0.97297033017346 |
| ebi-fl187-GCST90094848 | finngen_R11_O15_POOR_FETGRO | Poor fetal growth \|\| id:finngen_R11_O15_POOR_FETGRO | Spirits liking \|\| id:ebi-fl187-GCST90094848 | Inverse variance weighted | 2 | -0.017131117 | 0.292222406713979 | 0.953251945985639 | -0.589887035 | 0.555624799806288 | 0.983014785887869 | 0.554389908123531 | 1.74302968923968 | 0.994304114950957 |
| ebi-fl187-GCST90094849 | finngen_R11_O15_POOR_FETGRO | Poor fetal growth \|\| id:finngen_R11_O15_POOR_FETGRO | Strawberries liking \|\| id:ebi-fl187-GCST90094849 | Inverse variance weighted | 5 | -0.194255377 | 0.404614779700778 | 0.631156603062582 | -0.987300345 | 0.598789591633879 | 0.82344758818623 | 0.372581175241158 | 1.81991462679464 | 0.96571624475698 |
| ebi-fl187-GCST90094849 | finngen_R11_O15_POOR_FETGRO | Poor fetal growth \|\| id:finngen_R11_O15_POOR_FETGRO | Strawberries liking \|\| id:ebi-fl187-GCST90094849 | MR Egger | 5 | -1.957381076 | 4.63275958191137 | 0.701108137558942 | -11.03758986 | 7.12282770462817 | 0.141227801910992 | 1.60855395129588e-05 | 1239.95169801685 | 0.970850396659599 |
| ebi-fl187-GCST90094849 | finngen_R11_O15_POOR_FETGRO | Poor fetal growth \|\| id:finngen_R11_O15_POOR_FETGRO | Strawberries liking \|\| id:ebi-fl187-GCST90094849 | Weighted median | 5 | 0.0172685568111576 | 0.4386100466852 | 0.968594526709781 | -0.842407135 | 0.87694424831415 | 1.01741852031244 | 0.430672587777936 | 2.40354384014914 | 0.987966417243977 |
| ebi-fl187-GCST90094849 | finngen_R11_O15_POOR_FETGRO | Poor fetal growth \|\| id:finngen_R11_O15_POOR_FETGRO | Strawberries liking \|\| id:ebi-fl187-GCST90094849 | Weighted mode | 5 | 0.198942053662053 | 0.73346404929883 | 0.799631655863311 | -1.238647483 | 1.63653159028776 | 1.22011126287183 | 0.289775879828846 | 5.13732024441083 | 0.972577670507659 |
| ebi-fl187-GCST90094850 | finngen_R11_O15_POOR_FETGRO | Poor fetal growth \|\| id:finngen_R11_O15_POOR_FETGRO | F-strong alcohol liking (derived food-liking factor) \|\| id:ebi-fl187-GCST90094850 | Inverse variance weighted | 5 | 0.0622624365694401 | 0.167669179914119 | 0.710383583350755 | -0.266369156 | 0.390894029201113 | 1.06424160399812 | 0.766156244068049 | 1.47830184828447 | 0.96571624475698 |
| ebi-fl187-GCST90094850 | finngen_R11_O15_POOR_FETGRO | Poor fetal growth \|\| id:finngen_R11_O15_POOR_FETGRO | F-strong alcohol liking (derived food-liking factor) \|\| id:ebi-fl187-GCST90094850 | MR Egger | 5 | -1.299122202 | 1.87551006855341 | 0.538316501108487 | -4.975121936 | 2.37687753224158 | 0.272771125890896 | 0.00690767659027697 | 10.7712175211729 | 0.970850396659599 |
| ebi-fl187-GCST90094850 | finngen_R11_O15_POOR_FETGRO | Poor fetal growth \|\| id:finngen_R11_O15_POOR_FETGRO | F-strong alcohol liking (derived food-liking factor) \|\| id:ebi-fl187-GCST90094850 | Weighted median | 5 | -0.109184628 | 0.151361605359788 | 0.470694471371234 | -0.405853374 | 0.18748411895163 | 0.896564871637689 | 0.666407872943264 | 1.20621109337215 | 0.918328271889597 |
| ebi-fl187-GCST90094850 | finngen_R11_O15_POOR_FETGRO | Poor fetal growth \|\| id:finngen_R11_O15_POOR_FETGRO | F-strong alcohol liking (derived food-liking factor) \|\| id:ebi-fl187-GCST90094850 | Weighted mode | 5 | -0.168926321 | 0.184199653405717 | 0.410990110909407 | -0.529957642 | 0.192104999527568 | 0.844571128125141 | 0.588629902439985 | 1.21179774847626 | 0.972577670507659 |
| ebi-fl187-GCST90094851 | finngen_R11_O15_POOR_FETGRO | Poor fetal growth \|\| id:finngen_R11_O15_POOR_FETGRO | F-strong flavour liking (derived food-liking factor) \|\| id:ebi-fl187-GCST90094851 | Inverse variance weighted | 41 | -0.068117492 | 0.0615753850069406 | 0.268620276267244 | -0.188805247 | 0.0525702623699175 | 0.934150711666199 | 0.827947736417894 | 1.05397661437173 | 0.888224550184161 |
| ebi-fl187-GCST90094851 | finngen_R11_O15_POOR_FETGRO | Poor fetal growth \|\| id:finngen_R11_O15_POOR_FETGRO | F-strong flavour liking (derived food-liking factor) \|\| id:ebi-fl187-GCST90094851 | MR Egger | 41 | -0.281881546 | 0.35931301276369 | 0.437485384649995 | -0.986135051 | 0.422371958891178 | 0.754363036465276 | 0.373015594783039 | 1.52557587067131 | 0.970850396659599 |
| ebi-fl187-GCST90094851 | finngen_R11_O15_POOR_FETGRO | Poor fetal growth \|\| id:finngen_R11_O15_POOR_FETGRO | F-strong flavour liking (derived food-liking factor) \|\| id:ebi-fl187-GCST90094851 | Weighted median | 41 | -0.077854977 | 0.0855785987567827 | 0.362955312436082 | -0.245589031 | 0.0898790764352079 | 0.925098577268321 | 0.782243637002375 | 1.09404198024978 | 0.918328271889597 |
| ebi-fl187-GCST90094851 | finngen_R11_O15_POOR_FETGRO | Poor fetal growth \|\| id:finngen_R11_O15_POOR_FETGRO | F-strong flavour liking (derived food-liking factor) \|\| id:ebi-fl187-GCST90094851 | Weighted mode | 41 | -0.077371363 | 0.186678699023209 | 0.680749609891224 | -0.443261613 | 0.288518887039874 | 0.925546076167625 | 0.641939245382296 | 1.33444955308681 | 0.972577670507659 |
| ebi-fl187-GCST90094852 | finngen_R11_O15_POOR_FETGRO | Poor fetal growth \|\| id:finngen_R11_O15_POOR_FETGRO | F-strong vegetable liking (derived food-liking factor) \|\| id:ebi-fl187-GCST90094852 | Inverse variance weighted | 24 | -0.197712703 | 0.0724134427493705 | 0.0063270668812125 | -0.339643051 | -0.055782356 | 0.820605576444542 | 0.71202443366329 | 0.945744949548067 | 0.392515819825973 |
| ebi-fl187-GCST90094852 | finngen_R11_O15_POOR_FETGRO | Poor fetal growth \|\| id:finngen_R11_O15_POOR_FETGRO | F-strong vegetable liking (derived food-liking factor) \|\| id:ebi-fl187-GCST90094852 | MR Egger | 24 | -0.381944373 | 0.335138861395475 | 0.266684744402787 | -1.038816541 | 0.274927795665153 | 0.682533019638929 | 0.353873228699033 | 1.3164356190777 | 0.970850396659599 |
| ebi-fl187-GCST90094852 | finngen_R11_O15_POOR_FETGRO | Poor fetal growth \|\| id:finngen_R11_O15_POOR_FETGRO | F-strong vegetable liking (derived food-liking factor) \|\| id:ebi-fl187-GCST90094852 | Weighted median | 24 | -0.140764382 | 0.105152811283125 | 0.180679774620346 | -0.346863892 | 0.0653351282557875 | 0.86869396764403 | 0.706901536682923 | 1.0675167194602 | 0.918328271889597 |
| ebi-fl187-GCST90094852 | finngen_R11_O15_POOR_FETGRO | Poor fetal growth \|\| id:finngen_R11_O15_POOR_FETGRO | F-strong vegetable liking (derived food-liking factor) \|\| id:ebi-fl187-GCST90094852 | Weighted mode | 24 | -0.125662259 | 0.15471055130052 | 0.424980806199381 | -0.428894939 | 0.177570421796701 | 0.881912654769933 | 0.65122834409193 | 1.19431216054926 | 0.972577670507659 |
| ebi-fl187-GCST90094853 | finngen_R11_O15_POOR_FETGRO | Poor fetal growth \|\| id:finngen_R11_O15_POOR_FETGRO | F-sweetened coffee drink liking (derived food-liking factor) \|\| id:ebi-fl187-GCST90094853 | Inverse variance weighted | 4 | 0.0202433297104527 | 0.221391418921721 | 0.927145497572423 | -0.413683851 | 0.454170510797025 | 1.02044961552861 | 0.661209958844027 | 1.57486650632574 | 0.994304114950957 |
| ebi-fl187-GCST90094853 | finngen_R11_O15_POOR_FETGRO | Poor fetal growth \|\| id:finngen_R11_O15_POOR_FETGRO | F-sweetened coffee drink liking (derived food-liking factor) \|\| id:ebi-fl187-GCST90094853 | MR Egger | 4 | 1.55320761187245 | 1.31991583538106 | 0.360381581947049 | -1.033827425 | 4.14024264921932 | 4.72660701384664 | 0.3556431546314 | 62.8180623538193 | 0.970850396659599 |
| ebi-fl187-GCST90094853 | finngen_R11_O15_POOR_FETGRO | Poor fetal growth \|\| id:finngen_R11_O15_POOR_FETGRO | F-sweetened coffee drink liking (derived food-liking factor) \|\| id:ebi-fl187-GCST90094853 | Weighted median | 4 | -0.016601718 | 0.25153865253108 | 0.947377295557283 | -0.509617477 | 0.476414040475319 | 0.983535330577994 | 0.600725325853342 | 1.61028960302454 | 0.979383285272057 |
| ebi-fl187-GCST90094853 | finngen_R11_O15_POOR_FETGRO | Poor fetal growth \|\| id:finngen_R11_O15_POOR_FETGRO | F-sweetened coffee drink liking (derived food-liking factor) \|\| id:ebi-fl187-GCST90094853 | Weighted mode | 4 | -0.303150386 | 0.412791639388409 | 0.515905003457498 | -1.112222 | 0.505921226774619 | 0.738488029443332 | 0.328827494146871 | 1.65851268321106 | 0.972577670507659 |
| ebi-fl187-GCST90094854 | finngen_R11_O15_POOR_FETGRO | Poor fetal growth \|\| id:finngen_R11_O15_POOR_FETGRO | F-sweet food liking (derived food-liking factor) \|\| id:ebi-fl187-GCST90094854 | Inverse variance weighted | 13 | 0.115569493951386 | 0.139823691216627 | 0.408499377333804 | -0.158484941 | 0.389623928735975 | 1.12251251966828 | 0.853435815726464 | 1.47642544827985 | 0.888224550184161 |
| ebi-fl187-GCST90094854 | finngen_R11_O15_POOR_FETGRO | Poor fetal growth \|\| id:finngen_R11_O15_POOR_FETGRO | F-sweet food liking (derived food-liking factor) \|\| id:ebi-fl187-GCST90094854 | MR Egger | 13 | 0.393177317384777 | 0.574282007771955 | 0.507738974166072 | -0.732415418 | 1.51877005261781 | 1.48168109385417 | 0.4807463831703 | 4.56660505567901 | 0.970850396659599 |
| ebi-fl187-GCST90094854 | finngen_R11_O15_POOR_FETGRO | Poor fetal growth \|\| id:finngen_R11_O15_POOR_FETGRO | F-sweet food liking (derived food-liking factor) \|\| id:ebi-fl187-GCST90094854 | Weighted median | 13 | 0.304552789121926 | 0.157518378544216 | 0.053181661929113 | -0.004183233 | 0.61328881106859 | 1.35601844118804 | 0.995825504705742 | 1.84649419416648 | 0.81367942751543 |
| ebi-fl187-GCST90094854 | finngen_R11_O15_POOR_FETGRO | Poor fetal growth \|\| id:finngen_R11_O15_POOR_FETGRO | F-sweet food liking (derived food-liking factor) \|\| id:ebi-fl187-GCST90094854 | Weighted mode | 13 | 0.29081265316205 | 0.226660538862534 | 0.223707515400434 | -0.153442003 | 0.735067309332618 | 1.33751398146084 | 0.857750509705275 | 2.08562236963042 | 0.972577670507659 |
| ebi-fl187-GCST90094855 | finngen_R11_O15_POOR_FETGRO | Poor fetal growth \|\| id:finngen_R11_O15_POOR_FETGRO | Tea difference liking \|\| id:ebi-fl187-GCST90094855 | Inverse variance weighted | 2 | -0.178794751 | 0.135278095585044 | 0.186273751951194 | -0.443939818 | 0.0863503162958076 | 0.836277526866817 | 0.641504026405913 | 1.09018817210036 | 0.888224550184161 |
| ebi-fl187-GCST90094857 | finngen_R11_O15_POOR_FETGRO | Poor fetal growth \|\| id:finngen_R11_O15_POOR_FETGRO | Tea with sugar liking \|\| id:ebi-fl187-GCST90094857 | Inverse variance weighted | 6 | -0.176587018 | 0.190085951057323 | 0.352896802672441 | -0.549155482 | 0.19598144579371 | 0.838125843713666 | 0.577437260479077 | 1.21650433385256 | 0.888224550184161 |
| ebi-fl187-GCST90094857 | finngen_R11_O15_POOR_FETGRO | Poor fetal growth \|\| id:finngen_R11_O15_POOR_FETGRO | Tea with sugar liking \|\| id:ebi-fl187-GCST90094857 | MR Egger | 6 | -0.797378875 | 0.87057361297993 | 0.41152993665967 | -2.503703156 | 0.908945406518029 | 0.450508256394163 | 0.0817815871756639 | 2.48170396403977 | 0.970850396659599 |
| ebi-fl187-GCST90094857 | finngen_R11_O15_POOR_FETGRO | Poor fetal growth \|\| id:finngen_R11_O15_POOR_FETGRO | Tea with sugar liking \|\| id:ebi-fl187-GCST90094857 | Weighted median | 6 | -0.238054097 | 0.228867266515009 | 0.298274663102417 | -0.686633939 | 0.210525745665565 | 0.788160053079514 | 0.503267249385111 | 1.23432683137891 | 0.918328271889597 |
| ebi-fl187-GCST90094857 | finngen_R11_O15_POOR_FETGRO | Poor fetal growth \|\| id:finngen_R11_O15_POOR_FETGRO | Tea with sugar liking \|\| id:ebi-fl187-GCST90094857 | Weighted mode | 6 | -0.236781629 | 0.271352753307293 | 0.4228001819648 | -0.768633025 | 0.295069767894028 | 0.789163599972337 | 0.463646428362642 | 1.34322006905268 | 0.972577670507659 |
| ebi-fl187-GCST90094859 | finngen_R11_O15_POOR_FETGRO | Poor fetal growth \|\| id:finngen_R11_O15_POOR_FETGRO | Tinned tuna liking \|\| id:ebi-fl187-GCST90094859 | Inverse variance weighted | 3 | -0.009094244 | 0.303773433168507 | 0.976116829211079 | -0.604490173 | 0.586301685437118 | 0.990946983987444 | 0.546352901334883 | 1.79732902062859 | 0.994304114950957 |
| ebi-fl187-GCST90094859 | finngen_R11_O15_POOR_FETGRO | Poor fetal growth \|\| id:finngen_R11_O15_POOR_FETGRO | Tinned tuna liking \|\| id:ebi-fl187-GCST90094859 | MR Egger | 3 | 2.07002304340429 | 2.54273992610368 | 0.565013960675443 | -2.913747212 | 7.0537932985675 | 7.9250057348566 | 0.0542719797594668 | 1157.24018500642 | 0.970850396659599 |
| ebi-fl187-GCST90094859 | finngen_R11_O15_POOR_FETGRO | Poor fetal growth \|\| id:finngen_R11_O15_POOR_FETGRO | Tinned tuna liking \|\| id:ebi-fl187-GCST90094859 | Weighted median | 3 | 0.113193685227237 | 0.307338965657054 | 0.712647747250945 | -0.489190687 | 0.715578057915063 | 1.11984881011621 | 0.613122401092436 | 2.04536868214939 | 0.918328271889597 |
| ebi-fl187-GCST90094859 | finngen_R11_O15_POOR_FETGRO | Poor fetal growth \|\| id:finngen_R11_O15_POOR_FETGRO | Tinned tuna liking \|\| id:ebi-fl187-GCST90094859 | Weighted mode | 3 | 0.213375042209037 | 0.350109088717091 | 0.604237348779738 | -0.472838772 | 0.899588856094536 | 1.23784880975071 | 0.623230545493815 | 2.45859206818426 | 0.972577670507659 |
| ebi-fl187-GCST90094860 | finngen_R11_O15_POOR_FETGRO | Poor fetal growth \|\| id:finngen_R11_O15_POOR_FETGRO | Tomatoes liking \|\| id:ebi-fl187-GCST90094860 | Inverse variance weighted (multiplicative random effects) | 4 | 0.0202592704559525 | 0.57026541916208 | 0.971660284847478 | -1.097460951 | 1.13797949201363 | 1.02046588238588 | 0.33371733353664 | 3.12045708287805 | 0.994304114950957 |
| ebi-fl187-GCST90094860 | finngen_R11_O15_POOR_FETGRO | Poor fetal growth \|\| id:finngen_R11_O15_POOR_FETGRO | Tomatoes liking \|\| id:ebi-fl187-GCST90094860 | MR Egger | 4 | -7.485458427 | 5.3513501823301 | 0.296778764856307 | -17.97410478 | 3.00318792997768 | 0.000561185848031381 | 1.56295140357922e-08 | 20.1496703806336 | 0.970850396659599 |
| ebi-fl187-GCST90094860 | finngen_R11_O15_POOR_FETGRO | Poor fetal growth \|\| id:finngen_R11_O15_POOR_FETGRO | Tomatoes liking \|\| id:ebi-fl187-GCST90094860 | Weighted median | 4 | -0.063743221 | 0.403644343121689 | 0.874520411310687 | -0.854886134 | 0.72739969147725 | 0.938245890404332 | 0.425331619322456 | 2.06969176724488 | 0.969669326094948 |
| ebi-fl187-GCST90094860 | finngen_R11_O15_POOR_FETGRO | Poor fetal growth \|\| id:finngen_R11_O15_POOR_FETGRO | Tomatoes liking \|\| id:ebi-fl187-GCST90094860 | Weighted mode | 4 | -0.490872278 | 0.781154774748095 | 0.5743318724167 | -2.021935637 | 1.04019108043314 | 0.612092246610227 | 0.132398940658576 | 2.8297576740172 | 0.972577670507659 |
| ebi-fl187-GCST90094861 | finngen_R11_O15_POOR_FETGRO | Poor fetal growth \|\| id:finngen_R11_O15_POOR_FETGRO | Turnip liking \|\| id:ebi-fl187-GCST90094861 | Inverse variance weighted | 3 | -0.292851695 | 0.272860013206242 | 0.283151258964246 | -0.827657321 | 0.241953930625615 | 0.746132787522384 | 0.437072007250888 | 1.27373551126636 | 0.888224550184161 |
| ebi-fl187-GCST90094861 | finngen_R11_O15_POOR_FETGRO | Poor fetal growth \|\| id:finngen_R11_O15_POOR_FETGRO | Turnip liking \|\| id:ebi-fl187-GCST90094861 | MR Egger | 3 | -1.892746284 | 3.22605290670563 | 0.662217482527117 | -8.215809981 | 4.43031741280009 | 0.150657491869602 | 0.00027034544752013 | 83.9580620448554 | 0.970850396659599 |
| ebi-fl187-GCST90094861 | finngen_R11_O15_POOR_FETGRO | Poor fetal growth \|\| id:finngen_R11_O15_POOR_FETGRO | Turnip liking \|\| id:ebi-fl187-GCST90094861 | Weighted median | 3 | -0.261303248 | 0.312334126299003 | 0.402809311881203 | -0.873478136 | 0.350871639421099 | 0.77004736878424 | 0.417496910102383 | 1.4203050030385 | 0.918328271889597 |
| ebi-fl187-GCST90094861 | finngen_R11_O15_POOR_FETGRO | Poor fetal growth \|\| id:finngen_R11_O15_POOR_FETGRO | Turnip liking \|\| id:ebi-fl187-GCST90094861 | Weighted mode | 3 | -0.464726879 | 0.357594198229396 | 0.323361923957184 | -1.165611508 | 0.236157749219166 | 0.628306685312675 | 0.3117319771879 | 1.26637406393072 | 0.972577670507659 |
| ebi-fl187-GCST90094862 | finngen_R11_O15_POOR_FETGRO | Poor fetal growth \|\| id:finngen_R11_O15_POOR_FETGRO | Vegetables liking \|\| id:ebi-fl187-GCST90094862 | Inverse variance weighted (multiplicative random effects) | 3 | -1.609920179 | 0.896200606711904 | 0.0724335112918738 | -3.366473368 | 0.146633009833835 | 0.199903569876917 | 0.0345111310284089 | 1.15792893651153 | 0.706473758635168 |
| ebi-fl187-GCST90094862 | finngen_R11_O15_POOR_FETGRO | Poor fetal growth \|\| id:finngen_R11_O15_POOR_FETGRO | Vegetables liking \|\| id:ebi-fl187-GCST90094862 | MR Egger | 3 | -14.34017905 | 12.2254168800627 | 0.449428337921712 | -38.30199613 | 9.62163803566557 | 5.91751502629771e-07 | 2.32088933602702e-17 | 15087.743970767 | 0.970850396659599 |
| ebi-fl187-GCST90094862 | finngen_R11_O15_POOR_FETGRO | Poor fetal growth \|\| id:finngen_R11_O15_POOR_FETGRO | Vegetables liking \|\| id:ebi-fl187-GCST90094862 | Weighted median | 3 | -1.446473556 | 0.637512428910565 | 0.0232723463879032 | -2.695997916 | -0.196949195 | 0.235398947385071 | 0.0674750137410272 | 0.821232354878451 | 0.652429624709108 |
| ebi-fl187-GCST90094862 | finngen_R11_O15_POOR_FETGRO | Poor fetal growth \|\| id:finngen_R11_O15_POOR_FETGRO | Vegetables liking \|\| id:ebi-fl187-GCST90094862 | Weighted mode | 3 | -1.319000616 | 0.769108345133675 | 0.228485455415497 | -2.826452972 | 0.188451740713647 | 0.267402406227358 | 0.0592225455730124 | 1.20737881434069 | 0.972577670507659 |
| ebi-fl187-GCST90094863 | finngen_R11_O15_POOR_FETGRO | Poor fetal growth \|\| id:finngen_R11_O15_POOR_FETGRO | F-vegetarian liking (derived food-liking factor) \|\| id:ebi-fl187-GCST90094863 | Inverse variance weighted (multiplicative random effects) | 13 | 0.0724670764659077 | 0.118800100613098 | 0.54186729942736 | -0.160381121 | 0.305315273667581 | 1.07515740752587 | 0.851819081178749 | 1.3570527785761 | 0.927237411635561 |
| ebi-fl187-GCST90094863 | finngen_R11_O15_POOR_FETGRO | Poor fetal growth \|\| id:finngen_R11_O15_POOR_FETGRO | F-vegetarian liking (derived food-liking factor) \|\| id:ebi-fl187-GCST90094863 | MR Egger | 13 | -0.372426182 | 0.762990238017059 | 0.635053184041336 | -1.867887049 | 1.1230346841151 | 0.689060514475147 | 0.154449661863877 | 3.07416919453809 | 0.970850396659599 |
| ebi-fl187-GCST90094863 | finngen_R11_O15_POOR_FETGRO | Poor fetal growth \|\| id:finngen_R11_O15_POOR_FETGRO | F-vegetarian liking (derived food-liking factor) \|\| id:ebi-fl187-GCST90094863 | Weighted median | 13 | -0.044608001 | 0.131688367598321 | 0.73480627262085 | -0.302717201 | 0.213501199617344 | 0.956372305469601 | 0.738808000722019 | 1.23800498339944 | 0.918328271889597 |
| ebi-fl187-GCST90094863 | finngen_R11_O15_POOR_FETGRO | Poor fetal growth \|\| id:finngen_R11_O15_POOR_FETGRO | F-vegetarian liking (derived food-liking factor) \|\| id:ebi-fl187-GCST90094863 | Weighted mode | 13 | -0.018607726 | 0.176181614696707 | 0.917631412846021 | -0.363923691 | 0.326708238406923 | 0.981564328505945 | 0.694944223076454 | 1.38639692079192 | 0.988620775239461 |
| ebi-fl187-GCST90094864 | finngen_R11_O15_POOR_FETGRO | Poor fetal growth \|\| id:finngen_R11_O15_POOR_FETGRO | Vinegar liking \|\| id:ebi-fl187-GCST90094864 | Inverse variance weighted | 11 | 0.140054654700367 | 0.159467590150754 | 0.379800429722257 | -0.172501822 | 0.452611131395844 | 1.15033666844509 | 0.841556755498083 | 1.57241260571445 | 0.888224550184161 |
| ebi-fl187-GCST90094864 | finngen_R11_O15_POOR_FETGRO | Poor fetal growth \|\| id:finngen_R11_O15_POOR_FETGRO | Vinegar liking \|\| id:ebi-fl187-GCST90094864 | MR Egger | 11 | 1.00167573674274 | 0.886338143624658 | 0.287639980992926 | -0.735547025 | 2.73889849824707 | 2.72284077192382 | 0.479243229345106 | 15.4699355468869 | 0.970850396659599 |
| ebi-fl187-GCST90094864 | finngen_R11_O15_POOR_FETGRO | Poor fetal growth \|\| id:finngen_R11_O15_POOR_FETGRO | Vinegar liking \|\| id:ebi-fl187-GCST90094864 | Weighted median | 11 | 0.169384151129976 | 0.188309831587081 | 0.368387929295353 | -0.199703119 | 0.538471421040654 | 1.1845751073961 | 0.818973854946636 | 1.71338581395117 | 0.918328271889597 |
| ebi-fl187-GCST90094864 | finngen_R11_O15_POOR_FETGRO | Poor fetal growth \|\| id:finngen_R11_O15_POOR_FETGRO | Vinegar liking \|\| id:ebi-fl187-GCST90094864 | Weighted mode | 11 | 0.246007876117665 | 0.252664951136209 | 0.353192182111637 | -0.249215428 | 0.741231180344635 | 1.27890964634493 | 0.779412048033552 | 2.09851757827036 | 0.972577670507659 |
| ebi-fl187-GCST90094865 | finngen_R11_O15_POOR_FETGRO | Poor fetal growth \|\| id:finngen_R11_O15_POOR_FETGRO | White bread liking \|\| id:ebi-fl187-GCST90094865 | Inverse variance weighted | 4 | -0.403515036 | 0.251577304619639 | 0.10872716107788 | -0.896606553 | 0.0895764808300342 | 0.667967983006957 | 0.407951675792029 | 1.09371097803712 | 0.761090127545161 |
| ebi-fl187-GCST90094865 | finngen_R11_O15_POOR_FETGRO | Poor fetal growth \|\| id:finngen_R11_O15_POOR_FETGRO | White bread liking \|\| id:ebi-fl187-GCST90094865 | MR Egger | 4 | -1.809391887 | 1.94384215709362 | 0.450206640270741 | -5.619322515 | 2.00053874086501 | 0.163753687270395 | 0.00362709758117536 | 7.39303795790374 | 0.970850396659599 |
| ebi-fl187-GCST90094865 | finngen_R11_O15_POOR_FETGRO | Poor fetal growth \|\| id:finngen_R11_O15_POOR_FETGRO | White bread liking \|\| id:ebi-fl187-GCST90094865 | Weighted median | 4 | -0.455215834 | 0.309171262822917 | 0.140919818448594 | -1.061191509 | 0.150759841067838 | 0.634311047240833 | 0.34604325087835 | 1.1627173875823 | 0.918328271889597 |
| ebi-fl187-GCST90094865 | finngen_R11_O15_POOR_FETGRO | Poor fetal growth \|\| id:finngen_R11_O15_POOR_FETGRO | White bread liking \|\| id:ebi-fl187-GCST90094865 | Weighted mode | 4 | -0.46745462 | 0.360531831603839 | 0.285501255810846 | -1.17409701 | 0.239187769454126 | 0.626595162650786 | 0.309097965946677 | 1.27021702214985 | 0.972577670507659 |
| ebi-fl187-GCST90094867 | finngen_R11_O15_POOR_FETGRO | Poor fetal growth \|\| id:finngen_R11_O15_POOR_FETGRO | White wine liking \|\| id:ebi-fl187-GCST90094867 | Inverse variance weighted (multiplicative random effects) | 5 | 0.340265893279329 | 0.336567350317224 | 0.312021688437406 | -0.319406113 | 0.999937899901088 | 1.4053212063545 | 0.726580415380447 | 2.71811302812994 | 0.888224550184161 |
| ebi-fl187-GCST90094867 | finngen_R11_O15_POOR_FETGRO | Poor fetal growth \|\| id:finngen_R11_O15_POOR_FETGRO | White wine liking \|\| id:ebi-fl187-GCST90094867 | MR Egger | 5 | 1.13293543309228 | 0.867023023483998 | 0.282460988101968 | -0.566429693 | 2.83230055912092 | 3.10475694299339 | 0.567548146863985 | 16.9844897359445 | 0.970850396659599 |
| ebi-fl187-GCST90094867 | finngen_R11_O15_POOR_FETGRO | Poor fetal growth \|\| id:finngen_R11_O15_POOR_FETGRO | White wine liking \|\| id:ebi-fl187-GCST90094867 | Weighted median | 5 | 0.630070743634831 | 0.29473023300129 | 0.0325339566152649 | 0.0523994869523016 | 1.20774200031736 | 1.87774341296003 | 1.05379663644359 | 3.34592102781259 | 0.652429624709108 |
| ebi-fl187-GCST90094867 | finngen_R11_O15_POOR_FETGRO | Poor fetal growth \|\| id:finngen_R11_O15_POOR_FETGRO | White wine liking \|\| id:ebi-fl187-GCST90094867 | Weighted mode | 5 | 0.752380863279466 | 0.418063084085872 | 0.146292399447198 | -0.067022782 | 1.57178450808778 | 2.12204630909735 | 0.935173896403004 | 4.81523335421792 | 0.972577670507659 |
| ebi-fl187-GCST90094870 | finngen_R11_O15_POOR_FETGRO | Poor fetal growth \|\| id:finngen_R11_O15_POOR_FETGRO | Whole milk liking \|\| id:ebi-fl187-GCST90094870 | Inverse variance weighted | 4 | 0.0304644555135574 | 0.211822204344896 | 0.885641915621799 | -0.384707065 | 0.445635976029554 | 1.03093324540476 | 0.68064999320794 | 1.56148294584071 | 0.994304114950957 |
| ebi-fl187-GCST90094870 | finngen_R11_O15_POOR_FETGRO | Poor fetal growth \|\| id:finngen_R11_O15_POOR_FETGRO | Whole milk liking \|\| id:ebi-fl187-GCST90094870 | MR Egger | 4 | 0.365026794226201 | 1.26579690382851 | 0.800198261543199 | -2.115935137 | 2.84598872573008 | 1.4405526061245 | 0.120520533596534 | 17.2185747032882 | 0.970850396659599 |
| ebi-fl187-GCST90094870 | finngen_R11_O15_POOR_FETGRO | Poor fetal growth \|\| id:finngen_R11_O15_POOR_FETGRO | Whole milk liking \|\| id:ebi-fl187-GCST90094870 | Weighted median | 4 | -0.074139893 | 0.232935009082114 | 0.750268195988233 | -0.530692511 | 0.382412724783979 | 0.928541788242921 | 0.588197495475809 | 1.46581693928484 | 0.918328271889597 |
| ebi-fl187-GCST90094870 | finngen_R11_O15_POOR_FETGRO | Poor fetal growth \|\| id:finngen_R11_O15_POOR_FETGRO | Whole milk liking \|\| id:ebi-fl187-GCST90094870 | Weighted mode | 4 | -0.08834879 | 0.27194337035341 | 0.766609357537834 | -0.621357796 | 0.444660215504661 | 0.915441523828646 | 0.537214514293795 | 1.55996005553082 | 0.972577670507659 |
| ebi-fl187-GCST90094872 | finngen_R11_O15_POOR_FETGRO | Poor fetal growth \|\| id:finngen_R11_O15_POOR_FETGRO | F-wine liking (derived food-liking factor) \|\| id:ebi-fl187-GCST90094872 | Inverse variance weighted | 9 | 0.466721649605402 | 0.418806311464338 | 0.265103634161198 | -0.354138721 | 1.28758202007551 | 1.59475744026842 | 0.701777609405957 | 3.62401316201056 | 0.888224550184161 |
| ebi-fl187-GCST90094872 | finngen_R11_O15_POOR_FETGRO | Poor fetal growth \|\| id:finngen_R11_O15_POOR_FETGRO | F-wine liking (derived food-liking factor) \|\| id:ebi-fl187-GCST90094872 | MR Egger | 9 | 2.8565987828129 | 1.15661799732955 | 0.0428464262159965 | 0.58962750804699 | 5.12357005757881 | 17.4022373765421 | 1.80331656936619 | 167.933834166431 | 0.970850396659599 |
| ebi-fl187-GCST90094872 | finngen_R11_O15_POOR_FETGRO | Poor fetal growth \|\| id:finngen_R11_O15_POOR_FETGRO | F-wine liking (derived food-liking factor) \|\| id:ebi-fl187-GCST90094872 | Weighted median | 9 | 0.233475415400405 | 0.481502837156099 | 0.627755106008854 | -0.710270145 | 1.17722097622636 | 1.26298177760794 | 0.491511399968149 | 3.2453427747008 | 0.918328271889597 |
| ebi-fl187-GCST90094872 | finngen_R11_O15_POOR_FETGRO | Poor fetal growth \|\| id:finngen_R11_O15_POOR_FETGRO | F-wine liking (derived food-liking factor) \|\| id:ebi-fl187-GCST90094872 | Weighted mode | 9 | -0.048569118 | 0.887021820322688 | 0.957676173895638 | -1.787131885 | 1.68999365022747 | 0.952591496187645 | 0.167439717931974 | 5.41944629277077 | 0.988620775239461 |
| ebi-fl187-GCST90094873 | finngen_R11_O15_POOR_FETGRO | Poor fetal growth \|\| id:finngen_R11_O15_POOR_FETGRO | Whiskey liking \|\| id:ebi-fl187-GCST90094873 | Inverse variance weighted | 5 | 0.185048186924063 | 0.235071371934984 | 0.431164789928882 | -0.275691702 | 0.645788075916632 | 1.20327642092025 | 0.759046907283572 | 1.90748968377225 | 0.889015330589552 |
| ebi-fl187-GCST90094873 | finngen_R11_O15_POOR_FETGRO | Poor fetal growth \|\| id:finngen_R11_O15_POOR_FETGRO | Whiskey liking \|\| id:ebi-fl187-GCST90094873 | MR Egger | 5 | 0.843456400093856 | 6.09988048217049 | 0.898783296038343 | -11.11230934 | 12.799222145148 | 2.32438712024483 | 1.49274412389818e-05 | 361935.806563497 | 0.993285374120483 |
| ebi-fl187-GCST90094873 | finngen_R11_O15_POOR_FETGRO | Poor fetal growth \|\| id:finngen_R11_O15_POOR_FETGRO | Whiskey liking \|\| id:ebi-fl187-GCST90094873 | Weighted median | 5 | -0.006153667 | 0.24552278932217 | 0.980004293916992 | -0.487378334 | 0.475071000517718 | 0.993865228474524 | 0.614234603375232 | 1.60812837137946 | 0.989627362077292 |
| ebi-fl187-GCST90094873 | finngen_R11_O15_POOR_FETGRO | Poor fetal growth \|\| id:finngen_R11_O15_POOR_FETGRO | Whiskey liking \|\| id:ebi-fl187-GCST90094873 | Weighted mode | 5 | -0.027669661 | 0.278245331517337 | 0.925570708726821 | -0.573030511 | 0.517691188387539 | 0.972709637287278 | 0.563814201816957 | 1.67814864439815 | 0.988620775239461 |
